# Supplementary material for: Detection and characterization of the SARS-CoV-2 lineage B.1.526 in New York
Source: Nat Commun. 2021 Aug 9;12:4886. doi: 10.1038/s41467-021-25168-4 (PMC8352861; doi:10.1038/s41467-021-25168-4)
Supplement: Supplementary file 1 — Supplementary Information [file 41467_2021_25168_MOESM1_ESM.pdf]

Supplementary Information for

# Detection and characterization of the SARS-CoV-2 lineage B.1.526 in New York

Anthony P. West, Jr.[1*], Joel O. Wertheim[2], Jade C. Wang[3], Tetyana I. Vasylyeva[2], Jennifer L. Havens[4], Moinuddin A. Chowdhury[3], Edimarlyn Gonzalez[3], Courtney E. Fang[3], Steve S. Di Lonardo[3], Scott Hughes[3], Jennifer L. Rakeman[3], Henry H. Lee[5,6] Christopher O. Barnes[1], Priyanthi N. P. Gnanapragasam[1], Zhi Yang[1], Christian Gaebler[7], Marina Caskey[7], Michel C. Nussenzweig[7,8], Jennifer R. Keeffe[1], Pamela J. Bjorkman[1]

[1]Division of Biology and Biological Engineering, California Institute of Technology, Pasadena, CA 91125, USA.

[2]Department of Medicine, University of California San Diego, La Jolla, CA 92093

[3]New York City Public Health Laboratory, New York City Department of Health and Mental Hygiene, New York, NY, 10016 USA

[4]Bioinformatics and Systems Biology Graduate Program, University of California San Diego, La Jolla, CA 92093

[5]Pandemic Response Laboratory, Long Island City, NY 11101

[6]Department of Genetics, Harvard Medical School, Boston, MA 02115

[7]Laboratory of Molecular Immunology, The Rockefeller University, New York, NY 10065, USA.

[8]Howard Hughes Medical Institute, The Rockefeller University, New York, NY, 10065 USA.

*Corresponding author: Anthony P. West, Jr., apwest@caltech.edu

## Supplementary Methods.

Commands for the program **vdb**, implementing a mutation pattern query language:

Notation

| | | |
|---|---|---|
| cluster = group of viruses | < > = user input | n = an integer |
| pattern = group of mutations | [ ] = optional | ( ) = explanation of command |
| "world" = all viruses in database | -> result | |

To define a variable for a cluster or pattern: <name> = cluster or pattern
Set operations +, -, and * (intersection) can be applied to clusters or patterns
If no cluster is entered, all viruses will be used ("world")

Filter commands

| | | | |
|---|---|---|---|
| <cluster> from <country or state> | -> cluster | | |
| <cluster> containing [<n>] <pattern> | -> cluster | alias with | (matches for >=n mutations) |
| <cluster> not containing <pattern> | -> cluster | alias without | (considers whole pattern) |
| <cluster> before <date> | -> cluster | | |
| <cluster> after <date> | -> cluster | | |
| <cluster> > or < <n> | -> cluster | | (filter by number of mutations) |

Commands to find mutation patterns

| | | |
|---|---|---|
| consensus [for] <cluster or country or state> | -> pattern | |
| patterns [in] [<n>] <cluster> | -> pattern | (lists n patterns) |

Listing commands

| | | |
|---|---|---|
| list [<n>] <cluster> | | |
| [list] countries [for] <cluster> | | |
| [list] states [for] <cluster> | | |
| [list] frequencies [for] <cluster> | alias freq | (frequency of individual mutations) |
| [list] monthly [for] <cluster> [<cluster2>] | | (number of viruses per month or week) |
| [list] weekly [for] <cluster> [<cluster2>] | | (as a fraction of number of viruses in cluster2) |
| [list] patterns | | (list built-in and user-defined patterns) |
| [list] clusters | | (list built-in and user-defined clusters) |

Other commands

sort <cluster>    (by date)
help
history
quit

## Supplementary Fig. 1.

Phylogenetic tree of lineage B.1.526 indicating spike mutations. The inset lists non-spike mutations common in this lineage.

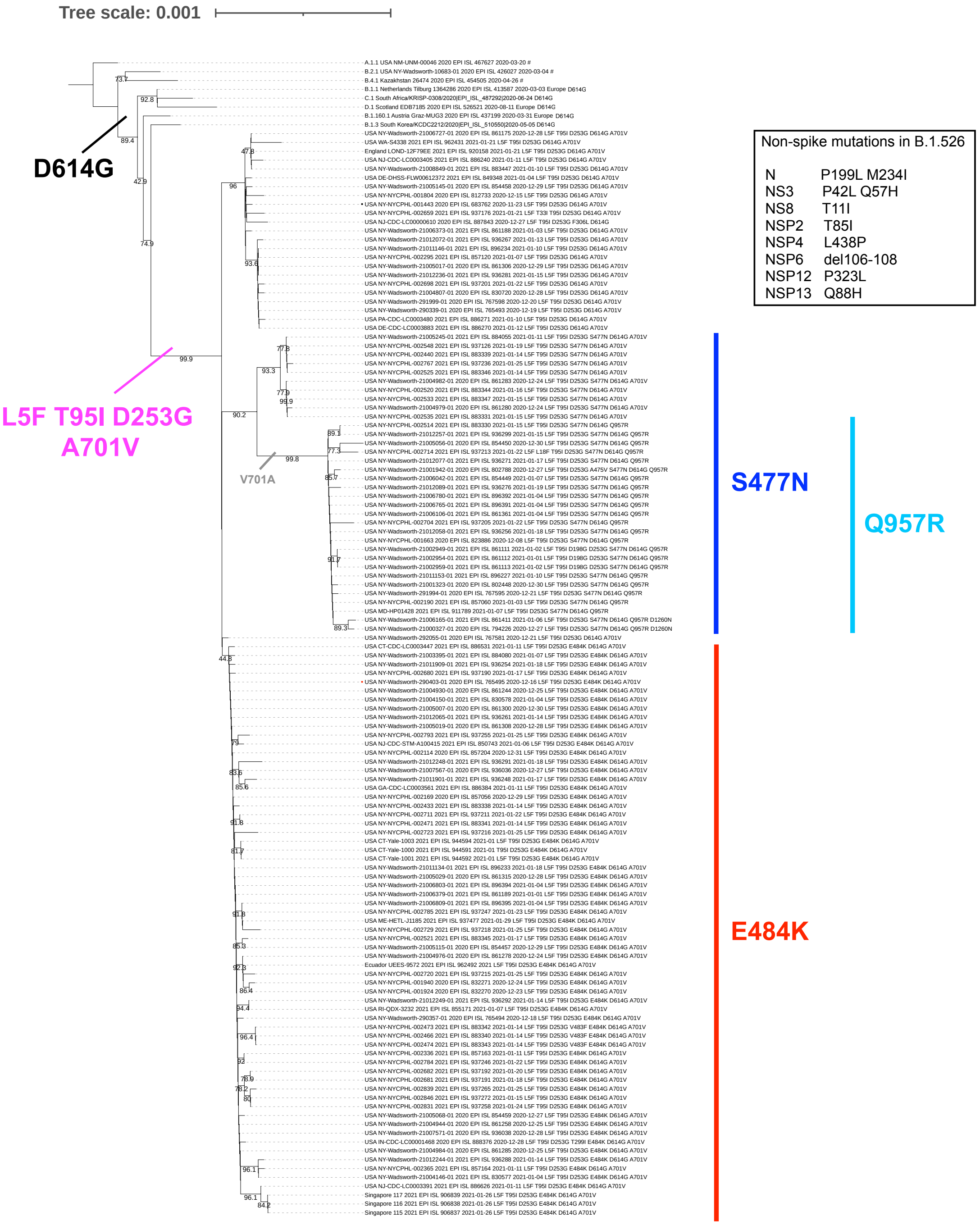

Supplementary Fig. 2.

Maximum likelihood phylogenetic tree of the B.1.526 lineage (including B.1.526.2) in relation to a sister clade defined by an L452R spike mutation (including B.1.526.1, shown in orange) and the 20C ancestral virus (shown in gray). Tree was rooted using the clade 20C ancestral viruses sampled in NYC. Amino acid substitutions in the spike protein occurring on internal branches are labeled. The B.1.526 lineage is colored blue, except for the clade defined by the E484K mutation, which is highlighted in red.

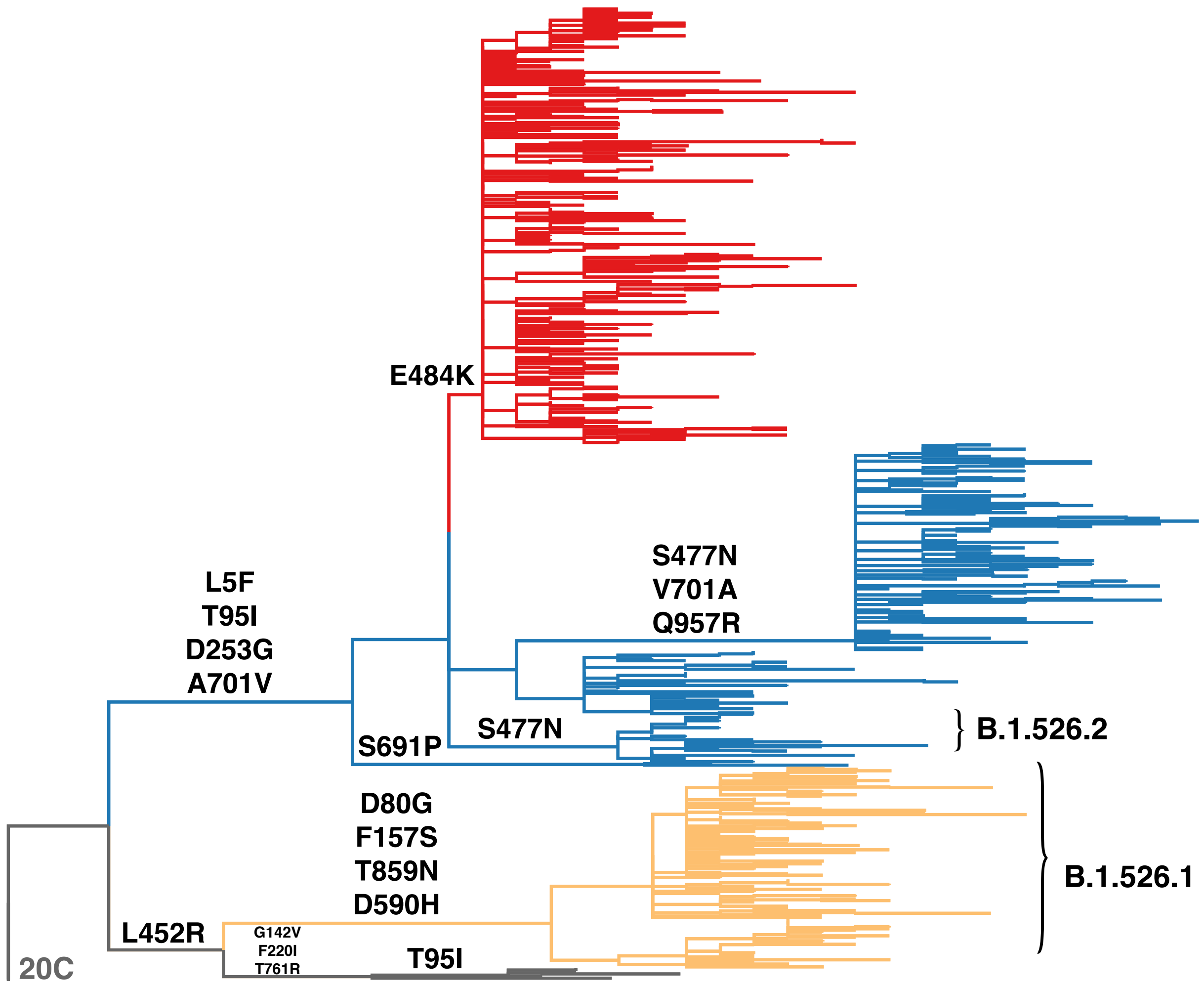

## Supplementary Fig. 3.

Bayesian skyline demographic reconstruction and time of most recent common ancestor (TMRCA) inference for SARS-CoV-2 variants in New York City, sampled December 2020 through April 2021. Thick solid lines represent the median estimate of the effective population size over time for each variant, the thinner solid lines represent the 95% Highest Posterior Density (HPD) intervals. The grey shaded areas represent the 95% HPD for the estimated TMRCAs, the solid grey lines represent the median estimated TMRCA values. B.1.526 (E484) estimate includes B.1.526.2 subclade.

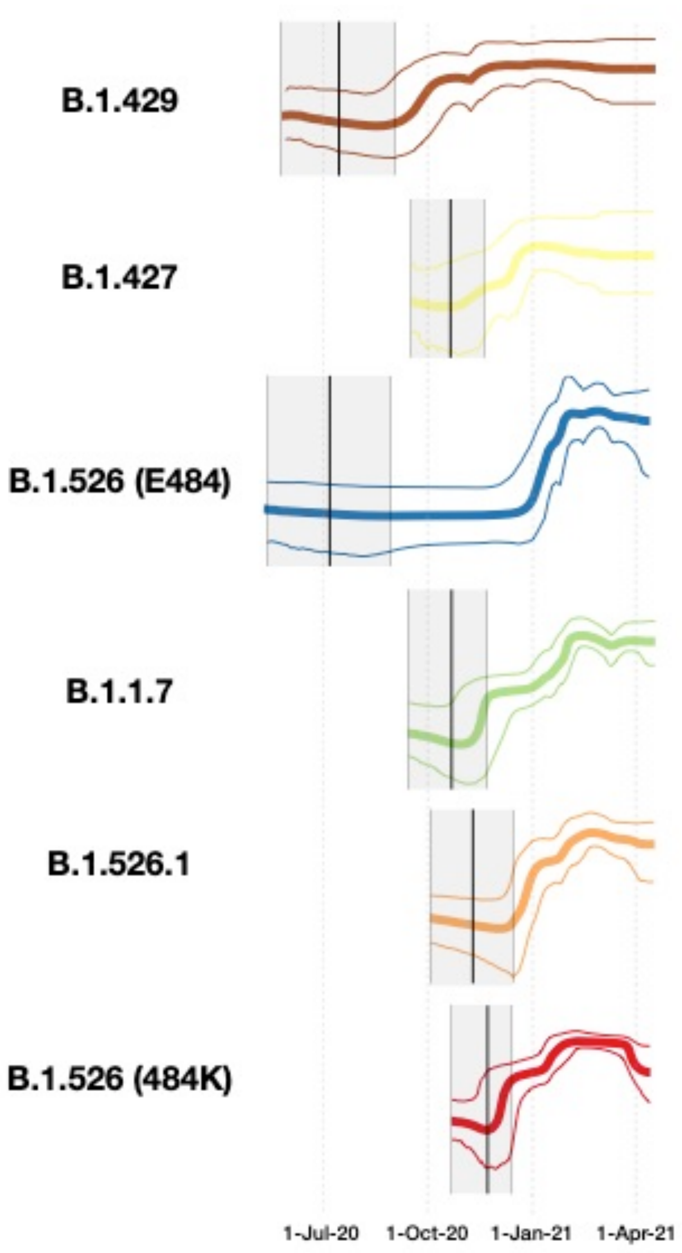

Supplementary Fig. 4.

Vaccinee plasma neutralizing activity against pseudoviruses with B.1.351 lineage spike mutations or D614G wildtype. SARS-CoV-2 pseudovirus neutralization assays were used to determine neutralization titer (NT50) for COVID-19 vaccinee plasma (n=10). B.1.351 pseudovirus containing spike mutations L18F, D80A, D215G, Δ242-244, R246I, K417N, E484K, N501Y, D614G, and A701V. Statistical significance was determined using paired two-tailed *t*-tests. Fold-difference of means is shown.

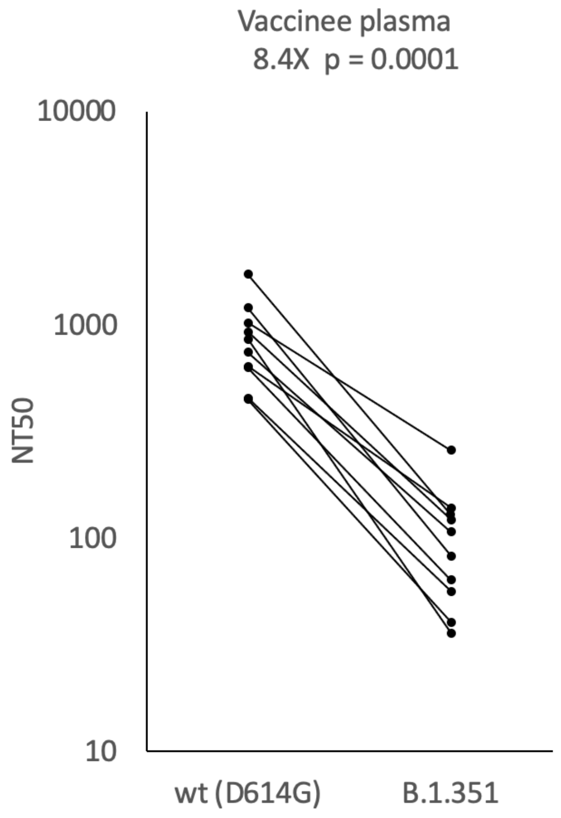

## Supplementary Table 1.

List of 124 viral genomes (with their accession number, location, collection date, and spike mutations) in lineage B.1.526. Mutations E484K, S477N, Q957R are highlighted in red, blue, and cyan, respectively.

EPI_ISL_683762, USA/NY-NYCPHL-001443/2020-11-23 : L5F T95I D253G D614G A701V
EPI_ISL_823886, USA/NY-NYCPHL-001663/2020-12-08 : L5F T95I D253G S477N D614G Q957R
EPI_ISL_812733, USA/NY-NYCPHL-001804/2020-12-15 : L5F T95I D253G D614G A701V
EPI_ISL_765495, USA/NY-Wadsworth-290403-01/2020-12-16 : L5F T95I D253G E484K D614G A701V
EPI_ISL_765494, USA/NY-Wadsworth-290357-01/2020-12-18 : L5F T95I D253G E484K D614G A701V
EPI_ISL_765493, USA/NY-Wadsworth-290339-01/2020-12-19 : L5F T95I D253G D614G A701V
EPI_ISL_767598, USA/NY-Wadsworth-291999-01/2020-12-20 : L5F T95I D253G D614G A701V
EPI_ISL_767581, USA/NY-Wadsworth-292055-01/2020-12-21 : L5F T95I D253G D614G A701V
EPI_ISL_767595, USA/NY-Wadsworth-291994-01/2020-12-21 : L5F T95I D253G S477N D614G Q957R
EPI_ISL_832270, USA/NY-NYCPHL-001924/2020-12-23 : L5F T95I D253G E484K D614G A701V
EPI_ISL_832271, USA/NY-NYCPHL-001940/2020-12-24 : L5F T95I D253G E484K D614G A701V
EPI_ISL_861278, USA/NY-Wadsworth-21004976-01/2020-12-24 : L5F T95I D253G E484K D614G A701V
EPI_ISL_861280, USA/NY-Wadsworth-21004979-01/2020-12-24 : L5F T95I D253G S477N D614G A701V
EPI_ISL_861283, USA/NY-Wadsworth-21004982-01/2020-12-24 : L5F T95I D253G S477N D614G A701V
EPI_ISL_861258, USA/NY-Wadsworth-21004944-01/2020-12-25 : L5F T95I D253G E484K D614G A701V
EPI_ISL_861285, USA/NY-Wadsworth-21004984-01/2020-12-25 : L5F T95I D253G E484K D614G A701V
EPI_ISL_861244, USA/NY-Wadsworth-21004930-01/2020-12-25 : L5F T95I D253G E484K D614G A701V
EPI_ISL_802788, USA/NY-Wadsworth-21001942-01/2020-12-27 : L5F T95I D253G A475V S477N D614G Q957R
EPI_ISL_794226, USA/NY-Wadsworth-21000327-01/2020-12-27 : L5F T95I D253G S477N D614G Q957R D1260N
EPI_ISL_936036, USA/NY-Wadsworth-21007567-01/2020-12-27 : L5F T95I D253G E484K D614G A701V
EPI_ISL_854459, USA/NY-Wadsworth-21005068-01/2020-12-27 : L5F T95I D253G E484K D614G A701V
EPI_ISL_887843, USA/NJ-CDC-LC00000610/2020-12-27 : L5F T95I D253G F306L D614G
EPI_ISL_861315, USA/NY-Wadsworth-21005029-01/2020-12-28 : L5F T95I D253G E484K D614G A701V
EPI_ISL_888376, USA/IN-CDC-LC00001468/2020-12-28 : L5F T95I D253G T299I E484K D614G A701V
EPI_ISL_830720, USA/NY-Wadsworth-21004807-01/2020-12-28 : L5F T95I D253G D614G A701V
EPI_ISL_861308, USA/NY-Wadsworth-21005019-01/2020-12-28 : L5F T95I D253G E484K D614G A701V
EPI_ISL_861175, USA/NY-Wadsworth-21006727-01/2020-12-28 : L5F T95I D253G D614G A701V
EPI_ISL_936038, USA/NY-Wadsworth-21007571-01/2020-12-28 : L5F T95I D253G E484K D614G A701V
EPI_ISL_854458, USA/NY-Wadsworth-21005145-01/2020-12-29 : L5F T95I D253G D614G A701V
EPI_ISL_854457, USA/NY-Wadsworth-21005115-01/2020-12-29 : L5F T95I D253G E484K D614G A701V
EPI_ISL_857056, USA/NY-NYCPHL-002169/2020-12-29 : L5F T95I D253G E484K D614G A701V
EPI_ISL_861306, USA/NY-Wadsworth-21005017-01/2020-12-29 : L5F T95I D253G D614G A701V
EPI_ISL_802448, USA/NY-Wadsworth-21001323-01/2020-12-30 : L5F T95I D253G S477N D614G Q957R
EPI_ISL_861300, USA/NY-Wadsworth-21005007-01/2020-12-30 : L5F T95I D253G E484K D614G A701V
EPI_ISL_854450, USA/NY-Wadsworth-21005056-01/2020-12-30 : L5F T95I D253G S477N D614G Q957R
EPI_ISL_857204, USA/NY-NYCPHL-002114/2020-12-31 : L5F T95I D253G E484K D614G A701V
EPI_ISL_861112, USA/NY-Wadsworth-21002954-01/2021-01-01 : L5F T95I D198G D253G S477N D614G Q957R
EPI_ISL_861189, USA/NY-Wadsworth-21006379-01/2021-01-01 : L5F T95I D253G E484K D614G A701V
EPI_ISL_944591, USA/CT-Yale-1000/2021-01-01 : T95I D253G E484K D614G A701V
EPI_ISL_944592, USA/CT-Yale-1001/2021-01-01 : L5F T95I D253G E484K D614G A701V
EPI_ISL_944594, USA/CT-Yale-1003/2021-01-01 : L5F T95I D253G E484K D614G A701V

EPI_ISL_962492, Ecuador/UEES-9572/2021-01-01 : L5F T95I D253G E484K D614G A701V
EPI_ISL_962493, Ecuador/UEES-9602/2021-01-01 : L5F T95I D253G E484K D614G A701V
EPI_ISL_861113, USA/NY-Wadsworth-21002959-01/2021-01-02 : L5F T95I D198G D253G S477N D614G Q957R
EPI_ISL_861111, USA/NY-Wadsworth-21002949-01/2021-01-02 : L5F T95I D198G D253G S477N D614G Q957R
EPI_ISL_857060, USA/NY-NYCPHL-002190/2021-01-03 : L5F T95I D253G S477N D614G Q957R
EPI_ISL_861188, USA/NY-Wadsworth-21006373-01/2021-01-03 : L5F T95I D253G D614G A701V
EPI_ISL_896394, USA/NY-Wadsworth-21006803-01/2021-01-04 : L5F T95I D253G E484K D614G A701V
EPI_ISL_849348, USA/DE-DHSS-FLW00612372/2021-01-04 : L5F T95I D253G D614G A701V
EPI_ISL_896391, USA/NY-Wadsworth-21006765-01/2021-01-04 : L5F T95I D253G S477N D614G Q957R
EPI_ISL_896392, USA/NY-Wadsworth-21006780-01/2021-01-04 : L5F T95I D253G S477N D614G Q957R
EPI_ISL_830577, USA/NY-Wadsworth-21004146-01/2021-01-04 : L5F T95I D253G E484K D614G A701V
EPI_ISL_896395, USA/NY-Wadsworth-21006809-01/2021-01-04 : L5F T95I D253G E484K D614G A701V
EPI_ISL_861361, USA/NY-Wadsworth-21006106-01/2021-01-04 : L5F T95I D253G S477N D614G Q957R
EPI_ISL_830578, USA/NY-Wadsworth-21004150-01/2021-01-04 : L5F T95I D253G E484K D614G A701V
EPI_ISL_861411, USA/NY-Wadsworth-21006165-01/2021-01-06 : L5F T95I D253G S477N D614G Q957R D1260N
EPI_ISL_850743, USA/NJ-CDC-STM-A100415/2021-01-06 : L5F T95I D253G E484K D614G A701V
EPI_ISL_854449, USA/NY-Wadsworth-21006042-01/2021-01-07 : L5F T95I D253G S477N D614G Q957R
EPI_ISL_911789, USA/MD-HP01428/2021-01-07 : L5F T95I D253G S477N D614G Q957R
EPI_ISL_857120, USA/NY-NYCPHL-002295/2021-01-07 : L5F T95I D253G D614G A701V
EPI_ISL_855171, USA/RI-QDX-3232/2021-01-07 : L5F T95I D253G E484K D614G A701V
EPI_ISL_884080, USA/NY-Wadsworth-21003395-01/2021-01-07 : L5F T95I D253G E484K D614G A701V
EPI_ISL_896234, USA/NY-Wadsworth-21011146-01/2021-01-10 : L5F T95I D253G D614G A701V
EPI_ISL_883447, USA/NY-Wadsworth-21008849-01/2021-01-10 : L5F T95I D253G D614G A701V
EPI_ISL_896227, USA/NY-Wadsworth-21011153-01/2021-01-10 : L5F T95I D253G S477N D614G Q957R
EPI_ISL_886271, USA/PA-CDC-LC0003480/2021-01-10 : L5F T95I D253G D614G A701V
EPI_ISL_886626, USA/NJ-CDC-LC0003391/2021-01-11 : L5F T95I D253G E484K D614G A701V
EPI_ISL_857164, USA/NY-NYCPHL-002365/2021-01-11 : L5F T95I D253G E484K D614G A701V
EPI_ISL_886531, USA/CT-CDC-LC0003447/2021-01-11 : L5F T95I D253G E484K D614G A701V
EPI_ISL_857163, USA/NY-NYCPHL-002336/2021-01-11 : L5F T95I D253G E484K D614G A701V
EPI_ISL_886240, USA/NJ-CDC-LC0003405/2021-01-11 : L5F T95I D253G D614G A701V
EPI_ISL_884055, USA/NY-Wadsworth-21005245-01/2021-01-11 : L5F T95I D253G S477N D614G A701V
EPI_ISL_886384, USA/GA-CDC-LC0003561/2021-01-11 : L5F T95I D253G E484K D614G A701V
EPI_ISL_886270, USA/DE-CDC-LC0003883/2021-01-12 : L5F T95I D253G D614G A701V
EPI_ISL_936267, USA/NY-Wadsworth-21012072-01/2021-01-13 : L5F T95I D253G D614G A701V
EPI_ISL_936292, USA/NY-Wadsworth-21012249-01/2021-01-14 : L5F T95I D253G E484K D614G A701V
EPI_ISL_936261, USA/NY-Wadsworth-21012065-01/2021-01-14 : L5F T95I D253G E484K D614G A701V
EPI_ISL_883338, USA/NY-NYCPHL-002433/2021-01-14 : L5F T95I D253G E484K D614G A701V
EPI_ISL_883342, USA/NY-NYCPHL-002473/2021-01-14 : L5F T95I D253G V483F E484K D614G A701V
EPI_ISL_883339, USA/NY-NYCPHL-002440/2021-01-14 : L5F T95I D253G S477N D614G A701V
EPI_ISL_883340, USA/NY-NYCPHL-002466/2021-01-14 : L5F T95I D253G V483F E484K D614G A701V
EPI_ISL_936288, USA/NY-Wadsworth-21012244-01/2021-01-14 : L5F T95I D253G E484K D614G A701V
EPI_ISL_883343, USA/NY-NYCPHL-002474/2021-01-14 : L5F T95I D253G V483F E484K D614G A701V
EPI_ISL_883346, USA/NY-NYCPHL-002525/2021-01-14 : L5F T95I D253G S477N D614G A701V
EPI_ISL_883341, USA/NY-NYCPHL-002471/2021-01-14 : L5F T95I D253G E484K D614G A701V
EPI_ISL_883330, USA/NY-NYCPHL-002514/2021-01-15 : L5F T95I D253G S477N D614G Q957R
EPI_ISL_883347, USA/NY-NYCPHL-002533/2021-01-15 : L5F T95I D253G S477N D614G A701V
EPI_ISL_936299, USA/NY-Wadsworth-21012257-01/2021-01-15 : L5F T95I D253G S477N D614G Q957R
EPI_ISL_883331, USA/NY-NYCPHL-002535/2021-01-15 : L5F T95I D253G S477N D614G A701V
EPI_ISL_937272, USA/NY-NYCPHL-002846/2021-01-15 : L5F T95I D253G E484K D614G A701V
EPI_ISL_936281, USA/NY-Wadsworth-21012236-01/2021-01-15 : L5F T95I D253G D614G A701V
EPI_ISL_883344, USA/NY-NYCPHL-002520/2021-01-16 : L5F T95I D253G S477N D614G A701V
EPI_ISL_937190, USA/NY-NYCPHL-002680/2021-01-17 : L5F T95I D253G E484K D614G A701V
EPI_ISL_883345, USA/NY-NYCPHL-002521/2021-01-17 : L5F T95I D253G E484K D614G A701V

EPI_ISL_936248, USA/NY-Wadsworth-21011901-01/2021-01-17 : L5F T95I D253G E484K D614G A701V
EPI_ISL_936271, USA/NY-Wadsworth-21012077-01/2021-01-17 : L5F T95I D253G S477N D614G Q957R
EPI_ISL_936256, USA/NY-Wadsworth-21012058-01/2021-01-18 : L5F T95I D253G S477N D614G Q957R
EPI_ISL_936254, USA/NY-Wadsworth-21011909-01/2021-01-18 : L5F T95I D253G E484K D614G A701V
EPI_ISL_936291, USA/NY-Wadsworth-21012248-01/2021-01-18 : L5F T95I D253G E484K D614G A701V
EPI_ISL_896233, USA/NY-Wadsworth-21011134-01/2021-01-18 : L5F T95I D253G E484K D614G A701V
EPI_ISL_937191, USA/NY-NYCPHL-002681/2021-01-18 : L5F T95I D253G E484K D614G A701V
EPI_ISL_936276, USA/NY-Wadsworth-21012089-01/2021-01-19 : L5F T95I D253G S477N D614G Q957R
EPI_ISL_937126, USA/NY-NYCPHL-002548/2021-01-19 : L5F T95I D253G S477N D614G A701V
EPI_ISL_937192, USA/NY-NYCPHL-002682/2021-01-20 : L5F T95I D253G E484K D614G A701V
EPI_ISL_937176, USA/NY-NYCPHL-002659/2021-01-21 : L5F T33I T95I D253G D614G A701V
EPI_ISL_920158, England/LOND-12F79EE/2021-01-21 : L5F T95I D253G D614G A701V
EPI_ISL_962431, USA/WA-S4338/2021-01-21 : L5F T95I D253G D614G A701V
EPI_ISL_937205, USA/NY-NYCPHL-002704/2021-01-22 : L5F T95I D253G S477N D614G Q957R
EPI_ISL_937211, USA/NY-NYCPHL-002711/2021-01-22 : L5F T95I D253G E484K D614G A701V
EPI_ISL_937201, USA/NY-NYCPHL-002698/2021-01-22 : L5F T95I D253G D614G A701V
EPI_ISL_937213, USA/NY-NYCPHL-002714/2021-01-22 : L5F L18F T95I D253G S477N D614G Q957R
EPI_ISL_937246, USA/NY-NYCPHL-002784/2021-01-22 : L5F T95I D253G E484K D614G A701V
EPI_ISL_937247, USA/NY-NYCPHL-002785/2021-01-23 : L5F T95I D253G E484K D614G A701V
EPI_ISL_937258, USA/NY-NYCPHL-002831/2021-01-24 : L5F T95I D253G E484K D614G A701V
EPI_ISL_937236, USA/NY-NYCPHL-002767/2021-01-25 : L5F T95I D253G S477N D614G A701V
EPI_ISL_937218, USA/NY-NYCPHL-002729/2021-01-25 : L5F T95I D253G E484K D614G A701V
EPI_ISL_937216, USA/NY-NYCPHL-002723/2021-01-25 : L5F T95I D253G E484K D614G A701V
EPI_ISL_937265, USA/NY-NYCPHL-002839/2021-01-25 : L5F T95I D253G E484K D614G A701V
EPI_ISL_937215, USA/NY-NYCPHL-002720/2021-01-25 : L5F T95I D253G E484K D614G A701V
EPI_ISL_937255, USA/NY-NYCPHL-002793/2021-01-25 : L5F T95I D253G E484K D614G A701V
EPI_ISL_906839, Singapore/117/2021-01-26 : L5F T95I D253G E484K D614G A701V
EPI_ISL_906838, Singapore/116/2021-01-26 : L5F T95I D253G E484K D614G A701V
EPI_ISL_906837, Singapore/115/2021-01-26 : L5F T95I D253G E484K D614G A701V
EPI_ISL_937477, USA/ME-HETL-J1185/2021-01-29 : L5F T95I D253G E484K D614G A701V

**Supplementary Table 2. Individual vaccinee characteristics and plasma titers**

| | Demographic characteristics | | | | Vaccination details | | | | | Neutralization titer (reciprocal dilution) NT50 | | | | | |
|---|---|---|---|---|---|---|---|---|---|---|---|---|---|---|---|
| | | | | | | | Time between (days) | | | Experiment 1 | | Experiment 2 | | Experiment 3 | |
| Participant ID | Age range (years) | Sex | Race § | Ethnicity | Vaccine platform | # of doses received | 1st & 2nd dose | 1st dose & blood draw | 2nd dose & blood draw | B.1.526 v.1† | D614G | B.1.526 v.2‡ | D614G | B.1.351* | D614G |
| MOD1 | 50-59 | Male | W | Hispanic | Moderna | 2 | 28 | 74 | 46 | 364 | 2176 | 497 | 917 | 64 | 630 |
| MOD2 | 30-39 | Male | W | Non-Hispanic | Moderna | 2 | 34 | 62 | 28 | 265 | 975 | 205 | 573 | 41 | 447 |
| MOD3 | 30-39 | Male | W | Non-Hispanic | Moderna | 2 | 31 | 78 | 47 | 269 | 1412 | 460 | 1762 | 107 | 744 |
| MOD6 | 40-49 | Female | W | Non-Hispanic | Moderna | 2 | 28 | 89 | 61 | 223 | 679 | 201 | 983 | 57 | 454 |
| MOD7 | 60-69 | Male | A | Non-Hispanic | Moderna | 2 | 35 | 84 | 49 | 563 | 2412 | 746 | 2981 | 122 | 927 |
| MOD8 | 60-69 | Female | W | Non-Hispanic | Moderna | 2 | 35 | 96 | 63 | 214 | 1713 | 381 | 1660 | 83 | 1203 |
| PFZ12 | 50-59 | Female | W | Non-Hispanic | Pfizer/BioNTech | 2 | 21 | 83 | 62 | 345 | 1516 | 733 | 1278 | 36 | 857 |
| PFZ16 | 60-69 | Female | W | Non-Hispanic | Pfizer/BioNTech | 2 | 22 | 119 | 97 | 109 | 566 | 188 | 718 | 139 | 639 |
| C001 | 30-39 | Female | W | Non-Hispanic | Pfizer/BioNTech | 2 | 22 | 57 | 35 | 884 | 2652 | 1035 | 1738 | 129 | 1726 |
| C004 | 30-39 | Male | W | Non-Hispanic | Pfizer/BioNTech | 2 | 21 | 91 | 70 | 254 | 1603 | 860 | 1991 | 258 | 1023 |

§ = White (W); Asian (A)

† = B.1.516 v.1 spike mutations L5F, T95I, D253G, E484K, D614G, and A701V

‡ = B.1.526 v.2 spike mutations: L5F, T95I, D253G, S477N, D614G, and Q957R

* = B.1.351 spike mutations: L18F D80A D215G Δ242-244 R246I K417N E484K N501Y D614G A701V

Characteristics from Supplementary Table 1 of Wang, et al., "mRNA vaccine-elicited antibodies to SARS-CoV-2 and circulating variants", Nature (2021)

**Supplementary Table 3. Individual convalescent participant characteristics and plasma titers**

| | | | | | Temporal dynamics (days) | | | | | | | Neutralization titer (reciprocal dilution) NT50 | | | | | | | |
|---|---|---|---|---|---|---|---|---|---|---|---|---|---|---|---|---|---|---|---|
| | | | | | | | | | | | | Timepoint T1 - 1.3 months | | | | Timepoint T2 - 6.2 months | | | |
| | | | | | | | | | | | | Experiment 1 | | Experiment 2 | | Experiment 1 | | Experiment 2 | |
| **ID** | **Age range (years)** | **Sex** | **Race** | **Ethnicity** | **Sx duration during acute disease** | **Sx onset to initial visit (T1)** | **Sx onset to follow-up visit (T2)** | **Time between visits** | **# of solicited comorbidities §** | **Acute disease severity by WHO (0-8) ¶** | **Post-acute Sx persistence #** | **B.1.526 v.1†** | **D614G** | **B.1.526 v.2‡** | **D614G** | **B.1.526 v.1†** | **D614G** | **B.1.526 v.2‡** | **D614G** |
| **38** | 50-59 | F | White | Non-Hispanic | 10 | 38 | 211 | 173 | 0 | 2 | N | 27 | 148 | 255 | 385 | 154 | 891 | 775 | 2597 |
| **40** | 40-49 | M | White | Non-Hispanic | 7 | 23 | 195 | 172 | 0 | 2 | N | 13 | 37 | 72 | 90 | N.N. | 10 | N.N. | N.N. |
| **76** | 40-49 | F | White | Non-Hispanic | 28 | 34 | 204 | 170 | 0 | 1 | Y | 33 | 105 | 105 | 184 | 10 | 23 | 13 | 24 |
| **99** | 30-39 | F | White | Non-Hispanic | 13 | 29 | 204 | 175 | 0 | 2 | N | 180 | 973 | 794 | 1055 | 22 | 358 | 176 | 240 |
| **233** | 50-59 | M | White | Non-Hispanic | 20 | 41 | 206 | 165 | 0 | 2 | N | 55 | 194 | 108 | 165 | 19 | 50 | 17 | 46 |
| **328** | 50-59 | F | White | Non-Hispanic | 22 | 62 | 203 | 141 | 0 | 2 | N | 24 | 347 | 50 | 131 | 22 | 135 | 29 | 77 |
| **352** | 40-49 | M | White | Non-Hispanic | 16 | 43 | 197 | 154 | 0 | 2 | N | 129 | 1684 | 587 | 744 | 27 | 148 | 40 | 107 |
| **403*** | 50-59 | M | Asian | Non-Hispanic | 18 | 39 | 174 | 135 | 1 | 4 | Y | 555 | 3223 | 1067 | 2433 | 89 | 463 | 45 | 183 |
| **437** | 40-49 | F | Asian | Non-Hispanic | 14 | 34 | 192 | 158 | 1 | 2 | N | 122 | 525 | 276 | 996 | 56 | 132 | 65 | 270 |
| **537** | 50-59 | M | White | Non-Hispanic | 15 | 45 | 178 | 133 | 2 | 2 | Y | 650 | 3528 | 1610 | 4291 | 240 | 853 | 334 | 957 |

Sx = symptoms, N.N = non-neutralizing
* = hospitalized
§ = Arterial hypertension (HTN), obesity (OB), diabetes mellitus (DM), asthma (A), chronic obstructive pulmonary disease (COPD), coronary artery disease (CAD), cancer (CX)
¶ = WHO Ordinal Scale for Clinical Improvement, COVID-19 Trial Design Synopsis
# = Persistent fatigue, dyspnea, athletic deficit, or ≥ 3 other solicited symptoms beyond 6 weeks from Sx onset
† = B.1.516 v.1 spike mutations L5F, T95I, D253G, E484K, D614G, and A701V
‡ = B.1.526 v.2 spike mutations: L5F, T95I, D253G, S477N, D614G, and Q957R
Characteristics from Supplementary Table 2 of Gaebler, et al., "Evolution of antibody immunity to SARS-CoV-2" Nature 591, 639–644 (2021)

**Supplementary Table 4. Monoclonal antibody neutralizataion of B.1.526**

| | | Neutralization IC50 (μg/mL) | |
|---|---|---|---|
| **Antibody** | **Antibody Class*** | **B.1.526 v.1†** | **D614G** |
| C105[a] | 1 | 0.112 | 0.035 |
| C002[a] | 2 | 53 | 0.006 |
| BG10-19[b] | 3 | 0.008 | 0.004 |
| BG7-15[b] | 2/3 | 0.0002 | 0.002 |

a = Robbiani, D.F. et al. "Convergent antibody responses to SARS-CoV-2 in convalescent individuals" Nature 584, 437–442 (2020)
b = Schied, J.F. et al. "B cell genomics behind cross-neutralization of SARS-CoV-2 variants and SARS-CoV" Cell April 24 (2021)
* = Barnes, C.O. et al. "SARS-CoV-2 neutralizing antibody structures inform therapeutic strategies" Nature 588, 682–687 (2020)
† = B.1.516 v.1 spike mutations L5F, T95I, D253G, E484K, D614G, and A701V

## Supplementary Table 5.

List of primers used to make B.1.526 v.1 and v.2 pseudovirus constructs

| Primer Name | Sequence |
|---|---|
| L5F_1 | GCCACCATGTTCGTGTTCTTCGTACTCCTTCCTTTGGTG |
| L5F_2 | CACCAAAGGAAGGAGTACGAAGAACACGAACATGGTGGC |
| T95I_1 | GGAGTGTACTTCGCGTCCATCGAGAAGAGCAATATCATTC |
| T95I_2 | GAATGATATTGCTCTTCTCGATGGACGCGAAGTACACTCC |
| D253G_1 | CTGACACCGGGCGGCTCAAGTTCCGGA |
| D253G_2 | TCCGGAACTTGAGCCGCCCGGTGTCAG |
| S477N_1 | GGAGATATATCAAGCCGGCAATACACCATGTAATGGTGTG |
| S477N_2 | CACACCATTACATGGTGTATTGCCGGCTTGATATATCTCC |
| E484K_1 | CACCATGTAATGGTGTGAAGGGCTTTAACTGCTAC |
| E484K_2 | GTAGCAGTTAAAGCCCTTCACACCATTACATGGTG |
| A701V_1 | CGATGAGCCTCGGAGTGGAGAACAGCGTCGCG |
| A701V_2 | CGCGACGCTGTTCTCCACTCCGAGGCTCATCG |
| Q957R_1 | CGTGGTGAATCAGAATGCACGGGCACTGAATACCTTGGTTAA |
| Q957R_2 | TTAACCAAGGTATTCAGTGCCCGTGCATTCTGATTCACCACG |
