# Supplementary material for: Detection and characterization of the SARS-CoV-2 lineage B.1.526 in New York
Source: Nat Commun. 2021 Aug 9;12:4886. doi: 10.1038/s41467-021-25168-4 (PMC8352861; doi:10.1038/s41467-021-25168-4)
Supplement: Supplementary file 3 — Reporting Summary [file 41467_2021_25168_MOESM3_ESM.pdf]

nature research

Corresponding author(s): Anthony West

Last updated by author(s): Jul 27, 2021

Reporting Summary

Nature Research wishes to improve the reproducibility of the work that we publish. This form provides structure for consistency and transparency in reporting. For further information on Nature Research policies, see our Editorial Policies and the Editorial Policy Checklist.

Statistics

For all statistical analyses, confirm that the following items are present in the figure legend, table legend, main text, or Methods section.

n/a	Confirmed	
☐	☒	The exact sample size (*n*) for each experimental group/condition, given as a discrete number and unit of measurement
☐	☒	A statement on whether measurements were taken from distinct samples or whether the same sample was measured repeatedly
☐	☒	The statistical test(s) used AND whether they are one- or two-sided<br>*Only common tests should be described solely by name; describe more complex techniques in the Methods section.*
☒	☐	A description of all covariates tested
☒	☐	A description of any assumptions or corrections, such as tests of normality and adjustment for multiple comparisons
☒	☐	A full description of the statistical parameters including central tendency (e.g. means) or other basic estimates (e.g. regression coefficient) AND variation (e.g. standard deviation) or associated estimates of uncertainty (e.g. confidence intervals)
☐	☒	For null hypothesis testing, the test statistic (e.g. *F*, *t*, *r*) with confidence intervals, effect sizes, degrees of freedom and *P* value noted<br>*Give P values as exact values whenever suitable.*
☐	☒	For Bayesian analysis, information on the choice of priors and Markov chain Monte Carlo settings
☒	☐	For hierarchical and complex designs, identification of the appropriate level for tests and full reporting of outcomes
☒	☐	Estimates of effect sizes (e.g. Cohen's *d*, Pearson's *r*), indicating how they were calculated

Our web collection on statistics for biologists contains articles on many of the points above.

Software and code

Policy information about availability of computer code

Data collection	Sequence reads were trimmed using Trim Galore version 0.6.4_dev and 0.6.6 (Krueger, 2015). The trimmed reads were assembled using the Burrows-Wheeler Aligner MEM algorithm (BWA-MEM) version 0.7.12 (Li and Durbin, 2009). Consensus and mutations were called using samtools version 1.9 (Li, 2011) and Intrahost variant analysis of replicates (iVar) (Grubaugh et al., 2019).
Data analysis	SARS-CoV-2 mutation patterns were analyzed with the custom program Variant Database, vdb, available at the Github repository: https://github.com/variant-database/vdb. Multiple sequence alignments were performed with MAFFT version 7.464 and 7.475 (Katoh and Standley, 2013). The phylogenetic tree was calculated by IQ-TREE version 1.6.12(Nguyen et al., 2015), and the tree diagram was generated using iTOL version 6.1.1 (Interactive Tree of Life) (Letunic and Bork, 2007). Pango lineage designations (Rambaut et al., 2020b) for variants were assigned using Pangolin v2.4.2 (O'Toole et al., 2020). Segmented regression analysis was performed using the segmented package in R version 3.6.0 (2019-04-26). Maximum likelihood trees were inferred using IQTree2 v2.0.4. Population growth rate inference in coalescence-based framework was calculated using an exponential growth model in BEAST 1.10.4 (Suchard et al., 2018). Fig. 3 was prepared using ggplot2 version 3.3.3 and epiMuller version 0.0.8. Geocoding was performed using the NYC Department of Health and Mental Hygiene's Geoportal application, and a map using these locations was created in ArcMap version 10.6.1. Pseudovirus neutralization data was fit using non-linear regression in Antibody database version 2.0 (West et al., 2013).

For manuscripts utilizing custom algorithms or software that are central to the research but not yet described in published literature, software must be made available to editors and reviewers. We strongly encourage code deposition in a community repository (e.g. GitHub). See the Nature Research guidelines for submitting code & software for further information.

Data

Policy information about availability of data

All manuscripts must include a data availability statement. This statement should provide the following information, where applicable:

- Accession codes, unique identifiers, or web links for publicly available datasets
- A list of figures that have associated raw data
- A description of any restrictions on data availability

SARS-CoV-2 genomes were deposited in GenBank under accession codes MZ637509-MZ642234 and with the Global Initiative on Sharing Avian Influenza Data (GISAID). Data for Figure 5 is given in Supplemental Tables 2 and 3.

Field-specific reporting

Please select the one below that is the best fit for your research. If you are not sure, read the appropriate sections before making your selection.

☒ Life sciences ☐ Behavioural & social sciences ☐ Ecological, evolutionary & environmental sciences

For a reference copy of the document with all sections, see nature.com/documents/nr-reporting-summary-flat.pdf

Life sciences study design

All studies must disclose on these points even when the disclosure is negative.

Sample size	Plasma samples sizes (10 each from vaccinees and convalescent individuals) were chosen to capture the variety of anti-SARS-CoV-2 neutralization titers across individuals as observed in Gaebler, et al., Nature 2021 and Wang, et al., Nature 2021.
Data exclusions	In the plasma neutralization experiment, one 6.2-month sample had neutralization titers below the limit of detection and was thus excluded from the analysis in Fig. 5. Pseudovirus neutralization curves consisted of 8 serial dilutions. Of these 8 points, individual data points were excluded if, at the end of the experiment, the cells in the wells had syncytia or had died (which sometime occurred for the lowest dilution), or if the plate had a severe edge effect. This is our standard procedure, and the remaining points of the neutralization curves were sufficient for neutralization titer determination.
Replication	Pseudovirus neutralization experiments included internal controls (D614G pseudovirus) and technical duplicates. The D614G control was replicated (but not the B.1.526 pseudoviruses), all attempts at replication were successful, and most of the corresponding neutralization titers were within 2-fold of each other. For the Fig. 5 analysis, the internal control (D614G) values were used to minimize systematic errors.
Randomization	This is not relevant as this is an observational study.
Blinding	Blinding is not relevant as this is an observational study.

Reporting for specific materials, systems and methods

We require information from authors about some types of materials, experimental systems and methods used in many studies. Here, indicate whether each material, system or method listed is relevant to your study. If you are not sure if a list item applies to your research, read the appropriate section before selecting a response.

Materials & experimental systems

n/a	Involved in the study	
☒	☐	Antibodies
☐	☒	Eukaryotic cell lines
☒	☐	Palaeontology and archaeology
☒	☐	Animals and other organisms
☐	☒	Human research participants
☒	☐	Clinical data
☒	☐	Dual use research of concern

Methods

n/a	Involved in the study	
☒	☐	ChIP-seq
☒	☐	Flow cytometry
☒	☐	MRI-based neuroimaging

Eukaryotic cell lines

Policy information about cell lines

Cell line source(s)	HEK293T cells from ATCC CRL-11268. HEK293TAce2 cells provided by Jesse Bloom's laboratory (Fred Hutchinson Cancer Research Center). This cell line is available from BEI: https://www.beiresources.org/Catalog/cellBanks/NR-52511.aspx
Authentication	Not authenticated after purchase from ATCC.
Mycoplasma contamination	The cell lines were not tested for mycoplasma contamination.
Commonly misidentified lines (See ICLAC register)	No commonly misidentified cell lines were used.

Human research participants

Policy information about studies involving human research participants

Population characteristics	Vaccinee plasma samples were a subset of those collected in Wang, et al. "mRNA vaccine-elicited antibodies to SARS-CoV-2 and circulating variants", Nature (2021).<br><br>Convalescent plasma samples were a subset of those collected in Gaelber, et al., "Evolution of antibody immunity to SARS-CoV-2" Nature 591, 639–644 (2021).<br><br>Please see Supplementary Tables 2 and 3 of this manuscript and the Nature Reporting Summaries for these studies for complete information on population characteristics.
Recruitment	Vaccinee plasma samples were a randomly selected subset of those collected in Wang, et al. "mRNA vaccine-elicited antibodies to SARS-CoV-2 and circulating variants", Nature (2021):<br>Between 19 October 2020 and 15 January 2021, 20 volunteers who received two doses of the Moderna (n=14) or Pfizer mRNA (n=6) vaccines were recruited for blood donation and analyzed. Ages of the analyzed volunteers ranged from 29-69 years (median 43); 12 (60%) were male and 8 (40%) female. 16 participants identified as Caucasian, 2 as Hispanic, and 1 as African American or Asian, respectively. The time from the second vaccination to sample collection varied between 3-14 weeks with an average of 8 weeks. None of the volunteers had a history of prior SARS-CoV-2 infection and none reported serious adverse events after vaccination.<br><br>Convalescent plasma samples were a randomly selected subset of those collected in Gaelber, et al., "Evolution of antibody immunity to SARS-CoV-2" Nature 591, 639–644 (2021):<br>Study participants were recruited at the Rockefeller University Hospital in New York between August 31 and October 16, 2020. Most study participants were residents of the Greater New York City tri-state region and were enrolled sequentially according to eligibility criteria. Participants were first interviewed by phone to collect information on their clinical presentation, and subsequently presented to the Rockefeller University Hospital for a single blood sample collection.<br>At Mount Sinai Hospital eligible participants included adults, 18-76 years of age who were previously diagnosed with SARS-CoV-2 by RT-PCR or through a combination of clinical symptoms consistent with COVID-19 plus evidence of seroconversion, and presented to the gastroenterology clinics of Mount Sinai Hospital.<br>Other than these criteria no other parameters were used to exclude or include patients. Therefore, we cannot identify any factors that would lead to self-selection bias.
Ethics oversight	The protocol for human participants was approved by the Institutional Review Board (IRB) of the Rockefeller University (protocol #DRO-1006). Informed consent was obtained from the participants.

Note that full information on the approval of the study protocol must also be provided in the manuscript.
