# Supplementary material for: Detection and characterization of the SARS-CoV-2 lineage B.1.526 in New York
Source: Nat Commun. 2021 Aug 9;12:4886. doi: 10.1038/s41467-021-25168-4 (PMC8352861; doi:10.1038/s41467-021-25168-4)
Supplement: Supplementary file 4 — Description of Additional Supplementary Files [file 41467_2021_25168_MOESM4_ESM.pdf]

Description of Additional Supplementary Files

File Name: Supplementary Data 1
Description: List of SARS-CoV-2 genomes with GenBank accession numbers

File Name: Supplementary Data 2
Description: List of SARS-CoV-2 genomes used in phylogenetic and phylodynamic analyses

File Name: Supplementary Data 3
Description: List of SARS-CoV-2 genomes used in mapping Fig. 4

File Name: Supplementary Data 4
Description: GISAID Contributors Acknowledgements
