# Supplementary material for: Detection and characterization of the SARS-CoV-2 lineage B.1.526 in New York
Source: Nat Commun. 2021 Aug 9;12:4886. doi: 10.1038/s41467-021-25168-4 (PMC8352861; doi:10.1038/s41467-021-25168-4)
Supplement: Supplementary file 8 — Supplementary Data 4 [file 41467_2021_25168_MOESM8_ESM.zip › GISAID_acknowledements_tables/gisaid_hcov-19_acknowledgement_table_2021_02_13_00-5.pdf]

We gratefully acknowledge the following Authors from the Originating laboratories responsible for obtaining the specimens, as well as the Submitting laboratories where the genome data were generated and shared via GISAID, on which this research is based.

All Submitters of data may be contacted directly via www.gisaid.org

Authors are sorted alphabetically.

Accession ID	Originating Laboratory	Submitting Laboratory	Authors
EPI_ISL_622786	Canterbury Health Laboratories	Institute of Environmental Science and Research (ESR)	Xiaoyun Ren, Matt Storey, Nikki Freed, Muhammad Faisal, Jing Wang, Hermes Perez, Anja Werno, Antje van der Linden, Arlo Upton, Chris Mansell, David Hammer, Dragana Drinkovic, Gary McAuliffe, Hana Sofia Andersson, James Ussher, Jill Sherwood, Josh Freeman, Julia Howard, Juliet Elvy, Mary DeAlmeida, Matt Blakiston, Matthew Rogers, Max Bloomfield, Michael Addidle, Michelle Balm, Sally Roberts, Sarah Jefferies, Sharmini Muttaiyah, Susan Morpeth, Susan Taylor, Timothy Blackmore, Vani Sathyendran, Veronica Playle, Virginia Hope, Erasmus Smit, Lauren Jelly, Olin Silander, Joep de Ligt
EPI_ISL_625695, EPI_ISL_625698, EPI_ISL_625706, EPI_ISL_625709, EPI_ISL_625742, EPI_ISL_625822, EPI_ISL_625823, EPI_ISL_625864, EPI_ISL_625883, EPI_ISL_625890, EPI_ISL_626124, EPI_ISL_626127, EPI_ISL_626129, EPI_ISL_626132, EPI_ISL_626133, EPI_ISL_626134, EPI_ISL_626137, EPI_ISL_626138, EPI_ISL_626139, EPI_ISL_626144, EPI_ISL_626146, EPI_ISL_626148, EPI_ISL_626149, EPI_ISL_626157, EPI_ISL_626162, EPI_ISL_626164			
see above	Department of Virus and Microbiological Special Diagnostics, Statens Serum Institut, Denmark	Albertsen lab, Department of Chemistry and Bioscience, Aalborg University, Denmark	Danish Covid-19 Genome Consortia
EPI_ISL_626356	LabPLUS	Institute of Environmental Science and Research (ESR)	Xiaoyun Ren, Matt Storey, Nikki Freed, Muhammad Faisal, Jing Wang, Hermes Perez, Anja Werno, Antje van der Linden, Arlo Upton, Chris Mansell, David Hammer, Dragana Drinkovic, Gary McAuliffe, Hana Sofia Andersson, James Ussher, Jill Sherwood, Josh Freeman, Julia Howard, Juliet Elvy, Mary DeAlmeida, Matt Blakiston, Matthew Rogers, Max Bloomfield, Michael Addidle, Michelle Balm, Sally Roberts, Sarah Jefferies, Sharmini Muttaiyah, Susan Morpeth, Susan Taylor, Timothy Blackmore, Vani Sathyendran, Veronica Playle, Virginia Hope, Erasmus Smit, Lauren Jelly, Olin Silander, Joep de Ligt
EPI_ISL_626646	National Public Health Laboratory, National Centre for Infectious Diseases	National Public Health Laboratory, National Centre for Infectious Diseases	Tze Minn Mak, Sophie Octavia, Zhenyang Zhou, Lin Cui, Raymond Tzer Pin Lin
EPI_ISL_626811, EPI_ISL_627216, EPI_ISL_627304, EPI_ISL_627711, EPI_ISL_627712, EPI_ISL_627713, EPI_ISL_627714, EPI_ISL_627715, EPI_ISL_627717, EPI_ISL_627718, EPI_ISL_627719, EPI_ISL_627720, EPI_ISL_627721, EPI_ISL_627727, EPI_ISL_627728, EPI_ISL_627729			
see above	Queens Medical Centre, Clinical Microbiology Department / DeepSeq Nottingham	COVID-19 Genomics UK (COG-UK) Consortium	Gemma Clark, Wendy Smith, Manjinder Khakh, Vicki M Fleming, Michelle M Lister, Hannah Howson-Wells, Jonathan Ball, Patrick McClure, Joseph Chappell, Theocharis Tsoleridis, Nadine Holmes, Matthew Carlisle, Christopher Moore, Fei Sang, Johnny Debebe, Victoria Wright, Matthew Loose
EPI_ISL_635059, EPI_ISL_635060, EPI_ISL_635061, EPI_ISL_635062	1-Laboratory of Microbiology, National Reference Lab, Charles Nicolle Hospital; 2-University of Tunis ElManar, Faculty of Medicine of Tunis, LR99ES09, Tunis, Tunisia	1-Clinical and Experimental Pharmacology Lab, LR16SP02, National Center of Pharmacovigilance, University of Tunis El Manar, Tunis, Tunisia. 2-Neurodegenerative diseases and psychiatric troubles, LR18SP03, Razi Hospital, University of Tunis El Manar, Tunis, Tunisia. 3- Ministry of Health, National Observatory of New and Emerging Diseases, 1006, Tunis, Tunisia	Ilhem Boutiba-Ben Boubaker, Sameh Trabelsi, Nissaf Ben Alaya, Maher Kharrat, Alia Ben Kahla, Jalila Ben Khelil, Salma Abid, Sana Ferjani, Mouna Ben Sassi, Mouna Safer, Imen Mkada, Imen Kacem, Gaies Emna, Soumaya Rammeh, Riadh Daghfous, Riadh Gouider.
EPI_ISL_637086	Middlemore Hospital	Institute of Environmental Science and Research (ESR)	Xiaoyun Ren, Matt Storey, Nikki Freed, Muhammad Faisal, Jing Wang, Hermes Perez, Anja Werno, Antje van der Linden, Arlo Upton, Chris Mansell, David Hammer, Dragana Drinkovic, Gary McAuliffe, Hana Sofia Andersson, James Ussher, Jill Sherwood, Josh Freeman, Julia Howard, Juliet Elvy, Mary DeAlmeida, Matt Blakiston, Matthew Rogers, Max Bloomfield, Michael Addidle, Michelle Balm, Sally Roberts, Sarah Jefferies, Sharmini Muttaiyah, Susan Morpeth, Susan Taylor, Timothy Blackmore, Vani Sathyendran, Veronica Playle, Virginia Hope, Erasmus Smit, Lauren Jelly, Olin Silander, Joep de Ligt
EPI_ISL_637180, EPI_ISL_637181, EPI_ISL_637182, EPI_ISL_637183, EPI_ISL_637184	Respiratory Virus Unit, Microbiology Services Colindale, Public Health England	COVID-19 Genomics UK (COG-UK) Consortium	PHE Covid Sequencing Team
EPI_ISL_637284	Quadram Institute Bioscience	COVID-19 Genomics UK (COG-UK) Consortium	Dave J. Baker, Gemma L. Kay, Alp Aydin, Thanh Le-Viet, Steven Rudder, Ana P. Tedim, Anastasia Kolyva, Maria Diaz, Leonardo de Oliveira Martins, Nabil-Fareed Alikhan, Lizzie Meadows, Rachael Stanley, Ngozi Elumogo, Muhammed Yasir, Nicholas M. Thomson, Alexander J Trotter, Rachel Gilroy, Samuel Bloomfield, Claire Stuart, Andrew Bell, Reenesh Prakash, Samir Dervisevic, Alison E. Mather, John Wain, Mark Webber, Andrew J. Page, Justin O'Grady
EPI_ISL_637332, EPI_ISL_637378, EPI_ISL_637379, EPI_ISL_637454, EPI_ISL_637587	Wales Specialist Virology Centre Sequencing lab: Pathogen Genomics Unit	COVID-19 Genomics UK (COG-UK) Consortium	Catherine Moore, Johnathan Evans, Laura Gifford, Malorie Perry, Simon Cottrell, Angela Marchbank, Alec Birchley, Alexander Adams, Amy Gaskin, Bree Gatica-Wilcox, Jason Coombes, Joel Southgate, Lauren Gilbert, Lee Graham, Nicole Pacchiarini, Sara Kumziene-Summerhayes, Sarah Taylor, Sophie Jones, Sara Rey, Matthew Bull, Joanne Watkins, Sally Corden, Tom Connor
EPI_ISL_637588, EPI_ISL_637602	Quadram Institute Bioscience	COVID-19 Genomics UK (COG-UK) Consortium	Dave J. Baker, Gemma L. Kay, Alp Aydin, Thanh Le-Viet, Steven Rudder, Ana P. Tedim, Anastasia Kolyva, Maria Diaz, Leonardo de Oliveira Martins, Nabil-Fareed Alikhan, Lizzie Meadows, Rachael Stanley, Ngozi Elumogo, Muhammed Yasir, Nicholas M. Thomson, Alexander J Trotter, Rachel Gilroy, Samuel Bloomfield, Claire Stuart, Andrew Bell, Reenesh Prakash, Samir Dervisevic, Alison E. Mather, John Wain, Mark Webber, Andrew J. Page, Justin O'Grady
EPI_ISL_637615, EPI_ISL_637915, EPI_ISL_637928, EPI_ISL_637971, EPI_ISL_638058, EPI_ISL_638059, EPI_ISL_638061, EPI_ISL_638062, EPI_ISL_638063, EPI_ISL_638064, EPI_ISL_638113, EPI_ISL_638123, EPI_ISL_638125, EPI_ISL_638128, EPI_ISL_638162, EPI_ISL_638163, EPI_ISL_638206, EPI_ISL_639396, EPI_ISL_639399, EPI_ISL_639402, EPI_ISL_639410, EPI_ISL_639413, EPI_ISL_639414, EPI_ISL_639416, EPI_ISL_639422, EPI_ISL_639424, EPI_ISL_639425, EPI_ISL_639431, EPI_ISL_639432, EPI_ISL_639436, EPI_ISL_639437, EPI_ISL_639438, EPI_ISL_639440, EPI_ISL_639445, EPI_ISL_639449, EPI_ISL_639450, EPI_ISL_639457, EPI_ISL_639459, EPI_ISL_639461, EPI_ISL_639463, EPI_ISL_639473, EPI_ISL_639474, EPI_ISL_639476, EPI_ISL_639477, EPI_ISL_639479, EPI_ISL_639483, EPI_ISL_639486, EPI_ISL_639489, EPI_ISL_639491, EPI_ISL_639494, EPI_ISL_639497, EPI_ISL_639501, EPI_ISL_639503, EPI_ISL_639507, EPI_ISL_639508, EPI_ISL_639509, EPI_ISL_639512, EPI_ISL_639514, EPI_ISL_639521, EPI_ISL_639523, EPI_ISL_639525, EPI_ISL_639527, EPI_ISL_639528, EPI_ISL_639530, EPI_ISL_639534, EPI_ISL_639539, EPI_ISL_639541, EPI_ISL_639542, EPI_ISL_639544, EPI_ISL_639549, EPI_ISL_639553, EPI_ISL_639557, EPI_ISL_639561, EPI_ISL_639563, EPI_ISL_639566, EPI_ISL_639567, EPI_ISL_639569, EPI_ISL_639571, EPI_ISL_639578, EPI_ISL_639580, EPI_ISL_639584, EPI_ISL_639588, EPI_ISL_639592, EPI_ISL_639593, EPI_ISL_639597, EPI_ISL_639603, EPI_ISL_639606, EPI_ISL_639607, EPI_ISL_639612, EPI_ISL_639615, EPI_ISL_639616, EPI_ISL_639617, EPI_ISL_639618			
see above	Wales Specialist Virology Centre Sequencing lab: Pathogen Genomics Unit	COVID-19 Genomics UK (COG-UK) Consortium	Catherine Moore, Johnathan Evans, Laura Gifford, Malorie Perry, Simon Cottrell, Angela Marchbank, Alec Birchley, Alexander Adams, Amy Gaskin, Bree Gatica-Wilcox, Jason Coombes, Joel Southgate, Lauren Gilbert, Lee Graham, Nicole Pacchiarini, Sara Kumziene-Summerhayes, Sarah Taylor, Sophie Jones, Sara Rey, Matthew Bull, Joanne Watkins, Sally Corden, Tom Connor
EPI_ISL_639733	Laverty Pathology	NSW Health Pathology - Institute of Clinical Pathology and Medical Research; Westmead Hospital; University of Sydney	CIDM-PH et al.
EPI_ISL_639734, EPI_ISL_639735	Sydney South West Pathology Service (SSWPS) - Liverpool Hospital - NSW Health Pathology	NSW Health Pathology - Institute of Clinical Pathology and Medical Research; Westmead Hospital; University of Sydney	CIDM-PH et al.
EPI_ISL_639983	CNR Virus des Infections Respiratoires - France SUD	CNR Virus des Infections Respiratoires - France SUD	Antonin Bal, Gregory Destras, Gwendolyne Burfin, Hadrien Règue, Alexandre Gaymard, Maude Bouscambert-Duchamp, Florence Morfin-Sherpa, Martine Valette, Bruno Lina, Laurence Josset
EPI_ISL_641607, EPI_ISL_641608, EPI_ISL_641609, EPI_ISL_641611, EPI_ISL_641612, EPI_ISL_641614, EPI_ISL_641615, EPI_ISL_641616, EPI_ISL_641617, EPI_ISL_641618, EPI_ISL_641619, EPI_ISL_641620, EPI_ISL_641621, EPI_ISL_641622, EPI_ISL_641623, EPI_ISL_641624, EPI_ISL_641625, EPI_ISL_641626, EPI_ISL_641627, EPI_ISL_641629, EPI_ISL_641630, EPI_ISL_641632, EPI_ISL_641633, EPI_ISL_641634, EPI_ISL_641636, EPI_ISL_641637, EPI_ISL_641638, EPI_ISL_641639, EPI_ISL_641640, EPI_ISL_641641, EPI_ISL_641642, EPI_ISL_641643, EPI_ISL_641644, EPI_ISL_641645, EPI_ISL_641646, EPI_ISL_641647, EPI_ISL_641648, EPI_ISL_641651, EPI_ISL_641653, EPI_ISL_641654, EPI_ISL_641655, EPI_ISL_641656, EPI_ISL_641657, EPI_ISL_641658, EPI_ISL_641659, EPI_ISL_641660, EPI_ISL_641661, EPI_ISL_641662, EPI_ISL_641663, EPI_ISL_641664, EPI_ISL_641665, EPI_ISL_641666, EPI_ISL_641669, EPI_ISL_641670, EPI_ISL_641672, EPI_ISL_641673, EPI_ISL_641674, EPI_ISL_641675, EPI_ISL_641676, EPI_ISL_641677, EPI_ISL_641678, EPI_ISL_641679, EPI_ISL_641680, EPI_ISL_641681, EPI_ISL_641682, EPI_ISL_641683, EPI_ISL_641684, EPI_ISL_641688, EPI_ISL_641689, EPI_ISL_641690, EPI_ISL_641691, EPI_ISL_641692, EPI_ISL_641693, EPI_ISL_641694, EPI_ISL_641695, EPI_ISL_641697, EPI_ISL_641698, EPI_ISL_641699, EPI_ISL_641700, EPI_ISL_641701, EPI_ISL_641702, EPI_ISL_641703, EPI_ISL_641704, EPI_ISL_641705, EPI_ISL_641706, EPI_ISL_641708, EPI_ISL_641709, EPI_ISL_641712, EPI_ISL_641713, EPI_ISL_641714, EPI_ISL_641716, EPI_ISL_641717, EPI_ISL_641718, EPI_ISL_641719, EPI_ISL_641721, EPI_ISL_641722, EPI_ISL_641723, EPI_ISL_641724, EPI_ISL_641725, EPI_ISL_641726, EPI_ISL_641727, EPI_ISL_641728, EPI_ISL_641729, EPI_ISL_641730, EPI_ISL_641731, EPI_ISL_641732, EPI_ISL_641735, EPI_ISL_641736, EPI_ISL_641737, EPI_ISL_641738, EPI_ISL_641739, EPI_ISL_641740, EPI_ISL_641741, EPI_ISL_641742, EPI_ISL_641743, EPI_ISL_641746, EPI_ISL_641748, EPI_ISL_641749, EPI_ISL_641750, EPI_ISL_641751, EPI_ISL_641752, EPI_ISL_641754, EPI_ISL_641755, EPI_ISL_641757, EPI_ISL_641760, EPI_ISL_641764, EPI_ISL_641765, EPI_ISL_641766, EPI_ISL_641767, EPI_ISL_641768, EPI_ISL_641770, EPI_ISL_641771, EPI_ISL_641772, EPI_ISL_641773, EPI_ISL_641774, EPI_ISL_641776, EPI_ISL_641777, EPI_ISL_641778, EPI_ISL_641779, EPI_ISL_641781, EPI_ISL_641783, EPI_ISL_641784, EPI_ISL_641786, EPI_ISL_641787, EPI_ISL_641789, EPI_ISL_641790, EPI_ISL_641791, EPI_ISL_641792, EPI_ISL_641793, EPI_ISL_641794, EPI_ISL_641797, EPI_ISL_641798, EPI_ISL_641800, EPI_ISL_641801, EPI_ISL_641803, EPI_ISL_641805, EPI_ISL_641806, EPI_ISL_641807, EPI_ISL_641808, EPI_ISL_641809, EPI_ISL_641810, EPI_ISL_641811,			

EPI_ISL_641812, EPI_ISL_641813, EPI_ISL_641817, EPI_ISL_641818, EPI_ISL_641819, EPI_ISL_641820, EPI_ISL_641821, EPI_ISL_641822, EPI_ISL_641823, EPI_ISL_641824, EPI_ISL_641825, EPI_ISL_641826, EPI_ISL_641827, EPI_ISL_641828, EPI_ISL_641829, EPI_ISL_641830, EPI_ISL_641831, EPI_ISL_641834, EPI_ISL_641835, EPI_ISL_641836, EPI_ISL_641837, EPI_ISL_641838, EPI_ISL_641839, EPI_ISL_641840, EPI_ISL_641841, EPI_ISL_641843, EPI_ISL_641844, EPI_ISL_641845, EPI_ISL_641846, EPI_ISL_641848, EPI_ISL_641849, EPI_ISL_641851, EPI_ISL_641852, EPI_ISL_641853, EPI_ISL_641854, EPI_ISL_641855, EPI_ISL_641856, EPI_ISL_641858, EPI_ISL_641859, EPI_ISL_641860, EPI_ISL_641862, EPI_ISL_641863, EPI_ISL_641865, EPI_ISL_641866, EPI_ISL_641867, EPI_ISL_641869, EPI_ISL_641870, EPI_ISL_641871, EPI_ISL_641872, EPI_ISL_641873, EPI_ISL_641874, EPI_ISL_641875, EPI_ISL_641876, EPI_ISL_641880, EPI_ISL_641881, EPI_ISL_641883, EPI_ISL_641885, EPI_ISL_641886, EPI_ISL_641887, EPI_ISL_641889, EPI_ISL_641890, EPI_ISL_641891, EPI_ISL_641892, EPI_ISL_641893, EPI_ISL_641894, EPI_ISL_641895, EPI_ISL_641896, EPI_ISL_641898, EPI_ISL_641899, EPI_ISL_641902, EPI_ISL_641903, EPI_ISL_641904, EPI_ISL_641905, EPI_ISL_641906, EPI_ISL_641907, EPI_ISL_641908, EPI_ISL_641909, EPI_ISL_641910, EPI_ISL_641912, EPI_ISL_641913, EPI_ISL_641914, EPI_ISL_641915, EPI_ISL_641916, EPI_ISL_641917, EPI_ISL_641918, EPI_ISL_641919, EPI_ISL_641920, EPI_ISL_641921, EPI_ISL_641922, EPI_ISL_641923, EPI_ISL_641924, EPI_ISL_641925, EPI_ISL_641926, EPI_ISL_641927, EPI_ISL_641928, EPI_ISL_641929, EPI_ISL_641930, EPI_ISL_641931, EPI_ISL_641932, EPI_ISL_641933, EPI_ISL_641934, EPI_ISL_641935, EPI_ISL_641936, EPI_ISL_641937, EPI_ISL_641938, EPI_ISL_641939, EPI_ISL_641940, EPI_ISL_641941, EPI_ISL_641942, EPI_ISL_641943, EPI_ISL_641944, EPI_ISL_641945, EPI_ISL_641946, EPI_ISL_641947, EPI_ISL_641948, EPI_ISL_641949, EPI_ISL_641950, EPI_ISL_641951, EPI_ISL_641952, EPI_ISL_641953, EPI_ISL_641954, EPI_ISL_641955, EPI_ISL_641956, EPI_ISL_641957, EPI_ISL_641958, EPI_ISL_641959, EPI_ISL_641961, EPI_ISL_641962, EPI_ISL_641963, EPI_ISL_641964, EPI_ISL_641965, EPI_ISL_641966, EPI_ISL_641967, EPI_ISL_641968, EPI_ISL_641969, EPI_ISL_641970, EPI_ISL_641971, EPI_ISL_641972, EPI_ISL_641973, EPI_ISL_641974, EPI_ISL_641975, EPI_ISL_641976, EPI_ISL_641977, EPI_ISL_641978, EPI_ISL_641980, EPI_ISL_641981, EPI_ISL_641982, EPI_ISL_641983, EPI_ISL_641984, EPI_ISL_641985, EPI_ISL_641986, EPI_ISL_641987, EPI_ISL_641988, EPI_ISL_641989, EPI_ISL_641990, EPI_ISL_641991, EPI_ISL_641992, EPI_ISL_641993, EPI_ISL_641995, EPI_ISL_641996, EPI_ISL_641997, EPI_ISL_641998, EPI_ISL_641999, EPI_ISL_642000, EPI_ISL_642001, EPI_ISL_642002, EPI_ISL_642003, EPI_ISL_642004, EPI_ISL_642005, EPI_ISL_642006, EPI_ISL_642007, EPI_ISL_642008, EPI_ISL_642009, EPI_ISL_642010, EPI_ISL_642011, EPI_ISL_642012, EPI_ISL_642013, EPI_ISL_642014, EPI_ISL_642015, EPI_ISL_642016, EPI_ISL_642017, EPI_ISL_642019, EPI_ISL_642020, EPI_ISL_642021, EPI_ISL_642022, EPI_ISL_642023, EPI_ISL_642024, EPI_ISL_642025, EPI_ISL_642026, EPI_ISL_642027, EPI_ISL_642028, EPI_ISL_642029, EPI_ISL_642030, EPI_ISL_642031, EPI_ISL_642032, EPI_ISL_642033, EPI_ISL_642034, EPI_ISL_642035, EPI_ISL_642036, EPI_ISL_642037, EPI_ISL_642039, EPI_ISL_642040, EPI_ISL_642041, EPI_ISL_642042, EPI_ISL_642043, EPI_ISL_642044, EPI_ISL_642045, EPI_ISL_642046, EPI_ISL_642047, EPI_ISL_642048, EPI_ISL_642049, EPI_ISL_642050, EPI_ISL_642051, EPI_ISL_642052, EPI_ISL_642053, EPI_ISL_642054, EPI_ISL_642055, EPI_ISL_642056, EPI_ISL_642057, EPI_ISL_642058, EPI_ISL_642059, EPI_ISL_642060, EPI_ISL_642061, EPI_ISL_642062, EPI_ISL_642063, EPI_ISL_642064, EPI_ISL_642065, EPI_ISL_642066, EPI_ISL_642067, EPI_ISL_642068, EPI_ISL_642070, EPI_ISL_642071, EPI_ISL_642072, EPI_ISL_642073, EPI_ISL_642075, EPI_ISL_642076, EPI_ISL_642077, EPI_ISL_642078, EPI_ISL_642079, EPI_ISL_642080, EPI_ISL_642081, EPI_ISL_642082, EPI_ISL_642083, EPI_ISL_642084, EPI_ISL_642085, EPI_ISL_642086, EPI_ISL_642087, EPI_ISL_642088, EPI_ISL_642089, EPI_ISL_642090, EPI_ISL_642091, EPI_ISL_642092, EPI_ISL_642093, EPI_ISL_642094, EPI_ISL_642095, EPI_ISL_642096, EPI_ISL_642097, EPI_ISL_642098, EPI_ISL_642099, EPI_ISL_642100, EPI_ISL_642101, EPI_ISL_642102, EPI_ISL_642103, EPI_ISL_642104, EPI_ISL_642105, EPI_ISL_642106, EPI_ISL_642107, EPI_ISL_642108, EPI_ISL_642109, EPI_ISL_642110, EPI_ISL_642111, EPI_ISL_642112, EPI_ISL_642113, EPI_ISL_642114, EPI_ISL_642115, EPI_ISL_642116, EPI_ISL_642117, EPI_ISL_642118, EPI_ISL_642119, EPI_ISL_642120, EPI_ISL_642121, EPI_ISL_642122, EPI_ISL_642123, EPI_ISL_642124, EPI_ISL_642125, EPI_ISL_642126, EPI_ISL_642127, EPI_ISL_642128, EPI_ISL_642129, EPI_ISL_642130, EPI_ISL_642131, EPI_ISL_642132, EPI_ISL_642133, EPI_ISL_642134, EPI_ISL_642135, EPI_ISL_642136, EPI_ISL_642137, EPI_ISL_642138, EPI_ISL_642139, EPI_ISL_642140, EPI_ISL_642141, EPI_ISL_642142, EPI_ISL_642143, EPI_ISL_642144, EPI_ISL_642145, EPI_ISL_642146, EPI_ISL_642147, EPI_ISL_642148, EPI_ISL_642149, EPI_ISL_642150, EPI_ISL_642151, EPI_ISL_642152, EPI_ISL_642153, EPI_ISL_642154, EPI_ISL_642156, EPI_ISL_642157, EPI_ISL_642158, EPI_ISL_642159, EPI_ISL_642160, EPI_ISL_642161, EPI_ISL_642162, EPI_ISL_642163, EPI_ISL_642164, EPI_ISL_642165, EPI_ISL_642167, EPI_ISL_642168, EPI_ISL_642171, EPI_ISL_642172, EPI_ISL_642173, EPI_ISL_642174, EPI_ISL_642175, EPI_ISL_642176, EPI_ISL_642177, EPI_ISL_642178, EPI_ISL_642179, EPI_ISL_642180, EPI_ISL_642181, EPI_ISL_642182, EPI_ISL_642183, EPI_ISL_642184, EPI_ISL_642185, EPI_ISL_642186, EPI_ISL_642187, EPI_ISL_642188, EPI_ISL_642189, EPI_ISL_642190, EPI_ISL_642191, EPI_ISL_642192, EPI_ISL_642193, EPI_ISL_642194, EPI_ISL_642195, EPI_ISL_642196, EPI_ISL_642197, EPI_ISL_642198, EPI_ISL_642199, EPI_ISL_642200, EPI_ISL_642201, EPI_ISL_642202, EPI_ISL_642204, EPI_ISL_642205, EPI_ISL_642206, EPI_ISL_642207, EPI_ISL_642208, EPI_ISL_642209, EPI_ISL_642210, EPI_ISL_642211, EPI_ISL_642212, EPI_ISL_642213, EPI_ISL_642214, EPI_ISL_642215, EPI_ISL_642216, EPI_ISL_642217, EPI_ISL_642218, EPI_ISL_642219, EPI_ISL_642220, EPI_ISL_642221, EPI_ISL_642223, EPI_ISL_642224, EPI_ISL_642225, EPI_ISL_642226, EPI_ISL_642227, EPI_ISL_642228, EPI_ISL_642229, EPI_ISL_642230, EPI_ISL_642231, EPI_ISL_642232, EPI_ISL_642233, EPI_ISL_642234, EPI_ISL_642235, EPI_ISL_642236, EPI_ISL_642237, EPI_ISL_642238, EPI_ISL_642239, EPI_ISL_642240, EPI_ISL_642241, EPI_ISL_642242, EPI_ISL_642244, EPI_ISL_642245, EPI_ISL_642246, EPI_ISL_642248, EPI_ISL_642249, EPI_ISL_642250, EPI_ISL_642252, EPI_ISL_642253, EPI_ISL_642254, EPI_ISL_642255, EPI_ISL_642256, EPI_ISL_642257, EPI_ISL_642258, EPI_ISL_642259, EPI_ISL_642260, EPI_ISL_642261, EPI_ISL_642262, EPI_ISL_642263, EPI_ISL_642264, EPI_ISL_642265, EPI_ISL_642266, EPI_ISL_642267, EPI_ISL_642268, EPI_ISL_642269, EPI_ISL_642270, EPI_ISL_642271, EPI_ISL_642272, EPI_ISL_642273, EPI_ISL_642274, EPI_ISL_642275, EPI_ISL_642276, EPI_ISL_642277, EPI_ISL_642278, EPI_ISL_642279, EPI_ISL_642280, EPI_ISL_642281, EPI_ISL_642282, EPI_ISL_642283, EPI_ISL_642285, EPI_ISL_642286, EPI_ISL_642287, EPI_ISL_642288, EPI_ISL_642289, EPI_ISL_642290, EPI_ISL_642291, EPI_ISL_642292, EPI_ISL_642293, EPI_ISL_642294, EPI_ISL_642295, EPI_ISL_642296, EPI_ISL_642297, EPI_ISL_642298, EPI_ISL_642299, EPI_ISL_642300, EPI_ISL_642301, EPI_ISL_642302, EPI_ISL_642303, EPI_ISL_642304, EPI_ISL_642305, EPI_ISL_642306, EPI_ISL_642307, EPI_ISL_642308, EPI_ISL_642309, EPI_ISL_642310, EPI_ISL_642311, EPI_ISL_642312, EPI_ISL_642313, EPI_ISL_642314, EPI_ISL_642315, EPI_ISL_642316, EPI_ISL_642317, EPI_ISL_642318, EPI_ISL_642319, EPI_ISL_642320, EPI_ISL_642321, EPI_ISL_642322, EPI_ISL_642323, EPI_ISL_642324, EPI_ISL_642325, EPI_ISL_642326, EPI_ISL_642327, EPI_ISL_642328, EPI_ISL_642329, EPI_ISL_642330, EPI_ISL_642331, EPI_ISL_642332, EPI_ISL_642333, EPI_ISL_642334, EPI_ISL_642335, EPI_ISL_642336, EPI_ISL_642337, EPI_ISL_642338, EPI_ISL_642339, EPI_ISL_642340, EPI_ISL_642341, EPI_ISL_642342, EPI_ISL_642343, EPI_ISL_642344, EPI_ISL_642345, EPI_ISL_642346, EPI_ISL_642347, EPI_ISL_642348, EPI_ISL_642349, EPI_ISL_642350, EPI_ISL_642351, EPI_ISL_642352, EPI_ISL_642353, EPI_ISL_642355, EPI_ISL_642356, EPI_ISL_642357, EPI_ISL_642358, EPI_ISL_642359, EPI_ISL_642360, EPI_ISL_642361, EPI_ISL_642362, EPI_ISL_642363, EPI_ISL_642364, EPI_ISL_642365, EPI_ISL_642367, EPI_ISL_642368, EPI_ISL_642369, EPI_ISL_642370, EPI_ISL_642371, EPI_ISL_642372, EPI_ISL_642373, EPI_ISL_642374, EPI_ISL_642375, EPI_ISL_642376, EPI_ISL_642377, EPI_ISL_642378, EPI_ISL_642380, EPI_ISL_642381, EPI_ISL_642382, EPI_ISL_642383, EPI_ISL_642384, EPI_ISL_642385, EPI_ISL_642386, EPI_ISL_642387, EPI_ISL_642388, EPI_ISL_642389, EPI_ISL_642390, EPI_ISL_642391, EPI_ISL_642392, EPI_ISL_642393, EPI_ISL_642394, EPI_ISL_642395, EPI_ISL_642396, EPI_ISL_642397, EPI_ISL_642398, EPI_ISL_642399, EPI_ISL_642400, EPI_ISL_642401, EPI_ISL_642402, EPI_ISL_642403, EPI_ISL_642404, EPI_ISL_642405, EPI_ISL_642406, EPI_ISL_642407, EPI_ISL_642408, EPI_ISL_642409, EPI_ISL_642410, EPI_ISL_642411, EPI_ISL_642412, EPI_ISL_642413, EPI_ISL_642414, EPI_ISL_642415, EPI_ISL_642416, EPI_ISL_642417, EPI_ISL_642418, EPI_ISL_642419, EPI_ISL_642421, EPI_ISL_642422, EPI_ISL_642423, EPI_ISL_642424, EPI_ISL_642425, EPI_ISL_642426, EPI_ISL_642427, EPI_ISL_642428, EPI_ISL_642429, EPI_ISL_642430, EPI_ISL_642431, EPI_ISL_642432, EPI_ISL_642433, EPI_ISL_642434, EPI_ISL_642435, EPI_ISL_642436, EPI_ISL_642438, EPI_ISL_642440, EPI_ISL_642441, EPI_ISL_642442, EPI_ISL_642443, EPI_ISL_642444, EPI_ISL_642445, EPI_ISL_642446, EPI_ISL_642447, EPI_ISL_642448, EPI_ISL_642449, EPI_ISL_642450, EPI_ISL_642451, EPI_ISL_642452, EPI_ISL_642453, EPI_ISL_642454, EPI_ISL_642455, EPI_ISL_642456, EPI_ISL_642457, EPI_ISL_642458, EPI_ISL_642459, EPI_ISL_642460, EPI_ISL_642461, EPI_ISL_642462, EPI_ISL_642463, EPI_ISL_642464, EPI_ISL_642465, EPI_ISL_642466, EPI_ISL_642467, EPI_ISL_642468, EPI_ISL_642469, EPI_ISL_642470, EPI_ISL_642471, EPI_ISL_642472, EPI_ISL_642473, EPI_ISL_642474, EPI_ISL_642475, EPI_ISL_642476, EPI_ISL_642478, EPI_ISL_642479, EPI_ISL_642480, EPI_ISL_642481, EPI_ISL_642482, EPI_ISL_642483, EPI_ISL_642484, EPI_ISL_642485, EPI_ISL_642486, EPI_ISL_642487, EPI_ISL_642488, EPI_ISL_642489, EPI_ISL_642490, EPI_ISL_642491, EPI_ISL_642492, EPI_ISL_642493, EPI_ISL_642494, EPI_ISL_642495, EPI_ISL_642496, EPI_ISL_642497, EPI_ISL_642498, EPI_ISL_642499, EPI_ISL_642500, EPI_ISL_642501, EPI_ISL_642502, EPI_ISL_642503, EPI_ISL_642504, EPI_ISL_642505, EPI_ISL_642507, EPI_ISL_642509, EPI_ISL_642510, EPI_ISL_642511, EPI_ISL_642512, EPI_ISL_642513, EPI_ISL_642514, EPI_ISL_642515, EPI_ISL_642516, EPI_ISL_642517, EPI_ISL_642518, EPI_ISL_642519, EPI_ISL_642520, EPI_ISL_642522, EPI_ISL_642523, EPI_ISL_642524, EPI_ISL_642525, EPI_ISL_642526, EPI_ISL_642527, EPI_ISL_642528, EPI_ISL_642529, EPI_ISL_642530, EPI_ISL_642531, EPI_ISL_642532, EPI_ISL_642533, EPI_ISL_642534, EPI_ISL_642535, EPI_ISL_642536, EPI_ISL_642537, EPI_ISL_642538, EPI_ISL_642539, EPI_ISL_642540, EPI_ISL_642541, EPI_ISL_642542, EPI_ISL_642543, EPI_ISL_642544, EPI_ISL_642545, EPI_ISL_642546, EPI_ISL_642547, EPI_ISL_642548, EPI_ISL_642549, EPI_ISL_642550, EPI_ISL_642551, EPI_ISL_642552, EPI_ISL_642553, EPI_ISL_642554, EPI_ISL_642555, EPI_ISL_642556, EPI_ISL_642557, EPI_ISL_642558, EPI_ISL_642559, EPI_ISL_642560, EPI_ISL_642561, EPI_ISL_642562, EPI_ISL_642563, EPI_ISL_642564, EPI_ISL_642565, EPI_ISL_642566, EPI_ISL_642567, EPI_ISL_642568, EPI_ISL_642570, EPI_ISL_642571, EPI_ISL_642572, EPI_ISL_642573, EPI_ISL_642574, EPI_ISL_642575, EPI_ISL_642576, EPI_ISL_642577, EPI_ISL_642578, EPI_ISL_642579, EPI_ISL_642580, EPI_ISL_642582, EPI_ISL_642583, EPI_ISL_642584, EPI_ISL_642585, EPI_ISL_642586, EPI_ISL_642587, EPI_ISL_642588, EPI_ISL_642589, EPI_ISL_642590, EPI_ISL_642591, EPI_ISL_642592, EPI_ISL_642593, EPI_ISL_642594, EPI_ISL_642595, EPI_ISL_642596, EPI_ISL_642597, EPI_ISL_642598, EPI_ISL_642600, EPI_ISL_642601, EPI_ISL_642602, EPI_ISL_642603, EPI_ISL_642604, EPI_ISL_642606, EPI_ISL_642607, EPI_ISL_642608, EPI_ISL_642609, EPI_ISL_642610, EPI_ISL_642611, EPI_ISL_642612, EPI_ISL_642613, EPI_ISL_642614, EPI_ISL_642615, EPI_ISL_642616, EPI_ISL_642617, EPI_ISL_642618, EPI_ISL_642619, EPI_ISL_642620, EPI_ISL_642621, EPI_ISL_642622, EPI_ISL_642623, EPI_ISL_642624, EPI_ISL_642625, EPI_ISL_642626, EPI_ISL_642627, EPI_ISL_642628, EPI_ISL_642629, EPI_ISL_642630, EPI_ISL_642631, EPI_ISL_642632, EPI_ISL_642633, EPI_ISL_642636, EPI_ISL_642637, EPI_ISL_642638, EPI_ISL_642639, EPI_ISL_642640, EPI_ISL_642641, EPI_ISL_642642, EPI_ISL_642643, EPI_ISL_642645, EPI_ISL_642646, EPI_ISL_642647, EPI_ISL_642648, EPI_ISL_642649, EPI_ISL_642650, EPI_ISL_642651, EPI_ISL_642652, EPI_ISL_642653, EPI_ISL_642654, EPI_ISL_642656, EPI_ISL_642657, EPI_ISL_642658, EPI_ISL_642659, EPI_ISL_642660, EPI_ISL_642661, EPI_ISL_642662, EPI_ISL_642665, EPI_ISL_642666, EPI_ISL_642667, EPI_ISL_642668, EPI_ISL_642669, EPI_ISL_642670, EPI_ISL_642671, EPI_ISL_642672, EPI_ISL_642673, EPI_ISL_642674, EPI_ISL_642675, EPI_ISL_642676, EPI_ISL_642677, EPI_ISL_642678, EPI_ISL_642679, EPI_ISL_642680, EPI_ISL_642681, EPI_ISL_642682, EPI_ISL_642683, EPI_ISL_642685, EPI_ISL_642686, EPI_ISL_642687, EPI_ISL_642688, EPI_ISL_642689, EPI_ISL_642690, EPI_ISL_642691, EPI_ISL_642692, EPI_ISL_642693, EPI_ISL_642694, EPI_ISL_642695, EPI_ISL_642696, EPI_ISL_642697, EPI_ISL_642698, EPI_ISL_642699, EPI_ISL_642700, EPI_ISL_642701, EPI_ISL_642704, EPI_ISL_642705, EPI_ISL_642706, EPI_ISL_642707, EPI_ISL_642708, EPI_ISL_642709, EPI_ISL_642710, EPI_ISL_642711, EPI_ISL_642712, EPI_ISL_642713, EPI_ISL_642714, EPI_ISL_642715, EPI_ISL_642716, EPI_ISL_642717, EPI_ISL_642718, EPI_ISL_642719, EPI_ISL_642720, EPI_ISL_642721, EPI_ISL_642722, EPI_ISL_642723, EPI_ISL_642724, EPI_ISL_642725, EPI_ISL_642726, EPI_ISL_642727, EPI_ISL_642728, EPI_ISL_642730, EPI_ISL_642731, EPI_ISL_642732, EPI_ISL_642733, EPI_ISL_642734, EPI_ISL_642735, EPI_ISL_642736, EPI_ISL_642737, EPI_ISL_642738, EPI_ISL_642739, EPI_ISL_642740, EPI_ISL_642741, EPI_ISL_642742, EPI_ISL_642743, EPI_ISL_642745, EPI_ISL_642746, EPI_ISL_642747, EPI_ISL_642748, EPI_ISL_642749, EPI_ISL_642750, EPI_ISL_642751, EPI_ISL_642752, EPI_ISL_642753, EPI_ISL_642754, EPI_ISL_642755, EPI_ISL_642756, EPI_ISL_642757, EPI_ISL_642758, EPI_ISL_642759, EPI_ISL_642760			
see above	Lighthouse Lab in Glasgow	Wellcome Sanger Institute for the COVID-19 Genomics UK (COG-UK) Consortium	Harper VanSteenhouse, Yumi Kasai, David Gray, Carol Clugston, Anna Dominiczak and Alex Alderton, Roberto Amato, Sonia Goncalves, Ewan Harrison, David K. Jackson, Ian Johnston, Dominic Kwiatkowski, Cordelia Langford, John Sillitoe on behalf of the Wellcome Sanger Institute COVID-19 Surveillance Team
EPI_ISL_644614, EPI_ISL_644615	Department of Clinical Microbiology	GIGA Medical Genomics	Keith Durkin, Maria Artesi, Sébastien Bontems, Raphaël Boreux, Bouchra Boujemla, Cécile Meex, Pierrette Melin, Marie-Pierre Hayette, Vincent Bours
EPI_ISL_645118	National Public Health Laboratory, National Centre for Infectious Diseases	National Public Health Laboratory, National Centre for Infectious Diseases	Tze Minn Mak, Sophie Octavia, Zhenyang Zhou, Lin Cui, Raymond Tzer Pin Lin
EPI_ISL_645264, EPI_ISL_645296, EPI_ISL_645302	Lighthouse Lab in Milton Keynes	Wellcome Sanger Institute for the COVID-19 Genomics UK (COG-UK) Consortium	The Lighthouse Lab in Milton Keynes and Alex Alderton, Roberto Amato, Sonia Goncalves, Ewan Harrison, David K. Jackson, Ian Johnston, Dominic Kwiatkowski, Cordelia Langford, John Sillitoe on behalf of the Wellcome Sanger Institute COVID-19 Surveillance Team
EPI_ISL_645322	Lighthouse Lab in Cambridge	Wellcome Sanger Institute for the COVID-19 Genomics UK (COG-UK) Consortium	Rob Howes, The Lighthouse Lab in Cambridge and Alex Alderton, Roberto Amato, Sonia Goncalves, Ewan Harrison, David K. Jackson, Ian Johnston, Dominic Kwiatkowski, Cordelia Langford, John Sillitoe on behalf of the Wellcome Sanger Institute COVID-19 Surveillance Team
EPI_ISL_645345, EPI_ISL_645354, EPI_ISL_645360, EPI_ISL_645375, EPI_ISL_645406	Lighthouse Lab in Milton Keynes	Wellcome Sanger Institute for the COVID-19 Genomics UK (COG-UK) Consortium	The Lighthouse Lab in Milton Keynes and Alex Alderton, Roberto Amato, Sonia Goncalves, Ewan Harrison, David K. Jackson, Ian Johnston, Dominic Kwiatkowski, Cordelia Langford, John Sillitoe on behalf of the Wellcome Sanger Institute COVID-19 Surveillance Team
EPI_ISL_645414	Lighthouse Lab in Cambridge	Wellcome Sanger Institute for the COVID-19 Genomics UK (COG-UK) Consortium	Rob Howes, The Lighthouse Lab in Cambridge and Alex Alderton, Roberto Amato, Sonia Goncalves, Ewan Harrison, David K. Jackson, Ian Johnston, Dominic Kwiatkowski, Cordelia Langford, John Sillitoe on behalf of the Wellcome Sanger Institute COVID-19 Surveillance Team
EPI_ISL_645421, EPI_ISL_645438, EPI_ISL_645489	Lighthouse Lab in Milton Keynes	Wellcome Sanger Institute for the COVID-19 Genomics UK (COG-UK) Consortium	The Lighthouse Lab in Milton Keynes and Alex Alderton, Roberto Amato, Sonia Goncalves, Ewan Harrison, David K. Jackson, Ian Johnston, Dominic Kwiatkowski, Cordelia Langford, John Sillitoe on behalf of the Wellcome Sanger Institute COVID-19 Surveillance Team
EPI_ISL_645538, EPI_ISL_645539, EPI_ISL_645545, EPI_ISL_645551, EPI_ISL_645572, EPI_ISL_645576, EPI_ISL_645584, EPI_ISL_645608, EPI_ISL_645610, EPI_ISL_645623, EPI_ISL_645631, EPI_ISL_645659, EPI_ISL_645665, EPI_ISL_645678, EPI_ISL_645686, EPI_ISL_645692, EPI_ISL_645731, EPI_ISL_645735, EPI_ISL_645755, EPI_ISL_645757, EPI_ISL_645758, EPI_ISL_645768, EPI_ISL_645791, EPI_ISL_645796, EPI_ISL_645812, EPI_ISL_645813, EPI_ISL_645825			
see above	Lighthouse Lab in Alderley Park	Wellcome Sanger Institute for the COVID-19 Genomics UK (COG-UK) Consortium	Jacquelyn Wynn, Mairead Hyland, The Lighthouse Lab in Alderley Park and Alex Alderton, Roberto Amato, Sonia Goncalves, Ewan Harrison, David K. Jackson, Ian Johnston, Dominic Kwiatkowski, Cordelia Langford, John Sillitoe on behalf of the Wellcome Sanger Institute COVID-19 Surveillance Team
EPI_ISL_645826, EPI_ISL_645828, EPI_ISL_645829, EPI_ISL_645832, EPI_ISL_645836, EPI_ISL_645838	Lighthouse Lab in Cambridge	Wellcome Sanger Institute for the COVID-19 Genomics UK (COG-UK) Consortium	Rob Howes, The Lighthouse Lab in Cambridge and Alex Alderton, Roberto Amato, Sonia Goncalves, Ewan Harrison, David K. Jackson, Ian Johnston, Dominic Kwiatkowski, Cordelia Langford, John Sillitoe on behalf of the Wellcome Sanger Institute COVID-19 Surveillance Team
EPI_ISL_645839	Lighthouse Lab in Alderley Park	Wellcome Sanger Institute for the COVID-19 Genomics UK (COG-UK) Consortium	Jacquelyn Wynn, Mairead Hyland, The Lighthouse Lab in Alderley Park and Alex Alderton, Roberto Amato, Sonia Goncalves, Ewan Harrison, David K. Jackson, Ian Johnston, Dominic Kwiatkowski, Cordelia Langford, John Sillitoe on behalf of the Wellcome Sanger Institute COVID-19 Surveillance Team
EPI_ISL_645850, EPI_ISL_645851, EPI_ISL_645852, EPI_ISL_645866, EPI_ISL_645868, EPI_ISL_645869	Lighthouse Lab in Cambridge	Wellcome Sanger Institute for the COVID-19 Genomics UK (COG-UK) Consortium	Rob Howes, The Lighthouse Lab in Cambridge and Alex Alderton, Roberto Amato, Sonia Goncalves, Ewan Harrison, David K. Jackson, Ian Johnston, Dominic Kwiatkowski, Cordelia Langford, John Sillitoe on behalf of the Wellcome Sanger Institute COVID-19 Surveillance Team
EPI_ISL_645875	Lighthouse Lab in Alderley Park	Wellcome Sanger Institute for the COVID-19 Genomics UK	Jacquelyn Wynn, Mairead Hyland, The Lighthouse Lab in Alderley Park and Alex Alderton, Roberto Amato, Sonia Goncalves, Ewan Harrison, David K.

		(COG-UK) Consortium	Jackson, Ian Johnston, Dominic Kwiatkowski, Cordelia Langford, John Sillitoe on behalf of the Wellcome Sanger Institute COVID-19 Surveillance Team
EPI_ISL_645877, EPI_ISL_645880	Lighthouse Lab in Cambridge	Wellcome Sanger Institute for the COVID-19 Genomics UK (COG-UK) Consortium	Rob Howes, The Lighthouse Lab in Cambridge and Alex Alderton, Roberto Amato, Sonia Goncalves, Ewan Harrison, David K. Jackson, Ian Johnston, Dominic Kwiatkowski, Cordelia Langford, John Sillitoe on behalf of the Wellcome Sanger Institute COVID-19 Surveillance Team
EPI_ISL_645881	Lighthouse Lab in Alderley Park	Wellcome Sanger Institute for the COVID-19 Genomics UK (COG-UK) Consortium	Jacquelyn Wynn, Mairead Hyland, The Lighthouse Lab in Alderley Park and Alex Alderton, Roberto Amato, Sonia Goncalves, Ewan Harrison, David K. Jackson, Ian Johnston, Dominic Kwiatkowski, Cordelia Langford, John Sillitoe on behalf of the Wellcome Sanger Institute COVID-19 Surveillance Team
EPI_ISL_645882, EPI_ISL_645885	Lighthouse Lab in Cambridge	Wellcome Sanger Institute for the COVID-19 Genomics UK (COG-UK) Consortium	Rob Howes, The Lighthouse Lab in Cambridge and Alex Alderton, Roberto Amato, Sonia Goncalves, Ewan Harrison, David K. Jackson, Ian Johnston, Dominic Kwiatkowski, Cordelia Langford, John Sillitoe on behalf of the Wellcome Sanger Institute COVID-19 Surveillance Team
EPI_ISL_645889	Lighthouse Lab in Alderley Park	Wellcome Sanger Institute for the COVID-19 Genomics UK (COG-UK) Consortium	Jacquelyn Wynn, Mairead Hyland, The Lighthouse Lab in Alderley Park and Alex Alderton, Roberto Amato, Sonia Goncalves, Ewan Harrison, David K. Jackson, Ian Johnston, Dominic Kwiatkowski, Cordelia Langford, John Sillitoe on behalf of the Wellcome Sanger Institute COVID-19 Surveillance Team
EPI_ISL_645899, EPI_ISL_645908, EPI_ISL_645917, EPI_ISL_645918, EPI_ISL_645920, EPI_ISL_645923, EPI_ISL_645925, EPI_ISL_645926, EPI_ISL_645927	Lighthouse Lab in Cambridge	Wellcome Sanger Institute for the COVID-19 Genomics UK (COG-UK) Consortium	Rob Howes, The Lighthouse Lab in Cambridge and Alex Alderton, Roberto Amato, Sonia Goncalves, Ewan Harrison, David K. Jackson, Ian Johnston, Dominic Kwiatkowski, Cordelia Langford, John Sillitoe on behalf of the Wellcome Sanger Institute COVID-19 Surveillance Team
EPI_ISL_645935	Lighthouse Lab in Alderley Park	Wellcome Sanger Institute for the COVID-19 Genomics UK (COG-UK) Consortium	Jacquelyn Wynn, Mairead Hyland, The Lighthouse Lab in Alderley Park and Alex Alderton, Roberto Amato, Sonia Goncalves, Ewan Harrison, David K. Jackson, Ian Johnston, Dominic Kwiatkowski, Cordelia Langford, John Sillitoe on behalf of the Wellcome Sanger Institute COVID-19 Surveillance Team
EPI_ISL_645949	Lighthouse Lab in Cambridge	Wellcome Sanger Institute for the COVID-19 Genomics UK (COG-UK) Consortium	Rob Howes, The Lighthouse Lab in Cambridge and Alex Alderton, Roberto Amato, Sonia Goncalves, Ewan Harrison, David K. Jackson, Ian Johnston, Dominic Kwiatkowski, Cordelia Langford, John Sillitoe on behalf of the Wellcome Sanger Institute COVID-19 Surveillance Team
EPI_ISL_645950	Lighthouse Lab in Alderley Park	Wellcome Sanger Institute for the COVID-19 Genomics UK (COG-UK) Consortium	Jacquelyn Wynn, Mairead Hyland, The Lighthouse Lab in Alderley Park and Alex Alderton, Roberto Amato, Sonia Goncalves, Ewan Harrison, David K. Jackson, Ian Johnston, Dominic Kwiatkowski, Cordelia Langford, John Sillitoe on behalf of the Wellcome Sanger Institute COVID-19 Surveillance Team
EPI_ISL_645958, EPI_ISL_645962	Lighthouse Lab in Cambridge	Wellcome Sanger Institute for the COVID-19 Genomics UK (COG-UK) Consortium	Rob Howes, The Lighthouse Lab in Cambridge and Alex Alderton, Roberto Amato, Sonia Goncalves, Ewan Harrison, David K. Jackson, Ian Johnston, Dominic Kwiatkowski, Cordelia Langford, John Sillitoe on behalf of the Wellcome Sanger Institute COVID-19 Surveillance Team
EPI_ISL_645963	Lighthouse Lab in Alderley Park	Wellcome Sanger Institute for the COVID-19 Genomics UK (COG-UK) Consortium	Jacquelyn Wynn, Mairead Hyland, The Lighthouse Lab in Alderley Park and Alex Alderton, Roberto Amato, Sonia Goncalves, Ewan Harrison, David K. Jackson, Ian Johnston, Dominic Kwiatkowski, Cordelia Langford, John Sillitoe on behalf of the Wellcome Sanger Institute COVID-19 Surveillance Team
EPI_ISL_645970	Lighthouse Lab in Cambridge	Wellcome Sanger Institute for the COVID-19 Genomics UK (COG-UK) Consortium	Rob Howes, The Lighthouse Lab in Cambridge and Alex Alderton, Roberto Amato, Sonia Goncalves, Ewan Harrison, David K. Jackson, Ian Johnston, Dominic Kwiatkowski, Cordelia Langford, John Sillitoe on behalf of the Wellcome Sanger Institute COVID-19 Surveillance Team
EPI_ISL_645974	Lighthouse Lab in Alderley Park	Wellcome Sanger Institute for the COVID-19 Genomics UK (COG-UK) Consortium	Jacquelyn Wynn, Mairead Hyland, The Lighthouse Lab in Alderley Park and Alex Alderton, Roberto Amato, Sonia Goncalves, Ewan Harrison, David K. Jackson, Ian Johnston, Dominic Kwiatkowski, Cordelia Langford, John Sillitoe on behalf of the Wellcome Sanger Institute COVID-19 Surveillance Team
EPI_ISL_645980, EPI_ISL_645981, EPI_ISL_645983, EPI_ISL_645989	Lighthouse Lab in Cambridge	Wellcome Sanger Institute for the COVID-19 Genomics UK (COG-UK) Consortium	Rob Howes, The Lighthouse Lab in Cambridge and Alex Alderton, Roberto Amato, Sonia Goncalves, Ewan Harrison, David K. Jackson, Ian Johnston, Dominic Kwiatkowski, Cordelia Langford, John Sillitoe on behalf of the Wellcome Sanger Institute COVID-19 Surveillance Team
EPI_ISL_645991	Lighthouse Lab in Alderley Park	Wellcome Sanger Institute for the COVID-19 Genomics UK (COG-UK) Consortium	Jacquelyn Wynn, Mairead Hyland, The Lighthouse Lab in Alderley Park and Alex Alderton, Roberto Amato, Sonia Goncalves, Ewan Harrison, David K. Jackson, Ian Johnston, Dominic Kwiatkowski, Cordelia Langford, John Sillitoe on behalf of the Wellcome Sanger Institute COVID-19 Surveillance Team
EPI_ISL_645992, EPI_ISL_645993, EPI_ISL_645995, EPI_ISL_645998, EPI_ISL_646009, EPI_ISL_646014, EPI_ISL_646017, EPI_ISL_646018, EPI_ISL_646022, EPI_ISL_646028	Lighthouse Lab in Cambridge	Wellcome Sanger Institute for the COVID-19 Genomics UK (COG-UK) Consortium	Rob Howes, The Lighthouse Lab in Cambridge and Alex Alderton, Roberto Amato, Sonia Goncalves, Ewan Harrison, David K. Jackson, Ian Johnston, Dominic Kwiatkowski, Cordelia Langford, John Sillitoe on behalf of the Wellcome Sanger Institute COVID-19 Surveillance Team
EPI_ISL_646029	Lighthouse Lab in Alderley Park	Wellcome Sanger Institute for the COVID-19 Genomics UK (COG-UK) Consortium	Jacquelyn Wynn, Mairead Hyland, The Lighthouse Lab in Alderley Park and Alex Alderton, Roberto Amato, Sonia Goncalves, Ewan Harrison, David K. Jackson, Ian Johnston, Dominic Kwiatkowski, Cordelia Langford, John Sillitoe on behalf of the Wellcome Sanger Institute COVID-19 Surveillance Team
EPI_ISL_646030, EPI_ISL_646031, EPI_ISL_646032, EPI_ISL_646034, EPI_ISL_646035, EPI_ISL_646041, EPI_ISL_646046, EPI_ISL_646052	Lighthouse Lab in Cambridge	Wellcome Sanger Institute for the COVID-19 Genomics UK (COG-UK) Consortium	Rob Howes, The Lighthouse Lab in Cambridge and Alex Alderton, Roberto Amato, Sonia Goncalves, Ewan Harrison, David K. Jackson, Ian Johnston, Dominic Kwiatkowski, Cordelia Langford, John Sillitoe on behalf of the Wellcome Sanger Institute COVID-19 Surveillance Team
EPI_ISL_646054	Lighthouse Lab in Alderley Park	Wellcome Sanger Institute for the COVID-19 Genomics UK (COG-UK) Consortium	Jacquelyn Wynn, Mairead Hyland, The Lighthouse Lab in Alderley Park and Alex Alderton, Roberto Amato, Sonia Goncalves, Ewan Harrison, David K. Jackson, Ian Johnston, Dominic Kwiatkowski, Cordelia Langford, John Sillitoe on behalf of the Wellcome Sanger Institute COVID-19 Surveillance Team
EPI_ISL_646056, EPI_ISL_646058	Lighthouse Lab in Cambridge	Wellcome Sanger Institute for the COVID-19 Genomics UK (COG-UK) Consortium	Rob Howes, The Lighthouse Lab in Cambridge and Alex Alderton, Roberto Amato, Sonia Goncalves, Ewan Harrison, David K. Jackson, Ian Johnston, Dominic Kwiatkowski, Cordelia Langford, John Sillitoe on behalf of the Wellcome Sanger Institute COVID-19 Surveillance Team
EPI_ISL_646062	Lighthouse Lab in Alderley Park	Wellcome Sanger Institute for the COVID-19 Genomics UK (COG-UK) Consortium	Jacquelyn Wynn, Mairead Hyland, The Lighthouse Lab in Alderley Park and Alex Alderton, Roberto Amato, Sonia Goncalves, Ewan Harrison, David K. Jackson, Ian Johnston, Dominic Kwiatkowski, Cordelia Langford, John Sillitoe on behalf of the Wellcome Sanger Institute COVID-19 Surveillance Team
EPI_ISL_646067	Lighthouse Lab in Cambridge	Wellcome Sanger Institute for the COVID-19 Genomics UK (COG-UK) Consortium	Rob Howes, The Lighthouse Lab in Cambridge and Alex Alderton, Roberto Amato, Sonia Goncalves, Ewan Harrison, David K. Jackson, Ian Johnston, Dominic Kwiatkowski, Cordelia Langford, John Sillitoe on behalf of the Wellcome Sanger Institute COVID-19 Surveillance Team
EPI_ISL_646069, EPI_ISL_646078	Lighthouse Lab in Alderley Park	Wellcome Sanger Institute for the COVID-19 Genomics UK (COG-UK) Consortium	Jacquelyn Wynn, Mairead Hyland, The Lighthouse Lab in Alderley Park and Alex Alderton, Roberto Amato, Sonia Goncalves, Ewan Harrison, David K. Jackson, Ian Johnston, Dominic Kwiatkowski, Cordelia Langford, John Sillitoe on behalf of the Wellcome Sanger Institute COVID-19 Surveillance Team
EPI_ISL_646083, EPI_ISL_646084, EPI_ISL_646085, EPI_ISL_646089, EPI_ISL_646091	Lighthouse Lab in Cambridge	Wellcome Sanger Institute for the COVID-19 Genomics UK (COG-UK) Consortium	Rob Howes, The Lighthouse Lab in Cambridge and Alex Alderton, Roberto Amato, Sonia Goncalves, Ewan Harrison, David K. Jackson, Ian Johnston, Dominic Kwiatkowski, Cordelia Langford, John Sillitoe on behalf of the Wellcome Sanger Institute COVID-19 Surveillance Team
EPI_ISL_646096, EPI_ISL_646098	Lighthouse Lab in Alderley Park	Wellcome Sanger Institute for the COVID-19 Genomics UK (COG-UK) Consortium	Jacquelyn Wynn, Mairead Hyland, The Lighthouse Lab in Alderley Park and Alex Alderton, Roberto Amato, Sonia Goncalves, Ewan Harrison, David K. Jackson, Ian Johnston, Dominic Kwiatkowski, Cordelia Langford, John Sillitoe on behalf of the Wellcome Sanger Institute COVID-19 Surveillance Team
EPI_ISL_646101, EPI_ISL_646102, EPI_ISL_646115, EPI_ISL_646122	Lighthouse Lab in Cambridge	Wellcome Sanger Institute for the COVID-19 Genomics UK (COG-UK) Consortium	Rob Howes, The Lighthouse Lab in Cambridge and Alex Alderton, Roberto Amato, Sonia Goncalves, Ewan Harrison, David K. Jackson, Ian Johnston, Dominic Kwiatkowski, Cordelia Langford, John Sillitoe on behalf of the Wellcome Sanger Institute COVID-19 Surveillance Team
EPI_ISL_646125	Lighthouse Lab in Alderley Park	Wellcome Sanger Institute for the COVID-19 Genomics UK (COG-UK) Consortium	Jacquelyn Wynn, Mairead Hyland, The Lighthouse Lab in Alderley Park and Alex Alderton, Roberto Amato, Sonia Goncalves, Ewan Harrison, David K. Jackson, Ian Johnston, Dominic Kwiatkowski, Cordelia Langford, John Sillitoe on behalf of the Wellcome Sanger Institute COVID-19 Surveillance Team
EPI_ISL_646128, EPI_ISL_646129, EPI_ISL_646135, EPI_ISL_646136, EPI_ISL_646139	Lighthouse Lab in Cambridge	Wellcome Sanger Institute for the COVID-19 Genomics UK (COG-UK) Consortium	Rob Howes, The Lighthouse Lab in Cambridge and Alex Alderton, Roberto Amato, Sonia Goncalves, Ewan Harrison, David K. Jackson, Ian Johnston, Dominic Kwiatkowski, Cordelia Langford, John Sillitoe on behalf of the Wellcome Sanger Institute COVID-19 Surveillance Team
EPI_ISL_646142, EPI_ISL_646152, EPI_ISL_646154	Lighthouse Lab in Glasgow	Wellcome Sanger Institute for the COVID-19 Genomics UK (COG-UK) Consortium	Harper VanSteenhouse, Yumi Kasai, David Gray, Carol Clugston, Anna Dominiczak and Alex Alderton, Roberto Amato, Sonia Goncalves, Ewan Harrison, David K. Jackson, Ian Johnston, Dominic Kwiatkowski, Cordelia Langford, John Sillitoe on behalf of the Wellcome Sanger Institute COVID-19 Surveillance Team
EPI_ISL_646162	Lighthouse Lab in Alderley Park	Wellcome Sanger Institute for the COVID-19 Genomics UK (COG-UK) Consortium	Jacquelyn Wynn, Mairead Hyland, The Lighthouse Lab in Alderley Park and Alex Alderton, Roberto Amato, Sonia Goncalves, Ewan Harrison, David K. Jackson, Ian Johnston, Dominic Kwiatkowski, Cordelia Langford, John Sillitoe on behalf of the Wellcome Sanger Institute COVID-19 Surveillance Team
EPI_ISL_646164, EPI_ISL_646168, EPI_ISL_646174, EPI_ISL_646179, EPI_ISL_646181	Lighthouse Lab in Glasgow	Wellcome Sanger Institute for the COVID-19 Genomics UK (COG-UK) Consortium	Harper VanSteenhouse, Yumi Kasai, David Gray, Carol Clugston, Anna Dominiczak and Alex Alderton, Roberto Amato, Sonia Goncalves, Ewan Harrison, David K. Jackson, Ian Johnston, Dominic Kwiatkowski, Cordelia Langford, John Sillitoe on behalf of the Wellcome Sanger Institute COVID-19 Surveillance Team

EPI_ISL_646182	Lighthouse Lab in Alderley Park	Wellcome Sanger Institute for the COVID-19 Genomics UK (COG-UK) Consortium	Jacquelyn Wynn, Mairead Hyland, The Lighthouse Lab in Alderley Park and Alex Alderton, Roberto Amato, Sonia Goncalves, Ewan Harrison, David K. Jackson, Ian Johnston, Dominic Kwiatkowski, Cordelia Langford, John Sillitoe on behalf of the Wellcome Sanger Institute COVID-19 Surveillance Team
EPI_ISL_646186, EPI_ISL_646188	Lighthouse Lab in Glasgow	Wellcome Sanger Institute for the COVID-19 Genomics UK (COG-UK) Consortium	Harper VanSteenhouse, Yumi Kasai, David Gray, Carol Clugston, Anna Dominiczak and Alex Alderton, Roberto Amato, Sonia Goncalves, Ewan Harrison, David K. Jackson, Ian Johnston, Dominic Kwiatkowski, Cordelia Langford, John Sillitoe on behalf of the Wellcome Sanger Institute COVID-19 Surveillance Team
EPI_ISL_646191	Lighthouse Lab in Alderley Park	Wellcome Sanger Institute for the COVID-19 Genomics UK (COG-UK) Consortium	Jacquelyn Wynn, Mairead Hyland, The Lighthouse Lab in Alderley Park and Alex Alderton, Roberto Amato, Sonia Goncalves, Ewan Harrison, David K. Jackson, Ian Johnston, Dominic Kwiatkowski, Cordelia Langford, John Sillitoe on behalf of the Wellcome Sanger Institute COVID-19 Surveillance Team
EPI_ISL_646192, EPI_ISL_646200, EPI_ISL_646225, EPI_ISL_646226, EPI_ISL_646237, EPI_ISL_646241, EPI_ISL_646243, EPI_ISL_646247, EPI_ISL_646254, EPI_ISL_646264, EPI_ISL_646266, EPI_ISL_646282, EPI_ISL_646284, EPI_ISL_646286, EPI_ISL_646292, EPI_ISL_646295, EPI_ISL_646298, EPI_ISL_646300			
see above	Lighthouse Lab in Glasgow	Wellcome Sanger Institute for the COVID-19 Genomics UK (COG-UK) Consortium	Harper VanSteenhouse, Yumi Kasai, David Gray, Carol Clugston, Anna Dominiczak and Alex Alderton, Roberto Amato, Sonia Goncalves, Ewan Harrison, David K. Jackson, Ian Johnston, Dominic Kwiatkowski, Cordelia Langford, John Sillitoe on behalf of the Wellcome Sanger Institute COVID-19 Surveillance Team
EPI_ISL_646303	Lighthouse Lab in Alderley Park	Wellcome Sanger Institute for the COVID-19 Genomics UK (COG-UK) Consortium	Jacquelyn Wynn, Mairead Hyland, The Lighthouse Lab in Alderley Park and Alex Alderton, Roberto Amato, Sonia Goncalves, Ewan Harrison, David K. Jackson, Ian Johnston, Dominic Kwiatkowski, Cordelia Langford, John Sillitoe on behalf of the Wellcome Sanger Institute COVID-19 Surveillance Team
EPI_ISL_646308, EPI_ISL_646310, EPI_ISL_646316, EPI_ISL_646321, EPI_ISL_646322, EPI_ISL_646330, EPI_ISL_646344, EPI_ISL_646351, EPI_ISL_646355, EPI_ISL_646357, EPI_ISL_646358, EPI_ISL_646359, EPI_ISL_646365, EPI_ISL_646368			
see above	Lighthouse Lab in Glasgow	Wellcome Sanger Institute for the COVID-19 Genomics UK (COG-UK) Consortium	Harper VanSteenhouse, Yumi Kasai, David Gray, Carol Clugston, Anna Dominiczak and Alex Alderton, Roberto Amato, Sonia Goncalves, Ewan Harrison, David K. Jackson, Ian Johnston, Dominic Kwiatkowski, Cordelia Langford, John Sillitoe on behalf of the Wellcome Sanger Institute COVID-19 Surveillance Team
EPI_ISL_646370	Lighthouse Lab in Alderley Park	Wellcome Sanger Institute for the COVID-19 Genomics UK (COG-UK) Consortium	Jacquelyn Wynn, Mairead Hyland, The Lighthouse Lab in Alderley Park and Alex Alderton, Roberto Amato, Sonia Goncalves, Ewan Harrison, David K. Jackson, Ian Johnston, Dominic Kwiatkowski, Cordelia Langford, John Sillitoe on behalf of the Wellcome Sanger Institute COVID-19 Surveillance Team
EPI_ISL_646371, EPI_ISL_646372, EPI_ISL_646380, EPI_ISL_646386, EPI_ISL_646387, EPI_ISL_646391, EPI_ISL_646394, EPI_ISL_646396, EPI_ISL_646401, EPI_ISL_646405, EPI_ISL_646407, EPI_ISL_646410, EPI_ISL_646416, EPI_ISL_646417, EPI_ISL_646422, EPI_ISL_646423, EPI_ISL_646424, EPI_ISL_646427, EPI_ISL_646436, EPI_ISL_646447, EPI_ISL_646451, EPI_ISL_646453, EPI_ISL_646455, EPI_ISL_646456, EPI_ISL_646457, EPI_ISL_646458, EPI_ISL_646459, EPI_ISL_646460, EPI_ISL_646462, EPI_ISL_646464, EPI_ISL_646465, EPI_ISL_646470, EPI_ISL_646471, EPI_ISL_646472, EPI_ISL_646474, EPI_ISL_646475, EPI_ISL_646480, EPI_ISL_646481, EPI_ISL_646482, EPI_ISL_646483, EPI_ISL_646484, EPI_ISL_646495, EPI_ISL_646496, EPI_ISL_646498, EPI_ISL_646499, EPI_ISL_646501, EPI_ISL_646504, EPI_ISL_646517, EPI_ISL_646523, EPI_ISL_646524, EPI_ISL_646530, EPI_ISL_646531, EPI_ISL_646536, EPI_ISL_646539, EPI_ISL_646547, EPI_ISL_646550, EPI_ISL_646552, EPI_ISL_646553, EPI_ISL_646554, EPI_ISL_646556, EPI_ISL_646562, EPI_ISL_646565, EPI_ISL_646570, EPI_ISL_646575, EPI_ISL_646578, EPI_ISL_646582, EPI_ISL_646584, EPI_ISL_646587, EPI_ISL_646588, EPI_ISL_646589, EPI_ISL_646596, EPI_ISL_646598, EPI_ISL_646604, EPI_ISL_646609, EPI_ISL_646611, EPI_ISL_646615, EPI_ISL_646617, EPI_ISL_646620, EPI_ISL_646622, EPI_ISL_646624, EPI_ISL_646627, EPI_ISL_646629, EPI_ISL_646630, EPI_ISL_646633, EPI_ISL_646634, EPI_ISL_646636, EPI_ISL_646637, EPI_ISL_646641, EPI_ISL_646643, EPI_ISL_646644, EPI_ISL_646646, EPI_ISL_646647, EPI_ISL_646649, EPI_ISL_646650, EPI_ISL_646654, EPI_ISL_646656, EPI_ISL_646657, EPI_ISL_646659, EPI_ISL_646661, EPI_ISL_646663, EPI_ISL_646667, EPI_ISL_646669, EPI_ISL_646670, EPI_ISL_646672, EPI_ISL_646674, EPI_ISL_646677, EPI_ISL_646680, EPI_ISL_646681, EPI_ISL_646682, EPI_ISL_646685, EPI_ISL_646688, EPI_ISL_646690, EPI_ISL_646694, EPI_ISL_646699, EPI_ISL_646700, EPI_ISL_646701, EPI_ISL_646703, EPI_ISL_646705, EPI_ISL_646709, EPI_ISL_646714, EPI_ISL_646717, EPI_ISL_646720, EPI_ISL_646723, EPI_ISL_646725, EPI_ISL_646726, EPI_ISL_646728, EPI_ISL_646729, EPI_ISL_646730, EPI_ISL_646732, EPI_ISL_646733, EPI_ISL_646735, EPI_ISL_646742, EPI_ISL_646745, EPI_ISL_646748, EPI_ISL_646750, EPI_ISL_646754, EPI_ISL_646759, EPI_ISL_646765, EPI_ISL_646766, EPI_ISL_646772, EPI_ISL_646774, EPI_ISL_646775, EPI_ISL_646777, EPI_ISL_646778, EPI_ISL_646780, EPI_ISL_646781, EPI_ISL_646783, EPI_ISL_646785, EPI_ISL_646786, EPI_ISL_646788, EPI_ISL_646792, EPI_ISL_646794, EPI_ISL_646798, EPI_ISL_646810, EPI_ISL_646818, EPI_ISL_646825, EPI_ISL_646826, EPI_ISL_646837, EPI_ISL_646840, EPI_ISL_646850, EPI_ISL_646853, EPI_ISL_646854, EPI_ISL_646868, EPI_ISL_646869, EPI_ISL_646870, EPI_ISL_646872, EPI_ISL_646877, EPI_ISL_646880, EPI_ISL_646885, EPI_ISL_646891, EPI_ISL_646892, EPI_ISL_646902			
see above	Lighthouse Lab in Glasgow	Wellcome Sanger Institute for the COVID-19 Genomics UK (COG-UK) Consortium	Harper VanSteenhouse, Yumi Kasai, David Gray, Carol Clugston, Anna Dominiczak and Alex Alderton, Roberto Amato, Sonia Goncalves, Ewan Harrison, David K. Jackson, Ian Johnston, Dominic Kwiatkowski, Cordelia Langford, John Sillitoe on behalf of the Wellcome Sanger Institute COVID-19 Surveillance Team
EPI_ISL_646903	Lighthouse Lab in Alderley Park	Wellcome Sanger Institute for the COVID-19 Genomics UK (COG-UK) Consortium	Jacquelyn Wynn, Mairead Hyland, The Lighthouse Lab in Alderley Park and Alex Alderton, Roberto Amato, Sonia Goncalves, Ewan Harrison, David K. Jackson, Ian Johnston, Dominic Kwiatkowski, Cordelia Langford, John Sillitoe on behalf of the Wellcome Sanger Institute COVID-19 Surveillance Team
EPI_ISL_646905, EPI_ISL_646912, EPI_ISL_646915, EPI_ISL_646921, EPI_ISL_646929, EPI_ISL_646936, EPI_ISL_646937, EPI_ISL_646939, EPI_ISL_646948, EPI_ISL_646955, EPI_ISL_646960, EPI_ISL_646964, EPI_ISL_646967, EPI_ISL_646974, EPI_ISL_646975, EPI_ISL_646976, EPI_ISL_646977, EPI_ISL_646983, EPI_ISL_646988, EPI_ISL_646992, EPI_ISL_646996, EPI_ISL_647001, EPI_ISL_647003, EPI_ISL_647006, EPI_ISL_647014, EPI_ISL_647023, EPI_ISL_647031, EPI_ISL_647032, EPI_ISL_647035, EPI_ISL_647038, EPI_ISL_647045, EPI_ISL_647050, EPI_ISL_647053, EPI_ISL_647059, EPI_ISL_647061, EPI_ISL_647067, EPI_ISL_647076, EPI_ISL_647081, EPI_ISL_647087, EPI_ISL_647090, EPI_ISL_647097, EPI_ISL_647102, EPI_ISL_647105, EPI_ISL_647109, EPI_ISL_647114, EPI_ISL_647115, EPI_ISL_647117, EPI_ISL_647121			
see above	Lighthouse Lab in Glasgow	Wellcome Sanger Institute for the COVID-19 Genomics UK (COG-UK) Consortium	Harper VanSteenhouse, Yumi Kasai, David Gray, Carol Clugston, Anna Dominiczak and Alex Alderton, Roberto Amato, Sonia Goncalves, Ewan Harrison, David K. Jackson, Ian Johnston, Dominic Kwiatkowski, Cordelia Langford, John Sillitoe on behalf of the Wellcome Sanger Institute COVID-19 Surveillance Team
EPI_ISL_647123, EPI_ISL_647124, EPI_ISL_647125, EPI_ISL_647126, EPI_ISL_647127, EPI_ISL_647128, EPI_ISL_647129, EPI_ISL_647130, EPI_ISL_647131, EPI_ISL_647132, EPI_ISL_647133, EPI_ISL_647136, EPI_ISL_647137, EPI_ISL_647138, EPI_ISL_647140, EPI_ISL_647141, EPI_ISL_647142, EPI_ISL_647143, EPI_ISL_647144, EPI_ISL_647146, EPI_ISL_647149, EPI_ISL_647150, EPI_ISL_647151, EPI_ISL_647154, EPI_ISL_647155, EPI_ISL_647156, EPI_ISL_647157, EPI_ISL_647158, EPI_ISL_647159, EPI_ISL_647162, EPI_ISL_647163, EPI_ISL_647164, EPI_ISL_647166, EPI_ISL_647167, EPI_ISL_647168, EPI_ISL_647169, EPI_ISL_647170, EPI_ISL_647171, EPI_ISL_647173, EPI_ISL_647174, EPI_ISL_647175, EPI_ISL_647176, EPI_ISL_647177, EPI_ISL_647179, EPI_ISL_647181, EPI_ISL_647182, EPI_ISL_647183, EPI_ISL_647184, EPI_ISL_647185, EPI_ISL_647186, EPI_ISL_647188, EPI_ISL_647189, EPI_ISL_647190, EPI_ISL_647191, EPI_ISL_647192, EPI_ISL_647193, EPI_ISL_647194, EPI_ISL_647195, EPI_ISL_647196, EPI_ISL_647198, EPI_ISL_647202, EPI_ISL_647203, EPI_ISL_647204, EPI_ISL_647206, EPI_ISL_647207, EPI_ISL_647208, EPI_ISL_647210, EPI_ISL_647211, EPI_ISL_647212, EPI_ISL_647215, EPI_ISL_647217, EPI_ISL_647218, EPI_ISL_647219, EPI_ISL_647220, EPI_ISL_647221, EPI_ISL_647222, EPI_ISL_647225, EPI_ISL_647226, EPI_ISL_647227, EPI_ISL_647228, EPI_ISL_647229, EPI_ISL_647231, EPI_ISL_647233, EPI_ISL_647235, EPI_ISL_647236, EPI_ISL_647237, EPI_ISL_647238, EPI_ISL_647239, EPI_ISL_647241, EPI_ISL_647242, EPI_ISL_647243, EPI_ISL_647244, EPI_ISL_647245, EPI_ISL_647246, EPI_ISL_647248, EPI_ISL_647249, EPI_ISL_647252, EPI_ISL_647253, EPI_ISL_647257, EPI_ISL_647258, EPI_ISL_647261, EPI_ISL_647262, EPI_ISL_647264, EPI_ISL_647265, EPI_ISL_647268, EPI_ISL_647270, EPI_ISL_647271, EPI_ISL_647272, EPI_ISL_647274, EPI_ISL_647276, EPI_ISL_647277, EPI_ISL_647278, EPI_ISL_647283, EPI_ISL_647284, EPI_ISL_647285, EPI_ISL_647286, EPI_ISL_647287, EPI_ISL_647288, EPI_ISL_647289, EPI_ISL_647293, EPI_ISL_647295, EPI_ISL_647297, EPI_ISL_647298, EPI_ISL_647299, EPI_ISL_647300, EPI_ISL_647301, EPI_ISL_647302, EPI_ISL_647303, EPI_ISL_647304, EPI_ISL_647305, EPI_ISL_647306, EPI_ISL_647307, EPI_ISL_647309, EPI_ISL_647310, EPI_ISL_647311, EPI_ISL_647312, EPI_ISL_647313, EPI_ISL_647316, EPI_ISL_647317, EPI_ISL_647318, EPI_ISL_647319, EPI_ISL_647320, EPI_ISL_647322, EPI_ISL_647325, EPI_ISL_647326, EPI_ISL_647327, EPI_ISL_647331, EPI_ISL_647333, EPI_ISL_647335, EPI_ISL_647336, EPI_ISL_647338, EPI_ISL_647340, EPI_ISL_647341, EPI_ISL_647344, EPI_ISL_647345, EPI_ISL_647346, EPI_ISL_647348, EPI_ISL_647349, EPI_ISL_647350, EPI_ISL_647351, EPI_ISL_647352, EPI_ISL_647353, EPI_ISL_647355, EPI_ISL_647356, EPI_ISL_647357, EPI_ISL_647358, EPI_ISL_647359, EPI_ISL_647360, EPI_ISL_647364, EPI_ISL_647365, EPI_ISL_647366, EPI_ISL_647367, EPI_ISL_647368, EPI_ISL_647369, EPI_ISL_647370, EPI_ISL_647371, EPI_ISL_647372, EPI_ISL_647373, EPI_ISL_647374, EPI_ISL_647375, EPI_ISL_647376, EPI_ISL_647377, EPI_ISL_647378, EPI_ISL_647379, EPI_ISL_647380, EPI_ISL_647381, EPI_ISL_647382, EPI_ISL_647383, EPI_ISL_647384, EPI_ISL_647385, EPI_ISL_647386, EPI_ISL_647387, EPI_ISL_647388, EPI_ISL_647389, EPI_ISL_647390, EPI_ISL_647391, EPI_ISL_647392, EPI_ISL_647393, EPI_ISL_647394, EPI_ISL_647395, EPI_ISL_647396, EPI_ISL_647397, EPI_ISL_647398, EPI_ISL_647400, EPI_ISL_647401, EPI_ISL_647402, EPI_ISL_647403, EPI_ISL_647404, EPI_ISL_647406, EPI_ISL_647407, EPI_ISL_647408, EPI_ISL_647409, EPI_ISL_647410, EPI_ISL_647412, EPI_ISL_647413, EPI_ISL_647414, EPI_ISL_647415, EPI_ISL_647416, EPI_ISL_647417, EPI_ISL_647418, EPI_ISL_647421, EPI_ISL_647422, EPI_ISL_647423, EPI_ISL_647424, EPI_ISL_647426, EPI_ISL_647430, EPI_ISL_647431, EPI_ISL_647432, EPI_ISL_647433, EPI_ISL_647434, EPI_ISL_647435, EPI_ISL_647437, EPI_ISL_647438, EPI_ISL_647439, EPI_ISL_647440, EPI_ISL_647441, EPI_ISL_647442, EPI_ISL_647444, EPI_ISL_647446, EPI_ISL_647447, EPI_ISL_647448, EPI_ISL_647449, EPI_ISL_647450, EPI_ISL_647451, EPI_ISL_647453, EPI_ISL_647454, EPI_ISL_647455, EPI_ISL_647456, EPI_ISL_647457, EPI_ISL_647458, EPI_ISL_647459, EPI_ISL_647461, EPI_ISL_647462, EPI_ISL_647463, EPI_ISL_647464, EPI_ISL_647465, EPI_ISL_647466, EPI_ISL_647467, EPI_ISL_647470, EPI_ISL_647471, EPI_ISL_647472, EPI_ISL_647475, EPI_ISL_647476, EPI_ISL_647480, EPI_ISL_647481, EPI_ISL_647482, EPI_ISL_647484, EPI_ISL_647487, EPI_ISL_647488, EPI_ISL_647489, EPI_ISL_647490, EPI_ISL_647491, EPI_ISL_647493, EPI_ISL_647494, EPI_ISL_647495, EPI_ISL_647496, EPI_ISL_647500, EPI_ISL_647501, EPI_ISL_647502, EPI_ISL_647504, EPI_ISL_647505, EPI_ISL_647507, EPI_ISL_647508, EPI_ISL_647510, EPI_ISL_647512, EPI_ISL_647516, EPI_ISL_647517, EPI_ISL_647520, EPI_ISL_647521, EPI_ISL_647523, EPI_ISL_647524, EPI_ISL_647525, EPI_ISL_647527, EPI_ISL_647529, EPI_ISL_647530, EPI_ISL_647532, EPI_ISL_647533, EPI_ISL_647537, EPI_ISL_647538, EPI_ISL_647539, EPI_ISL_647540, EPI_ISL_647542, EPI_ISL_647544, EPI_ISL_647545, EPI_ISL_647548, EPI_ISL_647549, EPI_ISL_647551, EPI_ISL_647552, EPI_ISL_647554, EPI_ISL_647555, EPI_ISL_647556, EPI_ISL_647557, EPI_ISL_647559, EPI_ISL_647561, EPI_ISL_647564, EPI_ISL_647565, EPI_ISL_647566, EPI_ISL_647567, EPI_ISL_647568, EPI_ISL_647571, EPI_ISL_647574, EPI_ISL_647575, EPI_ISL_647577, EPI_ISL_647578, EPI_ISL_647581, EPI_ISL_647582, EPI_ISL_647583, EPI_ISL_647585, EPI_ISL_647586, EPI_ISL_647587, EPI_ISL_647588, EPI_ISL_647589, EPI_ISL_647590, EPI_ISL_647593, EPI_ISL_647596, EPI_ISL_647597, EPI_ISL_647599, EPI_ISL_647601, EPI_ISL_647602, EPI_ISL_647607, EPI_ISL_647609, EPI_ISL_647610, EPI_ISL_647611, EPI_ISL_647613, EPI_ISL_647616, EPI_ISL_647617, EPI_ISL_647621, EPI_ISL_647622, EPI_ISL_647623, EPI_ISL_647625, EPI_ISL_647626, EPI_ISL_647627, EPI_ISL_647628, EPI_ISL_647629, EPI_ISL_647631, EPI_ISL_647636, EPI_ISL_647639, EPI_ISL_647641, EPI_ISL_647644, EPI_ISL_647645, EPI_ISL_647647, EPI_ISL_647649, EPI_ISL_647651, EPI_ISL_647652, EPI_ISL_647653, EPI_ISL_647654, EPI_ISL_647656, EPI_ISL_647657, EPI_ISL_647660, EPI_ISL_647661, EPI_ISL_647663, EPI_ISL_647664, EPI_ISL_647665, EPI_ISL_647666, EPI_ISL_647669, EPI_ISL_647670, EPI_ISL_647673, EPI_ISL_647674, EPI_ISL_647675, EPI_ISL_647676, EPI_ISL_647678, EPI_ISL_647679, EPI_ISL_647681, EPI_ISL_647684, EPI_ISL_647685, EPI_ISL_647688, EPI_ISL_647690, EPI_ISL_647691, EPI_ISL_647692, EPI_ISL_647696, EPI_ISL_647699, EPI_ISL_647701, EPI_ISL_647702, EPI_ISL_647703, EPI_ISL_647709, EPI_ISL_647712, EPI_ISL_647717, EPI_ISL_647718, EPI_ISL_647719, EPI_ISL_647721, EPI_ISL_647723, EPI_ISL_647728, EPI_ISL_647729, EPI_ISL_647730, EPI_ISL_647731, EPI_ISL_647734, EPI_ISL_647735, EPI_ISL_647736, EPI_ISL_647737, EPI_ISL_647740, EPI_ISL_647742, EPI_ISL_647743, EPI_ISL_647744, EPI_ISL_647745, EPI_ISL_647746, EPI_ISL_647747, EPI_ISL_647749, EPI_ISL_647751, EPI_ISL_647753, EPI_ISL_647754, EPI_ISL_647757, EPI_ISL_647759, EPI_ISL_647763, EPI_ISL_647765, EPI_ISL_647766, EPI_ISL_647767, EPI_ISL_647768, EPI_ISL_647769, EPI_ISL_647770, EPI_ISL_647772, EPI_ISL_647773, EPI_ISL_647777, EPI_ISL_647778, EPI_ISL_647781, EPI_ISL_647782, EPI_ISL_647783, EPI_ISL_647784, EPI_ISL_647785, EPI_ISL_647788, EPI_ISL_647790, EPI_ISL_647791, EPI_ISL_647793, EPI_ISL_647794			
see above	Lighthouse Lab in Cambridge	Wellcome Sanger Institute for the COVID-19 Genomics UK (COG-UK) Consortium	Rob Howes, The Lighthouse Lab in Cambridge and Alex Alderton, Roberto Amato, Sonia Goncalves, Ewan Harrison, David K. Jackson, Ian Johnston, Dominic Kwiatkowski, Cordelia Langford, John Sillitoe on behalf of the Wellcome Sanger Institute COVID-19 Surveillance Team
EPI_ISL_647797, EPI_ISL_647799, EPI_ISL_647802, EPI_ISL_647803, EPI_ISL_647805, EPI_ISL_647862, EPI_ISL_647866, EPI_ISL_647867, EPI_ISL_647868, EPI_ISL_647869, EPI_ISL_647870, EPI_ISL_647871, EPI_ISL_647872			
see above	Lighthouse Lab in Glasgow	Wellcome Sanger Institute for the COVID-19 Genomics UK (COG-UK) Consortium	Harper VanSteenhouse, Yumi Kasai, David Gray, Carol Clugston, Anna Dominiczak and Alex Alderton, Roberto Amato, Sonia Goncalves, Ewan Harrison, David K. Jackson, Ian Johnston, Dominic Kwiatkowski, Cordelia Langford, John Sillitoe on behalf of the Wellcome Sanger Institute COVID-19 Surveillance Team
EPI_ISL_649494, EPI_ISL_649495, EPI_ISL_649496, EPI_ISL_649497, EPI_ISL_649498, EPI_ISL_649500, EPI_ISL_649501, EPI_ISL_649502, EPI_ISL_649504, EPI_ISL_649505, EPI_ISL_649506, EPI_ISL_649507, EPI_ISL_649508, EPI_ISL_649509, EPI_ISL_649510, EPI_ISL_649511, EPI_ISL_649512, EPI_ISL_649513, EPI_ISL_649514, EPI_ISL_649515, EPI_ISL_649516, EPI_ISL_649517, EPI_ISL_649518, EPI_ISL_649519, EPI_ISL_649520, EPI_ISL_649521, EPI_ISL_649522, EPI_ISL_649523, EPI_ISL_649524, EPI_ISL_649525, EPI_ISL_649526, EPI_ISL_649527, EPI_ISL_649528, EPI_ISL_649529, EPI_ISL_649530, EPI_ISL_649531, EPI_ISL_649532, EPI_ISL_649533, EPI_ISL_649534, EPI_ISL_649535, EPI_ISL_649536, EPI_ISL_649537, EPI_ISL_649538, EPI_ISL_649539, EPI_ISL_649540, EPI_ISL_649541, EPI_ISL_649542, EPI_ISL_649543, EPI_ISL_649544, EPI_ISL_649545, EPI_ISL_649546, EPI_ISL_649547, EPI_ISL_649569, EPI_ISL_649570, EPI_ISL_649571, EPI_ISL_649572, EPI_ISL_649573, EPI_ISL_649574, EPI_ISL_649575, EPI_ISL_649576, EPI_ISL_649577, EPI_ISL_649578, EPI_ISL_649579, EPI_ISL_649580, EPI_ISL_649581, EPI_ISL_649582, EPI_ISL_649583, EPI_ISL_649584, EPI_ISL_649585, EPI_ISL_649586, EPI_ISL_649587, EPI_ISL_649588, EPI_ISL_649589, EPI_ISL_649590, EPI_ISL_649591, EPI_ISL_649592, EPI_ISL_649593, EPI_ISL_649594, EPI_ISL_649595, EPI_ISL_649596, EPI_ISL_649597, EPI_ISL_649598, EPI_ISL_649599, EPI_ISL_649600, EPI_ISL_649601, EPI_ISL_649602, EPI_ISL_649603, EPI_ISL_649604, EPI_ISL_649605, EPI_ISL_649606, EPI_ISL_649607, EPI_ISL_649608, EPI_ISL_649609, EPI_ISL_649610, EPI_ISL_649611, EPI_ISL_649612, EPI_ISL_649613, EPI_ISL_649614, EPI_ISL_649615, EPI_ISL_649616, EPI_ISL_649617, EPI_ISL_649618, EPI_ISL_649619, EPI_ISL_649620, EPI_ISL_649621, EPI_ISL_649622, EPI_ISL_649623, EPI_ISL_649624, EPI_ISL_649710			

see above	Lighthouse Lab in Glasgow	Wellcome Sanger Institute for the COVID-19 Genomics UK (COG-UK) Consortium	Harper VanSteenhouse, Yumi Kasai, David Gray, Carol Clugston, Anna Dominiczak and Alex Alderton, Roberto Amato, Sonia Goncalves, Ewan Harrison, David K. Jackson, Ian Johnston, Dominic Kwiatkowski, Cordelia Langford, John Sillitoe on behalf of the Wellcome Sanger Institute COVID-19 Surveillance Team (http://www.sanger.ac.uk/covid-team)
EPI_ISL_649786, EPI_ISL_649787, EPI_ISL_649788, EPI_ISL_649789, EPI_ISL_649790, EPI_ISL_649792, EPI_ISL_649793, EPI_ISL_649794, EPI_ISL_649795, EPI_ISL_649796, EPI_ISL_649797, EPI_ISL_649799, EPI_ISL_649800, EPI_ISL_649801, EPI_ISL_649802, EPI_ISL_649803, EPI_ISL_649806, EPI_ISL_649807			
see above	Respiratory Virus Unit, Microbiology Services Colindale, Public Health England	COVID-19 Genomics UK (COG-UK) Consortium	PHE Covid Sequencing Team
EPI_ISL_649999, EPI_ISL_650001	University of Michigan Clinical Microbiology Laboratory	Lauring Lab, University of Michigan, Department of Microbiology and Immunology	Valesano
EPI_ISL_650115	Liverpool Clinical Laboratories	COVID-19 Genomics UK (COG-UK) Consortium	Sam Haldenby, Anita Lucaci, Steve Paterson, Julian Hiscox, Alistair Darby, M Almsaud, A Alrezaihi, Muhannad Alruwaili, Stuart D Armstrong, Jones Benjamin, Eleanor G Bentley, Anu Chawla, Jordan J Clark, Angela Cowell, Richard Eccles, Isabel García-Dorival, Matthew Gemmell, Alessandro Gerada, PKF Gilmore, Richard Gregory, Ximeng Han, Catherine Hartley, Margaret Hughes, Miren Iturriza-Gomara, James Johnson, L Luu, Jenifer Manson, Charlotte Nelson, Elaine O'Toole, Cassie Olateju, Rebekah Penrice-Randal , Lucille Rainbow, N.P Randle, Trevor Ian Robinson, Parul Sharma, Ghada T Shawli, James P Stewart, Neil Swainston, Ecaterina Vamos, Joanne Watts, Mark Whitehead
EPI_ISL_650121	Virology Department, Sheffield Teaching Hospitals NHS Foundation Trust/Department of Infection, Immunity and Cardiovascular Disease, The Medical School, University of Sheffield	COVID-19 Genomics UK (COG-UK) Consortium	Thushan de Silva, Matthew Parker, Nikki Smith, Adri Angyal, Rebecca Brown, Luke Green, Rachel Tucker, Paul Parsons, Danielle Groves, Katie Johnson, Laura Carrilero, Alex Keeley, Dave Partridge, Matthew Wyles, Benjamin Lindsey, Mehmet Yavuz, Mohammad Raza, Cariad Evans
EPI_ISL_650139	Liverpool Clinical Laboratories	COVID-19 Genomics UK (COG-UK) Consortium	Sam Haldenby, Anita Lucaci, Steve Paterson, Julian Hiscox, Alistair Darby, M Almsaud, A Alrezaihi, Muhannad Alruwaili, Stuart D Armstrong, Jones Benjamin, Eleanor G Bentley, Anu Chawla, Jordan J Clark, Angela Cowell, Richard Eccles, Isabel García-Dorival, Matthew Gemmell, Alessandro Gerada, PKF Gilmore, Richard Gregory, Ximeng Han, Catherine Hartley, Margaret Hughes, Miren Iturriza-Gomara, James Johnson, L Luu, Jenifer Manson, Charlotte Nelson, Elaine O'Toole, Cassie Olateju, Rebekah Penrice-Randal , Lucille Rainbow, N.P Randle, Trevor Ian Robinson, Parul Sharma, Ghada T Shawli, James P Stewart, Neil Swainston, Ecaterina Vamos, Joanne Watts, Mark Whitehead
EPI_ISL_650146	West of Scotland Specialist Virology Centre, NHSGGC / MRC-University of Glasgow Centre for Virus Research	COVID-19 Genomics UK (COG-UK) Consortium	Ana da Silva Filipe, Natasha Johnson, Kathy Smollett, Daniel Mair, Stephen Carmichael, Alice Broos, Lily Tong, Jenna Nichols, Kyriaki Nomikou; Sarah McDonald; Richard Orton, Joseph Hughes, Sreenu Vattipally, David L Robertson; Alasdair MacLean, Rory Gunson; Sharif Shaaban, Matthew Holden; Rachel Blacow, Guy Mollett, Kathy Li, James Shepherd, Antonia Ho, Emma Thomson
EPI_ISL_650151	University of Exeter	COVID-19 Genomics UK (COG-UK) Consortium	Ben Temperton,Aaron Jeffries,Michelle Michelsen,Joanna Warwick-Dugdale,Audrey Farbos,Robyn Manley,Stephen Michell,Jane Masoli
EPI_ISL_650160, EPI_ISL_650161	Liverpool Clinical Laboratories	COVID-19 Genomics UK (COG-UK) Consortium	Sam Haldenby, Anita Lucaci, Steve Paterson, Julian Hiscox, Alistair Darby, M Almsaud, A Alrezaihi, Muhannad Alruwaili, Stuart D Armstrong, Jones Benjamin, Eleanor G Bentley, Anu Chawla, Jordan J Clark, Angela Cowell, Richard Eccles, Isabel García-Dorival, Matthew Gemmell, Alessandro Gerada, PKF Gilmore, Richard Gregory, Ximeng Han, Catherine Hartley, Margaret Hughes, Miren Iturriza-Gomara, James Johnson, L Luu, Jenifer Manson, Charlotte Nelson, Elaine O'Toole, Cassie Olateju, Rebekah Penrice-Randal , Lucille Rainbow, N.P Randle, Trevor Ian Robinson, Parul Sharma, Ghada T Shawli, James P Stewart, Neil Swainston, Ecaterina Vamos, Joanne Watts, Mark Whitehead
EPI_ISL_650163	University of Exeter	COVID-19 Genomics UK (COG-UK) Consortium	Ben Temperton,Aaron Jeffries,Michelle Michelsen,Joanna Warwick-Dugdale,Audrey Farbos,Robyn Manley,Stephen Michell,Jane Masoli
EPI_ISL_650189	Liverpool Clinical Laboratories	COVID-19 Genomics UK (COG-UK) Consortium	Sam Haldenby, Anita Lucaci, Steve Paterson, Julian Hiscox, Alistair Darby, M Almsaud, A Alrezaihi, Muhannad Alruwaili, Stuart D Armstrong, Jones Benjamin, Eleanor G Bentley, Anu Chawla, Jordan J Clark, Angela Cowell, Richard Eccles, Isabel García-Dorival, Matthew Gemmell, Alessandro Gerada, PKF Gilmore, Richard Gregory, Ximeng Han, Catherine Hartley, Margaret Hughes, Miren Iturriza-Gomara, James Johnson, L Luu, Jenifer Manson, Charlotte Nelson, Elaine O'Toole, Cassie Olateju, Rebekah Penrice-Randal , Lucille Rainbow, N.P Randle, Trevor Ian Robinson, Parul Sharma, Ghada T Shawli, James P Stewart, Neil Swainston, Ecaterina Vamos, Joanne Watts, Mark Whitehead
EPI_ISL_650204	Wales Specialist Virology Centre Sequencing lab: Pathogen Genomics Unit	COVID-19 Genomics UK (COG-UK) Consortium	Catherine Moore, Johnathan Evans, Laura Gifford, Malorie Perry, Simon Cottrell, Angela Marchbank, Alec Birchley, Alexander Adams, Amy Gaskin, Bree Gatica-Wilcox, Jason Coombes, Joel Southgate, Lauren Gilbert, Lee Graham, Nicole Pacchiarini, Sara Kumziene-Summerhayes, Sarah Taylor, Sophie Jones, Sara Rey, Matthew Bull, Joanne Watkins, Sally Corden, Tom Connor
EPI_ISL_650208	Virology Department, Sheffield Teaching Hospitals NHS Foundation Trust/Department of Infection, Immunity and Cardiovascular Disease, The Medical School, University of Sheffield	COVID-19 Genomics UK (COG-UK) Consortium	Thushan de Silva, Matthew Parker, Nikki Smith, Adri Angyal, Rebecca Brown, Luke Green, Rachel Tucker, Paul Parsons, Danielle Groves, Katie Johnson, Laura Carrilero, Alex Keeley, Dave Partridge, Matthew Wyles, Benjamin Lindsey, Mehmet Yavuz, Mohammad Raza, Cariad Evans
EPI_ISL_650219, EPI_ISL_650222, EPI_ISL_650226	University of Exeter	COVID-19 Genomics UK (COG-UK) Consortium	Ben Temperton,Aaron Jeffries,Michelle Michelsen,Joanna Warwick-Dugdale,Audrey Farbos,Robyn Manley,Stephen Michell,Jane Masoli
EPI_ISL_650232	Department of Pathology, University of Cambridge	COVID-19 Genomics UK (COG-UK) Consortium	Aminu S. Jahun, Yasmin Chaudhry, Grant Hall, Iliana Georgana, Myra Hosmillo, Martin D. Curran, Malte Pinckert, Surendra Parmar, Ian Goodfellow
EPI_ISL_650234	University of Birmingham	COVID-19 Genomics UK (COG-UK) Consortium	Institute of Microbiology, University of Birmingham: Claire McMurray, Joanne Stockton, Samuel Nicholls, Radoslaw Poplawski, Will Rowe, Josh Quick, Nicholas Loman. University of Birmingham Testing Laboratory: Celina M Whalley, Andrew Bosworth, Charlotte Poxon, Kasun Wanigasooriya, Oliver Pickles, Mike Kidd, Alex Richter, Andrew D Beggs PHE Heartlands Lab: Husam Osman, Andrew Bosworth. Queen Elizabeth Hospital: Anna Casey
EPI_ISL_650256	Liverpool Clinical Laboratories	COVID-19 Genomics UK (COG-UK) Consortium	Sam Haldenby, Anita Lucaci, Steve Paterson, Julian Hiscox, Alistair Darby, M Almsaud, A Alrezaihi, Muhannad Alruwaili, Stuart D Armstrong, Jones Benjamin, Eleanor G Bentley, Anu Chawla, Jordan J Clark, Angela Cowell, Richard Eccles, Isabel García-Dorival, Matthew Gemmell, Alessandro Gerada, PKF Gilmore, Richard Gregory, Ximeng Han, Catherine Hartley, Margaret Hughes, Miren Iturriza-Gomara, James Johnson, L Luu, Jenifer Manson, Charlotte Nelson, Elaine O'Toole, Cassie Olateju, Rebekah Penrice-Randal , Lucille Rainbow, N.P Randle, Trevor Ian Robinson, Parul Sharma, Ghada T Shawli, James P Stewart, Neil Swainston, Ecaterina Vamos, Joanne Watts, Mark Whitehead
EPI_ISL_650258	University of Exeter	COVID-19 Genomics UK (COG-UK) Consortium	Ben Temperton,Aaron Jeffries,Michelle Michelsen,Joanna Warwick-Dugdale,Audrey Farbos,Robyn Manley,Stephen Michell,Jane Masoli
EPI_ISL_650267	Virology Department, Royal Infirmary of Edinburgh, NHS Lothian / School of Biological Sciences, University of Edinburgh / Institute of Genetics and Molecular Medicine, University of Edinburgh	COVID-19 Genomics UK (COG-UK) Consortium	McHugh M, Dewar R, Rooke S, Gallagher M, Balcaza C, O'Toole Á, Scher E, Hill V, McCrone JT, Colquhoun R, Yu X, Jackson B, Rambaut A, Williams TC, Templeton K
EPI_ISL_650278	Liverpool Clinical Laboratories	COVID-19 Genomics UK (COG-UK) Consortium	Sam Haldenby, Anita Lucaci, Steve Paterson, Julian Hiscox, Alistair Darby, M Almsaud, A Alrezaihi, Muhannad Alruwaili, Stuart D Armstrong, Jones Benjamin, Eleanor G Bentley, Anu Chawla, Jordan J Clark, Angela Cowell, Richard Eccles, Isabel García-Dorival, Matthew Gemmell, Alessandro Gerada, PKF Gilmore, Richard Gregory, Ximeng Han, Catherine Hartley, Margaret Hughes, Miren Iturriza-Gomara, James Johnson, L Luu, Jenifer Manson, Charlotte Nelson, Elaine O'Toole, Cassie Olateju, Rebekah Penrice-Randal , Lucille Rainbow, N.P Randle, Trevor Ian Robinson, Parul Sharma, Ghada T Shawli, James P Stewart, Neil Swainston, Ecaterina Vamos, Joanne Watts, Mark Whitehead
EPI_ISL_650281	University of Birmingham	COVID-19 Genomics UK (COG-UK) Consortium	Institute of Microbiology, University of Birmingham: Claire McMurray, Joanne Stockton, Samuel Nicholls, Radoslaw Poplawski, Will Rowe, Josh Quick, Nicholas Loman. University of Birmingham Testing Laboratory: Celina M Whalley, Andrew Bosworth, Charlotte Poxon, Kasun Wanigasooriya, Oliver Pickles, Mike Kidd, Alex Richter, Andrew D Beggs PHE Heartlands Lab: Husam Osman, Andrew Bosworth. Queen Elizabeth Hospital: Anna Casey
EPI_ISL_650320	Wales Specialist Virology Centre Sequencing lab: Pathogen Genomics Unit	COVID-19 Genomics UK (COG-UK) Consortium	Catherine Moore, Johnathan Evans, Laura Gifford, Malorie Perry, Simon Cottrell, Angela Marchbank, Alec Birchley, Alexander Adams, Amy Gaskin, Bree Gatica-Wilcox, Jason Coombes, Joel Southgate, Lauren Gilbert, Lee Graham, Nicole Pacchiarini, Sara Kumziene-Summerhayes, Sarah Taylor, Sophie Jones, Sara Rey, Matthew Bull, Joanne Watkins, Sally Corden, Tom Connor
EPI_ISL_650323	Quadram Institute Bioscience	COVID-19 Genomics UK (COG-UK) Consortium	Dave J. Baker, Gemma L. Kay, Alp Aydin, Thanh Le-Viet, Steven Rudder, Ana P. Tedim, Anastasia Kolyva, Maria Diaz, Leonardo de Oliveira Martins, Nabil-Fareed Alikhan, Lizzie Meadows, Rachael Stanley, Ngozi Elumogo, Muhammed Yasir, Nicholas M. Thomson, Alexander J Trotter, Rachel Gilroy, Samuel Bloomfield, Claire Stuart, Andrew Bell, Reenesh Prakash, Samir Dervisevic, Alison E. Mather, John Wain, Mark Webber, Andrew J. Page, Justin O'Grady

EPI_ISL_650349	Virology Department, Sheffield Teaching Hospitals NHS Foundation Trust/Department of Infection, Immunity and Cardiovascular Disease, The Medical School, University of Sheffield	COVID-19 Genomics UK (COG-UK) Consortium	Thushan de Silva, Matthew Parker, Nikki Smith, Adri Angyal, Rebecca Brown, Luke Green, Rachel Tucker, Paul Parsons, Danielle Groves, Katie Johnson, Laura Carrilero, Alex Keeley, Dave Partridge, Matthew Wyles, Benjamin Lindsey, Mehmet Yavuz, Mohammad Raza, Cariad Evans
EPI_ISL_650352	University of Exeter	COVID-19 Genomics UK (COG-UK) Consortium	Ben Temperton,Aaron Jeffries,Michelle Michelsen,Joanna Warwick-Dugdale,Audrey Farbos,Robyn Manley,Stephen Michell,Jane Masoli
EPI_ISL_650353	Department of Pathology, University of Cambridge	COVID-19 Genomics UK (COG-UK) Consortium	Aminu S. Jahun, Yasmin Chaudhry, Grant Hall, Iliana Georgana, Myra Hosmillo, Martin D. Curran, Malte Pinckert, Surendra Parmar, Ian Goodfellow
EPI_ISL_650358, EPI_ISL_650377	University of Exeter	COVID-19 Genomics UK (COG-UK) Consortium	Ben Temperton,Aaron Jeffries,Michelle Michelsen,Joanna Warwick-Dugdale,Audrey Farbos,Robyn Manley,Stephen Michell,Jane Masoli
EPI_ISL_650388	Centre for Enzyme Innovation, University of Portsmouth / Translational Research Laboratory, Portsmouth Hospitals NHS Trust	COVID-19 Genomics UK (COG-UK) Consortium	Angela Beckett,Yann Bourgeois,Garry Scarlett,Sharon Glaysher,Scott Elliott,Kelly Bicknell,Robert Impey,Allyson Lloyd,Sarah Wyllie,Ethan Butcher,Anoop Chauhan,Samuel Robson
EPI_ISL_650395	Virology Department, Sheffield Teaching Hospitals NHS Foundation Trust/Department of Infection, Immunity and Cardiovascular Disease, The Medical School, University of Sheffield	COVID-19 Genomics UK (COG-UK) Consortium	Thushan de Silva, Matthew Parker, Nikki Smith, Adri Angyal, Rebecca Brown, Luke Green, Rachel Tucker, Paul Parsons, Danielle Groves, Katie Johnson, Laura Carrilero, Alex Keeley, Dave Partridge, Matthew Wyles, Benjamin Lindsey, Mehmet Yavuz, Mohammad Raza, Cariad Evans
EPI_ISL_650397	Department of Pathology, University of Cambridge	COVID-19 Genomics UK (COG-UK) Consortium	Aminu S. Jahun, Yasmin Chaudhry, Grant Hall, Iliana Georgana, Myra Hosmillo, Martin D. Curran, Malte Pinckert, Surendra Parmar, Ian Goodfellow
EPI_ISL_650399	University of Exeter	COVID-19 Genomics UK (COG-UK) Consortium	Ben Temperton,Aaron Jeffries,Michelle Michelsen,Joanna Warwick-Dugdale,Audrey Farbos,Robyn Manley,Stephen Michell,Jane Masoli
EPI_ISL_650405	University of Birmingham	COVID-19 Genomics UK (COG-UK) Consortium	Institute of Microbiology, University of Birmingham: Claire McMurray, Joanne Stockton, Samuel Nicholls, Radoslaw Poplawski, Will Rowe, Josh Quick, Nicholas Loman. University of Birmingham Testing Laboratory: Celina M Whalley, Andrew Bosworth, Charlotte Poxon, Kasun Wanigasooriya, Oliver Pickles, Mike Kidd, Alex Richter, Andrew D Beggs PHE Heartlands Lab: Husam Osman, Andrew Bosworth. Queen Elizabeth Hospital: Anna Casey
EPI_ISL_650408	Virology Department, Sheffield Teaching Hospitals NHS Foundation Trust/Department of Infection, Immunity and Cardiovascular Disease, The Medical School, University of Sheffield	COVID-19 Genomics UK (COG-UK) Consortium	Thushan de Silva, Matthew Parker, Nikki Smith, Adri Angyal, Rebecca Brown, Luke Green, Rachel Tucker, Paul Parsons, Danielle Groves, Katie Johnson, Laura Carrilero, Alex Keeley, Dave Partridge, Matthew Wyles, Benjamin Lindsey, Mehmet Yavuz, Mohammad Raza, Cariad Evans
EPI_ISL_650414	University of Exeter	COVID-19 Genomics UK (COG-UK) Consortium	Ben Temperton,Aaron Jeffries,Michelle Michelsen,Joanna Warwick-Dugdale,Audrey Farbos,Robyn Manley,Stephen Michell,Jane Masoli
EPI_ISL_650422	Virology Department, Sheffield Teaching Hospitals NHS Foundation Trust/Department of Infection, Immunity and Cardiovascular Disease, The Medical School, University of Sheffield	COVID-19 Genomics UK (COG-UK) Consortium	Thushan de Silva, Matthew Parker, Nikki Smith, Adri Angyal, Rebecca Brown, Luke Green, Rachel Tucker, Paul Parsons, Danielle Groves, Katie Johnson, Laura Carrilero, Alex Keeley, Dave Partridge, Matthew Wyles, Benjamin Lindsey, Mehmet Yavuz, Mohammad Raza, Cariad Evans
EPI_ISL_650433	Virology Department, Royal Infirmary of Edinburgh, NHS Lothian / School of Biological Sciences, University of Edinburgh / Institute of Genetics and Molecular Medicine, University of Edinburgh	COVID-19 Genomics UK (COG-UK) Consortium	McHugh M, Dewar R, Rooke S, Gallagher M, Balcaza C, O'Toole Á, Scher E, Hill V, McCrone JT, Colquhoun R, Yu X, Jackson B, Rambaut A, Williams TC, Templeton K
EPI_ISL_650453	Liverpool Clinical Laboratories	COVID-19 Genomics UK (COG-UK) Consortium	Sam Haldenby, Anita Lucaci, Steve Paterson, Julian Hiscox, Alistair Darby, M Almsaud, A Alrezaihi, Muhannad Alruwaili, Stuart D Armstrong, Jones Benjamin, Eleanor G Bentley, Anu Chawla, Jordan J Clark, Angela Cowell, Richard Eccles, Isabel García-Dorival, Matthew Gemmell, Alessandro Gerada, PKF Gilmore, Richard Gregory, Ximeng Han, Catherine Hartley, Margaret Hughes, Miren Iturriza-Gomara, James Johnson, L Luu, Jenifer Manson, Charlotte Nelson, Elaine O'Toole, Cassie Olateju, Rebekah Penrice-Randal , Lucille Rainbow, N.P Randle, Trevor Ian Robinson, Parul Sharma, Ghada T Shawli, James P Stewart, Neil Swainston, Ecaterina Vamos, Joanne Watts, Mark Whitehead
EPI_ISL_650501	Virology Department, Royal Infirmary of Edinburgh, NHS Lothian / School of Biological Sciences, University of Edinburgh / Institute of Genetics and Molecular Medicine, University of Edinburgh	COVID-19 Genomics UK (COG-UK) Consortium	McHugh M, Dewar R, Rooke S, Gallagher M, Balcaza C, O'Toole Á, Scher E, Hill V, McCrone JT, Colquhoun R, Yu X, Jackson B, Rambaut A, Williams TC, Templeton K
EPI_ISL_650551	Wales Specialist Virology Centre Sequencing lab: Pathogen Genomics Unit	COVID-19 Genomics UK (COG-UK) Consortium	Catherine Moore, Johnathan Evans, Laura Gifford, Malorie Perry, Simon Cottrell, Angela Marchbank, Alec Birchley, Alexander Adams, Amy Gaskin, Bree Gatica-Wilcox, Jason Coombes, Joel Southgate, Lauren Gilbert, Lee Graham, Nicole Pacchiarini, Sara Kumziene-Summerhayes, Sarah Taylor, Sophie Jones, Sara Rey, Matthew Bull, Joanne Watkins, Sally Corden, Tom Connor
EPI_ISL_650575, EPI_ISL_650576	Centre for Enzyme Innovation, University of Portsmouth / Translational Research Laboratory, Portsmouth Hospitals NHS Trust	COVID-19 Genomics UK (COG-UK) Consortium	Angela Beckett,Yann Bourgeois,Garry Scarlett,Sharon Glaysher,Scott Elliott,Kelly Bicknell,Robert Impey,Allyson Lloyd,Sarah Wyllie,Ethan Butcher,Anoop Chauhan,Samuel Robson
EPI_ISL_650583	Department of Pathology, University of Cambridge	COVID-19 Genomics UK (COG-UK) Consortium	Aminu S. Jahun, Yasmin Chaudhry, Grant Hall, Iliana Georgana, Myra Hosmillo, Martin D. Curran, Malte Pinckert, Surendra Parmar, Ian Goodfellow
EPI_ISL_650597, EPI_ISL_650607, EPI_ISL_650610	University of Exeter	COVID-19 Genomics UK (COG-UK) Consortium	Ben Temperton,Aaron Jeffries,Michelle Michelsen,Joanna Warwick-Dugdale,Audrey Farbos,Robyn Manley,Stephen Michell,Jane Masoli
EPI_ISL_650615, EPI_ISL_650616	Department of Pathology, University of Cambridge	COVID-19 Genomics UK (COG-UK) Consortium	Aminu S. Jahun, Yasmin Chaudhry, Grant Hall, Iliana Georgana, Myra Hosmillo, Martin D. Curran, Malte Pinckert, Surendra Parmar, Ian Goodfellow
EPI_ISL_650634, EPI_ISL_650635, EPI_ISL_650636, EPI_ISL_650637, EPI_ISL_650638, EPI_ISL_650639	University of Exeter	COVID-19 Genomics UK (COG-UK) Consortium	Ben Temperton,Aaron Jeffries,Michelle Michelsen,Joanna Warwick-Dugdale,Audrey Farbos,Robyn Manley,Stephen Michell,Jane Masoli
EPI_ISL_650646, EPI_ISL_650662	Virology Department, Sheffield Teaching Hospitals NHS Foundation Trust/Department of Infection, Immunity and Cardiovascular Disease, The Medical School, University of Sheffield	COVID-19 Genomics UK (COG-UK) Consortium	Thushan de Silva, Matthew Parker, Nikki Smith, Adri Angyal, Rebecca Brown, Luke Green, Rachel Tucker, Paul Parsons, Danielle Groves, Katie Johnson, Laura Carrilero, Alex Keeley, Dave Partridge, Matthew Wyles, Benjamin Lindsey, Mehmet Yavuz, Mohammad Raza, Cariad Evans
EPI_ISL_650667	University of Exeter	COVID-19 Genomics UK (COG-UK) Consortium	Ben Temperton,Aaron Jeffries,Michelle Michelsen,Joanna Warwick-Dugdale,Audrey Farbos,Robyn Manley,Stephen Michell,Jane Masoli
EPI_ISL_650670, EPI_ISL_650689	Virology Department, Sheffield Teaching Hospitals NHS Foundation Trust/Department of Infection, Immunity and Cardiovascular Disease, The Medical School, University of Sheffield	COVID-19 Genomics UK (COG-UK) Consortium	Thushan de Silva, Matthew Parker, Nikki Smith, Adri Angyal, Rebecca Brown, Luke Green, Rachel Tucker, Paul Parsons, Danielle Groves, Katie Johnson, Laura Carrilero, Alex Keeley, Dave Partridge, Matthew Wyles, Benjamin Lindsey, Mehmet Yavuz, Mohammad Raza, Cariad Evans
EPI_ISL_650692	University of Birmingham	COVID-19 Genomics UK (COG-UK) Consortium	Institute of Microbiology, University of Birmingham: Claire McMurray, Joanne Stockton, Samuel Nicholls, Radoslaw Poplawski, Will Rowe, Josh Quick, Nicholas Loman. University of Birmingham Testing Laboratory: Celina M Whalley, Andrew Bosworth, Charlotte Poxon, Kasun Wanigasooriya, Oliver Pickles, Mike Kidd, Alex Richter, Andrew D Beggs PHE Heartlands Lab: Husam Osman, Andrew Bosworth. Queen Elizabeth Hospital: Anna Casey
EPI_ISL_650702	University of Exeter	COVID-19 Genomics UK (COG-UK) Consortium	Ben Temperton,Aaron Jeffries,Michelle Michelsen,Joanna Warwick-Dugdale,Audrey Farbos,Robyn Manley,Stephen Michell,Jane Masoli
EPI_ISL_650709	West of Scotland Specialist Virology Centre, NHSGGC / MRC-University of Glasgow Centre for Virus Research	COVID-19 Genomics UK (COG-UK) Consortium	Ana da Silva Filipe, Natasha Johnson, Kathy Smollett, Daniel Mair, Stephen Carmichael, Alice Broos, Lily Tong, Jenna Nichols, Kyriaki Nomikou; Sarah McDonald; Richard Orton, Joseph Hughes, Sreenu Vattipally, David L Robertson; Alasdair MacLean, Rory Gunson; Sharif Shaaban, Matthew Holden; Rachel Blacow, Guy Mollett, Kathy Li, James Shepherd, Antonia Ho, Emma Thomson
EPI_ISL_650713, EPI_ISL_650715	Virology Department, Sheffield Teaching Hospitals NHS Foundation Trust/Department of Infection, Immunity and Cardiovascular Disease, The Medical School, University of Sheffield	COVID-19 Genomics UK (COG-UK) Consortium	Thushan de Silva, Matthew Parker, Nikki Smith, Adri Angyal, Rebecca Brown, Luke Green, Rachel Tucker, Paul Parsons, Danielle Groves, Katie Johnson, Laura Carrilero, Alex Keeley, Dave Partridge, Matthew Wyles, Benjamin Lindsey, Mehmet Yavuz, Mohammad Raza, Cariad Evans

EPI_ISL_650740	University of Birmingham	COVID-19 Genomics UK (COG-UK) Consortium	Institute of Microbiology, University of Birmingham: Claire McMurray, Joanne Stockton, Samuel Nicholls, Radoslaw Poplawski, Will Rowe, Josh Quick, Nicholas Loman. University of Birmingham Testing Laboratory: Celina M Whalley, Andrew Bosworth, Charlotte Poxon, Kasun Wanigasooriya, Oliver Pickles, Mike Kidd, Alex Richter, Andrew D Beggs PHE Heartlands Lab: Husam Osman, Andrew Bosworth. Queen Elizabeth Hospital: Anna Casey
EPI_ISL_650774	Department of Pathology, University of Cambridge	COVID-19 Genomics UK (COG-UK) Consortium	Aminu S. Jahun, Yasmin Chaudhry, Grant Hall, Iliana Georgana, Myra Hosmillo, Martin D. Curran, Malte Pinckert, Surendra Parmar, Ian Goodfellow
EPI_ISL_650781	Virology Department, Royal Infirmary of Edinburgh, NHS Lothian / School of Biological Sciences, University of Edinburgh / Institute of Genetics and Molecular Medicine, University of Edinburgh	COVID-19 Genomics UK (COG-UK) Consortium	McHugh M, Dewar R, Rooke S, Gallagher M, Balcaza C, O'Toole Á, Scher E, Hill V, McCrone JT, Colquhoun R, Yu X, Jackson B, Rambaut A, Williams TC, Templeton K
EPI_ISL_650787	Wales Specialist Virology Centre Sequencing lab: Pathogen Genomics Unit	COVID-19 Genomics UK (COG-UK) Consortium	Catherine Moore, Johnathan Evans, Laura Gifford, Malorie Perry, Simon Cottrell, Angela Marchbank, Alec Birchley, Alexander Adams, Amy Gaskin, Bree Gatica-Wilcox, Jason Coombes, Joel Southgate, Lauren Gilbert, Lee Graham, Nicole Pacchiarini, Sara Kumziene-Summerhayes, Sarah Taylor, Sophie Jones, Sara Rey, Matthew Bull, Joanne Watkins, Sally Corden, Tom Connor
EPI_ISL_650789	West of Scotland Specialist Virology Centre, NHSGGC / MRC-University of Glasgow Centre for Virus Research	COVID-19 Genomics UK (COG-UK) Consortium	Ana da Silva Filipe, Natasha Johnson, Kathy Smollett, Daniel Mair, Stephen Carmichael, Alice Broos, Lily Tong, Jenna Nichols, Kyriaki Nomikou; Sarah McDonald; Richard Orton, Joseph Hughes, Sreenu Vattipally, David L Robertson; Alasdair MacLean, Rory Gunson; Sharif Shaaban, Matthew Holden; Rachel Blacow, Guy Mollett, Kathy Li, James Shepherd, Antonia Ho, Emma Thomson
EPI_ISL_650799	Virology Department, Royal Infirmary of Edinburgh, NHS Lothian / School of Biological Sciences, University of Edinburgh / Institute of Genetics and Molecular Medicine, University of Edinburgh	COVID-19 Genomics UK (COG-UK) Consortium	McHugh M, Dewar R, Rooke S, Gallagher M, Balcaza C, O'Toole Á, Scher E, Hill V, McCrone JT, Colquhoun R, Yu X, Jackson B, Rambaut A, Williams TC, Templeton K
EPI_ISL_650805	West of Scotland Specialist Virology Centre, NHSGGC / MRC-University of Glasgow Centre for Virus Research	COVID-19 Genomics UK (COG-UK) Consortium	Ana da Silva Filipe, Natasha Johnson, Kathy Smollett, Daniel Mair, Stephen Carmichael, Alice Broos, Lily Tong, Jenna Nichols, Kyriaki Nomikou; Sarah McDonald; Richard Orton, Joseph Hughes, Sreenu Vattipally, David L Robertson; Alasdair MacLean, Rory Gunson; Sharif Shaaban, Matthew Holden; Rachel Blacow, Guy Mollett, Kathy Li, James Shepherd, Antonia Ho, Emma Thomson
EPI_ISL_650826	Virology Department, Royal Infirmary of Edinburgh, NHS Lothian / School of Biological Sciences, University of Edinburgh / Institute of Genetics and Molecular Medicine, University of Edinburgh	COVID-19 Genomics UK (COG-UK) Consortium	McHugh M, Dewar R, Rooke S, Gallagher M, Balcaza C, O'Toole Á, Scher E, Hill V, McCrone JT, Colquhoun R, Yu X, Jackson B, Rambaut A, Williams TC, Templeton K
EPI_ISL_650870, EPI_ISL_650871	West of Scotland Specialist Virology Centre, NHSGGC / MRC-University of Glasgow Centre for Virus Research	COVID-19 Genomics UK (COG-UK) Consortium	Ana da Silva Filipe, Natasha Johnson, Kathy Smollett, Daniel Mair, Stephen Carmichael, Alice Broos, Lily Tong, Jenna Nichols, Kyriaki Nomikou; Sarah McDonald; Richard Orton, Joseph Hughes, Sreenu Vattipally, David L Robertson; Alasdair MacLean, Rory Gunson; Sharif Shaaban, Matthew Holden; Rachel Blacow, Guy Mollett, Kathy Li, James Shepherd, Antonia Ho, Emma Thomson
EPI_ISL_650884, EPI_ISL_650905, EPI_ISL_650929	Wales Specialist Virology Centre Sequencing lab: Pathogen Genomics Unit	COVID-19 Genomics UK (COG-UK) Consortium	Catherine Moore, Johnathan Evans, Laura Gifford, Malorie Perry, Simon Cottrell, Angela Marchbank, Alec Birchley, Alexander Adams, Amy Gaskin, Bree Gatica-Wilcox, Jason Coombes, Joel Southgate, Lauren Gilbert, Lee Graham, Nicole Pacchiarini, Sara Kumziene-Summerhayes, Sarah Taylor, Sophie Jones, Sara Rey, Matthew Bull, Joanne Watkins, Sally Corden, Tom Connor
EPI_ISL_650941	Centre for Enzyme Innovation, University of Portsmouth / Translational Research Laboratory, Portsmouth Hospitals NHS Trust	COVID-19 Genomics UK (COG-UK) Consortium	Angela Beckett,Yann Bourgeois,Garry Scarlett,Sharon Glaysher,Scott Elliott,Kelly Bicknell,Robert Impey,Allyson Lloyd,Sarah Wyllie,Ethan Butcher,Anoop Chauhan,Samuel Robson
EPI_ISL_650990, EPI_ISL_650991, EPI_ISL_650993	Department of Pathology, University of Cambridge	COVID-19 Genomics UK (COG-UK) Consortium	Aminu S. Jahun, Yasmin Chaudhry, Grant Hall, Iliana Georgana, Myra Hosmillo, Martin D. Curran, Malte Pinckert, Surendra Parmar, Ian Goodfellow
EPI_ISL_650998	University of Birmingham	COVID-19 Genomics UK (COG-UK) Consortium	Institute of Microbiology, University of Birmingham: Claire McMurray, Joanne Stockton, Samuel Nicholls, Radoslaw Poplawski, Will Rowe, Josh Quick, Nicholas Loman. University of Birmingham Testing Laboratory: Celina M Whalley, Andrew Bosworth, Charlotte Poxon, Kasun Wanigasooriya, Oliver Pickles, Mike Kidd, Alex Richter, Andrew D Beggs PHE Heartlands Lab: Husam Osman, Andrew Bosworth. Queen Elizabeth Hospital: Anna Casey
EPI_ISL_651003, EPI_ISL_651004, EPI_ISL_651005, EPI_ISL_651006, EPI_ISL_651007, EPI_ISL_651008	Department of Pathology, University of Cambridge	COVID-19 Genomics UK (COG-UK) Consortium	Aminu S. Jahun, Yasmin Chaudhry, Grant Hall, Iliana Georgana, Myra Hosmillo, Martin D. Curran, Malte Pinckert, Surendra Parmar, Ian Goodfellow
EPI_ISL_651054, EPI_ISL_651056, EPI_ISL_651058	University of Exeter	COVID-19 Genomics UK (COG-UK) Consortium	Ben Temperton,Aaron Jeffries,Michelle Michelsen,Joanna Warwick-Dugdale,Audrey Farbos,Robyn Manley,Stephen Michell,Jane Masoli
EPI_ISL_651078	Virology Department, Sheffield Teaching Hospitals NHS Foundation Trust/Department of Infection, Immunity and Cardiovascular Disease, The Medical School, University of Sheffield	COVID-19 Genomics UK (COG-UK) Consortium	Thushan de Silva, Matthew Parker, Nikki Smith, Adri Angyal, Rebecca Brown, Luke Green, Rachel Tucker, Paul Parsons, Danielle Groves, Katie Johnson, Laura Carrilero, Alex Keeley, Dave Partridge, Matthew Wyles, Benjamin Lindsey, Mehmet Yavuz, Mohammad Raza, Cariad Evans
EPI_ISL_651084	University of Exeter	COVID-19 Genomics UK (COG-UK) Consortium	Ben Temperton,Aaron Jeffries,Michelle Michelsen,Joanna Warwick-Dugdale,Audrey Farbos,Robyn Manley,Stephen Michell,Jane Masoli
EPI_ISL_651109	Virology Department, Sheffield Teaching Hospitals NHS Foundation Trust/Department of Infection, Immunity and Cardiovascular Disease, The Medical School, University of Sheffield	COVID-19 Genomics UK (COG-UK) Consortium	Thushan de Silva, Matthew Parker, Nikki Smith, Adri Angyal, Rebecca Brown, Luke Green, Rachel Tucker, Paul Parsons, Danielle Groves, Katie Johnson, Laura Carrilero, Alex Keeley, Dave Partridge, Matthew Wyles, Benjamin Lindsey, Mehmet Yavuz, Mohammad Raza, Cariad Evans
EPI_ISL_651122	Department of Pathology, University of Cambridge	COVID-19 Genomics UK (COG-UK) Consortium	Aminu S. Jahun, Yasmin Chaudhry, Grant Hall, Iliana Georgana, Myra Hosmillo, Martin D. Curran, Malte Pinckert, Surendra Parmar, Ian Goodfellow
EPI_ISL_651125	West of Scotland Specialist Virology Centre, NHSGGC / MRC-University of Glasgow Centre for Virus Research	COVID-19 Genomics UK (COG-UK) Consortium	Ana da Silva Filipe, Natasha Johnson, Kathy Smollett, Daniel Mair, Stephen Carmichael, Alice Broos, Lily Tong, Jenna Nichols, Kyriaki Nomikou; Sarah McDonald; Richard Orton, Joseph Hughes, Sreenu Vattipally, David L Robertson; Alasdair MacLean, Rory Gunson; Sharif Shaaban, Matthew Holden; Rachel Blacow, Guy Mollett, Kathy Li, James Shepherd, Antonia Ho, Emma Thomson
EPI_ISL_651130	University of Exeter	COVID-19 Genomics UK (COG-UK) Consortium	Ben Temperton,Aaron Jeffries,Michelle Michelsen,Joanna Warwick-Dugdale,Audrey Farbos,Robyn Manley,Stephen Michell,Jane Masoli
EPI_ISL_651136	Department of Pathology, University of Cambridge	COVID-19 Genomics UK (COG-UK) Consortium	Aminu S. Jahun, Yasmin Chaudhry, Grant Hall, Iliana Georgana, Myra Hosmillo, Martin D. Curran, Malte Pinckert, Surendra Parmar, Ian Goodfellow
EPI_ISL_651140	University of Birmingham	COVID-19 Genomics UK (COG-UK) Consortium	Institute of Microbiology, University of Birmingham: Claire McMurray, Joanne Stockton, Samuel Nicholls, Radoslaw Poplawski, Will Rowe, Josh Quick, Nicholas Loman. University of Birmingham Testing Laboratory: Celina M Whalley, Andrew Bosworth, Charlotte Poxon, Kasun Wanigasooriya, Oliver Pickles, Mike Kidd, Alex Richter, Andrew D Beggs PHE Heartlands Lab: Husam Osman, Andrew Bosworth. Queen Elizabeth Hospital: Anna Casey
EPI_ISL_651148	Liverpool Clinical Laboratories	COVID-19 Genomics UK (COG-UK) Consortium	Sam Haldenby, Anita Lucaci, Steve Paterson, Julian Hiscox, Alistair Darby, M Almsaud, A Alrezaihi, Muhannad Alruwaili, Stuart D Armstrong, Jones Benjamin, Eleanor G Bentley, Anu Chawla, Jordan J Clark, Angela Cowell, Richard Eccles, Isabel García-Dorival, Matthew Gemmell, Alessandro Gerada, PKF Gilmore, Richard Gregory, Ximeng Han, Catherine Hartley, Margaret Hughes, Miren Iturriza-Gomara, James Johnson, L Luu, Jenifer Manson, Charlotte Nelson, Elaine O'Toole, Cassie Olateju, Rebekah Penrice-Randal , Lucille Rainbow, N.P Randle, Trevor Ian Robinson, Parul Sharma, Ghada T Shawli, James P Stewart, Neil Swainston, Ecaterina Vamos, Joanne Watts, Mark Whitehead
EPI_ISL_651152	Virology Department, Sheffield Teaching Hospitals NHS Foundation Trust/Department of Infection, Immunity and Cardiovascular Disease, The Medical School, University of Sheffield	COVID-19 Genomics UK (COG-UK) Consortium	Thushan de Silva, Matthew Parker, Nikki Smith, Adri Angyal, Rebecca Brown, Luke Green, Rachel Tucker, Paul Parsons, Danielle Groves, Katie Johnson, Laura Carrilero, Alex Keeley, Dave Partridge, Matthew Wyles, Benjamin Lindsey, Mehmet Yavuz, Mohammad Raza, Cariad Evans
EPI_ISL_651158	Virology Department, Royal Infirmary of Edinburgh, NHS Lothian / School of Biological Sciences, University of Edinburgh / Institute of Genetics and Molecular Medicine, University of Edinburgh	COVID-19 Genomics UK (COG-UK) Consortium	McHugh M, Dewar R, Rooke S, Gallagher M, Balcaza C, O'Toole Á, Scher E, Hill V, McCrone JT, Colquhoun R, Yu X, Jackson B, Rambaut A, Williams TC, Templeton K

EPI_ISL_651223	Liverpool Clinical Laboratories	COVID-19 Genomics UK (COG-UK) Consortium	Sam Haldenby, Anita Lucaci, Steve Paterson, Julian Hiscox, Alistair Darby, M Almsaud, A Alrezaihi, Muhannad Alruwaili, Stuart D Armstrong, Jones Benjamin, Eleanor G Bentley, Anu Chawla, Jordan J Clark, Angela Cowell, Richard Eccles, Isabel García-Dorival, Matthew Gemmell, Alessandro Gerada, PKF Gilmore, Richard Gregory, Ximeng Han, Catherine Hartley, Margaret Hughes, Miren Iturriza-Gomara, James Johnson, L Luu, Jenifer Manson, Charlotte Nelson, Elaine O'Toole, Cassie Olateju, Rebekah Penrice-Randal , Lucille Rainbow, N.P Randle, Trevor Ian Robinson, Parul Sharma, Ghada T Shawli, James P Stewart, Neil Swainston, Ecaterina Vamos, Joanne Watts, Mark Whitehead
EPI_ISL_651238	University of Exeter	COVID-19 Genomics UK (COG-UK) Consortium	Ben Temperton,Aaron Jeffries,Michelle Michelsen,Joanna Warwick-Dugdale,Audrey Farbos,Robyn Manley,Stephen Michell,Jane Masoli
EPI_ISL_651239	Liverpool Clinical Laboratories	COVID-19 Genomics UK (COG-UK) Consortium	Sam Haldenby, Anita Lucaci, Steve Paterson, Julian Hiscox, Alistair Darby, M Almsaud, A Alrezaihi, Muhannad Alruwaili, Stuart D Armstrong, Jones Benjamin, Eleanor G Bentley, Anu Chawla, Jordan J Clark, Angela Cowell, Richard Eccles, Isabel García-Dorival, Matthew Gemmell, Alessandro Gerada, PKF Gilmore, Richard Gregory, Ximeng Han, Catherine Hartley, Margaret Hughes, Miren Iturriza-Gomara, James Johnson, L Luu, Jenifer Manson, Charlotte Nelson, Elaine O'Toole, Cassie Olateju, Rebekah Penrice-Randal , Lucille Rainbow, N.P Randle, Trevor Ian Robinson, Parul Sharma, Ghada T Shawli, James P Stewart, Neil Swainston, Ecaterina Vamos, Joanne Watts, Mark Whitehead
EPI_ISL_651241	University of Exeter	COVID-19 Genomics UK (COG-UK) Consortium	Ben Temperton,Aaron Jeffries,Michelle Michelsen,Joanna Warwick-Dugdale,Audrey Farbos,Robyn Manley,Stephen Michell,Jane Masoli
EPI_ISL_651248, EPI_ISL_651251	Liverpool Clinical Laboratories	COVID-19 Genomics UK (COG-UK) Consortium	Sam Haldenby, Anita Lucaci, Steve Paterson, Julian Hiscox, Alistair Darby, M Almsaud, A Alrezaihi, Muhannad Alruwaili, Stuart D Armstrong, Jones Benjamin, Eleanor G Bentley, Anu Chawla, Jordan J Clark, Angela Cowell, Richard Eccles, Isabel García-Dorival, Matthew Gemmell, Alessandro Gerada, PKF Gilmore, Richard Gregory, Ximeng Han, Catherine Hartley, Margaret Hughes, Miren Iturriza-Gomara, James Johnson, L Luu, Jenifer Manson, Charlotte Nelson, Elaine O'Toole, Cassie Olateju, Rebekah Penrice-Randal , Lucille Rainbow, N.P Randle, Trevor Ian Robinson, Parul Sharma, Ghada T Shawli, James P Stewart, Neil Swainston, Ecaterina Vamos, Joanne Watts, Mark Whitehead
EPI_ISL_651267	Quadram Institute Bioscience	COVID-19 Genomics UK (COG-UK) Consortium	Dave J. Baker, Gemma L. Kay, Alp Aydin, Thanh Le-Viet, Steven Rudder, Ana P. Tedim, Anastasia Kolyva, Maria Diaz, Leonardo de Oliveira Martins, Nabil-Fareed Alikhan, Lizzie Meadows, Rachael Stanley, Ngozi Elumogo, Muhammed Yasir, Nicholas M. Thomson, Alexander J Trotter, Rachel Gilroy, Samuel Bloomfield, Claire Stuart, Andrew Bell, Reenesh Prakash, Samir Dervisevic, Alison E. Mather, John Wain, Mark Webber, Andrew J. Page, Justin O'Grady
EPI_ISL_651282	University of Birmingham	COVID-19 Genomics UK (COG-UK) Consortium	Institute of Microbiology, University of Birmingham: Claire McMurray, Joanne Stockton, Samuel Nicholls, Radoslaw Poplawski, Will Rowe, Josh Quick, Nicholas Loman. University of Birmingham Testing Laboratory: Celina M Whalley, Andrew Bosworth, Charlotte Poxon, Kasun Wanigasooriya, Oliver Pickles, Mike Kidd, Alex Richter, Andrew D Beggs PHE Heartlands Lab: Husam Osman, Andrew Bosworth. Queen Elizabeth Hospital: Anna Casey
EPI_ISL_651289	University of Exeter	COVID-19 Genomics UK (COG-UK) Consortium	Ben Temperton,Aaron Jeffries,Michelle Michelsen,Joanna Warwick-Dugdale,Audrey Farbos,Robyn Manley,Stephen Michell,Jane Masoli
EPI_ISL_651292	Department of Pathology, University of Cambridge	COVID-19 Genomics UK (COG-UK) Consortium	Aminu S. Jahun, Yasmin Chaudhry, Grant Hall, Iliana Georgana, Myra Hosmillo, Martin D. Curran, Malte Pinckert, Surendra Parmar, Ian Goodfellow
EPI_ISL_651304	University of Birmingham	COVID-19 Genomics UK (COG-UK) Consortium	Institute of Microbiology, University of Birmingham: Claire McMurray, Joanne Stockton, Samuel Nicholls, Radoslaw Poplawski, Will Rowe, Josh Quick, Nicholas Loman. University of Birmingham Testing Laboratory: Celina M Whalley, Andrew Bosworth, Charlotte Poxon, Kasun Wanigasooriya, Oliver Pickles, Mike Kidd, Alex Richter, Andrew D Beggs PHE Heartlands Lab: Husam Osman, Andrew Bosworth. Queen Elizabeth Hospital: Anna Casey
EPI_ISL_651355	Liverpool Clinical Laboratories	COVID-19 Genomics UK (COG-UK) Consortium	Sam Haldenby, Anita Lucaci, Steve Paterson, Julian Hiscox, Alistair Darby, M Almsaud, A Alrezaihi, Muhannad Alruwaili, Stuart D Armstrong, Jones Benjamin, Eleanor G Bentley, Anu Chawla, Jordan J Clark, Angela Cowell, Richard Eccles, Isabel García-Dorival, Matthew Gemmell, Alessandro Gerada, PKF Gilmore, Richard Gregory, Ximeng Han, Catherine Hartley, Margaret Hughes, Miren Iturriza-Gomara, James Johnson, L Luu, Jenifer Manson, Charlotte Nelson, Elaine O'Toole, Cassie Olateju, Rebekah Penrice-Randal , Lucille Rainbow, N.P Randle, Trevor Ian Robinson, Parul Sharma, Ghada T Shawli, James P Stewart, Neil Swainston, Ecaterina Vamos, Joanne Watts, Mark Whitehead
EPI_ISL_651359	West of Scotland Specialist Virology Centre, NHSGGC / MRC-University of Glasgow Centre for Virus Research	COVID-19 Genomics UK (COG-UK) Consortium	Ana da Silva Filipe, Natasha Johnson, Kathy Smollett, Daniel Mair, Stephen Carmichael, Alice Broos, Lily Tong, Jenna Nichols, Kyriaki Nomikou; Sarah McDonald; Richard Orton, Joseph Hughes, Sreenu Vattipally, David L Robertson; Alasdair MacLean, Rory Gunson; Sharif Shaaban, Matthew Holden; Rachel Blacow, Guy Mollett, Kathy Li, James Shepherd, Antonia Ho, Emma Thomson
EPI_ISL_651363	Department of Pathology, University of Cambridge	COVID-19 Genomics UK (COG-UK) Consortium	Aminu S. Jahun, Yasmin Chaudhry, Grant Hall, Iliana Georgana, Myra Hosmillo, Martin D. Curran, Malte Pinckert, Surendra Parmar, Ian Goodfellow
EPI_ISL_651373	University of Exeter	COVID-19 Genomics UK (COG-UK) Consortium	Ben Temperton,Aaron Jeffries,Michelle Michelsen,Joanna Warwick-Dugdale,Audrey Farbos,Robyn Manley,Stephen Michell,Jane Masoli
EPI_ISL_651383	Department of Pathology, University of Cambridge	COVID-19 Genomics UK (COG-UK) Consortium	Aminu S. Jahun, Yasmin Chaudhry, Grant Hall, Iliana Georgana, Myra Hosmillo, Martin D. Curran, Malte Pinckert, Surendra Parmar, Ian Goodfellow
EPI_ISL_651398	Queens Medical Centre, Clinical Microbiology Department / DeepSeq Nottingham	COVID-19 Genomics UK (COG-UK) Consortium	Gemma Clark, Wendy Smith, Manjinder Khakh, Vicki M Fleming, Michelle M Lister, Hannah Howson-Wells, Jonathan Ball, Patrick McClure, Joseph Chappell, Theocharis Tsoleridis, Nadine Holmes, Matthew Carlisle, Christopher Moore, Fei Sang, Johnny Debebe, Victoria Wright, Matthew Loose
EPI_ISL_651414	Virology Department, Sheffield Teaching Hospitals NHS Foundation Trust/Department of Infection, Immunity and Cardiovascular Disease, The Medical School, University of Sheffield	COVID-19 Genomics UK (COG-UK) Consortium	Thushan de Silva, Matthew Parker, Nikki Smith, Adri Angyal, Rebecca Brown, Luke Green, Rachel Tucker, Paul Parsons, Danielle Groves, Katie Johnson, Laura Carrilero, Alex Keeley, Dave Partridge, Matthew Wyles, Benjamin Lindsey, Mehmet Yavuz, Mohammad Raza, Cariad Evans
EPI_ISL_651416	Wales Specialist Virology Centre Sequencing lab: Pathogen Genomics Unit	COVID-19 Genomics UK (COG-UK) Consortium	Catherine Moore, Johnathan Evans, Laura Gifford, Malorie Perry, Simon Cottrell, Angela Marchbank, Alec Birchley, Alexander Adams, Amy Gaskin, Bree Gatica-Wilcox, Jason Coombes, Joel Southgate, Lauren Gilbert, Lee Graham, Nicole Pacchiarini, Sara Kumziene-Summerhayes, Sarah Taylor, Sophie Jones, Sara Rey, Matthew Bull, Joanne Watkins, Sally Corden, Tom Connor
EPI_ISL_651439	University of Exeter	COVID-19 Genomics UK (COG-UK) Consortium	Ben Temperton,Aaron Jeffries,Michelle Michelsen,Joanna Warwick-Dugdale,Audrey Farbos,Robyn Manley,Stephen Michell,Jane Masoli
EPI_ISL_651450, EPI_ISL_651452, EPI_ISL_651453, EPI_ISL_651454, EPI_ISL_651460, EPI_ISL_651461, EPI_ISL_651463, EPI_ISL_651464	Wales Specialist Virology Centre Sequencing lab: Pathogen Genomics Unit	COVID-19 Genomics UK (COG-UK) Consortium	Catherine Moore, Johnathan Evans, Laura Gifford, Malorie Perry, Simon Cottrell, Angela Marchbank, Alec Birchley, Alexander Adams, Amy Gaskin, Bree Gatica-Wilcox, Jason Coombes, Joel Southgate, Lauren Gilbert, Lee Graham, Nicole Pacchiarini, Sara Kumziene-Summerhayes, Sarah Taylor, Sophie Jones, Sara Rey, Matthew Bull, Joanne Watkins, Sally Corden, Tom Connor
EPI_ISL_651486	University of Birmingham	COVID-19 Genomics UK (COG-UK) Consortium	Institute of Microbiology, University of Birmingham: Claire McMurray, Joanne Stockton, Samuel Nicholls, Radoslaw Poplawski, Will Rowe, Josh Quick, Nicholas Loman. University of Birmingham Testing Laboratory: Celina M Whalley, Andrew Bosworth, Charlotte Poxon, Kasun Wanigasooriya, Oliver Pickles, Mike Kidd, Alex Richter, Andrew D Beggs PHE Heartlands Lab: Husam Osman, Andrew Bosworth. Queen Elizabeth Hospital: Anna Casey
EPI_ISL_651514, EPI_ISL_651518	Virology Department, Sheffield Teaching Hospitals NHS Foundation Trust/Department of Infection, Immunity and Cardiovascular Disease, The Medical School, University of Sheffield	COVID-19 Genomics UK (COG-UK) Consortium	Thushan de Silva, Matthew Parker, Nikki Smith, Adri Angyal, Rebecca Brown, Luke Green, Rachel Tucker, Paul Parsons, Danielle Groves, Katie Johnson, Laura Carrilero, Alex Keeley, Dave Partridge, Matthew Wyles, Benjamin Lindsey, Mehmet Yavuz, Mohammad Raza, Cariad Evans
EPI_ISL_651523, EPI_ISL_651524	Virology Department, Royal Infirmary of Edinburgh, NHS Lothian / School of Biological Sciences, University of Edinburgh / Institute of Genetics and Molecular Medicine, University of Edinburgh	COVID-19 Genomics UK (COG-UK) Consortium	McHugh M, Dewar R, Rooke S, Gallagher M, Balcaza C, O'Toole Á, Scher E, Hill V, McCrone JT, Colquhoun R, Yu X, Jackson B, Rambaut A, Williams TC, Templeton K
EPI_ISL_651527	University of Exeter	COVID-19 Genomics UK (COG-UK) Consortium	Ben Temperton,Aaron Jeffries,Michelle Michelsen,Joanna Warwick-Dugdale,Audrey Farbos,Robyn Manley,Stephen Michell,Jane Masoli
EPI_ISL_651546	Wales Specialist Virology Centre Sequencing lab: Pathogen Genomics Unit	COVID-19 Genomics UK (COG-UK) Consortium	Catherine Moore, Johnathan Evans, Laura Gifford, Malorie Perry, Simon Cottrell, Angela Marchbank, Alec Birchley, Alexander Adams, Amy Gaskin, Bree Gatica-Wilcox, Jason Coombes, Joel Southgate, Lauren Gilbert, Lee Graham, Nicole Pacchiarini, Sara Kumziene-Summerhayes, Sarah Taylor, Sophie Jones, Sara Rey, Matthew Bull, Joanne Watkins, Sally Corden, Tom Connor
EPI_ISL_651560, EPI_ISL_651562, EPI_ISL_651564, EPI_ISL_651581	Department of Pathology, University of Cambridge	COVID-19 Genomics UK (COG-UK) Consortium	Aminu S. Jahun, Yasmin Chaudhry, Grant Hall, Iliana Georgana, Myra Hosmillo, Martin D. Curran, Malte Pinckert, Surendra Parmar, Ian Goodfellow
EPI_ISL_651624	University of Birmingham	COVID-19 Genomics UK (COG-UK) Consortium	Institute of Microbiology, University of Birmingham: Claire McMurray, Joanne Stockton, Samuel Nicholls, Radoslaw Poplawski, Will Rowe, Josh Quick, Nicholas Loman. University of Birmingham Testing Laboratory: Celina M Whalley, Andrew Bosworth, Charlotte Poxon, Kasun Wanigasooriya, Oliver Pickles, Mike Kidd, Alex Richter, Andrew D Beggs PHE Heartlands Lab: Husam Osman, Andrew Bosworth. Queen Elizabeth Hospital: Anna Casey

EPI_ISL_651682, EPI_ISL_651683, EPI_ISL_651687, EPI_ISL_651688, EPI_ISL_651689, EPI_ISL_651690, EPI_ISL_651691, EPI_ISL_651692, EPI_ISL_651693, EPI_ISL_651694, EPI_ISL_651695, EPI_ISL_651696, EPI_ISL_651697, EPI_ISL_651698, EPI_ISL_651703, EPI_ISL_651704, EPI_ISL_651705, EPI_ISL_651706, EPI_ISL_651707			
see above	Department of Pathology, University of Cambridge	COVID-19 Genomics UK (COG-UK) Consortium	Aminu S. Jahun, Yasmin Chaudhry, Grant Hall, Iliana Georgana, Myra Hosmillo, Martin D. Curran, Malte Pinckert, Surendra Parmar, Ian Goodfellow
EPI_ISL_651715, EPI_ISL_651716, EPI_ISL_651718, EPI_ISL_651719, EPI_ISL_651720, EPI_ISL_651721, EPI_ISL_651722, EPI_ISL_651723, EPI_ISL_651724, EPI_ISL_651725, EPI_ISL_651726, EPI_ISL_651727, EPI_ISL_651728, EPI_ISL_651729, EPI_ISL_651730, EPI_ISL_651731, EPI_ISL_651732			
see above	Virology Department, Sheffield Teaching Hospitals NHS Foundation Trust/Department of Infection, Immunity and Cardiovascular Disease, The Medical School, University of Sheffield	COVID-19 Genomics UK (COG-UK) Consortium	Thushan de Silva, Matthew Parker, Nikki Smith, Adri Angyal, Rebecca Brown, Luke Green, Rachel Tucker, Paul Parsons, Danielle Groves, Katie Johnson, Laura Carrilero, Alex Keeley, Dave Partridge, Matthew Wyles, Benjamin Lindsey, Mehmet Yavuz, Mohammad Raza, Cariad Evans
EPI_ISL_651830, EPI_ISL_651831, EPI_ISL_651860, EPI_ISL_651861, EPI_ISL_651862, EPI_ISL_651864, EPI_ISL_651869, EPI_ISL_651870, EPI_ISL_651871, EPI_ISL_651872, EPI_ISL_651887, EPI_ISL_651892, EPI_ISL_651946			
see above	West of Scotland Specialist Virology Centre, NHSGGC / MRC-University of Glasgow Centre for Virus Research	COVID-19 Genomics UK (COG-UK) Consortium	Ana da Silva Filipe, Natasha Johnson, Kathy Smollett, Daniel Mair, Stephen Carmichael, Alice Broos, Lily Tong, Jenna Nichols, Kyriaki Nomikou; Sarah McDonald; Richard Orton, Joseph Hughes, Sreenu Vattipally, David L Robertson; Alasdair MacLean, Rory Gunson; Sharif Shaaban, Matthew Holden; Rachel Blacow, Guy Mollett, Kathy Li, James Shepherd, Antonia Ho, Emma Thomson
EPI_ISL_652037, EPI_ISL_652049, EPI_ISL_652050, EPI_ISL_652052, EPI_ISL_652061, EPI_ISL_652065, EPI_ISL_652066	Virology Department, Royal Infirmary of Edinburgh, NHS Lothian / School of Biological Sciences, University of Edinburgh / Institute of Genetics and Molecular Medicine, University of Edinburgh	COVID-19 Genomics UK (COG-UK) Consortium	McHugh M, Dewar R, Rooke S, Gallagher M, Balcaza C, O'Toole Á, Scher E, Hill V, McCrone JT, Colquhoun R, Yu X, Jackson B, Rambaut A, Williams TC, Templeton K
EPI_ISL_652290, EPI_ISL_652291, EPI_ISL_652292, EPI_ISL_652293, EPI_ISL_652294, EPI_ISL_652300, EPI_ISL_652302	University of Exeter	COVID-19 Genomics UK (COG-UK) Consortium	Ben Temperton,Aaron Jeffries,Michelle Michelsen,Joanna Warwick-Dugdale,Audrey Farbos,Robyn Manley,Stephen Michell,Jane Masoli
EPI_ISL_652362	Quadram Institute Bioscience	COVID-19 Genomics UK (COG-UK) Consortium	Dave J. Baker, Gemma L. Kay, Alp Aydin, Thanh Le-Viet, Steven Rudder, Ana P. Tedim, Anastasia Kolyva, Maria Diaz, Leonardo de Oliveira Martins, Nabil-Fareed Alikhan, Lizzie Meadows, Rachael Stanley, Ngozi Elumogo, Muhammed Yasir, Nicholas M. Thomson, Alexander J Trotter, Rachel Gilroy, Samuel Bloomfield, Claire Stuart, Andrew Bell, Reenesh Prakash, Samir Dervisevic, Alison E. Mather, John Wain, Mark Webber, Andrew J. Page, Justin O'Grady
EPI_ISL_652496, EPI_ISL_652497, EPI_ISL_652500, EPI_ISL_652501, EPI_ISL_652502, EPI_ISL_652508, EPI_ISL_652509, EPI_ISL_652510, EPI_ISL_652511, EPI_ISL_652513, EPI_ISL_652514, EPI_ISL_652516, EPI_ISL_652518, EPI_ISL_652519, EPI_ISL_652520, EPI_ISL_652525, EPI_ISL_652526, EPI_ISL_652528, EPI_ISL_652531, EPI_ISL_652532, EPI_ISL_652538, EPI_ISL_652540, EPI_ISL_652541, EPI_ISL_652542, EPI_ISL_652544, EPI_ISL_652547, EPI_ISL_652549, EPI_ISL_652558, EPI_ISL_652559, EPI_ISL_652563, EPI_ISL_652564, EPI_ISL_652565, EPI_ISL_652567, EPI_ISL_652569, EPI_ISL_652570, EPI_ISL_652577, EPI_ISL_652583, EPI_ISL_652588, EPI_ISL_652589, EPI_ISL_652597, EPI_ISL_652602, EPI_ISL_652604, EPI_ISL_652610, EPI_ISL_652611, EPI_ISL_652612, EPI_ISL_652614, EPI_ISL_652615, EPI_ISL_652618			
see above	Wales Specialist Virology Centre Sequencing lab: Pathogen Genomics Unit	COVID-19 Genomics UK (COG-UK) Consortium	Catherine Moore, Johnathan Evans, Laura Gifford, Malorie Perry, Simon Cottrell, Angela Marchbank, Alec Birchley, Alexander Adams, Amy Gaskin, Bree Gatica-Wilcox, Jason Coombes, Joel Southgate, Lauren Gilbert, Lee Graham, Nicole Pacchiarini, Sara Kumziene-Summerhayes, Sarah Taylor, Sophie Jones, Sara Rey, Matthew Bull, Joanne Watkins, Sally Corden, Tom Connor
EPI_ISL_653061, EPI_ISL_653064, EPI_ISL_653072, EPI_ISL_653081	Virology Department, Sheffield Teaching Hospitals NHS Foundation Trust/Department of Infection, Immunity and Cardiovascular Disease, The Medical School, University of Sheffield	COVID-19 Genomics UK (COG-UK) Consortium	Thushan de Silva, Matthew Parker, Nikki Smith, Adri Angyal, Rebecca Brown, Luke Green, Rachel Tucker, Paul Parsons, Danielle Groves, Katie Johnson, Laura Carrilero, Alex Keeley, Dave Partridge, Matthew Wyles, Benjamin Lindsey, Mehmet Yavuz, Mohammad Raza, Cariad Evans
EPI_ISL_653765, EPI_ISL_653766, EPI_ISL_653767, EPI_ISL_653768, EPI_ISL_653769, EPI_ISL_653770, EPI_ISL_653771, EPI_ISL_653772, EPI_ISL_653773, EPI_ISL_653774, EPI_ISL_653775, EPI_ISL_653776, EPI_ISL_653777, EPI_ISL_653778, EPI_ISL_653779, EPI_ISL_653780, EPI_ISL_653781, EPI_ISL_653783, EPI_ISL_653787, EPI_ISL_653788, EPI_ISL_653789, EPI_ISL_653790, EPI_ISL_653791, EPI_ISL_653792, EPI_ISL_653793			
see above	I.R.C.C.S. "S. De Bellis" - Ente Ospedaliero	Istituto Zooprofilattico Sperimentale della Puglia e della Basilicata	Parisi A., Bianco A., Capozzi L., Del Sambro L., Lippolis A., Notarnicola M., Manzulli V, Rondinone V., Pace L.
EPI_ISL_653794, EPI_ISL_653795, EPI_ISL_653796, EPI_ISL_653797, EPI_ISL_653798, EPI_ISL_653799, EPI_ISL_653800, EPI_ISL_653801, EPI_ISL_653804, EPI_ISL_653805, EPI_ISL_653806, EPI_ISL_653807, EPI_ISL_653808, EPI_ISL_653809, EPI_ISL_653811, EPI_ISL_653812			
see above	I.R.C.C.S. "S. De Bellis" - Ente Ospedaliero	Istituto Zooprofilattico Sperimentale della Puglia e della Basilicata	Parisi A., Bianco A., Capozzi L., Del Sambro L., Lippolis A., Notarnicola M., Cipolletta D., Galante D.
EPI_ISL_654017, EPI_ISL_654018, EPI_ISL_654019	Laboratory of Microbiology, National Reference Lab, Charles Nicolle Hospital; 2-University of Tunis ElManar, Faculty of Medicine of Tunis, LR99ES09, Tunis, Tunisia	1-Clinical and Experimental Pharmacology Lab, LR16SP02, National Center of Pharmacovigilance, University of Tunis El Manar, Tunis, Tunisia. 2-Neurodegenerative diseases and psychiatric troubles, LR18SP03, Razi Hospital, University of Tunis El Manar, Tunis, Tunisia. 3- Ministry of Health, National Observatory of New and Emerging Diseases, 1006, Tunis, Tunisia	Ilhem Boutiba-Ben Boubaker, Sameh Trabelsi, Nissaf Ben Alaya, Maher Kharrat, Alia Ben Kahla, Jalila Ben Khelil, Salma Abid, Sana Ferjani, Mouna Ben Sassi, Mouna Safer, Imen Mkada, Imen Kacem, Gaies Emna, Soumaya Rammeh, Riadh Daghfous, Riadh Gouider.
EPI_ISL_654703, EPI_ISL_654705, EPI_ISL_654706, EPI_ISL_654707, EPI_ISL_654708, EPI_ISL_654709, EPI_ISL_654710, EPI_ISL_654711, EPI_ISL_654712, EPI_ISL_654713, EPI_ISL_654714, EPI_ISL_654715, EPI_ISL_654716, EPI_ISL_654717, EPI_ISL_654718, EPI_ISL_654719, EPI_ISL_654720, EPI_ISL_654721, EPI_ISL_654722, EPI_ISL_654723, EPI_ISL_654724, EPI_ISL_654727			
see above	Minnesota Department of Health, Public Health Laboratory	Minnesota Department of Health, Public Health Laboratory	Matt Plumb, Jacob Garfin, Alexandra Lorentz, and Xiong Wang
EPI_ISL_657472, EPI_ISL_657861, EPI_ISL_657991, EPI_ISL_658261, EPI_ISL_658754, EPI_ISL_658755, EPI_ISL_658765, EPI_ISL_658772, EPI_ISL_658777, EPI_ISL_658780, EPI_ISL_658789, EPI_ISL_658791, EPI_ISL_658803, EPI_ISL_658804, EPI_ISL_658812, EPI_ISL_658821, EPI_ISL_658827, EPI_ISL_658832, EPI_ISL_658847, EPI_ISL_658851, EPI_ISL_658855			
see above	Lighthouse Lab in Milton Keynes	Wellcome Sanger Institute for the COVID-19 Genomics UK (COG-UK) Consortium	The Lighthouse Lab in Milton Keynes and Alex Alderton, Roberto Amato, Sonia Goncalves, Ewan Harrison, David K. Jackson, Ian Johnston, Dominic Kwiatkowski, Cordelia Langford, John Sillitoe on behalf of the Wellcome Sanger Institute COVID-19 Surveillance Team
EPI_ISL_658863, EPI_ISL_658865, EPI_ISL_658868, EPI_ISL_658870	Instituto de Diagnostico y Referencia Epidemiologicos (INDRE)	Instituto de Diagnostico y Referencia Epidemiologicos (INDRE)	Gisela Barrera-Badillo , Abril Rodriguez-Maldonado, Claudia Wong-Arambula , Natividad Cruz-Ortiz, Tatiana Nunez-Garcia, Dayanira Arellano-Suarez, Fabiola Garces-Ayala, Lucia Hernandez-Rivas, Irma Lopez-Martinez, Ernesto Ramirez-Gonzalez.
EPI_ISL_658873, EPI_ISL_658875	Instituto de Diagnostico y Referencia Epidemiologicos (INDRE)	Instituto de Diagnostico y Referencia Epidemiologicos (INDRE)	Gisela Barrera-Badillo , Abril Rodriguez-Maldonado, Claudia Wong-Arambula , Natividad Cruz-Ortiz, Tatiana Nunez-Garcia, Dayanira Arellano-Suarez, Fabiola Garces-Ayala, Lucia Hernandez-Rivas, Irma Lopez-Martinez, Ernesto Ramirez-Gonzalez.
EPI_ISL_658878	Instituto de Diagnostico y Referencia Epidemiologicos (INDRE)	Instituto de Diagnostico y Referencia Epidemiologicos (INDRE)	Ernesto Ramirez-Gonzalez, Abril Rodriguez-Maldonado, Claudia Wong-Arambula , Natividad Cruz-Ortiz, Tatiana Nunez-Garcia, Dayanira Arellano-Suarez, Fabiola Garces-Ayala, Lucia Hernandez-Rivas, Irma Lopez-Martinez, Gisela Barrera-Badillo.
EPI_ISL_658879	Lighthouse Lab in Milton Keynes	Wellcome Sanger Institute for the COVID-19 Genomics UK (COG-UK) Consortium	The Lighthouse Lab in Milton Keynes and Alex Alderton, Roberto Amato, Sonia Goncalves, Ewan Harrison, David K. Jackson, Ian Johnston, Dominic Kwiatkowski, Cordelia Langford, John Sillitoe on behalf of the Wellcome Sanger Institute COVID-19 Surveillance Team
EPI_ISL_658880, EPI_ISL_658883, EPI_ISL_658886, EPI_ISL_658888, EPI_ISL_658893	Instituto de Diagnostico y Referencia Epidemiologicos (INDRE)	Instituto de Diagnostico y Referencia Epidemiologicos (INDRE)	Ernesto Ramirez-Gonzalez, Abril Rodriguez-Maldonado, Claudia Wong-Arambula , Natividad Cruz-Ortiz, Tatiana Nunez-Garcia, Dayanira Arellano-Suarez, Fabiola Garces-Ayala, Lucia Hernandez-Rivas, Irma Lopez-Martinez, Gisela Barrera-Badillo.
EPI_ISL_658908, EPI_ISL_658922, EPI_ISL_658928, EPI_ISL_658935, EPI_ISL_658938, EPI_ISL_658951, EPI_ISL_658955, EPI_ISL_658985, EPI_ISL_658994, EPI_ISL_659001, EPI_ISL_659008, EPI_ISL_659009, EPI_ISL_659014, EPI_ISL_659021, EPI_ISL_659023, EPI_ISL_659026, EPI_ISL_659032, EPI_ISL_659033, EPI_ISL_659034, EPI_ISL_659037, EPI_ISL_659041, EPI_ISL_659056, EPI_ISL_659088			
see above	Lighthouse Lab in Milton Keynes	Wellcome Sanger Institute for the COVID-19 Genomics UK (COG-UK) Consortium	The Lighthouse Lab in Milton Keynes and Alex Alderton, Roberto Amato, Sonia Goncalves, Ewan Harrison, David K. Jackson, Ian Johnston, Dominic Kwiatkowski, Cordelia Langford, John Sillitoe on behalf of the Wellcome Sanger Institute COVID-19 Surveillance Team
EPI_ISL_659135, EPI_ISL_659163, EPI_ISL_659167, EPI_ISL_659345, EPI_ISL_659395	Lighthouse Lab in Cambridge	Wellcome Sanger Institute for the COVID-19 Genomics UK (COG-UK) Consortium	Rob Howes, The Lighthouse Lab in Cambridge and Alex Alderton, Roberto Amato, Sonia Goncalves, Ewan Harrison, David K. Jackson, Ian Johnston, Dominic Kwiatkowski, Cordelia Langford, John Sillitoe on behalf of the Wellcome Sanger Institute COVID-19 Surveillance Team

EPI_ISL_659745, EPI_ISL_659746, EPI_ISL_659747, EPI_ISL_659749, EPI_ISL_659751, EPI_ISL_659754, EPI_ISL_659758, EPI_ISL_659766, EPI_ISL_659773, EPI_ISL_659774, EPI_ISL_659775, EPI_ISL_659778, EPI_ISL_659780, EPI_ISL_659781, EPI_ISL_659782, EPI_ISL_659785, EPI_ISL_659786, EPI_ISL_659791, EPI_ISL_659796, EPI_ISL_659797, EPI_ISL_659799, EPI_ISL_659800, EPI_ISL_659803, EPI_ISL_659806, EPI_ISL_659810, EPI_ISL_659813, EPI_ISL_659814, EPI_ISL_659816, EPI_ISL_659817, EPI_ISL_659819, EPI_ISL_659822, EPI_ISL_659829, EPI_ISL_659831, EPI_ISL_659834, EPI_ISL_659838, EPI_ISL_659839, EPI_ISL_659841, EPI_ISL_659844, EPI_ISL_659847, EPI_ISL_659848, EPI_ISL_659849, EPI_ISL_659851, EPI_ISL_659854, EPI_ISL_659855, EPI_ISL_659859, EPI_ISL_659861, EPI_ISL_659862, EPI_ISL_659865, EPI_ISL_659866, EPI_ISL_659870, EPI_ISL_659873, EPI_ISL_659876, EPI_ISL_659878, EPI_ISL_659881, EPI_ISL_659885, EPI_ISL_659886, EPI_ISL_659887, EPI_ISL_659889, EPI_ISL_659893, EPI_ISL_659894, EPI_ISL_659895, EPI_ISL_659898, EPI_ISL_659899, EPI_ISL_659901, EPI_ISL_659902, EPI_ISL_659911, EPI_ISL_659912, EPI_ISL_659914, EPI_ISL_659915, EPI_ISL_659916, EPI_ISL_659917, EPI_ISL_659921, EPI_ISL_659922, EPI_ISL_659923, EPI_ISL_659925, EPI_ISL_659927, EPI_ISL_659929, EPI_ISL_659930, EPI_ISL_659932, EPI_ISL_659933, EPI_ISL_659936, EPI_ISL_659937, EPI_ISL_659938, EPI_ISL_659940, EPI_ISL_659943, EPI_ISL_659945, EPI_ISL_659946, EPI_ISL_659950, EPI_ISL_659952, EPI_ISL_659953, EPI_ISL_659958, EPI_ISL_659959, EPI_ISL_659960, EPI_ISL_659965, EPI_ISL_659969, EPI_ISL_659972, EPI_ISL_659976, EPI_ISL_659978, EPI_ISL_659982			
see above	Lighthouse Lab in Glasgow	Wellcome Sanger Institute for the COVID-19 Genomics UK (COG-UK) Consortium	Harper VanSteenhouse, Yumi Kasai, David Gray, Carol Clugston, Anna Dominiczak and Alex Alderton, Roberto Amato, Sonia Goncalves, Ewan Harrison, David K. Jackson, Ian Johnston, Dominic Kwiatkowski, Cordelia Langford, John Sillitoe on behalf of the Wellcome Sanger Institute COVID-19 Surveillance Team
EPI_ISL_659985, EPI_ISL_659991, EPI_ISL_659994, EPI_ISL_659996, EPI_ISL_659997, EPI_ISL_660000, EPI_ISL_660002, EPI_ISL_660003, EPI_ISL_660004, EPI_ISL_660005, EPI_ISL_660007, EPI_ISL_660009, EPI_ISL_660010, EPI_ISL_660017, EPI_ISL_660018, EPI_ISL_660021, EPI_ISL_660028, EPI_ISL_660030, EPI_ISL_660032, EPI_ISL_660033, EPI_ISL_660035, EPI_ISL_660039, EPI_ISL_660041, EPI_ISL_660044, EPI_ISL_660045, EPI_ISL_660047, EPI_ISL_660050, EPI_ISL_660051, EPI_ISL_660052, EPI_ISL_660055, EPI_ISL_660056, EPI_ISL_660057, EPI_ISL_660058, EPI_ISL_660059, EPI_ISL_660063, EPI_ISL_660065, EPI_ISL_660066			
see above	Lighthouse Lab in Glasgow	Wellcome Sanger Institute for the COVID-19 Genomics UK (COG-UK) Consortium	Harper VanSteenhouse, Yumi Kasai, David Gray, Carol Clugston, Anna Dominiczak and Alex Alderton, Roberto Amato, Sonia Goncalves, Ewan Harrison, David K. Jackson, Ian Johnston, Dominic Kwiatkowski, Cordelia Langford, John Sillitoe on behalf of the Wellcome Sanger Institute COVID-19 Surveillance Team (http://www.sanger.ac.uk/covid-team)
EPI_ISL_660086, EPI_ISL_660087, EPI_ISL_660088, EPI_ISL_660089, EPI_ISL_660090, EPI_ISL_660091, EPI_ISL_660092, EPI_ISL_660093, EPI_ISL_660097	MD PHL	MD PHL	Maryland Department of Health Laboratories Administration
EPI_ISL_660225	NHLS-IALCH	KRISP, KZN Research Innovation and Sequencing Platform	Giandhari J, Pillay S, Lessells R, Mdlalose K, York D, Khan S, Tegally H, Wilkinson E, de Oliveira T
EPI_ISL_660324, EPI_ISL_660325	General practitioner	National Reference Center for Viruses of Respiratory Infections, Institut Pasteur, Paris	Marion Barbet, Sylvie Behillil, Méline Bizard, Angela Brisebarre, Camille Capel, Etienne Simon-Lorière, Vincent Enouf, Maud Vanpeene, Sylvie van der Werf
EPI_ISL_660473, EPI_ISL_660519	Laboratoire de Microbiologie CHU Souro Sanou	Centre Muraz	Abdoul-Salam Ouedraogo, Yacouba Sawadogo, Essia Belarbi, Grit Schubert, Fabian Leendertz, Arsène Zongo, Soumeya Ouangraoua, Zekiba Tarnagda, Lassana Sangaré, Halidou Tinto
EPI_ISL_660637, EPI_ISL_660639, EPI_ISL_660642, EPI_ISL_660644, EPI_ISL_660645, EPI_ISL_660650, EPI_ISL_660651, EPI_ISL_660653, EPI_ISL_660654, EPI_ISL_660655, EPI_ISL_660656, EPI_ISL_660659, EPI_ISL_660660, EPI_ISL_660661, EPI_ISL_660663			
see above	NHLS-IALCH	KRISP, KZN Research Innovation and Sequencing Platform	Giandhari J, Pillay S, Lessells R, Mdlalose K, York D, Khan S, Tegally H, Wilkinson E, de Oliveira T
EPI_ISL_660782, EPI_ISL_660784, EPI_ISL_660785, EPI_ISL_660786, EPI_ISL_660787, EPI_ISL_660788, EPI_ISL_660789, EPI_ISL_660790, EPI_ISL_660791, EPI_ISL_660792, EPI_ISL_660793			
see above	Respiratory Virus Unit, Microbiology Services Colindale, Public Health England	COVID-19 Genomics UK (COG-UK) Consortium	PHE Covid Sequencing Team
EPI_ISL_661045, EPI_ISL_661046, EPI_ISL_661047, EPI_ISL_661048, EPI_ISL_661049, EPI_ISL_661050, EPI_ISL_661051, EPI_ISL_661052, EPI_ISL_661053, EPI_ISL_661054, EPI_ISL_661055, EPI_ISL_661056, EPI_ISL_661057, EPI_ISL_661058, EPI_ISL_661059, EPI_ISL_661060, EPI_ISL_661061, EPI_ISL_661062, EPI_ISL_661063, EPI_ISL_661064, EPI_ISL_661065, EPI_ISL_661066, EPI_ISL_661067, EPI_ISL_661068, EPI_ISL_661069, EPI_ISL_661070, EPI_ISL_661071, EPI_ISL_661072, EPI_ISL_661073, EPI_ISL_661074, EPI_ISL_661075, EPI_ISL_661076, EPI_ISL_661077, EPI_ISL_661078, EPI_ISL_661079, EPI_ISL_661080			
see above	Gundersen Molecular Diagnostics Laboratory	Kabara Cancer Research Institute	Craig S. Richmond, Paraic A. Kenny
EPI_ISL_661294	Unilabs	The Public Health Agency of Sweden	Department of Microbiology, The Public Health Agency of Sweden
EPI_ISL_661300	Laboratoriemedicin, Klinisk mikrobiologi	The Public Health Agency of Sweden	Department of Microbiology, The Public Health Agency of Sweden
EPI_ISL_662638, EPI_ISL_662642, EPI_ISL_662643, EPI_ISL_662645	Lighthouse Lab in Milton Keynes	Wellcome Sanger Institute for the COVID-19 Genomics UK (COG-UK) Consortium	The Lighthouse Lab in Milton Keynes and Alex Alderton, Roberto Amato, Sonia Goncalves, Ewan Harrison, David K. Jackson, Ian Johnston, Dominic Kwiatkowski, Cordelia Langford, John Sillitoe on behalf of the Wellcome Sanger Institute COVID-19 Surveillance Team
EPI_ISL_662654	Lighthouse Lab in Glasgow	Wellcome Sanger Institute for the COVID-19 Genomics UK (COG-UK) Consortium	Harper VanSteenhouse, Yumi Kasai, David Gray, Carol Clugston, Anna Dominiczak and Alex Alderton, Roberto Amato, Sonia Goncalves, Ewan Harrison, David K. Jackson, Ian Johnston, Dominic Kwiatkowski, Cordelia Langford, John Sillitoe on behalf of the Wellcome Sanger Institute COVID-19 Surveillance Team
EPI_ISL_664137	Centre for Enzyme Innovation, University of Portsmouth / Translational Research Laboratory, Portsmouth Hospitals NHS Trust	COVID-19 Genomics UK (COG-UK) Consortium	Angela Beckett,Yann Bourgeois,Garry Scarlett,Sharon Glaysher,Scott Elliott,Kelly Bicknell,Robert Impey,Allyson Lloyd,Sarah Wyllie,Ethan Butcher,Anoop Chauhan,Samuel Robson
EPI_ISL_664170	University of Exeter	COVID-19 Genomics UK (COG-UK) Consortium	Ben Temperton,Aaron Jeffries,Michelle Michelsen,Joanna Warwick-Dugdale,Audrey Farbos,Robyn Manley,Stephen Michell,Jane Masoli
EPI_ISL_664191	Centre for Enzyme Innovation, University of Portsmouth / Translational Research Laboratory, Portsmouth Hospitals NHS Trust	COVID-19 Genomics UK (COG-UK) Consortium	Angela Beckett,Yann Bourgeois,Garry Scarlett,Sharon Glaysher,Scott Elliott,Kelly Bicknell,Robert Impey,Allyson Lloyd,Sarah Wyllie,Ethan Butcher,Anoop Chauhan,Samuel Robson
EPI_ISL_664230	Liverpool Clinical Laboratories	COVID-19 Genomics UK (COG-UK) Consortium	Sam Haldenby, Anita Lucaci, Steve Paterson, Julian Hiscox, Alistair Darby, M Almsaud, A Alrezaihi, Muhannad Alruwaili, Stuart D Armstrong, Jones Benjamin, Eleanor G Bentley, Anu Chawla, Jordan J Clark, Angela Cowell, Richard Eccles, Isabel García-Dorival, Matthew Gemmell, Alessandro Gerada, PKF Gilmore, Richard Gregory, Ximeng Han, Catherine Hartley, Margaret Hughes, Miren Iturriza-Gomara, James Johnson, L Luu, Jenifer Manson, Charlotte Nelson, Elaine O'Toole, Cassie Olateju, Rebekah Penrice-Randal , Lucille Rainbow, N.P Randle, Trevor Ian Robinson, Parul Sharma, Ghada T Shawli, James P Stewart, Neil Swainston, Ecaterina Vamos, Joanne Watts, Mark Whitehead
EPI_ISL_664283	Virology Department, Sheffield Teaching Hospitals NHS Foundation Trust/Department of Infection, Immunity and Cardiovascular Disease, The Medical School, University of Sheffield	COVID-19 Genomics UK (COG-UK) Consortium	Thushan de Silva, Matthew Parker, Nikki Smith, Adri Angyal, Rebecca Brown, Luke Green, Rachel Tucker, Paul Parsons, Danielle Groves, Katie Johnson, Laura Carrilero, Alex Keeley, Dave Partridge, Matthew Wyles, Benjamin Lindsey, Mehmet Yavuz, Mohammad Raza, Cariad Evans
EPI_ISL_664295	University of Exeter	COVID-19 Genomics UK (COG-UK) Consortium	Ben Temperton,Aaron Jeffries,Michelle Michelsen,Joanna Warwick-Dugdale,Audrey Farbos,Robyn Manley,Stephen Michell,Jane Masoli
EPI_ISL_664298	Virology Department, Sheffield Teaching Hospitals NHS Foundation Trust/Department of Infection, Immunity and Cardiovascular Disease, The Medical School, University of Sheffield	COVID-19 Genomics UK (COG-UK) Consortium	Thushan de Silva, Matthew Parker, Nikki Smith, Adri Angyal, Rebecca Brown, Luke Green, Rachel Tucker, Paul Parsons, Danielle Groves, Katie Johnson, Laura Carrilero, Alex Keeley, Dave Partridge, Matthew Wyles, Benjamin Lindsey, Mehmet Yavuz, Mohammad Raza, Cariad Evans
EPI_ISL_664339	Liverpool Clinical Laboratories	COVID-19 Genomics UK (COG-UK) Consortium	Sam Haldenby, Anita Lucaci, Steve Paterson, Julian Hiscox, Alistair Darby, M Almsaud, A Alrezaihi, Muhannad Alruwaili, Stuart D Armstrong, Jones Benjamin, Eleanor G Bentley, Anu Chawla, Jordan J Clark, Angela Cowell, Richard Eccles, Isabel García-Dorival, Matthew Gemmell, Alessandro Gerada, PKF Gilmore, Richard Gregory, Ximeng Han, Catherine Hartley, Margaret Hughes, Miren Iturriza-Gomara, James Johnson, L Luu, Jenifer Manson, Charlotte Nelson, Elaine O'Toole, Cassie Olateju, Rebekah Penrice-Randal , Lucille Rainbow, N.P Randle, Trevor Ian Robinson, Parul Sharma, Ghada T Shawli, James P Stewart, Neil Swainston, Ecaterina Vamos, Joanne Watts, Mark Whitehead
EPI_ISL_664359	University College London Hospital	COVID-19 Genomics UK (COG-UK) Consortium	Judith Heaney, Matthew Byott, Catherine Houlihan, Dan Frampton, Stuart Kirk, Moira Spyer and Eleni Nastouli
EPI_ISL_664370	Virology Department, Sheffield Teaching Hospitals NHS Foundation Trust/Department of Infection, Immunity and Cardiovascular Disease, The Medical School, University of Sheffield	COVID-19 Genomics UK (COG-UK) Consortium	Thushan de Silva, Matthew Parker, Nikki Smith, Adri Angyal, Rebecca Brown, Luke Green, Rachel Tucker, Paul Parsons, Danielle Groves, Katie Johnson, Laura Carrilero, Alex Keeley, Dave Partridge, Matthew Wyles, Benjamin Lindsey, Mehmet Yavuz, Mohammad Raza, Cariad Evans
EPI_ISL_664373	Northumbria University / South Tees Hospitals NHS Foundation Trust / North Cumbria Integrated Care NHS	COVID-19 Genomics UK (COG-UK) Consortium	Darren L Smith,Andrew Nelson,Matthew Bashton,Greg R Young,Joshua Loh,John Allan,Mohammad A Tariq,Giles S Holt,Gary Black,Wen C Yew,Lynn Dover,Paul Baker,Steve Liggett,Sarah Essex,Jane Greenaway,Debra Padgett,Clive Graham,Garren Scott,Edward Barton,Emma Swindells,Brendan

	Foundation Trust / North Tees and Hartlepool NHS Foundation Trust / Newcastle Hospitals NHS Foundation Trust		Payne,Jennifer Collins,Yusri Taha,Gary Eltringham
EPI_ISL_664374, EPI_ISL_664375	Centre for Enzyme Innovation, University of Portsmouth / Translational Research Laboratory, Portsmouth Hospitals NHS Trust	COVID-19 Genomics UK (COG-UK) Consortium	Angela Beckett,Yann Bourgeois,Garry Scarlett,Sharon Glaysher,Scott Elliott,Kelly Bicknell,Robert Impey,Allyson Lloyd,Sarah Wyllie,Ethan Butcher,Anoop Chauhan,Samuel Robson
EPI_ISL_664398	University of Exeter	COVID-19 Genomics UK (COG-UK) Consortium	Ben Temperton,Aaron Jeffries,Michelle Michelsen,Joanna Warwick-Dugdale,Audrey Farbos,Robyn Manley,Stephen Michell,Jane Masoli
EPI_ISL_664425, EPI_ISL_664426, EPI_ISL_664431, EPI_ISL_664437	Virology Department, Sheffield Teaching Hospitals NHS Foundation Trust/Department of Infection, Immunity and Cardiovascular Disease, The Medical School, University of Sheffield	COVID-19 Genomics UK (COG-UK) Consortium	Thushan de Silva, Matthew Parker, Nikki Smith, Adri Angyal, Rebecca Brown, Luke Green, Rachel Tucker, Paul Parsons, Danielle Groves, Katie Johnson, Laura Carrilero, Alex Keeley, Dave Partridge, Matthew Wyles, Benjamin Lindsey, Mehmet Yavuz, Mohammad Raza, Cariad Evans
EPI_ISL_664526, EPI_ISL_664556	Centre for Enzyme Innovation, University of Portsmouth / Translational Research Laboratory, Portsmouth Hospitals NHS Trust	COVID-19 Genomics UK (COG-UK) Consortium	Angela Beckett,Yann Bourgeois,Garry Scarlett,Sharon Glaysher,Scott Elliott,Kelly Bicknell,Robert Impey,Allyson Lloyd,Sarah Wyllie,Ethan Butcher,Anoop Chauhan,Samuel Robson
EPI_ISL_664600, EPI_ISL_664602, EPI_ISL_664604	Virology Department, Sheffield Teaching Hospitals NHS Foundation Trust/Department of Infection, Immunity and Cardiovascular Disease, The Medical School, University of Sheffield	COVID-19 Genomics UK (COG-UK) Consortium	Thushan de Silva, Matthew Parker, Nikki Smith, Adri Angyal, Rebecca Brown, Luke Green, Rachel Tucker, Paul Parsons, Danielle Groves, Katie Johnson, Laura Carrilero, Alex Keeley, Dave Partridge, Matthew Wyles, Benjamin Lindsey, Mehmet Yavuz, Mohammad Raza, Cariad Evans
EPI_ISL_664614	Liverpool Clinical Laboratories	COVID-19 Genomics UK (COG-UK) Consortium	Sam Haldenby, Anita Lucaci, Steve Paterson, Julian Hiscox, Alistair Darby, M Almsaud, A Alrezaihi, Muhannad Alruwaili, Stuart D Armstrong, Jones Benjamin, Eleanor G Bentley, Anu Chawla, Jordan J Clark, Angela Cowell, Richard Eccles, Isabel García-Dorival, Matthew Gemmell, Alessandro Gerada, PKF Gilmore, Richard Gregory, Ximeng Han, Catherine Hartley, Margaret Hughes, Miren Iturriza-Gomara, James Johnson, L Luu, Jenifer Manson, Charlotte Nelson, Elaine O'Toole, Cassie Olateju, Rebekah Penrice-Randal , Lucille Rainbow, N.P Randle, Trevor Ian Robinson, Parul Sharma, Ghada T Shawli, James P Stewart, Neil Swainston, Ecaterina Vamos, Joanne Watts, Mark Whitehead
EPI_ISL_664630	Virology Department, Sheffield Teaching Hospitals NHS Foundation Trust/Department of Infection, Immunity and Cardiovascular Disease, The Medical School, University of Sheffield	COVID-19 Genomics UK (COG-UK) Consortium	Thushan de Silva, Matthew Parker, Nikki Smith, Adri Angyal, Rebecca Brown, Luke Green, Rachel Tucker, Paul Parsons, Danielle Groves, Katie Johnson, Laura Carrilero, Alex Keeley, Dave Partridge, Matthew Wyles, Benjamin Lindsey, Mehmet Yavuz, Mohammad Raza, Cariad Evans
EPI_ISL_664679	Centre for Enzyme Innovation, University of Portsmouth / Translational Research Laboratory, Portsmouth Hospitals NHS Trust	COVID-19 Genomics UK (COG-UK) Consortium	Angela Beckett,Yann Bourgeois,Garry Scarlett,Sharon Glaysher,Scott Elliott,Kelly Bicknell,Robert Impey,Allyson Lloyd,Sarah Wyllie,Ethan Butcher,Anoop Chauhan,Samuel Robson
EPI_ISL_664699	University of Exeter	COVID-19 Genomics UK (COG-UK) Consortium	Ben Temperton,Aaron Jeffries,Michelle Michelsen,Joanna Warwick-Dugdale,Audrey Farbos,Robyn Manley,Stephen Michell,Jane Masoli
EPI_ISL_664713	Virology Department, Sheffield Teaching Hospitals NHS Foundation Trust/Department of Infection, Immunity and Cardiovascular Disease, The Medical School, University of Sheffield	COVID-19 Genomics UK (COG-UK) Consortium	Thushan de Silva, Matthew Parker, Nikki Smith, Adri Angyal, Rebecca Brown, Luke Green, Rachel Tucker, Paul Parsons, Danielle Groves, Katie Johnson, Laura Carrilero, Alex Keeley, Dave Partridge, Matthew Wyles, Benjamin Lindsey, Mehmet Yavuz, Mohammad Raza, Cariad Evans
EPI_ISL_664734	University College London Hospital	COVID-19 Genomics UK (COG-UK) Consortium	Judith Heaney, Matthew Byott, Catherine Houlihan, Dan Frampton, Stuart Kirk, Moira Spyer and Eleni Nastouli
EPI_ISL_664740	University of Exeter	COVID-19 Genomics UK (COG-UK) Consortium	Ben Temperton,Aaron Jeffries,Michelle Michelsen,Joanna Warwick-Dugdale,Audrey Farbos,Robyn Manley,Stephen Michell,Jane Masoli
EPI_ISL_664746	Centre for Enzyme Innovation, University of Portsmouth / Translational Research Laboratory, Portsmouth Hospitals NHS Trust	COVID-19 Genomics UK (COG-UK) Consortium	Angela Beckett,Yann Bourgeois,Garry Scarlett,Sharon Glaysher,Scott Elliott,Kelly Bicknell,Robert Impey,Allyson Lloyd,Sarah Wyllie,Ethan Butcher,Anoop Chauhan,Samuel Robson
EPI_ISL_664755	University College London Hospital	COVID-19 Genomics UK (COG-UK) Consortium	Judith Heaney, Matthew Byott, Catherine Houlihan, Dan Frampton, Stuart Kirk, Moira Spyer and Eleni Nastouli
EPI_ISL_664840, EPI_ISL_664843, EPI_ISL_664844, EPI_ISL_664845, EPI_ISL_664847, EPI_ISL_664849	Centre for Enzyme Innovation, University of Portsmouth / Translational Research Laboratory, Portsmouth Hospitals NHS Trust	COVID-19 Genomics UK (COG-UK) Consortium	Angela Beckett,Yann Bourgeois,Garry Scarlett,Sharon Glaysher,Scott Elliott,Kelly Bicknell,Robert Impey,Allyson Lloyd,Sarah Wyllie,Ethan Butcher,Anoop Chauhan,Samuel Robson
EPI_ISL_665023, EPI_ISL_665024, EPI_ISL_665025, EPI_ISL_665026, EPI_ISL_665027, EPI_ISL_665028, EPI_ISL_665029, EPI_ISL_665030, EPI_ISL_665031, EPI_ISL_665032, EPI_ISL_665033, EPI_ISL_665034, EPI_ISL_665035, EPI_ISL_665036, EPI_ISL_665037, EPI_ISL_665038, EPI_ISL_665039, EPI_ISL_665040, EPI_ISL_665041, EPI_ISL_665042, EPI_ISL_665043, EPI_ISL_665044, EPI_ISL_665045			
see above	Virology Department, Sheffield Teaching Hospitals NHS Foundation Trust/Department of Infection, Immunity and Cardiovascular Disease, The Medical School, University of Sheffield	COVID-19 Genomics UK (COG-UK) Consortium	Thushan de Silva, Matthew Parker, Nikki Smith, Adri Angyal, Rebecca Brown, Luke Green, Rachel Tucker, Paul Parsons, Danielle Groves, Katie Johnson, Laura Carrilero, Alex Keeley, Dave Partridge, Matthew Wyles, Benjamin Lindsey, Mehmet Yavuz, Mohammad Raza, Cariad Evans
EPI_ISL_665243, EPI_ISL_665244, EPI_ISL_665245, EPI_ISL_665246, EPI_ISL_665247, EPI_ISL_665248, EPI_ISL_665249, EPI_ISL_665250	University College London Hospital	COVID-19 Genomics UK (COG-UK) Consortium	Judith Heaney, Matthew Byott, Catherine Houlihan, Dan Frampton, Stuart Kirk, Moira Spyer and Eleni Nastouli
EPI_ISL_665292	Quadram Institute Bioscience	COVID-19 Genomics UK (COG-UK) Consortium	Dave J. Baker, Gemma L. Kay, Alp Aydin, Thanh Le-Viet, Steven Rudder, Ana P. Tedim, Anastasia Kolyva, Maria Diaz, Leonardo de Oliveira Martins, Nabil-Fareed Alikhan, Lizzie Meadows, Rachael Stanley, Ngozi Elumogo, Muhammed Yasir, Nicholas M. Thomson, Alexander J Trotter, Rachel Gilroy, Samuel Bloomfield, Claire Stuart, Andrew Bell, Reenesh Prakash, Samir Dervisevic, Alison E. Mather, John Wain, Mark Webber, Andrew J. Page, Justin O'Grady
EPI_ISL_665294	Liverpool Clinical Laboratories	COVID-19 Genomics UK (COG-UK) Consortium	Sam Haldenby, Anita Lucaci, Steve Paterson, Julian Hiscox, Alistair Darby, M Almsaud, A Alrezaihi, Muhannad Alruwaili, Stuart D Armstrong, Jones Benjamin, Eleanor G Bentley, Anu Chawla, Jordan J Clark, Angela Cowell, Richard Eccles, Isabel García-Dorival, Matthew Gemmell, Alessandro Gerada, PKF Gilmore, Richard Gregory, Ximeng Han, Catherine Hartley, Margaret Hughes, Miren Iturriza-Gomara, James Johnson, L Luu, Jenifer Manson, Charlotte Nelson, Elaine O'Toole, Cassie Olateju, Rebekah Penrice-Randal , Lucille Rainbow, N.P Randle, Trevor Ian Robinson, Parul Sharma, Ghada T Shawli, James P Stewart, Neil Swainston, Ecaterina Vamos, Joanne Watts, Mark Whitehead
EPI_ISL_665315	Wales Specialist Virology Centre Sequencing lab: Pathogen Genomics Unit	COVID-19 Genomics UK (COG-UK) Consortium	Catherine Moore, Johnathan Evans, Laura Gifford, Malorie Perry, Simon Cottrell, Angela Marchbank, Alec Birchley, Alexander Adams, Amy Gaskin, Bree Gatica-Wilcox, Jason Coombes, Joel Southgate, Lauren Gilbert, Lee Graham, Nicole Pacchiarini, Sara Kumziene-Summerhayes, Sarah Taylor, Sophie Jones, Sara Rey, Matthew Bull, Joanne Watkins, Sally Corden, Tom Connor
EPI_ISL_665351, EPI_ISL_665371, EPI_ISL_665406	Quadram Institute Bioscience	COVID-19 Genomics UK (COG-UK) Consortium	Dave J. Baker, Gemma L. Kay, Alp Aydin, Thanh Le-Viet, Steven Rudder, Ana P. Tedim, Anastasia Kolyva, Maria Diaz, Leonardo de Oliveira Martins, Nabil-Fareed Alikhan, Lizzie Meadows, Rachael Stanley, Ngozi Elumogo, Muhammed Yasir, Nicholas M. Thomson, Alexander J Trotter, Rachel Gilroy, Samuel Bloomfield, Claire Stuart, Andrew Bell, Reenesh Prakash, Samir Dervisevic, Alison E. Mather, John Wain, Mark Webber, Andrew J. Page, Justin O'Grady
EPI_ISL_665414	Liverpool Clinical Laboratories	COVID-19 Genomics UK (COG-UK) Consortium	Sam Haldenby, Anita Lucaci, Steve Paterson, Julian Hiscox, Alistair Darby, M Almsaud, A Alrezaihi, Muhannad Alruwaili, Stuart D Armstrong, Jones Benjamin, Eleanor G Bentley, Anu Chawla, Jordan J Clark, Angela Cowell, Richard Eccles, Isabel García-Dorival, Matthew Gemmell, Alessandro Gerada, PKF Gilmore, Richard Gregory, Ximeng Han, Catherine Hartley, Margaret Hughes, Miren Iturriza-Gomara, James Johnson, L Luu, Jenifer Manson, Charlotte Nelson, Elaine O'Toole, Cassie Olateju, Rebekah Penrice-Randal , Lucille Rainbow, N.P Randle, Trevor Ian Robinson, Parul Sharma, Ghada T Shawli, James P Stewart, Neil Swainston, Ecaterina Vamos, Joanne Watts, Mark Whitehead
EPI_ISL_665454, EPI_ISL_665461	Quadram Institute Bioscience	COVID-19 Genomics UK (COG-UK) Consortium	Dave J. Baker, Gemma L. Kay, Alp Aydin, Thanh Le-Viet, Steven Rudder, Ana P. Tedim, Anastasia Kolyva, Maria Diaz, Leonardo de Oliveira Martins,

			Nabil-Fareed Alikhan, Lizzie Meadows, Rachael Stanley, Ngozi Elumogo, Muhammed Yasir, Nicholas M. Thomson, Alexander J Trotter, Rachel Gilroy, Samuel Bloomfield, Claire Stuart, Andrew Bell, Reenesh Prakash, Samir Dervisevic, Alison E. Mather, John Wain, Mark Webber, Andrew J. Page, Justin O'Grady
EPI_ISL_665468	Wales Specialist Virology Centre Sequencing lab: Pathogen Genomics Unit	COVID-19 Genomics UK (COG-UK) Consortium	Catherine Moore, Johnathan Evans, Laura Gifford, Malorie Perry, Simon Cottrell, Angela Marchbank, Alec Birchley, Alexander Adams, Amy Gaskin, Bree Gatica-Wilcox, Jason Coombes, Joel Southgate, Lauren Gilbert, Lee Graham, Nicole Pacchiarini, Sara Kumziene-Summerhayes, Sarah Taylor, Sophie Jones, Sara Rey, Matthew Bull, Joanne Watkins, Sally Corden, Tom Connor
EPI_ISL_665481	Quadram Institute Bioscience	COVID-19 Genomics UK (COG-UK) Consortium	Dave J. Baker, Gemma L. Kay, Alp Aydin, Thanh Le-Viet, Steven Rudder, Ana P. Tedim, Anastasia Kolyva, Maria Diaz, Leonardo de Oliveira Martins, Nabil-Fareed Alikhan, Lizzie Meadows, Rachael Stanley, Ngozi Elumogo, Muhammed Yasir, Nicholas M. Thomson, Alexander J Trotter, Rachel Gilroy, Samuel Bloomfield, Claire Stuart, Andrew Bell, Reenesh Prakash, Samir Dervisevic, Alison E. Mather, John Wain, Mark Webber, Andrew J. Page, Justin O'Grady
EPI_ISL_665504, EPI_ISL_665516, EPI_ISL_665526	Wales Specialist Virology Centre Sequencing lab: Pathogen Genomics Unit	COVID-19 Genomics UK (COG-UK) Consortium	Catherine Moore, Johnathan Evans, Laura Gifford, Malorie Perry, Simon Cottrell, Angela Marchbank, Alec Birchley, Alexander Adams, Amy Gaskin, Bree Gatica-Wilcox, Jason Coombes, Joel Southgate, Lauren Gilbert, Lee Graham, Nicole Pacchiarini, Sara Kumziene-Summerhayes, Sarah Taylor, Sophie Jones, Sara Rey, Matthew Bull, Joanne Watkins, Sally Corden, Tom Connor
EPI_ISL_665533	Quadram Institute Bioscience	COVID-19 Genomics UK (COG-UK) Consortium	Dave J. Baker, Gemma L. Kay, Alp Aydin, Thanh Le-Viet, Steven Rudder, Ana P. Tedim, Anastasia Kolyva, Maria Diaz, Leonardo de Oliveira Martins, Nabil-Fareed Alikhan, Lizzie Meadows, Rachael Stanley, Ngozi Elumogo, Muhammed Yasir, Nicholas M. Thomson, Alexander J Trotter, Rachel Gilroy, Samuel Bloomfield, Claire Stuart, Andrew Bell, Reenesh Prakash, Samir Dervisevic, Alison E. Mather, John Wain, Mark Webber, Andrew J. Page, Justin O'Grady
EPI_ISL_665552	Virology Department, Royal Infirmary of Edinburgh, NHS Lothian / School of Biological Sciences, University of Edinburgh / Institute of Genetics and Molecular Medicine, University of Edinburgh	COVID-19 Genomics UK (COG-UK) Consortium	McHugh M, Dewar R, Rooke S, Gallagher M, Balcaza C, O'Toole Á, Scher E, Hill V, McCrone JT, Colquhoun R, Yu X, Jackson B, Rambaut A, Williams TC, Templeton K
EPI_ISL_665573	Wales Specialist Virology Centre Sequencing lab: Pathogen Genomics Unit	COVID-19 Genomics UK (COG-UK) Consortium	Catherine Moore, Johnathan Evans, Laura Gifford, Malorie Perry, Simon Cottrell, Angela Marchbank, Alec Birchley, Alexander Adams, Amy Gaskin, Bree Gatica-Wilcox, Jason Coombes, Joel Southgate, Lauren Gilbert, Lee Graham, Nicole Pacchiarini, Sara Kumziene-Summerhayes, Sarah Taylor, Sophie Jones, Sara Rey, Matthew Bull, Joanne Watkins, Sally Corden, Tom Connor
EPI_ISL_665581, EPI_ISL_665583	Quadram Institute Bioscience	COVID-19 Genomics UK (COG-UK) Consortium	Dave J. Baker, Gemma L. Kay, Alp Aydin, Thanh Le-Viet, Steven Rudder, Ana P. Tedim, Anastasia Kolyva, Maria Diaz, Leonardo de Oliveira Martins, Nabil-Fareed Alikhan, Lizzie Meadows, Rachael Stanley, Ngozi Elumogo, Muhammed Yasir, Nicholas M. Thomson, Alexander J Trotter, Rachel Gilroy, Samuel Bloomfield, Claire Stuart, Andrew Bell, Reenesh Prakash, Samir Dervisevic, Alison E. Mather, John Wain, Mark Webber, Andrew J. Page, Justin O'Grady
EPI_ISL_665584	Wales Specialist Virology Centre Sequencing lab: Pathogen Genomics Unit	COVID-19 Genomics UK (COG-UK) Consortium	Catherine Moore, Johnathan Evans, Laura Gifford, Malorie Perry, Simon Cottrell, Angela Marchbank, Alec Birchley, Alexander Adams, Amy Gaskin, Bree Gatica-Wilcox, Jason Coombes, Joel Southgate, Lauren Gilbert, Lee Graham, Nicole Pacchiarini, Sara Kumziene-Summerhayes, Sarah Taylor, Sophie Jones, Sara Rey, Matthew Bull, Joanne Watkins, Sally Corden, Tom Connor
EPI_ISL_665591	Quadram Institute Bioscience	COVID-19 Genomics UK (COG-UK) Consortium	Dave J. Baker, Gemma L. Kay, Alp Aydin, Thanh Le-Viet, Steven Rudder, Ana P. Tedim, Anastasia Kolyva, Maria Diaz, Leonardo de Oliveira Martins, Nabil-Fareed Alikhan, Lizzie Meadows, Rachael Stanley, Ngozi Elumogo, Muhammed Yasir, Nicholas M. Thomson, Alexander J Trotter, Rachel Gilroy, Samuel Bloomfield, Claire Stuart, Andrew Bell, Reenesh Prakash, Samir Dervisevic, Alison E. Mather, John Wain, Mark Webber, Andrew J. Page, Justin O'Grady
EPI_ISL_665608, EPI_ISL_665660	Wales Specialist Virology Centre Sequencing lab: Pathogen Genomics Unit	COVID-19 Genomics UK (COG-UK) Consortium	Catherine Moore, Johnathan Evans, Laura Gifford, Malorie Perry, Simon Cottrell, Angela Marchbank, Alec Birchley, Alexander Adams, Amy Gaskin, Bree Gatica-Wilcox, Jason Coombes, Joel Southgate, Lauren Gilbert, Lee Graham, Nicole Pacchiarini, Sara Kumziene-Summerhayes, Sarah Taylor, Sophie Jones, Sara Rey, Matthew Bull, Joanne Watkins, Sally Corden, Tom Connor
EPI_ISL_665662	Liverpool Clinical Laboratories	COVID-19 Genomics UK (COG-UK) Consortium	Sam Haldenby, Anita Lucaci, Steve Paterson, Julian Hiscox, Alistair Darby, M Almsaud, A Alrezaihi, Muhannad Alruwaili, Stuart D Armstrong, Jones Benjamin, Eleanor G Bentley, Anu Chawla, Jordan J Clark, Angela Cowell, Richard Eccles, Isabel García-Dorival, Matthew Gemmell, Alessandro Gerada, PKF Gilmore, Richard Gregory, Ximeng Han, Catherine Hartley, Margaret Hughes, Miren Iturriza-Gomara, James Johnson, L Luu, Jenifer Manson, Charlotte Nelson, Elaine O'Toole, Cassie Olateju, Rebekah Penrice-Randal , Lucille Rainbow, N.P Randle, Trevor Ian Robinson, Parul Sharma, Ghada T Shawli, James P Stewart, Neil Swainston, Ecaterina Vamos, Joanne Watts, Mark Whitehead
EPI_ISL_665672	Quadram Institute Bioscience	COVID-19 Genomics UK (COG-UK) Consortium	Dave J. Baker, Gemma L. Kay, Alp Aydin, Thanh Le-Viet, Steven Rudder, Ana P. Tedim, Anastasia Kolyva, Maria Diaz, Leonardo de Oliveira Martins, Nabil-Fareed Alikhan, Lizzie Meadows, Rachael Stanley, Ngozi Elumogo, Muhammed Yasir, Nicholas M. Thomson, Alexander J Trotter, Rachel Gilroy, Samuel Bloomfield, Claire Stuart, Andrew Bell, Reenesh Prakash, Samir Dervisevic, Alison E. Mather, John Wain, Mark Webber, Andrew J. Page, Justin O'Grady
EPI_ISL_665677	Wales Specialist Virology Centre Sequencing lab: Pathogen Genomics Unit	COVID-19 Genomics UK (COG-UK) Consortium	Catherine Moore, Johnathan Evans, Laura Gifford, Malorie Perry, Simon Cottrell, Angela Marchbank, Alec Birchley, Alexander Adams, Amy Gaskin, Bree Gatica-Wilcox, Jason Coombes, Joel Southgate, Lauren Gilbert, Lee Graham, Nicole Pacchiarini, Sara Kumziene-Summerhayes, Sarah Taylor, Sophie Jones, Sara Rey, Matthew Bull, Joanne Watkins, Sally Corden, Tom Connor
EPI_ISL_665684	Quadram Institute Bioscience	COVID-19 Genomics UK (COG-UK) Consortium	Dave J. Baker, Gemma L. Kay, Alp Aydin, Thanh Le-Viet, Steven Rudder, Ana P. Tedim, Anastasia Kolyva, Maria Diaz, Leonardo de Oliveira Martins, Nabil-Fareed Alikhan, Lizzie Meadows, Rachael Stanley, Ngozi Elumogo, Muhammed Yasir, Nicholas M. Thomson, Alexander J Trotter, Rachel Gilroy, Samuel Bloomfield, Claire Stuart, Andrew Bell, Reenesh Prakash, Samir Dervisevic, Alison E. Mather, John Wain, Mark Webber, Andrew J. Page, Justin O'Grady
EPI_ISL_665703	University College London, Great Ormond Street Hospital for Children NHS Foundation Trust, Imperial College Healthcare NHS Trust	COVID-19 Genomics UK (COG-UK) Consortium	Sergi Castellano, Rachel Williams, Mark Kristiansen, Paola Resende Silva, Sunando Roy, Tony Brooks, Helena Tutill, Paola Niola, Patricia Dyal, Charlotte Williams, Leysa Forrest, Yasmin Panchbhaya, Jacqueline Findlay, Samuel Weeks, Julianne Brown, Kathryn Harris, Paul Randell, James Price, Alison Holmes, Judith Breuer
EPI_ISL_665704, EPI_ISL_665707	Liverpool Clinical Laboratories	COVID-19 Genomics UK (COG-UK) Consortium	Sam Haldenby, Anita Lucaci, Steve Paterson, Julian Hiscox, Alistair Darby, M Almsaud, A Alrezaihi, Muhannad Alruwaili, Stuart D Armstrong, Jones Benjamin, Eleanor G Bentley, Anu Chawla, Jordan J Clark, Angela Cowell, Richard Eccles, Isabel García-Dorival, Matthew Gemmell, Alessandro Gerada, PKF Gilmore, Richard Gregory, Ximeng Han, Catherine Hartley, Margaret Hughes, Miren Iturriza-Gomara, James Johnson, L Luu, Jenifer Manson, Charlotte Nelson, Elaine O'Toole, Cassie Olateju, Rebekah Penrice-Randal , Lucille Rainbow, N.P Randle, Trevor Ian Robinson, Parul Sharma, Ghada T Shawli, James P Stewart, Neil Swainston, Ecaterina Vamos, Joanne Watts, Mark Whitehead
EPI_ISL_665713	Wales Specialist Virology Centre Sequencing lab: Pathogen Genomics Unit	COVID-19 Genomics UK (COG-UK) Consortium	Catherine Moore, Johnathan Evans, Laura Gifford, Malorie Perry, Simon Cottrell, Angela Marchbank, Alec Birchley, Alexander Adams, Amy Gaskin, Bree Gatica-Wilcox, Jason Coombes, Joel Southgate, Lauren Gilbert, Lee Graham, Nicole Pacchiarini, Sara Kumziene-Summerhayes, Sarah Taylor, Sophie Jones, Sara Rey, Matthew Bull, Joanne Watkins, Sally Corden, Tom Connor
EPI_ISL_665720	University College London, Great Ormond Street Hospital for Children NHS Foundation Trust, Imperial College Healthcare NHS Trust	COVID-19 Genomics UK (COG-UK) Consortium	Sergi Castellano, Rachel Williams, Mark Kristiansen, Paola Resende Silva, Sunando Roy, Tony Brooks, Helena Tutill, Paola Niola, Patricia Dyal, Charlotte Williams, Leysa Forrest, Yasmin Panchbhaya, Jacqueline Findlay, Samuel Weeks, Julianne Brown, Kathryn Harris, Paul Randell, James Price, Alison Holmes, Judith Breuer
EPI_ISL_665743	Wales Specialist Virology Centre Sequencing lab: Pathogen Genomics Unit	COVID-19 Genomics UK (COG-UK) Consortium	Catherine Moore, Johnathan Evans, Laura Gifford, Malorie Perry, Simon Cottrell, Angela Marchbank, Alec Birchley, Alexander Adams, Amy Gaskin, Bree Gatica-Wilcox, Jason Coombes, Joel Southgate, Lauren Gilbert, Lee Graham, Nicole Pacchiarini, Sara Kumziene-Summerhayes, Sarah Taylor, Sophie Jones, Sara Rey, Matthew Bull, Joanne Watkins, Sally Corden, Tom Connor
EPI_ISL_665747	Quadram Institute Bioscience	COVID-19 Genomics UK (COG-UK) Consortium	Dave J. Baker, Gemma L. Kay, Alp Aydin, Thanh Le-Viet, Steven Rudder, Ana P. Tedim, Anastasia Kolyva, Maria Diaz, Leonardo de Oliveira Martins, Nabil-Fareed Alikhan, Lizzie Meadows, Rachael Stanley, Ngozi Elumogo, Muhammed Yasir, Nicholas M. Thomson, Alexander J Trotter, Rachel Gilroy, Samuel Bloomfield, Claire Stuart, Andrew Bell, Reenesh Prakash, Samir Dervisevic, Alison E. Mather, John Wain, Mark Webber, Andrew J. Page, Justin O'Grady
EPI_ISL_665756, EPI_ISL_665764, EPI_ISL_665771	Wales Specialist Virology Centre Sequencing lab: Pathogen Genomics Unit	COVID-19 Genomics UK (COG-UK) Consortium	Catherine Moore, Johnathan Evans, Laura Gifford, Malorie Perry, Simon Cottrell, Angela Marchbank, Alec Birchley, Alexander Adams, Amy Gaskin, Bree Gatica-Wilcox, Jason Coombes, Joel Southgate, Lauren Gilbert, Lee Graham, Nicole Pacchiarini, Sara Kumziene-Summerhayes, Sarah Taylor, Sophie

			Jones, Sara Rey, Matthew Bull, Joanne Watkins, Sally Corden, Tom Connor
EPI_ISL_665788	Quadram Institute Bioscience	COVID-19 Genomics UK (COG-UK) Consortium	Dave J. Baker, Gemma L. Kay, Alp Aydin, Thanh Le-Viet, Steven Rudder, Ana P. Tedim, Anastasia Kolyva, Maria Diaz, Leonardo de Oliveira Martins, Nabil-Fareed Alikhan, Lizzie Meadows, Rachael Stanley, Ngozi Elumogo, Muhammed Yasir, Nicholas M. Thomson, Alexander J Trotter, Rachel Gilroy, Samuel Bloomfield, Claire Stuart, Andrew Bell, Reenesh Prakash, Samir Dervisevic, Alison E. Mather, John Wain, Mark Webber, Andrew J. Page, Justin O'Grady
EPI_ISL_665794, EPI_ISL_665800	Wales Specialist Virology Centre Sequencing lab: Pathogen Genomics Unit	COVID-19 Genomics UK (COG-UK) Consortium	Catherine Moore, Johnathan Evans, Laura Gifford, Malorie Perry, Simon Cottrell, Angela Marchbank, Alec Birchley, Alexander Adams, Amy Gaskin, Bree Gatica-Wilcox, Jason Coombes, Joel Southgate, Lauren Gilbert, Lee Graham, Nicole Pacchiarini, Sara Kumziene-Summerhayes, Sarah Taylor, Sophie Jones, Sara Rey, Matthew Bull, Joanne Watkins, Sally Corden, Tom Connor
EPI_ISL_665878, EPI_ISL_665884, EPI_ISL_665885, EPI_ISL_665888, EPI_ISL_665890, EPI_ISL_665893, EPI_ISL_665896	Quadram Institute Bioscience	COVID-19 Genomics UK (COG-UK) Consortium	Dave J. Baker, Gemma L. Kay, Alp Aydin, Thanh Le-Viet, Steven Rudder, Ana P. Tedim, Anastasia Kolyva, Maria Diaz, Leonardo de Oliveira Martins, Nabil-Fareed Alikhan, Lizzie Meadows, Rachael Stanley, Ngozi Elumogo, Muhammed Yasir, Nicholas M. Thomson, Alexander J Trotter, Rachel Gilroy, Samuel Bloomfield, Claire Stuart, Andrew Bell, Reenesh Prakash, Samir Dervisevic, Alison E. Mather, John Wain, Mark Webber, Andrew J. Page, Justin O'Grady
EPI_ISL_666022	Liverpool Clinical Laboratories	COVID-19 Genomics UK (COG-UK) Consortium	Sam Haldenby, Anita Lucaci, Steve Paterson, Julian Hiscox, Alistair Darby, M Almsaud, A Alrezaihi, Muhannad Alruwaili, Stuart D Armstrong, Jones Benjamin, Eleanor G Bentley, Anu Chawla, Jordan J Clark, Angela Cowell, Richard Eccles, Isabel García-Dorival, Matthew Gemmell, Alessandro Gerada, PKF Gilmore, Richard Gregory, Ximeng Han, Catherine Hartley, Margaret Hughes, Miren Iturriza-Gomara, James Johnson, L Luu, Jenifer Manson, Charlotte Nelson, Elaine O'Toole, Cassie Olateju, Rebekah Penrice-Randal , Lucille Rainbow, N.P Randle, Trevor Ian Robinson, Parul Sharma, Ghada T Shawli, James P Stewart, Neil Swainston, Ecaterina Vamos, Joanne Watts, Mark Whitehead
EPI_ISL_666061, EPI_ISL_666062, EPI_ISL_666063, EPI_ISL_666066, EPI_ISL_666068, EPI_ISL_666069	Virology Department, Royal Infirmary of Edinburgh, NHS Lothian / School of Biological Sciences, University of Edinburgh / Institute of Genetics and Molecular Medicine, University of Edinburgh	COVID-19 Genomics UK (COG-UK) Consortium	McHugh M, Dewar R, Rooke S, Gallagher M, Balcaza C, O'Toole Á, Scher E, Hill V, McCrone JT, Colquhoun R, Yu X, Jackson B, Rambaut A, Williams TC, Templeton K
EPI_ISL_666111, EPI_ISL_666117, EPI_ISL_666118, EPI_ISL_666119, EPI_ISL_666123, EPI_ISL_666124, EPI_ISL_666128, EPI_ISL_666132, EPI_ISL_666134, EPI_ISL_666141, EPI_ISL_666148, EPI_ISL_666160, EPI_ISL_666162, EPI_ISL_666164, EPI_ISL_666168, EPI_ISL_666171, EPI_ISL_666174, EPI_ISL_666179, EPI_ISL_666180, EPI_ISL_666184, EPI_ISL_666193, EPI_ISL_666194, EPI_ISL_666199, EPI_ISL_666208, EPI_ISL_666209, EPI_ISL_666211, EPI_ISL_666212, EPI_ISL_666230, EPI_ISL_666234, EPI_ISL_666238, EPI_ISL_666247, EPI_ISL_666250, EPI_ISL_666254, EPI_ISL_666266, EPI_ISL_666268, EPI_ISL_666270, EPI_ISL_666285, EPI_ISL_666287, EPI_ISL_666301, EPI_ISL_666310, EPI_ISL_666312, EPI_ISL_666320, EPI_ISL_666328, EPI_ISL_666330, EPI_ISL_666332, EPI_ISL_666340, EPI_ISL_666345, EPI_ISL_666347, EPI_ISL_666349, EPI_ISL_666356, EPI_ISL_666365, EPI_ISL_666371, EPI_ISL_666376, EPI_ISL_666377, EPI_ISL_666383, EPI_ISL_666396, EPI_ISL_666397, EPI_ISL_666399, EPI_ISL_666405, EPI_ISL_666406, EPI_ISL_666407, EPI_ISL_666409, EPI_ISL_666411, EPI_ISL_666417, EPI_ISL_666424, EPI_ISL_666425, EPI_ISL_666437, EPI_ISL_666443, EPI_ISL_666451, EPI_ISL_666452, EPI_ISL_666454, EPI_ISL_666457, EPI_ISL_666458, EPI_ISL_666460, EPI_ISL_666461, EPI_ISL_666465, EPI_ISL_666467, EPI_ISL_666474, EPI_ISL_666476, EPI_ISL_666493, EPI_ISL_666498, EPI_ISL_666499, EPI_ISL_666504, EPI_ISL_666514, EPI_ISL_666516, EPI_ISL_666518, EPI_ISL_666520, EPI_ISL_666532, EPI_ISL_666536, EPI_ISL_666537, EPI_ISL_666539, EPI_ISL_666547, EPI_ISL_666548, EPI_ISL_666551, EPI_ISL_666554, EPI_ISL_666555, EPI_ISL_666580, EPI_ISL_666586, EPI_ISL_666589			
see above	Wales Specialist Virology Centre Sequencing lab: Pathogen Genomics Unit	COVID-19 Genomics UK (COG-UK) Consortium	Catherine Moore, Johnathan Evans, Laura Gifford, Malorie Perry, Simon Cottrell, Angela Marchbank, Alec Birchley, Alexander Adams, Amy Gaskin, Bree Gatica-Wilcox, Jason Coombes, Joel Southgate, Lauren Gilbert, Lee Graham, Nicole Pacchiarini, Sara Kumziene-Summerhayes, Sarah Taylor, Sophie Jones, Sara Rey, Matthew Bull, Joanne Watkins, Sally Corden, Tom Connor
EPI_ISL_666729, EPI_ISL_666732, EPI_ISL_666733, EPI_ISL_666734, EPI_ISL_666735, EPI_ISL_666736, EPI_ISL_666741	Respiratory Virus Unit, Microbiology Services Colindale, Public Health England	COVID-19 Genomics UK (COG-UK) Consortium	PHE Covid Sequencing Team
EPI_ISL_666792	Maryland Public Health Laboratory	Maryland Public Health Laboratory	Maryland Department of Health Laboratories Administration
EPI_ISL_666894, EPI_ISL_666899, EPI_ISL_666907, EPI_ISL_666909, EPI_ISL_666910, EPI_ISL_666919, EPI_ISL_666924, EPI_ISL_666928, EPI_ISL_666932, EPI_ISL_666933, EPI_ISL_666936, EPI_ISL_666941, EPI_ISL_666945, EPI_ISL_666949, EPI_ISL_666954, EPI_ISL_666957, EPI_ISL_666963			
see above	Michigan Department of Health and Human Services, Bureau of Laboratories	Michigan Department of Health and Human Services, Bureau of Laboratories	Blankenship HM, Riner D, Soehnlen MK
EPI_ISL_666972, EPI_ISL_666973, EPI_ISL_667042, EPI_ISL_667043, EPI_ISL_667060, EPI_ISL_667061, EPI_ISL_667076, EPI_ISL_667078	San Diego County Public Health Laboratory	Andersen lab at Scripps Research	SEARCH Alliance San Diego with Tracy Basler, Jovan Shephard, Brett Austin
EPI_ISL_667518, EPI_ISL_667529, EPI_ISL_667530, EPI_ISL_667531, EPI_ISL_667532, EPI_ISL_667533, EPI_ISL_667534, EPI_ISL_667535, EPI_ISL_667539, EPI_ISL_667540, EPI_ISL_667550			
see above	OHSU Lab Services Molecular Microbiology Lab	Oregon SARS-CoV-2 Genome Sequencing Center	Brendan L. O'Connell, Ruth V. Nichols, Sally Grindstaff, Alec J. Hirsch, Donna Hansel, Guang Fan, Daniel N. Streblow, William B. Messer, Andrew C. Adey, Benjamin N. Bimber, Brian J. O'Roak
EPI_ISL_667781, EPI_ISL_667806	South Eastern Area Laboratory Services (SEALS)	NSW Health Pathology - Institute of Clinical Pathology and Medical Research; Westmead Hospital; University of Sydney	CIDM-PH et al.
EPI_ISL_668447, EPI_ISL_668448, EPI_ISL_668453, EPI_ISL_668454, EPI_ISL_668455	Centre for Dengue Research, Department of Immunology and Molecular Medicine	Centre for Dengue Research, Department of Immunology and Molecular Medicine	Chandima Jeewandara, Deshni Jayathilaka, Dinuka Ariyaratne, Diyanath Ranasinghe, Laksiri Gomes, Gathsaurie Neelika Malavige
EPI_ISL_669458, EPI_ISL_669460, EPI_ISL_669461, EPI_ISL_669462, EPI_ISL_669464, EPI_ISL_669466, EPI_ISL_669467, EPI_ISL_669468, EPI_ISL_669469, EPI_ISL_669470, EPI_ISL_669471, EPI_ISL_669472, EPI_ISL_669473, EPI_ISL_669474, EPI_ISL_669475, EPI_ISL_669476, EPI_ISL_669477, EPI_ISL_669478, EPI_ISL_669479, EPI_ISL_669480, EPI_ISL_669481, EPI_ISL_669482, EPI_ISL_669483, EPI_ISL_669484, EPI_ISL_669485, EPI_ISL_669487, EPI_ISL_669488, EPI_ISL_669489, EPI_ISL_669490, EPI_ISL_669491, EPI_ISL_669492, EPI_ISL_669493, EPI_ISL_669494, EPI_ISL_669496, EPI_ISL_669497, EPI_ISL_669498, EPI_ISL_669499, EPI_ISL_669500, EPI_ISL_669501, EPI_ISL_669502, EPI_ISL_669505, EPI_ISL_669506, EPI_ISL_669507, EPI_ISL_669508, EPI_ISL_669509, EPI_ISL_669510, EPI_ISL_669511, EPI_ISL_669512, EPI_ISL_669513, EPI_ISL_669514, EPI_ISL_669515, EPI_ISL_669516, EPI_ISL_669517, EPI_ISL_669518, EPI_ISL_669519, EPI_ISL_669520, EPI_ISL_669522, EPI_ISL_669523, EPI_ISL_669524, EPI_ISL_669526, EPI_ISL_669527, EPI_ISL_669528, EPI_ISL_669529, EPI_ISL_669530, EPI_ISL_669531, EPI_ISL_669532, EPI_ISL_669533, EPI_ISL_669534, EPI_ISL_669535, EPI_ISL_669536, EPI_ISL_669538, EPI_ISL_669539, EPI_ISL_669540, EPI_ISL_669541, EPI_ISL_669542, EPI_ISL_669543, EPI_ISL_669544, EPI_ISL_669545, EPI_ISL_669546, EPI_ISL_669547, EPI_ISL_669549, EPI_ISL_669550, EPI_ISL_669551, EPI_ISL_669552, EPI_ISL_669553, EPI_ISL_669554, EPI_ISL_669555, EPI_ISL_669556, EPI_ISL_669557, EPI_ISL_669558, EPI_ISL_669559, EPI_ISL_669560, EPI_ISL_669561, EPI_ISL_669562, EPI_ISL_669563, EPI_ISL_669565, EPI_ISL_669566, EPI_ISL_669567, EPI_ISL_669568, EPI_ISL_669569, EPI_ISL_669570, EPI_ISL_669571, EPI_ISL_669572, EPI_ISL_669573, EPI_ISL_669574, EPI_ISL_669575, EPI_ISL_669576, EPI_ISL_669577, EPI_ISL_669578, EPI_ISL_669579, EPI_ISL_669580, EPI_ISL_669581, EPI_ISL_669582, EPI_ISL_669584, EPI_ISL_669585, EPI_ISL_669586, EPI_ISL_669587, EPI_ISL_669588, EPI_ISL_669589, EPI_ISL_669590, EPI_ISL_669591, EPI_ISL_669592, EPI_ISL_669593, EPI_ISL_669594, EPI_ISL_669595, EPI_ISL_669596, EPI_ISL_669597, EPI_ISL_669598, EPI_ISL_669599, EPI_ISL_669600, EPI_ISL_669601, EPI_ISL_669602, EPI_ISL_669603, EPI_ISL_669604, EPI_ISL_669605, EPI_ISL_669606, EPI_ISL_669607, EPI_ISL_669608, EPI_ISL_669609, EPI_ISL_669610, EPI_ISL_669611, EPI_ISL_669614, EPI_ISL_669615, EPI_ISL_669616, EPI_ISL_669617, EPI_ISL_669618, EPI_ISL_669619, EPI_ISL_669620, EPI_ISL_669621, EPI_ISL_669622, EPI_ISL_669623, EPI_ISL_669624, EPI_ISL_669625, EPI_ISL_669626, EPI_ISL_669627, EPI_ISL_669628, EPI_ISL_669629, EPI_ISL_669630, EPI_ISL_669631, EPI_ISL_669632, EPI_ISL_669633, EPI_ISL_669634, EPI_ISL_669635, EPI_ISL_669644, EPI_ISL_669645, EPI_ISL_669646, EPI_ISL_669647, EPI_ISL_669648, EPI_ISL_669649, EPI_ISL_669650, EPI_ISL_669651, EPI_ISL_669652, EPI_ISL_669653, EPI_ISL_669654, EPI_ISL_669655, EPI_ISL_669656, EPI_ISL_669657, EPI_ISL_669658, EPI_ISL_669659, EPI_ISL_669660, EPI_ISL_669661, EPI_ISL_669662, EPI_ISL_669663, EPI_ISL_669664, EPI_ISL_669665, EPI_ISL_669666, EPI_ISL_669667, EPI_ISL_669668, EPI_ISL_669669, EPI_ISL_669670, EPI_ISL_669671, EPI_ISL_669672, EPI_ISL_669673, EPI_ISL_669674, EPI_ISL_669675, EPI_ISL_669676, EPI_ISL_669677, EPI_ISL_669678, EPI_ISL_669679, EPI_ISL_669680, EPI_ISL_669681, EPI_ISL_669682, EPI_ISL_669683, EPI_ISL_669684, EPI_ISL_669685, EPI_ISL_669686, EPI_ISL_669687, EPI_ISL_669688, EPI_ISL_669689, EPI_ISL_669690, EPI_ISL_669691, EPI_ISL_669692, EPI_ISL_669693, EPI_ISL_669694, EPI_ISL_669695, EPI_ISL_669696, EPI_ISL_669697, EPI_ISL_669698, EPI_ISL_669699, EPI_ISL_669700, EPI_ISL_669701, EPI_ISL_669702, EPI_ISL_669703, EPI_ISL_669704, EPI_ISL_669705, EPI_ISL_669706, EPI_ISL_669707, EPI_ISL_669708, EPI_ISL_669709, EPI_ISL_669710, EPI_ISL_669711, EPI_ISL_669712, EPI_ISL_669713, EPI_ISL_669714, EPI_ISL_669715, EPI_ISL_669716, EPI_ISL_669717, EPI_ISL_669718, EPI_ISL_669719, EPI_ISL_669720, EPI_ISL_669721, EPI_ISL_669722, EPI_ISL_669723, EPI_ISL_669724, EPI_ISL_669725, EPI_ISL_669726, EPI_ISL_669727, EPI_ISL_669728, EPI_ISL_669729, EPI_ISL_669730, EPI_ISL_669732, EPI_ISL_669736, EPI_ISL_669737, EPI_ISL_669738, EPI_ISL_669739, EPI_ISL_669740, EPI_ISL_669741, EPI_ISL_669742, EPI_ISL_669743, EPI_ISL_669744, EPI_ISL_669745, EPI_ISL_669746, EPI_ISL_669747, EPI_ISL_669748, EPI_ISL_669749, EPI_ISL_669750, EPI_ISL_669751, EPI_ISL_669752, EPI_ISL_669753, EPI_ISL_669754, EPI_ISL_669755, EPI_ISL_669756, EPI_ISL_669758, EPI_ISL_669759, EPI_ISL_669761, EPI_ISL_669762, EPI_ISL_669763, EPI_ISL_669764, EPI_ISL_669765, EPI_ISL_669766, EPI_ISL_669767, EPI_ISL_669768, EPI_ISL_669769, EPI_ISL_669770, EPI_ISL_669771, EPI_ISL_669772, EPI_ISL_669775, EPI_ISL_669776, EPI_ISL_669777, EPI_ISL_669778, EPI_ISL_669779, EPI_ISL_669780, EPI_ISL_669781, EPI_ISL_669782, EPI_ISL_669783, EPI_ISL_669784, EPI_ISL_669788, EPI_ISL_669789, EPI_ISL_669790, EPI_ISL_669791, EPI_ISL_669792, EPI_ISL_669793, EPI_ISL_669794, EPI_ISL_669795, EPI_ISL_669796, EPI_ISL_669797, EPI_ISL_669798, EPI_ISL_669799, EPI_ISL_669800, EPI_ISL_669801, EPI_ISL_669802, EPI_ISL_669803, EPI_ISL_669804, EPI_ISL_669805, EPI_ISL_669806, EPI_ISL_669807, EPI_ISL_669808, EPI_ISL_669809, EPI_ISL_669810, EPI_ISL_669811, EPI_ISL_669812, EPI_ISL_669813, EPI_ISL_669814, EPI_ISL_669815, EPI_ISL_669816, EPI_ISL_669818, EPI_ISL_669819, EPI_ISL_669820, EPI_ISL_669821, EPI_ISL_669822, EPI_ISL_669823, EPI_ISL_669824, EPI_ISL_669825, EPI_ISL_669826, EPI_ISL_669827, EPI_ISL_669828, EPI_ISL_669829, EPI_ISL_669830, EPI_ISL_669831, EPI_ISL_669832, EPI_ISL_669882, EPI_ISL_669883, EPI_ISL_669884, EPI_ISL_669885, EPI_ISL_669886, EPI_ISL_669887, EPI_ISL_669888, EPI_ISL_669889, EPI_ISL_669890, EPI_ISL_669891, EPI_ISL_669892, EPI_ISL_669893, EPI_ISL_669894, EPI_ISL_669895, EPI_ISL_669896, EPI_ISL_669897, EPI_ISL_669898, EPI_ISL_669899, EPI_ISL_669900, EPI_ISL_669901, EPI_ISL_669902, EPI_ISL_669903, EPI_ISL_669904, EPI_ISL_669905, EPI_ISL_669906, EPI_ISL_669907, EPI_ISL_669908, EPI_ISL_669909, EPI_ISL_669910, EPI_ISL_669911, EPI_ISL_669912, EPI_ISL_669913, EPI_ISL_669914, EPI_ISL_669915, EPI_ISL_669916, EPI_ISL_669917, EPI_ISL_669918, EPI_ISL_669919, EPI_ISL_669920, EPI_ISL_669921, EPI_ISL_669922, EPI_ISL_669923, EPI_ISL_669924, EPI_ISL_669925, EPI_ISL_669926, EPI_ISL_669927, EPI_ISL_669928, EPI_ISL_669929, EPI_ISL_669930, EPI_ISL_669931, EPI_ISL_669932, EPI_ISL_669933, EPI_ISL_669934, EPI_ISL_669935, EPI_ISL_669936, EPI_ISL_669937, EPI_ISL_669938, EPI_ISL_669939, EPI_ISL_669940, EPI_ISL_669941, EPI_ISL_669942, EPI_ISL_669943, EPI_ISL_669944, EPI_ISL_669945, EPI_ISL_669946, EPI_ISL_669947, EPI_ISL_669948, EPI_ISL_669949, EPI_ISL_669950, EPI_ISL_669951, EPI_ISL_669952, EPI_ISL_669953, EPI_ISL_669954, EPI_ISL_669955, EPI_ISL_669956, EPI_ISL_669957, EPI_ISL_669958, EPI_ISL_669959, EPI_ISL_669960, EPI_ISL_669961, EPI_ISL_669962, EPI_ISL_669963, EPI_ISL_669964, EPI_ISL_669965, EPI_ISL_669966, EPI_ISL_669967, EPI_ISL_669968, EPI_ISL_669969, EPI_ISL_669970, EPI_ISL_669971, EPI_ISL_669972, EPI_ISL_669973, EPI_ISL_669974, EPI_ISL_669975, EPI_ISL_669976, EPI_ISL_669977, EPI_ISL_669978, EPI_ISL_669979, EPI_ISL_669980, EPI_ISL_669981, EPI_ISL_669982, EPI_ISL_669983, EPI_ISL_669984, EPI_ISL_669985, EPI_ISL_669986, EPI_ISL_669987, EPI_ISL_669988, EPI_ISL_669989, EPI_ISL_669990, EPI_ISL_669991, EPI_ISL_669992, EPI_ISL_669993, EPI_ISL_669994, EPI_ISL_669995, EPI_ISL_669996, EPI_ISL_669997, EPI_ISL_669998, EPI_ISL_669999, EPI_ISL_670000, EPI_ISL_670001, EPI_ISL_670002, EPI_ISL_670003, EPI_ISL_670004, EPI_ISL_670005, EPI_ISL_670006, EPI_ISL_670007, EPI_ISL_670008, EPI_ISL_670009, EPI_ISL_670010, EPI_ISL_670011, EPI_ISL_670012, EPI_ISL_670013, EPI_ISL_670014, EPI_ISL_670015, EPI_ISL_670016, EPI_ISL_670017, EPI_ISL_670018, EPI_ISL_670019, EPI_ISL_670020, EPI_ISL_670021, EPI_ISL_670022, EPI_ISL_670023, EPI_ISL_670024, EPI_ISL_670025, EPI_ISL_670026, EPI_ISL_670027, EPI_ISL_670028, EPI_ISL_670029, EPI_ISL_670030, EPI_ISL_670031, EPI_ISL_670032, EPI_ISL_670033, EPI_ISL_670034, EPI_ISL_670035, EPI_ISL_670036, EPI_ISL_670037, EPI_ISL_670038, EPI_ISL_670039, EPI_ISL_670040, EPI_ISL_670041, EPI_ISL_670042, EPI_ISL_670043, EPI_ISL_670044, EPI_ISL_670045, EPI_ISL_670046, EPI_ISL_670047, EPI_ISL_670048, EPI_ISL_670049, EPI_ISL_670050, EPI_ISL_670051, EPI_ISL_670052, EPI_ISL_670053, EPI_ISL_670054, EPI_ISL_670055, EPI_ISL_670056, EPI_ISL_670057, EPI_ISL_670058, EPI_ISL_670059, EPI_ISL_670060, EPI_ISL_670061, EPI_ISL_670062, EPI_ISL_670063,			

EPI_ISL_670064, EPI_ISL_670065, EPI_ISL_670066, EPI_ISL_670067, EPI_ISL_670068, EPI_ISL_670069, EPI_ISL_670070, EPI_ISL_670071, EPI_ISL_670072, EPI_ISL_670073, EPI_ISL_670074, EPI_ISL_670075, EPI_ISL_670076, EPI_ISL_670077, EPI_ISL_670078, EPI_ISL_670079, EPI_ISL_670080, EPI_ISL_670081, EPI_ISL_670082, EPI_ISL_670083, EPI_ISL_670084, EPI_ISL_670085, EPI_ISL_670086, EPI_ISL_670087, EPI_ISL_670088, EPI_ISL_670089, EPI_ISL_670090, EPI_ISL_670091, EPI_ISL_670092, EPI_ISL_670093, EPI_ISL_670094, EPI_ISL_670095, EPI_ISL_670096, EPI_ISL_670097, EPI_ISL_670098, EPI_ISL_670099, EPI_ISL_670100, EPI_ISL_670101, EPI_ISL_670102, EPI_ISL_670103, EPI_ISL_670104, EPI_ISL_670105, EPI_ISL_670106, EPI_ISL_670107, EPI_ISL_670108, EPI_ISL_670109, EPI_ISL_670110, EPI_ISL_670111, EPI_ISL_670112, EPI_ISL_670113, EPI_ISL_670114, EPI_ISL_670115, EPI_ISL_670116, EPI_ISL_670117, EPI_ISL_670118, EPI_ISL_670119, EPI_ISL_670120, EPI_ISL_670121, EPI_ISL_670122, EPI_ISL_670123, EPI_ISL_670124, EPI_ISL_670125, EPI_ISL_670126, EPI_ISL_670127, EPI_ISL_670128, EPI_ISL_670129, EPI_ISL_670130, EPI_ISL_670131, EPI_ISL_670132, EPI_ISL_670133, EPI_ISL_670134, EPI_ISL_670135, EPI_ISL_670136, EPI_ISL_670137, EPI_ISL_670138, EPI_ISL_670139, EPI_ISL_670140, EPI_ISL_670141, EPI_ISL_670142, EPI_ISL_670143, EPI_ISL_670144, EPI_ISL_670145, EPI_ISL_670146, EPI_ISL_670147, EPI_ISL_670148, EPI_ISL_670149, EPI_ISL_670150, EPI_ISL_670151, EPI_ISL_670152, EPI_ISL_670153, EPI_ISL_670154, EPI_ISL_670155, EPI_ISL_670156, EPI_ISL_670157, EPI_ISL_670158, EPI_ISL_670159, EPI_ISL_670160, EPI_ISL_670161, EPI_ISL_670162, EPI_ISL_670163, EPI_ISL_670164, EPI_ISL_670165, EPI_ISL_670166, EPI_ISL_670167, EPI_ISL_670168, EPI_ISL_670169, EPI_ISL_670170, EPI_ISL_670171, EPI_ISL_670172, EPI_ISL_670173, EPI_ISL_670174, EPI_ISL_670175, EPI_ISL_670176, EPI_ISL_670177, EPI_ISL_670178, EPI_ISL_670179, EPI_ISL_670180, EPI_ISL_670181, EPI_ISL_670182, EPI_ISL_670183, EPI_ISL_670184, EPI_ISL_670185, EPI_ISL_670186, EPI_ISL_670187, EPI_ISL_670188, EPI_ISL_670189, EPI_ISL_670190, EPI_ISL_670191, EPI_ISL_670192, EPI_ISL_670193, EPI_ISL_670194, EPI_ISL_670195, EPI_ISL_670196, EPI_ISL_670197, EPI_ISL_670198, EPI_ISL_670199, EPI_ISL_670200, EPI_ISL_670201, EPI_ISL_670202, EPI_ISL_670203, EPI_ISL_670204, EPI_ISL_670205, EPI_ISL_670206, EPI_ISL_670207, EPI_ISL_670208, EPI_ISL_670209, EPI_ISL_670210, EPI_ISL_670211, EPI_ISL_670212, EPI_ISL_670213, EPI_ISL_670214, EPI_ISL_670215, EPI_ISL_670216, EPI_ISL_670217, EPI_ISL_670218, EPI_ISL_670219, EPI_ISL_670220, EPI_ISL_670221, EPI_ISL_670222, EPI_ISL_670223, EPI_ISL_670224, EPI_ISL_670225, EPI_ISL_670226, EPI_ISL_670227, EPI_ISL_670228, EPI_ISL_670229, EPI_ISL_670230, EPI_ISL_670231, EPI_ISL_670232, EPI_ISL_670233, EPI_ISL_670234, EPI_ISL_670235, EPI_ISL_670236, EPI_ISL_670237, EPI_ISL_670238, EPI_ISL_670239, EPI_ISL_670240, EPI_ISL_670241, EPI_ISL_670242, EPI_ISL_670243, EPI_ISL_670244, EPI_ISL_670245, EPI_ISL_670246, EPI_ISL_670247, EPI_ISL_670248, EPI_ISL_670249, EPI_ISL_670250, EPI_ISL_670251, EPI_ISL_670252, EPI_ISL_670253, EPI_ISL_670254, EPI_ISL_670255, EPI_ISL_670256, EPI_ISL_670257, EPI_ISL_670258, EPI_ISL_670259, EPI_ISL_670260, EPI_ISL_670261, EPI_ISL_670262, EPI_ISL_670263, EPI_ISL_670264, EPI_ISL_670265, EPI_ISL_670266, EPI_ISL_670267, EPI_ISL_670268, EPI_ISL_670269, EPI_ISL_670270, EPI_ISL_670271, EPI_ISL_670272, EPI_ISL_670273, EPI_ISL_670274, EPI_ISL_670275, EPI_ISL_670276, EPI_ISL_670277, EPI_ISL_670278, EPI_ISL_670279, EPI_ISL_670280, EPI_ISL_670281, EPI_ISL_670282, EPI_ISL_670283, EPI_ISL_670284, EPI_ISL_670285, EPI_ISL_670286, EPI_ISL_670287, EPI_ISL_670288, EPI_ISL_670289, EPI_ISL_670290, EPI_ISL_670291, EPI_ISL_670292, EPI_ISL_670293, EPI_ISL_670294, EPI_ISL_670295, EPI_ISL_670296, EPI_ISL_670297, EPI_ISL_670298, EPI_ISL_670299, EPI_ISL_670300, EPI_ISL_670301, EPI_ISL_670302, EPI_ISL_670303, EPI_ISL_670304, EPI_ISL_670305, EPI_ISL_670306, EPI_ISL_670307, EPI_ISL_670308, EPI_ISL_670309, EPI_ISL_670310, EPI_ISL_670311, EPI_ISL_670312, EPI_ISL_670313, EPI_ISL_670314, EPI_ISL_670315, EPI_ISL_670316, EPI_ISL_670317, EPI_ISL_670318, EPI_ISL_670319, EPI_ISL_670320, EPI_ISL_670321, EPI_ISL_670322, EPI_ISL_670323, EPI_ISL_670324, EPI_ISL_670325, EPI_ISL_670326, EPI_ISL_670327, EPI_ISL_670328, EPI_ISL_670329, EPI_ISL_670330, EPI_ISL_670331, EPI_ISL_670332, EPI_ISL_670333, EPI_ISL_670334, EPI_ISL_670335, EPI_ISL_670336, EPI_ISL_670337, EPI_ISL_670338, EPI_ISL_670339, EPI_ISL_670340, EPI_ISL_670341, EPI_ISL_670342, EPI_ISL_670343, EPI_ISL_670344, EPI_ISL_670345, EPI_ISL_670346, EPI_ISL_670347, EPI_ISL_670348, EPI_ISL_670349, EPI_ISL_670350, EPI_ISL_670351, EPI_ISL_670352, EPI_ISL_670353, EPI_ISL_670354, EPI_ISL_670355, EPI_ISL_670356, EPI_ISL_670357, EPI_ISL_670358, EPI_ISL_670359, EPI_ISL_670360, EPI_ISL_670361, EPI_ISL_670362, EPI_ISL_670363, EPI_ISL_670364, EPI_ISL_670365, EPI_ISL_670366, EPI_ISL_670367, EPI_ISL_670368, EPI_ISL_670369, EPI_ISL_670370, EPI_ISL_670371, EPI_ISL_670372, EPI_ISL_670373, EPI_ISL_670374, EPI_ISL_670375, EPI_ISL_670376, EPI_ISL_670377, EPI_ISL_670378, EPI_ISL_670379, EPI_ISL_670380, EPI_ISL_670381, EPI_ISL_670382, EPI_ISL_670383, EPI_ISL_670384, EPI_ISL_670385, EPI_ISL_670386, EPI_ISL_670387, EPI_ISL_670388, EPI_ISL_670389, EPI_ISL_670390, EPI_ISL_670391, EPI_ISL_670392, EPI_ISL_670393, EPI_ISL_670394, EPI_ISL_670395, EPI_ISL_670396, EPI_ISL_670397, EPI_ISL_670398, EPI_ISL_670399, EPI_ISL_670400, EPI_ISL_670401, EPI_ISL_670402, EPI_ISL_670403, EPI_ISL_670404, EPI_ISL_670405, EPI_ISL_670406, EPI_ISL_670407, EPI_ISL_670408, EPI_ISL_670409, EPI_ISL_670410, EPI_ISL_670411, EPI_ISL_670412, EPI_ISL_670413, EPI_ISL_670414, EPI_ISL_670415, EPI_ISL_670416, EPI_ISL_670417, EPI_ISL_670418, EPI_ISL_670419, EPI_ISL_670420, EPI_ISL_670421, EPI_ISL_670422, EPI_ISL_670423, EPI_ISL_670424, EPI_ISL_670425, EPI_ISL_670426, EPI_ISL_670427, EPI_ISL_670428, EPI_ISL_670429, EPI_ISL_670430, EPI_ISL_670431, EPI_ISL_670432, EPI_ISL_670433, EPI_ISL_670434, EPI_ISL_670435, EPI_ISL_670436, EPI_ISL_670437, EPI_ISL_670438, EPI_ISL_670439, EPI_ISL_670440, EPI_ISL_670441, EPI_ISL_670442, EPI_ISL_670443, EPI_ISL_670444, EPI_ISL_670445, EPI_ISL_670446, EPI_ISL_670447, EPI_ISL_670448, EPI_ISL_670449, EPI_ISL_670450, EPI_ISL_670451, EPI_ISL_670452, EPI_ISL_670453, EPI_ISL_670454, EPI_ISL_670455, EPI_ISL_670456, EPI_ISL_670457, EPI_ISL_670459, EPI_ISL_670462, EPI_ISL_670463, EPI_ISL_670464, EPI_ISL_670465, EPI_ISL_670466, EPI_ISL_670467, EPI_ISL_670468, EPI_ISL_670469, EPI_ISL_670470, EPI_ISL_670474, EPI_ISL_670475, EPI_ISL_670476, EPI_ISL_670479, EPI_ISL_670480, EPI_ISL_670481, EPI_ISL_670483, EPI_ISL_670486, EPI_ISL_670487, EPI_ISL_670488, EPI_ISL_670492, EPI_ISL_670497, EPI_ISL_670499, EPI_ISL_670503, EPI_ISL_670504, EPI_ISL_670505, EPI_ISL_670506, EPI_ISL_670507, EPI_ISL_670508, EPI_ISL_670509, EPI_ISL_670519, EPI_ISL_670520, EPI_ISL_670521, EPI_ISL_670523, EPI_ISL_670529, EPI_ISL_670532, EPI_ISL_670533, EPI_ISL_670539, EPI_ISL_670542, EPI_ISL_670543, EPI_ISL_670548, EPI_ISL_670550, EPI_ISL_670551, EPI_ISL_670552, EPI_ISL_670553, EPI_ISL_670554, EPI_ISL_670555, EPI_ISL_670558, EPI_ISL_670559, EPI_ISL_670560, EPI_ISL_670561, EPI_ISL_670562, EPI_ISL_670563, EPI_ISL_670564, EPI_ISL_670565, EPI_ISL_670566, EPI_ISL_670567, EPI_ISL_670568, EPI_ISL_670576, EPI_ISL_670577, EPI_ISL_670578, EPI_ISL_670579, EPI_ISL_670580, EPI_ISL_670581, EPI_ISL_670582, EPI_ISL_670583, EPI_ISL_670584, EPI_ISL_670585, EPI_ISL_670586, EPI_ISL_670587, EPI_ISL_670588, EPI_ISL_670589, EPI_ISL_670590, EPI_ISL_670591, EPI_ISL_670592, EPI_ISL_670593, EPI_ISL_670594, EPI_ISL_670595, EPI_ISL_670596, EPI_ISL_670597, EPI_ISL_670598, EPI_ISL_670599, EPI_ISL_670600, EPI_ISL_670601, EPI_ISL_670602, EPI_ISL_670603, EPI_ISL_670604, EPI_ISL_670605, EPI_ISL_670606, EPI_ISL_670607, EPI_ISL_670608, EPI_ISL_670609, EPI_ISL_670610, EPI_ISL_670611, EPI_ISL_670612, EPI_ISL_670613, EPI_ISL_670614, EPI_ISL_670615, EPI_ISL_670616, EPI_ISL_670617, EPI_ISL_670618, EPI_ISL_670619, EPI_ISL_670620, EPI_ISL_670621, EPI_ISL_670622, EPI_ISL_670623, EPI_ISL_670624, EPI_ISL_670625, EPI_ISL_670666, EPI_ISL_670667, EPI_ISL_670668, EPI_ISL_670669, EPI_ISL_670676, EPI_ISL_670677, EPI_ISL_670678, EPI_ISL_670679, EPI_ISL_670680, EPI_ISL_670686, EPI_ISL_670687, EPI_ISL_670690, EPI_ISL_670691, EPI_ISL_670692, EPI_ISL_670693, EPI_ISL_670702, EPI_ISL_670703, EPI_ISL_670704, EPI_ISL_670705, EPI_ISL_670706, EPI_ISL_670707, EPI_ISL_670708, EPI_ISL_670709, EPI_ISL_670710, EPI_ISL_670711, EPI_ISL_670712, EPI_ISL_670713, EPI_ISL_670731, EPI_ISL_670732, EPI_ISL_670733, EPI_ISL_670734, EPI_ISL_670735, EPI_ISL_670736, EPI_ISL_670737, EPI_ISL_670738, EPI_ISL_670739, EPI_ISL_670740, EPI_ISL_670746, EPI_ISL_670747, EPI_ISL_670748, EPI_ISL_670749, EPI_ISL_670750, EPI_ISL_670751, EPI_ISL_670752, EPI_ISL_670753, EPI_ISL_670768, EPI_ISL_670772, EPI_ISL_670773, EPI_ISL_670774, EPI_ISL_670775, EPI_ISL_670776, EPI_ISL_670777, EPI_ISL_670778, EPI_ISL_670779, EPI_ISL_670780, EPI_ISL_670781, EPI_ISL_670782, EPI_ISL_670783, EPI_ISL_670784, EPI_ISL_670785, EPI_ISL_670786, EPI_ISL_670787, EPI_ISL_670788, EPI_ISL_670789, EPI_ISL_670790, EPI_ISL_670791, EPI_ISL_670792, EPI_ISL_670793, EPI_ISL_670794, EPI_ISL_670795, EPI_ISL_670796, EPI_ISL_670797, EPI_ISL_670798, EPI_ISL_670799, EPI_ISL_670800, EPI_ISL_670801, EPI_ISL_670802, EPI_ISL_670803, EPI_ISL_670804, EPI_ISL_670805, EPI_ISL_670806, EPI_ISL_670807, EPI_ISL_670808, EPI_ISL_670809, EPI_ISL_670810, EPI_ISL_670811, EPI_ISL_670812, EPI_ISL_670813, EPI_ISL_670814, EPI_ISL_670815, EPI_ISL_670816, EPI_ISL_670817, EPI_ISL_670818, EPI_ISL_670819, EPI_ISL_670820, EPI_ISL_670821, EPI_ISL_670822, EPI_ISL_670823, EPI_ISL_670824, EPI_ISL_670825, EPI_ISL_670826, EPI_ISL_670827, EPI_ISL_670828, EPI_ISL_670829, EPI_ISL_670830, EPI_ISL_670831, EPI_ISL_670832, EPI_ISL_670833, EPI_ISL_670834, EPI_ISL_670835, EPI_ISL_670836, EPI_ISL_670837, EPI_ISL_670838, EPI_ISL_670839, EPI_ISL_670840, EPI_ISL_670841, EPI_ISL_670842, EPI_ISL_670843, EPI_ISL_670844, EPI_ISL_670845, EPI_ISL_670846, EPI_ISL_670847, EPI_ISL_670848, EPI_ISL_670849, EPI_ISL_670850, EPI_ISL_670851, EPI_ISL_670852, EPI_ISL_670853, EPI_ISL_670854, EPI_ISL_670855, EPI_ISL_670856, EPI_ISL_670857, EPI_ISL_670858, EPI_ISL_670859, EPI_ISL_670860, EPI_ISL_670861, EPI_ISL_670862, EPI_ISL_670863, EPI_ISL_670864, EPI_ISL_670865, EPI_ISL_670866, EPI_ISL_670867, EPI_ISL_670868, EPI_ISL_670869, EPI_ISL_670870, EPI_ISL_670871, EPI_ISL_670872, EPI_ISL_670873, EPI_ISL_670874, EPI_ISL_670875, EPI_ISL_670876, EPI_ISL_670877, EPI_ISL_670878, EPI_ISL_670879, EPI_ISL_670880, EPI_ISL_670881, EPI_ISL_670882, EPI_ISL_670883, EPI_ISL_670884, EPI_ISL_670885, EPI_ISL_670886, EPI_ISL_670887, EPI_ISL_670888, EPI_ISL_670889, EPI_ISL_670890, EPI_ISL_670891, EPI_ISL_670892, EPI_ISL_670893, EPI_ISL_670894, EPI_ISL_670895, EPI_ISL_670896, EPI_ISL_670897, EPI_ISL_670898, EPI_ISL_670899, EPI_ISL_670900, EPI_ISL_670901, EPI_ISL_670902, EPI_ISL_670903, EPI_ISL_670904, EPI_ISL_670905, EPI_ISL_670906, EPI_ISL_670907, EPI_ISL_670908, EPI_ISL_670909, EPI_ISL_670910, EPI_ISL_670911, EPI_ISL_670912, EPI_ISL_670913, EPI_ISL_670914, EPI_ISL_670915, EPI_ISL_670916, EPI_ISL_670917, EPI_ISL_670918, EPI_ISL_670919, EPI_ISL_670920, EPI_ISL_670921, EPI_ISL_670922, EPI_ISL_670923, EPI_ISL_670924, EPI_ISL_670925, EPI_ISL_670926, EPI_ISL_670927, EPI_ISL_670928, EPI_ISL_670929, EPI_ISL_670930, EPI_ISL_670931, EPI_ISL_670932, EPI_ISL_670933, EPI_ISL_670934, EPI_ISL_670935, EPI_ISL_670936, EPI_ISL_670937, EPI_ISL_670938, EPI_ISL_670939, EPI_ISL_670940, EPI_ISL_670941, EPI_ISL_670942, EPI_ISL_670943, EPI_ISL_670944, EPI_ISL_670945, EPI_ISL_670946, EPI_ISL_670947, EPI_ISL_670948, EPI_ISL_670949, EPI_ISL_670950, EPI_ISL_670951, EPI_ISL_670952, EPI_ISL_670953, EPI_ISL_670954, EPI_ISL_670955, EPI_ISL_670956, EPI_ISL_670957, EPI_ISL_670958, EPI_ISL_670959, EPI_ISL_670960, EPI_ISL_670961, EPI_ISL_670962, EPI_ISL_670963, EPI_ISL_670964, EPI_ISL_670965, EPI_ISL_670966, EPI_ISL_670967, EPI_ISL_670968, EPI_ISL_670969, EPI_ISL_670970, EPI_ISL_670971, EPI_ISL_670972, EPI_ISL_670973, EPI_ISL_670974, EPI_ISL_670975, EPI_ISL_670976, EPI_ISL_670977, EPI_ISL_670978, EPI_ISL_670979, EPI_ISL_670980, EPI_ISL_670981, EPI_ISL_670982, EPI_ISL_670983, EPI_ISL_670984, EPI_ISL_670985, EPI_ISL_670986, EPI_ISL_670987, EPI_ISL_670988, EPI_ISL_670989, EPI_ISL_670990, EPI_ISL_670991, EPI_ISL_670992, EPI_ISL_670993, EPI_ISL_670994, EPI_ISL_670995, EPI_ISL_670996, EPI_ISL_670997, EPI_ISL_670998, EPI_ISL_670999, EPI_ISL_671000, EPI_ISL_671001, EPI_ISL_671002, EPI_ISL_671003, EPI_ISL_671004, EPI_ISL_671005, EPI_ISL_671006, EPI_ISL_671007, EPI_ISL_671008, EPI_ISL_671009, EPI_ISL_671010, EPI_ISL_671011, EPI_ISL_671012, EPI_ISL_671013, EPI_ISL_671014, EPI_ISL_671015, EPI_ISL_671016, EPI_ISL_671017, EPI_ISL_671018, EPI_ISL_671019, EPI_ISL_671020, EPI_ISL_671021, EPI_ISL_671022, EPI_ISL_671023, EPI_ISL_671024, EPI_ISL_671025, EPI_ISL_671026, EPI_ISL_671027, EPI_ISL_671028, EPI_ISL_671029, EPI_ISL_671030, EPI_ISL_671031, EPI_ISL_671032, EPI_ISL_671033, EPI_ISL_671034, EPI_ISL_671035, EPI_ISL_671036, EPI_ISL_671037, EPI_ISL_671038, EPI_ISL_671039			
see above	Department of Virus and Microbiological Special Diagnostics, Statens Serum Institut, Copenhagen, Denmark	Albertsen Lab, Department of Chemistry and Bioscience, Aalborg University, Denmark	Danish Covid-19 Genome Consortium
EPI_ISL_672048, EPI_ISL_672067	Madera County Department of Public Health	Chan-Zuckerberg Biohub	CZB Cliahub Consortium
EPI_ISL_672434, EPI_ISL_672435, EPI_ISL_672436, EPI_ISL_672437, EPI_ISL_672438	Santa Clara County Public Health Laboratory	Chan-Zuckerberg Biohub	CZB Cliahub Consortium
EPI_ISL_672481, EPI_ISL_672482, EPI_ISL_672483, EPI_ISL_672484, EPI_ISL_672485, EPI_ISL_672486, EPI_ISL_672487	Orange County Public Health Lab	Chan-Zuckerberg Biohub	CZB Cliahub Consortium
EPI_ISL_672504, EPI_ISL_672505	Madera County Department of Public Health	Chan-Zuckerberg Biohub	CZB Cliahub Consortium
EPI_ISL_676395, EPI_ISL_676396, EPI_ISL_676397, EPI_ISL_676398, EPI_ISL_676401, EPI_ISL_676402, EPI_ISL_676403, EPI_ISL_676404, EPI_ISL_676429, EPI_ISL_676430, EPI_ISL_676431, EPI_ISL_676432, EPI_ISL_676433, EPI_ISL_676435, EPI_ISL_676436, EPI_ISL_676437, EPI_ISL_676438, EPI_ISL_676439, EPI_ISL_676440			
see above	Lighthouse Lab in Glasgow	Wellcome Sanger Institute for the COVID-19 Genomics UK (COG-UK) Consortium	Harper VanSteenhouse, Yumi Kasai, David Gray, Carol Clugston, Anna Dominiczak and Alex Alderton, Roberto Amato, Sonia Goncalves, Ewan Harrison, David K. Jackson, Ian Johnston, Dominic Kwiatkowski, Cordelia Langford, John Sillitoe on behalf of the Wellcome Sanger Institute COVID-19 Surveillance Team
EPI_ISL_676499	Klinisk Mikrobiologi	The Public Health Agency of Sweden	Department of Microbiology, The Public Health Agency of Sweden
EPI_ISL_676539, EPI_ISL_676540	Laboratoriemedicin, Klinisk mikrobiologi	The Public Health Agency of Sweden	Department of Microbiology, The Public Health Agency of Sweden
EPI_ISL_677498, EPI_ISL_677499, EPI_ISL_677500, EPI_ISL_677632, EPI_ISL_677633	University of Wisconsin-Madison AIDS Vaccine Research Laboratories	University of Wisconsin-Madison AIDS Vaccine Research Laboratories	Gage Moreno, Katarina Braun, et al. AIDS Vaccine Research Laboratories
EPI_ISL_678376	Area of Virology, Serology and Virology Division (SAViD), New South Wales Health Pathology Randwick	Virology Research Laboratory; Area of Virology, Serology and Virology Division (SAViD), New South Wales Health Pathology Randwick	Foster, C.; Au, J.; Ruiz Silva, M.; Deveson, I.; Bull, R.; Van Hal, S.; Rawlinson, W.
EPI_ISL_678599	NHLS-IALCH	KRISP, KZN Research Innovation and Sequencing Platform	Giandhari J, Pillay S, Lessells R, ChimukangaraB, Mdlalose K, York D, Khan S, Tegally H, Wilkinson E, de Oliveira T
EPI_ISL_678644, EPI_ISL_678645, EPI_ISL_678646, EPI_ISL_678647, EPI_ISL_678648, EPI_ISL_678649, EPI_ISL_678651, EPI_ISL_678652, EPI_ISL_678653, EPI_ISL_678654, EPI_ISL_678655, EPI_ISL_678656, EPI_ISL_678657, EPI_ISL_678659, EPI_ISL_678661, EPI_ISL_678663, EPI_ISL_678664, EPI_ISL_678665, EPI_ISL_678667, EPI_ISL_678669, EPI_ISL_678671, EPI_ISL_678676, EPI_ISL_678680			
see above	Respiratory Virus Unit, Microbiology Services Colindale, Public Health England	COVID-19 Genomics UK (COG-UK) Consortium	PHE Covid Sequencing Team
EPI_ISL_678831	Centre for Enzyme Innovation, University of Portsmouth / Translational Research Laboratory, Portsmouth Hospitals NHS Trust	COVID-19 Genomics UK (COG-UK) Consortium	Angela Beckett,Yann Bourgeois,Garry Scarlett,Sharon Glaysher,Scott Elliott,Kelly Bicknell,Robert Impey,Allyson Lloyd,Sarah Wyllie,Ethan Butcher,Anoop Chauhan,Samuel Robson
EPI_ISL_679140	Department of Pathology, University of Cambridge	COVID-19 Genomics UK (COG-UK) Consortium	Aminu S. Jahun, Yasmin Chaudhry, Grant Hall, Iliana Georgana, Myra Hosmillo, Martin D. Curran, Malte Pinckert, Surendra Parmar, Ian Goodfellow

EPI_ISL_679450, EPI_ISL_679458, EPI_ISL_679459, EPI_ISL_679460, EPI_ISL_679461, EPI_ISL_679462, EPI_ISL_679463, EPI_ISL_679464, EPI_ISL_679465, EPI_ISL_679467, EPI_ISL_679468, EPI_ISL_679469, EPI_ISL_679470, EPI_ISL_679471, EPI_ISL_679472, EPI_ISL_679473, EPI_ISL_679477			
see above	University College London, Great Ormond Street Hospital for Children NHS Foundation Trust, Imperial College Healthcare NHS Trust	COVID-19 Genomics UK (COG-UK) Consortium	Sergi Castellano, Rachel Williams, Mark Kristiansen, Paola Resende Silva, Sunando Roy, Tony Brooks, Helena Tutill, Paola Niola, Patricia Dyal, Charlotte Williams, Leysa Forrest, Yasmin Panchbhaya, Jacqueline Findlay, Samuel Weeks, Julianne Brown, Kathryn Harris, Paul Randell, James Price, Alison Holmes, Judith Breuer
EPI_ISL_679629, EPI_ISL_679630, EPI_ISL_679632, EPI_ISL_679633, EPI_ISL_679642, EPI_ISL_679643, EPI_ISL_679644, EPI_ISL_679645, EPI_ISL_679648, EPI_ISL_679651, EPI_ISL_679652, EPI_ISL_679653, EPI_ISL_679655, EPI_ISL_679658, EPI_ISL_679659, EPI_ISL_679666, EPI_ISL_679669, EPI_ISL_679671, EPI_ISL_679673, EPI_ISL_679678, EPI_ISL_679679, EPI_ISL_679680, EPI_ISL_679682, EPI_ISL_679683, EPI_ISL_679684, EPI_ISL_679685, EPI_ISL_679688, EPI_ISL_679690, EPI_ISL_679692, EPI_ISL_679693, EPI_ISL_679694, EPI_ISL_679695, EPI_ISL_679696, EPI_ISL_679697, EPI_ISL_679698, EPI_ISL_679699, EPI_ISL_679700, EPI_ISL_679702, EPI_ISL_679703, EPI_ISL_679704, EPI_ISL_679705, EPI_ISL_679706, EPI_ISL_679707, EPI_ISL_679709, EPI_ISL_679712, EPI_ISL_679714, EPI_ISL_679716, EPI_ISL_679717, EPI_ISL_679718, EPI_ISL_679719, EPI_ISL_679720, EPI_ISL_679721, EPI_ISL_679723, EPI_ISL_679766, EPI_ISL_679767, EPI_ISL_679770, EPI_ISL_679773, EPI_ISL_679774, EPI_ISL_679777, EPI_ISL_679783, EPI_ISL_679784, EPI_ISL_679785, EPI_ISL_679787, EPI_ISL_679788, EPI_ISL_679806, EPI_ISL_679810, EPI_ISL_679811, EPI_ISL_679812, EPI_ISL_679814, EPI_ISL_679816, EPI_ISL_679818, EPI_ISL_679819			
see above	Oxford Viromics, NDM, University of Oxford; Oxford University Hospitals; Basingstoke and North Hampshire Hospital	COVID-19 Genomics UK (COG-UK) Consortium	Tanya Golubchik, David Bonsall, George Macintyre, Amy Trebes, Mariateresa de Cesare, Catrin Moore, Alex Mobbs, Anita Justice, Robert Shaw, Monique Andersson, Timothy Peto, Emma Wise, Nathan Moore, Jessica Lynch, Nick Cortes, Matilde Mori, Stephen Kidd, David Buck, John Todd, Christophe Fraser
EPI_ISL_679905	Virology Department, Sheffield Teaching Hospitals NHS Foundation Trust/Department of Infection, Immunity and Cardiovascular Disease, The Medical School, University of Sheffield	COVID-19 Genomics UK (COG-UK) Consortium	Thushan de Silva, Matthew Parker, Nikki Smith, Adri Angyal, Rebecca Brown, Luke Green, Rachel Tucker, Paul Parsons, Danielle Groves, Katie Johnson, Laura Carrilero, Alex Keeley, Dave Partridge, Matthew Wyles, Benjamin Lindsey, Mehmet Yavuz, Mohammad Raza, Cariad Evans
EPI_ISL_679928, EPI_ISL_679929, EPI_ISL_679930	Queens Medical Centre, Clinical Microbiology Department / DeepSeq Nottingham	COVID-19 Genomics UK (COG-UK) Consortium	Gemma Clark, Wendy Smith, Manjinder Khakh, Vicki M Fleming, Michelle M Lister, Hannah Howson-Wells, Jonathan Ball, Patrick McClure, Joseph Chappell, Theocharis Tsoleridis, Nadine Holmes, Matthew Carlisle, Christopher Moore, Fei Sang, Johnny Debebe, Victoria Wright, Matthew Loose
EPI_ISL_680049, EPI_ISL_680055, EPI_ISL_680059, EPI_ISL_680068, EPI_ISL_680069, EPI_ISL_680070, EPI_ISL_680073, EPI_ISL_680074, EPI_ISL_680075, EPI_ISL_680076, EPI_ISL_680077			
see above	Department of Pathology, University of Cambridge	COVID-19 Genomics UK (COG-UK) Consortium	Aminu S. Jahun, Yasmin Chaudhry, Grant Hall, Iliana Georgana, Myra Hosmillo, Martin D. Curran, Malte Pinckert, Surendra Parmar, Ian Goodfellow
EPI_ISL_680131	Virology Department, Sheffield Teaching Hospitals NHS Foundation Trust/Department of Infection, Immunity and Cardiovascular Disease, The Medical School, University of Sheffield	COVID-19 Genomics UK (COG-UK) Consortium	Thushan de Silva, Matthew Parker, Nikki Smith, Adri Angyal, Rebecca Brown, Luke Green, Rachel Tucker, Paul Parsons, Danielle Groves, Katie Johnson, Laura Carrilero, Alex Keeley, Dave Partridge, Matthew Wyles, Benjamin Lindsey, Mehmet Yavuz, Mohammad Raza, Cariad Evans
EPI_ISL_680544, EPI_ISL_680545	Virology Department, Royal Infirmary of Edinburgh, NHS Lothian / School of Biological Sciences, University of Edinburgh / Institute of Genetics and Molecular Medicine, University of Edinburgh	COVID-19 Genomics UK (COG-UK) Consortium	McHugh M, Dewar R, Rooke S, Gallagher M, Balcaza C, O'Toole Á, Scher E, Hill V, McCrone JT, Colquhoun R, Yu X, Jackson B, Rambaut A, Williams TC, Templeton K
EPI_ISL_680571, EPI_ISL_680572, EPI_ISL_680573	Department of Pathology, University of Cambridge	COVID-19 Genomics UK (COG-UK) Consortium	Aminu S. Jahun, Yasmin Chaudhry, Grant Hall, Iliana Georgana, Myra Hosmillo, Martin D. Curran, Malte Pinckert, Surendra Parmar, Ian Goodfellow
EPI_ISL_680585, EPI_ISL_680590, EPI_ISL_680685, EPI_ISL_680732, EPI_ISL_680733, EPI_ISL_680734, EPI_ISL_680735, EPI_ISL_680736, EPI_ISL_680737, EPI_ISL_680738, EPI_ISL_680740, EPI_ISL_680961, EPI_ISL_680962, EPI_ISL_680963, EPI_ISL_680964, EPI_ISL_680965, EPI_ISL_680966, EPI_ISL_680967, EPI_ISL_681028, EPI_ISL_681029, EPI_ISL_681030, EPI_ISL_681031, EPI_ISL_681032, EPI_ISL_681033, EPI_ISL_681034			
see above	Wales Specialist Virology Centre Sequencing lab: Pathogen Genomics Unit	COVID-19 Genomics UK (COG-UK) Consortium	Catherine Moore, Johnathan Evans, Laura Gifford, Malorie Perry, Simon Cottrell, Angela Marchbank, Alec Birchley, Alexander Adams, Amy Gaskin, Bree Gatica-Wilcox, Jason Coombes, Joel Southgate, Lauren Gilbert, Lee Graham, Nicole Pacchiarini, Sara Kumziene-Summerhayes, Sarah Taylor, Sophie Jones, Sara Rey, Matthew Bull, Joanne Watkins, Sally Corden, Tom Connor
EPI_ISL_681260	Department of Pathology, University of Cambridge	COVID-19 Genomics UK (COG-UK) Consortium	Aminu S. Jahun, Yasmin Chaudhry, Grant Hall, Iliana Georgana, Myra Hosmillo, Martin D. Curran, Malte Pinckert, Surendra Parmar, Ian Goodfellow
EPI_ISL_682105, EPI_ISL_682108, EPI_ISL_682111, EPI_ISL_682124, EPI_ISL_682125, EPI_ISL_682129, EPI_ISL_682133, EPI_ISL_682161, EPI_ISL_682162, EPI_ISL_682163, EPI_ISL_682164, EPI_ISL_682165, EPI_ISL_682166, EPI_ISL_682167, EPI_ISL_682168, EPI_ISL_682169, EPI_ISL_682170, EPI_ISL_682171, EPI_ISL_682172, EPI_ISL_682173, EPI_ISL_682174, EPI_ISL_682175, EPI_ISL_682176, EPI_ISL_682177, EPI_ISL_682178, EPI_ISL_682179, EPI_ISL_682180, EPI_ISL_682181, EPI_ISL_682182, EPI_ISL_682183, EPI_ISL_682184, EPI_ISL_682185, EPI_ISL_682186, EPI_ISL_682187, EPI_ISL_682188, EPI_ISL_682189, EPI_ISL_682190, EPI_ISL_682191, EPI_ISL_682192, EPI_ISL_682193, EPI_ISL_682194, EPI_ISL_682195, EPI_ISL_682196, EPI_ISL_682197, EPI_ISL_682198, EPI_ISL_682200, EPI_ISL_682201, EPI_ISL_682202, EPI_ISL_682203, EPI_ISL_682204, EPI_ISL_682205, EPI_ISL_682206, EPI_ISL_682207, EPI_ISL_682208, EPI_ISL_682209, EPI_ISL_682210, EPI_ISL_682211, EPI_ISL_682212, EPI_ISL_682213, EPI_ISL_682214, EPI_ISL_682215, EPI_ISL_682216, EPI_ISL_682217, EPI_ISL_682218, EPI_ISL_682219, EPI_ISL_682220, EPI_ISL_682221, EPI_ISL_682222, EPI_ISL_682223, EPI_ISL_682224, EPI_ISL_682225, EPI_ISL_682226, EPI_ISL_682227, EPI_ISL_682228, EPI_ISL_682229, EPI_ISL_682230, EPI_ISL_682231, EPI_ISL_682232, EPI_ISL_682233, EPI_ISL_682234			
see above	University of Michigan Clinical Microbiology Laboratory	Lauring Lab, University of Michigan, Department of Microbiology and Immunology	Valesano
EPI_ISL_682967, EPI_ISL_682968, EPI_ISL_682969, EPI_ISL_682970, EPI_ISL_682971, EPI_ISL_682972, EPI_ISL_682973, EPI_ISL_682974, EPI_ISL_682975, EPI_ISL_682976, EPI_ISL_682977, EPI_ISL_682978, EPI_ISL_682979, EPI_ISL_682980, EPI_ISL_682981, EPI_ISL_682982, EPI_ISL_683038, EPI_ISL_683039, EPI_ISL_683040, EPI_ISL_683041, EPI_ISL_683042, EPI_ISL_683043, EPI_ISL_683044, EPI_ISL_683045, EPI_ISL_683046, EPI_ISL_683047, EPI_ISL_683048, EPI_ISL_683049, EPI_ISL_683050, EPI_ISL_683051, EPI_ISL_683052, EPI_ISL_683053, EPI_ISL_683054, EPI_ISL_683055, EPI_ISL_683056, EPI_ISL_683057, EPI_ISL_683058, EPI_ISL_683059, EPI_ISL_683060, EPI_ISL_683061, EPI_ISL_683062, EPI_ISL_683063, EPI_ISL_683064, EPI_ISL_683065, EPI_ISL_683066, EPI_ISL_683067, EPI_ISL_683068, EPI_ISL_683069, EPI_ISL_683070, EPI_ISL_683071, EPI_ISL_683072, EPI_ISL_683073, EPI_ISL_683074, EPI_ISL_683075, EPI_ISL_683076, EPI_ISL_683077, EPI_ISL_683078, EPI_ISL_683079, EPI_ISL_683080, EPI_ISL_683081, EPI_ISL_683082, EPI_ISL_683083, EPI_ISL_683084, EPI_ISL_683085, EPI_ISL_683086			
see above	Department of Virus and Microbiological Special Diagnostics, Statens Serum Institut, Copenhagen, Denmark	Albertsen Lab, Department of Chemistry and Bioscience, Aalborg University, Denmark	Danish Covid-19 Genome Consortium
EPI_ISL_683334, EPI_ISL_683402	CNR Virus des Infections Respiratoires - France SUD	CNR Virus des Infections Respiratoires - France SUD	Antonin Bal, Gregory Destras, Gwendolyne Burfin, Quentin Semanas, Martine Valette, Bruno Lina, Laurence Josset
EPI_ISL_683611, EPI_ISL_683612, EPI_ISL_683613, EPI_ISL_683614, EPI_ISL_683615, EPI_ISL_683616, EPI_ISL_683617, EPI_ISL_683618, EPI_ISL_683647, EPI_ISL_683651, EPI_ISL_683656			
see above	Servicio de Microbiología, Laboratori Clínic Metropolitana Nord. Hospital Universitari Germans Trias i Pujol. Institut d'Investigació en Ciències de la Salut Germans Trias i Pujol (IGTP)	SeqCOVID-SPAIN consortium/IBV(CSIC)	Elisa Martró, Antoni E. Bordoy, Anna Not, Adrián Antuori, Anabel Fernández, Nona Romaní, Verónica Saludes, Cristina Casañ and SeqCOVID-SPAIN consortium
EPI_ISL_683698, EPI_ISL_683699, EPI_ISL_683700, EPI_ISL_683701, EPI_ISL_683709, EPI_ISL_683711	Essentia Health-St. Mary's Medical Center	Minnesota Department of Health, Public Health Laboratory	Alexandra Lorentz, Jacob Garfin, Matt Plumb, and Xiong Wang
EPI_ISL_683735, EPI_ISL_683736, EPI_ISL_683737, EPI_ISL_683738, EPI_ISL_683739, EPI_ISL_683740, EPI_ISL_683741, EPI_ISL_683742, EPI_ISL_683743	Minnesota Department of Health, Public Health Laboratory	Minnesota Department of Health, Public Health Laboratory	Alexandra Lorentz, Jacob Garfin, Matt Plumb, and Xiong Wang
EPI_ISL_683773	DOHMH Corona	New York City Public Health Laboratory	Jade Wang, et al.
EPI_ISL_683774	DOHMH PHL	New York City Public Health Laboratory	Jade Wang, et al.
EPI_ISL_683775, EPI_ISL_683776	DOHMH Jamaica	New York City Public Health Laboratory	Jade Wang, et al.
EPI_ISL_683777	DOHMH Corona	New York City Public Health Laboratory	Jade Wang, et al.
EPI_ISL_683778	DOHMH Morrisania	New York City Public Health Laboratory	Jade Wang, et al.
EPI_ISL_683859	DOHMH Central Harlem	New York City Public Health Laboratory	Jade Wang, et al.
EPI_ISL_686541, EPI_ISL_686542, EPI_ISL_686543	Respiratory Virus Unit, Microbiology Services Colindale, Public Health England	COVID-19 Genomics UK (COG-UK) Consortium	PHE Covid Sequencing Team
EPI_ISL_692802, EPI_ISL_692803, EPI_ISL_692804, EPI_ISL_692805, EPI_ISL_692808	Massachusetts State Public Health Laboratory	Massachusetts State Public Health Laboratory	Andrew Lang, Timelia Fink, Glen Gallagher, Sandra Smole

EPI_ISL_693661, EPI_ISL_693665, EPI_ISL_693666, EPI_ISL_693669, EPI_ISL_693672, EPI_ISL_693675, EPI_ISL_693677, EPI_ISL_693678, EPI_ISL_693679, EPI_ISL_693682, EPI_ISL_693684, EPI_ISL_693685, EPI_ISL_693686			
see above	The National Institute of Public Health	State Veterinary Institute Prague	Nagy,A;Jirincova,H;Trnka,D;Vecerova,J
EPI_ISL_693767, EPI_ISL_693769	General practitioner	National Reference Center for Viruses of Respiratory Infections, Institut Pasteur, Paris	Marion Barbet, Sylvie Behillil, Méline Bizard, Angela Brisebarre, Camille Capel, Etienne Simon-Lorière, Vincent Enouf, Maud Vanpeene, Sylvie van der Werf
EPI_ISL_693775, EPI_ISL_693776, EPI_ISL_693777, EPI_ISL_693778, EPI_ISL_693779, EPI_ISL_693780, EPI_ISL_693781, EPI_ISL_693782, EPI_ISL_693783, EPI_ISL_693784, EPI_ISL_693785, EPI_ISL_693786, EPI_ISL_693787, EPI_ISL_693788, EPI_ISL_693789, EPI_ISL_693790, EPI_ISL_693791, EPI_ISL_693792, EPI_ISL_693793, EPI_ISL_693794, EPI_ISL_693795, EPI_ISL_693796, EPI_ISL_693797, EPI_ISL_693798, EPI_ISL_693799, EPI_ISL_693800, EPI_ISL_693801, EPI_ISL_693802, EPI_ISL_693803, EPI_ISL_693804, EPI_ISL_693805, EPI_ISL_693806, EPI_ISL_693807, EPI_ISL_693808, EPI_ISL_693809, EPI_ISL_693810, EPI_ISL_693811, EPI_ISL_693812, EPI_ISL_693813, EPI_ISL_693814, EPI_ISL_693815, EPI_ISL_693816, EPI_ISL_693817, EPI_ISL_693818, EPI_ISL_693819, EPI_ISL_693820, EPI_ISL_693821, EPI_ISL_693822, EPI_ISL_693823, EPI_ISL_693824, EPI_ISL_693825			
see above	Viollier AG	Department of Biosystems Science and Engineering, ETH Zürich	Christian Beisel, Sarah Nadeau, Chaoran Chen, Ivan Topolsky, Pedro Ferreira, Philipp Jablonski, Susana Posada-Céspedes, Tobias Schär, Ina Nissen, Natascha Santacroce, Elodie Burcklen, Christiane Beckmann, Maurice Redondo, Olivier Kobel, Christoph Noppen, Sophie Seidel, Noemie Santamaria de Souza, Niko Beerenwinkel, Tanja Stadler
EPI_ISL_700431	Thembalethu CDC wc THC	NHLS/UCT	Houriiyah Tegally, Arash Iranzadeh, Deelan Doolabh, Lynn Tyers, Bruna Galvao, Innocent Mudau, Marvin Hsiao, Kruger Marais, Diana Hardie, Stephen Korsman, Carolyn Williamson
EPI_ISL_700439	Pacaltsdorp Clinic wc PAC	NHLS/UCT	Houriiyah Tegally, Arash Iranzadeh, Deelan Doolabh, Lynn Tyers, Bruna Galvao, Innocent Mudau, Marvin Hsiao, Kruger Marais, Diana Hardie, Stephen Korsman, Carolyn Williamson
EPI_ISL_700458	Hornlee Clinic wc HLC	NHLS/UCT	Houriiyah Tegally, Arash Iranzadeh, Deelan Doolabh, Lynn Tyers, Bruna Galvao, Innocent Mudau, Marvin Hsiao, Kruger Marais, Diana Hardie, Stephen Korsman, Carolyn Williamson
EPI_ISL_700467, EPI_ISL_700496	Conville CDC wc CVC	NHLS/UCT	Houriiyah Tegally, Arash Iranzadeh, Deelan Doolabh, Lynn Tyers, Bruna Galvao, Innocent Mudau, Marvin Hsiao, Kruger Marais, Diana Hardie, Stephen Korsman, Carolyn Williamson
EPI_ISL_700500	Plettenberg Bay Clinic wc PLC	NHLS/UCT	Houriiyah Tegally, Arash Iranzadeh, Deelan Doolabh, Lynn Tyers, Bruna Galvao, Innocent Mudau, Marvin Hsiao, Kruger Marais, Diana Hardie, Stephen Korsman, Carolyn Williamson
EPI_ISL_700548	Alma CDC wc AHC	NHLS/UCT	Arash Iranzadeh, Deelan Doolabh, Lynn Tyers, Bruna Galvao, Innocent Mudau, Marvin Hsiao, Kruger Marais, Diana Hardie, Stephen Korsman, Carolyn Williamson
EPI_ISL_700554	Conville CDC wc CVC	NHLS/UCT	Houriiyah Tegally, Arash Iranzadeh, Deelan Doolabh, Lynn Tyers, Bruna Galvao, Innocent Mudau, Marvin Hsiao, Kruger Marais, Diana Hardie, Stephen Korsman, Carolyn Williamson
EPI_ISL_702569, EPI_ISL_702667, EPI_ISL_702779	Liverpool Clinical Laboratories	COVID-19 Genomics UK (COG-UK) Consortium	Sam Haldenby, Anita Lucaci, Steve Paterson, Julian Hiscox, Alistair Darby, M Almsaud, A Alrezaihi, Muhannad Alruwaili, Stuart D Armstrong, Jones Benjamin, Eleanor G Bentley, Anu Chawla, Jordan J Clark, Angela Cowell, Richard Eccles, Isabel García-Dorival, Matthew Gemmell, Alessandro Gerada, PKF Gilmore, Richard Gregory, Ximeng Han, Catherine Hartley, Margaret Hughes, Miren Iturriza-Gomara, James Johnson, L Luu, Jenifer Manson, Charlotte Nelson, Elaine O'Toole, Cassie Olateju, Rebekah Penrice-Randal , Lucille Rainbow, N.P Randle, Trevor Ian Robinson, Parul Sharma, Ghada T Shawli, James P Stewart, Neil Swainston, Ecaterina Vamos, Joanne Watts, Mark Whitehead
EPI_ISL_702784	Northumbria University / South Tees Hospitals NHS Foundation Trust / North Cumbria Integrated Care NHS Foundation Trust / North Tees and Hartlepool NHS Foundation Trust / Newcastle Hospitals NHS Foundation Trust	COVID-19 Genomics UK (COG-UK) Consortium	Darren L Smith,Andrew Nelson,Matthew Bashton,Greg R Young,Joshua Loh,John Allan,Mohammad A Tariq,Giles S Holt,Gary Black,Wen C Yew,Lynn Dover,Paul Baker,Steve Liggett,Sarah Essex,Jane Greenaway,Debra Padgett,Clive Graham,Garren Scott,Edward Barton,Emma Swindells,Brendan Payne,Jennifer Collins,Yusri Taha,Gary Eltringham
EPI_ISL_702792, EPI_ISL_702877	Liverpool Clinical Laboratories	COVID-19 Genomics UK (COG-UK) Consortium	Sam Haldenby, Anita Lucaci, Steve Paterson, Julian Hiscox, Alistair Darby, M Almsaud, A Alrezaihi, Muhannad Alruwaili, Stuart D Armstrong, Jones Benjamin, Eleanor G Bentley, Anu Chawla, Jordan J Clark, Angela Cowell, Richard Eccles, Isabel García-Dorival, Matthew Gemmell, Alessandro Gerada, PKF Gilmore, Richard Gregory, Ximeng Han, Catherine Hartley, Margaret Hughes, Miren Iturriza-Gomara, James Johnson, L Luu, Jenifer Manson, Charlotte Nelson, Elaine O'Toole, Cassie Olateju, Rebekah Penrice-Randal , Lucille Rainbow, N.P Randle, Trevor Ian Robinson, Parul Sharma, Ghada T Shawli, James P Stewart, Neil Swainston, Ecaterina Vamos, Joanne Watts, Mark Whitehead
EPI_ISL_702937	Virology Department, Royal Infirmary of Edinburgh, NHS Lothian / School of Biological Sciences, University of Edinburgh / Institute of Genetics and Molecular Medicine, University of Edinburgh	COVID-19 Genomics UK (COG-UK) Consortium	McHugh M, Dewar R, Rooke S, Gallagher M, Balcaza C, O'Toole Á, Scher E, Hill V, McCrone JT, Colquhoun R, Yu X, Jackson B, Rambaut A, Williams TC, Templeton K
EPI_ISL_702953	Liverpool Clinical Laboratories	COVID-19 Genomics UK (COG-UK) Consortium	Sam Haldenby, Anita Lucaci, Steve Paterson, Julian Hiscox, Alistair Darby, M Almsaud, A Alrezaihi, Muhannad Alruwaili, Stuart D Armstrong, Jones Benjamin, Eleanor G Bentley, Anu Chawla, Jordan J Clark, Angela Cowell, Richard Eccles, Isabel García-Dorival, Matthew Gemmell, Alessandro Gerada, PKF Gilmore, Richard Gregory, Ximeng Han, Catherine Hartley, Margaret Hughes, Miren Iturriza-Gomara, James Johnson, L Luu, Jenifer Manson, Charlotte Nelson, Elaine O'Toole, Cassie Olateju, Rebekah Penrice-Randal , Lucille Rainbow, N.P Randle, Trevor Ian Robinson, Parul Sharma, Ghada T Shawli, James P Stewart, Neil Swainston, Ecaterina Vamos, Joanne Watts, Mark Whitehead
EPI_ISL_703048	Virology Department, Sheffield Teaching Hospitals NHS Foundation Trust/Department of Infection, Immunity and Cardiovascular Disease, The Medical School, University of Sheffield	COVID-19 Genomics UK (COG-UK) Consortium	Thushan de Silva, Matthew Parker, Nikki Smith, Adri Angyal, Rebecca Brown, Luke Green, Rachel Tucker, Paul Parsons, Danielle Groves, Katie Johnson, Laura Carrilero, Alex Keeley, Dave Partridge, Matthew Wyles, Benjamin Lindsey, Mehmet Yavuz, Mohammad Raza, Cariad Evans
EPI_ISL_703085, EPI_ISL_703881, EPI_ISL_704057, EPI_ISL_704129	Liverpool Clinical Laboratories	COVID-19 Genomics UK (COG-UK) Consortium	Sam Haldenby, Anita Lucaci, Steve Paterson, Julian Hiscox, Alistair Darby, M Almsaud, A Alrezaihi, Muhannad Alruwaili, Stuart D Armstrong, Jones Benjamin, Eleanor G Bentley, Anu Chawla, Jordan J Clark, Angela Cowell, Richard Eccles, Isabel García-Dorival, Matthew Gemmell, Alessandro Gerada, PKF Gilmore, Richard Gregory, Ximeng Han, Catherine Hartley, Margaret Hughes, Miren Iturriza-Gomara, James Johnson, L Luu, Jenifer Manson, Charlotte Nelson, Elaine O'Toole, Cassie Olateju, Rebekah Penrice-Randal , Lucille Rainbow, N.P Randle, Trevor Ian Robinson, Parul Sharma, Ghada T Shawli, James P Stewart, Neil Swainston, Ecaterina Vamos, Joanne Watts, Mark Whitehead
EPI_ISL_704676, EPI_ISL_704679, EPI_ISL_704692, EPI_ISL_704695, EPI_ISL_704706, EPI_ISL_704815, EPI_ISL_704836, EPI_ISL_704879, EPI_ISL_704885	Oxford Viromics, NDM, University of Oxford; Oxford University Hospitals; Basingstoke and North Hampshire Hospital	COVID-19 Genomics UK (COG-UK) Consortium	Tanya Golubchik, David Bonsall, George Macintyre, Amy Trebes, Mariateresa de Cesare, Catrin Moore, Alex Mobbs, Anita Justice, Robert Shaw, Monique Andersson, Timothy Peto, Emma Wise, Nathan Moore, Jessica Lynch, Nick Cortes, Matilde Mori, Stephen Kidd, David Buck, John Todd, Christophe Fraser
EPI_ISL_705224	Northumbria University / South Tees Hospitals NHS Foundation Trust / North Cumbria Integrated Care NHS Foundation Trust / North Tees and Hartlepool NHS Foundation Trust / Newcastle Hospitals NHS Foundation Trust	COVID-19 Genomics UK (COG-UK) Consortium	Darren L Smith,Andrew Nelson,Matthew Bashton,Greg R Young,Joshua Loh,John Allan,Mohammad A Tariq,Giles S Holt,Gary Black,Wen C Yew,Lynn Dover,Paul Baker,Steve Liggett,Sarah Essex,Jane Greenaway,Debra Padgett,Clive Graham,Garren Scott,Edward Barton,Emma Swindells,Brendan Payne,Jennifer Collins,Yusri Taha,Gary Eltringham
EPI_ISL_705236	University College London, Great Ormond Street Hospital for Children NHS Foundation Trust, Imperial College Healthcare NHS Trust	COVID-19 Genomics UK (COG-UK) Consortium	Sergi Castellano, Rachel Williams, Mark Kristiansen, Paola Resende Silva, Sunando Roy, Tony Brooks, Helena Tutill, Paola Niola, Patricia Dyal, Charlotte Williams, Leysa Forrest, Yasmin Panchbhaya, Jacqueline Findlay, Samuel Weeks, Julianne Brown, Kathryn Harris, Paul Randell, James Price, Alison Holmes, Judith Breuer
EPI_ISL_705259	Liverpool Clinical Laboratories	COVID-19 Genomics UK (COG-UK) Consortium	Sam Haldenby, Anita Lucaci, Steve Paterson, Julian Hiscox, Alistair Darby, M Almsaud, A Alrezaihi, Muhannad Alruwaili, Stuart D Armstrong, Jones Benjamin, Eleanor G Bentley, Anu Chawla, Jordan J Clark, Angela Cowell, Richard Eccles, Isabel García-Dorival, Matthew Gemmell, Alessandro Gerada, PKF Gilmore, Richard Gregory, Ximeng Han, Catherine Hartley, Margaret Hughes, Miren Iturriza-Gomara, James Johnson, L Luu, Jenifer Manson, Charlotte Nelson, Elaine O'Toole, Cassie Olateju, Rebekah Penrice-Randal , Lucille Rainbow, N.P Randle, Trevor Ian Robinson, Parul Sharma, Ghada T Shawli, James P Stewart, Neil Swainston, Ecaterina Vamos, Joanne Watts, Mark Whitehead
EPI_ISL_705280, EPI_ISL_705281	Northumbria University / South Tees Hospitals NHS Foundation Trust / North Cumbria Integrated Care NHS Foundation Trust / North Tees and Hartlepool NHS	COVID-19 Genomics UK (COG-UK) Consortium	Darren L Smith,Andrew Nelson,Matthew Bashton,Greg R Young,Joshua Loh,John Allan,Mohammad A Tariq,Giles S Holt,Gary Black,Wen C Yew,Lynn Dover,Paul Baker,Steve Liggett,Sarah Essex,Jane Greenaway,Debra Padgett,Clive Graham,Garren Scott,Edward Barton,Emma Swindells,Brendan Payne,Jennifer Collins,Yusri Taha,Gary Eltringham

	Foundation Trust / Newcastle Hospitals NHS Foundation Trust		
EPI_ISL_705284, EPI_ISL_705314, EPI_ISL_705322	Liverpool Clinical Laboratories	COVID-19 Genomics UK (COG-UK) Consortium	Sam Haldenby, Anita Lucaci, Steve Paterson, Julian Hiscox, Alistair Darby, M Almsaud, A Alrezaihi, Muhannad Alruwaili, Stuart D Armstrong, Jones Benjamin, Eleanor G Bentley, Anu Chawla, Jordan J Clark, Angela Cowell, Richard Eccles, Isabel García-Dorival, Matthew Gemmell, Alessandro Gerada, PKF Gilmore, Richard Gregory, Ximeng Han, Catherine Hartley, Margaret Hughes, Miren Iturriza-Gomara, James Johnson, L Luu, Jenifer Manson, Charlotte Nelson, Elaine O'Toole, Cassie Olateju, Rebekah Penrice-Randal , Lucille Rainbow, N.P Randle, Trevor Ian Robinson, Parul Sharma, Ghada T Shawli, James P Stewart, Neil Swainston, Ecaterina Vamos, Joanne Watts, Mark Whitehead
EPI_ISL_705338	University College London, Great Ormond Street Hospital for Children NHS Foundation Trust, Imperial College Healthcare NHS Trust	COVID-19 Genomics UK (COG-UK) Consortium	Sergi Castellano, Rachel Williams, Mark Kristiansen, Paola Resende Silva, Sunando Roy, Tony Brooks, Helena Tutill, Paola Niola, Patricia Dyal, Charlotte Williams, Leysa Forrest, Yasmin Panchbhaya, Jacqueline Findlay, Samuel Weeks, Julianne Brown, Kathryn Harris, Paul Randell, James Price, Alison Holmes, Judith Breuer
EPI_ISL_705340, EPI_ISL_705360, EPI_ISL_705362	Liverpool Clinical Laboratories	COVID-19 Genomics UK (COG-UK) Consortium	Sam Haldenby, Anita Lucaci, Steve Paterson, Julian Hiscox, Alistair Darby, M Almsaud, A Alrezaihi, Muhannad Alruwaili, Stuart D Armstrong, Jones Benjamin, Eleanor G Bentley, Anu Chawla, Jordan J Clark, Angela Cowell, Richard Eccles, Isabel García-Dorival, Matthew Gemmell, Alessandro Gerada, PKF Gilmore, Richard Gregory, Ximeng Han, Catherine Hartley, Margaret Hughes, Miren Iturriza-Gomara, James Johnson, L Luu, Jenifer Manson, Charlotte Nelson, Elaine O'Toole, Cassie Olateju, Rebekah Penrice-Randal , Lucille Rainbow, N.P Randle, Trevor Ian Robinson, Parul Sharma, Ghada T Shawli, James P Stewart, Neil Swainston, Ecaterina Vamos, Joanne Watts, Mark Whitehead
EPI_ISL_705395	University College London, Great Ormond Street Hospital for Children NHS Foundation Trust, Imperial College Healthcare NHS Trust	COVID-19 Genomics UK (COG-UK) Consortium	Sergi Castellano, Rachel Williams, Mark Kristiansen, Paola Resende Silva, Sunando Roy, Tony Brooks, Helena Tutill, Paola Niola, Patricia Dyal, Charlotte Williams, Leysa Forrest, Yasmin Panchbhaya, Jacqueline Findlay, Samuel Weeks, Julianne Brown, Kathryn Harris, Paul Randell, James Price, Alison Holmes, Judith Breuer
EPI_ISL_705407, EPI_ISL_705411	Liverpool Clinical Laboratories	COVID-19 Genomics UK (COG-UK) Consortium	Sam Haldenby, Anita Lucaci, Steve Paterson, Julian Hiscox, Alistair Darby, M Almsaud, A Alrezaihi, Muhannad Alruwaili, Stuart D Armstrong, Jones Benjamin, Eleanor G Bentley, Anu Chawla, Jordan J Clark, Angela Cowell, Richard Eccles, Isabel García-Dorival, Matthew Gemmell, Alessandro Gerada, PKF Gilmore, Richard Gregory, Ximeng Han, Catherine Hartley, Margaret Hughes, Miren Iturriza-Gomara, James Johnson, L Luu, Jenifer Manson, Charlotte Nelson, Elaine O'Toole, Cassie Olateju, Rebekah Penrice-Randal , Lucille Rainbow, N.P Randle, Trevor Ian Robinson, Parul Sharma, Ghada T Shawli, James P Stewart, Neil Swainston, Ecaterina Vamos, Joanne Watts, Mark Whitehead
EPI_ISL_705429, EPI_ISL_705430, EPI_ISL_705453, EPI_ISL_705454, EPI_ISL_705456, EPI_ISL_705467, EPI_ISL_705468, EPI_ISL_705470, EPI_ISL_705474, EPI_ISL_705481, EPI_ISL_705497			
see above	Oxford Viromics, NDM, University of Oxford; Oxford University Hospitals; Basingstoke and North Hampshire Hospital	COVID-19 Genomics UK (COG-UK) Consortium	Tanya Golubchik, David Bonsall, George Macintyre, Amy Trebes, Mariateresa de Cesare, Catrin Moore, Alex Mobbs, Anita Justice, Robert Shaw, Monique Andersson, Timothy Peto, Emma Wise, Nathan Moore, Jessica Lynch, Nick Cortes, Matilde Mori, Stephen Kidd, David Buck, John Todd, Christophe Fraser
EPI_ISL_705772, EPI_ISL_705799	Virology Department, Royal Infirmary of Edinburgh, NHS Lothian / School of Biological Sciences, University of Edinburgh / Institute of Genetics and Molecular Medicine, University of Edinburgh	COVID-19 Genomics UK (COG-UK) Consortium	McHugh M, Dewar R, Rooke S, Gallagher M, Balcaza C, O'Toole Á, Scher E, Hill V, McCrone JT, Colquhoun R, Yu X, Jackson B, Rambaut A, Williams TC, Templeton K
EPI_ISL_706025, EPI_ISL_706026, EPI_ISL_706027, EPI_ISL_706032, EPI_ISL_706033, EPI_ISL_706034, EPI_ISL_706037, EPI_ISL_706038, EPI_ISL_706058, EPI_ISL_706059, EPI_ISL_706060, EPI_ISL_706063, EPI_ISL_706065, EPI_ISL_706066, EPI_ISL_706068, EPI_ISL_706069, EPI_ISL_706070, EPI_ISL_706072, EPI_ISL_706073, EPI_ISL_706081, EPI_ISL_706085, EPI_ISL_706087, EPI_ISL_706088, EPI_ISL_706089, EPI_ISL_706090, EPI_ISL_706091, EPI_ISL_706093, EPI_ISL_706095, EPI_ISL_706096, EPI_ISL_706097, EPI_ISL_706098, EPI_ISL_706101, EPI_ISL_706102, EPI_ISL_706104, EPI_ISL_706105, EPI_ISL_706106, EPI_ISL_706110, EPI_ISL_706134, EPI_ISL_706136, EPI_ISL_706144, EPI_ISL_706145, EPI_ISL_706147, EPI_ISL_706149, EPI_ISL_706150, EPI_ISL_706156, EPI_ISL_706158, EPI_ISL_706160, EPI_ISL_706163, EPI_ISL_706166, EPI_ISL_706168, EPI_ISL_706169, EPI_ISL_706170, EPI_ISL_706185, EPI_ISL_706201, EPI_ISL_706202, EPI_ISL_706203, EPI_ISL_706204, EPI_ISL_706205, EPI_ISL_706206, EPI_ISL_706207, EPI_ISL_706208, EPI_ISL_706209, EPI_ISL_706210, EPI_ISL_706211, EPI_ISL_706212, EPI_ISL_706213, EPI_ISL_706214, EPI_ISL_706216, EPI_ISL_706218, EPI_ISL_706222, EPI_ISL_706223, EPI_ISL_706229, EPI_ISL_706230, EPI_ISL_706239, EPI_ISL_706240, EPI_ISL_706241, EPI_ISL_706243, EPI_ISL_706244, EPI_ISL_706247, EPI_ISL_706269, EPI_ISL_706270, EPI_ISL_706271, EPI_ISL_706272, EPI_ISL_706273, EPI_ISL_706274, EPI_ISL_706275			
see above	Oxford Viromics, NDM, University of Oxford; Oxford University Hospitals; Basingstoke and North Hampshire Hospital	COVID-19 Genomics UK (COG-UK) Consortium	Tanya Golubchik, David Bonsall, George Macintyre, Amy Trebes, Mariateresa de Cesare, Catrin Moore, Alex Mobbs, Anita Justice, Robert Shaw, Monique Andersson, Timothy Peto, Emma Wise, Nathan Moore, Jessica Lynch, Nick Cortes, Matilde Mori, Stephen Kidd, David Buck, John Todd, Christophe Fraser
EPI_ISL_706354	Northumbria University / South Tees Hospitals NHS Foundation Trust / North Cumbria Integrated Care NHS Foundation Trust / North Tees and Hartlepool NHS Foundation Trust / Newcastle Hospitals NHS Foundation Trust	COVID-19 Genomics UK (COG-UK) Consortium	Darren L Smith,Andrew Nelson,Matthew Bashton,Greg R Young,Joshua Loh,John Allan,Mohammad A Tariq,Giles S Holt,Gary Black,Wen C Yew,Lynn Dover,Paul Baker,Steve Liggett,Sarah Essex,Jane Greenaway,Debra Padgett,Clive Graham,Garren Scott,Edward Barton,Emma Swindells,Brendan Payne,Jennifer Collins,Yusri Taha,Gary Eltringham
EPI_ISL_706442, EPI_ISL_706483, EPI_ISL_706659	Wales Specialist Virology Centre Sequencing lab: Pathogen Genomics Unit	COVID-19 Genomics UK (COG-UK) Consortium	Catherine Moore, Johnathan Evans, Laura Gifford, Malorie Perry, Simon Cottrell, Angela Marchbank, Alec Birchley, Alexander Adams, Amy Gaskin, Bree Gatica-Wilcox, Jason Coombes, Joel Southgate, Lauren Gilbert, Lee Graham, Nicole Pacchiarini, Sara Kumziene-Summerhayes, Sarah Taylor, Sophie Jones, Sara Rey, Matthew Bull, Joanne Watkins, Sally Corden, Tom Connor
EPI_ISL_707808, EPI_ISL_707813, EPI_ISL_707819, EPI_ISL_707824, EPI_ISL_707825, EPI_ISL_707826	University of Michigan Clinical Microbiology Laboratory	Lauring Lab, University of Michigan, Department of Microbiology and Immunology	Valesano
EPI_ISL_707894	Area of Virology, Serology and Virology Division (SAViD), New South Wales Health Pathology Randwick	Virology Research Laboratory; Area of Virology, Serology and Virology Division (SAViD), New South Wales Health Pathology Randwick	Foster, C.; Au, J.; Ruiz Silva, M.; Deveson, I.; Bull, R.; Van Hal, S.; Rawlinson, W.
EPI_ISL_708040	Norwegian Institute of Public Health, Department of Virology	Norwegian Institute of Public Health, Department of Virology	Kathrine Stene-Johansen, Kamilla Heddeland Instefjord, Hilde Elshaug, Marie Paulsen Madsen, Rasmus Riis Kopperud, Hilde Vollan, Karoline Bragstad, Olav Hungnes
EPI_ISL_708060, EPI_ISL_708061, EPI_ISL_708062, EPI_ISL_708063, EPI_ISL_708064	Unilabs Laboratory Medicine	Norwegian Institute of Public Health, Department of Virology	Kathrine Stene-Johansen, Kamilla Heddeland Instefjord, Hilde Elshaug, Marie Paulsen Madsen, Rasmus Riis Kopperud, Hilde Vollan, Karoline Bragstad, Olav Hungnes
EPI_ISL_708065, EPI_ISL_708066, EPI_ISL_708067, EPI_ISL_708068	University Hospital of Northern Norway, Department for Microbiology and Infectious Disease Control	Norwegian Institute of Public Health, Department of Virology	Kathrine Stene-Johansen, Kamilla Heddeland Instefjord, Hilde Elshaug, Marie Paulsen Madsen, Rasmus Riis Kopperud, Hilde Vollan, Karoline Bragstad, Olav Hungnes
EPI_ISL_708078	Foerde Hospital, Department of Microbiology	Norwegian Institute of Public Health, Department of Virology	Kathrine Stene-Johansen, Kamilla Heddeland Instefjord, Hilde Elshaug, Marie Paulsen Madsen, Rasmus Riis Kopperud, Hilde Vollan, Karoline Bragstad, Olav Hungnes
EPI_ISL_708080	University Hospital of Northern Norway, Department for Microbiology and Infectious Disease Control	Norwegian Institute of Public Health, Department of Virology	Kathrine Stene-Johansen, Kamilla Heddeland Instefjord, Hilde Elshaug, Marie Paulsen Madsen, Rasmus Riis Kopperud, Hilde Vollan, Karoline Bragstad, Olav Hungnes
EPI_ISL_708081	Dept. of Medical Microbiology, Stavanger University Hospital, Helse Stavanger HF	Norwegian Institute of Public Health, Department of Virology	Kathrine Stene-Johansen, Kamilla Heddeland Instefjord, Hilde Elshaug, Marie Paulsen Madsen, Rasmus Riis Kopperud, Hilde Vollan, Karoline Bragstad, Olav Hungnes
EPI_ISL_708086, EPI_ISL_708087, EPI_ISL_708088, EPI_ISL_708089, EPI_ISL_708090, EPI_ISL_708092, EPI_ISL_708094	University Hospital of Northern Norway, Department for Microbiology and Infectious Disease Control	Norwegian Institute of Public Health, Department of Virology	Kathrine Stene-Johansen, Kamilla Heddeland Instefjord, Hilde Elshaug, Marie Paulsen Madsen, Rasmus Riis Kopperud, Hilde Vollan, Karoline Bragstad, Olav Hungnes
EPI_ISL_708096	Oslo University Hospital, Department of Medical Microbiology	Norwegian Institute of Public Health, Department of Virology	Kathrine Stene-Johansen, Kamilla Heddeland Instefjord, Hilde Elshaug, Marie Paulsen Madsen, Rasmus Riis Kopperud, Hilde Vollan, Karoline Bragstad, Olav Hungnes
EPI_ISL_708097, EPI_ISL_708101, EPI_ISL_708104, EPI_ISL_708105	Nordland Hospital - Bodo, Laboratory Department, Molecular Biology Unit	Norwegian Institute of Public Health, Department of Virology	Kathrine Stene-Johansen, Kamilla Heddeland Instefjord, Hilde Elshaug, Marie Paulsen Madsen, Rasmus Riis Kopperud, Hilde Vollan, Karoline Bragstad, Olav Hungnes
EPI_ISL_708110, EPI_ISL_708111, EPI_ISL_708112	Ostfold Hospital Trust - Kalnes, Centre for Laboratory Medicine, Section for gene technology and infection serology	Norwegian Institute of Public Health, Department of Virology	Kathrine Stene-Johansen, Kamilla Heddeland Instefjord, Hilde Elshaug, Marie Paulsen Madsen, Rasmus Riis Kopperud, Hilde Vollan, Karoline Bragstad, Olav Hungnes
EPI_ISL_708319, EPI_ISL_708347	Michigan Department of Health and Human Services, Bureau of Laboratories	Michigan Department of Health and Human Services, Bureau of Laboratories	Blankenship HM, Riner D, Soehnlen MK

EPI_ISL_708440, EPI_ISL_708444	Delaware Public Health Lab	Delaware Public Health Lab	Gregory Hovan
EPI_ISL_708462, EPI_ISL_708463	Minnesota Department of Health, Public Health Laboratory	Minnesota Department of Health, Public Health Laboratory	Alexandra Lorentz, Jacob Garfin, Matt Plumb, and Xiong Wang
EPI_ISL_708484	Essentia Health-St. Mary's Medical Center	Minnesota Department of Health, Public Health Laboratory	Alexandra Lorentz, Jacob Garfin, Matt Plumb, and Xiong Wang
EPI_ISL_708532, EPI_ISL_708533, EPI_ISL_708536, EPI_ISL_708542, EPI_ISL_708543, EPI_ISL_708547, EPI_ISL_708549, EPI_ISL_708553, EPI_ISL_708554, EPI_ISL_708556, EPI_ISL_708557, EPI_ISL_708559, EPI_ISL_708561, EPI_ISL_708566, EPI_ISL_708567, EPI_ISL_708568, EPI_ISL_708569, EPI_ISL_708570, EPI_ISL_708573, EPI_ISL_708576, EPI_ISL_708579, EPI_ISL_708581, EPI_ISL_708586, EPI_ISL_708588, EPI_ISL_708589, EPI_ISL_708590, EPI_ISL_708591, EPI_ISL_708592, EPI_ISL_708594, EPI_ISL_708596, EPI_ISL_708597, EPI_ISL_708598, EPI_ISL_708599, EPI_ISL_708600, EPI_ISL_708604, EPI_ISL_708605			
see above	Michigan Department of Health and Human Services, Bureau of Laboratories	Michigan Department of Health and Human Services, Bureau of Laboratories	Blankenship HM, Riner D, Soehnlen MK
EPI_ISL_710037, EPI_ISL_710038	University of Michigan Clinical Microbiology Laboratory	Lauring Lab, University of Michigan, Department of Microbiology and Immunology	Valesano
EPI_ISL_710121	South Eastern Area Laboratory Services (SEALS)	CIDM-PH et al.	CIDM-PH et al.
EPI_ISL_710592, EPI_ISL_710593	MLF Care AB	The Public Health Agency of Sweden	Department of Microbiology, The Public Health Agency of Sweden
EPI_ISL_710601, EPI_ISL_710602	Klinisk mikrobiologi	The Public Health Agency of Sweden	Department of Microbiology, The Public Health Agency of Sweden
EPI_ISL_710903, EPI_ISL_710904, EPI_ISL_710905, EPI_ISL_710906	Lighthouse Lab in Glasgow	Wellcome Sanger Institute for the COVID-19 Genomics UK (COG-UK) Consortium	Harper VanSteenhouse, Yumi Kasai, David Gray, Carol Clugston, Anna Dominiczak and Alex Alderton, Roberto Amato, Sonia Goncalves, Ewan Harrison, David K. Jackson, Ian Johnston, Dominic Kwiatkowski, Cordelia Langford, John Sillitoe on behalf of the Wellcome Sanger Institute COVID-19 Surveillance Team
EPI_ISL_710916, EPI_ISL_710924, EPI_ISL_710930	Lighthouse Lab in Alderley Park	Wellcome Sanger Institute for the COVID-19 Genomics UK (COG-UK) Consortium	Jacquelyn Wynn, Mairead Hyland, The Lighthouse Lab in Alderley Park and Alex Alderton, Roberto Amato, Sonia Goncalves, Ewan Harrison, David K. Jackson, Ian Johnston, Dominic Kwiatkowski, Cordelia Langford, John Sillitoe on behalf of the Wellcome Sanger Institute COVID-19 Surveillance Team
EPI_ISL_710995, EPI_ISL_710996, EPI_ISL_710997, EPI_ISL_710998, EPI_ISL_710999, EPI_ISL_711000, EPI_ISL_711001, EPI_ISL_711002, EPI_ISL_711003, EPI_ISL_711004, EPI_ISL_711005, EPI_ISL_711006, EPI_ISL_711007			
see above	Lighthouse Lab in Glasgow	Wellcome Sanger Institute for the COVID-19 Genomics UK (COG-UK) Consortium	Harper VanSteenhouse, Yumi Kasai, David Gray, Carol Clugston, Anna Dominiczak and Alex Alderton, Roberto Amato, Sonia Goncalves, Ewan Harrison, David K. Jackson, Ian Johnston, Dominic Kwiatkowski, Cordelia Langford, John Sillitoe on behalf of the Wellcome Sanger Institute COVID-19 Surveillance Team
EPI_ISL_711023, EPI_ISL_711024	Lighthouse Lab in Cambridge	Wellcome Sanger Institute for the COVID-19 Genomics UK (COG-UK) Consortium	Rob Howes, The Lighthouse Lab in Cambridge and Alex Alderton, Roberto Amato, Sonia Goncalves, Ewan Harrison, David K. Jackson, Ian Johnston, Dominic Kwiatkowski, Cordelia Langford, John Sillitoe on behalf of the Wellcome Sanger Institute COVID-19 Surveillance Team
EPI_ISL_712087, EPI_ISL_712088, EPI_ISL_712095	Port Elizabeth Provincial Hospital, National Health Laboratory Services, Eastern Cape, South Africa	National Institute for Communicable Diseases of the National Health Laboratory Service	Mohale T, Ntuli N, Mahlangu B, Allam M, Ismail A, Bhiman JN
EPI_ISL_714107, EPI_ISL_714227, EPI_ISL_714252, EPI_ISL_714269, EPI_ISL_714377, EPI_ISL_714476, EPI_ISL_714923, EPI_ISL_714924, EPI_ISL_714925, EPI_ISL_714926, EPI_ISL_714927			
see above	Department of Virus and Microbiological Special Diagnostics, Statens Serum Institut, Copenhagen, Denmark	Albertsen Lab, Department of Chemistry and Bioscience, Aalborg University, Denmark	Danish Covid-19 Genome Consortium
EPI_ISL_717808, EPI_ISL_717825, EPI_ISL_717826, EPI_ISL_717827, EPI_ISL_717890, EPI_ISL_717891, EPI_ISL_717892, EPI_ISL_717942, EPI_ISL_717943, EPI_ISL_717944, EPI_ISL_717945, EPI_ISL_717946, EPI_ISL_717947, EPI_ISL_717948, EPI_ISL_717949, EPI_ISL_717950			
see above	Laboratorio de Virologia Molecular / UFRJ	Bioinformatics Laboratory / LNCC	Carolina M Voloch, Ronaldo da Silva F Jr, Luiz G P de Almeida, Cynthia C Cardoso, Otavio Bustrolini, Alexandra L Gerber, Ana Paula de C Guimarães, Diana Mariani, Andréa Cony Cavalcanti, Claudia dos Santos Rodrigues, Terezinha M P P Castiñeira, Amilcar Tanuri, Ana Tereza R de Vasconcelos
EPI_ISL_717995, EPI_ISL_717996	Lab voor klinische biologie	Onderzoeksgroep Virologie	Laurens Lambrechts, Nick Vereecke, Marthe Pauwels, Bruno Verhasselt, Linos Vandekerckhove, Hans Nauwynck, Sebastiaan Theuns
EPI_ISL_718188	Ministry of Health Hospitals	Institute of Health and Community Medicine	David Perera, Ooi Mong How, Chua Hock Hin, Tonnii Sia Loong Loong, Wong Jyn Shan, Wong Kiing Aik, Chan Chia Jui
EPI_ISL_718191	Borneo Medical Centre	Institute of Health and Community Medicine	David Perera, Ooi Mong How, Chua Hock Hin, Tonnii Sia Loong Loong, Wong Jyn Shan, Wong Kiing Aik, Chan Chia Jui
EPI_ISL_718248	Hospital	National Reference Center for Viruses of Respiratory Infections, Institut Pasteur, Paris	Marion Barbet, Sylvie Behillil, Méline Bizard, Angela Brisebarre, Camille Capel, Etienne Simon-Lorière, Vincent Enouf, Maud Vanpeene, Sylvie van der Werf, Gisèle Lagathu
EPI_ISL_718262	National Institute for Infectious Diseases, INMI, "L. Spallanzani" IRCCS	National Institute for Infectious Diseases, INMI, "L. Spallanzani" IRCCS	C.E.M Gruber, F Messina, M Rueca, B Bartolini, E Giombini, MR Capobianchi, A Di Caro
EPI_ISL_718264	National Institute for Infectious Diseases, INMI, "L. Spallanzani" IRCCS	National Institute for Infectious Diseases, INMI, "L. Spallanzani" IRCCS	F Messina, M Rueca, B Bartolini, C.E.M Gruber, E Giombini, MR Capobianchi, A Di Caro
EPI_ISL_718265	National Institute for Infectious Diseases, INMI, "L. Spallanzani" IRCCS	National Institute for Infectious Diseases, INMI, "L. Spallanzani" IRCCS	B Bartolini, M Rueca, C.E.M Gruber, F Messina, E Giombini, A Di Caro, MR Capobianchi
EPI_ISL_721534	Lighthouse Lab in Glasgow	Wellcome Sanger Institute for the COVID-19 Genomics UK (COG-UK) Consortium	Harper VanSteenhouse, Yumi Kasai, David Gray, Carol Clugston, Anna Dominiczak and Alex Alderton, Roberto Amato, Sonia Goncalves, Ewan Harrison, David K. Jackson, Ian Johnston, Dominic Kwiatkowski, Cordelia Langford, John Sillitoe on behalf of the Wellcome Sanger Institute COVID-19 Surveillance Team
EPI_ISL_722275, EPI_ISL_722282, EPI_ISL_722295	Dutch COVID-19 response team	Erasmus Medical Center	Bas Oude Munnink, Reina Sikkema, David Nieuwenhuijse, Irina Chestakova, Anne van der Linden, Marjan Boter, Emmanuelle Munger, Corine GeurtsvanKessel, Annemiek van der Eijk, Richard Molenkamp, Marion Koopmans, on behalf of the Dutch national COVID-19 response team.
EPI_ISL_722320, EPI_ISL_722448, EPI_ISL_722449, EPI_ISL_722450, EPI_ISL_722451	Dutch COVID-19 response team	Erasmus Medical Center	OH consortium
EPI_ISL_722679, EPI_ISL_722680, EPI_ISL_722681, EPI_ISL_722698, EPI_ISL_722699, EPI_ISL_722700, EPI_ISL_722701, EPI_ISL_722702, EPI_ISL_722703, EPI_ISL_722704, EPI_ISL_722705, EPI_ISL_722706, EPI_ISL_722707, EPI_ISL_722708, EPI_ISL_722709, EPI_ISL_722710, EPI_ISL_722850			
see above	Dutch COVID-19 response team	Erasmus Medical Center	Bas Oude Munnink, Reina Sikkema, David Nieuwenhuijse, Irina Chestakova, Anne van der Linden, Marjan Boter, Emmanuelle Munger, Corine GeurtsvanKessel, Annemiek van der Eijk, Richard Molenkamp, Marion Koopmans, on behalf of the Dutch national COVID-19 response team.
EPI_ISL_723141	Mayo Clinic & Mayo Clinic Laboratories	Minnesota Department of Health, Public Health Laboratory	Alexandra Lorentz, Jacob Garfin, Matt Plumb, and Xiong Wang
EPI_ISL_723496, EPI_ISL_723497, EPI_ISL_723498	Virginia Division of Consolidated Laboratory Services (DCLS)	Virginia Division of Consolidated Laboratory Services (DCLS)	Virginia DCLS
EPI_ISL_723525, EPI_ISL_723526, EPI_ISL_723539, EPI_ISL_723547, EPI_ISL_723556	Centre for Enzyme Innovation, University of Portsmouth / Translational Research Laboratory, Portsmouth Hospitals NHS Trust	COVID-19 Genomics UK (COG-UK) Consortium	Angela Beckett,Yann Bourgeois,Garry Scarlett,Sharon Glaysher,Scott Elliott,Kelly Bicknell,Robert Impey,Allyson Lloyd,Sarah Wyllie,Ethan Butcher,Anoop Chauhan,Samuel Robson
EPI_ISL_724620, EPI_ISL_724629, EPI_ISL_724660, EPI_ISL_724662, EPI_ISL_724664, EPI_ISL_724675, EPI_ISL_724683, EPI_ISL_724684, EPI_ISL_724708, EPI_ISL_724716, EPI_ISL_724740			
see above	University College London, Great Ormond Street Hospital for Children NHS Foundation Trust, Imperial College Healthcare NHS Trust	COVID-19 Genomics UK (COG-UK) Consortium	Sergi Castellano, Rachel Williams, Mark Kristiansen, Paola Resende Silva, Sunando Roy, Tony Brooks, Helena Tutill, Paola Niola, Patricia Dyal, Charlotte Williams, Leysa Forrest, Yasmin Panchbhaya, Jacqueline Findlay, Samuel Weeks, Julianne Brown, Kathryn Harris, Paul Randell, James Price, Alison Holmes, Judith Breuer
EPI_ISL_724963, EPI_ISL_724980, EPI_ISL_724981, EPI_ISL_724982	Northumbria University / South Tees Hospitals NHS Foundation Trust / North Cumbria Integrated Care NHS Foundation Trust / North Tees and Hartlepool NHS Foundation Trust / Newcastle Hospitals NHS Foundation Trust	COVID-19 Genomics UK (COG-UK) Consortium	Darren L Smith,Andrew Nelson,Matthew Bashton,Greg R Young,Joshua Loh,John Allan,Mohammad A Tariq,Giles S Holt,Gary Black,Wen C Yew,Lynn Dover,Paul Baker,Steve Liggett,Sarah Essex,Jane Greenaway,Debra Padgett,Clive Graham,Garren Scott,Edward Barton,Emma Swindells,Brendan Payne,Jennifer Collins,Yusri Taha,Gary Eltringham
EPI_ISL_725271	Quadram Institute Bioscience	COVID-19 Genomics UK (COG-UK) Consortium	Dave J. Baker, Gemma L. Kay, Alp Aydin, Thanh Le-Viet, Steven Rudder, Ana P. Tedim, Anastasia Kolyva, Maria Diaz, Leonardo de Oliveira Martins, Nabil-Fareed Alikhan, Lizzie Meadows, Rachael Stanley, Ngozi Elumogo, Muhammed Yasir, Nicholas M. Thomson, Alexander J Trotter, Rachel Gilroy,

			Samuel Bloomfield, Claire Stuart, Andrew Bell, Reenesh Prakash, Samir Dervisevic, Alison E. Mather, John Wain, Mark Webber, Andrew J. Page, Justin O'Grady
EPI_ISL_727750	Centre for Enzyme Innovation, University of Portsmouth / Translational Research Laboratory, Portsmouth Hospitals NHS Trust	COVID-19 Genomics UK (COG-UK) Consortium	Angela Beckett,Yann Bourgeois,Garry Scarlett,Sharon Glaysher,Scott Elliott,Kelly Bicknell,Robert Impey,Allyson Lloyd,Sarah Wyllie,Ethan Butcher,Anoop Chauhan,Samuel Robson
EPI_ISL_728134, EPI_ISL_728135, EPI_ISL_728136, EPI_ISL_728137, EPI_ISL_728138, EPI_ISL_728139, EPI_ISL_728140	University of Wisconsin-Madison AIDS Vaccine Research Laboratories	University of Wisconsin-Madison AIDS Vaccine Research Laboratories	Gage Moreno, Katarina Braun, et al. AIDS Vaccine Research Laboratories
EPI_ISL_728169, EPI_ISL_728170	Institute for Medical Research, Infectious Disease Research Centre, National Institutes of Health, Ministry of Health Malaysia	Institute for Medical Research, Infectious Disease Research Centre, National Institutes of Health, Ministry of Health Malaysia	Suppiah J, Kamel K, Mohd-Zawawi Z, Thayan R
EPI_ISL_728773, EPI_ISL_728777, EPI_ISL_728781, EPI_ISL_728785, EPI_ISL_728788, EPI_ISL_728793, EPI_ISL_728804, EPI_ISL_728807, EPI_ISL_728810, EPI_ISL_728816, EPI_ISL_728821, EPI_ISL_728826, EPI_ISL_728830, EPI_ISL_728836, EPI_ISL_728840, EPI_ISL_728844, EPI_ISL_728855, EPI_ISL_728860, EPI_ISL_728861, EPI_ISL_728877, EPI_ISL_728915, EPI_ISL_728934, EPI_ISL_728935, EPI_ISL_728941, EPI_ISL_728946, EPI_ISL_728970, EPI_ISL_728971, EPI_ISL_728985, EPI_ISL_729192, EPI_ISL_729193, EPI_ISL_729194, EPI_ISL_729195, EPI_ISL_729196, EPI_ISL_729197, EPI_ISL_729198, EPI_ISL_729199, EPI_ISL_729200, EPI_ISL_729204, EPI_ISL_729205, EPI_ISL_729206, EPI_ISL_729207, EPI_ISL_729208, EPI_ISL_729209, EPI_ISL_729210, EPI_ISL_729256			
see above	Viollier AG	Department of Biosystems Science and Engineering, ETH Zürich	Chaoran Chen, Sarah Nadeau, Catharine Aquino, Ivan Topolsky, Pedro Ferreira, Philipp Jablonski, Susana Posada-Céspedes, Andreia Cabral de Gouvea, Maria Domenica Moccia, Simon Grüter, Timothy Sykes, Lennart Opitz, Ralph Schlapbach, Christiane Beckmann, Maurice Redondo, Olivier Kobel, Christoph Noppen, Sophie Seidel, Noemie Santamaria de Souza, Niko Beerenwinkel, Tanja Stadler
EPI_ISL_729341, EPI_ISL_729349, EPI_ISL_729369, EPI_ISL_729401, EPI_ISL_729414, EPI_ISL_729427, EPI_ISL_729428, EPI_ISL_729429, EPI_ISL_729430, EPI_ISL_729444, EPI_ISL_729462, EPI_ISL_729471, EPI_ISL_729620, EPI_ISL_729622, EPI_ISL_729623, EPI_ISL_729657, EPI_ISL_729660, EPI_ISL_729664			
see above	A. Krumbholz, Labor Dr. Krause und Kollegen MVZ GmbH, Kiel	Charité Universitätsmedizin Berlin, Institut für Virologie	Victor M Corman, Barbara Mühlemann, Jörn Beheim-Schwarzbach, Talitha Veith, Julia Schneider, Terry Jones, Christian Drosten
EPI_ISL_729767, EPI_ISL_729768	Yale Pathology Lab	Grubaugh Lab - Yale School of Public Health	Joseph Fauver, Tara Alpert, Anderson Brito, Annie Watkins, Anne Wyllie, Chantal Vogels, Mary Petrone, Chaney Kalinich, Isabel Ott, Arnau Casanovas, Catherine Muenker, Adam Moore, Alice Lu, Maria Tokuyama, Patrick Wong, Peiwen Lu, Saad Omer, Richard Martinello, Allison Nelson, Shelli Farhadian, Akiko Iwasaki, Charlese Dela Cruz, Albert Ko, Nathan Grubaugh
EPI_ISL_730055, EPI_ISL_730058, EPI_ISL_730060	Connecticut Department of Health	Grubaugh Lab - Yale School of Public Health	Joseph Fauver, Tara Alpert, Anderson Brito, Annie Watkins, Anne Wyllie, Chantal Vogels, Mary Petrone, Chaney Kalinich, Isabel Ott, Arnau Casanovas, Catherine Muenker, Adam Moore, Alice Lu, Maria Tokuyama, Patrick Wong, Peiwen Lu, Saad Omer, Richard Martinello, Allison Nelson, Shelli Farhadian, Akiko Iwasaki, Charlese Dela Cruz, Albert Ko, Nathan Grubaugh
EPI_ISL_730168, EPI_ISL_730177, EPI_ISL_730186, EPI_ISL_730188, EPI_ISL_730190, EPI_ISL_730191, EPI_ISL_730194, EPI_ISL_730196	San Diego County Public Health Laboratory	Andersen lab at Scripps Research	SEARCH Alliance San Diego with Tracy Basler, Jovan Shephard, Brett Austin
EPI_ISL_730517, EPI_ISL_730523, EPI_ISL_730528, EPI_ISL_730532, EPI_ISL_730538, EPI_ISL_730543, EPI_ISL_730544, EPI_ISL_730548, EPI_ISL_730549, EPI_ISL_730554, EPI_ISL_730555, EPI_ISL_730560, EPI_ISL_730562			
see above	Biolab Diagnostic Laboratories	Andersen lab at Scripps Research	Issa Abu-Dayyeh, Ahmad Tibi, Lama Hussein, Lina Mohammad, Zein Naber, Amid Abdelnour with SEARCH Alliance San Diego
EPI_ISL_731911	Instituto Nacional de Saude (INSA)	Instituto Nacional de Saude (INSA)	Borges et al
EPI_ISL_732374, EPI_ISL_732385, EPI_ISL_732456	National Virus Reference Laboratory	National Virus Reference Laboratory	Michael Carr, Gabriel Gonzalez, Jonathan Dean, Daniel Hare, Cillian F De Gascun
EPI_ISL_732677, EPI_ISL_732678	CNR Virus des Infections Respiratoires - France SUD	CNR Virus des Infections Respiratoires - France SUD	Antonin Bal, Gregory Destras, Claudia Gonzalez, Gwendolyne Burfin, Quentin Semanas, Martine Valette, Bruno Lina, Laurence Josset
EPI_ISL_732792	Centro de Investigación Biomédica de La Rioja - Hospital San Pedro Logroño	SeqCOVID-SPAIN consortium/IBV(CSIC)	María de Toro, José Manuel Azcona Gutiérrez, María Pilar Bea Escudero, Miriam Blasco Alberdi and SeqCOVID-SPAIN consortium
EPI_ISL_733483, EPI_ISL_733484	Omsk Research Institute of Natural Focal Infections	WHO National Influenza Centre Russian Federation	Andrey Komissarov, Artem Fadeev, Anna Ivanova, Kseniya Komissarova, Dmitry Bazhenov, Daria Danilenko, Ekaterina Gradoboeva, Elena Poleshchuk, Aleksei Vasilenko, Valery Yakimenko, Ksenia Safina, Elena Nabieva, Georgii Bazykin, Dmitry Lioznov
EPI_ISL_734207, EPI_ISL_734208, EPI_ISL_734209, EPI_ISL_734210, EPI_ISL_734211, EPI_ISL_734212, EPI_ISL_734214, EPI_ISL_734215, EPI_ISL_734219, EPI_ISL_734220, EPI_ISL_734221, EPI_ISL_734222, EPI_ISL_734223, EPI_ISL_734278			
see above	Virginia Division of Consolidated Laboratory Services (DCLS)	Virginia Division of Consolidated Laboratory Services (DCLS)	Virginia DCLS
EPI_ISL_734414	Wadsworth Center, New York State Department.of Health	Wadsworth Center, New York State Department.of Health	Kirsten St. George, Daryl M. Lamson, Alexis Russel, Jonathan Plitnick, Navjot Singh, John Kelly, Sara Griesemer, Erasmus Schneider, Erica Lasek-Nesselquist
EPI_ISL_734471, EPI_ISL_734472, EPI_ISL_734473, EPI_ISL_734474	Masonic Medical Research Institute	Wadsworth Center, New York State Department.of Health	Kirsten St. George, Nathan Tucker, Ryan D. Pfeiffer, Daryl M. Lamson, Alexis Russel, Jonathan Plitnick, Navjot Singh, John Kelly, Sara Griesemer, Erasmus Schneider, Erica Lasek-Nesselquist
EPI_ISL_734483, EPI_ISL_735215, EPI_ISL_735216, EPI_ISL_735217, EPI_ISL_735218, EPI_ISL_735219, EPI_ISL_735220	UZ Leuven, National Reference Laboratory for Coronaviruses, Laboratory Medicine, Leuven, Belgium	KU Leuven, Rega Institute, Clinical and Epidemiological Virology	Tony Wawina-Bokalanga, Joan Marti-Carerras, Bert Vanmechelen, Piet Maes
EPI_ISL_737014, EPI_ISL_737015	The National Institute of Public Health	State Veterinary Institute Prague	Nagy,A;Jirincova,H;Trnka,D;Vecerova,J
EPI_ISL_737140	Michigan Department of Health and Human Services, Bureau of Laboratories	Michigan Department of Health and Human Services, Bureau of Laboratories	Blankenship HM, Riner D, Soehnlen MK
EPI_ISL_738947, EPI_ISL_738953, EPI_ISL_739433, EPI_ISL_739442, EPI_ISL_739530	Humboldt County Public Health Laboratory	Chan-Zuckerberg Biohub	CZB Cliahub Consortium
EPI_ISL_739699, EPI_ISL_739701, EPI_ISL_739755, EPI_ISL_739809, EPI_ISL_739810, EPI_ISL_739816, EPI_ISL_739817, EPI_ISL_739829, EPI_ISL_739843, EPI_ISL_739844, EPI_ISL_739851, EPI_ISL_739892, EPI_ISL_739905, EPI_ISL_739916, EPI_ISL_739933, EPI_ISL_739963, EPI_ISL_739998, EPI_ISL_740007, EPI_ISL_740047, EPI_ISL_740123, EPI_ISL_740190, EPI_ISL_740231, EPI_ISL_740270, EPI_ISL_740295, EPI_ISL_740302, EPI_ISL_740332, EPI_ISL_740348, EPI_ISL_740364, EPI_ISL_740372, EPI_ISL_740434, EPI_ISL_740436, EPI_ISL_740450, EPI_ISL_740499, EPI_ISL_740504, EPI_ISL_740506			
see above	Laboratoire national de santé, Microbiology, Virology	Laboratoire national de santé, Microbiology, Microbial Genomics Platform	Anke Wienecke-Baldacchino, Catherine Ragimbeau,Jessica Tapp, Fatu Djabi, Lise Pignon, Raoul Salmon, Tamir Abdelrahman
EPI_ISL_741367, EPI_ISL_741368, EPI_ISL_741370, EPI_ISL_741373, EPI_ISL_741384, EPI_ISL_741385, EPI_ISL_741386, EPI_ISL_741387, EPI_ISL_741388, EPI_ISL_741390, EPI_ISL_741393, EPI_ISL_741394, EPI_ISL_741404, EPI_ISL_741405, EPI_ISL_741406, EPI_ISL_741409, EPI_ISL_741411, EPI_ISL_741412, EPI_ISL_741413, EPI_ISL_741415, EPI_ISL_741418			
see above	Oxford Viromics, NDM, University of Oxford; Oxford University Hospitals; Basingstoke and North Hampshire Hospital	COVID-19 Genomics UK (COG-UK) Consortium	Tanya Golubchik, David Bonsall, George Macintyre, Amy Trebes, Mariateresa de Cesare, Catrin Moore, Alex Mobbs, Anita Justice, Robert Shaw, Monique Andersson, Timothy Peto, Emma Wise, Nathan Moore, Jessica Lynch, Nick Cortes, Matilde Mori, Stephen Kidd, David Buck, John Todd, Christophe Fraser
EPI_ISL_741609	Queens Medical Centre, Clinical Microbiology Department / DeepSeq Nottingham	COVID-19 Genomics UK (COG-UK) Consortium	Gemma Clark, Wendy Smith, Manjinder Khakh, Vicki M Fleming, Michelle M Lister, Hannah Howson-Wells, Jonathan Ball, Patrick McClure, Joseph Chappell, Theocharis Tsoleridis, Nadine Holmes, Matthew Carlisle, Christopher Moore, Fei Sang, Johnny Debebe, Victoria Wright, Matthew Loose
EPI_ISL_744168, EPI_ISL_744172, EPI_ISL_744194, EPI_ISL_744217, EPI_ISL_744218, EPI_ISL_744258, EPI_ISL_744347, EPI_ISL_744359, EPI_ISL_744389, EPI_ISL_744419, EPI_ISL_744428, EPI_ISL_744440, EPI_ISL_744443, EPI_ISL_744460, EPI_ISL_744501, EPI_ISL_744534, EPI_ISL_744536, EPI_ISL_744565, EPI_ISL_744567, EPI_ISL_744572, EPI_ISL_744575, EPI_ISL_744581, EPI_ISL_744588, EPI_ISL_744624, EPI_ISL_744659, EPI_ISL_744680, EPI_ISL_744684, EPI_ISL_744686, EPI_ISL_744712, EPI_ISL_744732, EPI_ISL_744742, EPI_ISL_744788, EPI_ISL_744808, EPI_ISL_744829, EPI_ISL_744877, EPI_ISL_744907, EPI_ISL_744947			
see above	Laboratoire national de santé, Microbiology, Virology	Laboratoire national de santé, Microbiology, Microbial Genomics Platform	Anke Wienecke-Baldacchino, Catherine Ragimbeau,Jessica Tapp, Fatu Djabi, Lise Pignon, Raoul Salmon, Tamir Abdelrahman

EPI_ISL_745313	CHU Clermont-Ferrand	CNR Virus des Infections Respiratoires - France SUD	Antonin Bal, Gregory Destras, Gwendolyne Burfin, Hadrien Règue, Quentin Semanas, Martine Valette, Bruno Lina, Christine Archimbaud, Amélie Brebion, Hélène Chabrolles, Martine Chambon, Audrey Mirand, Christel Regagnon, Maxime Bisseux, Patricia Combes, Cécile Henquell, Laurence Josset
EPI_ISL_745441, EPI_ISL_745442, EPI_ISL_745443	DOHMH Morrisania	New York City Public Health Laboratory	Jade Wang, et al.
EPI_ISL_745444	DOHMH Jamaica	New York City Public Health Laboratory	Jade Wang, et al.
EPI_ISL_747093, EPI_ISL_747100, EPI_ISL_747101, EPI_ISL_747117, EPI_ISL_747120, EPI_ISL_747122, EPI_ISL_747123, EPI_ISL_747124, EPI_ISL_747125, EPI_ISL_747126, EPI_ISL_747136, EPI_ISL_747137, EPI_ISL_747138, EPI_ISL_747139, EPI_ISL_747140, EPI_ISL_747141, EPI_ISL_747142, EPI_ISL_747143			
see above	Respiratory Viruses Branch, Centers for Disease Control and Prevention	Respiratory Viruses Branch, Centers for Disease Control and Prevention	Queen,K., Li,Y., Tao,Y., Uehara,A., Montmayeur,A., Paden,C.R., Cook,P.W., Marine,R., Sheth,M., Wang,H., Lee,J., Tong,S.
EPI_ISL_748122, EPI_ISL_748123	Department of Virus and Microbiological Special Diagnostics, Statens Serum Institut, Copenhagen, Denmark	Albertsen Lab, Department of Chemistry and Bioscience, Aalborg University, Denmark	Danish Covid-19 Genome Consortium
EPI_ISL_751450, EPI_ISL_751451, EPI_ISL_751452, EPI_ISL_751453, EPI_ISL_751498	CHU Purpan - Laboratoire de Virologie - Institut Fédératif de Biologie	CHU Purpan - Laboratoire de Virologie - Institut Fédératif de Biologie	Latour J., Ranger N., Dubois M., Carcenac R., Harter A., Boyer P., Tremeaux P., Izopet J.
EPI_ISL_751545	NYSDOH Wadsworth Center, Virology Lab	Genomics and Discovery, Respiratory Viruses Branch, Division of Viral Diseases, Centers for Disease Control and Prevention	Krista Queen, Yan Li, Ying Tao, Jing Zhang, Anna Uehara, Anna Montmayeur, Clinton R. Paden, Peter W. Cook,Rachel Marine, Mili Sheth, Haibin Wang, Justin Lee, Suxiang Tong
EPI_ISL_751572	KS Health and Environmental Laboratories	Genomics and Discovery, Respiratory Viruses Branch, Division of Viral Diseases, Centers for Disease Control and Prevention	Krista Queen, Yan Li, Ying Tao, Jing Zhang, Anna Uehara, Anna Montmayeur, Clinton R. Paden, Peter W. Cook,Rachel Marine, Mili Sheth, Haibin Wang, Justin Lee, Suxiang Tong
EPI_ISL_751584, EPI_ISL_751587	NH Dept. of Health and Human Services Public Health Labs	Genomics and Discovery, Respiratory Viruses Branch, Division of Viral Diseases, Centers for Disease Control and Prevention	Krista Queen, Yan Li, Ying Tao, Jing Zhang, Anna Uehara, Anna Montmayeur, Clinton R. Paden, Peter W. Cook,Rachel Marine, Mili Sheth, Haibin Wang, Justin Lee, Suxiang Tong
EPI_ISL_751677	KS Health and Environmental Laboratories	Genomics and Discovery, Respiratory Viruses Branch, Division of Viral Diseases, Centers for Disease Control and Prevention	Krista Queen, Yan Li, Ying Tao, Jing Zhang, Anna Uehara, Anna Montmayeur, Clinton R. Paden, Peter W. Cook,Rachel Marine, Mili Sheth, Haibin Wang, Justin Lee, Suxiang Tong
EPI_ISL_751678	NH Dept. of Health and Human Services Public Health Labs	Genomics and Discovery, Respiratory Viruses Branch, Division of Viral Diseases, Centers for Disease Control and Prevention	Krista Queen, Yan Li, Ying Tao, Jing Zhang, Anna Uehara, Anna Montmayeur, Clinton R. Paden, Peter W. Cook,Rachel Marine, Mili Sheth, Haibin Wang, Justin Lee, Suxiang Tong
EPI_ISL_751681	NE Public Health Laboratory	Genomics and Discovery, Respiratory Viruses Branch, Division of Viral Diseases, Centers for Disease Control and Prevention	Krista Queen, Yan Li, Ying Tao, Jing Zhang, Anna Uehara, Anna Montmayeur, Clinton R. Paden, Peter W. Cook,Rachel Marine, Mili Sheth, Haibin Wang, Justin Lee, Suxiang Tong
EPI_ISL_751707	KS Health and Environmental Laboratories	Genomics and Discovery, Respiratory Viruses Branch, Division of Viral Diseases, Centers for Disease Control and Prevention	Krista Queen, Yan Li, Ying Tao, Jing Zhang, Anna Uehara, Anna Montmayeur, Clinton R. Paden, Peter W. Cook,Rachel Marine, Mili Sheth, Haibin Wang, Justin Lee, Suxiang Tong
EPI_ISL_751712	NH Dept. of Health and Human Services Public Health Labs	Genomics and Discovery, Respiratory Viruses Branch, Division of Viral Diseases, Centers for Disease Control and Prevention	Krista Queen, Yan Li, Ying Tao, Jing Zhang, Anna Uehara, Anna Montmayeur, Clinton R. Paden, Peter W. Cook,Rachel Marine, Mili Sheth, Haibin Wang, Justin Lee, Suxiang Tong
EPI_ISL_751724, EPI_ISL_751730	KS Health and Environmental Laboratories	Genomics and Discovery, Respiratory Viruses Branch, Division of Viral Diseases, Centers for Disease Control and Prevention	Krista Queen, Yan Li, Ying Tao, Jing Zhang, Anna Uehara, Anna Montmayeur, Clinton R. Paden, Peter W. Cook,Rachel Marine, Mili Sheth, Haibin Wang, Justin Lee, Suxiang Tong
EPI_ISL_753055, EPI_ISL_753056, EPI_ISL_753057, EPI_ISL_753058, EPI_ISL_753059, EPI_ISL_753060, EPI_ISL_753061, EPI_ISL_753062, EPI_ISL_753121, EPI_ISL_753122	State Laboratories Division, Hawaii State Department of Health	State Laboratories Division, Hawaii State Department of Health	Pamela O'Brien, Sabrina Diemert, Drew Kuwazaki, Razvan Sultana, Edward Desmond
EPI_ISL_753262, EPI_ISL_753263, EPI_ISL_753264, EPI_ISL_753265, EPI_ISL_753266, EPI_ISL_753267, EPI_ISL_753268, EPI_ISL_753269, EPI_ISL_753270, EPI_ISL_753271, EPI_ISL_753272, EPI_ISL_753276, EPI_ISL_753285			
see above	Clinical virology Laboratory, Children's Hospital Los Angeles	Center for Personalized Medicine, Children's Hospital Los Angeles	Gai et al
EPI_ISL_754400, EPI_ISL_754510, EPI_ISL_754511, EPI_ISL_754512	Wadsworth Center, New York State Department.of Health	Wadsworth Center, New York State Department.of Health	Kirsten St. George, Daryl M. Lamson, Alexis Russel, Matthew Shudt, Melissa A Leisner, Jonathan Plitnick, Navjot Singh, John Kelly, Sara Griesemer, Erasmus Schneider, Erica Lasek-Nesselquist
EPI_ISL_756276	State Laboratories Division, Hawaii State Department of Health	State Laboratories Division, Hawaii State Department of Health	Pamela O'Brien, Sabrina Diemert, Drew Kuwazaki, Razvan Sultana, Edward Desmond
EPI_ISL_757700	Department of Virus and Microbiological Special Diagnostics, Statens Serum Institut, Copenhagen, Denmark	Albertsen Lab, Department of Chemistry and Bioscience, Aalborg University, Denmark	Danish Covid-19 Genome Consortium
EPI_ISL_760148, EPI_ISL_760149, EPI_ISL_760153, EPI_ISL_760174, EPI_ISL_760215	Division of Emerging Infectious Diseases, Bureau of Infectious Diseases Diagnosis Control, Korea Disease Control and Prevention Agency	Division of Emerging Infectious Diseases, Bureau of Infectious Diseases Diagnosis Control, Korea Disease Control and Prevention Agency	Ae Kyung Park, Il-Hwan Kim, Heui Man Kim, Jeong-Min Kim, Namjoo Lee, Chaeyoung Lee, Sang Hee Woo, Eun-Jin Kim
EPI_ISL_764356	Virology Department, Royal Infirmary of Edinburgh, NHS Lothian / School of Biological Sciences, University of Edinburgh / Institute of Genetics and Molecular Medicine, University of Edinburgh	COVID-19 Genomics UK (COG-UK) Consortium	McHugh M, Dewar R, Rooke S, Gallagher M, Balcaza C, O'Toole Á, Scher E, Hill V, McCrone JT, Colquhoun R, Yu X, Jackson B, Rambaut A, Williams TC, Templeton K
EPI_ISL_764432, EPI_ISL_764435	Regional Virus Laboratory, Belfast Health and Social Care Trust	COVID-19 Genomics UK (COG-UK) Consortium	Conall McCaughey, James McKenna, Tanya Curran, Susan Feeney, Alison Watt, Ciara Cox, Mairead Connor, Zoltan Molnar, David Simpson, Derek Fairley
EPI_ISL_765786, EPI_ISL_765787, EPI_ISL_765788, EPI_ISL_765789, EPI_ISL_765790, EPI_ISL_765791	Massachusetts General Hospital	Infectious Disease Program, Broad Institute of Harvard and MIT	Lemieux,J.E., Siddle,K.J., Shaw,B., Adams,G., Pierce,V., Turbett,S., Anahtar,M., Branda,J., Slater,D., Harris,J., Lin,A.E., Gladden-Young,A., Lagerborg,K., Rudy,M., DeRuff,K., Carter,A., Normandin,E., Bauer,M., Reilly,S., Tomkins-Tinch,C., Loreth,C., Chaluvadi,S., Neumann,A., Cusick,C., Chapman,S.B., Gnirke,A., Flowers,K., Cerrato,F., Birren,B.W., Gallagher,G., Smole,S., Park,D.J., MacInnis,B.L., Ryan,E., LaRocque,R., Rosenberg,E. and Sabeti,P.C.
EPI_ISL_766623, EPI_ISL_766624	Klinisk mikrobiologi	The Public Health Agency of Sweden	Department of Microbiology, The Public Health Agency of Sweden
EPI_ISL_768726	Jena University Hospital, Institute for Infectious Diseases and Infection Control	Institute of infectious medicine & hospital hygiene, CaSe-Group	Spott, Riccardo; Marquet, Mike; Pletz, Matthias W.; Brandt, Christian
EPI_ISL_768784, EPI_ISL_768785, EPI_ISL_768787, EPI_ISL_768788, EPI_ISL_768833	AIID	Irish Coronavirus Sequencing Consortium - National Virus Reference Laboratory	Michael Carr, Gabriel Gonzalez, Alejandro Abner Garcia Leon, Patrick Mallon
EPI_ISL_769997	Area De Salud Catedral Noreste	Inciensa, Instituto Costarricense de Investigación y	Francisco Duarte, Hebleen Porras, Claudio Soto-Garita, Estela Cordero, Adriana Godínez, Melany Calderón & Mariel López

		Enseñanza en Nutrición y Salud	
EPI_ISL_770778, EPI_ISL_770779	Minnesota Department of Health, Public Health Laboratory	Minnesota Department of Health, Public Health Laboratory	Alexandra Lorentz, Jacob Garfin, Matt Plumb, and Xiong Wang
EPI_ISL_775295	Nordland Hospital - Bodo, Laboratory Department, Molecular Biology Unit	Norwegian Institute of Public Health, Department of Virology	Kathrine Stene-Johansen, Kamilla Heddeland Instefjord, Hilde Elshaug, Atiya R Ali,Marie Paulsen Madsen, Rasmus Riis Kopperud, Hilde Vollan, Karoline Bragstad, Olav Hungnes
EPI_ISL_775328, EPI_ISL_775329	Dept. of Medical Microbiology, Stavanger University Hospital, Helse Stavanger HF	Norwegian Institute of Public Health, Department of Virology	Kathrine Stene-Johansen, Kamilla Heddeland Instefjord, Hilde Elshaug, Atiya R Ali,Marie Paulsen Madsen, Rasmus Riis Kopperud, Hilde Vollan, Karoline Bragstad, Olav Hungnes
EPI_ISL_775455	University Hospital of Northern Norway, Department for Microbiology and Infectious Disease Control	Norwegian Institute of Public Health, Department of Virology	Kathrine Stene-Johansen, Kamilla Heddeland Instefjord, Hilde Elshaug, Atiya R Ali,Marie Paulsen Madsen, Rasmus Riis Kopperud, Hilde Vollan, Karoline Bragstad, Olav Hungnes
EPI_ISL_775486	Nordland Hospital - Bodo, Laboratory Department, Molecular Biology Unit	Norwegian Institute of Public Health, Department of Virology	Kathrine Stene-Johansen, Kamilla Heddeland Instefjord, Hilde Elshaug, Atiya R Ali,Marie Paulsen Madsen, Rasmus Riis Kopperud, Hilde Vollan, Karoline Bragstad, Olav Hungnes
EPI_ISL_775504	Vestfold Hospital, Toensberg Department of Microbiology	Norwegian Institute of Public Health, Department of Virology	Kathrine Stene-Johansen, Kamilla Heddeland Instefjord, Hilde Elshaug, Atiya R Ali,Marie Paulsen Madsen, Rasmus Riis Kopperud, Hilde Vollan, Karoline Bragstad, Olav Hungnes
EPI_ISL_779315	Utah Public Health Laboratory, Utah Public Health Laboratory Infectious Disease submission group	Utah Public Health Laboratory, Utah Public Health Laboratory Infectious Disease submission group	Gallagher,T., Young,E.L., Oakeson,K.F.
EPI_ISL_780048	Hospital General Universitario Gregorio Marañón	SeqCOVID-SPAIN consortium/IBV(CSIC)	Darío García de Viedma, Laura Pérez-Lago, Marta Herranz, Jon Sicilia, Julia Suárez, Pilar Catalán, Patricia Muñoz and SeqCOVID-SPAIN consortium
EPI_ISL_780407	Bermuda Government Molecular Diagnostics Laboratory (MDL)	Respiratory Virus Unit, National Infection Service, Public Health England	PHE Covid Sequencing Team, Dr Carika Weldon (Bermuda), Dr Ayoola Oyinloye (Bermuda)
EPI_ISL_786001, EPI_ISL_786002, EPI_ISL_786003, EPI_ISL_786004, EPI_ISL_786005, EPI_ISL_786006, EPI_ISL_786012, EPI_ISL_786013, EPI_ISL_786014, EPI_ISL_786015, EPI_ISL_786016, EPI_ISL_786017, EPI_ISL_786018, EPI_ISL_786024, EPI_ISL_786025, EPI_ISL_786026, EPI_ISL_786027, EPI_ISL_786028, EPI_ISL_786029, EPI_ISL_786030, EPI_ISL_786036, EPI_ISL_786037, EPI_ISL_786038, EPI_ISL_786039, EPI_ISL_786040, EPI_ISL_786041, EPI_ISL_786042, EPI_ISL_786048, EPI_ISL_786049, EPI_ISL_786050, EPI_ISL_786051, EPI_ISL_786052, EPI_ISL_786053, EPI_ISL_786054, EPI_ISL_786060, EPI_ISL_786061, EPI_ISL_786062, EPI_ISL_786063, EPI_ISL_786064, EPI_ISL_786065, EPI_ISL_786066, EPI_ISL_786072, EPI_ISL_786073, EPI_ISL_786074, EPI_ISL_786075, EPI_ISL_786076, EPI_ISL_786077, EPI_ISL_786078, EPI_ISL_786084, EPI_ISL_786085, EPI_ISL_786086, EPI_ISL_786087, EPI_ISL_786088, EPI_ISL_786089, EPI_ISL_786090			
see above	Houston Methodist Hospital	Houston Methodist Hospital	S. Wesley Long, Randall J. Olsen, Paul A. Christensen, David W. Bernard, James J. Davis, Maulik Shukla, Marcus Nguyen, Matthew Ojeda Saavedra, Prasanti Yerramilli, Layne Pruitt, Sishir Subedi, Heather Hendrickson, and James M. Musser
EPI_ISL_790568	Florida Bureau of Public Health Laboratories	Florida Bureau of Public Health Laboratories	Sarah Schmedes, Jason Blanton
EPI_ISL_791336, EPI_ISL_791355, EPI_ISL_791361, EPI_ISL_791362, EPI_ISL_791378	Johns Hopkins Hospital Department of Pathology	Johns Hopkins Hospital Department of Pathology	C. Paul Morris, Chun Huai Luo, Heba H. Mostafa
EPI_ISL_792017	Plateforme COVID IDF	National Reference Center for Viruses of Respiratory Infections, Institut Pasteur, Paris	Marion Barbet, Sylvie Behillil, Méline Bizard, Angela Brisebarre, Camille Capel, Etienne Simon-Lorière, Vincent Enouf, Maud Vanpeene, Sylvie van der Werf,Jacques Fourgeaud
EPI_ISL_792627	LACEN-PB	Laboratory of Respiratory Viruses and Measles, Oswaldo Cruz Institute, FIOCRUZ	Paola Resende, Luciana Appolinario, Fernando Motta, Anna Carolina Paixao, Ana Carolina Mendonca, João Felipe Bezerra, Romero Henrique Teixeira de Vasconcelos, Dalane Loudal Florentino Teixeira, Thiago Franco de Oliveira Carneiro, Marilda Siqueira
EPI_ISL_792646	LACEN-PR	Laboratory of Respiratory Viruses and Measles, Oswaldo Cruz Institute, FIOCRUZ	Paola Resende, Luciana Appolinario, Fernando Motta, Anna Carolina Paixao, Ana Carolina Mendonca, Maria do Carmo Debur, Irina Nastassja Riediger, Marilda Siqueira
EPI_ISL_794719	PathWest Laboratory Medicine WA	PathWest Laboratory Medicine WA Microbial Surveillance Unit	PathWest Laboratory Medicine WA Microbial Surveillance Unit
EPI_ISL_794828	ULSS 7 Pedemontana - Distretto 2	Istituto Zooprofilattico Sperimentale delle Venezie	Adelaide Milani, Alessia Schivo, Annalisa Salviato, Erika Giorgia Quaranta, Ambra Pastori, Bianca Zecchin, Alice Fusaro, Isabella Monne, Calogero Terregino, Antonia Ricci
EPI_ISL_796649	Akershus University Hospital, Department for Microbiology and Infectious Disease Control	Norwegian Institute of Public Health, Department of Virology	Kathrine Stene-Johansen, Kamilla Heddeland Instefjord, Hilde Elshaug, Atiya R Ali,Marie Paulsen Madsen, Rasmus Riis Kopperud, Hilde Vollan, Karoline Bragstad, Olav Hungnes
EPI_ISL_801395	Laboratorio de Ecologia de Doencas Transmissiveis na Amazonia, Instituto Leonidas e Maria Deane - Fiocruz Amazonia	Laboratorio de Ecologia de Doencas Transmissiveis na Amazonia, Instituto Leonidas e Maria Deane - Fiocruz Amazonia	Valdinete Nascimento, Victor Souza, André Corado, Fernanda Nascimento, George Silva, Ágatha Costa, Debora Duarte, Luciana Gonçalves, Maria Júlia Brandão, Michele Jesus, Felipe Naveca
EPI_ISL_801482, EPI_ISL_801489	Dutch COVID-19 response team	Erasmus Medical Center	Bas Oude Munnink, Reina Sikkema, David Nieuwenhuijse, Irina Chestakova, Anne van der Linden, Marjan Boter, Emmanuelle Munger, Corine GeurtsvanKessel, Annemiek van der Eijk, Richard Molenkamp, Marion Koopmans, on behalf of the Dutch national COVID-19 response team.
EPI_ISL_802502, EPI_ISL_802504	Connecticut Department of Health	Grubaugh Lab - Yale School of Public Health	Tara Alpert, Joseph Fauver, Anderson Brito, Mallery Breban, Anne Wyllie, Chantal Vogels, Mary Petrone, Chaney Kalinich, Isabel Ott, Nathan Grubaugh
EPI_ISL_802544	Dutch COVID-19 response team	Erasmus Medical Center	Bas Oude Munnink, Reina Sikkema, David Nieuwenhuijse, Irina Chestakova, Anne van der Linden, Marjan Boter, Emmanuelle Munger, Corine GeurtsvanKessel, Annemiek van der Eijk, Richard Molenkamp, Marion Koopmans, on behalf of the Dutch national COVID-19 response team.
EPI_ISL_803428, EPI_ISL_803429, EPI_ISL_803431, EPI_ISL_803432, EPI_ISL_803433, EPI_ISL_803434, EPI_ISL_803435, EPI_ISL_803436, EPI_ISL_803479, EPI_ISL_803480, EPI_ISL_803481, EPI_ISL_803482, EPI_ISL_803483, EPI_ISL_803484, EPI_ISL_803485			
see above	Wisconsin State Laboratory of Hygiene Communicable Disease Division	Wisconsin State Laboratory of Hygiene Communicable Disease Division	Kelsey R. Florek, Abigail C. Shockey
EPI_ISL_804570, EPI_ISL_804571, EPI_ISL_804572, EPI_ISL_804573	MEPHI, Aix Marseille University	MEPHI, Aix Marseille University	Anthony LEVASSEUR
EPI_ISL_812143, EPI_ISL_812144, EPI_ISL_812182, EPI_ISL_812183, EPI_ISL_812184, EPI_ISL_812185, EPI_ISL_812186, EPI_ISL_812187, EPI_ISL_812188, EPI_ISL_812189, EPI_ISL_812190, EPI_ISL_812191, EPI_ISL_812192, EPI_ISL_812193, EPI_ISL_812194			
see above	GA Department of Public Health Laboratory	Pathogen Discovery, Respiratory Viruses Branch, Division of Viral Diseases, Centers for Disease Control and Prevention	Yan Li, Ying Tao, Anna Montmayeur, Jing Zhang, Brian Lynch, Krista Queen, Anna Uehara, Rachel Marine, Peter Cook, Clinton R. Paden, Haibin Wang, Suxiang Tong
EPI_ISL_812997, EPI_ISL_812998, EPI_ISL_812999, EPI_ISL_813000, EPI_ISL_813001, EPI_ISL_813002, EPI_ISL_813003, EPI_ISL_813004, EPI_ISL_813005, EPI_ISL_813006	University of Birmingham	COVID-19 Genomics UK (COG-UK) Consortium	Institute of Microbiology, University of Birmingham: Claire McMurray, Joanne Stockton, Samuel Nicholls, Radoslaw Poplawski, Will Rowe, Josh Quick, Nicholas Loman. University of Birmingham Testing Laboratory: Celina M Whalley, Andrew Bosworth, Charlotte Poxon, Kasun Wanigasooriya, Oliver Pickles, Mike Kidd, Alex Richter, Andrew D Beggs PHE Heartlands Lab: Husam Osman, Andrew Bosworth. Queen Elizabeth Hospital: Anna Casey
EPI_ISL_814685, EPI_ISL_814686	Virology Department, Royal Infirmary of Edinburgh, NHS Lothian / School of Biological Sciences, University of Edinburgh / Institute of Genetics and Molecular Medicine, University of Edinburgh	COVID-19 Genomics UK (COG-UK) Consortium	McHugh M, Dewar R, Rooke S, Gallagher M, Balcaza C, O'Toole Á, Scher E, Hill V, McCrone JT, Colquhoun R, Yu X, Jackson B, Rambaut A, Williams TC, Templeton K
EPI_ISL_815479, EPI_ISL_815556, EPI_ISL_815604, EPI_ISL_816128, EPI_ISL_816129, EPI_ISL_816130, EPI_ISL_816131	Department of Virus and Microbiological Special Diagnostics, Statens Serum Institut, Copenhagen, Denmark	Albertsen Lab, Department of Chemistry and Bioscience, Aalborg University, Denmark	Danish Covid-19 Genome Consortium
EPI_ISL_819456	Northumbria University / South Tees Hospitals NHS Foundation Trust / North Cumbria Integrated Care NHS Foundation Trust / North Tees and Hartlepool NHS Foundation Trust / Newcastle Hospitals NHS Foundation Trust	COVID-19 Genomics UK (COG-UK) Consortium	Darren L Smith,Andrew Nelson,Matthew Bashton,Greg R Young,Joshua Loh,John Allan,Mohammad A Tariq,Giles S Holt,Gary Black,Wen C Yew,Lynn Dover,Paul Baker,Steve Liggett,Sarah Essex,Jane Greenaway,Debra Padgett,Clive Graham,Garren Scott,Edward Barton,Emma Swindells,Brendan Payne,Jennifer Collins,Yusri Taha,Gary Eltringham

EPI_ISL_825337, EPI_ISL_825349	Hospital Universitari Vall d'Hebron - Vall d'Hebron Institut de Rercerca	Hospital Universitari Vall d'Hebron	Cristina Andrés, Maria Piñana, Josep F Abril, Damir Garcia-Cehic, Ariadna Rando, Juliana Esperalba, Maria Gema Codina, Carla Castillo, Maria Carmen Martin, Tomàs Pumarola, Josep Quer, Andrés Antón
EPI_ISL_825549, EPI_ISL_825593, EPI_ISL_825594	Respiratory Virus Unit, National Infection Service, Public Health England	COVID-19 Genomics UK (COG-UK) Consortium	PHE Covid Sequencing Team
EPI_ISL_826944, EPI_ISL_826945, EPI_ISL_826946, EPI_ISL_826947, EPI_ISL_826948, EPI_ISL_826950, EPI_ISL_826951, EPI_ISL_826954, EPI_ISL_827211, EPI_ISL_827344, EPI_ISL_827508, EPI_ISL_827509, EPI_ISL_827512, EPI_ISL_827581, EPI_ISL_827832, EPI_ISL_827833, EPI_ISL_827834, EPI_ISL_827836, EPI_ISL_827837, EPI_ISL_827838, EPI_ISL_827839, EPI_ISL_827840, EPI_ISL_827901, EPI_ISL_827941, EPI_ISL_827944, EPI_ISL_827946, EPI_ISL_827947, EPI_ISL_827988, EPI_ISL_828024, EPI_ISL_828150, EPI_ISL_828274, EPI_ISL_828288			
see above	deCODE genetics	deCODE genetics	Daniel F Gudbjartsson; Agnar Helgason; Hakon Jonsson; Olafur T Magnusson; Pall Melsted; Gudmundur L Norddahl; Jona Saemundsdottir; Asgeir Sigurdsson; Patrick Sulem; Arna B Agustsdottir; Hannes Eggertsson; Berglind Eiriksdottir; Run Fridriksdottir; Elisabet E Gardarsdottir; Gudmundur Georgsson; Olafia S Gretarsdottir; Kjartan R Gudmundsson; Thora R Gunnarsdottir; Arnaldur Gylfason; Hilma Holm; Brynjar O Jensson; Aslaug Jonasdottir; Kamilla S Josefsdottir; Thordur Kristjansson; Droplaug N Magnusdottir; Solvi Rognvaldsson; Louise le Roux; Gudrun Sigmundsdottir; Gardar Sveinbjornsson; Kristin E Sveinsdottir; Maney Sveinsdottir; Emil A Thorarensen; Bjarni Thorbjornsson; Gisli Masson; Ingileif Jonsdottir; Alma Moller; Thorolfur Gudnason; Karl G Kristinsson; Unnur Thorsteinsdottir; Kari Stefansson
EPI_ISL_828554, EPI_ISL_828635	The National University Hospital of Iceland	deCODE genetics	Daniel F Gudbjartsson; Agnar Helgason; Hakon Jonsson; Olafur T Magnusson; Pall Melsted; Gudmundur L Norddahl; Jona Saemundsdottir; Asgeir Sigurdsson; Patrick Sulem; Arna B Agustsdottir; Hannes Eggertsson; Berglind Eiriksdottir; Run Fridriksdottir; Elisabet E Gardarsdottir; Gudmundur Georgsson; Olafia S Gretarsdottir; Kjartan R Gudmundsson; Thora R Gunnarsdottir; Arnaldur Gylfason; Hilma Holm; Brynjar O Jensson; Aslaug Jonasdottir; Kamilla S Josefsdottir; Thordur Kristjansson; Droplaug N Magnusdottir; Solvi Rognvaldsson; Louise le Roux; Gudrun Sigmundsdottir; Gardar Sveinbjornsson; Kristin E Sveinsdottir; Maney Sveinsdottir; Emil A Thorarensen; Bjarni Thorbjornsson; Gisli Masson; Ingileif Jonsdottir; Alma Moller; Thorolfur Gudnason; Karl G Kristinsson; Unnur Thorsteinsdottir; Kari Stefansson
EPI_ISL_828888, EPI_ISL_828899, EPI_ISL_828901, EPI_ISL_828903, EPI_ISL_829222, EPI_ISL_829404, EPI_ISL_829681, EPI_ISL_829717, EPI_ISL_829738, EPI_ISL_829781, EPI_ISL_829783			
see above	deCODE genetics	deCODE genetics	Daniel F Gudbjartsson; Agnar Helgason; Hakon Jonsson; Olafur T Magnusson; Pall Melsted; Gudmundur L Norddahl; Jona Saemundsdottir; Asgeir Sigurdsson; Patrick Sulem; Arna B Agustsdottir; Hannes Eggertsson; Berglind Eiriksdottir; Run Fridriksdottir; Elisabet E Gardarsdottir; Gudmundur Georgsson; Olafia S Gretarsdottir; Kjartan R Gudmundsson; Thora R Gunnarsdottir; Arnaldur Gylfason; Hilma Holm; Brynjar O Jensson; Aslaug Jonasdottir; Kamilla S Josefsdottir; Thordur Kristjansson; Droplaug N Magnusdottir; Solvi Rognvaldsson; Louise le Roux; Gudrun Sigmundsdottir; Gardar Sveinbjornsson; Kristin E Sveinsdottir; Maney Sveinsdottir; Emil A Thorarensen; Bjarni Thorbjornsson; Gisli Masson; Ingileif Jonsdottir; Alma Moller; Thorolfur Gudnason; Karl G Kristinsson; Unnur Thorsteinsdottir; Kari Stefansson
EPI_ISL_829793	The National University Hospital of Iceland	deCODE genetics	Daniel F Gudbjartsson; Agnar Helgason; Hakon Jonsson; Olafur T Magnusson; Pall Melsted; Gudmundur L Norddahl; Jona Saemundsdottir; Asgeir Sigurdsson; Patrick Sulem; Arna B Agustsdottir; Hannes Eggertsson; Berglind Eiriksdottir; Run Fridriksdottir; Elisabet E Gardarsdottir; Gudmundur Georgsson; Olafia S Gretarsdottir; Kjartan R Gudmundsson; Thora R Gunnarsdottir; Arnaldur Gylfason; Hilma Holm; Brynjar O Jensson; Aslaug Jonasdottir; Kamilla S Josefsdottir; Thordur Kristjansson; Droplaug N Magnusdottir; Solvi Rognvaldsson; Louise le Roux; Gudrun Sigmundsdottir; Gardar Sveinbjornsson; Kristin E Sveinsdottir; Maney Sveinsdottir; Emil A Thorarensen; Bjarni Thorbjornsson; Gisli Masson; Ingileif Jonsdottir; Alma Moller; Thorolfur Gudnason; Karl G Kristinsson; Unnur Thorsteinsdottir; Kari Stefansson
EPI_ISL_829828, EPI_ISL_829829, EPI_ISL_829835, EPI_ISL_829932, EPI_ISL_829950, EPI_ISL_829962, EPI_ISL_829978, EPI_ISL_829987, EPI_ISL_830070	deCODE genetics	deCODE genetics	Daniel F Gudbjartsson; Agnar Helgason; Hakon Jonsson; Olafur T Magnusson; Pall Melsted; Gudmundur L Norddahl; Jona Saemundsdottir; Asgeir Sigurdsson; Patrick Sulem; Arna B Agustsdottir; Hannes Eggertsson; Berglind Eiriksdottir; Run Fridriksdottir; Elisabet E Gardarsdottir; Gudmundur Georgsson; Olafia S Gretarsdottir; Kjartan R Gudmundsson; Thora R Gunnarsdottir; Arnaldur Gylfason; Hilma Holm; Brynjar O Jensson; Aslaug Jonasdottir; Kamilla S Josefsdottir; Thordur Kristjansson; Droplaug N Magnusdottir; Solvi Rognvaldsson; Louise le Roux; Gudrun Sigmundsdottir; Gardar Sveinbjornsson; Kristin E Sveinsdottir; Maney Sveinsdottir; Emil A Thorarensen; Bjarni Thorbjornsson; Gisli Masson; Ingileif Jonsdottir; Alma Moller; Thorolfur Gudnason; Karl G Kristinsson; Unnur Thorsteinsdottir; Kari Stefansson
EPI_ISL_830264	The National University Hospital of Iceland	deCODE genetics	Daniel F Gudbjartsson; Agnar Helgason; Hakon Jonsson; Olafur T Magnusson; Pall Melsted; Gudmundur L Norddahl; Jona Saemundsdottir; Asgeir Sigurdsson; Patrick Sulem; Arna B Agustsdottir; Hannes Eggertsson; Berglind Eiriksdottir; Run Fridriksdottir; Elisabet E Gardarsdottir; Gudmundur Georgsson; Olafia S Gretarsdottir; Kjartan R Gudmundsson; Thora R Gunnarsdottir; Arnaldur Gylfason; Hilma Holm; Brynjar O Jensson; Aslaug Jonasdottir; Kamilla S Josefsdottir; Thordur Kristjansson; Droplaug N Magnusdottir; Solvi Rognvaldsson; Louise le Roux; Gudrun Sigmundsdottir; Gardar Sveinbjornsson; Kristin E Sveinsdottir; Maney Sveinsdottir; Emil A Thorarensen; Bjarni Thorbjornsson; Gisli Masson; Ingileif Jonsdottir; Alma Moller; Thorolfur Gudnason; Karl G Kristinsson; Unnur Thorsteinsdottir; Kari Stefansson
EPI_ISL_831222, EPI_ISL_831223	Hospital Universitario La Paz (Madrid)	SeqCOVID-SPAIN consortium/IBV(CSIC)	María Rodríguez-Tejedor, Elias Dahdouh, Fernando Lázaro-Perona, Jesús Mingorance and SeqCOVID-SPAIN consortium
EPI_ISL_831784, EPI_ISL_831786, EPI_ISL_831788, EPI_ISL_831790, EPI_ISL_831792, EPI_ISL_831793, EPI_ISL_831795, EPI_ISL_831813	United States Air Force School of Aerospace Medicine	United States Air Force School of Aerospace Medicine	Anthony Fries, Jennifer Meyer, William Gruner, Amanda Javorina, Sarah Purves, Clarise Starr, Elizabeth Macias
EPI_ISL_832665, EPI_ISL_832666, EPI_ISL_832667, EPI_ISL_832668, EPI_ISL_832669, EPI_ISL_832670, EPI_ISL_832671, EPI_ISL_832672, EPI_ISL_832673, EPI_ISL_832674, EPI_ISL_832685, EPI_ISL_832686, EPI_ISL_832693			
see above	OHSU Lab Services Molecular Microbiology Lab	Oregon SARS-CoV-2 Genome Sequencing Center	Brendan L. O'Connell, Ruth V. Nichols, Sally Grindstaff, Alec J. Hirsch, Donna Hansel, Guang Fan, Daniel N. Streblow, William B. Messer, Andrew C. Adey, Benjamin N. Bimber, Brian J. O'Roak
EPI_ISL_833461, EPI_ISL_833469, EPI_ISL_833473	CHU Purpan - Laboratoire de Virologie - Institut Fédératif de Biologie	CHU Purpan - Laboratoire de Virologie - Institut Fédératif de Biologie	Latour J., Ranger N., Dubois M., Carcenac R., Harter A., Boyer P., Tremeaux P., Izopet J.
EPI_ISL_838313	Virology Department, Royal Infirmary of Edinburgh, NHS Lothian / School of Biological Sciences, University of Edinburgh / Institute of Genetics and Molecular Medicine, University of Edinburgh	COVID-19 Genomics UK (COG-UK) Consortium	McHugh M, Dewar R, Rooke S, Gallagher M, Balcaza C, O'Toole Á, Scher E, Hill V, McCrone JT, Colquhoun R, Yu X, Jackson B, Rambaut A, Williams TC, Templeton K
EPI_ISL_840202	Oxford Viromics, NDM, University of Oxford; Oxford University Hospitals; Basingstoke and North Hampshire Hospital	COVID-19 Genomics UK (COG-UK) Consortium	Tanya Golubchik, David Bonsall, George Macintyre, Amy Trebes, Mariateresa de Cesare, Catrin Moore, Alex Mobbs, Anita Justice, Robert Shaw, Monique Andersson, Timothy Peto, Emma Wise, Nathan Moore, Jessica Lynch, Nick Cortes, Matilde Mori, Stephen Kidd, David Buck, John Todd, Christophe Fraser
EPI_ISL_842353	Virology Department, Sheffield Teaching Hospitals NHS Foundation Trust/Department of Infection, Immunity and Cardiovascular Disease, The Medical School, University of Sheffield	COVID-19 Genomics UK (COG-UK) Consortium	Thushan de Silva, Matthew Parker, Nikki Smith, Adri Angyal, Rebecca Brown, Luke Green, Rachel Tucker, Paul Parsons, Danielle Groves, Katie Johnson, Laura Carrilero, Alex Keeley, Dave Partridge, Matthew Wyles, Benjamin Lindsey, Mehmet Yavuz, Mohammad Raza, Cariad Evans
EPI_ISL_842939	Barts Health NHS Trust	COVID-19 Genomics UK (COG-UK) Consortium	CUTINO-MOGUEL, Maria-Teresa; HARRINGTON, David; OWOYEMI, Dola; SHYLINI, Raghavendran; BROAD, Claire; KELE, Beatrix
EPI_ISL_844542	Department of Virus and Microbiological Special Diagnostics, Statens Serum Institut, Copenhagen, Denmark	Albertsen Lab, Department of Chemistry and Bioscience, Aalborg University, Denmark	Danish Covid-19 Genome Consortium
EPI_ISL_845755	Emory Molecular Diagnostics Laboratory, Emory Healthcare	Piantadosi Lab, Emory Department of Pathology	Ahmed Babiker, Anne Piantadosi
EPI_ISL_847704, EPI_ISL_847729	California Department of Public Health	Chiu Laboratory, University of California, San Francisco	Charles Chiu, Xianding (Wayne) Deng, Candace Wang, Brian Bushnell, Scot Federman, Jill Hacker, Debra Wadford
EPI_ISL_847831	Yale New Haven Hospital	Grubaugh Lab - Yale School of Public Health	Tara Alpert, Joseph Fauver, Anderson Brito, Mallery Breban, Anne Wyllie, Chantal Vogels, Mary Petrone, Chaney Kalinich, Isabel Ott, Nathan Grubaugh
EPI_ISL_848382, EPI_ISL_848383, EPI_ISL_848384	Illinois Department of Public Health	Gagnon Lab, Southern Illinois University	Keith Gagnon
EPI_ISL_848557	Evandro Chagas Institute	Evandro Chagas Institute	Santos, M.C.; Silva, A.M.; Junior, W.D.C.; Barbagelata, L.S.; Ferreira, J.A.; Sousa, E.M.A.; da Silva, P.S.; Pinheiro, K.C.; L.C.; Sousa Junior, E.C.
EPI_ISL_848871, EPI_ISL_848872, EPI_ISL_848873	Florida Bureau of Public Health Laboratories	Florida Bureau of Public Health Laboratories	Sarah Schmedes, Jason Blanton

EPI_ISL_849440, EPI_ISL_849500, EPI_ISL_849501, EPI_ISL_849502, EPI_ISL_849564	Seattle Flu Study	Seattle Flu Study	Deborah A. Nickerson, Chris D. Frazar, Jover Lee, Benjamin Pelle, Matthew Richardson, Amanda Adler, Elisabeth Brandstetter, Peter D. Han, Kairsten Fay, Misja Ilcisin, Kirsten Lacombe, Thomas R. Sibley, Melissa Truong, Caitlin R. Wolf, Michael Boeckh, Janet A. Englund, Michael Famulare, Barry R. Lutz, Mark J. Rieder, Lea M. Starita, Matthew Thompson, Jay Shendure, Trevor Bedford, Helen Y. Chu
EPI_ISL_849944	UC Davis- Department of Pathology and Laboratory Medicine	Chan-Zuckerberg Biohub	CZB Cliahub Consortium
EPI_ISL_852595, EPI_ISL_852596	Max von Pettenkofer Institute, Virology, National Reference Center for Retroviruses, LMU München	Laboratory for Functional Genome Analysis, Dept. Genomics, Gene Center of the LMU Munich	Max Muenchhoff, Stefan Krebs, Alexander Graf, Oliver Keppler, Helmut Blum
EPI_ISL_853325	UPMC Clinical Microbiology Laboratory	Microbial Genome Sequencing Center; Microbial Genomic Epidemiology Laboratory	Mustapha M. Mustapha, Jane W. Marsh, Dan Snyder, Marissa P. Griffith, Stephanie L. Mitchell, Vatsala R. Srinivasa, Kady D. Waggle, Chinelo Ezeonwuku, Vaughn S. Cooper, Lee H. Harrison
EPI_ISL_853752	Institute for Water Quality and Resource Management, Technical University Vienna	Bergthaler laboratory, CeMM Research Center for Molecular Medicine of the Austrian Academy of Sciences	Lukas Endler, Alexandra Popa, Benedikt Agerer, Jakob-Wendelin Genger, Alexander Lercher, Anna Schedl, Thomas Penz, Michael Schuster, Jan Laine, Martin Senekowitsch, Christoph Bock, Andreas Bergthaler
EPI_ISL_853941	Department of Microbiology, University Innsbruck	Bergthaler laboratory, CeMM Research Center for Molecular Medicine of the Austrian Academy of Sciences	Lukas Endler, Alexandra Popa, Benedikt Agerer, Jakob-Wendelin Genger, Alexander Lercher, Anna Schedl, Thomas Penz, Michael Schuster, Jan Laine, Martin Senekowitsch, Christoph Bock, Andreas Bergthaler
EPI_ISL_853975, EPI_ISL_853984, EPI_ISL_853985, EPI_ISL_853994, EPI_ISL_853995, EPI_ISL_854003, EPI_ISL_854004, EPI_ISL_854010, EPI_ISL_854017, EPI_ISL_854030, EPI_ISL_854031, EPI_ISL_854040, EPI_ISL_854228, EPI_ISL_854272, EPI_ISL_854280			
see above	Austrian Agency for Health and Food Safety (AGES)	Bergthaler laboratory, CeMM Research Center for Molecular Medicine of the Austrian Academy of Sciences	Lukas Endler, Alexandra Popa, Benedikt Agerer, Jakob-Wendelin Genger, Alexander Lercher, Anna Schedl, Thomas Penz, Michael Schuster, Jan Laine, Martin Senekowitsch, Christoph Bock, Andreas Bergthaler
EPI_ISL_855525, EPI_ISL_855537, EPI_ISL_855545	KEMRI-Wellcome Trust Research Programme/KEMRI-CGMR-C Kilifi	KEMRI-Wellcome Trust Research Programme/KEMRI-CGMR-C Kilifi	Githinji et al
EPI_ISL_856797, EPI_ISL_856798, EPI_ISL_856799	Servicio Virosis Respiratorias-Departamento Virología-INEI	Instituto Nacional Enfermedades Infecciosas C.G.Malbran	Baumeister E., Avaro M., Benedetti E., Russo M., Dattero ME, Pontoriero A., Cisterna D., Molina V., Perandones C., Tuduri E., Lorenzo F., Poklepovich T., Campos J.
EPI_ISL_860737, EPI_ISL_860738, EPI_ISL_860741, EPI_ISL_860742, EPI_ISL_860777, EPI_ISL_860778, EPI_ISL_860779, EPI_ISL_860780, EPI_ISL_860781	Swiss National Reference Centre for Influenza	Swiss National Reference Centre for Influenza	Ana Rita Gonçalves Cabecinhas, Samuel Cordey, Florian Laubscher,Christoph Grünig, Laurent Kaiser
EPI_ISL_860805, EPI_ISL_860806, EPI_ISL_860812	WHO/Minsk	Charité Universitätsmedizin Berlin, Institut für Virologie	Victor M Corman, Barbara Mühlemann, Jörn Beheim-Schwarzbach, Talitha Veith, Julia Tesch, Tobias Bleicker, Julia Schneider, Shmialiova Natallia, Sivets Natallia, Terry Jones, Christian Drosten
EPI_ISL_861672	Day Hospital de Ermelino Matarazzo	Instituto Adolfo Lutz, Interdiciplinary Procedures Center, Strategic Laboratory	Claudio Tavares Sacchi, Claudia Regina Gonçalves, Erica Valessa Ramos Gomes, Karoline Rodrigues Campos
EPI_ISL_861723	Department of Medical Microbiology, Hospital Pengajar Universiti Putra Malaysia	Malaysia Genome Institute	Mohd Noor Mat Isa, Syafinaz Amin-Nordin, Irni Suhayu Sapian, Hui-Yee Chee, Yusuf Muhammad Noor, Nurhezreen Md Iqbal, Enizza Kasim, Siti Noraini Othman, Mohd Faizal Abu Bakar, Shamsidar Sopie, Azrin Ahmad, Narcisse Joseph, Muhammad MI, Avisha Richards, Nor Zahrin Hasran, Nor Azfa Johari
EPI_ISL_861804, EPI_ISL_861854	Hospital General Universitario Gregorio Marañón	SeqCOVID-SPAIN consortium/IBV(CSIC)	Darío García de Viedma, Laura Pérez-Lago, Pedro J Sola-Campoy, Sergio Buenestado-Serrano, Marta Herranz, Victor Manuel de la Cueva, Julia Suárez, Pilar Catalán, Patricia Muñoz and SeqCOVID-SPAIN consortium
EPI_ISL_865164, EPI_ISL_865165, EPI_ISL_865166, EPI_ISL_865167, EPI_ISL_865168, EPI_ISL_865170	Liverpool Clinical Laboratories	COVID-19 Genomics UK (COG-UK) Consortium	Sam Haldenby, Anita Lucaci, Steve Paterson, Julian Hiscox, Alistair Darby, M Almsaud, A Alrezaihi, Muhannad Alruwaili, Stuart D Armstrong, Jones Benjamin, Eleanor G Bentley, Anu Chawla, Jordan J Clark, Angela Cowell, Richard Eccles, Isabel García-Dorival, Matthew Gemmell, Alessandro Gerada, PKF Gilmore, Richard Gregory, Ximeng Han, Catherine Hartley, Margaret Hughes, Miren Iturriza-Gomara, James Johnson, L Luu, Jenifer Manson, Charlotte Nelson, Elaine O'Toole, Cassie Olateju, Rebekah Penrice-Randal , Lucille Rainbow, N.P Randle, Trevor Ian Robinson, Parul Sharma, Ghada T Shawli, James P Stewart, Neil Swainston, Ecaterina Vamos, Joanne Watts, Mark Whitehead
EPI_ISL_867963	Centre for Enzyme Innovation, University of Portsmouth / Translational Research Laboratory, Portsmouth Hospitals NHS Trust	COVID-19 Genomics UK (COG-UK) Consortium	Angela Beckett,Yann Bourgeois,Garry Scarlett,Sharon Glaysher,Scott Elliott,Kelly Bicknell,Robert Impey,Allyson Lloyd,Sarah Wyllie,Ethan Butcher,Anoop Chauhan,Samuel Robson
EPI_ISL_871787	Department of Virus and Microbiological Special Diagnostics, Statens Serum Institut, Copenhagen, Denmark	Aalborg University	Danish Covid-19 Genome Consortium
EPI_ISL_872682, EPI_ISL_872683	Rhode Island Department of Health	Infectious Disease Program, Broad Institute of Harvard and MIT	Lemieux,J.E., Siddle,K.J., Huard,R., King,E., Azevedo,K., Miller,A., Adams,G., Gladden-Young,A., Lagerborg,K., Rudy,M., DeRuff,K., Carter,A., Normandin,E., Bauer,M., Reilly,S., Tomkins-Tinch,C., Loreth,C., Chaluvadi,S., Birren,B.W., Gallagher,G., Smole,S., Park,D.J., MacInnis,B.L., and Sabeti,P.C.
EPI_ISL_872746, EPI_ISL_872747, EPI_ISL_872748	Colorado Mesa University	Infectious Disease Program, Broad Institute of Harvard and MIT	Lemieux,J.E., Siddle,K.J., Marshall,J., ONeill,M., Bronson,A., Adams,G., Gladden-Young,A., Lagerborg,K., Rudy,M., DeRuff,K., Carter,A., Normandin,E., Bauer,M., Reilly,S., Tomkins-Tinch,C., Loreth,C., Chaluvadi,S., Birren,B.W., Gallagher,G., Smole,S., Park,D.J., MacInnis,B.L., and Sabeti,P.C.
EPI_ISL_875874, EPI_ISL_875877, EPI_ISL_875879, EPI_ISL_875882, EPI_ISL_875883, EPI_ISL_875884, EPI_ISL_875886, EPI_ISL_875890, EPI_ISL_875891, EPI_ISL_875892, EPI_ISL_875893, EPI_ISL_875894, EPI_ISL_875895, EPI_ISL_875899, EPI_ISL_875901, EPI_ISL_875906, EPI_ISL_875907, EPI_ISL_875908, EPI_ISL_875909, EPI_ISL_875910, EPI_ISL_875911, EPI_ISL_875912, EPI_ISL_875915, EPI_ISL_875921, EPI_ISL_875922, EPI_ISL_875924, EPI_ISL_875934, EPI_ISL_875938, EPI_ISL_875939, EPI_ISL_875945, EPI_ISL_875946, EPI_ISL_875949, EPI_ISL_875950, EPI_ISL_875952, EPI_ISL_875953, EPI_ISL_875958, EPI_ISL_876428, EPI_ISL_876429, EPI_ISL_876514, EPI_ISL_876515, EPI_ISL_876516, EPI_ISL_876517			
see above	Eurofins Diatherix	Hudsonalpha Genome Sequencing Center	Jane Grimwood, Melissa Williams, Lori H. Handley, Joshua Stough, Leslie Malone, Stefan Brzezinski, Ada Stewart, Teresa Jones, Jenell Webber, John Lovell, Jennifer Cart, and Jeremy Schmutz
EPI_ISL_876848, EPI_ISL_876849	Quest Diagnostics	Quest Diagnostics	Rosenthal,S.H., Gerasimova,A., Kagan,R.M., Anderson, B., Hua, M., Liu Y., Bernstein, L.E., Livingston, K.E., Perez, A., Shalhout, D.F., Shlyakhter, I.A., Owen, R., Tanpaiboon, P., Lacbawan, F.
EPI_ISL_882696, EPI_ISL_882733, EPI_ISL_882744, EPI_ISL_882745, EPI_ISL_882751, EPI_ISL_882753, EPI_ISL_882758, EPI_ISL_882759	1.AO Universitaria 'S. Giovanni di Dio e Ruggi D'Aragona, Scuola Medica Salernitana' Hospital / 2.UOC di Virologia e Microbiologia, Università della Campania 'L. Vanvitelli' / 3.AO Universitaria 'Federico II' Napoli Hospital / 4.AORN 'San Giuseppe Moscati' Avellino Hospital / 5.AO 'San Pio - presidio G. Rummo' Benevento Hospital / 6.AO 'Sant'Anna e San Sebastiano' Caserta Hospital / 7.PO 'Maria Santissima Addolorata' Eboli Hospital / 8.Biogem Istituto di Ricerche Genetiche	1. Genome Research Center for Health (CRGS) / 2. Laboratory of Molecular Medicine and Genomics(LMMGe) / 3. Center for Research in Pure and Applied Mathematics (CRMPA)	Giorgio Giurato, Francesca Rizzo, Alessandro Weisz, Gianluigi Franci, Giovanni Nassa, Pasquale Pagliano, Roberta Tarallo, Elena Alexandrova, Ylenia D'Agostino, Carlo Ferravante, Jessica Lamberti, Viola Melone, Domenico Memoli, Valeria Mirici Cappa, Domenico Palumbo, Giovanni Pecoraro, Assunta Sellitto, Oriana Strianese, Ilaria Terenzi, Giuseppe Fenza, Aniello Gentile, Antonello Saccomanno, Sonia Amabile, Teresa Rocco, Annamaria Salvati, Emilia Vaccaro, Massimiliano Galdiero, Michele Cennamo, Giuseppe Portella, Maria Grazia Foti, Mariarosaria Ingino, Maria Landi, Maurizio Fumi, Vincenzo Rocco, Rita Greco, Vittoria Letizia, Arnolfo Petruzziello, Maddalena Schioppa, Gregorio Goffredi, Francesca Marciano, Michele Caraglia, Alessia Cossu, Marianna Scrima
EPI_ISL_882961	Laboratorium Diagnostyi Mikrobiologicznej z Pracowni Prtka Grulicy SPSzW im. Jana Boego w Lublinie	National Institute of Public Health - National Institute of Hygiene	Wokowicz Tomasz, Zacharczuk Katarzyna, Gawor Jan
EPI_ISL_884400	Infectious Diseases, Quest Diagnostics	Infectious Diseases, Quest Diagnostics	Rosenthal,S.H., Gerasimova,A., Kagan,R.M., Anderson,B., Bernstein,L.E., Livingston,K.E., Hua,M., Liu,Y., Shalhout,D.F., Owen,R., Lacbawan,F.
EPI_ISL_887159	Massachusetts General Hospital	Infectious Disease Program, Broad Institute of Harvard and MIT	Lemieux,J.E., Siddle,K.J., Shaw,B., Adams,G., Pierce,V., Turbett,S., Anahtar,M., Branda,J., Slater,D., Harris,J., Lin,A.E., Gladden-Young,A., Lagerborg,K., Rudy,M., DeRuff,K., Carter,A., Normandin,E., Bauer,M., Reilly,S., Tomkins-Tinch,C., Loreth,C., Chaluvadi,S., Neumann,A., Cusick,C., Chapman,S.B., Gnirke,A., Flowers,K., Cerrato,F., Birren,B.W., Gallagher,G., Smole,S., Park,D.J., MacInnis,B.L., Ryan,E., LaRocque,R., Rosenberg,E. and Sabeti,P.C.
EPI_ISL_888835, EPI_ISL_888887	Michigan Department of Health and Human Services, Bureau of Laboratories	Michigan Department of Health and Human Services, Bureau of Laboratories	Blankenship HM, Riner D, Soehnlen MK
EPI_ISL_891190	DPH, Massachusetts State Public Health Lab	DPH, Massachusetts State Public Health Lab	Lang,A.S., Fink,T., Gallagher,G.R., Smole,S.C.
EPI_ISL_900430	MEPHI, Aix Marseille University	MEPHI, Aix Marseille University	Anthony LEVASSEUR

EPI_ISL_903977	NYSDOH Wadsworth Center, Virology Lab	Genomics and Discovery, Respiratory Viruses Branch, Division of Viral Diseases, Centers for Disease Control and Prevention	Krista Queen, Yan Li, Ying Tao, Jing Zhang, Anna Uehara, Anna Montmayeur, Clinton R. Paden, Peter W. Cook, Rachel Marine, Mili Sheth, Jasmine Padilla, Sarah Nobles, Mark Burroughs, Lori Rowe, Haibin Wang, Ben L. Rambo-Martin, Dhwani Batra, Justin Lee, Suxiang Tong
EPI_ISL_912473, EPI_ISL_912510, EPI_ISL_912531	NHLS Universitas Academic	UFS Virology	PA Bester, MM Nyaga, P Nthiga, MT Mogotsi, D Goedhals, T de Oliveira
EPI_ISL_913097	Center for Virology	Center for Virology	Jeremy V. Camp, Irene Goerzer, Monika Redlberger-Fritz, Stephan W. Aberle
EPI_ISL_924421, EPI_ISL_924429	Virology Department, Sheffield Teaching Hospitals NHS Foundation Trust/Department of Infection, Immunity and Cardiovascular Disease, The Medical School, University of Sheffield	COVID-19 Genomics UK (COG-UK) Consortium	Thushan de Silva, Matthew Parker, Nikki Smith, Adri Angyal, Rebecca Brown, Luke Green, Rachel Tucker, Paul Parsons, Danielle Groves, Katie Johnson, Laura Carrilero, Alex Keeley, Dave Partridge, Matthew Wyles, Benjamin Lindsey, Mehmet Yavuz, Mohammad Raza, Cariad Evans
EPI_ISL_925093, EPI_ISL_925096, EPI_ISL_925103, EPI_ISL_925106, EPI_ISL_925115, EPI_ISL_925118, EPI_ISL_925125, EPI_ISL_925128	1.AO Universitaria 'S. Giovanni di Dio e Ruggi D'Aragona, Scuola Medica Salernitana' Hospital / 2.UOC di Virologia e Microbiologia, Università della Campania 'L. Vanvitelli' / 3.AO Universitaria 'Federico II' Napoli Hospital / 4.AORN 'San Giuseppe Moscati' Avellino Hospital / 5.AO 'San Pio - presidio G. Rummo' Benevento Hospital / 6.AO 'Sant'Anna e San Sebastiano' Caserta Hospital / 7.PO 'Maria Santissima Addolorata' Eboli Hospital / 8.Biogem Istituto di Ricerche Genetiche	1. Genome Research Center for Health (CRGS) / 2. Laboratory of Molecular Medicine and Genomics(LMMGe) / 3. Center for Research in Pure and Applied Mathematics (CRMPA)	Giorgio Giurato, Francesca Rizzo, Alessandro Weisz, Gianluigi Franci, Giovanni Nassa, Pasquale Pagliano, Roberta Tarallo, Elena Alexandrova, Ylenia D'Agostino, Carlo Ferravante, Jessica Lamberti, Viola Melone, Domenico Memoli, Valeria Mirici Cappa, Domenico Palumbo, Giovanni Pecoraro, Assunta Sellitto, Oriana Strianese, Ilaria Terenzi, Giuseppe Fenza, Aniello Gentile, Antonello Saccomanno, Sonia Amabile, Teresa Rocco, Annamaria Salvati, Emilia Vaccaro, Massimiliano Galdiero, Michele Cennamo, Giuseppe Portella, Maria Grazia Foti, Mariarosaria Ingino, Maria Landi, Maurizio Fumi, Vincenzo Rocco, Rita Greco, Vittoria Letizia, Arnolfo Petruzziello, Maddalena Schioppa, Gregorio Goffredi, Francesca Marciano, Michele Caraglia, Alessia Cossu, Marianna Scrima
EPI_ISL_925142, EPI_ISL_925143, EPI_ISL_925144, EPI_ISL_925145, EPI_ISL_925146, EPI_ISL_925148, EPI_ISL_925149, EPI_ISL_925150	Virginia DCLS	Virginia DCLS	Virginia DCLS
EPI_ISL_925463, EPI_ISL_925464	Department of Clinical Microbiology	GIGA Medical Genomics	Keith Durkin, Maria Artesi, Sébastien Bontems, Raphaël Boreux, Bouchra Boujemla, Cécile Meex, Pierrette Melin, Marie-Pierre Hayette, Vincent Bours
EPI_ISL_925901, EPI_ISL_925902, EPI_ISL_925903, EPI_ISL_925904, EPI_ISL_925905, EPI_ISL_925906, EPI_ISL_925907	Nucleic Acid Testing, National Reference Laboratory	GIGA Medical Genomics	Yvan Butera, Keith Durkin, Maria Artesi, Bouchra Boujemla, Robert Rutayisire, Patrick Tuyisenge, Esperence Umumararungu, Sébastien Bontems, Marie-Pierre Hayette, Nathalie Renotte, Swaibu Gatare, Jacob Souopgui, Sabin Nsanzimana, Vincent Bours, Léon Mutesa
EPI_ISL_926075, EPI_ISL_926386, EPI_ISL_926465, EPI_ISL_926794, EPI_ISL_926833, EPI_ISL_926955, EPI_ISL_927091, EPI_ISL_927398, EPI_ISL_927621, EPI_ISL_927887, EPI_ISL_928043, EPI_ISL_928105, EPI_ISL_928305, EPI_ISL_928412, EPI_ISL_928550, EPI_ISL_928588, EPI_ISL_928629, EPI_ISL_928682, EPI_ISL_929197, EPI_ISL_929214, EPI_ISL_929229, EPI_ISL_930075, EPI_ISL_930222			
see above	Department of Virus and Microbiological Special Diagnostics, Statens Serum Institut, Copenhagen, Denmark	Aalborg University	Danish Covid-19 Genome Consortium
EPI_ISL_931072, EPI_ISL_931073	University Hospital Basel, Clinical Virology	University Hospital Basel, Clinical Bacteriology	Tim Roloff, Madlen Stange, Helena MB Seth-Smith, Alfredo Mari, Karoline Leuzinger, Julia Bielicki, Manuel Battegay, Hans Hirsch, Adrian Egli
EPI_ISL_933660	General Hospital - Veles	Research Center for Genetic Engineering and Biotechnology "Georgi D. Efremov" , Macedonian Academy of Sciences and Arts	RCGEB - MASA
EPI_ISL_933713	General Hospital - Prilep	Research Center for Genetic Engineering and Biotechnology "Georgi D. Efremov" , Macedonian Academy of Sciences and Arts	RCGEB - MASA
EPI_ISL_933717	General Hospital - Strumica	Research Center for Genetic Engineering and Biotechnology "Georgi D. Efremov" , Macedonian Academy of Sciences and Arts	RCGEB - MASA
EPI_ISL_933720	General Hospital - Tetovo	Research Center for Genetic Engineering and Biotechnology "Georgi D. Efremov" , Macedonian Academy of Sciences and Arts	RCGEB - MASA
EPI_ISL_933722	Gerontology Institute - Skopje	Research Center for Genetic Engineering and Biotechnology "Georgi D. Efremov" , Macedonian Academy of Sciences and Arts	RCGEB - MASA
EPI_ISL_933726	General Hospital - Strumica	Research Center for Genetic Engineering and Biotechnology "Georgi D. Efremov" , Macedonian Academy of Sciences and Arts	RCGEB - MASA
EPI_ISL_936601, EPI_ISL_936602, EPI_ISL_936603, EPI_ISL_936604, EPI_ISL_936605	Northwestern Memorial Hospital	Ozer Lab	Ramon Lorenzo-Redondo, Lacy M. Simons, Chad J. Achenbach, Lawrence J. Jennings, Michael G. Ison, Judd F. Hultquist, Egon A. Ozer
EPI_ISL_937024, EPI_ISL_937049	Quest Diagnostics	Quest Diagnostics	Rosenthal,S.H., Gerasimova,A., Kagan,R.M., Anderson, B., Livingston, K.E., Hua, M., Liu Y., Shalhout, D.F., Owen, R., Lacbawan, F.
EPI_ISL_940138	National Institute for Communicable Diseases,National Health Laboratory Services, Gauteng, South Africa	National Institute for Communicable Diseases of the National Health Laboratory Service	Amoako DG, Mohale T, Ntuli N, Mahlangu B, Allam M, Ismail A, Bhiman JN
EPI_ISL_940155, EPI_ISL_940156	NHLS Universitas Academic	UFS Virology	PA Bester, MM Nyaga, P Nthiga, MT Mogotsi, D Goedhals, T de Oliveira
EPI_ISL_940173, EPI_ISL_940175, EPI_ISL_940176, EPI_ISL_940177, EPI_ISL_940178, EPI_ISL_940179, EPI_ISL_940192	Hôpital Bichat Claude Bernard, Laboratoire de Virologie	IAME UMR1137 Inserm, Université de Paris, Hôpital Bichat	Antoine Bridier-Nahmias, Amélie Recoing, Quentin Le Hingrat, Lena Daniel, Siham Hamri, Gilles Collin, Alexandre Storto, Mélanie Bertine, Charlotte Charpentier, Nadhira Houhou-Fidouh, Diane Descamps, Benoit Visseaux
EPI_ISL_940872	Vaccines and Infectious Diseases Analytics Research Unit (VIDA)	KRISP, KZN Research Innovation and Sequencing Platform	Baillie Vicky, du Plessis Jeanine, Giandhari Jennifer, Pillay Sureshnee, Naidoo Yeshnee, Tegally Houriiyah, de Oliveira Tulio, Madhi Shabir
EPI_ISL_954190	1.AO Universitaria 'S. Giovanni di Dio e Ruggi D'Aragona, Scuola Medica Salernitana' Hospital / 2.UOC di Virologia e Microbiologia, Università della Campania 'L. Vanvitelli' / 3.AO Universitaria 'Federico II' Napoli Hospital / 4.AORN 'San Giuseppe Moscati' Avellino Hospital / 5.AO 'San Pio - presidio G. Rummo' Benevento Hospital / 6.AO 'Sant'Anna e San Sebastiano' Caserta Hospital / 7.PO 'Maria Santissima Addolorata' Eboli Hospital / 8.Biogem Istituto di Ricerche Genetiche	1. Genome Research Center for Health (CRGS) / 2. Laboratory of Molecular Medicine and Genomics(LMMGe) / 3. Center for Research in Pure and Applied Mathematics (CRMPA)	Giorgio Giurato, Francesca Rizzo, Alessandro Weisz, Gianluigi Franci, Giovanni Nassa, Pasquale Pagliano, Roberta Tarallo, Elena Alexandrova, Ylenia D'Agostino, Carlo Ferravante, Jessica Lamberti, Viola Melone, Domenico Memoli, Valeria Mirici Cappa, Domenico Palumbo, Giovanni Pecoraro, Assunta Sellitto, Oriana Strianese, Ilaria Terenzi, Giuseppe Fenza, Aniello Gentile, Antonello Saccomanno, Sonia Amabile, Teresa Rocco, Annamaria Salvati, Emilia Vaccaro, Massimiliano Galdiero, Michele Cennamo, Giuseppe Portella, Maria Grazia Foti, Mariarosaria Ingino, Maria Landi, Maurizio Fumi, Vincenzo Rocco, Rita Greco, Vittoria Letizia, Arnolfo Petruzziello, Maddalena Schioppa, Gregorio Goffredi, Francesca Marciano, Michele Caraglia, Alessia Cossu, Marianna Scrima, Edmondo Adorisio, Morena D'Avenia, Michela Iacobellis, Rosanna Piluscio, Giorgio Dirani, Vittorio Sambri, Simona Semprini, Silvia Zanoli, Francesco Curcio, Stefania Marzinotto, Andreina Baj, Fausto Sessa.
EPI_ISL_955689	Orange County Public Health Lab	Chan-Zuckerberg Biohub	CZB Cliahub Consortium
EPI_ISL_960120	Knysna Hospital wc KNY	National Health Laboratory Service/UCT	Arash Iranzadeh, Deelan Doolabh, Lynn Tyers, Bruna Galvao, Innocent Mudau, Marvin Hsiao, Kruger Marais, Diana Hardie, Stephen Korsman, Carolyn

			Williamson
EPI_ISL_960121	Mitchells Plain Hospital wc MPH	National Health Laboratory Service/UCT	Arash Iranzadeh, Deelan Doolabh, Lynn Tyers, Bruna Galvao, Innocent Mudau, Marvin Hsiao, Kruger Marais, Diana Hardie, Stephen Korsman, Carolyn Williamson
EPI_ISL_960122	Conville CDC wc CVC	National Health Laboratory Service/UCT	Arash Iranzadeh, Deelan Doolabh, Lynn Tyers, Bruna Galvao, Innocent Mudau, Marvin Hsiao, Kruger Marais, Diana Hardie, Stephen Korsman, Carolyn Williamson
EPI_ISL_960123	Kwanokuthula CDC wc KWA	National Health Laboratory Service/UCT	Arash Iranzadeh, Deelan Doolabh, Lynn Tyers, Bruna Galvao, Innocent Mudau, Marvin Hsiao, Kruger Marais, Diana Hardie, Stephen Korsman, Carolyn Williamson
EPI_ISL_960124	Heideveld Emergency Centre	National Health Laboratory Service/UCT	Arash Iranzadeh, Deelan Doolabh, Lynn Tyers, Bruna Galvao, Innocent Mudau, Marvin Hsiao, Kruger Marais, Diana Hardie, Stephen Korsman, Carolyn Williamson
EPI_ISL_961914, EPI_ISL_961958, EPI_ISL_962101, EPI_ISL_962122, EPI_ISL_962177	Illinois Department of Public Health	Gagnon Lab, Southern Illinois University	Keith Gagnon
EPI_ISL_965362, EPI_ISL_965377, EPI_ISL_965378, EPI_ISL_965379, EPI_ISL_965380	University of Liège COVID-19 testing center	GIGA Medical Genomics	Keith Durkin, Maria Artesi, Bouchra Boujemla, Emmanuel André, Marc Van Ranst, Fabrice Bureau, Laurent Gillet, Wouter Coppieters, Vincent Bours
EPI_ISL_965623	Dutch COVID-19 response team	Medical Microbiology, Maastricht University Medical Centre	Jozef Dingemans*, Brian van der Veer*, Erik Beuken, Carmen Reumkens, Lieke van Alphen, Christian Hoebe, Paul Savelkoul
EPI_ISL_968905, EPI_ISL_968907, EPI_ISL_968908, EPI_ISL_968909	KEMRI-Wellcome Trust Research Programme/KEMRI-CGMR-C Kilifi	KEMRI-Wellcome Trust Research Programme/KEMRI-CGMR-C Kilifi	Githinji et al
EPI_ISL_974825, EPI_ISL_974826, EPI_ISL_974827, EPI_ISL_974828, EPI_ISL_974829, EPI_ISL_974830, EPI_ISL_974831, EPI_ISL_974832, EPI_ISL_974833, EPI_ISL_974834, EPI_ISL_974835, EPI_ISL_974836, EPI_ISL_974837, EPI_ISL_974838, EPI_ISL_974839, EPI_ISL_974840, EPI_ISL_974841, EPI_ISL_974842, EPI_ISL_974843, EPI_ISL_974844, EPI_ISL_974845, EPI_ISL_974846, EPI_ISL_974847, EPI_ISL_974848, EPI_ISL_974849, EPI_ISL_974850, EPI_ISL_974851, EPI_ISL_974852, EPI_ISL_974854, EPI_ISL_974855, EPI_ISL_974856, EPI_ISL_974857, EPI_ISL_974858, EPI_ISL_974859, EPI_ISL_974860, EPI_ISL_974861, EPI_ISL_974862, EPI_ISL_974863, EPI_ISL_974864, EPI_ISL_974865, EPI_ISL_974866, EPI_ISL_974867, EPI_ISL_974868, EPI_ISL_974869, EPI_ISL_974870, EPI_ISL_974871, EPI_ISL_974872, EPI_ISL_974873, EPI_ISL_974874, EPI_ISL_974875, EPI_ISL_974876, EPI_ISL_974877, EPI_ISL_974878, EPI_ISL_974879, EPI_ISL_974880, EPI_ISL_974881, EPI_ISL_974882, EPI_ISL_974883, EPI_ISL_974884, EPI_ISL_974885, EPI_ISL_974886, EPI_ISL_974887, EPI_ISL_974888, EPI_ISL_974889			
see above	BCCDC Public Health Laboratory	BCCDC Public Health Laboratory	Prystajecky Natalie, Linda Hoang, Dan Fornika, John Tyson, Shannon Russell, Kim Macdonald, Kimia Kamelian, Ana Pacagnella, Corrinne Ng, Loretta Janz, Robert Azana Terry Snutch, Mel Krajden
EPI_ISL_977589	Department of Medical Microbiology, Hospital Pengajar Universiti Putra Malaysia	Malaysia Genome Institute	Mohd Noor Mat Isa, Syafinaz Amin-Nordin, Irni Suhayu Sapian, Hui-Yee Chee, Yusuf Muhammad Noor, Nurhezreen Md Iqbal, Enizza Kasim, Siti Noraini Othman, Mohd Faizal Abu Bakar, Shamsidar Sopie, Azrin Ahmad, Narcisse Joseph, Muhammad MI, Avisha Richards, Nor Zahrin Hasran, Nor Azfa Johari
