# Supplementary material for: Detection and characterization of the SARS-CoV-2 lineage B.1.526 in New York
Source: Nat Commun. 2021 Aug 9;12:4886. doi: 10.1038/s41467-021-25168-4 (PMC8352861; doi:10.1038/s41467-021-25168-4)
Supplement: Supplementary file 8 — Supplementary Data 4 [file 41467_2021_25168_MOESM8_ESM.zip › GISAID_acknowledements_tables/gisaid_hcov-19_acknowledgement_table_2021_02_13_00-4.pdf]

We gratefully acknowledge the following Authors from the Originating laboratories responsible for obtaining the specimens, as well as the Submitting laboratories where the genome data were generated and shared via GISAID, on which this research is based.

All Submitters of data may be contacted directly via www.gisaid.org

Authors are sorted alphabetically.

Accession ID	Originating Laboratory	Submitting Laboratory	Authors
EPI_ISL_622780	Canterbury Health Laboratories	Institute of Environmental Science and Research (ESR)	Xiaoyun Ren, Matt Storey, Nikki Freed, Muhammad Faisal, Jing Wang, Hermes Perez, Anja Werno, Antje van der Linden, Arlo Upton, Chris Mansell, David Hammer, Dragana Drinkovic, Gary McAuliffe, Hana Sofia Andersson, James Ussher, Jill Sherwood, Josh Freeman, Julia Howard, Juliet Elvy, Mary DeAlmeida, Matt Blakiston, Matthew Rogers, Max Bloomfield, Michael Addidle, Michelle Balm, Sally Roberts, Sarah Jefferies, Sharmini Muttaiyah, Susan Morpeth, Susan Taylor, Timothy Blackmore, Vani Sathyendran, Veronica Playle, Virginia Hope, Erasmus Smit, Lauren Jelly, Olin Silander, Joep de Ligt
EPI_ISL_622792	Middlemore Hospital	Institute of Environmental Science and Research (ESR)	Xiaoyun Ren, Matt Storey, Nikki Freed, Muhammad Faisal, Jing Wang, Hermes Perez, Anja Werno, Antje van der Linden, Arlo Upton, Chris Mansell, David Hammer, Dragana Drinkovic, Gary McAuliffe, Hana Sofia Andersson, James Ussher, Jill Sherwood, Josh Freeman, Julia Howard, Juliet Elvy, Mary DeAlmeida, Matt Blakiston, Matthew Rogers, Max Bloomfield, Michael Addidle, Michelle Balm, Sally Roberts, Sarah Jefferies, Sharmini Muttaiyah, Susan Morpeth, Susan Taylor, Timothy Blackmore, Vani Sathyendran, Veronica Playle, Virginia Hope, Erasmus Smit, Lauren Jelly, Olin Silander, Joep de Ligt
EPI_ISL_622824, EPI_ISL_622828, EPI_ISL_622829	Canterbury Health Laboratories	Institute of Environmental Science and Research (ESR)	Xiaoyun Ren, Matt Storey, Nikki Freed, Muhammad Faisal, Jing Wang, Hermes Perez, Anja Werno, Antje van der Linden, Arlo Upton, Chris Mansell, David Hammer, Dragana Drinkovic, Gary McAuliffe, Hana Sofia Andersson, James Ussher, Jill Sherwood, Josh Freeman, Julia Howard, Juliet Elvy, Mary DeAlmeida, Matt Blakiston, Matthew Rogers, Max Bloomfield, Michael Addidle, Michelle Balm, Sally Roberts, Sarah Jefferies, Sharmini Muttaiyah, Susan Morpeth, Susan Taylor, Timothy Blackmore, Vani Sathyendran, Veronica Playle, Virginia Hope, Erasmus Smit, Lauren Jelly, Olin Silander, Joep de Ligt
EPI_ISL_622831	Middlemore Hospital	Institute of Environmental Science and Research (ESR)	Xiaoyun Ren, Matt Storey, Nikki Freed, Muhammad Faisal, Jing Wang, Hermes Perez, Anja Werno, Antje van der Linden, Arlo Upton, Chris Mansell, David Hammer, Dragana Drinkovic, Gary McAuliffe, Hana Sofia Andersson, James Ussher, Jill Sherwood, Josh Freeman, Julia Howard, Juliet Elvy, Mary DeAlmeida, Matt Blakiston, Matthew Rogers, Max Bloomfield, Michael Addidle, Michelle Balm, Sally Roberts, Sarah Jefferies, Sharmini Muttaiyah, Susan Morpeth, Susan Taylor, Timothy Blackmore, Vani Sathyendran, Veronica Playle, Virginia Hope, Erasmus Smit, Lauren Jelly, Olin Silander, Joep de Ligt
EPI_ISL_626352, EPI_ISL_626353, EPI_ISL_626354	LabPLUS	Institute of Environmental Science and Research (ESR)	Xiaoyun Ren, Matt Storey, Nikki Freed, Muhammad Faisal, Jing Wang, Hermes Perez, Anja Werno, Antje van der Linden, Arlo Upton, Chris Mansell, David Hammer, Dragana Drinkovic, Gary McAuliffe, Hana Sofia Andersson, James Ussher, Jill Sherwood, Josh Freeman, Julia Howard, Juliet Elvy, Mary DeAlmeida, Matt Blakiston, Matthew Rogers, Max Bloomfield, Michael Addidle, Michelle Balm, Sally Roberts, Sarah Jefferies, Sharmini Muttaiyah, Susan Morpeth, Susan Taylor, Timothy Blackmore, Vani Sathyendran, Veronica Playle, Virginia Hope, Erasmus Smit, Lauren Jelly, Olin Silander, Joep de Ligt
EPI_ISL_626641, EPI_ISL_626642, EPI_ISL_626643, EPI_ISL_626644	National Public Health Laboratory, National Centre for Infectious Diseases	National Public Health Laboratory, National Centre for Infectious Diseases	Tze Minn Mak, Sophie Octavia, Zhenyang Zhou, Lin Cui, Raymond Tzer Pin Lin
EPI_ISL_626670, EPI_ISL_626740, EPI_ISL_626750, EPI_ISL_626770, EPI_ISL_627215, EPI_ISL_627310, EPI_ISL_627700, EPI_ISL_627701, EPI_ISL_627702, EPI_ISL_627703, EPI_ISL_627704, EPI_ISL_627705, EPI_ISL_627706, EPI_ISL_627707, EPI_ISL_627708, EPI_ISL_627709, EPI_ISL_627710, EPI_ISL_627716			
see above	Queens Medical Centre, Clinical Microbiology Department / DeepSeq Nottingham	COVID-19 Genomics UK (COG-UK) Consortium	Gemma Clark, Wendy Smith, Manjinder Khakh, Vicki M Fleming, Michelle M Lister, Hannah Howson-Wells, Jonathan Ball, Patrick McClure, Joseph Chappell, Theocharis Tsoleridis, Nadine Holmes, Matthew Carlisle, Christopher Moore, Fei Sang, Johnny Debebe, Victoria Wright, Matthew Loose
EPI_ISL_629009	Laverty Pathology	NSW Health Pathology - Institute of Clinical Pathology and Medical Research; Westmead Hospital; University of Sydney	CIDM-PH et al.
EPI_ISL_632310	1-Laboratory of Microbiology, National Reference Lab, Charles Nicolle Hospital; 2-University of Tunis ElManar, Faculty of Medicine of Tunis, LR99ES09, Tunis, Tunisia	1-Clinical and Experimental Pharmacology Lab, LR16SP02, National Center of Pharmacovigilance, University of Tunis El Manar, Tunis, Tunisia. 2-Neurodegenerative diseases and psychiatric troubles, LR18SP03, Razi Hospital, University of Tunis El Manar, Tunis, Tunisia. 3- Ministry of Health, National Observatory of New and Emerging Diseases, 1006, Tunis, Tunisia	Ilhem Boutiba-Ben Boubaker, Sameh Trabelsi, Nissaf Ben Alaya, Maher Kharrat, Alia Ben Kahla, Jalila Ben Khelil, Salma Abid, Sana Ferjani, Mouna Ben Sassi, Mouna Safer, Imen Mkada, Imen Kacem, Gaies Emna, Soumaya Rammeh, Riadh Daghfous, Riadh Gouider.
EPI_ISL_632398, EPI_ISL_632406, EPI_ISL_632449, EPI_ISL_632766, EPI_ISL_632767, EPI_ISL_632769, EPI_ISL_632793, EPI_ISL_632794	Dutch COVID-19 response team	Erasmus Medical Center	Bas Oude Munnink, David Nieuwenhuijse, Reina Sikkema, Claudia Schapendonk, Irina Chestakova, Anne van der Linden, Theo Bestebroer, Stefan van Nieuwkoop, Mark Pronk, Pascal Lexmond, Corien Swaan, Manon Haverkate, Madelief Mollers, Mart Stein, Sandra Kengne Kamga Mobou, Jeroen van Kampen, Jolanda Voermans, Aura Timen, Corine GeurtsvanKessel, Annemiek van der Eijk, Richard Molenkamp, Marion Koopmans, on behalf of the Dutch national COVID-19 response team.
EPI_ISL_634977	1-Laboratory of Microbiology, National Reference Lab, Charles Nicolle Hospital; 2-University of Tunis ElManar, Faculty of Medicine of Tunis, LR99ES09, Tunis, Tunisia	1-Clinical and Experimental Pharmacology Lab, LR16SP02, National Center of Pharmacovigilance, University of Tunis El Manar, Tunis, Tunisia. 2-Neurodegenerative diseases and psychiatric troubles, LR18SP03, Razi Hospital, University of Tunis El Manar, Tunis, Tunisia. 3- Ministry of Health, National Observatory of New and Emerging Diseases, 1006, Tunis, Tunisia	Ilhem Boutiba-Ben Boubaker, Sameh Trabelsi, Nissaf Ben Alaya, Maher Kharrat, Alia Ben Kahla, Jalila Ben Khelil, Salma Abid, Sana Ferjani, Mouna Ben Sassi, Mouna Safer, Imen Mkada, Imen Kacem, Gaies Emna, Soumaya Rammeh, Riadh Daghfous, Riadh Gouider.
EPI_ISL_635083	Haukeland University Hospital, Department of Medical Microbiology	Norwegian Institute of Public Health, Department of Virology	Kathrine Stene-Johansen, Kamilla Heddeland Instefjord, Hilde Elshaug, Marie Paulsen Madsen, Rasmus Riis Kopperud, Hilde Vollan, Karoline Bragstad, Olav Hungnes
EPI_ISL_637168, EPI_ISL_637169, EPI_ISL_637170, EPI_ISL_637172, EPI_ISL_637174, EPI_ISL_637176, EPI_ISL_637177	Respiratory Virus Unit, Microbiology Services Colindale, Public Health England	COVID-19 Genomics UK (COG-UK) Consortium	PHE Covid Sequencing Team
EPI_ISL_637257, EPI_ISL_637259, EPI_ISL_637263	Liverpool Clinical Laboratories	COVID-19 Genomics UK (COG-UK) Consortium	Sam Haldenby, Anita Lucaci, Steve Paterson, Julian Hiscox, Alistair Darby, M Almsaud, A Alrezaihi, Muhannad Alruwaili, Stuart D Armstrong, Jones Benjamin, Eleanor G Bentley, Anu Chawla, Jordan J Clark, Angela Cowell, Richard Eccles, Isabel García-Dorival, Matthew Gemmell, Alessandro Gerada, PKF Gilmore, Richard Gregory, Ximeng Han, Catherine Hartley, Margaret Hughes, Miren Iturriza-Gomara, James Johnson, L Luu, Jenifer Manson, Charlotte Nelson, Elaine O'Toole, Cassie Olateju, Rebekah Penrice-Randal , Lucille Rainbow, N.P Randle, Trevor Ian Robinson, Parul Sharma, Ghada T Shawli, James P Stewart, Neil Swainston, Ecaterina Vamos, Joanne Watts, Mark Whitehead
EPI_ISL_637273	Virology Department, Sheffield Teaching Hospitals NHS Foundation Trust/Department of Infection, Immunity and Cardiovascular Disease, The Medical School, University of Sheffield	COVID-19 Genomics UK (COG-UK) Consortium	Thushan de Silva, Matthew Parker, Nikki Smith, Adri Angyal, Rebecca Brown, Luke Green, Rachel Tucker, Paul Parsons, Danielle Groves, Katie Johnson, Laura Carrilero, Alex Keeley, Dave Partridge, Matthew Wyles, Benjamin Lindsey, Mehmet Yavuz, Mohammad Raza, Cariad Evans
EPI_ISL_637289	Wales Specialist Virology Centre Sequencing lab: Pathogen Genomics Unit	COVID-19 Genomics UK (COG-UK) Consortium	Catherine Moore, Johnathan Evans, Laura Gifford, Malorie Perry, Simon Cottrell, Angela Marchbank, Alec Birchley, Alexander Adams, Amy Gaskin, Bree Gatica-Wilcox, Jason Coombes, Joel Southgate, Lauren Gilbert, Lee Graham, Nicole Pacchiarini, Sara Kumziene-Summerhayes, Sarah Taylor, Sophie Jones, Sara Rey, Matthew Bull, Joanne Watkins, Sally Corden, Tom Connor
EPI_ISL_637300	Queens Medical Centre, Clinical Microbiology Department / DeepSeq Nottingham	COVID-19 Genomics UK (COG-UK) Consortium	Gemma Clark, Wendy Smith, Manjinder Khakh, Vicki M Fleming, Michelle M Lister, Hannah Howson-Wells, Jonathan Ball, Patrick McClure, Joseph Chappell, Theocharis Tsoleridis, Nadine Holmes, Matthew Carlisle, Christopher Moore, Fei Sang, Johnny Debebe, Victoria Wright, Matthew Loose
EPI_ISL_637301	Quadram Institute Bioscience	COVID-19 Genomics UK (COG-UK) Consortium	Dave J. Baker, Gemma L. Kay, Alp Aydin, Thanh Le-Viet, Steven Rudder, Ana P. Tedim, Anastasia Kolyva, Maria Diaz, Leonardo de Oliveira Martins,

			Nabil-Fareed Alikhan, Lizzie Meadows, Rachael Stanley, Ngozi Elumogo, Muhammed Yasir, Nicholas M. Thomson, Alexander J Trotter, Rachel Gilroy, Samuel Bloomfield, Claire Stuart, Andrew Bell, Reenesh Prakash, Samir Dervisevic, Alison E. Mather, John Wain, Mark Webber, Andrew J. Page, Justin O'Grady
EPI_ISL_637302, EPI_ISL_637319	Wales Specialist Virology Centre Sequencing lab: Pathogen Genomics Unit	COVID-19 Genomics UK (COG-UK) Consortium	Catherine Moore, Johnathan Evans, Laura Gifford, Malorie Perry, Simon Cottrell, Angela Marchbank, Alec Birchley, Alexander Adams, Amy Gaskin, Bree Gatica-Wilcox, Jason Coombes, Joel Southgate, Lauren Gilbert, Lee Graham, Nicole Pacchiarini, Sara Kumziene-Summerhayes, Sarah Taylor, Sophie Jones, Sara Rey, Matthew Bull, Joanne Watkins, Sally Corden, Tom Connor
EPI_ISL_637336	Liverpool Clinical Laboratories	COVID-19 Genomics UK (COG-UK) Consortium	Sam Haldenby, Anita Lucaci, Steve Paterson, Julian Hiscox, Alistair Darby, M Almsaud, A Alrezaihi, Muhannad Alruwaili, Stuart D Armstrong, Jones Benjamin, Eleanor G Bentley, Anu Chawla, Jordan J Clark, Angela Cowell, Richard Eccles, Isabel García-Dorival, Matthew Gemmell, Alessandro Gerada, PKF Gilmore, Richard Gregory, Ximeng Han, Catherine Hartley, Margaret Hughes, Miren Iturriza-Gomara, James Johnson, L Luu, Jenifer Manson, Charlotte Nelson, Elaine O'Toole, Cassie Olateju, Rebekah Penrice-Randal , Lucille Rainbow, N.P Randle, Trevor Ian Robinson, Parul Sharma, Ghada T Shawli, James P Stewart, Neil Swainston, Ecaterina Vamos, Joanne Watts, Mark Whitehead
EPI_ISL_637343	Queens Medical Centre, Clinical Microbiology Department / DeepSeq Nottingham	COVID-19 Genomics UK (COG-UK) Consortium	Gemma Clark, Wendy Smith, Manjinder Khakh, Vicki M Fleming, Michelle M Lister, Hannah Howson-Wells, Jonathan Ball, Patrick McClure, Joseph Chappell, Theocharis Tsoleridis, Nadine Holmes, Matthew Carlisle, Christopher Moore, Fei Sang, Johnny Debebe, Victoria Wright, Matthew Loose
EPI_ISL_637349	Wales Specialist Virology Centre Sequencing lab: Pathogen Genomics Unit	COVID-19 Genomics UK (COG-UK) Consortium	Catherine Moore, Johnathan Evans, Laura Gifford, Malorie Perry, Simon Cottrell, Angela Marchbank, Alec Birchley, Alexander Adams, Amy Gaskin, Bree Gatica-Wilcox, Jason Coombes, Joel Southgate, Lauren Gilbert, Lee Graham, Nicole Pacchiarini, Sara Kumziene-Summerhayes, Sarah Taylor, Sophie Jones, Sara Rey, Matthew Bull, Joanne Watkins, Sally Corden, Tom Connor
EPI_ISL_637358	Virology Department, Sheffield Teaching Hospitals NHS Foundation Trust/Department of Infection, Immunity and Cardiovascular Disease, The Medical School, University of Sheffield	COVID-19 Genomics UK (COG-UK) Consortium	Thushan de Silva, Matthew Parker, Nikki Smith, Adri Angyal, Rebecca Brown, Luke Green, Rachel Tucker, Paul Parsons, Danielle Groves, Katie Johnson, Laura Carrilero, Alex Keeley, Dave Partridge, Matthew Wyles, Benjamin Lindsey, Mehmet Yavuz, Mohammad Raza, Cariad Evans
EPI_ISL_637380, EPI_ISL_637395	Wales Specialist Virology Centre Sequencing lab: Pathogen Genomics Unit	COVID-19 Genomics UK (COG-UK) Consortium	Catherine Moore, Johnathan Evans, Laura Gifford, Malorie Perry, Simon Cottrell, Angela Marchbank, Alec Birchley, Alexander Adams, Amy Gaskin, Bree Gatica-Wilcox, Jason Coombes, Joel Southgate, Lauren Gilbert, Lee Graham, Nicole Pacchiarini, Sara Kumziene-Summerhayes, Sarah Taylor, Sophie Jones, Sara Rey, Matthew Bull, Joanne Watkins, Sally Corden, Tom Connor
EPI_ISL_637434	Liverpool Clinical Laboratories	COVID-19 Genomics UK (COG-UK) Consortium	Sam Haldenby, Anita Lucaci, Steve Paterson, Julian Hiscox, Alistair Darby, M Almsaud, A Alrezaihi, Muhannad Alruwaili, Stuart D Armstrong, Jones Benjamin, Eleanor G Bentley, Anu Chawla, Jordan J Clark, Angela Cowell, Richard Eccles, Isabel García-Dorival, Matthew Gemmell, Alessandro Gerada, PKF Gilmore, Richard Gregory, Ximeng Han, Catherine Hartley, Margaret Hughes, Miren Iturriza-Gomara, James Johnson, L Luu, Jenifer Manson, Charlotte Nelson, Elaine O'Toole, Cassie Olateju, Rebekah Penrice-Randal , Lucille Rainbow, N.P Randle, Trevor Ian Robinson, Parul Sharma, Ghada T Shawli, James P Stewart, Neil Swainston, Ecaterina Vamos, Joanne Watts, Mark Whitehead
EPI_ISL_637436	Virology Department, Sheffield Teaching Hospitals NHS Foundation Trust/Department of Infection, Immunity and Cardiovascular Disease, The Medical School, University of Sheffield	COVID-19 Genomics UK (COG-UK) Consortium	Thushan de Silva, Matthew Parker, Nikki Smith, Adri Angyal, Rebecca Brown, Luke Green, Rachel Tucker, Paul Parsons, Danielle Groves, Katie Johnson, Laura Carrilero, Alex Keeley, Dave Partridge, Matthew Wyles, Benjamin Lindsey, Mehmet Yavuz, Mohammad Raza, Cariad Evans
EPI_ISL_637442	Quadram Institute Bioscience	COVID-19 Genomics UK (COG-UK) Consortium	Dave J. Baker, Gemma L. Kay, Alp Aydin, Thanh Le-Viet, Steven Rudder, Ana P. Tedim, Anastasia Kolyva, Maria Diaz, Leonardo de Oliveira Martins, Nabil-Fareed Alikhan, Lizzie Meadows, Rachael Stanley, Ngozi Elumogo, Muhammed Yasir, Nicholas M. Thomson, Alexander J Trotter, Rachel Gilroy, Samuel Bloomfield, Claire Stuart, Andrew Bell, Reenesh Prakash, Samir Dervisevic, Alison E. Mather, John Wain, Mark Webber, Andrew J. Page, Justin O'Grady
EPI_ISL_637450, EPI_ISL_637452, EPI_ISL_637525	Wales Specialist Virology Centre Sequencing lab: Pathogen Genomics Unit	COVID-19 Genomics UK (COG-UK) Consortium	Catherine Moore, Johnathan Evans, Laura Gifford, Malorie Perry, Simon Cottrell, Angela Marchbank, Alec Birchley, Alexander Adams, Amy Gaskin, Bree Gatica-Wilcox, Jason Coombes, Joel Southgate, Lauren Gilbert, Lee Graham, Nicole Pacchiarini, Sara Kumziene-Summerhayes, Sarah Taylor, Sophie Jones, Sara Rey, Matthew Bull, Joanne Watkins, Sally Corden, Tom Connor
EPI_ISL_637533	Virology Department, Sheffield Teaching Hospitals NHS Foundation Trust/Department of Infection, Immunity and Cardiovascular Disease, The Medical School, University of Sheffield	COVID-19 Genomics UK (COG-UK) Consortium	Thushan de Silva, Matthew Parker, Nikki Smith, Adri Angyal, Rebecca Brown, Luke Green, Rachel Tucker, Paul Parsons, Danielle Groves, Katie Johnson, Laura Carrilero, Alex Keeley, Dave Partridge, Matthew Wyles, Benjamin Lindsey, Mehmet Yavuz, Mohammad Raza, Cariad Evans
EPI_ISL_637536	Quadram Institute Bioscience	COVID-19 Genomics UK (COG-UK) Consortium	Dave J. Baker, Gemma L. Kay, Alp Aydin, Thanh Le-Viet, Steven Rudder, Ana P. Tedim, Anastasia Kolyva, Maria Diaz, Leonardo de Oliveira Martins, Nabil-Fareed Alikhan, Lizzie Meadows, Rachael Stanley, Ngozi Elumogo, Muhammed Yasir, Nicholas M. Thomson, Alexander J Trotter, Rachel Gilroy, Samuel Bloomfield, Claire Stuart, Andrew Bell, Reenesh Prakash, Samir Dervisevic, Alison E. Mather, John Wain, Mark Webber, Andrew J. Page, Justin O'Grady
EPI_ISL_637542, EPI_ISL_637551	Wales Specialist Virology Centre Sequencing lab: Pathogen Genomics Unit	COVID-19 Genomics UK (COG-UK) Consortium	Catherine Moore, Johnathan Evans, Laura Gifford, Malorie Perry, Simon Cottrell, Angela Marchbank, Alec Birchley, Alexander Adams, Amy Gaskin, Bree Gatica-Wilcox, Jason Coombes, Joel Southgate, Lauren Gilbert, Lee Graham, Nicole Pacchiarini, Sara Kumziene-Summerhayes, Sarah Taylor, Sophie Jones, Sara Rey, Matthew Bull, Joanne Watkins, Sally Corden, Tom Connor
EPI_ISL_637557	Quadram Institute Bioscience	COVID-19 Genomics UK (COG-UK) Consortium	Dave J. Baker, Gemma L. Kay, Alp Aydin, Thanh Le-Viet, Steven Rudder, Ana P. Tedim, Anastasia Kolyva, Maria Diaz, Leonardo de Oliveira Martins, Nabil-Fareed Alikhan, Lizzie Meadows, Rachael Stanley, Ngozi Elumogo, Muhammed Yasir, Nicholas M. Thomson, Alexander J Trotter, Rachel Gilroy, Samuel Bloomfield, Claire Stuart, Andrew Bell, Reenesh Prakash, Samir Dervisevic, Alison E. Mather, John Wain, Mark Webber, Andrew J. Page, Justin O'Grady
EPI_ISL_637585	Virology Department, Sheffield Teaching Hospitals NHS Foundation Trust/Department of Infection, Immunity and Cardiovascular Disease, The Medical School, University of Sheffield	COVID-19 Genomics UK (COG-UK) Consortium	Thushan de Silva, Matthew Parker, Nikki Smith, Adri Angyal, Rebecca Brown, Luke Green, Rachel Tucker, Paul Parsons, Danielle Groves, Katie Johnson, Laura Carrilero, Alex Keeley, Dave Partridge, Matthew Wyles, Benjamin Lindsey, Mehmet Yavuz, Mohammad Raza, Cariad Evans
EPI_ISL_637586, EPI_ISL_637593, EPI_ISL_637600, EPI_ISL_637608, EPI_ISL_637609	Wales Specialist Virology Centre Sequencing lab: Pathogen Genomics Unit	COVID-19 Genomics UK (COG-UK) Consortium	Catherine Moore, Johnathan Evans, Laura Gifford, Malorie Perry, Simon Cottrell, Angela Marchbank, Alec Birchley, Alexander Adams, Amy Gaskin, Bree Gatica-Wilcox, Jason Coombes, Joel Southgate, Lauren Gilbert, Lee Graham, Nicole Pacchiarini, Sara Kumziene-Summerhayes, Sarah Taylor, Sophie Jones, Sara Rey, Matthew Bull, Joanne Watkins, Sally Corden, Tom Connor
EPI_ISL_637617	Liverpool Clinical Laboratories	COVID-19 Genomics UK (COG-UK) Consortium	Sam Haldenby, Anita Lucaci, Steve Paterson, Julian Hiscox, Alistair Darby, M Almsaud, A Alrezaihi, Muhannad Alruwaili, Stuart D Armstrong, Jones Benjamin, Eleanor G Bentley, Anu Chawla, Jordan J Clark, Angela Cowell, Richard Eccles, Isabel García-Dorival, Matthew Gemmell, Alessandro Gerada, PKF Gilmore, Richard Gregory, Ximeng Han, Catherine Hartley, Margaret Hughes, Miren Iturriza-Gomara, James Johnson, L Luu, Jenifer Manson, Charlotte Nelson, Elaine O'Toole, Cassie Olateju, Rebekah Penrice-Randal , Lucille Rainbow, N.P Randle, Trevor Ian Robinson, Parul Sharma, Ghada T Shawli, James P Stewart, Neil Swainston, Ecaterina Vamos, Joanne Watts, Mark Whitehead
EPI_ISL_637626	Wales Specialist Virology Centre Sequencing lab: Pathogen Genomics Unit	COVID-19 Genomics UK (COG-UK) Consortium	Catherine Moore, Johnathan Evans, Laura Gifford, Malorie Perry, Simon Cottrell, Angela Marchbank, Alec Birchley, Alexander Adams, Amy Gaskin, Bree Gatica-Wilcox, Jason Coombes, Joel Southgate, Lauren Gilbert, Lee Graham, Nicole Pacchiarini, Sara Kumziene-Summerhayes, Sarah Taylor, Sophie Jones, Sara Rey, Matthew Bull, Joanne Watkins, Sally Corden, Tom Connor
EPI_ISL_637660	Queens Medical Centre, Clinical Microbiology Department / DeepSeq Nottingham	COVID-19 Genomics UK (COG-UK) Consortium	Gemma Clark, Wendy Smith, Manjinder Khakh, Vicki M Fleming, Michelle M Lister, Hannah Howson-Wells, Jonathan Ball, Patrick McClure, Joseph Chappell, Theocharis Tsoleridis, Nadine Holmes, Matthew Carlisle, Christopher Moore, Fei Sang, Johnny Debebe, Victoria Wright, Matthew Loose
EPI_ISL_637675	Wales Specialist Virology Centre Sequencing lab: Pathogen Genomics Unit	COVID-19 Genomics UK (COG-UK) Consortium	Catherine Moore, Johnathan Evans, Laura Gifford, Malorie Perry, Simon Cottrell, Angela Marchbank, Alec Birchley, Alexander Adams, Amy Gaskin, Bree Gatica-Wilcox, Jason Coombes, Joel Southgate, Lauren Gilbert, Lee Graham, Nicole Pacchiarini, Sara Kumziene-Summerhayes, Sarah Taylor, Sophie Jones, Sara Rey, Matthew Bull, Joanne Watkins, Sally Corden, Tom Connor
EPI_ISL_637865, EPI_ISL_637866	Quadram Institute Bioscience	COVID-19 Genomics UK (COG-UK) Consortium	Dave J. Baker, Gemma L. Kay, Alp Aydin, Thanh Le-Viet, Steven Rudder, Ana P. Tedim, Anastasia Kolyva, Maria Diaz, Leonardo de Oliveira Martins, Nabil-Fareed Alikhan, Lizzie Meadows, Rachael Stanley, Ngozi Elumogo, Muhammed Yasir, Nicholas M. Thomson, Alexander J Trotter, Rachel Gilroy, Samuel Bloomfield, Claire Stuart, Andrew Bell, Reenesh Prakash, Samir Dervisevic, Alison E. Mather, John Wain, Mark Webber, Andrew J. Page, Justin

			O'Grady
EPI_ISL_637868, EPI_ISL_637869	Wales Specialist Virology Centre Sequencing lab: Pathogen Genomics Unit	COVID-19 Genomics UK (COG-UK) Consortium	Catherine Moore, Johnathan Evans, Laura Gifford, Malorie Perry, Simon Cottrell, Angela Marchbank, Alec Birchley, Alexander Adams, Amy Gaskin, Bree Gatica-Wilcox, Jason Coombes, Joel Southgate, Lauren Gilbert, Lee Graham, Nicole Pacchiarini, Sara Kumziene-Summerhayes, Sarah Taylor, Sophie Jones, Sara Rey, Matthew Bull, Joanne Watkins, Sally Corden, Tom Connor
EPI_ISL_637872	Virology Department, Sheffield Teaching Hospitals NHS Foundation Trust/Department of Infection, Immunity and Cardiovascular Disease, The Medical School, University of Sheffield	COVID-19 Genomics UK (COG-UK) Consortium	Thushan de Silva, Matthew Parker, Nikki Smith, Adri Angyal, Rebecca Brown, Luke Green, Rachel Tucker, Paul Parsons, Danielle Groves, Katie Johnson, Laura Carrilero, Alex Keeley, Dave Partridge, Matthew Wyles, Benjamin Lindsey, Mehmet Yavuz, Mohammad Raza, Cariad Evans
EPI_ISL_637878	Quadram Institute Bioscience	COVID-19 Genomics UK (COG-UK) Consortium	Dave J. Baker, Gemma L. Kay, Alp Aydin, Thanh Le-Viet, Steven Rudder, Ana P. Tedim, Anastasia Kolyva, Maria Diaz, Leonardo de Oliveira Martins, Nabil-Fareed Alikhan, Lizzie Meadows, Rachael Stanley, Ngozi Elumogo, Muhammed Yasir, Nicholas M. Thomson, Alexander J Trotter, Rachel Gilroy, Samuel Bloomfield, Claire Stuart, Andrew Bell, Reenesh Prakash, Samir Dervisevic, Alison E. Mather, John Wain, Mark Webber, Andrew J. Page, Justin O'Grady
EPI_ISL_637898	Liverpool Clinical Laboratories	COVID-19 Genomics UK (COG-UK) Consortium	Sam Haldenby, Anita Lucaci, Steve Paterson, Julian Hiscox, Alistair Darby, M Almsaud, A Alrezaihi, Muhannad Alruwaili, Stuart D Armstrong, Jones Benjamin, Eleanor G Bentley, Anu Chawla, Jordan J Clark, Angela Cowell, Richard Eccles, Isabel García-Dorival, Matthew Gemmell, Alessandro Gerada, PKF Gilmore, Richard Gregory, Ximeng Han, Catherine Hartley, Margaret Hughes, Miren Iturriza-Gomara, James Johnson, L Luu, Jenifer Manson, Charlotte Nelson, Elaine O'Toole, Cassie Olateju, Rebekah Penrice-Randal , Lucille Rainbow, N.P Randle, Trevor Ian Robinson, Parul Sharma, Ghada T Shawli, James P Stewart, Neil Swainston, Ecaterina Vamos, Joanne Watts, Mark Whitehead
EPI_ISL_637907, EPI_ISL_637909, EPI_ISL_637918	Wales Specialist Virology Centre Sequencing lab: Pathogen Genomics Unit	COVID-19 Genomics UK (COG-UK) Consortium	Catherine Moore, Johnathan Evans, Laura Gifford, Malorie Perry, Simon Cottrell, Angela Marchbank, Alec Birchley, Alexander Adams, Amy Gaskin, Bree Gatica-Wilcox, Jason Coombes, Joel Southgate, Lauren Gilbert, Lee Graham, Nicole Pacchiarini, Sara Kumziene-Summerhayes, Sarah Taylor, Sophie Jones, Sara Rey, Matthew Bull, Joanne Watkins, Sally Corden, Tom Connor
EPI_ISL_637922	Quadram Institute Bioscience	COVID-19 Genomics UK (COG-UK) Consortium	Dave J. Baker, Gemma L. Kay, Alp Aydin, Thanh Le-Viet, Steven Rudder, Ana P. Tedim, Anastasia Kolyva, Maria Diaz, Leonardo de Oliveira Martins, Nabil-Fareed Alikhan, Lizzie Meadows, Rachael Stanley, Ngozi Elumogo, Muhammed Yasir, Nicholas M. Thomson, Alexander J Trotter, Rachel Gilroy, Samuel Bloomfield, Claire Stuart, Andrew Bell, Reenesh Prakash, Samir Dervisevic, Alison E. Mather, John Wain, Mark Webber, Andrew J. Page, Justin O'Grady
EPI_ISL_637945, EPI_ISL_637976	Liverpool Clinical Laboratories	COVID-19 Genomics UK (COG-UK) Consortium	Sam Haldenby, Anita Lucaci, Steve Paterson, Julian Hiscox, Alistair Darby, M Almsaud, A Alrezaihi, Muhannad Alruwaili, Stuart D Armstrong, Jones Benjamin, Eleanor G Bentley, Anu Chawla, Jordan J Clark, Angela Cowell, Richard Eccles, Isabel García-Dorival, Matthew Gemmell, Alessandro Gerada, PKF Gilmore, Richard Gregory, Ximeng Han, Catherine Hartley, Margaret Hughes, Miren Iturriza-Gomara, James Johnson, L Luu, Jenifer Manson, Charlotte Nelson, Elaine O'Toole, Cassie Olateju, Rebekah Penrice-Randal , Lucille Rainbow, N.P Randle, Trevor Ian Robinson, Parul Sharma, Ghada T Shawli, James P Stewart, Neil Swainston, Ecaterina Vamos, Joanne Watts, Mark Whitehead
EPI_ISL_638007	Wales Specialist Virology Centre Sequencing lab: Pathogen Genomics Unit	COVID-19 Genomics UK (COG-UK) Consortium	Catherine Moore, Johnathan Evans, Laura Gifford, Malorie Perry, Simon Cottrell, Angela Marchbank, Alec Birchley, Alexander Adams, Amy Gaskin, Bree Gatica-Wilcox, Jason Coombes, Joel Southgate, Lauren Gilbert, Lee Graham, Nicole Pacchiarini, Sara Kumziene-Summerhayes, Sarah Taylor, Sophie Jones, Sara Rey, Matthew Bull, Joanne Watkins, Sally Corden, Tom Connor
EPI_ISL_638011	Liverpool Clinical Laboratories	COVID-19 Genomics UK (COG-UK) Consortium	Sam Haldenby, Anita Lucaci, Steve Paterson, Julian Hiscox, Alistair Darby, M Almsaud, A Alrezaihi, Muhannad Alruwaili, Stuart D Armstrong, Jones Benjamin, Eleanor G Bentley, Anu Chawla, Jordan J Clark, Angela Cowell, Richard Eccles, Isabel García-Dorival, Matthew Gemmell, Alessandro Gerada, PKF Gilmore, Richard Gregory, Ximeng Han, Catherine Hartley, Margaret Hughes, Miren Iturriza-Gomara, James Johnson, L Luu, Jenifer Manson, Charlotte Nelson, Elaine O'Toole, Cassie Olateju, Rebekah Penrice-Randal , Lucille Rainbow, N.P Randle, Trevor Ian Robinson, Parul Sharma, Ghada T Shawli, James P Stewart, Neil Swainston, Ecaterina Vamos, Joanne Watts, Mark Whitehead
EPI_ISL_638012	Wales Specialist Virology Centre Sequencing lab: Pathogen Genomics Unit	COVID-19 Genomics UK (COG-UK) Consortium	Catherine Moore, Johnathan Evans, Laura Gifford, Malorie Perry, Simon Cottrell, Angela Marchbank, Alec Birchley, Alexander Adams, Amy Gaskin, Bree Gatica-Wilcox, Jason Coombes, Joel Southgate, Lauren Gilbert, Lee Graham, Nicole Pacchiarini, Sara Kumziene-Summerhayes, Sarah Taylor, Sophie Jones, Sara Rey, Matthew Bull, Joanne Watkins, Sally Corden, Tom Connor
EPI_ISL_638025	Liverpool Clinical Laboratories	COVID-19 Genomics UK (COG-UK) Consortium	Sam Haldenby, Anita Lucaci, Steve Paterson, Julian Hiscox, Alistair Darby, M Almsaud, A Alrezaihi, Muhannad Alruwaili, Stuart D Armstrong, Jones Benjamin, Eleanor G Bentley, Anu Chawla, Jordan J Clark, Angela Cowell, Richard Eccles, Isabel García-Dorival, Matthew Gemmell, Alessandro Gerada, PKF Gilmore, Richard Gregory, Ximeng Han, Catherine Hartley, Margaret Hughes, Miren Iturriza-Gomara, James Johnson, L Luu, Jenifer Manson, Charlotte Nelson, Elaine O'Toole, Cassie Olateju, Rebekah Penrice-Randal , Lucille Rainbow, N.P Randle, Trevor Ian Robinson, Parul Sharma, Ghada T Shawli, James P Stewart, Neil Swainston, Ecaterina Vamos, Joanne Watts, Mark Whitehead
EPI_ISL_638033	Quadram Institute Bioscience	COVID-19 Genomics UK (COG-UK) Consortium	Dave J. Baker, Gemma L. Kay, Alp Aydin, Thanh Le-Viet, Steven Rudder, Ana P. Tedim, Anastasia Kolyva, Maria Diaz, Leonardo de Oliveira Martins, Nabil-Fareed Alikhan, Lizzie Meadows, Rachael Stanley, Ngozi Elumogo, Muhammed Yasir, Nicholas M. Thomson, Alexander J Trotter, Rachel Gilroy, Samuel Bloomfield, Claire Stuart, Andrew Bell, Reenesh Prakash, Samir Dervisevic, Alison E. Mather, John Wain, Mark Webber, Andrew J. Page, Justin O'Grady

EPI_ISL_638035, EPI_ISL_638044, EPI_ISL_638046, EPI_ISL_638047, EPI_ISL_638060, EPI_ISL_638091, EPI_ISL_638092, EPI_ISL_638098, EPI_ISL_638101, EPI_ISL_638104, EPI_ISL_638106, EPI_ISL_638107, EPI_ISL_638112, EPI_ISL_638120, EPI_ISL_638121, EPI_ISL_638129, EPI_ISL_638159, EPI_ISL_638172

see above	Wales Specialist Virology Centre Sequencing lab: Pathogen Genomics Unit	COVID-19 Genomics UK (COG-UK) Consortium	Catherine Moore, Johnathan Evans, Laura Gifford, Malorie Perry, Simon Cottrell, Angela Marchbank, Alec Birchley, Alexander Adams, Amy Gaskin, Bree Gatica-Wilcox, Jason Coombes, Joel Southgate, Lauren Gilbert, Lee Graham, Nicole Pacchiarini, Sara Kumziene-Summerhayes, Sarah Taylor, Sophie Jones, Sara Rey, Matthew Bull, Joanne Watkins, Sally Corden, Tom Connor
EPI_ISL_638449, EPI_ISL_638450, EPI_ISL_638451, EPI_ISL_638452, EPI_ISL_638453	Liverpool Clinical Laboratories	COVID-19 Genomics UK (COG-UK) Consortium	Sam Haldenby, Anita Lucaci, Steve Paterson, Julian Hiscox, Alistair Darby, M Almsaud, A Alrezaihi, Muhannad Alruwaili, Stuart D Armstrong, Jones Benjamin, Eleanor G Bentley, Anu Chawla, Jordan J Clark, Angela Cowell, Richard Eccles, Isabel García-Dorival, Matthew Gemmell, Alessandro Gerada, PKF Gilmore, Richard Gregory, Ximeng Han, Catherine Hartley, Margaret Hughes, Miren Iturriza-Gomara, James Johnson, L Luu, Jenifer Manson, Charlotte Nelson, Elaine O'Toole, Cassie Olateju, Rebekah Penrice-Randal , Lucille Rainbow, N.P Randle, Trevor Ian Robinson, Parul Sharma, Ghada T Shawli, James P Stewart, Neil Swainston, Ecaterina Vamos, Joanne Watts, Mark Whitehead
EPI_ISL_638582, EPI_ISL_638583	Quadram Institute Bioscience	COVID-19 Genomics UK (COG-UK) Consortium	Dave J. Baker, Gemma L. Kay, Alp Aydin, Thanh Le-Viet, Steven Rudder, Ana P. Tedim, Anastasia Kolyva, Maria Diaz, Leonardo de Oliveira Martins, Nabil-Fareed Alikhan, Lizzie Meadows, Rachael Stanley, Ngozi Elumogo, Muhammed Yasir, Nicholas M. Thomson, Alexander J Trotter, Rachel Gilroy, Samuel Bloomfield, Claire Stuart, Andrew Bell, Reenesh Prakash, Samir Dervisevic, Alison E. Mather, John Wain, Mark Webber, Andrew J. Page, Justin O'Grady
EPI_ISL_638586	Queens Medical Centre, Clinical Microbiology Department / DeepSeq Nottingham	COVID-19 Genomics UK (COG-UK) Consortium	Gemma Clark, Wendy Smith, Manjinder Khakh, Vicki M Fleming, Michelle M Lister, Hannah Howson-Wells, Jonathan Ball, Patrick McClure, Joseph Chappell, Theocharis Tsoleridis, Nadine Holmes, Matthew Carlisle, Christopher Moore, Fei Sang, Johnny Debebe, Victoria Wright, Matthew Loose
EPI_ISL_638877, EPI_ISL_638880, EPI_ISL_638881, EPI_ISL_638884, EPI_ISL_638887, EPI_ISL_638888, EPI_ISL_638889, EPI_ISL_638891, EPI_ISL_638893	Virology Department, Sheffield Teaching Hospitals NHS Foundation Trust/Department of Infection, Immunity and Cardiovascular Disease, The Medical School, University of Sheffield	COVID-19 Genomics UK (COG-UK) Consortium	Thushan de Silva, Matthew Parker, Nikki Smith, Adri Angyal, Rebecca Brown, Luke Green, Rachel Tucker, Paul Parsons, Danielle Groves, Katie Johnson, Laura Carrilero, Alex Keeley, Dave Partridge, Matthew Wyles, Benjamin Lindsey, Mehmet Yavuz, Mohammad Raza, Cariad Evans

EPI_ISL_639011, EPI_ISL_639017, EPI_ISL_639018, EPI_ISL_639021, EPI_ISL_639027, EPI_ISL_639031, EPI_ISL_639032, EPI_ISL_639034, EPI_ISL_639035, EPI_ISL_639037, EPI_ISL_639040, EPI_ISL_639041, EPI_ISL_639045, EPI_ISL_639053, EPI_ISL_639056, EPI_ISL_639057, EPI_ISL_639060, EPI_ISL_639067, EPI_ISL_639077, EPI_ISL_639080, EPI_ISL_639082, EPI_ISL_639085, EPI_ISL_639088, EPI_ISL_639090, EPI_ISL_639105, EPI_ISL_639108, EPI_ISL_639115, EPI_ISL_639119, EPI_ISL_639121, EPI_ISL_639122, EPI_ISL_639125, EPI_ISL_639146, EPI_ISL_639148, EPI_ISL_639155, EPI_ISL_639157, EPI_ISL_639158, EPI_ISL_639160, EPI_ISL_639161, EPI_ISL_639166, EPI_ISL_639173, EPI_ISL_639174, EPI_ISL_639175, EPI_ISL_639176, EPI_ISL_639183, EPI_ISL_639188, EPI_ISL_639193, EPI_ISL_639195, EPI_ISL_639197, EPI_ISL_639199, EPI_ISL_639203, EPI_ISL_639204, EPI_ISL_639205, EPI_ISL_639206, EPI_ISL_639211, EPI_ISL_639212, EPI_ISL_639213, EPI_ISL_639217, EPI_ISL_639222, EPI_ISL_639225, EPI_ISL_639227, EPI_ISL_639233, EPI_ISL_639234, EPI_ISL_639235, EPI_ISL_639238, EPI_ISL_639239, EPI_ISL_639240, EPI_ISL_639244, EPI_ISL_639249, EPI_ISL_639252, EPI_ISL_639253, EPI_ISL_639254, EPI_ISL_639258, EPI_ISL_639259, EPI_ISL_639261, EPI_ISL_639262, EPI_ISL_639267, EPI_ISL_639269, EPI_ISL_639271, EPI_ISL_639273, EPI_ISL_639275, EPI_ISL_639277, EPI_ISL_639280, EPI_ISL_639282, EPI_ISL_639288, EPI_ISL_639296, EPI_ISL_639308, EPI_ISL_639310, EPI_ISL_639319, EPI_ISL_639321, EPI_ISL_639322, EPI_ISL_639325, EPI_ISL_639327, EPI_ISL_639328, EPI_ISL_639330, EPI_ISL_639332, EPI_ISL_639334, EPI_ISL_639339, EPI_ISL_639344, EPI_ISL_639345, EPI_ISL_639351, EPI_ISL_639358, EPI_ISL_639359, EPI_ISL_639360, EPI_ISL_639362, EPI_ISL_639363, EPI_ISL_639365, EPI_ISL_639369, EPI_ISL_639379, EPI_ISL_639381, EPI_ISL_639384, EPI_ISL_639388, EPI_ISL_639390, EPI_ISL_639395, EPI_ISL_639397, EPI_ISL_639398, EPI_ISL_639403, EPI_ISL_639404, EPI_ISL_639405, EPI_ISL_639407, EPI_ISL_639418, EPI_ISL_639421, EPI_ISL_639426, EPI_ISL_639427, EPI_ISL_639428, EPI_ISL_639433, EPI_ISL_639434, EPI_ISL_639441, EPI_ISL_639443, EPI_ISL_639447, EPI_ISL_639448, EPI_ISL_639452, EPI_ISL_639453, EPI_ISL_639460, EPI_ISL_639462, EPI_ISL_639464, EPI_ISL_639467, EPI_ISL_639468, EPI_ISL_639469, EPI_ISL_639475, EPI_ISL_639481, EPI_ISL_639482, EPI_ISL_639484, EPI_ISL_639485, EPI_ISL_639490, EPI_ISL_639492, EPI_ISL_639495, EPI_ISL_639496, EPI_ISL_639498, EPI_ISL_639499, EPI_ISL_639500, EPI_ISL_639504, EPI_ISL_639506, EPI_ISL_639510, EPI_ISL_639515, EPI_ISL_639516, EPI_ISL_639518, EPI_ISL_639526, EPI_ISL_639529, EPI_ISL_639531, EPI_ISL_639532, EPI_ISL_639535, EPI_ISL_639536,

EPI_ISL_639537, EPI_ISL_639540, EPI_ISL_639545, EPI_ISL_639547, EPI_ISL_639548, EPI_ISL_639551, EPI_ISL_639556, EPI_ISL_639558, EPI_ISL_639560, EPI_ISL_639562, EPI_ISL_639565, EPI_ISL_639568, EPI_ISL_639572, EPI_ISL_639574, EPI_ISL_639576, EPI_ISL_639581, EPI_ISL_639586, EPI_ISL_639587, EPI_ISL_639591, EPI_ISL_639596, EPI_ISL_639599, EPI_ISL_639601, EPI_ISL_639602, EPI_ISL_639605, EPI_ISL_639609, EPI_ISL_639610, EPI_ISL_639614, EPI_ISL_639620, EPI_ISL_639621, EPI_ISL_639623			
see above	Wales Specialist Virology Centre Sequencing lab: Pathogen Genomics Unit	COVID-19 Genomics UK (COG-UK) Consortium	Catherine Moore, Johnathan Evans, Laura Gifford, Malorie Perry, Simon Cottrell, Angela Marchbank, Alec Birchley, Alexander Adams, Amy Gaskin, Bree Gatica-Wilcox, Jason Coombes, Joel Southgate, Lauren Gilbert, Lee Graham, Nicole Pacchiarini, Sara Kumziene-Summerhayes, Sarah Taylor, Sophie Jones, Sara Rey, Matthew Bull, Joanne Watkins, Sally Corden, Tom Connor
EPI_ISL_639822	Respiratory Virus Unit, Microbiology Services Colindale, Public Health England	COVID-19 Genomics UK (COG-UK) Consortium	PHE Covid Sequencing Team
EPI_ISL_639986, EPI_ISL_639988, EPI_ISL_640008	CNR Virus des Infections Respiratoires - France SUD	CNR Virus des Infections Respiratoires - France SUD	Antonin Bal, Gregory Destras, Gwendolyne Burfin, Hadrien Règue, Alexandre Gaymard, Maude Bouscambert-Duchamp, Florence Morfin-Sherpa, Martine Valette, Bruno Lina, Laurence Josset
EPI_ISL_641396	Department of Virus and Microbiological Special Diagnostics, Statens Serum Institut, Copenhagen, Denmark	Albertsen lab, Department of Chemistry and Bioscience, Aalborg University, Denmark	Thomas Bruun Rasmussen, Jannik Fonager, Morten Rasmussen
EPI_ISL_641610, EPI_ISL_641613, EPI_ISL_641628, EPI_ISL_641631, EPI_ISL_641635, EPI_ISL_641649, EPI_ISL_641650, EPI_ISL_641652, EPI_ISL_641667, EPI_ISL_641668, EPI_ISL_641671, EPI_ISL_641685, EPI_ISL_641686, EPI_ISL_641687, EPI_ISL_641696, EPI_ISL_641707, EPI_ISL_641710, EPI_ISL_641711, EPI_ISL_641715, EPI_ISL_641720, EPI_ISL_641733, EPI_ISL_641734, EPI_ISL_641744, EPI_ISL_641745, EPI_ISL_641747, EPI_ISL_641753, EPI_ISL_641756, EPI_ISL_641758, EPI_ISL_641759, EPI_ISL_641761, EPI_ISL_641762, EPI_ISL_641763, EPI_ISL_641769, EPI_ISL_641775, EPI_ISL_641780, EPI_ISL_641782, EPI_ISL_641785, EPI_ISL_641788, EPI_ISL_641795, EPI_ISL_641796, EPI_ISL_641799, EPI_ISL_641802, EPI_ISL_641804, EPI_ISL_641814, EPI_ISL_641815, EPI_ISL_641816, EPI_ISL_641832, EPI_ISL_641833, EPI_ISL_641842, EPI_ISL_641847, EPI_ISL_641850, EPI_ISL_641857, EPI_ISL_641861, EPI_ISL_641864, EPI_ISL_641868, EPI_ISL_641878, EPI_ISL_641879, EPI_ISL_641882, EPI_ISL_641884, EPI_ISL_641888, EPI_ISL_641897, EPI_ISL_641900, EPI_ISL_641901, EPI_ISL_641911, EPI_ISL_641960, EPI_ISL_641979, EPI_ISL_641994, EPI_ISL_642018, EPI_ISL_642038, EPI_ISL_642069, EPI_ISL_642074, EPI_ISL_642155, EPI_ISL_642166, EPI_ISL_642169, EPI_ISL_642170, EPI_ISL_642203, EPI_ISL_642222, EPI_ISL_642243, EPI_ISL_642247, EPI_ISL_642251, EPI_ISL_642284, EPI_ISL_642354, EPI_ISL_642366, EPI_ISL_642379, EPI_ISL_642420, EPI_ISL_642437, EPI_ISL_642439, EPI_ISL_642477, EPI_ISL_642506, EPI_ISL_642508, EPI_ISL_642521, EPI_ISL_642569, EPI_ISL_642581, EPI_ISL_642599, EPI_ISL_642605, EPI_ISL_642634, EPI_ISL_642635, EPI_ISL_642644, EPI_ISL_642655, EPI_ISL_642663, EPI_ISL_642664, EPI_ISL_642684, EPI_ISL_642702, EPI_ISL_642703, EPI_ISL_642729, EPI_ISL_642744			
see above	Lighthouse Lab in Glasgow	Wellcome Sanger Institute for the COVID-19 Genomics UK (COG-UK) Consortium	Harper VanSteenhouse, Yumi Kasai, David Gray, Carol Clugston, Anna Dominiczak and Alex Alderton, Roberto Amato, Sonia Goncalves, Ewan Harrison, David K. Jackson, Ian Johnston, Dominic Kwiatkowski, Cordelia Langford, John Sillitoe on behalf of the Wellcome Sanger Institute COVID-19 Surveillance Team
EPI_ISL_643315, EPI_ISL_643316, EPI_ISL_643317, EPI_ISL_643318, EPI_ISL_643319, EPI_ISL_643320, EPI_ISL_643321, EPI_ISL_643323, EPI_ISL_643326, EPI_ISL_643327, EPI_ISL_643328, EPI_ISL_643332, EPI_ISL_643333, EPI_ISL_643335, EPI_ISL_643336, EPI_ISL_643340, EPI_ISL_643342, EPI_ISL_643343, EPI_ISL_643344, EPI_ISL_643345, EPI_ISL_643346, EPI_ISL_643347, EPI_ISL_643348, EPI_ISL_643354, EPI_ISL_643357, EPI_ISL_643358, EPI_ISL_643359, EPI_ISL_643360, EPI_ISL_643361, EPI_ISL_643362, EPI_ISL_643363, EPI_ISL_643366, EPI_ISL_643367, EPI_ISL_643369, EPI_ISL_643371, EPI_ISL_643373, EPI_ISL_643377, EPI_ISL_643379, EPI_ISL_643381, EPI_ISL_643384, EPI_ISL_643385, EPI_ISL_643387, EPI_ISL_643388, EPI_ISL_643390, EPI_ISL_643394, EPI_ISL_643395, EPI_ISL_643397, EPI_ISL_643398, EPI_ISL_643400, EPI_ISL_643401, EPI_ISL_643402, EPI_ISL_643404, EPI_ISL_643405, EPI_ISL_643406, EPI_ISL_643407, EPI_ISL_643409, EPI_ISL_643410, EPI_ISL_643419, EPI_ISL_643420, EPI_ISL_643422, EPI_ISL_643425, EPI_ISL_643426, EPI_ISL_643427, EPI_ISL_643429, EPI_ISL_643431, EPI_ISL_643433, EPI_ISL_643436, EPI_ISL_643437, EPI_ISL_643438, EPI_ISL_643439, EPI_ISL_643440, EPI_ISL_643441, EPI_ISL_643443, EPI_ISL_643444, EPI_ISL_643445, EPI_ISL_643452, EPI_ISL_643453, EPI_ISL_643455, EPI_ISL_643456, EPI_ISL_643457, EPI_ISL_643460, EPI_ISL_643463, EPI_ISL_643464, EPI_ISL_643465, EPI_ISL_643466, EPI_ISL_643468, EPI_ISL_643469, EPI_ISL_643473, EPI_ISL_643476, EPI_ISL_643479, EPI_ISL_643483, EPI_ISL_643486, EPI_ISL_643487, EPI_ISL_643490, EPI_ISL_643493, EPI_ISL_643496, EPI_ISL_643497, EPI_ISL_643500, EPI_ISL_643501, EPI_ISL_643502, EPI_ISL_643503, EPI_ISL_643507, EPI_ISL_643508, EPI_ISL_643511, EPI_ISL_643515, EPI_ISL_643522, EPI_ISL_643523, EPI_ISL_643524, EPI_ISL_643525, EPI_ISL_643529, EPI_ISL_643530, EPI_ISL_643532, EPI_ISL_643535, EPI_ISL_643537, EPI_ISL_643538, EPI_ISL_643541, EPI_ISL_643547, EPI_ISL_643549, EPI_ISL_643550, EPI_ISL_643552, EPI_ISL_643555, EPI_ISL_643556, EPI_ISL_643558, EPI_ISL_643559, EPI_ISL_643560, EPI_ISL_643561, EPI_ISL_643562, EPI_ISL_643563, EPI_ISL_643564, EPI_ISL_643566, EPI_ISL_643567, EPI_ISL_643569, EPI_ISL_643570, EPI_ISL_643571, EPI_ISL_643573, EPI_ISL_643574, EPI_ISL_643578, EPI_ISL_643579, EPI_ISL_643580, EPI_ISL_643582, EPI_ISL_643583, EPI_ISL_643585, EPI_ISL_643587, EPI_ISL_643589, EPI_ISL_643591, EPI_ISL_643592, EPI_ISL_643596, EPI_ISL_643597, EPI_ISL_643599, EPI_ISL_643601, EPI_ISL_643604, EPI_ISL_643606, EPI_ISL_643607, EPI_ISL_643608, EPI_ISL_643611, EPI_ISL_643614, EPI_ISL_643618, EPI_ISL_643619, EPI_ISL_643620, EPI_ISL_643621, EPI_ISL_643622, EPI_ISL_643623, EPI_ISL_643624, EPI_ISL_643625, EPI_ISL_643627, EPI_ISL_643639, EPI_ISL_643645, EPI_ISL_643647, EPI_ISL_643648, EPI_ISL_643649, EPI_ISL_643652, EPI_ISL_643657, EPI_ISL_643659, EPI_ISL_643660, EPI_ISL_643661, EPI_ISL_643665, EPI_ISL_643666, EPI_ISL_643667, EPI_ISL_643670, EPI_ISL_643673, EPI_ISL_643674, EPI_ISL_643680, EPI_ISL_643683, EPI_ISL_643685, EPI_ISL_643687, EPI_ISL_643688, EPI_ISL_643697, EPI_ISL_643698, EPI_ISL_643701, EPI_ISL_643702, EPI_ISL_643704, EPI_ISL_643707, EPI_ISL_643709, EPI_ISL_643716, EPI_ISL_643717, EPI_ISL_643719, EPI_ISL_643722, EPI_ISL_643723, EPI_ISL_643724, EPI_ISL_643726, EPI_ISL_643727, EPI_ISL_643728, EPI_ISL_643736, EPI_ISL_643738, EPI_ISL_643739, EPI_ISL_643743, EPI_ISL_643747, EPI_ISL_643749, EPI_ISL_643752, EPI_ISL_643756, EPI_ISL_643758, EPI_ISL_643759, EPI_ISL_643761, EPI_ISL_643762, EPI_ISL_643765, EPI_ISL_643766, EPI_ISL_643767, EPI_ISL_643774, EPI_ISL_643775, EPI_ISL_643778, EPI_ISL_643779, EPI_ISL_643781, EPI_ISL_643782, EPI_ISL_643789, EPI_ISL_643792, EPI_ISL_643795, EPI_ISL_643797, EPI_ISL_643804, EPI_ISL_643805, EPI_ISL_643808, EPI_ISL_643809, EPI_ISL_643810, EPI_ISL_643812, EPI_ISL_643813, EPI_ISL_643817, EPI_ISL_643819, EPI_ISL_643820, EPI_ISL_643821, EPI_ISL_643822, EPI_ISL_643823, EPI_ISL_643824, EPI_ISL_643825, EPI_ISL_643826, EPI_ISL_643828, EPI_ISL_643831, EPI_ISL_643837, EPI_ISL_643840, EPI_ISL_643843, EPI_ISL_643859, EPI_ISL_643860, EPI_ISL_643861, EPI_ISL_643862, EPI_ISL_643863, EPI_ISL_643866, EPI_ISL_643868, EPI_ISL_643870, EPI_ISL_643876, EPI_ISL_643877, EPI_ISL_643879, EPI_ISL_643880, EPI_ISL_643883, EPI_ISL_643885, EPI_ISL_643886, EPI_ISL_643888, EPI_ISL_643890, EPI_ISL_643891, EPI_ISL_643892, EPI_ISL_643893, EPI_ISL_643894, EPI_ISL_643896, EPI_ISL_643897, EPI_ISL_643898, EPI_ISL_643900, EPI_ISL_643910, EPI_ISL_643913, EPI_ISL_643916, EPI_ISL_643918, EPI_ISL_643921, EPI_ISL_643924, EPI_ISL_643926, EPI_ISL_643934, EPI_ISL_643935, EPI_ISL_643936, EPI_ISL_643938, EPI_ISL_643941, EPI_ISL_643942, EPI_ISL_643946, EPI_ISL_643947, EPI_ISL_643951, EPI_ISL_643952, EPI_ISL_643953, EPI_ISL_643957, EPI_ISL_643959, EPI_ISL_643961, EPI_ISL_643963, EPI_ISL_643970, EPI_ISL_643973, EPI_ISL_643975, EPI_ISL_643980, EPI_ISL_643982			
see above	Lighthouse Lab in Cambridge	Wellcome Sanger Institute for the COVID-19 Genomics UK (COG-UK) Consortium	Rob Howes, The Lighthouse Lab in Cambridge and Alex Alderton, Roberto Amato, Sonia Goncalves, Ewan Harrison, David K. Jackson, Ian Johnston, Dominic Kwiatkowski, Cordelia Langford, John Sillitoe on behalf of the Wellcome Sanger Institute COVID-19 Surveillance Team
EPI_ISL_644611, EPI_ISL_644612, EPI_ISL_644613, EPI_ISL_644616, EPI_ISL_644617, EPI_ISL_644618, EPI_ISL_644619, EPI_ISL_644620, EPI_ISL_644621, EPI_ISL_644622, EPI_ISL_644623, EPI_ISL_644624, EPI_ISL_644625, EPI_ISL_644626			
see above	Department of Clinical Microbiology	GIGA Medical Genomics	Keith Durkin, Maria Artesi, Sébastien Bontems, Raphaël Boreux, Bouchra Boujemla, Cécile Meex, Pierrette Melin, Marie-Pierre Hayette, Vincent Bours
EPI_ISL_645221, EPI_ISL_645226, EPI_ISL_645241, EPI_ISL_645243, EPI_ISL_645244, EPI_ISL_645263, EPI_ISL_645285, EPI_ISL_645300, EPI_ISL_645308, EPI_ISL_645319, EPI_ISL_645324, EPI_ISL_645327, EPI_ISL_645329, EPI_ISL_645367, EPI_ISL_645369, EPI_ISL_645374, EPI_ISL_645383, EPI_ISL_645391, EPI_ISL_645393, EPI_ISL_645395, EPI_ISL_645399, EPI_ISL_645405, EPI_ISL_645424, EPI_ISL_645441, EPI_ISL_645453, EPI_ISL_645477, EPI_ISL_645513, EPI_ISL_645518, EPI_ISL_645520			
see above	Lighthouse Lab in Milton Keynes	Wellcome Sanger Institute for the COVID-19 Genomics UK (COG-UK) Consortium	The Lighthouse Lab in Milton Keynes and Alex Alderton, Roberto Amato, Sonia Goncalves, Ewan Harrison, David K. Jackson, Ian Johnston, Dominic Kwiatkowski, Cordelia Langford, John Sillitoe on behalf of the Wellcome Sanger Institute COVID-19 Surveillance Team
EPI_ISL_645530, EPI_ISL_645531, EPI_ISL_645532, EPI_ISL_645533, EPI_ISL_645534, EPI_ISL_645535, EPI_ISL_645536, EPI_ISL_645537, EPI_ISL_645540, EPI_ISL_645541, EPI_ISL_645542, EPI_ISL_645543, EPI_ISL_645544, EPI_ISL_645546, EPI_ISL_645547, EPI_ISL_645548, EPI_ISL_645549, EPI_ISL_645550, EPI_ISL_645552, EPI_ISL_645553, EPI_ISL_645554, EPI_ISL_645555, EPI_ISL_645556, EPI_ISL_645557, EPI_ISL_645558, EPI_ISL_645559, EPI_ISL_645560, EPI_ISL_645561, EPI_ISL_645562, EPI_ISL_645563, EPI_ISL_645564, EPI_ISL_645565, EPI_ISL_645566, EPI_ISL_645567, EPI_ISL_645568, EPI_ISL_645569, EPI_ISL_645570, EPI_ISL_645571, EPI_ISL_645573, EPI_ISL_645574, EPI_ISL_645575, EPI_ISL_645577, EPI_ISL_645578, EPI_ISL_645579, EPI_ISL_645580, EPI_ISL_645581, EPI_ISL_645582, EPI_ISL_645583, EPI_ISL_645585, EPI_ISL_645586, EPI_ISL_645587, EPI_ISL_645588, EPI_ISL_645589, EPI_ISL_645590, EPI_ISL_645591, EPI_ISL_645592, EPI_ISL_645593, EPI_ISL_645594, EPI_ISL_645595, EPI_ISL_645596, EPI_ISL_645597, EPI_ISL_645598, EPI_ISL_645599, EPI_ISL_645600, EPI_ISL_645601, EPI_ISL_645602, EPI_ISL_645603, EPI_ISL_645604, EPI_ISL_645605, EPI_ISL_645606, EPI_ISL_645607, EPI_ISL_645609, EPI_ISL_645611, EPI_ISL_645612, EPI_ISL_645613, EPI_ISL_645614, EPI_ISL_645615, EPI_ISL_645616, EPI_ISL_645617, EPI_ISL_645618, EPI_ISL_645619, EPI_ISL_645620, EPI_ISL_645621, EPI_ISL_645622, EPI_ISL_645624, EPI_ISL_645625, EPI_ISL_645626, EPI_ISL_645627, EPI_ISL_645628, EPI_ISL_645629, EPI_ISL_645630, EPI_ISL_645632, EPI_ISL_645633, EPI_ISL_645634, EPI_ISL_645635, EPI_ISL_645636, EPI_ISL_645637, EPI_ISL_645638, EPI_ISL_645639, EPI_ISL_645640, EPI_ISL_645641, EPI_ISL_645642, EPI_ISL_645643, EPI_ISL_645644, EPI_ISL_645645, EPI_ISL_645646, EPI_ISL_645647, EPI_ISL_645648, EPI_ISL_645649, EPI_ISL_645650, EPI_ISL_645651, EPI_ISL_645652, EPI_ISL_645653, EPI_ISL_645654, EPI_ISL_645655, EPI_ISL_645656, EPI_ISL_645657, EPI_ISL_645658, EPI_ISL_645660, EPI_ISL_645661, EPI_ISL_645662, EPI_ISL_645663, EPI_ISL_645664, EPI_ISL_645666, EPI_ISL_645667, EPI_ISL_645668, EPI_ISL_645669, EPI_ISL_645670, EPI_ISL_645671, EPI_ISL_645672, EPI_ISL_645673, EPI_ISL_645674, EPI_ISL_645675, EPI_ISL_645676, EPI_ISL_645677, EPI_ISL_645679, EPI_ISL_645680, EPI_ISL_645681, EPI_ISL_645682, EPI_ISL_645683, EPI_ISL_645684, EPI_ISL_645685, EPI_ISL_645687, EPI_ISL_645688, EPI_ISL_645689, EPI_ISL_645690, EPI_ISL_645691, EPI_ISL_645693, EPI_ISL_645694, EPI_ISL_645695, EPI_ISL_645696, EPI_ISL_645697, EPI_ISL_645698, EPI_ISL_645699, EPI_ISL_645700, EPI_ISL_645701, EPI_ISL_645702, EPI_ISL_645703, EPI_ISL_645704, EPI_ISL_645705, EPI_ISL_645706, EPI_ISL_645707, EPI_ISL_645708, EPI_ISL_645709, EPI_ISL_645710, EPI_ISL_645711, EPI_ISL_645712, EPI_ISL_645713, EPI_ISL_645714, EPI_ISL_645715, EPI_ISL_645716, EPI_ISL_645717, EPI_ISL_645718, EPI_ISL_645719, EPI_ISL_645720, EPI_ISL_645721, EPI_ISL_645722, EPI_ISL_645723, EPI_ISL_645724, EPI_ISL_645725, EPI_ISL_645726, EPI_ISL_645727, EPI_ISL_645728, EPI_ISL_645729, EPI_ISL_645730, EPI_ISL_645732, EPI_ISL_645733, EPI_ISL_645734, EPI_ISL_645736, EPI_ISL_645737, EPI_ISL_645738, EPI_ISL_645739, EPI_ISL_645740, EPI_ISL_645741, EPI_ISL_645742, EPI_ISL_645743, EPI_ISL_645744, EPI_ISL_645745, EPI_ISL_645746, EPI_ISL_645747, EPI_ISL_645748, EPI_ISL_645749, EPI_ISL_645750, EPI_ISL_645751, EPI_ISL_645752, EPI_ISL_645753, EPI_ISL_645754, EPI_ISL_645756, EPI_ISL_645759, EPI_ISL_645760, EPI_ISL_645761, EPI_ISL_645762, EPI_ISL_645763, EPI_ISL_645764, EPI_ISL_645765, EPI_ISL_645766, EPI_ISL_645767, EPI_ISL_645769, EPI_ISL_645770, EPI_ISL_645771, EPI_ISL_645772, EPI_ISL_645773, EPI_ISL_645774, EPI_ISL_645775, EPI_ISL_645776, EPI_ISL_645777, EPI_ISL_645778, EPI_ISL_645779, EPI_ISL_645780, EPI_ISL_645781, EPI_ISL_645782, EPI_ISL_645783, EPI_ISL_645784, EPI_ISL_645785, EPI_ISL_645786, EPI_ISL_645787, EPI_ISL_645788, EPI_ISL_645789, EPI_ISL_645790, EPI_ISL_645792, EPI_ISL_645793, EPI_ISL_645794, EPI_ISL_645795, EPI_ISL_645797, EPI_ISL_645798, EPI_ISL_645799, EPI_ISL_645800, EPI_ISL_645801, EPI_ISL_645802, EPI_ISL_645803, EPI_ISL_645804, EPI_ISL_645805, EPI_ISL_645806, EPI_ISL_645807, EPI_ISL_645808, EPI_ISL_645809, EPI_ISL_645810, EPI_ISL_645811, EPI_ISL_645814, EPI_ISL_645815, EPI_ISL_645816, EPI_ISL_645817, EPI_ISL_645818, EPI_ISL_645819, EPI_ISL_645820, EPI_ISL_645821, EPI_ISL_645822, EPI_ISL_645823, EPI_ISL_645824, EPI_ISL_645827, EPI_ISL_645830, EPI_ISL_645831, EPI_ISL_645833, EPI_ISL_645834, EPI_ISL_645835, EPI_ISL_645837			
see above	Lighthouse Lab in Alderley Park	Wellcome Sanger Institute for the COVID-19 Genomics UK (COG-UK) Consortium	Jacquelyn Wynn, Mairead Hyland, The Lighthouse Lab in Alderley Park and Alex Alderton, Roberto Amato, Sonia Goncalves, Ewan Harrison, David K. Jackson, Ian Johnston, Dominic Kwiatkowski, Cordelia Langford, John Sillitoe on behalf of the Wellcome Sanger Institute COVID-19 Surveillance Team
EPI_ISL_645840	Lighthouse Lab in Cambridge	Wellcome Sanger Institute for the COVID-19 Genomics UK (COG-UK) Consortium	Rob Howes, The Lighthouse Lab in Cambridge and Alex Alderton, Roberto Amato, Sonia Goncalves, Ewan Harrison, David K. Jackson, Ian Johnston, Dominic Kwiatkowski, Cordelia Langford, John Sillitoe on behalf of the Wellcome Sanger Institute COVID-19 Surveillance Team
EPI_ISL_645841, EPI_ISL_645842, EPI_ISL_645843, EPI_ISL_645844, EPI_ISL_645845, EPI_ISL_645846, EPI_ISL_645847, EPI_ISL_645848, EPI_ISL_645849, EPI_ISL_645853, EPI_ISL_645854, EPI_ISL_645855, EPI_ISL_645856, EPI_ISL_645857, EPI_ISL_645858, EPI_ISL_645859, EPI_ISL_645860, EPI_ISL_645861, EPI_ISL_645862, EPI_ISL_645863, EPI_ISL_645864, EPI_ISL_645865, EPI_ISL_645867, EPI_ISL_645870, EPI_ISL_645871, EPI_ISL_645872, EPI_ISL_645873, EPI_ISL_645874, EPI_ISL_645876, EPI_ISL_645878, EPI_ISL_645879, EPI_ISL_645883, EPI_ISL_645884			
see above	Lighthouse Lab in Alderley Park	Wellcome Sanger Institute for the COVID-19 Genomics UK (COG-UK) Consortium	Jacquelyn Wynn, Mairead Hyland, The Lighthouse Lab in Alderley Park and Alex Alderton, Roberto Amato, Sonia Goncalves, Ewan Harrison, David K. Jackson, Ian Johnston, Dominic Kwiatkowski, Cordelia Langford, John Sillitoe on behalf of the Wellcome Sanger Institute COVID-19 Surveillance Team
EPI_ISL_645886	Lighthouse Lab in Cambridge	Wellcome Sanger Institute for the COVID-19 Genomics UK (COG-UK) Consortium	Rob Howes, The Lighthouse Lab in Cambridge and Alex Alderton, Roberto Amato, Sonia Goncalves, Ewan Harrison, David K. Jackson, Ian Johnston, Dominic Kwiatkowski, Cordelia Langford, John Sillitoe on behalf of the Wellcome Sanger Institute COVID-19 Surveillance Team
EPI_ISL_645887, EPI_ISL_645888, EPI_ISL_645890, EPI_ISL_645891, EPI_ISL_645892, EPI_ISL_645893, EPI_ISL_645894, EPI_ISL_645895	Lighthouse Lab in Alderley Park	Wellcome Sanger Institute for the COVID-19 Genomics UK (COG-UK) Consortium	Jacquelyn Wynn, Mairead Hyland, The Lighthouse Lab in Alderley Park and Alex Alderton, Roberto Amato, Sonia Goncalves, Ewan Harrison, David K. Jackson, Ian Johnston, Dominic Kwiatkowski, Cordelia Langford, John Sillitoe on behalf of the Wellcome Sanger Institute COVID-19 Surveillance Team
EPI_ISL_645896, EPI_ISL_645897	Lighthouse Lab in Cambridge	Wellcome Sanger Institute for the COVID-19 Genomics UK (COG-UK) Consortium	Rob Howes, The Lighthouse Lab in Cambridge and Alex Alderton, Roberto Amato, Sonia Goncalves, Ewan Harrison, David K. Jackson, Ian Johnston, Dominic Kwiatkowski, Cordelia Langford, John Sillitoe on behalf of the Wellcome Sanger Institute COVID-19 Surveillance Team

EPI_ISL_645898, EPI_ISL_645900, EPI_ISL_645901	Lighthouse Lab in Alderley Park	Wellcome Sanger Institute for the COVID-19 Genomics UK (COG-UK) Consortium	Jacquelyn Wynn, Mairead Hyland, The Lighthouse Lab in Alderley Park and Alex Alderton, Roberto Amato, Sonia Goncalves, Ewan Harrison, David K. Jackson, Ian Johnston, Dominic Kwiatkowski, Cordelia Langford, John Sillitoe on behalf of the Wellcome Sanger Institute COVID-19 Surveillance Team
EPI_ISL_645902	Lighthouse Lab in Cambridge	Wellcome Sanger Institute for the COVID-19 Genomics UK (COG-UK) Consortium	Rob Howes, The Lighthouse Lab in Cambridge and Alex Alderton, Roberto Amato, Sonia Goncalves, Ewan Harrison, David K. Jackson, Ian Johnston, Dominic Kwiatkowski, Cordelia Langford, John Sillitoe on behalf of the Wellcome Sanger Institute COVID-19 Surveillance Team
EPI_ISL_645903, EPI_ISL_645904	Lighthouse Lab in Alderley Park	Wellcome Sanger Institute for the COVID-19 Genomics UK (COG-UK) Consortium	Jacquelyn Wynn, Mairead Hyland, The Lighthouse Lab in Alderley Park and Alex Alderton, Roberto Amato, Sonia Goncalves, Ewan Harrison, David K. Jackson, Ian Johnston, Dominic Kwiatkowski, Cordelia Langford, John Sillitoe on behalf of the Wellcome Sanger Institute COVID-19 Surveillance Team
EPI_ISL_645905	Lighthouse Lab in Cambridge	Wellcome Sanger Institute for the COVID-19 Genomics UK (COG-UK) Consortium	Rob Howes, The Lighthouse Lab in Cambridge and Alex Alderton, Roberto Amato, Sonia Goncalves, Ewan Harrison, David K. Jackson, Ian Johnston, Dominic Kwiatkowski, Cordelia Langford, John Sillitoe on behalf of the Wellcome Sanger Institute COVID-19 Surveillance Team
EPI_ISL_645906, EPI_ISL_645907, EPI_ISL_645909, EPI_ISL_645910, EPI_ISL_645911, EPI_ISL_645912, EPI_ISL_645913, EPI_ISL_645914, EPI_ISL_645915	Lighthouse Lab in Alderley Park	Wellcome Sanger Institute for the COVID-19 Genomics UK (COG-UK) Consortium	Jacquelyn Wynn, Mairead Hyland, The Lighthouse Lab in Alderley Park and Alex Alderton, Roberto Amato, Sonia Goncalves, Ewan Harrison, David K. Jackson, Ian Johnston, Dominic Kwiatkowski, Cordelia Langford, John Sillitoe on behalf of the Wellcome Sanger Institute COVID-19 Surveillance Team
EPI_ISL_645916	Lighthouse Lab in Cambridge	Wellcome Sanger Institute for the COVID-19 Genomics UK (COG-UK) Consortium	Rob Howes, The Lighthouse Lab in Cambridge and Alex Alderton, Roberto Amato, Sonia Goncalves, Ewan Harrison, David K. Jackson, Ian Johnston, Dominic Kwiatkowski, Cordelia Langford, John Sillitoe on behalf of the Wellcome Sanger Institute COVID-19 Surveillance Team
EPI_ISL_645919, EPI_ISL_645921, EPI_ISL_645922, EPI_ISL_645924, EPI_ISL_645928, EPI_ISL_645929	Lighthouse Lab in Alderley Park	Wellcome Sanger Institute for the COVID-19 Genomics UK (COG-UK) Consortium	Jacquelyn Wynn, Mairead Hyland, The Lighthouse Lab in Alderley Park and Alex Alderton, Roberto Amato, Sonia Goncalves, Ewan Harrison, David K. Jackson, Ian Johnston, Dominic Kwiatkowski, Cordelia Langford, John Sillitoe on behalf of the Wellcome Sanger Institute COVID-19 Surveillance Team
EPI_ISL_645930	Lighthouse Lab in Cambridge	Wellcome Sanger Institute for the COVID-19 Genomics UK (COG-UK) Consortium	Rob Howes, The Lighthouse Lab in Cambridge and Alex Alderton, Roberto Amato, Sonia Goncalves, Ewan Harrison, David K. Jackson, Ian Johnston, Dominic Kwiatkowski, Cordelia Langford, John Sillitoe on behalf of the Wellcome Sanger Institute COVID-19 Surveillance Team
EPI_ISL_645931, EPI_ISL_645932, EPI_ISL_645933, EPI_ISL_645934, EPI_ISL_645936, EPI_ISL_645937, EPI_ISL_645938, EPI_ISL_645939, EPI_ISL_645940, EPI_ISL_645941, EPI_ISL_645942, EPI_ISL_645943, EPI_ISL_645944			
see above	Lighthouse Lab in Alderley Park	Wellcome Sanger Institute for the COVID-19 Genomics UK (COG-UK) Consortium	Jacquelyn Wynn, Mairead Hyland, The Lighthouse Lab in Alderley Park and Alex Alderton, Roberto Amato, Sonia Goncalves, Ewan Harrison, David K. Jackson, Ian Johnston, Dominic Kwiatkowski, Cordelia Langford, John Sillitoe on behalf of the Wellcome Sanger Institute COVID-19 Surveillance Team
EPI_ISL_645945	Lighthouse Lab in Cambridge	Wellcome Sanger Institute for the COVID-19 Genomics UK (COG-UK) Consortium	Rob Howes, The Lighthouse Lab in Cambridge and Alex Alderton, Roberto Amato, Sonia Goncalves, Ewan Harrison, David K. Jackson, Ian Johnston, Dominic Kwiatkowski, Cordelia Langford, John Sillitoe on behalf of the Wellcome Sanger Institute COVID-19 Surveillance Team
EPI_ISL_645946, EPI_ISL_645947, EPI_ISL_645948, EPI_ISL_645951, EPI_ISL_645952, EPI_ISL_645953, EPI_ISL_645954, EPI_ISL_645955, EPI_ISL_645956, EPI_ISL_645957	Lighthouse Lab in Alderley Park	Wellcome Sanger Institute for the COVID-19 Genomics UK (COG-UK) Consortium	Jacquelyn Wynn, Mairead Hyland, The Lighthouse Lab in Alderley Park and Alex Alderton, Roberto Amato, Sonia Goncalves, Ewan Harrison, David K. Jackson, Ian Johnston, Dominic Kwiatkowski, Cordelia Langford, John Sillitoe on behalf of the Wellcome Sanger Institute COVID-19 Surveillance Team
EPI_ISL_645959	Lighthouse Lab in Cambridge	Wellcome Sanger Institute for the COVID-19 Genomics UK (COG-UK) Consortium	Rob Howes, The Lighthouse Lab in Cambridge and Alex Alderton, Roberto Amato, Sonia Goncalves, Ewan Harrison, David K. Jackson, Ian Johnston, Dominic Kwiatkowski, Cordelia Langford, John Sillitoe on behalf of the Wellcome Sanger Institute COVID-19 Surveillance Team
EPI_ISL_645960, EPI_ISL_645961, EPI_ISL_645964, EPI_ISL_645965, EPI_ISL_645966, EPI_ISL_645967	Lighthouse Lab in Alderley Park	Wellcome Sanger Institute for the COVID-19 Genomics UK (COG-UK) Consortium	Jacquelyn Wynn, Mairead Hyland, The Lighthouse Lab in Alderley Park and Alex Alderton, Roberto Amato, Sonia Goncalves, Ewan Harrison, David K. Jackson, Ian Johnston, Dominic Kwiatkowski, Cordelia Langford, John Sillitoe on behalf of the Wellcome Sanger Institute COVID-19 Surveillance Team
EPI_ISL_645968	Lighthouse Lab in Cambridge	Wellcome Sanger Institute for the COVID-19 Genomics UK (COG-UK) Consortium	Rob Howes, The Lighthouse Lab in Cambridge and Alex Alderton, Roberto Amato, Sonia Goncalves, Ewan Harrison, David K. Jackson, Ian Johnston, Dominic Kwiatkowski, Cordelia Langford, John Sillitoe on behalf of the Wellcome Sanger Institute COVID-19 Surveillance Team
EPI_ISL_645969, EPI_ISL_645971, EPI_ISL_645972, EPI_ISL_645973, EPI_ISL_645975, EPI_ISL_645976, EPI_ISL_645977, EPI_ISL_645978	Lighthouse Lab in Alderley Park	Wellcome Sanger Institute for the COVID-19 Genomics UK (COG-UK) Consortium	Jacquelyn Wynn, Mairead Hyland, The Lighthouse Lab in Alderley Park and Alex Alderton, Roberto Amato, Sonia Goncalves, Ewan Harrison, David K. Jackson, Ian Johnston, Dominic Kwiatkowski, Cordelia Langford, John Sillitoe on behalf of the Wellcome Sanger Institute COVID-19 Surveillance Team
EPI_ISL_645979	Lighthouse Lab in Cambridge	Wellcome Sanger Institute for the COVID-19 Genomics UK (COG-UK) Consortium	Rob Howes, The Lighthouse Lab in Cambridge and Alex Alderton, Roberto Amato, Sonia Goncalves, Ewan Harrison, David K. Jackson, Ian Johnston, Dominic Kwiatkowski, Cordelia Langford, John Sillitoe on behalf of the Wellcome Sanger Institute COVID-19 Surveillance Team
EPI_ISL_645982, EPI_ISL_645984, EPI_ISL_645985, EPI_ISL_645986	Lighthouse Lab in Alderley Park	Wellcome Sanger Institute for the COVID-19 Genomics UK (COG-UK) Consortium	Jacquelyn Wynn, Mairead Hyland, The Lighthouse Lab in Alderley Park and Alex Alderton, Roberto Amato, Sonia Goncalves, Ewan Harrison, David K. Jackson, Ian Johnston, Dominic Kwiatkowski, Cordelia Langford, John Sillitoe on behalf of the Wellcome Sanger Institute COVID-19 Surveillance Team
EPI_ISL_645987	Lighthouse Lab in Cambridge	Wellcome Sanger Institute for the COVID-19 Genomics UK (COG-UK) Consortium	Rob Howes, The Lighthouse Lab in Cambridge and Alex Alderton, Roberto Amato, Sonia Goncalves, Ewan Harrison, David K. Jackson, Ian Johnston, Dominic Kwiatkowski, Cordelia Langford, John Sillitoe on behalf of the Wellcome Sanger Institute COVID-19 Surveillance Team
EPI_ISL_645988, EPI_ISL_645990	Lighthouse Lab in Alderley Park	Wellcome Sanger Institute for the COVID-19 Genomics UK (COG-UK) Consortium	Jacquelyn Wynn, Mairead Hyland, The Lighthouse Lab in Alderley Park and Alex Alderton, Roberto Amato, Sonia Goncalves, Ewan Harrison, David K. Jackson, Ian Johnston, Dominic Kwiatkowski, Cordelia Langford, John Sillitoe on behalf of the Wellcome Sanger Institute COVID-19 Surveillance Team
EPI_ISL_645994	Lighthouse Lab in Cambridge	Wellcome Sanger Institute for the COVID-19 Genomics UK (COG-UK) Consortium	Rob Howes, The Lighthouse Lab in Cambridge and Alex Alderton, Roberto Amato, Sonia Goncalves, Ewan Harrison, David K. Jackson, Ian Johnston, Dominic Kwiatkowski, Cordelia Langford, John Sillitoe on behalf of the Wellcome Sanger Institute COVID-19 Surveillance Team
EPI_ISL_645996, EPI_ISL_645997, EPI_ISL_645999, EPI_ISL_646000, EPI_ISL_646001, EPI_ISL_646002, EPI_ISL_646003, EPI_ISL_646004, EPI_ISL_646005, EPI_ISL_646006, EPI_ISL_646007, EPI_ISL_646008, EPI_ISL_646010, EPI_ISL_646011, EPI_ISL_646012, EPI_ISL_646013, EPI_ISL_646015, EPI_ISL_646016, EPI_ISL_646019, EPI_ISL_646020, EPI_ISL_646021, EPI_ISL_646023, EPI_ISL_646024, EPI_ISL_646025, EPI_ISL_646026			
see above	Lighthouse Lab in Alderley Park	Wellcome Sanger Institute for the COVID-19 Genomics UK (COG-UK) Consortium	Jacquelyn Wynn, Mairead Hyland, The Lighthouse Lab in Alderley Park and Alex Alderton, Roberto Amato, Sonia Goncalves, Ewan Harrison, David K. Jackson, Ian Johnston, Dominic Kwiatkowski, Cordelia Langford, John Sillitoe on behalf of the Wellcome Sanger Institute COVID-19 Surveillance Team
EPI_ISL_646027	Lighthouse Lab in Cambridge	Wellcome Sanger Institute for the COVID-19 Genomics UK (COG-UK) Consortium	Rob Howes, The Lighthouse Lab in Cambridge and Alex Alderton, Roberto Amato, Sonia Goncalves, Ewan Harrison, David K. Jackson, Ian Johnston, Dominic Kwiatkowski, Cordelia Langford, John Sillitoe on behalf of the Wellcome Sanger Institute COVID-19 Surveillance Team
EPI_ISL_646033, EPI_ISL_646036, EPI_ISL_646037, EPI_ISL_646038	Lighthouse Lab in Alderley Park	Wellcome Sanger Institute for the COVID-19 Genomics UK (COG-UK) Consortium	Jacquelyn Wynn, Mairead Hyland, The Lighthouse Lab in Alderley Park and Alex Alderton, Roberto Amato, Sonia Goncalves, Ewan Harrison, David K. Jackson, Ian Johnston, Dominic Kwiatkowski, Cordelia Langford, John Sillitoe on behalf of the Wellcome Sanger Institute COVID-19 Surveillance Team
EPI_ISL_646039	Lighthouse Lab in Cambridge	Wellcome Sanger Institute for the COVID-19 Genomics UK (COG-UK) Consortium	Rob Howes, The Lighthouse Lab in Cambridge and Alex Alderton, Roberto Amato, Sonia Goncalves, Ewan Harrison, David K. Jackson, Ian Johnston, Dominic Kwiatkowski, Cordelia Langford, John Sillitoe on behalf of the Wellcome Sanger Institute COVID-19 Surveillance Team
EPI_ISL_646040, EPI_ISL_646042, EPI_ISL_646043, EPI_ISL_646044, EPI_ISL_646045	Lighthouse Lab in Alderley Park	Wellcome Sanger Institute for the COVID-19 Genomics UK (COG-UK) Consortium	Jacquelyn Wynn, Mairead Hyland, The Lighthouse Lab in Alderley Park and Alex Alderton, Roberto Amato, Sonia Goncalves, Ewan Harrison, David K. Jackson, Ian Johnston, Dominic Kwiatkowski, Cordelia Langford, John Sillitoe on behalf of the Wellcome Sanger Institute COVID-19 Surveillance Team
EPI_ISL_646047	Lighthouse Lab in Cambridge	Wellcome Sanger Institute for the COVID-19 Genomics UK (COG-UK) Consortium	Rob Howes, The Lighthouse Lab in Cambridge and Alex Alderton, Roberto Amato, Sonia Goncalves, Ewan Harrison, David K. Jackson, Ian Johnston, Dominic Kwiatkowski, Cordelia Langford, John Sillitoe on behalf of the Wellcome Sanger Institute COVID-19 Surveillance Team
EPI_ISL_646048, EPI_ISL_646049, EPI_ISL_646050, EPI_ISL_646051, EPI_ISL_646053, EPI_ISL_646055, EPI_ISL_646057, EPI_ISL_646059, EPI_ISL_646060, EPI_ISL_646061, EPI_ISL_646063, EPI_ISL_646064, EPI_ISL_646065, EPI_ISL_646066, EPI_ISL_646068			
see above	Lighthouse Lab in Alderley Park	Wellcome Sanger Institute for the COVID-19 Genomics UK (COG-UK) Consortium	Jacquelyn Wynn, Mairead Hyland, The Lighthouse Lab in Alderley Park and Alex Alderton, Roberto Amato, Sonia Goncalves, Ewan Harrison, David K. Jackson, Ian Johnston, Dominic Kwiatkowski, Cordelia Langford, John Sillitoe on behalf of the Wellcome Sanger Institute COVID-19 Surveillance Team
EPI_ISL_646070	Lighthouse Lab in Cambridge	Wellcome Sanger Institute for the COVID-19 Genomics UK (COG-UK) Consortium	Rob Howes, The Lighthouse Lab in Cambridge and Alex Alderton, Roberto Amato, Sonia Goncalves, Ewan Harrison, David K. Jackson, Ian Johnston, Dominic Kwiatkowski, Cordelia Langford, John Sillitoe on behalf of the Wellcome Sanger Institute COVID-19 Surveillance Team
EPI_ISL_646071, EPI_ISL_646072,	Lighthouse Lab in Alderley Park	Wellcome Sanger Institute for the COVID-19 Genomics UK	Jacquelyn Wynn, Mairead Hyland, The Lighthouse Lab in Alderley Park and Alex Alderton, Roberto Amato, Sonia Goncalves, Ewan Harrison, David K.

EPI_ISL_646073, EPI_ISL_646074, EPI_ISL_646075		(COG-UK) Consortium	Jackson, Ian Johnston, Dominic Kwiatkowski, Cordelia Langford, John Sillitoe on behalf of the Wellcome Sanger Institute COVID-19 Surveillance Team
EPI_ISL_646076	Lighthouse Lab in Cambridge	Wellcome Sanger Institute for the COVID-19 Genomics UK (COG-UK) Consortium	Rob Howes, The Lighthouse Lab in Cambridge and Alex Alderton, Roberto Amato, Sonia Goncalves, Ewan Harrison, David K. Jackson, Ian Johnston, Dominic Kwiatkowski, Cordelia Langford, John Sillitoe on behalf of the Wellcome Sanger Institute COVID-19 Surveillance Team
EPI_ISL_646077, EPI_ISL_646079, EPI_ISL_646080, EPI_ISL_646081, EPI_ISL_646082	Lighthouse Lab in Alderley Park	Wellcome Sanger Institute for the COVID-19 Genomics UK (COG-UK) Consortium	Jacquelyn Wynn, Mairead Hyland, The Lighthouse Lab in Alderley Park and Alex Alderton, Roberto Amato, Sonia Goncalves, Ewan Harrison, David K. Jackson, Ian Johnston, Dominic Kwiatkowski, Cordelia Langford, John Sillitoe on behalf of the Wellcome Sanger Institute COVID-19 Surveillance Team
EPI_ISL_646086	Lighthouse Lab in Cambridge	Wellcome Sanger Institute for the COVID-19 Genomics UK (COG-UK) Consortium	Rob Howes, The Lighthouse Lab in Cambridge and Alex Alderton, Roberto Amato, Sonia Goncalves, Ewan Harrison, David K. Jackson, Ian Johnston, Dominic Kwiatkowski, Cordelia Langford, John Sillitoe on behalf of the Wellcome Sanger Institute COVID-19 Surveillance Team
EPI_ISL_646087, EPI_ISL_646088, EPI_ISL_646090, EPI_ISL_646092, EPI_ISL_646093, EPI_ISL_646094, EPI_ISL_646095, EPI_ISL_646097, EPI_ISL_646099, EPI_ISL_646100, EPI_ISL_646103, EPI_ISL_646104			
see above	Lighthouse Lab in Alderley Park	Wellcome Sanger Institute for the COVID-19 Genomics UK (COG-UK) Consortium	Jacquelyn Wynn, Mairead Hyland, The Lighthouse Lab in Alderley Park and Alex Alderton, Roberto Amato, Sonia Goncalves, Ewan Harrison, David K. Jackson, Ian Johnston, Dominic Kwiatkowski, Cordelia Langford, John Sillitoe on behalf of the Wellcome Sanger Institute COVID-19 Surveillance Team
EPI_ISL_646105	Lighthouse Lab in Cambridge	Wellcome Sanger Institute for the COVID-19 Genomics UK (COG-UK) Consortium	Rob Howes, The Lighthouse Lab in Cambridge and Alex Alderton, Roberto Amato, Sonia Goncalves, Ewan Harrison, David K. Jackson, Ian Johnston, Dominic Kwiatkowski, Cordelia Langford, John Sillitoe on behalf of the Wellcome Sanger Institute COVID-19 Surveillance Team
EPI_ISL_646106, EPI_ISL_646107, EPI_ISL_646108, EPI_ISL_646109, EPI_ISL_646110, EPI_ISL_646111, EPI_ISL_646112, EPI_ISL_646113, EPI_ISL_646114, EPI_ISL_646116, EPI_ISL_646117, EPI_ISL_646118, EPI_ISL_646119, EPI_ISL_646120, EPI_ISL_646121, EPI_ISL_646123, EPI_ISL_646124, EPI_ISL_646126, EPI_ISL_646127, EPI_ISL_646130			
see above	Lighthouse Lab in Alderley Park	Wellcome Sanger Institute for the COVID-19 Genomics UK (COG-UK) Consortium	Jacquelyn Wynn, Mairead Hyland, The Lighthouse Lab in Alderley Park and Alex Alderton, Roberto Amato, Sonia Goncalves, Ewan Harrison, David K. Jackson, Ian Johnston, Dominic Kwiatkowski, Cordelia Langford, John Sillitoe on behalf of the Wellcome Sanger Institute COVID-19 Surveillance Team
EPI_ISL_646131	Lighthouse Lab in Cambridge	Wellcome Sanger Institute for the COVID-19 Genomics UK (COG-UK) Consortium	Rob Howes, The Lighthouse Lab in Cambridge and Alex Alderton, Roberto Amato, Sonia Goncalves, Ewan Harrison, David K. Jackson, Ian Johnston, Dominic Kwiatkowski, Cordelia Langford, John Sillitoe on behalf of the Wellcome Sanger Institute COVID-19 Surveillance Team
EPI_ISL_646132, EPI_ISL_646133, EPI_ISL_646134, EPI_ISL_646137, EPI_ISL_646138	Lighthouse Lab in Alderley Park	Wellcome Sanger Institute for the COVID-19 Genomics UK (COG-UK) Consortium	Jacquelyn Wynn, Mairead Hyland, The Lighthouse Lab in Alderley Park and Alex Alderton, Roberto Amato, Sonia Goncalves, Ewan Harrison, David K. Jackson, Ian Johnston, Dominic Kwiatkowski, Cordelia Langford, John Sillitoe on behalf of the Wellcome Sanger Institute COVID-19 Surveillance Team
EPI_ISL_646153, EPI_ISL_646222, EPI_ISL_646461, EPI_ISL_646503, EPI_ISL_646507, EPI_ISL_646519, EPI_ISL_646527, EPI_ISL_646534, EPI_ISL_646574, EPI_ISL_646579, EPI_ISL_646590, EPI_ISL_646593, EPI_ISL_646602, EPI_ISL_646608, EPI_ISL_646619, EPI_ISL_646632, EPI_ISL_646645, EPI_ISL_646679, EPI_ISL_646704, EPI_ISL_646764, EPI_ISL_646779, EPI_ISL_647073			
see above	Lighthouse Lab in Glasgow	Wellcome Sanger Institute for the COVID-19 Genomics UK (COG-UK) Consortium	Harper VanSteenhouse, Yumi Kasai, David Gray, Carol Clugston, Anna Dominiczak and Alex Alderton, Roberto Amato, Sonia Goncalves, Ewan Harrison, David K. Jackson, Ian Johnston, Dominic Kwiatkowski, Cordelia Langford, John Sillitoe on behalf of the Wellcome Sanger Institute COVID-19 Surveillance Team
EPI_ISL_647134, EPI_ISL_647135, EPI_ISL_647139, EPI_ISL_647145, EPI_ISL_647147, EPI_ISL_647148, EPI_ISL_647152, EPI_ISL_647153, EPI_ISL_647160, EPI_ISL_647161, EPI_ISL_647165, EPI_ISL_647172, EPI_ISL_647178, EPI_ISL_647180, EPI_ISL_647187, EPI_ISL_647197, EPI_ISL_647199, EPI_ISL_647200, EPI_ISL_647201, EPI_ISL_647205, EPI_ISL_647209, EPI_ISL_647214, EPI_ISL_647216, EPI_ISL_647223, EPI_ISL_647224, EPI_ISL_647230, EPI_ISL_647232, EPI_ISL_647234, EPI_ISL_647240, EPI_ISL_647247, EPI_ISL_647250, EPI_ISL_647251, EPI_ISL_647254, EPI_ISL_647255, EPI_ISL_647256, EPI_ISL_647259, EPI_ISL_647260, EPI_ISL_647263, EPI_ISL_647266, EPI_ISL_647267, EPI_ISL_647269, EPI_ISL_647273, EPI_ISL_647275, EPI_ISL_647279, EPI_ISL_647280, EPI_ISL_647281, EPI_ISL_647282, EPI_ISL_647290, EPI_ISL_647291, EPI_ISL_647292, EPI_ISL_647294, EPI_ISL_647296, EPI_ISL_647308, EPI_ISL_647314, EPI_ISL_647315, EPI_ISL_647321, EPI_ISL_647323, EPI_ISL_647324, EPI_ISL_647328, EPI_ISL_647329, EPI_ISL_647330, EPI_ISL_647332, EPI_ISL_647334, EPI_ISL_647337, EPI_ISL_647339, EPI_ISL_647342, EPI_ISL_647347, EPI_ISL_647354, EPI_ISL_647361, EPI_ISL_647362, EPI_ISL_647363, EPI_ISL_647399, EPI_ISL_647405, EPI_ISL_647411, EPI_ISL_647419, EPI_ISL_647420, EPI_ISL_647425, EPI_ISL_647427, EPI_ISL_647428, EPI_ISL_647429, EPI_ISL_647436, EPI_ISL_647443, EPI_ISL_647445, EPI_ISL_647452, EPI_ISL_647460			
see above	Lighthouse Lab in Cambridge	Wellcome Sanger Institute for the COVID-19 Genomics UK (COG-UK) Consortium	Rob Howes, The Lighthouse Lab in Cambridge and Alex Alderton, Roberto Amato, Sonia Goncalves, Ewan Harrison, David K. Jackson, Ian Johnston, Dominic Kwiatkowski, Cordelia Langford, John Sillitoe on behalf of the Wellcome Sanger Institute COVID-19 Surveillance Team
EPI_ISL_647468, EPI_ISL_647469, EPI_ISL_647473, EPI_ISL_647474, EPI_ISL_647477	Lighthouse Lab in Alderley Park	Wellcome Sanger Institute for the COVID-19 Genomics UK (COG-UK) Consortium	Jacquelyn Wynn, Mairead Hyland, The Lighthouse Lab in Alderley Park and Alex Alderton, Roberto Amato, Sonia Goncalves, Ewan Harrison, David K. Jackson, Ian Johnston, Dominic Kwiatkowski, Cordelia Langford, John Sillitoe on behalf of the Wellcome Sanger Institute COVID-19 Surveillance Team
EPI_ISL_647478, EPI_ISL_647479, EPI_ISL_647483, EPI_ISL_647485, EPI_ISL_647486, EPI_ISL_647492	Lighthouse Lab in Cambridge	Wellcome Sanger Institute for the COVID-19 Genomics UK (COG-UK) Consortium	Rob Howes, The Lighthouse Lab in Cambridge and Alex Alderton, Roberto Amato, Sonia Goncalves, Ewan Harrison, David K. Jackson, Ian Johnston, Dominic Kwiatkowski, Cordelia Langford, John Sillitoe on behalf of the Wellcome Sanger Institute COVID-19 Surveillance Team
EPI_ISL_647497	Lighthouse Lab in Alderley Park	Wellcome Sanger Institute for the COVID-19 Genomics UK (COG-UK) Consortium	Jacquelyn Wynn, Mairead Hyland, The Lighthouse Lab in Alderley Park and Alex Alderton, Roberto Amato, Sonia Goncalves, Ewan Harrison, David K. Jackson, Ian Johnston, Dominic Kwiatkowski, Cordelia Langford, John Sillitoe on behalf of the Wellcome Sanger Institute COVID-19 Surveillance Team
EPI_ISL_647498	Lighthouse Lab in Cambridge	Wellcome Sanger Institute for the COVID-19 Genomics UK (COG-UK) Consortium	Rob Howes, The Lighthouse Lab in Cambridge and Alex Alderton, Roberto Amato, Sonia Goncalves, Ewan Harrison, David K. Jackson, Ian Johnston, Dominic Kwiatkowski, Cordelia Langford, John Sillitoe on behalf of the Wellcome Sanger Institute COVID-19 Surveillance Team
EPI_ISL_647499, EPI_ISL_647503, EPI_ISL_647506	Lighthouse Lab in Alderley Park	Wellcome Sanger Institute for the COVID-19 Genomics UK (COG-UK) Consortium	Jacquelyn Wynn, Mairead Hyland, The Lighthouse Lab in Alderley Park and Alex Alderton, Roberto Amato, Sonia Goncalves, Ewan Harrison, David K. Jackson, Ian Johnston, Dominic Kwiatkowski, Cordelia Langford, John Sillitoe on behalf of the Wellcome Sanger Institute COVID-19 Surveillance Team
EPI_ISL_647509	Lighthouse Lab in Cambridge	Wellcome Sanger Institute for the COVID-19 Genomics UK (COG-UK) Consortium	Rob Howes, The Lighthouse Lab in Cambridge and Alex Alderton, Roberto Amato, Sonia Goncalves, Ewan Harrison, David K. Jackson, Ian Johnston, Dominic Kwiatkowski, Cordelia Langford, John Sillitoe on behalf of the Wellcome Sanger Institute COVID-19 Surveillance Team
EPI_ISL_647511, EPI_ISL_647513	Lighthouse Lab in Alderley Park	Wellcome Sanger Institute for the COVID-19 Genomics UK (COG-UK) Consortium	Jacquelyn Wynn, Mairead Hyland, The Lighthouse Lab in Alderley Park and Alex Alderton, Roberto Amato, Sonia Goncalves, Ewan Harrison, David K. Jackson, Ian Johnston, Dominic Kwiatkowski, Cordelia Langford, John Sillitoe on behalf of the Wellcome Sanger Institute COVID-19 Surveillance Team
EPI_ISL_647514, EPI_ISL_647515, EPI_ISL_647518, EPI_ISL_647519, EPI_ISL_647522	Lighthouse Lab in Cambridge	Wellcome Sanger Institute for the COVID-19 Genomics UK (COG-UK) Consortium	Rob Howes, The Lighthouse Lab in Cambridge and Alex Alderton, Roberto Amato, Sonia Goncalves, Ewan Harrison, David K. Jackson, Ian Johnston, Dominic Kwiatkowski, Cordelia Langford, John Sillitoe on behalf of the Wellcome Sanger Institute COVID-19 Surveillance Team
EPI_ISL_647526, EPI_ISL_647528	Lighthouse Lab in Alderley Park	Wellcome Sanger Institute for the COVID-19 Genomics UK (COG-UK) Consortium	Jacquelyn Wynn, Mairead Hyland, The Lighthouse Lab in Alderley Park and Alex Alderton, Roberto Amato, Sonia Goncalves, Ewan Harrison, David K. Jackson, Ian Johnston, Dominic Kwiatkowski, Cordelia Langford, John Sillitoe on behalf of the Wellcome Sanger Institute COVID-19 Surveillance Team
EPI_ISL_647531, EPI_ISL_647534	Lighthouse Lab in Cambridge	Wellcome Sanger Institute for the COVID-19 Genomics UK (COG-UK) Consortium	Rob Howes, The Lighthouse Lab in Cambridge and Alex Alderton, Roberto Amato, Sonia Goncalves, Ewan Harrison, David K. Jackson, Ian Johnston, Dominic Kwiatkowski, Cordelia Langford, John Sillitoe on behalf of the Wellcome Sanger Institute COVID-19 Surveillance Team
EPI_ISL_647535, EPI_ISL_647536, EPI_ISL_647541	Lighthouse Lab in Alderley Park	Wellcome Sanger Institute for the COVID-19 Genomics UK (COG-UK) Consortium	Jacquelyn Wynn, Mairead Hyland, The Lighthouse Lab in Alderley Park and Alex Alderton, Roberto Amato, Sonia Goncalves, Ewan Harrison, David K. Jackson, Ian Johnston, Dominic Kwiatkowski, Cordelia Langford, John Sillitoe on behalf of the Wellcome Sanger Institute COVID-19 Surveillance Team
EPI_ISL_647543	Lighthouse Lab in Cambridge	Wellcome Sanger Institute for the COVID-19 Genomics UK (COG-UK) Consortium	Rob Howes, The Lighthouse Lab in Cambridge and Alex Alderton, Roberto Amato, Sonia Goncalves, Ewan Harrison, David K. Jackson, Ian Johnston, Dominic Kwiatkowski, Cordelia Langford, John Sillitoe on behalf of the Wellcome Sanger Institute COVID-19 Surveillance Team
EPI_ISL_647546	Lighthouse Lab in Alderley Park	Wellcome Sanger Institute for the COVID-19 Genomics UK (COG-UK) Consortium	Jacquelyn Wynn, Mairead Hyland, The Lighthouse Lab in Alderley Park and Alex Alderton, Roberto Amato, Sonia Goncalves, Ewan Harrison, David K. Jackson, Ian Johnston, Dominic Kwiatkowski, Cordelia Langford, John Sillitoe on behalf of the Wellcome Sanger Institute COVID-19 Surveillance Team
EPI_ISL_647547	Lighthouse Lab in Cambridge	Wellcome Sanger Institute for the COVID-19 Genomics UK (COG-UK) Consortium	Rob Howes, The Lighthouse Lab in Cambridge and Alex Alderton, Roberto Amato, Sonia Goncalves, Ewan Harrison, David K. Jackson, Ian Johnston, Dominic Kwiatkowski, Cordelia Langford, John Sillitoe on behalf of the Wellcome Sanger Institute COVID-19 Surveillance Team
EPI_ISL_647550, EPI_ISL_647553, EPI_ISL_647558, EPI_ISL_647560	Lighthouse Lab in Alderley Park	Wellcome Sanger Institute for the COVID-19 Genomics UK (COG-UK) Consortium	Jacquelyn Wynn, Mairead Hyland, The Lighthouse Lab in Alderley Park and Alex Alderton, Roberto Amato, Sonia Goncalves, Ewan Harrison, David K. Jackson, Ian Johnston, Dominic Kwiatkowski, Cordelia Langford, John Sillitoe on behalf of the Wellcome Sanger Institute COVID-19 Surveillance Team
EPI_ISL_647562, EPI_ISL_647563, EPI_ISL_647569, EPI_ISL_647570	Lighthouse Lab in Cambridge	Wellcome Sanger Institute for the COVID-19 Genomics UK (COG-UK) Consortium	Rob Howes, The Lighthouse Lab in Cambridge and Alex Alderton, Roberto Amato, Sonia Goncalves, Ewan Harrison, David K. Jackson, Ian Johnston, Dominic Kwiatkowski, Cordelia Langford, John Sillitoe on behalf of the Wellcome Sanger Institute COVID-19 Surveillance Team
EPI_ISL_647572, EPI_ISL_647573, EPI_ISL_647576, EPI_ISL_647579,	Lighthouse Lab in Alderley Park	Wellcome Sanger Institute for the COVID-19 Genomics UK (COG-UK) Consortium	Jacquelyn Wynn, Mairead Hyland, The Lighthouse Lab in Alderley Park and Alex Alderton, Roberto Amato, Sonia Goncalves, Ewan Harrison, David K. Jackson, Ian Johnston, Dominic Kwiatkowski, Cordelia Langford, John Sillitoe on behalf of the Wellcome Sanger Institute COVID-19 Surveillance Team

EPI_ISL_647580, EPI_ISL_647584, EPI_ISL_647591			
EPI_ISL_647592	Lighthouse Lab in Cambridge	Wellcome Sanger Institute for the COVID-19 Genomics UK (COG-UK) Consortium	Rob Howes, The Lighthouse Lab in Cambridge and Alex Alderton, Roberto Amato, Sonia Goncalves, Ewan Harrison, David K. Jackson, Ian Johnston, Dominic Kwiatkowski, Cordelia Langford, John Sillitoe on behalf of the Wellcome Sanger Institute COVID-19 Surveillance Team
EPI_ISL_647594	Lighthouse Lab in Alderley Park	Wellcome Sanger Institute for the COVID-19 Genomics UK (COG-UK) Consortium	Jacquelyn Wynn, Mairead Hyland, The Lighthouse Lab in Alderley Park and Alex Alderton, Roberto Amato, Sonia Goncalves, Ewan Harrison, David K. Jackson, Ian Johnston, Dominic Kwiatkowski, Cordelia Langford, John Sillitoe on behalf of the Wellcome Sanger Institute COVID-19 Surveillance Team
EPI_ISL_647595, EPI_ISL_647598, EPI_ISL_647600	Lighthouse Lab in Cambridge	Wellcome Sanger Institute for the COVID-19 Genomics UK (COG-UK) Consortium	Rob Howes, The Lighthouse Lab in Cambridge and Alex Alderton, Roberto Amato, Sonia Goncalves, Ewan Harrison, David K. Jackson, Ian Johnston, Dominic Kwiatkowski, Cordelia Langford, John Sillitoe on behalf of the Wellcome Sanger Institute COVID-19 Surveillance Team
EPI_ISL_647603	Lighthouse Lab in Alderley Park	Wellcome Sanger Institute for the COVID-19 Genomics UK (COG-UK) Consortium	Jacquelyn Wynn, Mairead Hyland, The Lighthouse Lab in Alderley Park and Alex Alderton, Roberto Amato, Sonia Goncalves, Ewan Harrison, David K. Jackson, Ian Johnston, Dominic Kwiatkowski, Cordelia Langford, John Sillitoe on behalf of the Wellcome Sanger Institute COVID-19 Surveillance Team
EPI_ISL_647604, EPI_ISL_647605	Lighthouse Lab in Cambridge	Wellcome Sanger Institute for the COVID-19 Genomics UK (COG-UK) Consortium	Rob Howes, The Lighthouse Lab in Cambridge and Alex Alderton, Roberto Amato, Sonia Goncalves, Ewan Harrison, David K. Jackson, Ian Johnston, Dominic Kwiatkowski, Cordelia Langford, John Sillitoe on behalf of the Wellcome Sanger Institute COVID-19 Surveillance Team
EPI_ISL_647606, EPI_ISL_647608, EPI_ISL_647612	Lighthouse Lab in Alderley Park	Wellcome Sanger Institute for the COVID-19 Genomics UK (COG-UK) Consortium	Jacquelyn Wynn, Mairead Hyland, The Lighthouse Lab in Alderley Park and Alex Alderton, Roberto Amato, Sonia Goncalves, Ewan Harrison, David K. Jackson, Ian Johnston, Dominic Kwiatkowski, Cordelia Langford, John Sillitoe on behalf of the Wellcome Sanger Institute COVID-19 Surveillance Team
EPI_ISL_647614	Lighthouse Lab in Cambridge	Wellcome Sanger Institute for the COVID-19 Genomics UK (COG-UK) Consortium	Rob Howes, The Lighthouse Lab in Cambridge and Alex Alderton, Roberto Amato, Sonia Goncalves, Ewan Harrison, David K. Jackson, Ian Johnston, Dominic Kwiatkowski, Cordelia Langford, John Sillitoe on behalf of the Wellcome Sanger Institute COVID-19 Surveillance Team
EPI_ISL_647615, EPI_ISL_647618	Lighthouse Lab in Alderley Park	Wellcome Sanger Institute for the COVID-19 Genomics UK (COG-UK) Consortium	Jacquelyn Wynn, Mairead Hyland, The Lighthouse Lab in Alderley Park and Alex Alderton, Roberto Amato, Sonia Goncalves, Ewan Harrison, David K. Jackson, Ian Johnston, Dominic Kwiatkowski, Cordelia Langford, John Sillitoe on behalf of the Wellcome Sanger Institute COVID-19 Surveillance Team
EPI_ISL_647619, EPI_ISL_647620	Lighthouse Lab in Cambridge	Wellcome Sanger Institute for the COVID-19 Genomics UK (COG-UK) Consortium	Rob Howes, The Lighthouse Lab in Cambridge and Alex Alderton, Roberto Amato, Sonia Goncalves, Ewan Harrison, David K. Jackson, Ian Johnston, Dominic Kwiatkowski, Cordelia Langford, John Sillitoe on behalf of the Wellcome Sanger Institute COVID-19 Surveillance Team
EPI_ISL_647624, EPI_ISL_647630, EPI_ISL_647632	Lighthouse Lab in Alderley Park	Wellcome Sanger Institute for the COVID-19 Genomics UK (COG-UK) Consortium	Jacquelyn Wynn, Mairead Hyland, The Lighthouse Lab in Alderley Park and Alex Alderton, Roberto Amato, Sonia Goncalves, Ewan Harrison, David K. Jackson, Ian Johnston, Dominic Kwiatkowski, Cordelia Langford, John Sillitoe on behalf of the Wellcome Sanger Institute COVID-19 Surveillance Team
EPI_ISL_647633	Lighthouse Lab in Cambridge	Wellcome Sanger Institute for the COVID-19 Genomics UK (COG-UK) Consortium	Rob Howes, The Lighthouse Lab in Cambridge and Alex Alderton, Roberto Amato, Sonia Goncalves, Ewan Harrison, David K. Jackson, Ian Johnston, Dominic Kwiatkowski, Cordelia Langford, John Sillitoe on behalf of the Wellcome Sanger Institute COVID-19 Surveillance Team
EPI_ISL_647634, EPI_ISL_647635	Lighthouse Lab in Alderley Park	Wellcome Sanger Institute for the COVID-19 Genomics UK (COG-UK) Consortium	Jacquelyn Wynn, Mairead Hyland, The Lighthouse Lab in Alderley Park and Alex Alderton, Roberto Amato, Sonia Goncalves, Ewan Harrison, David K. Jackson, Ian Johnston, Dominic Kwiatkowski, Cordelia Langford, John Sillitoe on behalf of the Wellcome Sanger Institute COVID-19 Surveillance Team
EPI_ISL_647637	Lighthouse Lab in Cambridge	Wellcome Sanger Institute for the COVID-19 Genomics UK (COG-UK) Consortium	Rob Howes, The Lighthouse Lab in Cambridge and Alex Alderton, Roberto Amato, Sonia Goncalves, Ewan Harrison, David K. Jackson, Ian Johnston, Dominic Kwiatkowski, Cordelia Langford, John Sillitoe on behalf of the Wellcome Sanger Institute COVID-19 Surveillance Team
EPI_ISL_647638	Lighthouse Lab in Alderley Park	Wellcome Sanger Institute for the COVID-19 Genomics UK (COG-UK) Consortium	Jacquelyn Wynn, Mairead Hyland, The Lighthouse Lab in Alderley Park and Alex Alderton, Roberto Amato, Sonia Goncalves, Ewan Harrison, David K. Jackson, Ian Johnston, Dominic Kwiatkowski, Cordelia Langford, John Sillitoe on behalf of the Wellcome Sanger Institute COVID-19 Surveillance Team
EPI_ISL_647640, EPI_ISL_647642	Lighthouse Lab in Cambridge	Wellcome Sanger Institute for the COVID-19 Genomics UK (COG-UK) Consortium	Rob Howes, The Lighthouse Lab in Cambridge and Alex Alderton, Roberto Amato, Sonia Goncalves, Ewan Harrison, David K. Jackson, Ian Johnston, Dominic Kwiatkowski, Cordelia Langford, John Sillitoe on behalf of the Wellcome Sanger Institute COVID-19 Surveillance Team
EPI_ISL_647643	Lighthouse Lab in Alderley Park	Wellcome Sanger Institute for the COVID-19 Genomics UK (COG-UK) Consortium	Jacquelyn Wynn, Mairead Hyland, The Lighthouse Lab in Alderley Park and Alex Alderton, Roberto Amato, Sonia Goncalves, Ewan Harrison, David K. Jackson, Ian Johnston, Dominic Kwiatkowski, Cordelia Langford, John Sillitoe on behalf of the Wellcome Sanger Institute COVID-19 Surveillance Team
EPI_ISL_647646, EPI_ISL_647648	Lighthouse Lab in Cambridge	Wellcome Sanger Institute for the COVID-19 Genomics UK (COG-UK) Consortium	Rob Howes, The Lighthouse Lab in Cambridge and Alex Alderton, Roberto Amato, Sonia Goncalves, Ewan Harrison, David K. Jackson, Ian Johnston, Dominic Kwiatkowski, Cordelia Langford, John Sillitoe on behalf of the Wellcome Sanger Institute COVID-19 Surveillance Team
EPI_ISL_647650, EPI_ISL_647655	Lighthouse Lab in Alderley Park	Wellcome Sanger Institute for the COVID-19 Genomics UK (COG-UK) Consortium	Jacquelyn Wynn, Mairead Hyland, The Lighthouse Lab in Alderley Park and Alex Alderton, Roberto Amato, Sonia Goncalves, Ewan Harrison, David K. Jackson, Ian Johnston, Dominic Kwiatkowski, Cordelia Langford, John Sillitoe on behalf of the Wellcome Sanger Institute COVID-19 Surveillance Team
EPI_ISL_647658	Lighthouse Lab in Cambridge	Wellcome Sanger Institute for the COVID-19 Genomics UK (COG-UK) Consortium	Rob Howes, The Lighthouse Lab in Cambridge and Alex Alderton, Roberto Amato, Sonia Goncalves, Ewan Harrison, David K. Jackson, Ian Johnston, Dominic Kwiatkowski, Cordelia Langford, John Sillitoe on behalf of the Wellcome Sanger Institute COVID-19 Surveillance Team
EPI_ISL_647659	Lighthouse Lab in Alderley Park	Wellcome Sanger Institute for the COVID-19 Genomics UK (COG-UK) Consortium	Jacquelyn Wynn, Mairead Hyland, The Lighthouse Lab in Alderley Park and Alex Alderton, Roberto Amato, Sonia Goncalves, Ewan Harrison, David K. Jackson, Ian Johnston, Dominic Kwiatkowski, Cordelia Langford, John Sillitoe on behalf of the Wellcome Sanger Institute COVID-19 Surveillance Team
EPI_ISL_647662	Lighthouse Lab in Cambridge	Wellcome Sanger Institute for the COVID-19 Genomics UK (COG-UK) Consortium	Rob Howes, The Lighthouse Lab in Cambridge and Alex Alderton, Roberto Amato, Sonia Goncalves, Ewan Harrison, David K. Jackson, Ian Johnston, Dominic Kwiatkowski, Cordelia Langford, John Sillitoe on behalf of the Wellcome Sanger Institute COVID-19 Surveillance Team
EPI_ISL_647667, EPI_ISL_647668	Lighthouse Lab in Alderley Park	Wellcome Sanger Institute for the COVID-19 Genomics UK (COG-UK) Consortium	Jacquelyn Wynn, Mairead Hyland, The Lighthouse Lab in Alderley Park and Alex Alderton, Roberto Amato, Sonia Goncalves, Ewan Harrison, David K. Jackson, Ian Johnston, Dominic Kwiatkowski, Cordelia Langford, John Sillitoe on behalf of the Wellcome Sanger Institute COVID-19 Surveillance Team
EPI_ISL_647671	Lighthouse Lab in Cambridge	Wellcome Sanger Institute for the COVID-19 Genomics UK (COG-UK) Consortium	Rob Howes, The Lighthouse Lab in Cambridge and Alex Alderton, Roberto Amato, Sonia Goncalves, Ewan Harrison, David K. Jackson, Ian Johnston, Dominic Kwiatkowski, Cordelia Langford, John Sillitoe on behalf of the Wellcome Sanger Institute COVID-19 Surveillance Team
EPI_ISL_647672, EPI_ISL_647677	Lighthouse Lab in Alderley Park	Wellcome Sanger Institute for the COVID-19 Genomics UK (COG-UK) Consortium	Jacquelyn Wynn, Mairead Hyland, The Lighthouse Lab in Alderley Park and Alex Alderton, Roberto Amato, Sonia Goncalves, Ewan Harrison, David K. Jackson, Ian Johnston, Dominic Kwiatkowski, Cordelia Langford, John Sillitoe on behalf of the Wellcome Sanger Institute COVID-19 Surveillance Team
EPI_ISL_647680	Lighthouse Lab in Cambridge	Wellcome Sanger Institute for the COVID-19 Genomics UK (COG-UK) Consortium	Rob Howes, The Lighthouse Lab in Cambridge and Alex Alderton, Roberto Amato, Sonia Goncalves, Ewan Harrison, David K. Jackson, Ian Johnston, Dominic Kwiatkowski, Cordelia Langford, John Sillitoe on behalf of the Wellcome Sanger Institute COVID-19 Surveillance Team
EPI_ISL_647682, EPI_ISL_647683	Lighthouse Lab in Alderley Park	Wellcome Sanger Institute for the COVID-19 Genomics UK (COG-UK) Consortium	Jacquelyn Wynn, Mairead Hyland, The Lighthouse Lab in Alderley Park and Alex Alderton, Roberto Amato, Sonia Goncalves, Ewan Harrison, David K. Jackson, Ian Johnston, Dominic Kwiatkowski, Cordelia Langford, John Sillitoe on behalf of the Wellcome Sanger Institute COVID-19 Surveillance Team
EPI_ISL_647686, EPI_ISL_647687	Lighthouse Lab in Cambridge	Wellcome Sanger Institute for the COVID-19 Genomics UK (COG-UK) Consortium	Rob Howes, The Lighthouse Lab in Cambridge and Alex Alderton, Roberto Amato, Sonia Goncalves, Ewan Harrison, David K. Jackson, Ian Johnston, Dominic Kwiatkowski, Cordelia Langford, John Sillitoe on behalf of the Wellcome Sanger Institute COVID-19 Surveillance Team
EPI_ISL_647689, EPI_ISL_647693	Lighthouse Lab in Alderley Park	Wellcome Sanger Institute for the COVID-19 Genomics UK (COG-UK) Consortium	Jacquelyn Wynn, Mairead Hyland, The Lighthouse Lab in Alderley Park and Alex Alderton, Roberto Amato, Sonia Goncalves, Ewan Harrison, David K. Jackson, Ian Johnston, Dominic Kwiatkowski, Cordelia Langford, John Sillitoe on behalf of the Wellcome Sanger Institute COVID-19 Surveillance Team
EPI_ISL_647694	Lighthouse Lab in Cambridge	Wellcome Sanger Institute for the COVID-19 Genomics UK (COG-UK) Consortium	Rob Howes, The Lighthouse Lab in Cambridge and Alex Alderton, Roberto Amato, Sonia Goncalves, Ewan Harrison, David K. Jackson, Ian Johnston, Dominic Kwiatkowski, Cordelia Langford, John Sillitoe on behalf of the Wellcome Sanger Institute COVID-19 Surveillance Team
EPI_ISL_647695, EPI_ISL_647698, EPI_ISL_647700, EPI_ISL_647704, EPI_ISL_647705	Lighthouse Lab in Alderley Park	Wellcome Sanger Institute for the COVID-19 Genomics UK (COG-UK) Consortium	Jacquelyn Wynn, Mairead Hyland, The Lighthouse Lab in Alderley Park and Alex Alderton, Roberto Amato, Sonia Goncalves, Ewan Harrison, David K. Jackson, Ian Johnston, Dominic Kwiatkowski, Cordelia Langford, John Sillitoe on behalf of the Wellcome Sanger Institute COVID-19 Surveillance Team
EPI_ISL_647706, EPI_ISL_647707	Lighthouse Lab in Cambridge	Wellcome Sanger Institute for the COVID-19 Genomics UK (COG-UK) Consortium	Rob Howes, The Lighthouse Lab in Cambridge and Alex Alderton, Roberto Amato, Sonia Goncalves, Ewan Harrison, David K. Jackson, Ian Johnston, Dominic Kwiatkowski, Cordelia Langford, John Sillitoe on behalf of the Wellcome Sanger Institute COVID-19 Surveillance Team
EPI_ISL_647708	Lighthouse Lab in Alderley Park	Wellcome Sanger Institute for the COVID-19 Genomics UK (COG-UK) Consortium	Jacquelyn Wynn, Mairead Hyland, The Lighthouse Lab in Alderley Park and Alex Alderton, Roberto Amato, Sonia Goncalves, Ewan Harrison, David K. Jackson, Ian Johnston, Dominic Kwiatkowski, Cordelia Langford, John Sillitoe on behalf of the Wellcome Sanger Institute COVID-19 Surveillance Team
EPI_ISL_647710	Lighthouse Lab in Cambridge	Wellcome Sanger Institute for the COVID-19 Genomics UK (COG-UK) Consortium	Rob Howes, The Lighthouse Lab in Cambridge and Alex Alderton, Roberto Amato, Sonia Goncalves, Ewan Harrison, David K. Jackson, Ian Johnston, Dominic Kwiatkowski, Cordelia Langford, John Sillitoe on behalf of the Wellcome Sanger Institute COVID-19 Surveillance Team
EPI_ISL_647711	Lighthouse Lab in Alderley Park	Wellcome Sanger Institute for the COVID-19 Genomics UK (COG-UK) Consortium	Jacquelyn Wynn, Mairead Hyland, The Lighthouse Lab in Alderley Park and Alex Alderton, Roberto Amato, Sonia Goncalves, Ewan Harrison, David K. Jackson, Ian Johnston, Dominic Kwiatkowski, Cordelia Langford, John Sillitoe on behalf of the Wellcome Sanger Institute COVID-19 Surveillance Team

EPI_ISL_647713, EPI_ISL_647714, EPI_ISL_647715, EPI_ISL_647716, EPI_ISL_647720, EPI_ISL_647722, EPI_ISL_647724, EPI_ISL_647725	Lighthouse Lab in Cambridge	Wellcome Sanger Institute for the COVID-19 Genomics UK (COG-UK) Consortium	Rob Howes, The Lighthouse Lab in Cambridge and Alex Alderton, Roberto Amato, Sonia Goncalves, Ewan Harrison, David K. Jackson, Ian Johnston, Dominic Kwiatkowski, Cordelia Langford, John Sillitoe on behalf of the Wellcome Sanger Institute COVID-19 Surveillance Team
EPI_ISL_647726	Lighthouse Lab in Alderley Park	Wellcome Sanger Institute for the COVID-19 Genomics UK (COG-UK) Consortium	Jacquelyn Wynn, Mairead Hyland, The Lighthouse Lab in Alderley Park and Alex Alderton, Roberto Amato, Sonia Goncalves, Ewan Harrison, David K. Jackson, Ian Johnston, Dominic Kwiatkowski, Cordelia Langford, John Sillitoe on behalf of the Wellcome Sanger Institute COVID-19 Surveillance Team
EPI_ISL_647727, EPI_ISL_647732	Lighthouse Lab in Cambridge	Wellcome Sanger Institute for the COVID-19 Genomics UK (COG-UK) Consortium	Rob Howes, The Lighthouse Lab in Cambridge and Alex Alderton, Roberto Amato, Sonia Goncalves, Ewan Harrison, David K. Jackson, Ian Johnston, Dominic Kwiatkowski, Cordelia Langford, John Sillitoe on behalf of the Wellcome Sanger Institute COVID-19 Surveillance Team
EPI_ISL_647733	Lighthouse Lab in Alderley Park	Wellcome Sanger Institute for the COVID-19 Genomics UK (COG-UK) Consortium	Jacquelyn Wynn, Mairead Hyland, The Lighthouse Lab in Alderley Park and Alex Alderton, Roberto Amato, Sonia Goncalves, Ewan Harrison, David K. Jackson, Ian Johnston, Dominic Kwiatkowski, Cordelia Langford, John Sillitoe on behalf of the Wellcome Sanger Institute COVID-19 Surveillance Team
EPI_ISL_647738, EPI_ISL_647739	Lighthouse Lab in Cambridge	Wellcome Sanger Institute for the COVID-19 Genomics UK (COG-UK) Consortium	Rob Howes, The Lighthouse Lab in Cambridge and Alex Alderton, Roberto Amato, Sonia Goncalves, Ewan Harrison, David K. Jackson, Ian Johnston, Dominic Kwiatkowski, Cordelia Langford, John Sillitoe on behalf of the Wellcome Sanger Institute COVID-19 Surveillance Team
EPI_ISL_647741	Lighthouse Lab in Alderley Park	Wellcome Sanger Institute for the COVID-19 Genomics UK (COG-UK) Consortium	Jacquelyn Wynn, Mairead Hyland, The Lighthouse Lab in Alderley Park and Alex Alderton, Roberto Amato, Sonia Goncalves, Ewan Harrison, David K. Jackson, Ian Johnston, Dominic Kwiatkowski, Cordelia Langford, John Sillitoe on behalf of the Wellcome Sanger Institute COVID-19 Surveillance Team
EPI_ISL_647748	Lighthouse Lab in Cambridge	Wellcome Sanger Institute for the COVID-19 Genomics UK (COG-UK) Consortium	Rob Howes, The Lighthouse Lab in Cambridge and Alex Alderton, Roberto Amato, Sonia Goncalves, Ewan Harrison, David K. Jackson, Ian Johnston, Dominic Kwiatkowski, Cordelia Langford, John Sillitoe on behalf of the Wellcome Sanger Institute COVID-19 Surveillance Team
EPI_ISL_647750, EPI_ISL_647752	Lighthouse Lab in Alderley Park	Wellcome Sanger Institute for the COVID-19 Genomics UK (COG-UK) Consortium	Jacquelyn Wynn, Mairead Hyland, The Lighthouse Lab in Alderley Park and Alex Alderton, Roberto Amato, Sonia Goncalves, Ewan Harrison, David K. Jackson, Ian Johnston, Dominic Kwiatkowski, Cordelia Langford, John Sillitoe on behalf of the Wellcome Sanger Institute COVID-19 Surveillance Team
EPI_ISL_647755, EPI_ISL_647756, EPI_ISL_647758, EPI_ISL_647760, EPI_ISL_647761	Lighthouse Lab in Cambridge	Wellcome Sanger Institute for the COVID-19 Genomics UK (COG-UK) Consortium	Rob Howes, The Lighthouse Lab in Cambridge and Alex Alderton, Roberto Amato, Sonia Goncalves, Ewan Harrison, David K. Jackson, Ian Johnston, Dominic Kwiatkowski, Cordelia Langford, John Sillitoe on behalf of the Wellcome Sanger Institute COVID-19 Surveillance Team
EPI_ISL_647762	Lighthouse Lab in Alderley Park	Wellcome Sanger Institute for the COVID-19 Genomics UK (COG-UK) Consortium	Jacquelyn Wynn, Mairead Hyland, The Lighthouse Lab in Alderley Park and Alex Alderton, Roberto Amato, Sonia Goncalves, Ewan Harrison, David K. Jackson, Ian Johnston, Dominic Kwiatkowski, Cordelia Langford, John Sillitoe on behalf of the Wellcome Sanger Institute COVID-19 Surveillance Team
EPI_ISL_647764	Lighthouse Lab in Cambridge	Wellcome Sanger Institute for the COVID-19 Genomics UK (COG-UK) Consortium	Rob Howes, The Lighthouse Lab in Cambridge and Alex Alderton, Roberto Amato, Sonia Goncalves, Ewan Harrison, David K. Jackson, Ian Johnston, Dominic Kwiatkowski, Cordelia Langford, John Sillitoe on behalf of the Wellcome Sanger Institute COVID-19 Surveillance Team
EPI_ISL_647771	Lighthouse Lab in Alderley Park	Wellcome Sanger Institute for the COVID-19 Genomics UK (COG-UK) Consortium	Jacquelyn Wynn, Mairead Hyland, The Lighthouse Lab in Alderley Park and Alex Alderton, Roberto Amato, Sonia Goncalves, Ewan Harrison, David K. Jackson, Ian Johnston, Dominic Kwiatkowski, Cordelia Langford, John Sillitoe on behalf of the Wellcome Sanger Institute COVID-19 Surveillance Team
EPI_ISL_647774, EPI_ISL_647775	Lighthouse Lab in Cambridge	Wellcome Sanger Institute for the COVID-19 Genomics UK (COG-UK) Consortium	Rob Howes, The Lighthouse Lab in Cambridge and Alex Alderton, Roberto Amato, Sonia Goncalves, Ewan Harrison, David K. Jackson, Ian Johnston, Dominic Kwiatkowski, Cordelia Langford, John Sillitoe on behalf of the Wellcome Sanger Institute COVID-19 Surveillance Team
EPI_ISL_647776	Lighthouse Lab in Alderley Park	Wellcome Sanger Institute for the COVID-19 Genomics UK (COG-UK) Consortium	Jacquelyn Wynn, Mairead Hyland, The Lighthouse Lab in Alderley Park and Alex Alderton, Roberto Amato, Sonia Goncalves, Ewan Harrison, David K. Jackson, Ian Johnston, Dominic Kwiatkowski, Cordelia Langford, John Sillitoe on behalf of the Wellcome Sanger Institute COVID-19 Surveillance Team
EPI_ISL_647779	Lighthouse Lab in Cambridge	Wellcome Sanger Institute for the COVID-19 Genomics UK (COG-UK) Consortium	Rob Howes, The Lighthouse Lab in Cambridge and Alex Alderton, Roberto Amato, Sonia Goncalves, Ewan Harrison, David K. Jackson, Ian Johnston, Dominic Kwiatkowski, Cordelia Langford, John Sillitoe on behalf of the Wellcome Sanger Institute COVID-19 Surveillance Team
EPI_ISL_647780, EPI_ISL_647786, EPI_ISL_647787	Lighthouse Lab in Alderley Park	Wellcome Sanger Institute for the COVID-19 Genomics UK (COG-UK) Consortium	Jacquelyn Wynn, Mairead Hyland, The Lighthouse Lab in Alderley Park and Alex Alderton, Roberto Amato, Sonia Goncalves, Ewan Harrison, David K. Jackson, Ian Johnston, Dominic Kwiatkowski, Cordelia Langford, John Sillitoe on behalf of the Wellcome Sanger Institute COVID-19 Surveillance Team
EPI_ISL_647789	Lighthouse Lab in Cambridge	Wellcome Sanger Institute for the COVID-19 Genomics UK (COG-UK) Consortium	Rob Howes, The Lighthouse Lab in Cambridge and Alex Alderton, Roberto Amato, Sonia Goncalves, Ewan Harrison, David K. Jackson, Ian Johnston, Dominic Kwiatkowski, Cordelia Langford, John Sillitoe on behalf of the Wellcome Sanger Institute COVID-19 Surveillance Team
EPI_ISL_647792	Lighthouse Lab in Alderley Park	Wellcome Sanger Institute for the COVID-19 Genomics UK (COG-UK) Consortium	Jacquelyn Wynn, Mairead Hyland, The Lighthouse Lab in Alderley Park and Alex Alderton, Roberto Amato, Sonia Goncalves, Ewan Harrison, David K. Jackson, Ian Johnston, Dominic Kwiatkowski, Cordelia Langford, John Sillitoe on behalf of the Wellcome Sanger Institute COVID-19 Surveillance Team
EPI_ISL_647795, EPI_ISL_647796	Lighthouse Lab in Cambridge	Wellcome Sanger Institute for the COVID-19 Genomics UK (COG-UK) Consortium	Rob Howes, The Lighthouse Lab in Cambridge and Alex Alderton, Roberto Amato, Sonia Goncalves, Ewan Harrison, David K. Jackson, Ian Johnston, Dominic Kwiatkowski, Cordelia Langford, John Sillitoe on behalf of the Wellcome Sanger Institute COVID-19 Surveillance Team
EPI_ISL_647798, EPI_ISL_647800, EPI_ISL_647801, EPI_ISL_647804, EPI_ISL_647806, EPI_ISL_647861, EPI_ISL_647863, EPI_ISL_647864, EPI_ISL_647865	Lighthouse Lab in Glasgow	Wellcome Sanger Institute for the COVID-19 Genomics UK (COG-UK) Consortium	Harper VanSteenhouse, Yumi Kasai, David Gray, Carol Clugston, Anna Dominiczak and Alex Alderton, Roberto Amato, Sonia Goncalves, Ewan Harrison, David K. Jackson, Ian Johnston, Dominic Kwiatkowski, Cordelia Langford, John Sillitoe on behalf of the Wellcome Sanger Institute COVID-19 Surveillance Team
EPI_ISL_648151	Halmstad	The Public Health Agency of Sweden	Anna-Malin Linde, Maria Lind Karlberg, Mattias Haukland, Reza Advani, Olov Svartstrom, Oskar Karlsson Lindsjo, Sandra Broddesson, Petra Edquist, Mia Brytting, Anna Risberg, Karin Tegmark-Wisell
EPI_ISL_648157, EPI_ISL_648158	Sundsvall	The Public Health Agency of Sweden	Anna-Malin Linde, Maria Lind Karlberg, Mattias Haukland, Reza Advani, Olov Svartstrom, Oskar Karlsson Lindsjo, Sandra Broddesson, Petra Edquist, Mia Brytting, Anna Risberg, Karin Tegmark-Wisell
EPI_ISL_649018	San Diego County Public Health Laboratory	Andersen lab at Scripps Research	SEARCH Alliance San Diego with Tracy Basler, Jovan Shephard, Brett Austin
EPI_ISL_649058	University of Michigan Clinical Microbiology Laboratory	Lauring Lab, University of Michigan, Department of Microbiology and Immunology	Valesano
EPI_ISL_649483, EPI_ISL_649484, EPI_ISL_649485, EPI_ISL_649486, EPI_ISL_649490, EPI_ISL_649491	Lighthouse Lab in Alderley Park	Wellcome Sanger Institute for the COVID-19 Genomics UK (COG-UK) Consortium	Jacquelyn Wynn, Mairead Hyland, The Lighthouse Lab in Alderley Park and Alex Alderton, Roberto Amato, Sonia Goncalves, Ewan Harrison, David K. Jackson, Ian Johnston, Dominic Kwiatkowski, Cordelia Langford, John Sillitoe on behalf of the Wellcome Sanger Institute COVID-19 Surveillance Team (http://www.sanger.ac.uk/covid-team)
EPI_ISL_649492, EPI_ISL_649493	Lighthouse Lab in Cambridge	Wellcome Sanger Institute for the COVID-19 Genomics UK (COG-UK) Consortium	Rob Howes, The Lighthouse Lab in Cambridge and Alex Alderton, Roberto Amato, Sonia Goncalves, Ewan Harrison, David K. Jackson, Ian Johnston, Dominic Kwiatkowski, Cordelia Langford, John Sillitoe on behalf of the Wellcome Sanger Institute COVID-19 Surveillance Team (http://www.sanger.ac.uk/covid-team)
EPI_ISL_649499, EPI_ISL_649503	Lighthouse Lab in Glasgow	Wellcome Sanger Institute for the COVID-19 Genomics UK (COG-UK) Consortium	Harper VanSteenhouse, Yumi Kasai, David Gray, Carol Clugston, Anna Dominiczak and Alex Alderton, Roberto Amato, Sonia Goncalves, Ewan Harrison, David K. Jackson, Ian Johnston, Dominic Kwiatkowski, Cordelia Langford, John Sillitoe on behalf of the Wellcome Sanger Institute COVID-19 Surveillance Team (http://www.sanger.ac.uk/covid-team)
EPI_ISL_649629, EPI_ISL_649632	Lighthouse Lab in Cambridge	Wellcome Sanger Institute for the COVID-19 Genomics UK (COG-UK) Consortium	Rob Howes, The Lighthouse Lab in Cambridge and Alex Alderton, Roberto Amato, Sonia Goncalves, Ewan Harrison, David K. Jackson, Ian Johnston, Dominic Kwiatkowski, Cordelia Langford, John Sillitoe on behalf of the Wellcome Sanger Institute COVID-19 Surveillance Team (http://www.sanger.ac.uk/covid-team)
EPI_ISL_649890	Respiratory Virus Unit, Microbiology Services Colindale, Public Health England	COVID-19 Genomics UK (COG-UK) Consortium	PHE Covid Sequencing Team
EPI_ISL_650105	University of Michigan Clinical Microbiology Laboratory	Lauring Lab, University of Michigan, Department of Microbiology and Immunology	Valesano
EPI_ISL_650123	University College London, Great Ormond Street Hospital for Children NHS Foundation Trust, Imperial College Healthcare NHS Trust	COVID-19 Genomics UK (COG-UK) Consortium	Sergi Castellano, Rachel Williams, Mark Kristiansen, Paola Resende Silva, Sunando Roy, Tony Brooks, Helena Tutill, Paola Niola, Patricia Dyal, Charlotte Williams, Leysa Forrest, Yasmin Panchbhaya, Jacqueline Findlay, Samuel Weeks, Julianne Brown, Kathryn Harris, Paul Randell, James Price, Alison Holmes, Judith Breuer

EPI_ISL_650131	Virology Department, Sheffield Teaching Hospitals NHS Foundation Trust/Department of Infection, Immunity and Cardiovascular Disease, The Medical School, University of Sheffield	COVID-19 Genomics UK (COG-UK) Consortium	Thushan de Silva, Matthew Parker, Nikki Smith, Adri Angyal, Rebecca Brown, Luke Green, Rachel Tucker, Paul Parsons, Danielle Groves, Katie Johnson, Laura Carrilero, Alex Keeley, Dave Partridge, Matthew Wyles, Benjamin Lindsey, Mehmet Yavuz, Mohammad Raza, Cariad Evans
EPI_ISL_650135	Virology Department, Royal Infirmary of Edinburgh, NHS Lothian / School of Biological Sciences, University of Edinburgh / Institute of Genetics and Molecular Medicine, University of Edinburgh	COVID-19 Genomics UK (COG-UK) Consortium	McHugh M, Dewar R, Rooke S, Gallagher M, Balcaza C, O'Toole Á, Scher E, Hill V, McCrone JT, Colquhoun R, Yu X, Jackson B, Rambaut A, Williams TC, Templeton K
EPI_ISL_650141	University of Exeter	COVID-19 Genomics UK (COG-UK) Consortium	Ben Temperton,Aaron Jeffries,Michelle Michelsen,Joanna Warwick-Dugdale,Audrey Farbos,Robyn Manley,Stephen Michell,Jane Masoli
EPI_ISL_650143	Department of Pathology, University of Cambridge	COVID-19 Genomics UK (COG-UK) Consortium	Aminu S. Jahun, Yasmin Chaudhry, Grant Hall, Iliana Georgana, Myra Hosmillo, Martin D. Curran, Malte Pinckert, Surendra Parmar, Ian Goodfellow
EPI_ISL_650153, EPI_ISL_650158	University of Birmingham	COVID-19 Genomics UK (COG-UK) Consortium	Institute of Microbiology, University of Birmingham: Claire McMurray, Joanne Stockton, Samuel Nicholls, Radoslaw Poplawski, Will Rowe, Josh Quick, Nicholas Loman. University of Birmingham Testing Laboratory: Celina M Whalley, Andrew Bosworth, Charlotte Poxon, Kasun Wanigasooriya, Oliver Pickles, Mike Kidd, Alex Richter, Andrew D Beggs PHE Heartlands Lab: Husam Osman, Andrew Bosworth. Queen Elizabeth Hospital: Anna Casey
EPI_ISL_650162	Liverpool Clinical Laboratories	COVID-19 Genomics UK (COG-UK) Consortium	Sam Haldenby, Anita Lucaci, Steve Paterson, Julian Hiscox, Alistair Darby, M Almsaud, A Alrezaihi, Muhannad Alruwaili, Stuart D Armstrong, Jones Benjamin, Eleanor G Bentley, Anu Chawla, Jordan J Clark, Angela Cowell, Richard Eccles, Isabel García-Dorival, Matthew Gemmell, Alessandro Gerada, PKF Gilmore, Richard Gregory, Ximeng Han, Catherine Hartley, Margaret Hughes, Miren Iturriza-Gomara, James Johnson, L Luu, Jenifer Manson, Charlotte Nelson, Elaine O'Toole, Cassie Olateju, Rebekah Penrice-Randal , Lucille Rainbow, N.P Randle, Trevor Ian Robinson, Parul Sharma, Ghada T Shawli, James P Stewart, Neil Swainston, Ecaterina Vamos, Joanne Watts, Mark Whitehead
EPI_ISL_650164	West of Scotland Specialist Virology Centre, NHSGGC / MRC-University of Glasgow Centre for Virus Research	COVID-19 Genomics UK (COG-UK) Consortium	Ana da Silva Filipe, Natasha Johnson, Kathy Smollett, Daniel Mair, Stephen Carmichael, Alice Broos, Lily Tong, Jenna Nichols, Kyriaki Nomikou; Sarah McDonald; Richard Orton, Joseph Hughes, Sreenu Vattipally, David L Robertson; Alasdair MacLean, Rory Gunson; Sharif Shaaban, Matthew Holden; Rachel Blacow, Guy Mollett, Kathy Li, James Shepherd, Antonia Ho, Emma Thomson
EPI_ISL_650184	Liverpool Clinical Laboratories	COVID-19 Genomics UK (COG-UK) Consortium	Sam Haldenby, Anita Lucaci, Steve Paterson, Julian Hiscox, Alistair Darby, M Almsaud, A Alrezaihi, Muhannad Alruwaili, Stuart D Armstrong, Jones Benjamin, Eleanor G Bentley, Anu Chawla, Jordan J Clark, Angela Cowell, Richard Eccles, Isabel García-Dorival, Matthew Gemmell, Alessandro Gerada, PKF Gilmore, Richard Gregory, Ximeng Han, Catherine Hartley, Margaret Hughes, Miren Iturriza-Gomara, James Johnson, L Luu, Jenifer Manson, Charlotte Nelson, Elaine O'Toole, Cassie Olateju, Rebekah Penrice-Randal , Lucille Rainbow, N.P Randle, Trevor Ian Robinson, Parul Sharma, Ghada T Shawli, James P Stewart, Neil Swainston, Ecaterina Vamos, Joanne Watts, Mark Whitehead
EPI_ISL_650210	Virology Department, Sheffield Teaching Hospitals NHS Foundation Trust/Department of Infection, Immunity and Cardiovascular Disease, The Medical School, University of Sheffield	COVID-19 Genomics UK (COG-UK) Consortium	Thushan de Silva, Matthew Parker, Nikki Smith, Adri Angyal, Rebecca Brown, Luke Green, Rachel Tucker, Paul Parsons, Danielle Groves, Katie Johnson, Laura Carrilero, Alex Keeley, Dave Partridge, Matthew Wyles, Benjamin Lindsey, Mehmet Yavuz, Mohammad Raza, Cariad Evans
EPI_ISL_650215	University of Exeter	COVID-19 Genomics UK (COG-UK) Consortium	Ben Temperton,Aaron Jeffries,Michelle Michelsen,Joanna Warwick-Dugdale,Audrey Farbos,Robyn Manley,Stephen Michell,Jane Masoli
EPI_ISL_650235, EPI_ISL_650251, EPI_ISL_650252	University of Birmingham	COVID-19 Genomics UK (COG-UK) Consortium	Institute of Microbiology, University of Birmingham: Claire McMurray, Joanne Stockton, Samuel Nicholls, Radoslaw Poplawski, Will Rowe, Josh Quick, Nicholas Loman. University of Birmingham Testing Laboratory: Celina M Whalley, Andrew Bosworth, Charlotte Poxon, Kasun Wanigasooriya, Oliver Pickles, Mike Kidd, Alex Richter, Andrew D Beggs PHE Heartlands Lab: Husam Osman, Andrew Bosworth. Queen Elizabeth Hospital: Anna Casey
EPI_ISL_650269	Liverpool Clinical Laboratories	COVID-19 Genomics UK (COG-UK) Consortium	Sam Haldenby, Anita Lucaci, Steve Paterson, Julian Hiscox, Alistair Darby, M Almsaud, A Alrezaihi, Muhannad Alruwaili, Stuart D Armstrong, Jones Benjamin, Eleanor G Bentley, Anu Chawla, Jordan J Clark, Angela Cowell, Richard Eccles, Isabel García-Dorival, Matthew Gemmell, Alessandro Gerada, PKF Gilmore, Richard Gregory, Ximeng Han, Catherine Hartley, Margaret Hughes, Miren Iturriza-Gomara, James Johnson, L Luu, Jenifer Manson, Charlotte Nelson, Elaine O'Toole, Cassie Olateju, Rebekah Penrice-Randal , Lucille Rainbow, N.P Randle, Trevor Ian Robinson, Parul Sharma, Ghada T Shawli, James P Stewart, Neil Swainston, Ecaterina Vamos, Joanne Watts, Mark Whitehead
EPI_ISL_650273	Department of Pathology, University of Cambridge	COVID-19 Genomics UK (COG-UK) Consortium	Aminu S. Jahun, Yasmin Chaudhry, Grant Hall, Iliana Georgana, Myra Hosmillo, Martin D. Curran, Malte Pinckert, Surendra Parmar, Ian Goodfellow
EPI_ISL_650298	West of Scotland Specialist Virology Centre, NHSGGC / MRC-University of Glasgow Centre for Virus Research	COVID-19 Genomics UK (COG-UK) Consortium	Ana da Silva Filipe, Natasha Johnson, Kathy Smollett, Daniel Mair, Stephen Carmichael, Alice Broos, Lily Tong, Jenna Nichols, Kyriaki Nomikou; Sarah McDonald; Richard Orton, Joseph Hughes, Sreenu Vattipally, David L Robertson; Alasdair MacLean, Rory Gunson; Sharif Shaaban, Matthew Holden; Rachel Blacow, Guy Mollett, Kathy Li, James Shepherd, Antonia Ho, Emma Thomson
EPI_ISL_650299	Liverpool Clinical Laboratories	COVID-19 Genomics UK (COG-UK) Consortium	Sam Haldenby, Anita Lucaci, Steve Paterson, Julian Hiscox, Alistair Darby, M Almsaud, A Alrezaihi, Muhannad Alruwaili, Stuart D Armstrong, Jones Benjamin, Eleanor G Bentley, Anu Chawla, Jordan J Clark, Angela Cowell, Richard Eccles, Isabel García-Dorival, Matthew Gemmell, Alessandro Gerada, PKF Gilmore, Richard Gregory, Ximeng Han, Catherine Hartley, Margaret Hughes, Miren Iturriza-Gomara, James Johnson, L Luu, Jenifer Manson, Charlotte Nelson, Elaine O'Toole, Cassie Olateju, Rebekah Penrice-Randal , Lucille Rainbow, N.P Randle, Trevor Ian Robinson, Parul Sharma, Ghada T Shawli, James P Stewart, Neil Swainston, Ecaterina Vamos, Joanne Watts, Mark Whitehead
EPI_ISL_650310	West of Scotland Specialist Virology Centre, NHSGGC / MRC-University of Glasgow Centre for Virus Research	COVID-19 Genomics UK (COG-UK) Consortium	Ana da Silva Filipe, Natasha Johnson, Kathy Smollett, Daniel Mair, Stephen Carmichael, Alice Broos, Lily Tong, Jenna Nichols, Kyriaki Nomikou; Sarah McDonald; Richard Orton, Joseph Hughes, Sreenu Vattipally, David L Robertson; Alasdair MacLean, Rory Gunson; Sharif Shaaban, Matthew Holden; Rachel Blacow, Guy Mollett, Kathy Li, James Shepherd, Antonia Ho, Emma Thomson
EPI_ISL_650311	Virology Department, Royal Infirmary of Edinburgh, NHS Lothian / School of Biological Sciences, University of Edinburgh / Institute of Genetics and Molecular Medicine, University of Edinburgh	COVID-19 Genomics UK (COG-UK) Consortium	McHugh M, Dewar R, Rooke S, Gallagher M, Balcaza C, O'Toole Á, Scher E, Hill V, McCrone JT, Colquhoun R, Yu X, Jackson B, Rambaut A, Williams TC, Templeton K
EPI_ISL_650316	West of Scotland Specialist Virology Centre, NHSGGC / MRC-University of Glasgow Centre for Virus Research	COVID-19 Genomics UK (COG-UK) Consortium	Ana da Silva Filipe, Natasha Johnson, Kathy Smollett, Daniel Mair, Stephen Carmichael, Alice Broos, Lily Tong, Jenna Nichols, Kyriaki Nomikou; Sarah McDonald; Richard Orton, Joseph Hughes, Sreenu Vattipally, David L Robertson; Alasdair MacLean, Rory Gunson; Sharif Shaaban, Matthew Holden; Rachel Blacow, Guy Mollett, Kathy Li, James Shepherd, Antonia Ho, Emma Thomson
EPI_ISL_650335	Quadram Institute Bioscience	COVID-19 Genomics UK (COG-UK) Consortium	Dave J. Baker, Gemma L. Kay, Alp Aydin, Thanh Le-Viet, Steven Rudder, Ana P. Tedim, Anastasia Kolyva, Maria Diaz, Leonardo de Oliveira Martins, Nabil-Fareed Alikhan, Lizzie Meadows, Rachael Stanley, Ngozi Elumogo, Muhammed Yasir, Nicholas M. Thomson, Alexander J Trotter, Rachel Gilroy, Samuel Bloomfield, Claire Stuart, Andrew Bell, Reenesh Prakash, Samir Dervisevic, Alison E. Mather, John Wain, Mark Webber, Andrew J. Page, Justin O'Grady
EPI_ISL_650354	University of Birmingham	COVID-19 Genomics UK (COG-UK) Consortium	Institute of Microbiology, University of Birmingham: Claire McMurray, Joanne Stockton, Samuel Nicholls, Radoslaw Poplawski, Will Rowe, Josh Quick, Nicholas Loman. University of Birmingham Testing Laboratory: Celina M Whalley, Andrew Bosworth, Charlotte Poxon, Kasun Wanigasooriya, Oliver Pickles, Mike Kidd, Alex Richter, Andrew D Beggs PHE Heartlands Lab: Husam Osman, Andrew Bosworth. Queen Elizabeth Hospital: Anna Casey
EPI_ISL_650357	University of Exeter	COVID-19 Genomics UK (COG-UK) Consortium	Ben Temperton,Aaron Jeffries,Michelle Michelsen,Joanna Warwick-Dugdale,Audrey Farbos,Robyn Manley,Stephen Michell,Jane Masoli
EPI_ISL_650364, EPI_ISL_650365	Department of Pathology, University of Cambridge	COVID-19 Genomics UK (COG-UK) Consortium	Aminu S. Jahun, Yasmin Chaudhry, Grant Hall, Iliana Georgana, Myra Hosmillo, Martin D. Curran, Malte Pinckert, Surendra Parmar, Ian Goodfellow
EPI_ISL_650370	Virology Department, Sheffield Teaching Hospitals NHS Foundation Trust/Department of Infection, Immunity and Cardiovascular Disease, The Medical School, University of Sheffield	COVID-19 Genomics UK (COG-UK) Consortium	Thushan de Silva, Matthew Parker, Nikki Smith, Adri Angyal, Rebecca Brown, Luke Green, Rachel Tucker, Paul Parsons, Danielle Groves, Katie Johnson, Laura Carrilero, Alex Keeley, Dave Partridge, Matthew Wyles, Benjamin Lindsey, Mehmet Yavuz, Mohammad Raza, Cariad Evans
EPI_ISL_650390	Department of Pathology, University of Cambridge	COVID-19 Genomics UK (COG-UK) Consortium	Aminu S. Jahun, Yasmin Chaudhry, Grant Hall, Iliana Georgana, Myra Hosmillo, Martin D. Curran, Malte Pinckert, Surendra Parmar, Ian Goodfellow
EPI_ISL_650424	West of Scotland Specialist Virology Centre, NHSGGC / MRC-University of Glasgow Centre for Virus Research	COVID-19 Genomics UK (COG-UK) Consortium	Ana da Silva Filipe, Natasha Johnson, Kathy Smollett, Daniel Mair, Stephen Carmichael, Alice Broos, Lily Tong, Jenna Nichols, Kyriaki Nomikou; Sarah McDonald; Richard Orton, Joseph Hughes, Sreenu Vattipally, David L Robertson; Alasdair MacLean, Rory Gunson; Sharif Shaaban, Matthew Holden; Rachel Blacow, Guy Mollett, Kathy Li, James Shepherd, Antonia Ho, Emma Thomson

EPI_ISL_650436, EPI_ISL_650437	University of Birmingham	COVID-19 Genomics UK (COG-UK) Consortium	Institute of Microbiology, University of Birmingham: Claire McMurray, Joanne Stockton, Samuel Nicholls, Radoslaw Poplawski, Will Rowe, Josh Quick, Nicholas Loman. University of Birmingham Testing Laboratory: Celina M Whalley, Andrew Bosworth, Charlotte Poxon, Kasun Wanigasooriya, Oliver Pickles, Mike Kidd, Alex Richter, Andrew D Beggs PHE Heartlands Lab: Husam Osman, Andrew Bosworth. Queen Elizabeth Hospital: Anna Casey
EPI_ISL_650439	Virology Department, Royal Infirmary of Edinburgh, NHS Lothian / School of Biological Sciences, University of Edinburgh / Institute of Genetics and Molecular Medicine, University of Edinburgh	COVID-19 Genomics UK (COG-UK) Consortium	McHugh M, Dewar R, Rooke S, Gallagher M, Balcaza C, O'Toole Á, Scher E, Hill V, McCrone JT, Colquhoun R, Yu X, Jackson B, Rambaut A, Williams TC, Templeton K
EPI_ISL_650447	Department of Pathology, University of Cambridge	COVID-19 Genomics UK (COG-UK) Consortium	Aminu S. Jahun, Yasmin Chaudhry, Grant Hall, Iliana Georgana, Myra Hosmillo, Martin D. Curran, Malte Pinckert, Surendra Parmar, Ian Goodfellow
EPI_ISL_650476	Quadram Institute Bioscience	COVID-19 Genomics UK (COG-UK) Consortium	Dave J. Baker, Gemma L. Kay, Alp Aydin, Thanh Le-Viet, Steven Rudder, Ana P. Tedim, Anastasia Kolyva, Maria Diaz, Leonardo de Oliveira Martins, Nabil-Fareed Alikhan, Lizzie Meadows, Rachael Stanley, Ngozi Elumogo, Muhammed Yasir, Nicholas M. Thomson, Alexander J Trotter, Rachel Gilroy, Samuel Bloomfield, Claire Stuart, Andrew Bell, Reenesh Prakash, Samir Dervisevic, Alison E. Mather, John Wain, Mark Webber, Andrew J. Page, Justin O'Grady
EPI_ISL_650478	Virology Department, Royal Infirmary of Edinburgh, NHS Lothian / School of Biological Sciences, University of Edinburgh / Institute of Genetics and Molecular Medicine, University of Edinburgh	COVID-19 Genomics UK (COG-UK) Consortium	McHugh M, Dewar R, Rooke S, Gallagher M, Balcaza C, O'Toole Á, Scher E, Hill V, McCrone JT, Colquhoun R, Yu X, Jackson B, Rambaut A, Williams TC, Templeton K
EPI_ISL_650481	Department of Pathology, University of Cambridge	COVID-19 Genomics UK (COG-UK) Consortium	Aminu S. Jahun, Yasmin Chaudhry, Grant Hall, Iliana Georgana, Myra Hosmillo, Martin D. Curran, Malte Pinckert, Surendra Parmar, Ian Goodfellow
EPI_ISL_650482	West of Scotland Specialist Virology Centre, NHSGGC / MRC-University of Glasgow Centre for Virus Research	COVID-19 Genomics UK (COG-UK) Consortium	Ana da Silva Filipe, Natasha Johnson, Kathy Smollett, Daniel Mair, Stephen Carmichael, Alice Broos, Lily Tong, Jenna Nichols, Kyriaki Nomikou; Sarah McDonald; Richard Orton, Joseph Hughes, Sreenu Vattipally, David L Robertson; Alasdair MacLean, Rory Gunson; Sharif Shaaban, Matthew Holden; Rachel Blacow, Guy Mollett, Kathy Li, James Shepherd, Antonia Ho, Emma Thomson
EPI_ISL_650495	Virology Department, Royal Infirmary of Edinburgh, NHS Lothian / School of Biological Sciences, University of Edinburgh / Institute of Genetics and Molecular Medicine, University of Edinburgh	COVID-19 Genomics UK (COG-UK) Consortium	McHugh M, Dewar R, Rooke S, Gallagher M, Balcaza C, O'Toole Á, Scher E, Hill V, McCrone JT, Colquhoun R, Yu X, Jackson B, Rambaut A, Williams TC, Templeton K
EPI_ISL_650506, EPI_ISL_650507, EPI_ISL_650511, EPI_ISL_650514, EPI_ISL_650538, EPI_ISL_650550	West of Scotland Specialist Virology Centre, NHSGGC / MRC-University of Glasgow Centre for Virus Research	COVID-19 Genomics UK (COG-UK) Consortium	Ana da Silva Filipe, Natasha Johnson, Kathy Smollett, Daniel Mair, Stephen Carmichael, Alice Broos, Lily Tong, Jenna Nichols, Kyriaki Nomikou; Sarah McDonald; Richard Orton, Joseph Hughes, Sreenu Vattipally, David L Robertson; Alasdair MacLean, Rory Gunson; Sharif Shaaban, Matthew Holden; Rachel Blacow, Guy Mollett, Kathy Li, James Shepherd, Antonia Ho, Emma Thomson
EPI_ISL_650563	Liverpool Clinical Laboratories	COVID-19 Genomics UK (COG-UK) Consortium	Sam Haldenby, Anita Lucaci, Steve Paterson, Julian Hiscox, Alistair Darby, M Almsaud, A Alrezaihi, Muhannad Alruwaili, Stuart D Armstrong, Jones Benjamin, Eleanor G Bentley, Anu Chawla, Jordan J Clark, Angela Cowell, Richard Eccles, Isabel García-Dorival, Matthew Gemmell, Alessandro Gerada, PKF Gilmore, Richard Gregory, Ximeng Han, Catherine Hartley, Margaret Hughes, Miren Iturriza-Gomara, James Johnson, L Luu, Jenifer Manson, Charlotte Nelson, Elaine O'Toole, Cassie Olateju, Rebekah Penrice-Randal , Lucille Rainbow, N.P Randle, Trevor Ian Robinson, Parul Sharma, Ghada T Shawli, James P Stewart, Neil Swainston, Ecaterina Vamos, Joanne Watts, Mark Whitehead
EPI_ISL_650565	University of Exeter	COVID-19 Genomics UK (COG-UK) Consortium	Ben Temperton,Aaron Jeffries,Michelle Michelsen,Joanna Warwick-Dugdale,Audrey Farbos,Robyn Manley,Stephen Michell,Jane Masoli
EPI_ISL_650580	Department of Pathology, University of Cambridge	COVID-19 Genomics UK (COG-UK) Consortium	Aminu S. Jahun, Yasmin Chaudhry, Grant Hall, Iliana Georgana, Myra Hosmillo, Martin D. Curran, Malte Pinckert, Surendra Parmar, Ian Goodfellow
EPI_ISL_650606	University of Exeter	COVID-19 Genomics UK (COG-UK) Consortium	Ben Temperton,Aaron Jeffries,Michelle Michelsen,Joanna Warwick-Dugdale,Audrey Farbos,Robyn Manley,Stephen Michell,Jane Masoli
EPI_ISL_650608	Centre for Enzyme Innovation, University of Portsmouth / Translational Research Laboratory, Portsmouth Hospitals NHS Trust	COVID-19 Genomics UK (COG-UK) Consortium	Angela Beckett,Yann Bourgeois,Garry Scarlett,Sharon Glaysher,Scott Elliott,Kelly Bicknell,Robert Impey,Allyson Lloyd,Sarah Wyllie,Ethan Butcher,Anoop Chauhan,Samuel Robson
EPI_ISL_650614, EPI_ISL_650664	Department of Pathology, University of Cambridge	COVID-19 Genomics UK (COG-UK) Consortium	Aminu S. Jahun, Yasmin Chaudhry, Grant Hall, Iliana Georgana, Myra Hosmillo, Martin D. Curran, Malte Pinckert, Surendra Parmar, Ian Goodfellow
EPI_ISL_650665, EPI_ISL_650668	University of Birmingham	COVID-19 Genomics UK (COG-UK) Consortium	Institute of Microbiology, University of Birmingham: Claire McMurray, Joanne Stockton, Samuel Nicholls, Radoslaw Poplawski, Will Rowe, Josh Quick, Nicholas Loman. University of Birmingham Testing Laboratory: Celina M Whalley, Andrew Bosworth, Charlotte Poxon, Kasun Wanigasooriya, Oliver Pickles, Mike Kidd, Alex Richter, Andrew D Beggs PHE Heartlands Lab: Husam Osman, Andrew Bosworth. Queen Elizabeth Hospital: Anna Casey
EPI_ISL_650691	Virology Department, Sheffield Teaching Hospitals NHS Foundation Trust/Department of Infection, Immunity and Cardiovascular Disease, The Medical School, University of Sheffield	COVID-19 Genomics UK (COG-UK) Consortium	Thushan de Silva, Matthew Parker, Nikki Smith, Adri Angyal, Rebecca Brown, Luke Green, Rachel Tucker, Paul Parsons, Danielle Groves, Katie Johnson, Laura Carrilero, Alex Keeley, Dave Partridge, Matthew Wyles, Benjamin Lindsey, Mehmet Yavuz, Mohammad Raza, Cariad Evans
EPI_ISL_650724	University of Birmingham	COVID-19 Genomics UK (COG-UK) Consortium	Institute of Microbiology, University of Birmingham: Claire McMurray, Joanne Stockton, Samuel Nicholls, Radoslaw Poplawski, Will Rowe, Josh Quick, Nicholas Loman. University of Birmingham Testing Laboratory: Celina M Whalley, Andrew Bosworth, Charlotte Poxon, Kasun Wanigasooriya, Oliver Pickles, Mike Kidd, Alex Richter, Andrew D Beggs PHE Heartlands Lab: Husam Osman, Andrew Bosworth. Queen Elizabeth Hospital: Anna Casey
EPI_ISL_650727	West of Scotland Specialist Virology Centre, NHSGGC / MRC-University of Glasgow Centre for Virus Research	COVID-19 Genomics UK (COG-UK) Consortium	Ana da Silva Filipe, Natasha Johnson, Kathy Smollett, Daniel Mair, Stephen Carmichael, Alice Broos, Lily Tong, Jenna Nichols, Kyriaki Nomikou; Sarah McDonald; Richard Orton, Joseph Hughes, Sreenu Vattipally, David L Robertson; Alasdair MacLean, Rory Gunson; Sharif Shaaban, Matthew Holden; Rachel Blacow, Guy Mollett, Kathy Li, James Shepherd, Antonia Ho, Emma Thomson
EPI_ISL_650729	University of Birmingham	COVID-19 Genomics UK (COG-UK) Consortium	Institute of Microbiology, University of Birmingham: Claire McMurray, Joanne Stockton, Samuel Nicholls, Radoslaw Poplawski, Will Rowe, Josh Quick, Nicholas Loman. University of Birmingham Testing Laboratory: Celina M Whalley, Andrew Bosworth, Charlotte Poxon, Kasun Wanigasooriya, Oliver Pickles, Mike Kidd, Alex Richter, Andrew D Beggs PHE Heartlands Lab: Husam Osman, Andrew Bosworth. Queen Elizabeth Hospital: Anna Casey
EPI_ISL_650733, EPI_ISL_650735	West of Scotland Specialist Virology Centre, NHSGGC / MRC-University of Glasgow Centre for Virus Research	COVID-19 Genomics UK (COG-UK) Consortium	Ana da Silva Filipe, Natasha Johnson, Kathy Smollett, Daniel Mair, Stephen Carmichael, Alice Broos, Lily Tong, Jenna Nichols, Kyriaki Nomikou; Sarah McDonald; Richard Orton, Joseph Hughes, Sreenu Vattipally, David L Robertson; Alasdair MacLean, Rory Gunson; Sharif Shaaban, Matthew Holden; Rachel Blacow, Guy Mollett, Kathy Li, James Shepherd, Antonia Ho, Emma Thomson
EPI_ISL_650744	University of Birmingham	COVID-19 Genomics UK (COG-UK) Consortium	Institute of Microbiology, University of Birmingham: Claire McMurray, Joanne Stockton, Samuel Nicholls, Radoslaw Poplawski, Will Rowe, Josh Quick, Nicholas Loman. University of Birmingham Testing Laboratory: Celina M Whalley, Andrew Bosworth, Charlotte Poxon, Kasun Wanigasooriya, Oliver Pickles, Mike Kidd, Alex Richter, Andrew D Beggs PHE Heartlands Lab: Husam Osman, Andrew Bosworth. Queen Elizabeth Hospital: Anna Casey
EPI_ISL_650750, EPI_ISL_650751, EPI_ISL_650752, EPI_ISL_650753, EPI_ISL_650754, EPI_ISL_650764	West of Scotland Specialist Virology Centre, NHSGGC / MRC-University of Glasgow Centre for Virus Research	COVID-19 Genomics UK (COG-UK) Consortium	Ana da Silva Filipe, Natasha Johnson, Kathy Smollett, Daniel Mair, Stephen Carmichael, Alice Broos, Lily Tong, Jenna Nichols, Kyriaki Nomikou; Sarah McDonald; Richard Orton, Joseph Hughes, Sreenu Vattipally, David L Robertson; Alasdair MacLean, Rory Gunson; Sharif Shaaban, Matthew Holden; Rachel Blacow, Guy Mollett, Kathy Li, James Shepherd, Antonia Ho, Emma Thomson
EPI_ISL_650769, EPI_ISL_650770, EPI_ISL_650771, EPI_ISL_650773	University of Birmingham	COVID-19 Genomics UK (COG-UK) Consortium	Institute of Microbiology, University of Birmingham: Claire McMurray, Joanne Stockton, Samuel Nicholls, Radoslaw Poplawski, Will Rowe, Josh Quick, Nicholas Loman. University of Birmingham Testing Laboratory: Celina M Whalley, Andrew Bosworth, Charlotte Poxon, Kasun Wanigasooriya, Oliver Pickles, Mike Kidd, Alex Richter, Andrew D Beggs PHE Heartlands Lab: Husam Osman, Andrew Bosworth. Queen Elizabeth Hospital: Anna Casey
EPI_ISL_650780	Virology Department, Royal Infirmary of Edinburgh, NHS Lothian / School of Biological Sciences, University of Edinburgh / Institute of Genetics and Molecular Medicine, University of Edinburgh	COVID-19 Genomics UK (COG-UK) Consortium	McHugh M, Dewar R, Rooke S, Gallagher M, Balcaza C, O'Toole Á, Scher E, Hill V, McCrone JT, Colquhoun R, Yu X, Jackson B, Rambaut A, Williams TC, Templeton K
EPI_ISL_650783, EPI_ISL_650784	University of Exeter	COVID-19 Genomics UK (COG-UK) Consortium	Ben Temperton,Aaron Jeffries,Michelle Michelsen,Joanna Warwick-Dugdale,Audrey Farbos,Robyn Manley,Stephen Michell,Jane Masoli
EPI_ISL_650792	University of Birmingham	COVID-19 Genomics UK (COG-UK) Consortium	Institute of Microbiology, University of Birmingham: Claire McMurray, Joanne Stockton, Samuel Nicholls, Radoslaw Poplawski, Will Rowe, Josh Quick, Nicholas Loman. University of Birmingham Testing Laboratory: Celina M Whalley, Andrew Bosworth, Charlotte Poxon, Kasun Wanigasooriya, Oliver

			Pickles, Mike Kidd, Alex Richter, Andrew D Beggs PHE Heartlands Lab: Husam Osman, Andrew Bosworth. Queen Elizabeth Hospital: Anna Casey
EPI_ISL_650808	Virology Department, Sheffield Teaching Hospitals NHS Foundation Trust/Department of Infection, Immunity and Cardiovascular Disease, The Medical School, University of Sheffield	COVID-19 Genomics UK (COG-UK) Consortium	Thushan de Silva, Matthew Parker, Nikki Smith, Adri Angyal, Rebecca Brown, Luke Green, Rachel Tucker, Paul Parsons, Danielle Groves, Katie Johnson, Laura Carrilero, Alex Keeley, Dave Partridge, Matthew Wyles, Benjamin Lindsey, Mehmet Yavuz, Mohammad Raza, Cariad Evans
EPI_ISL_650837	West of Scotland Specialist Virology Centre, NHSGGC / MRC-University of Glasgow Centre for Virus Research	COVID-19 Genomics UK (COG-UK) Consortium	Ana da Silva Filipe, Natasha Johnson, Kathy Smollett, Daniel Mair, Stephen Carmichael, Alice Broos, Lily Tong, Jenna Nichols, Kyriaki Nomikou; Sarah McDonald; Richard Orton, Joseph Hughes, Sreenu Vattipally, David L Robertson; Alasdair MacLean, Rory Gunson; Sharif Shaaban, Matthew Holden; Rachel Blacow, Guy Mollett, Kathy Li, James Shepherd, Antonia Ho, Emma Thomson
EPI_ISL_650849	University of Birmingham	COVID-19 Genomics UK (COG-UK) Consortium	Institute of Microbiology, University of Birmingham: Claire McMurray, Joanne Stockton, Samuel Nicholls, Radoslaw Poplawski, Will Rowe, Josh Quick, Nicholas Loman. University of Birmingham Testing Laboratory: Celina M Whalley, Andrew Bosworth, Charlotte Poxon, Kasun Wanigasooriya, Oliver Pickles, Mike Kidd, Alex Richter, Andrew D Beggs PHE Heartlands Lab: Husam Osman, Andrew Bosworth. Queen Elizabeth Hospital: Anna Casey
EPI_ISL_650866, EPI_ISL_650867, EPI_ISL_650868, EPI_ISL_650869	West of Scotland Specialist Virology Centre, NHSGGC / MRC-University of Glasgow Centre for Virus Research	COVID-19 Genomics UK (COG-UK) Consortium	Ana da Silva Filipe, Natasha Johnson, Kathy Smollett, Daniel Mair, Stephen Carmichael, Alice Broos, Lily Tong, Jenna Nichols, Kyriaki Nomikou; Sarah McDonald; Richard Orton, Joseph Hughes, Sreenu Vattipally, David L Robertson; Alasdair MacLean, Rory Gunson; Sharif Shaaban, Matthew Holden; Rachel Blacow, Guy Mollett, Kathy Li, James Shepherd, Antonia Ho, Emma Thomson
EPI_ISL_650885	Quadram Institute Bioscience	COVID-19 Genomics UK (COG-UK) Consortium	Dave J. Baker, Gemma L. Kay, Alp Aydin, Thanh Le-Viet, Steven Rudder, Ana P. Tedim, Anastasia Kolyva, Maria Diaz, Leonardo de Oliveira Martins, Nabil-Fareed Alikhan, Lizzie Meadows, Rachael Stanley, Ngozi Elumogo, Muhammed Yasir, Nicholas M. Thomson, Alexander J Trotter, Rachel Gilroy, Samuel Bloomfield, Claire Stuart, Andrew Bell, Reenesh Prakash, Samir Dervisevic, Alison E. Mather, John Wain, Mark Webber, Andrew J. Page, Justin O'Grady
EPI_ISL_650906	Wales Specialist Virology Centre Sequencing lab: Pathogen Genomics Unit	COVID-19 Genomics UK (COG-UK) Consortium	Catherine Moore, Johnathan Evans, Laura Gifford, Malorie Perry, Simon Cottrell, Angela Marchbank, Alec Birchley, Alexander Adams, Amy Gaskin, Bree Gatica-Wilcox, Jason Coombes, Joel Southgate, Lauren Gilbert, Lee Graham, Nicole Pacchiarini, Sara Kumziene-Summerhayes, Sarah Taylor, Sophie Jones, Sara Rey, Matthew Bull, Joanne Watkins, Sally Corden, Tom Connor
EPI_ISL_650911	Quadram Institute Bioscience	COVID-19 Genomics UK (COG-UK) Consortium	Dave J. Baker, Gemma L. Kay, Alp Aydin, Thanh Le-Viet, Steven Rudder, Ana P. Tedim, Anastasia Kolyva, Maria Diaz, Leonardo de Oliveira Martins, Nabil-Fareed Alikhan, Lizzie Meadows, Rachael Stanley, Ngozi Elumogo, Muhammed Yasir, Nicholas M. Thomson, Alexander J Trotter, Rachel Gilroy, Samuel Bloomfield, Claire Stuart, Andrew Bell, Reenesh Prakash, Samir Dervisevic, Alison E. Mather, John Wain, Mark Webber, Andrew J. Page, Justin O'Grady
EPI_ISL_650916	Department of Pathology, University of Cambridge	COVID-19 Genomics UK (COG-UK) Consortium	Aminu S. Jahun, Yasmin Chaudhry, Grant Hall, Iliana Georgana, Myra Hosmillo, Martin D. Curran, Malte Pinckert, Surendra Parmar, Ian Goodfellow
EPI_ISL_650938, EPI_ISL_650940, EPI_ISL_650958	Quadram Institute Bioscience	COVID-19 Genomics UK (COG-UK) Consortium	Dave J. Baker, Gemma L. Kay, Alp Aydin, Thanh Le-Viet, Steven Rudder, Ana P. Tedim, Anastasia Kolyva, Maria Diaz, Leonardo de Oliveira Martins, Nabil-Fareed Alikhan, Lizzie Meadows, Rachael Stanley, Ngozi Elumogo, Muhammed Yasir, Nicholas M. Thomson, Alexander J Trotter, Rachel Gilroy, Samuel Bloomfield, Claire Stuart, Andrew Bell, Reenesh Prakash, Samir Dervisevic, Alison E. Mather, John Wain, Mark Webber, Andrew J. Page, Justin O'Grady
EPI_ISL_650996	University of Birmingham	COVID-19 Genomics UK (COG-UK) Consortium	Institute of Microbiology, University of Birmingham: Claire McMurray, Joanne Stockton, Samuel Nicholls, Radoslaw Poplawski, Will Rowe, Josh Quick, Nicholas Loman. University of Birmingham Testing Laboratory: Celina M Whalley, Andrew Bosworth, Charlotte Poxon, Kasun Wanigasooriya, Oliver Pickles, Mike Kidd, Alex Richter, Andrew D Beggs PHE Heartlands Lab: Husam Osman, Andrew Bosworth. Queen Elizabeth Hospital: Anna Casey
EPI_ISL_651002	Department of Pathology, University of Cambridge	COVID-19 Genomics UK (COG-UK) Consortium	Aminu S. Jahun, Yasmin Chaudhry, Grant Hall, Iliana Georgana, Myra Hosmillo, Martin D. Curran, Malte Pinckert, Surendra Parmar, Ian Goodfellow
EPI_ISL_651053, EPI_ISL_651055, EPI_ISL_651057, EPI_ISL_651059, EPI_ISL_651083, EPI_ISL_651085	University of Exeter	COVID-19 Genomics UK (COG-UK) Consortium	Ben Temperton,Aaron Jeffries,Michelle Michelsen,Joanna Warwick-Dugdale,Audrey Farbos,Robyn Manley,Stephen Michell,Jane Masoli
EPI_ISL_651123	Liverpool Clinical Laboratories	COVID-19 Genomics UK (COG-UK) Consortium	Sam Haldenby, Anita Lucaci, Steve Paterson, Julian Hiscox, Alistair Darby, M Almsaud, A Alrezaihi, Muhannad Alruwaili, Stuart D Armstrong, Jones Benjamin, Eleanor G Bentley, Anu Chawla, Jordan J Clark, Angela Cowell, Richard Eccles, Isabel García-Dorival, Matthew Gemmell, Alessandro Gerada, PKF Gilmore, Richard Gregory, Ximeng Han, Catherine Hartley, Margaret Hughes, Miren Iturriza-Gomara, James Johnson, L Luu, Jenifer Manson, Charlotte Nelson, Elaine O'Toole, Cassie Olateju, Rebekah Penrice-Randal , Lucille Rainbow, N.P Randle, Trevor Ian Robinson, Parul Sharma, Ghada T Shawli, James P Stewart, Neil Swainston, Ecaterina Vamos, Joanne Watts, Mark Whitehead
EPI_ISL_651127	University of Birmingham	COVID-19 Genomics UK (COG-UK) Consortium	Institute of Microbiology, University of Birmingham: Claire McMurray, Joanne Stockton, Samuel Nicholls, Radoslaw Poplawski, Will Rowe, Josh Quick, Nicholas Loman. University of Birmingham Testing Laboratory: Celina M Whalley, Andrew Bosworth, Charlotte Poxon, Kasun Wanigasooriya, Oliver Pickles, Mike Kidd, Alex Richter, Andrew D Beggs PHE Heartlands Lab: Husam Osman, Andrew Bosworth. Queen Elizabeth Hospital: Anna Casey
EPI_ISL_651133, EPI_ISL_651134	Quadram Institute Bioscience	COVID-19 Genomics UK (COG-UK) Consortium	Dave J. Baker, Gemma L. Kay, Alp Aydin, Thanh Le-Viet, Steven Rudder, Ana P. Tedim, Anastasia Kolyva, Maria Diaz, Leonardo de Oliveira Martins, Nabil-Fareed Alikhan, Lizzie Meadows, Rachael Stanley, Ngozi Elumogo, Muhammed Yasir, Nicholas M. Thomson, Alexander J Trotter, Rachel Gilroy, Samuel Bloomfield, Claire Stuart, Andrew Bell, Reenesh Prakash, Samir Dervisevic, Alison E. Mather, John Wain, Mark Webber, Andrew J. Page, Justin O'Grady
EPI_ISL_651137	University of Birmingham	COVID-19 Genomics UK (COG-UK) Consortium	Institute of Microbiology, University of Birmingham: Claire McMurray, Joanne Stockton, Samuel Nicholls, Radoslaw Poplawski, Will Rowe, Josh Quick, Nicholas Loman. University of Birmingham Testing Laboratory: Celina M Whalley, Andrew Bosworth, Charlotte Poxon, Kasun Wanigasooriya, Oliver Pickles, Mike Kidd, Alex Richter, Andrew D Beggs PHE Heartlands Lab: Husam Osman, Andrew Bosworth. Queen Elizabeth Hospital: Anna Casey
EPI_ISL_651141	Virology Department, Royal Infirmary of Edinburgh, NHS Lothian / School of Biological Sciences, University of Edinburgh / Institute of Genetics and Molecular Medicine, University of Edinburgh	COVID-19 Genomics UK (COG-UK) Consortium	McHugh M, Dewar R, Rooke S, Gallagher M, Balcaza C, O'Toole Á, Scher E, Hill V, McCrone JT, Colquhoun R, Yu X, Jackson B, Rambaut A, Williams TC, Templeton K
EPI_ISL_651142	Quadram Institute Bioscience	COVID-19 Genomics UK (COG-UK) Consortium	Dave J. Baker, Gemma L. Kay, Alp Aydin, Thanh Le-Viet, Steven Rudder, Ana P. Tedim, Anastasia Kolyva, Maria Diaz, Leonardo de Oliveira Martins, Nabil-Fareed Alikhan, Lizzie Meadows, Rachael Stanley, Ngozi Elumogo, Muhammed Yasir, Nicholas M. Thomson, Alexander J Trotter, Rachel Gilroy, Samuel Bloomfield, Claire Stuart, Andrew Bell, Reenesh Prakash, Samir Dervisevic, Alison E. Mather, John Wain, Mark Webber, Andrew J. Page, Justin O'Grady
EPI_ISL_651146	Liverpool Clinical Laboratories	COVID-19 Genomics UK (COG-UK) Consortium	Sam Haldenby, Anita Lucaci, Steve Paterson, Julian Hiscox, Alistair Darby, M Almsaud, A Alrezaihi, Muhannad Alruwaili, Stuart D Armstrong, Jones Benjamin, Eleanor G Bentley, Anu Chawla, Jordan J Clark, Angela Cowell, Richard Eccles, Isabel García-Dorival, Matthew Gemmell, Alessandro Gerada, PKF Gilmore, Richard Gregory, Ximeng Han, Catherine Hartley, Margaret Hughes, Miren Iturriza-Gomara, James Johnson, L Luu, Jenifer Manson, Charlotte Nelson, Elaine O'Toole, Cassie Olateju, Rebekah Penrice-Randal , Lucille Rainbow, N.P Randle, Trevor Ian Robinson, Parul Sharma, Ghada T Shawli, James P Stewart, Neil Swainston, Ecaterina Vamos, Joanne Watts, Mark Whitehead
EPI_ISL_651154	Wales Specialist Virology Centre Sequencing lab: Pathogen Genomics Unit	COVID-19 Genomics UK (COG-UK) Consortium	Catherine Moore, Johnathan Evans, Laura Gifford, Malorie Perry, Simon Cottrell, Angela Marchbank, Alec Birchley, Alexander Adams, Amy Gaskin, Bree Gatica-Wilcox, Jason Coombes, Joel Southgate, Lauren Gilbert, Lee Graham, Nicole Pacchiarini, Sara Kumziene-Summerhayes, Sarah Taylor, Sophie Jones, Sara Rey, Matthew Bull, Joanne Watkins, Sally Corden, Tom Connor
EPI_ISL_651177	University of Exeter	COVID-19 Genomics UK (COG-UK) Consortium	Ben Temperton,Aaron Jeffries,Michelle Michelsen,Joanna Warwick-Dugdale,Audrey Farbos,Robyn Manley,Stephen Michell,Jane Masoli
EPI_ISL_651179	University College London, Great Ormond Street Hospital for Children NHS Foundation Trust, Imperial College Healthcare NHS Trust	COVID-19 Genomics UK (COG-UK) Consortium	Sergi Castellano, Rachel Williams, Mark Kristiansen, Paola Resende Silva, Sunando Roy, Tony Brooks, Helena Tutill, Paola Niola, Patricia Dyal, Charlotte Williams, Leysa Forrest, Yasmin Panchbhaya, Jacqueline Findlay, Samuel Weeks, Julianne Brown, Kathryn Harris, Paul Randell, James Price, Alison Holmes, Judith Breuer
EPI_ISL_651183	University of Exeter	COVID-19 Genomics UK (COG-UK) Consortium	Ben Temperton,Aaron Jeffries,Michelle Michelsen,Joanna Warwick-Dugdale,Audrey Farbos,Robyn Manley,Stephen Michell,Jane Masoli
EPI_ISL_651190	University of Birmingham	COVID-19 Genomics UK (COG-UK) Consortium	Institute of Microbiology, University of Birmingham: Claire McMurray, Joanne Stockton, Samuel Nicholls, Radoslaw Poplawski, Will Rowe, Josh Quick, Nicholas Loman. University of Birmingham Testing Laboratory: Celina M Whalley, Andrew Bosworth, Charlotte Poxon, Kasun Wanigasooriya, Oliver

			Pickles, Mike Kidd, Alex Richter, Andrew D Beggs PHE Heartlands Lab: Husam Osman, Andrew Bosworth. Queen Elizabeth Hospital: Anna Casey
EPI_ISL_651197	Liverpool Clinical Laboratories	COVID-19 Genomics UK (COG-UK) Consortium	Sam Haldenby, Anita Lucaci, Steve Paterson, Julian Hiscox, Alistair Darby, M Almsaud, A Alrezaihi, Muhannad Alruwaili, Stuart D Armstrong, Jones Benjamin, Eleanor G Bentley, Anu Chawla, Jordan J Clark, Angela Cowell, Richard Eccles, Isabel García-Dorival, Matthew Gemmell, Alessandro Gerada, PKF Gilmore, Richard Gregory, Ximeng Han, Catherine Hartley, Margaret Hughes, Miren Iturriza-Gomara, James Johnson, L Luu, Jenifer Manson, Charlotte Nelson, Elaine O'Toole, Cassie Olateju, Rebekah Penrice-Randal , Lucille Rainbow, N.P Randle, Trevor Ian Robinson, Parul Sharma, Ghada T Shawli, James P Stewart, Neil Swainston, Ecaterina Vamos, Joanne Watts, Mark Whitehead
EPI_ISL_651224	Virology Department, Sheffield Teaching Hospitals NHS Foundation Trust/Department of Infection, Immunity and Cardiovascular Disease, The Medical School, University of Sheffield	COVID-19 Genomics UK (COG-UK) Consortium	Thushan de Silva, Matthew Parker, Nikki Smith, Adri Angyal, Rebecca Brown, Luke Green, Rachel Tucker, Paul Parsons, Danielle Groves, Katie Johnson, Laura Carrilero, Alex Keeley, Dave Partridge, Matthew Wyles, Benjamin Lindsey, Mehmet Yavuz, Mohammad Raza, Cariad Evans
EPI_ISL_651227	University of Birmingham	COVID-19 Genomics UK (COG-UK) Consortium	Institute of Microbiology, University of Birmingham: Claire McMurray, Joanne Stockton, Samuel Nicholls, Radoslaw Poplawski, Will Rowe, Josh Quick, Nicholas Loman. University of Birmingham Testing Laboratory: Celina M Whalley, Andrew Bosworth, Charlotte Poxon, Kasun Wanigasooriya, Oliver Pickles, Mike Kidd, Alex Richter, Andrew D Beggs PHE Heartlands Lab: Husam Osman, Andrew Bosworth. Queen Elizabeth Hospital: Anna Casey
EPI_ISL_651229, EPI_ISL_651230	Virology Department, Royal Infirmary of Edinburgh, NHS Lothian / School of Biological Sciences, University of Edinburgh / Institute of Genetics and Molecular Medicine, University of Edinburgh	COVID-19 Genomics UK (COG-UK) Consortium	McHugh M, Dewar R, Rooke S, Gallagher M, Balcaza C, O'Toole Á, Scher E, Hill V, McCrone JT, Colquhoun R, Yu X, Jackson B, Rambaut A, Williams TC, Templeton K
EPI_ISL_651233	Virology Department, Sheffield Teaching Hospitals NHS Foundation Trust/Department of Infection, Immunity and Cardiovascular Disease, The Medical School, University of Sheffield	COVID-19 Genomics UK (COG-UK) Consortium	Thushan de Silva, Matthew Parker, Nikki Smith, Adri Angyal, Rebecca Brown, Luke Green, Rachel Tucker, Paul Parsons, Danielle Groves, Katie Johnson, Laura Carrilero, Alex Keeley, Dave Partridge, Matthew Wyles, Benjamin Lindsey, Mehmet Yavuz, Mohammad Raza, Cariad Evans
EPI_ISL_651234	Liverpool Clinical Laboratories	COVID-19 Genomics UK (COG-UK) Consortium	Sam Haldenby, Anita Lucaci, Steve Paterson, Julian Hiscox, Alistair Darby, M Almsaud, A Alrezaihi, Muhannad Alruwaili, Stuart D Armstrong, Jones Benjamin, Eleanor G Bentley, Anu Chawla, Jordan J Clark, Angela Cowell, Richard Eccles, Isabel García-Dorival, Matthew Gemmell, Alessandro Gerada, PKF Gilmore, Richard Gregory, Ximeng Han, Catherine Hartley, Margaret Hughes, Miren Iturriza-Gomara, James Johnson, L Luu, Jenifer Manson, Charlotte Nelson, Elaine O'Toole, Cassie Olateju, Rebekah Penrice-Randal , Lucille Rainbow, N.P Randle, Trevor Ian Robinson, Parul Sharma, Ghada T Shawli, James P Stewart, Neil Swainston, Ecaterina Vamos, Joanne Watts, Mark Whitehead
EPI_ISL_651247, EPI_ISL_651255	Quadram Institute Bioscience	COVID-19 Genomics UK (COG-UK) Consortium	Dave J. Baker, Gemma L. Kay, Alp Aydin, Thanh Le-Viet, Steven Rudder, Ana P. Tedim, Anastasia Kolyva, Maria Diaz, Leonardo de Oliveira Martins, Nabil-Fareed Alikhan, Lizzie Meadows, Rachael Stanley, Ngozi Elumogo, Muhammed Yasir, Nicholas M. Thomson, Alexander J Trotter, Rachel Gilroy, Samuel Bloomfield, Claire Stuart, Andrew Bell, Reenesh Prakash, Samir Dervisevic, Alison E. Mather, John Wain, Mark Webber, Andrew J. Page, Justin O'Grady
EPI_ISL_651265	Liverpool Clinical Laboratories	COVID-19 Genomics UK (COG-UK) Consortium	Sam Haldenby, Anita Lucaci, Steve Paterson, Julian Hiscox, Alistair Darby, M Almsaud, A Alrezaihi, Muhannad Alruwaili, Stuart D Armstrong, Jones Benjamin, Eleanor G Bentley, Anu Chawla, Jordan J Clark, Angela Cowell, Richard Eccles, Isabel García-Dorival, Matthew Gemmell, Alessandro Gerada, PKF Gilmore, Richard Gregory, Ximeng Han, Catherine Hartley, Margaret Hughes, Miren Iturriza-Gomara, James Johnson, L Luu, Jenifer Manson, Charlotte Nelson, Elaine O'Toole, Cassie Olateju, Rebekah Penrice-Randal , Lucille Rainbow, N.P Randle, Trevor Ian Robinson, Parul Sharma, Ghada T Shawli, James P Stewart, Neil Swainston, Ecaterina Vamos, Joanne Watts, Mark Whitehead
EPI_ISL_651269	Department of Pathology, University of Cambridge	COVID-19 Genomics UK (COG-UK) Consortium	Aminu S. Jahun, Yasmin Chaudhry, Grant Hall, Iliana Georgana, Myra Hosmillo, Martin D. Curran, Malte Pinckert, Surendra Parmar, Ian Goodfellow
EPI_ISL_651271, EPI_ISL_651273	Liverpool Clinical Laboratories	COVID-19 Genomics UK (COG-UK) Consortium	Sam Haldenby, Anita Lucaci, Steve Paterson, Julian Hiscox, Alistair Darby, M Almsaud, A Alrezaihi, Muhannad Alruwaili, Stuart D Armstrong, Jones Benjamin, Eleanor G Bentley, Anu Chawla, Jordan J Clark, Angela Cowell, Richard Eccles, Isabel García-Dorival, Matthew Gemmell, Alessandro Gerada, PKF Gilmore, Richard Gregory, Ximeng Han, Catherine Hartley, Margaret Hughes, Miren Iturriza-Gomara, James Johnson, L Luu, Jenifer Manson, Charlotte Nelson, Elaine O'Toole, Cassie Olateju, Rebekah Penrice-Randal , Lucille Rainbow, N.P Randle, Trevor Ian Robinson, Parul Sharma, Ghada T Shawli, James P Stewart, Neil Swainston, Ecaterina Vamos, Joanne Watts, Mark Whitehead
EPI_ISL_651276	Department of Pathology, University of Cambridge	COVID-19 Genomics UK (COG-UK) Consortium	Aminu S. Jahun, Yasmin Chaudhry, Grant Hall, Iliana Georgana, Myra Hosmillo, Martin D. Curran, Malte Pinckert, Surendra Parmar, Ian Goodfellow
EPI_ISL_651290	University of Exeter	COVID-19 Genomics UK (COG-UK) Consortium	Ben Temperton,Aaron Jeffries,Michelle Michelsen,Joanna Warwick-Dugdale,Audrey Farbos,Robyn Manley,Stephen Michell,Jane Masoli
EPI_ISL_651294	Virology Department, Royal Infirmary of Edinburgh, NHS Lothian / School of Biological Sciences, University of Edinburgh / Institute of Genetics and Molecular Medicine, University of Edinburgh	COVID-19 Genomics UK (COG-UK) Consortium	McHugh M, Dewar R, Rooke S, Gallagher M, Balcaza C, O'Toole Á, Scher E, Hill V, McCrone JT, Colquhoun R, Yu X, Jackson B, Rambaut A, Williams TC, Templeton K
EPI_ISL_651303	West of Scotland Specialist Virology Centre, NHSGGC / MRC-University of Glasgow Centre for Virus Research	COVID-19 Genomics UK (COG-UK) Consortium	Ana da Silva Filipe, Natasha Johnson, Kathy Smollett, Daniel Mair, Stephen Carmichael, Alice Broos, Lily Tong, Jenna Nichols, Kyriaki Nomikou; Sarah McDonald; Richard Orton, Joseph Hughes, Sreenu Vattipally, David L Robertson; Alasdair MacLean, Rory Gunson; Sharif Shaaban, Matthew Holden; Rachel Blacow, Guy Mollett, Kathy Li, James Shepherd, Antonia Ho, Emma Thomson
EPI_ISL_651305	University of Birmingham	COVID-19 Genomics UK (COG-UK) Consortium	Institute of Microbiology, University of Birmingham: Claire McMurray, Joanne Stockton, Samuel Nicholls, Radoslaw Poplawski, Will Rowe, Josh Quick, Nicholas Loman. University of Birmingham Testing Laboratory: Celina M Whalley, Andrew Bosworth, Charlotte Poxon, Kasun Wanigasooriya, Oliver Pickles, Mike Kidd, Alex Richter, Andrew D Beggs PHE Heartlands Lab: Husam Osman, Andrew Bosworth. Queen Elizabeth Hospital: Anna Casey
EPI_ISL_651307	Liverpool Clinical Laboratories	COVID-19 Genomics UK (COG-UK) Consortium	Sam Haldenby, Anita Lucaci, Steve Paterson, Julian Hiscox, Alistair Darby, M Almsaud, A Alrezaihi, Muhannad Alruwaili, Stuart D Armstrong, Jones Benjamin, Eleanor G Bentley, Anu Chawla, Jordan J Clark, Angela Cowell, Richard Eccles, Isabel García-Dorival, Matthew Gemmell, Alessandro Gerada, PKF Gilmore, Richard Gregory, Ximeng Han, Catherine Hartley, Margaret Hughes, Miren Iturriza-Gomara, James Johnson, L Luu, Jenifer Manson, Charlotte Nelson, Elaine O'Toole, Cassie Olateju, Rebekah Penrice-Randal , Lucille Rainbow, N.P Randle, Trevor Ian Robinson, Parul Sharma, Ghada T Shawli, James P Stewart, Neil Swainston, Ecaterina Vamos, Joanne Watts, Mark Whitehead
EPI_ISL_651315	West of Scotland Specialist Virology Centre, NHSGGC / MRC-University of Glasgow Centre for Virus Research	COVID-19 Genomics UK (COG-UK) Consortium	Ana da Silva Filipe, Natasha Johnson, Kathy Smollett, Daniel Mair, Stephen Carmichael, Alice Broos, Lily Tong, Jenna Nichols, Kyriaki Nomikou; Sarah McDonald; Richard Orton, Joseph Hughes, Sreenu Vattipally, David L Robertson; Alasdair MacLean, Rory Gunson; Sharif Shaaban, Matthew Holden; Rachel Blacow, Guy Mollett, Kathy Li, James Shepherd, Antonia Ho, Emma Thomson
EPI_ISL_651317	Quadram Institute Bioscience	COVID-19 Genomics UK (COG-UK) Consortium	Dave J. Baker, Gemma L. Kay, Alp Aydin, Thanh Le-Viet, Steven Rudder, Ana P. Tedim, Anastasia Kolyva, Maria Diaz, Leonardo de Oliveira Martins, Nabil-Fareed Alikhan, Lizzie Meadows, Rachael Stanley, Ngozi Elumogo, Muhammed Yasir, Nicholas M. Thomson, Alexander J Trotter, Rachel Gilroy, Samuel Bloomfield, Claire Stuart, Andrew Bell, Reenesh Prakash, Samir Dervisevic, Alison E. Mather, John Wain, Mark Webber, Andrew J. Page, Justin O'Grady
EPI_ISL_651321	University of Birmingham	COVID-19 Genomics UK (COG-UK) Consortium	Institute of Microbiology, University of Birmingham: Claire McMurray, Joanne Stockton, Samuel Nicholls, Radoslaw Poplawski, Will Rowe, Josh Quick, Nicholas Loman. University of Birmingham Testing Laboratory: Celina M Whalley, Andrew Bosworth, Charlotte Poxon, Kasun Wanigasooriya, Oliver Pickles, Mike Kidd, Alex Richter, Andrew D Beggs PHE Heartlands Lab: Husam Osman, Andrew Bosworth. Queen Elizabeth Hospital: Anna Casey
EPI_ISL_651357	Quadram Institute Bioscience	COVID-19 Genomics UK (COG-UK) Consortium	Dave J. Baker, Gemma L. Kay, Alp Aydin, Thanh Le-Viet, Steven Rudder, Ana P. Tedim, Anastasia Kolyva, Maria Diaz, Leonardo de Oliveira Martins, Nabil-Fareed Alikhan, Lizzie Meadows, Rachael Stanley, Ngozi Elumogo, Muhammed Yasir, Nicholas M. Thomson, Alexander J Trotter, Rachel Gilroy, Samuel Bloomfield, Claire Stuart, Andrew Bell, Reenesh Prakash, Samir Dervisevic, Alison E. Mather, John Wain, Mark Webber, Andrew J. Page, Justin O'Grady
EPI_ISL_651367	Virology Department, Royal Infirmary of Edinburgh, NHS Lothian / School of Biological Sciences, University of Edinburgh / Institute of Genetics and Molecular Medicine, University of Edinburgh	COVID-19 Genomics UK (COG-UK) Consortium	McHugh M, Dewar R, Rooke S, Gallagher M, Balcaza C, O'Toole Á, Scher E, Hill V, McCrone JT, Colquhoun R, Yu X, Jackson B, Rambaut A, Williams TC, Templeton K

EPI_ISL_651378	University of Birmingham	COVID-19 Genomics UK (COG-UK) Consortium	Institute of Microbiology, University of Birmingham: Claire McMurray, Joanne Stockton, Samuel Nicholls, Radoslaw Poplawski, Will Rowe, Josh Quick, Nicholas Loman. University of Birmingham Testing Laboratory: Celina M Whalley, Andrew Bosworth, Charlotte Poxon, Kasun Wanigasooriya, Oliver Pickles, Mike Kidd, Alex Richter, Andrew D Beggs PHE Heartlands Lab: Husam Osman, Andrew Bosworth. Queen Elizabeth Hospital: Anna Casey
EPI_ISL_651394	Virology Department, Royal Infirmary of Edinburgh, NHS Lothian / School of Biological Sciences, University of Edinburgh / Institute of Genetics and Molecular Medicine, University of Edinburgh	COVID-19 Genomics UK (COG-UK) Consortium	McHugh M, Dewar R, Rooke S, Gallagher M, Balcaza C, O'Toole Á, Scher E, Hill V, McCrone JT, Colquhoun R, Yu X, Jackson B, Rambaut A, Williams TC, Templeton K
EPI_ISL_651418, EPI_ISL_651419	Quadram Institute Bioscience	COVID-19 Genomics UK (COG-UK) Consortium	Dave J. Baker, Gemma L. Kay, Alp Aydin, Thanh Le-Viet, Steven Rudder, Ana P. Tedim, Anastasia Kolyva, Maria Diaz, Leonardo de Oliveira Martins, Nabil-Fareed Alikhan, Lizzie Meadows, Rachael Stanley, Ngozi Elumogo, Muhammed Yasir, Nicholas M. Thomson, Alexander J Trotter, Rachel Gilroy, Samuel Bloomfield, Claire Stuart, Andrew Bell, Reenesh Prakash, Samir Dervisevic, Alison E. Mather, John Wain, Mark Webber, Andrew J. Page, Justin O'Grady
EPI_ISL_651423	Department of Pathology, University of Cambridge	COVID-19 Genomics UK (COG-UK) Consortium	Aminu S. Jahun, Yasmin Chaudhry, Grant Hall, Iliana Georgana, Myra Hosmillo, Martin D. Curran, Malte Pinckert, Surendra Parmar, Ian Goodfellow
EPI_ISL_651427	Virology Department, Royal Infirmary of Edinburgh, NHS Lothian / School of Biological Sciences, University of Edinburgh / Institute of Genetics and Molecular Medicine, University of Edinburgh	COVID-19 Genomics UK (COG-UK) Consortium	McHugh M, Dewar R, Rooke S, Gallagher M, Balcaza C, O'Toole Á, Scher E, Hill V, McCrone JT, Colquhoun R, Yu X, Jackson B, Rambaut A, Williams TC, Templeton K
EPI_ISL_651484	Quadram Institute Bioscience	COVID-19 Genomics UK (COG-UK) Consortium	Dave J. Baker, Gemma L. Kay, Alp Aydin, Thanh Le-Viet, Steven Rudder, Ana P. Tedim, Anastasia Kolyva, Maria Diaz, Leonardo de Oliveira Martins, Nabil-Fareed Alikhan, Lizzie Meadows, Rachael Stanley, Ngozi Elumogo, Muhammed Yasir, Nicholas M. Thomson, Alexander J Trotter, Rachel Gilroy, Samuel Bloomfield, Claire Stuart, Andrew Bell, Reenesh Prakash, Samir Dervisevic, Alison E. Mather, John Wain, Mark Webber, Andrew J. Page, Justin O'Grady
EPI_ISL_651502	Wales Specialist Virology Centre Sequencing lab: Pathogen Genomics Unit	COVID-19 Genomics UK (COG-UK) Consortium	Catherine Moore, Johnathan Evans, Laura Gifford, Malorie Perry, Simon Cottrell, Angela Marchbank, Alec Birchley, Alexander Adams, Amy Gaskin, Bree Gatica-Wilcox, Jason Coombes, Joel Southgate, Lauren Gilbert, Lee Graham, Nicole Pacchiarini, Sara Kumziene-Summerhayes, Sarah Taylor, Sophie Jones, Sara Rey, Matthew Bull, Joanne Watkins, Sally Corden, Tom Connor
EPI_ISL_651542, EPI_ISL_651556, EPI_ISL_651557	Department of Pathology, University of Cambridge	COVID-19 Genomics UK (COG-UK) Consortium	Aminu S. Jahun, Yasmin Chaudhry, Grant Hall, Iliana Georgana, Myra Hosmillo, Martin D. Curran, Malte Pinckert, Surendra Parmar, Ian Goodfellow
EPI_ISL_651592, EPI_ISL_651622, EPI_ISL_651623, EPI_ISL_651628	University of Birmingham	COVID-19 Genomics UK (COG-UK) Consortium	Institute of Microbiology, University of Birmingham: Claire McMurray, Joanne Stockton, Samuel Nicholls, Radoslaw Poplawski, Will Rowe, Josh Quick, Nicholas Loman. University of Birmingham Testing Laboratory: Celina M Whalley, Andrew Bosworth, Charlotte Poxon, Kasun Wanigasooriya, Oliver Pickles, Mike Kidd, Alex Richter, Andrew D Beggs PHE Heartlands Lab: Husam Osman, Andrew Bosworth. Queen Elizabeth Hospital: Anna Casey
EPI_ISL_651666, EPI_ISL_651667, EPI_ISL_651669, EPI_ISL_651670, EPI_ISL_651672, EPI_ISL_651679, EPI_ISL_651680, EPI_ISL_651684, EPI_ISL_651685, EPI_ISL_651686	Department of Pathology, University of Cambridge	COVID-19 Genomics UK (COG-UK) Consortium	Aminu S. Jahun, Yasmin Chaudhry, Grant Hall, Iliana Georgana, Myra Hosmillo, Martin D. Curran, Malte Pinckert, Surendra Parmar, Ian Goodfellow
EPI_ISL_651717	Virology Department, Sheffield Teaching Hospitals NHS Foundation Trust/Department of Infection, Immunity and Cardiovascular Disease, The Medical School, University of Sheffield	COVID-19 Genomics UK (COG-UK) Consortium	Thushan de Silva, Matthew Parker, Nikki Smith, Adri Angyal, Rebecca Brown, Luke Green, Rachel Tucker, Paul Parsons, Danielle Groves, Katie Johnson, Laura Carrilero, Alex Keeley, Dave Partridge, Matthew Wyles, Benjamin Lindsey, Mehmet Yavuz, Mohammad Raza, Cariad Evans
EPI_ISL_651811, EPI_ISL_651812, EPI_ISL_651813, EPI_ISL_651821, EPI_ISL_651822, EPI_ISL_651823, EPI_ISL_651824, EPI_ISL_651825, EPI_ISL_651826, EPI_ISL_651827, EPI_ISL_651828, EPI_ISL_651829, EPI_ISL_651850, EPI_ISL_651851, EPI_ISL_651852, EPI_ISL_651853, EPI_ISL_651854, EPI_ISL_651855, EPI_ISL_651856, EPI_ISL_651857, EPI_ISL_651858, EPI_ISL_651859, EPI_ISL_651863, EPI_ISL_651865, EPI_ISL_651866, EPI_ISL_651867, EPI_ISL_651868, EPI_ISL_651873, EPI_ISL_651883			
see above	West of Scotland Specialist Virology Centre, NHSGGC / MRC-University of Glasgow Centre for Virus Research	COVID-19 Genomics UK (COG-UK) Consortium	Ana da Silva Filipe, Natasha Johnson, Kathy Smollett, Daniel Mair, Stephen Carmichael, Alice Broos, Lily Tong, Jenna Nichols, Kyriaki Nomikou; Sarah McDonald; Richard Orton, Joseph Hughes, Sreenu Vattipally, David L Robertson; Alasdair MacLean, Rory Gunson; Sharif Shaaban, Matthew Holden; Rachel Blacow, Guy Mollett, Kathy Li, James Shepherd, Antonia Ho, Emma Thomson
EPI_ISL_652024, EPI_ISL_652025, EPI_ISL_652026, EPI_ISL_652027, EPI_ISL_652028, EPI_ISL_652029, EPI_ISL_652030, EPI_ISL_652031, EPI_ISL_652032, EPI_ISL_652033, EPI_ISL_652034, EPI_ISL_652035, EPI_ISL_652036, EPI_ISL_652038, EPI_ISL_652039, EPI_ISL_652040, EPI_ISL_652051, EPI_ISL_652057, EPI_ISL_652058, EPI_ISL_652059, EPI_ISL_652060			
see above	Virology Department, Royal Infirmary of Edinburgh, NHS Lothian / School of Biological Sciences, University of Edinburgh / Institute of Genetics and Molecular Medicine, University of Edinburgh	COVID-19 Genomics UK (COG-UK) Consortium	McHugh M, Dewar R, Rooke S, Gallagher M, Balcaza C, O'Toole Á, Scher E, Hill V, McCrone JT, Colquhoun R, Yu X, Jackson B, Rambaut A, Williams TC, Templeton K
EPI_ISL_652289, EPI_ISL_652295, EPI_ISL_652296, EPI_ISL_652297, EPI_ISL_652298, EPI_ISL_652299, EPI_ISL_652301, EPI_ISL_652303	University of Exeter	COVID-19 Genomics UK (COG-UK) Consortium	Ben Temperton,Aaron Jeffries,Michelle Michelsen,Joanna Warwick-Dugdale,Audrey Farbos,Robyn Manley,Stephen Michell,Jane Masoli
EPI_ISL_652530, EPI_ISL_652556, EPI_ISL_652566, EPI_ISL_652576, EPI_ISL_652601, EPI_ISL_652616	Wales Specialist Virology Centre Sequencing lab: Pathogen Genomics Unit	COVID-19 Genomics UK (COG-UK) Consortium	Catherine Moore, Johnathan Evans, Laura Gifford, Malorie Perry, Simon Cottrell, Angela Marchbank, Alec Birchley, Alexander Adams, Amy Gaskin, Bree Gatica-Wilcox, Jason Coombes, Joel Southgate, Lauren Gilbert, Lee Graham, Nicole Pacchiarini, Sara Kumziene-Summerhayes, Sarah Taylor, Sophie Jones, Sara Rey, Matthew Bull, Joanne Watkins, Sally Corden, Tom Connor
EPI_ISL_653027, EPI_ISL_653028, EPI_ISL_653029, EPI_ISL_653030, EPI_ISL_653031, EPI_ISL_653032, EPI_ISL_653033, EPI_ISL_653034, EPI_ISL_653035, EPI_ISL_653036, EPI_ISL_653037, EPI_ISL_653038, EPI_ISL_653039, EPI_ISL_653040, EPI_ISL_653041, EPI_ISL_653042, EPI_ISL_653043, EPI_ISL_653044, EPI_ISL_653045			
see above	Quadram Institute Bioscience	COVID-19 Genomics UK (COG-UK) Consortium	Dave J. Baker, Gemma L. Kay, Alp Aydin, Thanh Le-Viet, Steven Rudder, Ana P. Tedim, Anastasia Kolyva, Maria Diaz, Leonardo de Oliveira Martins, Nabil-Fareed Alikhan, Lizzie Meadows, Rachael Stanley, Ngozi Elumogo, Muhammed Yasir, Nicholas M. Thomson, Alexander J Trotter, Rachel Gilroy, Samuel Bloomfield, Claire Stuart, Andrew Bell, Reenesh Prakash, Samir Dervisevic, Alison E. Mather, John Wain, Mark Webber, Andrew J. Page, Justin O'Grady
EPI_ISL_653067, EPI_ISL_653068, EPI_ISL_653082, EPI_ISL_653091	Virology Department, Sheffield Teaching Hospitals NHS Foundation Trust/Department of Infection, Immunity and Cardiovascular Disease, The Medical School, University of Sheffield	COVID-19 Genomics UK (COG-UK) Consortium	Thushan de Silva, Matthew Parker, Nikki Smith, Adri Angyal, Rebecca Brown, Luke Green, Rachel Tucker, Paul Parsons, Danielle Groves, Katie Johnson, Laura Carrilero, Alex Keeley, Dave Partridge, Matthew Wyles, Benjamin Lindsey, Mehmet Yavuz, Mohammad Raza, Cariad Evans
EPI_ISL_653782	I.R.C.C.S. "S. De Bellis" - Ente Ospedaliero	Istituto Zooprofilattico Sperimentale della Puglia e della Basilicata	Parisi A., Bianco A., Capozzi L., Del Sambro L., Lippolis A., Notarnicola M., Manzulli V, Rondinone V., Pace L.
EPI_ISL_653810	I.R.C.C.S. "S. De Bellis" - Ente Ospedaliero	Istituto Zooprofilattico Sperimentale della Puglia e della Basilicata	Parisi A., Bianco A., Capozzi L., Del Sambro L., Lippolis A., Notarnicola M., Cipolletta D., Galante D.
EPI_ISL_653938, EPI_ISL_653947, EPI_ISL_653953, EPI_ISL_653954, EPI_ISL_653992, EPI_ISL_654002, EPI_ISL_654003, EPI_ISL_654004, EPI_ISL_654006, EPI_ISL_654008, EPI_ISL_654009, EPI_ISL_654011, EPI_ISL_654012, EPI_ISL_654013, EPI_ISL_654014, EPI_ISL_654015			
see above	Respiratory Virus Unit, Microbiology Services Colindale, Public Health England	COVID-19 Genomics UK (COG-UK) Consortium	PHE Covid Sequencing Team
EPI_ISL_654400	Hospital General Universitario Gregorio Marañón	SeqCOVID-SPAIN consortium/IBV(CSIC)	Darío García de Viedma, Laura Pérez-Lago, Marta Herranz, Jon Sicilia, Julia Suárez, Pilar Catalán, Patricia Muñoz and SeqCOVID-SPAIN consortium
EPI_ISL_654700, EPI_ISL_654701, EPI_ISL_654702, EPI_ISL_654704	Minnesota Department of Health, Public Health Laboratory	Minnesota Department of Health, Public Health Laboratory	Matt Plumb, Jacob Garfin, Alexandra Lorentz, and Xiong Wang

EPI_ISL_658741, EPI_ISL_658742, EPI_ISL_658743, EPI_ISL_658746, EPI_ISL_658747, EPI_ISL_658748, EPI_ISL_658749, EPI_ISL_658751, EPI_ISL_658757, EPI_ISL_658758, EPI_ISL_658761, EPI_ISL_658762, EPI_ISL_658766, EPI_ISL_658770, EPI_ISL_658771, EPI_ISL_658773, EPI_ISL_658774, EPI_ISL_658776, EPI_ISL_658778, EPI_ISL_658779, EPI_ISL_658782, EPI_ISL_658783, EPI_ISL_658784, EPI_ISL_658785, EPI_ISL_658786, EPI_ISL_658790, EPI_ISL_658792, EPI_ISL_658793, EPI_ISL_658794, EPI_ISL_658796, EPI_ISL_658797, EPI_ISL_658798, EPI_ISL_658799, EPI_ISL_658801, EPI_ISL_658802, EPI_ISL_658805, EPI_ISL_658806, EPI_ISL_658808, EPI_ISL_658810, EPI_ISL_658811, EPI_ISL_658813, EPI_ISL_658814, EPI_ISL_658815, EPI_ISL_658817, EPI_ISL_658818, EPI_ISL_658819, EPI_ISL_658820, EPI_ISL_658822, EPI_ISL_658823, EPI_ISL_658824, EPI_ISL_658825, EPI_ISL_658826, EPI_ISL_658828, EPI_ISL_658830, EPI_ISL_658831, EPI_ISL_658834, EPI_ISL_658835, EPI_ISL_658839, EPI_ISL_658840, EPI_ISL_658841, EPI_ISL_658842, EPI_ISL_658843, EPI_ISL_658844, EPI_ISL_658845, EPI_ISL_658848, EPI_ISL_658849, EPI_ISL_658850, EPI_ISL_658853, EPI_ISL_658854, EPI_ISL_658856, EPI_ISL_658857, EPI_ISL_658858, EPI_ISL_658859, EPI_ISL_658860, EPI_ISL_658861, EPI_ISL_658862, EPI_ISL_658867, EPI_ISL_658869, EPI_ISL_658871, EPI_ISL_658872, EPI_ISL_658874, EPI_ISL_658876, EPI_ISL_658882, EPI_ISL_658884, EPI_ISL_658887, EPI_ISL_658889, EPI_ISL_658890			
see above	Lighthouse Lab in Milton Keynes	Wellcome Sanger Institute for the COVID-19 Genomics UK (COG-UK) Consortium	The Lighthouse Lab in Milton Keynes and Alex Alderton, Roberto Amato, Sonia Goncalves, Ewan Harrison, David K. Jackson, Ian Johnston, Dominic Kwiatkowski, Cordelia Langford, John Sillitoe on behalf of the Wellcome Sanger Institute COVID-19 Surveillance Team
EPI_ISL_658891	Instituto de Diagnostico y Referencia Epidemiologicos (INDRE)	Instituto de Diagnostico y Referencia Epidemiologicos (INDRE)	Ernesto Ramirez-Gonzalez, Abril Rodriguez-Maldonado, Claudia Wong-Arambula , Natividad Cruz-Ortiz, Tatiana Nunez-Garcia, Dayanira Arellano-Suarez, Fabiola Garces-Ayala, Lucia Hernandez-Rivas, Irma Lopez-Martinez, Gisela Barrera-Badillo.
EPI_ISL_658892, EPI_ISL_658894, EPI_ISL_658895, EPI_ISL_658897, EPI_ISL_658898, EPI_ISL_658902, EPI_ISL_658903, EPI_ISL_658906, EPI_ISL_658907, EPI_ISL_658910, EPI_ISL_658911, EPI_ISL_658915, EPI_ISL_658917, EPI_ISL_658918, EPI_ISL_658920, EPI_ISL_658924, EPI_ISL_658926, EPI_ISL_658927, EPI_ISL_658929, EPI_ISL_658932, EPI_ISL_658934, EPI_ISL_658937, EPI_ISL_658939, EPI_ISL_658940, EPI_ISL_658941, EPI_ISL_658942, EPI_ISL_658943, EPI_ISL_658944, EPI_ISL_658945, EPI_ISL_658946, EPI_ISL_658947, EPI_ISL_658949, EPI_ISL_658950, EPI_ISL_658952, EPI_ISL_658953, EPI_ISL_658956, EPI_ISL_658957, EPI_ISL_658958, EPI_ISL_658960, EPI_ISL_658961, EPI_ISL_658962, EPI_ISL_658964, EPI_ISL_658965, EPI_ISL_658966, EPI_ISL_658967, EPI_ISL_658968, EPI_ISL_658969, EPI_ISL_658972, EPI_ISL_658973, EPI_ISL_658974, EPI_ISL_658975, EPI_ISL_658977, EPI_ISL_658978, EPI_ISL_658980, EPI_ISL_658981, EPI_ISL_658982, EPI_ISL_658983, EPI_ISL_658984, EPI_ISL_658986, EPI_ISL_658987, EPI_ISL_658988, EPI_ISL_658989, EPI_ISL_658990, EPI_ISL_658991, EPI_ISL_658992, EPI_ISL_658993, EPI_ISL_658997, EPI_ISL_658998, EPI_ISL_658999, EPI_ISL_659002, EPI_ISL_659003, EPI_ISL_659004, EPI_ISL_659006, EPI_ISL_659010, EPI_ISL_659012, EPI_ISL_659013, EPI_ISL_659015, EPI_ISL_659016, EPI_ISL_659020, EPI_ISL_659022, EPI_ISL_659024, EPI_ISL_659027, EPI_ISL_659029, EPI_ISL_659030, EPI_ISL_659031, EPI_ISL_659035, EPI_ISL_659036, EPI_ISL_659038, EPI_ISL_659039, EPI_ISL_659042, EPI_ISL_659043, EPI_ISL_659044, EPI_ISL_659046, EPI_ISL_659047, EPI_ISL_659048, EPI_ISL_659049, EPI_ISL_659051, EPI_ISL_659052, EPI_ISL_659055, EPI_ISL_659057, EPI_ISL_659058, EPI_ISL_659059, EPI_ISL_659060, EPI_ISL_659061, EPI_ISL_659062, EPI_ISL_659063, EPI_ISL_659064, EPI_ISL_659065, EPI_ISL_659066, EPI_ISL_659067, EPI_ISL_659068, EPI_ISL_659069, EPI_ISL_659074, EPI_ISL_659075, EPI_ISL_659078, EPI_ISL_659081, EPI_ISL_659085, EPI_ISL_659086, EPI_ISL_659087, EPI_ISL_659089, EPI_ISL_659092, EPI_ISL_659095, EPI_ISL_659096			
see above	Lighthouse Lab in Milton Keynes	Wellcome Sanger Institute for the COVID-19 Genomics UK (COG-UK) Consortium	The Lighthouse Lab in Milton Keynes and Alex Alderton, Roberto Amato, Sonia Goncalves, Ewan Harrison, David K. Jackson, Ian Johnston, Dominic Kwiatkowski, Cordelia Langford, John Sillitoe on behalf of the Wellcome Sanger Institute COVID-19 Surveillance Team
EPI_ISL_659772	Lighthouse Lab in Glasgow	Wellcome Sanger Institute for the COVID-19 Genomics UK (COG-UK) Consortium	Harper VanSteenhouse, Yumi Kasai, David Gray, Carol Clugston, Anna Dominiczak and Alex Alderton, Roberto Amato, Sonia Goncalves, Ewan Harrison, David K. Jackson, Ian Johnston, Dominic Kwiatkowski, Cordelia Langford, John Sillitoe on behalf of the Wellcome Sanger Institute COVID-19 Surveillance Team
EPI_ISL_660020	Lighthouse Lab in Glasgow	Wellcome Sanger Institute for the COVID-19 Genomics UK (COG-UK) Consortium	Harper VanSteenhouse, Yumi Kasai, David Gray, Carol Clugston, Anna Dominiczak and Alex Alderton, Roberto Amato, Sonia Goncalves, Ewan Harrison, David K. Jackson, Ian Johnston, Dominic Kwiatkowski, Cordelia Langford, John Sillitoe on behalf of the Wellcome Sanger Institute COVID-19 Surveillance Team (http://www.sanger.ac.uk/covid-team)
EPI_ISL_660068	Instituto de Diagnostico y Referencia Epidemiologicos (INDRE)	Instituto de diagnóstico y Referencia Epidemiologicos (INDRE) Departamento de Virología	Gisela Barrera-Badillo , Abril Rodriguez-Maldonado, Claudia Wong-Arambula , Natividad Cruz-Ortiz, Tatiana Nunez-Garcia, Dayanira Arellano-Suarez, Fabiola Garces-Ayala, Lucia Hernandez-Rivas, Irma Lopez-Martinez, Ernesto Ramirez-Gonzalez.
EPI_ISL_660094	MD PHL	MD PHL	Maryland Department of Health Laboratories Administration
EPI_ISL_660223, EPI_ISL_660224, EPI_ISL_660226, EPI_ISL_660227, EPI_ISL_660643, EPI_ISL_660652, EPI_ISL_660657	NHLS-IALCH	KRISP, KZN Research Innovation and Sequencing Platform	Giandhari J, Pillay S, Lessells R, Mdlalose K, York D, Khan S, Tegally H, Wilkinson E, de Oliveira T
EPI_ISL_660754, EPI_ISL_660755, EPI_ISL_660757, EPI_ISL_660758, EPI_ISL_660759, EPI_ISL_660760, EPI_ISL_660763, EPI_ISL_660764, EPI_ISL_660765, EPI_ISL_660766, EPI_ISL_660767, EPI_ISL_660768, EPI_ISL_660769, EPI_ISL_660770, EPI_ISL_660771, EPI_ISL_660772, EPI_ISL_660773, EPI_ISL_660774, EPI_ISL_660775, EPI_ISL_660776, EPI_ISL_660777, EPI_ISL_660778, EPI_ISL_660779, EPI_ISL_660780, EPI_ISL_660781, EPI_ISL_660783			
see above	Respiratory Virus Unit, Microbiology Services Colindale, Public Health England	COVID-19 Genomics UK (COG-UK) Consortium	PHE Covid Sequencing Team
EPI_ISL_661027, EPI_ISL_661028, EPI_ISL_661029, EPI_ISL_661030, EPI_ISL_661031, EPI_ISL_661032, EPI_ISL_661033, EPI_ISL_661034, EPI_ISL_661035, EPI_ISL_661036, EPI_ISL_661037, EPI_ISL_661038, EPI_ISL_661039, EPI_ISL_661040, EPI_ISL_661041, EPI_ISL_661042, EPI_ISL_661043, EPI_ISL_661044			
see above	Gundersen Molecular Diagnostics Laboratory	Kabara Cancer Research Institute	Craig S. Richmond, Paraic A. Kenny
EPI_ISL_661292	Mikrobiologen	The Public Health Agency of Sweden	Department of Microbiology, The Public Health Agency of Sweden
EPI_ISL_661293	Klinisk mikrobiologi	The Public Health Agency of Sweden	Department of Microbiology, The Public Health Agency of Sweden
EPI_ISL_661297	Unilabs	The Public Health Agency of Sweden	Department of Microbiology, The Public Health Agency of Sweden
EPI_ISL_662637, EPI_ISL_662639, EPI_ISL_662640, EPI_ISL_662641, EPI_ISL_662644, EPI_ISL_662646	Lighthouse Lab in Milton Keynes	Wellcome Sanger Institute for the COVID-19 Genomics UK (COG-UK) Consortium	The Lighthouse Lab in Milton Keynes and Alex Alderton, Roberto Amato, Sonia Goncalves, Ewan Harrison, David K. Jackson, Ian Johnston, Dominic Kwiatkowski, Cordelia Langford, John Sillitoe on behalf of the Wellcome Sanger Institute COVID-19 Surveillance Team
EPI_ISL_662656, EPI_ISL_662657, EPI_ISL_662659, EPI_ISL_662662, EPI_ISL_662663, EPI_ISL_662668, EPI_ISL_662673, EPI_ISL_662675, EPI_ISL_662677, EPI_ISL_662683, EPI_ISL_662687, EPI_ISL_662691, EPI_ISL_662693, EPI_ISL_662695, EPI_ISL_662700, EPI_ISL_662702, EPI_ISL_662708, EPI_ISL_662711, EPI_ISL_662712, EPI_ISL_662717, EPI_ISL_662722, EPI_ISL_662723, EPI_ISL_662727, EPI_ISL_662730, EPI_ISL_662731, EPI_ISL_662733, EPI_ISL_662734, EPI_ISL_662735, EPI_ISL_662737, EPI_ISL_662738, EPI_ISL_662739, EPI_ISL_662743, EPI_ISL_662744, EPI_ISL_662750, EPI_ISL_662753, EPI_ISL_662762, EPI_ISL_662765, EPI_ISL_662769, EPI_ISL_662775, EPI_ISL_662780, EPI_ISL_662782, EPI_ISL_662784, EPI_ISL_662793, EPI_ISL_662800, EPI_ISL_662802, EPI_ISL_662803, EPI_ISL_662804, EPI_ISL_662807, EPI_ISL_662810, EPI_ISL_662814, EPI_ISL_662819, EPI_ISL_662823, EPI_ISL_662825, EPI_ISL_662826, EPI_ISL_662827, EPI_ISL_662828, EPI_ISL_662831, EPI_ISL_662833, EPI_ISL_662837, EPI_ISL_662838, EPI_ISL_662840, EPI_ISL_662843, EPI_ISL_662848, EPI_ISL_662850, EPI_ISL_662851, EPI_ISL_662852, EPI_ISL_662862, EPI_ISL_662863, EPI_ISL_662866, EPI_ISL_662870, EPI_ISL_662874, EPI_ISL_662881, EPI_ISL_662883, EPI_ISL_662885, EPI_ISL_662890, EPI_ISL_662891, EPI_ISL_662892, EPI_ISL_662893, EPI_ISL_662894, EPI_ISL_662895, EPI_ISL_662896, EPI_ISL_662897, EPI_ISL_662898, EPI_ISL_662899, EPI_ISL_662900, EPI_ISL_662901, EPI_ISL_662902, EPI_ISL_662903, EPI_ISL_662904, EPI_ISL_662905, EPI_ISL_662906, EPI_ISL_662907, EPI_ISL_662908, EPI_ISL_662909, EPI_ISL_662910, EPI_ISL_662911, EPI_ISL_662912, EPI_ISL_662913, EPI_ISL_662914, EPI_ISL_662915, EPI_ISL_662916, EPI_ISL_662917, EPI_ISL_662918, EPI_ISL_662919, EPI_ISL_662920, EPI_ISL_662921, EPI_ISL_662922, EPI_ISL_662923, EPI_ISL_662924, EPI_ISL_662925, EPI_ISL_662926, EPI_ISL_662927, EPI_ISL_662928, EPI_ISL_662929, EPI_ISL_662930, EPI_ISL_662931, EPI_ISL_662932, EPI_ISL_662933, EPI_ISL_662934, EPI_ISL_662935, EPI_ISL_662936, EPI_ISL_662937, EPI_ISL_662938, EPI_ISL_662939, EPI_ISL_662940, EPI_ISL_662941, EPI_ISL_662942, EPI_ISL_662943, EPI_ISL_662944, EPI_ISL_662945, EPI_ISL_662946, EPI_ISL_662947, EPI_ISL_662948, EPI_ISL_662949, EPI_ISL_662950, EPI_ISL_662951, EPI_ISL_662952, EPI_ISL_662953, EPI_ISL_662954, EPI_ISL_662955, EPI_ISL_662956, EPI_ISL_662957, EPI_ISL_662958, EPI_ISL_662959, EPI_ISL_662960, EPI_ISL_662961, EPI_ISL_662962, EPI_ISL_662963, EPI_ISL_662964, EPI_ISL_662965, EPI_ISL_662966, EPI_ISL_662967, EPI_ISL_662968, EPI_ISL_662969, EPI_ISL_662970, EPI_ISL_662971, EPI_ISL_662972, EPI_ISL_662973, EPI_ISL_662974, EPI_ISL_662975, EPI_ISL_662976, EPI_ISL_662977, EPI_ISL_662978, EPI_ISL_662979, EPI_ISL_662980, EPI_ISL_662981, EPI_ISL_662982, EPI_ISL_662983, EPI_ISL_662984, EPI_ISL_662985, EPI_ISL_662986, EPI_ISL_662987, EPI_ISL_662988, EPI_ISL_662989, EPI_ISL_662990, EPI_ISL_662991, EPI_ISL_662992, EPI_ISL_662993, EPI_ISL_662994, EPI_ISL_662995, EPI_ISL_662996, EPI_ISL_662997, EPI_ISL_662998, EPI_ISL_662999, EPI_ISL_663000, EPI_ISL_663001, EPI_ISL_663002, EPI_ISL_663003, EPI_ISL_663004, EPI_ISL_663005, EPI_ISL_663006, EPI_ISL_663007, EPI_ISL_663008, EPI_ISL_663009, EPI_ISL_663010, EPI_ISL_663011, EPI_ISL_663012, EPI_ISL_663013, EPI_ISL_663014, EPI_ISL_663015, EPI_ISL_663016, EPI_ISL_663017, EPI_ISL_663018, EPI_ISL_663019, EPI_ISL_663020, EPI_ISL_663021, EPI_ISL_663022, EPI_ISL_663023, EPI_ISL_663024, EPI_ISL_663025, EPI_ISL_663026, EPI_ISL_663027, EPI_ISL_663028, EPI_ISL_663029, EPI_ISL_663030, EPI_ISL_663031, EPI_ISL_663032, EPI_ISL_663033, EPI_ISL_663034, EPI_ISL_663035, EPI_ISL_663036, EPI_ISL_663037, EPI_ISL_663038, EPI_ISL_663039, EPI_ISL_663040, EPI_ISL_663041, EPI_ISL_663042, EPI_ISL_663043, EPI_ISL_663044, EPI_ISL_663045, EPI_ISL_663046, EPI_ISL_663047, EPI_ISL_663048, EPI_ISL_663049, EPI_ISL_663050, EPI_ISL_663051, EPI_ISL_663052, EPI_ISL_663053, EPI_ISL_663054, EPI_ISL_663055, EPI_ISL_663056, EPI_ISL_663057, EPI_ISL_663058, EPI_ISL_663059, EPI_ISL_663060, EPI_ISL_663061, EPI_ISL_663062, EPI_ISL_663063, EPI_ISL_663064, EPI_ISL_663065, EPI_ISL_663066, EPI_ISL_663067, EPI_ISL_663068, EPI_ISL_663069, EPI_ISL_663070, EPI_ISL_663071, EPI_ISL_663072, EPI_ISL_663073, EPI_ISL_663074, EPI_ISL_663075, EPI_ISL_663076, EPI_ISL_663077, EPI_ISL_663078, EPI_ISL_663079, EPI_ISL_663080, EPI_ISL_663081, EPI_ISL_663082, EPI_ISL_663083, EPI_ISL_663084, EPI_ISL_663085, EPI_ISL_663086, EPI_ISL_663087, EPI_ISL_663088, EPI_ISL_663089, EPI_ISL_663090, EPI_ISL_663091, EPI_ISL_663092, EPI_ISL_663093, EPI_ISL_663094, EPI_ISL_663095, EPI_ISL_663096, EPI_ISL_663097, EPI_ISL_663098, EPI_ISL_663099, EPI_ISL_663100, EPI_ISL_663101, EPI_ISL_663102, EPI_ISL_663103, EPI_ISL_663104, EPI_ISL_663105, EPI_ISL_663106, EPI_ISL_663107, EPI_ISL_663108, EPI_ISL_663109, EPI_ISL_663110, EPI_ISL_663111, EPI_ISL_663112, EPI_ISL_663113, EPI_ISL_663114, EPI_ISL_663115, EPI_ISL_663116, EPI_ISL_663117, EPI_ISL_663118, EPI_ISL_663119, EPI_ISL_663120, EPI_ISL_663121, EPI_ISL_663122, EPI_ISL_663123, EPI_ISL_663124, EPI_ISL_663125, EPI_ISL_663126, EPI_ISL_663127, EPI_ISL_663128, EPI_ISL_663129, EPI_ISL_663130, EPI_ISL_663131, EPI_ISL_663132, EPI_ISL_663133, EPI_ISL_663135, EPI_ISL_663136, EPI_ISL_663137, EPI_ISL_663138, EPI_ISL_663139, EPI_ISL_663140, EPI_ISL_663141, EPI_ISL_663142, EPI_ISL_663143, EPI_ISL_663144, EPI_ISL_663145, EPI_ISL_663146, EPI_ISL_663147, EPI_ISL_663148, EPI_ISL_663149, EPI_ISL_663150, EPI_ISL_663151, EPI_ISL_663152, EPI_ISL_663153, EPI_ISL_663154, EPI_ISL_663155, EPI_ISL_663156, EPI_ISL_663157, EPI_ISL_663158, EPI_ISL_663159, EPI_ISL_663160, EPI_ISL_663161, EPI_ISL_663162, EPI_ISL_663163, EPI_ISL_663164, EPI_ISL_663165, EPI_ISL_663166, EPI_ISL_663167, EPI_ISL_663168, EPI_ISL_663169, EPI_ISL_663170, EPI_ISL_663171, EPI_ISL_663172, EPI_ISL_663173, EPI_ISL_663174, EPI_ISL_663175, EPI_ISL_663176, EPI_ISL_663177, EPI_ISL_663178, EPI_ISL_663179, EPI_ISL_663180, EPI_ISL_663181, EPI_ISL_663182, EPI_ISL_663183, EPI_ISL_663184, EPI_ISL_663185, EPI_ISL_663186, EPI_ISL_663187, EPI_ISL_663188, EPI_ISL_663189, EPI_ISL_663190, EPI_ISL_663191, EPI_ISL_663192, EPI_ISL_663193, EPI_ISL_663194, EPI_ISL_663195, EPI_ISL_663196, EPI_ISL_663197, EPI_ISL_663198, EPI_ISL_663199, EPI_ISL_663200, EPI_ISL_663201, EPI_ISL_663202, EPI_ISL_663203, EPI_ISL_663204, EPI_ISL_663205			
see above	Lighthouse Lab in Glasgow	Wellcome Sanger Institute for the COVID-19 Genomics UK (COG-UK) Consortium	Harper VanSteenhouse, Yumi Kasai, David Gray, Carol Clugston, Anna Dominiczak and Alex Alderton, Roberto Amato, Sonia Goncalves, Ewan Harrison, David K. Jackson, Ian Johnston, Dominic Kwiatkowski, Cordelia Langford, John Sillitoe on behalf of the Wellcome Sanger Institute COVID-19 Surveillance Team
EPI_ISL_664117, EPI_ISL_664162, EPI_ISL_664205, EPI_ISL_664206	University of Exeter	COVID-19 Genomics UK (COG-UK) Consortium	Ben Temperton,Aaron Jeffries,Michelle Michelsen,Joanna Warwick-Dugdale,Audrey Farbos,Robyn Manley,Stephen Michell,Jane Masoli
EPI_ISL_664228	University College London Hospital	COVID-19 Genomics UK (COG-UK) Consortium	Judith Heaney, Matthew Byott, Catherine Houlihan, Dan Frampton, Stuart Kirk, Moira Spyer and Eleni Nastouli
EPI_ISL_664289	University of Exeter	COVID-19 Genomics UK (COG-UK) Consortium	Ben Temperton,Aaron Jeffries,Michelle Michelsen,Joanna Warwick-Dugdale,Audrey Farbos,Robyn Manley,Stephen Michell,Jane Masoli
EPI_ISL_664302	Centre for Enzyme Innovation, University of Portsmouth / Translational Research Laboratory, Portsmouth Hospitals NHS Trust	COVID-19 Genomics UK (COG-UK) Consortium	Angela Beckett,Yann Bourgeois,Garry Scarlett,Sharon Glaysher,Scott Elliott,Kelly Bicknell,Robert Impey,Allyson Lloyd,Sarah Wyllie,Ethan Butcher,Anoop Chauhan,Samuel Robson

EPI_ISL_664327	University of Exeter	COVID-19 Genomics UK (COG-UK) Consortium	Ben Temperton,Aaron Jeffries,Michelle Michelsen,Joanna Warwick-Dugdale,Audrey Farbos,Robyn Manley,Stephen Michell,Jane Masoli
EPI_ISL_664343, EPI_ISL_664354	Liverpool Clinical Laboratories	COVID-19 Genomics UK (COG-UK) Consortium	Sam Haldenby, Anita Lucaci, Steve Paterson, Julian Hiscox, Alistair Darby, M Almsaud, A Alrezaihi, Muhannad Alruwaili, Stuart D Armstrong, Jones Benjamin, Eleanor G Bentley, Anu Chawla, Jordan J Clark, Angela Cowell, Richard Eccles, Isabel García-Dorival, Matthew Gemmell, Alessandro Gerada, PKF Gilmore, Richard Gregory, Ximeng Han, Catherine Hartley, Margaret Hughes, Miren Iturriza-Gomara, James Johnson, L Luu, Jenifer Manson, Charlotte Nelson, Elaine O'Toole, Cassie Olateju, Rebekah Penrice-Randal , Lucille Rainbow, N.P Randle, Trevor Ian Robinson, Parul Sharma, Ghada T Shawli, James P Stewart, Neil Swainston, Ecaterina Vamos, Joanne Watts, Mark Whitehead
EPI_ISL_664376, EPI_ISL_664377	Centre for Enzyme Innovation, University of Portsmouth / Translational Research Laboratory, Portsmouth Hospitals NHS Trust	COVID-19 Genomics UK (COG-UK) Consortium	Angela Beckett,Yann Bourgeois,Garry Scarlett,Sharon Glaysher,Scott Elliott,Kelly Bicknell,Robert Impey,Allyson Lloyd,Sarah Wyllie,Ethan Butcher,Anoop Chauhan,Samuel Robson
EPI_ISL_664399, EPI_ISL_664473	University of Exeter	COVID-19 Genomics UK (COG-UK) Consortium	Ben Temperton,Aaron Jeffries,Michelle Michelsen,Joanna Warwick-Dugdale,Audrey Farbos,Robyn Manley,Stephen Michell,Jane Masoli
EPI_ISL_664492, EPI_ISL_664557, EPI_ISL_664558, EPI_ISL_664559	Centre for Enzyme Innovation, University of Portsmouth / Translational Research Laboratory, Portsmouth Hospitals NHS Trust	COVID-19 Genomics UK (COG-UK) Consortium	Angela Beckett,Yann Bourgeois,Garry Scarlett,Sharon Glaysher,Scott Elliott,Kelly Bicknell,Robert Impey,Allyson Lloyd,Sarah Wyllie,Ethan Butcher,Anoop Chauhan,Samuel Robson
EPI_ISL_664592	University of Exeter	COVID-19 Genomics UK (COG-UK) Consortium	Ben Temperton,Aaron Jeffries,Michelle Michelsen,Joanna Warwick-Dugdale,Audrey Farbos,Robyn Manley,Stephen Michell,Jane Masoli
EPI_ISL_664606, EPI_ISL_664623, EPI_ISL_664637	Liverpool Clinical Laboratories	COVID-19 Genomics UK (COG-UK) Consortium	Sam Haldenby, Anita Lucaci, Steve Paterson, Julian Hiscox, Alistair Darby, M Almsaud, A Alrezaihi, Muhannad Alruwaili, Stuart D Armstrong, Jones Benjamin, Eleanor G Bentley, Anu Chawla, Jordan J Clark, Angela Cowell, Richard Eccles, Isabel García-Dorival, Matthew Gemmell, Alessandro Gerada, PKF Gilmore, Richard Gregory, Ximeng Han, Catherine Hartley, Margaret Hughes, Miren Iturriza-Gomara, James Johnson, L Luu, Jenifer Manson, Charlotte Nelson, Elaine O'Toole, Cassie Olateju, Rebekah Penrice-Randal , Lucille Rainbow, N.P Randle, Trevor Ian Robinson, Parul Sharma, Ghada T Shawli, James P Stewart, Neil Swainston, Ecaterina Vamos, Joanne Watts, Mark Whitehead
EPI_ISL_664689	Virology Department, Sheffield Teaching Hospitals NHS Foundation Trust/Department of Infection, Immunity and Cardiovascular Disease, The Medical School, University of Sheffield	COVID-19 Genomics UK (COG-UK) Consortium	Thushan de Silva, Matthew Parker, Nikki Smith, Adri Angyal, Rebecca Brown, Luke Green, Rachel Tucker, Paul Parsons, Danielle Groves, Katie Johnson, Laura Carrilero, Alex Keeley, Dave Partridge, Matthew Wyles, Benjamin Lindsey, Mehmet Yavuz, Mohammad Raza, Cariad Evans
EPI_ISL_664690	Centre for Enzyme Innovation, University of Portsmouth / Translational Research Laboratory, Portsmouth Hospitals NHS Trust	COVID-19 Genomics UK (COG-UK) Consortium	Angela Beckett,Yann Bourgeois,Garry Scarlett,Sharon Glaysher,Scott Elliott,Kelly Bicknell,Robert Impey,Allyson Lloyd,Sarah Wyllie,Ethan Butcher,Anoop Chauhan,Samuel Robson
EPI_ISL_664720	University of Exeter	COVID-19 Genomics UK (COG-UK) Consortium	Ben Temperton,Aaron Jeffries,Michelle Michelsen,Joanna Warwick-Dugdale,Audrey Farbos,Robyn Manley,Stephen Michell,Jane Masoli
EPI_ISL_664752	Virology Department, Sheffield Teaching Hospitals NHS Foundation Trust/Department of Infection, Immunity and Cardiovascular Disease, The Medical School, University of Sheffield	COVID-19 Genomics UK (COG-UK) Consortium	Thushan de Silva, Matthew Parker, Nikki Smith, Adri Angyal, Rebecca Brown, Luke Green, Rachel Tucker, Paul Parsons, Danielle Groves, Katie Johnson, Laura Carrilero, Alex Keeley, Dave Partridge, Matthew Wyles, Benjamin Lindsey, Mehmet Yavuz, Mohammad Raza, Cariad Evans
EPI_ISL_664763, EPI_ISL_664767, EPI_ISL_664841, EPI_ISL_664842, EPI_ISL_664846, EPI_ISL_664848, EPI_ISL_664850, EPI_ISL_664851, EPI_ISL_664852, EPI_ISL_664853, EPI_ISL_664854, EPI_ISL_664855			
see above	Centre for Enzyme Innovation, University of Portsmouth / Translational Research Laboratory, Portsmouth Hospitals NHS Trust	COVID-19 Genomics UK (COG-UK) Consortium	Angela Beckett,Yann Bourgeois,Garry Scarlett,Sharon Glaysher,Scott Elliott,Kelly Bicknell,Robert Impey,Allyson Lloyd,Sarah Wyllie,Ethan Butcher,Anoop Chauhan,Samuel Robson
EPI_ISL_665046, EPI_ISL_665067	University of Exeter	COVID-19 Genomics UK (COG-UK) Consortium	Ben Temperton,Aaron Jeffries,Michelle Michelsen,Joanna Warwick-Dugdale,Audrey Farbos,Robyn Manley,Stephen Michell,Jane Masoli
EPI_ISL_665140	Liverpool Clinical Laboratories	COVID-19 Genomics UK (COG-UK) Consortium	Sam Haldenby, Anita Lucaci, Steve Paterson, Julian Hiscox, Alistair Darby, M Almsaud, A Alrezaihi, Muhannad Alruwaili, Stuart D Armstrong, Jones Benjamin, Eleanor G Bentley, Anu Chawla, Jordan J Clark, Angela Cowell, Richard Eccles, Isabel García-Dorival, Matthew Gemmell, Alessandro Gerada, PKF Gilmore, Richard Gregory, Ximeng Han, Catherine Hartley, Margaret Hughes, Miren Iturriza-Gomara, James Johnson, L Luu, Jenifer Manson, Charlotte Nelson, Elaine O'Toole, Cassie Olateju, Rebekah Penrice-Randal , Lucille Rainbow, N.P Randle, Trevor Ian Robinson, Parul Sharma, Ghada T Shawli, James P Stewart, Neil Swainston, Ecaterina Vamos, Joanne Watts, Mark Whitehead
EPI_ISL_665239	University College London Hospital	COVID-19 Genomics UK (COG-UK) Consortium	Judith Heaney, Matthew Byott, Catherine Houlihan, Dan Frampton, Stuart Kirk, Moira Spyer and Eleni Nastouli
EPI_ISL_665263	Quadram Institute Bioscience	COVID-19 Genomics UK (COG-UK) Consortium	Dave J. Baker, Gemma L. Kay, Alp Aydin, Thanh Le-Viet, Steven Rudder, Ana P. Tedim, Anastasia Kolyva, Maria Diaz, Leonardo de Oliveira Martins, Nabil-Fareed Alikhan, Lizzie Meadows, Rachael Stanley, Ngozi Elumogo, Muhammed Yasir, Nicholas M. Thomson, Alexander J Trotter, Rachel Gilroy, Samuel Bloomfield, Claire Stuart, Andrew Bell, Reenesh Prakash, Samir Dervisevic, Alison E. Mather, John Wain, Mark Webber, Andrew J. Page, Justin O'Grady
EPI_ISL_665288, EPI_ISL_665302, EPI_ISL_665331	Wales Specialist Virology Centre Sequencing lab: Pathogen Genomics Unit	COVID-19 Genomics UK (COG-UK) Consortium	Catherine Moore, Johnathan Evans, Laura Gifford, Malorie Perry, Simon Cottrell, Angela Marchbank, Alec Birchley, Alexander Adams, Amy Gaskin, Bree Gatica-Wilcox, Jason Coombes, Joel Southgate, Lauren Gilbert, Lee Graham, Nicole Pacchiarini, Sara Kumziene-Summerhayes, Sarah Taylor, Sophie Jones, Sara Rey, Matthew Bull, Joanne Watkins, Sally Corden, Tom Connor
EPI_ISL_665335	University College London, Great Ormond Street Hospital for Children NHS Foundation Trust, Imperial College Healthcare NHS Trust	COVID-19 Genomics UK (COG-UK) Consortium	Sergi Castellano, Rachel Williams, Mark Kristiansen, Paola Resende Silva, Sunando Roy, Tony Brooks, Helena Tutill, Paola Niola, Patricia Dyal, Charlotte Williams, Leysa Forrest, Yasmin Panchbhaya, Jacqueline Findlay, Samuel Weeks, Julianne Brown, Kathryn Harris, Paul Randell, James Price, Alison Holmes, Judith Breuer
EPI_ISL_665337	Quadram Institute Bioscience	COVID-19 Genomics UK (COG-UK) Consortium	Dave J. Baker, Gemma L. Kay, Alp Aydin, Thanh Le-Viet, Steven Rudder, Ana P. Tedim, Anastasia Kolyva, Maria Diaz, Leonardo de Oliveira Martins, Nabil-Fareed Alikhan, Lizzie Meadows, Rachael Stanley, Ngozi Elumogo, Muhammed Yasir, Nicholas M. Thomson, Alexander J Trotter, Rachel Gilroy, Samuel Bloomfield, Claire Stuart, Andrew Bell, Reenesh Prakash, Samir Dervisevic, Alison E. Mather, John Wain, Mark Webber, Andrew J. Page, Justin O'Grady
EPI_ISL_665338, EPI_ISL_665347	Centre for Enzyme Innovation, University of Portsmouth / Translational Research Laboratory, Portsmouth Hospitals NHS Trust	COVID-19 Genomics UK (COG-UK) Consortium	Angela Beckett,Yann Bourgeois,Garry Scarlett,Sharon Glaysher,Scott Elliott,Kelly Bicknell,Robert Impey,Allyson Lloyd,Sarah Wyllie,Ethan Butcher,Anoop Chauhan,Samuel Robson
EPI_ISL_665428	University College London, Great Ormond Street Hospital for Children NHS Foundation Trust, Imperial College Healthcare NHS Trust	COVID-19 Genomics UK (COG-UK) Consortium	Sergi Castellano, Rachel Williams, Mark Kristiansen, Paola Resende Silva, Sunando Roy, Tony Brooks, Helena Tutill, Paola Niola, Patricia Dyal, Charlotte Williams, Leysa Forrest, Yasmin Panchbhaya, Jacqueline Findlay, Samuel Weeks, Julianne Brown, Kathryn Harris, Paul Randell, James Price, Alison Holmes, Judith Breuer
EPI_ISL_665457, EPI_ISL_665458, EPI_ISL_665467	Quadram Institute Bioscience	COVID-19 Genomics UK (COG-UK) Consortium	Dave J. Baker, Gemma L. Kay, Alp Aydin, Thanh Le-Viet, Steven Rudder, Ana P. Tedim, Anastasia Kolyva, Maria Diaz, Leonardo de Oliveira Martins, Nabil-Fareed Alikhan, Lizzie Meadows, Rachael Stanley, Ngozi Elumogo, Muhammed Yasir, Nicholas M. Thomson, Alexander J Trotter, Rachel Gilroy, Samuel Bloomfield, Claire Stuart, Andrew Bell, Reenesh Prakash, Samir Dervisevic, Alison E. Mather, John Wain, Mark Webber, Andrew J. Page, Justin O'Grady
EPI_ISL_665469, EPI_ISL_665474	Wales Specialist Virology Centre Sequencing lab: Pathogen Genomics Unit	COVID-19 Genomics UK (COG-UK) Consortium	Catherine Moore, Johnathan Evans, Laura Gifford, Malorie Perry, Simon Cottrell, Angela Marchbank, Alec Birchley, Alexander Adams, Amy Gaskin, Bree Gatica-Wilcox, Jason Coombes, Joel Southgate, Lauren Gilbert, Lee Graham, Nicole Pacchiarini, Sara Kumziene-Summerhayes, Sarah Taylor, Sophie Jones, Sara Rey, Matthew Bull, Joanne Watkins, Sally Corden, Tom Connor
EPI_ISL_665568	Quadram Institute Bioscience	COVID-19 Genomics UK (COG-UK) Consortium	Dave J. Baker, Gemma L. Kay, Alp Aydin, Thanh Le-Viet, Steven Rudder, Ana P. Tedim, Anastasia Kolyva, Maria Diaz, Leonardo de Oliveira Martins, Nabil-Fareed Alikhan, Lizzie Meadows, Rachael Stanley, Ngozi Elumogo, Muhammed Yasir, Nicholas M. Thomson, Alexander J Trotter, Rachel Gilroy, Samuel Bloomfield, Claire Stuart, Andrew Bell, Reenesh Prakash, Samir Dervisevic, Alison E. Mather, John Wain, Mark Webber, Andrew J. Page, Justin O'Grady
EPI_ISL_665576, EPI_ISL_665588	Wales Specialist Virology Centre Sequencing lab: Pathogen Genomics Unit	COVID-19 Genomics UK (COG-UK) Consortium	Catherine Moore, Johnathan Evans, Laura Gifford, Malorie Perry, Simon Cottrell, Angela Marchbank, Alec Birchley, Alexander Adams, Amy Gaskin, Bree Gatica-Wilcox, Jason Coombes, Joel Southgate, Lauren Gilbert, Lee Graham, Nicole Pacchiarini, Sara Kumziene-Summerhayes, Sarah Taylor, Sophie

			Jones, Sara Rey, Matthew Bull, Joanne Watkins, Sally Corden, Tom Connor
EPI_ISL_665657	Quadram Institute Bioscience	COVID-19 Genomics UK (COG-UK) Consortium	Dave J. Baker, Gemma L. Kay, Alp Aydin, Thanh Le-Viet, Steven Rudder, Ana P. Tedim, Anastasia Kolyva, Maria Diaz, Leonardo de Oliveira Martins, Nabil-Fareed Alikhan, Lizzie Meadows, Rachael Stanley, Ngozi Elumogo, Muhammed Yasir, Nicholas M. Thomson, Alexander J Trotter, Rachel Gilroy, Samuel Bloomfield, Claire Stuart, Andrew Bell, Reenesh Prakash, Samir Dervisevic, Alison E. Mather, John Wain, Mark Webber, Andrew J. Page, Justin O'Grady
EPI_ISL_665673	Northumbria University / South Tees Hospitals NHS Foundation Trust / North Cumbria Integrated Care NHS Foundation Trust / North Tees and Hartlepool NHS Foundation Trust / Newcastle Hospitals NHS Foundation Trust	COVID-19 Genomics UK (COG-UK) Consortium	Darren L Smith,Andrew Nelson,Matthew Bashton,Greg R Young,Joshua Loh,John Allan,Mohammad A Tariq,Giles S Holt,Gary Black,Wen C Yew,Lynn Dover,Paul Baker,Steve Liggett,Sarah Essex,Jane Greenaway,Debra Padgett,Clive Graham,Garren Scott,Edward Barton,Emma Swindells,Brendan Payne,Jennifer Collins,Yusri Taha,Gary Eltringham
EPI_ISL_665691	Wales Specialist Virology Centre Sequencing lab: Pathogen Genomics Unit	COVID-19 Genomics UK (COG-UK) Consortium	Catherine Moore, Johnathan Evans, Laura Gifford, Malorie Perry, Simon Cottrell, Angela Marchbank, Alec Birchley, Alexander Adams, Amy Gaskin, Bree Gatica-Wilcox, Jason Coombes, Joel Southgate, Lauren Gilbert, Lee Graham, Nicole Pacchiarini, Sara Kumziene-Summerhayes, Sarah Taylor, Sophie Jones, Sara Rey, Matthew Bull, Joanne Watkins, Sally Corden, Tom Connor
EPI_ISL_665723, EPI_ISL_665726	Centre for Enzyme Innovation, University of Portsmouth / Translational Research Laboratory, Portsmouth Hospitals NHS Trust	COVID-19 Genomics UK (COG-UK) Consortium	Angela Beckett,Yann Bourgeois,Garry Scarlett,Sharon Glaysher,Scott Elliott,Kelly Bicknell,Robert Impey,Allyson Lloyd,Sarah Wyllie,Ethan Butcher,Anoop Chauhan,Samuel Robson
EPI_ISL_665768	Wales Specialist Virology Centre Sequencing lab: Pathogen Genomics Unit	COVID-19 Genomics UK (COG-UK) Consortium	Catherine Moore, Johnathan Evans, Laura Gifford, Malorie Perry, Simon Cottrell, Angela Marchbank, Alec Birchley, Alexander Adams, Amy Gaskin, Bree Gatica-Wilcox, Jason Coombes, Joel Southgate, Lauren Gilbert, Lee Graham, Nicole Pacchiarini, Sara Kumziene-Summerhayes, Sarah Taylor, Sophie Jones, Sara Rey, Matthew Bull, Joanne Watkins, Sally Corden, Tom Connor
EPI_ISL_665877, EPI_ISL_665887, EPI_ISL_665889, EPI_ISL_665891, EPI_ISL_665895	Quadram Institute Bioscience	COVID-19 Genomics UK (COG-UK) Consortium	Dave J. Baker, Gemma L. Kay, Alp Aydin, Thanh Le-Viet, Steven Rudder, Ana P. Tedim, Anastasia Kolyva, Maria Diaz, Leonardo de Oliveira Martins, Nabil-Fareed Alikhan, Lizzie Meadows, Rachael Stanley, Ngozi Elumogo, Muhammed Yasir, Nicholas M. Thomson, Alexander J Trotter, Rachel Gilroy, Samuel Bloomfield, Claire Stuart, Andrew Bell, Reenesh Prakash, Samir Dervisevic, Alison E. Mather, John Wain, Mark Webber, Andrew J. Page, Justin O'Grady
EPI_ISL_666007, EPI_ISL_666009, EPI_ISL_666018, EPI_ISL_666019, EPI_ISL_666020, EPI_ISL_666021	Northumbria University / South Tees Hospitals NHS Foundation Trust / North Cumbria Integrated Care NHS Foundation Trust / North Tees and Hartlepool NHS Foundation Trust / Newcastle Hospitals NHS Foundation Trust	COVID-19 Genomics UK (COG-UK) Consortium	Darren L Smith,Andrew Nelson,Matthew Bashton,Greg R Young,Joshua Loh,John Allan,Mohammad A Tariq,Giles S Holt,Gary Black,Wen C Yew,Lynn Dover,Paul Baker,Steve Liggett,Sarah Essex,Jane Greenaway,Debra Padgett,Clive Graham,Garren Scott,Edward Barton,Emma Swindells,Brendan Payne,Jennifer Collins,Yusri Taha,Gary Eltringham
EPI_ISL_666060	Virology Department, Royal Infirmary of Edinburgh, NHS Lothian / School of Biological Sciences, University of Edinburgh / Institute of Genetics and Molecular Medicine, University of Edinburgh	COVID-19 Genomics UK (COG-UK) Consortium	McHugh M, Dewar R, Rooke S, Gallagher M, Balcaza C, O'Toole Á, Scher E, Hill V, McCrone JT, Colquhoun R, Yu X, Jackson B, Rambaut A, Williams TC, Templeton K
EPI_ISL_666107, EPI_ISL_666108, EPI_ISL_666109	Centre for Enzyme Innovation, University of Portsmouth / Translational Research Laboratory, Portsmouth Hospitals NHS Trust	COVID-19 Genomics UK (COG-UK) Consortium	Angela Beckett,Yann Bourgeois,Garry Scarlett,Sharon Glaysher,Scott Elliott,Kelly Bicknell,Robert Impey,Allyson Lloyd,Sarah Wyllie,Ethan Butcher,Anoop Chauhan,Samuel Robson
EPI_ISL_666112, EPI_ISL_666127, EPI_ISL_666136, EPI_ISL_666147, EPI_ISL_666149, EPI_ISL_666157, EPI_ISL_666165, EPI_ISL_666166, EPI_ISL_666172, EPI_ISL_666177, EPI_ISL_666185, EPI_ISL_666197, EPI_ISL_666204, EPI_ISL_666207, EPI_ISL_666214, EPI_ISL_666218, EPI_ISL_666221, EPI_ISL_666223, EPI_ISL_666227, EPI_ISL_666229, EPI_ISL_666235, EPI_ISL_666253, EPI_ISL_666261, EPI_ISL_666264, EPI_ISL_666265, EPI_ISL_666280, EPI_ISL_666283, EPI_ISL_666291, EPI_ISL_666292, EPI_ISL_666307, EPI_ISL_666321, EPI_ISL_666327, EPI_ISL_666338, EPI_ISL_666343, EPI_ISL_666344, EPI_ISL_666354, EPI_ISL_666358, EPI_ISL_666360, EPI_ISL_666367, EPI_ISL_666379, EPI_ISL_666418, EPI_ISL_666430, EPI_ISL_666470, EPI_ISL_666480, EPI_ISL_666483, EPI_ISL_666509, EPI_ISL_666513, EPI_ISL_666525, EPI_ISL_666565, EPI_ISL_666581			
see above	Wales Specialist Virology Centre Sequencing lab: Pathogen Genomics Unit	COVID-19 Genomics UK (COG-UK) Consortium	Catherine Moore, Johnathan Evans, Laura Gifford, Malorie Perry, Simon Cottrell, Angela Marchbank, Alec Birchley, Alexander Adams, Amy Gaskin, Bree Gatica-Wilcox, Jason Coombes, Joel Southgate, Lauren Gilbert, Lee Graham, Nicole Pacchiarini, Sara Kumziene-Summerhayes, Sarah Taylor, Sophie Jones, Sara Rey, Matthew Bull, Joanne Watkins, Sally Corden, Tom Connor
EPI_ISL_666726, EPI_ISL_666770	Respiratory Virus Unit, Microbiology Services Colindale, Public Health England	COVID-19 Genomics UK (COG-UK) Consortium	PHE Covid Sequencing Team
EPI_ISL_666903, EPI_ISL_666913, EPI_ISL_666917, EPI_ISL_666920, EPI_ISL_666931, EPI_ISL_666940, EPI_ISL_666944, EPI_ISL_666948, EPI_ISL_666953, EPI_ISL_666958	Michigan Department of Health and Human Services, Bureau of Laboratories	Michigan Department of Health and Human Services, Bureau of Laboratories	Blankenship HM, Riner D, Soehnlen MK
EPI_ISL_666966, EPI_ISL_666967, EPI_ISL_666971, EPI_ISL_666974	San Diego County Public Health Laboratory	Andersen lab at Scripps Research	SEARCH Alliance San Diego with Tracy Basler, Jovan Shephard, Brett Austin
EPI_ISL_667516, EPI_ISL_667517, EPI_ISL_667526, EPI_ISL_667527, EPI_ISL_667528, EPI_ISL_667536	OHSU Lab Services Molecular Microbiology Lab	Oregon SARS-CoV-2 Genome Sequencing Center	Brendan L. O'Connell, Ruth V. Nichols, Sally Grindstaff, Alec J. Hirsch, Donna Hansel, Guang Fan, Daniel N. Streblow, William B. Messer, Andrew C. Adey, Benjamin N. Bimber, Brian J. O'Roak
EPI_ISL_667694, EPI_ISL_667695, EPI_ISL_667696, EPI_ISL_667697, EPI_ISL_667698, EPI_ISL_667699, EPI_ISL_667700, EPI_ISL_667701, EPI_ISL_667702, EPI_ISL_667703, EPI_ISL_667704, EPI_ISL_667705, EPI_ISL_667706, EPI_ISL_667707, EPI_ISL_667708, EPI_ISL_667709, EPI_ISL_667710, EPI_ISL_667711, EPI_ISL_667712, EPI_ISL_667713, EPI_ISL_667714, EPI_ISL_667715, EPI_ISL_667716, EPI_ISL_667717, EPI_ISL_667718, EPI_ISL_667719, EPI_ISL_667720, EPI_ISL_667721, EPI_ISL_667722, EPI_ISL_667723, EPI_ISL_667724, EPI_ISL_667725, EPI_ISL_667726, EPI_ISL_667727, EPI_ISL_667728, EPI_ISL_667729, EPI_ISL_667730, EPI_ISL_667731, EPI_ISL_667732, EPI_ISL_667733, EPI_ISL_667734, EPI_ISL_667740, EPI_ISL_667741, EPI_ISL_667742, EPI_ISL_667743, EPI_ISL_667744, EPI_ISL_667745, EPI_ISL_667746, EPI_ISL_667747, EPI_ISL_667748, EPI_ISL_667749, EPI_ISL_667750, EPI_ISL_667751, EPI_ISL_667752, EPI_ISL_667753, EPI_ISL_667754			
see above	Pathogen Genomics Center, National Institute of Infectious Diseases	Pathogen Genomics Center, National Institute of Infectious Diseases	Tsuyoshi Sekizuka, Kentaro Itokawa, Rina Tanaka, Masanori Hashino, Makoto Kuroda
EPI_ISL_667780, EPI_ISL_667788, EPI_ISL_667790, EPI_ISL_667793, EPI_ISL_667794, EPI_ISL_667795	South Eastern Area Laboratory Services (SEALS)	NSW Health Pathology - Institute of Clinical Pathology and Medical Research; Westmead Hospital; University of Sydney	CIDM-PH et al.
EPI_ISL_671759	DOHMH Fort Greene	New York City Public Health Laboratory	Jade Wang, et al.
EPI_ISL_671760	DOHMH Crown Heights	New York City Public Health Laboratory	Jade Wang, et al.
EPI_ISL_671761	DOHMH Corona	New York City Public Health Laboratory	Jade Wang, et al.
EPI_ISL_671762	DOHMH Riverside	New York City Public Health Laboratory	Jade Wang, et al.
EPI_ISL_671763	DOHMH Chelsea	New York City Public Health Laboratory	Jade Wang, et al.
EPI_ISL_671866	National Virus Reference Laboratory	National Virus Reference Laboratory	Michael Carr, Gabriel Gonzalez, Jonathan Dean, Daniel Hare, Cillian F De Gascun
EPI_ISL_672072, EPI_ISL_672104, EPI_ISL_672106, EPI_ISL_672107, EPI_ISL_672108	Santa Clara County Public Health Laboratory	Chan-Zuckerberg Biohub	CZB Cliahub Consortium
EPI_ISL_672428	UCSF Clinical Microbiology Laboratory	Chan-Zuckerberg Biohub	CZB Cliahub Consortium
EPI_ISL_672506	Madera County Department of Public Health	Chan-Zuckerberg Biohub	CZB Cliahub Consortium
EPI_ISL_672913, EPI_ISL_672914, EPI_ISL_672915, EPI_ISL_672920, EPI_ISL_672921, EPI_ISL_672928, EPI_ISL_672929, EPI_ISL_672930, EPI_ISL_672931, EPI_ISL_672932, EPI_ISL_672933, EPI_ISL_672936, EPI_ISL_672937, EPI_ISL_672938, EPI_ISL_672940, EPI_ISL_672941, EPI_ISL_672942, EPI_ISL_672946, EPI_ISL_672948, EPI_ISL_672950, EPI_ISL_672952, EPI_ISL_672953, EPI_ISL_672954, EPI_ISL_672955, EPI_ISL_672957, EPI_ISL_672958, EPI_ISL_672960, EPI_ISL_672962, EPI_ISL_672966, EPI_ISL_672967, EPI_ISL_672969, EPI_ISL_672970, EPI_ISL_672973, EPI_ISL_672975, EPI_ISL_672976, EPI_ISL_672979,			

EPI_ISL_672981, EPI_ISL_672982, EPI_ISL_672986, EPI_ISL_672988, EPI_ISL_672994, EPI_ISL_672995, EPI_ISL_672996, EPI_ISL_672997, EPI_ISL_673000, EPI_ISL_673002, EPI_ISL_673003, EPI_ISL_673008, EPI_ISL_673010, EPI_ISL_673013, EPI_ISL_673014, EPI_ISL_673020, EPI_ISL_673021, EPI_ISL_673024, EPI_ISL_673026, EPI_ISL_673030, EPI_ISL_673031, EPI_ISL_673033, EPI_ISL_673034, EPI_ISL_673035, EPI_ISL_673036, EPI_ISL_673038, EPI_ISL_673042, EPI_ISL_673043, EPI_ISL_673044, EPI_ISL_673045, EPI_ISL_673046, EPI_ISL_673047, EPI_ISL_673049, EPI_ISL_673052, EPI_ISL_673058, EPI_ISL_673060, EPI_ISL_673063, EPI_ISL_673064, EPI_ISL_673066, EPI_ISL_673068, EPI_ISL_673072, EPI_ISL_673079, EPI_ISL_673081, EPI_ISL_673084, EPI_ISL_673087, EPI_ISL_673088, EPI_ISL_673089, EPI_ISL_673091, EPI_ISL_673093, EPI_ISL_673094, EPI_ISL_673095, EPI_ISL_673096, EPI_ISL_673097, EPI_ISL_673107, EPI_ISL_673108, EPI_ISL_673109, EPI_ISL_673111, EPI_ISL_673112, EPI_ISL_673114, EPI_ISL_673117, EPI_ISL_673119, EPI_ISL_673120, EPI_ISL_673121, EPI_ISL_673123, EPI_ISL_673124, EPI_ISL_673127, EPI_ISL_673128, EPI_ISL_673129, EPI_ISL_673134, EPI_ISL_673135, EPI_ISL_673136, EPI_ISL_673139, EPI_ISL_673140, EPI_ISL_673142, EPI_ISL_673143, EPI_ISL_673144, EPI_ISL_673147, EPI_ISL_673148, EPI_ISL_673150, EPI_ISL_673151, EPI_ISL_673153, EPI_ISL_673154, EPI_ISL_673156, EPI_ISL_673158, EPI_ISL_673161, EPI_ISL_673162, EPI_ISL_673164, EPI_ISL_673166, EPI_ISL_673167, EPI_ISL_673168, EPI_ISL_673169, EPI_ISL_673170, EPI_ISL_673171, EPI_ISL_673173, EPI_ISL_673175, EPI_ISL_673180, EPI_ISL_673188, EPI_ISL_673193, EPI_ISL_673196, EPI_ISL_673200, EPI_ISL_673201, EPI_ISL_673202, EPI_ISL_673204, EPI_ISL_673206, EPI_ISL_673207, EPI_ISL_673208, EPI_ISL_673210, EPI_ISL_673213, EPI_ISL_673214, EPI_ISL_673215, EPI_ISL_673217, EPI_ISL_673218, EPI_ISL_673221, EPI_ISL_673224, EPI_ISL_673225, EPI_ISL_673226, EPI_ISL_673227, EPI_ISL_673229, EPI_ISL_673230, EPI_ISL_673231, EPI_ISL_673232, EPI_ISL_673235			
see above	Lighthouse Lab in Cambridge	Wellcome Sanger Institute for the COVID-19 Genomics UK (COG-UK) Consortium	Rob Howes, The Lighthouse Lab in Cambridge and Alex Alderton, Roberto Amato, Sonia Goncalves, Ewan Harrison, David K. Jackson, Ian Johnston, Dominic Kwiatkowski, Cordelia Langford, John Sillitoe on behalf of the Wellcome Sanger Institute COVID-19 Surveillance Team
EPI_ISL_676434, EPI_ISL_676441, EPI_ISL_676442, EPI_ISL_676443, EPI_ISL_676445, EPI_ISL_676446, EPI_ISL_676447, EPI_ISL_676448, EPI_ISL_676449, EPI_ISL_676451, EPI_ISL_676452, EPI_ISL_676453, EPI_ISL_676454, EPI_ISL_676455, EPI_ISL_676456, EPI_ISL_676457, EPI_ISL_676458, EPI_ISL_676459, EPI_ISL_676460, EPI_ISL_676461, EPI_ISL_676462, EPI_ISL_676463, EPI_ISL_676464, EPI_ISL_676465, EPI_ISL_676466, EPI_ISL_676467, EPI_ISL_676468, EPI_ISL_676469, EPI_ISL_676470, EPI_ISL_676471, EPI_ISL_676472, EPI_ISL_676473, EPI_ISL_676474, EPI_ISL_676475, EPI_ISL_676476, EPI_ISL_676478, EPI_ISL_676479, EPI_ISL_676480, EPI_ISL_676481, EPI_ISL_676482, EPI_ISL_676483, EPI_ISL_676484, EPI_ISL_676485, EPI_ISL_676487, EPI_ISL_676488			
see above	Lighthouse Lab in Glasgow	Wellcome Sanger Institute for the COVID-19 Genomics UK (COG-UK) Consortium	Harper VanSteenhouse, Yumi Kasai, David Gray, Carol Clugston, Anna Dominiczak and Alex Alderton, Roberto Amato, Sonia Goncalves, Ewan Harrison, David K. Jackson, Ian Johnston, Dominic Kwiatkowski, Cordelia Langford, John Sillitoe on behalf of the Wellcome Sanger Institute COVID-19 Surveillance Team
EPI_ISL_677319	Colorado Department of Public Health and Environment	Colorado Department of Puplic Health and Environment	Laura Bankers, Molly Hetherington-Rauth, Shannon Ely, Shannon R. Matzinger, Sarah Elizabeth Totten, Emily A. Travanty
EPI_ISL_677330, EPI_ISL_677331, EPI_ISL_677332, EPI_ISL_677333, EPI_ISL_677334, EPI_ISL_677503, EPI_ISL_677504	University of Wisconsin-Madison AIDS Vaccine Research Laboratories	University of Wisconsin-Madison AIDS Vaccine Research Laboratories	Gage Moreno, Katarina Braun, et al. AIDS Vaccine Research Laboratories
EPI_ISL_678375, EPI_ISL_678377	Area of Virology, Serology and Virology Division (SAViD), New South Wales Health Pathology Randwick	Virology Research Laboratory; Area of Virology, Serology and Virology Division (SAViD), New South Wales Health Pathology Randwick	Foster, C.; Au, J.; Ruiz Silva, M.; Deveson, I.; Bull, R.; Van Hal, S.; Rawlinson, W.
EPI_ISL_678601, EPI_ISL_678602, EPI_ISL_678603	NHLS-IALCH	KRISP, KZN Research Innovation and Sequencing Platform	Giandhari J, Pillay S, Lessells R, ChimukangaraB, Mdlalose K, York D, Khan S, Tegally H, Wilkinson E, de Oliveira T
EPI_ISL_678787, EPI_ISL_678788, EPI_ISL_678789, EPI_ISL_678790, EPI_ISL_678791, EPI_ISL_678792, EPI_ISL_678793, EPI_ISL_678794, EPI_ISL_678795, EPI_ISL_678796, EPI_ISL_678797, EPI_ISL_678798, EPI_ISL_678799, EPI_ISL_678800, EPI_ISL_678801, EPI_ISL_678802, EPI_ISL_678803, EPI_ISL_678804, EPI_ISL_678805, EPI_ISL_678806, EPI_ISL_678807, EPI_ISL_678808, EPI_ISL_678809, EPI_ISL_678810, EPI_ISL_678811, EPI_ISL_678812, EPI_ISL_678813, EPI_ISL_678814, EPI_ISL_678815, EPI_ISL_678816, EPI_ISL_678817, EPI_ISL_678818, EPI_ISL_678819, EPI_ISL_678820, EPI_ISL_678821, EPI_ISL_678822, EPI_ISL_678823, EPI_ISL_678824, EPI_ISL_678825, EPI_ISL_678826, EPI_ISL_678827			
see above	Respiratory Virus Unit, Microbiology Services Colindale, Public Health England	COVID-19 Genomics UK (COG-UK) Consortium	PHE Covid Sequencing Team
EPI_ISL_679130, EPI_ISL_679131	University of Birmingham	COVID-19 Genomics UK (COG-UK) Consortium	Institute of Microbiology, University of Birmingham: Claire McMurray, Joanne Stockton, Samuel Nicholls, Radoslaw Poplawski, Will Rowe, Josh Quick, Nicholas Loman. University of Birmingham Testing Laboratory: Celina M Whalley, Andrew Bosworth, Charlotte Poxon, Kasun Wanigasooriya, Oliver Pickles, Mike Kidd, Alex Richter, Andrew D Beggs PHE Heartlands Lab: Husam Osman, Andrew Bosworth. Queen Elizabeth Hospital: Anna Casey
EPI_ISL_679474, EPI_ISL_679475, EPI_ISL_679476, EPI_ISL_679479	University College London, Great Ormond Street Hospital for Children NHS Foundation Trust, Imperial College Healthcare NHS Trust	COVID-19 Genomics UK (COG-UK) Consortium	Sergi Castellano, Rachel Williams, Mark Kristiansen, Paola Resende Silva, Sunando Roy, Tony Brooks, Helena Tutill, Paola Niola, Patricia Dyal, Charlotte Williams, Leysa Forrest, Yasmin Panchbhaya, Jacqueline Findlay, Samuel Weeks, Julianne Brown, Kathryn Harris, Paul Randell, James Price, Alison Holmes, Judith Breuer
EPI_ISL_679631, EPI_ISL_679634, EPI_ISL_679635, EPI_ISL_679636, EPI_ISL_679637, EPI_ISL_679638, EPI_ISL_679639, EPI_ISL_679640, EPI_ISL_679641, EPI_ISL_679646, EPI_ISL_679647, EPI_ISL_679649, EPI_ISL_679650, EPI_ISL_679654, EPI_ISL_679656, EPI_ISL_679657, EPI_ISL_679660, EPI_ISL_679661, EPI_ISL_679662, EPI_ISL_679663, EPI_ISL_679664, EPI_ISL_679665, EPI_ISL_679667, EPI_ISL_679668, EPI_ISL_679670, EPI_ISL_679672, EPI_ISL_679674, EPI_ISL_679675, EPI_ISL_679676, EPI_ISL_679677, EPI_ISL_679681, EPI_ISL_679686, EPI_ISL_679687, EPI_ISL_679689, EPI_ISL_679691, EPI_ISL_679701, EPI_ISL_679708, EPI_ISL_679710, EPI_ISL_679711, EPI_ISL_679713, EPI_ISL_679715, EPI_ISL_679722, EPI_ISL_679724, EPI_ISL_679725, EPI_ISL_679726, EPI_ISL_679729, EPI_ISL_679730, EPI_ISL_679731, EPI_ISL_679732, EPI_ISL_679733, EPI_ISL_679734, EPI_ISL_679735, EPI_ISL_679736, EPI_ISL_679737, EPI_ISL_679738, EPI_ISL_679739, EPI_ISL_679740, EPI_ISL_679741, EPI_ISL_679742, EPI_ISL_679743, EPI_ISL_679744, EPI_ISL_679745, EPI_ISL_679746, EPI_ISL_679747, EPI_ISL_679748, EPI_ISL_679749, EPI_ISL_679750, EPI_ISL_679751, EPI_ISL_679752, EPI_ISL_679753, EPI_ISL_679754, EPI_ISL_679755, EPI_ISL_679756, EPI_ISL_679757, EPI_ISL_679758, EPI_ISL_679759, EPI_ISL_679760, EPI_ISL_679761, EPI_ISL_679762, EPI_ISL_679763, EPI_ISL_679764, EPI_ISL_679765, EPI_ISL_679768, EPI_ISL_679769, EPI_ISL_679771, EPI_ISL_679772, EPI_ISL_679775, EPI_ISL_679776, EPI_ISL_679778, EPI_ISL_679779, EPI_ISL_679780, EPI_ISL_679781, EPI_ISL_679782, EPI_ISL_679786, EPI_ISL_679789, EPI_ISL_679790, EPI_ISL_679791, EPI_ISL_679792, EPI_ISL_679793, EPI_ISL_679794, EPI_ISL_679795, EPI_ISL_679796, EPI_ISL_679797, EPI_ISL_679798, EPI_ISL_679799, EPI_ISL_679800, EPI_ISL_679801, EPI_ISL_679802, EPI_ISL_679803, EPI_ISL_679804, EPI_ISL_679805, EPI_ISL_679807, EPI_ISL_679808, EPI_ISL_679809, EPI_ISL_679813, EPI_ISL_679815, EPI_ISL_679817, EPI_ISL_679820, EPI_ISL_679821, EPI_ISL_679822, EPI_ISL_679823, EPI_ISL_679824, EPI_ISL_679825, EPI_ISL_679826, EPI_ISL_679827, EPI_ISL_679828, EPI_ISL_679829, EPI_ISL_679830, EPI_ISL_679831, EPI_ISL_679832, EPI_ISL_679833, EPI_ISL_679834, EPI_ISL_679835, EPI_ISL_679836, EPI_ISL_679837, EPI_ISL_679838, EPI_ISL_679839, EPI_ISL_679840, EPI_ISL_679841, EPI_ISL_679842, EPI_ISL_679843, EPI_ISL_679844, EPI_ISL_679845, EPI_ISL_679846, EPI_ISL_679847, EPI_ISL_679848, EPI_ISL_679849, EPI_ISL_679850, EPI_ISL_679851			
see above	Oxford Viromics, NDM, University of Oxford; Oxford University Hospitals; Basingstoke and North Hampshire Hospital	COVID-19 Genomics UK (COG-UK) Consortium	Tanya Golubchik, David Bonsall, George Macintyre, Amy Trebes, Mariateresa de Cesare, Catrin Moore, Alex Mobbs, Anita Justice, Robert Shaw, Monique Andersson, Timothy Peto, Emma Wise, Nathan Moore, Jessica Lynch, Nick Cortes, Matilde Mori, Stephen Kidd, David Buck, John Todd, Christophe Fraser
EPI_ISL_679927	Queens Medical Centre, Clinical Microbiology Department / DeepSeq Nottingham	COVID-19 Genomics UK (COG-UK) Consortium	Gemma Clark, Wendy Smith, Manjinder Khakh, Vicki M Fleming, Michelle M Lister, Hannah Howson-Wells, Jonathan Ball, Patrick McClure, Joseph Chappell, Theocharis Tsoleridis, Nadine Holmes, Matthew Carlisle, Christopher Moore, Fei Sang, Johnny Debebe, Victoria Wright, Matthew Loose
EPI_ISL_680071	Department of Pathology, University of Cambridge	COVID-19 Genomics UK (COG-UK) Consortium	Aminu S. Jahun, Yasmin Chaudhry, Grant Hall, Iliana Georgana, Myra Hosmillo, Martin D. Curran, Malte Pinckert, Surendra Parmar, Ian Goodfellow
EPI_ISL_680508, EPI_ISL_680509, EPI_ISL_680515, EPI_ISL_680516, EPI_ISL_680517, EPI_ISL_680518, EPI_ISL_680519, EPI_ISL_680520, EPI_ISL_680521, EPI_ISL_680524, EPI_ISL_680525, EPI_ISL_680526, EPI_ISL_680527, EPI_ISL_680528, EPI_ISL_680529, EPI_ISL_680530, EPI_ISL_680531, EPI_ISL_680532, EPI_ISL_680533, EPI_ISL_680534, EPI_ISL_680535, EPI_ISL_680536, EPI_ISL_680537			
see above	Virology Department, Royal Infirmary of Edinburgh, NHS Lothian / School of Biological Sciences, University of Edinburgh / Institute of Genetics and Molecular Medicine, University of Edinburgh	COVID-19 Genomics UK (COG-UK) Consortium	McHugh M, Dewar R, Rooke S, Gallagher M, Balcaza C, O'Toole Á, Scher E, Hill V, McCrone JT, Colquhoun R, Yu X, Jackson B, Rambaut A, Williams TC, Templeton K
EPI_ISL_680578	Centre for Enzyme Innovation, University of Portsmouth / Translational Research Laboratory, Portsmouth Hospitals NHS Trust	COVID-19 Genomics UK (COG-UK) Consortium	Angela Beckett,Yann Bourgeois,Garry Scarlett,Sharon Glaysher,Scott Elliott,Kelly Bicknell,Robert Impey,Allyson Lloyd,Sarah Wyllie,Ethan Butcher,Anoop Chauhan,Samuel Robson
EPI_ISL_680579	Oxford Viromics, NDM, University of Oxford; Oxford University Hospitals; Basingstoke and North Hampshire Hospital	COVID-19 Genomics UK (COG-UK) Consortium	Tanya Golubchik, David Bonsall, George Macintyre, Amy Trebes, Mariateresa de Cesare, Catrin Moore, Alex Mobbs, Anita Justice, Robert Shaw, Monique Andersson, Timothy Peto, Emma Wise, Nathan Moore, Jessica Lynch, Nick Cortes, Matilde Mori, Stephen Kidd, David Buck, John Todd, Christophe Fraser
EPI_ISL_680582, EPI_ISL_680587, EPI_ISL_680622, EPI_ISL_680623, EPI_ISL_680624, EPI_ISL_680625, EPI_ISL_680626, EPI_ISL_680627, EPI_ISL_680628, EPI_ISL_680629, EPI_ISL_680630, EPI_ISL_680631, EPI_ISL_680632, EPI_ISL_680633, EPI_ISL_680684, EPI_ISL_680714, EPI_ISL_680715, EPI_ISL_680716, EPI_ISL_680717, EPI_ISL_680718, EPI_ISL_680719, EPI_ISL_680720, EPI_ISL_680721, EPI_ISL_680722, EPI_ISL_680723, EPI_ISL_680724, EPI_ISL_680725, EPI_ISL_680726, EPI_ISL_680727, EPI_ISL_680728, EPI_ISL_680729, EPI_ISL_680730, EPI_ISL_680731			
see above	Wales Specialist Virology Centre Sequencing lab: Pathogen Genomics Unit	COVID-19 Genomics UK (COG-UK) Consortium	Catherine Moore, Johnathan Evans, Laura Gifford, Malorie Perry, Simon Cottrell, Angela Marchbank, Alec Birchley, Alexander Adams, Amy Gaskin, Bree Gatica-Wilcox, Jason Coombes, Joel Southgate, Lauren Gilbert, Lee Graham, Nicole Pacchiarini, Sara Kumziene-Summerhayes, Sarah Taylor, Sophie Jones, Sara Rey, Matthew Bull, Joanne Watkins, Sally Corden, Tom Connor
EPI_ISL_681308, EPI_ISL_681311	Communicable Disease Laboratory, Public Health Directorate	Communicable Disease Laboratory, Public Health Directorate	Alwasti,H., Altaif,Z., AlHujairi,Z., AlAbbas,Z.
EPI_ISL_681323, EPI_ISL_681324	Respiratory Virus Unit, Microbiology Services Colindale, Public Health England	COVID-19 Genomics UK (COG-UK) Consortium	PHE Covid Sequencing Team
EPI_ISL_681657	Lighthouse Lab in Cambridge	Wellcome Sanger Institute for the COVID-19 Genomics UK (COG-UK) Consortium	Rob Howes, The Lighthouse Lab in Cambridge and Alex Alderton, Roberto Amato, Sonia Goncalves, Ewan Harrison, David K. Jackson, Ian Johnston, Dominic Kwiatkowski, Cordelia Langford, John Sillitoe on behalf of the Wellcome Sanger Institute COVID-19 Surveillance Team
EPI_ISL_682080, EPI_ISL_682081, EPI_ISL_682094, EPI_ISL_682095, EPI_ISL_682100, EPI_ISL_682103, EPI_ISL_682104, EPI_ISL_682106, EPI_ISL_682107, EPI_ISL_682109, EPI_ISL_682110, EPI_ISL_682113, EPI_ISL_682116, EPI_ISL_682117, EPI_ISL_682118, EPI_ISL_682121, EPI_ISL_682122, EPI_ISL_682123, EPI_ISL_682127, EPI_ISL_682128, EPI_ISL_682131, EPI_ISL_682132, EPI_ISL_682134, EPI_ISL_682135, EPI_ISL_682136, EPI_ISL_682137, EPI_ISL_682138, EPI_ISL_682139, EPI_ISL_682140, EPI_ISL_682141, EPI_ISL_682142, EPI_ISL_682143, EPI_ISL_682144, EPI_ISL_682145, EPI_ISL_682146, EPI_ISL_682147, EPI_ISL_682148, EPI_ISL_682149, EPI_ISL_682150, EPI_ISL_682151, EPI_ISL_682152, EPI_ISL_682153, EPI_ISL_682154, EPI_ISL_682155, EPI_ISL_682156, EPI_ISL_682157, EPI_ISL_682158, EPI_ISL_682159, EPI_ISL_682160			

see above	University of Michigan Clinical Microbiology Laboratory	Lauring Lab, University of Michigan, Department of Microbiology and Immunology	Valesano
EPI_ISL_682278	Middlemore Hospital	Institute of Environmental Science and Research (ESR)	Xiaoyun Ren, Matt Storey, Nikki Freed, Muhammad Faisal, Jing Wang, Hermes Perez, Anja Werno, Antje van der Linden, Arlo Upton, Chris Mansell, David Hammer, Dragana Drinkovic, Gary McAuliffe, Hana Sofia Andersson, James Ussher, Jill Sherwood, Josh Freeman, Julia Howard, Juliet Elvy, Mary DeAlmeida, Matt Blakiston, Matthew Rogers, Max Bloomfield, Michael Addidle, Michelle Balm, Sally Roberts, Sarah Jefferies, Sharmini Muttaiyah, Susan Morpeth, Susan Taylor, Timothy Blackmore, Vani Sathyendran, Veronica Playle, Virginia Hope, Erasmus Smit, Lauren Jelly, Olin Silander, Joep de Ligt
EPI_ISL_682299, EPI_ISL_682302	Communicable Disease Laboratory, Public Health Directorate	Communicable Disease Laboratory, Public Health Directorate	Alwasti,H., Altaif,Z., AlHujairi,Z., AlAbbas,Z.
EPI_ISL_683087, EPI_ISL_683088, EPI_ISL_683089, EPI_ISL_683090, EPI_ISL_683091	Department of Virus and Microbiological Special Diagnostics, Statens Serum Institut, Copenhagen, Denmark	Albertsen Lab, Department of Chemistry and Bioscience, Aalborg University, Denmark	Danish Covid-19 Genome Consortium
EPI_ISL_683400, EPI_ISL_683401	CNR Virus des Infections Respiratoires - France SUD	CNR Virus des Infections Respiratoires - France SUD	Antonin Bal, Gregory Destras, Gwendolyne Burfin, Quentin Semanas, Martine Valette, Bruno Lina, Laurence Josset
EPI_ISL_683646	Servicio de Microbiología, Laboratori Clínic Metropolitana Nord. Hospital Universitari Germans Trias i Pujol. Institut d'Investigació en Ciències de la Salut Germans Trias i Pujol (IGTP)	SeqCOVID-SPAIN consortium/IBV(CSIC)	Elisa Martró, Antoni E. Bordoy, Anna Not, Adrián Antuori, Anabel Fernández, Nona Romaní, Verónica Saludes, Cristina Casañ and SeqCOVID-SPAIN consortium
EPI_ISL_683696, EPI_ISL_683702, EPI_ISL_683703, EPI_ISL_683705, EPI_ISL_683706, EPI_ISL_683707, EPI_ISL_683708, EPI_ISL_683710, EPI_ISL_683712, EPI_ISL_683713	Essentia Health-St. Mary's Medical Center	Minnesota Department of Health, Public Health Laboratory	Alexandra Lorentz, Jacob Garfin, Matt Plumb, and Xiong Wang
EPI_ISL_683716, EPI_ISL_683717, EPI_ISL_683718	Mayo Clinic & Mayo Clinic Laboratories	Minnesota Department of Health, Public Health Laboratory	Alexandra Lorentz, Jacob Garfin, Matt Plumb, and Xiong Wang
EPI_ISL_683744, EPI_ISL_683745, EPI_ISL_683746, EPI_ISL_683747, EPI_ISL_683748, EPI_ISL_683749, EPI_ISL_683750	Minnesota Department of Health, Public Health Laboratory	Minnesota Department of Health, Public Health Laboratory	Alexandra Lorentz, Jacob Garfin, Matt Plumb, and Xiong Wang
EPI_ISL_683772, EPI_ISL_683884, EPI_ISL_683886	DOHMH Morrisania	New York City Public Health Laboratory	Jade Wang, et al.
EPI_ISL_683937	DOHMH Central Harlem	New York City Public Health Laboratory	Jade Wang, et al.
EPI_ISL_683938	DOHMH Morrisania	New York City Public Health Laboratory	Jade Wang, et al.
EPI_ISL_692809, EPI_ISL_692810, EPI_ISL_692811, EPI_ISL_692812, EPI_ISL_692814, EPI_ISL_692817, EPI_ISL_692818, EPI_ISL_692819	Massachusetts State Public Health Laboratory	Massachusetts State Public Health Laboratory	Andrew Lang, Timelia Fink, Glen Gallagher, Sandra Smole
EPI_ISL_693269, EPI_ISL_693297	unknown	Public Health Virology Laboratory, Forensic and Scientific Services (PHV-FSS)	Son Nguyen et al.
EPI_ISL_693667, EPI_ISL_693671, EPI_ISL_693676, EPI_ISL_693680	The National Institute of Public Health	State Veterinary Institute Prague	Nagy,A;Jirincova,H;Trnka,D;Vecerova,J
EPI_ISL_693768	General practitioner	National Reference Center for Viruses of Respiratory Infections, Institut Pasteur, Paris	Marion Barbet, Sylvie Behillil, Méline Bizard, Angela Brisebarre, Camille Capel, Etienne Simon-Lorière, Vincent Enouf, Maud Vanpeene, Sylvie van der Werf
EPI_ISL_693844, EPI_ISL_693845, EPI_ISL_693846, EPI_ISL_693847, EPI_ISL_693848, EPI_ISL_693849, EPI_ISL_693850, EPI_ISL_693851, EPI_ISL_693852, EPI_ISL_693853, EPI_ISL_693854, EPI_ISL_693855, EPI_ISL_693856, EPI_ISL_693857, EPI_ISL_693858, EPI_ISL_693859			
see above	Viollier AG	Department of Biosystems Science and Engineering, ETH Zürich	Christian Beisel, Sarah Nadeau, Chaoran Chen, Ivan Topolsky, Pedro Ferreira, Philipp Jablonski, Susana Posada-Céspedes, Tobias Schär, Ina Nissen, Natascha Santacroce, Elodie Burcklen, Christiane Beckmann, Maurice Redondo, Olivier Kobel, Christoph Noppen, Sophie Seidel, Noemie Santamaria de Souza, Niko Beerenwinkel, Tanja Stadler
EPI_ISL_700428	Thembalethu CDC wc THC	NHLS/UCT	Houriiyah Tegally, Arash Iranzadeh, Deelan Doolabh, Lynn Tyers, Bruna Galvao, Innocent Mudau, Marvin Hsiao, Kruger Marais, Diana Hardie, Stephen Korsman, Carolyn Williamson
EPI_ISL_702820	Northumbria University / South Tees Hospitals NHS Foundation Trust / North Cumbria Integrated Care NHS Foundation Trust / North Tees and Hartlepool NHS Foundation Trust / Newcastle Hospitals NHS Foundation Trust	COVID-19 Genomics UK (COG-UK) Consortium	Darren L Smith,Andrew Nelson,Matthew Bashton,Greg R Young,Joshua Loh,John Allan,Mohammad A Tariq,Giles S Holt,Gary Black,Wen C Yew,Lynn Dover,Paul Baker,Steve Liggett,Sarah Essex,Jane Greenaway,Debra Padgett,Clive Graham,Garren Scott,Edward Barton,Emma Swindells,Brendan Payne,Jennifer Collins,Yusri Taha,Gary Eltringham
EPI_ISL_702896	Wales Specialist Virology Centre Sequencing lab: Pathogen Genomics Unit	COVID-19 Genomics UK (COG-UK) Consortium	Catherine Moore, Johnathan Evans, Laura Gifford, Malorie Perry, Simon Cottrell, Angela Marchbank, Alec Birchley, Alexander Adams, Amy Gaskin, Bree Gatica-Wilcox, Jason Coombes, Joel Southgate, Lauren Gilbert, Lee Graham, Nicole Pacchiarini, Sara Kumziene-Summerhayes, Sarah Taylor, Sophie Jones, Sara Rey, Matthew Bull, Joanne Watkins, Sally Corden, Tom Connor
EPI_ISL_703002	Northumbria University / South Tees Hospitals NHS Foundation Trust / North Cumbria Integrated Care NHS Foundation Trust / North Tees and Hartlepool NHS Foundation Trust / Newcastle Hospitals NHS Foundation Trust	COVID-19 Genomics UK (COG-UK) Consortium	Darren L Smith,Andrew Nelson,Matthew Bashton,Greg R Young,Joshua Loh,John Allan,Mohammad A Tariq,Giles S Holt,Gary Black,Wen C Yew,Lynn Dover,Paul Baker,Steve Liggett,Sarah Essex,Jane Greenaway,Debra Padgett,Clive Graham,Garren Scott,Edward Barton,Emma Swindells,Brendan Payne,Jennifer Collins,Yusri Taha,Gary Eltringham
EPI_ISL_703642	Wales Specialist Virology Centre Sequencing lab: Pathogen Genomics Unit	COVID-19 Genomics UK (COG-UK) Consortium	Catherine Moore, Johnathan Evans, Laura Gifford, Malorie Perry, Simon Cottrell, Angela Marchbank, Alec Birchley, Alexander Adams, Amy Gaskin, Bree Gatica-Wilcox, Jason Coombes, Joel Southgate, Lauren Gilbert, Lee Graham, Nicole Pacchiarini, Sara Kumziene-Summerhayes, Sarah Taylor, Sophie Jones, Sara Rey, Matthew Bull, Joanne Watkins, Sally Corden, Tom Connor
EPI_ISL_703863	Virology Department, Royal Infirmary of Edinburgh, NHS Lothian / School of Biological Sciences, University of Edinburgh / Institute of Genetics and Molecular Medicine, University of Edinburgh	COVID-19 Genomics UK (COG-UK) Consortium	McHugh M, Dewar R, Rooke S, Gallagher M, Balcaza C, O'Toole Á, Scher E, Hill V, McCrone JT, Colquhoun R, Yu X, Jackson B, Rambaut A, Williams TC, Templeton K
EPI_ISL_704017	Wales Specialist Virology Centre Sequencing lab: Pathogen Genomics Unit	COVID-19 Genomics UK (COG-UK) Consortium	Catherine Moore, Johnathan Evans, Laura Gifford, Malorie Perry, Simon Cottrell, Angela Marchbank, Alec Birchley, Alexander Adams, Amy Gaskin, Bree Gatica-Wilcox, Jason Coombes, Joel Southgate, Lauren Gilbert, Lee Graham, Nicole Pacchiarini, Sara Kumziene-Summerhayes, Sarah Taylor, Sophie Jones, Sara Rey, Matthew Bull, Joanne Watkins, Sally Corden, Tom Connor
EPI_ISL_704089	West of Scotland Specialist Virology Centre, NHSGGC / MRC-University of Glasgow Centre for Virus Research	COVID-19 Genomics UK (COG-UK) Consortium	Ana da Silva Filipe, Natasha Johnson, Kathy Smollett, Daniel Mair, Stephen Carmichael, Alice Broos, Lily Tong, Jenna Nichols, Kyriaki Nomikou; Sarah McDonald; Richard Orton, Joseph Hughes, Sreenu Vattipally, David L Robertson; Alasdair MacLean, Rory Gunson; Sharif Shaaban, Matthew Holden; Rachel Blacow, Guy Mollett, Kathy Li, James Shepherd, Antonia Ho, Emma Thomson
EPI_ISL_704166, EPI_ISL_704579	Northumbria University / South Tees Hospitals NHS Foundation Trust / North Cumbria Integrated Care NHS Foundation Trust / North Tees and Hartlepool NHS Foundation Trust / Newcastle Hospitals NHS Foundation Trust	COVID-19 Genomics UK (COG-UK) Consortium	Darren L Smith,Andrew Nelson,Matthew Bashton,Greg R Young,Joshua Loh,John Allan,Mohammad A Tariq,Giles S Holt,Gary Black,Wen C Yew,Lynn Dover,Paul Baker,Steve Liggett,Sarah Essex,Jane Greenaway,Debra Padgett,Clive Graham,Garren Scott,Edward Barton,Emma Swindells,Brendan Payne,Jennifer Collins,Yusri Taha,Gary Eltringham

EPI_ISL_704682, EPI_ISL_704685, EPI_ISL_704687, EPI_ISL_704690, EPI_ISL_704698, EPI_ISL_704701, EPI_ISL_704703, EPI_ISL_704817, EPI_ISL_704839, EPI_ISL_704861, EPI_ISL_704864, EPI_ISL_704872, EPI_ISL_704874, EPI_ISL_704877, EPI_ISL_704882			
see above	Oxford Viromics, NDM, University of Oxford; Oxford University Hospitals; Basingstoke and North Hampshire Hospital	COVID-19 Genomics UK (COG-UK) Consortium	Tanya Golubchik, David Bonsall, George Macintyre, Amy Trebes, Mariateresa de Cesare, Catrin Moore, Alex Mobbs, Anita Justice, Robert Shaw, Monique Andersson, Timothy Peto, Emma Wise, Nathan Moore, Jessica Lynch, Nick Cortes, Matilde Mori, Stephen Kidd, David Buck, John Todd, Christophe Fraser
EPI_ISL_704952	West of Scotland Specialist Virology Centre, NHSGGC / MRC-University of Glasgow Centre for Virus Research	COVID-19 Genomics UK (COG-UK) Consortium	Ana da Silva Filipe, Natasha Johnson, Kathy Smollett, Daniel Mair, Stephen Carmichael, Alice Broos, Lily Tong, Jenna Nichols, Kyriaki Nomikou; Sarah McDonald; Richard Orton, Joseph Hughes, Sreenu Vattipally, David L Robertson; Alasdair MacLean, Rory Gunson; Sharif Shaaban, Matthew Holden; Rachel Blacow, Guy Mollett, Kathy Li, James Shepherd, Antonia Ho, Emma Thomson
EPI_ISL_705105, EPI_ISL_705219	Northumbria University / South Tees Hospitals NHS Foundation Trust / North Cumbria Integrated Care NHS Foundation Trust / North Tees and Hartlepool NHS Foundation Trust / Newcastle Hospitals NHS Foundation Trust	COVID-19 Genomics UK (COG-UK) Consortium	Darren L Smith,Andrew Nelson,Matthew Bashton,Greg R Young,Joshua Loh,John Allan,Mohammad A Tariq,Giles S Holt,Gary Black,Wen C Yew,Lynn Dover,Paul Baker,Steve Liggett,Sarah Essex,Jane Greenaway,Debra Padgett,Clive Graham,Garren Scott,Edward Barton,Emma Swindells,Brendan Payne,Jennifer Collins,Yusri Taha,Gary Eltringham
EPI_ISL_705265	Wales Specialist Virology Centre Sequencing lab: Pathogen Genomics Unit	COVID-19 Genomics UK (COG-UK) Consortium	Catherine Moore, Johnathan Evans, Laura Gifford, Malorie Perry, Simon Cottrell, Angela Marchbank, Alec Birchley, Alexander Adams, Amy Gaskin, Bree Gatica-Wilcox, Jason Coombes, Joel Southgate, Lauren Gilbert, Lee Graham, Nicole Pacchiarini, Sara Kumziene-Summerhayes, Sarah Taylor, Sophie Jones, Sara Rey, Matthew Bull, Joanne Watkins, Sally Corden, Tom Connor
EPI_ISL_705293	Northumbria University / South Tees Hospitals NHS Foundation Trust / North Cumbria Integrated Care NHS Foundation Trust / North Tees and Hartlepool NHS Foundation Trust / Newcastle Hospitals NHS Foundation Trust	COVID-19 Genomics UK (COG-UK) Consortium	Darren L Smith,Andrew Nelson,Matthew Bashton,Greg R Young,Joshua Loh,John Allan,Mohammad A Tariq,Giles S Holt,Gary Black,Wen C Yew,Lynn Dover,Paul Baker,Steve Liggett,Sarah Essex,Jane Greenaway,Debra Padgett,Clive Graham,Garren Scott,Edward Barton,Emma Swindells,Brendan Payne,Jennifer Collins,Yusri Taha,Gary Eltringham
EPI_ISL_705316	West of Scotland Specialist Virology Centre, NHSGGC / MRC-University of Glasgow Centre for Virus Research	COVID-19 Genomics UK (COG-UK) Consortium	Ana da Silva Filipe, Natasha Johnson, Kathy Smollett, Daniel Mair, Stephen Carmichael, Alice Broos, Lily Tong, Jenna Nichols, Kyriaki Nomikou; Sarah McDonald; Richard Orton, Joseph Hughes, Sreenu Vattipally, David L Robertson; Alasdair MacLean, Rory Gunson; Sharif Shaaban, Matthew Holden; Rachel Blacow, Guy Mollett, Kathy Li, James Shepherd, Antonia Ho, Emma Thomson
EPI_ISL_705390	Wales Specialist Virology Centre Sequencing lab: Pathogen Genomics Unit	COVID-19 Genomics UK (COG-UK) Consortium	Catherine Moore, Johnathan Evans, Laura Gifford, Malorie Perry, Simon Cottrell, Angela Marchbank, Alec Birchley, Alexander Adams, Amy Gaskin, Bree Gatica-Wilcox, Jason Coombes, Joel Southgate, Lauren Gilbert, Lee Graham, Nicole Pacchiarini, Sara Kumziene-Summerhayes, Sarah Taylor, Sophie Jones, Sara Rey, Matthew Bull, Joanne Watkins, Sally Corden, Tom Connor
EPI_ISL_705426, EPI_ISL_705427, EPI_ISL_705428, EPI_ISL_705450, EPI_ISL_705451, EPI_ISL_705452, EPI_ISL_705455	Oxford Viromics, NDM, University of Oxford; Oxford University Hospitals; Basingstoke and North Hampshire Hospital	COVID-19 Genomics UK (COG-UK) Consortium	Tanya Golubchik, David Bonsall, George Macintyre, Amy Trebes, Mariateresa de Cesare, Catrin Moore, Alex Mobbs, Anita Justice, Robert Shaw, Monique Andersson, Timothy Peto, Emma Wise, Nathan Moore, Jessica Lynch, Nick Cortes, Matilde Mori, Stephen Kidd, David Buck, John Todd, Christophe Fraser
EPI_ISL_705462	Wales Specialist Virology Centre Sequencing lab: Pathogen Genomics Unit	COVID-19 Genomics UK (COG-UK) Consortium	Catherine Moore, Johnathan Evans, Laura Gifford, Malorie Perry, Simon Cottrell, Angela Marchbank, Alec Birchley, Alexander Adams, Amy Gaskin, Bree Gatica-Wilcox, Jason Coombes, Joel Southgate, Lauren Gilbert, Lee Graham, Nicole Pacchiarini, Sara Kumziene-Summerhayes, Sarah Taylor, Sophie Jones, Sara Rey, Matthew Bull, Joanne Watkins, Sally Corden, Tom Connor
EPI_ISL_705469, EPI_ISL_705475, EPI_ISL_705477, EPI_ISL_705478, EPI_ISL_705479, EPI_ISL_705480, EPI_ISL_705482, EPI_ISL_705483, EPI_ISL_705484, EPI_ISL_705485, EPI_ISL_705493, EPI_ISL_705526			
see above	Oxford Viromics, NDM, University of Oxford; Oxford University Hospitals; Basingstoke and North Hampshire Hospital	COVID-19 Genomics UK (COG-UK) Consortium	Tanya Golubchik, David Bonsall, George Macintyre, Amy Trebes, Mariateresa de Cesare, Catrin Moore, Alex Mobbs, Anita Justice, Robert Shaw, Monique Andersson, Timothy Peto, Emma Wise, Nathan Moore, Jessica Lynch, Nick Cortes, Matilde Mori, Stephen Kidd, David Buck, John Todd, Christophe Fraser
EPI_ISL_705771, EPI_ISL_705798	Virology Department, Royal Infirmary of Edinburgh, NHS Lothian / School of Biological Sciences, University of Edinburgh / Institute of Genetics and Molecular Medicine, University of Edinburgh	COVID-19 Genomics UK (COG-UK) Consortium	McHugh M, Dewar R, Rooke S, Gallagher M, Balcaza C, O'Toole Á, Scher E, Hill V, McCrone JT, Colquhoun R, Yu X, Jackson B, Rambaut A, Williams TC, Templeton K
EPI_ISL_706013, EPI_ISL_706014, EPI_ISL_706015, EPI_ISL_706016, EPI_ISL_706017, EPI_ISL_706018, EPI_ISL_706019, EPI_ISL_706020, EPI_ISL_706021, EPI_ISL_706022, EPI_ISL_706023, EPI_ISL_706024, EPI_ISL_706028, EPI_ISL_706029, EPI_ISL_706030, EPI_ISL_706031, EPI_ISL_706035, EPI_ISL_706036, EPI_ISL_706039, EPI_ISL_706040, EPI_ISL_706041, EPI_ISL_706042, EPI_ISL_706043, EPI_ISL_706044, EPI_ISL_706045, EPI_ISL_706046, EPI_ISL_706047, EPI_ISL_706048, EPI_ISL_706049, EPI_ISL_706050, EPI_ISL_706051, EPI_ISL_706052, EPI_ISL_706053, EPI_ISL_706054, EPI_ISL_706055, EPI_ISL_706056, EPI_ISL_706057, EPI_ISL_706061, EPI_ISL_706062, EPI_ISL_706064, EPI_ISL_706067, EPI_ISL_706071, EPI_ISL_706074, EPI_ISL_706075, EPI_ISL_706076, EPI_ISL_706077, EPI_ISL_706078, EPI_ISL_706079, EPI_ISL_706080, EPI_ISL_706082, EPI_ISL_706083, EPI_ISL_706084, EPI_ISL_706086, EPI_ISL_706092, EPI_ISL_706094, EPI_ISL_706099, EPI_ISL_706100, EPI_ISL_706103, EPI_ISL_706107, EPI_ISL_706108, EPI_ISL_706109, EPI_ISL_706111, EPI_ISL_706112, EPI_ISL_706113, EPI_ISL_706114, EPI_ISL_706115, EPI_ISL_706116, EPI_ISL_706117, EPI_ISL_706118, EPI_ISL_706119, EPI_ISL_706120, EPI_ISL_706121, EPI_ISL_706122, EPI_ISL_706123, EPI_ISL_706124, EPI_ISL_706125, EPI_ISL_706126, EPI_ISL_706127, EPI_ISL_706129, EPI_ISL_706130, EPI_ISL_706131, EPI_ISL_706132, EPI_ISL_706133, EPI_ISL_706135, EPI_ISL_706137, EPI_ISL_706138, EPI_ISL_706139, EPI_ISL_706140, EPI_ISL_706141, EPI_ISL_706142, EPI_ISL_706143, EPI_ISL_706146, EPI_ISL_706148, EPI_ISL_706151, EPI_ISL_706152, EPI_ISL_706153, EPI_ISL_706154, EPI_ISL_706155, EPI_ISL_706157, EPI_ISL_706159, EPI_ISL_706161, EPI_ISL_706162, EPI_ISL_706164, EPI_ISL_706165, EPI_ISL_706167, EPI_ISL_706171, EPI_ISL_706172, EPI_ISL_706173, EPI_ISL_706174, EPI_ISL_706175, EPI_ISL_706176, EPI_ISL_706177, EPI_ISL_706178, EPI_ISL_706179, EPI_ISL_706180, EPI_ISL_706181, EPI_ISL_706182, EPI_ISL_706183, EPI_ISL_706184, EPI_ISL_706186, EPI_ISL_706187, EPI_ISL_706188, EPI_ISL_706189, EPI_ISL_706190, EPI_ISL_706191, EPI_ISL_706192, EPI_ISL_706193, EPI_ISL_706195, EPI_ISL_706196, EPI_ISL_706197, EPI_ISL_706198, EPI_ISL_706199, EPI_ISL_706200, EPI_ISL_706215, EPI_ISL_706217, EPI_ISL_706219, EPI_ISL_706220, EPI_ISL_706221, EPI_ISL_706224, EPI_ISL_706225, EPI_ISL_706226, EPI_ISL_706227, EPI_ISL_706228, EPI_ISL_706231, EPI_ISL_706232, EPI_ISL_706233, EPI_ISL_706234, EPI_ISL_706235, EPI_ISL_706236, EPI_ISL_706237, EPI_ISL_706238, EPI_ISL_706242, EPI_ISL_706245, EPI_ISL_706246, EPI_ISL_706248, EPI_ISL_706249, EPI_ISL_706250, EPI_ISL_706251, EPI_ISL_706252, EPI_ISL_706253, EPI_ISL_706254, EPI_ISL_706255, EPI_ISL_706256, EPI_ISL_706257, EPI_ISL_706258, EPI_ISL_706259, EPI_ISL_706260, EPI_ISL_706261, EPI_ISL_706262, EPI_ISL_706263, EPI_ISL_706264, EPI_ISL_706265, EPI_ISL_706266, EPI_ISL_706267, EPI_ISL_706268			
see above	Oxford Viromics, NDM, University of Oxford; Oxford University Hospitals; Basingstoke and North Hampshire Hospital	COVID-19 Genomics UK (COG-UK) Consortium	Tanya Golubchik, David Bonsall, George Macintyre, Amy Trebes, Mariateresa de Cesare, Catrin Moore, Alex Mobbs, Anita Justice, Robert Shaw, Monique Andersson, Timothy Peto, Emma Wise, Nathan Moore, Jessica Lynch, Nick Cortes, Matilde Mori, Stephen Kidd, David Buck, John Todd, Christophe Fraser
EPI_ISL_706276, EPI_ISL_706353	Northumbria University / South Tees Hospitals NHS Foundation Trust / North Cumbria Integrated Care NHS Foundation Trust / North Tees and Hartlepool NHS Foundation Trust / Newcastle Hospitals NHS Foundation Trust	COVID-19 Genomics UK (COG-UK) Consortium	Darren L Smith,Andrew Nelson,Matthew Bashton,Greg R Young,Joshua Loh,John Allan,Mohammad A Tariq,Giles S Holt,Gary Black,Wen C Yew,Lynn Dover,Paul Baker,Steve Liggett,Sarah Essex,Jane Greenaway,Debra Padgett,Clive Graham,Garren Scott,Edward Barton,Emma Swindells,Brendan Payne,Jennifer Collins,Yusri Taha,Gary Eltringham
EPI_ISL_706473, EPI_ISL_706474, EPI_ISL_706475, EPI_ISL_706476, EPI_ISL_706477, EPI_ISL_706478, EPI_ISL_706479, EPI_ISL_706480, EPI_ISL_706481, EPI_ISL_706482	Wales Specialist Virology Centre Sequencing lab: Pathogen Genomics Unit	COVID-19 Genomics UK (COG-UK) Consortium	Catherine Moore, Johnathan Evans, Laura Gifford, Malorie Perry, Simon Cottrell, Angela Marchbank, Alec Birchley, Alexander Adams, Amy Gaskin, Bree Gatica-Wilcox, Jason Coombes, Joel Southgate, Lauren Gilbert, Lee Graham, Nicole Pacchiarini, Sara Kumziene-Summerhayes, Sarah Taylor, Sophie Jones, Sara Rey, Matthew Bull, Joanne Watkins, Sally Corden, Tom Connor
EPI_ISL_708039	Innlandet Hospital Trust, Division Lillehammer, Department for Medical Microbiology	Norwegian Institute of Public Health, Department of Virology	Kathrine Stene-Johansen, Kamilla Heddeland Instefjord, Hilde Elshaug, Marie Paulsen Madsen, Rasmus Riis Kopperud, Hilde Vollan, Karoline Bragstad, Olav Hungnes
EPI_ISL_708050	Norwegian Institute of Public Health, Department of Virology	Norwegian Institute of Public Health, Department of Virology	Kathrine Stene-Johansen, Kamilla Heddeland Instefjord, Hilde Elshaug, Marie Paulsen Madsen, Rasmus Riis Kopperud, Hilde Vollan, Karoline Bragstad, Olav Hungnes
EPI_ISL_708055, EPI_ISL_708056	Hospital of Southern Norway - Kristiansand, Department of Medical Microbiology	Norwegian Institute of Public Health, Department of Virology	Kathrine Stene-Johansen, Kamilla Heddeland Instefjord, Hilde Elshaug, Marie Paulsen Madsen, Rasmus Riis Kopperud, Hilde Vollan, Karoline Bragstad, Olav Hungnes
EPI_ISL_708093	University Hospital of Northern Norway, Department for Microbiology and Infectious Disease Control	Norwegian Institute of Public Health, Department of Virology	Kathrine Stene-Johansen, Kamilla Heddeland Instefjord, Hilde Elshaug, Marie Paulsen Madsen, Rasmus Riis Kopperud, Hilde Vollan, Karoline Bragstad, Olav Hungnes
EPI_ISL_708102, EPI_ISL_708103	Nordland Hospital - Bodo, Laboratory Department, Molecular Biology Unit	Norwegian Institute of Public Health, Department of Virology	Kathrine Stene-Johansen, Kamilla Heddeland Instefjord, Hilde Elshaug, Marie Paulsen Madsen, Rasmus Riis Kopperud, Hilde Vollan, Karoline Bragstad, Olav Hungnes
EPI_ISL_708141	Norwegian Institute of Public Health, Department of Virology	Norwegian Institute of Public Health, Department of Virology	Kathrine Stene-Johansen, Kamilla Heddeland Instefjord, Hilde Elshaug, Marie Paulsen Madsen, Rasmus Riis Kopperud, Hilde Vollan, Karoline Bragstad, Olav Hungnes
EPI_ISL_708243	Michigan Department of Health and Human Services, Bureau of Laboratories	Michigan Department of Health and Human Services, Bureau of Laboratories	Blankenship HM, Riner D, Soehnlen MK
EPI_ISL_708315	University Hospitals of Geneva, Laboratory of Virology	University Hospitals of Geneva, Laboratory of Virology	Cordey Samuel, Laubscher Florian
EPI_ISL_708341, EPI_ISL_708367	Michigan Department of Health and Human Services, Bureau of Laboratories	Michigan Department of Health and Human Services, Bureau of Laboratories	Blankenship HM, Riner D, Soehnlen MK

EPI_ISL_708379, EPI_ISL_708380	University Hospitals of Geneva, Laboratory of Virology	University Hospitals of Geneva, Laboratory of Virology	Cordey Samuel, Laubscher Florian
EPI_ISL_708450, EPI_ISL_708452	Delaware Public Health Lab	Delaware Public Health Lab	Gregory Hovan
EPI_ISL_708460, EPI_ISL_708461	Minnesota Department of Health, Public Health Laboratory	Minnesota Department of Health, Public Health Laboratory	Alexandra Lorentz, Jacob Garfin, Matt Plumb, and Xiong Wang
EPI_ISL_708550, EPI_ISL_708552, EPI_ISL_708582	Michigan Department of Health and Human Services, Bureau of Laboratories	Michigan Department of Health and Human Services, Bureau of Laboratories	Blankenship HM, Riner D, Soehnlen MK
EPI_ISL_708747, EPI_ISL_708774	PathWest Laboratory Medicine WA	PathWest Laboratory Medicine WA Microbial Surveillance Unit	PathWest Laboratory Medicine WA Microbial Surveillance Unit
EPI_ISL_708819	Urban Institute for Disease Prevention and Control	National Institute of Health, Department of Medical Sciences, Ministry of Public Health, Thailand	Pilailuk Okada; Siripaporn Phuygun; Thanutsapa Thanadachakul; Sittiporn Parnmen; Pakorn Piromtong; Warawan Wongboot; Sunthareeya Waicharoen; Malinee Chittaganpitch
EPI_ISL_708823	Vibharam Hospital	National Institute of Health, Department of Medical Sciences, Ministry of Public Health, Thailand	Pilailuk Okada; Siripaporn Phuygun; Thanutsapa Thanadachakul; Sittiporn Parnmen; Pakorn Piromtong; Warawan Wongboot; Sunthareeya Waicharoen; Malinee Chittaganpitch
EPI_ISL_709903, EPI_ISL_709904, EPI_ISL_709905	Lighthouse Lab in Milton Keynes	Wellcome Sanger Institute for the COVID-19 Genomics UK (COG-UK) Consortium	The Lighthouse Lab in Milton Keynes and Alex Alderton, Roberto Amato, Sonia Goncalves, Ewan Harrison, David K. Jackson, Ian Johnston, Dominic Kwiatkowski, Cordelia Langford, John Sillitoe on behalf of the Wellcome Sanger Institute COVID-19 Surveillance Team
EPI_ISL_710039	University of Michigan Clinical Microbiology Laboratory	Lauring Lab, University of Michigan, Department of Microbiology and Immunology	Valesano
EPI_ISL_710157, EPI_ISL_710241, EPI_ISL_710242, EPI_ISL_710243, EPI_ISL_710244, EPI_ISL_710246, EPI_ISL_710369, EPI_ISL_710370, EPI_ISL_710371, EPI_ISL_710372, EPI_ISL_710373			
see above	Colorado Department of Public Health and Environment	Colorado Department of Puplic Health and Environment	Laura Bankers, Molly C. Hetherington-Rauth, Shannon Ely, Shannon R. Matzinger, Sarah Elizabeth Totten, Emily A. Travanty
EPI_ISL_710596, EPI_ISL_710598, EPI_ISL_710599, EPI_ISL_710600	Klinisk mikrobiologi	The Public Health Agency of Sweden	Department of Microbiology, The Public Health Agency of Sweden
EPI_ISL_710907	Lighthouse Lab in Milton Keynes	Wellcome Sanger Institute for the COVID-19 Genomics UK (COG-UK) Consortium	The Lighthouse Lab in Milton Keynes and Alex Alderton, Roberto Amato, Sonia Goncalves, Ewan Harrison, David K. Jackson, Ian Johnston, Dominic Kwiatkowski, Cordelia Langford, John Sillitoe on behalf of the Wellcome Sanger Institute COVID-19 Surveillance Team
EPI_ISL_710908, EPI_ISL_710909, EPI_ISL_710910, EPI_ISL_710911, EPI_ISL_710912, EPI_ISL_710913, EPI_ISL_710914, EPI_ISL_710915, EPI_ISL_710917, EPI_ISL_710918, EPI_ISL_710919, EPI_ISL_710920, EPI_ISL_710921, EPI_ISL_710922, EPI_ISL_710923, EPI_ISL_710925, EPI_ISL_710926, EPI_ISL_710927, EPI_ISL_710928, EPI_ISL_710929, EPI_ISL_710931, EPI_ISL_710932, EPI_ISL_710933, EPI_ISL_710934, EPI_ISL_710935, EPI_ISL_710936, EPI_ISL_710937, EPI_ISL_710938, EPI_ISL_710939, EPI_ISL_710940, EPI_ISL_710941, EPI_ISL_710942, EPI_ISL_710943, EPI_ISL_710944, EPI_ISL_710945, EPI_ISL_710946, EPI_ISL_710947, EPI_ISL_710948, EPI_ISL_710949, EPI_ISL_710950, EPI_ISL_710951, EPI_ISL_710952, EPI_ISL_710953, EPI_ISL_710954, EPI_ISL_710955, EPI_ISL_710956, EPI_ISL_710957, EPI_ISL_710958, EPI_ISL_710959, EPI_ISL_710960, EPI_ISL_710961, EPI_ISL_710962, EPI_ISL_710963, EPI_ISL_710964, EPI_ISL_710965, EPI_ISL_710966, EPI_ISL_710967, EPI_ISL_710968, EPI_ISL_710969, EPI_ISL_710970, EPI_ISL_710971, EPI_ISL_710972, EPI_ISL_710973, EPI_ISL_710974, EPI_ISL_710975, EPI_ISL_710976, EPI_ISL_710977, EPI_ISL_710978, EPI_ISL_710979, EPI_ISL_710980, EPI_ISL_710981, EPI_ISL_710982, EPI_ISL_710983, EPI_ISL_710984, EPI_ISL_710985, EPI_ISL_710986, EPI_ISL_710987, EPI_ISL_710988, EPI_ISL_710989, EPI_ISL_710990, EPI_ISL_710991, EPI_ISL_710992, EPI_ISL_710993, EPI_ISL_710994			
see above	Lighthouse Lab in Alderley Park	Wellcome Sanger Institute for the COVID-19 Genomics UK (COG-UK) Consortium	Jacquelyn Wynn, Mairead Hyland, The Lighthouse Lab in Alderley Park and Alex Alderton, Roberto Amato, Sonia Goncalves, Ewan Harrison, David K. Jackson, Ian Johnston, Dominic Kwiatkowski, Cordelia Langford, John Sillitoe on behalf of the Wellcome Sanger Institute COVID-19 Surveillance Team
EPI_ISL_711014	Lighthouse Lab in Glasgow	Wellcome Sanger Institute for the COVID-19 Genomics UK (COG-UK) Consortium	Harper VanSteenhouse, Yumi Kasai, David Gray, Carol Clugston, Anna Dominiczak and Alex Alderton, Roberto Amato, Sonia Goncalves, Ewan Harrison, David K. Jackson, Ian Johnston, Dominic Kwiatkowski, Cordelia Langford, John Sillitoe on behalf of the Wellcome Sanger Institute COVID-19 Surveillance Team
EPI_ISL_711025, EPI_ISL_711026	Lighthouse Lab in Cambridge	Wellcome Sanger Institute for the COVID-19 Genomics UK (COG-UK) Consortium	Rob Howes, The Lighthouse Lab in Cambridge and Alex Alderton, Roberto Amato, Sonia Goncalves, Ewan Harrison, David K. Jackson, Ian Johnston, Dominic Kwiatkowski, Cordelia Langford, John Sillitoe on behalf of the Wellcome Sanger Institute COVID-19 Surveillance Team
EPI_ISL_711027, EPI_ISL_711028, EPI_ISL_711029, EPI_ISL_711030, EPI_ISL_711031, EPI_ISL_711032, EPI_ISL_711033, EPI_ISL_711034, EPI_ISL_711035, EPI_ISL_711036, EPI_ISL_711037			
see above	Lighthouse Lab in Alderley Park	Wellcome Sanger Institute for the COVID-19 Genomics UK (COG-UK) Consortium	Jacquelyn Wynn, Mairead Hyland, The Lighthouse Lab in Alderley Park and Alex Alderton, Roberto Amato, Sonia Goncalves, Ewan Harrison, David K. Jackson, Ian Johnston, Dominic Kwiatkowski, Cordelia Langford, John Sillitoe on behalf of the Wellcome Sanger Institute COVID-19 Surveillance Team
EPI_ISL_711038	Lighthouse Lab in Cambridge	Wellcome Sanger Institute for the COVID-19 Genomics UK (COG-UK) Consortium	Rob Howes, The Lighthouse Lab in Cambridge and Alex Alderton, Roberto Amato, Sonia Goncalves, Ewan Harrison, David K. Jackson, Ian Johnston, Dominic Kwiatkowski, Cordelia Langford, John Sillitoe on behalf of the Wellcome Sanger Institute COVID-19 Surveillance Team
EPI_ISL_711039, EPI_ISL_711040, EPI_ISL_711041, EPI_ISL_711042, EPI_ISL_711043, EPI_ISL_711044, EPI_ISL_711045, EPI_ISL_711046, EPI_ISL_711047	Lighthouse Lab in Alderley Park	Wellcome Sanger Institute for the COVID-19 Genomics UK (COG-UK) Consortium	Jacquelyn Wynn, Mairead Hyland, The Lighthouse Lab in Alderley Park and Alex Alderton, Roberto Amato, Sonia Goncalves, Ewan Harrison, David K. Jackson, Ian Johnston, Dominic Kwiatkowski, Cordelia Langford, John Sillitoe on behalf of the Wellcome Sanger Institute COVID-19 Surveillance Team
EPI_ISL_712084, EPI_ISL_712085, EPI_ISL_712090	Port Elizabeth Provincial Hospital, National Health Laboratory Services, Eastern Cape, South Africa	National Institute for Communicable Diseases of the National Health Laboratory Service	Mohale T, Ntuli N, Mahlangu B, Allam M, Ismail A, Bhiman JN
EPI_ISL_717831, EPI_ISL_717889, EPI_ISL_717940, EPI_ISL_717941	Laboratorio de Virologia Molecular / UFRJ	Bioinformatics Laboratory / LNCC	Carolina M Voloch, Ronaldo da Silva F Jr, Luiz G P de Almeida, Cynthia C Cardoso, Otavio Bustrolini, Alexandra L Gerber, Ana Paula de C Guimarães, Diana Mariani, Andréa Cony Cavalcanti, Claudia dos Santos Rodrigues, Terezinha M P P Castiñeira, Amilcar Tanuri, Ana Tereza R de Vasconcelos
EPI_ISL_717994	Lab voor klinische biologie	Onderzoeksgroep Virologie	Laurens Lambrechts, Nick Vereecke, Marthe Pauwels, Bruno Verhasselt, Linos Vandekerckhove, Hans Nauwynck, Sebastiaan Theuns
EPI_ISL_718060, EPI_ISL_718061, EPI_ISL_718062, EPI_ISL_718063, EPI_ISL_718064, EPI_ISL_718065, EPI_ISL_718066, EPI_ISL_718067, EPI_ISL_718068, EPI_ISL_718069, EPI_ISL_718070, EPI_ISL_718071, EPI_ISL_718072, EPI_ISL_718073, EPI_ISL_718074, EPI_ISL_718075, EPI_ISL_718076, EPI_ISL_718077, EPI_ISL_718078, EPI_ISL_718079, EPI_ISL_718080, EPI_ISL_718081, EPI_ISL_718082, EPI_ISL_718083			
see above	ZOTZ KLIMAS MVZ Düsseldorf-Centrum GbR ÜBAG für Labormedizin, Genetik, Zytologie, Pathologie	Center of Medical Microbiology, Virology, and Hospital Hygiene, University of Duesseldorf	Maximilian Damagnez, Alexander Dilthey, Ashley-Jane Duplessis, Patrick Finzer, Katrin Hoffmann, Torsten Houwaart, Lisanna Hülse, Malte Kohns Vasconcelos, Marek Korencak, Nadine Lübke, Jessica Nicolai, Klaus Pfeffer, Daniel Strelow, Jörg Timm, Andreas Walker, Tobias Wienemann, Rainer Zotz
EPI_ISL_718167	Borneo Medical Centre	Institute of Health and Community Medicine	David Perera, Ooi Mong How, Chua Hock Hin, Tonnii Sia Loong Loong, Wong Jyn Shan, Wong Kiing Aik, Chan Chia Jui
EPI_ISL_718171, EPI_ISL_718177	Ministry of Health Hospitals	Institute of Health and Community Medicine	David Perera, Ooi Mong How, Chua Hock Hin, Tonnii Sia Loong Loong, Wong Jyn Shan, Wong Kiing Aik, Chan Chia Jui
EPI_ISL_718203, EPI_ISL_718205	Borneo Medical Centre	Institute of Health and Community Medicine	David Perera, Ooi Mong How, Chua Hock Hin, Tonnii Sia Loong Loong, Wong Jyn Shan, Wong Kiing Aik, Chan Chia Jui
EPI_ISL_718208, EPI_ISL_718209	Ministry of Health Hospitals	Institute of Health and Community Medicine	David Perera, Ooi Mong How, Chua Hock Hin, Tonnii Sia Loong Loong, Wong Jyn Shan, Wong Kiing Aik, Chan Chia Jui
EPI_ISL_718226, EPI_ISL_718227, EPI_ISL_718247	Hospital	National Reference Center for Viruses of Respiratory Infections, Institut Pasteur, Paris	Marion Barbet, Sylvie Behillil, Méline Bizard, Angela Brisebarre, Camille Capel, Etienne Simon-Lorière, Vincent Enouf, Maud Vanpeene, Sylvie van der Werf, Gisèle Lagathu
EPI_ISL_718314, EPI_ISL_718315	Institute for Medical Research, Infectious Disease Research Centre, National Institutes of Health, Ministry of Health Malaysia	Institute for Medical Research, Infectious Disease Research Centre, National Institutes of Health, Ministry of Health Malaysia	Suppiah J, Kamel K, Mohd-Zawawi Z, Thayan R
EPI_ISL_721535	Lighthouse Lab in Glasgow	Wellcome Sanger Institute for the COVID-19 Genomics UK (COG-UK) Consortium	Harper VanSteenhouse, Yumi Kasai, David Gray, Carol Clugston, Anna Dominiczak and Alex Alderton, Roberto Amato, Sonia Goncalves, Ewan Harrison, David K. Jackson, Ian Johnston, Dominic Kwiatkowski, Cordelia Langford, John Sillitoe on behalf of the Wellcome Sanger Institute COVID-19 Surveillance Team
EPI_ISL_721569, EPI_ISL_721570, EPI_ISL_721571, EPI_ISL_721572, EPI_ISL_721574, EPI_ISL_721576, EPI_ISL_721577, EPI_ISL_721578, EPI_ISL_721579, EPI_ISL_721580, EPI_ISL_721581, EPI_ISL_721582, EPI_ISL_721583, EPI_ISL_721584, EPI_ISL_721585, EPI_ISL_721586, EPI_ISL_721587, EPI_ISL_721588, EPI_ISL_721589, EPI_ISL_721590, EPI_ISL_721591, EPI_ISL_721592, EPI_ISL_721593, EPI_ISL_721594, EPI_ISL_721595, EPI_ISL_721596, EPI_ISL_721597, EPI_ISL_721601, EPI_ISL_721602, EPI_ISL_721603, EPI_ISL_721604, EPI_ISL_721605, EPI_ISL_721606, EPI_ISL_721607, EPI_ISL_721608, EPI_ISL_721609, EPI_ISL_721610, EPI_ISL_721618, EPI_ISL_721619, EPI_ISL_721620, EPI_ISL_721621			
see above	Pathogen Genomics Center, National Institute of Infectious Diseases	Pathogen Genomics Center, National Institute of Infectious Diseases	Tsuyoshi Sekizuka, Kentaro Itokawa, Rina Tanaka, Masanori Hashino, Makoto Kuroda
EPI_ISL_722374, EPI_ISL_722396, EPI_ISL_722405, EPI_ISL_722412, EPI_ISL_722571, EPI_ISL_722572, EPI_ISL_722573, EPI_ISL_722574, EPI_ISL_722575, EPI_ISL_722576, EPI_ISL_722577, EPI_ISL_722578, EPI_ISL_722579, EPI_ISL_722580, EPI_ISL_722581, EPI_ISL_722582, EPI_ISL_722583, EPI_ISL_722584, EPI_ISL_722585, EPI_ISL_722586, EPI_ISL_722587, EPI_ISL_722588, EPI_ISL_722589, EPI_ISL_722590, EPI_ISL_722597, EPI_ISL_722598, EPI_ISL_722599, EPI_ISL_722600, EPI_ISL_722601, EPI_ISL_722602, EPI_ISL_722603, EPI_ISL_722604, EPI_ISL_722605, EPI_ISL_722606, EPI_ISL_722607			

see above	Dutch COVID-19 response team	Erasmus Medical Center	Bas Oude Munnink, Reina Sikkema, David Nieuwenhuijse, Irina Chestakova, Anne van der Linden, Marjan Boter, Emmanuelle Munger, Corine GeurtsvanKessel, Annemiek van der Eijk, Richard Molenkamp, Marion Koopmans, on behalf of the Dutch national COVID-19 response team.
EPI_ISL_723469	Laboratoire Biolife	Laboratoire de Biotechnologie	Mouna Ouadghiri, Tarik Aanniz, Mohammed Walid Chemao Elfihri, Mohamed Chenaoui, Hanae Dakka, Afaf Alaoui, Otmane Touzani, Bouchra Belfquih, Lahecn Belyamani, Saaid Amzazi and Azeddine Ibrahimi
EPI_ISL_723518, EPI_ISL_723519	Virginia Division of Consolidated Laboratory Services (DCLS)	Virginia Division of Consolidated Laboratory Services (DCLS)	Virginia DCLS
EPI_ISL_723521, EPI_ISL_723522, EPI_ISL_723523, EPI_ISL_723524, EPI_ISL_723527, EPI_ISL_723528, EPI_ISL_723529, EPI_ISL_723530, EPI_ISL_723531, EPI_ISL_723532, EPI_ISL_723533, EPI_ISL_723534, EPI_ISL_723535, EPI_ISL_723536, EPI_ISL_723537, EPI_ISL_723538, EPI_ISL_723540, EPI_ISL_723541, EPI_ISL_723542, EPI_ISL_723543, EPI_ISL_723544, EPI_ISL_723545, EPI_ISL_723546, EPI_ISL_723548, EPI_ISL_723549, EPI_ISL_723550, EPI_ISL_723551, EPI_ISL_723552, EPI_ISL_723553, EPI_ISL_723554, EPI_ISL_723555, EPI_ISL_723557			
see above	Centre for Enzyme Innovation, University of Portsmouth / Translational Research Laboratory, Portsmouth Hospitals NHS Trust	COVID-19 Genomics UK (COG-UK) Consortium	Angela Beckett,Yann Bourgeois,Garry Scarlett,Sharon Glaysher,Scott Elliott,Kelly Bicknell,Robert Impey,Allyson Lloyd,Sarah Wyllie,Ethan Butcher,Anoop Chauhan,Samuel Robson
EPI_ISL_724673, EPI_ISL_724705, EPI_ISL_724719, EPI_ISL_724725	University College London, Great Ormond Street Hospital for Children NHS Foundation Trust, Imperial College Healthcare NHS Trust	COVID-19 Genomics UK (COG-UK) Consortium	Sergi Castellano, Rachel Williams, Mark Kristiansen, Paola Resende Silva, Sunando Roy, Tony Brooks, Helena Tutill, Paola Niola, Patricia Dyal, Charlotte Williams, Leysa Forrest, Yasmin Panchbhaya, Jacqueline Findlay, Samuel Weeks, Julianne Brown, Kathryn Harris, Paul Randell, James Price, Alison Holmes, Judith Breuer
EPI_ISL_726017	Wales Specialist Virology Centre Sequencing lab: Pathogen Genomics Unit	COVID-19 Genomics UK (COG-UK) Consortium	Catherine Moore, Johnathan Evans, Laura Gifford, Malorie Perry, Simon Cottrell, Angela Marchbank, Alec Birchley, Alexander Adams, Amy Gaskin, Bree Gatica-Wilcox, Jason Coombes, Joel Southgate, Lauren Gilbert, Lee Graham, Nicole Pacchiarini, Sara Kumziene-Summerhayes, Sarah Taylor, Sophie Jones, Sara Rey, Matthew Bull, Joanne Watkins, Sally Corden, Tom Connor
EPI_ISL_727990, EPI_ISL_727992, EPI_ISL_727993, EPI_ISL_727994, EPI_ISL_727995	Virology Department, Sheffield Teaching Hospitals NHS Foundation Trust/Department of Infection, Immunity and Cardiovascular Disease, The Medical School, University of Sheffield	COVID-19 Genomics UK (COG-UK) Consortium	Thushan de Silva, Matthew Parker, Nikki Smith, Adri Angyal, Rebecca Brown, Luke Green, Rachel Tucker, Paul Parsons, Danielle Groves, Katie Johnson, Laura Carrilero, Alex Keeley, Dave Partridge, Matthew Wyles, Benjamin Lindsey, Mehmet Yavuz, Mohammad Raza, Cariad Evans
EPI_ISL_728034, EPI_ISL_728053, EPI_ISL_728141, EPI_ISL_728142, EPI_ISL_728143, EPI_ISL_728144, EPI_ISL_728145	University of Wisconsin-Madison AIDS Vaccine Research Laboratories	University of Wisconsin-Madison AIDS Vaccine Research Laboratories	Gage Moreno, Katarina Braun, et al. AIDS Vaccine Research Laboratories
EPI_ISL_728167	Institute for Medical Research, Infectious Disease Research Centre, National Institutes of Health, Ministry of Health Malaysia	Institute for Medical Research, Infectious Disease Research Centre, National Institutes of Health, Ministry of Health Malaysia	Suppiah J, Kamel K, Mohd-Zawawi Z, Thayan R
EPI_ISL_728219	Laboratoire Biolife	Laboratoire de Biotechnologie	Mouna Ouadghiri, Tarik Aanniz, Mohammed Walid Chemao Elfihri, Mohamed Chenaoui, Hanae Dakka, Afaf Alaoui, Otmane Touzani, Bouchra Belfquih, Lahcen Belyamani, Saaid Amzazi and Azeddine Ibrahimi
EPI_ISL_728272, EPI_ISL_728273, EPI_ISL_728274, EPI_ISL_728275, EPI_ISL_728276, EPI_ISL_728277, EPI_ISL_728289, EPI_ISL_728290, EPI_ISL_728291, EPI_ISL_728295, EPI_ISL_728297, EPI_ISL_728301, EPI_ISL_728322, EPI_ISL_728332			
see above	Laboratoire Biolife	Laboratoire de Biotechnologie	Mouna Ouadghiri, Tarik Aanniz, Mohammed Walid Chemao Elfihri, Mohamed Chenaoui, Hanae Dakka, Afaf Alaoui, Otmane Touzani, Bouchra Belfquih, Lahcen belyamani, Saaid Amzazi and Azeddine Ibrahimi
EPI_ISL_728556	University of Wisconsin-Madison AIDS Vaccine Research Laboratories	University of Wisconsin-Madison AIDS Vaccine Research Laboratories	Gage Moreno, Katarina Braun, et al. AIDS Vaccine Research Laboratories
EPI_ISL_728726	Dutch COVID-19 response team	National Institute for Public Health and the Environment (RIVM)	Adam Meijer, Harry Vennema, Jeroen Cremer, Sharon van den Brink, Bas van der Veer, AnneMarie van den Brandt, Florian Zwagemaker, Dennis Schmitz, Chantal Reusken, on behalf of the national COVID-19 response team
EPI_ISL_728780, EPI_ISL_728784, EPI_ISL_728787, EPI_ISL_728789, EPI_ISL_728796, EPI_ISL_728797, EPI_ISL_728801, EPI_ISL_728803, EPI_ISL_728805, EPI_ISL_728812, EPI_ISL_728814, EPI_ISL_728815, EPI_ISL_728822, EPI_ISL_728823, EPI_ISL_728834, EPI_ISL_728845, EPI_ISL_728846, EPI_ISL_728847, EPI_ISL_728848, EPI_ISL_728849, EPI_ISL_728850, EPI_ISL_728851, EPI_ISL_728864, EPI_ISL_728867, EPI_ISL_728869, EPI_ISL_728870, EPI_ISL_728871, EPI_ISL_728872, EPI_ISL_728873, EPI_ISL_728874, EPI_ISL_728875, EPI_ISL_728931, EPI_ISL_728959, EPI_ISL_728965, EPI_ISL_728966, EPI_ISL_728976, EPI_ISL_728982, EPI_ISL_728983, EPI_ISL_729000, EPI_ISL_729004, EPI_ISL_729022, EPI_ISL_729025, EPI_ISL_729030, EPI_ISL_729190, EPI_ISL_729191			
see above	Viollier AG	Department of Biosystems Science and Engineering, ETH Zürich	Chaoran Chen, Sarah Nadeau, Catharine Aquino, Ivan Topolsky, Pedro Ferreira, Philipp Jablonski, Susana Posada-Céspedes, Andreia Cabral de Gouvea, Maria Domenica Moccia, Simon Grüter, Timothy Sykes, Lennart Opitz, Ralph Schlapbach, Christiane Beckmann, Maurice Redondo, Olivier Kobel, Christoph Noppen, Sophie Seidel, Noemie Santamaria de Souza, Niko Beerenwinkel, Tanja Stadler
EPI_ISL_729384, EPI_ISL_729424, EPI_ISL_729425, EPI_ISL_729426, EPI_ISL_729621, EPI_ISL_729683	A. Krumbholz, Labor Dr. Krause und Kollegen MVZ GmbH, Kiel	Charité Universitätsmedizin Berlin, Institut für Virologie	Victor M Corman, Barbara Mühlemann, Jörn Beheim-Schwarzbach, Talitha Veith, Julia Schneider, Terry Jones, Christian Drosten
EPI_ISL_730053	Connecticut Department of Health	Grubaugh Lab - Yale School of Public Health	Joseph Fauver, Tara Alpert, Anderson Brito, Annie Watkins, Anne Wyllie, Chantal Vogels, Mary Petrone, Chaney Kalinich, Isabel Ott, Arnau Casanovas, Catherine Muenker, Adam Moore, Alice Lu, Maria Tokuyama, Patrick Wong, Peiwen Lu, Saad Omer, Richard Martinello, Allison Nelson, Shelli Farhadian, Akiko Iwasaki, Charlese Dela Cruz, Albert Ko, Nathan Grubaugh
EPI_ISL_730093, EPI_ISL_730111, EPI_ISL_730117, EPI_ISL_730123	San Diego County Public Health Laboratory	Andersen lab at Scripps Research	SEARCH Alliance San Diego with Tracy Basler, Jovan Shephard, Brett Austin
EPI_ISL_730521, EPI_ISL_730527, EPI_ISL_730533, EPI_ISL_730537, EPI_ISL_730542, EPI_ISL_730547	Biolab Diagnostic Laboratories	Andersen lab at Scripps Research	Issa Abu-Dayyeh, Ahmad Tibi, Lama Hussein, Lina Mohammad, Zein Naber, Amid Abdelnour with SEARCH Alliance San Diego
EPI_ISL_732565, EPI_ISL_732566, EPI_ISL_732568, EPI_ISL_732569, EPI_ISL_732571, EPI_ISL_732573, EPI_ISL_732599, EPI_ISL_732601, EPI_ISL_732602, EPI_ISL_732603, EPI_ISL_732618, EPI_ISL_732625, EPI_ISL_732626, EPI_ISL_732628, EPI_ISL_732632, EPI_ISL_732633, EPI_ISL_732634, EPI_ISL_732636, EPI_ISL_732637, EPI_ISL_732638, EPI_ISL_732639, EPI_ISL_732640, EPI_ISL_732641, EPI_ISL_732642, EPI_ISL_732643, EPI_ISL_732644, EPI_ISL_732645, EPI_ISL_732646, EPI_ISL_732647, EPI_ISL_732648, EPI_ISL_732649, EPI_ISL_732650, EPI_ISL_732651, EPI_ISL_732653			
see above	Department of Virology and Immunology, University of Helsinki and Helsinki University Hospital, Huslab Finland	Department of Virology, Faculty of Medicine, University of Helsinki, Helsinki, Finland	Teemu Smura, Ravi Kant, Phuoc Truong, Hussein Alburkat, Hannimari Kallio-Kokko, Jenni Virtanen, Maija Suvanto, Sari Hannula, Harri Kangas, Pekka Ellonen, Olli Vapalahti
EPI_ISL_732703	CNR Virus des Infections Respiratoires - France SUD	CNR Virus des Infections Respiratoires - France SUD	Antonin Bal, Gregory Destras, Claudia Gonzalez, Gwendolyne Burfin, Quentin Semanas, Martine Valette, Bruno Lina, Laurence Josset
EPI_ISL_732787, EPI_ISL_732791	Centro de Investigación Biomédica de La Rioja - Hospital San Pedro Logroño	SeqCOVID-SPAIN consortium/IBV(CSIC)	María de Toro, José Manuel Azcona Gutiérrez, María Pilar Bea Escudero, Miriam Blasco Alberdi and SeqCOVID-SPAIN consortium
EPI_ISL_732827, EPI_ISL_732828, EPI_ISL_732829, EPI_ISL_732830, EPI_ISL_732831, EPI_ISL_732832, EPI_ISL_732833, EPI_ISL_732834, EPI_ISL_732835, EPI_ISL_732836, EPI_ISL_732837, EPI_ISL_732838, EPI_ISL_732839, EPI_ISL_732840, EPI_ISL_732841, EPI_ISL_732842, EPI_ISL_732843, EPI_ISL_732844, EPI_ISL_732845, EPI_ISL_732846, EPI_ISL_732847, EPI_ISL_732848, EPI_ISL_732849, EPI_ISL_732850, EPI_ISL_732851, EPI_ISL_732852, EPI_ISL_732853, EPI_ISL_732854, EPI_ISL_732855, EPI_ISL_732856, EPI_ISL_732857, EPI_ISL_732858, EPI_ISL_732859, EPI_ISL_732860, EPI_ISL_732861, EPI_ISL_732862, EPI_ISL_732863, EPI_ISL_732864, EPI_ISL_732865, EPI_ISL_732866, EPI_ISL_732867, EPI_ISL_732868, EPI_ISL_732869, EPI_ISL_732870, EPI_ISL_732871, EPI_ISL_732872, EPI_ISL_732873, EPI_ISL_732874, EPI_ISL_732875, EPI_ISL_732876, EPI_ISL_732877, EPI_ISL_732878, EPI_ISL_732879, EPI_ISL_732880, EPI_ISL_732881, EPI_ISL_732883, EPI_ISL_732884, EPI_ISL_732885, EPI_ISL_732886, EPI_ISL_732887, EPI_ISL_732888, EPI_ISL_732889, EPI_ISL_732890, EPI_ISL_732891, EPI_ISL_732892, EPI_ISL_732893, EPI_ISL_732894, EPI_ISL_732895, EPI_ISL_732896, EPI_ISL_732897, EPI_ISL_732898, EPI_ISL_732899, EPI_ISL_732900, EPI_ISL_732901, EPI_ISL_732902, EPI_ISL_732903, EPI_ISL_732904, EPI_ISL_732905, EPI_ISL_732906, EPI_ISL_732907, EPI_ISL_732908, EPI_ISL_732909, EPI_ISL_732910, EPI_ISL_732911, EPI_ISL_732912, EPI_ISL_732913, EPI_ISL_732914, EPI_ISL_732915, EPI_ISL_732916, EPI_ISL_732917, EPI_ISL_732918, EPI_ISL_732919, EPI_ISL_732920, EPI_ISL_732921, EPI_ISL_732922, EPI_ISL_732923, EPI_ISL_732924, EPI_ISL_732925, EPI_ISL_732926			
see above	genXone SA, Molecular Diagnostics Laboratory / NZOZ	genXone SA, Research & Development Laboratory	Maciej Sykulski, Grzegorz Nowicki, Monika Makowska-Woniak, Jakub Grabowski, Natalia Drwska-Matelska, ukasz Krych, Micha Kaszuba
EPI_ISL_734218, EPI_ISL_734245, EPI_ISL_734277	Virginia Division of Consolidated Laboratory Services (DCLS)	Virginia Division of Consolidated Laboratory Services (DCLS)	Virginia DCLS
EPI_ISL_734293, EPI_ISL_734410, EPI_ISL_734413	Wadsworth Center, New York State Department.of Health	Wadsworth Center, New York State Department.of Health	Kirsten St. George, Daryl M. Lamson, Alexis Russel, Jonathan Plitnick, Navjot Singh, John Kelly, Sara Griesemer, Erasmus Schneider, Erica Lasek-Nesselquist
EPI_ISL_734470	Masonic Medical Research Institute	Wadsworth Center, New York State Department.of Health	Kirsten St. George, Nathan Tucker, Ryan D. Pfeiffer, Daryl M. Lamson, Alexis Russel, Jonathan Plitnick, Navjot Singh, John Kelly, Sara Griesemer, Erasmus Schneider, Erica Lasek-Nesselquist

EPI_ISL_737016, EPI_ISL_737028, EPI_ISL_737030, EPI_ISL_737031, EPI_ISL_737032	The National Institute of Public Health	State Veterinary Institute Prague	Nagy,A;Jirincova,H;Trnka,D;Vecerova,J
EPI_ISL_739430	Humboldt County Public Health Laboratory	Chan-Zuckerberg Biohub	CZB Cliahub Consortium
EPI_ISL_739663, EPI_ISL_739666	Instituto Nacional de Salud, Bogotá, Colombia	Instituto Nacional de Salud, Bogotá, Colombia	Katherine Laiton-Donato, Diego A. Álvarez-Díaz, Carlos Franco-Muñoz, Mauricio Pacheco-Montealegre, Jonathan Reales, Diego Andrés Prada, Sheryl Corchuelo, Magdalena Weisner, Martha Lucia Ospina Martinez, Marcela Mercado-Reyes
EPI_ISL_739711, EPI_ISL_739718, EPI_ISL_739734, EPI_ISL_739743, EPI_ISL_739747, EPI_ISL_739791, EPI_ISL_739794, EPI_ISL_739800, EPI_ISL_739802, EPI_ISL_739803, EPI_ISL_739806, EPI_ISL_739822, EPI_ISL_739824, EPI_ISL_739825, EPI_ISL_739827, EPI_ISL_739831, EPI_ISL_739838, EPI_ISL_739886, EPI_ISL_739898, EPI_ISL_739917, EPI_ISL_739920, EPI_ISL_739927, EPI_ISL_739953, EPI_ISL_739955, EPI_ISL_739983, EPI_ISL_739992, EPI_ISL_739996, EPI_ISL_740004, EPI_ISL_740006, EPI_ISL_740008, EPI_ISL_740026, EPI_ISL_740031, EPI_ISL_740036, EPI_ISL_740041, EPI_ISL_740053, EPI_ISL_740055, EPI_ISL_740057, EPI_ISL_740060, EPI_ISL_740076, EPI_ISL_740080, EPI_ISL_740095, EPI_ISL_740101, EPI_ISL_740105, EPI_ISL_740106, EPI_ISL_740114, EPI_ISL_740116, EPI_ISL_740155, EPI_ISL_740169, EPI_ISL_740187, EPI_ISL_740224, EPI_ISL_740244, EPI_ISL_740266, EPI_ISL_740272, EPI_ISL_740298, EPI_ISL_740349, EPI_ISL_740361, EPI_ISL_740382, EPI_ISL_740390, EPI_ISL_740398, EPI_ISL_740399, EPI_ISL_740410, EPI_ISL_740423, EPI_ISL_740445, EPI_ISL_740449, EPI_ISL_740453, EPI_ISL_740455, EPI_ISL_740477, EPI_ISL_740498, EPI_ISL_740507, EPI_ISL_740512, EPI_ISL_740514, EPI_ISL_740516, EPI_ISL_740535			
see above	Laboratoire national de santé, Microbiology, Virology	Laboratoire national de santé, Microbiology, Microbial Genomics Platform	Anke Wienecke-Baldacchino, Catherine Ragimbeau,Jessica Tapp, Fatu Djabi, Lise Pignon, Raoul Salmon, Tamir Abdelrahman
EPI_ISL_740623, EPI_ISL_740624, EPI_ISL_740625, EPI_ISL_740626, EPI_ISL_740627, EPI_ISL_740628, EPI_ISL_740629, EPI_ISL_740630, EPI_ISL_740631, EPI_ISL_740632, EPI_ISL_740633, EPI_ISL_740634, EPI_ISL_740635, EPI_ISL_740636, EPI_ISL_740637, EPI_ISL_740638, EPI_ISL_740639, EPI_ISL_740640, EPI_ISL_740641, EPI_ISL_740642, EPI_ISL_740643, EPI_ISL_740644, EPI_ISL_740645, EPI_ISL_740646, EPI_ISL_740647, EPI_ISL_740648, EPI_ISL_740649, EPI_ISL_740650, EPI_ISL_740651, EPI_ISL_740652, EPI_ISL_740653, EPI_ISL_740654, EPI_ISL_740656, EPI_ISL_740657, EPI_ISL_740658, EPI_ISL_740659, EPI_ISL_740660, EPI_ISL_740661, EPI_ISL_740662, EPI_ISL_740665, EPI_ISL_740666, EPI_ISL_740667, EPI_ISL_740668, EPI_ISL_740669, EPI_ISL_740670, EPI_ISL_740675, EPI_ISL_740678, EPI_ISL_740679, EPI_ISL_740680, EPI_ISL_740683, EPI_ISL_740685, EPI_ISL_740686, EPI_ISL_740687, EPI_ISL_740688, EPI_ISL_740689, EPI_ISL_740690, EPI_ISL_740691, EPI_ISL_740692, EPI_ISL_740693, EPI_ISL_740694, EPI_ISL_740695, EPI_ISL_740696, EPI_ISL_740697, EPI_ISL_740698, EPI_ISL_740699, EPI_ISL_740700, EPI_ISL_740701, EPI_ISL_740702, EPI_ISL_740703, EPI_ISL_740704, EPI_ISL_740705, EPI_ISL_740706, EPI_ISL_740707, EPI_ISL_740708, EPI_ISL_740709, EPI_ISL_740710, EPI_ISL_740711, EPI_ISL_740712, EPI_ISL_740713, EPI_ISL_740714, EPI_ISL_740715, EPI_ISL_740716, EPI_ISL_740717, EPI_ISL_740718, EPI_ISL_740719, EPI_ISL_740720, EPI_ISL_740721, EPI_ISL_740722, EPI_ISL_740723, EPI_ISL_740724, EPI_ISL_740725, EPI_ISL_740726, EPI_ISL_740727, EPI_ISL_740728, EPI_ISL_740729, EPI_ISL_740730, EPI_ISL_740731, EPI_ISL_740732, EPI_ISL_740733, EPI_ISL_740734, EPI_ISL_740735, EPI_ISL_740736, EPI_ISL_740737, EPI_ISL_740738, EPI_ISL_740739, EPI_ISL_740740, EPI_ISL_740741, EPI_ISL_740742, EPI_ISL_740743, EPI_ISL_740744, EPI_ISL_740745, EPI_ISL_740746, EPI_ISL_740747, EPI_ISL_740748, EPI_ISL_740749, EPI_ISL_740750, EPI_ISL_740751, EPI_ISL_740752, EPI_ISL_740753, EPI_ISL_740754, EPI_ISL_740755, EPI_ISL_740756, EPI_ISL_740757, EPI_ISL_740758, EPI_ISL_740759, EPI_ISL_740760, EPI_ISL_740761, EPI_ISL_740762, EPI_ISL_740763, EPI_ISL_740764, EPI_ISL_740765, EPI_ISL_740766, EPI_ISL_740768, EPI_ISL_740769, EPI_ISL_740770, EPI_ISL_740771, EPI_ISL_740772, EPI_ISL_740773, EPI_ISL_740774, EPI_ISL_740775, EPI_ISL_740776, EPI_ISL_740782, EPI_ISL_740783, EPI_ISL_740790, EPI_ISL_740791, EPI_ISL_740792, EPI_ISL_740793, EPI_ISL_740794, EPI_ISL_740795, EPI_ISL_740796, EPI_ISL_740797, EPI_ISL_740798, EPI_ISL_740799, EPI_ISL_740800, EPI_ISL_740801, EPI_ISL_740802, EPI_ISL_740803, EPI_ISL_740804, EPI_ISL_740805, EPI_ISL_740806, EPI_ISL_740807, EPI_ISL_740808, EPI_ISL_740809, EPI_ISL_740810, EPI_ISL_740811, EPI_ISL_740812, EPI_ISL_740813, EPI_ISL_740814, EPI_ISL_740815, EPI_ISL_740816, EPI_ISL_740817, EPI_ISL_740818, EPI_ISL_740819, EPI_ISL_740820, EPI_ISL_740821, EPI_ISL_740822, EPI_ISL_740823, EPI_ISL_740824, EPI_ISL_740825, EPI_ISL_740826, EPI_ISL_740827, EPI_ISL_740828, EPI_ISL_740830, EPI_ISL_740831, EPI_ISL_740832, EPI_ISL_740833, EPI_ISL_740834, EPI_ISL_740835, EPI_ISL_740836, EPI_ISL_740837, EPI_ISL_740838, EPI_ISL_740839, EPI_ISL_740840, EPI_ISL_740841, EPI_ISL_740842, EPI_ISL_740843, EPI_ISL_740844, EPI_ISL_740845, EPI_ISL_740846, EPI_ISL_740847, EPI_ISL_740848, EPI_ISL_740849, EPI_ISL_740850, EPI_ISL_740851, EPI_ISL_740852, EPI_ISL_740853, EPI_ISL_740854, EPI_ISL_740855, EPI_ISL_740856, EPI_ISL_740857, EPI_ISL_740858, EPI_ISL_740859, EPI_ISL_740860, EPI_ISL_740861, EPI_ISL_740862			
see above	BCCDC Public Health Laboratory	BCCDC Public Health Laboratory	Prystajecky Natalie, Linda Hoang, Dan Fornika, Shannon Russell, Kim Macdonald, Kimia Kamelian, John Tyson, Inna Sekirov, Mel Krajden
EPI_ISL_741359, EPI_ISL_741360, EPI_ISL_741361	University of Exeter	COVID-19 Genomics UK (COG-UK) Consortium	Ben Temperton,Aaron Jeffries,Michelle Michelsen,Joanna Warwick-Dugdale,Audrey Farbos,Robyn Manley,Stephen Michell,Jane Masoli
EPI_ISL_741362, EPI_ISL_741363, EPI_ISL_741364, EPI_ISL_741365, EPI_ISL_741366, EPI_ISL_741369, EPI_ISL_741371, EPI_ISL_741372, EPI_ISL_741374, EPI_ISL_741375, EPI_ISL_741376, EPI_ISL_741377, EPI_ISL_741378, EPI_ISL_741379, EPI_ISL_741380, EPI_ISL_741381, EPI_ISL_741382, EPI_ISL_741383, EPI_ISL_741389, EPI_ISL_741391, EPI_ISL_741392, EPI_ISL_741395, EPI_ISL_741396, EPI_ISL_741397, EPI_ISL_741398, EPI_ISL_741399, EPI_ISL_741400, EPI_ISL_741401, EPI_ISL_741402, EPI_ISL_741403, EPI_ISL_741407, EPI_ISL_741408, EPI_ISL_741410, EPI_ISL_741414, EPI_ISL_741416, EPI_ISL_741417, EPI_ISL_741419, EPI_ISL_741420			
see above	Oxford Viromics, NDM, University of Oxford; Oxford University Hospitals; Basingstoke and North Hampshire Hospital	COVID-19 Genomics UK (COG-UK) Consortium	Tanya Golubchik, David Bonsall, George Macintyre, Amy Trebes, Mariateresa de Cesare, Catrin Moore, Alex Mobbs, Anita Justice, Robert Shaw, Monique Andersson, Timothy Peto, Emma Wise, Nathan Moore, Jessica Lynch, Nick Cortes, Matilde Mori, Stephen Kidd, David Buck, John Todd, Christophe Fraser
EPI_ISL_741567	Quadram Institute Bioscience	COVID-19 Genomics UK (COG-UK) Consortium	Dave J. Baker, Gemma L. Kay, Alp Aydin, Thanh Le-Viet, Steven Rudder, Ana P. Tedim, Anastasia Kolyva, Maria Diaz, Leonardo de Oliveira Martins, Nabil-Fareed Alikhan, Lizzie Meadows, Rachael Stanley, Ngozi Elumogo, Muhammed Yasir, Nicholas M. Thomson, Alexander J Trotter, Rachel Gilroy, Samuel Bloomfield, Claire Stuart, Andrew Bell, Reenesh Prakash, Samir Dervisevic, Alison E. Mather, John Wain, Mark Webber, Andrew J. Page, Justin O'Grady
EPI_ISL_742117	University of Exeter	COVID-19 Genomics UK (COG-UK) Consortium	Ben Temperton,Aaron Jeffries,Michelle Michelsen,Joanna Warwick-Dugdale,Audrey Farbos,Robyn Manley,Stephen Michell,Jane Masoli
EPI_ISL_742261	Oxford Viromics, NDM, University of Oxford; Oxford University Hospitals; Basingstoke and North Hampshire Hospital	COVID-19 Genomics UK (COG-UK) Consortium	Tanya Golubchik, David Bonsall, George Macintyre, Amy Trebes, Mariateresa de Cesare, Catrin Moore, Alex Mobbs, Anita Justice, Robert Shaw, Monique Andersson, Timothy Peto, Emma Wise, Nathan Moore, Jessica Lynch, Nick Cortes, Matilde Mori, Stephen Kidd, David Buck, John Todd, Christophe Fraser
EPI_ISL_742300, EPI_ISL_742301, EPI_ISL_742308, EPI_ISL_742309, EPI_ISL_742310, EPI_ISL_742311, EPI_ISL_742312, EPI_ISL_742313, EPI_ISL_742314, EPI_ISL_742315, EPI_ISL_742316			
see above	Wales Specialist Virology Centre Sequencing lab: Pathogen Genomics Unit	COVID-19 Genomics UK (COG-UK) Consortium	Catherine Moore, Johnathan Evans, Laura Gifford, Malorie Perry, Simon Cottrell, Angela Marchbank, Alec Birchley, Alexander Adams, Amy Gaskin, Bree Gatica-Wilcox, Jason Coombes, Joel Southgate, Lauren Gilbert, Lee Graham, Nicole Pacchiarini, Sara Kumziene-Summerhayes, Sarah Taylor, Sophie Jones, Sara Rey, Matthew Bull, Joanne Watkins, Sally Corden, Tom Connor
EPI_ISL_744136, EPI_ISL_744164, EPI_ISL_744191, EPI_ISL_744193, EPI_ISL_744201, EPI_ISL_744207, EPI_ISL_744215, EPI_ISL_744219, EPI_ISL_744220, EPI_ISL_744232, EPI_ISL_744261, EPI_ISL_744290, EPI_ISL_744324, EPI_ISL_744325, EPI_ISL_744332, EPI_ISL_744373, EPI_ISL_744374, EPI_ISL_744378, EPI_ISL_744381, EPI_ISL_744385, EPI_ISL_744401, EPI_ISL_744402, EPI_ISL_744405, EPI_ISL_744437, EPI_ISL_744467, EPI_ISL_744473, EPI_ISL_744512, EPI_ISL_744529, EPI_ISL_744556, EPI_ISL_744559, EPI_ISL_744604, EPI_ISL_744616, EPI_ISL_744617, EPI_ISL_744625, EPI_ISL_744627, EPI_ISL_744630, EPI_ISL_744649, EPI_ISL_744668, EPI_ISL_744675, EPI_ISL_744683, EPI_ISL_744701, EPI_ISL_744729, EPI_ISL_744733, EPI_ISL_744763, EPI_ISL_744783, EPI_ISL_744789, EPI_ISL_744800, EPI_ISL_744806, EPI_ISL_744818, EPI_ISL_744848, EPI_ISL_744872, EPI_ISL_744876, EPI_ISL_744882, EPI_ISL_744895, EPI_ISL_744902, EPI_ISL_744926, EPI_ISL_744955, EPI_ISL_744964, EPI_ISL_744978, EPI_ISL_744984, EPI_ISL_744988, EPI_ISL_745010, EPI_ISL_745025			
see above	Laboratoire national de santé, Microbiology, Virology	Laboratoire national de santé, Microbiology, Microbial Genomics Platform	Anke Wienecke-Baldacchino, Catherine Ragimbeau,Jessica Tapp, Fatu Djabi, Lise Pignon, Raoul Salmon, Tamir Abdelrahman
EPI_ISL_747032, EPI_ISL_747033, EPI_ISL_747034, EPI_ISL_747067, EPI_ISL_747099, EPI_ISL_747107, EPI_ISL_747108, EPI_ISL_747109, EPI_ISL_747119, EPI_ISL_747127	Respiratory Viruses Branch, Centers for Disease Control and Prevention	Respiratory Viruses Branch, Centers for Disease Control and Prevention	Queen,K., Li,Y., Tao,Y., Uehara,A., Montmayeur,A., Paden,C.R., Cook,P.W., Marine,R., Sheth,M., Wang,H., Lee,J., Tong,S.
EPI_ISL_751448, EPI_ISL_751490	CHU Purpan - Laboratoire de Virologie - Institut Fédératif de Biologie	CHU Purpan - Laboratoire de Virologie - Institut Fédératif de Biologie	Latour J., Ranger N., Dubois M., Carcenac R., Harter A., Boyer P., Tremeaux P., Izopet J.
EPI_ISL_751548	NYSDOH Wadsworth Center, Virology Lab	Genomics and Discovery, Respiratory Viruses Branch, Division of Viral Diseases, Centers for Disease Control and Prevention	Krista Queen, Yan Li, Ying Tao, Jing Zhang, Anna Uehara, Anna Montmayeur, Clinton R. Paden, Peter W. Cook,Rachel Marine, Mili Sheth, Haibin Wang, Justin Lee, Suxiang Tong
EPI_ISL_751732	NE Public Health Laboratory	Genomics and Discovery, Respiratory Viruses Branch, Division of Viral Diseases, Centers for Disease Control and Prevention	Krista Queen, Yan Li, Ying Tao, Jing Zhang, Anna Uehara, Anna Montmayeur, Clinton R. Paden, Peter W. Cook,Rachel Marine, Mili Sheth, Haibin Wang, Justin Lee, Suxiang Tong
EPI_ISL_753054	State Laboratories Division, Hawaii State Department of Health	State Laboratories Division, Hawaii State Department of Health	Pamela O'Brien, Sabrina Diemert, Drew Kuwazaki, Razvan Sultana, Edward Desmond
EPI_ISL_753324	Clinical virology Laboratory, Children's Hospital Los Angeles	Center for Personalized Medicine, Children's Hospital Los Angeles	Gai et al
EPI_ISL_754626, EPI_ISL_754627	University of Wisconsin-Madison AIDS Vaccine Research Laboratories	University of Wisconsin-Madison AIDS Vaccine Research Laboratories	Gage Moreno, Katarina Braun, et al. AIDS Vaccine Research Laboratories
EPI_ISL_757342	Department of Virology and Immunology, University of Helsinki and Helsinki University Hospital, Huslab Finland	Department of Virology, Faculty of Medicine, University of Helsinki, Helsinki, Finland	Teemu Smura, Ravi Kant, Phuoc Truong, Hussein Alburkat, Hannimari Kallio-Kokko, Jenni Virtanen, Maija Suvanto, Sari Hannula, Harri Kangas, Pekka Ellonen, Olli Vapalahti
EPI_ISL_760096, EPI_ISL_760097, EPI_ISL_760118, EPI_ISL_760122, EPI_ISL_760147, EPI_ISL_760175, EPI_ISL_760176, EPI_ISL_760177, EPI_ISL_760178, EPI_ISL_760179, EPI_ISL_760180, EPI_ISL_760199, EPI_ISL_760200, EPI_ISL_760216, EPI_ISL_760217			
see above	Division of Emerging Infectious Diseases, Bureau of Infectious Diseases Diagnosis Control, Korea Disease Control and	Division of Emerging Infectious Diseases, Bureau of Infectious Diseases Diagnosis Control, Korea Disease Control and	Ae Kyung Park, Il-Hwan Kim, Heui Man Kim, Jeong-Min Kim, Namjoo Lee, Chaeyoung Lee, Sang Hee Woo, Eun-Jin Kim

	Prevention Agency	Prevention Agency	
EPI_ISL_763232	Dutch COVID-19 response team	Erasmus Medical Center	Bas Oude Munnink, Reina Sikkema, David Nieuwenhuijse, Irina Chestakova, Anne van der Linden, Marjan Boter, Emmanuelle Munger, Corine GeurtsvanKessel, Annemiek van der Eijk, Richard Molenkamp, Marion Koopmans, on behalf of the Dutch national COVID-19 response team.
EPI_ISL_765778, EPI_ISL_765781, EPI_ISL_765782, EPI_ISL_765783, EPI_ISL_765784, EPI_ISL_765785	Massachusetts General Hospital	Infectious Disease Program, Broad Institute of Harvard and MIT	Lemieux,J.E., Siddle,K.J., Shaw,B., Adams,G., Pierce,V., Turbett,S., Anahtar,M., Branda,J., Slater,D., Harris,J., Lin,A.E., Gladden-Young,A., Lagerborg,K., Rudy,M., DeRuff,K., Carter,A., Normandin,E., Bauer,M., Reilly,S., Tomkins-Tinch,C., Loreth,C., Chaluvadi,S., Neumann,A., Cusick,C., Chapman,S.B., Gnirke,A., Flowers,K., Cerrato,F., Birren,B.W., Gallagher,G., Smole,S., Park,D.J., MacInnis,B.L., Ryan,E., LaRocque,R., Rosenberg,E. and Sabeti,P.C.
EPI_ISL_765942, EPI_ISL_765958	Worobey Lab, Department of Ecology and Evolutionary Biology, University of Arizona	Worobey Lab, Department of Ecology and Evolutionary Biology, University of Arizona	Brendan Larsen, Grace Quirk, Thomas Watts, David Baltrus, Michael Worobey
EPI_ISL_766615	Klinisk mikrobiologi	The Public Health Agency of Sweden	Department of Microbiology, The Public Health Agency of Sweden
EPI_ISL_768634, EPI_ISL_768635, EPI_ISL_768636, EPI_ISL_768637, EPI_ISL_768638, EPI_ISL_768639, EPI_ISL_768651, EPI_ISL_768652, EPI_ISL_768653, EPI_ISL_768654, EPI_ISL_768702			
see above	Pathogen Genomics Center, National Institute of Infectious Diseases	Pathogen Genomics Center, National Institute of Infectious Diseases	Tsuyoshi Sekizuka, Kentaro Itokawa, Rina Tanaka, Masanori Hashino, Makoto Kuroda
EPI_ISL_768753, EPI_ISL_768757, EPI_ISL_768786	AIID	Irish Coronavirus Sequencing Consortium - National Virus Reference Laboratory	Michael Carr, Gabriel Gonzalez, Alejandro Abner Garcia Leon, Patrick Mallon
EPI_ISL_768839	Laboratoire Biolife	Laboratoire de Biotechnologie	Mouna Ouadghiri, Tarik Aanniz, Mohammed Walid Chemao Elfihri, Mohamed Chenaoui, Hanae Dakka, Afaf Alaoui, Otmane Touzani, Amina Benouda, Bouchra Belfquih, Lahcen belyamani, Saaid Amzazi and Azeddine Ibrahimi
EPI_ISL_770029	Area De Salud Corredores	Inciensa, Instituto Costarricense de Investigación y Enseñanza en Nutrición y Salud	Francisco Duarte, Hebleen Porras, Claudio Soto-Garita, Estela Cordero, Adriana Godínez, Melany Calderón & Mariel López
EPI_ISL_770533, EPI_ISL_770534, EPI_ISL_770535, EPI_ISL_770536, EPI_ISL_770537, EPI_ISL_770538, EPI_ISL_770539, EPI_ISL_770540, EPI_ISL_770541	Private lab	Lithuanian University of Health Sciences, Molecular cardiology lab.	Lukas Zemaitis, Ingrida Olendrait, Arnoldas Pautienius, Kamile Tamusauskaite, Dovydas Gecys, Laura Pareckaite, Vaiva Lesauskaite, Astra Vitkauskiene
EPI_ISL_770810	Essentia Health-St. Mary's Medical Center	Minnesota Department of Health, Public Health Laboratory	Alexandra Lorentz, Jacob Garfin, Matt Plumb, and Xiong Wang
EPI_ISL_775021, EPI_ISL_775022, EPI_ISL_775023, EPI_ISL_775024, EPI_ISL_775025, EPI_ISL_775026, EPI_ISL_775027, EPI_ISL_775028, EPI_ISL_775029, EPI_ISL_775030, EPI_ISL_775031, EPI_ISL_775032, EPI_ISL_775033, EPI_ISL_775034, EPI_ISL_775035, EPI_ISL_775036, EPI_ISL_775037, EPI_ISL_775038, EPI_ISL_775039, EPI_ISL_775040, EPI_ISL_775041, EPI_ISL_775042, EPI_ISL_775043, EPI_ISL_775044, EPI_ISL_775045, EPI_ISL_775046, EPI_ISL_775047, EPI_ISL_775048, EPI_ISL_775049, EPI_ISL_775050, EPI_ISL_775051, EPI_ISL_775052, EPI_ISL_775053, EPI_ISL_775054, EPI_ISL_775055, EPI_ISL_775056, EPI_ISL_775057, EPI_ISL_775058, EPI_ISL_775059, EPI_ISL_775060, EPI_ISL_775061, EPI_ISL_775062, EPI_ISL_775063, EPI_ISL_775064, EPI_ISL_775065, EPI_ISL_775066, EPI_ISL_775067, EPI_ISL_775068, EPI_ISL_775069, EPI_ISL_775070, EPI_ISL_775071, EPI_ISL_775072, EPI_ISL_775073, EPI_ISL_775074, EPI_ISL_775075, EPI_ISL_775076, EPI_ISL_775077, EPI_ISL_775078, EPI_ISL_775079, EPI_ISL_775080, EPI_ISL_775081, EPI_ISL_775082, EPI_ISL_775083, EPI_ISL_775084, EPI_ISL_775085, EPI_ISL_775086, EPI_ISL_775087, EPI_ISL_775088, EPI_ISL_775089, EPI_ISL_775090, EPI_ISL_775091, EPI_ISL_775092, EPI_ISL_775093, EPI_ISL_775094, EPI_ISL_775095, EPI_ISL_775096, EPI_ISL_775097, EPI_ISL_775098, EPI_ISL_775099, EPI_ISL_775100, EPI_ISL_775101, EPI_ISL_775102, EPI_ISL_775103, EPI_ISL_775104, EPI_ISL_775105, EPI_ISL_775106, EPI_ISL_775107, EPI_ISL_775108, EPI_ISL_775109, EPI_ISL_775110, EPI_ISL_775111, EPI_ISL_775112, EPI_ISL_775113, EPI_ISL_775114, EPI_ISL_775115, EPI_ISL_775116, EPI_ISL_775117, EPI_ISL_775118, EPI_ISL_775119, EPI_ISL_775120, EPI_ISL_775121, EPI_ISL_775122, EPI_ISL_775123, EPI_ISL_775124, EPI_ISL_775125, EPI_ISL_775126, EPI_ISL_775127, EPI_ISL_775128, EPI_ISL_775129, EPI_ISL_775130, EPI_ISL_775131, EPI_ISL_775132, EPI_ISL_775133, EPI_ISL_775134, EPI_ISL_775135, EPI_ISL_775136, EPI_ISL_775137, EPI_ISL_775138, EPI_ISL_775139, EPI_ISL_775140, EPI_ISL_775141, EPI_ISL_775142, EPI_ISL_775143, EPI_ISL_775144, EPI_ISL_775145, EPI_ISL_775146, EPI_ISL_775147, EPI_ISL_775148, EPI_ISL_775149, EPI_ISL_775150, EPI_ISL_775151, EPI_ISL_775152, EPI_ISL_775153, EPI_ISL_775154, EPI_ISL_775155, EPI_ISL_775156, EPI_ISL_775157, EPI_ISL_775158, EPI_ISL_775159, EPI_ISL_775160, EPI_ISL_775161, EPI_ISL_775162, EPI_ISL_775163, EPI_ISL_775164, EPI_ISL_775165, EPI_ISL_775166, EPI_ISL_775167, EPI_ISL_775168, EPI_ISL_775169, EPI_ISL_775170, EPI_ISL_775171, EPI_ISL_775172, EPI_ISL_775173, EPI_ISL_775174, EPI_ISL_775175, EPI_ISL_775176, EPI_ISL_775177, EPI_ISL_775178, EPI_ISL_775179, EPI_ISL_775180, EPI_ISL_775181, EPI_ISL_775182, EPI_ISL_775183, EPI_ISL_775184, EPI_ISL_775185, EPI_ISL_775186, EPI_ISL_775187, EPI_ISL_775188, EPI_ISL_775189, EPI_ISL_775190, EPI_ISL_775191, EPI_ISL_775192, EPI_ISL_775193, EPI_ISL_775194, EPI_ISL_775195, EPI_ISL_775196, EPI_ISL_775197, EPI_ISL_775198, EPI_ISL_775199, EPI_ISL_775200, EPI_ISL_775201, EPI_ISL_775202, EPI_ISL_775203, EPI_ISL_775204, EPI_ISL_775205, EPI_ISL_775206, EPI_ISL_775207, EPI_ISL_775208, EPI_ISL_775209, EPI_ISL_775210, EPI_ISL_775211, EPI_ISL_775212			
see above	Public Health Ontario Laboratory	Public Health Ontario Laboratory	Vanessa G Allen, Philip Banh, Richard de Borja, Yao Chen, Alireza Eshaghi, Nahuel Fittipaldi, Christine Frantz, Jonathan B Gubbay, Jennifer L Guthrie, Lawrence Heisler, Esha Joshi, Michael Laszloffy, Aimin Li, Michael CY Li, Dean Maxwell, Sandeep Nagra, Samir N Patel, Heather Rilkoff, Jared Simpson, Karthikeyan Sivaraman, Yogi Sundaravadanam, Sarah Teatero, Andre Villegas, Sandra Zittermann
EPI_ISL_775250, EPI_ISL_775252, EPI_ISL_775264, EPI_ISL_775265	Laboratoire Biolife	Laboratoire de Biotechnologie	Mouna Ouadghiri, Tarik Aanniz, Mohammed Walid Chemao Elfihri, Mohamed Chenaoui, Hanae Dakka, Afaf Alaoui, Otmane Touzani, Amina Benouda, Bouchra Belfquih, Lahcen belyamani, Saaid Amzazi and Azeddine Ibrahimi
EPI_ISL_775327	Dept. of Medical Microbiology, Stavanger University Hospital, Helse Stavanger HF	Norwegian Institute of Public Health, Department of Virology	Kathrine Stene-Johansen, Kamilla Heddeland Instefjord, Hilde Elshaug, Atiya R Ali,Marie Paulsen Madsen, Rasmus Riis Kopperud, Hilde Vollan, Karoline Bragstad, Olav Hungnes
EPI_ISL_775330, EPI_ISL_775382	Nordland Hospital - Bodo, Laboratory Department, Molecular Biology Unit	Norwegian Institute of Public Health, Department of Virology	Kathrine Stene-Johansen, Kamilla Heddeland Instefjord, Hilde Elshaug, Atiya R Ali,Marie Paulsen Madsen, Rasmus Riis Kopperud, Hilde Vollan, Karoline Bragstad, Olav Hungnes
EPI_ISL_775393	Vestfold Hospital, Toensberg Department of Microbiology	Norwegian Institute of Public Health, Department of Virology	Kathrine Stene-Johansen, Kamilla Heddeland Instefjord, Hilde Elshaug, Atiya R Ali,Marie Paulsen Madsen, Rasmus Riis Kopperud, Hilde Vollan, Karoline Bragstad, Olav Hungnes
EPI_ISL_775460, EPI_ISL_775461	Norwegian Institute of Public Health, Department of Virology	Norwegian Institute of Public Health, Department of Virology	Kathrine Stene-Johansen, Kamilla Heddeland Instefjord, Hilde Elshaug, Atiya R Ali,Marie Paulsen Madsen, Rasmus Riis Kopperud, Hilde Vollan, Karoline Bragstad, Olav Hungnes
EPI_ISL_775501, EPI_ISL_775502, EPI_ISL_775503	Vestfold Hospital, Toensberg Department of Microbiology	Norwegian Institute of Public Health, Department of Virology	Kathrine Stene-Johansen, Kamilla Heddeland Instefjord, Hilde Elshaug, Atiya R Ali,Marie Paulsen Madsen, Rasmus Riis Kopperud, Hilde Vollan, Karoline Bragstad, Olav Hungnes
EPI_ISL_778845, EPI_ISL_778846, EPI_ISL_778847, EPI_ISL_778848, EPI_ISL_778849, EPI_ISL_778850, EPI_ISL_778851, EPI_ISL_778852, EPI_ISL_778853, EPI_ISL_778854	Public Health Ontario Laboratory	Public Health Ontario Laboratory	Vanessa G Allen, Philip Banh, Richard de Borja, Yao Chen, Alireza Eshaghi, Nahuel Fittipaldi, Christine Frantz, Jonathan B Gubbay, Jennifer L Guthrie, Lawrence Heisler, Esha Joshi, Michael Laszloffy, Aimin Li, Michael CY Li, Dean Maxwell, Sandeep Nagra, Samir N Patel, Heather Rilkoff, Jared Simpson, Karthikeyan Sivaraman, Yogi Sundaravadanam, Sarah Teatero, Andre Villegas, Sandra Zittermann
EPI_ISL_779228, EPI_ISL_779229, EPI_ISL_779230, EPI_ISL_779231, EPI_ISL_779670, EPI_ISL_779671, EPI_ISL_779672, EPI_ISL_779673, EPI_ISL_779674, EPI_ISL_779675, EPI_ISL_779676, EPI_ISL_779677, EPI_ISL_779678, EPI_ISL_779699, EPI_ISL_779700, EPI_ISL_779701, EPI_ISL_779702			
see above	Pathogen Genomics Center, National Institute of Infectious Diseases	Pathogen Genomics Center, National Institute of Infectious Diseases	Tsuyoshi Sekizuka, Kentaro Itokawa, Rina Tanaka, Masanori Hashino, Makoto Kuroda
EPI_ISL_780022, EPI_ISL_780025, EPI_ISL_780074	Hospital General Universitario Gregorio Marañón	SeqCOVID-SPAIN consortium/IBV(CSIC)	Darío García de Viedma, Laura Pérez-Lago, Marta Herranz, Jon Sicilia, Julia Suárez, Pilar Catalán, Patricia Muñoz and SeqCOVID-SPAIN consortium
EPI_ISL_780383, EPI_ISL_780385, EPI_ISL_780386, EPI_ISL_780404, EPI_ISL_780405, EPI_ISL_780406, EPI_ISL_780410	Bermuda Government Molecular Diagnostics Laboratory (MDL)	Respiratory Virus Unit, National Infection Service, Public Health England	PHE Covid Sequencing Team, Dr Carika Weldon (Bermuda), Dr Ayoola Oyinloye (Bermuda)
EPI_ISL_785709, EPI_ISL_785722, EPI_ISL_785741, EPI_ISL_785770, EPI_ISL_785771, EPI_ISL_785780, EPI_ISL_785812, EPI_ISL_785820, EPI_ISL_785824, EPI_ISL_785832, EPI_ISL_785836, EPI_ISL_785846, EPI_ISL_785848, EPI_ISL_785849, EPI_ISL_785858, EPI_ISL_785860, EPI_ISL_785862, EPI_ISL_785870, EPI_ISL_785871, EPI_ISL_785883, EPI_ISL_785894, EPI_ISL_785899, EPI_ISL_785900, EPI_ISL_785901, EPI_ISL_785902, EPI_ISL_785903, EPI_ISL_785905, EPI_ISL_785906, EPI_ISL_785907, EPI_ISL_785908, EPI_ISL_785911, EPI_ISL_785912, EPI_ISL_785913, EPI_ISL_785914, EPI_ISL_785916, EPI_ISL_785917, EPI_ISL_785918, EPI_ISL_785919, EPI_ISL_785920, EPI_ISL_785923, EPI_ISL_785924, EPI_ISL_785925, EPI_ISL_785926, EPI_ISL_785927, EPI_ISL_785928, EPI_ISL_785929, EPI_ISL_785930, EPI_ISL_785931, EPI_ISL_785932, EPI_ISL_785935, EPI_ISL_785936, EPI_ISL_785937, EPI_ISL_785938, EPI_ISL_785939, EPI_ISL_785940, EPI_ISL_785941, EPI_ISL_785942, EPI_ISL_785943, EPI_ISL_785944, EPI_ISL_785947, EPI_ISL_785948, EPI_ISL_785949, EPI_ISL_785950, EPI_ISL_785951, EPI_ISL_785952, EPI_ISL_785953, EPI_ISL_785954, EPI_ISL_785955, EPI_ISL_785956, EPI_ISL_785959, EPI_ISL_785960, EPI_ISL_785961, EPI_ISL_785962, EPI_ISL_785963, EPI_ISL_785964, EPI_ISL_785965, EPI_ISL_785966, EPI_ISL_785967, EPI_ISL_785968, EPI_ISL_785971, EPI_ISL_785972, EPI_ISL_785973, EPI_ISL_785974, EPI_ISL_785975, EPI_ISL_785976, EPI_ISL_785977, EPI_ISL_785978, EPI_ISL_785979, EPI_ISL_785983, EPI_ISL_785984, EPI_ISL_785985, EPI_ISL_785986, EPI_ISL_785987, EPI_ISL_785988, EPI_ISL_785989, EPI_ISL_785990, EPI_ISL_785991, EPI_ISL_785997, EPI_ISL_785998, EPI_ISL_785999, EPI_ISL_786000, EPI_ISL_786009, EPI_ISL_786010, EPI_ISL_786011, EPI_ISL_786021, EPI_ISL_786022, EPI_ISL_786023, EPI_ISL_786032, EPI_ISL_786033, EPI_ISL_786034, EPI_ISL_786035, EPI_ISL_786044, EPI_ISL_786045, EPI_ISL_786046, EPI_ISL_786047, EPI_ISL_786056, EPI_ISL_786057, EPI_ISL_786058, EPI_ISL_786059, EPI_ISL_786068, EPI_ISL_786069, EPI_ISL_786070, EPI_ISL_786071, EPI_ISL_786080, EPI_ISL_786081, EPI_ISL_786082, EPI_ISL_786083			
see above	Houston Methodist Hospital	Houston Methodist Hospital	S. Wesley Long, Randall J. Olsen, Paul A. Christensen, David W. Bernard, James J. Davis, Maulik Shukla, Marcus Nguyen, Matthew Ojeda Saavedra, Prasanti Yerramilli, Layne Pruitt, Sishir Subedi, Heather Hendrickson, and James M. Musser

EPI_ISL_791338, EPI_ISL_791347, EPI_ISL_791353	Johns Hopkins Hospital Department of Pathology	Johns Hopkins Hospital Department of Pathology	C. Paul Morris, Chun Huai Luo, Heba H. Mostafa
EPI_ISL_794078, EPI_ISL_794079, EPI_ISL_794080, EPI_ISL_794081, EPI_ISL_794082	Wadsworth Center, New York State Department.of Health	Wadsworth Center, New York State Department.of Health	Kirsten St. George, Daryl M. Lamson, Alexis Russel, Matthew Shudt, Melissa A Leisner, Jonathan Plitnick, Navjot Singh, John Kelly, Sara Griesemer, Erasmus Schneider, Erica Lasek-Nesselquist
EPI_ISL_801407, EPI_ISL_801430, EPI_ISL_801488	Dutch COVID-19 response team	Erasmus Medical Center	Bas Oude Munnink, Reina Sikkema, David Nieuwenhuijse, Irina Chestakova, Anne van der Linden, Marjan Boter, Emmanuelle Munger, Corine GeurtsvanKessel, Annemiek van der Eijk, Richard Molenkamp, Marion Koopmans, on behalf of the Dutch national COVID-19 response team.
EPI_ISL_803102	Respiratory Viruses Branch, Centers for Disease Control and Prevention	Respiratory Viruses Branch, Centers for Disease Control and Prevention	Queen,K., Li,Y., Tao,Y., Uehara,A., Montmayeur,A., Paden,C.R., Cook,P.W., Marine,R., Sheth,M., Wang,H., Lee,J., Tong,S.
EPI_ISL_803426, EPI_ISL_803427, EPI_ISL_803472, EPI_ISL_803473, EPI_ISL_803474, EPI_ISL_803475, EPI_ISL_803476, EPI_ISL_803477, EPI_ISL_803478	Wisconsin State Laboratory of Hygiene Communicable Disease Division	Wisconsin State Laboratory of Hygiene Communicable Disease Division	Kelsey R. Florek, Abigail C. Shockey
EPI_ISL_803866	Institute of Virology, University of Cologne	Institute of Virology, University of Cologne	Saleta Sierra, Gibran Rubio, Zevanya Tessalonica, Dominik Aschenmeier, Eva Heger, Elena Knops, Rolf Kaiser, Maximilian Damagnez, Andreas Walker, Jörg Timm, Alexander Dilthey, Martin Däumer, Alex Thielen
EPI_ISL_803879	Keio University School of Medicine	Keio University School of Medicine	Kenjiro Kosaki, Yuka Iwasaki, Hirotsugu Ishizu, Haruhiko Siomi, Kodai Abe
EPI_ISL_803917, EPI_ISL_803918, EPI_ISL_803921, EPI_ISL_803922, EPI_ISL_803923, EPI_ISL_803924, EPI_ISL_803925, EPI_ISL_803926, EPI_ISL_803927, EPI_ISL_803928, EPI_ISL_803929, EPI_ISL_803930, EPI_ISL_803931, EPI_ISL_803932, EPI_ISL_803933, EPI_ISL_803934, EPI_ISL_803935, EPI_ISL_803936, EPI_ISL_803937, EPI_ISL_803938, EPI_ISL_803939			
see above	Pathogen Genomics Center, National Institute of Infectious Diseases	Pathogen Genomics Center, National Institute of Infectious Diseases	Tsuyoshi Sekizuka, Kentaro Itokawa, Rina Tanaka, Masanori Hashino, Makoto Kuroda
EPI_ISL_804568, EPI_ISL_804569	MEPHI, Aix Marseille University	MEPHI, Aix Marseille University	Anthony LEVASSEUR
EPI_ISL_812133, EPI_ISL_812137, EPI_ISL_812180, EPI_ISL_812181	GA Department of Public Health Laboratory	Pathogen Discovery, Respiratory Viruses Branch, Division of Viral Diseases, Centers for Disease Control and Prevention	Yan Li, Ying Tao, Anna Montmayeur, Jing Zhang, Brian Lynch, Krista Queen, Anna Uehara, Rachel Marine, Peter Cook, Clinton R. Paden, Haibin Wang, Suxiang Tong
EPI_ISL_812991, EPI_ISL_812992, EPI_ISL_812993, EPI_ISL_812994, EPI_ISL_812995, EPI_ISL_812996	University of Birmingham	COVID-19 Genomics UK (COG-UK) Consortium	Institute of Microbiology, University of Birmingham: Claire McMurray, Joanne Stockton, Samuel Nicholls, Radoslaw Poplawski, Will Rowe, Josh Quick, Nicholas Loman. University of Birmingham Testing Laboratory: Celina M Whalley, Andrew Bosworth, Charlotte Poxon, Kasun Wanigasooriya, Oliver Pickles, Mike Kidd, Alex Richter, Andrew D Beggs PHE Heartlands Lab: Husam Osman, Andrew Bosworth. Queen Elizabeth Hospital: Anna Casey
EPI_ISL_814682, EPI_ISL_814683, EPI_ISL_814684, EPI_ISL_814687	Virology Department, Royal Infirmary of Edinburgh, NHS Lothian / School of Biological Sciences, University of Edinburgh / Institute of Genetics and Molecular Medicine, University of Edinburgh	COVID-19 Genomics UK (COG-UK) Consortium	McHugh M, Dewar R, Rooke S, Gallagher M, Balcaza C, O'Toole Á, Scher E, Hill V, McCrone JT, Colquhoun R, Yu X, Jackson B, Rambaut A, Williams TC, Templeton K
EPI_ISL_814836	Wales Specialist Virology Centre Sequencing lab: Pathogen Genomics Unit	COVID-19 Genomics UK (COG-UK) Consortium	Catherine Moore, Johnathan Evans, Laura Gifford, Malorie Perry, Simon Cottrell, Angela Marchbank, Alec Birchley, Alexander Adams, Amy Gaskin, Bree Gatica-Wilcox, Jason Coombes, Joel Southgate, Lauren Gilbert, Lee Graham, Nicole Pacchiarini, Sara Kumziene-Summerhayes, Sarah Taylor, Sophie Jones, Sara Rey, Matthew Bull, Joanne Watkins, Sally Corden, Tom Connor
EPI_ISL_819454, EPI_ISL_819455	Northumbria University / South Tees Hospitals NHS Foundation Trust / North Cumbria Integrated Care NHS Foundation Trust / North Tees and Hartlepool NHS Foundation Trust / Newcastle Hospitals NHS Foundation Trust	COVID-19 Genomics UK (COG-UK) Consortium	Darren L Smith,Andrew Nelson,Matthew Bashton,Greg R Young,Joshua Loh,John Allan,Mohammad A Tariq,Giles S Holt,Gary Black,Wen C Yew,Lynn Dover,Paul Baker,Steve Liggett,Sarah Essex,Jane Greenaway,Debra Padgett,Clive Graham,Garren Scott,Edward Barton,Emma Swindells,Brendan Payne,Jennifer Collins,Yusri Taha,Gary Eltringham
EPI_ISL_825326, EPI_ISL_825347, EPI_ISL_825348	Hospital Universitari Vall d'Hebron - Vall d'Hebron Institut de Rercerca	Hospital Universitari Vall d'Hebron	Cristina Andrés, Maria Piñana, Josep F Abril, Damir Garcia-Cehic, Ariadna Rando, Juliana Esperalba, Maria Gema Codina, Carla Castillo, Maria Carmen Martin, Tomàs Pumarola, Josep Quer, Andrés Antón
EPI_ISL_825591, EPI_ISL_825592	Respiratory Virus Unit, National Infection Service, Public Health England	COVID-19 Genomics UK (COG-UK) Consortium	PHE Covid Sequencing Team
EPI_ISL_826591, EPI_ISL_826669, EPI_ISL_826670	Montefiore Medical Center	Albert Einstein College of Medicine, Dept. of Microbiology & Immunology, Chandran lab	J. Maximilian Fels, Saad Khan, Ryan Forster, Karin A. Skalina, Surksha Sirichand, Amy S. Fox, Aviv Bergman, William B. Mitchell, Lucia R. Wolgast, Wendy Szymczak, Robert H. Bortz III, M. Eugenia Dieterle, Catalina Florez, Denise Haslwanter, Rohit K. Jangra, Ethan Laudermilch, Ariel S. Wirchnianski, Jason Barnhill, David L. Goldman, Hnin Khine, D. Yitzchak Goldstein, Johanna P. Daily, Kartik Chandran, Libusha Kelly
EPI_ISL_826991	deCODE genetics	deCODE genetics	Daniel F Gudbjartsson; Agnar Helgason; Hakon Jonsson; Olafur T Magnusson; Pall Melsted; Gudmundur L Norddahl; Jona Saemundsdottir; Asgeir Sigurdsson; Patrick Sulem; Arna B Agustsdottir; Hannes Eggertsson; Berglind Eiriksdottir; Run Fridriksdottir; Elisabet E Gardarsdottir; Gudmundur Georgsson; Olafia S Gretarsdottir; Kjartan R Gudmundsson; Thora R Gunnarsdottir; Arnaldur Gylfason; Hilma Holm; Brynjar O Jensson; Aslaug Jonasdottir; Kamilla S Josefsdottir; Thordur Kristjansson; Droplaug N Magnusdottir; Solvi Rognvaldsson; Louise le Roux; Gudrun Sigmundsdottir; Gardar Sveinbjornsson; Kristin E Sveinsdottir; Maney Sveinsdottir; Emil A Thorarensen; Bjarni Thorbjornsson; Gisli Masson; Ingileif Jonsdottir; Alma Moller; Thorolfur Gudnason; Karl G Kristinsson; Unnur Thorsteinsdottir; Kari Stefansson
EPI_ISL_827074	The National University Hospital of Iceland	deCODE genetics	Daniel F Gudbjartsson; Agnar Helgason; Hakon Jonsson; Olafur T Magnusson; Pall Melsted; Gudmundur L Norddahl; Jona Saemundsdottir; Asgeir Sigurdsson; Patrick Sulem; Arna B Agustsdottir; Hannes Eggertsson; Berglind Eiriksdottir; Run Fridriksdottir; Elisabet E Gardarsdottir; Gudmundur Georgsson; Olafia S Gretarsdottir; Kjartan R Gudmundsson; Thora R Gunnarsdottir; Arnaldur Gylfason; Hilma Holm; Brynjar O Jensson; Aslaug Jonasdottir; Kamilla S Josefsdottir; Thordur Kristjansson; Droplaug N Magnusdottir; Solvi Rognvaldsson; Louise le Roux; Gudrun Sigmundsdottir; Gardar Sveinbjornsson; Kristin E Sveinsdottir; Maney Sveinsdottir; Emil A Thorarensen; Bjarni Thorbjornsson; Gisli Masson; Ingileif Jonsdottir; Alma Moller; Thorolfur Gudnason; Karl G Kristinsson; Unnur Thorsteinsdottir; Kari Stefansson
EPI_ISL_827278, EPI_ISL_827280, EPI_ISL_827281, EPI_ISL_827282, EPI_ISL_827283, EPI_ISL_827284, EPI_ISL_827285, EPI_ISL_827305, EPI_ISL_827308, EPI_ISL_827311, EPI_ISL_827312, EPI_ISL_827314, EPI_ISL_827315, EPI_ISL_827316, EPI_ISL_827317, EPI_ISL_827318, EPI_ISL_827319, EPI_ISL_827321, EPI_ISL_827370, EPI_ISL_827401, EPI_ISL_827418, EPI_ISL_827627, EPI_ISL_827628, EPI_ISL_827726, EPI_ISL_827728, EPI_ISL_827729, EPI_ISL_827730, EPI_ISL_827731, EPI_ISL_827962, EPI_ISL_828019			
see above	deCODE genetics	deCODE genetics	Daniel F Gudbjartsson; Agnar Helgason; Hakon Jonsson; Olafur T Magnusson; Pall Melsted; Gudmundur L Norddahl; Jona Saemundsdottir; Asgeir Sigurdsson; Patrick Sulem; Arna B Agustsdottir; Hannes Eggertsson; Berglind Eiriksdottir; Run Fridriksdottir; Elisabet E Gardarsdottir; Gudmundur Georgsson; Olafia S Gretarsdottir; Kjartan R Gudmundsson; Thora R Gunnarsdottir; Arnaldur Gylfason; Hilma Holm; Brynjar O Jensson; Aslaug Jonasdottir; Kamilla S Josefsdottir; Thordur Kristjansson; Droplaug N Magnusdottir; Solvi Rognvaldsson; Louise le Roux; Gudrun Sigmundsdottir; Gardar Sveinbjornsson; Kristin E Sveinsdottir; Maney Sveinsdottir; Emil A Thorarensen; Bjarni Thorbjornsson; Gisli Masson; Ingileif Jonsdottir; Alma Moller; Thorolfur Gudnason; Karl G Kristinsson; Unnur Thorsteinsdottir; Kari Stefansson
EPI_ISL_828020, EPI_ISL_828022, EPI_ISL_828025, EPI_ISL_828027	The National University Hospital of Iceland	deCODE genetics	Daniel F Gudbjartsson; Agnar Helgason; Hakon Jonsson; Olafur T Magnusson; Pall Melsted; Gudmundur L Norddahl; Jona Saemundsdottir; Asgeir Sigurdsson; Patrick Sulem; Arna B Agustsdottir; Hannes Eggertsson; Berglind Eiriksdottir; Run Fridriksdottir; Elisabet E Gardarsdottir; Gudmundur Georgsson; Olafia S Gretarsdottir; Kjartan R Gudmundsson; Thora R Gunnarsdottir; Arnaldur Gylfason; Hilma Holm; Brynjar O Jensson; Aslaug Jonasdottir; Kamilla S Josefsdottir; Thordur Kristjansson; Droplaug N Magnusdottir; Solvi Rognvaldsson; Louise le Roux; Gudrun Sigmundsdottir; Gardar Sveinbjornsson; Kristin E Sveinsdottir; Maney Sveinsdottir; Emil A Thorarensen; Bjarni Thorbjornsson; Gisli Masson; Ingileif Jonsdottir; Alma Moller; Thorolfur Gudnason; Karl G Kristinsson; Unnur Thorsteinsdottir; Kari Stefansson
EPI_ISL_828028, EPI_ISL_828039, EPI_ISL_828047, EPI_ISL_828072, EPI_ISL_828295, EPI_ISL_828296, EPI_ISL_828298, EPI_ISL_828299, EPI_ISL_828301, EPI_ISL_828303, EPI_ISL_828468, EPI_ISL_828496, EPI_ISL_828512, EPI_ISL_828569, EPI_ISL_828570, EPI_ISL_828571, EPI_ISL_828573, EPI_ISL_828575, EPI_ISL_828675, EPI_ISL_828691, EPI_ISL_828717, EPI_ISL_828729, EPI_ISL_828760, EPI_ISL_828787, EPI_ISL_828900			
see above	deCODE genetics	deCODE genetics	Daniel F Gudbjartsson; Agnar Helgason; Hakon Jonsson; Olafur T Magnusson; Pall Melsted; Gudmundur L Norddahl; Jona Saemundsdottir; Asgeir Sigurdsson; Patrick Sulem; Arna B Agustsdottir; Hannes Eggertsson; Berglind Eiriksdottir; Run Fridriksdottir; Elisabet E Gardarsdottir; Gudmundur

			Georgsson; Olafia S Gretarsdottir; Kjartan R Gudmundsson; Thora R Gunnarsdottir; Arnaldur Gylfason; Hilma Holm; Brynjar O Jensson; Aslaug Jonasdottir; Kamilla S Josefsdottir; Thordur Kristjansson; Droplaug N Magnusdottir; Solvi Rognvaldsson; Louise le Roux; Gudrun Sigmundsdottir; Gardar Sveinbjornsson; Kristin E Sveinsdottir; Maney Sveinsdottir; Emil A Thorarensen; Bjarni Thorbjornsson; Gisli Masson; Ingileif Jonsdottir; Alma Moller; Thorolfur Gudnason; Karl G Kristinsson; Unnur Thorsteinsdottir; Kari Stefansson
EPI_ISL_829060	The National University Hospital of Iceland	deCODE genetics	Daniel F Gudbjartsson; Agnar Helgason; Hakon Jonsson; Olafur T Magnusson; Pall Melsted; Gudmundur L Norddahl; Jona Saemundsdottir; Asgeir Sigurdsson; Patrick Sulem; Arna B Agustsdottir; Hannes Eggertsson; Berglind Eiriksdottir; Run Fridriksdottir; Elisabet E Gardarsdottir; Gudmundur Georgsson; Olafia S Gretarsdottir; Kjartan R Gudmundsson; Thora R Gunnarsdottir; Arnaldur Gylfason; Hilma Holm; Brynjar O Jensson; Aslaug Jonasdottir; Kamilla S Josefsdottir; Thordur Kristjansson; Droplaug N Magnusdottir; Solvi Rognvaldsson; Louise le Roux; Gudrun Sigmundsdottir; Gardar Sveinbjornsson; Kristin E Sveinsdottir; Maney Sveinsdottir; Emil A Thorarensen; Bjarni Thorbjornsson; Gisli Masson; Ingileif Jonsdottir; Alma Moller; Thorolfur Gudnason; Karl G Kristinsson; Unnur Thorsteinsdottir; Kari Stefansson
EPI_ISL_829061, EPI_ISL_829063, EPI_ISL_829064, EPI_ISL_829065, EPI_ISL_829067, EPI_ISL_829068, EPI_ISL_829069, EPI_ISL_829070, EPI_ISL_829095, EPI_ISL_829163, EPI_ISL_829187			
see above	deCODE genetics	deCODE genetics	Daniel F Gudbjartsson; Agnar Helgason; Hakon Jonsson; Olafur T Magnusson; Pall Melsted; Gudmundur L Norddahl; Jona Saemundsdottir; Asgeir Sigurdsson; Patrick Sulem; Arna B Agustsdottir; Hannes Eggertsson; Berglind Eiriksdottir; Run Fridriksdottir; Elisabet E Gardarsdottir; Gudmundur Georgsson; Olafia S Gretarsdottir; Kjartan R Gudmundsson; Thora R Gunnarsdottir; Arnaldur Gylfason; Hilma Holm; Brynjar O Jensson; Aslaug Jonasdottir; Kamilla S Josefsdottir; Thordur Kristjansson; Droplaug N Magnusdottir; Solvi Rognvaldsson; Louise le Roux; Gudrun Sigmundsdottir; Gardar Sveinbjornsson; Kristin E Sveinsdottir; Maney Sveinsdottir; Emil A Thorarensen; Bjarni Thorbjornsson; Gisli Masson; Ingileif Jonsdottir; Alma Moller; Thorolfur Gudnason; Karl G Kristinsson; Unnur Thorsteinsdottir; Kari Stefansson
EPI_ISL_829314, EPI_ISL_829315, EPI_ISL_829317, EPI_ISL_829318	The National University Hospital of Iceland	deCODE genetics	Daniel F Gudbjartsson; Agnar Helgason; Hakon Jonsson; Olafur T Magnusson; Pall Melsted; Gudmundur L Norddahl; Jona Saemundsdottir; Asgeir Sigurdsson; Patrick Sulem; Arna B Agustsdottir; Hannes Eggertsson; Berglind Eiriksdottir; Run Fridriksdottir; Elisabet E Gardarsdottir; Gudmundur Georgsson; Olafia S Gretarsdottir; Kjartan R Gudmundsson; Thora R Gunnarsdottir; Arnaldur Gylfason; Hilma Holm; Brynjar O Jensson; Aslaug Jonasdottir; Kamilla S Josefsdottir; Thordur Kristjansson; Droplaug N Magnusdottir; Solvi Rognvaldsson; Louise le Roux; Gudrun Sigmundsdottir; Gardar Sveinbjornsson; Kristin E Sveinsdottir; Maney Sveinsdottir; Emil A Thorarensen; Bjarni Thorbjornsson; Gisli Masson; Ingileif Jonsdottir; Alma Moller; Thorolfur Gudnason; Karl G Kristinsson; Unnur Thorsteinsdottir; Kari Stefansson
EPI_ISL_829566, EPI_ISL_829569, EPI_ISL_829784, EPI_ISL_829785, EPI_ISL_829786, EPI_ISL_829787, EPI_ISL_829788, EPI_ISL_829789, EPI_ISL_829790, EPI_ISL_829791, EPI_ISL_829792, EPI_ISL_829822, EPI_ISL_829823, EPI_ISL_829826, EPI_ISL_829827, EPI_ISL_829960, EPI_ISL_829961, EPI_ISL_829984, EPI_ISL_829986, EPI_ISL_830006			
see above	deCODE genetics	deCODE genetics	Daniel F Gudbjartsson; Agnar Helgason; Hakon Jonsson; Olafur T Magnusson; Pall Melsted; Gudmundur L Norddahl; Jona Saemundsdottir; Asgeir Sigurdsson; Patrick Sulem; Arna B Agustsdottir; Hannes Eggertsson; Berglind Eiriksdottir; Run Fridriksdottir; Elisabet E Gardarsdottir; Gudmundur Georgsson; Olafia S Gretarsdottir; Kjartan R Gudmundsson; Thora R Gunnarsdottir; Arnaldur Gylfason; Hilma Holm; Brynjar O Jensson; Aslaug Jonasdottir; Kamilla S Josefsdottir; Thordur Kristjansson; Droplaug N Magnusdottir; Solvi Rognvaldsson; Louise le Roux; Gudrun Sigmundsdottir; Gardar Sveinbjornsson; Kristin E Sveinsdottir; Maney Sveinsdottir; Emil A Thorarensen; Bjarni Thorbjornsson; Gisli Masson; Ingileif Jonsdottir; Alma Moller; Thorolfur Gudnason; Karl G Kristinsson; Unnur Thorsteinsdottir; Kari Stefansson
EPI_ISL_830316, EPI_ISL_830327, EPI_ISL_830537	The National University Hospital of Iceland	deCODE genetics	Daniel F Gudbjartsson; Agnar Helgason; Hakon Jonsson; Olafur T Magnusson; Pall Melsted; Gudmundur L Norddahl; Jona Saemundsdottir; Asgeir Sigurdsson; Patrick Sulem; Arna B Agustsdottir; Hannes Eggertsson; Berglind Eiriksdottir; Run Fridriksdottir; Elisabet E Gardarsdottir; Gudmundur Georgsson; Olafia S Gretarsdottir; Kjartan R Gudmundsson; Thora R Gunnarsdottir; Arnaldur Gylfason; Hilma Holm; Brynjar O Jensson; Aslaug Jonasdottir; Kamilla S Josefsdottir; Thordur Kristjansson; Droplaug N Magnusdottir; Solvi Rognvaldsson; Louise le Roux; Gudrun Sigmundsdottir; Gardar Sveinbjornsson; Kristin E Sveinsdottir; Maney Sveinsdottir; Emil A Thorarensen; Bjarni Thorbjornsson; Gisli Masson; Ingileif Jonsdottir; Alma Moller; Thorolfur Gudnason; Karl G Kristinsson; Unnur Thorsteinsdottir; Kari Stefansson
EPI_ISL_830732	University Hospital Basel, Clinical Virology	University Hospital Basel, Clinical Bacteriology	Tim Roloff, Madlen Stange, Helena MB Seth-Smith, Alfredo Mari, Karoline Leuzinger, Julia Bielicki, Manuel Battegay, Hans Hirsch, Adrian Egli
EPI_ISL_831224, EPI_ISL_831225, EPI_ISL_831226, EPI_ISL_831227	Hospital Universitario La Paz (Madrid)	SeqCOVID-SPAIN consortium/IBV(CSIC)	María Rodríguez-Tejedor, Elias Dahdouh, Fernando Lázaro-Perona, Jesús Mingorance and SeqCOVID-SPAIN consortium
EPI_ISL_831763	United States Air Force School of Aerospace Medicine	United States Air Force School of Aerospace Medicine	Anthony Fries, Jennifer Meyer, William Gruner, Amanda Javorina, Sarah Purves, Clarise Starr, Elizabeth Macias
EPI_ISL_832597, EPI_ISL_832598, EPI_ISL_832599, EPI_ISL_832600, EPI_ISL_832601, EPI_ISL_832602, EPI_ISL_832603, EPI_ISL_832604, EPI_ISL_832605, EPI_ISL_832606, EPI_ISL_832607, EPI_ISL_832608, EPI_ISL_832655, EPI_ISL_832656, EPI_ISL_832657, EPI_ISL_832658, EPI_ISL_832659, EPI_ISL_832660, EPI_ISL_832661, EPI_ISL_832662, EPI_ISL_832663, EPI_ISL_832664, EPI_ISL_832679, EPI_ISL_832680, EPI_ISL_832681, EPI_ISL_832682, EPI_ISL_832683, EPI_ISL_832684, EPI_ISL_832694			
see above	OHSU Lab Services Molecular Microbiology Lab	Oregon SARS-CoV-2 Genome Sequencing Center	Brendan L. O'Connell, Ruth V. Nichols, Sally Grindstaff, Alec J. Hirsch, Donna Hansel, Guang Fan, Daniel N. Streblow, William B. Messer, Andrew C. Adey, Benjamin N. Bimber, Brian J. O'Roak
EPI_ISL_839985	Queens Medical Centre, Clinical Microbiology Department / DeepSeq Nottingham	COVID-19 Genomics UK (COG-UK) Consortium	Gemma Clark, Wendy Smith, Manjinder Khakh, Vicki M Fleming, Michelle M Lister, Hannah Howson-Wells, Jonathan Ball, Patrick McClure, Joseph Chappell, Theocharis Tsoleridis, Nadine Holmes, Matthew Carlisle, Christopher Moore, Fei Sang, Johnny Debebe, Victoria Wright, Matthew Loose
EPI_ISL_842347, EPI_ISL_842352	Virology Department, Sheffield Teaching Hospitals NHS Foundation Trust/Department of Infection, Immunity and Cardiovascular Disease, The Medical School, University of Sheffield	COVID-19 Genomics UK (COG-UK) Consortium	Thushan de Silva, Matthew Parker, Nikki Smith, Adri Angyal, Rebecca Brown, Luke Green, Rachel Tucker, Paul Parsons, Danielle Groves, Katie Johnson, Laura Carrilero, Alex Keeley, Dave Partridge, Matthew Wyles, Benjamin Lindsey, Mehmet Yavuz, Mohammad Raza, Cariad Evans
EPI_ISL_842867, EPI_ISL_842868	Barts Health NHS Trust	COVID-19 Genomics UK (COG-UK) Consortium	CUTINO-MOGUEL, Maria-Teresa; HARRINGTON, David; OWOYEMI, Dola; SHYLINI, Raghavendran; BROAD, Claire; KELE, Beatrix
EPI_ISL_849441, EPI_ISL_849499, EPI_ISL_849607	Seattle Flu Study	Seattle Flu Study	Deborah A. Nickerson, Chris D. Frazar, Jover Lee, Benjamin Pelle, Matthew Richardson, Amanda Adler, Elisabeth Brandstetter, Peter D. Han, Kairsten Fay, Misja Ilcisin, Kirsten Lacombe, Thomas R. Sibley, Melissa Truong, Caitlin R. Wolf, Michael Boeckh, Janet A. Englund, Michael Famulare, Barry R. Lutz, Mark J. Rieder, Lea M. Starita, Matthew Thompson, Jay Shendure, Trevor Bedford, Helen Y. Chu
EPI_ISL_850950	National Institute for Viral Disease Control and Prevention, China CDC	National Institute for Viral Disease Control and Prevention, China CDC	Xiang Zhao, Yenan Feng, Zhixiao Chen, Yao Meng, Yuchao Wu, Yang Song, Ji Wang, Kai Nie, Yong Zhang, Yanhai Wang, Weimin Zhou, Wenjie Tan, Jun Han, Shiwen Wang, Wenbo Xu, Cao Chen, Dayan Wang
EPI_ISL_851055, EPI_ISL_851056, EPI_ISL_851057, EPI_ISL_851058, EPI_ISL_851059, EPI_ISL_851060, EPI_ISL_851061	National Food and Veterinary Risk Assessment Institute	Lithuanian University of Health Sciences, Molecular cardiology lab.	Lukas Zemaitis, Arnoldas Pautienius, Ingrida Olendrait, Kamile Tamusauskaite, Dovydas Gecys, Laura Pareckaite, Vaiva Lesauskaite, Astra Vitkauskiene, Arunas Stankevicius, Algirdas Malakauskas
EPI_ISL_852983	Hospital General Universitario Gregorio Marañón	SeqCOVID-SPAIN consortium/IBV(CSIC)	Darío García de Viedma, Laura Pérez-Lago, Pedro J Sola-Campoy, Sergio Buenestado-Serrano, Marta Herranz, Victor Manuel de la Cueva, Julia Suárez, Pilar Catalán, Patricia Muñoz and SeqCOVID-SPAIN consortium
EPI_ISL_853316	UPMC Clinical Microbiology Laboratory	Microbial Genome Sequencing Center; Microbial Genomic Epidemiology Laboratory	Mustapha M. Mustapha, Jane W. Marsh, Dan Snyder, Marissa P. Griffith, Stephanie L. Mitchell, Vatsala R. Srinivasa, Kady D. Waggle, Chinelo Ezeonwuku, Vaughn S. Cooper, Lee H. Harrison
EPI_ISL_853936, EPI_ISL_853938, EPI_ISL_853944	Department of Microbiology, University Innsbruck	Bergthaler laboratory, CeMM Research Center for Molecular Medicine of the Austrian Academy of Sciences	Lukas Endler, Alexandra Popa, Benedikt Agerer, Jakob-Wendelin Genger, Alexander Lercher, Anna Schedl, Thomas Penz, Michael Schuster, Jan Laine, Martin Senekowitsch, Christoph Bock, Andreas Bergthaler
EPI_ISL_854613, EPI_ISL_854614, EPI_ISL_854615, EPI_ISL_854616, EPI_ISL_854617, EPI_ISL_854618, EPI_ISL_854619, EPI_ISL_854620, EPI_ISL_854621, EPI_ISL_854622, EPI_ISL_854623, EPI_ISL_854624, EPI_ISL_854715, EPI_ISL_854736			
see above	Public Health Ontario Laboratory	Public Health Ontario Laboratory	Vanessa G Allen, Philip Banh, Yao Chen, Richard de Borja, Alireza Eshaghi, Nahuel Fittipaldi, Christine Frantz, Jonathan B Gubbay, Jennifer L Guthrie, Lawrence Heisler, Esha Joshi, Michael Laszloffy, Aimin Li, Michael CY Li, Dean Maxwell, Sandeep Nagra, Samir N Patel, Jared Simpson, Karthikeyan Sivaraman, Ashleigh Sullivan, Yogi Sundaravadanam, Sarah Teatero, Matthew Watson, Andre Villegas, Sandra Zittermann
EPI_ISL_855500	KEMRI-Wellcome Trust Research Programme/KEMRI-CGMR-C Kilifi	KEMRI-Wellcome Trust Research Programme/KEMRI-CGMR-C Kilifi	Githinji et al
EPI_ISL_856794, EPI_ISL_856795, EPI_ISL_856796	Servicio Virosis Respiratorias-Departamento Virología-INEI	Instituto Nacional Enfermedades Infecciosas C.G.Malbran	Baumeister E., Avaro M., Benedetti E., Russo M., Dattero ME, Pontoriero A., Cisterna D., Molina V., Perandones C., Tuduri E., Lorenzo F., Poklepovich T., Campos J.

EPI_ISL_859797, EPI_ISL_859800	BTC, Khalifa University	BTC, Khalifa University	Al Safar et al
EPI_ISL_860160	Keio University School of Medicine	Keio University School of Medicine	Kenjiro Kosaki, Yuka Iwasaki, Hirotsugu Ishizu, Haruhiko Siomi, Kodai Abe
EPI_ISL_860732, EPI_ISL_860773, EPI_ISL_860774, EPI_ISL_860775, EPI_ISL_860776	Swiss National Reference Centre for Influenza	Swiss National Reference Centre for Influenza	Ana Rita Gonçalves Cabecinhas, Samuel Cordey, Florian Laubscher,Christoph Grünig, Laurent Kaiser
EPI_ISL_860814	WHO/Minsk	Charité Universitätsmedizin Berlin, Institut für Virologie	Victor M Corman, Barbara Mühlemann, Jörn Beheim-Schwarzbach, Talitha Veith, Julia Tesch, Tobias Bleicker, Julia Schneider, Shmialiova Natallia, Sivets Natallia, Terry Jones, Christian Drosten
EPI_ISL_861461	UHAS COVID-19 Lab	UHAS COVID-19 Lab	Kwabena O. Duedu, Jones Gyamfi, Reuben Ayivor-Djanie, John O. Gyapong and the UHAS COVID-19 Lab Team
EPI_ISL_861671	Hospital Municipal Prefeito Waldemar Costa Filho	Instituto Adolfo Lutz, Interdiciplinary Procedures Center, Strategic Laboratory	Claudio Tavares Sacchi, Claudia Regina Gonçalves, Erica Valessa Ramos Gomes, Karoline Rodrigues Campos
EPI_ISL_867964	Centre for Enzyme Innovation, University of Portsmouth / Translational Research Laboratory, Portsmouth Hospitals NHS Trust	COVID-19 Genomics UK (COG-UK) Consortium	Angela Beckett,Yann Bourgeois,Garry Scarlett,Sharon Glaysher,Scott Elliott,Kelly Bicknell,Robert Impey,Allyson Lloyd,Sarah Wyllie,Ethan Butcher,Anoop Chauhan,Samuel Robson
EPI_ISL_868794, EPI_ISL_868796, EPI_ISL_868803, EPI_ISL_868812, EPI_ISL_868814	Bioinformatics and Biostatistics Lab, Advanced Sequencing Facility	COVID-19 Genomics UK (COG-UK) Consortium	Aengus Stewart,Jerome Nicod,Chelsea Sawyer,Laura Cubitt,Harshil Patel,Margaret Crawford
EPI_ISL_872745	Colorado Mesa University	Infectious Disease Program, Broad Institute of Harvard and MIT	Lemieux,J.E., Siddle,K.J., Marshall,J., ONeill,M., Bronson,A., Adams,G., Gladden-Young,A., Lagerborg,K., Rudy,M., DeRuff,K., Carter,A., Normandin,E., Bauer,M., Reilly,S., Tomkins-Tinch,C., Loreth,C., Chaluvadi,S., Birren,B.W., Gallagher,G., Smole,S., Park,D.J., MacInnis,B.L., and Sabeti,P.C.
EPI_ISL_875814, EPI_ISL_875815	Public Health Ontario Laboratory	Public Health Ontario Laboratory	Vanessa G Allen, Philip Banh, Yao Chen, Richard de Borja, Alireza Eshaghi, Nahuel Fittipaldi, Christine Frantz, Jonathan B Gubbay, Jennifer L Guthrie, Lawrence Heisler, Esha Joshi, Michael Laszloffy, Aimin Li, Michael CY Li, Dean Maxwell, Sandeep Nagra, Samir N Patel, Jared Simpson, Karthikeyan Sivaraman, Ashleigh Sullivan, Yogi Sundaravadanam, Sarah Teatero, Matthew Watson, Andre Villegas, Sandra Zittermann
EPI_ISL_875876, EPI_ISL_875878, EPI_ISL_875880, EPI_ISL_875881, EPI_ISL_875885, EPI_ISL_875888, EPI_ISL_875889, EPI_ISL_875900, EPI_ISL_875902, EPI_ISL_875905, EPI_ISL_875913, EPI_ISL_875914, EPI_ISL_875916, EPI_ISL_875917, EPI_ISL_875918, EPI_ISL_875919, EPI_ISL_875920, EPI_ISL_875923, EPI_ISL_875925, EPI_ISL_875926, EPI_ISL_875928, EPI_ISL_875929, EPI_ISL_875930, EPI_ISL_875931, EPI_ISL_875932, EPI_ISL_875933, EPI_ISL_875935, EPI_ISL_875936, EPI_ISL_875937, EPI_ISL_875940, EPI_ISL_875941, EPI_ISL_875942, EPI_ISL_875943, EPI_ISL_875944, EPI_ISL_875947, EPI_ISL_875951, EPI_ISL_875955, EPI_ISL_875956, EPI_ISL_875957			
see above	Eurofins Diatherix	Hudsonalpha Genome Sequencing Center	Jane Grimwood, Melissa Williams, Lori H. Handley, Joshua Stough, Leslie Malone, Stefan Brzezinski, Ada Stewart, Teresa Jones, Jenell Webber, John Lovell, Jennifer Cart, and Jeremy Schmutz
EPI_ISL_876819, EPI_ISL_876829, EPI_ISL_876850, EPI_ISL_876851, EPI_ISL_876852, EPI_ISL_876853, EPI_ISL_876854, EPI_ISL_876855, EPI_ISL_876856, EPI_ISL_876857, EPI_ISL_876858, EPI_ISL_876859			
see above	Quest Diagnostics	Quest Diagnostics	Rosenthal,S.H., Gerasimova,A., Kagan,R.M., Anderson, B., Hua, M., Liu Y., Bernstein, L.E., Livingston, K.E., Perez, A., Shalhout, D.F., Shlyakhter, I.A., Owen, R., Tanpaiboon, P., Lacbawan, F.
EPI_ISL_877428, EPI_ISL_877429	Institute of Microbiology and Immunology, Faculty of Medicine, University of Ljubljana	Institute of Microbiology and Immunology, Faculty of Medicine, University of Ljubljana	Samo Zakotnik, Tomaž Mark Zorec, Matic Brvar, Miša Korva, Mario Poljak, Tatjana Avši - Županc
EPI_ISL_882687, EPI_ISL_882710, EPI_ISL_882761	1.AO Universitaria 'S. Giovanni di Dio e Ruggi D'Aragona, Scuola Medica Salernitana' Hospital / 2.UOC di Virologia e Microbiologia, Università della Campania 'L. Vanvitelli' / 3.AO Universitaria 'Federico II' Napoli Hospital / 4.AORN 'San Giuseppe Moscati' Avellino Hospital / 5.AO 'San Pio - presidio G. Rummo' Benevento Hospital / 6.AO 'Sant'Anna e San Sebastiano' Caserta Hospital / 7.PO 'Maria Santissima Addolorata' Eboli Hospital / 8.Biogem Istituto di Ricerche Genetiche	1. Genome Research Center for Health (CRGS) / 2. Laboratory of Molecular Medicine and Genomics(LMMGe) / 3. Center for Research in Pure and Applied Mathematics (CRMPA)	Giorgio Giurato, Francesca Rizzo, Alessandro Weisz, Gianluigi Franci, Giovanni Nassa, Pasquale Pagliano, Roberta Tarallo, Elena Alexandrova, Ylenia D'Agostino, Carlo Ferravante, Jessica Lamberti, Viola Melone, Domenico Memoli, Valeria Mirici Cappa, Domenico Palumbo, Giovanni Pecoraro, Assunta Sellitto, Oriana Strianese, Ilaria Terenzi, Giuseppe Fenza, Aniello Gentile, Antonello Saccomanno, Sonia Amabile, Teresa Rocco, Annamaria Salvati, Emilia Vaccaro, Massimiliano Galdiero, Michele Cennamo, Giuseppe Portella, Maria Grazia Foti, Mariarosaria Ingino, Maria Landi, Maurizio Fumi, Vincenzo Rocco, Rita Greco, Vittoria Letizia, Arnolfo Petruzziello, Maddalena Schioppa, Gregorio Goffredi, Francesca Marciano, Michele Caraglia, Alessia Cossu, Marianna Scrima
EPI_ISL_884318, EPI_ISL_884319, EPI_ISL_884370	Infectious Diseases, Quest Diagnostics	Infectious Diseases, Quest Diagnostics	Rosenthal,S.H., Gerasimova,A., Kagan,R.M., Anderson,B., Bernstein,L.E., Livingston,K.E., Hua,M., Liu,Y., Shalhout,D.F., Owen,R., Lacbawan,F.
EPI_ISL_885128	California Institute of Technology	Chan-Zuckerberg Biohub	CZB Cliahub Consortium
EPI_ISL_887158	Massachusetts General Hospital	Infectious Disease Program, Broad Institute of Harvard and MIT	Lemieux,J.E., Siddle,K.J., Shaw,B., Adams,G., Pierce,V., Turbett,S., Anahtar,M., Branda,J., Slater,D., Harris,J., Lin,A.E., Gladden-Young,A., Lagerborg,K., Rudy,M., DeRuff,K., Carter,A., Normandin,E., Bauer,M., Reilly,S., Tomkins-Tinch,C., Loreth,C., Chaluvadi,S., Neumann,A., Cusick,C., Chapman,S.B., Gnirke,A., Flowers,K., Cerrato,F., Birren,B.W., Gallagher,G., Smole,S., Park,D.J., MacInnis,B.L., Ryan,E., LaRocque,R., Rosenberg,E. and Sabeti,P.C.
EPI_ISL_892373, EPI_ISL_892374, EPI_ISL_892375, EPI_ISL_892376, EPI_ISL_892377, EPI_ISL_892407, EPI_ISL_892408, EPI_ISL_892409, EPI_ISL_892410, EPI_ISL_892411, EPI_ISL_892412, EPI_ISL_892413, EPI_ISL_892414, EPI_ISL_892566, EPI_ISL_892567, EPI_ISL_892568, EPI_ISL_892569, EPI_ISL_892570, EPI_ISL_892571, EPI_ISL_892572, EPI_ISL_892573, EPI_ISL_892574, EPI_ISL_892575, EPI_ISL_892576, EPI_ISL_892577, EPI_ISL_892578, EPI_ISL_892579, EPI_ISL_892580, EPI_ISL_892581, EPI_ISL_892582, EPI_ISL_892583, EPI_ISL_892584, EPI_ISL_892700, EPI_ISL_893252, EPI_ISL_893253, EPI_ISL_893254, EPI_ISL_893272, EPI_ISL_893273, EPI_ISL_893274, EPI_ISL_893275, EPI_ISL_893276, EPI_ISL_893277, EPI_ISL_893278, EPI_ISL_893279, EPI_ISL_893280			
see above	Pathogen Genomics Center, National Institute of Infectious Diseases	Pathogen Genomics Center, National Institute of Infectious Diseases	Tsuyoshi Sekizuka, Kentaro Itokawa, Rina Tanaka, Masanori Hashino, Makoto Kuroda
EPI_ISL_893369, EPI_ISL_893370, EPI_ISL_893372, EPI_ISL_893373, EPI_ISL_893374, EPI_ISL_893409, EPI_ISL_893410, EPI_ISL_893411, EPI_ISL_893412, EPI_ISL_893413, EPI_ISL_893414, EPI_ISL_893415, EPI_ISL_893416, EPI_ISL_893417, EPI_ISL_893418, EPI_ISL_893419, EPI_ISL_893443, EPI_ISL_893444, EPI_ISL_893445, EPI_ISL_893446, EPI_ISL_893447, EPI_ISL_893448, EPI_ISL_893449, EPI_ISL_893450, EPI_ISL_893451, EPI_ISL_893452, EPI_ISL_893453, EPI_ISL_893454, EPI_ISL_893455, EPI_ISL_893456, EPI_ISL_893457, EPI_ISL_893458, EPI_ISL_893459, EPI_ISL_893460, EPI_ISL_893461, EPI_ISL_893462, EPI_ISL_893463, EPI_ISL_893464, EPI_ISL_893465, EPI_ISL_893466, EPI_ISL_893467, EPI_ISL_893468, EPI_ISL_893469, EPI_ISL_893470, EPI_ISL_893471, EPI_ISL_893472, EPI_ISL_893473, EPI_ISL_893474, EPI_ISL_893475, EPI_ISL_893476, EPI_ISL_893477, EPI_ISL_893478, EPI_ISL_893479, EPI_ISL_893480, EPI_ISL_893481, EPI_ISL_893482, EPI_ISL_893483, EPI_ISL_893484, EPI_ISL_893485, EPI_ISL_893486, EPI_ISL_893487, EPI_ISL_893488, EPI_ISL_893489, EPI_ISL_893490, EPI_ISL_893491, EPI_ISL_893492, EPI_ISL_893493, EPI_ISL_893494, EPI_ISL_893495, EPI_ISL_893496, EPI_ISL_893497, EPI_ISL_893498, EPI_ISL_893499, EPI_ISL_893500, EPI_ISL_893501, EPI_ISL_893502, EPI_ISL_893503, EPI_ISL_893504, EPI_ISL_893505, EPI_ISL_893506, EPI_ISL_893507, EPI_ISL_893508, EPI_ISL_893509, EPI_ISL_893510, EPI_ISL_893511, EPI_ISL_893512, EPI_ISL_893513, EPI_ISL_893514, EPI_ISL_893515, EPI_ISL_893516, EPI_ISL_893517, EPI_ISL_893518, EPI_ISL_893519, EPI_ISL_893520, EPI_ISL_893521, EPI_ISL_893522, EPI_ISL_893523, EPI_ISL_893524, EPI_ISL_893525, EPI_ISL_893526, EPI_ISL_893527, EPI_ISL_893528, EPI_ISL_893529, EPI_ISL_893530, EPI_ISL_893531, EPI_ISL_893532, EPI_ISL_893533, EPI_ISL_893534, EPI_ISL_893535, EPI_ISL_893536, EPI_ISL_893537, EPI_ISL_893538, EPI_ISL_893539, EPI_ISL_893540, EPI_ISL_893541, EPI_ISL_893542, EPI_ISL_893543, EPI_ISL_893544, EPI_ISL_893545, EPI_ISL_893546, EPI_ISL_893547, EPI_ISL_893548, EPI_ISL_893549, EPI_ISL_893550, EPI_ISL_893551, EPI_ISL_893552, EPI_ISL_893553, EPI_ISL_893554, EPI_ISL_893555, EPI_ISL_893556, EPI_ISL_893557, EPI_ISL_893558, EPI_ISL_893559, EPI_ISL_893560, EPI_ISL_893561, EPI_ISL_893562, EPI_ISL_893563, EPI_ISL_893564, EPI_ISL_893565, EPI_ISL_893566, EPI_ISL_893567, EPI_ISL_893568, EPI_ISL_893569, EPI_ISL_893570, EPI_ISL_893571, EPI_ISL_893572, EPI_ISL_893573, EPI_ISL_893574, EPI_ISL_893575, EPI_ISL_893576, EPI_ISL_893577, EPI_ISL_893578, EPI_ISL_893579, EPI_ISL_893580, EPI_ISL_893581, EPI_ISL_893582, EPI_ISL_893583, EPI_ISL_893584, EPI_ISL_893585, EPI_ISL_893586, EPI_ISL_893587, EPI_ISL_893588, EPI_ISL_893589, EPI_ISL_893590, EPI_ISL_893591, EPI_ISL_893592, EPI_ISL_893593, EPI_ISL_893594, EPI_ISL_893595, EPI_ISL_893596, EPI_ISL_893597, EPI_ISL_893598, EPI_ISL_893599, EPI_ISL_893600, EPI_ISL_893601, EPI_ISL_893602, EPI_ISL_893603, EPI_ISL_893604, EPI_ISL_893605, EPI_ISL_893606, EPI_ISL_893607, EPI_ISL_893674, EPI_ISL_893675, EPI_ISL_893676			
see above	Department of Infectious Diseases, Kobe Institute of Health	Department of Infectious Diseases, Kobe Institute of Health	Ryohei Nomoto, Tomotada Iwamoto, Tsuyoshi Sekizuka, Kentaro Itokawa, Rina Tanaka, Masanori Hashino, Makoto Kuroda
EPI_ISL_894287	Fukuoka Institute of Health and Environmental Sciences	Pathogen Genomics Center, National Institute of Infectious Diseases	Tsuyoshi Sekizuka, Kentaro Itokawa, Rina Tanaka, Masanori Hashino, Makoto Kuroda
EPI_ISL_894297, EPI_ISL_894300, EPI_ISL_894303, EPI_ISL_894531, EPI_ISL_894532, EPI_ISL_894533, EPI_ISL_894534, EPI_ISL_894535, EPI_ISL_894536, EPI_ISL_894537, EPI_ISL_894538, EPI_ISL_894539, EPI_ISL_894540, EPI_ISL_894541, EPI_ISL_894542, EPI_ISL_894543, EPI_ISL_894544, EPI_ISL_894545, EPI_ISL_894546, EPI_ISL_894547, EPI_ISL_894548, EPI_ISL_894549, EPI_ISL_894550, EPI_ISL_894551, EPI_ISL_894552, EPI_ISL_894553, EPI_ISL_894554, EPI_ISL_894555, EPI_ISL_894556, EPI_ISL_894557, EPI_ISL_894558, EPI_ISL_894559, EPI_ISL_894560, EPI_ISL_894561, EPI_ISL_894562, EPI_ISL_894563, EPI_ISL_894564, EPI_ISL_894565, EPI_ISL_894566, EPI_ISL_894567, EPI_ISL_894568, EPI_ISL_894569, EPI_ISL_894570, EPI_ISL_894571, EPI_ISL_894572, EPI_ISL_894573, EPI_ISL_894574, EPI_ISL_894575, EPI_ISL_894576, EPI_ISL_894577, EPI_ISL_894578, EPI_ISL_894579, EPI_ISL_894580, EPI_ISL_894581, EPI_ISL_894582, EPI_ISL_894583, EPI_ISL_894584, EPI_ISL_894585, EPI_ISL_894586, EPI_ISL_894587, EPI_ISL_894588, EPI_ISL_894589, EPI_ISL_894590, EPI_ISL_894591, EPI_ISL_894592, EPI_ISL_894593, EPI_ISL_894594, EPI_ISL_894595, EPI_ISL_894596, EPI_ISL_894597, EPI_ISL_894598, EPI_ISL_894599, EPI_ISL_894600, EPI_ISL_894601, EPI_ISL_894602, EPI_ISL_894603, EPI_ISL_894604, EPI_ISL_894605, EPI_ISL_894606, EPI_ISL_894607, EPI_ISL_894608, EPI_ISL_894609, EPI_ISL_894610, EPI_ISL_894611, EPI_ISL_894612, EPI_ISL_894613, EPI_ISL_894614, EPI_ISL_894615, EPI_ISL_894616, EPI_ISL_894617, EPI_ISL_894618, EPI_ISL_894619, EPI_ISL_894620, EPI_ISL_894621, EPI_ISL_894622, EPI_ISL_894623			
see above	Pathogen Genomics Center, National Institute of Infectious Diseases	Pathogen Genomics Center, National Institute of Infectious Diseases	Tsuyoshi Sekizuka, Kentaro Itokawa, Rina Tanaka, Masanori Hashino, Makoto Kuroda
EPI_ISL_894672, EPI_ISL_894673, EPI_ISL_894674, EPI_ISL_894675, EPI_ISL_894676, EPI_ISL_894677, EPI_ISL_894678, EPI_ISL_894679, EPI_ISL_894680, EPI_ISL_894688, EPI_ISL_894689, EPI_ISL_894690, EPI_ISL_894691, EPI_ISL_894692, EPI_ISL_894693, EPI_ISL_894694			

see above	Fukuoka Institute of Health and Environmental Sciences	Pathogen Genomics Center, National Institute of Infectious Diseases	Tsuyoshi Sekizuka, Kentaro Itokawa, Rina Tanaka, Masanori Hashino, Makoto Kuroda
EPI_ISL_894817, EPI_ISL_894818, EPI_ISL_894819, EPI_ISL_894893, EPI_ISL_894894, EPI_ISL_894895, EPI_ISL_894896, EPI_ISL_894897, EPI_ISL_894898, EPI_ISL_894899, EPI_ISL_894900, EPI_ISL_894901, EPI_ISL_894902, EPI_ISL_894903, EPI_ISL_894904, EPI_ISL_894905, EPI_ISL_894906, EPI_ISL_894907, EPI_ISL_894908, EPI_ISL_894909, EPI_ISL_894910, EPI_ISL_894911, EPI_ISL_894912, EPI_ISL_894948, EPI_ISL_894949, EPI_ISL_894950, EPI_ISL_894951, EPI_ISL_894952, EPI_ISL_894953, EPI_ISL_894954, EPI_ISL_894955, EPI_ISL_894956, EPI_ISL_894957, EPI_ISL_894958, EPI_ISL_894967, EPI_ISL_894968, EPI_ISL_894969, EPI_ISL_894970, EPI_ISL_894971, EPI_ISL_894972, EPI_ISL_894973, EPI_ISL_894974, EPI_ISL_894975, EPI_ISL_894976, EPI_ISL_894977, EPI_ISL_894978, EPI_ISL_894979, EPI_ISL_894980, EPI_ISL_894981, EPI_ISL_894982, EPI_ISL_894983, EPI_ISL_894984, EPI_ISL_894985, EPI_ISL_894986, EPI_ISL_894987, EPI_ISL_894988, EPI_ISL_894989, EPI_ISL_894990, EPI_ISL_894991, EPI_ISL_894992, EPI_ISL_894993, EPI_ISL_894994, EPI_ISL_894995, EPI_ISL_894996, EPI_ISL_894997, EPI_ISL_894998, EPI_ISL_894999, EPI_ISL_895000, EPI_ISL_895001			
see above	Pathogen Genomics Center, National Institute of Infectious Diseases	Pathogen Genomics Center, National Institute of Infectious Diseases	Tsuyoshi Sekizuka, Kentaro Itokawa, Rina Tanaka, Masanori Hashino, Makoto Kuroda
EPI_ISL_895002, EPI_ISL_895003, EPI_ISL_895004, EPI_ISL_895005, EPI_ISL_895006, EPI_ISL_895007, EPI_ISL_895008, EPI_ISL_895009, EPI_ISL_895010, EPI_ISL_895011, EPI_ISL_895012			
see above	Niigata Prefectural Institute of Public Health and Environmental Sciences	Pathogen Genomics Center, National Institute of Infectious Diseases	Tsuyoshi Sekizuka, Kentaro Itokawa, Rina Tanaka, Masanori Hashino, Makoto Kuroda
EPI_ISL_895014, EPI_ISL_895015, EPI_ISL_895016, EPI_ISL_895017, EPI_ISL_895018, EPI_ISL_895019, EPI_ISL_895020, EPI_ISL_895021, EPI_ISL_895022, EPI_ISL_895070, EPI_ISL_895071, EPI_ISL_895072, EPI_ISL_895073, EPI_ISL_895074, EPI_ISL_895075, EPI_ISL_895076, EPI_ISL_895077, EPI_ISL_895078, EPI_ISL_895079, EPI_ISL_895080, EPI_ISL_895081, EPI_ISL_895082, EPI_ISL_895083, EPI_ISL_895084, EPI_ISL_895085, EPI_ISL_895086, EPI_ISL_895087, EPI_ISL_895088, EPI_ISL_895093			
see above	Department of Infectious Diseases, Kobe Institute of Health	Pathogen Genomics Center, National Institute of Infectious Diseases	Tsuyoshi Sekizuka, Kentaro Itokawa, Rina Tanaka, Masanori Hashino, Makoto Kuroda
EPI_ISL_895106, EPI_ISL_895107, EPI_ISL_895108, EPI_ISL_895109, EPI_ISL_895110, EPI_ISL_895111, EPI_ISL_895112, EPI_ISL_895113, EPI_ISL_895114, EPI_ISL_895115, EPI_ISL_895116, EPI_ISL_895117, EPI_ISL_895118, EPI_ISL_895119, EPI_ISL_895120, EPI_ISL_895121, EPI_ISL_895122, EPI_ISL_895123, EPI_ISL_895124, EPI_ISL_895125, EPI_ISL_895126, EPI_ISL_895127, EPI_ISL_895128, EPI_ISL_895129, EPI_ISL_895130, EPI_ISL_895131, EPI_ISL_895132, EPI_ISL_895133, EPI_ISL_895134, EPI_ISL_895135, EPI_ISL_895136, EPI_ISL_895137, EPI_ISL_895138, EPI_ISL_895139, EPI_ISL_895140, EPI_ISL_895141, EPI_ISL_895142, EPI_ISL_895143, EPI_ISL_895144, EPI_ISL_895145, EPI_ISL_895146, EPI_ISL_895147, EPI_ISL_895148, EPI_ISL_895149, EPI_ISL_895150, EPI_ISL_895179, EPI_ISL_895180, EPI_ISL_895181, EPI_ISL_895182, EPI_ISL_895183, EPI_ISL_895184, EPI_ISL_895185, EPI_ISL_895189, EPI_ISL_895190, EPI_ISL_895191, EPI_ISL_895192, EPI_ISL_895193, EPI_ISL_895194, EPI_ISL_895195, EPI_ISL_895196, EPI_ISL_895197, EPI_ISL_895198, EPI_ISL_895199, EPI_ISL_895200, EPI_ISL_895201, EPI_ISL_895202, EPI_ISL_895203, EPI_ISL_895204, EPI_ISL_895205, EPI_ISL_895206, EPI_ISL_895207, EPI_ISL_895208, EPI_ISL_895209, EPI_ISL_895210, EPI_ISL_895211, EPI_ISL_895212, EPI_ISL_895213, EPI_ISL_895214, EPI_ISL_895215, EPI_ISL_895216, EPI_ISL_895217, EPI_ISL_895218, EPI_ISL_895219, EPI_ISL_895220, EPI_ISL_895221, EPI_ISL_895222, EPI_ISL_895223, EPI_ISL_895224, EPI_ISL_895225, EPI_ISL_895226, EPI_ISL_895227, EPI_ISL_895228, EPI_ISL_895229, EPI_ISL_895230, EPI_ISL_895231, EPI_ISL_895232, EPI_ISL_895233, EPI_ISL_895234, EPI_ISL_895235, EPI_ISL_895236, EPI_ISL_895237, EPI_ISL_895238, EPI_ISL_895239, EPI_ISL_895240, EPI_ISL_895241, EPI_ISL_895242, EPI_ISL_895243, EPI_ISL_895244, EPI_ISL_895245, EPI_ISL_895246, EPI_ISL_895247, EPI_ISL_895248, EPI_ISL_895249, EPI_ISL_895250, EPI_ISL_895251, EPI_ISL_895252, EPI_ISL_895253, EPI_ISL_895254, EPI_ISL_895255, EPI_ISL_895256, EPI_ISL_895257, EPI_ISL_895258, EPI_ISL_895259, EPI_ISL_895260, EPI_ISL_895261, EPI_ISL_895262, EPI_ISL_895263, EPI_ISL_895264, EPI_ISL_895265, EPI_ISL_895396, EPI_ISL_895397, EPI_ISL_895398, EPI_ISL_895399, EPI_ISL_895400, EPI_ISL_895401, EPI_ISL_895402, EPI_ISL_895403, EPI_ISL_895404, EPI_ISL_895405, EPI_ISL_895406, EPI_ISL_895407, EPI_ISL_895408, EPI_ISL_895409, EPI_ISL_895410, EPI_ISL_895411, EPI_ISL_895412, EPI_ISL_895413, EPI_ISL_895414, EPI_ISL_895415, EPI_ISL_895416, EPI_ISL_895417, EPI_ISL_895418, EPI_ISL_895419, EPI_ISL_895420, EPI_ISL_895421, EPI_ISL_895422, EPI_ISL_895423, EPI_ISL_895424, EPI_ISL_895425, EPI_ISL_895426, EPI_ISL_895427, EPI_ISL_895428, EPI_ISL_895429, EPI_ISL_895430, EPI_ISL_895431, EPI_ISL_895432, EPI_ISL_895433, EPI_ISL_895434, EPI_ISL_895435, EPI_ISL_895436, EPI_ISL_895437, EPI_ISL_895438, EPI_ISL_895439, EPI_ISL_895440, EPI_ISL_895441, EPI_ISL_895442, EPI_ISL_895443, EPI_ISL_895444, EPI_ISL_895445, EPI_ISL_895446, EPI_ISL_895447, EPI_ISL_895448, EPI_ISL_895449, EPI_ISL_895450, EPI_ISL_895451, EPI_ISL_895452, EPI_ISL_895453, EPI_ISL_895454, EPI_ISL_895455, EPI_ISL_895456, EPI_ISL_895457, EPI_ISL_895458, EPI_ISL_895459, EPI_ISL_895460, EPI_ISL_895461, EPI_ISL_895462, EPI_ISL_895463, EPI_ISL_895464, EPI_ISL_895465, EPI_ISL_895466, EPI_ISL_895487, EPI_ISL_895490, EPI_ISL_895491, EPI_ISL_895492, EPI_ISL_895493, EPI_ISL_895494, EPI_ISL_895495, EPI_ISL_895496, EPI_ISL_895497, EPI_ISL_895498, EPI_ISL_895499, EPI_ISL_895500, EPI_ISL_895501, EPI_ISL_895502, EPI_ISL_895503, EPI_ISL_895504, EPI_ISL_895505, EPI_ISL_895506, EPI_ISL_895507, EPI_ISL_895518, EPI_ISL_895519, EPI_ISL_895520, EPI_ISL_895521, EPI_ISL_895522, EPI_ISL_895523, EPI_ISL_895524, EPI_ISL_895525, EPI_ISL_895526, EPI_ISL_895527, EPI_ISL_895528, EPI_ISL_895529, EPI_ISL_895530, EPI_ISL_895531, EPI_ISL_895532, EPI_ISL_895536, EPI_ISL_895537, EPI_ISL_895538, EPI_ISL_895539, EPI_ISL_895540, EPI_ISL_895541, EPI_ISL_895542, EPI_ISL_895543, EPI_ISL_895544, EPI_ISL_895545, EPI_ISL_895546, EPI_ISL_895547, EPI_ISL_895548, EPI_ISL_895549, EPI_ISL_895550, EPI_ISL_895551, EPI_ISL_895552, EPI_ISL_895553, EPI_ISL_895554, EPI_ISL_895555, EPI_ISL_895556, EPI_ISL_895557, EPI_ISL_895558, EPI_ISL_895559, EPI_ISL_895560, EPI_ISL_895561, EPI_ISL_895562, EPI_ISL_895563, EPI_ISL_895564, EPI_ISL_895565, EPI_ISL_895566, EPI_ISL_895639, EPI_ISL_895640, EPI_ISL_895641, EPI_ISL_895642, EPI_ISL_895643, EPI_ISL_895644, EPI_ISL_895645, EPI_ISL_895646, EPI_ISL_895647, EPI_ISL_895648, EPI_ISL_895649, EPI_ISL_895650, EPI_ISL_895651, EPI_ISL_895652, EPI_ISL_895653, EPI_ISL_895654, EPI_ISL_895655, EPI_ISL_895656, EPI_ISL_895657			
see above	Pathogen Genomics Center, National Institute of Infectious Diseases	Pathogen Genomics Center, National Institute of Infectious Diseases	Tsuyoshi Sekizuka, Kentaro Itokawa, Rina Tanaka, Masanori Hashino, Makoto Kuroda
EPI_ISL_895979, EPI_ISL_895980, EPI_ISL_895981, EPI_ISL_895982, EPI_ISL_895983, EPI_ISL_895984, EPI_ISL_895985, EPI_ISL_895986, EPI_ISL_895987, EPI_ISL_895988	Tokyo Metropolitan Institute of Public Health	Pathogen Genomics Center, National Institute of Infectious Diseases	Tsuyoshi Sekizuka, Kentaro Itokawa, Rina Tanaka, Masanori Hashino, Makoto Kuroda
EPI_ISL_896013, EPI_ISL_896014, EPI_ISL_896015, EPI_ISL_896016, EPI_ISL_896017, EPI_ISL_896018, EPI_ISL_896020, EPI_ISL_896021, EPI_ISL_896022, EPI_ISL_896023, EPI_ISL_896024, EPI_ISL_896025, EPI_ISL_896026, EPI_ISL_896027, EPI_ISL_896028, EPI_ISL_896029, EPI_ISL_896030, EPI_ISL_896031, EPI_ISL_896032, EPI_ISL_896033, EPI_ISL_896034, EPI_ISL_896035, EPI_ISL_896036, EPI_ISL_896037, EPI_ISL_896038, EPI_ISL_896039, EPI_ISL_896040, EPI_ISL_896041, EPI_ISL_896042, EPI_ISL_896043, EPI_ISL_896044, EPI_ISL_896045, EPI_ISL_896046, EPI_ISL_896047, EPI_ISL_896048, EPI_ISL_896049, EPI_ISL_896050, EPI_ISL_896051, EPI_ISL_896052, EPI_ISL_896053, EPI_ISL_896054, EPI_ISL_896055, EPI_ISL_896056, EPI_ISL_896057, EPI_ISL_896058, EPI_ISL_896059, EPI_ISL_896060, EPI_ISL_896061, EPI_ISL_896062, EPI_ISL_896063			
see above	Pathogen Genomics Center, National Institute of Infectious Diseases	Pathogen Genomics Center, National Institute of Infectious Diseases	Tsuyoshi Sekizuka, Kentaro Itokawa, Rina Tanaka, Masanori Hashino, Makoto Kuroda
EPI_ISL_896375	University of Medicine and Pharmacy of Craiova	"Stefan cel Mare" University Metagenomics Lab	Lobiuc Andrei, Gheorghita Roxana
EPI_ISL_896602, EPI_ISL_896603, EPI_ISL_896604, EPI_ISL_896605, EPI_ISL_896606, EPI_ISL_896607, EPI_ISL_896608, EPI_ISL_896609, EPI_ISL_896610, EPI_ISL_896611, EPI_ISL_896642, EPI_ISL_896643, EPI_ISL_896644, EPI_ISL_896645, EPI_ISL_896646, EPI_ISL_896659, EPI_ISL_896660, EPI_ISL_896661, EPI_ISL_896662, EPI_ISL_896663, EPI_ISL_896664, EPI_ISL_896665, EPI_ISL_896666, EPI_ISL_896667, EPI_ISL_896668, EPI_ISL_896669, EPI_ISL_896670, EPI_ISL_896671, EPI_ISL_896672, EPI_ISL_896673, EPI_ISL_896674, EPI_ISL_896675, EPI_ISL_896676, EPI_ISL_896677, EPI_ISL_896678, EPI_ISL_896679, EPI_ISL_896680, EPI_ISL_896681, EPI_ISL_896682, EPI_ISL_896683, EPI_ISL_896684, EPI_ISL_896685, EPI_ISL_896686, EPI_ISL_896687, EPI_ISL_896688, EPI_ISL_896689, EPI_ISL_896690, EPI_ISL_896691, EPI_ISL_896692, EPI_ISL_896699, EPI_ISL_896700, EPI_ISL_896701, EPI_ISL_896702, EPI_ISL_896703, EPI_ISL_896704, EPI_ISL_896705, EPI_ISL_896706, EPI_ISL_896707, EPI_ISL_896708, EPI_ISL_896709, EPI_ISL_896710, EPI_ISL_896711, EPI_ISL_896712, EPI_ISL_896713, EPI_ISL_896714, EPI_ISL_896715, EPI_ISL_896716, EPI_ISL_896717, EPI_ISL_896718, EPI_ISL_896719, EPI_ISL_896720, EPI_ISL_896721, EPI_ISL_896722, EPI_ISL_896723, EPI_ISL_896724, EPI_ISL_896725, EPI_ISL_896726, EPI_ISL_896727, EPI_ISL_896728, EPI_ISL_896729, EPI_ISL_896730, EPI_ISL_896731, EPI_ISL_896732, EPI_ISL_896733, EPI_ISL_896734, EPI_ISL_896735, EPI_ISL_896736, EPI_ISL_896737, EPI_ISL_896738, EPI_ISL_896739, EPI_ISL_896740, EPI_ISL_896741, EPI_ISL_896742, EPI_ISL_896743, EPI_ISL_896744, EPI_ISL_896745, EPI_ISL_896746, EPI_ISL_896747, EPI_ISL_896748, EPI_ISL_896749, EPI_ISL_896750, EPI_ISL_896751, EPI_ISL_896752, EPI_ISL_896753, EPI_ISL_896754, EPI_ISL_896755, EPI_ISL_896756, EPI_ISL_896757, EPI_ISL_896758, EPI_ISL_896759, EPI_ISL_896760, EPI_ISL_896761, EPI_ISL_896762, EPI_ISL_896763			
see above	Pathogen Genomics Center, National Institute of Infectious Diseases	Pathogen Genomics Center, National Institute of Infectious Diseases	Tsuyoshi Sekizuka, Kentaro Itokawa, Rina Tanaka, Masanori Hashino, Makoto Kuroda
EPI_ISL_896764, EPI_ISL_896765, EPI_ISL_896766, EPI_ISL_896767, EPI_ISL_896768, EPI_ISL_896769, EPI_ISL_896770, EPI_ISL_896771, EPI_ISL_896772, EPI_ISL_896773, EPI_ISL_896774, EPI_ISL_896775, EPI_ISL_896776, EPI_ISL_896777, EPI_ISL_896778, EPI_ISL_896779, EPI_ISL_896780, EPI_ISL_896781, EPI_ISL_896782, EPI_ISL_896783, EPI_ISL_896784, EPI_ISL_896785, EPI_ISL_896786, EPI_ISL_896787, EPI_ISL_896788, EPI_ISL_896789, EPI_ISL_896790, EPI_ISL_896791, EPI_ISL_896792, EPI_ISL_896793, EPI_ISL_896794			
see above	Gunma Prefectural Institute of Public Health and Environmental Sciences	Pathogen Genomics Center, National Institute of Infectious Diseases	Tsuyoshi Sekizuka, Kentaro Itokawa, Rina Tanaka, Masanori Hashino, Makoto Kuroda
EPI_ISL_896795, EPI_ISL_896796, EPI_ISL_896797, EPI_ISL_896798, EPI_ISL_896799, EPI_ISL_896800, EPI_ISL_896801, EPI_ISL_896802, EPI_ISL_896803, EPI_ISL_896804, EPI_ISL_896805, EPI_ISL_896806, EPI_ISL_896807, EPI_ISL_896808, EPI_ISL_896809, EPI_ISL_896810, EPI_ISL_896811, EPI_ISL_896812, EPI_ISL_896813, EPI_ISL_896814, EPI_ISL_896815, EPI_ISL_896816, EPI_ISL_896817, EPI_ISL_896818, EPI_ISL_896819, EPI_ISL_896820, EPI_ISL_896821, EPI_ISL_896822, EPI_ISL_896823, EPI_ISL_896824, EPI_ISL_896825, EPI_ISL_896826, EPI_ISL_896827, EPI_ISL_896828, EPI_ISL_896829, EPI_ISL_896830, EPI_ISL_896831, EPI_ISL_896832, EPI_ISL_896833, EPI_ISL_896834, EPI_ISL_896835, EPI_ISL_896836, EPI_ISL_896837, EPI_ISL_896838, EPI_ISL_896839, EPI_ISL_896840, EPI_ISL_896841, EPI_ISL_896842, EPI_ISL_896843, EPI_ISL_896844, EPI_ISL_896845, EPI_ISL_896846, EPI_ISL_896847, EPI_ISL_896848, EPI_ISL_896849, EPI_ISL_896850, EPI_ISL_896851, EPI_ISL_896852, EPI_ISL_896853, EPI_ISL_896854			
see above	Department of Infectious Diseases, Kobe Institute of Health	Pathogen Genomics Center, National Institute of Infectious Diseases	Tsuyoshi Sekizuka, Kentaro Itokawa, Rina Tanaka, Masanori Hashino, Makoto Kuroda
EPI_ISL_896940, EPI_ISL_896941, EPI_ISL_896942, EPI_ISL_896943, EPI_ISL_896944, EPI_ISL_896945, EPI_ISL_896946, EPI_ISL_896947, EPI_ISL_896948, EPI_ISL_896949, EPI_ISL_896950, EPI_ISL_896951, EPI_ISL_896952, EPI_ISL_896953, EPI_ISL_896954, EPI_ISL_896955, EPI_ISL_896956, EPI_ISL_896957, EPI_ISL_896958, EPI_ISL_896959, EPI_ISL_896960, EPI_ISL_896961, EPI_ISL_896962, EPI_ISL_896963, EPI_ISL_896964, EPI_ISL_896965, EPI_ISL_896966, EPI_ISL_896967, EPI_ISL_896968, EPI_ISL_896969, EPI_ISL_896970, EPI_ISL_896971, EPI_ISL_896972, EPI_ISL_896973, EPI_ISL_896974, EPI_ISL_896975, EPI_ISL_896976, EPI_ISL_896977, EPI_ISL_896978, EPI_ISL_896979, EPI_ISL_896980, EPI_ISL_896981, EPI_ISL_896982, EPI_ISL_896983, EPI_ISL_896984, EPI_ISL_896985, EPI_ISL_896986, EPI_ISL_896987, EPI_ISL_896988, EPI_ISL_896989, EPI_ISL_896990, EPI_ISL_896991, EPI_ISL_896992, EPI_ISL_896993, EPI_ISL_896994, EPI_ISL_896995, EPI_ISL_896996, EPI_ISL_896997, EPI_ISL_896998, EPI_ISL_896999, EPI_ISL_897000, EPI_ISL_897001, EPI_ISL_897002, EPI_ISL_897003, EPI_ISL_897004, EPI_ISL_897005, EPI_ISL_897006, EPI_ISL_897007, EPI_ISL_897008, EPI_ISL_897009, EPI_ISL_897010, EPI_ISL_897011, EPI_ISL_897012, EPI_ISL_897013, EPI_ISL_897014, EPI_ISL_897015, EPI_ISL_897016, EPI_ISL_897017, EPI_ISL_897018, EPI_ISL_897019, EPI_ISL_897020, EPI_ISL_897021, EPI_ISL_897022, EPI_ISL_897023, EPI_ISL_897034, EPI_ISL_897035, EPI_ISL_897036, EPI_ISL_897037, EPI_ISL_897038, EPI_ISL_897039, EPI_ISL_897040, EPI_ISL_897041, EPI_ISL_897042, EPI_ISL_897043, EPI_ISL_897044, EPI_ISL_897045, EPI_ISL_897046, EPI_ISL_897047, EPI_ISL_897048, EPI_ISL_897049, EPI_ISL_897050, EPI_ISL_897051, EPI_ISL_897052, EPI_ISL_897053, EPI_ISL_897054, EPI_ISL_897055			
see above	Pathogen Genomics Center, National Institute of Infectious Diseases	Pathogen Genomics Center, National Institute of Infectious Diseases	Tsuyoshi Sekizuka, Kentaro Itokawa, Rina Tanaka, Masanori Hashino, Makoto Kuroda
EPI_ISL_897061, EPI_ISL_897062, EPI_ISL_897063, EPI_ISL_897064, EPI_ISL_897065, EPI_ISL_897067, EPI_ISL_897068, EPI_ISL_897069, EPI_ISL_897070, EPI_ISL_897071, EPI_ISL_897072, EPI_ISL_897073, EPI_ISL_897074, EPI_ISL_897075, EPI_ISL_897076, EPI_ISL_897077, EPI_ISL_897078, EPI_ISL_897079, EPI_ISL_897080			
see above	Niigata Prefectural Institute of Public Health and Environmental Sciences	Pathogen Genomics Center, National Institute of Infectious Diseases	Tsuyoshi Sekizuka, Kentaro Itokawa, Rina Tanaka, Masanori Hashino, Makoto Kuroda
EPI_ISL_897088, EPI_ISL_897089, EPI_ISL_897090, EPI_ISL_897091, EPI_ISL_897092, EPI_ISL_897093, EPI_ISL_897094, EPI_ISL_897095, EPI_ISL_897096, EPI_ISL_897098, EPI_ISL_897099, EPI_ISL_897100, EPI_ISL_897101, EPI_ISL_897102, EPI_ISL_897103, EPI_ISL_897104, EPI_ISL_897105, EPI_ISL_897106,			

EPI_ISL_897107, EPI_ISL_897108, EPI_ISL_897109, EPI_ISL_897110, EPI_ISL_897111, EPI_ISL_897112, EPI_ISL_897113, EPI_ISL_897114, EPI_ISL_897115, EPI_ISL_897116, EPI_ISL_897117, EPI_ISL_897118, EPI_ISL_897119, EPI_ISL_897120, EPI_ISL_897121, EPI_ISL_897122, EPI_ISL_897123, EPI_ISL_897124, EPI_ISL_897125, EPI_ISL_897126, EPI_ISL_897127, EPI_ISL_897128, EPI_ISL_897129, EPI_ISL_897130, EPI_ISL_897131, EPI_ISL_897132, EPI_ISL_897133, EPI_ISL_897134, EPI_ISL_897139, EPI_ISL_897140, EPI_ISL_897141, EPI_ISL_897142, EPI_ISL_897143, EPI_ISL_897144, EPI_ISL_897145, EPI_ISL_897146, EPI_ISL_897147, EPI_ISL_897148, EPI_ISL_897151, EPI_ISL_897152, EPI_ISL_897153, EPI_ISL_897154, EPI_ISL_897155, EPI_ISL_897156, EPI_ISL_897157, EPI_ISL_897158, EPI_ISL_897159, EPI_ISL_897160, EPI_ISL_897161, EPI_ISL_897162, EPI_ISL_897163, EPI_ISL_897164, EPI_ISL_897165, EPI_ISL_897166, EPI_ISL_897167, EPI_ISL_897168, EPI_ISL_897169, EPI_ISL_897170			
see above	Pathogen Genomics Center, National Institute of Infectious Diseases	Pathogen Genomics Center, National Institute of Infectious Diseases	Tsuyoshi Sekizuka, Kentaro Itokawa, Rina Tanaka, Masanori Hashino, Makoto Kuroda
EPI_ISL_897171, EPI_ISL_897172, EPI_ISL_897173, EPI_ISL_897174, EPI_ISL_897175, EPI_ISL_897176, EPI_ISL_897177, EPI_ISL_897178, EPI_ISL_897179, EPI_ISL_897180, EPI_ISL_897181, EPI_ISL_897182, EPI_ISL_897183, EPI_ISL_897184, EPI_ISL_897185, EPI_ISL_897186, EPI_ISL_897187, EPI_ISL_897188, EPI_ISL_897189, EPI_ISL_897190, EPI_ISL_897191, EPI_ISL_897192, EPI_ISL_897193, EPI_ISL_897194, EPI_ISL_897195, EPI_ISL_897196, EPI_ISL_897197, EPI_ISL_897198, EPI_ISL_897199, EPI_ISL_897200, EPI_ISL_897201, EPI_ISL_897202, EPI_ISL_897203, EPI_ISL_897204, EPI_ISL_897205			
see above	Tokyo Metropolitan Institute of Public Health	Pathogen Genomics Center, National Institute of Infectious Diseases	Tsuyoshi Sekizuka, Kentaro Itokawa, Rina Tanaka, Masanori Hashino, Makoto Kuroda
EPI_ISL_897206, EPI_ISL_897207, EPI_ISL_897208, EPI_ISL_897209, EPI_ISL_897210, EPI_ISL_897211, EPI_ISL_897212, EPI_ISL_897213, EPI_ISL_897214, EPI_ISL_897215, EPI_ISL_897216, EPI_ISL_897217, EPI_ISL_897218, EPI_ISL_897219, EPI_ISL_897220, EPI_ISL_897221, EPI_ISL_897222, EPI_ISL_897223, EPI_ISL_897224, EPI_ISL_897225, EPI_ISL_897226, EPI_ISL_897227, EPI_ISL_897228, EPI_ISL_897229, EPI_ISL_897230, EPI_ISL_897231, EPI_ISL_897232, EPI_ISL_897233, EPI_ISL_897234, EPI_ISL_897235, EPI_ISL_897236, EPI_ISL_897237, EPI_ISL_897238, EPI_ISL_897239, EPI_ISL_897240, EPI_ISL_897241, EPI_ISL_897242, EPI_ISL_897243, EPI_ISL_897244, EPI_ISL_897245, EPI_ISL_897246, EPI_ISL_897247, EPI_ISL_897251, EPI_ISL_897283, EPI_ISL_897284, EPI_ISL_897285, EPI_ISL_897286, EPI_ISL_897287, EPI_ISL_897288, EPI_ISL_897289, EPI_ISL_897290, EPI_ISL_897291, EPI_ISL_897292, EPI_ISL_897293, EPI_ISL_897294, EPI_ISL_897295, EPI_ISL_897296, EPI_ISL_897297, EPI_ISL_897298, EPI_ISL_897299, EPI_ISL_897300, EPI_ISL_897301, EPI_ISL_897302, EPI_ISL_897303, EPI_ISL_897304, EPI_ISL_897305, EPI_ISL_897306, EPI_ISL_897307, EPI_ISL_897308, EPI_ISL_897309, EPI_ISL_897310, EPI_ISL_897311, EPI_ISL_897312, EPI_ISL_897340, EPI_ISL_897341, EPI_ISL_897342, EPI_ISL_897343, EPI_ISL_897344, EPI_ISL_897345, EPI_ISL_897346, EPI_ISL_897347, EPI_ISL_897348, EPI_ISL_897349, EPI_ISL_897350, EPI_ISL_897351, EPI_ISL_897352, EPI_ISL_897353, EPI_ISL_897354, EPI_ISL_897355, EPI_ISL_897356, EPI_ISL_897357, EPI_ISL_897358, EPI_ISL_897359, EPI_ISL_897360, EPI_ISL_897361, EPI_ISL_897362, EPI_ISL_897363, EPI_ISL_897364, EPI_ISL_897365, EPI_ISL_897366, EPI_ISL_897367, EPI_ISL_897368, EPI_ISL_897369, EPI_ISL_897370, EPI_ISL_897371, EPI_ISL_897372, EPI_ISL_897373, EPI_ISL_897374, EPI_ISL_897375, EPI_ISL_897376, EPI_ISL_897377, EPI_ISL_897378, EPI_ISL_897379, EPI_ISL_897380, EPI_ISL_897381, EPI_ISL_897382, EPI_ISL_897383, EPI_ISL_897384, EPI_ISL_897385, EPI_ISL_897386, EPI_ISL_897387, EPI_ISL_897388, EPI_ISL_897389, EPI_ISL_897390, EPI_ISL_897391, EPI_ISL_897392, EPI_ISL_897393, EPI_ISL_897394, EPI_ISL_897395, EPI_ISL_897396, EPI_ISL_897397, EPI_ISL_897398, EPI_ISL_897399, EPI_ISL_897400, EPI_ISL_897401, EPI_ISL_897402, EPI_ISL_897403, EPI_ISL_897404, EPI_ISL_897405, EPI_ISL_897406, EPI_ISL_897407, EPI_ISL_897408, EPI_ISL_897409, EPI_ISL_897410, EPI_ISL_897411, EPI_ISL_897412, EPI_ISL_897413, EPI_ISL_897414, EPI_ISL_897415, EPI_ISL_897416, EPI_ISL_897417, EPI_ISL_897418, EPI_ISL_897419, EPI_ISL_897420, EPI_ISL_897421, EPI_ISL_897422, EPI_ISL_897423, EPI_ISL_897424, EPI_ISL_897425, EPI_ISL_897426, EPI_ISL_897427, EPI_ISL_897428, EPI_ISL_897429, EPI_ISL_897430, EPI_ISL_897431, EPI_ISL_897432, EPI_ISL_897433, EPI_ISL_897453, EPI_ISL_897454, EPI_ISL_897455, EPI_ISL_897456, EPI_ISL_897457, EPI_ISL_897458, EPI_ISL_897459, EPI_ISL_897460, EPI_ISL_897461, EPI_ISL_897462, EPI_ISL_897463, EPI_ISL_897464, EPI_ISL_897465, EPI_ISL_897466, EPI_ISL_897467, EPI_ISL_897468, EPI_ISL_897469, EPI_ISL_897470, EPI_ISL_897471, EPI_ISL_897472, EPI_ISL_897473, EPI_ISL_897474, EPI_ISL_897475, EPI_ISL_897476, EPI_ISL_897477, EPI_ISL_897478, EPI_ISL_897479, EPI_ISL_897480, EPI_ISL_897481, EPI_ISL_897482, EPI_ISL_897483, EPI_ISL_897484, EPI_ISL_897485, EPI_ISL_897486, EPI_ISL_897487, EPI_ISL_897488, EPI_ISL_897489, EPI_ISL_897490, EPI_ISL_897491, EPI_ISL_897492, EPI_ISL_897493, EPI_ISL_897494, EPI_ISL_897495, EPI_ISL_897496, EPI_ISL_897497, EPI_ISL_897498, EPI_ISL_897499, EPI_ISL_897500, EPI_ISL_897501, EPI_ISL_897502, EPI_ISL_897503, EPI_ISL_897504, EPI_ISL_897505, EPI_ISL_897506, EPI_ISL_897507, EPI_ISL_897508, EPI_ISL_897509, EPI_ISL_897510, EPI_ISL_897511, EPI_ISL_897512, EPI_ISL_897513, EPI_ISL_897514, EPI_ISL_897515, EPI_ISL_897516, EPI_ISL_897517, EPI_ISL_897518, EPI_ISL_897519, EPI_ISL_897520, EPI_ISL_897521, EPI_ISL_897522, EPI_ISL_897523, EPI_ISL_897524, EPI_ISL_897525, EPI_ISL_897526, EPI_ISL_897527, EPI_ISL_897595, EPI_ISL_897596, EPI_ISL_897597			
see above	Pathogen Genomics Center, National Institute of Infectious Diseases	Pathogen Genomics Center, National Institute of Infectious Diseases	Tsuyoshi Sekizuka, Kentaro Itokawa, Rina Tanaka, Masanori Hashino, Makoto Kuroda
EPI_ISL_898054, EPI_ISL_898055, EPI_ISL_898056	Sakai City Institute of Public Health	Pathogen Genomics Center, National Institute of Infectious Diseases	Tsuyoshi Sekizuka, Kentaro Itokawa, Rina Tanaka, Masanori Hashino, Makoto Kuroda
EPI_ISL_898074, EPI_ISL_898075, EPI_ISL_898076, EPI_ISL_898086, EPI_ISL_898087, EPI_ISL_898089, EPI_ISL_898090, EPI_ISL_898091, EPI_ISL_898092, EPI_ISL_898093, EPI_ISL_898094, EPI_ISL_898095, EPI_ISL_898096, EPI_ISL_898097, EPI_ISL_898098, EPI_ISL_898099, EPI_ISL_898100, EPI_ISL_898101, EPI_ISL_898102, EPI_ISL_898103, EPI_ISL_898104, EPI_ISL_898105, EPI_ISL_898106, EPI_ISL_898107, EPI_ISL_898108, EPI_ISL_898109, EPI_ISL_898110, EPI_ISL_898111, EPI_ISL_898112, EPI_ISL_898113, EPI_ISL_898114, EPI_ISL_898115, EPI_ISL_898116, EPI_ISL_898117, EPI_ISL_898118, EPI_ISL_898119, EPI_ISL_898120, EPI_ISL_898121, EPI_ISL_898122, EPI_ISL_898123, EPI_ISL_898124, EPI_ISL_898125, EPI_ISL_898126, EPI_ISL_898127, EPI_ISL_898128, EPI_ISL_898129, EPI_ISL_898130, EPI_ISL_898131, EPI_ISL_898132, EPI_ISL_898133, EPI_ISL_898136, EPI_ISL_898137, EPI_ISL_898138, EPI_ISL_898139, EPI_ISL_898140, EPI_ISL_898141, EPI_ISL_898142, EPI_ISL_898146, EPI_ISL_898147, EPI_ISL_898148, EPI_ISL_898149, EPI_ISL_898150, EPI_ISL_898151, EPI_ISL_898152, EPI_ISL_898153, EPI_ISL_898154, EPI_ISL_898155, EPI_ISL_898156, EPI_ISL_898157, EPI_ISL_898158, EPI_ISL_898159, EPI_ISL_898160, EPI_ISL_898161, EPI_ISL_898162, EPI_ISL_898163, EPI_ISL_898164, EPI_ISL_898165, EPI_ISL_898166, EPI_ISL_898167, EPI_ISL_898168, EPI_ISL_898169, EPI_ISL_898170, EPI_ISL_898171, EPI_ISL_898172, EPI_ISL_898173, EPI_ISL_898174, EPI_ISL_898175, EPI_ISL_898176, EPI_ISL_898177, EPI_ISL_898178, EPI_ISL_898179, EPI_ISL_898180, EPI_ISL_898181, EPI_ISL_898182, EPI_ISL_898183, EPI_ISL_898184, EPI_ISL_898185, EPI_ISL_898186, EPI_ISL_898187, EPI_ISL_898188, EPI_ISL_898189, EPI_ISL_898190, EPI_ISL_898191, EPI_ISL_898192, EPI_ISL_898193, EPI_ISL_898194, EPI_ISL_898195, EPI_ISL_898196, EPI_ISL_898197, EPI_ISL_898198, EPI_ISL_898199, EPI_ISL_898200, EPI_ISL_898201, EPI_ISL_898202, EPI_ISL_898203, EPI_ISL_898204, EPI_ISL_898205, EPI_ISL_898206, EPI_ISL_898207, EPI_ISL_898208, EPI_ISL_898209, EPI_ISL_898210, EPI_ISL_898211, EPI_ISL_898212, EPI_ISL_898213, EPI_ISL_898214, EPI_ISL_898215, EPI_ISL_898216, EPI_ISL_898217, EPI_ISL_898218, EPI_ISL_898219, EPI_ISL_898220, EPI_ISL_898221, EPI_ISL_898222, EPI_ISL_898223, EPI_ISL_898224, EPI_ISL_898225, EPI_ISL_898226, EPI_ISL_898227, EPI_ISL_898228, EPI_ISL_898229, EPI_ISL_898230, EPI_ISL_898231, EPI_ISL_898232, EPI_ISL_898233, EPI_ISL_898234, EPI_ISL_898235, EPI_ISL_898236, EPI_ISL_898237, EPI_ISL_898238, EPI_ISL_898239, EPI_ISL_898240, EPI_ISL_898241, EPI_ISL_898242, EPI_ISL_898243, EPI_ISL_898244, EPI_ISL_898245, EPI_ISL_898246, EPI_ISL_898247, EPI_ISL_898248, EPI_ISL_898249, EPI_ISL_898250, EPI_ISL_898251, EPI_ISL_898252, EPI_ISL_898253, EPI_ISL_898254, EPI_ISL_898255, EPI_ISL_898256, EPI_ISL_898257, EPI_ISL_898258, EPI_ISL_898259, EPI_ISL_898260, EPI_ISL_898261, EPI_ISL_898262, EPI_ISL_898263, EPI_ISL_898264, EPI_ISL_898265, EPI_ISL_898266, EPI_ISL_898267, EPI_ISL_898268, EPI_ISL_898270, EPI_ISL_898271, EPI_ISL_898272, EPI_ISL_898273, EPI_ISL_898274, EPI_ISL_898275, EPI_ISL_898307, EPI_ISL_898308, EPI_ISL_898309, EPI_ISL_898310, EPI_ISL_898311, EPI_ISL_898312, EPI_ISL_898315, EPI_ISL_898316, EPI_ISL_898317, EPI_ISL_898318, EPI_ISL_898319, EPI_ISL_898320			
see above	Pathogen Genomics Center, National Institute of Infectious Diseases	Pathogen Genomics Center, National Institute of Infectious Diseases	Tsuyoshi Sekizuka, Kentaro Itokawa, Rina Tanaka, Masanori Hashino, Makoto Kuroda
EPI_ISL_898322, EPI_ISL_898323, EPI_ISL_898324	Toyama Institute of Health	Pathogen Genomics Center, National Institute of Infectious Diseases	Tsuyoshi Sekizuka, Kentaro Itokawa, Rina Tanaka, Masanori Hashino, Makoto Kuroda
EPI_ISL_898332, EPI_ISL_898333, EPI_ISL_898334, EPI_ISL_898335, EPI_ISL_898336, EPI_ISL_898337	Yamagata Prefectural Institute of Public Health	Pathogen Genomics Center, National Institute of Infectious Diseases	Tsuyoshi Sekizuka, Kentaro Itokawa, Rina Tanaka, Masanori Hashino, Makoto Kuroda
EPI_ISL_898554, EPI_ISL_898555, EPI_ISL_898556, EPI_ISL_898557, EPI_ISL_898558, EPI_ISL_898559, EPI_ISL_898560, EPI_ISL_898561, EPI_ISL_898562, EPI_ISL_898563, EPI_ISL_898564, EPI_ISL_898609, EPI_ISL_898610, EPI_ISL_898611, EPI_ISL_898612, EPI_ISL_898613, EPI_ISL_898614, EPI_ISL_898615, EPI_ISL_898625, EPI_ISL_898626, EPI_ISL_898627, EPI_ISL_898628, EPI_ISL_898637, EPI_ISL_898638, EPI_ISL_898639, EPI_ISL_898640, EPI_ISL_898641, EPI_ISL_898642, EPI_ISL_898643, EPI_ISL_898644, EPI_ISL_898645, EPI_ISL_898646, EPI_ISL_898647, EPI_ISL_898648, EPI_ISL_898649, EPI_ISL_898650, EPI_ISL_898651, EPI_ISL_898652, EPI_ISL_898653, EPI_ISL_898654, EPI_ISL_898655, EPI_ISL_898656, EPI_ISL_898657, EPI_ISL_898658, EPI_ISL_898659, EPI_ISL_898709, EPI_ISL_898710			
see above	Pathogen Genomics Center, National Institute of Infectious Diseases	Pathogen Genomics Center, National Institute of Infectious Diseases	Tsuyoshi Sekizuka, Kentaro Itokawa, Rina Tanaka, Masanori Hashino, Makoto Kuroda
EPI_ISL_898711, EPI_ISL_898712, EPI_ISL_898713, EPI_ISL_898714, EPI_ISL_898715, EPI_ISL_898716, EPI_ISL_898717, EPI_ISL_898718, EPI_ISL_898719, EPI_ISL_898720, EPI_ISL_898721, EPI_ISL_898722, EPI_ISL_898723, EPI_ISL_898724, EPI_ISL_898725, EPI_ISL_898726, EPI_ISL_898727, EPI_ISL_898728, EPI_ISL_898729, EPI_ISL_898730, EPI_ISL_898731, EPI_ISL_898732, EPI_ISL_898733, EPI_ISL_898734, EPI_ISL_898735, EPI_ISL_898736, EPI_ISL_898737, EPI_ISL_898738, EPI_ISL_898739, EPI_ISL_898740, EPI_ISL_898741, EPI_ISL_898742, EPI_ISL_898743, EPI_ISL_898744, EPI_ISL_898745, EPI_ISL_898746, EPI_ISL_898747, EPI_ISL_898748, EPI_ISL_898749, EPI_ISL_898750, EPI_ISL_898751, EPI_ISL_898752, EPI_ISL_898753, EPI_ISL_898754, EPI_ISL_898755, EPI_ISL_898756, EPI_ISL_898757, EPI_ISL_898758, EPI_ISL_898759, EPI_ISL_898760, EPI_ISL_898761, EPI_ISL_898762, EPI_ISL_898763, EPI_ISL_898764, EPI_ISL_898765, EPI_ISL_898766, EPI_ISL_898767, EPI_ISL_898768, EPI_ISL_898769, EPI_ISL_898770, EPI_ISL_898771, EPI_ISL_898772, EPI_ISL_898773, EPI_ISL_898774, EPI_ISL_898775, EPI_ISL_898776, EPI_ISL_898777, EPI_ISL_898778, EPI_ISL_898779, EPI_ISL_898780, EPI_ISL_898781, EPI_ISL_898782, EPI_ISL_898783, EPI_ISL_898784, EPI_ISL_898785, EPI_ISL_898786, EPI_ISL_898787, EPI_ISL_898788, EPI_ISL_898789, EPI_ISL_898790, EPI_ISL_898791, EPI_ISL_898792, EPI_ISL_898793, EPI_ISL_898794, EPI_ISL_898795, EPI_ISL_898796, EPI_ISL_898797, EPI_ISL_898798, EPI_ISL_898799, EPI_ISL_898800, EPI_ISL_898801, EPI_ISL_898802, EPI_ISL_898803, EPI_ISL_898804, EPI_ISL_898805, EPI_ISL_898806, EPI_ISL_898807, EPI_ISL_898808, EPI_ISL_898809			
see above	Department of Infectious Diseases, Kobe Institute of Health	Pathogen Genomics Center, National Institute of Infectious Diseases	Tsuyoshi Sekizuka, Kentaro Itokawa, Rina Tanaka, Masanori Hashino, Makoto Kuroda
EPI_ISL_898810, EPI_ISL_898811, EPI_ISL_898812, EPI_ISL_898813, EPI_ISL_898814, EPI_ISL_898815, EPI_ISL_898816, EPI_ISL_898817, EPI_ISL_898818, EPI_ISL_898819, EPI_ISL_898820, EPI_ISL_898821, EPI_ISL_898822, EPI_ISL_898823, EPI_ISL_898824, EPI_ISL_898825, EPI_ISL_898826, EPI_ISL_898827, EPI_ISL_898828, EPI_ISL_898829, EPI_ISL_898830, EPI_ISL_898831, EPI_ISL_898832, EPI_ISL_898833, EPI_ISL_898834, EPI_ISL_898835, EPI_ISL_898836, EPI_ISL_898837, EPI_ISL_898838, EPI_ISL_898839, EPI_ISL_898840, EPI_ISL_898841, EPI_ISL_898842, EPI_ISL_898843, EPI_ISL_898844, EPI_ISL_898845, EPI_ISL_898846, EPI_ISL_898847, EPI_ISL_898848, EPI_ISL_898849, EPI_ISL_898850, EPI_ISL_898851, EPI_ISL_898852, EPI_ISL_898853, EPI_ISL_898854, EPI_ISL_898855, EPI_ISL_898856, EPI_ISL_898857, EPI_ISL_898858, EPI_ISL_898872, EPI_ISL_898873, EPI_ISL_898874, EPI_ISL_898875, EPI_ISL_898876, EPI_ISL_898877, EPI_ISL_898878, EPI_ISL_898879, EPI_ISL_898880, EPI_ISL_898881, EPI_ISL_898882, EPI_ISL_898883, EPI_ISL_898884, EPI_ISL_898885, EPI_ISL_898886, EPI_ISL_898887, EPI_ISL_898888, EPI_ISL_898889, EPI_ISL_898890, EPI_ISL_898891, EPI_ISL_898892, EPI_ISL_898893, EPI_ISL_898915, EPI_ISL_898916, EPI_ISL_898917, EPI_ISL_898918, EPI_ISL_898919, EPI_ISL_898920, EPI_ISL_898921, EPI_ISL_898922, EPI_ISL_898923, EPI_ISL_898924, EPI_ISL_898925, EPI_ISL_898926, EPI_ISL_898927, EPI_ISL_898928, EPI_ISL_898929, EPI_ISL_898930, EPI_ISL_898931, EPI_ISL_898932, EPI_ISL_898933, EPI_ISL_898934, EPI_ISL_898935, EPI_ISL_898936, EPI_ISL_898937, EPI_ISL_898938, EPI_ISL_898939, EPI_ISL_898940, EPI_ISL_898941, EPI_ISL_898942, EPI_ISL_898943, EPI_ISL_898944, EPI_ISL_898945, EPI_ISL_898946, EPI_ISL_898947, EPI_ISL_898948, EPI_ISL_898949, EPI_ISL_898950, EPI_ISL_898951, EPI_ISL_898952, EPI_ISL_898953, EPI_ISL_898954, EPI_ISL_898955, EPI_ISL_898956, EPI_ISL_898957, EPI_ISL_898958, EPI_ISL_898959, EPI_ISL_898960, EPI_ISL_898961, EPI_ISL_898962, EPI_ISL_898963, EPI_ISL_898964, EPI_ISL_898965, EPI_ISL_898966, EPI_ISL_898967, EPI_ISL_898968, EPI_ISL_898969, EPI_ISL_898970, EPI_ISL_898971, EPI_ISL_898972, EPI_ISL_898973, EPI_ISL_898974, EPI_ISL_898975, EPI_ISL_898976, EPI_ISL_898977, EPI_ISL_898978, EPI_ISL_898979, EPI_ISL_898980, EPI_ISL_898981, EPI_ISL_898982, EPI_ISL_898983, EPI_ISL_898984, EPI_ISL_898985, EPI_ISL_898986, EPI_ISL_898987, EPI_ISL_898988, EPI_ISL_898989, EPI_ISL_898990, EPI_ISL_898991, EPI_ISL_898992, EPI_ISL_901433, EPI_ISL_901434, EPI_ISL_901435, EPI_ISL_901436, EPI_ISL_901437, EPI_ISL_901438, EPI_ISL_901439, EPI_ISL_901440, EPI_ISL_901441, EPI_ISL_901442, EPI_ISL_901443, EPI_ISL_901444, EPI_ISL_901445, EPI_ISL_901446, EPI_ISL_901447, EPI_ISL_901448, EPI_ISL_901449, EPI_ISL_901498			
see above	Pathogen Genomics Center, National Institute of Infectious Diseases	Pathogen Genomics Center, National Institute of Infectious Diseases	Tsuyoshi Sekizuka, Kentaro Itokawa, Rina Tanaka, Masanori Hashino, Makoto Kuroda
EPI_ISL_901499, EPI_ISL_901500	Tokyo Metropolitan Institute of Public Health	Pathogen Genomics Center, National Institute of Infectious Diseases	Tsuyoshi Sekizuka, Kentaro Itokawa, Rina Tanaka, Masanori Hashino, Makoto Kuroda
EPI_ISL_904004	Scientific Veterinary Institute Novi Sad	Veterinary Specialized Institute "Kraljevo", Serbia	Vidanovic,D., Tesovic,B., Knezevic,A., Jovanovic,T., Jankovic,M., Sekler,M., Banovic Djeri,B., Petrovic,T., Volkening,J., Afonso,C.
EPI_ISL_904241, EPI_ISL_904251, EPI_ISL_904282, EPI_ISL_904455, EPI_ISL_904456, EPI_ISL_904457,	Dutch COVID-19 response team	Erasmus Medical Center	Bas Oude Munnink, Reina Sikkema, David Nieuwenhuijse, Irina Chestakova, Anne van der Linden, Marjan Boter, Emmanuelle Munger, Corine GeurtsvanKessel, Annemiek van der Eijk, Richard Molenkamp, Marion Koopmans, on behalf of the Dutch national COVID-19 response team.

EPI_ISL_904458, EPI_ISL_904459, EPI_ISL_904460			
EPI_ISL_906516, EPI_ISL_906517, EPI_ISL_906518	Department of Infectious Diseases, Kobe Institute of Health	Pathogen Genomics Center, National Institute of Infectious Diseases	Tsuyoshi Sekizuka, Kentaro Itokawa, Rina Tanaka, Masanori Hashino, Makoto Kuroda
EPI_ISL_906519, EPI_ISL_906522, EPI_ISL_906524, EPI_ISL_906525	Pathogen Genomics Center, National Institute of Infectious Diseases	Pathogen Genomics Center, National Institute of Infectious Diseases	Tsuyoshi Sekizuka, Kentaro Itokawa, Rina Tanaka, Masanori Hashino, Makoto Kuroda
EPI_ISL_907076	Biology, MCL	Biology, MCL	Seadawy,M.G., Zekri,A.N., Shamel,M.D., Gad,A.F., Elhoseiny,M.F.
EPI_ISL_911675	Tanjungpura University Hospital	Tanjungpura University Hospital	Mahyarudin; Andriani; Virhan Novianry; Delima Fajar Liana ; Sofi Siti Shofiyah; Puji Astuti ; Muhammad Ibnu Kahtan ; Ambar Rialita; Eka Ardiani Putri; Wiwik Windarti; Helmi Sastriawan
EPI_ISL_912457, EPI_ISL_912503, EPI_ISL_912533	NHLS Universitas Academic	UFS Virology	PA Bester, MM Nyaga, P Nthiga, MT Mogotsi, D Goedhals, T de Oliveira
EPI_ISL_913091	Center for Virology	Center for Virology	Jeremy V. Camp, Irene Goerzer, Monika Redlberger-Fritz, Stephan W. Aberle
EPI_ISL_913352, EPI_ISL_913353	Klinisk mikrobiologi	The Public Health Agency of Sweden	Anna-Malin Linde, Maria Lind Karlberg, Carlo Berg, Oskar Karlsson Lindsjo, Sofia Stamouli, Reza Advani, Mattias Haukland, Petra Holmstrom, Noura Walai, Petra Edquist, Mia Brytting, Anna Risberg, Karin Tegmark-Wisell
EPI_ISL_914884, EPI_ISL_914886	Vilnius university hospital Santaros Klinikos, Center of Laboratory Medicine	Vilnius University Hospital Santaros Klinikos	Ingrida Olendraite, Daniel Naumovas, Rimvydas Norvilas, Dovil Ežerskyt, Justinas Šlikas
EPI_ISL_918377, EPI_ISL_918380, EPI_ISL_918381, EPI_ISL_918382, EPI_ISL_918383, EPI_ISL_918384, EPI_ISL_918385, EPI_ISL_918386, EPI_ISL_918387, EPI_ISL_918388, EPI_ISL_918389, EPI_ISL_918390, EPI_ISL_918391, EPI_ISL_918392, EPI_ISL_918393, EPI_ISL_918394, EPI_ISL_918395, EPI_ISL_918396, EPI_ISL_918397, EPI_ISL_918398, EPI_ISL_918399, EPI_ISL_918400, EPI_ISL_918401, EPI_ISL_918402, EPI_ISL_918403, EPI_ISL_918404, EPI_ISL_918405, EPI_ISL_918406			
see above	Ibaraki Prefectural Institute of Public Health	Ibaraki Prefectural Institute of Public Health	Keiko Goto, Tsuyoshi Sekizuka, Kentaro Itokawa, Rina Tanaka, Masanori Hashino, Makoto Kuroda
EPI_ISL_923224	Centre for Enzyme Innovation, University of Portsmouth / Translational Research Laboratory, Portsmouth Hospitals NHS Trust	COVID-19 Genomics UK (COG-UK) Consortium	Angela Beckett,Salman Goudarzi,Christopher Fearn,Kate Cook,Katie Loveson,Sharon Glaysher,Scott Elliott,Samuel Robson
EPI_ISL_925104, EPI_ISL_925126	1.AO Universitaria 'S. Giovanni di Dio e Ruggi D'Aragona, Scuola Medica Salernitana' Hospital / 2.UOC di Virologia e Microbiologia, Università della Campania 'L. Vanvitelli' / 3.AO Universitaria 'Federico II' Napoli Hospital / 4.AORN 'San Giuseppe Moscati' Avellino Hospital / 5.AO 'San Pio - presidio G. Rummo' Benevento Hospital / 6.AO 'Sant'Anna e San Sebastiano' Caserta Hospital / 7.PO 'Maria Santissima Addolorata' Eboli Hospital / 8.Biogem Istituto di Ricerche Genetiche	1. Genome Research Center for Health (CRGS) / 2. Laboratory of Molecular Medicine and Genomics(LMMGe) / 3. Center for Research in Pure and Applied Mathematics (CRMPA)	Giorgio Giurato, Francesca Rizzo, Alessandro Weisz, Gianluigi Franci, Giovanni Nassa, Pasquale Pagliano, Roberta Tarallo, Elena Alexandrova, Ylenia D'Agostino, Carlo Ferravante, Jessica Lamberti, Viola Melone, Domenico Memoli, Valeria Mirici Cappa, Domenico Palumbo, Giovanni Pecoraro, Assunta Sellitto, Oriana Strianese, Ilaria Terenzi, Giuseppe Fenza, Aniello Gentile, Antonello Saccomanno, Sonia Amabile, Teresa Rocco, Annamaria Salvati, Emilia Vaccaro, Massimiliano Galdiero, Michele Cennamo, Giuseppe Portella, Maria Grazia Foti, Mariarosaria Ingino, Maria Landi, Maurizio Fumi, Vincenzo Rocco, Rita Greco, Vittoria Letizia, Arnolfo Petruzziello, Maddalena Schioppa, Gregorio Goffredi, Francesca Marciano, Michele Caraglia, Alessia Cossu, Marianna Scrima
EPI_ISL_925147	Virginia DCLS	Virginia DCLS	Virginia DCLS
EPI_ISL_925538, EPI_ISL_925586, EPI_ISL_925587, EPI_ISL_925588, EPI_ISL_925589, EPI_ISL_925590, EPI_ISL_925591, EPI_ISL_925592, EPI_ISL_925593, EPI_ISL_925594, EPI_ISL_925595, EPI_ISL_925596, EPI_ISL_925698, EPI_ISL_925699, EPI_ISL_925700, EPI_ISL_925762			
see above	Public Health Ontario Laboratory	Public Health Ontario Laboratory	Vanessa G Allen, Philip Banh, Yao Chen, Richard de Borja, Alireza Eshaghi, Nahuel Fittipaldi, Christine Frantz, Jonathan B Gubbay, Jennifer L Guthrie, Lawrence Heisler, Esha Joshi, Michael Laszloffy, Aimin Li, Michael CY Li, Dean Maxwell, Sandeep Nagra, Samir N Patel, Jared Simpson, Karthikeyan Sivaraman, Ashleigh Sullivan, Yogi Sundaravadanam, Sarah Teatero, Matthew Watson, Andre Villegas, Sandra Zittermann
EPI_ISL_931311, EPI_ISL_931312, EPI_ISL_931313, EPI_ISL_931314, EPI_ISL_931315, EPI_ISL_931316, EPI_ISL_931317, EPI_ISL_931318, EPI_ISL_931319, EPI_ISL_931320, EPI_ISL_931321, EPI_ISL_931322, EPI_ISL_931323, EPI_ISL_931324, EPI_ISL_931325, EPI_ISL_931326, EPI_ISL_931327, EPI_ISL_931328, EPI_ISL_931329, EPI_ISL_931330, EPI_ISL_931331, EPI_ISL_931332, EPI_ISL_931333, EPI_ISL_931334, EPI_ISL_931335, EPI_ISL_931336, EPI_ISL_931337, EPI_ISL_931338, EPI_ISL_931339, EPI_ISL_931340, EPI_ISL_931341, EPI_ISL_931376, EPI_ISL_931377, EPI_ISL_931378, EPI_ISL_931379, EPI_ISL_931380, EPI_ISL_931381, EPI_ISL_931382, EPI_ISL_931383, EPI_ISL_931384, EPI_ISL_931385, EPI_ISL_931386, EPI_ISL_931387, EPI_ISL_931388, EPI_ISL_931395, EPI_ISL_931400, EPI_ISL_931401, EPI_ISL_931402, EPI_ISL_931403, EPI_ISL_931404, EPI_ISL_931405, EPI_ISL_931406, EPI_ISL_931407, EPI_ISL_931408, EPI_ISL_931409, EPI_ISL_931410, EPI_ISL_931411, EPI_ISL_931412, EPI_ISL_931413, EPI_ISL_931414, EPI_ISL_931415, EPI_ISL_931416, EPI_ISL_931417, EPI_ISL_931418, EPI_ISL_931419, EPI_ISL_931420, EPI_ISL_931421, EPI_ISL_931422, EPI_ISL_931423, EPI_ISL_931424, EPI_ISL_931425, EPI_ISL_931426, EPI_ISL_931427, EPI_ISL_931428, EPI_ISL_931429, EPI_ISL_931430, EPI_ISL_931431, EPI_ISL_931432, EPI_ISL_931433, EPI_ISL_931434, EPI_ISL_931435, EPI_ISL_931436, EPI_ISL_931437, EPI_ISL_931438, EPI_ISL_931439, EPI_ISL_931440, EPI_ISL_931441, EPI_ISL_931442, EPI_ISL_931443, EPI_ISL_931444, EPI_ISL_931445, EPI_ISL_931446, EPI_ISL_931447			
see above	University Hospital Basel, Clinical Virology	University Hospital Basel, Clinical Bacteriology	Tim Roloff, Madlen Stange, Helena MB Seth-Smith, Alfredo Mari, Karoline Leuzinger, Julia Bielicki, Manuel Battegay, Hans Hirsch, Adrian Egli
EPI_ISL_934335, EPI_ISL_934336	Klinisk mikrobiologi	The Public Health Agency of Sweden	Anna-Malin Linde, Maria Lind Karlberg, Carlo Berg, Oskar Karlsson Lindsjo, Sofia Stamouli, Reza Advani, Mattias Haukland, Petra Holmstrom, Noura Walai, Petra Edquist, Mia Brytting, Anna Risberg, Karin Tegmark-Wisell
EPI_ISL_936589, EPI_ISL_936590, EPI_ISL_936591, EPI_ISL_936592, EPI_ISL_936593, EPI_ISL_936594, EPI_ISL_936595, EPI_ISL_936596, EPI_ISL_936597, EPI_ISL_936598, EPI_ISL_936599, EPI_ISL_936600			
see above	Northwestern Memorial Hospital	Ozer Lab	Ramon Lorenzo-Redondo, Lacy M. Simons, Chad J. Achenbach, Lawrence J. Jennings, Michael G. Ison, Judd F. Hultquist, Egon A. Ozer
EPI_ISL_937048	Quest Diagnostics	Quest Diagnostics	Rosenthal,S.H., Gerasimova,A., Kagan,R.M., Anderson, B., Livingston, K.E., Hua, M., Liu Y., Shalhout, D.F., Owen, R., Lacbawan, F.
EPI_ISL_939642	Department of Infectious Diseases, Kobe Institute of Health	Department of Infectious Diseases, Kobe Institute of Health	Ryohei Nomoto, Tomotada Iwamoto, Tsuyoshi Sekizuka, Kentaro Itokawa, Rina Tanaka, Masanori Hashino, Makoto Kuroda
EPI_ISL_940162	Hôpital Bichat Claude Bernard, Laboratoire de Virologie	IAME UMR1137 Inserm, Université de Paris, Hôpital Bichat	Antoine Bridier-Nahmias, Amélie Recoing, Quentin Le Hingrat, Lena Daniel, Siham Hamri, Gilles Collin, Alexandre Storto, Mélanie Bertine, Charlotte Charpentier, Nadhira Houhou-Fidouh, Diane Descamps, Benoit Visseaux
EPI_ISL_949190	University Hospital Basel, Clinical Virology	University Hospital Basel, Clinical Bacteriology	Tim Roloff, Madlen Stange, Helena MB Seth-Smith, Alfredo Mari, Karoline Leuzinger, Julia Bielicki, Manuel Battegay, Hans Hirsch, Adrian Egli
EPI_ISL_949224	Departamento de Microbiología, CDB, Hospital Clínic, Barcelona	SeqCOVID-SPAIN consortium/IBV(CSIC)	Andrea Vergara, Mikel Martínez, Elisa Rubio, Jéssica Navero, Aida Peiró and SeqCOVID-SPAIN consortium
EPI_ISL_949649	Virology Department, Royal Infirmary of Edinburgh, NHS Lothian / School of Biological Sciences, University of Edinburgh / Institute of Genetics and Molecular Medicine, University of Edinburgh	COVID-19 Genomics UK (COG-UK) Consortium	McHugh M, Dewar R, Rooke S, Gallagher M, Balcaza C, O'Toole Á, Scher E, Hill V, McCrone JT, Colquhoun R, Yu X, Jackson B, Rambaut A, Williams TC, Templeton K
EPI_ISL_954189	1.AO Universitaria 'S. Giovanni di Dio e Ruggi D'Aragona, Scuola Medica Salernitana' Hospital / 2.UOC di Virologia e Microbiologia, Università della Campania 'L. Vanvitelli' / 3.AO Universitaria 'Federico II' Napoli Hospital / 4.AORN 'San Giuseppe Moscati' Avellino Hospital / 5.AO 'San Pio - presidio G. Rummo' Benevento Hospital / 6.AO 'Sant'Anna e San Sebastiano' Caserta Hospital / 7.PO 'Maria Santissima Addolorata' Eboli Hospital / 8.Biogem Istituto di Ricerche Genetiche	1. Genome Research Center for Health (CRGS) / 2. Laboratory of Molecular Medicine and Genomics(LMMGe) / 3. Center for Research in Pure and Applied Mathematics (CRMPA)	Giorgio Giurato, Francesca Rizzo, Alessandro Weisz, Gianluigi Franci, Giovanni Nassa, Pasquale Pagliano, Roberta Tarallo, Elena Alexandrova, Ylenia D'Agostino, Carlo Ferravante, Jessica Lamberti, Viola Melone, Domenico Memoli, Valeria Mirici Cappa, Domenico Palumbo, Giovanni Pecoraro, Assunta Sellitto, Oriana Strianese, Ilaria Terenzi, Giuseppe Fenza, Aniello Gentile, Antonello Saccomanno, Sonia Amabile, Teresa Rocco, Annamaria Salvati, Emilia Vaccaro, Massimiliano Galdiero, Michele Cennamo, Giuseppe Portella, Maria Grazia Foti, Mariarosaria Ingino, Maria Landi, Maurizio Fumi, Vincenzo Rocco, Rita Greco, Vittoria Letizia, Arnolfo Petruzziello, Maddalena Schioppa, Gregorio Goffredi, Francesca Marciano, Michele Caraglia, Alessia Cossu, Marianna Scrima, Edmondo Adorisio, Morena D'Avenia, Michela Iacobellis, Rosanna Piluscio, Giorgio Dirani, Vittorio Sambri, Simona Semprini, Silvia Zanoli, Francesco Curcio, Stefania Marzinotto, Andreina Baj, Fausto Sessa.
EPI_ISL_956318, EPI_ISL_956320	Laboratory Medicine	Department of Laboratory Medicine, Lin-Kou Chang Gung Memorial Hospital, Taoyuan, Taiwan	Kuo-Chien Tsao, Yu-Nong Gong, Shu-Li Yang, Yi-Chun Liu, Chung-Guei Huang, Mei-Jen Hsiao, Po-Wei Huang, Cheng-Ta Yang, Cheng-Hsun Chiu, Peng-Nien Huang, Kuo-Ming Lee, Guang-Wu Chen, Shin-Ru Shih
EPI_ISL_960125	Mitchells Plain Hospital wc MPH	National Health Laboratory Service/UCT	Arash Iranzadeh, Deelan Doolabh, Lynn Tyers, Bruna Galvao, Innocent Mudau, Marvin Hsiao, Kruger Marais, Diana Hardie, Stephen Korsman, Carolyn Williamson
EPI_ISL_960126	Groote Schuur Hospital wc GSH	National Health Laboratory Service/UCT	Arash Iranzadeh, Deelan Doolabh, Lynn Tyers, Bruna Galvao, Innocent Mudau, Marvin Hsiao, Kruger Marais, Diana Hardie, Stephen Korsman, Carolyn Williamson

EPI_ISL_960355	University of Wisconsin-Madison AIDS Vaccine Research Laboratories	University of Wisconsin-Madison AIDS Vaccine Research Laboratories	Gage Moreno, Katarina Braun, et al. AIDS Vaccine Research Laboratories
EPI_ISL_962102	Illinois Department of Public Health	Gagnon Lab, Southern Illinois University	Keith Gagnon
EPI_ISL_965339, EPI_ISL_965342, EPI_ISL_965346, EPI_ISL_965352, EPI_ISL_965382, EPI_ISL_965457	University of Liège COVID-19 testing center	GIGA Medical Genomics	Keith Durkin, Maria Artesi, Bouchra Boujemla, Emmanuel André, Marc Van Ranst, Fabrice Bureau, Laurent Gillet, Wouter Coppieters, Vincent Bours
EPI_ISL_974759, EPI_ISL_974760, EPI_ISL_974761, EPI_ISL_974762, EPI_ISL_974763, EPI_ISL_974764, EPI_ISL_974765, EPI_ISL_974766, EPI_ISL_974767, EPI_ISL_974768, EPI_ISL_974769, EPI_ISL_974770, EPI_ISL_974771, EPI_ISL_974772, EPI_ISL_974773, EPI_ISL_974774, EPI_ISL_974775, EPI_ISL_974776, EPI_ISL_974777, EPI_ISL_974778, EPI_ISL_974779, EPI_ISL_974780, EPI_ISL_974781, EPI_ISL_974782, EPI_ISL_974783, EPI_ISL_974784, EPI_ISL_974785, EPI_ISL_974786, EPI_ISL_974787, EPI_ISL_974788, EPI_ISL_974789, EPI_ISL_974790, EPI_ISL_974791, EPI_ISL_974792, EPI_ISL_974793, EPI_ISL_974794, EPI_ISL_974795, EPI_ISL_974796, EPI_ISL_974797, EPI_ISL_974798, EPI_ISL_974799, EPI_ISL_974800, EPI_ISL_974801, EPI_ISL_974802, EPI_ISL_974803, EPI_ISL_974804, EPI_ISL_974805, EPI_ISL_974806, EPI_ISL_974807, EPI_ISL_974808, EPI_ISL_974809, EPI_ISL_974810, EPI_ISL_974811, EPI_ISL_974812, EPI_ISL_974813, EPI_ISL_974814, EPI_ISL_974815, EPI_ISL_974816, EPI_ISL_974817, EPI_ISL_974818, EPI_ISL_974819, EPI_ISL_974820, EPI_ISL_974821, EPI_ISL_974822, EPI_ISL_974823, EPI_ISL_974824			
see above	BCCDC Public Health Laboratory	BCCDC Public Health Laboratory	Prystajecky Natalie, Linda Hoang, Dan Fornika, John Tyson, Shannon Russell, Kim Macdonald, Kimia Kamelian, Ana Pacagnella, Corrinne Ng, Loretta Janz, Robert Azana Terry Snutch, Mel Krajden
