# Supplementary material for: Detection and characterization of the SARS-CoV-2 lineage B.1.526 in New York
Source: Nat Commun. 2021 Aug 9;12:4886. doi: 10.1038/s41467-021-25168-4 (PMC8352861; doi:10.1038/s41467-021-25168-4)
Supplement: Supplementary file 8 — Supplementary Data 4 [file 41467_2021_25168_MOESM8_ESM.zip › GISAID_acknowledements_tables/gisaid_hcov-19_acknowledgement_table_2021_02_13_010-12.pdf]

We gratefully acknowledge the following Authors from the Originating laboratories responsible for obtaining the specimens, as well as the Submitting laboratories where the genome data were generated and shared via GISAID, on which this research is based.

All Submitters of data may be contacted directly via www.gisaid.org

Authors are sorted alphabetically.

Accession ID	Originating Laboratory	Submitting Laboratory	Authors
EPI_ISL_845802, EPI_ISL_845803, EPI_ISL_845804	Medlab Pathology	NSW Health Pathology - Institute of Clinical Pathology and Medical Research; Westmead Hospital; University of Sydney	CIDM-PH et al.
EPI_ISL_845805	Sydney South West Pathology Service (SSWPS) - Royal Prince Alfred Hospital - NSW Health Pathology	NSW Health Pathology - Institute of Clinical Pathology and Medical Research; Westmead Hospital; University of Sydney	CIDM-PH et al.
EPI_ISL_846774	Respiratory Virus Unit, National Infection Service, Public Health England	COVID-19 Genomics UK (COG-UK) Consortium	PHE Covid Sequencing Team
EPI_ISL_849762	unknown	PHV-FSS	Son Nguyen et al.
EPI_ISL_852014	Lighthouse Lab in Milton Keynes	Wellcome Sanger Institute for the COVID-19 Genomics UK (COG-UK) Consortium	The Lighthouse Lab in Milton Keynes and Alex Alderton, Roberto Amato, Sonia Goncalves, Ewan Harrison, David K. Jackson, Ian Johnston, Dominic Kwiatkowski, Cordelia Langford, John Sillitoe on behalf of the Wellcome Sanger Institute COVID-19 Surveillance Team
EPI_ISL_855604	Respiratory Virus Unit, National Infection Service, Public Health England	COVID-19 Genomics UK (COG-UK) Consortium	PHE Covid Sequencing Team
EPI_ISL_857310, EPI_ISL_857311, EPI_ISL_857312, EPI_ISL_857313	South Eastern Area Laboratory Services (SEALS)	NSW Health Pathology - Institute of Clinical Pathology and Medical Research; Westmead Hospital; University of Sydney	CIDM-PH et al.
EPI_ISL_857408, EPI_ISL_857409, EPI_ISL_857411, EPI_ISL_857414	Maine HETL	Tewhey Lab, The Jackson Laboratory	Matluk,N., Dewey,H., Iosue,F., Barter,M., Lynch,R., Munger,H. and Tewhey,R.
EPI_ISL_857469, EPI_ISL_857470, EPI_ISL_857471, EPI_ISL_857472, EPI_ISL_857474, EPI_ISL_857475, EPI_ISL_857476, EPI_ISL_857477, EPI_ISL_857479, EPI_ISL_857480, EPI_ISL_857481, EPI_ISL_857482, EPI_ISL_857483, EPI_ISL_857484			
see above	National Public Health Laboratory, National Centre for Infectious Diseases	National Public Health Laboratory, National Centre for Infectious Diseases	Tze Minn Mak, Sophie Octavia, Zhenyang Zhou, Lin Cui, Raymond Tzer Pin Lin
EPI_ISL_857487, EPI_ISL_857488	PathWest Laboratory Medicine WA	PathWest Laboratory Medicine WA Microbial Surveillance Unit	PathWest Laboratory Medicine WA Microbial Surveillance Unit
EPI_ISL_860691, EPI_ISL_860692, EPI_ISL_860693, EPI_ISL_860694, EPI_ISL_860696, EPI_ISL_860697, EPI_ISL_860698, EPI_ISL_860699, EPI_ISL_860700, EPI_ISL_860701, EPI_ISL_860702, EPI_ISL_860704, EPI_ISL_860705, EPI_ISL_860706, EPI_ISL_860707, EPI_ISL_860708, EPI_ISL_860783			
see above	Respiratory Virus Unit, National Infection Service, Public Health England	COVID-19 Genomics UK (COG-UK) Consortium	PHE Covid Sequencing Team
EPI_ISL_861003, EPI_ISL_861010, EPI_ISL_861011, EPI_ISL_861013, EPI_ISL_861036, EPI_ISL_861054, EPI_ISL_861063, EPI_ISL_861067, EPI_ISL_861075, EPI_ISL_861080, EPI_ISL_861085, EPI_ISL_861089, EPI_ISL_861090, EPI_ISL_861091, EPI_ISL_861095, EPI_ISL_861096			
see above	Johns Hopkins Hospital Department of Pathology	Johns Hopkins Hospital Department of Pathology	C. Paul Morris, Chun Huai Luo, Adannaya Amadi, Nicholas Gallagher, Heba H. Mostafa
EPI_ISL_861443, EPI_ISL_861444, EPI_ISL_861449, EPI_ISL_861450, EPI_ISL_861451, EPI_ISL_861454	Clinical Molecular Microbiology Laboratory, UNC Hospitals	Jeremy Wang	Jeremy Wang, Alexander Rubinsteyn, Colleen Rice, Jason Smedberg, Melissa Miller, Corbin Jones, Robert Hagan
EPI_ISL_861730, EPI_ISL_861731	UW Virology Lab	UW Virology Lab	Pavitra Roychoudhury, Hong Xie, Lasata Shrestha, Michelle Lin, Meei-Li Huang, Keith R Jerome, Alexander Greninger
EPI_ISL_861737, EPI_ISL_861746, EPI_ISL_861751, EPI_ISL_861754, EPI_ISL_861756, EPI_ISL_861772	Tempus	Grubaugh Lab - Yale School of Public Health	Tara Alpert, Joseph Fauver, Anderson Brito, Mallery Breban, Anne Wyllie, Chantal Vogels, Mary Petrone, Annie Watkins, Chaney Kalinich, Isabel Ott, Nathan Grubaugh
EPI_ISL_862191, EPI_ISL_862192, EPI_ISL_862259, EPI_ISL_862260, EPI_ISL_862261, EPI_ISL_862262, EPI_ISL_862263, EPI_ISL_862264	Respiratory Virus Unit, National Infection Service, Public Health England	COVID-19 Genomics UK (COG-UK) Consortium	PHE Covid Sequencing Team
EPI_ISL_862834	National Institute of Infectious Diseases-Prof. Dr. Matei Bals Molecular Diagnostics Laboratory	National Institute of Infectious Diseases-Prof. Dr. Matei Bals Molecular Diagnostics Laboratory	Leontina Banica, Marius Surleac, Corina Casangiu, Petre Milu, Andreea Tudor, Simona Paraschiv, Dan Otelea
EPI_ISL_864583, EPI_ISL_864585, EPI_ISL_864587	CHU Purpan - Laboratoire de Virologie - Institut Fédératif de Biologie	CHU Purpan - Laboratoire de Virologie - Institut Fédératif de Biologie	Latour J., Ranger N., Dubois M., Carcenac R., Harter A., Boyer P., Tremeaux P., Izopet J.
EPI_ISL_864927, EPI_ISL_864928, EPI_ISL_864929, EPI_ISL_864930, EPI_ISL_864931, EPI_ISL_864932, EPI_ISL_864934, EPI_ISL_864936, EPI_ISL_864945, EPI_ISL_864946, EPI_ISL_864953, EPI_ISL_864955, EPI_ISL_864956, EPI_ISL_864958, EPI_ISL_864959			
see above	Department of Pathology, University of Cambridge	COVID-19 Genomics UK (COG-UK) Consortium	Aminu S. Jahun, Yasmin Chaudhry, Grant Hall, Iliana Georgana, Myra Hosmillo, Martin D. Curran, Malte Pinckert, Surendra Parmar, Ian Goodfellow
EPI_ISL_865163	University of Exeter	COVID-19 Genomics UK (COG-UK) Consortium	Ben Temperton,Aaron Jeffries,Michelle Michelsen,Joanna Warwick-Dugdale,Audrey Farbos,Robyn Manley,Stephen Michell,Jane Masoli
EPI_ISL_865467, EPI_ISL_865468, EPI_ISL_865469, EPI_ISL_865470, EPI_ISL_865471, EPI_ISL_865477	Liverpool Clinical Laboratories	COVID-19 Genomics UK (COG-UK) Consortium	Sam Haldenby, Anita Lucaci, Steve Paterson, Julian Hiscox, Alistair Darby, M Almsaud, A Alrezaihi, Muhannad Alruwaili, Stuart D Armstrong, Jones Benjamin, Eleanor G Bentley, Anu Chawla, Jordan J Clark, Angela Cowell, Richard Eccles, Isabel García-Dorival, Matthew Gemmell, Alessandro Gerada, PKF Gilmore, Richard Gregory, Ximeng Han, Catherine Hartley, Margaret Hughes, Miren Iturriza-Gomara, James Johnson, L Luu, Jenifer Manson, Charlotte Nelson, Elaine O'Toole, Cassie Olateju, Rebekah Penrice-Randal , Lucille Rainbow, N.P Randle, Trevor Ian Robinson, Parul Sharma, Ghada T Shawli, James P Stewart, Neil Swainston, Ecaterina Vamos, Joanne Watts, Mark Whitehead
EPI_ISL_865597, EPI_ISL_865598, EPI_ISL_865601, EPI_ISL_865605, EPI_ISL_865606, EPI_ISL_865607, EPI_ISL_865608, EPI_ISL_865609, EPI_ISL_865610, EPI_ISL_865611, EPI_ISL_865612, EPI_ISL_865613, EPI_ISL_865619, EPI_ISL_865620, EPI_ISL_865621, EPI_ISL_865622, EPI_ISL_865624, EPI_ISL_865625, EPI_ISL_865626, EPI_ISL_865627, EPI_ISL_865628, EPI_ISL_865629, EPI_ISL_865632, EPI_ISL_865633, EPI_ISL_865635, EPI_ISL_865636, EPI_ISL_865637, EPI_ISL_865641, EPI_ISL_865642, EPI_ISL_865643, EPI_ISL_865644, EPI_ISL_865645, EPI_ISL_865646, EPI_ISL_865647, EPI_ISL_865649, EPI_ISL_865650, EPI_ISL_865651, EPI_ISL_865652, EPI_ISL_865653, EPI_ISL_865654, EPI_ISL_865655, EPI_ISL_865656, EPI_ISL_865657, EPI_ISL_865853, EPI_ISL_865855, EPI_ISL_865856, EPI_ISL_865857, EPI_ISL_865858, EPI_ISL_865859, EPI_ISL_865860, EPI_ISL_865861, EPI_ISL_865862, EPI_ISL_865863, EPI_ISL_865865, EPI_ISL_865866, EPI_ISL_865882, EPI_ISL_865884, EPI_ISL_865885, EPI_ISL_865886, EPI_ISL_865887, EPI_ISL_865888, EPI_ISL_865889, EPI_ISL_865890, EPI_ISL_865902, EPI_ISL_865904, EPI_ISL_865907, EPI_ISL_865908, EPI_ISL_865917, EPI_ISL_865920, EPI_ISL_865926, EPI_ISL_865927, EPI_ISL_865928, EPI_ISL_865935, EPI_ISL_865936, EPI_ISL_865937, EPI_ISL_865938, EPI_ISL_865939, EPI_ISL_865946, EPI_ISL_865947, EPI_ISL_865948, EPI_ISL_865951, EPI_ISL_865967, EPI_ISL_865969, EPI_ISL_865970, EPI_ISL_865975, EPI_ISL_865976, EPI_ISL_865977, EPI_ISL_865978, EPI_ISL_865980, EPI_ISL_865985, EPI_ISL_865986, EPI_ISL_865987, EPI_ISL_865993, EPI_ISL_865994, EPI_ISL_865995, EPI_ISL_865996, EPI_ISL_866001, EPI_ISL_866002, EPI_ISL_866003, EPI_ISL_866007, EPI_ISL_866008, EPI_ISL_866009, EPI_ISL_866011, EPI_ISL_866013, EPI_ISL_866014, EPI_ISL_866015, EPI_ISL_866020, EPI_ISL_866290, EPI_ISL_866291, EPI_ISL_866292, EPI_ISL_866293, EPI_ISL_866298, EPI_ISL_866301, EPI_ISL_866302, EPI_ISL_866303, EPI_ISL_866304, EPI_ISL_866305, EPI_ISL_866306, EPI_ISL_866307, EPI_ISL_866311, EPI_ISL_866312, EPI_ISL_866320, EPI_ISL_866321, EPI_ISL_866322, EPI_ISL_866323, EPI_ISL_866324, EPI_ISL_866326, EPI_ISL_866327, EPI_ISL_866329, EPI_ISL_866330, EPI_ISL_866331, EPI_ISL_866332, EPI_ISL_866333			
see above	University College London, Great Ormond Street Hospital for Children NHS Foundation Trust, Imperial College Healthcare NHS Trust	COVID-19 Genomics UK (COG-UK) Consortium	Sergi Castellano, Rachel Williams, Mark Kristiansen, Paola Resende Silva, Sunando Roy, Tony Brooks, Helena Tutill, Paola Niola, Patricia Dyal, Charlotte Williams, Leysa Forrest, Yasmin Panchbhaya, Jacqueline Findlay, Samuel Weeks, Julianne Brown, Kathryn Harris, Paul Randell, James Price, Alison Holmes, Judith Breuer
EPI_ISL_866432	Respiratory Virus Unit, National Infection Service, Public Health England	COVID-19 Genomics UK (COG-UK) Consortium	PHE Covid Sequencing Team
EPI_ISL_866943, EPI_ISL_866944, EPI_ISL_866945, EPI_ISL_866946, EPI_ISL_866947, EPI_ISL_866948, EPI_ISL_866949, EPI_ISL_866950, EPI_ISL_866951, EPI_ISL_866952, EPI_ISL_866953, EPI_ISL_866954, EPI_ISL_866955, EPI_ISL_866956, EPI_ISL_866957, EPI_ISL_866958, EPI_ISL_866959, EPI_ISL_866960,			

EPI_ISL_866961			
see above	Queens Medical Centre, Clinical Microbiology Department / DeepSeq Nottingham	COVID-19 Genomics UK (COG-UK) Consortium	Gemma Clark, Wendy Smith, Manjinder Khakh, Vicki M Fleming, Michelle M Lister, Hannah Howson-Wells, Jonathan Ball, Patrick McClure, Joseph Chappell, Theocharis Tsoleridis, Nadine Holmes, Matthew Carlisle, Christopher Moore, Fei Sang, Johnny Debebe, Victoria Wright, Matthew Loose
EPI_ISL_867993, EPI_ISL_867999, EPI_ISL_868226, EPI_ISL_868228, EPI_ISL_868229, EPI_ISL_868230, EPI_ISL_868231, EPI_ISL_868232, EPI_ISL_868233, EPI_ISL_868234, EPI_ISL_868235, EPI_ISL_868236, EPI_ISL_868237, EPI_ISL_868240, EPI_ISL_868241, EPI_ISL_868242, EPI_ISL_868243, EPI_ISL_868244, EPI_ISL_868245, EPI_ISL_868246, EPI_ISL_868247, EPI_ISL_868248, EPI_ISL_868249, EPI_ISL_868250, EPI_ISL_868252, EPI_ISL_868253, EPI_ISL_868254, EPI_ISL_868255, EPI_ISL_868256			
see above	Centre for Enzyme Innovation, University of Portsmouth / Translational Research Laboratory, Portsmouth Hospitals NHS Trust	COVID-19 Genomics UK (COG-UK) Consortium	Angela Beckett,Yann Bourgeois,Garry Scarlett,Sharon Glaysher,Scott Elliott,Kelly Bicknell,Robert Impey,Allyson Lloyd,Sarah Wyllie,Ethan Butcher,Anoop Chauhan,Samuel Robson
EPI_ISL_868363, EPI_ISL_868366, EPI_ISL_868371, EPI_ISL_868385, EPI_ISL_868387, EPI_ISL_868397, EPI_ISL_868417, EPI_ISL_868421, EPI_ISL_868426, EPI_ISL_868437, EPI_ISL_868439, EPI_ISL_868447, EPI_ISL_868456, EPI_ISL_868458, EPI_ISL_868461, EPI_ISL_868463, EPI_ISL_868489, EPI_ISL_868493, EPI_ISL_868497, EPI_ISL_868501, EPI_ISL_868504, EPI_ISL_868509, EPI_ISL_868510, EPI_ISL_868517, EPI_ISL_868529, EPI_ISL_868532, EPI_ISL_868544, EPI_ISL_868546, EPI_ISL_868562, EPI_ISL_868563, EPI_ISL_868568, EPI_ISL_868569, EPI_ISL_868580, EPI_ISL_868581, EPI_ISL_868582, EPI_ISL_868584, EPI_ISL_868591, EPI_ISL_868592, EPI_ISL_868595, EPI_ISL_868596, EPI_ISL_868611, EPI_ISL_868623, EPI_ISL_868634, EPI_ISL_868646, EPI_ISL_868652, EPI_ISL_868657, EPI_ISL_868666, EPI_ISL_868667, EPI_ISL_868668, EPI_ISL_868687, EPI_ISL_868689, EPI_ISL_868691, EPI_ISL_868695, EPI_ISL_868697, EPI_ISL_868705, EPI_ISL_868706, EPI_ISL_868707, EPI_ISL_868708, EPI_ISL_868714			
see above	Virology Department, Sheffield Teaching Hospitals NHS Foundation Trust/Department of Infection, Immunity and Cardiovascular Disease, The Medical School, University of Sheffield	COVID-19 Genomics UK (COG-UK) Consortium	Thushan de Silva, Matthew Parker, Nikki Smith, Adri Angyal, Rebecca Brown, Luke Green, Rachel Tucker, Paul Parsons, Danielle Groves, Katie Johnson, Laura Carrilero, Alex Keeley, Dave Partridge, Matthew Wyles, Benjamin Lindsey, Mehmet Yavuz, Mohammad Raza, Cariad Evans
EPI_ISL_869084, EPI_ISL_869102, EPI_ISL_869103, EPI_ISL_869104, EPI_ISL_869105, EPI_ISL_869106, EPI_ISL_869107, EPI_ISL_869108, EPI_ISL_869109, EPI_ISL_869110, EPI_ISL_869111, EPI_ISL_869112, EPI_ISL_869113, EPI_ISL_869114, EPI_ISL_869115, EPI_ISL_869116, EPI_ISL_869117			
see above	A. Krumbholz, Labor Dr. Krause und Kollegen MVZ GmbH, Kiel	Charité Universitätsmedizin Berlin, Institut für Virologie	Victor M Corman, Barbara Mühlemann, Jörn Beheim-Schwarzbach, Tobias Bleicker, Julia Tesch, Talitha Veith, Julia Schneider, Terry Jones, Christian Drosten
EPI_ISL_869119	Charité Universitätsmedizin Berlin, Institut für Virologie/Labor Berlin	Charité Universitätsmedizin Berlin, Institut für Virologie	Victor M Corman, Barbara Mühlemann, Jörn Beheim-Schwarzbach, Tobias Bleicker, Julia Tesch, Talitha Veith, Julia Schneider, Terry Jones, Christian Drosten
EPI_ISL_869120, EPI_ISL_869121, EPI_ISL_869122, EPI_ISL_869123, EPI_ISL_869137, EPI_ISL_869138, EPI_ISL_869139, EPI_ISL_869140, EPI_ISL_869141, EPI_ISL_869142	A. Krumbholz, Labor Dr. Krause und Kollegen MVZ GmbH, Kiel	Charité Universitätsmedizin Berlin, Institut für Virologie	Victor M Corman, Barbara Mühlemann, Jörn Beheim-Schwarzbach, Tobias Bleicker, Julia Tesch, Talitha Veith, Julia Schneider, Terry Jones, Christian Drosten
EPI_ISL_871842, EPI_ISL_871843, EPI_ISL_871850, EPI_ISL_871851, EPI_ISL_871852, EPI_ISL_871853, EPI_ISL_871854, EPI_ISL_871855, EPI_ISL_871856, EPI_ISL_871857, EPI_ISL_871858, EPI_ISL_871859, EPI_ISL_871860, EPI_ISL_871861, EPI_ISL_871862, EPI_ISL_871863, EPI_ISL_871864, EPI_ISL_871865, EPI_ISL_871866, EPI_ISL_871867, EPI_ISL_871868, EPI_ISL_871970, EPI_ISL_871971, EPI_ISL_872000			
see above	Wyoming Public Health Laboratory	Wyoming Public Health Laboratory	Noah Hull, Taylor Fearing, Lynette Gumbleton, Channing Weber, Ashley Norberg, Bailey Bowcutt, and Wanda Manley
EPI_ISL_872044, EPI_ISL_872045, EPI_ISL_872046, EPI_ISL_872047, EPI_ISL_872048	Department of Clinical Microbiology	GIGA Medical Genomics	Keith Durkin, Maria Artesi, Sébastien Bontems, Raphaël Boreux, Bouchra Boujemla, Cécile Meex, Pierrette Melin, Marie-Pierre Hayette, Vincent Bours
EPI_ISL_872051	CHC	GIGA Medical Genomics	Keith Durkin, Maria Artesi, Sébastien Bontems, Raphaël Boreux, Bouchra Boujemla, Cécile Meex, Pierrette Melin, Marie-Pierre Hayette, Vincent Bours
EPI_ISL_872073, EPI_ISL_872074, EPI_ISL_872075, EPI_ISL_872076, EPI_ISL_872077, EPI_ISL_872078, EPI_ISL_872079, EPI_ISL_872080	Department of Clinical Microbiology	GIGA Medical Genomics	Keith Durkin, Maria Artesi, Sébastien Bontems, Raphaël Boreux, Bouchra Boujemla, Cécile Meex, Pierrette Melin, Marie-Pierre Hayette, Vincent Bours
EPI_ISL_872151	CHR Citadelle	GIGA Medical Genomics	Keith Durkin, Maria Artesi, Sébastien Bontems, Raphaël Boreux, Bouchra Boujemla, Cécile Meex, Pierrette Melin, Marie-Pierre Hayette, Vincent Bours
EPI_ISL_872156, EPI_ISL_872157, EPI_ISL_872158, EPI_ISL_872159, EPI_ISL_872160, EPI_ISL_872161, EPI_ISL_872162, EPI_ISL_872163, EPI_ISL_872165, EPI_ISL_872166, EPI_ISL_872168, EPI_ISL_872169, EPI_ISL_872173, EPI_ISL_872174, EPI_ISL_872175, EPI_ISL_872178, EPI_ISL_872179, EPI_ISL_872182, EPI_ISL_872183, EPI_ISL_872184, EPI_ISL_872185, EPI_ISL_872186, EPI_ISL_872187			
see above	Wyoming Public Health Laboratory	Wyoming Public Health Laboratory	Noah Hull, Taylor Fearing, Lynette Gumbleton, Channing Weber, Ashley Norberg, Bailey Bowcutt, and Wanda Manley
EPI_ISL_872191	Conjunto Hospitalar do Mandaqui de Sao Paulo	Instituto Adolfo Lutz, Interdiciplinary Procedures Center, Strategic Laboratory	Claudio Tavares Sacchi, Claudia Regina Gonçalves, Erica Valessa Ramos Gomes, Karoline Rodrigues Campos, Katia Correa de Oliveira Santos, Ana Lucia de Carvalho Avelino, Fabiana Cristina Pereira dos Santos
EPI_ISL_872557, EPI_ISL_872558, EPI_ISL_872559, EPI_ISL_872560, EPI_ISL_872561, EPI_ISL_872564, EPI_ISL_872565, EPI_ISL_872566	New Mexico Department of Health Scientific Laboratory	Center for Global Health, University of New Mexico Health Sciences Center	Daryl Domman, Kurt Schwalm, Twila Kunde, Joseph Hicks, Anastacia Griego, Michael Edwards, Darrell Dinwiddie
EPI_ISL_872567	Centre for Dengue Research and AICBU, Department of Immunology and Molecular Medicine	Centre for Dengue Research and AICBU, Department of Immunology and Molecular Medicine	Chandima Jeewandara, Deshni Jayathilaka, Dinuka Ariyaratne, Tibutius Thanesh Pramanayagam, Diyanath Ranasinghe, Laksiri Gomes, Gathsaurie Neelika Malavige
EPI_ISL_873270, EPI_ISL_873271	Minnesota Department of Health, Public Health Laboratory	Minnesota Department of Health, Public Health Laboratory	Alexandra Lorentz, Jacob Garfin, Matt Plumb, and Xiong Wang
EPI_ISL_873283, EPI_ISL_873286, EPI_ISL_873291, EPI_ISL_873347, EPI_ISL_873348, EPI_ISL_873369, EPI_ISL_873370, EPI_ISL_873371, EPI_ISL_873381, EPI_ISL_873388, EPI_ISL_873399, EPI_ISL_873426, EPI_ISL_873428, EPI_ISL_873451, EPI_ISL_873482, EPI_ISL_873492, EPI_ISL_873501, EPI_ISL_873518, EPI_ISL_873537, EPI_ISL_873574, EPI_ISL_873625, EPI_ISL_873630, EPI_ISL_873674, EPI_ISL_873702, EPI_ISL_873737, EPI_ISL_873814, EPI_ISL_873820, EPI_ISL_873839, EPI_ISL_873876, EPI_ISL_873903			
see above	Lighthouse Lab in Cambridge	Wellcome Sanger Institute for the COVID-19 Genomics UK (COG-UK) Consortium	Rob Howes, The Lighthouse Lab in Cambridge and Alex Alderton, Roberto Amato, Sonia Goncalves, Ewan Harrison, David K. Jackson, Ian Johnston, Dominic Kwiatkowski, Cordelia Langford, John Sillitoe on behalf of the Wellcome Sanger Institute COVID-19 Surveillance Team
EPI_ISL_875344	Charité Universitätsmedizin Berlin, Institute of Virology, Charitéplatz 1, 10117 Berlin, Germany	Charité Universitätsmedizin Berlin, Institute of Virology, Charitéplatz 1, 10117 Berlin, Germany	Victor M Corman, Julia Schneider, Jörn Beheim-Schwarzbach, Tobias Bleicker, Barbara Mühlemann, Talitha Veith, Terry Jones, Christian Drosten
EPI_ISL_875349, EPI_ISL_875350, EPI_ISL_875351, EPI_ISL_875352	National Virus Reference Laboratory	National Virus Reference Laboratory	Michael Carr, Gabriel Gonzalez, Jonathan Dean, Cillian F De Gascun
EPI_ISL_875669, EPI_ISL_875670	CHU Purpan - Laboratoire de Virologie - Institut Fédératif de Biologie	CHU Purpan - Laboratoire de Virologie - Institut Fédératif de Biologie	Latour J., Ranger N., Dubois M., Carcenac R., Harter A., Boyer P., Tremeaux P., Izopet J.
EPI_ISL_875689	Hospital do Servidor Publico	Instituto Adolfo Lutz, Interdiciplinary Procedures Center, Strategic Laboratory	Claudio Tavares Sacchi, Claudia Regina Gonçalves, Erica Valessa Ramos Gomes, Karoline Rodrigues Campos
EPI_ISL_876231, EPI_ISL_876232, EPI_ISL_876233	Massachusetts State Public Health Laboratory	Massachusetts State Public Health Laboratory	Andrew Lang, Timelia Fink, Glen Gallagher, Sandra Smole
EPI_ISL_877037, EPI_ISL_877038, EPI_ISL_877039, EPI_ISL_877040, EPI_ISL_877041, EPI_ISL_877042, EPI_ISL_877043, EPI_ISL_877046, EPI_ISL_877047, EPI_ISL_877048, EPI_ISL_877049, EPI_ISL_877050, EPI_ISL_877059, EPI_ISL_877060, EPI_ISL_877061, EPI_ISL_877062, EPI_ISL_877063, EPI_ISL_877064, EPI_ISL_877065, EPI_ISL_877066, EPI_ISL_877067, EPI_ISL_877068, EPI_ISL_877069, EPI_ISL_877070, EPI_ISL_877071, EPI_ISL_877072, EPI_ISL_877073, EPI_ISL_877074, EPI_ISL_877075, EPI_ISL_877076, EPI_ISL_877077, EPI_ISL_877078, EPI_ISL_877079, EPI_ISL_877080, EPI_ISL_877081, EPI_ISL_877082, EPI_ISL_877083, EPI_ISL_877084, EPI_ISL_877085, EPI_ISL_877086, EPI_ISL_877087, EPI_ISL_877088, EPI_ISL_877089, EPI_ISL_877090, EPI_ISL_877091, EPI_ISL_877092, EPI_ISL_877093, EPI_ISL_877094, EPI_ISL_877095, EPI_ISL_877096, EPI_ISL_877097, EPI_ISL_877098, EPI_ISL_877099, EPI_ISL_877100, EPI_ISL_877101, EPI_ISL_877102, EPI_ISL_877103, EPI_ISL_877104, EPI_ISL_877105, EPI_ISL_877106, EPI_ISL_877107, EPI_ISL_877108, EPI_ISL_877109, EPI_ISL_877110, EPI_ISL_877111, EPI_ISL_877112, EPI_ISL_877113, EPI_ISL_877114, EPI_ISL_877115, EPI_ISL_877116, EPI_ISL_877117, EPI_ISL_877118			
see above	Quest Diagnostics	Quest Diagnostics	Rosenthal,S.H., Gerasimova,A., Kagan,R.M., Anderson, B., Hua, M., Liu Y., Bernstein, L.E., Livingston, K.E., Perez, A., Shalhout, D.F., Shlyakhter, I.A., Owen, R., Tanpaiboon, P., Lacbawan, F.
EPI_ISL_877146, EPI_ISL_877147, EPI_ISL_877148, EPI_ISL_877149, EPI_ISL_877150, EPI_ISL_877151, EPI_ISL_877152, EPI_ISL_877154, EPI_ISL_877164, EPI_ISL_877165, EPI_ISL_877166, EPI_ISL_877167, EPI_ISL_877168, EPI_ISL_877169, EPI_ISL_877170, EPI_ISL_877171, EPI_ISL_877172, EPI_ISL_877173, EPI_ISL_877174, EPI_ISL_877175, EPI_ISL_877176, EPI_ISL_877177, EPI_ISL_877178, EPI_ISL_877179, EPI_ISL_877180			

see above	Univeristy of New Mexico Hospital	Center for Global Health, University of New Mexico Health Sciences Center	Daryl Domman, Kurt Schwalm, Justin Bacca, Jon Femling, Darrell Dinwiddie
EPI_ISL_877220	Canterbury Health Laboratories	Institute of Environmental Science and Research (ESR)	Xiaoyun Ren, Matt Storey, Nikki Freed, Muhammad Faisal, Jing Wang, Hermes Perez, Anja Werno, Antje van der Linden, Arlo Upton, Chris Mansell, David Hammer, Dragana Drinkovic, Gary McAuliffe, Hana Sofia Andersson, James Ussher, Jill Sherwood, Josh Freeman, Julia Howard, Juliet Elvy, Mary DeAlmeida, Matt Blakiston, Matthew Rogers, Max Bloomfield, Michael Addidle, Michelle Balm, Sally Roberts, Sarah Jefferies, Sharmini Muttaiyah, Susan Morpeth, Susan Taylor, Timothy Blackmore, Vani Sathyendran, Veronica Playle, Virginia Hope, Erasmus Smit, Lauren Jelly, Olin Silander, Joep de Ligt
EPI_ISL_877552, EPI_ISL_877553, EPI_ISL_877554, EPI_ISL_877555	Institute of Microbiology, Universidad San Francisco de Quito	Institute of Microbiology, Universidad San Francisco de Quito	Belén Prado-Vivar, Sully Márquez, Juan José Guadalupe, Monica Becerra-Wong, Bernardo Gutiérrez, Kyllen Briones, Edmundo Encalada, Ninfa Hernandez, Francisco Cordova, Verónica Barragán, Patricio Rojas-Silva, Gabriel Trueba, Michelle Grunauer, Paúl Cárdenas
EPI_ISL_877556, EPI_ISL_877557, EPI_ISL_877558, EPI_ISL_877559, EPI_ISL_877560	Institute of Microbiology, Universidad San Francisco de Quito	Institute of Microbiology, Universidad San Francisco de Quito	Belén Prado-Vivar, Sully Márquez, Juan José Guadalupe, Monica Becerra-Wong, Bernardo Gutiérrez, Eulalia Pazmiño, Katalina Pacheco, Verónica Barragán, Patricio Rojas-Silva, Gabriel Trueba, Michelle Grunauer, Paúl Cárdenas
EPI_ISL_877564, EPI_ISL_877565, EPI_ISL_877566, EPI_ISL_877567, EPI_ISL_877568, EPI_ISL_877569	Victorian Infectious Diseases Reference Laboratory (VIDRL)	VIDRL and MDU-PHL	Caly L., Seemann T., Sait, M.L., Druce J., Sherry, N.L.
EPI_ISL_877571, EPI_ISL_877572, EPI_ISL_877573, EPI_ISL_877574, EPI_ISL_877575	Microbiological Diagnostic Unit - Public Health Laboratory (MDU-PHL)	MDU-PHL	Seemann T., Sait, M.L., Sherry, N.L.
EPI_ISL_877766	Thai Red Cross Emerging Infectious Diseases Health Science Centre, Chulalongkorn Hospital, Faculty of Medicine, Chulalongkorn University	Thai Red Cross Emerging Infectious Diseases Center and Faculty of Medicine, Chulalongkorn University	Rome Buathong, Sopon Iamsirithaworn, Sininat Petcharat, Yutthana Joyjinda, Weenassarin Ampoot, Apaporn Rodpan, Opass Putcharoen, Thiravat Hemachudha, Supaporn Wacharapluesadee
EPI_ISL_877770, EPI_ISL_877772, EPI_ISL_877773, EPI_ISL_877774, EPI_ISL_877775, EPI_ISL_877776, EPI_ISL_877777, EPI_ISL_877778, EPI_ISL_877779, EPI_ISL_877780, EPI_ISL_877781, EPI_ISL_877782, EPI_ISL_877783, EPI_ISL_877784, EPI_ISL_877785, EPI_ISL_877786, EPI_ISL_877787, EPI_ISL_877788, EPI_ISL_877789, EPI_ISL_877790, EPI_ISL_877791, EPI_ISL_877792, EPI_ISL_877793, EPI_ISL_877794, EPI_ISL_877795, EPI_ISL_877796, EPI_ISL_877797, EPI_ISL_877798, EPI_ISL_877799, EPI_ISL_877800, EPI_ISL_877801, EPI_ISL_877802, EPI_ISL_877803, EPI_ISL_877804, EPI_ISL_877805, EPI_ISL_877806, EPI_ISL_877808, EPI_ISL_877809, EPI_ISL_877810, EPI_ISL_877811, EPI_ISL_877812, EPI_ISL_877813, EPI_ISL_877814, EPI_ISL_877815, EPI_ISL_877816, EPI_ISL_877817, EPI_ISL_877818, EPI_ISL_877819, EPI_ISL_877820, EPI_ISL_877822, EPI_ISL_877823, EPI_ISL_877824, EPI_ISL_877825, EPI_ISL_877826, EPI_ISL_877827, EPI_ISL_877829, EPI_ISL_877830, EPI_ISL_877831, EPI_ISL_877832, EPI_ISL_877833, EPI_ISL_877834, EPI_ISL_877835, EPI_ISL_877836, EPI_ISL_877837, EPI_ISL_877838, EPI_ISL_877839, EPI_ISL_877840, EPI_ISL_877841, EPI_ISL_877842, EPI_ISL_877843, EPI_ISL_877845, EPI_ISL_877846, EPI_ISL_877847, EPI_ISL_877848, EPI_ISL_877849, EPI_ISL_877850, EPI_ISL_877851, EPI_ISL_877852, EPI_ISL_877853, EPI_ISL_877854, EPI_ISL_877855, EPI_ISL_877856, EPI_ISL_877857, EPI_ISL_877858, EPI_ISL_877859, EPI_ISL_877860, EPI_ISL_877861, EPI_ISL_877862, EPI_ISL_877863, EPI_ISL_877864, EPI_ISL_877865, EPI_ISL_877866, EPI_ISL_877867, EPI_ISL_877868, EPI_ISL_877869, EPI_ISL_877870, EPI_ISL_877871, EPI_ISL_877872, EPI_ISL_877873, EPI_ISL_877874, EPI_ISL_877875, EPI_ISL_877876, EPI_ISL_877877, EPI_ISL_877878, EPI_ISL_877879, EPI_ISL_877880, EPI_ISL_877881, EPI_ISL_877882, EPI_ISL_877883, EPI_ISL_877884, EPI_ISL_877885, EPI_ISL_877886, EPI_ISL_877887, EPI_ISL_877888, EPI_ISL_877889, EPI_ISL_877890, EPI_ISL_877891, EPI_ISL_877892, EPI_ISL_877893, EPI_ISL_877894, EPI_ISL_877895, EPI_ISL_877896, EPI_ISL_877897, EPI_ISL_877898, EPI_ISL_877899, EPI_ISL_877900, EPI_ISL_877901, EPI_ISL_877902, EPI_ISL_877904, EPI_ISL_877905, EPI_ISL_877906, EPI_ISL_877907, EPI_ISL_877908, EPI_ISL_877909, EPI_ISL_877910, EPI_ISL_877911, EPI_ISL_877912, EPI_ISL_877914, EPI_ISL_877915, EPI_ISL_877916, EPI_ISL_877917, EPI_ISL_877918, EPI_ISL_877919, EPI_ISL_877920, EPI_ISL_877921, EPI_ISL_877922, EPI_ISL_877923, EPI_ISL_877924, EPI_ISL_877925, EPI_ISL_877926, EPI_ISL_877927, EPI_ISL_877928, EPI_ISL_877929, EPI_ISL_877930, EPI_ISL_877931, EPI_ISL_877932, EPI_ISL_877933, EPI_ISL_877934, EPI_ISL_877935, EPI_ISL_877936, EPI_ISL_877937, EPI_ISL_877938, EPI_ISL_877939, EPI_ISL_877941, EPI_ISL_877942, EPI_ISL_877943, EPI_ISL_877944, EPI_ISL_877945, EPI_ISL_877946, EPI_ISL_877947, EPI_ISL_877948, EPI_ISL_877949, EPI_ISL_877950, EPI_ISL_877951, EPI_ISL_877952, EPI_ISL_877953, EPI_ISL_877954, EPI_ISL_877956, EPI_ISL_877957, EPI_ISL_877958, EPI_ISL_877959, EPI_ISL_877960, EPI_ISL_877961, EPI_ISL_877962, EPI_ISL_877963, EPI_ISL_877964, EPI_ISL_877965, EPI_ISL_877966, EPI_ISL_877967, EPI_ISL_877968, EPI_ISL_877969, EPI_ISL_877970, EPI_ISL_877971, EPI_ISL_877973, EPI_ISL_877974, EPI_ISL_877975, EPI_ISL_877976, EPI_ISL_877977, EPI_ISL_877978, EPI_ISL_877979, EPI_ISL_877980, EPI_ISL_877981, EPI_ISL_877982, EPI_ISL_877983, EPI_ISL_877984, EPI_ISL_877985, EPI_ISL_877986, EPI_ISL_877987, EPI_ISL_877988, EPI_ISL_877989, EPI_ISL_877990, EPI_ISL_877991, EPI_ISL_877992, EPI_ISL_877993, EPI_ISL_877994, EPI_ISL_877995, EPI_ISL_877996, EPI_ISL_877997, EPI_ISL_877998, EPI_ISL_877999, EPI_ISL_878000, EPI_ISL_878001, EPI_ISL_878002, EPI_ISL_878003, EPI_ISL_878004, EPI_ISL_878005, EPI_ISL_878006, EPI_ISL_878007, EPI_ISL_878008, EPI_ISL_878009, EPI_ISL_878010, EPI_ISL_878011, EPI_ISL_878012, EPI_ISL_878013, EPI_ISL_878014, EPI_ISL_878015, EPI_ISL_878016, EPI_ISL_878017, EPI_ISL_878018, EPI_ISL_878019, EPI_ISL_878020, EPI_ISL_878021, EPI_ISL_878022, EPI_ISL_878023, EPI_ISL_878024, EPI_ISL_878025, EPI_ISL_878026, EPI_ISL_878027, EPI_ISL_878028, EPI_ISL_878029, EPI_ISL_878030, EPI_ISL_878031, EPI_ISL_878032, EPI_ISL_878033, EPI_ISL_878034, EPI_ISL_878035, EPI_ISL_878036, EPI_ISL_878037, EPI_ISL_878038, EPI_ISL_878039, EPI_ISL_878040, EPI_ISL_878041, EPI_ISL_878042, EPI_ISL_878043, EPI_ISL_878044, EPI_ISL_878045, EPI_ISL_878046, EPI_ISL_878047, EPI_ISL_878048, EPI_ISL_878049, EPI_ISL_878050, EPI_ISL_878051, EPI_ISL_878052, EPI_ISL_878053, EPI_ISL_878054, EPI_ISL_878055, EPI_ISL_878056, EPI_ISL_878057, EPI_ISL_878058, EPI_ISL_878059, EPI_ISL_878060, EPI_ISL_878061, EPI_ISL_878062, EPI_ISL_878063, EPI_ISL_878064, EPI_ISL_878065, EPI_ISL_878066, EPI_ISL_878067, EPI_ISL_878068, EPI_ISL_878069, EPI_ISL_878070, EPI_ISL_878071, EPI_ISL_878072, EPI_ISL_878073, EPI_ISL_878074, EPI_ISL_878075, EPI_ISL_878076, EPI_ISL_878077, EPI_ISL_878078, EPI_ISL_878079, EPI_ISL_878080, EPI_ISL_878081, EPI_ISL_878082, EPI_ISL_878083, EPI_ISL_878084, EPI_ISL_878085, EPI_ISL_878086, EPI_ISL_878087, EPI_ISL_878088, EPI_ISL_878090, EPI_ISL_878091, EPI_ISL_878092, EPI_ISL_878093, EPI_ISL_878094, EPI_ISL_878095, EPI_ISL_878096, EPI_ISL_878097, EPI_ISL_878098, EPI_ISL_878099, EPI_ISL_878100, EPI_ISL_878101, EPI_ISL_878102, EPI_ISL_878103, EPI_ISL_878104, EPI_ISL_878105, EPI_ISL_878106, EPI_ISL_878107, EPI_ISL_878108, EPI_ISL_878109, EPI_ISL_878110, EPI_ISL_878111, EPI_ISL_878112, EPI_ISL_878113, EPI_ISL_878114, EPI_ISL_878115, EPI_ISL_878116, EPI_ISL_878117, EPI_ISL_878119, EPI_ISL_878120, EPI_ISL_878121, EPI_ISL_878122, EPI_ISL_878123, EPI_ISL_878125, EPI_ISL_878127, EPI_ISL_878128, EPI_ISL_878129, EPI_ISL_878130, EPI_ISL_878131, EPI_ISL_878132, EPI_ISL_878133, EPI_ISL_878134, EPI_ISL_878135, EPI_ISL_878136, EPI_ISL_878137, EPI_ISL_878138, EPI_ISL_878139, EPI_ISL_878140, EPI_ISL_878141, EPI_ISL_878142, EPI_ISL_878143, EPI_ISL_878144, EPI_ISL_878145, EPI_ISL_878146, EPI_ISL_878147, EPI_ISL_878148, EPI_ISL_878149, EPI_ISL_878150, EPI_ISL_878151, EPI_ISL_878153, EPI_ISL_878154, EPI_ISL_878156, EPI_ISL_878158, EPI_ISL_878160, EPI_ISL_878161, EPI_ISL_878164, EPI_ISL_878166, EPI_ISL_878168, EPI_ISL_878169, EPI_ISL_878171, EPI_ISL_878174, EPI_ISL_878177, EPI_ISL_878181, EPI_ISL_878182, EPI_ISL_878184, EPI_ISL_878185, EPI_ISL_878187, EPI_ISL_878188, EPI_ISL_878190, EPI_ISL_878193, EPI_ISL_878195, EPI_ISL_878196, EPI_ISL_878198, EPI_ISL_878200, EPI_ISL_878201, EPI_ISL_878203, EPI_ISL_878204, EPI_ISL_878206, EPI_ISL_878210, EPI_ISL_878211, EPI_ISL_878213, EPI_ISL_878214, EPI_ISL_878216, EPI_ISL_878218, EPI_ISL_878219, EPI_ISL_878220, EPI_ISL_878222, EPI_ISL_878224, EPI_ISL_878225, EPI_ISL_878227, EPI_ISL_878228, EPI_ISL_878230, EPI_ISL_878231, EPI_ISL_878233, EPI_ISL_878235, EPI_ISL_878236, EPI_ISL_878238, EPI_ISL_878240, EPI_ISL_878241, EPI_ISL_878243, EPI_ISL_878244, EPI_ISL_878246, EPI_ISL_878247, EPI_ISL_878251, EPI_ISL_878254, EPI_ISL_878255, EPI_ISL_878257, EPI_ISL_878259, EPI_ISL_878261, EPI_ISL_878262, EPI_ISL_878264, EPI_ISL_878267, EPI_ISL_878269, EPI_ISL_878270, EPI_ISL_878272, EPI_ISL_878273, EPI_ISL_878275, EPI_ISL_878278, EPI_ISL_878280, EPI_ISL_878282, EPI_ISL_878283, EPI_ISL_878286, EPI_ISL_878288, EPI_ISL_878289, EPI_ISL_878291, EPI_ISL_878293, EPI_ISL_878294, EPI_ISL_878296, EPI_ISL_878297, EPI_ISL_878299, EPI_ISL_878301, EPI_ISL_878302, EPI_ISL_878304, EPI_ISL_878306, EPI_ISL_878307, EPI_ISL_878309, EPI_ISL_878310, EPI_ISL_878312, EPI_ISL_878313, EPI_ISL_878315, EPI_ISL_878317, EPI_ISL_878318, EPI_ISL_878320, EPI_ISL_878321, EPI_ISL_878324, EPI_ISL_878326, EPI_ISL_878328, EPI_ISL_878329, EPI_ISL_878331, EPI_ISL_878332, EPI_ISL_878334, EPI_ISL_878335, EPI_ISL_878340, EPI_ISL_878409, EPI_ISL_878410, EPI_ISL_878412, EPI_ISL_878414, EPI_ISL_878415, EPI_ISL_878417, EPI_ISL_878419, EPI_ISL_878424, EPI_ISL_878425, EPI_ISL_878427, EPI_ISL_878428, EPI_ISL_878430, EPI_ISL_878432, EPI_ISL_878433, EPI_ISL_878435, EPI_ISL_878436, EPI_ISL_878438, EPI_ISL_878439, EPI_ISL_878441, EPI_ISL_878442, EPI_ISL_878444, EPI_ISL_878446, EPI_ISL_878447, EPI_ISL_878451, EPI_ISL_878453, EPI_ISL_878459, EPI_ISL_878461, EPI_ISL_878462, EPI_ISL_878464, EPI_ISL_878467, EPI_ISL_878469, EPI_ISL_878471, EPI_ISL_878473, EPI_ISL_878475, EPI_ISL_878476, EPI_ISL_878478, EPI_ISL_878479, EPI_ISL_878482, EPI_ISL_878487, EPI_ISL_878489, EPI_ISL_878490, EPI_ISL_878493, EPI_ISL_878494, EPI_ISL_878496, EPI_ISL_878497, EPI_ISL_878499, EPI_ISL_878501, EPI_ISL_878502, EPI_ISL_878504, EPI_ISL_878508, EPI_ISL_878510, EPI_ISL_878513, EPI_ISL_878515, EPI_ISL_878517, EPI_ISL_878518, EPI_ISL_878520, EPI_ISL_878521, EPI_ISL_878523, EPI_ISL_878525, EPI_ISL_878527, EPI_ISL_878528, EPI_ISL_878532, EPI_ISL_878533, EPI_ISL_878535, EPI_ISL_878536, EPI_ISL_878538, EPI_ISL_878540, EPI_ISL_878543, EPI_ISL_878545, EPI_ISL_878546, EPI_ISL_878548, EPI_ISL_878550, EPI_ISL_878551, EPI_ISL_878553, EPI_ISL_878555, EPI_ISL_878558, EPI_ISL_878559, EPI_ISL_878561, EPI_ISL_878562, EPI_ISL_878564, EPI_ISL_878566, EPI_ISL_878567, EPI_ISL_878569, EPI_ISL_878573, EPI_ISL_878575, EPI_ISL_878577, EPI_ISL_878578, EPI_ISL_878580, EPI_ISL_878582, EPI_ISL_878583, EPI_ISL_878585, EPI_ISL_878588, EPI_ISL_878590, EPI_ISL_878591, EPI_ISL_878593, EPI_ISL_878594, EPI_ISL_878596, EPI_ISL_878599, EPI_ISL_878601, EPI_ISL_878602, EPI_ISL_878604, EPI_ISL_878606, EPI_ISL_878607, EPI_ISL_878610, EPI_ISL_878612, EPI_ISL_878615, EPI_ISL_878617, EPI_ISL_878620, EPI_ISL_878621, EPI_ISL_878623, EPI_ISL_878625, EPI_ISL_878626, EPI_ISL_878628, EPI_ISL_878630, EPI_ISL_878631, EPI_ISL_878633, EPI_ISL_878634, EPI_ISL_878636, EPI_ISL_878638, EPI_ISL_878639, EPI_ISL_878641, EPI_ISL_878642, EPI_ISL_878644, EPI_ISL_878646, EPI_ISL_878647, EPI_ISL_878649, EPI_ISL_878650, EPI_ISL_878655, EPI_ISL_878657, EPI_ISL_878659, EPI_ISL_878660, EPI_ISL_878662, EPI_ISL_878663, EPI_ISL_878665, EPI_ISL_878667, EPI_ISL_878668, EPI_ISL_878670, EPI_ISL_878672, EPI_ISL_878673, EPI_ISL_878675, EPI_ISL_878676, EPI_ISL_878678, EPI_ISL_878680, EPI_ISL_878685, EPI_ISL_878686, EPI_ISL_878691, EPI_ISL_878692, EPI_ISL_878694, EPI_ISL_878696, EPI_ISL_878698, EPI_ISL_878699, EPI_ISL_878703, EPI_ISL_878708, EPI_ISL_878709, EPI_ISL_878711, EPI_ISL_878714, EPI_ISL_878715, EPI_ISL_878717, EPI_ISL_878718, EPI_ISL_878720, EPI_ISL_878722, EPI_ISL_878723, EPI_ISL_878725, EPI_ISL_878727			
see above	Lighthouse Lab in Alderley Park	Wellcome Sanger Institute for the COVID-19 Genomics UK (COG-UK) Consortium	Jacquelyn Wynn, Mairead Hyland, The Lighthouse Lab in Alderley Park and Alex Alderton, Roberto Amato, Sonia Goncalves, Ewan Harrison, David K. Jackson, Ian Johnston, Dominic Kwiatkowski, Cordelia Langford, John Sillitoe on behalf of the Wellcome Sanger Institute COVID-19 Surveillance Team
EPI_ISL_878728, EPI_ISL_878731, EPI_ISL_878733, EPI_ISL_878735, EPI_ISL_878737, EPI_ISL_878738, EPI_ISL_878740	Lighthouse Lab in Cambridge	Wellcome Sanger Institute for the COVID-19 Genomics UK (COG-UK) Consortium	Rob Howes, The Lighthouse Lab in Cambridge and Alex Alderton, Roberto Amato, Sonia Goncalves, Ewan Harrison, David K. Jackson, Ian Johnston, Dominic Kwiatkowski, Cordelia Langford, John Sillitoe on behalf of the Wellcome Sanger Institute COVID-19 Surveillance Team
EPI_ISL_878741	Lighthouse Lab in Alderley Park	Wellcome Sanger Institute for the COVID-19 Genomics UK (COG-UK) Consortium	Jacquelyn Wynn, Mairead Hyland, The Lighthouse Lab in Alderley Park and Alex Alderton, Roberto Amato, Sonia Goncalves, Ewan Harrison, David K. Jackson, Ian Johnston, Dominic Kwiatkowski, Cordelia Langford, John Sillitoe on behalf of the Wellcome Sanger Institute COVID-19 Surveillance Team
EPI_ISL_878743, EPI_ISL_878745	Lighthouse Lab in Cambridge	Wellcome Sanger Institute for the COVID-19 Genomics UK (COG-UK) Consortium	Rob Howes, The Lighthouse Lab in Cambridge and Alex Alderton, Roberto Amato, Sonia Goncalves, Ewan Harrison, David K. Jackson, Ian Johnston, Dominic Kwiatkowski, Cordelia Langford, John Sillitoe on behalf of the Wellcome Sanger Institute COVID-19 Surveillance Team
EPI_ISL_878746	Lighthouse Lab in Alderley Park	Wellcome Sanger Institute for the COVID-19 Genomics UK (COG-UK) Consortium	Jacquelyn Wynn, Mairead Hyland, The Lighthouse Lab in Alderley Park and Alex Alderton, Roberto Amato, Sonia Goncalves, Ewan Harrison, David K. Jackson, Ian Johnston, Dominic Kwiatkowski, Cordelia Langford, John Sillitoe on behalf of the Wellcome Sanger Institute COVID-19 Surveillance Team
EPI_ISL_878748, EPI_ISL_878749, EPI_ISL_878753	Lighthouse Lab in Cambridge	Wellcome Sanger Institute for the COVID-19 Genomics UK (COG-UK) Consortium	Rob Howes, The Lighthouse Lab in Cambridge and Alex Alderton, Roberto Amato, Sonia Goncalves, Ewan Harrison, David K. Jackson, Ian Johnston, Dominic Kwiatkowski, Cordelia Langford, John Sillitoe on behalf of the Wellcome Sanger Institute COVID-19 Surveillance Team
EPI_ISL_878754	Lighthouse Lab in Alderley Park	Wellcome Sanger Institute for the COVID-19 Genomics UK (COG-UK) Consortium	Jacquelyn Wynn, Mairead Hyland, The Lighthouse Lab in Alderley Park and Alex Alderton, Roberto Amato, Sonia Goncalves, Ewan Harrison, David K. Jackson, Ian Johnston, Dominic Kwiatkowski, Cordelia Langford, John Sillitoe on behalf of the Wellcome Sanger Institute COVID-19 Surveillance Team
EPI_ISL_878758, EPI_ISL_878761, EPI_ISL_878762, EPI_ISL_878764, EPI_ISL_878766, EPI_ISL_878767, EPI_ISL_878770, EPI_ISL_878774	Lighthouse Lab in Cambridge	Wellcome Sanger Institute for the COVID-19 Genomics UK (COG-UK) Consortium	Rob Howes, The Lighthouse Lab in Cambridge and Alex Alderton, Roberto Amato, Sonia Goncalves, Ewan Harrison, David K. Jackson, Ian Johnston, Dominic Kwiatkowski, Cordelia Langford, John Sillitoe on behalf of the Wellcome Sanger Institute COVID-19 Surveillance Team
EPI_ISL_878777	Lighthouse Lab in Alderley Park	Wellcome Sanger Institute for the COVID-19 Genomics UK (COG-UK) Consortium	Jacquelyn Wynn, Mairead Hyland, The Lighthouse Lab in Alderley Park and Alex Alderton, Roberto Amato, Sonia Goncalves, Ewan Harrison, David K. Jackson, Ian Johnston, Dominic Kwiatkowski, Cordelia Langford, John Sillitoe on behalf of the Wellcome Sanger Institute COVID-19 Surveillance Team
EPI_ISL_878783	Lighthouse Lab in Cambridge	Wellcome Sanger Institute for the COVID-19 Genomics UK	Rob Howes, The Lighthouse Lab in Cambridge and Alex Alderton, Roberto Amato, Sonia Goncalves, Ewan Harrison, David K. Jackson, Ian Johnston,

		(COG-UK) Consortium	Dominic Kwiatkowski, Cordelia Langford, John Sillitoe on behalf of the Wellcome Sanger Institute COVID-19 Surveillance Team
EPI_ISL_878785	Lighthouse Lab in Alderley Park	Wellcome Sanger Institute for the COVID-19 Genomics UK (COG-UK) Consortium	Jacquelyn Wynn, Mairead Hyland, The Lighthouse Lab in Alderley Park and Alex Alderton, Roberto Amato, Sonia Goncalves, Ewan Harrison, David K. Jackson, Ian Johnston, Dominic Kwiatkowski, Cordelia Langford, John Sillitoe on behalf of the Wellcome Sanger Institute COVID-19 Surveillance Team
EPI_ISL_878787, EPI_ISL_878788	Lighthouse Lab in Cambridge	Wellcome Sanger Institute for the COVID-19 Genomics UK (COG-UK) Consortium	Rob Howes, The Lighthouse Lab in Cambridge and Alex Alderton, Roberto Amato, Sonia Goncalves, Ewan Harrison, David K. Jackson, Ian Johnston, Dominic Kwiatkowski, Cordelia Langford, John Sillitoe on behalf of the Wellcome Sanger Institute COVID-19 Surveillance Team
EPI_ISL_878790	Lighthouse Lab in Alderley Park	Wellcome Sanger Institute for the COVID-19 Genomics UK (COG-UK) Consortium	Jacquelyn Wynn, Mairead Hyland, The Lighthouse Lab in Alderley Park and Alex Alderton, Roberto Amato, Sonia Goncalves, Ewan Harrison, David K. Jackson, Ian Johnston, Dominic Kwiatkowski, Cordelia Langford, John Sillitoe on behalf of the Wellcome Sanger Institute COVID-19 Surveillance Team
EPI_ISL_878795, EPI_ISL_878796, EPI_ISL_878799, EPI_ISL_878801, EPI_ISL_878802, EPI_ISL_878803	Lighthouse Lab in Cambridge	Wellcome Sanger Institute for the COVID-19 Genomics UK (COG-UK) Consortium	Rob Howes, The Lighthouse Lab in Cambridge and Alex Alderton, Roberto Amato, Sonia Goncalves, Ewan Harrison, David K. Jackson, Ian Johnston, Dominic Kwiatkowski, Cordelia Langford, John Sillitoe on behalf of the Wellcome Sanger Institute COVID-19 Surveillance Team
EPI_ISL_878804, EPI_ISL_878805, EPI_ISL_878808, EPI_ISL_878809	Lighthouse Lab in Alderley Park	Wellcome Sanger Institute for the COVID-19 Genomics UK (COG-UK) Consortium	Jacquelyn Wynn, Mairead Hyland, The Lighthouse Lab in Alderley Park and Alex Alderton, Roberto Amato, Sonia Goncalves, Ewan Harrison, David K. Jackson, Ian Johnston, Dominic Kwiatkowski, Cordelia Langford, John Sillitoe on behalf of the Wellcome Sanger Institute COVID-19 Surveillance Team
EPI_ISL_878810, EPI_ISL_878811	Lighthouse Lab in Cambridge	Wellcome Sanger Institute for the COVID-19 Genomics UK (COG-UK) Consortium	Rob Howes, The Lighthouse Lab in Cambridge and Alex Alderton, Roberto Amato, Sonia Goncalves, Ewan Harrison, David K. Jackson, Ian Johnston, Dominic Kwiatkowski, Cordelia Langford, John Sillitoe on behalf of the Wellcome Sanger Institute COVID-19 Surveillance Team
EPI_ISL_878812	Lighthouse Lab in Alderley Park	Wellcome Sanger Institute for the COVID-19 Genomics UK (COG-UK) Consortium	Jacquelyn Wynn, Mairead Hyland, The Lighthouse Lab in Alderley Park and Alex Alderton, Roberto Amato, Sonia Goncalves, Ewan Harrison, David K. Jackson, Ian Johnston, Dominic Kwiatkowski, Cordelia Langford, John Sillitoe on behalf of the Wellcome Sanger Institute COVID-19 Surveillance Team
EPI_ISL_878813, EPI_ISL_878815, EPI_ISL_878816	Lighthouse Lab in Cambridge	Wellcome Sanger Institute for the COVID-19 Genomics UK (COG-UK) Consortium	Rob Howes, The Lighthouse Lab in Cambridge and Alex Alderton, Roberto Amato, Sonia Goncalves, Ewan Harrison, David K. Jackson, Ian Johnston, Dominic Kwiatkowski, Cordelia Langford, John Sillitoe on behalf of the Wellcome Sanger Institute COVID-19 Surveillance Team
EPI_ISL_878817	Lighthouse Lab in Alderley Park	Wellcome Sanger Institute for the COVID-19 Genomics UK (COG-UK) Consortium	Jacquelyn Wynn, Mairead Hyland, The Lighthouse Lab in Alderley Park and Alex Alderton, Roberto Amato, Sonia Goncalves, Ewan Harrison, David K. Jackson, Ian Johnston, Dominic Kwiatkowski, Cordelia Langford, John Sillitoe on behalf of the Wellcome Sanger Institute COVID-19 Surveillance Team
EPI_ISL_878818	Lighthouse Lab in Cambridge	Wellcome Sanger Institute for the COVID-19 Genomics UK (COG-UK) Consortium	Rob Howes, The Lighthouse Lab in Cambridge and Alex Alderton, Roberto Amato, Sonia Goncalves, Ewan Harrison, David K. Jackson, Ian Johnston, Dominic Kwiatkowski, Cordelia Langford, John Sillitoe on behalf of the Wellcome Sanger Institute COVID-19 Surveillance Team
EPI_ISL_878820	Lighthouse Lab in Alderley Park	Wellcome Sanger Institute for the COVID-19 Genomics UK (COG-UK) Consortium	Jacquelyn Wynn, Mairead Hyland, The Lighthouse Lab in Alderley Park and Alex Alderton, Roberto Amato, Sonia Goncalves, Ewan Harrison, David K. Jackson, Ian Johnston, Dominic Kwiatkowski, Cordelia Langford, John Sillitoe on behalf of the Wellcome Sanger Institute COVID-19 Surveillance Team
EPI_ISL_878821	Lighthouse Lab in Cambridge	Wellcome Sanger Institute for the COVID-19 Genomics UK (COG-UK) Consortium	Rob Howes, The Lighthouse Lab in Cambridge and Alex Alderton, Roberto Amato, Sonia Goncalves, Ewan Harrison, David K. Jackson, Ian Johnston, Dominic Kwiatkowski, Cordelia Langford, John Sillitoe on behalf of the Wellcome Sanger Institute COVID-19 Surveillance Team
EPI_ISL_878822	Lighthouse Lab in Alderley Park	Wellcome Sanger Institute for the COVID-19 Genomics UK (COG-UK) Consortium	Jacquelyn Wynn, Mairead Hyland, The Lighthouse Lab in Alderley Park and Alex Alderton, Roberto Amato, Sonia Goncalves, Ewan Harrison, David K. Jackson, Ian Johnston, Dominic Kwiatkowski, Cordelia Langford, John Sillitoe on behalf of the Wellcome Sanger Institute COVID-19 Surveillance Team
EPI_ISL_878823	Lighthouse Lab in Cambridge	Wellcome Sanger Institute for the COVID-19 Genomics UK (COG-UK) Consortium	Rob Howes, The Lighthouse Lab in Cambridge and Alex Alderton, Roberto Amato, Sonia Goncalves, Ewan Harrison, David K. Jackson, Ian Johnston, Dominic Kwiatkowski, Cordelia Langford, John Sillitoe on behalf of the Wellcome Sanger Institute COVID-19 Surveillance Team
EPI_ISL_878825	Lighthouse Lab in Alderley Park	Wellcome Sanger Institute for the COVID-19 Genomics UK (COG-UK) Consortium	Jacquelyn Wynn, Mairead Hyland, The Lighthouse Lab in Alderley Park and Alex Alderton, Roberto Amato, Sonia Goncalves, Ewan Harrison, David K. Jackson, Ian Johnston, Dominic Kwiatkowski, Cordelia Langford, John Sillitoe on behalf of the Wellcome Sanger Institute COVID-19 Surveillance Team
EPI_ISL_878826, EPI_ISL_878827, EPI_ISL_878828, EPI_ISL_878829, EPI_ISL_878830, EPI_ISL_878831, EPI_ISL_878832, EPI_ISL_878834	Lighthouse Lab in Cambridge	Wellcome Sanger Institute for the COVID-19 Genomics UK (COG-UK) Consortium	Rob Howes, The Lighthouse Lab in Cambridge and Alex Alderton, Roberto Amato, Sonia Goncalves, Ewan Harrison, David K. Jackson, Ian Johnston, Dominic Kwiatkowski, Cordelia Langford, John Sillitoe on behalf of the Wellcome Sanger Institute COVID-19 Surveillance Team
EPI_ISL_878835, EPI_ISL_878836	Lighthouse Lab in Alderley Park	Wellcome Sanger Institute for the COVID-19 Genomics UK (COG-UK) Consortium	Jacquelyn Wynn, Mairead Hyland, The Lighthouse Lab in Alderley Park and Alex Alderton, Roberto Amato, Sonia Goncalves, Ewan Harrison, David K. Jackson, Ian Johnston, Dominic Kwiatkowski, Cordelia Langford, John Sillitoe on behalf of the Wellcome Sanger Institute COVID-19 Surveillance Team
EPI_ISL_878837, EPI_ISL_878838, EPI_ISL_878839	Lighthouse Lab in Cambridge	Wellcome Sanger Institute for the COVID-19 Genomics UK (COG-UK) Consortium	Rob Howes, The Lighthouse Lab in Cambridge and Alex Alderton, Roberto Amato, Sonia Goncalves, Ewan Harrison, David K. Jackson, Ian Johnston, Dominic Kwiatkowski, Cordelia Langford, John Sillitoe on behalf of the Wellcome Sanger Institute COVID-19 Surveillance Team
EPI_ISL_878840	Lighthouse Lab in Alderley Park	Wellcome Sanger Institute for the COVID-19 Genomics UK (COG-UK) Consortium	Jacquelyn Wynn, Mairead Hyland, The Lighthouse Lab in Alderley Park and Alex Alderton, Roberto Amato, Sonia Goncalves, Ewan Harrison, David K. Jackson, Ian Johnston, Dominic Kwiatkowski, Cordelia Langford, John Sillitoe on behalf of the Wellcome Sanger Institute COVID-19 Surveillance Team
EPI_ISL_878841	Lighthouse Lab in Cambridge	Wellcome Sanger Institute for the COVID-19 Genomics UK (COG-UK) Consortium	Rob Howes, The Lighthouse Lab in Cambridge and Alex Alderton, Roberto Amato, Sonia Goncalves, Ewan Harrison, David K. Jackson, Ian Johnston, Dominic Kwiatkowski, Cordelia Langford, John Sillitoe on behalf of the Wellcome Sanger Institute COVID-19 Surveillance Team
EPI_ISL_878843	Lighthouse Lab in Alderley Park	Wellcome Sanger Institute for the COVID-19 Genomics UK (COG-UK) Consortium	Jacquelyn Wynn, Mairead Hyland, The Lighthouse Lab in Alderley Park and Alex Alderton, Roberto Amato, Sonia Goncalves, Ewan Harrison, David K. Jackson, Ian Johnston, Dominic Kwiatkowski, Cordelia Langford, John Sillitoe on behalf of the Wellcome Sanger Institute COVID-19 Surveillance Team
EPI_ISL_878847	Lighthouse Lab in Cambridge	Wellcome Sanger Institute for the COVID-19 Genomics UK (COG-UK) Consortium	Rob Howes, The Lighthouse Lab in Cambridge and Alex Alderton, Roberto Amato, Sonia Goncalves, Ewan Harrison, David K. Jackson, Ian Johnston, Dominic Kwiatkowski, Cordelia Langford, John Sillitoe on behalf of the Wellcome Sanger Institute COVID-19 Surveillance Team
EPI_ISL_878849	Lighthouse Lab in Alderley Park	Wellcome Sanger Institute for the COVID-19 Genomics UK (COG-UK) Consortium	Jacquelyn Wynn, Mairead Hyland, The Lighthouse Lab in Alderley Park and Alex Alderton, Roberto Amato, Sonia Goncalves, Ewan Harrison, David K. Jackson, Ian Johnston, Dominic Kwiatkowski, Cordelia Langford, John Sillitoe on behalf of the Wellcome Sanger Institute COVID-19 Surveillance Team
EPI_ISL_878850	Lighthouse Lab in Cambridge	Wellcome Sanger Institute for the COVID-19 Genomics UK (COG-UK) Consortium	Rob Howes, The Lighthouse Lab in Cambridge and Alex Alderton, Roberto Amato, Sonia Goncalves, Ewan Harrison, David K. Jackson, Ian Johnston, Dominic Kwiatkowski, Cordelia Langford, John Sillitoe on behalf of the Wellcome Sanger Institute COVID-19 Surveillance Team
EPI_ISL_878853	Lighthouse Lab in Alderley Park	Wellcome Sanger Institute for the COVID-19 Genomics UK (COG-UK) Consortium	Jacquelyn Wynn, Mairead Hyland, The Lighthouse Lab in Alderley Park and Alex Alderton, Roberto Amato, Sonia Goncalves, Ewan Harrison, David K. Jackson, Ian Johnston, Dominic Kwiatkowski, Cordelia Langford, John Sillitoe on behalf of the Wellcome Sanger Institute COVID-19 Surveillance Team
EPI_ISL_878855	Lighthouse Lab in Cambridge	Wellcome Sanger Institute for the COVID-19 Genomics UK (COG-UK) Consortium	Rob Howes, The Lighthouse Lab in Cambridge and Alex Alderton, Roberto Amato, Sonia Goncalves, Ewan Harrison, David K. Jackson, Ian Johnston, Dominic Kwiatkowski, Cordelia Langford, John Sillitoe on behalf of the Wellcome Sanger Institute COVID-19 Surveillance Team
EPI_ISL_878856	Lighthouse Lab in Alderley Park	Wellcome Sanger Institute for the COVID-19 Genomics UK (COG-UK) Consortium	Jacquelyn Wynn, Mairead Hyland, The Lighthouse Lab in Alderley Park and Alex Alderton, Roberto Amato, Sonia Goncalves, Ewan Harrison, David K. Jackson, Ian Johnston, Dominic Kwiatkowski, Cordelia Langford, John Sillitoe on behalf of the Wellcome Sanger Institute COVID-19 Surveillance Team
EPI_ISL_878857	Lighthouse Lab in Cambridge	Wellcome Sanger Institute for the COVID-19 Genomics UK (COG-UK) Consortium	Rob Howes, The Lighthouse Lab in Cambridge and Alex Alderton, Roberto Amato, Sonia Goncalves, Ewan Harrison, David K. Jackson, Ian Johnston, Dominic Kwiatkowski, Cordelia Langford, John Sillitoe on behalf of the Wellcome Sanger Institute COVID-19 Surveillance Team
EPI_ISL_878859	Lighthouse Lab in Alderley Park	Wellcome Sanger Institute for the COVID-19 Genomics UK (COG-UK) Consortium	Jacquelyn Wynn, Mairead Hyland, The Lighthouse Lab in Alderley Park and Alex Alderton, Roberto Amato, Sonia Goncalves, Ewan Harrison, David K. Jackson, Ian Johnston, Dominic Kwiatkowski, Cordelia Langford, John Sillitoe on behalf of the Wellcome Sanger Institute COVID-19 Surveillance Team
EPI_ISL_878862, EPI_ISL_878863, EPI_ISL_878864	Lighthouse Lab in Cambridge	Wellcome Sanger Institute for the COVID-19 Genomics UK (COG-UK) Consortium	Rob Howes, The Lighthouse Lab in Cambridge and Alex Alderton, Roberto Amato, Sonia Goncalves, Ewan Harrison, David K. Jackson, Ian Johnston, Dominic Kwiatkowski, Cordelia Langford, John Sillitoe on behalf of the Wellcome Sanger Institute COVID-19 Surveillance Team
EPI_ISL_878865, EPI_ISL_878866	Lighthouse Lab in Alderley Park	Wellcome Sanger Institute for the COVID-19 Genomics UK (COG-UK) Consortium	Jacquelyn Wynn, Mairead Hyland, The Lighthouse Lab in Alderley Park and Alex Alderton, Roberto Amato, Sonia Goncalves, Ewan Harrison, David K. Jackson, Ian Johnston, Dominic Kwiatkowski, Cordelia Langford, John Sillitoe on behalf of the Wellcome Sanger Institute COVID-19 Surveillance Team
EPI_ISL_878867	Lighthouse Lab in Cambridge	Wellcome Sanger Institute for the COVID-19 Genomics UK (COG-UK) Consortium	Rob Howes, The Lighthouse Lab in Cambridge and Alex Alderton, Roberto Amato, Sonia Goncalves, Ewan Harrison, David K. Jackson, Ian Johnston, Dominic Kwiatkowski, Cordelia Langford, John Sillitoe on behalf of the Wellcome Sanger Institute COVID-19 Surveillance Team
EPI_ISL_878868	Lighthouse Lab in Alderley Park	Wellcome Sanger Institute for the COVID-19 Genomics UK (COG-UK) Consortium	Jacquelyn Wynn, Mairead Hyland, The Lighthouse Lab in Alderley Park and Alex Alderton, Roberto Amato, Sonia Goncalves, Ewan Harrison, David K. Jackson, Ian Johnston, Dominic Kwiatkowski, Cordelia Langford, John Sillitoe on behalf of the Wellcome Sanger Institute COVID-19 Surveillance Team
EPI_ISL_878869, EPI_ISL_878870,	Lighthouse Lab in Cambridge	Wellcome Sanger Institute for the COVID-19 Genomics UK	Rob Howes, The Lighthouse Lab in Cambridge and Alex Alderton, Roberto Amato, Sonia Goncalves, Ewan Harrison, David K. Jackson, Ian Johnston,

EPI_ISL_878871, EPI_ISL_878872, EPI_ISL_878873		(COG-UK) Consortium	Dominic Kwiatkowski, Cordelia Langford, John Sillitoe on behalf of the Wellcome Sanger Institute COVID-19 Surveillance Team
EPI_ISL_878875	Lighthouse Lab in Alderley Park	Wellcome Sanger Institute for the COVID-19 Genomics UK (COG-UK) Consortium	Jacquelyn Wynn, Mairead Hyland, The Lighthouse Lab in Alderley Park and Alex Alderton, Roberto Amato, Sonia Goncalves, Ewan Harrison, David K. Jackson, Ian Johnston, Dominic Kwiatkowski, Cordelia Langford, John Sillitoe on behalf of the Wellcome Sanger Institute COVID-19 Surveillance Team
EPI_ISL_878877	Lighthouse Lab in Cambridge	Wellcome Sanger Institute for the COVID-19 Genomics UK (COG-UK) Consortium	Rob Howes, The Lighthouse Lab in Cambridge and Alex Alderton, Roberto Amato, Sonia Goncalves, Ewan Harrison, David K. Jackson, Ian Johnston, Dominic Kwiatkowski, Cordelia Langford, John Sillitoe on behalf of the Wellcome Sanger Institute COVID-19 Surveillance Team
EPI_ISL_878878	Lighthouse Lab in Alderley Park	Wellcome Sanger Institute for the COVID-19 Genomics UK (COG-UK) Consortium	Jacquelyn Wynn, Mairead Hyland, The Lighthouse Lab in Alderley Park and Alex Alderton, Roberto Amato, Sonia Goncalves, Ewan Harrison, David K. Jackson, Ian Johnston, Dominic Kwiatkowski, Cordelia Langford, John Sillitoe on behalf of the Wellcome Sanger Institute COVID-19 Surveillance Team
EPI_ISL_878881	Lighthouse Lab in Cambridge	Wellcome Sanger Institute for the COVID-19 Genomics UK (COG-UK) Consortium	Rob Howes, The Lighthouse Lab in Cambridge and Alex Alderton, Roberto Amato, Sonia Goncalves, Ewan Harrison, David K. Jackson, Ian Johnston, Dominic Kwiatkowski, Cordelia Langford, John Sillitoe on behalf of the Wellcome Sanger Institute COVID-19 Surveillance Team
EPI_ISL_878883	Lighthouse Lab in Alderley Park	Wellcome Sanger Institute for the COVID-19 Genomics UK (COG-UK) Consortium	Jacquelyn Wynn, Mairead Hyland, The Lighthouse Lab in Alderley Park and Alex Alderton, Roberto Amato, Sonia Goncalves, Ewan Harrison, David K. Jackson, Ian Johnston, Dominic Kwiatkowski, Cordelia Langford, John Sillitoe on behalf of the Wellcome Sanger Institute COVID-19 Surveillance Team
EPI_ISL_878885	Lighthouse Lab in Cambridge	Wellcome Sanger Institute for the COVID-19 Genomics UK (COG-UK) Consortium	Rob Howes, The Lighthouse Lab in Cambridge and Alex Alderton, Roberto Amato, Sonia Goncalves, Ewan Harrison, David K. Jackson, Ian Johnston, Dominic Kwiatkowski, Cordelia Langford, John Sillitoe on behalf of the Wellcome Sanger Institute COVID-19 Surveillance Team
EPI_ISL_878886, EPI_ISL_878887, EPI_ISL_878888	Lighthouse Lab in Alderley Park	Wellcome Sanger Institute for the COVID-19 Genomics UK (COG-UK) Consortium	Jacquelyn Wynn, Mairead Hyland, The Lighthouse Lab in Alderley Park and Alex Alderton, Roberto Amato, Sonia Goncalves, Ewan Harrison, David K. Jackson, Ian Johnston, Dominic Kwiatkowski, Cordelia Langford, John Sillitoe on behalf of the Wellcome Sanger Institute COVID-19 Surveillance Team
EPI_ISL_878889, EPI_ISL_878890	Lighthouse Lab in Cambridge	Wellcome Sanger Institute for the COVID-19 Genomics UK (COG-UK) Consortium	Rob Howes, The Lighthouse Lab in Cambridge and Alex Alderton, Roberto Amato, Sonia Goncalves, Ewan Harrison, David K. Jackson, Ian Johnston, Dominic Kwiatkowski, Cordelia Langford, John Sillitoe on behalf of the Wellcome Sanger Institute COVID-19 Surveillance Team
EPI_ISL_878891	Lighthouse Lab in Alderley Park	Wellcome Sanger Institute for the COVID-19 Genomics UK (COG-UK) Consortium	Jacquelyn Wynn, Mairead Hyland, The Lighthouse Lab in Alderley Park and Alex Alderton, Roberto Amato, Sonia Goncalves, Ewan Harrison, David K. Jackson, Ian Johnston, Dominic Kwiatkowski, Cordelia Langford, John Sillitoe on behalf of the Wellcome Sanger Institute COVID-19 Surveillance Team
EPI_ISL_878893, EPI_ISL_878894, EPI_ISL_878895, EPI_ISL_878897, EPI_ISL_878898, EPI_ISL_878899, EPI_ISL_878900, EPI_ISL_878901, EPI_ISL_878903, EPI_ISL_878904, EPI_ISL_878905, EPI_ISL_878907, EPI_ISL_878908			
see above	Lighthouse Lab in Cambridge	Wellcome Sanger Institute for the COVID-19 Genomics UK (COG-UK) Consortium	Rob Howes, The Lighthouse Lab in Cambridge and Alex Alderton, Roberto Amato, Sonia Goncalves, Ewan Harrison, David K. Jackson, Ian Johnston, Dominic Kwiatkowski, Cordelia Langford, John Sillitoe on behalf of the Wellcome Sanger Institute COVID-19 Surveillance Team
EPI_ISL_878909	Lighthouse Lab in Alderley Park	Wellcome Sanger Institute for the COVID-19 Genomics UK (COG-UK) Consortium	Jacquelyn Wynn, Mairead Hyland, The Lighthouse Lab in Alderley Park and Alex Alderton, Roberto Amato, Sonia Goncalves, Ewan Harrison, David K. Jackson, Ian Johnston, Dominic Kwiatkowski, Cordelia Langford, John Sillitoe on behalf of the Wellcome Sanger Institute COVID-19 Surveillance Team
EPI_ISL_878910, EPI_ISL_878911, EPI_ISL_878912	Lighthouse Lab in Cambridge	Wellcome Sanger Institute for the COVID-19 Genomics UK (COG-UK) Consortium	Rob Howes, The Lighthouse Lab in Cambridge and Alex Alderton, Roberto Amato, Sonia Goncalves, Ewan Harrison, David K. Jackson, Ian Johnston, Dominic Kwiatkowski, Cordelia Langford, John Sillitoe on behalf of the Wellcome Sanger Institute COVID-19 Surveillance Team
EPI_ISL_878914, EPI_ISL_878915	Lighthouse Lab in Alderley Park	Wellcome Sanger Institute for the COVID-19 Genomics UK (COG-UK) Consortium	Jacquelyn Wynn, Mairead Hyland, The Lighthouse Lab in Alderley Park and Alex Alderton, Roberto Amato, Sonia Goncalves, Ewan Harrison, David K. Jackson, Ian Johnston, Dominic Kwiatkowski, Cordelia Langford, John Sillitoe on behalf of the Wellcome Sanger Institute COVID-19 Surveillance Team
EPI_ISL_878916	Lighthouse Lab in Cambridge	Wellcome Sanger Institute for the COVID-19 Genomics UK (COG-UK) Consortium	Rob Howes, The Lighthouse Lab in Cambridge and Alex Alderton, Roberto Amato, Sonia Goncalves, Ewan Harrison, David K. Jackson, Ian Johnston, Dominic Kwiatkowski, Cordelia Langford, John Sillitoe on behalf of the Wellcome Sanger Institute COVID-19 Surveillance Team
EPI_ISL_878917, EPI_ISL_878920	Lighthouse Lab in Alderley Park	Wellcome Sanger Institute for the COVID-19 Genomics UK (COG-UK) Consortium	Jacquelyn Wynn, Mairead Hyland, The Lighthouse Lab in Alderley Park and Alex Alderton, Roberto Amato, Sonia Goncalves, Ewan Harrison, David K. Jackson, Ian Johnston, Dominic Kwiatkowski, Cordelia Langford, John Sillitoe on behalf of the Wellcome Sanger Institute COVID-19 Surveillance Team
EPI_ISL_878921, EPI_ISL_878922, EPI_ISL_878923, EPI_ISL_878924	Lighthouse Lab in Cambridge	Wellcome Sanger Institute for the COVID-19 Genomics UK (COG-UK) Consortium	Rob Howes, The Lighthouse Lab in Cambridge and Alex Alderton, Roberto Amato, Sonia Goncalves, Ewan Harrison, David K. Jackson, Ian Johnston, Dominic Kwiatkowski, Cordelia Langford, John Sillitoe on behalf of the Wellcome Sanger Institute COVID-19 Surveillance Team
EPI_ISL_878925, EPI_ISL_878926, EPI_ISL_878927	Lighthouse Lab in Alderley Park	Wellcome Sanger Institute for the COVID-19 Genomics UK (COG-UK) Consortium	Jacquelyn Wynn, Mairead Hyland, The Lighthouse Lab in Alderley Park and Alex Alderton, Roberto Amato, Sonia Goncalves, Ewan Harrison, David K. Jackson, Ian Johnston, Dominic Kwiatkowski, Cordelia Langford, John Sillitoe on behalf of the Wellcome Sanger Institute COVID-19 Surveillance Team
EPI_ISL_878928	Lighthouse Lab in Cambridge	Wellcome Sanger Institute for the COVID-19 Genomics UK (COG-UK) Consortium	Rob Howes, The Lighthouse Lab in Cambridge and Alex Alderton, Roberto Amato, Sonia Goncalves, Ewan Harrison, David K. Jackson, Ian Johnston, Dominic Kwiatkowski, Cordelia Langford, John Sillitoe on behalf of the Wellcome Sanger Institute COVID-19 Surveillance Team
EPI_ISL_878929	Lighthouse Lab in Alderley Park	Wellcome Sanger Institute for the COVID-19 Genomics UK (COG-UK) Consortium	Jacquelyn Wynn, Mairead Hyland, The Lighthouse Lab in Alderley Park and Alex Alderton, Roberto Amato, Sonia Goncalves, Ewan Harrison, David K. Jackson, Ian Johnston, Dominic Kwiatkowski, Cordelia Langford, John Sillitoe on behalf of the Wellcome Sanger Institute COVID-19 Surveillance Team
EPI_ISL_878930, EPI_ISL_878931	Lighthouse Lab in Cambridge	Wellcome Sanger Institute for the COVID-19 Genomics UK (COG-UK) Consortium	Rob Howes, The Lighthouse Lab in Cambridge and Alex Alderton, Roberto Amato, Sonia Goncalves, Ewan Harrison, David K. Jackson, Ian Johnston, Dominic Kwiatkowski, Cordelia Langford, John Sillitoe on behalf of the Wellcome Sanger Institute COVID-19 Surveillance Team
EPI_ISL_878932	Lighthouse Lab in Alderley Park	Wellcome Sanger Institute for the COVID-19 Genomics UK (COG-UK) Consortium	Jacquelyn Wynn, Mairead Hyland, The Lighthouse Lab in Alderley Park and Alex Alderton, Roberto Amato, Sonia Goncalves, Ewan Harrison, David K. Jackson, Ian Johnston, Dominic Kwiatkowski, Cordelia Langford, John Sillitoe on behalf of the Wellcome Sanger Institute COVID-19 Surveillance Team
EPI_ISL_878933, EPI_ISL_878935	Lighthouse Lab in Cambridge	Wellcome Sanger Institute for the COVID-19 Genomics UK (COG-UK) Consortium	Rob Howes, The Lighthouse Lab in Cambridge and Alex Alderton, Roberto Amato, Sonia Goncalves, Ewan Harrison, David K. Jackson, Ian Johnston, Dominic Kwiatkowski, Cordelia Langford, John Sillitoe on behalf of the Wellcome Sanger Institute COVID-19 Surveillance Team
EPI_ISL_878936	Lighthouse Lab in Alderley Park	Wellcome Sanger Institute for the COVID-19 Genomics UK (COG-UK) Consortium	Jacquelyn Wynn, Mairead Hyland, The Lighthouse Lab in Alderley Park and Alex Alderton, Roberto Amato, Sonia Goncalves, Ewan Harrison, David K. Jackson, Ian Johnston, Dominic Kwiatkowski, Cordelia Langford, John Sillitoe on behalf of the Wellcome Sanger Institute COVID-19 Surveillance Team
EPI_ISL_878937	Lighthouse Lab in Cambridge	Wellcome Sanger Institute for the COVID-19 Genomics UK (COG-UK) Consortium	Rob Howes, The Lighthouse Lab in Cambridge and Alex Alderton, Roberto Amato, Sonia Goncalves, Ewan Harrison, David K. Jackson, Ian Johnston, Dominic Kwiatkowski, Cordelia Langford, John Sillitoe on behalf of the Wellcome Sanger Institute COVID-19 Surveillance Team
EPI_ISL_878938	Lighthouse Lab in Alderley Park	Wellcome Sanger Institute for the COVID-19 Genomics UK (COG-UK) Consortium	Jacquelyn Wynn, Mairead Hyland, The Lighthouse Lab in Alderley Park and Alex Alderton, Roberto Amato, Sonia Goncalves, Ewan Harrison, David K. Jackson, Ian Johnston, Dominic Kwiatkowski, Cordelia Langford, John Sillitoe on behalf of the Wellcome Sanger Institute COVID-19 Surveillance Team
EPI_ISL_878939, EPI_ISL_878940	Lighthouse Lab in Cambridge	Wellcome Sanger Institute for the COVID-19 Genomics UK (COG-UK) Consortium	Rob Howes, The Lighthouse Lab in Cambridge and Alex Alderton, Roberto Amato, Sonia Goncalves, Ewan Harrison, David K. Jackson, Ian Johnston, Dominic Kwiatkowski, Cordelia Langford, John Sillitoe on behalf of the Wellcome Sanger Institute COVID-19 Surveillance Team
EPI_ISL_878946, EPI_ISL_878947	Lighthouse Lab in Alderley Park	Wellcome Sanger Institute for the COVID-19 Genomics UK (COG-UK) Consortium	Jacquelyn Wynn, Mairead Hyland, The Lighthouse Lab in Alderley Park and Alex Alderton, Roberto Amato, Sonia Goncalves, Ewan Harrison, David K. Jackson, Ian Johnston, Dominic Kwiatkowski, Cordelia Langford, John Sillitoe on behalf of the Wellcome Sanger Institute COVID-19 Surveillance Team
EPI_ISL_878948, EPI_ISL_878949, EPI_ISL_878950, EPI_ISL_878953, EPI_ISL_878954	Lighthouse Lab in Cambridge	Wellcome Sanger Institute for the COVID-19 Genomics UK (COG-UK) Consortium	Rob Howes, The Lighthouse Lab in Cambridge and Alex Alderton, Roberto Amato, Sonia Goncalves, Ewan Harrison, David K. Jackson, Ian Johnston, Dominic Kwiatkowski, Cordelia Langford, John Sillitoe on behalf of the Wellcome Sanger Institute COVID-19 Surveillance Team
EPI_ISL_878955	Lighthouse Lab in Alderley Park	Wellcome Sanger Institute for the COVID-19 Genomics UK (COG-UK) Consortium	Jacquelyn Wynn, Mairead Hyland, The Lighthouse Lab in Alderley Park and Alex Alderton, Roberto Amato, Sonia Goncalves, Ewan Harrison, David K. Jackson, Ian Johnston, Dominic Kwiatkowski, Cordelia Langford, John Sillitoe on behalf of the Wellcome Sanger Institute COVID-19 Surveillance Team
EPI_ISL_878956, EPI_ISL_878957	Lighthouse Lab in Cambridge	Wellcome Sanger Institute for the COVID-19 Genomics UK (COG-UK) Consortium	Rob Howes, The Lighthouse Lab in Cambridge and Alex Alderton, Roberto Amato, Sonia Goncalves, Ewan Harrison, David K. Jackson, Ian Johnston, Dominic Kwiatkowski, Cordelia Langford, John Sillitoe on behalf of the Wellcome Sanger Institute COVID-19 Surveillance Team
EPI_ISL_878958, EPI_ISL_878959, EPI_ISL_878961	Lighthouse Lab in Alderley Park	Wellcome Sanger Institute for the COVID-19 Genomics UK (COG-UK) Consortium	Jacquelyn Wynn, Mairead Hyland, The Lighthouse Lab in Alderley Park and Alex Alderton, Roberto Amato, Sonia Goncalves, Ewan Harrison, David K. Jackson, Ian Johnston, Dominic Kwiatkowski, Cordelia Langford, John Sillitoe on behalf of the Wellcome Sanger Institute COVID-19 Surveillance Team
EPI_ISL_878963, EPI_ISL_878964	Lighthouse Lab in Cambridge	Wellcome Sanger Institute for the COVID-19 Genomics UK (COG-UK) Consortium	Rob Howes, The Lighthouse Lab in Cambridge and Alex Alderton, Roberto Amato, Sonia Goncalves, Ewan Harrison, David K. Jackson, Ian Johnston, Dominic Kwiatkowski, Cordelia Langford, John Sillitoe on behalf of the Wellcome Sanger Institute COVID-19 Surveillance Team
EPI_ISL_878965, EPI_ISL_878966	Lighthouse Lab in Alderley Park	Wellcome Sanger Institute for the COVID-19 Genomics UK (COG-UK) Consortium	Jacquelyn Wynn, Mairead Hyland, The Lighthouse Lab in Alderley Park and Alex Alderton, Roberto Amato, Sonia Goncalves, Ewan Harrison, David K. Jackson, Ian Johnston, Dominic Kwiatkowski, Cordelia Langford, John Sillitoe on behalf of the Wellcome Sanger Institute COVID-19 Surveillance Team

EPI_ISL_878968, EPI_ISL_878969	Lighthouse Lab in Cambridge	Wellcome Sanger Institute for the COVID-19 Genomics UK (COG-UK) Consortium	Rob Howes, The Lighthouse Lab in Cambridge and Alex Alderton, Roberto Amato, Sonia Goncalves, Ewan Harrison, David K. Jackson, Ian Johnston, Dominic Kwiatkowski, Cordelia Langford, John Sillitoe on behalf of the Wellcome Sanger Institute COVID-19 Surveillance Team
EPI_ISL_878970	Lighthouse Lab in Alderley Park	Wellcome Sanger Institute for the COVID-19 Genomics UK (COG-UK) Consortium	Jacquelyn Wynn, Mairead Hyland, The Lighthouse Lab in Alderley Park and Alex Alderton, Roberto Amato, Sonia Goncalves, Ewan Harrison, David K. Jackson, Ian Johnston, Dominic Kwiatkowski, Cordelia Langford, John Sillitoe on behalf of the Wellcome Sanger Institute COVID-19 Surveillance Team
EPI_ISL_878973, EPI_ISL_878974, EPI_ISL_878975, EPI_ISL_878976, EPI_ISL_878977, EPI_ISL_878978	Lighthouse Lab in Cambridge	Wellcome Sanger Institute for the COVID-19 Genomics UK (COG-UK) Consortium	Rob Howes, The Lighthouse Lab in Cambridge and Alex Alderton, Roberto Amato, Sonia Goncalves, Ewan Harrison, David K. Jackson, Ian Johnston, Dominic Kwiatkowski, Cordelia Langford, John Sillitoe on behalf of the Wellcome Sanger Institute COVID-19 Surveillance Team
EPI_ISL_878979	Lighthouse Lab in Alderley Park	Wellcome Sanger Institute for the COVID-19 Genomics UK (COG-UK) Consortium	Jacquelyn Wynn, Mairead Hyland, The Lighthouse Lab in Alderley Park and Alex Alderton, Roberto Amato, Sonia Goncalves, Ewan Harrison, David K. Jackson, Ian Johnston, Dominic Kwiatkowski, Cordelia Langford, John Sillitoe on behalf of the Wellcome Sanger Institute COVID-19 Surveillance Team
EPI_ISL_878980	Lighthouse Lab in Cambridge	Wellcome Sanger Institute for the COVID-19 Genomics UK (COG-UK) Consortium	Rob Howes, The Lighthouse Lab in Cambridge and Alex Alderton, Roberto Amato, Sonia Goncalves, Ewan Harrison, David K. Jackson, Ian Johnston, Dominic Kwiatkowski, Cordelia Langford, John Sillitoe on behalf of the Wellcome Sanger Institute COVID-19 Surveillance Team
EPI_ISL_878981, EPI_ISL_878983	Lighthouse Lab in Alderley Park	Wellcome Sanger Institute for the COVID-19 Genomics UK (COG-UK) Consortium	Jacquelyn Wynn, Mairead Hyland, The Lighthouse Lab in Alderley Park and Alex Alderton, Roberto Amato, Sonia Goncalves, Ewan Harrison, David K. Jackson, Ian Johnston, Dominic Kwiatkowski, Cordelia Langford, John Sillitoe on behalf of the Wellcome Sanger Institute COVID-19 Surveillance Team
EPI_ISL_878984	Lighthouse Lab in Cambridge	Wellcome Sanger Institute for the COVID-19 Genomics UK (COG-UK) Consortium	Rob Howes, The Lighthouse Lab in Cambridge and Alex Alderton, Roberto Amato, Sonia Goncalves, Ewan Harrison, David K. Jackson, Ian Johnston, Dominic Kwiatkowski, Cordelia Langford, John Sillitoe on behalf of the Wellcome Sanger Institute COVID-19 Surveillance Team
EPI_ISL_878985	Lighthouse Lab in Alderley Park	Wellcome Sanger Institute for the COVID-19 Genomics UK (COG-UK) Consortium	Jacquelyn Wynn, Mairead Hyland, The Lighthouse Lab in Alderley Park and Alex Alderton, Roberto Amato, Sonia Goncalves, Ewan Harrison, David K. Jackson, Ian Johnston, Dominic Kwiatkowski, Cordelia Langford, John Sillitoe on behalf of the Wellcome Sanger Institute COVID-19 Surveillance Team
EPI_ISL_878986, EPI_ISL_878987, EPI_ISL_878988, EPI_ISL_878989, EPI_ISL_878991	Lighthouse Lab in Cambridge	Wellcome Sanger Institute for the COVID-19 Genomics UK (COG-UK) Consortium	Rob Howes, The Lighthouse Lab in Cambridge and Alex Alderton, Roberto Amato, Sonia Goncalves, Ewan Harrison, David K. Jackson, Ian Johnston, Dominic Kwiatkowski, Cordelia Langford, John Sillitoe on behalf of the Wellcome Sanger Institute COVID-19 Surveillance Team
EPI_ISL_878992, EPI_ISL_878994	Lighthouse Lab in Alderley Park	Wellcome Sanger Institute for the COVID-19 Genomics UK (COG-UK) Consortium	Jacquelyn Wynn, Mairead Hyland, The Lighthouse Lab in Alderley Park and Alex Alderton, Roberto Amato, Sonia Goncalves, Ewan Harrison, David K. Jackson, Ian Johnston, Dominic Kwiatkowski, Cordelia Langford, John Sillitoe on behalf of the Wellcome Sanger Institute COVID-19 Surveillance Team
EPI_ISL_878995, EPI_ISL_878996, EPI_ISL_878997, EPI_ISL_878998	Lighthouse Lab in Cambridge	Wellcome Sanger Institute for the COVID-19 Genomics UK (COG-UK) Consortium	Rob Howes, The Lighthouse Lab in Cambridge and Alex Alderton, Roberto Amato, Sonia Goncalves, Ewan Harrison, David K. Jackson, Ian Johnston, Dominic Kwiatkowski, Cordelia Langford, John Sillitoe on behalf of the Wellcome Sanger Institute COVID-19 Surveillance Team
EPI_ISL_878999, EPI_ISL_879000	Lighthouse Lab in Alderley Park	Wellcome Sanger Institute for the COVID-19 Genomics UK (COG-UK) Consortium	Jacquelyn Wynn, Mairead Hyland, The Lighthouse Lab in Alderley Park and Alex Alderton, Roberto Amato, Sonia Goncalves, Ewan Harrison, David K. Jackson, Ian Johnston, Dominic Kwiatkowski, Cordelia Langford, John Sillitoe on behalf of the Wellcome Sanger Institute COVID-19 Surveillance Team
EPI_ISL_879002	Lighthouse Lab in Cambridge	Wellcome Sanger Institute for the COVID-19 Genomics UK (COG-UK) Consortium	Rob Howes, The Lighthouse Lab in Cambridge and Alex Alderton, Roberto Amato, Sonia Goncalves, Ewan Harrison, David K. Jackson, Ian Johnston, Dominic Kwiatkowski, Cordelia Langford, John Sillitoe on behalf of the Wellcome Sanger Institute COVID-19 Surveillance Team
EPI_ISL_879003	Lighthouse Lab in Alderley Park	Wellcome Sanger Institute for the COVID-19 Genomics UK (COG-UK) Consortium	Jacquelyn Wynn, Mairead Hyland, The Lighthouse Lab in Alderley Park and Alex Alderton, Roberto Amato, Sonia Goncalves, Ewan Harrison, David K. Jackson, Ian Johnston, Dominic Kwiatkowski, Cordelia Langford, John Sillitoe on behalf of the Wellcome Sanger Institute COVID-19 Surveillance Team
EPI_ISL_879004, EPI_ISL_879005, EPI_ISL_879006	Lighthouse Lab in Cambridge	Wellcome Sanger Institute for the COVID-19 Genomics UK (COG-UK) Consortium	Rob Howes, The Lighthouse Lab in Cambridge and Alex Alderton, Roberto Amato, Sonia Goncalves, Ewan Harrison, David K. Jackson, Ian Johnston, Dominic Kwiatkowski, Cordelia Langford, John Sillitoe on behalf of the Wellcome Sanger Institute COVID-19 Surveillance Team
EPI_ISL_879007, EPI_ISL_879008	Lighthouse Lab in Alderley Park	Wellcome Sanger Institute for the COVID-19 Genomics UK (COG-UK) Consortium	Jacquelyn Wynn, Mairead Hyland, The Lighthouse Lab in Alderley Park and Alex Alderton, Roberto Amato, Sonia Goncalves, Ewan Harrison, David K. Jackson, Ian Johnston, Dominic Kwiatkowski, Cordelia Langford, John Sillitoe on behalf of the Wellcome Sanger Institute COVID-19 Surveillance Team
EPI_ISL_879010, EPI_ISL_879011, EPI_ISL_879012	Lighthouse Lab in Cambridge	Wellcome Sanger Institute for the COVID-19 Genomics UK (COG-UK) Consortium	Rob Howes, The Lighthouse Lab in Cambridge and Alex Alderton, Roberto Amato, Sonia Goncalves, Ewan Harrison, David K. Jackson, Ian Johnston, Dominic Kwiatkowski, Cordelia Langford, John Sillitoe on behalf of the Wellcome Sanger Institute COVID-19 Surveillance Team
EPI_ISL_879015	Lighthouse Lab in Alderley Park	Wellcome Sanger Institute for the COVID-19 Genomics UK (COG-UK) Consortium	Jacquelyn Wynn, Mairead Hyland, The Lighthouse Lab in Alderley Park and Alex Alderton, Roberto Amato, Sonia Goncalves, Ewan Harrison, David K. Jackson, Ian Johnston, Dominic Kwiatkowski, Cordelia Langford, John Sillitoe on behalf of the Wellcome Sanger Institute COVID-19 Surveillance Team
EPI_ISL_879017	Lighthouse Lab in Cambridge	Wellcome Sanger Institute for the COVID-19 Genomics UK (COG-UK) Consortium	Rob Howes, The Lighthouse Lab in Cambridge and Alex Alderton, Roberto Amato, Sonia Goncalves, Ewan Harrison, David K. Jackson, Ian Johnston, Dominic Kwiatkowski, Cordelia Langford, John Sillitoe on behalf of the Wellcome Sanger Institute COVID-19 Surveillance Team
EPI_ISL_879018	Lighthouse Lab in Alderley Park	Wellcome Sanger Institute for the COVID-19 Genomics UK (COG-UK) Consortium	Jacquelyn Wynn, Mairead Hyland, The Lighthouse Lab in Alderley Park and Alex Alderton, Roberto Amato, Sonia Goncalves, Ewan Harrison, David K. Jackson, Ian Johnston, Dominic Kwiatkowski, Cordelia Langford, John Sillitoe on behalf of the Wellcome Sanger Institute COVID-19 Surveillance Team
EPI_ISL_879020, EPI_ISL_879022, EPI_ISL_879024	Lighthouse Lab in Cambridge	Wellcome Sanger Institute for the COVID-19 Genomics UK (COG-UK) Consortium	Rob Howes, The Lighthouse Lab in Cambridge and Alex Alderton, Roberto Amato, Sonia Goncalves, Ewan Harrison, David K. Jackson, Ian Johnston, Dominic Kwiatkowski, Cordelia Langford, John Sillitoe on behalf of the Wellcome Sanger Institute COVID-19 Surveillance Team
EPI_ISL_879026	Lighthouse Lab in Alderley Park	Wellcome Sanger Institute for the COVID-19 Genomics UK (COG-UK) Consortium	Jacquelyn Wynn, Mairead Hyland, The Lighthouse Lab in Alderley Park and Alex Alderton, Roberto Amato, Sonia Goncalves, Ewan Harrison, David K. Jackson, Ian Johnston, Dominic Kwiatkowski, Cordelia Langford, John Sillitoe on behalf of the Wellcome Sanger Institute COVID-19 Surveillance Team
EPI_ISL_879027, EPI_ISL_879029, EPI_ISL_879030, EPI_ISL_879031	Lighthouse Lab in Cambridge	Wellcome Sanger Institute for the COVID-19 Genomics UK (COG-UK) Consortium	Rob Howes, The Lighthouse Lab in Cambridge and Alex Alderton, Roberto Amato, Sonia Goncalves, Ewan Harrison, David K. Jackson, Ian Johnston, Dominic Kwiatkowski, Cordelia Langford, John Sillitoe on behalf of the Wellcome Sanger Institute COVID-19 Surveillance Team
EPI_ISL_879033	Lighthouse Lab in Alderley Park	Wellcome Sanger Institute for the COVID-19 Genomics UK (COG-UK) Consortium	Jacquelyn Wynn, Mairead Hyland, The Lighthouse Lab in Alderley Park and Alex Alderton, Roberto Amato, Sonia Goncalves, Ewan Harrison, David K. Jackson, Ian Johnston, Dominic Kwiatkowski, Cordelia Langford, John Sillitoe on behalf of the Wellcome Sanger Institute COVID-19 Surveillance Team
EPI_ISL_879035, EPI_ISL_879036, EPI_ISL_879039, EPI_ISL_879041, EPI_ISL_879045, EPI_ISL_879046, EPI_ISL_879048, EPI_ISL_879051, EPI_ISL_879053, EPI_ISL_879054, EPI_ISL_879058, EPI_ISL_879059, EPI_ISL_879060			
see above	Lighthouse Lab in Cambridge	Wellcome Sanger Institute for the COVID-19 Genomics UK (COG-UK) Consortium	Rob Howes, The Lighthouse Lab in Cambridge and Alex Alderton, Roberto Amato, Sonia Goncalves, Ewan Harrison, David K. Jackson, Ian Johnston, Dominic Kwiatkowski, Cordelia Langford, John Sillitoe on behalf of the Wellcome Sanger Institute COVID-19 Surveillance Team
EPI_ISL_879061	Lighthouse Lab in Alderley Park	Wellcome Sanger Institute for the COVID-19 Genomics UK (COG-UK) Consortium	Jacquelyn Wynn, Mairead Hyland, The Lighthouse Lab in Alderley Park and Alex Alderton, Roberto Amato, Sonia Goncalves, Ewan Harrison, David K. Jackson, Ian Johnston, Dominic Kwiatkowski, Cordelia Langford, John Sillitoe on behalf of the Wellcome Sanger Institute COVID-19 Surveillance Team
EPI_ISL_879062	Lighthouse Lab in Cambridge	Wellcome Sanger Institute for the COVID-19 Genomics UK (COG-UK) Consortium	Rob Howes, The Lighthouse Lab in Cambridge and Alex Alderton, Roberto Amato, Sonia Goncalves, Ewan Harrison, David K. Jackson, Ian Johnston, Dominic Kwiatkowski, Cordelia Langford, John Sillitoe on behalf of the Wellcome Sanger Institute COVID-19 Surveillance Team
EPI_ISL_879066	Lighthouse Lab in Alderley Park	Wellcome Sanger Institute for the COVID-19 Genomics UK (COG-UK) Consortium	Jacquelyn Wynn, Mairead Hyland, The Lighthouse Lab in Alderley Park and Alex Alderton, Roberto Amato, Sonia Goncalves, Ewan Harrison, David K. Jackson, Ian Johnston, Dominic Kwiatkowski, Cordelia Langford, John Sillitoe on behalf of the Wellcome Sanger Institute COVID-19 Surveillance Team
EPI_ISL_879067, EPI_ISL_879068, EPI_ISL_879069	Lighthouse Lab in Cambridge	Wellcome Sanger Institute for the COVID-19 Genomics UK (COG-UK) Consortium	Rob Howes, The Lighthouse Lab in Cambridge and Alex Alderton, Roberto Amato, Sonia Goncalves, Ewan Harrison, David K. Jackson, Ian Johnston, Dominic Kwiatkowski, Cordelia Langford, John Sillitoe on behalf of the Wellcome Sanger Institute COVID-19 Surveillance Team
EPI_ISL_879070	Lighthouse Lab in Alderley Park	Wellcome Sanger Institute for the COVID-19 Genomics UK (COG-UK) Consortium	Jacquelyn Wynn, Mairead Hyland, The Lighthouse Lab in Alderley Park and Alex Alderton, Roberto Amato, Sonia Goncalves, Ewan Harrison, David K. Jackson, Ian Johnston, Dominic Kwiatkowski, Cordelia Langford, John Sillitoe on behalf of the Wellcome Sanger Institute COVID-19 Surveillance Team
EPI_ISL_879071	Lighthouse Lab in Cambridge	Wellcome Sanger Institute for the COVID-19 Genomics UK (COG-UK) Consortium	Rob Howes, The Lighthouse Lab in Cambridge and Alex Alderton, Roberto Amato, Sonia Goncalves, Ewan Harrison, David K. Jackson, Ian Johnston, Dominic Kwiatkowski, Cordelia Langford, John Sillitoe on behalf of the Wellcome Sanger Institute COVID-19 Surveillance Team
EPI_ISL_879072	Lighthouse Lab in Alderley Park	Wellcome Sanger Institute for the COVID-19 Genomics UK (COG-UK) Consortium	Jacquelyn Wynn, Mairead Hyland, The Lighthouse Lab in Alderley Park and Alex Alderton, Roberto Amato, Sonia Goncalves, Ewan Harrison, David K. Jackson, Ian Johnston, Dominic Kwiatkowski, Cordelia Langford, John Sillitoe on behalf of the Wellcome Sanger Institute COVID-19 Surveillance Team
EPI_ISL_879074, EPI_ISL_879076	Lighthouse Lab in Cambridge	Wellcome Sanger Institute for the COVID-19 Genomics UK (COG-UK) Consortium	Rob Howes, The Lighthouse Lab in Cambridge and Alex Alderton, Roberto Amato, Sonia Goncalves, Ewan Harrison, David K. Jackson, Ian Johnston, Dominic Kwiatkowski, Cordelia Langford, John Sillitoe on behalf of the Wellcome Sanger Institute COVID-19 Surveillance Team
EPI_ISL_879077	Lighthouse Lab in Alderley Park	Wellcome Sanger Institute for the COVID-19 Genomics UK (COG-UK) Consortium	Jacquelyn Wynn, Mairead Hyland, The Lighthouse Lab in Alderley Park and Alex Alderton, Roberto Amato, Sonia Goncalves, Ewan Harrison, David K. Jackson, Ian Johnston, Dominic Kwiatkowski, Cordelia Langford, John Sillitoe on behalf of the Wellcome Sanger Institute COVID-19 Surveillance Team

EPI_ISL_879078	Lighthouse Lab in Cambridge	Wellcome Sanger Institute for the COVID-19 Genomics UK (COG-UK) Consortium	Rob Howes, The Lighthouse Lab in Cambridge and Alex Alderton, Roberto Amato, Sonia Goncalves, Ewan Harrison, David K. Jackson, Ian Johnston, Dominic Kwiatkowski, Cordelia Langford, John Sillitoe on behalf of the Wellcome Sanger Institute COVID-19 Surveillance Team
EPI_ISL_879079	Lighthouse Lab in Alderley Park	Wellcome Sanger Institute for the COVID-19 Genomics UK (COG-UK) Consortium	Jacquelyn Wynn, Mairead Hyland, The Lighthouse Lab in Alderley Park and Alex Alderton, Roberto Amato, Sonia Goncalves, Ewan Harrison, David K. Jackson, Ian Johnston, Dominic Kwiatkowski, Cordelia Langford, John Sillitoe on behalf of the Wellcome Sanger Institute COVID-19 Surveillance Team
EPI_ISL_879080	Lighthouse Lab in Cambridge	Wellcome Sanger Institute for the COVID-19 Genomics UK (COG-UK) Consortium	Rob Howes, The Lighthouse Lab in Cambridge and Alex Alderton, Roberto Amato, Sonia Goncalves, Ewan Harrison, David K. Jackson, Ian Johnston, Dominic Kwiatkowski, Cordelia Langford, John Sillitoe on behalf of the Wellcome Sanger Institute COVID-19 Surveillance Team
EPI_ISL_879081	Lighthouse Lab in Alderley Park	Wellcome Sanger Institute for the COVID-19 Genomics UK (COG-UK) Consortium	Jacquelyn Wynn, Mairead Hyland, The Lighthouse Lab in Alderley Park and Alex Alderton, Roberto Amato, Sonia Goncalves, Ewan Harrison, David K. Jackson, Ian Johnston, Dominic Kwiatkowski, Cordelia Langford, John Sillitoe on behalf of the Wellcome Sanger Institute COVID-19 Surveillance Team
EPI_ISL_879082	Lighthouse Lab in Cambridge	Wellcome Sanger Institute for the COVID-19 Genomics UK (COG-UK) Consortium	Rob Howes, The Lighthouse Lab in Cambridge and Alex Alderton, Roberto Amato, Sonia Goncalves, Ewan Harrison, David K. Jackson, Ian Johnston, Dominic Kwiatkowski, Cordelia Langford, John Sillitoe on behalf of the Wellcome Sanger Institute COVID-19 Surveillance Team
EPI_ISL_879083	Lighthouse Lab in Alderley Park	Wellcome Sanger Institute for the COVID-19 Genomics UK (COG-UK) Consortium	Jacquelyn Wynn, Mairead Hyland, The Lighthouse Lab in Alderley Park and Alex Alderton, Roberto Amato, Sonia Goncalves, Ewan Harrison, David K. Jackson, Ian Johnston, Dominic Kwiatkowski, Cordelia Langford, John Sillitoe on behalf of the Wellcome Sanger Institute COVID-19 Surveillance Team
EPI_ISL_879084, EPI_ISL_879085	Lighthouse Lab in Cambridge	Wellcome Sanger Institute for the COVID-19 Genomics UK (COG-UK) Consortium	Rob Howes, The Lighthouse Lab in Cambridge and Alex Alderton, Roberto Amato, Sonia Goncalves, Ewan Harrison, David K. Jackson, Ian Johnston, Dominic Kwiatkowski, Cordelia Langford, John Sillitoe on behalf of the Wellcome Sanger Institute COVID-19 Surveillance Team
EPI_ISL_879088	Lighthouse Lab in Alderley Park	Wellcome Sanger Institute for the COVID-19 Genomics UK (COG-UK) Consortium	Jacquelyn Wynn, Mairead Hyland, The Lighthouse Lab in Alderley Park and Alex Alderton, Roberto Amato, Sonia Goncalves, Ewan Harrison, David K. Jackson, Ian Johnston, Dominic Kwiatkowski, Cordelia Langford, John Sillitoe on behalf of the Wellcome Sanger Institute COVID-19 Surveillance Team
EPI_ISL_879090	Lighthouse Lab in Cambridge	Wellcome Sanger Institute for the COVID-19 Genomics UK (COG-UK) Consortium	Rob Howes, The Lighthouse Lab in Cambridge and Alex Alderton, Roberto Amato, Sonia Goncalves, Ewan Harrison, David K. Jackson, Ian Johnston, Dominic Kwiatkowski, Cordelia Langford, John Sillitoe on behalf of the Wellcome Sanger Institute COVID-19 Surveillance Team
EPI_ISL_879091	Lighthouse Lab in Alderley Park	Wellcome Sanger Institute for the COVID-19 Genomics UK (COG-UK) Consortium	Jacquelyn Wynn, Mairead Hyland, The Lighthouse Lab in Alderley Park and Alex Alderton, Roberto Amato, Sonia Goncalves, Ewan Harrison, David K. Jackson, Ian Johnston, Dominic Kwiatkowski, Cordelia Langford, John Sillitoe on behalf of the Wellcome Sanger Institute COVID-19 Surveillance Team
EPI_ISL_879095	Lighthouse Lab in Cambridge	Wellcome Sanger Institute for the COVID-19 Genomics UK (COG-UK) Consortium	Rob Howes, The Lighthouse Lab in Cambridge and Alex Alderton, Roberto Amato, Sonia Goncalves, Ewan Harrison, David K. Jackson, Ian Johnston, Dominic Kwiatkowski, Cordelia Langford, John Sillitoe on behalf of the Wellcome Sanger Institute COVID-19 Surveillance Team
EPI_ISL_879097, EPI_ISL_879098, EPI_ISL_879099, EPI_ISL_879100, EPI_ISL_879101	Lighthouse Lab in Alderley Park	Wellcome Sanger Institute for the COVID-19 Genomics UK (COG-UK) Consortium	Jacquelyn Wynn, Mairead Hyland, The Lighthouse Lab in Alderley Park and Alex Alderton, Roberto Amato, Sonia Goncalves, Ewan Harrison, David K. Jackson, Ian Johnston, Dominic Kwiatkowski, Cordelia Langford, John Sillitoe on behalf of the Wellcome Sanger Institute COVID-19 Surveillance Team
EPI_ISL_879102	Lighthouse Lab in Cambridge	Wellcome Sanger Institute for the COVID-19 Genomics UK (COG-UK) Consortium	Rob Howes, The Lighthouse Lab in Cambridge and Alex Alderton, Roberto Amato, Sonia Goncalves, Ewan Harrison, David K. Jackson, Ian Johnston, Dominic Kwiatkowski, Cordelia Langford, John Sillitoe on behalf of the Wellcome Sanger Institute COVID-19 Surveillance Team
EPI_ISL_879103	Lighthouse Lab in Alderley Park	Wellcome Sanger Institute for the COVID-19 Genomics UK (COG-UK) Consortium	Jacquelyn Wynn, Mairead Hyland, The Lighthouse Lab in Alderley Park and Alex Alderton, Roberto Amato, Sonia Goncalves, Ewan Harrison, David K. Jackson, Ian Johnston, Dominic Kwiatkowski, Cordelia Langford, John Sillitoe on behalf of the Wellcome Sanger Institute COVID-19 Surveillance Team
EPI_ISL_879104, EPI_ISL_879105, EPI_ISL_879106, EPI_ISL_879107	Lighthouse Lab in Cambridge	Wellcome Sanger Institute for the COVID-19 Genomics UK (COG-UK) Consortium	Rob Howes, The Lighthouse Lab in Cambridge and Alex Alderton, Roberto Amato, Sonia Goncalves, Ewan Harrison, David K. Jackson, Ian Johnston, Dominic Kwiatkowski, Cordelia Langford, John Sillitoe on behalf of the Wellcome Sanger Institute COVID-19 Surveillance Team
EPI_ISL_879108, EPI_ISL_879109, EPI_ISL_879110, EPI_ISL_879111	Lighthouse Lab in Alderley Park	Wellcome Sanger Institute for the COVID-19 Genomics UK (COG-UK) Consortium	Jacquelyn Wynn, Mairead Hyland, The Lighthouse Lab in Alderley Park and Alex Alderton, Roberto Amato, Sonia Goncalves, Ewan Harrison, David K. Jackson, Ian Johnston, Dominic Kwiatkowski, Cordelia Langford, John Sillitoe on behalf of the Wellcome Sanger Institute COVID-19 Surveillance Team
EPI_ISL_879112	Lighthouse Lab in Cambridge	Wellcome Sanger Institute for the COVID-19 Genomics UK (COG-UK) Consortium	Rob Howes, The Lighthouse Lab in Cambridge and Alex Alderton, Roberto Amato, Sonia Goncalves, Ewan Harrison, David K. Jackson, Ian Johnston, Dominic Kwiatkowski, Cordelia Langford, John Sillitoe on behalf of the Wellcome Sanger Institute COVID-19 Surveillance Team
EPI_ISL_879113, EPI_ISL_879114	Lighthouse Lab in Alderley Park	Wellcome Sanger Institute for the COVID-19 Genomics UK (COG-UK) Consortium	Jacquelyn Wynn, Mairead Hyland, The Lighthouse Lab in Alderley Park and Alex Alderton, Roberto Amato, Sonia Goncalves, Ewan Harrison, David K. Jackson, Ian Johnston, Dominic Kwiatkowski, Cordelia Langford, John Sillitoe on behalf of the Wellcome Sanger Institute COVID-19 Surveillance Team
EPI_ISL_879115	Lighthouse Lab in Cambridge	Wellcome Sanger Institute for the COVID-19 Genomics UK (COG-UK) Consortium	Rob Howes, The Lighthouse Lab in Cambridge and Alex Alderton, Roberto Amato, Sonia Goncalves, Ewan Harrison, David K. Jackson, Ian Johnston, Dominic Kwiatkowski, Cordelia Langford, John Sillitoe on behalf of the Wellcome Sanger Institute COVID-19 Surveillance Team
EPI_ISL_879118	Lighthouse Lab in Alderley Park	Wellcome Sanger Institute for the COVID-19 Genomics UK (COG-UK) Consortium	Jacquelyn Wynn, Mairead Hyland, The Lighthouse Lab in Alderley Park and Alex Alderton, Roberto Amato, Sonia Goncalves, Ewan Harrison, David K. Jackson, Ian Johnston, Dominic Kwiatkowski, Cordelia Langford, John Sillitoe on behalf of the Wellcome Sanger Institute COVID-19 Surveillance Team
EPI_ISL_879119	Lighthouse Lab in Cambridge	Wellcome Sanger Institute for the COVID-19 Genomics UK (COG-UK) Consortium	Rob Howes, The Lighthouse Lab in Cambridge and Alex Alderton, Roberto Amato, Sonia Goncalves, Ewan Harrison, David K. Jackson, Ian Johnston, Dominic Kwiatkowski, Cordelia Langford, John Sillitoe on behalf of the Wellcome Sanger Institute COVID-19 Surveillance Team
EPI_ISL_879121	Lighthouse Lab in Alderley Park	Wellcome Sanger Institute for the COVID-19 Genomics UK (COG-UK) Consortium	Jacquelyn Wynn, Mairead Hyland, The Lighthouse Lab in Alderley Park and Alex Alderton, Roberto Amato, Sonia Goncalves, Ewan Harrison, David K. Jackson, Ian Johnston, Dominic Kwiatkowski, Cordelia Langford, John Sillitoe on behalf of the Wellcome Sanger Institute COVID-19 Surveillance Team
EPI_ISL_879122, EPI_ISL_879123	Lighthouse Lab in Cambridge	Wellcome Sanger Institute for the COVID-19 Genomics UK (COG-UK) Consortium	Rob Howes, The Lighthouse Lab in Cambridge and Alex Alderton, Roberto Amato, Sonia Goncalves, Ewan Harrison, David K. Jackson, Ian Johnston, Dominic Kwiatkowski, Cordelia Langford, John Sillitoe on behalf of the Wellcome Sanger Institute COVID-19 Surveillance Team
EPI_ISL_879124, EPI_ISL_879125, EPI_ISL_879126	Lighthouse Lab in Alderley Park	Wellcome Sanger Institute for the COVID-19 Genomics UK (COG-UK) Consortium	Jacquelyn Wynn, Mairead Hyland, The Lighthouse Lab in Alderley Park and Alex Alderton, Roberto Amato, Sonia Goncalves, Ewan Harrison, David K. Jackson, Ian Johnston, Dominic Kwiatkowski, Cordelia Langford, John Sillitoe on behalf of the Wellcome Sanger Institute COVID-19 Surveillance Team
EPI_ISL_879127	Lighthouse Lab in Cambridge	Wellcome Sanger Institute for the COVID-19 Genomics UK (COG-UK) Consortium	Rob Howes, The Lighthouse Lab in Cambridge and Alex Alderton, Roberto Amato, Sonia Goncalves, Ewan Harrison, David K. Jackson, Ian Johnston, Dominic Kwiatkowski, Cordelia Langford, John Sillitoe on behalf of the Wellcome Sanger Institute COVID-19 Surveillance Team
EPI_ISL_879128, EPI_ISL_879129, EPI_ISL_879130, EPI_ISL_879131, EPI_ISL_879132	Lighthouse Lab in Alderley Park	Wellcome Sanger Institute for the COVID-19 Genomics UK (COG-UK) Consortium	Jacquelyn Wynn, Mairead Hyland, The Lighthouse Lab in Alderley Park and Alex Alderton, Roberto Amato, Sonia Goncalves, Ewan Harrison, David K. Jackson, Ian Johnston, Dominic Kwiatkowski, Cordelia Langford, John Sillitoe on behalf of the Wellcome Sanger Institute COVID-19 Surveillance Team
EPI_ISL_879133	Lighthouse Lab in Cambridge	Wellcome Sanger Institute for the COVID-19 Genomics UK (COG-UK) Consortium	Rob Howes, The Lighthouse Lab in Cambridge and Alex Alderton, Roberto Amato, Sonia Goncalves, Ewan Harrison, David K. Jackson, Ian Johnston, Dominic Kwiatkowski, Cordelia Langford, John Sillitoe on behalf of the Wellcome Sanger Institute COVID-19 Surveillance Team
EPI_ISL_879134	Lighthouse Lab in Alderley Park	Wellcome Sanger Institute for the COVID-19 Genomics UK (COG-UK) Consortium	Jacquelyn Wynn, Mairead Hyland, The Lighthouse Lab in Alderley Park and Alex Alderton, Roberto Amato, Sonia Goncalves, Ewan Harrison, David K. Jackson, Ian Johnston, Dominic Kwiatkowski, Cordelia Langford, John Sillitoe on behalf of the Wellcome Sanger Institute COVID-19 Surveillance Team
EPI_ISL_879135, EPI_ISL_879136	Lighthouse Lab in Cambridge	Wellcome Sanger Institute for the COVID-19 Genomics UK (COG-UK) Consortium	Rob Howes, The Lighthouse Lab in Cambridge and Alex Alderton, Roberto Amato, Sonia Goncalves, Ewan Harrison, David K. Jackson, Ian Johnston, Dominic Kwiatkowski, Cordelia Langford, John Sillitoe on behalf of the Wellcome Sanger Institute COVID-19 Surveillance Team
EPI_ISL_879137	Lighthouse Lab in Alderley Park	Wellcome Sanger Institute for the COVID-19 Genomics UK (COG-UK) Consortium	Jacquelyn Wynn, Mairead Hyland, The Lighthouse Lab in Alderley Park and Alex Alderton, Roberto Amato, Sonia Goncalves, Ewan Harrison, David K. Jackson, Ian Johnston, Dominic Kwiatkowski, Cordelia Langford, John Sillitoe on behalf of the Wellcome Sanger Institute COVID-19 Surveillance Team
EPI_ISL_879139	Lighthouse Lab in Cambridge	Wellcome Sanger Institute for the COVID-19 Genomics UK (COG-UK) Consortium	Rob Howes, The Lighthouse Lab in Cambridge and Alex Alderton, Roberto Amato, Sonia Goncalves, Ewan Harrison, David K. Jackson, Ian Johnston, Dominic Kwiatkowski, Cordelia Langford, John Sillitoe on behalf of the Wellcome Sanger Institute COVID-19 Surveillance Team
EPI_ISL_879140	Lighthouse Lab in Alderley Park	Wellcome Sanger Institute for the COVID-19 Genomics UK (COG-UK) Consortium	Jacquelyn Wynn, Mairead Hyland, The Lighthouse Lab in Alderley Park and Alex Alderton, Roberto Amato, Sonia Goncalves, Ewan Harrison, David K. Jackson, Ian Johnston, Dominic Kwiatkowski, Cordelia Langford, John Sillitoe on behalf of the Wellcome Sanger Institute COVID-19 Surveillance Team
EPI_ISL_879141, EPI_ISL_879142, EPI_ISL_879143	Lighthouse Lab in Cambridge	Wellcome Sanger Institute for the COVID-19 Genomics UK (COG-UK) Consortium	Rob Howes, The Lighthouse Lab in Cambridge and Alex Alderton, Roberto Amato, Sonia Goncalves, Ewan Harrison, David K. Jackson, Ian Johnston, Dominic Kwiatkowski, Cordelia Langford, John Sillitoe on behalf of the Wellcome Sanger Institute COVID-19 Surveillance Team
EPI_ISL_879144, EPI_ISL_879145, EPI_ISL_879147	Lighthouse Lab in Alderley Park	Wellcome Sanger Institute for the COVID-19 Genomics UK (COG-UK) Consortium	Jacquelyn Wynn, Mairead Hyland, The Lighthouse Lab in Alderley Park and Alex Alderton, Roberto Amato, Sonia Goncalves, Ewan Harrison, David K. Jackson, Ian Johnston, Dominic Kwiatkowski, Cordelia Langford, John Sillitoe on behalf of the Wellcome Sanger Institute COVID-19 Surveillance Team

EPI_ISL_879148	Lighthouse Lab in Cambridge	Wellcome Sanger Institute for the COVID-19 Genomics UK (COG-UK) Consortium	Rob Howes, The Lighthouse Lab in Cambridge and Alex Alderton, Roberto Amato, Sonia Goncalves, Ewan Harrison, David K. Jackson, Ian Johnston, Dominic Kwiatkowski, Cordelia Langford, John Sillitoe on behalf of the Wellcome Sanger Institute COVID-19 Surveillance Team
EPI_ISL_879149	Lighthouse Lab in Alderley Park	Wellcome Sanger Institute for the COVID-19 Genomics UK (COG-UK) Consortium	Jacquelyn Wynn, Mairead Hyland, The Lighthouse Lab in Alderley Park and Alex Alderton, Roberto Amato, Sonia Goncalves, Ewan Harrison, David K. Jackson, Ian Johnston, Dominic Kwiatkowski, Cordelia Langford, John Sillitoe on behalf of the Wellcome Sanger Institute COVID-19 Surveillance Team
EPI_ISL_879150	Lighthouse Lab in Cambridge	Wellcome Sanger Institute for the COVID-19 Genomics UK (COG-UK) Consortium	Rob Howes, The Lighthouse Lab in Cambridge and Alex Alderton, Roberto Amato, Sonia Goncalves, Ewan Harrison, David K. Jackson, Ian Johnston, Dominic Kwiatkowski, Cordelia Langford, John Sillitoe on behalf of the Wellcome Sanger Institute COVID-19 Surveillance Team
EPI_ISL_879151, EPI_ISL_879152	Lighthouse Lab in Alderley Park	Wellcome Sanger Institute for the COVID-19 Genomics UK (COG-UK) Consortium	Jacquelyn Wynn, Mairead Hyland, The Lighthouse Lab in Alderley Park and Alex Alderton, Roberto Amato, Sonia Goncalves, Ewan Harrison, David K. Jackson, Ian Johnston, Dominic Kwiatkowski, Cordelia Langford, John Sillitoe on behalf of the Wellcome Sanger Institute COVID-19 Surveillance Team
EPI_ISL_879153, EPI_ISL_879155, EPI_ISL_879156	Lighthouse Lab in Cambridge	Wellcome Sanger Institute for the COVID-19 Genomics UK (COG-UK) Consortium	Rob Howes, The Lighthouse Lab in Cambridge and Alex Alderton, Roberto Amato, Sonia Goncalves, Ewan Harrison, David K. Jackson, Ian Johnston, Dominic Kwiatkowski, Cordelia Langford, John Sillitoe on behalf of the Wellcome Sanger Institute COVID-19 Surveillance Team
EPI_ISL_879157	Lighthouse Lab in Alderley Park	Wellcome Sanger Institute for the COVID-19 Genomics UK (COG-UK) Consortium	Jacquelyn Wynn, Mairead Hyland, The Lighthouse Lab in Alderley Park and Alex Alderton, Roberto Amato, Sonia Goncalves, Ewan Harrison, David K. Jackson, Ian Johnston, Dominic Kwiatkowski, Cordelia Langford, John Sillitoe on behalf of the Wellcome Sanger Institute COVID-19 Surveillance Team
EPI_ISL_879158	Lighthouse Lab in Cambridge	Wellcome Sanger Institute for the COVID-19 Genomics UK (COG-UK) Consortium	Rob Howes, The Lighthouse Lab in Cambridge and Alex Alderton, Roberto Amato, Sonia Goncalves, Ewan Harrison, David K. Jackson, Ian Johnston, Dominic Kwiatkowski, Cordelia Langford, John Sillitoe on behalf of the Wellcome Sanger Institute COVID-19 Surveillance Team
EPI_ISL_879159, EPI_ISL_879160, EPI_ISL_879161, EPI_ISL_879162	Lighthouse Lab in Alderley Park	Wellcome Sanger Institute for the COVID-19 Genomics UK (COG-UK) Consortium	Jacquelyn Wynn, Mairead Hyland, The Lighthouse Lab in Alderley Park and Alex Alderton, Roberto Amato, Sonia Goncalves, Ewan Harrison, David K. Jackson, Ian Johnston, Dominic Kwiatkowski, Cordelia Langford, John Sillitoe on behalf of the Wellcome Sanger Institute COVID-19 Surveillance Team
EPI_ISL_879163	Lighthouse Lab in Cambridge	Wellcome Sanger Institute for the COVID-19 Genomics UK (COG-UK) Consortium	Rob Howes, The Lighthouse Lab in Cambridge and Alex Alderton, Roberto Amato, Sonia Goncalves, Ewan Harrison, David K. Jackson, Ian Johnston, Dominic Kwiatkowski, Cordelia Langford, John Sillitoe on behalf of the Wellcome Sanger Institute COVID-19 Surveillance Team
EPI_ISL_879164	Lighthouse Lab in Alderley Park	Wellcome Sanger Institute for the COVID-19 Genomics UK (COG-UK) Consortium	Jacquelyn Wynn, Mairead Hyland, The Lighthouse Lab in Alderley Park and Alex Alderton, Roberto Amato, Sonia Goncalves, Ewan Harrison, David K. Jackson, Ian Johnston, Dominic Kwiatkowski, Cordelia Langford, John Sillitoe on behalf of the Wellcome Sanger Institute COVID-19 Surveillance Team
EPI_ISL_879165, EPI_ISL_879166	Lighthouse Lab in Cambridge	Wellcome Sanger Institute for the COVID-19 Genomics UK (COG-UK) Consortium	Rob Howes, The Lighthouse Lab in Cambridge and Alex Alderton, Roberto Amato, Sonia Goncalves, Ewan Harrison, David K. Jackson, Ian Johnston, Dominic Kwiatkowski, Cordelia Langford, John Sillitoe on behalf of the Wellcome Sanger Institute COVID-19 Surveillance Team
EPI_ISL_879167	Lighthouse Lab in Alderley Park	Wellcome Sanger Institute for the COVID-19 Genomics UK (COG-UK) Consortium	Jacquelyn Wynn, Mairead Hyland, The Lighthouse Lab in Alderley Park and Alex Alderton, Roberto Amato, Sonia Goncalves, Ewan Harrison, David K. Jackson, Ian Johnston, Dominic Kwiatkowski, Cordelia Langford, John Sillitoe on behalf of the Wellcome Sanger Institute COVID-19 Surveillance Team
EPI_ISL_879168	Lighthouse Lab in Cambridge	Wellcome Sanger Institute for the COVID-19 Genomics UK (COG-UK) Consortium	Rob Howes, The Lighthouse Lab in Cambridge and Alex Alderton, Roberto Amato, Sonia Goncalves, Ewan Harrison, David K. Jackson, Ian Johnston, Dominic Kwiatkowski, Cordelia Langford, John Sillitoe on behalf of the Wellcome Sanger Institute COVID-19 Surveillance Team
EPI_ISL_879169	Lighthouse Lab in Alderley Park	Wellcome Sanger Institute for the COVID-19 Genomics UK (COG-UK) Consortium	Jacquelyn Wynn, Mairead Hyland, The Lighthouse Lab in Alderley Park and Alex Alderton, Roberto Amato, Sonia Goncalves, Ewan Harrison, David K. Jackson, Ian Johnston, Dominic Kwiatkowski, Cordelia Langford, John Sillitoe on behalf of the Wellcome Sanger Institute COVID-19 Surveillance Team
EPI_ISL_879170	Lighthouse Lab in Cambridge	Wellcome Sanger Institute for the COVID-19 Genomics UK (COG-UK) Consortium	Rob Howes, The Lighthouse Lab in Cambridge and Alex Alderton, Roberto Amato, Sonia Goncalves, Ewan Harrison, David K. Jackson, Ian Johnston, Dominic Kwiatkowski, Cordelia Langford, John Sillitoe on behalf of the Wellcome Sanger Institute COVID-19 Surveillance Team
EPI_ISL_879171	Lighthouse Lab in Alderley Park	Wellcome Sanger Institute for the COVID-19 Genomics UK (COG-UK) Consortium	Jacquelyn Wynn, Mairead Hyland, The Lighthouse Lab in Alderley Park and Alex Alderton, Roberto Amato, Sonia Goncalves, Ewan Harrison, David K. Jackson, Ian Johnston, Dominic Kwiatkowski, Cordelia Langford, John Sillitoe on behalf of the Wellcome Sanger Institute COVID-19 Surveillance Team
EPI_ISL_879172	Lighthouse Lab in Cambridge	Wellcome Sanger Institute for the COVID-19 Genomics UK (COG-UK) Consortium	Rob Howes, The Lighthouse Lab in Cambridge and Alex Alderton, Roberto Amato, Sonia Goncalves, Ewan Harrison, David K. Jackson, Ian Johnston, Dominic Kwiatkowski, Cordelia Langford, John Sillitoe on behalf of the Wellcome Sanger Institute COVID-19 Surveillance Team
EPI_ISL_879173	Lighthouse Lab in Alderley Park	Wellcome Sanger Institute for the COVID-19 Genomics UK (COG-UK) Consortium	Jacquelyn Wynn, Mairead Hyland, The Lighthouse Lab in Alderley Park and Alex Alderton, Roberto Amato, Sonia Goncalves, Ewan Harrison, David K. Jackson, Ian Johnston, Dominic Kwiatkowski, Cordelia Langford, John Sillitoe on behalf of the Wellcome Sanger Institute COVID-19 Surveillance Team
EPI_ISL_879174, EPI_ISL_879175, EPI_ISL_879176, EPI_ISL_879177, EPI_ISL_879178, EPI_ISL_879179, EPI_ISL_879180	Lighthouse Lab in Cambridge	Wellcome Sanger Institute for the COVID-19 Genomics UK (COG-UK) Consortium	Rob Howes, The Lighthouse Lab in Cambridge and Alex Alderton, Roberto Amato, Sonia Goncalves, Ewan Harrison, David K. Jackson, Ian Johnston, Dominic Kwiatkowski, Cordelia Langford, John Sillitoe on behalf of the Wellcome Sanger Institute COVID-19 Surveillance Team
EPI_ISL_879181	Lighthouse Lab in Alderley Park	Wellcome Sanger Institute for the COVID-19 Genomics UK (COG-UK) Consortium	Jacquelyn Wynn, Mairead Hyland, The Lighthouse Lab in Alderley Park and Alex Alderton, Roberto Amato, Sonia Goncalves, Ewan Harrison, David K. Jackson, Ian Johnston, Dominic Kwiatkowski, Cordelia Langford, John Sillitoe on behalf of the Wellcome Sanger Institute COVID-19 Surveillance Team
EPI_ISL_879182, EPI_ISL_879183, EPI_ISL_879184, EPI_ISL_879186	Lighthouse Lab in Cambridge	Wellcome Sanger Institute for the COVID-19 Genomics UK (COG-UK) Consortium	Rob Howes, The Lighthouse Lab in Cambridge and Alex Alderton, Roberto Amato, Sonia Goncalves, Ewan Harrison, David K. Jackson, Ian Johnston, Dominic Kwiatkowski, Cordelia Langford, John Sillitoe on behalf of the Wellcome Sanger Institute COVID-19 Surveillance Team
EPI_ISL_879187, EPI_ISL_879188	Lighthouse Lab in Alderley Park	Wellcome Sanger Institute for the COVID-19 Genomics UK (COG-UK) Consortium	Jacquelyn Wynn, Mairead Hyland, The Lighthouse Lab in Alderley Park and Alex Alderton, Roberto Amato, Sonia Goncalves, Ewan Harrison, David K. Jackson, Ian Johnston, Dominic Kwiatkowski, Cordelia Langford, John Sillitoe on behalf of the Wellcome Sanger Institute COVID-19 Surveillance Team
EPI_ISL_879189, EPI_ISL_879190	Lighthouse Lab in Cambridge	Wellcome Sanger Institute for the COVID-19 Genomics UK (COG-UK) Consortium	Rob Howes, The Lighthouse Lab in Cambridge and Alex Alderton, Roberto Amato, Sonia Goncalves, Ewan Harrison, David K. Jackson, Ian Johnston, Dominic Kwiatkowski, Cordelia Langford, John Sillitoe on behalf of the Wellcome Sanger Institute COVID-19 Surveillance Team
EPI_ISL_879191	Lighthouse Lab in Alderley Park	Wellcome Sanger Institute for the COVID-19 Genomics UK (COG-UK) Consortium	Jacquelyn Wynn, Mairead Hyland, The Lighthouse Lab in Alderley Park and Alex Alderton, Roberto Amato, Sonia Goncalves, Ewan Harrison, David K. Jackson, Ian Johnston, Dominic Kwiatkowski, Cordelia Langford, John Sillitoe on behalf of the Wellcome Sanger Institute COVID-19 Surveillance Team
EPI_ISL_879192	Lighthouse Lab in Cambridge	Wellcome Sanger Institute for the COVID-19 Genomics UK (COG-UK) Consortium	Rob Howes, The Lighthouse Lab in Cambridge and Alex Alderton, Roberto Amato, Sonia Goncalves, Ewan Harrison, David K. Jackson, Ian Johnston, Dominic Kwiatkowski, Cordelia Langford, John Sillitoe on behalf of the Wellcome Sanger Institute COVID-19 Surveillance Team
EPI_ISL_879194, EPI_ISL_879195	Lighthouse Lab in Alderley Park	Wellcome Sanger Institute for the COVID-19 Genomics UK (COG-UK) Consortium	Jacquelyn Wynn, Mairead Hyland, The Lighthouse Lab in Alderley Park and Alex Alderton, Roberto Amato, Sonia Goncalves, Ewan Harrison, David K. Jackson, Ian Johnston, Dominic Kwiatkowski, Cordelia Langford, John Sillitoe on behalf of the Wellcome Sanger Institute COVID-19 Surveillance Team
EPI_ISL_879196, EPI_ISL_879197, EPI_ISL_879198	Lighthouse Lab in Cambridge	Wellcome Sanger Institute for the COVID-19 Genomics UK (COG-UK) Consortium	Rob Howes, The Lighthouse Lab in Cambridge and Alex Alderton, Roberto Amato, Sonia Goncalves, Ewan Harrison, David K. Jackson, Ian Johnston, Dominic Kwiatkowski, Cordelia Langford, John Sillitoe on behalf of the Wellcome Sanger Institute COVID-19 Surveillance Team
EPI_ISL_879199	Lighthouse Lab in Alderley Park	Wellcome Sanger Institute for the COVID-19 Genomics UK (COG-UK) Consortium	Jacquelyn Wynn, Mairead Hyland, The Lighthouse Lab in Alderley Park and Alex Alderton, Roberto Amato, Sonia Goncalves, Ewan Harrison, David K. Jackson, Ian Johnston, Dominic Kwiatkowski, Cordelia Langford, John Sillitoe on behalf of the Wellcome Sanger Institute COVID-19 Surveillance Team
EPI_ISL_879200	Lighthouse Lab in Cambridge	Wellcome Sanger Institute for the COVID-19 Genomics UK (COG-UK) Consortium	Rob Howes, The Lighthouse Lab in Cambridge and Alex Alderton, Roberto Amato, Sonia Goncalves, Ewan Harrison, David K. Jackson, Ian Johnston, Dominic Kwiatkowski, Cordelia Langford, John Sillitoe on behalf of the Wellcome Sanger Institute COVID-19 Surveillance Team
EPI_ISL_879201, EPI_ISL_879202, EPI_ISL_879203	Lighthouse Lab in Alderley Park	Wellcome Sanger Institute for the COVID-19 Genomics UK (COG-UK) Consortium	Jacquelyn Wynn, Mairead Hyland, The Lighthouse Lab in Alderley Park and Alex Alderton, Roberto Amato, Sonia Goncalves, Ewan Harrison, David K. Jackson, Ian Johnston, Dominic Kwiatkowski, Cordelia Langford, John Sillitoe on behalf of the Wellcome Sanger Institute COVID-19 Surveillance Team
EPI_ISL_879204, EPI_ISL_879205	Lighthouse Lab in Cambridge	Wellcome Sanger Institute for the COVID-19 Genomics UK (COG-UK) Consortium	Rob Howes, The Lighthouse Lab in Cambridge and Alex Alderton, Roberto Amato, Sonia Goncalves, Ewan Harrison, David K. Jackson, Ian Johnston, Dominic Kwiatkowski, Cordelia Langford, John Sillitoe on behalf of the Wellcome Sanger Institute COVID-19 Surveillance Team
EPI_ISL_879206, EPI_ISL_879207	Lighthouse Lab in Alderley Park	Wellcome Sanger Institute for the COVID-19 Genomics UK (COG-UK) Consortium	Jacquelyn Wynn, Mairead Hyland, The Lighthouse Lab in Alderley Park and Alex Alderton, Roberto Amato, Sonia Goncalves, Ewan Harrison, David K. Jackson, Ian Johnston, Dominic Kwiatkowski, Cordelia Langford, John Sillitoe on behalf of the Wellcome Sanger Institute COVID-19 Surveillance Team
EPI_ISL_879208, EPI_ISL_879209, EPI_ISL_879210, EPI_ISL_879211	Lighthouse Lab in Cambridge	Wellcome Sanger Institute for the COVID-19 Genomics UK (COG-UK) Consortium	Rob Howes, The Lighthouse Lab in Cambridge and Alex Alderton, Roberto Amato, Sonia Goncalves, Ewan Harrison, David K. Jackson, Ian Johnston, Dominic Kwiatkowski, Cordelia Langford, John Sillitoe on behalf of the Wellcome Sanger Institute COVID-19 Surveillance Team
EPI_ISL_879212	Lighthouse Lab in Alderley Park	Wellcome Sanger Institute for the COVID-19 Genomics UK (COG-UK) Consortium	Jacquelyn Wynn, Mairead Hyland, The Lighthouse Lab in Alderley Park and Alex Alderton, Roberto Amato, Sonia Goncalves, Ewan Harrison, David K. Jackson, Ian Johnston, Dominic Kwiatkowski, Cordelia Langford, John Sillitoe on behalf of the Wellcome Sanger Institute COVID-19 Surveillance Team
EPI_ISL_879213	Lighthouse Lab in Cambridge	Wellcome Sanger Institute for the COVID-19 Genomics UK	Rob Howes, The Lighthouse Lab in Cambridge and Alex Alderton, Roberto Amato, Sonia Goncalves, Ewan Harrison, David K. Jackson, Ian Johnston,

		(COG-UK) Consortium	Dominic Kwiatkowski, Cordelia Langford, John Sillitoe on behalf of the Wellcome Sanger Institute COVID-19 Surveillance Team
EPI_ISL_879214	Lighthouse Lab in Alderley Park	Wellcome Sanger Institute for the COVID-19 Genomics UK (COG-UK) Consortium	Jacquelyn Wynn, Mairead Hyland, The Lighthouse Lab in Alderley Park and Alex Alderton, Roberto Amato, Sonia Goncalves, Ewan Harrison, David K. Jackson, Ian Johnston, Dominic Kwiatkowski, Cordelia Langford, John Sillitoe on behalf of the Wellcome Sanger Institute COVID-19 Surveillance Team
EPI_ISL_879215, EPI_ISL_879216, EPI_ISL_879217	Lighthouse Lab in Cambridge	Wellcome Sanger Institute for the COVID-19 Genomics UK (COG-UK) Consortium	Rob Howes, The Lighthouse Lab in Cambridge and Alex Alderton, Roberto Amato, Sonia Goncalves, Ewan Harrison, David K. Jackson, Ian Johnston, Dominic Kwiatkowski, Cordelia Langford, John Sillitoe on behalf of the Wellcome Sanger Institute COVID-19 Surveillance Team
EPI_ISL_879218, EPI_ISL_879219	Lighthouse Lab in Alderley Park	Wellcome Sanger Institute for the COVID-19 Genomics UK (COG-UK) Consortium	Jacquelyn Wynn, Mairead Hyland, The Lighthouse Lab in Alderley Park and Alex Alderton, Roberto Amato, Sonia Goncalves, Ewan Harrison, David K. Jackson, Ian Johnston, Dominic Kwiatkowski, Cordelia Langford, John Sillitoe on behalf of the Wellcome Sanger Institute COVID-19 Surveillance Team
EPI_ISL_879221, EPI_ISL_879222, EPI_ISL_879223	Lighthouse Lab in Cambridge	Wellcome Sanger Institute for the COVID-19 Genomics UK (COG-UK) Consortium	Rob Howes, The Lighthouse Lab in Cambridge and Alex Alderton, Roberto Amato, Sonia Goncalves, Ewan Harrison, David K. Jackson, Ian Johnston, Dominic Kwiatkowski, Cordelia Langford, John Sillitoe on behalf of the Wellcome Sanger Institute COVID-19 Surveillance Team
EPI_ISL_879224, EPI_ISL_879225, EPI_ISL_879226, EPI_ISL_879227	Lighthouse Lab in Alderley Park	Wellcome Sanger Institute for the COVID-19 Genomics UK (COG-UK) Consortium	Jacquelyn Wynn, Mairead Hyland, The Lighthouse Lab in Alderley Park and Alex Alderton, Roberto Amato, Sonia Goncalves, Ewan Harrison, David K. Jackson, Ian Johnston, Dominic Kwiatkowski, Cordelia Langford, John Sillitoe on behalf of the Wellcome Sanger Institute COVID-19 Surveillance Team
EPI_ISL_879228	Lighthouse Lab in Cambridge	Wellcome Sanger Institute for the COVID-19 Genomics UK (COG-UK) Consortium	Rob Howes, The Lighthouse Lab in Cambridge and Alex Alderton, Roberto Amato, Sonia Goncalves, Ewan Harrison, David K. Jackson, Ian Johnston, Dominic Kwiatkowski, Cordelia Langford, John Sillitoe on behalf of the Wellcome Sanger Institute COVID-19 Surveillance Team
EPI_ISL_879229, EPI_ISL_879230, EPI_ISL_879231	Lighthouse Lab in Alderley Park	Wellcome Sanger Institute for the COVID-19 Genomics UK (COG-UK) Consortium	Jacquelyn Wynn, Mairead Hyland, The Lighthouse Lab in Alderley Park and Alex Alderton, Roberto Amato, Sonia Goncalves, Ewan Harrison, David K. Jackson, Ian Johnston, Dominic Kwiatkowski, Cordelia Langford, John Sillitoe on behalf of the Wellcome Sanger Institute COVID-19 Surveillance Team
EPI_ISL_879232, EPI_ISL_879233	Lighthouse Lab in Cambridge	Wellcome Sanger Institute for the COVID-19 Genomics UK (COG-UK) Consortium	Rob Howes, The Lighthouse Lab in Cambridge and Alex Alderton, Roberto Amato, Sonia Goncalves, Ewan Harrison, David K. Jackson, Ian Johnston, Dominic Kwiatkowski, Cordelia Langford, John Sillitoe on behalf of the Wellcome Sanger Institute COVID-19 Surveillance Team
EPI_ISL_879234	Lighthouse Lab in Alderley Park	Wellcome Sanger Institute for the COVID-19 Genomics UK (COG-UK) Consortium	Jacquelyn Wynn, Mairead Hyland, The Lighthouse Lab in Alderley Park and Alex Alderton, Roberto Amato, Sonia Goncalves, Ewan Harrison, David K. Jackson, Ian Johnston, Dominic Kwiatkowski, Cordelia Langford, John Sillitoe on behalf of the Wellcome Sanger Institute COVID-19 Surveillance Team
EPI_ISL_879236	Lighthouse Lab in Cambridge	Wellcome Sanger Institute for the COVID-19 Genomics UK (COG-UK) Consortium	Rob Howes, The Lighthouse Lab in Cambridge and Alex Alderton, Roberto Amato, Sonia Goncalves, Ewan Harrison, David K. Jackson, Ian Johnston, Dominic Kwiatkowski, Cordelia Langford, John Sillitoe on behalf of the Wellcome Sanger Institute COVID-19 Surveillance Team
EPI_ISL_879237	Lighthouse Lab in Alderley Park	Wellcome Sanger Institute for the COVID-19 Genomics UK (COG-UK) Consortium	Jacquelyn Wynn, Mairead Hyland, The Lighthouse Lab in Alderley Park and Alex Alderton, Roberto Amato, Sonia Goncalves, Ewan Harrison, David K. Jackson, Ian Johnston, Dominic Kwiatkowski, Cordelia Langford, John Sillitoe on behalf of the Wellcome Sanger Institute COVID-19 Surveillance Team
EPI_ISL_879238	Lighthouse Lab in Cambridge	Wellcome Sanger Institute for the COVID-19 Genomics UK (COG-UK) Consortium	Rob Howes, The Lighthouse Lab in Cambridge and Alex Alderton, Roberto Amato, Sonia Goncalves, Ewan Harrison, David K. Jackson, Ian Johnston, Dominic Kwiatkowski, Cordelia Langford, John Sillitoe on behalf of the Wellcome Sanger Institute COVID-19 Surveillance Team
EPI_ISL_879239	Lighthouse Lab in Alderley Park	Wellcome Sanger Institute for the COVID-19 Genomics UK (COG-UK) Consortium	Jacquelyn Wynn, Mairead Hyland, The Lighthouse Lab in Alderley Park and Alex Alderton, Roberto Amato, Sonia Goncalves, Ewan Harrison, David K. Jackson, Ian Johnston, Dominic Kwiatkowski, Cordelia Langford, John Sillitoe on behalf of the Wellcome Sanger Institute COVID-19 Surveillance Team
EPI_ISL_879240	Lighthouse Lab in Cambridge	Wellcome Sanger Institute for the COVID-19 Genomics UK (COG-UK) Consortium	Rob Howes, The Lighthouse Lab in Cambridge and Alex Alderton, Roberto Amato, Sonia Goncalves, Ewan Harrison, David K. Jackson, Ian Johnston, Dominic Kwiatkowski, Cordelia Langford, John Sillitoe on behalf of the Wellcome Sanger Institute COVID-19 Surveillance Team
EPI_ISL_879241	Lighthouse Lab in Alderley Park	Wellcome Sanger Institute for the COVID-19 Genomics UK (COG-UK) Consortium	Jacquelyn Wynn, Mairead Hyland, The Lighthouse Lab in Alderley Park and Alex Alderton, Roberto Amato, Sonia Goncalves, Ewan Harrison, David K. Jackson, Ian Johnston, Dominic Kwiatkowski, Cordelia Langford, John Sillitoe on behalf of the Wellcome Sanger Institute COVID-19 Surveillance Team
EPI_ISL_879242	Lighthouse Lab in Cambridge	Wellcome Sanger Institute for the COVID-19 Genomics UK (COG-UK) Consortium	Rob Howes, The Lighthouse Lab in Cambridge and Alex Alderton, Roberto Amato, Sonia Goncalves, Ewan Harrison, David K. Jackson, Ian Johnston, Dominic Kwiatkowski, Cordelia Langford, John Sillitoe on behalf of the Wellcome Sanger Institute COVID-19 Surveillance Team
EPI_ISL_879243, EPI_ISL_879244	Lighthouse Lab in Alderley Park	Wellcome Sanger Institute for the COVID-19 Genomics UK (COG-UK) Consortium	Jacquelyn Wynn, Mairead Hyland, The Lighthouse Lab in Alderley Park and Alex Alderton, Roberto Amato, Sonia Goncalves, Ewan Harrison, David K. Jackson, Ian Johnston, Dominic Kwiatkowski, Cordelia Langford, John Sillitoe on behalf of the Wellcome Sanger Institute COVID-19 Surveillance Team
EPI_ISL_879245, EPI_ISL_879246	Lighthouse Lab in Cambridge	Wellcome Sanger Institute for the COVID-19 Genomics UK (COG-UK) Consortium	Rob Howes, The Lighthouse Lab in Cambridge and Alex Alderton, Roberto Amato, Sonia Goncalves, Ewan Harrison, David K. Jackson, Ian Johnston, Dominic Kwiatkowski, Cordelia Langford, John Sillitoe on behalf of the Wellcome Sanger Institute COVID-19 Surveillance Team
EPI_ISL_879247, EPI_ISL_879248, EPI_ISL_879249, EPI_ISL_879250	Lighthouse Lab in Alderley Park	Wellcome Sanger Institute for the COVID-19 Genomics UK (COG-UK) Consortium	Jacquelyn Wynn, Mairead Hyland, The Lighthouse Lab in Alderley Park and Alex Alderton, Roberto Amato, Sonia Goncalves, Ewan Harrison, David K. Jackson, Ian Johnston, Dominic Kwiatkowski, Cordelia Langford, John Sillitoe on behalf of the Wellcome Sanger Institute COVID-19 Surveillance Team
EPI_ISL_879251	Lighthouse Lab in Cambridge	Wellcome Sanger Institute for the COVID-19 Genomics UK (COG-UK) Consortium	Rob Howes, The Lighthouse Lab in Cambridge and Alex Alderton, Roberto Amato, Sonia Goncalves, Ewan Harrison, David K. Jackson, Ian Johnston, Dominic Kwiatkowski, Cordelia Langford, John Sillitoe on behalf of the Wellcome Sanger Institute COVID-19 Surveillance Team
EPI_ISL_879252, EPI_ISL_879254	Lighthouse Lab in Alderley Park	Wellcome Sanger Institute for the COVID-19 Genomics UK (COG-UK) Consortium	Jacquelyn Wynn, Mairead Hyland, The Lighthouse Lab in Alderley Park and Alex Alderton, Roberto Amato, Sonia Goncalves, Ewan Harrison, David K. Jackson, Ian Johnston, Dominic Kwiatkowski, Cordelia Langford, John Sillitoe on behalf of the Wellcome Sanger Institute COVID-19 Surveillance Team
EPI_ISL_879255	Lighthouse Lab in Cambridge	Wellcome Sanger Institute for the COVID-19 Genomics UK (COG-UK) Consortium	Rob Howes, The Lighthouse Lab in Cambridge and Alex Alderton, Roberto Amato, Sonia Goncalves, Ewan Harrison, David K. Jackson, Ian Johnston, Dominic Kwiatkowski, Cordelia Langford, John Sillitoe on behalf of the Wellcome Sanger Institute COVID-19 Surveillance Team
EPI_ISL_879256	Lighthouse Lab in Alderley Park	Wellcome Sanger Institute for the COVID-19 Genomics UK (COG-UK) Consortium	Jacquelyn Wynn, Mairead Hyland, The Lighthouse Lab in Alderley Park and Alex Alderton, Roberto Amato, Sonia Goncalves, Ewan Harrison, David K. Jackson, Ian Johnston, Dominic Kwiatkowski, Cordelia Langford, John Sillitoe on behalf of the Wellcome Sanger Institute COVID-19 Surveillance Team
EPI_ISL_879257	Lighthouse Lab in Cambridge	Wellcome Sanger Institute for the COVID-19 Genomics UK (COG-UK) Consortium	Rob Howes, The Lighthouse Lab in Cambridge and Alex Alderton, Roberto Amato, Sonia Goncalves, Ewan Harrison, David K. Jackson, Ian Johnston, Dominic Kwiatkowski, Cordelia Langford, John Sillitoe on behalf of the Wellcome Sanger Institute COVID-19 Surveillance Team
EPI_ISL_879258, EPI_ISL_879259	Lighthouse Lab in Alderley Park	Wellcome Sanger Institute for the COVID-19 Genomics UK (COG-UK) Consortium	Jacquelyn Wynn, Mairead Hyland, The Lighthouse Lab in Alderley Park and Alex Alderton, Roberto Amato, Sonia Goncalves, Ewan Harrison, David K. Jackson, Ian Johnston, Dominic Kwiatkowski, Cordelia Langford, John Sillitoe on behalf of the Wellcome Sanger Institute COVID-19 Surveillance Team
EPI_ISL_879260, EPI_ISL_879261	Lighthouse Lab in Cambridge	Wellcome Sanger Institute for the COVID-19 Genomics UK (COG-UK) Consortium	Rob Howes, The Lighthouse Lab in Cambridge and Alex Alderton, Roberto Amato, Sonia Goncalves, Ewan Harrison, David K. Jackson, Ian Johnston, Dominic Kwiatkowski, Cordelia Langford, John Sillitoe on behalf of the Wellcome Sanger Institute COVID-19 Surveillance Team
EPI_ISL_879262, EPI_ISL_879263	Lighthouse Lab in Alderley Park	Wellcome Sanger Institute for the COVID-19 Genomics UK (COG-UK) Consortium	Jacquelyn Wynn, Mairead Hyland, The Lighthouse Lab in Alderley Park and Alex Alderton, Roberto Amato, Sonia Goncalves, Ewan Harrison, David K. Jackson, Ian Johnston, Dominic Kwiatkowski, Cordelia Langford, John Sillitoe on behalf of the Wellcome Sanger Institute COVID-19 Surveillance Team
EPI_ISL_879264	Lighthouse Lab in Cambridge	Wellcome Sanger Institute for the COVID-19 Genomics UK (COG-UK) Consortium	Rob Howes, The Lighthouse Lab in Cambridge and Alex Alderton, Roberto Amato, Sonia Goncalves, Ewan Harrison, David K. Jackson, Ian Johnston, Dominic Kwiatkowski, Cordelia Langford, John Sillitoe on behalf of the Wellcome Sanger Institute COVID-19 Surveillance Team
EPI_ISL_879265, EPI_ISL_879266	Lighthouse Lab in Alderley Park	Wellcome Sanger Institute for the COVID-19 Genomics UK (COG-UK) Consortium	Jacquelyn Wynn, Mairead Hyland, The Lighthouse Lab in Alderley Park and Alex Alderton, Roberto Amato, Sonia Goncalves, Ewan Harrison, David K. Jackson, Ian Johnston, Dominic Kwiatkowski, Cordelia Langford, John Sillitoe on behalf of the Wellcome Sanger Institute COVID-19 Surveillance Team
EPI_ISL_879267	Lighthouse Lab in Cambridge	Wellcome Sanger Institute for the COVID-19 Genomics UK (COG-UK) Consortium	Rob Howes, The Lighthouse Lab in Cambridge and Alex Alderton, Roberto Amato, Sonia Goncalves, Ewan Harrison, David K. Jackson, Ian Johnston, Dominic Kwiatkowski, Cordelia Langford, John Sillitoe on behalf of the Wellcome Sanger Institute COVID-19 Surveillance Team
EPI_ISL_879268	Lighthouse Lab in Alderley Park	Wellcome Sanger Institute for the COVID-19 Genomics UK (COG-UK) Consortium	Jacquelyn Wynn, Mairead Hyland, The Lighthouse Lab in Alderley Park and Alex Alderton, Roberto Amato, Sonia Goncalves, Ewan Harrison, David K. Jackson, Ian Johnston, Dominic Kwiatkowski, Cordelia Langford, John Sillitoe on behalf of the Wellcome Sanger Institute COVID-19 Surveillance Team
EPI_ISL_879269	Lighthouse Lab in Cambridge	Wellcome Sanger Institute for the COVID-19 Genomics UK (COG-UK) Consortium	Rob Howes, The Lighthouse Lab in Cambridge and Alex Alderton, Roberto Amato, Sonia Goncalves, Ewan Harrison, David K. Jackson, Ian Johnston, Dominic Kwiatkowski, Cordelia Langford, John Sillitoe on behalf of the Wellcome Sanger Institute COVID-19 Surveillance Team
EPI_ISL_879270, EPI_ISL_879271	Lighthouse Lab in Alderley Park	Wellcome Sanger Institute for the COVID-19 Genomics UK (COG-UK) Consortium	Jacquelyn Wynn, Mairead Hyland, The Lighthouse Lab in Alderley Park and Alex Alderton, Roberto Amato, Sonia Goncalves, Ewan Harrison, David K. Jackson, Ian Johnston, Dominic Kwiatkowski, Cordelia Langford, John Sillitoe on behalf of the Wellcome Sanger Institute COVID-19 Surveillance Team
EPI_ISL_879272	Lighthouse Lab in Cambridge	Wellcome Sanger Institute for the COVID-19 Genomics UK (COG-UK) Consortium	Rob Howes, The Lighthouse Lab in Cambridge and Alex Alderton, Roberto Amato, Sonia Goncalves, Ewan Harrison, David K. Jackson, Ian Johnston, Dominic Kwiatkowski, Cordelia Langford, John Sillitoe on behalf of the Wellcome Sanger Institute COVID-19 Surveillance Team
EPI_ISL_879273	Lighthouse Lab in Alderley Park	Wellcome Sanger Institute for the COVID-19 Genomics UK	Jacquelyn Wynn, Mairead Hyland, The Lighthouse Lab in Alderley Park and Alex Alderton, Roberto Amato, Sonia Goncalves, Ewan Harrison, David K.

		(COG-UK) Consortium	Jackson, Ian Johnston, Dominic Kwiatkowski, Cordelia Langford, John Sillitoe on behalf of the Wellcome Sanger Institute COVID-19 Surveillance Team
EPI_ISL_879275	Lighthouse Lab in Cambridge	Wellcome Sanger Institute for the COVID-19 Genomics UK (COG-UK) Consortium	Rob Howes, The Lighthouse Lab in Cambridge and Alex Alderton, Roberto Amato, Sonia Goncalves, Ewan Harrison, David K. Jackson, Ian Johnston, Dominic Kwiatkowski, Cordelia Langford, John Sillitoe on behalf of the Wellcome Sanger Institute COVID-19 Surveillance Team
EPI_ISL_879276, EPI_ISL_879277, EPI_ISL_879279	Lighthouse Lab in Alderley Park	Wellcome Sanger Institute for the COVID-19 Genomics UK (COG-UK) Consortium	Jacquelyn Wynn, Mairead Hyland, The Lighthouse Lab in Alderley Park and Alex Alderton, Roberto Amato, Sonia Goncalves, Ewan Harrison, David K. Jackson, Ian Johnston, Dominic Kwiatkowski, Cordelia Langford, John Sillitoe on behalf of the Wellcome Sanger Institute COVID-19 Surveillance Team
EPI_ISL_879280, EPI_ISL_879281, EPI_ISL_879282, EPI_ISL_879283	Lighthouse Lab in Cambridge	Wellcome Sanger Institute for the COVID-19 Genomics UK (COG-UK) Consortium	Rob Howes, The Lighthouse Lab in Cambridge and Alex Alderton, Roberto Amato, Sonia Goncalves, Ewan Harrison, David K. Jackson, Ian Johnston, Dominic Kwiatkowski, Cordelia Langford, John Sillitoe on behalf of the Wellcome Sanger Institute COVID-19 Surveillance Team
EPI_ISL_879284	Lighthouse Lab in Alderley Park	Wellcome Sanger Institute for the COVID-19 Genomics UK (COG-UK) Consortium	Jacquelyn Wynn, Mairead Hyland, The Lighthouse Lab in Alderley Park and Alex Alderton, Roberto Amato, Sonia Goncalves, Ewan Harrison, David K. Jackson, Ian Johnston, Dominic Kwiatkowski, Cordelia Langford, John Sillitoe on behalf of the Wellcome Sanger Institute COVID-19 Surveillance Team
EPI_ISL_879285	Lighthouse Lab in Cambridge	Wellcome Sanger Institute for the COVID-19 Genomics UK (COG-UK) Consortium	Rob Howes, The Lighthouse Lab in Cambridge and Alex Alderton, Roberto Amato, Sonia Goncalves, Ewan Harrison, David K. Jackson, Ian Johnston, Dominic Kwiatkowski, Cordelia Langford, John Sillitoe on behalf of the Wellcome Sanger Institute COVID-19 Surveillance Team
EPI_ISL_879286	Lighthouse Lab in Alderley Park	Wellcome Sanger Institute for the COVID-19 Genomics UK (COG-UK) Consortium	Jacquelyn Wynn, Mairead Hyland, The Lighthouse Lab in Alderley Park and Alex Alderton, Roberto Amato, Sonia Goncalves, Ewan Harrison, David K. Jackson, Ian Johnston, Dominic Kwiatkowski, Cordelia Langford, John Sillitoe on behalf of the Wellcome Sanger Institute COVID-19 Surveillance Team
EPI_ISL_879287, EPI_ISL_879288, EPI_ISL_879289, EPI_ISL_879291, EPI_ISL_879292, EPI_ISL_879293, EPI_ISL_879294, EPI_ISL_879295	Lighthouse Lab in Cambridge	Wellcome Sanger Institute for the COVID-19 Genomics UK (COG-UK) Consortium	Rob Howes, The Lighthouse Lab in Cambridge and Alex Alderton, Roberto Amato, Sonia Goncalves, Ewan Harrison, David K. Jackson, Ian Johnston, Dominic Kwiatkowski, Cordelia Langford, John Sillitoe on behalf of the Wellcome Sanger Institute COVID-19 Surveillance Team
EPI_ISL_879296, EPI_ISL_879297, EPI_ISL_879298, EPI_ISL_879299, EPI_ISL_879300	Lighthouse Lab in Alderley Park	Wellcome Sanger Institute for the COVID-19 Genomics UK (COG-UK) Consortium	Jacquelyn Wynn, Mairead Hyland, The Lighthouse Lab in Alderley Park and Alex Alderton, Roberto Amato, Sonia Goncalves, Ewan Harrison, David K. Jackson, Ian Johnston, Dominic Kwiatkowski, Cordelia Langford, John Sillitoe on behalf of the Wellcome Sanger Institute COVID-19 Surveillance Team
EPI_ISL_879301	Lighthouse Lab in Cambridge	Wellcome Sanger Institute for the COVID-19 Genomics UK (COG-UK) Consortium	Rob Howes, The Lighthouse Lab in Cambridge and Alex Alderton, Roberto Amato, Sonia Goncalves, Ewan Harrison, David K. Jackson, Ian Johnston, Dominic Kwiatkowski, Cordelia Langford, John Sillitoe on behalf of the Wellcome Sanger Institute COVID-19 Surveillance Team
EPI_ISL_879302, EPI_ISL_879303	Lighthouse Lab in Alderley Park	Wellcome Sanger Institute for the COVID-19 Genomics UK (COG-UK) Consortium	Jacquelyn Wynn, Mairead Hyland, The Lighthouse Lab in Alderley Park and Alex Alderton, Roberto Amato, Sonia Goncalves, Ewan Harrison, David K. Jackson, Ian Johnston, Dominic Kwiatkowski, Cordelia Langford, John Sillitoe on behalf of the Wellcome Sanger Institute COVID-19 Surveillance Team
EPI_ISL_879304	Lighthouse Lab in Cambridge	Wellcome Sanger Institute for the COVID-19 Genomics UK (COG-UK) Consortium	Rob Howes, The Lighthouse Lab in Cambridge and Alex Alderton, Roberto Amato, Sonia Goncalves, Ewan Harrison, David K. Jackson, Ian Johnston, Dominic Kwiatkowski, Cordelia Langford, John Sillitoe on behalf of the Wellcome Sanger Institute COVID-19 Surveillance Team
EPI_ISL_879305, EPI_ISL_879306, EPI_ISL_879307, EPI_ISL_879308	Lighthouse Lab in Alderley Park	Wellcome Sanger Institute for the COVID-19 Genomics UK (COG-UK) Consortium	Jacquelyn Wynn, Mairead Hyland, The Lighthouse Lab in Alderley Park and Alex Alderton, Roberto Amato, Sonia Goncalves, Ewan Harrison, David K. Jackson, Ian Johnston, Dominic Kwiatkowski, Cordelia Langford, John Sillitoe on behalf of the Wellcome Sanger Institute COVID-19 Surveillance Team
EPI_ISL_879309, EPI_ISL_879310, EPI_ISL_879311, EPI_ISL_879312	Lighthouse Lab in Cambridge	Wellcome Sanger Institute for the COVID-19 Genomics UK (COG-UK) Consortium	Rob Howes, The Lighthouse Lab in Cambridge and Alex Alderton, Roberto Amato, Sonia Goncalves, Ewan Harrison, David K. Jackson, Ian Johnston, Dominic Kwiatkowski, Cordelia Langford, John Sillitoe on behalf of the Wellcome Sanger Institute COVID-19 Surveillance Team
EPI_ISL_879313	Lighthouse Lab in Alderley Park	Wellcome Sanger Institute for the COVID-19 Genomics UK (COG-UK) Consortium	Jacquelyn Wynn, Mairead Hyland, The Lighthouse Lab in Alderley Park and Alex Alderton, Roberto Amato, Sonia Goncalves, Ewan Harrison, David K. Jackson, Ian Johnston, Dominic Kwiatkowski, Cordelia Langford, John Sillitoe on behalf of the Wellcome Sanger Institute COVID-19 Surveillance Team
EPI_ISL_879315	Lighthouse Lab in Cambridge	Wellcome Sanger Institute for the COVID-19 Genomics UK (COG-UK) Consortium	Rob Howes, The Lighthouse Lab in Cambridge and Alex Alderton, Roberto Amato, Sonia Goncalves, Ewan Harrison, David K. Jackson, Ian Johnston, Dominic Kwiatkowski, Cordelia Langford, John Sillitoe on behalf of the Wellcome Sanger Institute COVID-19 Surveillance Team
EPI_ISL_879316	Lighthouse Lab in Alderley Park	Wellcome Sanger Institute for the COVID-19 Genomics UK (COG-UK) Consortium	Jacquelyn Wynn, Mairead Hyland, The Lighthouse Lab in Alderley Park and Alex Alderton, Roberto Amato, Sonia Goncalves, Ewan Harrison, David K. Jackson, Ian Johnston, Dominic Kwiatkowski, Cordelia Langford, John Sillitoe on behalf of the Wellcome Sanger Institute COVID-19 Surveillance Team
EPI_ISL_879317	Lighthouse Lab in Cambridge	Wellcome Sanger Institute for the COVID-19 Genomics UK (COG-UK) Consortium	Rob Howes, The Lighthouse Lab in Cambridge and Alex Alderton, Roberto Amato, Sonia Goncalves, Ewan Harrison, David K. Jackson, Ian Johnston, Dominic Kwiatkowski, Cordelia Langford, John Sillitoe on behalf of the Wellcome Sanger Institute COVID-19 Surveillance Team
EPI_ISL_879318	Lighthouse Lab in Alderley Park	Wellcome Sanger Institute for the COVID-19 Genomics UK (COG-UK) Consortium	Jacquelyn Wynn, Mairead Hyland, The Lighthouse Lab in Alderley Park and Alex Alderton, Roberto Amato, Sonia Goncalves, Ewan Harrison, David K. Jackson, Ian Johnston, Dominic Kwiatkowski, Cordelia Langford, John Sillitoe on behalf of the Wellcome Sanger Institute COVID-19 Surveillance Team
EPI_ISL_879319, EPI_ISL_879320, EPI_ISL_879321	Lighthouse Lab in Cambridge	Wellcome Sanger Institute for the COVID-19 Genomics UK (COG-UK) Consortium	Rob Howes, The Lighthouse Lab in Cambridge and Alex Alderton, Roberto Amato, Sonia Goncalves, Ewan Harrison, David K. Jackson, Ian Johnston, Dominic Kwiatkowski, Cordelia Langford, John Sillitoe on behalf of the Wellcome Sanger Institute COVID-19 Surveillance Team
EPI_ISL_879323, EPI_ISL_879324	Lighthouse Lab in Alderley Park	Wellcome Sanger Institute for the COVID-19 Genomics UK (COG-UK) Consortium	Jacquelyn Wynn, Mairead Hyland, The Lighthouse Lab in Alderley Park and Alex Alderton, Roberto Amato, Sonia Goncalves, Ewan Harrison, David K. Jackson, Ian Johnston, Dominic Kwiatkowski, Cordelia Langford, John Sillitoe on behalf of the Wellcome Sanger Institute COVID-19 Surveillance Team
EPI_ISL_879325, EPI_ISL_879326, EPI_ISL_879327, EPI_ISL_879328	Lighthouse Lab in Cambridge	Wellcome Sanger Institute for the COVID-19 Genomics UK (COG-UK) Consortium	Rob Howes, The Lighthouse Lab in Cambridge and Alex Alderton, Roberto Amato, Sonia Goncalves, Ewan Harrison, David K. Jackson, Ian Johnston, Dominic Kwiatkowski, Cordelia Langford, John Sillitoe on behalf of the Wellcome Sanger Institute COVID-19 Surveillance Team
EPI_ISL_879329	Lighthouse Lab in Alderley Park	Wellcome Sanger Institute for the COVID-19 Genomics UK (COG-UK) Consortium	Jacquelyn Wynn, Mairead Hyland, The Lighthouse Lab in Alderley Park and Alex Alderton, Roberto Amato, Sonia Goncalves, Ewan Harrison, David K. Jackson, Ian Johnston, Dominic Kwiatkowski, Cordelia Langford, John Sillitoe on behalf of the Wellcome Sanger Institute COVID-19 Surveillance Team
EPI_ISL_879330, EPI_ISL_879332	Lighthouse Lab in Cambridge	Wellcome Sanger Institute for the COVID-19 Genomics UK (COG-UK) Consortium	Rob Howes, The Lighthouse Lab in Cambridge and Alex Alderton, Roberto Amato, Sonia Goncalves, Ewan Harrison, David K. Jackson, Ian Johnston, Dominic Kwiatkowski, Cordelia Langford, John Sillitoe on behalf of the Wellcome Sanger Institute COVID-19 Surveillance Team
EPI_ISL_879333	Lighthouse Lab in Alderley Park	Wellcome Sanger Institute for the COVID-19 Genomics UK (COG-UK) Consortium	Jacquelyn Wynn, Mairead Hyland, The Lighthouse Lab in Alderley Park and Alex Alderton, Roberto Amato, Sonia Goncalves, Ewan Harrison, David K. Jackson, Ian Johnston, Dominic Kwiatkowski, Cordelia Langford, John Sillitoe on behalf of the Wellcome Sanger Institute COVID-19 Surveillance Team
EPI_ISL_879334, EPI_ISL_879335, EPI_ISL_879336, EPI_ISL_879337	Lighthouse Lab in Cambridge	Wellcome Sanger Institute for the COVID-19 Genomics UK (COG-UK) Consortium	Rob Howes, The Lighthouse Lab in Cambridge and Alex Alderton, Roberto Amato, Sonia Goncalves, Ewan Harrison, David K. Jackson, Ian Johnston, Dominic Kwiatkowski, Cordelia Langford, John Sillitoe on behalf of the Wellcome Sanger Institute COVID-19 Surveillance Team
EPI_ISL_879339, EPI_ISL_879340, EPI_ISL_879341, EPI_ISL_879342, EPI_ISL_879343, EPI_ISL_879345, EPI_ISL_879346, EPI_ISL_879347, EPI_ISL_879348	Lighthouse Lab in Alderley Park	Wellcome Sanger Institute for the COVID-19 Genomics UK (COG-UK) Consortium	Jacquelyn Wynn, Mairead Hyland, The Lighthouse Lab in Alderley Park and Alex Alderton, Roberto Amato, Sonia Goncalves, Ewan Harrison, David K. Jackson, Ian Johnston, Dominic Kwiatkowski, Cordelia Langford, John Sillitoe on behalf of the Wellcome Sanger Institute COVID-19 Surveillance Team
EPI_ISL_879349, EPI_ISL_879350	Lighthouse Lab in Cambridge	Wellcome Sanger Institute for the COVID-19 Genomics UK (COG-UK) Consortium	Rob Howes, The Lighthouse Lab in Cambridge and Alex Alderton, Roberto Amato, Sonia Goncalves, Ewan Harrison, David K. Jackson, Ian Johnston, Dominic Kwiatkowski, Cordelia Langford, John Sillitoe on behalf of the Wellcome Sanger Institute COVID-19 Surveillance Team
EPI_ISL_879351	Lighthouse Lab in Alderley Park	Wellcome Sanger Institute for the COVID-19 Genomics UK (COG-UK) Consortium	Jacquelyn Wynn, Mairead Hyland, The Lighthouse Lab in Alderley Park and Alex Alderton, Roberto Amato, Sonia Goncalves, Ewan Harrison, David K. Jackson, Ian Johnston, Dominic Kwiatkowski, Cordelia Langford, John Sillitoe on behalf of the Wellcome Sanger Institute COVID-19 Surveillance Team
EPI_ISL_879352, EPI_ISL_879353	Lighthouse Lab in Cambridge	Wellcome Sanger Institute for the COVID-19 Genomics UK (COG-UK) Consortium	Rob Howes, The Lighthouse Lab in Cambridge and Alex Alderton, Roberto Amato, Sonia Goncalves, Ewan Harrison, David K. Jackson, Ian Johnston, Dominic Kwiatkowski, Cordelia Langford, John Sillitoe on behalf of the Wellcome Sanger Institute COVID-19 Surveillance Team
EPI_ISL_879354	Lighthouse Lab in Alderley Park	Wellcome Sanger Institute for the COVID-19 Genomics UK (COG-UK) Consortium	Jacquelyn Wynn, Mairead Hyland, The Lighthouse Lab in Alderley Park and Alex Alderton, Roberto Amato, Sonia Goncalves, Ewan Harrison, David K. Jackson, Ian Johnston, Dominic Kwiatkowski, Cordelia Langford, John Sillitoe on behalf of the Wellcome Sanger Institute COVID-19 Surveillance Team
EPI_ISL_879355	Lighthouse Lab in Cambridge	Wellcome Sanger Institute for the COVID-19 Genomics UK (COG-UK) Consortium	Rob Howes, The Lighthouse Lab in Cambridge and Alex Alderton, Roberto Amato, Sonia Goncalves, Ewan Harrison, David K. Jackson, Ian Johnston, Dominic Kwiatkowski, Cordelia Langford, John Sillitoe on behalf of the Wellcome Sanger Institute COVID-19 Surveillance Team
EPI_ISL_879356, EPI_ISL_879357, EPI_ISL_879358, EPI_ISL_879359, EPI_ISL_879360, EPI_ISL_879361,	Lighthouse Lab in Alderley Park	Wellcome Sanger Institute for the COVID-19 Genomics UK (COG-UK) Consortium	Jacquelyn Wynn, Mairead Hyland, The Lighthouse Lab in Alderley Park and Alex Alderton, Roberto Amato, Sonia Goncalves, Ewan Harrison, David K. Jackson, Ian Johnston, Dominic Kwiatkowski, Cordelia Langford, John Sillitoe on behalf of the Wellcome Sanger Institute COVID-19 Surveillance Team

EPI_ISL_879362			
EPI_ISL_879364, EPI_ISL_879365, EPI_ISL_879366	Lighthouse Lab in Cambridge	Wellcome Sanger Institute for the COVID-19 Genomics UK (COG-UK) Consortium	Rob Howes, The Lighthouse Lab in Cambridge and Alex Alderton, Roberto Amato, Sonia Goncalves, Ewan Harrison, David K. Jackson, Ian Johnston, Dominic Kwiatkowski, Cordelia Langford, John Sillitoe on behalf of the Wellcome Sanger Institute COVID-19 Surveillance Team
EPI_ISL_879367	Lighthouse Lab in Alderley Park	Wellcome Sanger Institute for the COVID-19 Genomics UK (COG-UK) Consortium	Jacquelyn Wynn, Mairead Hyland, The Lighthouse Lab in Alderley Park and Alex Alderton, Roberto Amato, Sonia Goncalves, Ewan Harrison, David K. Jackson, Ian Johnston, Dominic Kwiatkowski, Cordelia Langford, John Sillitoe on behalf of the Wellcome Sanger Institute COVID-19 Surveillance Team
EPI_ISL_879368, EPI_ISL_879369, EPI_ISL_879370	Lighthouse Lab in Cambridge	Wellcome Sanger Institute for the COVID-19 Genomics UK (COG-UK) Consortium	Rob Howes, The Lighthouse Lab in Cambridge and Alex Alderton, Roberto Amato, Sonia Goncalves, Ewan Harrison, David K. Jackson, Ian Johnston, Dominic Kwiatkowski, Cordelia Langford, John Sillitoe on behalf of the Wellcome Sanger Institute COVID-19 Surveillance Team
EPI_ISL_879371	Lighthouse Lab in Alderley Park	Wellcome Sanger Institute for the COVID-19 Genomics UK (COG-UK) Consortium	Jacquelyn Wynn, Mairead Hyland, The Lighthouse Lab in Alderley Park and Alex Alderton, Roberto Amato, Sonia Goncalves, Ewan Harrison, David K. Jackson, Ian Johnston, Dominic Kwiatkowski, Cordelia Langford, John Sillitoe on behalf of the Wellcome Sanger Institute COVID-19 Surveillance Team
EPI_ISL_879372, EPI_ISL_879373	Lighthouse Lab in Cambridge	Wellcome Sanger Institute for the COVID-19 Genomics UK (COG-UK) Consortium	Rob Howes, The Lighthouse Lab in Cambridge and Alex Alderton, Roberto Amato, Sonia Goncalves, Ewan Harrison, David K. Jackson, Ian Johnston, Dominic Kwiatkowski, Cordelia Langford, John Sillitoe on behalf of the Wellcome Sanger Institute COVID-19 Surveillance Team
EPI_ISL_879374, EPI_ISL_879375	Lighthouse Lab in Alderley Park	Wellcome Sanger Institute for the COVID-19 Genomics UK (COG-UK) Consortium	Jacquelyn Wynn, Mairead Hyland, The Lighthouse Lab in Alderley Park and Alex Alderton, Roberto Amato, Sonia Goncalves, Ewan Harrison, David K. Jackson, Ian Johnston, Dominic Kwiatkowski, Cordelia Langford, John Sillitoe on behalf of the Wellcome Sanger Institute COVID-19 Surveillance Team
EPI_ISL_879376, EPI_ISL_879381	Lighthouse Lab in Cambridge	Wellcome Sanger Institute for the COVID-19 Genomics UK (COG-UK) Consortium	Rob Howes, The Lighthouse Lab in Cambridge and Alex Alderton, Roberto Amato, Sonia Goncalves, Ewan Harrison, David K. Jackson, Ian Johnston, Dominic Kwiatkowski, Cordelia Langford, John Sillitoe on behalf of the Wellcome Sanger Institute COVID-19 Surveillance Team
EPI_ISL_879382	Lighthouse Lab in Alderley Park	Wellcome Sanger Institute for the COVID-19 Genomics UK (COG-UK) Consortium	Jacquelyn Wynn, Mairead Hyland, The Lighthouse Lab in Alderley Park and Alex Alderton, Roberto Amato, Sonia Goncalves, Ewan Harrison, David K. Jackson, Ian Johnston, Dominic Kwiatkowski, Cordelia Langford, John Sillitoe on behalf of the Wellcome Sanger Institute COVID-19 Surveillance Team
EPI_ISL_879383, EPI_ISL_879385	Lighthouse Lab in Cambridge	Wellcome Sanger Institute for the COVID-19 Genomics UK (COG-UK) Consortium	Rob Howes, The Lighthouse Lab in Cambridge and Alex Alderton, Roberto Amato, Sonia Goncalves, Ewan Harrison, David K. Jackson, Ian Johnston, Dominic Kwiatkowski, Cordelia Langford, John Sillitoe on behalf of the Wellcome Sanger Institute COVID-19 Surveillance Team
EPI_ISL_879386	Lighthouse Lab in Alderley Park	Wellcome Sanger Institute for the COVID-19 Genomics UK (COG-UK) Consortium	Jacquelyn Wynn, Mairead Hyland, The Lighthouse Lab in Alderley Park and Alex Alderton, Roberto Amato, Sonia Goncalves, Ewan Harrison, David K. Jackson, Ian Johnston, Dominic Kwiatkowski, Cordelia Langford, John Sillitoe on behalf of the Wellcome Sanger Institute COVID-19 Surveillance Team
EPI_ISL_879387, EPI_ISL_879389, EPI_ISL_879390, EPI_ISL_879391	Lighthouse Lab in Cambridge	Wellcome Sanger Institute for the COVID-19 Genomics UK (COG-UK) Consortium	Rob Howes, The Lighthouse Lab in Cambridge and Alex Alderton, Roberto Amato, Sonia Goncalves, Ewan Harrison, David K. Jackson, Ian Johnston, Dominic Kwiatkowski, Cordelia Langford, John Sillitoe on behalf of the Wellcome Sanger Institute COVID-19 Surveillance Team
EPI_ISL_879392, EPI_ISL_879393	Lighthouse Lab in Alderley Park	Wellcome Sanger Institute for the COVID-19 Genomics UK (COG-UK) Consortium	Jacquelyn Wynn, Mairead Hyland, The Lighthouse Lab in Alderley Park and Alex Alderton, Roberto Amato, Sonia Goncalves, Ewan Harrison, David K. Jackson, Ian Johnston, Dominic Kwiatkowski, Cordelia Langford, John Sillitoe on behalf of the Wellcome Sanger Institute COVID-19 Surveillance Team
EPI_ISL_879394, EPI_ISL_879398, EPI_ISL_879400, EPI_ISL_879401, EPI_ISL_879402, EPI_ISL_879403, EPI_ISL_879405, EPI_ISL_879406, EPI_ISL_879407, EPI_ISL_879408, EPI_ISL_879410, EPI_ISL_879411, EPI_ISL_879414			
see above	Lighthouse Lab in Cambridge	Wellcome Sanger Institute for the COVID-19 Genomics UK (COG-UK) Consortium	Rob Howes, The Lighthouse Lab in Cambridge and Alex Alderton, Roberto Amato, Sonia Goncalves, Ewan Harrison, David K. Jackson, Ian Johnston, Dominic Kwiatkowski, Cordelia Langford, John Sillitoe on behalf of the Wellcome Sanger Institute COVID-19 Surveillance Team
EPI_ISL_879415, EPI_ISL_879416, EPI_ISL_879417, EPI_ISL_879418	Lighthouse Lab in Alderley Park	Wellcome Sanger Institute for the COVID-19 Genomics UK (COG-UK) Consortium	Jacquelyn Wynn, Mairead Hyland, The Lighthouse Lab in Alderley Park and Alex Alderton, Roberto Amato, Sonia Goncalves, Ewan Harrison, David K. Jackson, Ian Johnston, Dominic Kwiatkowski, Cordelia Langford, John Sillitoe on behalf of the Wellcome Sanger Institute COVID-19 Surveillance Team
EPI_ISL_879419	Lighthouse Lab in Cambridge	Wellcome Sanger Institute for the COVID-19 Genomics UK (COG-UK) Consortium	Rob Howes, The Lighthouse Lab in Cambridge and Alex Alderton, Roberto Amato, Sonia Goncalves, Ewan Harrison, David K. Jackson, Ian Johnston, Dominic Kwiatkowski, Cordelia Langford, John Sillitoe on behalf of the Wellcome Sanger Institute COVID-19 Surveillance Team
EPI_ISL_879420	Lighthouse Lab in Alderley Park	Wellcome Sanger Institute for the COVID-19 Genomics UK (COG-UK) Consortium	Jacquelyn Wynn, Mairead Hyland, The Lighthouse Lab in Alderley Park and Alex Alderton, Roberto Amato, Sonia Goncalves, Ewan Harrison, David K. Jackson, Ian Johnston, Dominic Kwiatkowski, Cordelia Langford, John Sillitoe on behalf of the Wellcome Sanger Institute COVID-19 Surveillance Team
EPI_ISL_879421, EPI_ISL_879422, EPI_ISL_879423	Lighthouse Lab in Cambridge	Wellcome Sanger Institute for the COVID-19 Genomics UK (COG-UK) Consortium	Rob Howes, The Lighthouse Lab in Cambridge and Alex Alderton, Roberto Amato, Sonia Goncalves, Ewan Harrison, David K. Jackson, Ian Johnston, Dominic Kwiatkowski, Cordelia Langford, John Sillitoe on behalf of the Wellcome Sanger Institute COVID-19 Surveillance Team
EPI_ISL_879427	Lighthouse Lab in Alderley Park	Wellcome Sanger Institute for the COVID-19 Genomics UK (COG-UK) Consortium	Jacquelyn Wynn, Mairead Hyland, The Lighthouse Lab in Alderley Park and Alex Alderton, Roberto Amato, Sonia Goncalves, Ewan Harrison, David K. Jackson, Ian Johnston, Dominic Kwiatkowski, Cordelia Langford, John Sillitoe on behalf of the Wellcome Sanger Institute COVID-19 Surveillance Team
EPI_ISL_879428, EPI_ISL_879430, EPI_ISL_879431, EPI_ISL_879432	Lighthouse Lab in Cambridge	Wellcome Sanger Institute for the COVID-19 Genomics UK (COG-UK) Consortium	Rob Howes, The Lighthouse Lab in Cambridge and Alex Alderton, Roberto Amato, Sonia Goncalves, Ewan Harrison, David K. Jackson, Ian Johnston, Dominic Kwiatkowski, Cordelia Langford, John Sillitoe on behalf of the Wellcome Sanger Institute COVID-19 Surveillance Team
EPI_ISL_879433	Lighthouse Lab in Alderley Park	Wellcome Sanger Institute for the COVID-19 Genomics UK (COG-UK) Consortium	Jacquelyn Wynn, Mairead Hyland, The Lighthouse Lab in Alderley Park and Alex Alderton, Roberto Amato, Sonia Goncalves, Ewan Harrison, David K. Jackson, Ian Johnston, Dominic Kwiatkowski, Cordelia Langford, John Sillitoe on behalf of the Wellcome Sanger Institute COVID-19 Surveillance Team
EPI_ISL_879435, EPI_ISL_879436, EPI_ISL_879437, EPI_ISL_879438, EPI_ISL_879439, EPI_ISL_879440, EPI_ISL_879441, EPI_ISL_879443	Lighthouse Lab in Cambridge	Wellcome Sanger Institute for the COVID-19 Genomics UK (COG-UK) Consortium	Rob Howes, The Lighthouse Lab in Cambridge and Alex Alderton, Roberto Amato, Sonia Goncalves, Ewan Harrison, David K. Jackson, Ian Johnston, Dominic Kwiatkowski, Cordelia Langford, John Sillitoe on behalf of the Wellcome Sanger Institute COVID-19 Surveillance Team
EPI_ISL_879444	Lighthouse Lab in Alderley Park	Wellcome Sanger Institute for the COVID-19 Genomics UK (COG-UK) Consortium	Jacquelyn Wynn, Mairead Hyland, The Lighthouse Lab in Alderley Park and Alex Alderton, Roberto Amato, Sonia Goncalves, Ewan Harrison, David K. Jackson, Ian Johnston, Dominic Kwiatkowski, Cordelia Langford, John Sillitoe on behalf of the Wellcome Sanger Institute COVID-19 Surveillance Team
EPI_ISL_879445	Lighthouse Lab in Cambridge	Wellcome Sanger Institute for the COVID-19 Genomics UK (COG-UK) Consortium	Rob Howes, The Lighthouse Lab in Cambridge and Alex Alderton, Roberto Amato, Sonia Goncalves, Ewan Harrison, David K. Jackson, Ian Johnston, Dominic Kwiatkowski, Cordelia Langford, John Sillitoe on behalf of the Wellcome Sanger Institute COVID-19 Surveillance Team
EPI_ISL_879446	Lighthouse Lab in Alderley Park	Wellcome Sanger Institute for the COVID-19 Genomics UK (COG-UK) Consortium	Jacquelyn Wynn, Mairead Hyland, The Lighthouse Lab in Alderley Park and Alex Alderton, Roberto Amato, Sonia Goncalves, Ewan Harrison, David K. Jackson, Ian Johnston, Dominic Kwiatkowski, Cordelia Langford, John Sillitoe on behalf of the Wellcome Sanger Institute COVID-19 Surveillance Team
EPI_ISL_879447	Lighthouse Lab in Cambridge	Wellcome Sanger Institute for the COVID-19 Genomics UK (COG-UK) Consortium	Rob Howes, The Lighthouse Lab in Cambridge and Alex Alderton, Roberto Amato, Sonia Goncalves, Ewan Harrison, David K. Jackson, Ian Johnston, Dominic Kwiatkowski, Cordelia Langford, John Sillitoe on behalf of the Wellcome Sanger Institute COVID-19 Surveillance Team
EPI_ISL_879451	Lighthouse Lab in Alderley Park	Wellcome Sanger Institute for the COVID-19 Genomics UK (COG-UK) Consortium	Jacquelyn Wynn, Mairead Hyland, The Lighthouse Lab in Alderley Park and Alex Alderton, Roberto Amato, Sonia Goncalves, Ewan Harrison, David K. Jackson, Ian Johnston, Dominic Kwiatkowski, Cordelia Langford, John Sillitoe on behalf of the Wellcome Sanger Institute COVID-19 Surveillance Team
EPI_ISL_879453, EPI_ISL_879454	Lighthouse Lab in Cambridge	Wellcome Sanger Institute for the COVID-19 Genomics UK (COG-UK) Consortium	Rob Howes, The Lighthouse Lab in Cambridge and Alex Alderton, Roberto Amato, Sonia Goncalves, Ewan Harrison, David K. Jackson, Ian Johnston, Dominic Kwiatkowski, Cordelia Langford, John Sillitoe on behalf of the Wellcome Sanger Institute COVID-19 Surveillance Team
EPI_ISL_879456, EPI_ISL_879460	Lighthouse Lab in Alderley Park	Wellcome Sanger Institute for the COVID-19 Genomics UK (COG-UK) Consortium	Jacquelyn Wynn, Mairead Hyland, The Lighthouse Lab in Alderley Park and Alex Alderton, Roberto Amato, Sonia Goncalves, Ewan Harrison, David K. Jackson, Ian Johnston, Dominic Kwiatkowski, Cordelia Langford, John Sillitoe on behalf of the Wellcome Sanger Institute COVID-19 Surveillance Team
EPI_ISL_879463, EPI_ISL_879465, EPI_ISL_879468, EPI_ISL_879469, EPI_ISL_879470, EPI_ISL_879472, EPI_ISL_879474	Lighthouse Lab in Cambridge	Wellcome Sanger Institute for the COVID-19 Genomics UK (COG-UK) Consortium	Rob Howes, The Lighthouse Lab in Cambridge and Alex Alderton, Roberto Amato, Sonia Goncalves, Ewan Harrison, David K. Jackson, Ian Johnston, Dominic Kwiatkowski, Cordelia Langford, John Sillitoe on behalf of the Wellcome Sanger Institute COVID-19 Surveillance Team
EPI_ISL_879476	Lighthouse Lab in Alderley Park	Wellcome Sanger Institute for the COVID-19 Genomics UK (COG-UK) Consortium	Jacquelyn Wynn, Mairead Hyland, The Lighthouse Lab in Alderley Park and Alex Alderton, Roberto Amato, Sonia Goncalves, Ewan Harrison, David K. Jackson, Ian Johnston, Dominic Kwiatkowski, Cordelia Langford, John Sillitoe on behalf of the Wellcome Sanger Institute COVID-19 Surveillance Team
EPI_ISL_879478	Lighthouse Lab in Cambridge	Wellcome Sanger Institute for the COVID-19 Genomics UK (COG-UK) Consortium	Rob Howes, The Lighthouse Lab in Cambridge and Alex Alderton, Roberto Amato, Sonia Goncalves, Ewan Harrison, David K. Jackson, Ian Johnston, Dominic Kwiatkowski, Cordelia Langford, John Sillitoe on behalf of the Wellcome Sanger Institute COVID-19 Surveillance Team
EPI_ISL_879480	Lighthouse Lab in Alderley Park	Wellcome Sanger Institute for the COVID-19 Genomics UK (COG-UK) Consortium	Jacquelyn Wynn, Mairead Hyland, The Lighthouse Lab in Alderley Park and Alex Alderton, Roberto Amato, Sonia Goncalves, Ewan Harrison, David K. Jackson, Ian Johnston, Dominic Kwiatkowski, Cordelia Langford, John Sillitoe on behalf of the Wellcome Sanger Institute COVID-19 Surveillance Team
EPI_ISL_879481, EPI_ISL_879482	Lighthouse Lab in Cambridge	Wellcome Sanger Institute for the COVID-19 Genomics UK	Rob Howes, The Lighthouse Lab in Cambridge and Alex Alderton, Roberto Amato, Sonia Goncalves, Ewan Harrison, David K. Jackson, Ian Johnston,

		(COG-UK) Consortium	Dominic Kwiatkowski, Cordelia Langford, John Sillitoe on behalf of the Wellcome Sanger Institute COVID-19 Surveillance Team
EPI_ISL_879483	Lighthouse Lab in Alderley Park	Wellcome Sanger Institute for the COVID-19 Genomics UK (COG-UK) Consortium	Jacquelyn Wynn, Mairead Hyland, The Lighthouse Lab in Alderley Park and Alex Alderton, Roberto Amato, Sonia Goncalves, Ewan Harrison, David K. Jackson, Ian Johnston, Dominic Kwiatkowski, Cordelia Langford, John Sillitoe on behalf of the Wellcome Sanger Institute COVID-19 Surveillance Team
EPI_ISL_879484, EPI_ISL_879485, EPI_ISL_879488, EPI_ISL_879489, EPI_ISL_879490, EPI_ISL_879491	Lighthouse Lab in Cambridge	Wellcome Sanger Institute for the COVID-19 Genomics UK (COG-UK) Consortium	Rob Howes, The Lighthouse Lab in Cambridge and Alex Alderton, Roberto Amato, Sonia Goncalves, Ewan Harrison, David K. Jackson, Ian Johnston, Dominic Kwiatkowski, Cordelia Langford, John Sillitoe on behalf of the Wellcome Sanger Institute COVID-19 Surveillance Team
EPI_ISL_879493	Lighthouse Lab in Alderley Park	Wellcome Sanger Institute for the COVID-19 Genomics UK (COG-UK) Consortium	Jacquelyn Wynn, Mairead Hyland, The Lighthouse Lab in Alderley Park and Alex Alderton, Roberto Amato, Sonia Goncalves, Ewan Harrison, David K. Jackson, Ian Johnston, Dominic Kwiatkowski, Cordelia Langford, John Sillitoe on behalf of the Wellcome Sanger Institute COVID-19 Surveillance Team
EPI_ISL_879494, EPI_ISL_879496	Lighthouse Lab in Cambridge	Wellcome Sanger Institute for the COVID-19 Genomics UK (COG-UK) Consortium	Rob Howes, The Lighthouse Lab in Cambridge and Alex Alderton, Roberto Amato, Sonia Goncalves, Ewan Harrison, David K. Jackson, Ian Johnston, Dominic Kwiatkowski, Cordelia Langford, John Sillitoe on behalf of the Wellcome Sanger Institute COVID-19 Surveillance Team
EPI_ISL_879497	Lighthouse Lab in Alderley Park	Wellcome Sanger Institute for the COVID-19 Genomics UK (COG-UK) Consortium	Jacquelyn Wynn, Mairead Hyland, The Lighthouse Lab in Alderley Park and Alex Alderton, Roberto Amato, Sonia Goncalves, Ewan Harrison, David K. Jackson, Ian Johnston, Dominic Kwiatkowski, Cordelia Langford, John Sillitoe on behalf of the Wellcome Sanger Institute COVID-19 Surveillance Team
EPI_ISL_879498, EPI_ISL_879500	Lighthouse Lab in Cambridge	Wellcome Sanger Institute for the COVID-19 Genomics UK (COG-UK) Consortium	Rob Howes, The Lighthouse Lab in Cambridge and Alex Alderton, Roberto Amato, Sonia Goncalves, Ewan Harrison, David K. Jackson, Ian Johnston, Dominic Kwiatkowski, Cordelia Langford, John Sillitoe on behalf of the Wellcome Sanger Institute COVID-19 Surveillance Team
EPI_ISL_879501	Lighthouse Lab in Alderley Park	Wellcome Sanger Institute for the COVID-19 Genomics UK (COG-UK) Consortium	Jacquelyn Wynn, Mairead Hyland, The Lighthouse Lab in Alderley Park and Alex Alderton, Roberto Amato, Sonia Goncalves, Ewan Harrison, David K. Jackson, Ian Johnston, Dominic Kwiatkowski, Cordelia Langford, John Sillitoe on behalf of the Wellcome Sanger Institute COVID-19 Surveillance Team
EPI_ISL_879503	Lighthouse Lab in Cambridge	Wellcome Sanger Institute for the COVID-19 Genomics UK (COG-UK) Consortium	Rob Howes, The Lighthouse Lab in Cambridge and Alex Alderton, Roberto Amato, Sonia Goncalves, Ewan Harrison, David K. Jackson, Ian Johnston, Dominic Kwiatkowski, Cordelia Langford, John Sillitoe on behalf of the Wellcome Sanger Institute COVID-19 Surveillance Team
EPI_ISL_879505	Lighthouse Lab in Alderley Park	Wellcome Sanger Institute for the COVID-19 Genomics UK (COG-UK) Consortium	Jacquelyn Wynn, Mairead Hyland, The Lighthouse Lab in Alderley Park and Alex Alderton, Roberto Amato, Sonia Goncalves, Ewan Harrison, David K. Jackson, Ian Johnston, Dominic Kwiatkowski, Cordelia Langford, John Sillitoe on behalf of the Wellcome Sanger Institute COVID-19 Surveillance Team
EPI_ISL_879506, EPI_ISL_879507, EPI_ISL_879508, EPI_ISL_879509, EPI_ISL_879511, EPI_ISL_879512, EPI_ISL_879513	Lighthouse Lab in Cambridge	Wellcome Sanger Institute for the COVID-19 Genomics UK (COG-UK) Consortium	Rob Howes, The Lighthouse Lab in Cambridge and Alex Alderton, Roberto Amato, Sonia Goncalves, Ewan Harrison, David K. Jackson, Ian Johnston, Dominic Kwiatkowski, Cordelia Langford, John Sillitoe on behalf of the Wellcome Sanger Institute COVID-19 Surveillance Team
EPI_ISL_879515	Lighthouse Lab in Alderley Park	Wellcome Sanger Institute for the COVID-19 Genomics UK (COG-UK) Consortium	Jacquelyn Wynn, Mairead Hyland, The Lighthouse Lab in Alderley Park and Alex Alderton, Roberto Amato, Sonia Goncalves, Ewan Harrison, David K. Jackson, Ian Johnston, Dominic Kwiatkowski, Cordelia Langford, John Sillitoe on behalf of the Wellcome Sanger Institute COVID-19 Surveillance Team
EPI_ISL_879516, EPI_ISL_879517	Lighthouse Lab in Cambridge	Wellcome Sanger Institute for the COVID-19 Genomics UK (COG-UK) Consortium	Rob Howes, The Lighthouse Lab in Cambridge and Alex Alderton, Roberto Amato, Sonia Goncalves, Ewan Harrison, David K. Jackson, Ian Johnston, Dominic Kwiatkowski, Cordelia Langford, John Sillitoe on behalf of the Wellcome Sanger Institute COVID-19 Surveillance Team
EPI_ISL_879518	Lighthouse Lab in Alderley Park	Wellcome Sanger Institute for the COVID-19 Genomics UK (COG-UK) Consortium	Jacquelyn Wynn, Mairead Hyland, The Lighthouse Lab in Alderley Park and Alex Alderton, Roberto Amato, Sonia Goncalves, Ewan Harrison, David K. Jackson, Ian Johnston, Dominic Kwiatkowski, Cordelia Langford, John Sillitoe on behalf of the Wellcome Sanger Institute COVID-19 Surveillance Team
EPI_ISL_879521	Lighthouse Lab in Cambridge	Wellcome Sanger Institute for the COVID-19 Genomics UK (COG-UK) Consortium	Rob Howes, The Lighthouse Lab in Cambridge and Alex Alderton, Roberto Amato, Sonia Goncalves, Ewan Harrison, David K. Jackson, Ian Johnston, Dominic Kwiatkowski, Cordelia Langford, John Sillitoe on behalf of the Wellcome Sanger Institute COVID-19 Surveillance Team
EPI_ISL_879522	Lighthouse Lab in Alderley Park	Wellcome Sanger Institute for the COVID-19 Genomics UK (COG-UK) Consortium	Jacquelyn Wynn, Mairead Hyland, The Lighthouse Lab in Alderley Park and Alex Alderton, Roberto Amato, Sonia Goncalves, Ewan Harrison, David K. Jackson, Ian Johnston, Dominic Kwiatkowski, Cordelia Langford, John Sillitoe on behalf of the Wellcome Sanger Institute COVID-19 Surveillance Team
EPI_ISL_879523	Lighthouse Lab in Cambridge	Wellcome Sanger Institute for the COVID-19 Genomics UK (COG-UK) Consortium	Rob Howes, The Lighthouse Lab in Cambridge and Alex Alderton, Roberto Amato, Sonia Goncalves, Ewan Harrison, David K. Jackson, Ian Johnston, Dominic Kwiatkowski, Cordelia Langford, John Sillitoe on behalf of the Wellcome Sanger Institute COVID-19 Surveillance Team
EPI_ISL_879524	Lighthouse Lab in Alderley Park	Wellcome Sanger Institute for the COVID-19 Genomics UK (COG-UK) Consortium	Jacquelyn Wynn, Mairead Hyland, The Lighthouse Lab in Alderley Park and Alex Alderton, Roberto Amato, Sonia Goncalves, Ewan Harrison, David K. Jackson, Ian Johnston, Dominic Kwiatkowski, Cordelia Langford, John Sillitoe on behalf of the Wellcome Sanger Institute COVID-19 Surveillance Team
EPI_ISL_879525, EPI_ISL_879527, EPI_ISL_879528, EPI_ISL_879529, EPI_ISL_879530, EPI_ISL_879531, EPI_ISL_879533	Lighthouse Lab in Cambridge	Wellcome Sanger Institute for the COVID-19 Genomics UK (COG-UK) Consortium	Rob Howes, The Lighthouse Lab in Cambridge and Alex Alderton, Roberto Amato, Sonia Goncalves, Ewan Harrison, David K. Jackson, Ian Johnston, Dominic Kwiatkowski, Cordelia Langford, John Sillitoe on behalf of the Wellcome Sanger Institute COVID-19 Surveillance Team
EPI_ISL_879534, EPI_ISL_879535	Lighthouse Lab in Alderley Park	Wellcome Sanger Institute for the COVID-19 Genomics UK (COG-UK) Consortium	Jacquelyn Wynn, Mairead Hyland, The Lighthouse Lab in Alderley Park and Alex Alderton, Roberto Amato, Sonia Goncalves, Ewan Harrison, David K. Jackson, Ian Johnston, Dominic Kwiatkowski, Cordelia Langford, John Sillitoe on behalf of the Wellcome Sanger Institute COVID-19 Surveillance Team
EPI_ISL_879536, EPI_ISL_879537, EPI_ISL_879541	Lighthouse Lab in Cambridge	Wellcome Sanger Institute for the COVID-19 Genomics UK (COG-UK) Consortium	Rob Howes, The Lighthouse Lab in Cambridge and Alex Alderton, Roberto Amato, Sonia Goncalves, Ewan Harrison, David K. Jackson, Ian Johnston, Dominic Kwiatkowski, Cordelia Langford, John Sillitoe on behalf of the Wellcome Sanger Institute COVID-19 Surveillance Team
EPI_ISL_879542	Lighthouse Lab in Alderley Park	Wellcome Sanger Institute for the COVID-19 Genomics UK (COG-UK) Consortium	Jacquelyn Wynn, Mairead Hyland, The Lighthouse Lab in Alderley Park and Alex Alderton, Roberto Amato, Sonia Goncalves, Ewan Harrison, David K. Jackson, Ian Johnston, Dominic Kwiatkowski, Cordelia Langford, John Sillitoe on behalf of the Wellcome Sanger Institute COVID-19 Surveillance Team
EPI_ISL_879543, EPI_ISL_879544, EPI_ISL_879545, EPI_ISL_879551, EPI_ISL_879555, EPI_ISL_879557, EPI_ISL_879558, EPI_ISL_879559, EPI_ISL_879562	Lighthouse Lab in Cambridge	Wellcome Sanger Institute for the COVID-19 Genomics UK (COG-UK) Consortium	Rob Howes, The Lighthouse Lab in Cambridge and Alex Alderton, Roberto Amato, Sonia Goncalves, Ewan Harrison, David K. Jackson, Ian Johnston, Dominic Kwiatkowski, Cordelia Langford, John Sillitoe on behalf of the Wellcome Sanger Institute COVID-19 Surveillance Team
EPI_ISL_879563, EPI_ISL_879564	Lighthouse Lab in Alderley Park	Wellcome Sanger Institute for the COVID-19 Genomics UK (COG-UK) Consortium	Jacquelyn Wynn, Mairead Hyland, The Lighthouse Lab in Alderley Park and Alex Alderton, Roberto Amato, Sonia Goncalves, Ewan Harrison, David K. Jackson, Ian Johnston, Dominic Kwiatkowski, Cordelia Langford, John Sillitoe on behalf of the Wellcome Sanger Institute COVID-19 Surveillance Team
EPI_ISL_879567, EPI_ISL_879568, EPI_ISL_879569, EPI_ISL_879570, EPI_ISL_879573, EPI_ISL_879574, EPI_ISL_879577	Lighthouse Lab in Cambridge	Wellcome Sanger Institute for the COVID-19 Genomics UK (COG-UK) Consortium	Rob Howes, The Lighthouse Lab in Cambridge and Alex Alderton, Roberto Amato, Sonia Goncalves, Ewan Harrison, David K. Jackson, Ian Johnston, Dominic Kwiatkowski, Cordelia Langford, John Sillitoe on behalf of the Wellcome Sanger Institute COVID-19 Surveillance Team
EPI_ISL_879578	Lighthouse Lab in Alderley Park	Wellcome Sanger Institute for the COVID-19 Genomics UK (COG-UK) Consortium	Jacquelyn Wynn, Mairead Hyland, The Lighthouse Lab in Alderley Park and Alex Alderton, Roberto Amato, Sonia Goncalves, Ewan Harrison, David K. Jackson, Ian Johnston, Dominic Kwiatkowski, Cordelia Langford, John Sillitoe on behalf of the Wellcome Sanger Institute COVID-19 Surveillance Team
EPI_ISL_879579, EPI_ISL_879580	Lighthouse Lab in Cambridge	Wellcome Sanger Institute for the COVID-19 Genomics UK (COG-UK) Consortium	Rob Howes, The Lighthouse Lab in Cambridge and Alex Alderton, Roberto Amato, Sonia Goncalves, Ewan Harrison, David K. Jackson, Ian Johnston, Dominic Kwiatkowski, Cordelia Langford, John Sillitoe on behalf of the Wellcome Sanger Institute COVID-19 Surveillance Team
EPI_ISL_879582	Lighthouse Lab in Alderley Park	Wellcome Sanger Institute for the COVID-19 Genomics UK (COG-UK) Consortium	Jacquelyn Wynn, Mairead Hyland, The Lighthouse Lab in Alderley Park and Alex Alderton, Roberto Amato, Sonia Goncalves, Ewan Harrison, David K. Jackson, Ian Johnston, Dominic Kwiatkowski, Cordelia Langford, John Sillitoe on behalf of the Wellcome Sanger Institute COVID-19 Surveillance Team
EPI_ISL_879583, EPI_ISL_879584, EPI_ISL_879587, EPI_ISL_879588	Lighthouse Lab in Cambridge	Wellcome Sanger Institute for the COVID-19 Genomics UK (COG-UK) Consortium	Rob Howes, The Lighthouse Lab in Cambridge and Alex Alderton, Roberto Amato, Sonia Goncalves, Ewan Harrison, David K. Jackson, Ian Johnston, Dominic Kwiatkowski, Cordelia Langford, John Sillitoe on behalf of the Wellcome Sanger Institute COVID-19 Surveillance Team
EPI_ISL_879589	Lighthouse Lab in Alderley Park	Wellcome Sanger Institute for the COVID-19 Genomics UK (COG-UK) Consortium	Jacquelyn Wynn, Mairead Hyland, The Lighthouse Lab in Alderley Park and Alex Alderton, Roberto Amato, Sonia Goncalves, Ewan Harrison, David K. Jackson, Ian Johnston, Dominic Kwiatkowski, Cordelia Langford, John Sillitoe on behalf of the Wellcome Sanger Institute COVID-19 Surveillance Team
EPI_ISL_879590	Lighthouse Lab in Cambridge	Wellcome Sanger Institute for the COVID-19 Genomics UK (COG-UK) Consortium	Rob Howes, The Lighthouse Lab in Cambridge and Alex Alderton, Roberto Amato, Sonia Goncalves, Ewan Harrison, David K. Jackson, Ian Johnston, Dominic Kwiatkowski, Cordelia Langford, John Sillitoe on behalf of the Wellcome Sanger Institute COVID-19 Surveillance Team
EPI_ISL_879593	Lighthouse Lab in Alderley Park	Wellcome Sanger Institute for the COVID-19 Genomics UK	Jacquelyn Wynn, Mairead Hyland, The Lighthouse Lab in Alderley Park and Alex Alderton, Roberto Amato, Sonia Goncalves, Ewan Harrison, David K.

		(COG-UK) Consortium	Jackson, Ian Johnston, Dominic Kwiatkowski, Cordelia Langford, John Sillitoe on behalf of the Wellcome Sanger Institute COVID-19 Surveillance Team
EPI_ISL_879595	Lighthouse Lab in Cambridge	Wellcome Sanger Institute for the COVID-19 Genomics UK (COG-UK) Consortium	Rob Howes, The Lighthouse Lab in Cambridge and Alex Alderton, Roberto Amato, Sonia Goncalves, Ewan Harrison, David K. Jackson, Ian Johnston, Dominic Kwiatkowski, Cordelia Langford, John Sillitoe on behalf of the Wellcome Sanger Institute COVID-19 Surveillance Team
EPI_ISL_879596	Lighthouse Lab in Alderley Park	Wellcome Sanger Institute for the COVID-19 Genomics UK (COG-UK) Consortium	Jacquelyn Wynn, Mairead Hyland, The Lighthouse Lab in Alderley Park and Alex Alderton, Roberto Amato, Sonia Goncalves, Ewan Harrison, David K. Jackson, Ian Johnston, Dominic Kwiatkowski, Cordelia Langford, John Sillitoe on behalf of the Wellcome Sanger Institute COVID-19 Surveillance Team
EPI_ISL_879598, EPI_ISL_879599	Lighthouse Lab in Cambridge	Wellcome Sanger Institute for the COVID-19 Genomics UK (COG-UK) Consortium	Rob Howes, The Lighthouse Lab in Cambridge and Alex Alderton, Roberto Amato, Sonia Goncalves, Ewan Harrison, David K. Jackson, Ian Johnston, Dominic Kwiatkowski, Cordelia Langford, John Sillitoe on behalf of the Wellcome Sanger Institute COVID-19 Surveillance Team
EPI_ISL_879602, EPI_ISL_879603, EPI_ISL_879604	Lighthouse Lab in Alderley Park	Wellcome Sanger Institute for the COVID-19 Genomics UK (COG-UK) Consortium	Jacquelyn Wynn, Mairead Hyland, The Lighthouse Lab in Alderley Park and Alex Alderton, Roberto Amato, Sonia Goncalves, Ewan Harrison, David K. Jackson, Ian Johnston, Dominic Kwiatkowski, Cordelia Langford, John Sillitoe on behalf of the Wellcome Sanger Institute COVID-19 Surveillance Team
EPI_ISL_879605, EPI_ISL_879606, EPI_ISL_879607, EPI_ISL_879609, EPI_ISL_879610, EPI_ISL_879612	Lighthouse Lab in Cambridge	Wellcome Sanger Institute for the COVID-19 Genomics UK (COG-UK) Consortium	Rob Howes, The Lighthouse Lab in Cambridge and Alex Alderton, Roberto Amato, Sonia Goncalves, Ewan Harrison, David K. Jackson, Ian Johnston, Dominic Kwiatkowski, Cordelia Langford, John Sillitoe on behalf of the Wellcome Sanger Institute COVID-19 Surveillance Team
EPI_ISL_879614	Lighthouse Lab in Alderley Park	Wellcome Sanger Institute for the COVID-19 Genomics UK (COG-UK) Consortium	Jacquelyn Wynn, Mairead Hyland, The Lighthouse Lab in Alderley Park and Alex Alderton, Roberto Amato, Sonia Goncalves, Ewan Harrison, David K. Jackson, Ian Johnston, Dominic Kwiatkowski, Cordelia Langford, John Sillitoe on behalf of the Wellcome Sanger Institute COVID-19 Surveillance Team
EPI_ISL_879615	Lighthouse Lab in Cambridge	Wellcome Sanger Institute for the COVID-19 Genomics UK (COG-UK) Consortium	Rob Howes, The Lighthouse Lab in Cambridge and Alex Alderton, Roberto Amato, Sonia Goncalves, Ewan Harrison, David K. Jackson, Ian Johnston, Dominic Kwiatkowski, Cordelia Langford, John Sillitoe on behalf of the Wellcome Sanger Institute COVID-19 Surveillance Team
EPI_ISL_879616, EPI_ISL_879619	Lighthouse Lab in Alderley Park	Wellcome Sanger Institute for the COVID-19 Genomics UK (COG-UK) Consortium	Jacquelyn Wynn, Mairead Hyland, The Lighthouse Lab in Alderley Park and Alex Alderton, Roberto Amato, Sonia Goncalves, Ewan Harrison, David K. Jackson, Ian Johnston, Dominic Kwiatkowski, Cordelia Langford, John Sillitoe on behalf of the Wellcome Sanger Institute COVID-19 Surveillance Team
EPI_ISL_879620, EPI_ISL_879622, EPI_ISL_879624, EPI_ISL_879625, EPI_ISL_879626	Lighthouse Lab in Cambridge	Wellcome Sanger Institute for the COVID-19 Genomics UK (COG-UK) Consortium	Rob Howes, The Lighthouse Lab in Cambridge and Alex Alderton, Roberto Amato, Sonia Goncalves, Ewan Harrison, David K. Jackson, Ian Johnston, Dominic Kwiatkowski, Cordelia Langford, John Sillitoe on behalf of the Wellcome Sanger Institute COVID-19 Surveillance Team
EPI_ISL_879627	Lighthouse Lab in Alderley Park	Wellcome Sanger Institute for the COVID-19 Genomics UK (COG-UK) Consortium	Jacquelyn Wynn, Mairead Hyland, The Lighthouse Lab in Alderley Park and Alex Alderton, Roberto Amato, Sonia Goncalves, Ewan Harrison, David K. Jackson, Ian Johnston, Dominic Kwiatkowski, Cordelia Langford, John Sillitoe on behalf of the Wellcome Sanger Institute COVID-19 Surveillance Team
EPI_ISL_879630, EPI_ISL_879632, EPI_ISL_879633, EPI_ISL_879634, EPI_ISL_879635, EPI_ISL_879636, EPI_ISL_879637, EPI_ISL_879638, EPI_ISL_879639, EPI_ISL_879640	Lighthouse Lab in Cambridge	Wellcome Sanger Institute for the COVID-19 Genomics UK (COG-UK) Consortium	Rob Howes, The Lighthouse Lab in Cambridge and Alex Alderton, Roberto Amato, Sonia Goncalves, Ewan Harrison, David K. Jackson, Ian Johnston, Dominic Kwiatkowski, Cordelia Langford, John Sillitoe on behalf of the Wellcome Sanger Institute COVID-19 Surveillance Team
EPI_ISL_879641	Lighthouse Lab in Alderley Park	Wellcome Sanger Institute for the COVID-19 Genomics UK (COG-UK) Consortium	Jacquelyn Wynn, Mairead Hyland, The Lighthouse Lab in Alderley Park and Alex Alderton, Roberto Amato, Sonia Goncalves, Ewan Harrison, David K. Jackson, Ian Johnston, Dominic Kwiatkowski, Cordelia Langford, John Sillitoe on behalf of the Wellcome Sanger Institute COVID-19 Surveillance Team
EPI_ISL_879644, EPI_ISL_879645	Lighthouse Lab in Cambridge	Wellcome Sanger Institute for the COVID-19 Genomics UK (COG-UK) Consortium	Rob Howes, The Lighthouse Lab in Cambridge and Alex Alderton, Roberto Amato, Sonia Goncalves, Ewan Harrison, David K. Jackson, Ian Johnston, Dominic Kwiatkowski, Cordelia Langford, John Sillitoe on behalf of the Wellcome Sanger Institute COVID-19 Surveillance Team
EPI_ISL_879647	Lighthouse Lab in Alderley Park	Wellcome Sanger Institute for the COVID-19 Genomics UK (COG-UK) Consortium	Jacquelyn Wynn, Mairead Hyland, The Lighthouse Lab in Alderley Park and Alex Alderton, Roberto Amato, Sonia Goncalves, Ewan Harrison, David K. Jackson, Ian Johnston, Dominic Kwiatkowski, Cordelia Langford, John Sillitoe on behalf of the Wellcome Sanger Institute COVID-19 Surveillance Team
EPI_ISL_879648	Lighthouse Lab in Cambridge	Wellcome Sanger Institute for the COVID-19 Genomics UK (COG-UK) Consortium	Rob Howes, The Lighthouse Lab in Cambridge and Alex Alderton, Roberto Amato, Sonia Goncalves, Ewan Harrison, David K. Jackson, Ian Johnston, Dominic Kwiatkowski, Cordelia Langford, John Sillitoe on behalf of the Wellcome Sanger Institute COVID-19 Surveillance Team
EPI_ISL_879649	Lighthouse Lab in Alderley Park	Wellcome Sanger Institute for the COVID-19 Genomics UK (COG-UK) Consortium	Jacquelyn Wynn, Mairead Hyland, The Lighthouse Lab in Alderley Park and Alex Alderton, Roberto Amato, Sonia Goncalves, Ewan Harrison, David K. Jackson, Ian Johnston, Dominic Kwiatkowski, Cordelia Langford, John Sillitoe on behalf of the Wellcome Sanger Institute COVID-19 Surveillance Team
EPI_ISL_879650, EPI_ISL_879651, EPI_ISL_879652, EPI_ISL_879657, EPI_ISL_879658, EPI_ISL_879659, EPI_ISL_879660, EPI_ISL_879661, EPI_ISL_879662	Lighthouse Lab in Cambridge	Wellcome Sanger Institute for the COVID-19 Genomics UK (COG-UK) Consortium	Rob Howes, The Lighthouse Lab in Cambridge and Alex Alderton, Roberto Amato, Sonia Goncalves, Ewan Harrison, David K. Jackson, Ian Johnston, Dominic Kwiatkowski, Cordelia Langford, John Sillitoe on behalf of the Wellcome Sanger Institute COVID-19 Surveillance Team
EPI_ISL_879665, EPI_ISL_879666	Lighthouse Lab in Alderley Park	Wellcome Sanger Institute for the COVID-19 Genomics UK (COG-UK) Consortium	Jacquelyn Wynn, Mairead Hyland, The Lighthouse Lab in Alderley Park and Alex Alderton, Roberto Amato, Sonia Goncalves, Ewan Harrison, David K. Jackson, Ian Johnston, Dominic Kwiatkowski, Cordelia Langford, John Sillitoe on behalf of the Wellcome Sanger Institute COVID-19 Surveillance Team
EPI_ISL_879667, EPI_ISL_879668, EPI_ISL_879669, EPI_ISL_879670, EPI_ISL_879671	Lighthouse Lab in Cambridge	Wellcome Sanger Institute for the COVID-19 Genomics UK (COG-UK) Consortium	Rob Howes, The Lighthouse Lab in Cambridge and Alex Alderton, Roberto Amato, Sonia Goncalves, Ewan Harrison, David K. Jackson, Ian Johnston, Dominic Kwiatkowski, Cordelia Langford, John Sillitoe on behalf of the Wellcome Sanger Institute COVID-19 Surveillance Team
EPI_ISL_879672	Lighthouse Lab in Alderley Park	Wellcome Sanger Institute for the COVID-19 Genomics UK (COG-UK) Consortium	Jacquelyn Wynn, Mairead Hyland, The Lighthouse Lab in Alderley Park and Alex Alderton, Roberto Amato, Sonia Goncalves, Ewan Harrison, David K. Jackson, Ian Johnston, Dominic Kwiatkowski, Cordelia Langford, John Sillitoe on behalf of the Wellcome Sanger Institute COVID-19 Surveillance Team
EPI_ISL_879673	Lighthouse Lab in Cambridge	Wellcome Sanger Institute for the COVID-19 Genomics UK (COG-UK) Consortium	Rob Howes, The Lighthouse Lab in Cambridge and Alex Alderton, Roberto Amato, Sonia Goncalves, Ewan Harrison, David K. Jackson, Ian Johnston, Dominic Kwiatkowski, Cordelia Langford, John Sillitoe on behalf of the Wellcome Sanger Institute COVID-19 Surveillance Team
EPI_ISL_879674	Lighthouse Lab in Alderley Park	Wellcome Sanger Institute for the COVID-19 Genomics UK (COG-UK) Consortium	Jacquelyn Wynn, Mairead Hyland, The Lighthouse Lab in Alderley Park and Alex Alderton, Roberto Amato, Sonia Goncalves, Ewan Harrison, David K. Jackson, Ian Johnston, Dominic Kwiatkowski, Cordelia Langford, John Sillitoe on behalf of the Wellcome Sanger Institute COVID-19 Surveillance Team
EPI_ISL_879676, EPI_ISL_879677, EPI_ISL_879680	Lighthouse Lab in Cambridge	Wellcome Sanger Institute for the COVID-19 Genomics UK (COG-UK) Consortium	Rob Howes, The Lighthouse Lab in Cambridge and Alex Alderton, Roberto Amato, Sonia Goncalves, Ewan Harrison, David K. Jackson, Ian Johnston, Dominic Kwiatkowski, Cordelia Langford, John Sillitoe on behalf of the Wellcome Sanger Institute COVID-19 Surveillance Team
EPI_ISL_879682, EPI_ISL_879683	Lighthouse Lab in Alderley Park	Wellcome Sanger Institute for the COVID-19 Genomics UK (COG-UK) Consortium	Jacquelyn Wynn, Mairead Hyland, The Lighthouse Lab in Alderley Park and Alex Alderton, Roberto Amato, Sonia Goncalves, Ewan Harrison, David K. Jackson, Ian Johnston, Dominic Kwiatkowski, Cordelia Langford, John Sillitoe on behalf of the Wellcome Sanger Institute COVID-19 Surveillance Team
EPI_ISL_879684, EPI_ISL_879685	Lighthouse Lab in Cambridge	Wellcome Sanger Institute for the COVID-19 Genomics UK (COG-UK) Consortium	Rob Howes, The Lighthouse Lab in Cambridge and Alex Alderton, Roberto Amato, Sonia Goncalves, Ewan Harrison, David K. Jackson, Ian Johnston, Dominic Kwiatkowski, Cordelia Langford, John Sillitoe on behalf of the Wellcome Sanger Institute COVID-19 Surveillance Team
EPI_ISL_879687	Lighthouse Lab in Alderley Park	Wellcome Sanger Institute for the COVID-19 Genomics UK (COG-UK) Consortium	Jacquelyn Wynn, Mairead Hyland, The Lighthouse Lab in Alderley Park and Alex Alderton, Roberto Amato, Sonia Goncalves, Ewan Harrison, David K. Jackson, Ian Johnston, Dominic Kwiatkowski, Cordelia Langford, John Sillitoe on behalf of the Wellcome Sanger Institute COVID-19 Surveillance Team
EPI_ISL_879688, EPI_ISL_879690, EPI_ISL_879691, EPI_ISL_879694	Lighthouse Lab in Cambridge	Wellcome Sanger Institute for the COVID-19 Genomics UK (COG-UK) Consortium	Rob Howes, The Lighthouse Lab in Cambridge and Alex Alderton, Roberto Amato, Sonia Goncalves, Ewan Harrison, David K. Jackson, Ian Johnston, Dominic Kwiatkowski, Cordelia Langford, John Sillitoe on behalf of the Wellcome Sanger Institute COVID-19 Surveillance Team
EPI_ISL_879695	Lighthouse Lab in Alderley Park	Wellcome Sanger Institute for the COVID-19 Genomics UK (COG-UK) Consortium	Jacquelyn Wynn, Mairead Hyland, The Lighthouse Lab in Alderley Park and Alex Alderton, Roberto Amato, Sonia Goncalves, Ewan Harrison, David K. Jackson, Ian Johnston, Dominic Kwiatkowski, Cordelia Langford, John Sillitoe on behalf of the Wellcome Sanger Institute COVID-19 Surveillance Team
EPI_ISL_879696, EPI_ISL_879697, EPI_ISL_879698, EPI_ISL_879699, EPI_ISL_879701	Lighthouse Lab in Cambridge	Wellcome Sanger Institute for the COVID-19 Genomics UK (COG-UK) Consortium	Rob Howes, The Lighthouse Lab in Cambridge and Alex Alderton, Roberto Amato, Sonia Goncalves, Ewan Harrison, David K. Jackson, Ian Johnston, Dominic Kwiatkowski, Cordelia Langford, John Sillitoe on behalf of the Wellcome Sanger Institute COVID-19 Surveillance Team
EPI_ISL_879702	Lighthouse Lab in Alderley Park	Wellcome Sanger Institute for the COVID-19 Genomics UK (COG-UK) Consortium	Jacquelyn Wynn, Mairead Hyland, The Lighthouse Lab in Alderley Park and Alex Alderton, Roberto Amato, Sonia Goncalves, Ewan Harrison, David K. Jackson, Ian Johnston, Dominic Kwiatkowski, Cordelia Langford, John Sillitoe on behalf of the Wellcome Sanger Institute COVID-19 Surveillance Team
EPI_ISL_879704, EPI_ISL_879705,	Lighthouse Lab in Cambridge	Wellcome Sanger Institute for the COVID-19 Genomics UK	Rob Howes, The Lighthouse Lab in Cambridge and Alex Alderton, Roberto Amato, Sonia Goncalves, Ewan Harrison, David K. Jackson, Ian Johnston,

EPI_ISL_879706, EPI_ISL_879708, EPI_ISL_879709, EPI_ISL_879716		(COG-UK) Consortium	Dominic Kwiatkowski, Cordelia Langford, John Sillitoe on behalf of the Wellcome Sanger Institute COVID-19 Surveillance Team
EPI_ISL_879718	Lighthouse Lab in Alderley Park	Wellcome Sanger Institute for the COVID-19 Genomics UK (COG-UK) Consortium	Jacquelyn Wynn, Mairead Hyland, The Lighthouse Lab in Alderley Park and Alex Alderton, Roberto Amato, Sonia Goncalves, Ewan Harrison, David K. Jackson, Ian Johnston, Dominic Kwiatkowski, Cordelia Langford, John Sillitoe on behalf of the Wellcome Sanger Institute COVID-19 Surveillance Team
EPI_ISL_879722, EPI_ISL_879733, EPI_ISL_879735, EPI_ISL_879743, EPI_ISL_879745, EPI_ISL_879746, EPI_ISL_879770, EPI_ISL_879782, EPI_ISL_879786, EPI_ISL_879796, EPI_ISL_879799, EPI_ISL_879804, EPI_ISL_879805, EPI_ISL_879807, EPI_ISL_879812, EPI_ISL_879815, EPI_ISL_879824, EPI_ISL_879830, EPI_ISL_879840, EPI_ISL_879847, EPI_ISL_879853, EPI_ISL_879857, EPI_ISL_879881, EPI_ISL_879883, EPI_ISL_879893, EPI_ISL_879899, EPI_ISL_879900, EPI_ISL_879902, EPI_ISL_879910, EPI_ISL_879917, EPI_ISL_879943, EPI_ISL_879951, EPI_ISL_879952, EPI_ISL_879983, EPI_ISL_879992, EPI_ISL_880001, EPI_ISL_880026, EPI_ISL_880051, EPI_ISL_880073, EPI_ISL_880080, EPI_ISL_880081			
see above	Lighthouse Lab in Cambridge	Wellcome Sanger Institute for the COVID-19 Genomics UK (COG-UK) Consortium	Rob Howes, The Lighthouse Lab in Cambridge and Alex Alderton, Roberto Amato, Sonia Goncalves, Ewan Harrison, David K. Jackson, Ian Johnston, Dominic Kwiatkowski, Cordelia Langford, John Sillitoe on behalf of the Wellcome Sanger Institute COVID-19 Surveillance Team
EPI_ISL_880091, EPI_ISL_880100, EPI_ISL_880112, EPI_ISL_880113, EPI_ISL_880120, EPI_ISL_880123, EPI_ISL_880124, EPI_ISL_880126, EPI_ISL_880136, EPI_ISL_880216, EPI_ISL_880222, EPI_ISL_880258, EPI_ISL_880260, EPI_ISL_880264, EPI_ISL_880265, EPI_ISL_880286, EPI_ISL_880288, EPI_ISL_880291, EPI_ISL_880298, EPI_ISL_880301, EPI_ISL_880306, EPI_ISL_880332, EPI_ISL_880335, EPI_ISL_880337, EPI_ISL_880340, EPI_ISL_880343, EPI_ISL_880348, EPI_ISL_880352, EPI_ISL_880356, EPI_ISL_880361, EPI_ISL_880366, EPI_ISL_880375, EPI_ISL_880376, EPI_ISL_880379, EPI_ISL_880387, EPI_ISL_880390, EPI_ISL_880392, EPI_ISL_880400, EPI_ISL_880402, EPI_ISL_880417, EPI_ISL_880418, EPI_ISL_880426, EPI_ISL_880427, EPI_ISL_880433, EPI_ISL_880435, EPI_ISL_880436, EPI_ISL_880443, EPI_ISL_880446, EPI_ISL_880456, EPI_ISL_880458, EPI_ISL_880464, EPI_ISL_880467, EPI_ISL_880475, EPI_ISL_880477, EPI_ISL_880482, EPI_ISL_880493, EPI_ISL_880498, EPI_ISL_880502, EPI_ISL_880508, EPI_ISL_880509, EPI_ISL_880518, EPI_ISL_880519, EPI_ISL_880529, EPI_ISL_880531, EPI_ISL_880575, EPI_ISL_880583			
see above	Lighthouse Lab in Alderley Park	Wellcome Sanger Institute for the COVID-19 Genomics UK (COG-UK) Consortium	Jacquelyn Wynn, Mairead Hyland, The Lighthouse Lab in Alderley Park and Alex Alderton, Roberto Amato, Sonia Goncalves, Ewan Harrison, David K. Jackson, Ian Johnston, Dominic Kwiatkowski, Cordelia Langford, John Sillitoe on behalf of the Wellcome Sanger Institute COVID-19 Surveillance Team
EPI_ISL_882673	Hospital Municipal Dr. Guido Guida	Instituto Adolfo Lutz, Interdiciplinary Procedures Center, Strategic Laboratory	Claudio Tavares Sacchi, Claudia Regina Gonçalves, Erica Valessa Ramos Gomes, Karoline Rodrigues Campos
EPI_ISL_883307, EPI_ISL_883308, EPI_ISL_883311, EPI_ISL_883317	Pandemic Response Laboratory	New York City Public Health Laboratory	Jade Wang, et al.
EPI_ISL_883325, EPI_ISL_883330	OCME Office Of Chief Medical Examiner	New York City Public Health Laboratory	Jade Wang, et al.
EPI_ISL_883331	DOHMH Morrisania	New York City Public Health Laboratory	Jade Wang, et al.
EPI_ISL_883337	DOHMH Crown Heights	New York City Public Health Laboratory	Jade Wang, et al.
EPI_ISL_883344, EPI_ISL_883345	OCME Office Of Chief Medical Examiner	New York City Public Health Laboratory	Jade Wang, et al.
EPI_ISL_883347	DOHMH Morrisania	New York City Public Health Laboratory	Jade Wang, et al.
EPI_ISL_883349	DOHMH Jamaica	New York City Public Health Laboratory	Jade Wang, et al.
EPI_ISL_883383, EPI_ISL_883384, EPI_ISL_883385, EPI_ISL_883386, EPI_ISL_883387, EPI_ISL_883388, EPI_ISL_883389	Pandemic Response Laboratory	New York City Public Health Laboratory	Jade Wang, et al.
EPI_ISL_883403	Department of Homeless Services	New York City Public Health Laboratory	Jade Wang, et al.
EPI_ISL_883404, EPI_ISL_883405, EPI_ISL_883406, EPI_ISL_883407, EPI_ISL_883408	OCME Office Of Chief Medical Examiner	New York City Public Health Laboratory	Jade Wang, et al.
EPI_ISL_883410, EPI_ISL_883411	DOHMH Corona	New York City Public Health Laboratory	Jade Wang, et al.
EPI_ISL_883412	DOHMH Crown Heights	New York City Public Health Laboratory	Jade Wang, et al.
EPI_ISL_883413, EPI_ISL_883414, EPI_ISL_883415	DOHMH Central Harlem	New York City Public Health Laboratory	Jade Wang, et al.
EPI_ISL_883416, EPI_ISL_883417	DOHMH Fort Greene	New York City Public Health Laboratory	Jade Wang, et al.
EPI_ISL_883418, EPI_ISL_883419, EPI_ISL_883420, EPI_ISL_883421, EPI_ISL_883422	DOHMH Jamaica	New York City Public Health Laboratory	Jade Wang, et al.
EPI_ISL_883424	DOHMH PHL	New York City Public Health Laboratory	Jade Wang, et al.
EPI_ISL_883430	BOSTON HEART DIAGNOSTICS CORP	Wadsworth Center, New York State Department of Health	Kirsten St. George, Daryl M. Lamson, Alexis Russel, Matthew Shudt, Melissa A Leisner, Jonathan Plitnick, Navjot Singh, John Kelly, Erasmus Schneider, Erica Lasek-Nesselquist
EPI_ISL_884008, EPI_ISL_884009, EPI_ISL_884010, EPI_ISL_884011, EPI_ISL_884012	ALBANY MEDICAL CENTER HOSPITAL CLINICAL LABORATORIES	Wadsworth Center, New York State Department of Health	Kirsten St. George, Daryl M. Lamson, Alexis Russel, Matthew Shudt, Melissa A Leisner, Jonathan Plitnick, Navjot Singh, John Kelly, Erasmus Schneider, Erica Lasek-Nesselquist
EPI_ISL_884013, EPI_ISL_884014, EPI_ISL_884015, EPI_ISL_884016	Wadsworth Center, New York State Department of Health	Wadsworth Center, New York State Department of Health	Kirsten St. George, Daryl M. Lamson, Alexis Russel, Matthew Shudt, Melissa A Leisner, Jonathan Plitnick, Navjot Singh, John Kelly, Erasmus Schneider, Erica Lasek-Nesselquist
EPI_ISL_884017, EPI_ISL_884018, EPI_ISL_884019, EPI_ISL_884020	ALBANY MEDICAL CENTER HOSPITAL CLINICAL LABORATORIES	Wadsworth Center, New York State Department of Health	Kirsten St. George, Daryl M. Lamson, Alexis Russel, Matthew Shudt, Melissa A Leisner, Jonathan Plitnick, Navjot Singh, John Kelly, Erasmus Schneider, Erica Lasek-Nesselquist
EPI_ISL_884194, EPI_ISL_884195	Wyoming Public Health Laboratory	Wyoming Public Health Laboratory	Noah Hull, Taylor Fearing, Lynette Gumbleton, Channing Weber, Ashley Norberg, Bailey Bowcutt, and Wanda Manley
EPI_ISL_884580, EPI_ISL_884581, EPI_ISL_884582, EPI_ISL_884583, EPI_ISL_884584, EPI_ISL_884585, EPI_ISL_884586, EPI_ISL_884587, EPI_ISL_884588, EPI_ISL_884589	Respiratory Viruses Branch, Centers for Disease Control and Prevention	Respiratory Viruses Branch, Centers for Disease Control and Prevention	Cook,P.W., Batra,D., Rambo-Martin,B.L., de Feo,E., Antico,J., Tran,C., Tolentino,M., Wickline,S., Gietzen,K., Sickler,B., Liu,J., Allen,E., Febbo,P., Galloway,S., Washington,N.L., White,S., Levan,G., Barret,K.S., Cirulli,E., Bolze,A., Ascencio,A., Rivera-Garcia,C., Cho,R., Nguyen,J., Wang,S., Ramirez,J., Cassens,T., Sandoval,E., Isaksson,M., Lee,W., Becker,D., Laurent,M., Lu,J., Paden,C.R., Tong,S., MacCannell,D.
EPI_ISL_885155, EPI_ISL_885156, EPI_ISL_885157, EPI_ISL_885158, EPI_ISL_885159, EPI_ISL_885160, EPI_ISL_885161, EPI_ISL_885162, EPI_ISL_885163, EPI_ISL_885164, EPI_ISL_885165, EPI_ISL_885166, EPI_ISL_885167, EPI_ISL_885168, EPI_ISL_885169, EPI_ISL_885170, EPI_ISL_885171, EPI_ISL_885172, EPI_ISL_885173, EPI_ISL_885174, EPI_ISL_885175, EPI_ISL_885176, EPI_ISL_885177, EPI_ISL_885178, EPI_ISL_885179, EPI_ISL_885180, EPI_ISL_885181, EPI_ISL_885182, EPI_ISL_885184, EPI_ISL_885185, EPI_ISL_885186, EPI_ISL_885187, EPI_ISL_885188, EPI_ISL_885189, EPI_ISL_885190, EPI_ISL_885191, EPI_ISL_885192, EPI_ISL_885193, EPI_ISL_885194, EPI_ISL_885195, EPI_ISL_885196, EPI_ISL_885197, EPI_ISL_885198, EPI_ISL_885199, EPI_ISL_885200, EPI_ISL_885201, EPI_ISL_885202, EPI_ISL_885203, EPI_ISL_885205, EPI_ISL_885206, EPI_ISL_885207, EPI_ISL_885208, EPI_ISL_885209, EPI_ISL_885210, EPI_ISL_885212, EPI_ISL_885213, EPI_ISL_885214, EPI_ISL_885215, EPI_ISL_885216, EPI_ISL_885217, EPI_ISL_885218, EPI_ISL_885219, EPI_ISL_885220, EPI_ISL_885221, EPI_ISL_885222, EPI_ISL_885223, EPI_ISL_885224, EPI_ISL_885225, EPI_ISL_885226, EPI_ISL_885227, EPI_ISL_885228, EPI_ISL_885229, EPI_ISL_885230, EPI_ISL_885231, EPI_ISL_885232, EPI_ISL_885233, EPI_ISL_885234, EPI_ISL_885235, EPI_ISL_885236, EPI_ISL_885237, EPI_ISL_885238, EPI_ISL_885239, EPI_ISL_885241, EPI_ISL_885242, EPI_ISL_885243, EPI_ISL_885245, EPI_ISL_885246, EPI_ISL_885247, EPI_ISL_885248, EPI_ISL_885249, EPI_ISL_885250, EPI_ISL_885251, EPI_ISL_885252, EPI_ISL_885253, EPI_ISL_885254, EPI_ISL_885255, EPI_ISL_885256, EPI_ISL_885257, EPI_ISL_885258, EPI_ISL_885259, EPI_ISL_885260, EPI_ISL_885261, EPI_ISL_885262, EPI_ISL_885263, EPI_ISL_885264, EPI_ISL_885265, EPI_ISL_885266, EPI_ISL_885267, EPI_ISL_885268, EPI_ISL_885269, EPI_ISL_885270, EPI_ISL_885271, EPI_ISL_885272, EPI_ISL_885273, EPI_ISL_885274, EPI_ISL_885275, EPI_ISL_885276, EPI_ISL_885277, EPI_ISL_885278, EPI_ISL_885279, EPI_ISL_885280, EPI_ISL_885281, EPI_ISL_885282, EPI_ISL_885283, EPI_ISL_885284, EPI_ISL_885285, EPI_ISL_885286, EPI_ISL_885287, EPI_ISL_885288, EPI_ISL_885289, EPI_ISL_885290, EPI_ISL_885292, EPI_ISL_885293, EPI_ISL_885294, EPI_ISL_885295, EPI_ISL_885296, EPI_ISL_885297, EPI_ISL_885298, EPI_ISL_885299, EPI_ISL_885300, EPI_ISL_885301, EPI_ISL_885302, EPI_ISL_885303, EPI_ISL_885304, EPI_ISL_885305, EPI_ISL_885306, EPI_ISL_885307, EPI_ISL_885308, EPI_ISL_885309, EPI_ISL_885310, EPI_ISL_885311, EPI_ISL_885312, EPI_ISL_885313, EPI_ISL_885314, EPI_ISL_885315, EPI_ISL_885316, EPI_ISL_885317, EPI_ISL_885318, EPI_ISL_885319, EPI_ISL_885320, EPI_ISL_885322, EPI_ISL_885323, EPI_ISL_885324, EPI_ISL_885325, EPI_ISL_885326, EPI_ISL_885328, EPI_ISL_885329, EPI_ISL_885330, EPI_ISL_885331, EPI_ISL_885332, EPI_ISL_885333, EPI_ISL_885334, EPI_ISL_885335, EPI_ISL_885336, EPI_ISL_885337, EPI_ISL_885338, EPI_ISL_885339, EPI_ISL_885340, EPI_ISL_885341, EPI_ISL_885342, EPI_ISL_885343, EPI_ISL_885344, EPI_ISL_885345, EPI_ISL_885346, EPI_ISL_885347, EPI_ISL_885348, EPI_ISL_885350, EPI_ISL_885351, EPI_ISL_885352, EPI_ISL_885353, EPI_ISL_885354, EPI_ISL_885355, EPI_ISL_885356, EPI_ISL_885357, EPI_ISL_885358, EPI_ISL_885360, EPI_ISL_885361, EPI_ISL_885362, EPI_ISL_885363, EPI_ISL_885364, EPI_ISL_885365, EPI_ISL_885366, EPI_ISL_885367, EPI_ISL_885368, EPI_ISL_885369, EPI_ISL_885370, EPI_ISL_885371, EPI_ISL_885372, EPI_ISL_885373, EPI_ISL_885374, EPI_ISL_885375, EPI_ISL_885376, EPI_ISL_885377, EPI_ISL_885378, EPI_ISL_885379, EPI_ISL_885380, EPI_ISL_885381, EPI_ISL_885382, EPI_ISL_885383, EPI_ISL_885384, EPI_ISL_885385, EPI_ISL_885386, EPI_ISL_885387, EPI_ISL_885388, EPI_ISL_885389, EPI_ISL_885390, EPI_ISL_885391, EPI_ISL_885393, EPI_ISL_885394, EPI_ISL_885395, EPI_ISL_885396, EPI_ISL_885397, EPI_ISL_885398, EPI_ISL_885399, EPI_ISL_885400, EPI_ISL_885401, EPI_ISL_885402, EPI_ISL_885403, EPI_ISL_885404, EPI_ISL_885405, EPI_ISL_885406, EPI_ISL_885407, EPI_ISL_885408, EPI_ISL_885410, EPI_ISL_885412, EPI_ISL_885413, EPI_ISL_885414, EPI_ISL_885415, EPI_ISL_885416, EPI_ISL_885417, EPI_ISL_885418, EPI_ISL_885419, EPI_ISL_885420, EPI_ISL_885421, EPI_ISL_885422, EPI_ISL_885423, EPI_ISL_885424, EPI_ISL_885425, EPI_ISL_885426, EPI_ISL_885427, EPI_ISL_885428, EPI_ISL_885429, EPI_ISL_885430, EPI_ISL_885431, EPI_ISL_885432, EPI_ISL_885433, EPI_ISL_885435, EPI_ISL_885436, EPI_ISL_885437, EPI_ISL_885438, EPI_ISL_885439, EPI_ISL_885440, EPI_ISL_885441, EPI_ISL_885442, EPI_ISL_885443, EPI_ISL_885444, EPI_ISL_885445, EPI_ISL_885446, EPI_ISL_885447, EPI_ISL_885448, EPI_ISL_885449, EPI_ISL_885450, EPI_ISL_885451, EPI_ISL_885452, EPI_ISL_885453, EPI_ISL_885454, EPI_ISL_885455, EPI_ISL_885456,			

EPI_ISL_885457, EPI_ISL_885458, EPI_ISL_885459, EPI_ISL_885460, EPI_ISL_885461, EPI_ISL_885462, EPI_ISL_885463, EPI_ISL_885464, EPI_ISL_885465, EPI_ISL_885466, EPI_ISL_885467, EPI_ISL_885468, EPI_ISL_885469, EPI_ISL_885470, EPI_ISL_885471, EPI_ISL_885472, EPI_ISL_885473, EPI_ISL_885474, EPI_ISL_885475, EPI_ISL_885476, EPI_ISL_885477, EPI_ISL_885478, EPI_ISL_885479, EPI_ISL_885480, EPI_ISL_885481, EPI_ISL_885482, EPI_ISL_885483, EPI_ISL_885484, EPI_ISL_885485, EPI_ISL_885486, EPI_ISL_885487, EPI_ISL_885488, EPI_ISL_885489, EPI_ISL_885490, EPI_ISL_885493, EPI_ISL_885495, EPI_ISL_885498, EPI_ISL_885499, EPI_ISL_885500, EPI_ISL_885501, EPI_ISL_885505, EPI_ISL_885506, EPI_ISL_885509, EPI_ISL_885510, EPI_ISL_885513, EPI_ISL_885515, EPI_ISL_885518, EPI_ISL_885521, EPI_ISL_885522, EPI_ISL_885523, EPI_ISL_885525, EPI_ISL_885527, EPI_ISL_885529, EPI_ISL_885530, EPI_ISL_885531, EPI_ISL_885532, EPI_ISL_885533, EPI_ISL_885536, EPI_ISL_885537, EPI_ISL_885539, EPI_ISL_885540, EPI_ISL_885541, EPI_ISL_885542, EPI_ISL_885543, EPI_ISL_885544, EPI_ISL_885547, EPI_ISL_885548, EPI_ISL_885549, EPI_ISL_885552, EPI_ISL_885553, EPI_ISL_885554, EPI_ISL_885555, EPI_ISL_885556, EPI_ISL_885557, EPI_ISL_885558, EPI_ISL_885559, EPI_ISL_885560, EPI_ISL_885561, EPI_ISL_885565, EPI_ISL_885566, EPI_ISL_885568, EPI_ISL_885569, EPI_ISL_885570, EPI_ISL_885571, EPI_ISL_885572, EPI_ISL_885573, EPI_ISL_885576, EPI_ISL_885577, EPI_ISL_885578, EPI_ISL_885581, EPI_ISL_885583, EPI_ISL_885584, EPI_ISL_885586, EPI_ISL_885587, EPI_ISL_885590, EPI_ISL_885591, EPI_ISL_885592, EPI_ISL_885594, EPI_ISL_885595, EPI_ISL_885596, EPI_ISL_885597, EPI_ISL_885598, EPI_ISL_885599, EPI_ISL_885600, EPI_ISL_885601, EPI_ISL_885603, EPI_ISL_885605, EPI_ISL_885606, EPI_ISL_885610, EPI_ISL_885611, EPI_ISL_885612, EPI_ISL_885613, EPI_ISL_885615, EPI_ISL_885616, EPI_ISL_885617, EPI_ISL_885618, EPI_ISL_885619, EPI_ISL_885620, EPI_ISL_885622, EPI_ISL_885623, EPI_ISL_885624, EPI_ISL_885629, EPI_ISL_885630, EPI_ISL_885631, EPI_ISL_885632, EPI_ISL_885633, EPI_ISL_885635, EPI_ISL_885637, EPI_ISL_885638, EPI_ISL_885640, EPI_ISL_885641, EPI_ISL_885642, EPI_ISL_885643, EPI_ISL_885644, EPI_ISL_885645, EPI_ISL_885646, EPI_ISL_885648, EPI_ISL_885649, EPI_ISL_885650, EPI_ISL_885651, EPI_ISL_885652, EPI_ISL_885653, EPI_ISL_885654, EPI_ISL_885655, EPI_ISL_885656, EPI_ISL_885657, EPI_ISL_885659, EPI_ISL_885661, EPI_ISL_885662, EPI_ISL_885663, EPI_ISL_885664, EPI_ISL_885667, EPI_ISL_885668, EPI_ISL_885671, EPI_ISL_885673, EPI_ISL_885674, EPI_ISL_885676, EPI_ISL_885677, EPI_ISL_885679, EPI_ISL_885680, EPI_ISL_885682, EPI_ISL_885683, EPI_ISL_885686, EPI_ISL_885690, EPI_ISL_885691, EPI_ISL_885694, EPI_ISL_885695, EPI_ISL_885697, EPI_ISL_885698, EPI_ISL_885700, EPI_ISL_885701, EPI_ISL_885702, EPI_ISL_885703, EPI_ISL_885705, EPI_ISL_885706, EPI_ISL_885707, EPI_ISL_885708, EPI_ISL_885709, EPI_ISL_885711, EPI_ISL_885716, EPI_ISL_885717, EPI_ISL_885718, EPI_ISL_885719, EPI_ISL_885721, EPI_ISL_885723, EPI_ISL_885725, EPI_ISL_885726, EPI_ISL_885727, EPI_ISL_885728, EPI_ISL_885729, EPI_ISL_885730, EPI_ISL_885731, EPI_ISL_885732, EPI_ISL_885735, EPI_ISL_885736, EPI_ISL_885739, EPI_ISL_885740, EPI_ISL_885741, EPI_ISL_885744, EPI_ISL_885746, EPI_ISL_885747, EPI_ISL_885749, EPI_ISL_885751, EPI_ISL_885752, EPI_ISL_885753, EPI_ISL_885754, EPI_ISL_885757, EPI_ISL_885758, EPI_ISL_885759, EPI_ISL_885760, EPI_ISL_885762, EPI_ISL_885763, EPI_ISL_885766, EPI_ISL_885767, EPI_ISL_885769, EPI_ISL_885771, EPI_ISL_885772, EPI_ISL_885774, EPI_ISL_885777, EPI_ISL_885778, EPI_ISL_885781, EPI_ISL_885782, EPI_ISL_885783, EPI_ISL_885784, EPI_ISL_885786, EPI_ISL_885787, EPI_ISL_885789, EPI_ISL_885790, EPI_ISL_885791, EPI_ISL_885796, EPI_ISL_885797, EPI_ISL_885798, EPI_ISL_885799, EPI_ISL_885801, EPI_ISL_885802, EPI_ISL_885803, EPI_ISL_885805, EPI_ISL_885806, EPI_ISL_885808, EPI_ISL_885809, EPI_ISL_885811, EPI_ISL_885814, EPI_ISL_885816, EPI_ISL_885817, EPI_ISL_885818, EPI_ISL_885819, EPI_ISL_885821, EPI_ISL_885823, EPI_ISL_885827, EPI_ISL_885828, EPI_ISL_885833, EPI_ISL_885835, EPI_ISL_885838, EPI_ISL_885843, EPI_ISL_885844, EPI_ISL_885846, EPI_ISL_885848, EPI_ISL_885855, EPI_ISL_885856, EPI_ISL_885860, EPI_ISL_885864, EPI_ISL_885867, EPI_ISL_885868, EPI_ISL_885870, EPI_ISL_885871, EPI_ISL_885874, EPI_ISL_885875, EPI_ISL_885879, EPI_ISL_885883, EPI_ISL_885885, EPI_ISL_885888, EPI_ISL_885894, EPI_ISL_885898, EPI_ISL_885899, EPI_ISL_885902, EPI_ISL_885906, EPI_ISL_885910, EPI_ISL_885912, EPI_ISL_885915, EPI_ISL_885917, EPI_ISL_885918, EPI_ISL_885919, EPI_ISL_885924, EPI_ISL_885926, EPI_ISL_885927, EPI_ISL_885928, EPI_ISL_885929, EPI_ISL_885931, EPI_ISL_885934, EPI_ISL_885935, EPI_ISL_885937, EPI_ISL_885939, EPI_ISL_885946, EPI_ISL_885951, EPI_ISL_885954, EPI_ISL_885958, EPI_ISL_885959, EPI_ISL_885961, EPI_ISL_885962, EPI_ISL_885963, EPI_ISL_885966, EPI_ISL_885967, EPI_ISL_885970, EPI_ISL_885974, EPI_ISL_885976, EPI_ISL_885980, EPI_ISL_885982, EPI_ISL_885983, EPI_ISL_885984, EPI_ISL_885987, EPI_ISL_885988, EPI_ISL_885990, EPI_ISL_885992, EPI_ISL_885993, EPI_ISL_885995, EPI_ISL_885996, EPI_ISL_885997, EPI_ISL_885998, EPI_ISL_885999, EPI_ISL_886001, EPI_ISL_886002, EPI_ISL_886004, EPI_ISL_886005, EPI_ISL_886006, EPI_ISL_886009, EPI_ISL_886014, EPI_ISL_886015, EPI_ISL_886018, EPI_ISL_886020, EPI_ISL_886021, EPI_ISL_886027, EPI_ISL_886029, EPI_ISL_886032, EPI_ISL_886037, EPI_ISL_886038, EPI_ISL_886039, EPI_ISL_886040, EPI_ISL_886043, EPI_ISL_886044, EPI_ISL_886046, EPI_ISL_886047, EPI_ISL_886049, EPI_ISL_886050, EPI_ISL_886053, EPI_ISL_886056, EPI_ISL_886057, EPI_ISL_886060, EPI_ISL_886063, EPI_ISL_886064, EPI_ISL_886066, EPI_ISL_886067, EPI_ISL_886070, EPI_ISL_886071, EPI_ISL_886074, EPI_ISL_886075, EPI_ISL_886076, EPI_ISL_886077, EPI_ISL_886078, EPI_ISL_886081, EPI_ISL_886083, EPI_ISL_886089, EPI_ISL_886090, EPI_ISL_886091, EPI_ISL_886096, EPI_ISL_886097, EPI_ISL_886098, EPI_ISL_886099, EPI_ISL_886101, EPI_ISL_886103, EPI_ISL_886106, EPI_ISL_886110, EPI_ISL_886112, EPI_ISL_886116, EPI_ISL_886117, EPI_ISL_886118, EPI_ISL_886119, EPI_ISL_886120, EPI_ISL_886121, EPI_ISL_886127, EPI_ISL_886129, EPI_ISL_886132, EPI_ISL_886133, EPI_ISL_886134, EPI_ISL_886136, EPI_ISL_886137, EPI_ISL_886140, EPI_ISL_886141, EPI_ISL_886142, EPI_ISL_886143, EPI_ISL_886144, EPI_ISL_886145, EPI_ISL_886146, EPI_ISL_886148, EPI_ISL_886149, EPI_ISL_886150, EPI_ISL_886154			
see above	Lighthouse Lab in Alderley Park	Wellcome Sanger Institute for the COVID-19 Genomics UK (COG-UK) Consortium	Jacquelyn Wynn, Mairead Hyland, The Lighthouse Lab in Alderley Park and Alex Alderton, Roberto Amato, Sonia Goncalves, Ewan Harrison, David K. Jackson, Ian Johnston, Dominic Kwiatkowski, Cordelia Langford, John Sillitoe on behalf of the Wellcome Sanger Institute COVID-19 Surveillance Team
EPI_ISL_887116, EPI_ISL_887119, EPI_ISL_887122, EPI_ISL_887128, EPI_ISL_887138, EPI_ISL_887139	Institute of Medical Microbiology and Hospital Hygiene	Institute of Medical Microbiology and Hospital Hygiene	Prof. Dr. Achim Kaasch, Aljoscha Tersteegen
EPI_ISL_887512, EPI_ISL_887516, EPI_ISL_887517, EPI_ISL_887518, EPI_ISL_887524, EPI_ISL_887528, EPI_ISL_887539, EPI_ISL_887540, EPI_ISL_887541, EPI_ISL_887542, EPI_ISL_887543, EPI_ISL_887545, EPI_ISL_887546, EPI_ISL_887547, EPI_ISL_887548, EPI_ISL_887549, EPI_ISL_887551, EPI_ISL_887553, EPI_ISL_887554, EPI_ISL_887555, EPI_ISL_887556, EPI_ISL_887557, EPI_ISL_887569, EPI_ISL_887570, EPI_ISL_887571, EPI_ISL_887572, EPI_ISL_887573, EPI_ISL_887574, EPI_ISL_887575, EPI_ISL_887576, EPI_ISL_887578, EPI_ISL_887579			
see above	Johns Hopkins Hospital Department of Pathology	Johns Hopkins Hospital Department of Pathology	C. Paul Morris, Chun Huai Luo, Adannaya Amadi, Matthew Schwartz, Nicholas Gallagher, Heba H. Mostafa
EPI_ISL_888850, EPI_ISL_888906	Michigan Department of Health and Human Services, Bureau of Laboratories	Michigan Department of Health and Human Services, Bureau of Laboratories	Blankenship HM, Riner D, Soehnlen MK
EPI_ISL_888909, EPI_ISL_888919, EPI_ISL_888930, EPI_ISL_888945, EPI_ISL_888946	Wyoming Public Health Laboratory	Wyoming Public Health Laboratory	Noah Hull, Taylor Fearing, Lynette Gumbleton, Channing Weber, Ashley Norberg, Bailey Bowcutt, and Wanda Manley
EPI_ISL_889743, EPI_ISL_889744, EPI_ISL_889746, EPI_ISL_889747, EPI_ISL_889748, EPI_ISL_889749, EPI_ISL_889750, EPI_ISL_889751, EPI_ISL_889752, EPI_ISL_889753, EPI_ISL_889754, EPI_ISL_889755, EPI_ISL_889756, EPI_ISL_889757, EPI_ISL_889758, EPI_ISL_889759, EPI_ISL_889760, EPI_ISL_889761			
see above	LSUHS Emerging Viral Threat Laboratory	Microbial Genome Sequencing Center	Jeremy P. Kamil, Jennifer L. Carroll, Camille F. Abshire, Maarten Van Diest, Mohammed N.A. Siddiquey, Andrew D. Yurochko, Martin J. Sapp, Rona S. Scott, Christopher G. Kevil, Daniel J. Snyder, Vaughn S. Cooper, John A. Vanchiere
EPI_ISL_891070, EPI_ISL_891071, EPI_ISL_891072	Seattle Flu Study	Seattle Flu Study	Deborah A. Nickerson, Chris D. Frazar, Jover Lee, Benjamin Pelle, Erica Ryke, Matthew Richardson, Amanda Adler, Elisabeth Brandstetter, Peter D. Han, Kairsten Fay, Misja Ilcisin, Kirsten Lacombe, Thomas R. Sibley, Melissa Truong, Caitlin R. Wolf, Michael Boeckh, Janet A. Englund, Michael Famulare, Barry R. Lutz, Mark J. Rieder, Lea M. Starita, Matthew Thompson, Jay Shendure, Trevor Bedford, Helen Y. Chu
EPI_ISL_891073, EPI_ISL_891074	Seattle Flu Study	Seattle Flu Study	Deborah A. Nickerson, Chris D. Frazar, Jover Lee, Benjamin Pelle, Erica Ryke, Matthew Richardson, Amanda Adler, Elisabeth Brandstetter, Peter D. Han, Kairsten Fay, Misja Ilcisin, Kirsten Lacombe, Thomas R. Sibley, Melissa Truong, Caitlin R. Wolf, Karen Cowgill, Stephanie Schrag, Jeff Duchin, Michael Boeckh, Janet A. Englund, Michael Famulare, Barry R. Lutz, Mark J. Rieder, Lea M. Starita, Matthew Thompson, Helen Y. Chu, Trevor Bedford, Jay Shendure
EPI_ISL_891075, EPI_ISL_891076	Seattle Flu Study	Seattle Flu Study	Deborah A. Nickerson, Chris D. Frazar, Jover Lee, Benjamin Pelle, Erica Ryke, Matthew Richardson, Amanda Adler, Elisabeth Brandstetter, Peter D. Han, Kairsten Fay, Misja Ilcisin, Kirsten Lacombe, Thomas R. Sibley, Melissa Truong, Caitlin R. Wolf, Michael Boeckh, Janet A. Englund, Michael Famulare, Barry R. Lutz, Mark J. Rieder, Lea M. Starita, Matthew Thompson, Jay Shendure, Trevor Bedford, Helen Y. Chu
EPI_ISL_891077, EPI_ISL_891078, EPI_ISL_891079, EPI_ISL_891080, EPI_ISL_891081	Seattle Flu Study	Seattle Flu Study	Deborah A. Nickerson, Chris D. Frazar, Jover Lee, Benjamin Pelle, Erica Ryke, Matthew Richardson, Amanda Adler, Elisabeth Brandstetter, Peter D. Han, Kairsten Fay, Misja Ilcisin, Kirsten Lacombe, Thomas R. Sibley, Melissa Truong, Caitlin R. Wolf, Karen Cowgill, Stephanie Schrag, Jeff Duchin, Michael Boeckh, Janet A. Englund, Michael Famulare, Barry R. Lutz, Mark J. Rieder, Lea M. Starita, Matthew Thompson, Helen Y. Chu, Trevor Bedford, Jay Shendure
EPI_ISL_891082, EPI_ISL_891083	Seattle Flu Study	Seattle Flu Study	Deborah A. Nickerson, Chris D. Frazar, Jover Lee, Benjamin Pelle, Erica Ryke, Matthew Richardson, Amanda Adler, Elisabeth Brandstetter, Peter D. Han, Kairsten Fay, Misja Ilcisin, Kirsten Lacombe, Thomas R. Sibley, Melissa Truong, Caitlin R. Wolf, Michael Boeckh, Janet A. Englund, Michael Famulare, Barry R. Lutz, Mark J. Rieder, Lea M. Starita, Matthew Thompson, Jay Shendure, Trevor Bedford, Helen Y. Chu
EPI_ISL_891092	Seattle Flu Study	Seattle Flu Study	Deborah A. Nickerson, Chris D. Frazar, Jover Lee, Benjamin Pelle, Erica Ryke, Matthew Richardson, Amanda Adler, Elisabeth Brandstetter, Peter D. Han, Kairsten Fay, Misja Ilcisin, Kirsten Lacombe, Thomas R. Sibley, Melissa Truong, Caitlin R. Wolf, Karen Cowgill, Stephanie Schrag, Jeff Duchin, Michael Boeckh, Janet A. Englund, Michael Famulare, Barry R. Lutz, Mark J. Rieder, Lea M. Starita, Matthew Thompson, Helen Y. Chu, Trevor Bedford, Jay Shendure
EPI_ISL_891274, EPI_ISL_891277, EPI_ISL_891287, EPI_ISL_891291, EPI_ISL_891302, EPI_ISL_891303, EPI_ISL_891321, EPI_ISL_891336, EPI_ISL_891341, EPI_ISL_891343, EPI_ISL_891348, EPI_ISL_891362, EPI_ISL_891368, EPI_ISL_891381, EPI_ISL_891390, EPI_ISL_891407, EPI_ISL_891408, EPI_ISL_891414, EPI_ISL_891418, EPI_ISL_891425, EPI_ISL_891428, EPI_ISL_891429, EPI_ISL_891432, EPI_ISL_891443			
see above	Lighthouse Lab in Cambridge	Wellcome Sanger Institute for the COVID-19 Genomics UK (COG-UK) Consortium	Rob Howes, The Lighthouse Lab in Cambridge and Alex Alderton, Roberto Amato, Sonia Goncalves, Ewan Harrison, David K. Jackson, Ian Johnston, Dominic Kwiatkowski, Cordelia Langford, John Sillitoe on behalf of the Wellcome Sanger Institute COVID-19 Surveillance Team
EPI_ISL_891445	Lighthouse Lab in Glasgow	Wellcome Sanger Institute for the COVID-19 Genomics UK (COG-UK) Consortium	Harper VanSteenhouse, Yumi Kasai, David Gray, Carol Clugston, Anna Dominiczak and Alex Alderton, Roberto Amato, Sonia Goncalves, Ewan Harrison, David K. Jackson, Ian Johnston, Dominic Kwiatkowski, Cordelia Langford, John Sillitoe on behalf of the Wellcome Sanger Institute COVID-19 Surveillance Team
EPI_ISL_891449	Lighthouse Lab in Cambridge	Wellcome Sanger Institute for the COVID-19 Genomics UK (COG-UK) Consortium	Rob Howes, The Lighthouse Lab in Cambridge and Alex Alderton, Roberto Amato, Sonia Goncalves, Ewan Harrison, David K. Jackson, Ian Johnston, Dominic Kwiatkowski, Cordelia Langford, John Sillitoe on behalf of the Wellcome Sanger Institute COVID-19 Surveillance Team
EPI_ISL_891458	Lighthouse Lab in Alderley Park	Wellcome Sanger Institute for the COVID-19 Genomics UK (COG-UK) Consortium	Jacquelyn Wynn, Mairead Hyland, The Lighthouse Lab in Alderley Park and Alex Alderton, Roberto Amato, Sonia Goncalves, Ewan Harrison, David K. Jackson, Ian Johnston, Dominic Kwiatkowski, Cordelia Langford, John Sillitoe on behalf of the Wellcome Sanger Institute COVID-19 Surveillance Team
EPI_ISL_891468, EPI_ISL_891471, EPI_ISL_891474, EPI_ISL_891482	Lighthouse Lab in Cambridge	Wellcome Sanger Institute for the COVID-19 Genomics UK (COG-UK) Consortium	Rob Howes, The Lighthouse Lab in Cambridge and Alex Alderton, Roberto Amato, Sonia Goncalves, Ewan Harrison, David K. Jackson, Ian Johnston, Dominic Kwiatkowski, Cordelia Langford, John Sillitoe on behalf of the Wellcome Sanger Institute COVID-19 Surveillance Team
EPI_ISL_891507	Lighthouse Lab in Alderley Park	Wellcome Sanger Institute for the COVID-19 Genomics UK (COG-UK) Consortium	Jacquelyn Wynn, Mairead Hyland, The Lighthouse Lab in Alderley Park and Alex Alderton, Roberto Amato, Sonia Goncalves, Ewan Harrison, David K. Jackson, Ian Johnston, Dominic Kwiatkowski, Cordelia Langford, John Sillitoe on behalf of the Wellcome Sanger Institute COVID-19 Surveillance Team
EPI_ISL_891523, EPI_ISL_891524, EPI_ISL_891533, EPI_ISL_891546,	Lighthouse Lab in Cambridge	Wellcome Sanger Institute for the COVID-19 Genomics UK (COG-UK) Consortium	Rob Howes, The Lighthouse Lab in Cambridge and Alex Alderton, Roberto Amato, Sonia Goncalves, Ewan Harrison, David K. Jackson, Ian Johnston, Dominic Kwiatkowski, Cordelia Langford, John Sillitoe on behalf of the Wellcome Sanger Institute COVID-19 Surveillance Team

EPI_ISL_891552, EPI_ISL_891573, EPI_ISL_891576, EPI_ISL_891655			
EPI_ISL_891700	Lighthouse Lab in Glasgow	Wellcome Sanger Institute for the COVID-19 Genomics UK (COG-UK) Consortium	Harper VanSteenhouse, Yumi Kasai, David Gray, Carol Clugston, Anna Dominiczak and Alex Alderton, Roberto Amato, Sonia Goncalves, Ewan Harrison, David K. Jackson, Ian Johnston, Dominic Kwiatkowski, Cordelia Langford, John Sillitoe on behalf of the Wellcome Sanger Institute COVID-19 Surveillance Team
EPI_ISL_891709, EPI_ISL_891755, EPI_ISL_891799	Lighthouse Lab in Cambridge	Wellcome Sanger Institute for the COVID-19 Genomics UK (COG-UK) Consortium	Rob Howes, The Lighthouse Lab in Cambridge and Alex Alderton, Roberto Amato, Sonia Goncalves, Ewan Harrison, David K. Jackson, Ian Johnston, Dominic Kwiatkowski, Cordelia Langford, John Sillitoe on behalf of the Wellcome Sanger Institute COVID-19 Surveillance Team
EPI_ISL_891822	Lighthouse Lab in Alderley Park	Wellcome Sanger Institute for the COVID-19 Genomics UK (COG-UK) Consortium	Jacquelyn Wynn, Mairead Hyland, The Lighthouse Lab in Alderley Park and Alex Alderton, Roberto Amato, Sonia Goncalves, Ewan Harrison, David K. Jackson, Ian Johnston, Dominic Kwiatkowski, Cordelia Langford, John Sillitoe on behalf of the Wellcome Sanger Institute COVID-19 Surveillance Team
EPI_ISL_891832	Lighthouse Lab in Cambridge	Wellcome Sanger Institute for the COVID-19 Genomics UK (COG-UK) Consortium	Rob Howes, The Lighthouse Lab in Cambridge and Alex Alderton, Roberto Amato, Sonia Goncalves, Ewan Harrison, David K. Jackson, Ian Johnston, Dominic Kwiatkowski, Cordelia Langford, John Sillitoe on behalf of the Wellcome Sanger Institute COVID-19 Surveillance Team
EPI_ISL_891850, EPI_ISL_891860, EPI_ISL_891867	Lighthouse Lab in Alderley Park	Wellcome Sanger Institute for the COVID-19 Genomics UK (COG-UK) Consortium	Jacquelyn Wynn, Mairead Hyland, The Lighthouse Lab in Alderley Park and Alex Alderton, Roberto Amato, Sonia Goncalves, Ewan Harrison, David K. Jackson, Ian Johnston, Dominic Kwiatkowski, Cordelia Langford, John Sillitoe on behalf of the Wellcome Sanger Institute COVID-19 Surveillance Team
EPI_ISL_891882, EPI_ISL_891896	Lighthouse Lab in Cambridge	Wellcome Sanger Institute for the COVID-19 Genomics UK (COG-UK) Consortium	Rob Howes, The Lighthouse Lab in Cambridge and Alex Alderton, Roberto Amato, Sonia Goncalves, Ewan Harrison, David K. Jackson, Ian Johnston, Dominic Kwiatkowski, Cordelia Langford, John Sillitoe on behalf of the Wellcome Sanger Institute COVID-19 Surveillance Team
EPI_ISL_891899, EPI_ISL_891900, EPI_ISL_891901, EPI_ISL_891902, EPI_ISL_891904, EPI_ISL_891905, EPI_ISL_891906, EPI_ISL_891907, EPI_ISL_891908, EPI_ISL_891910, EPI_ISL_891911, EPI_ISL_891912, EPI_ISL_891913, EPI_ISL_891914, EPI_ISL_891915, EPI_ISL_891916, EPI_ISL_891917, EPI_ISL_891919, EPI_ISL_891920, EPI_ISL_891921, EPI_ISL_891922, EPI_ISL_891923, EPI_ISL_891924, EPI_ISL_891925, EPI_ISL_891926, EPI_ISL_891927, EPI_ISL_891929, EPI_ISL_891930, EPI_ISL_891931, EPI_ISL_891932, EPI_ISL_891933, EPI_ISL_891934, EPI_ISL_891935, EPI_ISL_891936, EPI_ISL_891937, EPI_ISL_891938, EPI_ISL_891939, EPI_ISL_891940, EPI_ISL_891941, EPI_ISL_891942, EPI_ISL_891943, EPI_ISL_891944, EPI_ISL_891946, EPI_ISL_891947, EPI_ISL_891948, EPI_ISL_891949, EPI_ISL_891950, EPI_ISL_891951, EPI_ISL_891952, EPI_ISL_891953, EPI_ISL_891954, EPI_ISL_891955, EPI_ISL_891956, EPI_ISL_891957, EPI_ISL_891959, EPI_ISL_891960, EPI_ISL_891961, EPI_ISL_891962, EPI_ISL_891963, EPI_ISL_891964, EPI_ISL_891965, EPI_ISL_891966, EPI_ISL_891968, EPI_ISL_891969, EPI_ISL_891970, EPI_ISL_891971, EPI_ISL_892002, EPI_ISL_892005, EPI_ISL_892006, EPI_ISL_892009, EPI_ISL_892010, EPI_ISL_892011, EPI_ISL_892012			
see above	Lighthouse Lab in Alderley Park	Wellcome Sanger Institute for the COVID-19 Genomics UK (COG-UK) Consortium	Jacquelyn Wynn, Mairead Hyland, The Lighthouse Lab in Alderley Park and Alex Alderton, Roberto Amato, Sonia Goncalves, Ewan Harrison, David K. Jackson, Ian Johnston, Dominic Kwiatkowski, Cordelia Langford, John Sillitoe on behalf of the Wellcome Sanger Institute COVID-19 Surveillance Team
EPI_ISL_896109	Abklärungs- und _Teststation BL	University Hospital Basel, Clinical Bacteriology	Tim Roloff, Madlen Stange, Helena MB Seth-Smith, Alfredo Mari, Karoline Leuzinger, Julia Bielicki, Samuel Erny, Thomas Goetz, Juerg Sommer, Manuel Battegay, Hans Hirsch, Adrian Egli
EPI_ISL_896218, EPI_ISL_896224, EPI_ISL_896229	SUNY UPSTATE MEDICAL UNIVERSITY	Wadsworth Center, New York State Department of Health	Kirsten St. George, Daryl M. Lamson, Alexis Russel, Matthew Shudt, Melissa A Leisner, Jonathan Plitnick, Navjot Singh, John Kelly, Erasmus Schneider, Erica Lasek-Nesselquist
EPI_ISL_896236, EPI_ISL_896237, EPI_ISL_896238, EPI_ISL_896239	Columbia University Irving Medical Center	Wadsworth Center, New York State Department of Health	Kirsten St. George, Daryl M. Lamson, Alexis Russel, Matthew Shudt, Melissa A Leisner, Jonathan Plitnick, Navjot Singh, John Kelly, Erasmus Schneider, Erica Lasek-Nesselquist
EPI_ISL_896278, EPI_ISL_896281, EPI_ISL_896282, EPI_ISL_896286, EPI_ISL_896289, EPI_ISL_896291	SUNY UPSTATE MEDICAL UNIVERSITY	Wadsworth Center, New York State Department of Health	Kirsten St. George, Daryl M. Lamson, Alexis Russel, Matthew Shudt, Melissa A Leisner, Jonathan Plitnick, Navjot Singh, John Kelly, Erasmus Schneider, Erica Lasek-Nesselquist
EPI_ISL_896307, EPI_ISL_896308, EPI_ISL_896309	Wadsworth Center, New York State Department of Health	Wadsworth Center, New York State Department of Health	Kirsten St. George, Daryl M. Lamson, Alexis Russel, Matthew Shudt, Melissa A Leisner, Jonathan Plitnick, Navjot Singh, John Kelly, Erasmus Schneider, Erica Lasek-Nesselquist
EPI_ISL_896381	Suceava County Emergency Hospital	"Stefan cel Mare" University Metagenomics Lab	Lobiuc Andrei, Gheorghita Roxana
EPI_ISL_896447, EPI_ISL_896448, EPI_ISL_896449, EPI_ISL_896450, EPI_ISL_896454, EPI_ISL_896458, EPI_ISL_896459, EPI_ISL_896460, EPI_ISL_896466, EPI_ISL_896467, EPI_ISL_896468, EPI_ISL_896473, EPI_ISL_896476, EPI_ISL_896477, EPI_ISL_896478, EPI_ISL_896479, EPI_ISL_896480, EPI_ISL_896481, EPI_ISL_896482, EPI_ISL_896483, EPI_ISL_896484, EPI_ISL_896485, EPI_ISL_896486, EPI_ISL_896489, EPI_ISL_896490, EPI_ISL_896491, EPI_ISL_896492			
see above	KU Leuven, Rega Institute, Clinical and Epidemiological Virology	KU Leuven, Rega Institute, Clinical and Epidemiological Virology	Tony Wawina-Bokalanga, Bert Vanmechelen, Joan Marti-Carerras, Piet Maes
EPI_ISL_896503, EPI_ISL_896514, EPI_ISL_896516, EPI_ISL_896517, EPI_ISL_896518, EPI_ISL_896519	URMC LABS	Wadsworth Center, New York State Department of Health	Kirsten St. George, Daryl M. Lamson, Alexis Russel, Matthew Shudt, Melissa A Leisner, Jonathan Plitnick, Navjot Singh, John Kelly, Erasmus Schneider, Erica Lasek-Nesselquist
EPI_ISL_897599, EPI_ISL_897600, EPI_ISL_897601, EPI_ISL_897602, EPI_ISL_897607, EPI_ISL_897608, EPI_ISL_897609, EPI_ISL_897610, EPI_ISL_897612, EPI_ISL_897613, EPI_ISL_897614, EPI_ISL_897615, EPI_ISL_897619, EPI_ISL_897620, EPI_ISL_897626, EPI_ISL_897627, EPI_ISL_897628, EPI_ISL_897629, EPI_ISL_897630, EPI_ISL_897631, EPI_ISL_897632, EPI_ISL_897633, EPI_ISL_897634, EPI_ISL_897635, EPI_ISL_897636, EPI_ISL_897637, EPI_ISL_897638, EPI_ISL_897639, EPI_ISL_897640, EPI_ISL_897641, EPI_ISL_897642, EPI_ISL_897643, EPI_ISL_897644, EPI_ISL_897645, EPI_ISL_897646, EPI_ISL_897678, EPI_ISL_897683, EPI_ISL_897686, EPI_ISL_897696, EPI_ISL_897698, EPI_ISL_897701, EPI_ISL_897702, EPI_ISL_897703, EPI_ISL_897709, EPI_ISL_897710, EPI_ISL_897715, EPI_ISL_897720, EPI_ISL_897771, EPI_ISL_897772, EPI_ISL_897773, EPI_ISL_897774, EPI_ISL_897775, EPI_ISL_897776, EPI_ISL_897777, EPI_ISL_897778, EPI_ISL_897779, EPI_ISL_897780, EPI_ISL_897781, EPI_ISL_897782, EPI_ISL_897783, EPI_ISL_897784, EPI_ISL_897785, EPI_ISL_897786, EPI_ISL_897787, EPI_ISL_897788, EPI_ISL_897789, EPI_ISL_897790, EPI_ISL_897791, EPI_ISL_897792, EPI_ISL_897793, EPI_ISL_897794, EPI_ISL_897795, EPI_ISL_897796, EPI_ISL_897797, EPI_ISL_897798, EPI_ISL_897799, EPI_ISL_897800, EPI_ISL_897801, EPI_ISL_897802, EPI_ISL_897803, EPI_ISL_897804, EPI_ISL_897805, EPI_ISL_897806, EPI_ISL_897807, EPI_ISL_897809, EPI_ISL_897810, EPI_ISL_897811, EPI_ISL_897812, EPI_ISL_897813, EPI_ISL_897814, EPI_ISL_897815, EPI_ISL_897816, EPI_ISL_897817, EPI_ISL_897818, EPI_ISL_897819, EPI_ISL_897820, EPI_ISL_897821, EPI_ISL_897822, EPI_ISL_897823, EPI_ISL_897824, EPI_ISL_897825, EPI_ISL_897826, EPI_ISL_897827, EPI_ISL_897828, EPI_ISL_897829, EPI_ISL_897830, EPI_ISL_897831, EPI_ISL_897832, EPI_ISL_897833, EPI_ISL_897834, EPI_ISL_897835, EPI_ISL_897836, EPI_ISL_897837, EPI_ISL_897838, EPI_ISL_897839, EPI_ISL_897840, EPI_ISL_897841, EPI_ISL_897842, EPI_ISL_897843, EPI_ISL_897844, EPI_ISL_897845, EPI_ISL_897859, EPI_ISL_897905, EPI_ISL_897920, EPI_ISL_897921, EPI_ISL_897922, EPI_ISL_897923, EPI_ISL_897929, EPI_ISL_897930, EPI_ISL_897953, EPI_ISL_897967, EPI_ISL_897968, EPI_ISL_897969, EPI_ISL_897970, EPI_ISL_897971, EPI_ISL_897972, EPI_ISL_897973, EPI_ISL_897974, EPI_ISL_897975, EPI_ISL_897976, EPI_ISL_897977, EPI_ISL_897978, EPI_ISL_897979			
see above	University Hospitals of Geneva, Laboratory of Virology	HUG, Laboratory of Virology and the Health2030 Genome Center	Samuel Cordey, Ana Rita Goncalves, Laurent Kaiser, Lorenzo Cerutti, Henri Pegeot, Melyssa Elies, Deborah Penet, Keith Harshman, Ioannis Xenarios, Emmanouil Dermitzakis
EPI_ISL_900491, EPI_ISL_900493, EPI_ISL_900494, EPI_ISL_900495, EPI_ISL_900497, EPI_ISL_900498, EPI_ISL_900500	Althaia. Xarxa Assistencial Universitària de Manresa	IrsiCaixa - Can Ruti CovidSeq	Fundació irsiCaixa. Hospital Universitari Germans Trias i Pujol(HUGTiP), 2a planta, maternal Ctra Canyet s/n, Badalona Gloria Trujillo, Rafel Perez Vidal, Jaume Trape Pujol, Carolina Gonzalez Fernandez, Roger Paredes, Eulalia Grau, Bonaventura Clotet
EPI_ISL_902754	University Hospital Sant'Andrea-Sapienza	INMI Lazzaro Spallanzani IRCCS	B Bartolini, E Giombini, M Rueca, O Butera, F Messina, C.E.M Gruber, M Simmaco, I Santino, A Di Caro, MR Capobianchi
EPI_ISL_902755	IRCCS San Raffaele	INMI Lazzaro Spallanzani IRCCS	E Giombini, M Rueca, O Butera, F Messina, C.E.M Gruber, B Bartolini, D Russo, D Limongi, MR Capobianchi, A Di Caro
EPI_ISL_902942	Maryland Public Health Laboratory	Maryland Public Health Laboratory	Maryland Department of Health Laboratories Administration
EPI_ISL_903366	University Medical Center Hamburg Eppendorf	Heinrich Pette Institute, Leibniz Institute for Experimental Virology	Alexis Robitaille, Thomas Günther, Johannes Knobloch, Martin Aepfelbacher, Nicole Fischer, Adam Grundhoff
EPI_ISL_904071, EPI_ISL_904072, EPI_ISL_904073, EPI_ISL_904074, EPI_ISL_904075, EPI_ISL_904076, EPI_ISL_904077, EPI_ISL_904078, EPI_ISL_904079, EPI_ISL_904080, EPI_ISL_904081, EPI_ISL_904082, EPI_ISL_904083, EPI_ISL_904084, EPI_ISL_904085			
see above	New Mexico Department of Health Scientific Laboratory	New Mexico Department of Health Scientific Laboratory	Ellie Johnson, Anastacia Griego-Fisher, D'eldra Malone
EPI_ISL_904217, EPI_ISL_904218, EPI_ISL_904254, EPI_ISL_904603, EPI_ISL_904604, EPI_ISL_904605, EPI_ISL_904606	Dutch COVID-19 response team	Erasmus Medical Center	Bas Oude Munnink, Reina Sikkema, David Nieuwenhuijse, Irina Chestakova, Anne van der Linden, Marjan Boter, Emmanuelle Munger, Corine GeurtsvanKessel, Annemiek van der Eijk, Richard Molenkamp, Marion Koopmans, on behalf of the Dutch national COVID-19 response team.
EPI_ISL_904661, EPI_ISL_904662, EPI_ISL_904663, EPI_ISL_904675, EPI_ISL_904676, EPI_ISL_904679, EPI_ISL_904680, EPI_ISL_904681, EPI_ISL_904685, EPI_ISL_904713, EPI_ISL_904734, EPI_ISL_904739, EPI_ISL_904745, EPI_ISL_904758, EPI_ISL_904763, EPI_ISL_904772, EPI_ISL_904773, EPI_ISL_904774, EPI_ISL_904777, EPI_ISL_904780, EPI_ISL_904790, EPI_ISL_904793, EPI_ISL_904808, EPI_ISL_904809, EPI_ISL_904811, EPI_ISL_904813, EPI_ISL_904830, EPI_ISL_904831, EPI_ISL_904836, EPI_ISL_904837, EPI_ISL_904838, EPI_ISL_904839, EPI_ISL_904842, EPI_ISL_904868, EPI_ISL_904881, EPI_ISL_904884, EPI_ISL_905179, EPI_ISL_905184, EPI_ISL_905189, EPI_ISL_905198, EPI_ISL_905199, EPI_ISL_905205, EPI_ISL_905206, EPI_ISL_905207, EPI_ISL_905208, EPI_ISL_905209, EPI_ISL_905210, EPI_ISL_905234, EPI_ISL_905236, EPI_ISL_905292, EPI_ISL_905293, EPI_ISL_905311, EPI_ISL_905319, EPI_ISL_905320, EPI_ISL_905330, EPI_ISL_905331, EPI_ISL_905332, EPI_ISL_905338, EPI_ISL_905341, EPI_ISL_905342, EPI_ISL_905343, EPI_ISL_905344, EPI_ISL_905346, EPI_ISL_905347, EPI_ISL_905348			
see above	Dutch COVID-19 response team	National Institute for Public Health and the Environment (RIVM)	Adam Meijer, Harry Vennema, Dirk Eggink, Jeroen Cremer, Sharon van den Brink, Bas van der Veer, AnneMarie van den Brandt, Florian Zwagemaker, Dennis Schmitz, Chantal Reusken, on behalf of the national COVID-19 response team
EPI_ISL_905788, EPI_ISL_905789,	UCLA Clinical Micro Lab	Los Angeles County PHL	P. Hemarajata et al.

EPI_ISL_905790			
EPI_ISL_906068, EPI_ISL_906069	Instituto Adolfo Lutz - Regional de Campinas	Instituto Adolfo Lutz, Interdiciplinary Procedures Center, Strategic Laboratory	Claudio Tavares Sacchi, Claudia Regina Gonçalves, Erica Valessa Ramos Gomes, Karoline Rodrigues Campos
EPI_ISL_906190, EPI_ISL_906191, EPI_ISL_906192, EPI_ISL_906193, EPI_ISL_906194, EPI_ISL_906195, EPI_ISL_906196, EPI_ISL_906197, EPI_ISL_906198, EPI_ISL_906199, EPI_ISL_906201, EPI_ISL_906202, EPI_ISL_906203, EPI_ISL_906204, EPI_ISL_906206, EPI_ISL_906208, EPI_ISL_906209, EPI_ISL_906211, EPI_ISL_906213, EPI_ISL_906214, EPI_ISL_906216, EPI_ISL_906217, EPI_ISL_906219, EPI_ISL_906227, EPI_ISL_906229, EPI_ISL_906233, EPI_ISL_906238, EPI_ISL_906240, EPI_ISL_906241, EPI_ISL_906242, EPI_ISL_906246, EPI_ISL_906249, EPI_ISL_906266, EPI_ISL_906268, EPI_ISL_906269, EPI_ISL_906270, EPI_ISL_906271, EPI_ISL_906272			
see above	University of Wisconsin-Madison AIDS Vaccine Research Laboratories	University of Wisconsin-Madison AIDS Vaccine Research Laboratories	Gage Moreno, Katarina Braun, et al. AIDS Vaccine Research Laboratories
EPI_ISL_906558	Laboratorio de Salud Publica de Amazonas	Instituto Nacional de Salud- Dirección de Investigación en Salud Pública, Universidad de los Andes- Applied genomics research group, Vicerrectoria de Investigación y Creación, Universidad de los Andes- Systems and Computing Engineering Department	Katherine Laiton-Donato, Diego A. Álvarez-Díaz, Carlos Franco-Muñoz, Mauricio Pacheco-Montealegre, Héctor Alejandro Ruiz-Moreno, Maria T. Herrera-Sepúlveda, Diego Andrés Prada, Jhonnatan Reales-González, Sheryll Corchuelo, Julian Naizaque, Gerardo Santamaría Jorge Duitama, Laura Natalia Gonzalez, Jorge Ivan Diaz, Silvia Restrepo-Restrepo, Magdalena Wiesner, Martha Lucia Ospina Martinez, Marcela Mercado-Reyes
EPI_ISL_906652, EPI_ISL_906653, EPI_ISL_906654	Maine Health and Environmental Testing Laboratory (Maine HETL)	Tewhey Lab, The Jackson Laboratory	Matluk,N., Dewey,H., Iosue,F., Barter,M., Lynch,R., Munger,H. and Tewhey,R.
EPI_ISL_906794, EPI_ISL_906795	Norwegian Institute of Public Health, Department of Virology	Norwegian Institute of Public Health, Department of Virology	Kathrine Stene-Johansen, Kamilla Heddeland Instefjord, Hilde Elshaug, Atiya R Ali,Marie Paulsen Madsen, Rasmus Riis Kopperud, Hilde Vollan, Karoline Bragstad, Olav Hungnes
EPI_ISL_906797	Dept. of Medical Microbiology, Stavanger University Hospital, Helse Stavanger HF	Norwegian Institute of Public Health, Department of Virology	Kathrine Stene-Johansen, Kamilla Heddeland Instefjord, Hilde Elshaug, Atiya R Ali,Marie Paulsen Madsen, Rasmus Riis Kopperud, Hilde Vollan, Karoline Bragstad, Olav Hungnes
EPI_ISL_906815, EPI_ISL_906816	Medical Microbiology Unit, Department for Laboratory Medicine, Drammen Hospital, Vestre Viken Health Trust,	Norwegian Institute of Public Health, Department of Virology	Kathrine Stene-Johansen, Kamilla Heddeland Instefjord, Hilde Elshaug, Atiya R Ali,Marie Paulsen Madsen, Rasmus Riis Kopperud, Hilde Vollan, Karoline Bragstad, Olav Hungnes
EPI_ISL_907725, EPI_ISL_907775, EPI_ISL_907779, EPI_ISL_907783, EPI_ISL_907841, EPI_ISL_907884, EPI_ISL_907909, EPI_ISL_907933, EPI_ISL_907978, EPI_ISL_908001	Lighthouse Lab in Alderley Park	Wellcome Sanger Institute for the COVID-19 Genomics UK (COG-UK) Consortium	Jacquelyn Wynn, Mairead Hyland, The Lighthouse Lab in Alderley Park and Alex Alderton, Roberto Amato, Sonia Goncalves, Ewan Harrison, David K. Jackson, Ian Johnston, Dominic Kwiatkowski, Cordelia Langford, John Sillitoe on behalf of the Wellcome Sanger Institute COVID-19 Surveillance Team
EPI_ISL_908035	Lighthouse Lab in Cambridge	Wellcome Sanger Institute for the COVID-19 Genomics UK (COG-UK) Consortium	Rob Howes, The Lighthouse Lab in Cambridge and Alex Alderton, Roberto Amato, Sonia Goncalves, Ewan Harrison, David K. Jackson, Ian Johnston, Dominic Kwiatkowski, Cordelia Langford, John Sillitoe on behalf of the Wellcome Sanger Institute COVID-19 Surveillance Team
EPI_ISL_908036	Lighthouse Lab in Glasgow	Wellcome Sanger Institute for the COVID-19 Genomics UK (COG-UK) Consortium	Harper VanSteenhouse, Yumi Kasai, David Gray, Carol Clugston, Anna Dominiczak and Alex Alderton, Roberto Amato, Sonia Goncalves, Ewan Harrison, David K. Jackson, Ian Johnston, Dominic Kwiatkowski, Cordelia Langford, John Sillitoe on behalf of the Wellcome Sanger Institute COVID-19 Surveillance Team
EPI_ISL_908037, EPI_ISL_908038, EPI_ISL_908039, EPI_ISL_908040, EPI_ISL_908041, EPI_ISL_908042, EPI_ISL_908043, EPI_ISL_908044, EPI_ISL_908045, EPI_ISL_908046, EPI_ISL_908047, EPI_ISL_908048, EPI_ISL_908049, EPI_ISL_908050			
see above	Lighthouse Lab in Cambridge	Wellcome Sanger Institute for the COVID-19 Genomics UK (COG-UK) Consortium	Rob Howes, The Lighthouse Lab in Cambridge and Alex Alderton, Roberto Amato, Sonia Goncalves, Ewan Harrison, David K. Jackson, Ian Johnston, Dominic Kwiatkowski, Cordelia Langford, John Sillitoe on behalf of the Wellcome Sanger Institute COVID-19 Surveillance Team
EPI_ISL_908051	Lighthouse Lab in Glasgow	Wellcome Sanger Institute for the COVID-19 Genomics UK (COG-UK) Consortium	Harper VanSteenhouse, Yumi Kasai, David Gray, Carol Clugston, Anna Dominiczak and Alex Alderton, Roberto Amato, Sonia Goncalves, Ewan Harrison, David K. Jackson, Ian Johnston, Dominic Kwiatkowski, Cordelia Langford, John Sillitoe on behalf of the Wellcome Sanger Institute COVID-19 Surveillance Team
EPI_ISL_908052, EPI_ISL_908053	Lighthouse Lab in Cambridge	Wellcome Sanger Institute for the COVID-19 Genomics UK (COG-UK) Consortium	Rob Howes, The Lighthouse Lab in Cambridge and Alex Alderton, Roberto Amato, Sonia Goncalves, Ewan Harrison, David K. Jackson, Ian Johnston, Dominic Kwiatkowski, Cordelia Langford, John Sillitoe on behalf of the Wellcome Sanger Institute COVID-19 Surveillance Team
EPI_ISL_908054	Lighthouse Lab in Glasgow	Wellcome Sanger Institute for the COVID-19 Genomics UK (COG-UK) Consortium	Harper VanSteenhouse, Yumi Kasai, David Gray, Carol Clugston, Anna Dominiczak and Alex Alderton, Roberto Amato, Sonia Goncalves, Ewan Harrison, David K. Jackson, Ian Johnston, Dominic Kwiatkowski, Cordelia Langford, John Sillitoe on behalf of the Wellcome Sanger Institute COVID-19 Surveillance Team
EPI_ISL_908055	Lighthouse Lab in Cambridge	Wellcome Sanger Institute for the COVID-19 Genomics UK (COG-UK) Consortium	Rob Howes, The Lighthouse Lab in Cambridge and Alex Alderton, Roberto Amato, Sonia Goncalves, Ewan Harrison, David K. Jackson, Ian Johnston, Dominic Kwiatkowski, Cordelia Langford, John Sillitoe on behalf of the Wellcome Sanger Institute COVID-19 Surveillance Team
EPI_ISL_908056	Lighthouse Lab in Glasgow	Wellcome Sanger Institute for the COVID-19 Genomics UK (COG-UK) Consortium	Harper VanSteenhouse, Yumi Kasai, David Gray, Carol Clugston, Anna Dominiczak and Alex Alderton, Roberto Amato, Sonia Goncalves, Ewan Harrison, David K. Jackson, Ian Johnston, Dominic Kwiatkowski, Cordelia Langford, John Sillitoe on behalf of the Wellcome Sanger Institute COVID-19 Surveillance Team
EPI_ISL_908057, EPI_ISL_908058	Lighthouse Lab in Cambridge	Wellcome Sanger Institute for the COVID-19 Genomics UK (COG-UK) Consortium	Rob Howes, The Lighthouse Lab in Cambridge and Alex Alderton, Roberto Amato, Sonia Goncalves, Ewan Harrison, David K. Jackson, Ian Johnston, Dominic Kwiatkowski, Cordelia Langford, John Sillitoe on behalf of the Wellcome Sanger Institute COVID-19 Surveillance Team
EPI_ISL_908059, EPI_ISL_908060, EPI_ISL_908061	Lighthouse Lab in Glasgow	Wellcome Sanger Institute for the COVID-19 Genomics UK (COG-UK) Consortium	Harper VanSteenhouse, Yumi Kasai, David Gray, Carol Clugston, Anna Dominiczak and Alex Alderton, Roberto Amato, Sonia Goncalves, Ewan Harrison, David K. Jackson, Ian Johnston, Dominic Kwiatkowski, Cordelia Langford, John Sillitoe on behalf of the Wellcome Sanger Institute COVID-19 Surveillance Team
EPI_ISL_908062, EPI_ISL_908063	Lighthouse Lab in Cambridge	Wellcome Sanger Institute for the COVID-19 Genomics UK (COG-UK) Consortium	Rob Howes, The Lighthouse Lab in Cambridge and Alex Alderton, Roberto Amato, Sonia Goncalves, Ewan Harrison, David K. Jackson, Ian Johnston, Dominic Kwiatkowski, Cordelia Langford, John Sillitoe on behalf of the Wellcome Sanger Institute COVID-19 Surveillance Team
EPI_ISL_908064	Lighthouse Lab in Glasgow	Wellcome Sanger Institute for the COVID-19 Genomics UK (COG-UK) Consortium	Harper VanSteenhouse, Yumi Kasai, David Gray, Carol Clugston, Anna Dominiczak and Alex Alderton, Roberto Amato, Sonia Goncalves, Ewan Harrison, David K. Jackson, Ian Johnston, Dominic Kwiatkowski, Cordelia Langford, John Sillitoe on behalf of the Wellcome Sanger Institute COVID-19 Surveillance Team
EPI_ISL_908065, EPI_ISL_908066, EPI_ISL_908067, EPI_ISL_908068, EPI_ISL_908069	Lighthouse Lab in Cambridge	Wellcome Sanger Institute for the COVID-19 Genomics UK (COG-UK) Consortium	Rob Howes, The Lighthouse Lab in Cambridge and Alex Alderton, Roberto Amato, Sonia Goncalves, Ewan Harrison, David K. Jackson, Ian Johnston, Dominic Kwiatkowski, Cordelia Langford, John Sillitoe on behalf of the Wellcome Sanger Institute COVID-19 Surveillance Team
EPI_ISL_908070	Lighthouse Lab in Glasgow	Wellcome Sanger Institute for the COVID-19 Genomics UK (COG-UK) Consortium	Harper VanSteenhouse, Yumi Kasai, David Gray, Carol Clugston, Anna Dominiczak and Alex Alderton, Roberto Amato, Sonia Goncalves, Ewan Harrison, David K. Jackson, Ian Johnston, Dominic Kwiatkowski, Cordelia Langford, John Sillitoe on behalf of the Wellcome Sanger Institute COVID-19 Surveillance Team
EPI_ISL_908071	Lighthouse Lab in Cambridge	Wellcome Sanger Institute for the COVID-19 Genomics UK (COG-UK) Consortium	Rob Howes, The Lighthouse Lab in Cambridge and Alex Alderton, Roberto Amato, Sonia Goncalves, Ewan Harrison, David K. Jackson, Ian Johnston, Dominic Kwiatkowski, Cordelia Langford, John Sillitoe on behalf of the Wellcome Sanger Institute COVID-19 Surveillance Team
EPI_ISL_908072, EPI_ISL_908073, EPI_ISL_908074, EPI_ISL_908075	Lighthouse Lab in Glasgow	Wellcome Sanger Institute for the COVID-19 Genomics UK (COG-UK) Consortium	Harper VanSteenhouse, Yumi Kasai, David Gray, Carol Clugston, Anna Dominiczak and Alex Alderton, Roberto Amato, Sonia Goncalves, Ewan Harrison, David K. Jackson, Ian Johnston, Dominic Kwiatkowski, Cordelia Langford, John Sillitoe on behalf of the Wellcome Sanger Institute COVID-19 Surveillance Team
EPI_ISL_908076, EPI_ISL_908077, EPI_ISL_908078, EPI_ISL_908079, EPI_ISL_908080	Lighthouse Lab in Cambridge	Wellcome Sanger Institute for the COVID-19 Genomics UK (COG-UK) Consortium	Rob Howes, The Lighthouse Lab in Cambridge and Alex Alderton, Roberto Amato, Sonia Goncalves, Ewan Harrison, David K. Jackson, Ian Johnston, Dominic Kwiatkowski, Cordelia Langford, John Sillitoe on behalf of the Wellcome Sanger Institute COVID-19 Surveillance Team
EPI_ISL_908081	Lighthouse Lab in Glasgow	Wellcome Sanger Institute for the COVID-19 Genomics UK (COG-UK) Consortium	Harper VanSteenhouse, Yumi Kasai, David Gray, Carol Clugston, Anna Dominiczak and Alex Alderton, Roberto Amato, Sonia Goncalves, Ewan Harrison, David K. Jackson, Ian Johnston, Dominic Kwiatkowski, Cordelia Langford, John Sillitoe on behalf of the Wellcome Sanger Institute COVID-19 Surveillance

			Team
EPI_ISL_908082, EPI_ISL_908083	Lighthouse Lab in Cambridge	Wellcome Sanger Institute for the COVID-19 Genomics UK (COG-UK) Consortium	Rob Howes, The Lighthouse Lab in Cambridge and Alex Alderton, Roberto Amato, Sonia Goncalves, Ewan Harrison, David K. Jackson, Ian Johnston, Dominic Kwiatkowski, Cordelia Langford, John Sillitoe on behalf of the Wellcome Sanger Institute COVID-19 Surveillance Team
EPI_ISL_908084, EPI_ISL_908085, EPI_ISL_908086, EPI_ISL_908087	Lighthouse Lab in Glasgow	Wellcome Sanger Institute for the COVID-19 Genomics UK (COG-UK) Consortium	Harper VanSteenhouse, Yumi Kasai, David Gray, Carol Clugston, Anna Dominiczak and Alex Alderton, Roberto Amato, Sonia Goncalves, Ewan Harrison, David K. Jackson, Ian Johnston, Dominic Kwiatkowski, Cordelia Langford, John Sillitoe on behalf of the Wellcome Sanger Institute COVID-19 Surveillance Team
EPI_ISL_908088	Lighthouse Lab in Cambridge	Wellcome Sanger Institute for the COVID-19 Genomics UK (COG-UK) Consortium	Rob Howes, The Lighthouse Lab in Cambridge and Alex Alderton, Roberto Amato, Sonia Goncalves, Ewan Harrison, David K. Jackson, Ian Johnston, Dominic Kwiatkowski, Cordelia Langford, John Sillitoe on behalf of the Wellcome Sanger Institute COVID-19 Surveillance Team
EPI_ISL_908089	Lighthouse Lab in Glasgow	Wellcome Sanger Institute for the COVID-19 Genomics UK (COG-UK) Consortium	Harper VanSteenhouse, Yumi Kasai, David Gray, Carol Clugston, Anna Dominiczak and Alex Alderton, Roberto Amato, Sonia Goncalves, Ewan Harrison, David K. Jackson, Ian Johnston, Dominic Kwiatkowski, Cordelia Langford, John Sillitoe on behalf of the Wellcome Sanger Institute COVID-19 Surveillance Team
EPI_ISL_908090, EPI_ISL_908091	Lighthouse Lab in Cambridge	Wellcome Sanger Institute for the COVID-19 Genomics UK (COG-UK) Consortium	Rob Howes, The Lighthouse Lab in Cambridge and Alex Alderton, Roberto Amato, Sonia Goncalves, Ewan Harrison, David K. Jackson, Ian Johnston, Dominic Kwiatkowski, Cordelia Langford, John Sillitoe on behalf of the Wellcome Sanger Institute COVID-19 Surveillance Team
EPI_ISL_908092	Lighthouse Lab in Glasgow	Wellcome Sanger Institute for the COVID-19 Genomics UK (COG-UK) Consortium	Harper VanSteenhouse, Yumi Kasai, David Gray, Carol Clugston, Anna Dominiczak and Alex Alderton, Roberto Amato, Sonia Goncalves, Ewan Harrison, David K. Jackson, Ian Johnston, Dominic Kwiatkowski, Cordelia Langford, John Sillitoe on behalf of the Wellcome Sanger Institute COVID-19 Surveillance Team
EPI_ISL_908093	Lighthouse Lab in Cambridge	Wellcome Sanger Institute for the COVID-19 Genomics UK (COG-UK) Consortium	Rob Howes, The Lighthouse Lab in Cambridge and Alex Alderton, Roberto Amato, Sonia Goncalves, Ewan Harrison, David K. Jackson, Ian Johnston, Dominic Kwiatkowski, Cordelia Langford, John Sillitoe on behalf of the Wellcome Sanger Institute COVID-19 Surveillance Team
EPI_ISL_908094	Lighthouse Lab in Glasgow	Wellcome Sanger Institute for the COVID-19 Genomics UK (COG-UK) Consortium	Harper VanSteenhouse, Yumi Kasai, David Gray, Carol Clugston, Anna Dominiczak and Alex Alderton, Roberto Amato, Sonia Goncalves, Ewan Harrison, David K. Jackson, Ian Johnston, Dominic Kwiatkowski, Cordelia Langford, John Sillitoe on behalf of the Wellcome Sanger Institute COVID-19 Surveillance Team
EPI_ISL_908095	Lighthouse Lab in Cambridge	Wellcome Sanger Institute for the COVID-19 Genomics UK (COG-UK) Consortium	Rob Howes, The Lighthouse Lab in Cambridge and Alex Alderton, Roberto Amato, Sonia Goncalves, Ewan Harrison, David K. Jackson, Ian Johnston, Dominic Kwiatkowski, Cordelia Langford, John Sillitoe on behalf of the Wellcome Sanger Institute COVID-19 Surveillance Team
EPI_ISL_908096, EPI_ISL_908097	Lighthouse Lab in Glasgow	Wellcome Sanger Institute for the COVID-19 Genomics UK (COG-UK) Consortium	Harper VanSteenhouse, Yumi Kasai, David Gray, Carol Clugston, Anna Dominiczak and Alex Alderton, Roberto Amato, Sonia Goncalves, Ewan Harrison, David K. Jackson, Ian Johnston, Dominic Kwiatkowski, Cordelia Langford, John Sillitoe on behalf of the Wellcome Sanger Institute COVID-19 Surveillance Team
EPI_ISL_908098, EPI_ISL_908099, EPI_ISL_908100, EPI_ISL_908101, EPI_ISL_908102, EPI_ISL_908103	Lighthouse Lab in Cambridge	Wellcome Sanger Institute for the COVID-19 Genomics UK (COG-UK) Consortium	Rob Howes, The Lighthouse Lab in Cambridge and Alex Alderton, Roberto Amato, Sonia Goncalves, Ewan Harrison, David K. Jackson, Ian Johnston, Dominic Kwiatkowski, Cordelia Langford, John Sillitoe on behalf of the Wellcome Sanger Institute COVID-19 Surveillance Team
EPI_ISL_908104	Lighthouse Lab in Glasgow	Wellcome Sanger Institute for the COVID-19 Genomics UK (COG-UK) Consortium	Harper VanSteenhouse, Yumi Kasai, David Gray, Carol Clugston, Anna Dominiczak and Alex Alderton, Roberto Amato, Sonia Goncalves, Ewan Harrison, David K. Jackson, Ian Johnston, Dominic Kwiatkowski, Cordelia Langford, John Sillitoe on behalf of the Wellcome Sanger Institute COVID-19 Surveillance Team
EPI_ISL_908105, EPI_ISL_908106, EPI_ISL_908107, EPI_ISL_908108, EPI_ISL_908109, EPI_ISL_908110, EPI_ISL_908111, EPI_ISL_908112	Lighthouse Lab in Cambridge	Wellcome Sanger Institute for the COVID-19 Genomics UK (COG-UK) Consortium	Rob Howes, The Lighthouse Lab in Cambridge and Alex Alderton, Roberto Amato, Sonia Goncalves, Ewan Harrison, David K. Jackson, Ian Johnston, Dominic Kwiatkowski, Cordelia Langford, John Sillitoe on behalf of the Wellcome Sanger Institute COVID-19 Surveillance Team
EPI_ISL_908113	Lighthouse Lab in Glasgow	Wellcome Sanger Institute for the COVID-19 Genomics UK (COG-UK) Consortium	Harper VanSteenhouse, Yumi Kasai, David Gray, Carol Clugston, Anna Dominiczak and Alex Alderton, Roberto Amato, Sonia Goncalves, Ewan Harrison, David K. Jackson, Ian Johnston, Dominic Kwiatkowski, Cordelia Langford, John Sillitoe on behalf of the Wellcome Sanger Institute COVID-19 Surveillance Team
EPI_ISL_908114, EPI_ISL_908115	Lighthouse Lab in Cambridge	Wellcome Sanger Institute for the COVID-19 Genomics UK (COG-UK) Consortium	Rob Howes, The Lighthouse Lab in Cambridge and Alex Alderton, Roberto Amato, Sonia Goncalves, Ewan Harrison, David K. Jackson, Ian Johnston, Dominic Kwiatkowski, Cordelia Langford, John Sillitoe on behalf of the Wellcome Sanger Institute COVID-19 Surveillance Team
EPI_ISL_908116	Lighthouse Lab in Glasgow	Wellcome Sanger Institute for the COVID-19 Genomics UK (COG-UK) Consortium	Harper VanSteenhouse, Yumi Kasai, David Gray, Carol Clugston, Anna Dominiczak and Alex Alderton, Roberto Amato, Sonia Goncalves, Ewan Harrison, David K. Jackson, Ian Johnston, Dominic Kwiatkowski, Cordelia Langford, John Sillitoe on behalf of the Wellcome Sanger Institute COVID-19 Surveillance Team
EPI_ISL_908117, EPI_ISL_908118	Lighthouse Lab in Cambridge	Wellcome Sanger Institute for the COVID-19 Genomics UK (COG-UK) Consortium	Rob Howes, The Lighthouse Lab in Cambridge and Alex Alderton, Roberto Amato, Sonia Goncalves, Ewan Harrison, David K. Jackson, Ian Johnston, Dominic Kwiatkowski, Cordelia Langford, John Sillitoe on behalf of the Wellcome Sanger Institute COVID-19 Surveillance Team
EPI_ISL_908119, EPI_ISL_908120	Lighthouse Lab in Glasgow	Wellcome Sanger Institute for the COVID-19 Genomics UK (COG-UK) Consortium	Harper VanSteenhouse, Yumi Kasai, David Gray, Carol Clugston, Anna Dominiczak and Alex Alderton, Roberto Amato, Sonia Goncalves, Ewan Harrison, David K. Jackson, Ian Johnston, Dominic Kwiatkowski, Cordelia Langford, John Sillitoe on behalf of the Wellcome Sanger Institute COVID-19 Surveillance Team
EPI_ISL_908121	Lighthouse Lab in Cambridge	Wellcome Sanger Institute for the COVID-19 Genomics UK (COG-UK) Consortium	Rob Howes, The Lighthouse Lab in Cambridge and Alex Alderton, Roberto Amato, Sonia Goncalves, Ewan Harrison, David K. Jackson, Ian Johnston, Dominic Kwiatkowski, Cordelia Langford, John Sillitoe on behalf of the Wellcome Sanger Institute COVID-19 Surveillance Team
EPI_ISL_908122	Lighthouse Lab in Glasgow	Wellcome Sanger Institute for the COVID-19 Genomics UK (COG-UK) Consortium	Harper VanSteenhouse, Yumi Kasai, David Gray, Carol Clugston, Anna Dominiczak and Alex Alderton, Roberto Amato, Sonia Goncalves, Ewan Harrison, David K. Jackson, Ian Johnston, Dominic Kwiatkowski, Cordelia Langford, John Sillitoe on behalf of the Wellcome Sanger Institute COVID-19 Surveillance Team
EPI_ISL_908123, EPI_ISL_908124, EPI_ISL_908125	Lighthouse Lab in Cambridge	Wellcome Sanger Institute for the COVID-19 Genomics UK (COG-UK) Consortium	Rob Howes, The Lighthouse Lab in Cambridge and Alex Alderton, Roberto Amato, Sonia Goncalves, Ewan Harrison, David K. Jackson, Ian Johnston, Dominic Kwiatkowski, Cordelia Langford, John Sillitoe on behalf of the Wellcome Sanger Institute COVID-19 Surveillance Team
EPI_ISL_908126	Lighthouse Lab in Glasgow	Wellcome Sanger Institute for the COVID-19 Genomics UK (COG-UK) Consortium	Harper VanSteenhouse, Yumi Kasai, David Gray, Carol Clugston, Anna Dominiczak and Alex Alderton, Roberto Amato, Sonia Goncalves, Ewan Harrison, David K. Jackson, Ian Johnston, Dominic Kwiatkowski, Cordelia Langford, John Sillitoe on behalf of the Wellcome Sanger Institute COVID-19 Surveillance Team
EPI_ISL_908127, EPI_ISL_908128, EPI_ISL_908129, EPI_ISL_908130	Lighthouse Lab in Cambridge	Wellcome Sanger Institute for the COVID-19 Genomics UK (COG-UK) Consortium	Rob Howes, The Lighthouse Lab in Cambridge and Alex Alderton, Roberto Amato, Sonia Goncalves, Ewan Harrison, David K. Jackson, Ian Johnston, Dominic Kwiatkowski, Cordelia Langford, John Sillitoe on behalf of the Wellcome Sanger Institute COVID-19 Surveillance Team
EPI_ISL_908131	Lighthouse Lab in Glasgow	Wellcome Sanger Institute for the COVID-19 Genomics UK (COG-UK) Consortium	Harper VanSteenhouse, Yumi Kasai, David Gray, Carol Clugston, Anna Dominiczak and Alex Alderton, Roberto Amato, Sonia Goncalves, Ewan Harrison, David K. Jackson, Ian Johnston, Dominic Kwiatkowski, Cordelia Langford, John Sillitoe on behalf of the Wellcome Sanger Institute COVID-19 Surveillance Team
EPI_ISL_908132, EPI_ISL_908133, EPI_ISL_908134, EPI_ISL_908135, EPI_ISL_908136, EPI_ISL_908137, EPI_ISL_908138	Lighthouse Lab in Cambridge	Wellcome Sanger Institute for the COVID-19 Genomics UK (COG-UK) Consortium	Rob Howes, The Lighthouse Lab in Cambridge and Alex Alderton, Roberto Amato, Sonia Goncalves, Ewan Harrison, David K. Jackson, Ian Johnston, Dominic Kwiatkowski, Cordelia Langford, John Sillitoe on behalf of the Wellcome Sanger Institute COVID-19 Surveillance Team
EPI_ISL_908139	Lighthouse Lab in Glasgow	Wellcome Sanger Institute for the COVID-19 Genomics UK (COG-UK) Consortium	Harper VanSteenhouse, Yumi Kasai, David Gray, Carol Clugston, Anna Dominiczak and Alex Alderton, Roberto Amato, Sonia Goncalves, Ewan Harrison, David K. Jackson, Ian Johnston, Dominic Kwiatkowski, Cordelia Langford, John Sillitoe on behalf of the Wellcome Sanger Institute COVID-19 Surveillance Team

EPI_ISL_908140, EPI_ISL_908141, EPI_ISL_908142, EPI_ISL_908143, EPI_ISL_908144, EPI_ISL_908145, EPI_ISL_908146, EPI_ISL_908147, EPI_ISL_908148, EPI_ISL_908149, EPI_ISL_908150

see above	Lighthouse Lab in Cambridge	Wellcome Sanger Institute for the COVID-19 Genomics UK	Rob Howes, The Lighthouse Lab in Cambridge and Alex Alderton, Roberto Amato, Sonia Goncalves, Ewan Harrison, David K. Jackson, Ian Johnston,

		(COG-UK) Consortium	Dominic Kwiatkowski, Cordelia Langford, John Sillitoe on behalf of the Wellcome Sanger Institute COVID-19 Surveillance Team
EPI_ISL_908151, EPI_ISL_908152	Lighthouse Lab in Glasgow	Wellcome Sanger Institute for the COVID-19 Genomics UK (COG-UK) Consortium	Harper VanSteenhouse, Yumi Kasai, David Gray, Carol Clugston, Anna Dominiczak and Alex Alderton, Roberto Amato, Sonia Goncalves, Ewan Harrison, David K. Jackson, Ian Johnston, Dominic Kwiatkowski, Cordelia Langford, John Sillitoe on behalf of the Wellcome Sanger Institute COVID-19 Surveillance Team
EPI_ISL_908153, EPI_ISL_908154, EPI_ISL_908155, EPI_ISL_908156, EPI_ISL_908157, EPI_ISL_908158, EPI_ISL_908159, EPI_ISL_908160, EPI_ISL_908161, EPI_ISL_908162, EPI_ISL_908163, EPI_ISL_908164, EPI_ISL_908165, EPI_ISL_908166, EPI_ISL_908167			
see above	Lighthouse Lab in Cambridge	Wellcome Sanger Institute for the COVID-19 Genomics UK (COG-UK) Consortium	Rob Howes, The Lighthouse Lab in Cambridge and Alex Alderton, Roberto Amato, Sonia Goncalves, Ewan Harrison, David K. Jackson, Ian Johnston, Dominic Kwiatkowski, Cordelia Langford, John Sillitoe on behalf of the Wellcome Sanger Institute COVID-19 Surveillance Team
EPI_ISL_908168	Lighthouse Lab in Glasgow	Wellcome Sanger Institute for the COVID-19 Genomics UK (COG-UK) Consortium	Harper VanSteenhouse, Yumi Kasai, David Gray, Carol Clugston, Anna Dominiczak and Alex Alderton, Roberto Amato, Sonia Goncalves, Ewan Harrison, David K. Jackson, Ian Johnston, Dominic Kwiatkowski, Cordelia Langford, John Sillitoe on behalf of the Wellcome Sanger Institute COVID-19 Surveillance Team
EPI_ISL_908169, EPI_ISL_908170	Lighthouse Lab in Cambridge	Wellcome Sanger Institute for the COVID-19 Genomics UK (COG-UK) Consortium	Rob Howes, The Lighthouse Lab in Cambridge and Alex Alderton, Roberto Amato, Sonia Goncalves, Ewan Harrison, David K. Jackson, Ian Johnston, Dominic Kwiatkowski, Cordelia Langford, John Sillitoe on behalf of the Wellcome Sanger Institute COVID-19 Surveillance Team
EPI_ISL_908171	Lighthouse Lab in Glasgow	Wellcome Sanger Institute for the COVID-19 Genomics UK (COG-UK) Consortium	Harper VanSteenhouse, Yumi Kasai, David Gray, Carol Clugston, Anna Dominiczak and Alex Alderton, Roberto Amato, Sonia Goncalves, Ewan Harrison, David K. Jackson, Ian Johnston, Dominic Kwiatkowski, Cordelia Langford, John Sillitoe on behalf of the Wellcome Sanger Institute COVID-19 Surveillance Team
EPI_ISL_908172, EPI_ISL_908173	Lighthouse Lab in Cambridge	Wellcome Sanger Institute for the COVID-19 Genomics UK (COG-UK) Consortium	Rob Howes, The Lighthouse Lab in Cambridge and Alex Alderton, Roberto Amato, Sonia Goncalves, Ewan Harrison, David K. Jackson, Ian Johnston, Dominic Kwiatkowski, Cordelia Langford, John Sillitoe on behalf of the Wellcome Sanger Institute COVID-19 Surveillance Team
EPI_ISL_908174	Lighthouse Lab in Glasgow	Wellcome Sanger Institute for the COVID-19 Genomics UK (COG-UK) Consortium	Harper VanSteenhouse, Yumi Kasai, David Gray, Carol Clugston, Anna Dominiczak and Alex Alderton, Roberto Amato, Sonia Goncalves, Ewan Harrison, David K. Jackson, Ian Johnston, Dominic Kwiatkowski, Cordelia Langford, John Sillitoe on behalf of the Wellcome Sanger Institute COVID-19 Surveillance Team
EPI_ISL_908175, EPI_ISL_908176	Lighthouse Lab in Cambridge	Wellcome Sanger Institute for the COVID-19 Genomics UK (COG-UK) Consortium	Rob Howes, The Lighthouse Lab in Cambridge and Alex Alderton, Roberto Amato, Sonia Goncalves, Ewan Harrison, David K. Jackson, Ian Johnston, Dominic Kwiatkowski, Cordelia Langford, John Sillitoe on behalf of the Wellcome Sanger Institute COVID-19 Surveillance Team
EPI_ISL_908177	Lighthouse Lab in Glasgow	Wellcome Sanger Institute for the COVID-19 Genomics UK (COG-UK) Consortium	Harper VanSteenhouse, Yumi Kasai, David Gray, Carol Clugston, Anna Dominiczak and Alex Alderton, Roberto Amato, Sonia Goncalves, Ewan Harrison, David K. Jackson, Ian Johnston, Dominic Kwiatkowski, Cordelia Langford, John Sillitoe on behalf of the Wellcome Sanger Institute COVID-19 Surveillance Team
EPI_ISL_908178	Lighthouse Lab in Cambridge	Wellcome Sanger Institute for the COVID-19 Genomics UK (COG-UK) Consortium	Rob Howes, The Lighthouse Lab in Cambridge and Alex Alderton, Roberto Amato, Sonia Goncalves, Ewan Harrison, David K. Jackson, Ian Johnston, Dominic Kwiatkowski, Cordelia Langford, John Sillitoe on behalf of the Wellcome Sanger Institute COVID-19 Surveillance Team
EPI_ISL_908179	Lighthouse Lab in Glasgow	Wellcome Sanger Institute for the COVID-19 Genomics UK (COG-UK) Consortium	Harper VanSteenhouse, Yumi Kasai, David Gray, Carol Clugston, Anna Dominiczak and Alex Alderton, Roberto Amato, Sonia Goncalves, Ewan Harrison, David K. Jackson, Ian Johnston, Dominic Kwiatkowski, Cordelia Langford, John Sillitoe on behalf of the Wellcome Sanger Institute COVID-19 Surveillance Team
EPI_ISL_908180	Lighthouse Lab in Cambridge	Wellcome Sanger Institute for the COVID-19 Genomics UK (COG-UK) Consortium	Rob Howes, The Lighthouse Lab in Cambridge and Alex Alderton, Roberto Amato, Sonia Goncalves, Ewan Harrison, David K. Jackson, Ian Johnston, Dominic Kwiatkowski, Cordelia Langford, John Sillitoe on behalf of the Wellcome Sanger Institute COVID-19 Surveillance Team
EPI_ISL_908181	Lighthouse Lab in Glasgow	Wellcome Sanger Institute for the COVID-19 Genomics UK (COG-UK) Consortium	Harper VanSteenhouse, Yumi Kasai, David Gray, Carol Clugston, Anna Dominiczak and Alex Alderton, Roberto Amato, Sonia Goncalves, Ewan Harrison, David K. Jackson, Ian Johnston, Dominic Kwiatkowski, Cordelia Langford, John Sillitoe on behalf of the Wellcome Sanger Institute COVID-19 Surveillance Team
EPI_ISL_908182	Lighthouse Lab in Cambridge	Wellcome Sanger Institute for the COVID-19 Genomics UK (COG-UK) Consortium	Rob Howes, The Lighthouse Lab in Cambridge and Alex Alderton, Roberto Amato, Sonia Goncalves, Ewan Harrison, David K. Jackson, Ian Johnston, Dominic Kwiatkowski, Cordelia Langford, John Sillitoe on behalf of the Wellcome Sanger Institute COVID-19 Surveillance Team
EPI_ISL_908183	Lighthouse Lab in Glasgow	Wellcome Sanger Institute for the COVID-19 Genomics UK (COG-UK) Consortium	Harper VanSteenhouse, Yumi Kasai, David Gray, Carol Clugston, Anna Dominiczak and Alex Alderton, Roberto Amato, Sonia Goncalves, Ewan Harrison, David K. Jackson, Ian Johnston, Dominic Kwiatkowski, Cordelia Langford, John Sillitoe on behalf of the Wellcome Sanger Institute COVID-19 Surveillance Team
EPI_ISL_908184, EPI_ISL_908185, EPI_ISL_908186	Lighthouse Lab in Cambridge	Wellcome Sanger Institute for the COVID-19 Genomics UK (COG-UK) Consortium	Rob Howes, The Lighthouse Lab in Cambridge and Alex Alderton, Roberto Amato, Sonia Goncalves, Ewan Harrison, David K. Jackson, Ian Johnston, Dominic Kwiatkowski, Cordelia Langford, John Sillitoe on behalf of the Wellcome Sanger Institute COVID-19 Surveillance Team
EPI_ISL_908187	Lighthouse Lab in Glasgow	Wellcome Sanger Institute for the COVID-19 Genomics UK (COG-UK) Consortium	Harper VanSteenhouse, Yumi Kasai, David Gray, Carol Clugston, Anna Dominiczak and Alex Alderton, Roberto Amato, Sonia Goncalves, Ewan Harrison, David K. Jackson, Ian Johnston, Dominic Kwiatkowski, Cordelia Langford, John Sillitoe on behalf of the Wellcome Sanger Institute COVID-19 Surveillance Team
EPI_ISL_908188, EPI_ISL_908189, EPI_ISL_908190, EPI_ISL_908191, EPI_ISL_908192, EPI_ISL_908193	Lighthouse Lab in Cambridge	Wellcome Sanger Institute for the COVID-19 Genomics UK (COG-UK) Consortium	Rob Howes, The Lighthouse Lab in Cambridge and Alex Alderton, Roberto Amato, Sonia Goncalves, Ewan Harrison, David K. Jackson, Ian Johnston, Dominic Kwiatkowski, Cordelia Langford, John Sillitoe on behalf of the Wellcome Sanger Institute COVID-19 Surveillance Team
EPI_ISL_908194	Lighthouse Lab in Glasgow	Wellcome Sanger Institute for the COVID-19 Genomics UK (COG-UK) Consortium	Harper VanSteenhouse, Yumi Kasai, David Gray, Carol Clugston, Anna Dominiczak and Alex Alderton, Roberto Amato, Sonia Goncalves, Ewan Harrison, David K. Jackson, Ian Johnston, Dominic Kwiatkowski, Cordelia Langford, John Sillitoe on behalf of the Wellcome Sanger Institute COVID-19 Surveillance Team
EPI_ISL_908195, EPI_ISL_908196, EPI_ISL_908197, EPI_ISL_908198, EPI_ISL_908199	Lighthouse Lab in Cambridge	Wellcome Sanger Institute for the COVID-19 Genomics UK (COG-UK) Consortium	Rob Howes, The Lighthouse Lab in Cambridge and Alex Alderton, Roberto Amato, Sonia Goncalves, Ewan Harrison, David K. Jackson, Ian Johnston, Dominic Kwiatkowski, Cordelia Langford, John Sillitoe on behalf of the Wellcome Sanger Institute COVID-19 Surveillance Team
EPI_ISL_908200	Lighthouse Lab in Glasgow	Wellcome Sanger Institute for the COVID-19 Genomics UK (COG-UK) Consortium	Harper VanSteenhouse, Yumi Kasai, David Gray, Carol Clugston, Anna Dominiczak and Alex Alderton, Roberto Amato, Sonia Goncalves, Ewan Harrison, David K. Jackson, Ian Johnston, Dominic Kwiatkowski, Cordelia Langford, John Sillitoe on behalf of the Wellcome Sanger Institute COVID-19 Surveillance Team
EPI_ISL_908201, EPI_ISL_908202, EPI_ISL_908203, EPI_ISL_908204, EPI_ISL_908205	Lighthouse Lab in Cambridge	Wellcome Sanger Institute for the COVID-19 Genomics UK (COG-UK) Consortium	Rob Howes, The Lighthouse Lab in Cambridge and Alex Alderton, Roberto Amato, Sonia Goncalves, Ewan Harrison, David K. Jackson, Ian Johnston, Dominic Kwiatkowski, Cordelia Langford, John Sillitoe on behalf of the Wellcome Sanger Institute COVID-19 Surveillance Team
EPI_ISL_908206	Lighthouse Lab in Glasgow	Wellcome Sanger Institute for the COVID-19 Genomics UK (COG-UK) Consortium	Harper VanSteenhouse, Yumi Kasai, David Gray, Carol Clugston, Anna Dominiczak and Alex Alderton, Roberto Amato, Sonia Goncalves, Ewan Harrison, David K. Jackson, Ian Johnston, Dominic Kwiatkowski, Cordelia Langford, John Sillitoe on behalf of the Wellcome Sanger Institute COVID-19 Surveillance Team
EPI_ISL_908207	Lighthouse Lab in Cambridge	Wellcome Sanger Institute for the COVID-19 Genomics UK (COG-UK) Consortium	Rob Howes, The Lighthouse Lab in Cambridge and Alex Alderton, Roberto Amato, Sonia Goncalves, Ewan Harrison, David K. Jackson, Ian Johnston, Dominic Kwiatkowski, Cordelia Langford, John Sillitoe on behalf of the Wellcome Sanger Institute COVID-19 Surveillance Team
EPI_ISL_908208	Lighthouse Lab in Glasgow	Wellcome Sanger Institute for the COVID-19 Genomics UK (COG-UK) Consortium	Harper VanSteenhouse, Yumi Kasai, David Gray, Carol Clugston, Anna Dominiczak and Alex Alderton, Roberto Amato, Sonia Goncalves, Ewan Harrison, David K. Jackson, Ian Johnston, Dominic Kwiatkowski, Cordelia Langford, John Sillitoe on behalf of the Wellcome Sanger Institute COVID-19 Surveillance Team
EPI_ISL_908209, EPI_ISL_908210, EPI_ISL_908211, EPI_ISL_908212, EPI_ISL_908213	Lighthouse Lab in Cambridge	Wellcome Sanger Institute for the COVID-19 Genomics UK (COG-UK) Consortium	Rob Howes, The Lighthouse Lab in Cambridge and Alex Alderton, Roberto Amato, Sonia Goncalves, Ewan Harrison, David K. Jackson, Ian Johnston, Dominic Kwiatkowski, Cordelia Langford, John Sillitoe on behalf of the Wellcome Sanger Institute COVID-19 Surveillance Team
EPI_ISL_908214	Lighthouse Lab in Glasgow	Wellcome Sanger Institute for the COVID-19 Genomics UK (COG-UK) Consortium	Harper VanSteenhouse, Yumi Kasai, David Gray, Carol Clugston, Anna Dominiczak and Alex Alderton, Roberto Amato, Sonia Goncalves, Ewan Harrison, David K. Jackson, Ian Johnston, Dominic Kwiatkowski, Cordelia Langford, John Sillitoe on behalf of the Wellcome Sanger Institute COVID-19 Surveillance

			Team
EPI_ISL_908215, EPI_ISL_908216, EPI_ISL_908217	Lighthouse Lab in Cambridge	Wellcome Sanger Institute for the COVID-19 Genomics UK (COG-UK) Consortium	Rob Howes, The Lighthouse Lab in Cambridge and Alex Alderton, Roberto Amato, Sonia Goncalves, Ewan Harrison, David K. Jackson, Ian Johnston, Dominic Kwiatkowski, Cordelia Langford, John Sillitoe on behalf of the Wellcome Sanger Institute COVID-19 Surveillance Team
EPI_ISL_908218	Lighthouse Lab in Glasgow	Wellcome Sanger Institute for the COVID-19 Genomics UK (COG-UK) Consortium	Harper VanSteenhouse, Yumi Kasai, David Gray, Carol Clugston, Anna Dominiczak and Alex Alderton, Roberto Amato, Sonia Goncalves, Ewan Harrison, David K. Jackson, Ian Johnston, Dominic Kwiatkowski, Cordelia Langford, John Sillitoe on behalf of the Wellcome Sanger Institute COVID-19 Surveillance Team
EPI_ISL_908219, EPI_ISL_908220, EPI_ISL_908221, EPI_ISL_908222, EPI_ISL_908223, EPI_ISL_908224, EPI_ISL_908225, EPI_ISL_908226, EPI_ISL_908227, EPI_ISL_908228	Lighthouse Lab in Cambridge	Wellcome Sanger Institute for the COVID-19 Genomics UK (COG-UK) Consortium	Rob Howes, The Lighthouse Lab in Cambridge and Alex Alderton, Roberto Amato, Sonia Goncalves, Ewan Harrison, David K. Jackson, Ian Johnston, Dominic Kwiatkowski, Cordelia Langford, John Sillitoe on behalf of the Wellcome Sanger Institute COVID-19 Surveillance Team
EPI_ISL_908229	Lighthouse Lab in Glasgow	Wellcome Sanger Institute for the COVID-19 Genomics UK (COG-UK) Consortium	Harper VanSteenhouse, Yumi Kasai, David Gray, Carol Clugston, Anna Dominiczak and Alex Alderton, Roberto Amato, Sonia Goncalves, Ewan Harrison, David K. Jackson, Ian Johnston, Dominic Kwiatkowski, Cordelia Langford, John Sillitoe on behalf of the Wellcome Sanger Institute COVID-19 Surveillance Team
EPI_ISL_908230	Lighthouse Lab in Cambridge	Wellcome Sanger Institute for the COVID-19 Genomics UK (COG-UK) Consortium	Rob Howes, The Lighthouse Lab in Cambridge and Alex Alderton, Roberto Amato, Sonia Goncalves, Ewan Harrison, David K. Jackson, Ian Johnston, Dominic Kwiatkowski, Cordelia Langford, John Sillitoe on behalf of the Wellcome Sanger Institute COVID-19 Surveillance Team
EPI_ISL_908231	Lighthouse Lab in Glasgow	Wellcome Sanger Institute for the COVID-19 Genomics UK (COG-UK) Consortium	Harper VanSteenhouse, Yumi Kasai, David Gray, Carol Clugston, Anna Dominiczak and Alex Alderton, Roberto Amato, Sonia Goncalves, Ewan Harrison, David K. Jackson, Ian Johnston, Dominic Kwiatkowski, Cordelia Langford, John Sillitoe on behalf of the Wellcome Sanger Institute COVID-19 Surveillance Team
EPI_ISL_908232, EPI_ISL_908233, EPI_ISL_908234, EPI_ISL_908235, EPI_ISL_908236	Lighthouse Lab in Cambridge	Wellcome Sanger Institute for the COVID-19 Genomics UK (COG-UK) Consortium	Rob Howes, The Lighthouse Lab in Cambridge and Alex Alderton, Roberto Amato, Sonia Goncalves, Ewan Harrison, David K. Jackson, Ian Johnston, Dominic Kwiatkowski, Cordelia Langford, John Sillitoe on behalf of the Wellcome Sanger Institute COVID-19 Surveillance Team
EPI_ISL_908237, EPI_ISL_908238	Lighthouse Lab in Glasgow	Wellcome Sanger Institute for the COVID-19 Genomics UK (COG-UK) Consortium	Harper VanSteenhouse, Yumi Kasai, David Gray, Carol Clugston, Anna Dominiczak and Alex Alderton, Roberto Amato, Sonia Goncalves, Ewan Harrison, David K. Jackson, Ian Johnston, Dominic Kwiatkowski, Cordelia Langford, John Sillitoe on behalf of the Wellcome Sanger Institute COVID-19 Surveillance Team
EPI_ISL_908239, EPI_ISL_908240	Lighthouse Lab in Cambridge	Wellcome Sanger Institute for the COVID-19 Genomics UK (COG-UK) Consortium	Rob Howes, The Lighthouse Lab in Cambridge and Alex Alderton, Roberto Amato, Sonia Goncalves, Ewan Harrison, David K. Jackson, Ian Johnston, Dominic Kwiatkowski, Cordelia Langford, John Sillitoe on behalf of the Wellcome Sanger Institute COVID-19 Surveillance Team
EPI_ISL_908241, EPI_ISL_908242, EPI_ISL_908243	Lighthouse Lab in Glasgow	Wellcome Sanger Institute for the COVID-19 Genomics UK (COG-UK) Consortium	Harper VanSteenhouse, Yumi Kasai, David Gray, Carol Clugston, Anna Dominiczak and Alex Alderton, Roberto Amato, Sonia Goncalves, Ewan Harrison, David K. Jackson, Ian Johnston, Dominic Kwiatkowski, Cordelia Langford, John Sillitoe on behalf of the Wellcome Sanger Institute COVID-19 Surveillance Team
EPI_ISL_908244, EPI_ISL_908245, EPI_ISL_908246, EPI_ISL_908247, EPI_ISL_908248, EPI_ISL_908249, EPI_ISL_908250, EPI_ISL_908251	Lighthouse Lab in Cambridge	Wellcome Sanger Institute for the COVID-19 Genomics UK (COG-UK) Consortium	Rob Howes, The Lighthouse Lab in Cambridge and Alex Alderton, Roberto Amato, Sonia Goncalves, Ewan Harrison, David K. Jackson, Ian Johnston, Dominic Kwiatkowski, Cordelia Langford, John Sillitoe on behalf of the Wellcome Sanger Institute COVID-19 Surveillance Team
EPI_ISL_908252	Lighthouse Lab in Glasgow	Wellcome Sanger Institute for the COVID-19 Genomics UK (COG-UK) Consortium	Harper VanSteenhouse, Yumi Kasai, David Gray, Carol Clugston, Anna Dominiczak and Alex Alderton, Roberto Amato, Sonia Goncalves, Ewan Harrison, David K. Jackson, Ian Johnston, Dominic Kwiatkowski, Cordelia Langford, John Sillitoe on behalf of the Wellcome Sanger Institute COVID-19 Surveillance Team
EPI_ISL_908253	Lighthouse Lab in Cambridge	Wellcome Sanger Institute for the COVID-19 Genomics UK (COG-UK) Consortium	Rob Howes, The Lighthouse Lab in Cambridge and Alex Alderton, Roberto Amato, Sonia Goncalves, Ewan Harrison, David K. Jackson, Ian Johnston, Dominic Kwiatkowski, Cordelia Langford, John Sillitoe on behalf of the Wellcome Sanger Institute COVID-19 Surveillance Team
EPI_ISL_908254	Lighthouse Lab in Glasgow	Wellcome Sanger Institute for the COVID-19 Genomics UK (COG-UK) Consortium	Harper VanSteenhouse, Yumi Kasai, David Gray, Carol Clugston, Anna Dominiczak and Alex Alderton, Roberto Amato, Sonia Goncalves, Ewan Harrison, David K. Jackson, Ian Johnston, Dominic Kwiatkowski, Cordelia Langford, John Sillitoe on behalf of the Wellcome Sanger Institute COVID-19 Surveillance Team
EPI_ISL_908255	Lighthouse Lab in Cambridge	Wellcome Sanger Institute for the COVID-19 Genomics UK (COG-UK) Consortium	Rob Howes, The Lighthouse Lab in Cambridge and Alex Alderton, Roberto Amato, Sonia Goncalves, Ewan Harrison, David K. Jackson, Ian Johnston, Dominic Kwiatkowski, Cordelia Langford, John Sillitoe on behalf of the Wellcome Sanger Institute COVID-19 Surveillance Team
EPI_ISL_908256	Lighthouse Lab in Glasgow	Wellcome Sanger Institute for the COVID-19 Genomics UK (COG-UK) Consortium	Harper VanSteenhouse, Yumi Kasai, David Gray, Carol Clugston, Anna Dominiczak and Alex Alderton, Roberto Amato, Sonia Goncalves, Ewan Harrison, David K. Jackson, Ian Johnston, Dominic Kwiatkowski, Cordelia Langford, John Sillitoe on behalf of the Wellcome Sanger Institute COVID-19 Surveillance Team
EPI_ISL_908257, EPI_ISL_908258, EPI_ISL_908259	Lighthouse Lab in Cambridge	Wellcome Sanger Institute for the COVID-19 Genomics UK (COG-UK) Consortium	Rob Howes, The Lighthouse Lab in Cambridge and Alex Alderton, Roberto Amato, Sonia Goncalves, Ewan Harrison, David K. Jackson, Ian Johnston, Dominic Kwiatkowski, Cordelia Langford, John Sillitoe on behalf of the Wellcome Sanger Institute COVID-19 Surveillance Team
EPI_ISL_908260, EPI_ISL_908261, EPI_ISL_908262, EPI_ISL_908263	Lighthouse Lab in Glasgow	Wellcome Sanger Institute for the COVID-19 Genomics UK (COG-UK) Consortium	Harper VanSteenhouse, Yumi Kasai, David Gray, Carol Clugston, Anna Dominiczak and Alex Alderton, Roberto Amato, Sonia Goncalves, Ewan Harrison, David K. Jackson, Ian Johnston, Dominic Kwiatkowski, Cordelia Langford, John Sillitoe on behalf of the Wellcome Sanger Institute COVID-19 Surveillance Team
EPI_ISL_908264	Lighthouse Lab in Cambridge	Wellcome Sanger Institute for the COVID-19 Genomics UK (COG-UK) Consortium	Rob Howes, The Lighthouse Lab in Cambridge and Alex Alderton, Roberto Amato, Sonia Goncalves, Ewan Harrison, David K. Jackson, Ian Johnston, Dominic Kwiatkowski, Cordelia Langford, John Sillitoe on behalf of the Wellcome Sanger Institute COVID-19 Surveillance Team
EPI_ISL_908265, EPI_ISL_908266	Lighthouse Lab in Glasgow	Wellcome Sanger Institute for the COVID-19 Genomics UK (COG-UK) Consortium	Harper VanSteenhouse, Yumi Kasai, David Gray, Carol Clugston, Anna Dominiczak and Alex Alderton, Roberto Amato, Sonia Goncalves, Ewan Harrison, David K. Jackson, Ian Johnston, Dominic Kwiatkowski, Cordelia Langford, John Sillitoe on behalf of the Wellcome Sanger Institute COVID-19 Surveillance Team
EPI_ISL_908267	Lighthouse Lab in Cambridge	Wellcome Sanger Institute for the COVID-19 Genomics UK (COG-UK) Consortium	Rob Howes, The Lighthouse Lab in Cambridge and Alex Alderton, Roberto Amato, Sonia Goncalves, Ewan Harrison, David K. Jackson, Ian Johnston, Dominic Kwiatkowski, Cordelia Langford, John Sillitoe on behalf of the Wellcome Sanger Institute COVID-19 Surveillance Team
EPI_ISL_908376, EPI_ISL_908465, EPI_ISL_908474, EPI_ISL_908513, EPI_ISL_908570, EPI_ISL_908592	Lighthouse Lab in Alderley Park	Wellcome Sanger Institute for the COVID-19 Genomics UK (COG-UK) Consortium	Jacquelyn Wynn, Mairead Hyland, The Lighthouse Lab in Alderley Park and Alex Alderton, Roberto Amato, Sonia Goncalves, Ewan Harrison, David K. Jackson, Ian Johnston, Dominic Kwiatkowski, Cordelia Langford, John Sillitoe on behalf of the Wellcome Sanger Institute COVID-19 Surveillance Team
EPI_ISL_908932, EPI_ISL_908936, EPI_ISL_908937, EPI_ISL_908938, EPI_ISL_908939, EPI_ISL_908940, EPI_ISL_908941, EPI_ISL_908943, EPI_ISL_908944, EPI_ISL_908947, EPI_ISL_908948, EPI_ISL_908950, EPI_ISL_908954, EPI_ISL_908956, EPI_ISL_908960, EPI_ISL_908964, EPI_ISL_908970, EPI_ISL_908976			
see above	Lighthouse Lab in Milton Keynes	Wellcome Sanger Institute for the COVID-19 Genomics UK (COG-UK) Consortium	The Lighthouse Lab in Milton Keynes and Alex Alderton, Roberto Amato, Sonia Goncalves, Ewan Harrison, David K. Jackson, Ian Johnston, Dominic Kwiatkowski, Cordelia Langford, John Sillitoe on behalf of the Wellcome Sanger Institute COVID-19 Surveillance Team
EPI_ISL_908982	Lighthouse Lab in Glasgow	Wellcome Sanger Institute for the COVID-19 Genomics UK (COG-UK) Consortium	Harper VanSteenhouse, Yumi Kasai, David Gray, Carol Clugston, Anna Dominiczak and Alex Alderton, Roberto Amato, Sonia Goncalves, Ewan Harrison, David K. Jackson, Ian Johnston, Dominic Kwiatkowski, Cordelia Langford, John Sillitoe on behalf of the Wellcome Sanger Institute COVID-19 Surveillance Team
EPI_ISL_908984, EPI_ISL_908987, EPI_ISL_908988, EPI_ISL_908994, EPI_ISL_908995, EPI_ISL_908996, EPI_ISL_908997, EPI_ISL_909003, EPI_ISL_909012, EPI_ISL_909013, EPI_ISL_909014, EPI_ISL_909016, EPI_ISL_909018, EPI_ISL_909020, EPI_ISL_909022, EPI_ISL_909023, EPI_ISL_909025, EPI_ISL_909026, EPI_ISL_909027, EPI_ISL_909028, EPI_ISL_909031, EPI_ISL_909032, EPI_ISL_909037, EPI_ISL_909039, EPI_ISL_909041, EPI_ISL_909042, EPI_ISL_909043, EPI_ISL_909044, EPI_ISL_909051, EPI_ISL_909054, EPI_ISL_909055, EPI_ISL_909059, EPI_ISL_909062, EPI_ISL_909064, EPI_ISL_909065, EPI_ISL_909070, EPI_ISL_909073, EPI_ISL_909076, EPI_ISL_909077, EPI_ISL_909078, EPI_ISL_909082, EPI_ISL_909084, EPI_ISL_909091, EPI_ISL_909092, EPI_ISL_909099, EPI_ISL_909100, EPI_ISL_909104, EPI_ISL_909107, EPI_ISL_909108, EPI_ISL_909110, EPI_ISL_909120, EPI_ISL_909123, EPI_ISL_909124, EPI_ISL_909125, EPI_ISL_909126, EPI_ISL_909127, EPI_ISL_909129, EPI_ISL_909130, EPI_ISL_909132, EPI_ISL_909133, EPI_ISL_909134, EPI_ISL_909135, EPI_ISL_909136, EPI_ISL_909137, EPI_ISL_909138, EPI_ISL_909139, EPI_ISL_909141, EPI_ISL_909143, EPI_ISL_909146, EPI_ISL_909147, EPI_ISL_909149, EPI_ISL_909152,			

EPI_ISL_909153, EPI_ISL_909154, EPI_ISL_909159, EPI_ISL_909160, EPI_ISL_909161, EPI_ISL_909163, EPI_ISL_909166, EPI_ISL_909167, EPI_ISL_909172, EPI_ISL_909174, EPI_ISL_909179, EPI_ISL_909181, EPI_ISL_909183, EPI_ISL_909185, EPI_ISL_909186, EPI_ISL_909191, EPI_ISL_909195, EPI_ISL_909196, EPI_ISL_909197, EPI_ISL_909198, EPI_ISL_909199, EPI_ISL_909201, EPI_ISL_909205, EPI_ISL_909207, EPI_ISL_909209, EPI_ISL_909212, EPI_ISL_909213, EPI_ISL_909214, EPI_ISL_909215, EPI_ISL_909216, EPI_ISL_909221, EPI_ISL_909222, EPI_ISL_909224, EPI_ISL_909226, EPI_ISL_909228, EPI_ISL_909229, EPI_ISL_909231, EPI_ISL_909232, EPI_ISL_909241, EPI_ISL_909244, EPI_ISL_909245, EPI_ISL_909246, EPI_ISL_909247, EPI_ISL_909248, EPI_ISL_909250, EPI_ISL_909252, EPI_ISL_909256, EPI_ISL_909258, EPI_ISL_909261, EPI_ISL_909264, EPI_ISL_909265, EPI_ISL_909266, EPI_ISL_909267, EPI_ISL_909268			
see above	Lighthouse Lab in Milton Keynes	Wellcome Sanger Institute for the COVID-19 Genomics UK (COG-UK) Consortium	The Lighthouse Lab in Milton Keynes and Alex Alderton, Roberto Amato, Sonia Goncalves, Ewan Harrison, David K. Jackson, Ian Johnston, Dominic Kwiatkowski, Cordelia Langford, John Sillitoe on behalf of the Wellcome Sanger Institute COVID-19 Surveillance Team
EPI_ISL_909273	Lighthouse Lab in Alderley Park	Wellcome Sanger Institute for the COVID-19 Genomics UK (COG-UK) Consortium	Jacquelyn Wynn, Mairead Hyland, The Lighthouse Lab in Alderley Park and Alex Alderton, Roberto Amato, Sonia Goncalves, Ewan Harrison, David K. Jackson, Ian Johnston, Dominic Kwiatkowski, Cordelia Langford, John Sillitoe on behalf of the Wellcome Sanger Institute COVID-19 Surveillance Team
EPI_ISL_909274, EPI_ISL_909279, EPI_ISL_909280	Lighthouse Lab in Milton Keynes	Wellcome Sanger Institute for the COVID-19 Genomics UK (COG-UK) Consortium	The Lighthouse Lab in Milton Keynes and Alex Alderton, Roberto Amato, Sonia Goncalves, Ewan Harrison, David K. Jackson, Ian Johnston, Dominic Kwiatkowski, Cordelia Langford, John Sillitoe on behalf of the Wellcome Sanger Institute COVID-19 Surveillance Team
EPI_ISL_909281	Lighthouse Lab in Alderley Park	Wellcome Sanger Institute for the COVID-19 Genomics UK (COG-UK) Consortium	Jacquelyn Wynn, Mairead Hyland, The Lighthouse Lab in Alderley Park and Alex Alderton, Roberto Amato, Sonia Goncalves, Ewan Harrison, David K. Jackson, Ian Johnston, Dominic Kwiatkowski, Cordelia Langford, John Sillitoe on behalf of the Wellcome Sanger Institute COVID-19 Surveillance Team
EPI_ISL_909282, EPI_ISL_909286, EPI_ISL_909288, EPI_ISL_909290, EPI_ISL_909295, EPI_ISL_909299, EPI_ISL_909300, EPI_ISL_909305, EPI_ISL_909307, EPI_ISL_909308	Lighthouse Lab in Milton Keynes	Wellcome Sanger Institute for the COVID-19 Genomics UK (COG-UK) Consortium	The Lighthouse Lab in Milton Keynes and Alex Alderton, Roberto Amato, Sonia Goncalves, Ewan Harrison, David K. Jackson, Ian Johnston, Dominic Kwiatkowski, Cordelia Langford, John Sillitoe on behalf of the Wellcome Sanger Institute COVID-19 Surveillance Team
EPI_ISL_909311	Lighthouse Lab in Alderley Park	Wellcome Sanger Institute for the COVID-19 Genomics UK (COG-UK) Consortium	Jacquelyn Wynn, Mairead Hyland, The Lighthouse Lab in Alderley Park and Alex Alderton, Roberto Amato, Sonia Goncalves, Ewan Harrison, David K. Jackson, Ian Johnston, Dominic Kwiatkowski, Cordelia Langford, John Sillitoe on behalf of the Wellcome Sanger Institute COVID-19 Surveillance Team
EPI_ISL_909313, EPI_ISL_909315	Lighthouse Lab in Milton Keynes	Wellcome Sanger Institute for the COVID-19 Genomics UK (COG-UK) Consortium	The Lighthouse Lab in Milton Keynes and Alex Alderton, Roberto Amato, Sonia Goncalves, Ewan Harrison, David K. Jackson, Ian Johnston, Dominic Kwiatkowski, Cordelia Langford, John Sillitoe on behalf of the Wellcome Sanger Institute COVID-19 Surveillance Team
EPI_ISL_909318	Lighthouse Lab in Alderley Park	Wellcome Sanger Institute for the COVID-19 Genomics UK (COG-UK) Consortium	Jacquelyn Wynn, Mairead Hyland, The Lighthouse Lab in Alderley Park and Alex Alderton, Roberto Amato, Sonia Goncalves, Ewan Harrison, David K. Jackson, Ian Johnston, Dominic Kwiatkowski, Cordelia Langford, John Sillitoe on behalf of the Wellcome Sanger Institute COVID-19 Surveillance Team
EPI_ISL_909319, EPI_ISL_909320, EPI_ISL_909323, EPI_ISL_909324	Lighthouse Lab in Milton Keynes	Wellcome Sanger Institute for the COVID-19 Genomics UK (COG-UK) Consortium	The Lighthouse Lab in Milton Keynes and Alex Alderton, Roberto Amato, Sonia Goncalves, Ewan Harrison, David K. Jackson, Ian Johnston, Dominic Kwiatkowski, Cordelia Langford, John Sillitoe on behalf of the Wellcome Sanger Institute COVID-19 Surveillance Team
EPI_ISL_909328	Lighthouse Lab in Alderley Park	Wellcome Sanger Institute for the COVID-19 Genomics UK (COG-UK) Consortium	Jacquelyn Wynn, Mairead Hyland, The Lighthouse Lab in Alderley Park and Alex Alderton, Roberto Amato, Sonia Goncalves, Ewan Harrison, David K. Jackson, Ian Johnston, Dominic Kwiatkowski, Cordelia Langford, John Sillitoe on behalf of the Wellcome Sanger Institute COVID-19 Surveillance Team
EPI_ISL_909330, EPI_ISL_909332, EPI_ISL_909336, EPI_ISL_909337	Lighthouse Lab in Milton Keynes	Wellcome Sanger Institute for the COVID-19 Genomics UK (COG-UK) Consortium	The Lighthouse Lab in Milton Keynes and Alex Alderton, Roberto Amato, Sonia Goncalves, Ewan Harrison, David K. Jackson, Ian Johnston, Dominic Kwiatkowski, Cordelia Langford, John Sillitoe on behalf of the Wellcome Sanger Institute COVID-19 Surveillance Team
EPI_ISL_909344	Lighthouse Lab in Alderley Park	Wellcome Sanger Institute for the COVID-19 Genomics UK (COG-UK) Consortium	Jacquelyn Wynn, Mairead Hyland, The Lighthouse Lab in Alderley Park and Alex Alderton, Roberto Amato, Sonia Goncalves, Ewan Harrison, David K. Jackson, Ian Johnston, Dominic Kwiatkowski, Cordelia Langford, John Sillitoe on behalf of the Wellcome Sanger Institute COVID-19 Surveillance Team
EPI_ISL_909346, EPI_ISL_909347, EPI_ISL_909357, EPI_ISL_909360, EPI_ISL_909362, EPI_ISL_909363	Lighthouse Lab in Milton Keynes	Wellcome Sanger Institute for the COVID-19 Genomics UK (COG-UK) Consortium	The Lighthouse Lab in Milton Keynes and Alex Alderton, Roberto Amato, Sonia Goncalves, Ewan Harrison, David K. Jackson, Ian Johnston, Dominic Kwiatkowski, Cordelia Langford, John Sillitoe on behalf of the Wellcome Sanger Institute COVID-19 Surveillance Team
EPI_ISL_909364, EPI_ISL_909373	Lighthouse Lab in Alderley Park	Wellcome Sanger Institute for the COVID-19 Genomics UK (COG-UK) Consortium	Jacquelyn Wynn, Mairead Hyland, The Lighthouse Lab in Alderley Park and Alex Alderton, Roberto Amato, Sonia Goncalves, Ewan Harrison, David K. Jackson, Ian Johnston, Dominic Kwiatkowski, Cordelia Langford, John Sillitoe on behalf of the Wellcome Sanger Institute COVID-19 Surveillance Team
EPI_ISL_909377, EPI_ISL_909378, EPI_ISL_909384, EPI_ISL_909395, EPI_ISL_909398, EPI_ISL_909399, EPI_ISL_909400	Lighthouse Lab in Milton Keynes	Wellcome Sanger Institute for the COVID-19 Genomics UK (COG-UK) Consortium	The Lighthouse Lab in Milton Keynes and Alex Alderton, Roberto Amato, Sonia Goncalves, Ewan Harrison, David K. Jackson, Ian Johnston, Dominic Kwiatkowski, Cordelia Langford, John Sillitoe on behalf of the Wellcome Sanger Institute COVID-19 Surveillance Team
EPI_ISL_909401	Lighthouse Lab in Alderley Park	Wellcome Sanger Institute for the COVID-19 Genomics UK (COG-UK) Consortium	Jacquelyn Wynn, Mairead Hyland, The Lighthouse Lab in Alderley Park and Alex Alderton, Roberto Amato, Sonia Goncalves, Ewan Harrison, David K. Jackson, Ian Johnston, Dominic Kwiatkowski, Cordelia Langford, John Sillitoe on behalf of the Wellcome Sanger Institute COVID-19 Surveillance Team
EPI_ISL_909402, EPI_ISL_909404	Lighthouse Lab in Milton Keynes	Wellcome Sanger Institute for the COVID-19 Genomics UK (COG-UK) Consortium	The Lighthouse Lab in Milton Keynes and Alex Alderton, Roberto Amato, Sonia Goncalves, Ewan Harrison, David K. Jackson, Ian Johnston, Dominic Kwiatkowski, Cordelia Langford, John Sillitoe on behalf of the Wellcome Sanger Institute COVID-19 Surveillance Team
EPI_ISL_909414	Lighthouse Lab in Alderley Park	Wellcome Sanger Institute for the COVID-19 Genomics UK (COG-UK) Consortium	Jacquelyn Wynn, Mairead Hyland, The Lighthouse Lab in Alderley Park and Alex Alderton, Roberto Amato, Sonia Goncalves, Ewan Harrison, David K. Jackson, Ian Johnston, Dominic Kwiatkowski, Cordelia Langford, John Sillitoe on behalf of the Wellcome Sanger Institute COVID-19 Surveillance Team
EPI_ISL_909419, EPI_ISL_909420, EPI_ISL_909423	Lighthouse Lab in Milton Keynes	Wellcome Sanger Institute for the COVID-19 Genomics UK (COG-UK) Consortium	The Lighthouse Lab in Milton Keynes and Alex Alderton, Roberto Amato, Sonia Goncalves, Ewan Harrison, David K. Jackson, Ian Johnston, Dominic Kwiatkowski, Cordelia Langford, John Sillitoe on behalf of the Wellcome Sanger Institute COVID-19 Surveillance Team
EPI_ISL_909425, EPI_ISL_909426	Lighthouse Lab in Alderley Park	Wellcome Sanger Institute for the COVID-19 Genomics UK (COG-UK) Consortium	Jacquelyn Wynn, Mairead Hyland, The Lighthouse Lab in Alderley Park and Alex Alderton, Roberto Amato, Sonia Goncalves, Ewan Harrison, David K. Jackson, Ian Johnston, Dominic Kwiatkowski, Cordelia Langford, John Sillitoe on behalf of the Wellcome Sanger Institute COVID-19 Surveillance Team
EPI_ISL_909430, EPI_ISL_909432, EPI_ISL_909433, EPI_ISL_909434, EPI_ISL_909436, EPI_ISL_909437, EPI_ISL_909439, EPI_ISL_909441, EPI_ISL_909445	Lighthouse Lab in Milton Keynes	Wellcome Sanger Institute for the COVID-19 Genomics UK (COG-UK) Consortium	The Lighthouse Lab in Milton Keynes and Alex Alderton, Roberto Amato, Sonia Goncalves, Ewan Harrison, David K. Jackson, Ian Johnston, Dominic Kwiatkowski, Cordelia Langford, John Sillitoe on behalf of the Wellcome Sanger Institute COVID-19 Surveillance Team
EPI_ISL_909447	Lighthouse Lab in Alderley Park	Wellcome Sanger Institute for the COVID-19 Genomics UK (COG-UK) Consortium	Jacquelyn Wynn, Mairead Hyland, The Lighthouse Lab in Alderley Park and Alex Alderton, Roberto Amato, Sonia Goncalves, Ewan Harrison, David K. Jackson, Ian Johnston, Dominic Kwiatkowski, Cordelia Langford, John Sillitoe on behalf of the Wellcome Sanger Institute COVID-19 Surveillance Team
EPI_ISL_909448, EPI_ISL_909449, EPI_ISL_909451, EPI_ISL_909454, EPI_ISL_909455, EPI_ISL_909458, EPI_ISL_909459, EPI_ISL_909461, EPI_ISL_909462, EPI_ISL_909466	Lighthouse Lab in Milton Keynes	Wellcome Sanger Institute for the COVID-19 Genomics UK (COG-UK) Consortium	The Lighthouse Lab in Milton Keynes and Alex Alderton, Roberto Amato, Sonia Goncalves, Ewan Harrison, David K. Jackson, Ian Johnston, Dominic Kwiatkowski, Cordelia Langford, John Sillitoe on behalf of the Wellcome Sanger Institute COVID-19 Surveillance Team
EPI_ISL_909467	Lighthouse Lab in Alderley Park	Wellcome Sanger Institute for the COVID-19 Genomics UK (COG-UK) Consortium	Jacquelyn Wynn, Mairead Hyland, The Lighthouse Lab in Alderley Park and Alex Alderton, Roberto Amato, Sonia Goncalves, Ewan Harrison, David K. Jackson, Ian Johnston, Dominic Kwiatkowski, Cordelia Langford, John Sillitoe on behalf of the Wellcome Sanger Institute COVID-19 Surveillance Team
EPI_ISL_909469, EPI_ISL_909470, EPI_ISL_909472, EPI_ISL_909473, EPI_ISL_909477, EPI_ISL_909483, EPI_ISL_909485, EPI_ISL_909486	Lighthouse Lab in Milton Keynes	Wellcome Sanger Institute for the COVID-19 Genomics UK (COG-UK) Consortium	The Lighthouse Lab in Milton Keynes and Alex Alderton, Roberto Amato, Sonia Goncalves, Ewan Harrison, David K. Jackson, Ian Johnston, Dominic Kwiatkowski, Cordelia Langford, John Sillitoe on behalf of the Wellcome Sanger Institute COVID-19 Surveillance Team
EPI_ISL_909488	Lighthouse Lab in Alderley Park	Wellcome Sanger Institute for the COVID-19 Genomics UK (COG-UK) Consortium	Jacquelyn Wynn, Mairead Hyland, The Lighthouse Lab in Alderley Park and Alex Alderton, Roberto Amato, Sonia Goncalves, Ewan Harrison, David K. Jackson, Ian Johnston, Dominic Kwiatkowski, Cordelia Langford, John Sillitoe on behalf of the Wellcome Sanger Institute COVID-19 Surveillance Team
EPI_ISL_909489, EPI_ISL_909491, EPI_ISL_909494, EPI_ISL_909495, EPI_ISL_909498, EPI_ISL_909499, EPI_ISL_909500, EPI_ISL_909501, EPI_ISL_909502, EPI_ISL_909507, EPI_ISL_909509, EPI_ISL_909516, EPI_ISL_909517, EPI_ISL_909519, EPI_ISL_909520, EPI_ISL_909521, EPI_ISL_909523, EPI_ISL_909527, EPI_ISL_909530, EPI_ISL_909535			
see above	Lighthouse Lab in Milton Keynes	Wellcome Sanger Institute for the COVID-19 Genomics UK (COG-UK) Consortium	The Lighthouse Lab in Milton Keynes and Alex Alderton, Roberto Amato, Sonia Goncalves, Ewan Harrison, David K. Jackson, Ian Johnston, Dominic Kwiatkowski, Cordelia Langford, John Sillitoe on behalf of the Wellcome Sanger Institute COVID-19 Surveillance Team

EPI_ISL_909536	Lighthouse Lab in Alderley Park	Wellcome Sanger Institute for the COVID-19 Genomics UK (COG-UK) Consortium	Jacquelyn Wynn, Mairead Hyland, The Lighthouse Lab in Alderley Park and Alex Alderton, Roberto Amato, Sonia Goncalves, Ewan Harrison, David K. Jackson, Ian Johnston, Dominic Kwiatkowski, Cordelia Langford, John Sillitoe on behalf of the Wellcome Sanger Institute COVID-19 Surveillance Team
EPI_ISL_909542, EPI_ISL_909543, EPI_ISL_909547, EPI_ISL_909548, EPI_ISL_909550, EPI_ISL_909551, EPI_ISL_909555, EPI_ISL_909558, EPI_ISL_909559, EPI_ISL_909563, EPI_ISL_909565, EPI_ISL_909567, EPI_ISL_909574, EPI_ISL_909579, EPI_ISL_909585, EPI_ISL_909587, EPI_ISL_909591, EPI_ISL_909592			
see above	Lighthouse Lab in Milton Keynes	Wellcome Sanger Institute for the COVID-19 Genomics UK (COG-UK) Consortium	The Lighthouse Lab in Milton Keynes and Alex Alderton, Roberto Amato, Sonia Goncalves, Ewan Harrison, David K. Jackson, Ian Johnston, Dominic Kwiatkowski, Cordelia Langford, John Sillitoe on behalf of the Wellcome Sanger Institute COVID-19 Surveillance Team
EPI_ISL_909594	Lighthouse Lab in Alderley Park	Wellcome Sanger Institute for the COVID-19 Genomics UK (COG-UK) Consortium	Jacquelyn Wynn, Mairead Hyland, The Lighthouse Lab in Alderley Park and Alex Alderton, Roberto Amato, Sonia Goncalves, Ewan Harrison, David K. Jackson, Ian Johnston, Dominic Kwiatkowski, Cordelia Langford, John Sillitoe on behalf of the Wellcome Sanger Institute COVID-19 Surveillance Team
EPI_ISL_909655, EPI_ISL_909658, EPI_ISL_909663, EPI_ISL_909664, EPI_ISL_909669, EPI_ISL_909670, EPI_ISL_909671, EPI_ISL_909672, EPI_ISL_909673	Labo Analyses med	National Reference Center for Viruses of Respiratory Infections, Institut Pasteur, Paris	Marion Barbet, Sylvie Behillil, Méline Bizard, Angela Brisebarre, Camille Capel, Etienne Simon-Lorière, Vincent Enouf, Maud Vanpeene, Sylvie van der Werf,Amzalag Jonas
EPI_ISL_909688, EPI_ISL_909689, EPI_ISL_909690, EPI_ISL_909691, EPI_ISL_909692, EPI_ISL_909693, EPI_ISL_909694, EPI_ISL_909695, EPI_ISL_909696, EPI_ISL_909698, EPI_ISL_909699, EPI_ISL_909700, EPI_ISL_909701, EPI_ISL_909710, EPI_ISL_909711, EPI_ISL_909712, EPI_ISL_909713, EPI_ISL_909715, EPI_ISL_909716, EPI_ISL_909717, EPI_ISL_909718, EPI_ISL_909719, EPI_ISL_909720, EPI_ISL_909721, EPI_ISL_909722			
see above	CH de Mayotte - Laboratoire de Biologie	National Reference Center for Viruses of Respiratory Infections, Institut Pasteur, Paris	Marion Barbet, Sylvie Behillil, Méline Bizard, Angela Brisebarre, Camille Capel, Etienne Simon-Lorière, Vincent Enouf, Maud Vanpeene, Sylvie van der Werf,Combe Patrice
EPI_ISL_909755, EPI_ISL_909756	Charité Universitätsmedizin Berlin, Institut für Virologie/Labor Berlin	Charité Universitätsmedizin Berlin, Institut für Virologie	Victor M Corman, Barbara Mühlemann, Jörn Beheim-Schwarzbach, Tobias Bleicker, Julia Tesch, Talitha Veith, Julia Schneider, Terry Jones, Christian Drosten
EPI_ISL_909832, EPI_ISL_909850, EPI_ISL_909917, EPI_ISL_909932	National Virus Reference Laboratory	National Virus Reference Laboratory	Michael Carr, Gabriel Gonzalez, Jonathan Dean, Cillian F De Gascun
EPI_ISL_911175, EPI_ISL_911176, EPI_ISL_911177, EPI_ISL_911178, EPI_ISL_911179, EPI_ISL_911180, EPI_ISL_911181, EPI_ISL_911182, EPI_ISL_911183, EPI_ISL_911184, EPI_ISL_911185, EPI_ISL_911186, EPI_ISL_911187, EPI_ISL_911188, EPI_ISL_911189, EPI_ISL_911190, EPI_ISL_911191, EPI_ISL_911192, EPI_ISL_911193, EPI_ISL_911194, EPI_ISL_911195, EPI_ISL_911196, EPI_ISL_911197, EPI_ISL_911198, EPI_ISL_911199, EPI_ISL_911200, EPI_ISL_911201, EPI_ISL_911202, EPI_ISL_911203, EPI_ISL_911204, EPI_ISL_911205, EPI_ISL_911206, EPI_ISL_911207, EPI_ISL_911208			
see above	Laboratoire national de sante, Microbiology, Virology	Laboratoire national de sante, Microbiology, Microbial Genomics Platform	Anke Wienecke-Baldacchino, Catherine Ragimbeau,Jessica Tapp, Fatu Djabi, Lise Pignon, Raoul Salmon, Tamir Abdelrahman
EPI_ISL_911285, EPI_ISL_911287, EPI_ISL_911288, EPI_ISL_911291, EPI_ISL_911293, EPI_ISL_911294, EPI_ISL_911296, EPI_ISL_911301, EPI_ISL_911303, EPI_ISL_911304, EPI_ISL_911306, EPI_ISL_911312, EPI_ISL_911313, EPI_ISL_911322, EPI_ISL_911323, EPI_ISL_911326, EPI_ISL_911328, EPI_ISL_911330, EPI_ISL_911333, EPI_ISL_911338, EPI_ISL_911339, EPI_ISL_911347			
see above	Servicio de Microbiología, Hospital Universitario Son Espases	SeqCOVID-SPAIN consortium/IBV(CSIC)	Carla López-Causapé, Jordi Reina, Antonio Oliver and SeqCOVID-SPAIN consortium
EPI_ISL_911599	Clinical Molecular Microbiology Laboratory, UNC Hospitals	Jeremy Wang	Jeremy Wang, Alexander Rubinsteyn, Colleen Rice, Jason Smedberg, Melissa Miller, Corbin Jones, Robert Hagan
EPI_ISL_911759, EPI_ISL_911760, EPI_ISL_911761, EPI_ISL_911769, EPI_ISL_911770, EPI_ISL_911771, EPI_ISL_911773, EPI_ISL_911783, EPI_ISL_911823, EPI_ISL_911904	Johns Hopkins Hospital Department of Pathology	Johns Hopkins Hospital Department of Pathology	C. Paul Morris, Chun Huai Luo, Adannaya Amadi, Matthew Schwartz, Nicholas Gallagher, Heba H. Mostafa
EPI_ISL_912160	Yale Clinical Virology Lab	Grubaugh Lab - Yale School of Public Health	Tara Alpert, Joseph Fauver, Anderson Brito, Mallery Breban, Anne Wyllie, Chantal Vogels, Mary Petrone, Annie Watkins, Chaney Kalinich, Isabel Ott, Nathan Grubaugh
EPI_ISL_912161	Tempus	Grubaugh Lab - Yale School of Public Health	Tara Alpert, Joseph Fauver, Anderson Brito, Mallery Breban, Anne Wyllie, Chantal Vogels, Mary Petrone, Annie Watkins, Chaney Kalinich, Isabel Ott, Nathan Grubaugh
EPI_ISL_912168	Yale Pathology Lab	Grubaugh Lab - Yale School of Public Health	Tara Alpert, Joseph Fauver, Chen Liu, Pei Hui, Jianhui Wang, Susan Bell and Han Zhou, Anderson Brito, Mallery Breban, Anne Wyllie, Chantal Vogels, Mary Petrone, Chaney Kalinich, Isabel Ott, Arnau Casanovas, Catherine Muenker, Adam Moore, Alice Lu, Maria Tokuyama, Patrick Wong, Peiwen Lu, Saad Omer, Richard Martinello, Allison Nelson, Shelli Farhadian, Akiko Iwasaki, Charlese Dela Cruz, Albert Ko, Nathan Grubaugh
EPI_ISL_912224, EPI_ISL_912225	Lighthouse Lab in Milton Keynes	Wellcome Sanger Institute for the COVID-19 Genomics UK (COG-UK) Consortium	The Lighthouse Lab in Milton Keynes and Alex Alderton, Roberto Amato, Sonia Goncalves, Ewan Harrison, David K. Jackson, Ian Johnston, Dominic Kwiatkowski, Cordelia Langford, John Sillitoe on behalf of the Wellcome Sanger Institute COVID-19 Surveillance Team (http://www.sanger.ac.uk/covid-team)
EPI_ISL_912236, EPI_ISL_912237, EPI_ISL_912238, EPI_ISL_912239, EPI_ISL_912240, EPI_ISL_912241, EPI_ISL_912242, EPI_ISL_912243, EPI_ISL_912244	Charité Universitätsmedizin Berlin, Institut für Virologie/Labor Berlin	Charité Universitätsmedizin Berlin, Institut für Virologie	Victor M Corman, Barbara Mühlemann, Jörn Beheim-Schwarzbach, Tobias Bleicker, Julia Tesch, Talitha Veith, Julia Schneider, Terry Jones, Christian Drosten
EPI_ISL_912268, EPI_ISL_912271, EPI_ISL_912282, EPI_ISL_912285, EPI_ISL_912287, EPI_ISL_912289, EPI_ISL_912291, EPI_ISL_912292, EPI_ISL_912294, EPI_ISL_912295, EPI_ISL_912296, EPI_ISL_912300, EPI_ISL_912301, EPI_ISL_912302, EPI_ISL_912304, EPI_ISL_912307, EPI_ISL_912308, EPI_ISL_912322, EPI_ISL_912326, EPI_ISL_912327, EPI_ISL_912334, EPI_ISL_912341, EPI_ISL_912345, EPI_ISL_912346, EPI_ISL_912347			
see above	Hospital General Universitario Gregorio Marañón	SeqCOVID-SPAIN consortium / IBV (CSIC)	Darío García de Viedma, Laura Pérez-Lago, Pedro J Sola-Campoy, Sergio Buenestado-Serrano, Marta Herranz, Victor Manuel de la Cueva, Julia Suárez, Pilar Catalán, Patricia Muñoz and SeqCOVID-SPAIN consortium
EPI_ISL_912403	CH de Mayotte - Laboratoire de Biologie	National Reference Center for Viruses of Respiratory Infections, Institut Pasteur, Paris	Marion Barbet, Sylvie Behillil, Méline Bizard, Angela Brisebarre, Camille Capel, Etienne Simon-Lorière, Vincent Enouf, Maud Vanpeene, Sylvie van der Werf,Combe Patrice
EPI_ISL_912545, EPI_ISL_912549	Labo Analyses Med	National Reference Center for Viruses of Respiratory Infections, Institut Pasteur, Paris	Marion Barbet, Sylvie Behillil, Méline Bizard, Angela Brisebarre, Camille Capel, Etienne Simon-Lorière, Vincent Enouf, Maud Vanpeene, Sylvie van der Werf
EPI_ISL_912557, EPI_ISL_912558	Centre Hospitalier de Gisors Route de Rouen	National Reference Center for Viruses of Respiratory Infections, Institut Pasteur, Paris	Marion Barbet, Sylvie Behillil, Méline Bizard, Angela Brisebarre, Camille Capel, Etienne Simon-Lorière, Vincent Enouf, Maud Vanpeene, Sylvie van der Werf
EPI_ISL_912561, EPI_ISL_912562	HOPITAL SIMONE VEIL	National Reference Center for Viruses of Respiratory Infections, Institut Pasteur, Paris	Marion Barbet, Sylvie Behillil, Méline Bizard, Angela Brisebarre, Camille Capel, Etienne Simon-Lorière, Vincent Enouf, Maud Vanpeene, Sylvie van der Werf,Moreau Farida
EPI_ISL_912565, EPI_ISL_912568, EPI_ISL_912577, EPI_ISL_912578	Laboratoire de Virologie du CHU de Lille Bâtiment Paul Boulanger	National Reference Center for Viruses of Respiratory Infections, Institut Pasteur, Paris	Marion Barbet, Sylvie Behillil, Méline Bizard, Angela Brisebarre, Camille Capel, Etienne Simon-Lorière, Vincent Enouf, Maud Vanpeene, Sylvie van der Werf,Guigon Aurélie
EPI_ISL_912598	Hopital Avicenne Laboratoire central de bactériologie virologie hygiène	National Reference Center for Viruses of Respiratory Infections, Institut Pasteur, Paris	Marion Barbet, Sylvie Behillil, Méline Bizard, Angela Brisebarre, Camille Capel, Etienne Simon-Lorière, Vincent Enouf, Maud Vanpeene, Sylvie van der Werf,Brichler Ségolène
EPI_ISL_912601, EPI_ISL_912603, EPI_ISL_912605	Labo Analyses Med	National Reference Center for Viruses of Respiratory Infections, Institut Pasteur, Paris	Marion Barbet, Sylvie Behillil, Méline Bizard, Angela Brisebarre, Camille Capel, Etienne Simon-Lorière, Vincent Enouf, Maud Vanpeene, Sylvie van der Werf
EPI_ISL_912614	CENTRE HOSPITALIER JACQUES LACARIN LABORATOIRE	National Reference Center for Viruses of Respiratory Infections, Institut Pasteur, Paris	Marion Barbet, Sylvie Behillil, Méline Bizard, Angela Brisebarre, Camille Capel, Etienne Simon-Lorière, Vincent Enouf, Maud Vanpeene, Sylvie van der Werf
EPI_ISL_912621	HOPITAL YVES LE FOLL Laboratoire de Biologie Médicale	National Reference Center for Viruses of Respiratory Infections, Institut Pasteur, Paris	Marion Barbet, Sylvie Behillil, Méline Bizard, Angela Brisebarre, Camille Capel, Etienne Simon-Lorière, Vincent Enouf, Maud Vanpeene, Sylvie van der Werf,Chrevet Laurent
EPI_ISL_912623	Labo Analyses Med	National Reference Center for Viruses of Respiratory Infections, Institut Pasteur, Paris	Marion Barbet, Sylvie Behillil, Méline Bizard, Angela Brisebarre, Camille Capel, Etienne Simon-Lorière, Vincent Enouf, Maud Vanpeene, Sylvie van der Werf

EPI_ISL_912636	Hôpital Henri Mondor	Department of Virology, Henri Mondor University Hospital, Assistance Publique Hôpitaux de Paris, Université Paris-Est Créteil, INSERM U955	Christophe Rodriguez, Slim Fourati, Vanessa Demontant, Guillaume Gricourt, Melissa N'Debi, Alexandre Soulier, Elisabeth Trawinski, Jean-Michel Pawlotsky
EPI_ISL_912637	Hôpital Pitié-Salpêtrière	Department of Virology, Henri Mondor University Hospital, Assistance Publique Hôpitaux de Paris, Université Paris-Est Créteil, INSERM U955	Christophe Rodriguez, Slim Fourati, Vanessa Demontant, Guillaume Gricourt, Melissa N'Debi, Alexandre Soulier, Elisabeth Trawinski, Jean-Michel Pawlotsky
EPI_ISL_912638	Hôpital Henri Mondor	Department of Virology, Henri Mondor University Hospital, Assistance Publique Hôpitaux de Paris, Université Paris-Est Créteil, INSERM U955	Christophe Rodriguez, Slim Fourati, Vanessa Demontant, Guillaume Gricourt, Melissa N'Debi, Alexandre Soulier, Elisabeth Trawinski, Jean-Michel Pawlotsky
EPI_ISL_912644	Hôpital Pitié-Salpêtrière	Department of Virology, Henri Mondor University Hospital, Assistance Publique Hôpitaux de Paris, Université Paris-Est Créteil, INSERM U955	Christophe Rodriguez, Slim Fourati, Vanessa Demontant, Guillaume Gricourt, Melissa N'Debi, Alexandre Soulier, Elisabeth Trawinski, Jean-Michel Pawlotsky
EPI_ISL_912653, EPI_ISL_912657, EPI_ISL_912659	Hôpital Henri Mondor	Department of Virology, Henri Mondor University Hospital, Assistance Publique Hôpitaux de Paris, Université Paris-Est Créteil, INSERM U955	Christophe Rodriguez, Slim Fourati, Vanessa Demontant, Guillaume Gricourt, Melissa N'Debi, Alexandre Soulier, Elisabeth Trawinski, Jean-Michel Pawlotsky
EPI_ISL_912662	Hôpital Pitié-Salpêtrière	Department of Virology, Henri Mondor University Hospital, Assistance Publique Hôpitaux de Paris, Université Paris-Est Créteil, INSERM U955	Christophe Rodriguez, Slim Fourati, Vanessa Demontant, Guillaume Gricourt, Melissa N'Debi, Alexandre Soulier, Elisabeth Trawinski, Jean-Michel Pawlotsky
EPI_ISL_912663	Hôpital Henri Mondor	Department of Virology, Henri Mondor University Hospital, Assistance Publique Hôpitaux de Paris, Université Paris-Est Créteil, INSERM U955	Christophe Rodriguez, Slim Fourati, Vanessa Demontant, Guillaume Gricourt, Melissa N'Debi, Alexandre Soulier, Elisabeth Trawinski, Jean-Michel Pawlotsky
EPI_ISL_912667	Hôpital Pitié-Salpêtrière	Department of Virology, Henri Mondor University Hospital, Assistance Publique Hôpitaux de Paris, Université Paris-Est Créteil, INSERM U955	Christophe Rodriguez, Slim Fourati, Vanessa Demontant, Guillaume Gricourt, Melissa N'Debi, Alexandre Soulier, Elisabeth Trawinski, Jean-Michel Pawlotsky
EPI_ISL_912668, EPI_ISL_912671, EPI_ISL_912694, EPI_ISL_912776, EPI_ISL_912777, EPI_ISL_912778, EPI_ISL_912779, EPI_ISL_912780	Hôpital Henri Mondor	Department of Virology, Henri Mondor University Hospital, Assistance Publique Hôpitaux de Paris, Université Paris-Est Créteil, INSERM U955	Christophe Rodriguez, Slim Fourati, Vanessa Demontant, Guillaume Gricourt, Melissa N'Debi, Alexandre Soulier, Elisabeth Trawinski, Jean-Michel Pawlotsky
EPI_ISL_912788, EPI_ISL_912793, EPI_ISL_912794	Hôpital Pitié-Salpêtrière	Department of Virology, Henri Mondor University Hospital, Assistance Publique Hôpitaux de Paris, Université Paris-Est Créteil, INSERM U955	Christophe Rodriguez, Slim Fourati, Vanessa Demontant, Guillaume Gricourt, Melissa N'Debi, Alexandre Soulier, Elisabeth Trawinski, Jean-Michel Pawlotsky
EPI_ISL_912804, EPI_ISL_912805, EPI_ISL_912806, EPI_ISL_912807, EPI_ISL_912808, EPI_ISL_912809, EPI_ISL_912810, EPI_ISL_912863	Hôpital Henri Mondor	Department of Virology, Henri Mondor University Hospital, Assistance Publique Hôpitaux de Paris, Université Paris-Est Créteil, INSERM U955	Christophe Rodriguez, Slim Fourati, Vanessa Demontant, Guillaume Gricourt, Melissa N'Debi, Alexandre Soulier, Elisabeth Trawinski, Jean-Michel Pawlotsky
EPI_ISL_912891	CH.INTERCOMMUNAL DE CRETEIL	Department of Virology, Henri Mondor University Hospital, Assistance Publique Hôpitaux de Paris, Université Paris-Est Créteil, INSERM U955	Christophe Rodriguez, Slim Fourati, Vanessa Demontant, Guillaume Gricourt, Melissa N'Debi, Alexandre Soulier, Elisabeth Trawinski, Jean-Michel Pawlotsky
EPI_ISL_912897, EPI_ISL_912898	Hôpital Henri Mondor	Department of Virology, Henri Mondor University Hospital, Assistance Publique Hôpitaux de Paris, Université Paris-Est Créteil, INSERM U955	Christophe Rodriguez, Slim Fourati, Vanessa Demontant, Guillaume Gricourt, Melissa N'Debi, Alexandre Soulier, Elisabeth Trawinski, Jean-Michel Pawlotsky
EPI_ISL_912907	CH.INTERCOMMUNAL DE CRETEIL	Department of Virology, Henri Mondor University Hospital, Assistance Publique Hôpitaux de Paris, Université Paris-Est Créteil, INSERM U955	Christophe Rodriguez, Slim Fourati, Vanessa Demontant, Guillaume Gricourt, Melissa N'Debi, Alexandre Soulier, Elisabeth Trawinski, Jean-Michel Pawlotsky
EPI_ISL_912933, EPI_ISL_912936, EPI_ISL_912940	Hôpital Pitié-Salpêtrière	Department of Virology, Henri Mondor University Hospital, Assistance Publique Hôpitaux de Paris, Université Paris-Est Créteil, INSERM U955	Christophe Rodriguez, Slim Fourati, Vanessa Demontant, Guillaume Gricourt, Melissa N'Debi, Alexandre Soulier, Elisabeth Trawinski, Jean-Michel Pawlotsky
EPI_ISL_912942, EPI_ISL_912944, EPI_ISL_912945, EPI_ISL_912946, EPI_ISL_912947, EPI_ISL_912948, EPI_ISL_912949, EPI_ISL_912950, EPI_ISL_912951, EPI_ISL_912952, EPI_ISL_912960			
see above	Hôpital Henri Mondor	Department of Virology, Henri Mondor University Hospital, Assistance Publique Hôpitaux de Paris, Université Paris-Est Créteil, INSERM U955	Christophe Rodriguez, Slim Fourati, Vanessa Demontant, Guillaume Gricourt, Melissa N'Debi, Alexandre Soulier, Elisabeth Trawinski, Jean-Michel Pawlotsky
EPI_ISL_912961	CH.INTERCOMMUNAL DE CRETEIL	Department of Virology, Henri Mondor University Hospital, Assistance Publique Hôpitaux de Paris, Université Paris-Est Créteil, INSERM U955	Christophe Rodriguez, Slim Fourati, Vanessa Demontant, Guillaume Gricourt, Melissa N'Debi, Alexandre Soulier, Elisabeth Trawinski, Jean-Michel Pawlotsky
EPI_ISL_913025, EPI_ISL_913026, EPI_ISL_913027, EPI_ISL_913029	Hospital los Arcos	Instituto de Salud Carlos III	Iglesias-Caballero, M. Camarero, S. Sandonís,V. Vázquez, S. Pozo, F. Casas, I. Jiménez, P. Zaballos, A. Monzón, S. Varona, S. Cuesta, I. Cámara, M.
EPI_ISL_913283	Klinisk mikrobiologi	The Public Health Agency of Sweden	Anna-Malin Linde, Maria Lind Karlberg, Carlo Berg, Oskar Karlsson Lindsjo, Sofia Stamouli, Reza Advani, Mattias Haukland, Petra Holmstrom, Noura Walai, Petra Edquist, Mia Brytting, Anna Risberg, Karin Tegmark-Wisell
EPI_ISL_913393, EPI_ISL_913397	Massachusetts State Public Health Laboratory	Massachusetts State Public Health Laboratory	Andrew Lang, Timelia Fink, Glen Gallagher, Sandra Smole
EPI_ISL_913446	Laboratorio Genzano - ASL RM 6	INMI Lazzaro Spallanzani IRCCS	Emanuela Giombini, Martina Rueca, Barbara Bartolini, Ornella Butera, Cesare E.M. Gruber, Francesco Messina, Grazia Tramini, Emanuela Conti, Antonino Di Caro, Maria R. Capobianchi
EPI_ISL_913447	Laboratorio Genzano - ASL RM 6	INMI Lazzaro Spallanzani IRCCS	Francesco Messina, Emanuela Giombini, Ornella Butera, Cesare EM Gruber, Martina Rueca, Barbara Bartolini, Grazia Tramini, Emanuela Conti, Maria R Capobianchi, Antonino Di Caro
EPI_ISL_913457, EPI_ISL_913458	Klinisk mikrobiologi	The Public Health Agency of Sweden	Anna-Malin Linde, Maria Lind Karlberg, Carlo Berg, Oskar Karlsson Lindsjo, Sofia Stamouli, Reza Advani, Mattias Haukland, Petra Holmstrom, Noura Walai, Petra Edquist, Mia Brytting, Anna Risberg, Karin Tegmark-Wisell
EPI_ISL_913594, EPI_ISL_913595, EPI_ISL_913596, EPI_ISL_913597, EPI_ISL_913598, EPI_ISL_913599, EPI_ISL_913600, EPI_ISL_913601, EPI_ISL_913602, EPI_ISL_913603, EPI_ISL_913604, EPI_ISL_913605			
see above	AZDelta	AZDelta	Geert Martens; Dieter De Smet
EPI_ISL_913607	Hopital Avicenne Laboratoire central de bactériologie virologie hygiène	National Reference Center for Viruses of Respiratory Infections, Institut Pasteur, Paris	Marion Barbet, Sylvie Behillil, Méline Bizard, Angela Brisebarre, Camille Capel, Etienne Simon-Lorière, Vincent Enouf, Maud Vanpeene, Sylvie van der Werf,Brichler Ségolène
EPI_ISL_913608, EPI_ISL_913609, EPI_ISL_913610	Laboratoire de Virologie du CHU de Lille Bâtiment Paul Boulanger	National Reference Center for Viruses of Respiratory Infections, Institut Pasteur, Paris	Marion Barbet, Sylvie Behillil, Méline Bizard, Angela Brisebarre, Camille Capel, Etienne Simon-Lorière, Vincent Enouf, Maud Vanpeene, Sylvie van der Werf,Guigon Aurélie
EPI_ISL_913615	Michigan Department of Health and Human Services, Bureau of Laboratories	Michigan Department of Health and Human Services, Bureau of Laboratories	Blankenship HM, Riner D, Soehnlen MK

EPI_ISL_913767	KU Leuven, Rega Institute, Clinical and Epidemiological Virology	KU Leuven, Rega Institute, Clinical and Epidemiological Virology	Tony Wawina-Bokalanga, Bert Vanmechelen, Joan Marti-Carerras, Piet Maes
EPI_ISL_913991, EPI_ISL_913995, EPI_ISL_913996	IL Dept. of Public Health Springfield Laboratory	Pathogen Discovery, Respiratory Viruses Branch, Division of Viral Diseases, Centers for Disease Control and Prevention	Ying Tao, Yan Li, Jing Zhang, Krista Queen, Anna Uehara, Peter Cook, Clinton R. Paden, Haibin Wang, Suxiang Tong
EPI_ISL_914005	Clinical Pathology Lab	Pathogen Discovery, Respiratory Viruses Branch, Division of Viral Diseases, Centers for Disease Control and Prevention	Ying Tao, Yan Li, Jing Zhang, Krista Queen, Anna Uehara, Peter Cook, Clinton R. Paden, Haibin Wang, Suxiang Tong
EPI_ISL_914035	IN State Department of Health Laboratory Service	Pathogen Discovery, Respiratory Viruses Branch, Division of Viral Diseases, Centers for Disease Control and Prevention	Ying Tao, Yan Li, Jing Zhang, Krista Queen, Anna Uehara, Peter Cook, Clinton R. Paden, Haibin Wang, Suxiang Tong
EPI_ISL_914642	Santa Clara County Public Health Laboratory	Santa Clara County Public Health Laboratory	Santa Clara County Public Health Department
EPI_ISL_914745, EPI_ISL_914755, EPI_ISL_914765, EPI_ISL_914773, EPI_ISL_914776	Wyoming Public Health Laboratory	Wyoming Public Health Laboratory	Noah Hull, Taylor Fearing, Lynette Gumbleton, Channing Weber, Ashley Norberg, Bailey Bowcutt, and Wanda Manley
EPI_ISL_914914, EPI_ISL_914944, EPI_ISL_915193, EPI_ISL_915194, EPI_ISL_915206, EPI_ISL_915207, EPI_ISL_915211, EPI_ISL_915212, EPI_ISL_915213, EPI_ISL_915215, EPI_ISL_915224, EPI_ISL_915226, EPI_ISL_915227, EPI_ISL_915228, EPI_ISL_915230, EPI_ISL_915231, EPI_ISL_915232, EPI_ISL_915234, EPI_ISL_915264, EPI_ISL_915267, EPI_ISL_915268, EPI_ISL_915269, EPI_ISL_915270, EPI_ISL_915271, EPI_ISL_915272, EPI_ISL_915273, EPI_ISL_915274, EPI_ISL_915276, EPI_ISL_915278, EPI_ISL_915279, EPI_ISL_915280, EPI_ISL_915281, EPI_ISL_915282, EPI_ISL_915283, EPI_ISL_915302, EPI_ISL_915306, EPI_ISL_915308, EPI_ISL_915310, EPI_ISL_915311, EPI_ISL_915312, EPI_ISL_915313, EPI_ISL_915314, EPI_ISL_915316, EPI_ISL_915318, EPI_ISL_915319, EPI_ISL_915320, EPI_ISL_915321, EPI_ISL_915322, EPI_ISL_915324, EPI_ISL_915328, EPI_ISL_915329, EPI_ISL_915330, EPI_ISL_915331, EPI_ISL_915332, EPI_ISL_915335, EPI_ISL_915336, EPI_ISL_915337, EPI_ISL_915338, EPI_ISL_915339, EPI_ISL_915340, EPI_ISL_915344, EPI_ISL_915346, EPI_ISL_915347, EPI_ISL_915348, EPI_ISL_915349, EPI_ISL_915351, EPI_ISL_915353, EPI_ISL_915355, EPI_ISL_915356, EPI_ISL_915357			
see above	Quest Diagnostics	Quest Diagnostics	Rosenthal,S.H., Gerasimova,A., Kagan,R.M., Anderson, B., Hua, M., Liu Y., Bernstein, L.E., Livingston, K.E., Perez, A., Shalhout, D.F., Shlyakhter, I.A., Owen, R., Tanpaiboon, P., Lacbawan, F.
EPI_ISL_915421	MRCG at LSHTM Genomics lab	MRCG at LSHTM Genomics lab	Abdul Karim sesay, Abdoulie Kanteh, Jarra Manneh, Mariama Kujabi, Bakary Sanyang
EPI_ISL_916633, EPI_ISL_916649, EPI_ISL_916661, EPI_ISL_916675, EPI_ISL_916682, EPI_ISL_916688, EPI_ISL_916694, EPI_ISL_916696, EPI_ISL_916704, EPI_ISL_916705, EPI_ISL_916713, EPI_ISL_916728, EPI_ISL_916730, EPI_ISL_916738, EPI_ISL_916771, EPI_ISL_916782, EPI_ISL_916784, EPI_ISL_916800, EPI_ISL_916802, EPI_ISL_916809, EPI_ISL_916810, EPI_ISL_916812, EPI_ISL_916815, EPI_ISL_916821, EPI_ISL_916825, EPI_ISL_916833, EPI_ISL_916834, EPI_ISL_916838, EPI_ISL_916845, EPI_ISL_916847, EPI_ISL_916850, EPI_ISL_916882, EPI_ISL_916887, EPI_ISL_916896, EPI_ISL_916898, EPI_ISL_916905, EPI_ISL_916907, EPI_ISL_916908, EPI_ISL_916909, EPI_ISL_916911, EPI_ISL_916912, EPI_ISL_916916, EPI_ISL_916918, EPI_ISL_916923, EPI_ISL_916940, EPI_ISL_916945, EPI_ISL_916946, EPI_ISL_916948, EPI_ISL_916949, EPI_ISL_916953, EPI_ISL_916954, EPI_ISL_916956, EPI_ISL_916961, EPI_ISL_916968, EPI_ISL_916970, EPI_ISL_916974, EPI_ISL_916976, EPI_ISL_916977, EPI_ISL_916981, EPI_ISL_916984, EPI_ISL_916985, EPI_ISL_916987, EPI_ISL_916991, EPI_ISL_916999, EPI_ISL_917009, EPI_ISL_917012, EPI_ISL_917013, EPI_ISL_917014, EPI_ISL_917019, EPI_ISL_917021, EPI_ISL_917022, EPI_ISL_917024, EPI_ISL_917025, EPI_ISL_917026, EPI_ISL_917029, EPI_ISL_917031, EPI_ISL_917032, EPI_ISL_917036, EPI_ISL_917038, EPI_ISL_917042, EPI_ISL_917047, EPI_ISL_917054, EPI_ISL_917055, EPI_ISL_917063, EPI_ISL_917067, EPI_ISL_917070, EPI_ISL_917071, EPI_ISL_917074, EPI_ISL_917075, EPI_ISL_917077, EPI_ISL_917079, EPI_ISL_917081, EPI_ISL_917082, EPI_ISL_917083, EPI_ISL_917086, EPI_ISL_917088, EPI_ISL_917091, EPI_ISL_917093, EPI_ISL_917096, EPI_ISL_917099, EPI_ISL_917105, EPI_ISL_917107, EPI_ISL_917110, EPI_ISL_917116, EPI_ISL_917120, EPI_ISL_917125, EPI_ISL_917128, EPI_ISL_917129, EPI_ISL_917133, EPI_ISL_917139, EPI_ISL_917141, EPI_ISL_917143, EPI_ISL_917145, EPI_ISL_917148, EPI_ISL_917149, EPI_ISL_917151, EPI_ISL_917156, EPI_ISL_917159, EPI_ISL_917160, EPI_ISL_917163, EPI_ISL_917849, EPI_ISL_917850, EPI_ISL_917851, EPI_ISL_917852, EPI_ISL_917853, EPI_ISL_917854, EPI_ISL_917855, EPI_ISL_917856, EPI_ISL_917857, EPI_ISL_917858, EPI_ISL_917859, EPI_ISL_917860, EPI_ISL_917861, EPI_ISL_917862, EPI_ISL_917863, EPI_ISL_917864, EPI_ISL_917865, EPI_ISL_917866, EPI_ISL_917867, EPI_ISL_917868, EPI_ISL_917869, EPI_ISL_917870, EPI_ISL_917871, EPI_ISL_917872, EPI_ISL_917873, EPI_ISL_917874, EPI_ISL_917875, EPI_ISL_917876, EPI_ISL_917877, EPI_ISL_917878, EPI_ISL_917879, EPI_ISL_917880, EPI_ISL_917881, EPI_ISL_917882, EPI_ISL_917883, EPI_ISL_917884, EPI_ISL_917885, EPI_ISL_917886, EPI_ISL_917887, EPI_ISL_917888, EPI_ISL_917889, EPI_ISL_917890, EPI_ISL_917891, EPI_ISL_917892, EPI_ISL_917893, EPI_ISL_917894, EPI_ISL_917895, EPI_ISL_917896, EPI_ISL_917897, EPI_ISL_917898, EPI_ISL_917899, EPI_ISL_917900, EPI_ISL_917901, EPI_ISL_917902, EPI_ISL_917903, EPI_ISL_917904, EPI_ISL_917905, EPI_ISL_917906, EPI_ISL_917907, EPI_ISL_917908, EPI_ISL_917909, EPI_ISL_917910, EPI_ISL_917911, EPI_ISL_917912, EPI_ISL_917913, EPI_ISL_917914, EPI_ISL_917915, EPI_ISL_917916, EPI_ISL_917917, EPI_ISL_917918, EPI_ISL_917919, EPI_ISL_917920, EPI_ISL_917921, EPI_ISL_917922, EPI_ISL_917923, EPI_ISL_917924, EPI_ISL_917925, EPI_ISL_917926, EPI_ISL_917927, EPI_ISL_917928, EPI_ISL_917929, EPI_ISL_917930, EPI_ISL_917931, EPI_ISL_917932, EPI_ISL_917933, EPI_ISL_917934, EPI_ISL_917935, EPI_ISL_917937, EPI_ISL_917939, EPI_ISL_917940, EPI_ISL_917942, EPI_ISL_917956, EPI_ISL_917957, EPI_ISL_917958, EPI_ISL_917959, EPI_ISL_917960, EPI_ISL_917961, EPI_ISL_917962, EPI_ISL_917963, EPI_ISL_917964, EPI_ISL_917965, EPI_ISL_917966, EPI_ISL_917967, EPI_ISL_917968, EPI_ISL_917969, EPI_ISL_917970, EPI_ISL_917971, EPI_ISL_917972, EPI_ISL_917973, EPI_ISL_917974, EPI_ISL_917975, EPI_ISL_917976, EPI_ISL_917977, EPI_ISL_917978, EPI_ISL_917979, EPI_ISL_917980, EPI_ISL_917981, EPI_ISL_917982, EPI_ISL_917983, EPI_ISL_917984			
see above	Lighthouse Lab in Glasgow	Wellcome Sanger Institute for the COVID-19 Genomics UK (COG-UK) Consortium	Harper VanSteenhouse, Yumi Kasai, David Gray, Carol Clugston, Anna Dominiczak and Alex Alderton, Roberto Amato, Sonia Goncalves, Ewan Harrison, David K. Jackson, Ian Johnston, Dominic Kwiatkowski, Cordelia Langford, John Sillitoe on behalf of the Wellcome Sanger Institute COVID-19 Surveillance Team
EPI_ISL_918060	Lighthouse Lab in Alderley Park	Wellcome Sanger Institute for the COVID-19 Genomics UK (COG-UK) Consortium	Jacquelyn Wynn, Mairead Hyland, The Lighthouse Lab in Alderley Park and Alex Alderton, Roberto Amato, Sonia Goncalves, Ewan Harrison, David K. Jackson, Ian Johnston, Dominic Kwiatkowski, Cordelia Langford, John Sillitoe on behalf of the Wellcome Sanger Institute COVID-19 Surveillance Team
EPI_ISL_918230	Innovative Genomics Institute, UC Berkeley	Innovative Genomics Institute, UC Berkeley	Stacia Wyman, Haridha Shivram, Phil Frankino, Liana Lareau, Shana McDevitt, Justin Choi
EPI_ISL_918289, EPI_ISL_918290	Hospital Universitari Vall d'Hebron - Vall d'Hebron Institut de Rercerca	Hospital Universitari Vall d'Hebron	Cristina Andrés, Maria Piñana, Josep F Abril, Damir Garcia-Cehic, Ariadna Rando, Juliana Esperalba, Maria Gema Codina, Carla Castillo, Maria Carmen Martin, Tomàs Pumarola, Josep Quer, Andrés Antón
EPI_ISL_918493	Florida Bureau of Public Health Laboratories	Florida Bureau of Public Health Laboratories	Sarah Schmedes, Jason Blanton
EPI_ISL_918986, EPI_ISL_918987, EPI_ISL_918988, EPI_ISL_918989, EPI_ISL_918994, EPI_ISL_918995, EPI_ISL_918996	Department of Pathology, University of Cambridge	COVID-19 Genomics UK (COG-UK) Consortium	Aminu S. Jahun, Yasmin Chaudhry, Iliana Georgana, Myra Hosmillo, Rhys Izu, Martin D. Curran, Surendra Parmar, Ian Goodfellow
EPI_ISL_919205, EPI_ISL_919206, EPI_ISL_919207, EPI_ISL_919208, EPI_ISL_919209, EPI_ISL_919210, EPI_ISL_919211, EPI_ISL_919212, EPI_ISL_919213, EPI_ISL_919214, EPI_ISL_919215, EPI_ISL_919216, EPI_ISL_919217, EPI_ISL_919218, EPI_ISL_919219, EPI_ISL_919220, EPI_ISL_919221, EPI_ISL_919222, EPI_ISL_919223, EPI_ISL_919224, EPI_ISL_919225, EPI_ISL_919226, EPI_ISL_919227, EPI_ISL_919228, EPI_ISL_919229, EPI_ISL_919230			
see above	West of Scotland Specialist Virology Centre, NHSGGC / MRC-University of Glasgow Centre for Virus Research	COVID-19 Genomics UK (COG-UK) Consortium	Ana da Silva Filipe, Natasha Johnson, Kathy Smollett, Daniel Mair, Stephen Carmichael, Alice Broos, Lily Tong, Jenna Nichols, Kyriaki Nomikou; Sarah McDonald; Richard Orton, Joseph Hughes, Sreenu Vattipally, David L Robertson; Alasdair MacLean, Rory Gunson; Sharif Shaaban, Matthew Holden; Rachel Blacow, Guy Mollett, Kathy Li, James Shepherd, Antonia Ho, Emma Thomson
EPI_ISL_919342, EPI_ISL_919345	Virology Department, Royal Infirmary of Edinburgh, NHS Lothian / School of Biological Sciences, University of Edinburgh / Institute of Genetics and Molecular Medicine, University of Edinburgh	COVID-19 Genomics UK (COG-UK) Consortium	McHugh M, Dewar R, Rooke S, Gallagher M, Balcaza C, O'Toole Á, Scher E, Hill V, McCrone JT, Colquhoun R, Yu X, Jackson B, Rambaut A, Williams TC, Templeton K
EPI_ISL_919488, EPI_ISL_919495, EPI_ISL_919500, EPI_ISL_919502, EPI_ISL_919503, EPI_ISL_919504, EPI_ISL_919505, EPI_ISL_919507, EPI_ISL_919508, EPI_ISL_919509, EPI_ISL_919510, EPI_ISL_919529, EPI_ISL_919530, EPI_ISL_919531, EPI_ISL_919532, EPI_ISL_919533, EPI_ISL_919535, EPI_ISL_919536, EPI_ISL_919537, EPI_ISL_919538, EPI_ISL_919541, EPI_ISL_919542, EPI_ISL_919544, EPI_ISL_919545, EPI_ISL_919547, EPI_ISL_919548, EPI_ISL_919549, EPI_ISL_919550, EPI_ISL_919551, EPI_ISL_919552, EPI_ISL_919554, EPI_ISL_919556, EPI_ISL_919557, EPI_ISL_919558, EPI_ISL_919559, EPI_ISL_919561, EPI_ISL_919562, EPI_ISL_919563, EPI_ISL_919564, EPI_ISL_919565, EPI_ISL_919568, EPI_ISL_919570, EPI_ISL_919571, EPI_ISL_919572, EPI_ISL_919573, EPI_ISL_919575, EPI_ISL_919576, EPI_ISL_919577, EPI_ISL_919578, EPI_ISL_919581, EPI_ISL_919585, EPI_ISL_919587, EPI_ISL_919589, EPI_ISL_919590, EPI_ISL_919614, EPI_ISL_919647, EPI_ISL_919648, EPI_ISL_919649, EPI_ISL_919650, EPI_ISL_919652, EPI_ISL_919653, EPI_ISL_919654, EPI_ISL_919655, EPI_ISL_919656, EPI_ISL_919657, EPI_ISL_919659, EPI_ISL_919661, EPI_ISL_919662			
see above	Liverpool Clinical Laboratories	COVID-19 Genomics UK (COG-UK) Consortium	Sam Haldenby, Anita Lucaci, Steve Paterson, Julian Hiscox, Alistair Darby, M Almsaud, A Alrezaihi, Muhannad Alruwaili, Stuart D Armstrong, Jones Benjamin, Eleanor G Bentley, Anu Chawla, Jordan J Clark, Angela Cowell, Richard Eccles, Isabel García-Dorival, Matthew Gemmell, Alessandro Gerada, PKF Gilmore, Richard Gregory, Ximeng Han, Catherine Hartley, Margaret Hughes, Miren Iturriza-Gomara, James Johnson, L Luu, Jenifer Manson, Charlotte Nelson, Elaine O'Toole, Cassie Olateju, Rebekah Penrice-Randal , Lucille Rainbow, N.P Randle, Trevor Ian Robinson, Parul Sharma, Ghada T Shawli, James P Stewart, Neil Swainston, Ecaterina Vamos, Joanne Watts, Mark Whitehead
EPI_ISL_919854	Barts Health NHS Trust	COVID-19 Genomics UK (COG-UK) Consortium	CUTINO-MOGUEL, Maria-Teresa; HARRINGTON, David; OWOYEMI, Dola; KULASEGARAN-SHYLINI, Raghavendran; BROAD, Claire; KELE, Beatrix
EPI_ISL_920052, EPI_ISL_920053, EPI_ISL_920054, EPI_ISL_920055, EPI_ISL_920056, EPI_ISL_920057, EPI_ISL_920058, EPI_ISL_920059, EPI_ISL_920060, EPI_ISL_920079, EPI_ISL_920080			
see above	University College London, Great Ormond Street Hospital for Children NHS Foundation Trust, Imperial College Healthcare NHS Trust	COVID-19 Genomics UK (COG-UK) Consortium	Sergi Castellano, Rachel Williams, Mark Kristiansen, Paola Resende Silva, Sunando Roy, Tony Brooks, Helena Tutill, Paola Niola, Patricia Dyal, Charlotte Williams, Leysa Forrest, Yasmin Panchbhaya, Jacqueline Findlay, Samuel Weeks, Julianne Brown, Kathryn Harris, Paul Randell, James Price, Alison Holmes, Judith Breuer
EPI_ISL_920195, EPI_ISL_920214, EPI_ISL_920218, EPI_ISL_920231, EPI_ISL_920234, EPI_ISL_920235, EPI_ISL_920236, EPI_ISL_920239, EPI_ISL_920244, EPI_ISL_920247, EPI_ISL_920252, EPI_ISL_920255, EPI_ISL_920261, EPI_ISL_920262, EPI_ISL_920266, EPI_ISL_920271, EPI_ISL_920272, EPI_ISL_920276, EPI_ISL_920283, EPI_ISL_920284, EPI_ISL_920287, EPI_ISL_920289, EPI_ISL_920293, EPI_ISL_920300, EPI_ISL_920307, EPI_ISL_920308, EPI_ISL_920312, EPI_ISL_920313, EPI_ISL_920314, EPI_ISL_920316, EPI_ISL_920317, EPI_ISL_920323, EPI_ISL_920324, EPI_ISL_920325, EPI_ISL_920326, EPI_ISL_920332, EPI_ISL_920333, EPI_ISL_920334, EPI_ISL_920335, EPI_ISL_920336, EPI_ISL_920340, EPI_ISL_920341, EPI_ISL_920342, EPI_ISL_920343, EPI_ISL_920347, EPI_ISL_920348, EPI_ISL_920349, EPI_ISL_920350, EPI_ISL_920351, EPI_ISL_920352, EPI_ISL_920358, EPI_ISL_920359, EPI_ISL_920361, EPI_ISL_920366, EPI_ISL_920367, EPI_ISL_920368, EPI_ISL_920369, EPI_ISL_920374, EPI_ISL_920375, EPI_ISL_920400, EPI_ISL_920409, EPI_ISL_920456, EPI_ISL_920464, EPI_ISL_920481, EPI_ISL_920489, EPI_ISL_920490, EPI_ISL_920497, EPI_ISL_920498, EPI_ISL_920507, EPI_ISL_920514, EPI_ISL_920670, EPI_ISL_920683, EPI_ISL_920684, EPI_ISL_920691, EPI_ISL_920700, EPI_ISL_920705, EPI_ISL_920714, EPI_ISL_920738, EPI_ISL_920747, EPI_ISL_920748, EPI_ISL_920755, EPI_ISL_920770, EPI_ISL_920775			
see above	University College London Hospital	COVID-19 Genomics UK (COG-UK) Consortium	Judith Heaney, Matthew Byott, Catherine Houlihan, Dan Frampton, Stuart Kirk, Moira Spyer and Eleni Nastouli

EPI_ISL_920782, EPI_ISL_920784, EPI_ISL_920785, EPI_ISL_920786, EPI_ISL_920787, EPI_ISL_920792, EPI_ISL_920793, EPI_ISL_920796	University College London, Great Ormond Street Hospital for Children NHS Foundation Trust, Imperial College Healthcare NHS Trust	COVID-19 Genomics UK (COG-UK) Consortium	Sergi Castellano, Rachel Williams, Mark Kristiansen, Paola Resende Silva, Sunando Roy, Tony Brooks, Helena Tutill, Paola Niola, Patricia Dyal, Charlotte Williams, Leysa Forrest, Yasmin Panchbhaya, Jacqueline Findlay, Samuel Weeks, Julianne Brown, Kathryn Harris, Paul Randell, James Price, Alison Holmes, Judith Breuer
EPI_ISL_921563, EPI_ISL_921564, EPI_ISL_921578, EPI_ISL_921579, EPI_ISL_921580, EPI_ISL_921582, EPI_ISL_921583, EPI_ISL_921584, EPI_ISL_921585, EPI_ISL_921586, EPI_ISL_921587, EPI_ISL_921589, EPI_ISL_921591, EPI_ISL_921596, EPI_ISL_921597, EPI_ISL_921598, EPI_ISL_921599, EPI_ISL_921600, EPI_ISL_921602, EPI_ISL_921604, EPI_ISL_921610			
see above	Northumbria University / South Tees Hospitals NHS Foundation Trust / North Cumbria Integrated Care NHS Foundation Trust / North Tees and Hartlepool NHS Foundation Trust / Newcastle Hospitals NHS Foundation Trust	COVID-19 Genomics UK (COG-UK) Consortium	Darren L Smith,Andrew Nelson,Matthew Bashton,Greg R Young,Joshua Loh,John Allan,Mohammad A Tariq,Giles S Holt,Gary Black,Wen C Yew,Lynn Dover,Paul Baker,Steve Liggett,Sarah Essex,Jane Greenaway,Debra Padgett,Clive Graham,Garren Scott,Edward Barton,Emma Swindells,Brendan Payne,Jennifer Collins,Yusri Taha,Gary Eltringham
EPI_ISL_922135, EPI_ISL_922136, EPI_ISL_922137, EPI_ISL_922138, EPI_ISL_922139, EPI_ISL_922140, EPI_ISL_922141, EPI_ISL_922142, EPI_ISL_922143, EPI_ISL_922144, EPI_ISL_922145, EPI_ISL_922146, EPI_ISL_922147, EPI_ISL_922148, EPI_ISL_922149, EPI_ISL_922150, EPI_ISL_922151, EPI_ISL_922152, EPI_ISL_922153, EPI_ISL_922154, EPI_ISL_922155, EPI_ISL_922156, EPI_ISL_922157, EPI_ISL_922158, EPI_ISL_922159, EPI_ISL_922160, EPI_ISL_922161, EPI_ISL_922162, EPI_ISL_922163			
see above	Lincolnshire Hospitals and DeepSeq Nottingham	COVID-19 Genomics UK (COG-UK) Consortium	Nichola Duckworth, Tim Sloan, Sarah Walsh, Jonathan Ball, Patrick McClure, Joeseph Chappell, Nadine Holmes, Matthew Carlisle, Christopher Moore, Fei Sang, Johnny Debebe, Victoria Wright, Matthew Loose
EPI_ISL_922353, EPI_ISL_922356, EPI_ISL_922357, EPI_ISL_922358, EPI_ISL_922359, EPI_ISL_922367, EPI_ISL_922368, EPI_ISL_922369, EPI_ISL_922370, EPI_ISL_922371, EPI_ISL_922372, EPI_ISL_922373, EPI_ISL_922436, EPI_ISL_922437, EPI_ISL_922439, EPI_ISL_922443, EPI_ISL_922483, EPI_ISL_922485, EPI_ISL_922488, EPI_ISL_922491, EPI_ISL_922494, EPI_ISL_922495, EPI_ISL_922497, EPI_ISL_922498, EPI_ISL_922499, EPI_ISL_922500, EPI_ISL_922502, EPI_ISL_922504, EPI_ISL_922505, EPI_ISL_922507, EPI_ISL_922510, EPI_ISL_922511, EPI_ISL_922512, EPI_ISL_922513, EPI_ISL_922514, EPI_ISL_922515, EPI_ISL_922516, EPI_ISL_922518, EPI_ISL_922520, EPI_ISL_922521, EPI_ISL_922522, EPI_ISL_922530, EPI_ISL_922536, EPI_ISL_922537, EPI_ISL_922538, EPI_ISL_922542, EPI_ISL_922546, EPI_ISL_922552, EPI_ISL_922555, EPI_ISL_922556, EPI_ISL_922559, EPI_ISL_922560, EPI_ISL_922561, EPI_ISL_922564, EPI_ISL_922575, EPI_ISL_922576, EPI_ISL_922577, EPI_ISL_922585, EPI_ISL_922586, EPI_ISL_922587, EPI_ISL_922600, EPI_ISL_922602, EPI_ISL_922603, EPI_ISL_922604, EPI_ISL_922605, EPI_ISL_922606, EPI_ISL_922607, EPI_ISL_922608, EPI_ISL_922609, EPI_ISL_922610, EPI_ISL_922611, EPI_ISL_922616, EPI_ISL_922618, EPI_ISL_922626, EPI_ISL_922627, EPI_ISL_922628, EPI_ISL_922629, EPI_ISL_922656, EPI_ISL_922692, EPI_ISL_922697, EPI_ISL_922699, EPI_ISL_922700, EPI_ISL_922703, EPI_ISL_922704, EPI_ISL_922708, EPI_ISL_922709, EPI_ISL_922712, EPI_ISL_922713, EPI_ISL_922714, EPI_ISL_922716, EPI_ISL_922717, EPI_ISL_922719, EPI_ISL_922721, EPI_ISL_922722, EPI_ISL_922723, EPI_ISL_922724, EPI_ISL_922726, EPI_ISL_922727, EPI_ISL_922728, EPI_ISL_922731, EPI_ISL_922732, EPI_ISL_922733, EPI_ISL_922734, EPI_ISL_922735, EPI_ISL_922737, EPI_ISL_922739, EPI_ISL_922740, EPI_ISL_922741, EPI_ISL_922743, EPI_ISL_922744, EPI_ISL_922746, EPI_ISL_922747, EPI_ISL_922748, EPI_ISL_922749, EPI_ISL_922751, EPI_ISL_922752, EPI_ISL_922753, EPI_ISL_922754, EPI_ISL_922755, EPI_ISL_922756, EPI_ISL_922757, EPI_ISL_922758, EPI_ISL_922759, EPI_ISL_922760, EPI_ISL_922761, EPI_ISL_922762, EPI_ISL_922763, EPI_ISL_922764, EPI_ISL_922765, EPI_ISL_922766, EPI_ISL_922767, EPI_ISL_922768, EPI_ISL_922770, EPI_ISL_922771, EPI_ISL_922772, EPI_ISL_922773, EPI_ISL_922774, EPI_ISL_922775, EPI_ISL_922776, EPI_ISL_922777, EPI_ISL_922779, EPI_ISL_922781, EPI_ISL_922782, EPI_ISL_922783, EPI_ISL_922784, EPI_ISL_922785, EPI_ISL_922786, EPI_ISL_922787, EPI_ISL_922788, EPI_ISL_922789, EPI_ISL_922790, EPI_ISL_922791, EPI_ISL_922792, EPI_ISL_922793, EPI_ISL_922794, EPI_ISL_922796, EPI_ISL_922798, EPI_ISL_922800, EPI_ISL_922806, EPI_ISL_922810, EPI_ISL_922811, EPI_ISL_922812, EPI_ISL_922813, EPI_ISL_922814, EPI_ISL_922815, EPI_ISL_922816, EPI_ISL_922817, EPI_ISL_922819, EPI_ISL_922820, EPI_ISL_922821, EPI_ISL_922822, EPI_ISL_922823, EPI_ISL_922824, EPI_ISL_922875, EPI_ISL_922906, EPI_ISL_922907, EPI_ISL_922908, EPI_ISL_922909, EPI_ISL_922910, EPI_ISL_922911, EPI_ISL_922912, EPI_ISL_922914, EPI_ISL_922915, EPI_ISL_922932, EPI_ISL_922933, EPI_ISL_922934, EPI_ISL_922935, EPI_ISL_922956, EPI_ISL_922966, EPI_ISL_923016, EPI_ISL_923017, EPI_ISL_923018, EPI_ISL_923019, EPI_ISL_923020, EPI_ISL_923021, EPI_ISL_923022, EPI_ISL_923023, EPI_ISL_923025, EPI_ISL_923026, EPI_ISL_923027, EPI_ISL_923028, EPI_ISL_923045, EPI_ISL_923046, EPI_ISL_923049, EPI_ISL_923050			
see above	Wales Specialist Virology Centre Sequencing lab: Pathogen Genomics Unit	Public Health Wales Microbiology Cardiff Wales Specialist Virology Centre	Catherine Moore, Johnathan Evans, Laura Gifford, Malorie Perry, Simon Cottrell, Angela Marchbank, Alec Birchley, Alexander Adams, Amy Gaskin, Bree Gatica-Wilcox, Jason Coombes, Joel Southgate, Lauren Gilbert, Lee Graham, Nicole Pacchiarini, Sara Kumziene-Summerhayes, Sarah Taylor, Sophie Jones, Sara Rey, Matthew Bull, Joanne Watkins, Sally Corden, Tom Connor
EPI_ISL_923315, EPI_ISL_923316, EPI_ISL_923318, EPI_ISL_923319, EPI_ISL_923357, EPI_ISL_923360, EPI_ISL_923362, EPI_ISL_923363, EPI_ISL_923366, EPI_ISL_923367, EPI_ISL_923368, EPI_ISL_923369, EPI_ISL_923370, EPI_ISL_923415, EPI_ISL_923417, EPI_ISL_923418, EPI_ISL_923420, EPI_ISL_923421, EPI_ISL_923591, EPI_ISL_923592, EPI_ISL_923595, EPI_ISL_923596, EPI_ISL_923597, EPI_ISL_923680, EPI_ISL_923682			
see above	Centre for Enzyme Innovation, University of Portsmouth / Translational Research Laboratory, Portsmouth Hospitals NHS Trust	COVID-19 Genomics UK (COG-UK) Consortium	Angela Beckett,Salman Goudarzi,Christopher Fearn,Kate Cook,Katie Loveson,Sharon Glaysher,Scott Elliott,Samuel Robson
EPI_ISL_923686, EPI_ISL_923687, EPI_ISL_923688, EPI_ISL_923689, EPI_ISL_923691, EPI_ISL_923693, EPI_ISL_923694, EPI_ISL_923696, EPI_ISL_923697, EPI_ISL_923698, EPI_ISL_923699, EPI_ISL_923700, EPI_ISL_923701, EPI_ISL_923702, EPI_ISL_923703, EPI_ISL_923704, EPI_ISL_923705, EPI_ISL_923706, EPI_ISL_923707, EPI_ISL_923708, EPI_ISL_923709, EPI_ISL_923710, EPI_ISL_923711, EPI_ISL_923717, EPI_ISL_923719, EPI_ISL_923720, EPI_ISL_923721, EPI_ISL_923722, EPI_ISL_923723, EPI_ISL_923724, EPI_ISL_923725, EPI_ISL_923727, EPI_ISL_923728, EPI_ISL_923729, EPI_ISL_923730, EPI_ISL_923731, EPI_ISL_923732, EPI_ISL_923733, EPI_ISL_923734, EPI_ISL_923735, EPI_ISL_923736, EPI_ISL_923737, EPI_ISL_923739, EPI_ISL_923740, EPI_ISL_923741, EPI_ISL_923743, EPI_ISL_923744, EPI_ISL_923745, EPI_ISL_923746, EPI_ISL_923747, EPI_ISL_923749, EPI_ISL_923750, EPI_ISL_923751, EPI_ISL_923753, EPI_ISL_923754, EPI_ISL_923755, EPI_ISL_923756, EPI_ISL_923757, EPI_ISL_923759, EPI_ISL_923761, EPI_ISL_923762, EPI_ISL_923763, EPI_ISL_923764, EPI_ISL_923765, EPI_ISL_923766, EPI_ISL_923769, EPI_ISL_923770, EPI_ISL_923771, EPI_ISL_923772, EPI_ISL_923773, EPI_ISL_923774, EPI_ISL_923775, EPI_ISL_923779, EPI_ISL_923782, EPI_ISL_923783, EPI_ISL_923784, EPI_ISL_923785, EPI_ISL_923786, EPI_ISL_923787, EPI_ISL_923788, EPI_ISL_923789, EPI_ISL_923790, EPI_ISL_923791, EPI_ISL_923792, EPI_ISL_923793, EPI_ISL_923794, EPI_ISL_923795, EPI_ISL_923796, EPI_ISL_923797, EPI_ISL_923799, EPI_ISL_923800, EPI_ISL_923802, EPI_ISL_923803, EPI_ISL_923805, EPI_ISL_923807, EPI_ISL_923808, EPI_ISL_923811, EPI_ISL_923812, EPI_ISL_923813, EPI_ISL_923814, EPI_ISL_923815, EPI_ISL_923816, EPI_ISL_923818, EPI_ISL_923820, EPI_ISL_923821, EPI_ISL_923822, EPI_ISL_923824, EPI_ISL_923825, EPI_ISL_923826, EPI_ISL_923828, EPI_ISL_923829, EPI_ISL_923830, EPI_ISL_923831, EPI_ISL_923832, EPI_ISL_923833, EPI_ISL_923834, EPI_ISL_923835, EPI_ISL_923836, EPI_ISL_923837, EPI_ISL_923841, EPI_ISL_923844, EPI_ISL_923845, EPI_ISL_923847, EPI_ISL_923848, EPI_ISL_923849, EPI_ISL_923850, EPI_ISL_923851, EPI_ISL_923853, EPI_ISL_923854, EPI_ISL_923856, EPI_ISL_923857, EPI_ISL_923858, EPI_ISL_923859, EPI_ISL_923860, EPI_ISL_923861, EPI_ISL_923862, EPI_ISL_923863, EPI_ISL_923865, EPI_ISL_923866, EPI_ISL_923867, EPI_ISL_923868, EPI_ISL_923869, EPI_ISL_923870, EPI_ISL_923871, EPI_ISL_923872, EPI_ISL_923873, EPI_ISL_923874, EPI_ISL_923876, EPI_ISL_923878, EPI_ISL_923879, EPI_ISL_923880, EPI_ISL_923881, EPI_ISL_923883, EPI_ISL_923884, EPI_ISL_923885, EPI_ISL_923886, EPI_ISL_923887, EPI_ISL_923888, EPI_ISL_923890, EPI_ISL_923891, EPI_ISL_923892, EPI_ISL_923893, EPI_ISL_923894, EPI_ISL_923895, EPI_ISL_923896, EPI_ISL_923897, EPI_ISL_923898, EPI_ISL_923900, EPI_ISL_923901, EPI_ISL_923904, EPI_ISL_923905, EPI_ISL_923906, EPI_ISL_923907, EPI_ISL_923908, EPI_ISL_923909, EPI_ISL_923910, EPI_ISL_923911, EPI_ISL_923912, EPI_ISL_923913, EPI_ISL_923914, EPI_ISL_923915, EPI_ISL_923917, EPI_ISL_923919, EPI_ISL_923920, EPI_ISL_923921, EPI_ISL_923922, EPI_ISL_923923, EPI_ISL_923926, EPI_ISL_923927, EPI_ISL_923928, EPI_ISL_923929, EPI_ISL_923930, EPI_ISL_923931, EPI_ISL_923932, EPI_ISL_923933, EPI_ISL_923934, EPI_ISL_923935, EPI_ISL_923936, EPI_ISL_923937, EPI_ISL_923938, EPI_ISL_923940, EPI_ISL_923941, EPI_ISL_923942, EPI_ISL_923943, EPI_ISL_923944, EPI_ISL_923945, EPI_ISL_923946, EPI_ISL_923947, EPI_ISL_923948, EPI_ISL_923949, EPI_ISL_923954, EPI_ISL_923955, EPI_ISL_923956, EPI_ISL_923957, EPI_ISL_923958, EPI_ISL_923961, EPI_ISL_923964, EPI_ISL_923965, EPI_ISL_923966, EPI_ISL_923967, EPI_ISL_923968, EPI_ISL_923969, EPI_ISL_923970, EPI_ISL_923971, EPI_ISL_923972, EPI_ISL_923973, EPI_ISL_923974, EPI_ISL_923975, EPI_ISL_923976, EPI_ISL_923977, EPI_ISL_923978, EPI_ISL_923979, EPI_ISL_923980, EPI_ISL_923981, EPI_ISL_923984, EPI_ISL_923985, EPI_ISL_923986, EPI_ISL_923989, EPI_ISL_923991, EPI_ISL_923992, EPI_ISL_923993, EPI_ISL_923994, EPI_ISL_923997, EPI_ISL_923999, EPI_ISL_924002, EPI_ISL_924003, EPI_ISL_924004, EPI_ISL_924005, EPI_ISL_924006, EPI_ISL_924008, EPI_ISL_924011, EPI_ISL_924012, EPI_ISL_924013, EPI_ISL_924015, EPI_ISL_924016, EPI_ISL_924017, EPI_ISL_924018, EPI_ISL_924019, EPI_ISL_924020, EPI_ISL_924021, EPI_ISL_924022, EPI_ISL_924023, EPI_ISL_924024, EPI_ISL_924025, EPI_ISL_924027, EPI_ISL_924028, EPI_ISL_924029, EPI_ISL_924030, EPI_ISL_924031, EPI_ISL_924032, EPI_ISL_924033, EPI_ISL_924034, EPI_ISL_924035, EPI_ISL_924036, EPI_ISL_924039, EPI_ISL_924042, EPI_ISL_924044, EPI_ISL_924045, EPI_ISL_924046, EPI_ISL_924049, EPI_ISL_924050, EPI_ISL_924052, EPI_ISL_924054, EPI_ISL_924055, EPI_ISL_924056, EPI_ISL_924057, EPI_ISL_924058, EPI_ISL_924059, EPI_ISL_924060, EPI_ISL_924062, EPI_ISL_924063, EPI_ISL_924064, EPI_ISL_924065, EPI_ISL_924066, EPI_ISL_924068, EPI_ISL_924069, EPI_ISL_924071, EPI_ISL_924072, EPI_ISL_924073, EPI_ISL_924074, EPI_ISL_924075			
see above	Department of Pathology, University of Cambridge	COVID-19 Genomics UK (COG-UK) Consortium	Aminu S. Jahun, Yasmin Chaudhry, Iliana Georgana, Myra Hosmillo, Rhys Izu, Martin D. Curran, Surendra Parmar, Ian Goodfellow
EPI_ISL_924096, EPI_ISL_924121, EPI_ISL_924199, EPI_ISL_924218, EPI_ISL_924295, EPI_ISL_924319, EPI_ISL_924324, EPI_ISL_924354, EPI_ISL_924408	Virology Department, Sheffield Teaching Hospitals NHS Foundation Trust/Department of Infection, Immunity and Cardiovascular Disease, The Medical School, University of Sheffield	COVID-19 Genomics UK (COG-UK) Consortium	Thushan de Silva, Matthew Parker, Nikki Smith, Adri Angyal, Rebecca Brown, Luke Green, Rachel Tucker, Paul Parsons, Danielle Groves, Katie Johnson, Laura Carrilero, Alex Keeley, Dave Partridge, Matthew Wyles, Benjamin Lindsey, Mehmet Yavuz, Mohammad Raza, Cariad Evans
EPI_ISL_924858, EPI_ISL_924869, EPI_ISL_924873, EPI_ISL_924882, EPI_ISL_924884	Bioinformatics and Biostatistics Lab, Advanced Sequencing Facility	COVID-19 Genomics UK (COG-UK) Consortium	Aengus Stewart,Jerome Nicod,Chelsea Sawyer,Laura Cubitt,Harshil Patel,Margaret Crawford
EPI_ISL_925208	Baylor Scott & White-Temple	Baylor Scott & White-Temple	Ari Rao, Linden Morales, Kimberly Walker, Marcus Volz, Shelby Hendrickson
EPI_ISL_925278	Wyoming Public Health Laboratory	Wyoming Public Health Laboratory	Noah Hull, Taylor Fearing, Lynette Gumbleton, Channing Weber, Ashley Norberg, Bailey Bowcutt, and Wanda Manley
EPI_ISL_930588, EPI_ISL_930597	Synlab	GIGA Medical Genomics	Keith Durkin, Maria Artesi, Sébastien Bontems, Raphaël Boreux, Bouchra Boujemla, Cécile Meex, Pierrette Melin, Marie-Pierre Hayette, Vincent Bours
EPI_ISL_930792	Monterey County Public Health Laboratory	Monterey County Public Health Laboratory	C. Anaya et al.,
EPI_ISL_930794, EPI_ISL_930796	Monterey County Public Health Laboratory	Monterey County Public Health Laboratory	C. Anaya et al.
EPI_ISL_930797	Monterey County Public Health Laboratory	Monterey County Public Health Laboratory	C. Anaya et al.,
EPI_ISL_930800	Monterey County Public Health Laboratory	Monterey County Public Health Laboratory	C. Anaya et al.
EPI_ISL_930804	Monterey County Public Health Laboratory	Monterey County Public Health Laboratory	C. Anaya et al.,
EPI_ISL_930805	Monterey County Public Health Laboratory	Monterey County Public Health Laboratory	C. Anaya et al.
EPI_ISL_930830	Monterey County Public Health Laboratory	Monterey County Public Health Laboratory	C. Anaya et al.,
EPI_ISL_931451, EPI_ISL_931452, EPI_ISL_931453, EPI_ISL_931454	Maryland Public Health Laboratory (MD PHL)	Maryland Public Health Laboratory (MD PHL)	Maryland Department of Health Laboratories Administration
EPI_ISL_933723	Servicio de Microbiología Hospital Ramón y Cajal	Servicio de Microbiología Hospital Ramón y Cajal	José M Gonzalez-Alba, Concepción Rodríguez, Melanie Abreu, Laura Martínez, Val F Lanza, Luz Leticia Olavarrieta, Rafael Cantón, JC Galán
EPI_ISL_933724	Botswana Harvard HIV Reference Laboratory	Botswana Harvard HIV Reference Laboratory	Sikhulile Moyo, Dorcas Maruapula, Wonderful T. Choga, Botshelo Radibe, Boitumelo Zuze, David Lawrence, Roger Shapiro, Shahin Lockman, Mosepele Mosepele, Joseph Makhema, Simani Gaseitsiwe

EPI_ISL_933725, EPI_ISL_933727, EPI_ISL_933728, EPI_ISL_933729	Servicio de Microbiología Hospital Ramón y Cajal	Servicio de Microbiología Hospital Ramón y Cajal	José M Gonzalez-Alba, Concepción Rodríguez, Melanie Abreu, Laura Martínez, Val F Lanza, Luz Leticia Olavarrieta, Rafael Cantón, JC Galán
EPI_ISL_933739, EPI_ISL_933741, EPI_ISL_933742, EPI_ISL_933743, EPI_ISL_933744, EPI_ISL_933745, EPI_ISL_933746, EPI_ISL_933747, EPI_ISL_933748, EPI_ISL_933749, EPI_ISL_933750, EPI_ISL_933751, EPI_ISL_933752, EPI_ISL_933753, EPI_ISL_933754, EPI_ISL_933755, EPI_ISL_933756, EPI_ISL_933757, EPI_ISL_933758, EPI_ISL_933759, EPI_ISL_933764, EPI_ISL_933765			
see above	DPHL	Delaware Public Health Lab	Gregory Hovan
EPI_ISL_933771, EPI_ISL_933773, EPI_ISL_933774	Servicio de Microbiología Hospital Ramón y Cajal	Servicio de Microbiología Hospital Ramón y Cajal	José M Gonzalez-Alba, Concepción Rodríguez, Melanie Abreu, Laura Martínez, Val F Lanza, Luz Leticia Olavarrieta, Rafael Cantón, JC Galán
EPI_ISL_934280, EPI_ISL_934281, EPI_ISL_934282, EPI_ISL_934283, EPI_ISL_934284, EPI_ISL_934285, EPI_ISL_934286, EPI_ISL_934287, EPI_ISL_934288, EPI_ISL_934289, EPI_ISL_934290, EPI_ISL_934291, EPI_ISL_934292, EPI_ISL_934293, EPI_ISL_934294, EPI_ISL_934295, EPI_ISL_934296, EPI_ISL_934297, EPI_ISL_934298, EPI_ISL_934299, EPI_ISL_934300, EPI_ISL_934301, EPI_ISL_934302, EPI_ISL_934303, EPI_ISL_934304, EPI_ISL_934305, EPI_ISL_934306, EPI_ISL_934307			
see above	Vilnius university hospital Santaros Klinikos, Center of Laboratory Medicine	Vilnius university hospital Santaros Klinikos, Center of Laboratory Medicine	Ingrida Olendraite, Daniel Naumovas, Rimvydas Norvilas, Dovile Ezerskyte, Justinas Slikas, Gytis Dudas
EPI_ISL_934381	Klinisk mikrobiologi	The Public Health Agency of Sweden	Anna-Malin Linde, Maria Lind Karlberg, Carlo Berg, Oskar Karlsson Lindsjo, Sofia Stamouli, Reza Advani, Mattias Haukland, Petra Holmstrom, Noura Walai, Petra Edquist, Mia Brytting, Anna Risberg, Karin Tegmark-Wisell
EPI_ISL_934397, EPI_ISL_934398, EPI_ISL_934400	Synlab Medilab, Mikrobiologi	The Public Health Agency of Sweden	Anna-Malin Linde, Maria Lind Karlberg, Carlo Berg, Oskar Karlsson Lindsjo, Sofia Stamouli, Reza Advani, Mattias Haukland, Petra Holmstrom, Noura Walai, Petra Edquist, Mia Brytting, Anna Risberg, Karin Tegmark-Wisell
EPI_ISL_934406	The Public Health Agency of Sweden	The Public Health Agency of Sweden	Anna-Malin Linde, Maria Lind Karlberg, Carlo Berg, Oskar Karlsson Lindsjo, Sofia Stamouli, Reza Advani, Mattias Haukland, Petra Holmstrom, Noura Walai, Petra Edquist, Mia Brytting, Anna Risberg, Karin Tegmark-Wisell
EPI_ISL_934655, EPI_ISL_934656, EPI_ISL_934657, EPI_ISL_934658, EPI_ISL_934659, EPI_ISL_934660, EPI_ISL_934661, EPI_ISL_934662, EPI_ISL_934663, EPI_ISL_934664, EPI_ISL_934665, EPI_ISL_934666, EPI_ISL_934667, EPI_ISL_934668, EPI_ISL_934669, EPI_ISL_934670, EPI_ISL_934671, EPI_ISL_934672, EPI_ISL_934673, EPI_ISL_934674, EPI_ISL_934675, EPI_ISL_934676, EPI_ISL_934677, EPI_ISL_934678, EPI_ISL_934679, EPI_ISL_934680, EPI_ISL_934681			
see above	Department of Laboratory Medicine, Division of Clinical Virology, University of Medicine, Vienna	Bergthaler laboratory, CeMM Research Center for Molecular Medicine of the Austrian Academy of Sciences	Lukas Endler, Anna Schedl, Thomas Penz, Benedikt Agerer, Maelle Le Moing, Michael Schuster, Bekir Erguner, Jan Laine, Martin Senekowitsch, Christoph Bock, Andreas Bergthaler
EPI_ISL_934974	Novabio	CNR Virus des Infections Respiratoires - France SUD	Antonin Bal, Gregory Destras, Gwendolyne Burfin, Hadrien Règue, Quentin Semanas, Martine Valette, Bruno Lina, Laurence Josset
EPI_ISL_934978, EPI_ISL_934981, EPI_ISL_934982, EPI_ISL_934983, EPI_ISL_934991, EPI_ISL_935011	ADMED Microbiologie	Genomics and Transcriptomics, Philip Morris International	Reto Lienhard, Marie-Lise Tritten, Emmanuel Guedj, Nicolas Sierro, Rémi Dulize, David Bornand, Mehdi Auberson, Maxime Berthouzoz, Nikolai Ivanov, Manuel Peitsch
EPI_ISL_935040	Botswana Harvard HIV Reference Laboratory	Botswana Harvard HIV Reference Laboratory	Sikhulile Moyo, Wonderful T. Choga, Dorcas Maruapula, Botshelo Radibe, Boitumelo Zuze, David Lawrence, Roger Shapiro, Shahin Lockman, Mosepele Mosepele, Joseph, Makhema, Simani Gaseitsiwe
EPI_ISL_935042	Botswana Harvard HIV Reference Laboratory	Botswana Harvard HIV Reference Laboratory	Sikhulile Moyo, Dorcas Maruapula, Wonderful Choga, Botshelo Radibe, Boitumelo Zuze, David Lawrence, Roger Shapiro, Shahin Lockman, Mosepele Mosepele, Joseph Makhema, Simani Gaseitsiwe
EPI_ISL_935045	Botswana Harvard HIV Reference Laboratory	Botswana Harvard HIV Reference Laboratory	Sikhulile Moyo, Dorcas Maruapula, Wonderful T. Choga, Botshelo Radibe, Boitumelo Zuze, David Lawrence, Roger Shapiro, Shahin Lockman, Mosepele Mosepele, Joseph Makhema, Simani Gaseitsiwe
EPI_ISL_935196, EPI_ISL_935197, EPI_ISL_935200, EPI_ISL_935202, EPI_ISL_935203, EPI_ISL_935204, EPI_ISL_935205	KU Leuven, Rega Institute, Clinical and Epidemiological Virology	KU Leuven, Rega Institute, Clinical and Epidemiological Virology	Tony Wawina-Bokalanga, Bert Vanmechelen, Joan Marti-Carerras, Piet Maes
EPI_ISL_935538	Labo Analyses Med	National Reference Center for Viruses of Respiratory Infections, Institut Pasteur, Paris	Marion Barbet, Sylvie Behillil, Méline Bizard, Angela Brisebarre, Camille Capel, Etienne Simon-Lorière, Vincent Enouf, Maud Vanpeene, Sylvie van der Werf,Bonnaudet GéRaldine
EPI_ISL_935539	Labo Analyses Med	National Reference Center for Viruses of Respiratory Infections, Institut Pasteur, Paris	Marion Barbet, Sylvie Behillil, Méline Bizard, Angela Brisebarre, Camille Capel, Etienne Simon-Lorière, Vincent Enouf, Maud Vanpeene, Sylvie van der Werf,Tomasi Florent
EPI_ISL_935550	Labo Analyses Med	National Reference Center for Viruses of Respiratory Infections, Institut Pasteur, Paris	Marion Barbet, Sylvie Behillil, Méline Bizard, Angela Brisebarre, Camille Capel, Etienne Simon-Lorière, Vincent Enouf, Maud Vanpeene, Sylvie van der Werf,Lefaure Brieuc
EPI_ISL_935556, EPI_ISL_935557	Hopital	National Reference Center for Viruses of Respiratory Infections, Institut Pasteur, Paris	Marion Barbet, Sylvie Behillil, Méline Bizard, Angela Brisebarre, Camille Capel, Etienne Simon-Lorière, Vincent Enouf, Maud Vanpeene, Sylvie van der Werf,Goudeau Alain
EPI_ISL_935564	Labo Analyses Med	National Reference Center for Viruses of Respiratory Infections, Institut Pasteur, Paris	Marion Barbet, Sylvie Behillil, Méline Bizard, Angela Brisebarre, Camille Capel, Etienne Simon-Lorière, Vincent Enouf, Maud Vanpeene, Sylvie van der Werf
EPI_ISL_935565, EPI_ISL_935566	Hopital	National Reference Center for Viruses of Respiratory Infections, Institut Pasteur, Paris	Marion Barbet, Sylvie Behillil, Méline Bizard, Angela Brisebarre, Camille Capel, Etienne Simon-Lorière, Vincent Enouf, Maud Vanpeene, Sylvie van der Werf,Bret Laurent
EPI_ISL_935570, EPI_ISL_935571, EPI_ISL_935572, EPI_ISL_935573, EPI_ISL_935574, EPI_ISL_935575	Hopital	National Reference Center for Viruses of Respiratory Infections, Institut Pasteur, Paris	Marion Barbet, Sylvie Behillil, Méline Bizard, Angela Brisebarre, Camille Capel, Etienne Simon-Lorière, Vincent Enouf, Maud Vanpeene, Sylvie van der Werf,Castelain Sandrine
EPI_ISL_935582	Hopital	National Reference Center for Viruses of Respiratory Infections, Institut Pasteur, Paris	Marion Barbet, Sylvie Behillil, Méline Bizard, Angela Brisebarre, Camille Capel, Etienne Simon-Lorière, Vincent Enouf, Maud Vanpeene, Sylvie van der Werf,Blazejewski C.
EPI_ISL_935583, EPI_ISL_935584, EPI_ISL_935585	Hopital	National Reference Center for Viruses of Respiratory Infections, Institut Pasteur, Paris	Marion Barbet, Sylvie Behillil, Méline Bizard, Angela Brisebarre, Camille Capel, Etienne Simon-Lorière, Vincent Enouf, Maud Vanpeene, Sylvie van der Werf,Fourgeaud Jacques
EPI_ISL_935587, EPI_ISL_935588, EPI_ISL_935589, EPI_ISL_935595, EPI_ISL_935596, EPI_ISL_935597, EPI_ISL_935598, EPI_ISL_935600, EPI_ISL_935601, EPI_ISL_935621, EPI_ISL_935622, EPI_ISL_935623, EPI_ISL_935624, EPI_ISL_935625, EPI_ISL_935626, EPI_ISL_935627, EPI_ISL_935628			
see above	Labo Analyses med	National Reference Center for Viruses of Respiratory Infections, Institut Pasteur, Paris	Marion Barbet, Sylvie Behillil, Méline Bizard, Angela Brisebarre, Camille Capel, Etienne Simon-Lorière, Vincent Enouf, Maud Vanpeene, Sylvie van der Werf,Amzalag Jonas
EPI_ISL_936242, EPI_ISL_936243, EPI_ISL_936244, EPI_ISL_936245, EPI_ISL_936246, EPI_ISL_936247	Wadsworth Center, New York State Department of Health	Wadsworth Center, New York State Department of Health	Kirsten St. George, Daryl M. Lamson, Alexis Russel, Matthew Shudt, Melissa A Leisner, Jonathan Plitnick, Navjot Singh, John Kelly, Erasmus Schneider, Erica Lasek-Nesselquist
EPI_ISL_936248, EPI_ISL_936250, EPI_ISL_936251, EPI_ISL_936253, EPI_ISL_936259, EPI_ISL_936265, EPI_ISL_936271, EPI_ISL_936275, EPI_ISL_936281, EPI_ISL_936282, EPI_ISL_936286, EPI_ISL_936287, EPI_ISL_936290, EPI_ISL_936297, EPI_ISL_936299			
see above	MONTEFIORE MEDICAL CENTER LABORATORIES	Wadsworth Center, New York State Department of Health	Kirsten St. George, Daryl M. Lamson, Alexis Russel, Matthew Shudt, Melissa A Leisner, Jonathan Plitnick, Navjot Singh, John Kelly, Erasmus Schneider, Erica Lasek-Nesselquist
EPI_ISL_936467, EPI_ISL_936472	DPH, Massachusetts State Public Health Lab	DPH, Massachusetts State Public Health Lab	Lang,A.S., Fink,T., Gallagher,G.R., Smole,S.C.
EPI_ISL_937121	Quest Diagnostics	Quest Diagnostics	Rosenthal,S.H., Gerasimova,A., Kagan,R.M., Anderson, B., Hua, M., Liu Y., Bernstein, L.E., Livingston, K.E., Perez, A., Shalhout, D.F., Shlyakhter, I.A., Owen, R., Tanpaiboon, P., Lacbawan, F.
EPI_ISL_937154, EPI_ISL_937155, EPI_ISL_937156	DOHMH PHL	New York City Public Health Laboratory	Jade Wang, et al.
EPI_ISL_937186, EPI_ISL_937187, EPI_ISL_937189, EPI_ISL_937190	OCME Office Of Chief Medical Examiner	New York City Public Health Laboratory	Jade Wang, et al.

EPI_ISL_937266, EPI_ISL_937269, EPI_ISL_937272	NYC HH Elmhurst Hospital Medical Center	New York City Public Health Laboratory	Jade Wang, et al.
EPI_ISL_937392, EPI_ISL_937394, EPI_ISL_937395, EPI_ISL_937396, EPI_ISL_937397, EPI_ISL_937398, EPI_ISL_937399, EPI_ISL_937400, EPI_ISL_937401, EPI_ISL_937402, EPI_ISL_937403, EPI_ISL_937404, EPI_ISL_937405, EPI_ISL_937406, EPI_ISL_937407, EPI_ISL_937409, EPI_ISL_937410, EPI_ISL_937411, EPI_ISL_937412, EPI_ISL_937413			
see above	Maine Health and Environmental Testing Laboratory (Maine HETL)	Tewhey Lab, The Jackson Laboratory	Matluk,N., Dewey,H., Iosue,F., Barter,M., Lynch,R., Munger,H. and Tewhey,R.
EPI_ISL_939608	Lighthouse Lab in Alderley Park	Wellcome Sanger Institute for the COVID-19 Genomics UK (COG-UK) Consortium	Jacquelyn Wynn, Mairead Hyland, The Lighthouse Lab in Alderley Park and Alex Alderton, Roberto Amato, Sonia Goncalves, Ewan Harrison, David K. Jackson, Ian Johnston, Dominic Kwiatkowski, Cordelia Langford, John Sillitoe on behalf of the Wellcome Sanger Institute COVID-19 Surveillance Team
EPI_ISL_939636, EPI_ISL_939640, EPI_ISL_939641	MEPHI, Aix Marseille University	MEPHI, Aix Marseille University	Anthony LEVASSEUR
EPI_ISL_940074	University Hospitals of Geneva, Laboratory of Virology	HUG, Laboratory of Virology and the Health2030 Genome Center	Samuel Cordey, Ana Rita Goncalves, Laurent Kaiser, Lorenzo Cerutti, Henri Pegeot, Melyssa Elies, Deborah Penet, Keith Harshman, Ioannis Xenarios, Emmanouil Dermitzakis
EPI_ISL_940612	Hospital de Campanha para Enfrentamento do Coronavirus - Goiania	Instituto Adolfo Lutz, Interdiciplinary Procedures Center, Strategic Laboratory	Claudio Tavares Sacchi, Claudia Regina Gonçalves, Erica Valessa Ramos Gomes, Karoline Rodrigues Campos
EPI_ISL_940619, EPI_ISL_940622, EPI_ISL_940624	Hospital Sao Joaquim - Beneficiencia Portuguesa	Instituto Adolfo Lutz, Interdiciplinary Procedures Center, Strategic Laboratory	Claudio Tavares Sacchi, Claudia Regina Gonçalves, Erica Valessa Ramos Gomes, Karoline Rodrigues Campos
EPI_ISL_940772	Platform BIS UZA/UAntwerpen	UAntwerp, Laboratory of Medical Microbiology, Campus Drie Eiken S6.26, Universiteitsplein 1, 2610, Wilrijk, Belgium	Basil Britto Xavier, Jasmine Coppens, Marie Le Mercier, Christine Lammens, Veerle Matheeussen, Herman Goossens
EPI_ISL_940890	Vaccines and Infectious Diseases Analytics Research Unit (VIDA)	KRISP, KZN Research Innovation and Sequencing Platform	Baillie Vicky, du Plessis Jeanine, Giandhari Jennifer, Pillay Sureshnee, Naidoo Yeshnee, Tegally Houriiyah, de Oliveira Tulio, Madhi Shabir
EPI_ISL_941001, EPI_ISL_941002, EPI_ISL_941003	Hopital	National Reference Center for Viruses of Respiratory Infections, Institut Pasteur, Paris	Marion Barbet, Sylvie Behillil, Méline Bizard, Angela Brisebarre, Camille Capel, Etienne Simon-Lorière, Vincent Enouf, Maud Vanpeene, Sylvie van der Werf, Cron Sophie
EPI_ISL_941028, EPI_ISL_941029, EPI_ISL_941030, EPI_ISL_941031, EPI_ISL_941032, EPI_ISL_941033, EPI_ISL_941034, EPI_ISL_941038, EPI_ISL_941039, EPI_ISL_941042, EPI_ISL_941043, EPI_ISL_941044, EPI_ISL_941045, EPI_ISL_941046, EPI_ISL_941047, EPI_ISL_941048, EPI_ISL_941049, EPI_ISL_941050, EPI_ISL_941051, EPI_ISL_941052, EPI_ISL_941053, EPI_ISL_941054, EPI_ISL_941055, EPI_ISL_941056, EPI_ISL_941057, EPI_ISL_941059, EPI_ISL_941060, EPI_ISL_941061, EPI_ISL_941062, EPI_ISL_941064, EPI_ISL_941066, EPI_ISL_941067, EPI_ISL_941068, EPI_ISL_941069, EPI_ISL_941070, EPI_ISL_941072, EPI_ISL_941073, EPI_ISL_941074, EPI_ISL_941075, EPI_ISL_941076, EPI_ISL_941077, EPI_ISL_941078			
see above	Labo Analyses Med	National Reference Center for Viruses of Respiratory Infections, Institut Pasteur, Paris	Marion Barbet, Sylvie Behillil, Méline Bizard, Angela Brisebarre, Camille Capel, Etienne Simon-Lorière, Vincent Enouf, Maud Vanpeene, Sylvie van der Werf, Merah Kader
EPI_ISL_941212, EPI_ISL_941213, EPI_ISL_941214, EPI_ISL_941215, EPI_ISL_941216, EPI_ISL_941217, EPI_ISL_941218, EPI_ISL_941219	Servicio de Microbiología. Hospital Clínico Universitario de Valencia	SeqCOVID-SPAIN consortium/IBV(CSIC)	David Navarro Ortega, Eliseo Albert Vicent, Ignacio Torres and SeqCOVID-SPAIN consortium
EPI_ISL_941294, EPI_ISL_941295	Nigeria Centre for Disease Control (NCDC)	African Centre of Excellence for Genomics of Infectious Diseases (ACEGID), Redeemer's University	Oluniyi P.E. et al
EPI_ISL_941360, EPI_ISL_941361, EPI_ISL_941362, EPI_ISL_941363, EPI_ISL_941364, EPI_ISL_941365, EPI_ISL_941366, EPI_ISL_941367, EPI_ISL_941368, EPI_ISL_941392, EPI_ISL_941393, EPI_ISL_941402, EPI_ISL_941405, EPI_ISL_941406, EPI_ISL_941407, EPI_ISL_941408, EPI_ISL_941409, EPI_ISL_941410, EPI_ISL_941413, EPI_ISL_941423, EPI_ISL_941424, EPI_ISL_941425, EPI_ISL_941430, EPI_ISL_941431, EPI_ISL_941432, EPI_ISL_941486, EPI_ISL_941487, EPI_ISL_941488, EPI_ISL_941489, EPI_ISL_941490, EPI_ISL_941491, EPI_ISL_941492, EPI_ISL_941493, EPI_ISL_941494, EPI_ISL_941495, EPI_ISL_941496, EPI_ISL_941497, EPI_ISL_941498, EPI_ISL_941499, EPI_ISL_941500, EPI_ISL_941501, EPI_ISL_941502, EPI_ISL_941503, EPI_ISL_941504, EPI_ISL_941505, EPI_ISL_941506, EPI_ISL_941507, EPI_ISL_941509, EPI_ISL_941514, EPI_ISL_941516, EPI_ISL_941518, EPI_ISL_941519, EPI_ISL_941523, EPI_ISL_941524, EPI_ISL_941525, EPI_ISL_941526, EPI_ISL_941536, EPI_ISL_941553, EPI_ISL_941554, EPI_ISL_941555, EPI_ISL_941556, EPI_ISL_941557, EPI_ISL_941558, EPI_ISL_941559, EPI_ISL_941560, EPI_ISL_941561, EPI_ISL_941562, EPI_ISL_941563, EPI_ISL_941564, EPI_ISL_941565, EPI_ISL_941566, EPI_ISL_941567, EPI_ISL_941568, EPI_ISL_941569, EPI_ISL_941570, EPI_ISL_941596, EPI_ISL_941597, EPI_ISL_941598, EPI_ISL_941599, EPI_ISL_941604, EPI_ISL_941605, EPI_ISL_941606, EPI_ISL_941607, EPI_ISL_941608, EPI_ISL_941609, EPI_ISL_941635, EPI_ISL_941639, EPI_ISL_941643, EPI_ISL_941644, EPI_ISL_941645, EPI_ISL_941646			
see above	Instituto Nacional de Saude (INSA)	Instituto Nacional de Saude (INSA)	Borges et al
EPI_ISL_941653, EPI_ISL_941654, EPI_ISL_941655, EPI_ISL_941656, EPI_ISL_941657, EPI_ISL_941658, EPI_ISL_941660, EPI_ISL_941661, EPI_ISL_941668, EPI_ISL_941669, EPI_ISL_941679, EPI_ISL_941684, EPI_ISL_941696, EPI_ISL_941697, EPI_ISL_941698, EPI_ISL_941699, EPI_ISL_941700, EPI_ISL_941701, EPI_ISL_941708, EPI_ISL_941720, EPI_ISL_941721, EPI_ISL_941722, EPI_ISL_941723, EPI_ISL_941724, EPI_ISL_941725, EPI_ISL_941756, EPI_ISL_941757, EPI_ISL_941758, EPI_ISL_941763, EPI_ISL_941764, EPI_ISL_941789, EPI_ISL_941790, EPI_ISL_941791, EPI_ISL_941792, EPI_ISL_941793, EPI_ISL_941794, EPI_ISL_941795, EPI_ISL_941803, EPI_ISL_941804, EPI_ISL_941805, EPI_ISL_941806, EPI_ISL_941807, EPI_ISL_941814, EPI_ISL_941815, EPI_ISL_941816, EPI_ISL_941817, EPI_ISL_941832, EPI_ISL_941833, EPI_ISL_941834, EPI_ISL_941843, EPI_ISL_941844, EPI_ISL_941847, EPI_ISL_941848, EPI_ISL_941851, EPI_ISL_941861, EPI_ISL_941872, EPI_ISL_941876, EPI_ISL_941879, EPI_ISL_941880, EPI_ISL_941885, EPI_ISL_941886, EPI_ISL_941887, EPI_ISL_941888, EPI_ISL_941889, EPI_ISL_941898, EPI_ISL_941899, EPI_ISL_941900			
see above	Instituto Nacional de Saude (INSA) and Instituto Gulbenkian de Ciencia (IGC)	Instituto Nacional de Saude (INSA) and Instituto Gulbenkian de Ciencia (IGC)	Borges et al
EPI_ISL_942835, EPI_ISL_942836, EPI_ISL_942837, EPI_ISL_942838, EPI_ISL_942839, EPI_ISL_942840, EPI_ISL_942841, EPI_ISL_942842, EPI_ISL_942843, EPI_ISL_942844, EPI_ISL_942845, EPI_ISL_942846, EPI_ISL_942847, EPI_ISL_942848, EPI_ISL_942849, EPI_ISL_942850			
see above	Gundersen Molecular Diagnostics Laboratory	Kabara Cancer Research Institute	Craig S. Richmond, Paraic A. Kenny
EPI_ISL_942978, EPI_ISL_942994, EPI_ISL_942995, EPI_ISL_943024, EPI_ISL_943059, EPI_ISL_943079, EPI_ISL_943080, EPI_ISL_943092, EPI_ISL_943093, EPI_ISL_943094, EPI_ISL_943095, EPI_ISL_943096, EPI_ISL_943097, EPI_ISL_943098, EPI_ISL_943103, EPI_ISL_943114, EPI_ISL_943115, EPI_ISL_943116, EPI_ISL_943124, EPI_ISL_943125, EPI_ISL_943137, EPI_ISL_943138, EPI_ISL_943139, EPI_ISL_943164, EPI_ISL_943165, EPI_ISL_943166, EPI_ISL_943167, EPI_ISL_943231, EPI_ISL_943272, EPI_ISL_943276, EPI_ISL_943281, EPI_ISL_943289, EPI_ISL_943293, EPI_ISL_943301, EPI_ISL_943304, EPI_ISL_943307, EPI_ISL_943308, EPI_ISL_943309, EPI_ISL_943310, EPI_ISL_943322, EPI_ISL_943330, EPI_ISL_943338, EPI_ISL_943342, EPI_ISL_943349, EPI_ISL_943354, EPI_ISL_943384, EPI_ISL_943426, EPI_ISL_943429, EPI_ISL_943441, EPI_ISL_943442, EPI_ISL_943482, EPI_ISL_943516, EPI_ISL_943521, EPI_ISL_943524, EPI_ISL_943527, EPI_ISL_943528			
see above	Dutch COVID-19 response team	National Institute for Public Health and the Environment (RIVM)	Adam Meijer, Harry Vennema, Dirk Eggink, Jeroen Cremer, Sharon van den Brink, Bas van der Veer, AnneMarie van den Brandt, Florian Zwagemaker, Dennis Schmitz, Chantal Reusken, on behalf of the national COVID-19 response team
EPI_ISL_943550	Baylor Scott & White-Temple	Baylor Scott & White-Temple	Ari Rao, Linden Morales, Kimberly Walker, Marcus Volz, Shelby Hendrickson
EPI_ISL_943813, EPI_ISL_943848, EPI_ISL_943849, EPI_ISL_943850, EPI_ISL_943851, EPI_ISL_943852, EPI_ISL_943853, EPI_ISL_943854, EPI_ISL_943855, EPI_ISL_943856, EPI_ISL_943857, EPI_ISL_943858, EPI_ISL_943859, EPI_ISL_943860, EPI_ISL_943861, EPI_ISL_943862, EPI_ISL_943863, EPI_ISL_943864, EPI_ISL_943865, EPI_ISL_943866, EPI_ISL_943867, EPI_ISL_943869, EPI_ISL_943870, EPI_ISL_943871, EPI_ISL_943872, EPI_ISL_943873, EPI_ISL_943874, EPI_ISL_943875, EPI_ISL_943876, EPI_ISL_943877, EPI_ISL_943878, EPI_ISL_943879, EPI_ISL_943880, EPI_ISL_943881, EPI_ISL_943882, EPI_ISL_943883, EPI_ISL_943884, EPI_ISL_943885, EPI_ISL_943886, EPI_ISL_943887, EPI_ISL_943888, EPI_ISL_943889, EPI_ISL_943890, EPI_ISL_943891, EPI_ISL_943892, EPI_ISL_943893, EPI_ISL_943894, EPI_ISL_943895, EPI_ISL_943896, EPI_ISL_943897, EPI_ISL_943898, EPI_ISL_943899, EPI_ISL_943900, EPI_ISL_943901, EPI_ISL_943902, EPI_ISL_943903, EPI_ISL_943904, EPI_ISL_943905, EPI_ISL_943906, EPI_ISL_943907, EPI_ISL_943908, EPI_ISL_943909, EPI_ISL_943910, EPI_ISL_943911, EPI_ISL_943912, EPI_ISL_943913, EPI_ISL_943914, EPI_ISL_943915, EPI_ISL_943916, EPI_ISL_943922, EPI_ISL_943923, EPI_ISL_943924, EPI_ISL_943929, EPI_ISL_943932, EPI_ISL_943936, EPI_ISL_943938, EPI_ISL_943939, EPI_ISL_943941, EPI_ISL_943942, EPI_ISL_943943, EPI_ISL_943944, EPI_ISL_943946, EPI_ISL_943947, EPI_ISL_943951, EPI_ISL_943952, EPI_ISL_943955, EPI_ISL_943956, EPI_ISL_943957, EPI_ISL_943958, EPI_ISL_943964, EPI_ISL_943966			
see above	Utah Public Health Laboratory	Utah Public Health Laboratory	Erin L. Young, Kelly F. Oakeson, Tara Gallagher
EPI_ISL_943992	Botswana Harvard HIV Reference Laboratory	Botswana Harvard HIV Reference Laboratory	Sikhulile Moyo, Wonderful T. Choga, Dorcas Maruapula, Botshelo Radibe, Boitumelo Zuze, David Lawrence, Roger Shapiro, Shahin Lockman, Mosepele Mosepele, Joseph, Makhema, Simani Gaseitsiwe
EPI_ISL_944229, EPI_ISL_944366, EPI_ISL_944380, EPI_ISL_944387, EPI_ISL_944412, EPI_ISL_944566, EPI_ISL_944567, EPI_ISL_944568, EPI_ISL_944569, EPI_ISL_944570	Israel Central Virology laboratory	Israel National Consortium for SARS-CoV-2 sequencing	Neta Zuckerman, Efrat Dahan Bucris, Michal Mandelboim, Dana Bar-Ilan, Oran Erster, Tzvia Mann, Omer Murik, David A. Zeevi, Assaf Rokney, Joseph Jaffe, Eva Nachum, Maya Davidovich Cohen, Ephraim Fass, Gal Zizelski Valenci, Mor Rubinstein, Efrat Rorman, Israel Nissan, Efrat Glick-Saar, Omri Nayshool, Gideon Rechavi, Ella Mendelson, Orna Mor
EPI_ISL_944739	unknown	Public Health Virology-Forensic and Scientific Services (PHV-FSS)	Son Nguyen et al.
EPI_ISL_948967, EPI_ISL_948968, EPI_ISL_948969, EPI_ISL_948970, EPI_ISL_948971, EPI_ISL_948972, EPI_ISL_948974, EPI_ISL_948976, EPI_ISL_948977, EPI_ISL_948979, EPI_ISL_948980, EPI_ISL_948982, EPI_ISL_948983, EPI_ISL_948985			
see above	Lighthouse Lab in Glasgow	Wellcome Sanger Institute for the COVID-19 Genomics UK	Harper VanSteenhouse, Yumi Kasai, David Gray, Carol Clugston, Anna Dominiczak and Alex Alderton, Roberto Amato, Sonia Goncalves, Ewan Harrison,

		(COG-UK) Consortium	David K. Jackson, Ian Johnston, Dominic Kwiatkowski, Cordelia Langford, John Sillitoe on behalf of the Wellcome Sanger Institute COVID-19 Surveillance Team
EPI_ISL_948986	Jessa	Jessa	Jessa_cmdLab
EPI_ISL_948987, EPI_ISL_948988, EPI_ISL_948990, EPI_ISL_948992, EPI_ISL_948993, EPI_ISL_948995, EPI_ISL_948996, EPI_ISL_948998, EPI_ISL_948999, EPI_ISL_949001, EPI_ISL_949002, EPI_ISL_949004, EPI_ISL_949006, EPI_ISL_949007, EPI_ISL_949009, EPI_ISL_949010, EPI_ISL_949012, EPI_ISL_949014, EPI_ISL_949015, EPI_ISL_949017, EPI_ISL_949018, EPI_ISL_949020, EPI_ISL_949021, EPI_ISL_949023, EPI_ISL_949025, EPI_ISL_949026, EPI_ISL_949027, EPI_ISL_949028, EPI_ISL_949029, EPI_ISL_949030, EPI_ISL_949031, EPI_ISL_949032, EPI_ISL_949033, EPI_ISL_949034, EPI_ISL_949035, EPI_ISL_949036, EPI_ISL_949037, EPI_ISL_949038, EPI_ISL_949039, EPI_ISL_949040, EPI_ISL_949041, EPI_ISL_949042, EPI_ISL_949043, EPI_ISL_949044, EPI_ISL_949045, EPI_ISL_949046, EPI_ISL_949047, EPI_ISL_949048, EPI_ISL_949049, EPI_ISL_949050, EPI_ISL_949051, EPI_ISL_949052, EPI_ISL_949053, EPI_ISL_949054, EPI_ISL_949055, EPI_ISL_949056, EPI_ISL_949057, EPI_ISL_949058, EPI_ISL_949059, EPI_ISL_949060, EPI_ISL_949061, EPI_ISL_949062, EPI_ISL_949063, EPI_ISL_949064, EPI_ISL_949065, EPI_ISL_949066			
see above	Lighthouse Lab in Glasgow	Wellcome Sanger Institute for the COVID-19 Genomics UK (COG-UK) Consortium	Harper VanSteenhouse, Yumi Kasai, David Gray, Carol Clugston, Anna Dominiczak and Alex Alderton, Roberto Amato, Sonia Goncalves, Ewan Harrison, David K. Jackson, Ian Johnston, Dominic Kwiatkowski, Cordelia Langford, John Sillitoe on behalf of the Wellcome Sanger Institute COVID-19 Surveillance Team
EPI_ISL_949255	Jessa	Jessa	Jessa_cmdLab
EPI_ISL_949256, EPI_ISL_949258, EPI_ISL_949259, EPI_ISL_949260, EPI_ISL_949261, EPI_ISL_949262, EPI_ISL_949263, EPI_ISL_949265, EPI_ISL_949266, EPI_ISL_949267, EPI_ISL_949268, EPI_ISL_949269, EPI_ISL_949270, EPI_ISL_949272, EPI_ISL_949273, EPI_ISL_949274, EPI_ISL_949275, EPI_ISL_949276, EPI_ISL_949277, EPI_ISL_949278, EPI_ISL_949279, EPI_ISL_949280, EPI_ISL_949281, EPI_ISL_949282, EPI_ISL_949284, EPI_ISL_949285, EPI_ISL_949286, EPI_ISL_949287, EPI_ISL_949288, EPI_ISL_949289, EPI_ISL_949291, EPI_ISL_949293, EPI_ISL_949294, EPI_ISL_949295, EPI_ISL_949296, EPI_ISL_949297, EPI_ISL_949298, EPI_ISL_949299, EPI_ISL_949300, EPI_ISL_949301, EPI_ISL_949302, EPI_ISL_949303, EPI_ISL_949304, EPI_ISL_949305, EPI_ISL_949306, EPI_ISL_949307, EPI_ISL_949308, EPI_ISL_949309, EPI_ISL_949311, EPI_ISL_949312, EPI_ISL_949313, EPI_ISL_949314, EPI_ISL_949315, EPI_ISL_949316, EPI_ISL_949317, EPI_ISL_949319, EPI_ISL_949320, EPI_ISL_949321, EPI_ISL_949322, EPI_ISL_949323, EPI_ISL_949324, EPI_ISL_949325, EPI_ISL_949326			
see above	Department of Pathology, University of Cambridge	COVID-19 Genomics UK (COG-UK) Consortium	Aminu S. Jahun, Yasmin Chaudhry, Iliana Georgana, Myra Hosmillo, Rhys Izu, Martin D. Curran, Surendra Parmar, Ian Goodfellow
EPI_ISL_949407, EPI_ISL_949417, EPI_ISL_949418, EPI_ISL_949419, EPI_ISL_949420, EPI_ISL_949421, EPI_ISL_949422, EPI_ISL_949423, EPI_ISL_949424, EPI_ISL_949425	University of Birmingham	COVID-19 Genomics UK (COG-UK) Consortium	Institute of Microbiology, University of Birmingham: Claire McMurray, Joanne Stockton, Samuel Nicholls, Radoslaw Poplawski, Will Rowe, Josh Quick, Nicholas Loman. University of Birmingham Testing Laboratory: Celina M Whalley, Andrew Bosworth, Charlotte Poxon, Kasun Wanigasooriya, Oliver Pickles, Mike Kidd, Alex Richter, Andrew D Beggs PHE Heartlands Lab: Husam Osman, Andrew Bosworth. Queen Elizabeth Hospital: Anna Casey
EPI_ISL_949492, EPI_ISL_949494, EPI_ISL_949496, EPI_ISL_949498, EPI_ISL_949504, EPI_ISL_949506	Department of Pathology, University of Cambridge	COVID-19 Genomics UK (COG-UK) Consortium	Aminu S. Jahun, Yasmin Chaudhry, Iliana Georgana, Myra Hosmillo, Rhys Izu, Martin D. Curran, Surendra Parmar, Ian Goodfellow
EPI_ISL_949637, EPI_ISL_949638, EPI_ISL_949641	Virology Department, Royal Infirmary of Edinburgh, NHS Lothian / School of Biological Sciences, University of Edinburgh / Institute of Genetics and Molecular Medicine, University of Edinburgh	COVID-19 Genomics UK (COG-UK) Consortium	McHugh M, Dewar R, Rooke S, Gallagher M, Balcaza C, O'Toole Á, Scher E, Hill V, McCrone JT, Colquhoun R, Yu X, Jackson B, Rambaut A, Williams TC, Templeton K
EPI_ISL_949905, EPI_ISL_950156, EPI_ISL_950157, EPI_ISL_950159, EPI_ISL_950160, EPI_ISL_950161, EPI_ISL_950222, EPI_ISL_950223, EPI_ISL_950224, EPI_ISL_950225, EPI_ISL_950226, EPI_ISL_950227, EPI_ISL_950228			
see above	University College London, Great Ormond Street Hospital for Children NHS Foundation Trust, Imperial College Healthcare NHS Trust	COVID-19 Genomics UK (COG-UK) Consortium	Sergi Castellano, Rachel Williams, Mark Kristiansen, Paola Resende Silva, Sunando Roy, Tony Brooks, Helena Tutill, Paola Niola, Patricia Dyal, Charlotte Williams, Leysa Forrest, Yasmin Panchbhaya, Jacqueline Findlay, Samuel Weeks, Julianne Brown, Kathryn Harris, Paul Randell, James Price, Alison Holmes, Judith Breuer
EPI_ISL_950715	Lincolnshire Hospitals and DeepSeq Nottingham	COVID-19 Genomics UK (COG-UK) Consortium	Nichola Duckworth, Tim Sloan, Sarah Walsh, Jonathan Ball, Patrick McClure, Joeseph Chappell, Nadine Holmes, Matthew Carlisle, Christopher Moore, Fei Sang, Johnny Debebe, Victoria Wright, Matthew Loose
EPI_ISL_951359, EPI_ISL_951361, EPI_ISL_951368, EPI_ISL_951372, EPI_ISL_951373, EPI_ISL_951449, EPI_ISL_951458, EPI_ISL_951459, EPI_ISL_951461, EPI_ISL_951462, EPI_ISL_951463, EPI_ISL_951464, EPI_ISL_951469, EPI_ISL_951475, EPI_ISL_951477, EPI_ISL_951478			
see above	Oxford Viromics, NDM, University of Oxford; Oxford University Hospitals; Basingstoke and North Hampshire Hospital	COVID-19 Genomics UK (COG-UK) Consortium	Tanya Golubchik, David Bonsall, George Macintyre, Amy Trebes, Mariateresa de Cesare, Catrin Moore, Alex Mobbs, Anita Justice, Robert Shaw, Monique Andersson, Timothy Peto, Emma Wise, Nathan Moore, Jessica Lynch, Nick Cortes, Matilde Mori, Stephen Kidd, David Buck, John Todd, Christophe Fraser
EPI_ISL_951819	Originating lab: Wales Specialist Virology Centre Sequencing lab: Pathogen Genomics Unit	Public Health Wales Microbiology Cardiff Wales Specialist Virology Centre	Catherine Moore, Johnathan Evans, Laura Gifford, Malorie Perry, Simon Cottrell, Angela Marchbank, Alec Birchley, Alexander Adams, Amy Gaskin, Bree Gatica-Wilcox, Jason Coombes, Joel Southgate, Lauren Gilbert, Lee Graham, Nicole Pacchiarini, Sara Kumziene-Summerhayes, Sarah Taylor, Sophie Jones, Sara Rey, Matthew Bull, Joanne Watkins, Sally Corden, Tom Connor
EPI_ISL_952378, EPI_ISL_952386, EPI_ISL_952387, EPI_ISL_952389, EPI_ISL_952393, EPI_ISL_952554, EPI_ISL_952601, EPI_ISL_952602, EPI_ISL_952624, EPI_ISL_952783, EPI_ISL_952821, EPI_ISL_952833, EPI_ISL_952834, EPI_ISL_952835, EPI_ISL_952854			
see above	Centre for Enzyme Innovation, University of Portsmouth / Translational Research Laboratory, Portsmouth Hospitals NHS Trust	COVID-19 Genomics UK (COG-UK) Consortium	Angela Beckett,Salman Goudarzi,Christopher Fearn,Kate Cook,Katie Loveson,Sharon Glaysher,Scott Elliott,Samuel Robson
EPI_ISL_952858, EPI_ISL_952859, EPI_ISL_952860, EPI_ISL_952862, EPI_ISL_952863, EPI_ISL_952865, EPI_ISL_952866, EPI_ISL_952867, EPI_ISL_952868, EPI_ISL_952871, EPI_ISL_952874, EPI_ISL_952876, EPI_ISL_952877, EPI_ISL_952878, EPI_ISL_952879, EPI_ISL_952880, EPI_ISL_952881, EPI_ISL_952882, EPI_ISL_952883, EPI_ISL_952884, EPI_ISL_952885, EPI_ISL_952886, EPI_ISL_952888, EPI_ISL_952889, EPI_ISL_952890, EPI_ISL_952891, EPI_ISL_952892, EPI_ISL_952893, EPI_ISL_952894, EPI_ISL_952896, EPI_ISL_952897, EPI_ISL_952900, EPI_ISL_952901, EPI_ISL_952902, EPI_ISL_952903, EPI_ISL_952904, EPI_ISL_952905, EPI_ISL_952906, EPI_ISL_952907, EPI_ISL_952910, EPI_ISL_952913, EPI_ISL_952914, EPI_ISL_952916, EPI_ISL_952917, EPI_ISL_952919, EPI_ISL_952920, EPI_ISL_952923, EPI_ISL_952924, EPI_ISL_952925, EPI_ISL_952926, EPI_ISL_952929, EPI_ISL_952932, EPI_ISL_952933, EPI_ISL_952934, EPI_ISL_952935, EPI_ISL_952937, EPI_ISL_952938, EPI_ISL_952939, EPI_ISL_952940, EPI_ISL_952941			
see above	Department of Pathology, University of Cambridge	COVID-19 Genomics UK (COG-UK) Consortium	Aminu S. Jahun, Yasmin Chaudhry, Iliana Georgana, Myra Hosmillo, Rhys Izu, Martin D. Curran, Surendra Parmar, Ian Goodfellow
EPI_ISL_953065, EPI_ISL_953076, EPI_ISL_953077, EPI_ISL_953078, EPI_ISL_953079, EPI_ISL_953080, EPI_ISL_953081, EPI_ISL_953082, EPI_ISL_953083, EPI_ISL_953085, EPI_ISL_953086, EPI_ISL_953087, EPI_ISL_953088, EPI_ISL_953089, EPI_ISL_953090, EPI_ISL_953091, EPI_ISL_953092, EPI_ISL_953093, EPI_ISL_953094, EPI_ISL_953095, EPI_ISL_953096, EPI_ISL_953097, EPI_ISL_953098, EPI_ISL_953099, EPI_ISL_953100, EPI_ISL_953101, EPI_ISL_953102, EPI_ISL_953103, EPI_ISL_953104, EPI_ISL_953105, EPI_ISL_953106, EPI_ISL_953107, EPI_ISL_953108, EPI_ISL_953109, EPI_ISL_953111, EPI_ISL_953113, EPI_ISL_953114, EPI_ISL_953115, EPI_ISL_953116, EPI_ISL_953117			
see above	Bioinformatics and Biostatistics Lab, Advanced Sequencing Facility	COVID-19 Genomics UK (COG-UK) Consortium	Aengus Stewart,Jerome Nicod,Chelsea Sawyer,Laura Cubitt,Harshil Patel,Margaret Crawford
EPI_ISL_953498, EPI_ISL_953668, EPI_ISL_953669, EPI_ISL_953670	University Hospitals of Geneva, Laboratory of Virology	HUG, Laboratory of Virology and the Health2030 Genome Center	Samuel Cordey, Ana Rita Goncalves, Laurent Kaiser, Lorenzo Cerutti, Henri Pegeot, Melyssa Elies, Deborah Penet, Keith Harshman, Ioannis Xenarios, Emmanouil Dermitzakis
EPI_ISL_953949, EPI_ISL_953950	Hopital	National Reference Center for Viruses of Respiratory Infections, Institut Pasteur, Paris	Marion Barbet, Sylvie Behillil, Méline Bizard, Angela Brisebarre, Camille Capel, Etienne Simon-Lorière, Vincent Enouf, Maud Vanpeene, Sylvie van der Werf,Cady Anne
EPI_ISL_953957, EPI_ISL_953991, EPI_ISL_953992, EPI_ISL_953993	Hopital	National Reference Center for Viruses of Respiratory Infections, Institut Pasteur, Paris	Marion Barbet, Sylvie Behillil, Méline Bizard, Angela Brisebarre, Camille Capel, Etienne Simon-Lorière, Vincent Enouf, Maud Vanpeene, Sylvie van der Werf,Lagathu GisèLe
EPI_ISL_954112	Hopital	National Reference Center for Viruses of Respiratory Infections, Institut Pasteur, Paris	Marion Barbet, Sylvie Behillil, Méline Bizard, Angela Brisebarre, Camille Capel, Etienne Simon-Lorière, Vincent Enouf, Maud Vanpeene, Sylvie van der Werf,Lesimple BéAtrice
EPI_ISL_954113	Hopital	National Reference Center for Viruses of Respiratory Infections, Institut Pasteur, Paris	Marion Barbet, Sylvie Behillil, Méline Bizard, Angela Brisebarre, Camille Capel, Etienne Simon-Lorière, Vincent Enouf, Maud Vanpeene, Sylvie van der Werf,Lagathu GisèLe
EPI_ISL_954114	Labo Analyses Med	National Reference Center for Viruses of Respiratory Infections, Institut Pasteur, Paris	Marion Barbet, Sylvie Behillil, Méline Bizard, Angela Brisebarre, Camille Capel, Etienne Simon-Lorière, Vincent Enouf, Maud Vanpeene, Sylvie van der Werf,Selas Labomaine
EPI_ISL_954115	Hopital	National Reference Center for Viruses of Respiratory Infections, Institut Pasteur, Paris	Marion Barbet, Sylvie Behillil, Méline Bizard, Angela Brisebarre, Camille Capel, Etienne Simon-Lorière, Vincent Enouf, Maud Vanpeene, Sylvie van der Werf,Lagathu GisèLe
EPI_ISL_954116, EPI_ISL_954117, EPI_ISL_954118	Outre Mer	National Reference Center for Viruses of Respiratory Infections, Institut Pasteur, Paris	Marion Barbet, Sylvie Behillil, Méline Bizard, Angela Brisebarre, Camille Capel, Etienne Simon-Lorière, Vincent Enouf, Maud Vanpeene, Sylvie van der Werf,Rousset Dominique
EPI_ISL_954787, EPI_ISL_954790	Hospital J.M. Morales Meseguer	Instituto de Salud Carlos III	Iglesias-Caballero, M. Camarero, S. Sandonís,V. Vázquez, S. Pozo, F. Casas, I. Jiménez, P. Zaballos, A. Monzón, S. Varona, S. Cuesta, I. Navarro, D.
EPI_ISL_954792	HOSPITAL COMARCAL DE MELILLA	Instituto de Salud Carlos III	Iglesias-Caballero, M. Camarero, S. Sandonís,V. Vázquez, S. Pozo, F. Casas, I. Jiménez, P. Zaballos, A. Monzón, S. Varona, S. Cuesta, I. Roman, S.

EPI_ISL_954795	HOSPITAL GENERAL RIO CARRION	Instituto de Salud Carlos III	Iglesias-Caballero, M. Camarero, S. Sandonís,V. Vázquez, S. Pozo, F. Casas, I. Jiménez, P. Zaballos, A. Monzón, S. Varona, S. Cuesta, I. García, A.
EPI_ISL_954812	Hospital Universitario Virgen de la Arrixaca	Instituto de Salud Carlos III	Iglesias-Caballero, M. Camarero, S. Sandonís,V. Vázquez, S. Pozo, F. Casas, I. Jiménez, P. Zaballos, A. Monzón, S. Varona, S. Cuesta, I. Moreno, L.
EPI_ISL_954819, EPI_ISL_954822, EPI_ISL_954823, EPI_ISL_954824, EPI_ISL_954826, EPI_ISL_954827, EPI_ISL_954833, EPI_ISL_954834, EPI_ISL_954836, EPI_ISL_954837, EPI_ISL_954839, EPI_ISL_954841, EPI_ISL_954843, EPI_ISL_954849, EPI_ISL_954850, EPI_ISL_954851, EPI_ISL_954853, EPI_ISL_954859, EPI_ISL_954860, EPI_ISL_954861, EPI_ISL_954866, EPI_ISL_954870, EPI_ISL_954874, EPI_ISL_954880, EPI_ISL_954882, EPI_ISL_954888, EPI_ISL_954889, EPI_ISL_954890, EPI_ISL_954891, EPI_ISL_954951, EPI_ISL_954952, EPI_ISL_954953, EPI_ISL_954954, EPI_ISL_954955, EPI_ISL_954956, EPI_ISL_954957, EPI_ISL_954958, EPI_ISL_954959, EPI_ISL_954960, EPI_ISL_954961, EPI_ISL_954962, EPI_ISL_954963, EPI_ISL_954964, EPI_ISL_954965, EPI_ISL_954966, EPI_ISL_954967, EPI_ISL_954968, EPI_ISL_954969, EPI_ISL_954970, EPI_ISL_954971, EPI_ISL_954972, EPI_ISL_954973, EPI_ISL_954974, EPI_ISL_954975, EPI_ISL_954976, EPI_ISL_954977, EPI_ISL_954978, EPI_ISL_954979, EPI_ISL_954980, EPI_ISL_954981, EPI_ISL_954982, EPI_ISL_954983, EPI_ISL_954984, EPI_ISL_954985, EPI_ISL_954986, EPI_ISL_954987, EPI_ISL_954988, EPI_ISL_954989, EPI_ISL_954990, EPI_ISL_954991, EPI_ISL_954992, EPI_ISL_954993, EPI_ISL_954994, EPI_ISL_954995, EPI_ISL_954996, EPI_ISL_954997, EPI_ISL_954998, EPI_ISL_955131, EPI_ISL_955132, EPI_ISL_955133, EPI_ISL_955134, EPI_ISL_955135			
see above	Colorado Department of Public Health and Environment	Colorado Department of Puplic Health and Environment	Laura Bankers, Molly C. Hetherington-Rauth, Diana Ir, Shannon Ely, Shannon R. Matzinger, Sarah Elizabeth Totten, Emily A. Travanty
EPI_ISL_955174	Hopital	National Reference Center for Viruses of Respiratory Infections, Institut Pasteur, Paris	Marion Barbet, Sylvie Behillil, Méline Bizard, Angela Brisebarre, Camille Capel, Etienne Simon-Lorière, Vincent Enouf, Maud Vanpeene, Sylvie van der Werf,Goudeau Alain
EPI_ISL_955175	Hopital	National Reference Center for Viruses of Respiratory Infections, Institut Pasteur, Paris	Marion Barbet, Sylvie Behillil, Méline Bizard, Angela Brisebarre, Camille Capel, Etienne Simon-Lorière, Vincent Enouf, Maud Vanpeene, Sylvie van der Werf
EPI_ISL_955186	University of Sarajevo, Veterinary Faculty, Laboratory for Molecular Diagnostic and Research Laboratory	University of Sarajevo, Veterinary Faculty, Laboratory for Molecular Diagnostic and Research Laboratory	Goleti Š., Goleti T., Ali-Šeho A., Softi A., Terzi I., Jaži A., Nicevi M., Hodži A., Šabi E.
EPI_ISL_955187	University of Sarajevo, Veterinary Faculty, Laboratory for Molecular Diagnostic and Research Laboratory	University of Sarajevo, Veterinary Faculty, Laboratory for Molecular Diagnostic and Research Laboratory	Goleti Š., Goleti T., Ali-Šeho A., Softi A., Hodži A., Terzi I., Šabi E., Jaži A., Nicevi M.
EPI_ISL_955273	American Esoteric Laboratory	Pathogen Discovery, Respiratory Viruses Branch, Division of Viral Diseases, Centers for Disease Control and Prevention	Ying Tao, Jing Zhang, Yan Li, Krista Queen, Anna Uehara, Peter Cook, Clinton R. Paden, Haibin Wang, Suxiang Tong
EPI_ISL_955391	Alameda County Public Health Lab	Chan-Zuckerberg Biohub	CZB Cliahub Consortium
EPI_ISL_955778, EPI_ISL_955780, EPI_ISL_955783, EPI_ISL_955784, EPI_ISL_955788, EPI_ISL_955791, EPI_ISL_955792, EPI_ISL_955793, EPI_ISL_955802, EPI_ISL_955840, EPI_ISL_955841, EPI_ISL_955842, EPI_ISL_955843, EPI_ISL_955844			
see above	University of Michigan Clinical Microbiology Laboratory	Lauring Lab, University of Michigan, Department of Microbiology and Immunology	Valesano
EPI_ISL_956283	Instituto Nacional de Salud- Dirección de Redes de Laboratorios de Salud Pública	Instituto Nacional de Salud- Dirección de Investigación en Salud Pública	Katherine Laiton-Donato, Diego A. Álvarez-Díaz, Carlos Franco-Muñoz, Mauricio Pacheco-Montealegre, Hector Alejandro Ruiz-Moreno, Maria T. Herrera-Sepúlveda, Diego Andrés Prada, Jhonnatan Reales-González, Sheryll Corchuelo, Julian Naizaque, Gerardo Santamaría, Magdalena Wiesner, Martha Lucia Ospina Martinez, Marcela Mercado-Reyes
EPI_ISL_956303	LABORATORIO ANALIZAR SYNLAB	Instituto Nacional de Salud- Dirección de Investigación en Salud Pública	Katherine Laiton-Donato, Diego A. Álvarez-Díaz, Carlos Franco-Muñoz, Mauricio Pacheco-Montealegre, Hector Alejandro Ruiz-Moreno, Maria T. Herrera-Sepúlveda, Diego Andrés Prada, Jhonnatan Reales-González, Sheryll Corchuelo, Julian Naizaque, Gerardo Santamaría, Magdalena Wiesner, Martha Lucia Ospina Martinez, Marcela Mercado-Reyes
EPI_ISL_956334, EPI_ISL_956336	Utah Public Health Laboratory, Utah Public Health Laboratory Infectious Disease submission group	Utah Public Health Laboratory, Utah Public Health Laboratory Infectious Disease submission group	Gallagher,T., Young,E.L., Oakeson,K.F.
EPI_ISL_959444, EPI_ISL_959468, EPI_ISL_959469, EPI_ISL_959470, EPI_ISL_959471	Servicio de Microbiología, Hospital Universitario Son Espases	SeqCOVID-SPAIN consortium/IBV(CSIC)	Carla López-Causapé, Jordi Reina, Antonio Oliver and SeqCOVID-SPAIN consortium
EPI_ISL_959542	University of Michigan Clinical Microbiology Laboratory	Lauring Lab, University of Michigan, Department of Microbiology and Immunology	Valesano
EPI_ISL_959546	Laboratory of Virology and Molecular Diagnostics	Institute of Public Health of Republic of North Macedonia Laboratory of Virology and Molecular Diagnostics	Kuzmanovska M., Boshevska G.
EPI_ISL_959563	University of Liège COVID-19 testing center	GIGA Medical Genomics	Keith Durkin, Maria Artesi, Sébastien Bontems, Raphaël Boreux, Bouchra Boujemla, Cécile Meex, Pierrette Melin, Marie-Pierre Hayette, Vincent Bours
EPI_ISL_959564, EPI_ISL_959565	Vivalia - Clinique Saint-Joseph	GIGA Medical Genomics	Keith Durkin, Maria Artesi, Sébastien Bontems, Raphaël Boreux, Bouchra Boujemla, Cécile Meex, Pierrette Melin, Marie-Pierre Hayette, Vincent Bours
EPI_ISL_959566	University of Liège COVID-19 testing center	GIGA Medical Genomics	Keith Durkin, Maria Artesi, Sébastien Bontems, Raphaël Boreux, Bouchra Boujemla, Cécile Meex, Pierrette Melin, Marie-Pierre Hayette, Vincent Bours
EPI_ISL_959839, EPI_ISL_959856	National Virus Reference Laboratory	National Virus Reference Laboratory	Michael Carr, Gabriel Gonzalez, Jonathan Dean, Cillian F De Gascun
EPI_ISL_960226	Botswana Harvard HIV Reference Laboratory	Botswana Harvard HIV Reference Laboratory	Sikhulile Moyo,Dorcas Maruapula, Wonderful T. Choga, Botshelo Radibe, Boitumelo Zuze, David Lawrence, Roger Shapiro, Shahin Lockman, Mosepele Mosepele, Joseph Makhema, Simani Gaseitsiwe
EPI_ISL_960303	Botswana Harvard HIV Reference Laboratory	Botswana Harvard HIV Reference Laboratory	Sikhulile Moyo, Dorcas Maruapula, Wonderful Choga, Botshelo Radibe, Boitumelo Zuze, David Lawrence, Roger Shapiro, Shahin Lockman, Mosepele Mosepele, Joseph Makhema, Simani Gaseitsiwe
EPI_ISL_960314, EPI_ISL_960315, EPI_ISL_960316, EPI_ISL_960317	University of Wisconsin-Madison AIDS Vaccine Research Laboratories	University of Wisconsin-Madison AIDS Vaccine Research Laboratories	Gage Moreno, Katarina Braun, et al. AIDS Vaccine Research Laboratories
EPI_ISL_960406, EPI_ISL_960420, EPI_ISL_960421, EPI_ISL_960428	The National Institute of Public Health	State Veterinary Institute Prague	Nagy,A;Vecerova,J;Cernikova,L;Stara,M;Jirincova,H;Trnka,D
EPI_ISL_960986, EPI_ISL_960987, EPI_ISL_960988, EPI_ISL_960991, EPI_ISL_960994, EPI_ISL_960999, EPI_ISL_961000, EPI_ISL_961002	AIID	Irish Coronavirus Sequencing Consortium-Teagasc Grange	Matthew McCabe, Aljandro Abner Garcia Leon, Fiona Crispie, Calum Walsh, Michael Carr, John Kenny, Paul Cotter, Patrick Mallon, Gabriel Gonzalez
EPI_ISL_961134, EPI_ISL_961166	Texas Department of State Health Services	Texas Department of State Health Services	Bonnie Oh, Anita Pokharel, James Daniel Bonser, Myong Koag, Chung Wang, Rachel Lee, Grace Kubin, Rashmi Tuladhar, Mayela Pedrueza, Maliha Rahman, Jenny Zhang
EPI_ISL_961228, EPI_ISL_961229	Hospital General Universitario de Alicante - Instituto de Investigación Sanitaria y Biomédica de Alicante	SeqCOVID-SPAIN consortium/IBV(CSIC)	Maripaz Ventero Martín, Carmen Molina Pardines and SeqCOVID-SPAIN consortium
EPI_ISL_961471, EPI_ISL_961484, EPI_ISL_961494, EPI_ISL_961497, EPI_ISL_961502, EPI_ISL_961503, EPI_ISL_961511, EPI_ISL_961520, EPI_ISL_961526, EPI_ISL_961544, EPI_ISL_961545, EPI_ISL_961546			
see above	Michigan Department of Health and Human Services, Bureau of Laboratories	Michigan Department of Health and Human Services, Bureau of Laboratories	Blankenship HM, Riner D, Soehnlen MK
EPI_ISL_961591, EPI_ISL_961592, EPI_ISL_961593, EPI_ISL_961594, EPI_ISL_961595, EPI_ISL_961596, EPI_ISL_961630, EPI_ISL_961631, EPI_ISL_961632, EPI_ISL_961633, EPI_ISL_961634, EPI_ISL_961635, EPI_ISL_961636			
see above	Hôpital Georges L. Dumont	National Microbiology Laboratory (NML)	Anna Majer, Shari Tyson, Grace Seo, Philip Mabon, Elsie Grudeski, Rhiannon Huzarewich, Russell Mandes, Anneliese Landgraff, Jennifer Tanner, Natalie Knox, Morag Graham, Gary Van Domselaar, Richard Garceau, Guillaume Desnoyers, Nathalie Bastien, Yan Li, Timothy Booth, Darian Hole, Madison Chapel, Kirsten Biggar, CanCOGeN's metadata curation team, Public Health Agency of Canada CanCOGeN team
EPI_ISL_961873, EPI_ISL_961874	E. Gulbja laboratorija	Latvian Biomedical Research and Study Centre	Janis Pjalkovskis, Nikita Zrelovs, Monta Ustinova, Ivars Silamikelis, Liga Birzniece, Kaspars Megnis, Vita Rovite, Lauma Freimane, Laila Silamikele, Laura Ansone, Davids Fridmanis, Mikus Gavars, Dmitrijs Perminovs, Jurijs Perevoscikovs, Uga Dumpis, Janis Klovins
EPI_ISL_961972, EPI_ISL_962176	Illinois Department of Public Health	Gagnon Lab, Southern Illinois University	Keith Gagnon
EPI_ISL_962203, EPI_ISL_962204, EPI_ISL_962207	Institute for Medical Research, Infectious Disease Research Centre, National Institutes of Health, Ministry of Health Malaysia	Institute for Medical Research, Infectious Disease Research Centre, National Institutes of Health, Ministry of Health Malaysia	Suppiah J, Kamel K, Azizan MA, Thayan R

EPI_ISL_962278, EPI_ISL_962279, EPI_ISL_962280, EPI_ISL_962281, EPI_ISL_962282, EPI_ISL_962283, EPI_ISL_962284, EPI_ISL_962287	Seattle Flu Study	Seattle Flu Study	Deborah A. Nickerson, Chris D. Frazar, Jover Lee, Benjamin Pelle, Erica Ryke, Matthew Richardson, Amanda Adler, Elisabeth Brandstetter, Peter D. Han, Kairsten Fay, Misja Ilcisin, Kirsten Lacombe, Thomas R. Sibley, Melissa Truong, Caitlin R. Wolf, Karen Cowgill, Stephanie Schrag, Jeff Duchin, Michael Boeckh, Janet A. Englund, Michael Famulare, Barry R. Lutz, Mark J. Rieder, Lea M. Starita, Matthew Thompson, Helen Y. Chu, Trevor Bedford, Jay Shendure
EPI_ISL_962389, EPI_ISL_962390, EPI_ISL_962391, EPI_ISL_962392, EPI_ISL_962393, EPI_ISL_962394, EPI_ISL_962395, EPI_ISL_962396, EPI_ISL_962397, EPI_ISL_962398, EPI_ISL_962399, EPI_ISL_962400, EPI_ISL_962401, EPI_ISL_962402			
see above	Washington State Department of Health	Seattle Flu Study	Deborah A. Nickerson, Chris D. Frazar, Jover Lee, Benjamin Pelle, Erica Ryke, Matthew Richardson, Amanda Adler, Elisabeth Brandstetter, Peter D. Han, Kairsten Fay, Misja Ilcisin, Kirsten Lacombe, Thomas R. Sibley, Melissa Truong, Caitlin R. Wolf, Romesh Gautom, Geoff Melly, Brian Hiatt, Philip Dykema, Scott Lindquist, Michael Boeckh, Janet A. Englund, Michael Famulare, Barry R. Lutz, Mark J. Rieder, Lea M. Starita, Matthew Thompson, Helen Y. Chu, Jay Shendure, Trevor Bedford
EPI_ISL_962525, EPI_ISL_962526	Institute for Medical Research, Infectious Disease Research Centre, National Institutes of Health, Ministry of Health Malaysia	Institute for Medical Research, Infectious Disease Research Centre, National Institutes of Health, Ministry of Health Malaysia	Suppiah J, Kamel K, Azizan MA, Thayan R
EPI_ISL_962621, EPI_ISL_962622, EPI_ISL_962623, EPI_ISL_962625, EPI_ISL_962628, EPI_ISL_962630, EPI_ISL_962631, EPI_ISL_962634, EPI_ISL_962635, EPI_ISL_962637, EPI_ISL_962638, EPI_ISL_962639, EPI_ISL_962640, EPI_ISL_962642, EPI_ISL_962644, EPI_ISL_962646, EPI_ISL_962647			
see above	Scripps Medical Laboratory	Andersen lab at Scripps Research	SEARCH Alliance San Diego with Michael Quigley, Ellen Stefanski, Ian Mchardy
EPI_ISL_962710, EPI_ISL_962711, EPI_ISL_962712, EPI_ISL_962713, EPI_ISL_962714, EPI_ISL_962715, EPI_ISL_962716, EPI_ISL_962717, EPI_ISL_962718, EPI_ISL_962720, EPI_ISL_962721, EPI_ISL_962722, EPI_ISL_962723, EPI_ISL_962724, EPI_ISL_962725, EPI_ISL_962726, EPI_ISL_962728, EPI_ISL_962729, EPI_ISL_962731, EPI_ISL_962732, EPI_ISL_962733, EPI_ISL_962734, EPI_ISL_962735, EPI_ISL_962736, EPI_ISL_962737, EPI_ISL_962738, EPI_ISL_962740, EPI_ISL_962741, EPI_ISL_962742, EPI_ISL_962743, EPI_ISL_962746, EPI_ISL_962747, EPI_ISL_962748, EPI_ISL_962749, EPI_ISL_962750, EPI_ISL_962751, EPI_ISL_962752, EPI_ISL_962753, EPI_ISL_962755, EPI_ISL_962757, EPI_ISL_962760, EPI_ISL_962761, EPI_ISL_962762, EPI_ISL_962763, EPI_ISL_962764			
see above	Sharp HealthCare Laboratory	Andersen lab at Scripps Research	SEARCH Alliance San Diego with Aaron Harding, Jacquelyn Berumen, Cathy Woerle, Liam McGinnis, Art Mendoza, Omid Bakhtar
EPI_ISL_962813	Robert Garry lab	Andersen lab at Scripps Research	Allison Smither, Gilberto Sabino-Santos, Patricia Snarski, Lilia Melnik, Antoinette Bell, Kaylynn Genemaras, Arnaud Drouin, Dahlene Fusco, Robert Garry with SEARCH Alliance San Diego
EPI_ISL_962887, EPI_ISL_962890	Laboratory of Virology and Molecular Diagnostics	Institute of Public Health of Republic of North Macedonia Laboratory of Virology and Molecular Diagnostics	Kuzmanovska M., Boshevska G.
EPI_ISL_962891	Laboratory of Virology and Molecular Diagnostics	Laboratory of Virology and Molecular Diagnostics, Institute of Public Health of Republic of North Macedonia	Kuzmanovska M., Boshevska G.
EPI_ISL_962893, EPI_ISL_962894	Laboratory of Virology and Molecular Diagnostics	Institute of Public Health of Republic of North Macedonia Laboratory of Virology and Molecular Diagnostics	Kuzmanovska M., Boshevska G.
EPI_ISL_962944, EPI_ISL_962954, EPI_ISL_962955	Hospital Universitario de Gran Canaria Dr. Negrín	SeqCOVID-SPAIN consortium/IBV(CSIC)	M. Carmen Pérez González, Francisco J. Chamizo López, Ana Bordes Benítez and SeqCOVID-SPAIN consortium
EPI_ISL_964272, EPI_ISL_964275	Oslo University Hospital, Department of Medical Microbiology	Norwegian Institute of Public Health, Department of Virology	Kathrine Stene-Johansen, Kamilla Heddeland Instefjord, Hilde Elshaug, Ignacio Garcia Llorente, Serina B Engebretsen, Atiya R Ali, Marie Paulsen Madsen, Rasmus Riis Kopperud, Hilde Vollan, Karoline Bragstad, Olav Hungnes
EPI_ISL_964881, EPI_ISL_964883	Laboratory of Virology and Molecular Diagnostics	Institute of Public Health of Republic of North Macedonia Laboratory of Virology and Molecular Diagnostics	Kuzmanovska M., Boshevska G.
EPI_ISL_964919, EPI_ISL_964920, EPI_ISL_964922, EPI_ISL_964923, EPI_ISL_964925, EPI_ISL_964926, EPI_ISL_964927, EPI_ISL_964928, EPI_ISL_964929, EPI_ISL_964930, EPI_ISL_964931, EPI_ISL_964934, EPI_ISL_964935, EPI_ISL_964936, EPI_ISL_964937, EPI_ISL_964939, EPI_ISL_964942, EPI_ISL_964943, EPI_ISL_964944, EPI_ISL_964945, EPI_ISL_964946, EPI_ISL_964947			
see above	Instituto Nacional de Saude (INS), Mozambique	KRISP, KZN Research Innovation and Sequencing Platform	Nalia Ismael, Nadia Sitoe, Paulo Arnaldo, Nedio Mabunda, Giandhari J, Pillay S, Emmanuel S, Tegally H, Wilkinson E, de Oliveira T
EPI_ISL_964995	Foerde Hospital, Department of Microbiology	Norwegian Institute of Public Health, Department of Virology	Kathrine Stene-Johansen, Kamilla Heddeland Instefjord, Hilde Elshaug, Ignacio Garcia Llorente, Serina B Engebretsen, Atiya R Ali, Marie Paulsen Madsen, Rasmus Riis Kopperud, Hilde Vollan, Karoline Bragstad, Olav Hungnes
EPI_ISL_965021, EPI_ISL_965022, EPI_ISL_965023, EPI_ISL_965024	Florida Bureau of Public Health Laboratories	Florida Bureau of Public Health Laboratories	Sarah Schmedes, Jason Blanton
EPI_ISL_965101, EPI_ISL_965102, EPI_ISL_965103, EPI_ISL_965104, EPI_ISL_965105, EPI_ISL_965106, EPI_ISL_965107, EPI_ISL_965108, EPI_ISL_965109, EPI_ISL_965110, EPI_ISL_965111, EPI_ISL_965112			
see above	Wyoming Public Health Laboratory	Wyoming Public Health Laboratory	Noah Hull, Taylor Fearing, Lynette Gumbleton, Channing Weber, Ashley Norberg, Bailey Bowcutt, and Wanda Manley
EPI_ISL_965127	INMI Lazzaro Spallanzani IRCCS	INMI Lazzaro Spallanzani IRCCS	CEM Gruber, B Bartolini, E Giombini, M Rueca, O Butera, F Messina, A Di Caro, MR Capobianchi
EPI_ISL_965550, EPI_ISL_965738, EPI_ISL_965745, EPI_ISL_965746	Dutch COVID-19 response team	Medical Microbiology, Maastricht University Medical Centre	Jozef Dingemans*, Brian van der Veer*, Erik Beuken, Carmen Reumkens, Lieke van Alphen, Christian Hoebe, Paul Savelkoul
EPI_ISL_965847, EPI_ISL_965848, EPI_ISL_965849, EPI_ISL_965850, EPI_ISL_965851, EPI_ISL_965852	GA Department of Public Health	GA Department of Public Health	Stacy Reeves, Jonathan Edwards, Cynthia Dixey, Tonia Parrott
EPI_ISL_965863	Massachusetts State Public Health Laboratory	Massachusetts State Public Health Laboratory	Andrew Lang, Timelia Fink, Glen Gallagher, Sandra Smole
EPI_ISL_965912	Servicio Murciano de Salud	Instituto de Salud Carlos III	Vázquez, S. Iglesias-Caballero, M. Sandonís,V. Camarero, S. Pozo, F. Casas, I. Jiménez, P. Zaballos, A. Monzón, S. Varona, S. Cuesta, I. Blázquez, A.
EPI_ISL_965921	Consejería de Sanidad y Asuntos Sociales de Castilla La Mancha	Instituto de Salud Carlos III	Vázquez, S. Iglesias-Caballero, M. Sandonís,V. Camarero, S. Pozo, F. Casas, I. Jiménez, P. Zaballos, A. Monzón, S. Varona, S. Cuesta, I. Gutiérrez, G.
EPI_ISL_965922, EPI_ISL_965925	Servicio Murciano de Salud	Instituto de Salud Carlos III	Vázquez, S. Iglesias-Caballero, M. Sandonís,V. Camarero, S. Pozo, F. Casas, I. Jiménez, P. Zaballos, A. Monzón, S. Varona, S. Cuesta, I. Blázquez, A.
EPI_ISL_965927, EPI_ISL_965931	Servicio Murciano de Salud	Instituto de Salud Carlos III	Sandonís,V. Vázquez, S. Iglesias-Caballero, M. Camarero, S. Pozo, F. Casas, I. Jiménez, P. Zaballos, A. Monzón, S. Varona, S. Cuesta, I. Blázquez, A.
EPI_ISL_965953	Servicio Murciano de Salud	Instituto de Salud Carlos III	Iglesias-Caballero, M. Sandonís,V. Vázquez, S. Camarero, S. Pozo, F. Casas, I. Jiménez, P. Zaballos, A. Monzón, S. Varona, S. Cuesta, I. Blázquez, A.
EPI_ISL_966500, EPI_ISL_966501, EPI_ISL_966502, EPI_ISL_966503, EPI_ISL_966504, EPI_ISL_966505, EPI_ISL_966506, EPI_ISL_966507, EPI_ISL_966508, EPI_ISL_966509, EPI_ISL_966510, EPI_ISL_966511, EPI_ISL_966512, EPI_ISL_966514, EPI_ISL_966515, EPI_ISL_966516, EPI_ISL_966517, EPI_ISL_966518, EPI_ISL_966519, EPI_ISL_966520, EPI_ISL_966521, EPI_ISL_966522, EPI_ISL_966523, EPI_ISL_966524, EPI_ISL_966525, EPI_ISL_966526, EPI_ISL_966527, EPI_ISL_966528, EPI_ISL_966529, EPI_ISL_966530, EPI_ISL_966531, EPI_ISL_966577, EPI_ISL_966578, EPI_ISL_966579, EPI_ISL_966580, EPI_ISL_966581, EPI_ISL_966582, EPI_ISL_966583, EPI_ISL_966584, EPI_ISL_966585, EPI_ISL_966586, EPI_ISL_966587, EPI_ISL_966588, EPI_ISL_966589, EPI_ISL_966590, EPI_ISL_966600, EPI_ISL_966601, EPI_ISL_966604, EPI_ISL_967170, EPI_ISL_967172, EPI_ISL_967173, EPI_ISL_967176, EPI_ISL_967177, EPI_ISL_967178, EPI_ISL_967185, EPI_ISL_967202, EPI_ISL_967203, EPI_ISL_967204, EPI_ISL_967205, EPI_ISL_967206, EPI_ISL_967207, EPI_ISL_967208, EPI_ISL_967209, EPI_ISL_967210, EPI_ISL_967211, EPI_ISL_967212, EPI_ISL_967215, EPI_ISL_967217, EPI_ISL_967219, EPI_ISL_967327, EPI_ISL_967328, EPI_ISL_967329, EPI_ISL_967330, EPI_ISL_967331, EPI_ISL_967332, EPI_ISL_967333, EPI_ISL_967334, EPI_ISL_967335, EPI_ISL_967336, EPI_ISL_967337, EPI_ISL_967338, EPI_ISL_967339, EPI_ISL_967340, EPI_ISL_967341, EPI_ISL_967342, EPI_ISL_967343, EPI_ISL_967344, EPI_ISL_967345, EPI_ISL_967346, EPI_ISL_967347, EPI_ISL_967348, EPI_ISL_967349, EPI_ISL_967350, EPI_ISL_967351, EPI_ISL_967352, EPI_ISL_967353, EPI_ISL_967354, EPI_ISL_967355, EPI_ISL_967356, EPI_ISL_967357, EPI_ISL_967358, EPI_ISL_967359, EPI_ISL_967360, EPI_ISL_967361, EPI_ISL_967362, EPI_ISL_967363, EPI_ISL_967364, EPI_ISL_967365, EPI_ISL_967366, EPI_ISL_967367, EPI_ISL_967368, EPI_ISL_967369, EPI_ISL_967370, EPI_ISL_967371, EPI_ISL_967372, EPI_ISL_967373, EPI_ISL_967374, EPI_ISL_967375, EPI_ISL_967376, EPI_ISL_967377, EPI_ISL_967378, EPI_ISL_967379, EPI_ISL_967380, EPI_ISL_967381, EPI_ISL_967382, EPI_ISL_967383, EPI_ISL_967384, EPI_ISL_967385, EPI_ISL_967386, EPI_ISL_967387, EPI_ISL_967388, EPI_ISL_967389, EPI_ISL_967390, EPI_ISL_967391, EPI_ISL_967392, EPI_ISL_967393, EPI_ISL_967394, EPI_ISL_967395, EPI_ISL_967396, EPI_ISL_967397, EPI_ISL_967398, EPI_ISL_967399, EPI_ISL_967400, EPI_ISL_967401, EPI_ISL_967402, EPI_ISL_967403, EPI_ISL_967404, EPI_ISL_967405, EPI_ISL_967406, EPI_ISL_967407, EPI_ISL_967408, EPI_ISL_967409, EPI_ISL_967410, EPI_ISL_967411, EPI_ISL_967412, EPI_ISL_967413, EPI_ISL_967414, EPI_ISL_967415, EPI_ISL_967416, EPI_ISL_967417, EPI_ISL_967418, EPI_ISL_967419, EPI_ISL_967420, EPI_ISL_967421, EPI_ISL_967422, EPI_ISL_967423, EPI_ISL_967424			
see above	Helix/Illumina	Respiratory Viruses Branch, Division of Viral Diseases, Centers for Disease Control and Prevention	Peter W. Cook,Dakota Howard,Dhwani Batra,Ben L. Rambo-Martin,Eileen de Feo,Jan Antico,Christine Tran,Matthew Tolentino,Shannon Wickline,Kim Gietzen,Brad Sickler,Jingtao Liu,Eric Allen,Phil Febbo,Summer Galloway,Nicole L. Washington,Simon White,Geraint Levan,Kelly Schiabor Barrett,Elizabeth Cirulli,Alexandre Bolze,Ary Ascencio,Charlotte Rivera-Garcia,Ryan Cho,Jason Nguyen,Sherry Wang,Jimmy Ramirez,Tyler Cassens,Efren Sandoval,Magnus Isaksson,William Lee,David Becker,Marc Laurent,James Lu,Clinton R. Paden,Suxiang Tong,Duncan MacCannell,
EPI_ISL_967704, EPI_ISL_967715, EPI_ISL_967720, EPI_ISL_967721, EPI_ISL_967746, EPI_ISL_967757	State Laboratories Division, Hawaii State Department of Health	State Laboratories Division, Hawaii State Department of Health	Pamela O'Brien, Drew Kuwazaki, Ayana Garnet, Razvan Sultana, Edward Desmond

EPI_ISL_967781, EPI_ISL_967782, EPI_ISL_967783, EPI_ISL_967784, EPI_ISL_967785, EPI_ISL_967786, EPI_ISL_967787, EPI_ISL_967788, EPI_ISL_967789, EPI_ISL_967790, EPI_ISL_967791, EPI_ISL_967792, EPI_ISL_967793, EPI_ISL_967794, EPI_ISL_967795, EPI_ISL_967796, EPI_ISL_967797, EPI_ISL_967798, EPI_ISL_967799, EPI_ISL_967800, EPI_ISL_967801, EPI_ISL_967802, EPI_ISL_967803, EPI_ISL_967804, EPI_ISL_967805, EPI_ISL_967806, EPI_ISL_967807, EPI_ISL_967808, EPI_ISL_967809, EPI_ISL_967810, EPI_ISL_967811, EPI_ISL_967812, EPI_ISL_967813, EPI_ISL_967814, EPI_ISL_967815, EPI_ISL_967816, EPI_ISL_967817, EPI_ISL_967818, EPI_ISL_967819, EPI_ISL_967820, EPI_ISL_967821, EPI_ISL_967822, EPI_ISL_967823, EPI_ISL_967824, EPI_ISL_967825, EPI_ISL_967826, EPI_ISL_967827, EPI_ISL_967828, EPI_ISL_967829, EPI_ISL_967830, EPI_ISL_967831, EPI_ISL_967832, EPI_ISL_967833, EPI_ISL_967834, EPI_ISL_967835, EPI_ISL_967836, EPI_ISL_967837, EPI_ISL_967838, EPI_ISL_967839, EPI_ISL_967840, EPI_ISL_967841, EPI_ISL_967842			
see above	Helix/Illumina	Respiratory Viruses Branch, Division of Viral Diseases, Centers for Disease Control and Prevention	Peter W. Cook,Dakota Howard,Dhwani Batra,Ben L. Rambo-Martin,Eileen de Feo,Jan Antico,Christine Tran,Matthew Tolentino,Shannon Wickline,Kim Gietzen,Brad Sickler,Jingtao Liu,Eric Allen,Phil Febbo,Summer Galloway,Nicole L. Washington,Simon White,Geraint Levan,Kelly Schiabor Barrett,Elizabeth Cirulli,Alexandre Bolze,Ary Ascencio,Charlotte Rivera-Garcia,Ryan Cho,Jason Nguyen,Sherry Wang,Jimmy Ramirez,Tyler Cassens,Efren Sandoval,Magnus Isaksson,William Lee,David Becker,Marc Laurent,James Lu,Clinton R. Paden,Suxiang Tong,Duncan MacCannell,
EPI_ISL_967887	TGen North	TGen North	"Jolene Bowers, Megan Folkerts, Chris French, Hayley Yaglom, Ashlyn Pfeiffer, Darrin Lemmer, Dave Engelthaler, The Arizona COVID Genomics Union (ACGU)"
EPI_ISL_967891, EPI_ISL_967899, EPI_ISL_967900, EPI_ISL_967907, EPI_ISL_967926, EPI_ISL_967932, EPI_ISL_967933, EPI_ISL_967934, EPI_ISL_967942, EPI_ISL_967943, EPI_ISL_967965, EPI_ISL_967969, EPI_ISL_967978			
see above	TGen North	Sonora Quest Laboratories	"Jolene Bowers, Megan Folkerts, Chris French, Hayley Yaglom, Ashlyn Pfeiffer, Darrin Lemmer, Dave Engelthaler, The Arizona COVID Genomics Union (ACGU)"
EPI_ISL_967979	TGen North	TGen North	"Jolene Bowers, Megan Folkerts, Chris French, Hayley Yaglom, Ashlyn Pfeiffer, Darrin Lemmer, Dave Engelthaler, The Arizona COVID Genomics Union (ACGU)"
EPI_ISL_967980, EPI_ISL_967989, EPI_ISL_967993, EPI_ISL_967994, EPI_ISL_968000, EPI_ISL_968004, EPI_ISL_968014, EPI_ISL_968023, EPI_ISL_968024, EPI_ISL_968028, EPI_ISL_968031, EPI_ISL_968033, EPI_ISL_968036, EPI_ISL_968052, EPI_ISL_968056			
see above	TGen North	Sonora Quest Laboratories	"Jolene Bowers, Megan Folkerts, Chris French, Hayley Yaglom, Ashlyn Pfeiffer, Darrin Lemmer, Dave Engelthaler, The Arizona COVID Genomics Union (ACGU)"
EPI_ISL_968057	TGen North	TGen North	"Jolene Bowers, Megan Folkerts, Chris French, Hayley Yaglom, Ashlyn Pfeiffer, Darrin Lemmer, Dave Engelthaler, The Arizona COVID Genomics Union (ACGU)"
EPI_ISL_968062	TGen North	Sonora Quest Laboratories	"Jolene Bowers, Megan Folkerts, Chris French, Hayley Yaglom, Ashlyn Pfeiffer, Darrin Lemmer, Dave Engelthaler, The Arizona COVID Genomics Union (ACGU)"
EPI_ISL_968065	TGen North	TGen North	"Jolene Bowers, Megan Folkerts, Chris French, Hayley Yaglom, Ashlyn Pfeiffer, Darrin Lemmer, Dave Engelthaler, The Arizona COVID Genomics Union (ACGU)"
EPI_ISL_968998, EPI_ISL_968999, EPI_ISL_969003, EPI_ISL_969050, EPI_ISL_969051	KEMRI-Wellcome Trust Research Programme/KEMRI-CGMR-C Kilifi	KEMRI-Wellcome Trust Research Programme/KEMRI-CGMR-C Kilifi	Githinji et al
EPI_ISL_977102, EPI_ISL_977103, EPI_ISL_977104, EPI_ISL_977105, EPI_ISL_977106, EPI_ISL_977107, EPI_ISL_977108, EPI_ISL_977109, EPI_ISL_977110, EPI_ISL_977111, EPI_ISL_977115, EPI_ISL_977116			
see above	Massachusetts General Hospital	Infectious Disease Program, Broad Institute of Harvard and MIT	Lemieux,J.E., Siddle,K.J., Shaw,B., Adams,G., Pierce,V., Turbett,S., Anahtar,M., Branda,J., Slater,D., Harris,J., Lin,A.E., Gladden-Young,A., Lagerborg,K., Rudy,M., DeRuff,K., Carter,A., Normandin,E., Bauer,M., Reilly,S., Tomkins-Tinch,C., Loreth,C., Chaluvadi,S., Neumann,A., Cusick,C., Chapman,S.B., Gnirke,A., Flowers,K., Cerrato,F., Birren,B.W., Gallagher,G., Smole,S., Park,D.J., MacInnis,B.L., Ryan,E., LaRocque,R., Rosenberg,E. and Sabeti,P.C.
EPI_ISL_977131, EPI_ISL_977132, EPI_ISL_977138, EPI_ISL_977139, EPI_ISL_977140, EPI_ISL_977141, EPI_ISL_977142, EPI_ISL_977143	Flow Health	Infectious Disease Program, Broad Institute of Harvard and MIT	Lemieux,J.E., Siddle,K.J., Adams,G., Gladden-Young,A., Lagerborg,K., Rudy,M., DeRuff,K., Carter,A., Normandin,E., Bauer,M., Reilly,S., Tomkins-Tinch,C., Loreth,C., Chaluvadi,S., Birren,B.W., Gallagher,G., Smole,S., Park,D.J., MacInnis,B.L., and Sabeti,P.C.
EPI_ISL_977583	Caribbean Public Health Agency	Carrington Lab, Department of PreClinical Sciences	Nikita S. D. Sahadeo, Arianne Brown-Jordan, Vernie Ramkissoon, Sarah Hill, Naresh Nandram, Avery Hinds, Dr. Sharon Belmar-George, Jerome Foster, Stanley Giddings, Karla Georges, Marsha Ivey, Rahul Naidu, Risha Singh, SueMin Nathaniel, Rajini Haraksingh, Jaya Jayaraman, Chinna Chinnadurai, Adesh Ramsubhag, Nuno Faria, Oliver Pybus, Christopher Oura, Gabriel Escobar, Christine V. F. Carrington
EPI_ISL_977597	Servicio Murciano de Salud	Instituto de Salud Carlos III	Vázquez, S. Iglesias-Caballero, M. Sandonís,V. Camarero, S. Pozo, F. Casas, I. Jiménez, P. Zaballos, A. Monzón, S. Varona, S. Cuesta, I. Blázquez, A.
EPI_ISL_977654	Michigan Department of Health and Human Services, Bureau of Laboratories	Michigan Department of Health and Human Services, Bureau of Laboratories	Blankenship HM, Riner D, Soehnlen MK
EPI_ISL_977666	NYU Langone Health	Departments of Pathology and Medicine, New York University School of Medicine	Adriana Heguy, Dacia Dimartino, Emily Guzman, Christian Marier, Peter Meyn, Sitharam Ramaswami, Gael Westby, Paul Zappile, Yutong Zhang, Paolo Cotzia, Guiqing Wang
EPI_ISL_977918, EPI_ISL_977919, EPI_ISL_977920, EPI_ISL_977921, EPI_ISL_977922, EPI_ISL_977923, EPI_ISL_977924, EPI_ISL_977925, EPI_ISL_977926, EPI_ISL_977927, EPI_ISL_977928, EPI_ISL_977929, EPI_ISL_977930, EPI_ISL_977931, EPI_ISL_977932, EPI_ISL_977933, EPI_ISL_977934, EPI_ISL_977935, EPI_ISL_977936, EPI_ISL_977937, EPI_ISL_977938, EPI_ISL_977939, EPI_ISL_977979			
see above	Chiu Laboratory, University of California, San Francisco	Chiu Laboratory, University of California, San Francisco	Charles Chiu, Xianding (Wayne) Deng, Candace Wang, Venice Servellita, Jill Hacker, Debra Wadford
EPI_ISL_978222, EPI_ISL_978223, EPI_ISL_978224, EPI_ISL_978225	Virginia Division of Consolidated Laboratory Services	Virginia Division of Consolidated Laboratory Services	Virginia DCLS
EPI_ISL_978280, EPI_ISL_978281	Texas Department of State Health Services	Texas Department of State Health Services	Bonnie Oh, Anita Pokharel, James Daniel Bonser, Myong Koag, Chung Wang, Rachel Lee, Grace Kubin, Rashmi Tuladhar, Mayela Pedrueza, Maliha Rahman, Jenny Zhang
EPI_ISL_978391, EPI_ISL_978392, EPI_ISL_978393, EPI_ISL_978394, EPI_ISL_978395, EPI_ISL_978462, EPI_ISL_978463, EPI_ISL_978464, EPI_ISL_978465, EPI_ISL_978466, EPI_ISL_978467			
see above	Arizona State Public Health Laboratory	Arizona State Public Health Laboratory	Trung Huynh, Jessica Escobar, Katherine Fullerton, Nobuko Fukushima, Stacy White, Linda Getsinger, Victor Waddell
EPI_ISL_978867, EPI_ISL_978930	Centre for Dengue Research and AICBU, Department of Immunology and Molecular Medicine	Centre for Dengue Research and AICBU, Department of Immunology and Molecular Medicine	Chandima Jeewandara, Deshni Jayathilaka, Dinuka Ariyaratne, Tibutius Thanesh Pramanayagam, Diyanath Ranasinghe, Laksiri Gomes, Gathsaurie Neelika Malavige
EPI_ISL_979103, EPI_ISL_979104, EPI_ISL_979105, EPI_ISL_979106, EPI_ISL_979107, EPI_ISL_979110, EPI_ISL_979112, EPI_ISL_979115, EPI_ISL_979116, EPI_ISL_979117, EPI_ISL_979118, EPI_ISL_979119, EPI_ISL_979120, EPI_ISL_979121, EPI_ISL_979122, EPI_ISL_979124, EPI_ISL_979125, EPI_ISL_979126, EPI_ISL_979129, EPI_ISL_979130, EPI_ISL_979131, EPI_ISL_979132, EPI_ISL_979135			
see above	Santa Clara County Public Health Laboratory	Chan-Zuckerberg Biohub	CZB Cliahub Consortium
EPI_ISL_979251, EPI_ISL_979268	Institute of Microbiology and Immunology, Faculty of Medicine, University of Ljubljana	Institute of Microbiology and Immunology, Faculty of Medicine, University of Ljubljana	Samo Zakotnik, Tomaž Mark Zorec, Matic Brvar, Doroteja Vlaj, Patricija Pozvek,Špela Pleh, Miša Korva, Mario Poljak, Tatjana Avši - Županc
EPI_ISL_979315	Cadham Provincial laboratory	National Microbiology Laboratory (NML)	Anna Majer, Shari Tyson, Grace Seo, Philip Mabon, Elsie Grudeski, Rhiannon Huzarewich, Russell Mandes, Anneliese Landgraff, Jennifer Tanner, Natalie Knox, Morag Graham, Gary Van Domselaar, Paul Van Caeseele, Jared Bullard, David Alexander, Kerry Dust, Nathalie Bastien, Yan Li, Timothy Booth, Darian Hole, Madison Chapel, Kirsten Biggar, CanCOGeN's metadata curation team, Public Health Agency of Canada CanCOGeN team
EPI_ISL_979453	University of Iowa Hospitals and Clinics	State Hygienic Laboratory at the University of Iowa	Valerie Reeb, Wes Hottel, Alankar Kampoowale
EPI_ISL_980905, EPI_ISL_980933, EPI_ISL_980938, EPI_ISL_980939, EPI_ISL_980982, EPI_ISL_980983	Innovative Genomics Institute, UC Berkeley	Innovative Genomics Institute, UC Berkeley	Stacia Wyman, Haridha Shivram, Phil Frankino, Liana Lareau
EPI_ISL_981371	AZ Klina	AZ Klina	Dr. C. Vael
EPI_ISL_981967, EPI_ISL_981970, EPI_ISL_981974	Microbiology Service, Hospital Universitario Clinico San Cecilio, Granada	Microbiology Service, Hospital Universitario Clinico San Cecilio, Granada	Adolfo de Salazar, Natalia Chueca, Laura Viñuela, Ana Fuentes, Federico García
EPI_ISL_982081	TGen North	Sonora Quest Laboratories	"Jolene Bowers, Megan Folkerts, Chris French, Hayley Yaglom, Ashlyn Pfeiffer, Darrin Lemmer, Dave Engelthaler, The Arizona COVID Genomics Union (ACGU)"

EPI_ISL_982259, EPI_ISL_982268, EPI_ISL_982269	Lab voor klinische biologie	Lab voor klinische biologie	Hannelore Hamerlinck, Marija Janevska, Bruno Verhasselt
EPI_ISL_982420, EPI_ISL_982421, EPI_ISL_982422, EPI_ISL_982423, EPI_ISL_982424	M Health Fairview	Minnesota Department of Health, Public Health Laboratory	Alexandra Lorentz, Jacob Garfin, Matt Plumb, and Xiong Wang
EPI_ISL_982538, EPI_ISL_982553, EPI_ISL_982579, EPI_ISL_982611, EPI_ISL_982625, EPI_ISL_982643, EPI_ISL_982675, EPI_ISL_982679, EPI_ISL_982680, EPI_ISL_982730, EPI_ISL_982734, EPI_ISL_982735, EPI_ISL_982747, EPI_ISL_982761			
see above	US Air Force School of Aerospace Medicine	US Air Force School of Aerospace Medicine	Anthony Fries, Jennifer Meyer, William Gruner, William Buggele, Amanda Javorina, Sarah Purves, Clarise Starr, Elizabeth Macias
EPI_ISL_982860	Botswana Harvard HIV Reference Laboratory	Botswana Harvard HIV Reference Laboratory	Sikhulile Moyo, Dorcas Maruapula, Wonderful T. Choga, Botshelo Radibe, Boitumelo Zuze, David Lawrence, Roger Shapiro, Shahin Lockman, Mosepele Mosepele, Joseph Makhema, Simani Gaseitsiwe
EPI_ISL_983083	Gravity Diagnostics	Kentucky State Public Health Lab	Stephanie Lunn, Karim George, Joshua Tobias, William Grooms, Vaneet Arora, Matthew Johnson, Rachel Zinner, Rhonda Lucas
