# Supplementary material for: Detection and characterization of the SARS-CoV-2 lineage B.1.526 in New York
Source: Nat Commun. 2021 Aug 9;12:4886. doi: 10.1038/s41467-021-25168-4 (PMC8352861; doi:10.1038/s41467-021-25168-4)
Supplement: Supplementary file 8 — Supplementary Data 4 [file 41467_2021_25168_MOESM8_ESM.zip › GISAID_acknowledements_tables/gisaid_hcov-19_acknowledgement_table_2021_02_13_01.pdf]

We gratefully acknowledge the following Authors from the Originating laboratories responsible for obtaining the specimens, as well as the Submitting laboratories where the genome data were generated and shared via GISAID, on which this research is based.

All Submitters of data may be contacted directly via www.gisaid.org

Authors are sorted alphabetically.

Accession ID	Originating Laboratory	Submitting Laboratory	Authors
EPI_ISL_888653, EPI_ISL_888654, EPI_ISL_888655, EPI_ISL_888656, EPI_ISL_888657, EPI_ISL_888658, EPI_ISL_888659, EPI_ISL_888660, EPI_ISL_888661, EPI_ISL_888662	University of Michigan Clinical Microbiology Laboratory	Lauring Lab, University of Michigan, Department of Microbiology and Immunology	Valesano
EPI_ISL_892365, EPI_ISL_892367	National Laboratory for Health, Environment and Food	National Laboratory for Health, Environment and Food	Aleksander Mahnic, Sandra Janezic, Maja Rupnik
EPI_ISL_903209, EPI_ISL_903210, EPI_ISL_903211, EPI_ISL_903212, EPI_ISL_903213, EPI_ISL_903214, EPI_ISL_903215, EPI_ISL_903216, EPI_ISL_903217	University of Michigan Clinical Microbiology Laboratory	Lauring Lab, University of Michigan, Department of Microbiology and Immunology	Valesano
EPI_ISL_903349, EPI_ISL_903350, EPI_ISL_903351, EPI_ISL_903352, EPI_ISL_903354, EPI_ISL_903355, EPI_ISL_903356, EPI_ISL_903359, EPI_ISL_903360	Wyoming Public Health Laboratory	Wyoming Public Health Laboratory	Noah Hull, Taylor Fearing, Lynette Gumbleton, Channing Weber, Ashley Norberg, Bailey Bowcutt, and Wanda Manley
EPI_ISL_905756, EPI_ISL_905757	National Institute of Public Health - National Institute of Hygiene	National Institute of Public Health - National Institute of Hygiene	Wokowicz Tomasz, Zacharczuk Katarzyna
EPI_ISL_906146	Gundersen Molecular Diagnostics Laboratory	Kabara Cancer Research Institute	Craig S. Richmond, Paraic A. Kenny
EPI_ISL_906189	University of Wisconsin-Madison AIDS Vaccine Research Laboratories	University of Wisconsin-Madison AIDS Vaccine Research Laboratories	Gage Moreno, Katarina Braun, et al. AIDS Vaccine Research Laboratories
EPI_ISL_906833, EPI_ISL_906834, EPI_ISL_906835, EPI_ISL_906836, EPI_ISL_906837, EPI_ISL_906838, EPI_ISL_906839, EPI_ISL_906840, EPI_ISL_906841, EPI_ISL_906842, EPI_ISL_906843, EPI_ISL_906844, EPI_ISL_906845, EPI_ISL_906846			
see above	National Public Health Laboratory, National Centre for Infectious Diseases	National Public Health Laboratory, National Centre for Infectious Diseases	Tze Minn Mak, Zhenyang Zhou, Lin Cui, Raymond Tzer Pin Lin
EPI_ISL_911373, EPI_ISL_911376, EPI_ISL_911377, EPI_ISL_911381, EPI_ISL_911382	University of Michigan Clinical Microbiology Laboratory	Lauring Lab, University of Michigan, Department of Microbiology and Immunology	Valesano
EPI_ISL_911874, EPI_ISL_911875, EPI_ISL_911876, EPI_ISL_911877, EPI_ISL_911878, EPI_ISL_911879, EPI_ISL_911880, EPI_ISL_911881, EPI_ISL_911882, EPI_ISL_911883, EPI_ISL_911884, EPI_ISL_911886, EPI_ISL_911887, EPI_ISL_911888, EPI_ISL_911889, EPI_ISL_911893, EPI_ISL_911895, EPI_ISL_911896, EPI_ISL_911897, EPI_ISL_911898, EPI_ISL_911899, EPI_ISL_911912, EPI_ISL_911913, EPI_ISL_911914, EPI_ISL_911915, EPI_ISL_911916, EPI_ISL_911917			
see above	Johns Hopkins Hospital Department of Pathology	Johns Hopkins Hospital Department of Pathology	C. Paul Morris, Chun Huai Luo, Adannaya Amadi, Matthew Schwartz, Nicholas Gallagher, Heba H. Mostafa
EPI_ISL_912178	Tempus	Grubaugh Lab - Yale School of Public Health	Tara Alpert, Joseph Fauver, Anderson Brito, Mallery Breban, Anne Wyllie, Chantal Vogels, Mary Petrone, Annie Watkins, Chaney Kalinich, Isabel Ott, Nathan Grubaugh
EPI_ISL_912179, EPI_ISL_912180, EPI_ISL_912181, EPI_ISL_912182, EPI_ISL_912183, EPI_ISL_912184	Yale Pathology Lab	Grubaugh Lab - Yale School of Public Health	Tara Alpert, Joseph Fauver, Chen Liu, Pei Hui, Jianhui Wang, Susan Bell and Han Zhou, Anderson Brito, Mallery Breban, Anne Wyllie, Chantal Vogels, Mary Petrone, Chaney Kalinich, Isabel Ott, Arnau Casanovas, Catherine Muenker, Adam Moore, Alice Lu, Maria Tokuyama, Patrick Wong, Peiwen Lu, Saad Omer, Richard Martinello, Allison Nelson, Shelli Farhadian, Akiko Iwasaki, Charlese Dela Cruz, Albert Ko, Nathan Grubaugh
EPI_ISL_912186	Sydney South West Pathology Service (SSWPS) - Concord Repatriation General Hospital - NSW Health Pathology	NSW Health Pathology - Institute of Clinical Pathology and Medical Research; Westmead Hospital; University of Sydney	CIDM-PH et al.
EPI_ISL_912677, EPI_ISL_912811, EPI_ISL_912813, EPI_ISL_912815, EPI_ISL_912816, EPI_ISL_912817, EPI_ISL_912819, EPI_ISL_912820, EPI_ISL_912824, EPI_ISL_912825, EPI_ISL_912826, EPI_ISL_912829, EPI_ISL_912832, EPI_ISL_912833, EPI_ISL_912834, EPI_ISL_912835, EPI_ISL_912836, EPI_ISL_912839, EPI_ISL_912842, EPI_ISL_912843, EPI_ISL_912846, EPI_ISL_912847, EPI_ISL_912848, EPI_ISL_912849, EPI_ISL_912864, EPI_ISL_912865, EPI_ISL_912866, EPI_ISL_912867, EPI_ISL_912868, EPI_ISL_912869, EPI_ISL_912870, EPI_ISL_912871, EPI_ISL_912872, EPI_ISL_912873, EPI_ISL_912874, EPI_ISL_912875, EPI_ISL_912876, EPI_ISL_912877, EPI_ISL_912878, EPI_ISL_912879, EPI_ISL_912880, EPI_ISL_912881, EPI_ISL_912882, EPI_ISL_912883, EPI_ISL_912884, EPI_ISL_912885, EPI_ISL_912886, EPI_ISL_912887, EPI_ISL_912888, EPI_ISL_912889, EPI_ISL_912890			
see above	Hôpital Henri Mondor	Department of Virology, Henri Mondor University Hospital, Assistance Publique Hôpitaux de Paris, Université Paris-Est Créteil, INSERM U955	Christophe Rodriguez, Slim Fourati, Vanessa Demontant, Guillaume Gricourt, Melissa N'Debi, Alexandre Soulier, Elisabeth Trawinski, Jean-Michel Pawlotsky
EPI_ISL_913398, EPI_ISL_913408, EPI_ISL_913418	Massachusetts State Public Health Laboratory	Massachusetts State Public Health Laboratory	Andrew Lang, Timelia Fink, Glen Gallagher, Sandra Smole
EPI_ISL_913440, EPI_ISL_913441, EPI_ISL_913442, EPI_ISL_913443, EPI_ISL_913444	Institute for Infectious Diseases, University of Bern, Switzerland	Institute for Infectious Diseases, University of Bern, Switzerland	Michel C Koch, Christian Baumann, Miguel A Terrazos Miani, Cora Sägesser, Pascal Bittel, Stephen L Leib, Peter Keller, Franziska Suter-Riniker, Alban Ramette
EPI_ISL_915429	Bundeswehr Institute of Microbiology	Bundeswehr Institute of Microbiology	Markus Antwerpen, Alexandra Rehn, Mathias Walter, Malena Bestehorn-Willmann, Sabine Zange, Enrico Georgi, Roman Wölfel
EPI_ISL_915430, EPI_ISL_915431, EPI_ISL_915432, EPI_ISL_915433	Bundeswehrkrankenhaus Westerstede	Bundeswehr Institute of Microbiology	Markus Antwerpen, Klaus Peter Ebert, Alexandra Rehn, Mathias Walter, Malena Bestehorn-Willmann, Sabine Zange, Enrico Georgi, Roman Wölfel
EPI_ISL_918166	Thai Red Cross Emerging Infectious Diseases Health Science Centre, Chulalongkorn Hospital, Faculty of Medicine, Chulalongkorn University	Thai Red Cross Emerging Infectious Diseases Center and Faculty of Medicine, Chulalongkorn University	Rome Buathong, Sopon Iamsirithaworn, Sininat Petcharat, Yutthana Joyjinda, Weenassarin Ampoot, Apaporn Rodpan, Opass Putcharoen, Thiravat Hemachudha, Supaporn Wacharapluesadee
EPI_ISL_918269	SIESP DIPARTIMENTO DI PREVENZIONE TERAMO	Istituto Zooprofilattico Sperimentale dell'Abruzzo e Molise "G. Caporale"	Lorusso A, Marcacci M, Di Domenico M, Ancora M, Curini V, Mangone I, Rinaldi A, Scialabba S, Di Pasquale A, Cammà C, Puglia I, Calistri P, Savini G
EPI_ISL_918358	Institute of Virology, Medical Center, University of Freiburg, Freiburg, Germany	Institute of Virology, Clinial Virus Genomics, Medical Center, University of Freiburg, Freiburg, Germany	Jonas Fuchs, Lisa Kern, Sandra Reuter, Hajo Grundmann, Marcus Panning
EPI_ISL_918423	München Klinik GmbH	Bundeswehr Institute of Microbiology	Markus Antwerpen, Hans-Ulrich Schmidt, Alexandra Rehn, Mathias Walter, Malena Bestehorn-Willmann, Sabine Zange, Enrico Georgi, Roman Wölfel
EPI_ISL_918424, EPI_ISL_918425	Bundeswehrzentralkrankenhaus Koblenz	Bundeswehr Institute of Microbiology	Markus Antwerpen, Ralf Hagen, Ingo Fengler, Alexandra Rehn, Mathias Walter, Malena Bestehorn-Willmann, Sabine Zange, Enrico Georgi, Roman Wölfel
EPI_ISL_918426, EPI_ISL_918427	München Klinik GmbH	Bundeswehr Institute of Microbiology	Markus Antwerpen, Hans-Ulrich Schmidt, Alexandra Rehn, Mathias Walter, Malena Bestehorn-Willmann, Sabine Zange, Enrico Georgi, Roman Wölfel
EPI_ISL_918428	Isar-Amper-Klinikum Haar	Bundeswehr Institute of Microbiology	Markus Antwerpen, Alexandra Rehn, Mathias Walter, Malena Bestehorn-Willmann, Sabine Zange, Enrico Georgi, Roman Wölfel

EPI_ISL_918429	München Klinik GmbH	Bundeswehr Institute of Microbiology	Markus Antwerpen, Hans-Ulrich Schmidt, Alexandra Rehn, Mathias Walter, Malena Bestehorn-Willmann, Sabine Zange, Enrico Georgi, Roman Wölfel
EPI_ISL_918496	BSWMC-Marble Falls	BSWMC-Temple Molecular	Ari Rao, Linden Morales, Kimberly Walker, Shelby Hendrickson
EPI_ISL_919149, EPI_ISL_919150, EPI_ISL_919151, EPI_ISL_919152, EPI_ISL_919153, EPI_ISL_919154, EPI_ISL_919155, EPI_ISL_919156, EPI_ISL_919157, EPI_ISL_919158, EPI_ISL_919159, EPI_ISL_919160, EPI_ISL_919161, EPI_ISL_919162, EPI_ISL_919163, EPI_ISL_919164, EPI_ISL_919165, EPI_ISL_919166, EPI_ISL_919167, EPI_ISL_919168, EPI_ISL_919169, EPI_ISL_919170, EPI_ISL_919171, EPI_ISL_919172, EPI_ISL_919173, EPI_ISL_919174, EPI_ISL_919175, EPI_ISL_919176, EPI_ISL_919177, EPI_ISL_919179, EPI_ISL_919180, EPI_ISL_919181, EPI_ISL_919182, EPI_ISL_919183, EPI_ISL_919184, EPI_ISL_919185, EPI_ISL_919186, EPI_ISL_919187, EPI_ISL_919188, EPI_ISL_919189			
see above	Department of Pathology, University of Cambridge	COVID-19 Genomics UK (COG-UK) Consortium	Aminu S. Jahun, Yasmin Chaudhry, Iliana Georgana, Myra Hosmillo, Rhys Izu, Martin D. Curran, Surendra Parmar, Ian Goodfellow
EPI_ISL_919405	Virology Department, Royal Infirmary of Edinburgh, NHS Lothian / School of Biological Sciences, University of Edinburgh / Institute of Genetics and Molecular Medicine, University of Edinburgh	COVID-19 Genomics UK (COG-UK) Consortium	McHugh M, Dewar R, Rooke S, Gallagher M, Balcaza C, O'Toole Á, Scher E, Hill V, McCrone JT, Colquhoun R, Yu X, Jackson B, Rambaut A, Williams TC, Templeton K
EPI_ISL_919629, EPI_ISL_919630, EPI_ISL_919663, EPI_ISL_919706, EPI_ISL_919707, EPI_ISL_919708, EPI_ISL_919709, EPI_ISL_919710, EPI_ISL_919712, EPI_ISL_919713, EPI_ISL_919714, EPI_ISL_919715, EPI_ISL_919716, EPI_ISL_919717, EPI_ISL_919718, EPI_ISL_919719, EPI_ISL_919720, EPI_ISL_919721, EPI_ISL_919722, EPI_ISL_919723, EPI_ISL_919724			
see above	Liverpool Clinical Laboratories	COVID-19 Genomics UK (COG-UK) Consortium	Sam Haldenby, Anita Lucaci, Steve Paterson, Julian Hiscox, Alistair Darby, M Almsaud, A Alrezaihi, Muhannad Alruwaili, Stuart D Armstrong, Jones Benjamin, Eleanor G Bentley, Anu Chawla, Jordan J Clark, Angela Cowell, Richard Eccles, Isabel García-Dorival, Matthew Gemmell, Alessandro Gerada, PKF Gilmore, Richard Gregory, Ximeng Han, Catherine Hartley, Margaret Hughes, Miren Iturriza-Gomara, James Johnson, L Luu, Jenifer Manson, Charlotte Nelson, Elaine O'Toole, Cassie Olateju, Rebekah Penrice-Randal , Lucille Rainbow, N.P Randle, Trevor Ian Robinson, Parul Sharma, Ghada T Shawli, James P Stewart, Neil Swainston, Ecaterina Vamos, Joanne Watts, Mark Whitehead
EPI_ISL_919871, EPI_ISL_919872, EPI_ISL_919873, EPI_ISL_919881, EPI_ISL_919903, EPI_ISL_919904, EPI_ISL_919905, EPI_ISL_919906, EPI_ISL_919907, EPI_ISL_919908, EPI_ISL_919909, EPI_ISL_919910, EPI_ISL_919911, EPI_ISL_919912, EPI_ISL_919913, EPI_ISL_919914, EPI_ISL_919915, EPI_ISL_919916, EPI_ISL_919917, EPI_ISL_919918, EPI_ISL_919919, EPI_ISL_919927, EPI_ISL_919928, EPI_ISL_919929, EPI_ISL_919930, EPI_ISL_919931, EPI_ISL_919932, EPI_ISL_919934, EPI_ISL_919935, EPI_ISL_919936, EPI_ISL_919937, EPI_ISL_919938, EPI_ISL_919939, EPI_ISL_919940, EPI_ISL_919941, EPI_ISL_919942, EPI_ISL_919943, EPI_ISL_919944, EPI_ISL_919946, EPI_ISL_919947, EPI_ISL_919949, EPI_ISL_919950, EPI_ISL_919951, EPI_ISL_919953, EPI_ISL_919954, EPI_ISL_919956, EPI_ISL_919958, EPI_ISL_919959, EPI_ISL_919960, EPI_ISL_920092, EPI_ISL_920093, EPI_ISL_920094, EPI_ISL_920096, EPI_ISL_920097, EPI_ISL_920098, EPI_ISL_920099, EPI_ISL_920100, EPI_ISL_920101, EPI_ISL_920102, EPI_ISL_920103, EPI_ISL_920104, EPI_ISL_920105, EPI_ISL_920106, EPI_ISL_920107, EPI_ISL_920108, EPI_ISL_920109, EPI_ISL_920110, EPI_ISL_920111, EPI_ISL_920112, EPI_ISL_920113, EPI_ISL_920114, EPI_ISL_920115, EPI_ISL_920116, EPI_ISL_920152			
see above	University College London, Great Ormond Street Hospital for Children NHS Foundation Trust, Imperial College Healthcare NHS Trust	COVID-19 Genomics UK (COG-UK) Consortium	Sergi Castellano, Rachel Williams, Mark Kristiansen, Paola Resende Silva, Sunando Roy, Tony Brooks, Helena Tutill, Paola Niola, Patricia Dyal, Charlotte Williams, Leysa Forrest, Yasmin Panchbhaya, Jacqueline Findlay, Samuel Weeks, Julianne Brown, Kathryn Harris, Paul Randell, James Price, Alison Holmes, Judith Breuer
EPI_ISL_920671, EPI_ISL_920685, EPI_ISL_920693, EPI_ISL_920701, EPI_ISL_920702, EPI_ISL_920723, EPI_ISL_920724, EPI_ISL_920725, EPI_ISL_920728, EPI_ISL_920731, EPI_ISL_920732, EPI_ISL_920733, EPI_ISL_920734, EPI_ISL_920752, EPI_ISL_920756, EPI_ISL_920757, EPI_ISL_920758, EPI_ISL_920761, EPI_ISL_920763, EPI_ISL_920771, EPI_ISL_920776, EPI_ISL_920777, EPI_ISL_920778, EPI_ISL_920779, EPI_ISL_920780			
see above	University College London Hospital	COVID-19 Genomics UK (COG-UK) Consortium	Judith Heaney, Matthew Byott, Catherine Houlihan, Dan Frampton, Stuart Kirk, Moira Spyer and Eleni Nastouli
EPI_ISL_921659, EPI_ISL_921660, EPI_ISL_921661	Northumbria University / South Tees Hospitals NHS Foundation Trust / North Cumbria Integrated Care NHS Foundation Trust / North Tees and Hartlepool NHS Foundation Trust / Newcastle Hospitals NHS Foundation Trust	COVID-19 Genomics UK (COG-UK) Consortium	Darren L Smith,Andrew Nelson,Matthew Bashton,Greg R Young,Joshua Loh,John Allan,Mohammad A Tariq,Giles S Holt,Gary Black,Wen C Yew,Lynn Dover,Paul Baker,Steve Liggett,Sarah Essex,Jane Greenaway,Debra Padgett,Clive Graham,Garren Scott,Edward Barton,Emma Swindells,Brendan Payne,Jennifer Collins,Yusri Taha,Gary Eltringham
EPI_ISL_922037, EPI_ISL_922038, EPI_ISL_922039, EPI_ISL_922040	Queens Medical Centre, Clinical Microbiology Department / DeepSeq Nottingham	COVID-19 Genomics UK (COG-UK) Consortium	Gemma Clark, Wendy Smith, Manjinder Khakh, Vicki M Fleming, Michelle M Lister, Hannah Howson-Wells, Jonathan Ball, Patrick McClure, Joseph Chappell, Theocharis Tsoleridis, Nadine Holmes, Matthew Carlisle, Christopher Moore, Fei Sang, Johnny Debebe, Victoria Wright, Matthew Loose
EPI_ISL_923349	Centre for Enzyme Innovation, University of Portsmouth / Translational Research Laboratory, Portsmouth Hospitals NHS Trust	COVID-19 Genomics UK (COG-UK) Consortium	Angela Beckett,Salman Goudarzi,Christopher Fearn,Kate Cook,Katie Loveson,Sharon Glaysher,Scott Elliott,Samuel Robson
EPI_ISL_924082, EPI_ISL_924085, EPI_ISL_924087, EPI_ISL_924089, EPI_ISL_924092, EPI_ISL_924097, EPI_ISL_924099, EPI_ISL_924103, EPI_ISL_924105, EPI_ISL_924109, EPI_ISL_924111, EPI_ISL_924112, EPI_ISL_924117, EPI_ISL_924118, EPI_ISL_924125, EPI_ISL_924126, EPI_ISL_924129, EPI_ISL_924131, EPI_ISL_924136, EPI_ISL_924142, EPI_ISL_924144, EPI_ISL_924146, EPI_ISL_924153, EPI_ISL_924160, EPI_ISL_924163, EPI_ISL_924165, EPI_ISL_924173, EPI_ISL_924183, EPI_ISL_924184, EPI_ISL_924185, EPI_ISL_924186, EPI_ISL_924191, EPI_ISL_924193, EPI_ISL_924198, EPI_ISL_924200, EPI_ISL_924201, EPI_ISL_924209, EPI_ISL_924212, EPI_ISL_924216, EPI_ISL_924217, EPI_ISL_924223, EPI_ISL_924227, EPI_ISL_924228, EPI_ISL_924229, EPI_ISL_924235, EPI_ISL_924238, EPI_ISL_924243, EPI_ISL_924249, EPI_ISL_924251, EPI_ISL_924258, EPI_ISL_924263, EPI_ISL_924269, EPI_ISL_924273, EPI_ISL_924276, EPI_ISL_924277, EPI_ISL_924282, EPI_ISL_924287, EPI_ISL_924293, EPI_ISL_924297, EPI_ISL_924298, EPI_ISL_924304, EPI_ISL_924305, EPI_ISL_924306, EPI_ISL_924308, EPI_ISL_924313, EPI_ISL_924330, EPI_ISL_924335, EPI_ISL_924339, EPI_ISL_924340, EPI_ISL_924350, EPI_ISL_924352, EPI_ISL_924356, EPI_ISL_924359, EPI_ISL_924361, EPI_ISL_924366, EPI_ISL_924368, EPI_ISL_924374, EPI_ISL_924377, EPI_ISL_924379, EPI_ISL_924382, EPI_ISL_924390, EPI_ISL_924410, EPI_ISL_924414			
see above	Virology Department, Sheffield Teaching Hospitals NHS Foundation Trust/Department of Infection, Immunity and Cardiovascular Disease, The Medical School, University of Sheffield	COVID-19 Genomics UK (COG-UK) Consortium	Thushan de Silva, Matthew Parker, Nikki Smith, Adri Angyal, Rebecca Brown, Luke Green, Rachel Tucker, Paul Parsons, Danielle Groves, Katie Johnson, Laura Carrilero, Alex Keeley, Dave Partridge, Matthew Wyles, Benjamin Lindsey, Mehmet Yavuz, Mohammad Raza, Cariad Evans
EPI_ISL_925031, EPI_ISL_925032	Mirialis	CNR Virus des Infections Respiratoires - France SUD	Antonin Bal, Gregory Destras, Gwendolyne Burfin, Hadrien Règue, Quentin Semanas, Martine Valette, Bruno Lina, Laurence Josset
EPI_ISL_925259, EPI_ISL_925260, EPI_ISL_925272, EPI_ISL_925289, EPI_ISL_925291, EPI_ISL_925300, EPI_ISL_925303	Wyoming Public Health Laboratory	Wyoming Public Health Laboratory	Noah Hull, Taylor Fearing, Lynette Gumbleton, Channing Weber, Ashley Norberg, Bailey Bowcutt, and Wanda Manley
EPI_ISL_925918, EPI_ISL_925929, EPI_ISL_925939, EPI_ISL_925940, EPI_ISL_925944, EPI_ISL_925955, EPI_ISL_925958, EPI_ISL_925965, EPI_ISL_925968, EPI_ISL_925970, EPI_ISL_925985, EPI_ISL_925987, EPI_ISL_925990, EPI_ISL_926003, EPI_ISL_926011, EPI_ISL_926012, EPI_ISL_926017, EPI_ISL_926018, EPI_ISL_926029, EPI_ISL_926031, EPI_ISL_926037, EPI_ISL_926044, EPI_ISL_926045, EPI_ISL_926071, EPI_ISL_926079, EPI_ISL_926086, EPI_ISL_926088, EPI_ISL_926090, EPI_ISL_926099, EPI_ISL_926107, EPI_ISL_926113, EPI_ISL_926117, EPI_ISL_926118, EPI_ISL_926120, EPI_ISL_926126, EPI_ISL_926140, EPI_ISL_926142, EPI_ISL_926147, EPI_ISL_926156, EPI_ISL_926157, EPI_ISL_926158, EPI_ISL_926162, EPI_ISL_926163, EPI_ISL_926164, EPI_ISL_926174, EPI_ISL_926183, EPI_ISL_926199, EPI_ISL_926210, EPI_ISL_926211, EPI_ISL_926216, EPI_ISL_926222, EPI_ISL_926225, EPI_ISL_926227, EPI_ISL_926243, EPI_ISL_926245, EPI_ISL_926247, EPI_ISL_926257, EPI_ISL_926259, EPI_ISL_926261, EPI_ISL_926272, EPI_ISL_926274, EPI_ISL_926291, EPI_ISL_926295, EPI_ISL_926297, EPI_ISL_926303, EPI_ISL_926304, EPI_ISL_926308, EPI_ISL_926311, EPI_ISL_926320, EPI_ISL_926327, EPI_ISL_926328, EPI_ISL_926337, EPI_ISL_926342, EPI_ISL_926346, EPI_ISL_926347, EPI_ISL_926348, EPI_ISL_926360, EPI_ISL_926364, EPI_ISL_926367, EPI_ISL_926372, EPI_ISL_926374, EPI_ISL_926389, EPI_ISL_926390, EPI_ISL_926397, EPI_ISL_926398, EPI_ISL_926399, EPI_ISL_926406, EPI_ISL_926416, EPI_ISL_926422, EPI_ISL_926423, EPI_ISL_926435, EPI_ISL_926437, EPI_ISL_926440, EPI_ISL_926455, EPI_ISL_926461, EPI_ISL_926476, EPI_ISL_926478, EPI_ISL_926479, EPI_ISL_926483, EPI_ISL_926489, EPI_ISL_926492, EPI_ISL_926499, EPI_ISL_926512, EPI_ISL_926520, EPI_ISL_926521, EPI_ISL_926535, EPI_ISL_926549, EPI_ISL_926552, EPI_ISL_926558, EPI_ISL_926560, EPI_ISL_926567, EPI_ISL_926586, EPI_ISL_926601, EPI_ISL_926603, EPI_ISL_926616, EPI_ISL_926622, EPI_ISL_926628, EPI_ISL_926642, EPI_ISL_926643, EPI_ISL_926649, EPI_ISL_926655, EPI_ISL_926656, EPI_ISL_926666, EPI_ISL_926668, EPI_ISL_926674, EPI_ISL_926676, EPI_ISL_926687, EPI_ISL_926693, EPI_ISL_926710, EPI_ISL_926721, EPI_ISL_926722, EPI_ISL_926735, EPI_ISL_926752, EPI_ISL_926757, EPI_ISL_926758, EPI_ISL_926760, EPI_ISL_926763, EPI_ISL_926765, EPI_ISL_926778, EPI_ISL_926797, EPI_ISL_926799, EPI_ISL_926801, EPI_ISL_926805, EPI_ISL_926806, EPI_ISL_926810, EPI_ISL_926813, EPI_ISL_926817, EPI_ISL_926821, EPI_ISL_926825, EPI_ISL_926836, EPI_ISL_926853, EPI_ISL_926855, EPI_ISL_926872, EPI_ISL_926880, EPI_ISL_926899, EPI_ISL_926900, EPI_ISL_926909, EPI_ISL_926911, EPI_ISL_926915, EPI_ISL_926917, EPI_ISL_926923, EPI_ISL_926931, EPI_ISL_926934, EPI_ISL_926962, EPI_ISL_926967, EPI_ISL_926968, EPI_ISL_926973, EPI_ISL_926982, EPI_ISL_926989, EPI_ISL_927002, EPI_ISL_927004, EPI_ISL_927011, EPI_ISL_927014, EPI_ISL_927018, EPI_ISL_927020, EPI_ISL_927024, EPI_ISL_927032, EPI_ISL_927036, EPI_ISL_927066, EPI_ISL_927069, EPI_ISL_927070, EPI_ISL_927081, EPI_ISL_927082, EPI_ISL_927084, EPI_ISL_927097, EPI_ISL_927100, EPI_ISL_927106, EPI_ISL_927114, EPI_ISL_927122, EPI_ISL_927127, EPI_ISL_927132, EPI_ISL_927133, EPI_ISL_927134, EPI_ISL_927140, EPI_ISL_927141, EPI_ISL_927144, EPI_ISL_927148, EPI_ISL_927153, EPI_ISL_927167, EPI_ISL_927174, EPI_ISL_927177, EPI_ISL_927181, EPI_ISL_927182, EPI_ISL_927184, EPI_ISL_927189, EPI_ISL_927196, EPI_ISL_927199, EPI_ISL_927203, EPI_ISL_927213, EPI_ISL_927222, EPI_ISL_927236, EPI_ISL_927240, EPI_ISL_927241, EPI_ISL_927243, EPI_ISL_927248, EPI_ISL_927255, EPI_ISL_927263, EPI_ISL_927266, EPI_ISL_927278, EPI_ISL_927284, EPI_ISL_927288, EPI_ISL_927298, EPI_ISL_927300, EPI_ISL_927302, EPI_ISL_927319, EPI_ISL_927327, EPI_ISL_927341, EPI_ISL_927384, EPI_ISL_927385, EPI_ISL_927389, EPI_ISL_927397, EPI_ISL_927402, EPI_ISL_927408, EPI_ISL_927420, EPI_ISL_927424, EPI_ISL_927433, EPI_ISL_927441, EPI_ISL_927447, EPI_ISL_927455, EPI_ISL_927464, EPI_ISL_927470, EPI_ISL_927472, EPI_ISL_927473, EPI_ISL_927479, EPI_ISL_927484, EPI_ISL_927498, EPI_ISL_927522, EPI_ISL_927525, EPI_ISL_927534, EPI_ISL_927536, EPI_ISL_927550, EPI_ISL_927554, EPI_ISL_927555, EPI_ISL_927568, EPI_ISL_927577, EPI_ISL_927578, EPI_ISL_927587, EPI_ISL_927589, EPI_ISL_927598, EPI_ISL_927599, EPI_ISL_927600, EPI_ISL_927608, EPI_ISL_927610, EPI_ISL_927612, EPI_ISL_927615, EPI_ISL_927616, EPI_ISL_927617, EPI_ISL_927623, EPI_ISL_927628, EPI_ISL_927633, EPI_ISL_927634, EPI_ISL_927636, EPI_ISL_927641, EPI_ISL_927643, EPI_ISL_927644, EPI_ISL_927648, EPI_ISL_927653, EPI_ISL_927658, EPI_ISL_927669, EPI_ISL_927683, EPI_ISL_927711, EPI_ISL_927716, EPI_ISL_927723, EPI_ISL_927726, EPI_ISL_927733, EPI_ISL_927751, EPI_ISL_927758, EPI_ISL_927763, EPI_ISL_927769, EPI_ISL_927788, EPI_ISL_927794, EPI_ISL_927820, EPI_ISL_927822, EPI_ISL_927831, EPI_ISL_927833, EPI_ISL_927839, EPI_ISL_927849, EPI_ISL_927856, EPI_ISL_927861, EPI_ISL_927862, EPI_ISL_927864, EPI_ISL_927866, EPI_ISL_927868, EPI_ISL_927873, EPI_ISL_927876, EPI_ISL_927880, EPI_ISL_927905, EPI_ISL_927918, EPI_ISL_927919, EPI_ISL_927926, EPI_ISL_927927, EPI_ISL_927928, EPI_ISL_927943, EPI_ISL_927945, EPI_ISL_927952, EPI_ISL_927953, EPI_ISL_927956, EPI_ISL_927957, EPI_ISL_927964, EPI_ISL_927967, EPI_ISL_927972, EPI_ISL_927976, EPI_ISL_927980, EPI_ISL_927982, EPI_ISL_927984, EPI_ISL_927988, EPI_ISL_927998, EPI_ISL_928022, EPI_ISL_928025, EPI_ISL_928044, EPI_ISL_928049, EPI_ISL_928070, EPI_ISL_928072, EPI_ISL_928089, EPI_ISL_928096, EPI_ISL_928109, EPI_ISL_928125, EPI_ISL_928131, EPI_ISL_928135, EPI_ISL_928136, EPI_ISL_928141, EPI_ISL_928150, EPI_ISL_928154, EPI_ISL_928157, EPI_ISL_928163, EPI_ISL_928166, EPI_ISL_928167, EPI_ISL_928175, EPI_ISL_928191, EPI_ISL_928197, EPI_ISL_928200, EPI_ISL_928210, EPI_ISL_928212, EPI_ISL_928217, EPI_ISL_928225, EPI_ISL_928247, EPI_ISL_928249, EPI_ISL_928251, EPI_ISL_928259, EPI_ISL_928263, EPI_ISL_928273, EPI_ISL_928299, EPI_ISL_928315, EPI_ISL_928340, EPI_ISL_928362, EPI_ISL_928367, EPI_ISL_928383, EPI_ISL_928392, EPI_ISL_928417, EPI_ISL_928424, EPI_ISL_928425, EPI_ISL_928426, EPI_ISL_928428, EPI_ISL_928430, EPI_ISL_928431, EPI_ISL_928439, EPI_ISL_928441, EPI_ISL_928442, EPI_ISL_928444, EPI_ISL_928445, EPI_ISL_928454, EPI_ISL_928455, EPI_ISL_928456, EPI_ISL_928459, EPI_ISL_928462, EPI_ISL_928466, EPI_ISL_928470, EPI_ISL_928491, EPI_ISL_928494, EPI_ISL_928499, EPI_ISL_928504, EPI_ISL_928524, EPI_ISL_928525, EPI_ISL_928528, EPI_ISL_928548, EPI_ISL_928565, EPI_ISL_928568, EPI_ISL_928569, EPI_ISL_928586, EPI_ISL_928587, EPI_ISL_928595, EPI_ISL_928614, EPI_ISL_928621, EPI_ISL_928646, EPI_ISL_928648, EPI_ISL_928650, EPI_ISL_928652, EPI_ISL_928657, EPI_ISL_928660, EPI_ISL_928678, EPI_ISL_928688, EPI_ISL_928690, EPI_ISL_928705, EPI_ISL_928706, EPI_ISL_928711, EPI_ISL_928714, EPI_ISL_928716, EPI_ISL_928719, EPI_ISL_928723, EPI_ISL_928735, EPI_ISL_928743, EPI_ISL_928755, EPI_ISL_928757, EPI_ISL_928769, EPI_ISL_928780, EPI_ISL_928786, EPI_ISL_928788, EPI_ISL_928797, EPI_ISL_928808, EPI_ISL_928823, EPI_ISL_928833, EPI_ISL_928842, EPI_ISL_928853, EPI_ISL_928859, EPI_ISL_928860, EPI_ISL_928861, EPI_ISL_928863, EPI_ISL_928872, EPI_ISL_928878, EPI_ISL_928881, EPI_ISL_928885, EPI_ISL_928892, EPI_ISL_928895, EPI_ISL_928918, EPI_ISL_928926, EPI_ISL_928943, EPI_ISL_928944, EPI_ISL_928946, EPI_ISL_928965, EPI_ISL_928980, EPI_ISL_928987, EPI_ISL_928989, EPI_ISL_928994, EPI_ISL_928995, EPI_ISL_929010, EPI_ISL_929026, EPI_ISL_929039, EPI_ISL_929046, EPI_ISL_929055, EPI_ISL_929056, EPI_ISL_929058, EPI_ISL_929061, EPI_ISL_929062, EPI_ISL_929075, EPI_ISL_929076, EPI_ISL_929083, EPI_ISL_929088, EPI_ISL_929090, EPI_ISL_929097, EPI_ISL_929112, EPI_ISL_929120, EPI_ISL_929124, EPI_ISL_929140, EPI_ISL_929142, EPI_ISL_929145, EPI_ISL_929153, EPI_ISL_929159, EPI_ISL_929162, EPI_ISL_929165, EPI_ISL_929166, EPI_ISL_929168, EPI_ISL_929180, EPI_ISL_929182, EPI_ISL_929188, EPI_ISL_929194, EPI_ISL_929198,			

EPI_ISL_929200, EPI_ISL_929203, EPI_ISL_929205, EPI_ISL_929207, EPI_ISL_929222, EPI_ISL_929223, EPI_ISL_929224, EPI_ISL_929226, EPI_ISL_929230, EPI_ISL_929235, EPI_ISL_929243, EPI_ISL_929250, EPI_ISL_929261, EPI_ISL_929268, EPI_ISL_929270, EPI_ISL_929308, EPI_ISL_929314, EPI_ISL_929323, EPI_ISL_929326, EPI_ISL_929331, EPI_ISL_929332, EPI_ISL_929338, EPI_ISL_929340, EPI_ISL_929349, EPI_ISL_929360, EPI_ISL_929362, EPI_ISL_929372, EPI_ISL_929389, EPI_ISL_929418, EPI_ISL_929425, EPI_ISL_929435, EPI_ISL_929444, EPI_ISL_929451, EPI_ISL_929461, EPI_ISL_929462, EPI_ISL_929463, EPI_ISL_929477, EPI_ISL_929486, EPI_ISL_929495, EPI_ISL_929515, EPI_ISL_929518, EPI_ISL_929524, EPI_ISL_929527, EPI_ISL_929538, EPI_ISL_929540, EPI_ISL_929546, EPI_ISL_929547, EPI_ISL_929560, EPI_ISL_929568, EPI_ISL_929575, EPI_ISL_929579, EPI_ISL_929600, EPI_ISL_929601, EPI_ISL_929607, EPI_ISL_929609, EPI_ISL_929610, EPI_ISL_929614, EPI_ISL_929617, EPI_ISL_929618, EPI_ISL_929623, EPI_ISL_929631, EPI_ISL_929637, EPI_ISL_929643, EPI_ISL_929645, EPI_ISL_929653, EPI_ISL_929660, EPI_ISL_929661, EPI_ISL_929673, EPI_ISL_929686, EPI_ISL_929687, EPI_ISL_929688, EPI_ISL_929693, EPI_ISL_929709, EPI_ISL_929711, EPI_ISL_929720, EPI_ISL_929723, EPI_ISL_929725, EPI_ISL_929741, EPI_ISL_929755, EPI_ISL_929756, EPI_ISL_929762, EPI_ISL_929783, EPI_ISL_929788, EPI_ISL_929807, EPI_ISL_929808, EPI_ISL_929810, EPI_ISL_929816, EPI_ISL_929823, EPI_ISL_929856, EPI_ISL_929860, EPI_ISL_929866, EPI_ISL_929868, EPI_ISL_929872, EPI_ISL_929873, EPI_ISL_929890, EPI_ISL_929899, EPI_ISL_929907, EPI_ISL_929908, EPI_ISL_929910, EPI_ISL_929929, EPI_ISL_929931, EPI_ISL_929936, EPI_ISL_929945, EPI_ISL_929951, EPI_ISL_929956, EPI_ISL_929957, EPI_ISL_929958, EPI_ISL_929960, EPI_ISL_929964, EPI_ISL_929976, EPI_ISL_929980, EPI_ISL_929987, EPI_ISL_929988, EPI_ISL_929993, EPI_ISL_929994, EPI_ISL_930008, EPI_ISL_930010, EPI_ISL_930011, EPI_ISL_930016, EPI_ISL_930034, EPI_ISL_930043, EPI_ISL_930049, EPI_ISL_930054, EPI_ISL_930074, EPI_ISL_930079, EPI_ISL_930080, EPI_ISL_930083, EPI_ISL_930100, EPI_ISL_930107, EPI_ISL_930109, EPI_ISL_930115, EPI_ISL_930117, EPI_ISL_930128, EPI_ISL_930137, EPI_ISL_930138, EPI_ISL_930140, EPI_ISL_930153, EPI_ISL_930157, EPI_ISL_930160, EPI_ISL_930166, EPI_ISL_930172, EPI_ISL_930181, EPI_ISL_930184, EPI_ISL_930185, EPI_ISL_930186, EPI_ISL_930188, EPI_ISL_930189, EPI_ISL_930210, EPI_ISL_930211, EPI_ISL_930215, EPI_ISL_930221, EPI_ISL_930224, EPI_ISL_930239, EPI_ISL_930247, EPI_ISL_930249, EPI_ISL_930254, EPI_ISL_930255, EPI_ISL_930258, EPI_ISL_930263, EPI_ISL_930268, EPI_ISL_930274, EPI_ISL_930286, EPI_ISL_930288, EPI_ISL_930289, EPI_ISL_930292, EPI_ISL_930296, EPI_ISL_930314, EPI_ISL_930315, EPI_ISL_930319, EPI_ISL_930322, EPI_ISL_930333, EPI_ISL_930334, EPI_ISL_930335, EPI_ISL_930342, EPI_ISL_930360, EPI_ISL_930368, EPI_ISL_930372, EPI_ISL_930387, EPI_ISL_930396, EPI_ISL_930409, EPI_ISL_930412, EPI_ISL_930419, EPI_ISL_930421, EPI_ISL_930422, EPI_ISL_930423, EPI_ISL_930442, EPI_ISL_930450, EPI_ISL_930452, EPI_ISL_930453, EPI_ISL_930454, EPI_ISL_930460, EPI_ISL_930478, EPI_ISL_930485, EPI_ISL_930488, EPI_ISL_930504, EPI_ISL_930511, EPI_ISL_930513, EPI_ISL_930519, EPI_ISL_930522, EPI_ISL_930524, EPI_ISL_930526, EPI_ISL_930527, EPI_ISL_930546, EPI_ISL_930547, EPI_ISL_930552, EPI_ISL_930554, EPI_ISL_930563			
see above	Department of Virus and Microbiological Special Diagnostics, Statens Serum Institut, Copenhagen, Denmark	Aalborg University	Danish Covid-19 Genome Consortium
EPI_ISL_930568, EPI_ISL_930572, EPI_ISL_930573, EPI_ISL_930574, EPI_ISL_930576	University of Liège COVID-19 testing center	GIGA Medical Genomics	Keith Durkin, Maria Artesi, Bouchra Boujemla, Emmanuel André, Marc Van Ranst, Fabrice Bureau, Laurent Gillet, Wouter Coppieters, Vincent Bours
EPI_ISL_930578	St. Nikolaus-Hospital Eupen	GIGA Medical Genomics	Keith Durkin, Maria Artesi, Sébastien Bontems, Raphaël Boreux, Bouchra Boujemla, Cécile Meex, Pierrette Melin, Marie-Pierre Hayette, Vincent Bours
EPI_ISL_930580, EPI_ISL_930581, EPI_ISL_930582, EPI_ISL_930583, EPI_ISL_930584	CHC	GIGA Medical Genomics	Keith Durkin, Maria Artesi, Sébastien Bontems, Raphaël Boreux, Bouchra Boujemla, Cécile Meex, Pierrette Melin, Marie-Pierre Hayette, Vincent Bours
EPI_ISL_930594, EPI_ISL_930595	Labo Luc Olivier	GIGA Medical Genomics	Keith Durkin, Maria Artesi, Sébastien Bontems, Raphaël Boreux, Bouchra Boujemla, Cécile Meex, Pierrette Melin, Marie-Pierre Hayette, Vincent Bours
EPI_ISL_930596	Synlab	GIGA Medical Genomics	Keith Durkin, Maria Artesi, Sébastien Bontems, Raphaël Boreux, Bouchra Boujemla, Cécile Meex, Pierrette Melin, Marie-Pierre Hayette, Vincent Bours
EPI_ISL_930604	CHC	GIGA Medical Genomics	Keith Durkin, Maria Artesi, Sébastien Bontems, Raphaël Boreux, Bouchra Boujemla, Cécile Meex, Pierrette Melin, Marie-Pierre Hayette, Vincent Bours
EPI_ISL_930611, EPI_ISL_930614	University of Liège COVID-19 testing center	GIGA Medical Genomics	Keith Durkin, Maria Artesi, Bouchra Boujemla, Emmanuel André, Marc Van Ranst, Fabrice Bureau, Laurent Gillet, Wouter Coppieters, Vincent Bours
EPI_ISL_930621	St. Nikolaus-Hospital Eupen	GIGA Medical Genomics	Keith Durkin, Maria Artesi, Sébastien Bontems, Raphaël Boreux, Bouchra Boujemla, Cécile Meex, Pierrette Melin, Marie-Pierre Hayette, Vincent Bours
EPI_ISL_930622	Synlab	GIGA Medical Genomics	Keith Durkin, Maria Artesi, Sébastien Bontems, Raphaël Boreux, Bouchra Boujemla, Cécile Meex, Pierrette Melin, Marie-Pierre Hayette, Vincent Bours
EPI_ISL_930623	CHC	GIGA Medical Genomics	Keith Durkin, Maria Artesi, Sébastien Bontems, Raphaël Boreux, Bouchra Boujemla, Cécile Meex, Pierrette Melin, Marie-Pierre Hayette, Vincent Bours
EPI_ISL_930626, EPI_ISL_930632, EPI_ISL_930633	University of Liège COVID-19 testing center	GIGA Medical Genomics	Keith Durkin, Maria Artesi, Bouchra Boujemla, Emmanuel André, Marc Van Ranst, Fabrice Bureau, Laurent Gillet, Wouter Coppieters, Vincent Bours
EPI_ISL_931490, EPI_ISL_931494, EPI_ISL_931495, EPI_ISL_931501, EPI_ISL_931502, EPI_ISL_931503, EPI_ISL_931504, EPI_ISL_931505, EPI_ISL_931506	Maryland Public Health Laboratory (MD PHL)	Maryland Public Health Laboratory (MD PHL)	Maryland Department of Health Laboratories Administration
EPI_ISL_931586, EPI_ISL_931587, EPI_ISL_931588, EPI_ISL_931590, EPI_ISL_931591, EPI_ISL_931592, EPI_ISL_931593, EPI_ISL_931594, EPI_ISL_931596, EPI_ISL_931597, EPI_ISL_931598, EPI_ISL_931599, EPI_ISL_931600, EPI_ISL_931601, EPI_ISL_931602, EPI_ISL_931603, EPI_ISL_931604, EPI_ISL_931605, EPI_ISL_931606, EPI_ISL_931607, EPI_ISL_931608, EPI_ISL_931609, EPI_ISL_931610, EPI_ISL_931611, EPI_ISL_931612, EPI_ISL_931613, EPI_ISL_931614, EPI_ISL_931615, EPI_ISL_931616, EPI_ISL_931617, EPI_ISL_931618, EPI_ISL_931619, EPI_ISL_931620, EPI_ISL_931621, EPI_ISL_931622, EPI_ISL_931623, EPI_ISL_931624, EPI_ISL_931625, EPI_ISL_931626, EPI_ISL_931627, EPI_ISL_931628, EPI_ISL_931629, EPI_ISL_931630, EPI_ISL_931631, EPI_ISL_931632, EPI_ISL_931633, EPI_ISL_931634, EPI_ISL_931635, EPI_ISL_931636, EPI_ISL_931637, EPI_ISL_931638, EPI_ISL_931639, EPI_ISL_931640, EPI_ISL_931641, EPI_ISL_931642, EPI_ISL_931643, EPI_ISL_931644, EPI_ISL_931645, EPI_ISL_931646, EPI_ISL_931647, EPI_ISL_931648, EPI_ISL_931649, EPI_ISL_931650, EPI_ISL_931651, EPI_ISL_931652, EPI_ISL_931653, EPI_ISL_931654, EPI_ISL_931655, EPI_ISL_931656, EPI_ISL_931657, EPI_ISL_931658, EPI_ISL_931659, EPI_ISL_931660, EPI_ISL_931661, EPI_ISL_931662, EPI_ISL_931663, EPI_ISL_931664, EPI_ISL_931665, EPI_ISL_931666, EPI_ISL_931667, EPI_ISL_931668, EPI_ISL_931669, EPI_ISL_931670, EPI_ISL_931671, EPI_ISL_931672, EPI_ISL_931673, EPI_ISL_931674, EPI_ISL_931675, EPI_ISL_931676, EPI_ISL_931677, EPI_ISL_931678, EPI_ISL_931679, EPI_ISL_931680, EPI_ISL_931681, EPI_ISL_931682, EPI_ISL_931683, EPI_ISL_931684, EPI_ISL_931685, EPI_ISL_931686, EPI_ISL_931687, EPI_ISL_931688, EPI_ISL_931689, EPI_ISL_931691, EPI_ISL_931692, EPI_ISL_931693, EPI_ISL_931694, EPI_ISL_931695, EPI_ISL_931696, EPI_ISL_931697, EPI_ISL_931698, EPI_ISL_931699, EPI_ISL_931700, EPI_ISL_931701, EPI_ISL_931702, EPI_ISL_931703, EPI_ISL_931704, EPI_ISL_931705, EPI_ISL_931706, EPI_ISL_931707, EPI_ISL_931708, EPI_ISL_931709, EPI_ISL_931710, EPI_ISL_931711, EPI_ISL_931712, EPI_ISL_931713, EPI_ISL_931714, EPI_ISL_931715, EPI_ISL_931716, EPI_ISL_931717, EPI_ISL_931718, EPI_ISL_931719, EPI_ISL_931720, EPI_ISL_931721, EPI_ISL_931722, EPI_ISL_931723, EPI_ISL_931724, EPI_ISL_931725, EPI_ISL_931727, EPI_ISL_931728, EPI_ISL_931729, EPI_ISL_931730, EPI_ISL_931731, EPI_ISL_931732, EPI_ISL_931733, EPI_ISL_931734, EPI_ISL_931735, EPI_ISL_931736, EPI_ISL_931737, EPI_ISL_931738, EPI_ISL_931739, EPI_ISL_931740, EPI_ISL_931741, EPI_ISL_931742, EPI_ISL_931743, EPI_ISL_931744, EPI_ISL_931745, EPI_ISL_931746, EPI_ISL_931747, EPI_ISL_931748, EPI_ISL_931749, EPI_ISL_931750, EPI_ISL_931751, EPI_ISL_931752, EPI_ISL_931753, EPI_ISL_931754, EPI_ISL_931755, EPI_ISL_931756, EPI_ISL_931757, EPI_ISL_931758, EPI_ISL_931759, EPI_ISL_931760, EPI_ISL_931761, EPI_ISL_931762, EPI_ISL_931763, EPI_ISL_931764, EPI_ISL_931765, EPI_ISL_931766, EPI_ISL_931767, EPI_ISL_931768, EPI_ISL_931769, EPI_ISL_931770, EPI_ISL_931771, EPI_ISL_931772, EPI_ISL_931773, EPI_ISL_931774, EPI_ISL_931775, EPI_ISL_931776, EPI_ISL_931777, EPI_ISL_931778, EPI_ISL_931779, EPI_ISL_931780, EPI_ISL_931781, EPI_ISL_931782, EPI_ISL_931783, EPI_ISL_931784, EPI_ISL_931785, EPI_ISL_931786, EPI_ISL_931787, EPI_ISL_931788, EPI_ISL_931789, EPI_ISL_931790, EPI_ISL_931791, EPI_ISL_931792, EPI_ISL_931793, EPI_ISL_931794, EPI_ISL_931795, EPI_ISL_931796, EPI_ISL_931797, EPI_ISL_931798, EPI_ISL_931799, EPI_ISL_931800, EPI_ISL_931801, EPI_ISL_931802, EPI_ISL_931803, EPI_ISL_931804, EPI_ISL_931805, EPI_ISL_931806, EPI_ISL_931807, EPI_ISL_931808, EPI_ISL_931809, EPI_ISL_931810, EPI_ISL_931811, EPI_ISL_931812, EPI_ISL_931813, EPI_ISL_931814, EPI_ISL_931815, EPI_ISL_931816, EPI_ISL_931817, EPI_ISL_931819, EPI_ISL_931820, EPI_ISL_931821, EPI_ISL_931823, EPI_ISL_931824, EPI_ISL_931825, EPI_ISL_931826, EPI_ISL_931828, EPI_ISL_931829, EPI_ISL_931830, EPI_ISL_931831, EPI_ISL_931832, EPI_ISL_931833, EPI_ISL_931834, EPI_ISL_931835, EPI_ISL_931836, EPI_ISL_931837, EPI_ISL_931838, EPI_ISL_931839, EPI_ISL_931840, EPI_ISL_931841, EPI_ISL_931842, EPI_ISL_931843, EPI_ISL_931844, EPI_ISL_931845, EPI_ISL_931846, EPI_ISL_931847, EPI_ISL_931848, EPI_ISL_931849, EPI_ISL_931850, EPI_ISL_931851, EPI_ISL_931852, EPI_ISL_931853, EPI_ISL_931854, EPI_ISL_931855, EPI_ISL_931856, EPI_ISL_931857, EPI_ISL_931858, EPI_ISL_931859, EPI_ISL_931860, EPI_ISL_931861, EPI_ISL_931862, EPI_ISL_931863, EPI_ISL_931864, EPI_ISL_931865, EPI_ISL_931866, EPI_ISL_931867, EPI_ISL_931868, EPI_ISL_931869, EPI_ISL_931870, EPI_ISL_931871, EPI_ISL_931872, EPI_ISL_931873, EPI_ISL_931875, EPI_ISL_931876, EPI_ISL_931877, EPI_ISL_931878, EPI_ISL_931879, EPI_ISL_931880, EPI_ISL_931881, EPI_ISL_931882, EPI_ISL_931883, EPI_ISL_931884, EPI_ISL_931885, EPI_ISL_931886, EPI_ISL_931887, EPI_ISL_931888, EPI_ISL_931889, EPI_ISL_931890, EPI_ISL_931891, EPI_ISL_931892, EPI_ISL_931893, EPI_ISL_931894, EPI_ISL_931895, EPI_ISL_931896, EPI_ISL_931897, EPI_ISL_931898, EPI_ISL_931899, EPI_ISL_931900, EPI_ISL_931901, EPI_ISL_931902, EPI_ISL_931903, EPI_ISL_931904, EPI_ISL_931905, EPI_ISL_931906, EPI_ISL_931907, EPI_ISL_931908, EPI_ISL_931909, EPI_ISL_931910, EPI_ISL_931911, EPI_ISL_931912, EPI_ISL_931913, EPI_ISL_931914, EPI_ISL_931915, EPI_ISL_931916, EPI_ISL_931917, EPI_ISL_931919, EPI_ISL_931920, EPI_ISL_931922, EPI_ISL_931923, EPI_ISL_931924, EPI_ISL_931925, EPI_ISL_931926, EPI_ISL_931927, EPI_ISL_931928, EPI_ISL_931929, EPI_ISL_931930, EPI_ISL_931931, EPI_ISL_931932, EPI_ISL_931933, EPI_ISL_931934, EPI_ISL_931935, EPI_ISL_931936, EPI_ISL_931938, EPI_ISL_931940, EPI_ISL_931941, EPI_ISL_931942, EPI_ISL_931944, EPI_ISL_931945, EPI_ISL_931946, EPI_ISL_931947, EPI_ISL_931948, EPI_ISL_931949, EPI_ISL_931950, EPI_ISL_931951, EPI_ISL_931952, EPI_ISL_931954, EPI_ISL_931955, EPI_ISL_931956, EPI_ISL_931958, EPI_ISL_931960, EPI_ISL_931961, EPI_ISL_931962, EPI_ISL_931963, EPI_ISL_931964, EPI_ISL_931965, EPI_ISL_931966, EPI_ISL_931967, EPI_ISL_931968, EPI_ISL_931969, EPI_ISL_931970, EPI_ISL_931971, EPI_ISL_931972, EPI_ISL_931973, EPI_ISL_931974, EPI_ISL_931975, EPI_ISL_931976, EPI_ISL_931977, EPI_ISL_931978, EPI_ISL_931980, EPI_ISL_931981, EPI_ISL_931982, EPI_ISL_931983, EPI_ISL_931984, EPI_ISL_931985, EPI_ISL_931986, EPI_ISL_931987, EPI_ISL_931988, EPI_ISL_931989, EPI_ISL_931990, EPI_ISL_931991, EPI_ISL_931992, EPI_ISL_931993, EPI_ISL_931994, EPI_ISL_931995, EPI_ISL_931996, EPI_ISL_931997, EPI_ISL_931998, EPI_ISL_931999, EPI_ISL_932000, EPI_ISL_932001, EPI_ISL_932002, EPI_ISL_932003, EPI_ISL_932004, EPI_ISL_932005, EPI_ISL_932006, EPI_ISL_932007, EPI_ISL_932008, EPI_ISL_932009, EPI_ISL_932010, EPI_ISL_932011, EPI_ISL_932012, EPI_ISL_932013, EPI_ISL_932014, EPI_ISL_932015, EPI_ISL_932016, EPI_ISL_932018, EPI_ISL_932019, EPI_ISL_932020, EPI_ISL_932021, EPI_ISL_932022, EPI_ISL_932023, EPI_ISL_932024, EPI_ISL_932025, EPI_ISL_932026, EPI_ISL_932027, EPI_ISL_932028, EPI_ISL_932029, EPI_ISL_932030, EPI_ISL_932031, EPI_ISL_932032, EPI_ISL_932033, EPI_ISL_932034, EPI_ISL_932035, EPI_ISL_932036, EPI_ISL_932037, EPI_ISL_932038, EPI_ISL_932040, EPI_ISL_932041, EPI_ISL_932042, EPI_ISL_932043, EPI_ISL_932044, EPI_ISL_932045, EPI_ISL_932047, EPI_ISL_932048, EPI_ISL_932049, EPI_ISL_932050, EPI_ISL_932051, EPI_ISL_932052, EPI_ISL_932053, EPI_ISL_932054, EPI_ISL_932055, EPI_ISL_932056, EPI_ISL_932058, EPI_ISL_932059, EPI_ISL_932060, EPI_ISL_932062, EPI_ISL_932063, EPI_ISL_932064, EPI_ISL_932065, EPI_ISL_932066, EPI_ISL_932067, EPI_ISL_932069, EPI_ISL_932070, EPI_ISL_932071, EPI_ISL_932072, EPI_ISL_932073, EPI_ISL_932074, EPI_ISL_932075, EPI_ISL_932076, EPI_ISL_932077, EPI_ISL_932078, EPI_ISL_932079, EPI_ISL_932080, EPI_ISL_932081, EPI_ISL_932082, EPI_ISL_932083, EPI_ISL_932084, EPI_ISL_932085, EPI_ISL_932087, EPI_ISL_932088, EPI_ISL_932089, EPI_ISL_932090, EPI_ISL_932091, EPI_ISL_932092, EPI_ISL_932093, EPI_ISL_932094, EPI_ISL_932095, EPI_ISL_932096, EPI_ISL_932097, EPI_ISL_932098, EPI_ISL_932099, EPI_ISL_932100, EPI_ISL_932101, EPI_ISL_932102, EPI_ISL_932103, EPI_ISL_932104, EPI_ISL_932105, EPI_ISL_932106, EPI_ISL_932107, EPI_ISL_932109, EPI_ISL_932110, EPI_ISL_932111, EPI_ISL_932112, EPI_ISL_932113, EPI_ISL_932115, EPI_ISL_932116, EPI_ISL_932117, EPI_ISL_932118, EPI_ISL_932119, EPI_ISL_932120, EPI_ISL_932121, EPI_ISL_932122, EPI_ISL_932123, EPI_ISL_932124, EPI_ISL_932125, EPI_ISL_932126, EPI_ISL_932127, EPI_ISL_932128, EPI_ISL_932129, EPI_ISL_932130, EPI_ISL_932131, EPI_ISL_932132, EPI_ISL_932133, EPI_ISL_932134, EPI_ISL_932135, EPI_ISL_932136, EPI_ISL_932137, EPI_ISL_932138, EPI_ISL_932139, EPI_ISL_932140, EPI_ISL_932141, EPI_ISL_932142, EPI_ISL_932143, EPI_ISL_932144, EPI_ISL_932145, EPI_ISL_932146, EPI_ISL_932147, EPI_ISL_932148, EPI_ISL_932149, EPI_ISL_932150, EPI_ISL_932151, EPI_ISL_932152, EPI_ISL_932153, EPI_ISL_932154, EPI_ISL_932155, EPI_ISL_932156, EPI_ISL_932158, EPI_ISL_932159, EPI_ISL_932160, EPI_ISL_932161, EPI_ISL_932162, EPI_ISL_932163, EPI_ISL_932164, EPI_ISL_932165, EPI_ISL_932166, EPI_ISL_932167, EPI_ISL_932168, EPI_ISL_932169, EPI_ISL_932170, EPI_ISL_932171, EPI_ISL_932172, EPI_ISL_932173, EPI_ISL_932174, EPI_ISL_932175, EPI_ISL_932176, EPI_ISL_932177, EPI_ISL_932178, EPI_ISL_932179, EPI_ISL_932180, EPI_ISL_932181, EPI_ISL_932182, EPI_ISL_932183, EPI_ISL_932184, EPI_ISL_932185, EPI_ISL_932186, EPI_ISL_932187, EPI_ISL_932188, EPI_ISL_932189, EPI_ISL_932190, EPI_ISL_932191, EPI_ISL_932192, EPI_ISL_932193, EPI_ISL_932194, EPI_ISL_932195, EPI_ISL_932196, EPI_ISL_932197, EPI_ISL_932198, EPI_ISL_932199, EPI_ISL_932200, EPI_ISL_932202, EPI_ISL_932203, EPI_ISL_932204, EPI_ISL_932205, EPI_ISL_932206, EPI_ISL_932207, EPI_ISL_932208, EPI_ISL_932209, EPI_ISL_932210, EPI_ISL_932211, EPI_ISL_932212, EPI_ISL_932213, EPI_ISL_932214, EPI_ISL_932215, EPI_ISL_932216, EPI_ISL_932217, EPI_ISL_932218, EPI_ISL_932219, EPI_ISL_932220, EPI_ISL_932221, EPI_ISL_932223, EPI_ISL_932224, EPI_ISL_932225, EPI_ISL_932226, EPI_ISL_932227, EPI_ISL_932228, EPI_ISL_932229, EPI_ISL_932230, EPI_ISL_932232, EPI_ISL_932233, EPI_ISL_932234, EPI_ISL_932235, EPI_ISL_932236, EPI_ISL_932237, EPI_ISL_932238, EPI_ISL_932239, EPI_ISL_932242, EPI_ISL_932243, EPI_ISL_932244, EPI_ISL_932245, EPI_ISL_932246, EPI_ISL_932247, EPI_ISL_932248, EPI_ISL_932249, EPI_ISL_932250			
see above	Lighthouse Lab in Alderley Park	Wellcome Sanger Institute for the COVID-19 Genomics UK (COG-UK) Consortium	Jacquelyn Wynn, Mairead Hyland, The Lighthouse Lab in Alderley Park and Alex Alderton, Roberto Amato, Sonia Goncalves, Ewan Harrison, David K. Jackson, Ian Johnston, Dominic Kwiatkowski, Cordelia Langford, John Sillitoe on behalf of the Wellcome Sanger Institute COVID-19 Surveillance Team
EPI_ISL_932850, EPI_ISL_932853, EPI_ISL_932855, EPI_ISL_932856, EPI_ISL_932857, EPI_ISL_932858, EPI_ISL_932859, EPI_ISL_932860, EPI_ISL_932863, EPI_ISL_932864, EPI_ISL_932865, EPI_ISL_932870, EPI_ISL_932871, EPI_ISL_932879, EPI_ISL_932884, EPI_ISL_932885, EPI_ISL_932894, EPI_ISL_932896, EPI_ISL_932897, EPI_ISL_932899, EPI_ISL_932903, EPI_ISL_932905, EPI_ISL_932908, EPI_ISL_932914, EPI_ISL_932915, EPI_ISL_932917, EPI_ISL_932918, EPI_ISL_932919, EPI_ISL_932920, EPI_ISL_932921, EPI_ISL_932925, EPI_ISL_932929, EPI_ISL_932930, EPI_ISL_932931, EPI_ISL_932932, EPI_ISL_932936, EPI_ISL_932939, EPI_ISL_932944, EPI_ISL_932948, EPI_ISL_932949, EPI_ISL_932955, EPI_ISL_932957, EPI_ISL_932958, EPI_ISL_932965, EPI_ISL_932966, EPI_ISL_932967, EPI_ISL_932970, EPI_ISL_932980, EPI_ISL_932986, EPI_ISL_932987, EPI_ISL_932991, EPI_ISL_932994, EPI_ISL_932995, EPI_ISL_932996, EPI_ISL_933000, EPI_ISL_933002, EPI_ISL_933004, EPI_ISL_933006, EPI_ISL_933007, EPI_ISL_933009, EPI_ISL_933011, EPI_ISL_933018, EPI_ISL_933020, EPI_ISL_933021, EPI_ISL_933028, EPI_ISL_933033, EPI_ISL_933035, EPI_ISL_933036, EPI_ISL_933037, EPI_ISL_933038, EPI_ISL_933040, EPI_ISL_933042, EPI_ISL_933044, EPI_ISL_933050, EPI_ISL_933051, EPI_ISL_933053, EPI_ISL_933054, EPI_ISL_933056, EPI_ISL_933058, EPI_ISL_933065, EPI_ISL_933067, EPI_ISL_933069, EPI_ISL_933070, EPI_ISL_933072, EPI_ISL_933073, EPI_ISL_933074, EPI_ISL_933079, EPI_ISL_933080, EPI_ISL_933096, EPI_ISL_933099, EPI_ISL_933100, EPI_ISL_933109, EPI_ISL_933110			

see above	Lighthouse Lab in Glasgow	Wellcome Sanger Institute for the COVID-19 Genomics UK (COG-UK) Consortium	Harper VanSteenhouse, Yumi Kasai, David Gray, Carol Clugston, Anna Dominiczak and Alex Alderton, Roberto Amato, Sonia Goncalves, Ewan Harrison, David K. Jackson, Ian Johnston, Dominic Kwiatkowski, Cordelia Langford, John Sillitoe on behalf of the Wellcome Sanger Institute COVID-19 Surveillance Team
EPI_ISL_933777	Victorian Infectious Diseases Reference Laboratory (VIDRL)	VIDRL and MDU-PHL	Caly L., Seemann T., Sait, M.L., Druce J., Sherry, N.L.
EPI_ISL_933778	Microbiological Diagnostic Unit - Public Health Laboratory (MDU-PHL)	MDU-PHL	Seemann T., Sait, M.L., Sherry, N.L.
EPI_ISL_933779	Victorian Infectious Diseases Reference Laboratory (VIDRL)	VIDRL and MDU-PHL	Caly L., Seemann T., Sait, M.L., Druce J., Sherry, N.L.
EPI_ISL_933794, EPI_ISL_933795, EPI_ISL_933796	PathWest Laboratory Medicine WA	PathWest Laboratory Medicine WA Microbial Surveillance Unit	PathWest Laboratory Medicine WA Microbial Surveillance Unit
EPI_ISL_935012, EPI_ISL_935013, EPI_ISL_935014, EPI_ISL_935015, EPI_ISL_935016	University of Michigan Clinical Microbiology Laboratory	Lauring Lab, University of Michigan, Department of Microbiology and Immunology	Valesano
EPI_ISL_935155	Cytocheck Laboratory	Kansas Health and Environmental Lab	Mike Grose, Paige Drury, Carissa Robertson, Ben Olsen, and Phil Adam
EPI_ISL_935171, EPI_ISL_935172, EPI_ISL_935173, EPI_ISL_935174, EPI_ISL_935175, EPI_ISL_935176, EPI_ISL_935177, EPI_ISL_935178, EPI_ISL_935179, EPI_ISL_935180	Wyoming Public Health Laboratory	Wyoming Public Health Laboratory	Noah Hull, Taylor Fearing, Lynette Gumbleton, Channing Weber, Ashley Norberg, Bailey Bowcutt, and Wanda Manley
EPI_ISL_935602, EPI_ISL_935603	Labo Analyses med	National Reference Center for Viruses of Respiratory Infections, Institut Pasteur, Paris	Marion Barbet, Sylvie Behillil, Méline Bizard, Angela Brisebarre, Camille Capel, Etienne Simon-Lorière, Vincent Enouf, Maud Vanpeene, Sylvie van der Werf,Amzalag Jonas
EPI_ISL_935655, EPI_ISL_935656, EPI_ISL_935657, EPI_ISL_935658, EPI_ISL_935659, EPI_ISL_935660, EPI_ISL_935661, EPI_ISL_935662, EPI_ISL_935663, EPI_ISL_935664, EPI_ISL_935665, EPI_ISL_935666, EPI_ISL_935667, EPI_ISL_935668, EPI_ISL_935669, EPI_ISL_935670, EPI_ISL_935671, EPI_ISL_935672, EPI_ISL_935673			
see above	Department of Clinical Microbiology	GIGA Medical Genomics	Keith Durkin, Maria Artesi, Sébastien Bontems, Raphaël Boreux, Bouchra Boujemla, Cécile Meex, Pierrette Melin, Marie-Pierre Hayette, Vincent Bours
EPI_ISL_935674, EPI_ISL_935675, EPI_ISL_935676, EPI_ISL_935677, EPI_ISL_935678, EPI_ISL_935679, EPI_ISL_935680, EPI_ISL_935681, EPI_ISL_935682, EPI_ISL_935683, EPI_ISL_935684, EPI_ISL_935685, EPI_ISL_935686, EPI_ISL_935687, EPI_ISL_935688, EPI_ISL_935689, EPI_ISL_935690, EPI_ISL_935691, EPI_ISL_935692, EPI_ISL_935693, EPI_ISL_935694, EPI_ISL_935695			
see above	CHR de la Citadelle	GIGA Medical Genomics	Keith Durkin, Maria Artesi, Sébastien Bontems, Raphaël Boreux, Bouchra Boujemla, Cécile Meex, Pierrette Melin, Marie-Pierre Hayette, Vincent Bours
EPI_ISL_936485	DPH, Massachusetts State Public Health Lab	DPH, Massachusetts State Public Health Lab	Lang,A.S., Fink,T., Gallagher,G.R., Smole,S.C.
EPI_ISL_937215	DOHMH PHL	New York City Public Health Laboratory	Jade Wang, et al.
EPI_ISL_937216	DOHMH Central Harlem	New York City Public Health Laboratory	Jade Wang, et al.
EPI_ISL_937217, EPI_ISL_937218	DOHMH Chelsea	New York City Public Health Laboratory	Jade Wang, et al.
EPI_ISL_937219, EPI_ISL_937220, EPI_ISL_937221, EPI_ISL_937222, EPI_ISL_937223, EPI_ISL_937224, EPI_ISL_937225, EPI_ISL_937226	DOHMH Jamaica	New York City Public Health Laboratory	Jade Wang, et al.
EPI_ISL_937227, EPI_ISL_937228, EPI_ISL_937229, EPI_ISL_937230, EPI_ISL_937231	DOHMH Corona	New York City Public Health Laboratory	Jade Wang, et al.
EPI_ISL_937232, EPI_ISL_937233, EPI_ISL_937234, EPI_ISL_937235, EPI_ISL_937236	DOHMH Morrisania	New York City Public Health Laboratory	Jade Wang, et al.
EPI_ISL_937237	DOHMH Riverside	New York City Public Health Laboratory	Jade Wang, et al.
EPI_ISL_937238, EPI_ISL_937239	DOHMH Morrisania	New York City Public Health Laboratory	Jade Wang, et al.
EPI_ISL_937251, EPI_ISL_937252, EPI_ISL_937253, EPI_ISL_937254, EPI_ISL_937255	OCME Office Of Chief Medical Examiner	New York City Public Health Laboratory	Jade Wang, et al.
EPI_ISL_937265, EPI_ISL_937268	NYC HH Elmhurst Hospital Medical Center	New York City Public Health Laboratory	Jade Wang, et al.
EPI_ISL_937459, EPI_ISL_937460, EPI_ISL_937461, EPI_ISL_937462, EPI_ISL_937463, EPI_ISL_937464, EPI_ISL_937465, EPI_ISL_937466, EPI_ISL_937467, EPI_ISL_937495, EPI_ISL_937496, EPI_ISL_937497, EPI_ISL_937498, EPI_ISL_937499, EPI_ISL_937500, EPI_ISL_937501, EPI_ISL_937502, EPI_ISL_937503, EPI_ISL_937504, EPI_ISL_937505, EPI_ISL_937507, EPI_ISL_937510, EPI_ISL_937512			
see above	Maine Health and Environmental Testing Laboratory (Maine HETL)	Tewhey Lab, The Jackson Laboratory	Matluk,N., Dewey,H., Iosue,F., Barter,M., Lynch,R., Munger,H. and Tewhey,R.
EPI_ISL_937545, EPI_ISL_937546, EPI_ISL_937547, EPI_ISL_937548, EPI_ISL_937552, EPI_ISL_937555, EPI_ISL_937556, EPI_ISL_937560, EPI_ISL_937561, EPI_ISL_937563, EPI_ISL_937565, EPI_ISL_937566, EPI_ISL_937567, EPI_ISL_937572, EPI_ISL_937574, EPI_ISL_937575, EPI_ISL_937577, EPI_ISL_937582, EPI_ISL_937584, EPI_ISL_937585, EPI_ISL_937589, EPI_ISL_937591, EPI_ISL_937593, EPI_ISL_937597, EPI_ISL_937598, EPI_ISL_937600, EPI_ISL_937602, EPI_ISL_937603, EPI_ISL_937606, EPI_ISL_937607, EPI_ISL_937608, EPI_ISL_937611, EPI_ISL_937613, EPI_ISL_937615, EPI_ISL_937617, EPI_ISL_937619, EPI_ISL_937622, EPI_ISL_937624, EPI_ISL_937625, EPI_ISL_937626, EPI_ISL_937628, EPI_ISL_937629, EPI_ISL_937631, EPI_ISL_937632, EPI_ISL_937633, EPI_ISL_937635, EPI_ISL_937637, EPI_ISL_937642, EPI_ISL_937644, EPI_ISL_937645, EPI_ISL_937646, EPI_ISL_937647, EPI_ISL_937652, EPI_ISL_937654, EPI_ISL_937655, EPI_ISL_937656, EPI_ISL_937657, EPI_ISL_937659, EPI_ISL_937660, EPI_ISL_937661, EPI_ISL_937662, EPI_ISL_937667, EPI_ISL_937671, EPI_ISL_937673, EPI_ISL_937676, EPI_ISL_937677, EPI_ISL_937678, EPI_ISL_937679, EPI_ISL_937681, EPI_ISL_937683, EPI_ISL_937684, EPI_ISL_937686, EPI_ISL_937690, EPI_ISL_937691, EPI_ISL_937692, EPI_ISL_937694, EPI_ISL_937695, EPI_ISL_937696, EPI_ISL_937698, EPI_ISL_937701, EPI_ISL_937702, EPI_ISL_937705, EPI_ISL_937706, EPI_ISL_937708, EPI_ISL_937713, EPI_ISL_937718, EPI_ISL_937719, EPI_ISL_937722, EPI_ISL_937723, EPI_ISL_937725, EPI_ISL_937727, EPI_ISL_937728, EPI_ISL_937729, EPI_ISL_937731, EPI_ISL_937732, EPI_ISL_937733, EPI_ISL_937735, EPI_ISL_937736, EPI_ISL_937739, EPI_ISL_937740, EPI_ISL_937743, EPI_ISL_937747, EPI_ISL_937750, EPI_ISL_937754, EPI_ISL_937755, EPI_ISL_937756, EPI_ISL_937757, EPI_ISL_937758, EPI_ISL_937759, EPI_ISL_937760, EPI_ISL_937764, EPI_ISL_937771, EPI_ISL_937772, EPI_ISL_937773, EPI_ISL_937774, EPI_ISL_937775, EPI_ISL_937776, EPI_ISL_937778, EPI_ISL_937779, EPI_ISL_937782, EPI_ISL_937783, EPI_ISL_937785, EPI_ISL_937786, EPI_ISL_937788, EPI_ISL_937789, EPI_ISL_937791, EPI_ISL_937793, EPI_ISL_937795, EPI_ISL_937796, EPI_ISL_937797, EPI_ISL_937804, EPI_ISL_937807, EPI_ISL_937809, EPI_ISL_937810, EPI_ISL_937812, EPI_ISL_937813, EPI_ISL_937814, EPI_ISL_937816, EPI_ISL_937819, EPI_ISL_937821, EPI_ISL_937823, EPI_ISL_937825, EPI_ISL_937827, EPI_ISL_937828, EPI_ISL_937832, EPI_ISL_937833, EPI_ISL_937834, EPI_ISL_937836, EPI_ISL_937838, EPI_ISL_937841, EPI_ISL_937842, EPI_ISL_937844, EPI_ISL_937845, EPI_ISL_937846, EPI_ISL_937847, EPI_ISL_937848, EPI_ISL_937851, EPI_ISL_937855, EPI_ISL_937856, EPI_ISL_937857, EPI_ISL_937858, EPI_ISL_937859, EPI_ISL_937861, EPI_ISL_937866, EPI_ISL_937868, EPI_ISL_937869, EPI_ISL_937870, EPI_ISL_937871, EPI_ISL_937876, EPI_ISL_937878, EPI_ISL_937879, EPI_ISL_937880, EPI_ISL_937882, EPI_ISL_937884, EPI_ISL_937885			
see above	Lighthouse Lab in Alderley Park	Wellcome Sanger Institute for the COVID-19 Genomics UK (COG-UK) Consortium	Jacquelyn Wynn, Mairead Hyland, The Lighthouse Lab in Alderley Park and Alex Alderton, Roberto Amato, Sonia Goncalves, Ewan Harrison, David K. Jackson, Ian Johnston, Dominic Kwiatkowski, Cordelia Langford, John Sillitoe on behalf of the Wellcome Sanger Institute COVID-19 Surveillance Team
EPI_ISL_938579, EPI_ISL_938580, EPI_ISL_938582, EPI_ISL_938583, EPI_ISL_938584, EPI_ISL_938585, EPI_ISL_938586, EPI_ISL_938588, EPI_ISL_938589, EPI_ISL_938590, EPI_ISL_938591, EPI_ISL_938593, EPI_ISL_938594, EPI_ISL_938595, EPI_ISL_938597, EPI_ISL_938598, EPI_ISL_938600, EPI_ISL_938601, EPI_ISL_938604, EPI_ISL_938605, EPI_ISL_938606, EPI_ISL_938608, EPI_ISL_938609, EPI_ISL_938611, EPI_ISL_938615, EPI_ISL_938616, EPI_ISL_938617, EPI_ISL_938618, EPI_ISL_938620, EPI_ISL_938621, EPI_ISL_938622, EPI_ISL_938623, EPI_ISL_938624, EPI_ISL_938625, EPI_ISL_938626, EPI_ISL_938627, EPI_ISL_938628, EPI_ISL_938629, EPI_ISL_938634, EPI_ISL_938635, EPI_ISL_938638, EPI_ISL_938639, EPI_ISL_938640, EPI_ISL_938641, EPI_ISL_938642, EPI_ISL_938644, EPI_ISL_938646, EPI_ISL_938647, EPI_ISL_938649, EPI_ISL_938651, EPI_ISL_938653, EPI_ISL_938654, EPI_ISL_938655, EPI_ISL_938656, EPI_ISL_938658, EPI_ISL_938659, EPI_ISL_938660, EPI_ISL_938661, EPI_ISL_938662, EPI_ISL_938663, EPI_ISL_938664, EPI_ISL_938665, EPI_ISL_938668, EPI_ISL_938669, EPI_ISL_938670, EPI_ISL_938671, EPI_ISL_938672, EPI_ISL_938673, EPI_ISL_938674, EPI_ISL_938675, EPI_ISL_938677, EPI_ISL_938678, EPI_ISL_938679, EPI_ISL_938680, EPI_ISL_938681, EPI_ISL_938682, EPI_ISL_938683, EPI_ISL_938685, EPI_ISL_938686, EPI_ISL_938687, EPI_ISL_938688, EPI_ISL_938689, EPI_ISL_938690, EPI_ISL_938691, EPI_ISL_938693, EPI_ISL_938695, EPI_ISL_938696, EPI_ISL_938697, EPI_ISL_938698, EPI_ISL_938699, EPI_ISL_938702, EPI_ISL_938703, EPI_ISL_938704, EPI_ISL_938705, EPI_ISL_938706, EPI_ISL_938708, EPI_ISL_938709, EPI_ISL_938710, EPI_ISL_938711, EPI_ISL_938712, EPI_ISL_938713, EPI_ISL_938714, EPI_ISL_938715, EPI_ISL_938716, EPI_ISL_938717, EPI_ISL_938718, EPI_ISL_938719, EPI_ISL_938720, EPI_ISL_938722, EPI_ISL_938723, EPI_ISL_938724, EPI_ISL_938725, EPI_ISL_938726, EPI_ISL_938727, EPI_ISL_938728, EPI_ISL_938730, EPI_ISL_938734, EPI_ISL_938735, EPI_ISL_938736, EPI_ISL_938738, EPI_ISL_938739, EPI_ISL_938740, EPI_ISL_938742, EPI_ISL_938745, EPI_ISL_938748, EPI_ISL_938749, EPI_ISL_938754, EPI_ISL_938755, EPI_ISL_938759, EPI_ISL_938760, EPI_ISL_938761, EPI_ISL_938763, EPI_ISL_938765, EPI_ISL_938766, EPI_ISL_938767, EPI_ISL_938768, EPI_ISL_938770, EPI_ISL_938772, EPI_ISL_938773, EPI_ISL_938774, EPI_ISL_938775, EPI_ISL_938776, EPI_ISL_938778, EPI_ISL_938779, EPI_ISL_938780, EPI_ISL_938781, EPI_ISL_938782, EPI_ISL_938784, EPI_ISL_938786, EPI_ISL_938788, EPI_ISL_938789, EPI_ISL_938790, EPI_ISL_938791, EPI_ISL_938792, EPI_ISL_938793, EPI_ISL_938795, EPI_ISL_938798, EPI_ISL_938799, EPI_ISL_938802, EPI_ISL_938805, EPI_ISL_938808, EPI_ISL_938809, EPI_ISL_938812, EPI_ISL_938813, EPI_ISL_938814, EPI_ISL_938816, EPI_ISL_938817, EPI_ISL_938818, EPI_ISL_938819, EPI_ISL_938820, EPI_ISL_938821, EPI_ISL_938822, EPI_ISL_938823, EPI_ISL_938824, EPI_ISL_938825, EPI_ISL_938826, EPI_ISL_938827, EPI_ISL_938828, EPI_ISL_938830, EPI_ISL_938831, EPI_ISL_938832, EPI_ISL_938833, EPI_ISL_938834, EPI_ISL_938835, EPI_ISL_938836, EPI_ISL_938838, EPI_ISL_938839, EPI_ISL_938841, EPI_ISL_938842, EPI_ISL_938843, EPI_ISL_938844, EPI_ISL_938847, EPI_ISL_938848, EPI_ISL_938849, EPI_ISL_938850, EPI_ISL_938851, EPI_ISL_938852, EPI_ISL_938853,			

EPI_ISL_938855, EPI_ISL_938856, EPI_ISL_938857, EPI_ISL_938858, EPI_ISL_938859, EPI_ISL_938860, EPI_ISL_938861, EPI_ISL_938862, EPI_ISL_938863, EPI_ISL_938864, EPI_ISL_938866, EPI_ISL_938867, EPI_ISL_938868, EPI_ISL_938869, EPI_ISL_938870, EPI_ISL_938871, EPI_ISL_938872, EPI_ISL_938873, EPI_ISL_938874, EPI_ISL_938877, EPI_ISL_938878, EPI_ISL_938879, EPI_ISL_938880, EPI_ISL_938881, EPI_ISL_938883, EPI_ISL_938884, EPI_ISL_938887, EPI_ISL_938888, EPI_ISL_938889, EPI_ISL_938890, EPI_ISL_938891, EPI_ISL_938892, EPI_ISL_938893, EPI_ISL_938894, EPI_ISL_938895, EPI_ISL_938896, EPI_ISL_938897, EPI_ISL_938898, EPI_ISL_938899, EPI_ISL_938902, EPI_ISL_938904, EPI_ISL_938905, EPI_ISL_938906, EPI_ISL_938907, EPI_ISL_938909, EPI_ISL_938910, EPI_ISL_938912, EPI_ISL_938914, EPI_ISL_938915, EPI_ISL_938917, EPI_ISL_938918, EPI_ISL_938919, EPI_ISL_938920, EPI_ISL_938922, EPI_ISL_938923, EPI_ISL_938924, EPI_ISL_938925, EPI_ISL_938926, EPI_ISL_938927, EPI_ISL_938928, EPI_ISL_938929, EPI_ISL_938930, EPI_ISL_938932			
see above	Lighthouse Lab in Milton Keynes	Wellcome Sanger Institute for the COVID-19 Genomics UK (COG-UK) Consortium	The Lighthouse Lab in Milton Keynes and Alex Alderton, Roberto Amato, Sonia Goncalves, Ewan Harrison, David K. Jackson, Ian Johnston, Dominic Kwiatkowski, Cordelia Langford, John Sillitoe on behalf of the Wellcome Sanger Institute COVID-19 Surveillance Team
EPI_ISL_938935, EPI_ISL_938936, EPI_ISL_938937, EPI_ISL_938938, EPI_ISL_938939, EPI_ISL_938941, EPI_ISL_938942, EPI_ISL_938943	Lighthouse Lab in Alderley Park	Wellcome Sanger Institute for the COVID-19 Genomics UK (COG-UK) Consortium	Jacquelyn Wynn, Mairead Hyland, The Lighthouse Lab in Alderley Park and Alex Alderton, Roberto Amato, Sonia Goncalves, Ewan Harrison, David K. Jackson, Ian Johnston, Dominic Kwiatkowski, Cordelia Langford, John Sillitoe on behalf of the Wellcome Sanger Institute COVID-19 Surveillance Team
EPI_ISL_939283, EPI_ISL_939284, EPI_ISL_939285, EPI_ISL_939287, EPI_ISL_939289, EPI_ISL_939295, EPI_ISL_939297, EPI_ISL_939298, EPI_ISL_939299, EPI_ISL_939303, EPI_ISL_939304, EPI_ISL_939305, EPI_ISL_939308, EPI_ISL_939309, EPI_ISL_939310, EPI_ISL_939311, EPI_ISL_939312, EPI_ISL_939316, EPI_ISL_939317, EPI_ISL_939322, EPI_ISL_939323, EPI_ISL_939324, EPI_ISL_939325, EPI_ISL_939326, EPI_ISL_939328, EPI_ISL_939330, EPI_ISL_939331, EPI_ISL_939333, EPI_ISL_939335, EPI_ISL_939336, EPI_ISL_939338, EPI_ISL_939341, EPI_ISL_939342, EPI_ISL_939344, EPI_ISL_939345, EPI_ISL_939347, EPI_ISL_939349, EPI_ISL_939353, EPI_ISL_939360, EPI_ISL_939362, EPI_ISL_939363, EPI_ISL_939366, EPI_ISL_939367, EPI_ISL_939371, EPI_ISL_939374, EPI_ISL_939375, EPI_ISL_939376, EPI_ISL_939377, EPI_ISL_939379, EPI_ISL_939381, EPI_ISL_939382, EPI_ISL_939383, EPI_ISL_939384, EPI_ISL_939385, EPI_ISL_939386, EPI_ISL_939387, EPI_ISL_939392, EPI_ISL_939394, EPI_ISL_939400, EPI_ISL_939408, EPI_ISL_939409, EPI_ISL_939410, EPI_ISL_939411, EPI_ISL_939412, EPI_ISL_939417, EPI_ISL_939419, EPI_ISL_939421, EPI_ISL_939423, EPI_ISL_939424, EPI_ISL_939426, EPI_ISL_939427, EPI_ISL_939430, EPI_ISL_939433, EPI_ISL_939434, EPI_ISL_939435, EPI_ISL_939436, EPI_ISL_939437, EPI_ISL_939440, EPI_ISL_939441, EPI_ISL_939442, EPI_ISL_939444, EPI_ISL_939445, EPI_ISL_939446, EPI_ISL_939447, EPI_ISL_939448, EPI_ISL_939449, EPI_ISL_939451, EPI_ISL_939452, EPI_ISL_939453, EPI_ISL_939455, EPI_ISL_939457, EPI_ISL_939463, EPI_ISL_939464, EPI_ISL_939465, EPI_ISL_939467, EPI_ISL_939470, EPI_ISL_939471, EPI_ISL_939474, EPI_ISL_939475, EPI_ISL_939476, EPI_ISL_939479, EPI_ISL_939480, EPI_ISL_939482, EPI_ISL_939484, EPI_ISL_939486, EPI_ISL_939488, EPI_ISL_939489, EPI_ISL_939490, EPI_ISL_939491, EPI_ISL_939495, EPI_ISL_939496, EPI_ISL_939499, EPI_ISL_939501, EPI_ISL_939502, EPI_ISL_939503, EPI_ISL_939511, EPI_ISL_939515, EPI_ISL_939516, EPI_ISL_939518, EPI_ISL_939525, EPI_ISL_939527, EPI_ISL_939529, EPI_ISL_939530, EPI_ISL_939531, EPI_ISL_939534, EPI_ISL_939538, EPI_ISL_939539, EPI_ISL_939540, EPI_ISL_939543, EPI_ISL_939544, EPI_ISL_939545, EPI_ISL_939546, EPI_ISL_939549, EPI_ISL_939553, EPI_ISL_939557, EPI_ISL_939558, EPI_ISL_939560, EPI_ISL_939564, EPI_ISL_939568, EPI_ISL_939569, EPI_ISL_939570, EPI_ISL_939575, EPI_ISL_939580, EPI_ISL_939583, EPI_ISL_939584, EPI_ISL_939586, EPI_ISL_939587, EPI_ISL_939589, EPI_ISL_939590, EPI_ISL_939591, EPI_ISL_939592, EPI_ISL_939593, EPI_ISL_939597, EPI_ISL_939602			
see above	Lighthouse Lab in Glasgow	Wellcome Sanger Institute for the COVID-19 Genomics UK (COG-UK) Consortium	Harper VanSteenhouse, Yumi Kasai, David Gray, Carol Clugston, Anna Dominiczak and Alex Alderton, Roberto Amato, Sonia Goncalves, Ewan Harrison, David K. Jackson, Ian Johnston, Dominic Kwiatkowski, Cordelia Langford, John Sillitoe on behalf of the Wellcome Sanger Institute COVID-19 Surveillance Team
EPI_ISL_939618	Los Angeles County PHL	Los Angeles County PHL	P. Hemarajata et al.
EPI_ISL_940554	Laboratory for Respiratory Viruses, Cantacuzino National Military-Medical Institute for Research and Development	Cantacuzino Institute Virology	Luiza Ustea, Nicoleta Paraschiv, Mihaela Lazar
EPI_ISL_940566, EPI_ISL_940567, EPI_ISL_940568, EPI_ISL_940569, EPI_ISL_940570, EPI_ISL_940571, EPI_ISL_940572	Jessa	Jessa	Jessa_cmdLab
EPI_ISL_940573, EPI_ISL_940574, EPI_ISL_940575	AZT	Jessa	Jessa_cmdLab
EPI_ISL_940576, EPI_ISL_940587, EPI_ISL_940588, EPI_ISL_940589, EPI_ISL_940590, EPI_ISL_940591, EPI_ISL_940592, EPI_ISL_940593, EPI_ISL_940594, EPI_ISL_940595, EPI_ISL_940596, EPI_ISL_940597, EPI_ISL_940598, EPI_ISL_940599, EPI_ISL_940600, EPI_ISL_940601, EPI_ISL_940602, EPI_ISL_940603, EPI_ISL_940604, EPI_ISL_940605, EPI_ISL_940606			
see above	Jessa	Jessa	Jessa_cmdLab
EPI_ISL_940629	Hospital Municipal Josanias Castanha Braga	Instituto Adolfo Lutz, Interdiciplinary Procedures Center, Strategic Laboratory	Claudio Tavares Sacchi, Claudia Regina Gonçalves, Erica Valessa Ramos Gomes, Karoline Rodrigues Campos
EPI_ISL_940630	Hospital Geral de Sao Paulo	Instituto Adolfo Lutz, Interdiciplinary Procedures Center, Strategic Laboratory	Claudio Tavares Sacchi, Claudia Regina Gonçalves, Erica Valessa Ramos Gomes, Karoline Rodrigues Campos
EPI_ISL_940739	University of Bari Biomedical Sciences and Human Oncology	University of Bari Biomedical Sciences and Human Oncology	Chironna M., Sallustio A., Loconsole D., Accogli M.
EPI_ISL_941300, EPI_ISL_941301, EPI_ISL_941302, EPI_ISL_941303, EPI_ISL_941304, EPI_ISL_941305, EPI_ISL_941306, EPI_ISL_941307, EPI_ISL_941308, EPI_ISL_941309, EPI_ISL_941310, EPI_ISL_941311, EPI_ISL_941312, EPI_ISL_941313, EPI_ISL_941314, EPI_ISL_941315, EPI_ISL_941316, EPI_ISL_941317, EPI_ISL_941318, EPI_ISL_941319, EPI_ISL_941320, EPI_ISL_941321, EPI_ISL_941322, EPI_ISL_941323, EPI_ISL_941324, EPI_ISL_941325, EPI_ISL_941326, EPI_ISL_941327, EPI_ISL_941328, EPI_ISL_941329, EPI_ISL_941330, EPI_ISL_941331, EPI_ISL_941332, EPI_ISL_941333, EPI_ISL_941334			
see above	New Mexico Department of Health Scientific Laboratory	New Mexico Department of Health Scientific Laboratory	Ellie Johnson, Anastacia Griego-Fisher, D'eldra Malone, Jennifer Benoit
EPI_ISL_941601, EPI_ISL_941620, EPI_ISL_941621, EPI_ISL_941622	Instituto Nacional de Saude (INSA)	Instituto Nacional de Saude (INSA)	Borges et al
EPI_ISL_942326, EPI_ISL_942327, EPI_ISL_942328, EPI_ISL_942329, EPI_ISL_942330, EPI_ISL_942331, EPI_ISL_942332, EPI_ISL_942333, EPI_ISL_942334, EPI_ISL_942335, EPI_ISL_942336, EPI_ISL_942337, EPI_ISL_942338, EPI_ISL_942339, EPI_ISL_942340, EPI_ISL_942341, EPI_ISL_942342, EPI_ISL_942343, EPI_ISL_942344, EPI_ISL_942345, EPI_ISL_942346, EPI_ISL_942347, EPI_ISL_942348, EPI_ISL_942349, EPI_ISL_942350, EPI_ISL_942351, EPI_ISL_942352, EPI_ISL_942353, EPI_ISL_942355, EPI_ISL_942356, EPI_ISL_942357, EPI_ISL_942358, EPI_ISL_942359, EPI_ISL_942360, EPI_ISL_942361, EPI_ISL_942362, EPI_ISL_942363, EPI_ISL_942364, EPI_ISL_942365, EPI_ISL_942367, EPI_ISL_942368			
see above	MD PHL	MD PHL	Maryland Department of Health Laboratories Administration
EPI_ISL_942880, EPI_ISL_942881, EPI_ISL_942882, EPI_ISL_942883, EPI_ISL_942884, EPI_ISL_942885, EPI_ISL_942886, EPI_ISL_942887, EPI_ISL_942888, EPI_ISL_942889, EPI_ISL_942890, EPI_ISL_942891, EPI_ISL_942892, EPI_ISL_942893, EPI_ISL_942949, EPI_ISL_942950			
see above	Gundersen Molecular Diagnostics Laboratory	Kabara Cancer Research Institute	Craig S. Richmond, Paraic A. Kenny
EPI_ISL_943016, EPI_ISL_943023, EPI_ISL_943026, EPI_ISL_943029, EPI_ISL_943037, EPI_ISL_943038, EPI_ISL_943041, EPI_ISL_943045, EPI_ISL_943046, EPI_ISL_943047, EPI_ISL_943054, EPI_ISL_943126, EPI_ISL_943127, EPI_ISL_943128, EPI_ISL_943129, EPI_ISL_943130, EPI_ISL_943131, EPI_ISL_943132, EPI_ISL_943133, EPI_ISL_943134, EPI_ISL_943135, EPI_ISL_943136, EPI_ISL_943158, EPI_ISL_943159, EPI_ISL_943160, EPI_ISL_943161, EPI_ISL_943162, EPI_ISL_943221, EPI_ISL_943222, EPI_ISL_943223, EPI_ISL_943224, EPI_ISL_943225, EPI_ISL_943226, EPI_ISL_943227, EPI_ISL_943253, EPI_ISL_943254, EPI_ISL_943255, EPI_ISL_943297, EPI_ISL_943305, EPI_ISL_943306, EPI_ISL_943320, EPI_ISL_943326, EPI_ISL_943327, EPI_ISL_943332, EPI_ISL_943365, EPI_ISL_943366, EPI_ISL_943367, EPI_ISL_943368, EPI_ISL_943369, EPI_ISL_943370, EPI_ISL_943372, EPI_ISL_943373, EPI_ISL_943374, EPI_ISL_943375, EPI_ISL_943411, EPI_ISL_943412, EPI_ISL_943413, EPI_ISL_943414, EPI_ISL_943415, EPI_ISL_943416, EPI_ISL_943417, EPI_ISL_943418, EPI_ISL_943419, EPI_ISL_943436, EPI_ISL_943439, EPI_ISL_943445, EPI_ISL_943448, EPI_ISL_943486, EPI_ISL_943490, EPI_ISL_943498, EPI_ISL_943509, EPI_ISL_943525, EPI_ISL_943526, EPI_ISL_943542			
see above	Dutch COVID-19 response team	National Institute for Public Health and the Environment (RIVM)	Adam Meijer, Harry Vennema, Dirk Eggink, Jeroen Cremer, Sharon van den Brink, Bas van der Veer, AnneMarie van den Brandt, Florian Zwagemaker, Dennis Schmitz, Chantal Reusken, on behalf of the national COVID-19 response team
EPI_ISL_943569	Arizona State University	Arizona State University	Peter T. Skidmore, Emily A. Kaelin, LaRinda A. Holland, Nicholas J. Mellor, Kristina Buss, Joy M. Blain, Valerie Harris, Joshua LaBaer, Vel Murugan, Efrem S. Lim
EPI_ISL_943967, EPI_ISL_943968, EPI_ISL_943969, EPI_ISL_943970, EPI_ISL_943971, EPI_ISL_943972	Hospital Geral de Sao Paulo	Instituto Adolfo Lutz, Interdiciplinary Procedures Center, Strategic Laboratory	Claudio Tavares Sacchi, Claudia Regina Gonçalves, Erica Valessa Ramos Gomes, Karoline Rodrigues Campos
EPI_ISL_944117	Platform BIS UZA/UAntwerpen	UAntwerp, Laboratory of Medical Microbiology	Basil Britto Xavier, Jasmine Coppens, Marie Le Mercier, Christine Lammens, Veerle Matheeussen, Herman Goossens
EPI_ISL_944573	National Institute of Infectious Diseases-Prof. Dr. Matei Bals Molecular Diagnostics Laboratory	National Institute of Infectious Diseases-Prof. Dr. Matei Bals Molecular Diagnostics Laboratory	Leontina Banica, Marius Surleac, Corina Casangiu, Petre Milu, Andreea Tudor, Simona Paraschiv, Dan Otelea
EPI_ISL_944746	unknown	Public Health Virology-Forensic and Scientific Services (PHV-FSS)	Son Nguyen et al.
EPI_ISL_944770	National Institute of Infectious Diseases-Prof. Dr. Matei Bals Molecular Diagnostics Laboratory	National Institute of Infectious Diseases-Prof. Dr. Matei Bals Molecular Diagnostics Laboratory	Leontina Banica, Marius Surleac, Corina Casangiu, Petre Milu, Andreea Tudor, Simona Paraschiv, Dan Otelea
EPI_ISL_944799, EPI_ISL_944805, EPI_ISL_944824, EPI_ISL_944863, EPI_ISL_944883, EPI_ISL_944908, EPI_ISL_944914, EPI_ISL_944923, EPI_ISL_944953, EPI_ISL_944976, EPI_ISL_944978, EPI_ISL_945023, EPI_ISL_945032, EPI_ISL_945034			
see above	Lighthouse Lab in Glasgow	Wellcome Sanger Institute for the COVID-19 Genomics UK (COG-UK) Consortium	Harper VanSteenhouse, Yumi Kasai, David Gray, Carol Clugston, Anna Dominiczak and Alex Alderton, Roberto Amato, Sonia Goncalves, Ewan Harrison, David K. Jackson, Ian Johnston, Dominic Kwiatkowski, Cordelia Langford, John Sillitoe on behalf of the Wellcome Sanger Institute COVID-19 Surveillance Team

EPI_ISL_945377, EPI_ISL_945378, EPI_ISL_945380, EPI_ISL_945381, EPI_ISL_945382, EPI_ISL_945383, EPI_ISL_945384, EPI_ISL_945385, EPI_ISL_945388, EPI_ISL_945390, EPI_ISL_945391, EPI_ISL_945392, EPI_ISL_945393, EPI_ISL_945394, EPI_ISL_945395, EPI_ISL_945396, EPI_ISL_945397, EPI_ISL_945398, EPI_ISL_945399, EPI_ISL_945400, EPI_ISL_945401, EPI_ISL_945403, EPI_ISL_945404, EPI_ISL_945405, EPI_ISL_945406, EPI_ISL_945407, EPI_ISL_945410, EPI_ISL_945411, EPI_ISL_945412, EPI_ISL_945413, EPI_ISL_945414, EPI_ISL_945415, EPI_ISL_945416, EPI_ISL_945417, EPI_ISL_945418, EPI_ISL_945421, EPI_ISL_945424, EPI_ISL_945425, EPI_ISL_945426, EPI_ISL_945427, EPI_ISL_945428, EPI_ISL_945429, EPI_ISL_945430, EPI_ISL_945431, EPI_ISL_945432, EPI_ISL_945434, EPI_ISL_945435, EPI_ISL_945436, EPI_ISL_945437, EPI_ISL_945438, EPI_ISL_945439, EPI_ISL_945440, EPI_ISL_945441, EPI_ISL_945442, EPI_ISL_945443, EPI_ISL_945445, EPI_ISL_945447, EPI_ISL_945448, EPI_ISL_945450, EPI_ISL_945451, EPI_ISL_945452, EPI_ISL_945453, EPI_ISL_945454, EPI_ISL_945455, EPI_ISL_945456, EPI_ISL_945457, EPI_ISL_945460, EPI_ISL_945461, EPI_ISL_945462, EPI_ISL_945463, EPI_ISL_945464, EPI_ISL_945465, EPI_ISL_945470, EPI_ISL_945471, EPI_ISL_945472, EPI_ISL_945473, EPI_ISL_945474, EPI_ISL_945476, EPI_ISL_945477, EPI_ISL_945478, EPI_ISL_945479, EPI_ISL_945481, EPI_ISL_945482, EPI_ISL_945483, EPI_ISL_945484, EPI_ISL_945486, EPI_ISL_945487, EPI_ISL_945488, EPI_ISL_945489, EPI_ISL_945491, EPI_ISL_945492, EPI_ISL_945493, EPI_ISL_945496, EPI_ISL_945497, EPI_ISL_945499, EPI_ISL_945500, EPI_ISL_945501, EPI_ISL_945503, EPI_ISL_945504, EPI_ISL_945505, EPI_ISL_945507, EPI_ISL_945509, EPI_ISL_945510, EPI_ISL_945511, EPI_ISL_945512, EPI_ISL_945513, EPI_ISL_945515, EPI_ISL_945516, EPI_ISL_945517, EPI_ISL_945519, EPI_ISL_945520, EPI_ISL_945521, EPI_ISL_945523, EPI_ISL_945524, EPI_ISL_945526, EPI_ISL_945527, EPI_ISL_945528, EPI_ISL_945530, EPI_ISL_945531, EPI_ISL_945532, EPI_ISL_945533, EPI_ISL_945534, EPI_ISL_945535, EPI_ISL_945536, EPI_ISL_945537, EPI_ISL_945540, EPI_ISL_945541, EPI_ISL_945542, EPI_ISL_945544, EPI_ISL_945550, EPI_ISL_945551, EPI_ISL_945552, EPI_ISL_945553, EPI_ISL_945554, EPI_ISL_945556, EPI_ISL_945557, EPI_ISL_945558, EPI_ISL_945559, EPI_ISL_945560, EPI_ISL_945562, EPI_ISL_945564, EPI_ISL_945565, EPI_ISL_945566, EPI_ISL_945567, EPI_ISL_945568, EPI_ISL_945570, EPI_ISL_945571, EPI_ISL_945572, EPI_ISL_945573, EPI_ISL_945574, EPI_ISL_945575, EPI_ISL_945576, EPI_ISL_945577, EPI_ISL_945578, EPI_ISL_945580, EPI_ISL_945581, EPI_ISL_945583, EPI_ISL_945584, EPI_ISL_945585, EPI_ISL_945586, EPI_ISL_945588, EPI_ISL_945590, EPI_ISL_945592, EPI_ISL_945593, EPI_ISL_945594, EPI_ISL_945595, EPI_ISL_945596, EPI_ISL_945598, EPI_ISL_945599, EPI_ISL_945600, EPI_ISL_945601, EPI_ISL_945602, EPI_ISL_945603, EPI_ISL_945604, EPI_ISL_945605, EPI_ISL_945606, EPI_ISL_945607, EPI_ISL_945608, EPI_ISL_945609, EPI_ISL_945610, EPI_ISL_945611, EPI_ISL_945612, EPI_ISL_945613, EPI_ISL_945614, EPI_ISL_945615, EPI_ISL_945616, EPI_ISL_945617, EPI_ISL_945618, EPI_ISL_945620, EPI_ISL_945622, EPI_ISL_945623, EPI_ISL_945624, EPI_ISL_945625, EPI_ISL_945626, EPI_ISL_945628, EPI_ISL_945629, EPI_ISL_945630, EPI_ISL_945631, EPI_ISL_945632, EPI_ISL_945633, EPI_ISL_945634, EPI_ISL_945637, EPI_ISL_945638, EPI_ISL_945639, EPI_ISL_945640, EPI_ISL_945641, EPI_ISL_945642, EPI_ISL_945643, EPI_ISL_945644, EPI_ISL_945645, EPI_ISL_945646, EPI_ISL_945647, EPI_ISL_945649, EPI_ISL_945651, EPI_ISL_945652, EPI_ISL_945653, EPI_ISL_945654, EPI_ISL_945655, EPI_ISL_945657, EPI_ISL_945658, EPI_ISL_945659, EPI_ISL_945660, EPI_ISL_945661, EPI_ISL_945662, EPI_ISL_945663, EPI_ISL_945665, EPI_ISL_945666, EPI_ISL_945667, EPI_ISL_945669, EPI_ISL_945670, EPI_ISL_945671, EPI_ISL_945673, EPI_ISL_945675, EPI_ISL_945676, EPI_ISL_945679, EPI_ISL_945680, EPI_ISL_945683, EPI_ISL_945684, EPI_ISL_945685, EPI_ISL_945686, EPI_ISL_945687, EPI_ISL_945688, EPI_ISL_945689, EPI_ISL_945690, EPI_ISL_945691, EPI_ISL_945693, EPI_ISL_945694, EPI_ISL_945695, EPI_ISL_945699, EPI_ISL_945700, EPI_ISL_945702, EPI_ISL_945703, EPI_ISL_945705, EPI_ISL_945706, EPI_ISL_945707, EPI_ISL_945708, EPI_ISL_945709, EPI_ISL_945710, EPI_ISL_945711, EPI_ISL_945712, EPI_ISL_945713, EPI_ISL_945714			
see above	Lighthouse Lab in Alderley Park	Wellcome Sanger Institute for the COVID-19 Genomics UK (COG-UK) Consortium	Jacquelyn Wynn, Mairead Hyland, The Lighthouse Lab in Alderley Park and Alex Alderton, Roberto Amato, Sonia Goncalves, Ewan Harrison, David K. Jackson, Ian Johnston, Dominic Kwiatkowski, Cordelia Langford, John Sillitoe on behalf of the Wellcome Sanger Institute COVID-19 Surveillance Team
EPI_ISL_945715, EPI_ISL_945717, EPI_ISL_945719, EPI_ISL_945720, EPI_ISL_945721, EPI_ISL_945723, EPI_ISL_945724, EPI_ISL_945727, EPI_ISL_945731, EPI_ISL_945734, EPI_ISL_945739, EPI_ISL_945740, EPI_ISL_945745, EPI_ISL_945746, EPI_ISL_945748, EPI_ISL_945749, EPI_ISL_945750, EPI_ISL_945751, EPI_ISL_945754, EPI_ISL_945756, EPI_ISL_945760			
see above	Lighthouse Lab in Milton Keynes	Wellcome Sanger Institute for the COVID-19 Genomics UK (COG-UK) Consortium	The Lighthouse Lab in Milton Keynes and Alex Alderton, Roberto Amato, Sonia Goncalves, Ewan Harrison, David K. Jackson, Ian Johnston, Dominic Kwiatkowski, Cordelia Langford, John Sillitoe on behalf of the Wellcome Sanger Institute COVID-19 Surveillance Team
EPI_ISL_945761	Lighthouse Lab in Glasgow	Wellcome Sanger Institute for the COVID-19 Genomics UK (COG-UK) Consortium	Harper VanSteenhouse, Yumi Kasai, David Gray, Carol Clugston, Anna Dominiczak and Alex Alderton, Roberto Amato, Sonia Goncalves, Ewan Harrison, David K. Jackson, Ian Johnston, Dominic Kwiatkowski, Cordelia Langford, John Sillitoe on behalf of the Wellcome Sanger Institute COVID-19 Surveillance Team
EPI_ISL_945764, EPI_ISL_945765, EPI_ISL_945766, EPI_ISL_945767, EPI_ISL_945769, EPI_ISL_945770, EPI_ISL_945773, EPI_ISL_945774, EPI_ISL_945775, EPI_ISL_945778, EPI_ISL_945779, EPI_ISL_945780, EPI_ISL_945783, EPI_ISL_945784, EPI_ISL_945785, EPI_ISL_945786, EPI_ISL_945787, EPI_ISL_945789, EPI_ISL_945790, EPI_ISL_945791, EPI_ISL_945793, EPI_ISL_945794, EPI_ISL_945797, EPI_ISL_945798, EPI_ISL_945801, EPI_ISL_945805, EPI_ISL_945810, EPI_ISL_945811, EPI_ISL_945814, EPI_ISL_945819, EPI_ISL_945820, EPI_ISL_945823, EPI_ISL_945825, EPI_ISL_945827, EPI_ISL_945829, EPI_ISL_945830, EPI_ISL_945832, EPI_ISL_945833, EPI_ISL_945834, EPI_ISL_945838, EPI_ISL_945840, EPI_ISL_945842, EPI_ISL_945845, EPI_ISL_945847, EPI_ISL_945852, EPI_ISL_945854, EPI_ISL_945855, EPI_ISL_945856, EPI_ISL_945857			
see above	Lighthouse Lab in Milton Keynes	Wellcome Sanger Institute for the COVID-19 Genomics UK (COG-UK) Consortium	The Lighthouse Lab in Milton Keynes and Alex Alderton, Roberto Amato, Sonia Goncalves, Ewan Harrison, David K. Jackson, Ian Johnston, Dominic Kwiatkowski, Cordelia Langford, John Sillitoe on behalf of the Wellcome Sanger Institute COVID-19 Surveillance Team
EPI_ISL_945858	Lighthouse Lab in Glasgow	Wellcome Sanger Institute for the COVID-19 Genomics UK (COG-UK) Consortium	Harper VanSteenhouse, Yumi Kasai, David Gray, Carol Clugston, Anna Dominiczak and Alex Alderton, Roberto Amato, Sonia Goncalves, Ewan Harrison, David K. Jackson, Ian Johnston, Dominic Kwiatkowski, Cordelia Langford, John Sillitoe on behalf of the Wellcome Sanger Institute COVID-19 Surveillance Team
EPI_ISL_945859, EPI_ISL_945861, EPI_ISL_945864, EPI_ISL_945866	Lighthouse Lab in Milton Keynes	Wellcome Sanger Institute for the COVID-19 Genomics UK (COG-UK) Consortium	The Lighthouse Lab in Milton Keynes and Alex Alderton, Roberto Amato, Sonia Goncalves, Ewan Harrison, David K. Jackson, Ian Johnston, Dominic Kwiatkowski, Cordelia Langford, John Sillitoe on behalf of the Wellcome Sanger Institute COVID-19 Surveillance Team
EPI_ISL_945875	Lighthouse Lab in Glasgow	Wellcome Sanger Institute for the COVID-19 Genomics UK (COG-UK) Consortium	Harper VanSteenhouse, Yumi Kasai, David Gray, Carol Clugston, Anna Dominiczak and Alex Alderton, Roberto Amato, Sonia Goncalves, Ewan Harrison, David K. Jackson, Ian Johnston, Dominic Kwiatkowski, Cordelia Langford, John Sillitoe on behalf of the Wellcome Sanger Institute COVID-19 Surveillance Team
EPI_ISL_945876, EPI_ISL_945880, EPI_ISL_945881, EPI_ISL_945883, EPI_ISL_945886, EPI_ISL_945887, EPI_ISL_945888, EPI_ISL_945890, EPI_ISL_945891, EPI_ISL_945892, EPI_ISL_945893, EPI_ISL_945894, EPI_ISL_945896			
see above	Lighthouse Lab in Milton Keynes	Wellcome Sanger Institute for the COVID-19 Genomics UK (COG-UK) Consortium	The Lighthouse Lab in Milton Keynes and Alex Alderton, Roberto Amato, Sonia Goncalves, Ewan Harrison, David K. Jackson, Ian Johnston, Dominic Kwiatkowski, Cordelia Langford, John Sillitoe on behalf of the Wellcome Sanger Institute COVID-19 Surveillance Team
EPI_ISL_945897	Lighthouse Lab in Glasgow	Wellcome Sanger Institute for the COVID-19 Genomics UK (COG-UK) Consortium	Harper VanSteenhouse, Yumi Kasai, David Gray, Carol Clugston, Anna Dominiczak and Alex Alderton, Roberto Amato, Sonia Goncalves, Ewan Harrison, David K. Jackson, Ian Johnston, Dominic Kwiatkowski, Cordelia Langford, John Sillitoe on behalf of the Wellcome Sanger Institute COVID-19 Surveillance Team
EPI_ISL_945902, EPI_ISL_945903, EPI_ISL_945905, EPI_ISL_945912, EPI_ISL_945913, EPI_ISL_945915, EPI_ISL_945918, EPI_ISL_945919, EPI_ISL_945922	Lighthouse Lab in Milton Keynes	Wellcome Sanger Institute for the COVID-19 Genomics UK (COG-UK) Consortium	The Lighthouse Lab in Milton Keynes and Alex Alderton, Roberto Amato, Sonia Goncalves, Ewan Harrison, David K. Jackson, Ian Johnston, Dominic Kwiatkowski, Cordelia Langford, John Sillitoe on behalf of the Wellcome Sanger Institute COVID-19 Surveillance Team
EPI_ISL_945923	Lighthouse Lab in Glasgow	Wellcome Sanger Institute for the COVID-19 Genomics UK (COG-UK) Consortium	Harper VanSteenhouse, Yumi Kasai, David Gray, Carol Clugston, Anna Dominiczak and Alex Alderton, Roberto Amato, Sonia Goncalves, Ewan Harrison, David K. Jackson, Ian Johnston, Dominic Kwiatkowski, Cordelia Langford, John Sillitoe on behalf of the Wellcome Sanger Institute COVID-19 Surveillance Team
EPI_ISL_945924, EPI_ISL_945925, EPI_ISL_945927, EPI_ISL_945928, EPI_ISL_945929, EPI_ISL_945930, EPI_ISL_945931, EPI_ISL_945932, EPI_ISL_945934, EPI_ISL_945936, EPI_ISL_945937, EPI_ISL_945941, EPI_ISL_945942, EPI_ISL_945943, EPI_ISL_945946, EPI_ISL_945947, EPI_ISL_945948, EPI_ISL_945949, EPI_ISL_945952, EPI_ISL_945953, EPI_ISL_945955, EPI_ISL_945957, EPI_ISL_945959, EPI_ISL_945964, EPI_ISL_945966, EPI_ISL_945968, EPI_ISL_945973, EPI_ISL_945974, EPI_ISL_945975			
see above	Lighthouse Lab in Milton Keynes	Wellcome Sanger Institute for the COVID-19 Genomics UK (COG-UK) Consortium	The Lighthouse Lab in Milton Keynes and Alex Alderton, Roberto Amato, Sonia Goncalves, Ewan Harrison, David K. Jackson, Ian Johnston, Dominic Kwiatkowski, Cordelia Langford, John Sillitoe on behalf of the Wellcome Sanger Institute COVID-19 Surveillance Team
EPI_ISL_945977	Lighthouse Lab in Glasgow	Wellcome Sanger Institute for the COVID-19 Genomics UK (COG-UK) Consortium	Harper VanSteenhouse, Yumi Kasai, David Gray, Carol Clugston, Anna Dominiczak and Alex Alderton, Roberto Amato, Sonia Goncalves, Ewan Harrison, David K. Jackson, Ian Johnston, Dominic Kwiatkowski, Cordelia Langford, John Sillitoe on behalf of the Wellcome Sanger Institute COVID-19 Surveillance Team
EPI_ISL_945978, EPI_ISL_945981, EPI_ISL_945982, EPI_ISL_945985, EPI_ISL_945986, EPI_ISL_945987, EPI_ISL_945989, EPI_ISL_945991, EPI_ISL_945992, EPI_ISL_945995, EPI_ISL_945996, EPI_ISL_946001			
see above	Lighthouse Lab in Milton Keynes	Wellcome Sanger Institute for the COVID-19 Genomics UK (COG-UK) Consortium	The Lighthouse Lab in Milton Keynes and Alex Alderton, Roberto Amato, Sonia Goncalves, Ewan Harrison, David K. Jackson, Ian Johnston, Dominic Kwiatkowski, Cordelia Langford, John Sillitoe on behalf of the Wellcome Sanger Institute COVID-19 Surveillance Team
EPI_ISL_946003	Lighthouse Lab in Glasgow	Wellcome Sanger Institute for the COVID-19 Genomics UK (COG-UK) Consortium	Harper VanSteenhouse, Yumi Kasai, David Gray, Carol Clugston, Anna Dominiczak and Alex Alderton, Roberto Amato, Sonia Goncalves, Ewan Harrison, David K. Jackson, Ian Johnston, Dominic Kwiatkowski, Cordelia Langford, John Sillitoe on behalf of the Wellcome Sanger Institute COVID-19 Surveillance Team
EPI_ISL_946004, EPI_ISL_946012, EPI_ISL_946013, EPI_ISL_946015, EPI_ISL_946016, EPI_ISL_946017, EPI_ISL_946018, EPI_ISL_946019, EPI_ISL_946020, EPI_ISL_946022, EPI_ISL_946024, EPI_ISL_946025, EPI_ISL_946027, EPI_ISL_946029, EPI_ISL_946031			
see above	Lighthouse Lab in Milton Keynes	Wellcome Sanger Institute for the COVID-19 Genomics UK (COG-UK) Consortium	The Lighthouse Lab in Milton Keynes and Alex Alderton, Roberto Amato, Sonia Goncalves, Ewan Harrison, David K. Jackson, Ian Johnston, Dominic Kwiatkowski, Cordelia Langford, John Sillitoe on behalf of the Wellcome Sanger Institute COVID-19 Surveillance Team
EPI_ISL_946032	Lighthouse Lab in Glasgow	Wellcome Sanger Institute for the COVID-19 Genomics UK (COG-UK) Consortium	Harper VanSteenhouse, Yumi Kasai, David Gray, Carol Clugston, Anna Dominiczak and Alex Alderton, Roberto Amato, Sonia Goncalves, Ewan Harrison, David K. Jackson, Ian Johnston, Dominic Kwiatkowski, Cordelia Langford, John Sillitoe on behalf of the Wellcome Sanger Institute COVID-19 Surveillance Team
EPI_ISL_946033, EPI_ISL_946034, EPI_ISL_946036, EPI_ISL_946038, EPI_ISL_946039, EPI_ISL_946040, EPI_ISL_946042, EPI_ISL_946043, EPI_ISL_946044, EPI_ISL_946045, EPI_ISL_946046			
see above	Lighthouse Lab in Milton Keynes	Wellcome Sanger Institute for the COVID-19 Genomics UK (COG-UK) Consortium	The Lighthouse Lab in Milton Keynes and Alex Alderton, Roberto Amato, Sonia Goncalves, Ewan Harrison, David K. Jackson, Ian Johnston, Dominic Kwiatkowski, Cordelia Langford, John Sillitoe on behalf of the Wellcome Sanger Institute COVID-19 Surveillance Team
EPI_ISL_946047	Lighthouse Lab in Glasgow	Wellcome Sanger Institute for the COVID-19 Genomics UK	Harper VanSteenhouse, Yumi Kasai, David Gray, Carol Clugston, Anna Dominiczak and Alex Alderton, Roberto Amato, Sonia Goncalves, Ewan Harrison,

		(COG-UK) Consortium	David K. Jackson, Ian Johnston, Dominic Kwiatkowski, Cordelia Langford, John Sillitoe on behalf of the Wellcome Sanger Institute COVID-19 Surveillance Team
EPI_ISL_946050, EPI_ISL_946051, EPI_ISL_946053, EPI_ISL_946054, EPI_ISL_946056, EPI_ISL_946060, EPI_ISL_946061, EPI_ISL_946063, EPI_ISL_946064, EPI_ISL_946065, EPI_ISL_946074, EPI_ISL_946075, EPI_ISL_946077, EPI_ISL_946078, EPI_ISL_946079, EPI_ISL_946081, EPI_ISL_946082, EPI_ISL_946083, EPI_ISL_946084, EPI_ISL_946085, EPI_ISL_946089, EPI_ISL_946091, EPI_ISL_946092, EPI_ISL_946095, EPI_ISL_946097, EPI_ISL_946098, EPI_ISL_946099, EPI_ISL_946100, EPI_ISL_946101, EPI_ISL_946102, EPI_ISL_946103, EPI_ISL_946104, EPI_ISL_946105, EPI_ISL_946107, EPI_ISL_946108, EPI_ISL_946111, EPI_ISL_946114, EPI_ISL_946116			
see above	Lighthouse Lab in Milton Keynes	Wellcome Sanger Institute for the COVID-19 Genomics UK (COG-UK) Consortium	The Lighthouse Lab in Milton Keynes and Alex Alderton, Roberto Amato, Sonia Goncalves, Ewan Harrison, David K. Jackson, Ian Johnston, Dominic Kwiatkowski, Cordelia Langford, John Sillitoe on behalf of the Wellcome Sanger Institute COVID-19 Surveillance Team
EPI_ISL_946117	Lighthouse Lab in Glasgow	Wellcome Sanger Institute for the COVID-19 Genomics UK (COG-UK) Consortium	Harper VanSteenhouse, Yumi Kasai, David Gray, Carol Clugston, Anna Dominiczak and Alex Alderton, Roberto Amato, Sonia Goncalves, Ewan Harrison, David K. Jackson, Ian Johnston, Dominic Kwiatkowski, Cordelia Langford, John Sillitoe on behalf of the Wellcome Sanger Institute COVID-19 Surveillance Team
EPI_ISL_946118, EPI_ISL_946119, EPI_ISL_946120, EPI_ISL_946121, EPI_ISL_946122, EPI_ISL_946125, EPI_ISL_946128, EPI_ISL_946130, EPI_ISL_946131, EPI_ISL_946132, EPI_ISL_946133, EPI_ISL_946134, EPI_ISL_946136, EPI_ISL_946138, EPI_ISL_946139, EPI_ISL_946140, EPI_ISL_946141, EPI_ISL_946142, EPI_ISL_946143, EPI_ISL_946144, EPI_ISL_946146, EPI_ISL_946148, EPI_ISL_946151, EPI_ISL_946152, EPI_ISL_946154, EPI_ISL_946155, EPI_ISL_946156, EPI_ISL_946158, EPI_ISL_946159, EPI_ISL_946161, EPI_ISL_946163, EPI_ISL_946164, EPI_ISL_946165, EPI_ISL_946166, EPI_ISL_946167, EPI_ISL_946168, EPI_ISL_946170, EPI_ISL_946171, EPI_ISL_946172, EPI_ISL_946175, EPI_ISL_946178, EPI_ISL_946179, EPI_ISL_946183, EPI_ISL_946184, EPI_ISL_946185, EPI_ISL_946186, EPI_ISL_946187, EPI_ISL_946189, EPI_ISL_946191, EPI_ISL_946193, EPI_ISL_946194, EPI_ISL_946195, EPI_ISL_946197, EPI_ISL_946198, EPI_ISL_946200, EPI_ISL_946201, EPI_ISL_946203, EPI_ISL_946204, EPI_ISL_946205, EPI_ISL_946207, EPI_ISL_946209, EPI_ISL_946212, EPI_ISL_946214, EPI_ISL_946216, EPI_ISL_946217			
see above	Lighthouse Lab in Milton Keynes	Wellcome Sanger Institute for the COVID-19 Genomics UK (COG-UK) Consortium	The Lighthouse Lab in Milton Keynes and Alex Alderton, Roberto Amato, Sonia Goncalves, Ewan Harrison, David K. Jackson, Ian Johnston, Dominic Kwiatkowski, Cordelia Langford, John Sillitoe on behalf of the Wellcome Sanger Institute COVID-19 Surveillance Team
EPI_ISL_946219	Lighthouse Lab in Glasgow	Wellcome Sanger Institute for the COVID-19 Genomics UK (COG-UK) Consortium	Harper VanSteenhouse, Yumi Kasai, David Gray, Carol Clugston, Anna Dominiczak and Alex Alderton, Roberto Amato, Sonia Goncalves, Ewan Harrison, David K. Jackson, Ian Johnston, Dominic Kwiatkowski, Cordelia Langford, John Sillitoe on behalf of the Wellcome Sanger Institute COVID-19 Surveillance Team
EPI_ISL_946222, EPI_ISL_946223, EPI_ISL_946226, EPI_ISL_946228, EPI_ISL_946230, EPI_ISL_946235, EPI_ISL_946236, EPI_ISL_946237, EPI_ISL_946238, EPI_ISL_946239, EPI_ISL_946240, EPI_ISL_946241, EPI_ISL_946242, EPI_ISL_946244, EPI_ISL_946245, EPI_ISL_946246, EPI_ISL_946247, EPI_ISL_946248, EPI_ISL_946249, EPI_ISL_946251, EPI_ISL_946252, EPI_ISL_946255, EPI_ISL_946256, EPI_ISL_946257, EPI_ISL_946258			
see above	Lighthouse Lab in Milton Keynes	Wellcome Sanger Institute for the COVID-19 Genomics UK (COG-UK) Consortium	The Lighthouse Lab in Milton Keynes and Alex Alderton, Roberto Amato, Sonia Goncalves, Ewan Harrison, David K. Jackson, Ian Johnston, Dominic Kwiatkowski, Cordelia Langford, John Sillitoe on behalf of the Wellcome Sanger Institute COVID-19 Surveillance Team
EPI_ISL_946259	Lighthouse Lab in Glasgow	Wellcome Sanger Institute for the COVID-19 Genomics UK (COG-UK) Consortium	Harper VanSteenhouse, Yumi Kasai, David Gray, Carol Clugston, Anna Dominiczak and Alex Alderton, Roberto Amato, Sonia Goncalves, Ewan Harrison, David K. Jackson, Ian Johnston, Dominic Kwiatkowski, Cordelia Langford, John Sillitoe on behalf of the Wellcome Sanger Institute COVID-19 Surveillance Team
EPI_ISL_946261, EPI_ISL_946262, EPI_ISL_946263, EPI_ISL_946265, EPI_ISL_946266, EPI_ISL_946267, EPI_ISL_946268, EPI_ISL_946272, EPI_ISL_946273, EPI_ISL_946275, EPI_ISL_946276, EPI_ISL_946277, EPI_ISL_946278, EPI_ISL_946279, EPI_ISL_946280, EPI_ISL_946282, EPI_ISL_946283, EPI_ISL_946287, EPI_ISL_946288			
see above	Lighthouse Lab in Milton Keynes	Wellcome Sanger Institute for the COVID-19 Genomics UK (COG-UK) Consortium	The Lighthouse Lab in Milton Keynes and Alex Alderton, Roberto Amato, Sonia Goncalves, Ewan Harrison, David K. Jackson, Ian Johnston, Dominic Kwiatkowski, Cordelia Langford, John Sillitoe on behalf of the Wellcome Sanger Institute COVID-19 Surveillance Team
EPI_ISL_946289, EPI_ISL_946290	Lighthouse Lab in Glasgow	Wellcome Sanger Institute for the COVID-19 Genomics UK (COG-UK) Consortium	Harper VanSteenhouse, Yumi Kasai, David Gray, Carol Clugston, Anna Dominiczak and Alex Alderton, Roberto Amato, Sonia Goncalves, Ewan Harrison, David K. Jackson, Ian Johnston, Dominic Kwiatkowski, Cordelia Langford, John Sillitoe on behalf of the Wellcome Sanger Institute COVID-19 Surveillance Team
EPI_ISL_946291, EPI_ISL_946293, EPI_ISL_946294, EPI_ISL_946295, EPI_ISL_946296, EPI_ISL_946297, EPI_ISL_946299, EPI_ISL_946300	Lighthouse Lab in Milton Keynes	Wellcome Sanger Institute for the COVID-19 Genomics UK (COG-UK) Consortium	The Lighthouse Lab in Milton Keynes and Alex Alderton, Roberto Amato, Sonia Goncalves, Ewan Harrison, David K. Jackson, Ian Johnston, Dominic Kwiatkowski, Cordelia Langford, John Sillitoe on behalf of the Wellcome Sanger Institute COVID-19 Surveillance Team
EPI_ISL_946303	Lighthouse Lab in Glasgow	Wellcome Sanger Institute for the COVID-19 Genomics UK (COG-UK) Consortium	Harper VanSteenhouse, Yumi Kasai, David Gray, Carol Clugston, Anna Dominiczak and Alex Alderton, Roberto Amato, Sonia Goncalves, Ewan Harrison, David K. Jackson, Ian Johnston, Dominic Kwiatkowski, Cordelia Langford, John Sillitoe on behalf of the Wellcome Sanger Institute COVID-19 Surveillance Team
EPI_ISL_946305, EPI_ISL_946306, EPI_ISL_946307, EPI_ISL_946308, EPI_ISL_946309, EPI_ISL_946310, EPI_ISL_946311, EPI_ISL_946312, EPI_ISL_946313, EPI_ISL_946314, EPI_ISL_946315, EPI_ISL_946316, EPI_ISL_946317, EPI_ISL_946320, EPI_ISL_946321, EPI_ISL_946322, EPI_ISL_946324, EPI_ISL_946325, EPI_ISL_946327, EPI_ISL_946329, EPI_ISL_946330			
see above	Lighthouse Lab in Milton Keynes	Wellcome Sanger Institute for the COVID-19 Genomics UK (COG-UK) Consortium	The Lighthouse Lab in Milton Keynes and Alex Alderton, Roberto Amato, Sonia Goncalves, Ewan Harrison, David K. Jackson, Ian Johnston, Dominic Kwiatkowski, Cordelia Langford, John Sillitoe on behalf of the Wellcome Sanger Institute COVID-19 Surveillance Team
EPI_ISL_946331	Lighthouse Lab in Glasgow	Wellcome Sanger Institute for the COVID-19 Genomics UK (COG-UK) Consortium	Harper VanSteenhouse, Yumi Kasai, David Gray, Carol Clugston, Anna Dominiczak and Alex Alderton, Roberto Amato, Sonia Goncalves, Ewan Harrison, David K. Jackson, Ian Johnston, Dominic Kwiatkowski, Cordelia Langford, John Sillitoe on behalf of the Wellcome Sanger Institute COVID-19 Surveillance Team
EPI_ISL_946332, EPI_ISL_946334, EPI_ISL_946336, EPI_ISL_946337, EPI_ISL_946340, EPI_ISL_946341, EPI_ISL_946342, EPI_ISL_946343, EPI_ISL_946344, EPI_ISL_946345, EPI_ISL_946346, EPI_ISL_946347, EPI_ISL_946348, EPI_ISL_946349, EPI_ISL_946350, EPI_ISL_946352, EPI_ISL_946353, EPI_ISL_946354, EPI_ISL_946355, EPI_ISL_946358, EPI_ISL_946359, EPI_ISL_946362, EPI_ISL_946364, EPI_ISL_946368, EPI_ISL_946369, EPI_ISL_946370, EPI_ISL_946372			
see above	Lighthouse Lab in Milton Keynes	Wellcome Sanger Institute for the COVID-19 Genomics UK (COG-UK) Consortium	The Lighthouse Lab in Milton Keynes and Alex Alderton, Roberto Amato, Sonia Goncalves, Ewan Harrison, David K. Jackson, Ian Johnston, Dominic Kwiatkowski, Cordelia Langford, John Sillitoe on behalf of the Wellcome Sanger Institute COVID-19 Surveillance Team
EPI_ISL_946373	Lighthouse Lab in Glasgow	Wellcome Sanger Institute for the COVID-19 Genomics UK (COG-UK) Consortium	Harper VanSteenhouse, Yumi Kasai, David Gray, Carol Clugston, Anna Dominiczak and Alex Alderton, Roberto Amato, Sonia Goncalves, Ewan Harrison, David K. Jackson, Ian Johnston, Dominic Kwiatkowski, Cordelia Langford, John Sillitoe on behalf of the Wellcome Sanger Institute COVID-19 Surveillance Team
EPI_ISL_946376, EPI_ISL_946377, EPI_ISL_946382, EPI_ISL_946383, EPI_ISL_946384, EPI_ISL_946386	Lighthouse Lab in Milton Keynes	Wellcome Sanger Institute for the COVID-19 Genomics UK (COG-UK) Consortium	The Lighthouse Lab in Milton Keynes and Alex Alderton, Roberto Amato, Sonia Goncalves, Ewan Harrison, David K. Jackson, Ian Johnston, Dominic Kwiatkowski, Cordelia Langford, John Sillitoe on behalf of the Wellcome Sanger Institute COVID-19 Surveillance Team
EPI_ISL_946387	Lighthouse Lab in Glasgow	Wellcome Sanger Institute for the COVID-19 Genomics UK (COG-UK) Consortium	Harper VanSteenhouse, Yumi Kasai, David Gray, Carol Clugston, Anna Dominiczak and Alex Alderton, Roberto Amato, Sonia Goncalves, Ewan Harrison, David K. Jackson, Ian Johnston, Dominic Kwiatkowski, Cordelia Langford, John Sillitoe on behalf of the Wellcome Sanger Institute COVID-19 Surveillance Team
EPI_ISL_946388, EPI_ISL_946389, EPI_ISL_946390, EPI_ISL_946392, EPI_ISL_946393, EPI_ISL_946394	Lighthouse Lab in Milton Keynes	Wellcome Sanger Institute for the COVID-19 Genomics UK (COG-UK) Consortium	The Lighthouse Lab in Milton Keynes and Alex Alderton, Roberto Amato, Sonia Goncalves, Ewan Harrison, David K. Jackson, Ian Johnston, Dominic Kwiatkowski, Cordelia Langford, John Sillitoe on behalf of the Wellcome Sanger Institute COVID-19 Surveillance Team
EPI_ISL_946395	Lighthouse Lab in Glasgow	Wellcome Sanger Institute for the COVID-19 Genomics UK (COG-UK) Consortium	Harper VanSteenhouse, Yumi Kasai, David Gray, Carol Clugston, Anna Dominiczak and Alex Alderton, Roberto Amato, Sonia Goncalves, Ewan Harrison, David K. Jackson, Ian Johnston, Dominic Kwiatkowski, Cordelia Langford, John Sillitoe on behalf of the Wellcome Sanger Institute COVID-19 Surveillance Team
EPI_ISL_946396	Lighthouse Lab in Milton Keynes	Wellcome Sanger Institute for the COVID-19 Genomics UK (COG-UK) Consortium	The Lighthouse Lab in Milton Keynes and Alex Alderton, Roberto Amato, Sonia Goncalves, Ewan Harrison, David K. Jackson, Ian Johnston, Dominic Kwiatkowski, Cordelia Langford, John Sillitoe on behalf of the Wellcome Sanger Institute COVID-19 Surveillance Team
EPI_ISL_946398	Lighthouse Lab in Glasgow	Wellcome Sanger Institute for the COVID-19 Genomics UK (COG-UK) Consortium	Harper VanSteenhouse, Yumi Kasai, David Gray, Carol Clugston, Anna Dominiczak and Alex Alderton, Roberto Amato, Sonia Goncalves, Ewan Harrison, David K. Jackson, Ian Johnston, Dominic Kwiatkowski, Cordelia Langford, John Sillitoe on behalf of the Wellcome Sanger Institute COVID-19 Surveillance Team
EPI_ISL_946399, EPI_ISL_946401, EPI_ISL_946402, EPI_ISL_946404, EPI_ISL_946406	Lighthouse Lab in Milton Keynes	Wellcome Sanger Institute for the COVID-19 Genomics UK (COG-UK) Consortium	The Lighthouse Lab in Milton Keynes and Alex Alderton, Roberto Amato, Sonia Goncalves, Ewan Harrison, David K. Jackson, Ian Johnston, Dominic Kwiatkowski, Cordelia Langford, John Sillitoe on behalf of the Wellcome Sanger Institute COVID-19 Surveillance Team

EPI_ISL_946408	Lighthouse Lab in Glasgow	Wellcome Sanger Institute for the COVID-19 Genomics UK (COG-UK) Consortium	Harper VanSteenhouse, Yumi Kasai, David Gray, Carol Clugston, Anna Dominiczak and Alex Alderton, Roberto Amato, Sonia Goncalves, Ewan Harrison, David K. Jackson, Ian Johnston, Dominic Kwiatkowski, Cordelia Langford, John Sillitoe on behalf of the Wellcome Sanger Institute COVID-19 Surveillance Team
EPI_ISL_946409, EPI_ISL_946412, EPI_ISL_946413, EPI_ISL_946414, EPI_ISL_946415, EPI_ISL_946416, EPI_ISL_946417, EPI_ISL_946418	Lighthouse Lab in Milton Keynes	Wellcome Sanger Institute for the COVID-19 Genomics UK (COG-UK) Consortium	The Lighthouse Lab in Milton Keynes and Alex Alderton, Roberto Amato, Sonia Goncalves, Ewan Harrison, David K. Jackson, Ian Johnston, Dominic Kwiatkowski, Cordelia Langford, John Sillitoe on behalf of the Wellcome Sanger Institute COVID-19 Surveillance Team
EPI_ISL_946419	Lighthouse Lab in Glasgow	Wellcome Sanger Institute for the COVID-19 Genomics UK (COG-UK) Consortium	Harper VanSteenhouse, Yumi Kasai, David Gray, Carol Clugston, Anna Dominiczak and Alex Alderton, Roberto Amato, Sonia Goncalves, Ewan Harrison, David K. Jackson, Ian Johnston, Dominic Kwiatkowski, Cordelia Langford, John Sillitoe on behalf of the Wellcome Sanger Institute COVID-19 Surveillance Team
EPI_ISL_946420	Lighthouse Lab in Milton Keynes	Wellcome Sanger Institute for the COVID-19 Genomics UK (COG-UK) Consortium	The Lighthouse Lab in Milton Keynes and Alex Alderton, Roberto Amato, Sonia Goncalves, Ewan Harrison, David K. Jackson, Ian Johnston, Dominic Kwiatkowski, Cordelia Langford, John Sillitoe on behalf of the Wellcome Sanger Institute COVID-19 Surveillance Team
EPI_ISL_946422, EPI_ISL_946423, EPI_ISL_946424, EPI_ISL_946425, EPI_ISL_946426, EPI_ISL_946428, EPI_ISL_946429, EPI_ISL_946430, EPI_ISL_946431, EPI_ISL_946432, EPI_ISL_946433, EPI_ISL_946434, EPI_ISL_946435, EPI_ISL_946436, EPI_ISL_946437, EPI_ISL_946438, EPI_ISL_946439, EPI_ISL_946440, EPI_ISL_946441, EPI_ISL_946442, EPI_ISL_946443, EPI_ISL_946444, EPI_ISL_946446, EPI_ISL_946447, EPI_ISL_946448, EPI_ISL_946449, EPI_ISL_946450, EPI_ISL_946451, EPI_ISL_946452, EPI_ISL_946453, EPI_ISL_946455, EPI_ISL_946456, EPI_ISL_946457, EPI_ISL_946458, EPI_ISL_946459, EPI_ISL_946460, EPI_ISL_946461, EPI_ISL_946462, EPI_ISL_946463, EPI_ISL_946464, EPI_ISL_946465, EPI_ISL_946466, EPI_ISL_946467, EPI_ISL_946468, EPI_ISL_946469, EPI_ISL_946470, EPI_ISL_946472, EPI_ISL_946473, EPI_ISL_946474, EPI_ISL_946475, EPI_ISL_946476, EPI_ISL_946477, EPI_ISL_946478, EPI_ISL_946479, EPI_ISL_946480, EPI_ISL_946481, EPI_ISL_946482, EPI_ISL_946483, EPI_ISL_946484, EPI_ISL_946485, EPI_ISL_946486, EPI_ISL_946487, EPI_ISL_946488, EPI_ISL_946489, EPI_ISL_946490, EPI_ISL_946491, EPI_ISL_946492, EPI_ISL_946494, EPI_ISL_946495, EPI_ISL_946496, EPI_ISL_946497, EPI_ISL_946498, EPI_ISL_946499, EPI_ISL_946500, EPI_ISL_946501, EPI_ISL_946502, EPI_ISL_946503, EPI_ISL_946504, EPI_ISL_946505, EPI_ISL_946506, EPI_ISL_946507, EPI_ISL_946508, EPI_ISL_946509, EPI_ISL_946510, EPI_ISL_946511, EPI_ISL_946512, EPI_ISL_946513, EPI_ISL_946514, EPI_ISL_946515, EPI_ISL_946516, EPI_ISL_946517, EPI_ISL_946518, EPI_ISL_946519, EPI_ISL_946520, EPI_ISL_946521, EPI_ISL_946522, EPI_ISL_946523, EPI_ISL_946524, EPI_ISL_946525, EPI_ISL_946526, EPI_ISL_946527, EPI_ISL_946528, EPI_ISL_946529, EPI_ISL_946530, EPI_ISL_946531, EPI_ISL_946532, EPI_ISL_946533, EPI_ISL_946534, EPI_ISL_946535, EPI_ISL_946536, EPI_ISL_946537, EPI_ISL_946538, EPI_ISL_946539, EPI_ISL_946540, EPI_ISL_946541, EPI_ISL_946542, EPI_ISL_946543, EPI_ISL_946544, EPI_ISL_946545, EPI_ISL_946546, EPI_ISL_946548, EPI_ISL_946549, EPI_ISL_946550, EPI_ISL_946551, EPI_ISL_946552, EPI_ISL_946553, EPI_ISL_946554, EPI_ISL_946555, EPI_ISL_946556, EPI_ISL_946557, EPI_ISL_946558, EPI_ISL_946559, EPI_ISL_946560, EPI_ISL_946561, EPI_ISL_946562, EPI_ISL_946563, EPI_ISL_946564, EPI_ISL_946565, EPI_ISL_946566, EPI_ISL_946567, EPI_ISL_946568, EPI_ISL_946569, EPI_ISL_946570, EPI_ISL_946571, EPI_ISL_946572, EPI_ISL_946573, EPI_ISL_946574, EPI_ISL_946575, EPI_ISL_946576, EPI_ISL_946577, EPI_ISL_946578, EPI_ISL_946579, EPI_ISL_946581, EPI_ISL_946582, EPI_ISL_946583, EPI_ISL_946584, EPI_ISL_946586, EPI_ISL_946587, EPI_ISL_946588, EPI_ISL_946589, EPI_ISL_946590, EPI_ISL_946591, EPI_ISL_946592, EPI_ISL_946593, EPI_ISL_946594, EPI_ISL_946595, EPI_ISL_946596, EPI_ISL_946597, EPI_ISL_946598, EPI_ISL_946599, EPI_ISL_946600, EPI_ISL_946601, EPI_ISL_946602, EPI_ISL_946603, EPI_ISL_946604, EPI_ISL_946605, EPI_ISL_946606, EPI_ISL_946607, EPI_ISL_946608, EPI_ISL_946609, EPI_ISL_946610, EPI_ISL_946611, EPI_ISL_946612, EPI_ISL_946613, EPI_ISL_946614, EPI_ISL_946615, EPI_ISL_946616, EPI_ISL_946617, EPI_ISL_946618, EPI_ISL_946619, EPI_ISL_946620, EPI_ISL_946621, EPI_ISL_946622, EPI_ISL_946623, EPI_ISL_946624, EPI_ISL_946625, EPI_ISL_946626, EPI_ISL_946627, EPI_ISL_946628, EPI_ISL_946629, EPI_ISL_946630, EPI_ISL_946631, EPI_ISL_946632, EPI_ISL_946633, EPI_ISL_946634, EPI_ISL_946635, EPI_ISL_946636, EPI_ISL_946637, EPI_ISL_946638, EPI_ISL_946639, EPI_ISL_946640, EPI_ISL_946641, EPI_ISL_946642, EPI_ISL_946643, EPI_ISL_946644, EPI_ISL_946645, EPI_ISL_946647, EPI_ISL_946648, EPI_ISL_946649, EPI_ISL_946650, EPI_ISL_946651, EPI_ISL_946652, EPI_ISL_946653, EPI_ISL_946654, EPI_ISL_946655, EPI_ISL_946656, EPI_ISL_946657, EPI_ISL_946658, EPI_ISL_946659, EPI_ISL_946660, EPI_ISL_946661, EPI_ISL_946662, EPI_ISL_946663, EPI_ISL_946664, EPI_ISL_946665, EPI_ISL_946666, EPI_ISL_946667, EPI_ISL_946668, EPI_ISL_946669, EPI_ISL_946670, EPI_ISL_946671, EPI_ISL_946673, EPI_ISL_946674, EPI_ISL_946675, EPI_ISL_946676, EPI_ISL_946677, EPI_ISL_946678, EPI_ISL_946679, EPI_ISL_946680, EPI_ISL_946681, EPI_ISL_946682, EPI_ISL_946683, EPI_ISL_946684, EPI_ISL_946685, EPI_ISL_946686, EPI_ISL_946687, EPI_ISL_946688, EPI_ISL_946689, EPI_ISL_946690, EPI_ISL_946691, EPI_ISL_946692, EPI_ISL_946693, EPI_ISL_946694, EPI_ISL_946695, EPI_ISL_946696, EPI_ISL_946697, EPI_ISL_946698, EPI_ISL_946699, EPI_ISL_946700, EPI_ISL_946702, EPI_ISL_946703, EPI_ISL_946704, EPI_ISL_946705, EPI_ISL_946706, EPI_ISL_946707, EPI_ISL_946708, EPI_ISL_946709, EPI_ISL_946710, EPI_ISL_946711, EPI_ISL_946712, EPI_ISL_946713, EPI_ISL_946714, EPI_ISL_946715, EPI_ISL_946716, EPI_ISL_946717, EPI_ISL_946718, EPI_ISL_946719, EPI_ISL_946720, EPI_ISL_946721, EPI_ISL_946722, EPI_ISL_946723, EPI_ISL_946724, EPI_ISL_946725, EPI_ISL_946726, EPI_ISL_946727, EPI_ISL_946728, EPI_ISL_946729, EPI_ISL_946730, EPI_ISL_946731, EPI_ISL_946732, EPI_ISL_946733, EPI_ISL_946734, EPI_ISL_946735, EPI_ISL_946736, EPI_ISL_946737, EPI_ISL_946738, EPI_ISL_946739, EPI_ISL_946740, EPI_ISL_946741, EPI_ISL_946742, EPI_ISL_946743, EPI_ISL_946744, EPI_ISL_946745, EPI_ISL_946747, EPI_ISL_946748, EPI_ISL_946749, EPI_ISL_946750, EPI_ISL_946751, EPI_ISL_946752, EPI_ISL_946753, EPI_ISL_946754, EPI_ISL_946755, EPI_ISL_946756, EPI_ISL_946757, EPI_ISL_946758, EPI_ISL_946759, EPI_ISL_946760, EPI_ISL_946761, EPI_ISL_946762, EPI_ISL_946763, EPI_ISL_946764, EPI_ISL_946765, EPI_ISL_946766, EPI_ISL_946767, EPI_ISL_946768, EPI_ISL_946769, EPI_ISL_946770, EPI_ISL_946771, EPI_ISL_946772, EPI_ISL_946773, EPI_ISL_946774, EPI_ISL_946775, EPI_ISL_946776, EPI_ISL_946777, EPI_ISL_946778, EPI_ISL_946779, EPI_ISL_946781, EPI_ISL_946782, EPI_ISL_946783, EPI_ISL_946784, EPI_ISL_946785, EPI_ISL_946786, EPI_ISL_946787, EPI_ISL_946788, EPI_ISL_946789, EPI_ISL_946790, EPI_ISL_946791, EPI_ISL_946792, EPI_ISL_946793, EPI_ISL_946794, EPI_ISL_946795, EPI_ISL_946796, EPI_ISL_946797, EPI_ISL_946798, EPI_ISL_946799, EPI_ISL_946800, EPI_ISL_946801, EPI_ISL_946802, EPI_ISL_946803, EPI_ISL_946804, EPI_ISL_946805, EPI_ISL_946806, EPI_ISL_946807, EPI_ISL_946808, EPI_ISL_946809, EPI_ISL_946810, EPI_ISL_946811, EPI_ISL_946812, EPI_ISL_946813, EPI_ISL_946814, EPI_ISL_946815, EPI_ISL_946816, EPI_ISL_946817, EPI_ISL_946818, EPI_ISL_946819, EPI_ISL_946820, EPI_ISL_946821, EPI_ISL_946822, EPI_ISL_946823, EPI_ISL_946824, EPI_ISL_946825, EPI_ISL_946826, EPI_ISL_946827, EPI_ISL_946828, EPI_ISL_946829, EPI_ISL_946830, EPI_ISL_946831, EPI_ISL_946832, EPI_ISL_946833, EPI_ISL_946834, EPI_ISL_946835, EPI_ISL_946836, EPI_ISL_946837, EPI_ISL_946838, EPI_ISL_946839, EPI_ISL_946840, EPI_ISL_946841, EPI_ISL_946842, EPI_ISL_946843, EPI_ISL_946844, EPI_ISL_946845, EPI_ISL_946846, EPI_ISL_946847, EPI_ISL_946848, EPI_ISL_946849, EPI_ISL_946850, EPI_ISL_946851, EPI_ISL_946852, EPI_ISL_946853, EPI_ISL_946854, EPI_ISL_946855, EPI_ISL_946856, EPI_ISL_946857, EPI_ISL_946858, EPI_ISL_946859, EPI_ISL_946860, EPI_ISL_946861, EPI_ISL_946862, EPI_ISL_946863, EPI_ISL_946864, EPI_ISL_946865, EPI_ISL_946866, EPI_ISL_946867, EPI_ISL_946868, EPI_ISL_946869, EPI_ISL_946870, EPI_ISL_946871, EPI_ISL_946872, EPI_ISL_946873, EPI_ISL_946874, EPI_ISL_946875, EPI_ISL_946876, EPI_ISL_946877, EPI_ISL_946878, EPI_ISL_946879, EPI_ISL_946880, EPI_ISL_946881, EPI_ISL_946882, EPI_ISL_946883, EPI_ISL_946884, EPI_ISL_946885, EPI_ISL_946886, EPI_ISL_946887, EPI_ISL_946888, EPI_ISL_946889, EPI_ISL_946890, EPI_ISL_946891, EPI_ISL_946892, EPI_ISL_946893, EPI_ISL_946894, EPI_ISL_946895, EPI_ISL_946896, EPI_ISL_946897, EPI_ISL_946898, EPI_ISL_946899, EPI_ISL_946900, EPI_ISL_946901, EPI_ISL_946902, EPI_ISL_946903, EPI_ISL_946904, EPI_ISL_946905, EPI_ISL_946906, EPI_ISL_946907, EPI_ISL_946908, EPI_ISL_946909, EPI_ISL_946910, EPI_ISL_946911, EPI_ISL_946912, EPI_ISL_946914, EPI_ISL_946915, EPI_ISL_946916, EPI_ISL_946917, EPI_ISL_946918, EPI_ISL_946919, EPI_ISL_946920, EPI_ISL_946921, EPI_ISL_946922, EPI_ISL_946923, EPI_ISL_946924, EPI_ISL_946925, EPI_ISL_946926, EPI_ISL_946927, EPI_ISL_946928, EPI_ISL_946929, EPI_ISL_946930, EPI_ISL_946931, EPI_ISL_946932, EPI_ISL_946933, EPI_ISL_946934, EPI_ISL_946935, EPI_ISL_946936, EPI_ISL_946937, EPI_ISL_946938, EPI_ISL_946939, EPI_ISL_946940, EPI_ISL_946941, EPI_ISL_946942, EPI_ISL_946943, EPI_ISL_946944, EPI_ISL_946945, EPI_ISL_946946, EPI_ISL_946947, EPI_ISL_946948, EPI_ISL_946949, EPI_ISL_946950, EPI_ISL_946951, EPI_ISL_946952, EPI_ISL_946953, EPI_ISL_946954, EPI_ISL_946955, EPI_ISL_946957, EPI_ISL_946958, EPI_ISL_946959, EPI_ISL_946960, EPI_ISL_946961, EPI_ISL_946962, EPI_ISL_946964, EPI_ISL_946965, EPI_ISL_946966, EPI_ISL_946967, EPI_ISL_946968, EPI_ISL_946969, EPI_ISL_946970, EPI_ISL_946971, EPI_ISL_946972, EPI_ISL_946973, EPI_ISL_946974, EPI_ISL_946975, EPI_ISL_946976, EPI_ISL_946977, EPI_ISL_946978, EPI_ISL_946979, EPI_ISL_946980, EPI_ISL_946981, EPI_ISL_946982, EPI_ISL_946983, EPI_ISL_946984, EPI_ISL_946985, EPI_ISL_946986, EPI_ISL_946987, EPI_ISL_946988, EPI_ISL_946989, EPI_ISL_946990, EPI_ISL_946991, EPI_ISL_946993, EPI_ISL_946994, EPI_ISL_946995, EPI_ISL_946996, EPI_ISL_946997, EPI_ISL_946998, EPI_ISL_946999, EPI_ISL_947000, EPI_ISL_947001, EPI_ISL_947002, EPI_ISL_947003, EPI_ISL_947004, EPI_ISL_947005, EPI_ISL_947006, EPI_ISL_947007, EPI_ISL_947008, EPI_ISL_947009, EPI_ISL_947010, EPI_ISL_947011, EPI_ISL_947012, EPI_ISL_947013, EPI_ISL_947014, EPI_ISL_947015, EPI_ISL_947016, EPI_ISL_947017, EPI_ISL_947018, EPI_ISL_947019, EPI_ISL_947020, EPI_ISL_947021, EPI_ISL_947022, EPI_ISL_947023, EPI_ISL_947024, EPI_ISL_947025, EPI_ISL_947026, EPI_ISL_947027, EPI_ISL_947028, EPI_ISL_947029, EPI_ISL_947030, EPI_ISL_947031, EPI_ISL_947032, EPI_ISL_947033, EPI_ISL_947034, EPI_ISL_947035, EPI_ISL_947036, EPI_ISL_947037, EPI_ISL_947038, EPI_ISL_947039, EPI_ISL_947040, EPI_ISL_947041, EPI_ISL_947042, EPI_ISL_947043, EPI_ISL_947044, EPI_ISL_947045, EPI_ISL_947046, EPI_ISL_947047, EPI_ISL_947048, EPI_ISL_947049, EPI_ISL_947050, EPI_ISL_947051, EPI_ISL_947052, EPI_ISL_947053, EPI_ISL_947054, EPI_ISL_947055, EPI_ISL_947056, EPI_ISL_947057, EPI_ISL_947058, EPI_ISL_947059, EPI_ISL_947060, EPI_ISL_947061, EPI_ISL_947062, EPI_ISL_947063, EPI_ISL_947064, EPI_ISL_947065, EPI_ISL_947066, EPI_ISL_947067, EPI_ISL_947068, EPI_ISL_947069, EPI_ISL_947070, EPI_ISL_947071, EPI_ISL_947072, EPI_ISL_947073, EPI_ISL_947074, EPI_ISL_947075, EPI_ISL_947076, EPI_ISL_947077, EPI_ISL_947078, EPI_ISL_947079, EPI_ISL_947080, EPI_ISL_947081, EPI_ISL_947082, EPI_ISL_947083, EPI_ISL_947084, EPI_ISL_947085, EPI_ISL_947086, EPI_ISL_947087, EPI_ISL_947088, EPI_ISL_947089, EPI_ISL_947090, EPI_ISL_947091, EPI_ISL_947092, EPI_ISL_947093, EPI_ISL_947094, EPI_ISL_947095, EPI_ISL_947096, EPI_ISL_947097, EPI_ISL_947098, EPI_ISL_947099, EPI_ISL_947100, EPI_ISL_947101, EPI_ISL_947102, EPI_ISL_947103, EPI_ISL_947104, EPI_ISL_947105, EPI_ISL_947106			
see above	Lighthouse Lab in Alderley Park	Wellcome Sanger Institute for the COVID-19 Genomics UK (COG-UK) Consortium	Jacquelyn Wynn, Mairead Hyland, The Lighthouse Lab in Alderley Park and Alex Alderton, Roberto Amato, Sonia Goncalves, Ewan Harrison, David K. Jackson, Ian Johnston, Dominic Kwiatkowski, Cordelia Langford, John Sillitoe on behalf of the Wellcome Sanger Institute COVID-19 Surveillance Team
EPI_ISL_947107, EPI_ISL_947108	Lighthouse Lab in Glasgow	Wellcome Sanger Institute for the COVID-19 Genomics UK (COG-UK) Consortium	Harper VanSteenhouse, Yumi Kasai, David Gray, Carol Clugston, Anna Dominiczak and Alex Alderton, Roberto Amato, Sonia Goncalves, Ewan Harrison, David K. Jackson, Ian Johnston, Dominic Kwiatkowski, Cordelia Langford, John Sillitoe on behalf of the Wellcome Sanger Institute COVID-19 Surveillance Team
EPI_ISL_947109, EPI_ISL_947111, EPI_ISL_947112, EPI_ISL_947114, EPI_ISL_947115, EPI_ISL_947116, EPI_ISL_947117	Lighthouse Lab in Milton Keynes	Wellcome Sanger Institute for the COVID-19 Genomics UK (COG-UK) Consortium	The Lighthouse Lab in Milton Keynes and Alex Alderton, Roberto Amato, Sonia Goncalves, Ewan Harrison, David K. Jackson, Ian Johnston, Dominic Kwiatkowski, Cordelia Langford, John Sillitoe on behalf of the Wellcome Sanger Institute COVID-19 Surveillance Team
EPI_ISL_947118, EPI_ISL_947119, EPI_ISL_947121	Lighthouse Lab in Glasgow	Wellcome Sanger Institute for the COVID-19 Genomics UK (COG-UK) Consortium	Harper VanSteenhouse, Yumi Kasai, David Gray, Carol Clugston, Anna Dominiczak and Alex Alderton, Roberto Amato, Sonia Goncalves, Ewan Harrison, David K. Jackson, Ian Johnston, Dominic Kwiatkowski, Cordelia Langford, John Sillitoe on behalf of the Wellcome Sanger Institute COVID-19 Surveillance Team
EPI_ISL_947122, EPI_ISL_947123, EPI_ISL_947124, EPI_ISL_947125	Lighthouse Lab in Milton Keynes	Wellcome Sanger Institute for the COVID-19 Genomics UK (COG-UK) Consortium	The Lighthouse Lab in Milton Keynes and Alex Alderton, Roberto Amato, Sonia Goncalves, Ewan Harrison, David K. Jackson, Ian Johnston, Dominic Kwiatkowski, Cordelia Langford, John Sillitoe on behalf of the Wellcome Sanger Institute COVID-19 Surveillance Team
EPI_ISL_947126, EPI_ISL_947127	Lighthouse Lab in Glasgow	Wellcome Sanger Institute for the COVID-19 Genomics UK (COG-UK) Consortium	Harper VanSteenhouse, Yumi Kasai, David Gray, Carol Clugston, Anna Dominiczak and Alex Alderton, Roberto Amato, Sonia Goncalves, Ewan Harrison, David K. Jackson, Ian Johnston, Dominic Kwiatkowski, Cordelia Langford, John Sillitoe on behalf of the Wellcome Sanger Institute COVID-19 Surveillance Team
EPI_ISL_947128, EPI_ISL_947129, EPI_ISL_947130	Lighthouse Lab in Milton Keynes	Wellcome Sanger Institute for the COVID-19 Genomics UK (COG-UK) Consortium	The Lighthouse Lab in Milton Keynes and Alex Alderton, Roberto Amato, Sonia Goncalves, Ewan Harrison, David K. Jackson, Ian Johnston, Dominic Kwiatkowski, Cordelia Langford, John Sillitoe on behalf of the Wellcome Sanger Institute COVID-19 Surveillance Team
EPI_ISL_947131	Lighthouse Lab in Glasgow	Wellcome Sanger Institute for the COVID-19 Genomics UK (COG-UK) Consortium	Harper VanSteenhouse, Yumi Kasai, David Gray, Carol Clugston, Anna Dominiczak and Alex Alderton, Roberto Amato, Sonia Goncalves, Ewan Harrison, David K. Jackson, Ian Johnston, Dominic Kwiatkowski, Cordelia Langford, John Sillitoe on behalf of the Wellcome Sanger Institute COVID-19 Surveillance Team
EPI_ISL_947132, EPI_ISL_947133	Lighthouse Lab in Milton Keynes	Wellcome Sanger Institute for the COVID-19 Genomics UK (COG-UK) Consortium	The Lighthouse Lab in Milton Keynes and Alex Alderton, Roberto Amato, Sonia Goncalves, Ewan Harrison, David K. Jackson, Ian Johnston, Dominic Kwiatkowski, Cordelia Langford, John Sillitoe on behalf of the Wellcome Sanger Institute COVID-19 Surveillance Team
EPI_ISL_947134	Lighthouse Lab in Glasgow	Wellcome Sanger Institute for the COVID-19 Genomics UK (COG-UK) Consortium	Harper VanSteenhouse, Yumi Kasai, David Gray, Carol Clugston, Anna Dominiczak and Alex Alderton, Roberto Amato, Sonia Goncalves, Ewan Harrison, David K. Jackson, Ian Johnston, Dominic Kwiatkowski, Cordelia Langford, John Sillitoe on behalf of the Wellcome Sanger Institute COVID-19 Surveillance Team
EPI_ISL_947135, EPI_ISL_947136	Lighthouse Lab in Milton Keynes	Wellcome Sanger Institute for the COVID-19 Genomics UK (COG-UK) Consortium	The Lighthouse Lab in Milton Keynes and Alex Alderton, Roberto Amato, Sonia Goncalves, Ewan Harrison, David K. Jackson, Ian Johnston, Dominic Kwiatkowski, Cordelia Langford, John Sillitoe on behalf of the Wellcome Sanger Institute COVID-19 Surveillance Team

EPI_ISL_947137	Lighthouse Lab in Glasgow	Wellcome Sanger Institute for the COVID-19 Genomics UK (COG-UK) Consortium	Harper VanSteenhouse, Yumi Kasai, David Gray, Carol Clugston, Anna Dominiczak and Alex Alderton, Roberto Amato, Sonia Goncalves, Ewan Harrison, David K. Jackson, Ian Johnston, Dominic Kwiatkowski, Cordelia Langford, John Sillitoe on behalf of the Wellcome Sanger Institute COVID-19 Surveillance Team
EPI_ISL_947138	Lighthouse Lab in Milton Keynes	Wellcome Sanger Institute for the COVID-19 Genomics UK (COG-UK) Consortium	The Lighthouse Lab in Milton Keynes and Alex Alderton, Roberto Amato, Sonia Goncalves, Ewan Harrison, David K. Jackson, Ian Johnston, Dominic Kwiatkowski, Cordelia Langford, John Sillitoe on behalf of the Wellcome Sanger Institute COVID-19 Surveillance Team
EPI_ISL_947139, EPI_ISL_947141	Lighthouse Lab in Glasgow	Wellcome Sanger Institute for the COVID-19 Genomics UK (COG-UK) Consortium	Harper VanSteenhouse, Yumi Kasai, David Gray, Carol Clugston, Anna Dominiczak and Alex Alderton, Roberto Amato, Sonia Goncalves, Ewan Harrison, David K. Jackson, Ian Johnston, Dominic Kwiatkowski, Cordelia Langford, John Sillitoe on behalf of the Wellcome Sanger Institute COVID-19 Surveillance Team
EPI_ISL_947142, EPI_ISL_947143, EPI_ISL_947144, EPI_ISL_947145, EPI_ISL_947146	Lighthouse Lab in Milton Keynes	Wellcome Sanger Institute for the COVID-19 Genomics UK (COG-UK) Consortium	The Lighthouse Lab in Milton Keynes and Alex Alderton, Roberto Amato, Sonia Goncalves, Ewan Harrison, David K. Jackson, Ian Johnston, Dominic Kwiatkowski, Cordelia Langford, John Sillitoe on behalf of the Wellcome Sanger Institute COVID-19 Surveillance Team
EPI_ISL_947147	Lighthouse Lab in Glasgow	Wellcome Sanger Institute for the COVID-19 Genomics UK (COG-UK) Consortium	Harper VanSteenhouse, Yumi Kasai, David Gray, Carol Clugston, Anna Dominiczak and Alex Alderton, Roberto Amato, Sonia Goncalves, Ewan Harrison, David K. Jackson, Ian Johnston, Dominic Kwiatkowski, Cordelia Langford, John Sillitoe on behalf of the Wellcome Sanger Institute COVID-19 Surveillance Team
EPI_ISL_947148	Lighthouse Lab in Milton Keynes	Wellcome Sanger Institute for the COVID-19 Genomics UK (COG-UK) Consortium	The Lighthouse Lab in Milton Keynes and Alex Alderton, Roberto Amato, Sonia Goncalves, Ewan Harrison, David K. Jackson, Ian Johnston, Dominic Kwiatkowski, Cordelia Langford, John Sillitoe on behalf of the Wellcome Sanger Institute COVID-19 Surveillance Team
EPI_ISL_947150	Lighthouse Lab in Glasgow	Wellcome Sanger Institute for the COVID-19 Genomics UK (COG-UK) Consortium	Harper VanSteenhouse, Yumi Kasai, David Gray, Carol Clugston, Anna Dominiczak and Alex Alderton, Roberto Amato, Sonia Goncalves, Ewan Harrison, David K. Jackson, Ian Johnston, Dominic Kwiatkowski, Cordelia Langford, John Sillitoe on behalf of the Wellcome Sanger Institute COVID-19 Surveillance Team
EPI_ISL_947151, EPI_ISL_947152	Lighthouse Lab in Milton Keynes	Wellcome Sanger Institute for the COVID-19 Genomics UK (COG-UK) Consortium	The Lighthouse Lab in Milton Keynes and Alex Alderton, Roberto Amato, Sonia Goncalves, Ewan Harrison, David K. Jackson, Ian Johnston, Dominic Kwiatkowski, Cordelia Langford, John Sillitoe on behalf of the Wellcome Sanger Institute COVID-19 Surveillance Team
EPI_ISL_947153	Lighthouse Lab in Glasgow	Wellcome Sanger Institute for the COVID-19 Genomics UK (COG-UK) Consortium	Harper VanSteenhouse, Yumi Kasai, David Gray, Carol Clugston, Anna Dominiczak and Alex Alderton, Roberto Amato, Sonia Goncalves, Ewan Harrison, David K. Jackson, Ian Johnston, Dominic Kwiatkowski, Cordelia Langford, John Sillitoe on behalf of the Wellcome Sanger Institute COVID-19 Surveillance Team
EPI_ISL_947154, EPI_ISL_947155, EPI_ISL_947156, EPI_ISL_947157, EPI_ISL_947158, EPI_ISL_947159, EPI_ISL_947160, EPI_ISL_947161, EPI_ISL_947162	Lighthouse Lab in Milton Keynes	Wellcome Sanger Institute for the COVID-19 Genomics UK (COG-UK) Consortium	The Lighthouse Lab in Milton Keynes and Alex Alderton, Roberto Amato, Sonia Goncalves, Ewan Harrison, David K. Jackson, Ian Johnston, Dominic Kwiatkowski, Cordelia Langford, John Sillitoe on behalf of the Wellcome Sanger Institute COVID-19 Surveillance Team
EPI_ISL_947163	Lighthouse Lab in Glasgow	Wellcome Sanger Institute for the COVID-19 Genomics UK (COG-UK) Consortium	Harper VanSteenhouse, Yumi Kasai, David Gray, Carol Clugston, Anna Dominiczak and Alex Alderton, Roberto Amato, Sonia Goncalves, Ewan Harrison, David K. Jackson, Ian Johnston, Dominic Kwiatkowski, Cordelia Langford, John Sillitoe on behalf of the Wellcome Sanger Institute COVID-19 Surveillance Team
EPI_ISL_947164	Lighthouse Lab in Milton Keynes	Wellcome Sanger Institute for the COVID-19 Genomics UK (COG-UK) Consortium	The Lighthouse Lab in Milton Keynes and Alex Alderton, Roberto Amato, Sonia Goncalves, Ewan Harrison, David K. Jackson, Ian Johnston, Dominic Kwiatkowski, Cordelia Langford, John Sillitoe on behalf of the Wellcome Sanger Institute COVID-19 Surveillance Team
EPI_ISL_947165	Lighthouse Lab in Glasgow	Wellcome Sanger Institute for the COVID-19 Genomics UK (COG-UK) Consortium	Harper VanSteenhouse, Yumi Kasai, David Gray, Carol Clugston, Anna Dominiczak and Alex Alderton, Roberto Amato, Sonia Goncalves, Ewan Harrison, David K. Jackson, Ian Johnston, Dominic Kwiatkowski, Cordelia Langford, John Sillitoe on behalf of the Wellcome Sanger Institute COVID-19 Surveillance Team
EPI_ISL_947166, EPI_ISL_947167, EPI_ISL_947168	Lighthouse Lab in Milton Keynes	Wellcome Sanger Institute for the COVID-19 Genomics UK (COG-UK) Consortium	The Lighthouse Lab in Milton Keynes and Alex Alderton, Roberto Amato, Sonia Goncalves, Ewan Harrison, David K. Jackson, Ian Johnston, Dominic Kwiatkowski, Cordelia Langford, John Sillitoe on behalf of the Wellcome Sanger Institute COVID-19 Surveillance Team
EPI_ISL_947169	Lighthouse Lab in Glasgow	Wellcome Sanger Institute for the COVID-19 Genomics UK (COG-UK) Consortium	Harper VanSteenhouse, Yumi Kasai, David Gray, Carol Clugston, Anna Dominiczak and Alex Alderton, Roberto Amato, Sonia Goncalves, Ewan Harrison, David K. Jackson, Ian Johnston, Dominic Kwiatkowski, Cordelia Langford, John Sillitoe on behalf of the Wellcome Sanger Institute COVID-19 Surveillance Team
EPI_ISL_947170, EPI_ISL_947171, EPI_ISL_947173, EPI_ISL_947174, EPI_ISL_947175, EPI_ISL_947176, EPI_ISL_947177, EPI_ISL_947178	Lighthouse Lab in Milton Keynes	Wellcome Sanger Institute for the COVID-19 Genomics UK (COG-UK) Consortium	The Lighthouse Lab in Milton Keynes and Alex Alderton, Roberto Amato, Sonia Goncalves, Ewan Harrison, David K. Jackson, Ian Johnston, Dominic Kwiatkowski, Cordelia Langford, John Sillitoe on behalf of the Wellcome Sanger Institute COVID-19 Surveillance Team
EPI_ISL_947179	Lighthouse Lab in Glasgow	Wellcome Sanger Institute for the COVID-19 Genomics UK (COG-UK) Consortium	Harper VanSteenhouse, Yumi Kasai, David Gray, Carol Clugston, Anna Dominiczak and Alex Alderton, Roberto Amato, Sonia Goncalves, Ewan Harrison, David K. Jackson, Ian Johnston, Dominic Kwiatkowski, Cordelia Langford, John Sillitoe on behalf of the Wellcome Sanger Institute COVID-19 Surveillance Team
EPI_ISL_947180, EPI_ISL_947181	Lighthouse Lab in Milton Keynes	Wellcome Sanger Institute for the COVID-19 Genomics UK (COG-UK) Consortium	The Lighthouse Lab in Milton Keynes and Alex Alderton, Roberto Amato, Sonia Goncalves, Ewan Harrison, David K. Jackson, Ian Johnston, Dominic Kwiatkowski, Cordelia Langford, John Sillitoe on behalf of the Wellcome Sanger Institute COVID-19 Surveillance Team
EPI_ISL_947182, EPI_ISL_947183	Lighthouse Lab in Glasgow	Wellcome Sanger Institute for the COVID-19 Genomics UK (COG-UK) Consortium	Harper VanSteenhouse, Yumi Kasai, David Gray, Carol Clugston, Anna Dominiczak and Alex Alderton, Roberto Amato, Sonia Goncalves, Ewan Harrison, David K. Jackson, Ian Johnston, Dominic Kwiatkowski, Cordelia Langford, John Sillitoe on behalf of the Wellcome Sanger Institute COVID-19 Surveillance Team
EPI_ISL_947185	Lighthouse Lab in Milton Keynes	Wellcome Sanger Institute for the COVID-19 Genomics UK (COG-UK) Consortium	The Lighthouse Lab in Milton Keynes and Alex Alderton, Roberto Amato, Sonia Goncalves, Ewan Harrison, David K. Jackson, Ian Johnston, Dominic Kwiatkowski, Cordelia Langford, John Sillitoe on behalf of the Wellcome Sanger Institute COVID-19 Surveillance Team
EPI_ISL_947186, EPI_ISL_947187	Lighthouse Lab in Glasgow	Wellcome Sanger Institute for the COVID-19 Genomics UK (COG-UK) Consortium	Harper VanSteenhouse, Yumi Kasai, David Gray, Carol Clugston, Anna Dominiczak and Alex Alderton, Roberto Amato, Sonia Goncalves, Ewan Harrison, David K. Jackson, Ian Johnston, Dominic Kwiatkowski, Cordelia Langford, John Sillitoe on behalf of the Wellcome Sanger Institute COVID-19 Surveillance Team
EPI_ISL_947188, EPI_ISL_947190	Lighthouse Lab in Milton Keynes	Wellcome Sanger Institute for the COVID-19 Genomics UK (COG-UK) Consortium	The Lighthouse Lab in Milton Keynes and Alex Alderton, Roberto Amato, Sonia Goncalves, Ewan Harrison, David K. Jackson, Ian Johnston, Dominic Kwiatkowski, Cordelia Langford, John Sillitoe on behalf of the Wellcome Sanger Institute COVID-19 Surveillance Team
EPI_ISL_947191	Lighthouse Lab in Glasgow	Wellcome Sanger Institute for the COVID-19 Genomics UK (COG-UK) Consortium	Harper VanSteenhouse, Yumi Kasai, David Gray, Carol Clugston, Anna Dominiczak and Alex Alderton, Roberto Amato, Sonia Goncalves, Ewan Harrison, David K. Jackson, Ian Johnston, Dominic Kwiatkowski, Cordelia Langford, John Sillitoe on behalf of the Wellcome Sanger Institute COVID-19 Surveillance Team
EPI_ISL_947192, EPI_ISL_947193, EPI_ISL_947194, EPI_ISL_947195, EPI_ISL_947196	Lighthouse Lab in Milton Keynes	Wellcome Sanger Institute for the COVID-19 Genomics UK (COG-UK) Consortium	The Lighthouse Lab in Milton Keynes and Alex Alderton, Roberto Amato, Sonia Goncalves, Ewan Harrison, David K. Jackson, Ian Johnston, Dominic Kwiatkowski, Cordelia Langford, John Sillitoe on behalf of the Wellcome Sanger Institute COVID-19 Surveillance Team
EPI_ISL_947197	Lighthouse Lab in Glasgow	Wellcome Sanger Institute for the COVID-19 Genomics UK (COG-UK) Consortium	Harper VanSteenhouse, Yumi Kasai, David Gray, Carol Clugston, Anna Dominiczak and Alex Alderton, Roberto Amato, Sonia Goncalves, Ewan Harrison, David K. Jackson, Ian Johnston, Dominic Kwiatkowski, Cordelia Langford, John Sillitoe on behalf of the Wellcome Sanger Institute COVID-19 Surveillance Team
EPI_ISL_947198, EPI_ISL_947199, EPI_ISL_947200	Lighthouse Lab in Milton Keynes	Wellcome Sanger Institute for the COVID-19 Genomics UK (COG-UK) Consortium	The Lighthouse Lab in Milton Keynes and Alex Alderton, Roberto Amato, Sonia Goncalves, Ewan Harrison, David K. Jackson, Ian Johnston, Dominic Kwiatkowski, Cordelia Langford, John Sillitoe on behalf of the Wellcome Sanger Institute COVID-19 Surveillance Team
EPI_ISL_947202	Lighthouse Lab in Glasgow	Wellcome Sanger Institute for the COVID-19 Genomics UK (COG-UK) Consortium	Harper VanSteenhouse, Yumi Kasai, David Gray, Carol Clugston, Anna Dominiczak and Alex Alderton, Roberto Amato, Sonia Goncalves, Ewan Harrison, David K. Jackson, Ian Johnston, Dominic Kwiatkowski, Cordelia Langford, John Sillitoe on behalf of the Wellcome Sanger Institute COVID-19 Surveillance

			Team
EPI_ISL_947203	Lighthouse Lab in Milton Keynes	Wellcome Sanger Institute for the COVID-19 Genomics UK (COG-UK) Consortium	The Lighthouse Lab in Milton Keynes and Alex Alderton, Roberto Amato, Sonia Goncalves, Ewan Harrison, David K. Jackson, Ian Johnston, Dominic Kwiatkowski, Cordelia Langford, John Sillitoe on behalf of the Wellcome Sanger Institute COVID-19 Surveillance Team
EPI_ISL_947204	Lighthouse Lab in Glasgow	Wellcome Sanger Institute for the COVID-19 Genomics UK (COG-UK) Consortium	Harper VanSteenhouse, Yumi Kasai, David Gray, Carol Clugston, Anna Dominiczak and Alex Alderton, Roberto Amato, Sonia Goncalves, Ewan Harrison, David K. Jackson, Ian Johnston, Dominic Kwiatkowski, Cordelia Langford, John Sillitoe on behalf of the Wellcome Sanger Institute COVID-19 Surveillance Team
EPI_ISL_947205, EPI_ISL_947206, EPI_ISL_947207, EPI_ISL_947209, EPI_ISL_947210	Lighthouse Lab in Milton Keynes	Wellcome Sanger Institute for the COVID-19 Genomics UK (COG-UK) Consortium	The Lighthouse Lab in Milton Keynes and Alex Alderton, Roberto Amato, Sonia Goncalves, Ewan Harrison, David K. Jackson, Ian Johnston, Dominic Kwiatkowski, Cordelia Langford, John Sillitoe on behalf of the Wellcome Sanger Institute COVID-19 Surveillance Team
EPI_ISL_947211, EPI_ISL_947212, EPI_ISL_947213, EPI_ISL_947214	Lighthouse Lab in Glasgow	Wellcome Sanger Institute for the COVID-19 Genomics UK (COG-UK) Consortium	Harper VanSteenhouse, Yumi Kasai, David Gray, Carol Clugston, Anna Dominiczak and Alex Alderton, Roberto Amato, Sonia Goncalves, Ewan Harrison, David K. Jackson, Ian Johnston, Dominic Kwiatkowski, Cordelia Langford, John Sillitoe on behalf of the Wellcome Sanger Institute COVID-19 Surveillance Team
EPI_ISL_947215, EPI_ISL_947216, EPI_ISL_947217, EPI_ISL_947218, EPI_ISL_947219, EPI_ISL_947220, EPI_ISL_947221, EPI_ISL_947222, EPI_ISL_947223, EPI_ISL_947224, EPI_ISL_947225, EPI_ISL_947226, EPI_ISL_947227			
see above	Lighthouse Lab in Milton Keynes	Wellcome Sanger Institute for the COVID-19 Genomics UK (COG-UK) Consortium	The Lighthouse Lab in Milton Keynes and Alex Alderton, Roberto Amato, Sonia Goncalves, Ewan Harrison, David K. Jackson, Ian Johnston, Dominic Kwiatkowski, Cordelia Langford, John Sillitoe on behalf of the Wellcome Sanger Institute COVID-19 Surveillance Team
EPI_ISL_947228, EPI_ISL_947229, EPI_ISL_947230	Lighthouse Lab in Glasgow	Wellcome Sanger Institute for the COVID-19 Genomics UK (COG-UK) Consortium	Harper VanSteenhouse, Yumi Kasai, David Gray, Carol Clugston, Anna Dominiczak and Alex Alderton, Roberto Amato, Sonia Goncalves, Ewan Harrison, David K. Jackson, Ian Johnston, Dominic Kwiatkowski, Cordelia Langford, John Sillitoe on behalf of the Wellcome Sanger Institute COVID-19 Surveillance Team
EPI_ISL_947231, EPI_ISL_947232	Lighthouse Lab in Milton Keynes	Wellcome Sanger Institute for the COVID-19 Genomics UK (COG-UK) Consortium	The Lighthouse Lab in Milton Keynes and Alex Alderton, Roberto Amato, Sonia Goncalves, Ewan Harrison, David K. Jackson, Ian Johnston, Dominic Kwiatkowski, Cordelia Langford, John Sillitoe on behalf of the Wellcome Sanger Institute COVID-19 Surveillance Team
EPI_ISL_947233	Lighthouse Lab in Glasgow	Wellcome Sanger Institute for the COVID-19 Genomics UK (COG-UK) Consortium	Harper VanSteenhouse, Yumi Kasai, David Gray, Carol Clugston, Anna Dominiczak and Alex Alderton, Roberto Amato, Sonia Goncalves, Ewan Harrison, David K. Jackson, Ian Johnston, Dominic Kwiatkowski, Cordelia Langford, John Sillitoe on behalf of the Wellcome Sanger Institute COVID-19 Surveillance Team
EPI_ISL_947234, EPI_ISL_947235	Lighthouse Lab in Milton Keynes	Wellcome Sanger Institute for the COVID-19 Genomics UK (COG-UK) Consortium	The Lighthouse Lab in Milton Keynes and Alex Alderton, Roberto Amato, Sonia Goncalves, Ewan Harrison, David K. Jackson, Ian Johnston, Dominic Kwiatkowski, Cordelia Langford, John Sillitoe on behalf of the Wellcome Sanger Institute COVID-19 Surveillance Team
EPI_ISL_947236	Lighthouse Lab in Glasgow	Wellcome Sanger Institute for the COVID-19 Genomics UK (COG-UK) Consortium	Harper VanSteenhouse, Yumi Kasai, David Gray, Carol Clugston, Anna Dominiczak and Alex Alderton, Roberto Amato, Sonia Goncalves, Ewan Harrison, David K. Jackson, Ian Johnston, Dominic Kwiatkowski, Cordelia Langford, John Sillitoe on behalf of the Wellcome Sanger Institute COVID-19 Surveillance Team
EPI_ISL_947237	Lighthouse Lab in Milton Keynes	Wellcome Sanger Institute for the COVID-19 Genomics UK (COG-UK) Consortium	The Lighthouse Lab in Milton Keynes and Alex Alderton, Roberto Amato, Sonia Goncalves, Ewan Harrison, David K. Jackson, Ian Johnston, Dominic Kwiatkowski, Cordelia Langford, John Sillitoe on behalf of the Wellcome Sanger Institute COVID-19 Surveillance Team
EPI_ISL_947238, EPI_ISL_947239	Lighthouse Lab in Glasgow	Wellcome Sanger Institute for the COVID-19 Genomics UK (COG-UK) Consortium	Harper VanSteenhouse, Yumi Kasai, David Gray, Carol Clugston, Anna Dominiczak and Alex Alderton, Roberto Amato, Sonia Goncalves, Ewan Harrison, David K. Jackson, Ian Johnston, Dominic Kwiatkowski, Cordelia Langford, John Sillitoe on behalf of the Wellcome Sanger Institute COVID-19 Surveillance Team
EPI_ISL_947241, EPI_ISL_947242, EPI_ISL_947243, EPI_ISL_947244	Lighthouse Lab in Milton Keynes	Wellcome Sanger Institute for the COVID-19 Genomics UK (COG-UK) Consortium	The Lighthouse Lab in Milton Keynes and Alex Alderton, Roberto Amato, Sonia Goncalves, Ewan Harrison, David K. Jackson, Ian Johnston, Dominic Kwiatkowski, Cordelia Langford, John Sillitoe on behalf of the Wellcome Sanger Institute COVID-19 Surveillance Team
EPI_ISL_947245	Lighthouse Lab in Glasgow	Wellcome Sanger Institute for the COVID-19 Genomics UK (COG-UK) Consortium	Harper VanSteenhouse, Yumi Kasai, David Gray, Carol Clugston, Anna Dominiczak and Alex Alderton, Roberto Amato, Sonia Goncalves, Ewan Harrison, David K. Jackson, Ian Johnston, Dominic Kwiatkowski, Cordelia Langford, John Sillitoe on behalf of the Wellcome Sanger Institute COVID-19 Surveillance Team
EPI_ISL_947246, EPI_ISL_947248	Lighthouse Lab in Milton Keynes	Wellcome Sanger Institute for the COVID-19 Genomics UK (COG-UK) Consortium	The Lighthouse Lab in Milton Keynes and Alex Alderton, Roberto Amato, Sonia Goncalves, Ewan Harrison, David K. Jackson, Ian Johnston, Dominic Kwiatkowski, Cordelia Langford, John Sillitoe on behalf of the Wellcome Sanger Institute COVID-19 Surveillance Team
EPI_ISL_947250	Lighthouse Lab in Glasgow	Wellcome Sanger Institute for the COVID-19 Genomics UK (COG-UK) Consortium	Harper VanSteenhouse, Yumi Kasai, David Gray, Carol Clugston, Anna Dominiczak and Alex Alderton, Roberto Amato, Sonia Goncalves, Ewan Harrison, David K. Jackson, Ian Johnston, Dominic Kwiatkowski, Cordelia Langford, John Sillitoe on behalf of the Wellcome Sanger Institute COVID-19 Surveillance Team
EPI_ISL_947251	Lighthouse Lab in Milton Keynes	Wellcome Sanger Institute for the COVID-19 Genomics UK (COG-UK) Consortium	The Lighthouse Lab in Milton Keynes and Alex Alderton, Roberto Amato, Sonia Goncalves, Ewan Harrison, David K. Jackson, Ian Johnston, Dominic Kwiatkowski, Cordelia Langford, John Sillitoe on behalf of the Wellcome Sanger Institute COVID-19 Surveillance Team
EPI_ISL_947253	Lighthouse Lab in Glasgow	Wellcome Sanger Institute for the COVID-19 Genomics UK (COG-UK) Consortium	Harper VanSteenhouse, Yumi Kasai, David Gray, Carol Clugston, Anna Dominiczak and Alex Alderton, Roberto Amato, Sonia Goncalves, Ewan Harrison, David K. Jackson, Ian Johnston, Dominic Kwiatkowski, Cordelia Langford, John Sillitoe on behalf of the Wellcome Sanger Institute COVID-19 Surveillance Team
EPI_ISL_947254, EPI_ISL_947258, EPI_ISL_947259	Lighthouse Lab in Milton Keynes	Wellcome Sanger Institute for the COVID-19 Genomics UK (COG-UK) Consortium	The Lighthouse Lab in Milton Keynes and Alex Alderton, Roberto Amato, Sonia Goncalves, Ewan Harrison, David K. Jackson, Ian Johnston, Dominic Kwiatkowski, Cordelia Langford, John Sillitoe on behalf of the Wellcome Sanger Institute COVID-19 Surveillance Team
EPI_ISL_947263	Lighthouse Lab in Glasgow	Wellcome Sanger Institute for the COVID-19 Genomics UK (COG-UK) Consortium	Harper VanSteenhouse, Yumi Kasai, David Gray, Carol Clugston, Anna Dominiczak and Alex Alderton, Roberto Amato, Sonia Goncalves, Ewan Harrison, David K. Jackson, Ian Johnston, Dominic Kwiatkowski, Cordelia Langford, John Sillitoe on behalf of the Wellcome Sanger Institute COVID-19 Surveillance Team
EPI_ISL_947264, EPI_ISL_947266	Lighthouse Lab in Milton Keynes	Wellcome Sanger Institute for the COVID-19 Genomics UK (COG-UK) Consortium	The Lighthouse Lab in Milton Keynes and Alex Alderton, Roberto Amato, Sonia Goncalves, Ewan Harrison, David K. Jackson, Ian Johnston, Dominic Kwiatkowski, Cordelia Langford, John Sillitoe on behalf of the Wellcome Sanger Institute COVID-19 Surveillance Team
EPI_ISL_947267, EPI_ISL_947269, EPI_ISL_947271, EPI_ISL_947272	Lighthouse Lab in Glasgow	Wellcome Sanger Institute for the COVID-19 Genomics UK (COG-UK) Consortium	Harper VanSteenhouse, Yumi Kasai, David Gray, Carol Clugston, Anna Dominiczak and Alex Alderton, Roberto Amato, Sonia Goncalves, Ewan Harrison, David K. Jackson, Ian Johnston, Dominic Kwiatkowski, Cordelia Langford, John Sillitoe on behalf of the Wellcome Sanger Institute COVID-19 Surveillance Team
EPI_ISL_947276, EPI_ISL_947277, EPI_ISL_947279	Lighthouse Lab in Milton Keynes	Wellcome Sanger Institute for the COVID-19 Genomics UK (COG-UK) Consortium	The Lighthouse Lab in Milton Keynes and Alex Alderton, Roberto Amato, Sonia Goncalves, Ewan Harrison, David K. Jackson, Ian Johnston, Dominic Kwiatkowski, Cordelia Langford, John Sillitoe on behalf of the Wellcome Sanger Institute COVID-19 Surveillance Team
EPI_ISL_947280	Lighthouse Lab in Glasgow	Wellcome Sanger Institute for the COVID-19 Genomics UK (COG-UK) Consortium	Harper VanSteenhouse, Yumi Kasai, David Gray, Carol Clugston, Anna Dominiczak and Alex Alderton, Roberto Amato, Sonia Goncalves, Ewan Harrison, David K. Jackson, Ian Johnston, Dominic Kwiatkowski, Cordelia Langford, John Sillitoe on behalf of the Wellcome Sanger Institute COVID-19 Surveillance Team
EPI_ISL_947282	Lighthouse Lab in Milton Keynes	Wellcome Sanger Institute for the COVID-19 Genomics UK (COG-UK) Consortium	The Lighthouse Lab in Milton Keynes and Alex Alderton, Roberto Amato, Sonia Goncalves, Ewan Harrison, David K. Jackson, Ian Johnston, Dominic Kwiatkowski, Cordelia Langford, John Sillitoe on behalf of the Wellcome Sanger Institute COVID-19 Surveillance Team
EPI_ISL_947283	Lighthouse Lab in Glasgow	Wellcome Sanger Institute for the COVID-19 Genomics UK (COG-UK) Consortium	Harper VanSteenhouse, Yumi Kasai, David Gray, Carol Clugston, Anna Dominiczak and Alex Alderton, Roberto Amato, Sonia Goncalves, Ewan Harrison, David K. Jackson, Ian Johnston, Dominic Kwiatkowski, Cordelia Langford, John Sillitoe on behalf of the Wellcome Sanger Institute COVID-19 Surveillance Team
EPI_ISL_947285, EPI_ISL_947286, EPI_ISL_947288, EPI_ISL_947289, EPI_ISL_947291, EPI_ISL_947292	Lighthouse Lab in Milton Keynes	Wellcome Sanger Institute for the COVID-19 Genomics UK (COG-UK) Consortium	The Lighthouse Lab in Milton Keynes and Alex Alderton, Roberto Amato, Sonia Goncalves, Ewan Harrison, David K. Jackson, Ian Johnston, Dominic Kwiatkowski, Cordelia Langford, John Sillitoe on behalf of the Wellcome Sanger Institute COVID-19 Surveillance Team
EPI_ISL_947294, EPI_ISL_947295	Lighthouse Lab in Glasgow	Wellcome Sanger Institute for the COVID-19 Genomics UK (COG-UK) Consortium	Harper VanSteenhouse, Yumi Kasai, David Gray, Carol Clugston, Anna Dominiczak and Alex Alderton, Roberto Amato, Sonia Goncalves, Ewan Harrison, David K. Jackson, Ian Johnston, Dominic Kwiatkowski, Cordelia Langford, John Sillitoe on behalf of the Wellcome Sanger Institute COVID-19 Surveillance

			Team
EPI_ISL_947297, EPI_ISL_947299, EPI_ISL_947300, EPI_ISL_947302, EPI_ISL_947303	Lighthouse Lab in Milton Keynes	Wellcome Sanger Institute for the COVID-19 Genomics UK (COG-UK) Consortium	The Lighthouse Lab in Milton Keynes and Alex Alderton, Roberto Amato, Sonia Goncalves, Ewan Harrison, David K. Jackson, Ian Johnston, Dominic Kwiatkowski, Cordelia Langford, John Sillitoe on behalf of the Wellcome Sanger Institute COVID-19 Surveillance Team
EPI_ISL_947305	Lighthouse Lab in Glasgow	Wellcome Sanger Institute for the COVID-19 Genomics UK (COG-UK) Consortium	Harper VanSteenhouse, Yumi Kasai, David Gray, Carol Clugston, Anna Dominiczak and Alex Alderton, Roberto Amato, Sonia Goncalves, Ewan Harrison, David K. Jackson, Ian Johnston, Dominic Kwiatkowski, Cordelia Langford, John Sillitoe on behalf of the Wellcome Sanger Institute COVID-19 Surveillance Team
EPI_ISL_947306	Lighthouse Lab in Milton Keynes	Wellcome Sanger Institute for the COVID-19 Genomics UK (COG-UK) Consortium	The Lighthouse Lab in Milton Keynes and Alex Alderton, Roberto Amato, Sonia Goncalves, Ewan Harrison, David K. Jackson, Ian Johnston, Dominic Kwiatkowski, Cordelia Langford, John Sillitoe on behalf of the Wellcome Sanger Institute COVID-19 Surveillance Team
EPI_ISL_947308	Lighthouse Lab in Glasgow	Wellcome Sanger Institute for the COVID-19 Genomics UK (COG-UK) Consortium	Harper VanSteenhouse, Yumi Kasai, David Gray, Carol Clugston, Anna Dominiczak and Alex Alderton, Roberto Amato, Sonia Goncalves, Ewan Harrison, David K. Jackson, Ian Johnston, Dominic Kwiatkowski, Cordelia Langford, John Sillitoe on behalf of the Wellcome Sanger Institute COVID-19 Surveillance Team
EPI_ISL_947310, EPI_ISL_947311, EPI_ISL_947313	Lighthouse Lab in Milton Keynes	Wellcome Sanger Institute for the COVID-19 Genomics UK (COG-UK) Consortium	The Lighthouse Lab in Milton Keynes and Alex Alderton, Roberto Amato, Sonia Goncalves, Ewan Harrison, David K. Jackson, Ian Johnston, Dominic Kwiatkowski, Cordelia Langford, John Sillitoe on behalf of the Wellcome Sanger Institute COVID-19 Surveillance Team
EPI_ISL_947314, EPI_ISL_947316	Lighthouse Lab in Glasgow	Wellcome Sanger Institute for the COVID-19 Genomics UK (COG-UK) Consortium	Harper VanSteenhouse, Yumi Kasai, David Gray, Carol Clugston, Anna Dominiczak and Alex Alderton, Roberto Amato, Sonia Goncalves, Ewan Harrison, David K. Jackson, Ian Johnston, Dominic Kwiatkowski, Cordelia Langford, John Sillitoe on behalf of the Wellcome Sanger Institute COVID-19 Surveillance Team
EPI_ISL_947319, EPI_ISL_947321	Lighthouse Lab in Milton Keynes	Wellcome Sanger Institute for the COVID-19 Genomics UK (COG-UK) Consortium	The Lighthouse Lab in Milton Keynes and Alex Alderton, Roberto Amato, Sonia Goncalves, Ewan Harrison, David K. Jackson, Ian Johnston, Dominic Kwiatkowski, Cordelia Langford, John Sillitoe on behalf of the Wellcome Sanger Institute COVID-19 Surveillance Team
EPI_ISL_947331, EPI_ISL_947332	Lighthouse Lab in Glasgow	Wellcome Sanger Institute for the COVID-19 Genomics UK (COG-UK) Consortium	Harper VanSteenhouse, Yumi Kasai, David Gray, Carol Clugston, Anna Dominiczak and Alex Alderton, Roberto Amato, Sonia Goncalves, Ewan Harrison, David K. Jackson, Ian Johnston, Dominic Kwiatkowski, Cordelia Langford, John Sillitoe on behalf of the Wellcome Sanger Institute COVID-19 Surveillance Team
EPI_ISL_947333, EPI_ISL_947334	Lighthouse Lab in Milton Keynes	Wellcome Sanger Institute for the COVID-19 Genomics UK (COG-UK) Consortium	The Lighthouse Lab in Milton Keynes and Alex Alderton, Roberto Amato, Sonia Goncalves, Ewan Harrison, David K. Jackson, Ian Johnston, Dominic Kwiatkowski, Cordelia Langford, John Sillitoe on behalf of the Wellcome Sanger Institute COVID-19 Surveillance Team
EPI_ISL_947335	Lighthouse Lab in Glasgow	Wellcome Sanger Institute for the COVID-19 Genomics UK (COG-UK) Consortium	Harper VanSteenhouse, Yumi Kasai, David Gray, Carol Clugston, Anna Dominiczak and Alex Alderton, Roberto Amato, Sonia Goncalves, Ewan Harrison, David K. Jackson, Ian Johnston, Dominic Kwiatkowski, Cordelia Langford, John Sillitoe on behalf of the Wellcome Sanger Institute COVID-19 Surveillance Team
EPI_ISL_947336	Lighthouse Lab in Milton Keynes	Wellcome Sanger Institute for the COVID-19 Genomics UK (COG-UK) Consortium	The Lighthouse Lab in Milton Keynes and Alex Alderton, Roberto Amato, Sonia Goncalves, Ewan Harrison, David K. Jackson, Ian Johnston, Dominic Kwiatkowski, Cordelia Langford, John Sillitoe on behalf of the Wellcome Sanger Institute COVID-19 Surveillance Team
EPI_ISL_947338	Lighthouse Lab in Glasgow	Wellcome Sanger Institute for the COVID-19 Genomics UK (COG-UK) Consortium	Harper VanSteenhouse, Yumi Kasai, David Gray, Carol Clugston, Anna Dominiczak and Alex Alderton, Roberto Amato, Sonia Goncalves, Ewan Harrison, David K. Jackson, Ian Johnston, Dominic Kwiatkowski, Cordelia Langford, John Sillitoe on behalf of the Wellcome Sanger Institute COVID-19 Surveillance Team
EPI_ISL_947339, EPI_ISL_947340	Lighthouse Lab in Milton Keynes	Wellcome Sanger Institute for the COVID-19 Genomics UK (COG-UK) Consortium	The Lighthouse Lab in Milton Keynes and Alex Alderton, Roberto Amato, Sonia Goncalves, Ewan Harrison, David K. Jackson, Ian Johnston, Dominic Kwiatkowski, Cordelia Langford, John Sillitoe on behalf of the Wellcome Sanger Institute COVID-19 Surveillance Team
EPI_ISL_947341	Lighthouse Lab in Glasgow	Wellcome Sanger Institute for the COVID-19 Genomics UK (COG-UK) Consortium	Harper VanSteenhouse, Yumi Kasai, David Gray, Carol Clugston, Anna Dominiczak and Alex Alderton, Roberto Amato, Sonia Goncalves, Ewan Harrison, David K. Jackson, Ian Johnston, Dominic Kwiatkowski, Cordelia Langford, John Sillitoe on behalf of the Wellcome Sanger Institute COVID-19 Surveillance Team
EPI_ISL_947342	Lighthouse Lab in Milton Keynes	Wellcome Sanger Institute for the COVID-19 Genomics UK (COG-UK) Consortium	The Lighthouse Lab in Milton Keynes and Alex Alderton, Roberto Amato, Sonia Goncalves, Ewan Harrison, David K. Jackson, Ian Johnston, Dominic Kwiatkowski, Cordelia Langford, John Sillitoe on behalf of the Wellcome Sanger Institute COVID-19 Surveillance Team
EPI_ISL_947343, EPI_ISL_947344	Lighthouse Lab in Glasgow	Wellcome Sanger Institute for the COVID-19 Genomics UK (COG-UK) Consortium	Harper VanSteenhouse, Yumi Kasai, David Gray, Carol Clugston, Anna Dominiczak and Alex Alderton, Roberto Amato, Sonia Goncalves, Ewan Harrison, David K. Jackson, Ian Johnston, Dominic Kwiatkowski, Cordelia Langford, John Sillitoe on behalf of the Wellcome Sanger Institute COVID-19 Surveillance Team
EPI_ISL_947345, EPI_ISL_947346	Lighthouse Lab in Milton Keynes	Wellcome Sanger Institute for the COVID-19 Genomics UK (COG-UK) Consortium	The Lighthouse Lab in Milton Keynes and Alex Alderton, Roberto Amato, Sonia Goncalves, Ewan Harrison, David K. Jackson, Ian Johnston, Dominic Kwiatkowski, Cordelia Langford, John Sillitoe on behalf of the Wellcome Sanger Institute COVID-19 Surveillance Team
EPI_ISL_947347	Lighthouse Lab in Glasgow	Wellcome Sanger Institute for the COVID-19 Genomics UK (COG-UK) Consortium	Harper VanSteenhouse, Yumi Kasai, David Gray, Carol Clugston, Anna Dominiczak and Alex Alderton, Roberto Amato, Sonia Goncalves, Ewan Harrison, David K. Jackson, Ian Johnston, Dominic Kwiatkowski, Cordelia Langford, John Sillitoe on behalf of the Wellcome Sanger Institute COVID-19 Surveillance Team
EPI_ISL_947348, EPI_ISL_947349, EPI_ISL_947351	Lighthouse Lab in Milton Keynes	Wellcome Sanger Institute for the COVID-19 Genomics UK (COG-UK) Consortium	The Lighthouse Lab in Milton Keynes and Alex Alderton, Roberto Amato, Sonia Goncalves, Ewan Harrison, David K. Jackson, Ian Johnston, Dominic Kwiatkowski, Cordelia Langford, John Sillitoe on behalf of the Wellcome Sanger Institute COVID-19 Surveillance Team
EPI_ISL_947353	Lighthouse Lab in Glasgow	Wellcome Sanger Institute for the COVID-19 Genomics UK (COG-UK) Consortium	Harper VanSteenhouse, Yumi Kasai, David Gray, Carol Clugston, Anna Dominiczak and Alex Alderton, Roberto Amato, Sonia Goncalves, Ewan Harrison, David K. Jackson, Ian Johnston, Dominic Kwiatkowski, Cordelia Langford, John Sillitoe on behalf of the Wellcome Sanger Institute COVID-19 Surveillance Team
EPI_ISL_947355	Lighthouse Lab in Milton Keynes	Wellcome Sanger Institute for the COVID-19 Genomics UK (COG-UK) Consortium	The Lighthouse Lab in Milton Keynes and Alex Alderton, Roberto Amato, Sonia Goncalves, Ewan Harrison, David K. Jackson, Ian Johnston, Dominic Kwiatkowski, Cordelia Langford, John Sillitoe on behalf of the Wellcome Sanger Institute COVID-19 Surveillance Team
EPI_ISL_947356, EPI_ISL_947357	Lighthouse Lab in Glasgow	Wellcome Sanger Institute for the COVID-19 Genomics UK (COG-UK) Consortium	Harper VanSteenhouse, Yumi Kasai, David Gray, Carol Clugston, Anna Dominiczak and Alex Alderton, Roberto Amato, Sonia Goncalves, Ewan Harrison, David K. Jackson, Ian Johnston, Dominic Kwiatkowski, Cordelia Langford, John Sillitoe on behalf of the Wellcome Sanger Institute COVID-19 Surveillance Team
EPI_ISL_947358, EPI_ISL_947359	Lighthouse Lab in Milton Keynes	Wellcome Sanger Institute for the COVID-19 Genomics UK (COG-UK) Consortium	The Lighthouse Lab in Milton Keynes and Alex Alderton, Roberto Amato, Sonia Goncalves, Ewan Harrison, David K. Jackson, Ian Johnston, Dominic Kwiatkowski, Cordelia Langford, John Sillitoe on behalf of the Wellcome Sanger Institute COVID-19 Surveillance Team
EPI_ISL_947360, EPI_ISL_947362, EPI_ISL_947363, EPI_ISL_947364, EPI_ISL_947365	Lighthouse Lab in Glasgow	Wellcome Sanger Institute for the COVID-19 Genomics UK (COG-UK) Consortium	Harper VanSteenhouse, Yumi Kasai, David Gray, Carol Clugston, Anna Dominiczak and Alex Alderton, Roberto Amato, Sonia Goncalves, Ewan Harrison, David K. Jackson, Ian Johnston, Dominic Kwiatkowski, Cordelia Langford, John Sillitoe on behalf of the Wellcome Sanger Institute COVID-19 Surveillance Team
EPI_ISL_947366, EPI_ISL_947367, EPI_ISL_947369	Lighthouse Lab in Milton Keynes	Wellcome Sanger Institute for the COVID-19 Genomics UK (COG-UK) Consortium	The Lighthouse Lab in Milton Keynes and Alex Alderton, Roberto Amato, Sonia Goncalves, Ewan Harrison, David K. Jackson, Ian Johnston, Dominic Kwiatkowski, Cordelia Langford, John Sillitoe on behalf of the Wellcome Sanger Institute COVID-19 Surveillance Team
EPI_ISL_947370	Lighthouse Lab in Glasgow	Wellcome Sanger Institute for the COVID-19 Genomics UK (COG-UK) Consortium	Harper VanSteenhouse, Yumi Kasai, David Gray, Carol Clugston, Anna Dominiczak and Alex Alderton, Roberto Amato, Sonia Goncalves, Ewan Harrison, David K. Jackson, Ian Johnston, Dominic Kwiatkowski, Cordelia Langford, John Sillitoe on behalf of the Wellcome Sanger Institute COVID-19 Surveillance Team
EPI_ISL_947371, EPI_ISL_947372, EPI_ISL_947373, EPI_ISL_947375, EPI_ISL_947376	Lighthouse Lab in Milton Keynes	Wellcome Sanger Institute for the COVID-19 Genomics UK (COG-UK) Consortium	The Lighthouse Lab in Milton Keynes and Alex Alderton, Roberto Amato, Sonia Goncalves, Ewan Harrison, David K. Jackson, Ian Johnston, Dominic Kwiatkowski, Cordelia Langford, John Sillitoe on behalf of the Wellcome Sanger Institute COVID-19 Surveillance Team
EPI_ISL_947377	Lighthouse Lab in Glasgow	Wellcome Sanger Institute for the COVID-19 Genomics UK (COG-UK) Consortium	Harper VanSteenhouse, Yumi Kasai, David Gray, Carol Clugston, Anna Dominiczak and Alex Alderton, Roberto Amato, Sonia Goncalves, Ewan Harrison, David K. Jackson, Ian Johnston, Dominic Kwiatkowski, Cordelia Langford, John Sillitoe on behalf of the Wellcome Sanger Institute COVID-19 Surveillance Team

EPI_ISL_947378, EPI_ISL_947379	Lighthouse Lab in Milton Keynes	Wellcome Sanger Institute for the COVID-19 Genomics UK (COG-UK) Consortium	The Lighthouse Lab in Milton Keynes and Alex Alderton, Roberto Amato, Sonia Goncalves, Ewan Harrison, David K. Jackson, Ian Johnston, Dominic Kwiatkowski, Cordelia Langford, John Sillitoe on behalf of the Wellcome Sanger Institute COVID-19 Surveillance Team
EPI_ISL_947380	Lighthouse Lab in Glasgow	Wellcome Sanger Institute for the COVID-19 Genomics UK (COG-UK) Consortium	Harper VanSteenhouse, Yumi Kasai, David Gray, Carol Clugston, Anna Dominiczak and Alex Alderton, Roberto Amato, Sonia Goncalves, Ewan Harrison, David K. Jackson, Ian Johnston, Dominic Kwiatkowski, Cordelia Langford, John Sillitoe on behalf of the Wellcome Sanger Institute COVID-19 Surveillance Team
EPI_ISL_947381, EPI_ISL_947382	Lighthouse Lab in Milton Keynes	Wellcome Sanger Institute for the COVID-19 Genomics UK (COG-UK) Consortium	The Lighthouse Lab in Milton Keynes and Alex Alderton, Roberto Amato, Sonia Goncalves, Ewan Harrison, David K. Jackson, Ian Johnston, Dominic Kwiatkowski, Cordelia Langford, John Sillitoe on behalf of the Wellcome Sanger Institute COVID-19 Surveillance Team
EPI_ISL_947383	Lighthouse Lab in Glasgow	Wellcome Sanger Institute for the COVID-19 Genomics UK (COG-UK) Consortium	Harper VanSteenhouse, Yumi Kasai, David Gray, Carol Clugston, Anna Dominiczak and Alex Alderton, Roberto Amato, Sonia Goncalves, Ewan Harrison, David K. Jackson, Ian Johnston, Dominic Kwiatkowski, Cordelia Langford, John Sillitoe on behalf of the Wellcome Sanger Institute COVID-19 Surveillance Team
EPI_ISL_947384, EPI_ISL_947385	Lighthouse Lab in Milton Keynes	Wellcome Sanger Institute for the COVID-19 Genomics UK (COG-UK) Consortium	The Lighthouse Lab in Milton Keynes and Alex Alderton, Roberto Amato, Sonia Goncalves, Ewan Harrison, David K. Jackson, Ian Johnston, Dominic Kwiatkowski, Cordelia Langford, John Sillitoe on behalf of the Wellcome Sanger Institute COVID-19 Surveillance Team
EPI_ISL_947386	Lighthouse Lab in Glasgow	Wellcome Sanger Institute for the COVID-19 Genomics UK (COG-UK) Consortium	Harper VanSteenhouse, Yumi Kasai, David Gray, Carol Clugston, Anna Dominiczak and Alex Alderton, Roberto Amato, Sonia Goncalves, Ewan Harrison, David K. Jackson, Ian Johnston, Dominic Kwiatkowski, Cordelia Langford, John Sillitoe on behalf of the Wellcome Sanger Institute COVID-19 Surveillance Team
EPI_ISL_947387, EPI_ISL_947388	Lighthouse Lab in Milton Keynes	Wellcome Sanger Institute for the COVID-19 Genomics UK (COG-UK) Consortium	The Lighthouse Lab in Milton Keynes and Alex Alderton, Roberto Amato, Sonia Goncalves, Ewan Harrison, David K. Jackson, Ian Johnston, Dominic Kwiatkowski, Cordelia Langford, John Sillitoe on behalf of the Wellcome Sanger Institute COVID-19 Surveillance Team
EPI_ISL_947389	Lighthouse Lab in Glasgow	Wellcome Sanger Institute for the COVID-19 Genomics UK (COG-UK) Consortium	Harper VanSteenhouse, Yumi Kasai, David Gray, Carol Clugston, Anna Dominiczak and Alex Alderton, Roberto Amato, Sonia Goncalves, Ewan Harrison, David K. Jackson, Ian Johnston, Dominic Kwiatkowski, Cordelia Langford, John Sillitoe on behalf of the Wellcome Sanger Institute COVID-19 Surveillance Team
EPI_ISL_947390	Lighthouse Lab in Milton Keynes	Wellcome Sanger Institute for the COVID-19 Genomics UK (COG-UK) Consortium	The Lighthouse Lab in Milton Keynes and Alex Alderton, Roberto Amato, Sonia Goncalves, Ewan Harrison, David K. Jackson, Ian Johnston, Dominic Kwiatkowski, Cordelia Langford, John Sillitoe on behalf of the Wellcome Sanger Institute COVID-19 Surveillance Team
EPI_ISL_947391	Lighthouse Lab in Glasgow	Wellcome Sanger Institute for the COVID-19 Genomics UK (COG-UK) Consortium	Harper VanSteenhouse, Yumi Kasai, David Gray, Carol Clugston, Anna Dominiczak and Alex Alderton, Roberto Amato, Sonia Goncalves, Ewan Harrison, David K. Jackson, Ian Johnston, Dominic Kwiatkowski, Cordelia Langford, John Sillitoe on behalf of the Wellcome Sanger Institute COVID-19 Surveillance Team
EPI_ISL_947392, EPI_ISL_947393	Lighthouse Lab in Milton Keynes	Wellcome Sanger Institute for the COVID-19 Genomics UK (COG-UK) Consortium	The Lighthouse Lab in Milton Keynes and Alex Alderton, Roberto Amato, Sonia Goncalves, Ewan Harrison, David K. Jackson, Ian Johnston, Dominic Kwiatkowski, Cordelia Langford, John Sillitoe on behalf of the Wellcome Sanger Institute COVID-19 Surveillance Team
EPI_ISL_947394	Lighthouse Lab in Glasgow	Wellcome Sanger Institute for the COVID-19 Genomics UK (COG-UK) Consortium	Harper VanSteenhouse, Yumi Kasai, David Gray, Carol Clugston, Anna Dominiczak and Alex Alderton, Roberto Amato, Sonia Goncalves, Ewan Harrison, David K. Jackson, Ian Johnston, Dominic Kwiatkowski, Cordelia Langford, John Sillitoe on behalf of the Wellcome Sanger Institute COVID-19 Surveillance Team
EPI_ISL_947395	Lighthouse Lab in Milton Keynes	Wellcome Sanger Institute for the COVID-19 Genomics UK (COG-UK) Consortium	The Lighthouse Lab in Milton Keynes and Alex Alderton, Roberto Amato, Sonia Goncalves, Ewan Harrison, David K. Jackson, Ian Johnston, Dominic Kwiatkowski, Cordelia Langford, John Sillitoe on behalf of the Wellcome Sanger Institute COVID-19 Surveillance Team
EPI_ISL_947396	Lighthouse Lab in Glasgow	Wellcome Sanger Institute for the COVID-19 Genomics UK (COG-UK) Consortium	Harper VanSteenhouse, Yumi Kasai, David Gray, Carol Clugston, Anna Dominiczak and Alex Alderton, Roberto Amato, Sonia Goncalves, Ewan Harrison, David K. Jackson, Ian Johnston, Dominic Kwiatkowski, Cordelia Langford, John Sillitoe on behalf of the Wellcome Sanger Institute COVID-19 Surveillance Team
EPI_ISL_947397	Lighthouse Lab in Milton Keynes	Wellcome Sanger Institute for the COVID-19 Genomics UK (COG-UK) Consortium	The Lighthouse Lab in Milton Keynes and Alex Alderton, Roberto Amato, Sonia Goncalves, Ewan Harrison, David K. Jackson, Ian Johnston, Dominic Kwiatkowski, Cordelia Langford, John Sillitoe on behalf of the Wellcome Sanger Institute COVID-19 Surveillance Team
EPI_ISL_947398, EPI_ISL_947399	Lighthouse Lab in Glasgow	Wellcome Sanger Institute for the COVID-19 Genomics UK (COG-UK) Consortium	Harper VanSteenhouse, Yumi Kasai, David Gray, Carol Clugston, Anna Dominiczak and Alex Alderton, Roberto Amato, Sonia Goncalves, Ewan Harrison, David K. Jackson, Ian Johnston, Dominic Kwiatkowski, Cordelia Langford, John Sillitoe on behalf of the Wellcome Sanger Institute COVID-19 Surveillance Team
EPI_ISL_947401	Lighthouse Lab in Milton Keynes	Wellcome Sanger Institute for the COVID-19 Genomics UK (COG-UK) Consortium	The Lighthouse Lab in Milton Keynes and Alex Alderton, Roberto Amato, Sonia Goncalves, Ewan Harrison, David K. Jackson, Ian Johnston, Dominic Kwiatkowski, Cordelia Langford, John Sillitoe on behalf of the Wellcome Sanger Institute COVID-19 Surveillance Team
EPI_ISL_947402	Lighthouse Lab in Glasgow	Wellcome Sanger Institute for the COVID-19 Genomics UK (COG-UK) Consortium	Harper VanSteenhouse, Yumi Kasai, David Gray, Carol Clugston, Anna Dominiczak and Alex Alderton, Roberto Amato, Sonia Goncalves, Ewan Harrison, David K. Jackson, Ian Johnston, Dominic Kwiatkowski, Cordelia Langford, John Sillitoe on behalf of the Wellcome Sanger Institute COVID-19 Surveillance Team
EPI_ISL_947403	Lighthouse Lab in Milton Keynes	Wellcome Sanger Institute for the COVID-19 Genomics UK (COG-UK) Consortium	The Lighthouse Lab in Milton Keynes and Alex Alderton, Roberto Amato, Sonia Goncalves, Ewan Harrison, David K. Jackson, Ian Johnston, Dominic Kwiatkowski, Cordelia Langford, John Sillitoe on behalf of the Wellcome Sanger Institute COVID-19 Surveillance Team
EPI_ISL_947404, EPI_ISL_947405, EPI_ISL_948100	Lighthouse Lab in Glasgow	Wellcome Sanger Institute for the COVID-19 Genomics UK (COG-UK) Consortium	Harper VanSteenhouse, Yumi Kasai, David Gray, Carol Clugston, Anna Dominiczak and Alex Alderton, Roberto Amato, Sonia Goncalves, Ewan Harrison, David K. Jackson, Ian Johnston, Dominic Kwiatkowski, Cordelia Langford, John Sillitoe on behalf of the Wellcome Sanger Institute COVID-19 Surveillance Team
EPI_ISL_948102	Lighthouse Lab in Cambridge	Wellcome Sanger Institute for the COVID-19 Genomics UK (COG-UK) Consortium	Rob Howes, The Lighthouse Lab in Cambridge and Alex Alderton, Roberto Amato, Sonia Goncalves, Ewan Harrison, David K. Jackson, Ian Johnston, Dominic Kwiatkowski, Cordelia Langford, John Sillitoe on behalf of the Wellcome Sanger Institute COVID-19 Surveillance Team
EPI_ISL_948103	Lighthouse Lab in Glasgow	Wellcome Sanger Institute for the COVID-19 Genomics UK (COG-UK) Consortium	Harper VanSteenhouse, Yumi Kasai, David Gray, Carol Clugston, Anna Dominiczak and Alex Alderton, Roberto Amato, Sonia Goncalves, Ewan Harrison, David K. Jackson, Ian Johnston, Dominic Kwiatkowski, Cordelia Langford, John Sillitoe on behalf of the Wellcome Sanger Institute COVID-19 Surveillance Team
EPI_ISL_948104	Lighthouse Lab in Cambridge	Wellcome Sanger Institute for the COVID-19 Genomics UK (COG-UK) Consortium	Rob Howes, The Lighthouse Lab in Cambridge and Alex Alderton, Roberto Amato, Sonia Goncalves, Ewan Harrison, David K. Jackson, Ian Johnston, Dominic Kwiatkowski, Cordelia Langford, John Sillitoe on behalf of the Wellcome Sanger Institute COVID-19 Surveillance Team
EPI_ISL_948105, EPI_ISL_948106	Lighthouse Lab in Glasgow	Wellcome Sanger Institute for the COVID-19 Genomics UK (COG-UK) Consortium	Harper VanSteenhouse, Yumi Kasai, David Gray, Carol Clugston, Anna Dominiczak and Alex Alderton, Roberto Amato, Sonia Goncalves, Ewan Harrison, David K. Jackson, Ian Johnston, Dominic Kwiatkowski, Cordelia Langford, John Sillitoe on behalf of the Wellcome Sanger Institute COVID-19 Surveillance Team
EPI_ISL_948107	Lighthouse Lab in Cambridge	Wellcome Sanger Institute for the COVID-19 Genomics UK (COG-UK) Consortium	Rob Howes, The Lighthouse Lab in Cambridge and Alex Alderton, Roberto Amato, Sonia Goncalves, Ewan Harrison, David K. Jackson, Ian Johnston, Dominic Kwiatkowski, Cordelia Langford, John Sillitoe on behalf of the Wellcome Sanger Institute COVID-19 Surveillance Team
EPI_ISL_948108, EPI_ISL_948109	Lighthouse Lab in Glasgow	Wellcome Sanger Institute for the COVID-19 Genomics UK (COG-UK) Consortium	Harper VanSteenhouse, Yumi Kasai, David Gray, Carol Clugston, Anna Dominiczak and Alex Alderton, Roberto Amato, Sonia Goncalves, Ewan Harrison, David K. Jackson, Ian Johnston, Dominic Kwiatkowski, Cordelia Langford, John Sillitoe on behalf of the Wellcome Sanger Institute COVID-19 Surveillance Team
EPI_ISL_948110	Lighthouse Lab in Cambridge	Wellcome Sanger Institute for the COVID-19 Genomics UK (COG-UK) Consortium	Rob Howes, The Lighthouse Lab in Cambridge and Alex Alderton, Roberto Amato, Sonia Goncalves, Ewan Harrison, David K. Jackson, Ian Johnston, Dominic Kwiatkowski, Cordelia Langford, John Sillitoe on behalf of the Wellcome Sanger Institute COVID-19 Surveillance Team
EPI_ISL_948111, EPI_ISL_948112, EPI_ISL_948113	Lighthouse Lab in Glasgow	Wellcome Sanger Institute for the COVID-19 Genomics UK (COG-UK) Consortium	Harper VanSteenhouse, Yumi Kasai, David Gray, Carol Clugston, Anna Dominiczak and Alex Alderton, Roberto Amato, Sonia Goncalves, Ewan Harrison, David K. Jackson, Ian Johnston, Dominic Kwiatkowski, Cordelia Langford, John Sillitoe on behalf of the Wellcome Sanger Institute COVID-19 Surveillance Team
EPI_ISL_948114	Lighthouse Lab in Cambridge	Wellcome Sanger Institute for the COVID-19 Genomics UK (COG-UK) Consortium	Rob Howes, The Lighthouse Lab in Cambridge and Alex Alderton, Roberto Amato, Sonia Goncalves, Ewan Harrison, David K. Jackson, Ian Johnston, Dominic Kwiatkowski, Cordelia Langford, John Sillitoe on behalf of the Wellcome Sanger Institute COVID-19 Surveillance Team
EPI_ISL_948115	Lighthouse Lab in Glasgow	Wellcome Sanger Institute for the COVID-19 Genomics UK	Harper VanSteenhouse, Yumi Kasai, David Gray, Carol Clugston, Anna Dominiczak and Alex Alderton, Roberto Amato, Sonia Goncalves, Ewan Harrison,

		(COG-UK) Consortium	David K. Jackson, Ian Johnston, Dominic Kwiatkowski, Cordelia Langford, John Sillitoe on behalf of the Wellcome Sanger Institute COVID-19 Surveillance Team
EPI_ISL_948117, EPI_ISL_948118	Lighthouse Lab in Cambridge	Wellcome Sanger Institute for the COVID-19 Genomics UK (COG-UK) Consortium	Rob Howes, The Lighthouse Lab in Cambridge and Alex Alderton, Roberto Amato, Sonia Goncalves, Ewan Harrison, David K. Jackson, Ian Johnston, Dominic Kwiatkowski, Cordelia Langford, John Sillitoe on behalf of the Wellcome Sanger Institute COVID-19 Surveillance Team
EPI_ISL_948119	Lighthouse Lab in Glasgow	Wellcome Sanger Institute for the COVID-19 Genomics UK (COG-UK) Consortium	Harper VanSteenhouse, Yumi Kasai, David Gray, Carol Clugston, Anna Dominiczak and Alex Alderton, Roberto Amato, Sonia Goncalves, Ewan Harrison, David K. Jackson, Ian Johnston, Dominic Kwiatkowski, Cordelia Langford, John Sillitoe on behalf of the Wellcome Sanger Institute COVID-19 Surveillance Team
EPI_ISL_948120	Lighthouse Lab in Cambridge	Wellcome Sanger Institute for the COVID-19 Genomics UK (COG-UK) Consortium	Rob Howes, The Lighthouse Lab in Cambridge and Alex Alderton, Roberto Amato, Sonia Goncalves, Ewan Harrison, David K. Jackson, Ian Johnston, Dominic Kwiatkowski, Cordelia Langford, John Sillitoe on behalf of the Wellcome Sanger Institute COVID-19 Surveillance Team
EPI_ISL_948121	Lighthouse Lab in Glasgow	Wellcome Sanger Institute for the COVID-19 Genomics UK (COG-UK) Consortium	Harper VanSteenhouse, Yumi Kasai, David Gray, Carol Clugston, Anna Dominiczak and Alex Alderton, Roberto Amato, Sonia Goncalves, Ewan Harrison, David K. Jackson, Ian Johnston, Dominic Kwiatkowski, Cordelia Langford, John Sillitoe on behalf of the Wellcome Sanger Institute COVID-19 Surveillance Team
EPI_ISL_948122, EPI_ISL_948123, EPI_ISL_948124	Lighthouse Lab in Cambridge	Wellcome Sanger Institute for the COVID-19 Genomics UK (COG-UK) Consortium	Rob Howes, The Lighthouse Lab in Cambridge and Alex Alderton, Roberto Amato, Sonia Goncalves, Ewan Harrison, David K. Jackson, Ian Johnston, Dominic Kwiatkowski, Cordelia Langford, John Sillitoe on behalf of the Wellcome Sanger Institute COVID-19 Surveillance Team
EPI_ISL_948126, EPI_ISL_948127, EPI_ISL_948129, EPI_ISL_948130	Lighthouse Lab in Glasgow	Wellcome Sanger Institute for the COVID-19 Genomics UK (COG-UK) Consortium	Harper VanSteenhouse, Yumi Kasai, David Gray, Carol Clugston, Anna Dominiczak and Alex Alderton, Roberto Amato, Sonia Goncalves, Ewan Harrison, David K. Jackson, Ian Johnston, Dominic Kwiatkowski, Cordelia Langford, John Sillitoe on behalf of the Wellcome Sanger Institute COVID-19 Surveillance Team
EPI_ISL_948131	Lighthouse Lab in Cambridge	Wellcome Sanger Institute for the COVID-19 Genomics UK (COG-UK) Consortium	Rob Howes, The Lighthouse Lab in Cambridge and Alex Alderton, Roberto Amato, Sonia Goncalves, Ewan Harrison, David K. Jackson, Ian Johnston, Dominic Kwiatkowski, Cordelia Langford, John Sillitoe on behalf of the Wellcome Sanger Institute COVID-19 Surveillance Team
EPI_ISL_948132	Lighthouse Lab in Glasgow	Wellcome Sanger Institute for the COVID-19 Genomics UK (COG-UK) Consortium	Harper VanSteenhouse, Yumi Kasai, David Gray, Carol Clugston, Anna Dominiczak and Alex Alderton, Roberto Amato, Sonia Goncalves, Ewan Harrison, David K. Jackson, Ian Johnston, Dominic Kwiatkowski, Cordelia Langford, John Sillitoe on behalf of the Wellcome Sanger Institute COVID-19 Surveillance Team
EPI_ISL_948134	Lighthouse Lab in Cambridge	Wellcome Sanger Institute for the COVID-19 Genomics UK (COG-UK) Consortium	Rob Howes, The Lighthouse Lab in Cambridge and Alex Alderton, Roberto Amato, Sonia Goncalves, Ewan Harrison, David K. Jackson, Ian Johnston, Dominic Kwiatkowski, Cordelia Langford, John Sillitoe on behalf of the Wellcome Sanger Institute COVID-19 Surveillance Team
EPI_ISL_948135	Lighthouse Lab in Milton Keynes	Wellcome Sanger Institute for the COVID-19 Genomics UK (COG-UK) Consortium	The Lighthouse Lab in Milton Keynes and Alex Alderton, Roberto Amato, Sonia Goncalves, Ewan Harrison, David K. Jackson, Ian Johnston, Dominic Kwiatkowski, Cordelia Langford, John Sillitoe on behalf of the Wellcome Sanger Institute COVID-19 Surveillance Team
EPI_ISL_948136, EPI_ISL_948137	Lighthouse Lab in Cambridge	Wellcome Sanger Institute for the COVID-19 Genomics UK (COG-UK) Consortium	Rob Howes, The Lighthouse Lab in Cambridge and Alex Alderton, Roberto Amato, Sonia Goncalves, Ewan Harrison, David K. Jackson, Ian Johnston, Dominic Kwiatkowski, Cordelia Langford, John Sillitoe on behalf of the Wellcome Sanger Institute COVID-19 Surveillance Team
EPI_ISL_948138	Lighthouse Lab in Glasgow	Wellcome Sanger Institute for the COVID-19 Genomics UK (COG-UK) Consortium	Harper VanSteenhouse, Yumi Kasai, David Gray, Carol Clugston, Anna Dominiczak and Alex Alderton, Roberto Amato, Sonia Goncalves, Ewan Harrison, David K. Jackson, Ian Johnston, Dominic Kwiatkowski, Cordelia Langford, John Sillitoe on behalf of the Wellcome Sanger Institute COVID-19 Surveillance Team
EPI_ISL_948139	Lighthouse Lab in Cambridge	Wellcome Sanger Institute for the COVID-19 Genomics UK (COG-UK) Consortium	Rob Howes, The Lighthouse Lab in Cambridge and Alex Alderton, Roberto Amato, Sonia Goncalves, Ewan Harrison, David K. Jackson, Ian Johnston, Dominic Kwiatkowski, Cordelia Langford, John Sillitoe on behalf of the Wellcome Sanger Institute COVID-19 Surveillance Team
EPI_ISL_948140, EPI_ISL_948141, EPI_ISL_948142, EPI_ISL_948143, EPI_ISL_948144	Lighthouse Lab in Glasgow	Wellcome Sanger Institute for the COVID-19 Genomics UK (COG-UK) Consortium	Harper VanSteenhouse, Yumi Kasai, David Gray, Carol Clugston, Anna Dominiczak and Alex Alderton, Roberto Amato, Sonia Goncalves, Ewan Harrison, David K. Jackson, Ian Johnston, Dominic Kwiatkowski, Cordelia Langford, John Sillitoe on behalf of the Wellcome Sanger Institute COVID-19 Surveillance Team
EPI_ISL_948145	Lighthouse Lab in Cambridge	Wellcome Sanger Institute for the COVID-19 Genomics UK (COG-UK) Consortium	Rob Howes, The Lighthouse Lab in Cambridge and Alex Alderton, Roberto Amato, Sonia Goncalves, Ewan Harrison, David K. Jackson, Ian Johnston, Dominic Kwiatkowski, Cordelia Langford, John Sillitoe on behalf of the Wellcome Sanger Institute COVID-19 Surveillance Team
EPI_ISL_948146	Lighthouse Lab in Glasgow	Wellcome Sanger Institute for the COVID-19 Genomics UK (COG-UK) Consortium	Harper VanSteenhouse, Yumi Kasai, David Gray, Carol Clugston, Anna Dominiczak and Alex Alderton, Roberto Amato, Sonia Goncalves, Ewan Harrison, David K. Jackson, Ian Johnston, Dominic Kwiatkowski, Cordelia Langford, John Sillitoe on behalf of the Wellcome Sanger Institute COVID-19 Surveillance Team
EPI_ISL_948147	Lighthouse Lab in Cambridge	Wellcome Sanger Institute for the COVID-19 Genomics UK (COG-UK) Consortium	Rob Howes, The Lighthouse Lab in Cambridge and Alex Alderton, Roberto Amato, Sonia Goncalves, Ewan Harrison, David K. Jackson, Ian Johnston, Dominic Kwiatkowski, Cordelia Langford, John Sillitoe on behalf of the Wellcome Sanger Institute COVID-19 Surveillance Team
EPI_ISL_948148	Lighthouse Lab in Glasgow	Wellcome Sanger Institute for the COVID-19 Genomics UK (COG-UK) Consortium	Harper VanSteenhouse, Yumi Kasai, David Gray, Carol Clugston, Anna Dominiczak and Alex Alderton, Roberto Amato, Sonia Goncalves, Ewan Harrison, David K. Jackson, Ian Johnston, Dominic Kwiatkowski, Cordelia Langford, John Sillitoe on behalf of the Wellcome Sanger Institute COVID-19 Surveillance Team
EPI_ISL_948149, EPI_ISL_948151	Lighthouse Lab in Cambridge	Wellcome Sanger Institute for the COVID-19 Genomics UK (COG-UK) Consortium	Rob Howes, The Lighthouse Lab in Cambridge and Alex Alderton, Roberto Amato, Sonia Goncalves, Ewan Harrison, David K. Jackson, Ian Johnston, Dominic Kwiatkowski, Cordelia Langford, John Sillitoe on behalf of the Wellcome Sanger Institute COVID-19 Surveillance Team
EPI_ISL_948152	Lighthouse Lab in Glasgow	Wellcome Sanger Institute for the COVID-19 Genomics UK (COG-UK) Consortium	Harper VanSteenhouse, Yumi Kasai, David Gray, Carol Clugston, Anna Dominiczak and Alex Alderton, Roberto Amato, Sonia Goncalves, Ewan Harrison, David K. Jackson, Ian Johnston, Dominic Kwiatkowski, Cordelia Langford, John Sillitoe on behalf of the Wellcome Sanger Institute COVID-19 Surveillance Team
EPI_ISL_948153	Lighthouse Lab in Cambridge	Wellcome Sanger Institute for the COVID-19 Genomics UK (COG-UK) Consortium	Rob Howes, The Lighthouse Lab in Cambridge and Alex Alderton, Roberto Amato, Sonia Goncalves, Ewan Harrison, David K. Jackson, Ian Johnston, Dominic Kwiatkowski, Cordelia Langford, John Sillitoe on behalf of the Wellcome Sanger Institute COVID-19 Surveillance Team
EPI_ISL_948154, EPI_ISL_948156, EPI_ISL_948157, EPI_ISL_948158, EPI_ISL_948161, EPI_ISL_948162	Lighthouse Lab in Glasgow	Wellcome Sanger Institute for the COVID-19 Genomics UK (COG-UK) Consortium	Harper VanSteenhouse, Yumi Kasai, David Gray, Carol Clugston, Anna Dominiczak and Alex Alderton, Roberto Amato, Sonia Goncalves, Ewan Harrison, David K. Jackson, Ian Johnston, Dominic Kwiatkowski, Cordelia Langford, John Sillitoe on behalf of the Wellcome Sanger Institute COVID-19 Surveillance Team
EPI_ISL_948163, EPI_ISL_948164, EPI_ISL_948165, EPI_ISL_948166	Lighthouse Lab in Cambridge	Wellcome Sanger Institute for the COVID-19 Genomics UK (COG-UK) Consortium	Rob Howes, The Lighthouse Lab in Cambridge and Alex Alderton, Roberto Amato, Sonia Goncalves, Ewan Harrison, David K. Jackson, Ian Johnston, Dominic Kwiatkowski, Cordelia Langford, John Sillitoe on behalf of the Wellcome Sanger Institute COVID-19 Surveillance Team
EPI_ISL_948167, EPI_ISL_948168, EPI_ISL_948169, EPI_ISL_948170, EPI_ISL_948171	Lighthouse Lab in Glasgow	Wellcome Sanger Institute for the COVID-19 Genomics UK (COG-UK) Consortium	Harper VanSteenhouse, Yumi Kasai, David Gray, Carol Clugston, Anna Dominiczak and Alex Alderton, Roberto Amato, Sonia Goncalves, Ewan Harrison, David K. Jackson, Ian Johnston, Dominic Kwiatkowski, Cordelia Langford, John Sillitoe on behalf of the Wellcome Sanger Institute COVID-19 Surveillance Team
EPI_ISL_948172	Lighthouse Lab in Cambridge	Wellcome Sanger Institute for the COVID-19 Genomics UK (COG-UK) Consortium	Rob Howes, The Lighthouse Lab in Cambridge and Alex Alderton, Roberto Amato, Sonia Goncalves, Ewan Harrison, David K. Jackson, Ian Johnston, Dominic Kwiatkowski, Cordelia Langford, John Sillitoe on behalf of the Wellcome Sanger Institute COVID-19 Surveillance Team
EPI_ISL_948173	Lighthouse Lab in Glasgow	Wellcome Sanger Institute for the COVID-19 Genomics UK (COG-UK) Consortium	Harper VanSteenhouse, Yumi Kasai, David Gray, Carol Clugston, Anna Dominiczak and Alex Alderton, Roberto Amato, Sonia Goncalves, Ewan Harrison, David K. Jackson, Ian Johnston, Dominic Kwiatkowski, Cordelia Langford, John Sillitoe on behalf of the Wellcome Sanger Institute COVID-19 Surveillance Team
EPI_ISL_948174	Lighthouse Lab in Cambridge	Wellcome Sanger Institute for the COVID-19 Genomics UK (COG-UK) Consortium	Rob Howes, The Lighthouse Lab in Cambridge and Alex Alderton, Roberto Amato, Sonia Goncalves, Ewan Harrison, David K. Jackson, Ian Johnston, Dominic Kwiatkowski, Cordelia Langford, John Sillitoe on behalf of the Wellcome Sanger Institute COVID-19 Surveillance Team
EPI_ISL_948175	Lighthouse Lab in Glasgow	Wellcome Sanger Institute for the COVID-19 Genomics UK (COG-UK) Consortium	Harper VanSteenhouse, Yumi Kasai, David Gray, Carol Clugston, Anna Dominiczak and Alex Alderton, Roberto Amato, Sonia Goncalves, Ewan Harrison, David K. Jackson, Ian Johnston, Dominic Kwiatkowski, Cordelia Langford, John Sillitoe on behalf of the Wellcome Sanger Institute COVID-19 Surveillance Team
EPI_ISL_948176	Lighthouse Lab in Cambridge	Wellcome Sanger Institute for the COVID-19 Genomics UK (COG-UK) Consortium	Rob Howes, The Lighthouse Lab in Cambridge and Alex Alderton, Roberto Amato, Sonia Goncalves, Ewan Harrison, David K. Jackson, Ian Johnston, Dominic Kwiatkowski, Cordelia Langford, John Sillitoe on behalf of the Wellcome Sanger Institute COVID-19 Surveillance Team

EPI_ISL_948179	Lighthouse Lab in Glasgow	Wellcome Sanger Institute for the COVID-19 Genomics UK (COG-UK) Consortium	Harper VanSteenhouse, Yumi Kasai, David Gray, Carol Clugston, Anna Dominiczak and Alex Alderton, Roberto Amato, Sonia Goncalves, Ewan Harrison, David K. Jackson, Ian Johnston, Dominic Kwiatkowski, Cordelia Langford, John Sillitoe on behalf of the Wellcome Sanger Institute COVID-19 Surveillance Team
EPI_ISL_948180, EPI_ISL_948181	Lighthouse Lab in Cambridge	Wellcome Sanger Institute for the COVID-19 Genomics UK (COG-UK) Consortium	Rob Howes, The Lighthouse Lab in Cambridge and Alex Alderton, Roberto Amato, Sonia Goncalves, Ewan Harrison, David K. Jackson, Ian Johnston, Dominic Kwiatkowski, Cordelia Langford, John Sillitoe on behalf of the Wellcome Sanger Institute COVID-19 Surveillance Team
EPI_ISL_948182	Lighthouse Lab in Glasgow	Wellcome Sanger Institute for the COVID-19 Genomics UK (COG-UK) Consortium	Harper VanSteenhouse, Yumi Kasai, David Gray, Carol Clugston, Anna Dominiczak and Alex Alderton, Roberto Amato, Sonia Goncalves, Ewan Harrison, David K. Jackson, Ian Johnston, Dominic Kwiatkowski, Cordelia Langford, John Sillitoe on behalf of the Wellcome Sanger Institute COVID-19 Surveillance Team
EPI_ISL_948183	Lighthouse Lab in Cambridge	Wellcome Sanger Institute for the COVID-19 Genomics UK (COG-UK) Consortium	Rob Howes, The Lighthouse Lab in Cambridge and Alex Alderton, Roberto Amato, Sonia Goncalves, Ewan Harrison, David K. Jackson, Ian Johnston, Dominic Kwiatkowski, Cordelia Langford, John Sillitoe on behalf of the Wellcome Sanger Institute COVID-19 Surveillance Team
EPI_ISL_948184	Lighthouse Lab in Glasgow	Wellcome Sanger Institute for the COVID-19 Genomics UK (COG-UK) Consortium	Harper VanSteenhouse, Yumi Kasai, David Gray, Carol Clugston, Anna Dominiczak and Alex Alderton, Roberto Amato, Sonia Goncalves, Ewan Harrison, David K. Jackson, Ian Johnston, Dominic Kwiatkowski, Cordelia Langford, John Sillitoe on behalf of the Wellcome Sanger Institute COVID-19 Surveillance Team
EPI_ISL_948187, EPI_ISL_948188, EPI_ISL_948189	Lighthouse Lab in Cambridge	Wellcome Sanger Institute for the COVID-19 Genomics UK (COG-UK) Consortium	Rob Howes, The Lighthouse Lab in Cambridge and Alex Alderton, Roberto Amato, Sonia Goncalves, Ewan Harrison, David K. Jackson, Ian Johnston, Dominic Kwiatkowski, Cordelia Langford, John Sillitoe on behalf of the Wellcome Sanger Institute COVID-19 Surveillance Team
EPI_ISL_948190	Lighthouse Lab in Glasgow	Wellcome Sanger Institute for the COVID-19 Genomics UK (COG-UK) Consortium	Harper VanSteenhouse, Yumi Kasai, David Gray, Carol Clugston, Anna Dominiczak and Alex Alderton, Roberto Amato, Sonia Goncalves, Ewan Harrison, David K. Jackson, Ian Johnston, Dominic Kwiatkowski, Cordelia Langford, John Sillitoe on behalf of the Wellcome Sanger Institute COVID-19 Surveillance Team
EPI_ISL_948191, EPI_ISL_948192, EPI_ISL_948193	Lighthouse Lab in Cambridge	Wellcome Sanger Institute for the COVID-19 Genomics UK (COG-UK) Consortium	Rob Howes, The Lighthouse Lab in Cambridge and Alex Alderton, Roberto Amato, Sonia Goncalves, Ewan Harrison, David K. Jackson, Ian Johnston, Dominic Kwiatkowski, Cordelia Langford, John Sillitoe on behalf of the Wellcome Sanger Institute COVID-19 Surveillance Team
EPI_ISL_948195, EPI_ISL_948196	Lighthouse Lab in Glasgow	Wellcome Sanger Institute for the COVID-19 Genomics UK (COG-UK) Consortium	Harper VanSteenhouse, Yumi Kasai, David Gray, Carol Clugston, Anna Dominiczak and Alex Alderton, Roberto Amato, Sonia Goncalves, Ewan Harrison, David K. Jackson, Ian Johnston, Dominic Kwiatkowski, Cordelia Langford, John Sillitoe on behalf of the Wellcome Sanger Institute COVID-19 Surveillance Team
EPI_ISL_948197, EPI_ISL_948198, EPI_ISL_948199, EPI_ISL_948200, EPI_ISL_948201	Lighthouse Lab in Cambridge	Wellcome Sanger Institute for the COVID-19 Genomics UK (COG-UK) Consortium	Rob Howes, The Lighthouse Lab in Cambridge and Alex Alderton, Roberto Amato, Sonia Goncalves, Ewan Harrison, David K. Jackson, Ian Johnston, Dominic Kwiatkowski, Cordelia Langford, John Sillitoe on behalf of the Wellcome Sanger Institute COVID-19 Surveillance Team
EPI_ISL_948202	Lighthouse Lab in Glasgow	Wellcome Sanger Institute for the COVID-19 Genomics UK (COG-UK) Consortium	Harper VanSteenhouse, Yumi Kasai, David Gray, Carol Clugston, Anna Dominiczak and Alex Alderton, Roberto Amato, Sonia Goncalves, Ewan Harrison, David K. Jackson, Ian Johnston, Dominic Kwiatkowski, Cordelia Langford, John Sillitoe on behalf of the Wellcome Sanger Institute COVID-19 Surveillance Team
EPI_ISL_948203, EPI_ISL_948205	Lighthouse Lab in Cambridge	Wellcome Sanger Institute for the COVID-19 Genomics UK (COG-UK) Consortium	Rob Howes, The Lighthouse Lab in Cambridge and Alex Alderton, Roberto Amato, Sonia Goncalves, Ewan Harrison, David K. Jackson, Ian Johnston, Dominic Kwiatkowski, Cordelia Langford, John Sillitoe on behalf of the Wellcome Sanger Institute COVID-19 Surveillance Team
EPI_ISL_948206	Lighthouse Lab in Glasgow	Wellcome Sanger Institute for the COVID-19 Genomics UK (COG-UK) Consortium	Harper VanSteenhouse, Yumi Kasai, David Gray, Carol Clugston, Anna Dominiczak and Alex Alderton, Roberto Amato, Sonia Goncalves, Ewan Harrison, David K. Jackson, Ian Johnston, Dominic Kwiatkowski, Cordelia Langford, John Sillitoe on behalf of the Wellcome Sanger Institute COVID-19 Surveillance Team
EPI_ISL_948207, EPI_ISL_948208, EPI_ISL_948209, EPI_ISL_948211, EPI_ISL_948212, EPI_ISL_948213, EPI_ISL_948214, EPI_ISL_948216	Lighthouse Lab in Cambridge	Wellcome Sanger Institute for the COVID-19 Genomics UK (COG-UK) Consortium	Rob Howes, The Lighthouse Lab in Cambridge and Alex Alderton, Roberto Amato, Sonia Goncalves, Ewan Harrison, David K. Jackson, Ian Johnston, Dominic Kwiatkowski, Cordelia Langford, John Sillitoe on behalf of the Wellcome Sanger Institute COVID-19 Surveillance Team
EPI_ISL_948217, EPI_ISL_948218	Lighthouse Lab in Glasgow	Wellcome Sanger Institute for the COVID-19 Genomics UK (COG-UK) Consortium	Harper VanSteenhouse, Yumi Kasai, David Gray, Carol Clugston, Anna Dominiczak and Alex Alderton, Roberto Amato, Sonia Goncalves, Ewan Harrison, David K. Jackson, Ian Johnston, Dominic Kwiatkowski, Cordelia Langford, John Sillitoe on behalf of the Wellcome Sanger Institute COVID-19 Surveillance Team
EPI_ISL_948219, EPI_ISL_948220	Lighthouse Lab in Cambridge	Wellcome Sanger Institute for the COVID-19 Genomics UK (COG-UK) Consortium	Rob Howes, The Lighthouse Lab in Cambridge and Alex Alderton, Roberto Amato, Sonia Goncalves, Ewan Harrison, David K. Jackson, Ian Johnston, Dominic Kwiatkowski, Cordelia Langford, John Sillitoe on behalf of the Wellcome Sanger Institute COVID-19 Surveillance Team
EPI_ISL_948222	Lighthouse Lab in Glasgow	Wellcome Sanger Institute for the COVID-19 Genomics UK (COG-UK) Consortium	Harper VanSteenhouse, Yumi Kasai, David Gray, Carol Clugston, Anna Dominiczak and Alex Alderton, Roberto Amato, Sonia Goncalves, Ewan Harrison, David K. Jackson, Ian Johnston, Dominic Kwiatkowski, Cordelia Langford, John Sillitoe on behalf of the Wellcome Sanger Institute COVID-19 Surveillance Team
EPI_ISL_948223, EPI_ISL_948224	Lighthouse Lab in Cambridge	Wellcome Sanger Institute for the COVID-19 Genomics UK (COG-UK) Consortium	Rob Howes, The Lighthouse Lab in Cambridge and Alex Alderton, Roberto Amato, Sonia Goncalves, Ewan Harrison, David K. Jackson, Ian Johnston, Dominic Kwiatkowski, Cordelia Langford, John Sillitoe on behalf of the Wellcome Sanger Institute COVID-19 Surveillance Team
EPI_ISL_948225, EPI_ISL_948226	Lighthouse Lab in Glasgow	Wellcome Sanger Institute for the COVID-19 Genomics UK (COG-UK) Consortium	Harper VanSteenhouse, Yumi Kasai, David Gray, Carol Clugston, Anna Dominiczak and Alex Alderton, Roberto Amato, Sonia Goncalves, Ewan Harrison, David K. Jackson, Ian Johnston, Dominic Kwiatkowski, Cordelia Langford, John Sillitoe on behalf of the Wellcome Sanger Institute COVID-19 Surveillance Team
EPI_ISL_948227, EPI_ISL_948228	Lighthouse Lab in Cambridge	Wellcome Sanger Institute for the COVID-19 Genomics UK (COG-UK) Consortium	Rob Howes, The Lighthouse Lab in Cambridge and Alex Alderton, Roberto Amato, Sonia Goncalves, Ewan Harrison, David K. Jackson, Ian Johnston, Dominic Kwiatkowski, Cordelia Langford, John Sillitoe on behalf of the Wellcome Sanger Institute COVID-19 Surveillance Team
EPI_ISL_948229	Lighthouse Lab in Glasgow	Wellcome Sanger Institute for the COVID-19 Genomics UK (COG-UK) Consortium	Harper VanSteenhouse, Yumi Kasai, David Gray, Carol Clugston, Anna Dominiczak and Alex Alderton, Roberto Amato, Sonia Goncalves, Ewan Harrison, David K. Jackson, Ian Johnston, Dominic Kwiatkowski, Cordelia Langford, John Sillitoe on behalf of the Wellcome Sanger Institute COVID-19 Surveillance Team
EPI_ISL_948230	Lighthouse Lab in Cambridge	Wellcome Sanger Institute for the COVID-19 Genomics UK (COG-UK) Consortium	Rob Howes, The Lighthouse Lab in Cambridge and Alex Alderton, Roberto Amato, Sonia Goncalves, Ewan Harrison, David K. Jackson, Ian Johnston, Dominic Kwiatkowski, Cordelia Langford, John Sillitoe on behalf of the Wellcome Sanger Institute COVID-19 Surveillance Team
EPI_ISL_948232	Lighthouse Lab in Glasgow	Wellcome Sanger Institute for the COVID-19 Genomics UK (COG-UK) Consortium	Harper VanSteenhouse, Yumi Kasai, David Gray, Carol Clugston, Anna Dominiczak and Alex Alderton, Roberto Amato, Sonia Goncalves, Ewan Harrison, David K. Jackson, Ian Johnston, Dominic Kwiatkowski, Cordelia Langford, John Sillitoe on behalf of the Wellcome Sanger Institute COVID-19 Surveillance Team
EPI_ISL_948233, EPI_ISL_948234, EPI_ISL_948235	Lighthouse Lab in Cambridge	Wellcome Sanger Institute for the COVID-19 Genomics UK (COG-UK) Consortium	Rob Howes, The Lighthouse Lab in Cambridge and Alex Alderton, Roberto Amato, Sonia Goncalves, Ewan Harrison, David K. Jackson, Ian Johnston, Dominic Kwiatkowski, Cordelia Langford, John Sillitoe on behalf of the Wellcome Sanger Institute COVID-19 Surveillance Team
EPI_ISL_948236	Lighthouse Lab in Glasgow	Wellcome Sanger Institute for the COVID-19 Genomics UK (COG-UK) Consortium	Harper VanSteenhouse, Yumi Kasai, David Gray, Carol Clugston, Anna Dominiczak and Alex Alderton, Roberto Amato, Sonia Goncalves, Ewan Harrison, David K. Jackson, Ian Johnston, Dominic Kwiatkowski, Cordelia Langford, John Sillitoe on behalf of the Wellcome Sanger Institute COVID-19 Surveillance Team
EPI_ISL_948237	Lighthouse Lab in Cambridge	Wellcome Sanger Institute for the COVID-19 Genomics UK (COG-UK) Consortium	Rob Howes, The Lighthouse Lab in Cambridge and Alex Alderton, Roberto Amato, Sonia Goncalves, Ewan Harrison, David K. Jackson, Ian Johnston, Dominic Kwiatkowski, Cordelia Langford, John Sillitoe on behalf of the Wellcome Sanger Institute COVID-19 Surveillance Team
EPI_ISL_948238	Lighthouse Lab in Glasgow	Wellcome Sanger Institute for the COVID-19 Genomics UK (COG-UK) Consortium	Harper VanSteenhouse, Yumi Kasai, David Gray, Carol Clugston, Anna Dominiczak and Alex Alderton, Roberto Amato, Sonia Goncalves, Ewan Harrison, David K. Jackson, Ian Johnston, Dominic Kwiatkowski, Cordelia Langford, John Sillitoe on behalf of the Wellcome Sanger Institute COVID-19 Surveillance Team
EPI_ISL_948239, EPI_ISL_948240, EPI_ISL_948241	Lighthouse Lab in Cambridge	Wellcome Sanger Institute for the COVID-19 Genomics UK (COG-UK) Consortium	Rob Howes, The Lighthouse Lab in Cambridge and Alex Alderton, Roberto Amato, Sonia Goncalves, Ewan Harrison, David K. Jackson, Ian Johnston, Dominic Kwiatkowski, Cordelia Langford, John Sillitoe on behalf of the Wellcome Sanger Institute COVID-19 Surveillance Team

EPI_ISL_948242, EPI_ISL_948243	Lighthouse Lab in Glasgow	Wellcome Sanger Institute for the COVID-19 Genomics UK (COG-UK) Consortium	Harper VanSteenhouse, Yumi Kasai, David Gray, Carol Clugston, Anna Dominiczak and Alex Alderton, Roberto Amato, Sonia Goncalves, Ewan Harrison, David K. Jackson, Ian Johnston, Dominic Kwiatkowski, Cordelia Langford, John Sillitoe on behalf of the Wellcome Sanger Institute COVID-19 Surveillance Team
EPI_ISL_948244	Lighthouse Lab in Cambridge	Wellcome Sanger Institute for the COVID-19 Genomics UK (COG-UK) Consortium	Rob Howes, The Lighthouse Lab in Cambridge and Alex Alderton, Roberto Amato, Sonia Goncalves, Ewan Harrison, David K. Jackson, Ian Johnston, Dominic Kwiatkowski, Cordelia Langford, John Sillitoe on behalf of the Wellcome Sanger Institute COVID-19 Surveillance Team
EPI_ISL_948245, EPI_ISL_948246	Lighthouse Lab in Glasgow	Wellcome Sanger Institute for the COVID-19 Genomics UK (COG-UK) Consortium	Harper VanSteenhouse, Yumi Kasai, David Gray, Carol Clugston, Anna Dominiczak and Alex Alderton, Roberto Amato, Sonia Goncalves, Ewan Harrison, David K. Jackson, Ian Johnston, Dominic Kwiatkowski, Cordelia Langford, John Sillitoe on behalf of the Wellcome Sanger Institute COVID-19 Surveillance Team
EPI_ISL_948247	Lighthouse Lab in Cambridge	Wellcome Sanger Institute for the COVID-19 Genomics UK (COG-UK) Consortium	Rob Howes, The Lighthouse Lab in Cambridge and Alex Alderton, Roberto Amato, Sonia Goncalves, Ewan Harrison, David K. Jackson, Ian Johnston, Dominic Kwiatkowski, Cordelia Langford, John Sillitoe on behalf of the Wellcome Sanger Institute COVID-19 Surveillance Team
EPI_ISL_948248	Lighthouse Lab in Glasgow	Wellcome Sanger Institute for the COVID-19 Genomics UK (COG-UK) Consortium	Harper VanSteenhouse, Yumi Kasai, David Gray, Carol Clugston, Anna Dominiczak and Alex Alderton, Roberto Amato, Sonia Goncalves, Ewan Harrison, David K. Jackson, Ian Johnston, Dominic Kwiatkowski, Cordelia Langford, John Sillitoe on behalf of the Wellcome Sanger Institute COVID-19 Surveillance Team
EPI_ISL_948249, EPI_ISL_948250, EPI_ISL_948251	Lighthouse Lab in Cambridge	Wellcome Sanger Institute for the COVID-19 Genomics UK (COG-UK) Consortium	Rob Howes, The Lighthouse Lab in Cambridge and Alex Alderton, Roberto Amato, Sonia Goncalves, Ewan Harrison, David K. Jackson, Ian Johnston, Dominic Kwiatkowski, Cordelia Langford, John Sillitoe on behalf of the Wellcome Sanger Institute COVID-19 Surveillance Team
EPI_ISL_948252	Lighthouse Lab in Milton Keynes	Wellcome Sanger Institute for the COVID-19 Genomics UK (COG-UK) Consortium	The Lighthouse Lab in Milton Keynes and Alex Alderton, Roberto Amato, Sonia Goncalves, Ewan Harrison, David K. Jackson, Ian Johnston, Dominic Kwiatkowski, Cordelia Langford, John Sillitoe on behalf of the Wellcome Sanger Institute COVID-19 Surveillance Team
EPI_ISL_948253	Lighthouse Lab in Cambridge	Wellcome Sanger Institute for the COVID-19 Genomics UK (COG-UK) Consortium	Rob Howes, The Lighthouse Lab in Cambridge and Alex Alderton, Roberto Amato, Sonia Goncalves, Ewan Harrison, David K. Jackson, Ian Johnston, Dominic Kwiatkowski, Cordelia Langford, John Sillitoe on behalf of the Wellcome Sanger Institute COVID-19 Surveillance Team
EPI_ISL_948254	Lighthouse Lab in Glasgow	Wellcome Sanger Institute for the COVID-19 Genomics UK (COG-UK) Consortium	Harper VanSteenhouse, Yumi Kasai, David Gray, Carol Clugston, Anna Dominiczak and Alex Alderton, Roberto Amato, Sonia Goncalves, Ewan Harrison, David K. Jackson, Ian Johnston, Dominic Kwiatkowski, Cordelia Langford, John Sillitoe on behalf of the Wellcome Sanger Institute COVID-19 Surveillance Team
EPI_ISL_948255	Lighthouse Lab in Cambridge	Wellcome Sanger Institute for the COVID-19 Genomics UK (COG-UK) Consortium	Rob Howes, The Lighthouse Lab in Cambridge and Alex Alderton, Roberto Amato, Sonia Goncalves, Ewan Harrison, David K. Jackson, Ian Johnston, Dominic Kwiatkowski, Cordelia Langford, John Sillitoe on behalf of the Wellcome Sanger Institute COVID-19 Surveillance Team
EPI_ISL_948256	Lighthouse Lab in Glasgow	Wellcome Sanger Institute for the COVID-19 Genomics UK (COG-UK) Consortium	Harper VanSteenhouse, Yumi Kasai, David Gray, Carol Clugston, Anna Dominiczak and Alex Alderton, Roberto Amato, Sonia Goncalves, Ewan Harrison, David K. Jackson, Ian Johnston, Dominic Kwiatkowski, Cordelia Langford, John Sillitoe on behalf of the Wellcome Sanger Institute COVID-19 Surveillance Team
EPI_ISL_948257, EPI_ISL_948258, EPI_ISL_948259	Lighthouse Lab in Cambridge	Wellcome Sanger Institute for the COVID-19 Genomics UK (COG-UK) Consortium	Rob Howes, The Lighthouse Lab in Cambridge and Alex Alderton, Roberto Amato, Sonia Goncalves, Ewan Harrison, David K. Jackson, Ian Johnston, Dominic Kwiatkowski, Cordelia Langford, John Sillitoe on behalf of the Wellcome Sanger Institute COVID-19 Surveillance Team
EPI_ISL_948260	Lighthouse Lab in Glasgow	Wellcome Sanger Institute for the COVID-19 Genomics UK (COG-UK) Consortium	Harper VanSteenhouse, Yumi Kasai, David Gray, Carol Clugston, Anna Dominiczak and Alex Alderton, Roberto Amato, Sonia Goncalves, Ewan Harrison, David K. Jackson, Ian Johnston, Dominic Kwiatkowski, Cordelia Langford, John Sillitoe on behalf of the Wellcome Sanger Institute COVID-19 Surveillance Team
EPI_ISL_948261	Lighthouse Lab in Cambridge	Wellcome Sanger Institute for the COVID-19 Genomics UK (COG-UK) Consortium	Rob Howes, The Lighthouse Lab in Cambridge and Alex Alderton, Roberto Amato, Sonia Goncalves, Ewan Harrison, David K. Jackson, Ian Johnston, Dominic Kwiatkowski, Cordelia Langford, John Sillitoe on behalf of the Wellcome Sanger Institute COVID-19 Surveillance Team
EPI_ISL_948262, EPI_ISL_948263	Lighthouse Lab in Glasgow	Wellcome Sanger Institute for the COVID-19 Genomics UK (COG-UK) Consortium	Harper VanSteenhouse, Yumi Kasai, David Gray, Carol Clugston, Anna Dominiczak and Alex Alderton, Roberto Amato, Sonia Goncalves, Ewan Harrison, David K. Jackson, Ian Johnston, Dominic Kwiatkowski, Cordelia Langford, John Sillitoe on behalf of the Wellcome Sanger Institute COVID-19 Surveillance Team
EPI_ISL_948264, EPI_ISL_948265	Lighthouse Lab in Cambridge	Wellcome Sanger Institute for the COVID-19 Genomics UK (COG-UK) Consortium	Rob Howes, The Lighthouse Lab in Cambridge and Alex Alderton, Roberto Amato, Sonia Goncalves, Ewan Harrison, David K. Jackson, Ian Johnston, Dominic Kwiatkowski, Cordelia Langford, John Sillitoe on behalf of the Wellcome Sanger Institute COVID-19 Surveillance Team
EPI_ISL_948267, EPI_ISL_948268	Lighthouse Lab in Glasgow	Wellcome Sanger Institute for the COVID-19 Genomics UK (COG-UK) Consortium	Harper VanSteenhouse, Yumi Kasai, David Gray, Carol Clugston, Anna Dominiczak and Alex Alderton, Roberto Amato, Sonia Goncalves, Ewan Harrison, David K. Jackson, Ian Johnston, Dominic Kwiatkowski, Cordelia Langford, John Sillitoe on behalf of the Wellcome Sanger Institute COVID-19 Surveillance Team
EPI_ISL_948269	Lighthouse Lab in Cambridge	Wellcome Sanger Institute for the COVID-19 Genomics UK (COG-UK) Consortium	Rob Howes, The Lighthouse Lab in Cambridge and Alex Alderton, Roberto Amato, Sonia Goncalves, Ewan Harrison, David K. Jackson, Ian Johnston, Dominic Kwiatkowski, Cordelia Langford, John Sillitoe on behalf of the Wellcome Sanger Institute COVID-19 Surveillance Team
EPI_ISL_948270, EPI_ISL_948271, EPI_ISL_948272, EPI_ISL_948273	Lighthouse Lab in Glasgow	Wellcome Sanger Institute for the COVID-19 Genomics UK (COG-UK) Consortium	Harper VanSteenhouse, Yumi Kasai, David Gray, Carol Clugston, Anna Dominiczak and Alex Alderton, Roberto Amato, Sonia Goncalves, Ewan Harrison, David K. Jackson, Ian Johnston, Dominic Kwiatkowski, Cordelia Langford, John Sillitoe on behalf of the Wellcome Sanger Institute COVID-19 Surveillance Team
EPI_ISL_948274	Lighthouse Lab in Cambridge	Wellcome Sanger Institute for the COVID-19 Genomics UK (COG-UK) Consortium	Rob Howes, The Lighthouse Lab in Cambridge and Alex Alderton, Roberto Amato, Sonia Goncalves, Ewan Harrison, David K. Jackson, Ian Johnston, Dominic Kwiatkowski, Cordelia Langford, John Sillitoe on behalf of the Wellcome Sanger Institute COVID-19 Surveillance Team
EPI_ISL_948275, EPI_ISL_948276	Lighthouse Lab in Glasgow	Wellcome Sanger Institute for the COVID-19 Genomics UK (COG-UK) Consortium	Harper VanSteenhouse, Yumi Kasai, David Gray, Carol Clugston, Anna Dominiczak and Alex Alderton, Roberto Amato, Sonia Goncalves, Ewan Harrison, David K. Jackson, Ian Johnston, Dominic Kwiatkowski, Cordelia Langford, John Sillitoe on behalf of the Wellcome Sanger Institute COVID-19 Surveillance Team
EPI_ISL_948277, EPI_ISL_948278, EPI_ISL_948279, EPI_ISL_948280, EPI_ISL_948281, EPI_ISL_948282, EPI_ISL_948283, EPI_ISL_948284	Lighthouse Lab in Cambridge	Wellcome Sanger Institute for the COVID-19 Genomics UK (COG-UK) Consortium	Rob Howes, The Lighthouse Lab in Cambridge and Alex Alderton, Roberto Amato, Sonia Goncalves, Ewan Harrison, David K. Jackson, Ian Johnston, Dominic Kwiatkowski, Cordelia Langford, John Sillitoe on behalf of the Wellcome Sanger Institute COVID-19 Surveillance Team
EPI_ISL_948285	Lighthouse Lab in Glasgow	Wellcome Sanger Institute for the COVID-19 Genomics UK (COG-UK) Consortium	Harper VanSteenhouse, Yumi Kasai, David Gray, Carol Clugston, Anna Dominiczak and Alex Alderton, Roberto Amato, Sonia Goncalves, Ewan Harrison, David K. Jackson, Ian Johnston, Dominic Kwiatkowski, Cordelia Langford, John Sillitoe on behalf of the Wellcome Sanger Institute COVID-19 Surveillance Team
EPI_ISL_948286	Lighthouse Lab in Cambridge	Wellcome Sanger Institute for the COVID-19 Genomics UK (COG-UK) Consortium	Rob Howes, The Lighthouse Lab in Cambridge and Alex Alderton, Roberto Amato, Sonia Goncalves, Ewan Harrison, David K. Jackson, Ian Johnston, Dominic Kwiatkowski, Cordelia Langford, John Sillitoe on behalf of the Wellcome Sanger Institute COVID-19 Surveillance Team
EPI_ISL_948287	Lighthouse Lab in Glasgow	Wellcome Sanger Institute for the COVID-19 Genomics UK (COG-UK) Consortium	Harper VanSteenhouse, Yumi Kasai, David Gray, Carol Clugston, Anna Dominiczak and Alex Alderton, Roberto Amato, Sonia Goncalves, Ewan Harrison, David K. Jackson, Ian Johnston, Dominic Kwiatkowski, Cordelia Langford, John Sillitoe on behalf of the Wellcome Sanger Institute COVID-19 Surveillance Team
EPI_ISL_948288	Lighthouse Lab in Cambridge	Wellcome Sanger Institute for the COVID-19 Genomics UK (COG-UK) Consortium	Rob Howes, The Lighthouse Lab in Cambridge and Alex Alderton, Roberto Amato, Sonia Goncalves, Ewan Harrison, David K. Jackson, Ian Johnston, Dominic Kwiatkowski, Cordelia Langford, John Sillitoe on behalf of the Wellcome Sanger Institute COVID-19 Surveillance Team
EPI_ISL_948290, EPI_ISL_948291, EPI_ISL_948293, EPI_ISL_948295	Lighthouse Lab in Glasgow	Wellcome Sanger Institute for the COVID-19 Genomics UK (COG-UK) Consortium	Harper VanSteenhouse, Yumi Kasai, David Gray, Carol Clugston, Anna Dominiczak and Alex Alderton, Roberto Amato, Sonia Goncalves, Ewan Harrison, David K. Jackson, Ian Johnston, Dominic Kwiatkowski, Cordelia Langford, John Sillitoe on behalf of the Wellcome Sanger Institute COVID-19 Surveillance Team
EPI_ISL_948296	Lighthouse Lab in Cambridge	Wellcome Sanger Institute for the COVID-19 Genomics UK (COG-UK) Consortium	Rob Howes, The Lighthouse Lab in Cambridge and Alex Alderton, Roberto Amato, Sonia Goncalves, Ewan Harrison, David K. Jackson, Ian Johnston, Dominic Kwiatkowski, Cordelia Langford, John Sillitoe on behalf of the Wellcome Sanger Institute COVID-19 Surveillance Team
EPI_ISL_948297	Lighthouse Lab in Glasgow	Wellcome Sanger Institute for the COVID-19 Genomics UK (COG-UK) Consortium	Harper VanSteenhouse, Yumi Kasai, David Gray, Carol Clugston, Anna Dominiczak and Alex Alderton, Roberto Amato, Sonia Goncalves, Ewan Harrison, David K. Jackson, Ian Johnston, Dominic Kwiatkowski, Cordelia Langford, John Sillitoe on behalf of the Wellcome Sanger Institute COVID-19 Surveillance

			Team
EPI_ISL_948298	Lighthouse Lab in Cambridge	Wellcome Sanger Institute for the COVID-19 Genomics UK (COG-UK) Consortium	Rob Howes, The Lighthouse Lab in Cambridge and Alex Alderton, Roberto Amato, Sonia Goncalves, Ewan Harrison, David K. Jackson, Ian Johnston, Dominic Kwiatkowski, Cordelia Langford, John Sillitoe on behalf of the Wellcome Sanger Institute COVID-19 Surveillance Team
EPI_ISL_948299	Lighthouse Lab in Glasgow	Wellcome Sanger Institute for the COVID-19 Genomics UK (COG-UK) Consortium	Harper VanSteenhouse, Yumi Kasai, David Gray, Carol Clugston, Anna Dominiczak and Alex Alderton, Roberto Amato, Sonia Goncalves, Ewan Harrison, David K. Jackson, Ian Johnston, Dominic Kwiatkowski, Cordelia Langford, John Sillitoe on behalf of the Wellcome Sanger Institute COVID-19 Surveillance Team
EPI_ISL_948300, EPI_ISL_948301, EPI_ISL_948302	Lighthouse Lab in Cambridge	Wellcome Sanger Institute for the COVID-19 Genomics UK (COG-UK) Consortium	Rob Howes, The Lighthouse Lab in Cambridge and Alex Alderton, Roberto Amato, Sonia Goncalves, Ewan Harrison, David K. Jackson, Ian Johnston, Dominic Kwiatkowski, Cordelia Langford, John Sillitoe on behalf of the Wellcome Sanger Institute COVID-19 Surveillance Team
EPI_ISL_948303, EPI_ISL_948304	Lighthouse Lab in Glasgow	Wellcome Sanger Institute for the COVID-19 Genomics UK (COG-UK) Consortium	Harper VanSteenhouse, Yumi Kasai, David Gray, Carol Clugston, Anna Dominiczak and Alex Alderton, Roberto Amato, Sonia Goncalves, Ewan Harrison, David K. Jackson, Ian Johnston, Dominic Kwiatkowski, Cordelia Langford, John Sillitoe on behalf of the Wellcome Sanger Institute COVID-19 Surveillance Team
EPI_ISL_948305, EPI_ISL_948306	Lighthouse Lab in Cambridge	Wellcome Sanger Institute for the COVID-19 Genomics UK (COG-UK) Consortium	Rob Howes, The Lighthouse Lab in Cambridge and Alex Alderton, Roberto Amato, Sonia Goncalves, Ewan Harrison, David K. Jackson, Ian Johnston, Dominic Kwiatkowski, Cordelia Langford, John Sillitoe on behalf of the Wellcome Sanger Institute COVID-19 Surveillance Team
EPI_ISL_948307	Lighthouse Lab in Glasgow	Wellcome Sanger Institute for the COVID-19 Genomics UK (COG-UK) Consortium	Harper VanSteenhouse, Yumi Kasai, David Gray, Carol Clugston, Anna Dominiczak and Alex Alderton, Roberto Amato, Sonia Goncalves, Ewan Harrison, David K. Jackson, Ian Johnston, Dominic Kwiatkowski, Cordelia Langford, John Sillitoe on behalf of the Wellcome Sanger Institute COVID-19 Surveillance Team
EPI_ISL_948308	Lighthouse Lab in Cambridge	Wellcome Sanger Institute for the COVID-19 Genomics UK (COG-UK) Consortium	Rob Howes, The Lighthouse Lab in Cambridge and Alex Alderton, Roberto Amato, Sonia Goncalves, Ewan Harrison, David K. Jackson, Ian Johnston, Dominic Kwiatkowski, Cordelia Langford, John Sillitoe on behalf of the Wellcome Sanger Institute COVID-19 Surveillance Team
EPI_ISL_948309	Lighthouse Lab in Glasgow	Wellcome Sanger Institute for the COVID-19 Genomics UK (COG-UK) Consortium	Harper VanSteenhouse, Yumi Kasai, David Gray, Carol Clugston, Anna Dominiczak and Alex Alderton, Roberto Amato, Sonia Goncalves, Ewan Harrison, David K. Jackson, Ian Johnston, Dominic Kwiatkowski, Cordelia Langford, John Sillitoe on behalf of the Wellcome Sanger Institute COVID-19 Surveillance Team
EPI_ISL_948310, EPI_ISL_948311, EPI_ISL_948312, EPI_ISL_948313, EPI_ISL_948314	Lighthouse Lab in Cambridge	Wellcome Sanger Institute for the COVID-19 Genomics UK (COG-UK) Consortium	Rob Howes, The Lighthouse Lab in Cambridge and Alex Alderton, Roberto Amato, Sonia Goncalves, Ewan Harrison, David K. Jackson, Ian Johnston, Dominic Kwiatkowski, Cordelia Langford, John Sillitoe on behalf of the Wellcome Sanger Institute COVID-19 Surveillance Team
EPI_ISL_948315	Lighthouse Lab in Glasgow	Wellcome Sanger Institute for the COVID-19 Genomics UK (COG-UK) Consortium	Harper VanSteenhouse, Yumi Kasai, David Gray, Carol Clugston, Anna Dominiczak and Alex Alderton, Roberto Amato, Sonia Goncalves, Ewan Harrison, David K. Jackson, Ian Johnston, Dominic Kwiatkowski, Cordelia Langford, John Sillitoe on behalf of the Wellcome Sanger Institute COVID-19 Surveillance Team
EPI_ISL_948316	Lighthouse Lab in Cambridge	Wellcome Sanger Institute for the COVID-19 Genomics UK (COG-UK) Consortium	Rob Howes, The Lighthouse Lab in Cambridge and Alex Alderton, Roberto Amato, Sonia Goncalves, Ewan Harrison, David K. Jackson, Ian Johnston, Dominic Kwiatkowski, Cordelia Langford, John Sillitoe on behalf of the Wellcome Sanger Institute COVID-19 Surveillance Team
EPI_ISL_948317, EPI_ISL_948318, EPI_ISL_948319, EPI_ISL_948320	Lighthouse Lab in Glasgow	Wellcome Sanger Institute for the COVID-19 Genomics UK (COG-UK) Consortium	Harper VanSteenhouse, Yumi Kasai, David Gray, Carol Clugston, Anna Dominiczak and Alex Alderton, Roberto Amato, Sonia Goncalves, Ewan Harrison, David K. Jackson, Ian Johnston, Dominic Kwiatkowski, Cordelia Langford, John Sillitoe on behalf of the Wellcome Sanger Institute COVID-19 Surveillance Team
EPI_ISL_948321, EPI_ISL_948322, EPI_ISL_948323	Lighthouse Lab in Cambridge	Wellcome Sanger Institute for the COVID-19 Genomics UK (COG-UK) Consortium	Rob Howes, The Lighthouse Lab in Cambridge and Alex Alderton, Roberto Amato, Sonia Goncalves, Ewan Harrison, David K. Jackson, Ian Johnston, Dominic Kwiatkowski, Cordelia Langford, John Sillitoe on behalf of the Wellcome Sanger Institute COVID-19 Surveillance Team
EPI_ISL_948324, EPI_ISL_948325	Lighthouse Lab in Glasgow	Wellcome Sanger Institute for the COVID-19 Genomics UK (COG-UK) Consortium	Harper VanSteenhouse, Yumi Kasai, David Gray, Carol Clugston, Anna Dominiczak and Alex Alderton, Roberto Amato, Sonia Goncalves, Ewan Harrison, David K. Jackson, Ian Johnston, Dominic Kwiatkowski, Cordelia Langford, John Sillitoe on behalf of the Wellcome Sanger Institute COVID-19 Surveillance Team
EPI_ISL_948326	Lighthouse Lab in Cambridge	Wellcome Sanger Institute for the COVID-19 Genomics UK (COG-UK) Consortium	Rob Howes, The Lighthouse Lab in Cambridge and Alex Alderton, Roberto Amato, Sonia Goncalves, Ewan Harrison, David K. Jackson, Ian Johnston, Dominic Kwiatkowski, Cordelia Langford, John Sillitoe on behalf of the Wellcome Sanger Institute COVID-19 Surveillance Team
EPI_ISL_948327	Lighthouse Lab in Milton Keynes	Wellcome Sanger Institute for the COVID-19 Genomics UK (COG-UK) Consortium	The Lighthouse Lab in Milton Keynes and Alex Alderton, Roberto Amato, Sonia Goncalves, Ewan Harrison, David K. Jackson, Ian Johnston, Dominic Kwiatkowski, Cordelia Langford, John Sillitoe on behalf of the Wellcome Sanger Institute COVID-19 Surveillance Team
EPI_ISL_948328	Lighthouse Lab in Cambridge	Wellcome Sanger Institute for the COVID-19 Genomics UK (COG-UK) Consortium	Rob Howes, The Lighthouse Lab in Cambridge and Alex Alderton, Roberto Amato, Sonia Goncalves, Ewan Harrison, David K. Jackson, Ian Johnston, Dominic Kwiatkowski, Cordelia Langford, John Sillitoe on behalf of the Wellcome Sanger Institute COVID-19 Surveillance Team
EPI_ISL_948329	Lighthouse Lab in Glasgow	Wellcome Sanger Institute for the COVID-19 Genomics UK (COG-UK) Consortium	Harper VanSteenhouse, Yumi Kasai, David Gray, Carol Clugston, Anna Dominiczak and Alex Alderton, Roberto Amato, Sonia Goncalves, Ewan Harrison, David K. Jackson, Ian Johnston, Dominic Kwiatkowski, Cordelia Langford, John Sillitoe on behalf of the Wellcome Sanger Institute COVID-19 Surveillance Team
EPI_ISL_948331, EPI_ISL_948332	Lighthouse Lab in Cambridge	Wellcome Sanger Institute for the COVID-19 Genomics UK (COG-UK) Consortium	Rob Howes, The Lighthouse Lab in Cambridge and Alex Alderton, Roberto Amato, Sonia Goncalves, Ewan Harrison, David K. Jackson, Ian Johnston, Dominic Kwiatkowski, Cordelia Langford, John Sillitoe on behalf of the Wellcome Sanger Institute COVID-19 Surveillance Team
EPI_ISL_948333, EPI_ISL_948334, EPI_ISL_948335, EPI_ISL_948336	Lighthouse Lab in Glasgow	Wellcome Sanger Institute for the COVID-19 Genomics UK (COG-UK) Consortium	Harper VanSteenhouse, Yumi Kasai, David Gray, Carol Clugston, Anna Dominiczak and Alex Alderton, Roberto Amato, Sonia Goncalves, Ewan Harrison, David K. Jackson, Ian Johnston, Dominic Kwiatkowski, Cordelia Langford, John Sillitoe on behalf of the Wellcome Sanger Institute COVID-19 Surveillance Team
EPI_ISL_948337, EPI_ISL_948338	Lighthouse Lab in Cambridge	Wellcome Sanger Institute for the COVID-19 Genomics UK (COG-UK) Consortium	Rob Howes, The Lighthouse Lab in Cambridge and Alex Alderton, Roberto Amato, Sonia Goncalves, Ewan Harrison, David K. Jackson, Ian Johnston, Dominic Kwiatkowski, Cordelia Langford, John Sillitoe on behalf of the Wellcome Sanger Institute COVID-19 Surveillance Team
EPI_ISL_948339	Lighthouse Lab in Glasgow	Wellcome Sanger Institute for the COVID-19 Genomics UK (COG-UK) Consortium	Harper VanSteenhouse, Yumi Kasai, David Gray, Carol Clugston, Anna Dominiczak and Alex Alderton, Roberto Amato, Sonia Goncalves, Ewan Harrison, David K. Jackson, Ian Johnston, Dominic Kwiatkowski, Cordelia Langford, John Sillitoe on behalf of the Wellcome Sanger Institute COVID-19 Surveillance Team
EPI_ISL_948340, EPI_ISL_948341, EPI_ISL_948342	Lighthouse Lab in Cambridge	Wellcome Sanger Institute for the COVID-19 Genomics UK (COG-UK) Consortium	Rob Howes, The Lighthouse Lab in Cambridge and Alex Alderton, Roberto Amato, Sonia Goncalves, Ewan Harrison, David K. Jackson, Ian Johnston, Dominic Kwiatkowski, Cordelia Langford, John Sillitoe on behalf of the Wellcome Sanger Institute COVID-19 Surveillance Team
EPI_ISL_948343	Lighthouse Lab in Glasgow	Wellcome Sanger Institute for the COVID-19 Genomics UK (COG-UK) Consortium	Harper VanSteenhouse, Yumi Kasai, David Gray, Carol Clugston, Anna Dominiczak and Alex Alderton, Roberto Amato, Sonia Goncalves, Ewan Harrison, David K. Jackson, Ian Johnston, Dominic Kwiatkowski, Cordelia Langford, John Sillitoe on behalf of the Wellcome Sanger Institute COVID-19 Surveillance Team
EPI_ISL_948344, EPI_ISL_948345, EPI_ISL_948346	Lighthouse Lab in Cambridge	Wellcome Sanger Institute for the COVID-19 Genomics UK (COG-UK) Consortium	Rob Howes, The Lighthouse Lab in Cambridge and Alex Alderton, Roberto Amato, Sonia Goncalves, Ewan Harrison, David K. Jackson, Ian Johnston, Dominic Kwiatkowski, Cordelia Langford, John Sillitoe on behalf of the Wellcome Sanger Institute COVID-19 Surveillance Team
EPI_ISL_948347, EPI_ISL_948348, EPI_ISL_948349	Lighthouse Lab in Glasgow	Wellcome Sanger Institute for the COVID-19 Genomics UK (COG-UK) Consortium	Harper VanSteenhouse, Yumi Kasai, David Gray, Carol Clugston, Anna Dominiczak and Alex Alderton, Roberto Amato, Sonia Goncalves, Ewan Harrison, David K. Jackson, Ian Johnston, Dominic Kwiatkowski, Cordelia Langford, John Sillitoe on behalf of the Wellcome Sanger Institute COVID-19 Surveillance Team
EPI_ISL_948350	Lighthouse Lab in Cambridge	Wellcome Sanger Institute for the COVID-19 Genomics UK (COG-UK) Consortium	Rob Howes, The Lighthouse Lab in Cambridge and Alex Alderton, Roberto Amato, Sonia Goncalves, Ewan Harrison, David K. Jackson, Ian Johnston, Dominic Kwiatkowski, Cordelia Langford, John Sillitoe on behalf of the Wellcome Sanger Institute COVID-19 Surveillance Team
EPI_ISL_948351, EPI_ISL_948352, EPI_ISL_948353, EPI_ISL_948354	Lighthouse Lab in Glasgow	Wellcome Sanger Institute for the COVID-19 Genomics UK (COG-UK) Consortium	Harper VanSteenhouse, Yumi Kasai, David Gray, Carol Clugston, Anna Dominiczak and Alex Alderton, Roberto Amato, Sonia Goncalves, Ewan Harrison, David K. Jackson, Ian Johnston, Dominic Kwiatkowski, Cordelia Langford, John Sillitoe on behalf of the Wellcome Sanger Institute COVID-19 Surveillance Team
EPI_ISL_948355	Lighthouse Lab in Cambridge	Wellcome Sanger Institute for the COVID-19 Genomics UK (COG-UK) Consortium	Rob Howes, The Lighthouse Lab in Cambridge and Alex Alderton, Roberto Amato, Sonia Goncalves, Ewan Harrison, David K. Jackson, Ian Johnston, Dominic Kwiatkowski, Cordelia Langford, John Sillitoe on behalf of the Wellcome Sanger Institute COVID-19 Surveillance Team

EPI_ISL_948356, EPI_ISL_948357, EPI_ISL_948358	Lighthouse Lab in Glasgow	Wellcome Sanger Institute for the COVID-19 Genomics UK (COG-UK) Consortium	Harper VanSteenhouse, Yumi Kasai, David Gray, Carol Clugston, Anna Dominiczak and Alex Alderton, Roberto Amato, Sonia Goncalves, Ewan Harrison, David K. Jackson, Ian Johnston, Dominic Kwiatkowski, Cordelia Langford, John Sillitoe on behalf of the Wellcome Sanger Institute COVID-19 Surveillance Team
EPI_ISL_948359	Lighthouse Lab in Cambridge	Wellcome Sanger Institute for the COVID-19 Genomics UK (COG-UK) Consortium	Rob Howes, The Lighthouse Lab in Cambridge and Alex Alderton, Roberto Amato, Sonia Goncalves, Ewan Harrison, David K. Jackson, Ian Johnston, Dominic Kwiatkowski, Cordelia Langford, John Sillitoe on behalf of the Wellcome Sanger Institute COVID-19 Surveillance Team
EPI_ISL_948360	Lighthouse Lab in Glasgow	Wellcome Sanger Institute for the COVID-19 Genomics UK (COG-UK) Consortium	Harper VanSteenhouse, Yumi Kasai, David Gray, Carol Clugston, Anna Dominiczak and Alex Alderton, Roberto Amato, Sonia Goncalves, Ewan Harrison, David K. Jackson, Ian Johnston, Dominic Kwiatkowski, Cordelia Langford, John Sillitoe on behalf of the Wellcome Sanger Institute COVID-19 Surveillance Team
EPI_ISL_948361	Lighthouse Lab in Cambridge	Wellcome Sanger Institute for the COVID-19 Genomics UK (COG-UK) Consortium	Rob Howes, The Lighthouse Lab in Cambridge and Alex Alderton, Roberto Amato, Sonia Goncalves, Ewan Harrison, David K. Jackson, Ian Johnston, Dominic Kwiatkowski, Cordelia Langford, John Sillitoe on behalf of the Wellcome Sanger Institute COVID-19 Surveillance Team
EPI_ISL_948362, EPI_ISL_948363	Lighthouse Lab in Glasgow	Wellcome Sanger Institute for the COVID-19 Genomics UK (COG-UK) Consortium	Harper VanSteenhouse, Yumi Kasai, David Gray, Carol Clugston, Anna Dominiczak and Alex Alderton, Roberto Amato, Sonia Goncalves, Ewan Harrison, David K. Jackson, Ian Johnston, Dominic Kwiatkowski, Cordelia Langford, John Sillitoe on behalf of the Wellcome Sanger Institute COVID-19 Surveillance Team
EPI_ISL_948364	Lighthouse Lab in Cambridge	Wellcome Sanger Institute for the COVID-19 Genomics UK (COG-UK) Consortium	Rob Howes, The Lighthouse Lab in Cambridge and Alex Alderton, Roberto Amato, Sonia Goncalves, Ewan Harrison, David K. Jackson, Ian Johnston, Dominic Kwiatkowski, Cordelia Langford, John Sillitoe on behalf of the Wellcome Sanger Institute COVID-19 Surveillance Team
EPI_ISL_948365	Lighthouse Lab in Glasgow	Wellcome Sanger Institute for the COVID-19 Genomics UK (COG-UK) Consortium	Harper VanSteenhouse, Yumi Kasai, David Gray, Carol Clugston, Anna Dominiczak and Alex Alderton, Roberto Amato, Sonia Goncalves, Ewan Harrison, David K. Jackson, Ian Johnston, Dominic Kwiatkowski, Cordelia Langford, John Sillitoe on behalf of the Wellcome Sanger Institute COVID-19 Surveillance Team
EPI_ISL_948366	Lighthouse Lab in Cambridge	Wellcome Sanger Institute for the COVID-19 Genomics UK (COG-UK) Consortium	Rob Howes, The Lighthouse Lab in Cambridge and Alex Alderton, Roberto Amato, Sonia Goncalves, Ewan Harrison, David K. Jackson, Ian Johnston, Dominic Kwiatkowski, Cordelia Langford, John Sillitoe on behalf of the Wellcome Sanger Institute COVID-19 Surveillance Team
EPI_ISL_948367, EPI_ISL_948369, EPI_ISL_948370, EPI_ISL_948371	Lighthouse Lab in Glasgow	Wellcome Sanger Institute for the COVID-19 Genomics UK (COG-UK) Consortium	Harper VanSteenhouse, Yumi Kasai, David Gray, Carol Clugston, Anna Dominiczak and Alex Alderton, Roberto Amato, Sonia Goncalves, Ewan Harrison, David K. Jackson, Ian Johnston, Dominic Kwiatkowski, Cordelia Langford, John Sillitoe on behalf of the Wellcome Sanger Institute COVID-19 Surveillance Team
EPI_ISL_948372	Lighthouse Lab in Cambridge	Wellcome Sanger Institute for the COVID-19 Genomics UK (COG-UK) Consortium	Rob Howes, The Lighthouse Lab in Cambridge and Alex Alderton, Roberto Amato, Sonia Goncalves, Ewan Harrison, David K. Jackson, Ian Johnston, Dominic Kwiatkowski, Cordelia Langford, John Sillitoe on behalf of the Wellcome Sanger Institute COVID-19 Surveillance Team
EPI_ISL_948373, EPI_ISL_948375	Lighthouse Lab in Glasgow	Wellcome Sanger Institute for the COVID-19 Genomics UK (COG-UK) Consortium	Harper VanSteenhouse, Yumi Kasai, David Gray, Carol Clugston, Anna Dominiczak and Alex Alderton, Roberto Amato, Sonia Goncalves, Ewan Harrison, David K. Jackson, Ian Johnston, Dominic Kwiatkowski, Cordelia Langford, John Sillitoe on behalf of the Wellcome Sanger Institute COVID-19 Surveillance Team
EPI_ISL_948378	Lighthouse Lab in Cambridge	Wellcome Sanger Institute for the COVID-19 Genomics UK (COG-UK) Consortium	Rob Howes, The Lighthouse Lab in Cambridge and Alex Alderton, Roberto Amato, Sonia Goncalves, Ewan Harrison, David K. Jackson, Ian Johnston, Dominic Kwiatkowski, Cordelia Langford, John Sillitoe on behalf of the Wellcome Sanger Institute COVID-19 Surveillance Team
EPI_ISL_948379	Lighthouse Lab in Glasgow	Wellcome Sanger Institute for the COVID-19 Genomics UK (COG-UK) Consortium	Harper VanSteenhouse, Yumi Kasai, David Gray, Carol Clugston, Anna Dominiczak and Alex Alderton, Roberto Amato, Sonia Goncalves, Ewan Harrison, David K. Jackson, Ian Johnston, Dominic Kwiatkowski, Cordelia Langford, John Sillitoe on behalf of the Wellcome Sanger Institute COVID-19 Surveillance Team
EPI_ISL_948380, EPI_ISL_948381, EPI_ISL_948382, EPI_ISL_948383	Lighthouse Lab in Cambridge	Wellcome Sanger Institute for the COVID-19 Genomics UK (COG-UK) Consortium	Rob Howes, The Lighthouse Lab in Cambridge and Alex Alderton, Roberto Amato, Sonia Goncalves, Ewan Harrison, David K. Jackson, Ian Johnston, Dominic Kwiatkowski, Cordelia Langford, John Sillitoe on behalf of the Wellcome Sanger Institute COVID-19 Surveillance Team
EPI_ISL_948384, EPI_ISL_948385, EPI_ISL_948386, EPI_ISL_948387, EPI_ISL_948389, EPI_ISL_948390	Lighthouse Lab in Glasgow	Wellcome Sanger Institute for the COVID-19 Genomics UK (COG-UK) Consortium	Harper VanSteenhouse, Yumi Kasai, David Gray, Carol Clugston, Anna Dominiczak and Alex Alderton, Roberto Amato, Sonia Goncalves, Ewan Harrison, David K. Jackson, Ian Johnston, Dominic Kwiatkowski, Cordelia Langford, John Sillitoe on behalf of the Wellcome Sanger Institute COVID-19 Surveillance Team
EPI_ISL_948391, EPI_ISL_948392, EPI_ISL_948393	Lighthouse Lab in Cambridge	Wellcome Sanger Institute for the COVID-19 Genomics UK (COG-UK) Consortium	Rob Howes, The Lighthouse Lab in Cambridge and Alex Alderton, Roberto Amato, Sonia Goncalves, Ewan Harrison, David K. Jackson, Ian Johnston, Dominic Kwiatkowski, Cordelia Langford, John Sillitoe on behalf of the Wellcome Sanger Institute COVID-19 Surveillance Team
EPI_ISL_948394	Lighthouse Lab in Glasgow	Wellcome Sanger Institute for the COVID-19 Genomics UK (COG-UK) Consortium	Harper VanSteenhouse, Yumi Kasai, David Gray, Carol Clugston, Anna Dominiczak and Alex Alderton, Roberto Amato, Sonia Goncalves, Ewan Harrison, David K. Jackson, Ian Johnston, Dominic Kwiatkowski, Cordelia Langford, John Sillitoe on behalf of the Wellcome Sanger Institute COVID-19 Surveillance Team
EPI_ISL_948395, EPI_ISL_948396, EPI_ISL_948397	Lighthouse Lab in Cambridge	Wellcome Sanger Institute for the COVID-19 Genomics UK (COG-UK) Consortium	Rob Howes, The Lighthouse Lab in Cambridge and Alex Alderton, Roberto Amato, Sonia Goncalves, Ewan Harrison, David K. Jackson, Ian Johnston, Dominic Kwiatkowski, Cordelia Langford, John Sillitoe on behalf of the Wellcome Sanger Institute COVID-19 Surveillance Team
EPI_ISL_948398, EPI_ISL_948399, EPI_ISL_948400, EPI_ISL_948401	Lighthouse Lab in Glasgow	Wellcome Sanger Institute for the COVID-19 Genomics UK (COG-UK) Consortium	Harper VanSteenhouse, Yumi Kasai, David Gray, Carol Clugston, Anna Dominiczak and Alex Alderton, Roberto Amato, Sonia Goncalves, Ewan Harrison, David K. Jackson, Ian Johnston, Dominic Kwiatkowski, Cordelia Langford, John Sillitoe on behalf of the Wellcome Sanger Institute COVID-19 Surveillance Team
EPI_ISL_948402	Lighthouse Lab in Cambridge	Wellcome Sanger Institute for the COVID-19 Genomics UK (COG-UK) Consortium	Rob Howes, The Lighthouse Lab in Cambridge and Alex Alderton, Roberto Amato, Sonia Goncalves, Ewan Harrison, David K. Jackson, Ian Johnston, Dominic Kwiatkowski, Cordelia Langford, John Sillitoe on behalf of the Wellcome Sanger Institute COVID-19 Surveillance Team
EPI_ISL_948403, EPI_ISL_948404, EPI_ISL_948405, EPI_ISL_948406	Lighthouse Lab in Glasgow	Wellcome Sanger Institute for the COVID-19 Genomics UK (COG-UK) Consortium	Harper VanSteenhouse, Yumi Kasai, David Gray, Carol Clugston, Anna Dominiczak and Alex Alderton, Roberto Amato, Sonia Goncalves, Ewan Harrison, David K. Jackson, Ian Johnston, Dominic Kwiatkowski, Cordelia Langford, John Sillitoe on behalf of the Wellcome Sanger Institute COVID-19 Surveillance Team
EPI_ISL_948408	Lighthouse Lab in Cambridge	Wellcome Sanger Institute for the COVID-19 Genomics UK (COG-UK) Consortium	Rob Howes, The Lighthouse Lab in Cambridge and Alex Alderton, Roberto Amato, Sonia Goncalves, Ewan Harrison, David K. Jackson, Ian Johnston, Dominic Kwiatkowski, Cordelia Langford, John Sillitoe on behalf of the Wellcome Sanger Institute COVID-19 Surveillance Team
EPI_ISL_948409, EPI_ISL_948410, EPI_ISL_948411	Lighthouse Lab in Glasgow	Wellcome Sanger Institute for the COVID-19 Genomics UK (COG-UK) Consortium	Harper VanSteenhouse, Yumi Kasai, David Gray, Carol Clugston, Anna Dominiczak and Alex Alderton, Roberto Amato, Sonia Goncalves, Ewan Harrison, David K. Jackson, Ian Johnston, Dominic Kwiatkowski, Cordelia Langford, John Sillitoe on behalf of the Wellcome Sanger Institute COVID-19 Surveillance Team
EPI_ISL_948412, EPI_ISL_948413, EPI_ISL_948414	Lighthouse Lab in Cambridge	Wellcome Sanger Institute for the COVID-19 Genomics UK (COG-UK) Consortium	Rob Howes, The Lighthouse Lab in Cambridge and Alex Alderton, Roberto Amato, Sonia Goncalves, Ewan Harrison, David K. Jackson, Ian Johnston, Dominic Kwiatkowski, Cordelia Langford, John Sillitoe on behalf of the Wellcome Sanger Institute COVID-19 Surveillance Team
EPI_ISL_948415, EPI_ISL_948416, EPI_ISL_948417, EPI_ISL_948418, EPI_ISL_948419	Lighthouse Lab in Glasgow	Wellcome Sanger Institute for the COVID-19 Genomics UK (COG-UK) Consortium	Harper VanSteenhouse, Yumi Kasai, David Gray, Carol Clugston, Anna Dominiczak and Alex Alderton, Roberto Amato, Sonia Goncalves, Ewan Harrison, David K. Jackson, Ian Johnston, Dominic Kwiatkowski, Cordelia Langford, John Sillitoe on behalf of the Wellcome Sanger Institute COVID-19 Surveillance Team
EPI_ISL_948420, EPI_ISL_948421, EPI_ISL_948422, EPI_ISL_948423, EPI_ISL_948424	Lighthouse Lab in Cambridge	Wellcome Sanger Institute for the COVID-19 Genomics UK (COG-UK) Consortium	Rob Howes, The Lighthouse Lab in Cambridge and Alex Alderton, Roberto Amato, Sonia Goncalves, Ewan Harrison, David K. Jackson, Ian Johnston, Dominic Kwiatkowski, Cordelia Langford, John Sillitoe on behalf of the Wellcome Sanger Institute COVID-19 Surveillance Team
EPI_ISL_948425, EPI_ISL_948426, EPI_ISL_948427, EPI_ISL_948428, EPI_ISL_948429, EPI_ISL_948430	Lighthouse Lab in Glasgow	Wellcome Sanger Institute for the COVID-19 Genomics UK (COG-UK) Consortium	Harper VanSteenhouse, Yumi Kasai, David Gray, Carol Clugston, Anna Dominiczak and Alex Alderton, Roberto Amato, Sonia Goncalves, Ewan Harrison, David K. Jackson, Ian Johnston, Dominic Kwiatkowski, Cordelia Langford, John Sillitoe on behalf of the Wellcome Sanger Institute COVID-19 Surveillance Team
EPI_ISL_948431, EPI_ISL_948433, EPI_ISL_948434, EPI_ISL_948435	Lighthouse Lab in Cambridge	Wellcome Sanger Institute for the COVID-19 Genomics UK (COG-UK) Consortium	Rob Howes, The Lighthouse Lab in Cambridge and Alex Alderton, Roberto Amato, Sonia Goncalves, Ewan Harrison, David K. Jackson, Ian Johnston, Dominic Kwiatkowski, Cordelia Langford, John Sillitoe on behalf of the Wellcome Sanger Institute COVID-19 Surveillance Team
EPI_ISL_948436	Lighthouse Lab in Glasgow	Wellcome Sanger Institute for the COVID-19 Genomics UK (COG-UK) Consortium	Harper VanSteenhouse, Yumi Kasai, David Gray, Carol Clugston, Anna Dominiczak and Alex Alderton, Roberto Amato, Sonia Goncalves, Ewan Harrison, David K. Jackson, Ian Johnston, Dominic Kwiatkowski, Cordelia Langford, John Sillitoe on behalf of the Wellcome Sanger Institute COVID-19 Surveillance

			Team
EPI_ISL_948437	Lighthouse Lab in Cambridge	Wellcome Sanger Institute for the COVID-19 Genomics UK (COG-UK) Consortium	Rob Howes, The Lighthouse Lab in Cambridge and Alex Alderton, Roberto Amato, Sonia Goncalves, Ewan Harrison, David K. Jackson, Ian Johnston, Dominic Kwiatkowski, Cordelia Langford, John Sillitoe on behalf of the Wellcome Sanger Institute COVID-19 Surveillance Team
EPI_ISL_948438, EPI_ISL_948439, EPI_ISL_948440	Lighthouse Lab in Glasgow	Wellcome Sanger Institute for the COVID-19 Genomics UK (COG-UK) Consortium	Harper VanSteenhouse, Yumi Kasai, David Gray, Carol Clugston, Anna Dominiczak and Alex Alderton, Roberto Amato, Sonia Goncalves, Ewan Harrison, David K. Jackson, Ian Johnston, Dominic Kwiatkowski, Cordelia Langford, John Sillitoe on behalf of the Wellcome Sanger Institute COVID-19 Surveillance Team
EPI_ISL_948441, EPI_ISL_948442	Lighthouse Lab in Cambridge	Wellcome Sanger Institute for the COVID-19 Genomics UK (COG-UK) Consortium	Rob Howes, The Lighthouse Lab in Cambridge and Alex Alderton, Roberto Amato, Sonia Goncalves, Ewan Harrison, David K. Jackson, Ian Johnston, Dominic Kwiatkowski, Cordelia Langford, John Sillitoe on behalf of the Wellcome Sanger Institute COVID-19 Surveillance Team
EPI_ISL_948443, EPI_ISL_948444	Lighthouse Lab in Glasgow	Wellcome Sanger Institute for the COVID-19 Genomics UK (COG-UK) Consortium	Harper VanSteenhouse, Yumi Kasai, David Gray, Carol Clugston, Anna Dominiczak and Alex Alderton, Roberto Amato, Sonia Goncalves, Ewan Harrison, David K. Jackson, Ian Johnston, Dominic Kwiatkowski, Cordelia Langford, John Sillitoe on behalf of the Wellcome Sanger Institute COVID-19 Surveillance Team
EPI_ISL_948445	Lighthouse Lab in Cambridge	Wellcome Sanger Institute for the COVID-19 Genomics UK (COG-UK) Consortium	Rob Howes, The Lighthouse Lab in Cambridge and Alex Alderton, Roberto Amato, Sonia Goncalves, Ewan Harrison, David K. Jackson, Ian Johnston, Dominic Kwiatkowski, Cordelia Langford, John Sillitoe on behalf of the Wellcome Sanger Institute COVID-19 Surveillance Team
EPI_ISL_948446, EPI_ISL_948447, EPI_ISL_948448, EPI_ISL_948787, EPI_ISL_948788, EPI_ISL_948789, EPI_ISL_948790, EPI_ISL_948791, EPI_ISL_948792, EPI_ISL_948793, EPI_ISL_948794, EPI_ISL_948795, EPI_ISL_948796, EPI_ISL_948797, EPI_ISL_948798, EPI_ISL_948799, EPI_ISL_948800, EPI_ISL_948801, EPI_ISL_948802, EPI_ISL_948803, EPI_ISL_948804, EPI_ISL_948805, EPI_ISL_948806, EPI_ISL_948807, EPI_ISL_948808, EPI_ISL_948809, EPI_ISL_948810, EPI_ISL_948811, EPI_ISL_948812, EPI_ISL_948813, EPI_ISL_948814, EPI_ISL_948815, EPI_ISL_948816, EPI_ISL_948817, EPI_ISL_948818, EPI_ISL_948819, EPI_ISL_948820, EPI_ISL_948821, EPI_ISL_948822, EPI_ISL_948823, EPI_ISL_948824, EPI_ISL_948825, EPI_ISL_948826, EPI_ISL_948827, EPI_ISL_948828, EPI_ISL_948831, EPI_ISL_948832, EPI_ISL_948833, EPI_ISL_948835, EPI_ISL_948836, EPI_ISL_948837, EPI_ISL_948838, EPI_ISL_948839, EPI_ISL_948840, EPI_ISL_948841, EPI_ISL_948842, EPI_ISL_948843, EPI_ISL_948844, EPI_ISL_948846, EPI_ISL_948847, EPI_ISL_948849, EPI_ISL_948850, EPI_ISL_948851, EPI_ISL_948852, EPI_ISL_948853, EPI_ISL_948854, EPI_ISL_948855, EPI_ISL_948856, EPI_ISL_948857, EPI_ISL_948858, EPI_ISL_948859, EPI_ISL_948860, EPI_ISL_948861, EPI_ISL_948862, EPI_ISL_948863, EPI_ISL_948864, EPI_ISL_948865, EPI_ISL_948866, EPI_ISL_948867, EPI_ISL_948868, EPI_ISL_948869, EPI_ISL_948870, EPI_ISL_948871, EPI_ISL_948872, EPI_ISL_948873, EPI_ISL_948874, EPI_ISL_948875, EPI_ISL_948876, EPI_ISL_948877, EPI_ISL_948878, EPI_ISL_948879, EPI_ISL_948880, EPI_ISL_948881, EPI_ISL_948882, EPI_ISL_948883, EPI_ISL_948884, EPI_ISL_948885, EPI_ISL_948886, EPI_ISL_948887, EPI_ISL_948888, EPI_ISL_948889, EPI_ISL_948890, EPI_ISL_948891, EPI_ISL_948892, EPI_ISL_948893, EPI_ISL_948894, EPI_ISL_948895, EPI_ISL_948896, EPI_ISL_948897, EPI_ISL_948898, EPI_ISL_948899, EPI_ISL_948900, EPI_ISL_948901, EPI_ISL_948902, EPI_ISL_948903, EPI_ISL_948904, EPI_ISL_948905, EPI_ISL_948906, EPI_ISL_948907, EPI_ISL_948908, EPI_ISL_948909, EPI_ISL_948910, EPI_ISL_948911, EPI_ISL_948912, EPI_ISL_948913, EPI_ISL_948914, EPI_ISL_948915, EPI_ISL_948916, EPI_ISL_948917, EPI_ISL_948918, EPI_ISL_948919, EPI_ISL_948920, EPI_ISL_948921, EPI_ISL_948922, EPI_ISL_948923, EPI_ISL_948924, EPI_ISL_948925, EPI_ISL_948926, EPI_ISL_948927, EPI_ISL_948928, EPI_ISL_948929, EPI_ISL_948930, EPI_ISL_948931, EPI_ISL_948932, EPI_ISL_948933, EPI_ISL_948934, EPI_ISL_948935, EPI_ISL_948936, EPI_ISL_948937, EPI_ISL_948938, EPI_ISL_948939, EPI_ISL_948940, EPI_ISL_948941, EPI_ISL_948942, EPI_ISL_948943, EPI_ISL_948944			
see above	Lighthouse Lab in Glasgow	Wellcome Sanger Institute for the COVID-19 Genomics UK (COG-UK) Consortium	Harper VanSteenhouse, Yumi Kasai, David Gray, Carol Clugston, Anna Dominiczak and Alex Alderton, Roberto Amato, Sonia Goncalves, Ewan Harrison, David K. Jackson, Ian Johnston, Dominic Kwiatkowski, Cordelia Langford, John Sillitoe on behalf of the Wellcome Sanger Institute COVID-19 Surveillance Team
EPI_ISL_949138, EPI_ISL_949139, EPI_ISL_949158, EPI_ISL_949159, EPI_ISL_949160, EPI_ISL_949161, EPI_ISL_949162, EPI_ISL_949163, EPI_ISL_949164, EPI_ISL_949166, EPI_ISL_949167, EPI_ISL_949168, EPI_ISL_949169, EPI_ISL_949170, EPI_ISL_949171, EPI_ISL_949172, EPI_ISL_949173, EPI_ISL_949174, EPI_ISL_949175			
see above	Jessa	Jessa	Jessa_cmdLab
EPI_ISL_949181	Laboratory of Virology and Molecular Diagnostics	Institute of Public Health of Republic of North Macedonia Laboratory of Virology and Molecular Diagnostics	M. Kuzmanovska, G. Boshevska
EPI_ISL_949184	University of Bari Biomedical Sciences and Human Oncology	University of Bari Biomedical Sciences and Human Oncology	Chironna M., Sallustio A., Loconsole D., Accogli A.
EPI_ISL_949185, EPI_ISL_949191	University of Bari Biomedical Sciences and Human Oncology	University of Bari Biomedical Sciences and Human Oncology	Chironna M., Sallustio A., Loconsole D., Accogli M.
EPI_ISL_949410	University of Birmingham	COVID-19 Genomics UK (COG-UK) Consortium	Institute of Microbiology, University of Birmingham: Claire McMurray, Joanne Stockton, Samuel Nicholls, Radoslaw Poplawski, Will Rowe, Josh Quick, Nicholas Loman. University of Birmingham Testing Laboratory: Celina M Whalley, Andrew Bosworth, Charlotte Poxon, Kasun Wanigasooriya, Oliver Pickles, Mike Kidd, Alex Richter, Andrew D Beggs PHE Heartlands Lab: Husam Osman, Andrew Bosworth. Queen Elizabeth Hospital: Anna Casey
EPI_ISL_949462, EPI_ISL_949463, EPI_ISL_949464, EPI_ISL_949465, EPI_ISL_949467, EPI_ISL_949468, EPI_ISL_949469, EPI_ISL_949473, EPI_ISL_949475, EPI_ISL_949476, EPI_ISL_949477, EPI_ISL_949524, EPI_ISL_949525, EPI_ISL_949526, EPI_ISL_949527, EPI_ISL_949528, EPI_ISL_949529, EPI_ISL_949530, EPI_ISL_949531, EPI_ISL_949532, EPI_ISL_949533, EPI_ISL_949534, EPI_ISL_949535, EPI_ISL_949536, EPI_ISL_949537, EPI_ISL_949538, EPI_ISL_949539, EPI_ISL_949540, EPI_ISL_949542, EPI_ISL_949544, EPI_ISL_949546, EPI_ISL_949548, EPI_ISL_949549, EPI_ISL_949550, EPI_ISL_949553, EPI_ISL_949554, EPI_ISL_949555, EPI_ISL_949556			
see above	Department of Pathology, University of Cambridge	COVID-19 Genomics UK (COG-UK) Consortium	Aminu S. Jahun, Yasmin Chaudhry, Iliana Georgana, Myra Hosmillo, Rhys Izu, Martin D. Curran, Surendra Parmar, Ian Goodfellow
EPI_ISL_949611, EPI_ISL_949615	West of Scotland Specialist Virology Centre, NHSGGC / MRC-University of Glasgow Centre for Virus Research	COVID-19 Genomics UK (COG-UK) Consortium	Ana da Silva Filipe, Natasha Johnson, Kathy Smollett, Daniel Mair, Stephen Carmichael, Alice Broos, Lily Tong, Jenna Nichols, Kyriaki Nomikou; Sarah McDonald; Richard Orton, Joseph Hughes, Sreenu Vattipally, David L Robertson; Alasdair MacLean, Rory Gunson; Sharif Shaaban, Matthew Holden; Rachel Blacow, Guy Mollett, Kathy Li, James Shepherd, Antonia Ho, Emma Thomson
EPI_ISL_949645, EPI_ISL_949646	Virology Department, Royal Infirmary of Edinburgh, NHS Lothian / School of Biological Sciences, University of Edinburgh / Institute of Genetics and Molecular Medicine, University of Edinburgh	COVID-19 Genomics UK (COG-UK) Consortium	McHugh M, Dewar R, Rooke S, Gallagher M, Balcaza C, O'Toole Á, Scher E, Hill V, McCrone JT, Colquhoun R, Yu X, Jackson B, Rambaut A, Williams TC, Templeton K
EPI_ISL_949655, EPI_ISL_949656, EPI_ISL_949657, EPI_ISL_949658, EPI_ISL_949659, EPI_ISL_949660, EPI_ISL_949661, EPI_ISL_949665, EPI_ISL_949667, EPI_ISL_949673, EPI_ISL_949674, EPI_ISL_949675, EPI_ISL_949676, EPI_ISL_949677, EPI_ISL_949678, EPI_ISL_949679, EPI_ISL_949680, EPI_ISL_949681, EPI_ISL_949682, EPI_ISL_949683, EPI_ISL_949688, EPI_ISL_949689, EPI_ISL_949690, EPI_ISL_949691, EPI_ISL_949692, EPI_ISL_949693, EPI_ISL_949694, EPI_ISL_949695, EPI_ISL_949696, EPI_ISL_949697, EPI_ISL_949698, EPI_ISL_949699, EPI_ISL_949700, EPI_ISL_949701, EPI_ISL_949702, EPI_ISL_949703, EPI_ISL_949704, EPI_ISL_949705, EPI_ISL_949706, EPI_ISL_949707			
see above	Liverpool Clinical Laboratories	COVID-19 Genomics UK (COG-UK) Consortium	Sam Haldenby, Anita Lucaci, Steve Paterson, Julian Hiscox, Alistair Darby, M Almsaud, A Alrezaihi, Muhannad Alruwaili, Stuart D Armstrong, Jones Benjamin, Eleanor G Bentley, Anu Chawla, Jordan J Clark, Angela Cowell, Richard Eccles, Isabel García-Dorival, Matthew Gemmell, Alessandro Gerada, PKF Gilmore, Richard Gregory, Ximeng Han, Catherine Hartley, Margaret Hughes, Miren Iturriza-Gomara, James Johnson, L Luu, Jenifer Manson, Charlotte Nelson, Elaine O'Toole, Cassie Olateju, Rebekah Penrice-Randal , Lucille Rainbow, N.P Randle, Trevor Ian Robinson, Parul Sharma, Ghada T Shawli, James P Stewart, Neil Swainston, Ecaterina Vamos, Joanne Watts, Mark Whitehead
EPI_ISL_949793, EPI_ISL_949794, EPI_ISL_949802, EPI_ISL_949909, EPI_ISL_949910, EPI_ISL_949911, EPI_ISL_949912, EPI_ISL_949913, EPI_ISL_949914, EPI_ISL_949917, EPI_ISL_949919, EPI_ISL_949948, EPI_ISL_950019, EPI_ISL_950028, EPI_ISL_950034, EPI_ISL_950039, EPI_ISL_950040, EPI_ISL_950041, EPI_ISL_950042, EPI_ISL_950043, EPI_ISL_950044, EPI_ISL_950045, EPI_ISL_950046, EPI_ISL_950047, EPI_ISL_950049, EPI_ISL_950050, EPI_ISL_950051, EPI_ISL_950052, EPI_ISL_950053, EPI_ISL_950054, EPI_ISL_950055, EPI_ISL_950056, EPI_ISL_950057, EPI_ISL_950058, EPI_ISL_950059, EPI_ISL_950060, EPI_ISL_950061, EPI_ISL_950062, EPI_ISL_950063, EPI_ISL_950064, EPI_ISL_950065, EPI_ISL_950066, EPI_ISL_950067, EPI_ISL_950171, EPI_ISL_950172, EPI_ISL_950174, EPI_ISL_950175, EPI_ISL_950176, EPI_ISL_950178, EPI_ISL_950179, EPI_ISL_950180, EPI_ISL_950181, EPI_ISL_950182, EPI_ISL_950183, EPI_ISL_950184, EPI_ISL_950185, EPI_ISL_950186, EPI_ISL_950187, EPI_ISL_950188, EPI_ISL_950189, EPI_ISL_950190, EPI_ISL_950191, EPI_ISL_950192, EPI_ISL_950193, EPI_ISL_950194, EPI_ISL_950196, EPI_ISL_950199			
see above	University College London, Great Ormond Street Hospital for Children NHS Foundation Trust, Imperial College Healthcare NHS Trust	COVID-19 Genomics UK (COG-UK) Consortium	Sergi Castellano, Rachel Williams, Mark Kristiansen, Paola Resende Silva, Sunando Roy, Tony Brooks, Helena Tutill, Paola Niola, Patricia Dyal, Charlotte Williams, Leysa Forrest, Yasmin Panchbhaya, Jacqueline Findlay, Samuel Weeks, Julianne Brown, Kathryn Harris, Paul Randell, James Price, Alison Holmes, Judith Breuer
EPI_ISL_950373, EPI_ISL_950376, EPI_ISL_950382, EPI_ISL_950391, EPI_ISL_950394, EPI_ISL_950396, EPI_ISL_950397, EPI_ISL_950399, EPI_ISL_950523, EPI_ISL_950525, EPI_ISL_950526, EPI_ISL_950527, EPI_ISL_950529, EPI_ISL_950531, EPI_ISL_950532, EPI_ISL_950533, EPI_ISL_950534, EPI_ISL_950535, EPI_ISL_950536, EPI_ISL_950537, EPI_ISL_950538, EPI_ISL_950539, EPI_ISL_950540, EPI_ISL_950541, EPI_ISL_950542, EPI_ISL_950543			
see above	Northumbria University / South Tees Hospitals NHS Foundation Trust / North Cumbria Integrated Care NHS Foundation Trust / North Tees and Hartlepool NHS Foundation Trust / Newcastle Hospitals NHS Foundation Trust	COVID-19 Genomics UK (COG-UK) Consortium	Darren L Smith,Andrew Nelson,Matthew Bashton,Greg R Young,Joshua Loh,John Allan,Mohammad A Tariq,Giles S Holt,Gary Black,Wen C Yew,Lynn Dover,Paul Baker,Steve Liggett,Sarah Essex,Jane Greenaway,Debra Padgett,Clive Graham,Garren Scott,Edward Barton,Emma Swindells,Brendan Payne,Jennifer Collins,Yusri Taha,Gary Eltringham
EPI_ISL_950639, EPI_ISL_950640, EPI_ISL_950641, EPI_ISL_950642, EPI_ISL_950643, EPI_ISL_950644, EPI_ISL_950645	Queens Medical Centre, Clinical Microbiology Department / DeepSeq Nottingham	COVID-19 Genomics UK (COG-UK) Consortium	Gemma Clark, Wendy Smith, Manjinder Khakh, Vicki M Fleming, Michelle M Lister, Hannah Howson-Wells, Jonathan Ball, Patrick McClure, Joseph Chappell, Theocharis Tsoleridis, Nadine Holmes, Matthew Carlisle, Christopher Moore, Fei Sang, Johnny Debebe, Victoria Wright, Matthew Loose
EPI_ISL_950755, EPI_ISL_950756, EPI_ISL_950757, EPI_ISL_950758, EPI_ISL_950759, EPI_ISL_950760, EPI_ISL_950761	Lincolnshire Hospitals and DeepSeq Nottingham	COVID-19 Genomics UK (COG-UK) Consortium	Nichola Duckworth, Tim Sloan, Sarah Walsh, Jonathan Ball, Patrick McClure, Joeseph Chappell, Nadine Holmes, Matthew Carlisle, Christopher Moore, Fei Sang, Johnny Debebe, Victoria Wright, Matthew Loose

EPI_ISL_951492, EPI_ISL_951493, EPI_ISL_951494, EPI_ISL_951495, EPI_ISL_951496, EPI_ISL_951497, EPI_ISL_951498, EPI_ISL_951499, EPI_ISL_951501, EPI_ISL_951502, EPI_ISL_951503, EPI_ISL_951504, EPI_ISL_951505, EPI_ISL_951506, EPI_ISL_951508, EPI_ISL_951512, EPI_ISL_951513, EPI_ISL_951514, EPI_ISL_951515, EPI_ISL_951517, EPI_ISL_951518, EPI_ISL_951519, EPI_ISL_951521, EPI_ISL_951522, EPI_ISL_951523, EPI_ISL_951524, EPI_ISL_951525, EPI_ISL_951527, EPI_ISL_951528, EPI_ISL_951529, EPI_ISL_951530, EPI_ISL_951531, EPI_ISL_951532, EPI_ISL_951533, EPI_ISL_951534, EPI_ISL_951535, EPI_ISL_951536, EPI_ISL_951537, EPI_ISL_951538, EPI_ISL_951541, EPI_ISL_951542, EPI_ISL_951543, EPI_ISL_951544, EPI_ISL_951545, EPI_ISL_951546, EPI_ISL_951547, EPI_ISL_951548, EPI_ISL_951549, EPI_ISL_951550, EPI_ISL_951552, EPI_ISL_951553, EPI_ISL_951554, EPI_ISL_951556, EPI_ISL_951557, EPI_ISL_951558, EPI_ISL_951559, EPI_ISL_951560, EPI_ISL_951561, EPI_ISL_951562, EPI_ISL_951564, EPI_ISL_951567, EPI_ISL_951569, EPI_ISL_951570, EPI_ISL_951571, EPI_ISL_951572, EPI_ISL_951573, EPI_ISL_951574, EPI_ISL_951575, EPI_ISL_951576, EPI_ISL_951577, EPI_ISL_951578, EPI_ISL_951579, EPI_ISL_951580, EPI_ISL_951581, EPI_ISL_951582, EPI_ISL_951583, EPI_ISL_951585, EPI_ISL_951738, EPI_ISL_951739, EPI_ISL_951742, EPI_ISL_951744, EPI_ISL_951745, EPI_ISL_951753, EPI_ISL_951758, EPI_ISL_951759, EPI_ISL_951760, EPI_ISL_951761, EPI_ISL_951765, EPI_ISL_951766, EPI_ISL_951767, EPI_ISL_951769, EPI_ISL_951778, EPI_ISL_951779, EPI_ISL_951780, EPI_ISL_951781, EPI_ISL_951782, EPI_ISL_951785, EPI_ISL_951786, EPI_ISL_951787, EPI_ISL_951820, EPI_ISL_951821, EPI_ISL_951843, EPI_ISL_951844, EPI_ISL_951851, EPI_ISL_951852, EPI_ISL_951853, EPI_ISL_951855, EPI_ISL_951856, EPI_ISL_951857, EPI_ISL_951858, EPI_ISL_951859, EPI_ISL_951861, EPI_ISL_951862, EPI_ISL_951863, EPI_ISL_951866, EPI_ISL_951867, EPI_ISL_951869, EPI_ISL_951875, EPI_ISL_951876, EPI_ISL_951877, EPI_ISL_951878, EPI_ISL_951881, EPI_ISL_951882, EPI_ISL_951884, EPI_ISL_951886, EPI_ISL_951887, EPI_ISL_951888, EPI_ISL_951890, EPI_ISL_951893, EPI_ISL_951894, EPI_ISL_951895, EPI_ISL_951896, EPI_ISL_951897, EPI_ISL_951898, EPI_ISL_951900, EPI_ISL_951901, EPI_ISL_951902, EPI_ISL_951903, EPI_ISL_951904, EPI_ISL_951905, EPI_ISL_951906, EPI_ISL_951907, EPI_ISL_951908, EPI_ISL_951909, EPI_ISL_951910, EPI_ISL_951911, EPI_ISL_951912, EPI_ISL_951913, EPI_ISL_951916, EPI_ISL_951917, EPI_ISL_951918, EPI_ISL_951919, EPI_ISL_951920, EPI_ISL_951921, EPI_ISL_951922, EPI_ISL_951923, EPI_ISL_951924, EPI_ISL_951925, EPI_ISL_951926, EPI_ISL_951927, EPI_ISL_951928, EPI_ISL_951929, EPI_ISL_951930, EPI_ISL_951931, EPI_ISL_951932, EPI_ISL_951933, EPI_ISL_951934, EPI_ISL_951935, EPI_ISL_951936, EPI_ISL_951937, EPI_ISL_951938, EPI_ISL_951939, EPI_ISL_951940, EPI_ISL_951941, EPI_ISL_951942, EPI_ISL_951943, EPI_ISL_951944, EPI_ISL_951945, EPI_ISL_951946, EPI_ISL_951947, EPI_ISL_951948, EPI_ISL_951949, EPI_ISL_951950, EPI_ISL_951951, EPI_ISL_951952, EPI_ISL_951953, EPI_ISL_951954, EPI_ISL_951955, EPI_ISL_951956, EPI_ISL_951957, EPI_ISL_951958, EPI_ISL_951959, EPI_ISL_951960, EPI_ISL_951961, EPI_ISL_951962, EPI_ISL_951963, EPI_ISL_951964, EPI_ISL_951965, EPI_ISL_951966, EPI_ISL_951968, EPI_ISL_951969, EPI_ISL_951972, EPI_ISL_951973, EPI_ISL_952006, EPI_ISL_952007, EPI_ISL_952008, EPI_ISL_952009, EPI_ISL_952010, EPI_ISL_952011, EPI_ISL_952012, EPI_ISL_952013, EPI_ISL_952014, EPI_ISL_952015, EPI_ISL_952018, EPI_ISL_952019, EPI_ISL_952020, EPI_ISL_952022, EPI_ISL_952025, EPI_ISL_952027, EPI_ISL_952029, EPI_ISL_952030, EPI_ISL_952031, EPI_ISL_952032, EPI_ISL_952033, EPI_ISL_952035, EPI_ISL_952036, EPI_ISL_952037, EPI_ISL_952038, EPI_ISL_952039, EPI_ISL_952040, EPI_ISL_952041, EPI_ISL_952061, EPI_ISL_952062, EPI_ISL_952063, EPI_ISL_952064, EPI_ISL_952065, EPI_ISL_952066, EPI_ISL_952067, EPI_ISL_952069, EPI_ISL_952070, EPI_ISL_952071, EPI_ISL_952072, EPI_ISL_952073, EPI_ISL_952074, EPI_ISL_952075, EPI_ISL_952076, EPI_ISL_952077, EPI_ISL_952078, EPI_ISL_952079, EPI_ISL_952082, EPI_ISL_952083, EPI_ISL_952084, EPI_ISL_952085, EPI_ISL_952086, EPI_ISL_952087, EPI_ISL_952088, EPI_ISL_952089, EPI_ISL_952090, EPI_ISL_952091, EPI_ISL_952092, EPI_ISL_952093, EPI_ISL_952095, EPI_ISL_952096, EPI_ISL_952097, EPI_ISL_952100, EPI_ISL_952102, EPI_ISL_952104, EPI_ISL_952108, EPI_ISL_952109, EPI_ISL_952110, EPI_ISL_952111, EPI_ISL_952112, EPI_ISL_952113, EPI_ISL_952114, EPI_ISL_952119, EPI_ISL_952120, EPI_ISL_952122, EPI_ISL_952123, EPI_ISL_952124, EPI_ISL_952126, EPI_ISL_952128, EPI_ISL_952129, EPI_ISL_952130, EPI_ISL_952131, EPI_ISL_952132, EPI_ISL_952133, EPI_ISL_952134, EPI_ISL_952136, EPI_ISL_952137, EPI_ISL_952138, EPI_ISL_952139, EPI_ISL_952140, EPI_ISL_952141, EPI_ISL_952143, EPI_ISL_952144, EPI_ISL_952145, EPI_ISL_952146, EPI_ISL_952147, EPI_ISL_952148, EPI_ISL_952149, EPI_ISL_952150, EPI_ISL_952151, EPI_ISL_952152, EPI_ISL_952153, EPI_ISL_952154, EPI_ISL_952156, EPI_ISL_952157, EPI_ISL_952158, EPI_ISL_952159, EPI_ISL_952161, EPI_ISL_952162, EPI_ISL_952163, EPI_ISL_952164, EPI_ISL_952165, EPI_ISL_952166, EPI_ISL_952167			
see above	Originating lab: Wales Specialist Virology Centre Sequencing lab: Pathogen Genomics Unit	Public Health Wales Microbiology Cardiff Wales Specialist Virology Centre	Catherine Moore, Johnathan Evans, Laura Gifford, Malorie Perry, Simon Cottrell, Angela Marchbank, Alec Birchley, Alexander Adams, Amy Gaskin, Bree Gatica-Wilcox, Jason Coombes, Joel Southgate, Lauren Gilbert, Lee Graham, Nicole Pacchiarini, Sara Kumziene-Summerhayes, Sarah Taylor, Sophie Jones, Sara Rey, Matthew Bull, Joanne Watkins, Sally Corden, Tom Connor
EPI_ISL_952453, EPI_ISL_952454, EPI_ISL_952456, EPI_ISL_952460, EPI_ISL_952462, EPI_ISL_952464, EPI_ISL_952465, EPI_ISL_952466, EPI_ISL_952467, EPI_ISL_952469, EPI_ISL_952470, EPI_ISL_952477, EPI_ISL_952478, EPI_ISL_952479, EPI_ISL_952480, EPI_ISL_952481, EPI_ISL_952482, EPI_ISL_952483, EPI_ISL_952484, EPI_ISL_952485, EPI_ISL_952558			
see above	Centre for Enzyme Innovation, University of Portsmouth / Translational Research Laboratory, Portsmouth Hospitals NHS Trust	COVID-19 Genomics UK (COG-UK) Consortium	Angela Beckett,Salman Goudarzi,Christopher Fearn,Kate Cook,Katie Loveson,Sharon Glaysher,Scott Elliott,Samuel Robson
EPI_ISL_952953, EPI_ISL_952978	Virology Department, Sheffield Teaching Hospitals NHS Foundation Trust/Department of Infection, Immunity and Cardiovascular Disease, The Medical School, University of Sheffield	COVID-19 Genomics UK (COG-UK) Consortium	Thushan de Silva, Matthew Parker, Nikki Smith, Adri Angyal, Rebecca Brown, Luke Green, Rachel Tucker, Paul Parsons, Danielle Groves, Katie Johnson, Laura Carrilero, Alex Keeley, Dave Partridge, Matthew Wyles, Benjamin Lindsey, Mehmet Yavuz, Mohammad Raza, Cariad Evans
EPI_ISL_953241, EPI_ISL_953243, EPI_ISL_953244, EPI_ISL_953246, EPI_ISL_953247, EPI_ISL_953251, EPI_ISL_953254, EPI_ISL_953259, EPI_ISL_953261, EPI_ISL_953262, EPI_ISL_953263, EPI_ISL_953267, EPI_ISL_953272, EPI_ISL_953281			
see above	Bioinformatics and Biostatistics Lab, Advanced Sequencing Facility	COVID-19 Genomics UK (COG-UK) Consortium	Aengus Stewart,Jerome Nicod,Chelsea Sawyer,Laura Cubitt,Harshil Patel,Margaret Crawford
EPI_ISL_953426	Laboratory of Virology and Molecular Diagnostics	Institute of Public Health of Republic of North Macedonia Laboratory of Virology and Molecular Diagnostics	M. Kuzmanovska, G. Boshevska
EPI_ISL_953452	Jessa	Jessa	Jessa_cmdLab
EPI_ISL_953490, EPI_ISL_953491, EPI_ISL_953492, EPI_ISL_953494, EPI_ISL_953495, EPI_ISL_953496, EPI_ISL_953497, EPI_ISL_953500, EPI_ISL_953505, EPI_ISL_953506, EPI_ISL_953509, EPI_ISL_953510, EPI_ISL_953511, EPI_ISL_953514, EPI_ISL_953519, EPI_ISL_953520, EPI_ISL_953560, EPI_ISL_953561, EPI_ISL_953562, EPI_ISL_953563, EPI_ISL_953564, EPI_ISL_953565, EPI_ISL_953566, EPI_ISL_953567, EPI_ISL_953568, EPI_ISL_953569, EPI_ISL_953570, EPI_ISL_953571, EPI_ISL_953572, EPI_ISL_953573, EPI_ISL_953574, EPI_ISL_953575, EPI_ISL_953576, EPI_ISL_953577, EPI_ISL_953578, EPI_ISL_953579, EPI_ISL_953580, EPI_ISL_953581, EPI_ISL_953582, EPI_ISL_953583, EPI_ISL_953584, EPI_ISL_953585, EPI_ISL_953586, EPI_ISL_953587, EPI_ISL_953611, EPI_ISL_953612, EPI_ISL_953613, EPI_ISL_953614, EPI_ISL_953615, EPI_ISL_953616, EPI_ISL_953617, EPI_ISL_953618, EPI_ISL_953619, EPI_ISL_953620, EPI_ISL_953631, EPI_ISL_953634, EPI_ISL_953635, EPI_ISL_953642, EPI_ISL_953643, EPI_ISL_953644, EPI_ISL_953645, EPI_ISL_953646, EPI_ISL_953647, EPI_ISL_953648, EPI_ISL_953653, EPI_ISL_953658, EPI_ISL_953664, EPI_ISL_953854, EPI_ISL_953855, EPI_ISL_953856, EPI_ISL_953857, EPI_ISL_953858, EPI_ISL_953859, EPI_ISL_953860, EPI_ISL_953861, EPI_ISL_953862, EPI_ISL_953863, EPI_ISL_953864, EPI_ISL_953865, EPI_ISL_953866, EPI_ISL_953867, EPI_ISL_953868, EPI_ISL_953869, EPI_ISL_953870, EPI_ISL_953871, EPI_ISL_953872, EPI_ISL_953873, EPI_ISL_953874, EPI_ISL_953875, EPI_ISL_953876, EPI_ISL_953877, EPI_ISL_953878, EPI_ISL_953879, EPI_ISL_953880, EPI_ISL_953881, EPI_ISL_953882, EPI_ISL_953883, EPI_ISL_953884, EPI_ISL_953885, EPI_ISL_953886, EPI_ISL_953887, EPI_ISL_953888, EPI_ISL_953889, EPI_ISL_953890, EPI_ISL_953891, EPI_ISL_953892, EPI_ISL_953893, EPI_ISL_953894, EPI_ISL_953895, EPI_ISL_953896, EPI_ISL_953897, EPI_ISL_953898, EPI_ISL_953899, EPI_ISL_953900, EPI_ISL_953901, EPI_ISL_953902, EPI_ISL_953903, EPI_ISL_953904, EPI_ISL_953905, EPI_ISL_953906, EPI_ISL_953907, EPI_ISL_953908, EPI_ISL_953909, EPI_ISL_953910, EPI_ISL_953911, EPI_ISL_953912, EPI_ISL_953913, EPI_ISL_953914, EPI_ISL_953915, EPI_ISL_953916, EPI_ISL_953917, EPI_ISL_953918, EPI_ISL_953919, EPI_ISL_953920, EPI_ISL_953921			
see above	University Hospitals of Geneva, Laboratory of Virology	HUG, Laboratory of Virology and the Health2030 Genome Center	Samuel Cordey, Ana Rita Goncalves, Laurent Kaiser, Lorenzo Cerutti, Henri Pegeot, Melyssa Elies, Deborah Penet, Keith Harshman, Ioannis Xenarios, Emmanouil Dermitzakis
EPI_ISL_953959	Hopital	National Reference Center for Viruses of Respiratory Infections, Institut Pasteur, Paris	Marion Barbet, Sylvie Behillil, Méline Bizard, Angela Brisebarre, Camille Capel, Etienne Simon-Lorière, Vincent Enouf, Maud Vanpeene, Sylvie van der Werf,Ramirez José-Manuel
EPI_ISL_954009, EPI_ISL_954010	Hopital	National Reference Center for Viruses of Respiratory Infections, Institut Pasteur, Paris	Marion Barbet, Sylvie Behillil, Méline Bizard, Angela Brisebarre, Camille Capel, Etienne Simon-Lorière, Vincent Enouf, Maud Vanpeene, Sylvie van der Werf,Fourgeaud Jacques
EPI_ISL_954030, EPI_ISL_954038	Labo Analyses Med	National Reference Center for Viruses of Respiratory Infections, Institut Pasteur, Paris	Marion Barbet, Sylvie Behillil, Méline Bizard, Angela Brisebarre, Camille Capel, Etienne Simon-Lorière, Vincent Enouf, Maud Vanpeene, Sylvie van der Werf
EPI_ISL_954039	Recherche	National Reference Center for Viruses of Respiratory Infections, Institut Pasteur, Paris	Marion Barbet, Sylvie Behillil, Méline Bizard, Angela Brisebarre, Camille Capel, Etienne Simon-Lorière, Vincent Enouf, Maud Vanpeene, Sylvie van der Werf,Leruez-Ville Marianne
EPI_ISL_954040	Labo Analyses Med	National Reference Center for Viruses of Respiratory Infections, Institut Pasteur, Paris	Marion Barbet, Sylvie Behillil, Méline Bizard, Angela Brisebarre, Camille Capel, Etienne Simon-Lorière, Vincent Enouf, Maud Vanpeene, Sylvie van der Werf,Labm Hignard
EPI_ISL_954144, EPI_ISL_954180	Hospital Universitari Vall d'Hebron - Vall d'Hebron Institut de Rercerca	Hospital Universitari Vall d'Hebron	Cristina Andrés, Maria Piñana, Josep F Abril, Damir Garcia-Cehic, Ariadna Rando, Juliana Esperalba, Maria Gema Codina, Carla Castillo, Maria Carmen Martin, Tomàs Pumarola, Josep Quer, Andrés Antón
EPI_ISL_954748	National Institute of Laboratory Medicine and Referral Center	Genomic Research Lab, BCSIR	Md. Murshed Hasan Sarkar, Mohammad Samir Uzzaman, Eshrar Osman, Md. Ahashan Habib, Shahina Akter, Tanjina Akhtar Banu, Abu Sayeed Mohammad Mahmud, Barna Goswami, Iffat Jahan, Md. Saddam Hossain, Tasnim Nafisa, Md. Maruf Ahmed Molla, Mahmuda Yeasmin, Asish Kumar Ghosh, Arifa Akram, A.K.M.Shamsuzzaman, Md. Salim Khan
EPI_ISL_954749	National Institute of Laboratory Medicine and Referral Center	Genomic Research Lab, BCSIR	Barna Goswami, Mohammad Samir Uzzaman, Eshrar Osman, Md. Ahashan Habib, Shahina Akter,Tanjina Akhtar Banu, Abu Sayeed Mohammad Mahmud, Md. Murshed Hasan Sarkar, Iffat Jahan, Md. Saddam Hossain, Tasnim Nafisa, Md. Maruf Ahmed Molla, Mahmuda Yeasmin, Asish Kumar Ghosh, Arifa Akram, A. K. M. Shamsuzzaman, Md. Salim Khan
EPI_ISL_954751	National Institute of Laboratory Medicine and Referral Center	Genomic Research Lab, BCSIR	Iffat Jahan, Mohammad Samir Uzzaman, Eshrar Osman, Md. Ahashan Habib, Shahina Akter, Tanjina Akhtar Banu, Abu Sayeed Mohammad Mahmud, Md. Murshed Hasan Sarkar, Barna Goswami, Md. Saddam Hossain, Tasnim Nafisa, Md. Maruf Ahmed Molla, Mahmuda Yeasmin, Asish Kumar Ghosh, Arifa Akram, A. K. M. Shamsuzzaman, Md. Salim Khan
EPI_ISL_954814, EPI_ISL_954815, EPI_ISL_954816, EPI_ISL_954817	AZDelta	AZDelta	Geert Martens; Dieter De Smet
EPI_ISL_955060	Colorado Department of Public Health and Environment	Colorado Department of Puplic Health and Environment	Laura Bankers, Molly C. Hetherington-Rauth, Diana Ir, Shannon Ely, Shannon R. Matzinger, Sarah Elizabeth Totten, Emily A. Travanty
EPI_ISL_955061	Hopital	National Reference Center for Viruses of Respiratory Infections, Institut Pasteur, Paris	Marion Barbet, Sylvie Behillil, Méline Bizard, Angela Brisebarre, Camille Capel, Etienne Simon-Lorière, Vincent Enouf, Maud Vanpeene, Sylvie van der Werf,Fourgeaud Jacques
EPI_ISL_955062, EPI_ISL_955063	Labo analyses Med	National Reference Center for Viruses of Respiratory Infections, Institut Pasteur, Paris	Marion Barbet, Sylvie Behillil, Méline Bizard, Angela Brisebarre, Camille Capel, Etienne Simon-Lorière, Vincent Enouf, Maud Vanpeene, Sylvie van der Werf,Potiron GréGoire
EPI_ISL_955065	Hopital	National Reference Center for Viruses of Respiratory	Marion Barbet, Sylvie Behillil, Méline Bizard, Angela Brisebarre, Camille Capel, Etienne Simon-Lorière, Vincent Enouf, Maud Vanpeene, Sylvie van der

		Infections, Institut Pasteur, Paris	Werf,Fourgeaud Jacques
EPI_ISL_955066, EPI_ISL_955067	Labo analyses med	National Reference Center for Viruses of Respiratory Infections, Institut Pasteur, Paris	Marion Barbet, Sylvie Behillil, Méline Bizard, Angela Brisebarre, Camille Capel, Etienne Simon-Lorière, Vincent Enouf, Maud Vanpeene, Sylvie van der Werf,Amzalag Jonas
EPI_ISL_955068, EPI_ISL_955069, EPI_ISL_955070, EPI_ISL_955071, EPI_ISL_955072	Labo analyses Med	National Reference Center for Viruses of Respiratory Infections, Institut Pasteur, Paris	Marion Barbet, Sylvie Behillil, Méline Bizard, Angela Brisebarre, Camille Capel, Etienne Simon-Lorière, Vincent Enouf, Maud Vanpeene, Sylvie van der Werf,Potiron GréGoire
EPI_ISL_955073	Labo Analyses Med	National Reference Center for Viruses of Respiratory Infections, Institut Pasteur, Paris	Marion Barbet, Sylvie Behillil, Méline Bizard, Angela Brisebarre, Camille Capel, Etienne Simon-Lorière, Vincent Enouf, Maud Vanpeene, Sylvie van der Werf,Jacques Alexandra
EPI_ISL_955076	Labo analyses Med	National Reference Center for Viruses of Respiratory Infections, Institut Pasteur, Paris	Marion Barbet, Sylvie Behillil, Méline Bizard, Angela Brisebarre, Camille Capel, Etienne Simon-Lorière, Vincent Enouf, Maud Vanpeene, Sylvie van der Werf,Potiron GréGoire
EPI_ISL_955078	Hopital	National Reference Center for Viruses of Respiratory Infections, Institut Pasteur, Paris	Marion Barbet, Sylvie Behillil, Méline Bizard, Angela Brisebarre, Camille Capel, Etienne Simon-Lorière, Vincent Enouf, Maud Vanpeene, Sylvie van der Werf,Fourgeaud Jacques
EPI_ISL_955079, EPI_ISL_955080, EPI_ISL_955082	Labo analyses Med	National Reference Center for Viruses of Respiratory Infections, Institut Pasteur, Paris	Marion Barbet, Sylvie Behillil, Méline Bizard, Angela Brisebarre, Camille Capel, Etienne Simon-Lorière, Vincent Enouf, Maud Vanpeene, Sylvie van der Werf,Potiron GréGoire
EPI_ISL_955083, EPI_ISL_955084	Hopital	National Reference Center for Viruses of Respiratory Infections, Institut Pasteur, Paris	Marion Barbet, Sylvie Behillil, Méline Bizard, Angela Brisebarre, Camille Capel, Etienne Simon-Lorière, Vincent Enouf, Maud Vanpeene, Sylvie van der Werf,Fourgeaud Jacques
EPI_ISL_955085	Hopital	National Reference Center for Viruses of Respiratory Infections, Institut Pasteur, Paris	Marion Barbet, Sylvie Behillil, Méline Bizard, Angela Brisebarre, Camille Capel, Etienne Simon-Lorière, Vincent Enouf, Maud Vanpeene, Sylvie van der Werf,Joly Isabelle
EPI_ISL_955086, EPI_ISL_955087, EPI_ISL_955088, EPI_ISL_955089, EPI_ISL_955090, EPI_ISL_955091, EPI_ISL_955092, EPI_ISL_955093	Labo analyses Med	National Reference Center for Viruses of Respiratory Infections, Institut Pasteur, Paris	Marion Barbet, Sylvie Behillil, Méline Bizard, Angela Brisebarre, Camille Capel, Etienne Simon-Lorière, Vincent Enouf, Maud Vanpeene, Sylvie van der Werf,Potiron GréGoire
EPI_ISL_955094	Labo Analyses Med	National Reference Center for Viruses of Respiratory Infections, Institut Pasteur, Paris	Marion Barbet, Sylvie Behillil, Méline Bizard, Angela Brisebarre, Camille Capel, Etienne Simon-Lorière, Vincent Enouf, Maud Vanpeene, Sylvie van der Werf,Potiron GréGoire
EPI_ISL_955112, EPI_ISL_955113, EPI_ISL_955114, EPI_ISL_955115	Maryland Public Health Laboratory	Maryland Public Health Laboratory	Maryland Department of Health Laboratories Administration
EPI_ISL_955172	National Institute of Laboratory Medicine and Referral Center	Genomic Research Lab, BCSIR	Tasnim Nafisa, Mohammad Samir Uzzaman, Eshrar Osman, Md. Ahashan Habib, Shahina Akter,Tanjina Akhtar Banu, Abu Sayeed Mohammad Mahmud, Md. Murshed Hasan Sarkar, Barna Goswami, Iffat Jahan, Md. Saddam Hossain, Md. Maruf Ahmed Molla, ,Mahmuda Yeasmin, Asish Kumar Ghosh, Arifa Akram, A. K. M. Shamsuzzaman, Md. Salim Khan.
EPI_ISL_955215, EPI_ISL_955222	Indiana Animal Disease Diagnostic Laboratory	Carpi Laboratory - Purdue University	Jack Dorman, Ilinca I Ciubotariu, Lev Gorenstein, Abebe A Fola, G Kenitra Hendrix, Rebecca P Wilkes, Giovanna Carpi
EPI_ISL_955267	Clinical Pathology Lab	Pathogen Discovery, Respiratory Viruses Branch, Division of Viral Diseases, Centers for Disease Control and Prevention	Ying Tao, Jing Zhang, Yan Li, Krista Queen, Anna Uehara, Peter Cook, Clinton R. Paden, Haibin Wang, Suxiang Tong
EPI_ISL_955272	AR Dept. of Health-PHL, Molecular Diagnostics	Pathogen Discovery, Respiratory Viruses Branch, Division of Viral Diseases, Centers for Disease Control and Prevention	Ying Tao, Jing Zhang, Yan Li, Krista Queen, Anna Uehara, Peter Cook, Clinton R. Paden, Haibin Wang, Suxiang Tong
EPI_ISL_955280, EPI_ISL_955282	Clinical Pathology Lab	Pathogen Discovery, Respiratory Viruses Branch, Division of Viral Diseases, Centers for Disease Control and Prevention	Ying Tao, Jing Zhang, Yan Li, Krista Queen, Anna Uehara, Peter Cook, Clinton R. Paden, Haibin Wang, Suxiang Tong
EPI_ISL_955284	NC State Laboratory of Public Health	Pathogen Discovery, Respiratory Viruses Branch, Division of Viral Diseases, Centers for Disease Control and Prevention	Ying Tao, Jing Zhang, Yan Li, Krista Queen, Anna Uehara, Peter Cook, Clinton R. Paden, Haibin Wang, Suxiang Tong
EPI_ISL_955287	Clinical Pathology Lab	Pathogen Discovery, Respiratory Viruses Branch, Division of Viral Diseases, Centers for Disease Control and Prevention	Ying Tao, Jing Zhang, Yan Li, Krista Queen, Anna Uehara, Peter Cook, Clinton R. Paden, Haibin Wang, Suxiang Tong
EPI_ISL_955288	GA Department of Public Health Laboratory	Pathogen Discovery, Respiratory Viruses Branch, Division of Viral Diseases, Centers for Disease Control and Prevention	Ying Tao, Jing Zhang, Yan Li, Krista Queen, Anna Uehara, Peter Cook, Clinton R. Paden, Haibin Wang, Suxiang Tong
EPI_ISL_955291	IL Dept. of Public Health Springfield Laboratory	Pathogen Discovery, Respiratory Viruses Branch, Division of Viral Diseases, Centers for Disease Control and Prevention	Ying Tao, Jing Zhang, Yan Li, Krista Queen, Anna Uehara, Peter Cook, Clinton R. Paden, Haibin Wang, Suxiang Tong
EPI_ISL_955293	TMC/Pathology	Pathogen Discovery, Respiratory Viruses Branch, Division of Viral Diseases, Centers for Disease Control and Prevention	Ying Tao, Jing Zhang, Yan Li, Krista Queen, Anna Uehara, Peter Cook, Clinton R. Paden, Haibin Wang, Suxiang Tong
EPI_ISL_955294	Clinical Pathology Lab	Pathogen Discovery, Respiratory Viruses Branch, Division of Viral Diseases, Centers for Disease Control and Prevention	Ying Tao, Jing Zhang, Yan Li, Krista Queen, Anna Uehara, Peter Cook, Clinton R. Paden, Haibin Wang, Suxiang Tong
EPI_ISL_955298	NC State Laboratory of Public Health	Pathogen Discovery, Respiratory Viruses Branch, Division of Viral Diseases, Centers for Disease Control and Prevention	Ying Tao, Jing Zhang, Yan Li, Krista Queen, Anna Uehara, Peter Cook, Clinton R. Paden, Haibin Wang, Suxiang Tong
EPI_ISL_955301	Clinical Pathology Lab	Pathogen Discovery, Respiratory Viruses Branch, Division of Viral Diseases, Centers for Disease Control and Prevention	Ying Tao, Jing Zhang, Yan Li, Krista Queen, Anna Uehara, Peter Cook, Clinton R. Paden, Haibin Wang, Suxiang Tong
EPI_ISL_955307	OK Public Health Laboratory, Oklahoma State DOH	Pathogen Discovery, Respiratory Viruses Branch, Division of Viral Diseases, Centers for Disease Control and Prevention	Ying Tao, Jing Zhang, Yan Li, Krista Queen, Anna Uehara, Peter Cook, Clinton R. Paden, Haibin Wang, Suxiang Tong
EPI_ISL_955309	IL Dept. of Public Health Springfield Laboratory	Pathogen Discovery, Respiratory Viruses Branch, Division of Viral Diseases, Centers for Disease Control and Prevention	Ying Tao, Jing Zhang, Yan Li, Krista Queen, Anna Uehara, Peter Cook, Clinton R. Paden, Haibin Wang, Suxiang Tong
EPI_ISL_955313, EPI_ISL_955315, EPI_ISL_955331, EPI_ISL_955332	AR Dept. of Health-PHL, Molecular Diagnostics	Pathogen Discovery, Respiratory Viruses Branch, Division of Viral Diseases, Centers for Disease Control and Prevention	Ying Tao, Jing Zhang, Yan Li, Krista Queen, Anna Uehara, Peter Cook, Clinton R. Paden, Haibin Wang, Suxiang Tong
EPI_ISL_955830, EPI_ISL_955862, EPI_ISL_955863, EPI_ISL_955864, EPI_ISL_955865, EPI_ISL_955866, EPI_ISL_955867, EPI_ISL_955869, EPI_ISL_955870, EPI_ISL_955871, EPI_ISL_955872, EPI_ISL_955873, EPI_ISL_955874, EPI_ISL_955876, EPI_ISL_955877, EPI_ISL_955878, EPI_ISL_955879, EPI_ISL_955881, EPI_ISL_955882, EPI_ISL_955883, EPI_ISL_955884, EPI_ISL_955885, EPI_ISL_955886, EPI_ISL_955887, EPI_ISL_955888, EPI_ISL_955889, EPI_ISL_955891, EPI_ISL_955892, EPI_ISL_955893, EPI_ISL_955894, EPI_ISL_955895, EPI_ISL_955896, EPI_ISL_955898, EPI_ISL_955899, EPI_ISL_955900, EPI_ISL_955901, EPI_ISL_955902, EPI_ISL_955903, EPI_ISL_955904, EPI_ISL_955918, EPI_ISL_955919, EPI_ISL_955920, EPI_ISL_955921			
see above	University of Michigan Clinical Microbiology Laboratory	Lauring Lab, University of Michigan, Department of Microbiology and Immunology	Valesano
EPI_ISL_956275	Thai Red Cross Emerging Infectious Diseases Health Science Centre, Chulalongkorn Hospital, Faculty of Medicine, Chulalongkorn University	Thai Red Cross Emerging Infectious Diseases Center and Faculty of Medicine, Chulalongkorn University	Ratima samorn, Panpit Suwangool, Sininat Petcharat, Yutthana Joyjinda, Weenassarin Ampoot, Apaporn Rodpan, Opass Putcharoen, Thiravat Hemachudha, Supaporn Wacharapluesadee
EPI_ISL_956331	Laboratory Medicine	Department of Laboratory Medicine, Lin-Kou Chang Gung Memorial Hospital, Taoyuan, Taiwan	Kuo-Chien Tsao, Yu-Nong Gong, Shu-Li Yang, Yi-Chun Liu, Chung-Guei Huang, Mei-Jen Hsiao, Po-Wei Huang, Cheng-Ta Yang, Cheng-Hsun Chiu, Peng-Nien Huang, Kuo-Ming Lee, Guang-Wu Chen, Shin-Ru Shih
EPI_ISL_956446, EPI_ISL_956487, EPI_ISL_956508, EPI_ISL_956562, EPI_ISL_956576, EPI_ISL_956585, EPI_ISL_956587, EPI_ISL_956592, EPI_ISL_956601, EPI_ISL_956602, EPI_ISL_956606, EPI_ISL_956618, EPI_ISL_956625, EPI_ISL_956626, EPI_ISL_956631, EPI_ISL_956636, EPI_ISL_956651, EPI_ISL_956690, EPI_ISL_956691, EPI_ISL_956694, EPI_ISL_956699, EPI_ISL_956708, EPI_ISL_956729			
see above	Lighthouse Lab in Alderley Park	Wellcome Sanger Institute for the COVID-19 Genomics UK	Jacquelyn Wynn, Mairead Hyland, The Lighthouse Lab in Alderley Park and Alex Alderton, Roberto Amato, Sonia Goncalves, Ewan Harrison, David K.

		(COG-UK) Consortium	Jackson, Ian Johnston, Dominic Kwiatkowski, Cordelia Langford, John Sillitoe on behalf of the Wellcome Sanger Institute COVID-19 Surveillance Team
EPI_ISL_957613, EPI_ISL_957616, EPI_ISL_957620, EPI_ISL_957621, EPI_ISL_957624, EPI_ISL_957627, EPI_ISL_957630, EPI_ISL_957635, EPI_ISL_957641, EPI_ISL_957643, EPI_ISL_957645, EPI_ISL_957650, EPI_ISL_957652, EPI_ISL_957654, EPI_ISL_957655, EPI_ISL_957656, EPI_ISL_957660, EPI_ISL_957670, EPI_ISL_957672, EPI_ISL_957675, EPI_ISL_957677, EPI_ISL_957680, EPI_ISL_957683, EPI_ISL_957686, EPI_ISL_957687, EPI_ISL_957691, EPI_ISL_957698, EPI_ISL_957700, EPI_ISL_957701, EPI_ISL_957707, EPI_ISL_957721, EPI_ISL_957722, EPI_ISL_957723, EPI_ISL_957726, EPI_ISL_957735, EPI_ISL_957740, EPI_ISL_957741, EPI_ISL_957745, EPI_ISL_957747, EPI_ISL_957748, EPI_ISL_957753, EPI_ISL_957755, EPI_ISL_957757, EPI_ISL_957759, EPI_ISL_957766, EPI_ISL_957769, EPI_ISL_957771, EPI_ISL_957772, EPI_ISL_957780, EPI_ISL_957783, EPI_ISL_957787, EPI_ISL_957797, EPI_ISL_957799, EPI_ISL_957813, EPI_ISL_957817, EPI_ISL_957822, EPI_ISL_957826, EPI_ISL_957828, EPI_ISL_957829, EPI_ISL_957830, EPI_ISL_957832, EPI_ISL_957838, EPI_ISL_957841, EPI_ISL_957844, EPI_ISL_957856, EPI_ISL_957859, EPI_ISL_957862, EPI_ISL_957863, EPI_ISL_957866, EPI_ISL_957867, EPI_ISL_957868, EPI_ISL_957872, EPI_ISL_957877, EPI_ISL_957878, EPI_ISL_957886, EPI_ISL_957892, EPI_ISL_957895, EPI_ISL_957897, EPI_ISL_957904, EPI_ISL_957907, EPI_ISL_957921, EPI_ISL_957961, EPI_ISL_957962, EPI_ISL_957971, EPI_ISL_958044, EPI_ISL_958194, EPI_ISL_958206, EPI_ISL_958215, EPI_ISL_958216, EPI_ISL_958217, EPI_ISL_958218, EPI_ISL_958219, EPI_ISL_958220, EPI_ISL_958221, EPI_ISL_958222, EPI_ISL_958223, EPI_ISL_958224, EPI_ISL_958225, EPI_ISL_958226, EPI_ISL_958227, EPI_ISL_958228, EPI_ISL_958229, EPI_ISL_958230, EPI_ISL_958231, EPI_ISL_958232, EPI_ISL_958233, EPI_ISL_958234, EPI_ISL_958235, EPI_ISL_958236, EPI_ISL_958237, EPI_ISL_958238, EPI_ISL_958239, EPI_ISL_958240, EPI_ISL_958241, EPI_ISL_958242, EPI_ISL_958243, EPI_ISL_958244, EPI_ISL_958245, EPI_ISL_958246, EPI_ISL_958247, EPI_ISL_958248, EPI_ISL_958249, EPI_ISL_958250, EPI_ISL_958251, EPI_ISL_958252, EPI_ISL_958253, EPI_ISL_958254, EPI_ISL_958255, EPI_ISL_958256, EPI_ISL_958257, EPI_ISL_958258, EPI_ISL_958259, EPI_ISL_958260, EPI_ISL_958261, EPI_ISL_958262, EPI_ISL_958263, EPI_ISL_958264, EPI_ISL_958265, EPI_ISL_958266, EPI_ISL_958267, EPI_ISL_958268, EPI_ISL_958269, EPI_ISL_958270, EPI_ISL_958271, EPI_ISL_958272, EPI_ISL_958273, EPI_ISL_958274, EPI_ISL_958275, EPI_ISL_958277, EPI_ISL_958278, EPI_ISL_958279, EPI_ISL_958280, EPI_ISL_958281, EPI_ISL_958282, EPI_ISL_958283, EPI_ISL_958284, EPI_ISL_958285, EPI_ISL_958286, EPI_ISL_958287, EPI_ISL_958288, EPI_ISL_958289, EPI_ISL_958290, EPI_ISL_958291, EPI_ISL_958292, EPI_ISL_958293, EPI_ISL_958294, EPI_ISL_958295, EPI_ISL_958296, EPI_ISL_958297, EPI_ISL_958298, EPI_ISL_958299, EPI_ISL_958300, EPI_ISL_958301, EPI_ISL_958302, EPI_ISL_958303, EPI_ISL_958304, EPI_ISL_958305, EPI_ISL_958306, EPI_ISL_958307, EPI_ISL_958308, EPI_ISL_958309, EPI_ISL_958310, EPI_ISL_958311, EPI_ISL_958312, EPI_ISL_958313, EPI_ISL_958314, EPI_ISL_958315, EPI_ISL_958316, EPI_ISL_958317, EPI_ISL_958318, EPI_ISL_958319, EPI_ISL_958320, EPI_ISL_958321, EPI_ISL_958322, EPI_ISL_958323, EPI_ISL_958324, EPI_ISL_958325, EPI_ISL_958326, EPI_ISL_958327, EPI_ISL_958328, EPI_ISL_958329, EPI_ISL_958330, EPI_ISL_958331, EPI_ISL_958332, EPI_ISL_958333, EPI_ISL_958334, EPI_ISL_958335, EPI_ISL_958336, EPI_ISL_958337, EPI_ISL_958338, EPI_ISL_958339, EPI_ISL_958340, EPI_ISL_958341, EPI_ISL_958342, EPI_ISL_958343, EPI_ISL_958344, EPI_ISL_958345, EPI_ISL_958346, EPI_ISL_958347, EPI_ISL_958348, EPI_ISL_958349, EPI_ISL_958350, EPI_ISL_958351, EPI_ISL_958352, EPI_ISL_958353, EPI_ISL_958354, EPI_ISL_958355, EPI_ISL_958356, EPI_ISL_958357, EPI_ISL_958358, EPI_ISL_958359, EPI_ISL_958360, EPI_ISL_958361, EPI_ISL_958362, EPI_ISL_958363, EPI_ISL_958364, EPI_ISL_958365, EPI_ISL_958366, EPI_ISL_958367, EPI_ISL_958368, EPI_ISL_958369, EPI_ISL_958370, EPI_ISL_958371, EPI_ISL_958372, EPI_ISL_958373, EPI_ISL_958374, EPI_ISL_958375, EPI_ISL_958376, EPI_ISL_958377, EPI_ISL_958378, EPI_ISL_958379, EPI_ISL_958380, EPI_ISL_958381, EPI_ISL_958382, EPI_ISL_958383, EPI_ISL_958384, EPI_ISL_958385, EPI_ISL_958386, EPI_ISL_958387, EPI_ISL_958388, EPI_ISL_958389, EPI_ISL_958390, EPI_ISL_958391, EPI_ISL_958392, EPI_ISL_958393, EPI_ISL_958394, EPI_ISL_958395, EPI_ISL_958396, EPI_ISL_958397, EPI_ISL_958399, EPI_ISL_958400, EPI_ISL_958401, EPI_ISL_958402, EPI_ISL_958403, EPI_ISL_958404, EPI_ISL_958405, EPI_ISL_958406, EPI_ISL_958407, EPI_ISL_958408, EPI_ISL_958409, EPI_ISL_958410, EPI_ISL_958411, EPI_ISL_958412, EPI_ISL_958413, EPI_ISL_958414, EPI_ISL_958415, EPI_ISL_958416, EPI_ISL_958417, EPI_ISL_958418, EPI_ISL_958419, EPI_ISL_958420, EPI_ISL_958421, EPI_ISL_958422, EPI_ISL_958423, EPI_ISL_958424, EPI_ISL_958425, EPI_ISL_958426, EPI_ISL_958427, EPI_ISL_958428, EPI_ISL_958429, EPI_ISL_958430, EPI_ISL_958431, EPI_ISL_958432, EPI_ISL_958433, EPI_ISL_958434, EPI_ISL_958435, EPI_ISL_958436, EPI_ISL_958437, EPI_ISL_958438, EPI_ISL_958439, EPI_ISL_958440, EPI_ISL_958441, EPI_ISL_958442, EPI_ISL_958443, EPI_ISL_958444, EPI_ISL_958445, EPI_ISL_958446, EPI_ISL_958447, EPI_ISL_958448, EPI_ISL_958449, EPI_ISL_958450, EPI_ISL_958451, EPI_ISL_958452, EPI_ISL_958453, EPI_ISL_958454, EPI_ISL_958455, EPI_ISL_958456, EPI_ISL_958457, EPI_ISL_958458, EPI_ISL_958459, EPI_ISL_958460, EPI_ISL_958461, EPI_ISL_958462, EPI_ISL_958463, EPI_ISL_958464, EPI_ISL_958465, EPI_ISL_958466, EPI_ISL_958467, EPI_ISL_958468, EPI_ISL_958469, EPI_ISL_958470, EPI_ISL_958471, EPI_ISL_958472, EPI_ISL_958473, EPI_ISL_958474, EPI_ISL_958475, EPI_ISL_958476, EPI_ISL_958477, EPI_ISL_958478, EPI_ISL_958479, EPI_ISL_958480, EPI_ISL_958481, EPI_ISL_958482, EPI_ISL_958483, EPI_ISL_958484, EPI_ISL_958485, EPI_ISL_958486, EPI_ISL_958487, EPI_ISL_958488, EPI_ISL_958489, EPI_ISL_958490, EPI_ISL_958491, EPI_ISL_958492, EPI_ISL_958493, EPI_ISL_958494, EPI_ISL_958495, EPI_ISL_958496, EPI_ISL_958497, EPI_ISL_958498, EPI_ISL_958499, EPI_ISL_958500, EPI_ISL_958501, EPI_ISL_958502, EPI_ISL_958503, EPI_ISL_958504, EPI_ISL_958505, EPI_ISL_958506, EPI_ISL_958507, EPI_ISL_958508, EPI_ISL_958509, EPI_ISL_958510, EPI_ISL_958511, EPI_ISL_958512, EPI_ISL_958513, EPI_ISL_958514, EPI_ISL_958515, EPI_ISL_958516, EPI_ISL_958517, EPI_ISL_958518, EPI_ISL_958519, EPI_ISL_958520, EPI_ISL_958521, EPI_ISL_958522, EPI_ISL_958523, EPI_ISL_958524, EPI_ISL_958525, EPI_ISL_958526, EPI_ISL_958527, EPI_ISL_958528, EPI_ISL_958529, EPI_ISL_958530, EPI_ISL_958531, EPI_ISL_958532, EPI_ISL_958533, EPI_ISL_958534, EPI_ISL_958535, EPI_ISL_958536, EPI_ISL_958537, EPI_ISL_958538, EPI_ISL_958539, EPI_ISL_958540, EPI_ISL_958541, EPI_ISL_958542, EPI_ISL_958543, EPI_ISL_958544, EPI_ISL_958545, EPI_ISL_958546, EPI_ISL_958547, EPI_ISL_958548, EPI_ISL_958549, EPI_ISL_958550, EPI_ISL_958551, EPI_ISL_958552, EPI_ISL_958553, EPI_ISL_958554, EPI_ISL_958555, EPI_ISL_958556, EPI_ISL_958557, EPI_ISL_958558, EPI_ISL_958559, EPI_ISL_958560, EPI_ISL_958561, EPI_ISL_958562, EPI_ISL_958563			
see above	Lighthouse Lab in Milton Keynes	Wellcome Sanger Institute for the COVID-19 Genomics UK (COG-UK) Consortium	The Lighthouse Lab in Milton Keynes and Alex Alderton, Roberto Amato, Sonia Goncalves, Ewan Harrison, David K. Jackson, Ian Johnston, Dominic Kwiatkowski, Cordelia Langford, John Sillitoe on behalf of the Wellcome Sanger Institute COVID-19 Surveillance Team
EPI_ISL_958566	Lighthouse Lab in Alderley Park	Wellcome Sanger Institute for the COVID-19 Genomics UK (COG-UK) Consortium	Jacquelyn Wynn, Mairead Hyland, The Lighthouse Lab in Alderley Park and Alex Alderton, Roberto Amato, Sonia Goncalves, Ewan Harrison, David K. Jackson, Ian Johnston, Dominic Kwiatkowski, Cordelia Langford, John Sillitoe on behalf of the Wellcome Sanger Institute COVID-19 Surveillance Team
EPI_ISL_958569, EPI_ISL_958572, EPI_ISL_958578, EPI_ISL_958579, EPI_ISL_958586, EPI_ISL_958587, EPI_ISL_958590, EPI_ISL_958591, EPI_ISL_958595, EPI_ISL_958600, EPI_ISL_958602, EPI_ISL_958610, EPI_ISL_958624, EPI_ISL_958632, EPI_ISL_958633, EPI_ISL_958634, EPI_ISL_958636, EPI_ISL_958638, EPI_ISL_958639, EPI_ISL_958641, EPI_ISL_958646, EPI_ISL_958650, EPI_ISL_958659, EPI_ISL_958662, EPI_ISL_958663, EPI_ISL_958670, EPI_ISL_958671, EPI_ISL_958673, EPI_ISL_958680, EPI_ISL_958684, EPI_ISL_958690, EPI_ISL_958691, EPI_ISL_958696, EPI_ISL_958705, EPI_ISL_958706, EPI_ISL_958713, EPI_ISL_958715, EPI_ISL_958716, EPI_ISL_958723, EPI_ISL_958725, EPI_ISL_958726, EPI_ISL_958731, EPI_ISL_958733, EPI_ISL_958735, EPI_ISL_958745, EPI_ISL_958747, EPI_ISL_958748, EPI_ISL_958754, EPI_ISL_958756, EPI_ISL_958760, EPI_ISL_958763, EPI_ISL_958767, EPI_ISL_958769, EPI_ISL_958775, EPI_ISL_958779, EPI_ISL_958784, EPI_ISL_958787, EPI_ISL_958789, EPI_ISL_958791, EPI_ISL_958792, EPI_ISL_958793, EPI_ISL_958803, EPI_ISL_958807, EPI_ISL_958808, EPI_ISL_958811, EPI_ISL_958815, EPI_ISL_958823, EPI_ISL_958826, EPI_ISL_958828, EPI_ISL_958833, EPI_ISL_958836, EPI_ISL_958840, EPI_ISL_958842, EPI_ISL_958849, EPI_ISL_958851, EPI_ISL_958863, EPI_ISL_958866, EPI_ISL_958873, EPI_ISL_958878, EPI_ISL_958884, EPI_ISL_958885, EPI_ISL_958887, EPI_ISL_958889, EPI_ISL_958891, EPI_ISL_958893, EPI_ISL_958895, EPI_ISL_958898, EPI_ISL_958902, EPI_ISL_958903, EPI_ISL_958904, EPI_ISL_958908, EPI_ISL_958911, EPI_ISL_958912, EPI_ISL_958915, EPI_ISL_958916			
see above	Lighthouse Lab in Milton Keynes	Wellcome Sanger Institute for the COVID-19 Genomics UK (COG-UK) Consortium	The Lighthouse Lab in Milton Keynes and Alex Alderton, Roberto Amato, Sonia Goncalves, Ewan Harrison, David K. Jackson, Ian Johnston, Dominic Kwiatkowski, Cordelia Langford, John Sillitoe on behalf of the Wellcome Sanger Institute COVID-19 Surveillance Team
EPI_ISL_958917, EPI_ISL_958918, EPI_ISL_958919, EPI_ISL_958920, EPI_ISL_958921, EPI_ISL_958922, EPI_ISL_958923, EPI_ISL_958924, EPI_ISL_958925, EPI_ISL_958926, EPI_ISL_958927, EPI_ISL_958928, EPI_ISL_958929, EPI_ISL_958930, EPI_ISL_958931, EPI_ISL_958932, EPI_ISL_958933, EPI_ISL_958934, EPI_ISL_958935, EPI_ISL_958936, EPI_ISL_958937, EPI_ISL_958938, EPI_ISL_958939, EPI_ISL_958940, EPI_ISL_958941, EPI_ISL_958942, EPI_ISL_958943, EPI_ISL_958944, EPI_ISL_958945, EPI_ISL_958946, EPI_ISL_958947, EPI_ISL_958948, EPI_ISL_958949, EPI_ISL_958950, EPI_ISL_958951, EPI_ISL_958952, EPI_ISL_958953, EPI_ISL_958954, EPI_ISL_958955, EPI_ISL_958956, EPI_ISL_958957, EPI_ISL_958958, EPI_ISL_958959, EPI_ISL_958960, EPI_ISL_958961, EPI_ISL_958962, EPI_ISL_958963, EPI_ISL_958964, EPI_ISL_958965, EPI_ISL_958966, EPI_ISL_958967, EPI_ISL_958968, EPI_ISL_958969, EPI_ISL_958970, EPI_ISL_958971, EPI_ISL_958972, EPI_ISL_958973, EPI_ISL_958974, EPI_ISL_958975, EPI_ISL_958976, EPI_ISL_958977, EPI_ISL_958978, EPI_ISL_958979, EPI_ISL_958980, EPI_ISL_958981, EPI_ISL_958982, EPI_ISL_958983, EPI_ISL_958984, EPI_ISL_958985, EPI_ISL_958986, EPI_ISL_958987, EPI_ISL_958988, EPI_ISL_958989, EPI_ISL_958990, EPI_ISL_958991, EPI_ISL_958992, EPI_ISL_958993, EPI_ISL_958994, EPI_ISL_958995, EPI_ISL_958996, EPI_ISL_958997, EPI_ISL_958998, EPI_ISL_958999, EPI_ISL_959000, EPI_ISL_959001, EPI_ISL_959002, EPI_ISL_959003, EPI_ISL_959004, EPI_ISL_959005, EPI_ISL_959006, EPI_ISL_959007, EPI_ISL_959008, EPI_ISL_959009, EPI_ISL_959010, EPI_ISL_959011, EPI_ISL_959012, EPI_ISL_959013, EPI_ISL_959014, EPI_ISL_959015, EPI_ISL_959016, EPI_ISL_959017, EPI_ISL_959018, EPI_ISL_959019, EPI_ISL_959020, EPI_ISL_959021, EPI_ISL_959022, EPI_ISL_959023, EPI_ISL_959024, EPI_ISL_959025, EPI_ISL_959026, EPI_ISL_959027, EPI_ISL_959028, EPI_ISL_959029, EPI_ISL_959030, EPI_ISL_959031, EPI_ISL_959032, EPI_ISL_959033, EPI_ISL_959034, EPI_ISL_959035, EPI_ISL_959036, EPI_ISL_959037, EPI_ISL_959038, EPI_ISL_959039, EPI_ISL_959040, EPI_ISL_959041, EPI_ISL_959042, EPI_ISL_959043, EPI_ISL_959044, EPI_ISL_959045, EPI_ISL_959046, EPI_ISL_959047, EPI_ISL_959048, EPI_ISL_959049, EPI_ISL_959050, EPI_ISL_959051, EPI_ISL_959052, EPI_ISL_959053, EPI_ISL_959054, EPI_ISL_959055, EPI_ISL_959056, EPI_ISL_959057, EPI_ISL_959058, EPI_ISL_959059, EPI_ISL_959060, EPI_ISL_959061, EPI_ISL_959062, EPI_ISL_959063, EPI_ISL_959064, EPI_ISL_959065, EPI_ISL_959066, EPI_ISL_959067, EPI_ISL_959068, EPI_ISL_959069, EPI_ISL_959070, EPI_ISL_959071, EPI_ISL_959072, EPI_ISL_959073, EPI_ISL_959074, EPI_ISL_959075, EPI_ISL_959076, EPI_ISL_959077, EPI_ISL_959078, EPI_ISL_959079, EPI_ISL_959080, EPI_ISL_959081, EPI_ISL_959082, EPI_ISL_959083, EPI_ISL_959084, EPI_ISL_959085, EPI_ISL_959086, EPI_ISL_959087, EPI_ISL_959088, EPI_ISL_959089, EPI_ISL_959090, EPI_ISL_959091, EPI_ISL_959092, EPI_ISL_959093, EPI_ISL_959094, EPI_ISL_959095, EPI_ISL_959096, EPI_ISL_959097, EPI_ISL_959098, EPI_ISL_959099, EPI_ISL_959100, EPI_ISL_959101, EPI_ISL_959102, EPI_ISL_959103, EPI_ISL_959104, EPI_ISL_959105, EPI_ISL_959106, EPI_ISL_959107, EPI_ISL_959108, EPI_ISL_959109, EPI_ISL_959110, EPI_ISL_959111, EPI_ISL_959112, EPI_ISL_959113, EPI_ISL_959114, EPI_ISL_959115, EPI_ISL_959116, EPI_ISL_959117, EPI_ISL_959118, EPI_ISL_959119, EPI_ISL_959120, EPI_ISL_959121, EPI_ISL_959122, EPI_ISL_959123, EPI_ISL_959124, EPI_ISL_959125, EPI_ISL_959126, EPI_ISL_959127, EPI_ISL_959128, EPI_ISL_959129, EPI_ISL_959130, EPI_ISL_959131, EPI_ISL_959132, EPI_ISL_959133, EPI_ISL_959134, EPI_ISL_959135, EPI_ISL_959136, EPI_ISL_959137, EPI_ISL_959138, EPI_ISL_959139, EPI_ISL_959140, EPI_ISL_959141, EPI_ISL_959142, EPI_ISL_959143, EPI_ISL_959144, EPI_ISL_959145, EPI_ISL_959146, EPI_ISL_959147, EPI_ISL_959148, EPI_ISL_959149, EPI_ISL_959150, EPI_ISL_959151, EPI_ISL_959152, EPI_ISL_959153, EPI_ISL_959154, EPI_ISL_959155, EPI_ISL_959156, EPI_ISL_959157, EPI_ISL_959158, EPI_ISL_959159, EPI_ISL_959160, EPI_ISL_959161, EPI_ISL_959162, EPI_ISL_959163, EPI_ISL_959164, EPI_ISL_959165, EPI_ISL_959166, EPI_ISL_959167, EPI_ISL_959168, EPI_ISL_959169, EPI_ISL_959170, EPI_ISL_959171, EPI_ISL_959172, EPI_ISL_959173, EPI_ISL_959174, EPI_ISL_959175, EPI_ISL_959176, EPI_ISL_959177, EPI_ISL_959178, EPI_ISL_959179, EPI_ISL_959180, EPI_ISL_959181, EPI_ISL_959182, EPI_ISL_959183, EPI_ISL_959184, EPI_ISL_959185, EPI_ISL_959186, EPI_ISL_959187, EPI_ISL_959188, EPI_ISL_959189, EPI_ISL_959190, EPI_ISL_959191, EPI_ISL_959192, EPI_ISL_959193, EPI_ISL_959194, EPI_ISL_959195, EPI_ISL_959196, EPI_ISL_959197, EPI_ISL_959198, EPI_ISL_959199, EPI_ISL_959200, EPI_ISL_959201, EPI_ISL_959202, EPI_ISL_959203, EPI_ISL_959204, EPI_ISL_959205, EPI_ISL_959206, EPI_ISL_959207, EPI_ISL_959208, EPI_ISL_959209, EPI_ISL_959210, EPI_ISL_959211, EPI_ISL_959212, EPI_ISL_959213, EPI_ISL_959214, EPI_ISL_959215, EPI_ISL_959216, EPI_ISL_959217, EPI_ISL_959218, EPI_ISL_959219, EPI_ISL_959220, EPI_ISL_959221, EPI_ISL_959222, EPI_ISL_959223, EPI_ISL_959224, EPI_ISL_959225, EPI_ISL_959226, EPI_ISL_959227, EPI_ISL_959228, EPI_ISL_959229, EPI_ISL_959230, EPI_ISL_959231, EPI_ISL_959232, EPI_ISL_959233, EPI_ISL_959234, EPI_ISL_959235, EPI_ISL_959236, EPI_ISL_959237, EPI_ISL_959238, EPI_ISL_959239, EPI_ISL_959240, EPI_ISL_959241, EPI_ISL_959242, EPI_ISL_959243, EPI_ISL_959244, EPI_ISL_959245, EPI_ISL_959246, EPI_ISL_959247, EPI_ISL_959248, EPI_ISL_959249, EPI_ISL_959250, EPI_ISL_959251, EPI_ISL_959252, EPI_ISL_959253, EPI_ISL_959254, EPI_ISL_959255, EPI_ISL_959256, EPI_ISL_959257, EPI_ISL_959258, EPI_ISL_959259, EPI_ISL_959260, EPI_ISL_959261, EPI_ISL_959262, EPI_ISL_959263, EPI_ISL_959264, EPI_ISL_959265, EPI_ISL_959266			
see above	Lighthouse Lab in Alderley Park	Wellcome Sanger Institute for the COVID-19 Genomics UK (COG-UK) Consortium	Jacquelyn Wynn, Mairead Hyland, The Lighthouse Lab in Alderley Park and Alex Alderton, Roberto Amato, Sonia Goncalves, Ewan Harrison, David K. Jackson, Ian Johnston, Dominic Kwiatkowski, Cordelia Langford, John Sillitoe on behalf of the Wellcome Sanger Institute COVID-19 Surveillance Team
EPI_ISL_959267	Lighthouse Lab in Cambridge	Wellcome Sanger Institute for the COVID-19 Genomics UK (COG-UK) Consortium	Rob Howes, The Lighthouse Lab in Cambridge and Alex Alderton, Roberto Amato, Sonia Goncalves, Ewan Harrison, David K. Jackson, Ian Johnston, Dominic Kwiatkowski, Cordelia Langford, John Sillitoe on behalf of the Wellcome Sanger Institute COVID-19 Surveillance Team
EPI_ISL_959268, EPI_ISL_959269, EPI_ISL_959270	Lighthouse Lab in Alderley Park	Wellcome Sanger Institute for the COVID-19 Genomics UK (COG-UK) Consortium	Jacquelyn Wynn, Mairead Hyland, The Lighthouse Lab in Alderley Park and Alex Alderton, Roberto Amato, Sonia Goncalves, Ewan Harrison, David K. Jackson, Ian Johnston, Dominic Kwiatkowski, Cordelia Langford, John Sillitoe on behalf of the Wellcome Sanger Institute COVID-19 Surveillance Team
EPI_ISL_959271, EPI_ISL_959272	Lighthouse Lab in Milton Keynes	Wellcome Sanger Institute for the COVID-19 Genomics UK (COG-UK) Consortium	The Lighthouse Lab in Milton Keynes and Alex Alderton, Roberto Amato, Sonia Goncalves, Ewan Harrison, David K. Jackson, Ian Johnston, Dominic Kwiatkowski, Cordelia Langford, John Sillitoe on behalf of the Wellcome Sanger Institute COVID-19 Surveillance Team
EPI_ISL_959367	National Institute of Laboratory Medicine and Referral Center	Genomic Research Lab, BCSIR	Md. Murshed Hasan Sarkar, Mohammad Samir Uzzaman, Eshrar Osman, Md. Ashahan Habib, Shahina Akter, Tanjina Akhtar Banu, Abu Sayeed Mohammad Mahmud, Barna Goswami, Iffat Jahan, Md. Saddam Hossain, Tasnim Nafisa, Md. Maruf Ahmed Molla, Mahmuda Yeasmin, Asish Kumar Ghosh, Arifa Akram, A. K. M.Shamsuzzaman, Md. Salim Khan
EPI_ISL_959394, EPI_ISL_959395, EPI_ISL_959396, EPI_ISL_959397, EPI_ISL_959398, EPI_ISL_959399, EPI_ISL_959400, EPI_ISL_959401, EPI_ISL_959402, EPI_ISL_959413	genXone SA, Molecular Diagnostics Laboratory / NZOZ	genXone SA, Research & Development Laboratory	Maciej Sykulski, Grzegorz Nowicki, Jakub Grabowski, Natalia Drwska-Matelska, Anna Brylak-Baszków, Aleksandra Gidlewicz, Karol Szeszko, ukasz Krych, Micha Kaszuba
EPI_ISL_959418, EPI_ISL_959422, EPI_ISL_959435, EPI_ISL_959436, EPI_ISL_959437, EPI_ISL_959443, EPI_ISL_959445, EPI_ISL_959450, EPI_ISL_959451, EPI_ISL_959452, EPI_ISL_959453, EPI_ISL_959454			
see above	Servicio de Microbiología, Hospital Universitario Son Espases	SeqCOVID-SPAIN consortium/IBV(CSIC)	Carla López-Causapé, Jordi Reina, Antonio Oliver and SeqCOVID-SPAIN consortium
EPI_ISL_959543	University of Michigan Clinical Microbiology Laboratory	Lauring Lab, University of Michigan, Department of Microbiology and Immunology	Valesano
EPI_ISL_959598	Vivalia - Clinique Saint-Joseph	GIGA Medical Genomics	Keith Durkin, Maria Artesi, Sébastien Bontems, Raphaël Boreux, Bouchra Boujemla, Cécile Meex, Pierrette Melin, Marie-Pierre Hayette, Vincent Bours

EPI_ISL_959599	Synlab	GIGA Medical Genomics	Keith Durkin, Maria Artesi, Sébastien Bontems, Raphaël Boreux, Bouchra Boujemla, Cécile Meex, Pierrette Melin, Marie-Pierre Hayette, Vincent Bours
EPI_ISL_959600, EPI_ISL_959601, EPI_ISL_959602	CHR de la Citadelle	GIGA Medical Genomics	Keith Durkin, Maria Artesi, Sébastien Bontems, Raphaël Boreux, Bouchra Boujemla, Cécile Meex, Pierrette Melin, Marie-Pierre Hayette, Vincent Bours
EPI_ISL_959603	Synlab	GIGA Medical Genomics	Keith Durkin, Maria Artesi, Sébastien Bontems, Raphaël Boreux, Bouchra Boujemla, Cécile Meex, Pierrette Melin, Marie-Pierre Hayette, Vincent Bours
EPI_ISL_959621	Institute of Virology, Biomedical Research Center of the Slovak Academy of Sciences, Bratislava	Faculty of Natural Sciences, Comenius University, Bratislava	Kristína Boršová, Viktória abanová, Broa Brejová, Viktória Hodorová, Sabina Fumaová Havlíková, Juraj Kopáek, Martina Liková, ubomíra Lukáiková, Martina Neboháová, Monika Sláviková, Tomáš Vina, Boris Klempa, Jozef Nosek
EPI_ISL_959632	Institute of Virology, Biomedical Research Center of the Slovak Academy of Sciences, Bratislava	Faculty of Natural Sciences, Comenius University, Bratislava	Broa Brejová, Viktória abanová, Kristína Boršová, Viktória Hodorová, Sabina Fumaová Havlíková, Juraj Kopáek, Martina Liková, ubomíra Lukáiková, Martina Neboháová, Monika Sláviková, Tomáš Vina, Jozef Nosek, Boris Klempa
EPI_ISL_959633	Institute of Virology, Biomedical Research Center of the Slovak Academy of Sciences, Bratislava	Faculty of Natural Sciences, Comenius University, Bratislava	Viktória abanová, Kristína Boršová, Broa Brejová, Viktória Hodorová, Sabina Fumaová Havlíková, Juraj Kopáek, Martina Liková, ubomíra Lukáiková, Martina Neboháová, Monika Sláviková, Tomáš Vina, Jozef Nosek, Boris Klempa
EPI_ISL_959634	Institute of Virology, Biomedical Research Center of the Slovak Academy of Sciences, Bratislava	Faculty of Natural Sciences, Comenius University, Bratislava	Kristína Boršová, Viktória abanová, Broa Brejová, Viktória Hodorová, Sabina Fumaová Havlíková, Juraj Kopáek, Martina Liková, ubomíra Lukáiková, Martina Neboháová, Monika Sláviková, Tomáš Vina, Boris Klempa, Jozef Nosek
EPI_ISL_959635	Institute of Virology, Biomedical Research Center of the Slovak Academy of Sciences, Bratislava	Faculty of Natural Sciences, Comenius University, Bratislava	Broa Brejová, Viktória abanová, Kristína Boršová, Viktória Hodorová, Sabina Fumaová Havlíková, Juraj Kopáek, Martina Liková, ubomíra Lukáiková, Martina Neboháová, Monika Sláviková, Tomáš Vina, Jozef Nosek, Boris Klempa
EPI_ISL_959636	Institute of Virology, Biomedical Research Center of the Slovak Academy of Sciences, Bratislava	Faculty of Natural Sciences, Comenius University, Bratislava	Viktória abanová, Kristína Boršová, Broa Brejová, Viktória Hodorová, Sabina Fumaová Havlíková, Juraj Kopáek, Martina Liková, ubomíra Lukáiková, Martina Neboháová, Monika Sláviková, Tomáš Vina, Jozef Nosek, Boris Klempa
EPI_ISL_959713, EPI_ISL_959714, EPI_ISL_959715, EPI_ISL_959716, EPI_ISL_959717, EPI_ISL_959718, EPI_ISL_959719, EPI_ISL_959720, EPI_ISL_959721, EPI_ISL_959722, EPI_ISL_959723, EPI_ISL_959724, EPI_ISL_959725, EPI_ISL_959726, EPI_ISL_959727, EPI_ISL_959728, EPI_ISL_959729, EPI_ISL_959730, EPI_ISL_959731, EPI_ISL_959732, EPI_ISL_959733, EPI_ISL_959734, EPI_ISL_959735, EPI_ISL_959736, EPI_ISL_959737, EPI_ISL_959738, EPI_ISL_959739, EPI_ISL_959740, EPI_ISL_959741, EPI_ISL_959742, EPI_ISL_959743, EPI_ISL_959744, EPI_ISL_959745, EPI_ISL_959746, EPI_ISL_959747			
see above	National Virus Reference Laboratory	National Virus Reference Laboratory	Michael Carr, Gabriel Gonzalez, Jonathan Dean, Cillian F De Gascun
EPI_ISL_960319, EPI_ISL_960323, EPI_ISL_960326, EPI_ISL_960328, EPI_ISL_960329, EPI_ISL_960331, EPI_ISL_960332, EPI_ISL_960337, EPI_ISL_960338, EPI_ISL_960345, EPI_ISL_960346, EPI_ISL_960350, EPI_ISL_960351, EPI_ISL_960371, EPI_ISL_960372, EPI_ISL_960373, EPI_ISL_960374, EPI_ISL_960377, EPI_ISL_960379, EPI_ISL_960384, EPI_ISL_960387, EPI_ISL_960400, EPI_ISL_960401, EPI_ISL_960402			
see above	University of Wisconsin-Madison AIDS Vaccine Research Laboratories	University of Wisconsin-Madison AIDS Vaccine Research Laboratories	Gage Moreno, Katarina Braun, et al. AIDS Vaccine Research Laboratories
EPI_ISL_960838, EPI_ISL_960839, EPI_ISL_960840, EPI_ISL_960841	Medizinisches Labor Prof. Dr. Schenk/ Dr. Ansorge & Kollegen, Magdeburg	Institute of Medical Microbiology and Hospital Hygiene	Prof. Dr. Achim Kaasch, Aljoscha Tersteegen
EPI_ISL_960842	Institute of Medical Microbiology and Hospital Hygiene	Institute of Medical Microbiology and Hospital Hygiene	Prof. Dr. Achim Kaasch, Aljoscha Tersteegen
EPI_ISL_960844	Medizinisches Labor Prof. Dr. Schenk/ Dr. Ansorge & Kollegen, Magdeburg	Institute of Medical Microbiology and Hospital Hygiene	Prof. Dr. Achim Kaasch, Aljoscha Tersteegen
EPI_ISL_960845, EPI_ISL_960847, EPI_ISL_960850, EPI_ISL_960858, EPI_ISL_960861, EPI_ISL_960864, EPI_ISL_960874, EPI_ISL_960875	Institute of Medical Microbiology and Hospital Hygiene	Institute of Medical Microbiology and Hospital Hygiene	Prof. Dr. Achim Kaasch, Aljoscha Tersteegen
EPI_ISL_960883, EPI_ISL_960886, EPI_ISL_960904, EPI_ISL_960905, EPI_ISL_960906, EPI_ISL_960907, EPI_ISL_960908, EPI_ISL_960909, EPI_ISL_960910, EPI_ISL_960911, EPI_ISL_960912, EPI_ISL_960913, EPI_ISL_960914, EPI_ISL_960915, EPI_ISL_960916, EPI_ISL_960917, EPI_ISL_960918, EPI_ISL_960919, EPI_ISL_960920, EPI_ISL_960921, EPI_ISL_960922, EPI_ISL_960923, EPI_ISL_960924, EPI_ISL_960925, EPI_ISL_960926, EPI_ISL_960927, EPI_ISL_960928, EPI_ISL_960929, EPI_ISL_960930, EPI_ISL_960931, EPI_ISL_960932, EPI_ISL_960933, EPI_ISL_960934, EPI_ISL_960943, EPI_ISL_960944, EPI_ISL_960945, EPI_ISL_960946, EPI_ISL_960947, EPI_ISL_960948, EPI_ISL_960949, EPI_ISL_960950, EPI_ISL_960954, EPI_ISL_960955, EPI_ISL_960956, EPI_ISL_960958, EPI_ISL_960959, EPI_ISL_960960, EPI_ISL_960961, EPI_ISL_960962, EPI_ISL_960963, EPI_ISL_960964, EPI_ISL_960965, EPI_ISL_960966, EPI_ISL_960967, EPI_ISL_960968, EPI_ISL_960969, EPI_ISL_960970, EPI_ISL_960971, EPI_ISL_960972, EPI_ISL_960973, EPI_ISL_960974, EPI_ISL_960975, EPI_ISL_960976, EPI_ISL_960977, EPI_ISL_960978, EPI_ISL_960979, EPI_ISL_960980, EPI_ISL_960981			
see above	Medizinisches Labor Prof. Dr. Schenk/ Dr. Ansorge & Kollegen, Magdeburg	Institute of Medical Microbiology and Hospital Hygiene	Prof. Dr. Achim Kaasch, Aljoscha Tersteegen
EPI_ISL_961204, EPI_ISL_961205, EPI_ISL_961206, EPI_ISL_961208, EPI_ISL_961209, EPI_ISL_961210, EPI_ISL_961211, EPI_ISL_961212, EPI_ISL_961213, EPI_ISL_961214, EPI_ISL_961216, EPI_ISL_961218, EPI_ISL_961226, EPI_ISL_961232, EPI_ISL_961233, EPI_ISL_961235, EPI_ISL_961236, EPI_ISL_961237, EPI_ISL_961239, EPI_ISL_961240, EPI_ISL_961241, EPI_ISL_961242, EPI_ISL_961243, EPI_ISL_961244, EPI_ISL_961245, EPI_ISL_961246, EPI_ISL_961247, EPI_ISL_961248, EPI_ISL_961249, EPI_ISL_961250, EPI_ISL_961251, EPI_ISL_961252, EPI_ISL_961254, EPI_ISL_961255, EPI_ISL_961256, EPI_ISL_961257, EPI_ISL_961258, EPI_ISL_961259, EPI_ISL_961260, EPI_ISL_961261, EPI_ISL_961262, EPI_ISL_961263, EPI_ISL_961264, EPI_ISL_961265, EPI_ISL_961266, EPI_ISL_961267, EPI_ISL_961268, EPI_ISL_961269, EPI_ISL_961272, EPI_ISL_961273, EPI_ISL_961274, EPI_ISL_961275, EPI_ISL_961276, EPI_ISL_961277, EPI_ISL_961278, EPI_ISL_961279, EPI_ISL_961280, EPI_ISL_961281, EPI_ISL_961282, EPI_ISL_961283, EPI_ISL_961284, EPI_ISL_961285			
see above	Hospital General Universitario de Alicante - Instituto de Investigación Sanitaria y Biomédica de Alicante	SeqCOVID-SPAIN consortium/IBV(CSIC)	Maripaz Ventero Martín, Carmen Molina Pardines and SeqCOVID-SPAIN consortium
EPI_ISL_961355	Centrum voor Medische Analyse	UAntwerp, Laboratory of Medical Microbiology	Basil Britto Xavier, Jasmine Coppens, Marie Le Mercier, Christine Lammens, Veerle Matheeussen, Herman Goossens
EPI_ISL_961359, EPI_ISL_961362	Toronto Invasive Bacterial Diseases Network	McMaster University	Allison McGeer, Patryk Aftanas, Hooman Derakhshani, Angel Li, Kuganya Nirmalarajah, Emily Panousis, Ahmed Draia, Jalees Nasir, Michael Surette, Samira Mubareka, Andrew G. McArthur
EPI_ISL_961508, EPI_ISL_961522, EPI_ISL_961528	Michigan Department of Health and Human Services, Bureau of Laboratories	Michigan Department of Health and Human Services, Bureau of Laboratories	Blankenship HM, Riner D, Soehnlen MK
EPI_ISL_961752, EPI_ISL_961753	SIESP SULMONA	Istituto Zooprofilattico Sperimentale dell'Abruzzo e Molise "G. Caporale"	Lorusso A, Marcacci M, Di Domenico M, Ancora M, Curini V, Mangone I, Rinaldi A, Scialabba S, Di Pasquale A, Cammà C, Puglia I, Calistri P, Savini G
EPI_ISL_962189, EPI_ISL_962197, EPI_ISL_962198, EPI_ISL_962199, EPI_ISL_962200, EPI_ISL_962201, EPI_ISL_962202	Toronto Invasive Bacterial Diseases Network	McMaster University	Allison McGeer, Patryk Aftanas, Hooman Derakhshani, Angel Li, Kuganya Nirmalarajah, Emily Panousis, Ahmed Draia, Jalees Nasir, Michael Surette, Samira Mubareka, Andrew G. McArthur
EPI_ISL_962303, EPI_ISL_962304	Seattle Flu Study	Seattle Flu Study	Deborah A. Nickerson, Chris D. Frazar, Jover Lee, Benjamin Pelle, Erica Ryke, Matthew Richardson, Amanda Adler, Elisabeth Brandstetter, Peter D. Han, Kairsten Fay, Misja Ilcisin, Kirsten Lacombe, Thomas R. Sibley, Melissa Truong, Caitlin R. Wolf, Michael Boeckh, Janet A. Englund, Michael Famulare, Barry R. Lutz, Mark J. Rieder, Lea M. Starita, Matthew Thompson, Jay Shendure, Trevor Bedford, Helen Y. Chu
EPI_ISL_962372, EPI_ISL_962373, EPI_ISL_962374	Seattle Flu Study	Seattle Flu Study	Deborah A. Nickerson, Chris D. Frazar, Jover Lee, Benjamin Pelle, Erica Ryke, Matthew Richardson, Amanda Adler, Elisabeth Brandstetter, Peter D. Han, Kairsten Fay, Misja Ilcisin, Kirsten Lacombe, Thomas R. Sibley, Melissa Truong, Caitlin R. Wolf, Karen Cowgill, Stephanie Schrag, Jeff Duchin, Michael Boeckh, Janet A. Englund, Michael Famulare, Barry R. Lutz, Mark J. Rieder, Lea M. Starita, Matthew Thompson, Helen Y. Chu, Trevor Bedford, Jay Shendure
EPI_ISL_962436, EPI_ISL_962437, EPI_ISL_962438, EPI_ISL_962439, EPI_ISL_962440, EPI_ISL_962441	Washington State Department of Health	Seattle Flu Study	Deborah A. Nickerson, Chris D. Frazar, Jover Lee, Benjamin Pelle, Erica Ryke, Matthew Richardson, Amanda Adler, Elisabeth Brandstetter, Peter D. Han, Kairsten Fay, Misja Ilcisin, Kirsten Lacombe, Thomas R. Sibley, Melissa Truong, Caitlin R. Wolf, Romesh Gautom, Geoff Melly, Brian Hiatt, Philip Dykema, Scott Lindquist, Michael Boeckh, Janet A. Englund, Michael Famulare, Barry R. Lutz, Mark J. Rieder, Lea M. Starita, Matthew Thompson, Helen Y. Chu, Jay Shendure, Trevor Bedford
EPI_ISL_962443, EPI_ISL_962444, EPI_ISL_962465, EPI_ISL_962466, EPI_ISL_962468, EPI_ISL_962469, EPI_ISL_962470, EPI_ISL_962471, EPI_ISL_962472, EPI_ISL_962473	Seattle Flu Study	Seattle Flu Study	Deborah A. Nickerson, Chris D. Frazar, Jover Lee, Benjamin Pelle, Erica Ryke, Matthew Richardson, Amanda Adler, Elisabeth Brandstetter, Peter D. Han, Kairsten Fay, Misja Ilcisin, Kirsten Lacombe, Thomas R. Sibley, Melissa Truong, Caitlin R. Wolf, Karen Cowgill, Stephanie Schrag, Jeff Duchin, Michael Boeckh, Janet A. Englund, Michael Famulare, Barry R. Lutz, Mark J. Rieder, Lea M. Starita, Matthew Thompson, Helen Y. Chu, Trevor Bedford, Jay Shendure
EPI_ISL_962517, EPI_ISL_962518, EPI_ISL_962519, EPI_ISL_962520	VA Medical Center	Los Angeles County PHL	P. Hemarajata et al.

EPI_ISL_962523, EPI_ISL_962524	Institute for Medical Research, Infectious Disease Research Centre, National Institutes of Health, Ministry of Health Malaysia	Institute for Medical Research, Infectious Disease Research Centre, National Institutes of Health, Ministry of Health Malaysia	Suppiah J, Kamel K, Azizan MA, Thayan R
EPI_ISL_962833, EPI_ISL_962834, EPI_ISL_962835, EPI_ISL_962836, EPI_ISL_962841, EPI_ISL_962842, EPI_ISL_962843, EPI_ISL_962844, EPI_ISL_962845	UCLA Clinical Micro Lab	Los Angeles County PHL	P. Hemarajata et al.
EPI_ISL_962852, EPI_ISL_962853, EPI_ISL_962854, EPI_ISL_962855, EPI_ISL_962856, EPI_ISL_962857, EPI_ISL_962858, EPI_ISL_962859, EPI_ISL_962860, EPI_ISL_962861, EPI_ISL_962862, EPI_ISL_962863, EPI_ISL_962864, EPI_ISL_962865, EPI_ISL_962866, EPI_ISL_962867			
see above	Los Angeles County PHL	Los Angeles County PHL	P. Hemarajata et al.
EPI_ISL_962879	UCLA Clinical Micro Lab	Los Angeles County PHL	P. Hemarajata et al.
EPI_ISL_963325, EPI_ISL_963408, EPI_ISL_963471, EPI_ISL_963524, EPI_ISL_963555	Lighthouse Lab in Milton Keynes	Wellcome Sanger Institute for the COVID-19 Genomics UK (COG-UK) Consortium	The Lighthouse Lab in Milton Keynes and Alex Alderton, Roberto Amato, Sonia Goncalves, Ewan Harrison, David K. Jackson, Ian Johnston, Dominic Kwiatkowski, Cordelia Langford, John Sillitoe on behalf of the Wellcome Sanger Institute COVID-19 Surveillance Team
EPI_ISL_963651, EPI_ISL_963654, EPI_ISL_963658, EPI_ISL_963659, EPI_ISL_963665, EPI_ISL_963670, EPI_ISL_963671, EPI_ISL_963672, EPI_ISL_963673, EPI_ISL_963676, EPI_ISL_963677, EPI_ISL_963679, EPI_ISL_963681, EPI_ISL_963685, EPI_ISL_963686, EPI_ISL_963687, EPI_ISL_963689, EPI_ISL_963690, EPI_ISL_963694, EPI_ISL_963695, EPI_ISL_963700, EPI_ISL_963701, EPI_ISL_963703, EPI_ISL_963704, EPI_ISL_963706, EPI_ISL_963707, EPI_ISL_963709, EPI_ISL_963718, EPI_ISL_963720, EPI_ISL_963721, EPI_ISL_963724, EPI_ISL_963726, EPI_ISL_963727, EPI_ISL_963731, EPI_ISL_963732, EPI_ISL_963733, EPI_ISL_963734, EPI_ISL_963735, EPI_ISL_963736, EPI_ISL_963737, EPI_ISL_963738, EPI_ISL_963740, EPI_ISL_963741, EPI_ISL_963747, EPI_ISL_963750, EPI_ISL_963753, EPI_ISL_963754, EPI_ISL_963758, EPI_ISL_963759, EPI_ISL_963761, EPI_ISL_963764, EPI_ISL_963765, EPI_ISL_963766, EPI_ISL_963768, EPI_ISL_963769, EPI_ISL_963772, EPI_ISL_963773, EPI_ISL_963774, EPI_ISL_963775, EPI_ISL_963779, EPI_ISL_963780, EPI_ISL_963781, EPI_ISL_963783, EPI_ISL_963786, EPI_ISL_963789, EPI_ISL_963790, EPI_ISL_963794, EPI_ISL_963798, EPI_ISL_963800, EPI_ISL_963803, EPI_ISL_963805, EPI_ISL_963806, EPI_ISL_963807, EPI_ISL_963808, EPI_ISL_963810, EPI_ISL_963813, EPI_ISL_963814, EPI_ISL_963815, EPI_ISL_963816, EPI_ISL_963819, EPI_ISL_963821, EPI_ISL_963822, EPI_ISL_963823, EPI_ISL_963824, EPI_ISL_963825, EPI_ISL_963826, EPI_ISL_963831, EPI_ISL_963832, EPI_ISL_963833, EPI_ISL_963835, EPI_ISL_963838, EPI_ISL_963839, EPI_ISL_963841, EPI_ISL_963844, EPI_ISL_963845, EPI_ISL_963846, EPI_ISL_963849, EPI_ISL_963850, EPI_ISL_963851, EPI_ISL_963852, EPI_ISL_963853, EPI_ISL_963855, EPI_ISL_963857, EPI_ISL_963859, EPI_ISL_963861, EPI_ISL_963863, EPI_ISL_963864, EPI_ISL_963865, EPI_ISL_963866, EPI_ISL_963867, EPI_ISL_963868, EPI_ISL_963869, EPI_ISL_963870, EPI_ISL_963872, EPI_ISL_963873, EPI_ISL_963874, EPI_ISL_963875, EPI_ISL_963877, EPI_ISL_963878, EPI_ISL_963879, EPI_ISL_963880, EPI_ISL_963881, EPI_ISL_963882, EPI_ISL_963883, EPI_ISL_963884, EPI_ISL_963889, EPI_ISL_963890, EPI_ISL_963891, EPI_ISL_963893, EPI_ISL_963895, EPI_ISL_963896, EPI_ISL_963900, EPI_ISL_963901, EPI_ISL_963902, EPI_ISL_963903, EPI_ISL_963904, EPI_ISL_963906, EPI_ISL_963908, EPI_ISL_963909, EPI_ISL_963911, EPI_ISL_963914, EPI_ISL_963915, EPI_ISL_963916, EPI_ISL_963921, EPI_ISL_963922, EPI_ISL_963923, EPI_ISL_963925, EPI_ISL_963927, EPI_ISL_963932, EPI_ISL_963934, EPI_ISL_963936, EPI_ISL_963938, EPI_ISL_963941, EPI_ISL_963942, EPI_ISL_963945, EPI_ISL_963947, EPI_ISL_963949, EPI_ISL_963952, EPI_ISL_963953, EPI_ISL_963958, EPI_ISL_963960, EPI_ISL_963962, EPI_ISL_963964, EPI_ISL_963968, EPI_ISL_963970, EPI_ISL_963971, EPI_ISL_963973, EPI_ISL_963976, EPI_ISL_963977, EPI_ISL_963978, EPI_ISL_963979, EPI_ISL_963981, EPI_ISL_963984, EPI_ISL_964067, EPI_ISL_964087, EPI_ISL_964236, EPI_ISL_964710, EPI_ISL_964726, EPI_ISL_964749, EPI_ISL_964759, EPI_ISL_964817, EPI_ISL_964877			
see above	Lighthouse Lab in Alderley Park	Wellcome Sanger Institute for the COVID-19 Genomics UK (COG-UK) Consortium	Jacquelyn Wynn, Mairead Hyland, The Lighthouse Lab in Alderley Park and Alex Alderton, Roberto Amato, Sonia Goncalves, Ewan Harrison, David K. Jackson, Ian Johnston, Dominic Kwiatkowski, Cordelia Langford, John Sillitoe on behalf of the Wellcome Sanger Institute COVID-19 Surveillance Team
EPI_ISL_964917	National Institute of Laboratory Medicine and Referral Center	Genomic Research Lab, BCSIR	Md. Maruf Ahmed Molla, Mohammad Samir Uzzaman, Eshrar Osman, Md. Ahashan Habib, Shahina Akter,Tanjina Akhtar Banu, Abu Sayeed Mohammad Mahmud, Md. Murshed Hasan Sarkar, Barna Goswami, Iffat Jahan, Md. Saddam Hossain, Tasnim Nafisa, Mahmuda Yeasmin, Asish Kumar Ghosh, Arifa Akram, A. K. M. Shamsuzzaman, Md. Salim Khan
EPI_ISL_964956, EPI_ISL_964957	Nordland Hospital - Bodo, Laboratory Department, Molecular Biology Unit	Norwegian Institute of Public Health, Department of Virology	Kathrine Stene-Johansen, Kamilla Heddeland Instefjord, Hilde Elshaug, Ignacio Garcia Llorente, Serina B Engebretsen, Atiya R Ali,Marie Paulsen Madsen, Rasmus Riis Kopperud, Hilde Vollan, Karoline Bragstad, Olav Hungnes
EPI_ISL_964962	Norwegian Institute of Public Health, Department of Virology	Norwegian Institute of Public Health, Department of Virology	Kathrine Stene-Johansen, Kamilla Heddeland Instefjord, Hilde Elshaug, Ignacio Garcia Llorente, Serina B Engebretsen, Atiya R Ali,Marie Paulsen Madsen, Rasmus Riis Kopperud, Hilde Vollan, Karoline Bragstad, Olav Hungnes
EPI_ISL_964965	Akershus University Hospital, Department for Microbiology and Infectious Disease Control	Norwegian Institute of Public Health, Department of Virology	Kathrine Stene-Johansen, Kamilla Heddeland Instefjord, Hilde Elshaug, Ignacio Garcia Llorente, Serina B Engebretsen, Atiya R Ali,Marie Paulsen Madsen, Rasmus Riis Kopperud, Hilde Vollan, Karoline Bragstad, Olav Hungnes
EPI_ISL_964972	Ostfold Hospital Trust - Kalnes, Centre for Laboratory Medicine, Section for gene technology and infection serology	Norwegian Institute of Public Health, Department of Virology	Kathrine Stene-Johansen, Kamilla Heddeland Instefjord, Hilde Elshaug, Ignacio Garcia Llorente, Serina B Engebretsen, Atiya R Ali,Marie Paulsen Madsen, Rasmus Riis Kopperud, Hilde Vollan, Karoline Bragstad, Olav Hungnes
EPI_ISL_964975	Akershus University Hospital, Department for Microbiology and Infectious Disease Control	Norwegian Institute of Public Health, Department of Virology	Kathrine Stene-Johansen, Kamilla Heddeland Instefjord, Hilde Elshaug, Ignacio Garcia Llorente, Serina B Engebretsen, Atiya R Ali,Marie Paulsen Madsen, Rasmus Riis Kopperud, Hilde Vollan, Karoline Bragstad, Olav Hungnes
EPI_ISL_964977, EPI_ISL_964978	Vestfold Hospital, Toensberg Department of Microbiology	Norwegian Institute of Public Health, Department of Virology	Kathrine Stene-Johansen, Kamilla Heddeland Instefjord, Hilde Elshaug, Ignacio Garcia Llorente, Serina B Engebretsen, Atiya R Ali,Marie Paulsen Madsen, Rasmus Riis Kopperud, Hilde Vollan, Karoline Bragstad, Olav Hungnes
EPI_ISL_964979	Furst Medical Laboratory	Norwegian Institute of Public Health, Department of Virology	Kathrine Stene-Johansen, Kamilla Heddeland Instefjord, Hilde Elshaug, Ignacio Garcia Llorente, Serina B Engebretsen, Atiya R Ali,Marie Paulsen Madsen, Rasmus Riis Kopperud, Hilde Vollan, Karoline Bragstad, Olav Hungnes
EPI_ISL_964984, EPI_ISL_964985, EPI_ISL_964986	Hospital of Southern Norway - Kristiansand, Department of Medical Microbiology	Norwegian Institute of Public Health, Department of Virology	Kathrine Stene-Johansen, Kamilla Heddeland Instefjord, Hilde Elshaug, Ignacio Garcia Llorente, Serina B Engebretsen, Atiya R Ali,Marie Paulsen Madsen, Rasmus Riis Kopperud, Hilde Vollan, Karoline Bragstad, Olav Hungnes
EPI_ISL_964989	Furst Medical Laboratory	Norwegian Institute of Public Health, Department of Virology	Kathrine Stene-Johansen, Kamilla Heddeland Instefjord, Hilde Elshaug, Ignacio Garcia Llorente, Serina B Engebretsen, Atiya R Ali,Marie Paulsen Madsen, Rasmus Riis Kopperud, Hilde Vollan, Karoline Bragstad, Olav Hungnes
EPI_ISL_964991	Nordland Hospital - Bodo, Laboratory Department, Molecular Biology Unit	Norwegian Institute of Public Health, Department of Virology	Kathrine Stene-Johansen, Kamilla Heddeland Instefjord, Hilde Elshaug, Ignacio Garcia Llorente, Serina B Engebretsen, Atiya R Ali,Marie Paulsen Madsen, Rasmus Riis Kopperud, Hilde Vollan, Karoline Bragstad, Olav Hungnes
EPI_ISL_964992, EPI_ISL_964993	Akershus University Hospital, Department for Microbiology and Infectious Disease Control	Norwegian Institute of Public Health, Department of Virology	Kathrine Stene-Johansen, Kamilla Heddeland Instefjord, Hilde Elshaug, Ignacio Garcia Llorente, Serina B Engebretsen, Atiya R Ali,Marie Paulsen Madsen, Rasmus Riis Kopperud, Hilde Vollan, Karoline Bragstad, Olav Hungnes
EPI_ISL_964998, EPI_ISL_964999	Oslo University Hospital, Department of Medical Microbiology	Norwegian Institute of Public Health, Department of Virology	Kathrine Stene-Johansen, Kamilla Heddeland Instefjord, Hilde Elshaug, Ignacio Garcia Llorente, Serina B Engebretsen, Atiya R Ali, Marie Paulsen Madsen, Rasmus Riis Kopperud, Hilde Vollan, Karoline Bragstad, Olav Hungnes
EPI_ISL_965017, EPI_ISL_965018	Florida Bureau of Public Health Laboratories	Florida Bureau of Public Health Laboratories	Sarah Schmedes, Jason Blanton
EPI_ISL_965045, EPI_ISL_965046, EPI_ISL_965047, EPI_ISL_965048, EPI_ISL_965049, EPI_ISL_965050, EPI_ISL_965051, EPI_ISL_965052	Wyoming Public Health Laboratory	Wyoming Public Health Laboratory	Noah Hull, Taylor Fearing, Lynette Gumbleton, Channing Weber, Ashley Norberg, Bailey Bowcutt, and Wanda Manley
EPI_ISL_965157, EPI_ISL_965158, EPI_ISL_965159, EPI_ISL_965160, EPI_ISL_965161, EPI_ISL_965162, EPI_ISL_965163, EPI_ISL_965164, EPI_ISL_965165, EPI_ISL_965166, EPI_ISL_965167, EPI_ISL_965169, EPI_ISL_965171, EPI_ISL_965172, EPI_ISL_965173, EPI_ISL_965174, EPI_ISL_965175, EPI_ISL_965176, EPI_ISL_965177			
see above	Clinical Molecular Microbiology Laboratory, UNC Hospitals	Jeremy Wang	Jeremy Wang, Alexander Rubinsteyn, Colleen Rice, Jason Smedberg, Shawn Hawken, Melissa Miller, Corbin Jones, Robert Hagan
EPI_ISL_965306, EPI_ISL_965307, EPI_ISL_965308	Labo Luc Olivier	GIGA Medical Genomics	Keith Durkin, Maria Artesi, Sébastien Bontems, Raphaël Boreux, Bouchra Boujemla, Cécile Meex, Pierrette Melin, Marie-Pierre Hayette, Vincent Bours
EPI_ISL_965311, EPI_ISL_965312, EPI_ISL_965313, EPI_ISL_965316, EPI_ISL_965317	Synlab	GIGA Medical Genomics	Keith Durkin, Maria Artesi, Sébastien Bontems, Raphaël Boreux, Bouchra Boujemla, Cécile Meex, Pierrette Melin, Marie-Pierre Hayette, Vincent Bours
EPI_ISL_965318, EPI_ISL_965319	Labo Luc Olivier	GIGA Medical Genomics	Keith Durkin, Maria Artesi, Sébastien Bontems, Raphaël Boreux, Bouchra Boujemla, Cécile Meex, Pierrette Melin, Marie-Pierre Hayette, Vincent Bours
EPI_ISL_965320, EPI_ISL_965325, EPI_ISL_965327	Synlab	GIGA Medical Genomics	Keith Durkin, Maria Artesi, Sébastien Bontems, Raphaël Boreux, Bouchra Boujemla, Cécile Meex, Pierrette Melin, Marie-Pierre Hayette, Vincent Bours
EPI_ISL_965328, EPI_ISL_965330, EPI_ISL_965331	Labo Luc Olivier	GIGA Medical Genomics	Keith Durkin, Maria Artesi, Sébastien Bontems, Raphaël Boreux, Bouchra Boujemla, Cécile Meex, Pierrette Melin, Marie-Pierre Hayette, Vincent Bours

EPI_ISL_965333, EPI_ISL_965334, EPI_ISL_965336, EPI_ISL_965337	Synlab	GIGA Medical Genomics	Keith Durkin, Maria Artesi, Sébastien Bontems, Raphaël Boreux, Bouchra Boujemla, Cécile Meex, Pierrette Melin, Marie-Pierre Hayette, Vincent Bours
EPI_ISL_965343	Labo Luc Olivier	GIGA Medical Genomics	Keith Durkin, Maria Artesi, Sébastien Bontems, Raphaël Boreux, Bouchra Boujemla, Cécile Meex, Pierrette Melin, Marie-Pierre Hayette, Vincent Bours
EPI_ISL_965393	Clinique N.D de Grâce Gosselies	GIGA Medical Genomics	Keith Durkin, Maria Artesi, Sébastien Bontems, Raphaël Boreux, Bouchra Boujemla, Cécile Meex, Pierrette Melin, Marie-Pierre Hayette, Vincent Bours
EPI_ISL_965398	Synlab	GIGA Medical Genomics	Keith Durkin, Maria Artesi, Sébastien Bontems, Raphaël Boreux, Bouchra Boujemla, Cécile Meex, Pierrette Melin, Marie-Pierre Hayette, Vincent Bours
EPI_ISL_965399	Labo Luc Olivier	GIGA Medical Genomics	Keith Durkin, Maria Artesi, Sébastien Bontems, Raphaël Boreux, Bouchra Boujemla, Cécile Meex, Pierrette Melin, Marie-Pierre Hayette, Vincent Bours
EPI_ISL_965401	Synlab	GIGA Medical Genomics	Keith Durkin, Maria Artesi, Sébastien Bontems, Raphaël Boreux, Bouchra Boujemla, Cécile Meex, Pierrette Melin, Marie-Pierre Hayette, Vincent Bours
EPI_ISL_965402	Clinique N.D de Grâce Gosselies	GIGA Medical Genomics	Keith Durkin, Maria Artesi, Sébastien Bontems, Raphaël Boreux, Bouchra Boujemla, Cécile Meex, Pierrette Melin, Marie-Pierre Hayette, Vincent Bours
EPI_ISL_965405	Synlab	GIGA Medical Genomics	Keith Durkin, Maria Artesi, Sébastien Bontems, Raphaël Boreux, Bouchra Boujemla, Cécile Meex, Pierrette Melin, Marie-Pierre Hayette, Vincent Bours
EPI_ISL_965406, EPI_ISL_965407	Labo Luc Olivier	GIGA Medical Genomics	Keith Durkin, Maria Artesi, Sébastien Bontems, Raphaël Boreux, Bouchra Boujemla, Cécile Meex, Pierrette Melin, Marie-Pierre Hayette, Vincent Bours
EPI_ISL_965408	Synlab	GIGA Medical Genomics	Keith Durkin, Maria Artesi, Sébastien Bontems, Raphaël Boreux, Bouchra Boujemla, Cécile Meex, Pierrette Melin, Marie-Pierre Hayette, Vincent Bours
EPI_ISL_965409	Labo Luc Olivier	GIGA Medical Genomics	Keith Durkin, Maria Artesi, Sébastien Bontems, Raphaël Boreux, Bouchra Boujemla, Cécile Meex, Pierrette Melin, Marie-Pierre Hayette, Vincent Bours
EPI_ISL_965410	Synlab	GIGA Medical Genomics	Keith Durkin, Maria Artesi, Sébastien Bontems, Raphaël Boreux, Bouchra Boujemla, Cécile Meex, Pierrette Melin, Marie-Pierre Hayette, Vincent Bours
EPI_ISL_965411, EPI_ISL_965412, EPI_ISL_965413	Labo Luc Olivier	GIGA Medical Genomics	Keith Durkin, Maria Artesi, Sébastien Bontems, Raphaël Boreux, Bouchra Boujemla, Cécile Meex, Pierrette Melin, Marie-Pierre Hayette, Vincent Bours
EPI_ISL_965414, EPI_ISL_965415	CHR de la Citadelle	GIGA Medical Genomics	Keith Durkin, Maria Artesi, Sébastien Bontems, Raphaël Boreux, Bouchra Boujemla, Cécile Meex, Pierrette Melin, Marie-Pierre Hayette, Vincent Bours
EPI_ISL_965416, EPI_ISL_965418	Synlab	GIGA Medical Genomics	Keith Durkin, Maria Artesi, Sébastien Bontems, Raphaël Boreux, Bouchra Boujemla, Cécile Meex, Pierrette Melin, Marie-Pierre Hayette, Vincent Bours
EPI_ISL_965419, EPI_ISL_965420	Labo Luc Olivier	GIGA Medical Genomics	Keith Durkin, Maria Artesi, Sébastien Bontems, Raphaël Boreux, Bouchra Boujemla, Cécile Meex, Pierrette Melin, Marie-Pierre Hayette, Vincent Bours
EPI_ISL_965421, EPI_ISL_965422, EPI_ISL_965423, EPI_ISL_965424, EPI_ISL_965425, EPI_ISL_965426, EPI_ISL_965427, EPI_ISL_965428, EPI_ISL_965429, EPI_ISL_965430	CHR de la Citadelle	GIGA Medical Genomics	Keith Durkin, Maria Artesi, Sébastien Bontems, Raphaël Boreux, Bouchra Boujemla, Cécile Meex, Pierrette Melin, Marie-Pierre Hayette, Vincent Bours
EPI_ISL_965431	Synlab	GIGA Medical Genomics	Keith Durkin, Maria Artesi, Sébastien Bontems, Raphaël Boreux, Bouchra Boujemla, Cécile Meex, Pierrette Melin, Marie-Pierre Hayette, Vincent Bours
EPI_ISL_965432	CHR de la Citadelle	GIGA Medical Genomics	Keith Durkin, Maria Artesi, Sébastien Bontems, Raphaël Boreux, Bouchra Boujemla, Cécile Meex, Pierrette Melin, Marie-Pierre Hayette, Vincent Bours
EPI_ISL_965434, EPI_ISL_965435, EPI_ISL_965436	Synlab	GIGA Medical Genomics	Keith Durkin, Maria Artesi, Sébastien Bontems, Raphaël Boreux, Bouchra Boujemla, Cécile Meex, Pierrette Melin, Marie-Pierre Hayette, Vincent Bours
EPI_ISL_965437, EPI_ISL_965438, EPI_ISL_965439, EPI_ISL_965440, EPI_ISL_965441, EPI_ISL_965442, EPI_ISL_965443, EPI_ISL_965444, EPI_ISL_965445, EPI_ISL_965446, EPI_ISL_965447, EPI_ISL_965448, EPI_ISL_965449, EPI_ISL_965450			
see above	CHR de la Citadelle	GIGA Medical Genomics	Keith Durkin, Maria Artesi, Sébastien Bontems, Raphaël Boreux, Bouchra Boujemla, Cécile Meex, Pierrette Melin, Marie-Pierre Hayette, Vincent Bours
EPI_ISL_965452, EPI_ISL_965453, EPI_ISL_965454, EPI_ISL_965456	Synlab	GIGA Medical Genomics	Keith Durkin, Maria Artesi, Sébastien Bontems, Raphaël Boreux, Bouchra Boujemla, Cécile Meex, Pierrette Melin, Marie-Pierre Hayette, Vincent Bours
EPI_ISL_965460	St. Nikolaus-Hospital Eupen	GIGA Medical Genomics	Keith Durkin, Maria Artesi, Sébastien Bontems, Raphaël Boreux, Bouchra Boujemla, Cécile Meex, Pierrette Melin, Marie-Pierre Hayette, Vincent Bours
EPI_ISL_965467	Synlab	GIGA Medical Genomics	Keith Durkin, Maria Artesi, Sébastien Bontems, Raphaël Boreux, Bouchra Boujemla, Cécile Meex, Pierrette Melin, Marie-Pierre Hayette, Vincent Bours
EPI_ISL_965469	Labo Luc Olivier	GIGA Medical Genomics	Keith Durkin, Maria Artesi, Sébastien Bontems, Raphaël Boreux, Bouchra Boujemla, Cécile Meex, Pierrette Melin, Marie-Pierre Hayette, Vincent Bours
EPI_ISL_965472, EPI_ISL_965473	Synlab	GIGA Medical Genomics	Keith Durkin, Maria Artesi, Sébastien Bontems, Raphaël Boreux, Bouchra Boujemla, Cécile Meex, Pierrette Melin, Marie-Pierre Hayette, Vincent Bours
EPI_ISL_965474, EPI_ISL_965476, EPI_ISL_965477, EPI_ISL_965478	Labo Luc Olivier	GIGA Medical Genomics	Keith Durkin, Maria Artesi, Sébastien Bontems, Raphaël Boreux, Bouchra Boujemla, Cécile Meex, Pierrette Melin, Marie-Pierre Hayette, Vincent Bours
EPI_ISL_965479, EPI_ISL_965480, EPI_ISL_965482, EPI_ISL_965483	Synlab	GIGA Medical Genomics	Keith Durkin, Maria Artesi, Sébastien Bontems, Raphaël Boreux, Bouchra Boujemla, Cécile Meex, Pierrette Melin, Marie-Pierre Hayette, Vincent Bours
EPI_ISL_965487, EPI_ISL_965488, EPI_ISL_965489, EPI_ISL_965490, EPI_ISL_965491, EPI_ISL_965492	St. Nikolaus-Hospital Eupen	GIGA Medical Genomics	Keith Durkin, Maria Artesi, Sébastien Bontems, Raphaël Boreux, Bouchra Boujemla, Cécile Meex, Pierrette Melin, Marie-Pierre Hayette, Vincent Bours
EPI_ISL_965494, EPI_ISL_965497	Synlab	GIGA Medical Genomics	Keith Durkin, Maria Artesi, Sébastien Bontems, Raphaël Boreux, Bouchra Boujemla, Cécile Meex, Pierrette Melin, Marie-Pierre Hayette, Vincent Bours
EPI_ISL_965498, EPI_ISL_965500	St. Nikolaus-Hospital Eupen	GIGA Medical Genomics	Keith Durkin, Maria Artesi, Sébastien Bontems, Raphaël Boreux, Bouchra Boujemla, Cécile Meex, Pierrette Melin, Marie-Pierre Hayette, Vincent Bours
EPI_ISL_965501, EPI_ISL_965502	Synlab	GIGA Medical Genomics	Keith Durkin, Maria Artesi, Sébastien Bontems, Raphaël Boreux, Bouchra Boujemla, Cécile Meex, Pierrette Melin, Marie-Pierre Hayette, Vincent Bours
EPI_ISL_965504	Labo Luc Olivier	GIGA Medical Genomics	Keith Durkin, Maria Artesi, Sébastien Bontems, Raphaël Boreux, Bouchra Boujemla, Cécile Meex, Pierrette Melin, Marie-Pierre Hayette, Vincent Bours
EPI_ISL_965507, EPI_ISL_965514, EPI_ISL_965516, EPI_ISL_965519	Synlab	GIGA Medical Genomics	Keith Durkin, Maria Artesi, Sébastien Bontems, Raphaël Boreux, Bouchra Boujemla, Cécile Meex, Pierrette Melin, Marie-Pierre Hayette, Vincent Bours
EPI_ISL_965520, EPI_ISL_965522, EPI_ISL_965523	St. Nikolaus-Hospital Eupen	GIGA Medical Genomics	Keith Durkin, Maria Artesi, Sébastien Bontems, Raphaël Boreux, Bouchra Boujemla, Cécile Meex, Pierrette Melin, Marie-Pierre Hayette, Vincent Bours
EPI_ISL_965526	Synlab	GIGA Medical Genomics	Keith Durkin, Maria Artesi, Sébastien Bontems, Raphaël Boreux, Bouchra Boujemla, Cécile Meex, Pierrette Melin, Marie-Pierre Hayette, Vincent Bours
EPI_ISL_965643, EPI_ISL_965711	Dutch COVID-19 response team	Medical Microbiology, Maastricht University Medical Centre	Jozef Dingemans*, Brian van der Veer*, Erik Beuken, Carmen Reumkens, Lieke van Alphen, Christian Hoebe, Paul Savelkoul
EPI_ISL_965877, EPI_ISL_965878, EPI_ISL_965885, EPI_ISL_965895, EPI_ISL_965896, EPI_ISL_965898, EPI_ISL_965899, EPI_ISL_965901, EPI_ISL_965902	Massachusetts State Public Health Laboratory	Massachusetts State Public Health Laboratory	Andrew Lang, Timelia Fink, Glen Gallagher, Sandra Smole
EPI_ISL_965907, EPI_ISL_965908	Clinical Molecular Microbiology Laboratory, UNC Hospitals	Jeremy Wang	Jeremy Wang, Alexander Rubinsteyn, Colleen Rice, Jason Smedberg, Shawn Hawken, Melissa Miller, Corbin Jones, Robert Hagan
EPI_ISL_965917, EPI_ISL_965918, EPI_ISL_965919, EPI_ISL_965923	Hospital Los Arcos del Mar Menor	Instituto de Salud Carlos III	Vázquez, S. Iglesias-Caballero, M. Sandonís,V. Camarero, S. Pozo, F. Casas, I. Jiménez, P. Zaballos, A. Monzón, S. Varona, S. Cuesta, I. Cámara, M.
EPI_ISL_965930	Hospital Comarcal de Melilla	Instituto de Salud Carlos III	Sandonís,V. Vázquez, S. Iglesias-Caballero, M. Camarero, S. Pozo, F. Casas, I. Jiménez, P. Zaballos, A. Monzón, S. Varona, S. Cuesta, I. Román, S.
EPI_ISL_965935, EPI_ISL_965939, EPI_ISL_965941	Hospital Los Arcos del Mar Menor	Instituto de Salud Carlos III	Sandonís,V. Vázquez, S. Iglesias-Caballero, M. Camarero, S. Pozo, F. Casas, I. Jiménez, P. Zaballos, A. Monzón, S. Varona, S. Cuesta, I. Cámara, M.
EPI_ISL_965945, EPI_ISL_965947, EPI_ISL_965951, EPI_ISL_965958	Hospital Los Arcos del Mar Menor	Instituto de Salud Carlos III	Iglesias-Caballero, M. Sandonís,V. Vázquez, S. Camarero, S. Pozo, F. Casas, I. Jiménez, P. Zaballos, A. Monzón, S. Varona, S. Cuesta, I. Cámara, M.
EPI_ISL_966379, EPI_ISL_966381,	OCME Office Of Chief Medical Examiner	New York City Public Health Laboratory	Jade Wang, et al.

EPI_ISL_966383			
EPI_ISL_966385	Department of Homeless Services	New York City Public Health Laboratory	Jade Wang, et al.
EPI_ISL_966389	DOHMH Central Harlem	New York City Public Health Laboratory	Jade Wang, et al.
EPI_ISL_966390	OCME Office Of Chief Medical Examiner	New York City Public Health Laboratory	Jade Wang, et al.
EPI_ISL_966392	DOHMH Corona	New York City Public Health Laboratory	Jade Wang, et al.
EPI_ISL_966393	OCME Office Of Chief Medical Examiner	New York City Public Health Laboratory	Jade Wang, et al.
EPI_ISL_966394	DOHMH Corona	New York City Public Health Laboratory	Jade Wang, et al.
EPI_ISL_966396	OCME Office Of Chief Medical Examiner	New York City Public Health Laboratory	Jade Wang, et al.
EPI_ISL_966398	DOHMH Jamaica	New York City Public Health Laboratory	Jade Wang, et al.
EPI_ISL_966399	DOHMH Corona	New York City Public Health Laboratory	Jade Wang, et al.
EPI_ISL_966407, EPI_ISL_966408	DOHMH Morrisania	New York City Public Health Laboratory	Jade Wang, et al.
EPI_ISL_966409, EPI_ISL_966410	DOHMH Corona	New York City Public Health Laboratory	Jade Wang, et al.
EPI_ISL_966420	DOHMH Morrisania	New York City Public Health Laboratory	Jade Wang, et al.
EPI_ISL_966421, EPI_ISL_966422	DOHMH Jamaica	New York City Public Health Laboratory	Jade Wang, et al.
EPI_ISL_966423	DOHMH Morrisania	New York City Public Health Laboratory	Jade Wang, et al.
EPI_ISL_966424	DOHMH Jamaica	New York City Public Health Laboratory	Jade Wang, et al.
EPI_ISL_966425	DOHMH Corona	New York City Public Health Laboratory	Jade Wang, et al.
EPI_ISL_966426	DOHMH PHL	New York City Public Health Laboratory	Jade Wang, et al.
EPI_ISL_966461, EPI_ISL_966462, EPI_ISL_966463	DOHMH Jamaica	New York City Public Health Laboratory	Jade Wang, et al.
EPI_ISL_966464, EPI_ISL_966465, EPI_ISL_966466	DOHMH PHL	New York City Public Health Laboratory	Jade Wang, et al.
EPI_ISL_966467	DOHMH Corona	New York City Public Health Laboratory	Jade Wang, et al.
EPI_ISL_966468	DOHMH Chelsea	New York City Public Health Laboratory	Jade Wang, et al.
EPI_ISL_966469	Department of Homeless Services	New York City Public Health Laboratory	Jade Wang, et al.
EPI_ISL_966470, EPI_ISL_966471, EPI_ISL_966473	OCME Office Of Chief Medical Examiner	New York City Public Health Laboratory	Jade Wang, et al.
EPI_ISL_966474	DOHMH Corona	New York City Public Health Laboratory	Jade Wang, et al.
EPI_ISL_966475	DOHMH Central Harlem	New York City Public Health Laboratory	Jade Wang, et al.
EPI_ISL_966476, EPI_ISL_966477, EPI_ISL_966478	DOHMH Corona	New York City Public Health Laboratory	Jade Wang, et al.
EPI_ISL_966479	DOHMH Jamaica	New York City Public Health Laboratory	Jade Wang, et al.
EPI_ISL_966480	DOHMH Corona	New York City Public Health Laboratory	Jade Wang, et al.
EPI_ISL_966481	DOHMH Jamaica	New York City Public Health Laboratory	Jade Wang, et al.
EPI_ISL_966482	DOHMH Corona	New York City Public Health Laboratory	Jade Wang, et al.
EPI_ISL_966483	DOHMH Chelsea	New York City Public Health Laboratory	Jade Wang, et al.
EPI_ISL_966484	DOHMH Crown Heights	New York City Public Health Laboratory	Jade Wang, et al.
EPI_ISL_966485, EPI_ISL_966486, EPI_ISL_966487, EPI_ISL_966488, EPI_ISL_966490	OCME Office Of Chief Medical Examiner	New York City Public Health Laboratory	Jade Wang, et al.
EPI_ISL_966491	DOHMH Corona	New York City Public Health Laboratory	Jade Wang, et al.
EPI_ISL_966492, EPI_ISL_966493, EPI_ISL_966494	OCME Office Of Chief Medical Examiner	New York City Public Health Laboratory	Jade Wang, et al.
EPI_ISL_966786, EPI_ISL_966787	Maine HETL	Tewhey Lab, The Jackson Laboratory	Matluk,N., Dewey,H., Iosue,F., Barter,M., Lynch,R., Munger,H. and Tewhey,R.
EPI_ISL_966929, EPI_ISL_966930, EPI_ISL_966931, EPI_ISL_966932, EPI_ISL_966933, EPI_ISL_966934, EPI_ISL_966935, EPI_ISL_966936, EPI_ISL_966937, EPI_ISL_966938, EPI_ISL_966939, EPI_ISL_966941, EPI_ISL_966942, EPI_ISL_966943, EPI_ISL_966944, EPI_ISL_966945, EPI_ISL_966946, EPI_ISL_966947, EPI_ISL_966948, EPI_ISL_966949, EPI_ISL_966950, EPI_ISL_966951, EPI_ISL_966952, EPI_ISL_966953, EPI_ISL_966954, EPI_ISL_966955, EPI_ISL_966956, EPI_ISL_966957, EPI_ISL_966958, EPI_ISL_966959, EPI_ISL_966960, EPI_ISL_966961, EPI_ISL_966962, EPI_ISL_966963, EPI_ISL_966964, EPI_ISL_966965, EPI_ISL_966966, EPI_ISL_966967, EPI_ISL_966968, EPI_ISL_966969, EPI_ISL_966970, EPI_ISL_966971, EPI_ISL_966972, EPI_ISL_966973, EPI_ISL_966974, EPI_ISL_966975, EPI_ISL_966976, EPI_ISL_966977, EPI_ISL_966978, EPI_ISL_966979, EPI_ISL_966980, EPI_ISL_966981, EPI_ISL_966982, EPI_ISL_966983, EPI_ISL_966984, EPI_ISL_966985, EPI_ISL_966986, EPI_ISL_966987, EPI_ISL_966988, EPI_ISL_966989, EPI_ISL_966990, EPI_ISL_966991, EPI_ISL_966992, EPI_ISL_966993, EPI_ISL_966994, EPI_ISL_966995, EPI_ISL_966996, EPI_ISL_966997, EPI_ISL_966998, EPI_ISL_966999, EPI_ISL_967000, EPI_ISL_967001, EPI_ISL_967002, EPI_ISL_967003, EPI_ISL_967004, EPI_ISL_967005, EPI_ISL_967006, EPI_ISL_967007, EPI_ISL_967008, EPI_ISL_967009, EPI_ISL_967010, EPI_ISL_967011, EPI_ISL_967012, EPI_ISL_967013, EPI_ISL_967014, EPI_ISL_967015, EPI_ISL_967016, EPI_ISL_967017, EPI_ISL_967018, EPI_ISL_967019, EPI_ISL_967020, EPI_ISL_967021, EPI_ISL_967022, EPI_ISL_967023, EPI_ISL_967024, EPI_ISL_967025, EPI_ISL_967026, EPI_ISL_967027, EPI_ISL_967028, EPI_ISL_967029, EPI_ISL_967030, EPI_ISL_967031, EPI_ISL_967032, EPI_ISL_967033, EPI_ISL_967034, EPI_ISL_967035, EPI_ISL_967036, EPI_ISL_967037, EPI_ISL_967038, EPI_ISL_967039, EPI_ISL_967040, EPI_ISL_967041, EPI_ISL_967042, EPI_ISL_967043, EPI_ISL_967044, EPI_ISL_967045, EPI_ISL_967046, EPI_ISL_967047, EPI_ISL_967048, EPI_ISL_967049, EPI_ISL_967050, EPI_ISL_967051, EPI_ISL_967052, EPI_ISL_967053, EPI_ISL_967054, EPI_ISL_967055, EPI_ISL_967056, EPI_ISL_967057			
see above	Helix/Illumina	Respiratory Viruses Branch, Division of Viral Diseases, Centers for Disease Control and Prevention	Peter W. Cook,Dakota Howard,Dhwani Batra,Ben L. Rambo-Martin,Eileen de Feo,Jan Antico,Christine Tran,Matthew Tolentino,Shannon Wickline,Kim Gietzen,Brad Sickler,Jingtao Liu,Eric Allen,Phil Febbo,Summer Galloway,Nicole L. Washington,Simon White,Geraint Levan,Kelly Schiabor Barrett,Elizabeth Cirulli,Alexandre Bolze,Ary Ascencio,Charlotte Rivera-Garcia,Ryan Cho,Jason Nguyen,Sherry Wang,Jimmy Ramirez,Tyler Cassens,Efren Sandoval,Magnus Isaksson,William Lee,David Becker,Marc Laurent,James Lu,Clinton R. Paden,Suxiang Tong,Duncan MacCannell,
EPI_ISL_967764	State Laboratories Division, Hawaii State Department of Health	State Laboratories Division, Hawaii State Department of Health	Pamela O'Brien, Drew Kuwazaki, Ayana Garnet, Razvan Sultana, Edward Desmond
EPI_ISL_969007, EPI_ISL_969008, EPI_ISL_969012, EPI_ISL_969013, EPI_ISL_969014, EPI_ISL_969016, EPI_ISL_969017, EPI_ISL_969025, EPI_ISL_969026, EPI_ISL_969030, EPI_ISL_969031, EPI_ISL_969043, EPI_ISL_969044, EPI_ISL_969046, EPI_ISL_969053, EPI_ISL_969056, EPI_ISL_969061, EPI_ISL_969063, EPI_ISL_969064, EPI_ISL_969065, EPI_ISL_969066, EPI_ISL_969067, EPI_ISL_969071, EPI_ISL_969245			
see above	KEMRI-Wellcome Trust Research Programme/KEMRI-CGMR-C Kilifi	KEMRI-Wellcome Trust Research Programme/KEMRI-CGMR-C Kilifi	Githinji et al
EPI_ISL_969454, EPI_ISL_970032, EPI_ISL_970055, EPI_ISL_970142	Lighthouse Lab in Alderley Park	Wellcome Sanger Institute for the COVID-19 Genomics UK (COG-UK) Consortium	Jacquelyn Wynn, Mairead Hyland, The Lighthouse Lab in Alderley Park and Alex Alderton, Roberto Amato, Sonia Goncalves, Ewan Harrison, David K. Jackson, Ian Johnston, Dominic Kwiatkowski, Cordelia Langford, John Sillitoe on behalf of the Wellcome Sanger Institute COVID-19 Surveillance Team
EPI_ISL_970469	EHNV	Laboratory of genomics and metagenomics, Institute of Microbiology, University Hospital Centre and University of	Trestan Pillonel, Damien Jacot, Sébastien Aeby, Gilbert Greub, Claire Bertelli

		Lausanne, Switzerland	
EPI_ISL_970589, EPI_ISL_970591, EPI_ISL_970593, EPI_ISL_970595, EPI_ISL_970599, EPI_ISL_970601, EPI_ISL_970602, EPI_ISL_970605, EPI_ISL_970607, EPI_ISL_970609, EPI_ISL_970612, EPI_ISL_970614, EPI_ISL_970618, EPI_ISL_970619, EPI_ISL_970623, EPI_ISL_970625, EPI_ISL_970630, EPI_ISL_970632, EPI_ISL_970633, EPI_ISL_970637, EPI_ISL_970641, EPI_ISL_970643, EPI_ISL_970648, EPI_ISL_970650, EPI_ISL_970652, EPI_ISL_970654, EPI_ISL_970656, EPI_ISL_970658, EPI_ISL_970660, EPI_ISL_970661, EPI_ISL_970666, EPI_ISL_970668, EPI_ISL_970670, EPI_ISL_970675, EPI_ISL_970677, EPI_ISL_970683, EPI_ISL_970685, EPI_ISL_970689, EPI_ISL_970690, EPI_ISL_970692, EPI_ISL_970694, EPI_ISL_970697, EPI_ISL_970699, EPI_ISL_970703, EPI_ISL_970704, EPI_ISL_970706, EPI_ISL_970711, EPI_ISL_970713, EPI_ISL_970717, EPI_ISL_970720, EPI_ISL_970722, EPI_ISL_970726, EPI_ISL_970727, EPI_ISL_970729, EPI_ISL_970734, EPI_ISL_970736, EPI_ISL_970738, EPI_ISL_970739, EPI_ISL_970741, EPI_ISL_970746, EPI_ISL_970749, EPI_ISL_970755, EPI_ISL_970757, EPI_ISL_970758, EPI_ISL_970760, EPI_ISL_970762, EPI_ISL_970764, EPI_ISL_970766, EPI_ISL_970770, EPI_ISL_970772, EPI_ISL_970774, EPI_ISL_970777, EPI_ISL_970779, EPI_ISL_970781, EPI_ISL_970782, EPI_ISL_970784, EPI_ISL_970786, EPI_ISL_970788, EPI_ISL_970792, EPI_ISL_970794, EPI_ISL_970796, EPI_ISL_970797, EPI_ISL_970800, EPI_ISL_970802, EPI_ISL_970803, EPI_ISL_970805, EPI_ISL_970807, EPI_ISL_970808			
see above	Lighthouse Lab in Milton Keynes	Wellcome Sanger Institute for the COVID-19 Genomics UK (COG-UK) Consortium	The Lighthouse Lab in Milton Keynes and Alex Alderton, Roberto Amato, Sonia Goncalves, Ewan Harrison, David K. Jackson, Ian Johnston, Dominic Kwiatkowski, Cordelia Langford, John Sillitoe on behalf of the Wellcome Sanger Institute COVID-19 Surveillance Team
EPI_ISL_970810, EPI_ISL_970813, EPI_ISL_970814, EPI_ISL_970815, EPI_ISL_970816, EPI_ISL_970817, EPI_ISL_970822, EPI_ISL_970823, EPI_ISL_970825, EPI_ISL_970828, EPI_ISL_970833, EPI_ISL_970835, EPI_ISL_970836, EPI_ISL_970838, EPI_ISL_970839, EPI_ISL_970841, EPI_ISL_970842, EPI_ISL_970843			
see above	Department of Virus and Microbiological Special Diagnostics, Statens Serum Institut, Copenhagen, Denmark	Aalborg University	Danish Covid-19 Genome Consortium
EPI_ISL_970845	Lighthouse Lab in Milton Keynes	Wellcome Sanger Institute for the COVID-19 Genomics UK (COG-UK) Consortium	The Lighthouse Lab in Milton Keynes and Alex Alderton, Roberto Amato, Sonia Goncalves, Ewan Harrison, David K. Jackson, Ian Johnston, Dominic Kwiatkowski, Cordelia Langford, John Sillitoe on behalf of the Wellcome Sanger Institute COVID-19 Surveillance Team
EPI_ISL_970847, EPI_ISL_970848, EPI_ISL_970852, EPI_ISL_970853, EPI_ISL_970854, EPI_ISL_970855, EPI_ISL_970857, EPI_ISL_970858, EPI_ISL_970861, EPI_ISL_970864, EPI_ISL_970865, EPI_ISL_970866, EPI_ISL_970869, EPI_ISL_970870, EPI_ISL_970871, EPI_ISL_970872, EPI_ISL_970873, EPI_ISL_970876, EPI_ISL_970877, EPI_ISL_970881, EPI_ISL_970883, EPI_ISL_970884, EPI_ISL_970887, EPI_ISL_970888, EPI_ISL_970889, EPI_ISL_970890, EPI_ISL_970893, EPI_ISL_970894, EPI_ISL_970896, EPI_ISL_970897, EPI_ISL_970898			
see above	Department of Virus and Microbiological Special Diagnostics, Statens Serum Institut, Copenhagen, Denmark	Aalborg University	Danish Covid-19 Genome Consortium
EPI_ISL_970899	Lighthouse Lab in Milton Keynes	Wellcome Sanger Institute for the COVID-19 Genomics UK (COG-UK) Consortium	The Lighthouse Lab in Milton Keynes and Alex Alderton, Roberto Amato, Sonia Goncalves, Ewan Harrison, David K. Jackson, Ian Johnston, Dominic Kwiatkowski, Cordelia Langford, John Sillitoe on behalf of the Wellcome Sanger Institute COVID-19 Surveillance Team
EPI_ISL_970900, EPI_ISL_970903, EPI_ISL_970904, EPI_ISL_970906, EPI_ISL_970909, EPI_ISL_970911, EPI_ISL_970912, EPI_ISL_970913, EPI_ISL_970914, EPI_ISL_970915, EPI_ISL_970916, EPI_ISL_970917, EPI_ISL_970918, EPI_ISL_970919, EPI_ISL_970920, EPI_ISL_970921, EPI_ISL_970922, EPI_ISL_970923, EPI_ISL_970924			
see above	Department of Virus and Microbiological Special Diagnostics, Statens Serum Institut, Copenhagen, Denmark	Aalborg University	Danish Covid-19 Genome Consortium
EPI_ISL_970925	Lighthouse Lab in Milton Keynes	Wellcome Sanger Institute for the COVID-19 Genomics UK (COG-UK) Consortium	The Lighthouse Lab in Milton Keynes and Alex Alderton, Roberto Amato, Sonia Goncalves, Ewan Harrison, David K. Jackson, Ian Johnston, Dominic Kwiatkowski, Cordelia Langford, John Sillitoe on behalf of the Wellcome Sanger Institute COVID-19 Surveillance Team
EPI_ISL_970926, EPI_ISL_970933, EPI_ISL_970934, EPI_ISL_970935, EPI_ISL_970937, EPI_ISL_970940, EPI_ISL_970941	Department of Virus and Microbiological Special Diagnostics, Statens Serum Institut, Copenhagen, Denmark	Aalborg University	Danish Covid-19 Genome Consortium
EPI_ISL_970944	Lighthouse Lab in Milton Keynes	Wellcome Sanger Institute for the COVID-19 Genomics UK (COG-UK) Consortium	The Lighthouse Lab in Milton Keynes and Alex Alderton, Roberto Amato, Sonia Goncalves, Ewan Harrison, David K. Jackson, Ian Johnston, Dominic Kwiatkowski, Cordelia Langford, John Sillitoe on behalf of the Wellcome Sanger Institute COVID-19 Surveillance Team
EPI_ISL_970945, EPI_ISL_970948, EPI_ISL_970951, EPI_ISL_970954, EPI_ISL_970955, EPI_ISL_970956, EPI_ISL_970957, EPI_ISL_970958, EPI_ISL_970962, EPI_ISL_970963, EPI_ISL_970964, EPI_ISL_970967			
see above	Department of Virus and Microbiological Special Diagnostics, Statens Serum Institut, Copenhagen, Denmark	Aalborg University	Danish Covid-19 Genome Consortium
EPI_ISL_970968	Lighthouse Lab in Milton Keynes	Wellcome Sanger Institute for the COVID-19 Genomics UK (COG-UK) Consortium	The Lighthouse Lab in Milton Keynes and Alex Alderton, Roberto Amato, Sonia Goncalves, Ewan Harrison, David K. Jackson, Ian Johnston, Dominic Kwiatkowski, Cordelia Langford, John Sillitoe on behalf of the Wellcome Sanger Institute COVID-19 Surveillance Team
EPI_ISL_970971, EPI_ISL_970972, EPI_ISL_970974, EPI_ISL_970977, EPI_ISL_970979, EPI_ISL_970981, EPI_ISL_970983, EPI_ISL_970985, EPI_ISL_970988, EPI_ISL_970993, EPI_ISL_970997, EPI_ISL_970998, EPI_ISL_971001, EPI_ISL_971002, EPI_ISL_971003, EPI_ISL_971005, EPI_ISL_971007			
see above	Department of Virus and Microbiological Special Diagnostics, Statens Serum Institut, Copenhagen, Denmark	Aalborg University	Danish Covid-19 Genome Consortium
EPI_ISL_971008	Lighthouse Lab in Milton Keynes	Wellcome Sanger Institute for the COVID-19 Genomics UK (COG-UK) Consortium	The Lighthouse Lab in Milton Keynes and Alex Alderton, Roberto Amato, Sonia Goncalves, Ewan Harrison, David K. Jackson, Ian Johnston, Dominic Kwiatkowski, Cordelia Langford, John Sillitoe on behalf of the Wellcome Sanger Institute COVID-19 Surveillance Team
EPI_ISL_971010, EPI_ISL_971011, EPI_ISL_971013, EPI_ISL_971015, EPI_ISL_971016, EPI_ISL_971017, EPI_ISL_971018, EPI_ISL_971019, EPI_ISL_971020	Department of Virus and Microbiological Special Diagnostics, Statens Serum Institut, Copenhagen, Denmark	Aalborg University	Danish Covid-19 Genome Consortium
EPI_ISL_971023	Lighthouse Lab in Milton Keynes	Wellcome Sanger Institute for the COVID-19 Genomics UK (COG-UK) Consortium	The Lighthouse Lab in Milton Keynes and Alex Alderton, Roberto Amato, Sonia Goncalves, Ewan Harrison, David K. Jackson, Ian Johnston, Dominic Kwiatkowski, Cordelia Langford, John Sillitoe on behalf of the Wellcome Sanger Institute COVID-19 Surveillance Team
EPI_ISL_971024, EPI_ISL_971025, EPI_ISL_971026, EPI_ISL_971027, EPI_ISL_971029, EPI_ISL_971031, EPI_ISL_971034, EPI_ISL_971038	Department of Virus and Microbiological Special Diagnostics, Statens Serum Institut, Copenhagen, Denmark	Aalborg University	Danish Covid-19 Genome Consortium
EPI_ISL_971039	Lighthouse Lab in Milton Keynes	Wellcome Sanger Institute for the COVID-19 Genomics UK (COG-UK) Consortium	The Lighthouse Lab in Milton Keynes and Alex Alderton, Roberto Amato, Sonia Goncalves, Ewan Harrison, David K. Jackson, Ian Johnston, Dominic Kwiatkowski, Cordelia Langford, John Sillitoe on behalf of the Wellcome Sanger Institute COVID-19 Surveillance Team
EPI_ISL_971041, EPI_ISL_971048	Department of Virus and Microbiological Special Diagnostics, Statens Serum Institut, Copenhagen, Denmark	Aalborg University	Danish Covid-19 Genome Consortium
EPI_ISL_971050, EPI_ISL_971055	Lighthouse Lab in Milton Keynes	Wellcome Sanger Institute for the COVID-19 Genomics UK (COG-UK) Consortium	The Lighthouse Lab in Milton Keynes and Alex Alderton, Roberto Amato, Sonia Goncalves, Ewan Harrison, David K. Jackson, Ian Johnston, Dominic Kwiatkowski, Cordelia Langford, John Sillitoe on behalf of the Wellcome Sanger Institute COVID-19 Surveillance Team
EPI_ISL_971056, EPI_ISL_971058	Department of Virus and Microbiological Special Diagnostics, Statens Serum Institut, Copenhagen, Denmark	Aalborg University	Danish Covid-19 Genome Consortium
EPI_ISL_971059	Lighthouse Lab in Milton Keynes	Wellcome Sanger Institute for the COVID-19 Genomics UK (COG-UK) Consortium	The Lighthouse Lab in Milton Keynes and Alex Alderton, Roberto Amato, Sonia Goncalves, Ewan Harrison, David K. Jackson, Ian Johnston, Dominic Kwiatkowski, Cordelia Langford, John Sillitoe on behalf of the Wellcome Sanger Institute COVID-19 Surveillance Team
EPI_ISL_971062	Department of Virus and Microbiological Special Diagnostics, Statens Serum Institut, Copenhagen, Denmark	Aalborg University	Danish Covid-19 Genome Consortium
EPI_ISL_971063, EPI_ISL_971066	Lighthouse Lab in Milton Keynes	Wellcome Sanger Institute for the COVID-19 Genomics UK (COG-UK) Consortium	The Lighthouse Lab in Milton Keynes and Alex Alderton, Roberto Amato, Sonia Goncalves, Ewan Harrison, David K. Jackson, Ian Johnston, Dominic Kwiatkowski, Cordelia Langford, John Sillitoe on behalf of the Wellcome Sanger Institute COVID-19 Surveillance Team
EPI_ISL_971067	Department of Virus and Microbiological Special Diagnostics, Statens Serum Institut, Copenhagen, Denmark	Aalborg University	Danish Covid-19 Genome Consortium
EPI_ISL_971069	Lighthouse Lab in Milton Keynes	Wellcome Sanger Institute for the COVID-19 Genomics UK (COG-UK) Consortium	The Lighthouse Lab in Milton Keynes and Alex Alderton, Roberto Amato, Sonia Goncalves, Ewan Harrison, David K. Jackson, Ian Johnston, Dominic Kwiatkowski, Cordelia Langford, John Sillitoe on behalf of the Wellcome Sanger Institute COVID-19 Surveillance Team
EPI_ISL_971070, EPI_ISL_971073	Department of Virus and Microbiological Special Diagnostics, Statens Serum Institut, Copenhagen, Denmark	Aalborg University	Danish Covid-19 Genome Consortium

EPI_ISL_971074	Lighthouse Lab in Milton Keynes	Wellcome Sanger Institute for the COVID-19 Genomics UK (COG-UK) Consortium	The Lighthouse Lab in Milton Keynes and Alex Alderton, Roberto Amato, Sonia Goncalves, Ewan Harrison, David K. Jackson, Ian Johnston, Dominic Kwiatkowski, Cordelia Langford, John Sillitoe on behalf of the Wellcome Sanger Institute COVID-19 Surveillance Team
EPI_ISL_971078	Department of Virus and Microbiological Special Diagnostics, Statens Serum Institut, Copenhagen, Denmark	Aalborg University	Danish Covid-19 Genome Consortium
EPI_ISL_971079	Lighthouse Lab in Milton Keynes	Wellcome Sanger Institute for the COVID-19 Genomics UK (COG-UK) Consortium	The Lighthouse Lab in Milton Keynes and Alex Alderton, Roberto Amato, Sonia Goncalves, Ewan Harrison, David K. Jackson, Ian Johnston, Dominic Kwiatkowski, Cordelia Langford, John Sillitoe on behalf of the Wellcome Sanger Institute COVID-19 Surveillance Team
EPI_ISL_971082	Department of Virus and Microbiological Special Diagnostics, Statens Serum Institut, Copenhagen, Denmark	Aalborg University	Danish Covid-19 Genome Consortium
EPI_ISL_971083, EPI_ISL_971087	Lighthouse Lab in Milton Keynes	Wellcome Sanger Institute for the COVID-19 Genomics UK (COG-UK) Consortium	The Lighthouse Lab in Milton Keynes and Alex Alderton, Roberto Amato, Sonia Goncalves, Ewan Harrison, David K. Jackson, Ian Johnston, Dominic Kwiatkowski, Cordelia Langford, John Sillitoe on behalf of the Wellcome Sanger Institute COVID-19 Surveillance Team
EPI_ISL_971088	Department of Virus and Microbiological Special Diagnostics, Statens Serum Institut, Copenhagen, Denmark	Aalborg University	Danish Covid-19 Genome Consortium
EPI_ISL_971090	Lighthouse Lab in Milton Keynes	Wellcome Sanger Institute for the COVID-19 Genomics UK (COG-UK) Consortium	The Lighthouse Lab in Milton Keynes and Alex Alderton, Roberto Amato, Sonia Goncalves, Ewan Harrison, David K. Jackson, Ian Johnston, Dominic Kwiatkowski, Cordelia Langford, John Sillitoe on behalf of the Wellcome Sanger Institute COVID-19 Surveillance Team
EPI_ISL_971095, EPI_ISL_971098	Department of Virus and Microbiological Special Diagnostics, Statens Serum Institut, Copenhagen, Denmark	Aalborg University	Danish Covid-19 Genome Consortium
EPI_ISL_971099, EPI_ISL_971102	Lighthouse Lab in Milton Keynes	Wellcome Sanger Institute for the COVID-19 Genomics UK (COG-UK) Consortium	The Lighthouse Lab in Milton Keynes and Alex Alderton, Roberto Amato, Sonia Goncalves, Ewan Harrison, David K. Jackson, Ian Johnston, Dominic Kwiatkowski, Cordelia Langford, John Sillitoe on behalf of the Wellcome Sanger Institute COVID-19 Surveillance Team
EPI_ISL_971103, EPI_ISL_971106	Department of Virus and Microbiological Special Diagnostics, Statens Serum Institut, Copenhagen, Denmark	Aalborg University	Danish Covid-19 Genome Consortium
EPI_ISL_971108, EPI_ISL_971109	Lighthouse Lab in Milton Keynes	Wellcome Sanger Institute for the COVID-19 Genomics UK (COG-UK) Consortium	The Lighthouse Lab in Milton Keynes and Alex Alderton, Roberto Amato, Sonia Goncalves, Ewan Harrison, David K. Jackson, Ian Johnston, Dominic Kwiatkowski, Cordelia Langford, John Sillitoe on behalf of the Wellcome Sanger Institute COVID-19 Surveillance Team
EPI_ISL_971110	Department of Virus and Microbiological Special Diagnostics, Statens Serum Institut, Copenhagen, Denmark	Aalborg University	Danish Covid-19 Genome Consortium
EPI_ISL_971113, EPI_ISL_971117	Lighthouse Lab in Milton Keynes	Wellcome Sanger Institute for the COVID-19 Genomics UK (COG-UK) Consortium	The Lighthouse Lab in Milton Keynes and Alex Alderton, Roberto Amato, Sonia Goncalves, Ewan Harrison, David K. Jackson, Ian Johnston, Dominic Kwiatkowski, Cordelia Langford, John Sillitoe on behalf of the Wellcome Sanger Institute COVID-19 Surveillance Team
EPI_ISL_971118	Department of Virus and Microbiological Special Diagnostics, Statens Serum Institut, Copenhagen, Denmark	Aalborg University	Danish Covid-19 Genome Consortium
EPI_ISL_971119, EPI_ISL_971123	Lighthouse Lab in Milton Keynes	Wellcome Sanger Institute for the COVID-19 Genomics UK (COG-UK) Consortium	The Lighthouse Lab in Milton Keynes and Alex Alderton, Roberto Amato, Sonia Goncalves, Ewan Harrison, David K. Jackson, Ian Johnston, Dominic Kwiatkowski, Cordelia Langford, John Sillitoe on behalf of the Wellcome Sanger Institute COVID-19 Surveillance Team
EPI_ISL_971127	Department of Virus and Microbiological Special Diagnostics, Statens Serum Institut, Copenhagen, Denmark	Aalborg University	Danish Covid-19 Genome Consortium
EPI_ISL_971128, EPI_ISL_971130	Lighthouse Lab in Milton Keynes	Wellcome Sanger Institute for the COVID-19 Genomics UK (COG-UK) Consortium	The Lighthouse Lab in Milton Keynes and Alex Alderton, Roberto Amato, Sonia Goncalves, Ewan Harrison, David K. Jackson, Ian Johnston, Dominic Kwiatkowski, Cordelia Langford, John Sillitoe on behalf of the Wellcome Sanger Institute COVID-19 Surveillance Team
EPI_ISL_971131	Department of Virus and Microbiological Special Diagnostics, Statens Serum Institut, Copenhagen, Denmark	Aalborg University	Danish Covid-19 Genome Consortium
EPI_ISL_971134	Lighthouse Lab in Milton Keynes	Wellcome Sanger Institute for the COVID-19 Genomics UK (COG-UK) Consortium	The Lighthouse Lab in Milton Keynes and Alex Alderton, Roberto Amato, Sonia Goncalves, Ewan Harrison, David K. Jackson, Ian Johnston, Dominic Kwiatkowski, Cordelia Langford, John Sillitoe on behalf of the Wellcome Sanger Institute COVID-19 Surveillance Team
EPI_ISL_971136	Department of Virus and Microbiological Special Diagnostics, Statens Serum Institut, Copenhagen, Denmark	Aalborg University	Danish Covid-19 Genome Consortium
EPI_ISL_971138, EPI_ISL_971146, EPI_ISL_971149	Lighthouse Lab in Milton Keynes	Wellcome Sanger Institute for the COVID-19 Genomics UK (COG-UK) Consortium	The Lighthouse Lab in Milton Keynes and Alex Alderton, Roberto Amato, Sonia Goncalves, Ewan Harrison, David K. Jackson, Ian Johnston, Dominic Kwiatkowski, Cordelia Langford, John Sillitoe on behalf of the Wellcome Sanger Institute COVID-19 Surveillance Team
EPI_ISL_971152	Department of Virus and Microbiological Special Diagnostics, Statens Serum Institut, Copenhagen, Denmark	Aalborg University	Danish Covid-19 Genome Consortium
EPI_ISL_971153, EPI_ISL_971156	Lighthouse Lab in Milton Keynes	Wellcome Sanger Institute for the COVID-19 Genomics UK (COG-UK) Consortium	The Lighthouse Lab in Milton Keynes and Alex Alderton, Roberto Amato, Sonia Goncalves, Ewan Harrison, David K. Jackson, Ian Johnston, Dominic Kwiatkowski, Cordelia Langford, John Sillitoe on behalf of the Wellcome Sanger Institute COVID-19 Surveillance Team
EPI_ISL_971159	Department of Virus and Microbiological Special Diagnostics, Statens Serum Institut, Copenhagen, Denmark	Aalborg University	Danish Covid-19 Genome Consortium
EPI_ISL_971161, EPI_ISL_971164, EPI_ISL_971165	Lighthouse Lab in Milton Keynes	Wellcome Sanger Institute for the COVID-19 Genomics UK (COG-UK) Consortium	The Lighthouse Lab in Milton Keynes and Alex Alderton, Roberto Amato, Sonia Goncalves, Ewan Harrison, David K. Jackson, Ian Johnston, Dominic Kwiatkowski, Cordelia Langford, John Sillitoe on behalf of the Wellcome Sanger Institute COVID-19 Surveillance Team
EPI_ISL_971169, EPI_ISL_971172, EPI_ISL_971175, EPI_ISL_971178	Department of Virus and Microbiological Special Diagnostics, Statens Serum Institut, Copenhagen, Denmark	Aalborg University	Danish Covid-19 Genome Consortium
EPI_ISL_971180	Lighthouse Lab in Milton Keynes	Wellcome Sanger Institute for the COVID-19 Genomics UK (COG-UK) Consortium	The Lighthouse Lab in Milton Keynes and Alex Alderton, Roberto Amato, Sonia Goncalves, Ewan Harrison, David K. Jackson, Ian Johnston, Dominic Kwiatkowski, Cordelia Langford, John Sillitoe on behalf of the Wellcome Sanger Institute COVID-19 Surveillance Team
EPI_ISL_971181	Department of Virus and Microbiological Special Diagnostics, Statens Serum Institut, Copenhagen, Denmark	Aalborg University	Danish Covid-19 Genome Consortium
EPI_ISL_971185	Lighthouse Lab in Milton Keynes	Wellcome Sanger Institute for the COVID-19 Genomics UK (COG-UK) Consortium	The Lighthouse Lab in Milton Keynes and Alex Alderton, Roberto Amato, Sonia Goncalves, Ewan Harrison, David K. Jackson, Ian Johnston, Dominic Kwiatkowski, Cordelia Langford, John Sillitoe on behalf of the Wellcome Sanger Institute COVID-19 Surveillance Team
EPI_ISL_971186	Department of Virus and Microbiological Special Diagnostics, Statens Serum Institut, Copenhagen, Denmark	Aalborg University	Danish Covid-19 Genome Consortium
EPI_ISL_971190	Lighthouse Lab in Milton Keynes	Wellcome Sanger Institute for the COVID-19 Genomics UK (COG-UK) Consortium	The Lighthouse Lab in Milton Keynes and Alex Alderton, Roberto Amato, Sonia Goncalves, Ewan Harrison, David K. Jackson, Ian Johnston, Dominic Kwiatkowski, Cordelia Langford, John Sillitoe on behalf of the Wellcome Sanger Institute COVID-19 Surveillance Team
EPI_ISL_971195	Department of Virus and Microbiological Special Diagnostics, Statens Serum Institut, Copenhagen, Denmark	Aalborg University	Danish Covid-19 Genome Consortium
EPI_ISL_971197, EPI_ISL_971200	Lighthouse Lab in Milton Keynes	Wellcome Sanger Institute for the COVID-19 Genomics UK (COG-UK) Consortium	The Lighthouse Lab in Milton Keynes and Alex Alderton, Roberto Amato, Sonia Goncalves, Ewan Harrison, David K. Jackson, Ian Johnston, Dominic Kwiatkowski, Cordelia Langford, John Sillitoe on behalf of the Wellcome Sanger Institute COVID-19 Surveillance Team
EPI_ISL_971201, EPI_ISL_971203	Department of Virus and Microbiological Special Diagnostics, Statens Serum Institut, Copenhagen, Denmark	Aalborg University	Danish Covid-19 Genome Consortium
EPI_ISL_971204	Lighthouse Lab in Milton Keynes	Wellcome Sanger Institute for the COVID-19 Genomics UK (COG-UK) Consortium	The Lighthouse Lab in Milton Keynes and Alex Alderton, Roberto Amato, Sonia Goncalves, Ewan Harrison, David K. Jackson, Ian Johnston, Dominic Kwiatkowski, Cordelia Langford, John Sillitoe on behalf of the Wellcome Sanger Institute COVID-19 Surveillance Team

EPI_ISL_971207	Department of Virus and Microbiological Special Diagnostics, Statens Serum Institut, Copenhagen, Denmark	Aalborg University	Danish Covid-19 Genome Consortium
EPI_ISL_971209, EPI_ISL_971211	Lighthouse Lab in Milton Keynes	Wellcome Sanger Institute for the COVID-19 Genomics UK (COG-UK) Consortium	The Lighthouse Lab in Milton Keynes and Alex Alderton, Roberto Amato, Sonia Goncalves, Ewan Harrison, David K. Jackson, Ian Johnston, Dominic Kwiatkowski, Cordelia Langford, John Sillitoe on behalf of the Wellcome Sanger Institute COVID-19 Surveillance Team
EPI_ISL_971213	Department of Virus and Microbiological Special Diagnostics, Statens Serum Institut, Copenhagen, Denmark	Aalborg University	Danish Covid-19 Genome Consortium
EPI_ISL_971215, EPI_ISL_971218, EPI_ISL_971221	Lighthouse Lab in Milton Keynes	Wellcome Sanger Institute for the COVID-19 Genomics UK (COG-UK) Consortium	The Lighthouse Lab in Milton Keynes and Alex Alderton, Roberto Amato, Sonia Goncalves, Ewan Harrison, David K. Jackson, Ian Johnston, Dominic Kwiatkowski, Cordelia Langford, John Sillitoe on behalf of the Wellcome Sanger Institute COVID-19 Surveillance Team
EPI_ISL_971223	Department of Virus and Microbiological Special Diagnostics, Statens Serum Institut, Copenhagen, Denmark	Aalborg University	Danish Covid-19 Genome Consortium
EPI_ISL_971224, EPI_ISL_971228, EPI_ISL_971234, EPI_ISL_971236	Lighthouse Lab in Milton Keynes	Wellcome Sanger Institute for the COVID-19 Genomics UK (COG-UK) Consortium	The Lighthouse Lab in Milton Keynes and Alex Alderton, Roberto Amato, Sonia Goncalves, Ewan Harrison, David K. Jackson, Ian Johnston, Dominic Kwiatkowski, Cordelia Langford, John Sillitoe on behalf of the Wellcome Sanger Institute COVID-19 Surveillance Team
EPI_ISL_971237, EPI_ISL_971244	Department of Virus and Microbiological Special Diagnostics, Statens Serum Institut, Copenhagen, Denmark	Aalborg University	Danish Covid-19 Genome Consortium
EPI_ISL_971246	Lighthouse Lab in Milton Keynes	Wellcome Sanger Institute for the COVID-19 Genomics UK (COG-UK) Consortium	The Lighthouse Lab in Milton Keynes and Alex Alderton, Roberto Amato, Sonia Goncalves, Ewan Harrison, David K. Jackson, Ian Johnston, Dominic Kwiatkowski, Cordelia Langford, John Sillitoe on behalf of the Wellcome Sanger Institute COVID-19 Surveillance Team
EPI_ISL_971247	Department of Virus and Microbiological Special Diagnostics, Statens Serum Institut, Copenhagen, Denmark	Aalborg University	Danish Covid-19 Genome Consortium
EPI_ISL_971248, EPI_ISL_971251	Lighthouse Lab in Milton Keynes	Wellcome Sanger Institute for the COVID-19 Genomics UK (COG-UK) Consortium	The Lighthouse Lab in Milton Keynes and Alex Alderton, Roberto Amato, Sonia Goncalves, Ewan Harrison, David K. Jackson, Ian Johnston, Dominic Kwiatkowski, Cordelia Langford, John Sillitoe on behalf of the Wellcome Sanger Institute COVID-19 Surveillance Team
EPI_ISL_971252	Department of Virus and Microbiological Special Diagnostics, Statens Serum Institut, Copenhagen, Denmark	Aalborg University	Danish Covid-19 Genome Consortium
EPI_ISL_971254	Lighthouse Lab in Milton Keynes	Wellcome Sanger Institute for the COVID-19 Genomics UK (COG-UK) Consortium	The Lighthouse Lab in Milton Keynes and Alex Alderton, Roberto Amato, Sonia Goncalves, Ewan Harrison, David K. Jackson, Ian Johnston, Dominic Kwiatkowski, Cordelia Langford, John Sillitoe on behalf of the Wellcome Sanger Institute COVID-19 Surveillance Team
EPI_ISL_971257	Department of Virus and Microbiological Special Diagnostics, Statens Serum Institut, Copenhagen, Denmark	Aalborg University	Danish Covid-19 Genome Consortium
EPI_ISL_971258	Lighthouse Lab in Milton Keynes	Wellcome Sanger Institute for the COVID-19 Genomics UK (COG-UK) Consortium	The Lighthouse Lab in Milton Keynes and Alex Alderton, Roberto Amato, Sonia Goncalves, Ewan Harrison, David K. Jackson, Ian Johnston, Dominic Kwiatkowski, Cordelia Langford, John Sillitoe on behalf of the Wellcome Sanger Institute COVID-19 Surveillance Team
EPI_ISL_971263	Department of Virus and Microbiological Special Diagnostics, Statens Serum Institut, Copenhagen, Denmark	Aalborg University	Danish Covid-19 Genome Consortium
EPI_ISL_971264, EPI_ISL_971267	Lighthouse Lab in Milton Keynes	Wellcome Sanger Institute for the COVID-19 Genomics UK (COG-UK) Consortium	The Lighthouse Lab in Milton Keynes and Alex Alderton, Roberto Amato, Sonia Goncalves, Ewan Harrison, David K. Jackson, Ian Johnston, Dominic Kwiatkowski, Cordelia Langford, John Sillitoe on behalf of the Wellcome Sanger Institute COVID-19 Surveillance Team
EPI_ISL_971270, EPI_ISL_971272	Department of Virus and Microbiological Special Diagnostics, Statens Serum Institut, Copenhagen, Denmark	Aalborg University	Danish Covid-19 Genome Consortium
EPI_ISL_971273	Lighthouse Lab in Milton Keynes	Wellcome Sanger Institute for the COVID-19 Genomics UK (COG-UK) Consortium	The Lighthouse Lab in Milton Keynes and Alex Alderton, Roberto Amato, Sonia Goncalves, Ewan Harrison, David K. Jackson, Ian Johnston, Dominic Kwiatkowski, Cordelia Langford, John Sillitoe on behalf of the Wellcome Sanger Institute COVID-19 Surveillance Team
EPI_ISL_971275	Department of Virus and Microbiological Special Diagnostics, Statens Serum Institut, Copenhagen, Denmark	Aalborg University	Danish Covid-19 Genome Consortium
EPI_ISL_971277	Lighthouse Lab in Milton Keynes	Wellcome Sanger Institute for the COVID-19 Genomics UK (COG-UK) Consortium	The Lighthouse Lab in Milton Keynes and Alex Alderton, Roberto Amato, Sonia Goncalves, Ewan Harrison, David K. Jackson, Ian Johnston, Dominic Kwiatkowski, Cordelia Langford, John Sillitoe on behalf of the Wellcome Sanger Institute COVID-19 Surveillance Team
EPI_ISL_971283	Department of Virus and Microbiological Special Diagnostics, Statens Serum Institut, Copenhagen, Denmark	Aalborg University	Danish Covid-19 Genome Consortium
EPI_ISL_971284, EPI_ISL_971286	Lighthouse Lab in Milton Keynes	Wellcome Sanger Institute for the COVID-19 Genomics UK (COG-UK) Consortium	The Lighthouse Lab in Milton Keynes and Alex Alderton, Roberto Amato, Sonia Goncalves, Ewan Harrison, David K. Jackson, Ian Johnston, Dominic Kwiatkowski, Cordelia Langford, John Sillitoe on behalf of the Wellcome Sanger Institute COVID-19 Surveillance Team
EPI_ISL_971287	Department of Virus and Microbiological Special Diagnostics, Statens Serum Institut, Copenhagen, Denmark	Aalborg University	Danish Covid-19 Genome Consortium
EPI_ISL_971289, EPI_ISL_971295	Lighthouse Lab in Milton Keynes	Wellcome Sanger Institute for the COVID-19 Genomics UK (COG-UK) Consortium	The Lighthouse Lab in Milton Keynes and Alex Alderton, Roberto Amato, Sonia Goncalves, Ewan Harrison, David K. Jackson, Ian Johnston, Dominic Kwiatkowski, Cordelia Langford, John Sillitoe on behalf of the Wellcome Sanger Institute COVID-19 Surveillance Team
EPI_ISL_971296	Department of Virus and Microbiological Special Diagnostics, Statens Serum Institut, Copenhagen, Denmark	Aalborg University	Danish Covid-19 Genome Consortium
EPI_ISL_971301	Lighthouse Lab in Milton Keynes	Wellcome Sanger Institute for the COVID-19 Genomics UK (COG-UK) Consortium	The Lighthouse Lab in Milton Keynes and Alex Alderton, Roberto Amato, Sonia Goncalves, Ewan Harrison, David K. Jackson, Ian Johnston, Dominic Kwiatkowski, Cordelia Langford, John Sillitoe on behalf of the Wellcome Sanger Institute COVID-19 Surveillance Team
EPI_ISL_971302	Department of Virus and Microbiological Special Diagnostics, Statens Serum Institut, Copenhagen, Denmark	Aalborg University	Danish Covid-19 Genome Consortium
EPI_ISL_971304, EPI_ISL_971307	Lighthouse Lab in Milton Keynes	Wellcome Sanger Institute for the COVID-19 Genomics UK (COG-UK) Consortium	The Lighthouse Lab in Milton Keynes and Alex Alderton, Roberto Amato, Sonia Goncalves, Ewan Harrison, David K. Jackson, Ian Johnston, Dominic Kwiatkowski, Cordelia Langford, John Sillitoe on behalf of the Wellcome Sanger Institute COVID-19 Surveillance Team
EPI_ISL_971309	Department of Virus and Microbiological Special Diagnostics, Statens Serum Institut, Copenhagen, Denmark	Aalborg University	Danish Covid-19 Genome Consortium
EPI_ISL_971310	Lighthouse Lab in Milton Keynes	Wellcome Sanger Institute for the COVID-19 Genomics UK (COG-UK) Consortium	The Lighthouse Lab in Milton Keynes and Alex Alderton, Roberto Amato, Sonia Goncalves, Ewan Harrison, David K. Jackson, Ian Johnston, Dominic Kwiatkowski, Cordelia Langford, John Sillitoe on behalf of the Wellcome Sanger Institute COVID-19 Surveillance Team
EPI_ISL_971312	Department of Virus and Microbiological Special Diagnostics, Statens Serum Institut, Copenhagen, Denmark	Aalborg University	Danish Covid-19 Genome Consortium
EPI_ISL_971313, EPI_ISL_971316	Lighthouse Lab in Milton Keynes	Wellcome Sanger Institute for the COVID-19 Genomics UK (COG-UK) Consortium	The Lighthouse Lab in Milton Keynes and Alex Alderton, Roberto Amato, Sonia Goncalves, Ewan Harrison, David K. Jackson, Ian Johnston, Dominic Kwiatkowski, Cordelia Langford, John Sillitoe on behalf of the Wellcome Sanger Institute COVID-19 Surveillance Team
EPI_ISL_971318	Department of Virus and Microbiological Special Diagnostics, Statens Serum Institut, Copenhagen, Denmark	Aalborg University	Danish Covid-19 Genome Consortium
EPI_ISL_971319, EPI_ISL_971320	Lighthouse Lab in Milton Keynes	Wellcome Sanger Institute for the COVID-19 Genomics UK (COG-UK) Consortium	The Lighthouse Lab in Milton Keynes and Alex Alderton, Roberto Amato, Sonia Goncalves, Ewan Harrison, David K. Jackson, Ian Johnston, Dominic Kwiatkowski, Cordelia Langford, John Sillitoe on behalf of the Wellcome Sanger Institute COVID-19 Surveillance Team
EPI_ISL_971322, EPI_ISL_971323	Department of Virus and Microbiological Special Diagnostics, Statens Serum Institut, Copenhagen, Denmark	Aalborg University	Danish Covid-19 Genome Consortium

EPI_ISL_971324	Lighthouse Lab in Milton Keynes	Wellcome Sanger Institute for the COVID-19 Genomics UK (COG-UK) Consortium	The Lighthouse Lab in Milton Keynes and Alex Alderton, Roberto Amato, Sonia Goncalves, Ewan Harrison, David K. Jackson, Ian Johnston, Dominic Kwiatkowski, Cordelia Langford, John Sillitoe on behalf of the Wellcome Sanger Institute COVID-19 Surveillance Team
EPI_ISL_971329	Department of Virus and Microbiological Special Diagnostics, Statens Serum Institut, Copenhagen, Denmark	Aalborg University	Danish Covid-19 Genome Consortium
EPI_ISL_971330	Lighthouse Lab in Milton Keynes	Wellcome Sanger Institute for the COVID-19 Genomics UK (COG-UK) Consortium	The Lighthouse Lab in Milton Keynes and Alex Alderton, Roberto Amato, Sonia Goncalves, Ewan Harrison, David K. Jackson, Ian Johnston, Dominic Kwiatkowski, Cordelia Langford, John Sillitoe on behalf of the Wellcome Sanger Institute COVID-19 Surveillance Team
EPI_ISL_971332	Department of Virus and Microbiological Special Diagnostics, Statens Serum Institut, Copenhagen, Denmark	Aalborg University	Danish Covid-19 Genome Consortium
EPI_ISL_971336	Lighthouse Lab in Milton Keynes	Wellcome Sanger Institute for the COVID-19 Genomics UK (COG-UK) Consortium	The Lighthouse Lab in Milton Keynes and Alex Alderton, Roberto Amato, Sonia Goncalves, Ewan Harrison, David K. Jackson, Ian Johnston, Dominic Kwiatkowski, Cordelia Langford, John Sillitoe on behalf of the Wellcome Sanger Institute COVID-19 Surveillance Team
EPI_ISL_971338	Department of Virus and Microbiological Special Diagnostics, Statens Serum Institut, Copenhagen, Denmark	Aalborg University	Danish Covid-19 Genome Consortium
EPI_ISL_971343, EPI_ISL_971350, EPI_ISL_971352, EPI_ISL_971357	Lighthouse Lab in Milton Keynes	Wellcome Sanger Institute for the COVID-19 Genomics UK (COG-UK) Consortium	The Lighthouse Lab in Milton Keynes and Alex Alderton, Roberto Amato, Sonia Goncalves, Ewan Harrison, David K. Jackson, Ian Johnston, Dominic Kwiatkowski, Cordelia Langford, John Sillitoe on behalf of the Wellcome Sanger Institute COVID-19 Surveillance Team
EPI_ISL_971358, EPI_ISL_971363, EPI_ISL_971368, EPI_ISL_971371	Department of Virus and Microbiological Special Diagnostics, Statens Serum Institut, Copenhagen, Denmark	Aalborg University	Danish Covid-19 Genome Consortium
EPI_ISL_971373, EPI_ISL_971376, EPI_ISL_971378	Lighthouse Lab in Milton Keynes	Wellcome Sanger Institute for the COVID-19 Genomics UK (COG-UK) Consortium	The Lighthouse Lab in Milton Keynes and Alex Alderton, Roberto Amato, Sonia Goncalves, Ewan Harrison, David K. Jackson, Ian Johnston, Dominic Kwiatkowski, Cordelia Langford, John Sillitoe on behalf of the Wellcome Sanger Institute COVID-19 Surveillance Team
EPI_ISL_971379	Department of Virus and Microbiological Special Diagnostics, Statens Serum Institut, Copenhagen, Denmark	Aalborg University	Danish Covid-19 Genome Consortium
EPI_ISL_971382	Lighthouse Lab in Milton Keynes	Wellcome Sanger Institute for the COVID-19 Genomics UK (COG-UK) Consortium	The Lighthouse Lab in Milton Keynes and Alex Alderton, Roberto Amato, Sonia Goncalves, Ewan Harrison, David K. Jackson, Ian Johnston, Dominic Kwiatkowski, Cordelia Langford, John Sillitoe on behalf of the Wellcome Sanger Institute COVID-19 Surveillance Team
EPI_ISL_971384	Department of Virus and Microbiological Special Diagnostics, Statens Serum Institut, Copenhagen, Denmark	Aalborg University	Danish Covid-19 Genome Consortium
EPI_ISL_971388	Lighthouse Lab in Milton Keynes	Wellcome Sanger Institute for the COVID-19 Genomics UK (COG-UK) Consortium	The Lighthouse Lab in Milton Keynes and Alex Alderton, Roberto Amato, Sonia Goncalves, Ewan Harrison, David K. Jackson, Ian Johnston, Dominic Kwiatkowski, Cordelia Langford, John Sillitoe on behalf of the Wellcome Sanger Institute COVID-19 Surveillance Team
EPI_ISL_971389	Department of Virus and Microbiological Special Diagnostics, Statens Serum Institut, Copenhagen, Denmark	Aalborg University	Danish Covid-19 Genome Consortium
EPI_ISL_971392, EPI_ISL_971397	Lighthouse Lab in Milton Keynes	Wellcome Sanger Institute for the COVID-19 Genomics UK (COG-UK) Consortium	The Lighthouse Lab in Milton Keynes and Alex Alderton, Roberto Amato, Sonia Goncalves, Ewan Harrison, David K. Jackson, Ian Johnston, Dominic Kwiatkowski, Cordelia Langford, John Sillitoe on behalf of the Wellcome Sanger Institute COVID-19 Surveillance Team
EPI_ISL_971398	Department of Virus and Microbiological Special Diagnostics, Statens Serum Institut, Copenhagen, Denmark	Aalborg University	Danish Covid-19 Genome Consortium
EPI_ISL_971400	Lighthouse Lab in Milton Keynes	Wellcome Sanger Institute for the COVID-19 Genomics UK (COG-UK) Consortium	The Lighthouse Lab in Milton Keynes and Alex Alderton, Roberto Amato, Sonia Goncalves, Ewan Harrison, David K. Jackson, Ian Johnston, Dominic Kwiatkowski, Cordelia Langford, John Sillitoe on behalf of the Wellcome Sanger Institute COVID-19 Surveillance Team
EPI_ISL_971401	Department of Virus and Microbiological Special Diagnostics, Statens Serum Institut, Copenhagen, Denmark	Aalborg University	Danish Covid-19 Genome Consortium
EPI_ISL_971402, EPI_ISL_971405, EPI_ISL_971407	Lighthouse Lab in Milton Keynes	Wellcome Sanger Institute for the COVID-19 Genomics UK (COG-UK) Consortium	The Lighthouse Lab in Milton Keynes and Alex Alderton, Roberto Amato, Sonia Goncalves, Ewan Harrison, David K. Jackson, Ian Johnston, Dominic Kwiatkowski, Cordelia Langford, John Sillitoe on behalf of the Wellcome Sanger Institute COVID-19 Surveillance Team
EPI_ISL_971408	Department of Virus and Microbiological Special Diagnostics, Statens Serum Institut, Copenhagen, Denmark	Aalborg University	Danish Covid-19 Genome Consortium
EPI_ISL_971410	Lighthouse Lab in Milton Keynes	Wellcome Sanger Institute for the COVID-19 Genomics UK (COG-UK) Consortium	The Lighthouse Lab in Milton Keynes and Alex Alderton, Roberto Amato, Sonia Goncalves, Ewan Harrison, David K. Jackson, Ian Johnston, Dominic Kwiatkowski, Cordelia Langford, John Sillitoe on behalf of the Wellcome Sanger Institute COVID-19 Surveillance Team
EPI_ISL_971411	Department of Virus and Microbiological Special Diagnostics, Statens Serum Institut, Copenhagen, Denmark	Aalborg University	Danish Covid-19 Genome Consortium
EPI_ISL_971413	Lighthouse Lab in Milton Keynes	Wellcome Sanger Institute for the COVID-19 Genomics UK (COG-UK) Consortium	The Lighthouse Lab in Milton Keynes and Alex Alderton, Roberto Amato, Sonia Goncalves, Ewan Harrison, David K. Jackson, Ian Johnston, Dominic Kwiatkowski, Cordelia Langford, John Sillitoe on behalf of the Wellcome Sanger Institute COVID-19 Surveillance Team
EPI_ISL_971415	Department of Virus and Microbiological Special Diagnostics, Statens Serum Institut, Copenhagen, Denmark	Aalborg University	Danish Covid-19 Genome Consortium
EPI_ISL_971417	Lighthouse Lab in Milton Keynes	Wellcome Sanger Institute for the COVID-19 Genomics UK (COG-UK) Consortium	The Lighthouse Lab in Milton Keynes and Alex Alderton, Roberto Amato, Sonia Goncalves, Ewan Harrison, David K. Jackson, Ian Johnston, Dominic Kwiatkowski, Cordelia Langford, John Sillitoe on behalf of the Wellcome Sanger Institute COVID-19 Surveillance Team
EPI_ISL_971419	Department of Virus and Microbiological Special Diagnostics, Statens Serum Institut, Copenhagen, Denmark	Aalborg University	Danish Covid-19 Genome Consortium
EPI_ISL_971420	Lighthouse Lab in Milton Keynes	Wellcome Sanger Institute for the COVID-19 Genomics UK (COG-UK) Consortium	The Lighthouse Lab in Milton Keynes and Alex Alderton, Roberto Amato, Sonia Goncalves, Ewan Harrison, David K. Jackson, Ian Johnston, Dominic Kwiatkowski, Cordelia Langford, John Sillitoe on behalf of the Wellcome Sanger Institute COVID-19 Surveillance Team
EPI_ISL_971422	Department of Virus and Microbiological Special Diagnostics, Statens Serum Institut, Copenhagen, Denmark	Aalborg University	Danish Covid-19 Genome Consortium
EPI_ISL_971423	Lighthouse Lab in Milton Keynes	Wellcome Sanger Institute for the COVID-19 Genomics UK (COG-UK) Consortium	The Lighthouse Lab in Milton Keynes and Alex Alderton, Roberto Amato, Sonia Goncalves, Ewan Harrison, David K. Jackson, Ian Johnston, Dominic Kwiatkowski, Cordelia Langford, John Sillitoe on behalf of the Wellcome Sanger Institute COVID-19 Surveillance Team
EPI_ISL_971425	Lighthouse Lab in Cambridge	Wellcome Sanger Institute for the COVID-19 Genomics UK (COG-UK) Consortium	Rob Howes, The Lighthouse Lab in Cambridge and Alex Alderton, Roberto Amato, Sonia Goncalves, Ewan Harrison, David K. Jackson, Ian Johnston, Dominic Kwiatkowski, Cordelia Langford, John Sillitoe on behalf of the Wellcome Sanger Institute COVID-19 Surveillance Team
EPI_ISL_971426	Department of Virus and Microbiological Special Diagnostics, Statens Serum Institut, Copenhagen, Denmark	Aalborg University	Danish Covid-19 Genome Consortium
EPI_ISL_971428	Lighthouse Lab in Milton Keynes	Wellcome Sanger Institute for the COVID-19 Genomics UK (COG-UK) Consortium	The Lighthouse Lab in Milton Keynes and Alex Alderton, Roberto Amato, Sonia Goncalves, Ewan Harrison, David K. Jackson, Ian Johnston, Dominic Kwiatkowski, Cordelia Langford, John Sillitoe on behalf of the Wellcome Sanger Institute COVID-19 Surveillance Team
EPI_ISL_971429, EPI_ISL_971434, EPI_ISL_971441, EPI_ISL_971443, EPI_ISL_971445, EPI_ISL_971450, EPI_ISL_971452, EPI_ISL_971455, EPI_ISL_971456, EPI_ISL_971458, EPI_ISL_971460, EPI_ISL_971462, EPI_ISL_971463, EPI_ISL_971464, EPI_ISL_971465, EPI_ISL_971466, EPI_ISL_971468, EPI_ISL_971469, EPI_ISL_971471, EPI_ISL_971472, EPI_ISL_971474, EPI_ISL_971475, EPI_ISL_971476, EPI_ISL_971479, EPI_ISL_971481, EPI_ISL_971482, EPI_ISL_971483, EPI_ISL_971484, EPI_ISL_971486, EPI_ISL_971487, EPI_ISL_971488, EPI_ISL_971489, EPI_ISL_971490, EPI_ISL_971491, EPI_ISL_971492, EPI_ISL_971493, EPI_ISL_971495, EPI_ISL_971498, EPI_ISL_971499, EPI_ISL_971501, EPI_ISL_971505, EPI_ISL_971506, EPI_ISL_971507, EPI_ISL_971510, EPI_ISL_971511, EPI_ISL_971512, EPI_ISL_971514, EPI_ISL_971517, EPI_ISL_971520, EPI_ISL_971521, EPI_ISL_971522, EPI_ISL_971523, EPI_ISL_971524, EPI_ISL_971525, EPI_ISL_971526, EPI_ISL_971528, EPI_ISL_971530, EPI_ISL_971531, EPI_ISL_971533, EPI_ISL_971535, EPI_ISL_971536, EPI_ISL_971537, EPI_ISL_971538, EPI_ISL_971540, EPI_ISL_971541, EPI_ISL_971543, EPI_ISL_971544, EPI_ISL_971545, EPI_ISL_971546, EPI_ISL_971551, EPI_ISL_971552, EPI_ISL_971554, EPI_ISL_971555, EPI_ISL_971559, EPI_ISL_971561, EPI_ISL_971562, EPI_ISL_971563, EPI_ISL_971564, EPI_ISL_971567, EPI_ISL_971568, EPI_ISL_971569, EPI_ISL_971570, EPI_ISL_971571, EPI_ISL_971572, EPI_ISL_971573, EPI_ISL_971574, EPI_ISL_971576, EPI_ISL_971577, EPI_ISL_971582, EPI_ISL_971583, EPI_ISL_971584, EPI_ISL_971585, EPI_ISL_971587, EPI_ISL_971588, EPI_ISL_971589, EPI_ISL_971590, EPI_ISL_971592, EPI_ISL_971593, EPI_ISL_971594, EPI_ISL_971595, EPI_ISL_971596, EPI_ISL_971597, EPI_ISL_971598, EPI_ISL_971599, EPI_ISL_971600, EPI_ISL_971601, EPI_ISL_971602, EPI_ISL_971603, EPI_ISL_971604, EPI_ISL_971605, EPI_ISL_971606, EPI_ISL_971607, EPI_ISL_971609, EPI_ISL_971611, EPI_ISL_971612, EPI_ISL_971618, EPI_ISL_971619, EPI_ISL_971620, EPI_ISL_971621, EPI_ISL_971622, EPI_ISL_971624, EPI_ISL_971626, EPI_ISL_971627, EPI_ISL_971629, EPI_ISL_971630, EPI_ISL_971631, EPI_ISL_971634, EPI_ISL_971636, EPI_ISL_971637, EPI_ISL_971639, EPI_ISL_971641, EPI_ISL_971644, EPI_ISL_971645, EPI_ISL_971646, EPI_ISL_971647, EPI_ISL_971651, EPI_ISL_971652, EPI_ISL_971653, EPI_ISL_971654, EPI_ISL_971656, EPI_ISL_971657, EPI_ISL_971658, EPI_ISL_971659, EPI_ISL_971660,			

EPI_ISL_971661, EPI_ISL_971662, EPI_ISL_971663, EPI_ISL_971664, EPI_ISL_971665, EPI_ISL_971666, EPI_ISL_971667, EPI_ISL_971668, EPI_ISL_971669, EPI_ISL_971670, EPI_ISL_971671, EPI_ISL_971672, EPI_ISL_971676, EPI_ISL_971677, EPI_ISL_971678, EPI_ISL_971679, EPI_ISL_971681, EPI_ISL_971682, EPI_ISL_971683, EPI_ISL_971685, EPI_ISL_971687, EPI_ISL_971688, EPI_ISL_971689, EPI_ISL_971691, EPI_ISL_971692, EPI_ISL_971693, EPI_ISL_971694, EPI_ISL_971696, EPI_ISL_971698, EPI_ISL_971700, EPI_ISL_971702, EPI_ISL_971703, EPI_ISL_971704, EPI_ISL_971707, EPI_ISL_971709, EPI_ISL_971712, EPI_ISL_971713, EPI_ISL_971717, EPI_ISL_971718, EPI_ISL_971720, EPI_ISL_971721, EPI_ISL_971722, EPI_ISL_971725, EPI_ISL_971726, EPI_ISL_971727, EPI_ISL_971728, EPI_ISL_971729, EPI_ISL_971731, EPI_ISL_971732, EPI_ISL_971734, EPI_ISL_971735, EPI_ISL_971740, EPI_ISL_971741, EPI_ISL_971744, EPI_ISL_971746, EPI_ISL_971747, EPI_ISL_971748, EPI_ISL_971750, EPI_ISL_971752, EPI_ISL_971753, EPI_ISL_971754, EPI_ISL_971756, EPI_ISL_971758, EPI_ISL_971759, EPI_ISL_971760, EPI_ISL_971762, EPI_ISL_971763, EPI_ISL_971764, EPI_ISL_971765, EPI_ISL_971766, EPI_ISL_971770, EPI_ISL_971771, EPI_ISL_971772, EPI_ISL_971774, EPI_ISL_971775, EPI_ISL_971776, EPI_ISL_971777, EPI_ISL_971778, EPI_ISL_971779, EPI_ISL_971780, EPI_ISL_971781, EPI_ISL_971782, EPI_ISL_971783, EPI_ISL_971786, EPI_ISL_971787, EPI_ISL_971788, EPI_ISL_971789, EPI_ISL_971792, EPI_ISL_971794, EPI_ISL_971795, EPI_ISL_971796, EPI_ISL_971797, EPI_ISL_971799, EPI_ISL_971800, EPI_ISL_971801, EPI_ISL_971802, EPI_ISL_971803, EPI_ISL_971804, EPI_ISL_971806, EPI_ISL_971807, EPI_ISL_971809, EPI_ISL_971810, EPI_ISL_971812, EPI_ISL_971813, EPI_ISL_971814, EPI_ISL_971816, EPI_ISL_971817, EPI_ISL_971818, EPI_ISL_971820, EPI_ISL_971821, EPI_ISL_971823, EPI_ISL_971824, EPI_ISL_971825, EPI_ISL_971826, EPI_ISL_971827, EPI_ISL_971828, EPI_ISL_971829, EPI_ISL_971830, EPI_ISL_971831, EPI_ISL_971833, EPI_ISL_971835, EPI_ISL_971836, EPI_ISL_971837, EPI_ISL_971838, EPI_ISL_971840, EPI_ISL_971841, EPI_ISL_971842, EPI_ISL_971843, EPI_ISL_971845, EPI_ISL_971847, EPI_ISL_971848, EPI_ISL_971850, EPI_ISL_971851, EPI_ISL_971852, EPI_ISL_971853, EPI_ISL_971855, EPI_ISL_971858, EPI_ISL_971860, EPI_ISL_971863, EPI_ISL_971864, EPI_ISL_971866, EPI_ISL_971868, EPI_ISL_971869, EPI_ISL_971870, EPI_ISL_971871, EPI_ISL_971873, EPI_ISL_971874, EPI_ISL_971878, EPI_ISL_971879, EPI_ISL_971881, EPI_ISL_971882, EPI_ISL_971883, EPI_ISL_971884, EPI_ISL_971887, EPI_ISL_971888, EPI_ISL_971889, EPI_ISL_971890, EPI_ISL_971891, EPI_ISL_971892, EPI_ISL_971893, EPI_ISL_971894, EPI_ISL_971895, EPI_ISL_971896, EPI_ISL_971897, EPI_ISL_971898, EPI_ISL_971900, EPI_ISL_971901, EPI_ISL_971902, EPI_ISL_971903, EPI_ISL_971904, EPI_ISL_971905, EPI_ISL_971907, EPI_ISL_971909, EPI_ISL_971912, EPI_ISL_971913, EPI_ISL_971916, EPI_ISL_971917, EPI_ISL_971918, EPI_ISL_971922, EPI_ISL_971923, EPI_ISL_971924, EPI_ISL_971926, EPI_ISL_971927, EPI_ISL_971928, EPI_ISL_971929, EPI_ISL_971930, EPI_ISL_971932, EPI_ISL_971935, EPI_ISL_971937, EPI_ISL_971940, EPI_ISL_971941, EPI_ISL_971944, EPI_ISL_971947, EPI_ISL_971948, EPI_ISL_971950, EPI_ISL_971952, EPI_ISL_971953, EPI_ISL_971955, EPI_ISL_971956, EPI_ISL_971957, EPI_ISL_971958, EPI_ISL_971959, EPI_ISL_971961, EPI_ISL_971964, EPI_ISL_971965, EPI_ISL_971967, EPI_ISL_971969, EPI_ISL_971972, EPI_ISL_971974, EPI_ISL_971976, EPI_ISL_971979, EPI_ISL_971980, EPI_ISL_971982, EPI_ISL_971983, EPI_ISL_971984, EPI_ISL_971989, EPI_ISL_971992, EPI_ISL_971993, EPI_ISL_971994, EPI_ISL_971998, EPI_ISL_972000, EPI_ISL_972001, EPI_ISL_972002, EPI_ISL_972003, EPI_ISL_972004, EPI_ISL_972005, EPI_ISL_972007, EPI_ISL_972009, EPI_ISL_972010, EPI_ISL_972011, EPI_ISL_972012, EPI_ISL_972013, EPI_ISL_972014, EPI_ISL_972017, EPI_ISL_972019, EPI_ISL_972020, EPI_ISL_972021, EPI_ISL_972022, EPI_ISL_972024, EPI_ISL_972026, EPI_ISL_972028, EPI_ISL_972029, EPI_ISL_972030, EPI_ISL_972031, EPI_ISL_972033, EPI_ISL_972035, EPI_ISL_972036, EPI_ISL_972037, EPI_ISL_972038, EPI_ISL_972039, EPI_ISL_972040, EPI_ISL_972041, EPI_ISL_972042, EPI_ISL_972043, EPI_ISL_972045, EPI_ISL_972047, EPI_ISL_972048, EPI_ISL_972052, EPI_ISL_972056, EPI_ISL_972057, EPI_ISL_972059, EPI_ISL_972061, EPI_ISL_972062, EPI_ISL_972063, EPI_ISL_972064, EPI_ISL_972065, EPI_ISL_972066, EPI_ISL_972067, EPI_ISL_972069, EPI_ISL_972071, EPI_ISL_972073, EPI_ISL_972075, EPI_ISL_972076, EPI_ISL_972077, EPI_ISL_972078, EPI_ISL_972079, EPI_ISL_972081, EPI_ISL_972083, EPI_ISL_972084, EPI_ISL_972086, EPI_ISL_972087, EPI_ISL_972095, EPI_ISL_972096, EPI_ISL_972097, EPI_ISL_972099, EPI_ISL_972100, EPI_ISL_972103, EPI_ISL_972104, EPI_ISL_972106, EPI_ISL_972107, EPI_ISL_972108, EPI_ISL_972110, EPI_ISL_972112, EPI_ISL_972113, EPI_ISL_972115, EPI_ISL_972120, EPI_ISL_972121, EPI_ISL_972122, EPI_ISL_972124, EPI_ISL_972125, EPI_ISL_972126, EPI_ISL_972127, EPI_ISL_972128, EPI_ISL_972129, EPI_ISL_972133, EPI_ISL_972134, EPI_ISL_972140, EPI_ISL_972141, EPI_ISL_972142, EPI_ISL_972144, EPI_ISL_972145, EPI_ISL_972146, EPI_ISL_972147, EPI_ISL_972148, EPI_ISL_972149, EPI_ISL_972151, EPI_ISL_972152, EPI_ISL_972154, EPI_ISL_972155, EPI_ISL_972157, EPI_ISL_972158, EPI_ISL_972159, EPI_ISL_972160, EPI_ISL_972161, EPI_ISL_972162, EPI_ISL_972163, EPI_ISL_972165, EPI_ISL_972166, EPI_ISL_972167, EPI_ISL_972168, EPI_ISL_972171, EPI_ISL_972173, EPI_ISL_972174, EPI_ISL_972175, EPI_ISL_972177, EPI_ISL_972179, EPI_ISL_972180, EPI_ISL_972181, EPI_ISL_972182, EPI_ISL_972183, EPI_ISL_972185, EPI_ISL_972186, EPI_ISL_972187, EPI_ISL_972188, EPI_ISL_972189, EPI_ISL_972191, EPI_ISL_972192, EPI_ISL_972199, EPI_ISL_972200, EPI_ISL_972203, EPI_ISL_972204, EPI_ISL_972205, EPI_ISL_972206, EPI_ISL_972208, EPI_ISL_972209, EPI_ISL_972210, EPI_ISL_972211, EPI_ISL_972214, EPI_ISL_972215, EPI_ISL_972219, EPI_ISL_972220, EPI_ISL_972224, EPI_ISL_972227, EPI_ISL_972228, EPI_ISL_972229, EPI_ISL_972230, EPI_ISL_972231, EPI_ISL_972233, EPI_ISL_972234, EPI_ISL_972235, EPI_ISL_972237, EPI_ISL_972239, EPI_ISL_972242, EPI_ISL_972243, EPI_ISL_972245, EPI_ISL_972246, EPI_ISL_972247, EPI_ISL_972248, EPI_ISL_972249, EPI_ISL_972250, EPI_ISL_972252, EPI_ISL_972253, EPI_ISL_972254, EPI_ISL_972255, EPI_ISL_972256, EPI_ISL_972257, EPI_ISL_972259, EPI_ISL_972260, EPI_ISL_972262, EPI_ISL_972264, EPI_ISL_972265, EPI_ISL_972268, EPI_ISL_972269, EPI_ISL_972272, EPI_ISL_972276, EPI_ISL_972278, EPI_ISL_972279, EPI_ISL_972281, EPI_ISL_972283, EPI_ISL_972284, EPI_ISL_972285, EPI_ISL_972286, EPI_ISL_972287, EPI_ISL_972289, EPI_ISL_972292, EPI_ISL_972294, EPI_ISL_972295, EPI_ISL_972296, EPI_ISL_972298, EPI_ISL_972299, EPI_ISL_972300, EPI_ISL_972301, EPI_ISL_972303, EPI_ISL_972304, EPI_ISL_972305, EPI_ISL_972306, EPI_ISL_972308, EPI_ISL_972310, EPI_ISL_972311, EPI_ISL_972315, EPI_ISL_972316, EPI_ISL_972317, EPI_ISL_972318, EPI_ISL_972319, EPI_ISL_972320, EPI_ISL_972321, EPI_ISL_972322, EPI_ISL_972323, EPI_ISL_972325, EPI_ISL_972326, EPI_ISL_972327, EPI_ISL_972329, EPI_ISL_972330, EPI_ISL_972331, EPI_ISL_972333, EPI_ISL_972334, EPI_ISL_972335, EPI_ISL_972337, EPI_ISL_972339, EPI_ISL_972340, EPI_ISL_972343, EPI_ISL_972344, EPI_ISL_972345, EPI_ISL_972347, EPI_ISL_972350, EPI_ISL_972355, EPI_ISL_972356, EPI_ISL_972357, EPI_ISL_972359, EPI_ISL_972360, EPI_ISL_972361, EPI_ISL_972362, EPI_ISL_972364, EPI_ISL_972365, EPI_ISL_972366, EPI_ISL_972367, EPI_ISL_972368, EPI_ISL_972372, EPI_ISL_972373, EPI_ISL_972374, EPI_ISL_972379, EPI_ISL_972380, EPI_ISL_972383, EPI_ISL_972384, EPI_ISL_972385, EPI_ISL_972386, EPI_ISL_972388, EPI_ISL_972389, EPI_ISL_972390, EPI_ISL_972395, EPI_ISL_972396, EPI_ISL_972400, EPI_ISL_972401, EPI_ISL_972402, EPI_ISL_972403, EPI_ISL_972404, EPI_ISL_972405, EPI_ISL_972408, EPI_ISL_972409, EPI_ISL_972411, EPI_ISL_972412, EPI_ISL_972414, EPI_ISL_972415, EPI_ISL_972416, EPI_ISL_972417, EPI_ISL_972419, EPI_ISL_972420, EPI_ISL_972421, EPI_ISL_972422, EPI_ISL_972424, EPI_ISL_972425, EPI_ISL_972427, EPI_ISL_972428, EPI_ISL_972429, EPI_ISL_972430, EPI_ISL_972432, EPI_ISL_972433, EPI_ISL_972434, EPI_ISL_972439, EPI_ISL_972440, EPI_ISL_972441, EPI_ISL_972444, EPI_ISL_972445, EPI_ISL_972446, EPI_ISL_972447, EPI_ISL_972449, EPI_ISL_972450, EPI_ISL_972451, EPI_ISL_972453, EPI_ISL_972454, EPI_ISL_972455, EPI_ISL_972457, EPI_ISL_972458, EPI_ISL_972460, EPI_ISL_972461, EPI_ISL_972462, EPI_ISL_972463, EPI_ISL_972464, EPI_ISL_972467, EPI_ISL_972469, EPI_ISL_972470, EPI_ISL_972473, EPI_ISL_972475, EPI_ISL_972476, EPI_ISL_972480, EPI_ISL_972481, EPI_ISL_972482, EPI_ISL_972483, EPI_ISL_972485, EPI_ISL_972487, EPI_ISL_972489, EPI_ISL_972490, EPI_ISL_972492, EPI_ISL_972494, EPI_ISL_972495, EPI_ISL_972497, EPI_ISL_972498, EPI_ISL_972499, EPI_ISL_972500, EPI_ISL_972503, EPI_ISL_972504, EPI_ISL_972505, EPI_ISL_972506, EPI_ISL_972507, EPI_ISL_972508, EPI_ISL_972509, EPI_ISL_972510, EPI_ISL_972511, EPI_ISL_972515, EPI_ISL_972516, EPI_ISL_972518, EPI_ISL_972519, EPI_ISL_972520, EPI_ISL_972521, EPI_ISL_972523, EPI_ISL_972524, EPI_ISL_972526, EPI_ISL_972527, EPI_ISL_972531, EPI_ISL_972532, EPI_ISL_972534, EPI_ISL_972535, EPI_ISL_972537, EPI_ISL_972538, EPI_ISL_972540, EPI_ISL_972542, EPI_ISL_972543, EPI_ISL_972548, EPI_ISL_972550, EPI_ISL_972551, EPI_ISL_972553, EPI_ISL_972558, EPI_ISL_972561, EPI_ISL_972562, EPI_ISL_972563, EPI_ISL_972564, EPI_ISL_972567, EPI_ISL_972568, EPI_ISL_972570, EPI_ISL_972571, EPI_ISL_972572, EPI_ISL_972573, EPI_ISL_972578, EPI_ISL_972581, EPI_ISL_972582, EPI_ISL_972583, EPI_ISL_972584, EPI_ISL_972588, EPI_ISL_972589, EPI_ISL_972593, EPI_ISL_972594, EPI_ISL_972595, EPI_ISL_972596, EPI_ISL_972598, EPI_ISL_972599, EPI_ISL_972600, EPI_ISL_972603, EPI_ISL_972604, EPI_ISL_972605, EPI_ISL_972606, EPI_ISL_972611, EPI_ISL_972613, EPI_ISL_972616, EPI_ISL_972617, EPI_ISL_972619, EPI_ISL_972620, EPI_ISL_972621, EPI_ISL_972622, EPI_ISL_972623, EPI_ISL_972626, EPI_ISL_972628, EPI_ISL_972629, EPI_ISL_972630, EPI_ISL_972631, EPI_ISL_972632, EPI_ISL_972636, EPI_ISL_972637, EPI_ISL_972638, EPI_ISL_972640, EPI_ISL_972641, EPI_ISL_972642, EPI_ISL_972643, EPI_ISL_972645, EPI_ISL_972646, EPI_ISL_972647, EPI_ISL_972648, EPI_ISL_972649, EPI_ISL_972650, EPI_ISL_972651, EPI_ISL_972652, EPI_ISL_972654, EPI_ISL_972655, EPI_ISL_972656, EPI_ISL_972657, EPI_ISL_972658, EPI_ISL_972659, EPI_ISL_972660, EPI_ISL_972664, EPI_ISL_972665, EPI_ISL_972666, EPI_ISL_972667, EPI_ISL_972669, EPI_ISL_972670, EPI_ISL_972672, EPI_ISL_972675, EPI_ISL_972676, EPI_ISL_972677, EPI_ISL_972678, EPI_ISL_972679, EPI_ISL_972681, EPI_ISL_972682, EPI_ISL_972683, EPI_ISL_972684, EPI_ISL_972685, EPI_ISL_972691, EPI_ISL_972693, EPI_ISL_972694, EPI_ISL_972695, EPI_ISL_972696, EPI_ISL_972698, EPI_ISL_972699, EPI_ISL_972700, EPI_ISL_972701, EPI_ISL_972703, EPI_ISL_972704, EPI_ISL_972705, EPI_ISL_972706, EPI_ISL_972708, EPI_ISL_972710, EPI_ISL_972711, EPI_ISL_972716, EPI_ISL_972717, EPI_ISL_972719, EPI_ISL_972720, EPI_ISL_972721, EPI_ISL_972723, EPI_ISL_972724, EPI_ISL_972725, EPI_ISL_972726, EPI_ISL_972729, EPI_ISL_972731, EPI_ISL_972732, EPI_ISL_972733, EPI_ISL_972735, EPI_ISL_972736, EPI_ISL_972739, EPI_ISL_972740, EPI_ISL_972745, EPI_ISL_972746, EPI_ISL_972747, EPI_ISL_972748, EPI_ISL_972749, EPI_ISL_972753, EPI_ISL_972754, EPI_ISL_972756, EPI_ISL_972757, EPI_ISL_972760, EPI_ISL_972762, EPI_ISL_972764, EPI_ISL_972765, EPI_ISL_972767, EPI_ISL_972770, EPI_ISL_972775, EPI_ISL_972780, EPI_ISL_972781, EPI_ISL_972782, EPI_ISL_972783, EPI_ISL_972784, EPI_ISL_972786, EPI_ISL_972788, EPI_ISL_972789, EPI_ISL_972792, EPI_ISL_972794, EPI_ISL_972795, EPI_ISL_972796, EPI_ISL_972797, EPI_ISL_972798, EPI_ISL_972799, EPI_ISL_972800, EPI_ISL_972801, EPI_ISL_972802, EPI_ISL_972803, EPI_ISL_972804, EPI_ISL_972806, EPI_ISL_972808, EPI_ISL_972814, EPI_ISL_972815, EPI_ISL_972817, EPI_ISL_972818, EPI_ISL_972820, EPI_ISL_972823, EPI_ISL_972824, EPI_ISL_972826, EPI_ISL_972827, EPI_ISL_972828, EPI_ISL_972829, EPI_ISL_972830, EPI_ISL_972831, EPI_ISL_972832, EPI_ISL_972836, EPI_ISL_972837, EPI_ISL_972838, EPI_ISL_972839, EPI_ISL_972840, EPI_ISL_972841, EPI_ISL_972842, EPI_ISL_972844, EPI_ISL_972845, EPI_ISL_972847, EPI_ISL_972849, EPI_ISL_972850, EPI_ISL_972852, EPI_ISL_972854, EPI_ISL_972856, EPI_ISL_972857, EPI_ISL_972859, EPI_ISL_972862, EPI_ISL_972863, EPI_ISL_972864, EPI_ISL_972865, EPI_ISL_972866, EPI_ISL_972867, EPI_ISL_972868, EPI_ISL_972869, EPI_ISL_972870, EPI_ISL_972873, EPI_ISL_972878, EPI_ISL_972879, EPI_ISL_972880, EPI_ISL_972883, EPI_ISL_972886, EPI_ISL_972889, EPI_ISL_972890, EPI_ISL_972893, EPI_ISL_972894, EPI_ISL_972896, EPI_ISL_972901, EPI_ISL_972902, EPI_ISL_972904, EPI_ISL_972906, EPI_ISL_972909, EPI_ISL_972910, EPI_ISL_972912, EPI_ISL_972914, EPI_ISL_972915, EPI_ISL_972919, EPI_ISL_972920, EPI_ISL_972921, EPI_ISL_972923, EPI_ISL_972925, EPI_ISL_972926, EPI_ISL_972927, EPI_ISL_972928, EPI_ISL_972930, EPI_ISL_972931, EPI_ISL_972932, EPI_ISL_972934, EPI_ISL_972935, EPI_ISL_972937, EPI_ISL_972939, EPI_ISL_972942, EPI_ISL_972943, EPI_ISL_972946, EPI_ISL_972947, EPI_ISL_972951, EPI_ISL_972952, EPI_ISL_972953, EPI_ISL_972955, EPI_ISL_972956, EPI_ISL_972958, EPI_ISL_972959, EPI_ISL_972961, EPI_ISL_972964, EPI_ISL_972966, EPI_ISL_972967, EPI_ISL_972968, EPI_ISL_972971, EPI_ISL_972974, EPI_ISL_972976, EPI_ISL_972977, EPI_ISL_972978, EPI_ISL_972979, EPI_ISL_972980, EPI_ISL_972981, EPI_ISL_972982, EPI_ISL_972983, EPI_ISL_972984, EPI_ISL_972985, EPI_ISL_972986, EPI_ISL_972987, EPI_ISL_972988, EPI_ISL_972989, EPI_ISL_972990, EPI_ISL_972991, EPI_ISL_972992, EPI_ISL_972993, EPI_ISL_972994, EPI_ISL_972997, EPI_ISL_972999, EPI_ISL_973000, EPI_ISL_973003, EPI_ISL_973004, EPI_ISL_973005, EPI_ISL_973006, EPI_ISL_973007, EPI_ISL_973008, EPI_ISL_973010, EPI_ISL_973011, EPI_ISL_973012, EPI_ISL_973013, EPI_ISL_973015, EPI_ISL_973016, EPI_ISL_973017, EPI_ISL_973018, EPI_ISL_973021, EPI_ISL_973023, EPI_ISL_973025, EPI_ISL_973029, EPI_ISL_973031, EPI_ISL_973032, EPI_ISL_973033, EPI_ISL_973034, EPI_ISL_973036, EPI_ISL_973037, EPI_ISL_973039, EPI_ISL_973041, EPI_ISL_973042, EPI_ISL_973043, EPI_ISL_973044, EPI_ISL_973045, EPI_ISL_973046, EPI_ISL_973049, EPI_ISL_973050, EPI_ISL_973051, EPI_ISL_973054, EPI_ISL_973056, EPI_ISL_973057, EPI_ISL_973058, EPI_ISL_973059, EPI_ISL_973061, EPI_ISL_973062, EPI_ISL_973065, EPI_ISL_973066, EPI_ISL_973067, EPI_ISL_973068, EPI_ISL_973070, EPI_ISL_973072, EPI_ISL_973074, EPI_ISL_973075, EPI_ISL_973079, EPI_ISL_973081, EPI_ISL_973083, EPI_ISL_973084, EPI_ISL_973087, EPI_ISL_973089, EPI_ISL_973090, EPI_ISL_973091, EPI_ISL_973092, EPI_ISL_973093, EPI_ISL_973095, EPI_ISL_973098, EPI_ISL_973099, EPI_ISL_973100, EPI_ISL_973102, EPI_ISL_973103, EPI_ISL_973104, EPI_ISL_973106, EPI_ISL_973107, EPI_ISL_973108, EPI_ISL_973109, EPI_ISL_973110, EPI_ISL_973112, EPI_ISL_973114, EPI_ISL_973117, EPI_ISL_973118, EPI_ISL_973119, EPI_ISL_973121, EPI_ISL_973125, EPI_ISL_973126, EPI_ISL_973129, EPI_ISL_973133, EPI_ISL_973134, EPI_ISL_973136, EPI_ISL_973139, EPI_ISL_973140, EPI_ISL_973141, EPI_ISL_973142, EPI_ISL_973144, EPI_ISL_973146, EPI_ISL_973147, EPI_ISL_973149, EPI_ISL_973150, EPI_ISL_973151, EPI_ISL_973152, EPI_ISL_973153, EPI_ISL_973154, EPI_ISL_973155, EPI_ISL_973156, EPI_ISL_973158, EPI_ISL_973159, EPI_ISL_973161, EPI_ISL_973162, EPI_ISL_973163, EPI_ISL_973164, EPI_ISL_973166, EPI_ISL_973168, EPI_ISL_973169, EPI_ISL_973170, EPI_ISL_973171, EPI_ISL_973172, EPI_ISL_973173, EPI_ISL_973174, EPI_ISL_973176, EPI_ISL_973177, EPI_ISL_973181, EPI_ISL_973183, EPI_ISL_973184, EPI_ISL_973185, EPI_ISL_973187, EPI_ISL_973188, EPI_ISL_973190, EPI_ISL_973192, EPI_ISL_973193, EPI_ISL_973198, EPI_ISL_973200, EPI_ISL_973201, EPI_ISL_973202, EPI_ISL_973203, EPI_ISL_973204, EPI_ISL_973205, EPI_ISL_973209, EPI_ISL_973210, EPI_ISL_973211, EPI_ISL_973212, EPI_ISL_973214, EPI_ISL_973215, EPI_ISL_973217, EPI_ISL_973218, EPI_ISL_973219, EPI_ISL_973220, EPI_ISL_973221, EPI_ISL_973222, EPI_ISL_973223, EPI_ISL_973226, EPI_ISL_973228, EPI_ISL_973231, EPI_ISL_973235, EPI_ISL_973237, EPI_ISL_973240, EPI_ISL_973241, EPI_ISL_973242, EPI_ISL_973243, EPI_ISL_973244, EPI_ISL_973245, EPI_ISL_973246, EPI_ISL_973247, EPI_ISL_973248, EPI_ISL_973253, EPI_ISL_973254, EPI_ISL_973255, EPI_ISL_973257, EPI_ISL_973258, EPI_ISL_973259, EPI_ISL_973261, EPI_ISL_973262, EPI_ISL_973267, EPI_ISL_973268, EPI_ISL_973269, EPI_ISL_973270, EPI_ISL_973272, EPI_ISL_973274, EPI_ISL_973275, EPI_ISL_973279, EPI_ISL_973280, EPI_ISL_973282, EPI_ISL_973283, EPI_ISL_973284, EPI_ISL_973285, EPI_ISL_973286, EPI_ISL_973287, EPI_ISL_973290, EPI_ISL_973291, EPI_ISL_973292, EPI_ISL_973293, EPI_ISL_973294, EPI_ISL_973295, EPI_ISL_973297, EPI_ISL_973299, EPI_ISL_973300, EPI_ISL_973301, EPI_ISL_973303, EPI_ISL_973305, EPI_ISL_973306, EPI_ISL_973307, EPI_ISL_973308, EPI_ISL_973309, EPI_ISL_973311, EPI_ISL_973313, EPI_ISL_973314, EPI_ISL_973315, EPI_ISL_973316, EPI_ISL_973318, EPI_ISL_973319, EPI_ISL_973320, EPI_ISL_973321, EPI_ISL_973322, EPI_ISL_973323, EPI_ISL_973326, EPI_ISL_973327, EPI_ISL_973329, EPI_ISL_973330, EPI_ISL_973331, EPI_ISL_973334, EPI_ISL_973335, EPI_ISL_973336, EPI_ISL_973337, EPI_ISL_973343, EPI_ISL_973344, EPI_ISL_973347, EPI_ISL_973348, EPI_ISL_973352, EPI_ISL_973353, EPI_ISL_973355, EPI_ISL_973356, EPI_ISL_973360, EPI_ISL_973361, EPI_ISL_973362, EPI_ISL_973364, EPI_ISL_973366, EPI_ISL_973367, EPI_ISL_973370, EPI_ISL_973371, EPI_ISL_973372, EPI_ISL_973374, EPI_ISL_973375, EPI_ISL_973376, EPI_ISL_973377, EPI_ISL_973378, EPI_ISL_973380, EPI_ISL_973381, EPI_ISL_973382, EPI_ISL_973385, EPI_ISL_973386, EPI_ISL_973387, EPI_ISL_973388, EPI_ISL_973391, EPI_ISL_973392, EPI_ISL_973393, EPI_ISL_973394, EPI_ISL_973395, EPI_ISL_973400, EPI_ISL_973401, EPI_ISL_973402, EPI_ISL_973403, EPI_ISL_973405, EPI_ISL_973408, EPI_ISL_973409, EPI_ISL_973411, EPI_ISL_973413, EPI_ISL_973414, EPI_ISL_973416, EPI_ISL_973418, EPI_ISL_973419, EPI_ISL_973421, EPI_ISL_973424, EPI_ISL_973425, EPI_ISL_973426, EPI_ISL_973427, EPI_ISL_973428, EPI_ISL_973429, EPI_ISL_973431, EPI_ISL_973433, EPI_ISL_973434, EPI_ISL_973435, EPI_ISL_973436, EPI_ISL_973437, EPI_ISL_973439, EPI_ISL_973440, EPI_ISL_973441, EPI_ISL_973442, EPI_ISL_973444, EPI_ISL_973446, EPI_ISL_973448, EPI_ISL_973449, EPI_ISL_973450, EPI_ISL_973451, EPI_ISL_973452, EPI_ISL_973454, EPI_ISL_973455, EPI_ISL_973457, EPI_ISL_973458, EPI_ISL_973460, EPI_ISL_973462, EPI_ISL_973464, EPI_ISL_973466, EPI_ISL_973469, EPI_ISL_973470, EPI_ISL_973472, EPI_ISL_973473, EPI_ISL_973475, EPI_ISL_973476, EPI_ISL_973480, EPI_ISL_973482, EPI_ISL_973489, EPI_ISL_973493, EPI_ISL_973497, EPI_ISL_973501, EPI_ISL_973503, EPI_ISL_973510, EPI_ISL_973512, EPI_ISL_973519, EPI_ISL_973521, EPI_ISL_973525, EPI_ISL_973527, EPI_ISL_973529, EPI_ISL_973532, EPI_ISL_973533, EPI_ISL_973538, EPI_ISL_973542, EPI_ISL_973546, EPI_ISL_973548, EPI_ISL_973550, EPI_ISL_973555, EPI_ISL_973557, EPI_ISL_973559, EPI_ISL_973561, EPI_ISL_973563, EPI_ISL_973566, EPI_ISL_973572, EPI_ISL_973574, EPI_ISL_973576, EPI_ISL_973589, EPI_ISL_973593, EPI_ISL_973597, EPI_ISL_973603, EPI_ISL_973605, EPI_ISL_973609, EPI_ISL_973611, EPI_ISL_973616, EPI_ISL_973618, EPI_ISL_973621, EPI_ISL_973623, EPI_ISL_973624, EPI_ISL_973626, EPI_ISL_973628, EPI_ISL_973629, EPI_ISL_973631, EPI_ISL_973640, EPI_ISL_973645, EPI_ISL_973647, EPI_ISL_973648, EPI_ISL_973651, EPI_ISL_973652, EPI_ISL_973658, EPI_ISL_973660, EPI_ISL_973662, EPI_ISL_973668, EPI_ISL_973670, EPI_ISL_973680, EPI_ISL_973682, EPI_ISL_973684, EPI_ISL_973686, EPI_ISL_973688, EPI_ISL_973689, EPI_ISL_973691, EPI_ISL_973696, EPI_ISL_973698, EPI_ISL_973703, EPI_ISL_973708, EPI_ISL_973710, EPI_ISL_973715, EPI_ISL_973717, EPI_ISL_973720, EPI_ISL_973726, EPI_ISL_973728, EPI_ISL_973730, EPI_ISL_973734, EPI_ISL_973735, EPI_ISL_973738, EPI_ISL_973739, EPI_ISL_973743, EPI_ISL_973744, EPI_ISL_973746, EPI_ISL_973750, EPI_ISL_973754, EPI_ISL_973756, EPI_ISL_973757, EPI_ISL_973759, EPI_ISL_973761, EPI_ISL_973763, EPI_ISL_973765, EPI_ISL_973768, EPI_ISL_973770, EPI_ISL_973773, EPI_ISL_973777, EPI_ISL_973778, EPI_ISL_973782, EPI_ISL_973786, EPI_ISL_973787, EPI_ISL_973789, EPI_ISL_973790, EPI_ISL_973792, EPI_ISL_973793, EPI_ISL_973798, EPI_ISL_973801, EPI_ISL_973803, EPI_ISL_973806, EPI_ISL_973808, EPI_ISL_973809, EPI_ISL_973811, EPI_ISL_973812, EPI_ISL_973814, EPI_ISL_973816, EPI_ISL_973819, EPI_ISL_973822, EPI_ISL_973825, EPI_ISL_973835, EPI_ISL_973836			
see above	Department of Virus and Microbiological Special Diagnostics, Statens Serum Institut, Copenhagen, Denmark	Aalborg University	Danish Covid-19 Genome Consortium
EPI_ISL_977490	Hospital Municipal Guido Guida	Instituto Adolfo Lutz, Interdiciplinary Procedures Center, Strategic Laboratory	Claudio Tavares Sacchi, Claudia Regina Gonçalves, Erica Valessa Ramos Gomes, Karoline Rodrigues Campos
EPI_ISL_977493	Lab voor klinische biologie	Lab voor klinische biologie	Hannelore Hamerlinck, Marija Janevska, Bruno Verhasselt
EPI_ISL_977503	Szpital Miejski im. Franciszka Raszei w Poznaniu, Oddzia Chirurgii Ogólnej i Minimalnie Inwazyjnej	genXone SA, Research & Development Laboratory	Maciej Sykulski, Grzegorz Nowicki, Jakub Grabowski, Natalia Drwska-Matelska, Anna Brylak-Baszków, Aleksandra Gidlewicz, Karol Szeszko, ukasz Krych, Micha Kaszuba
EPI_ISL_977600	Biolab Diagnostic Laboratories	Biolab Diagnostic Laboratories	Issa Abu-Dayyeh, Ahmad Tibi, Lama Hussein, Shayma Ali, Badia Saddedin, Amid Abdelnour
EPI_ISL_978169, EPI_ISL_978170, EPI_ISL_978171, EPI_ISL_978172, EPI_ISL_978173, EPI_ISL_978174, EPI_ISL_978175, EPI_ISL_978176, EPI_ISL_978177, EPI_ISL_978178, EPI_ISL_978179, EPI_ISL_978180			
see above	Virginia Division of Consolidated Laboratory Services	Virginia Division of Consolidated Laboratory Services	Virginia DCLS
EPI_ISL_978310, EPI_ISL_978319, EPI_ISL_978320, EPI_ISL_978321, EPI_ISL_978322, EPI_ISL_978323,	Texas Department of State Health Services	Texas Department of State Health Services	Bonnie Oh, Anita Pokharel, James Daniel Bonser, Myong Koag, Chung Wang, Rachel Lee, Grace Kubin, Rashmi Tuladhar, Mayela Pedrueza, Maliha Rahman, Jenny Zhang

EPI_ISL_978324, EPI_ISL_978325			
EPI_ISL_978429, EPI_ISL_978430, EPI_ISL_978431, EPI_ISL_978477, EPI_ISL_978478, EPI_ISL_978479, EPI_ISL_978480	Arizona State Public Health Laboratory	Arizona State Public Health Laboratory	Trung Huynh, Jessica Escobar, Katherine Fullerton, Nobuko Fukushima, Stacy White, Linda Getsinger, Victor Waddell
EPI_ISL_978553	Kansas Health and Environmental Lab	Kansas Health and Environmental Lab	Mike Grose, Paige Drury, Carissa Robertson, Ben Olsen, and Phil Adam
EPI_ISL_978621	Helix/Illumina	Respiratory Viruses Branch, Division of Viral Diseases, Centers for Disease Control and Prevention	Peter W. Cook,Dakota Howard,Dhwani Batra,Ben L. Rambo-Martin,Eileen de Feo,Jan Antico,Christine Tran,Matthew Tolentino,Shannon Wickline,Kim Gietzen,Brad Sickler,Jingtao Liu,Eric Allen,Phil Febbo,Summer Galloway,Nicole L. Washington,Simon White,Geraint Levan,Kelly Schiabor Barrett,Elizabeth Cirulli,Alexandre Bolze,Ary Ascencio,Charlotte Rivera-Garcia,Ryan Cho,Jason Nguyen,Sherry Wang,Jimmy Ramirez,Tyler Cassens,Efren Sandoval,Magnus Isaksson,William Lee,David Becker,Marc Laurent,James Lu,Clinton R. Paden,Suxiang Tong,Duncan MacCannell,
EPI_ISL_978887, EPI_ISL_978888, EPI_ISL_978952, EPI_ISL_978953, EPI_ISL_978985, EPI_ISL_978986, EPI_ISL_978987	Centre for Dengue Research and AICBU, Department of Immunology and Molecular Medicine	Centre for Dengue Research and AICBU, Department of Immunology and Molecular Medicine	Chandima Jeewandara, Deshni Jayathilaka, Dinuka Ariyaratne, Tibutius Thanesh Pramanayagam, Diyanath Ranasinghe, Laksiri Gomes, Gathsaurie Neelika Malavige
EPI_ISL_979253	Institute of Microbiology and Immunology, Faculty of Medicine, University of Ljubljana	Institute of Microbiology and Immunology, Faculty of Medicine, University of Ljubljana	Samo Zakotnik, Tomaž Mark Zorec, Matic Brvar, Doroteja Vlaj, Patricija Pozvek,Špela Pleh, Miša Korva, Mario Poljak, Tatjana Avši - Županc
EPI_ISL_979313, EPI_ISL_979318, EPI_ISL_979326	Cadham Provincial laboratory	National Microbiology Laboratory (NML)	Anna Majer, Shari Tyson, Grace Seo, Philip Mabon, Elsie Grudeski, Rhiannon Huzarewich, Russell Mandes, Anneliese Landgraff, Jennifer Tanner, Natalie Knox, Morag Graham, Gary Van Domselaar, Paul Van Caeseele, Jared Bullard, David Alexander, Kerry Dust, Nathalie Bastien, Yan Li, Timothy Booth, Darian Hole, Madison Chapel, Kirsten Biggar, CanCOGeN's metadata curation team, Public Health Agency of Canada CanCOGeN team
EPI_ISL_979365, EPI_ISL_979366, EPI_ISL_979367, EPI_ISL_979369	The Jackson Laboratory	The Jackson Laboratory	Lloyd M, Sanderson B, Srivastava A, Maurya R, Renzette N, Omerza G, Kelly K, Li L, Wei C L, Adams M
EPI_ISL_979410, EPI_ISL_979411, EPI_ISL_979412, EPI_ISL_979413, EPI_ISL_979414, EPI_ISL_979415, EPI_ISL_979416, EPI_ISL_979417, EPI_ISL_979418, EPI_ISL_979419, EPI_ISL_979420, EPI_ISL_979421, EPI_ISL_979422, EPI_ISL_979423, EPI_ISL_979424, EPI_ISL_979425, EPI_ISL_979426, EPI_ISL_979427, EPI_ISL_979428			
see above	New Mexico Department of Health Scientific Laboratory	New Mexico Department of Health Scientific Laboratory	Ellie Johnson, Anastacia Griego-Fisher, D'eldra Malone, Jennifer Benoit
EPI_ISL_979458	Test Iowa	State Hygienic Laboratory at the University of Iowa	Valerie Reeb, Erik Twait, Wes Hottel, Alankar Kampoowale
EPI_ISL_979469, EPI_ISL_979470, EPI_ISL_979471, EPI_ISL_979472, EPI_ISL_979473, EPI_ISL_979474, EPI_ISL_979475, EPI_ISL_979476, EPI_ISL_979477, EPI_ISL_979478, EPI_ISL_979479, EPI_ISL_979480, EPI_ISL_979481, EPI_ISL_979482, EPI_ISL_979483, EPI_ISL_979484, EPI_ISL_979485, EPI_ISL_979486, EPI_ISL_979487, EPI_ISL_979488, EPI_ISL_979489, EPI_ISL_979490, EPI_ISL_979491, EPI_ISL_979492, EPI_ISL_979493, EPI_ISL_979494, EPI_ISL_979495, EPI_ISL_979496, EPI_ISL_979497, EPI_ISL_979498, EPI_ISL_979499, EPI_ISL_979500, EPI_ISL_979501, EPI_ISL_979502, EPI_ISL_979503, EPI_ISL_979504, EPI_ISL_979505, EPI_ISL_979506, EPI_ISL_979507, EPI_ISL_979508, EPI_ISL_979509, EPI_ISL_979510, EPI_ISL_979511, EPI_ISL_979512, EPI_ISL_979513, EPI_ISL_979514, EPI_ISL_979515, EPI_ISL_979516, EPI_ISL_979517, EPI_ISL_979518, EPI_ISL_979519, EPI_ISL_979520, EPI_ISL_979521, EPI_ISL_979522, EPI_ISL_979523, EPI_ISL_979524, EPI_ISL_979525, EPI_ISL_979526, EPI_ISL_979527, EPI_ISL_979528, EPI_ISL_979529, EPI_ISL_979530, EPI_ISL_979531, EPI_ISL_979532, EPI_ISL_979533, EPI_ISL_979534, EPI_ISL_979535, EPI_ISL_979536, EPI_ISL_979537, EPI_ISL_979538, EPI_ISL_979539, EPI_ISL_979540, EPI_ISL_979541, EPI_ISL_979542, EPI_ISL_979543			
see above	Eurofins Diatherix	Hudsonalpha Genome Sequencing Center	Jane Grimwood, Melissa Williams, Lori H. Handley, Joshua Stough, Leslie Malone, Stefan Brzezinski, Ada Stewart, Teresa Jones, Jenell Webber, John Lovell, Jennifer Cart, and Jeremy Schmutz
EPI_ISL_979799, EPI_ISL_979800, EPI_ISL_979801, EPI_ISL_979802	National Institute of Infectious Diseases-Prof. Dr. Matei Bals Molecular Diagnostics Laboratory	National Institute of Infectious Diseases-Prof. Dr. Matei Bals Molecular Diagnostics Laboratory	Leontina Banica, Marius Surleac, Corina Casangiu, Petre Milu, Andreea Tudor, Simona Paraschiv, Dan Otelea
EPI_ISL_980807	genXone SA, Molecular Diagnostics Laboratory / NZOZ	genXone SA, Research & Development Laboratory	Maciej Sykulski, Grzegorz Nowicki, Jakub Grabowski, Natalia Drwska-Matelska, Anna Brylak-Baszków, Aleksandra Gidlewicz, Karol Szeszko, ukasz Krych, Micha Kaszuba
EPI_ISL_980841, EPI_ISL_980842, EPI_ISL_980844, EPI_ISL_980846, EPI_ISL_980847, EPI_ISL_980848, EPI_ISL_980849, EPI_ISL_980850, EPI_ISL_980853, EPI_ISL_980856, EPI_ISL_980862, EPI_ISL_980865, EPI_ISL_980866, EPI_ISL_980867, EPI_ISL_980872, EPI_ISL_980874, EPI_ISL_980875, EPI_ISL_980877, EPI_ISL_980878, EPI_ISL_980879, EPI_ISL_980882, EPI_ISL_980883, EPI_ISL_980886, EPI_ISL_980889, EPI_ISL_980890, EPI_ISL_980891, EPI_ISL_980892, EPI_ISL_980894, EPI_ISL_980895, EPI_ISL_980906, EPI_ISL_980909, EPI_ISL_980912, EPI_ISL_980913, EPI_ISL_980915, EPI_ISL_980918, EPI_ISL_980919, EPI_ISL_980946, EPI_ISL_980947, EPI_ISL_980948, EPI_ISL_980949, EPI_ISL_980950, EPI_ISL_980951, EPI_ISL_980952, EPI_ISL_980953, EPI_ISL_980954, EPI_ISL_980955, EPI_ISL_980956, EPI_ISL_980957, EPI_ISL_980958, EPI_ISL_980959, EPI_ISL_980960, EPI_ISL_980961, EPI_ISL_980962, EPI_ISL_980963, EPI_ISL_980964, EPI_ISL_980984, EPI_ISL_980985, EPI_ISL_980986, EPI_ISL_980987, EPI_ISL_980988			
see above	Innovative Genomics Institute, UC Berkeley	Innovative Genomics Institute, UC Berkeley	Stacia Wyman, Haridha Shivram, Phil Frankino, Liana Lareau
EPI_ISL_981070, EPI_ISL_981076, EPI_ISL_981081, EPI_ISL_981083, EPI_ISL_981084, EPI_ISL_981086, EPI_ISL_981101, EPI_ISL_981102, EPI_ISL_981108, EPI_ISL_981109, EPI_ISL_981118, EPI_ISL_981122, EPI_ISL_981133, EPI_ISL_981134, EPI_ISL_981139, EPI_ISL_981140, EPI_ISL_981141, EPI_ISL_981142, EPI_ISL_981143, EPI_ISL_981144, EPI_ISL_981145, EPI_ISL_981146			
see above	Johns Hopkins Hospital Department of Pathology	Johns Hopkins Hospital Department of Pathology	C. Paul Morris, Chun Huai Luo, Adannaya Amadi, Matthew Schwartz, Nicholas Gallagher, Heba H. Mostafa
EPI_ISL_981308, EPI_ISL_981309, EPI_ISL_981310, EPI_ISL_981311, EPI_ISL_981312, EPI_ISL_981313, EPI_ISL_981314, EPI_ISL_981315, EPI_ISL_981316, EPI_ISL_981319, EPI_ISL_981320, EPI_ISL_981321, EPI_ISL_981324, EPI_ISL_981326, EPI_ISL_981327, EPI_ISL_981328, EPI_ISL_981329, EPI_ISL_981330, EPI_ISL_981331, EPI_ISL_981332, EPI_ISL_981333, EPI_ISL_981334, EPI_ISL_981335, EPI_ISL_981336, EPI_ISL_981337, EPI_ISL_981338, EPI_ISL_981339, EPI_ISL_981340, EPI_ISL_981341, EPI_ISL_981342, EPI_ISL_981343, EPI_ISL_981344, EPI_ISL_981350, EPI_ISL_981352, EPI_ISL_981354, EPI_ISL_981355, EPI_ISL_981356, EPI_ISL_981357, EPI_ISL_981358, EPI_ISL_981359, EPI_ISL_981360, EPI_ISL_981361, EPI_ISL_981362, EPI_ISL_981363, EPI_ISL_981364, EPI_ISL_981365, EPI_ISL_981366, EPI_ISL_981367			
see above	Hospital Universitari Vall d'Hebron - Vall d'Hebron Institut de Rercerca	Hospital Universitari Vall d'Hebron - Vall d'Hebron Institut de Rercerca	Cristina Andrés, Maria Piñana, Josep F Abril, Damir Garcia-Cehic, Ariadna Rando, Juliana Esperalba, Maria Gema Codina, Carla Castillo, Maria Carmen Martin, Tomàs Pumarola, Josep Quer, Andrés Antón
EPI_ISL_981376	AZ Klina	AZ Klina	Dr. C. Vael
EPI_ISL_981385	IAL Regional de Bauru	Instituto Adolfo Lutz, Interdiciplinary Procedures Center, Strategic Laboratory	Claudio Tavares Sacchi, Claudia Regina Gonçalves, Erica Valessa Ramos Gomes, Karoline Rodrigues Campos
EPI_ISL_981389	Hospital Geral de Sao Paulo	Instituto Adolfo Lutz, Interdiciplinary Procedures Center, Strategic Laboratory	Claudio Tavares Sacchi, Claudia Regina Gonçalves, Erica Valessa Ramos Gomes, Karoline Rodrigues Campos
EPI_ISL_981395, EPI_ISL_981462, EPI_ISL_981463, EPI_ISL_981464, EPI_ISL_981465	CH.INTERCOMMUNAL DE CRETEIL	Department of Virology, Henri Mondor University Hospital, Assistance Publique Hôpitaux de Paris, Université Paris-Est Créteil, INSERM U955	Christophe Rodriguez, Slim Fourati, Vanessa Demontant, Guillaume Gricourt, Melissa N'Debi, Alexandre Soulier, Elisabeth Trawinski, Jean-Michel Pawlotsky
EPI_ISL_981466, EPI_ISL_981467, EPI_ISL_981468, EPI_ISL_981469, EPI_ISL_981470, EPI_ISL_981471, EPI_ISL_981472, EPI_ISL_981473, EPI_ISL_981474, EPI_ISL_981475, EPI_ISL_981478, EPI_ISL_981479, EPI_ISL_981480, EPI_ISL_981481			
see above	CHI VILLENEUVE ST GEORGES	Department of Virology, Henri Mondor University Hospital, Assistance Publique Hôpitaux de Paris, Université Paris-Est Créteil, INSERM U955	Christophe Rodriguez, Slim Fourati, Vanessa Demontant, Guillaume Gricourt, Melissa N'Debi, Alexandre Soulier, Elisabeth Trawinski, Jean-Michel Pawlotsky
EPI_ISL_981486, EPI_ISL_981488, EPI_ISL_981538, EPI_ISL_981539, EPI_ISL_981540, EPI_ISL_981541, EPI_ISL_981542, EPI_ISL_981543, EPI_ISL_981544, EPI_ISL_981545, EPI_ISL_981546			
see above	CH.INTERCOMMUNAL DE CRETEIL	Department of Virology, Henri Mondor University Hospital, Assistance Publique Hôpitaux de Paris, Université Paris-Est Créteil, INSERM U955	Christophe Rodriguez, Slim Fourati, Vanessa Demontant, Guillaume Gricourt, Melissa N'Debi, Alexandre Soulier, Elisabeth Trawinski, Jean-Michel Pawlotsky
EPI_ISL_981547	G.H.E.F.Grand Hôpital EST Francilien	Department of Virology, Henri Mondor University Hospital, Assistance Publique Hôpitaux de Paris, Université Paris-Est Créteil, INSERM U955	Christophe Rodriguez, Slim Fourati, Vanessa Demontant, Guillaume Gricourt, Melissa N'Debi, Alexandre Soulier, Elisabeth Trawinski, Jean-Michel Pawlotsky
EPI_ISL_981650, EPI_ISL_981654, EPI_ISL_981659, EPI_ISL_981746, EPI_ISL_981813	University Hospitals of Geneva, Laboratory of Virology	HUG, Laboratory of Virology and the Health2030 Genome Center	Samuel Cordey, Ana Rita Goncalves, Laurent Kaiser, Lorenzo Cerutti, Henri Pegeot, Melyssa Elies, Deborah Penet, Keith Harshman, Ioannis Xenarios, Emmanouil Dermitzakis
EPI_ISL_981977	National Institute of Laboratory Medicine and Referral Center	Genomic Research Lab, BCSIR	Md. Murshed Hasan Sarkar, Mohammad Samir Uzzaman, Eshrar Osman, Md. Ahashan Habib, Shahina Akter, Tanjina Akhtar Banu, Abu Sayeed

			Mohammad Mahmud, Barna Goswami, Iffat Jahan, Md. Saddam Hossain, Tasnim Nafisa, Md. Maruf Ahmed Molla, Mahmuda Yeasmin, Asish Kumar Ghosh, Arifa Akram, A. K. M.Shamsuzzaman, Md. Salim Khan
EPI_ISL_982021, EPI_ISL_982026, EPI_ISL_982027, EPI_ISL_982043, EPI_ISL_982044, EPI_ISL_982063, EPI_ISL_982086	TGen North	TGen North	"Jolene Bowers, Megan Folkerts, Chris French, Hayley Yaglom, Ashlyn Pfeiffer, Darrin Lemmer, Dave Engelthaler, The Arizona COVID Genomics Union (ACGU)"
EPI_ISL_982123	EFS ILE DE FRANCE BANQUE DE TISSUS	Department of Virology, Henri Mondor University Hospital, Assistance Publique Hôpitaux de Paris, Université Paris-Est Créteil, INSERM U955	Christophe Rodriguez, Slim Fourati, Vanessa Demontant, Guillaume Gricourt, Melissa N'Debi, Alexandre Soulier, Elisabeth Trawinski, Jean-Michel Pawlotsky
EPI_ISL_982153	Hôpital Henri Mondor	Department of Virology, Henri Mondor University Hospital, Assistance Publique Hôpitaux de Paris, Université Paris-Est Créteil, INSERM U955	Christophe Rodriguez, Slim Fourati, Vanessa Demontant, Guillaume Gricourt, Melissa N'Debi, Alexandre Soulier, Elisabeth Trawinski, Jean-Michel Pawlotsky
EPI_ISL_982156	Hôpital Pitié-Salpêtrière	Department of Virology, Henri Mondor University Hospital, Assistance Publique Hôpitaux de Paris, Université Paris-Est Créteil, INSERM U955	Christophe Rodriguez, Slim Fourati, Vanessa Demontant, Guillaume Gricourt, Melissa N'Debi, Alexandre Soulier, Elisabeth Trawinski, Jean-Michel Pawlotsky
EPI_ISL_982161	Hôpital Henri Mondor	Department of Virology, Henri Mondor University Hospital, Assistance Publique Hôpitaux de Paris, Université Paris-Est Créteil, INSERM U955	Christophe Rodriguez, Slim Fourati, Vanessa Demontant, Guillaume Gricourt, Melissa N'Debi, Alexandre Soulier, Elisabeth Trawinski, Jean-Michel Pawlotsky
EPI_ISL_982162	G.H.E.F.Grand Hôpital EST Francilien	Department of Virology, Henri Mondor University Hospital, Assistance Publique Hôpitaux de Paris, Université Paris-Est Créteil, INSERM U955	Christophe Rodriguez, Slim Fourati, Vanessa Demontant, Guillaume Gricourt, Melissa N'Debi, Alexandre Soulier, Elisabeth Trawinski, Jean-Michel Pawlotsky
EPI_ISL_982234, EPI_ISL_982235	MEPHI, Aix Marseille University	MEPHI, Aix Marseille University	Anthony LEVASSEUR
EPI_ISL_982237, EPI_ISL_982238, EPI_ISL_982270, EPI_ISL_982275, EPI_ISL_982277, EPI_ISL_982278, EPI_ISL_982295, EPI_ISL_982296	Lab voor klinische biologie	Lab voor klinische biologie	Hannelore Hamerlinck, Marija Janevska, Bruno Verhasselt
EPI_ISL_982303, EPI_ISL_982305, EPI_ISL_982306	CH.INTERCOMMUNAL DE CRETEIL	Department of Virology, Henri Mondor University Hospital, Assistance Publique Hôpitaux de Paris, Université Paris-Est Créteil, INSERM U955	Christophe Rodriguez, Slim Fourati, Vanessa Demontant, Guillaume Gricourt, Melissa N'Debi, Alexandre Soulier, Elisabeth Trawinski, Jean-Michel Pawlotsky
EPI_ISL_982310	G.H.E.F.Grand Hôpital EST Francilien	Department of Virology, Henri Mondor University Hospital, Assistance Publique Hôpitaux de Paris, Université Paris-Est Créteil, INSERM U955	Christophe Rodriguez, Slim Fourati, Vanessa Demontant, Guillaume Gricourt, Melissa N'Debi, Alexandre Soulier, Elisabeth Trawinski, Jean-Michel Pawlotsky
EPI_ISL_982312, EPI_ISL_982314, EPI_ISL_982315, EPI_ISL_982316, EPI_ISL_982317, EPI_ISL_982318, EPI_ISL_982319, EPI_ISL_982320	CH.INTERCOMMUNAL DE CRETEIL	Department of Virology, Henri Mondor University Hospital, Assistance Publique Hôpitaux de Paris, Université Paris-Est Créteil, INSERM U955	Christophe Rodriguez, Slim Fourati, Vanessa Demontant, Guillaume Gricourt, Melissa N'Debi, Alexandre Soulier, Elisabeth Trawinski, Jean-Michel Pawlotsky
EPI_ISL_982861, EPI_ISL_982866, EPI_ISL_982867, EPI_ISL_982869, EPI_ISL_982871, EPI_ISL_982877, EPI_ISL_982878, EPI_ISL_982879, EPI_ISL_982880, EPI_ISL_982881, EPI_ISL_982882, EPI_ISL_982889, EPI_ISL_982894, EPI_ISL_982896, EPI_ISL_982898, EPI_ISL_982909, EPI_ISL_982922, EPI_ISL_982923			
see above	MEPHI Aix Marseille University (AMU)	MEPHI Aix Marseille University (AMU)	Anthony LEVASSEUR
EPI_ISL_983001	National Institute of Laboratory Medicine and Referral Center	Genomic Research Lab, BCSIR	Tanjina Akhtar Banu, Mohammad Samir Uzzaman, Eshrar Osman, Md. Ahashan Habib, Shahina Akter, Abu Sayeed Mohammad Mahmud, Md. Murshed Hasan Sarkar, Barna Goswami, Iffat Jahan, Md. Saddam Hossain, Tasnim Nafisa, Md. Maruf Ahmed Molla, Mahmuda Yeasmin, Asish Kumar Ghosh, Arifa Akram, A. K. M.Shamsuzzaman, Md. Salim Khan
EPI_ISL_983097	Microbiology and Virology Unit, Florence Careggi University Hospital	Microbiology and Virology Unit, Florence Careggi University Hospital	Vincenzo Di Pilato, Marco Coppi, Fabio Morecchiato, Noemi Aiezza, Ilaria Baccani, Alberto Antonelli, Emanuele Gori, Gian Maria Rossolini
EPI_ISL_983344	National Institute of Laboratory Medicine and Referral Center	Genomic Research Lab, BCSIR	Tasnim Nafisa, Mohammad Samir Uzzaman, Eshrar Osman, Md. Ahashan Habib, Shahina Akter, Tanjina Akhtar Banu, Abu Sayeed Mohammad Mahmud, Md. Murshed Hasan Sarkar, Barna Goswami, Iffat Jahan, Md. Saddam Hossain, Md. Maruf Ahmed Molla, Mahmuda Yeasmin, Asish Kumar Ghosh, Arifa Akram, A. K.M.Shamsuzzaman, Md. Salim Khan
EPI_ISL_983371	Utah Public Health Laboratory	Utah Public Health Laboratory	Erin L. Young, Kelly F. Oakeson, Tara Gallagher
EPI_ISL_983438, EPI_ISL_983440, EPI_ISL_983441, EPI_ISL_983442, EPI_ISL_983444	URMC LABS	Wadsworth Center, New York State Department of Health	Kirsten St. George, Daryl M. Lamson, Alexis Russel, Matthew Shudt, Melissa A Leisner, Jonathan Plitnick, Navjot Singh, John Kelly, Erasmus Schneider, Erica Lasek-Nesselquist
EPI_ISL_983446	THE MARY IMOGENE BASSETT HOSPITAL	Wadsworth Center, New York State Department of Health	Kirsten St. George, Daryl M. Lamson, Alexis Russel, Matthew Shudt, Melissa A Leisner, Jonathan Plitnick, Navjot Singh, John Kelly, Erasmus Schneider, Erica Lasek-Nesselquist
EPI_ISL_983461, EPI_ISL_983462, EPI_ISL_983463, EPI_ISL_983464, EPI_ISL_983465, EPI_ISL_983466, EPI_ISL_983467, EPI_ISL_983468, EPI_ISL_983469, EPI_ISL_983470, EPI_ISL_983471, EPI_ISL_983472, EPI_ISL_983473, EPI_ISL_983474, EPI_ISL_983475			
see above	URMC LABS	Wadsworth Center, New York State Department of Health	Kirsten St. George, Daryl M. Lamson, Alexis Russel, Matthew Shudt, Melissa A Leisner, Jonathan Plitnick, Navjot Singh, John Kelly, Erasmus Schneider, Erica Lasek-Nesselquist
EPI_ISL_983476	Wadsworth Center, New York State Department of Health	Wadsworth Center, New York State Department of Health	Kirsten St. George, Daryl M. Lamson, Alexis Russel, Matthew Shudt, Melissa A Leisner, Jonathan Plitnick, Navjot Singh, John Kelly, Erasmus Schneider, Erica Lasek-Nesselquist
EPI_ISL_983477	THE MARY IMOGENE BASSETT HOSPITAL	Wadsworth Center, New York State Department of Health	Kirsten St. George, Daryl M. Lamson, Alexis Russel, Matthew Shudt, Melissa A Leisner, Jonathan Plitnick, Navjot Singh, John Kelly, Erasmus Schneider, Erica Lasek-Nesselquist
EPI_ISL_983478, EPI_ISL_983479, EPI_ISL_983480, EPI_ISL_983481, EPI_ISL_983482, EPI_ISL_983483, EPI_ISL_983484	URMC LABS	Wadsworth Center, New York State Department of Health	Kirsten St. George, Daryl M. Lamson, Alexis Russel, Matthew Shudt, Melissa A Leisner, Jonathan Plitnick, Navjot Singh, John Kelly, Erasmus Schneider, Erica Lasek-Nesselquist
EPI_ISL_983485, EPI_ISL_983486, EPI_ISL_983487	THE MARY IMOGENE BASSETT HOSPITAL	Wadsworth Center, New York State Department of Health	Kirsten St. George, Daryl M. Lamson, Alexis Russel, Matthew Shudt, Melissa A Leisner, Jonathan Plitnick, Navjot Singh, John Kelly, Erasmus Schneider, Erica Lasek-Nesselquist
EPI_ISL_983494, EPI_ISL_983495, EPI_ISL_983496, EPI_ISL_983497, EPI_ISL_983498, EPI_ISL_983499	Kansas Health and Environmental Lab	Kansas Health and Environmental Lab	Mike Grose, Paige Drury, Carissa Robertson, Ben Olsen, and Phil Adam
EPI_ISL_983599, EPI_ISL_983600, EPI_ISL_983601, EPI_ISL_983602, EPI_ISL_983603, EPI_ISL_983604	Texas Department of State Health Services	Texas Department of State Health Services	Bonnie Oh, Anita Pokharel, James Daniel Bonser, Myong Koag, Chung Wang, Rachel Lee, Grace Kubin, Rashmi Tuladhar, Mayela Pedrueza, Maliha Rahman, Jenny Zhang
EPI_ISL_983652, EPI_ISL_983692, EPI_ISL_983693, EPI_ISL_983694,	Vault Health	Minnesota Department of Health, Public Health Laboratory	Alexandra Lorentz, Jacob Garfin, Matt Plumb, and Xiong Wang

EPI_ISL_983695, EPI_ISL_983696, EPI_ISL_983697			
EPI_ISL_983705, EPI_ISL_983706, EPI_ISL_983718, EPI_ISL_983719, EPI_ISL_983726, EPI_ISL_983727, EPI_ISL_983731, EPI_ISL_983733, EPI_ISL_983734, EPI_ISL_983747, EPI_ISL_983748, EPI_ISL_983749, EPI_ISL_983750, EPI_ISL_983751, EPI_ISL_983752, EPI_ISL_983753, EPI_ISL_983754, EPI_ISL_983755, EPI_ISL_983756, EPI_ISL_983757, EPI_ISL_983758, EPI_ISL_983759, EPI_ISL_983760, EPI_ISL_983761, EPI_ISL_983762, EPI_ISL_983763, EPI_ISL_983764, EPI_ISL_983765, EPI_ISL_983766, EPI_ISL_983767, EPI_ISL_983768, EPI_ISL_983769, EPI_ISL_983770, EPI_ISL_983771, EPI_ISL_983772, EPI_ISL_983773, EPI_ISL_983774, EPI_ISL_983775, EPI_ISL_983776, EPI_ISL_983777, EPI_ISL_983778, EPI_ISL_983779, EPI_ISL_983780, EPI_ISL_983781, EPI_ISL_983782			
see above	Colorado Department of Public Health and Environment	Colorado Department of Puplic Health and Environment	Laura Bankers, Molly C. Hetherington-Rauth, Diana Ir, Shannon Ely, Shannon R. Matzinger, Sarah Elizabeth Totten, Emily A. Travanty
