# Supplementary material for: Detection and characterization of the SARS-CoV-2 lineage B.1.526 in New York
Source: Nat Commun. 2021 Aug 9;12:4886. doi: 10.1038/s41467-021-25168-4 (PMC8352861; doi:10.1038/s41467-021-25168-4)
Supplement: Supplementary file 8 — Supplementary Data 4 [file 41467_2021_25168_MOESM8_ESM.zip › GISAID_acknowledements_tables/gisaid_hcov-19_acknowledgement_table_2021_02_13_00_.pdf]

We gratefully acknowledge the following Authors from the Originating laboratories responsible for obtaining the specimens, as well as the Submitting laboratories where the genome data were generated and shared via GISAID, on which this research is based.

All Submitters of data may be contacted directly via www.gisaid.org

Authors are sorted alphabetically.

Accession ID	Originating Laboratory	Submitting Laboratory	Authors
EPI_ISL_595221, EPI_ISL_595223, EPI_ISL_595225, EPI_ISL_595226, EPI_ISL_595229	Quadram Institute Bioscience	COVID-19 Genomics UK (COG-UK) Consortium	Dave J. Baker, Gemma L. Kay, Alp Aydin, Thanh Le-Viet, Steven Rudder, Ana P. Tedim, Anastasia Kolyva, Maria Diaz, Leonardo de Oliveira Martins, Nabil-Fareed Alikhan, Lizzie Meadows, Rachael Stanley, Ngozi Elumogo, Muhammed Yasir, Nicholas M. Thomson, Alexander J Trotter, Rachel Gilroy, Samuel Bloomfield, Claire Stuart, Andrew Bell, Reenesh Prakash, Samir Dervisevic, Alison E. Mather, John Wain, Mark Webber, Andrew J. Page, Justin O'Grady
EPI_ISL_595307, EPI_ISL_595308, EPI_ISL_595309, EPI_ISL_595310, EPI_ISL_595311, EPI_ISL_595312, EPI_ISL_595313, EPI_ISL_595314, EPI_ISL_595315, EPI_ISL_595316, EPI_ISL_595323, EPI_ISL_595324, EPI_ISL_595325			
see above	Queens Medical Centre, Clinical Microbiology Department / DeepSeq Nottingham	COVID-19 Genomics UK (COG-UK) Consortium	Gemma Clark, Wendy Smith, Manjinder Khakh, Vicki M Fleming, Michelle M Lister, Hannah Howson-Wells, Jonathan Ball, Patrick McClure, Joseph Chappell, Theocharis Tsoleridis, Nadine Holmes, Matthew Carlisle, Christopher Moore, Fei Sang, Johnny Debebe, Victoria Wright, Matthew Loose
EPI_ISL_605818, EPI_ISL_605820, EPI_ISL_605821, EPI_ISL_605822, EPI_ISL_605823, EPI_ISL_605824	National Public Health Laboratory, National Centre for Infectious Diseases	National Public Health Laboratory, National Centre for Infectious Diseases	Tze Minn Mak, Sophie Octavia, Zhenyang Zhou, Lin Cui, Raymond Tzer Pin Lin
EPI_ISL_605825, EPI_ISL_605836, EPI_ISL_605842, EPI_ISL_605850, EPI_ISL_605853, EPI_ISL_605854, EPI_ISL_605857, EPI_ISL_605867	PathWest Laboratory Medicine WA	PathWest Laboratory Medicine WA Microbial Surveillance Unit	PathWest Laboratory Medicine WA Microbial Surveillance Unit
EPI_ISL_609904	Respiratory Virus Unit, Microbiology Services Colindale, Public Health England	Respiratory Virus Unit, Microbiology Services Colindale, Public Health England	PHE Covid Sequencing Team
EPI_ISL_609998	INMI Lazzaro Spallanzani IRCCS	INMI Lazzaro Spallanzani IRCCS	F Messina, B Bartolini, M Rueca, C.E.M Gruber, E Giombini, A Di Caro, MR Capobianchi
EPI_ISL_609999	INMI Lazzaro Spallanzani IRCCS	INMI Lazzaro Spallanzani IRCCS	B Bartolini, M Rueca, C.E.M Gruber, F Messina, E Giombini, MR Capobianchi, A Di Caro
EPI_ISL_610922, EPI_ISL_610923, EPI_ISL_610924, EPI_ISL_610925, EPI_ISL_610926, EPI_ISL_610927, EPI_ISL_610928, EPI_ISL_610929, EPI_ISL_610930, EPI_ISL_610931, EPI_ISL_610932, EPI_ISL_610933, EPI_ISL_610934, EPI_ISL_610935, EPI_ISL_610936, EPI_ISL_610937, EPI_ISL_610938, EPI_ISL_610939, EPI_ISL_610940, EPI_ISL_610941, EPI_ISL_610942, EPI_ISL_610943, EPI_ISL_610944, EPI_ISL_610945, EPI_ISL_610946, EPI_ISL_610947, EPI_ISL_610948, EPI_ISL_610949, EPI_ISL_610950, EPI_ISL_610951, EPI_ISL_610952, EPI_ISL_610953, EPI_ISL_610954, EPI_ISL_610955, EPI_ISL_610956, EPI_ISL_610957, EPI_ISL_610958, EPI_ISL_610959, EPI_ISL_610960, EPI_ISL_610961, EPI_ISL_610962, EPI_ISL_610963, EPI_ISL_610964, EPI_ISL_610965, EPI_ISL_610966, EPI_ISL_610967, EPI_ISL_610968, EPI_ISL_610969, EPI_ISL_610970, EPI_ISL_610971, EPI_ISL_610972, EPI_ISL_610973, EPI_ISL_610974, EPI_ISL_610975, EPI_ISL_610976, EPI_ISL_610977, EPI_ISL_610978, EPI_ISL_610979, EPI_ISL_610980, EPI_ISL_610981, EPI_ISL_610982, EPI_ISL_610983, EPI_ISL_610984, EPI_ISL_610985, EPI_ISL_610986, EPI_ISL_610987, EPI_ISL_610988, EPI_ISL_610989, EPI_ISL_610990, EPI_ISL_610991, EPI_ISL_610992, EPI_ISL_610993, EPI_ISL_610994, EPI_ISL_610995, EPI_ISL_610996, EPI_ISL_610997, EPI_ISL_610998, EPI_ISL_610999, EPI_ISL_611000, EPI_ISL_611001, EPI_ISL_611002, EPI_ISL_611003, EPI_ISL_611004, EPI_ISL_611005, EPI_ISL_611006, EPI_ISL_611007, EPI_ISL_611008, EPI_ISL_611009, EPI_ISL_611010, EPI_ISL_611011, EPI_ISL_611012, EPI_ISL_611013, EPI_ISL_611014, EPI_ISL_611015, EPI_ISL_611016, EPI_ISL_611017, EPI_ISL_611018, EPI_ISL_611019, EPI_ISL_611020, EPI_ISL_611021, EPI_ISL_611022, EPI_ISL_611023, EPI_ISL_611024, EPI_ISL_611025, EPI_ISL_611026, EPI_ISL_611027, EPI_ISL_611028, EPI_ISL_611029, EPI_ISL_611030, EPI_ISL_611031, EPI_ISL_611032, EPI_ISL_611033, EPI_ISL_611034, EPI_ISL_611035, EPI_ISL_611036, EPI_ISL_611037, EPI_ISL_611038, EPI_ISL_611039, EPI_ISL_611040, EPI_ISL_611041, EPI_ISL_611042, EPI_ISL_611043, EPI_ISL_611044, EPI_ISL_611045, EPI_ISL_611046, EPI_ISL_611047, EPI_ISL_611048, EPI_ISL_611049, EPI_ISL_611050, EPI_ISL_611051, EPI_ISL_611052, EPI_ISL_611053, EPI_ISL_611054, EPI_ISL_611055, EPI_ISL_611056, EPI_ISL_611057, EPI_ISL_611058, EPI_ISL_611059, EPI_ISL_611060, EPI_ISL_611061, EPI_ISL_611062, EPI_ISL_611063, EPI_ISL_611064, EPI_ISL_611065, EPI_ISL_611066, EPI_ISL_611067, EPI_ISL_611068, EPI_ISL_611069, EPI_ISL_611070, EPI_ISL_611071, EPI_ISL_611072, EPI_ISL_611073, EPI_ISL_611074, EPI_ISL_611075, EPI_ISL_611076, EPI_ISL_611077, EPI_ISL_611078, EPI_ISL_611079, EPI_ISL_611080, EPI_ISL_611081, EPI_ISL_611082, EPI_ISL_611083, EPI_ISL_611084, EPI_ISL_611085, EPI_ISL_611086, EPI_ISL_611087, EPI_ISL_611088, EPI_ISL_611089, EPI_ISL_611090, EPI_ISL_611091, EPI_ISL_611092			
see above	Lighthouse Lab in Alderley Park	Wellcome Sanger Institute for the COVID-19 Genomics UK (COG-UK) consortium	Jacquelyn Wynn, Mairead Hyland, The Lighthouse Lab in Alderley Park and Alex Alderton, Roberto Amato, Sonia Goncalves, Ewan Harrison, David K. Jackson, Ian Johnston, Dominic Kwiatkowski, Cordelia Langford, John Sillitoe on behalf of the Wellcome Sanger Institute COVID-19 Surveillance Team (http://www.sanger.ac.uk/covid-team)
EPI_ISL_611093	Lighthouse Lab in Alderley Park	Wellcome Sanger Institute for the COVID-19 Genomics UK (COG-UK) Consortium	Jacquelyn Wynn, Mairead Hyland, The Lighthouse Lab in Alderley Park and Alex Alderton, Roberto Amato, Sonia Goncalves, Ewan Harrison, David K. Jackson, Ian Johnston, Dominic Kwiatkowski, Cordelia Langford, John Sillitoe on behalf of the Wellcome Sanger Institute COVID-19 Surveillance Team (http://www.sanger.ac.uk/covid-team)
EPI_ISL_611094, EPI_ISL_611095, EPI_ISL_611096, EPI_ISL_611097, EPI_ISL_611098, EPI_ISL_611099, EPI_ISL_611100, EPI_ISL_611101, EPI_ISL_611102, EPI_ISL_611103, EPI_ISL_611104, EPI_ISL_611105, EPI_ISL_611106, EPI_ISL_611107, EPI_ISL_611108, EPI_ISL_611109, EPI_ISL_611110, EPI_ISL_611111, EPI_ISL_611112, EPI_ISL_611113, EPI_ISL_611114, EPI_ISL_611115, EPI_ISL_611116, EPI_ISL_611117, EPI_ISL_611118, EPI_ISL_611119, EPI_ISL_611120, EPI_ISL_611121, EPI_ISL_611122, EPI_ISL_611123, EPI_ISL_611124, EPI_ISL_611125, EPI_ISL_611126, EPI_ISL_611127, EPI_ISL_611128, EPI_ISL_611129, EPI_ISL_611130, EPI_ISL_611131, EPI_ISL_611132, EPI_ISL_611133, EPI_ISL_611134, EPI_ISL_611135, EPI_ISL_611136, EPI_ISL_611137, EPI_ISL_611138, EPI_ISL_611139, EPI_ISL_611140, EPI_ISL_611141, EPI_ISL_611142, EPI_ISL_611143, EPI_ISL_611144, EPI_ISL_611145, EPI_ISL_611146, EPI_ISL_611147, EPI_ISL_611148, EPI_ISL_611149, EPI_ISL_611150, EPI_ISL_611151, EPI_ISL_611152, EPI_ISL_611153, EPI_ISL_611154, EPI_ISL_611155, EPI_ISL_611156, EPI_ISL_611157, EPI_ISL_611158, EPI_ISL_611159, EPI_ISL_611160, EPI_ISL_611161, EPI_ISL_611162, EPI_ISL_611163, EPI_ISL_611164, EPI_ISL_611165, EPI_ISL_611166, EPI_ISL_611167, EPI_ISL_611168, EPI_ISL_611169, EPI_ISL_611170, EPI_ISL_611171, EPI_ISL_611172, EPI_ISL_611173, EPI_ISL_611174, EPI_ISL_611175, EPI_ISL_611176, EPI_ISL_611177, EPI_ISL_611178, EPI_ISL_611179, EPI_ISL_611180, EPI_ISL_611181, EPI_ISL_611182, EPI_ISL_611183, EPI_ISL_611184, EPI_ISL_611185, EPI_ISL_611186, EPI_ISL_611187, EPI_ISL_611188, EPI_ISL_611189, EPI_ISL_611190, EPI_ISL_611191, EPI_ISL_611192, EPI_ISL_611193, EPI_ISL_611194, EPI_ISL_611195, EPI_ISL_611196, EPI_ISL_611197, EPI_ISL_611198, EPI_ISL_611199, EPI_ISL_611200, EPI_ISL_611201, EPI_ISL_611202, EPI_ISL_611203, EPI_ISL_611204, EPI_ISL_611205, EPI_ISL_611206, EPI_ISL_611207, EPI_ISL_611208, EPI_ISL_611209, EPI_ISL_611210, EPI_ISL_611211, EPI_ISL_611212, EPI_ISL_611213, EPI_ISL_611214, EPI_ISL_611215, EPI_ISL_611216, EPI_ISL_611217, EPI_ISL_611218, EPI_ISL_611219, EPI_ISL_611220, EPI_ISL_611221, EPI_ISL_611222, EPI_ISL_611223, EPI_ISL_611224, EPI_ISL_611225, EPI_ISL_611226, EPI_ISL_611227, EPI_ISL_611228, EPI_ISL_611229, EPI_ISL_611230, EPI_ISL_611231, EPI_ISL_611232, EPI_ISL_611233, EPI_ISL_611234, EPI_ISL_611235, EPI_ISL_611236, EPI_ISL_611237, EPI_ISL_611238, EPI_ISL_611239, EPI_ISL_611240, EPI_ISL_611241, EPI_ISL_611242, EPI_ISL_611243, EPI_ISL_611244, EPI_ISL_611245, EPI_ISL_611246, EPI_ISL_611247, EPI_ISL_611248, EPI_ISL_611249, EPI_ISL_611250, EPI_ISL_611251, EPI_ISL_611252, EPI_ISL_611253, EPI_ISL_611254, EPI_ISL_611255, EPI_ISL_611256, EPI_ISL_611257, EPI_ISL_611258, EPI_ISL_611259, EPI_ISL_611260, EPI_ISL_611261, EPI_ISL_611262, EPI_ISL_611263, EPI_ISL_611264, EPI_ISL_611265, EPI_ISL_611266, EPI_ISL_611267, EPI_ISL_611268, EPI_ISL_611269, EPI_ISL_611270, EPI_ISL_611271, EPI_ISL_611272, EPI_ISL_611273, EPI_ISL_611274, EPI_ISL_611275, EPI_ISL_611276, EPI_ISL_611277, EPI_ISL_611278, EPI_ISL_611279, EPI_ISL_611280, EPI_ISL_611281, EPI_ISL_611282, EPI_ISL_611283, EPI_ISL_611284, EPI_ISL_611285, EPI_ISL_611286			
see above	Lighthouse Lab in Alderley Park	Wellcome Sanger Institute for the COVID-19 Genomics UK (COG-UK) consortium	Jacquelyn Wynn, Mairead Hyland, The Lighthouse Lab in Alderley Park and Alex Alderton, Roberto Amato, Sonia Goncalves, Ewan Harrison, David K. Jackson, Ian Johnston, Dominic Kwiatkowski, Cordelia Langford, John Sillitoe on behalf of the Wellcome Sanger Institute COVID-19 Surveillance Team (http://www.sanger.ac.uk/covid-team)
EPI_ISL_611287, EPI_ISL_611288, EPI_ISL_611289, EPI_ISL_611290, EPI_ISL_611291, EPI_ISL_611292, EPI_ISL_611293, EPI_ISL_611294, EPI_ISL_611295, EPI_ISL_611296, EPI_ISL_611297, EPI_ISL_611298, EPI_ISL_611299, EPI_ISL_611300, EPI_ISL_611301, EPI_ISL_611302, EPI_ISL_611303			
see above	Lighthouse Lab in Glasgow	Wellcome Sanger Institute for the COVID-19 Genomics UK (COG-UK) consortium	Harper VanSteenhouse, Yumi Kasai, David Gray, Carol Clugston, Anna Dominiczak and Alex Alderton, Roberto Amato, Sonia Goncalves, Ewan Harrison, David K. Jackson, Ian Johnston, Dominic Kwiatkowski, Cordelia Langford, John Sillitoe on behalf of the Wellcome Sanger Institute COVID-19 Surveillance Team (http://www.sanger.ac.uk/covid-team)
EPI_ISL_611304	Lighthouse Lab in Glasgow	Wellcome Sanger Institute for the COVID-19 Genomics UK (COG-UK) Consortium	Harper VanSteenhouse, Yumi Kasai, David Gray, Carol Clugston, Anna Dominiczak and Alex Alderton, Roberto Amato, Sonia Goncalves, Ewan Harrison, David K. Jackson, Ian Johnston, Dominic Kwiatkowski, Cordelia Langford, John Sillitoe on behalf of the Wellcome Sanger Institute COVID-19 Surveillance Team (http://www.sanger.ac.uk/covid-team)
EPI_ISL_611305, EPI_ISL_611306, EPI_ISL_611307, EPI_ISL_611308, EPI_ISL_611309, EPI_ISL_611310, EPI_ISL_611311, EPI_ISL_611312, EPI_ISL_611313, EPI_ISL_611314, EPI_ISL_611315, EPI_ISL_611316, EPI_ISL_611317, EPI_ISL_611318, EPI_ISL_611319, EPI_ISL_611320, EPI_ISL_611321, EPI_ISL_611322, EPI_ISL_611323, EPI_ISL_611324, EPI_ISL_611325, EPI_ISL_611326, EPI_ISL_611327, EPI_ISL_611328, EPI_ISL_611329, EPI_ISL_611330, EPI_ISL_611331, EPI_ISL_611332, EPI_ISL_611333, EPI_ISL_611334, EPI_ISL_611335, EPI_ISL_611336, EPI_ISL_611337, EPI_ISL_611338, EPI_ISL_611339, EPI_ISL_611340, EPI_ISL_611341, EPI_ISL_611342, EPI_ISL_611343, EPI_ISL_611344, EPI_ISL_611345, EPI_ISL_611346, EPI_ISL_611347, EPI_ISL_611348, EPI_ISL_611349, EPI_ISL_611350, EPI_ISL_611351, EPI_ISL_611352, EPI_ISL_611353, EPI_ISL_611354, EPI_ISL_611355, EPI_ISL_611356, EPI_ISL_611357, EPI_ISL_611358, EPI_ISL_611359, EPI_ISL_611360, EPI_ISL_611361, EPI_ISL_611362, EPI_ISL_611363, EPI_ISL_611364, EPI_ISL_611365, EPI_ISL_611366, EPI_ISL_611367, EPI_ISL_611368, EPI_ISL_611369, EPI_ISL_611370, EPI_ISL_611371, EPI_ISL_611372, EPI_ISL_611373, EPI_ISL_611374, EPI_ISL_611375, EPI_ISL_611376, EPI_ISL_611377			
see above	Lighthouse Lab in Glasgow	Wellcome Sanger Institute for the COVID-19 Genomics UK (COG-UK) consortium	Harper VanSteenhouse, Yumi Kasai, David Gray, Carol Clugston, Anna Dominiczak and Alex Alderton, Roberto Amato, Sonia Goncalves, Ewan Harrison, David K. Jackson, Ian Johnston, Dominic Kwiatkowski, Cordelia Langford, John Sillitoe on behalf of the Wellcome Sanger Institute COVID-19 Surveillance Team (http://www.sanger.ac.uk/covid-team)
EPI_ISL_611378	Lighthouse Lab in Glasgow	Wellcome Sanger Institute for the COVID-19 Genomics UK (COG-UK) Consortium	Harper VanSteenhouse, Yumi Kasai, David Gray, Carol Clugston, Anna Dominiczak and Alex Alderton, Roberto Amato, Sonia Goncalves, Ewan Harrison, David K. Jackson, Ian Johnston, Dominic Kwiatkowski, Cordelia Langford, John Sillitoe on behalf of the Wellcome Sanger Institute COVID-19 Surveillance Team (http://www.sanger.ac.uk/covid-team)

EPI_ISL_611379, EPI_ISL_611380, EPI_ISL_611381, EPI_ISL_611382, EPI_ISL_611383, EPI_ISL_611384, EPI_ISL_611385, EPI_ISL_611386, EPI_ISL_611387, EPI_ISL_611388, EPI_ISL_611389, EPI_ISL_611390, EPI_ISL_611391, EPI_ISL_611392, EPI_ISL_611393, EPI_ISL_611394, EPI_ISL_611395, EPI_ISL_611396, EPI_ISL_611397, EPI_ISL_611398, EPI_ISL_611399, EPI_ISL_611400, EPI_ISL_611401, EPI_ISL_611402, EPI_ISL_611403, EPI_ISL_611404, EPI_ISL_611405, EPI_ISL_611406, EPI_ISL_611407, EPI_ISL_611408, EPI_ISL_611409, EPI_ISL_611410, EPI_ISL_611411, EPI_ISL_611412, EPI_ISL_611413, EPI_ISL_611414, EPI_ISL_611415, EPI_ISL_611416, EPI_ISL_611417, EPI_ISL_611418, EPI_ISL_611419, EPI_ISL_611420, EPI_ISL_611421, EPI_ISL_611423, EPI_ISL_611424, EPI_ISL_611425, EPI_ISL_611427, EPI_ISL_611428, EPI_ISL_611429, EPI_ISL_611430, EPI_ISL_611431, EPI_ISL_611432, EPI_ISL_611433, EPI_ISL_611434, EPI_ISL_611435, EPI_ISL_611436, EPI_ISL_611437, EPI_ISL_611438, EPI_ISL_611439, EPI_ISL_611440, EPI_ISL_611441, EPI_ISL_611442, EPI_ISL_611443, EPI_ISL_611444, EPI_ISL_611445, EPI_ISL_611446, EPI_ISL_611447, EPI_ISL_611448, EPI_ISL_611449, EPI_ISL_611450, EPI_ISL_611451, EPI_ISL_611452, EPI_ISL_611453, EPI_ISL_611454, EPI_ISL_611455, EPI_ISL_611456, EPI_ISL_611457, EPI_ISL_611458, EPI_ISL_611459, EPI_ISL_611460, EPI_ISL_611461, EPI_ISL_611462, EPI_ISL_611463, EPI_ISL_611464, EPI_ISL_611465, EPI_ISL_611466, EPI_ISL_611467, EPI_ISL_611468, EPI_ISL_611469, EPI_ISL_611470, EPI_ISL_611471, EPI_ISL_611472, EPI_ISL_611473, EPI_ISL_611474, EPI_ISL_611475, EPI_ISL_611476, EPI_ISL_611477, EPI_ISL_611478, EPI_ISL_611479, EPI_ISL_611480, EPI_ISL_611481, EPI_ISL_611482, EPI_ISL_611483, EPI_ISL_611484

see above	Lighthouse Lab in Glasgow	Wellcome Sanger Institute for the COVID-19 Genomics UK (COG-UK) consortium	Harper VanSteenhouse, Yumi Kasai, David Gray, Carol Clugston, Anna Dominiczak and Alex Alderton, Roberto Amato, Sonia Goncalves, Ewan Harrison, David K. Jackson, Ian Johnston, Dominic Kwiatkowski, Cordelia Langford, John Sillitoe on behalf of the Wellcome Sanger Institute COVID-19 Surveillance Team (http://www.sanger.ac.uk/covid-team)
EPI_ISL_611519	Wales Specialist Virology Centre Sequencing lab: Pathogen Genomics Unit	COVID-19 Genomics UK (COG-UK) Consortium	Catherine Moore, Johnathan Evans, Laura Gifford, Malorie Perry, Simon Cottrell, Angela Marchbank, Alec Birchley, Alexander Adams, Amy Gaskin, Bree Gatica-Wilcox, Jason Coombes, Joel Southgate, Lauren Gilbert, Lee Graham, Nicole Pacchiarini, Sara Kumziene-Summerhayes, Sarah Taylor, Sophie Jones, Sara Rey, Matthew Bull, Joanne Watkins, Sally Corden, Tom Connor
EPI_ISL_611520	Virology Department, Sheffield Teaching Hospitals NHS Foundation Trust/Department of Infection, Immunity and Cardiovascular Disease, The Medical School, University of Sheffield	COVID-19 Genomics UK (COG-UK) Consortium	Thushan de Silva, Matthew Parker, Nikki Smith, Adri Angyal, Rebecca Brown, Luke Green, Rachel Tucker, Paul Parsons, Danielle Groves, Katie Johnson, Laura Carrilero, Alex Keeley, Dave Partridge, Matthew Wyles, Benjamin Lindsey, Mehmet Yavuz, Mohammad Raza, Cariad Evans
EPI_ISL_611527	University College London, Great Ormond Street Hospital for Children NHS Foundation Trust, Imperial College Healthcare NHS Trust	COVID-19 Genomics UK (COG-UK) Consortium	Sergi Castellano, Rachel Williams, Mark Kristiansen, Paola Resende Silva, Sunando Roy, Tony Brooks, Helena Tutill, Paola Niola, Patricia Dyal, Charlotte Williams, Leysa Forrest, Yasmin Panchbhaya, Jacqueline Findlay, Samuel Weeks, Julianne Brown, Kathryn Harris, Paul Randell, James Price, Alison Holmes, Judith Breuer
EPI_ISL_611529, EPI_ISL_611530, EPI_ISL_611534	Wales Specialist Virology Centre Sequencing lab: Pathogen Genomics Unit	COVID-19 Genomics UK (COG-UK) Consortium	Catherine Moore, Johnathan Evans, Laura Gifford, Malorie Perry, Simon Cottrell, Angela Marchbank, Alec Birchley, Alexander Adams, Amy Gaskin, Bree Gatica-Wilcox, Jason Coombes, Joel Southgate, Lauren Gilbert, Lee Graham, Nicole Pacchiarini, Sara Kumziene-Summerhayes, Sarah Taylor, Sophie Jones, Sara Rey, Matthew Bull, Joanne Watkins, Sally Corden, Tom Connor
EPI_ISL_611546	Queens Medical Centre, Clinical Microbiology Department / DeepSeq Nottingham	COVID-19 Genomics UK (COG-UK) Consortium	Gemma Clark, Wendy Smith, Manjinder Khakh, Vicki M Fleming, Michelle M Lister, Hannah Howson-Wells, Jonathan Ball, Patrick McClure, Joseph Chappell, Theocharis Tsoleridis, Nadine Holmes, Matthew Carlisle, Christopher Moore, Fei Sang, Johnny Debebe, Victoria Wright, Matthew Loose
EPI_ISL_611558	University College London, Great Ormond Street Hospital for Children NHS Foundation Trust, Imperial College Healthcare NHS Trust	COVID-19 Genomics UK (COG-UK) Consortium	Sergi Castellano, Rachel Williams, Mark Kristiansen, Paola Resende Silva, Sunando Roy, Tony Brooks, Helena Tutill, Paola Niola, Patricia Dyal, Charlotte Williams, Leysa Forrest, Yasmin Panchbhaya, Jacqueline Findlay, Samuel Weeks, Julianne Brown, Kathryn Harris, Paul Randell, James Price, Alison Holmes, Judith Breuer
EPI_ISL_611561, EPI_ISL_611563, EPI_ISL_611566, EPI_ISL_611581	Wales Specialist Virology Centre Sequencing lab: Pathogen Genomics Unit	COVID-19 Genomics UK (COG-UK) Consortium	Catherine Moore, Johnathan Evans, Laura Gifford, Malorie Perry, Simon Cottrell, Angela Marchbank, Alec Birchley, Alexander Adams, Amy Gaskin, Bree Gatica-Wilcox, Jason Coombes, Joel Southgate, Lauren Gilbert, Lee Graham, Nicole Pacchiarini, Sara Kumziene-Summerhayes, Sarah Taylor, Sophie Jones, Sara Rey, Matthew Bull, Joanne Watkins, Sally Corden, Tom Connor
EPI_ISL_611587, EPI_ISL_611598	Virology Department, Sheffield Teaching Hospitals NHS Foundation Trust/Department of Infection, Immunity and Cardiovascular Disease, The Medical School, University of Sheffield	COVID-19 Genomics UK (COG-UK) Consortium	Thushan de Silva, Matthew Parker, Nikki Smith, Adri Angyal, Rebecca Brown, Luke Green, Rachel Tucker, Paul Parsons, Danielle Groves, Katie Johnson, Laura Carrilero, Alex Keeley, Dave Partridge, Matthew Wyles, Benjamin Lindsey, Mehmet Yavuz, Mohammad Raza, Cariad Evans
EPI_ISL_611601	Wales Specialist Virology Centre Sequencing lab: Pathogen Genomics Unit	COVID-19 Genomics UK (COG-UK) Consortium	Catherine Moore, Johnathan Evans, Laura Gifford, Malorie Perry, Simon Cottrell, Angela Marchbank, Alec Birchley, Alexander Adams, Amy Gaskin, Bree Gatica-Wilcox, Jason Coombes, Joel Southgate, Lauren Gilbert, Lee Graham, Nicole Pacchiarini, Sara Kumziene-Summerhayes, Sarah Taylor, Sophie Jones, Sara Rey, Matthew Bull, Joanne Watkins, Sally Corden, Tom Connor
EPI_ISL_611606	Virology Department, Sheffield Teaching Hospitals NHS Foundation Trust/Department of Infection, Immunity and Cardiovascular Disease, The Medical School, University of Sheffield	COVID-19 Genomics UK (COG-UK) Consortium	Thushan de Silva, Matthew Parker, Nikki Smith, Adri Angyal, Rebecca Brown, Luke Green, Rachel Tucker, Paul Parsons, Danielle Groves, Katie Johnson, Laura Carrilero, Alex Keeley, Dave Partridge, Matthew Wyles, Benjamin Lindsey, Mehmet Yavuz, Mohammad Raza, Cariad Evans
EPI_ISL_611624, EPI_ISL_611625, EPI_ISL_611640, EPI_ISL_611652	Wales Specialist Virology Centre Sequencing lab: Pathogen Genomics Unit	COVID-19 Genomics UK (COG-UK) Consortium	Catherine Moore, Johnathan Evans, Laura Gifford, Malorie Perry, Simon Cottrell, Angela Marchbank, Alec Birchley, Alexander Adams, Amy Gaskin, Bree Gatica-Wilcox, Jason Coombes, Joel Southgate, Lauren Gilbert, Lee Graham, Nicole Pacchiarini, Sara Kumziene-Summerhayes, Sarah Taylor, Sophie Jones, Sara Rey, Matthew Bull, Joanne Watkins, Sally Corden, Tom Connor
EPI_ISL_611665	University College London, Great Ormond Street Hospital for Children NHS Foundation Trust, Imperial College Healthcare NHS Trust	COVID-19 Genomics UK (COG-UK) Consortium	Sergi Castellano, Rachel Williams, Mark Kristiansen, Paola Resende Silva, Sunando Roy, Tony Brooks, Helena Tutill, Paola Niola, Patricia Dyal, Charlotte Williams, Leysa Forrest, Yasmin Panchbhaya, Jacqueline Findlay, Samuel Weeks, Julianne Brown, Kathryn Harris, Paul Randell, James Price, Alison Holmes, Judith Breuer
EPI_ISL_611666	Wales Specialist Virology Centre Sequencing lab: Pathogen Genomics Unit	COVID-19 Genomics UK (COG-UK) Consortium	Catherine Moore, Johnathan Evans, Laura Gifford, Malorie Perry, Simon Cottrell, Angela Marchbank, Alec Birchley, Alexander Adams, Amy Gaskin, Bree Gatica-Wilcox, Jason Coombes, Joel Southgate, Lauren Gilbert, Lee Graham, Nicole Pacchiarini, Sara Kumziene-Summerhayes, Sarah Taylor, Sophie Jones, Sara Rey, Matthew Bull, Joanne Watkins, Sally Corden, Tom Connor
EPI_ISL_611676, EPI_ISL_611677	Queens Medical Centre, Clinical Microbiology Department / DeepSeq Nottingham	COVID-19 Genomics UK (COG-UK) Consortium	Gemma Clark, Wendy Smith, Manjinder Khakh, Vicki M Fleming, Michelle M Lister, Hannah Howson-Wells, Jonathan Ball, Patrick McClure, Joseph Chappell, Theocharis Tsoleridis, Nadine Holmes, Matthew Carlisle, Christopher Moore, Fei Sang, Johnny Debebe, Victoria Wright, Matthew Loose
EPI_ISL_611678	Virology Department, Sheffield Teaching Hospitals NHS Foundation Trust/Department of Infection, Immunity and Cardiovascular Disease, The Medical School, University of Sheffield	COVID-19 Genomics UK (COG-UK) Consortium	Thushan de Silva, Matthew Parker, Nikki Smith, Adri Angyal, Rebecca Brown, Luke Green, Rachel Tucker, Paul Parsons, Danielle Groves, Katie Johnson, Laura Carrilero, Alex Keeley, Dave Partridge, Matthew Wyles, Benjamin Lindsey, Mehmet Yavuz, Mohammad Raza, Cariad Evans
EPI_ISL_611687, EPI_ISL_611697, EPI_ISL_611706, EPI_ISL_611722, EPI_ISL_611726, EPI_ISL_611732	Wales Specialist Virology Centre Sequencing lab: Pathogen Genomics Unit	COVID-19 Genomics UK (COG-UK) Consortium	Catherine Moore, Johnathan Evans, Laura Gifford, Malorie Perry, Simon Cottrell, Angela Marchbank, Alec Birchley, Alexander Adams, Amy Gaskin, Bree Gatica-Wilcox, Jason Coombes, Joel Southgate, Lauren Gilbert, Lee Graham, Nicole Pacchiarini, Sara Kumziene-Summerhayes, Sarah Taylor, Sophie Jones, Sara Rey, Matthew Bull, Joanne Watkins, Sally Corden, Tom Connor
EPI_ISL_611744, EPI_ISL_611794, EPI_ISL_611799, EPI_ISL_611803	Virology Department, Sheffield Teaching Hospitals NHS Foundation Trust/Department of Infection, Immunity and Cardiovascular Disease, The Medical School, University of Sheffield	COVID-19 Genomics UK (COG-UK) Consortium	Thushan de Silva, Matthew Parker, Nikki Smith, Adri Angyal, Rebecca Brown, Luke Green, Rachel Tucker, Paul Parsons, Danielle Groves, Katie Johnson, Laura Carrilero, Alex Keeley, Dave Partridge, Matthew Wyles, Benjamin Lindsey, Mehmet Yavuz, Mohammad Raza, Cariad Evans
EPI_ISL_611823	University College London, Great Ormond Street Hospital for Children NHS Foundation Trust, Imperial College Healthcare NHS Trust	COVID-19 Genomics UK (COG-UK) Consortium	Sergi Castellano, Rachel Williams, Mark Kristiansen, Paola Resende Silva, Sunando Roy, Tony Brooks, Helena Tutill, Paola Niola, Patricia Dyal, Charlotte Williams, Leysa Forrest, Yasmin Panchbhaya, Jacqueline Findlay, Samuel Weeks, Julianne Brown, Kathryn Harris, Paul Randell, James Price, Alison Holmes, Judith Breuer
EPI_ISL_611830	Virology Department, Sheffield Teaching Hospitals NHS Foundation Trust/Department of Infection, Immunity and Cardiovascular Disease, The Medical School, University of Sheffield	COVID-19 Genomics UK (COG-UK) Consortium	Thushan de Silva, Matthew Parker, Nikki Smith, Adri Angyal, Rebecca Brown, Luke Green, Rachel Tucker, Paul Parsons, Danielle Groves, Katie Johnson, Laura Carrilero, Alex Keeley, Dave Partridge, Matthew Wyles, Benjamin Lindsey, Mehmet Yavuz, Mohammad Raza, Cariad Evans
EPI_ISL_611865	Wales Specialist Virology Centre Sequencing lab: Pathogen Genomics Unit	COVID-19 Genomics UK (COG-UK) Consortium	Catherine Moore, Johnathan Evans, Laura Gifford, Malorie Perry, Simon Cottrell, Angela Marchbank, Alec Birchley, Alexander Adams, Amy Gaskin, Bree Gatica-Wilcox, Jason Coombes, Joel Southgate, Lauren Gilbert, Lee Graham, Nicole Pacchiarini, Sara Kumziene-Summerhayes, Sarah Taylor, Sophie Jones, Sara Rey, Matthew Bull, Joanne Watkins, Sally Corden, Tom Connor
EPI_ISL_611869	Virology Department, Sheffield Teaching Hospitals NHS Foundation Trust/Department of Infection, Immunity and Cardiovascular Disease, The Medical School, University of	COVID-19 Genomics UK (COG-UK) Consortium	Thushan de Silva, Matthew Parker, Nikki Smith, Adri Angyal, Rebecca Brown, Luke Green, Rachel Tucker, Paul Parsons, Danielle Groves, Katie Johnson, Laura Carrilero, Alex Keeley, Dave Partridge, Matthew Wyles, Benjamin Lindsey, Mehmet Yavuz, Mohammad Raza, Cariad Evans

	Sheffield		
EPI_ISL_611903, EPI_ISL_611904, EPI_ISL_611907, EPI_ISL_611923, EPI_ISL_611932	Wales Specialist Virology Centre Sequencing lab: Pathogen Genomics Unit	COVID-19 Genomics UK (COG-UK) Consortium	Catherine Moore, Johnathan Evans, Laura Gifford, Malorie Perry, Simon Cottrell, Angela Marchbank, Alec Birchley, Alexander Adams, Amy Gaskin, Bree Gatica-Wilcox, Jason Coombes, Joel Southgate, Lauren Gilbert, Lee Graham, Nicole Pacchiarini, Sara Kumziene-Summerhayes, Sarah Taylor, Sophie Jones, Sara Rey, Matthew Bull, Joanne Watkins, Sally Corden, Tom Connor
EPI_ISL_611936	Virology Department, Sheffield Teaching Hospitals NHS Foundation Trust/Department of Infection, Immunity and Cardiovascular Disease, The Medical School, University of Sheffield	COVID-19 Genomics UK (COG-UK) Consortium	Thushan de Silva, Matthew Parker, Nikki Smith, Adri Angyal, Rebecca Brown, Luke Green, Rachel Tucker, Paul Parsons, Danielle Groves, Katie Johnson, Laura Carrilero, Alex Keeley, Dave Partridge, Matthew Wyles, Benjamin Lindsey, Mehmet Yavuz, Mohammad Raza, Cariad Evans
EPI_ISL_611954	Queens Medical Centre, Clinical Microbiology Department / DeepSeq Nottingham	COVID-19 Genomics UK (COG-UK) Consortium	Gemma Clark, Wendy Smith, Manjinder Khakh, Vicki M Fleming, Michelle M Lister, Hannah Howson-Wells, Jonathan Ball, Patrick McClure, Joseph Chappell, Theocharis Tsoleridis, Nadine Holmes, Matthew Carlisle, Christopher Moore, Fei Sang, Johnny Debebe, Victoria Wright, Matthew Loose
EPI_ISL_611973, EPI_ISL_611982, EPI_ISL_612013, EPI_ISL_612081, EPI_ISL_612083, EPI_ISL_612093	Wales Specialist Virology Centre Sequencing lab: Pathogen Genomics Unit	COVID-19 Genomics UK (COG-UK) Consortium	Catherine Moore, Johnathan Evans, Laura Gifford, Malorie Perry, Simon Cottrell, Angela Marchbank, Alec Birchley, Alexander Adams, Amy Gaskin, Bree Gatica-Wilcox, Jason Coombes, Joel Southgate, Lauren Gilbert, Lee Graham, Nicole Pacchiarini, Sara Kumziene-Summerhayes, Sarah Taylor, Sophie Jones, Sara Rey, Matthew Bull, Joanne Watkins, Sally Corden, Tom Connor
EPI_ISL_612105	University College London, Great Ormond Street Hospital for Children NHS Foundation Trust, Imperial College Healthcare NHS Trust	COVID-19 Genomics UK (COG-UK) Consortium	Sergi Castellano, Rachel Williams, Mark Kristiansen, Paola Resende Silva, Sunando Roy, Tony Brooks, Helena Tutill, Paola Niola, Patricia Dyal, Charlotte Williams, Leysa Forrest, Yasmin Panchbhaya, Jacqueline Findlay, Samuel Weeks, Julianne Brown, Kathryn Harris, Paul Randell, James Price, Alison Holmes, Judith Breuer
EPI_ISL_612116, EPI_ISL_612120, EPI_ISL_612146, EPI_ISL_612147, EPI_ISL_612152, EPI_ISL_612153, EPI_ISL_612154, EPI_ISL_612155, EPI_ISL_612156, EPI_ISL_612157, EPI_ISL_612177, EPI_ISL_612193, EPI_ISL_612199, EPI_ISL_612202			
see above	Wales Specialist Virology Centre Sequencing lab: Pathogen Genomics Unit	COVID-19 Genomics UK (COG-UK) Consortium	Catherine Moore, Johnathan Evans, Laura Gifford, Malorie Perry, Simon Cottrell, Angela Marchbank, Alec Birchley, Alexander Adams, Amy Gaskin, Bree Gatica-Wilcox, Jason Coombes, Joel Southgate, Lauren Gilbert, Lee Graham, Nicole Pacchiarini, Sara Kumziene-Summerhayes, Sarah Taylor, Sophie Jones, Sara Rey, Matthew Bull, Joanne Watkins, Sally Corden, Tom Connor
EPI_ISL_612373	Virology Department, Royal Infirmary of Edinburgh, NHS Lothian / School of Biological Sciences, University of Edinburgh / Institute of Genetics and Molecular Medicine, University of Edinburgh	COVID-19 Genomics UK (COG-UK) Consortium	McHugh M, Dewar R, Rooke S, Gallagher M, Balcaza C, O'Toole Á, Scher E, Hill V, McCrone JT, Colquhoun R, Yu X, Jackson B, Rambaut A, Williams TC, Templeton K
EPI_ISL_612451, EPI_ISL_612454	University College London, Great Ormond Street Hospital for Children NHS Foundation Trust, Imperial College Healthcare NHS Trust	COVID-19 Genomics UK (COG-UK) Consortium	Sergi Castellano, Rachel Williams, Mark Kristiansen, Paola Resende Silva, Sunando Roy, Tony Brooks, Helena Tutill, Paola Niola, Patricia Dyal, Charlotte Williams, Leysa Forrest, Yasmin Panchbhaya, Jacqueline Findlay, Samuel Weeks, Julianne Brown, Kathryn Harris, Paul Randell, James Price, Alison Holmes, Judith Breuer
EPI_ISL_612574, EPI_ISL_612575, EPI_ISL_612576	Queens Medical Centre, Clinical Microbiology Department / DeepSeq Nottingham	COVID-19 Genomics UK (COG-UK) Consortium	Gemma Clark, Wendy Smith, Manjinder Khakh, Vicki M Fleming, Michelle M Lister, Hannah Howson-Wells, Jonathan Ball, Patrick McClure, Joseph Chappell, Theocharis Tsoleridis, Nadine Holmes, Matthew Carlisle, Christopher Moore, Fei Sang, Johnny Debebe, Victoria Wright, Matthew Loose
EPI_ISL_612849, EPI_ISL_612861, EPI_ISL_612864, EPI_ISL_612865, EPI_ISL_612893, EPI_ISL_612904, EPI_ISL_612917, EPI_ISL_612918, EPI_ISL_612946, EPI_ISL_612951, EPI_ISL_612956, EPI_ISL_612971, EPI_ISL_612987, EPI_ISL_612999, EPI_ISL_613018, EPI_ISL_613019, EPI_ISL_613021, EPI_ISL_613025, EPI_ISL_613026, EPI_ISL_613032, EPI_ISL_613035, EPI_ISL_613042, EPI_ISL_613043, EPI_ISL_613044, EPI_ISL_613046, EPI_ISL_613048, EPI_ISL_613049, EPI_ISL_613057, EPI_ISL_613059, EPI_ISL_613066, EPI_ISL_613067, EPI_ISL_613068, EPI_ISL_613076, EPI_ISL_613077, EPI_ISL_613089, EPI_ISL_613090, EPI_ISL_613091, EPI_ISL_613094, EPI_ISL_613101, EPI_ISL_613107, EPI_ISL_613116, EPI_ISL_613125, EPI_ISL_613134, EPI_ISL_613135, EPI_ISL_613137, EPI_ISL_613138, EPI_ISL_613139, EPI_ISL_613141, EPI_ISL_613147, EPI_ISL_613148, EPI_ISL_613153, EPI_ISL_613155, EPI_ISL_613162, EPI_ISL_613163, EPI_ISL_613164, EPI_ISL_613166, EPI_ISL_613173, EPI_ISL_613178, EPI_ISL_613185, EPI_ISL_613186, EPI_ISL_613189, EPI_ISL_613190, EPI_ISL_613191, EPI_ISL_613192, EPI_ISL_613193, EPI_ISL_613194, EPI_ISL_613195, EPI_ISL_613196, EPI_ISL_613197, EPI_ISL_613198, EPI_ISL_613199, EPI_ISL_613200, EPI_ISL_613201, EPI_ISL_613202, EPI_ISL_613203, EPI_ISL_613204, EPI_ISL_613205, EPI_ISL_613206, EPI_ISL_613207, EPI_ISL_613208, EPI_ISL_613209, EPI_ISL_613210, EPI_ISL_613211, EPI_ISL_613212, EPI_ISL_613213, EPI_ISL_613214, EPI_ISL_613215, EPI_ISL_613217, EPI_ISL_613218, EPI_ISL_613219, EPI_ISL_613220, EPI_ISL_613221, EPI_ISL_613222, EPI_ISL_613223, EPI_ISL_613224, EPI_ISL_613225, EPI_ISL_613226, EPI_ISL_613227, EPI_ISL_613228, EPI_ISL_613229, EPI_ISL_613230, EPI_ISL_613231, EPI_ISL_613232, EPI_ISL_613233, EPI_ISL_613234, EPI_ISL_613235, EPI_ISL_613236, EPI_ISL_613237, EPI_ISL_613238, EPI_ISL_613239, EPI_ISL_613240, EPI_ISL_613241, EPI_ISL_613242, EPI_ISL_613243, EPI_ISL_613244, EPI_ISL_613245, EPI_ISL_613246, EPI_ISL_613247, EPI_ISL_613248, EPI_ISL_613249, EPI_ISL_613250, EPI_ISL_613251, EPI_ISL_613252, EPI_ISL_613253, EPI_ISL_613254, EPI_ISL_613255, EPI_ISL_613256, EPI_ISL_613257, EPI_ISL_613258, EPI_ISL_613259, EPI_ISL_613260, EPI_ISL_613261, EPI_ISL_613262, EPI_ISL_613263, EPI_ISL_613264, EPI_ISL_613266, EPI_ISL_613267, EPI_ISL_613268, EPI_ISL_613269, EPI_ISL_613270, EPI_ISL_613272, EPI_ISL_613273, EPI_ISL_613274, EPI_ISL_613276, EPI_ISL_613277, EPI_ISL_613279, EPI_ISL_613281			
see above	Wales Specialist Virology Centre Sequencing lab: Pathogen Genomics Unit	COVID-19 Genomics UK (COG-UK) Consortium	Catherine Moore, Johnathan Evans, Laura Gifford, Malorie Perry, Simon Cottrell, Angela Marchbank, Alec Birchley, Alexander Adams, Amy Gaskin, Bree Gatica-Wilcox, Jason Coombes, Joel Southgate, Lauren Gilbert, Lee Graham, Nicole Pacchiarini, Sara Kumziene-Summerhayes, Sarah Taylor, Sophie Jones, Sara Rey, Matthew Bull, Joanne Watkins, Sally Corden, Tom Connor
EPI_ISL_613366, EPI_ISL_613368, EPI_ISL_613383, EPI_ISL_613391, EPI_ISL_613400	Virology Department, Sheffield Teaching Hospitals NHS Foundation Trust/Department of Infection, Immunity and Cardiovascular Disease, The Medical School, University of Sheffield	COVID-19 Genomics UK (COG-UK) Consortium	Thushan de Silva, Matthew Parker, Nikki Smith, Adri Angyal, Rebecca Brown, Luke Green, Rachel Tucker, Paul Parsons, Danielle Groves, Katie Johnson, Laura Carrilero, Alex Keeley, Dave Partridge, Matthew Wyles, Benjamin Lindsey, Mehmet Yavuz, Mohammad Raza, Cariad Evans
EPI_ISL_613523	Public Health Laboratory - Infectious Disease Lab, Minnesota Department of Health Infectious Disease Laboratory Submission Group	Minnesota Department of Health, Public Health Laboratory	Plumb,M., Garfin,J., Lorentz,A., Wang,X.
EPI_ISL_613634	Respiratory Virus Unit, Microbiology Services Colindale, Public Health England	Respiratory Virus Unit, Microbiology Services Colindale, Public Health England	PHE Covid Sequencing Team
EPI_ISL_614182, EPI_ISL_614185, EPI_ISL_614188, EPI_ISL_614189, EPI_ISL_614192, EPI_ISL_614194, EPI_ISL_614195, EPI_ISL_614201, EPI_ISL_614205, EPI_ISL_614207, EPI_ISL_614209, EPI_ISL_614215, EPI_ISL_614221, EPI_ISL_614222, EPI_ISL_614226, EPI_ISL_614230, EPI_ISL_614237, EPI_ISL_614239, EPI_ISL_614240, EPI_ISL_614244, EPI_ISL_614245, EPI_ISL_614246			
see above	Michigan Department of Health and Human Services, Bureau of Laboratories	Michigan Department of Health and Human Services, Bureau of Laboratories	Blankenship HM, Riner D, Soehnlen MK
EPI_ISL_614287, EPI_ISL_614288, EPI_ISL_614289, EPI_ISL_614291	General practitioner	National Reference Center for Viruses of Respiratory Infections, Institut Pasteur, Paris	Marion Barbet, Sylvie Behillil, Méline Bizard, Angela Brisebarre, Camille Capel, Etienne Simon-Lorière, Vincent Enouf, Maud Vanpeene, Sylvie van der Werf
EPI_ISL_614893, EPI_ISL_614894, EPI_ISL_614912, EPI_ISL_614913, EPI_ISL_614914, EPI_ISL_614915, EPI_ISL_614916, EPI_ISL_614917, EPI_ISL_614918, EPI_ISL_614919, EPI_ISL_614920, EPI_ISL_614921, EPI_ISL_614922, EPI_ISL_614923, EPI_ISL_614924, EPI_ISL_614925, EPI_ISL_614926, EPI_ISL_614927, EPI_ISL_614937, EPI_ISL_614938, EPI_ISL_614939, EPI_ISL_614940, EPI_ISL_614941, EPI_ISL_614942, EPI_ISL_614943, EPI_ISL_614944, EPI_ISL_614945, EPI_ISL_614946, EPI_ISL_614965, EPI_ISL_614966, EPI_ISL_614967, EPI_ISL_614968, EPI_ISL_614969, EPI_ISL_614970, EPI_ISL_614971, EPI_ISL_614972, EPI_ISL_614973, EPI_ISL_614974, EPI_ISL_614975, EPI_ISL_614976, EPI_ISL_614977			
see above	Viollier AG	Department of Biosystems Science and Engineering, ETH Zürich	Christian Beisel, Sarah Nadeau, Ivan Topolsky, Pedro Ferreira, Philipp Jablonski, Susana Posada-Céspedes, Tobias Schär, Ina Nissen, Natascha Santacroce, Elodie Burcklen, Christiane Beckmann, Maurice Redondo, Olivier Kobel, Christoph Noppen, Sophie Seidel, Noemie Santamaria de Souza, Chaoran Chen, Niko Beerenwinkel, Tanja Stadler
EPI_ISL_617026, EPI_ISL_617251, EPI_ISL_617257, EPI_ISL_618499, EPI_ISL_618500, EPI_ISL_618501, EPI_ISL_618502, EPI_ISL_618503, EPI_ISL_618504, EPI_ISL_618505, EPI_ISL_618506, EPI_ISL_618507, EPI_ISL_618508, EPI_ISL_618509, EPI_ISL_618510, EPI_ISL_618512, EPI_ISL_618513, EPI_ISL_618514, EPI_ISL_618515, EPI_ISL_618516, EPI_ISL_618518, EPI_ISL_618519, EPI_ISL_618520, EPI_ISL_618521, EPI_ISL_618522, EPI_ISL_618523, EPI_ISL_618524, EPI_ISL_618526, EPI_ISL_618527, EPI_ISL_618528, EPI_ISL_618529, EPI_ISL_618530, EPI_ISL_618531, EPI_ISL_618532, EPI_ISL_618533, EPI_ISL_618534, EPI_ISL_618535, EPI_ISL_618536, EPI_ISL_618537, EPI_ISL_618538, EPI_ISL_618539, EPI_ISL_618540, EPI_ISL_618541, EPI_ISL_618542, EPI_ISL_618543, EPI_ISL_618544, EPI_ISL_618545, EPI_ISL_618546, EPI_ISL_618547, EPI_ISL_618548, EPI_ISL_618549, EPI_ISL_618550, EPI_ISL_618551, EPI_ISL_618552, EPI_ISL_618553, EPI_ISL_618554, EPI_ISL_618555, EPI_ISL_618556, EPI_ISL_618557, EPI_ISL_618558, EPI_ISL_618559, EPI_ISL_618560, EPI_ISL_618561, EPI_ISL_618562, EPI_ISL_618563, EPI_ISL_618564, EPI_ISL_618565, EPI_ISL_618566, EPI_ISL_618567, EPI_ISL_618568, EPI_ISL_618569, EPI_ISL_618570, EPI_ISL_618571, EPI_ISL_618572, EPI_ISL_618573, EPI_ISL_618574, EPI_ISL_618575, EPI_ISL_618576, EPI_ISL_618577, EPI_ISL_618578, EPI_ISL_618579, EPI_ISL_618580, EPI_ISL_618581, EPI_ISL_618582, EPI_ISL_618583, EPI_ISL_618584, EPI_ISL_618585, EPI_ISL_618586, EPI_ISL_618587, EPI_ISL_618588, EPI_ISL_618589, EPI_ISL_618590, EPI_ISL_618591, EPI_ISL_618592, EPI_ISL_618593, EPI_ISL_618594, EPI_ISL_618596, EPI_ISL_618597, EPI_ISL_618598, EPI_ISL_618599, EPI_ISL_618600, EPI_ISL_618601, EPI_ISL_618602, EPI_ISL_618603, EPI_ISL_618604, EPI_ISL_618605, EPI_ISL_618606, EPI_ISL_618607, EPI_ISL_618608, EPI_ISL_618610, EPI_ISL_618611, EPI_ISL_618612, EPI_ISL_618613, EPI_ISL_618614, EPI_ISL_618615, EPI_ISL_618616, EPI_ISL_618617, EPI_ISL_618618, EPI_ISL_618619, EPI_ISL_618620, EPI_ISL_618621, EPI_ISL_618622, EPI_ISL_618623, EPI_ISL_618624, EPI_ISL_618625, EPI_ISL_618663, EPI_ISL_618664, EPI_ISL_618665, EPI_ISL_618666, EPI_ISL_618667, EPI_ISL_618668, EPI_ISL_618669, EPI_ISL_618670, EPI_ISL_618671, EPI_ISL_618672, EPI_ISL_618673, EPI_ISL_618674, EPI_ISL_618675, EPI_ISL_618676, EPI_ISL_618677, EPI_ISL_618678, EPI_ISL_618679, EPI_ISL_618680, EPI_ISL_618681, EPI_ISL_618682, EPI_ISL_618683, EPI_ISL_618684, EPI_ISL_618685, EPI_ISL_618686, EPI_ISL_618687, EPI_ISL_618688, EPI_ISL_618689, EPI_ISL_618690, EPI_ISL_618691, EPI_ISL_618692, EPI_ISL_618693, EPI_ISL_618694, EPI_ISL_618695, EPI_ISL_618696, EPI_ISL_618697, EPI_ISL_618698, EPI_ISL_618699, EPI_ISL_618700, EPI_ISL_618701, EPI_ISL_618702, EPI_ISL_618703, EPI_ISL_618704, EPI_ISL_618705, EPI_ISL_618706, EPI_ISL_618707, EPI_ISL_618708, EPI_ISL_618709, EPI_ISL_618710, EPI_ISL_618711, EPI_ISL_618712, EPI_ISL_618713, EPI_ISL_618714, EPI_ISL_618715, EPI_ISL_618716, EPI_ISL_618717, EPI_ISL_618718, EPI_ISL_618719, EPI_ISL_618721, EPI_ISL_618722, EPI_ISL_618723, EPI_ISL_618724, EPI_ISL_618725, EPI_ISL_618726, EPI_ISL_618727, EPI_ISL_618728, EPI_ISL_618729, EPI_ISL_618731, EPI_ISL_618732, EPI_ISL_618733, EPI_ISL_618734, EPI_ISL_618735, EPI_ISL_618736, EPI_ISL_618737, EPI_ISL_618738, EPI_ISL_618739, EPI_ISL_618741, EPI_ISL_618742, EPI_ISL_618743, EPI_ISL_618744, EPI_ISL_618745, EPI_ISL_618746, EPI_ISL_618747, EPI_ISL_618748, EPI_ISL_618749, EPI_ISL_618750, EPI_ISL_618751, EPI_ISL_618752, EPI_ISL_618753, EPI_ISL_618754, EPI_ISL_618755, EPI_ISL_618756, EPI_ISL_618757, EPI_ISL_618758, EPI_ISL_618759, EPI_ISL_618760, EPI_ISL_618761, EPI_ISL_618762, EPI_ISL_618763, EPI_ISL_618764, EPI_ISL_618765, EPI_ISL_618766, EPI_ISL_618767, EPI_ISL_618769, EPI_ISL_618770, EPI_ISL_618771, EPI_ISL_618772, EPI_ISL_618773, EPI_ISL_618774, EPI_ISL_618775, EPI_ISL_618776, EPI_ISL_618777, EPI_ISL_618778, EPI_ISL_618779, EPI_ISL_618780, EPI_ISL_618781, EPI_ISL_618782, EPI_ISL_618783, EPI_ISL_618784, EPI_ISL_618785, EPI_ISL_618786, EPI_ISL_618787, EPI_ISL_618788, EPI_ISL_618789, EPI_ISL_618790, EPI_ISL_618791, EPI_ISL_618792, EPI_ISL_618793, EPI_ISL_618794, EPI_ISL_618795, EPI_ISL_618796, EPI_ISL_618797, EPI_ISL_618798, EPI_ISL_618799, EPI_ISL_618813, EPI_ISL_618822, EPI_ISL_618830, EPI_ISL_618833, EPI_ISL_618837, EPI_ISL_618844, EPI_ISL_618846, EPI_ISL_618847, EPI_ISL_618852, EPI_ISL_618855, EPI_ISL_618857, EPI_ISL_618872			

see above	Department of Virus and Microbiological Special Diagnostics, Statens Serum Institut, Denmark	Albertsen lab, Department of Chemistry and Bioscience, Aalborg University, Denmark	Danish Covid-19 Genome Consortia
EPI_ISL_622769, EPI_ISL_622770, EPI_ISL_622771	Canterbury Health Laboratories	Institute of Environmental Science and Research (ESR)	Xiaoyun Ren, Matt Storey, Nikki Freed, Muhammad Faisal, Jing Wang, Hermes Perez, Anja Werno, Antje van der Linden, Arlo Upton, Chris Mansell, David Hammer, Dragana Drinkovic, Gary McAuliffe, Hana Sofia Andersson, James Ussher, Jill Sherwood, Josh Freeman, Julia Howard, Juliet Elvy, Mary DeAlmeida, Matt Blakiston, Matthew Rogers, Max Bloomfield, Michael Addidle, Michelle Balm, Sally Roberts, Sarah Jefferies, Sharmini Muttaiyah, Susan Morpeth, Susan Taylor, Timothy Blackmore, Vani Sathyendran, Veronica Playle, Virginia Hope, Erasmus Smit, Lauren Jelly, Olin Silander, Joep de Ligt
EPI_ISL_622772	Middlemore Hospital	Institute of Environmental Science and Research (ESR)	Xiaoyun Ren, Matt Storey, Nikki Freed, Muhammad Faisal, Jing Wang, Hermes Perez, Anja Werno, Antje van der Linden, Arlo Upton, Chris Mansell, David Hammer, Dragana Drinkovic, Gary McAuliffe, Hana Sofia Andersson, James Ussher, Jill Sherwood, Josh Freeman, Julia Howard, Juliet Elvy, Mary DeAlmeida, Matt Blakiston, Matthew Rogers, Max Bloomfield, Michael Addidle, Michelle Balm, Sally Roberts, Sarah Jefferies, Sharmini Muttaiyah, Susan Morpeth, Susan Taylor, Timothy Blackmore, Vani Sathyendran, Veronica Playle, Virginia Hope, Erasmus Smit, Lauren Jelly, Olin Silander, Joep de Ligt
EPI_ISL_622774	LabPLUS	Institute of Environmental Science and Research (ESR)	Xiaoyun Ren, Matt Storey, Nikki Freed, Muhammad Faisal, Jing Wang, Hermes Perez, Anja Werno, Antje van der Linden, Arlo Upton, Chris Mansell, David Hammer, Dragana Drinkovic, Gary McAuliffe, Hana Sofia Andersson, James Ussher, Jill Sherwood, Josh Freeman, Julia Howard, Juliet Elvy, Mary DeAlmeida, Matt Blakiston, Matthew Rogers, Max Bloomfield, Michael Addidle, Michelle Balm, Sally Roberts, Sarah Jefferies, Sharmini Muttaiyah, Susan Morpeth, Susan Taylor, Timothy Blackmore, Vani Sathyendran, Veronica Playle, Virginia Hope, Erasmus Smit, Lauren Jelly, Olin Silander, Joep de Ligt
EPI_ISL_622775	Canterbury Health Laboratories	Institute of Environmental Science and Research (ESR)	Xiaoyun Ren, Matt Storey, Nikki Freed, Muhammad Faisal, Jing Wang, Hermes Perez, Anja Werno, Antje van der Linden, Arlo Upton, Chris Mansell, David Hammer, Dragana Drinkovic, Gary McAuliffe, Hana Sofia Andersson, James Ussher, Jill Sherwood, Josh Freeman, Julia Howard, Juliet Elvy, Mary DeAlmeida, Matt Blakiston, Matthew Rogers, Max Bloomfield, Michael Addidle, Michelle Balm, Sally Roberts, Sarah Jefferies, Sharmini Muttaiyah, Susan Morpeth, Susan Taylor, Timothy Blackmore, Vani Sathyendran, Veronica Playle, Virginia Hope, Erasmus Smit, Lauren Jelly, Olin Silander, Joep de Ligt
EPI_ISL_622776	Middlemore Hospital	Institute of Environmental Science and Research (ESR)	Xiaoyun Ren, Matt Storey, Nikki Freed, Muhammad Faisal, Jing Wang, Hermes Perez, Anja Werno, Antje van der Linden, Arlo Upton, Chris Mansell, David Hammer, Dragana Drinkovic, Gary McAuliffe, Hana Sofia Andersson, James Ussher, Jill Sherwood, Josh Freeman, Julia Howard, Juliet Elvy, Mary DeAlmeida, Matt Blakiston, Matthew Rogers, Max Bloomfield, Michael Addidle, Michelle Balm, Sally Roberts, Sarah Jefferies, Sharmini Muttaiyah, Susan Morpeth, Susan Taylor, Timothy Blackmore, Vani Sathyendran, Veronica Playle, Virginia Hope, Erasmus Smit, Lauren Jelly, Olin Silander, Joep de Ligt
EPI_ISL_622778	LabTests	Institute of Environmental Science and Research (ESR)	Xiaoyun Ren, Matt Storey, Nikki Freed, Muhammad Faisal, Jing Wang, Hermes Perez, Anja Werno, Antje van der Linden, Arlo Upton, Chris Mansell, David Hammer, Dragana Drinkovic, Gary McAuliffe, Hana Sofia Andersson, James Ussher, Jill Sherwood, Josh Freeman, Julia Howard, Juliet Elvy, Mary DeAlmeida, Matt Blakiston, Matthew Rogers, Max Bloomfield, Michael Addidle, Michelle Balm, Sally Roberts, Sarah Jefferies, Sharmini Muttaiyah, Susan Morpeth, Susan Taylor, Timothy Blackmore, Vani Sathyendran, Veronica Playle, Virginia Hope, Erasmus Smit, Lauren Jelly, Olin Silander, Joep de Ligt
EPI_ISL_622781, EPI_ISL_622782, EPI_ISL_622789, EPI_ISL_622791, EPI_ISL_622809	Canterbury Health Laboratories	Institute of Environmental Science and Research (ESR)	Xiaoyun Ren, Matt Storey, Nikki Freed, Muhammad Faisal, Jing Wang, Hermes Perez, Anja Werno, Antje van der Linden, Arlo Upton, Chris Mansell, David Hammer, Dragana Drinkovic, Gary McAuliffe, Hana Sofia Andersson, James Ussher, Jill Sherwood, Josh Freeman, Julia Howard, Juliet Elvy, Mary DeAlmeida, Matt Blakiston, Matthew Rogers, Max Bloomfield, Michael Addidle, Michelle Balm, Sally Roberts, Sarah Jefferies, Sharmini Muttaiyah, Susan Morpeth, Susan Taylor, Timothy Blackmore, Vani Sathyendran, Veronica Playle, Virginia Hope, Erasmus Smit, Lauren Jelly, Olin Silander, Joep de Ligt
EPI_ISL_622818	Middlemore Hospital	Institute of Environmental Science and Research (ESR)	Xiaoyun Ren, Matt Storey, Nikki Freed, Muhammad Faisal, Jing Wang, Hermes Perez, Anja Werno, Antje van der Linden, Arlo Upton, Chris Mansell, David Hammer, Dragana Drinkovic, Gary McAuliffe, Hana Sofia Andersson, James Ussher, Jill Sherwood, Josh Freeman, Julia Howard, Juliet Elvy, Mary DeAlmeida, Matt Blakiston, Matthew Rogers, Max Bloomfield, Michael Addidle, Michelle Balm, Sally Roberts, Sarah Jefferies, Sharmini Muttaiyah, Susan Morpeth, Susan Taylor, Timothy Blackmore, Vani Sathyendran, Veronica Playle, Virginia Hope, Erasmus Smit, Lauren Jelly, Olin Silander, Joep de Ligt
EPI_ISL_622879, EPI_ISL_622880, EPI_ISL_622881, EPI_ISL_622882, EPI_ISL_622883, EPI_ISL_622886, EPI_ISL_622887, EPI_ISL_622889	Respiratory Virus Unit, Microbiology Services Colindale, Public Health England	Respiratory Virus Unit, Microbiology Services Colindale, Public Health England	PHE Covid Sequencing Team
EPI_ISL_623097	General practitioner	National Reference Center for Viruses of Respiratory Infections, Institut Pasteur, Paris	Marion Barbet, Sylvie Behillil, Méline Bizard, Angela Brisebarre, Camille Capel, Etienne Simon-Lorière, Vincent Enouf, Maud Vanpeene, Sylvie van der Werf
EPI_ISL_623211, EPI_ISL_623212	Lighthouse Lab in Milton Keynes	Wellcome Sanger Institute for the COVID-19 Genomics UK (COG-UK) consortium	The Lighthouse Lab in Milton Keynes and Alex Alderton, Roberto Amato, Sonia Goncalves, Ewan Harrison, David K. Jackson, Ian Johnston, Dominic Kwiatkowski, Cordelia Langford, John Sillitoe on behalf of the Wellcome Sanger Institute COVID-19 Surveillance Team (http://www.sanger.ac.uk/covid-team)
EPI_ISL_623214	Lighthouse Lab in Milton Keynes	Wellcome Sanger Institute for the COVID-19 Genomics UK (COG-UK) Consortium	The Lighthouse Lab in Milton Keynes and Alex Alderton, Roberto Amato, Sonia Goncalves, Ewan Harrison, David K. Jackson, Ian Johnston, Dominic Kwiatkowski, Cordelia Langford, John Sillitoe on behalf of the Wellcome Sanger Institute COVID-19 Surveillance Team (http://www.sanger.ac.uk/covid-team)
EPI_ISL_623215, EPI_ISL_623216, EPI_ISL_623217, EPI_ISL_623218, EPI_ISL_623219, EPI_ISL_623221	Lighthouse Lab in Milton Keynes	Wellcome Sanger Institute for the COVID-19 Genomics UK (COG-UK) consortium	The Lighthouse Lab in Milton Keynes and Alex Alderton, Roberto Amato, Sonia Goncalves, Ewan Harrison, David K. Jackson, Ian Johnston, Dominic Kwiatkowski, Cordelia Langford, John Sillitoe on behalf of the Wellcome Sanger Institute COVID-19 Surveillance Team (http://www.sanger.ac.uk/covid-team)
EPI_ISL_623222	Lighthouse Lab in Cambridge	Wellcome Sanger Institute for the COVID-19 Genomics UK (COG-UK) consortium	Rob Howes, The Lighthouse Lab in Cambridge and Alex Alderton, Roberto Amato, Sonia Goncalves, Ewan Harrison, David K. Jackson, Ian Johnston, Dominic Kwiatkowski, Cordelia Langford, John Sillitoe on behalf of the Wellcome Sanger Institute COVID-19 Surveillance Team (http://www.sanger.ac.uk/covid-team)
EPI_ISL_623223, EPI_ISL_623224, EPI_ISL_623225, EPI_ISL_623226, EPI_ISL_623227, EPI_ISL_623228, EPI_ISL_623229, EPI_ISL_623231, EPI_ISL_623232, EPI_ISL_623233	Lighthouse Lab in Milton Keynes	Wellcome Sanger Institute for the COVID-19 Genomics UK (COG-UK) consortium	The Lighthouse Lab in Milton Keynes and Alex Alderton, Roberto Amato, Sonia Goncalves, Ewan Harrison, David K. Jackson, Ian Johnston, Dominic Kwiatkowski, Cordelia Langford, John Sillitoe on behalf of the Wellcome Sanger Institute COVID-19 Surveillance Team (http://www.sanger.ac.uk/covid-team)
EPI_ISL_623234	Lighthouse Lab in Cambridge	Wellcome Sanger Institute for the COVID-19 Genomics UK (COG-UK) consortium	Rob Howes, The Lighthouse Lab in Cambridge and Alex Alderton, Roberto Amato, Sonia Goncalves, Ewan Harrison, David K. Jackson, Ian Johnston, Dominic Kwiatkowski, Cordelia Langford, John Sillitoe on behalf of the Wellcome Sanger Institute COVID-19 Surveillance Team (http://www.sanger.ac.uk/covid-team)
EPI_ISL_623236, EPI_ISL_623237, EPI_ISL_623238, EPI_ISL_623239, EPI_ISL_623240, EPI_ISL_623241, EPI_ISL_623243	Lighthouse Lab in Milton Keynes	Wellcome Sanger Institute for the COVID-19 Genomics UK (COG-UK) consortium	The Lighthouse Lab in Milton Keynes and Alex Alderton, Roberto Amato, Sonia Goncalves, Ewan Harrison, David K. Jackson, Ian Johnston, Dominic Kwiatkowski, Cordelia Langford, John Sillitoe on behalf of the Wellcome Sanger Institute COVID-19 Surveillance Team (http://www.sanger.ac.uk/covid-team)
EPI_ISL_623247	Lighthouse Lab in Cambridge	Wellcome Sanger Institute for the COVID-19 Genomics UK (COG-UK) consortium	Rob Howes, The Lighthouse Lab in Cambridge and Alex Alderton, Roberto Amato, Sonia Goncalves, Ewan Harrison, David K. Jackson, Ian Johnston, Dominic Kwiatkowski, Cordelia Langford, John Sillitoe on behalf of the Wellcome Sanger Institute COVID-19 Surveillance Team (http://www.sanger.ac.uk/covid-team)
EPI_ISL_623249, EPI_ISL_623250, EPI_ISL_623251, EPI_ISL_623253, EPI_ISL_623254, EPI_ISL_623255	Lighthouse Lab in Milton Keynes	Wellcome Sanger Institute for the COVID-19 Genomics UK (COG-UK) consortium	The Lighthouse Lab in Milton Keynes and Alex Alderton, Roberto Amato, Sonia Goncalves, Ewan Harrison, David K. Jackson, Ian Johnston, Dominic Kwiatkowski, Cordelia Langford, John Sillitoe on behalf of the Wellcome Sanger Institute COVID-19 Surveillance Team (http://www.sanger.ac.uk/covid-team)
EPI_ISL_623256	Lighthouse Lab in Cambridge	Wellcome Sanger Institute for the COVID-19 Genomics UK (COG-UK) consortium	Rob Howes, The Lighthouse Lab in Cambridge and Alex Alderton, Roberto Amato, Sonia Goncalves, Ewan Harrison, David K. Jackson, Ian Johnston, Dominic Kwiatkowski, Cordelia Langford, John Sillitoe on behalf of the Wellcome Sanger Institute COVID-19 Surveillance Team (http://www.sanger.ac.uk/covid-team)
EPI_ISL_623257, EPI_ISL_623258	Lighthouse Lab in Milton Keynes	Wellcome Sanger Institute for the COVID-19 Genomics UK (COG-UK) consortium	The Lighthouse Lab in Milton Keynes and Alex Alderton, Roberto Amato, Sonia Goncalves, Ewan Harrison, David K. Jackson, Ian Johnston, Dominic Kwiatkowski, Cordelia Langford, John Sillitoe on behalf of the Wellcome Sanger Institute COVID-19 Surveillance Team (http://www.sanger.ac.uk/covid-team)
EPI_ISL_623259	Lighthouse Lab in Milton Keynes	Wellcome Sanger Institute for the COVID-19 Genomics UK (COG-UK) Consortium	The Lighthouse Lab in Milton Keynes and Alex Alderton, Roberto Amato, Sonia Goncalves, Ewan Harrison, David K. Jackson, Ian Johnston, Dominic Kwiatkowski, Cordelia Langford, John Sillitoe on behalf of the Wellcome Sanger Institute COVID-19 Surveillance Team (http://www.sanger.ac.uk/covid-team)

EPI_ISL_623260, EPI_ISL_623261, EPI_ISL_623262, EPI_ISL_623264, EPI_ISL_623266, EPI_ISL_623267, EPI_ISL_623269, EPI_ISL_623270, EPI_ISL_623271, EPI_ISL_623272, EPI_ISL_623274, EPI_ISL_623275

see above	Lighthouse Lab in Milton Keynes	Wellcome Sanger Institute for the COVID-19 Genomics UK (COG-UK) consortium	The Lighthouse Lab in Milton Keynes and Alex Alderton, Roberto Amato, Sonia Goncalves, Ewan Harrison, David K. Jackson, Ian Johnston, Dominic Kwiatkowski, Cordelia Langford, John Sillitoe on behalf of the Wellcome Sanger Institute COVID-19 Surveillance Team (http://www.sanger.ac.uk/covid-team)
EPI_ISL_623276	Lighthouse Lab in Cambridge	Wellcome Sanger Institute for the COVID-19 Genomics UK (COG-UK) consortium	Rob Howes, The Lighthouse Lab in Cambridge and Alex Alderton, Roberto Amato, Sonia Goncalves, Ewan Harrison, David K. Jackson, Ian Johnston, Dominic Kwiatkowski, Cordelia Langford, John Sillitoe on behalf of the Wellcome Sanger Institute COVID-19 Surveillance Team (http://www.sanger.ac.uk/covid-team)
EPI_ISL_623277, EPI_ISL_623278, EPI_ISL_623279, EPI_ISL_623280, EPI_ISL_623282, EPI_ISL_623283, EPI_ISL_623284, EPI_ISL_623285	Lighthouse Lab in Milton Keynes	Wellcome Sanger Institute for the COVID-19 Genomics UK (COG-UK) consortium	The Lighthouse Lab in Milton Keynes and Alex Alderton, Roberto Amato, Sonia Goncalves, Ewan Harrison, David K. Jackson, Ian Johnston, Dominic Kwiatkowski, Cordelia Langford, John Sillitoe on behalf of the Wellcome Sanger Institute COVID-19 Surveillance Team (http://www.sanger.ac.uk/covid-team)
EPI_ISL_623286	Lighthouse Lab in Cambridge	Wellcome Sanger Institute for the COVID-19 Genomics UK (COG-UK) consortium	Rob Howes, The Lighthouse Lab in Cambridge and Alex Alderton, Roberto Amato, Sonia Goncalves, Ewan Harrison, David K. Jackson, Ian Johnston, Dominic Kwiatkowski, Cordelia Langford, John Sillitoe on behalf of the Wellcome Sanger Institute COVID-19 Surveillance Team (http://www.sanger.ac.uk/covid-team)
EPI_ISL_623287	Lighthouse Lab in Milton Keynes	Wellcome Sanger Institute for the COVID-19 Genomics UK (COG-UK) consortium	The Lighthouse Lab in Milton Keynes and Alex Alderton, Roberto Amato, Sonia Goncalves, Ewan Harrison, David K. Jackson, Ian Johnston, Dominic Kwiatkowski, Cordelia Langford, John Sillitoe on behalf of the Wellcome Sanger Institute COVID-19 Surveillance Team (http://www.sanger.ac.uk/covid-team)
EPI_ISL_623288	Lighthouse Lab in Cambridge	Wellcome Sanger Institute for the COVID-19 Genomics UK (COG-UK) consortium	Rob Howes, The Lighthouse Lab in Cambridge and Alex Alderton, Roberto Amato, Sonia Goncalves, Ewan Harrison, David K. Jackson, Ian Johnston, Dominic Kwiatkowski, Cordelia Langford, John Sillitoe on behalf of the Wellcome Sanger Institute COVID-19 Surveillance Team (http://www.sanger.ac.uk/covid-team)
EPI_ISL_623289, EPI_ISL_623290, EPI_ISL_623291, EPI_ISL_623292, EPI_ISL_623293, EPI_ISL_623294, EPI_ISL_623295, EPI_ISL_623296	Lighthouse Lab in Milton Keynes	Wellcome Sanger Institute for the COVID-19 Genomics UK (COG-UK) consortium	The Lighthouse Lab in Milton Keynes and Alex Alderton, Roberto Amato, Sonia Goncalves, Ewan Harrison, David K. Jackson, Ian Johnston, Dominic Kwiatkowski, Cordelia Langford, John Sillitoe on behalf of the Wellcome Sanger Institute COVID-19 Surveillance Team (http://www.sanger.ac.uk/covid-team)
EPI_ISL_623298	Lighthouse Lab in Milton Keynes	Wellcome Sanger Institute for the COVID-19 Genomics UK (COG-UK) Consortium	The Lighthouse Lab in Milton Keynes and Alex Alderton, Roberto Amato, Sonia Goncalves, Ewan Harrison, David K. Jackson, Ian Johnston, Dominic Kwiatkowski, Cordelia Langford, John Sillitoe on behalf of the Wellcome Sanger Institute COVID-19 Surveillance Team (http://www.sanger.ac.uk/covid-team)
EPI_ISL_623299	Lighthouse Lab in Milton Keynes	Wellcome Sanger Institute for the COVID-19 Genomics UK (COG-UK) consortium	The Lighthouse Lab in Milton Keynes and Alex Alderton, Roberto Amato, Sonia Goncalves, Ewan Harrison, David K. Jackson, Ian Johnston, Dominic Kwiatkowski, Cordelia Langford, John Sillitoe on behalf of the Wellcome Sanger Institute COVID-19 Surveillance Team (http://www.sanger.ac.uk/covid-team)
EPI_ISL_623301	Lighthouse Lab in Cambridge	Wellcome Sanger Institute for the COVID-19 Genomics UK (COG-UK) consortium	Rob Howes, The Lighthouse Lab in Cambridge and Alex Alderton, Roberto Amato, Sonia Goncalves, Ewan Harrison, David K. Jackson, Ian Johnston, Dominic Kwiatkowski, Cordelia Langford, John Sillitoe on behalf of the Wellcome Sanger Institute COVID-19 Surveillance Team (http://www.sanger.ac.uk/covid-team)
EPI_ISL_623302, EPI_ISL_623303, EPI_ISL_623304, EPI_ISL_623305, EPI_ISL_623306, EPI_ISL_623307, EPI_ISL_623309, EPI_ISL_623310	Lighthouse Lab in Milton Keynes	Wellcome Sanger Institute for the COVID-19 Genomics UK (COG-UK) consortium	The Lighthouse Lab in Milton Keynes and Alex Alderton, Roberto Amato, Sonia Goncalves, Ewan Harrison, David K. Jackson, Ian Johnston, Dominic Kwiatkowski, Cordelia Langford, John Sillitoe on behalf of the Wellcome Sanger Institute COVID-19 Surveillance Team (http://www.sanger.ac.uk/covid-team)
EPI_ISL_623313	Lighthouse Lab in Milton Keynes	Wellcome Sanger Institute for the COVID-19 Genomics UK (COG-UK) Consortium	The Lighthouse Lab in Milton Keynes and Alex Alderton, Roberto Amato, Sonia Goncalves, Ewan Harrison, David K. Jackson, Ian Johnston, Dominic Kwiatkowski, Cordelia Langford, John Sillitoe on behalf of the Wellcome Sanger Institute COVID-19 Surveillance Team (http://www.sanger.ac.uk/covid-team)
EPI_ISL_623314, EPI_ISL_623315, EPI_ISL_623316, EPI_ISL_623317, EPI_ISL_623318, EPI_ISL_623319, EPI_ISL_623320	Lighthouse Lab in Milton Keynes	Wellcome Sanger Institute for the COVID-19 Genomics UK (COG-UK) consortium	The Lighthouse Lab in Milton Keynes and Alex Alderton, Roberto Amato, Sonia Goncalves, Ewan Harrison, David K. Jackson, Ian Johnston, Dominic Kwiatkowski, Cordelia Langford, John Sillitoe on behalf of the Wellcome Sanger Institute COVID-19 Surveillance Team (http://www.sanger.ac.uk/covid-team)
EPI_ISL_623321	Lighthouse Lab in Cambridge	Wellcome Sanger Institute for the COVID-19 Genomics UK (COG-UK) consortium	Rob Howes, The Lighthouse Lab in Cambridge and Alex Alderton, Roberto Amato, Sonia Goncalves, Ewan Harrison, David K. Jackson, Ian Johnston, Dominic Kwiatkowski, Cordelia Langford, John Sillitoe on behalf of the Wellcome Sanger Institute COVID-19 Surveillance Team (http://www.sanger.ac.uk/covid-team)
EPI_ISL_623322	Lighthouse Lab in Milton Keynes	Wellcome Sanger Institute for the COVID-19 Genomics UK (COG-UK) consortium	The Lighthouse Lab in Milton Keynes and Alex Alderton, Roberto Amato, Sonia Goncalves, Ewan Harrison, David K. Jackson, Ian Johnston, Dominic Kwiatkowski, Cordelia Langford, John Sillitoe on behalf of the Wellcome Sanger Institute COVID-19 Surveillance Team (http://www.sanger.ac.uk/covid-team)
EPI_ISL_623323	Lighthouse Lab in Cambridge	Wellcome Sanger Institute for the COVID-19 Genomics UK (COG-UK) consortium	Rob Howes, The Lighthouse Lab in Cambridge and Alex Alderton, Roberto Amato, Sonia Goncalves, Ewan Harrison, David K. Jackson, Ian Johnston, Dominic Kwiatkowski, Cordelia Langford, John Sillitoe on behalf of the Wellcome Sanger Institute COVID-19 Surveillance Team (http://www.sanger.ac.uk/covid-team)
EPI_ISL_623324, EPI_ISL_623325, EPI_ISL_623326, EPI_ISL_623327, EPI_ISL_623328, EPI_ISL_623329, EPI_ISL_623331, EPI_ISL_623332	Lighthouse Lab in Milton Keynes	Wellcome Sanger Institute for the COVID-19 Genomics UK (COG-UK) consortium	The Lighthouse Lab in Milton Keynes and Alex Alderton, Roberto Amato, Sonia Goncalves, Ewan Harrison, David K. Jackson, Ian Johnston, Dominic Kwiatkowski, Cordelia Langford, John Sillitoe on behalf of the Wellcome Sanger Institute COVID-19 Surveillance Team (http://www.sanger.ac.uk/covid-team)
EPI_ISL_623334	Lighthouse Lab in Cambridge	Wellcome Sanger Institute for the COVID-19 Genomics UK (COG-UK) consortium	Rob Howes, The Lighthouse Lab in Cambridge and Alex Alderton, Roberto Amato, Sonia Goncalves, Ewan Harrison, David K. Jackson, Ian Johnston, Dominic Kwiatkowski, Cordelia Langford, John Sillitoe on behalf of the Wellcome Sanger Institute COVID-19 Surveillance Team (http://www.sanger.ac.uk/covid-team)
EPI_ISL_623335, EPI_ISL_623336, EPI_ISL_623338, EPI_ISL_623339, EPI_ISL_623340, EPI_ISL_623341, EPI_ISL_623342	Lighthouse Lab in Milton Keynes	Wellcome Sanger Institute for the COVID-19 Genomics UK (COG-UK) consortium	The Lighthouse Lab in Milton Keynes and Alex Alderton, Roberto Amato, Sonia Goncalves, Ewan Harrison, David K. Jackson, Ian Johnston, Dominic Kwiatkowski, Cordelia Langford, John Sillitoe on behalf of the Wellcome Sanger Institute COVID-19 Surveillance Team (http://www.sanger.ac.uk/covid-team)
EPI_ISL_623344, EPI_ISL_623346	Lighthouse Lab in Cambridge	Wellcome Sanger Institute for the COVID-19 Genomics UK (COG-UK) consortium	Rob Howes, The Lighthouse Lab in Cambridge and Alex Alderton, Roberto Amato, Sonia Goncalves, Ewan Harrison, David K. Jackson, Ian Johnston, Dominic Kwiatkowski, Cordelia Langford, John Sillitoe on behalf of the Wellcome Sanger Institute COVID-19 Surveillance Team (http://www.sanger.ac.uk/covid-team)
EPI_ISL_623348, EPI_ISL_623349, EPI_ISL_623350, EPI_ISL_623351, EPI_ISL_623352, EPI_ISL_623353, EPI_ISL_623355, EPI_ISL_623356, EPI_ISL_623357, EPI_ISL_623358	Lighthouse Lab in Milton Keynes	Wellcome Sanger Institute for the COVID-19 Genomics UK (COG-UK) consortium	The Lighthouse Lab in Milton Keynes and Alex Alderton, Roberto Amato, Sonia Goncalves, Ewan Harrison, David K. Jackson, Ian Johnston, Dominic Kwiatkowski, Cordelia Langford, John Sillitoe on behalf of the Wellcome Sanger Institute COVID-19 Surveillance Team (http://www.sanger.ac.uk/covid-team)
EPI_ISL_623360	Lighthouse Lab in Cambridge	Wellcome Sanger Institute for the COVID-19 Genomics UK (COG-UK) consortium	Rob Howes, The Lighthouse Lab in Cambridge and Alex Alderton, Roberto Amato, Sonia Goncalves, Ewan Harrison, David K. Jackson, Ian Johnston, Dominic Kwiatkowski, Cordelia Langford, John Sillitoe on behalf of the Wellcome Sanger Institute COVID-19 Surveillance Team (http://www.sanger.ac.uk/covid-team)
EPI_ISL_623361, EPI_ISL_623362	Lighthouse Lab in Milton Keynes	Wellcome Sanger Institute for the COVID-19 Genomics UK (COG-UK) consortium	The Lighthouse Lab in Milton Keynes and Alex Alderton, Roberto Amato, Sonia Goncalves, Ewan Harrison, David K. Jackson, Ian Johnston, Dominic Kwiatkowski, Cordelia Langford, John Sillitoe on behalf of the Wellcome Sanger Institute COVID-19 Surveillance Team (http://www.sanger.ac.uk/covid-team)
EPI_ISL_623364	Lighthouse Lab in Cambridge	Wellcome Sanger Institute for the COVID-19 Genomics UK (COG-UK) consortium	Rob Howes, The Lighthouse Lab in Cambridge and Alex Alderton, Roberto Amato, Sonia Goncalves, Ewan Harrison, David K. Jackson, Ian Johnston, Dominic Kwiatkowski, Cordelia Langford, John Sillitoe on behalf of the Wellcome Sanger Institute COVID-19 Surveillance Team (http://www.sanger.ac.uk/covid-team)
EPI_ISL_623365	Lighthouse Lab in Milton Keynes	Wellcome Sanger Institute for the COVID-19 Genomics UK (COG-UK) consortium	The Lighthouse Lab in Milton Keynes and Alex Alderton, Roberto Amato, Sonia Goncalves, Ewan Harrison, David K. Jackson, Ian Johnston, Dominic Kwiatkowski, Cordelia Langford, John Sillitoe on behalf of the Wellcome Sanger Institute COVID-19 Surveillance Team (http://www.sanger.ac.uk/covid-team)

EPI_ISL_623366	Lighthouse Lab in Cambridge	Wellcome Sanger Institute for the COVID-19 Genomics UK (COG-UK) consortium	Rob Howes, The Lighthouse Lab in Cambridge and Alex Alderton, Roberto Amato, Sonia Goncalves, Ewan Harrison, David K. Jackson, Ian Johnston, Dominic Kwiatkowski, Cordelia Langford, John Sillitoe on behalf of the Wellcome Sanger Institute COVID-19 Surveillance Team (http://www.sanger.ac.uk/covid-team)
EPI_ISL_623367, EPI_ISL_623368, EPI_ISL_623369, EPI_ISL_623371, EPI_ISL_623373, EPI_ISL_623374, EPI_ISL_623375, EPI_ISL_623376, EPI_ISL_623378, EPI_ISL_623379, EPI_ISL_623380, EPI_ISL_623381, EPI_ISL_623382, EPI_ISL_623383			
see above	Lighthouse Lab in Milton Keynes	Wellcome Sanger Institute for the COVID-19 Genomics UK (COG-UK) consortium	The Lighthouse Lab in Milton Keynes and Alex Alderton, Roberto Amato, Sonia Goncalves, Ewan Harrison, David K. Jackson, Ian Johnston, Dominic Kwiatkowski, Cordelia Langford, John Sillitoe on behalf of the Wellcome Sanger Institute COVID-19 Surveillance Team (http://www.sanger.ac.uk/covid-team)
EPI_ISL_623385, EPI_ISL_623386	Lighthouse Lab in Cambridge	Wellcome Sanger Institute for the COVID-19 Genomics UK (COG-UK) consortium	Rob Howes, The Lighthouse Lab in Cambridge and Alex Alderton, Roberto Amato, Sonia Goncalves, Ewan Harrison, David K. Jackson, Ian Johnston, Dominic Kwiatkowski, Cordelia Langford, John Sillitoe on behalf of the Wellcome Sanger Institute COVID-19 Surveillance Team (http://www.sanger.ac.uk/covid-team)
EPI_ISL_623387, EPI_ISL_623388, EPI_ISL_623389, EPI_ISL_623391	Lighthouse Lab in Milton Keynes	Wellcome Sanger Institute for the COVID-19 Genomics UK (COG-UK) consortium	The Lighthouse Lab in Milton Keynes and Alex Alderton, Roberto Amato, Sonia Goncalves, Ewan Harrison, David K. Jackson, Ian Johnston, Dominic Kwiatkowski, Cordelia Langford, John Sillitoe on behalf of the Wellcome Sanger Institute COVID-19 Surveillance Team (http://www.sanger.ac.uk/covid-team)
EPI_ISL_623392, EPI_ISL_623393	Lighthouse Lab in Cambridge	Wellcome Sanger Institute for the COVID-19 Genomics UK (COG-UK) consortium	Rob Howes, The Lighthouse Lab in Cambridge and Alex Alderton, Roberto Amato, Sonia Goncalves, Ewan Harrison, David K. Jackson, Ian Johnston, Dominic Kwiatkowski, Cordelia Langford, John Sillitoe on behalf of the Wellcome Sanger Institute COVID-19 Surveillance Team (http://www.sanger.ac.uk/covid-team)
EPI_ISL_623394, EPI_ISL_623396	Lighthouse Lab in Milton Keynes	Wellcome Sanger Institute for the COVID-19 Genomics UK (COG-UK) consortium	The Lighthouse Lab in Milton Keynes and Alex Alderton, Roberto Amato, Sonia Goncalves, Ewan Harrison, David K. Jackson, Ian Johnston, Dominic Kwiatkowski, Cordelia Langford, John Sillitoe on behalf of the Wellcome Sanger Institute COVID-19 Surveillance Team (http://www.sanger.ac.uk/covid-team)
EPI_ISL_623398	Lighthouse Lab in Cambridge	Wellcome Sanger Institute for the COVID-19 Genomics UK (COG-UK) consortium	Rob Howes, The Lighthouse Lab in Cambridge and Alex Alderton, Roberto Amato, Sonia Goncalves, Ewan Harrison, David K. Jackson, Ian Johnston, Dominic Kwiatkowski, Cordelia Langford, John Sillitoe on behalf of the Wellcome Sanger Institute COVID-19 Surveillance Team (http://www.sanger.ac.uk/covid-team)
EPI_ISL_623399, EPI_ISL_623401, EPI_ISL_623402, EPI_ISL_623403	Lighthouse Lab in Milton Keynes	Wellcome Sanger Institute for the COVID-19 Genomics UK (COG-UK) consortium	The Lighthouse Lab in Milton Keynes and Alex Alderton, Roberto Amato, Sonia Goncalves, Ewan Harrison, David K. Jackson, Ian Johnston, Dominic Kwiatkowski, Cordelia Langford, John Sillitoe on behalf of the Wellcome Sanger Institute COVID-19 Surveillance Team (http://www.sanger.ac.uk/covid-team)
EPI_ISL_623404	Lighthouse Lab in Cambridge	Wellcome Sanger Institute for the COVID-19 Genomics UK (COG-UK) consortium	Rob Howes, The Lighthouse Lab in Cambridge and Alex Alderton, Roberto Amato, Sonia Goncalves, Ewan Harrison, David K. Jackson, Ian Johnston, Dominic Kwiatkowski, Cordelia Langford, John Sillitoe on behalf of the Wellcome Sanger Institute COVID-19 Surveillance Team (http://www.sanger.ac.uk/covid-team)
EPI_ISL_623405, EPI_ISL_623406, EPI_ISL_623407	Lighthouse Lab in Milton Keynes	Wellcome Sanger Institute for the COVID-19 Genomics UK (COG-UK) consortium	The Lighthouse Lab in Milton Keynes and Alex Alderton, Roberto Amato, Sonia Goncalves, Ewan Harrison, David K. Jackson, Ian Johnston, Dominic Kwiatkowski, Cordelia Langford, John Sillitoe on behalf of the Wellcome Sanger Institute COVID-19 Surveillance Team (http://www.sanger.ac.uk/covid-team)
EPI_ISL_623409	Lighthouse Lab in Cambridge	Wellcome Sanger Institute for the COVID-19 Genomics UK (COG-UK) consortium	Rob Howes, The Lighthouse Lab in Cambridge and Alex Alderton, Roberto Amato, Sonia Goncalves, Ewan Harrison, David K. Jackson, Ian Johnston, Dominic Kwiatkowski, Cordelia Langford, John Sillitoe on behalf of the Wellcome Sanger Institute COVID-19 Surveillance Team (http://www.sanger.ac.uk/covid-team)
EPI_ISL_623410	Lighthouse Lab in Milton Keynes	Wellcome Sanger Institute for the COVID-19 Genomics UK (COG-UK) consortium	The Lighthouse Lab in Milton Keynes and Alex Alderton, Roberto Amato, Sonia Goncalves, Ewan Harrison, David K. Jackson, Ian Johnston, Dominic Kwiatkowski, Cordelia Langford, John Sillitoe on behalf of the Wellcome Sanger Institute COVID-19 Surveillance Team (http://www.sanger.ac.uk/covid-team)
EPI_ISL_623411	Lighthouse Lab in Cambridge	Wellcome Sanger Institute for the COVID-19 Genomics UK (COG-UK) consortium	Rob Howes, The Lighthouse Lab in Cambridge and Alex Alderton, Roberto Amato, Sonia Goncalves, Ewan Harrison, David K. Jackson, Ian Johnston, Dominic Kwiatkowski, Cordelia Langford, John Sillitoe on behalf of the Wellcome Sanger Institute COVID-19 Surveillance Team (http://www.sanger.ac.uk/covid-team)
EPI_ISL_623412, EPI_ISL_623413, EPI_ISL_623414, EPI_ISL_623416, EPI_ISL_623417, EPI_ISL_623418, EPI_ISL_623419, EPI_ISL_623420, EPI_ISL_623422, EPI_ISL_623423	Lighthouse Lab in Milton Keynes	Wellcome Sanger Institute for the COVID-19 Genomics UK (COG-UK) consortium	The Lighthouse Lab in Milton Keynes and Alex Alderton, Roberto Amato, Sonia Goncalves, Ewan Harrison, David K. Jackson, Ian Johnston, Dominic Kwiatkowski, Cordelia Langford, John Sillitoe on behalf of the Wellcome Sanger Institute COVID-19 Surveillance Team (http://www.sanger.ac.uk/covid-team)
EPI_ISL_623425	Lighthouse Lab in Cambridge	Wellcome Sanger Institute for the COVID-19 Genomics UK (COG-UK) consortium	Rob Howes, The Lighthouse Lab in Cambridge and Alex Alderton, Roberto Amato, Sonia Goncalves, Ewan Harrison, David K. Jackson, Ian Johnston, Dominic Kwiatkowski, Cordelia Langford, John Sillitoe on behalf of the Wellcome Sanger Institute COVID-19 Surveillance Team (http://www.sanger.ac.uk/covid-team)
EPI_ISL_623428	Lighthouse Lab in Milton Keynes	Wellcome Sanger Institute for the COVID-19 Genomics UK (COG-UK) Consortium	The Lighthouse Lab in Milton Keynes and Alex Alderton, Roberto Amato, Sonia Goncalves, Ewan Harrison, David K. Jackson, Ian Johnston, Dominic Kwiatkowski, Cordelia Langford, John Sillitoe on behalf of the Wellcome Sanger Institute COVID-19 Surveillance Team (http://www.sanger.ac.uk/covid-team)
EPI_ISL_623429, EPI_ISL_623430, EPI_ISL_623431, EPI_ISL_623432, EPI_ISL_623434, EPI_ISL_623435, EPI_ISL_623436, EPI_ISL_623438, EPI_ISL_623439, EPI_ISL_623441, EPI_ISL_623446, EPI_ISL_623447			
see above	Lighthouse Lab in Milton Keynes	Wellcome Sanger Institute for the COVID-19 Genomics UK (COG-UK) consortium	The Lighthouse Lab in Milton Keynes and Alex Alderton, Roberto Amato, Sonia Goncalves, Ewan Harrison, David K. Jackson, Ian Johnston, Dominic Kwiatkowski, Cordelia Langford, John Sillitoe on behalf of the Wellcome Sanger Institute COVID-19 Surveillance Team (http://www.sanger.ac.uk/covid-team)
EPI_ISL_623448, EPI_ISL_623449	Lighthouse Lab in Cambridge	Wellcome Sanger Institute for the COVID-19 Genomics UK (COG-UK) consortium	Rob Howes, The Lighthouse Lab in Cambridge and Alex Alderton, Roberto Amato, Sonia Goncalves, Ewan Harrison, David K. Jackson, Ian Johnston, Dominic Kwiatkowski, Cordelia Langford, John Sillitoe on behalf of the Wellcome Sanger Institute COVID-19 Surveillance Team (http://www.sanger.ac.uk/covid-team)
EPI_ISL_623450	Lighthouse Lab in Milton Keynes	Wellcome Sanger Institute for the COVID-19 Genomics UK (COG-UK) consortium	The Lighthouse Lab in Milton Keynes and Alex Alderton, Roberto Amato, Sonia Goncalves, Ewan Harrison, David K. Jackson, Ian Johnston, Dominic Kwiatkowski, Cordelia Langford, John Sillitoe on behalf of the Wellcome Sanger Institute COVID-19 Surveillance Team (http://www.sanger.ac.uk/covid-team)
EPI_ISL_623451	Lighthouse Lab in Cambridge	Wellcome Sanger Institute for the COVID-19 Genomics UK (COG-UK) consortium	Rob Howes, The Lighthouse Lab in Cambridge and Alex Alderton, Roberto Amato, Sonia Goncalves, Ewan Harrison, David K. Jackson, Ian Johnston, Dominic Kwiatkowski, Cordelia Langford, John Sillitoe on behalf of the Wellcome Sanger Institute COVID-19 Surveillance Team (http://www.sanger.ac.uk/covid-team)
EPI_ISL_623452, EPI_ISL_623453, EPI_ISL_623454, EPI_ISL_623455	Lighthouse Lab in Milton Keynes	Wellcome Sanger Institute for the COVID-19 Genomics UK (COG-UK) consortium	The Lighthouse Lab in Milton Keynes and Alex Alderton, Roberto Amato, Sonia Goncalves, Ewan Harrison, David K. Jackson, Ian Johnston, Dominic Kwiatkowski, Cordelia Langford, John Sillitoe on behalf of the Wellcome Sanger Institute COVID-19 Surveillance Team (http://www.sanger.ac.uk/covid-team)
EPI_ISL_623456	Lighthouse Lab in Milton Keynes	Wellcome Sanger Institute for the COVID-19 Genomics UK (COG-UK) Consortium	The Lighthouse Lab in Milton Keynes and Alex Alderton, Roberto Amato, Sonia Goncalves, Ewan Harrison, David K. Jackson, Ian Johnston, Dominic Kwiatkowski, Cordelia Langford, John Sillitoe on behalf of the Wellcome Sanger Institute COVID-19 Surveillance Team (http://www.sanger.ac.uk/covid-team)
EPI_ISL_623458, EPI_ISL_623459, EPI_ISL_623460, EPI_ISL_623461, EPI_ISL_623462, EPI_ISL_623463, EPI_ISL_623464, EPI_ISL_623465, EPI_ISL_623466, EPI_ISL_623467, EPI_ISL_623469, EPI_ISL_623470, EPI_ISL_623471, EPI_ISL_623472, EPI_ISL_623473			
see above	Lighthouse Lab in Milton Keynes	Wellcome Sanger Institute for the COVID-19 Genomics UK (COG-UK) consortium	The Lighthouse Lab in Milton Keynes and Alex Alderton, Roberto Amato, Sonia Goncalves, Ewan Harrison, David K. Jackson, Ian Johnston, Dominic Kwiatkowski, Cordelia Langford, John Sillitoe on behalf of the Wellcome Sanger Institute COVID-19 Surveillance Team (http://www.sanger.ac.uk/covid-team)
EPI_ISL_623474	Lighthouse Lab in Cambridge	Wellcome Sanger Institute for the COVID-19 Genomics UK (COG-UK) consortium	Rob Howes, The Lighthouse Lab in Cambridge and Alex Alderton, Roberto Amato, Sonia Goncalves, Ewan Harrison, David K. Jackson, Ian Johnston, Dominic Kwiatkowski, Cordelia Langford, John Sillitoe on behalf of the Wellcome Sanger Institute COVID-19 Surveillance Team (http://www.sanger.ac.uk/covid-team)
EPI_ISL_623476, EPI_ISL_623478, EPI_ISL_623479, EPI_ISL_623480, EPI_ISL_623481	Lighthouse Lab in Milton Keynes	Wellcome Sanger Institute for the COVID-19 Genomics UK (COG-UK) consortium	The Lighthouse Lab in Milton Keynes and Alex Alderton, Roberto Amato, Sonia Goncalves, Ewan Harrison, David K. Jackson, Ian Johnston, Dominic Kwiatkowski, Cordelia Langford, John Sillitoe on behalf of the Wellcome Sanger Institute COVID-19 Surveillance Team (http://www.sanger.ac.uk/covid-team)
EPI_ISL_623482	Lighthouse Lab in Cambridge	Wellcome Sanger Institute for the COVID-19 Genomics UK (COG-UK) consortium	Rob Howes, The Lighthouse Lab in Cambridge and Alex Alderton, Roberto Amato, Sonia Goncalves, Ewan Harrison, David K. Jackson, Ian Johnston, Dominic Kwiatkowski, Cordelia Langford, John Sillitoe on behalf of the Wellcome Sanger Institute COVID-19 Surveillance Team (http://www.sanger.ac.uk/covid-team)
EPI_ISL_623483	Lighthouse Lab in Milton Keynes	Wellcome Sanger Institute for the COVID-19 Genomics UK (COG-UK) consortium	The Lighthouse Lab in Milton Keynes and Alex Alderton, Roberto Amato, Sonia Goncalves, Ewan Harrison, David K. Jackson, Ian Johnston, Dominic Kwiatkowski, Cordelia Langford, John Sillitoe on behalf of the Wellcome Sanger Institute COVID-19 Surveillance Team (http://www.sanger.ac.uk/covid-team)

EPI_ISL_623484	Lighthouse Lab in Milton Keynes	Wellcome Sanger Institute for the COVID-19 Genomics UK (COG-UK) Consortium	The Lighthouse Lab in Milton Keynes and Alex Alderton, Roberto Amato, Sonia Goncalves, Ewan Harrison, David K. Jackson, Ian Johnston, Dominic Kwiatkowski, Cordelia Langford, John Sillitoe on behalf of the Wellcome Sanger Institute COVID-19 Surveillance Team (http://www.sanger.ac.uk/covid-team)
EPI_ISL_623485	Lighthouse Lab in Cambridge	Wellcome Sanger Institute for the COVID-19 Genomics UK (COG-UK) consortium	Rob Howes, The Lighthouse Lab in Cambridge and Alex Alderton, Roberto Amato, Sonia Goncalves, Ewan Harrison, David K. Jackson, Ian Johnston, Dominic Kwiatkowski, Cordelia Langford, John Sillitoe on behalf of the Wellcome Sanger Institute COVID-19 Surveillance Team (http://www.sanger.ac.uk/covid-team)
EPI_ISL_623487	Lighthouse Lab in Milton Keynes	Wellcome Sanger Institute for the COVID-19 Genomics UK (COG-UK) consortium	The Lighthouse Lab in Milton Keynes and Alex Alderton, Roberto Amato, Sonia Goncalves, Ewan Harrison, David K. Jackson, Ian Johnston, Dominic Kwiatkowski, Cordelia Langford, John Sillitoe on behalf of the Wellcome Sanger Institute COVID-19 Surveillance Team (http://www.sanger.ac.uk/covid-team)
EPI_ISL_623488, EPI_ISL_623489	Lighthouse Lab in Cambridge	Wellcome Sanger Institute for the COVID-19 Genomics UK (COG-UK) consortium	Rob Howes, The Lighthouse Lab in Cambridge and Alex Alderton, Roberto Amato, Sonia Goncalves, Ewan Harrison, David K. Jackson, Ian Johnston, Dominic Kwiatkowski, Cordelia Langford, John Sillitoe on behalf of the Wellcome Sanger Institute COVID-19 Surveillance Team (http://www.sanger.ac.uk/covid-team)
EPI_ISL_623491, EPI_ISL_623492	Lighthouse Lab in Milton Keynes	Wellcome Sanger Institute for the COVID-19 Genomics UK (COG-UK) consortium	The Lighthouse Lab in Milton Keynes and Alex Alderton, Roberto Amato, Sonia Goncalves, Ewan Harrison, David K. Jackson, Ian Johnston, Dominic Kwiatkowski, Cordelia Langford, John Sillitoe on behalf of the Wellcome Sanger Institute COVID-19 Surveillance Team (http://www.sanger.ac.uk/covid-team)
EPI_ISL_623494	Lighthouse Lab in Cambridge	Wellcome Sanger Institute for the COVID-19 Genomics UK (COG-UK) consortium	Rob Howes, The Lighthouse Lab in Cambridge and Alex Alderton, Roberto Amato, Sonia Goncalves, Ewan Harrison, David K. Jackson, Ian Johnston, Dominic Kwiatkowski, Cordelia Langford, John Sillitoe on behalf of the Wellcome Sanger Institute COVID-19 Surveillance Team (http://www.sanger.ac.uk/covid-team)
EPI_ISL_623497, EPI_ISL_623498, EPI_ISL_623499, EPI_ISL_623500, EPI_ISL_623501, EPI_ISL_623502, EPI_ISL_623503, EPI_ISL_623504, EPI_ISL_623505, EPI_ISL_623506, EPI_ISL_623507, EPI_ISL_623508, EPI_ISL_623509, EPI_ISL_623510, EPI_ISL_623511, EPI_ISL_623513, EPI_ISL_623514			
see above	Lighthouse Lab in Milton Keynes	Wellcome Sanger Institute for the COVID-19 Genomics UK (COG-UK) consortium	The Lighthouse Lab in Milton Keynes and Alex Alderton, Roberto Amato, Sonia Goncalves, Ewan Harrison, David K. Jackson, Ian Johnston, Dominic Kwiatkowski, Cordelia Langford, John Sillitoe on behalf of the Wellcome Sanger Institute COVID-19 Surveillance Team (http://www.sanger.ac.uk/covid-team)
EPI_ISL_623515	Lighthouse Lab in Milton Keynes	Wellcome Sanger Institute for the COVID-19 Genomics UK (COG-UK) Consortium	The Lighthouse Lab in Milton Keynes and Alex Alderton, Roberto Amato, Sonia Goncalves, Ewan Harrison, David K. Jackson, Ian Johnston, Dominic Kwiatkowski, Cordelia Langford, John Sillitoe on behalf of the Wellcome Sanger Institute COVID-19 Surveillance Team (http://www.sanger.ac.uk/covid-team)
EPI_ISL_623516, EPI_ISL_623517, EPI_ISL_623518, EPI_ISL_623519	Lighthouse Lab in Milton Keynes	Wellcome Sanger Institute for the COVID-19 Genomics UK (COG-UK) consortium	The Lighthouse Lab in Milton Keynes and Alex Alderton, Roberto Amato, Sonia Goncalves, Ewan Harrison, David K. Jackson, Ian Johnston, Dominic Kwiatkowski, Cordelia Langford, John Sillitoe on behalf of the Wellcome Sanger Institute COVID-19 Surveillance Team (http://www.sanger.ac.uk/covid-team)
EPI_ISL_623520	Lighthouse Lab in Cambridge	Wellcome Sanger Institute for the COVID-19 Genomics UK (COG-UK) consortium	Rob Howes, The Lighthouse Lab in Cambridge and Alex Alderton, Roberto Amato, Sonia Goncalves, Ewan Harrison, David K. Jackson, Ian Johnston, Dominic Kwiatkowski, Cordelia Langford, John Sillitoe on behalf of the Wellcome Sanger Institute COVID-19 Surveillance Team (http://www.sanger.ac.uk/covid-team)
EPI_ISL_623521	Lighthouse Lab in Milton Keynes	Wellcome Sanger Institute for the COVID-19 Genomics UK (COG-UK) consortium	The Lighthouse Lab in Milton Keynes and Alex Alderton, Roberto Amato, Sonia Goncalves, Ewan Harrison, David K. Jackson, Ian Johnston, Dominic Kwiatkowski, Cordelia Langford, John Sillitoe on behalf of the Wellcome Sanger Institute COVID-19 Surveillance Team (http://www.sanger.ac.uk/covid-team)
EPI_ISL_623522	Lighthouse Lab in Milton Keynes	Wellcome Sanger Institute for the COVID-19 Genomics UK (COG-UK) Consortium	The Lighthouse Lab in Milton Keynes and Alex Alderton, Roberto Amato, Sonia Goncalves, Ewan Harrison, David K. Jackson, Ian Johnston, Dominic Kwiatkowski, Cordelia Langford, John Sillitoe on behalf of the Wellcome Sanger Institute COVID-19 Surveillance Team (http://www.sanger.ac.uk/covid-team)
EPI_ISL_623523, EPI_ISL_623524, EPI_ISL_623526	Lighthouse Lab in Milton Keynes	Wellcome Sanger Institute for the COVID-19 Genomics UK (COG-UK) consortium	The Lighthouse Lab in Milton Keynes and Alex Alderton, Roberto Amato, Sonia Goncalves, Ewan Harrison, David K. Jackson, Ian Johnston, Dominic Kwiatkowski, Cordelia Langford, John Sillitoe on behalf of the Wellcome Sanger Institute COVID-19 Surveillance Team (http://www.sanger.ac.uk/covid-team)
EPI_ISL_623527	Lighthouse Lab in Cambridge	Wellcome Sanger Institute for the COVID-19 Genomics UK (COG-UK) consortium	Rob Howes, The Lighthouse Lab in Cambridge and Alex Alderton, Roberto Amato, Sonia Goncalves, Ewan Harrison, David K. Jackson, Ian Johnston, Dominic Kwiatkowski, Cordelia Langford, John Sillitoe on behalf of the Wellcome Sanger Institute COVID-19 Surveillance Team (http://www.sanger.ac.uk/covid-team)
EPI_ISL_623530, EPI_ISL_623533, EPI_ISL_623534, EPI_ISL_623535, EPI_ISL_623536	Lighthouse Lab in Milton Keynes	Wellcome Sanger Institute for the COVID-19 Genomics UK (COG-UK) consortium	The Lighthouse Lab in Milton Keynes and Alex Alderton, Roberto Amato, Sonia Goncalves, Ewan Harrison, David K. Jackson, Ian Johnston, Dominic Kwiatkowski, Cordelia Langford, John Sillitoe on behalf of the Wellcome Sanger Institute COVID-19 Surveillance Team (http://www.sanger.ac.uk/covid-team)
EPI_ISL_623537	Lighthouse Lab in Cambridge	Wellcome Sanger Institute for the COVID-19 Genomics UK (COG-UK) consortium	Rob Howes, The Lighthouse Lab in Cambridge and Alex Alderton, Roberto Amato, Sonia Goncalves, Ewan Harrison, David K. Jackson, Ian Johnston, Dominic Kwiatkowski, Cordelia Langford, John Sillitoe on behalf of the Wellcome Sanger Institute COVID-19 Surveillance Team (http://www.sanger.ac.uk/covid-team)
EPI_ISL_623538	Lighthouse Lab in Milton Keynes	Wellcome Sanger Institute for the COVID-19 Genomics UK (COG-UK) consortium	The Lighthouse Lab in Milton Keynes and Alex Alderton, Roberto Amato, Sonia Goncalves, Ewan Harrison, David K. Jackson, Ian Johnston, Dominic Kwiatkowski, Cordelia Langford, John Sillitoe on behalf of the Wellcome Sanger Institute COVID-19 Surveillance Team (http://www.sanger.ac.uk/covid-team)
EPI_ISL_623539	Lighthouse Lab in Cambridge	Wellcome Sanger Institute for the COVID-19 Genomics UK (COG-UK) consortium	Rob Howes, The Lighthouse Lab in Cambridge and Alex Alderton, Roberto Amato, Sonia Goncalves, Ewan Harrison, David K. Jackson, Ian Johnston, Dominic Kwiatkowski, Cordelia Langford, John Sillitoe on behalf of the Wellcome Sanger Institute COVID-19 Surveillance Team (http://www.sanger.ac.uk/covid-team)
EPI_ISL_623540, EPI_ISL_623541, EPI_ISL_623543	Lighthouse Lab in Milton Keynes	Wellcome Sanger Institute for the COVID-19 Genomics UK (COG-UK) consortium	The Lighthouse Lab in Milton Keynes and Alex Alderton, Roberto Amato, Sonia Goncalves, Ewan Harrison, David K. Jackson, Ian Johnston, Dominic Kwiatkowski, Cordelia Langford, John Sillitoe on behalf of the Wellcome Sanger Institute COVID-19 Surveillance Team (http://www.sanger.ac.uk/covid-team)
EPI_ISL_623545	Lighthouse Lab in Cambridge	Wellcome Sanger Institute for the COVID-19 Genomics UK (COG-UK) consortium	Rob Howes, The Lighthouse Lab in Cambridge and Alex Alderton, Roberto Amato, Sonia Goncalves, Ewan Harrison, David K. Jackson, Ian Johnston, Dominic Kwiatkowski, Cordelia Langford, John Sillitoe on behalf of the Wellcome Sanger Institute COVID-19 Surveillance Team (http://www.sanger.ac.uk/covid-team)
EPI_ISL_623546, EPI_ISL_623549, EPI_ISL_623552, EPI_ISL_623554	Lighthouse Lab in Milton Keynes	Wellcome Sanger Institute for the COVID-19 Genomics UK (COG-UK) consortium	The Lighthouse Lab in Milton Keynes and Alex Alderton, Roberto Amato, Sonia Goncalves, Ewan Harrison, David K. Jackson, Ian Johnston, Dominic Kwiatkowski, Cordelia Langford, John Sillitoe on behalf of the Wellcome Sanger Institute COVID-19 Surveillance Team (http://www.sanger.ac.uk/covid-team)
EPI_ISL_623558	Lighthouse Lab in Cambridge	Wellcome Sanger Institute for the COVID-19 Genomics UK (COG-UK) consortium	Rob Howes, The Lighthouse Lab in Cambridge and Alex Alderton, Roberto Amato, Sonia Goncalves, Ewan Harrison, David K. Jackson, Ian Johnston, Dominic Kwiatkowski, Cordelia Langford, John Sillitoe on behalf of the Wellcome Sanger Institute COVID-19 Surveillance Team (http://www.sanger.ac.uk/covid-team)
EPI_ISL_623561, EPI_ISL_623563, EPI_ISL_623564, EPI_ISL_623565, EPI_ISL_623566, EPI_ISL_623571, EPI_ISL_623573, EPI_ISL_623577, EPI_ISL_623579, EPI_ISL_623580, EPI_ISL_623582, EPI_ISL_623583, EPI_ISL_623586, EPI_ISL_623588, EPI_ISL_623589, EPI_ISL_623590, EPI_ISL_623594, EPI_ISL_623596, EPI_ISL_623598, EPI_ISL_623599			
see above	Lighthouse Lab in Milton Keynes	Wellcome Sanger Institute for the COVID-19 Genomics UK (COG-UK) consortium	The Lighthouse Lab in Milton Keynes and Alex Alderton, Roberto Amato, Sonia Goncalves, Ewan Harrison, David K. Jackson, Ian Johnston, Dominic Kwiatkowski, Cordelia Langford, John Sillitoe on behalf of the Wellcome Sanger Institute COVID-19 Surveillance Team (http://www.sanger.ac.uk/covid-team)
EPI_ISL_623602	Lighthouse Lab in Milton Keynes	Wellcome Sanger Institute for the COVID-19 Genomics UK (COG-UK) Consortium	The Lighthouse Lab in Milton Keynes and Alex Alderton, Roberto Amato, Sonia Goncalves, Ewan Harrison, David K. Jackson, Ian Johnston, Dominic Kwiatkowski, Cordelia Langford, John Sillitoe on behalf of the Wellcome Sanger Institute COVID-19 Surveillance Team (http://www.sanger.ac.uk/covid-team)
EPI_ISL_623603, EPI_ISL_623604, EPI_ISL_623606, EPI_ISL_623607	Lighthouse Lab in Milton Keynes	Wellcome Sanger Institute for the COVID-19 Genomics UK (COG-UK) consortium	The Lighthouse Lab in Milton Keynes and Alex Alderton, Roberto Amato, Sonia Goncalves, Ewan Harrison, David K. Jackson, Ian Johnston, Dominic Kwiatkowski, Cordelia Langford, John Sillitoe on behalf of the Wellcome Sanger Institute COVID-19 Surveillance Team (http://www.sanger.ac.uk/covid-team)
EPI_ISL_623609	Lighthouse Lab in Cambridge	Wellcome Sanger Institute for the COVID-19 Genomics UK (COG-UK) consortium	Rob Howes, The Lighthouse Lab in Cambridge and Alex Alderton, Roberto Amato, Sonia Goncalves, Ewan Harrison, David K. Jackson, Ian Johnston, Dominic Kwiatkowski, Cordelia Langford, John Sillitoe on behalf of the Wellcome Sanger Institute COVID-19 Surveillance Team (http://www.sanger.ac.uk/covid-team)
EPI_ISL_623610, EPI_ISL_623614, EPI_ISL_623615, EPI_ISL_623618, EPI_ISL_623621, EPI_ISL_623622, EPI_ISL_623623, EPI_ISL_623624, EPI_ISL_623625, EPI_ISL_623627, EPI_ISL_623628, EPI_ISL_623629, EPI_ISL_623631, EPI_ISL_623632, EPI_ISL_623634, EPI_ISL_623636, EPI_ISL_623637, EPI_ISL_623638			
see above	Lighthouse Lab in Milton Keynes	Wellcome Sanger Institute for the COVID-19 Genomics UK (COG-UK) consortium	The Lighthouse Lab in Milton Keynes and Alex Alderton, Roberto Amato, Sonia Goncalves, Ewan Harrison, David K. Jackson, Ian Johnston, Dominic Kwiatkowski, Cordelia Langford, John Sillitoe on behalf of the Wellcome Sanger Institute COVID-19 Surveillance Team (http://www.sanger.ac.uk/covid-team)
EPI_ISL_623639	Lighthouse Lab in Cambridge	Wellcome Sanger Institute for the COVID-19 Genomics UK (COG-UK) consortium	Rob Howes, The Lighthouse Lab in Cambridge and Alex Alderton, Roberto Amato, Sonia Goncalves, Ewan Harrison, David K. Jackson, Ian Johnston, Dominic Kwiatkowski, Cordelia Langford, John Sillitoe on behalf of the Wellcome Sanger Institute COVID-19 Surveillance Team (http://www.sanger.ac.uk/covid-team)

EPI_ISL_623642, EPI_ISL_623646, EPI_ISL_623648, EPI_ISL_623649, EPI_ISL_623656, EPI_ISL_623659, EPI_ISL_623660, EPI_ISL_623668, EPI_ISL_623669, EPI_ISL_623670, EPI_ISL_623671, EPI_ISL_623676, EPI_ISL_623678, EPI_ISL_623681, EPI_ISL_623682, EPI_ISL_623684, EPI_ISL_623685, EPI_ISL_623687, EPI_ISL_623689, EPI_ISL_623692			
see above	Lighthouse Lab in Milton Keynes	Wellcome Sanger Institute for the COVID-19 Genomics UK (COG-UK) consortium	The Lighthouse Lab in Milton Keynes and Alex Alderton, Roberto Amato, Sonia Goncalves, Ewan Harrison, David K. Jackson, Ian Johnston, Dominic Kwiatkowski, Cordelia Langford, John Sillitoe on behalf of the Wellcome Sanger Institute COVID-19 Surveillance Team (http://www.sanger.ac.uk/covid-team)
EPI_ISL_623694	Lighthouse Lab in Cambridge	Wellcome Sanger Institute for the COVID-19 Genomics UK (COG-UK) consortium	Rob Howes, The Lighthouse Lab in Cambridge and Alex Alderton, Roberto Amato, Sonia Goncalves, Ewan Harrison, David K. Jackson, Ian Johnston, Dominic Kwiatkowski, Cordelia Langford, John Sillitoe on behalf of the Wellcome Sanger Institute COVID-19 Surveillance Team (http://www.sanger.ac.uk/covid-team)
EPI_ISL_623695, EPI_ISL_623696, EPI_ISL_623698, EPI_ISL_623701, EPI_ISL_623707, EPI_ISL_623709, EPI_ISL_623711	Lighthouse Lab in Milton Keynes	Wellcome Sanger Institute for the COVID-19 Genomics UK (COG-UK) consortium	The Lighthouse Lab in Milton Keynes and Alex Alderton, Roberto Amato, Sonia Goncalves, Ewan Harrison, David K. Jackson, Ian Johnston, Dominic Kwiatkowski, Cordelia Langford, John Sillitoe on behalf of the Wellcome Sanger Institute COVID-19 Surveillance Team (http://www.sanger.ac.uk/covid-team)
EPI_ISL_623712	Lighthouse Lab in Cambridge	Wellcome Sanger Institute for the COVID-19 Genomics UK (COG-UK) consortium	Rob Howes, The Lighthouse Lab in Cambridge and Alex Alderton, Roberto Amato, Sonia Goncalves, Ewan Harrison, David K. Jackson, Ian Johnston, Dominic Kwiatkowski, Cordelia Langford, John Sillitoe on behalf of the Wellcome Sanger Institute COVID-19 Surveillance Team (http://www.sanger.ac.uk/covid-team)
EPI_ISL_623714, EPI_ISL_623715, EPI_ISL_623718, EPI_ISL_623719, EPI_ISL_623720, EPI_ISL_623722, EPI_ISL_623727, EPI_ISL_623728, EPI_ISL_623729, EPI_ISL_623730, EPI_ISL_623732, EPI_ISL_623734, EPI_ISL_623737, EPI_ISL_623738, EPI_ISL_623739, EPI_ISL_623744, EPI_ISL_623745, EPI_ISL_623748, EPI_ISL_623753, EPI_ISL_623755, EPI_ISL_623756, EPI_ISL_623757, EPI_ISL_623760, EPI_ISL_623761, EPI_ISL_623766, EPI_ISL_623767, EPI_ISL_623769, EPI_ISL_623771, EPI_ISL_623778, EPI_ISL_623779, EPI_ISL_623780, EPI_ISL_623782, EPI_ISL_623783, EPI_ISL_623784, EPI_ISL_623786, EPI_ISL_623787			
see above	Lighthouse Lab in Milton Keynes	Wellcome Sanger Institute for the COVID-19 Genomics UK (COG-UK) consortium	The Lighthouse Lab in Milton Keynes and Alex Alderton, Roberto Amato, Sonia Goncalves, Ewan Harrison, David K. Jackson, Ian Johnston, Dominic Kwiatkowski, Cordelia Langford, John Sillitoe on behalf of the Wellcome Sanger Institute COVID-19 Surveillance Team (http://www.sanger.ac.uk/covid-team)
EPI_ISL_623790	Lighthouse Lab in Cambridge	Wellcome Sanger Institute for the COVID-19 Genomics UK (COG-UK) consortium	Rob Howes, The Lighthouse Lab in Cambridge and Alex Alderton, Roberto Amato, Sonia Goncalves, Ewan Harrison, David K. Jackson, Ian Johnston, Dominic Kwiatkowski, Cordelia Langford, John Sillitoe on behalf of the Wellcome Sanger Institute COVID-19 Surveillance Team (http://www.sanger.ac.uk/covid-team)
EPI_ISL_623792, EPI_ISL_623795, EPI_ISL_623801, EPI_ISL_623803, EPI_ISL_623804, EPI_ISL_623805, EPI_ISL_623807, EPI_ISL_623814, EPI_ISL_623817, EPI_ISL_623818, EPI_ISL_623819, EPI_ISL_623821, EPI_ISL_623826, EPI_ISL_623827, EPI_ISL_623830, EPI_ISL_623836, EPI_ISL_623837, EPI_ISL_623842, EPI_ISL_623853			
see above	Lighthouse Lab in Milton Keynes	Wellcome Sanger Institute for the COVID-19 Genomics UK (COG-UK) consortium	The Lighthouse Lab in Milton Keynes and Alex Alderton, Roberto Amato, Sonia Goncalves, Ewan Harrison, David K. Jackson, Ian Johnston, Dominic Kwiatkowski, Cordelia Langford, John Sillitoe on behalf of the Wellcome Sanger Institute COVID-19 Surveillance Team (http://www.sanger.ac.uk/covid-team)
EPI_ISL_623856	Lighthouse Lab in Cambridge	Wellcome Sanger Institute for the COVID-19 Genomics UK (COG-UK) consortium	Rob Howes, The Lighthouse Lab in Cambridge and Alex Alderton, Roberto Amato, Sonia Goncalves, Ewan Harrison, David K. Jackson, Ian Johnston, Dominic Kwiatkowski, Cordelia Langford, John Sillitoe on behalf of the Wellcome Sanger Institute COVID-19 Surveillance Team (http://www.sanger.ac.uk/covid-team)
EPI_ISL_623862	Lighthouse Lab in Alderley Park	Wellcome Sanger Institute for the COVID-19 Genomics UK (COG-UK) consortium	Jacquelyn Wynn, Mairead Hyland, The Lighthouse Lab in Alderley Park and Alex Alderton, Roberto Amato, Sonia Goncalves, Ewan Harrison, David K. Jackson, Ian Johnston, Dominic Kwiatkowski, Cordelia Langford, John Sillitoe on behalf of the Wellcome Sanger Institute COVID-19 Surveillance Team (http://www.sanger.ac.uk/covid-team)
EPI_ISL_623863, EPI_ISL_623873	Lighthouse Lab in Glasgow	Wellcome Sanger Institute for the COVID-19 Genomics UK (COG-UK) consortium	Harper VanSteenhouse, Yumi Kasai, David Gray, Carol Clugston, Anna Dominiczak and Alex Alderton, Roberto Amato, Sonia Goncalves, Ewan Harrison, David K. Jackson, Ian Johnston, Dominic Kwiatkowski, Cordelia Langford, John Sillitoe on behalf of the Wellcome Sanger Institute COVID-19 Surveillance Team (http://www.sanger.ac.uk/covid-team)
EPI_ISL_623879	Lighthouse Lab in Alderley Park	Wellcome Sanger Institute for the COVID-19 Genomics UK (COG-UK) consortium	Jacquelyn Wynn, Mairead Hyland, The Lighthouse Lab in Alderley Park and Alex Alderton, Roberto Amato, Sonia Goncalves, Ewan Harrison, David K. Jackson, Ian Johnston, Dominic Kwiatkowski, Cordelia Langford, John Sillitoe on behalf of the Wellcome Sanger Institute COVID-19 Surveillance Team (http://www.sanger.ac.uk/covid-team)
EPI_ISL_623908	Lighthouse Lab in Glasgow	Wellcome Sanger Institute for the COVID-19 Genomics UK (COG-UK) consortium	Harper VanSteenhouse, Yumi Kasai, David Gray, Carol Clugston, Anna Dominiczak and Alex Alderton, Roberto Amato, Sonia Goncalves, Ewan Harrison, David K. Jackson, Ian Johnston, Dominic Kwiatkowski, Cordelia Langford, John Sillitoe on behalf of the Wellcome Sanger Institute COVID-19 Surveillance Team (http://www.sanger.ac.uk/covid-team)
EPI_ISL_623910	Lighthouse Lab in Alderley Park	Wellcome Sanger Institute for the COVID-19 Genomics UK (COG-UK) consortium	Jacquelyn Wynn, Mairead Hyland, The Lighthouse Lab in Alderley Park and Alex Alderton, Roberto Amato, Sonia Goncalves, Ewan Harrison, David K. Jackson, Ian Johnston, Dominic Kwiatkowski, Cordelia Langford, John Sillitoe on behalf of the Wellcome Sanger Institute COVID-19 Surveillance Team (http://www.sanger.ac.uk/covid-team)
EPI_ISL_623911	Lighthouse Lab in Glasgow	Wellcome Sanger Institute for the COVID-19 Genomics UK (COG-UK) consortium	Harper VanSteenhouse, Yumi Kasai, David Gray, Carol Clugston, Anna Dominiczak and Alex Alderton, Roberto Amato, Sonia Goncalves, Ewan Harrison, David K. Jackson, Ian Johnston, Dominic Kwiatkowski, Cordelia Langford, John Sillitoe on behalf of the Wellcome Sanger Institute COVID-19 Surveillance Team (http://www.sanger.ac.uk/covid-team)
EPI_ISL_623918	Lighthouse Lab in Alderley Park	Wellcome Sanger Institute for the COVID-19 Genomics UK (COG-UK) consortium	Jacquelyn Wynn, Mairead Hyland, The Lighthouse Lab in Alderley Park and Alex Alderton, Roberto Amato, Sonia Goncalves, Ewan Harrison, David K. Jackson, Ian Johnston, Dominic Kwiatkowski, Cordelia Langford, John Sillitoe on behalf of the Wellcome Sanger Institute COVID-19 Surveillance Team (http://www.sanger.ac.uk/covid-team)
EPI_ISL_623924	Lighthouse Lab in Glasgow	Wellcome Sanger Institute for the COVID-19 Genomics UK (COG-UK) consortium	Harper VanSteenhouse, Yumi Kasai, David Gray, Carol Clugston, Anna Dominiczak and Alex Alderton, Roberto Amato, Sonia Goncalves, Ewan Harrison, David K. Jackson, Ian Johnston, Dominic Kwiatkowski, Cordelia Langford, John Sillitoe on behalf of the Wellcome Sanger Institute COVID-19 Surveillance Team (http://www.sanger.ac.uk/covid-team)
EPI_ISL_623925, EPI_ISL_623935, EPI_ISL_623939	Lighthouse Lab in Alderley Park	Wellcome Sanger Institute for the COVID-19 Genomics UK (COG-UK) consortium	Jacquelyn Wynn, Mairead Hyland, The Lighthouse Lab in Alderley Park and Alex Alderton, Roberto Amato, Sonia Goncalves, Ewan Harrison, David K. Jackson, Ian Johnston, Dominic Kwiatkowski, Cordelia Langford, John Sillitoe on behalf of the Wellcome Sanger Institute COVID-19 Surveillance Team (http://www.sanger.ac.uk/covid-team)
EPI_ISL_623942, EPI_ISL_623954	Lighthouse Lab in Glasgow	Wellcome Sanger Institute for the COVID-19 Genomics UK (COG-UK) consortium	Harper VanSteenhouse, Yumi Kasai, David Gray, Carol Clugston, Anna Dominiczak and Alex Alderton, Roberto Amato, Sonia Goncalves, Ewan Harrison, David K. Jackson, Ian Johnston, Dominic Kwiatkowski, Cordelia Langford, John Sillitoe on behalf of the Wellcome Sanger Institute COVID-19 Surveillance Team (http://www.sanger.ac.uk/covid-team)
EPI_ISL_623955	Lighthouse Lab in Alderley Park	Wellcome Sanger Institute for the COVID-19 Genomics UK (COG-UK) consortium	Jacquelyn Wynn, Mairead Hyland, The Lighthouse Lab in Alderley Park and Alex Alderton, Roberto Amato, Sonia Goncalves, Ewan Harrison, David K. Jackson, Ian Johnston, Dominic Kwiatkowski, Cordelia Langford, John Sillitoe on behalf of the Wellcome Sanger Institute COVID-19 Surveillance Team (http://www.sanger.ac.uk/covid-team)
EPI_ISL_623956	Lighthouse Lab in Glasgow	Wellcome Sanger Institute for the COVID-19 Genomics UK (COG-UK) consortium	Harper VanSteenhouse, Yumi Kasai, David Gray, Carol Clugston, Anna Dominiczak and Alex Alderton, Roberto Amato, Sonia Goncalves, Ewan Harrison, David K. Jackson, Ian Johnston, Dominic Kwiatkowski, Cordelia Langford, John Sillitoe on behalf of the Wellcome Sanger Institute COVID-19 Surveillance Team (http://www.sanger.ac.uk/covid-team)
EPI_ISL_623965, EPI_ISL_623971, EPI_ISL_623981	Lighthouse Lab in Alderley Park	Wellcome Sanger Institute for the COVID-19 Genomics UK (COG-UK) consortium	Jacquelyn Wynn, Mairead Hyland, The Lighthouse Lab in Alderley Park and Alex Alderton, Roberto Amato, Sonia Goncalves, Ewan Harrison, David K. Jackson, Ian Johnston, Dominic Kwiatkowski, Cordelia Langford, John Sillitoe on behalf of the Wellcome Sanger Institute COVID-19 Surveillance Team (http://www.sanger.ac.uk/covid-team)
EPI_ISL_623991	Lighthouse Lab in Glasgow	Wellcome Sanger Institute for the COVID-19 Genomics UK (COG-UK) consortium	Harper VanSteenhouse, Yumi Kasai, David Gray, Carol Clugston, Anna Dominiczak and Alex Alderton, Roberto Amato, Sonia Goncalves, Ewan Harrison, David K. Jackson, Ian Johnston, Dominic Kwiatkowski, Cordelia Langford, John Sillitoe on behalf of the Wellcome Sanger Institute COVID-19 Surveillance Team (http://www.sanger.ac.uk/covid-team)
EPI_ISL_624013	Lighthouse Lab in Alderley Park	Wellcome Sanger Institute for the COVID-19 Genomics UK (COG-UK) consortium	Jacquelyn Wynn, Mairead Hyland, The Lighthouse Lab in Alderley Park and Alex Alderton, Roberto Amato, Sonia Goncalves, Ewan Harrison, David K. Jackson, Ian Johnston, Dominic Kwiatkowski, Cordelia Langford, John Sillitoe on behalf of the Wellcome Sanger Institute COVID-19 Surveillance Team (http://www.sanger.ac.uk/covid-team)
EPI_ISL_624019, EPI_ISL_624027,	Lighthouse Lab in Glasgow	Wellcome Sanger Institute for the COVID-19 Genomics UK	Harper VanSteenhouse, Yumi Kasai, David Gray, Carol Clugston, Anna Dominiczak and Alex Alderton, Roberto Amato, Sonia Goncalves, Ewan Harrison,

EPI_ISL_624028		(COG-UK) consortium	David K. Jackson, Ian Johnston, Dominic Kwiatkowski, Cordelia Langford, John Sillitoe on behalf of the Wellcome Sanger Institute COVID-19 Surveillance Team (http://www.sanger.ac.uk/covid-team)
EPI_ISL_624037, EPI_ISL_624038, EPI_ISL_624047, EPI_ISL_624048, EPI_ISL_624059, EPI_ISL_624062, EPI_ISL_624066	Lighthouse Lab in Alderley Park	Wellcome Sanger Institute for the COVID-19 Genomics UK (COG-UK) consortium	Jacquelyn Wynn, Mairead Hyland, The Lighthouse Lab in Alderley Park and Alex Alderton, Roberto Amato, Sonia Goncalves, Ewan Harrison, David K. Jackson, Ian Johnston, Dominic Kwiatkowski, Cordelia Langford, John Sillitoe on behalf of the Wellcome Sanger Institute COVID-19 Surveillance Team (http://www.sanger.ac.uk/covid-team)
EPI_ISL_624073	Lighthouse Lab in Glasgow	Wellcome Sanger Institute for the COVID-19 Genomics UK (COG-UK) consortium	Harper VanSteenhouse, Yumi Kasai, David Gray, Carol Clugston, Anna Dominiczak and Alex Alderton, Roberto Amato, Sonia Goncalves, Ewan Harrison, David K. Jackson, Ian Johnston, Dominic Kwiatkowski, Cordelia Langford, John Sillitoe on behalf of the Wellcome Sanger Institute COVID-19 Surveillance Team (http://www.sanger.ac.uk/covid-team)
EPI_ISL_624074	Lighthouse Lab in Alderley Park	Wellcome Sanger Institute for the COVID-19 Genomics UK (COG-UK) consortium	Jacquelyn Wynn, Mairead Hyland, The Lighthouse Lab in Alderley Park and Alex Alderton, Roberto Amato, Sonia Goncalves, Ewan Harrison, David K. Jackson, Ian Johnston, Dominic Kwiatkowski, Cordelia Langford, John Sillitoe on behalf of the Wellcome Sanger Institute COVID-19 Surveillance Team (http://www.sanger.ac.uk/covid-team)
EPI_ISL_624077	Lighthouse Lab in Glasgow	Wellcome Sanger Institute for the COVID-19 Genomics UK (COG-UK) consortium	Harper VanSteenhouse, Yumi Kasai, David Gray, Carol Clugston, Anna Dominiczak and Alex Alderton, Roberto Amato, Sonia Goncalves, Ewan Harrison, David K. Jackson, Ian Johnston, Dominic Kwiatkowski, Cordelia Langford, John Sillitoe on behalf of the Wellcome Sanger Institute COVID-19 Surveillance Team (http://www.sanger.ac.uk/covid-team)
EPI_ISL_624081, EPI_ISL_624083, EPI_ISL_624106, EPI_ISL_624117, EPI_ISL_624125, EPI_ISL_624126	Lighthouse Lab in Alderley Park	Wellcome Sanger Institute for the COVID-19 Genomics UK (COG-UK) consortium	Jacquelyn Wynn, Mairead Hyland, The Lighthouse Lab in Alderley Park and Alex Alderton, Roberto Amato, Sonia Goncalves, Ewan Harrison, David K. Jackson, Ian Johnston, Dominic Kwiatkowski, Cordelia Langford, John Sillitoe on behalf of the Wellcome Sanger Institute COVID-19 Surveillance Team (http://www.sanger.ac.uk/covid-team)
EPI_ISL_624128	Lighthouse Lab in Glasgow	Wellcome Sanger Institute for the COVID-19 Genomics UK (COG-UK) consortium	Harper VanSteenhouse, Yumi Kasai, David Gray, Carol Clugston, Anna Dominiczak and Alex Alderton, Roberto Amato, Sonia Goncalves, Ewan Harrison, David K. Jackson, Ian Johnston, Dominic Kwiatkowski, Cordelia Langford, John Sillitoe on behalf of the Wellcome Sanger Institute COVID-19 Surveillance Team (http://www.sanger.ac.uk/covid-team)
EPI_ISL_624134, EPI_ISL_624136	Lighthouse Lab in Alderley Park	Wellcome Sanger Institute for the COVID-19 Genomics UK (COG-UK) consortium	Jacquelyn Wynn, Mairead Hyland, The Lighthouse Lab in Alderley Park and Alex Alderton, Roberto Amato, Sonia Goncalves, Ewan Harrison, David K. Jackson, Ian Johnston, Dominic Kwiatkowski, Cordelia Langford, John Sillitoe on behalf of the Wellcome Sanger Institute COVID-19 Surveillance Team (http://www.sanger.ac.uk/covid-team)
EPI_ISL_624147	Lighthouse Lab in Glasgow	Wellcome Sanger Institute for the COVID-19 Genomics UK (COG-UK) consortium	Harper VanSteenhouse, Yumi Kasai, David Gray, Carol Clugston, Anna Dominiczak and Alex Alderton, Roberto Amato, Sonia Goncalves, Ewan Harrison, David K. Jackson, Ian Johnston, Dominic Kwiatkowski, Cordelia Langford, John Sillitoe on behalf of the Wellcome Sanger Institute COVID-19 Surveillance Team (http://www.sanger.ac.uk/covid-team)
EPI_ISL_624153	Lighthouse Lab in Alderley Park	Wellcome Sanger Institute for the COVID-19 Genomics UK (COG-UK) consortium	Jacquelyn Wynn, Mairead Hyland, The Lighthouse Lab in Alderley Park and Alex Alderton, Roberto Amato, Sonia Goncalves, Ewan Harrison, David K. Jackson, Ian Johnston, Dominic Kwiatkowski, Cordelia Langford, John Sillitoe on behalf of the Wellcome Sanger Institute COVID-19 Surveillance Team (http://www.sanger.ac.uk/covid-team)
EPI_ISL_624158	Lighthouse Lab in Glasgow	Wellcome Sanger Institute for the COVID-19 Genomics UK (COG-UK) consortium	Harper VanSteenhouse, Yumi Kasai, David Gray, Carol Clugston, Anna Dominiczak and Alex Alderton, Roberto Amato, Sonia Goncalves, Ewan Harrison, David K. Jackson, Ian Johnston, Dominic Kwiatkowski, Cordelia Langford, John Sillitoe on behalf of the Wellcome Sanger Institute COVID-19 Surveillance Team (http://www.sanger.ac.uk/covid-team)
EPI_ISL_624161	Lighthouse Lab in Alderley Park	Wellcome Sanger Institute for the COVID-19 Genomics UK (COG-UK) consortium	Jacquelyn Wynn, Mairead Hyland, The Lighthouse Lab in Alderley Park and Alex Alderton, Roberto Amato, Sonia Goncalves, Ewan Harrison, David K. Jackson, Ian Johnston, Dominic Kwiatkowski, Cordelia Langford, John Sillitoe on behalf of the Wellcome Sanger Institute COVID-19 Surveillance Team (http://www.sanger.ac.uk/covid-team)
EPI_ISL_624163	Lighthouse Lab in Glasgow	Wellcome Sanger Institute for the COVID-19 Genomics UK (COG-UK) consortium	Harper VanSteenhouse, Yumi Kasai, David Gray, Carol Clugston, Anna Dominiczak and Alex Alderton, Roberto Amato, Sonia Goncalves, Ewan Harrison, David K. Jackson, Ian Johnston, Dominic Kwiatkowski, Cordelia Langford, John Sillitoe on behalf of the Wellcome Sanger Institute COVID-19 Surveillance Team (http://www.sanger.ac.uk/covid-team)
EPI_ISL_624166, EPI_ISL_624170, EPI_ISL_624172, EPI_ISL_624177, EPI_ISL_624179, EPI_ISL_624182	Lighthouse Lab in Alderley Park	Wellcome Sanger Institute for the COVID-19 Genomics UK (COG-UK) consortium	Jacquelyn Wynn, Mairead Hyland, The Lighthouse Lab in Alderley Park and Alex Alderton, Roberto Amato, Sonia Goncalves, Ewan Harrison, David K. Jackson, Ian Johnston, Dominic Kwiatkowski, Cordelia Langford, John Sillitoe on behalf of the Wellcome Sanger Institute COVID-19 Surveillance Team (http://www.sanger.ac.uk/covid-team)
EPI_ISL_624188, EPI_ISL_624189	Lighthouse Lab in Glasgow	Wellcome Sanger Institute for the COVID-19 Genomics UK (COG-UK) consortium	Harper VanSteenhouse, Yumi Kasai, David Gray, Carol Clugston, Anna Dominiczak and Alex Alderton, Roberto Amato, Sonia Goncalves, Ewan Harrison, David K. Jackson, Ian Johnston, Dominic Kwiatkowski, Cordelia Langford, John Sillitoe on behalf of the Wellcome Sanger Institute COVID-19 Surveillance Team (http://www.sanger.ac.uk/covid-team)
EPI_ISL_624197	Lighthouse Lab in Glasgow	Wellcome Sanger Institute for the COVID-19 Genomics UK (COG-UK) Consortium	Harper VanSteenhouse, Yumi Kasai, David Gray, Carol Clugston, Anna Dominiczak and Alex Alderton, Roberto Amato, Sonia Goncalves, Ewan Harrison, David K. Jackson, Ian Johnston, Dominic Kwiatkowski, Cordelia Langford, John Sillitoe on behalf of the Wellcome Sanger Institute COVID-19 Surveillance Team (http://www.sanger.ac.uk/covid-team)
EPI_ISL_624206, EPI_ISL_624208, EPI_ISL_624221, EPI_ISL_624230, EPI_ISL_624231, EPI_ISL_624234, EPI_ISL_624241, EPI_ISL_624243	Lighthouse Lab in Alderley Park	Wellcome Sanger Institute for the COVID-19 Genomics UK (COG-UK) consortium	Jacquelyn Wynn, Mairead Hyland, The Lighthouse Lab in Alderley Park and Alex Alderton, Roberto Amato, Sonia Goncalves, Ewan Harrison, David K. Jackson, Ian Johnston, Dominic Kwiatkowski, Cordelia Langford, John Sillitoe on behalf of the Wellcome Sanger Institute COVID-19 Surveillance Team (http://www.sanger.ac.uk/covid-team)
EPI_ISL_624244	Lighthouse Lab in Alderley Park	Wellcome Sanger Institute for the COVID-19 Genomics UK (COG-UK) Consortium	Jacquelyn Wynn, Mairead Hyland, The Lighthouse Lab in Alderley Park and Alex Alderton, Roberto Amato, Sonia Goncalves, Ewan Harrison, David K. Jackson, Ian Johnston, Dominic Kwiatkowski, Cordelia Langford, John Sillitoe on behalf of the Wellcome Sanger Institute COVID-19 Surveillance Team (http://www.sanger.ac.uk/covid-team)
EPI_ISL_624256, EPI_ISL_624261, EPI_ISL_624268, EPI_ISL_624278, EPI_ISL_624280, EPI_ISL_624284, EPI_ISL_624290, EPI_ISL_624295, EPI_ISL_624316, EPI_ISL_624319, EPI_ISL_624323, EPI_ISL_624328, EPI_ISL_624329, EPI_ISL_624334, EPI_ISL_624337, EPI_ISL_624344, EPI_ISL_624348, EPI_ISL_624352, EPI_ISL_624358, EPI_ISL_624359, EPI_ISL_624362, EPI_ISL_624364, EPI_ISL_624370, EPI_ISL_624372, EPI_ISL_624381, EPI_ISL_624385, EPI_ISL_624386, EPI_ISL_624397, EPI_ISL_624399, EPI_ISL_624403, EPI_ISL_624405, EPI_ISL_624416, EPI_ISL_624426, EPI_ISL_624427, EPI_ISL_624431, EPI_ISL_624439, EPI_ISL_624443, EPI_ISL_624447, EPI_ISL_624451, EPI_ISL_624465, EPI_ISL_624472, EPI_ISL_624473, EPI_ISL_624476, EPI_ISL_624479, EPI_ISL_624484, EPI_ISL_624488, EPI_ISL_624489, EPI_ISL_624492, EPI_ISL_624493, EPI_ISL_624501, EPI_ISL_624504, EPI_ISL_624509, EPI_ISL_624515, EPI_ISL_624517, EPI_ISL_624521, EPI_ISL_624522, EPI_ISL_624523, EPI_ISL_624536, EPI_ISL_624540, EPI_ISL_624548, EPI_ISL_624554, EPI_ISL_624559, EPI_ISL_624560, EPI_ISL_624561, EPI_ISL_624562, EPI_ISL_624563, EPI_ISL_624564, EPI_ISL_624565, EPI_ISL_624566, EPI_ISL_624567, EPI_ISL_624568, EPI_ISL_624569, EPI_ISL_624570, EPI_ISL_624571, EPI_ISL_624572, EPI_ISL_624573, EPI_ISL_624574, EPI_ISL_624575, EPI_ISL_624576, EPI_ISL_624577, EPI_ISL_624578, EPI_ISL_624579, EPI_ISL_624580, EPI_ISL_624581, EPI_ISL_624582, EPI_ISL_624583, EPI_ISL_624584, EPI_ISL_624585, EPI_ISL_624586, EPI_ISL_624587, EPI_ISL_624588, EPI_ISL_624589, EPI_ISL_624590, EPI_ISL_624591, EPI_ISL_624592, EPI_ISL_624593, EPI_ISL_624594, EPI_ISL_624595, EPI_ISL_624596, EPI_ISL_624597, EPI_ISL_624598, EPI_ISL_624599, EPI_ISL_624600, EPI_ISL_624601, EPI_ISL_624602, EPI_ISL_624603, EPI_ISL_624604, EPI_ISL_624605, EPI_ISL_624606, EPI_ISL_624607, EPI_ISL_624608, EPI_ISL_624609, EPI_ISL_624610, EPI_ISL_624611, EPI_ISL_624612, EPI_ISL_624613, EPI_ISL_624614, EPI_ISL_624615, EPI_ISL_624616, EPI_ISL_624617, EPI_ISL_624618, EPI_ISL_624619, EPI_ISL_624620, EPI_ISL_624621, EPI_ISL_624622, EPI_ISL_624623, EPI_ISL_624624, EPI_ISL_624625, EPI_ISL_624626, EPI_ISL_624627, EPI_ISL_624628, EPI_ISL_624629, EPI_ISL_624630, EPI_ISL_624631, EPI_ISL_624632, EPI_ISL_624633, EPI_ISL_624634, EPI_ISL_624635, EPI_ISL_624636, EPI_ISL_624637, EPI_ISL_624638, EPI_ISL_624639, EPI_ISL_624640, EPI_ISL_624641, EPI_ISL_624642, EPI_ISL_624643, EPI_ISL_624644, EPI_ISL_624645, EPI_ISL_624646, EPI_ISL_624647, EPI_ISL_624648, EPI_ISL_624649, EPI_ISL_624650, EPI_ISL_624651, EPI_ISL_624652, EPI_ISL_624653, EPI_ISL_624654, EPI_ISL_624655, EPI_ISL_624656, EPI_ISL_624657, EPI_ISL_624658, EPI_ISL_624659, EPI_ISL_624660, EPI_ISL_624661, EPI_ISL_624662, EPI_ISL_624663, EPI_ISL_624664, EPI_ISL_624665, EPI_ISL_624666, EPI_ISL_624667, EPI_ISL_624668, EPI_ISL_624669, EPI_ISL_624670, EPI_ISL_624671, EPI_ISL_624672, EPI_ISL_624673, EPI_ISL_624674, EPI_ISL_624675, EPI_ISL_624676, EPI_ISL_624677, EPI_ISL_624678, EPI_ISL_624679, EPI_ISL_624680, EPI_ISL_624681, EPI_ISL_624682, EPI_ISL_624683, EPI_ISL_624684, EPI_ISL_624685, EPI_ISL_624686, EPI_ISL_624687, EPI_ISL_624688, EPI_ISL_624689, EPI_ISL_624690, EPI_ISL_624691, EPI_ISL_624692, EPI_ISL_624693, EPI_ISL_624694, EPI_ISL_624695, EPI_ISL_624696, EPI_ISL_624697, EPI_ISL_624698, EPI_ISL_624699, EPI_ISL_624700, EPI_ISL_624701, EPI_ISL_624702, EPI_ISL_624703, EPI_ISL_624704, EPI_ISL_624705, EPI_ISL_624706, EPI_ISL_624707, EPI_ISL_624708, EPI_ISL_624709, EPI_ISL_624710, EPI_ISL_624711, EPI_ISL_624712, EPI_ISL_624713, EPI_ISL_624714, EPI_ISL_624715, EPI_ISL_624716, EPI_ISL_624717, EPI_ISL_624718, EPI_ISL_624719, EPI_ISL_624720, EPI_ISL_624721, EPI_ISL_624722, EPI_ISL_624723, EPI_ISL_624724, EPI_ISL_624725, EPI_ISL_624726, EPI_ISL_624727, EPI_ISL_624728, EPI_ISL_624729, EPI_ISL_624730, EPI_ISL_624731, EPI_ISL_624732, EPI_ISL_624733, EPI_ISL_624734, EPI_ISL_624735, EPI_ISL_624736, EPI_ISL_624737, EPI_ISL_624738, EPI_ISL_624739, EPI_ISL_624740, EPI_ISL_624741, EPI_ISL_624742, EPI_ISL_624743, EPI_ISL_624744, EPI_ISL_624745, EPI_ISL_624746, EPI_ISL_624747, EPI_ISL_624748, EPI_ISL_624749, EPI_ISL_624750, EPI_ISL_624751, EPI_ISL_624752, EPI_ISL_624753, EPI_ISL_624754, EPI_ISL_624755, EPI_ISL_624756, EPI_ISL_624757, EPI_ISL_624758, EPI_ISL_624759, EPI_ISL_624760, EPI_ISL_624761, EPI_ISL_624762, EPI_ISL_624763, EPI_ISL_624764, EPI_ISL_624765, EPI_ISL_624766, EPI_ISL_624767, EPI_ISL_624768, EPI_ISL_624769, EPI_ISL_624770, EPI_ISL_624771, EPI_ISL_624772, EPI_ISL_624773, EPI_ISL_624774, EPI_ISL_624775, EPI_ISL_624776, EPI_ISL_624777, EPI_ISL_624778, EPI_ISL_624779, EPI_ISL_624780, EPI_ISL_624781, EPI_ISL_624782, EPI_ISL_624783, EPI_ISL_624784, EPI_ISL_624785, EPI_ISL_624786, EPI_ISL_624787, EPI_ISL_624788, EPI_ISL_624789, EPI_ISL_624790, EPI_ISL_624791, EPI_ISL_624792, EPI_ISL_624793, EPI_ISL_624794, EPI_ISL_624795, EPI_ISL_624796, EPI_ISL_624797, EPI_ISL_624798, EPI_ISL_624799, EPI_ISL_624800, EPI_ISL_624801, EPI_ISL_624802, EPI_ISL_624803, EPI_ISL_624804, EPI_ISL_624805, EPI_ISL_624806, EPI_ISL_624807, EPI_ISL_624808, EPI_ISL_624809, EPI_ISL_624810, EPI_ISL_624811, EPI_ISL_624812, EPI_ISL_624813, EPI_ISL_624814, EPI_ISL_624815, EPI_ISL_624816, EPI_ISL_624817, EPI_ISL_624818, EPI_ISL_624819, EPI_ISL_624820, EPI_ISL_624821, EPI_ISL_624822, EPI_ISL_624823, EPI_ISL_624824, EPI_ISL_624825, EPI_ISL_624826, EPI_ISL_624827, EPI_ISL_624828, EPI_ISL_624829, EPI_ISL_624830, EPI_ISL_624831, EPI_ISL_624832, EPI_ISL_624833, EPI_ISL_624834, EPI_ISL_624835, EPI_ISL_624836, EPI_ISL_624837, EPI_ISL_624838, EPI_ISL_624839, EPI_ISL_624840, EPI_ISL_624841, EPI_ISL_624842, EPI_ISL_624843, EPI_ISL_624844, EPI_ISL_624845, EPI_ISL_624846, EPI_ISL_624847, EPI_ISL_624848, EPI_ISL_624849, EPI_ISL_624850, EPI_ISL_624851, EPI_ISL_624852, EPI_ISL_624853, EPI_ISL_624854, EPI_ISL_624855, EPI_ISL_624856, EPI_ISL_624857, EPI_ISL_624858, EPI_ISL_624859, EPI_ISL_624860, EPI_ISL_624861, EPI_ISL_624862, EPI_ISL_624863, EPI_ISL_624864, EPI_ISL_624865, EPI_ISL_624866, EPI_ISL_624867, EPI_ISL_624868, EPI_ISL_624869, EPI_ISL_624870, EPI_ISL_624871, EPI_ISL_624872, EPI_ISL_624873, EPI_ISL_624874, EPI_ISL_624875, EPI_ISL_624876, EPI_ISL_624877, EPI_ISL_624878, EPI_ISL_624879, EPI_ISL_624880, EPI_ISL_624881, EPI_ISL_624882, EPI_ISL_624883, EPI_ISL_624884, EPI_ISL_624885, EPI_ISL_624886, EPI_ISL_624887, EPI_ISL_624888, EPI_ISL_624889, EPI_ISL_624890, EPI_ISL_624891, EPI_ISL_624892, EPI_ISL_624893, EPI_ISL_624894, EPI_ISL_624895, EPI_ISL_624896, EPI_ISL_624897, EPI_ISL_624898, EPI_ISL_624899, EPI_ISL_624900, EPI_ISL_624901, EPI_ISL_624902, EPI_ISL_624903, EPI_ISL_624904, EPI_ISL_624905, EPI_ISL_624906, EPI_ISL_624907, EPI_ISL_624913, EPI_ISL_624923, EPI_ISL_624925, EPI_ISL_624934,			

EPI_ISL_624939, EPI_ISL_624941, EPI_ISL_624942, EPI_ISL_624947, EPI_ISL_624950, EPI_ISL_624953, EPI_ISL_624954, EPI_ISL_624965, EPI_ISL_624966, EPI_ISL_624967, EPI_ISL_624972, EPI_ISL_624975, EPI_ISL_624991, EPI_ISL_624992, EPI_ISL_625006, EPI_ISL_625007, EPI_ISL_625008, EPI_ISL_625012, EPI_ISL_625015, EPI_ISL_625021, EPI_ISL_625024, EPI_ISL_625026, EPI_ISL_625029, EPI_ISL_625033, EPI_ISL_625034, EPI_ISL_625044, EPI_ISL_625057, EPI_ISL_625058, EPI_ISL_625060, EPI_ISL_625063, EPI_ISL_625065, EPI_ISL_625066, EPI_ISL_625081, EPI_ISL_625085, EPI_ISL_625089, EPI_ISL_625090, EPI_ISL_625091, EPI_ISL_625095, EPI_ISL_625102, EPI_ISL_625110, EPI_ISL_625114, EPI_ISL_625116, EPI_ISL_625119, EPI_ISL_625124, EPI_ISL_625125, EPI_ISL_625128, EPI_ISL_625133, EPI_ISL_625137, EPI_ISL_625138, EPI_ISL_625143, EPI_ISL_625145, EPI_ISL_625146, EPI_ISL_625149, EPI_ISL_625153, EPI_ISL_625155, EPI_ISL_625157, EPI_ISL_625161, EPI_ISL_625163, EPI_ISL_625165, EPI_ISL_625168, EPI_ISL_625169, EPI_ISL_625172, EPI_ISL_625175, EPI_ISL_625180, EPI_ISL_625183, EPI_ISL_625185, EPI_ISL_625191, EPI_ISL_625197, EPI_ISL_625201, EPI_ISL_625210, EPI_ISL_625212, EPI_ISL_625215, EPI_ISL_625216, EPI_ISL_625217, EPI_ISL_625225, EPI_ISL_625230, EPI_ISL_625233, EPI_ISL_625234, EPI_ISL_625238, EPI_ISL_625245, EPI_ISL_625249, EPI_ISL_625252, EPI_ISL_625255, EPI_ISL_625257, EPI_ISL_625259, EPI_ISL_625266			
see above	Lighthouse Lab in Alderley Park	Wellcome Sanger Institute for the COVID-19 Genomics UK (COG-UK) consortium	Jacquelyn Wynn, Mairead Hyland, The Lighthouse Lab in Alderley Park and Alex Alderton, Roberto Amato, Sonia Goncalves, Ewan Harrison, David K. Jackson, Ian Johnston, Dominic Kwiatkowski, Cordelia Langford, John Sillitoe on behalf of the Wellcome Sanger Institute COVID-19 Surveillance Team (http://www.sanger.ac.uk/covid-team)
EPI_ISL_625315, EPI_ISL_625316, EPI_ISL_625317, EPI_ISL_625318, EPI_ISL_625319, EPI_ISL_625320, EPI_ISL_625321, EPI_ISL_625322, EPI_ISL_625323, EPI_ISL_625324, EPI_ISL_625325, EPI_ISL_625326, EPI_ISL_625327, EPI_ISL_625328, EPI_ISL_625329, EPI_ISL_625330, EPI_ISL_625331, EPI_ISL_625332, EPI_ISL_625333, EPI_ISL_625334, EPI_ISL_625335, EPI_ISL_625336, EPI_ISL_625337, EPI_ISL_625338, EPI_ISL_625339, EPI_ISL_625340, EPI_ISL_625341, EPI_ISL_625342, EPI_ISL_625343, EPI_ISL_625344, EPI_ISL_625345, EPI_ISL_625346, EPI_ISL_625347, EPI_ISL_625348, EPI_ISL_625349, EPI_ISL_625350, EPI_ISL_625351, EPI_ISL_625352, EPI_ISL_625353, EPI_ISL_625354, EPI_ISL_625355, EPI_ISL_625356, EPI_ISL_625357, EPI_ISL_625358, EPI_ISL_625359, EPI_ISL_625360, EPI_ISL_625361, EPI_ISL_625362, EPI_ISL_625363, EPI_ISL_625364, EPI_ISL_625365, EPI_ISL_625366, EPI_ISL_625367, EPI_ISL_625368, EPI_ISL_625369, EPI_ISL_625370, EPI_ISL_625371, EPI_ISL_625372, EPI_ISL_625373, EPI_ISL_625374, EPI_ISL_625375, EPI_ISL_625376, EPI_ISL_625377, EPI_ISL_625378, EPI_ISL_625379, EPI_ISL_625380, EPI_ISL_625381, EPI_ISL_625382, EPI_ISL_625383, EPI_ISL_625384, EPI_ISL_625385, EPI_ISL_625386, EPI_ISL_625387, EPI_ISL_625388, EPI_ISL_625389, EPI_ISL_625390, EPI_ISL_625391, EPI_ISL_625392, EPI_ISL_625393, EPI_ISL_625394, EPI_ISL_625395			
see above	Lighthouse Lab in Glasgow	Wellcome Sanger Institute for the COVID-19 Genomics UK (COG-UK) consortium	Harper VanSteenhouse, Yumi Kasai, David Gray, Carol Clugston, Anna Dominiczak and Alex Alderton, Roberto Amato, Sonia Goncalves, Ewan Harrison, David K. Jackson, Ian Johnston, Dominic Kwiatkowski, Cordelia Langford, John Sillitoe on behalf of the Wellcome Sanger Institute COVID-19 Surveillance Team (http://www.sanger.ac.uk/covid-team)
EPI_ISL_626624	The National Institute of Public Health	State Veterinary Institute Prague	Nagy,A;Jirincova,H;Novakova,L;Trnka,D;Vecerova,J
EPI_ISL_626631	National Public Health Laboratory, National Centre for Infectious Diseases	National Public Health Laboratory, National Centre for Infectious Diseases	Tze Minn Mak, Sophie Octavia, Zhenyang Zhou, Lin Cui, Raymond Tzer Pin Lin
EPI_ISL_626663	Quadram Institute Bioscience	COVID-19 Genomics UK (COG-UK) Consortium	Dave J. Baker, Gemma L. Kay, Alp Aydin, Thanh Le-Viet, Steven Rudder, Ana P. Tedim, Anastasia Kolyva, Maria Diaz, Leonardo de Oliveira Martins, Nabil-Fareed Alikhan, Lizzie Meadows, Rachael Stanley, Ngozi Elumogo, Muhammed Yasir, Nicholas M. Thomson, Alexander J Trotter, Rachel Gilroy, Samuel Bloomfield, Claire Stuart, Andrew Bell, Reenesh Prakash, Samir Dervisevic, Alison E. Mather, John Wain, Mark Webber, Andrew J. Page, Justin O'Grady
EPI_ISL_626666	University of Exeter	COVID-19 Genomics UK (COG-UK) Consortium	Ben Temperton,Aaron Jeffries,Michelle Michelsen,Joanna Warwick-Dugdale,Audrey Farbos,Robyn Manley,Stephen Michell,Jane Masoli
EPI_ISL_626667	Quadram Institute Bioscience	COVID-19 Genomics UK (COG-UK) Consortium	Dave J. Baker, Gemma L. Kay, Alp Aydin, Thanh Le-Viet, Steven Rudder, Ana P. Tedim, Anastasia Kolyva, Maria Diaz, Leonardo de Oliveira Martins, Nabil-Fareed Alikhan, Lizzie Meadows, Rachael Stanley, Ngozi Elumogo, Muhammed Yasir, Nicholas M. Thomson, Alexander J Trotter, Rachel Gilroy, Samuel Bloomfield, Claire Stuart, Andrew Bell, Reenesh Prakash, Samir Dervisevic, Alison E. Mather, John Wain, Mark Webber, Andrew J. Page, Justin O'Grady
EPI_ISL_626672	University of Exeter	COVID-19 Genomics UK (COG-UK) Consortium	Ben Temperton,Aaron Jeffries,Michelle Michelsen,Joanna Warwick-Dugdale,Audrey Farbos,Robyn Manley,Stephen Michell,Jane Masoli
EPI_ISL_626684	Centre for Enzyme Innovation, University of Portsmouth / Translational Research Laboratory, Portsmouth Hospitals NHS Trust	COVID-19 Genomics UK (COG-UK) Consortium	Angela Beckett,Yann Bourgeois,Garry Scarlett,Sharon Glaysher,Scott Elliott,Kelly Bicknell,Robert Impey,Allyson Lloyd,Sarah Wyllie,Ethan Butcher,Anoop Chauhan,Samuel Robson
EPI_ISL_626691, EPI_ISL_626692	West of Scotland Specialist Virology Centre, NHSGGC / MRC-University of Glasgow Centre for Virus Research	COVID-19 Genomics UK (COG-UK) Consortium	Ana da Silva Filipe, Natasha Johnson, Kathy Smollett, Daniel Mair, Stephen Carmichael, Lily Tong, Jenna Nichols, Elihu Aranday-Cortes, Kyriaki Nomikou; Sarah McDonald, Marc Niebel, Patawee Asamaphan; Richard Orton, Joseph Hughes, Sreenu Vattipally, David L Robertson; Alasdair MacLean, Rory Gunson; Kathy Li, Igor Starinskij, Natasha Jesudason, Rajiv Shah, James Shepherd, Antonia Ho, Emma Thomson
EPI_ISL_626693, EPI_ISL_626699, EPI_ISL_626702	Quadram Institute Bioscience	COVID-19 Genomics UK (COG-UK) Consortium	Dave J. Baker, Gemma L. Kay, Alp Aydin, Thanh Le-Viet, Steven Rudder, Ana P. Tedim, Anastasia Kolyva, Maria Diaz, Leonardo de Oliveira Martins, Nabil-Fareed Alikhan, Lizzie Meadows, Rachael Stanley, Ngozi Elumogo, Muhammed Yasir, Nicholas M. Thomson, Alexander J Trotter, Rachel Gilroy, Samuel Bloomfield, Claire Stuart, Andrew Bell, Reenesh Prakash, Samir Dervisevic, Alison E. Mather, John Wain, Mark Webber, Andrew J. Page, Justin O'Grady
EPI_ISL_626703, EPI_ISL_626711	Wales Specialist Virology Centre Sequencing lab: Pathogen Genomics Unit	COVID-19 Genomics UK (COG-UK) Consortium	Catherine Moore, Johnathan Evans, Laura Gifford, Malorie Perry, Simon Cottrell, Angela Marchbank, Alec Birchley, Alexander Adams, Amy Gaskin, Bree Gatica-Wilcox, Jason Coombes, Joel Southgate, Lauren Gilbert, Lee Graham, Nicole Pacchiarini, Sara Kumziene-Summerhayes, Sarah Taylor, Sophie Jones, Sara Rey, Matthew Bull, Joanne Watkins, Sally Corden, Tom Connor
EPI_ISL_626713, EPI_ISL_626730	University of Exeter	COVID-19 Genomics UK (COG-UK) Consortium	Ben Temperton,Aaron Jeffries,Michelle Michelsen,Joanna Warwick-Dugdale,Audrey Farbos,Robyn Manley,Stephen Michell,Jane Masoli
EPI_ISL_626749	West of Scotland Specialist Virology Centre, NHSGGC / MRC-University of Glasgow Centre for Virus Research	COVID-19 Genomics UK (COG-UK) Consortium	Ana da Silva Filipe, Natasha Johnson, Kathy Smollett, Daniel Mair, Stephen Carmichael, Lily Tong, Jenna Nichols, Elihu Aranday-Cortes, Kyriaki Nomikou; Sarah McDonald, Marc Niebel, Patawee Asamaphan; Richard Orton, Joseph Hughes, Sreenu Vattipally, David L Robertson; Alasdair MacLean, Rory Gunson; Kathy Li, Igor Starinskij, Natasha Jesudason, Rajiv Shah, James Shepherd, Antonia Ho, Emma Thomson
EPI_ISL_626764	Quadram Institute Bioscience	COVID-19 Genomics UK (COG-UK) Consortium	Dave J. Baker, Gemma L. Kay, Alp Aydin, Thanh Le-Viet, Steven Rudder, Ana P. Tedim, Anastasia Kolyva, Maria Diaz, Leonardo de Oliveira Martins, Nabil-Fareed Alikhan, Lizzie Meadows, Rachael Stanley, Ngozi Elumogo, Muhammed Yasir, Nicholas M. Thomson, Alexander J Trotter, Rachel Gilroy, Samuel Bloomfield, Claire Stuart, Andrew Bell, Reenesh Prakash, Samir Dervisevic, Alison E. Mather, John Wain, Mark Webber, Andrew J. Page, Justin O'Grady
EPI_ISL_626777	University of Exeter	COVID-19 Genomics UK (COG-UK) Consortium	Ben Temperton,Aaron Jeffries,Michelle Michelsen,Joanna Warwick-Dugdale,Audrey Farbos,Robyn Manley,Stephen Michell,Jane Masoli
EPI_ISL_626787, EPI_ISL_626796	Wales Specialist Virology Centre Sequencing lab: Pathogen Genomics Unit	COVID-19 Genomics UK (COG-UK) Consortium	Catherine Moore, Johnathan Evans, Laura Gifford, Malorie Perry, Simon Cottrell, Angela Marchbank, Alec Birchley, Alexander Adams, Amy Gaskin, Bree Gatica-Wilcox, Jason Coombes, Joel Southgate, Lauren Gilbert, Lee Graham, Nicole Pacchiarini, Sara Kumziene-Summerhayes, Sarah Taylor, Sophie Jones, Sara Rey, Matthew Bull, Joanne Watkins, Sally Corden, Tom Connor
EPI_ISL_626802, EPI_ISL_626813	University of Exeter	COVID-19 Genomics UK (COG-UK) Consortium	Ben Temperton,Aaron Jeffries,Michelle Michelsen,Joanna Warwick-Dugdale,Audrey Farbos,Robyn Manley,Stephen Michell,Jane Masoli
EPI_ISL_626829	Wales Specialist Virology Centre Sequencing lab: Pathogen Genomics Unit	COVID-19 Genomics UK (COG-UK) Consortium	Catherine Moore, Johnathan Evans, Laura Gifford, Malorie Perry, Simon Cottrell, Angela Marchbank, Alec Birchley, Alexander Adams, Amy Gaskin, Bree Gatica-Wilcox, Jason Coombes, Joel Southgate, Lauren Gilbert, Lee Graham, Nicole Pacchiarini, Sara Kumziene-Summerhayes, Sarah Taylor, Sophie Jones, Sara Rey, Matthew Bull, Joanne Watkins, Sally Corden, Tom Connor
EPI_ISL_626831	West of Scotland Specialist Virology Centre, NHSGGC / MRC-University of Glasgow Centre for Virus Research	COVID-19 Genomics UK (COG-UK) Consortium	Ana da Silva Filipe, Natasha Johnson, Kathy Smollett, Daniel Mair, Stephen Carmichael, Lily Tong, Jenna Nichols, Elihu Aranday-Cortes, Kyriaki Nomikou; Sarah McDonald, Marc Niebel, Patawee Asamaphan; Richard Orton, Joseph Hughes, Sreenu Vattipally, David L Robertson; Alasdair MacLean, Rory Gunson; Kathy Li, Igor Starinskij, Natasha Jesudason, Rajiv Shah, James Shepherd, Antonia Ho, Emma Thomson
EPI_ISL_626832	Liverpool Clinical Laboratories	COVID-19 Genomics UK (COG-UK) Consortium	Sam Haldenby, Anita Lucaci, Steve Paterson, Julian Hiscox, Alistair Darby, M Almsaud, A Alrezaihi, Muhannad Alruwaili, Stuart D Armstrong, Jones Benjamin, Eleanor G Bentley, Anu Chawla, Jordan J Clark, Angela Cowell, Richard Eccles, Isabel García-Dorival, Matthew Gemmell, Alessandro Gerada, PKF Gilmore, Richard Gregory, Ximeng Han, Catherine Hartley, Margaret Hughes, Miren Iturriza-Gomara, James Johnson, L Luu, Jenifer Manson, Charlotte Nelson, Elaine O'Toole, Cassie Olateju, Rebekah Penrice-Randal , Lucille Rainbow, N.P Randle, Trevor Ian Robinson, Parul Sharma, Ghada T Shawli, James P Stewart, Neil Swainston, Ecaterina Vamos, Joanne Watts, Mark Whitehead
EPI_ISL_626835, EPI_ISL_626846, EPI_ISL_626849, EPI_ISL_626851	West of Scotland Specialist Virology Centre, NHSGGC / MRC-University of Glasgow Centre for Virus Research	COVID-19 Genomics UK (COG-UK) Consortium	Ana da Silva Filipe, Natasha Johnson, Kathy Smollett, Daniel Mair, Stephen Carmichael, Lily Tong, Jenna Nichols, Elihu Aranday-Cortes, Kyriaki Nomikou; Sarah McDonald, Marc Niebel, Patawee Asamaphan; Richard Orton, Joseph Hughes, Sreenu Vattipally, David L Robertson; Alasdair MacLean, Rory Gunson; Kathy Li, Igor Starinskij, Natasha Jesudason, Rajiv Shah, James Shepherd, Antonia Ho, Emma Thomson
EPI_ISL_626854	Quadram Institute Bioscience	COVID-19 Genomics UK (COG-UK) Consortium	Dave J. Baker, Gemma L. Kay, Alp Aydin, Thanh Le-Viet, Steven Rudder, Ana P. Tedim, Anastasia Kolyva, Maria Diaz, Leonardo de Oliveira Martins, Nabil-Fareed Alikhan, Lizzie Meadows, Rachael Stanley, Ngozi Elumogo, Muhammed Yasir, Nicholas M. Thomson, Alexander J Trotter, Rachel Gilroy, Samuel Bloomfield, Claire Stuart, Andrew Bell, Reenesh Prakash, Samir Dervisevic, Alison E. Mather, John Wain, Mark Webber, Andrew J. Page, Justin O'Grady
EPI_ISL_626855	West of Scotland Specialist Virology Centre, NHSGGC / MRC-University of Glasgow Centre for Virus Research	COVID-19 Genomics UK (COG-UK) Consortium	Ana da Silva Filipe, Natasha Johnson, Kathy Smollett, Daniel Mair, Stephen Carmichael, Lily Tong, Jenna Nichols, Elihu Aranday-Cortes, Kyriaki Nomikou; Sarah McDonald, Marc Niebel, Patawee Asamaphan; Richard Orton, Joseph Hughes, Sreenu Vattipally, David L Robertson; Alasdair MacLean, Rory Gunson;

			Kathy Li, Igor Starinskij, Natasha Jesudason, Rajiv Shah, James Shepherd, Antonia Ho, Emma Thomson
EPI_ISL_626867	Wales Specialist Virology Centre Sequencing lab: Pathogen Genomics Unit	COVID-19 Genomics UK (COG-UK) Consortium	Catherine Moore, Johnathan Evans, Laura Gifford, Malorie Perry, Simon Cottrell, Angela Marchbank, Alec Birchley, Alexander Adams, Amy Gaskin, Bree Gatica-Wilcox, Jason Coombes, Joel Southgate, Lauren Gilbert, Lee Graham, Nicole Pacchiarini, Sara Kumziene-Summerhayes, Sarah Taylor, Sophie Jones, Sara Rey, Matthew Bull, Joanne Watkins, Sally Corden, Tom Connor
EPI_ISL_626889	University of Exeter	COVID-19 Genomics UK (COG-UK) Consortium	Ben Temperton,Aaron Jeffries,Michelle Michelsen,Joanna Warwick-Dugdale,Audrey Farbos,Robyn Manley,Stephen Michell,Jane Masoli
EPI_ISL_626895	Centre for Enzyme Innovation, University of Portsmouth / Translational Research Laboratory, Portsmouth Hospitals NHS Trust	COVID-19 Genomics UK (COG-UK) Consortium	Angela Beckett,Yann Bourgeois,Garry Scarlett,Sharon Glaysher,Scott Elliott,Kelly Bicknell,Robert Impey,Allyson Lloyd,Sarah Wyllie,Ethan Butcher,Anoop Chauhan,Samuel Robson
EPI_ISL_626921	Wales Specialist Virology Centre Sequencing lab: Pathogen Genomics Unit	COVID-19 Genomics UK (COG-UK) Consortium	Catherine Moore, Johnathan Evans, Laura Gifford, Malorie Perry, Simon Cottrell, Angela Marchbank, Alec Birchley, Alexander Adams, Amy Gaskin, Bree Gatica-Wilcox, Jason Coombes, Joel Southgate, Lauren Gilbert, Lee Graham, Nicole Pacchiarini, Sara Kumziene-Summerhayes, Sarah Taylor, Sophie Jones, Sara Rey, Matthew Bull, Joanne Watkins, Sally Corden, Tom Connor
EPI_ISL_626943, EPI_ISL_626945, EPI_ISL_626946	University of Exeter	COVID-19 Genomics UK (COG-UK) Consortium	Ben Temperton,Aaron Jeffries,Michelle Michelsen,Joanna Warwick-Dugdale,Audrey Farbos,Robyn Manley,Stephen Michell,Jane Masoli
EPI_ISL_626957, EPI_ISL_626972, EPI_ISL_626978, EPI_ISL_626982, EPI_ISL_626983, EPI_ISL_626988, EPI_ISL_626991, EPI_ISL_626995	West of Scotland Specialist Virology Centre, NHSGGC / MRC-University of Glasgow Centre for Virus Research	COVID-19 Genomics UK (COG-UK) Consortium	Ana da Silva Filipe, Natasha Johnson, Kathy Smollett, Daniel Mair, Stephen Carmichael, Lily Tong, Jenna Nichols, Elihu Aranday-Cortes, Kyriaki Nomikou; Sarah McDonald, Marc Niebel, Patawee Asamaphan; Richard Orton, Joseph Hughes, Sreenu Vattipally, David L Robertson; Alasdair MacLean, Rory Gunson; Kathy Li, Igor Starinskij, Natasha Jesudason, Rajiv Shah, James Shepherd, Antonia Ho, Emma Thomson
EPI_ISL_627016, EPI_ISL_627024	Quadram Institute Bioscience	COVID-19 Genomics UK (COG-UK) Consortium	Dave J. Baker, Gemma L. Kay, Alp Aydin, Thanh Le-Viet, Steven Rudder, Ana P. Tedim, Anastasia Kolyva, Maria Diaz, Leonardo de Oliveira Martins, Nabil-Fareed Alikhan, Lizzie Meadows, Rachael Stanley, Ngozi Elumogo, Muhammed Yasir, Nicholas M. Thomson, Alexander J Trotter, Rachel Gilroy, Samuel Bloomfield, Claire Stuart, Andrew Bell, Reenesh Prakash, Samir Dervisevic, Alison E. Mather, John Wain, Mark Webber, Andrew J. Page, Justin O'Grady
EPI_ISL_627030	West of Scotland Specialist Virology Centre, NHSGGC / MRC-University of Glasgow Centre for Virus Research	COVID-19 Genomics UK (COG-UK) Consortium	Ana da Silva Filipe, Natasha Johnson, Kathy Smollett, Daniel Mair, Stephen Carmichael, Lily Tong, Jenna Nichols, Elihu Aranday-Cortes, Kyriaki Nomikou; Sarah McDonald, Marc Niebel, Patawee Asamaphan; Richard Orton, Joseph Hughes, Sreenu Vattipally, David L Robertson; Alasdair MacLean, Rory Gunson; Kathy Li, Igor Starinskij, Natasha Jesudason, Rajiv Shah, James Shepherd, Antonia Ho, Emma Thomson
EPI_ISL_627033	Wales Specialist Virology Centre Sequencing lab: Pathogen Genomics Unit	COVID-19 Genomics UK (COG-UK) Consortium	Catherine Moore, Johnathan Evans, Laura Gifford, Malorie Perry, Simon Cottrell, Angela Marchbank, Alec Birchley, Alexander Adams, Amy Gaskin, Bree Gatica-Wilcox, Jason Coombes, Joel Southgate, Lauren Gilbert, Lee Graham, Nicole Pacchiarini, Sara Kumziene-Summerhayes, Sarah Taylor, Sophie Jones, Sara Rey, Matthew Bull, Joanne Watkins, Sally Corden, Tom Connor
EPI_ISL_627041	Quadram Institute Bioscience	COVID-19 Genomics UK (COG-UK) Consortium	Dave J. Baker, Gemma L. Kay, Alp Aydin, Thanh Le-Viet, Steven Rudder, Ana P. Tedim, Anastasia Kolyva, Maria Diaz, Leonardo de Oliveira Martins, Nabil-Fareed Alikhan, Lizzie Meadows, Rachael Stanley, Ngozi Elumogo, Muhammed Yasir, Nicholas M. Thomson, Alexander J Trotter, Rachel Gilroy, Samuel Bloomfield, Claire Stuart, Andrew Bell, Reenesh Prakash, Samir Dervisevic, Alison E. Mather, John Wain, Mark Webber, Andrew J. Page, Justin O'Grady
EPI_ISL_627042, EPI_ISL_627043	Wales Specialist Virology Centre Sequencing lab: Pathogen Genomics Unit	COVID-19 Genomics UK (COG-UK) Consortium	Catherine Moore, Johnathan Evans, Laura Gifford, Malorie Perry, Simon Cottrell, Angela Marchbank, Alec Birchley, Alexander Adams, Amy Gaskin, Bree Gatica-Wilcox, Jason Coombes, Joel Southgate, Lauren Gilbert, Lee Graham, Nicole Pacchiarini, Sara Kumziene-Summerhayes, Sarah Taylor, Sophie Jones, Sara Rey, Matthew Bull, Joanne Watkins, Sally Corden, Tom Connor
EPI_ISL_627063	West of Scotland Specialist Virology Centre, NHSGGC / MRC-University of Glasgow Centre for Virus Research	COVID-19 Genomics UK (COG-UK) Consortium	Ana da Silva Filipe, Natasha Johnson, Kathy Smollett, Daniel Mair, Stephen Carmichael, Lily Tong, Jenna Nichols, Elihu Aranday-Cortes, Kyriaki Nomikou; Sarah McDonald, Marc Niebel, Patawee Asamaphan; Richard Orton, Joseph Hughes, Sreenu Vattipally, David L Robertson; Alasdair MacLean, Rory Gunson; Kathy Li, Igor Starinskij, Natasha Jesudason, Rajiv Shah, James Shepherd, Antonia Ho, Emma Thomson
EPI_ISL_627072, EPI_ISL_627074, EPI_ISL_627075, EPI_ISL_627076, EPI_ISL_627077	Quadram Institute Bioscience	COVID-19 Genomics UK (COG-UK) Consortium	Dave J. Baker, Gemma L. Kay, Alp Aydin, Thanh Le-Viet, Steven Rudder, Ana P. Tedim, Anastasia Kolyva, Maria Diaz, Leonardo de Oliveira Martins, Nabil-Fareed Alikhan, Lizzie Meadows, Rachael Stanley, Ngozi Elumogo, Muhammed Yasir, Nicholas M. Thomson, Alexander J Trotter, Rachel Gilroy, Samuel Bloomfield, Claire Stuart, Andrew Bell, Reenesh Prakash, Samir Dervisevic, Alison E. Mather, John Wain, Mark Webber, Andrew J. Page, Justin O'Grady
EPI_ISL_627091, EPI_ISL_627095	Wales Specialist Virology Centre Sequencing lab: Pathogen Genomics Unit	COVID-19 Genomics UK (COG-UK) Consortium	Catherine Moore, Johnathan Evans, Laura Gifford, Malorie Perry, Simon Cottrell, Angela Marchbank, Alec Birchley, Alexander Adams, Amy Gaskin, Bree Gatica-Wilcox, Jason Coombes, Joel Southgate, Lauren Gilbert, Lee Graham, Nicole Pacchiarini, Sara Kumziene-Summerhayes, Sarah Taylor, Sophie Jones, Sara Rey, Matthew Bull, Joanne Watkins, Sally Corden, Tom Connor
EPI_ISL_627098	Quadram Institute Bioscience	COVID-19 Genomics UK (COG-UK) Consortium	Dave J. Baker, Gemma L. Kay, Alp Aydin, Thanh Le-Viet, Steven Rudder, Ana P. Tedim, Anastasia Kolyva, Maria Diaz, Leonardo de Oliveira Martins, Nabil-Fareed Alikhan, Lizzie Meadows, Rachael Stanley, Ngozi Elumogo, Muhammed Yasir, Nicholas M. Thomson, Alexander J Trotter, Rachel Gilroy, Samuel Bloomfield, Claire Stuart, Andrew Bell, Reenesh Prakash, Samir Dervisevic, Alison E. Mather, John Wain, Mark Webber, Andrew J. Page, Justin O'Grady
EPI_ISL_627109	Liverpool Clinical Laboratories	COVID-19 Genomics UK (COG-UK) Consortium	Sam Haldenby, Anita Lucaci, Steve Paterson, Julian Hiscox, Alistair Darby, M Almsaud, A Alrezaihi, Muhannad Alruwaili, Stuart D Armstrong, Jones Benjamin, Eleanor G Bentley, Anu Chawla, Jordan J Clark, Angela Cowell, Richard Eccles, Isabel García-Dorival, Matthew Gemmell, Alessandro Gerada, PKF Gilmore, Richard Gregory, Ximeng Han, Catherine Hartley, Margaret Hughes, Miren Iturriza-Gomara, James Johnson, L Luu, Jenifer Manson, Charlotte Nelson, Elaine O'Toole, Cassie Olateju, Rebekah Penrice-Randal , Lucille Rainbow, N.P Randle, Trevor Ian Robinson, Parul Sharma, Ghada T Shawli, James P Stewart, Neil Swainston, Ecaterina Vamos, Joanne Watts, Mark Whitehead
EPI_ISL_627120, EPI_ISL_627124	Wales Specialist Virology Centre Sequencing lab: Pathogen Genomics Unit	COVID-19 Genomics UK (COG-UK) Consortium	Catherine Moore, Johnathan Evans, Laura Gifford, Malorie Perry, Simon Cottrell, Angela Marchbank, Alec Birchley, Alexander Adams, Amy Gaskin, Bree Gatica-Wilcox, Jason Coombes, Joel Southgate, Lauren Gilbert, Lee Graham, Nicole Pacchiarini, Sara Kumziene-Summerhayes, Sarah Taylor, Sophie Jones, Sara Rey, Matthew Bull, Joanne Watkins, Sally Corden, Tom Connor
EPI_ISL_627128	Centre for Enzyme Innovation, University of Portsmouth / Translational Research Laboratory, Portsmouth Hospitals NHS Trust	COVID-19 Genomics UK (COG-UK) Consortium	Angela Beckett,Yann Bourgeois,Garry Scarlett,Sharon Glaysher,Scott Elliott,Kelly Bicknell,Robert Impey,Allyson Lloyd,Sarah Wyllie,Ethan Butcher,Anoop Chauhan,Samuel Robson
EPI_ISL_627145	University of Exeter	COVID-19 Genomics UK (COG-UK) Consortium	Ben Temperton,Aaron Jeffries,Michelle Michelsen,Joanna Warwick-Dugdale,Audrey Farbos,Robyn Manley,Stephen Michell,Jane Masoli
EPI_ISL_627154	Wales Specialist Virology Centre Sequencing lab: Pathogen Genomics Unit	COVID-19 Genomics UK (COG-UK) Consortium	Catherine Moore, Johnathan Evans, Laura Gifford, Malorie Perry, Simon Cottrell, Angela Marchbank, Alec Birchley, Alexander Adams, Amy Gaskin, Bree Gatica-Wilcox, Jason Coombes, Joel Southgate, Lauren Gilbert, Lee Graham, Nicole Pacchiarini, Sara Kumziene-Summerhayes, Sarah Taylor, Sophie Jones, Sara Rey, Matthew Bull, Joanne Watkins, Sally Corden, Tom Connor
EPI_ISL_627160	Quadram Institute Bioscience	COVID-19 Genomics UK (COG-UK) Consortium	Dave J. Baker, Gemma L. Kay, Alp Aydin, Thanh Le-Viet, Steven Rudder, Ana P. Tedim, Anastasia Kolyva, Maria Diaz, Leonardo de Oliveira Martins, Nabil-Fareed Alikhan, Lizzie Meadows, Rachael Stanley, Ngozi Elumogo, Muhammed Yasir, Nicholas M. Thomson, Alexander J Trotter, Rachel Gilroy, Samuel Bloomfield, Claire Stuart, Andrew Bell, Reenesh Prakash, Samir Dervisevic, Alison E. Mather, John Wain, Mark Webber, Andrew J. Page, Justin O'Grady
EPI_ISL_627161	Wales Specialist Virology Centre Sequencing lab: Pathogen Genomics Unit	COVID-19 Genomics UK (COG-UK) Consortium	Catherine Moore, Johnathan Evans, Laura Gifford, Malorie Perry, Simon Cottrell, Angela Marchbank, Alec Birchley, Alexander Adams, Amy Gaskin, Bree Gatica-Wilcox, Jason Coombes, Joel Southgate, Lauren Gilbert, Lee Graham, Nicole Pacchiarini, Sara Kumziene-Summerhayes, Sarah Taylor, Sophie Jones, Sara Rey, Matthew Bull, Joanne Watkins, Sally Corden, Tom Connor
EPI_ISL_627165	West of Scotland Specialist Virology Centre, NHSGGC / MRC-University of Glasgow Centre for Virus Research	COVID-19 Genomics UK (COG-UK) Consortium	Ana da Silva Filipe, Natasha Johnson, Kathy Smollett, Daniel Mair, Stephen Carmichael, Lily Tong, Jenna Nichols, Elihu Aranday-Cortes, Kyriaki Nomikou; Sarah McDonald, Marc Niebel, Patawee Asamaphan; Richard Orton, Joseph Hughes, Sreenu Vattipally, David L Robertson; Alasdair MacLean, Rory Gunson; Kathy Li, Igor Starinskij, Natasha Jesudason, Rajiv Shah, James Shepherd, Antonia Ho, Emma Thomson
EPI_ISL_627182	University of Exeter	COVID-19 Genomics UK (COG-UK) Consortium	Ben Temperton,Aaron Jeffries,Michelle Michelsen,Joanna Warwick-Dugdale,Audrey Farbos,Robyn Manley,Stephen Michell,Jane Masoli
EPI_ISL_627187	Quadram Institute Bioscience	COVID-19 Genomics UK (COG-UK) Consortium	Dave J. Baker, Gemma L. Kay, Alp Aydin, Thanh Le-Viet, Steven Rudder, Ana P. Tedim, Anastasia Kolyva, Maria Diaz, Leonardo de Oliveira Martins,

			Nabil-Fareed Alikhan, Lizzie Meadows, Rachael Stanley, Ngozi Elumogo, Muhammed Yasir, Nicholas M. Thomson, Alexander J Trotter, Rachel Gilroy, Samuel Bloomfield, Claire Stuart, Andrew Bell, Reenesh Prakash, Samir Dervisevic, Alison E. Mather, John Wain, Mark Webber, Andrew J. Page, Justin O'Grady
EPI_ISL_627192	Wales Specialist Virology Centre Sequencing lab: Pathogen Genomics Unit	COVID-19 Genomics UK (COG-UK) Consortium	Catherine Moore, Johnathan Evans, Laura Gifford, Malorie Perry, Simon Cottrell, Angela Marchbank, Alec Birchley, Alexander Adams, Amy Gaskin, Bree Gatica-Wilcox, Jason Coombes, Joel Southgate, Lauren Gilbert, Lee Graham, Nicole Pacchiarini, Sara Kumziene-Summerhayes, Sarah Taylor, Sophie Jones, Sara Rey, Matthew Bull, Joanne Watkins, Sally Corden, Tom Connor
EPI_ISL_627194	University of Exeter	COVID-19 Genomics UK (COG-UK) Consortium	Ben Temperton,Aaron Jeffries,Michelle Michelsen,Joanna Warwick-Dugdale,Audrey Farbos,Robyn Manley,Stephen Michell,Jane Masoli
EPI_ISL_627209	West of Scotland Specialist Virology Centre, NHSGGC / MRC-University of Glasgow Centre for Virus Research	COVID-19 Genomics UK (COG-UK) Consortium	Ana da Silva Filipe, Natasha Johnson, Kathy Smollett, Daniel Mair, Stephen Carmichael, Lily Tong, Jenna Nichols, Elihu Aranday-Cortes, Kyriaki Nomikou; Sarah McDonald, Marc Niebel, Patawee Asamaphan; Richard Orton, Joseph Hughes, Sreenu Vattipally, David L Robertson; Alasdair MacLean, Rory Gunson; Kathy Li, Igor Starinskij, Natasha Jesudason, Rajiv Shah, James Shepherd, Antonia Ho, Emma Thomson
EPI_ISL_627210	University of Exeter	COVID-19 Genomics UK (COG-UK) Consortium	Ben Temperton,Aaron Jeffries,Michelle Michelsen,Joanna Warwick-Dugdale,Audrey Farbos,Robyn Manley,Stephen Michell,Jane Masoli
EPI_ISL_627213	Quadram Institute Bioscience	COVID-19 Genomics UK (COG-UK) Consortium	Dave J. Baker, Gemma L. Kay, Alp Aydin, Thanh Le-Viet, Steven Rudder, Ana P. Tedim, Anastasia Kolyva, Maria Diaz, Leonardo de Oliveira Martins, Nabil-Fareed Alikhan, Lizzie Meadows, Rachael Stanley, Ngozi Elumogo, Muhammed Yasir, Nicholas M. Thomson, Alexander J Trotter, Rachel Gilroy, Samuel Bloomfield, Claire Stuart, Andrew Bell, Reenesh Prakash, Samir Dervisevic, Alison E. Mather, John Wain, Mark Webber, Andrew J. Page, Justin O'Grady
EPI_ISL_627218, EPI_ISL_627221, EPI_ISL_627222, EPI_ISL_627223, EPI_ISL_627225, EPI_ISL_627226, EPI_ISL_627238, EPI_ISL_627239, EPI_ISL_627241, EPI_ISL_627265, EPI_ISL_627268, EPI_ISL_627270, EPI_ISL_627271, EPI_ISL_627272, EPI_ISL_627274, EPI_ISL_627278			
see above	Wales Specialist Virology Centre Sequencing lab: Pathogen Genomics Unit	COVID-19 Genomics UK (COG-UK) Consortium	Catherine Moore, Johnathan Evans, Laura Gifford, Malorie Perry, Simon Cottrell, Angela Marchbank, Alec Birchley, Alexander Adams, Amy Gaskin, Bree Gatica-Wilcox, Jason Coombes, Joel Southgate, Lauren Gilbert, Lee Graham, Nicole Pacchiarini, Sara Kumziene-Summerhayes, Sarah Taylor, Sophie Jones, Sara Rey, Matthew Bull, Joanne Watkins, Sally Corden, Tom Connor
EPI_ISL_627377, EPI_ISL_627379, EPI_ISL_627380, EPI_ISL_627384, EPI_ISL_627388, EPI_ISL_627391, EPI_ISL_627396, EPI_ISL_627397, EPI_ISL_627398, EPI_ISL_627404, EPI_ISL_627409, EPI_ISL_627410, EPI_ISL_627418, EPI_ISL_627419			
see above	West of Scotland Specialist Virology Centre, NHSGGC / MRC-University of Glasgow Centre for Virus Research	COVID-19 Genomics UK (COG-UK) Consortium	Ana da Silva Filipe, Natasha Johnson, Kathy Smollett, Daniel Mair, Stephen Carmichael, Lily Tong, Jenna Nichols, Elihu Aranday-Cortes, Kyriaki Nomikou; Sarah McDonald, Marc Niebel, Patawee Asamaphan; Richard Orton, Joseph Hughes, Sreenu Vattipally, David L Robertson; Alasdair MacLean, Rory Gunson; Kathy Li, Igor Starinskij, Natasha Jesudason, Rajiv Shah, James Shepherd, Antonia Ho, Emma Thomson
EPI_ISL_627424, EPI_ISL_627428, EPI_ISL_627430, EPI_ISL_627431, EPI_ISL_627432, EPI_ISL_627433, EPI_ISL_627434	University of Exeter	COVID-19 Genomics UK (COG-UK) Consortium	Ben Temperton,Aaron Jeffries,Michelle Michelsen,Joanna Warwick-Dugdale,Audrey Farbos,Robyn Manley,Stephen Michell,Jane Masoli
EPI_ISL_627597, EPI_ISL_627598, EPI_ISL_627599, EPI_ISL_627600, EPI_ISL_627604, EPI_ISL_627606, EPI_ISL_627611, EPI_ISL_627617, EPI_ISL_627618, EPI_ISL_627620, EPI_ISL_627625, EPI_ISL_627627, EPI_ISL_627628, EPI_ISL_627630, EPI_ISL_627632, EPI_ISL_627633, EPI_ISL_627634, EPI_ISL_627637, EPI_ISL_627638, EPI_ISL_627639, EPI_ISL_627642, EPI_ISL_627643, EPI_ISL_627644, EPI_ISL_627648, EPI_ISL_627652, EPI_ISL_627654, EPI_ISL_627656, EPI_ISL_627664, EPI_ISL_627665			
see above	Quadram Institute Bioscience	COVID-19 Genomics UK (COG-UK) Consortium	Dave J. Baker, Gemma L. Kay, Alp Aydin, Thanh Le-Viet, Steven Rudder, Ana P. Tedim, Anastasia Kolyva, Maria Diaz, Leonardo de Oliveira Martins, Nabil-Fareed Alikhan, Lizzie Meadows, Rachael Stanley, Ngozi Elumogo, Muhammed Yasir, Nicholas M. Thomson, Alexander J Trotter, Rachel Gilroy, Samuel Bloomfield, Claire Stuart, Andrew Bell, Reenesh Prakash, Samir Dervisevic, Alison E. Mather, John Wain, Mark Webber, Andrew J. Page, Justin O'Grady
EPI_ISL_627667, EPI_ISL_627668, EPI_ISL_627669	Queens Medical Centre, Clinical Microbiology Department / DeepSeq Nottingham	COVID-19 Genomics UK (COG-UK) Consortium	Gemma Clark, Wendy Smith, Manjinder Khakh, Vicki M Fleming, Michelle M Lister, Hannah Howson-Wells, Jonathan Ball, Patrick McClure, Joseph Chappell, Theocharis Tsoleridis, Nadine Holmes, Matthew Carlisle, Christopher Moore, Fei Sang, Johnny Debebe, Victoria Wright, Matthew Loose
EPI_ISL_627732, EPI_ISL_627733, EPI_ISL_627734, EPI_ISL_627736, EPI_ISL_627737, EPI_ISL_627742, EPI_ISL_627745, EPI_ISL_627747, EPI_ISL_627753, EPI_ISL_627756, EPI_ISL_627757, EPI_ISL_627758, EPI_ISL_627764, EPI_ISL_627765, EPI_ISL_627766, EPI_ISL_627768, EPI_ISL_627771, EPI_ISL_627773, EPI_ISL_627776, EPI_ISL_627779, EPI_ISL_627782, EPI_ISL_627787, EPI_ISL_627795, EPI_ISL_627796, EPI_ISL_627797, EPI_ISL_627800, EPI_ISL_627801, EPI_ISL_627804, EPI_ISL_627812, EPI_ISL_627815, EPI_ISL_627816, EPI_ISL_627819, EPI_ISL_627827, EPI_ISL_627831, EPI_ISL_627835, EPI_ISL_627844, EPI_ISL_627852, EPI_ISL_627853, EPI_ISL_627857, EPI_ISL_627860, EPI_ISL_627861, EPI_ISL_627862, EPI_ISL_627863, EPI_ISL_627866, EPI_ISL_627867, EPI_ISL_627870, EPI_ISL_627876, EPI_ISL_627881, EPI_ISL_627882, EPI_ISL_627883, EPI_ISL_627884, EPI_ISL_627885, EPI_ISL_627886, EPI_ISL_627889, EPI_ISL_627895, EPI_ISL_627900, EPI_ISL_627911, EPI_ISL_627912, EPI_ISL_627913, EPI_ISL_627915, EPI_ISL_627920, EPI_ISL_627923, EPI_ISL_627924, EPI_ISL_627927, EPI_ISL_627930, EPI_ISL_627935, EPI_ISL_627937, EPI_ISL_627952, EPI_ISL_627955, EPI_ISL_627959, EPI_ISL_627964, EPI_ISL_627968, EPI_ISL_627970, EPI_ISL_627975, EPI_ISL_627979, EPI_ISL_627980, EPI_ISL_627981, EPI_ISL_627986, EPI_ISL_627990, EPI_ISL_627993, EPI_ISL_627994, EPI_ISL_627995, EPI_ISL_627996, EPI_ISL_627997, EPI_ISL_628006, EPI_ISL_628010, EPI_ISL_628011, EPI_ISL_628013, EPI_ISL_628022, EPI_ISL_628038, EPI_ISL_628039, EPI_ISL_628044, EPI_ISL_628045, EPI_ISL_628050, EPI_ISL_628052, EPI_ISL_628057, EPI_ISL_628066, EPI_ISL_628067, EPI_ISL_628070, EPI_ISL_628073, EPI_ISL_628074, EPI_ISL_628081, EPI_ISL_628082, EPI_ISL_628083, EPI_ISL_628090, EPI_ISL_628094, EPI_ISL_628095, EPI_ISL_628098, EPI_ISL_628099, EPI_ISL_628102, EPI_ISL_628103, EPI_ISL_628106, EPI_ISL_628109, EPI_ISL_628111, EPI_ISL_628112, EPI_ISL_628113, EPI_ISL_628121, EPI_ISL_628129, EPI_ISL_628133, EPI_ISL_628134, EPI_ISL_628142, EPI_ISL_628143, EPI_ISL_628144, EPI_ISL_628148, EPI_ISL_628150, EPI_ISL_628171, EPI_ISL_628172, EPI_ISL_628175, EPI_ISL_628176, EPI_ISL_628184, EPI_ISL_628186, EPI_ISL_628191, EPI_ISL_628195, EPI_ISL_628197, EPI_ISL_628199, EPI_ISL_628210, EPI_ISL_628213, EPI_ISL_628222, EPI_ISL_628223, EPI_ISL_628228, EPI_ISL_628229, EPI_ISL_628235, EPI_ISL_628238, EPI_ISL_628239, EPI_ISL_628248, EPI_ISL_628249, EPI_ISL_628252, EPI_ISL_628257, EPI_ISL_628259, EPI_ISL_628261, EPI_ISL_628262, EPI_ISL_628267, EPI_ISL_628270, EPI_ISL_628274, EPI_ISL_628275, EPI_ISL_628276, EPI_ISL_628277, EPI_ISL_628280, EPI_ISL_628284, EPI_ISL_628285, EPI_ISL_628290, EPI_ISL_628293, EPI_ISL_628299, EPI_ISL_628300, EPI_ISL_628302, EPI_ISL_628305, EPI_ISL_628306			
see above	Wales Specialist Virology Centre Sequencing lab: Pathogen Genomics Unit	COVID-19 Genomics UK (COG-UK) Consortium	Catherine Moore, Johnathan Evans, Laura Gifford, Malorie Perry, Simon Cottrell, Angela Marchbank, Alec Birchley, Alexander Adams, Amy Gaskin, Bree Gatica-Wilcox, Jason Coombes, Joel Southgate, Lauren Gilbert, Lee Graham, Nicole Pacchiarini, Sara Kumziene-Summerhayes, Sarah Taylor, Sophie Jones, Sara Rey, Matthew Bull, Joanne Watkins, Sally Corden, Tom Connor
EPI_ISL_628332, EPI_ISL_628333	Centre for Enzyme Innovation, University of Portsmouth / Translational Research Laboratory, Portsmouth Hospitals NHS Trust	COVID-19 Genomics UK (COG-UK) Consortium	Angela Beckett,Yann Bourgeois,Garry Scarlett,Sharon Glaysher,Scott Elliott,Kelly Bicknell,Robert Impey,Allyson Lloyd,Sarah Wyllie,Ethan Butcher,Anoop Chauhan,Samuel Robson
EPI_ISL_628806, EPI_ISL_628858, EPI_ISL_628867, EPI_ISL_628868, EPI_ISL_628869, EPI_ISL_628870, EPI_ISL_628871, EPI_ISL_628872, EPI_ISL_628873, EPI_ISL_628874, EPI_ISL_628875, EPI_ISL_628876, EPI_ISL_628877, EPI_ISL_628878, EPI_ISL_628879, EPI_ISL_628880, EPI_ISL_628881, EPI_ISL_628882, EPI_ISL_628883, EPI_ISL_628884, EPI_ISL_628885, EPI_ISL_628886			
see above	University of Michigan Clinical Microbiology Laboratory	Lauring Lab, University of Michigan, Department of Microbiology and Immunology	Valesano
EPI_ISL_629109, EPI_ISL_629110, EPI_ISL_629111, EPI_ISL_629112, EPI_ISL_629113, EPI_ISL_629114, EPI_ISL_629115, EPI_ISL_629116, EPI_ISL_629117, EPI_ISL_629118, EPI_ISL_629119, EPI_ISL_629120, EPI_ISL_629121, EPI_ISL_629122, EPI_ISL_629124, EPI_ISL_629125, EPI_ISL_629126, EPI_ISL_629127, EPI_ISL_629128, EPI_ISL_629129, EPI_ISL_629130, EPI_ISL_629131, EPI_ISL_629132, EPI_ISL_629133, EPI_ISL_629134, EPI_ISL_629136, EPI_ISL_629137			
see above	Lighthouse Lab in Milton Keynes	Wellcome Sanger Institute for the COVID-19 Genomics UK (COG-UK) consortium	The Lighthouse Lab in Milton Keynes and Alex Alderton, Roberto Amato, Sonia Goncalves, Ewan Harrison, David K. Jackson, Ian Johnston, Dominic Kwiatkowski, Cordelia Langford, John Sillitoe on behalf of the Wellcome Sanger Institute COVID-19 Surveillance Team
EPI_ISL_629138	Lighthouse Lab in Milton Keynes	Wellcome Sanger Institute for the COVID-19 Genomics UK (COG-UK) Consortium	The Lighthouse Lab in Milton Keynes and Alex Alderton, Roberto Amato, Sonia Goncalves, Ewan Harrison, David K. Jackson, Ian Johnston, Dominic Kwiatkowski, Cordelia Langford, John Sillitoe on behalf of the Wellcome Sanger Institute COVID-19 Surveillance Team
EPI_ISL_629139, EPI_ISL_629140, EPI_ISL_629141, EPI_ISL_629142, EPI_ISL_629143, EPI_ISL_629144, EPI_ISL_629145, EPI_ISL_629146, EPI_ISL_629147, EPI_ISL_629148, EPI_ISL_629149, EPI_ISL_629150, EPI_ISL_629151, EPI_ISL_629152, EPI_ISL_629153, EPI_ISL_629154, EPI_ISL_629155, EPI_ISL_629156, EPI_ISL_629157, EPI_ISL_629158, EPI_ISL_629159, EPI_ISL_629160, EPI_ISL_629161, EPI_ISL_629162, EPI_ISL_629163, EPI_ISL_629164, EPI_ISL_629165, EPI_ISL_629166, EPI_ISL_629167, EPI_ISL_629169, EPI_ISL_629170, EPI_ISL_629171, EPI_ISL_629172, EPI_ISL_629173, EPI_ISL_629174, EPI_ISL_629175, EPI_ISL_629176, EPI_ISL_629177, EPI_ISL_629178, EPI_ISL_629179, EPI_ISL_629180, EPI_ISL_629181, EPI_ISL_629182, EPI_ISL_629183, EPI_ISL_629184, EPI_ISL_629185, EPI_ISL_629186, EPI_ISL_629187, EPI_ISL_629188, EPI_ISL_629190, EPI_ISL_629191, EPI_ISL_629192, EPI_ISL_629193, EPI_ISL_629194, EPI_ISL_629196, EPI_ISL_629197, EPI_ISL_629198			
see above	Lighthouse Lab in Milton Keynes	Wellcome Sanger Institute for the COVID-19 Genomics UK (COG-UK) consortium	The Lighthouse Lab in Milton Keynes and Alex Alderton, Roberto Amato, Sonia Goncalves, Ewan Harrison, David K. Jackson, Ian Johnston, Dominic Kwiatkowski, Cordelia Langford, John Sillitoe on behalf of the Wellcome Sanger Institute COVID-19 Surveillance Team
EPI_ISL_629199	Lighthouse Lab in Milton Keynes	Wellcome Sanger Institute for the COVID-19 Genomics UK (COG-UK) Consortium	The Lighthouse Lab in Milton Keynes and Alex Alderton, Roberto Amato, Sonia Goncalves, Ewan Harrison, David K. Jackson, Ian Johnston, Dominic Kwiatkowski, Cordelia Langford, John Sillitoe on behalf of the Wellcome Sanger Institute COVID-19 Surveillance Team
EPI_ISL_629200, EPI_ISL_629201, EPI_ISL_629202, EPI_ISL_629203, EPI_ISL_629204, EPI_ISL_629205, EPI_ISL_629206, EPI_ISL_629207, EPI_ISL_629208, EPI_ISL_629209, EPI_ISL_629210, EPI_ISL_629211, EPI_ISL_629212, EPI_ISL_629213, EPI_ISL_629214, EPI_ISL_629215, EPI_ISL_629216, EPI_ISL_629217, EPI_ISL_629218			
see above	Lighthouse Lab in Milton Keynes	Wellcome Sanger Institute for the COVID-19 Genomics UK (COG-UK) consortium	The Lighthouse Lab in Milton Keynes and Alex Alderton, Roberto Amato, Sonia Goncalves, Ewan Harrison, David K. Jackson, Ian Johnston, Dominic Kwiatkowski, Cordelia Langford, John Sillitoe on behalf of the Wellcome Sanger Institute COVID-19 Surveillance Team
EPI_ISL_629219	Lighthouse Lab in Milton Keynes	Wellcome Sanger Institute for the COVID-19 Genomics UK	The Lighthouse Lab in Milton Keynes and Alex Alderton, Roberto Amato, Sonia Goncalves, Ewan Harrison, David K. Jackson, Ian Johnston, Dominic

		(COG-UK) Consortium	Kwiatkowski, Cordelia Langford, John Sillitoe on behalf of the Wellcome Sanger Institute COVID-19 Surveillance Team
EPI_ISL_629220, EPI_ISL_629221, EPI_ISL_629222, EPI_ISL_629223, EPI_ISL_629224, EPI_ISL_629225, EPI_ISL_629226, EPI_ISL_629227, EPI_ISL_629228, EPI_ISL_629229, EPI_ISL_629230, EPI_ISL_629231, EPI_ISL_629232, EPI_ISL_629233, EPI_ISL_629234, EPI_ISL_629235, EPI_ISL_629236, EPI_ISL_629237, EPI_ISL_629238, EPI_ISL_629239, EPI_ISL_629240, EPI_ISL_629241, EPI_ISL_629242, EPI_ISL_629243, EPI_ISL_629244			
see above	Lighthouse Lab in Milton Keynes	Wellcome Sanger Institute for the COVID-19 Genomics UK (COG-UK) consortium	The Lighthouse Lab in Milton Keynes and Alex Alderton, Roberto Amato, Sonia Goncalves, Ewan Harrison, David K. Jackson, Ian Johnston, Dominic Kwiatkowski, Cordelia Langford, John Sillitoe on behalf of the Wellcome Sanger Institute COVID-19 Surveillance Team
EPI_ISL_629245	Lighthouse Lab in Milton Keynes	Wellcome Sanger Institute for the COVID-19 Genomics UK (COG-UK) Consortium	The Lighthouse Lab in Milton Keynes and Alex Alderton, Roberto Amato, Sonia Goncalves, Ewan Harrison, David K. Jackson, Ian Johnston, Dominic Kwiatkowski, Cordelia Langford, John Sillitoe on behalf of the Wellcome Sanger Institute COVID-19 Surveillance Team
EPI_ISL_629247, EPI_ISL_629248, EPI_ISL_629249, EPI_ISL_629250, EPI_ISL_629251, EPI_ISL_629252, EPI_ISL_629253, EPI_ISL_629254, EPI_ISL_629255, EPI_ISL_629256, EPI_ISL_629257, EPI_ISL_629258, EPI_ISL_629259, EPI_ISL_629260, EPI_ISL_629261, EPI_ISL_629262, EPI_ISL_629263, EPI_ISL_629264, EPI_ISL_629265, EPI_ISL_629266, EPI_ISL_629267, EPI_ISL_629268, EPI_ISL_629269, EPI_ISL_629270, EPI_ISL_629271, EPI_ISL_629272, EPI_ISL_629273, EPI_ISL_629274, EPI_ISL_629275, EPI_ISL_629276, EPI_ISL_629277, EPI_ISL_629278, EPI_ISL_629280, EPI_ISL_629281, EPI_ISL_629282, EPI_ISL_629283, EPI_ISL_629284, EPI_ISL_629285, EPI_ISL_629286, EPI_ISL_629287, EPI_ISL_629288, EPI_ISL_629289, EPI_ISL_629290, EPI_ISL_629292, EPI_ISL_629294, EPI_ISL_629295, EPI_ISL_629296, EPI_ISL_629297, EPI_ISL_629298, EPI_ISL_629299, EPI_ISL_629300, EPI_ISL_629301, EPI_ISL_629302, EPI_ISL_629303, EPI_ISL_629304, EPI_ISL_629305, EPI_ISL_629306, EPI_ISL_629307, EPI_ISL_629308, EPI_ISL_629309, EPI_ISL_629310, EPI_ISL_629311, EPI_ISL_629312, EPI_ISL_629313, EPI_ISL_629314, EPI_ISL_629315, EPI_ISL_629316, EPI_ISL_629317, EPI_ISL_629318, EPI_ISL_629319, EPI_ISL_629320, EPI_ISL_629321, EPI_ISL_629322, EPI_ISL_629323, EPI_ISL_629324, EPI_ISL_629325, EPI_ISL_629326, EPI_ISL_629327, EPI_ISL_629328, EPI_ISL_629329, EPI_ISL_629330, EPI_ISL_629331, EPI_ISL_629332, EPI_ISL_629333, EPI_ISL_629334, EPI_ISL_629335, EPI_ISL_629336, EPI_ISL_629337, EPI_ISL_629338, EPI_ISL_629339, EPI_ISL_629340, EPI_ISL_629342, EPI_ISL_629343, EPI_ISL_629344, EPI_ISL_629345, EPI_ISL_629346, EPI_ISL_629347, EPI_ISL_629348, EPI_ISL_629349, EPI_ISL_629350, EPI_ISL_629351, EPI_ISL_629352, EPI_ISL_629353, EPI_ISL_629354, EPI_ISL_629355, EPI_ISL_629356, EPI_ISL_629357, EPI_ISL_629358, EPI_ISL_629361, EPI_ISL_629363, EPI_ISL_629364, EPI_ISL_629365, EPI_ISL_629366, EPI_ISL_629367, EPI_ISL_629368, EPI_ISL_629369, EPI_ISL_629371, EPI_ISL_629372, EPI_ISL_629373, EPI_ISL_629374, EPI_ISL_629375, EPI_ISL_629376, EPI_ISL_629377, EPI_ISL_629378, EPI_ISL_629379, EPI_ISL_629381, EPI_ISL_629382, EPI_ISL_629383, EPI_ISL_629384, EPI_ISL_629385, EPI_ISL_629386, EPI_ISL_629387, EPI_ISL_629388, EPI_ISL_629389, EPI_ISL_629390, EPI_ISL_629391, EPI_ISL_629393, EPI_ISL_629394, EPI_ISL_629395, EPI_ISL_629396, EPI_ISL_629397, EPI_ISL_629398, EPI_ISL_629399, EPI_ISL_629400, EPI_ISL_629401, EPI_ISL_629402, EPI_ISL_629403, EPI_ISL_629404, EPI_ISL_629405, EPI_ISL_629406, EPI_ISL_629407, EPI_ISL_629408			
see above	Lighthouse Lab in Milton Keynes	Wellcome Sanger Institute for the COVID-19 Genomics UK (COG-UK) consortium	The Lighthouse Lab in Milton Keynes and Alex Alderton, Roberto Amato, Sonia Goncalves, Ewan Harrison, David K. Jackson, Ian Johnston, Dominic Kwiatkowski, Cordelia Langford, John Sillitoe on behalf of the Wellcome Sanger Institute COVID-19 Surveillance Team
EPI_ISL_629409	Lighthouse Lab in Milton Keynes	Wellcome Sanger Institute for the COVID-19 Genomics UK (COG-UK) Consortium	The Lighthouse Lab in Milton Keynes and Alex Alderton, Roberto Amato, Sonia Goncalves, Ewan Harrison, David K. Jackson, Ian Johnston, Dominic Kwiatkowski, Cordelia Langford, John Sillitoe on behalf of the Wellcome Sanger Institute COVID-19 Surveillance Team
EPI_ISL_629410, EPI_ISL_629411, EPI_ISL_629412, EPI_ISL_629413, EPI_ISL_629415, EPI_ISL_629416, EPI_ISL_629417, EPI_ISL_629418, EPI_ISL_629419, EPI_ISL_629420, EPI_ISL_629421, EPI_ISL_629422, EPI_ISL_629423, EPI_ISL_629424, EPI_ISL_629425, EPI_ISL_629426, EPI_ISL_629427, EPI_ISL_629428, EPI_ISL_629429, EPI_ISL_629430, EPI_ISL_629431, EPI_ISL_629432, EPI_ISL_629433, EPI_ISL_629434, EPI_ISL_629436, EPI_ISL_629439, EPI_ISL_629441, EPI_ISL_629444, EPI_ISL_629448, EPI_ISL_629450, EPI_ISL_629452, EPI_ISL_629454, EPI_ISL_629456, EPI_ISL_629458, EPI_ISL_629460, EPI_ISL_629462, EPI_ISL_629465, EPI_ISL_629466, EPI_ISL_629468, EPI_ISL_629470, EPI_ISL_629471, EPI_ISL_629473, EPI_ISL_629474, EPI_ISL_629475, EPI_ISL_629476, EPI_ISL_629482, EPI_ISL_629483, EPI_ISL_629485, EPI_ISL_629486, EPI_ISL_629487, EPI_ISL_629489			
see above	Lighthouse Lab in Milton Keynes	Wellcome Sanger Institute for the COVID-19 Genomics UK (COG-UK) consortium	The Lighthouse Lab in Milton Keynes and Alex Alderton, Roberto Amato, Sonia Goncalves, Ewan Harrison, David K. Jackson, Ian Johnston, Dominic Kwiatkowski, Cordelia Langford, John Sillitoe on behalf of the Wellcome Sanger Institute COVID-19 Surveillance Team
EPI_ISL_629490	Lighthouse Lab in Milton Keynes	Wellcome Sanger Institute for the COVID-19 Genomics UK (COG-UK) Consortium	The Lighthouse Lab in Milton Keynes and Alex Alderton, Roberto Amato, Sonia Goncalves, Ewan Harrison, David K. Jackson, Ian Johnston, Dominic Kwiatkowski, Cordelia Langford, John Sillitoe on behalf of the Wellcome Sanger Institute COVID-19 Surveillance Team
EPI_ISL_629494, EPI_ISL_629495, EPI_ISL_629499, EPI_ISL_629501, EPI_ISL_629502, EPI_ISL_629503, EPI_ISL_629506, EPI_ISL_629510, EPI_ISL_629514, EPI_ISL_629515, EPI_ISL_629517, EPI_ISL_629518, EPI_ISL_629519, EPI_ISL_629520, EPI_ISL_629522, EPI_ISL_629523, EPI_ISL_629524, EPI_ISL_629528, EPI_ISL_629531, EPI_ISL_629532, EPI_ISL_629534, EPI_ISL_629535, EPI_ISL_629537, EPI_ISL_629539, EPI_ISL_629541, EPI_ISL_629544, EPI_ISL_629545, EPI_ISL_629549, EPI_ISL_629555, EPI_ISL_629565, EPI_ISL_629569, EPI_ISL_629572, EPI_ISL_629577, EPI_ISL_629581, EPI_ISL_629587, EPI_ISL_629591			
see above	Lighthouse Lab in Milton Keynes	Wellcome Sanger Institute for the COVID-19 Genomics UK (COG-UK) consortium	The Lighthouse Lab in Milton Keynes and Alex Alderton, Roberto Amato, Sonia Goncalves, Ewan Harrison, David K. Jackson, Ian Johnston, Dominic Kwiatkowski, Cordelia Langford, John Sillitoe on behalf of the Wellcome Sanger Institute COVID-19 Surveillance Team
EPI_ISL_629592	Lighthouse Lab in Milton Keynes	Wellcome Sanger Institute for the COVID-19 Genomics UK (COG-UK) Consortium	The Lighthouse Lab in Milton Keynes and Alex Alderton, Roberto Amato, Sonia Goncalves, Ewan Harrison, David K. Jackson, Ian Johnston, Dominic Kwiatkowski, Cordelia Langford, John Sillitoe on behalf of the Wellcome Sanger Institute COVID-19 Surveillance Team
EPI_ISL_629594, EPI_ISL_629595, EPI_ISL_629596, EPI_ISL_629597, EPI_ISL_629598, EPI_ISL_629599, EPI_ISL_629603, EPI_ISL_629608, EPI_ISL_629614, EPI_ISL_629615, EPI_ISL_629617, EPI_ISL_629618, EPI_ISL_629619, EPI_ISL_629621, EPI_ISL_629622, EPI_ISL_629624, EPI_ISL_629625, EPI_ISL_629626, EPI_ISL_629628, EPI_ISL_629630, EPI_ISL_629631, EPI_ISL_629633, EPI_ISL_629635, EPI_ISL_629636, EPI_ISL_629637, EPI_ISL_629641, EPI_ISL_629644, EPI_ISL_629645, EPI_ISL_629648, EPI_ISL_629649, EPI_ISL_629651, EPI_ISL_629656, EPI_ISL_629657, EPI_ISL_629662, EPI_ISL_629663, EPI_ISL_629664, EPI_ISL_629665, EPI_ISL_629669, EPI_ISL_629671, EPI_ISL_629672, EPI_ISL_629675, EPI_ISL_629677, EPI_ISL_629678, EPI_ISL_629679, EPI_ISL_629683, EPI_ISL_629685, EPI_ISL_629689, EPI_ISL_629690, EPI_ISL_629692, EPI_ISL_629693, EPI_ISL_629696, EPI_ISL_629701, EPI_ISL_629702, EPI_ISL_629703, EPI_ISL_629709, EPI_ISL_629710, EPI_ISL_629711, EPI_ISL_629720, EPI_ISL_629721, EPI_ISL_629724, EPI_ISL_629725, EPI_ISL_629729, EPI_ISL_629730, EPI_ISL_629733, EPI_ISL_629734, EPI_ISL_629735, EPI_ISL_629736, EPI_ISL_629737, EPI_ISL_629738, EPI_ISL_629741, EPI_ISL_629745, EPI_ISL_629747, EPI_ISL_629748, EPI_ISL_629750			
see above	Lighthouse Lab in Milton Keynes	Wellcome Sanger Institute for the COVID-19 Genomics UK (COG-UK) consortium	The Lighthouse Lab in Milton Keynes and Alex Alderton, Roberto Amato, Sonia Goncalves, Ewan Harrison, David K. Jackson, Ian Johnston, Dominic Kwiatkowski, Cordelia Langford, John Sillitoe on behalf of the Wellcome Sanger Institute COVID-19 Surveillance Team
EPI_ISL_629754	Lighthouse Lab in Glasgow	Wellcome Sanger Institute for the COVID-19 Genomics UK (COG-UK) consortium	Harper VanSteenhouse, Yumi Kasai, David Gray, Carol Clugston, Anna Dominiczak and Alex Alderton, Roberto Amato, Sonia Goncalves, Ewan Harrison, David K. Jackson, Ian Johnston, Dominic Kwiatkowski, Cordelia Langford, John Sillitoe on behalf of the Wellcome Sanger Institute COVID-19 Surveillance Team
EPI_ISL_630007, EPI_ISL_630008, EPI_ISL_630009, EPI_ISL_630010, EPI_ISL_630012, EPI_ISL_630013, EPI_ISL_630014, EPI_ISL_630015, EPI_ISL_630016, EPI_ISL_630018, EPI_ISL_630019, EPI_ISL_630020, EPI_ISL_630021, EPI_ISL_630022, EPI_ISL_630025, EPI_ISL_630026, EPI_ISL_630027, EPI_ISL_630028, EPI_ISL_630029, EPI_ISL_630030, EPI_ISL_630033, EPI_ISL_630034, EPI_ISL_630035, EPI_ISL_630036, EPI_ISL_630039, EPI_ISL_630041, EPI_ISL_630042, EPI_ISL_630043, EPI_ISL_630045, EPI_ISL_630046, EPI_ISL_630048, EPI_ISL_630049, EPI_ISL_630051, EPI_ISL_630052, EPI_ISL_630053, EPI_ISL_630054, EPI_ISL_630056, EPI_ISL_630058, EPI_ISL_630059, EPI_ISL_630060, EPI_ISL_630061, EPI_ISL_630062, EPI_ISL_630063, EPI_ISL_630064, EPI_ISL_630065, EPI_ISL_630068, EPI_ISL_630069, EPI_ISL_630071, EPI_ISL_630072, EPI_ISL_630073, EPI_ISL_630074, EPI_ISL_630076, EPI_ISL_630077, EPI_ISL_630078, EPI_ISL_630079, EPI_ISL_630082, EPI_ISL_630083, EPI_ISL_630084, EPI_ISL_630085, EPI_ISL_630087, EPI_ISL_630088, EPI_ISL_630089, EPI_ISL_630090, EPI_ISL_630095, EPI_ISL_630097, EPI_ISL_630098, EPI_ISL_630099, EPI_ISL_630100, EPI_ISL_630101, EPI_ISL_630102, EPI_ISL_630103, EPI_ISL_630106, EPI_ISL_630107, EPI_ISL_630108, EPI_ISL_630110, EPI_ISL_630111, EPI_ISL_630112, EPI_ISL_630113, EPI_ISL_630114, EPI_ISL_630115, EPI_ISL_630116, EPI_ISL_630117, EPI_ISL_630118, EPI_ISL_630120, EPI_ISL_630121, EPI_ISL_630122, EPI_ISL_630123, EPI_ISL_630124, EPI_ISL_630125, EPI_ISL_630127, EPI_ISL_630128, EPI_ISL_630129, EPI_ISL_630130, EPI_ISL_630131, EPI_ISL_630133, EPI_ISL_630135, EPI_ISL_630136, EPI_ISL_630137, EPI_ISL_630138, EPI_ISL_630139, EPI_ISL_630140, EPI_ISL_630141, EPI_ISL_630142, EPI_ISL_630145, EPI_ISL_630146, EPI_ISL_630147, EPI_ISL_630148, EPI_ISL_630149, EPI_ISL_630150, EPI_ISL_630151, EPI_ISL_630152, EPI_ISL_630154, EPI_ISL_630155, EPI_ISL_630156, EPI_ISL_630157, EPI_ISL_630158, EPI_ISL_630159, EPI_ISL_630160, EPI_ISL_630161, EPI_ISL_630162, EPI_ISL_630163, EPI_ISL_630165, EPI_ISL_630166, EPI_ISL_630167, EPI_ISL_630168, EPI_ISL_630169, EPI_ISL_630170, EPI_ISL_630171, EPI_ISL_630172, EPI_ISL_630173, EPI_ISL_630175, EPI_ISL_630176, EPI_ISL_630178, EPI_ISL_630180, EPI_ISL_630181, EPI_ISL_630183, EPI_ISL_630184, EPI_ISL_630185, EPI_ISL_630186, EPI_ISL_630187, EPI_ISL_630188, EPI_ISL_630189, EPI_ISL_630190			
see above	Lighthouse Lab in Milton Keynes	Wellcome Sanger Institute for the COVID-19 Genomics UK (COG-UK) consortium	The Lighthouse Lab in Milton Keynes and Alex Alderton, Roberto Amato, Sonia Goncalves, Ewan Harrison, David K. Jackson, Ian Johnston, Dominic Kwiatkowski, Cordelia Langford, John Sillitoe on behalf of the Wellcome Sanger Institute COVID-19 Surveillance Team
EPI_ISL_630191	Lighthouse Lab in Milton Keynes	Wellcome Sanger Institute for the COVID-19 Genomics UK (COG-UK) Consortium	The Lighthouse Lab in Milton Keynes and Alex Alderton, Roberto Amato, Sonia Goncalves, Ewan Harrison, David K. Jackson, Ian Johnston, Dominic Kwiatkowski, Cordelia Langford, John Sillitoe on behalf of the Wellcome Sanger Institute COVID-19 Surveillance Team
EPI_ISL_630192, EPI_ISL_630193, EPI_ISL_630194, EPI_ISL_630195, EPI_ISL_630197, EPI_ISL_630198, EPI_ISL_630201, EPI_ISL_630202, EPI_ISL_630203, EPI_ISL_630204, EPI_ISL_630205, EPI_ISL_630207, EPI_ISL_630209, EPI_ISL_630210, EPI_ISL_630212, EPI_ISL_630213, EPI_ISL_630214, EPI_ISL_630215, EPI_ISL_630216, EPI_ISL_630218			
see above	Lighthouse Lab in Milton Keynes	Wellcome Sanger Institute for the COVID-19 Genomics UK (COG-UK) consortium	The Lighthouse Lab in Milton Keynes and Alex Alderton, Roberto Amato, Sonia Goncalves, Ewan Harrison, David K. Jackson, Ian Johnston, Dominic Kwiatkowski, Cordelia Langford, John Sillitoe on behalf of the Wellcome Sanger Institute COVID-19 Surveillance Team
EPI_ISL_630219	Lighthouse Lab in Milton Keynes	Wellcome Sanger Institute for the COVID-19 Genomics UK (COG-UK) Consortium	The Lighthouse Lab in Milton Keynes and Alex Alderton, Roberto Amato, Sonia Goncalves, Ewan Harrison, David K. Jackson, Ian Johnston, Dominic Kwiatkowski, Cordelia Langford, John Sillitoe on behalf of the Wellcome Sanger Institute COVID-19 Surveillance Team
EPI_ISL_630220, EPI_ISL_630221, EPI_ISL_630222, EPI_ISL_630225, EPI_ISL_630226, EPI_ISL_630228, EPI_ISL_630229, EPI_ISL_630230, EPI_ISL_630231, EPI_ISL_630232, EPI_ISL_630234, EPI_ISL_630235, EPI_ISL_630236, EPI_ISL_630237, EPI_ISL_630239, EPI_ISL_630240, EPI_ISL_630242, EPI_ISL_630243, EPI_ISL_630244, EPI_ISL_630245, EPI_ISL_630246, EPI_ISL_630247, EPI_ISL_630248			
see above	Lighthouse Lab in Milton Keynes	Wellcome Sanger Institute for the COVID-19 Genomics UK (COG-UK) consortium	The Lighthouse Lab in Milton Keynes and Alex Alderton, Roberto Amato, Sonia Goncalves, Ewan Harrison, David K. Jackson, Ian Johnston, Dominic Kwiatkowski, Cordelia Langford, John Sillitoe on behalf of the Wellcome Sanger Institute COVID-19 Surveillance Team
EPI_ISL_630249	Lighthouse Lab in Milton Keynes	Wellcome Sanger Institute for the COVID-19 Genomics UK (COG-UK) Consortium	The Lighthouse Lab in Milton Keynes and Alex Alderton, Roberto Amato, Sonia Goncalves, Ewan Harrison, David K. Jackson, Ian Johnston, Dominic Kwiatkowski, Cordelia Langford, John Sillitoe on behalf of the Wellcome Sanger Institute COVID-19 Surveillance Team
EPI_ISL_630250, EPI_ISL_630251, EPI_ISL_630252, EPI_ISL_630253, EPI_ISL_630254, EPI_ISL_630255, EPI_ISL_630256, EPI_ISL_630257, EPI_ISL_630258, EPI_ISL_630259, EPI_ISL_630260, EPI_ISL_630261, EPI_ISL_630263, EPI_ISL_630264, EPI_ISL_630265, EPI_ISL_630266, EPI_ISL_630267, EPI_ISL_630268, EPI_ISL_630270, EPI_ISL_630271, EPI_ISL_630273, EPI_ISL_630274, EPI_ISL_630275, EPI_ISL_630278, EPI_ISL_630280, EPI_ISL_630281, EPI_ISL_630282, EPI_ISL_630283, EPI_ISL_630284, EPI_ISL_630286, EPI_ISL_630287, EPI_ISL_630288, EPI_ISL_630289, EPI_ISL_630290, EPI_ISL_630291, EPI_ISL_630292, EPI_ISL_630293, EPI_ISL_630295, EPI_ISL_630296, EPI_ISL_630297, EPI_ISL_630298, EPI_ISL_630299, EPI_ISL_630300, EPI_ISL_630301, EPI_ISL_630302, EPI_ISL_630303, EPI_ISL_630305, EPI_ISL_630306, EPI_ISL_630309, EPI_ISL_630310, EPI_ISL_630311, EPI_ISL_630312, EPI_ISL_630314, EPI_ISL_630315, EPI_ISL_630317, EPI_ISL_630318, EPI_ISL_630319, EPI_ISL_630321, EPI_ISL_630322, EPI_ISL_630323, EPI_ISL_630324, EPI_ISL_630326, EPI_ISL_630327, EPI_ISL_630329, EPI_ISL_630330, EPI_ISL_630334, EPI_ISL_630335, EPI_ISL_630336, EPI_ISL_630337, EPI_ISL_630338, EPI_ISL_630339, EPI_ISL_630340, EPI_ISL_630341, EPI_ISL_630343, EPI_ISL_630344, EPI_ISL_630345			
see above	Lighthouse Lab in Milton Keynes	Wellcome Sanger Institute for the COVID-19 Genomics UK (COG-UK) consortium	The Lighthouse Lab in Milton Keynes and Alex Alderton, Roberto Amato, Sonia Goncalves, Ewan Harrison, David K. Jackson, Ian Johnston, Dominic Kwiatkowski, Cordelia Langford, John Sillitoe on behalf of the Wellcome Sanger Institute COVID-19 Surveillance Team

EPI_ISL_630702, EPI_ISL_630704, EPI_ISL_630705	Lighthouse Lab in Glasgow	Wellcome Sanger Institute for the COVID-19 Genomics UK (COG-UK) consortium	Harper VanSteenhouse, Yumi Kasai, David Gray, Carol Clugston, Anna Dominiczak and Alex Alderton, Roberto Amato, Sonia Goncalves, Ewan Harrison, David K. Jackson, Ian Johnston, Dominic Kwiatkowski, Cordelia Langford, John Sillitoe on behalf of the Wellcome Sanger Institute COVID-19 Surveillance Team
EPI_ISL_630708, EPI_ISL_630710, EPI_ISL_630711, EPI_ISL_630716, EPI_ISL_630717, EPI_ISL_630720, EPI_ISL_630722, EPI_ISL_630723, EPI_ISL_630724, EPI_ISL_630729	Lighthouse Lab in Alderley Park	Wellcome Sanger Institute for the COVID-19 Genomics UK (COG-UK) consortium	Jacquelyn Wynn, Mairead Hyland, The Lighthouse Lab in Alderley Park and Alex Alderton, Roberto Amato, Sonia Goncalves, Ewan Harrison, David K. Jackson, Ian Johnston, Dominic Kwiatkowski, Cordelia Langford, John Sillitoe on behalf of the Wellcome Sanger Institute COVID-19 Surveillance Team
EPI_ISL_630731	Lighthouse Lab in Glasgow	Wellcome Sanger Institute for the COVID-19 Genomics UK (COG-UK) consortium	Harper VanSteenhouse, Yumi Kasai, David Gray, Carol Clugston, Anna Dominiczak and Alex Alderton, Roberto Amato, Sonia Goncalves, Ewan Harrison, David K. Jackson, Ian Johnston, Dominic Kwiatkowski, Cordelia Langford, John Sillitoe on behalf of the Wellcome Sanger Institute COVID-19 Surveillance Team
EPI_ISL_630732, EPI_ISL_630736, EPI_ISL_630737, EPI_ISL_630746, EPI_ISL_630747, EPI_ISL_630748	Lighthouse Lab in Alderley Park	Wellcome Sanger Institute for the COVID-19 Genomics UK (COG-UK) consortium	Jacquelyn Wynn, Mairead Hyland, The Lighthouse Lab in Alderley Park and Alex Alderton, Roberto Amato, Sonia Goncalves, Ewan Harrison, David K. Jackson, Ian Johnston, Dominic Kwiatkowski, Cordelia Langford, John Sillitoe on behalf of the Wellcome Sanger Institute COVID-19 Surveillance Team
EPI_ISL_630749	Lighthouse Lab in Glasgow	Wellcome Sanger Institute for the COVID-19 Genomics UK (COG-UK) consortium	Harper VanSteenhouse, Yumi Kasai, David Gray, Carol Clugston, Anna Dominiczak and Alex Alderton, Roberto Amato, Sonia Goncalves, Ewan Harrison, David K. Jackson, Ian Johnston, Dominic Kwiatkowski, Cordelia Langford, John Sillitoe on behalf of the Wellcome Sanger Institute COVID-19 Surveillance Team
EPI_ISL_630753, EPI_ISL_630754, EPI_ISL_630755, EPI_ISL_630756, EPI_ISL_630758, EPI_ISL_630762	Lighthouse Lab in Alderley Park	Wellcome Sanger Institute for the COVID-19 Genomics UK (COG-UK) consortium	Jacquelyn Wynn, Mairead Hyland, The Lighthouse Lab in Alderley Park and Alex Alderton, Roberto Amato, Sonia Goncalves, Ewan Harrison, David K. Jackson, Ian Johnston, Dominic Kwiatkowski, Cordelia Langford, John Sillitoe on behalf of the Wellcome Sanger Institute COVID-19 Surveillance Team
EPI_ISL_630764	Lighthouse Lab in Glasgow	Wellcome Sanger Institute for the COVID-19 Genomics UK (COG-UK) consortium	Harper VanSteenhouse, Yumi Kasai, David Gray, Carol Clugston, Anna Dominiczak and Alex Alderton, Roberto Amato, Sonia Goncalves, Ewan Harrison, David K. Jackson, Ian Johnston, Dominic Kwiatkowski, Cordelia Langford, John Sillitoe on behalf of the Wellcome Sanger Institute COVID-19 Surveillance Team
EPI_ISL_630766, EPI_ISL_630767, EPI_ISL_630771	Lighthouse Lab in Alderley Park	Wellcome Sanger Institute for the COVID-19 Genomics UK (COG-UK) consortium	Jacquelyn Wynn, Mairead Hyland, The Lighthouse Lab in Alderley Park and Alex Alderton, Roberto Amato, Sonia Goncalves, Ewan Harrison, David K. Jackson, Ian Johnston, Dominic Kwiatkowski, Cordelia Langford, John Sillitoe on behalf of the Wellcome Sanger Institute COVID-19 Surveillance Team
EPI_ISL_630775	Lighthouse Lab in Glasgow	Wellcome Sanger Institute for the COVID-19 Genomics UK (COG-UK) consortium	Harper VanSteenhouse, Yumi Kasai, David Gray, Carol Clugston, Anna Dominiczak and Alex Alderton, Roberto Amato, Sonia Goncalves, Ewan Harrison, David K. Jackson, Ian Johnston, Dominic Kwiatkowski, Cordelia Langford, John Sillitoe on behalf of the Wellcome Sanger Institute COVID-19 Surveillance Team
EPI_ISL_630777, EPI_ISL_630781, EPI_ISL_630784, EPI_ISL_630786, EPI_ISL_630788, EPI_ISL_630790, EPI_ISL_630793, EPI_ISL_630803, EPI_ISL_630807, EPI_ISL_630808, EPI_ISL_630810, EPI_ISL_630812			
see above	Lighthouse Lab in Alderley Park	Wellcome Sanger Institute for the COVID-19 Genomics UK (COG-UK) consortium	Jacquelyn Wynn, Mairead Hyland, The Lighthouse Lab in Alderley Park and Alex Alderton, Roberto Amato, Sonia Goncalves, Ewan Harrison, David K. Jackson, Ian Johnston, Dominic Kwiatkowski, Cordelia Langford, John Sillitoe on behalf of the Wellcome Sanger Institute COVID-19 Surveillance Team
EPI_ISL_630813	Lighthouse Lab in Glasgow	Wellcome Sanger Institute for the COVID-19 Genomics UK (COG-UK) consortium	Harper VanSteenhouse, Yumi Kasai, David Gray, Carol Clugston, Anna Dominiczak and Alex Alderton, Roberto Amato, Sonia Goncalves, Ewan Harrison, David K. Jackson, Ian Johnston, Dominic Kwiatkowski, Cordelia Langford, John Sillitoe on behalf of the Wellcome Sanger Institute COVID-19 Surveillance Team
EPI_ISL_630822, EPI_ISL_630823, EPI_ISL_630830, EPI_ISL_630832, EPI_ISL_630834, EPI_ISL_630835, EPI_ISL_630839, EPI_ISL_630843, EPI_ISL_630844, EPI_ISL_630845, EPI_ISL_630855, EPI_ISL_630856, EPI_ISL_630858, EPI_ISL_630861, EPI_ISL_630863, EPI_ISL_630867, EPI_ISL_630868, EPI_ISL_630869			
see above	Lighthouse Lab in Alderley Park	Wellcome Sanger Institute for the COVID-19 Genomics UK (COG-UK) consortium	Jacquelyn Wynn, Mairead Hyland, The Lighthouse Lab in Alderley Park and Alex Alderton, Roberto Amato, Sonia Goncalves, Ewan Harrison, David K. Jackson, Ian Johnston, Dominic Kwiatkowski, Cordelia Langford, John Sillitoe on behalf of the Wellcome Sanger Institute COVID-19 Surveillance Team
EPI_ISL_630876	Lighthouse Lab in Glasgow	Wellcome Sanger Institute for the COVID-19 Genomics UK (COG-UK) consortium	Harper VanSteenhouse, Yumi Kasai, David Gray, Carol Clugston, Anna Dominiczak and Alex Alderton, Roberto Amato, Sonia Goncalves, Ewan Harrison, David K. Jackson, Ian Johnston, Dominic Kwiatkowski, Cordelia Langford, John Sillitoe on behalf of the Wellcome Sanger Institute COVID-19 Surveillance Team
EPI_ISL_630877, EPI_ISL_630879, EPI_ISL_630880, EPI_ISL_630882	Lighthouse Lab in Alderley Park	Wellcome Sanger Institute for the COVID-19 Genomics UK (COG-UK) consortium	Jacquelyn Wynn, Mairead Hyland, The Lighthouse Lab in Alderley Park and Alex Alderton, Roberto Amato, Sonia Goncalves, Ewan Harrison, David K. Jackson, Ian Johnston, Dominic Kwiatkowski, Cordelia Langford, John Sillitoe on behalf of the Wellcome Sanger Institute COVID-19 Surveillance Team
EPI_ISL_630883	Lighthouse Lab in Glasgow	Wellcome Sanger Institute for the COVID-19 Genomics UK (COG-UK) consortium	Harper VanSteenhouse, Yumi Kasai, David Gray, Carol Clugston, Anna Dominiczak and Alex Alderton, Roberto Amato, Sonia Goncalves, Ewan Harrison, David K. Jackson, Ian Johnston, Dominic Kwiatkowski, Cordelia Langford, John Sillitoe on behalf of the Wellcome Sanger Institute COVID-19 Surveillance Team
EPI_ISL_630884	Lighthouse Lab in Alderley Park	Wellcome Sanger Institute for the COVID-19 Genomics UK (COG-UK) consortium	Jacquelyn Wynn, Mairead Hyland, The Lighthouse Lab in Alderley Park and Alex Alderton, Roberto Amato, Sonia Goncalves, Ewan Harrison, David K. Jackson, Ian Johnston, Dominic Kwiatkowski, Cordelia Langford, John Sillitoe on behalf of the Wellcome Sanger Institute COVID-19 Surveillance Team
EPI_ISL_630891	Lighthouse Lab in Alderley Park	Wellcome Sanger Institute for the COVID-19 Genomics UK (COG-UK) Consortium	Jacquelyn Wynn, Mairead Hyland, The Lighthouse Lab in Alderley Park and Alex Alderton, Roberto Amato, Sonia Goncalves, Ewan Harrison, David K. Jackson, Ian Johnston, Dominic Kwiatkowski, Cordelia Langford, John Sillitoe on behalf of the Wellcome Sanger Institute COVID-19 Surveillance Team
EPI_ISL_630892, EPI_ISL_630901, EPI_ISL_630902, EPI_ISL_630904, EPI_ISL_630905, EPI_ISL_630912, EPI_ISL_630914	Lighthouse Lab in Alderley Park	Wellcome Sanger Institute for the COVID-19 Genomics UK (COG-UK) consortium	Jacquelyn Wynn, Mairead Hyland, The Lighthouse Lab in Alderley Park and Alex Alderton, Roberto Amato, Sonia Goncalves, Ewan Harrison, David K. Jackson, Ian Johnston, Dominic Kwiatkowski, Cordelia Langford, John Sillitoe on behalf of the Wellcome Sanger Institute COVID-19 Surveillance Team
EPI_ISL_630915	Lighthouse Lab in Glasgow	Wellcome Sanger Institute for the COVID-19 Genomics UK (COG-UK) consortium	Harper VanSteenhouse, Yumi Kasai, David Gray, Carol Clugston, Anna Dominiczak and Alex Alderton, Roberto Amato, Sonia Goncalves, Ewan Harrison, David K. Jackson, Ian Johnston, Dominic Kwiatkowski, Cordelia Langford, John Sillitoe on behalf of the Wellcome Sanger Institute COVID-19 Surveillance Team
EPI_ISL_630917, EPI_ISL_630918, EPI_ISL_630928, EPI_ISL_630929, EPI_ISL_630932, EPI_ISL_630934, EPI_ISL_630936, EPI_ISL_630939, EPI_ISL_630940, EPI_ISL_630943, EPI_ISL_630944, EPI_ISL_630945, EPI_ISL_630947, EPI_ISL_630950, EPI_ISL_630953, EPI_ISL_630956, EPI_ISL_630958, EPI_ISL_630963, EPI_ISL_630964			
see above	Lighthouse Lab in Alderley Park	Wellcome Sanger Institute for the COVID-19 Genomics UK (COG-UK) consortium	Jacquelyn Wynn, Mairead Hyland, The Lighthouse Lab in Alderley Park and Alex Alderton, Roberto Amato, Sonia Goncalves, Ewan Harrison, David K. Jackson, Ian Johnston, Dominic Kwiatkowski, Cordelia Langford, John Sillitoe on behalf of the Wellcome Sanger Institute COVID-19 Surveillance Team
EPI_ISL_630965	Lighthouse Lab in Glasgow	Wellcome Sanger Institute for the COVID-19 Genomics UK (COG-UK) consortium	Harper VanSteenhouse, Yumi Kasai, David Gray, Carol Clugston, Anna Dominiczak and Alex Alderton, Roberto Amato, Sonia Goncalves, Ewan Harrison, David K. Jackson, Ian Johnston, Dominic Kwiatkowski, Cordelia Langford, John Sillitoe on behalf of the Wellcome Sanger Institute COVID-19 Surveillance Team
EPI_ISL_630966, EPI_ISL_630967	Lighthouse Lab in Alderley Park	Wellcome Sanger Institute for the COVID-19 Genomics UK (COG-UK) consortium	Jacquelyn Wynn, Mairead Hyland, The Lighthouse Lab in Alderley Park and Alex Alderton, Roberto Amato, Sonia Goncalves, Ewan Harrison, David K. Jackson, Ian Johnston, Dominic Kwiatkowski, Cordelia Langford, John Sillitoe on behalf of the Wellcome Sanger Institute COVID-19 Surveillance Team
EPI_ISL_630973, EPI_ISL_630980, EPI_ISL_630981, EPI_ISL_630986, EPI_ISL_630987, EPI_ISL_630993, EPI_ISL_630995, EPI_ISL_631002, EPI_ISL_631004, EPI_ISL_631007, EPI_ISL_631009, EPI_ISL_631010, EPI_ISL_631015			
see above	Lighthouse Lab in Milton Keynes	Wellcome Sanger Institute for the COVID-19 Genomics UK (COG-UK) consortium	The Lighthouse Lab in Milton Keynes and Alex Alderton, Roberto Amato, Sonia Goncalves, Ewan Harrison, David K. Jackson, Ian Johnston, Dominic Kwiatkowski, Cordelia Langford, John Sillitoe on behalf of the Wellcome Sanger Institute COVID-19 Surveillance Team
EPI_ISL_631016	Lighthouse Lab in Cambridge	Wellcome Sanger Institute for the COVID-19 Genomics UK (COG-UK) consortium	Rob Howes, The Lighthouse Lab in Cambridge and Alex Alderton, Roberto Amato, Sonia Goncalves, Ewan Harrison, David K. Jackson, Ian Johnston, Dominic Kwiatkowski, Cordelia Langford, John Sillitoe on behalf of the Wellcome Sanger Institute COVID-19 Surveillance Team
EPI_ISL_631019, EPI_ISL_631020, EPI_ISL_631021, EPI_ISL_631022, EPI_ISL_631026, EPI_ISL_631029, EPI_ISL_631030, EPI_ISL_631036, EPI_ISL_631037, EPI_ISL_631042, EPI_ISL_631047, EPI_ISL_631048, EPI_ISL_631051, EPI_ISL_631054, EPI_ISL_631061, EPI_ISL_631067, EPI_ISL_631070, EPI_ISL_631073, EPI_ISL_631075, EPI_ISL_631082, EPI_ISL_631083, EPI_ISL_631087, EPI_ISL_631088, EPI_ISL_631091, EPI_ISL_631097, EPI_ISL_631098, EPI_ISL_631104			
see above	Lighthouse Lab in Milton Keynes	Wellcome Sanger Institute for the COVID-19 Genomics UK	The Lighthouse Lab in Milton Keynes and Alex Alderton, Roberto Amato, Sonia Goncalves, Ewan Harrison, David K. Jackson, Ian Johnston, Dominic

		(COG-UK) consortium	Kwiatkowski, Cordelia Langford, John Sillitoe on behalf of the Wellcome Sanger Institute COVID-19 Surveillance Team
EPI_ISL_631105	Lighthouse Lab in Cambridge	Wellcome Sanger Institute for the COVID-19 Genomics UK (COG-UK) consortium	Rob Howes, The Lighthouse Lab in Cambridge and Alex Alderton, Roberto Amato, Sonia Goncalves, Ewan Harrison, David K. Jackson, Ian Johnston, Dominic Kwiatkowski, Cordelia Langford, John Sillitoe on behalf of the Wellcome Sanger Institute COVID-19 Surveillance Team
EPI_ISL_631106, EPI_ISL_631108, EPI_ISL_631110, EPI_ISL_631112, EPI_ISL_631114, EPI_ISL_631115, EPI_ISL_631119, EPI_ISL_631122, EPI_ISL_631124, EPI_ISL_631125, EPI_ISL_631130, EPI_ISL_631134, EPI_ISL_631136, EPI_ISL_631138, EPI_ISL_631141, EPI_ISL_631143, EPI_ISL_631145, EPI_ISL_631146, EPI_ISL_631147, EPI_ISL_631152, EPI_ISL_631153, EPI_ISL_631156, EPI_ISL_631157, EPI_ISL_631158, EPI_ISL_631162, EPI_ISL_631166, EPI_ISL_631168, EPI_ISL_631172, EPI_ISL_631176, EPI_ISL_631178, EPI_ISL_631179			
see above	Lighthouse Lab in Milton Keynes	Wellcome Sanger Institute for the COVID-19 Genomics UK (COG-UK) consortium	The Lighthouse Lab in Milton Keynes and Alex Alderton, Roberto Amato, Sonia Goncalves, Ewan Harrison, David K. Jackson, Ian Johnston, Dominic Kwiatkowski, Cordelia Langford, John Sillitoe on behalf of the Wellcome Sanger Institute COVID-19 Surveillance Team
EPI_ISL_631180, EPI_ISL_631181	Lighthouse Lab in Cambridge	Wellcome Sanger Institute for the COVID-19 Genomics UK (COG-UK) consortium	Rob Howes, The Lighthouse Lab in Cambridge and Alex Alderton, Roberto Amato, Sonia Goncalves, Ewan Harrison, David K. Jackson, Ian Johnston, Dominic Kwiatkowski, Cordelia Langford, John Sillitoe on behalf of the Wellcome Sanger Institute COVID-19 Surveillance Team
EPI_ISL_631183, EPI_ISL_631188, EPI_ISL_631193, EPI_ISL_631198, EPI_ISL_631201, EPI_ISL_631213, EPI_ISL_631216, EPI_ISL_631218, EPI_ISL_631223, EPI_ISL_631226, EPI_ISL_631229, EPI_ISL_631230, EPI_ISL_631233, EPI_ISL_631234, EPI_ISL_631236, EPI_ISL_631239, EPI_ISL_631240, EPI_ISL_631242, EPI_ISL_631246, EPI_ISL_631247, EPI_ISL_631251, EPI_ISL_631252, EPI_ISL_631253, EPI_ISL_631255, EPI_ISL_631256, EPI_ISL_631258, EPI_ISL_631259, EPI_ISL_631263, EPI_ISL_631267			
see above	Lighthouse Lab in Milton Keynes	Wellcome Sanger Institute for the COVID-19 Genomics UK (COG-UK) consortium	The Lighthouse Lab in Milton Keynes and Alex Alderton, Roberto Amato, Sonia Goncalves, Ewan Harrison, David K. Jackson, Ian Johnston, Dominic Kwiatkowski, Cordelia Langford, John Sillitoe on behalf of the Wellcome Sanger Institute COVID-19 Surveillance Team
EPI_ISL_632322, EPI_ISL_632339, EPI_ISL_632342, EPI_ISL_632402, EPI_ISL_632425, EPI_ISL_632447, EPI_ISL_632482, EPI_ISL_632710, EPI_ISL_632715, EPI_ISL_632721, EPI_ISL_632722, EPI_ISL_632732, EPI_ISL_632742, EPI_ISL_632743, EPI_ISL_632744, EPI_ISL_632745, EPI_ISL_632746, EPI_ISL_632761, EPI_ISL_632788			
see above	Dutch COVID-19 response team	Erasmus Medical Center	Bas Oude Munnink, David Nieuwenhuijse, Reina Sikkema, Claudia Schapendonk, Irina Chestakova, Anne van der Linden, Theo Bestebroer, Stefan van Nieuwkoop, Mark Pronk, Pascal Lexmond, Corien Swaan, Manon Haverkate, Madelief Mollers, Mart Stein, Sandra Kengne Kamga Mobou, Jeroen van Kampen, Jolanda Voermans, Aura Timen, Corine GeurtsvanKessel, Annemiek van der Eijk, Richard Molenkamp, Marion Koopmans, on behalf of the Dutch national COVID-19 response team.
EPI_ISL_633091, EPI_ISL_633092, EPI_ISL_633093, EPI_ISL_633095, EPI_ISL_633096, EPI_ISL_633097, EPI_ISL_633098, EPI_ISL_633099	MD PHL	MD PHL	Maryland Department of Health Laboratories Administration
EPI_ISL_633841, EPI_ISL_633918	Lighthouse Lab in Glasgow	Wellcome Sanger Institute for the COVID-19 Genomics UK (COG-UK) consortium	Harper VanSteenhouse, Yumi Kasai, David Gray, Carol Clugston, Anna Dominiczak and Alex Alderton, Roberto Amato, Sonia Goncalves, Ewan Harrison, David K. Jackson, Ian Johnston, Dominic Kwiatkowski, Cordelia Langford, John Sillitoe on behalf of the Wellcome Sanger Institute COVID-19 Surveillance Team
EPI_ISL_634256, EPI_ISL_634257, EPI_ISL_634258, EPI_ISL_634259, EPI_ISL_634260, EPI_ISL_634261, EPI_ISL_634262, EPI_ISL_634263, EPI_ISL_634264, EPI_ISL_634265	Lighthouse Lab in Milton Keynes	Wellcome Sanger Institute for the COVID-19 Genomics UK (COG-UK) consortium	The Lighthouse Lab in Milton Keynes and Alex Alderton, Roberto Amato, Sonia Goncalves, Ewan Harrison, David K. Jackson, Ian Johnston, Dominic Kwiatkowski, Cordelia Langford, John Sillitoe on behalf of the Wellcome Sanger Institute COVID-19 Surveillance Team
EPI_ISL_634266	Lighthouse Lab in Milton Keynes	Wellcome Sanger Institute for the COVID-19 Genomics UK (COG-UK) Consortium	The Lighthouse Lab in Milton Keynes and Alex Alderton, Roberto Amato, Sonia Goncalves, Ewan Harrison, David K. Jackson, Ian Johnston, Dominic Kwiatkowski, Cordelia Langford, John Sillitoe on behalf of the Wellcome Sanger Institute COVID-19 Surveillance Team
EPI_ISL_634267, EPI_ISL_634269, EPI_ISL_634270, EPI_ISL_634271, EPI_ISL_634272, EPI_ISL_634273, EPI_ISL_634274, EPI_ISL_634276, EPI_ISL_634278, EPI_ISL_634279, EPI_ISL_634280, EPI_ISL_634281, EPI_ISL_634283, EPI_ISL_634284, EPI_ISL_634285, EPI_ISL_634286, EPI_ISL_634287, EPI_ISL_634288, EPI_ISL_634289, EPI_ISL_634291, EPI_ISL_634292, EPI_ISL_634293, EPI_ISL_634294, EPI_ISL_634297, EPI_ISL_634298, EPI_ISL_634299, EPI_ISL_634300, EPI_ISL_634301, EPI_ISL_634302, EPI_ISL_634303, EPI_ISL_634304, EPI_ISL_634306, EPI_ISL_634307, EPI_ISL_634308, EPI_ISL_634309, EPI_ISL_634310, EPI_ISL_634311, EPI_ISL_634312, EPI_ISL_634313, EPI_ISL_634315, EPI_ISL_634316, EPI_ISL_634317, EPI_ISL_634319, EPI_ISL_634321, EPI_ISL_634322, EPI_ISL_634323, EPI_ISL_634324, EPI_ISL_634325, EPI_ISL_634326, EPI_ISL_634327, EPI_ISL_634328, EPI_ISL_634329, EPI_ISL_634330, EPI_ISL_634331, EPI_ISL_634332, EPI_ISL_634333, EPI_ISL_634334, EPI_ISL_634335, EPI_ISL_634336, EPI_ISL_634337, EPI_ISL_634338, EPI_ISL_634340			
see above	Lighthouse Lab in Milton Keynes	Wellcome Sanger Institute for the COVID-19 Genomics UK (COG-UK) consortium	The Lighthouse Lab in Milton Keynes and Alex Alderton, Roberto Amato, Sonia Goncalves, Ewan Harrison, David K. Jackson, Ian Johnston, Dominic Kwiatkowski, Cordelia Langford, John Sillitoe on behalf of the Wellcome Sanger Institute COVID-19 Surveillance Team
EPI_ISL_634341	Lighthouse Lab in Milton Keynes	Wellcome Sanger Institute for the COVID-19 Genomics UK (COG-UK) Consortium	The Lighthouse Lab in Milton Keynes and Alex Alderton, Roberto Amato, Sonia Goncalves, Ewan Harrison, David K. Jackson, Ian Johnston, Dominic Kwiatkowski, Cordelia Langford, John Sillitoe on behalf of the Wellcome Sanger Institute COVID-19 Surveillance Team
EPI_ISL_634342, EPI_ISL_634343, EPI_ISL_634344, EPI_ISL_634345, EPI_ISL_634346, EPI_ISL_634347, EPI_ISL_634348, EPI_ISL_634349, EPI_ISL_634350, EPI_ISL_634351, EPI_ISL_634352, EPI_ISL_634353, EPI_ISL_634354, EPI_ISL_634355, EPI_ISL_634357, EPI_ISL_634360, EPI_ISL_634361, EPI_ISL_634362, EPI_ISL_634364, EPI_ISL_634365, EPI_ISL_634366, EPI_ISL_634367, EPI_ISL_634368, EPI_ISL_634369, EPI_ISL_634371, EPI_ISL_634372, EPI_ISL_634373, EPI_ISL_634374, EPI_ISL_634375			
see above	Lighthouse Lab in Milton Keynes	Wellcome Sanger Institute for the COVID-19 Genomics UK (COG-UK) consortium	The Lighthouse Lab in Milton Keynes and Alex Alderton, Roberto Amato, Sonia Goncalves, Ewan Harrison, David K. Jackson, Ian Johnston, Dominic Kwiatkowski, Cordelia Langford, John Sillitoe on behalf of the Wellcome Sanger Institute COVID-19 Surveillance Team
EPI_ISL_634376	Lighthouse Lab in Milton Keynes	Wellcome Sanger Institute for the COVID-19 Genomics UK (COG-UK) Consortium	The Lighthouse Lab in Milton Keynes and Alex Alderton, Roberto Amato, Sonia Goncalves, Ewan Harrison, David K. Jackson, Ian Johnston, Dominic Kwiatkowski, Cordelia Langford, John Sillitoe on behalf of the Wellcome Sanger Institute COVID-19 Surveillance Team
EPI_ISL_634377, EPI_ISL_634378, EPI_ISL_634379, EPI_ISL_634381, EPI_ISL_634382, EPI_ISL_634383	Lighthouse Lab in Milton Keynes	Wellcome Sanger Institute for the COVID-19 Genomics UK (COG-UK) consortium	The Lighthouse Lab in Milton Keynes and Alex Alderton, Roberto Amato, Sonia Goncalves, Ewan Harrison, David K. Jackson, Ian Johnston, Dominic Kwiatkowski, Cordelia Langford, John Sillitoe on behalf of the Wellcome Sanger Institute COVID-19 Surveillance Team
EPI_ISL_634384	Lighthouse Lab in Milton Keynes	Wellcome Sanger Institute for the COVID-19 Genomics UK (COG-UK) Consortium	The Lighthouse Lab in Milton Keynes and Alex Alderton, Roberto Amato, Sonia Goncalves, Ewan Harrison, David K. Jackson, Ian Johnston, Dominic Kwiatkowski, Cordelia Langford, John Sillitoe on behalf of the Wellcome Sanger Institute COVID-19 Surveillance Team
EPI_ISL_634386, EPI_ISL_634387, EPI_ISL_634388, EPI_ISL_634389, EPI_ISL_634390, EPI_ISL_634391, EPI_ISL_634392, EPI_ISL_634393, EPI_ISL_634394, EPI_ISL_634395, EPI_ISL_634396, EPI_ISL_634397, EPI_ISL_634398, EPI_ISL_634399, EPI_ISL_634401, EPI_ISL_634402, EPI_ISL_634403, EPI_ISL_634404, EPI_ISL_634405, EPI_ISL_634407, EPI_ISL_634408, EPI_ISL_634410, EPI_ISL_634411, EPI_ISL_634412, EPI_ISL_634414, EPI_ISL_634416, EPI_ISL_634417, EPI_ISL_634418, EPI_ISL_634419, EPI_ISL_634420, EPI_ISL_634421, EPI_ISL_634422, EPI_ISL_634423, EPI_ISL_634424, EPI_ISL_634425, EPI_ISL_634426, EPI_ISL_634427			
see above	Lighthouse Lab in Milton Keynes	Wellcome Sanger Institute for the COVID-19 Genomics UK (COG-UK) consortium	The Lighthouse Lab in Milton Keynes and Alex Alderton, Roberto Amato, Sonia Goncalves, Ewan Harrison, David K. Jackson, Ian Johnston, Dominic Kwiatkowski, Cordelia Langford, John Sillitoe on behalf of the Wellcome Sanger Institute COVID-19 Surveillance Team
EPI_ISL_634428	Lighthouse Lab in Milton Keynes	Wellcome Sanger Institute for the COVID-19 Genomics UK (COG-UK) Consortium	The Lighthouse Lab in Milton Keynes and Alex Alderton, Roberto Amato, Sonia Goncalves, Ewan Harrison, David K. Jackson, Ian Johnston, Dominic Kwiatkowski, Cordelia Langford, John Sillitoe on behalf of the Wellcome Sanger Institute COVID-19 Surveillance Team
EPI_ISL_634430, EPI_ISL_634431, EPI_ISL_634432, EPI_ISL_634434, EPI_ISL_634435, EPI_ISL_634436, EPI_ISL_634437, EPI_ISL_634438, EPI_ISL_634439, EPI_ISL_634440, EPI_ISL_634441, EPI_ISL_634442, EPI_ISL_634443, EPI_ISL_634444, EPI_ISL_634445, EPI_ISL_634446, EPI_ISL_634447, EPI_ISL_634448, EPI_ISL_634449, EPI_ISL_634450, EPI_ISL_634451, EPI_ISL_634452, EPI_ISL_634453, EPI_ISL_634454, EPI_ISL_634455, EPI_ISL_634457, EPI_ISL_634458, EPI_ISL_634459, EPI_ISL_634460, EPI_ISL_634461, EPI_ISL_634462, EPI_ISL_634463, EPI_ISL_634464			
see above	Lighthouse Lab in Milton Keynes	Wellcome Sanger Institute for the COVID-19 Genomics UK (COG-UK) consortium	The Lighthouse Lab in Milton Keynes and Alex Alderton, Roberto Amato, Sonia Goncalves, Ewan Harrison, David K. Jackson, Ian Johnston, Dominic Kwiatkowski, Cordelia Langford, John Sillitoe on behalf of the Wellcome Sanger Institute COVID-19 Surveillance Team
EPI_ISL_634465	Lighthouse Lab in Milton Keynes	Wellcome Sanger Institute for the COVID-19 Genomics UK (COG-UK) Consortium	The Lighthouse Lab in Milton Keynes and Alex Alderton, Roberto Amato, Sonia Goncalves, Ewan Harrison, David K. Jackson, Ian Johnston, Dominic Kwiatkowski, Cordelia Langford, John Sillitoe on behalf of the Wellcome Sanger Institute COVID-19 Surveillance Team
EPI_ISL_634466, EPI_ISL_634467, EPI_ISL_634468, EPI_ISL_634469, EPI_ISL_634470, EPI_ISL_634471, EPI_ISL_634474, EPI_ISL_634475, EPI_ISL_634476, EPI_ISL_634477, EPI_ISL_634478, EPI_ISL_634479, EPI_ISL_634480, EPI_ISL_634481			
see above	Lighthouse Lab in Milton Keynes	Wellcome Sanger Institute for the COVID-19 Genomics UK (COG-UK) consortium	The Lighthouse Lab in Milton Keynes and Alex Alderton, Roberto Amato, Sonia Goncalves, Ewan Harrison, David K. Jackson, Ian Johnston, Dominic Kwiatkowski, Cordelia Langford, John Sillitoe on behalf of the Wellcome Sanger Institute COVID-19 Surveillance Team
EPI_ISL_634483	Lighthouse Lab in Milton Keynes	Wellcome Sanger Institute for the COVID-19 Genomics UK (COG-UK) Consortium	The Lighthouse Lab in Milton Keynes and Alex Alderton, Roberto Amato, Sonia Goncalves, Ewan Harrison, David K. Jackson, Ian Johnston, Dominic Kwiatkowski, Cordelia Langford, John Sillitoe on behalf of the Wellcome Sanger Institute COVID-19 Surveillance Team
EPI_ISL_634484, EPI_ISL_634485, EPI_ISL_634487, EPI_ISL_634488, EPI_ISL_634489, EPI_ISL_634490, EPI_ISL_634491, EPI_ISL_634492, EPI_ISL_634493, EPI_ISL_634495, EPI_ISL_634496, EPI_ISL_634500, EPI_ISL_634501, EPI_ISL_634502, EPI_ISL_634503, EPI_ISL_634505, EPI_ISL_634506, EPI_ISL_634507, EPI_ISL_634511, EPI_ISL_634513, EPI_ISL_634514, EPI_ISL_634515, EPI_ISL_634516, EPI_ISL_634517, EPI_ISL_634518, EPI_ISL_634519			

see above	Lighthouse Lab in Milton Keynes	Wellcome Sanger Institute for the COVID-19 Genomics UK (COG-UK) consortium	The Lighthouse Lab in Milton Keynes and Alex Alderton, Roberto Amato, Sonia Goncalves, Ewan Harrison, David K. Jackson, Ian Johnston, Dominic Kwiatkowski, Cordelia Langford, John Sillitoe on behalf of the Wellcome Sanger Institute COVID-19 Surveillance Team
EPI_ISL_634774, EPI_ISL_634786, EPI_ISL_634787	Lighthouse Lab in Alderley Park	Wellcome Sanger Institute for the COVID-19 Genomics UK (COG-UK) consortium	Jacquelyn Wynn, Mairead Hyland, The Lighthouse Lab in Alderley Park and Alex Alderton, Roberto Amato, Sonia Goncalves, Ewan Harrison, David K. Jackson, Ian Johnston, Dominic Kwiatkowski, Cordelia Langford, John Sillitoe on behalf of the Wellcome Sanger Institute COVID-19 Surveillance Team (http://www.sanger.ac.uk/covid-team)
EPI_ISL_634790, EPI_ISL_634791, EPI_ISL_634792, EPI_ISL_634793, EPI_ISL_634794, EPI_ISL_634795, EPI_ISL_634796, EPI_ISL_634797, EPI_ISL_634799, EPI_ISL_634800, EPI_ISL_634802, EPI_ISL_634803, EPI_ISL_634804, EPI_ISL_634805, EPI_ISL_634806, EPI_ISL_634814			
see above	Lighthouse Lab in Cambridge	Wellcome Sanger Institute for the COVID-19 Genomics UK (COG-UK) consortium	Rob Howes, The Lighthouse Lab in Cambridge and Alex Alderton, Roberto Amato, Sonia Goncalves, Ewan Harrison, David K. Jackson, Ian Johnston, Dominic Kwiatkowski, Cordelia Langford, John Sillitoe on behalf of the Wellcome Sanger Institute COVID-19 Surveillance Team (http://www.sanger.ac.uk/covid-team)
EPI_ISL_634857	Minneapolis VA Health Care System	Minnesota Department of Health, Public Health Laboratory	Matt Plumb, Jacob Garfin, Alexandra Lorentz, and Xiong Wang
EPI_ISL_634881, EPI_ISL_634883	Lab voor klinische biologie	Onderzoeksgroep Virologie	Laurens Lambrechts, Nick Vereecke, Marthe Pauwels, Bruno Verhasselt, Linos Vandekerckhove, Hans Nauwynck, Sebastiaan Theuns
EPI_ISL_634895	Lab voor klinische biologie	Onderzoeksgroep Virologie	Nick Vereecke, Laurens Lambrechts, Marthe Pauwels, Bruno Verhasselt, Linos Vandekerckhove, Hans Nauwynck, Sebastiaan Theuns
EPI_ISL_635026, EPI_ISL_635027, EPI_ISL_635028, EPI_ISL_635029, EPI_ISL_635030, EPI_ISL_635031, EPI_ISL_635033, EPI_ISL_635034, EPI_ISL_635035, EPI_ISL_635039, EPI_ISL_635041, EPI_ISL_635042, EPI_ISL_635043, EPI_ISL_635044, EPI_ISL_635045, EPI_ISL_635046, EPI_ISL_635047, EPI_ISL_635048, EPI_ISL_635049, EPI_ISL_635050, EPI_ISL_635051, EPI_ISL_635054, EPI_ISL_635055, EPI_ISL_635056, EPI_ISL_635057, EPI_ISL_635058			
see above	National Health Laboratory Service - Inkosi Albert Luthuli Central Hospital (NHLS-IALCH)	KRISP, KZN Research Innovation and Sequencing Platform	Giandhari J, Pillay S, Lessells R, Mdlalose K, York D, Khan S, Tegally H, Wilkinson E, de Oliveira T
EPI_ISL_635073	University Hospital of Northern Norway, Department for Microbiology and Infectious Disease Control	Norwegian Institute of Public Health, Department of Virology	Kathrine Stene-Johansen, Kamilla Heddeland Instefjord, Hilde Elshaug, Marie Paulsen Madsen, Rasmus Riis Kopperud, Hilde Vollan, Karoline Bragstad, Olav Hungnes
EPI_ISL_635090	Haukeland University Hospital, Department of Medical Microbiology	Norwegian Institute of Public Health, Department of Virology	Kathrine Stene-Johansen, Kamilla Heddeland Instefjord, Hilde Elshaug, Marie Paulsen Madsen, Rasmus Riis Kopperud, Hilde Vollan, Karoline Bragstad, Olav Hungnes
EPI_ISL_635102	Akershus University Hospital, Department for Microbiology and Infectious Disease Control	Norwegian Institute of Public Health, Department of Virology	Kathrine Stene-Johansen, Kamilla Heddeland Instefjord, Hilde Elshaug, Marie Paulsen Madsen, Rasmus Riis Kopperud, Hilde Vollan, Karoline Bragstad, Olav Hungnes
EPI_ISL_635181	University Hospital of Northern Norway, Department for Microbiology and Infectious Disease Control	Norwegian Institute of Public Health, Department of Virology	Kathrine Stene-Johansen, Kamilla Heddeland Instefjord, Hilde Elshaug, Marie Paulsen Madsen, Rasmus Riis Kopperud, Hilde Vollan, Karoline Bragstad, Olav Hungnes
EPI_ISL_635194	Akershus University Hospital, Department for Microbiology and Infectious Disease Control	Norwegian Institute of Public Health, Department of Virology	Kathrine Stene-Johansen, Kamilla Heddeland Instefjord, Hilde Elshaug, Marie Paulsen Madsen, Rasmus Riis Kopperud, Hilde Vollan, Karoline Bragstad, Olav Hungnes
EPI_ISL_635198	University Hospital of Northern Norway, Department for Microbiology and Infectious Disease Control	Norwegian Institute of Public Health, Department of Virology	Kathrine Stene-Johansen, Kamilla Heddeland Instefjord, Hilde Elshaug, Marie Paulsen Madsen, Rasmus Riis Kopperud, Hilde Vollan, Karoline Bragstad, Olav Hungnes
EPI_ISL_636537, EPI_ISL_636551, EPI_ISL_636559, EPI_ISL_636560, EPI_ISL_636591	Dutch COVID-19 response team	National Institute for Public Health and the Environment (RIVM)	Adam Meijer, Harry Vennema, Jeroen Cremer, Sharon van den Brink, Bas van der Veer, AnneMarie van den Brandt, Florian Zwagemaker, Dennis Schmitz, Chantal Reusken, on behalf of the national COVID-19 response team
EPI_ISL_636716, EPI_ISL_636717, EPI_ISL_636719, EPI_ISL_636720, EPI_ISL_636721, EPI_ISL_636722, EPI_ISL_636723	Respiratory Virus Unit, Microbiology Services Colindale, Public Health England	Respiratory Virus Unit, Microbiology Services Colindale, Public Health England	PHE Covid Sequencing Team
EPI_ISL_637118, EPI_ISL_637119	Respiratory Virus Unit, Microbiology Services Colindale, Public Health England	COVID-19 Genomics UK (COG-UK) Consortium	PHE Covid Sequencing Team
EPI_ISL_637256	Wales Specialist Virology Centre Sequencing lab: Pathogen Genomics Unit	COVID-19 Genomics UK (COG-UK) Consortium	Catherine Moore, Johnathan Evans, Laura Gifford, Malorie Perry, Simon Cottrell, Angela Marchbank, Alec Birchley, Alexander Adams, Amy Gaskin, Bree Gatica-Wilcox, Jason Coombes, Joel Southgate, Lauren Gilbert, Lee Graham, Nicole Pacchiarini, Sara Kumziene-Summerhayes, Sarah Taylor, Sophie Jones, Sara Rey, Matthew Bull, Joanne Watkins, Sally Corden, Tom Connor
EPI_ISL_637262, EPI_ISL_637265	Liverpool Clinical Laboratories	COVID-19 Genomics UK (COG-UK) Consortium	Sam Haldenby, Anita Lucaci, Steve Paterson, Julian Hiscox, Alistair Darby, M Almsaud, A Alrezaihi, Muhannad Alruwaili, Stuart D Armstrong, Jones Benjamin, Eleanor G Bentley, Anu Chawla, Jordan J Clark, Angela Cowell, Richard Eccles, Isabel García-Dorival, Matthew Gemmell, Alessandro Gerada, PKF Gilmore, Richard Gregory, Ximeng Han, Catherine Hartley, Margaret Hughes, Miren Iturriza-Gomara, James Johnson, L Luu, Jenifer Manson, Charlotte Nelson, Elaine O'Toole, Cassie Olateju, Rebekah Penrice-Randal , Lucille Rainbow, N.P Randle, Trevor Ian Robinson, Parul Sharma, Ghada T Shawli, James P Stewart, Neil Swainston, Ecaterina Vamos, Joanne Watts, Mark Whitehead
EPI_ISL_637283	Northumbria University / South Tees Hospitals NHS Foundation Trust / North Cumbria Integrated Care NHS Foundation Trust / North Tees and Hartlepool NHS Foundation Trust / Newcastle Hospitals NHS Foundation Trust	COVID-19 Genomics UK (COG-UK) Consortium	Darren L Smith,Andrew Nelson,Matthew Bashton,Greg R Young,Joshua Loh,John Allan,Mohammad A Tariq,Giles S Holt,Gary Black,Wen C Yew,Lynn Dover,Paul Baker,Steve Liggett,Sarah Essex,Jane Greenaway,Debra Padgett,Clive Graham,Garren Scott,Edward Barton,Emma Swindells,Brendan Payne,Jennifer Collins,Yusri Taha,Gary Eltringham
EPI_ISL_637285	Virology Department, Sheffield Teaching Hospitals NHS Foundation Trust/Department of Infection, Immunity and Cardiovascular Disease, The Medical School, University of Sheffield	COVID-19 Genomics UK (COG-UK) Consortium	Thushan de Silva, Matthew Parker, Nikki Smith, Adri Angyal, Rebecca Brown, Luke Green, Rachel Tucker, Paul Parsons, Danielle Groves, Katie Johnson, Laura Carrilero, Alex Keeley, Dave Partridge, Matthew Wyles, Benjamin Lindsey, Mehmet Yavuz, Mohammad Raza, Cariad Evans
EPI_ISL_637294	Department of Pathology, University of Cambridge	COVID-19 Genomics UK (COG-UK) Consortium	Aminu S. Jahun, Yasmin Chaudhry, Grant Hall, Iliana Georgana, Myra Hosmillo, Martin D. Curran, Malte Pinckert, Surendra Parmar, Ian Goodfellow
EPI_ISL_637295, EPI_ISL_637329, EPI_ISL_637350	Liverpool Clinical Laboratories	COVID-19 Genomics UK (COG-UK) Consortium	Sam Haldenby, Anita Lucaci, Steve Paterson, Julian Hiscox, Alistair Darby, M Almsaud, A Alrezaihi, Muhannad Alruwaili, Stuart D Armstrong, Jones Benjamin, Eleanor G Bentley, Anu Chawla, Jordan J Clark, Angela Cowell, Richard Eccles, Isabel García-Dorival, Matthew Gemmell, Alessandro Gerada, PKF Gilmore, Richard Gregory, Ximeng Han, Catherine Hartley, Margaret Hughes, Miren Iturriza-Gomara, James Johnson, L Luu, Jenifer Manson, Charlotte Nelson, Elaine O'Toole, Cassie Olateju, Rebekah Penrice-Randal , Lucille Rainbow, N.P Randle, Trevor Ian Robinson, Parul Sharma, Ghada T Shawli, James P Stewart, Neil Swainston, Ecaterina Vamos, Joanne Watts, Mark Whitehead
EPI_ISL_637355	Department of Pathology, University of Cambridge	COVID-19 Genomics UK (COG-UK) Consortium	Aminu S. Jahun, Yasmin Chaudhry, Grant Hall, Iliana Georgana, Myra Hosmillo, Martin D. Curran, Malte Pinckert, Surendra Parmar, Ian Goodfellow
EPI_ISL_637377	Northumbria University / South Tees Hospitals NHS Foundation Trust / North Cumbria Integrated Care NHS Foundation Trust / North Tees and Hartlepool NHS Foundation Trust / Newcastle Hospitals NHS Foundation Trust	COVID-19 Genomics UK (COG-UK) Consortium	Darren L Smith,Andrew Nelson,Matthew Bashton,Greg R Young,Joshua Loh,John Allan,Mohammad A Tariq,Giles S Holt,Gary Black,Wen C Yew,Lynn Dover,Paul Baker,Steve Liggett,Sarah Essex,Jane Greenaway,Debra Padgett,Clive Graham,Garren Scott,Edward Barton,Emma Swindells,Brendan Payne,Jennifer Collins,Yusri Taha,Gary Eltringham
EPI_ISL_637394, EPI_ISL_637410, EPI_ISL_637418, EPI_ISL_637420, EPI_ISL_637433	Department of Pathology, University of Cambridge	COVID-19 Genomics UK (COG-UK) Consortium	Aminu S. Jahun, Yasmin Chaudhry, Grant Hall, Iliana Georgana, Myra Hosmillo, Martin D. Curran, Malte Pinckert, Surendra Parmar, Ian Goodfellow
EPI_ISL_637444	Liverpool Clinical Laboratories	COVID-19 Genomics UK (COG-UK) Consortium	Sam Haldenby, Anita Lucaci, Steve Paterson, Julian Hiscox, Alistair Darby, M Almsaud, A Alrezaihi, Muhannad Alruwaili, Stuart D Armstrong, Jones Benjamin, Eleanor G Bentley, Anu Chawla, Jordan J Clark, Angela Cowell, Richard Eccles, Isabel García-Dorival, Matthew Gemmell, Alessandro Gerada, PKF Gilmore, Richard Gregory, Ximeng Han, Catherine Hartley, Margaret Hughes, Miren Iturriza-Gomara, James Johnson, L Luu, Jenifer Manson, Charlotte Nelson, Elaine O'Toole, Cassie Olateju, Rebekah Penrice-Randal , Lucille Rainbow, N.P Randle, Trevor Ian Robinson, Parul Sharma, Ghada T Shawli, James P Stewart, Neil Swainston, Ecaterina Vamos, Joanne Watts, Mark Whitehead

EPI_ISL_637455	Northumbria University / South Tees Hospitals NHS Foundation Trust / North Cumbria Integrated Care NHS Foundation Trust / North Tees and Hartlepool NHS Foundation Trust / Newcastle Hospitals NHS Foundation Trust	COVID-19 Genomics UK (COG-UK) Consortium	Darren L Smith,Andrew Nelson,Matthew Bashton,Greg R Young,Joshua Loh,John Allan,Mohammad A Tariq,Giles S Holt,Gary Black,Wen C Yew,Lynn Dover,Paul Baker,Steve Liggett,Sarah Essex,Jane Greenaway,Debra Padgett,Clive Graham,Garren Scott,Edward Barton,Emma Swindells,Brendan Payne,Jennifer Collins,Yusri Taha,Gary Eltringham
EPI_ISL_637468, EPI_ISL_637469, EPI_ISL_637509, EPI_ISL_637510, EPI_ISL_637511, EPI_ISL_637512, EPI_ISL_637529, EPI_ISL_637530	Department of Pathology, University of Cambridge	COVID-19 Genomics UK (COG-UK) Consortium	Aminu S. Jahun, Yasmin Chaudhry, Grant Hall, Iliana Georgana, Myra Hosmillo, Martin D. Curran, Malte Pinckert, Surendra Parmar, Ian Goodfellow
EPI_ISL_637541	Virology Department, Sheffield Teaching Hospitals NHS Foundation Trust/Department of Infection, Immunity and Cardiovascular Disease, The Medical School, University of Sheffield	COVID-19 Genomics UK (COG-UK) Consortium	Thushan de Silva, Matthew Parker, Nikki Smith, Adri Angyal, Rebecca Brown, Luke Green, Rachel Tucker, Paul Parsons, Danielle Groves, Katie Johnson, Laura Carrilero, Alex Keeley, Dave Partridge, Matthew Wyles, Benjamin Lindsey, Mehmet Yavuz, Mohammad Raza, Cariad Evans
EPI_ISL_637567	Department of Pathology, University of Cambridge	COVID-19 Genomics UK (COG-UK) Consortium	Aminu S. Jahun, Yasmin Chaudhry, Grant Hall, Iliana Georgana, Myra Hosmillo, Martin D. Curran, Malte Pinckert, Surendra Parmar, Ian Goodfellow
EPI_ISL_637599	Wales Specialist Virology Centre Sequencing lab: Pathogen Genomics Unit	COVID-19 Genomics UK (COG-UK) Consortium	Catherine Moore, Johnathan Evans, Laura Gifford, Malorie Perry, Simon Cottrell, Angela Marchbank, Alec Birchley, Alexander Adams, Amy Gaskin, Bree Gatica-Wilcox, Jason Coombes, Joel Southgate, Lauren Gilbert, Lee Graham, Nicole Pacchiarini, Sara Kumziene-Summerhayes, Sarah Taylor, Sophie Jones, Sara Rey, Matthew Bull, Joanne Watkins, Sally Corden, Tom Connor
EPI_ISL_637624, EPI_ISL_637640, EPI_ISL_637642, EPI_ISL_637695, EPI_ISL_637697, EPI_ISL_637700, EPI_ISL_637701, EPI_ISL_637702, EPI_ISL_637703, EPI_ISL_637705, EPI_ISL_637723, EPI_ISL_637724, EPI_ISL_637725, EPI_ISL_637737, EPI_ISL_637739, EPI_ISL_637740, EPI_ISL_637741, EPI_ISL_637742, EPI_ISL_637743, EPI_ISL_637744, EPI_ISL_637745, EPI_ISL_637746, EPI_ISL_637747			
see above	Department of Pathology, University of Cambridge	COVID-19 Genomics UK (COG-UK) Consortium	Aminu S. Jahun, Yasmin Chaudhry, Grant Hall, Iliana Georgana, Myra Hosmillo, Martin D. Curran, Malte Pinckert, Surendra Parmar, Ian Goodfellow
EPI_ISL_637874	Liverpool Clinical Laboratories	COVID-19 Genomics UK (COG-UK) Consortium	Sam Haldenby, Anita Lucaci, Steve Paterson, Julian Hiscox, Alistair Darby, M Almsaud, A Alrezaihi, Muhannad Alruwaili, Stuart D Armstrong, Jones Benjamin, Eleanor G Bentley, Anu Chawla, Jordan J Clark, Angela Cowell, Richard Eccles, Isabel García-Dorival, Matthew Gemmell, Alessandro Gerada, PKF Gilmore, Richard Gregory, Ximeng Han, Catherine Hartley, Margaret Hughes, Miren Iturriza-Gomara, James Johnson, L Luu, Jenifer Manson, Charlotte Nelson, Elaine O'Toole, Cassie Olateju, Rebekah Penrice-Randal , Lucille Rainbow, N.P Randle, Trevor Ian Robinson, Parul Sharma, Ghada T Shawli, James P Stewart, Neil Swainston, Ecaterina Vamos, Joanne Watts, Mark Whitehead
EPI_ISL_637876, EPI_ISL_637879, EPI_ISL_637881	Virology Department, Sheffield Teaching Hospitals NHS Foundation Trust/Department of Infection, Immunity and Cardiovascular Disease, The Medical School, University of Sheffield	COVID-19 Genomics UK (COG-UK) Consortium	Thushan de Silva, Matthew Parker, Nikki Smith, Adri Angyal, Rebecca Brown, Luke Green, Rachel Tucker, Paul Parsons, Danielle Groves, Katie Johnson, Laura Carrilero, Alex Keeley, Dave Partridge, Matthew Wyles, Benjamin Lindsey, Mehmet Yavuz, Mohammad Raza, Cariad Evans
EPI_ISL_637893	Liverpool Clinical Laboratories	COVID-19 Genomics UK (COG-UK) Consortium	Sam Haldenby, Anita Lucaci, Steve Paterson, Julian Hiscox, Alistair Darby, M Almsaud, A Alrezaihi, Muhannad Alruwaili, Stuart D Armstrong, Jones Benjamin, Eleanor G Bentley, Anu Chawla, Jordan J Clark, Angela Cowell, Richard Eccles, Isabel García-Dorival, Matthew Gemmell, Alessandro Gerada, PKF Gilmore, Richard Gregory, Ximeng Han, Catherine Hartley, Margaret Hughes, Miren Iturriza-Gomara, James Johnson, L Luu, Jenifer Manson, Charlotte Nelson, Elaine O'Toole, Cassie Olateju, Rebekah Penrice-Randal , Lucille Rainbow, N.P Randle, Trevor Ian Robinson, Parul Sharma, Ghada T Shawli, James P Stewart, Neil Swainston, Ecaterina Vamos, Joanne Watts, Mark Whitehead
EPI_ISL_637894	Wales Specialist Virology Centre Sequencing lab: Pathogen Genomics Unit	COVID-19 Genomics UK (COG-UK) Consortium	Catherine Moore, Johnathan Evans, Laura Gifford, Malorie Perry, Simon Cottrell, Angela Marchbank, Alec Birchley, Alexander Adams, Amy Gaskin, Bree Gatica-Wilcox, Jason Coombes, Joel Southgate, Lauren Gilbert, Lee Graham, Nicole Pacchiarini, Sara Kumziene-Summerhayes, Sarah Taylor, Sophie Jones, Sara Rey, Matthew Bull, Joanne Watkins, Sally Corden, Tom Connor
EPI_ISL_637897, EPI_ISL_637905	Virology Department, Sheffield Teaching Hospitals NHS Foundation Trust/Department of Infection, Immunity and Cardiovascular Disease, The Medical School, University of Sheffield	COVID-19 Genomics UK (COG-UK) Consortium	Thushan de Silva, Matthew Parker, Nikki Smith, Adri Angyal, Rebecca Brown, Luke Green, Rachel Tucker, Paul Parsons, Danielle Groves, Katie Johnson, Laura Carrilero, Alex Keeley, Dave Partridge, Matthew Wyles, Benjamin Lindsey, Mehmet Yavuz, Mohammad Raza, Cariad Evans
EPI_ISL_637914	Liverpool Clinical Laboratories	COVID-19 Genomics UK (COG-UK) Consortium	Sam Haldenby, Anita Lucaci, Steve Paterson, Julian Hiscox, Alistair Darby, M Almsaud, A Alrezaihi, Muhannad Alruwaili, Stuart D Armstrong, Jones Benjamin, Eleanor G Bentley, Anu Chawla, Jordan J Clark, Angela Cowell, Richard Eccles, Isabel García-Dorival, Matthew Gemmell, Alessandro Gerada, PKF Gilmore, Richard Gregory, Ximeng Han, Catherine Hartley, Margaret Hughes, Miren Iturriza-Gomara, James Johnson, L Luu, Jenifer Manson, Charlotte Nelson, Elaine O'Toole, Cassie Olateju, Rebekah Penrice-Randal , Lucille Rainbow, N.P Randle, Trevor Ian Robinson, Parul Sharma, Ghada T Shawli, James P Stewart, Neil Swainston, Ecaterina Vamos, Joanne Watts, Mark Whitehead
EPI_ISL_637919	Wales Specialist Virology Centre Sequencing lab: Pathogen Genomics Unit	COVID-19 Genomics UK (COG-UK) Consortium	Catherine Moore, Johnathan Evans, Laura Gifford, Malorie Perry, Simon Cottrell, Angela Marchbank, Alec Birchley, Alexander Adams, Amy Gaskin, Bree Gatica-Wilcox, Jason Coombes, Joel Southgate, Lauren Gilbert, Lee Graham, Nicole Pacchiarini, Sara Kumziene-Summerhayes, Sarah Taylor, Sophie Jones, Sara Rey, Matthew Bull, Joanne Watkins, Sally Corden, Tom Connor
EPI_ISL_637933	Liverpool Clinical Laboratories	COVID-19 Genomics UK (COG-UK) Consortium	Sam Haldenby, Anita Lucaci, Steve Paterson, Julian Hiscox, Alistair Darby, M Almsaud, A Alrezaihi, Muhannad Alruwaili, Stuart D Armstrong, Jones Benjamin, Eleanor G Bentley, Anu Chawla, Jordan J Clark, Angela Cowell, Richard Eccles, Isabel García-Dorival, Matthew Gemmell, Alessandro Gerada, PKF Gilmore, Richard Gregory, Ximeng Han, Catherine Hartley, Margaret Hughes, Miren Iturriza-Gomara, James Johnson, L Luu, Jenifer Manson, Charlotte Nelson, Elaine O'Toole, Cassie Olateju, Rebekah Penrice-Randal , Lucille Rainbow, N.P Randle, Trevor Ian Robinson, Parul Sharma, Ghada T Shawli, James P Stewart, Neil Swainston, Ecaterina Vamos, Joanne Watts, Mark Whitehead
EPI_ISL_637943	Northumbria University / South Tees Hospitals NHS Foundation Trust / North Cumbria Integrated Care NHS Foundation Trust / North Tees and Hartlepool NHS Foundation Trust / Newcastle Hospitals NHS Foundation Trust	COVID-19 Genomics UK (COG-UK) Consortium	Darren L Smith,Andrew Nelson,Matthew Bashton,Greg R Young,Joshua Loh,John Allan,Mohammad A Tariq,Giles S Holt,Gary Black,Wen C Yew,Lynn Dover,Paul Baker,Steve Liggett,Sarah Essex,Jane Greenaway,Debra Padgett,Clive Graham,Garren Scott,Edward Barton,Emma Swindells,Brendan Payne,Jennifer Collins,Yusri Taha,Gary Eltringham
EPI_ISL_637944	Virology Department, Sheffield Teaching Hospitals NHS Foundation Trust/Department of Infection, Immunity and Cardiovascular Disease, The Medical School, University of Sheffield	COVID-19 Genomics UK (COG-UK) Consortium	Thushan de Silva, Matthew Parker, Nikki Smith, Adri Angyal, Rebecca Brown, Luke Green, Rachel Tucker, Paul Parsons, Danielle Groves, Katie Johnson, Laura Carrilero, Alex Keeley, Dave Partridge, Matthew Wyles, Benjamin Lindsey, Mehmet Yavuz, Mohammad Raza, Cariad Evans
EPI_ISL_637948	Liverpool Clinical Laboratories	COVID-19 Genomics UK (COG-UK) Consortium	Sam Haldenby, Anita Lucaci, Steve Paterson, Julian Hiscox, Alistair Darby, M Almsaud, A Alrezaihi, Muhannad Alruwaili, Stuart D Armstrong, Jones Benjamin, Eleanor G Bentley, Anu Chawla, Jordan J Clark, Angela Cowell, Richard Eccles, Isabel García-Dorival, Matthew Gemmell, Alessandro Gerada, PKF Gilmore, Richard Gregory, Ximeng Han, Catherine Hartley, Margaret Hughes, Miren Iturriza-Gomara, James Johnson, L Luu, Jenifer Manson, Charlotte Nelson, Elaine O'Toole, Cassie Olateju, Rebekah Penrice-Randal , Lucille Rainbow, N.P Randle, Trevor Ian Robinson, Parul Sharma, Ghada T Shawli, James P Stewart, Neil Swainston, Ecaterina Vamos, Joanne Watts, Mark Whitehead
EPI_ISL_637957, EPI_ISL_637962, EPI_ISL_637964	Wales Specialist Virology Centre Sequencing lab: Pathogen Genomics Unit	COVID-19 Genomics UK (COG-UK) Consortium	Catherine Moore, Johnathan Evans, Laura Gifford, Malorie Perry, Simon Cottrell, Angela Marchbank, Alec Birchley, Alexander Adams, Amy Gaskin, Bree Gatica-Wilcox, Jason Coombes, Joel Southgate, Lauren Gilbert, Lee Graham, Nicole Pacchiarini, Sara Kumziene-Summerhayes, Sarah Taylor, Sophie Jones, Sara Rey, Matthew Bull, Joanne Watkins, Sally Corden, Tom Connor
EPI_ISL_637968	Liverpool Clinical Laboratories	COVID-19 Genomics UK (COG-UK) Consortium	Sam Haldenby, Anita Lucaci, Steve Paterson, Julian Hiscox, Alistair Darby, M Almsaud, A Alrezaihi, Muhannad Alruwaili, Stuart D Armstrong, Jones Benjamin, Eleanor G Bentley, Anu Chawla, Jordan J Clark, Angela Cowell, Richard Eccles, Isabel García-Dorival, Matthew Gemmell, Alessandro Gerada, PKF Gilmore, Richard Gregory, Ximeng Han, Catherine Hartley, Margaret Hughes, Miren Iturriza-Gomara, James Johnson, L Luu, Jenifer Manson, Charlotte Nelson, Elaine O'Toole, Cassie Olateju, Rebekah Penrice-Randal , Lucille Rainbow, N.P Randle, Trevor Ian Robinson, Parul Sharma, Ghada T Shawli, James P Stewart, Neil Swainston, Ecaterina Vamos, Joanne Watts, Mark Whitehead
EPI_ISL_637973	Virology Department, Sheffield Teaching Hospitals NHS	COVID-19 Genomics UK (COG-UK) Consortium	Thushan de Silva, Matthew Parker, Nikki Smith, Adri Angyal, Rebecca Brown, Luke Green, Rachel Tucker, Paul Parsons, Danielle Groves, Katie Johnson,

	Foundation Trust/Department of Infection, Immunity and Cardiovascular Disease, The Medical School, University of Sheffield		Laura Carrilero, Alex Keeley, Dave Partridge, Matthew Wyles, Benjamin Lindsey, Mehmet Yavuz, Mohammad Raza, Cariad Evans
EPI_ISL_637981, EPI_ISL_637984, EPI_ISL_637989	Liverpool Clinical Laboratories	COVID-19 Genomics UK (COG-UK) Consortium	Sam Haldenby, Anita Lucaci, Steve Paterson, Julian Hiscox, Alistair Darby, M Almsaud, A Alrezaihi, Muhannad Alruwaili, Stuart D Armstrong, Jones Benjamin, Eleanor G Bentley, Anu Chawla, Jordan J Clark, Angela Cowell, Richard Eccles, Isabel García-Dorival, Matthew Gemmell, Alessandro Gerada, PKF Gilmore, Richard Gregory, Ximeng Han, Catherine Hartley, Margaret Hughes, Miren Iturriza-Gomara, James Johnson, L Luu, Jenifer Manson, Charlotte Nelson, Elaine O'Toole, Cassie Olateju, Rebekah Penrice-Randal , Lucille Rainbow, N.P Randle, Trevor Ian Robinson, Parul Sharma, Ghada T Shawli, James P Stewart, Neil Swainston, Ecaterina Vamos, Joanne Watts, Mark Whitehead
EPI_ISL_637992	Virology Department, Sheffield Teaching Hospitals NHS Foundation Trust/Department of Infection, Immunity and Cardiovascular Disease, The Medical School, University of Sheffield	COVID-19 Genomics UK (COG-UK) Consortium	Thushan de Silva, Matthew Parker, Nikki Smith, Adri Angyal, Rebecca Brown, Luke Green, Rachel Tucker, Paul Parsons, Danielle Groves, Katie Johnson, Laura Carrilero, Alex Keeley, Dave Partridge, Matthew Wyles, Benjamin Lindsey, Mehmet Yavuz, Mohammad Raza, Cariad Evans
EPI_ISL_638001	Department of Pathology, University of Cambridge	COVID-19 Genomics UK (COG-UK) Consortium	Aminu S. Jahun, Yasmin Chaudhry, Grant Hall, Iliana Georgana, Myra Hosmillo, Martin D. Curran, Malte Pinckert, Surendra Parmar, Ian Goodfellow
EPI_ISL_638009	Northumbria University / South Tees Hospitals NHS Foundation Trust / North Cumbria Integrated Care NHS Foundation Trust / North Tees and Hartlepool NHS Foundation Trust / Newcastle Hospitals NHS Foundation Trust	COVID-19 Genomics UK (COG-UK) Consortium	Darren L Smith,Andrew Nelson,Matthew Bashton,Greg R Young,Joshua Loh,John Allan,Mohammad A Tariq,Giles S Holt,Gary Black,Wen C Yew,Lynn Dover,Paul Baker,Steve Liggett,Sarah Essex,Jane Greenaway,Debra Padgett,Clive Graham,Garren Scott,Edward Barton,Emma Swindells,Brendan Payne,Jennifer Collins,Yusri Taha,Gary Eltringham
EPI_ISL_638027	Department of Pathology, University of Cambridge	COVID-19 Genomics UK (COG-UK) Consortium	Aminu S. Jahun, Yasmin Chaudhry, Grant Hall, Iliana Georgana, Myra Hosmillo, Martin D. Curran, Malte Pinckert, Surendra Parmar, Ian Goodfellow
EPI_ISL_638118, EPI_ISL_638122, EPI_ISL_638126, EPI_ISL_638130, EPI_ISL_638147, EPI_ISL_638151, EPI_ISL_638152, EPI_ISL_638154, EPI_ISL_638156	Wales Specialist Virology Centre Sequencing lab: Pathogen Genomics Unit	COVID-19 Genomics UK (COG-UK) Consortium	Catherine Moore, Johnathan Evans, Laura Gifford, Malorie Perry, Simon Cottrell, Angela Marchbank, Alec Birchley, Alexander Adams, Amy Gaskin, Bree Gatica-Wilcox, Jason Coombes, Joel Southgate, Lauren Gilbert, Lee Graham, Nicole Pacchiarini, Sara Kumziene-Summerhayes, Sarah Taylor, Sophie Jones, Sara Rey, Matthew Bull, Joanne Watkins, Sally Corden, Tom Connor
EPI_ISL_638165, EPI_ISL_638195, EPI_ISL_638197, EPI_ISL_638198, EPI_ISL_638199, EPI_ISL_638200, EPI_ISL_638201, EPI_ISL_638203, EPI_ISL_638313, EPI_ISL_638314, EPI_ISL_638316, EPI_ISL_638320, EPI_ISL_638321, EPI_ISL_638322, EPI_ISL_638323, EPI_ISL_638324, EPI_ISL_638325, EPI_ISL_638327, EPI_ISL_638339, EPI_ISL_638340, EPI_ISL_638341, EPI_ISL_638342, EPI_ISL_638343, EPI_ISL_638344, EPI_ISL_638345, EPI_ISL_638346, EPI_ISL_638347, EPI_ISL_638348, EPI_ISL_638349, EPI_ISL_638350, EPI_ISL_638351, EPI_ISL_638352, EPI_ISL_638353, EPI_ISL_638354, EPI_ISL_638355, EPI_ISL_638356, EPI_ISL_638357, EPI_ISL_638358, EPI_ISL_638359, EPI_ISL_638360, EPI_ISL_638362, EPI_ISL_638363, EPI_ISL_638383, EPI_ISL_638384, EPI_ISL_638386, EPI_ISL_638387, EPI_ISL_638388, EPI_ISL_638390, EPI_ISL_638392, EPI_ISL_638394			
see above	Department of Pathology, University of Cambridge	COVID-19 Genomics UK (COG-UK) Consortium	Aminu S. Jahun, Yasmin Chaudhry, Grant Hall, Iliana Georgana, Myra Hosmillo, Martin D. Curran, Malte Pinckert, Surendra Parmar, Ian Goodfellow
EPI_ISL_638531	Oxford Viromics, NDM, University of Oxford; Oxford University Hospitals; Basingstoke and North Hampshire Hospital	COVID-19 Genomics UK (COG-UK) Consortium	Tanya Golubchik, David Bonsall, George Macintyre, Amy Trebes, Mariateresa de Cesare, Catrin Moore, Alex Mobbs, Anita Justice, Robert Shaw, Monique Andersson, Timothy Peto, Emma Wise, Nathan Moore, Jessica Lynch, Nick Cortes, Matilde Mori, Stephen Kidd, David Buck, John Todd, Christophe Fraser
EPI_ISL_638557	Northumbria University / South Tees Hospitals NHS Foundation Trust / North Cumbria Integrated Care NHS Foundation Trust / North Tees and Hartlepool NHS Foundation Trust / Newcastle Hospitals NHS Foundation Trust	COVID-19 Genomics UK (COG-UK) Consortium	Darren L Smith,Andrew Nelson,Matthew Bashton,Greg R Young,Joshua Loh,John Allan,Mohammad A Tariq,Giles S Holt,Gary Black,Wen C Yew,Lynn Dover,Paul Baker,Steve Liggett,Sarah Essex,Jane Greenaway,Debra Padgett,Clive Graham,Garren Scott,Edward Barton,Emma Swindells,Brendan Payne,Jennifer Collins,Yusri Taha,Gary Eltringham
EPI_ISL_638585	Queens Medical Centre, Clinical Microbiology Department / DeepSeq Nottingham	COVID-19 Genomics UK (COG-UK) Consortium	Gemma Clark, Wendy Smith, Manjinder Khakh, Vicki M Fleming, Michelle M Lister, Hannah Howson-Wells, Jonathan Ball, Patrick McClure, Joseph Chappell, Theocharis Tsoleridis, Nadine Holmes, Matthew Carlisle, Christopher Moore, Fei Sang, Johnny Debebe, Victoria Wright, Matthew Loose
EPI_ISL_638890	Virology Department, Sheffield Teaching Hospitals NHS Foundation Trust/Department of Infection, Immunity and Cardiovascular Disease, The Medical School, University of Sheffield	COVID-19 Genomics UK (COG-UK) Consortium	Thushan de Silva, Matthew Parker, Nikki Smith, Adri Angyal, Rebecca Brown, Luke Green, Rachel Tucker, Paul Parsons, Danielle Groves, Katie Johnson, Laura Carrilero, Alex Keeley, Dave Partridge, Matthew Wyles, Benjamin Lindsey, Mehmet Yavuz, Mohammad Raza, Cariad Evans
EPI_ISL_639036, EPI_ISL_639048, EPI_ISL_639049, EPI_ISL_639059, EPI_ISL_639117, EPI_ISL_639124, EPI_ISL_639129, EPI_ISL_639180, EPI_ISL_639429, EPI_ISL_639446, EPI_ISL_639455, EPI_ISL_639458, EPI_ISL_639470, EPI_ISL_639478, EPI_ISL_639511, EPI_ISL_639522, EPI_ISL_639554, EPI_ISL_639589			
see above	Wales Specialist Virology Centre Sequencing lab: Pathogen Genomics Unit	COVID-19 Genomics UK (COG-UK) Consortium	Catherine Moore, Johnathan Evans, Laura Gifford, Malorie Perry, Simon Cottrell, Angela Marchbank, Alec Birchley, Alexander Adams, Amy Gaskin, Bree Gatica-Wilcox, Jason Coombes, Joel Southgate, Lauren Gilbert, Lee Graham, Nicole Pacchiarini, Sara Kumziene-Summerhayes, Sarah Taylor, Sophie Jones, Sara Rey, Matthew Bull, Joanne Watkins, Sally Corden, Tom Connor
EPI_ISL_639736, EPI_ISL_639737	South Eastern Area Laboratory Services (SEALS)	NSW Health Pathology - Institute of Clinical Pathology and Medical Research; Westmead Hospital; University of Sydney	CIDM-PH et al.
EPI_ISL_639750, EPI_ISL_639818	unknown	Public Health Virology Laboratory, Forensic and Scientific Services (PHV-FSS)	Son Nguyen et al.
EPI_ISL_639853, EPI_ISL_639909	National Virus Reference Laboratory	National Virus Reference Laboratory	Michael Carr, Gabriel Gonzalez, Jonathan Dean, Daniel Hare, Cillian F De Gascun
EPI_ISL_639997, EPI_ISL_640002	CNR Virus des Infections Respiratoires - France SUD	CNR Virus des Infections Respiratoires - France SUD	Antonin Bal, Gregory Destras, Gwendolyne Burfin, Hadrien Règue, Alexandre Gaymard, Maude Bouscambert-Duchamp, Florence Morfin-Sherpa, Martine Valette, Bruno Lina, Laurence Josset
EPI_ISL_640142, EPI_ISL_640143, EPI_ISL_640144, EPI_ISL_640145, EPI_ISL_640146, EPI_ISL_640147, EPI_ISL_640148, EPI_ISL_640149, EPI_ISL_640150, EPI_ISL_640151, EPI_ISL_640152, EPI_ISL_640153, EPI_ISL_640154, EPI_ISL_640155, EPI_ISL_640156, EPI_ISL_640157, EPI_ISL_640159, EPI_ISL_640160, EPI_ISL_640162, EPI_ISL_640164, EPI_ISL_640165, EPI_ISL_640166, EPI_ISL_640168, EPI_ISL_640170, EPI_ISL_640171, EPI_ISL_640172, EPI_ISL_640173, EPI_ISL_640175, EPI_ISL_640176, EPI_ISL_640178, EPI_ISL_640179, EPI_ISL_640180, EPI_ISL_640181, EPI_ISL_640182, EPI_ISL_640184, EPI_ISL_640185, EPI_ISL_640187, EPI_ISL_640189, EPI_ISL_640191, EPI_ISL_640192, EPI_ISL_640193, EPI_ISL_640194, EPI_ISL_640195, EPI_ISL_640196, EPI_ISL_640199, EPI_ISL_640201, EPI_ISL_640202, EPI_ISL_640203, EPI_ISL_640204, EPI_ISL_640205, EPI_ISL_640206, EPI_ISL_640207, EPI_ISL_640209, EPI_ISL_640213			
see above	University of Michigan Clinical Microbiology Laboratory	Lauring Lab, University of Michigan, Department of Microbiology and Immunology	Valesano
EPI_ISL_641304	Microbiological Diagnostic Unit - Public Health Laboratory (MDU-PHL)	MDU-PHL	Seemann T., Schultz M.B., Sait, M.L., Sherry, N.L.
EPI_ISL_641404, EPI_ISL_641405, EPI_ISL_641406, EPI_ISL_641407, EPI_ISL_641408	Department of Virus and Microbiological Special Diagnostics, Statens Serum Institut, Copenhagen, Denmark	Albertsen lab, Department of Chemistry and Bioscience, Aalborg University, Denmark	Thomas Bruun Rasmussen, Jannik Fonager, Morten Rasmussen
EPI_ISL_642851, EPI_ISL_642852, EPI_ISL_642882, EPI_ISL_642917, EPI_ISL_642926, EPI_ISL_642970	Lighthouse Lab in Milton Keynes	Wellcome Sanger Institute for the COVID-19 Genomics UK (COG-UK) Consortium	The Lighthouse Lab in Milton Keynes and Alex Alderton, Roberto Amato, Sonia Goncalves, Ewan Harrison, David K. Jackson, Ian Johnston, Dominic Kwiatkowski, Cordelia Langford, John Sillitoe on behalf of the Wellcome Sanger Institute COVID-19 Surveillance Team
EPI_ISL_643117	Lighthouse Lab in Glasgow	Wellcome Sanger Institute for the COVID-19 Genomics UK (COG-UK) Consortium	Harper VanSteenhouse, Yumi Kasai, David Gray, Carol Clugston, Anna Dominiczak and Alex Alderton, Roberto Amato, Sonia Goncalves, Ewan Harrison, David K. Jackson, Ian Johnston, Dominic Kwiatkowski, Cordelia Langford, John Sillitoe on behalf of the Wellcome Sanger Institute COVID-19 Surveillance Team
EPI_ISL_643985, EPI_ISL_643991, EPI_ISL_644052, EPI_ISL_644053, EPI_ISL_644054, EPI_ISL_644055, EPI_ISL_644056, EPI_ISL_644057, EPI_ISL_644058, EPI_ISL_644059, EPI_ISL_644060, EPI_ISL_644061, EPI_ISL_644063, EPI_ISL_644065, EPI_ISL_644067, EPI_ISL_644068, EPI_ISL_644069, EPI_ISL_644070, EPI_ISL_644071, EPI_ISL_644072, EPI_ISL_644073, EPI_ISL_644074, EPI_ISL_644076, EPI_ISL_644077, EPI_ISL_644078, EPI_ISL_644079, EPI_ISL_644080, EPI_ISL_644082, EPI_ISL_644083, EPI_ISL_644084, EPI_ISL_644085, EPI_ISL_644086, EPI_ISL_644087, EPI_ISL_644088, EPI_ISL_644089, EPI_ISL_644090, EPI_ISL_644091, EPI_ISL_644092			
see above	Lighthouse Lab in Milton Keynes	Wellcome Sanger Institute for the COVID-19 Genomics UK (COG-UK) Consortium	The Lighthouse Lab in Milton Keynes and Alex Alderton, Roberto Amato, Sonia Goncalves, Ewan Harrison, David K. Jackson, Ian Johnston, Dominic Kwiatkowski, Cordelia Langford, John Sillitoe on behalf of the Wellcome Sanger Institute COVID-19 Surveillance Team

EPI_ISL_644096, EPI_ISL_644097, EPI_ISL_644100	Lighthouse Lab in Alderley Park	Wellcome Sanger Institute for the COVID-19 Genomics UK (COG-UK) Consortium	Jacquelyn Wynn, Mairead Hyland, The Lighthouse Lab in Alderley Park and Alex Alderton, Roberto Amato, Sonia Goncalves, Ewan Harrison, David K. Jackson, Ian Johnston, Dominic Kwiatkowski, Cordelia Langford, John Sillitoe on behalf of the Wellcome Sanger Institute COVID-19 Surveillance Team
EPI_ISL_644102	Lighthouse Lab in Glasgow	Wellcome Sanger Institute for the COVID-19 Genomics UK (COG-UK) Consortium	Harper VanSteenhouse, Yumi Kasai, David Gray, Carol Clugston, Anna Dominiczak and Alex Alderton, Roberto Amato, Sonia Goncalves, Ewan Harrison, David K. Jackson, Ian Johnston, Dominic Kwiatkowski, Cordelia Langford, John Sillitoe on behalf of the Wellcome Sanger Institute COVID-19 Surveillance Team
EPI_ISL_644105, EPI_ISL_644106	Lighthouse Lab in Alderley Park	Wellcome Sanger Institute for the COVID-19 Genomics UK (COG-UK) Consortium	Jacquelyn Wynn, Mairead Hyland, The Lighthouse Lab in Alderley Park and Alex Alderton, Roberto Amato, Sonia Goncalves, Ewan Harrison, David K. Jackson, Ian Johnston, Dominic Kwiatkowski, Cordelia Langford, John Sillitoe on behalf of the Wellcome Sanger Institute COVID-19 Surveillance Team
EPI_ISL_644107	Lighthouse Lab in Glasgow	Wellcome Sanger Institute for the COVID-19 Genomics UK (COG-UK) Consortium	Harper VanSteenhouse, Yumi Kasai, David Gray, Carol Clugston, Anna Dominiczak and Alex Alderton, Roberto Amato, Sonia Goncalves, Ewan Harrison, David K. Jackson, Ian Johnston, Dominic Kwiatkowski, Cordelia Langford, John Sillitoe on behalf of the Wellcome Sanger Institute COVID-19 Surveillance Team
EPI_ISL_644109, EPI_ISL_644110, EPI_ISL_644113	Lighthouse Lab in Alderley Park	Wellcome Sanger Institute for the COVID-19 Genomics UK (COG-UK) Consortium	Jacquelyn Wynn, Mairead Hyland, The Lighthouse Lab in Alderley Park and Alex Alderton, Roberto Amato, Sonia Goncalves, Ewan Harrison, David K. Jackson, Ian Johnston, Dominic Kwiatkowski, Cordelia Langford, John Sillitoe on behalf of the Wellcome Sanger Institute COVID-19 Surveillance Team
EPI_ISL_644114	Lighthouse Lab in Milton Keynes	Wellcome Sanger Institute for the COVID-19 Genomics UK (COG-UK) Consortium	The Lighthouse Lab in Milton Keynes and Alex Alderton, Roberto Amato, Sonia Goncalves, Ewan Harrison, David K. Jackson, Ian Johnston, Dominic Kwiatkowski, Cordelia Langford, John Sillitoe on behalf of the Wellcome Sanger Institute COVID-19 Surveillance Team
EPI_ISL_644115	Lighthouse Lab in Cambridge	Wellcome Sanger Institute for the COVID-19 Genomics UK (COG-UK) Consortium	Rob Howes, The Lighthouse Lab in Cambridge and Alex Alderton, Roberto Amato, Sonia Goncalves, Ewan Harrison, David K. Jackson, Ian Johnston, Dominic Kwiatkowski, Cordelia Langford, John Sillitoe on behalf of the Wellcome Sanger Institute COVID-19 Surveillance Team
EPI_ISL_644116, EPI_ISL_644119	Lighthouse Lab in Milton Keynes	Wellcome Sanger Institute for the COVID-19 Genomics UK (COG-UK) Consortium	The Lighthouse Lab in Milton Keynes and Alex Alderton, Roberto Amato, Sonia Goncalves, Ewan Harrison, David K. Jackson, Ian Johnston, Dominic Kwiatkowski, Cordelia Langford, John Sillitoe on behalf of the Wellcome Sanger Institute COVID-19 Surveillance Team
EPI_ISL_644346, EPI_ISL_644348, EPI_ISL_644352, EPI_ISL_644357, EPI_ISL_644372	Michigan Department of Health and Human Services, Bureau of Laboratories	Michigan Department of Health and Human Services, Bureau of Laboratories	Blankenship HM, Riner D, Soehnlen MK
EPI_ISL_645297, EPI_ISL_645418, EPI_ISL_645452, EPI_ISL_645459, EPI_ISL_645464, EPI_ISL_645502, EPI_ISL_645503	Lighthouse Lab in Alderley Park	Wellcome Sanger Institute for the COVID-19 Genomics UK (COG-UK) Consortium	Jacquelyn Wynn, Mairead Hyland, The Lighthouse Lab in Alderley Park and Alex Alderton, Roberto Amato, Sonia Goncalves, Ewan Harrison, David K. Jackson, Ian Johnston, Dominic Kwiatkowski, Cordelia Langford, John Sillitoe on behalf of the Wellcome Sanger Institute COVID-19 Surveillance Team
EPI_ISL_648153	Stockholm	The Public Health Agency of Sweden	Anna-Malin Linde, Maria Lind Karlberg, Mattias Haukland, Reza Advani, Olov Svartstrom, Oskar Karlsson Lindsjo, Sandra Broddesson, Petra Edquist, Mia Brytting, Anna Risberg, Karin Tegmark-Wisell
EPI_ISL_648167	Eskilstuna	The Public Health Agency of Sweden	Anna-Malin Linde, Maria Lind Karlberg, Mattias Haukland, Reza Advani, Olov Svartstrom, Oskar Karlsson Lindsjo, Sandra Broddesson, Petra Edquist, Mia Brytting, Anna Risberg, Karin Tegmark-Wisell
EPI_ISL_648176, EPI_ISL_648177, EPI_ISL_648178, EPI_ISL_648179	The Public Health Agency of Sweden	The Public Health Agency of Sweden	Anna-Malin Linde, Maria Lind Karlberg, Mattias Haukland, Reza Advani, Olov Svartstrom, Oskar Karlsson Lindsjo, Sandra Broddesson, Petra Edquist, Mia Brytting, Anna Risberg, Karin Tegmark-Wisell
EPI_ISL_648192	Sundsvall	The Public Health Agency of Sweden	Anna-Malin Linde, Maria Lind Karlberg, Mattias Haukland, Reza Advani, Olov Svartstrom, Oskar Karlsson Lindsjo, Sandra Broddesson, Petra Edquist, Mia Brytting, Anna Risberg, Karin Tegmark-Wisell
EPI_ISL_648287, EPI_ISL_648288, EPI_ISL_648289, EPI_ISL_648290, EPI_ISL_648291, EPI_ISL_648292, EPI_ISL_648293, EPI_ISL_648294, EPI_ISL_648295, EPI_ISL_648296	MD PHL	MD PHL	Maryland Department of Health Laboratories Administration
EPI_ISL_648480, EPI_ISL_648481, EPI_ISL_648482, EPI_ISL_648483, EPI_ISL_648484, EPI_ISL_648485, EPI_ISL_648486, EPI_ISL_648487, EPI_ISL_648488, EPI_ISL_648489, EPI_ISL_648490, EPI_ISL_648491, EPI_ISL_648492, EPI_ISL_648493, EPI_ISL_648494, EPI_ISL_648495			
see above	Santa Clara County Public Health Laboratory	Chan-Zuckerberg Biohub	CZB Cliahub Consortium
EPI_ISL_648923, EPI_ISL_648940, EPI_ISL_648947, EPI_ISL_648948, EPI_ISL_648961, EPI_ISL_649006, EPI_ISL_649010, EPI_ISL_649014, EPI_ISL_649030, EPI_ISL_649031, EPI_ISL_649038, EPI_ISL_649042, EPI_ISL_649044, EPI_ISL_649046, EPI_ISL_649049, EPI_ISL_649050, EPI_ISL_649053			
see above	San Diego County Public Health Laboratory	Andersen lab at Scripps Research	SEARCH Alliance San Diego with Tracy Basler, Jovan Shephard, Brett Austin
EPI_ISL_649192, EPI_ISL_649205	Lighthouse Lab in Milton Keynes	Wellcome Sanger Institute for the COVID-19 Genomics UK (COG-UK) Consortium	The Lighthouse Lab in Milton Keynes and Alex Alderton, Roberto Amato, Sonia Goncalves, Ewan Harrison, David K. Jackson, Ian Johnston, Dominic Kwiatkowski, Cordelia Langford, John Sillitoe on behalf of the Wellcome Sanger Institute COVID-19 Surveillance Team (http://www.sanger.ac.uk/covid-team)
EPI_ISL_649220	Lighthouse Lab in Alderley Park	Wellcome Sanger Institute for the COVID-19 Genomics UK (COG-UK) Consortium	Jacquelyn Wynn, Mairead Hyland, The Lighthouse Lab in Alderley Park and Alex Alderton, Roberto Amato, Sonia Goncalves, Ewan Harrison, David K. Jackson, Ian Johnston, Dominic Kwiatkowski, Cordelia Langford, John Sillitoe on behalf of the Wellcome Sanger Institute COVID-19 Surveillance Team (http://www.sanger.ac.uk/covid-team)
EPI_ISL_649255, EPI_ISL_649283, EPI_ISL_649320, EPI_ISL_649362	Lighthouse Lab in Milton Keynes	Wellcome Sanger Institute for the COVID-19 Genomics UK (COG-UK) Consortium	The Lighthouse Lab in Milton Keynes and Alex Alderton, Roberto Amato, Sonia Goncalves, Ewan Harrison, David K. Jackson, Ian Johnston, Dominic Kwiatkowski, Cordelia Langford, John Sillitoe on behalf of the Wellcome Sanger Institute COVID-19 Surveillance Team (http://www.sanger.ac.uk/covid-team)
EPI_ISL_649369, EPI_ISL_649370	Lighthouse Lab in Alderley Park	Wellcome Sanger Institute for the COVID-19 Genomics UK (COG-UK) Consortium	Jacquelyn Wynn, Mairead Hyland, The Lighthouse Lab in Alderley Park and Alex Alderton, Roberto Amato, Sonia Goncalves, Ewan Harrison, David K. Jackson, Ian Johnston, Dominic Kwiatkowski, Cordelia Langford, John Sillitoe on behalf of the Wellcome Sanger Institute COVID-19 Surveillance Team (http://www.sanger.ac.uk/covid-team)
EPI_ISL_649386, EPI_ISL_649405, EPI_ISL_649424, EPI_ISL_649479, EPI_ISL_649552, EPI_ISL_649554	Lighthouse Lab in Milton Keynes	Wellcome Sanger Institute for the COVID-19 Genomics UK (COG-UK) Consortium	The Lighthouse Lab in Milton Keynes and Alex Alderton, Roberto Amato, Sonia Goncalves, Ewan Harrison, David K. Jackson, Ian Johnston, Dominic Kwiatkowski, Cordelia Langford, John Sillitoe on behalf of the Wellcome Sanger Institute COVID-19 Surveillance Team (http://www.sanger.ac.uk/covid-team)
EPI_ISL_649813, EPI_ISL_649814, EPI_ISL_649815, EPI_ISL_649816, EPI_ISL_649818, EPI_ISL_649819, EPI_ISL_649820, EPI_ISL_649823, EPI_ISL_649824, EPI_ISL_649825, EPI_ISL_649826, EPI_ISL_649827, EPI_ISL_649828, EPI_ISL_649829, EPI_ISL_649830, EPI_ISL_649831, EPI_ISL_649832, EPI_ISL_649833, EPI_ISL_649834, EPI_ISL_649835, EPI_ISL_649836, EPI_ISL_649837, EPI_ISL_649838, EPI_ISL_649839, EPI_ISL_649840, EPI_ISL_649841, EPI_ISL_649842, EPI_ISL_649843, EPI_ISL_649847, EPI_ISL_649848, EPI_ISL_649850, EPI_ISL_649852, EPI_ISL_649854, EPI_ISL_649855, EPI_ISL_649859, EPI_ISL_649862, EPI_ISL_649865			
see above	Respiratory Virus Unit, Microbiology Services Colindale, Public Health England	COVID-19 Genomics UK (COG-UK) Consortium	PHE Covid Sequencing Team
EPI_ISL_650114	Liverpool Clinical Laboratories	COVID-19 Genomics UK (COG-UK) Consortium	Sam Haldenby, Anita Lucaci, Steve Paterson, Julian Hiscox, Alistair Darby, M Almsaud, A Alrezaihi, Muhannad Alruwaili, Stuart D Armstrong, Jones Benjamin, Eleanor G Bentley, Anu Chawla, Jordan J Clark, Angela Cowell, Richard Eccles, Isabel García-Dorival, Matthew Gemmell, Alessandro Gerada, PKF Gilmore, Richard Gregory, Ximeng Han, Catherine Hartley, Margaret Hughes, Miren Iturriza-Gomara, James Johnson, L Luu, Jenifer Manson, Charlotte Nelson, Elaine O'Toole, Cassie Olateju, Rebekah Penrice-Randal , Lucille Rainbow, N.P Randle, Trevor Ian Robinson, Parul Sharma, Ghada T Shawli, James P Stewart, Neil Swainston, Ecaterina Vamos, Joanne Watts, Mark Whitehead
EPI_ISL_650136	Queens Medical Centre, Clinical Microbiology Department / DeepSeq Nottingham	COVID-19 Genomics UK (COG-UK) Consortium	Gemma Clark, Wendy Smith, Manjinder Khakh, Vicki M Fleming, Michelle M Lister, Hannah Howson-Wells, Jonathan Ball, Patrick McClure, Joseph Chappell, Theocharis Tsoleridis, Nadine Holmes, Matthew Carlisle, Christopher Moore, Fei Sang, Johnny Debebe, Victoria Wright, Matthew Loose
EPI_ISL_650138	Virology Department, Royal Infirmary of Edinburgh, NHS Lothian / School of Biological Sciences, University of Edinburgh / Institute of Genetics and Molecular Medicine, University of Edinburgh	COVID-19 Genomics UK (COG-UK) Consortium	McHugh M, Dewar R, Rooke S, Gallagher M, Balcaza C, O'Toole Á, Scher E, Hill V, McCrone JT, Colquhoun R, Yu X, Jackson B, Rambaut A, Williams TC, Templeton K
EPI_ISL_650142	Northumbria University / South Tees Hospitals NHS Foundation Trust / North Cumbria Integrated Care NHS	COVID-19 Genomics UK (COG-UK) Consortium	Darren L Smith,Andrew Nelson,Matthew Bashton,Greg R Young,Joshua Loh,John Allan,Mohammad A Tariq,Giles S Holt,Gary Black,Wen C Yew,Lynn Dover,Paul Baker,Steve Liggett,Sarah Essex,Jane Greenaway,Debra Padgett,Clive Graham,Garren Scott,Edward Barton,Emma Swindells,Brendan

	Foundation Trust / North Tees and Hartlepool NHS Foundation Trust / Newcastle Hospitals NHS Foundation Trust		Payne,Jennifer Collins,Yusri Taha,Gary Eltringham
EPI_ISL_650150	Queens Medical Centre, Clinical Microbiology Department / DeepSeq Nottingham	COVID-19 Genomics UK (COG-UK) Consortium	Gemma Clark, Wendy Smith, Manjinder Khakh, Vicki M Fleming, Michelle M Lister, Hannah Howson-Wells, Jonathan Ball, Patrick McClure, Joseph Chappell, Theocharis Tsoleridis, Nadine Holmes, Matthew Carlisle, Christopher Moore, Fei Sang, Johnny Debebe, Victoria Wright, Matthew Loose
EPI_ISL_650156	Northumbria University / South Tees Hospitals NHS Foundation Trust / North Cumbria Integrated Care NHS Foundation Trust / North Tees and Hartlepool NHS Foundation Trust / Newcastle Hospitals NHS Foundation Trust	COVID-19 Genomics UK (COG-UK) Consortium	Darren L Smith,Andrew Nelson,Matthew Bashton,Greg R Young,Joshua Loh,John Allan,Mohammad A Tariq,Giles S Holt,Gary Black,Wen C Yew,Lynn Dover,Paul Baker,Steve Liggett,Sarah Essex,Jane Greenaway,Debra Padgett,Clive Graham,Garren Scott,Edward Barton,Emma Swindells,Brendan Payne,Jennifer Collins,Yusri Taha,Gary Eltringham
EPI_ISL_650166	Liverpool Clinical Laboratories	COVID-19 Genomics UK (COG-UK) Consortium	Sam Haldenby, Anita Lucaci, Steve Paterson, Julian Hiscox, Alistair Darby, M Almsaud, A Alrezaihi, Muhannad Alruwaili, Stuart D Armstrong, Jones Benjamin, Eleanor G Bentley, Anu Chawla, Jordan J Clark, Angela Cowell, Richard Eccles, Isabel García-Dorival, Matthew Gemmell, Alessandro Gerada, PKF Gilmore, Richard Gregory, Ximeng Han, Catherine Hartley, Margaret Hughes, Miren Iturriza-Gomara, James Johnson, L Luu, Jenifer Manson, Charlotte Nelson, Elaine O'Toole, Cassie Olateju, Rebekah Penrice-Randal , Lucille Rainbow, N.P Randle, Trevor Ian Robinson, Parul Sharma, Ghada T Shawli, James P Stewart, Neil Swainston, Ecaterina Vamos, Joanne Watts, Mark Whitehead
EPI_ISL_650175	West of Scotland Specialist Virology Centre, NHSGGC / MRC-University of Glasgow Centre for Virus Research	COVID-19 Genomics UK (COG-UK) Consortium	Ana da Silva Filipe, Natasha Johnson, Kathy Smollett, Daniel Mair, Stephen Carmichael, Alice Broos, Lily Tong, Jenna Nichols, Kyriaki Nomikou; Sarah McDonald; Richard Orton, Joseph Hughes, Sreenu Vattipally, David L Robertson; Alasdair MacLean, Rory Gunson; Sharif Shaaban, Matthew Holden; Rachel Blacow, Guy Mollett, Kathy Li, James Shepherd, Antonia Ho, Emma Thomson
EPI_ISL_650192, EPI_ISL_650194, EPI_ISL_650196, EPI_ISL_650198, EPI_ISL_650199, EPI_ISL_650202	Liverpool Clinical Laboratories	COVID-19 Genomics UK (COG-UK) Consortium	Sam Haldenby, Anita Lucaci, Steve Paterson, Julian Hiscox, Alistair Darby, M Almsaud, A Alrezaihi, Muhannad Alruwaili, Stuart D Armstrong, Jones Benjamin, Eleanor G Bentley, Anu Chawla, Jordan J Clark, Angela Cowell, Richard Eccles, Isabel García-Dorival, Matthew Gemmell, Alessandro Gerada, PKF Gilmore, Richard Gregory, Ximeng Han, Catherine Hartley, Margaret Hughes, Miren Iturriza-Gomara, James Johnson, L Luu, Jenifer Manson, Charlotte Nelson, Elaine O'Toole, Cassie Olateju, Rebekah Penrice-Randal , Lucille Rainbow, N.P Randle, Trevor Ian Robinson, Parul Sharma, Ghada T Shawli, James P Stewart, Neil Swainston, Ecaterina Vamos, Joanne Watts, Mark Whitehead
EPI_ISL_650225	University of Exeter	COVID-19 Genomics UK (COG-UK) Consortium	Ben Temperton,Aaron Jeffries,Michelle Michelsen,Joanna Warwick-Dugdale,Audrey Farbos,Robyn Manley,Stephen Michell,Jane Masoli
EPI_ISL_650255	Virology Department, Royal Infirmary of Edinburgh, NHS Lothian / School of Biological Sciences, University of Edinburgh / Institute of Genetics and Molecular Medicine, University of Edinburgh	COVID-19 Genomics UK (COG-UK) Consortium	McHugh M, Dewar R, Rooke S, Gallagher M, Balcaza C, O'Toole Á, Scher E, Hill V, McCrone JT, Colquhoun R, Yu X, Jackson B, Rambaut A, Williams TC, Templeton K
EPI_ISL_650259, EPI_ISL_650260, EPI_ISL_650261	University of Exeter	COVID-19 Genomics UK (COG-UK) Consortium	Ben Temperton,Aaron Jeffries,Michelle Michelsen,Joanna Warwick-Dugdale,Audrey Farbos,Robyn Manley,Stephen Michell,Jane Masoli
EPI_ISL_650270	Regional Virus Laboratory, Belfast Health and Social Care Trust	COVID-19 Genomics UK (COG-UK) Consortium	Conall McCaughey, James McKenna, Tanya Curran, Susan Feeney, Alison Watt, Ciara Cox, Mairead Connor, Zoltan Molnar, David Simpson, Derek Fairley
EPI_ISL_650284	Liverpool Clinical Laboratories	COVID-19 Genomics UK (COG-UK) Consortium	Sam Haldenby, Anita Lucaci, Steve Paterson, Julian Hiscox, Alistair Darby, M Almsaud, A Alrezaihi, Muhannad Alruwaili, Stuart D Armstrong, Jones Benjamin, Eleanor G Bentley, Anu Chawla, Jordan J Clark, Angela Cowell, Richard Eccles, Isabel García-Dorival, Matthew Gemmell, Alessandro Gerada, PKF Gilmore, Richard Gregory, Ximeng Han, Catherine Hartley, Margaret Hughes, Miren Iturriza-Gomara, James Johnson, L Luu, Jenifer Manson, Charlotte Nelson, Elaine O'Toole, Cassie Olateju, Rebekah Penrice-Randal , Lucille Rainbow, N.P Randle, Trevor Ian Robinson, Parul Sharma, Ghada T Shawli, James P Stewart, Neil Swainston, Ecaterina Vamos, Joanne Watts, Mark Whitehead
EPI_ISL_650296	University of Exeter	COVID-19 Genomics UK (COG-UK) Consortium	Ben Temperton,Aaron Jeffries,Michelle Michelsen,Joanna Warwick-Dugdale,Audrey Farbos,Robyn Manley,Stephen Michell,Jane Masoli
EPI_ISL_650318, EPI_ISL_650324, EPI_ISL_650326	Liverpool Clinical Laboratories	COVID-19 Genomics UK (COG-UK) Consortium	Sam Haldenby, Anita Lucaci, Steve Paterson, Julian Hiscox, Alistair Darby, M Almsaud, A Alrezaihi, Muhannad Alruwaili, Stuart D Armstrong, Jones Benjamin, Eleanor G Bentley, Anu Chawla, Jordan J Clark, Angela Cowell, Richard Eccles, Isabel García-Dorival, Matthew Gemmell, Alessandro Gerada, PKF Gilmore, Richard Gregory, Ximeng Han, Catherine Hartley, Margaret Hughes, Miren Iturriza-Gomara, James Johnson, L Luu, Jenifer Manson, Charlotte Nelson, Elaine O'Toole, Cassie Olateju, Rebekah Penrice-Randal , Lucille Rainbow, N.P Randle, Trevor Ian Robinson, Parul Sharma, Ghada T Shawli, James P Stewart, Neil Swainston, Ecaterina Vamos, Joanne Watts, Mark Whitehead
EPI_ISL_650329	Regional Virus Laboratory, Belfast Health and Social Care Trust	COVID-19 Genomics UK (COG-UK) Consortium	Conall McCaughey, James McKenna, Tanya Curran, Susan Feeney, Alison Watt, Ciara Cox, Mairead Connor, Zoltan Molnar, David Simpson, Derek Fairley
EPI_ISL_650331	Liverpool Clinical Laboratories	COVID-19 Genomics UK (COG-UK) Consortium	Sam Haldenby, Anita Lucaci, Steve Paterson, Julian Hiscox, Alistair Darby, M Almsaud, A Alrezaihi, Muhannad Alruwaili, Stuart D Armstrong, Jones Benjamin, Eleanor G Bentley, Anu Chawla, Jordan J Clark, Angela Cowell, Richard Eccles, Isabel García-Dorival, Matthew Gemmell, Alessandro Gerada, PKF Gilmore, Richard Gregory, Ximeng Han, Catherine Hartley, Margaret Hughes, Miren Iturriza-Gomara, James Johnson, L Luu, Jenifer Manson, Charlotte Nelson, Elaine O'Toole, Cassie Olateju, Rebekah Penrice-Randal , Lucille Rainbow, N.P Randle, Trevor Ian Robinson, Parul Sharma, Ghada T Shawli, James P Stewart, Neil Swainston, Ecaterina Vamos, Joanne Watts, Mark Whitehead
EPI_ISL_650347	Queens Medical Centre, Clinical Microbiology Department / DeepSeq Nottingham	COVID-19 Genomics UK (COG-UK) Consortium	Gemma Clark, Wendy Smith, Manjinder Khakh, Vicki M Fleming, Michelle M Lister, Hannah Howson-Wells, Jonathan Ball, Patrick McClure, Joseph Chappell, Theocharis Tsoleridis, Nadine Holmes, Matthew Carlisle, Christopher Moore, Fei Sang, Johnny Debebe, Victoria Wright, Matthew Loose
EPI_ISL_650351, EPI_ISL_650359	Northumbria University / South Tees Hospitals NHS Foundation Trust / North Cumbria Integrated Care NHS Foundation Trust / North Tees and Hartlepool NHS Foundation Trust / Newcastle Hospitals NHS Foundation Trust	COVID-19 Genomics UK (COG-UK) Consortium	Darren L Smith,Andrew Nelson,Matthew Bashton,Greg R Young,Joshua Loh,John Allan,Mohammad A Tariq,Giles S Holt,Gary Black,Wen C Yew,Lynn Dover,Paul Baker,Steve Liggett,Sarah Essex,Jane Greenaway,Debra Padgett,Clive Graham,Garren Scott,Edward Barton,Emma Swindells,Brendan Payne,Jennifer Collins,Yusri Taha,Gary Eltringham
EPI_ISL_650360, EPI_ISL_650361	University of Exeter	COVID-19 Genomics UK (COG-UK) Consortium	Ben Temperton,Aaron Jeffries,Michelle Michelsen,Joanna Warwick-Dugdale,Audrey Farbos,Robyn Manley,Stephen Michell,Jane Masoli
EPI_ISL_650366	Liverpool Clinical Laboratories	COVID-19 Genomics UK (COG-UK) Consortium	Sam Haldenby, Anita Lucaci, Steve Paterson, Julian Hiscox, Alistair Darby, M Almsaud, A Alrezaihi, Muhannad Alruwaili, Stuart D Armstrong, Jones Benjamin, Eleanor G Bentley, Anu Chawla, Jordan J Clark, Angela Cowell, Richard Eccles, Isabel García-Dorival, Matthew Gemmell, Alessandro Gerada, PKF Gilmore, Richard Gregory, Ximeng Han, Catherine Hartley, Margaret Hughes, Miren Iturriza-Gomara, James Johnson, L Luu, Jenifer Manson, Charlotte Nelson, Elaine O'Toole, Cassie Olateju, Rebekah Penrice-Randal , Lucille Rainbow, N.P Randle, Trevor Ian Robinson, Parul Sharma, Ghada T Shawli, James P Stewart, Neil Swainston, Ecaterina Vamos, Joanne Watts, Mark Whitehead
EPI_ISL_650378	Regional Virus Laboratory, Belfast Health and Social Care Trust	COVID-19 Genomics UK (COG-UK) Consortium	Conall McCaughey, James McKenna, Tanya Curran, Susan Feeney, Alison Watt, Ciara Cox, Mairead Connor, Zoltan Molnar, David Simpson, Derek Fairley
EPI_ISL_650386	Queens Medical Centre, Clinical Microbiology Department / DeepSeq Nottingham	COVID-19 Genomics UK (COG-UK) Consortium	Gemma Clark, Wendy Smith, Manjinder Khakh, Vicki M Fleming, Michelle M Lister, Hannah Howson-Wells, Jonathan Ball, Patrick McClure, Joseph Chappell, Theocharis Tsoleridis, Nadine Holmes, Matthew Carlisle, Christopher Moore, Fei Sang, Johnny Debebe, Victoria Wright, Matthew Loose
EPI_ISL_650393, EPI_ISL_650428	Northumbria University / South Tees Hospitals NHS Foundation Trust / North Cumbria Integrated Care NHS Foundation Trust / North Tees and Hartlepool NHS Foundation Trust / Newcastle Hospitals NHS Foundation Trust	COVID-19 Genomics UK (COG-UK) Consortium	Darren L Smith,Andrew Nelson,Matthew Bashton,Greg R Young,Joshua Loh,John Allan,Mohammad A Tariq,Giles S Holt,Gary Black,Wen C Yew,Lynn Dover,Paul Baker,Steve Liggett,Sarah Essex,Jane Greenaway,Debra Padgett,Clive Graham,Garren Scott,Edward Barton,Emma Swindells,Brendan Payne,Jennifer Collins,Yusri Taha,Gary Eltringham
EPI_ISL_650444, EPI_ISL_650445	University of Exeter	COVID-19 Genomics UK (COG-UK) Consortium	Ben Temperton,Aaron Jeffries,Michelle Michelsen,Joanna Warwick-Dugdale,Audrey Farbos,Robyn Manley,Stephen Michell,Jane Masoli
EPI_ISL_650479	Liverpool Clinical Laboratories	COVID-19 Genomics UK (COG-UK) Consortium	Sam Haldenby, Anita Lucaci, Steve Paterson, Julian Hiscox, Alistair Darby, M Almsaud, A Alrezaihi, Muhannad Alruwaili, Stuart D Armstrong, Jones Benjamin, Eleanor G Bentley, Anu Chawla, Jordan J Clark, Angela Cowell, Richard Eccles, Isabel García-Dorival, Matthew Gemmell, Alessandro Gerada, PKF Gilmore, Richard Gregory, Ximeng Han, Catherine Hartley, Margaret Hughes, Miren Iturriza-Gomara, James Johnson, L Luu, Jenifer Manson, Charlotte

			Nelson, Elaine O'Toole, Cassie Olateju, Rebekah Penrice-Randal , Lucille Rainbow, N.P Randle, Trevor Ian Robinson, Parul Sharma, Ghada T Shawli, James P Stewart, Neil Swainston, Ecaterina Vamos, Joanne Watts, Mark Whitehead
EPI_ISL_650491, EPI_ISL_650492, EPI_ISL_650509	West of Scotland Specialist Virology Centre, NHSGGC / MRC-University of Glasgow Centre for Virus Research	COVID-19 Genomics UK (COG-UK) Consortium	Ana da Silva Filipe, Natasha Johnson, Kathy Smollett, Daniel Mair, Stephen Carmichael, Alice Broos, Lily Tong, Jenna Nichols, Kyriaki Nomikou; Sarah McDonald; Richard Orton, Joseph Hughes, Sreenu Vattipally, David L Robertson; Alasdair MacLean, Rory Gunson; Sharif Shaaban, Matthew Holden; Rachel Blacow, Guy Mollett, Kathy Li, James Shepherd, Antonia Ho, Emma Thomson
EPI_ISL_650513, EPI_ISL_650524	Regional Virus Laboratory, Belfast Health and Social Care Trust	COVID-19 Genomics UK (COG-UK) Consortium	Conall McCaughey, James McKenna, Tanya Curran, Susan Feeney, Alison Watt, Ciara Cox, Mairead Connor, Zoltan Molnar, David Simpson, Derek Fairley
EPI_ISL_650529	Liverpool Clinical Laboratories	COVID-19 Genomics UK (COG-UK) Consortium	Sam Haldenby, Anita Lucaci, Steve Paterson, Julian Hiscox, Alistair Darby, M Almsaud, A Alrezaihi, Muhannad Alruwaili, Stuart D Armstrong, Jones Benjamin, Eleanor G Bentley, Anu Chawla, Jordan J Clark, Angela Cowell, Richard Eccles, Isabel García-Dorival, Matthew Gemmell, Alessandro Gerada, PKF Gilmore, Richard Gregory, Ximeng Han, Catherine Hartley, Margaret Hughes, Miren Iturriza-Gomara, James Johnson, L Luu, Jenifer Manson, Charlotte Nelson, Elaine O'Toole, Cassie Olateju, Rebekah Penrice-Randal , Lucille Rainbow, N.P Randle, Trevor Ian Robinson, Parul Sharma, Ghada T Shawli, James P Stewart, Neil Swainston, Ecaterina Vamos, Joanne Watts, Mark Whitehead
EPI_ISL_650531	Virology Department, Royal Infirmary of Edinburgh, NHS Lothian / School of Biological Sciences, University of Edinburgh / Institute of Genetics and Molecular Medicine, University of Edinburgh	COVID-19 Genomics UK (COG-UK) Consortium	McHugh M, Dewar R, Rooke S, Gallagher M, Balcaza C, O'Toole Á, Scher E, Hill V, McCrone JT, Colquhoun R, Yu X, Jackson B, Rambaut A, Williams TC, Templeton K
EPI_ISL_650544, EPI_ISL_650547, EPI_ISL_650561	Liverpool Clinical Laboratories	COVID-19 Genomics UK (COG-UK) Consortium	Sam Haldenby, Anita Lucaci, Steve Paterson, Julian Hiscox, Alistair Darby, M Almsaud, A Alrezaihi, Muhannad Alruwaili, Stuart D Armstrong, Jones Benjamin, Eleanor G Bentley, Anu Chawla, Jordan J Clark, Angela Cowell, Richard Eccles, Isabel García-Dorival, Matthew Gemmell, Alessandro Gerada, PKF Gilmore, Richard Gregory, Ximeng Han, Catherine Hartley, Margaret Hughes, Miren Iturriza-Gomara, James Johnson, L Luu, Jenifer Manson, Charlotte Nelson, Elaine O'Toole, Cassie Olateju, Rebekah Penrice-Randal , Lucille Rainbow, N.P Randle, Trevor Ian Robinson, Parul Sharma, Ghada T Shawli, James P Stewart, Neil Swainston, Ecaterina Vamos, Joanne Watts, Mark Whitehead
EPI_ISL_650586	Northumbria University / South Tees Hospitals NHS Foundation Trust / North Cumbria Integrated Care NHS Foundation Trust / North Tees and Hartlepool NHS Foundation Trust / Newcastle Hospitals NHS Foundation Trust	COVID-19 Genomics UK (COG-UK) Consortium	Darren L Smith,Andrew Nelson,Matthew Bashton,Greg R Young,Joshua Loh,John Allan,Mohammad A Tariq,Giles S Holt,Gary Black,Wen C Yew,Lynn Dover,Paul Baker,Steve Liggett,Sarah Essex,Jane Greenaway,Debra Padgett,Clive Graham,Garren Scott,Edward Barton,Emma Swindells,Brendan Payne,Jennifer Collins,Yusri Taha,Gary Eltringham
EPI_ISL_650611	Queens Medical Centre, Clinical Microbiology Department / DeepSeq Nottingham	COVID-19 Genomics UK (COG-UK) Consortium	Gemma Clark, Wendy Smith, Manjinder Khakh, Vicki M Fleming, Michelle M Lister, Hannah Howson-Wells, Jonathan Ball, Patrick McClure, Joseph Chappell, Theocharis Tsoleridis, Nadine Holmes, Matthew Carlisle, Christopher Moore, Fei Sang, Johnny Debebe, Victoria Wright, Matthew Loose
EPI_ISL_650619	Liverpool Clinical Laboratories	COVID-19 Genomics UK (COG-UK) Consortium	Sam Haldenby, Anita Lucaci, Steve Paterson, Julian Hiscox, Alistair Darby, M Almsaud, A Alrezaihi, Muhannad Alruwaili, Stuart D Armstrong, Jones Benjamin, Eleanor G Bentley, Anu Chawla, Jordan J Clark, Angela Cowell, Richard Eccles, Isabel García-Dorival, Matthew Gemmell, Alessandro Gerada, PKF Gilmore, Richard Gregory, Ximeng Han, Catherine Hartley, Margaret Hughes, Miren Iturriza-Gomara, James Johnson, L Luu, Jenifer Manson, Charlotte Nelson, Elaine O'Toole, Cassie Olateju, Rebekah Penrice-Randal , Lucille Rainbow, N.P Randle, Trevor Ian Robinson, Parul Sharma, Ghada T Shawli, James P Stewart, Neil Swainston, Ecaterina Vamos, Joanne Watts, Mark Whitehead
EPI_ISL_650625	Northumbria University / South Tees Hospitals NHS Foundation Trust / North Cumbria Integrated Care NHS Foundation Trust / North Tees and Hartlepool NHS Foundation Trust / Newcastle Hospitals NHS Foundation Trust	COVID-19 Genomics UK (COG-UK) Consortium	Darren L Smith,Andrew Nelson,Matthew Bashton,Greg R Young,Joshua Loh,John Allan,Mohammad A Tariq,Giles S Holt,Gary Black,Wen C Yew,Lynn Dover,Paul Baker,Steve Liggett,Sarah Essex,Jane Greenaway,Debra Padgett,Clive Graham,Garren Scott,Edward Barton,Emma Swindells,Brendan Payne,Jennifer Collins,Yusri Taha,Gary Eltringham
EPI_ISL_650641	Queens Medical Centre, Clinical Microbiology Department / DeepSeq Nottingham	COVID-19 Genomics UK (COG-UK) Consortium	Gemma Clark, Wendy Smith, Manjinder Khakh, Vicki M Fleming, Michelle M Lister, Hannah Howson-Wells, Jonathan Ball, Patrick McClure, Joseph Chappell, Theocharis Tsoleridis, Nadine Holmes, Matthew Carlisle, Christopher Moore, Fei Sang, Johnny Debebe, Victoria Wright, Matthew Loose
EPI_ISL_650642, EPI_ISL_650643, EPI_ISL_650644, EPI_ISL_650645	University of Exeter	COVID-19 Genomics UK (COG-UK) Consortium	Ben Temperton,Aaron Jeffries,Michelle Michelsen,Joanna Warwick-Dugdale,Audrey Farbos,Robyn Manley,Stephen Michell,Jane Masoli
EPI_ISL_650654, EPI_ISL_650656, EPI_ISL_650657, EPI_ISL_650658, EPI_ISL_650671, EPI_ISL_650688, EPI_ISL_650694, EPI_ISL_650710, EPI_ISL_650714, EPI_ISL_650755	Liverpool Clinical Laboratories	COVID-19 Genomics UK (COG-UK) Consortium	Sam Haldenby, Anita Lucaci, Steve Paterson, Julian Hiscox, Alistair Darby, M Almsaud, A Alrezaihi, Muhannad Alruwaili, Stuart D Armstrong, Jones Benjamin, Eleanor G Bentley, Anu Chawla, Jordan J Clark, Angela Cowell, Richard Eccles, Isabel García-Dorival, Matthew Gemmell, Alessandro Gerada, PKF Gilmore, Richard Gregory, Ximeng Han, Catherine Hartley, Margaret Hughes, Miren Iturriza-Gomara, James Johnson, L Luu, Jenifer Manson, Charlotte Nelson, Elaine O'Toole, Cassie Olateju, Rebekah Penrice-Randal , Lucille Rainbow, N.P Randle, Trevor Ian Robinson, Parul Sharma, Ghada T Shawli, James P Stewart, Neil Swainston, Ecaterina Vamos, Joanne Watts, Mark Whitehead
EPI_ISL_650775	Virology Department, Royal Infirmary of Edinburgh, NHS Lothian / School of Biological Sciences, University of Edinburgh / Institute of Genetics and Molecular Medicine, University of Edinburgh	COVID-19 Genomics UK (COG-UK) Consortium	McHugh M, Dewar R, Rooke S, Gallagher M, Balcaza C, O'Toole Á, Scher E, Hill V, McCrone JT, Colquhoun R, Yu X, Jackson B, Rambaut A, Williams TC, Templeton K
EPI_ISL_650782	Liverpool Clinical Laboratories	COVID-19 Genomics UK (COG-UK) Consortium	Sam Haldenby, Anita Lucaci, Steve Paterson, Julian Hiscox, Alistair Darby, M Almsaud, A Alrezaihi, Muhannad Alruwaili, Stuart D Armstrong, Jones Benjamin, Eleanor G Bentley, Anu Chawla, Jordan J Clark, Angela Cowell, Richard Eccles, Isabel García-Dorival, Matthew Gemmell, Alessandro Gerada, PKF Gilmore, Richard Gregory, Ximeng Han, Catherine Hartley, Margaret Hughes, Miren Iturriza-Gomara, James Johnson, L Luu, Jenifer Manson, Charlotte Nelson, Elaine O'Toole, Cassie Olateju, Rebekah Penrice-Randal , Lucille Rainbow, N.P Randle, Trevor Ian Robinson, Parul Sharma, Ghada T Shawli, James P Stewart, Neil Swainston, Ecaterina Vamos, Joanne Watts, Mark Whitehead
EPI_ISL_650785	Northumbria University / South Tees Hospitals NHS Foundation Trust / North Cumbria Integrated Care NHS Foundation Trust / North Tees and Hartlepool NHS Foundation Trust / Newcastle Hospitals NHS Foundation Trust	COVID-19 Genomics UK (COG-UK) Consortium	Darren L Smith,Andrew Nelson,Matthew Bashton,Greg R Young,Joshua Loh,John Allan,Mohammad A Tariq,Giles S Holt,Gary Black,Wen C Yew,Lynn Dover,Paul Baker,Steve Liggett,Sarah Essex,Jane Greenaway,Debra Padgett,Clive Graham,Garren Scott,Edward Barton,Emma Swindells,Brendan Payne,Jennifer Collins,Yusri Taha,Gary Eltringham
EPI_ISL_650788	University of Exeter	COVID-19 Genomics UK (COG-UK) Consortium	Ben Temperton,Aaron Jeffries,Michelle Michelsen,Joanna Warwick-Dugdale,Audrey Farbos,Robyn Manley,Stephen Michell,Jane Masoli
EPI_ISL_650816	West of Scotland Specialist Virology Centre, NHSGGC / MRC-University of Glasgow Centre for Virus Research	COVID-19 Genomics UK (COG-UK) Consortium	Ana da Silva Filipe, Natasha Johnson, Kathy Smollett, Daniel Mair, Stephen Carmichael, Alice Broos, Lily Tong, Jenna Nichols, Kyriaki Nomikou; Sarah McDonald; Richard Orton, Joseph Hughes, Sreenu Vattipally, David L Robertson; Alasdair MacLean, Rory Gunson; Sharif Shaaban, Matthew Holden; Rachel Blacow, Guy Mollett, Kathy Li, James Shepherd, Antonia Ho, Emma Thomson
EPI_ISL_650819, EPI_ISL_650833	Virology Department, Royal Infirmary of Edinburgh, NHS Lothian / School of Biological Sciences, University of Edinburgh / Institute of Genetics and Molecular Medicine, University of Edinburgh	COVID-19 Genomics UK (COG-UK) Consortium	McHugh M, Dewar R, Rooke S, Gallagher M, Balcaza C, O'Toole Á, Scher E, Hill V, McCrone JT, Colquhoun R, Yu X, Jackson B, Rambaut A, Williams TC, Templeton K
EPI_ISL_650840	West of Scotland Specialist Virology Centre, NHSGGC / MRC-University of Glasgow Centre for Virus Research	COVID-19 Genomics UK (COG-UK) Consortium	Ana da Silva Filipe, Natasha Johnson, Kathy Smollett, Daniel Mair, Stephen Carmichael, Alice Broos, Lily Tong, Jenna Nichols, Kyriaki Nomikou; Sarah McDonald; Richard Orton, Joseph Hughes, Sreenu Vattipally, David L Robertson; Alasdair MacLean, Rory Gunson; Sharif Shaaban, Matthew Holden; Rachel Blacow, Guy Mollett, Kathy Li, James Shepherd, Antonia Ho, Emma Thomson
EPI_ISL_650847	Liverpool Clinical Laboratories	COVID-19 Genomics UK (COG-UK) Consortium	Sam Haldenby, Anita Lucaci, Steve Paterson, Julian Hiscox, Alistair Darby, M Almsaud, A Alrezaihi, Muhannad Alruwaili, Stuart D Armstrong, Jones Benjamin, Eleanor G Bentley, Anu Chawla, Jordan J Clark, Angela Cowell, Richard Eccles, Isabel García-Dorival, Matthew Gemmell, Alessandro Gerada, PKF Gilmore, Richard Gregory, Ximeng Han, Catherine Hartley, Margaret Hughes, Miren Iturriza-Gomara, James Johnson, L Luu, Jenifer Manson, Charlotte Nelson, Elaine O'Toole, Cassie Olateju, Rebekah Penrice-Randal , Lucille Rainbow, N.P Randle, Trevor Ian Robinson, Parul Sharma, Ghada T Shawli, James P Stewart, Neil Swainston, Ecaterina Vamos, Joanne Watts, Mark Whitehead

EPI_ISL_650854, EPI_ISL_650855, EPI_ISL_650856, EPI_ISL_650858	West of Scotland Specialist Virology Centre, NHSGGC / MRC-University of Glasgow Centre for Virus Research	COVID-19 Genomics UK (COG-UK) Consortium	Ana da Silva Filipe, Natasha Johnson, Kathy Smollett, Daniel Mair, Stephen Carmichael, Alice Broos, Lily Tong, Jenna Nichols, Kyriaki Nomikou; Sarah McDonald; Richard Orton, Joseph Hughes, Sreenu Vattipally, David L Robertson; Alasdair MacLean, Rory Gunson; Sharif Shaaban, Matthew Holden; Rachel Blacow, Guy Mollett, Kathy Li, James Shepherd, Antonia Ho, Emma Thomson
EPI_ISL_650881, EPI_ISL_650886, EPI_ISL_650887, EPI_ISL_650889, EPI_ISL_650891, EPI_ISL_650892	Liverpool Clinical Laboratories	COVID-19 Genomics UK (COG-UK) Consortium	Sam Haldenby, Anita Lucaci, Steve Paterson, Julian Hiscox, Alistair Darby, M Almsaud, A Alrezaihi, Muhannad Alruwaili, Stuart D Armstrong, Jones Benjamin, Eleanor G Bentley, Anu Chawla, Jordan J Clark, Angela Cowell, Richard Eccles, Isabel García-Dorival, Matthew Gemmell, Alessandro Gerada, PKF Gilmore, Richard Gregory, Ximeng Han, Catherine Hartley, Margaret Hughes, Miren Iturriza-Gomara, James Johnson, L Luu, Jenifer Manson, Charlotte Nelson, Elaine O'Toole, Cassie Olateju, Rebekah Penrice-Randal , Lucille Rainbow, N.P Randle, Trevor Ian Robinson, Parul Sharma, Ghada T Shawli, James P Stewart, Neil Swainston, Ecaterina Vamos, Joanne Watts, Mark Whitehead
EPI_ISL_650898	Regional Virus Laboratory, Belfast Health and Social Care Trust	COVID-19 Genomics UK (COG-UK) Consortium	Conall McCaughey, James McKenna, Tanya Curran, Susan Feeney, Alison Watt, Ciara Cox, Mairead Connor, Zoltan Molnar, David Simpson, Derek Fairley
EPI_ISL_650904	Liverpool Clinical Laboratories	COVID-19 Genomics UK (COG-UK) Consortium	Sam Haldenby, Anita Lucaci, Steve Paterson, Julian Hiscox, Alistair Darby, M Almsaud, A Alrezaihi, Muhannad Alruwaili, Stuart D Armstrong, Jones Benjamin, Eleanor G Bentley, Anu Chawla, Jordan J Clark, Angela Cowell, Richard Eccles, Isabel García-Dorival, Matthew Gemmell, Alessandro Gerada, PKF Gilmore, Richard Gregory, Ximeng Han, Catherine Hartley, Margaret Hughes, Miren Iturriza-Gomara, James Johnson, L Luu, Jenifer Manson, Charlotte Nelson, Elaine O'Toole, Cassie Olateju, Rebekah Penrice-Randal , Lucille Rainbow, N.P Randle, Trevor Ian Robinson, Parul Sharma, Ghada T Shawli, James P Stewart, Neil Swainston, Ecaterina Vamos, Joanne Watts, Mark Whitehead
EPI_ISL_650923	Regional Virus Laboratory, Belfast Health and Social Care Trust	COVID-19 Genomics UK (COG-UK) Consortium	Conall McCaughey, James McKenna, Tanya Curran, Susan Feeney, Alison Watt, Ciara Cox, Mairead Connor, Zoltan Molnar, David Simpson, Derek Fairley
EPI_ISL_650926, EPI_ISL_650928	Liverpool Clinical Laboratories	COVID-19 Genomics UK (COG-UK) Consortium	Sam Haldenby, Anita Lucaci, Steve Paterson, Julian Hiscox, Alistair Darby, M Almsaud, A Alrezaihi, Muhannad Alruwaili, Stuart D Armstrong, Jones Benjamin, Eleanor G Bentley, Anu Chawla, Jordan J Clark, Angela Cowell, Richard Eccles, Isabel García-Dorival, Matthew Gemmell, Alessandro Gerada, PKF Gilmore, Richard Gregory, Ximeng Han, Catherine Hartley, Margaret Hughes, Miren Iturriza-Gomara, James Johnson, L Luu, Jenifer Manson, Charlotte Nelson, Elaine O'Toole, Cassie Olateju, Rebekah Penrice-Randal , Lucille Rainbow, N.P Randle, Trevor Ian Robinson, Parul Sharma, Ghada T Shawli, James P Stewart, Neil Swainston, Ecaterina Vamos, Joanne Watts, Mark Whitehead
EPI_ISL_650930	Regional Virus Laboratory, Belfast Health and Social Care Trust	COVID-19 Genomics UK (COG-UK) Consortium	Conall McCaughey, James McKenna, Tanya Curran, Susan Feeney, Alison Watt, Ciara Cox, Mairead Connor, Zoltan Molnar, David Simpson, Derek Fairley
EPI_ISL_650932, EPI_ISL_650933, EPI_ISL_650934, EPI_ISL_650935, EPI_ISL_650936, EPI_ISL_650939, EPI_ISL_650945, EPI_ISL_650946	Liverpool Clinical Laboratories	COVID-19 Genomics UK (COG-UK) Consortium	Sam Haldenby, Anita Lucaci, Steve Paterson, Julian Hiscox, Alistair Darby, M Almsaud, A Alrezaihi, Muhannad Alruwaili, Stuart D Armstrong, Jones Benjamin, Eleanor G Bentley, Anu Chawla, Jordan J Clark, Angela Cowell, Richard Eccles, Isabel García-Dorival, Matthew Gemmell, Alessandro Gerada, PKF Gilmore, Richard Gregory, Ximeng Han, Catherine Hartley, Margaret Hughes, Miren Iturriza-Gomara, James Johnson, L Luu, Jenifer Manson, Charlotte Nelson, Elaine O'Toole, Cassie Olateju, Rebekah Penrice-Randal , Lucille Rainbow, N.P Randle, Trevor Ian Robinson, Parul Sharma, Ghada T Shawli, James P Stewart, Neil Swainston, Ecaterina Vamos, Joanne Watts, Mark Whitehead
EPI_ISL_651014, EPI_ISL_651015, EPI_ISL_651016, EPI_ISL_651017, EPI_ISL_651018	Department of Pathology, University of Cambridge	COVID-19 Genomics UK (COG-UK) Consortium	Aminu S. Jahun, Yasmin Chaudhry, Grant Hall, Iliana Georgana, Myra Hosmillo, Martin D. Curran, Malte Pinckert, Surendra Parmar, Ian Goodfellow
EPI_ISL_651071, EPI_ISL_651072, EPI_ISL_651073	Queens Medical Centre, Clinical Microbiology Department / DeepSeq Nottingham	COVID-19 Genomics UK (COG-UK) Consortium	Gemma Clark, Wendy Smith, Manjinder Khakh, Vicki M Fleming, Michelle M Lister, Hannah Howson-Wells, Jonathan Ball, Patrick McClure, Joseph Chappell, Theocharis Tsoleridis, Nadine Holmes, Matthew Carlisle, Christopher Moore, Fei Sang, Johnny Debebe, Victoria Wright, Matthew Loose
EPI_ISL_651074, EPI_ISL_651075	University of Exeter	COVID-19 Genomics UK (COG-UK) Consortium	Ben Temperton,Aaron Jeffries,Michelle Michelsen,Joanna Warwick-Dugdale,Audrey Farbos,Robyn Manley,Stephen Michell,Jane Masoli
EPI_ISL_651086, EPI_ISL_651087, EPI_ISL_651088	Oxford Viromics, NDM, University of Oxford; Oxford University Hospitals; Basingstoke and North Hampshire Hospital	COVID-19 Genomics UK (COG-UK) Consortium	Tanya Golubchik, David Bonsall, George Macintyre, Amy Trebes, Mariateresa de Cesare, Catrin Moore, Alex Mobbs, Anita Justice, Robert Shaw, Monique Andersson, Timothy Peto, Emma Wise, Nathan Moore, Jessica Lynch, Nick Cortes, Matilde Mori, Stephen Kidd, David Buck, John Todd, Christophe Fraser
EPI_ISL_651095	Liverpool Clinical Laboratories	COVID-19 Genomics UK (COG-UK) Consortium	Sam Haldenby, Anita Lucaci, Steve Paterson, Julian Hiscox, Alistair Darby, M Almsaud, A Alrezaihi, Muhannad Alruwaili, Stuart D Armstrong, Jones Benjamin, Eleanor G Bentley, Anu Chawla, Jordan J Clark, Angela Cowell, Richard Eccles, Isabel García-Dorival, Matthew Gemmell, Alessandro Gerada, PKF Gilmore, Richard Gregory, Ximeng Han, Catherine Hartley, Margaret Hughes, Miren Iturriza-Gomara, James Johnson, L Luu, Jenifer Manson, Charlotte Nelson, Elaine O'Toole, Cassie Olateju, Rebekah Penrice-Randal , Lucille Rainbow, N.P Randle, Trevor Ian Robinson, Parul Sharma, Ghada T Shawli, James P Stewart, Neil Swainston, Ecaterina Vamos, Joanne Watts, Mark Whitehead
EPI_ISL_651097	Regional Virus Laboratory, Belfast Health and Social Care Trust	COVID-19 Genomics UK (COG-UK) Consortium	Conall McCaughey, James McKenna, Tanya Curran, Susan Feeney, Alison Watt, Ciara Cox, Mairead Connor, Zoltan Molnar, David Simpson, Derek Fairley
EPI_ISL_651103, EPI_ISL_651104, EPI_ISL_651105, EPI_ISL_651106	Oxford Viromics, NDM, University of Oxford; Oxford University Hospitals; Basingstoke and North Hampshire Hospital	COVID-19 Genomics UK (COG-UK) Consortium	Tanya Golubchik, David Bonsall, George Macintyre, Amy Trebes, Mariateresa de Cesare, Catrin Moore, Alex Mobbs, Anita Justice, Robert Shaw, Monique Andersson, Timothy Peto, Emma Wise, Nathan Moore, Jessica Lynch, Nick Cortes, Matilde Mori, Stephen Kidd, David Buck, John Todd, Christophe Fraser
EPI_ISL_651129, EPI_ISL_651131, EPI_ISL_651138	Liverpool Clinical Laboratories	COVID-19 Genomics UK (COG-UK) Consortium	Sam Haldenby, Anita Lucaci, Steve Paterson, Julian Hiscox, Alistair Darby, M Almsaud, A Alrezaihi, Muhannad Alruwaili, Stuart D Armstrong, Jones Benjamin, Eleanor G Bentley, Anu Chawla, Jordan J Clark, Angela Cowell, Richard Eccles, Isabel García-Dorival, Matthew Gemmell, Alessandro Gerada, PKF Gilmore, Richard Gregory, Ximeng Han, Catherine Hartley, Margaret Hughes, Miren Iturriza-Gomara, James Johnson, L Luu, Jenifer Manson, Charlotte Nelson, Elaine O'Toole, Cassie Olateju, Rebekah Penrice-Randal , Lucille Rainbow, N.P Randle, Trevor Ian Robinson, Parul Sharma, Ghada T Shawli, James P Stewart, Neil Swainston, Ecaterina Vamos, Joanne Watts, Mark Whitehead
EPI_ISL_651143	University of Exeter	COVID-19 Genomics UK (COG-UK) Consortium	Ben Temperton,Aaron Jeffries,Michelle Michelsen,Joanna Warwick-Dugdale,Audrey Farbos,Robyn Manley,Stephen Michell,Jane Masoli
EPI_ISL_651145, EPI_ISL_651161, EPI_ISL_651162, EPI_ISL_651192, EPI_ISL_651196, EPI_ISL_651199, EPI_ISL_651205, EPI_ISL_651225	Liverpool Clinical Laboratories	COVID-19 Genomics UK (COG-UK) Consortium	Sam Haldenby, Anita Lucaci, Steve Paterson, Julian Hiscox, Alistair Darby, M Almsaud, A Alrezaihi, Muhannad Alruwaili, Stuart D Armstrong, Jones Benjamin, Eleanor G Bentley, Anu Chawla, Jordan J Clark, Angela Cowell, Richard Eccles, Isabel García-Dorival, Matthew Gemmell, Alessandro Gerada, PKF Gilmore, Richard Gregory, Ximeng Han, Catherine Hartley, Margaret Hughes, Miren Iturriza-Gomara, James Johnson, L Luu, Jenifer Manson, Charlotte Nelson, Elaine O'Toole, Cassie Olateju, Rebekah Penrice-Randal , Lucille Rainbow, N.P Randle, Trevor Ian Robinson, Parul Sharma, Ghada T Shawli, James P Stewart, Neil Swainston, Ecaterina Vamos, Joanne Watts, Mark Whitehead
EPI_ISL_651231	University of Exeter	COVID-19 Genomics UK (COG-UK) Consortium	Ben Temperton,Aaron Jeffries,Michelle Michelsen,Joanna Warwick-Dugdale,Audrey Farbos,Robyn Manley,Stephen Michell,Jane Masoli
EPI_ISL_651236, EPI_ISL_651270	Liverpool Clinical Laboratories	COVID-19 Genomics UK (COG-UK) Consortium	Sam Haldenby, Anita Lucaci, Steve Paterson, Julian Hiscox, Alistair Darby, M Almsaud, A Alrezaihi, Muhannad Alruwaili, Stuart D Armstrong, Jones Benjamin, Eleanor G Bentley, Anu Chawla, Jordan J Clark, Angela Cowell, Richard Eccles, Isabel García-Dorival, Matthew Gemmell, Alessandro Gerada, PKF Gilmore, Richard Gregory, Ximeng Han, Catherine Hartley, Margaret Hughes, Miren Iturriza-Gomara, James Johnson, L Luu, Jenifer Manson, Charlotte Nelson, Elaine O'Toole, Cassie Olateju, Rebekah Penrice-Randal , Lucille Rainbow, N.P Randle, Trevor Ian Robinson, Parul Sharma, Ghada T Shawli, James P Stewart, Neil Swainston, Ecaterina Vamos, Joanne Watts, Mark Whitehead
EPI_ISL_651285, EPI_ISL_651293	Queens Medical Centre, Clinical Microbiology Department / DeepSeq Nottingham	COVID-19 Genomics UK (COG-UK) Consortium	Gemma Clark, Wendy Smith, Manjinder Khakh, Vicki M Fleming, Michelle M Lister, Hannah Howson-Wells, Jonathan Ball, Patrick McClure, Joseph Chappell, Theocharis Tsoleridis, Nadine Holmes, Matthew Carlisle, Christopher Moore, Fei Sang, Johnny Debebe, Victoria Wright, Matthew Loose
EPI_ISL_651300	University of Exeter	COVID-19 Genomics UK (COG-UK) Consortium	Ben Temperton,Aaron Jeffries,Michelle Michelsen,Joanna Warwick-Dugdale,Audrey Farbos,Robyn Manley,Stephen Michell,Jane Masoli
EPI_ISL_651309	Queens Medical Centre, Clinical Microbiology Department / DeepSeq Nottingham	COVID-19 Genomics UK (COG-UK) Consortium	Gemma Clark, Wendy Smith, Manjinder Khakh, Vicki M Fleming, Michelle M Lister, Hannah Howson-Wells, Jonathan Ball, Patrick McClure, Joseph Chappell, Theocharis Tsoleridis, Nadine Holmes, Matthew Carlisle, Christopher Moore, Fei Sang, Johnny Debebe, Victoria Wright, Matthew Loose
EPI_ISL_651311	Liverpool Clinical Laboratories	COVID-19 Genomics UK (COG-UK) Consortium	Sam Haldenby, Anita Lucaci, Steve Paterson, Julian Hiscox, Alistair Darby, M Almsaud, A Alrezaihi, Muhannad Alruwaili, Stuart D Armstrong, Jones Benjamin, Eleanor G Bentley, Anu Chawla, Jordan J Clark, Angela Cowell, Richard Eccles, Isabel García-Dorival, Matthew Gemmell, Alessandro Gerada, PKF Gilmore, Richard Gregory, Ximeng Han, Catherine Hartley, Margaret Hughes, Miren Iturriza-Gomara, James Johnson, L Luu, Jenifer Manson, Charlotte Nelson, Elaine O'Toole, Cassie Olateju, Rebekah Penrice-Randal , Lucille Rainbow, N.P Randle, Trevor Ian Robinson, Parul Sharma, Ghada T Shawli, James P Stewart, Neil Swainston, Ecaterina Vamos, Joanne Watts, Mark Whitehead

EPI_ISL_651314	Northumbria University / South Tees Hospitals NHS Foundation Trust / North Cumbria Integrated Care NHS Foundation Trust / North Tees and Hartlepool NHS Foundation Trust / Newcastle Hospitals NHS Foundation Trust	COVID-19 Genomics UK (COG-UK) Consortium	Darren L Smith,Andrew Nelson,Matthew Bashton,Greg R Young,Joshua Loh,John Allan,Mohammad A Tariq,Giles S Holt,Gary Black,Wen C Yew,Lynn Dover,Paul Baker,Steve Liggett,Sarah Essex,Jane Greenaway,Debra Padgett,Clive Graham,Garren Scott,Edward Barton,Emma Swindells,Brendan Payne,Jennifer Collins,Yusri Taha,Gary Eltringham
EPI_ISL_651318	Regional Virus Laboratory, Belfast Health and Social Care Trust	COVID-19 Genomics UK (COG-UK) Consortium	Conall McCaughey, James McKenna, Tanya Curran, Susan Feeney, Alison Watt, Ciara Cox, Mairead Connor, Zoltan Molnar, David Simpson, Derek Fairley
EPI_ISL_651320	Virology Department, Royal Infirmary of Edinburgh, NHS Lothian / School of Biological Sciences, University of Edinburgh / Institute of Genetics and Molecular Medicine, University of Edinburgh	COVID-19 Genomics UK (COG-UK) Consortium	McHugh M, Dewar R, Rooke S, Gallagher M, Balcaza C, O'Toole Á, Scher E, Hill V, McCrone JT, Colquhoun R, Yu X, Jackson B, Rambaut A, Williams TC, Templeton K
EPI_ISL_651344	Liverpool Clinical Laboratories	COVID-19 Genomics UK (COG-UK) Consortium	Sam Haldenby, Anita Lucaci, Steve Paterson, Julian Hiscox, Alistair Darby, M Almsaud, A Alrezaihi, Muhannad Alruwaili, Stuart D Armstrong, Jones Benjamin, Eleanor G Bentley, Anu Chawla, Jordan J Clark, Angela Cowell, Richard Eccles, Isabel García-Dorival, Matthew Gemmell, Alessandro Gerada, PKF Gilmore, Richard Gregory, Ximeng Han, Catherine Hartley, Margaret Hughes, Miren Iturriza-Gomara, James Johnson, L Luu, Jenifer Manson, Charlotte Nelson, Elaine O'Toole, Cassie Olateju, Rebekah Penrice-Randal , Lucille Rainbow, N.P Randle, Trevor Ian Robinson, Parul Sharma, Ghada T Shawli, James P Stewart, Neil Swainston, Ecaterina Vamos, Joanne Watts, Mark Whitehead
EPI_ISL_651354	Northumbria University / South Tees Hospitals NHS Foundation Trust / North Cumbria Integrated Care NHS Foundation Trust / North Tees and Hartlepool NHS Foundation Trust / Newcastle Hospitals NHS Foundation Trust	COVID-19 Genomics UK (COG-UK) Consortium	Darren L Smith,Andrew Nelson,Matthew Bashton,Greg R Young,Joshua Loh,John Allan,Mohammad A Tariq,Giles S Holt,Gary Black,Wen C Yew,Lynn Dover,Paul Baker,Steve Liggett,Sarah Essex,Jane Greenaway,Debra Padgett,Clive Graham,Garren Scott,Edward Barton,Emma Swindells,Brendan Payne,Jennifer Collins,Yusri Taha,Gary Eltringham
EPI_ISL_651358	Liverpool Clinical Laboratories	COVID-19 Genomics UK (COG-UK) Consortium	Sam Haldenby, Anita Lucaci, Steve Paterson, Julian Hiscox, Alistair Darby, M Almsaud, A Alrezaihi, Muhannad Alruwaili, Stuart D Armstrong, Jones Benjamin, Eleanor G Bentley, Anu Chawla, Jordan J Clark, Angela Cowell, Richard Eccles, Isabel García-Dorival, Matthew Gemmell, Alessandro Gerada, PKF Gilmore, Richard Gregory, Ximeng Han, Catherine Hartley, Margaret Hughes, Miren Iturriza-Gomara, James Johnson, L Luu, Jenifer Manson, Charlotte Nelson, Elaine O'Toole, Cassie Olateju, Rebekah Penrice-Randal , Lucille Rainbow, N.P Randle, Trevor Ian Robinson, Parul Sharma, Ghada T Shawli, James P Stewart, Neil Swainston, Ecaterina Vamos, Joanne Watts, Mark Whitehead
EPI_ISL_651369	Northumbria University / South Tees Hospitals NHS Foundation Trust / North Cumbria Integrated Care NHS Foundation Trust / North Tees and Hartlepool NHS Foundation Trust / Newcastle Hospitals NHS Foundation Trust	COVID-19 Genomics UK (COG-UK) Consortium	Darren L Smith,Andrew Nelson,Matthew Bashton,Greg R Young,Joshua Loh,John Allan,Mohammad A Tariq,Giles S Holt,Gary Black,Wen C Yew,Lynn Dover,Paul Baker,Steve Liggett,Sarah Essex,Jane Greenaway,Debra Padgett,Clive Graham,Garren Scott,Edward Barton,Emma Swindells,Brendan Payne,Jennifer Collins,Yusri Taha,Gary Eltringham
EPI_ISL_651372	University of Exeter	COVID-19 Genomics UK (COG-UK) Consortium	Ben Temperton,Aaron Jeffries,Michelle Michelsen,Joanna Warwick-Dugdale,Audrey Farbos,Robyn Manley,Stephen Michell,Jane Masoli
EPI_ISL_651375	Liverpool Clinical Laboratories	COVID-19 Genomics UK (COG-UK) Consortium	Sam Haldenby, Anita Lucaci, Steve Paterson, Julian Hiscox, Alistair Darby, M Almsaud, A Alrezaihi, Muhannad Alruwaili, Stuart D Armstrong, Jones Benjamin, Eleanor G Bentley, Anu Chawla, Jordan J Clark, Angela Cowell, Richard Eccles, Isabel García-Dorival, Matthew Gemmell, Alessandro Gerada, PKF Gilmore, Richard Gregory, Ximeng Han, Catherine Hartley, Margaret Hughes, Miren Iturriza-Gomara, James Johnson, L Luu, Jenifer Manson, Charlotte Nelson, Elaine O'Toole, Cassie Olateju, Rebekah Penrice-Randal , Lucille Rainbow, N.P Randle, Trevor Ian Robinson, Parul Sharma, Ghada T Shawli, James P Stewart, Neil Swainston, Ecaterina Vamos, Joanne Watts, Mark Whitehead
EPI_ISL_651384	University of Exeter	COVID-19 Genomics UK (COG-UK) Consortium	Ben Temperton,Aaron Jeffries,Michelle Michelsen,Joanna Warwick-Dugdale,Audrey Farbos,Robyn Manley,Stephen Michell,Jane Masoli
EPI_ISL_651387	Regional Virus Laboratory, Belfast Health and Social Care Trust	COVID-19 Genomics UK (COG-UK) Consortium	Conall McCaughey, James McKenna, Tanya Curran, Susan Feeney, Alison Watt, Ciara Cox, Mairead Connor, Zoltan Molnar, David Simpson, Derek Fairley
EPI_ISL_651396, EPI_ISL_651403	Liverpool Clinical Laboratories	COVID-19 Genomics UK (COG-UK) Consortium	Sam Haldenby, Anita Lucaci, Steve Paterson, Julian Hiscox, Alistair Darby, M Almsaud, A Alrezaihi, Muhannad Alruwaili, Stuart D Armstrong, Jones Benjamin, Eleanor G Bentley, Anu Chawla, Jordan J Clark, Angela Cowell, Richard Eccles, Isabel García-Dorival, Matthew Gemmell, Alessandro Gerada, PKF Gilmore, Richard Gregory, Ximeng Han, Catherine Hartley, Margaret Hughes, Miren Iturriza-Gomara, James Johnson, L Luu, Jenifer Manson, Charlotte Nelson, Elaine O'Toole, Cassie Olateju, Rebekah Penrice-Randal , Lucille Rainbow, N.P Randle, Trevor Ian Robinson, Parul Sharma, Ghada T Shawli, James P Stewart, Neil Swainston, Ecaterina Vamos, Joanne Watts, Mark Whitehead
EPI_ISL_651404	Regional Virus Laboratory, Belfast Health and Social Care Trust	COVID-19 Genomics UK (COG-UK) Consortium	Conall McCaughey, James McKenna, Tanya Curran, Susan Feeney, Alison Watt, Ciara Cox, Mairead Connor, Zoltan Molnar, David Simpson, Derek Fairley
EPI_ISL_651407	Liverpool Clinical Laboratories	COVID-19 Genomics UK (COG-UK) Consortium	Sam Haldenby, Anita Lucaci, Steve Paterson, Julian Hiscox, Alistair Darby, M Almsaud, A Alrezaihi, Muhannad Alruwaili, Stuart D Armstrong, Jones Benjamin, Eleanor G Bentley, Anu Chawla, Jordan J Clark, Angela Cowell, Richard Eccles, Isabel García-Dorival, Matthew Gemmell, Alessandro Gerada, PKF Gilmore, Richard Gregory, Ximeng Han, Catherine Hartley, Margaret Hughes, Miren Iturriza-Gomara, James Johnson, L Luu, Jenifer Manson, Charlotte Nelson, Elaine O'Toole, Cassie Olateju, Rebekah Penrice-Randal , Lucille Rainbow, N.P Randle, Trevor Ian Robinson, Parul Sharma, Ghada T Shawli, James P Stewart, Neil Swainston, Ecaterina Vamos, Joanne Watts, Mark Whitehead
EPI_ISL_651410	Regional Virus Laboratory, Belfast Health and Social Care Trust	COVID-19 Genomics UK (COG-UK) Consortium	Conall McCaughey, James McKenna, Tanya Curran, Susan Feeney, Alison Watt, Ciara Cox, Mairead Connor, Zoltan Molnar, David Simpson, Derek Fairley
EPI_ISL_651417, EPI_ISL_651421	Oxford Viromics, NDM, University of Oxford; Oxford University Hospitals; Basingstoke and North Hampshire Hospital	COVID-19 Genomics UK (COG-UK) Consortium	Tanya Golubchik, David Bonsall, George Macintyre, Amy Trebes, Mariateresa de Cesare, Catrin Moore, Alex Mobbs, Anita Justice, Robert Shaw, Monique Andersson, Timothy Peto, Emma Wise, Nathan Moore, Jessica Lynch, Nick Cortes, Matilde Mori, Stephen Kidd, David Buck, John Todd, Christophe Fraser
EPI_ISL_651466	Queens Medical Centre, Clinical Microbiology Department / DeepSeq Nottingham	COVID-19 Genomics UK (COG-UK) Consortium	Gemma Clark, Wendy Smith, Manjinder Khakh, Vicki M Fleming, Michelle M Lister, Hannah Howson-Wells, Jonathan Ball, Patrick McClure, Joseph Chappell, Theocharis Tsoleridis, Nadine Holmes, Matthew Carlisle, Christopher Moore, Fei Sang, Johnny Debebe, Victoria Wright, Matthew Loose
EPI_ISL_651467, EPI_ISL_651468	University of Exeter	COVID-19 Genomics UK (COG-UK) Consortium	Ben Temperton,Aaron Jeffries,Michelle Michelsen,Joanna Warwick-Dugdale,Audrey Farbos,Robyn Manley,Stephen Michell,Jane Masoli
EPI_ISL_651469, EPI_ISL_651470, EPI_ISL_651471, EPI_ISL_651472, EPI_ISL_651473, EPI_ISL_651474, EPI_ISL_651475, EPI_ISL_651476, EPI_ISL_651477, EPI_ISL_651479, EPI_ISL_651480, EPI_ISL_651481, EPI_ISL_651485			
see above	Oxford Viromics, NDM, University of Oxford; Oxford University Hospitals; Basingstoke and North Hampshire Hospital	COVID-19 Genomics UK (COG-UK) Consortium	Tanya Golubchik, David Bonsall, George Macintyre, Amy Trebes, Mariateresa de Cesare, Catrin Moore, Alex Mobbs, Anita Justice, Robert Shaw, Monique Andersson, Timothy Peto, Emma Wise, Nathan Moore, Jessica Lynch, Nick Cortes, Matilde Mori, Stephen Kidd, David Buck, John Todd, Christophe Fraser
EPI_ISL_651499	Liverpool Clinical Laboratories	COVID-19 Genomics UK (COG-UK) Consortium	Sam Haldenby, Anita Lucaci, Steve Paterson, Julian Hiscox, Alistair Darby, M Almsaud, A Alrezaihi, Muhannad Alruwaili, Stuart D Armstrong, Jones Benjamin, Eleanor G Bentley, Anu Chawla, Jordan J Clark, Angela Cowell, Richard Eccles, Isabel García-Dorival, Matthew Gemmell, Alessandro Gerada, PKF Gilmore, Richard Gregory, Ximeng Han, Catherine Hartley, Margaret Hughes, Miren Iturriza-Gomara, James Johnson, L Luu, Jenifer Manson, Charlotte Nelson, Elaine O'Toole, Cassie Olateju, Rebekah Penrice-Randal , Lucille Rainbow, N.P Randle, Trevor Ian Robinson, Parul Sharma, Ghada T Shawli, James P Stewart, Neil Swainston, Ecaterina Vamos, Joanne Watts, Mark Whitehead
EPI_ISL_651504	Queens Medical Centre, Clinical Microbiology Department / DeepSeq Nottingham	COVID-19 Genomics UK (COG-UK) Consortium	Gemma Clark, Wendy Smith, Manjinder Khakh, Vicki M Fleming, Michelle M Lister, Hannah Howson-Wells, Jonathan Ball, Patrick McClure, Joseph Chappell, Theocharis Tsoleridis, Nadine Holmes, Matthew Carlisle, Christopher Moore, Fei Sang, Johnny Debebe, Victoria Wright, Matthew Loose
EPI_ISL_651505, EPI_ISL_651506, EPI_ISL_651507, EPI_ISL_651509, EPI_ISL_651510, EPI_ISL_651513, EPI_ISL_651517	Oxford Viromics, NDM, University of Oxford; Oxford University Hospitals; Basingstoke and North Hampshire Hospital	COVID-19 Genomics UK (COG-UK) Consortium	Tanya Golubchik, David Bonsall, George Macintyre, Amy Trebes, Mariateresa de Cesare, Catrin Moore, Alex Mobbs, Anita Justice, Robert Shaw, Monique Andersson, Timothy Peto, Emma Wise, Nathan Moore, Jessica Lynch, Nick Cortes, Matilde Mori, Stephen Kidd, David Buck, John Todd, Christophe Fraser
EPI_ISL_651521, EPI_ISL_651522	Virology Department, Royal Infirmary of Edinburgh, NHS	COVID-19 Genomics UK (COG-UK) Consortium	McHugh M, Dewar R, Rooke S, Gallagher M, Balcaza C, O'Toole Á, Scher E, Hill V, McCrone JT, Colquhoun R, Yu X, Jackson B, Rambaut A, Williams TC,

	Lothian / School of Biological Sciences, University of Edinburgh / Institute of Genetics and Molecular Medicine, University of Edinburgh		Templeton K
EPI_ISL_651528	Queens Medical Centre, Clinical Microbiology Department / DeepSeq Nottingham	COVID-19 Genomics UK (COG-UK) Consortium	Gemma Clark, Wendy Smith, Manjinder Khakh, Vicki M Fleming, Michelle M Lister, Hannah Howson-Wells, Jonathan Ball, Patrick McClure, Joseph Chappell, Theocharis Tsoleridis, Nadine Holmes, Matthew Carlisle, Christopher Moore, Fei Sang, Johnny Debebe, Victoria Wright, Matthew Loose
EPI_ISL_651529, EPI_ISL_651541, EPI_ISL_651544	Oxford Viromics, NDM, University of Oxford; Oxford University Hospitals; Basingstoke and North Hampshire Hospital	COVID-19 Genomics UK (COG-UK) Consortium	Tanya Golubchik, David Bonsall, George Macintyre, Amy Trebes, Mariateresa de Cesare, Catrin Moore, Alex Mobbs, Anita Justice, Robert Shaw, Monique Andersson, Timothy Peto, Emma Wise, Nathan Moore, Jessica Lynch, Nick Cortes, Matilde Mori, Stephen Kidd, David Buck, John Todd, Christophe Fraser
EPI_ISL_651547	University of Exeter	COVID-19 Genomics UK (COG-UK) Consortium	Ben Temperton,Aaron Jeffries,Michelle Michelsen,Joanna Warwick-Dugdale,Audrey Farbos,Robyn Manley,Stephen Michell,Jane Masoli
EPI_ISL_651548, EPI_ISL_651549, EPI_ISL_651586, EPI_ISL_651587	Oxford Viromics, NDM, University of Oxford; Oxford University Hospitals; Basingstoke and North Hampshire Hospital	COVID-19 Genomics UK (COG-UK) Consortium	Tanya Golubchik, David Bonsall, George Macintyre, Amy Trebes, Mariateresa de Cesare, Catrin Moore, Alex Mobbs, Anita Justice, Robert Shaw, Monique Andersson, Timothy Peto, Emma Wise, Nathan Moore, Jessica Lynch, Nick Cortes, Matilde Mori, Stephen Kidd, David Buck, John Todd, Christophe Fraser
EPI_ISL_651591	University of Birmingham	COVID-19 Genomics UK (COG-UK) Consortium	Institute of Microbiology, University of Birmingham: Claire McMurray, Joanne Stockton, Samuel Nicholls, Radoslaw Poplawski, Will Rowe, Josh Quick, Nicholas Loman. University of Birmingham Testing Laboratory: Celina M Whalley, Andrew Bosworth, Charlotte Poxon, Kasun Wanigasooriya, Oliver Pickles, Mike Kidd, Alex Richter, Andrew D Beggs PHE Heartlands Lab: Husam Osman, Andrew Bosworth. Queen Elizabeth Hospital: Anna Casey
EPI_ISL_651741, EPI_ISL_651742, EPI_ISL_651817, EPI_ISL_651818, EPI_ISL_651819, EPI_ISL_651820, EPI_ISL_651884	West of Scotland Specialist Virology Centre, NHSGGC / MRC-University of Glasgow Centre for Virus Research	COVID-19 Genomics UK (COG-UK) Consortium	Ana da Silva Filipe, Natasha Johnson, Kathy Smollett, Daniel Mair, Stephen Carmichael, Alice Broos, Lily Tong, Jenna Nichols, Kyriaki Nomikou; Sarah McDonald; Richard Orton, Joseph Hughes, Sreenu Vattipally, David L Robertson; Alasdair MacLean, Rory Gunson; Sharif Shaaban, Matthew Holden; Rachel Blacow, Guy Mollett, Kathy Li, James Shepherd, Antonia Ho, Emma Thomson
EPI_ISL_651960, EPI_ISL_651961, EPI_ISL_651962, EPI_ISL_651963, EPI_ISL_651964, EPI_ISL_651965, EPI_ISL_651966, EPI_ISL_651969, EPI_ISL_651970, EPI_ISL_651971, EPI_ISL_651972, EPI_ISL_651973, EPI_ISL_651974, EPI_ISL_651981, EPI_ISL_652062, EPI_ISL_652063			
see above	Virology Department, Royal Infirmary of Edinburgh, NHS Lothian / School of Biological Sciences, University of Edinburgh / Institute of Genetics and Molecular Medicine, University of Edinburgh	COVID-19 Genomics UK (COG-UK) Consortium	McHugh M, Dewar R, Rooke S, Gallagher M, Balcaza C, O'Toole Á, Scher E, Hill V, McCrone JT, Colquhoun R, Yu X, Jackson B, Rambaut A, Williams TC, Templeton K
EPI_ISL_652073, EPI_ISL_652074, EPI_ISL_652075, EPI_ISL_652076, EPI_ISL_652077, EPI_ISL_652078, EPI_ISL_652079, EPI_ISL_652080, EPI_ISL_652081, EPI_ISL_652082, EPI_ISL_652083, EPI_ISL_652085, EPI_ISL_652086			
see above	Liverpool Clinical Laboratories	COVID-19 Genomics UK (COG-UK) Consortium	Sam Haldenby, Anita Lucaci, Steve Paterson, Julian Hiscox, Alistair Darby, M Almsaud, A Alrezaihi, Muhannad Alruwaili, Stuart D Armstrong, Jones Benjamin, Eleanor G Bentley, Anu Chawla, Jordan J Clark, Angela Cowell, Richard Eccles, Isabel García-Dorival, Matthew Gemmell, Alessandro Gerada, PKF Gilmore, Richard Gregory, Ximeng Han, Catherine Hartley, Margaret Hughes, Miren Iturriza-Gomara, James Johnson, L Luu, Jenifer Manson, Charlotte Nelson, Elaine O'Toole, Cassie Olateju, Rebekah Penrice-Randal , Lucille Rainbow, N.P Randle, Trevor Ian Robinson, Parul Sharma, Ghada T Shawli, James P Stewart, Neil Swainston, Ecaterina Vamos, Joanne Watts, Mark Whitehead
EPI_ISL_652111, EPI_ISL_652113	Northumbria University / South Tees Hospitals NHS Foundation Trust / North Cumbria Integrated Care NHS Foundation Trust / North Tees and Hartlepool NHS Foundation Trust / Newcastle Hospitals NHS Foundation Trust	COVID-19 Genomics UK (COG-UK) Consortium	Darren L Smith,Andrew Nelson,Matthew Bashton,Greg R Young,Joshua Loh,John Allan,Mohammad A Tariq,Giles S Holt,Gary Black,Wen C Yew,Lynn Dover,Paul Baker,Steve Liggett,Sarah Essex,Jane Greenaway,Debra Padgett,Clive Graham,Garren Scott,Edward Barton,Emma Swindells,Brendan Payne,Jennifer Collins,Yusri Taha,Gary Eltringham
EPI_ISL_652157, EPI_ISL_652158, EPI_ISL_652159, EPI_ISL_652160	Department of Pathology, University of Cambridge	COVID-19 Genomics UK (COG-UK) Consortium	Aminu S. Jahun, Yasmin Chaudhry, Grant Hall, Iliana Georgana, Myra Hosmillo, Martin D. Curran, Malte Pinckert, Surendra Parmar, Ian Goodfellow
EPI_ISL_652304, EPI_ISL_652306, EPI_ISL_652308, EPI_ISL_652311, EPI_ISL_652312, EPI_ISL_652313, EPI_ISL_652314, EPI_ISL_652315, EPI_ISL_652316, EPI_ISL_652317, EPI_ISL_652318, EPI_ISL_652319, EPI_ISL_652320, EPI_ISL_652321, EPI_ISL_652322, EPI_ISL_652323, EPI_ISL_652324, EPI_ISL_652325, EPI_ISL_652326, EPI_ISL_652327, EPI_ISL_652328, EPI_ISL_652329			
see above	Regional Virus Laboratory, Belfast Health and Social Care Trust	COVID-19 Genomics UK (COG-UK) Consortium	Conall McCaughey, James McKenna, Tanya Curran, Susan Feeney, Alison Watt, Ciara Cox, Mairead Connor, Zoltan Molnar, David Simpson, Derek Fairley
EPI_ISL_652342	Northumbria University / South Tees Hospitals NHS Foundation Trust / North Cumbria Integrated Care NHS Foundation Trust / North Tees and Hartlepool NHS Foundation Trust / Newcastle Hospitals NHS Foundation Trust	COVID-19 Genomics UK (COG-UK) Consortium	Darren L Smith,Andrew Nelson,Matthew Bashton,Greg R Young,Joshua Loh,John Allan,Mohammad A Tariq,Giles S Holt,Gary Black,Wen C Yew,Lynn Dover,Paul Baker,Steve Liggett,Sarah Essex,Jane Greenaway,Debra Padgett,Clive Graham,Garren Scott,Edward Barton,Emma Swindells,Brendan Payne,Jennifer Collins,Yusri Taha,Gary Eltringham
EPI_ISL_652414	Wales Specialist Virology Centre Sequencing lab: Pathogen Genomics Unit	COVID-19 Genomics UK (COG-UK) Consortium	Catherine Moore, Johnathan Evans, Laura Gifford, Malorie Perry, Simon Cottrell, Angela Marchbank, Alec Birchley, Alexander Adams, Amy Gaskin, Bree Gatica-Wilcox, Jason Coombes, Joel Southgate, Lauren Gilbert, Lee Graham, Nicole Pacchiarini, Sara Kumziene-Summerhayes, Sarah Taylor, Sophie Jones, Sara Rey, Matthew Bull, Joanne Watkins, Sally Corden, Tom Connor
EPI_ISL_652628, EPI_ISL_652629, EPI_ISL_652630, EPI_ISL_652631, EPI_ISL_652632, EPI_ISL_652633, EPI_ISL_652634, EPI_ISL_652635, EPI_ISL_652636, EPI_ISL_652637, EPI_ISL_652638, EPI_ISL_652639, EPI_ISL_652640, EPI_ISL_652641, EPI_ISL_652642, EPI_ISL_652643, EPI_ISL_652644, EPI_ISL_652645, EPI_ISL_652646, EPI_ISL_652647, EPI_ISL_652648, EPI_ISL_652649, EPI_ISL_652650, EPI_ISL_652651, EPI_ISL_652652, EPI_ISL_652653, EPI_ISL_652654, EPI_ISL_652655, EPI_ISL_652656, EPI_ISL_652657, EPI_ISL_652658, EPI_ISL_652659, EPI_ISL_652660, EPI_ISL_652661, EPI_ISL_652662, EPI_ISL_652663, EPI_ISL_652664, EPI_ISL_652665, EPI_ISL_652666, EPI_ISL_652667, EPI_ISL_652668, EPI_ISL_652669, EPI_ISL_652670, EPI_ISL_652671, EPI_ISL_652672, EPI_ISL_652673, EPI_ISL_652674			
see above	Queens Medical Centre, Clinical Microbiology Department / DeepSeq Nottingham	COVID-19 Genomics UK (COG-UK) Consortium	Gemma Clark, Wendy Smith, Manjinder Khakh, Vicki M Fleming, Michelle M Lister, Hannah Howson-Wells, Jonathan Ball, Patrick McClure, Joseph Chappell, Theocharis Tsoleridis, Nadine Holmes, Matthew Carlisle, Christopher Moore, Fei Sang, Johnny Debebe, Victoria Wright, Matthew Loose
EPI_ISL_652675, EPI_ISL_652676, EPI_ISL_652677, EPI_ISL_652678, EPI_ISL_652679, EPI_ISL_652680, EPI_ISL_652681, EPI_ISL_652682, EPI_ISL_652683, EPI_ISL_652684, EPI_ISL_652685, EPI_ISL_652686, EPI_ISL_652687, EPI_ISL_652688, EPI_ISL_652689, EPI_ISL_652690, EPI_ISL_652691, EPI_ISL_652692, EPI_ISL_652693, EPI_ISL_652694, EPI_ISL_652695, EPI_ISL_652696, EPI_ISL_652697, EPI_ISL_652698, EPI_ISL_652699, EPI_ISL_652700, EPI_ISL_652702, EPI_ISL_652703, EPI_ISL_652704, EPI_ISL_652705, EPI_ISL_652706, EPI_ISL_652707, EPI_ISL_652708, EPI_ISL_652709, EPI_ISL_652710			
see above	University of Exeter	COVID-19 Genomics UK (COG-UK) Consortium	Ben Temperton,Aaron Jeffries,Michelle Michelsen,Joanna Warwick-Dugdale,Audrey Farbos,Robyn Manley,Stephen Michell,Jane Masoli
EPI_ISL_652711, EPI_ISL_652712, EPI_ISL_652713, EPI_ISL_652714, EPI_ISL_652715, EPI_ISL_652716, EPI_ISL_652717, EPI_ISL_652718, EPI_ISL_652719, EPI_ISL_652720, EPI_ISL_652721, EPI_ISL_652722, EPI_ISL_652723, EPI_ISL_652724, EPI_ISL_652725, EPI_ISL_652726, EPI_ISL_652727, EPI_ISL_652728, EPI_ISL_652729, EPI_ISL_652730, EPI_ISL_652731, EPI_ISL_652732, EPI_ISL_652733, EPI_ISL_652734, EPI_ISL_652735, EPI_ISL_652736, EPI_ISL_652737, EPI_ISL_652738, EPI_ISL_652739, EPI_ISL_652740, EPI_ISL_652741, EPI_ISL_652742, EPI_ISL_652743, EPI_ISL_652744, EPI_ISL_652745, EPI_ISL_652746, EPI_ISL_652747, EPI_ISL_652748, EPI_ISL_652749, EPI_ISL_652750, EPI_ISL_652751, EPI_ISL_652752, EPI_ISL_652753, EPI_ISL_652754, EPI_ISL_652755, EPI_ISL_652756, EPI_ISL_652757, EPI_ISL_652758, EPI_ISL_652759, EPI_ISL_652760, EPI_ISL_652761, EPI_ISL_652762, EPI_ISL_652763, EPI_ISL_652764, EPI_ISL_652765, EPI_ISL_652766, EPI_ISL_652767, EPI_ISL_652768, EPI_ISL_652769, EPI_ISL_652770, EPI_ISL_652771, EPI_ISL_652772, EPI_ISL_652773, EPI_ISL_652774, EPI_ISL_652775, EPI_ISL_652776, EPI_ISL_652777, EPI_ISL_652778, EPI_ISL_652779, EPI_ISL_652780, EPI_ISL_652781, EPI_ISL_652782, EPI_ISL_652783, EPI_ISL_652784, EPI_ISL_652785, EPI_ISL_652786, EPI_ISL_652787, EPI_ISL_652788, EPI_ISL_652789, EPI_ISL_652790, EPI_ISL_652791, EPI_ISL_652792, EPI_ISL_652793, EPI_ISL_652794, EPI_ISL_652795, EPI_ISL_652796, EPI_ISL_652797, EPI_ISL_652798, EPI_ISL_652799, EPI_ISL_652800, EPI_ISL_652801, EPI_ISL_652802, EPI_ISL_652803, EPI_ISL_652804, EPI_ISL_652805, EPI_ISL_652806, EPI_ISL_652807, EPI_ISL_652808, EPI_ISL_652809, EPI_ISL_652810, EPI_ISL_652811, EPI_ISL_652812, EPI_ISL_652813, EPI_ISL_652814, EPI_ISL_652815, EPI_ISL_652816, EPI_ISL_652817, EPI_ISL_652818, EPI_ISL_652819, EPI_ISL_652820, EPI_ISL_652821, EPI_ISL_652822, EPI_ISL_652823, EPI_ISL_652824, EPI_ISL_652825, EPI_ISL_652826, EPI_ISL_652827, EPI_ISL_652828, EPI_ISL_652829, EPI_ISL_652830, EPI_ISL_652831, EPI_ISL_652832, EPI_ISL_652833, EPI_ISL_652834, EPI_ISL_652835, EPI_ISL_652836, EPI_ISL_652837, EPI_ISL_652838, EPI_ISL_652839, EPI_ISL_652840, EPI_ISL_652841, EPI_ISL_652842, EPI_ISL_652843, EPI_ISL_652844, EPI_ISL_652845, EPI_ISL_652846, EPI_ISL_652847, EPI_ISL_652848, EPI_ISL_652849, EPI_ISL_652850, EPI_ISL_652851, EPI_ISL_652852, EPI_ISL_652853, EPI_ISL_652854, EPI_ISL_652855, EPI_ISL_652856, EPI_ISL_652857, EPI_ISL_652858, EPI_ISL_652859, EPI_ISL_652860, EPI_ISL_652861, EPI_ISL_652862, EPI_ISL_652863, EPI_ISL_652864, EPI_ISL_652865, EPI_ISL_652866, EPI_ISL_652867, EPI_ISL_652868, EPI_ISL_652869, EPI_ISL_652870, EPI_ISL_652871, EPI_ISL_652872, EPI_ISL_652873, EPI_ISL_652874, EPI_ISL_652875, EPI_ISL_652876, EPI_ISL_652877, EPI_ISL_652878, EPI_ISL_652879, EPI_ISL_652880, EPI_ISL_652881, EPI_ISL_652882, EPI_ISL_652883, EPI_ISL_652884, EPI_ISL_652885, EPI_ISL_652886, EPI_ISL_652887, EPI_ISL_652888, EPI_ISL_652889, EPI_ISL_652890, EPI_ISL_652891, EPI_ISL_652892, EPI_ISL_652893, EPI_ISL_652894, EPI_ISL_652895, EPI_ISL_652896, EPI_ISL_652897, EPI_ISL_652898, EPI_ISL_652899, EPI_ISL_652900, EPI_ISL_652901, EPI_ISL_652902, EPI_ISL_652903, EPI_ISL_652904, EPI_ISL_652905, EPI_ISL_652906, EPI_ISL_652907, EPI_ISL_652908, EPI_ISL_652909, EPI_ISL_652910, EPI_ISL_652911, EPI_ISL_652912, EPI_ISL_652913, EPI_ISL_652914, EPI_ISL_652915, EPI_ISL_652916, EPI_ISL_652917, EPI_ISL_652918, EPI_ISL_652919, EPI_ISL_652920, EPI_ISL_652921, EPI_ISL_652922, EPI_ISL_652923, EPI_ISL_652924, EPI_ISL_652925, EPI_ISL_652926, EPI_ISL_652927, EPI_ISL_652928, EPI_ISL_652929, EPI_ISL_652930, EPI_ISL_652931, EPI_ISL_652932, EPI_ISL_652933, EPI_ISL_652934, EPI_ISL_652935, EPI_ISL_652936, EPI_ISL_652937, EPI_ISL_652938, EPI_ISL_652939, EPI_ISL_652940, EPI_ISL_652941, EPI_ISL_652942, EPI_ISL_652943, EPI_ISL_652944, EPI_ISL_652945, EPI_ISL_652946, EPI_ISL_652947, EPI_ISL_652948, EPI_ISL_652959, EPI_ISL_652965, EPI_ISL_652974, EPI_ISL_652977, EPI_ISL_652978, EPI_ISL_652979, EPI_ISL_652980, EPI_ISL_652981, EPI_ISL_652982, EPI_ISL_652983, EPI_ISL_652984, EPI_ISL_652985, EPI_ISL_652986, EPI_ISL_652987, EPI_ISL_652988, EPI_ISL_652989, EPI_ISL_652990, EPI_ISL_652991, EPI_ISL_652999, EPI_ISL_653000, EPI_ISL_653002, EPI_ISL_653004, EPI_ISL_653007, EPI_ISL_653010, EPI_ISL_653011, EPI_ISL_653012, EPI_ISL_653013, EPI_ISL_653022			
see above	Oxford Viromics, NDM, University of Oxford; Oxford University Hospitals; Basingstoke and North Hampshire Hospital	COVID-19 Genomics UK (COG-UK) Consortium	Tanya Golubchik, David Bonsall, George Macintyre, Amy Trebes, Mariateresa de Cesare, Catrin Moore, Alex Mobbs, Anita Justice, Robert Shaw, Monique Andersson, Timothy Peto, Emma Wise, Nathan Moore, Jessica Lynch, Nick Cortes, Matilde Mori, Stephen Kidd, David Buck, John Todd, Christophe Fraser
EPI_ISL_654248, EPI_ISL_654249, EPI_ISL_654251, EPI_ISL_654252, EPI_ISL_654253, EPI_ISL_654256, EPI_ISL_654257, EPI_ISL_654260, EPI_ISL_654262, EPI_ISL_654267, EPI_ISL_654269, EPI_ISL_654270, EPI_ISL_654271, EPI_ISL_654272, EPI_ISL_654273, EPI_ISL_654274, EPI_ISL_654275, EPI_ISL_654312,			

EPI_ISL_654313, EPI_ISL_654314, EPI_ISL_654315, EPI_ISL_654316, EPI_ISL_654317, EPI_ISL_654318, EPI_ISL_654319, EPI_ISL_654320, EPI_ISL_654321, EPI_ISL_654322, EPI_ISL_654323, EPI_ISL_654324, EPI_ISL_654325, EPI_ISL_654326, EPI_ISL_654327, EPI_ISL_654342, EPI_ISL_654343, EPI_ISL_654359			
see above	Hospital General Universitario Gregorio Marañón	SeqCOVID-SPAIN consortium/IBV(CSIC)	Darío García de Viedma, Laura Pérez-Lago, Marta Herranz, Jon Sicilia, Julia Suárez, Pilar Catalán, Patricia Muñoz and SeqCOVID-SPAIN consortium
EPI_ISL_654499, EPI_ISL_654500, EPI_ISL_654501	The Public Health Agency of Sweden	The Public Health Agency of Sweden	Anna-Malin Linde, Maria Lind Karlberg, Mattias Haukland, Reza Advani, Olov Svartstrom, Oskar Karlsson Lindsjo, Sandra Broddesson, Petra Edquist, Mia Brytting, Anna Risberg, Karin Tegmark-Wisell
EPI_ISL_654592, EPI_ISL_654593, EPI_ISL_654594, EPI_ISL_654597, EPI_ISL_654598, EPI_ISL_654599, EPI_ISL_654600, EPI_ISL_654601, EPI_ISL_654602, EPI_ISL_654603, EPI_ISL_654604			
see above	Servicio de Microbiología. Hospital Universitario Donostia. OSI Donostialdea. Área de Enfermedades Infecciosas, Grupo de Infección Respiratoria y Resistencia Antimicrobiana. Instituto de Investigación Sanitaria Biodonostia	SeqCOVID-SPAIN consortium/IBV(CSIC)	Gustavo Cilla Eguiluz, Milagrosa Montes Ros, Luis Piñeiro Vázquez, Ane Sorrarain, Jose Maria Marimón and SeqCOVID-SPAIN consortium
EPI_ISL_660252, EPI_ISL_660253, EPI_ISL_660254, EPI_ISL_660255	NHLS-IALCH	KRISP, KZN Research Innovation and Sequencing Platform	Giandhari J, Pillay S, Lessells R, Mdlalose K, York D, Khan S, Tegally H, Wilkinson E, de Oliveira T
EPI_ISL_660259, EPI_ISL_660260, EPI_ISL_660261, EPI_ISL_660262, EPI_ISL_660263	Molecualr Diagnostic Services (MDS)	KRISP, KZN Research Innovation and Sequencing Platform	Giandhari J, Pillay S, Lessells R, Mdlalose K, York D, Khan S, Tegally H, Wilkinson E, de Oliveira T
EPI_ISL_660489, EPI_ISL_660497	Laboratoire de Microbiologie CHU Souro Sanou	Centre Muraz	Abdoul-Salam Ouedraogo, Yacouba Sawadogo, Essia Belarbi, Grit Schubert, Fabian Leendertz, Arsène Zongo, Soumeya Ouangraoua, Zekiba Tarnagda, Lassana Sangaré, Halidou Tinto
EPI_ISL_660546, EPI_ISL_660547	Respiratory Virus Unit, Microbiology Services Colindale, Public Health England	COVID-19 Genomics UK (COG-UK) Consortium	PHE Covid Sequencing Team
EPI_ISL_660553, EPI_ISL_660566, EPI_ISL_660567, EPI_ISL_660569, EPI_ISL_660570, EPI_ISL_660571, EPI_ISL_660572, EPI_ISL_660573, EPI_ISL_660577, EPI_ISL_660580, EPI_ISL_660583, EPI_ISL_660584, EPI_ISL_660585, EPI_ISL_660586, EPI_ISL_660587, EPI_ISL_660588			
see above	The National Institute of Public Health	State Veterinary Institute Prague	Nagy,A;Jirincova,H;Novakova,L;Trnka,D;Vecerova,J
EPI_ISL_660956, EPI_ISL_660957, EPI_ISL_660958, EPI_ISL_660959, EPI_ISL_660960, EPI_ISL_660961, EPI_ISL_660962, EPI_ISL_660963, EPI_ISL_660964, EPI_ISL_660965, EPI_ISL_660966, EPI_ISL_660967, EPI_ISL_660968			
see above	Gundersen Molecular Diagnostics Laboratory	Kabara Cancer Research Institute	Craig S. Richmond, Paraic A. Kenny
EPI_ISL_661288	Klinisk mikrobiologi	The Public Health Agency of Sweden	Department of Microbiology, The Public Health Agency of Sweden
EPI_ISL_661290	Klinisk mikrobiologi, Viruslab	The Public Health Agency of Sweden	Department of Microbiology, The Public Health Agency of Sweden
EPI_ISL_661299	Laboratoriemedicin, Klinisk mikrobiologi	The Public Health Agency of Sweden	Department of Microbiology, The Public Health Agency of Sweden
EPI_ISL_664342, EPI_ISL_664672, EPI_ISL_664724, EPI_ISL_664753, EPI_ISL_665202, EPI_ISL_665209, EPI_ISL_665210, EPI_ISL_665211, EPI_ISL_665212, EPI_ISL_665213, EPI_ISL_665214, EPI_ISL_665215, EPI_ISL_665216, EPI_ISL_665217, EPI_ISL_665224, EPI_ISL_665225, EPI_ISL_665226, EPI_ISL_665227			
see above	University College London Hospital	COVID-19 Genomics UK (COG-UK) Consortium	Judith Heaney, Matthew Byott, Catherine Houlihan, Dan Frampton, Stuart Kirk, Moira Spyer and Eleni Nastouli
EPI_ISL_665275, EPI_ISL_665280, EPI_ISL_665300, EPI_ISL_665310, EPI_ISL_665317, EPI_ISL_665322, EPI_ISL_665329, EPI_ISL_665350, EPI_ISL_665368, EPI_ISL_665388, EPI_ISL_665389, EPI_ISL_665397, EPI_ISL_665398, EPI_ISL_665405, EPI_ISL_665407			
see above	Northumbria University / South Tees Hospitals NHS Foundation Trust / North Cumbria Integrated Care NHS Foundation Trust / North Tees and Hartlepool NHS Foundation Trust / Newcastle Hospitals NHS Foundation Trust	COVID-19 Genomics UK (COG-UK) Consortium	Darren L Smith,Andrew Nelson,Matthew Bashton,Greg R Young,Joshua Loh,John Allan,Mohammad A Tariq,Giles S Holt,Gary Black,Wen C Yew,Lynn Dover,Paul Baker,Steve Liggett,Sarah Essex,Jane Greenaway,Debra Padgett,Clive Graham,Garren Scott,Edward Barton,Emma Swindells,Brendan Payne,Jennifer Collins,Yusri Taha,Gary Eltringham
EPI_ISL_665422	University of Exeter	COVID-19 Genomics UK (COG-UK) Consortium	Ben Temperton,Aaron Jeffries,Michelle Michelsen,Joanna Warwick-Dugdale,Audrey Farbos,Robyn Manley,Stephen Michell,Jane Masoli
EPI_ISL_665489, EPI_ISL_665620, EPI_ISL_665627, EPI_ISL_665628, EPI_ISL_665631, EPI_ISL_665667, EPI_ISL_665681	Northumbria University / South Tees Hospitals NHS Foundation Trust / North Cumbria Integrated Care NHS Foundation Trust / North Tees and Hartlepool NHS Foundation Trust / Newcastle Hospitals NHS Foundation Trust	COVID-19 Genomics UK (COG-UK) Consortium	Darren L Smith,Andrew Nelson,Matthew Bashton,Greg R Young,Joshua Loh,John Allan,Mohammad A Tariq,Giles S Holt,Gary Black,Wen C Yew,Lynn Dover,Paul Baker,Steve Liggett,Sarah Essex,Jane Greenaway,Debra Padgett,Clive Graham,Garren Scott,Edward Barton,Emma Swindells,Brendan Payne,Jennifer Collins,Yusri Taha,Gary Eltringham
EPI_ISL_665699	Queens Medical Centre, Clinical Microbiology Department / DeepSeq Nottingham	COVID-19 Genomics UK (COG-UK) Consortium	Gemma Clark, Wendy Smith, Manjinder Khakh, Vicki M Fleming, Michelle M Lister, Hannah Howson-Wells, Jonathan Ball, Patrick McClure, Joseph Chappell, Theocharis Tsoleridis, Nadine Holmes, Matthew Carlisle, Christopher Moore, Fei Sang, Johnny Debebe, Victoria Wright, Matthew Loose
EPI_ISL_665706	Northumbria University / South Tees Hospitals NHS Foundation Trust / North Cumbria Integrated Care NHS Foundation Trust / North Tees and Hartlepool NHS Foundation Trust / Newcastle Hospitals NHS Foundation Trust	COVID-19 Genomics UK (COG-UK) Consortium	Darren L Smith,Andrew Nelson,Matthew Bashton,Greg R Young,Joshua Loh,John Allan,Mohammad A Tariq,Giles S Holt,Gary Black,Wen C Yew,Lynn Dover,Paul Baker,Steve Liggett,Sarah Essex,Jane Greenaway,Debra Padgett,Clive Graham,Garren Scott,Edward Barton,Emma Swindells,Brendan Payne,Jennifer Collins,Yusri Taha,Gary Eltringham
EPI_ISL_665719	University of Exeter	COVID-19 Genomics UK (COG-UK) Consortium	Ben Temperton,Aaron Jeffries,Michelle Michelsen,Joanna Warwick-Dugdale,Audrey Farbos,Robyn Manley,Stephen Michell,Jane Masoli
EPI_ISL_665739	Northumbria University / South Tees Hospitals NHS Foundation Trust / North Cumbria Integrated Care NHS Foundation Trust / North Tees and Hartlepool NHS Foundation Trust / Newcastle Hospitals NHS Foundation Trust	COVID-19 Genomics UK (COG-UK) Consortium	Darren L Smith,Andrew Nelson,Matthew Bashton,Greg R Young,Joshua Loh,John Allan,Mohammad A Tariq,Giles S Holt,Gary Black,Wen C Yew,Lynn Dover,Paul Baker,Steve Liggett,Sarah Essex,Jane Greenaway,Debra Padgett,Clive Graham,Garren Scott,Edward Barton,Emma Swindells,Brendan Payne,Jennifer Collins,Yusri Taha,Gary Eltringham
EPI_ISL_665761	Queens Medical Centre, Clinical Microbiology Department / DeepSeq Nottingham	COVID-19 Genomics UK (COG-UK) Consortium	Gemma Clark, Wendy Smith, Manjinder Khakh, Vicki M Fleming, Michelle M Lister, Hannah Howson-Wells, Jonathan Ball, Patrick McClure, Joseph Chappell, Theocharis Tsoleridis, Nadine Holmes, Matthew Carlisle, Christopher Moore, Fei Sang, Johnny Debebe, Victoria Wright, Matthew Loose
EPI_ISL_665784	Northumbria University / South Tees Hospitals NHS Foundation Trust / North Cumbria Integrated Care NHS Foundation Trust / North Tees and Hartlepool NHS Foundation Trust / Newcastle Hospitals NHS Foundation Trust	COVID-19 Genomics UK (COG-UK) Consortium	Darren L Smith,Andrew Nelson,Matthew Bashton,Greg R Young,Joshua Loh,John Allan,Mohammad A Tariq,Giles S Holt,Gary Black,Wen C Yew,Lynn Dover,Paul Baker,Steve Liggett,Sarah Essex,Jane Greenaway,Debra Padgett,Clive Graham,Garren Scott,Edward Barton,Emma Swindells,Brendan Payne,Jennifer Collins,Yusri Taha,Gary Eltringham
EPI_ISL_665977, EPI_ISL_665978, EPI_ISL_665979, EPI_ISL_665980, EPI_ISL_665981, EPI_ISL_665982	Queens Medical Centre, Clinical Microbiology Department / DeepSeq Nottingham	COVID-19 Genomics UK (COG-UK) Consortium	Gemma Clark, Wendy Smith, Manjinder Khakh, Vicki M Fleming, Michelle M Lister, Hannah Howson-Wells, Jonathan Ball, Patrick McClure, Joseph Chappell, Theocharis Tsoleridis, Nadine Holmes, Matthew Carlisle, Christopher Moore, Fei Sang, Johnny Debebe, Victoria Wright, Matthew Loose
EPI_ISL_665983, EPI_ISL_665984, EPI_ISL_665985, EPI_ISL_665986, EPI_ISL_665987, EPI_ISL_665988, EPI_ISL_665989, EPI_ISL_665990, EPI_ISL_665991, EPI_ISL_665992, EPI_ISL_665993, EPI_ISL_665994, EPI_ISL_665995, EPI_ISL_665996, EPI_ISL_665997, EPI_ISL_665998, EPI_ISL_665999, EPI_ISL_666000, EPI_ISL_666001, EPI_ISL_666002, EPI_ISL_666088, EPI_ISL_666089, EPI_ISL_666090, EPI_ISL_666091, EPI_ISL_666092, EPI_ISL_666093, EPI_ISL_666094, EPI_ISL_666095, EPI_ISL_666096, EPI_ISL_666097, EPI_ISL_666098, EPI_ISL_666099, EPI_ISL_666100			
see above	Northumbria University / South Tees Hospitals NHS Foundation Trust / North Cumbria Integrated Care NHS Foundation Trust / North Tees and Hartlepool NHS Foundation Trust / Newcastle Hospitals NHS Foundation Trust	COVID-19 Genomics UK (COG-UK) Consortium	Darren L Smith,Andrew Nelson,Matthew Bashton,Greg R Young,Joshua Loh,John Allan,Mohammad A Tariq,Giles S Holt,Gary Black,Wen C Yew,Lynn Dover,Paul Baker,Steve Liggett,Sarah Essex,Jane Greenaway,Debra Padgett,Clive Graham,Garren Scott,Edward Barton,Emma Swindells,Brendan Payne,Jennifer Collins,Yusri Taha,Gary Eltringham
EPI_ISL_666140, EPI_ISL_666181, EPI_ISL_666364	Wales Specialist Virology Centre Sequencing lab: Pathogen Genomics Unit	COVID-19 Genomics UK (COG-UK) Consortium	Catherine Moore, Johnathan Evans, Laura Gifford, Malorie Perry, Simon Cottrell, Angela Marchbank, Alec Birchley, Alexander Adams, Amy Gaskin, Bree Gatica-Wilcox, Jason Coombes, Joel Southgate, Lauren Gilbert, Lee Graham, Nicole Pacchiarini, Sara Kumziene-Summerhayes, Sarah Taylor, Sophie Jones, Sara Rey, Matthew Bull, Joanne Watkins, Sally Corden, Tom Connor

EPI_ISL_666891, EPI_ISL_666900, EPI_ISL_666905, EPI_ISL_666918, EPI_ISL_666935, EPI_ISL_666937, EPI_ISL_666943, EPI_ISL_666947	Michigan Department of Health and Human Services, Bureau of Laboratories	Michigan Department of Health and Human Services, Bureau of Laboratories	Blankenship HM, Riner D, Soehnlen MK
EPI_ISL_666979, EPI_ISL_666980, EPI_ISL_666982, EPI_ISL_666984, EPI_ISL_666986, EPI_ISL_666988, EPI_ISL_666989, EPI_ISL_666990, EPI_ISL_667045, EPI_ISL_667048, EPI_ISL_667052, EPI_ISL_667053			
see above	San Diego County Public Health Laboratory	Andersen lab at Scripps Research	SEARCH Alliance San Diego with Tracy Basler, Jovan Shephard, Brett Austin
EPI_ISL_667505, EPI_ISL_667506, EPI_ISL_667507, EPI_ISL_667509, EPI_ISL_667510, EPI_ISL_667511	OHSU Lab Services Molecular Microbiology Lab	Oregon SARS-CoV-2 Genome Sequencing Center	Brendan L. O'Connell, Ruth V. Nichols, Sally Grindstaff, Alec J. Hirsch, Donna Hansel, Guang Fan, Daniel N. Streblow, William B. Messer, Andrew C. Adey, Benjamin N. Bimber, Brian J. O'Roak
EPI_ISL_667787, EPI_ISL_667792	South Eastern Area Laboratory Services (SEALS)	NSW Health Pathology - Institute of Clinical Pathology and Medical Research; Westmead Hospital; University of Sydney	CIDM-PH et al.
EPI_ISL_668391	Akershus University Hospital, Department for Microbiology and Infectious Disease Control	Norwegian Institute of Public Health, Department of Virology	Kathrine Stene-Johansen, Kamilla Heddeland Instefjord, Hilde Elshaug, Marie Paulsen Madsen, Rasmus Riis Kopperud, Hilde Vollan, Karoline Bragstad, Olav Hungnes
EPI_ISL_668394, EPI_ISL_668395	Hospital of Southern Norway - Kristiansand, Department of Medical Microbiology	Norwegian Institute of Public Health, Department of Virology	Kathrine Stene-Johansen, Kamilla Heddeland Instefjord, Hilde Elshaug, Marie Paulsen Madsen, Rasmus Riis Kopperud, Hilde Vollan, Karoline Bragstad, Olav Hungnes
EPI_ISL_668399	Nordland Hospital - Bodo, Laboratory Department, Molecular Biology Unit	Norwegian Institute of Public Health, Department of Virology	Kathrine Stene-Johansen, Kamilla Heddeland Instefjord, Hilde Elshaug, Marie Paulsen Madsen, Rasmus Riis Kopperud, Hilde Vollan, Karoline Bragstad, Olav Hungnes
EPI_ISL_668401	Oslo University Hospital, Department of Medical Microbiology	Norwegian Institute of Public Health, Department of Virology	Kathrine Stene-Johansen, Kamilla Heddeland Instefjord, Hilde Elshaug, Marie Paulsen Madsen, Rasmus Riis Kopperud, Hilde Vollan, Karoline Bragstad, Olav Hungnes
EPI_ISL_668412, EPI_ISL_668414, EPI_ISL_668416	Innlandet Hospital Trust, Division Lillehammer, Department for Medical Microbiology	Norwegian Institute of Public Health, Department of Virology	Kathrine Stene-Johansen, Kamilla Heddeland Instefjord, Hilde Elshaug, Marie Paulsen Madsen, Rasmus Riis Kopperud, Hilde Vollan, Karoline Bragstad, Olav Hungnes
EPI_ISL_668417, EPI_ISL_668420, EPI_ISL_668421	Department of Medical Microbiology, St. Olavs hospital	Norwegian Institute of Public Health, Department of Virology	Kathrine Stene-Johansen, Kamilla Heddeland Instefjord, Hilde Elshaug, Marie Paulsen Madsen, Rasmus Riis Kopperud, Hilde Vollan, Karoline Bragstad, Olav Hungnes
EPI_ISL_668445	Respiratory Virus Unit, Microbiology Services Colindale, Public Health England	COVID-19 Genomics UK (COG-UK) Consortium	PHE Covid Sequencing Team
EPI_ISL_670491, EPI_ISL_670501, EPI_ISL_670536, EPI_ISL_670537, EPI_ISL_670644, EPI_ISL_670645, EPI_ISL_670646, EPI_ISL_671219, EPI_ISL_671220, EPI_ISL_671221, EPI_ISL_671222, EPI_ISL_671223			
see above	Department of Virus and Microbiological Special Diagnostics, Statens Serum Institut, Copenhagen, Denmark	Albertsen Lab, Department of Chemistry and Bioscience, Aalborg University, Denmark	Danish Covid-19 Genome Consortium
EPI_ISL_671415, EPI_ISL_671417	National Laboratory of Virology, Szentágothai Research Centre	National Laboratory of Virology, Szentágothai Research Centre	Endre Gábor Tóth, Balázs Somogyi, Brigitta, Ferenc Jakab, Gábor Kemenesi
EPI_ISL_671673	South Eastern Area Laboratory Services (SEALS)	NSW Health Pathology - Institute of Clinical Pathology and Medical Research; Westmead Hospital; University of Sydney	CIDM-PH et al.
EPI_ISL_671677	DOHMH Corona	New York City Public Health Laboratory	Jade Wang, et al.
EPI_ISL_671678	DOHMH Fort Greene	New York City Public Health Laboratory	Jade Wang, et al.
EPI_ISL_671679, EPI_ISL_671680	DOHMH Morrisania	New York City Public Health Laboratory	Jade Wang, et al.
EPI_ISL_671681	DOHMH Corona	New York City Public Health Laboratory	Jade Wang, et al.
EPI_ISL_671689	DOHMH Central Harlem	New York City Public Health Laboratory	Jade Wang, et al.
EPI_ISL_671690	DOHMH Crown Heights	New York City Public Health Laboratory	Jade Wang, et al.
EPI_ISL_671691	DOHMH Corona	New York City Public Health Laboratory	Jade Wang, et al.
EPI_ISL_671692	DOHMH Jamaica	New York City Public Health Laboratory	Jade Wang, et al.
EPI_ISL_671693	DOHMH Chelsea	New York City Public Health Laboratory	Jade Wang, et al.
EPI_ISL_671694	DOHMH Riverside	New York City Public Health Laboratory	Jade Wang, et al.
EPI_ISL_671695	DOHMH Corona	New York City Public Health Laboratory	Jade Wang, et al.
EPI_ISL_671696	DOHMH PHL	New York City Public Health Laboratory	Jade Wang, et al.
EPI_ISL_671697	DOHMH Corona	New York City Public Health Laboratory	Jade Wang, et al.
EPI_ISL_671698	DOHMH Jamaica	New York City Public Health Laboratory	Jade Wang, et al.
EPI_ISL_671699, EPI_ISL_671700	DOHMH Corona	New York City Public Health Laboratory	Jade Wang, et al.
EPI_ISL_671701	DOHMH Riverside	New York City Public Health Laboratory	Jade Wang, et al.
EPI_ISL_671702	DOHMH Chelsea	New York City Public Health Laboratory	Jade Wang, et al.
EPI_ISL_671863	Hospital Universitari i Politècnic la Fe, València, Spain	SeqCOVID-SPAIN consortium/IBV(CSIC)	Manuel Guerreiro, Marta Montero-Alonso, María Dolores Gómez and SeqCOVID-SPAIN consortium
EPI_ISL_672013, EPI_ISL_672035, EPI_ISL_672057, EPI_ISL_672069, EPI_ISL_672088, EPI_ISL_672292, EPI_ISL_672293, EPI_ISL_672294, EPI_ISL_672295, EPI_ISL_672296, EPI_ISL_672297			
see above	Santa Clara County Public Health Laboratory	Chan-Zuckerberg Biohub	CZB Cliahub Consortium
EPI_ISL_672366, EPI_ISL_672367, EPI_ISL_672368, EPI_ISL_672369, EPI_ISL_672370	Orange County Public Health Lab	Chan-Zuckerberg Biohub	CZB Cliahub Consortium
EPI_ISL_672525	Madera County Department of Public Health	Chan-Zuckerberg Biohub	CZB Cliahub Consortium
EPI_ISL_677134, EPI_ISL_677135, EPI_ISL_677136, EPI_ISL_677137, EPI_ISL_677138, EPI_ISL_677139, EPI_ISL_677140, EPI_ISL_677141, EPI_ISL_677142, EPI_ISL_677144, EPI_ISL_677146, EPI_ISL_677179, EPI_ISL_677180, EPI_ISL_677181, EPI_ISL_677182, EPI_ISL_677183, EPI_ISL_677184, EPI_ISL_677185, EPI_ISL_677186, EPI_ISL_677187, EPI_ISL_677188, EPI_ISL_677189, EPI_ISL_677190, EPI_ISL_677191, EPI_ISL_677192, EPI_ISL_677193, EPI_ISL_677229, EPI_ISL_677230, EPI_ISL_677231, EPI_ISL_677232, EPI_ISL_677233, EPI_ISL_677236			
see above	Virginia Division of Consolidated Laboratory Services	Virginia Division of Consolidated Laboratory Services	Virginia DCLS
EPI_ISL_677318	Colorado Department of Public Health and Environment	Colorado Department of Puplic Health and Environment	Laura Bankers, Molly Hetherington-Rauth, Shannon Ely, Shannon R. Matzinger, Sarah Elizabeth Totten, Emily A. Travanty
EPI_ISL_677320, EPI_ISL_677477, EPI_ISL_677478, EPI_ISL_677484, EPI_ISL_677485, EPI_ISL_677586, EPI_ISL_677587, EPI_ISL_677588, EPI_ISL_677589, EPI_ISL_677590, EPI_ISL_677591, EPI_ISL_677592, EPI_ISL_677626, EPI_ISL_677663			
see above	University of Wisconsin-Madison AIDS Vaccine Research Laboratories	University of Wisconsin-Madison AIDS Vaccine Research Laboratories	Gage Moreno, Katarina Braun, et al. AIDS Vaccine Research Laboratories
EPI_ISL_678380	Area of Virology, Serology and Virology Division (SAViD), New South Wales Health Pathology Randwick	Virology Research Laboratory; Area of Virology, Serology and Virology Division (SAViD), New South Wales Health Pathology Randwick	Foster, C.; Au, J.; Ruiz Silva, M.; Deveson, I.; Bull, R.; Van Hal, S.; Rawlinson, W.

EPI_ISL_679924, EPI_ISL_679925	Queens Medical Centre, Clinical Microbiology Department / DeepSeq Nottingham	COVID-19 Genomics UK (COG-UK) Consortium	Gemma Clark, Wendy Smith, Manjinder Khakh, Vicki M Fleming, Michelle M Lister, Hannah Howson-Wells, Jonathan Ball, Patrick McClure, Joseph Chappell, Theocharis Tsoleridis, Nadine Holmes, Matthew Carlisle, Christopher Moore, Fei Sang, Johnny Debebe, Victoria Wright, Matthew Loose
EPI_ISL_680016, EPI_ISL_680017, EPI_ISL_680018, EPI_ISL_680019, EPI_ISL_680020, EPI_ISL_680021, EPI_ISL_680022, EPI_ISL_680023, EPI_ISL_680024, EPI_ISL_680025, EPI_ISL_680026, EPI_ISL_680027, EPI_ISL_680028, EPI_ISL_680029, EPI_ISL_680030, EPI_ISL_680031, EPI_ISL_680032, EPI_ISL_680033, EPI_ISL_680034, EPI_ISL_680035, EPI_ISL_680036			
see above	Centre for Enzyme Innovation, University of Portsmouth / Translational Research Laboratory, Portsmouth Hospitals NHS Trust	COVID-19 Genomics UK (COG-UK) Consortium	Angela Beckett,Yann Bourgeois,Garry Scarlett,Sharon Glaysher,Scott Elliott,Kelly Bicknell,Robert Impey,Allyson Lloyd,Sarah Wyllie,Ethan Butcher,Anoop Chauhan,Samuel Robson
EPI_ISL_680202, EPI_ISL_680203, EPI_ISL_680217, EPI_ISL_680330, EPI_ISL_680388, EPI_ISL_680389, EPI_ISL_680390, EPI_ISL_680391, EPI_ISL_680392, EPI_ISL_680393, EPI_ISL_680394, EPI_ISL_680395, EPI_ISL_680396, EPI_ISL_680397, EPI_ISL_680398, EPI_ISL_680399, EPI_ISL_680400, EPI_ISL_680401, EPI_ISL_680402, EPI_ISL_680403, EPI_ISL_680404, EPI_ISL_680405, EPI_ISL_680406, EPI_ISL_680407, EPI_ISL_680408			
see above	Regional Virus Laboratory, Belfast Health and Social Care Trust	COVID-19 Genomics UK (COG-UK) Consortium	Conall McCaughey, James McKenna, Tanya Curran, Susan Feeney, Alison Watt, Ciara Cox, Mairead Connor, Zoltan Molnar, David Simpson, Derek Fairley
EPI_ISL_680485, EPI_ISL_680498, EPI_ISL_680499, EPI_ISL_680500, EPI_ISL_680501, EPI_ISL_680502	Virology Department, Royal Infirmary of Edinburgh, NHS Lothian / School of Biological Sciences, University of Edinburgh / Institute of Genetics and Molecular Medicine, University of Edinburgh	COVID-19 Genomics UK (COG-UK) Consortium	McHugh M, Dewar R, Rooke S, Gallagher M, Balcaza C, O'Toole Á, Scher E, Hill V, McCrone JT, Colquhoun R, Yu X, Jackson B, Rambaut A, Williams TC, Templeton K
EPI_ISL_680562, EPI_ISL_680563, EPI_ISL_680564, EPI_ISL_680565, EPI_ISL_680566, EPI_ISL_680567, EPI_ISL_680568, EPI_ISL_680569, EPI_ISL_680570	Centre for Enzyme Innovation, University of Portsmouth / Translational Research Laboratory, Portsmouth Hospitals NHS Trust	COVID-19 Genomics UK (COG-UK) Consortium	Angela Beckett,Yann Bourgeois,Garry Scarlett,Sharon Glaysher,Scott Elliott,Kelly Bicknell,Robert Impey,Allyson Lloyd,Sarah Wyllie,Ethan Butcher,Anoop Chauhan,Samuel Robson
EPI_ISL_682050, EPI_ISL_682052	UPMC Clinical Microbiology Laboratory	Microbial Genomic Epidemiology Laboratory, University of Pittsburgh	Mustapha M. Mustapha, Jane W. Marsh, Dan Snyder, Marissa P. Griffith, Stephanie L. Mitchell, Vatsala R. Srinivasa, Kady D. Waggle, Chinelo Ezeonwuku, Vaughn S. Cooper, Lee H. Harrison
EPI_ISL_683002, EPI_ISL_683003, EPI_ISL_683204, EPI_ISL_683205, EPI_ISL_683206, EPI_ISL_683207, EPI_ISL_683208, EPI_ISL_683209, EPI_ISL_683210, EPI_ISL_683211, EPI_ISL_683235, EPI_ISL_683236, EPI_ISL_683237, EPI_ISL_683238, EPI_ISL_683239, EPI_ISL_683240, EPI_ISL_683241, EPI_ISL_683242, EPI_ISL_683243, EPI_ISL_683244, EPI_ISL_683245, EPI_ISL_683246, EPI_ISL_683247, EPI_ISL_683248, EPI_ISL_683249, EPI_ISL_683250, EPI_ISL_683251, EPI_ISL_683252, EPI_ISL_683253, EPI_ISL_683254, EPI_ISL_683261, EPI_ISL_683262, EPI_ISL_683263, EPI_ISL_683264, EPI_ISL_683265, EPI_ISL_683266, EPI_ISL_683272, EPI_ISL_683273, EPI_ISL_683274, EPI_ISL_683277, EPI_ISL_683278, EPI_ISL_683284, EPI_ISL_683285, EPI_ISL_683286, EPI_ISL_683287, EPI_ISL_683288, EPI_ISL_683289, EPI_ISL_683290, EPI_ISL_683291, EPI_ISL_683292, EPI_ISL_683293, EPI_ISL_683294, EPI_ISL_683295, EPI_ISL_683296, EPI_ISL_683297			
see above	Department of Virus and Microbiological Special Diagnostics, Statens Serum Institut, Copenhagen, Denmark	Albertsen Lab, Department of Chemistry and Bioscience, Aalborg University, Denmark	Danish Covid-19 Genome Consortium
EPI_ISL_683726	Mayo Clinic & Mayo Clinic Laboratories	Minnesota Department of Health, Public Health Laboratory	Alexandra Lorentz, Jacob Garfin, Matt Plumb, and Xiong Wang
EPI_ISL_692835, EPI_ISL_692836, EPI_ISL_692837, EPI_ISL_692838, EPI_ISL_692839, EPI_ISL_692840, EPI_ISL_692841, EPI_ISL_692842, EPI_ISL_692843, EPI_ISL_692846	Massachusetts State Public Health Laboratory	Massachusetts State Public Health Laboratory	Andrew Lang, Timelia Fink, Glen Gallagher, Sandra Smole
EPI_ISL_700451, EPI_ISL_700452	Pacaltsdorp Clinic wc PAC	NHLS/UCT	Houriiyah Tegally, Arash Iranzadeh, Deelan Doolabh, Lynn Tyers, Bruna Galvao, Innocent Mudau, Marvin Hsiao, Kruger Marais, Diana Hardie, Stephen Korsman, Carolyn Williamson
EPI_ISL_700469	Knysna CDC wc WLC	NHLS/UCT	Houriiyah Tegally, Arash Iranzadeh, Deelan Doolabh, Lynn Tyers, Bruna Galvao, Innocent Mudau, Marvin Hsiao, Kruger Marais, Diana Hardie, Stephen Korsman, Carolyn Williamson
EPI_ISL_700476	Alma CDC wc AHC	NHLS/UCT	Arash Iranzadeh, Deelan Doolabh, Lynn Tyers, Bruna Galvao, Innocent Mudau, Marvin Hsiao, Kruger Marais, Diana Hardie, Stephen Korsman, Carolyn Williamson
EPI_ISL_700478	Pacaltsdorp Clinic wc PAC	NHLS/UCT	Houriiyah Tegally, Arash Iranzadeh, Deelan Doolabh, Lynn Tyers, Bruna Galvao, Innocent Mudau, Marvin Hsiao, Kruger Marais, Diana Hardie, Stephen Korsman, Carolyn Williamson
EPI_ISL_700484	Knysna Hospital wc KNY	NHLS/UCT	Arash Iranzadeh, Deelan Doolabh, Lynn Tyers, Bruna Galvao, Innocent Mudau, Marvin Hsiao, Kruger Marais, Diana Hardie, Stephen Korsman, Carolyn Williamson
EPI_ISL_700510	Knysna Hospital wc KNY	NHLS/UCT	Houriiyah Tegally, Arash Iranzadeh, Deelan Doolabh, Lynn Tyers, Bruna Galvao, Innocent Mudau, Marvin Hsiao, Kruger Marais, Diana Hardie, Stephen Korsman, Carolyn Williamson
EPI_ISL_700536, EPI_ISL_700556	Conville CDC wc CVC	NHLS/UCT	Arash Iranzadeh, Deelan Doolabh, Lynn Tyers, Bruna Galvao, Innocent Mudau, Marvin Hsiao, Kruger Marais, Diana Hardie, Stephen Korsman, Carolyn Williamson
EPI_ISL_700703, EPI_ISL_700708	Texas Department of State Health Services	Texas Department of State Health Services	Rashmi Tuladhar, Bonnie Oh, Jenny Zhang, Maliha Rahman, Anita Pokharel, Myong Koag, Chung Wang, Rachel Lee, Grace Kubin, Mayela Pedrueza, James Daniel Bonser
EPI_ISL_703144	Virology Department, Royal Infirmary of Edinburgh, NHS Lothian / School of Biological Sciences, University of Edinburgh / Institute of Genetics and Molecular Medicine, University of Edinburgh	COVID-19 Genomics UK (COG-UK) Consortium	McHugh M, Dewar R, Rooke S, Gallagher M, Balcaza C, O'Toole Á, Scher E, Hill V, McCrone JT, Colquhoun R, Yu X, Jackson B, Rambaut A, Williams TC, Templeton K
EPI_ISL_703674	Virology Department, Sheffield Teaching Hospitals NHS Foundation Trust/Department of Infection, Immunity and Cardiovascular Disease, The Medical School, University of Sheffield	COVID-19 Genomics UK (COG-UK) Consortium	Thushan de Silva, Matthew Parker, Nikki Smith, Adri Angyal, Rebecca Brown, Luke Green, Rachel Tucker, Paul Parsons, Danielle Groves, Katie Johnson, Laura Carrilero, Alex Keeley, Dave Partridge, Matthew Wyles, Benjamin Lindsey, Mehmet Yavuz, Mohammad Raza, Cariad Evans
EPI_ISL_704845, EPI_ISL_704866, EPI_ISL_704869	Oxford Viromics, NDM, University of Oxford; Oxford University Hospitals; Basingstoke and North Hampshire Hospital	COVID-19 Genomics UK (COG-UK) Consortium	Tanya Golubchik, David Bonsall, George Macintyre, Amy Trebes, Mariateresa de Cesare, Catrin Moore, Alex Mobbs, Anita Justice, Robert Shaw, Monique Andersson, Timothy Peto, Emma Wise, Nathan Moore, Jessica Lynch, Nick Cortes, Matilde Mori, Stephen Kidd, David Buck, John Todd, Christophe Fraser
EPI_ISL_705225, EPI_ISL_705226, EPI_ISL_705302	Northumbria University / South Tees Hospitals NHS Foundation Trust / North Cumbria Integrated Care NHS Foundation Trust / North Tees and Hartlepool NHS Foundation Trust / Newcastle Hospitals NHS Foundation Trust	COVID-19 Genomics UK (COG-UK) Consortium	Darren L Smith,Andrew Nelson,Matthew Bashton,Greg R Young,Joshua Loh,John Allan,Mohammad A Tariq,Giles S Holt,Gary Black,Wen C Yew,Lynn Dover,Paul Baker,Steve Liggett,Sarah Essex,Jane Greenaway,Debra Padgett,Clive Graham,Garren Scott,Edward Barton,Emma Swindells,Brendan Payne,Jennifer Collins,Yusri Taha,Gary Eltringham
EPI_ISL_705414, EPI_ISL_705417, EPI_ISL_705498	Oxford Viromics, NDM, University of Oxford; Oxford University Hospitals; Basingstoke and North Hampshire Hospital	COVID-19 Genomics UK (COG-UK) Consortium	Tanya Golubchik, David Bonsall, George Macintyre, Amy Trebes, Mariateresa de Cesare, Catrin Moore, Alex Mobbs, Anita Justice, Robert Shaw, Monique Andersson, Timothy Peto, Emma Wise, Nathan Moore, Jessica Lynch, Nick Cortes, Matilde Mori, Stephen Kidd, David Buck, John Todd, Christophe Fraser
EPI_ISL_705537, EPI_ISL_705541	Department of Pathology, University of Cambridge	COVID-19 Genomics UK (COG-UK) Consortium	Aminu S. Jahun, Yasmin Chaudhry, Grant Hall, Iliana Georgana, Myra Hosmillo, Martin D. Curran, Malte Pinckert, Surendra Parmar, Ian Goodfellow
EPI_ISL_705976, EPI_ISL_705977, EPI_ISL_705978, EPI_ISL_705979, EPI_ISL_705980, EPI_ISL_705981, EPI_ISL_705982, EPI_ISL_705983, EPI_ISL_705984, EPI_ISL_705985, EPI_ISL_705986, EPI_ISL_705987, EPI_ISL_705988, EPI_ISL_705989, EPI_ISL_705990, EPI_ISL_705991, EPI_ISL_705992, EPI_ISL_705993, EPI_ISL_705994, EPI_ISL_705995, EPI_ISL_705996, EPI_ISL_705997, EPI_ISL_705998, EPI_ISL_705999, EPI_ISL_706000, EPI_ISL_706001, EPI_ISL_706002, EPI_ISL_706003, EPI_ISL_706004, EPI_ISL_706005, EPI_ISL_706006, EPI_ISL_706007, EPI_ISL_706008, EPI_ISL_706009, EPI_ISL_706010, EPI_ISL_706011, EPI_ISL_706012			
see above	Oxford Viromics, NDM, University of Oxford; Oxford	COVID-19 Genomics UK (COG-UK) Consortium	Tanya Golubchik, David Bonsall, George Macintyre, Amy Trebes, Mariateresa de Cesare, Catrin Moore, Alex Mobbs, Anita Justice, Robert Shaw, Monique

	University Hospitals; Basingstoke and North Hampshire Hospital		Andersson, Timothy Peto, Emma Wise, Nathan Moore, Jessica Lynch, Nick Cortes, Matilde Mori, Stephen Kidd, David Buck, John Todd, Christophe Fraser
EPI_ISL_707789	Rwanda National Reference Laboratory	Rwanda National Reference Laboratory	Enatha Mukantwari,Jeanne d'Arc
EPI_ISL_707895, EPI_ISL_707899	Area of Virology, Serology and Virology Division (SAViD), New South Wales Health Pathology Randwick	Virology Research Laboratory; Area of Virology, Serology and Virology Division (SAViD), New South Wales Health Pathology Randwick	Foster, C.; Au, J.; Ruiz Silva, M.; Deveson, I.; Bull, R.; Van Hal, S.; Rawlinson, W.
EPI_ISL_707999, EPI_ISL_708000, EPI_ISL_708001, EPI_ISL_708002, EPI_ISL_708015	Virology, Universitätsklinikum des Saarlandes	Epigenetics, Saarland University	Kathrin Kattler, Markus Vogelgesang, Stefan Lohse, Sascha Tierling, Sigrun Smola, Jörn Walter
EPI_ISL_708033	Oslo University Hospital, Department of Medical Microbiology	Norwegian Institute of Public Health, Department of Virology	Kathrine Stene-Johansen, Kamilla Heddeland Instefjord, Hilde Elshaug, Marie Paulsen Madsen, Rasmus Riis Kopperud, Hilde Vollan, Karoline Bragstad, Olav Hungnes
EPI_ISL_708043	Department of Medical Microbiology, St. Olavs hospital	Norwegian Institute of Public Health, Department of Virology	Kathrine Stene-Johansen, Kamilla Heddeland Instefjord, Hilde Elshaug, Marie Paulsen Madsen, Rasmus Riis Kopperud, Hilde Vollan, Karoline Bragstad, Olav Hungnes
EPI_ISL_708163	Vestfold Hospital, Toensberg Department of Microbiology	Norwegian Institute of Public Health, Department of Virology	Kathrine Stene-Johansen, Kamilla Heddeland Instefjord, Hilde Elshaug, Marie Paulsen Madsen, Rasmus Riis Kopperud, Hilde Vollan, Karoline Bragstad, Olav Hungnes
EPI_ISL_708431	Delaware Public Health Lab	Delaware Public Health Lab	Gregory Hovan
EPI_ISL_708804, EPI_ISL_708806	Regional medical sciences center 6 chonburi	National Institute of Health, Department of Medical Sciences, Ministry of Public Health, Thailand	Pilailuk Okada; Siripaporn Phuygun; Thanutsapa Thanadachakul; Sittiporn Parnmen; Pakorn Piromtong; Warawan Wongboot; Sunthareeya Waicharoen; Malinee Chittaganpitch
EPI_ISL_709913, EPI_ISL_709914, EPI_ISL_709915, EPI_ISL_709919, EPI_ISL_709921, EPI_ISL_709924, EPI_ISL_709925, EPI_ISL_709926, EPI_ISL_709927, EPI_ISL_709928, EPI_ISL_709929, EPI_ISL_709930, EPI_ISL_709931			
see above	Lighthouse Lab in Milton Keynes	Wellcome Sanger Institute for the COVID-19 Genomics UK (COG-UK) Consortium	The Lighthouse Lab in Milton Keynes and Alex Alderton, Roberto Amato, Sonia Goncalves, Ewan Harrison, David K. Jackson, Ian Johnston, Dominic Kwiatkowski, Cordelia Langford, John Sillitoe on behalf of the Wellcome Sanger Institute COVID-19 Surveillance Team
EPI_ISL_710155, EPI_ISL_710162, EPI_ISL_710174, EPI_ISL_710237, EPI_ISL_710238, EPI_ISL_710239, EPI_ISL_710269, EPI_ISL_710277, EPI_ISL_710355, EPI_ISL_710356, EPI_ISL_710357, EPI_ISL_710358, EPI_ISL_710359			
see above	Colorado Department of Public Health and Environment	Colorado Department of Puplic Health and Environment	Laura Bankers, Molly C. Hetherington-Rauth, Shannon Ely, Shannon R. Matzinger, Sarah Elizabeth Totten, Emily A. Travanty
EPI_ISL_710503	Laboratorio specialistico UOC Ematologia - Ospedale "San Francesco" - ATS-ASSL Nuoro	Laboratorio specialistico UOC Ematologia - Ospedale "San Francesco" - ATS-ASSL Nuoro	Giovanna Piras
EPI_ISL_710610	Ektorps Vardcentral	The Public Health Agency of Sweden	Department of Microbiology, The Public Health Agency of Sweden
EPI_ISL_711057, EPI_ISL_712060	Hôpital Fattouma-Bourguiba de Monastir	Laboratoire des Procédés de Criblage Moléculaire et Cellulaire-Centre de Biotechnologie de Sfax	Souissi,A., Abid,N., Ben Ayed,I., Gargouri,S., Abdelmoulah,F.,Elargoubi,A., Smeti,I., Bensaid,M., Stambouli,N., Kharat,N., Ajili,F., Fki-berrajah,L., Mhalla,S., Chtourou,A., Gaaloul,I., Nabli,A., Turki,M., Aouni,M., Hammami,A., Mastouri,M., Karray Hakim,H., Kamoun,S., Rebai,A. and Masmoudi,S.
EPI_ISL_714423, EPI_ISL_714424, EPI_ISL_714425, EPI_ISL_714426, EPI_ISL_714427, EPI_ISL_714428, EPI_ISL_714429, EPI_ISL_714430, EPI_ISL_714431, EPI_ISL_714432, EPI_ISL_714433, EPI_ISL_714434, EPI_ISL_714435, EPI_ISL_714436, EPI_ISL_714437, EPI_ISL_714438, EPI_ISL_714439, EPI_ISL_714440, EPI_ISL_714441, EPI_ISL_714442, EPI_ISL_714443, EPI_ISL_714928			
see above	Department of Virus and Microbiological Special Diagnostics, Statens Serum Institut, Copenhagen, Denmark	Albertsen Lab, Department of Chemistry and Bioscience, Aalborg University, Denmark	Danish Covid-19 Genome Consortium
EPI_ISL_717574, EPI_ISL_717576	Lab voor klinische biologie	Onderzoeksgroep Virologie	Laurens Lambrechts, Nick Vereecke, Marthe Pauwels, Bruno Verhasselt, Linos Vandekerckhove, Hans Nauwynck, Sebastiaan Theuns
EPI_ISL_717580	Lab voor klinische biologie	Onderzoeksgroep Virologie	Nick Vereecke, Laurens Lambrechts, Marthe Pauwels, Bruno Verhasselt, Linos Vandekerckhove, Hans Nauwynck, Sebastiaan Theuns
EPI_ISL_717593, EPI_ISL_717595, EPI_ISL_717598	Lab voor klinische biologie	Onderzoeksgroep Virologie	Laurens Lambrechts, Nick Vereecke, Marthe Pauwels, Bruno Verhasselt, Linos Vandekerckhove, Hans Nauwynck, Sebastiaan Theuns
EPI_ISL_717602, EPI_ISL_717604, EPI_ISL_717605	Lab voor klinische biologie	Onderzoeksgroep Virologie	Nick Vereecke, Laurens Lambrechts, Marthe Pauwels, Bruno Verhasselt, Linos Vandekerckhove, Hans Nauwynck, Sebastiaan Theuns
EPI_ISL_717607, EPI_ISL_717608, EPI_ISL_717609, EPI_ISL_717616	Lab voor klinische biologie	Onderzoeksgroep Virologie	Laurens Lambrechts, Nick Vereecke, Marthe Pauwels, Bruno Verhasselt, Linos Vandekerckhove, Hans Nauwynck, Sebastiaan Theuns
EPI_ISL_717617, EPI_ISL_717618, EPI_ISL_717619	Lab voor klinische biologie	Onderzoeksgroep Virologie	Nick Vereecke, Laurens Lambrechts, Marthe Pauwels, Bruno Verhasselt, Linos Vandekerckhove, Hans Nauwynck, Sebastiaan Theuns
EPI_ISL_721542, EPI_ISL_721543, EPI_ISL_721545, EPI_ISL_721546, EPI_ISL_721547, EPI_ISL_721548, EPI_ISL_721549, EPI_ISL_721550, EPI_ISL_721551, EPI_ISL_721552, EPI_ISL_721553, EPI_ISL_721554, EPI_ISL_721555, EPI_ISL_721556, EPI_ISL_721557, EPI_ISL_721558, EPI_ISL_721559, EPI_ISL_721560, EPI_ISL_721561, EPI_ISL_721562, EPI_ISL_721563, EPI_ISL_721564, EPI_ISL_721565, EPI_ISL_721566, EPI_ISL_721567			
see above	Lighthouse Lab in Glasgow	Wellcome Sanger Institute for the COVID-19 Genomics UK (COG-UK) Consortium	Harper VanSteenhouse, Yumi Kasai, David Gray, Carol Clugston, Anna Dominiczak and Alex Alderton, Roberto Amato, Sonia Goncalves, Ewan Harrison, David K. Jackson, Ian Johnston, Dominic Kwiatkowski, Cordelia Langford, John Sillitoe on behalf of the Wellcome Sanger Institute COVID-19 Surveillance Team
EPI_ISL_722273, EPI_ISL_722296, EPI_ISL_722308, EPI_ISL_722348, EPI_ISL_722373, EPI_ISL_722398	Dutch COVID-19 response team	Erasmus Medical Center	Bas Oude Munnink, Reina Sikkema, David Nieuwenhuijse, Irina Chestakova, Anne van der Linden, Marjan Boter, Emmanuelle Munger, Corine GeurtsvanKessel, Annemiek van der Eijk, Richard Molenkamp, Marion Koopmans, on behalf of the Dutch national COVID-19 response team.
EPI_ISL_722414, EPI_ISL_722415, EPI_ISL_722416, EPI_ISL_722417, EPI_ISL_722418	Dutch COVID-19 response team	Erasmus Medical Center	OH consortium
EPI_ISL_722514, EPI_ISL_722515, EPI_ISL_722516, EPI_ISL_722518, EPI_ISL_722519, EPI_ISL_722520, EPI_ISL_722521, EPI_ISL_722522, EPI_ISL_722523, EPI_ISL_722524, EPI_ISL_722525, EPI_ISL_722526, EPI_ISL_722527			
see above	Dutch COVID-19 response team	Erasmus Medical Center	Bas Oude Munnink, Reina Sikkema, David Nieuwenhuijse, Irina Chestakova, Anne van der Linden, Marjan Boter, Emmanuelle Munger, Corine GeurtsvanKessel, Annemiek van der Eijk, Richard Molenkamp, Marion Koopmans, on behalf of the Dutch national COVID-19 response team.
EPI_ISL_722885, EPI_ISL_722886, EPI_ISL_722907, EPI_ISL_722908, EPI_ISL_722921, EPI_ISL_722924, EPI_ISL_722925	Istituto Zooprofilattico Sperimentale della Puglia e della Basilicata	Istituto Zooprofilattico Sperimentale della Puglia e della Basilicata	Parisi A., Bianco A., Capozzi L., Del Sambro L., Manzulli V, Rondinone V., Pace L., Cipolletta D., Galante D.
EPI_ISL_724975	Northumbria University / South Tees Hospitals NHS Foundation Trust / North Cumbria Integrated Care NHS Foundation Trust / North Tees and Hartlepool NHS Foundation Trust / Newcastle Hospitals NHS Foundation Trust	COVID-19 Genomics UK (COG-UK) Consortium	Darren L Smith,Andrew Nelson,Matthew Bashton,Greg R Young,Joshua Loh,John Allan,Mohammad A Tariq,Giles S Holt,Gary Black,Wen C Yew,Lynn Dover,Paul Baker,Steve Liggett,Sarah Essex,Jane Greenaway,Debra Padgett,Clive Graham,Garren Scott,Edward Barton,Emma Swindells,Brendan Payne,Jennifer Collins,Yusri Taha,Gary Eltringham
EPI_ISL_727710, EPI_ISL_727711, EPI_ISL_727774, EPI_ISL_727775, EPI_ISL_727776, EPI_ISL_727777, EPI_ISL_727778, EPI_ISL_727779, EPI_ISL_727780, EPI_ISL_727781, EPI_ISL_727782, EPI_ISL_727783, EPI_ISL_727784, EPI_ISL_727785, EPI_ISL_727786, EPI_ISL_727787, EPI_ISL_727788, EPI_ISL_727789, EPI_ISL_727790, EPI_ISL_727791, EPI_ISL_727792, EPI_ISL_727793, EPI_ISL_727794, EPI_ISL_727795, EPI_ISL_727796, EPI_ISL_727797, EPI_ISL_727798, EPI_ISL_727799, EPI_ISL_727800, EPI_ISL_727801, EPI_ISL_727802, EPI_ISL_727803, EPI_ISL_727804, EPI_ISL_727805, EPI_ISL_727806			
see above	Centre for Enzyme Innovation, University of Portsmouth / Translational Research Laboratory, Portsmouth Hospitals NHS Trust	COVID-19 Genomics UK (COG-UK) Consortium	Angela Beckett,Yann Bourgeois,Garry Scarlett,Sharon Glaysher,Scott Elliott,Kelly Bicknell,Robert Impey,Allyson Lloyd,Sarah Wyllie,Ethan Butcher,Anoop Chauhan,Samuel Robson
EPI_ISL_728051	University of Wisconsin-Madison AIDS Vaccine Research	University of Wisconsin-Madison AIDS Vaccine Research	Gage Moreno, Katarina Braun, et al. AIDS Vaccine Research Laboratories

	Laboratories	Laboratories	
EPI_ISL_730170	San Diego County Public Health Laboratory	Andersen lab at Scripps Research	SEARCH Alliance San Diego with Tracy Basler, Jovan Shephard, Brett Austin
EPI_ISL_730376, EPI_ISL_730378, EPI_ISL_730389, EPI_ISL_730399, EPI_ISL_730403, EPI_ISL_730417, EPI_ISL_730422, EPI_ISL_730428, EPI_ISL_730431, EPI_ISL_730434, EPI_ISL_730442, EPI_ISL_730446, EPI_ISL_730452, EPI_ISL_730458, EPI_ISL_730471			
see above	Biolab Diagnostic Laboratories	Andersen lab at Scripps Research	Issa Abu-Dayyeh, Ahmad Tibi, Lama Hussein, Lina Mohammad, Zein Naber, Amid Abdelnour with SEARCH Alliance San Diego
EPI_ISL_730616	United Christian Hospital	Hong Kong Department of Health	Mak Gannon C.K., Lam Edman T.K., Chan Rickjason C.W., Tsang Dominic N.C.
EPI_ISL_732396, EPI_ISL_732489	National Virus Reference Laboratory	National Virus Reference Laboratory	Michael Carr, Gabriel Gonzalez, Jonathan Dean, Daniel Hare, Cillian F De Gascun
EPI_ISL_732795	Centro de Investigación Biomédica de La Rioja - Hospital San Pedro Logroño	SeqCOVID-SPAIN consortium/IBV(CSIC)	María de Toro, José Manuel Azcona Gutiérrez, María Pilar Bea Escudero, Miriam Blasco Alberdi and SeqCOVID-SPAIN consortium
EPI_ISL_733240	UMMC-Health	WHO National Influenza Centre Russian Federation	Andrey Komissarov, Artem Fadeev, Anna Ivanova, Kseniya Komissarova, Dmitry Bazhenov, Tatiana Platonova, Daria Danilenko, Ksenia Safina, Elena Nabieva, Georgii Bazykin, Dmitry Lioznov
EPI_ISL_733254, EPI_ISL_733255, EPI_ISL_733256, EPI_ISL_733277, EPI_ISL_733278	WHO National Influenza Centre Russian Federation	WHO National Influenza Centre Russian Federation	Andrey Komissarov, Artem Fadeev, Anna Ivanova, Kseniya Komissarova, Dmitry Bazhenov, Daria Danilenko, Ksenia Safina, Elena Nabieva, Georgii Bazykin, Dmitry Lioznov
EPI_ISL_734296, EPI_ISL_734297, EPI_ISL_734298, EPI_ISL_734299, EPI_ISL_734300, EPI_ISL_734301, EPI_ISL_734302, EPI_ISL_734369, EPI_ISL_734370, EPI_ISL_734371, EPI_ISL_734372, EPI_ISL_734373, EPI_ISL_734374, EPI_ISL_734375, EPI_ISL_734376, EPI_ISL_734377, EPI_ISL_734378, EPI_ISL_734379, EPI_ISL_734380, EPI_ISL_734381, EPI_ISL_734382, EPI_ISL_734383, EPI_ISL_734384, EPI_ISL_734385, EPI_ISL_734386, EPI_ISL_734387			
see above	Wadsworth Center, New York State Department.of Health	Wadsworth Center, New York State Department.of Health	Kirsten St. George, Daryl M. Lamson, Alexis Russel, Jonathan Plitnick, Navjot Singh, John Kelly, Sara Griesemer, Erasmus Schneider, Erica Lasek-Nesselquist
EPI_ISL_734453, EPI_ISL_734454, EPI_ISL_734455, EPI_ISL_734456, EPI_ISL_734457, EPI_ISL_734458, EPI_ISL_734459, EPI_ISL_734461	Masonic Medical Research Institute	Wadsworth Center, New York State Department.of Health	Kirsten St. George, Nathan Tucker, Ryan D. Pfeiffer, Daryl M. Lamson, Alexis Russel, Jonathan Plitnick, Navjot Singh, John Kelly, Sara Griesemer, Erasmus Schneider, Erica Lasek-Nesselquist
EPI_ISL_734482, EPI_ISL_734484, EPI_ISL_734490, EPI_ISL_734491, EPI_ISL_734496, EPI_ISL_734498, EPI_ISL_734499, EPI_ISL_734501, EPI_ISL_734502, EPI_ISL_735140, EPI_ISL_735141, EPI_ISL_735142, EPI_ISL_735143, EPI_ISL_735144, EPI_ISL_735145, EPI_ISL_735146, EPI_ISL_735147, EPI_ISL_735148, EPI_ISL_735149, EPI_ISL_735150, EPI_ISL_735151, EPI_ISL_735152, EPI_ISL_735153, EPI_ISL_735154, EPI_ISL_735155, EPI_ISL_735156, EPI_ISL_735157, EPI_ISL_735158, EPI_ISL_735159, EPI_ISL_735160, EPI_ISL_735162, EPI_ISL_735163, EPI_ISL_735164, EPI_ISL_735165, EPI_ISL_735166, EPI_ISL_735167, EPI_ISL_735168, EPI_ISL_735169, EPI_ISL_735170, EPI_ISL_735171, EPI_ISL_735172, EPI_ISL_735173, EPI_ISL_735174, EPI_ISL_735175, EPI_ISL_735176, EPI_ISL_735177, EPI_ISL_735178, EPI_ISL_735179, EPI_ISL_735180, EPI_ISL_735181, EPI_ISL_735182, EPI_ISL_735183, EPI_ISL_735184, EPI_ISL_735185, EPI_ISL_735186, EPI_ISL_735187, EPI_ISL_735188, EPI_ISL_735189, EPI_ISL_735190, EPI_ISL_735191, EPI_ISL_735192, EPI_ISL_735193, EPI_ISL_735194, EPI_ISL_735195, EPI_ISL_735196, EPI_ISL_735197, EPI_ISL_735198, EPI_ISL_735199, EPI_ISL_735200, EPI_ISL_735201, EPI_ISL_735202, EPI_ISL_735203, EPI_ISL_735204, EPI_ISL_735205, EPI_ISL_735206, EPI_ISL_735207, EPI_ISL_735208, EPI_ISL_735209, EPI_ISL_735210, EPI_ISL_735211, EPI_ISL_735212, EPI_ISL_735213, EPI_ISL_735214			
see above	UZ Leuven, National Reference Laboratory for Coronaviruses, Laboratory Medicine, Leuven, Belgium	KU Leuven, Rega Institute, Clinical and Epidemiological Virology	Tony Wawina-Bokalanga, Joan Marti-Carerras, Bert Vanmechelen, Piet Maes
EPI_ISL_735444, EPI_ISL_735448	Nucleic Acid Testing - Rwanda National Reference Laboratory	GIGA Medical Genomics	Yvan Butera, Keith Durkin, Maria Artesi, Bouchra Boujemla, Robert Rutayisire, Patrick Tuyisenge, Esperence Umumararungu, Sébastien Bontems, Marie-Pierre Hayette, Swaibu Gatare, Jacob Souopgui, Sabin Nsanzimana, Vincent Bours, Léon Mutesa
EPI_ISL_735488, EPI_ISL_737081, EPI_ISL_737082, EPI_ISL_737083, EPI_ISL_737084, EPI_ISL_737085, EPI_ISL_737086, EPI_ISL_737087	UW Virology Lab	UW Virology Lab	Pavitra Roychoudhury, Hong Xie, Lasata Shrestha, Meei-Li Huang, Keith R Jerome, Alexander Greninger
EPI_ISL_738195	UZ Leuven, National Reference Laboratory for Coronaviruses, Laboratory Medicine, Leuven, Belgium	KU Leuven, Rega Institute, Clinical and Epidemiological Virology	Tony Wawina-Bokalanga, Joan Marti-Carerras, Bert Vanmechelen, Piet Maes
EPI_ISL_738756	Humboldt County Public Health Laboratory	Chan-Zuckerberg Biohub	CZB Cliahub Consortium
EPI_ISL_739674, EPI_ISL_739675, EPI_ISL_739676, EPI_ISL_739677	Instituto Nacional de Salud, Bogotá, Colombia	Instituto Nacional de Salud, Bogotá, Colombia	Katherine Laiton-Donato, Diego A. Álvarez-Díaz, Carlos Franco-Muñoz, Mauricio Pacheco-Montealegre, Jonathan Reales, Diego Andrés Prada, Sheryl Corchuelo, Magdalena Weisner, Martha Lucia Ospina Martinez, Marcela Mercado-Reyes
EPI_ISL_739726, EPI_ISL_739729, EPI_ISL_739749, EPI_ISL_739766, EPI_ISL_739778, EPI_ISL_739781, EPI_ISL_739782, EPI_ISL_739789, EPI_ISL_739790, EPI_ISL_739804, EPI_ISL_739841, EPI_ISL_739856, EPI_ISL_739862, EPI_ISL_739867, EPI_ISL_739868, EPI_ISL_739877, EPI_ISL_739902, EPI_ISL_739919, EPI_ISL_739958, EPI_ISL_739987, EPI_ISL_740014, EPI_ISL_740049, EPI_ISL_740064, EPI_ISL_740070, EPI_ISL_740086, EPI_ISL_740090, EPI_ISL_740108, EPI_ISL_740138, EPI_ISL_740161, EPI_ISL_740191, EPI_ISL_740202, EPI_ISL_740256, EPI_ISL_740210, EPI_ISL_740219, EPI_ISL_740220, EPI_ISL_740256, EPI_ISL_740279, EPI_ISL_740288, EPI_ISL_740316, EPI_ISL_740317, EPI_ISL_740327, EPI_ISL_740331, EPI_ISL_740333, EPI_ISL_740336, EPI_ISL_740342, EPI_ISL_740357, EPI_ISL_740359, EPI_ISL_740370, EPI_ISL_740406, EPI_ISL_740408, EPI_ISL_740409, EPI_ISL_740464, EPI_ISL_740473, EPI_ISL_740492, EPI_ISL_740500, EPI_ISL_740509, EPI_ISL_740521			
see above	Laboratoire national de santé, Microbiology, Virology	Laboratoire national de santé, Microbiology, Microbial Genomics Platform	Anke Wienecke-Baldacchino, Catherine Ragimbeau,Jessica Tapp, Fatu Djabi, Lise Pignon, Raoul Salmon, Tamir Abdelrahman
EPI_ISL_741772	Centre for Enzyme Innovation, University of Portsmouth / Translational Research Laboratory, Portsmouth Hospitals NHS Trust	COVID-19 Genomics UK (COG-UK) Consortium	Angela Beckett,Yann Bourgeois,Garry Scarlett,Sharon Glaysher,Scott Elliott,Kelly Bicknell,Robert Impey,Allyson Lloyd,Sarah Wyllie,Ethan Butcher,Anoop Chauhan,Samuel Robson
EPI_ISL_741773, EPI_ISL_741774, EPI_ISL_741775, EPI_ISL_741776	Oxford Viromics, NDM, University of Oxford; Oxford University Hospitals; Basingstoke and North Hampshire Hospital	COVID-19 Genomics UK (COG-UK) Consortium	Tanya Golubchik, David Bonsall, George Macintyre, Amy Trebes, Mariateresa de Cesare, Catrin Moore, Alex Mobbs, Anita Justice, Robert Shaw, Monique Andersson, Timothy Peto, Emma Wise, Nathan Moore, Jessica Lynch, Nick Cortes, Matilde Mori, Stephen Kidd, David Buck, John Todd, Christophe Fraser
EPI_ISL_744135, EPI_ISL_744153, EPI_ISL_744157, EPI_ISL_744161, EPI_ISL_744174, EPI_ISL_744175, EPI_ISL_744181, EPI_ISL_744224, EPI_ISL_744246, EPI_ISL_744275, EPI_ISL_744296, EPI_ISL_744364, EPI_ISL_744380, EPI_ISL_744388, EPI_ISL_744391, EPI_ISL_744392, EPI_ISL_744411, EPI_ISL_744416, EPI_ISL_744423, EPI_ISL_744458, EPI_ISL_744463, EPI_ISL_744464, EPI_ISL_744474, EPI_ISL_744475, EPI_ISL_744481, EPI_ISL_744502, EPI_ISL_744506, EPI_ISL_744516, EPI_ISL_744538, EPI_ISL_744551, EPI_ISL_744555, EPI_ISL_744574, EPI_ISL_744578, EPI_ISL_744579, EPI_ISL_744591, EPI_ISL_744595, EPI_ISL_744601, EPI_ISL_744609, EPI_ISL_744612, EPI_ISL_744646, EPI_ISL_744660, EPI_ISL_744679, EPI_ISL_744682, EPI_ISL_744690, EPI_ISL_744693, EPI_ISL_744700, EPI_ISL_744703, EPI_ISL_744716, EPI_ISL_744738, EPI_ISL_744741, EPI_ISL_744754, EPI_ISL_744755, EPI_ISL_744774, EPI_ISL_744784, EPI_ISL_744785, EPI_ISL_744790, EPI_ISL_744814, EPI_ISL_744816, EPI_ISL_744824, EPI_ISL_744842, EPI_ISL_744859, EPI_ISL_744866, EPI_ISL_744870, EPI_ISL_744917, EPI_ISL_744925, EPI_ISL_744928, EPI_ISL_744942, EPI_ISL_744951, EPI_ISL_744971, EPI_ISL_744990, EPI_ISL_745002, EPI_ISL_745004, EPI_ISL_745015, EPI_ISL_745019			
see above	Laboratoire national de santé, Microbiology, Virology	Laboratoire national de santé, Microbiology, Microbial Genomics Platform	Anke Wienecke-Baldacchino, Catherine Ragimbeau,Jessica Tapp, Fatu Djabi, Lise Pignon, Raoul Salmon, Tamir Abdelrahman
EPI_ISL_745520, EPI_ISL_745590, EPI_ISL_745648, EPI_ISL_746205, EPI_ISL_746209, EPI_ISL_746212, EPI_ISL_746216, EPI_ISL_746217, EPI_ISL_746230, EPI_ISL_746232, EPI_ISL_746236, EPI_ISL_746238, EPI_ISL_746240, EPI_ISL_746245, EPI_ISL_746252, EPI_ISL_746257, EPI_ISL_746260, EPI_ISL_746262, EPI_ISL_746271, EPI_ISL_746277, EPI_ISL_746279, EPI_ISL_746286, EPI_ISL_746291, EPI_ISL_746300, EPI_ISL_746301			
see above	Ginkgo Bioworks Clinical Laboratory	Utah Public Health Laboratory	Erin L. Young, Kelly Oakeson, Tara Gallagher, Michael T. Pyne, E. Susan Slechta, Melanie A. Mallory, Jeffrey B. Stevenson, Salika M. Shakir, David R. Hillyard, Malaika McKenzie-Bennett, James McGann, Jim Griffin, Keith Robison, Alex Plocik, Becky Schilling, Martha Pierson, Rebecca Littlefield, Michelle Spencer, Birgitte Simen
EPI_ISL_747036, EPI_ISL_747037, EPI_ISL_747038, EPI_ISL_747115	Respiratory Viruses Branch, Centers for Disease Control and Prevention	Respiratory Viruses Branch, Centers for Disease Control and Prevention	Queen,K., Li,Y., Tao,Y., Uehara,A., Montmayeur,A., Paden,C.R., Cook,P.W., Marine,R., Sheth,M., Wang,H., Lee,J., Tong,S.
EPI_ISL_747235	Sukabumi Health Laboratory	West Java Health Laboratory; School of Life Sciences and Technology, Institut Teknologi Bandung	Azzania Fibriani, Ema Rahmawati, Ryan Bayusantika Ristandi, Rifky Waluyajati Rachman, Cut Nur Cinthia Alamanda, Isak Solihin, Rini Robiani, Miftahul Faridl, Karimatu Khoirunnisa
EPI_ISL_747379, EPI_ISL_747380, EPI_ISL_747381, EPI_ISL_747382	Division of Emerging Infectious Diseases, Bureau of Infectious Diseases Diagnosis Control, Korea Disease Control and Prevention Agency	Division of Emerging Infectious Diseases, Bureau of Infectious Diseases Diagnosis Control, Korea Disease Control and Prevention Agency	Ae Kyung Park, Il-Hwan Kim, Heui Man Kim, Jeong-Min Kim, Namjoo Lee, Chaeyoung Lee, Sang Hee Woo, Eun-Jin Kim
EPI_ISL_748125, EPI_ISL_748126, EPI_ISL_748127, EPI_ISL_748128	Department of Virus and Microbiological Special Diagnostics, Statens Serum Institut, Copenhagen, Denmark	Albertsen Lab, Department of Chemistry and Bioscience, Aalborg University, Denmark	Danish Covid-19 Genome Consortium

EPI_ISL_751488	CHU Purpan - Laboratoire de Virologie - Institut Fédératif de Biologie	CHU Purpan - Laboratoire de Virologie - Institut Fédératif de Biologie	Latour J., Ranger N., Dubois M., Carcenac R., Harter A., Boyer P., Tremeaux P., Izopet J.
EPI_ISL_751543	DE Public Health Laboratory	Genomics and Discovery, Respiratory Viruses Branch, Division of Viral Diseases, Centers for Disease Control and Prevention	Krista Queen, Yan Li, Ying Tao, Jing Zhang, Anna Uehara, Anna Montmayeur, Clinton R. Paden, Peter W. Cook,Rachel Marine, Mili Sheth, Haibin Wang, Justin Lee, Suxiang Tong
EPI_ISL_751622	MI - Michigan Department of Health and Human Services - Bureau of Laboratories	Genomics and Discovery, Respiratory Viruses Branch, Division of Viral Diseases, Centers for Disease Control and Prevention	Krista Queen, Yan Li, Ying Tao, Jing Zhang, Anna Uehara, Anna Montmayeur, Clinton R. Paden, Peter W. Cook,Rachel Marine, Mili Sheth, Haibin Wang, Justin Lee, Suxiang Tong
EPI_ISL_751726	DE Public Health Laboratory	Genomics and Discovery, Respiratory Viruses Branch, Division of Viral Diseases, Centers for Disease Control and Prevention	Krista Queen, Yan Li, Ying Tao, Jing Zhang, Anna Uehara, Anna Montmayeur, Clinton R. Paden, Peter W. Cook,Rachel Marine, Mili Sheth, Haibin Wang, Justin Lee, Suxiang Tong
EPI_ISL_753017, EPI_ISL_753018	State Laboratories Division, Hawaii State Department of Health	State Laboratories Division, Hawaii State Department of Health	Pamela O'Brien, Sabrina Diemert, Drew Kuwazaki, Razvan Sultana, Edward Desmond
EPI_ISL_753790, EPI_ISL_753796, EPI_ISL_753804, EPI_ISL_753894, EPI_ISL_753902, EPI_ISL_754194	Charité Universitätsmedizin Berlin, Institut für Virologie/Labor Berlin	Charité Universitätsmedizin Berlin, Institut für Virologie	Victor M Corman, Jörn Beheim-Schwarzbach, Barbara Mühlemann, Julia Schneider, Talitha Veith, Terry Jones, Christian Drosten
EPI_ISL_754691, EPI_ISL_754692, EPI_ISL_754693, EPI_ISL_754694, EPI_ISL_754713, EPI_ISL_754714, EPI_ISL_754715, EPI_ISL_754716, EPI_ISL_754717, EPI_ISL_754718, EPI_ISL_754719, EPI_ISL_754720, EPI_ISL_754721, EPI_ISL_754722, EPI_ISL_754723, EPI_ISL_754724, EPI_ISL_754725, EPI_ISL_754726, EPI_ISL_754727, EPI_ISL_754728, EPI_ISL_754729, EPI_ISL_754730, EPI_ISL_754731, EPI_ISL_754732, EPI_ISL_754734, EPI_ISL_754735, EPI_ISL_754736, EPI_ISL_754737, EPI_ISL_754738, EPI_ISL_754739, EPI_ISL_754740, EPI_ISL_754741, EPI_ISL_754742, EPI_ISL_754743, EPI_ISL_754779, EPI_ISL_754780, EPI_ISL_754801, EPI_ISL_754802, EPI_ISL_754803, EPI_ISL_754804, EPI_ISL_754805, EPI_ISL_754806, EPI_ISL_754807, EPI_ISL_754808, EPI_ISL_754809, EPI_ISL_754810, EPI_ISL_754811, EPI_ISL_754812, EPI_ISL_754813, EPI_ISL_754814, EPI_ISL_754815, EPI_ISL_754816, EPI_ISL_754817, EPI_ISL_754818, EPI_ISL_754819, EPI_ISL_754820, EPI_ISL_754821, EPI_ISL_754822, EPI_ISL_754823, EPI_ISL_754824, EPI_ISL_754825, EPI_ISL_754826, EPI_ISL_754827, EPI_ISL_754828, EPI_ISL_754829, EPI_ISL_754830, EPI_ISL_754831, EPI_ISL_754832, EPI_ISL_754833, EPI_ISL_754834, EPI_ISL_754835, EPI_ISL_754836, EPI_ISL_754837, EPI_ISL_754838, EPI_ISL_754839			
see above	Wadsworth Center, New York State Department.of Health	Wadsworth Center, New York State Department.of Health	Kirsten St. George, Daryl M. Lamson, Alexis Russel, Matthew Shudt, Melissa A Leisner, Jonathan Plitnick, Navjot Singh, John Kelly, Sara Griesemer, Erasmus Schneider, Erica Lasek-Nesselquist
EPI_ISL_755137, EPI_ISL_755197	UCSD EXCITE lab	Andersen lab at Scripps Research	SEARCH Alliance San Diego
EPI_ISL_755619	Canterbury Health Laboratories	Institute of Environmental Science and Research (ESR)	Xiaoyun Ren, Matt Storey, Nikki Freed, Muhammad Faisal, Jing Wang, Hermes Perez, Anja Werno, Antje van der Linden, Arlo Upton, Chris Mansell, David Hammer, Dragana Drinkovic, Gary McAuliffe, Hana Sofia Andersson, James Ussher, Jill Sherwood, Josh Freeman, Julia Howard, Juliet Elvy, Mary DeAlmeida, Matt Blakiston, Matthew Rogers, Max Bloomfield, Michael Addidle, Michelle Balm, Sally Roberts, Sarah Jefferies, Sharmini Muttaiyah, Susan Morpeth, Susan Taylor, Timothy Blackmore, Vani Sathyendran, Veronica Playle, Virginia Hope, Erasmus Smit, Lauren Jelly, Olin Silander, Joep de Ligt
EPI_ISL_755742, EPI_ISL_755743, EPI_ISL_755744, EPI_ISL_755749	Toronto Invasive Bacterial Diseases Network	McMaster University	Allison McGeer, Patryk Aftanas, Hooman Derakhshani, Angel Li, Kuganya Nirmalarajah, Emily Panousis, Ahmed Draia, Jalees Nasir, Michael Surette, Samira Mubareka, Andrew G. McArthur
EPI_ISL_756377	Lighthouse Lab in Milton Keynes	Wellcome Sanger Institute for the COVID-19 Genomics UK (COG-UK) Consortium	The Lighthouse Lab in Milton Keynes and Alex Alderton, Roberto Amato, Sonia Goncalves, Ewan Harrison, David K. Jackson, Ian Johnston, Dominic Kwiatkowski, Cordelia Langford, John Sillitoe on behalf of the Wellcome Sanger Institute COVID-19 Surveillance Team
EPI_ISL_759703	RSUD Bung Karno	Universitas Sebelas Maret (UNS), Rumah sakit Universitas Sebelas Maret (RS-UNS) Surakarta, National Institute of Health Research and Development, Indonesian Ministry of Health	Betty Suryawati, Yulia sari, hartono, maryani, Afif Avicena Gufron, Hana Apsari Pawestri, Kartika, Dewi Puspa, Hartanti Dian Ika, Arie Ardiansyah Nugraha, Vivi Setiawaty
EPI_ISL_759842	Rumah Sakit Universitas Sebelas Maret (RS-UNS)	Universitas Sebelas Maret (UNS); Rumah Sakit Universitas Sebelas Maret (RS-UNS), Surakarta; National Institute of Health Research and Development, Indonesian Ministry of Health, Jakarta.	Betty Suryawati, Yulia Sari, Hartono, Maryani, Afif Avicena Ghufron, Hana Apsari Pawestri, Kartika, Dewi Puspa, Hartanti Dian Ika, Arie Ardiansyah Nugraha, Vivi Setiawaty.
EPI_ISL_760066, EPI_ISL_760106, EPI_ISL_760107, EPI_ISL_760108, EPI_ISL_760109, EPI_ISL_760110, EPI_ISL_760111, EPI_ISL_760137, EPI_ISL_760138, EPI_ISL_760139, EPI_ISL_760140, EPI_ISL_760141, EPI_ISL_760142, EPI_ISL_760143, EPI_ISL_760144, EPI_ISL_760189			
see above	Division of Emerging Infectious Diseases, Bureau of Infectious Diseases Diagnosis Control, Korea Disease Control and Prevention Agency	Division of Emerging Infectious Diseases, Bureau of Infectious Diseases Diagnosis Control, Korea Disease Control and Prevention Agency	Ae Kyung Park, Il-Hwan Kim, Heui Man Kim, Jeong-Min Kim, Namjoo Lee, Chaeyoung Lee, Sang Hee Woo, Eun-Jin Kim
EPI_ISL_763068	RSUD Kota Surakarta	Universitas Sebelas Maret (UNS); Rumah Sakit UNS (RS-UNS); National Institute of Health Research and Development, Indonesian Ministry of Health.	Maryani, Yulia Sari, Hartono, Afif A Ghufron, Revi G H Novika, Hana A Pawestri, Kartika, Dewi Puspa, Hartanti Dian Ika, Arie A Nugraha, Vivi Setiawaty, Betty Suryawati.
EPI_ISL_763070	Puskesmas Jambu Kulon, Klaten	Universitas Sebelas Maret (UNS); Rumah Sakit UNS (RS-UNS); National Institute of Health Research and Development, Indonesian Ministry of Health.	Yulia Sari, Hartono, Maryani, Afif A Ghufron, Hana A Pawestri, Kartika, Dewi Puspa, Hartanti Dian Ika, Arie A Nugraha, Vivi Setiawaty, Betty Suryawati.
EPI_ISL_763095, EPI_ISL_763138, EPI_ISL_763323	Istituto Zooprofilattico Sperimentale dell' Umbria e delle Marche -Togo Rosati	Istituto Superiore di Sanità	Massimo Biagetti , Monica Giammarioli, Luca De Sabato, Gabriele Vaccari, Ilaria Di Bartolo, Giovanni Ianiro
EPI_ISL_763395, EPI_ISL_763450, EPI_ISL_763524, EPI_ISL_763719, EPI_ISL_764127, EPI_ISL_764263, EPI_ISL_764395, EPI_ISL_764426, EPI_ISL_764427, EPI_ISL_764428, EPI_ISL_764429, EPI_ISL_764430			
see above	Regional Virus Laboratory, Belfast Health and Social Care Trust	COVID-19 Genomics UK (COG-UK) Consortium	Conall McCaughey, James McKenna, Tanya Curran, Susan Feeney, Alison Watt, Ciara Cox, Mairead Connor, Zoltan Molnar, David Simpson, Derek Fairley
EPI_ISL_765652, EPI_ISL_765655, EPI_ISL_765657, EPI_ISL_765659, EPI_ISL_765762, EPI_ISL_765763, EPI_ISL_765764, EPI_ISL_765765, EPI_ISL_765766, EPI_ISL_765767	Massachusetts General Hospital	Infectious Disease Program, Broad Institute of Harvard and MIT	Lemieux,J.E., Siddle,K.J., Shaw,B., Adams,G., Pierce,V., Turbett,S., Anahtar,M., Branda,J., Slater,D., Harris,J., Lin,A.E., Gladden-Young,A., Lagerborg,K., Rudy,M., DeRuff,K., Carter,A., Normandin,E., Bauer,M., Reilly,S., Tomkins-Tinch,C., Loreth,C., Chaluvadi,S., Neumann,A., Cusick,C., Chapman,S.B., Gnirke,A., Flowers,K., Cerrato,F., Birren,B.W., Gallagher,G., Smole,S., Park,D.J., MacInnis,B.L., Ryan,E., LaRocque,R., Rosenberg,E. and Sabeti,P.C.
EPI_ISL_766083	Respiratory Virus Unit, National Infection Service, Public Health England	COVID-19 Genomics UK (COG-UK) Consortium	PHE Covid Sequencing Team
EPI_ISL_766619	Klinisk mikrobiologi	The Public Health Agency of Sweden	Department of Microbiology, The Public Health Agency of Sweden
EPI_ISL_768780, EPI_ISL_768781, EPI_ISL_768782, EPI_ISL_768783	AIID	Irish Coronavirus Sequencing Consortium - National Virus Reference Laboratory	Michael Carr, Gabriel Gonzalez, Alejandro Abner Garcia Leon, Patrick Mallon
EPI_ISL_770008	Area De Salud Corredores	Inciensa, Instituto Costarricense de Investigación y Enseñanza en Nutrición y Salud	Francisco Duarte, Hebleen Porras, Claudio Soto-Garita, Estela Cordero, Adriana Godínez, Melany Calderón & Mariel López
EPI_ISL_770016	Area De Salud Goicoechea 2 - Clinica Dr. Jimenez Nuñez	Inciensa, Instituto Costarricense de Investigación y Enseñanza en Nutrición y Salud	Francisco Duarte, Hebleen Porras, Claudio Soto-Garita, Estela Cordero, Adriana Godínez, Melany Calderón & Mariel López
EPI_ISL_770017	Area De Salud Perez Zeledon	Inciensa, Instituto Costarricense de Investigación y Enseñanza en Nutrición y Salud	Francisco Duarte, Hebleen Porras, Claudio Soto-Garita, Estela Cordero, Adriana Godínez, Melany Calderón & Mariel López
EPI_ISL_770025	Area De Salud San Juan-San Diego-Concepcion 2	Inciensa, Instituto Costarricense de Investigación y Enseñanza en Nutrición y Salud	Francisco Duarte, Hebleen Porras, Claudio Soto-Garita, Estela Cordero, Adriana Godínez, Melany Calderón & Mariel López

EPI_ISL_775319, EPI_ISL_775320	Nordland Hospital - Bodo, Laboratory Department, Molecular Biology Unit	Norwegian Institute of Public Health, Department of Virology	Kathrine Stene-Johansen, Kamilla Heddeland Instefjord, Hilde Elshaug, Atiya R Ali,Marie Paulsen Madsen, Rasmus Riis Kopperud, Hilde Vollan, Karoline Bragstad, Olav Hungnes
EPI_ISL_775325, EPI_ISL_775326	Foerde Hospital, Department of Microbiology	Norwegian Institute of Public Health, Department of Virology	Kathrine Stene-Johansen, Kamilla Heddeland Instefjord, Hilde Elshaug, Atiya R Ali,Marie Paulsen Madsen, Rasmus Riis Kopperud, Hilde Vollan, Karoline Bragstad, Olav Hungnes
EPI_ISL_775441	Innlandet Hospital Trust, Division Lillehammer, Department for Medical Microbiology	Norwegian Institute of Public Health, Department of Virology	Kathrine Stene-Johansen, Kamilla Heddeland Instefjord, Hilde Elshaug, Atiya R Ali,Marie Paulsen Madsen, Rasmus Riis Kopperud, Hilde Vollan, Karoline Bragstad, Olav Hungnes
EPI_ISL_775480	Nordland Hospital - Bodo, Laboratory Department, Molecular Biology Unit	Norwegian Institute of Public Health, Department of Virology	Kathrine Stene-Johansen, Kamilla Heddeland Instefjord, Hilde Elshaug, Atiya R Ali,Marie Paulsen Madsen, Rasmus Riis Kopperud, Hilde Vollan, Karoline Bragstad, Olav Hungnes
EPI_ISL_779933	Center of Medical Microbiology, Virology, and Hospital Hygiene, University of Duesseldorf	Center of Medical Microbiology, Virology, and Hospital Hygiene, University of Duesseldorf	Maximilian Damagnez, Alexander Dilthey, Ashley-Jane Duplessis, Torsten Houwaart, Lisanna Hülse, Malte Kohns Vasconcelos, Nadine Lübke, Jessica Nicolai, Klaus Pfeffer, Daniel Strelow, Teresa Tamayo, Jörg Timm, Andreas Walker, Tobias Wienemann
EPI_ISL_780036	Hospital General Universitario Gregorio Marañón	SeqCOVID-SPAIN consortium/IBV(CSIC)	Darío García de Viedma, Laura Pérez-Lago, Marta Herranz, Jon Sicilia, Julia Suárez, Pilar Catalán, Patricia Muñoz and SeqCOVID-SPAIN consortium
EPI_ISL_785904, EPI_ISL_785934, EPI_ISL_785945, EPI_ISL_785994, EPI_ISL_786557, EPI_ISL_786558, EPI_ISL_786569, EPI_ISL_786570, EPI_ISL_786581, EPI_ISL_786582, EPI_ISL_786593, EPI_ISL_786594, EPI_ISL_786605, EPI_ISL_786606, EPI_ISL_786617, EPI_ISL_786618, EPI_ISL_786623, EPI_ISL_786629, EPI_ISL_786630, EPI_ISL_786640, EPI_ISL_786641, EPI_ISL_786642, EPI_ISL_786643, EPI_ISL_786644, EPI_ISL_786645, EPI_ISL_786646, EPI_ISL_786647, EPI_ISL_786648, EPI_ISL_786649, EPI_ISL_786650, EPI_ISL_786651, EPI_ISL_786652, EPI_ISL_786653, EPI_ISL_786654, EPI_ISL_786655, EPI_ISL_786656, EPI_ISL_786657, EPI_ISL_786658, EPI_ISL_786659, EPI_ISL_786660, EPI_ISL_786661, EPI_ISL_786662, EPI_ISL_786663, EPI_ISL_786664, EPI_ISL_786665, EPI_ISL_786666, EPI_ISL_786667, EPI_ISL_786668, EPI_ISL_786669, EPI_ISL_786670, EPI_ISL_786671, EPI_ISL_786672, EPI_ISL_786673, EPI_ISL_786674, EPI_ISL_786675, EPI_ISL_786676, EPI_ISL_786677, EPI_ISL_786678, EPI_ISL_786679, EPI_ISL_786680, EPI_ISL_786681, EPI_ISL_786682, EPI_ISL_786683, EPI_ISL_786684, EPI_ISL_786685, EPI_ISL_786686, EPI_ISL_786687, EPI_ISL_786688, EPI_ISL_786689, EPI_ISL_786690, EPI_ISL_786691, EPI_ISL_786692, EPI_ISL_786693, EPI_ISL_786694, EPI_ISL_786695, EPI_ISL_786696, EPI_ISL_786697, EPI_ISL_786698, EPI_ISL_786699, EPI_ISL_786700, EPI_ISL_786701, EPI_ISL_786702, EPI_ISL_786703, EPI_ISL_786704, EPI_ISL_786705, EPI_ISL_786706, EPI_ISL_786707, EPI_ISL_786708, EPI_ISL_786709, EPI_ISL_786710, EPI_ISL_786711, EPI_ISL_786712, EPI_ISL_786713, EPI_ISL_786714, EPI_ISL_786715, EPI_ISL_786716, EPI_ISL_786717, EPI_ISL_786718, EPI_ISL_786719, EPI_ISL_786720, EPI_ISL_786721, EPI_ISL_786722, EPI_ISL_786723, EPI_ISL_786724, EPI_ISL_786725, EPI_ISL_786726, EPI_ISL_786727, EPI_ISL_786728, EPI_ISL_786729, EPI_ISL_786730, EPI_ISL_786731, EPI_ISL_786732, EPI_ISL_786733, EPI_ISL_786734, EPI_ISL_786735, EPI_ISL_786736, EPI_ISL_786737, EPI_ISL_786738, EPI_ISL_786739, EPI_ISL_786740, EPI_ISL_786741, EPI_ISL_786742, EPI_ISL_786743, EPI_ISL_786744, EPI_ISL_786745, EPI_ISL_786746, EPI_ISL_786747, EPI_ISL_786749, EPI_ISL_786750, EPI_ISL_786751, EPI_ISL_786752, EPI_ISL_786753, EPI_ISL_786754, EPI_ISL_786755, EPI_ISL_786756, EPI_ISL_786757, EPI_ISL_786758, EPI_ISL_786759, EPI_ISL_786762, EPI_ISL_786763, EPI_ISL_786764, EPI_ISL_786765, EPI_ISL_786766, EPI_ISL_786767, EPI_ISL_786768, EPI_ISL_786770, EPI_ISL_786772, EPI_ISL_786773, EPI_ISL_786774, EPI_ISL_786775, EPI_ISL_786776, EPI_ISL_786777, EPI_ISL_786778, EPI_ISL_786779, EPI_ISL_786780, EPI_ISL_786781, EPI_ISL_786782, EPI_ISL_786784, EPI_ISL_786785, EPI_ISL_786786, EPI_ISL_786787, EPI_ISL_786788, EPI_ISL_786789, EPI_ISL_786790, EPI_ISL_786791, EPI_ISL_786792, EPI_ISL_786793, EPI_ISL_786794, EPI_ISL_786796, EPI_ISL_786797, EPI_ISL_786798, EPI_ISL_786799, EPI_ISL_786800, EPI_ISL_786801, EPI_ISL_786802, EPI_ISL_786803, EPI_ISL_786804, EPI_ISL_786805, EPI_ISL_786806, EPI_ISL_786807, EPI_ISL_786808, EPI_ISL_786809, EPI_ISL_786810, EPI_ISL_786811, EPI_ISL_786812, EPI_ISL_786813, EPI_ISL_786814, EPI_ISL_786815, EPI_ISL_786816, EPI_ISL_786817, EPI_ISL_786818, EPI_ISL_786819, EPI_ISL_786821, EPI_ISL_786822, EPI_ISL_786823, EPI_ISL_786824, EPI_ISL_786825, EPI_ISL_786826, EPI_ISL_786827, EPI_ISL_786828, EPI_ISL_786829, EPI_ISL_786830, EPI_ISL_786832, EPI_ISL_786834, EPI_ISL_786835, EPI_ISL_786836, EPI_ISL_786839, EPI_ISL_786841, EPI_ISL_786842, EPI_ISL_786848, EPI_ISL_786851, EPI_ISL_786852, EPI_ISL_786854, EPI_ISL_786855, EPI_ISL_786860, EPI_ISL_786861, EPI_ISL_786863, EPI_ISL_786864, EPI_ISL_786865, EPI_ISL_786869, EPI_ISL_786871, EPI_ISL_786873, EPI_ISL_786875, EPI_ISL_786877, EPI_ISL_786879, EPI_ISL_786883, EPI_ISL_786884, EPI_ISL_786887, EPI_ISL_786888, EPI_ISL_786889, EPI_ISL_786895, EPI_ISL_786896, EPI_ISL_786897, EPI_ISL_786899, EPI_ISL_786900, EPI_ISL_786901, EPI_ISL_786902, EPI_ISL_786903, EPI_ISL_786905, EPI_ISL_786910, EPI_ISL_786914, EPI_ISL_786919, EPI_ISL_786920, EPI_ISL_786921, EPI_ISL_786922, EPI_ISL_786923, EPI_ISL_786925			
see above	Houston Methodist Hospital	Houston Methodist Hospital	S. Wesley Long, Randall J. Olsen, Paul A. Christensen, David W. Bernard, James J. Davis, Maulik Shukla, Marcus Nguyen, Matthew Ojeda Saavedra, Prasanti Yerramilli, Layne Pruitt, Sishir Subedi, Heather Hendrickson, and James M. Musser
EPI_ISL_791979	RSUD Curup Bengkulu	National Institute of Health Research and Development	Nugraha,AA;Subangkit;Pawestri,HA;Ikawati,HD;Puspa,KD;Mardiani;Pangesti,KNA;Soekarso,T;Puspandari,N;Setiawaty,V
EPI_ISL_792018, EPI_ISL_792020	Plateforme COVID IDF	National Reference Center for Viruses of Respiratory Infections, Institut Pasteur, Paris	Marion Barbet, Sylvie Behillil, Méline Bizard, Angela Brisebarre, Camille Capel, Etienne Simon-Lorière, Vincent Enouf, Maud Vanpeene, Sylvie van der Werf,Jacques Fourgeaud
EPI_ISL_792599, EPI_ISL_792625, EPI_ISL_792626	LACEN-PB	Laboratory of Respiratory Viruses and Measles, Oswaldo Cruz Institute, FIOCRUZ	Paola Resende, Luciana Appolinario, Fernando Motta, Anna Carolina Paixao, Ana Carolina Mendonca, João Felipe Bezerra, Romero Henrique Teixeira de Vasconcelos, Dalane Loudal Florentino Teixeira, Thiago Franco de Oliveira Carneiro, Marilda Siqueira
EPI_ISL_794685	PathWest Laboratory Medicine WA	PathWest Laboratory Medicine WA Microbial Surveillance Unit	PathWest Laboratory Medicine WA Microbial Surveillance Unit
EPI_ISL_796711	Department of Medical Microbiology, St. Olavs hospital	Norwegian Institute of Public Health, Department of Virology	Kathrine Stene-Johansen, Kamilla Heddeland Instefjord, Hilde Elshaug, Atiya R Ali,Marie Paulsen Madsen, Rasmus Riis Kopperud, Hilde Vollan, Karoline Bragstad, Olav Hungnes
EPI_ISL_801406, EPI_ISL_801429, EPI_ISL_801486	Dutch COVID-19 response team	Erasmus Medical Center	Bas Oude Munnink, Reina Sikkema, David Nieuwenhuijse, Irina Chestakova, Anne van der Linden, Marjan Boter, Emmanuelle Munger, Corine GeurtsvanKessel, Annemiek van der Eijk, Richard Molenkamp, Marion Koopmans, on behalf of the Dutch national COVID-19 response team.
EPI_ISL_802750, EPI_ISL_802751, EPI_ISL_802752, EPI_ISL_802753, EPI_ISL_802754, EPI_ISL_802755, EPI_ISL_802756, EPI_ISL_802757, EPI_ISL_802758, EPI_ISL_802759, EPI_ISL_802760, EPI_ISL_802761, EPI_ISL_802762, EPI_ISL_802763, EPI_ISL_802764, EPI_ISL_802766, EPI_ISL_802767, EPI_ISL_802768, EPI_ISL_802769, EPI_ISL_802770, EPI_ISL_802771, EPI_ISL_802772, EPI_ISL_802773, EPI_ISL_802774, EPI_ISL_802775, EPI_ISL_802776			
see above	Hospital Santa María Nai	Instituto de Salud Carlos III	Iglesias-Caballero, M. Molinero Calamita, M. González-Esguevillas, M. Camarero, S. Pozo, F. Casas, I. Jiménez, P. Jiménez, M. Zaballos, A. Monzón, S. Varona, S. Juliá, M. Cuesta, I, J. García.
EPI_ISL_803105, EPI_ISL_803106	Respiratory Viruses Branch, Centers for Disease Control and Prevention	Respiratory Viruses Branch, Centers for Disease Control and Prevention	Queen,K., Li,Y., Tao,Y., Uehara,A., Montmayeur,A., Paden,C.R., Cook,P.W., Marine,R., Sheth,M., Wang,H., Lee,J., Tong,S.
EPI_ISL_803401, EPI_ISL_803402, EPI_ISL_803403, EPI_ISL_803404, EPI_ISL_803405, EPI_ISL_803406, EPI_ISL_803407, EPI_ISL_803408, EPI_ISL_803409, EPI_ISL_803412, EPI_ISL_803440, EPI_ISL_803441, EPI_ISL_803442, EPI_ISL_803443, EPI_ISL_803444, EPI_ISL_803445, EPI_ISL_803446, EPI_ISL_803447, EPI_ISL_803448, EPI_ISL_803449, EPI_ISL_803450, EPI_ISL_803451, EPI_ISL_803452, EPI_ISL_803453, EPI_ISL_803454, EPI_ISL_803455, EPI_ISL_803456, EPI_ISL_803700, EPI_ISL_803701, EPI_ISL_803702, EPI_ISL_803703, EPI_ISL_803704, EPI_ISL_803705, EPI_ISL_803809, EPI_ISL_803810, EPI_ISL_803811, EPI_ISL_803812, EPI_ISL_803813, EPI_ISL_803814, EPI_ISL_803815, EPI_ISL_803816, EPI_ISL_803818, EPI_ISL_803819, EPI_ISL_803820, EPI_ISL_803821, EPI_ISL_803822, EPI_ISL_803823, EPI_ISL_803824, EPI_ISL_803825, EPI_ISL_803826, EPI_ISL_803827, EPI_ISL_803831, EPI_ISL_803832, EPI_ISL_803833, EPI_ISL_803834, EPI_ISL_803835, EPI_ISL_803836, EPI_ISL_803837, EPI_ISL_803838, EPI_ISL_803839, EPI_ISL_803840, EPI_ISL_803841, EPI_ISL_803842, EPI_ISL_803843, EPI_ISL_803844, EPI_ISL_803845, EPI_ISL_803846, EPI_ISL_803847, EPI_ISL_803848, EPI_ISL_803849			
see above	Wisconsin State Laboratory of Hygiene Communicable Disease Division	Wisconsin State Laboratory of Hygiene Communicable Disease Division	Kelsey R. Florek, Abigail C. Shockey
EPI_ISL_804371, EPI_ISL_804372	CHU Purpan - Laboratoire de Virologie - Institut Fédératif de Biologie	CHU Purpan - Laboratoire de Virologie - Institut Fédératif de Biologie	Latour J., Ranger N., Dubois M., Carcenac R., Harter A., Boyer P., Tremeaux P., Izopet J.
EPI_ISL_804472, EPI_ISL_804480, EPI_ISL_804481, EPI_ISL_804514, EPI_ISL_804515, EPI_ISL_804516, EPI_ISL_804517, EPI_ISL_804518, EPI_ISL_804519, EPI_ISL_804520, EPI_ISL_804521, EPI_ISL_804522, EPI_ISL_804523, EPI_ISL_804524, EPI_ISL_804539, EPI_ISL_804540, EPI_ISL_804541, EPI_ISL_804542, EPI_ISL_804543, EPI_ISL_804544, EPI_ISL_804545, EPI_ISL_804546, EPI_ISL_804547, EPI_ISL_804548, EPI_ISL_804549, EPI_ISL_804550, EPI_ISL_804551, EPI_ISL_804552, EPI_ISL_804553, EPI_ISL_804554, EPI_ISL_804555, EPI_ISL_804556			
see above	MEPHI, Aix Marseille University	MEPHI, Aix Marseille University	Anthony LEVASSEUR
EPI_ISL_804598	Maryland Public Health Laboratory	Maryland Public Health Laboratory	Maryland Department of Health Laboratories Administration
EPI_ISL_819450	Northumbria University / South Tees Hospitals NHS Foundation Trust / North Cumbria Integrated Care NHS Foundation Trust / North Tees and Hartlepool NHS Foundation Trust / Newcastle Hospitals NHS Foundation Trust	COVID-19 Genomics UK (COG-UK) Consortium	Darren L Smith,Andrew Nelson,Matthew Bashton,Greg R Young,Joshua Loh,John Allan,Mohammad A Tariq,Giles S Holt,Gary Black,Wen C Yew,Lynn Dover,Paul Baker,Steve Liggett,Sarah Essex,Jane Greenaway,Debra Padgett,Clive Graham,Garren Scott,Edward Barton,Emma Swindells,Brendan Payne,Jennifer Collins,Yusri Taha,Gary Eltringham
EPI_ISL_825548, EPI_ISL_825583, EPI_ISL_825584, EPI_ISL_825585, EPI_ISL_825587	Respiratory Virus Unit, National Infection Service, Public Health England	COVID-19 Genomics UK (COG-UK) Consortium	PHE Covid Sequencing Team
EPI_ISL_826480, EPI_ISL_826481, EPI_ISL_826482, EPI_ISL_826483, EPI_ISL_826484, EPI_ISL_826485, EPI_ISL_826489, EPI_ISL_826490, EPI_ISL_826493, EPI_ISL_826497, EPI_ISL_826501, EPI_ISL_826502, EPI_ISL_826503, EPI_ISL_826505, EPI_ISL_826509, EPI_ISL_826510, EPI_ISL_826511, EPI_ISL_826512, EPI_ISL_826514, EPI_ISL_826515			
see above	Lighthouse Lab in Alderley Park	Wellcome Sanger Institute for the COVID-19 Genomics UK (COG-UK) Consortium	Jacquelyn Wynn, Mairead Hyland, The Lighthouse Lab in Alderley Park and Alex Alderton, Roberto Amato, Sonia Goncalves, Ewan Harrison, David K. Jackson, Ian Johnston, Dominic Kwiatkowski, Cordelia Langford, John Sillitoe on behalf of the Wellcome Sanger Institute COVID-19 Surveillance Team
EPI_ISL_826516	Lighthouse Lab in Glasgow	Wellcome Sanger Institute for the COVID-19 Genomics UK (COG-UK) Consortium	Harper VanSteenhouse, Yumi Kasai, David Gray, Carol Clugston, Anna Dominiczak and Alex Alderton, Roberto Amato, Sonia Goncalves, Ewan Harrison, David K. Jackson, Ian Johnston, Dominic Kwiatkowski, Cordelia Langford, John Sillitoe on behalf of the Wellcome Sanger Institute COVID-19 Surveillance Team
EPI_ISL_826674, EPI_ISL_826675, EPI_ISL_826676, EPI_ISL_826680, EPI_ISL_826682, EPI_ISL_826684, EPI_ISL_826696, EPI_ISL_826701, EPI_ISL_826702, EPI_ISL_826703, EPI_ISL_826705, EPI_ISL_826706, EPI_ISL_826707, EPI_ISL_826708, EPI_ISL_826709, EPI_ISL_826710, EPI_ISL_826713, EPI_ISL_826714, EPI_ISL_826743, EPI_ISL_826744, EPI_ISL_826766, EPI_ISL_826847, EPI_ISL_826848, EPI_ISL_826853, EPI_ISL_826857, EPI_ISL_826859, EPI_ISL_826860, EPI_ISL_826863, EPI_ISL_826876, EPI_ISL_827013, EPI_ISL_827057, EPI_ISL_827201			

see above	deCODE genetics	deCODE genetics	Daniel F Gudbjartsson; Agnar Helgason; Hakon Jonsson; Olafur T Magnusson; Pall Melsted; Gudmundur L Norddahl; Jona Saemundsdottir; Asgeir Sigurdsson; Patrick Sulem; Arna B Agustsdottir; Hannes Eggertsson; Berglind Eiriksdottir; Run Fridriksdottir; Elisabet E Gardarsdottir; Gudmundur Georgsson; Olafia S Gretarsdottir; Kjartan R Gudmundsson; Thora R Gunnarsdottir; Arnaldur Gylfason; Hilma Holm; Brynjar O Jensson; Aslaug Jonasdottir; Kamilla S Josefsdottir; Thordur Kristjansson; Droplaug N Magnusdottir; Solvi Rognvaldsson; Louise le Roux; Gudrun Sigmundsdottir; Gardar Sveinbjornsson; Kristin E Sveinsdottir; Maney Sveinsdottir; Emil A Thorarensen; Bjarni Thorbjornsson; Gisli Masson; Ingileif Jonsdottir; Alma Moller; Thorolfur Gudnason; Karl G Kristinsson; Unnur Thorsteinsdottir; Kari Stefansson
EPI_ISL_827212, EPI_ISL_827229, EPI_ISL_827252, EPI_ISL_827253, EPI_ISL_827254, EPI_ISL_827256, EPI_ISL_827257, EPI_ISL_827266, EPI_ISL_827363, EPI_ISL_827419	The National University Hospital of Iceland	deCODE genetics	Daniel F Gudbjartsson; Agnar Helgason; Hakon Jonsson; Olafur T Magnusson; Pall Melsted; Gudmundur L Norddahl; Jona Saemundsdottir; Asgeir Sigurdsson; Patrick Sulem; Arna B Agustsdottir; Hannes Eggertsson; Berglind Eiriksdottir; Run Fridriksdottir; Elisabet E Gardarsdottir; Gudmundur Georgsson; Olafia S Gretarsdottir; Kjartan R Gudmundsson; Thora R Gunnarsdottir; Arnaldur Gylfason; Hilma Holm; Brynjar O Jensson; Aslaug Jonasdottir; Kamilla S Josefsdottir; Thordur Kristjansson; Droplaug N Magnusdottir; Solvi Rognvaldsson; Louise le Roux; Gudrun Sigmundsdottir; Gardar Sveinbjornsson; Kristin E Sveinsdottir; Maney Sveinsdottir; Emil A Thorarensen; Bjarni Thorbjornsson; Gisli Masson; Ingileif Jonsdottir; Alma Moller; Thorolfur Gudnason; Karl G Kristinsson; Unnur Thorsteinsdottir; Kari Stefansson
EPI_ISL_827522, EPI_ISL_827526, EPI_ISL_827530	deCODE genetics	deCODE genetics	Daniel F Gudbjartsson; Agnar Helgason; Hakon Jonsson; Olafur T Magnusson; Pall Melsted; Gudmundur L Norddahl; Jona Saemundsdottir; Asgeir Sigurdsson; Patrick Sulem; Arna B Agustsdottir; Hannes Eggertsson; Berglind Eiriksdottir; Run Fridriksdottir; Elisabet E Gardarsdottir; Gudmundur Georgsson; Olafia S Gretarsdottir; Kjartan R Gudmundsson; Thora R Gunnarsdottir; Arnaldur Gylfason; Hilma Holm; Brynjar O Jensson; Aslaug Jonasdottir; Kamilla S Josefsdottir; Thordur Kristjansson; Droplaug N Magnusdottir; Solvi Rognvaldsson; Louise le Roux; Gudrun Sigmundsdottir; Gardar Sveinbjornsson; Kristin E Sveinsdottir; Maney Sveinsdottir; Emil A Thorarensen; Bjarni Thorbjornsson; Gisli Masson; Ingileif Jonsdottir; Alma Moller; Thorolfur Gudnason; Karl G Kristinsson; Unnur Thorsteinsdottir; Kari Stefansson
EPI_ISL_827531, EPI_ISL_827534, EPI_ISL_827535	The National University Hospital of Iceland	deCODE genetics	Daniel F Gudbjartsson; Agnar Helgason; Hakon Jonsson; Olafur T Magnusson; Pall Melsted; Gudmundur L Norddahl; Jona Saemundsdottir; Asgeir Sigurdsson; Patrick Sulem; Arna B Agustsdottir; Hannes Eggertsson; Berglind Eiriksdottir; Run Fridriksdottir; Elisabet E Gardarsdottir; Gudmundur Georgsson; Olafia S Gretarsdottir; Kjartan R Gudmundsson; Thora R Gunnarsdottir; Arnaldur Gylfason; Hilma Holm; Brynjar O Jensson; Aslaug Jonasdottir; Kamilla S Josefsdottir; Thordur Kristjansson; Droplaug N Magnusdottir; Solvi Rognvaldsson; Louise le Roux; Gudrun Sigmundsdottir; Gardar Sveinbjornsson; Kristin E Sveinsdottir; Maney Sveinsdottir; Emil A Thorarensen; Bjarni Thorbjornsson; Gisli Masson; Ingileif Jonsdottir; Alma Moller; Thorolfur Gudnason; Karl G Kristinsson; Unnur Thorsteinsdottir; Kari Stefansson
EPI_ISL_827538	deCODE genetics	deCODE genetics	Daniel F Gudbjartsson; Agnar Helgason; Hakon Jonsson; Olafur T Magnusson; Pall Melsted; Gudmundur L Norddahl; Jona Saemundsdottir; Asgeir Sigurdsson; Patrick Sulem; Arna B Agustsdottir; Hannes Eggertsson; Berglind Eiriksdottir; Run Fridriksdottir; Elisabet E Gardarsdottir; Gudmundur Georgsson; Olafia S Gretarsdottir; Kjartan R Gudmundsson; Thora R Gunnarsdottir; Arnaldur Gylfason; Hilma Holm; Brynjar O Jensson; Aslaug Jonasdottir; Kamilla S Josefsdottir; Thordur Kristjansson; Droplaug N Magnusdottir; Solvi Rognvaldsson; Louise le Roux; Gudrun Sigmundsdottir; Gardar Sveinbjornsson; Kristin E Sveinsdottir; Maney Sveinsdottir; Emil A Thorarensen; Bjarni Thorbjornsson; Gisli Masson; Ingileif Jonsdottir; Alma Moller; Thorolfur Gudnason; Karl G Kristinsson; Unnur Thorsteinsdottir; Kari Stefansson
EPI_ISL_827548, EPI_ISL_827550, EPI_ISL_827553, EPI_ISL_827587, EPI_ISL_827589, EPI_ISL_827821	The National University Hospital of Iceland	deCODE genetics	Daniel F Gudbjartsson; Agnar Helgason; Hakon Jonsson; Olafur T Magnusson; Pall Melsted; Gudmundur L Norddahl; Jona Saemundsdottir; Asgeir Sigurdsson; Patrick Sulem; Arna B Agustsdottir; Hannes Eggertsson; Berglind Eiriksdottir; Run Fridriksdottir; Elisabet E Gardarsdottir; Gudmundur Georgsson; Olafia S Gretarsdottir; Kjartan R Gudmundsson; Thora R Gunnarsdottir; Arnaldur Gylfason; Hilma Holm; Brynjar O Jensson; Aslaug Jonasdottir; Kamilla S Josefsdottir; Thordur Kristjansson; Droplaug N Magnusdottir; Solvi Rognvaldsson; Louise le Roux; Gudrun Sigmundsdottir; Gardar Sveinbjornsson; Kristin E Sveinsdottir; Maney Sveinsdottir; Emil A Thorarensen; Bjarni Thorbjornsson; Gisli Masson; Ingileif Jonsdottir; Alma Moller; Thorolfur Gudnason; Karl G Kristinsson; Unnur Thorsteinsdottir; Kari Stefansson
EPI_ISL_827953, EPI_ISL_827963	deCODE genetics	deCODE genetics	Daniel F Gudbjartsson; Agnar Helgason; Hakon Jonsson; Olafur T Magnusson; Pall Melsted; Gudmundur L Norddahl; Jona Saemundsdottir; Asgeir Sigurdsson; Patrick Sulem; Arna B Agustsdottir; Hannes Eggertsson; Berglind Eiriksdottir; Run Fridriksdottir; Elisabet E Gardarsdottir; Gudmundur Georgsson; Olafia S Gretarsdottir; Kjartan R Gudmundsson; Thora R Gunnarsdottir; Arnaldur Gylfason; Hilma Holm; Brynjar O Jensson; Aslaug Jonasdottir; Kamilla S Josefsdottir; Thordur Kristjansson; Droplaug N Magnusdottir; Solvi Rognvaldsson; Louise le Roux; Gudrun Sigmundsdottir; Gardar Sveinbjornsson; Kristin E Sveinsdottir; Maney Sveinsdottir; Emil A Thorarensen; Bjarni Thorbjornsson; Gisli Masson; Ingileif Jonsdottir; Alma Moller; Thorolfur Gudnason; Karl G Kristinsson; Unnur Thorsteinsdottir; Kari Stefansson
EPI_ISL_828008, EPI_ISL_828205	The National University Hospital of Iceland	deCODE genetics	Daniel F Gudbjartsson; Agnar Helgason; Hakon Jonsson; Olafur T Magnusson; Pall Melsted; Gudmundur L Norddahl; Jona Saemundsdottir; Asgeir Sigurdsson; Patrick Sulem; Arna B Agustsdottir; Hannes Eggertsson; Berglind Eiriksdottir; Run Fridriksdottir; Elisabet E Gardarsdottir; Gudmundur Georgsson; Olafia S Gretarsdottir; Kjartan R Gudmundsson; Thora R Gunnarsdottir; Arnaldur Gylfason; Hilma Holm; Brynjar O Jensson; Aslaug Jonasdottir; Kamilla S Josefsdottir; Thordur Kristjansson; Droplaug N Magnusdottir; Solvi Rognvaldsson; Louise le Roux; Gudrun Sigmundsdottir; Gardar Sveinbjornsson; Kristin E Sveinsdottir; Maney Sveinsdottir; Emil A Thorarensen; Bjarni Thorbjornsson; Gisli Masson; Ingileif Jonsdottir; Alma Moller; Thorolfur Gudnason; Karl G Kristinsson; Unnur Thorsteinsdottir; Kari Stefansson
EPI_ISL_828347, EPI_ISL_828382, EPI_ISL_828449, EPI_ISL_828456, EPI_ISL_828457, EPI_ISL_828458, EPI_ISL_828459, EPI_ISL_828460, EPI_ISL_828461, EPI_ISL_828462, EPI_ISL_828463			
see above	deCODE genetics	deCODE genetics	Daniel F Gudbjartsson; Agnar Helgason; Hakon Jonsson; Olafur T Magnusson; Pall Melsted; Gudmundur L Norddahl; Jona Saemundsdottir; Asgeir Sigurdsson; Patrick Sulem; Arna B Agustsdottir; Hannes Eggertsson; Berglind Eiriksdottir; Run Fridriksdottir; Elisabet E Gardarsdottir; Gudmundur Georgsson; Olafia S Gretarsdottir; Kjartan R Gudmundsson; Thora R Gunnarsdottir; Arnaldur Gylfason; Hilma Holm; Brynjar O Jensson; Aslaug Jonasdottir; Kamilla S Josefsdottir; Thordur Kristjansson; Droplaug N Magnusdottir; Solvi Rognvaldsson; Louise le Roux; Gudrun Sigmundsdottir; Gardar Sveinbjornsson; Kristin E Sveinsdottir; Maney Sveinsdottir; Emil A Thorarensen; Bjarni Thorbjornsson; Gisli Masson; Ingileif Jonsdottir; Alma Moller; Thorolfur Gudnason; Karl G Kristinsson; Unnur Thorsteinsdottir; Kari Stefansson
EPI_ISL_828464, EPI_ISL_828465, EPI_ISL_828466, EPI_ISL_828467, EPI_ISL_828469	The National University Hospital of Iceland	deCODE genetics	Daniel F Gudbjartsson; Agnar Helgason; Hakon Jonsson; Olafur T Magnusson; Pall Melsted; Gudmundur L Norddahl; Jona Saemundsdottir; Asgeir Sigurdsson; Patrick Sulem; Arna B Agustsdottir; Hannes Eggertsson; Berglind Eiriksdottir; Run Fridriksdottir; Elisabet E Gardarsdottir; Gudmundur Georgsson; Olafia S Gretarsdottir; Kjartan R Gudmundsson; Thora R Gunnarsdottir; Arnaldur Gylfason; Hilma Holm; Brynjar O Jensson; Aslaug Jonasdottir; Kamilla S Josefsdottir; Thordur Kristjansson; Droplaug N Magnusdottir; Solvi Rognvaldsson; Louise le Roux; Gudrun Sigmundsdottir; Gardar Sveinbjornsson; Kristin E Sveinsdottir; Maney Sveinsdottir; Emil A Thorarensen; Bjarni Thorbjornsson; Gisli Masson; Ingileif Jonsdottir; Alma Moller; Thorolfur Gudnason; Karl G Kristinsson; Unnur Thorsteinsdottir; Kari Stefansson
EPI_ISL_828481, EPI_ISL_828482	deCODE genetics	deCODE genetics	Daniel F Gudbjartsson; Agnar Helgason; Hakon Jonsson; Olafur T Magnusson; Pall Melsted; Gudmundur L Norddahl; Jona Saemundsdottir; Asgeir Sigurdsson; Patrick Sulem; Arna B Agustsdottir; Hannes Eggertsson; Berglind Eiriksdottir; Run Fridriksdottir; Elisabet E Gardarsdottir; Gudmundur Georgsson; Olafia S Gretarsdottir; Kjartan R Gudmundsson; Thora R Gunnarsdottir; Arnaldur Gylfason; Hilma Holm; Brynjar O Jensson; Aslaug Jonasdottir; Kamilla S Josefsdottir; Thordur Kristjansson; Droplaug N Magnusdottir; Solvi Rognvaldsson; Louise le Roux; Gudrun Sigmundsdottir; Gardar Sveinbjornsson; Kristin E Sveinsdottir; Maney Sveinsdottir; Emil A Thorarensen; Bjarni Thorbjornsson; Gisli Masson; Ingileif Jonsdottir; Alma Moller; Thorolfur Gudnason; Karl G Kristinsson; Unnur Thorsteinsdottir; Kari Stefansson
EPI_ISL_828493, EPI_ISL_828495	The National University Hospital of Iceland	deCODE genetics	Daniel F Gudbjartsson; Agnar Helgason; Hakon Jonsson; Olafur T Magnusson; Pall Melsted; Gudmundur L Norddahl; Jona Saemundsdottir; Asgeir Sigurdsson; Patrick Sulem; Arna B Agustsdottir; Hannes Eggertsson; Berglind Eiriksdottir; Run Fridriksdottir; Elisabet E Gardarsdottir; Gudmundur Georgsson; Olafia S Gretarsdottir; Kjartan R Gudmundsson; Thora R Gunnarsdottir; Arnaldur Gylfason; Hilma Holm; Brynjar O Jensson; Aslaug Jonasdottir; Kamilla S Josefsdottir; Thordur Kristjansson; Droplaug N Magnusdottir; Solvi Rognvaldsson; Louise le Roux; Gudrun Sigmundsdottir; Gardar Sveinbjornsson; Kristin E Sveinsdottir; Maney Sveinsdottir; Emil A Thorarensen; Bjarni Thorbjornsson; Gisli Masson; Ingileif Jonsdottir; Alma Moller; Thorolfur Gudnason; Karl G Kristinsson; Unnur Thorsteinsdottir; Kari Stefansson
EPI_ISL_828696, EPI_ISL_828698, EPI_ISL_828700, EPI_ISL_828701, EPI_ISL_828704, EPI_ISL_828718, EPI_ISL_828720, EPI_ISL_828721, EPI_ISL_828722, EPI_ISL_828723, EPI_ISL_828724, EPI_ISL_828727, EPI_ISL_828728, EPI_ISL_828927, EPI_ISL_828933, EPI_ISL_828935, EPI_ISL_828936, EPI_ISL_828937, EPI_ISL_829077, EPI_ISL_829078, EPI_ISL_829081, EPI_ISL_829091, EPI_ISL_829104, EPI_ISL_829117, EPI_ISL_829255, EPI_ISL_829256, EPI_ISL_829257, EPI_ISL_829258, EPI_ISL_829259, EPI_ISL_829260, EPI_ISL_829420, EPI_ISL_829458			
see above	deCODE genetics	deCODE genetics	Daniel F Gudbjartsson; Agnar Helgason; Hakon Jonsson; Olafur T Magnusson; Pall Melsted; Gudmundur L Norddahl; Jona Saemundsdottir; Asgeir Sigurdsson; Patrick Sulem; Arna B Agustsdottir; Hannes Eggertsson; Berglind Eiriksdottir; Run Fridriksdottir; Elisabet E Gardarsdottir; Gudmundur Georgsson; Olafia S Gretarsdottir; Kjartan R Gudmundsson; Thora R Gunnarsdottir; Arnaldur Gylfason; Hilma Holm; Brynjar O Jensson; Aslaug Jonasdottir;

			Kamilla S Josefsdottir; Thordur Kristjansson; Droplaug N Magnusdottir; Solvi Rognvaldsson; Louise le Roux; Gudrun Sigmundsdottir; Gardar Sveinbjornsson; Kristin E Sveinsdottir; Maney Sveinsdottir; Emil A Thorarensen; Bjarni Thorbjornsson; Gisli Masson; Ingileif Jonsdottir; Alma Moller; Thorolfur Gudnason; Karl G Kristinsson; Unnur Thorsteinsdottir; Kari Stefansson
EPI_ISL_829476	The National University Hospital of Iceland	deCODE genetics	Daniel F Gudbjartsson; Agnar Helgason; Hakon Jonsson; Olafur T Magnusson; Pall Melsted; Gudmundur L Norddahl; Jona Saemundsdottir; Asgeir Sigurdsson; Patrick Sulem; Arna B Agustsdottir; Hannes Eggertsson; Berglind Eiriksdottir; Run Fridriksdottir; Elisabet E Gardarsdottir; Gudmundur Georgsson; Olafia S Gretarsdottir; Kjartan R Gudmundsson; Thora R Gunnarsdottir; Arnaldur Gylfason; Hilma Holm; Brynjar O Jensson; Aslaug Jonasdottir; Kamilla S Josefsdottir; Thordur Kristjansson; Droplaug N Magnusdottir; Solvi Rognvaldsson; Louise le Roux; Gudrun Sigmundsdottir; Gardar Sveinbjornsson; Kristin E Sveinsdottir; Maney Sveinsdottir; Emil A Thorarensen; Bjarni Thorbjornsson; Gisli Masson; Ingileif Jonsdottir; Alma Moller; Thorolfur Gudnason; Karl G Kristinsson; Unnur Thorsteinsdottir; Kari Stefansson
EPI_ISL_829507, EPI_ISL_829508, EPI_ISL_829509, EPI_ISL_829510, EPI_ISL_829511, EPI_ISL_829513, EPI_ISL_829650, EPI_ISL_829736, EPI_ISL_829758	deCODE genetics	deCODE genetics	Daniel F Gudbjartsson; Agnar Helgason; Hakon Jonsson; Olafur T Magnusson; Pall Melsted; Gudmundur L Norddahl; Jona Saemundsdottir; Asgeir Sigurdsson; Patrick Sulem; Arna B Agustsdottir; Hannes Eggertsson; Berglind Eiriksdottir; Run Fridriksdottir; Elisabet E Gardarsdottir; Gudmundur Georgsson; Olafia S Gretarsdottir; Kjartan R Gudmundsson; Thora R Gunnarsdottir; Arnaldur Gylfason; Hilma Holm; Brynjar O Jensson; Aslaug Jonasdottir; Kamilla S Josefsdottir; Thordur Kristjansson; Droplaug N Magnusdottir; Solvi Rognvaldsson; Louise le Roux; Gudrun Sigmundsdottir; Gardar Sveinbjornsson; Kristin E Sveinsdottir; Maney Sveinsdottir; Emil A Thorarensen; Bjarni Thorbjornsson; Gisli Masson; Ingileif Jonsdottir; Alma Moller; Thorolfur Gudnason; Karl G Kristinsson; Unnur Thorsteinsdottir; Kari Stefansson
EPI_ISL_829948, EPI_ISL_830105	The National University Hospital of Iceland	deCODE genetics	Daniel F Gudbjartsson; Agnar Helgason; Hakon Jonsson; Olafur T Magnusson; Pall Melsted; Gudmundur L Norddahl; Jona Saemundsdottir; Asgeir Sigurdsson; Patrick Sulem; Arna B Agustsdottir; Hannes Eggertsson; Berglind Eiriksdottir; Run Fridriksdottir; Elisabet E Gardarsdottir; Gudmundur Georgsson; Olafia S Gretarsdottir; Kjartan R Gudmundsson; Thora R Gunnarsdottir; Arnaldur Gylfason; Hilma Holm; Brynjar O Jensson; Aslaug Jonasdottir; Kamilla S Josefsdottir; Thordur Kristjansson; Droplaug N Magnusdottir; Solvi Rognvaldsson; Louise le Roux; Gudrun Sigmundsdottir; Gardar Sveinbjornsson; Kristin E Sveinsdottir; Maney Sveinsdottir; Emil A Thorarensen; Bjarni Thorbjornsson; Gisli Masson; Ingileif Jonsdottir; Alma Moller; Thorolfur Gudnason; Karl G Kristinsson; Unnur Thorsteinsdottir; Kari Stefansson
EPI_ISL_830148, EPI_ISL_830149, EPI_ISL_830153, EPI_ISL_830154, EPI_ISL_830155, EPI_ISL_830156, EPI_ISL_830207, EPI_ISL_830271, EPI_ISL_830483, EPI_ISL_830484, EPI_ISL_830486, EPI_ISL_830488, EPI_ISL_830489, EPI_ISL_830490			
see above	deCODE genetics	deCODE genetics	Daniel F Gudbjartsson; Agnar Helgason; Hakon Jonsson; Olafur T Magnusson; Pall Melsted; Gudmundur L Norddahl; Jona Saemundsdottir; Asgeir Sigurdsson; Patrick Sulem; Arna B Agustsdottir; Hannes Eggertsson; Berglind Eiriksdottir; Run Fridriksdottir; Elisabet E Gardarsdottir; Gudmundur Georgsson; Olafia S Gretarsdottir; Kjartan R Gudmundsson; Thora R Gunnarsdottir; Arnaldur Gylfason; Hilma Holm; Brynjar O Jensson; Aslaug Jonasdottir; Kamilla S Josefsdottir; Thordur Kristjansson; Droplaug N Magnusdottir; Solvi Rognvaldsson; Louise le Roux; Gudrun Sigmundsdottir; Gardar Sveinbjornsson; Kristin E Sveinsdottir; Maney Sveinsdottir; Emil A Thorarensen; Bjarni Thorbjornsson; Gisli Masson; Ingileif Jonsdottir; Alma Moller; Thorolfur Gudnason; Karl G Kristinsson; Unnur Thorsteinsdottir; Kari Stefansson
EPI_ISL_830753	University Hospital Basel, Clinical Virology	University Hospital Basel, Clinical Bacteriology	Tim Roloff, Madlen Stange, Helena MB Seth-Smith, Alfredo Mari, Karoline Leuzinger, Julia Bielicki, Manuel Battegay, Hans Hirsch, Adrian Egli
EPI_ISL_831020	INSPI-CRN DE INFLUENZA Y OTROS VIRUS RESPIRATORIOS	Instituto de Salud Publica de Chile	Javier Tognarelli, Barbara Parra, Loredana Arata, Jaime Lagos, Gisselle Barra, Alfredo Bruno, Domenica de Mora, Solon Narvaez, Jimmy Garcez, Michelle Paez, Martiza Olmedo, Manuel Gonzalez, Patricia Bustos, Rodrigo Fasce, Andres Castillo, Jorge Fernandez
EPI_ISL_831106	Hospital Universitario La Paz (Madrid)	SeqCOVID-SPAIN consortium/IBV(CSIC)	María Rodríguez-Tejedor, Elias Dahdouh, Fernando Lázaro-Perona, Jesús Mingorance and SeqCOVID-SPAIN consortium
EPI_ISL_831778, EPI_ISL_831779, EPI_ISL_831780, EPI_ISL_831781	United States Air Force School of Aerospace Medicine	United States Air Force School of Aerospace Medicine	Anthony Fries, Jennifer Meyer, William Gruner, Amanda Javorina, Sarah Purves, Clarise Starr, Elizabeth Macias
EPI_ISL_832144, EPI_ISL_832145	Hospital	National Reference Center for Viruses of Respiratory Infections, Institut Pasteur, Paris	Marion Barbet, Sylvie Behillil, Méline Bizard, Angela Brisebarre, Camille Capel, Etienne Simon-Lorière, Vincent Enouf, Maud Vanpeene, Sylvie van der Werf, Léa Pilorge
EPI_ISL_832497, EPI_ISL_832498, EPI_ISL_832499, EPI_ISL_832500, EPI_ISL_832501, EPI_ISL_832502, EPI_ISL_832503, EPI_ISL_832504, EPI_ISL_832505, EPI_ISL_832506, EPI_ISL_832507, EPI_ISL_832508, EPI_ISL_832509, EPI_ISL_832510, EPI_ISL_832511, EPI_ISL_832512, EPI_ISL_832513, EPI_ISL_832514, EPI_ISL_832515, EPI_ISL_832516, EPI_ISL_832517, EPI_ISL_832518, EPI_ISL_832519, EPI_ISL_832539, EPI_ISL_832546, EPI_ISL_832550, EPI_ISL_832551, EPI_ISL_832552, EPI_ISL_832553, EPI_ISL_832554, EPI_ISL_832555, EPI_ISL_832556, EPI_ISL_832557, EPI_ISL_832558, EPI_ISL_832559, EPI_ISL_832560, EPI_ISL_832561, EPI_ISL_832562			
see above	OHSU Lab Services Molecular Microbiology Lab	Oregon SARS-CoV-2 Genome Sequencing Center	Brendan L. O'Connell, Ruth V. Nichols, Sally Grindstaff, Alec J. Hirsch, Donna Hansel, Guang Fan, Daniel N. Streblow, William B. Messer, Andrew C. Adey, Benjamin N. Bimber, Brian J. O'Roak
EPI_ISL_833467	CHU Purpan - Laboratoire de Virologie - Institut Fédératif de Biologie	CHU Purpan - Laboratoire de Virologie - Institut Fédératif de Biologie	Latour J., Ranger N., Dubois M., Carcenac R., Harter A., Boyer P., Tremeaux P., Izopet J.
EPI_ISL_842348, EPI_ISL_842350	Virology Department, Sheffield Teaching Hospitals NHS Foundation Trust/Department of Infection, Immunity and Cardiovascular Disease, The Medical School, University of Sheffield	COVID-19 Genomics UK (COG-UK) Consortium	Thushan de Silva, Matthew Parker, Nikki Smith, Adri Angyal, Rebecca Brown, Luke Green, Rachel Tucker, Paul Parsons, Danielle Groves, Katie Johnson, Laura Carrilero, Alex Keeley, Dave Partridge, Matthew Wyles, Benjamin Lindsey, Mehmet Yavuz, Mohammad Raza, Cariad Evans
EPI_ISL_848083	Laboratory of Clinical Research on Dermatozoonoses in Domestic Animals, Evandro Chagas National Institute of Infectious Diseases, Oswaldo Cruz Foundation (Fiocruz), Rio de Janeiro, RJ, Brazil.	Laboratory of Respiratory Viruses and Measles, Oswaldo Cruz Institute, FIOCRUZ	Guilherme Amaral Calvet, Michelle Fernanda Borges da Silva, Anielle de Pina Costa, Ezequias Batista Martins, Isabella Campos Vargas de Moraes, Lusiele Guaraldo, Patrícia Brasil, Sandro Antônio Pereira, Rodrigo Caldas Menezes, Isabella Dib Ferreira Gremião, Lucas Oliveira Keidel, Shanna Araujo dos Santos, Artur Augusto Velho Mendes Junior, Renato Orsini Ornellas, Maria Ogrzewalska, PaolaCristina Resende, Alex Pauvolid-Corrêa, Fernando do Couto Motta, Alice Sampaio Barreto da Rocha, Thiago C. Souza, Marilda Mendonça Siqueira
EPI_ISL_848270, EPI_ISL_848376, EPI_ISL_848522	Illinois Department of Public Health	Gagnon Lab, Southern Illinois University	Keith Gagnon
EPI_ISL_848558, EPI_ISL_848559, EPI_ISL_848560	Evandro Chagas Institute	Evandro Chagas Institute	Santos, M.C.; Silva, A.M.; Junior, W.D.C.; Barbagelata, L.S.; Ferreira, J.A.; Sousa, E.M.A.; da Silva, P.S.; Pinheiro, K.C.; L.C.; Sousa Junior, E.C.
EPI_ISL_848881, EPI_ISL_848882, EPI_ISL_848883, EPI_ISL_848884, EPI_ISL_848885, EPI_ISL_848886, EPI_ISL_848887, EPI_ISL_848888, EPI_ISL_848889, EPI_ISL_848890, EPI_ISL_848891, EPI_ISL_848892, EPI_ISL_848893, EPI_ISL_848894, EPI_ISL_848895, EPI_ISL_848896, EPI_ISL_848897, EPI_ISL_848898, EPI_ISL_848899, EPI_ISL_848900, EPI_ISL_848901, EPI_ISL_848902, EPI_ISL_848903, EPI_ISL_848904, EPI_ISL_848905, EPI_ISL_848906, EPI_ISL_848907, EPI_ISL_848908, EPI_ISL_848909, EPI_ISL_848910, EPI_ISL_848911, EPI_ISL_848912, EPI_ISL_848913, EPI_ISL_848914, EPI_ISL_848915, EPI_ISL_848916, EPI_ISL_848917, EPI_ISL_848918, EPI_ISL_848919, EPI_ISL_848920, EPI_ISL_848921, EPI_ISL_848922, EPI_ISL_848923, EPI_ISL_848924, EPI_ISL_848925, EPI_ISL_848926, EPI_ISL_848927, EPI_ISL_848928, EPI_ISL_848929, EPI_ISL_848930, EPI_ISL_848931			
see above	Florida Bureau of Public Health Laboratories	Florida Bureau of Public Health Laboratories	Sarah Schmedes, Jason Blanton
EPI_ISL_849352, EPI_ISL_849355, EPI_ISL_849356, EPI_ISL_849358, EPI_ISL_849359, EPI_ISL_849365, EPI_ISL_849366	Servicio Virosis Respiratorias-Departamento Virología-INEI	Instituto Nacional Enfermedades Infecciosas C.G.Malbran	Baumeister E., Avaro M., Benedetti E., Russo M., Dattero ME, Pontoriero A., Cisterna D., Molina V., Perandones C., Tuduri E., Lorenzo F., Poklepovich T., Campos J.
EPI_ISL_849492, EPI_ISL_849493, EPI_ISL_849494, EPI_ISL_849584, EPI_ISL_849603, EPI_ISL_849604	Seattle Flu Study	Seattle Flu Study	Deborah A. Nickerson, Chris D. Frazar, Jover Lee, Benjamin Pelle, Matthew Richardson, Amanda Adler, Elisabeth Brandstetter, Peter D. Han, Kairsten Fay, Misja Ilcisin, Kirsten Lacombe, Thomas R. Sibley, Melissa Truong, Caitlin R. Wolf, Michael Boeckh, Janet A. Englund, Michael Famulare, Barry R. Lutz, Mark J. Rieder, Lea M. Starita, Matthew Thompson, Jay Shendure, Trevor Bedford, Helen Y. Chu
EPI_ISL_852599	Max von Pettenkofer Institute, Virology, National Reference Center for Retroviruses, LMU München	Laboratory for Functional Genome Analysis, Dept. Genomics, Gene Center of the LMU Munich	Max Muenchhoff, Stefan Krebs, Alexander Graf, Oliver Keppler, Helmut Blum
EPI_ISL_852650, EPI_ISL_852651, EPI_ISL_852652	Institute of Virology, Medical Center, University of Freiburg, Freiburg, Germany	Institute of Virology, Clinial Virus Genomics, Medical Center, University of Freiburg, Freiburg, Germany	Jonas Fuchs, Lisa Kern, Sandra Reuter, Hajo Grundmann, Marcus Panning
EPI_ISL_853718, EPI_ISL_853761	Department of Microbiology, University Innsbruck	Bergthaler laboratory, CeMM Research Center for Molecular Medicine of the Austrian Academy of Sciences	Lukas Endler, Alexandra Popa, Benedikt Agerer, Jakob-Wendelin Genger, Alexander Lercher, Anna Schedl, Thomas Penz, Michael Schuster, Jan Laine, Martin Senekowitsch, Christoph Bock, Andreas Bergthaler
EPI_ISL_853870, EPI_ISL_853871, EPI_ISL_853872, EPI_ISL_853873	Institute for Laboratory Diagnostics and Microbiology, Klinikum Klagenfurt am Worthersee	Bergthaler laboratory, CeMM Research Center for Molecular Medicine of the Austrian Academy of Sciences	Lukas Endler, Alexandra Popa, Benedikt Agerer, Jakob-Wendelin Genger, Alexander Lercher, Anna Schedl, Thomas Penz, Michael Schuster, Jan Laine, Martin Senekowitsch, Christoph Bock, Andreas Bergthaler

EPI_ISL_854222, EPI_ISL_854225	Department of Microbiology, University Innsbruck	Bergthaler laboratory, CeMM Research Center for Molecular Medicine of the Austrian Academy of Sciences	Lukas Endler, Alexandra Popa, Benedikt Agerer, Jakob-Wendelin Genger, Alexander Lercher, Anna Schedl, Thomas Penz, Michael Schuster, Jan Laine, Martin Senekowitsch, Christoph Bock, Andreas Bergthaler
EPI_ISL_855495, EPI_ISL_855506, EPI_ISL_855510, EPI_ISL_855511	KEMRI-Wellcome Trust Research Programme/KEMRI-CGMR-C Kilifi	KEMRI-Wellcome Trust Research Programme/KEMRI-CGMR-C Kilifi	Githinji et al
EPI_ISL_859768, EPI_ISL_859769, EPI_ISL_859770, EPI_ISL_859771, EPI_ISL_859772, EPI_ISL_859773, EPI_ISL_859774, EPI_ISL_859776, EPI_ISL_859777, EPI_ISL_859782, EPI_ISL_859783, EPI_ISL_859784, EPI_ISL_859785, EPI_ISL_859791, EPI_ISL_859808, EPI_ISL_859809, EPI_ISL_859811, EPI_ISL_859812, EPI_ISL_859813, EPI_ISL_859818, EPI_ISL_859820, EPI_ISL_859821, EPI_ISL_859822, EPI_ISL_859824, EPI_ISL_859826, EPI_ISL_859828, EPI_ISL_859829, EPI_ISL_859830, EPI_ISL_859832, EPI_ISL_859834, EPI_ISL_859835, EPI_ISL_859836, EPI_ISL_859837, EPI_ISL_859838, EPI_ISL_859839, EPI_ISL_859840, EPI_ISL_859841, EPI_ISL_859842			
see above	BTC, Khalifa University	BTC, Khalifa University	Al Safar et al
EPI_ISL_860130	Keio University School of Medicine	Keio University School of Medicine	Kenjiro Kosaki, Yuka Iwasaki, Hirotsugu Ishizu, Haruhiko Siomi, Kodai Abe
EPI_ISL_860733, EPI_ISL_860734, EPI_ISL_860739, EPI_ISL_860752, EPI_ISL_860753, EPI_ISL_860754, EPI_ISL_860755, EPI_ISL_860756, EPI_ISL_860757, EPI_ISL_860758, EPI_ISL_860759			
see above	Swiss National Reference Centre for Influenza	Swiss National Reference Centre for Influenza	Ana Rita Gonçalves Cabecinhas, Samuel Cordey, Florian Laubscher,Christoph Grünig, Laurent Kaiser
EPI_ISL_861457	WHO/Minsk	Charité Universitätsmedizin Berlin, Institut für Virologie	Victor M Corman, Barbara Mühlemann, Jörn Beheim-Schwarzbach, Talitha Veith, Julia Tesch, Tobias Bleicker, Julia Schneider, Shmialiova Natallia, Sivets Natallia, Terry Jones, Christian Drosten
EPI_ISL_861718, EPI_ISL_861719, EPI_ISL_861720	Department of Medical Microbiology, Hospital Pengajar Universiti Putra Malaysia	Malaysia Genome Institute	Mohd Noor Mat Isa, Syafinaz Amin-Nordin, Irni Suhayu Sapian, Hui-Yee Chee, Yusuf Muhammad Noor, Nurhezreen Md Iqbal, Enizza Kasim, Siti Noraini Othman, Mohd Faizal Abu Bakar, Shamsidar Sopie, Azrin Ahmad, Narcisse Joseph, Muhammad MI, Avisha Richards, Nor Zahrin Hasran, Nor Azfa Johari
EPI_ISL_861876	LATE - Laboratório de Técnicas Especiais - Hospital Israelita Albert Einstein	LATE - Laboratório de Técnicas Especiais - Hospital Israelita Albert Einstein	Deyvid Amgarten, Fernanda de Mello Malta, Raquel Riyuzo, Ana Paula Moreira Salles, Pedro Henrique Sebe Rodrigues, João Renato Rebello Pinho
EPI_ISL_867934	Centre for Enzyme Innovation, University of Portsmouth / Translational Research Laboratory, Portsmouth Hospitals NHS Trust	COVID-19 Genomics UK (COG-UK) Consortium	Angela Beckett,Yann Bourgeois,Garry Scarlett,Sharon Glaysher,Scott Elliott,Kelly Bicknell,Robert Impey,Allyson Lloyd,Sarah Wyllie,Ethan Butcher,Anoop Chauhan,Samuel Robson
EPI_ISL_868938, EPI_ISL_868939, EPI_ISL_868940, EPI_ISL_868941, EPI_ISL_868942, EPI_ISL_868943, EPI_ISL_868944, EPI_ISL_868945, EPI_ISL_868946, EPI_ISL_868947, EPI_ISL_868948			
see above	Bioinformatics and Biostatistics Lab, Advanced Sequencing Facility	COVID-19 Genomics UK (COG-UK) Consortium	Aengus Stewart,Jerome Nicod,Chelsea Sawyer,Laura Cubitt,Harshil Patel,Margaret Crawford
EPI_ISL_872469, EPI_ISL_872470, EPI_ISL_872471, EPI_ISL_872472, EPI_ISL_872473, EPI_ISL_872474, EPI_ISL_872475, EPI_ISL_872476, EPI_ISL_872477	Eurofins Diatherix	Hudsonalpha Genome Sequencing Center	Jane Grimwood, Melissa Williams, Lori H. Handley, Joshua Stough, Leslie Malone, Stefan Brzezinski, Ada Stewart, Teresa Jones, Jenell Webber, John Lovell, Jennifer Cart, and Jeremy Schmutz
EPI_ISL_872903	WHO National Influenza Centre Russian Federation	WHO National Influenza Centre Russian Federation	Andrey Komissarov, Artem Fadeev, Anna Ivanova, Kseniya Komissarova, Dmitry Bazhenov, Mikhail Bakaev, Daria Danilenko, Ksenia Safina, Elena Nabieva, Georgii Bazykin, Dmitry Lioznov
EPI_ISL_876524	Florida Bureau of Public Health Laboratories	Florida Bureau of Public Health Laboratories	Sarah Schmedes, Jason Blanton
EPI_ISL_877411, EPI_ISL_877412, EPI_ISL_877413, EPI_ISL_877414, EPI_ISL_877415, EPI_ISL_877416, EPI_ISL_877417, EPI_ISL_877418	Eurofins Diatherix	Hudsonalpha Genome Sequencing Center	Jane Grimwood, Melissa Williams, Lori H. Handley, Joshua Stough, Leslie Malone, Stefan Brzezinski, Ada Stewart, Teresa Jones, Jenell Webber, John Lovell, Jennifer Cart, and Jeremy Schmutz
EPI_ISL_878565	Robert Garry lab	Andersen lab at Scripps Research	Allison Smither, Gilberto Sabino-Santos, Patricia Snarski, Lilia Melnik, Antoinette Bell, Kaylynn Genemaras, Arnaud Drouin, Dahlene Fusco, Robert Garry with SEARCH Alliance San Diego
EPI_ISL_878687, EPI_ISL_878693	Rady's Childrens Hospital	Andersen lab at Scripps Research	SEARCH Alliance San Diego with Nanda Radamchar, David Dimmock, Linda Luo, Christina Clarke, Kathryn Bouic, Teresa Mueller, Denise Malicki
EPI_ISL_882682, EPI_ISL_882690, EPI_ISL_882725, EPI_ISL_882726, EPI_ISL_882752	1.AO Universitaria 'S. Giovanni di Dio e Ruggi D'Aragona, Scuola Medica Salernitana' Hospital / 2.UOC di Virologia e Microbiologia, Università della Campania 'L. Vanvitelli' / 3.AO Universitaria 'Federico II' Napoli Hospital / 4.AORN 'San Giuseppe Moscati' Avellino Hospital / 5.AO 'San Pio - presidio G. Rummo' Benevento Hospital / 6.AO 'Sant'Anna e San Sebastiano' Caserta Hospital / 7.PO 'Maria Santissima Addolorata' Eboli Hospital / 8.Biogem Istituto di Ricerche Genetiche	1. Genome Research Center for Health (CRGS) / 2. Laboratory of Molecular Medicine and Genomics(LMMGe) / 3. Center for Research in Pure and Applied Mathematics (CRMPA)	Giorgio Giurato, Francesca Rizzo, Alessandro Weisz, Gianluigi Franci, Giovanni Nassa, Pasquale Pagliano, Roberta Tarallo, Elena Alexandrova, Ylenia D'Agostino, Carlo Ferravante, Jessica Lamberti, Viola Melone, Domenico Memoli, Valeria Mirici Cappa, Domenico Palumbo, Giovanni Pecoraro, Assunta Sellitto, Oriana Strianese, Ilaria Terenzi, Giuseppe Fenza, Aniello Gentile, Antonello Saccomanno, Sonia Amabile, Teresa Rocco, Annamaria Salvati, Emilia Vaccaro, Massimiliano Galdiero, Michele Cennamo, Giuseppe Portella, Maria Grazia Foti, Mariarosaria Ingino, Maria Landi, Maurizio Fumi, Vincenzo Rocco, Rita Greco, Vittoria Letizia, Arnolfo Petruzziello, Maddalena Schioppa, Gregorio Goffredi, Francesca Marciano, Michele Caraglia, Alessia Cossu, Marianna Scrima
EPI_ISL_882954	State Veterinary Institute Prague	State Veterinary Institute Prague	Nagy,A;Cernikova,L;Stara,M
EPI_ISL_884342, EPI_ISL_884353, EPI_ISL_884368, EPI_ISL_884418, EPI_ISL_884419, EPI_ISL_884444	Infectious Diseases, Quest Diagnostics	Infectious Diseases, Quest Diagnostics	Rosenthal,S.H., Gerasimova,A., Kagan,R.M., Anderson,B., Bernstein,L.E., Livingston,K.E., Hua,M., Liu,Y., Shalhout,D.F., Owen,R., Lacbawan,F.
EPI_ISL_888881, EPI_ISL_888882	Michigan Department of Health and Human Services, Bureau of Laboratories	Michigan Department of Health and Human Services, Bureau of Laboratories	Blankenship HM, Riner D, Soehnlen MK
EPI_ISL_892221, EPI_ISL_892222, EPI_ISL_892223	Lighthouse Lab in Alderley Park	Wellcome Sanger Institute for the COVID-19 Genomics UK (COG-UK) Consortium	Jacquelyn Wynn, Mairead Hyland, The Lighthouse Lab in Alderley Park and Alex Alderton, Roberto Amato, Sonia Goncalves, Ewan Harrison, David K. Jackson, Ian Johnston, Dominic Kwiatkowski, Cordelia Langford, John Sillitoe on behalf of the Wellcome Sanger Institute COVID-19 Surveillance Team
EPI_ISL_896118, EPI_ISL_896120, EPI_ISL_896171	MEPHI, Aix Marseille University	MEPHI, Aix Marseille University	Anthony LEVASSEUR
EPI_ISL_896373	University of Medicine and Pharmacy of Craiova	"Stefan cel Mare" University Metagenomics Lab	Lobiuc Andrei, Gheorghita Roxana
EPI_ISL_900097, EPI_ISL_900099, EPI_ISL_900122, EPI_ISL_900125, EPI_ISL_900154, EPI_ISL_900190, EPI_ISL_900231, EPI_ISL_900375, EPI_ISL_900388, EPI_ISL_900419, EPI_ISL_900435			
see above	MEPHI, Aix Marseille University	MEPHI, Aix Marseille University	Anthony LEVASSEUR
EPI_ISL_904136, EPI_ISL_904154, EPI_ISL_904239, EPI_ISL_904437, EPI_ISL_904438, EPI_ISL_904439, EPI_ISL_904440, EPI_ISL_904441, EPI_ISL_904442	Dutch COVID-19 response team	Erasmus Medical Center	Bas Oude Munnink, Reina Sikkema, David Nieuwenhuijse, Irina Chestakova, Anne van der Linden, Marjan Boter, Emmanuelle Munger, Corine GeurtsvanKessel, Annemiek van der Eijk, Richard Molenkamp, Marion Koopmans, on behalf of the Dutch national COVID-19 response team.
EPI_ISL_905383	Dutch COVID-19 response team	National Institute for Public Health and the Environment (RIVM)	Adam Meijer, Harry Vennema, Dirk Eggink, Jeroen Cremer, Sharon van den Brink, Bas van der Veer, AnneMarie van den Brandt, Florian Zwagemaker, Dennis Schmitz, Chantal Reusken, on behalf of the national COVID-19 response team
EPI_ISL_906118	Institute of Microbiology and Immunology, Faculty of Medicine, University of Ljubljana	Institute of Microbiology and Immunology, Faculty of Medicine, University of Ljubljana	Samo Zakotnik, Tomaž Mark Zorec, Matic Brvar, Miša Korva, Mario Poljak, Tatjana Avši - Županc
EPI_ISL_910330	General Hospital - Veles	Research Center for Genetic Engineering and Biotechnology "Georgi D. Efremov" , Macedonian Academy of Sciences and Arts	RCGEB - MASA

EPI_ISL_910333	General Hospital - Ohrid	Research Center for Genetic Engineering and Biotechnology "Georgi D. Efremov" , Macedoni	RCGEB - MASA
EPI_ISL_912456, EPI_ISL_912485, EPI_ISL_912488, EPI_ISL_912497, EPI_ISL_912504, EPI_ISL_912509, EPI_ISL_912513, EPI_ISL_912522	NHLS Universitas Academic	UFS Virology	PA Bester, MM Nyaga, P Nthiga, MT Mogotsi, D Goedhals, T de Oliveira
EPI_ISL_913079, EPI_ISL_913087	Center for Virology	Center for Virology	Jeremy V. Camp, Irene Goerzer, Monika Redlberger-Fritz, Stephan W. Aberle
EPI_ISL_913351	Klinisk mikrobiologi	The Public Health Agency of Sweden	Anna-Malin Linde, Maria Lind Karlberg, Carlo Berg, Oskar Karlsson Lindsjo, Sofia Stamouli, Reza Advani, Mattias Haukland, Petra Holmstrom, Noura Walai, Petra Edquist, Mia Brytting, Anna Risberg, Karin Tegmark-Wisell
EPI_ISL_913925, EPI_ISL_913927, EPI_ISL_913928, EPI_ISL_913929, EPI_ISL_913930, EPI_ISL_913931, EPI_ISL_913932, EPI_ISL_913957	Instituto de Diagnostico y Referencia Epidemiologicos INDRE_RNLSP	Instituto de Diagnostico y Referencia Epidemiologicos (INDRE)	Claudia Wong-Arambula, Abril Rodriguez-Maldonado, Fabiola Garces-Ayala, Adnan Araiza-Rodriguez, David Fragoso-Fonseca, Sergio Rangel-Guerrero, Mayra Jimenez-Morales, Nancy Munoz-Hernandez, Natividad Cruz-Ortiz, Tatiana Nunez-Garcia, Gisela Barrera-Badillo, Lucia Hernandez-Rivas, Irma Lopez-Martinez, Ernesto Ramirez-Gonzalez.
EPI_ISL_918484	Gerontology Institute - Skopje	Research Center for Genetic Engineering and Biotechnology "Georgi D. Efremov" , Macedonian Academy of Sciences and Arts	RCGEB - MASA
EPI_ISL_918486	General Hospital - Struga	Research Center for Genetic Engineering and Biotechnology "Georgi D. Efremov" , Macedonian Academy of Sciences and Arts	RCGEB - MASA
EPI_ISL_921025	Regional Virus Laboratory, Belfast Health and Social Care Trust	COVID-19 Genomics UK (COG-UK) Consortium	Conall McCaughey, James McKenna, Tanya Curran, Susan Feeney, Alison Watt, Ciara Cox, Mairead Connor, Zoltan Molnar, David Simpson, Derek Fairley
EPI_ISL_924419, EPI_ISL_924423	Virology Department, Sheffield Teaching Hospitals NHS Foundation Trust/Department of Infection, Immunity and Cardiovascular Disease, The Medical School, University of Sheffield	COVID-19 Genomics UK (COG-UK) Consortium	Thushan de Silva, Matthew Parker, Nikki Smith, Adri Angyal, Rebecca Brown, Luke Green, Rachel Tucker, Paul Parsons, Danielle Groves, Katie Johnson, Laura Carrilero, Alex Keeley, Dave Partridge, Matthew Wyles, Benjamin Lindsey, Mehmet Yavuz, Mohammad Raza, Cariad Evans
EPI_ISL_924613, EPI_ISL_925022	Bioinformatics and Biostatistics Lab, Advanced Sequencing Facility	COVID-19 Genomics UK (COG-UK) Consortium	Aengus Stewart,Jerome Nicod,Chelsea Sawyer,Laura Cubitt,Harshil Patel,Margaret Crawford
EPI_ISL_925107, EPI_ISL_925108, EPI_ISL_925129, EPI_ISL_925130	1.AO Universitaria 'S. Giovanni di Dio e Ruggi D'Aragona, Scuola Medica Salernitana' Hospital / 2.UOC di Virologia e Microbiologia, Università della Campania 'L. Vanvitelli' / 3.AO Universitaria 'Federico II' Napoli Hospital / 4.AORN 'San Giuseppe Moscati' Avellino Hospital / 5.AO 'San Pio - presidio G. Rummo' Benevento Hospital / 6.AO 'Sant'Anna e San Sebastiano' Caserta Hospital / 7.PO 'Maria Santissima Addolorata' Eboli Hospital / 8.Biogem Istituto di Ricerche Genetiche	1. Genome Research Center for Health (CRGS) / 2. Laboratory of Molecular Medicine and Genomics(LMMGe) / 3. Center for Research in Pure and Applied Mathematics (CRMPA)	Giorgio Giurato, Francesca Rizzo, Alessandro Weisz, Gianluigi Franci, Giovanni Nassa, Pasquale Pagliano, Roberta Tarallo, Elena Alexandrova, Ylenia D'Agostino, Carlo Ferravante, Jessica Lamberti, Viola Melone, Domenico Memoli, Valeria Mirici Cappa, Domenico Palumbo, Giovanni Pecoraro, Assunta Sellitto, Oriana Strianese, Ilaria Terenzi, Giuseppe Fenza, Aniello Gentile, Antonello Saccomanno, Sonia Amabile, Teresa Rocco, Annamaria Salvati, Emilia Vaccaro, Massimiliano Galdiero, Michele Cennamo, Giuseppe Portella, Maria Grazia Foti, Mariarosaria Ingino, Maria Landi, Maurizio Fumi, Vincenzo Rocco, Rita Greco, Vittoria Letizia, Arnolfo Petruzziello, Maddalena Schioppa, Gregorio Goffredi, Francesca Marciano, Michele Caraglia, Alessia Cossu, Marianna Scrima
EPI_ISL_925207	Virginia DCLS	Virginia DCLS	Virginia DCLS
EPI_ISL_925442, EPI_ISL_925443, EPI_ISL_925444, EPI_ISL_925445, EPI_ISL_925479, EPI_ISL_925480, EPI_ISL_925481, EPI_ISL_925482	Department of Clinical Microbiology	GIGA Medical Genomics	Keith Durkin, Maria Artesi, Sébastien Bontems, Raphaël Boreux, Bouchra Boujemla, Cécile Meex, Pierrette Melin, Marie-Pierre Hayette, Vincent Bours
EPI_ISL_925883	Nucleic Acid Testing, National Reference Laboratory	GIGA Medical Genomics	Yvan Butera, Keith Durkin, Maria Artesi, Bouchra Boujemla, Robert Rutayisire, Patrick Tuyisenge, Esperence Umumararungu, Sébastien Bontems, Marie-Pierre Hayette, Nathalie Renotte, Swaibu Gatare, Jacob Souopgui, Sabin Nsanzimana, Vincent Bours, Léon Mutesa
EPI_ISL_930920, EPI_ISL_930921, EPI_ISL_930922, EPI_ISL_930923, EPI_ISL_930924, EPI_ISL_930925, EPI_ISL_930926, EPI_ISL_930927, EPI_ISL_930928, EPI_ISL_930929, EPI_ISL_930931, EPI_ISL_930934, EPI_ISL_930935, EPI_ISL_930936, EPI_ISL_930937, EPI_ISL_930938, EPI_ISL_930939, EPI_ISL_930940, EPI_ISL_930941, EPI_ISL_930942, EPI_ISL_930943, EPI_ISL_930944, EPI_ISL_930945, EPI_ISL_930946, EPI_ISL_930947, EPI_ISL_930948, EPI_ISL_930949, EPI_ISL_930950, EPI_ISL_930951, EPI_ISL_930952, EPI_ISL_930953, EPI_ISL_930954, EPI_ISL_930955, EPI_ISL_930956, EPI_ISL_930957, EPI_ISL_930958, EPI_ISL_930959, EPI_ISL_930960, EPI_ISL_930961, EPI_ISL_930962, EPI_ISL_930963, EPI_ISL_930966, EPI_ISL_930967, EPI_ISL_930968, EPI_ISL_930969, EPI_ISL_930970, EPI_ISL_930971, EPI_ISL_930972, EPI_ISL_930973, EPI_ISL_930974, EPI_ISL_930975, EPI_ISL_930976, EPI_ISL_930977, EPI_ISL_930978, EPI_ISL_930979, EPI_ISL_930980, EPI_ISL_930981, EPI_ISL_930982, EPI_ISL_930983, EPI_ISL_930984, EPI_ISL_930985, EPI_ISL_930986, EPI_ISL_930987, EPI_ISL_930988, EPI_ISL_930989, EPI_ISL_930990, EPI_ISL_930991, EPI_ISL_930992, EPI_ISL_930993, EPI_ISL_931396			
see above	University Hospital Basel, Clinical Virology	University Hospital Basel, Clinical Bacteriology	Tim Roloff, Madlen Stange, Helena MB Seth-Smith, Alfredo Mari, Karoline Leuzinger, Julia Bielicki, Manuel Battegay, Hans Hirsch, Adrian Egli
EPI_ISL_933666, EPI_ISL_933667	Instituto de Diagnostico y Referencia Epidemiologicos INDRE_RNLSP	Instituto de Diagnostico y Referencia Epidemiologicos (INDRE)	Claudia Wong-Arambula, Abril Rodriguez-Maldonado, Fabiola Garces-Ayala, Adnan Araiza-Rodriguez, David Fragoso-Fonseca, Sergio Rangel-Guerrero, Mayra Jimenez-Morales, Nancy Munoz-Hernandez, Natividad Cruz-Ortiz, Tatiana Nunez-Garcia, Gisela Barrera-Badillo, Lucia Hernandez-Rivas, Irma Lopez-Martinez, Ernesto Ramirez-Gonzalez.
EPI_ISL_935143, EPI_ISL_935144, EPI_ISL_935145, EPI_ISL_935146, EPI_ISL_935147, EPI_ISL_935148	1.AO Universitaria 'S. Giovanni di Dio e Ruggi D'Aragona, Scuola Medica Salernitana' Hospital / 2.UOC di Virologia e Microbiologia, Università della Campania 'L. Vanvitelli' / 3.AO Universitaria 'Federico II' Napoli Hospital / 4.AORN 'San Giuseppe Moscati' Avellino Hospital / 5.AO 'San Pio - presidio G. Rummo' Benevento Hospital / 6.AO 'Sant'Anna e San Sebastiano' Caserta Hospital / 7.PO 'Maria Santissima Addolorata' Eboli Hospital / 8.Biogem Istituto di Ricerche Genetiche	1. Genome Research Center for Health (CRGS) / 2. Laboratory of Molecular Medicine and Genomics(LMMGe) / 3. Center for Research in Pure and Applied Mathematics (CRMPA)	Giorgio Giurato (Corresponding Author), Francesca Rizzo (Corresponding Author), Alessandro Weisz (Corresponding Author), Gianluigi Franci, Giovanni Nassa, Pasquale Pagliano, Roberta Tarallo, Elena Alexandrova, Ylenia D'Agostino, Carlo Ferravante, Jessica Lamberti, Viola Melone, Domenico Memoli, Valeria Mirici Cappa, Domenico Palumbo, Giovanni Pecoraro, Assunta Sellitto, Oriana Strianese, Ilaria Terenzi, Giuseppe Fenza, Aniello Gentile, Antonello Saccomanno, Sonia Amabile, Teresa Rocco, Annamaria Salvati, Emilia Vaccaro, Massimiliano Galdiero, Michele Cennamo, Giuseppe Portella, Maria Grazia Foti, Mariarosaria Ingino, Maria Landi, Maurizio Fumi, Vincenzo Rocco, Rita Greco, Vittoria Letizia, Arnolfo Petruzziello, Maddalena Schioppa, Gregorio Goffredi, Francesca Marciano, Michele Caraglia, Alessia Cossu, Marianna Scrima
EPI_ISL_937105, EPI_ISL_937119	Quest Diagnostics	Quest Diagnostics	Rosenthal,S.H., Gerasimova,A., Kagan,R.M., Anderson, B., Livingston, K.E., Hua, M., Liu Y., Shalhout, D.F., Owen, R., Lacbawan, F.
EPI_ISL_940487	Hôpital Bichat Claude Bernard, Laboratoire de Virologie	IAME UMR1137 Inserm, Université de Paris, Hôpital Bichat	Antoine Bridier-Nahmias, Amélie Recoing, Quentin Le Hingrat, Lena Daniel, Siham Hamri, Gilles Collin, Alexandre Storto, Mélanie Bertine, Charlotte Charpentier, Nadhira Houhou-Fidouh, Diane Descamps, Benoit Visseaux
EPI_ISL_940942, EPI_ISL_940943, EPI_ISL_940944, EPI_ISL_940945, EPI_ISL_940946, EPI_ISL_940947, EPI_ISL_940948, EPI_ISL_940949, EPI_ISL_940994	Centers for Disease Control and Prevention, Dengue Branch	Centers for Disease Control and Prevention, Dengue Branch	Gilberto A. Santiago, Glenda Gonzalez, Betzabel Flores, Keyla Charriez, Gabriela Paz-Bailey, Jorge L. Munoz-Jordan
EPI_ISL_941199	Servicio de Microbiología. Hospital Clínico Universitario de Valencia	SeqCOVID-SPAIN consortium/IBV(CSIC)	David Navarro Ortega, Eliseo Albert Vicent, Ignacio Torres and SeqCOVID-SPAIN consortium
EPI_ISL_942014, EPI_ISL_942015, EPI_ISL_942016, EPI_ISL_942017, EPI_ISL_942018, EPI_ISL_942019, EPI_ISL_942020, EPI_ISL_942021, EPI_ISL_942022, EPI_ISL_942023, EPI_ISL_942048, EPI_ISL_942049, EPI_ISL_942050, EPI_ISL_942051, EPI_ISL_942052, EPI_ISL_942053, EPI_ISL_942054, EPI_ISL_942055, EPI_ISL_942056, EPI_ISL_942057, EPI_ISL_942058, EPI_ISL_942059, EPI_ISL_942060, EPI_ISL_942061, EPI_ISL_942062, EPI_ISL_942063, EPI_ISL_942064, EPI_ISL_942065, EPI_ISL_942066, EPI_ISL_942067, EPI_ISL_942068			
see above	Wisconsin State Laboratory of Hygiene Communicable Disease Division	Wisconsin State Laboratory of Hygiene Communicable Disease Division	Kelsey R. Florek, Abigail C. Shockey
EPI_ISL_949234	Departamento de Microbiología, CDB, Hospital Clínic,	SeqCOVID-SPAIN consortium/IBV(CSIC)	Andrea Vergara, Mikel Martínez, Elisa Rubio, Jéssica Navero, Aida Peiró and SeqCOVID-SPAIN consortium

	Barcelona		
EPI_ISL_952951, EPI_ISL_952952	Liverpool Clinical Laboratories	COVID-19 Genomics UK (COG-UK) Consortium	Sam Haldenby, Anita Lucaci, Steve Paterson, Julian Hiscox, Alistair Darby, M Almsaud, A Alrezaihi, Muhannad Alruwaili, Stuart D Armstrong, Jones Benjamin, Eleanor G Bentley, Anu Chawla, Jordan J Clark, Angela Cowell, Richard Eccles, Isabel García-Dorival, Matthew Gemmell, Alessandro Gerada, PKF Gilmore, Richard Gregory, Ximeng Han, Catherine Hartley, Margaret Hughes, Miren Iturriza-Gomara, James Johnson, L Luu, Jenifer Manson, Charlotte Nelson, Elaine O'Toole, Cassie Olateju, Rebekah Penrice-Randal , Lucille Rainbow, N.P Randle, Trevor Ian Robinson, Parul Sharma, Ghada T Shawli, James P Stewart, Neil Swainston, Ecaterina Vamos, Joanne Watts, Mark Whitehead
EPI_ISL_954187, EPI_ISL_954188	1.AO Universitaria 'S. Giovanni di Dio e Ruggi D'Aragona, Scuola Medica Salernitana' Hospital / 2.UOC di Virologia e Microbiologia, Università della Campania 'L. Vanvitelli' / 3.AO Universitaria 'Federico II' Napoli Hospital / 4.AORN 'San Giuseppe Moscati' Avellino Hospital / 5.AO 'San Pio - presidio G. Rummo' Benevento Hospital / 6.AO 'Sant'Anna e San Sebastiano' Caserta Hospital / 7.PO 'Maria Santissima Addolorata' Eboli Hospital / 8.Biogem Istituto di Ricerche Genetiche	1. Genome Research Center for Health (CRGS) / 2. Laboratory of Molecular Medicine and Genomics(LMMGe) / 3. Center for Research in Pure and Applied Mathematics (CRMPA)	Giorgio Giurato, Francesca Rizzo, Alessandro Weisz, Gianluigi Franci, Giovanni Nassa, Pasquale Pagliano, Roberta Tarallo, Elena Alexandrova, Ylenia D'Agostino, Carlo Ferravante, Jessica Lamberti, Viola Melone, Domenico Memoli, Valeria Mirici Cappa, Domenico Palumbo, Giovanni Pecoraro, Assunta Sellitto, Oriana Strianese, Ilaria Terenzi, Giuseppe Fenza, Aniello Gentile, Antonello Saccomanno, Sonia Amabile, Teresa Rocco, Annamaria Salvati, Emilia Vaccaro, Massimiliano Galdiero, Michele Cennamo, Giuseppe Portella, Maria Grazia Foti, Mariarosaria Ingino, Maria Landi, Maurizio Fumi, Vincenzo Rocco, Rita Greco, Vittoria Letizia, Arnolfo Petruzziello, Maddalena Schioppa, Gregorio Goffredi, Francesca Marciano, Michele Caraglia, Alessia Cossu, Marianna Scrima, Edmondo Adorisio, Morena D'Avenia, Michela Iacobellis, Rosanna Piluscio, Giorgio Dirani, Vittorio Sambri, Simona Semprini, Silvia Zanoli, Francesco Curcio, Stefania Marzinotto, Andreina Baj, Fausto Sessa.
EPI_ISL_960131	Victoria Hospital wc VHW	National Health Laboratory Service/UCT	Arash Iranzadeh, Deelan Doolabh, Lynn Tyers, Bruna Galvao, Innocent Mudau, Marvin Hsiao, Kruger Marais, Diana Hardie, Stephen Korsman, Carolyn Williamson
EPI_ISL_960132, EPI_ISL_960133	Groote Schuur Hospital wc GSH	National Health Laboratory Service/UCT	Arash Iranzadeh, Deelan Doolabh, Lynn Tyers, Bruna Galvao, Innocent Mudau, Marvin Hsiao, Kruger Marais, Diana Hardie, Stephen Korsman, Carolyn Williamson
EPI_ISL_960134	Conville CDC wc CVC	National Health Laboratory Service/UCT	Arash Iranzadeh, Deelan Doolabh, Lynn Tyers, Bruna Galvao, Innocent Mudau, Marvin Hsiao, Kruger Marais, Diana Hardie, Stephen Korsman, Carolyn Williamson
EPI_ISL_960135	Groote Schuur Hospital wc GSH	National Health Laboratory Service/UCT	Arash Iranzadeh, Deelan Doolabh, Lynn Tyers, Bruna Galvao, Innocent Mudau, Marvin Hsiao, Kruger Marais, Diana Hardie, Stephen Korsman, Carolyn Williamson
EPI_ISL_960237	Nucleic Acid Testing, National Reference Laboratory	GIGA Medical Genomics	Yvan Butera, Keith Durkin, Maria Artesi, Bouchra Boujemla, Robert Rutayisire, Patrick Tuyisenge, Esperence Umumararungu, Sébastien Bontems, Marie-Pierre Hayette, Nathalie Renotte, Corinne Fasquelle, Swaibu Gatare, Jacob Souopgui, Sabin Nsanzimana, Vincent Bours, Léon Mutesa
EPI_ISL_965578, EPI_ISL_965619	Dutch COVID-19 response team	Medical Microbiology, Maastricht University Medical Centre	Jozef Dingemans*, Brian van der Veer*, Erik Beuken, Carmen Reumkens, Lieke van Alphen, Christian Hoebe, Paul Savelkoul
EPI_ISL_968894, EPI_ISL_968898, EPI_ISL_968899, EPI_ISL_968961	KEMRI-Wellcome Trust Research Programme/KEMRI-CGMR-C Kilifi	KEMRI-Wellcome Trust Research Programme/KEMRI-CGMR-C Kilifi	Githinji et al
EPI_ISL_969397	Indian Council of Medical Research-National Institute of Virology, Pune	Indian Council of Medical Research-National Institute of Virology, Maximum Containment Laboratory	Pragya D. Yadav, Anita Shete-Aich, Dimpal A. Nyayanit, Abhinendra Kumar, Savita Patil, Triparna D. Majumdar
EPI_ISL_974465, EPI_ISL_974466, EPI_ISL_974467, EPI_ISL_974468, EPI_ISL_974469, EPI_ISL_974470, EPI_ISL_974471, EPI_ISL_974472, EPI_ISL_974473, EPI_ISL_974474, EPI_ISL_974475, EPI_ISL_974476, EPI_ISL_974477, EPI_ISL_974478, EPI_ISL_974479, EPI_ISL_974480, EPI_ISL_974481, EPI_ISL_974482, EPI_ISL_974483, EPI_ISL_974484, EPI_ISL_974485, EPI_ISL_974486, EPI_ISL_974487, EPI_ISL_974488, EPI_ISL_974489, EPI_ISL_974490, EPI_ISL_974491, EPI_ISL_974492, EPI_ISL_974493, EPI_ISL_974494, EPI_ISL_974495, EPI_ISL_974496, EPI_ISL_974497, EPI_ISL_974498, EPI_ISL_974499, EPI_ISL_974500, EPI_ISL_974501, EPI_ISL_974502, EPI_ISL_974503, EPI_ISL_974504, EPI_ISL_974505, EPI_ISL_974506, EPI_ISL_974507, EPI_ISL_974508, EPI_ISL_974509, EPI_ISL_974510, EPI_ISL_974511, EPI_ISL_974512, EPI_ISL_974513, EPI_ISL_974514, EPI_ISL_974515, EPI_ISL_974516, EPI_ISL_974517, EPI_ISL_974518, EPI_ISL_974519, EPI_ISL_974520, EPI_ISL_974521, EPI_ISL_974522, EPI_ISL_974523, EPI_ISL_974524, EPI_ISL_974525, EPI_ISL_974526, EPI_ISL_974527, EPI_ISL_974528, EPI_ISL_974529, EPI_ISL_974530, EPI_ISL_974531, EPI_ISL_974532, EPI_ISL_974533, EPI_ISL_974534, EPI_ISL_974535, EPI_ISL_974536, EPI_ISL_974537, EPI_ISL_974538, EPI_ISL_974539, EPI_ISL_974540, EPI_ISL_974541, EPI_ISL_974542, EPI_ISL_974543, EPI_ISL_974544, EPI_ISL_974545, EPI_ISL_974546, EPI_ISL_974547, EPI_ISL_974548, EPI_ISL_974549, EPI_ISL_974550, EPI_ISL_974551, EPI_ISL_974552, EPI_ISL_974553, EPI_ISL_974554, EPI_ISL_974555, EPI_ISL_974556			
see above	BCCDC Public Health Laboratory	BCCDC Public Health Laboratory	Prystajecky Natalie, Linda Hoang, Dan Fornika, John Tyson, Shannon Russell, Kim Macdonald, Kimia Kamelian, Ana Pacagnella, Corrinne Ng, Loretta Janz, Robert Azana Terry Snutch, Mel Krajden
EPI_ISL_977586, EPI_ISL_977587, EPI_ISL_977588	Department of Medical Microbiology, Hospital Pengajar Universiti Putra Malaysia	Malaysia Genome Institute	Mohd Noor Mat Isa, Syafinaz Amin-Nordin, Irni Suhayu Sapian, Hui-Yee Chee, Yusuf Muhammad Noor, Nurhezreen Md Iqbal, Enizza Kasim, Siti Noraini Othman, Mohd Faizal Abu Bakar, Shamsidar Sopie, Azrin Ahmad, Narcisse Joseph, Muhammad MI, Avisha Richards, Nor Zahrin Hasran, Nor Azfa Johari
EPI_ISL_977915, EPI_ISL_977916	Chiu Laboratory, University of California, San Francisco	Chiu Laboratory, University of California, San Francisco	Charles Chiu, Xianding (Wayne) Deng, Candace Wang, Venice Servellita, Jill Hacker, Debra Wadford
EPI_ISL_982506	Kentucky State Public Health Lab	Kentucky State Public Health Lab	Stephanie Lunn, Karim George, Joshua Tobias, William Grooms, Vaneet Arora, Matthew Johnson, Rachel Zinner, Rhonda Lucas
EPI_ISL_982930	MEPHI Aix Marseille University (AMU)	MEPHI Aix Marseille University (AMU)	Anthony LEVASSEUR
