# Supplementary material for: Detection and characterization of the SARS-CoV-2 lineage B.1.526 in New York
Source: Nat Commun. 2021 Aug 9;12:4886. doi: 10.1038/s41467-021-25168-4 (PMC8352861; doi:10.1038/s41467-021-25168-4)
Supplement: Supplementary file 8 — Supplementary Data 4 [file 41467_2021_25168_MOESM8_ESM.zip › GISAID_acknowledements_tables/gisaid_hcov-19_acknowledgement_table_2021_02_13_010-13.pdf]

We gratefully acknowledge the following Authors from the Originating laboratories responsible for obtaining the specimens, as well as the Submitting laboratories where the genome data were generated and shared via GISAID, on which this research is based.

All Submitters of data may be contacted directly via www.gisaid.org

Authors are sorted alphabetically.

Accession ID	Originating Laboratory	Submitting Laboratory	Authors
EPI_ISL_857473	National Public Health Laboratory, National Centre for Infectious Diseases	National Public Health Laboratory, National Centre for Infectious Diseases	Tze Minn Mak, Sophie Octavia, Zhenyang Zhou, Lin Cui, Raymond Tzer Pin Lin
EPI_ISL_860727, EPI_ISL_860728	University of Michigan Clinical Microbiology Laboratory	Lauring Lab, University of Michigan, Department of Microbiology and Immunology	Valesano
EPI_ISL_861035, EPI_ISL_861055, EPI_ISL_861070	Johns Hopkins Hospital Department of Pathology	Johns Hopkins Hospital Department of Pathology	C. Paul Morris, Chun Huai Luo, Adannaya Amadi, Nicholas Gallagher, Heba H. Mostafa
EPI_ISL_861488	Cerballiance Côte d'Azur	CERBA LAB	Prots.L; Pichon R; Barrieu-Moussat S.
EPI_ISL_861489	Cerballiance Côte d'Azur	CERBA LAB	Adjtoutah Z; Mangiarotti T; Prots L.
EPI_ISL_861490	Cerballiance Côte d'Azur	CERBA LAB	De La Chapelle A; Prots.L;
EPI_ISL_861491	Cerballiance Côte d'Azur	CERBA LAB	Chobert F; Prots.L
EPI_ISL_861492	Cerballiance Côte d'Azur	CERBA LAB	Barrieu-Moussat S;Adoul O;Boudjahem Z; Roman E; Chaudet L; Martin L; Aankour N; Jaugin P; Gastaud S; Prots L.
EPI_ISL_861493	Cerballiance Côte d'Azur	CERBA LAB	Marius M; Barsanti M; Maltese S; Francois J; Sinsoulieu T; Van Der Rhyn I; Morello V; Marcadal M; Gandillet A; Prots L.
EPI_ISL_861494	Cerballiance Côte d'Azur	CERBA LAB	Prots L. Raimondi V.
EPI_ISL_861495	Cerballiance Côte d'Azur	CERBA LAB	Pichon R. Quintle S; Sanza O; Mangiarotti T; Viltard B; Prots L.
EPI_ISL_861497	Cerballiance Centre Est	CERBA LAB	Chanard C; Gazzano V; Munier C; Prots L.
EPI_ISL_861500, EPI_ISL_861501, EPI_ISL_861502	Cerballiance Centre Est	CERBA LAB	Chanard E; Gazzano V; Munier C; Prots L.
EPI_ISL_861736, EPI_ISL_861767	Yale Pathology Lab	Grubaugh Lab - Yale School of Public Health	Tara Alpert, Joseph Fauver, Chen Liu, Pei Hui, Jianhui Wang, Susan Bell and Han Zhou, Anderson Brito, Mallery Breban, Anne Wyllie, Chantal Vogels, Mary Petrone, Chaney Kalinich, Isabel Ott, Arnau Casanovas, Catherine Muenker, Adam Moore, Alice Lu, Maria Tokuyama, Patrick Wong, Peiwen Lu, Saad Omer, Richard Martinello, Allison Nelson, Shelli Farhadian, Akiko Iwasaki, Charlese Dela Cruz, Albert Ko, Nathan Grubaugh
EPI_ISL_862048	Respiratory Virus Unit, National Infection Service, Public Health England	COVID-19 Genomics UK (COG-UK) Consortium	PHE Covid Sequencing Team
EPI_ISL_862049	Department of Microbiology, National Influenza Reference Laboratory for Northern Greece	Department of Microbiology, National Influenza Reference Laboratory for Northern Greece	Anna Papa, Styliani Pappa
EPI_ISL_862188, EPI_ISL_862189, EPI_ISL_862194, EPI_ISL_862195, EPI_ISL_862196, EPI_ISL_862197, EPI_ISL_862198, EPI_ISL_862199, EPI_ISL_862200, EPI_ISL_862201, EPI_ISL_862202, EPI_ISL_862203, EPI_ISL_862204, EPI_ISL_862205, EPI_ISL_862206, EPI_ISL_862207, EPI_ISL_862208, EPI_ISL_862209, EPI_ISL_862210, EPI_ISL_862211, EPI_ISL_862212, EPI_ISL_862213, EPI_ISL_862214, EPI_ISL_862215, EPI_ISL_862216, EPI_ISL_862217, EPI_ISL_862218, EPI_ISL_862219, EPI_ISL_862220, EPI_ISL_862221, EPI_ISL_862222, EPI_ISL_862223, EPI_ISL_862224, EPI_ISL_862225, EPI_ISL_862226, EPI_ISL_862227, EPI_ISL_862228, EPI_ISL_862229, EPI_ISL_862230, EPI_ISL_862231, EPI_ISL_862232, EPI_ISL_862233, EPI_ISL_862234, EPI_ISL_862235, EPI_ISL_862236, EPI_ISL_862237, EPI_ISL_862238, EPI_ISL_862239, EPI_ISL_862240, EPI_ISL_862241, EPI_ISL_862242, EPI_ISL_862243, EPI_ISL_862244, EPI_ISL_862245, EPI_ISL_862246, EPI_ISL_862247, EPI_ISL_862248, EPI_ISL_862249, EPI_ISL_862250, EPI_ISL_862252, EPI_ISL_862253, EPI_ISL_862254, EPI_ISL_862255, EPI_ISL_862256, EPI_ISL_862257, EPI_ISL_862258, EPI_ISL_862265, EPI_ISL_862266			
see above	Respiratory Virus Unit, National Infection Service, Public Health England	COVID-19 Genomics UK (COG-UK) Consortium	PHE Covid Sequencing Team
EPI_ISL_862716, EPI_ISL_862717, EPI_ISL_862720, EPI_ISL_862721	Centre for Dengue Research and AICBU, Department of Immunology and Molecular Medicine	Centre for Dengue Research and AICBU, Department of Immunology and Molecular Medicine	Chandima Jeewandara, Deshni Jayathilaka, Dinuka Ariyaratne, Diyanath Ranasinghe, Laksiri Gomes, Gathsaurie Neelika Malavige
EPI_ISL_862724, EPI_ISL_862726, EPI_ISL_862727	Centre for Dengue Research and AICBU, Department of Immunology and Molecular Medicine	Centre for Dengue Research and AICBU, Department of Immunology and Molecular Medicine	Chandima Jeewandara, Deshni Jayathilaka, Dinuka Ariyaratne, Tibutius Thanesh Pramanayagam, Diyanath Ranasinghe, Laksiri Gomes, Gathsaurie Neelika Malavige
EPI_ISL_862816, EPI_ISL_862817, EPI_ISL_862818, EPI_ISL_862820, EPI_ISL_862821, EPI_ISL_862822, EPI_ISL_862823, EPI_ISL_862824, EPI_ISL_862826, EPI_ISL_862827, EPI_ISL_862828, EPI_ISL_862829, EPI_ISL_862830			
see above	National Public Health Laboratory, National Centre for Infectious Diseases	National Public Health Laboratory, National Centre for Infectious Diseases	Tze Minn Mak, Zhenyang Zhou, Lin Cui, Raymond Tzer Pin Lin
EPI_ISL_862835, EPI_ISL_862836	National Institute of Infectious Diseases-Prof. Dr. Matei Bals Molecular Diagnostics Laboratory	National Institute of Infectious Diseases-Prof. Dr. Matei Bals Molecular Diagnostics Laboratory	Leontina Banica, Marius Surleac, Corina Casangiu, Petre Milu, Andreea Tudor, Simona Paraschiv, Dan Otelea
EPI_ISL_864579	Institute of Medical Microbiology and Hospital Hygiene	Institute of Medical Microbiology and Hospital Hygiene	Prof. Dr. Achim Kaasch, Aljoscha Tersteegen
EPI_ISL_865631, EPI_ISL_865634, EPI_ISL_865638, EPI_ISL_865640, EPI_ISL_865648, EPI_ISL_865658, EPI_ISL_865659, EPI_ISL_865660, EPI_ISL_865661, EPI_ISL_865663, EPI_ISL_865664, EPI_ISL_865665, EPI_ISL_865666, EPI_ISL_865667, EPI_ISL_865668, EPI_ISL_865671, EPI_ISL_865672, EPI_ISL_865673, EPI_ISL_865674, EPI_ISL_865675, EPI_ISL_865676, EPI_ISL_865677, EPI_ISL_865678, EPI_ISL_865679, EPI_ISL_865680, EPI_ISL_865681, EPI_ISL_865971, EPI_ISL_865972, EPI_ISL_865973, EPI_ISL_865974, EPI_ISL_865981, EPI_ISL_865982, EPI_ISL_865983, EPI_ISL_865984, EPI_ISL_865988, EPI_ISL_865989, EPI_ISL_865990, EPI_ISL_865992, EPI_ISL_865997, EPI_ISL_865998, EPI_ISL_865999, EPI_ISL_866004, EPI_ISL_866005, EPI_ISL_866006, EPI_ISL_866010, EPI_ISL_866016, EPI_ISL_866018, EPI_ISL_866022, EPI_ISL_866023, EPI_ISL_866025, EPI_ISL_866026, EPI_ISL_866029, EPI_ISL_866038, EPI_ISL_866040, EPI_ISL_866043, EPI_ISL_866044			
see above	University College London, Great Ormond Street Hospital for Children NHS Foundation Trust, Imperial College Healthcare NHS Trust	COVID-19 Genomics UK (COG-UK) Consortium	Sergi Castellano, Rachel Williams, Mark Kristiansen, Paola Resende Silva, Sunando Roy, Tony Brooks, Helena Tutill, Paola Niola, Patricia Dyal, Charlotte Williams, Leysa Forrest, Yasmin Panchbhaya, Jacqueline Findlay, Samuel Weeks, Julianne Brown, Kathryn Harris, Paul Randell, James Price, Alison Holmes, Judith Breuer
EPI_ISL_866619, EPI_ISL_866793	Respiratory Virus Unit, National Infection Service, Public Health England	COVID-19 Genomics UK (COG-UK) Consortium	PHE Covid Sequencing Team
EPI_ISL_866962, EPI_ISL_866963, EPI_ISL_866964, EPI_ISL_866965, EPI_ISL_866966, EPI_ISL_866967, EPI_ISL_866968, EPI_ISL_866969, EPI_ISL_866970	Queens Medical Centre, Clinical Microbiology Department / DeepSeq Nottingham	COVID-19 Genomics UK (COG-UK) Consortium	Gemma Clark, Wendy Smith, Manjinder Khakh, Vicki M Fleming, Michelle M Lister, Hannah Howson-Wells, Jonathan Ball, Patrick McClure, Joseph Chappell, Theocharis Tsoleridis, Nadine Holmes, Matthew Carlisle, Christopher Moore, Fei Sang, Johnny Debebe, Victoria Wright, Matthew Loose
EPI_ISL_866985, EPI_ISL_867156	Respiratory Virus Unit, National Infection Service, Public Health England	COVID-19 Genomics UK (COG-UK) Consortium	PHE Covid Sequencing Team
EPI_ISL_868257	Centre for Enzyme Innovation, University of Portsmouth / Translational Research Laboratory, Portsmouth Hospitals NHS Trust	COVID-19 Genomics UK (COG-UK) Consortium	Angela Beckett,Yann Bourgeois,Garry Scarlett,Sharon Glaysher,Scott Elliott,Kelly Bicknell,Robert Impey,Allyson Lloyd,Sarah Wyllie,Ethan Butcher,Anoop Chauhan,Samuel Robson
EPI_ISL_868369, EPI_ISL_868374, EPI_ISL_868375, EPI_ISL_868376, EPI_ISL_868380, EPI_ISL_868383, EPI_ISL_868384, EPI_ISL_868394, EPI_ISL_868396, EPI_ISL_868404, EPI_ISL_868405, EPI_ISL_868410, EPI_ISL_868412, EPI_ISL_868435, EPI_ISL_868457, EPI_ISL_868460, EPI_ISL_868462, EPI_ISL_868473,			

EPI_ISL_868477, EPI_ISL_868490, EPI_ISL_868494, EPI_ISL_868506, EPI_ISL_868519, EPI_ISL_868528, EPI_ISL_868550, EPI_ISL_868551, EPI_ISL_868553, EPI_ISL_868555, EPI_ISL_868571, EPI_ISL_868583, EPI_ISL_868585, EPI_ISL_868587, EPI_ISL_868600, EPI_ISL_868604, EPI_ISL_868605, EPI_ISL_868610, EPI_ISL_868616, EPI_ISL_868619, EPI_ISL_868624, EPI_ISL_868633, EPI_ISL_868636, EPI_ISL_868637, EPI_ISL_868640, EPI_ISL_868641, EPI_ISL_868650, EPI_ISL_868672, EPI_ISL_868676, EPI_ISL_868682, EPI_ISL_868685, EPI_ISL_868694, EPI_ISL_868709, EPI_ISL_868711, EPI_ISL_868712			
see above	Virology Department, Sheffield Teaching Hospitals NHS Foundation Trust/Department of Infection, Immunity and Cardiovascular Disease, The Medical School, University of Sheffield	COVID-19 Genomics UK (COG-UK) Consortium	Thushan de Silva, Matthew Parker, Nikki Smith, Adri Angyal, Rebecca Brown, Luke Green, Rachel Tucker, Paul Parsons, Danielle Groves, Katie Johnson, Laura Carrilero, Alex Keeley, Dave Partridge, Matthew Wyles, Benjamin Lindsey, Mehmet Yavuz, Mohammad Raza, Cariad Evans
EPI_ISL_869135	Charité Universitätsmedizin Berlin, Institut für Virologie/Labor Berlin	Charité Universitätsmedizin Berlin, Institut für Virologie	Victor M Corman, Barbara Mühlemann, Jörn Beheim-Schwarzbach, Tobias Bleicker, Julia Tesch, Talitha Veith, Julia Schneider, Terry Jones, Christian Drosten
EPI_ISL_869265, EPI_ISL_869266, EPI_ISL_869267, EPI_ISL_869268, EPI_ISL_869269, EPI_ISL_869270, EPI_ISL_869271, EPI_ISL_869272, EPI_ISL_869273, EPI_ISL_869274, EPI_ISL_869275, EPI_ISL_869276, EPI_ISL_869277, EPI_ISL_869278, EPI_ISL_869279, EPI_ISL_869280, EPI_ISL_869281, EPI_ISL_869282, EPI_ISL_869283, EPI_ISL_869284, EPI_ISL_869285, EPI_ISL_869286, EPI_ISL_869287, EPI_ISL_869288, EPI_ISL_869289, EPI_ISL_869290, EPI_ISL_869291, EPI_ISL_869292, EPI_ISL_869293, EPI_ISL_869294, EPI_ISL_869295, EPI_ISL_869296, EPI_ISL_869297, EPI_ISL_869298, EPI_ISL_869299, EPI_ISL_869300, EPI_ISL_869301, EPI_ISL_869302, EPI_ISL_869303, EPI_ISL_869304, EPI_ISL_869305, EPI_ISL_869306, EPI_ISL_869307, EPI_ISL_869308, EPI_ISL_869309, EPI_ISL_869310, EPI_ISL_869311, EPI_ISL_869312, EPI_ISL_869313, EPI_ISL_869314, EPI_ISL_869315, EPI_ISL_869316, EPI_ISL_869317, EPI_ISL_869318, EPI_ISL_869319, EPI_ISL_869320, EPI_ISL_869321, EPI_ISL_869322, EPI_ISL_869323, EPI_ISL_869324, EPI_ISL_869325, EPI_ISL_869326, EPI_ISL_869327, EPI_ISL_869328, EPI_ISL_869329, EPI_ISL_869330, EPI_ISL_869331, EPI_ISL_869332, EPI_ISL_869333, EPI_ISL_869334, EPI_ISL_869335, EPI_ISL_869336, EPI_ISL_869337, EPI_ISL_869338, EPI_ISL_869339, EPI_ISL_869340, EPI_ISL_869341, EPI_ISL_869342, EPI_ISL_869343, EPI_ISL_869344, EPI_ISL_869345, EPI_ISL_869346, EPI_ISL_869347, EPI_ISL_869348, EPI_ISL_869349, EPI_ISL_869350, EPI_ISL_869351, EPI_ISL_869352, EPI_ISL_869353, EPI_ISL_869354, EPI_ISL_869355, EPI_ISL_869356, EPI_ISL_869357, EPI_ISL_869358, EPI_ISL_869359, EPI_ISL_869360, EPI_ISL_869361, EPI_ISL_869362, EPI_ISL_869363, EPI_ISL_869364, EPI_ISL_869365, EPI_ISL_869366, EPI_ISL_869367, EPI_ISL_869368, EPI_ISL_869369, EPI_ISL_869370, EPI_ISL_869371, EPI_ISL_869372, EPI_ISL_869373, EPI_ISL_869374, EPI_ISL_869375, EPI_ISL_869376, EPI_ISL_869377, EPI_ISL_869378, EPI_ISL_869379, EPI_ISL_869380, EPI_ISL_869381, EPI_ISL_869382, EPI_ISL_869383, EPI_ISL_869384, EPI_ISL_869385, EPI_ISL_869386, EPI_ISL_869387, EPI_ISL_869388, EPI_ISL_869389, EPI_ISL_869390, EPI_ISL_869391, EPI_ISL_869392, EPI_ISL_869393, EPI_ISL_869394, EPI_ISL_869395, EPI_ISL_869396, EPI_ISL_869397, EPI_ISL_869398, EPI_ISL_869399, EPI_ISL_869400, EPI_ISL_869401, EPI_ISL_869402, EPI_ISL_869403, EPI_ISL_869404, EPI_ISL_869405, EPI_ISL_869406, EPI_ISL_869407, EPI_ISL_869408, EPI_ISL_869409, EPI_ISL_869410, EPI_ISL_869411, EPI_ISL_869412, EPI_ISL_869413, EPI_ISL_869414, EPI_ISL_869415, EPI_ISL_869416, EPI_ISL_869417, EPI_ISL_869418, EPI_ISL_869419, EPI_ISL_869420, EPI_ISL_869421, EPI_ISL_869422, EPI_ISL_869423, EPI_ISL_869424, EPI_ISL_869425, EPI_ISL_869426, EPI_ISL_869427, EPI_ISL_869428, EPI_ISL_869429, EPI_ISL_869430, EPI_ISL_869431, EPI_ISL_869432, EPI_ISL_869433, EPI_ISL_869434, EPI_ISL_869435, EPI_ISL_869436, EPI_ISL_869437, EPI_ISL_869438, EPI_ISL_869439, EPI_ISL_869440, EPI_ISL_869441, EPI_ISL_869442, EPI_ISL_869443, EPI_ISL_869444, EPI_ISL_869445, EPI_ISL_869446, EPI_ISL_869447, EPI_ISL_869448, EPI_ISL_869449, EPI_ISL_869450, EPI_ISL_869451, EPI_ISL_869452, EPI_ISL_869453, EPI_ISL_869454, EPI_ISL_869455, EPI_ISL_869456, EPI_ISL_869457, EPI_ISL_869458, EPI_ISL_869459, EPI_ISL_869460, EPI_ISL_869461, EPI_ISL_869462, EPI_ISL_869463, EPI_ISL_869464, EPI_ISL_869465, EPI_ISL_869466, EPI_ISL_869467, EPI_ISL_869468, EPI_ISL_869469, EPI_ISL_869470, EPI_ISL_869471, EPI_ISL_869472, EPI_ISL_869473, EPI_ISL_869474, EPI_ISL_869475, EPI_ISL_869476, EPI_ISL_869477, EPI_ISL_869478, EPI_ISL_869479, EPI_ISL_869480, EPI_ISL_869481, EPI_ISL_869482, EPI_ISL_869483, EPI_ISL_869484, EPI_ISL_869485, EPI_ISL_869486, EPI_ISL_869487, EPI_ISL_869488, EPI_ISL_869489, EPI_ISL_869490, EPI_ISL_869491, EPI_ISL_869492, EPI_ISL_869493, EPI_ISL_869494, EPI_ISL_869495, EPI_ISL_869496, EPI_ISL_869497, EPI_ISL_869498, EPI_ISL_869499, EPI_ISL_869500, EPI_ISL_869501, EPI_ISL_869502, EPI_ISL_869503, EPI_ISL_869504, EPI_ISL_869505, EPI_ISL_869506, EPI_ISL_869507, EPI_ISL_869508, EPI_ISL_869509, EPI_ISL_869510, EPI_ISL_869511, EPI_ISL_869512, EPI_ISL_869513, EPI_ISL_869514, EPI_ISL_869515, EPI_ISL_869516, EPI_ISL_869517, EPI_ISL_869518, EPI_ISL_869519, EPI_ISL_869520, EPI_ISL_869521, EPI_ISL_869522, EPI_ISL_869523, EPI_ISL_869524, EPI_ISL_869525, EPI_ISL_869526, EPI_ISL_869527, EPI_ISL_869528, EPI_ISL_869529, EPI_ISL_869530, EPI_ISL_869531, EPI_ISL_869532, EPI_ISL_869533, EPI_ISL_869534, EPI_ISL_869535, EPI_ISL_869536, EPI_ISL_869537, EPI_ISL_869538, EPI_ISL_869539, EPI_ISL_869540, EPI_ISL_869541, EPI_ISL_869542, EPI_ISL_869543, EPI_ISL_869544, EPI_ISL_869545, EPI_ISL_869546, EPI_ISL_869547, EPI_ISL_869548, EPI_ISL_869549, EPI_ISL_869550, EPI_ISL_869551, EPI_ISL_869552, EPI_ISL_869553, EPI_ISL_869554, EPI_ISL_869555, EPI_ISL_869556, EPI_ISL_869557, EPI_ISL_869558, EPI_ISL_869559, EPI_ISL_869560, EPI_ISL_869561, EPI_ISL_869562, EPI_ISL_869563, EPI_ISL_869564, EPI_ISL_869565, EPI_ISL_869566, EPI_ISL_869567, EPI_ISL_869568, EPI_ISL_869569, EPI_ISL_869570, EPI_ISL_869571, EPI_ISL_869572, EPI_ISL_869573, EPI_ISL_869574, EPI_ISL_869575, EPI_ISL_869576, EPI_ISL_869577, EPI_ISL_869578, EPI_ISL_869579, EPI_ISL_869580, EPI_ISL_869581, EPI_ISL_869582, EPI_ISL_869583, EPI_ISL_869584, EPI_ISL_869585, EPI_ISL_869586, EPI_ISL_869587, EPI_ISL_869588, EPI_ISL_869589, EPI_ISL_869590, EPI_ISL_869591, EPI_ISL_869592, EPI_ISL_869593, EPI_ISL_869594, EPI_ISL_869595, EPI_ISL_869596, EPI_ISL_869597, EPI_ISL_869598, EPI_ISL_869599, EPI_ISL_869600, EPI_ISL_869601, EPI_ISL_869602, EPI_ISL_869603, EPI_ISL_869604, EPI_ISL_869605, EPI_ISL_869606, EPI_ISL_869607, EPI_ISL_869608, EPI_ISL_869609, EPI_ISL_869610, EPI_ISL_869611, EPI_ISL_869612, EPI_ISL_869613, EPI_ISL_869614, EPI_ISL_869615, EPI_ISL_869616, EPI_ISL_869617, EPI_ISL_869618, EPI_ISL_869619, EPI_ISL_869620, EPI_ISL_869621, EPI_ISL_869622, EPI_ISL_869623, EPI_ISL_869624, EPI_ISL_869625, EPI_ISL_869626, EPI_ISL_869627, EPI_ISL_869628, EPI_ISL_869629, EPI_ISL_869630, EPI_ISL_869631, EPI_ISL_869632, EPI_ISL_869633, EPI_ISL_869634, EPI_ISL_869635, EPI_ISL_869636, EPI_ISL_869637, EPI_ISL_869638, EPI_ISL_869639, EPI_ISL_869640, EPI_ISL_869641, EPI_ISL_869642, EPI_ISL_869643			
see above	Department of Virus and Microbiological Special Diagnostics, Statens Serum Institut, Copenhagen, Denmark	Aalborg University	Danish Covid-19 Genome Consortium
EPI_ISL_870315, EPI_ISL_870317, EPI_ISL_870319	University of Michigan Clinical Microbiology Laboratory	Lauring Lab, University of Michigan, Department of Microbiology and Immunology	Valesano
EPI_ISL_871872	Botswana Harvard HIV Reference Laboratory	Botswana Harvard HIV Reference Laboratory	Sikhulile Moyo, Dorcas Maruapula, Wonderful Choga, Botshelo Radibe, Boitumelo Zuze, David Lawrence, Roger Shapiro, Shahin Lockman, Mosepele Mosepele, Joseph, Makhema, Simani Gaseitsiwe
EPI_ISL_872052, EPI_ISL_872053, EPI_ISL_872054, EPI_ISL_872055, EPI_ISL_872056	CHR Citadelle	GIGA Medical Genomics	Keith Durkin, Maria Artesi, Sébastien Bontems, Raphaël Boreux, Bouchra Boujemla, Cécile Meex, Pierrette Melin, Marie-Pierre Hayette, Vincent Bours
EPI_ISL_872057, EPI_ISL_872081, EPI_ISL_872082, EPI_ISL_872083, EPI_ISL_872084, EPI_ISL_872085, EPI_ISL_872086, EPI_ISL_872087, EPI_ISL_872105, EPI_ISL_872106, EPI_ISL_872107, EPI_ISL_872108			
see above	Department of Clinical Microbiology	GIGA Medical Genomics	Keith Durkin, Maria Artesi, Sébastien Bontems, Raphaël Boreux, Bouchra Boujemla, Cécile Meex, Pierrette Melin, Marie-Pierre Hayette, Vincent Bours
EPI_ISL_872110, EPI_ISL_872111	CHC	GIGA Medical Genomics	Keith Durkin, Maria Artesi, Sébastien Bontems, Raphaël Boreux, Bouchra Boujemla, Cécile Meex, Pierrette Melin, Marie-Pierre Hayette, Vincent Bours
EPI_ISL_872112, EPI_ISL_872115, EPI_ISL_872116, EPI_ISL_872122	Department of Clinical Microbiology	GIGA Medical Genomics	Keith Durkin, Maria Artesi, Sébastien Bontems, Raphaël Boreux, Bouchra Boujemla, Cécile Meex, Pierrette Melin, Marie-Pierre Hayette, Vincent Bours
EPI_ISL_872123, EPI_ISL_872124, EPI_ISL_872125, EPI_ISL_872126	CHR Citadelle	GIGA Medical Genomics	Keith Durkin, Maria Artesi, Sébastien Bontems, Raphaël Boreux, Bouchra Boujemla, Cécile Meex, Pierrette Melin, Marie-Pierre Hayette, Vincent Bours
EPI_ISL_872127, EPI_ISL_872128, EPI_ISL_872129, EPI_ISL_872130, EPI_ISL_872135, EPI_ISL_872136, EPI_ISL_872137, EPI_ISL_872138, EPI_ISL_872139, EPI_ISL_872140, EPI_ISL_872141, EPI_ISL_872142, EPI_ISL_872143, EPI_ISL_872144, EPI_ISL_872145			
see above	Department of Clinical Microbiology	GIGA Medical Genomics	Keith Durkin, Maria Artesi, Sébastien Bontems, Raphaël Boreux, Bouchra Boujemla, Cécile Meex, Pierrette Melin, Marie-Pierre Hayette, Vincent Bours
EPI_ISL_872146, EPI_ISL_872147	CHC	GIGA Medical Genomics	Keith Durkin, Maria Artesi, Sébastien Bontems, Raphaël Boreux, Bouchra Boujemla, Cécile Meex, Pierrette Melin, Marie-Pierre Hayette, Vincent Bours
EPI_ISL_872148, EPI_ISL_872149	Department of Clinical Microbiology	GIGA Medical Genomics	Keith Durkin, Maria Artesi, Sébastien Bontems, Raphaël Boreux, Bouchra Boujemla, Cécile Meex, Pierrette Melin, Marie-Pierre Hayette, Vincent Bours
EPI_ISL_872150	CHR Citadelle	GIGA Medical Genomics	Keith Durkin, Maria Artesi, Sébastien Bontems, Raphaël Boreux, Bouchra Boujemla, Cécile Meex, Pierrette Melin, Marie-Pierre Hayette, Vincent Bours
EPI_ISL_872164, EPI_ISL_872167, EPI_ISL_872170, EPI_ISL_872171, EPI_ISL_872172	Wyoming Public Health Laboratory	Wyoming Public Health Laboratory	Noah Hull, Taylor Fearing, Lynette Gumbleton, Channing Weber, Ashley Norberg, Bailey Bowcutt, and Wanda Manley
EPI_ISL_872192	Conjunto Hospitalar do Mandaqui de Sao Paulo	Instituto Adolfo Lutz, Interdiciplinary Procedures Center, Strategic Laboratory	Claudio Tavares Sacchi, Claudia Regina Gonçalves, Erica Valessa Ramos Gomes, Karoline Rodrigues Campos, Katia Correa de Oliveira Santos, Ana Lucia de Carvalho Avelino, Fabiana Cristina Pereira dos Santos
EPI_ISL_872568	Centre for Dengue Research and AICBU, Department of Immunology and Molecular Medicine	Centre for Dengue Research and AICBU, Department of Immunology and Molecular Medicine	Chandima Jeewandara, Deshni Jayathilaka, Dinuka Ariyaratne, Tibutius Thanesh Pramanayagam, Diyanath Ranasinghe, Laksiri Gomes, Gathsaurie Neelika Malavige
EPI_ISL_872583, EPI_ISL_872584	South Eastern Area Laboratory Services (SEALS)	NSW Health Pathology - Institute of Clinical Pathology and Medical Research; Westmead Hospital; University of Sydney	CIDM-PH et al.
EPI_ISL_872586	Sydney South West Pathology Service (SSWPS) - Royal Prince Alfred Hospital - NSW Health Pathology	NSW Health Pathology - Institute of Clinical Pathology and Medical Research; Westmead Hospital; University of Sydney	CIDM-PH et al.
EPI_ISL_875566, EPI_ISL_875567, EPI_ISL_875568	SIESP L'AQUILA	Istituto Zooprofilattico Sperimentale dell'Abruzzo e Molise "G. Caporale"	Lorusso A, Marcacci M, Di Domenico M, Ancora M, Curini V, Mangone I, Rinaldi A, Scialabba S, Di Pasquale A, Cammà C, Puglia I, Calistri P, Savini G
EPI_ISL_875672, EPI_ISL_875673	CHU Purpan - Laboratoire de Virologie - Institut Fédératif de Biologie	CHU Purpan - Laboratoire de Virologie - Institut Fédératif de Biologie	Latour J., Ranger N., Dubois M., Carcenac R., Harter A., Boyer P., Tremeaux P., Izopet J.
EPI_ISL_876061, EPI_ISL_876118, EPI_ISL_876136, EPI_ISL_876143, EPI_ISL_876153, EPI_ISL_876161, EPI_ISL_876218, EPI_ISL_876219, EPI_ISL_876220, EPI_ISL_876221, EPI_ISL_876222, EPI_ISL_876223, EPI_ISL_876224, EPI_ISL_876225, EPI_ISL_876226, EPI_ISL_876227, EPI_ISL_876228, EPI_ISL_876229, EPI_ISL_876230			
see above	Massachusetts State Public Health Laboratory	Massachusetts State Public Health Laboratory	Andrew Lang, Timelia Fink, Glen Gallagher, Sandra Smole
EPI_ISL_877155, EPI_ISL_877156, EPI_ISL_877157, EPI_ISL_877158, EPI_ISL_877159, EPI_ISL_877160, EPI_ISL_877161, EPI_ISL_877162, EPI_ISL_877163, EPI_ISL_877181, EPI_ISL_877182, EPI_ISL_877183, EPI_ISL_877184, EPI_ISL_877185			
see above	Univeristy of New Mexico Hospital	Center for Global Health, University of New Mexico Health Sciences Center	Daryl Domman, Kurt Schwalm, Justin Bacca, Jon Femling, Darrell Dinwiddie
EPI_ISL_877454	National laboratory of health, environment and food Kranj	Institute of Microbiology and Immunology, Faculty of Medicine,	Samo Zakotnik, Tomaž Mark Zorec, Matic Brvar, Miša Korva, Mario Poljak, Tatjana Avši - Županc

		University of Ljubljana	
EPI_ISL_877570	Microbiological Diagnostic Unit - Public Health Laboratory (MDU-PHL)	MDU-PHL	Seemann T., Sait, M.L., Sherry, N.L.
EPI_ISL_877576, EPI_ISL_877578	Victorian Infectious Diseases Reference Laboratory (VIDRL)	VIDRL and MDU-PHL	Caly L., Seemann T., Sait, M.L., Druce J., Sherry, N.L.
EPI_ISL_879146	Lighthouse Lab in Alderley Park	Wellcome Sanger Institute for the COVID-19 Genomics UK (COG-UK) Consortium	Jacquelyn Wynn, Mairead Hyland, The Lighthouse Lab in Alderley Park and Alex Alderton, Roberto Amato, Sonia Goncalves, Ewan Harrison, David K. Jackson, Ian Johnston, Dominic Kwiatkowski, Cordelia Langford, John Sillitoe on behalf of the Wellcome Sanger Institute COVID-19 Surveillance Team
EPI_ISL_882997, EPI_ISL_882998	Motion Picture Television Fund Hospital Lab	Los Angeles County PHL	P. Hemarajata et al.
EPI_ISL_884182, EPI_ISL_884183, EPI_ISL_884184, EPI_ISL_884185, EPI_ISL_884186, EPI_ISL_884187, EPI_ISL_884188, EPI_ISL_884190, EPI_ISL_884191, EPI_ISL_884193	Wyoming Public Health Laboratory	Wyoming Public Health Laboratory	Noah Hull, Taylor Fearing, Lynette Gumbleton, Channing Weber, Ashley Norberg, Bailey Bowcutt, and Wanda Manley
EPI_ISL_885152	National Institute of Infectious Diseases-Prof. Dr. Matei Bals Molecular Diagnostics Laboratory	National Institute of Infectious Diseases-Prof. Dr. Matei Bals Molecular Diagnostics Laboratory	Leontina Banica, Marius Surleac, Corina Casangiu, Petre Milu, Andreea Tudor, Simona Paraschiv, Dan Otelea
EPI_ISL_885491, EPI_ISL_885492, EPI_ISL_885494, EPI_ISL_885496, EPI_ISL_885497, EPI_ISL_885502, EPI_ISL_885504, EPI_ISL_885507, EPI_ISL_885508, EPI_ISL_885511, EPI_ISL_885512, EPI_ISL_885516, EPI_ISL_885517, EPI_ISL_885519, EPI_ISL_885520, EPI_ISL_885524, EPI_ISL_885526, EPI_ISL_885528, EPI_ISL_885534, EPI_ISL_885535, EPI_ISL_885538, EPI_ISL_885545, EPI_ISL_885546, EPI_ISL_885550, EPI_ISL_885551, EPI_ISL_885562, EPI_ISL_885563, EPI_ISL_885564, EPI_ISL_885567, EPI_ISL_885574, EPI_ISL_885575, EPI_ISL_885579, EPI_ISL_885582, EPI_ISL_885585, EPI_ISL_885588, EPI_ISL_885593, EPI_ISL_885602, EPI_ISL_885604, EPI_ISL_885607, EPI_ISL_885608, EPI_ISL_885609, EPI_ISL_885614, EPI_ISL_885621, EPI_ISL_885625, EPI_ISL_885626, EPI_ISL_885627, EPI_ISL_885628, EPI_ISL_885634, EPI_ISL_885636, EPI_ISL_885639, EPI_ISL_885647, EPI_ISL_885658, EPI_ISL_885660, EPI_ISL_885665, EPI_ISL_885666, EPI_ISL_885669, EPI_ISL_885670, EPI_ISL_885672, EPI_ISL_885675, EPI_ISL_885678, EPI_ISL_885681, EPI_ISL_885684, EPI_ISL_885685, EPI_ISL_885687, EPI_ISL_885688, EPI_ISL_885689, EPI_ISL_885692, EPI_ISL_885693, EPI_ISL_885696, EPI_ISL_885699, EPI_ISL_885704, EPI_ISL_885710, EPI_ISL_885712, EPI_ISL_885713, EPI_ISL_885714, EPI_ISL_885715, EPI_ISL_885720, EPI_ISL_885722, EPI_ISL_885724, EPI_ISL_885733, EPI_ISL_885734, EPI_ISL_885737, EPI_ISL_885738, EPI_ISL_885742, EPI_ISL_885743, EPI_ISL_885745, EPI_ISL_885748, EPI_ISL_885750, EPI_ISL_885755, EPI_ISL_885756, EPI_ISL_885761, EPI_ISL_885764, EPI_ISL_885765, EPI_ISL_885768, EPI_ISL_885770, EPI_ISL_885773, EPI_ISL_885775, EPI_ISL_885776, EPI_ISL_885779, EPI_ISL_885780, EPI_ISL_885785, EPI_ISL_885788, EPI_ISL_885792, EPI_ISL_885793, EPI_ISL_885794, EPI_ISL_885795, EPI_ISL_885800, EPI_ISL_885804, EPI_ISL_885807, EPI_ISL_885810, EPI_ISL_885812, EPI_ISL_885815, EPI_ISL_885820, EPI_ISL_885822, EPI_ISL_885824, EPI_ISL_885825, EPI_ISL_885826, EPI_ISL_885829, EPI_ISL_885830, EPI_ISL_885831, EPI_ISL_885832, EPI_ISL_885834, EPI_ISL_885836, EPI_ISL_885837, EPI_ISL_885839, EPI_ISL_885840, EPI_ISL_885841, EPI_ISL_885842, EPI_ISL_885845, EPI_ISL_885847, EPI_ISL_885849, EPI_ISL_885850, EPI_ISL_885851, EPI_ISL_885852, EPI_ISL_885853, EPI_ISL_885854, EPI_ISL_885857, EPI_ISL_885858, EPI_ISL_885859, EPI_ISL_885861, EPI_ISL_885862, EPI_ISL_885863, EPI_ISL_885865, EPI_ISL_885866, EPI_ISL_885869, EPI_ISL_885872, EPI_ISL_885873, EPI_ISL_885876, EPI_ISL_885877, EPI_ISL_885878, EPI_ISL_885881, EPI_ISL_885882, EPI_ISL_885884, EPI_ISL_885886, EPI_ISL_885887, EPI_ISL_885890, EPI_ISL_885891, EPI_ISL_885892, EPI_ISL_885893, EPI_ISL_885895, EPI_ISL_885896, EPI_ISL_885897, EPI_ISL_885900, EPI_ISL_885901, EPI_ISL_885903, EPI_ISL_885904, EPI_ISL_885905, EPI_ISL_885907, EPI_ISL_885908, EPI_ISL_885909, EPI_ISL_885911, EPI_ISL_885913, EPI_ISL_885914, EPI_ISL_885920, EPI_ISL_885921, EPI_ISL_885922, EPI_ISL_885923, EPI_ISL_885925, EPI_ISL_885930, EPI_ISL_885932, EPI_ISL_885933, EPI_ISL_885936, EPI_ISL_885938, EPI_ISL_885940, EPI_ISL_885941, EPI_ISL_885942, EPI_ISL_885943, EPI_ISL_885944, EPI_ISL_885945, EPI_ISL_885947, EPI_ISL_885948, EPI_ISL_885949, EPI_ISL_885950, EPI_ISL_885952, EPI_ISL_885953, EPI_ISL_885955, EPI_ISL_885956, EPI_ISL_885957, EPI_ISL_885960, EPI_ISL_885964, EPI_ISL_885965, EPI_ISL_885968, EPI_ISL_885969, EPI_ISL_885971, EPI_ISL_885972, EPI_ISL_885973, EPI_ISL_885975, EPI_ISL_885977, EPI_ISL_885978, EPI_ISL_885979, EPI_ISL_885981, EPI_ISL_885985, EPI_ISL_885986, EPI_ISL_885989, EPI_ISL_885991, EPI_ISL_885994, EPI_ISL_886000, EPI_ISL_886003, EPI_ISL_886007, EPI_ISL_886008, EPI_ISL_886010, EPI_ISL_886011, EPI_ISL_886012, EPI_ISL_886013, EPI_ISL_886017, EPI_ISL_886019, EPI_ISL_886022, EPI_ISL_886023, EPI_ISL_886024, EPI_ISL_886025, EPI_ISL_886026, EPI_ISL_886028, EPI_ISL_886030, EPI_ISL_886031, EPI_ISL_886033, EPI_ISL_886034, EPI_ISL_886035, EPI_ISL_886036, EPI_ISL_886041, EPI_ISL_886042, EPI_ISL_886045, EPI_ISL_886048, EPI_ISL_886051, EPI_ISL_886052, EPI_ISL_886054, EPI_ISL_886055, EPI_ISL_886058, EPI_ISL_886059, EPI_ISL_886061, EPI_ISL_886062, EPI_ISL_886065, EPI_ISL_886068, EPI_ISL_886069, EPI_ISL_886072, EPI_ISL_886073, EPI_ISL_886079, EPI_ISL_886080, EPI_ISL_886082, EPI_ISL_886084, EPI_ISL_886085, EPI_ISL_886086, EPI_ISL_886087, EPI_ISL_886088, EPI_ISL_886092, EPI_ISL_886093, EPI_ISL_886094, EPI_ISL_886095, EPI_ISL_886100, EPI_ISL_886102, EPI_ISL_886104, EPI_ISL_886105, EPI_ISL_886107, EPI_ISL_886108, EPI_ISL_886109, EPI_ISL_886111, EPI_ISL_886113, EPI_ISL_886114, EPI_ISL_886115, EPI_ISL_886122, EPI_ISL_886123, EPI_ISL_886124, EPI_ISL_886125, EPI_ISL_886126, EPI_ISL_886128, EPI_ISL_886130, EPI_ISL_886131, EPI_ISL_886135, EPI_ISL_886138, EPI_ISL_886139, EPI_ISL_886147, EPI_ISL_886151, EPI_ISL_886152, EPI_ISL_886153, EPI_ISL_886155, EPI_ISL_886156			
see above	Lighthouse Lab in Alderley Park	Wellcome Sanger Institute for the COVID-19 Genomics UK (COG-UK) Consortium	Jacquelyn Wynn, Mairead Hyland, The Lighthouse Lab in Alderley Park and Alex Alderton, Roberto Amato, Sonia Goncalves, Ewan Harrison, David K. Jackson, Ian Johnston, Dominic Kwiatkowski, Cordelia Langford, John Sillitoe on behalf of the Wellcome Sanger Institute COVID-19 Surveillance Team
EPI_ISL_887120, EPI_ISL_887124, EPI_ISL_887125, EPI_ISL_887132	Institute of Medical Microbiology and Hospital Hygiene	Institute of Medical Microbiology and Hospital Hygiene	Prof. Dr. Achim Kaasch, Aljoscha Tersteegen
EPI_ISL_888663, EPI_ISL_888664, EPI_ISL_888665	University of Michigan Clinical Microbiology Laboratory	Lauring Lab, University of Michigan, Department of Microbiology and Immunology	Valesano
EPI_ISL_888673	Montana Public Health Laboratory	Wyoming Public Health Laboratory	Noah Hull, Joy Ritter, Taylor Fearing, Lynette Gumbleton, Channing Weber, Ashley Norberg, Bailey Bowcutt, Wanda Manley, Deborah Gibson
EPI_ISL_888824, EPI_ISL_888825	GZA Sint-Augustinus Hospital	UAntwerp, Laboratory of Medical Microbiology, Campus Drie Eiken S6.26, Universiteitsplein 1, 2610, Wilrijk, Antwerp, Belgium	Basil Britto Xavier, Jasmine Coppens, Christine Lammens, Veerle Matheeussen, Herman Goossens
EPI_ISL_888907, EPI_ISL_888912, EPI_ISL_888913, EPI_ISL_888915, EPI_ISL_888916, EPI_ISL_888917, EPI_ISL_888918, EPI_ISL_888920, EPI_ISL_888921, EPI_ISL_888922, EPI_ISL_888923, EPI_ISL_888924, EPI_ISL_888925, EPI_ISL_888926, EPI_ISL_888927, EPI_ISL_888928, EPI_ISL_888929, EPI_ISL_888931, EPI_ISL_888932, EPI_ISL_888933, EPI_ISL_888965, EPI_ISL_888967, EPI_ISL_888969, EPI_ISL_888971, EPI_ISL_888972, EPI_ISL_888973, EPI_ISL_888974, EPI_ISL_888975, EPI_ISL_888976, EPI_ISL_888977			
see above	Wyoming Public Health Laboratory	Wyoming Public Health Laboratory	Noah Hull, Taylor Fearing, Lynette Gumbleton, Channing Weber, Ashley Norberg, Bailey Bowcutt, and Wanda Manley
EPI_ISL_889762, EPI_ISL_889763, EPI_ISL_889764, EPI_ISL_889765, EPI_ISL_889766, EPI_ISL_889767, EPI_ISL_889768, EPI_ISL_889769, EPI_ISL_889770, EPI_ISL_889771, EPI_ISL_889772			
see above	LSUHS Emerging Viral Threat Laboratory	Microbial Genome Sequencing Center	Jeremy P. Kamil, Jennifer L. Carroll, Camille F. Abshire, Maarten Van Diest, Mohammed N.A. Siddiquey, Andrew D. Yurochko, Martin J. Sapp, Rona S. Scott, Christopher G. Kevil, Daniel J. Snyder, Vaughn S. Cooper, John A. Vanchiere
EPI_ISL_889773, EPI_ISL_889774, EPI_ISL_889775	LSUHS Emerging Viral Threat Laboratory	Microbial Genome Sequencing Center	Mohammed N.A. Siddiquey, Jennifer L. Carroll, Camille F. Abshire, Maarten Van Diest, Andrew D. Yurochko, Martin J. Sapp, Rona S. Scott, Christopher G. Kevil, Daniel J. Snyder, Vaughn S. Cooper, John A. Vanchiere, Jeremy P. Kamil,
EPI_ISL_889776, EPI_ISL_889777, EPI_ISL_889778, EPI_ISL_889779, EPI_ISL_889780, EPI_ISL_889781, EPI_ISL_889782, EPI_ISL_889783, EPI_ISL_889784, EPI_ISL_889785	LSUHS Emerging Viral Threat Laboratory	Microbial Genome Sequencing Center	Maarten Van Diest, Jennifer L. Carroll, Camille F. Abshire, Mohammed N.A. Siddiquey, Andrew D. Yurochko, Martin J. Sapp, Rona S. Scott, Christopher G. Kevil, Daniel J. Snyder, Vaughn S. Cooper, John A. Vanchiere, Jeremy P. Kamil
EPI_ISL_889786, EPI_ISL_889787, EPI_ISL_889788, EPI_ISL_889789, EPI_ISL_889790, EPI_ISL_889791	LSUHS Emerging Viral Threat Laboratory	Microbial Genome Sequencing Center	Jennifer L. Carroll, Rona S. Scott, Camille F. Abshire, Maarten Van Diest, Mohammed N.A. Siddiquey, Andrew D. Yurochko, Martin J. Sapp, Christopher G. Kevil, Daniel J. Snyder, Vaughn S. Cooper, John A. Vanchiere, Jeremy P. Kamil,
EPI_ISL_889792, EPI_ISL_889793, EPI_ISL_889794, EPI_ISL_889795, EPI_ISL_889796, EPI_ISL_889797, EPI_ISL_889798, EPI_ISL_889799, EPI_ISL_889800, EPI_ISL_889801	LSUHS Emerging Viral Threat Laboratory	Microbial Genome Sequencing Center	Rona S. Scott, Jennifer L. Carroll, Camille F. Abshire, Maarten Van Diest, Mohammed N.A. Siddiquey, Andrew D. Yurochko, Martin J. Sapp, Christopher G. Kevil, Daniel J. Snyder, Vaughn S. Cooper, John A. Vanchiere, Jeremy P. Kamil,
EPI_ISL_890233	School of Pharmacy, Shenandoah University	School of Pharmacy, Shenandoah University	Adams,S.M., Harralson,A.F., Kidd,R.S., Sawyer,G.W.
EPI_ISL_890367, EPI_ISL_890368, EPI_ISL_890369, EPI_ISL_890370	LSUHS Emerging Viral Threat Laboratory	Microbial Genome Sequencing Center	Jennifer L. Carroll, Rona S. Scott, Camille F. Abshire, Maarten Van Diest, Mohammed N.A. Siddiquey, Andrew D. Yurochko, Martin J. Sapp, Christopher G. Kevil, Daniel J. Snyder, Vaughn S. Cooper, John A. Vanchiere, Jeremy P. Kamil,
EPI_ISL_891093	Seattle Flu Study	Seattle Flu Study	Deborah A. Nickerson, Chris D. Frazar, Jover Lee, Benjamin Pelle, Erica Ryke, Matthew Richardson, Amanda Adler, Elisabeth Brandstetter, Peter D. Han, Kairsten Fay, Misja Ilcisin, Kirsten Lacombe, Thomas R. Sibley, Melissa Truong, Caitlin R. Wolf, Michael Boeckh, Janet A. Englund, Michael Famulare, Barry R. Lutz, Mark J. Rieder, Lea M. Starita, Matthew Thompson, Jay Shendure, Trevor Bedford, Helen Y. Chu
EPI_ISL_891205	DPH, Massachusetts State Public Health Lab	DPH, Massachusetts State Public Health Lab	Lang,A.S., Fink,T., Gallagher,G.R., Smole,S.C.
EPI_ISL_891253	Singapore General Hospital	Department of Microbiology	Nurdyana Abdul Rahman, Kun Lee Lim, Chenhao Li, Sui Sin Goh, Kenneth Xin Long Chan, Kian Sing Chan, Lynette Oon, Kern Rei Chng, Niranjan Nagarajan, Karrie Ko
EPI_ISL_891273, EPI_ISL_891275, EPI_ISL_891276	Lighthouse Lab in Cambridge	Wellcome Sanger Institute for the COVID-19 Genomics UK (COG-UK) Consortium	Rob Howes, The Lighthouse Lab in Cambridge and Alex Alderton, Roberto Amato, Sonia Goncalves, Ewan Harrison, David K. Jackson, Ian Johnston, Dominic Kwiatkowski, Cordelia Langford, John Sillitoe on behalf of the Wellcome Sanger Institute COVID-19 Surveillance Team
EPI_ISL_891278, EPI_ISL_891280	Lighthouse Lab in Alderley Park	Wellcome Sanger Institute for the COVID-19 Genomics UK	Jacquelyn Wynn, Mairead Hyland, The Lighthouse Lab in Alderley Park and Alex Alderton, Roberto Amato, Sonia Goncalves, Ewan Harrison, David K.

		(COG-UK) Consortium	Jackson, Ian Johnston, Dominic Kwiatkowski, Cordelia Langford, John Sillitoe on behalf of the Wellcome Sanger Institute COVID-19 Surveillance Team
EPI_ISL_891281	Lighthouse Lab in Glasgow	Wellcome Sanger Institute for the COVID-19 Genomics UK (COG-UK) Consortium	Harper VanSteenhouse, Yumi Kasai, David Gray, Carol Clugston, Anna Dominiczak and Alex Alderton, Roberto Amato, Sonia Goncalves, Ewan Harrison, David K. Jackson, Ian Johnston, Dominic Kwiatkowski, Cordelia Langford, John Sillitoe on behalf of the Wellcome Sanger Institute COVID-19 Surveillance Team
EPI_ISL_891282	Lighthouse Lab in Cambridge	Wellcome Sanger Institute for the COVID-19 Genomics UK (COG-UK) Consortium	Rob Howes, The Lighthouse Lab in Cambridge and Alex Alderton, Roberto Amato, Sonia Goncalves, Ewan Harrison, David K. Jackson, Ian Johnston, Dominic Kwiatkowski, Cordelia Langford, John Sillitoe on behalf of the Wellcome Sanger Institute COVID-19 Surveillance Team
EPI_ISL_891283	Lighthouse Lab in Glasgow	Wellcome Sanger Institute for the COVID-19 Genomics UK (COG-UK) Consortium	Harper VanSteenhouse, Yumi Kasai, David Gray, Carol Clugston, Anna Dominiczak and Alex Alderton, Roberto Amato, Sonia Goncalves, Ewan Harrison, David K. Jackson, Ian Johnston, Dominic Kwiatkowski, Cordelia Langford, John Sillitoe on behalf of the Wellcome Sanger Institute COVID-19 Surveillance Team
EPI_ISL_891284	Lighthouse Lab in Cambridge	Wellcome Sanger Institute for the COVID-19 Genomics UK (COG-UK) Consortium	Rob Howes, The Lighthouse Lab in Cambridge and Alex Alderton, Roberto Amato, Sonia Goncalves, Ewan Harrison, David K. Jackson, Ian Johnston, Dominic Kwiatkowski, Cordelia Langford, John Sillitoe on behalf of the Wellcome Sanger Institute COVID-19 Surveillance Team
EPI_ISL_891285	Lighthouse Lab in Alderley Park	Wellcome Sanger Institute for the COVID-19 Genomics UK (COG-UK) Consortium	Jacquelyn Wynn, Mairead Hyland, The Lighthouse Lab in Alderley Park and Alex Alderton, Roberto Amato, Sonia Goncalves, Ewan Harrison, David K. Jackson, Ian Johnston, Dominic Kwiatkowski, Cordelia Langford, John Sillitoe on behalf of the Wellcome Sanger Institute COVID-19 Surveillance Team
EPI_ISL_891286, EPI_ISL_891288	Lighthouse Lab in Glasgow	Wellcome Sanger Institute for the COVID-19 Genomics UK (COG-UK) Consortium	Harper VanSteenhouse, Yumi Kasai, David Gray, Carol Clugston, Anna Dominiczak and Alex Alderton, Roberto Amato, Sonia Goncalves, Ewan Harrison, David K. Jackson, Ian Johnston, Dominic Kwiatkowski, Cordelia Langford, John Sillitoe on behalf of the Wellcome Sanger Institute COVID-19 Surveillance Team
EPI_ISL_891289, EPI_ISL_891290, EPI_ISL_891292, EPI_ISL_891293, EPI_ISL_891294	Lighthouse Lab in Cambridge	Wellcome Sanger Institute for the COVID-19 Genomics UK (COG-UK) Consortium	Rob Howes, The Lighthouse Lab in Cambridge and Alex Alderton, Roberto Amato, Sonia Goncalves, Ewan Harrison, David K. Jackson, Ian Johnston, Dominic Kwiatkowski, Cordelia Langford, John Sillitoe on behalf of the Wellcome Sanger Institute COVID-19 Surveillance Team
EPI_ISL_891295	Lighthouse Lab in Alderley Park	Wellcome Sanger Institute for the COVID-19 Genomics UK (COG-UK) Consortium	Jacquelyn Wynn, Mairead Hyland, The Lighthouse Lab in Alderley Park and Alex Alderton, Roberto Amato, Sonia Goncalves, Ewan Harrison, David K. Jackson, Ian Johnston, Dominic Kwiatkowski, Cordelia Langford, John Sillitoe on behalf of the Wellcome Sanger Institute COVID-19 Surveillance Team
EPI_ISL_891296	Lighthouse Lab in Cambridge	Wellcome Sanger Institute for the COVID-19 Genomics UK (COG-UK) Consortium	Rob Howes, The Lighthouse Lab in Cambridge and Alex Alderton, Roberto Amato, Sonia Goncalves, Ewan Harrison, David K. Jackson, Ian Johnston, Dominic Kwiatkowski, Cordelia Langford, John Sillitoe on behalf of the Wellcome Sanger Institute COVID-19 Surveillance Team
EPI_ISL_891297, EPI_ISL_891298	Lighthouse Lab in Glasgow	Wellcome Sanger Institute for the COVID-19 Genomics UK (COG-UK) Consortium	Harper VanSteenhouse, Yumi Kasai, David Gray, Carol Clugston, Anna Dominiczak and Alex Alderton, Roberto Amato, Sonia Goncalves, Ewan Harrison, David K. Jackson, Ian Johnston, Dominic Kwiatkowski, Cordelia Langford, John Sillitoe on behalf of the Wellcome Sanger Institute COVID-19 Surveillance Team
EPI_ISL_891299	Lighthouse Lab in Alderley Park	Wellcome Sanger Institute for the COVID-19 Genomics UK (COG-UK) Consortium	Jacquelyn Wynn, Mairead Hyland, The Lighthouse Lab in Alderley Park and Alex Alderton, Roberto Amato, Sonia Goncalves, Ewan Harrison, David K. Jackson, Ian Johnston, Dominic Kwiatkowski, Cordelia Langford, John Sillitoe on behalf of the Wellcome Sanger Institute COVID-19 Surveillance Team
EPI_ISL_891300, EPI_ISL_891301	Lighthouse Lab in Cambridge	Wellcome Sanger Institute for the COVID-19 Genomics UK (COG-UK) Consortium	Rob Howes, The Lighthouse Lab in Cambridge and Alex Alderton, Roberto Amato, Sonia Goncalves, Ewan Harrison, David K. Jackson, Ian Johnston, Dominic Kwiatkowski, Cordelia Langford, John Sillitoe on behalf of the Wellcome Sanger Institute COVID-19 Surveillance Team
EPI_ISL_891304	Lighthouse Lab in Glasgow	Wellcome Sanger Institute for the COVID-19 Genomics UK (COG-UK) Consortium	Harper VanSteenhouse, Yumi Kasai, David Gray, Carol Clugston, Anna Dominiczak and Alex Alderton, Roberto Amato, Sonia Goncalves, Ewan Harrison, David K. Jackson, Ian Johnston, Dominic Kwiatkowski, Cordelia Langford, John Sillitoe on behalf of the Wellcome Sanger Institute COVID-19 Surveillance Team
EPI_ISL_891305	Lighthouse Lab in Alderley Park	Wellcome Sanger Institute for the COVID-19 Genomics UK (COG-UK) Consortium	Jacquelyn Wynn, Mairead Hyland, The Lighthouse Lab in Alderley Park and Alex Alderton, Roberto Amato, Sonia Goncalves, Ewan Harrison, David K. Jackson, Ian Johnston, Dominic Kwiatkowski, Cordelia Langford, John Sillitoe on behalf of the Wellcome Sanger Institute COVID-19 Surveillance Team
EPI_ISL_891306, EPI_ISL_891307, EPI_ISL_891308, EPI_ISL_891309, EPI_ISL_891310	Lighthouse Lab in Cambridge	Wellcome Sanger Institute for the COVID-19 Genomics UK (COG-UK) Consortium	Rob Howes, The Lighthouse Lab in Cambridge and Alex Alderton, Roberto Amato, Sonia Goncalves, Ewan Harrison, David K. Jackson, Ian Johnston, Dominic Kwiatkowski, Cordelia Langford, John Sillitoe on behalf of the Wellcome Sanger Institute COVID-19 Surveillance Team
EPI_ISL_891311, EPI_ISL_891312, EPI_ISL_891313	Lighthouse Lab in Alderley Park	Wellcome Sanger Institute for the COVID-19 Genomics UK (COG-UK) Consortium	Jacquelyn Wynn, Mairead Hyland, The Lighthouse Lab in Alderley Park and Alex Alderton, Roberto Amato, Sonia Goncalves, Ewan Harrison, David K. Jackson, Ian Johnston, Dominic Kwiatkowski, Cordelia Langford, John Sillitoe on behalf of the Wellcome Sanger Institute COVID-19 Surveillance Team
EPI_ISL_891314, EPI_ISL_891316	Lighthouse Lab in Cambridge	Wellcome Sanger Institute for the COVID-19 Genomics UK (COG-UK) Consortium	Rob Howes, The Lighthouse Lab in Cambridge and Alex Alderton, Roberto Amato, Sonia Goncalves, Ewan Harrison, David K. Jackson, Ian Johnston, Dominic Kwiatkowski, Cordelia Langford, John Sillitoe on behalf of the Wellcome Sanger Institute COVID-19 Surveillance Team
EPI_ISL_891317, EPI_ISL_891318, EPI_ISL_891319	Lighthouse Lab in Alderley Park	Wellcome Sanger Institute for the COVID-19 Genomics UK (COG-UK) Consortium	Jacquelyn Wynn, Mairead Hyland, The Lighthouse Lab in Alderley Park and Alex Alderton, Roberto Amato, Sonia Goncalves, Ewan Harrison, David K. Jackson, Ian Johnston, Dominic Kwiatkowski, Cordelia Langford, John Sillitoe on behalf of the Wellcome Sanger Institute COVID-19 Surveillance Team
EPI_ISL_891320	Lighthouse Lab in Glasgow	Wellcome Sanger Institute for the COVID-19 Genomics UK (COG-UK) Consortium	Harper VanSteenhouse, Yumi Kasai, David Gray, Carol Clugston, Anna Dominiczak and Alex Alderton, Roberto Amato, Sonia Goncalves, Ewan Harrison, David K. Jackson, Ian Johnston, Dominic Kwiatkowski, Cordelia Langford, John Sillitoe on behalf of the Wellcome Sanger Institute COVID-19 Surveillance Team
EPI_ISL_891322	Lighthouse Lab in Cambridge	Wellcome Sanger Institute for the COVID-19 Genomics UK (COG-UK) Consortium	Rob Howes, The Lighthouse Lab in Cambridge and Alex Alderton, Roberto Amato, Sonia Goncalves, Ewan Harrison, David K. Jackson, Ian Johnston, Dominic Kwiatkowski, Cordelia Langford, John Sillitoe on behalf of the Wellcome Sanger Institute COVID-19 Surveillance Team
EPI_ISL_891323, EPI_ISL_891324	Lighthouse Lab in Alderley Park	Wellcome Sanger Institute for the COVID-19 Genomics UK (COG-UK) Consortium	Jacquelyn Wynn, Mairead Hyland, The Lighthouse Lab in Alderley Park and Alex Alderton, Roberto Amato, Sonia Goncalves, Ewan Harrison, David K. Jackson, Ian Johnston, Dominic Kwiatkowski, Cordelia Langford, John Sillitoe on behalf of the Wellcome Sanger Institute COVID-19 Surveillance Team
EPI_ISL_891325	Lighthouse Lab in Cambridge	Wellcome Sanger Institute for the COVID-19 Genomics UK (COG-UK) Consortium	Rob Howes, The Lighthouse Lab in Cambridge and Alex Alderton, Roberto Amato, Sonia Goncalves, Ewan Harrison, David K. Jackson, Ian Johnston, Dominic Kwiatkowski, Cordelia Langford, John Sillitoe on behalf of the Wellcome Sanger Institute COVID-19 Surveillance Team
EPI_ISL_891326	Lighthouse Lab in Alderley Park	Wellcome Sanger Institute for the COVID-19 Genomics UK (COG-UK) Consortium	Jacquelyn Wynn, Mairead Hyland, The Lighthouse Lab in Alderley Park and Alex Alderton, Roberto Amato, Sonia Goncalves, Ewan Harrison, David K. Jackson, Ian Johnston, Dominic Kwiatkowski, Cordelia Langford, John Sillitoe on behalf of the Wellcome Sanger Institute COVID-19 Surveillance Team
EPI_ISL_891327	Lighthouse Lab in Glasgow	Wellcome Sanger Institute for the COVID-19 Genomics UK (COG-UK) Consortium	Harper VanSteenhouse, Yumi Kasai, David Gray, Carol Clugston, Anna Dominiczak and Alex Alderton, Roberto Amato, Sonia Goncalves, Ewan Harrison, David K. Jackson, Ian Johnston, Dominic Kwiatkowski, Cordelia Langford, John Sillitoe on behalf of the Wellcome Sanger Institute COVID-19 Surveillance Team
EPI_ISL_891328, EPI_ISL_891329	Lighthouse Lab in Cambridge	Wellcome Sanger Institute for the COVID-19 Genomics UK (COG-UK) Consortium	Rob Howes, The Lighthouse Lab in Cambridge and Alex Alderton, Roberto Amato, Sonia Goncalves, Ewan Harrison, David K. Jackson, Ian Johnston, Dominic Kwiatkowski, Cordelia Langford, John Sillitoe on behalf of the Wellcome Sanger Institute COVID-19 Surveillance Team
EPI_ISL_891330	Lighthouse Lab in Glasgow	Wellcome Sanger Institute for the COVID-19 Genomics UK (COG-UK) Consortium	Harper VanSteenhouse, Yumi Kasai, David Gray, Carol Clugston, Anna Dominiczak and Alex Alderton, Roberto Amato, Sonia Goncalves, Ewan Harrison, David K. Jackson, Ian Johnston, Dominic Kwiatkowski, Cordelia Langford, John Sillitoe on behalf of the Wellcome Sanger Institute COVID-19 Surveillance Team
EPI_ISL_891331, EPI_ISL_891332, EPI_ISL_891333	Lighthouse Lab in Cambridge	Wellcome Sanger Institute for the COVID-19 Genomics UK (COG-UK) Consortium	Rob Howes, The Lighthouse Lab in Cambridge and Alex Alderton, Roberto Amato, Sonia Goncalves, Ewan Harrison, David K. Jackson, Ian Johnston, Dominic Kwiatkowski, Cordelia Langford, John Sillitoe on behalf of the Wellcome Sanger Institute COVID-19 Surveillance Team
EPI_ISL_891334	Lighthouse Lab in Alderley Park	Wellcome Sanger Institute for the COVID-19 Genomics UK (COG-UK) Consortium	Jacquelyn Wynn, Mairead Hyland, The Lighthouse Lab in Alderley Park and Alex Alderton, Roberto Amato, Sonia Goncalves, Ewan Harrison, David K. Jackson, Ian Johnston, Dominic Kwiatkowski, Cordelia Langford, John Sillitoe on behalf of the Wellcome Sanger Institute COVID-19 Surveillance Team
EPI_ISL_891335	Lighthouse Lab in Glasgow	Wellcome Sanger Institute for the COVID-19 Genomics UK (COG-UK) Consortium	Harper VanSteenhouse, Yumi Kasai, David Gray, Carol Clugston, Anna Dominiczak and Alex Alderton, Roberto Amato, Sonia Goncalves, Ewan Harrison, David K. Jackson, Ian Johnston, Dominic Kwiatkowski, Cordelia Langford, John Sillitoe on behalf of the Wellcome Sanger Institute COVID-19 Surveillance Team
EPI_ISL_891337	Lighthouse Lab in Alderley Park	Wellcome Sanger Institute for the COVID-19 Genomics UK (COG-UK) Consortium	Jacquelyn Wynn, Mairead Hyland, The Lighthouse Lab in Alderley Park and Alex Alderton, Roberto Amato, Sonia Goncalves, Ewan Harrison, David K. Jackson, Ian Johnston, Dominic Kwiatkowski, Cordelia Langford, John Sillitoe on behalf of the Wellcome Sanger Institute COVID-19 Surveillance Team

EPI_ISL_891338	Lighthouse Lab in Glasgow	Wellcome Sanger Institute for the COVID-19 Genomics UK (COG-UK) Consortium	Harper VanSteenhouse, Yumi Kasai, David Gray, Carol Clugston, Anna Dominiczak and Alex Alderton, Roberto Amato, Sonia Goncalves, Ewan Harrison, David K. Jackson, Ian Johnston, Dominic Kwiatkowski, Cordelia Langford, John Sillitoe on behalf of the Wellcome Sanger Institute COVID-19 Surveillance Team
EPI_ISL_891339	Lighthouse Lab in Cambridge	Wellcome Sanger Institute for the COVID-19 Genomics UK (COG-UK) Consortium	Rob Howes, The Lighthouse Lab in Cambridge and Alex Alderton, Roberto Amato, Sonia Goncalves, Ewan Harrison, David K. Jackson, Ian Johnston, Dominic Kwiatkowski, Cordelia Langford, John Sillitoe on behalf of the Wellcome Sanger Institute COVID-19 Surveillance Team
EPI_ISL_891340, EPI_ISL_891342, EPI_ISL_891344	Lighthouse Lab in Alderley Park	Wellcome Sanger Institute for the COVID-19 Genomics UK (COG-UK) Consortium	Jacquelyn Wynn, Mairead Hyland, The Lighthouse Lab in Alderley Park and Alex Alderton, Roberto Amato, Sonia Goncalves, Ewan Harrison, David K. Jackson, Ian Johnston, Dominic Kwiatkowski, Cordelia Langford, John Sillitoe on behalf of the Wellcome Sanger Institute COVID-19 Surveillance Team
EPI_ISL_891345	Lighthouse Lab in Glasgow	Wellcome Sanger Institute for the COVID-19 Genomics UK (COG-UK) Consortium	Harper VanSteenhouse, Yumi Kasai, David Gray, Carol Clugston, Anna Dominiczak and Alex Alderton, Roberto Amato, Sonia Goncalves, Ewan Harrison, David K. Jackson, Ian Johnston, Dominic Kwiatkowski, Cordelia Langford, John Sillitoe on behalf of the Wellcome Sanger Institute COVID-19 Surveillance Team
EPI_ISL_891346, EPI_ISL_891347	Lighthouse Lab in Cambridge	Wellcome Sanger Institute for the COVID-19 Genomics UK (COG-UK) Consortium	Rob Howes, The Lighthouse Lab in Cambridge and Alex Alderton, Roberto Amato, Sonia Goncalves, Ewan Harrison, David K. Jackson, Ian Johnston, Dominic Kwiatkowski, Cordelia Langford, John Sillitoe on behalf of the Wellcome Sanger Institute COVID-19 Surveillance Team
EPI_ISL_891349	Lighthouse Lab in Alderley Park	Wellcome Sanger Institute for the COVID-19 Genomics UK (COG-UK) Consortium	Jacquelyn Wynn, Mairead Hyland, The Lighthouse Lab in Alderley Park and Alex Alderton, Roberto Amato, Sonia Goncalves, Ewan Harrison, David K. Jackson, Ian Johnston, Dominic Kwiatkowski, Cordelia Langford, John Sillitoe on behalf of the Wellcome Sanger Institute COVID-19 Surveillance Team
EPI_ISL_891350, EPI_ISL_891351	Lighthouse Lab in Cambridge	Wellcome Sanger Institute for the COVID-19 Genomics UK (COG-UK) Consortium	Rob Howes, The Lighthouse Lab in Cambridge and Alex Alderton, Roberto Amato, Sonia Goncalves, Ewan Harrison, David K. Jackson, Ian Johnston, Dominic Kwiatkowski, Cordelia Langford, John Sillitoe on behalf of the Wellcome Sanger Institute COVID-19 Surveillance Team
EPI_ISL_891352	Lighthouse Lab in Alderley Park	Wellcome Sanger Institute for the COVID-19 Genomics UK (COG-UK) Consortium	Jacquelyn Wynn, Mairead Hyland, The Lighthouse Lab in Alderley Park and Alex Alderton, Roberto Amato, Sonia Goncalves, Ewan Harrison, David K. Jackson, Ian Johnston, Dominic Kwiatkowski, Cordelia Langford, John Sillitoe on behalf of the Wellcome Sanger Institute COVID-19 Surveillance Team
EPI_ISL_891354, EPI_ISL_891355, EPI_ISL_891356	Lighthouse Lab in Cambridge	Wellcome Sanger Institute for the COVID-19 Genomics UK (COG-UK) Consortium	Rob Howes, The Lighthouse Lab in Cambridge and Alex Alderton, Roberto Amato, Sonia Goncalves, Ewan Harrison, David K. Jackson, Ian Johnston, Dominic Kwiatkowski, Cordelia Langford, John Sillitoe on behalf of the Wellcome Sanger Institute COVID-19 Surveillance Team
EPI_ISL_891357	Lighthouse Lab in Alderley Park	Wellcome Sanger Institute for the COVID-19 Genomics UK (COG-UK) Consortium	Jacquelyn Wynn, Mairead Hyland, The Lighthouse Lab in Alderley Park and Alex Alderton, Roberto Amato, Sonia Goncalves, Ewan Harrison, David K. Jackson, Ian Johnston, Dominic Kwiatkowski, Cordelia Langford, John Sillitoe on behalf of the Wellcome Sanger Institute COVID-19 Surveillance Team
EPI_ISL_891359	Lighthouse Lab in Cambridge	Wellcome Sanger Institute for the COVID-19 Genomics UK (COG-UK) Consortium	Rob Howes, The Lighthouse Lab in Cambridge and Alex Alderton, Roberto Amato, Sonia Goncalves, Ewan Harrison, David K. Jackson, Ian Johnston, Dominic Kwiatkowski, Cordelia Langford, John Sillitoe on behalf of the Wellcome Sanger Institute COVID-19 Surveillance Team
EPI_ISL_891360	Lighthouse Lab in Alderley Park	Wellcome Sanger Institute for the COVID-19 Genomics UK (COG-UK) Consortium	Jacquelyn Wynn, Mairead Hyland, The Lighthouse Lab in Alderley Park and Alex Alderton, Roberto Amato, Sonia Goncalves, Ewan Harrison, David K. Jackson, Ian Johnston, Dominic Kwiatkowski, Cordelia Langford, John Sillitoe on behalf of the Wellcome Sanger Institute COVID-19 Surveillance Team
EPI_ISL_891361	Lighthouse Lab in Glasgow	Wellcome Sanger Institute for the COVID-19 Genomics UK (COG-UK) Consortium	Harper VanSteenhouse, Yumi Kasai, David Gray, Carol Clugston, Anna Dominiczak and Alex Alderton, Roberto Amato, Sonia Goncalves, Ewan Harrison, David K. Jackson, Ian Johnston, Dominic Kwiatkowski, Cordelia Langford, John Sillitoe on behalf of the Wellcome Sanger Institute COVID-19 Surveillance Team
EPI_ISL_891363	Lighthouse Lab in Cambridge	Wellcome Sanger Institute for the COVID-19 Genomics UK (COG-UK) Consortium	Rob Howes, The Lighthouse Lab in Cambridge and Alex Alderton, Roberto Amato, Sonia Goncalves, Ewan Harrison, David K. Jackson, Ian Johnston, Dominic Kwiatkowski, Cordelia Langford, John Sillitoe on behalf of the Wellcome Sanger Institute COVID-19 Surveillance Team
EPI_ISL_891364	Lighthouse Lab in Alderley Park	Wellcome Sanger Institute for the COVID-19 Genomics UK (COG-UK) Consortium	Jacquelyn Wynn, Mairead Hyland, The Lighthouse Lab in Alderley Park and Alex Alderton, Roberto Amato, Sonia Goncalves, Ewan Harrison, David K. Jackson, Ian Johnston, Dominic Kwiatkowski, Cordelia Langford, John Sillitoe on behalf of the Wellcome Sanger Institute COVID-19 Surveillance Team
EPI_ISL_891366, EPI_ISL_891367, EPI_ISL_891369, EPI_ISL_891370	Lighthouse Lab in Cambridge	Wellcome Sanger Institute for the COVID-19 Genomics UK (COG-UK) Consortium	Rob Howes, The Lighthouse Lab in Cambridge and Alex Alderton, Roberto Amato, Sonia Goncalves, Ewan Harrison, David K. Jackson, Ian Johnston, Dominic Kwiatkowski, Cordelia Langford, John Sillitoe on behalf of the Wellcome Sanger Institute COVID-19 Surveillance Team
EPI_ISL_891371	Lighthouse Lab in Alderley Park	Wellcome Sanger Institute for the COVID-19 Genomics UK (COG-UK) Consortium	Jacquelyn Wynn, Mairead Hyland, The Lighthouse Lab in Alderley Park and Alex Alderton, Roberto Amato, Sonia Goncalves, Ewan Harrison, David K. Jackson, Ian Johnston, Dominic Kwiatkowski, Cordelia Langford, John Sillitoe on behalf of the Wellcome Sanger Institute COVID-19 Surveillance Team
EPI_ISL_891372	Lighthouse Lab in Glasgow	Wellcome Sanger Institute for the COVID-19 Genomics UK (COG-UK) Consortium	Harper VanSteenhouse, Yumi Kasai, David Gray, Carol Clugston, Anna Dominiczak and Alex Alderton, Roberto Amato, Sonia Goncalves, Ewan Harrison, David K. Jackson, Ian Johnston, Dominic Kwiatkowski, Cordelia Langford, John Sillitoe on behalf of the Wellcome Sanger Institute COVID-19 Surveillance Team
EPI_ISL_891373	Lighthouse Lab in Cambridge	Wellcome Sanger Institute for the COVID-19 Genomics UK (COG-UK) Consortium	Rob Howes, The Lighthouse Lab in Cambridge and Alex Alderton, Roberto Amato, Sonia Goncalves, Ewan Harrison, David K. Jackson, Ian Johnston, Dominic Kwiatkowski, Cordelia Langford, John Sillitoe on behalf of the Wellcome Sanger Institute COVID-19 Surveillance Team
EPI_ISL_891374, EPI_ISL_891376, EPI_ISL_891377, EPI_ISL_891378	Lighthouse Lab in Alderley Park	Wellcome Sanger Institute for the COVID-19 Genomics UK (COG-UK) Consortium	Jacquelyn Wynn, Mairead Hyland, The Lighthouse Lab in Alderley Park and Alex Alderton, Roberto Amato, Sonia Goncalves, Ewan Harrison, David K. Jackson, Ian Johnston, Dominic Kwiatkowski, Cordelia Langford, John Sillitoe on behalf of the Wellcome Sanger Institute COVID-19 Surveillance Team
EPI_ISL_891379	Lighthouse Lab in Cambridge	Wellcome Sanger Institute for the COVID-19 Genomics UK (COG-UK) Consortium	Rob Howes, The Lighthouse Lab in Cambridge and Alex Alderton, Roberto Amato, Sonia Goncalves, Ewan Harrison, David K. Jackson, Ian Johnston, Dominic Kwiatkowski, Cordelia Langford, John Sillitoe on behalf of the Wellcome Sanger Institute COVID-19 Surveillance Team
EPI_ISL_891380	Lighthouse Lab in Glasgow	Wellcome Sanger Institute for the COVID-19 Genomics UK (COG-UK) Consortium	Harper VanSteenhouse, Yumi Kasai, David Gray, Carol Clugston, Anna Dominiczak and Alex Alderton, Roberto Amato, Sonia Goncalves, Ewan Harrison, David K. Jackson, Ian Johnston, Dominic Kwiatkowski, Cordelia Langford, John Sillitoe on behalf of the Wellcome Sanger Institute COVID-19 Surveillance Team
EPI_ISL_891383, EPI_ISL_891384, EPI_ISL_891385, EPI_ISL_891386	Lighthouse Lab in Alderley Park	Wellcome Sanger Institute for the COVID-19 Genomics UK (COG-UK) Consortium	Jacquelyn Wynn, Mairead Hyland, The Lighthouse Lab in Alderley Park and Alex Alderton, Roberto Amato, Sonia Goncalves, Ewan Harrison, David K. Jackson, Ian Johnston, Dominic Kwiatkowski, Cordelia Langford, John Sillitoe on behalf of the Wellcome Sanger Institute COVID-19 Surveillance Team
EPI_ISL_891387, EPI_ISL_891388, EPI_ISL_891389	Lighthouse Lab in Cambridge	Wellcome Sanger Institute for the COVID-19 Genomics UK (COG-UK) Consortium	Rob Howes, The Lighthouse Lab in Cambridge and Alex Alderton, Roberto Amato, Sonia Goncalves, Ewan Harrison, David K. Jackson, Ian Johnston, Dominic Kwiatkowski, Cordelia Langford, John Sillitoe on behalf of the Wellcome Sanger Institute COVID-19 Surveillance Team
EPI_ISL_891391	Lighthouse Lab in Glasgow	Wellcome Sanger Institute for the COVID-19 Genomics UK (COG-UK) Consortium	Harper VanSteenhouse, Yumi Kasai, David Gray, Carol Clugston, Anna Dominiczak and Alex Alderton, Roberto Amato, Sonia Goncalves, Ewan Harrison, David K. Jackson, Ian Johnston, Dominic Kwiatkowski, Cordelia Langford, John Sillitoe on behalf of the Wellcome Sanger Institute COVID-19 Surveillance Team
EPI_ISL_891392	Lighthouse Lab in Alderley Park	Wellcome Sanger Institute for the COVID-19 Genomics UK (COG-UK) Consortium	Jacquelyn Wynn, Mairead Hyland, The Lighthouse Lab in Alderley Park and Alex Alderton, Roberto Amato, Sonia Goncalves, Ewan Harrison, David K. Jackson, Ian Johnston, Dominic Kwiatkowski, Cordelia Langford, John Sillitoe on behalf of the Wellcome Sanger Institute COVID-19 Surveillance Team
EPI_ISL_891393	Lighthouse Lab in Glasgow	Wellcome Sanger Institute for the COVID-19 Genomics UK (COG-UK) Consortium	Harper VanSteenhouse, Yumi Kasai, David Gray, Carol Clugston, Anna Dominiczak and Alex Alderton, Roberto Amato, Sonia Goncalves, Ewan Harrison, David K. Jackson, Ian Johnston, Dominic Kwiatkowski, Cordelia Langford, John Sillitoe on behalf of the Wellcome Sanger Institute COVID-19 Surveillance Team
EPI_ISL_891394, EPI_ISL_891395	Lighthouse Lab in Cambridge	Wellcome Sanger Institute for the COVID-19 Genomics UK (COG-UK) Consortium	Rob Howes, The Lighthouse Lab in Cambridge and Alex Alderton, Roberto Amato, Sonia Goncalves, Ewan Harrison, David K. Jackson, Ian Johnston, Dominic Kwiatkowski, Cordelia Langford, John Sillitoe on behalf of the Wellcome Sanger Institute COVID-19 Surveillance Team
EPI_ISL_891396	Lighthouse Lab in Glasgow	Wellcome Sanger Institute for the COVID-19 Genomics UK (COG-UK) Consortium	Harper VanSteenhouse, Yumi Kasai, David Gray, Carol Clugston, Anna Dominiczak and Alex Alderton, Roberto Amato, Sonia Goncalves, Ewan Harrison, David K. Jackson, Ian Johnston, Dominic Kwiatkowski, Cordelia Langford, John Sillitoe on behalf of the Wellcome Sanger Institute COVID-19 Surveillance Team
EPI_ISL_891397, EPI_ISL_891398, EPI_ISL_891399	Lighthouse Lab in Cambridge	Wellcome Sanger Institute for the COVID-19 Genomics UK (COG-UK) Consortium	Rob Howes, The Lighthouse Lab in Cambridge and Alex Alderton, Roberto Amato, Sonia Goncalves, Ewan Harrison, David K. Jackson, Ian Johnston, Dominic Kwiatkowski, Cordelia Langford, John Sillitoe on behalf of the Wellcome Sanger Institute COVID-19 Surveillance Team
EPI_ISL_891400, EPI_ISL_891401, EPI_ISL_891403, EPI_ISL_891404	Lighthouse Lab in Alderley Park	Wellcome Sanger Institute for the COVID-19 Genomics UK (COG-UK) Consortium	Jacquelyn Wynn, Mairead Hyland, The Lighthouse Lab in Alderley Park and Alex Alderton, Roberto Amato, Sonia Goncalves, Ewan Harrison, David K. Jackson, Ian Johnston, Dominic Kwiatkowski, Cordelia Langford, John Sillitoe on behalf of the Wellcome Sanger Institute COVID-19 Surveillance Team
EPI_ISL_891406, EPI_ISL_891409	Lighthouse Lab in Cambridge	Wellcome Sanger Institute for the COVID-19 Genomics UK (COG-UK) Consortium	Rob Howes, The Lighthouse Lab in Cambridge and Alex Alderton, Roberto Amato, Sonia Goncalves, Ewan Harrison, David K. Jackson, Ian Johnston, Dominic Kwiatkowski, Cordelia Langford, John Sillitoe on behalf of the Wellcome Sanger Institute COVID-19 Surveillance Team

EPI_ISL_891410	Lighthouse Lab in Glasgow	Wellcome Sanger Institute for the COVID-19 Genomics UK (COG-UK) Consortium	Harper VanSteenhouse, Yumi Kasai, David Gray, Carol Clugston, Anna Dominiczak and Alex Alderton, Roberto Amato, Sonia Goncalves, Ewan Harrison, David K. Jackson, Ian Johnston, Dominic Kwiatkowski, Cordelia Langford, John Sillitoe on behalf of the Wellcome Sanger Institute COVID-19 Surveillance Team
EPI_ISL_891411	Lighthouse Lab in Alderley Park	Wellcome Sanger Institute for the COVID-19 Genomics UK (COG-UK) Consortium	Jacquelyn Wynn, Mairead Hyland, The Lighthouse Lab in Alderley Park and Alex Alderton, Roberto Amato, Sonia Goncalves, Ewan Harrison, David K. Jackson, Ian Johnston, Dominic Kwiatkowski, Cordelia Langford, John Sillitoe on behalf of the Wellcome Sanger Institute COVID-19 Surveillance Team
EPI_ISL_891412, EPI_ISL_891413	Lighthouse Lab in Cambridge	Wellcome Sanger Institute for the COVID-19 Genomics UK (COG-UK) Consortium	Rob Howes, The Lighthouse Lab in Cambridge and Alex Alderton, Roberto Amato, Sonia Goncalves, Ewan Harrison, David K. Jackson, Ian Johnston, Dominic Kwiatkowski, Cordelia Langford, John Sillitoe on behalf of the Wellcome Sanger Institute COVID-19 Surveillance Team
EPI_ISL_891415	Lighthouse Lab in Glasgow	Wellcome Sanger Institute for the COVID-19 Genomics UK (COG-UK) Consortium	Harper VanSteenhouse, Yumi Kasai, David Gray, Carol Clugston, Anna Dominiczak and Alex Alderton, Roberto Amato, Sonia Goncalves, Ewan Harrison, David K. Jackson, Ian Johnston, Dominic Kwiatkowski, Cordelia Langford, John Sillitoe on behalf of the Wellcome Sanger Institute COVID-19 Surveillance Team
EPI_ISL_891416, EPI_ISL_891417, EPI_ISL_891419	Lighthouse Lab in Alderley Park	Wellcome Sanger Institute for the COVID-19 Genomics UK (COG-UK) Consortium	Jacquelyn Wynn, Mairead Hyland, The Lighthouse Lab in Alderley Park and Alex Alderton, Roberto Amato, Sonia Goncalves, Ewan Harrison, David K. Jackson, Ian Johnston, Dominic Kwiatkowski, Cordelia Langford, John Sillitoe on behalf of the Wellcome Sanger Institute COVID-19 Surveillance Team
EPI_ISL_891420	Lighthouse Lab in Glasgow	Wellcome Sanger Institute for the COVID-19 Genomics UK (COG-UK) Consortium	Harper VanSteenhouse, Yumi Kasai, David Gray, Carol Clugston, Anna Dominiczak and Alex Alderton, Roberto Amato, Sonia Goncalves, Ewan Harrison, David K. Jackson, Ian Johnston, Dominic Kwiatkowski, Cordelia Langford, John Sillitoe on behalf of the Wellcome Sanger Institute COVID-19 Surveillance Team
EPI_ISL_891421	Lighthouse Lab in Alderley Park	Wellcome Sanger Institute for the COVID-19 Genomics UK (COG-UK) Consortium	Jacquelyn Wynn, Mairead Hyland, The Lighthouse Lab in Alderley Park and Alex Alderton, Roberto Amato, Sonia Goncalves, Ewan Harrison, David K. Jackson, Ian Johnston, Dominic Kwiatkowski, Cordelia Langford, John Sillitoe on behalf of the Wellcome Sanger Institute COVID-19 Surveillance Team
EPI_ISL_891422	Lighthouse Lab in Cambridge	Wellcome Sanger Institute for the COVID-19 Genomics UK (COG-UK) Consortium	Rob Howes, The Lighthouse Lab in Cambridge and Alex Alderton, Roberto Amato, Sonia Goncalves, Ewan Harrison, David K. Jackson, Ian Johnston, Dominic Kwiatkowski, Cordelia Langford, John Sillitoe on behalf of the Wellcome Sanger Institute COVID-19 Surveillance Team
EPI_ISL_891423	Lighthouse Lab in Alderley Park	Wellcome Sanger Institute for the COVID-19 Genomics UK (COG-UK) Consortium	Jacquelyn Wynn, Mairead Hyland, The Lighthouse Lab in Alderley Park and Alex Alderton, Roberto Amato, Sonia Goncalves, Ewan Harrison, David K. Jackson, Ian Johnston, Dominic Kwiatkowski, Cordelia Langford, John Sillitoe on behalf of the Wellcome Sanger Institute COVID-19 Surveillance Team
EPI_ISL_891426	Lighthouse Lab in Glasgow	Wellcome Sanger Institute for the COVID-19 Genomics UK (COG-UK) Consortium	Harper VanSteenhouse, Yumi Kasai, David Gray, Carol Clugston, Anna Dominiczak and Alex Alderton, Roberto Amato, Sonia Goncalves, Ewan Harrison, David K. Jackson, Ian Johnston, Dominic Kwiatkowski, Cordelia Langford, John Sillitoe on behalf of the Wellcome Sanger Institute COVID-19 Surveillance Team
EPI_ISL_891427, EPI_ISL_891431, EPI_ISL_891433, EPI_ISL_891434	Lighthouse Lab in Cambridge	Wellcome Sanger Institute for the COVID-19 Genomics UK (COG-UK) Consortium	Rob Howes, The Lighthouse Lab in Cambridge and Alex Alderton, Roberto Amato, Sonia Goncalves, Ewan Harrison, David K. Jackson, Ian Johnston, Dominic Kwiatkowski, Cordelia Langford, John Sillitoe on behalf of the Wellcome Sanger Institute COVID-19 Surveillance Team
EPI_ISL_891435	Lighthouse Lab in Glasgow	Wellcome Sanger Institute for the COVID-19 Genomics UK (COG-UK) Consortium	Harper VanSteenhouse, Yumi Kasai, David Gray, Carol Clugston, Anna Dominiczak and Alex Alderton, Roberto Amato, Sonia Goncalves, Ewan Harrison, David K. Jackson, Ian Johnston, Dominic Kwiatkowski, Cordelia Langford, John Sillitoe on behalf of the Wellcome Sanger Institute COVID-19 Surveillance Team
EPI_ISL_891436, EPI_ISL_891437	Lighthouse Lab in Cambridge	Wellcome Sanger Institute for the COVID-19 Genomics UK (COG-UK) Consortium	Rob Howes, The Lighthouse Lab in Cambridge and Alex Alderton, Roberto Amato, Sonia Goncalves, Ewan Harrison, David K. Jackson, Ian Johnston, Dominic Kwiatkowski, Cordelia Langford, John Sillitoe on behalf of the Wellcome Sanger Institute COVID-19 Surveillance Team
EPI_ISL_891438	Lighthouse Lab in Alderley Park	Wellcome Sanger Institute for the COVID-19 Genomics UK (COG-UK) Consortium	Jacquelyn Wynn, Mairead Hyland, The Lighthouse Lab in Alderley Park and Alex Alderton, Roberto Amato, Sonia Goncalves, Ewan Harrison, David K. Jackson, Ian Johnston, Dominic Kwiatkowski, Cordelia Langford, John Sillitoe on behalf of the Wellcome Sanger Institute COVID-19 Surveillance Team
EPI_ISL_891439, EPI_ISL_891441, EPI_ISL_891442	Lighthouse Lab in Cambridge	Wellcome Sanger Institute for the COVID-19 Genomics UK (COG-UK) Consortium	Rob Howes, The Lighthouse Lab in Cambridge and Alex Alderton, Roberto Amato, Sonia Goncalves, Ewan Harrison, David K. Jackson, Ian Johnston, Dominic Kwiatkowski, Cordelia Langford, John Sillitoe on behalf of the Wellcome Sanger Institute COVID-19 Surveillance Team
EPI_ISL_891444	Lighthouse Lab in Alderley Park	Wellcome Sanger Institute for the COVID-19 Genomics UK (COG-UK) Consortium	Jacquelyn Wynn, Mairead Hyland, The Lighthouse Lab in Alderley Park and Alex Alderton, Roberto Amato, Sonia Goncalves, Ewan Harrison, David K. Jackson, Ian Johnston, Dominic Kwiatkowski, Cordelia Langford, John Sillitoe on behalf of the Wellcome Sanger Institute COVID-19 Surveillance Team
EPI_ISL_891446	Lighthouse Lab in Glasgow	Wellcome Sanger Institute for the COVID-19 Genomics UK (COG-UK) Consortium	Harper VanSteenhouse, Yumi Kasai, David Gray, Carol Clugston, Anna Dominiczak and Alex Alderton, Roberto Amato, Sonia Goncalves, Ewan Harrison, David K. Jackson, Ian Johnston, Dominic Kwiatkowski, Cordelia Langford, John Sillitoe on behalf of the Wellcome Sanger Institute COVID-19 Surveillance Team
EPI_ISL_891447	Lighthouse Lab in Cambridge	Wellcome Sanger Institute for the COVID-19 Genomics UK (COG-UK) Consortium	Rob Howes, The Lighthouse Lab in Cambridge and Alex Alderton, Roberto Amato, Sonia Goncalves, Ewan Harrison, David K. Jackson, Ian Johnston, Dominic Kwiatkowski, Cordelia Langford, John Sillitoe on behalf of the Wellcome Sanger Institute COVID-19 Surveillance Team
EPI_ISL_891448	Lighthouse Lab in Alderley Park	Wellcome Sanger Institute for the COVID-19 Genomics UK (COG-UK) Consortium	Jacquelyn Wynn, Mairead Hyland, The Lighthouse Lab in Alderley Park and Alex Alderton, Roberto Amato, Sonia Goncalves, Ewan Harrison, David K. Jackson, Ian Johnston, Dominic Kwiatkowski, Cordelia Langford, John Sillitoe on behalf of the Wellcome Sanger Institute COVID-19 Surveillance Team
EPI_ISL_891450	Lighthouse Lab in Cambridge	Wellcome Sanger Institute for the COVID-19 Genomics UK (COG-UK) Consortium	Rob Howes, The Lighthouse Lab in Cambridge and Alex Alderton, Roberto Amato, Sonia Goncalves, Ewan Harrison, David K. Jackson, Ian Johnston, Dominic Kwiatkowski, Cordelia Langford, John Sillitoe on behalf of the Wellcome Sanger Institute COVID-19 Surveillance Team
EPI_ISL_891451	Lighthouse Lab in Alderley Park	Wellcome Sanger Institute for the COVID-19 Genomics UK (COG-UK) Consortium	Jacquelyn Wynn, Mairead Hyland, The Lighthouse Lab in Alderley Park and Alex Alderton, Roberto Amato, Sonia Goncalves, Ewan Harrison, David K. Jackson, Ian Johnston, Dominic Kwiatkowski, Cordelia Langford, John Sillitoe on behalf of the Wellcome Sanger Institute COVID-19 Surveillance Team
EPI_ISL_891452	Lighthouse Lab in Cambridge	Wellcome Sanger Institute for the COVID-19 Genomics UK (COG-UK) Consortium	Rob Howes, The Lighthouse Lab in Cambridge and Alex Alderton, Roberto Amato, Sonia Goncalves, Ewan Harrison, David K. Jackson, Ian Johnston, Dominic Kwiatkowski, Cordelia Langford, John Sillitoe on behalf of the Wellcome Sanger Institute COVID-19 Surveillance Team
EPI_ISL_891453	Lighthouse Lab in Glasgow	Wellcome Sanger Institute for the COVID-19 Genomics UK (COG-UK) Consortium	Harper VanSteenhouse, Yumi Kasai, David Gray, Carol Clugston, Anna Dominiczak and Alex Alderton, Roberto Amato, Sonia Goncalves, Ewan Harrison, David K. Jackson, Ian Johnston, Dominic Kwiatkowski, Cordelia Langford, John Sillitoe on behalf of the Wellcome Sanger Institute COVID-19 Surveillance Team
EPI_ISL_891454, EPI_ISL_891455	Lighthouse Lab in Alderley Park	Wellcome Sanger Institute for the COVID-19 Genomics UK (COG-UK) Consortium	Jacquelyn Wynn, Mairead Hyland, The Lighthouse Lab in Alderley Park and Alex Alderton, Roberto Amato, Sonia Goncalves, Ewan Harrison, David K. Jackson, Ian Johnston, Dominic Kwiatkowski, Cordelia Langford, John Sillitoe on behalf of the Wellcome Sanger Institute COVID-19 Surveillance Team
EPI_ISL_891457	Lighthouse Lab in Cambridge	Wellcome Sanger Institute for the COVID-19 Genomics UK (COG-UK) Consortium	Rob Howes, The Lighthouse Lab in Cambridge and Alex Alderton, Roberto Amato, Sonia Goncalves, Ewan Harrison, David K. Jackson, Ian Johnston, Dominic Kwiatkowski, Cordelia Langford, John Sillitoe on behalf of the Wellcome Sanger Institute COVID-19 Surveillance Team
EPI_ISL_891459, EPI_ISL_891460	Lighthouse Lab in Alderley Park	Wellcome Sanger Institute for the COVID-19 Genomics UK (COG-UK) Consortium	Jacquelyn Wynn, Mairead Hyland, The Lighthouse Lab in Alderley Park and Alex Alderton, Roberto Amato, Sonia Goncalves, Ewan Harrison, David K. Jackson, Ian Johnston, Dominic Kwiatkowski, Cordelia Langford, John Sillitoe on behalf of the Wellcome Sanger Institute COVID-19 Surveillance Team
EPI_ISL_891461	Lighthouse Lab in Cambridge	Wellcome Sanger Institute for the COVID-19 Genomics UK (COG-UK) Consortium	Rob Howes, The Lighthouse Lab in Cambridge and Alex Alderton, Roberto Amato, Sonia Goncalves, Ewan Harrison, David K. Jackson, Ian Johnston, Dominic Kwiatkowski, Cordelia Langford, John Sillitoe on behalf of the Wellcome Sanger Institute COVID-19 Surveillance Team
EPI_ISL_891462	Lighthouse Lab in Glasgow	Wellcome Sanger Institute for the COVID-19 Genomics UK (COG-UK) Consortium	Harper VanSteenhouse, Yumi Kasai, David Gray, Carol Clugston, Anna Dominiczak and Alex Alderton, Roberto Amato, Sonia Goncalves, Ewan Harrison, David K. Jackson, Ian Johnston, Dominic Kwiatkowski, Cordelia Langford, John Sillitoe on behalf of the Wellcome Sanger Institute COVID-19 Surveillance Team
EPI_ISL_891463	Lighthouse Lab in Alderley Park	Wellcome Sanger Institute for the COVID-19 Genomics UK (COG-UK) Consortium	Jacquelyn Wynn, Mairead Hyland, The Lighthouse Lab in Alderley Park and Alex Alderton, Roberto Amato, Sonia Goncalves, Ewan Harrison, David K. Jackson, Ian Johnston, Dominic Kwiatkowski, Cordelia Langford, John Sillitoe on behalf of the Wellcome Sanger Institute COVID-19 Surveillance Team
EPI_ISL_891464, EPI_ISL_891465	Lighthouse Lab in Glasgow	Wellcome Sanger Institute for the COVID-19 Genomics UK (COG-UK) Consortium	Harper VanSteenhouse, Yumi Kasai, David Gray, Carol Clugston, Anna Dominiczak and Alex Alderton, Roberto Amato, Sonia Goncalves, Ewan Harrison, David K. Jackson, Ian Johnston, Dominic Kwiatkowski, Cordelia Langford, John Sillitoe on behalf of the Wellcome Sanger Institute COVID-19 Surveillance Team
EPI_ISL_891466, EPI_ISL_891467, EPI_ISL_891470, EPI_ISL_891472	Lighthouse Lab in Cambridge	Wellcome Sanger Institute for the COVID-19 Genomics UK (COG-UK) Consortium	Rob Howes, The Lighthouse Lab in Cambridge and Alex Alderton, Roberto Amato, Sonia Goncalves, Ewan Harrison, David K. Jackson, Ian Johnston, Dominic Kwiatkowski, Cordelia Langford, John Sillitoe on behalf of the Wellcome Sanger Institute COVID-19 Surveillance Team
EPI_ISL_891473	Lighthouse Lab in Glasgow	Wellcome Sanger Institute for the COVID-19 Genomics UK	Harper VanSteenhouse, Yumi Kasai, David Gray, Carol Clugston, Anna Dominiczak and Alex Alderton, Roberto Amato, Sonia Goncalves, Ewan Harrison,

		(COG-UK) Consortium	David K. Jackson, Ian Johnston, Dominic Kwiatkowski, Cordelia Langford, John Sillitoe on behalf of the Wellcome Sanger Institute COVID-19 Surveillance Team
EPI_ISL_891475, EPI_ISL_891476, EPI_ISL_891477	Lighthouse Lab in Cambridge	Wellcome Sanger Institute for the COVID-19 Genomics UK (COG-UK) Consortium	Rob Howes, The Lighthouse Lab in Cambridge and Alex Alderton, Roberto Amato, Sonia Goncalves, Ewan Harrison, David K. Jackson, Ian Johnston, Dominic Kwiatkowski, Cordelia Langford, John Sillitoe on behalf of the Wellcome Sanger Institute COVID-19 Surveillance Team
EPI_ISL_891478	Lighthouse Lab in Alderley Park	Wellcome Sanger Institute for the COVID-19 Genomics UK (COG-UK) Consortium	Jacquelyn Wynn, Mairead Hyland, The Lighthouse Lab in Alderley Park and Alex Alderton, Roberto Amato, Sonia Goncalves, Ewan Harrison, David K. Jackson, Ian Johnston, Dominic Kwiatkowski, Cordelia Langford, John Sillitoe on behalf of the Wellcome Sanger Institute COVID-19 Surveillance Team
EPI_ISL_891480, EPI_ISL_891481, EPI_ISL_891483	Lighthouse Lab in Cambridge	Wellcome Sanger Institute for the COVID-19 Genomics UK (COG-UK) Consortium	Rob Howes, The Lighthouse Lab in Cambridge and Alex Alderton, Roberto Amato, Sonia Goncalves, Ewan Harrison, David K. Jackson, Ian Johnston, Dominic Kwiatkowski, Cordelia Langford, John Sillitoe on behalf of the Wellcome Sanger Institute COVID-19 Surveillance Team
EPI_ISL_891484, EPI_ISL_891485	Lighthouse Lab in Alderley Park	Wellcome Sanger Institute for the COVID-19 Genomics UK (COG-UK) Consortium	Jacquelyn Wynn, Mairead Hyland, The Lighthouse Lab in Alderley Park and Alex Alderton, Roberto Amato, Sonia Goncalves, Ewan Harrison, David K. Jackson, Ian Johnston, Dominic Kwiatkowski, Cordelia Langford, John Sillitoe on behalf of the Wellcome Sanger Institute COVID-19 Surveillance Team
EPI_ISL_891486, EPI_ISL_891488	Lighthouse Lab in Cambridge	Wellcome Sanger Institute for the COVID-19 Genomics UK (COG-UK) Consortium	Rob Howes, The Lighthouse Lab in Cambridge and Alex Alderton, Roberto Amato, Sonia Goncalves, Ewan Harrison, David K. Jackson, Ian Johnston, Dominic Kwiatkowski, Cordelia Langford, John Sillitoe on behalf of the Wellcome Sanger Institute COVID-19 Surveillance Team
EPI_ISL_891489, EPI_ISL_891490	Lighthouse Lab in Alderley Park	Wellcome Sanger Institute for the COVID-19 Genomics UK (COG-UK) Consortium	Jacquelyn Wynn, Mairead Hyland, The Lighthouse Lab in Alderley Park and Alex Alderton, Roberto Amato, Sonia Goncalves, Ewan Harrison, David K. Jackson, Ian Johnston, Dominic Kwiatkowski, Cordelia Langford, John Sillitoe on behalf of the Wellcome Sanger Institute COVID-19 Surveillance Team
EPI_ISL_891491	Lighthouse Lab in Glasgow	Wellcome Sanger Institute for the COVID-19 Genomics UK (COG-UK) Consortium	Harper VanSteenhouse, Yumi Kasai, David Gray, Carol Clugston, Anna Dominiczak and Alex Alderton, Roberto Amato, Sonia Goncalves, Ewan Harrison, David K. Jackson, Ian Johnston, Dominic Kwiatkowski, Cordelia Langford, John Sillitoe on behalf of the Wellcome Sanger Institute COVID-19 Surveillance Team
EPI_ISL_891492, EPI_ISL_891493, EPI_ISL_891494	Lighthouse Lab in Cambridge	Wellcome Sanger Institute for the COVID-19 Genomics UK (COG-UK) Consortium	Rob Howes, The Lighthouse Lab in Cambridge and Alex Alderton, Roberto Amato, Sonia Goncalves, Ewan Harrison, David K. Jackson, Ian Johnston, Dominic Kwiatkowski, Cordelia Langford, John Sillitoe on behalf of the Wellcome Sanger Institute COVID-19 Surveillance Team
EPI_ISL_891495	Lighthouse Lab in Glasgow	Wellcome Sanger Institute for the COVID-19 Genomics UK (COG-UK) Consortium	Harper VanSteenhouse, Yumi Kasai, David Gray, Carol Clugston, Anna Dominiczak and Alex Alderton, Roberto Amato, Sonia Goncalves, Ewan Harrison, David K. Jackson, Ian Johnston, Dominic Kwiatkowski, Cordelia Langford, John Sillitoe on behalf of the Wellcome Sanger Institute COVID-19 Surveillance Team
EPI_ISL_891496	Lighthouse Lab in Alderley Park	Wellcome Sanger Institute for the COVID-19 Genomics UK (COG-UK) Consortium	Jacquelyn Wynn, Mairead Hyland, The Lighthouse Lab in Alderley Park and Alex Alderton, Roberto Amato, Sonia Goncalves, Ewan Harrison, David K. Jackson, Ian Johnston, Dominic Kwiatkowski, Cordelia Langford, John Sillitoe on behalf of the Wellcome Sanger Institute COVID-19 Surveillance Team
EPI_ISL_891497	Lighthouse Lab in Glasgow	Wellcome Sanger Institute for the COVID-19 Genomics UK (COG-UK) Consortium	Harper VanSteenhouse, Yumi Kasai, David Gray, Carol Clugston, Anna Dominiczak and Alex Alderton, Roberto Amato, Sonia Goncalves, Ewan Harrison, David K. Jackson, Ian Johnston, Dominic Kwiatkowski, Cordelia Langford, John Sillitoe on behalf of the Wellcome Sanger Institute COVID-19 Surveillance Team
EPI_ISL_891498	Lighthouse Lab in Cambridge	Wellcome Sanger Institute for the COVID-19 Genomics UK (COG-UK) Consortium	Rob Howes, The Lighthouse Lab in Cambridge and Alex Alderton, Roberto Amato, Sonia Goncalves, Ewan Harrison, David K. Jackson, Ian Johnston, Dominic Kwiatkowski, Cordelia Langford, John Sillitoe on behalf of the Wellcome Sanger Institute COVID-19 Surveillance Team
EPI_ISL_891499	Lighthouse Lab in Alderley Park	Wellcome Sanger Institute for the COVID-19 Genomics UK (COG-UK) Consortium	Jacquelyn Wynn, Mairead Hyland, The Lighthouse Lab in Alderley Park and Alex Alderton, Roberto Amato, Sonia Goncalves, Ewan Harrison, David K. Jackson, Ian Johnston, Dominic Kwiatkowski, Cordelia Langford, John Sillitoe on behalf of the Wellcome Sanger Institute COVID-19 Surveillance Team
EPI_ISL_891500, EPI_ISL_891501	Lighthouse Lab in Cambridge	Wellcome Sanger Institute for the COVID-19 Genomics UK (COG-UK) Consortium	Rob Howes, The Lighthouse Lab in Cambridge and Alex Alderton, Roberto Amato, Sonia Goncalves, Ewan Harrison, David K. Jackson, Ian Johnston, Dominic Kwiatkowski, Cordelia Langford, John Sillitoe on behalf of the Wellcome Sanger Institute COVID-19 Surveillance Team
EPI_ISL_891502	Lighthouse Lab in Glasgow	Wellcome Sanger Institute for the COVID-19 Genomics UK (COG-UK) Consortium	Harper VanSteenhouse, Yumi Kasai, David Gray, Carol Clugston, Anna Dominiczak and Alex Alderton, Roberto Amato, Sonia Goncalves, Ewan Harrison, David K. Jackson, Ian Johnston, Dominic Kwiatkowski, Cordelia Langford, John Sillitoe on behalf of the Wellcome Sanger Institute COVID-19 Surveillance Team
EPI_ISL_891503	Lighthouse Lab in Alderley Park	Wellcome Sanger Institute for the COVID-19 Genomics UK (COG-UK) Consortium	Jacquelyn Wynn, Mairead Hyland, The Lighthouse Lab in Alderley Park and Alex Alderton, Roberto Amato, Sonia Goncalves, Ewan Harrison, David K. Jackson, Ian Johnston, Dominic Kwiatkowski, Cordelia Langford, John Sillitoe on behalf of the Wellcome Sanger Institute COVID-19 Surveillance Team
EPI_ISL_891504	Lighthouse Lab in Cambridge	Wellcome Sanger Institute for the COVID-19 Genomics UK (COG-UK) Consortium	Rob Howes, The Lighthouse Lab in Cambridge and Alex Alderton, Roberto Amato, Sonia Goncalves, Ewan Harrison, David K. Jackson, Ian Johnston, Dominic Kwiatkowski, Cordelia Langford, John Sillitoe on behalf of the Wellcome Sanger Institute COVID-19 Surveillance Team
EPI_ISL_891505	Lighthouse Lab in Glasgow	Wellcome Sanger Institute for the COVID-19 Genomics UK (COG-UK) Consortium	Harper VanSteenhouse, Yumi Kasai, David Gray, Carol Clugston, Anna Dominiczak and Alex Alderton, Roberto Amato, Sonia Goncalves, Ewan Harrison, David K. Jackson, Ian Johnston, Dominic Kwiatkowski, Cordelia Langford, John Sillitoe on behalf of the Wellcome Sanger Institute COVID-19 Surveillance Team
EPI_ISL_891506	Lighthouse Lab in Cambridge	Wellcome Sanger Institute for the COVID-19 Genomics UK (COG-UK) Consortium	Rob Howes, The Lighthouse Lab in Cambridge and Alex Alderton, Roberto Amato, Sonia Goncalves, Ewan Harrison, David K. Jackson, Ian Johnston, Dominic Kwiatkowski, Cordelia Langford, John Sillitoe on behalf of the Wellcome Sanger Institute COVID-19 Surveillance Team
EPI_ISL_891508, EPI_ISL_891509	Lighthouse Lab in Glasgow	Wellcome Sanger Institute for the COVID-19 Genomics UK (COG-UK) Consortium	Harper VanSteenhouse, Yumi Kasai, David Gray, Carol Clugston, Anna Dominiczak and Alex Alderton, Roberto Amato, Sonia Goncalves, Ewan Harrison, David K. Jackson, Ian Johnston, Dominic Kwiatkowski, Cordelia Langford, John Sillitoe on behalf of the Wellcome Sanger Institute COVID-19 Surveillance Team
EPI_ISL_891510	Lighthouse Lab in Cambridge	Wellcome Sanger Institute for the COVID-19 Genomics UK (COG-UK) Consortium	Rob Howes, The Lighthouse Lab in Cambridge and Alex Alderton, Roberto Amato, Sonia Goncalves, Ewan Harrison, David K. Jackson, Ian Johnston, Dominic Kwiatkowski, Cordelia Langford, John Sillitoe on behalf of the Wellcome Sanger Institute COVID-19 Surveillance Team
EPI_ISL_891511	Lighthouse Lab in Alderley Park	Wellcome Sanger Institute for the COVID-19 Genomics UK (COG-UK) Consortium	Jacquelyn Wynn, Mairead Hyland, The Lighthouse Lab in Alderley Park and Alex Alderton, Roberto Amato, Sonia Goncalves, Ewan Harrison, David K. Jackson, Ian Johnston, Dominic Kwiatkowski, Cordelia Langford, John Sillitoe on behalf of the Wellcome Sanger Institute COVID-19 Surveillance Team
EPI_ISL_891513	Lighthouse Lab in Cambridge	Wellcome Sanger Institute for the COVID-19 Genomics UK (COG-UK) Consortium	Rob Howes, The Lighthouse Lab in Cambridge and Alex Alderton, Roberto Amato, Sonia Goncalves, Ewan Harrison, David K. Jackson, Ian Johnston, Dominic Kwiatkowski, Cordelia Langford, John Sillitoe on behalf of the Wellcome Sanger Institute COVID-19 Surveillance Team
EPI_ISL_891514	Lighthouse Lab in Alderley Park	Wellcome Sanger Institute for the COVID-19 Genomics UK (COG-UK) Consortium	Jacquelyn Wynn, Mairead Hyland, The Lighthouse Lab in Alderley Park and Alex Alderton, Roberto Amato, Sonia Goncalves, Ewan Harrison, David K. Jackson, Ian Johnston, Dominic Kwiatkowski, Cordelia Langford, John Sillitoe on behalf of the Wellcome Sanger Institute COVID-19 Surveillance Team
EPI_ISL_891515	Lighthouse Lab in Cambridge	Wellcome Sanger Institute for the COVID-19 Genomics UK (COG-UK) Consortium	Rob Howes, The Lighthouse Lab in Cambridge and Alex Alderton, Roberto Amato, Sonia Goncalves, Ewan Harrison, David K. Jackson, Ian Johnston, Dominic Kwiatkowski, Cordelia Langford, John Sillitoe on behalf of the Wellcome Sanger Institute COVID-19 Surveillance Team
EPI_ISL_891516	Lighthouse Lab in Glasgow	Wellcome Sanger Institute for the COVID-19 Genomics UK (COG-UK) Consortium	Harper VanSteenhouse, Yumi Kasai, David Gray, Carol Clugston, Anna Dominiczak and Alex Alderton, Roberto Amato, Sonia Goncalves, Ewan Harrison, David K. Jackson, Ian Johnston, Dominic Kwiatkowski, Cordelia Langford, John Sillitoe on behalf of the Wellcome Sanger Institute COVID-19 Surveillance Team
EPI_ISL_891517, EPI_ISL_891518, EPI_ISL_891519	Lighthouse Lab in Cambridge	Wellcome Sanger Institute for the COVID-19 Genomics UK (COG-UK) Consortium	Rob Howes, The Lighthouse Lab in Cambridge and Alex Alderton, Roberto Amato, Sonia Goncalves, Ewan Harrison, David K. Jackson, Ian Johnston, Dominic Kwiatkowski, Cordelia Langford, John Sillitoe on behalf of the Wellcome Sanger Institute COVID-19 Surveillance Team
EPI_ISL_891520	Lighthouse Lab in Alderley Park	Wellcome Sanger Institute for the COVID-19 Genomics UK (COG-UK) Consortium	Jacquelyn Wynn, Mairead Hyland, The Lighthouse Lab in Alderley Park and Alex Alderton, Roberto Amato, Sonia Goncalves, Ewan Harrison, David K. Jackson, Ian Johnston, Dominic Kwiatkowski, Cordelia Langford, John Sillitoe on behalf of the Wellcome Sanger Institute COVID-19 Surveillance Team
EPI_ISL_891521	Lighthouse Lab in Cambridge	Wellcome Sanger Institute for the COVID-19 Genomics UK (COG-UK) Consortium	Rob Howes, The Lighthouse Lab in Cambridge and Alex Alderton, Roberto Amato, Sonia Goncalves, Ewan Harrison, David K. Jackson, Ian Johnston, Dominic Kwiatkowski, Cordelia Langford, John Sillitoe on behalf of the Wellcome Sanger Institute COVID-19 Surveillance Team
EPI_ISL_891522, EPI_ISL_891525	Lighthouse Lab in Alderley Park	Wellcome Sanger Institute for the COVID-19 Genomics UK (COG-UK) Consortium	Jacquelyn Wynn, Mairead Hyland, The Lighthouse Lab in Alderley Park and Alex Alderton, Roberto Amato, Sonia Goncalves, Ewan Harrison, David K. Jackson, Ian Johnston, Dominic Kwiatkowski, Cordelia Langford, John Sillitoe on behalf of the Wellcome Sanger Institute COVID-19 Surveillance Team
EPI_ISL_891526	Lighthouse Lab in Cambridge	Wellcome Sanger Institute for the COVID-19 Genomics UK (COG-UK) Consortium	Rob Howes, The Lighthouse Lab in Cambridge and Alex Alderton, Roberto Amato, Sonia Goncalves, Ewan Harrison, David K. Jackson, Ian Johnston, Dominic Kwiatkowski, Cordelia Langford, John Sillitoe on behalf of the Wellcome Sanger Institute COVID-19 Surveillance Team
EPI_ISL_891527, EPI_ISL_891528,	Lighthouse Lab in Alderley Park	Wellcome Sanger Institute for the COVID-19 Genomics UK	Jacquelyn Wynn, Mairead Hyland, The Lighthouse Lab in Alderley Park and Alex Alderton, Roberto Amato, Sonia Goncalves, Ewan Harrison, David K.

EPI_ISL_891529		(COG-UK) Consortium	Jackson, Ian Johnston, Dominic Kwiatkowski, Cordelia Langford, John Sillitoe on behalf of the Wellcome Sanger Institute COVID-19 Surveillance Team
EPI_ISL_891530	Lighthouse Lab in Cambridge	Wellcome Sanger Institute for the COVID-19 Genomics UK (COG-UK) Consortium	Rob Howes, The Lighthouse Lab in Cambridge and Alex Alderton, Roberto Amato, Sonia Goncalves, Ewan Harrison, David K. Jackson, Ian Johnston, Dominic Kwiatkowski, Cordelia Langford, John Sillitoe on behalf of the Wellcome Sanger Institute COVID-19 Surveillance Team
EPI_ISL_891531	Lighthouse Lab in Alderley Park	Wellcome Sanger Institute for the COVID-19 Genomics UK (COG-UK) Consortium	Jacquelyn Wynn, Mairead Hyland, The Lighthouse Lab in Alderley Park and Alex Alderton, Roberto Amato, Sonia Goncalves, Ewan Harrison, David K. Jackson, Ian Johnston, Dominic Kwiatkowski, Cordelia Langford, John Sillitoe on behalf of the Wellcome Sanger Institute COVID-19 Surveillance Team
EPI_ISL_891534, EPI_ISL_891535	Lighthouse Lab in Cambridge	Wellcome Sanger Institute for the COVID-19 Genomics UK (COG-UK) Consortium	Rob Howes, The Lighthouse Lab in Cambridge and Alex Alderton, Roberto Amato, Sonia Goncalves, Ewan Harrison, David K. Jackson, Ian Johnston, Dominic Kwiatkowski, Cordelia Langford, John Sillitoe on behalf of the Wellcome Sanger Institute COVID-19 Surveillance Team
EPI_ISL_891536	Lighthouse Lab in Alderley Park	Wellcome Sanger Institute for the COVID-19 Genomics UK (COG-UK) Consortium	Jacquelyn Wynn, Mairead Hyland, The Lighthouse Lab in Alderley Park and Alex Alderton, Roberto Amato, Sonia Goncalves, Ewan Harrison, David K. Jackson, Ian Johnston, Dominic Kwiatkowski, Cordelia Langford, John Sillitoe on behalf of the Wellcome Sanger Institute COVID-19 Surveillance Team
EPI_ISL_891537	Lighthouse Lab in Cambridge	Wellcome Sanger Institute for the COVID-19 Genomics UK (COG-UK) Consortium	Rob Howes, The Lighthouse Lab in Cambridge and Alex Alderton, Roberto Amato, Sonia Goncalves, Ewan Harrison, David K. Jackson, Ian Johnston, Dominic Kwiatkowski, Cordelia Langford, John Sillitoe on behalf of the Wellcome Sanger Institute COVID-19 Surveillance Team
EPI_ISL_891539	Lighthouse Lab in Glasgow	Wellcome Sanger Institute for the COVID-19 Genomics UK (COG-UK) Consortium	Harper VanSteenhouse, Yumi Kasai, David Gray, Carol Clugston, Anna Dominiczak and Alex Alderton, Roberto Amato, Sonia Goncalves, Ewan Harrison, David K. Jackson, Ian Johnston, Dominic Kwiatkowski, Cordelia Langford, John Sillitoe on behalf of the Wellcome Sanger Institute COVID-19 Surveillance Team
EPI_ISL_891540, EPI_ISL_891541, EPI_ISL_891543	Lighthouse Lab in Cambridge	Wellcome Sanger Institute for the COVID-19 Genomics UK (COG-UK) Consortium	Rob Howes, The Lighthouse Lab in Cambridge and Alex Alderton, Roberto Amato, Sonia Goncalves, Ewan Harrison, David K. Jackson, Ian Johnston, Dominic Kwiatkowski, Cordelia Langford, John Sillitoe on behalf of the Wellcome Sanger Institute COVID-19 Surveillance Team
EPI_ISL_891544, EPI_ISL_891545, EPI_ISL_891547, EPI_ISL_891548	Lighthouse Lab in Alderley Park	Wellcome Sanger Institute for the COVID-19 Genomics UK (COG-UK) Consortium	Jacquelyn Wynn, Mairead Hyland, The Lighthouse Lab in Alderley Park and Alex Alderton, Roberto Amato, Sonia Goncalves, Ewan Harrison, David K. Jackson, Ian Johnston, Dominic Kwiatkowski, Cordelia Langford, John Sillitoe on behalf of the Wellcome Sanger Institute COVID-19 Surveillance Team
EPI_ISL_891549	Lighthouse Lab in Glasgow	Wellcome Sanger Institute for the COVID-19 Genomics UK (COG-UK) Consortium	Harper VanSteenhouse, Yumi Kasai, David Gray, Carol Clugston, Anna Dominiczak and Alex Alderton, Roberto Amato, Sonia Goncalves, Ewan Harrison, David K. Jackson, Ian Johnston, Dominic Kwiatkowski, Cordelia Langford, John Sillitoe on behalf of the Wellcome Sanger Institute COVID-19 Surveillance Team
EPI_ISL_891550, EPI_ISL_891551, EPI_ISL_891553, EPI_ISL_891554	Lighthouse Lab in Cambridge	Wellcome Sanger Institute for the COVID-19 Genomics UK (COG-UK) Consortium	Rob Howes, The Lighthouse Lab in Cambridge and Alex Alderton, Roberto Amato, Sonia Goncalves, Ewan Harrison, David K. Jackson, Ian Johnston, Dominic Kwiatkowski, Cordelia Langford, John Sillitoe on behalf of the Wellcome Sanger Institute COVID-19 Surveillance Team
EPI_ISL_891555	Lighthouse Lab in Alderley Park	Wellcome Sanger Institute for the COVID-19 Genomics UK (COG-UK) Consortium	Jacquelyn Wynn, Mairead Hyland, The Lighthouse Lab in Alderley Park and Alex Alderton, Roberto Amato, Sonia Goncalves, Ewan Harrison, David K. Jackson, Ian Johnston, Dominic Kwiatkowski, Cordelia Langford, John Sillitoe on behalf of the Wellcome Sanger Institute COVID-19 Surveillance Team
EPI_ISL_891556, EPI_ISL_891557, EPI_ISL_891558	Lighthouse Lab in Cambridge	Wellcome Sanger Institute for the COVID-19 Genomics UK (COG-UK) Consortium	Rob Howes, The Lighthouse Lab in Cambridge and Alex Alderton, Roberto Amato, Sonia Goncalves, Ewan Harrison, David K. Jackson, Ian Johnston, Dominic Kwiatkowski, Cordelia Langford, John Sillitoe on behalf of the Wellcome Sanger Institute COVID-19 Surveillance Team
EPI_ISL_891560	Lighthouse Lab in Alderley Park	Wellcome Sanger Institute for the COVID-19 Genomics UK (COG-UK) Consortium	Jacquelyn Wynn, Mairead Hyland, The Lighthouse Lab in Alderley Park and Alex Alderton, Roberto Amato, Sonia Goncalves, Ewan Harrison, David K. Jackson, Ian Johnston, Dominic Kwiatkowski, Cordelia Langford, John Sillitoe on behalf of the Wellcome Sanger Institute COVID-19 Surveillance Team
EPI_ISL_891561	Lighthouse Lab in Cambridge	Wellcome Sanger Institute for the COVID-19 Genomics UK (COG-UK) Consortium	Rob Howes, The Lighthouse Lab in Cambridge and Alex Alderton, Roberto Amato, Sonia Goncalves, Ewan Harrison, David K. Jackson, Ian Johnston, Dominic Kwiatkowski, Cordelia Langford, John Sillitoe on behalf of the Wellcome Sanger Institute COVID-19 Surveillance Team
EPI_ISL_891562	Lighthouse Lab in Glasgow	Wellcome Sanger Institute for the COVID-19 Genomics UK (COG-UK) Consortium	Harper VanSteenhouse, Yumi Kasai, David Gray, Carol Clugston, Anna Dominiczak and Alex Alderton, Roberto Amato, Sonia Goncalves, Ewan Harrison, David K. Jackson, Ian Johnston, Dominic Kwiatkowski, Cordelia Langford, John Sillitoe on behalf of the Wellcome Sanger Institute COVID-19 Surveillance Team
EPI_ISL_891563, EPI_ISL_891564	Lighthouse Lab in Alderley Park	Wellcome Sanger Institute for the COVID-19 Genomics UK (COG-UK) Consortium	Jacquelyn Wynn, Mairead Hyland, The Lighthouse Lab in Alderley Park and Alex Alderton, Roberto Amato, Sonia Goncalves, Ewan Harrison, David K. Jackson, Ian Johnston, Dominic Kwiatkowski, Cordelia Langford, John Sillitoe on behalf of the Wellcome Sanger Institute COVID-19 Surveillance Team
EPI_ISL_891565, EPI_ISL_891566, EPI_ISL_891567	Lighthouse Lab in Cambridge	Wellcome Sanger Institute for the COVID-19 Genomics UK (COG-UK) Consortium	Rob Howes, The Lighthouse Lab in Cambridge and Alex Alderton, Roberto Amato, Sonia Goncalves, Ewan Harrison, David K. Jackson, Ian Johnston, Dominic Kwiatkowski, Cordelia Langford, John Sillitoe on behalf of the Wellcome Sanger Institute COVID-19 Surveillance Team
EPI_ISL_891568	Lighthouse Lab in Glasgow	Wellcome Sanger Institute for the COVID-19 Genomics UK (COG-UK) Consortium	Harper VanSteenhouse, Yumi Kasai, David Gray, Carol Clugston, Anna Dominiczak and Alex Alderton, Roberto Amato, Sonia Goncalves, Ewan Harrison, David K. Jackson, Ian Johnston, Dominic Kwiatkowski, Cordelia Langford, John Sillitoe on behalf of the Wellcome Sanger Institute COVID-19 Surveillance Team
EPI_ISL_891569	Lighthouse Lab in Alderley Park	Wellcome Sanger Institute for the COVID-19 Genomics UK (COG-UK) Consortium	Jacquelyn Wynn, Mairead Hyland, The Lighthouse Lab in Alderley Park and Alex Alderton, Roberto Amato, Sonia Goncalves, Ewan Harrison, David K. Jackson, Ian Johnston, Dominic Kwiatkowski, Cordelia Langford, John Sillitoe on behalf of the Wellcome Sanger Institute COVID-19 Surveillance Team
EPI_ISL_891570	Lighthouse Lab in Cambridge	Wellcome Sanger Institute for the COVID-19 Genomics UK (COG-UK) Consortium	Rob Howes, The Lighthouse Lab in Cambridge and Alex Alderton, Roberto Amato, Sonia Goncalves, Ewan Harrison, David K. Jackson, Ian Johnston, Dominic Kwiatkowski, Cordelia Langford, John Sillitoe on behalf of the Wellcome Sanger Institute COVID-19 Surveillance Team
EPI_ISL_891571	Lighthouse Lab in Glasgow	Wellcome Sanger Institute for the COVID-19 Genomics UK (COG-UK) Consortium	Harper VanSteenhouse, Yumi Kasai, David Gray, Carol Clugston, Anna Dominiczak and Alex Alderton, Roberto Amato, Sonia Goncalves, Ewan Harrison, David K. Jackson, Ian Johnston, Dominic Kwiatkowski, Cordelia Langford, John Sillitoe on behalf of the Wellcome Sanger Institute COVID-19 Surveillance Team
EPI_ISL_891572	Lighthouse Lab in Alderley Park	Wellcome Sanger Institute for the COVID-19 Genomics UK (COG-UK) Consortium	Jacquelyn Wynn, Mairead Hyland, The Lighthouse Lab in Alderley Park and Alex Alderton, Roberto Amato, Sonia Goncalves, Ewan Harrison, David K. Jackson, Ian Johnston, Dominic Kwiatkowski, Cordelia Langford, John Sillitoe on behalf of the Wellcome Sanger Institute COVID-19 Surveillance Team
EPI_ISL_891574	Lighthouse Lab in Glasgow	Wellcome Sanger Institute for the COVID-19 Genomics UK (COG-UK) Consortium	Harper VanSteenhouse, Yumi Kasai, David Gray, Carol Clugston, Anna Dominiczak and Alex Alderton, Roberto Amato, Sonia Goncalves, Ewan Harrison, David K. Jackson, Ian Johnston, Dominic Kwiatkowski, Cordelia Langford, John Sillitoe on behalf of the Wellcome Sanger Institute COVID-19 Surveillance Team
EPI_ISL_891575, EPI_ISL_891577, EPI_ISL_891578, EPI_ISL_891579, EPI_ISL_891580, EPI_ISL_891581	Lighthouse Lab in Cambridge	Wellcome Sanger Institute for the COVID-19 Genomics UK (COG-UK) Consortium	Rob Howes, The Lighthouse Lab in Cambridge and Alex Alderton, Roberto Amato, Sonia Goncalves, Ewan Harrison, David K. Jackson, Ian Johnston, Dominic Kwiatkowski, Cordelia Langford, John Sillitoe on behalf of the Wellcome Sanger Institute COVID-19 Surveillance Team
EPI_ISL_891582	Lighthouse Lab in Alderley Park	Wellcome Sanger Institute for the COVID-19 Genomics UK (COG-UK) Consortium	Jacquelyn Wynn, Mairead Hyland, The Lighthouse Lab in Alderley Park and Alex Alderton, Roberto Amato, Sonia Goncalves, Ewan Harrison, David K. Jackson, Ian Johnston, Dominic Kwiatkowski, Cordelia Langford, John Sillitoe on behalf of the Wellcome Sanger Institute COVID-19 Surveillance Team
EPI_ISL_891583, EPI_ISL_891584	Lighthouse Lab in Cambridge	Wellcome Sanger Institute for the COVID-19 Genomics UK (COG-UK) Consortium	Rob Howes, The Lighthouse Lab in Cambridge and Alex Alderton, Roberto Amato, Sonia Goncalves, Ewan Harrison, David K. Jackson, Ian Johnston, Dominic Kwiatkowski, Cordelia Langford, John Sillitoe on behalf of the Wellcome Sanger Institute COVID-19 Surveillance Team
EPI_ISL_891585	Lighthouse Lab in Glasgow	Wellcome Sanger Institute for the COVID-19 Genomics UK (COG-UK) Consortium	Harper VanSteenhouse, Yumi Kasai, David Gray, Carol Clugston, Anna Dominiczak and Alex Alderton, Roberto Amato, Sonia Goncalves, Ewan Harrison, David K. Jackson, Ian Johnston, Dominic Kwiatkowski, Cordelia Langford, John Sillitoe on behalf of the Wellcome Sanger Institute COVID-19 Surveillance Team
EPI_ISL_891586	Lighthouse Lab in Alderley Park	Wellcome Sanger Institute for the COVID-19 Genomics UK (COG-UK) Consortium	Jacquelyn Wynn, Mairead Hyland, The Lighthouse Lab in Alderley Park and Alex Alderton, Roberto Amato, Sonia Goncalves, Ewan Harrison, David K. Jackson, Ian Johnston, Dominic Kwiatkowski, Cordelia Langford, John Sillitoe on behalf of the Wellcome Sanger Institute COVID-19 Surveillance Team
EPI_ISL_891587, EPI_ISL_891588	Lighthouse Lab in Glasgow	Wellcome Sanger Institute for the COVID-19 Genomics UK (COG-UK) Consortium	Harper VanSteenhouse, Yumi Kasai, David Gray, Carol Clugston, Anna Dominiczak and Alex Alderton, Roberto Amato, Sonia Goncalves, Ewan Harrison, David K. Jackson, Ian Johnston, Dominic Kwiatkowski, Cordelia Langford, John Sillitoe on behalf of the Wellcome Sanger Institute COVID-19 Surveillance Team
EPI_ISL_891589, EPI_ISL_891590, EPI_ISL_891591, EPI_ISL_891592, EPI_ISL_891593, EPI_ISL_891594, EPI_ISL_891595	Lighthouse Lab in Cambridge	Wellcome Sanger Institute for the COVID-19 Genomics UK (COG-UK) Consortium	Rob Howes, The Lighthouse Lab in Cambridge and Alex Alderton, Roberto Amato, Sonia Goncalves, Ewan Harrison, David K. Jackson, Ian Johnston, Dominic Kwiatkowski, Cordelia Langford, John Sillitoe on behalf of the Wellcome Sanger Institute COVID-19 Surveillance Team

EPI_ISL_891596	Lighthouse Lab in Alderley Park	Wellcome Sanger Institute for the COVID-19 Genomics UK (COG-UK) Consortium	Jacquelyn Wynn, Mairead Hyland, The Lighthouse Lab in Alderley Park and Alex Alderton, Roberto Amato, Sonia Goncalves, Ewan Harrison, David K. Jackson, Ian Johnston, Dominic Kwiatkowski, Cordelia Langford, John Sillitoe on behalf of the Wellcome Sanger Institute COVID-19 Surveillance Team
EPI_ISL_891597, EPI_ISL_891598	Lighthouse Lab in Cambridge	Wellcome Sanger Institute for the COVID-19 Genomics UK (COG-UK) Consortium	Rob Howes, The Lighthouse Lab in Cambridge and Alex Alderton, Roberto Amato, Sonia Goncalves, Ewan Harrison, David K. Jackson, Ian Johnston, Dominic Kwiatkowski, Cordelia Langford, John Sillitoe on behalf of the Wellcome Sanger Institute COVID-19 Surveillance Team
EPI_ISL_891599, EPI_ISL_891600	Lighthouse Lab in Alderley Park	Wellcome Sanger Institute for the COVID-19 Genomics UK (COG-UK) Consortium	Jacquelyn Wynn, Mairead Hyland, The Lighthouse Lab in Alderley Park and Alex Alderton, Roberto Amato, Sonia Goncalves, Ewan Harrison, David K. Jackson, Ian Johnston, Dominic Kwiatkowski, Cordelia Langford, John Sillitoe on behalf of the Wellcome Sanger Institute COVID-19 Surveillance Team
EPI_ISL_891601	Lighthouse Lab in Cambridge	Wellcome Sanger Institute for the COVID-19 Genomics UK (COG-UK) Consortium	Rob Howes, The Lighthouse Lab in Cambridge and Alex Alderton, Roberto Amato, Sonia Goncalves, Ewan Harrison, David K. Jackson, Ian Johnston, Dominic Kwiatkowski, Cordelia Langford, John Sillitoe on behalf of the Wellcome Sanger Institute COVID-19 Surveillance Team
EPI_ISL_891602	Lighthouse Lab in Alderley Park	Wellcome Sanger Institute for the COVID-19 Genomics UK (COG-UK) Consortium	Jacquelyn Wynn, Mairead Hyland, The Lighthouse Lab in Alderley Park and Alex Alderton, Roberto Amato, Sonia Goncalves, Ewan Harrison, David K. Jackson, Ian Johnston, Dominic Kwiatkowski, Cordelia Langford, John Sillitoe on behalf of the Wellcome Sanger Institute COVID-19 Surveillance Team
EPI_ISL_891604, EPI_ISL_891605	Lighthouse Lab in Glasgow	Wellcome Sanger Institute for the COVID-19 Genomics UK (COG-UK) Consortium	Harper VanSteenhouse, Yumi Kasai, David Gray, Carol Clugston, Anna Dominiczak and Alex Alderton, Roberto Amato, Sonia Goncalves, Ewan Harrison, David K. Jackson, Ian Johnston, Dominic Kwiatkowski, Cordelia Langford, John Sillitoe on behalf of the Wellcome Sanger Institute COVID-19 Surveillance Team
EPI_ISL_891606	Lighthouse Lab in Alderley Park	Wellcome Sanger Institute for the COVID-19 Genomics UK (COG-UK) Consortium	Jacquelyn Wynn, Mairead Hyland, The Lighthouse Lab in Alderley Park and Alex Alderton, Roberto Amato, Sonia Goncalves, Ewan Harrison, David K. Jackson, Ian Johnston, Dominic Kwiatkowski, Cordelia Langford, John Sillitoe on behalf of the Wellcome Sanger Institute COVID-19 Surveillance Team
EPI_ISL_891607, EPI_ISL_891608	Lighthouse Lab in Cambridge	Wellcome Sanger Institute for the COVID-19 Genomics UK (COG-UK) Consortium	Rob Howes, The Lighthouse Lab in Cambridge and Alex Alderton, Roberto Amato, Sonia Goncalves, Ewan Harrison, David K. Jackson, Ian Johnston, Dominic Kwiatkowski, Cordelia Langford, John Sillitoe on behalf of the Wellcome Sanger Institute COVID-19 Surveillance Team
EPI_ISL_891610, EPI_ISL_891612	Lighthouse Lab in Glasgow	Wellcome Sanger Institute for the COVID-19 Genomics UK (COG-UK) Consortium	Harper VanSteenhouse, Yumi Kasai, David Gray, Carol Clugston, Anna Dominiczak and Alex Alderton, Roberto Amato, Sonia Goncalves, Ewan Harrison, David K. Jackson, Ian Johnston, Dominic Kwiatkowski, Cordelia Langford, John Sillitoe on behalf of the Wellcome Sanger Institute COVID-19 Surveillance Team
EPI_ISL_891614	Lighthouse Lab in Cambridge	Wellcome Sanger Institute for the COVID-19 Genomics UK (COG-UK) Consortium	Rob Howes, The Lighthouse Lab in Cambridge and Alex Alderton, Roberto Amato, Sonia Goncalves, Ewan Harrison, David K. Jackson, Ian Johnston, Dominic Kwiatkowski, Cordelia Langford, John Sillitoe on behalf of the Wellcome Sanger Institute COVID-19 Surveillance Team
EPI_ISL_891615, EPI_ISL_891616	Lighthouse Lab in Alderley Park	Wellcome Sanger Institute for the COVID-19 Genomics UK (COG-UK) Consortium	Jacquelyn Wynn, Mairead Hyland, The Lighthouse Lab in Alderley Park and Alex Alderton, Roberto Amato, Sonia Goncalves, Ewan Harrison, David K. Jackson, Ian Johnston, Dominic Kwiatkowski, Cordelia Langford, John Sillitoe on behalf of the Wellcome Sanger Institute COVID-19 Surveillance Team
EPI_ISL_891617	Lighthouse Lab in Cambridge	Wellcome Sanger Institute for the COVID-19 Genomics UK (COG-UK) Consortium	Rob Howes, The Lighthouse Lab in Cambridge and Alex Alderton, Roberto Amato, Sonia Goncalves, Ewan Harrison, David K. Jackson, Ian Johnston, Dominic Kwiatkowski, Cordelia Langford, John Sillitoe on behalf of the Wellcome Sanger Institute COVID-19 Surveillance Team
EPI_ISL_891618	Lighthouse Lab in Glasgow	Wellcome Sanger Institute for the COVID-19 Genomics UK (COG-UK) Consortium	Harper VanSteenhouse, Yumi Kasai, David Gray, Carol Clugston, Anna Dominiczak and Alex Alderton, Roberto Amato, Sonia Goncalves, Ewan Harrison, David K. Jackson, Ian Johnston, Dominic Kwiatkowski, Cordelia Langford, John Sillitoe on behalf of the Wellcome Sanger Institute COVID-19 Surveillance Team
EPI_ISL_891619, EPI_ISL_891620, EPI_ISL_891621	Lighthouse Lab in Cambridge	Wellcome Sanger Institute for the COVID-19 Genomics UK (COG-UK) Consortium	Rob Howes, The Lighthouse Lab in Cambridge and Alex Alderton, Roberto Amato, Sonia Goncalves, Ewan Harrison, David K. Jackson, Ian Johnston, Dominic Kwiatkowski, Cordelia Langford, John Sillitoe on behalf of the Wellcome Sanger Institute COVID-19 Surveillance Team
EPI_ISL_891623	Lighthouse Lab in Alderley Park	Wellcome Sanger Institute for the COVID-19 Genomics UK (COG-UK) Consortium	Jacquelyn Wynn, Mairead Hyland, The Lighthouse Lab in Alderley Park and Alex Alderton, Roberto Amato, Sonia Goncalves, Ewan Harrison, David K. Jackson, Ian Johnston, Dominic Kwiatkowski, Cordelia Langford, John Sillitoe on behalf of the Wellcome Sanger Institute COVID-19 Surveillance Team
EPI_ISL_891624	Lighthouse Lab in Glasgow	Wellcome Sanger Institute for the COVID-19 Genomics UK (COG-UK) Consortium	Harper VanSteenhouse, Yumi Kasai, David Gray, Carol Clugston, Anna Dominiczak and Alex Alderton, Roberto Amato, Sonia Goncalves, Ewan Harrison, David K. Jackson, Ian Johnston, Dominic Kwiatkowski, Cordelia Langford, John Sillitoe on behalf of the Wellcome Sanger Institute COVID-19 Surveillance Team
EPI_ISL_891625	Lighthouse Lab in Alderley Park	Wellcome Sanger Institute for the COVID-19 Genomics UK (COG-UK) Consortium	Jacquelyn Wynn, Mairead Hyland, The Lighthouse Lab in Alderley Park and Alex Alderton, Roberto Amato, Sonia Goncalves, Ewan Harrison, David K. Jackson, Ian Johnston, Dominic Kwiatkowski, Cordelia Langford, John Sillitoe on behalf of the Wellcome Sanger Institute COVID-19 Surveillance Team
EPI_ISL_891626	Lighthouse Lab in Glasgow	Wellcome Sanger Institute for the COVID-19 Genomics UK (COG-UK) Consortium	Harper VanSteenhouse, Yumi Kasai, David Gray, Carol Clugston, Anna Dominiczak and Alex Alderton, Roberto Amato, Sonia Goncalves, Ewan Harrison, David K. Jackson, Ian Johnston, Dominic Kwiatkowski, Cordelia Langford, John Sillitoe on behalf of the Wellcome Sanger Institute COVID-19 Surveillance Team
EPI_ISL_891627	Lighthouse Lab in Cambridge	Wellcome Sanger Institute for the COVID-19 Genomics UK (COG-UK) Consortium	Rob Howes, The Lighthouse Lab in Cambridge and Alex Alderton, Roberto Amato, Sonia Goncalves, Ewan Harrison, David K. Jackson, Ian Johnston, Dominic Kwiatkowski, Cordelia Langford, John Sillitoe on behalf of the Wellcome Sanger Institute COVID-19 Surveillance Team
EPI_ISL_891628	Lighthouse Lab in Glasgow	Wellcome Sanger Institute for the COVID-19 Genomics UK (COG-UK) Consortium	Harper VanSteenhouse, Yumi Kasai, David Gray, Carol Clugston, Anna Dominiczak and Alex Alderton, Roberto Amato, Sonia Goncalves, Ewan Harrison, David K. Jackson, Ian Johnston, Dominic Kwiatkowski, Cordelia Langford, John Sillitoe on behalf of the Wellcome Sanger Institute COVID-19 Surveillance Team
EPI_ISL_891629, EPI_ISL_891630	Lighthouse Lab in Alderley Park	Wellcome Sanger Institute for the COVID-19 Genomics UK (COG-UK) Consortium	Jacquelyn Wynn, Mairead Hyland, The Lighthouse Lab in Alderley Park and Alex Alderton, Roberto Amato, Sonia Goncalves, Ewan Harrison, David K. Jackson, Ian Johnston, Dominic Kwiatkowski, Cordelia Langford, John Sillitoe on behalf of the Wellcome Sanger Institute COVID-19 Surveillance Team
EPI_ISL_891631, EPI_ISL_891632	Lighthouse Lab in Cambridge	Wellcome Sanger Institute for the COVID-19 Genomics UK (COG-UK) Consortium	Rob Howes, The Lighthouse Lab in Cambridge and Alex Alderton, Roberto Amato, Sonia Goncalves, Ewan Harrison, David K. Jackson, Ian Johnston, Dominic Kwiatkowski, Cordelia Langford, John Sillitoe on behalf of the Wellcome Sanger Institute COVID-19 Surveillance Team
EPI_ISL_891633, EPI_ISL_891634	Lighthouse Lab in Alderley Park	Wellcome Sanger Institute for the COVID-19 Genomics UK (COG-UK) Consortium	Jacquelyn Wynn, Mairead Hyland, The Lighthouse Lab in Alderley Park and Alex Alderton, Roberto Amato, Sonia Goncalves, Ewan Harrison, David K. Jackson, Ian Johnston, Dominic Kwiatkowski, Cordelia Langford, John Sillitoe on behalf of the Wellcome Sanger Institute COVID-19 Surveillance Team
EPI_ISL_891635	Lighthouse Lab in Cambridge	Wellcome Sanger Institute for the COVID-19 Genomics UK (COG-UK) Consortium	Rob Howes, The Lighthouse Lab in Cambridge and Alex Alderton, Roberto Amato, Sonia Goncalves, Ewan Harrison, David K. Jackson, Ian Johnston, Dominic Kwiatkowski, Cordelia Langford, John Sillitoe on behalf of the Wellcome Sanger Institute COVID-19 Surveillance Team
EPI_ISL_891636	Lighthouse Lab in Glasgow	Wellcome Sanger Institute for the COVID-19 Genomics UK (COG-UK) Consortium	Harper VanSteenhouse, Yumi Kasai, David Gray, Carol Clugston, Anna Dominiczak and Alex Alderton, Roberto Amato, Sonia Goncalves, Ewan Harrison, David K. Jackson, Ian Johnston, Dominic Kwiatkowski, Cordelia Langford, John Sillitoe on behalf of the Wellcome Sanger Institute COVID-19 Surveillance Team
EPI_ISL_891637, EPI_ISL_891638	Lighthouse Lab in Cambridge	Wellcome Sanger Institute for the COVID-19 Genomics UK (COG-UK) Consortium	Rob Howes, The Lighthouse Lab in Cambridge and Alex Alderton, Roberto Amato, Sonia Goncalves, Ewan Harrison, David K. Jackson, Ian Johnston, Dominic Kwiatkowski, Cordelia Langford, John Sillitoe on behalf of the Wellcome Sanger Institute COVID-19 Surveillance Team
EPI_ISL_891640	Lighthouse Lab in Alderley Park	Wellcome Sanger Institute for the COVID-19 Genomics UK (COG-UK) Consortium	Jacquelyn Wynn, Mairead Hyland, The Lighthouse Lab in Alderley Park and Alex Alderton, Roberto Amato, Sonia Goncalves, Ewan Harrison, David K. Jackson, Ian Johnston, Dominic Kwiatkowski, Cordelia Langford, John Sillitoe on behalf of the Wellcome Sanger Institute COVID-19 Surveillance Team
EPI_ISL_891641, EPI_ISL_891642	Lighthouse Lab in Cambridge	Wellcome Sanger Institute for the COVID-19 Genomics UK (COG-UK) Consortium	Rob Howes, The Lighthouse Lab in Cambridge and Alex Alderton, Roberto Amato, Sonia Goncalves, Ewan Harrison, David K. Jackson, Ian Johnston, Dominic Kwiatkowski, Cordelia Langford, John Sillitoe on behalf of the Wellcome Sanger Institute COVID-19 Surveillance Team
EPI_ISL_891643	Lighthouse Lab in Alderley Park	Wellcome Sanger Institute for the COVID-19 Genomics UK (COG-UK) Consortium	Jacquelyn Wynn, Mairead Hyland, The Lighthouse Lab in Alderley Park and Alex Alderton, Roberto Amato, Sonia Goncalves, Ewan Harrison, David K. Jackson, Ian Johnston, Dominic Kwiatkowski, Cordelia Langford, John Sillitoe on behalf of the Wellcome Sanger Institute COVID-19 Surveillance Team
EPI_ISL_891644, EPI_ISL_891645, EPI_ISL_891646	Lighthouse Lab in Cambridge	Wellcome Sanger Institute for the COVID-19 Genomics UK (COG-UK) Consortium	Rob Howes, The Lighthouse Lab in Cambridge and Alex Alderton, Roberto Amato, Sonia Goncalves, Ewan Harrison, David K. Jackson, Ian Johnston, Dominic Kwiatkowski, Cordelia Langford, John Sillitoe on behalf of the Wellcome Sanger Institute COVID-19 Surveillance Team
EPI_ISL_891647	Lighthouse Lab in Alderley Park	Wellcome Sanger Institute for the COVID-19 Genomics UK (COG-UK) Consortium	Jacquelyn Wynn, Mairead Hyland, The Lighthouse Lab in Alderley Park and Alex Alderton, Roberto Amato, Sonia Goncalves, Ewan Harrison, David K. Jackson, Ian Johnston, Dominic Kwiatkowski, Cordelia Langford, John Sillitoe on behalf of the Wellcome Sanger Institute COVID-19 Surveillance Team
EPI_ISL_891648, EPI_ISL_891649	Lighthouse Lab in Cambridge	Wellcome Sanger Institute for the COVID-19 Genomics UK (COG-UK) Consortium	Rob Howes, The Lighthouse Lab in Cambridge and Alex Alderton, Roberto Amato, Sonia Goncalves, Ewan Harrison, David K. Jackson, Ian Johnston, Dominic Kwiatkowski, Cordelia Langford, John Sillitoe on behalf of the Wellcome Sanger Institute COVID-19 Surveillance Team
EPI_ISL_891650	Lighthouse Lab in Alderley Park	Wellcome Sanger Institute for the COVID-19 Genomics UK	Jacquelyn Wynn, Mairead Hyland, The Lighthouse Lab in Alderley Park and Alex Alderton, Roberto Amato, Sonia Goncalves, Ewan Harrison, David K.

		(COG-UK) Consortium	Jackson, Ian Johnston, Dominic Kwiatkowski, Cordelia Langford, John Sillitoe on behalf of the Wellcome Sanger Institute COVID-19 Surveillance Team
EPI_ISL_891651, EPI_ISL_891652, EPI_ISL_891653, EPI_ISL_891654	Lighthouse Lab in Cambridge	Wellcome Sanger Institute for the COVID-19 Genomics UK (COG-UK) Consortium	Rob Howes, The Lighthouse Lab in Cambridge and Alex Alderton, Roberto Amato, Sonia Goncalves, Ewan Harrison, David K. Jackson, Ian Johnston, Dominic Kwiatkowski, Cordelia Langford, John Sillitoe on behalf of the Wellcome Sanger Institute COVID-19 Surveillance Team
EPI_ISL_891656	Lighthouse Lab in Glasgow	Wellcome Sanger Institute for the COVID-19 Genomics UK (COG-UK) Consortium	Harper VanSteenhouse, Yumi Kasai, David Gray, Carol Clugston, Anna Dominiczak and Alex Alderton, Roberto Amato, Sonia Goncalves, Ewan Harrison, David K. Jackson, Ian Johnston, Dominic Kwiatkowski, Cordelia Langford, John Sillitoe on behalf of the Wellcome Sanger Institute COVID-19 Surveillance Team
EPI_ISL_891657	Lighthouse Lab in Cambridge	Wellcome Sanger Institute for the COVID-19 Genomics UK (COG-UK) Consortium	Rob Howes, The Lighthouse Lab in Cambridge and Alex Alderton, Roberto Amato, Sonia Goncalves, Ewan Harrison, David K. Jackson, Ian Johnston, Dominic Kwiatkowski, Cordelia Langford, John Sillitoe on behalf of the Wellcome Sanger Institute COVID-19 Surveillance Team
EPI_ISL_891658	Lighthouse Lab in Glasgow	Wellcome Sanger Institute for the COVID-19 Genomics UK (COG-UK) Consortium	Harper VanSteenhouse, Yumi Kasai, David Gray, Carol Clugston, Anna Dominiczak and Alex Alderton, Roberto Amato, Sonia Goncalves, Ewan Harrison, David K. Jackson, Ian Johnston, Dominic Kwiatkowski, Cordelia Langford, John Sillitoe on behalf of the Wellcome Sanger Institute COVID-19 Surveillance Team
EPI_ISL_891659	Lighthouse Lab in Alderley Park	Wellcome Sanger Institute for the COVID-19 Genomics UK (COG-UK) Consortium	Jacquelyn Wynn, Mairead Hyland, The Lighthouse Lab in Alderley Park and Alex Alderton, Roberto Amato, Sonia Goncalves, Ewan Harrison, David K. Jackson, Ian Johnston, Dominic Kwiatkowski, Cordelia Langford, John Sillitoe on behalf of the Wellcome Sanger Institute COVID-19 Surveillance Team
EPI_ISL_891660	Lighthouse Lab in Cambridge	Wellcome Sanger Institute for the COVID-19 Genomics UK (COG-UK) Consortium	Rob Howes, The Lighthouse Lab in Cambridge and Alex Alderton, Roberto Amato, Sonia Goncalves, Ewan Harrison, David K. Jackson, Ian Johnston, Dominic Kwiatkowski, Cordelia Langford, John Sillitoe on behalf of the Wellcome Sanger Institute COVID-19 Surveillance Team
EPI_ISL_891661, EPI_ISL_891662	Lighthouse Lab in Alderley Park	Wellcome Sanger Institute for the COVID-19 Genomics UK (COG-UK) Consortium	Jacquelyn Wynn, Mairead Hyland, The Lighthouse Lab in Alderley Park and Alex Alderton, Roberto Amato, Sonia Goncalves, Ewan Harrison, David K. Jackson, Ian Johnston, Dominic Kwiatkowski, Cordelia Langford, John Sillitoe on behalf of the Wellcome Sanger Institute COVID-19 Surveillance Team
EPI_ISL_891663, EPI_ISL_891665, EPI_ISL_891666, EPI_ISL_891667, EPI_ISL_891669	Lighthouse Lab in Cambridge	Wellcome Sanger Institute for the COVID-19 Genomics UK (COG-UK) Consortium	Rob Howes, The Lighthouse Lab in Cambridge and Alex Alderton, Roberto Amato, Sonia Goncalves, Ewan Harrison, David K. Jackson, Ian Johnston, Dominic Kwiatkowski, Cordelia Langford, John Sillitoe on behalf of the Wellcome Sanger Institute COVID-19 Surveillance Team
EPI_ISL_891670	Lighthouse Lab in Alderley Park	Wellcome Sanger Institute for the COVID-19 Genomics UK (COG-UK) Consortium	Jacquelyn Wynn, Mairead Hyland, The Lighthouse Lab in Alderley Park and Alex Alderton, Roberto Amato, Sonia Goncalves, Ewan Harrison, David K. Jackson, Ian Johnston, Dominic Kwiatkowski, Cordelia Langford, John Sillitoe on behalf of the Wellcome Sanger Institute COVID-19 Surveillance Team
EPI_ISL_891671	Lighthouse Lab in Cambridge	Wellcome Sanger Institute for the COVID-19 Genomics UK (COG-UK) Consortium	Rob Howes, The Lighthouse Lab in Cambridge and Alex Alderton, Roberto Amato, Sonia Goncalves, Ewan Harrison, David K. Jackson, Ian Johnston, Dominic Kwiatkowski, Cordelia Langford, John Sillitoe on behalf of the Wellcome Sanger Institute COVID-19 Surveillance Team
EPI_ISL_891672, EPI_ISL_891673	Lighthouse Lab in Alderley Park	Wellcome Sanger Institute for the COVID-19 Genomics UK (COG-UK) Consortium	Jacquelyn Wynn, Mairead Hyland, The Lighthouse Lab in Alderley Park and Alex Alderton, Roberto Amato, Sonia Goncalves, Ewan Harrison, David K. Jackson, Ian Johnston, Dominic Kwiatkowski, Cordelia Langford, John Sillitoe on behalf of the Wellcome Sanger Institute COVID-19 Surveillance Team
EPI_ISL_891674, EPI_ISL_891675, EPI_ISL_891676	Lighthouse Lab in Cambridge	Wellcome Sanger Institute for the COVID-19 Genomics UK (COG-UK) Consortium	Rob Howes, The Lighthouse Lab in Cambridge and Alex Alderton, Roberto Amato, Sonia Goncalves, Ewan Harrison, David K. Jackson, Ian Johnston, Dominic Kwiatkowski, Cordelia Langford, John Sillitoe on behalf of the Wellcome Sanger Institute COVID-19 Surveillance Team
EPI_ISL_891677	Lighthouse Lab in Alderley Park	Wellcome Sanger Institute for the COVID-19 Genomics UK (COG-UK) Consortium	Jacquelyn Wynn, Mairead Hyland, The Lighthouse Lab in Alderley Park and Alex Alderton, Roberto Amato, Sonia Goncalves, Ewan Harrison, David K. Jackson, Ian Johnston, Dominic Kwiatkowski, Cordelia Langford, John Sillitoe on behalf of the Wellcome Sanger Institute COVID-19 Surveillance Team
EPI_ISL_891678, EPI_ISL_891679	Lighthouse Lab in Cambridge	Wellcome Sanger Institute for the COVID-19 Genomics UK (COG-UK) Consortium	Rob Howes, The Lighthouse Lab in Cambridge and Alex Alderton, Roberto Amato, Sonia Goncalves, Ewan Harrison, David K. Jackson, Ian Johnston, Dominic Kwiatkowski, Cordelia Langford, John Sillitoe on behalf of the Wellcome Sanger Institute COVID-19 Surveillance Team
EPI_ISL_891680, EPI_ISL_891681	Lighthouse Lab in Alderley Park	Wellcome Sanger Institute for the COVID-19 Genomics UK (COG-UK) Consortium	Jacquelyn Wynn, Mairead Hyland, The Lighthouse Lab in Alderley Park and Alex Alderton, Roberto Amato, Sonia Goncalves, Ewan Harrison, David K. Jackson, Ian Johnston, Dominic Kwiatkowski, Cordelia Langford, John Sillitoe on behalf of the Wellcome Sanger Institute COVID-19 Surveillance Team
EPI_ISL_891682	Lighthouse Lab in Cambridge	Wellcome Sanger Institute for the COVID-19 Genomics UK (COG-UK) Consortium	Rob Howes, The Lighthouse Lab in Cambridge and Alex Alderton, Roberto Amato, Sonia Goncalves, Ewan Harrison, David K. Jackson, Ian Johnston, Dominic Kwiatkowski, Cordelia Langford, John Sillitoe on behalf of the Wellcome Sanger Institute COVID-19 Surveillance Team
EPI_ISL_891683	Lighthouse Lab in Alderley Park	Wellcome Sanger Institute for the COVID-19 Genomics UK (COG-UK) Consortium	Jacquelyn Wynn, Mairead Hyland, The Lighthouse Lab in Alderley Park and Alex Alderton, Roberto Amato, Sonia Goncalves, Ewan Harrison, David K. Jackson, Ian Johnston, Dominic Kwiatkowski, Cordelia Langford, John Sillitoe on behalf of the Wellcome Sanger Institute COVID-19 Surveillance Team
EPI_ISL_891684	Lighthouse Lab in Cambridge	Wellcome Sanger Institute for the COVID-19 Genomics UK (COG-UK) Consortium	Rob Howes, The Lighthouse Lab in Cambridge and Alex Alderton, Roberto Amato, Sonia Goncalves, Ewan Harrison, David K. Jackson, Ian Johnston, Dominic Kwiatkowski, Cordelia Langford, John Sillitoe on behalf of the Wellcome Sanger Institute COVID-19 Surveillance Team
EPI_ISL_891685	Lighthouse Lab in Glasgow	Wellcome Sanger Institute for the COVID-19 Genomics UK (COG-UK) Consortium	Harper VanSteenhouse, Yumi Kasai, David Gray, Carol Clugston, Anna Dominiczak and Alex Alderton, Roberto Amato, Sonia Goncalves, Ewan Harrison, David K. Jackson, Ian Johnston, Dominic Kwiatkowski, Cordelia Langford, John Sillitoe on behalf of the Wellcome Sanger Institute COVID-19 Surveillance Team
EPI_ISL_891686	Lighthouse Lab in Cambridge	Wellcome Sanger Institute for the COVID-19 Genomics UK (COG-UK) Consortium	Rob Howes, The Lighthouse Lab in Cambridge and Alex Alderton, Roberto Amato, Sonia Goncalves, Ewan Harrison, David K. Jackson, Ian Johnston, Dominic Kwiatkowski, Cordelia Langford, John Sillitoe on behalf of the Wellcome Sanger Institute COVID-19 Surveillance Team
EPI_ISL_891687	Lighthouse Lab in Alderley Park	Wellcome Sanger Institute for the COVID-19 Genomics UK (COG-UK) Consortium	Jacquelyn Wynn, Mairead Hyland, The Lighthouse Lab in Alderley Park and Alex Alderton, Roberto Amato, Sonia Goncalves, Ewan Harrison, David K. Jackson, Ian Johnston, Dominic Kwiatkowski, Cordelia Langford, John Sillitoe on behalf of the Wellcome Sanger Institute COVID-19 Surveillance Team
EPI_ISL_891688	Lighthouse Lab in Cambridge	Wellcome Sanger Institute for the COVID-19 Genomics UK (COG-UK) Consortium	Rob Howes, The Lighthouse Lab in Cambridge and Alex Alderton, Roberto Amato, Sonia Goncalves, Ewan Harrison, David K. Jackson, Ian Johnston, Dominic Kwiatkowski, Cordelia Langford, John Sillitoe on behalf of the Wellcome Sanger Institute COVID-19 Surveillance Team
EPI_ISL_891689	Lighthouse Lab in Glasgow	Wellcome Sanger Institute for the COVID-19 Genomics UK (COG-UK) Consortium	Harper VanSteenhouse, Yumi Kasai, David Gray, Carol Clugston, Anna Dominiczak and Alex Alderton, Roberto Amato, Sonia Goncalves, Ewan Harrison, David K. Jackson, Ian Johnston, Dominic Kwiatkowski, Cordelia Langford, John Sillitoe on behalf of the Wellcome Sanger Institute COVID-19 Surveillance Team
EPI_ISL_891690, EPI_ISL_891691, EPI_ISL_891692, EPI_ISL_891693, EPI_ISL_891695, EPI_ISL_891696, EPI_ISL_891697, EPI_ISL_891698, EPI_ISL_891699, EPI_ISL_891701, EPI_ISL_891702, EPI_ISL_891703			
see above	Lighthouse Lab in Cambridge	Wellcome Sanger Institute for the COVID-19 Genomics UK (COG-UK) Consortium	Rob Howes, The Lighthouse Lab in Cambridge and Alex Alderton, Roberto Amato, Sonia Goncalves, Ewan Harrison, David K. Jackson, Ian Johnston, Dominic Kwiatkowski, Cordelia Langford, John Sillitoe on behalf of the Wellcome Sanger Institute COVID-19 Surveillance Team
EPI_ISL_891704	Lighthouse Lab in Alderley Park	Wellcome Sanger Institute for the COVID-19 Genomics UK (COG-UK) Consortium	Jacquelyn Wynn, Mairead Hyland, The Lighthouse Lab in Alderley Park and Alex Alderton, Roberto Amato, Sonia Goncalves, Ewan Harrison, David K. Jackson, Ian Johnston, Dominic Kwiatkowski, Cordelia Langford, John Sillitoe on behalf of the Wellcome Sanger Institute COVID-19 Surveillance Team
EPI_ISL_891705	Lighthouse Lab in Glasgow	Wellcome Sanger Institute for the COVID-19 Genomics UK (COG-UK) Consortium	Harper VanSteenhouse, Yumi Kasai, David Gray, Carol Clugston, Anna Dominiczak and Alex Alderton, Roberto Amato, Sonia Goncalves, Ewan Harrison, David K. Jackson, Ian Johnston, Dominic Kwiatkowski, Cordelia Langford, John Sillitoe on behalf of the Wellcome Sanger Institute COVID-19 Surveillance Team
EPI_ISL_891706, EPI_ISL_891707, EPI_ISL_891708	Lighthouse Lab in Cambridge	Wellcome Sanger Institute for the COVID-19 Genomics UK (COG-UK) Consortium	Rob Howes, The Lighthouse Lab in Cambridge and Alex Alderton, Roberto Amato, Sonia Goncalves, Ewan Harrison, David K. Jackson, Ian Johnston, Dominic Kwiatkowski, Cordelia Langford, John Sillitoe on behalf of the Wellcome Sanger Institute COVID-19 Surveillance Team
EPI_ISL_891710	Lighthouse Lab in Alderley Park	Wellcome Sanger Institute for the COVID-19 Genomics UK (COG-UK) Consortium	Jacquelyn Wynn, Mairead Hyland, The Lighthouse Lab in Alderley Park and Alex Alderton, Roberto Amato, Sonia Goncalves, Ewan Harrison, David K. Jackson, Ian Johnston, Dominic Kwiatkowski, Cordelia Langford, John Sillitoe on behalf of the Wellcome Sanger Institute COVID-19 Surveillance Team
EPI_ISL_891712	Lighthouse Lab in Cambridge	Wellcome Sanger Institute for the COVID-19 Genomics UK (COG-UK) Consortium	Rob Howes, The Lighthouse Lab in Cambridge and Alex Alderton, Roberto Amato, Sonia Goncalves, Ewan Harrison, David K. Jackson, Ian Johnston, Dominic Kwiatkowski, Cordelia Langford, John Sillitoe on behalf of the Wellcome Sanger Institute COVID-19 Surveillance Team
EPI_ISL_891713, EPI_ISL_891714	Lighthouse Lab in Alderley Park	Wellcome Sanger Institute for the COVID-19 Genomics UK (COG-UK) Consortium	Jacquelyn Wynn, Mairead Hyland, The Lighthouse Lab in Alderley Park and Alex Alderton, Roberto Amato, Sonia Goncalves, Ewan Harrison, David K. Jackson, Ian Johnston, Dominic Kwiatkowski, Cordelia Langford, John Sillitoe on behalf of the Wellcome Sanger Institute COVID-19 Surveillance Team
EPI_ISL_891715, EPI_ISL_891716	Lighthouse Lab in Cambridge	Wellcome Sanger Institute for the COVID-19 Genomics UK (COG-UK) Consortium	Rob Howes, The Lighthouse Lab in Cambridge and Alex Alderton, Roberto Amato, Sonia Goncalves, Ewan Harrison, David K. Jackson, Ian Johnston, Dominic Kwiatkowski, Cordelia Langford, John Sillitoe on behalf of the Wellcome Sanger Institute COVID-19 Surveillance Team
EPI_ISL_891717	Lighthouse Lab in Glasgow	Wellcome Sanger Institute for the COVID-19 Genomics UK	Harper VanSteenhouse, Yumi Kasai, David Gray, Carol Clugston, Anna Dominiczak and Alex Alderton, Roberto Amato, Sonia Goncalves, Ewan Harrison,

		(COG-UK) Consortium	David K. Jackson, Ian Johnston, Dominic Kwiatkowski, Cordelia Langford, John Sillitoe on behalf of the Wellcome Sanger Institute COVID-19 Surveillance Team
EPI_ISL_891718, EPI_ISL_891719	Lighthouse Lab in Alderley Park	Wellcome Sanger Institute for the COVID-19 Genomics UK (COG-UK) Consortium	Jacquelyn Wynn, Mairead Hyland, The Lighthouse Lab in Alderley Park and Alex Alderton, Roberto Amato, Sonia Goncalves, Ewan Harrison, David K. Jackson, Ian Johnston, Dominic Kwiatkowski, Cordelia Langford, John Sillitoe on behalf of the Wellcome Sanger Institute COVID-19 Surveillance Team
EPI_ISL_891720	Lighthouse Lab in Cambridge	Wellcome Sanger Institute for the COVID-19 Genomics UK (COG-UK) Consortium	Rob Howes, The Lighthouse Lab in Cambridge and Alex Alderton, Roberto Amato, Sonia Goncalves, Ewan Harrison, David K. Jackson, Ian Johnston, Dominic Kwiatkowski, Cordelia Langford, John Sillitoe on behalf of the Wellcome Sanger Institute COVID-19 Surveillance Team
EPI_ISL_891721, EPI_ISL_891722	Lighthouse Lab in Glasgow	Wellcome Sanger Institute for the COVID-19 Genomics UK (COG-UK) Consortium	Harper VanSteenhouse, Yumi Kasai, David Gray, Carol Clugston, Anna Dominiczak and Alex Alderton, Roberto Amato, Sonia Goncalves, Ewan Harrison, David K. Jackson, Ian Johnston, Dominic Kwiatkowski, Cordelia Langford, John Sillitoe on behalf of the Wellcome Sanger Institute COVID-19 Surveillance Team
EPI_ISL_891723, EPI_ISL_891724	Lighthouse Lab in Alderley Park	Wellcome Sanger Institute for the COVID-19 Genomics UK (COG-UK) Consortium	Jacquelyn Wynn, Mairead Hyland, The Lighthouse Lab in Alderley Park and Alex Alderton, Roberto Amato, Sonia Goncalves, Ewan Harrison, David K. Jackson, Ian Johnston, Dominic Kwiatkowski, Cordelia Langford, John Sillitoe on behalf of the Wellcome Sanger Institute COVID-19 Surveillance Team
EPI_ISL_891725	Lighthouse Lab in Glasgow	Wellcome Sanger Institute for the COVID-19 Genomics UK (COG-UK) Consortium	Harper VanSteenhouse, Yumi Kasai, David Gray, Carol Clugston, Anna Dominiczak and Alex Alderton, Roberto Amato, Sonia Goncalves, Ewan Harrison, David K. Jackson, Ian Johnston, Dominic Kwiatkowski, Cordelia Langford, John Sillitoe on behalf of the Wellcome Sanger Institute COVID-19 Surveillance Team
EPI_ISL_891726	Lighthouse Lab in Alderley Park	Wellcome Sanger Institute for the COVID-19 Genomics UK (COG-UK) Consortium	Jacquelyn Wynn, Mairead Hyland, The Lighthouse Lab in Alderley Park and Alex Alderton, Roberto Amato, Sonia Goncalves, Ewan Harrison, David K. Jackson, Ian Johnston, Dominic Kwiatkowski, Cordelia Langford, John Sillitoe on behalf of the Wellcome Sanger Institute COVID-19 Surveillance Team
EPI_ISL_891727	Lighthouse Lab in Glasgow	Wellcome Sanger Institute for the COVID-19 Genomics UK (COG-UK) Consortium	Harper VanSteenhouse, Yumi Kasai, David Gray, Carol Clugston, Anna Dominiczak and Alex Alderton, Roberto Amato, Sonia Goncalves, Ewan Harrison, David K. Jackson, Ian Johnston, Dominic Kwiatkowski, Cordelia Langford, John Sillitoe on behalf of the Wellcome Sanger Institute COVID-19 Surveillance Team
EPI_ISL_891728	Lighthouse Lab in Cambridge	Wellcome Sanger Institute for the COVID-19 Genomics UK (COG-UK) Consortium	Rob Howes, The Lighthouse Lab in Cambridge and Alex Alderton, Roberto Amato, Sonia Goncalves, Ewan Harrison, David K. Jackson, Ian Johnston, Dominic Kwiatkowski, Cordelia Langford, John Sillitoe on behalf of the Wellcome Sanger Institute COVID-19 Surveillance Team
EPI_ISL_891729	Lighthouse Lab in Alderley Park	Wellcome Sanger Institute for the COVID-19 Genomics UK (COG-UK) Consortium	Jacquelyn Wynn, Mairead Hyland, The Lighthouse Lab in Alderley Park and Alex Alderton, Roberto Amato, Sonia Goncalves, Ewan Harrison, David K. Jackson, Ian Johnston, Dominic Kwiatkowski, Cordelia Langford, John Sillitoe on behalf of the Wellcome Sanger Institute COVID-19 Surveillance Team
EPI_ISL_891730, EPI_ISL_891731, EPI_ISL_891732, EPI_ISL_891733, EPI_ISL_891734	Lighthouse Lab in Cambridge	Wellcome Sanger Institute for the COVID-19 Genomics UK (COG-UK) Consortium	Rob Howes, The Lighthouse Lab in Cambridge and Alex Alderton, Roberto Amato, Sonia Goncalves, Ewan Harrison, David K. Jackson, Ian Johnston, Dominic Kwiatkowski, Cordelia Langford, John Sillitoe on behalf of the Wellcome Sanger Institute COVID-19 Surveillance Team
EPI_ISL_891735, EPI_ISL_891736	Lighthouse Lab in Alderley Park	Wellcome Sanger Institute for the COVID-19 Genomics UK (COG-UK) Consortium	Jacquelyn Wynn, Mairead Hyland, The Lighthouse Lab in Alderley Park and Alex Alderton, Roberto Amato, Sonia Goncalves, Ewan Harrison, David K. Jackson, Ian Johnston, Dominic Kwiatkowski, Cordelia Langford, John Sillitoe on behalf of the Wellcome Sanger Institute COVID-19 Surveillance Team
EPI_ISL_891737	Lighthouse Lab in Cambridge	Wellcome Sanger Institute for the COVID-19 Genomics UK (COG-UK) Consortium	Rob Howes, The Lighthouse Lab in Cambridge and Alex Alderton, Roberto Amato, Sonia Goncalves, Ewan Harrison, David K. Jackson, Ian Johnston, Dominic Kwiatkowski, Cordelia Langford, John Sillitoe on behalf of the Wellcome Sanger Institute COVID-19 Surveillance Team
EPI_ISL_891738	Lighthouse Lab in Alderley Park	Wellcome Sanger Institute for the COVID-19 Genomics UK (COG-UK) Consortium	Jacquelyn Wynn, Mairead Hyland, The Lighthouse Lab in Alderley Park and Alex Alderton, Roberto Amato, Sonia Goncalves, Ewan Harrison, David K. Jackson, Ian Johnston, Dominic Kwiatkowski, Cordelia Langford, John Sillitoe on behalf of the Wellcome Sanger Institute COVID-19 Surveillance Team
EPI_ISL_891739	Lighthouse Lab in Cambridge	Wellcome Sanger Institute for the COVID-19 Genomics UK (COG-UK) Consortium	Rob Howes, The Lighthouse Lab in Cambridge and Alex Alderton, Roberto Amato, Sonia Goncalves, Ewan Harrison, David K. Jackson, Ian Johnston, Dominic Kwiatkowski, Cordelia Langford, John Sillitoe on behalf of the Wellcome Sanger Institute COVID-19 Surveillance Team
EPI_ISL_891740, EPI_ISL_891741	Lighthouse Lab in Glasgow	Wellcome Sanger Institute for the COVID-19 Genomics UK (COG-UK) Consortium	Harper VanSteenhouse, Yumi Kasai, David Gray, Carol Clugston, Anna Dominiczak and Alex Alderton, Roberto Amato, Sonia Goncalves, Ewan Harrison, David K. Jackson, Ian Johnston, Dominic Kwiatkowski, Cordelia Langford, John Sillitoe on behalf of the Wellcome Sanger Institute COVID-19 Surveillance Team
EPI_ISL_891742	Lighthouse Lab in Alderley Park	Wellcome Sanger Institute for the COVID-19 Genomics UK (COG-UK) Consortium	Jacquelyn Wynn, Mairead Hyland, The Lighthouse Lab in Alderley Park and Alex Alderton, Roberto Amato, Sonia Goncalves, Ewan Harrison, David K. Jackson, Ian Johnston, Dominic Kwiatkowski, Cordelia Langford, John Sillitoe on behalf of the Wellcome Sanger Institute COVID-19 Surveillance Team
EPI_ISL_891743, EPI_ISL_891744, EPI_ISL_891745	Lighthouse Lab in Cambridge	Wellcome Sanger Institute for the COVID-19 Genomics UK (COG-UK) Consortium	Rob Howes, The Lighthouse Lab in Cambridge and Alex Alderton, Roberto Amato, Sonia Goncalves, Ewan Harrison, David K. Jackson, Ian Johnston, Dominic Kwiatkowski, Cordelia Langford, John Sillitoe on behalf of the Wellcome Sanger Institute COVID-19 Surveillance Team
EPI_ISL_891746	Lighthouse Lab in Alderley Park	Wellcome Sanger Institute for the COVID-19 Genomics UK (COG-UK) Consortium	Jacquelyn Wynn, Mairead Hyland, The Lighthouse Lab in Alderley Park and Alex Alderton, Roberto Amato, Sonia Goncalves, Ewan Harrison, David K. Jackson, Ian Johnston, Dominic Kwiatkowski, Cordelia Langford, John Sillitoe on behalf of the Wellcome Sanger Institute COVID-19 Surveillance Team
EPI_ISL_891748, EPI_ISL_891750	Lighthouse Lab in Cambridge	Wellcome Sanger Institute for the COVID-19 Genomics UK (COG-UK) Consortium	Rob Howes, The Lighthouse Lab in Cambridge and Alex Alderton, Roberto Amato, Sonia Goncalves, Ewan Harrison, David K. Jackson, Ian Johnston, Dominic Kwiatkowski, Cordelia Langford, John Sillitoe on behalf of the Wellcome Sanger Institute COVID-19 Surveillance Team
EPI_ISL_891751	Lighthouse Lab in Alderley Park	Wellcome Sanger Institute for the COVID-19 Genomics UK (COG-UK) Consortium	Jacquelyn Wynn, Mairead Hyland, The Lighthouse Lab in Alderley Park and Alex Alderton, Roberto Amato, Sonia Goncalves, Ewan Harrison, David K. Jackson, Ian Johnston, Dominic Kwiatkowski, Cordelia Langford, John Sillitoe on behalf of the Wellcome Sanger Institute COVID-19 Surveillance Team
EPI_ISL_891752	Lighthouse Lab in Cambridge	Wellcome Sanger Institute for the COVID-19 Genomics UK (COG-UK) Consortium	Rob Howes, The Lighthouse Lab in Cambridge and Alex Alderton, Roberto Amato, Sonia Goncalves, Ewan Harrison, David K. Jackson, Ian Johnston, Dominic Kwiatkowski, Cordelia Langford, John Sillitoe on behalf of the Wellcome Sanger Institute COVID-19 Surveillance Team
EPI_ISL_891753	Lighthouse Lab in Alderley Park	Wellcome Sanger Institute for the COVID-19 Genomics UK (COG-UK) Consortium	Jacquelyn Wynn, Mairead Hyland, The Lighthouse Lab in Alderley Park and Alex Alderton, Roberto Amato, Sonia Goncalves, Ewan Harrison, David K. Jackson, Ian Johnston, Dominic Kwiatkowski, Cordelia Langford, John Sillitoe on behalf of the Wellcome Sanger Institute COVID-19 Surveillance Team
EPI_ISL_891754, EPI_ISL_891756	Lighthouse Lab in Cambridge	Wellcome Sanger Institute for the COVID-19 Genomics UK (COG-UK) Consortium	Rob Howes, The Lighthouse Lab in Cambridge and Alex Alderton, Roberto Amato, Sonia Goncalves, Ewan Harrison, David K. Jackson, Ian Johnston, Dominic Kwiatkowski, Cordelia Langford, John Sillitoe on behalf of the Wellcome Sanger Institute COVID-19 Surveillance Team
EPI_ISL_891757	Lighthouse Lab in Alderley Park	Wellcome Sanger Institute for the COVID-19 Genomics UK (COG-UK) Consortium	Jacquelyn Wynn, Mairead Hyland, The Lighthouse Lab in Alderley Park and Alex Alderton, Roberto Amato, Sonia Goncalves, Ewan Harrison, David K. Jackson, Ian Johnston, Dominic Kwiatkowski, Cordelia Langford, John Sillitoe on behalf of the Wellcome Sanger Institute COVID-19 Surveillance Team
EPI_ISL_891758, EPI_ISL_891759	Lighthouse Lab in Cambridge	Wellcome Sanger Institute for the COVID-19 Genomics UK (COG-UK) Consortium	Rob Howes, The Lighthouse Lab in Cambridge and Alex Alderton, Roberto Amato, Sonia Goncalves, Ewan Harrison, David K. Jackson, Ian Johnston, Dominic Kwiatkowski, Cordelia Langford, John Sillitoe on behalf of the Wellcome Sanger Institute COVID-19 Surveillance Team
EPI_ISL_891760	Lighthouse Lab in Alderley Park	Wellcome Sanger Institute for the COVID-19 Genomics UK (COG-UK) Consortium	Jacquelyn Wynn, Mairead Hyland, The Lighthouse Lab in Alderley Park and Alex Alderton, Roberto Amato, Sonia Goncalves, Ewan Harrison, David K. Jackson, Ian Johnston, Dominic Kwiatkowski, Cordelia Langford, John Sillitoe on behalf of the Wellcome Sanger Institute COVID-19 Surveillance Team
EPI_ISL_891761	Lighthouse Lab in Glasgow	Wellcome Sanger Institute for the COVID-19 Genomics UK (COG-UK) Consortium	Harper VanSteenhouse, Yumi Kasai, David Gray, Carol Clugston, Anna Dominiczak and Alex Alderton, Roberto Amato, Sonia Goncalves, Ewan Harrison, David K. Jackson, Ian Johnston, Dominic Kwiatkowski, Cordelia Langford, John Sillitoe on behalf of the Wellcome Sanger Institute COVID-19 Surveillance Team
EPI_ISL_891762	Lighthouse Lab in Alderley Park	Wellcome Sanger Institute for the COVID-19 Genomics UK (COG-UK) Consortium	Jacquelyn Wynn, Mairead Hyland, The Lighthouse Lab in Alderley Park and Alex Alderton, Roberto Amato, Sonia Goncalves, Ewan Harrison, David K. Jackson, Ian Johnston, Dominic Kwiatkowski, Cordelia Langford, John Sillitoe on behalf of the Wellcome Sanger Institute COVID-19 Surveillance Team
EPI_ISL_891763, EPI_ISL_891764	Lighthouse Lab in Cambridge	Wellcome Sanger Institute for the COVID-19 Genomics UK (COG-UK) Consortium	Rob Howes, The Lighthouse Lab in Cambridge and Alex Alderton, Roberto Amato, Sonia Goncalves, Ewan Harrison, David K. Jackson, Ian Johnston, Dominic Kwiatkowski, Cordelia Langford, John Sillitoe on behalf of the Wellcome Sanger Institute COVID-19 Surveillance Team
EPI_ISL_891765, EPI_ISL_891766	Lighthouse Lab in Alderley Park	Wellcome Sanger Institute for the COVID-19 Genomics UK (COG-UK) Consortium	Jacquelyn Wynn, Mairead Hyland, The Lighthouse Lab in Alderley Park and Alex Alderton, Roberto Amato, Sonia Goncalves, Ewan Harrison, David K. Jackson, Ian Johnston, Dominic Kwiatkowski, Cordelia Langford, John Sillitoe on behalf of the Wellcome Sanger Institute COVID-19 Surveillance Team
EPI_ISL_891767, EPI_ISL_891768, EPI_ISL_891770	Lighthouse Lab in Cambridge	Wellcome Sanger Institute for the COVID-19 Genomics UK (COG-UK) Consortium	Rob Howes, The Lighthouse Lab in Cambridge and Alex Alderton, Roberto Amato, Sonia Goncalves, Ewan Harrison, David K. Jackson, Ian Johnston, Dominic Kwiatkowski, Cordelia Langford, John Sillitoe on behalf of the Wellcome Sanger Institute COVID-19 Surveillance Team
EPI_ISL_891771, EPI_ISL_891772	Lighthouse Lab in Glasgow	Wellcome Sanger Institute for the COVID-19 Genomics UK (COG-UK) Consortium	Harper VanSteenhouse, Yumi Kasai, David Gray, Carol Clugston, Anna Dominiczak and Alex Alderton, Roberto Amato, Sonia Goncalves, Ewan Harrison, David K. Jackson, Ian Johnston, Dominic Kwiatkowski, Cordelia Langford, John Sillitoe on behalf of the Wellcome Sanger Institute COVID-19 Surveillance

			Team
EPI_ISL_891773, EPI_ISL_891774, EPI_ISL_891775, EPI_ISL_891776, EPI_ISL_891777	Lighthouse Lab in Alderley Park	Wellcome Sanger Institute for the COVID-19 Genomics UK (COG-UK) Consortium	Jacquelyn Wynn, Mairead Hyland, The Lighthouse Lab in Alderley Park and Alex Alderton, Roberto Amato, Sonia Goncalves, Ewan Harrison, David K. Jackson, Ian Johnston, Dominic Kwiatkowski, Cordelia Langford, John Sillitoe on behalf of the Wellcome Sanger Institute COVID-19 Surveillance Team
EPI_ISL_891778, EPI_ISL_891779	Lighthouse Lab in Cambridge	Wellcome Sanger Institute for the COVID-19 Genomics UK (COG-UK) Consortium	Rob Howes, The Lighthouse Lab in Cambridge and Alex Alderton, Roberto Amato, Sonia Goncalves, Ewan Harrison, David K. Jackson, Ian Johnston, Dominic Kwiatkowski, Cordelia Langford, John Sillitoe on behalf of the Wellcome Sanger Institute COVID-19 Surveillance Team
EPI_ISL_891780	Lighthouse Lab in Alderley Park	Wellcome Sanger Institute for the COVID-19 Genomics UK (COG-UK) Consortium	Jacquelyn Wynn, Mairead Hyland, The Lighthouse Lab in Alderley Park and Alex Alderton, Roberto Amato, Sonia Goncalves, Ewan Harrison, David K. Jackson, Ian Johnston, Dominic Kwiatkowski, Cordelia Langford, John Sillitoe on behalf of the Wellcome Sanger Institute COVID-19 Surveillance Team
EPI_ISL_891781, EPI_ISL_891782, EPI_ISL_891783, EPI_ISL_891784, EPI_ISL_891785	Lighthouse Lab in Cambridge	Wellcome Sanger Institute for the COVID-19 Genomics UK (COG-UK) Consortium	Rob Howes, The Lighthouse Lab in Cambridge and Alex Alderton, Roberto Amato, Sonia Goncalves, Ewan Harrison, David K. Jackson, Ian Johnston, Dominic Kwiatkowski, Cordelia Langford, John Sillitoe on behalf of the Wellcome Sanger Institute COVID-19 Surveillance Team
EPI_ISL_891786, EPI_ISL_891787, EPI_ISL_891788, EPI_ISL_891789	Lighthouse Lab in Alderley Park	Wellcome Sanger Institute for the COVID-19 Genomics UK (COG-UK) Consortium	Jacquelyn Wynn, Mairead Hyland, The Lighthouse Lab in Alderley Park and Alex Alderton, Roberto Amato, Sonia Goncalves, Ewan Harrison, David K. Jackson, Ian Johnston, Dominic Kwiatkowski, Cordelia Langford, John Sillitoe on behalf of the Wellcome Sanger Institute COVID-19 Surveillance Team
EPI_ISL_891790, EPI_ISL_891791	Lighthouse Lab in Glasgow	Wellcome Sanger Institute for the COVID-19 Genomics UK (COG-UK) Consortium	Harper VanSteenhouse, Yumi Kasai, David Gray, Carol Clugston, Anna Dominiczak and Alex Alderton, Roberto Amato, Sonia Goncalves, Ewan Harrison, David K. Jackson, Ian Johnston, Dominic Kwiatkowski, Cordelia Langford, John Sillitoe on behalf of the Wellcome Sanger Institute COVID-19 Surveillance Team
EPI_ISL_891792	Lighthouse Lab in Cambridge	Wellcome Sanger Institute for the COVID-19 Genomics UK (COG-UK) Consortium	Rob Howes, The Lighthouse Lab in Cambridge and Alex Alderton, Roberto Amato, Sonia Goncalves, Ewan Harrison, David K. Jackson, Ian Johnston, Dominic Kwiatkowski, Cordelia Langford, John Sillitoe on behalf of the Wellcome Sanger Institute COVID-19 Surveillance Team
EPI_ISL_891793	Lighthouse Lab in Glasgow	Wellcome Sanger Institute for the COVID-19 Genomics UK (COG-UK) Consortium	Harper VanSteenhouse, Yumi Kasai, David Gray, Carol Clugston, Anna Dominiczak and Alex Alderton, Roberto Amato, Sonia Goncalves, Ewan Harrison, David K. Jackson, Ian Johnston, Dominic Kwiatkowski, Cordelia Langford, John Sillitoe on behalf of the Wellcome Sanger Institute COVID-19 Surveillance Team
EPI_ISL_891794	Lighthouse Lab in Alderley Park	Wellcome Sanger Institute for the COVID-19 Genomics UK (COG-UK) Consortium	Jacquelyn Wynn, Mairead Hyland, The Lighthouse Lab in Alderley Park and Alex Alderton, Roberto Amato, Sonia Goncalves, Ewan Harrison, David K. Jackson, Ian Johnston, Dominic Kwiatkowski, Cordelia Langford, John Sillitoe on behalf of the Wellcome Sanger Institute COVID-19 Surveillance Team
EPI_ISL_891795, EPI_ISL_891796, EPI_ISL_891797, EPI_ISL_891798, EPI_ISL_891800, EPI_ISL_891801	Lighthouse Lab in Cambridge	Wellcome Sanger Institute for the COVID-19 Genomics UK (COG-UK) Consortium	Rob Howes, The Lighthouse Lab in Cambridge and Alex Alderton, Roberto Amato, Sonia Goncalves, Ewan Harrison, David K. Jackson, Ian Johnston, Dominic Kwiatkowski, Cordelia Langford, John Sillitoe on behalf of the Wellcome Sanger Institute COVID-19 Surveillance Team
EPI_ISL_891802	Lighthouse Lab in Glasgow	Wellcome Sanger Institute for the COVID-19 Genomics UK (COG-UK) Consortium	Harper VanSteenhouse, Yumi Kasai, David Gray, Carol Clugston, Anna Dominiczak and Alex Alderton, Roberto Amato, Sonia Goncalves, Ewan Harrison, David K. Jackson, Ian Johnston, Dominic Kwiatkowski, Cordelia Langford, John Sillitoe on behalf of the Wellcome Sanger Institute COVID-19 Surveillance Team
EPI_ISL_891803	Lighthouse Lab in Cambridge	Wellcome Sanger Institute for the COVID-19 Genomics UK (COG-UK) Consortium	Rob Howes, The Lighthouse Lab in Cambridge and Alex Alderton, Roberto Amato, Sonia Goncalves, Ewan Harrison, David K. Jackson, Ian Johnston, Dominic Kwiatkowski, Cordelia Langford, John Sillitoe on behalf of the Wellcome Sanger Institute COVID-19 Surveillance Team
EPI_ISL_891804	Lighthouse Lab in Alderley Park	Wellcome Sanger Institute for the COVID-19 Genomics UK (COG-UK) Consortium	Jacquelyn Wynn, Mairead Hyland, The Lighthouse Lab in Alderley Park and Alex Alderton, Roberto Amato, Sonia Goncalves, Ewan Harrison, David K. Jackson, Ian Johnston, Dominic Kwiatkowski, Cordelia Langford, John Sillitoe on behalf of the Wellcome Sanger Institute COVID-19 Surveillance Team
EPI_ISL_891805, EPI_ISL_891806	Lighthouse Lab in Cambridge	Wellcome Sanger Institute for the COVID-19 Genomics UK (COG-UK) Consortium	Rob Howes, The Lighthouse Lab in Cambridge and Alex Alderton, Roberto Amato, Sonia Goncalves, Ewan Harrison, David K. Jackson, Ian Johnston, Dominic Kwiatkowski, Cordelia Langford, John Sillitoe on behalf of the Wellcome Sanger Institute COVID-19 Surveillance Team
EPI_ISL_891807	Lighthouse Lab in Alderley Park	Wellcome Sanger Institute for the COVID-19 Genomics UK (COG-UK) Consortium	Jacquelyn Wynn, Mairead Hyland, The Lighthouse Lab in Alderley Park and Alex Alderton, Roberto Amato, Sonia Goncalves, Ewan Harrison, David K. Jackson, Ian Johnston, Dominic Kwiatkowski, Cordelia Langford, John Sillitoe on behalf of the Wellcome Sanger Institute COVID-19 Surveillance Team
EPI_ISL_891808	Lighthouse Lab in Cambridge	Wellcome Sanger Institute for the COVID-19 Genomics UK (COG-UK) Consortium	Rob Howes, The Lighthouse Lab in Cambridge and Alex Alderton, Roberto Amato, Sonia Goncalves, Ewan Harrison, David K. Jackson, Ian Johnston, Dominic Kwiatkowski, Cordelia Langford, John Sillitoe on behalf of the Wellcome Sanger Institute COVID-19 Surveillance Team
EPI_ISL_891809	Lighthouse Lab in Glasgow	Wellcome Sanger Institute for the COVID-19 Genomics UK (COG-UK) Consortium	Harper VanSteenhouse, Yumi Kasai, David Gray, Carol Clugston, Anna Dominiczak and Alex Alderton, Roberto Amato, Sonia Goncalves, Ewan Harrison, David K. Jackson, Ian Johnston, Dominic Kwiatkowski, Cordelia Langford, John Sillitoe on behalf of the Wellcome Sanger Institute COVID-19 Surveillance Team
EPI_ISL_891810	Lighthouse Lab in Cambridge	Wellcome Sanger Institute for the COVID-19 Genomics UK (COG-UK) Consortium	Rob Howes, The Lighthouse Lab in Cambridge and Alex Alderton, Roberto Amato, Sonia Goncalves, Ewan Harrison, David K. Jackson, Ian Johnston, Dominic Kwiatkowski, Cordelia Langford, John Sillitoe on behalf of the Wellcome Sanger Institute COVID-19 Surveillance Team
EPI_ISL_891811	Lighthouse Lab in Alderley Park	Wellcome Sanger Institute for the COVID-19 Genomics UK (COG-UK) Consortium	Jacquelyn Wynn, Mairead Hyland, The Lighthouse Lab in Alderley Park and Alex Alderton, Roberto Amato, Sonia Goncalves, Ewan Harrison, David K. Jackson, Ian Johnston, Dominic Kwiatkowski, Cordelia Langford, John Sillitoe on behalf of the Wellcome Sanger Institute COVID-19 Surveillance Team
EPI_ISL_891812	Lighthouse Lab in Cambridge	Wellcome Sanger Institute for the COVID-19 Genomics UK (COG-UK) Consortium	Rob Howes, The Lighthouse Lab in Cambridge and Alex Alderton, Roberto Amato, Sonia Goncalves, Ewan Harrison, David K. Jackson, Ian Johnston, Dominic Kwiatkowski, Cordelia Langford, John Sillitoe on behalf of the Wellcome Sanger Institute COVID-19 Surveillance Team
EPI_ISL_891813	Lighthouse Lab in Alderley Park	Wellcome Sanger Institute for the COVID-19 Genomics UK (COG-UK) Consortium	Jacquelyn Wynn, Mairead Hyland, The Lighthouse Lab in Alderley Park and Alex Alderton, Roberto Amato, Sonia Goncalves, Ewan Harrison, David K. Jackson, Ian Johnston, Dominic Kwiatkowski, Cordelia Langford, John Sillitoe on behalf of the Wellcome Sanger Institute COVID-19 Surveillance Team
EPI_ISL_891814, EPI_ISL_891815, EPI_ISL_891816, EPI_ISL_891817, EPI_ISL_891818, EPI_ISL_891820	Lighthouse Lab in Cambridge	Wellcome Sanger Institute for the COVID-19 Genomics UK (COG-UK) Consortium	Rob Howes, The Lighthouse Lab in Cambridge and Alex Alderton, Roberto Amato, Sonia Goncalves, Ewan Harrison, David K. Jackson, Ian Johnston, Dominic Kwiatkowski, Cordelia Langford, John Sillitoe on behalf of the Wellcome Sanger Institute COVID-19 Surveillance Team
EPI_ISL_891824	Lighthouse Lab in Alderley Park	Wellcome Sanger Institute for the COVID-19 Genomics UK (COG-UK) Consortium	Jacquelyn Wynn, Mairead Hyland, The Lighthouse Lab in Alderley Park and Alex Alderton, Roberto Amato, Sonia Goncalves, Ewan Harrison, David K. Jackson, Ian Johnston, Dominic Kwiatkowski, Cordelia Langford, John Sillitoe on behalf of the Wellcome Sanger Institute COVID-19 Surveillance Team
EPI_ISL_891825, EPI_ISL_891827	Lighthouse Lab in Cambridge	Wellcome Sanger Institute for the COVID-19 Genomics UK (COG-UK) Consortium	Rob Howes, The Lighthouse Lab in Cambridge and Alex Alderton, Roberto Amato, Sonia Goncalves, Ewan Harrison, David K. Jackson, Ian Johnston, Dominic Kwiatkowski, Cordelia Langford, John Sillitoe on behalf of the Wellcome Sanger Institute COVID-19 Surveillance Team
EPI_ISL_891828	Lighthouse Lab in Glasgow	Wellcome Sanger Institute for the COVID-19 Genomics UK (COG-UK) Consortium	Harper VanSteenhouse, Yumi Kasai, David Gray, Carol Clugston, Anna Dominiczak and Alex Alderton, Roberto Amato, Sonia Goncalves, Ewan Harrison, David K. Jackson, Ian Johnston, Dominic Kwiatkowski, Cordelia Langford, John Sillitoe on behalf of the Wellcome Sanger Institute COVID-19 Surveillance Team
EPI_ISL_891829, EPI_ISL_891830, EPI_ISL_891831	Lighthouse Lab in Cambridge	Wellcome Sanger Institute for the COVID-19 Genomics UK (COG-UK) Consortium	Rob Howes, The Lighthouse Lab in Cambridge and Alex Alderton, Roberto Amato, Sonia Goncalves, Ewan Harrison, David K. Jackson, Ian Johnston, Dominic Kwiatkowski, Cordelia Langford, John Sillitoe on behalf of the Wellcome Sanger Institute COVID-19 Surveillance Team
EPI_ISL_891833	Lighthouse Lab in Alderley Park	Wellcome Sanger Institute for the COVID-19 Genomics UK (COG-UK) Consortium	Jacquelyn Wynn, Mairead Hyland, The Lighthouse Lab in Alderley Park and Alex Alderton, Roberto Amato, Sonia Goncalves, Ewan Harrison, David K. Jackson, Ian Johnston, Dominic Kwiatkowski, Cordelia Langford, John Sillitoe on behalf of the Wellcome Sanger Institute COVID-19 Surveillance Team
EPI_ISL_891835	Lighthouse Lab in Cambridge	Wellcome Sanger Institute for the COVID-19 Genomics UK (COG-UK) Consortium	Rob Howes, The Lighthouse Lab in Cambridge and Alex Alderton, Roberto Amato, Sonia Goncalves, Ewan Harrison, David K. Jackson, Ian Johnston, Dominic Kwiatkowski, Cordelia Langford, John Sillitoe on behalf of the Wellcome Sanger Institute COVID-19 Surveillance Team
EPI_ISL_891836	Lighthouse Lab in Glasgow	Wellcome Sanger Institute for the COVID-19 Genomics UK (COG-UK) Consortium	Harper VanSteenhouse, Yumi Kasai, David Gray, Carol Clugston, Anna Dominiczak and Alex Alderton, Roberto Amato, Sonia Goncalves, Ewan Harrison, David K. Jackson, Ian Johnston, Dominic Kwiatkowski, Cordelia Langford, John Sillitoe on behalf of the Wellcome Sanger Institute COVID-19 Surveillance Team
EPI_ISL_891837	Lighthouse Lab in Cambridge	Wellcome Sanger Institute for the COVID-19 Genomics UK (COG-UK) Consortium	Rob Howes, The Lighthouse Lab in Cambridge and Alex Alderton, Roberto Amato, Sonia Goncalves, Ewan Harrison, David K. Jackson, Ian Johnston, Dominic Kwiatkowski, Cordelia Langford, John Sillitoe on behalf of the Wellcome Sanger Institute COVID-19 Surveillance Team
EPI_ISL_891838, EPI_ISL_891839	Lighthouse Lab in Alderley Park	Wellcome Sanger Institute for the COVID-19 Genomics UK	Jacquelyn Wynn, Mairead Hyland, The Lighthouse Lab in Alderley Park and Alex Alderton, Roberto Amato, Sonia Goncalves, Ewan Harrison, David K.

		(COG-UK) Consortium	Jackson, Ian Johnston, Dominic Kwiatkowski, Cordelia Langford, John Sillitoe on behalf of the Wellcome Sanger Institute COVID-19 Surveillance Team
EPI_ISL_891840, EPI_ISL_891841, EPI_ISL_891842	Lighthouse Lab in Cambridge	Wellcome Sanger Institute for the COVID-19 Genomics UK (COG-UK) Consortium	Rob Howes, The Lighthouse Lab in Cambridge and Alex Alderton, Roberto Amato, Sonia Goncalves, Ewan Harrison, David K. Jackson, Ian Johnston, Dominic Kwiatkowski, Cordelia Langford, John Sillitoe on behalf of the Wellcome Sanger Institute COVID-19 Surveillance Team
EPI_ISL_891843	Lighthouse Lab in Alderley Park	Wellcome Sanger Institute for the COVID-19 Genomics UK (COG-UK) Consortium	Jacquelyn Wynn, Mairead Hyland, The Lighthouse Lab in Alderley Park and Alex Alderton, Roberto Amato, Sonia Goncalves, Ewan Harrison, David K. Jackson, Ian Johnston, Dominic Kwiatkowski, Cordelia Langford, John Sillitoe on behalf of the Wellcome Sanger Institute COVID-19 Surveillance Team
EPI_ISL_891844	Lighthouse Lab in Cambridge	Wellcome Sanger Institute for the COVID-19 Genomics UK (COG-UK) Consortium	Rob Howes, The Lighthouse Lab in Cambridge and Alex Alderton, Roberto Amato, Sonia Goncalves, Ewan Harrison, David K. Jackson, Ian Johnston, Dominic Kwiatkowski, Cordelia Langford, John Sillitoe on behalf of the Wellcome Sanger Institute COVID-19 Surveillance Team
EPI_ISL_891846	Lighthouse Lab in Glasgow	Wellcome Sanger Institute for the COVID-19 Genomics UK (COG-UK) Consortium	Harper VanSteenhouse, Yumi Kasai, David Gray, Carol Clugston, Anna Dominiczak and Alex Alderton, Roberto Amato, Sonia Goncalves, Ewan Harrison, David K. Jackson, Ian Johnston, Dominic Kwiatkowski, Cordelia Langford, John Sillitoe on behalf of the Wellcome Sanger Institute COVID-19 Surveillance Team
EPI_ISL_891847, EPI_ISL_891848	Lighthouse Lab in Alderley Park	Wellcome Sanger Institute for the COVID-19 Genomics UK (COG-UK) Consortium	Jacquelyn Wynn, Mairead Hyland, The Lighthouse Lab in Alderley Park and Alex Alderton, Roberto Amato, Sonia Goncalves, Ewan Harrison, David K. Jackson, Ian Johnston, Dominic Kwiatkowski, Cordelia Langford, John Sillitoe on behalf of the Wellcome Sanger Institute COVID-19 Surveillance Team
EPI_ISL_891849	Lighthouse Lab in Cambridge	Wellcome Sanger Institute for the COVID-19 Genomics UK (COG-UK) Consortium	Rob Howes, The Lighthouse Lab in Cambridge and Alex Alderton, Roberto Amato, Sonia Goncalves, Ewan Harrison, David K. Jackson, Ian Johnston, Dominic Kwiatkowski, Cordelia Langford, John Sillitoe on behalf of the Wellcome Sanger Institute COVID-19 Surveillance Team
EPI_ISL_891851, EPI_ISL_891852, EPI_ISL_891853	Lighthouse Lab in Glasgow	Wellcome Sanger Institute for the COVID-19 Genomics UK (COG-UK) Consortium	Harper VanSteenhouse, Yumi Kasai, David Gray, Carol Clugston, Anna Dominiczak and Alex Alderton, Roberto Amato, Sonia Goncalves, Ewan Harrison, David K. Jackson, Ian Johnston, Dominic Kwiatkowski, Cordelia Langford, John Sillitoe on behalf of the Wellcome Sanger Institute COVID-19 Surveillance Team
EPI_ISL_891854	Lighthouse Lab in Cambridge	Wellcome Sanger Institute for the COVID-19 Genomics UK (COG-UK) Consortium	Rob Howes, The Lighthouse Lab in Cambridge and Alex Alderton, Roberto Amato, Sonia Goncalves, Ewan Harrison, David K. Jackson, Ian Johnston, Dominic Kwiatkowski, Cordelia Langford, John Sillitoe on behalf of the Wellcome Sanger Institute COVID-19 Surveillance Team
EPI_ISL_891855	Lighthouse Lab in Glasgow	Wellcome Sanger Institute for the COVID-19 Genomics UK (COG-UK) Consortium	Harper VanSteenhouse, Yumi Kasai, David Gray, Carol Clugston, Anna Dominiczak and Alex Alderton, Roberto Amato, Sonia Goncalves, Ewan Harrison, David K. Jackson, Ian Johnston, Dominic Kwiatkowski, Cordelia Langford, John Sillitoe on behalf of the Wellcome Sanger Institute COVID-19 Surveillance Team
EPI_ISL_891856	Lighthouse Lab in Alderley Park	Wellcome Sanger Institute for the COVID-19 Genomics UK (COG-UK) Consortium	Jacquelyn Wynn, Mairead Hyland, The Lighthouse Lab in Alderley Park and Alex Alderton, Roberto Amato, Sonia Goncalves, Ewan Harrison, David K. Jackson, Ian Johnston, Dominic Kwiatkowski, Cordelia Langford, John Sillitoe on behalf of the Wellcome Sanger Institute COVID-19 Surveillance Team
EPI_ISL_891857	Lighthouse Lab in Glasgow	Wellcome Sanger Institute for the COVID-19 Genomics UK (COG-UK) Consortium	Harper VanSteenhouse, Yumi Kasai, David Gray, Carol Clugston, Anna Dominiczak and Alex Alderton, Roberto Amato, Sonia Goncalves, Ewan Harrison, David K. Jackson, Ian Johnston, Dominic Kwiatkowski, Cordelia Langford, John Sillitoe on behalf of the Wellcome Sanger Institute COVID-19 Surveillance Team
EPI_ISL_891858	Lighthouse Lab in Alderley Park	Wellcome Sanger Institute for the COVID-19 Genomics UK (COG-UK) Consortium	Jacquelyn Wynn, Mairead Hyland, The Lighthouse Lab in Alderley Park and Alex Alderton, Roberto Amato, Sonia Goncalves, Ewan Harrison, David K. Jackson, Ian Johnston, Dominic Kwiatkowski, Cordelia Langford, John Sillitoe on behalf of the Wellcome Sanger Institute COVID-19 Surveillance Team
EPI_ISL_891859	Lighthouse Lab in Cambridge	Wellcome Sanger Institute for the COVID-19 Genomics UK (COG-UK) Consortium	Rob Howes, The Lighthouse Lab in Cambridge and Alex Alderton, Roberto Amato, Sonia Goncalves, Ewan Harrison, David K. Jackson, Ian Johnston, Dominic Kwiatkowski, Cordelia Langford, John Sillitoe on behalf of the Wellcome Sanger Institute COVID-19 Surveillance Team
EPI_ISL_891861	Lighthouse Lab in Glasgow	Wellcome Sanger Institute for the COVID-19 Genomics UK (COG-UK) Consortium	Harper VanSteenhouse, Yumi Kasai, David Gray, Carol Clugston, Anna Dominiczak and Alex Alderton, Roberto Amato, Sonia Goncalves, Ewan Harrison, David K. Jackson, Ian Johnston, Dominic Kwiatkowski, Cordelia Langford, John Sillitoe on behalf of the Wellcome Sanger Institute COVID-19 Surveillance Team
EPI_ISL_891862	Lighthouse Lab in Cambridge	Wellcome Sanger Institute for the COVID-19 Genomics UK (COG-UK) Consortium	Rob Howes, The Lighthouse Lab in Cambridge and Alex Alderton, Roberto Amato, Sonia Goncalves, Ewan Harrison, David K. Jackson, Ian Johnston, Dominic Kwiatkowski, Cordelia Langford, John Sillitoe on behalf of the Wellcome Sanger Institute COVID-19 Surveillance Team
EPI_ISL_891863, EPI_ISL_891864	Lighthouse Lab in Alderley Park	Wellcome Sanger Institute for the COVID-19 Genomics UK (COG-UK) Consortium	Jacquelyn Wynn, Mairead Hyland, The Lighthouse Lab in Alderley Park and Alex Alderton, Roberto Amato, Sonia Goncalves, Ewan Harrison, David K. Jackson, Ian Johnston, Dominic Kwiatkowski, Cordelia Langford, John Sillitoe on behalf of the Wellcome Sanger Institute COVID-19 Surveillance Team
EPI_ISL_891865	Lighthouse Lab in Glasgow	Wellcome Sanger Institute for the COVID-19 Genomics UK (COG-UK) Consortium	Harper VanSteenhouse, Yumi Kasai, David Gray, Carol Clugston, Anna Dominiczak and Alex Alderton, Roberto Amato, Sonia Goncalves, Ewan Harrison, David K. Jackson, Ian Johnston, Dominic Kwiatkowski, Cordelia Langford, John Sillitoe on behalf of the Wellcome Sanger Institute COVID-19 Surveillance Team
EPI_ISL_891866	Lighthouse Lab in Alderley Park	Wellcome Sanger Institute for the COVID-19 Genomics UK (COG-UK) Consortium	Jacquelyn Wynn, Mairead Hyland, The Lighthouse Lab in Alderley Park and Alex Alderton, Roberto Amato, Sonia Goncalves, Ewan Harrison, David K. Jackson, Ian Johnston, Dominic Kwiatkowski, Cordelia Langford, John Sillitoe on behalf of the Wellcome Sanger Institute COVID-19 Surveillance Team
EPI_ISL_891868	Lighthouse Lab in Cambridge	Wellcome Sanger Institute for the COVID-19 Genomics UK (COG-UK) Consortium	Rob Howes, The Lighthouse Lab in Cambridge and Alex Alderton, Roberto Amato, Sonia Goncalves, Ewan Harrison, David K. Jackson, Ian Johnston, Dominic Kwiatkowski, Cordelia Langford, John Sillitoe on behalf of the Wellcome Sanger Institute COVID-19 Surveillance Team
EPI_ISL_891869	Lighthouse Lab in Glasgow	Wellcome Sanger Institute for the COVID-19 Genomics UK (COG-UK) Consortium	Harper VanSteenhouse, Yumi Kasai, David Gray, Carol Clugston, Anna Dominiczak and Alex Alderton, Roberto Amato, Sonia Goncalves, Ewan Harrison, David K. Jackson, Ian Johnston, Dominic Kwiatkowski, Cordelia Langford, John Sillitoe on behalf of the Wellcome Sanger Institute COVID-19 Surveillance Team
EPI_ISL_891870	Lighthouse Lab in Alderley Park	Wellcome Sanger Institute for the COVID-19 Genomics UK (COG-UK) Consortium	Jacquelyn Wynn, Mairead Hyland, The Lighthouse Lab in Alderley Park and Alex Alderton, Roberto Amato, Sonia Goncalves, Ewan Harrison, David K. Jackson, Ian Johnston, Dominic Kwiatkowski, Cordelia Langford, John Sillitoe on behalf of the Wellcome Sanger Institute COVID-19 Surveillance Team
EPI_ISL_891871, EPI_ISL_891872	Lighthouse Lab in Cambridge	Wellcome Sanger Institute for the COVID-19 Genomics UK (COG-UK) Consortium	Rob Howes, The Lighthouse Lab in Cambridge and Alex Alderton, Roberto Amato, Sonia Goncalves, Ewan Harrison, David K. Jackson, Ian Johnston, Dominic Kwiatkowski, Cordelia Langford, John Sillitoe on behalf of the Wellcome Sanger Institute COVID-19 Surveillance Team
EPI_ISL_891873	Lighthouse Lab in Glasgow	Wellcome Sanger Institute for the COVID-19 Genomics UK (COG-UK) Consortium	Harper VanSteenhouse, Yumi Kasai, David Gray, Carol Clugston, Anna Dominiczak and Alex Alderton, Roberto Amato, Sonia Goncalves, Ewan Harrison, David K. Jackson, Ian Johnston, Dominic Kwiatkowski, Cordelia Langford, John Sillitoe on behalf of the Wellcome Sanger Institute COVID-19 Surveillance Team
EPI_ISL_891874	Lighthouse Lab in Cambridge	Wellcome Sanger Institute for the COVID-19 Genomics UK (COG-UK) Consortium	Rob Howes, The Lighthouse Lab in Cambridge and Alex Alderton, Roberto Amato, Sonia Goncalves, Ewan Harrison, David K. Jackson, Ian Johnston, Dominic Kwiatkowski, Cordelia Langford, John Sillitoe on behalf of the Wellcome Sanger Institute COVID-19 Surveillance Team
EPI_ISL_891875	Lighthouse Lab in Glasgow	Wellcome Sanger Institute for the COVID-19 Genomics UK (COG-UK) Consortium	Harper VanSteenhouse, Yumi Kasai, David Gray, Carol Clugston, Anna Dominiczak and Alex Alderton, Roberto Amato, Sonia Goncalves, Ewan Harrison, David K. Jackson, Ian Johnston, Dominic Kwiatkowski, Cordelia Langford, John Sillitoe on behalf of the Wellcome Sanger Institute COVID-19 Surveillance Team
EPI_ISL_891876, EPI_ISL_891877	Lighthouse Lab in Cambridge	Wellcome Sanger Institute for the COVID-19 Genomics UK (COG-UK) Consortium	Rob Howes, The Lighthouse Lab in Cambridge and Alex Alderton, Roberto Amato, Sonia Goncalves, Ewan Harrison, David K. Jackson, Ian Johnston, Dominic Kwiatkowski, Cordelia Langford, John Sillitoe on behalf of the Wellcome Sanger Institute COVID-19 Surveillance Team
EPI_ISL_891878	Lighthouse Lab in Alderley Park	Wellcome Sanger Institute for the COVID-19 Genomics UK (COG-UK) Consortium	Jacquelyn Wynn, Mairead Hyland, The Lighthouse Lab in Alderley Park and Alex Alderton, Roberto Amato, Sonia Goncalves, Ewan Harrison, David K. Jackson, Ian Johnston, Dominic Kwiatkowski, Cordelia Langford, John Sillitoe on behalf of the Wellcome Sanger Institute COVID-19 Surveillance Team
EPI_ISL_891879, EPI_ISL_891880	Lighthouse Lab in Cambridge	Wellcome Sanger Institute for the COVID-19 Genomics UK (COG-UK) Consortium	Rob Howes, The Lighthouse Lab in Cambridge and Alex Alderton, Roberto Amato, Sonia Goncalves, Ewan Harrison, David K. Jackson, Ian Johnston, Dominic Kwiatkowski, Cordelia Langford, John Sillitoe on behalf of the Wellcome Sanger Institute COVID-19 Surveillance Team
EPI_ISL_891881	Lighthouse Lab in Glasgow	Wellcome Sanger Institute for the COVID-19 Genomics UK (COG-UK) Consortium	Harper VanSteenhouse, Yumi Kasai, David Gray, Carol Clugston, Anna Dominiczak and Alex Alderton, Roberto Amato, Sonia Goncalves, Ewan Harrison, David K. Jackson, Ian Johnston, Dominic Kwiatkowski, Cordelia Langford, John Sillitoe on behalf of the Wellcome Sanger Institute COVID-19 Surveillance Team
EPI_ISL_891883	Lighthouse Lab in Cambridge	Wellcome Sanger Institute for the COVID-19 Genomics UK (COG-UK) Consortium	Rob Howes, The Lighthouse Lab in Cambridge and Alex Alderton, Roberto Amato, Sonia Goncalves, Ewan Harrison, David K. Jackson, Ian Johnston, Dominic Kwiatkowski, Cordelia Langford, John Sillitoe on behalf of the Wellcome Sanger Institute COVID-19 Surveillance Team
EPI_ISL_891884, EPI_ISL_891885	Lighthouse Lab in Glasgow	Wellcome Sanger Institute for the COVID-19 Genomics UK	Harper VanSteenhouse, Yumi Kasai, David Gray, Carol Clugston, Anna Dominiczak and Alex Alderton, Roberto Amato, Sonia Goncalves, Ewan Harrison,

		(COG-UK) Consortium	David K. Jackson, Ian Johnston, Dominic Kwiatkowski, Cordelia Langford, John Sillitoe on behalf of the Wellcome Sanger Institute COVID-19 Surveillance Team
EPI_ISL_891886	Lighthouse Lab in Cambridge	Wellcome Sanger Institute for the COVID-19 Genomics UK (COG-UK) Consortium	Rob Howes, The Lighthouse Lab in Cambridge and Alex Alderton, Roberto Amato, Sonia Goncalves, Ewan Harrison, David K. Jackson, Ian Johnston, Dominic Kwiatkowski, Cordelia Langford, John Sillitoe on behalf of the Wellcome Sanger Institute COVID-19 Surveillance Team
EPI_ISL_891887	Lighthouse Lab in Alderley Park	Wellcome Sanger Institute for the COVID-19 Genomics UK (COG-UK) Consortium	Jacquelyn Wynn, Mairead Hyland, The Lighthouse Lab in Alderley Park and Alex Alderton, Roberto Amato, Sonia Goncalves, Ewan Harrison, David K. Jackson, Ian Johnston, Dominic Kwiatkowski, Cordelia Langford, John Sillitoe on behalf of the Wellcome Sanger Institute COVID-19 Surveillance Team
EPI_ISL_891888, EPI_ISL_891889, EPI_ISL_891890, EPI_ISL_891891, EPI_ISL_891892, EPI_ISL_891893, EPI_ISL_891894	Lighthouse Lab in Cambridge	Wellcome Sanger Institute for the COVID-19 Genomics UK (COG-UK) Consortium	Rob Howes, The Lighthouse Lab in Cambridge and Alex Alderton, Roberto Amato, Sonia Goncalves, Ewan Harrison, David K. Jackson, Ian Johnston, Dominic Kwiatkowski, Cordelia Langford, John Sillitoe on behalf of the Wellcome Sanger Institute COVID-19 Surveillance Team
EPI_ISL_891895	Lighthouse Lab in Alderley Park	Wellcome Sanger Institute for the COVID-19 Genomics UK (COG-UK) Consortium	Jacquelyn Wynn, Mairead Hyland, The Lighthouse Lab in Alderley Park and Alex Alderton, Roberto Amato, Sonia Goncalves, Ewan Harrison, David K. Jackson, Ian Johnston, Dominic Kwiatkowski, Cordelia Langford, John Sillitoe on behalf of the Wellcome Sanger Institute COVID-19 Surveillance Team
EPI_ISL_891897, EPI_ISL_891898	Lighthouse Lab in Cambridge	Wellcome Sanger Institute for the COVID-19 Genomics UK (COG-UK) Consortium	Rob Howes, The Lighthouse Lab in Cambridge and Alex Alderton, Roberto Amato, Sonia Goncalves, Ewan Harrison, David K. Jackson, Ian Johnston, Dominic Kwiatkowski, Cordelia Langford, John Sillitoe on behalf of the Wellcome Sanger Institute COVID-19 Surveillance Team
EPI_ISL_896217	Columbia University Irving Medical Center	Wadsworth Center, New York State Department of Health	Kirsten St. George, Daryl M. Lamson, Alexis Russel, Matthew Shudt, Melissa A Leisner, Jonathan Plitnick, Navjot Singh, John Kelly, Erasmus Schneider, Erica Lasek-Nesselquist
EPI_ISL_896226	TEMPUS LABS INC	Wadsworth Center, New York State Department of Health	Kirsten St. George, Daryl M. Lamson, Alexis Russel, Matthew Shudt, Melissa A Leisner, Jonathan Plitnick, Navjot Singh, John Kelly, Erasmus Schneider, Erica Lasek-Nesselquist
EPI_ISL_896231, EPI_ISL_896232, EPI_ISL_896233, EPI_ISL_896240, EPI_ISL_896241, EPI_ISL_896242, EPI_ISL_896243	Columbia University Irving Medical Center	Wadsworth Center, New York State Department of Health	Kirsten St. George, Daryl M. Lamson, Alexis Russel, Matthew Shudt, Melissa A Leisner, Jonathan Plitnick, Navjot Singh, John Kelly, Erasmus Schneider, Erica Lasek-Nesselquist
EPI_ISL_896495, EPI_ISL_896496, EPI_ISL_896497, EPI_ISL_896502, EPI_ISL_896508, EPI_ISL_896509, EPI_ISL_896510, EPI_ISL_896511, EPI_ISL_896512, EPI_ISL_896513, EPI_ISL_896515, EPI_ISL_896563, EPI_ISL_896564, EPI_ISL_896565, EPI_ISL_896566, EPI_ISL_896567, EPI_ISL_896568, EPI_ISL_896569			
see above	URMC LABS	Wadsworth Center, New York State Department of Health	Kirsten St. George, Daryl M. Lamson, Alexis Russel, Matthew Shudt, Melissa A Leisner, Jonathan Plitnick, Navjot Singh, John Kelly, Erasmus Schneider, Erica Lasek-Nesselquist
EPI_ISL_897606, EPI_ISL_897647, EPI_ISL_897648, EPI_ISL_897649, EPI_ISL_897650, EPI_ISL_897651, EPI_ISL_897652, EPI_ISL_897653, EPI_ISL_897654, EPI_ISL_897655, EPI_ISL_897656, EPI_ISL_897657, EPI_ISL_897658, EPI_ISL_897659, EPI_ISL_897660, EPI_ISL_897661, EPI_ISL_897662, EPI_ISL_897663, EPI_ISL_897664, EPI_ISL_897665, EPI_ISL_897666, EPI_ISL_897667, EPI_ISL_897668, EPI_ISL_897669, EPI_ISL_897671, EPI_ISL_897672, EPI_ISL_897675, EPI_ISL_897679, EPI_ISL_897684, EPI_ISL_897690, EPI_ISL_897691, EPI_ISL_897695, EPI_ISL_897706, EPI_ISL_897707, EPI_ISL_897712, EPI_ISL_897717, EPI_ISL_897718, EPI_ISL_897719, EPI_ISL_897846, EPI_ISL_897847, EPI_ISL_897848, EPI_ISL_897849, EPI_ISL_897850, EPI_ISL_897851, EPI_ISL_897852, EPI_ISL_897853, EPI_ISL_897854, EPI_ISL_897855, EPI_ISL_897856, EPI_ISL_897857, EPI_ISL_897858, EPI_ISL_897860, EPI_ISL_897861, EPI_ISL_897862, EPI_ISL_897863, EPI_ISL_897864, EPI_ISL_897865, EPI_ISL_897866, EPI_ISL_897867, EPI_ISL_897868, EPI_ISL_897869, EPI_ISL_897870, EPI_ISL_897871, EPI_ISL_897872, EPI_ISL_897873, EPI_ISL_897874, EPI_ISL_897875, EPI_ISL_897876, EPI_ISL_897877, EPI_ISL_897878, EPI_ISL_897879, EPI_ISL_897880, EPI_ISL_897881, EPI_ISL_897882, EPI_ISL_897883, EPI_ISL_897884, EPI_ISL_897885, EPI_ISL_897886, EPI_ISL_897887, EPI_ISL_897888, EPI_ISL_897889, EPI_ISL_897890, EPI_ISL_897891, EPI_ISL_897892, EPI_ISL_897893, EPI_ISL_897894, EPI_ISL_897895, EPI_ISL_897896, EPI_ISL_897897, EPI_ISL_897898, EPI_ISL_897899, EPI_ISL_897900, EPI_ISL_897901, EPI_ISL_897902, EPI_ISL_897924, EPI_ISL_897925, EPI_ISL_897926, EPI_ISL_897927, EPI_ISL_897928, EPI_ISL_897931, EPI_ISL_897932, EPI_ISL_897933, EPI_ISL_897966			
see above	University Hospitals of Geneva, Laboratory of Virology	HUG, Laboratory of Virology and the Health2030 Genome Center	Samuel Cordey, Ana Rita Goncalves, Laurent Kaiser, Lorenzo Cerutti, Henri Pegeot, Melyssa Elies, Deborah Penet, Keith Harshman, Ioannis Xenarios, Emmanouil Dermitzakis
EPI_ISL_902730, EPI_ISL_902731, EPI_ISL_902733	Santa Clara County Public Health Laboratory	Santa Clara County Public Health Laboratory	Santa Clara County Public Health Department
EPI_ISL_902756	Fondazione Policlinico Universitario "A. Gemelli" IRCCS	INMI Lazzaro Spallanzani IRCCS	M Rueca, O Butera, F Messina, C.E.M Gruber, B Bartolini, E Giombini, P Cattani, M Sanguinetti, MR Capobianchi, A Di Caro
EPI_ISL_903208	University of Michigan Clinical Microbiology Laboratory	Lauring Lab, University of Michigan, Department of Microbiology and Immunology	Valesano
EPI_ISL_903364, EPI_ISL_903365	University Medical Center Hamburg Eppendorf	Heinrich Pette Institute, Leibniz Institute for Experimental Virology	Alexis Robitaille, Thomas Günther, Johannes Knobloch, Martin Aepfelbacher, Nicole Fischer, Adam Grundhoff
EPI_ISL_904086, EPI_ISL_904087, EPI_ISL_904088, EPI_ISL_904089, EPI_ISL_904090, EPI_ISL_904091, EPI_ISL_904092, EPI_ISL_904093, EPI_ISL_904094, EPI_ISL_904095, EPI_ISL_904096, EPI_ISL_904097, EPI_ISL_904098, EPI_ISL_904099, EPI_ISL_904100, EPI_ISL_904101, EPI_ISL_904102, EPI_ISL_904103, EPI_ISL_904104, EPI_ISL_904105, EPI_ISL_904106, EPI_ISL_904107, EPI_ISL_904108, EPI_ISL_904109, EPI_ISL_904110, EPI_ISL_904111, EPI_ISL_904112, EPI_ISL_904113, EPI_ISL_904114, EPI_ISL_904115, EPI_ISL_904116			
see above	New Mexico Department of Health Scientific Laboratory	New Mexico Department of Health Scientific Laboratory	Ellie Johnson, Anastacia Griego-Fisher, D'eldra Malone
EPI_ISL_904179, EPI_ISL_904222, EPI_ISL_904607, EPI_ISL_904608, EPI_ISL_904609	Dutch COVID-19 response team	Erasmus Medical Center	Bas Oude Munnink, Reina Sikkema, David Nieuwenhuijse, Irina Chestakova, Anne van der Linden, Marjan Boter, Emmanuelle Munger, Corine GeurtsvanKessel, Annemiek van der Eijk, Richard Molenkamp, Marion Koopmans, on behalf of the Dutch national COVID-19 response team.
EPI_ISL_904669, EPI_ISL_904670, EPI_ISL_904671, EPI_ISL_904672, EPI_ISL_904674, EPI_ISL_904677, EPI_ISL_904689, EPI_ISL_904716, EPI_ISL_904721, EPI_ISL_904733, EPI_ISL_904756, EPI_ISL_904759, EPI_ISL_904775, EPI_ISL_904846, EPI_ISL_904847, EPI_ISL_904848, EPI_ISL_904849, EPI_ISL_904871, EPI_ISL_904893, EPI_ISL_904914, EPI_ISL_904915, EPI_ISL_904916, EPI_ISL_904917, EPI_ISL_904918, EPI_ISL_904919, EPI_ISL_904920, EPI_ISL_904921, EPI_ISL_904922, EPI_ISL_904923, EPI_ISL_904924			
see above	Dutch COVID-19 response team	National Institute for Public Health and the Environment (RIVM)	Adam Meijer, Harry Vennema, Dirk Eggink, Jeroen Cremer, Sharon van den Brink, Bas van der Veer, AnneMarie van den Brandt, Florian Zwagemaker, Dennis Schmitz, Chantal Reusken, on behalf of the national COVID-19 response team
EPI_ISL_905764	Zakad Diagnostyki Laboratoryjnej i Mikrobiologii, Wojewódzki Szpital Podkarpacki w Kronie	National Institute of Public Health - National Institute of Hygiene	Wokowicz Tomasz, Zacharczuk Katarzyna
EPI_ISL_905773, EPI_ISL_905774, EPI_ISL_905775, EPI_ISL_905776, EPI_ISL_905777, EPI_ISL_905778, EPI_ISL_905779, EPI_ISL_905780, EPI_ISL_905781, EPI_ISL_905782, EPI_ISL_905783, EPI_ISL_905784, EPI_ISL_905785, EPI_ISL_905786, EPI_ISL_905787, EPI_ISL_905791			
see above	UCLA Clinical Micro Lab	Los Angeles County PHL	P. Hemarajata et al.
EPI_ISL_906053	Tilia Laboratories s.r.o.	Tilia Laboratories s.r.o.	Sona Pekova, MD, PhD.
EPI_ISL_906070	UPA Dr. Akira Tada	Instituto Adolfo Lutz, Interdiciplinary Procedures Center, Strategic Laboratory	Claudio Tavares Sacchi, Claudia Regina Gonçalves, Erica Valessa Ramos Gomes, Karoline Rodrigues Campos
EPI_ISL_906071	LACEN-PI DR. Costa Alvarenga	Instituto Adolfo Lutz, Interdiciplinary Procedures Center, Strategic Laboratory	Claudio Tavares Sacchi, Claudia Regina Gonçalves, Erica Valessa Ramos Gomes, Karoline Rodrigues Campos
EPI_ISL_906072	UPA Dr. Akira Tada	Instituto Adolfo Lutz, Interdiciplinary Procedures Center, Strategic Laboratory	Claudio Tavares Sacchi, Claudia Regina Gonçalves, Erica Valessa Ramos Gomes, Karoline Rodrigues Campos
EPI_ISL_906073	Hospital Vila Lobos	Instituto Adolfo Lutz, Interdiciplinary Procedures Center, Strategic Laboratory	Claudio Tavares Sacchi, Claudia Regina Gonçalves, Erica Valessa Ramos Gomes, Karoline Rodrigues Campos
EPI_ISL_906074	Searom Laboratorio Diagnostico	Instituto Adolfo Lutz, Interdiciplinary Procedures Center, Strategic Laboratory	Claudio Tavares Sacchi, Claudia Regina Gonçalves, Erica Valessa Ramos Gomes, Karoline Rodrigues Campos
EPI_ISL_906075	Hospital Geral de Vila Penteado Dr Jose Pangella Sao Paulo	Instituto Adolfo Lutz, Interdiciplinary Procedures Center, Strategic Laboratory	Claudio Tavares Sacchi, Claudia Regina Gonçalves, Erica Valessa Ramos Gomes, Karoline Rodrigues Campos
EPI_ISL_906076, EPI_ISL_906077	Hospital Sao Luiz Sao Caetano	Instituto Adolfo Lutz, Interdiciplinary Procedures Center, Strategic Laboratory	Claudio Tavares Sacchi, Claudia Regina Gonçalves, Erica Valessa Ramos Gomes, Karoline Rodrigues Campos
EPI_ISL_906131	National laboratory of health, environment and food Kranj	Institute of Microbiology and Immunology, Faculty of Medicine, University of Ljubljana	Samo Zakotnik, Tomaž Mark Zorec, Matic Brvar, Miša Korva, Mario Poljak, Tatjana Avši - Županc

EPI_ISL_906200, EPI_ISL_906205, EPI_ISL_906207, EPI_ISL_906210, EPI_ISL_906221, EPI_ISL_906223, EPI_ISL_906224, EPI_ISL_906228, EPI_ISL_906231, EPI_ISL_906232, EPI_ISL_906234, EPI_ISL_906235, EPI_ISL_906237, EPI_ISL_906239, EPI_ISL_906243, EPI_ISL_906244, EPI_ISL_906245, EPI_ISL_906247, EPI_ISL_906251, EPI_ISL_906253, EPI_ISL_906265, EPI_ISL_906267			
see above	University of Wisconsin-Madison AIDS Vaccine Research Laboratories	University of Wisconsin-Madison AIDS Vaccine Research Laboratories	Gage Moreno, Katarina Braun, et al. AIDS Vaccine Research Laboratories
EPI_ISL_906538	Laboratorio de Virologia-Instituto Nacional de Salud	Instituto Nacional de Salud- Dirección de Investigación en Salud Pública, Universidad de los Andes- Applied genomics research group, Vicerrectoria de Investigación y Creación, Universidad de los Andes- Systems and Computing Engineering Department	Katherine Laiton-Donato, Diego A. Álvarez-Díaz, Carlos Franco-Muñoz, Mauricio Pacheco-Montealegre, Héctor Alejandro Ruiz-Moreno, Maria T. Herrera-Sepúlveda, Diego Andrés Prada, Jhonnatan Reales-González, Sheryll Corchuelo, Julian Naizaque, Gerardo Santamaría Jorge Duitama, Laura Natalia Gonzalez, Jorge Ivan Diaz, Silvia Restrepo-Restrepo, Magdalena Wiesner, Martha Lucia Ospina Martinez, Marcela Mercado-Reyes
EPI_ISL_906655, EPI_ISL_906656, EPI_ISL_906657, EPI_ISL_906658, EPI_ISL_906659, EPI_ISL_906660, EPI_ISL_906661, EPI_ISL_906662, EPI_ISL_906663, EPI_ISL_906664, EPI_ISL_906665, EPI_ISL_906666, EPI_ISL_906667, EPI_ISL_906668			
see above	Maine Health and Environmental Testing Laboratory (Maine HETL)	Tewhey Lab, The Jackson Laboratory	Matluk,N., Dewey,H., Iosue,F., Barter,M., Lynch,R., Munger,H. and Tewhey,R.
EPI_ISL_906804	Norwegian Institute of Public Health, Department of Virology	Norwegian Institute of Public Health, Department of Virology	Kathrine Stene-Johansen, Kamilla Heddeland Instefjord, Hilde Elshaug, Atiya R Ali,Marie Paulsen Madsen, Rasmus Riis Kopperud, Hilde Vollan, Karoline Bragstad, Olav Hungnes
EPI_ISL_906813, EPI_ISL_906814	Medical Microbiology Unit, Department for Laboratory Medicine, Drammen Hospital, Vestre Viken Health Trust,	Norwegian Institute of Public Health, Department of Virology	Kathrine Stene-Johansen, Kamilla Heddeland Instefjord, Hilde Elshaug, Atiya R Ali,Marie Paulsen Madsen, Rasmus Riis Kopperud, Hilde Vollan, Karoline Bragstad, Olav Hungnes
EPI_ISL_907119, EPI_ISL_907133, EPI_ISL_907136, EPI_ISL_907139, EPI_ISL_907144, EPI_ISL_907148, EPI_ISL_907152, EPI_ISL_907155, EPI_ISL_907160, EPI_ISL_907164, EPI_ISL_907167, EPI_ISL_907173, EPI_ISL_907179, EPI_ISL_907182, EPI_ISL_907186, EPI_ISL_907187, EPI_ISL_907190, EPI_ISL_907193, EPI_ISL_907195, EPI_ISL_907200, EPI_ISL_907206, EPI_ISL_907209, EPI_ISL_907212, EPI_ISL_907214, EPI_ISL_907219, EPI_ISL_907221, EPI_ISL_907223, EPI_ISL_907224, EPI_ISL_907225, EPI_ISL_907238, EPI_ISL_907241, EPI_ISL_907250, EPI_ISL_907254, EPI_ISL_907256, EPI_ISL_907260, EPI_ISL_907263, EPI_ISL_907264, EPI_ISL_907267, EPI_ISL_907269, EPI_ISL_907279, EPI_ISL_907284, EPI_ISL_907287, EPI_ISL_907288, EPI_ISL_907292, EPI_ISL_907300, EPI_ISL_907304, EPI_ISL_907308, EPI_ISL_907315, EPI_ISL_907318, EPI_ISL_907321, EPI_ISL_907322, EPI_ISL_907323, EPI_ISL_907325, EPI_ISL_907328, EPI_ISL_907336, EPI_ISL_907341, EPI_ISL_907347, EPI_ISL_907351, EPI_ISL_907352, EPI_ISL_907353, EPI_ISL_907355, EPI_ISL_907362, EPI_ISL_907363, EPI_ISL_907367			
see above	Lighthouse Lab in Cambridge	Wellcome Sanger Institute for the COVID-19 Genomics UK (COG-UK) Consortium	Rob Howes, The Lighthouse Lab in Cambridge and Alex Alderton, Roberto Amato, Sonia Goncalves, Ewan Harrison, David K. Jackson, Ian Johnston, Dominic Kwiatkowski, Cordelia Langford, John Sillitoe on behalf of the Wellcome Sanger Institute COVID-19 Surveillance Team
EPI_ISL_907479, EPI_ISL_907492, EPI_ISL_907636, EPI_ISL_907700, EPI_ISL_907701, EPI_ISL_907704, EPI_ISL_907709, EPI_ISL_907710, EPI_ISL_907711, EPI_ISL_907712, EPI_ISL_907713, EPI_ISL_907714, EPI_ISL_907715, EPI_ISL_907717, EPI_ISL_907718, EPI_ISL_907719, EPI_ISL_907720, EPI_ISL_907724, EPI_ISL_907726, EPI_ISL_907727, EPI_ISL_907728, EPI_ISL_907729, EPI_ISL_907730, EPI_ISL_907732, EPI_ISL_907733, EPI_ISL_907734, EPI_ISL_907737, EPI_ISL_907739, EPI_ISL_907740, EPI_ISL_907741, EPI_ISL_907742, EPI_ISL_907743, EPI_ISL_907744, EPI_ISL_907746, EPI_ISL_907748, EPI_ISL_907751, EPI_ISL_907752, EPI_ISL_907753, EPI_ISL_907754, EPI_ISL_907758, EPI_ISL_907759, EPI_ISL_907761, EPI_ISL_907762, EPI_ISL_907764, EPI_ISL_907766, EPI_ISL_907768, EPI_ISL_907769, EPI_ISL_907770, EPI_ISL_907774, EPI_ISL_907777, EPI_ISL_907780, EPI_ISL_907781, EPI_ISL_907782, EPI_ISL_907784, EPI_ISL_907785			
see above	Lighthouse Lab in Alderley Park	Wellcome Sanger Institute for the COVID-19 Genomics UK (COG-UK) Consortium	Jacquelyn Wynn, Mairead Hyland, The Lighthouse Lab in Alderley Park and Alex Alderton, Roberto Amato, Sonia Goncalves, Ewan Harrison, David K. Jackson, Ian Johnston, Dominic Kwiatkowski, Cordelia Langford, John Sillitoe on behalf of the Wellcome Sanger Institute COVID-19 Surveillance Team
EPI_ISL_907788	Lighthouse Lab in Glasgow	Wellcome Sanger Institute for the COVID-19 Genomics UK (COG-UK) Consortium	Harper VanSteenhouse, Yumi Kasai, David Gray, Carol Clugston, Anna Dominiczak and Alex Alderton, Roberto Amato, Sonia Goncalves, Ewan Harrison, David K. Jackson, Ian Johnston, Dominic Kwiatkowski, Cordelia Langford, John Sillitoe on behalf of the Wellcome Sanger Institute COVID-19 Surveillance Team
EPI_ISL_907790, EPI_ISL_907791, EPI_ISL_907792, EPI_ISL_907798, EPI_ISL_907799, EPI_ISL_907803, EPI_ISL_907804, EPI_ISL_907806, EPI_ISL_907807, EPI_ISL_907808, EPI_ISL_907810, EPI_ISL_907815, EPI_ISL_907816, EPI_ISL_907818, EPI_ISL_907819, EPI_ISL_907822, EPI_ISL_907823, EPI_ISL_907825, EPI_ISL_907827, EPI_ISL_907828, EPI_ISL_907829, EPI_ISL_907830, EPI_ISL_907831, EPI_ISL_907836, EPI_ISL_907839, EPI_ISL_907840, EPI_ISL_907842, EPI_ISL_907843, EPI_ISL_907844, EPI_ISL_907845, EPI_ISL_907846, EPI_ISL_907848, EPI_ISL_907849, EPI_ISL_907850, EPI_ISL_907853, EPI_ISL_907856, EPI_ISL_907857, EPI_ISL_907858, EPI_ISL_907859, EPI_ISL_907861, EPI_ISL_907862, EPI_ISL_907864, EPI_ISL_907866, EPI_ISL_907869, EPI_ISL_907870, EPI_ISL_907871, EPI_ISL_907873, EPI_ISL_907876, EPI_ISL_907880, EPI_ISL_907883, EPI_ISL_907885, EPI_ISL_907886, EPI_ISL_907887, EPI_ISL_907888, EPI_ISL_907892, EPI_ISL_907896, EPI_ISL_907897, EPI_ISL_907900, EPI_ISL_907901, EPI_ISL_907904, EPI_ISL_907905, EPI_ISL_907906, EPI_ISL_907907, EPI_ISL_907908, EPI_ISL_907910, EPI_ISL_907913, EPI_ISL_907916, EPI_ISL_907917, EPI_ISL_907918, EPI_ISL_907919, EPI_ISL_907920, EPI_ISL_907922, EPI_ISL_907924, EPI_ISL_907927, EPI_ISL_907928, EPI_ISL_907929, EPI_ISL_907930, EPI_ISL_907932, EPI_ISL_907935, EPI_ISL_907937, EPI_ISL_907938, EPI_ISL_907939, EPI_ISL_907941, EPI_ISL_907942, EPI_ISL_907947, EPI_ISL_907950, EPI_ISL_907951, EPI_ISL_907952, EPI_ISL_907954, EPI_ISL_907955, EPI_ISL_907957, EPI_ISL_907958, EPI_ISL_907959, EPI_ISL_907961, EPI_ISL_907962, EPI_ISL_907963, EPI_ISL_907964, EPI_ISL_907965, EPI_ISL_907966, EPI_ISL_907968, EPI_ISL_907971, EPI_ISL_907977, EPI_ISL_907980, EPI_ISL_907983, EPI_ISL_907985, EPI_ISL_907986, EPI_ISL_907990, EPI_ISL_907991, EPI_ISL_907992, EPI_ISL_907993, EPI_ISL_907996, EPI_ISL_907997, EPI_ISL_907999, EPI_ISL_908000, EPI_ISL_908004, EPI_ISL_908005, EPI_ISL_908006, EPI_ISL_908008, EPI_ISL_908012, EPI_ISL_908014, EPI_ISL_908015, EPI_ISL_908018, EPI_ISL_908019, EPI_ISL_908020, EPI_ISL_908023, EPI_ISL_908024, EPI_ISL_908029, EPI_ISL_908031, EPI_ISL_908032, EPI_ISL_908268, EPI_ISL_908271, EPI_ISL_908272, EPI_ISL_908273, EPI_ISL_908276, EPI_ISL_908278, EPI_ISL_908280, EPI_ISL_908281, EPI_ISL_908283, EPI_ISL_908286, EPI_ISL_908287, EPI_ISL_908288, EPI_ISL_908294, EPI_ISL_908297			
see above	Lighthouse Lab in Alderley Park	Wellcome Sanger Institute for the COVID-19 Genomics UK (COG-UK) Consortium	Jacquelyn Wynn, Mairead Hyland, The Lighthouse Lab in Alderley Park and Alex Alderton, Roberto Amato, Sonia Goncalves, Ewan Harrison, David K. Jackson, Ian Johnston, Dominic Kwiatkowski, Cordelia Langford, John Sillitoe on behalf of the Wellcome Sanger Institute COVID-19 Surveillance Team
EPI_ISL_908298	Lighthouse Lab in Glasgow	Wellcome Sanger Institute for the COVID-19 Genomics UK (COG-UK) Consortium	Harper VanSteenhouse, Yumi Kasai, David Gray, Carol Clugston, Anna Dominiczak and Alex Alderton, Roberto Amato, Sonia Goncalves, Ewan Harrison, David K. Jackson, Ian Johnston, Dominic Kwiatkowski, Cordelia Langford, John Sillitoe on behalf of the Wellcome Sanger Institute COVID-19 Surveillance Team
EPI_ISL_908299, EPI_ISL_908300, EPI_ISL_908304, EPI_ISL_908305, EPI_ISL_908311, EPI_ISL_908312, EPI_ISL_908313, EPI_ISL_908314, EPI_ISL_908318, EPI_ISL_908319, EPI_ISL_908320, EPI_ISL_908324, EPI_ISL_908325, EPI_ISL_908327, EPI_ISL_908328, EPI_ISL_908330, EPI_ISL_908331, EPI_ISL_908332, EPI_ISL_908336, EPI_ISL_908339, EPI_ISL_908342, EPI_ISL_908345, EPI_ISL_908346, EPI_ISL_908347, EPI_ISL_908349, EPI_ISL_908350, EPI_ISL_908352, EPI_ISL_908354, EPI_ISL_908355, EPI_ISL_908356, EPI_ISL_908358, EPI_ISL_908359, EPI_ISL_908361, EPI_ISL_908362, EPI_ISL_908363, EPI_ISL_908364, EPI_ISL_908368, EPI_ISL_908370, EPI_ISL_908371, EPI_ISL_908373, EPI_ISL_908374, EPI_ISL_908375, EPI_ISL_908377, EPI_ISL_908378, EPI_ISL_908379, EPI_ISL_908381, EPI_ISL_908382, EPI_ISL_908386, EPI_ISL_908388, EPI_ISL_908390, EPI_ISL_908391, EPI_ISL_908392, EPI_ISL_908395, EPI_ISL_908396, EPI_ISL_908398, EPI_ISL_908399, EPI_ISL_908400, EPI_ISL_908404, EPI_ISL_908406, EPI_ISL_908409, EPI_ISL_908410, EPI_ISL_908414, EPI_ISL_908417, EPI_ISL_908418			
see above	Lighthouse Lab in Alderley Park	Wellcome Sanger Institute for the COVID-19 Genomics UK (COG-UK) Consortium	Jacquelyn Wynn, Mairead Hyland, The Lighthouse Lab in Alderley Park and Alex Alderton, Roberto Amato, Sonia Goncalves, Ewan Harrison, David K. Jackson, Ian Johnston, Dominic Kwiatkowski, Cordelia Langford, John Sillitoe on behalf of the Wellcome Sanger Institute COVID-19 Surveillance Team
EPI_ISL_908419	Lighthouse Lab in Glasgow	Wellcome Sanger Institute for the COVID-19 Genomics UK (COG-UK) Consortium	Harper VanSteenhouse, Yumi Kasai, David Gray, Carol Clugston, Anna Dominiczak and Alex Alderton, Roberto Amato, Sonia Goncalves, Ewan Harrison, David K. Jackson, Ian Johnston, Dominic Kwiatkowski, Cordelia Langford, John Sillitoe on behalf of the Wellcome Sanger Institute COVID-19 Surveillance Team
EPI_ISL_908420, EPI_ISL_908423, EPI_ISL_908424, EPI_ISL_908425, EPI_ISL_908426, EPI_ISL_908427, EPI_ISL_908428, EPI_ISL_908430, EPI_ISL_908432, EPI_ISL_908434, EPI_ISL_908436, EPI_ISL_908437, EPI_ISL_908438, EPI_ISL_908440, EPI_ISL_908441, EPI_ISL_908445, EPI_ISL_908448, EPI_ISL_908449, EPI_ISL_908450, EPI_ISL_908454, EPI_ISL_908457, EPI_ISL_908458, EPI_ISL_908459, EPI_ISL_908460, EPI_ISL_908461, EPI_ISL_908463, EPI_ISL_908464, EPI_ISL_908466, EPI_ISL_908468, EPI_ISL_908469, EPI_ISL_908472, EPI_ISL_908473, EPI_ISL_908475, EPI_ISL_908476, EPI_ISL_908477, EPI_ISL_908480, EPI_ISL_908482, EPI_ISL_908483, EPI_ISL_908484, EPI_ISL_908485, EPI_ISL_908487, EPI_ISL_908490, EPI_ISL_908491, EPI_ISL_908492, EPI_ISL_908493, EPI_ISL_908497, EPI_ISL_908498, EPI_ISL_908499, EPI_ISL_908501, EPI_ISL_908502, EPI_ISL_908503, EPI_ISL_908504, EPI_ISL_908505, EPI_ISL_908510, EPI_ISL_908514, EPI_ISL_908515, EPI_ISL_908517, EPI_ISL_908523, EPI_ISL_908531, EPI_ISL_908532, EPI_ISL_908533, EPI_ISL_908537, EPI_ISL_908539, EPI_ISL_908540, EPI_ISL_908541, EPI_ISL_908543, EPI_ISL_908544, EPI_ISL_908545, EPI_ISL_908548, EPI_ISL_908549, EPI_ISL_908552, EPI_ISL_908554, EPI_ISL_908555, EPI_ISL_908556, EPI_ISL_908562, EPI_ISL_908563, EPI_ISL_908565, EPI_ISL_908566, EPI_ISL_908567, EPI_ISL_908569, EPI_ISL_908572, EPI_ISL_908574, EPI_ISL_908575, EPI_ISL_908577, EPI_ISL_908578, EPI_ISL_908579, EPI_ISL_908583, EPI_ISL_908584, EPI_ISL_908586, EPI_ISL_908587, EPI_ISL_908588, EPI_ISL_908593, EPI_ISL_908594			
see above	Lighthouse Lab in Alderley Park	Wellcome Sanger Institute for the COVID-19 Genomics UK (COG-UK) Consortium	Jacquelyn Wynn, Mairead Hyland, The Lighthouse Lab in Alderley Park and Alex Alderton, Roberto Amato, Sonia Goncalves, Ewan Harrison, David K. Jackson, Ian Johnston, Dominic Kwiatkowski, Cordelia Langford, John Sillitoe on behalf of the Wellcome Sanger Institute COVID-19 Surveillance Team
EPI_ISL_908597, EPI_ISL_908598, EPI_ISL_908599, EPI_ISL_908600, EPI_ISL_908601, EPI_ISL_908602, EPI_ISL_908603, EPI_ISL_908604, EPI_ISL_908605, EPI_ISL_908606, EPI_ISL_908607, EPI_ISL_908608, EPI_ISL_908609, EPI_ISL_908610, EPI_ISL_908611, EPI_ISL_908612, EPI_ISL_908613, EPI_ISL_908614, EPI_ISL_908615, EPI_ISL_908616, EPI_ISL_908617, EPI_ISL_908618, EPI_ISL_908619, EPI_ISL_908620, EPI_ISL_908621, EPI_ISL_908622, EPI_ISL_908623, EPI_ISL_908624, EPI_ISL_908625, EPI_ISL_908626, EPI_ISL_908627, EPI_ISL_908628, EPI_ISL_908629, EPI_ISL_908630, EPI_ISL_908631, EPI_ISL_908632, EPI_ISL_908633, EPI_ISL_908634, EPI_ISL_908635, EPI_ISL_908636, EPI_ISL_908637, EPI_ISL_908638, EPI_ISL_908639, EPI_ISL_908640, EPI_ISL_908641, EPI_ISL_908642, EPI_ISL_908643, EPI_ISL_908644, EPI_ISL_908645, EPI_ISL_908646, EPI_ISL_908647, EPI_ISL_908648, EPI_ISL_908649, EPI_ISL_908650, EPI_ISL_908651, EPI_ISL_908652, EPI_ISL_908653, EPI_ISL_908654, EPI_ISL_908655, EPI_ISL_908656, EPI_ISL_908657, EPI_ISL_908658, EPI_ISL_908659, EPI_ISL_908660, EPI_ISL_908661, EPI_ISL_908662, EPI_ISL_908663, EPI_ISL_908664, EPI_ISL_908665, EPI_ISL_908666, EPI_ISL_908667, EPI_ISL_908668, EPI_ISL_908669, EPI_ISL_908670, EPI_ISL_908671, EPI_ISL_908672, EPI_ISL_908673, EPI_ISL_908674, EPI_ISL_908675, EPI_ISL_908676, EPI_ISL_908677, EPI_ISL_908678, EPI_ISL_908679, EPI_ISL_908680, EPI_ISL_908681, EPI_ISL_908682, EPI_ISL_908683, EPI_ISL_908684, EPI_ISL_908685, EPI_ISL_908686, EPI_ISL_908687, EPI_ISL_908688, EPI_ISL_908689, EPI_ISL_908690, EPI_ISL_908691, EPI_ISL_908692, EPI_ISL_908693, EPI_ISL_908694, EPI_ISL_908695, EPI_ISL_908696, EPI_ISL_908697, EPI_ISL_908698, EPI_ISL_908699, EPI_ISL_908700, EPI_ISL_908701, EPI_ISL_908702, EPI_ISL_908703, EPI_ISL_908704, EPI_ISL_908705, EPI_ISL_908706, EPI_ISL_908707, EPI_ISL_908708, EPI_ISL_908709, EPI_ISL_908710, EPI_ISL_908711, EPI_ISL_908712, EPI_ISL_908713, EPI_ISL_908714, EPI_ISL_908715, EPI_ISL_908716, EPI_ISL_908717, EPI_ISL_908718, EPI_ISL_908719, EPI_ISL_908720, EPI_ISL_908721, EPI_ISL_908722, EPI_ISL_908723, EPI_ISL_908724, EPI_ISL_908725, EPI_ISL_908726, EPI_ISL_908727, EPI_ISL_908728, EPI_ISL_908729, EPI_ISL_908730, EPI_ISL_908731, EPI_ISL_908732, EPI_ISL_908733, EPI_ISL_908734, EPI_ISL_908735, EPI_ISL_908736, EPI_ISL_908737, EPI_ISL_908738, EPI_ISL_908739, EPI_ISL_908740, EPI_ISL_908741, EPI_ISL_908742, EPI_ISL_908743, EPI_ISL_908744, EPI_ISL_908745, EPI_ISL_908746, EPI_ISL_908747, EPI_ISL_908748, EPI_ISL_908749, EPI_ISL_908750, EPI_ISL_908751, EPI_ISL_908752, EPI_ISL_908753, EPI_ISL_908754, EPI_ISL_908755, EPI_ISL_908756, EPI_ISL_908757, EPI_ISL_908758, EPI_ISL_908759, EPI_ISL_908760, EPI_ISL_908762, EPI_ISL_908763, EPI_ISL_908764, EPI_ISL_908765, EPI_ISL_908766, EPI_ISL_908767, EPI_ISL_908768, EPI_ISL_908769, EPI_ISL_908770, EPI_ISL_908771, EPI_ISL_908772, EPI_ISL_908773, EPI_ISL_908774, EPI_ISL_908775, EPI_ISL_908776, EPI_ISL_908777, EPI_ISL_908778, EPI_ISL_908779, EPI_ISL_908780, EPI_ISL_908781, EPI_ISL_908782, EPI_ISL_908783, EPI_ISL_908784, EPI_ISL_908785, EPI_ISL_908786, EPI_ISL_908787, EPI_ISL_908788, EPI_ISL_908789, EPI_ISL_908790, EPI_ISL_908791, EPI_ISL_908792, EPI_ISL_908793, EPI_ISL_908794, EPI_ISL_908795, EPI_ISL_908796, EPI_ISL_908797, EPI_ISL_908798, EPI_ISL_908799, EPI_ISL_908800, EPI_ISL_908801, EPI_ISL_908802, EPI_ISL_908803, EPI_ISL_908804, EPI_ISL_908805, EPI_ISL_908806, EPI_ISL_908807, EPI_ISL_908808, EPI_ISL_908809, EPI_ISL_908810, EPI_ISL_908811, EPI_ISL_908812, EPI_ISL_908813, EPI_ISL_908814, EPI_ISL_908815, EPI_ISL_908816, EPI_ISL_908817, EPI_ISL_908818, EPI_ISL_908819, EPI_ISL_908820, EPI_ISL_908821, EPI_ISL_908822, EPI_ISL_908823, EPI_ISL_908824, EPI_ISL_908825, EPI_ISL_908826, EPI_ISL_908827, EPI_ISL_908828, EPI_ISL_908829, EPI_ISL_908830, EPI_ISL_908831, EPI_ISL_908832, EPI_ISL_908833, EPI_ISL_908834, EPI_ISL_908835, EPI_ISL_908836, EPI_ISL_908837, EPI_ISL_908838, EPI_ISL_908839, EPI_ISL_908840, EPI_ISL_908841, EPI_ISL_908842, EPI_ISL_908843, EPI_ISL_908844, EPI_ISL_908845, EPI_ISL_908846, EPI_ISL_908847, EPI_ISL_908848, EPI_ISL_908849, EPI_ISL_908850, EPI_ISL_908851, EPI_ISL_908852, EPI_ISL_908853, EPI_ISL_908854, EPI_ISL_908855, EPI_ISL_908856, EPI_ISL_908857, EPI_ISL_908858, EPI_ISL_908859, EPI_ISL_908860, EPI_ISL_908861, EPI_ISL_908862, EPI_ISL_908863, EPI_ISL_908864, EPI_ISL_908865, EPI_ISL_908866, EPI_ISL_908867, EPI_ISL_908868, EPI_ISL_908869, EPI_ISL_908870, EPI_ISL_908871, EPI_ISL_908872, EPI_ISL_908873, EPI_ISL_908874, EPI_ISL_908875, EPI_ISL_908876, EPI_ISL_908877, EPI_ISL_908878, EPI_ISL_908879, EPI_ISL_908880, EPI_ISL_908881, EPI_ISL_908882, EPI_ISL_908883, EPI_ISL_908884, EPI_ISL_908885,			

EPI_ISL_908886, EPI_ISL_908887, EPI_ISL_908888, EPI_ISL_908889, EPI_ISL_908890, EPI_ISL_908891, EPI_ISL_908892, EPI_ISL_908893, EPI_ISL_908894, EPI_ISL_908895, EPI_ISL_908896, EPI_ISL_908897, EPI_ISL_908898, EPI_ISL_908899, EPI_ISL_908900, EPI_ISL_908901, EPI_ISL_908902, EPI_ISL_908903, EPI_ISL_908904, EPI_ISL_908905, EPI_ISL_908906, EPI_ISL_908907, EPI_ISL_908908, EPI_ISL_908909, EPI_ISL_908910, EPI_ISL_908911, EPI_ISL_908912, EPI_ISL_908913, EPI_ISL_908914, EPI_ISL_908915, EPI_ISL_908916, EPI_ISL_908917, EPI_ISL_908918, EPI_ISL_908919, EPI_ISL_908920, EPI_ISL_908921, EPI_ISL_908922, EPI_ISL_908923, EPI_ISL_908924, EPI_ISL_908925, EPI_ISL_908926, EPI_ISL_908927, EPI_ISL_908928, EPI_ISL_908929, EPI_ISL_908930, EPI_ISL_908931, EPI_ISL_908933, EPI_ISL_908935, EPI_ISL_908942			
see above	Lighthouse Lab in Cambridge	Wellcome Sanger Institute for the COVID-19 Genomics UK (COG-UK) Consortium	Rob Howes, The Lighthouse Lab in Cambridge and Alex Alderton, Roberto Amato, Sonia Goncalves, Ewan Harrison, David K. Jackson, Ian Johnston, Dominic Kwiatkowski, Cordelia Langford, John Sillitoe on behalf of the Wellcome Sanger Institute COVID-19 Surveillance Team
EPI_ISL_908945	Lighthouse Lab in Glasgow	Wellcome Sanger Institute for the COVID-19 Genomics UK (COG-UK) Consortium	Harper VanSteenhouse, Yumi Kasai, David Gray, Carol Clugston, Anna Dominiczak and Alex Alderton, Roberto Amato, Sonia Goncalves, Ewan Harrison, David K. Jackson, Ian Johnston, Dominic Kwiatkowski, Cordelia Langford, John Sillitoe on behalf of the Wellcome Sanger Institute COVID-19 Surveillance Team
EPI_ISL_908946, EPI_ISL_908949, EPI_ISL_908951, EPI_ISL_908952	Lighthouse Lab in Cambridge	Wellcome Sanger Institute for the COVID-19 Genomics UK (COG-UK) Consortium	Rob Howes, The Lighthouse Lab in Cambridge and Alex Alderton, Roberto Amato, Sonia Goncalves, Ewan Harrison, David K. Jackson, Ian Johnston, Dominic Kwiatkowski, Cordelia Langford, John Sillitoe on behalf of the Wellcome Sanger Institute COVID-19 Surveillance Team
EPI_ISL_908953	Lighthouse Lab in Glasgow	Wellcome Sanger Institute for the COVID-19 Genomics UK (COG-UK) Consortium	Harper VanSteenhouse, Yumi Kasai, David Gray, Carol Clugston, Anna Dominiczak and Alex Alderton, Roberto Amato, Sonia Goncalves, Ewan Harrison, David K. Jackson, Ian Johnston, Dominic Kwiatkowski, Cordelia Langford, John Sillitoe on behalf of the Wellcome Sanger Institute COVID-19 Surveillance Team
EPI_ISL_908955, EPI_ISL_908957	Lighthouse Lab in Cambridge	Wellcome Sanger Institute for the COVID-19 Genomics UK (COG-UK) Consortium	Rob Howes, The Lighthouse Lab in Cambridge and Alex Alderton, Roberto Amato, Sonia Goncalves, Ewan Harrison, David K. Jackson, Ian Johnston, Dominic Kwiatkowski, Cordelia Langford, John Sillitoe on behalf of the Wellcome Sanger Institute COVID-19 Surveillance Team
EPI_ISL_908958	Lighthouse Lab in Glasgow	Wellcome Sanger Institute for the COVID-19 Genomics UK (COG-UK) Consortium	Harper VanSteenhouse, Yumi Kasai, David Gray, Carol Clugston, Anna Dominiczak and Alex Alderton, Roberto Amato, Sonia Goncalves, Ewan Harrison, David K. Jackson, Ian Johnston, Dominic Kwiatkowski, Cordelia Langford, John Sillitoe on behalf of the Wellcome Sanger Institute COVID-19 Surveillance Team
EPI_ISL_908959, EPI_ISL_908961	Lighthouse Lab in Cambridge	Wellcome Sanger Institute for the COVID-19 Genomics UK (COG-UK) Consortium	Rob Howes, The Lighthouse Lab in Cambridge and Alex Alderton, Roberto Amato, Sonia Goncalves, Ewan Harrison, David K. Jackson, Ian Johnston, Dominic Kwiatkowski, Cordelia Langford, John Sillitoe on behalf of the Wellcome Sanger Institute COVID-19 Surveillance Team
EPI_ISL_908962	Lighthouse Lab in Glasgow	Wellcome Sanger Institute for the COVID-19 Genomics UK (COG-UK) Consortium	Harper VanSteenhouse, Yumi Kasai, David Gray, Carol Clugston, Anna Dominiczak and Alex Alderton, Roberto Amato, Sonia Goncalves, Ewan Harrison, David K. Jackson, Ian Johnston, Dominic Kwiatkowski, Cordelia Langford, John Sillitoe on behalf of the Wellcome Sanger Institute COVID-19 Surveillance Team
EPI_ISL_908963	Lighthouse Lab in Cambridge	Wellcome Sanger Institute for the COVID-19 Genomics UK (COG-UK) Consortium	Rob Howes, The Lighthouse Lab in Cambridge and Alex Alderton, Roberto Amato, Sonia Goncalves, Ewan Harrison, David K. Jackson, Ian Johnston, Dominic Kwiatkowski, Cordelia Langford, John Sillitoe on behalf of the Wellcome Sanger Institute COVID-19 Surveillance Team
EPI_ISL_908965	Lighthouse Lab in Glasgow	Wellcome Sanger Institute for the COVID-19 Genomics UK (COG-UK) Consortium	Harper VanSteenhouse, Yumi Kasai, David Gray, Carol Clugston, Anna Dominiczak and Alex Alderton, Roberto Amato, Sonia Goncalves, Ewan Harrison, David K. Jackson, Ian Johnston, Dominic Kwiatkowski, Cordelia Langford, John Sillitoe on behalf of the Wellcome Sanger Institute COVID-19 Surveillance Team
EPI_ISL_908967, EPI_ISL_908968	Lighthouse Lab in Cambridge	Wellcome Sanger Institute for the COVID-19 Genomics UK (COG-UK) Consortium	Rob Howes, The Lighthouse Lab in Cambridge and Alex Alderton, Roberto Amato, Sonia Goncalves, Ewan Harrison, David K. Jackson, Ian Johnston, Dominic Kwiatkowski, Cordelia Langford, John Sillitoe on behalf of the Wellcome Sanger Institute COVID-19 Surveillance Team
EPI_ISL_908974	Lighthouse Lab in Glasgow	Wellcome Sanger Institute for the COVID-19 Genomics UK (COG-UK) Consortium	Harper VanSteenhouse, Yumi Kasai, David Gray, Carol Clugston, Anna Dominiczak and Alex Alderton, Roberto Amato, Sonia Goncalves, Ewan Harrison, David K. Jackson, Ian Johnston, Dominic Kwiatkowski, Cordelia Langford, John Sillitoe on behalf of the Wellcome Sanger Institute COVID-19 Surveillance Team
EPI_ISL_908977, EPI_ISL_908978, EPI_ISL_908979, EPI_ISL_908983, EPI_ISL_908985	Lighthouse Lab in Cambridge	Wellcome Sanger Institute for the COVID-19 Genomics UK (COG-UK) Consortium	Rob Howes, The Lighthouse Lab in Cambridge and Alex Alderton, Roberto Amato, Sonia Goncalves, Ewan Harrison, David K. Jackson, Ian Johnston, Dominic Kwiatkowski, Cordelia Langford, John Sillitoe on behalf of the Wellcome Sanger Institute COVID-19 Surveillance Team
EPI_ISL_908986, EPI_ISL_908989	Lighthouse Lab in Glasgow	Wellcome Sanger Institute for the COVID-19 Genomics UK (COG-UK) Consortium	Harper VanSteenhouse, Yumi Kasai, David Gray, Carol Clugston, Anna Dominiczak and Alex Alderton, Roberto Amato, Sonia Goncalves, Ewan Harrison, David K. Jackson, Ian Johnston, Dominic Kwiatkowski, Cordelia Langford, John Sillitoe on behalf of the Wellcome Sanger Institute COVID-19 Surveillance Team
EPI_ISL_908990, EPI_ISL_908991, EPI_ISL_908992	Lighthouse Lab in Cambridge	Wellcome Sanger Institute for the COVID-19 Genomics UK (COG-UK) Consortium	Rob Howes, The Lighthouse Lab in Cambridge and Alex Alderton, Roberto Amato, Sonia Goncalves, Ewan Harrison, David K. Jackson, Ian Johnston, Dominic Kwiatkowski, Cordelia Langford, John Sillitoe on behalf of the Wellcome Sanger Institute COVID-19 Surveillance Team
EPI_ISL_908993	Lighthouse Lab in Glasgow	Wellcome Sanger Institute for the COVID-19 Genomics UK (COG-UK) Consortium	Harper VanSteenhouse, Yumi Kasai, David Gray, Carol Clugston, Anna Dominiczak and Alex Alderton, Roberto Amato, Sonia Goncalves, Ewan Harrison, David K. Jackson, Ian Johnston, Dominic Kwiatkowski, Cordelia Langford, John Sillitoe on behalf of the Wellcome Sanger Institute COVID-19 Surveillance Team
EPI_ISL_908998, EPI_ISL_908999	Lighthouse Lab in Cambridge	Wellcome Sanger Institute for the COVID-19 Genomics UK (COG-UK) Consortium	Rob Howes, The Lighthouse Lab in Cambridge and Alex Alderton, Roberto Amato, Sonia Goncalves, Ewan Harrison, David K. Jackson, Ian Johnston, Dominic Kwiatkowski, Cordelia Langford, John Sillitoe on behalf of the Wellcome Sanger Institute COVID-19 Surveillance Team
EPI_ISL_909002, EPI_ISL_909004, EPI_ISL_909005	Lighthouse Lab in Glasgow	Wellcome Sanger Institute for the COVID-19 Genomics UK (COG-UK) Consortium	Harper VanSteenhouse, Yumi Kasai, David Gray, Carol Clugston, Anna Dominiczak and Alex Alderton, Roberto Amato, Sonia Goncalves, Ewan Harrison, David K. Jackson, Ian Johnston, Dominic Kwiatkowski, Cordelia Langford, John Sillitoe on behalf of the Wellcome Sanger Institute COVID-19 Surveillance Team
EPI_ISL_909006, EPI_ISL_909007	Lighthouse Lab in Cambridge	Wellcome Sanger Institute for the COVID-19 Genomics UK (COG-UK) Consortium	Rob Howes, The Lighthouse Lab in Cambridge and Alex Alderton, Roberto Amato, Sonia Goncalves, Ewan Harrison, David K. Jackson, Ian Johnston, Dominic Kwiatkowski, Cordelia Langford, John Sillitoe on behalf of the Wellcome Sanger Institute COVID-19 Surveillance Team
EPI_ISL_909008	Lighthouse Lab in Glasgow	Wellcome Sanger Institute for the COVID-19 Genomics UK (COG-UK) Consortium	Harper VanSteenhouse, Yumi Kasai, David Gray, Carol Clugston, Anna Dominiczak and Alex Alderton, Roberto Amato, Sonia Goncalves, Ewan Harrison, David K. Jackson, Ian Johnston, Dominic Kwiatkowski, Cordelia Langford, John Sillitoe on behalf of the Wellcome Sanger Institute COVID-19 Surveillance Team
EPI_ISL_909009	Lighthouse Lab in Cambridge	Wellcome Sanger Institute for the COVID-19 Genomics UK (COG-UK) Consortium	Rob Howes, The Lighthouse Lab in Cambridge and Alex Alderton, Roberto Amato, Sonia Goncalves, Ewan Harrison, David K. Jackson, Ian Johnston, Dominic Kwiatkowski, Cordelia Langford, John Sillitoe on behalf of the Wellcome Sanger Institute COVID-19 Surveillance Team
EPI_ISL_909010	Lighthouse Lab in Glasgow	Wellcome Sanger Institute for the COVID-19 Genomics UK (COG-UK) Consortium	Harper VanSteenhouse, Yumi Kasai, David Gray, Carol Clugston, Anna Dominiczak and Alex Alderton, Roberto Amato, Sonia Goncalves, Ewan Harrison, David K. Jackson, Ian Johnston, Dominic Kwiatkowski, Cordelia Langford, John Sillitoe on behalf of the Wellcome Sanger Institute COVID-19 Surveillance Team
EPI_ISL_909011, EPI_ISL_909015	Lighthouse Lab in Cambridge	Wellcome Sanger Institute for the COVID-19 Genomics UK (COG-UK) Consortium	Rob Howes, The Lighthouse Lab in Cambridge and Alex Alderton, Roberto Amato, Sonia Goncalves, Ewan Harrison, David K. Jackson, Ian Johnston, Dominic Kwiatkowski, Cordelia Langford, John Sillitoe on behalf of the Wellcome Sanger Institute COVID-19 Surveillance Team
EPI_ISL_909017	Lighthouse Lab in Glasgow	Wellcome Sanger Institute for the COVID-19 Genomics UK (COG-UK) Consortium	Harper VanSteenhouse, Yumi Kasai, David Gray, Carol Clugston, Anna Dominiczak and Alex Alderton, Roberto Amato, Sonia Goncalves, Ewan Harrison, David K. Jackson, Ian Johnston, Dominic Kwiatkowski, Cordelia Langford, John Sillitoe on behalf of the Wellcome Sanger Institute COVID-19 Surveillance Team
EPI_ISL_909019	Lighthouse Lab in Cambridge	Wellcome Sanger Institute for the COVID-19 Genomics UK (COG-UK) Consortium	Rob Howes, The Lighthouse Lab in Cambridge and Alex Alderton, Roberto Amato, Sonia Goncalves, Ewan Harrison, David K. Jackson, Ian Johnston, Dominic Kwiatkowski, Cordelia Langford, John Sillitoe on behalf of the Wellcome Sanger Institute COVID-19 Surveillance Team
EPI_ISL_909021, EPI_ISL_909024, EPI_ISL_909029, EPI_ISL_909030, EPI_ISL_909034	Lighthouse Lab in Glasgow	Wellcome Sanger Institute for the COVID-19 Genomics UK (COG-UK) Consortium	Harper VanSteenhouse, Yumi Kasai, David Gray, Carol Clugston, Anna Dominiczak and Alex Alderton, Roberto Amato, Sonia Goncalves, Ewan Harrison, David K. Jackson, Ian Johnston, Dominic Kwiatkowski, Cordelia Langford, John Sillitoe on behalf of the Wellcome Sanger Institute COVID-19 Surveillance Team
EPI_ISL_909035, EPI_ISL_909036	Lighthouse Lab in Cambridge	Wellcome Sanger Institute for the COVID-19 Genomics UK (COG-UK) Consortium	Rob Howes, The Lighthouse Lab in Cambridge and Alex Alderton, Roberto Amato, Sonia Goncalves, Ewan Harrison, David K. Jackson, Ian Johnston, Dominic Kwiatkowski, Cordelia Langford, John Sillitoe on behalf of the Wellcome Sanger Institute COVID-19 Surveillance Team
EPI_ISL_909038	Lighthouse Lab in Glasgow	Wellcome Sanger Institute for the COVID-19 Genomics UK (COG-UK) Consortium	Harper VanSteenhouse, Yumi Kasai, David Gray, Carol Clugston, Anna Dominiczak and Alex Alderton, Roberto Amato, Sonia Goncalves, Ewan Harrison, David K. Jackson, Ian Johnston, Dominic Kwiatkowski, Cordelia Langford, John Sillitoe on behalf of the Wellcome Sanger Institute COVID-19 Surveillance

			Team
EPI_ISL_909040, EPI_ISL_909046, EPI_ISL_909047, EPI_ISL_909048, EPI_ISL_909049, EPI_ISL_909050, EPI_ISL_909052	Lighthouse Lab in Cambridge	Wellcome Sanger Institute for the COVID-19 Genomics UK (COG-UK) Consortium	Rob Howes, The Lighthouse Lab in Cambridge and Alex Alderton, Roberto Amato, Sonia Goncalves, Ewan Harrison, David K. Jackson, Ian Johnston, Dominic Kwiatkowski, Cordelia Langford, John Sillitoe on behalf of the Wellcome Sanger Institute COVID-19 Surveillance Team
EPI_ISL_909053, EPI_ISL_909056, EPI_ISL_909057, EPI_ISL_909058	Lighthouse Lab in Glasgow	Wellcome Sanger Institute for the COVID-19 Genomics UK (COG-UK) Consortium	Harper VanSteenhouse, Yumi Kasai, David Gray, Carol Clugston, Anna Dominiczak and Alex Alderton, Roberto Amato, Sonia Goncalves, Ewan Harrison, David K. Jackson, Ian Johnston, Dominic Kwiatkowski, Cordelia Langford, John Sillitoe on behalf of the Wellcome Sanger Institute COVID-19 Surveillance Team
EPI_ISL_909060	Lighthouse Lab in Cambridge	Wellcome Sanger Institute for the COVID-19 Genomics UK (COG-UK) Consortium	Rob Howes, The Lighthouse Lab in Cambridge and Alex Alderton, Roberto Amato, Sonia Goncalves, Ewan Harrison, David K. Jackson, Ian Johnston, Dominic Kwiatkowski, Cordelia Langford, John Sillitoe on behalf of the Wellcome Sanger Institute COVID-19 Surveillance Team
EPI_ISL_909061, EPI_ISL_909063	Lighthouse Lab in Glasgow	Wellcome Sanger Institute for the COVID-19 Genomics UK (COG-UK) Consortium	Harper VanSteenhouse, Yumi Kasai, David Gray, Carol Clugston, Anna Dominiczak and Alex Alderton, Roberto Amato, Sonia Goncalves, Ewan Harrison, David K. Jackson, Ian Johnston, Dominic Kwiatkowski, Cordelia Langford, John Sillitoe on behalf of the Wellcome Sanger Institute COVID-19 Surveillance Team
EPI_ISL_909066, EPI_ISL_909067	Lighthouse Lab in Cambridge	Wellcome Sanger Institute for the COVID-19 Genomics UK (COG-UK) Consortium	Rob Howes, The Lighthouse Lab in Cambridge and Alex Alderton, Roberto Amato, Sonia Goncalves, Ewan Harrison, David K. Jackson, Ian Johnston, Dominic Kwiatkowski, Cordelia Langford, John Sillitoe on behalf of the Wellcome Sanger Institute COVID-19 Surveillance Team
EPI_ISL_909068, EPI_ISL_909069	Lighthouse Lab in Glasgow	Wellcome Sanger Institute for the COVID-19 Genomics UK (COG-UK) Consortium	Harper VanSteenhouse, Yumi Kasai, David Gray, Carol Clugston, Anna Dominiczak and Alex Alderton, Roberto Amato, Sonia Goncalves, Ewan Harrison, David K. Jackson, Ian Johnston, Dominic Kwiatkowski, Cordelia Langford, John Sillitoe on behalf of the Wellcome Sanger Institute COVID-19 Surveillance Team
EPI_ISL_909071, EPI_ISL_909072, EPI_ISL_909075, EPI_ISL_909079, EPI_ISL_909080, EPI_ISL_909081	Lighthouse Lab in Cambridge	Wellcome Sanger Institute for the COVID-19 Genomics UK (COG-UK) Consortium	Rob Howes, The Lighthouse Lab in Cambridge and Alex Alderton, Roberto Amato, Sonia Goncalves, Ewan Harrison, David K. Jackson, Ian Johnston, Dominic Kwiatkowski, Cordelia Langford, John Sillitoe on behalf of the Wellcome Sanger Institute COVID-19 Surveillance Team
EPI_ISL_909083, EPI_ISL_909085, EPI_ISL_909086, EPI_ISL_909087	Lighthouse Lab in Glasgow	Wellcome Sanger Institute for the COVID-19 Genomics UK (COG-UK) Consortium	Harper VanSteenhouse, Yumi Kasai, David Gray, Carol Clugston, Anna Dominiczak and Alex Alderton, Roberto Amato, Sonia Goncalves, Ewan Harrison, David K. Jackson, Ian Johnston, Dominic Kwiatkowski, Cordelia Langford, John Sillitoe on behalf of the Wellcome Sanger Institute COVID-19 Surveillance Team
EPI_ISL_909088	Lighthouse Lab in Cambridge	Wellcome Sanger Institute for the COVID-19 Genomics UK (COG-UK) Consortium	Rob Howes, The Lighthouse Lab in Cambridge and Alex Alderton, Roberto Amato, Sonia Goncalves, Ewan Harrison, David K. Jackson, Ian Johnston, Dominic Kwiatkowski, Cordelia Langford, John Sillitoe on behalf of the Wellcome Sanger Institute COVID-19 Surveillance Team
EPI_ISL_909089, EPI_ISL_909090	Lighthouse Lab in Glasgow	Wellcome Sanger Institute for the COVID-19 Genomics UK (COG-UK) Consortium	Harper VanSteenhouse, Yumi Kasai, David Gray, Carol Clugston, Anna Dominiczak and Alex Alderton, Roberto Amato, Sonia Goncalves, Ewan Harrison, David K. Jackson, Ian Johnston, Dominic Kwiatkowski, Cordelia Langford, John Sillitoe on behalf of the Wellcome Sanger Institute COVID-19 Surveillance Team
EPI_ISL_909093	Lighthouse Lab in Cambridge	Wellcome Sanger Institute for the COVID-19 Genomics UK (COG-UK) Consortium	Rob Howes, The Lighthouse Lab in Cambridge and Alex Alderton, Roberto Amato, Sonia Goncalves, Ewan Harrison, David K. Jackson, Ian Johnston, Dominic Kwiatkowski, Cordelia Langford, John Sillitoe on behalf of the Wellcome Sanger Institute COVID-19 Surveillance Team
EPI_ISL_909094	Lighthouse Lab in Glasgow	Wellcome Sanger Institute for the COVID-19 Genomics UK (COG-UK) Consortium	Harper VanSteenhouse, Yumi Kasai, David Gray, Carol Clugston, Anna Dominiczak and Alex Alderton, Roberto Amato, Sonia Goncalves, Ewan Harrison, David K. Jackson, Ian Johnston, Dominic Kwiatkowski, Cordelia Langford, John Sillitoe on behalf of the Wellcome Sanger Institute COVID-19 Surveillance Team
EPI_ISL_909095, EPI_ISL_909096, EPI_ISL_909097	Lighthouse Lab in Cambridge	Wellcome Sanger Institute for the COVID-19 Genomics UK (COG-UK) Consortium	Rob Howes, The Lighthouse Lab in Cambridge and Alex Alderton, Roberto Amato, Sonia Goncalves, Ewan Harrison, David K. Jackson, Ian Johnston, Dominic Kwiatkowski, Cordelia Langford, John Sillitoe on behalf of the Wellcome Sanger Institute COVID-19 Surveillance Team
EPI_ISL_909101	Lighthouse Lab in Glasgow	Wellcome Sanger Institute for the COVID-19 Genomics UK (COG-UK) Consortium	Harper VanSteenhouse, Yumi Kasai, David Gray, Carol Clugston, Anna Dominiczak and Alex Alderton, Roberto Amato, Sonia Goncalves, Ewan Harrison, David K. Jackson, Ian Johnston, Dominic Kwiatkowski, Cordelia Langford, John Sillitoe on behalf of the Wellcome Sanger Institute COVID-19 Surveillance Team
EPI_ISL_909102, EPI_ISL_909103, EPI_ISL_909105	Lighthouse Lab in Cambridge	Wellcome Sanger Institute for the COVID-19 Genomics UK (COG-UK) Consortium	Rob Howes, The Lighthouse Lab in Cambridge and Alex Alderton, Roberto Amato, Sonia Goncalves, Ewan Harrison, David K. Jackson, Ian Johnston, Dominic Kwiatkowski, Cordelia Langford, John Sillitoe on behalf of the Wellcome Sanger Institute COVID-19 Surveillance Team
EPI_ISL_909106	Lighthouse Lab in Glasgow	Wellcome Sanger Institute for the COVID-19 Genomics UK (COG-UK) Consortium	Harper VanSteenhouse, Yumi Kasai, David Gray, Carol Clugston, Anna Dominiczak and Alex Alderton, Roberto Amato, Sonia Goncalves, Ewan Harrison, David K. Jackson, Ian Johnston, Dominic Kwiatkowski, Cordelia Langford, John Sillitoe on behalf of the Wellcome Sanger Institute COVID-19 Surveillance Team
EPI_ISL_909109	Lighthouse Lab in Cambridge	Wellcome Sanger Institute for the COVID-19 Genomics UK (COG-UK) Consortium	Rob Howes, The Lighthouse Lab in Cambridge and Alex Alderton, Roberto Amato, Sonia Goncalves, Ewan Harrison, David K. Jackson, Ian Johnston, Dominic Kwiatkowski, Cordelia Langford, John Sillitoe on behalf of the Wellcome Sanger Institute COVID-19 Surveillance Team
EPI_ISL_909111, EPI_ISL_909113, EPI_ISL_909114, EPI_ISL_909116, EPI_ISL_909117, EPI_ISL_909118	Lighthouse Lab in Glasgow	Wellcome Sanger Institute for the COVID-19 Genomics UK (COG-UK) Consortium	Harper VanSteenhouse, Yumi Kasai, David Gray, Carol Clugston, Anna Dominiczak and Alex Alderton, Roberto Amato, Sonia Goncalves, Ewan Harrison, David K. Jackson, Ian Johnston, Dominic Kwiatkowski, Cordelia Langford, John Sillitoe on behalf of the Wellcome Sanger Institute COVID-19 Surveillance Team
EPI_ISL_909119	Lighthouse Lab in Cambridge	Wellcome Sanger Institute for the COVID-19 Genomics UK (COG-UK) Consortium	Rob Howes, The Lighthouse Lab in Cambridge and Alex Alderton, Roberto Amato, Sonia Goncalves, Ewan Harrison, David K. Jackson, Ian Johnston, Dominic Kwiatkowski, Cordelia Langford, John Sillitoe on behalf of the Wellcome Sanger Institute COVID-19 Surveillance Team
EPI_ISL_909121	Lighthouse Lab in Glasgow	Wellcome Sanger Institute for the COVID-19 Genomics UK (COG-UK) Consortium	Harper VanSteenhouse, Yumi Kasai, David Gray, Carol Clugston, Anna Dominiczak and Alex Alderton, Roberto Amato, Sonia Goncalves, Ewan Harrison, David K. Jackson, Ian Johnston, Dominic Kwiatkowski, Cordelia Langford, John Sillitoe on behalf of the Wellcome Sanger Institute COVID-19 Surveillance Team
EPI_ISL_909122, EPI_ISL_909128	Lighthouse Lab in Cambridge	Wellcome Sanger Institute for the COVID-19 Genomics UK (COG-UK) Consortium	Rob Howes, The Lighthouse Lab in Cambridge and Alex Alderton, Roberto Amato, Sonia Goncalves, Ewan Harrison, David K. Jackson, Ian Johnston, Dominic Kwiatkowski, Cordelia Langford, John Sillitoe on behalf of the Wellcome Sanger Institute COVID-19 Surveillance Team
EPI_ISL_909131	Lighthouse Lab in Glasgow	Wellcome Sanger Institute for the COVID-19 Genomics UK (COG-UK) Consortium	Harper VanSteenhouse, Yumi Kasai, David Gray, Carol Clugston, Anna Dominiczak and Alex Alderton, Roberto Amato, Sonia Goncalves, Ewan Harrison, David K. Jackson, Ian Johnston, Dominic Kwiatkowski, Cordelia Langford, John Sillitoe on behalf of the Wellcome Sanger Institute COVID-19 Surveillance Team
EPI_ISL_909144, EPI_ISL_909145, EPI_ISL_909148	Lighthouse Lab in Cambridge	Wellcome Sanger Institute for the COVID-19 Genomics UK (COG-UK) Consortium	Rob Howes, The Lighthouse Lab in Cambridge and Alex Alderton, Roberto Amato, Sonia Goncalves, Ewan Harrison, David K. Jackson, Ian Johnston, Dominic Kwiatkowski, Cordelia Langford, John Sillitoe on behalf of the Wellcome Sanger Institute COVID-19 Surveillance Team
EPI_ISL_909150	Lighthouse Lab in Glasgow	Wellcome Sanger Institute for the COVID-19 Genomics UK (COG-UK) Consortium	Harper VanSteenhouse, Yumi Kasai, David Gray, Carol Clugston, Anna Dominiczak and Alex Alderton, Roberto Amato, Sonia Goncalves, Ewan Harrison, David K. Jackson, Ian Johnston, Dominic Kwiatkowski, Cordelia Langford, John Sillitoe on behalf of the Wellcome Sanger Institute COVID-19 Surveillance Team
EPI_ISL_909155, EPI_ISL_909156, EPI_ISL_909157	Lighthouse Lab in Cambridge	Wellcome Sanger Institute for the COVID-19 Genomics UK (COG-UK) Consortium	Rob Howes, The Lighthouse Lab in Cambridge and Alex Alderton, Roberto Amato, Sonia Goncalves, Ewan Harrison, David K. Jackson, Ian Johnston, Dominic Kwiatkowski, Cordelia Langford, John Sillitoe on behalf of the Wellcome Sanger Institute COVID-19 Surveillance Team
EPI_ISL_909158, EPI_ISL_909162, EPI_ISL_909165, EPI_ISL_909169, EPI_ISL_909170, EPI_ISL_909171	Lighthouse Lab in Glasgow	Wellcome Sanger Institute for the COVID-19 Genomics UK (COG-UK) Consortium	Harper VanSteenhouse, Yumi Kasai, David Gray, Carol Clugston, Anna Dominiczak and Alex Alderton, Roberto Amato, Sonia Goncalves, Ewan Harrison, David K. Jackson, Ian Johnston, Dominic Kwiatkowski, Cordelia Langford, John Sillitoe on behalf of the Wellcome Sanger Institute COVID-19 Surveillance Team
EPI_ISL_909173	Lighthouse Lab in Cambridge	Wellcome Sanger Institute for the COVID-19 Genomics UK (COG-UK) Consortium	Rob Howes, The Lighthouse Lab in Cambridge and Alex Alderton, Roberto Amato, Sonia Goncalves, Ewan Harrison, David K. Jackson, Ian Johnston, Dominic Kwiatkowski, Cordelia Langford, John Sillitoe on behalf of the Wellcome Sanger Institute COVID-19 Surveillance Team
EPI_ISL_909176, EPI_ISL_909177	Lighthouse Lab in Glasgow	Wellcome Sanger Institute for the COVID-19 Genomics UK (COG-UK) Consortium	Harper VanSteenhouse, Yumi Kasai, David Gray, Carol Clugston, Anna Dominiczak and Alex Alderton, Roberto Amato, Sonia Goncalves, Ewan Harrison, David K. Jackson, Ian Johnston, Dominic Kwiatkowski, Cordelia Langford, John Sillitoe on behalf of the Wellcome Sanger Institute COVID-19 Surveillance

			Team
EPI_ISL_909178, EPI_ISL_909180, EPI_ISL_909182	Lighthouse Lab in Cambridge	Wellcome Sanger Institute for the COVID-19 Genomics UK (COG-UK) Consortium	Rob Howes, The Lighthouse Lab in Cambridge and Alex Alderton, Roberto Amato, Sonia Goncalves, Ewan Harrison, David K. Jackson, Ian Johnston, Dominic Kwiatkowski, Cordelia Langford, John Sillitoe on behalf of the Wellcome Sanger Institute COVID-19 Surveillance Team
EPI_ISL_909184	Lighthouse Lab in Glasgow	Wellcome Sanger Institute for the COVID-19 Genomics UK (COG-UK) Consortium	Harper VanSteenhouse, Yumi Kasai, David Gray, Carol Clugston, Anna Dominiczak and Alex Alderton, Roberto Amato, Sonia Goncalves, Ewan Harrison, David K. Jackson, Ian Johnston, Dominic Kwiatkowski, Cordelia Langford, John Sillitoe on behalf of the Wellcome Sanger Institute COVID-19 Surveillance Team
EPI_ISL_909187, EPI_ISL_909189, EPI_ISL_909192, EPI_ISL_909193	Lighthouse Lab in Cambridge	Wellcome Sanger Institute for the COVID-19 Genomics UK (COG-UK) Consortium	Rob Howes, The Lighthouse Lab in Cambridge and Alex Alderton, Roberto Amato, Sonia Goncalves, Ewan Harrison, David K. Jackson, Ian Johnston, Dominic Kwiatkowski, Cordelia Langford, John Sillitoe on behalf of the Wellcome Sanger Institute COVID-19 Surveillance Team
EPI_ISL_909194, EPI_ISL_909200	Lighthouse Lab in Glasgow	Wellcome Sanger Institute for the COVID-19 Genomics UK (COG-UK) Consortium	Harper VanSteenhouse, Yumi Kasai, David Gray, Carol Clugston, Anna Dominiczak and Alex Alderton, Roberto Amato, Sonia Goncalves, Ewan Harrison, David K. Jackson, Ian Johnston, Dominic Kwiatkowski, Cordelia Langford, John Sillitoe on behalf of the Wellcome Sanger Institute COVID-19 Surveillance Team
EPI_ISL_909202, EPI_ISL_909203, EPI_ISL_909204	Lighthouse Lab in Cambridge	Wellcome Sanger Institute for the COVID-19 Genomics UK (COG-UK) Consortium	Rob Howes, The Lighthouse Lab in Cambridge and Alex Alderton, Roberto Amato, Sonia Goncalves, Ewan Harrison, David K. Jackson, Ian Johnston, Dominic Kwiatkowski, Cordelia Langford, John Sillitoe on behalf of the Wellcome Sanger Institute COVID-19 Surveillance Team
EPI_ISL_909206, EPI_ISL_909208, EPI_ISL_909210, EPI_ISL_909211	Lighthouse Lab in Glasgow	Wellcome Sanger Institute for the COVID-19 Genomics UK (COG-UK) Consortium	Harper VanSteenhouse, Yumi Kasai, David Gray, Carol Clugston, Anna Dominiczak and Alex Alderton, Roberto Amato, Sonia Goncalves, Ewan Harrison, David K. Jackson, Ian Johnston, Dominic Kwiatkowski, Cordelia Langford, John Sillitoe on behalf of the Wellcome Sanger Institute COVID-19 Surveillance Team
EPI_ISL_909217	Lighthouse Lab in Cambridge	Wellcome Sanger Institute for the COVID-19 Genomics UK (COG-UK) Consortium	Rob Howes, The Lighthouse Lab in Cambridge and Alex Alderton, Roberto Amato, Sonia Goncalves, Ewan Harrison, David K. Jackson, Ian Johnston, Dominic Kwiatkowski, Cordelia Langford, John Sillitoe on behalf of the Wellcome Sanger Institute COVID-19 Surveillance Team
EPI_ISL_909218, EPI_ISL_909219	Lighthouse Lab in Glasgow	Wellcome Sanger Institute for the COVID-19 Genomics UK (COG-UK) Consortium	Harper VanSteenhouse, Yumi Kasai, David Gray, Carol Clugston, Anna Dominiczak and Alex Alderton, Roberto Amato, Sonia Goncalves, Ewan Harrison, David K. Jackson, Ian Johnston, Dominic Kwiatkowski, Cordelia Langford, John Sillitoe on behalf of the Wellcome Sanger Institute COVID-19 Surveillance Team
EPI_ISL_909220, EPI_ISL_909223	Lighthouse Lab in Cambridge	Wellcome Sanger Institute for the COVID-19 Genomics UK (COG-UK) Consortium	Rob Howes, The Lighthouse Lab in Cambridge and Alex Alderton, Roberto Amato, Sonia Goncalves, Ewan Harrison, David K. Jackson, Ian Johnston, Dominic Kwiatkowski, Cordelia Langford, John Sillitoe on behalf of the Wellcome Sanger Institute COVID-19 Surveillance Team
EPI_ISL_909227	Lighthouse Lab in Glasgow	Wellcome Sanger Institute for the COVID-19 Genomics UK (COG-UK) Consortium	Harper VanSteenhouse, Yumi Kasai, David Gray, Carol Clugston, Anna Dominiczak and Alex Alderton, Roberto Amato, Sonia Goncalves, Ewan Harrison, David K. Jackson, Ian Johnston, Dominic Kwiatkowski, Cordelia Langford, John Sillitoe on behalf of the Wellcome Sanger Institute COVID-19 Surveillance Team
EPI_ISL_909230	Lighthouse Lab in Cambridge	Wellcome Sanger Institute for the COVID-19 Genomics UK (COG-UK) Consortium	Rob Howes, The Lighthouse Lab in Cambridge and Alex Alderton, Roberto Amato, Sonia Goncalves, Ewan Harrison, David K. Jackson, Ian Johnston, Dominic Kwiatkowski, Cordelia Langford, John Sillitoe on behalf of the Wellcome Sanger Institute COVID-19 Surveillance Team
EPI_ISL_909233	Lighthouse Lab in Glasgow	Wellcome Sanger Institute for the COVID-19 Genomics UK (COG-UK) Consortium	Harper VanSteenhouse, Yumi Kasai, David Gray, Carol Clugston, Anna Dominiczak and Alex Alderton, Roberto Amato, Sonia Goncalves, Ewan Harrison, David K. Jackson, Ian Johnston, Dominic Kwiatkowski, Cordelia Langford, John Sillitoe on behalf of the Wellcome Sanger Institute COVID-19 Surveillance Team
EPI_ISL_909234	Lighthouse Lab in Cambridge	Wellcome Sanger Institute for the COVID-19 Genomics UK (COG-UK) Consortium	Rob Howes, The Lighthouse Lab in Cambridge and Alex Alderton, Roberto Amato, Sonia Goncalves, Ewan Harrison, David K. Jackson, Ian Johnston, Dominic Kwiatkowski, Cordelia Langford, John Sillitoe on behalf of the Wellcome Sanger Institute COVID-19 Surveillance Team
EPI_ISL_909235, EPI_ISL_909236, EPI_ISL_909237	Lighthouse Lab in Glasgow	Wellcome Sanger Institute for the COVID-19 Genomics UK (COG-UK) Consortium	Harper VanSteenhouse, Yumi Kasai, David Gray, Carol Clugston, Anna Dominiczak and Alex Alderton, Roberto Amato, Sonia Goncalves, Ewan Harrison, David K. Jackson, Ian Johnston, Dominic Kwiatkowski, Cordelia Langford, John Sillitoe on behalf of the Wellcome Sanger Institute COVID-19 Surveillance Team
EPI_ISL_909238, EPI_ISL_909240	Lighthouse Lab in Cambridge	Wellcome Sanger Institute for the COVID-19 Genomics UK (COG-UK) Consortium	Rob Howes, The Lighthouse Lab in Cambridge and Alex Alderton, Roberto Amato, Sonia Goncalves, Ewan Harrison, David K. Jackson, Ian Johnston, Dominic Kwiatkowski, Cordelia Langford, John Sillitoe on behalf of the Wellcome Sanger Institute COVID-19 Surveillance Team
EPI_ISL_909242	Lighthouse Lab in Glasgow	Wellcome Sanger Institute for the COVID-19 Genomics UK (COG-UK) Consortium	Harper VanSteenhouse, Yumi Kasai, David Gray, Carol Clugston, Anna Dominiczak and Alex Alderton, Roberto Amato, Sonia Goncalves, Ewan Harrison, David K. Jackson, Ian Johnston, Dominic Kwiatkowski, Cordelia Langford, John Sillitoe on behalf of the Wellcome Sanger Institute COVID-19 Surveillance Team
EPI_ISL_909243, EPI_ISL_909251, EPI_ISL_909254	Lighthouse Lab in Cambridge	Wellcome Sanger Institute for the COVID-19 Genomics UK (COG-UK) Consortium	Rob Howes, The Lighthouse Lab in Cambridge and Alex Alderton, Roberto Amato, Sonia Goncalves, Ewan Harrison, David K. Jackson, Ian Johnston, Dominic Kwiatkowski, Cordelia Langford, John Sillitoe on behalf of the Wellcome Sanger Institute COVID-19 Surveillance Team
EPI_ISL_909255, EPI_ISL_909257	Lighthouse Lab in Glasgow	Wellcome Sanger Institute for the COVID-19 Genomics UK (COG-UK) Consortium	Harper VanSteenhouse, Yumi Kasai, David Gray, Carol Clugston, Anna Dominiczak and Alex Alderton, Roberto Amato, Sonia Goncalves, Ewan Harrison, David K. Jackson, Ian Johnston, Dominic Kwiatkowski, Cordelia Langford, John Sillitoe on behalf of the Wellcome Sanger Institute COVID-19 Surveillance Team
EPI_ISL_909259	Lighthouse Lab in Milton Keynes	Wellcome Sanger Institute for the COVID-19 Genomics UK (COG-UK) Consortium	The Lighthouse Lab in Milton Keynes and Alex Alderton, Roberto Amato, Sonia Goncalves, Ewan Harrison, David K. Jackson, Ian Johnston, Dominic Kwiatkowski, Cordelia Langford, John Sillitoe on behalf of the Wellcome Sanger Institute COVID-19 Surveillance Team
EPI_ISL_909260	Lighthouse Lab in Alderley Park	Wellcome Sanger Institute for the COVID-19 Genomics UK (COG-UK) Consortium	Jacquelyn Wynn, Mairead Hyland, The Lighthouse Lab in Alderley Park and Alex Alderton, Roberto Amato, Sonia Goncalves, Ewan Harrison, David K. Jackson, Ian Johnston, Dominic Kwiatkowski, Cordelia Langford, John Sillitoe on behalf of the Wellcome Sanger Institute COVID-19 Surveillance Team
EPI_ISL_909263, EPI_ISL_909269	Lighthouse Lab in Milton Keynes	Wellcome Sanger Institute for the COVID-19 Genomics UK (COG-UK) Consortium	The Lighthouse Lab in Milton Keynes and Alex Alderton, Roberto Amato, Sonia Goncalves, Ewan Harrison, David K. Jackson, Ian Johnston, Dominic Kwiatkowski, Cordelia Langford, John Sillitoe on behalf of the Wellcome Sanger Institute COVID-19 Surveillance Team
EPI_ISL_909270	Lighthouse Lab in Alderley Park	Wellcome Sanger Institute for the COVID-19 Genomics UK (COG-UK) Consortium	Jacquelyn Wynn, Mairead Hyland, The Lighthouse Lab in Alderley Park and Alex Alderton, Roberto Amato, Sonia Goncalves, Ewan Harrison, David K. Jackson, Ian Johnston, Dominic Kwiatkowski, Cordelia Langford, John Sillitoe on behalf of the Wellcome Sanger Institute COVID-19 Surveillance Team
EPI_ISL_909271	Lighthouse Lab in Milton Keynes	Wellcome Sanger Institute for the COVID-19 Genomics UK (COG-UK) Consortium	The Lighthouse Lab in Milton Keynes and Alex Alderton, Roberto Amato, Sonia Goncalves, Ewan Harrison, David K. Jackson, Ian Johnston, Dominic Kwiatkowski, Cordelia Langford, John Sillitoe on behalf of the Wellcome Sanger Institute COVID-19 Surveillance Team
EPI_ISL_909275	Lighthouse Lab in Alderley Park	Wellcome Sanger Institute for the COVID-19 Genomics UK (COG-UK) Consortium	Jacquelyn Wynn, Mairead Hyland, The Lighthouse Lab in Alderley Park and Alex Alderton, Roberto Amato, Sonia Goncalves, Ewan Harrison, David K. Jackson, Ian Johnston, Dominic Kwiatkowski, Cordelia Langford, John Sillitoe on behalf of the Wellcome Sanger Institute COVID-19 Surveillance Team
EPI_ISL_909276, EPI_ISL_909277, EPI_ISL_909284	Lighthouse Lab in Milton Keynes	Wellcome Sanger Institute for the COVID-19 Genomics UK (COG-UK) Consortium	The Lighthouse Lab in Milton Keynes and Alex Alderton, Roberto Amato, Sonia Goncalves, Ewan Harrison, David K. Jackson, Ian Johnston, Dominic Kwiatkowski, Cordelia Langford, John Sillitoe on behalf of the Wellcome Sanger Institute COVID-19 Surveillance Team
EPI_ISL_909285	Lighthouse Lab in Alderley Park	Wellcome Sanger Institute for the COVID-19 Genomics UK (COG-UK) Consortium	Jacquelyn Wynn, Mairead Hyland, The Lighthouse Lab in Alderley Park and Alex Alderton, Roberto Amato, Sonia Goncalves, Ewan Harrison, David K. Jackson, Ian Johnston, Dominic Kwiatkowski, Cordelia Langford, John Sillitoe on behalf of the Wellcome Sanger Institute COVID-19 Surveillance Team
EPI_ISL_909287, EPI_ISL_909291	Lighthouse Lab in Milton Keynes	Wellcome Sanger Institute for the COVID-19 Genomics UK (COG-UK) Consortium	The Lighthouse Lab in Milton Keynes and Alex Alderton, Roberto Amato, Sonia Goncalves, Ewan Harrison, David K. Jackson, Ian Johnston, Dominic Kwiatkowski, Cordelia Langford, John Sillitoe on behalf of the Wellcome Sanger Institute COVID-19 Surveillance Team
EPI_ISL_909292	Lighthouse Lab in Alderley Park	Wellcome Sanger Institute for the COVID-19 Genomics UK (COG-UK) Consortium	Jacquelyn Wynn, Mairead Hyland, The Lighthouse Lab in Alderley Park and Alex Alderton, Roberto Amato, Sonia Goncalves, Ewan Harrison, David K. Jackson, Ian Johnston, Dominic Kwiatkowski, Cordelia Langford, John Sillitoe on behalf of the Wellcome Sanger Institute COVID-19 Surveillance Team
EPI_ISL_909293	Lighthouse Lab in Milton Keynes	Wellcome Sanger Institute for the COVID-19 Genomics UK (COG-UK) Consortium	The Lighthouse Lab in Milton Keynes and Alex Alderton, Roberto Amato, Sonia Goncalves, Ewan Harrison, David K. Jackson, Ian Johnston, Dominic Kwiatkowski, Cordelia Langford, John Sillitoe on behalf of the Wellcome Sanger Institute COVID-19 Surveillance Team
EPI_ISL_909294, EPI_ISL_909297	Lighthouse Lab in Alderley Park	Wellcome Sanger Institute for the COVID-19 Genomics UK (COG-UK) Consortium	Jacquelyn Wynn, Mairead Hyland, The Lighthouse Lab in Alderley Park and Alex Alderton, Roberto Amato, Sonia Goncalves, Ewan Harrison, David K. Jackson, Ian Johnston, Dominic Kwiatkowski, Cordelia Langford, John Sillitoe on behalf of the Wellcome Sanger Institute COVID-19 Surveillance Team
EPI_ISL_909301	Lighthouse Lab in Milton Keynes	Wellcome Sanger Institute for the COVID-19 Genomics UK (COG-UK) Consortium	The Lighthouse Lab in Milton Keynes and Alex Alderton, Roberto Amato, Sonia Goncalves, Ewan Harrison, David K. Jackson, Ian Johnston, Dominic Kwiatkowski, Cordelia Langford, John Sillitoe on behalf of the Wellcome Sanger Institute COVID-19 Surveillance Team

EPI_ISL_909302, EPI_ISL_909309	Lighthouse Lab in Alderley Park	Wellcome Sanger Institute for the COVID-19 Genomics UK (COG-UK) Consortium	Jacquelyn Wynn, Mairead Hyland, The Lighthouse Lab in Alderley Park and Alex Alderton, Roberto Amato, Sonia Goncalves, Ewan Harrison, David K. Jackson, Ian Johnston, Dominic Kwiatkowski, Cordelia Langford, John Sillitoe on behalf of the Wellcome Sanger Institute COVID-19 Surveillance Team
EPI_ISL_909310	Lighthouse Lab in Milton Keynes	Wellcome Sanger Institute for the COVID-19 Genomics UK (COG-UK) Consortium	The Lighthouse Lab in Milton Keynes and Alex Alderton, Roberto Amato, Sonia Goncalves, Ewan Harrison, David K. Jackson, Ian Johnston, Dominic Kwiatkowski, Cordelia Langford, John Sillitoe on behalf of the Wellcome Sanger Institute COVID-19 Surveillance Team
EPI_ISL_909312, EPI_ISL_909314, EPI_ISL_909316, EPI_ISL_909321, EPI_ISL_909322, EPI_ISL_909326	Lighthouse Lab in Alderley Park	Wellcome Sanger Institute for the COVID-19 Genomics UK (COG-UK) Consortium	Jacquelyn Wynn, Mairead Hyland, The Lighthouse Lab in Alderley Park and Alex Alderton, Roberto Amato, Sonia Goncalves, Ewan Harrison, David K. Jackson, Ian Johnston, Dominic Kwiatkowski, Cordelia Langford, John Sillitoe on behalf of the Wellcome Sanger Institute COVID-19 Surveillance Team
EPI_ISL_909327	Lighthouse Lab in Glasgow	Wellcome Sanger Institute for the COVID-19 Genomics UK (COG-UK) Consortium	Harper VanSteenhouse, Yumi Kasai, David Gray, Carol Clugston, Anna Dominiczak and Alex Alderton, Roberto Amato, Sonia Goncalves, Ewan Harrison, David K. Jackson, Ian Johnston, Dominic Kwiatkowski, Cordelia Langford, John Sillitoe on behalf of the Wellcome Sanger Institute COVID-19 Surveillance Team
EPI_ISL_909329, EPI_ISL_909331	Lighthouse Lab in Milton Keynes	Wellcome Sanger Institute for the COVID-19 Genomics UK (COG-UK) Consortium	The Lighthouse Lab in Milton Keynes and Alex Alderton, Roberto Amato, Sonia Goncalves, Ewan Harrison, David K. Jackson, Ian Johnston, Dominic Kwiatkowski, Cordelia Langford, John Sillitoe on behalf of the Wellcome Sanger Institute COVID-19 Surveillance Team
EPI_ISL_909334	Lighthouse Lab in Alderley Park	Wellcome Sanger Institute for the COVID-19 Genomics UK (COG-UK) Consortium	Jacquelyn Wynn, Mairead Hyland, The Lighthouse Lab in Alderley Park and Alex Alderton, Roberto Amato, Sonia Goncalves, Ewan Harrison, David K. Jackson, Ian Johnston, Dominic Kwiatkowski, Cordelia Langford, John Sillitoe on behalf of the Wellcome Sanger Institute COVID-19 Surveillance Team
EPI_ISL_909335, EPI_ISL_909338	Lighthouse Lab in Milton Keynes	Wellcome Sanger Institute for the COVID-19 Genomics UK (COG-UK) Consortium	The Lighthouse Lab in Milton Keynes and Alex Alderton, Roberto Amato, Sonia Goncalves, Ewan Harrison, David K. Jackson, Ian Johnston, Dominic Kwiatkowski, Cordelia Langford, John Sillitoe on behalf of the Wellcome Sanger Institute COVID-19 Surveillance Team
EPI_ISL_909339	Lighthouse Lab in Alderley Park	Wellcome Sanger Institute for the COVID-19 Genomics UK (COG-UK) Consortium	Jacquelyn Wynn, Mairead Hyland, The Lighthouse Lab in Alderley Park and Alex Alderton, Roberto Amato, Sonia Goncalves, Ewan Harrison, David K. Jackson, Ian Johnston, Dominic Kwiatkowski, Cordelia Langford, John Sillitoe on behalf of the Wellcome Sanger Institute COVID-19 Surveillance Team
EPI_ISL_909340	Lighthouse Lab in Milton Keynes	Wellcome Sanger Institute for the COVID-19 Genomics UK (COG-UK) Consortium	The Lighthouse Lab in Milton Keynes and Alex Alderton, Roberto Amato, Sonia Goncalves, Ewan Harrison, David K. Jackson, Ian Johnston, Dominic Kwiatkowski, Cordelia Langford, John Sillitoe on behalf of the Wellcome Sanger Institute COVID-19 Surveillance Team
EPI_ISL_909341, EPI_ISL_909343	Lighthouse Lab in Alderley Park	Wellcome Sanger Institute for the COVID-19 Genomics UK (COG-UK) Consortium	Jacquelyn Wynn, Mairead Hyland, The Lighthouse Lab in Alderley Park and Alex Alderton, Roberto Amato, Sonia Goncalves, Ewan Harrison, David K. Jackson, Ian Johnston, Dominic Kwiatkowski, Cordelia Langford, John Sillitoe on behalf of the Wellcome Sanger Institute COVID-19 Surveillance Team
EPI_ISL_909345, EPI_ISL_909350	Lighthouse Lab in Milton Keynes	Wellcome Sanger Institute for the COVID-19 Genomics UK (COG-UK) Consortium	The Lighthouse Lab in Milton Keynes and Alex Alderton, Roberto Amato, Sonia Goncalves, Ewan Harrison, David K. Jackson, Ian Johnston, Dominic Kwiatkowski, Cordelia Langford, John Sillitoe on behalf of the Wellcome Sanger Institute COVID-19 Surveillance Team
EPI_ISL_909351	Lighthouse Lab in Glasgow	Wellcome Sanger Institute for the COVID-19 Genomics UK (COG-UK) Consortium	Harper VanSteenhouse, Yumi Kasai, David Gray, Carol Clugston, Anna Dominiczak and Alex Alderton, Roberto Amato, Sonia Goncalves, Ewan Harrison, David K. Jackson, Ian Johnston, Dominic Kwiatkowski, Cordelia Langford, John Sillitoe on behalf of the Wellcome Sanger Institute COVID-19 Surveillance Team
EPI_ISL_909352, EPI_ISL_909354, EPI_ISL_909355	Lighthouse Lab in Milton Keynes	Wellcome Sanger Institute for the COVID-19 Genomics UK (COG-UK) Consortium	The Lighthouse Lab in Milton Keynes and Alex Alderton, Roberto Amato, Sonia Goncalves, Ewan Harrison, David K. Jackson, Ian Johnston, Dominic Kwiatkowski, Cordelia Langford, John Sillitoe on behalf of the Wellcome Sanger Institute COVID-19 Surveillance Team
EPI_ISL_909356	Lighthouse Lab in Glasgow	Wellcome Sanger Institute for the COVID-19 Genomics UK (COG-UK) Consortium	Harper VanSteenhouse, Yumi Kasai, David Gray, Carol Clugston, Anna Dominiczak and Alex Alderton, Roberto Amato, Sonia Goncalves, Ewan Harrison, David K. Jackson, Ian Johnston, Dominic Kwiatkowski, Cordelia Langford, John Sillitoe on behalf of the Wellcome Sanger Institute COVID-19 Surveillance Team
EPI_ISL_909358, EPI_ISL_909359	Lighthouse Lab in Alderley Park	Wellcome Sanger Institute for the COVID-19 Genomics UK (COG-UK) Consortium	Jacquelyn Wynn, Mairead Hyland, The Lighthouse Lab in Alderley Park and Alex Alderton, Roberto Amato, Sonia Goncalves, Ewan Harrison, David K. Jackson, Ian Johnston, Dominic Kwiatkowski, Cordelia Langford, John Sillitoe on behalf of the Wellcome Sanger Institute COVID-19 Surveillance Team
EPI_ISL_909365	Lighthouse Lab in Milton Keynes	Wellcome Sanger Institute for the COVID-19 Genomics UK (COG-UK) Consortium	The Lighthouse Lab in Milton Keynes and Alex Alderton, Roberto Amato, Sonia Goncalves, Ewan Harrison, David K. Jackson, Ian Johnston, Dominic Kwiatkowski, Cordelia Langford, John Sillitoe on behalf of the Wellcome Sanger Institute COVID-19 Surveillance Team
EPI_ISL_909366, EPI_ISL_909367, EPI_ISL_909368	Lighthouse Lab in Alderley Park	Wellcome Sanger Institute for the COVID-19 Genomics UK (COG-UK) Consortium	Jacquelyn Wynn, Mairead Hyland, The Lighthouse Lab in Alderley Park and Alex Alderton, Roberto Amato, Sonia Goncalves, Ewan Harrison, David K. Jackson, Ian Johnston, Dominic Kwiatkowski, Cordelia Langford, John Sillitoe on behalf of the Wellcome Sanger Institute COVID-19 Surveillance Team
EPI_ISL_909369	Lighthouse Lab in Glasgow	Wellcome Sanger Institute for the COVID-19 Genomics UK (COG-UK) Consortium	Harper VanSteenhouse, Yumi Kasai, David Gray, Carol Clugston, Anna Dominiczak and Alex Alderton, Roberto Amato, Sonia Goncalves, Ewan Harrison, David K. Jackson, Ian Johnston, Dominic Kwiatkowski, Cordelia Langford, John Sillitoe on behalf of the Wellcome Sanger Institute COVID-19 Surveillance Team
EPI_ISL_909370, EPI_ISL_909372	Lighthouse Lab in Alderley Park	Wellcome Sanger Institute for the COVID-19 Genomics UK (COG-UK) Consortium	Jacquelyn Wynn, Mairead Hyland, The Lighthouse Lab in Alderley Park and Alex Alderton, Roberto Amato, Sonia Goncalves, Ewan Harrison, David K. Jackson, Ian Johnston, Dominic Kwiatkowski, Cordelia Langford, John Sillitoe on behalf of the Wellcome Sanger Institute COVID-19 Surveillance Team
EPI_ISL_909375	Lighthouse Lab in Glasgow	Wellcome Sanger Institute for the COVID-19 Genomics UK (COG-UK) Consortium	Harper VanSteenhouse, Yumi Kasai, David Gray, Carol Clugston, Anna Dominiczak and Alex Alderton, Roberto Amato, Sonia Goncalves, Ewan Harrison, David K. Jackson, Ian Johnston, Dominic Kwiatkowski, Cordelia Langford, John Sillitoe on behalf of the Wellcome Sanger Institute COVID-19 Surveillance Team
EPI_ISL_909380, EPI_ISL_909381	Lighthouse Lab in Alderley Park	Wellcome Sanger Institute for the COVID-19 Genomics UK (COG-UK) Consortium	Jacquelyn Wynn, Mairead Hyland, The Lighthouse Lab in Alderley Park and Alex Alderton, Roberto Amato, Sonia Goncalves, Ewan Harrison, David K. Jackson, Ian Johnston, Dominic Kwiatkowski, Cordelia Langford, John Sillitoe on behalf of the Wellcome Sanger Institute COVID-19 Surveillance Team
EPI_ISL_909382	Lighthouse Lab in Glasgow	Wellcome Sanger Institute for the COVID-19 Genomics UK (COG-UK) Consortium	Harper VanSteenhouse, Yumi Kasai, David Gray, Carol Clugston, Anna Dominiczak and Alex Alderton, Roberto Amato, Sonia Goncalves, Ewan Harrison, David K. Jackson, Ian Johnston, Dominic Kwiatkowski, Cordelia Langford, John Sillitoe on behalf of the Wellcome Sanger Institute COVID-19 Surveillance Team
EPI_ISL_909383	Lighthouse Lab in Milton Keynes	Wellcome Sanger Institute for the COVID-19 Genomics UK (COG-UK) Consortium	The Lighthouse Lab in Milton Keynes and Alex Alderton, Roberto Amato, Sonia Goncalves, Ewan Harrison, David K. Jackson, Ian Johnston, Dominic Kwiatkowski, Cordelia Langford, John Sillitoe on behalf of the Wellcome Sanger Institute COVID-19 Surveillance Team
EPI_ISL_909385	Lighthouse Lab in Alderley Park	Wellcome Sanger Institute for the COVID-19 Genomics UK (COG-UK) Consortium	Jacquelyn Wynn, Mairead Hyland, The Lighthouse Lab in Alderley Park and Alex Alderton, Roberto Amato, Sonia Goncalves, Ewan Harrison, David K. Jackson, Ian Johnston, Dominic Kwiatkowski, Cordelia Langford, John Sillitoe on behalf of the Wellcome Sanger Institute COVID-19 Surveillance Team
EPI_ISL_909386	Lighthouse Lab in Milton Keynes	Wellcome Sanger Institute for the COVID-19 Genomics UK (COG-UK) Consortium	The Lighthouse Lab in Milton Keynes and Alex Alderton, Roberto Amato, Sonia Goncalves, Ewan Harrison, David K. Jackson, Ian Johnston, Dominic Kwiatkowski, Cordelia Langford, John Sillitoe on behalf of the Wellcome Sanger Institute COVID-19 Surveillance Team
EPI_ISL_909388, EPI_ISL_909389	Lighthouse Lab in Alderley Park	Wellcome Sanger Institute for the COVID-19 Genomics UK (COG-UK) Consortium	Jacquelyn Wynn, Mairead Hyland, The Lighthouse Lab in Alderley Park and Alex Alderton, Roberto Amato, Sonia Goncalves, Ewan Harrison, David K. Jackson, Ian Johnston, Dominic Kwiatkowski, Cordelia Langford, John Sillitoe on behalf of the Wellcome Sanger Institute COVID-19 Surveillance Team
EPI_ISL_909390	Lighthouse Lab in Glasgow	Wellcome Sanger Institute for the COVID-19 Genomics UK (COG-UK) Consortium	Harper VanSteenhouse, Yumi Kasai, David Gray, Carol Clugston, Anna Dominiczak and Alex Alderton, Roberto Amato, Sonia Goncalves, Ewan Harrison, David K. Jackson, Ian Johnston, Dominic Kwiatkowski, Cordelia Langford, John Sillitoe on behalf of the Wellcome Sanger Institute COVID-19 Surveillance Team
EPI_ISL_909394, EPI_ISL_909396	Lighthouse Lab in Milton Keynes	Wellcome Sanger Institute for the COVID-19 Genomics UK (COG-UK) Consortium	The Lighthouse Lab in Milton Keynes and Alex Alderton, Roberto Amato, Sonia Goncalves, Ewan Harrison, David K. Jackson, Ian Johnston, Dominic Kwiatkowski, Cordelia Langford, John Sillitoe on behalf of the Wellcome Sanger Institute COVID-19 Surveillance Team
EPI_ISL_909397	Lighthouse Lab in Alderley Park	Wellcome Sanger Institute for the COVID-19 Genomics UK (COG-UK) Consortium	Jacquelyn Wynn, Mairead Hyland, The Lighthouse Lab in Alderley Park and Alex Alderton, Roberto Amato, Sonia Goncalves, Ewan Harrison, David K. Jackson, Ian Johnston, Dominic Kwiatkowski, Cordelia Langford, John Sillitoe on behalf of the Wellcome Sanger Institute COVID-19 Surveillance Team
EPI_ISL_909405	Lighthouse Lab in Glasgow	Wellcome Sanger Institute for the COVID-19 Genomics UK (COG-UK) Consortium	Harper VanSteenhouse, Yumi Kasai, David Gray, Carol Clugston, Anna Dominiczak and Alex Alderton, Roberto Amato, Sonia Goncalves, Ewan Harrison, David K. Jackson, Ian Johnston, Dominic Kwiatkowski, Cordelia Langford, John Sillitoe on behalf of the Wellcome Sanger Institute COVID-19 Surveillance Team
EPI_ISL_909409	Lighthouse Lab in Alderley Park	Wellcome Sanger Institute for the COVID-19 Genomics UK (COG-UK) Consortium	Jacquelyn Wynn, Mairead Hyland, The Lighthouse Lab in Alderley Park and Alex Alderton, Roberto Amato, Sonia Goncalves, Ewan Harrison, David K. Jackson, Ian Johnston, Dominic Kwiatkowski, Cordelia Langford, John Sillitoe on behalf of the Wellcome Sanger Institute COVID-19 Surveillance Team
EPI_ISL_909411, EPI_ISL_909412,	Lighthouse Lab in Milton Keynes	Wellcome Sanger Institute for the COVID-19 Genomics UK	The Lighthouse Lab in Milton Keynes and Alex Alderton, Roberto Amato, Sonia Goncalves, Ewan Harrison, David K. Jackson, Ian Johnston, Dominic

EPI_ISL_909413, EPI_ISL_909415		(COG-UK) Consortium	Kwiatkowski, Cordelia Langford, John Sillitoe on behalf of the Wellcome Sanger Institute COVID-19 Surveillance Team
EPI_ISL_909416, EPI_ISL_909417	Lighthouse Lab in Alderley Park	Wellcome Sanger Institute for the COVID-19 Genomics UK (COG-UK) Consortium	Jacquelyn Wynn, Mairead Hyland, The Lighthouse Lab in Alderley Park and Alex Alderton, Roberto Amato, Sonia Goncalves, Ewan Harrison, David K. Jackson, Ian Johnston, Dominic Kwiatkowski, Cordelia Langford, John Sillitoe on behalf of the Wellcome Sanger Institute COVID-19 Surveillance Team
EPI_ISL_909418	Lighthouse Lab in Milton Keynes	Wellcome Sanger Institute for the COVID-19 Genomics UK (COG-UK) Consortium	The Lighthouse Lab in Milton Keynes and Alex Alderton, Roberto Amato, Sonia Goncalves, Ewan Harrison, David K. Jackson, Ian Johnston, Dominic Kwiatkowski, Cordelia Langford, John Sillitoe on behalf of the Wellcome Sanger Institute COVID-19 Surveillance Team
EPI_ISL_909421, EPI_ISL_909424	Lighthouse Lab in Alderley Park	Wellcome Sanger Institute for the COVID-19 Genomics UK (COG-UK) Consortium	Jacquelyn Wynn, Mairead Hyland, The Lighthouse Lab in Alderley Park and Alex Alderton, Roberto Amato, Sonia Goncalves, Ewan Harrison, David K. Jackson, Ian Johnston, Dominic Kwiatkowski, Cordelia Langford, John Sillitoe on behalf of the Wellcome Sanger Institute COVID-19 Surveillance Team
EPI_ISL_909427, EPI_ISL_909431	Lighthouse Lab in Milton Keynes	Wellcome Sanger Institute for the COVID-19 Genomics UK (COG-UK) Consortium	The Lighthouse Lab in Milton Keynes and Alex Alderton, Roberto Amato, Sonia Goncalves, Ewan Harrison, David K. Jackson, Ian Johnston, Dominic Kwiatkowski, Cordelia Langford, John Sillitoe on behalf of the Wellcome Sanger Institute COVID-19 Surveillance Team
EPI_ISL_909435	Lighthouse Lab in Glasgow	Wellcome Sanger Institute for the COVID-19 Genomics UK (COG-UK) Consortium	Harper VanSteenhouse, Yumi Kasai, David Gray, Carol Clugston, Anna Dominiczak and Alex Alderton, Roberto Amato, Sonia Goncalves, Ewan Harrison, David K. Jackson, Ian Johnston, Dominic Kwiatkowski, Cordelia Langford, John Sillitoe on behalf of the Wellcome Sanger Institute COVID-19 Surveillance Team
EPI_ISL_909440	Lighthouse Lab in Alderley Park	Wellcome Sanger Institute for the COVID-19 Genomics UK (COG-UK) Consortium	Jacquelyn Wynn, Mairead Hyland, The Lighthouse Lab in Alderley Park and Alex Alderton, Roberto Amato, Sonia Goncalves, Ewan Harrison, David K. Jackson, Ian Johnston, Dominic Kwiatkowski, Cordelia Langford, John Sillitoe on behalf of the Wellcome Sanger Institute COVID-19 Surveillance Team
EPI_ISL_909442, EPI_ISL_909443, EPI_ISL_909444	Lighthouse Lab in Milton Keynes	Wellcome Sanger Institute for the COVID-19 Genomics UK (COG-UK) Consortium	The Lighthouse Lab in Milton Keynes and Alex Alderton, Roberto Amato, Sonia Goncalves, Ewan Harrison, David K. Jackson, Ian Johnston, Dominic Kwiatkowski, Cordelia Langford, John Sillitoe on behalf of the Wellcome Sanger Institute COVID-19 Surveillance Team
EPI_ISL_909446, EPI_ISL_909450	Lighthouse Lab in Alderley Park	Wellcome Sanger Institute for the COVID-19 Genomics UK (COG-UK) Consortium	Jacquelyn Wynn, Mairead Hyland, The Lighthouse Lab in Alderley Park and Alex Alderton, Roberto Amato, Sonia Goncalves, Ewan Harrison, David K. Jackson, Ian Johnston, Dominic Kwiatkowski, Cordelia Langford, John Sillitoe on behalf of the Wellcome Sanger Institute COVID-19 Surveillance Team
EPI_ISL_909452	Lighthouse Lab in Milton Keynes	Wellcome Sanger Institute for the COVID-19 Genomics UK (COG-UK) Consortium	The Lighthouse Lab in Milton Keynes and Alex Alderton, Roberto Amato, Sonia Goncalves, Ewan Harrison, David K. Jackson, Ian Johnston, Dominic Kwiatkowski, Cordelia Langford, John Sillitoe on behalf of the Wellcome Sanger Institute COVID-19 Surveillance Team
EPI_ISL_909453, EPI_ISL_909456, EPI_ISL_909457	Lighthouse Lab in Alderley Park	Wellcome Sanger Institute for the COVID-19 Genomics UK (COG-UK) Consortium	Jacquelyn Wynn, Mairead Hyland, The Lighthouse Lab in Alderley Park and Alex Alderton, Roberto Amato, Sonia Goncalves, Ewan Harrison, David K. Jackson, Ian Johnston, Dominic Kwiatkowski, Cordelia Langford, John Sillitoe on behalf of the Wellcome Sanger Institute COVID-19 Surveillance Team
EPI_ISL_909460, EPI_ISL_909463, EPI_ISL_909464	Lighthouse Lab in Milton Keynes	Wellcome Sanger Institute for the COVID-19 Genomics UK (COG-UK) Consortium	The Lighthouse Lab in Milton Keynes and Alex Alderton, Roberto Amato, Sonia Goncalves, Ewan Harrison, David K. Jackson, Ian Johnston, Dominic Kwiatkowski, Cordelia Langford, John Sillitoe on behalf of the Wellcome Sanger Institute COVID-19 Surveillance Team
EPI_ISL_909465, EPI_ISL_909471	Lighthouse Lab in Alderley Park	Wellcome Sanger Institute for the COVID-19 Genomics UK (COG-UK) Consortium	Jacquelyn Wynn, Mairead Hyland, The Lighthouse Lab in Alderley Park and Alex Alderton, Roberto Amato, Sonia Goncalves, Ewan Harrison, David K. Jackson, Ian Johnston, Dominic Kwiatkowski, Cordelia Langford, John Sillitoe on behalf of the Wellcome Sanger Institute COVID-19 Surveillance Team
EPI_ISL_909474, EPI_ISL_909475	Lighthouse Lab in Milton Keynes	Wellcome Sanger Institute for the COVID-19 Genomics UK (COG-UK) Consortium	The Lighthouse Lab in Milton Keynes and Alex Alderton, Roberto Amato, Sonia Goncalves, Ewan Harrison, David K. Jackson, Ian Johnston, Dominic Kwiatkowski, Cordelia Langford, John Sillitoe on behalf of the Wellcome Sanger Institute COVID-19 Surveillance Team
EPI_ISL_909476, EPI_ISL_909478	Lighthouse Lab in Alderley Park	Wellcome Sanger Institute for the COVID-19 Genomics UK (COG-UK) Consortium	Jacquelyn Wynn, Mairead Hyland, The Lighthouse Lab in Alderley Park and Alex Alderton, Roberto Amato, Sonia Goncalves, Ewan Harrison, David K. Jackson, Ian Johnston, Dominic Kwiatkowski, Cordelia Langford, John Sillitoe on behalf of the Wellcome Sanger Institute COVID-19 Surveillance Team
EPI_ISL_909479, EPI_ISL_909480	Lighthouse Lab in Milton Keynes	Wellcome Sanger Institute for the COVID-19 Genomics UK (COG-UK) Consortium	The Lighthouse Lab in Milton Keynes and Alex Alderton, Roberto Amato, Sonia Goncalves, Ewan Harrison, David K. Jackson, Ian Johnston, Dominic Kwiatkowski, Cordelia Langford, John Sillitoe on behalf of the Wellcome Sanger Institute COVID-19 Surveillance Team
EPI_ISL_909482	Lighthouse Lab in Alderley Park	Wellcome Sanger Institute for the COVID-19 Genomics UK (COG-UK) Consortium	Jacquelyn Wynn, Mairead Hyland, The Lighthouse Lab in Alderley Park and Alex Alderton, Roberto Amato, Sonia Goncalves, Ewan Harrison, David K. Jackson, Ian Johnston, Dominic Kwiatkowski, Cordelia Langford, John Sillitoe on behalf of the Wellcome Sanger Institute COVID-19 Surveillance Team
EPI_ISL_909484	Lighthouse Lab in Milton Keynes	Wellcome Sanger Institute for the COVID-19 Genomics UK (COG-UK) Consortium	The Lighthouse Lab in Milton Keynes and Alex Alderton, Roberto Amato, Sonia Goncalves, Ewan Harrison, David K. Jackson, Ian Johnston, Dominic Kwiatkowski, Cordelia Langford, John Sillitoe on behalf of the Wellcome Sanger Institute COVID-19 Surveillance Team
EPI_ISL_909487	Lighthouse Lab in Alderley Park	Wellcome Sanger Institute for the COVID-19 Genomics UK (COG-UK) Consortium	Jacquelyn Wynn, Mairead Hyland, The Lighthouse Lab in Alderley Park and Alex Alderton, Roberto Amato, Sonia Goncalves, Ewan Harrison, David K. Jackson, Ian Johnston, Dominic Kwiatkowski, Cordelia Langford, John Sillitoe on behalf of the Wellcome Sanger Institute COVID-19 Surveillance Team
EPI_ISL_909492, EPI_ISL_909493	Lighthouse Lab in Milton Keynes	Wellcome Sanger Institute for the COVID-19 Genomics UK (COG-UK) Consortium	The Lighthouse Lab in Milton Keynes and Alex Alderton, Roberto Amato, Sonia Goncalves, Ewan Harrison, David K. Jackson, Ian Johnston, Dominic Kwiatkowski, Cordelia Langford, John Sillitoe on behalf of the Wellcome Sanger Institute COVID-19 Surveillance Team
EPI_ISL_909497	Lighthouse Lab in Glasgow	Wellcome Sanger Institute for the COVID-19 Genomics UK (COG-UK) Consortium	Harper VanSteenhouse, Yumi Kasai, David Gray, Carol Clugston, Anna Dominiczak and Alex Alderton, Roberto Amato, Sonia Goncalves, Ewan Harrison, David K. Jackson, Ian Johnston, Dominic Kwiatkowski, Cordelia Langford, John Sillitoe on behalf of the Wellcome Sanger Institute COVID-19 Surveillance Team
EPI_ISL_909503, EPI_ISL_909504	Lighthouse Lab in Alderley Park	Wellcome Sanger Institute for the COVID-19 Genomics UK (COG-UK) Consortium	Jacquelyn Wynn, Mairead Hyland, The Lighthouse Lab in Alderley Park and Alex Alderton, Roberto Amato, Sonia Goncalves, Ewan Harrison, David K. Jackson, Ian Johnston, Dominic Kwiatkowski, Cordelia Langford, John Sillitoe on behalf of the Wellcome Sanger Institute COVID-19 Surveillance Team
EPI_ISL_909505, EPI_ISL_909506	Lighthouse Lab in Milton Keynes	Wellcome Sanger Institute for the COVID-19 Genomics UK (COG-UK) Consortium	The Lighthouse Lab in Milton Keynes and Alex Alderton, Roberto Amato, Sonia Goncalves, Ewan Harrison, David K. Jackson, Ian Johnston, Dominic Kwiatkowski, Cordelia Langford, John Sillitoe on behalf of the Wellcome Sanger Institute COVID-19 Surveillance Team
EPI_ISL_909508	Lighthouse Lab in Glasgow	Wellcome Sanger Institute for the COVID-19 Genomics UK (COG-UK) Consortium	Harper VanSteenhouse, Yumi Kasai, David Gray, Carol Clugston, Anna Dominiczak and Alex Alderton, Roberto Amato, Sonia Goncalves, Ewan Harrison, David K. Jackson, Ian Johnston, Dominic Kwiatkowski, Cordelia Langford, John Sillitoe on behalf of the Wellcome Sanger Institute COVID-19 Surveillance Team
EPI_ISL_909510, EPI_ISL_909511	Lighthouse Lab in Milton Keynes	Wellcome Sanger Institute for the COVID-19 Genomics UK (COG-UK) Consortium	The Lighthouse Lab in Milton Keynes and Alex Alderton, Roberto Amato, Sonia Goncalves, Ewan Harrison, David K. Jackson, Ian Johnston, Dominic Kwiatkowski, Cordelia Langford, John Sillitoe on behalf of the Wellcome Sanger Institute COVID-19 Surveillance Team
EPI_ISL_909513	Lighthouse Lab in Alderley Park	Wellcome Sanger Institute for the COVID-19 Genomics UK (COG-UK) Consortium	Jacquelyn Wynn, Mairead Hyland, The Lighthouse Lab in Alderley Park and Alex Alderton, Roberto Amato, Sonia Goncalves, Ewan Harrison, David K. Jackson, Ian Johnston, Dominic Kwiatkowski, Cordelia Langford, John Sillitoe on behalf of the Wellcome Sanger Institute COVID-19 Surveillance Team
EPI_ISL_909514, EPI_ISL_909515	Lighthouse Lab in Milton Keynes	Wellcome Sanger Institute for the COVID-19 Genomics UK (COG-UK) Consortium	The Lighthouse Lab in Milton Keynes and Alex Alderton, Roberto Amato, Sonia Goncalves, Ewan Harrison, David K. Jackson, Ian Johnston, Dominic Kwiatkowski, Cordelia Langford, John Sillitoe on behalf of the Wellcome Sanger Institute COVID-19 Surveillance Team
EPI_ISL_909518, EPI_ISL_909526	Lighthouse Lab in Alderley Park	Wellcome Sanger Institute for the COVID-19 Genomics UK (COG-UK) Consortium	Jacquelyn Wynn, Mairead Hyland, The Lighthouse Lab in Alderley Park and Alex Alderton, Roberto Amato, Sonia Goncalves, Ewan Harrison, David K. Jackson, Ian Johnston, Dominic Kwiatkowski, Cordelia Langford, John Sillitoe on behalf of the Wellcome Sanger Institute COVID-19 Surveillance Team
EPI_ISL_909529	Lighthouse Lab in Milton Keynes	Wellcome Sanger Institute for the COVID-19 Genomics UK (COG-UK) Consortium	The Lighthouse Lab in Milton Keynes and Alex Alderton, Roberto Amato, Sonia Goncalves, Ewan Harrison, David K. Jackson, Ian Johnston, Dominic Kwiatkowski, Cordelia Langford, John Sillitoe on behalf of the Wellcome Sanger Institute COVID-19 Surveillance Team
EPI_ISL_909531	Lighthouse Lab in Alderley Park	Wellcome Sanger Institute for the COVID-19 Genomics UK (COG-UK) Consortium	Jacquelyn Wynn, Mairead Hyland, The Lighthouse Lab in Alderley Park and Alex Alderton, Roberto Amato, Sonia Goncalves, Ewan Harrison, David K. Jackson, Ian Johnston, Dominic Kwiatkowski, Cordelia Langford, John Sillitoe on behalf of the Wellcome Sanger Institute COVID-19 Surveillance Team
EPI_ISL_909533	Lighthouse Lab in Milton Keynes	Wellcome Sanger Institute for the COVID-19 Genomics UK (COG-UK) Consortium	The Lighthouse Lab in Milton Keynes and Alex Alderton, Roberto Amato, Sonia Goncalves, Ewan Harrison, David K. Jackson, Ian Johnston, Dominic Kwiatkowski, Cordelia Langford, John Sillitoe on behalf of the Wellcome Sanger Institute COVID-19 Surveillance Team
EPI_ISL_909538	Lighthouse Lab in Glasgow	Wellcome Sanger Institute for the COVID-19 Genomics UK (COG-UK) Consortium	Harper VanSteenhouse, Yumi Kasai, David Gray, Carol Clugston, Anna Dominiczak and Alex Alderton, Roberto Amato, Sonia Goncalves, Ewan Harrison, David K. Jackson, Ian Johnston, Dominic Kwiatkowski, Cordelia Langford, John Sillitoe on behalf of the Wellcome Sanger Institute COVID-19 Surveillance Team
EPI_ISL_909539	Lighthouse Lab in Alderley Park	Wellcome Sanger Institute for the COVID-19 Genomics UK (COG-UK) Consortium	Jacquelyn Wynn, Mairead Hyland, The Lighthouse Lab in Alderley Park and Alex Alderton, Roberto Amato, Sonia Goncalves, Ewan Harrison, David K. Jackson, Ian Johnston, Dominic Kwiatkowski, Cordelia Langford, John Sillitoe on behalf of the Wellcome Sanger Institute COVID-19 Surveillance Team
EPI_ISL_909540, EPI_ISL_909541	Lighthouse Lab in Milton Keynes	Wellcome Sanger Institute for the COVID-19 Genomics UK (COG-UK) Consortium	The Lighthouse Lab in Milton Keynes and Alex Alderton, Roberto Amato, Sonia Goncalves, Ewan Harrison, David K. Jackson, Ian Johnston, Dominic Kwiatkowski, Cordelia Langford, John Sillitoe on behalf of the Wellcome Sanger Institute COVID-19 Surveillance Team

EPI_ISL_909545, EPI_ISL_909546	Lighthouse Lab in Alderley Park	Wellcome Sanger Institute for the COVID-19 Genomics UK (COG-UK) Consortium	Jacquelyn Wynn, Mairead Hyland, The Lighthouse Lab in Alderley Park and Alex Alderton, Roberto Amato, Sonia Goncalves, Ewan Harrison, David K. Jackson, Ian Johnston, Dominic Kwiatkowski, Cordelia Langford, John Sillitoe on behalf of the Wellcome Sanger Institute COVID-19 Surveillance Team
EPI_ISL_909549	Lighthouse Lab in Glasgow	Wellcome Sanger Institute for the COVID-19 Genomics UK (COG-UK) Consortium	Harper VanSteenhouse, Yumi Kasai, David Gray, Carol Clugston, Anna Dominiczak and Alex Alderton, Roberto Amato, Sonia Goncalves, Ewan Harrison, David K. Jackson, Ian Johnston, Dominic Kwiatkowski, Cordelia Langford, John Sillitoe on behalf of the Wellcome Sanger Institute COVID-19 Surveillance Team
EPI_ISL_909552, EPI_ISL_909553	Lighthouse Lab in Alderley Park	Wellcome Sanger Institute for the COVID-19 Genomics UK (COG-UK) Consortium	Jacquelyn Wynn, Mairead Hyland, The Lighthouse Lab in Alderley Park and Alex Alderton, Roberto Amato, Sonia Goncalves, Ewan Harrison, David K. Jackson, Ian Johnston, Dominic Kwiatkowski, Cordelia Langford, John Sillitoe on behalf of the Wellcome Sanger Institute COVID-19 Surveillance Team
EPI_ISL_909556, EPI_ISL_909560	Lighthouse Lab in Milton Keynes	Wellcome Sanger Institute for the COVID-19 Genomics UK (COG-UK) Consortium	The Lighthouse Lab in Milton Keynes and Alex Alderton, Roberto Amato, Sonia Goncalves, Ewan Harrison, David K. Jackson, Ian Johnston, Dominic Kwiatkowski, Cordelia Langford, John Sillitoe on behalf of the Wellcome Sanger Institute COVID-19 Surveillance Team
EPI_ISL_909561	Lighthouse Lab in Alderley Park	Wellcome Sanger Institute for the COVID-19 Genomics UK (COG-UK) Consortium	Jacquelyn Wynn, Mairead Hyland, The Lighthouse Lab in Alderley Park and Alex Alderton, Roberto Amato, Sonia Goncalves, Ewan Harrison, David K. Jackson, Ian Johnston, Dominic Kwiatkowski, Cordelia Langford, John Sillitoe on behalf of the Wellcome Sanger Institute COVID-19 Surveillance Team
EPI_ISL_909562	Lighthouse Lab in Milton Keynes	Wellcome Sanger Institute for the COVID-19 Genomics UK (COG-UK) Consortium	The Lighthouse Lab in Milton Keynes and Alex Alderton, Roberto Amato, Sonia Goncalves, Ewan Harrison, David K. Jackson, Ian Johnston, Dominic Kwiatkowski, Cordelia Langford, John Sillitoe on behalf of the Wellcome Sanger Institute COVID-19 Surveillance Team
EPI_ISL_909564	Lighthouse Lab in Alderley Park	Wellcome Sanger Institute for the COVID-19 Genomics UK (COG-UK) Consortium	Jacquelyn Wynn, Mairead Hyland, The Lighthouse Lab in Alderley Park and Alex Alderton, Roberto Amato, Sonia Goncalves, Ewan Harrison, David K. Jackson, Ian Johnston, Dominic Kwiatkowski, Cordelia Langford, John Sillitoe on behalf of the Wellcome Sanger Institute COVID-19 Surveillance Team
EPI_ISL_909566	Lighthouse Lab in Milton Keynes	Wellcome Sanger Institute for the COVID-19 Genomics UK (COG-UK) Consortium	The Lighthouse Lab in Milton Keynes and Alex Alderton, Roberto Amato, Sonia Goncalves, Ewan Harrison, David K. Jackson, Ian Johnston, Dominic Kwiatkowski, Cordelia Langford, John Sillitoe on behalf of the Wellcome Sanger Institute COVID-19 Surveillance Team
EPI_ISL_909568	Lighthouse Lab in Glasgow	Wellcome Sanger Institute for the COVID-19 Genomics UK (COG-UK) Consortium	Harper VanSteenhouse, Yumi Kasai, David Gray, Carol Clugston, Anna Dominiczak and Alex Alderton, Roberto Amato, Sonia Goncalves, Ewan Harrison, David K. Jackson, Ian Johnston, Dominic Kwiatkowski, Cordelia Langford, John Sillitoe on behalf of the Wellcome Sanger Institute COVID-19 Surveillance Team
EPI_ISL_909569	Lighthouse Lab in Milton Keynes	Wellcome Sanger Institute for the COVID-19 Genomics UK (COG-UK) Consortium	The Lighthouse Lab in Milton Keynes and Alex Alderton, Roberto Amato, Sonia Goncalves, Ewan Harrison, David K. Jackson, Ian Johnston, Dominic Kwiatkowski, Cordelia Langford, John Sillitoe on behalf of the Wellcome Sanger Institute COVID-19 Surveillance Team
EPI_ISL_909571, EPI_ISL_909572, EPI_ISL_909573, EPI_ISL_909575	Lighthouse Lab in Alderley Park	Wellcome Sanger Institute for the COVID-19 Genomics UK (COG-UK) Consortium	Jacquelyn Wynn, Mairead Hyland, The Lighthouse Lab in Alderley Park and Alex Alderton, Roberto Amato, Sonia Goncalves, Ewan Harrison, David K. Jackson, Ian Johnston, Dominic Kwiatkowski, Cordelia Langford, John Sillitoe on behalf of the Wellcome Sanger Institute COVID-19 Surveillance Team
EPI_ISL_909576	Lighthouse Lab in Milton Keynes	Wellcome Sanger Institute for the COVID-19 Genomics UK (COG-UK) Consortium	The Lighthouse Lab in Milton Keynes and Alex Alderton, Roberto Amato, Sonia Goncalves, Ewan Harrison, David K. Jackson, Ian Johnston, Dominic Kwiatkowski, Cordelia Langford, John Sillitoe on behalf of the Wellcome Sanger Institute COVID-19 Surveillance Team
EPI_ISL_909577, EPI_ISL_909578, EPI_ISL_909580, EPI_ISL_909581	Lighthouse Lab in Alderley Park	Wellcome Sanger Institute for the COVID-19 Genomics UK (COG-UK) Consortium	Jacquelyn Wynn, Mairead Hyland, The Lighthouse Lab in Alderley Park and Alex Alderton, Roberto Amato, Sonia Goncalves, Ewan Harrison, David K. Jackson, Ian Johnston, Dominic Kwiatkowski, Cordelia Langford, John Sillitoe on behalf of the Wellcome Sanger Institute COVID-19 Surveillance Team
EPI_ISL_909584	Lighthouse Lab in Milton Keynes	Wellcome Sanger Institute for the COVID-19 Genomics UK (COG-UK) Consortium	The Lighthouse Lab in Milton Keynes and Alex Alderton, Roberto Amato, Sonia Goncalves, Ewan Harrison, David K. Jackson, Ian Johnston, Dominic Kwiatkowski, Cordelia Langford, John Sillitoe on behalf of the Wellcome Sanger Institute COVID-19 Surveillance Team
EPI_ISL_909586	Lighthouse Lab in Alderley Park	Wellcome Sanger Institute for the COVID-19 Genomics UK (COG-UK) Consortium	Jacquelyn Wynn, Mairead Hyland, The Lighthouse Lab in Alderley Park and Alex Alderton, Roberto Amato, Sonia Goncalves, Ewan Harrison, David K. Jackson, Ian Johnston, Dominic Kwiatkowski, Cordelia Langford, John Sillitoe on behalf of the Wellcome Sanger Institute COVID-19 Surveillance Team
EPI_ISL_909588, EPI_ISL_909590	Lighthouse Lab in Glasgow	Wellcome Sanger Institute for the COVID-19 Genomics UK (COG-UK) Consortium	Harper VanSteenhouse, Yumi Kasai, David Gray, Carol Clugston, Anna Dominiczak and Alex Alderton, Roberto Amato, Sonia Goncalves, Ewan Harrison, David K. Jackson, Ian Johnston, Dominic Kwiatkowski, Cordelia Langford, John Sillitoe on behalf of the Wellcome Sanger Institute COVID-19 Surveillance Team
EPI_ISL_909745, EPI_ISL_909746, EPI_ISL_909748, EPI_ISL_909749	A. Krumbholz, Labor Dr. Krause und Kollegen MVZ GmbH, Kiel	Charité Universitätsmedizin Berlin, Institut für Virologie	Victor M Corman, Barbara Mühlemann, Jörn Beheim-Schwarzbach, Tobias Bleicker, Julia Tesch, Talitha Veith, Julia Schneider, Terry Jones, Christian Drosten
EPI_ISL_909750, EPI_ISL_909757	Charité Universitätsmedizin Berlin, Institut für Virologie/Labor Berlin	Charité Universitätsmedizin Berlin, Institut für Virologie	Victor M Corman, Barbara Mühlemann, Jörn Beheim-Schwarzbach, Tobias Bleicker, Julia Tesch, Talitha Veith, Julia Schneider, Terry Jones, Christian Drosten
EPI_ISL_909773, EPI_ISL_909782, EPI_ISL_909791, EPI_ISL_909792, EPI_ISL_909793, EPI_ISL_909795, EPI_ISL_909796, EPI_ISL_909800, EPI_ISL_909805, EPI_ISL_909807, EPI_ISL_909808, EPI_ISL_909810, EPI_ISL_909812, EPI_ISL_909823, EPI_ISL_909824, EPI_ISL_909825, EPI_ISL_909876, EPI_ISL_909880, EPI_ISL_909882, EPI_ISL_909885, EPI_ISL_909891, EPI_ISL_909892, EPI_ISL_909893, EPI_ISL_909894, EPI_ISL_909902, EPI_ISL_909922, EPI_ISL_909926, EPI_ISL_909927, EPI_ISL_909930, EPI_ISL_909935			
see above	National Virus Reference Laboratory	National Virus Reference Laboratory	Michael Carr, Gabriel Gonzalez, Jonathan Dean, Cillian F De Gascun
EPI_ISL_911209, EPI_ISL_911210, EPI_ISL_911211, EPI_ISL_911212, EPI_ISL_911213, EPI_ISL_911214, EPI_ISL_911215, EPI_ISL_911216, EPI_ISL_911217, EPI_ISL_911218, EPI_ISL_911219, EPI_ISL_911220, EPI_ISL_911221, EPI_ISL_911222, EPI_ISL_911223, EPI_ISL_911224, EPI_ISL_911225, EPI_ISL_911226, EPI_ISL_911227, EPI_ISL_911228, EPI_ISL_911229, EPI_ISL_911230, EPI_ISL_911231			
see above	Laboratoire national de sante, Microbiology, Virology	Laboratoire national de sante, Microbiology, Microbial Genomics Platform	Anke Wienecke-Baldacchino, Catherine Ragimbeau,Jessica Tapp, Fatu Djabi, Lise Pignon, Raoul Salmon, Tamir Abdelrahman
EPI_ISL_911314, EPI_ISL_911324, EPI_ISL_911341	Servicio de Microbiología, Hospital Universitario Son Espases	SeqCOVID-SPAIN consortium/IBV(CSIC)	Carla López-Causapé, Jordi Reina, Antonio Oliver and SeqCOVID-SPAIN consortium
EPI_ISL_911386, EPI_ISL_911387, EPI_ISL_911388, EPI_ISL_911389, EPI_ISL_911430, EPI_ISL_911431, EPI_ISL_911432, EPI_ISL_911433, EPI_ISL_911434, EPI_ISL_911435, EPI_ISL_911436, EPI_ISL_911437, EPI_ISL_911438, EPI_ISL_911439, EPI_ISL_911440, EPI_ISL_911441, EPI_ISL_911442			
see above	AZDelta	AZDelta	Geert Martens; Dieter De Smet
EPI_ISL_911525	Microbiology and Virology Unit, Florence Careggi University Hospital	Microbiology and Virology Unit, Florence Careggi University Hospital	Vincenzo Di Pilato, Marco Coppi, Fabio Morecchiato, Noemi Aiezza, Ilaria Baccani, Alberto Antonelli, Emanuele Gori, Gian Maria Rossolini
EPI_ISL_911582, EPI_ISL_911584, EPI_ISL_911585, EPI_ISL_911587, EPI_ISL_911588, EPI_ISL_911593, EPI_ISL_911595, EPI_ISL_911597, EPI_ISL_911598, EPI_ISL_911602, EPI_ISL_911674			
see above	Clinical Molecular Microbiology Laboratory, UNC Hospitals	Jeremy Wang	Jeremy Wang, Alexander Rubinsteyn, Colleen Rice, Jason Smedberg, Melissa Miller, Corbin Jones, Robert Hagan
EPI_ISL_911748, EPI_ISL_911749	Toronto Invasive Bacterial Diseases Network	McMaster University	Allison McGeer, Patryk Aftanas, Hooman Derakhshani, Angel Li, Kuganya Nirmalarajah, Emily Panousis, Ahmed Draia, Jalees Nasir, Michael Surette, Samira Mubareka, Andrew G. McArthur
EPI_ISL_911755, EPI_ISL_911757, EPI_ISL_911758, EPI_ISL_911762, EPI_ISL_911763, EPI_ISL_911764, EPI_ISL_911765, EPI_ISL_911766, EPI_ISL_911768, EPI_ISL_911772, EPI_ISL_911775, EPI_ISL_911776, EPI_ISL_911777, EPI_ISL_911778, EPI_ISL_911779, EPI_ISL_911780, EPI_ISL_911781, EPI_ISL_911782, EPI_ISL_911791, EPI_ISL_911865, EPI_ISL_911900, EPI_ISL_911901, EPI_ISL_911905, EPI_ISL_911906			
see above	Johns Hopkins Hospital Department of Pathology	Johns Hopkins Hospital Department of Pathology	C. Paul Morris, Chun Huai Luo, Adannaya Amadi, Matthew Schwartz, Nicholas Gallagher, Heba H. Mostafa
EPI_ISL_912109, EPI_ISL_912110, EPI_ISL_912111, EPI_ISL_912112, EPI_ISL_912113, EPI_ISL_912114, EPI_ISL_912115, EPI_ISL_912116, EPI_ISL_912117, EPI_ISL_912118, EPI_ISL_912119, EPI_ISL_912120, EPI_ISL_912121, EPI_ISL_912122, EPI_ISL_912123, EPI_ISL_912124, EPI_ISL_912125, EPI_ISL_912126, EPI_ISL_912127, EPI_ISL_912128, EPI_ISL_912129, EPI_ISL_912130, EPI_ISL_912131, EPI_ISL_912132, EPI_ISL_912133, EPI_ISL_912134, EPI_ISL_912135, EPI_ISL_912136, EPI_ISL_912137, EPI_ISL_912138, EPI_ISL_912139, EPI_ISL_912140, EPI_ISL_912141, EPI_ISL_912142, EPI_ISL_912143, EPI_ISL_912144, EPI_ISL_912145, EPI_ISL_912146, EPI_ISL_912147, EPI_ISL_912148, EPI_ISL_912149, EPI_ISL_912150, EPI_ISL_912151, EPI_ISL_912152, EPI_ISL_912153, EPI_ISL_912154, EPI_ISL_912155, EPI_ISL_912156, EPI_ISL_912157, EPI_ISL_912158, EPI_ISL_912159			
see above	Altius Institute for Biomedical Sciences	Seattle Flu Study	Deborah A. Nickerson, Chris D. Frazar, Jover Lee, Benjamin Pelle, Erica Ryke, Matthew Richardson, Amanda Adler, Elisabeth Brandstetter, Peter D. Han, Kairsten Fay, Misja Ilcisin, Kirsten Lacombe, Thomas R. Sibley, Melissa Truong, Caitlin R. Wolf, Ryan Alexander, Daniel Bates, Rebecca Bruders, Stephanie DeBaun, Clem Green, Muhammad Halimun, Jessica Halow, Kneshay Harper, Matt Hartman, Andrew Meuser, Alex Nguyen, Truong Nguyen, Sofia Olsson, Sadie Patraw, Hannah Petersen, Tobias Ragoczy, Joshua Richards, Jacob Rodriguez, John Stamatoyannopoulos, Julia Wald, Olivia Waltner, Michael Boeckh, Janet A. Englund, Michael Famulare, Barry R. Lutz, Mark J. Rieder, Lea M. Starita, Matthew Thompson, Helen Y. Chu, Jay Shendure, Trevor Bedford
EPI_ISL_912171, EPI_ISL_912172, EPI_ISL_912173, EPI_ISL_912174	Tempus	Grubaugh Lab - Yale School of Public Health	Tara Alpert, Joseph Fauver, Anderson Brito, Mallery Breban, Anne Wyllie, Chantal Vogels, Mary Petrone, Annie Watkins, Chaney Kalinich, Isabel Ott, Nathan Grubaugh
EPI_ISL_912214	Lighthouse Lab in Cambridge	Wellcome Sanger Institute for the COVID-19 Genomics UK	Rob Howes, The Lighthouse Lab in Cambridge and Alex Alderton, Roberto Amato, Sonia Goncalves, Ewan Harrison, David K. Jackson, Ian Johnston,

		(COG-UK) Consortium	Dominic Kwiatkowski, Cordelia Langford, John Sillitoe on behalf of the Wellcome Sanger Institute COVID-19 Surveillance Team (http://www.sanger.ac.uk/covid-team)
EPI_ISL_912220, EPI_ISL_912221, EPI_ISL_912222, EPI_ISL_912223	Lighthouse Lab in Alderley Park	Wellcome Sanger Institute for the COVID-19 Genomics UK (COG-UK) Consortium	Jacquelyn Wynn, Mairead Hyland, The Lighthouse Lab in Alderley Park and Alex Alderton, Roberto Amato, Sonia Goncalves, Ewan Harrison, David K. Jackson, Ian Johnston, Dominic Kwiatkowski, Cordelia Langford, John Sillitoe on behalf of the Wellcome Sanger Institute COVID-19 Surveillance Team (http://www.sanger.ac.uk/covid-team)
EPI_ISL_912245, EPI_ISL_912246, EPI_ISL_912250, EPI_ISL_912251, EPI_ISL_912252, EPI_ISL_912253, EPI_ISL_912254, EPI_ISL_912257, EPI_ISL_912258, EPI_ISL_912260, EPI_ISL_912263, EPI_ISL_912264			
see above	Charité Universitätsmedizin Berlin, Institut für Virologie/Labor Berlin	Charité Universitätsmedizin Berlin, Institut für Virologie	Victor M Corman, Barbara Mühlemann, Jörn Beheim-Schwarzbach, Tobias Bleicker, Julia Tesch, Talitha Veith, Julia Schneider, Terry Jones, Christian Drosten
EPI_ISL_912579	Service Biologie -CH de Versailles	National Reference Center for Viruses of Respiratory Infections, Institut Pasteur, Paris	Marion Barbet, Sylvie Behillil, Méline Bizard, Angela Brisebarre, Camille Capel, Etienne Simon-Lorière, Vincent Enouf, Maud Vanpeene, Sylvie van der Werf,Marque Juillet StéPhanie
EPI_ISL_912639	Hôpital Henri Mondor	Department of Virology, Henri Mondor University Hospital, Assistance Publique Hôpitaux de Paris, Université Paris-Est Créteil, INSERM U955	Christophe Rodriguez, Slim Fourati, Vanessa Demontant, Guillaume Gricourt, Melissa N'Debi, Alexandre Soulier, Elisabeth Trawinski, Jean-Michel Pawlotsky
EPI_ISL_912649, EPI_ISL_912655	Hôpital Pitié-Salpêtrière	Department of Virology, Henri Mondor University Hospital, Assistance Publique Hôpitaux de Paris, Université Paris-Est Créteil, INSERM U955	Christophe Rodriguez, Slim Fourati, Vanessa Demontant, Guillaume Gricourt, Melissa N'Debi, Alexandre Soulier, Elisabeth Trawinski, Jean-Michel Pawlotsky
EPI_ISL_912665, EPI_ISL_912666, EPI_ISL_912696, EPI_ISL_912697, EPI_ISL_912698, EPI_ISL_912699, EPI_ISL_912700, EPI_ISL_912703, EPI_ISL_912704, EPI_ISL_912716, EPI_ISL_912717			
see above	Hôpital Henri Mondor	Department of Virology, Henri Mondor University Hospital, Assistance Publique Hôpitaux de Paris, Université Paris-Est Créteil, INSERM U955	Christophe Rodriguez, Slim Fourati, Vanessa Demontant, Guillaume Gricourt, Melissa N'Debi, Alexandre Soulier, Elisabeth Trawinski, Jean-Michel Pawlotsky
EPI_ISL_912719	Hôpital Pitié-Salpêtrière	Department of Virology, Henri Mondor University Hospital, Assistance Publique Hôpitaux de Paris, Université Paris-Est Créteil, INSERM U955	Christophe Rodriguez, Slim Fourati, Vanessa Demontant, Guillaume Gricourt, Melissa N'Debi, Alexandre Soulier, Elisabeth Trawinski, Jean-Michel Pawlotsky
EPI_ISL_912750, EPI_ISL_912751, EPI_ISL_912752, EPI_ISL_912753, EPI_ISL_912754, EPI_ISL_912755, EPI_ISL_912756, EPI_ISL_912757, EPI_ISL_912758, EPI_ISL_912759, EPI_ISL_912760, EPI_ISL_912761			
see above	Hôpital Henri Mondor	Department of Virology, Henri Mondor University Hospital, Assistance Publique Hôpitaux de Paris, Université Paris-Est Créteil, INSERM U955	Christophe Rodriguez, Slim Fourati, Vanessa Demontant, Guillaume Gricourt, Melissa N'Debi, Alexandre Soulier, Elisabeth Trawinski, Jean-Michel Pawlotsky
EPI_ISL_912786, EPI_ISL_912787	Hôpital Pitié-Salpêtrière	Department of Virology, Henri Mondor University Hospital, Assistance Publique Hôpitaux de Paris, Université Paris-Est Créteil, INSERM U955	Christophe Rodriguez, Slim Fourati, Vanessa Demontant, Guillaume Gricourt, Melissa N'Debi, Alexandre Soulier, Elisabeth Trawinski, Jean-Michel Pawlotsky
EPI_ISL_913402, EPI_ISL_913403, EPI_ISL_913413, EPI_ISL_913419, EPI_ISL_913422, EPI_ISL_913423, EPI_ISL_913432, EPI_ISL_913433	Massachusetts State Public Health Laboratory	Massachusetts State Public Health Laboratory	Andrew Lang, Timelia Fink, Glen Gallagher, Sandra Smole
EPI_ISL_913445	Institute for Infectious Diseases, University of Bern, Switzerland	Institute for Infectious Diseases, University of Bern, Switzerland	Michel C Koch, Christian Baumann, Miguel A Terrazos Miani, Cora Sägesser, Pascal Bittel, Stephen L Leib, Peter Keller, Franziska Suter-Riniker, Alban Ramette
EPI_ISL_913496, EPI_ISL_913497, EPI_ISL_913498, EPI_ISL_913499, EPI_ISL_913500, EPI_ISL_913501, EPI_ISL_913502, EPI_ISL_913503, EPI_ISL_913504, EPI_ISL_913505, EPI_ISL_913506, EPI_ISL_913507, EPI_ISL_913508, EPI_ISL_913509, EPI_ISL_913510, EPI_ISL_913511, EPI_ISL_913512, EPI_ISL_913513			
see above	AZDelta	AZDelta	Geert Martens; Dieter De Smet
EPI_ISL_913617, EPI_ISL_913618, EPI_ISL_913619, EPI_ISL_913620	Michigan Department of Health and Human Services, Bureau of Laboratories	Michigan Department of Health and Human Services, Bureau of Laboratories	Blankenship HM, Riner D, Soehnlen MK
EPI_ISL_914003, EPI_ISL_914004, EPI_ISL_914006, EPI_ISL_914007, EPI_ISL_914008, EPI_ISL_914009	Clinical Pathology Lab	Pathogen Discovery, Respiratory Viruses Branch, Division of Viral Diseases, Centers for Disease Control and Prevention	Ying Tao, Yan Li, Jing Zhang, Krista Queen, Anna Uehara, Peter Cook, Clinton R. Paden, Haibin Wang, Suxiang Tong
EPI_ISL_914640	Santa Clara County Public Health Laboratory	Santa Clara County Public Health Laboratory	Santa Clara County Public Health Department
EPI_ISL_914702, EPI_ISL_914703, EPI_ISL_914704, EPI_ISL_914705, EPI_ISL_914706, EPI_ISL_914707, EPI_ISL_914708, EPI_ISL_914709, EPI_ISL_914710, EPI_ISL_914711, EPI_ISL_914712, EPI_ISL_914713, EPI_ISL_914714, EPI_ISL_914716, EPI_ISL_914717, EPI_ISL_914718, EPI_ISL_914719, EPI_ISL_914720, EPI_ISL_914721, EPI_ISL_914722, EPI_ISL_914723, EPI_ISL_914724			
see above	Maryland Public Health Laboratory (MD PHL)	Maryland Public Health Laboratory (MD PHL)	Maryland Department of Health Laboratories Administration
EPI_ISL_914725, EPI_ISL_914726, EPI_ISL_914727, EPI_ISL_914728, EPI_ISL_914730, EPI_ISL_914731, EPI_ISL_914732, EPI_ISL_914733, EPI_ISL_914734, EPI_ISL_914735, EPI_ISL_914736, EPI_ISL_914737, EPI_ISL_914739, EPI_ISL_914740, EPI_ISL_914743, EPI_ISL_914744, EPI_ISL_914748, EPI_ISL_914749, EPI_ISL_914750, EPI_ISL_914751, EPI_ISL_914753, EPI_ISL_914779, EPI_ISL_914781, EPI_ISL_914784, EPI_ISL_914785, EPI_ISL_914786, EPI_ISL_914787, EPI_ISL_914788, EPI_ISL_914789, EPI_ISL_914792			
see above	Wyoming Public Health Laboratory	Wyoming Public Health Laboratory	Noah Hull, Taylor Fearing, Lynette Gumbleton, Channing Weber, Ashley Norberg, Bailey Bowcutt, and Wanda Manley
EPI_ISL_914840	HOSPITAL DR. RAUL BLANCO CERVANTES	Inciensa, Instituto Costarricense de Investigación y Enseñanza en Nutrición y Salud	Francisco Duarte, Hebleen Porras, Claudio Soto-Garita, Estela Cordero, Adriana Godínez, Melany Calderón & Rebeca Ruiz-Sánchez
EPI_ISL_916191, EPI_ISL_916192, EPI_ISL_916196, EPI_ISL_916210, EPI_ISL_916211, EPI_ISL_916212, EPI_ISL_916229, EPI_ISL_916231, EPI_ISL_916237, EPI_ISL_916242, EPI_ISL_916243, EPI_ISL_916247, EPI_ISL_916249, EPI_ISL_916252, EPI_ISL_916260, EPI_ISL_916262, EPI_ISL_916269, EPI_ISL_916270, EPI_ISL_916271, EPI_ISL_916274, EPI_ISL_916277, EPI_ISL_916280, EPI_ISL_916292, EPI_ISL_916297, EPI_ISL_916304, EPI_ISL_916313, EPI_ISL_916331, EPI_ISL_916334, EPI_ISL_916346, EPI_ISL_916348, EPI_ISL_916354, EPI_ISL_916355, EPI_ISL_916362, EPI_ISL_916363, EPI_ISL_916376, EPI_ISL_916382, EPI_ISL_916391, EPI_ISL_916392, EPI_ISL_916414, EPI_ISL_916416, EPI_ISL_916420, EPI_ISL_916427, EPI_ISL_916435, EPI_ISL_916444, EPI_ISL_916445, EPI_ISL_916450, EPI_ISL_916461, EPI_ISL_916470, EPI_ISL_916475, EPI_ISL_916495, EPI_ISL_916496, EPI_ISL_916497			
see above	Lighthouse Lab in Cambridge	Wellcome Sanger Institute for the COVID-19 Genomics UK (COG-UK) Consortium	Rob Howes, The Lighthouse Lab in Cambridge and Alex Alderton, Roberto Amato, Sonia Goncalves, Ewan Harrison, David K. Jackson, Ian Johnston, Dominic Kwiatkowski, Cordelia Langford, John Sillitoe on behalf of the Wellcome Sanger Institute COVID-19 Surveillance Team
EPI_ISL_917214, EPI_ISL_917501	Lighthouse Lab in Alderley Park	Wellcome Sanger Institute for the COVID-19 Genomics UK (COG-UK) Consortium	Jacquelyn Wynn, Mairead Hyland, The Lighthouse Lab in Alderley Park and Alex Alderton, Roberto Amato, Sonia Goncalves, Ewan Harrison, David K. Jackson, Ian Johnston, Dominic Kwiatkowski, Cordelia Langford, John Sillitoe on behalf of the Wellcome Sanger Institute COVID-19 Surveillance Team
EPI_ISL_917951, EPI_ISL_917952, EPI_ISL_917953	Los Angeles County PHL	Los Angeles County PHL	P. Hemarajata et al.
EPI_ISL_918020, EPI_ISL_918021, EPI_ISL_918022, EPI_ISL_918023, EPI_ISL_918024, EPI_ISL_918025, EPI_ISL_918026, EPI_ISL_918027, EPI_ISL_918028, EPI_ISL_918029, EPI_ISL_918030, EPI_ISL_918031, EPI_ISL_918032, EPI_ISL_918033, EPI_ISL_918034, EPI_ISL_918035, EPI_ISL_918036, EPI_ISL_918037, EPI_ISL_918038			
see above	Lighthouse Lab in Glasgow	Wellcome Sanger Institute for the COVID-19 Genomics UK (COG-UK) Consortium	Harper VanSteenhouse, Yumi Kasai, David Gray, Carol Clugston, Anna Dominiczak and Alex Alderton, Roberto Amato, Sonia Goncalves, Ewan Harrison, David K. Jackson, Ian Johnston, Dominic Kwiatkowski, Cordelia Langford, John Sillitoe on behalf of the Wellcome Sanger Institute COVID-19 Surveillance Team
EPI_ISL_918191	Innovative Genomics Institute, UC Berkeley	Innovative Genomics Institute, UC Berkeley	Stacia Wyman, Haridha Shivram, Phil Frankino, Liana Lareau, Shana McDevitt, Justin Choi
EPI_ISL_918292, EPI_ISL_918293, EPI_ISL_918298, EPI_ISL_918299, EPI_ISL_918300, EPI_ISL_918301, EPI_ISL_918303, EPI_ISL_918304, EPI_ISL_918305, EPI_ISL_918306, EPI_ISL_918307, EPI_ISL_918308, EPI_ISL_918309, EPI_ISL_918310, EPI_ISL_918311, EPI_ISL_918312, EPI_ISL_918314, EPI_ISL_918318, EPI_ISL_918319, EPI_ISL_918321, EPI_ISL_918322, EPI_ISL_918323, EPI_ISL_918325, EPI_ISL_918326, EPI_ISL_918330, EPI_ISL_918332, EPI_ISL_918333, EPI_ISL_918334, EPI_ISL_918335, EPI_ISL_918337, EPI_ISL_918338			
see above	Hospital Universitari Vall d'Hebron - Vall d'Hebron Institut de Rercerca	Hospital Universitari Vall d'Hebron	Cristina Andrés, Maria Piñana, Josep F Abril, Damir Garcia-Cehic, Ariadna Rando, Juliana Esperalba, Maria Gema Codina, Carla Castillo, Maria Carmen Martin, Tomàs Pumarola, Josep Quer, Andrés Antón

EPI_ISL_918495	Florida Bureau of Public Health Laboratories	Florida Bureau of Public Health Laboratories	Sarah Schmedes, Jason Blanton
EPI_ISL_918985, EPI_ISL_918990, EPI_ISL_918991, EPI_ISL_918992, EPI_ISL_918993, EPI_ISL_918998, EPI_ISL_918999, EPI_ISL_919000, EPI_ISL_919001, EPI_ISL_919002, EPI_ISL_919003, EPI_ISL_919004, EPI_ISL_919006, EPI_ISL_919008, EPI_ISL_919009, EPI_ISL_919010, EPI_ISL_919013, EPI_ISL_919014, EPI_ISL_919015, EPI_ISL_919016, EPI_ISL_919115, EPI_ISL_919148			
see above	Department of Pathology, University of Cambridge	COVID-19 Genomics UK (COG-UK) Consortium	Aminu S. Jahun, Yasmin Chaudhry, Iliana Georgana, Myra Hosmillo, Rhys Izu, Martin D. Curran, Surendra Parmar, Ian Goodfellow
EPI_ISL_919231, EPI_ISL_919277, EPI_ISL_919278, EPI_ISL_919279, EPI_ISL_919280	West of Scotland Specialist Virology Centre, NHSGGC / MRC-University of Glasgow Centre for Virus Research	COVID-19 Genomics UK (COG-UK) Consortium	Ana da Silva Filipe, Natasha Johnson, Kathy Smollett, Daniel Mair, Stephen Carmichael, Alice Broos, Lily Tong, Jenna Nichols, Kyriaki Nomikou; Sarah McDonald; Richard Orton, Joseph Hughes, Sreenu Vattipally, David L Robertson; Alasdair MacLean, Rory Gunson; Sharif Shaaban, Matthew Holden; Rachel Blacow, Guy Mollett, Kathy Li, James Shepherd, Antonia Ho, Emma Thomson
EPI_ISL_919346	Virology Department, Royal Infirmary of Edinburgh, NHS Lothian / School of Biological Sciences, University of Edinburgh / Institute of Genetics and Molecular Medicine, University of Edinburgh	COVID-19 Genomics UK (COG-UK) Consortium	McHugh M, Dewar R, Rooke S, Gallagher M, Balcaza C, O'Toole Á, Scher E, Hill V, McCrone JT, Colquhoun R, Yu X, Jackson B, Rambaut A, Williams TC, Templeton K
EPI_ISL_919489, EPI_ISL_919490, EPI_ISL_919491, EPI_ISL_919492, EPI_ISL_919493, EPI_ISL_919494, EPI_ISL_919496, EPI_ISL_919497, EPI_ISL_919498, EPI_ISL_919499, EPI_ISL_919501, EPI_ISL_919512, EPI_ISL_919514, EPI_ISL_919518, EPI_ISL_919525, EPI_ISL_919543, EPI_ISL_919574, EPI_ISL_919579, EPI_ISL_919582, EPI_ISL_919588, EPI_ISL_919604, EPI_ISL_919605, EPI_ISL_919606, EPI_ISL_919607, EPI_ISL_919608, EPI_ISL_919609, EPI_ISL_919611, EPI_ISL_919612, EPI_ISL_919613, EPI_ISL_919615, EPI_ISL_919617, EPI_ISL_919618, EPI_ISL_919619, EPI_ISL_919635, EPI_ISL_919638, EPI_ISL_919639, EPI_ISL_919691			
see above	Liverpool Clinical Laboratories	COVID-19 Genomics UK (COG-UK) Consortium	Sam Haldenby, Anita Lucaci, Steve Paterson, Julian Hiscox, Alistair Darby, M Almsaud, A Alrezaihi, Muhannad Alruwaili, Stuart D Armstrong, Jones Benjamin, Eleanor G Bentley, Anu Chawla, Jordan J Clark, Angela Cowell, Richard Eccles, Isabel García-Dorival, Matthew Gemmell, Alessandro Gerada, PKF Gilmore, Richard Gregory, Ximeng Han, Catherine Hartley, Margaret Hughes, Miren Iturriza-Gomara, James Johnson, L Luu, Jenifer Manson, Charlotte Nelson, Elaine O'Toole, Cassie Olateju, Rebekah Penrice-Randal , Lucille Rainbow, N.P Randle, Trevor Ian Robinson, Parul Sharma, Ghada T Shawli, James P Stewart, Neil Swainston, Ecaterina Vamos, Joanne Watts, Mark Whitehead
EPI_ISL_919855	Barts Health NHS Trust	COVID-19 Genomics UK (COG-UK) Consortium	CUTINO-MOGUEL, Maria-Teresa; HARRINGTON, David; OWOYEMI, Dola; KULASEGARAN-SHYLINI, Raghavendran; BROAD, Claire; KELE, Beatrix
EPI_ISL_920061, EPI_ISL_920062, EPI_ISL_920063, EPI_ISL_920064, EPI_ISL_920066, EPI_ISL_920068, EPI_ISL_920071, EPI_ISL_920072	University College London, Great Ormond Street Hospital for Children NHS Foundation Trust, Imperial College Healthcare NHS Trust	COVID-19 Genomics UK (COG-UK) Consortium	Sergi Castellano, Rachel Williams, Mark Kristiansen, Paola Resende Silva, Sunando Roy, Tony Brooks, Helena Tutill, Paola Niola, Patricia Dyal, Charlotte Williams, Leysa Forrest, Yasmin Panchbhaya, Jacqueline Findlay, Samuel Weeks, Julianne Brown, Kathryn Harris, Paul Randell, James Price, Alison Holmes, Judith Breuer
EPI_ISL_920379, EPI_ISL_920380, EPI_ISL_920381, EPI_ISL_920383, EPI_ISL_920384, EPI_ISL_920385, EPI_ISL_920386, EPI_ISL_920387, EPI_ISL_920388, EPI_ISL_920389, EPI_ISL_920390, EPI_ISL_920391, EPI_ISL_920392, EPI_ISL_920393, EPI_ISL_920394, EPI_ISL_920395, EPI_ISL_920396, EPI_ISL_920397, EPI_ISL_920398, EPI_ISL_920399, EPI_ISL_920401, EPI_ISL_920403, EPI_ISL_920404, EPI_ISL_920405, EPI_ISL_920406, EPI_ISL_920407, EPI_ISL_920408, EPI_ISL_920411, EPI_ISL_920412, EPI_ISL_920414, EPI_ISL_920415, EPI_ISL_920416, EPI_ISL_920418, EPI_ISL_920419, EPI_ISL_920420, EPI_ISL_920421, EPI_ISL_920422, EPI_ISL_920423, EPI_ISL_920425, EPI_ISL_920426, EPI_ISL_920428, EPI_ISL_920429, EPI_ISL_920430, EPI_ISL_920431, EPI_ISL_920432, EPI_ISL_920433, EPI_ISL_920434, EPI_ISL_920435, EPI_ISL_920436, EPI_ISL_920438, EPI_ISL_920439, EPI_ISL_920441, EPI_ISL_920442, EPI_ISL_920443, EPI_ISL_920444, EPI_ISL_920445, EPI_ISL_920446, EPI_ISL_920448, EPI_ISL_920449, EPI_ISL_920450, EPI_ISL_920451, EPI_ISL_920452, EPI_ISL_920453, EPI_ISL_920627, EPI_ISL_920739			
see above	University College London Hospital	COVID-19 Genomics UK (COG-UK) Consortium	Judith Heaney, Matthew Byott, Catherine Houlihan, Dan Frampton, Stuart Kirk, Moira Spyer and Eleni Nastouli
EPI_ISL_920783, EPI_ISL_920788, EPI_ISL_920789, EPI_ISL_920790, EPI_ISL_920791, EPI_ISL_920794, EPI_ISL_920795, EPI_ISL_920797, EPI_ISL_920798, EPI_ISL_920799, EPI_ISL_920800, EPI_ISL_920801, EPI_ISL_920802, EPI_ISL_920803, EPI_ISL_920804, EPI_ISL_920805, EPI_ISL_920806, EPI_ISL_920807, EPI_ISL_920809, EPI_ISL_920811, EPI_ISL_920812, EPI_ISL_920813, EPI_ISL_920816, EPI_ISL_920817, EPI_ISL_920831, EPI_ISL_920832			
see above	University College London, Great Ormond Street Hospital for Children NHS Foundation Trust, Imperial College Healthcare NHS Trust	COVID-19 Genomics UK (COG-UK) Consortium	Sergi Castellano, Rachel Williams, Mark Kristiansen, Paola Resende Silva, Sunando Roy, Tony Brooks, Helena Tutill, Paola Niola, Patricia Dyal, Charlotte Williams, Leysa Forrest, Yasmin Panchbhaya, Jacqueline Findlay, Samuel Weeks, Julianne Brown, Kathryn Harris, Paul Randell, James Price, Alison Holmes, Judith Breuer
EPI_ISL_921608, EPI_ISL_921609, EPI_ISL_921617, EPI_ISL_921618, EPI_ISL_921620, EPI_ISL_921621, EPI_ISL_921622, EPI_ISL_921623, EPI_ISL_921624, EPI_ISL_921625, EPI_ISL_921626, EPI_ISL_921627, EPI_ISL_921628, EPI_ISL_921629, EPI_ISL_921630			
see above	Northumbria University / South Tees Hospitals NHS Foundation Trust / North Cumbria Integrated Care NHS Foundation Trust / North Tees and Hartlepool NHS Foundation Trust / Newcastle Hospitals NHS Foundation Trust	COVID-19 Genomics UK (COG-UK) Consortium	Darren L Smith,Andrew Nelson,Matthew Bashton,Greg R Young,Joshua Loh,John Allan,Mohammad A Tariq,Giles S Holt,Gary Black,Wen C Yew,Lynn Dover,Paul Baker,Steve Liggett,Sarah Essex,Jane Greenaway,Debra Padgett,Clive Graham,Garren Scott,Edward Barton,Emma Swindells,Brendan Payne,Jennifer Collins,Yusri Taha,Gary Eltringham
EPI_ISL_921854, EPI_ISL_921855, EPI_ISL_921856, EPI_ISL_921860, EPI_ISL_921862, EPI_ISL_921869, EPI_ISL_921871, EPI_ISL_921873, EPI_ISL_921878, EPI_ISL_921880, EPI_ISL_921881, EPI_ISL_921882, EPI_ISL_921883, EPI_ISL_921884, EPI_ISL_921885, EPI_ISL_921886, EPI_ISL_921887, EPI_ISL_921888, EPI_ISL_921889, EPI_ISL_921890, EPI_ISL_921891, EPI_ISL_921892, EPI_ISL_921893, EPI_ISL_921894, EPI_ISL_921896, EPI_ISL_921897, EPI_ISL_921898, EPI_ISL_921899, EPI_ISL_921900, EPI_ISL_921901, EPI_ISL_921902, EPI_ISL_921903, EPI_ISL_921905, EPI_ISL_921906, EPI_ISL_921907, EPI_ISL_921908, EPI_ISL_921911, EPI_ISL_921915, EPI_ISL_921916, EPI_ISL_921918, EPI_ISL_921919, EPI_ISL_921920, EPI_ISL_921921, EPI_ISL_921922, EPI_ISL_921924, EPI_ISL_921926, EPI_ISL_921927, EPI_ISL_921928, EPI_ISL_921929, EPI_ISL_921930, EPI_ISL_921932, EPI_ISL_921936, EPI_ISL_921937, EPI_ISL_921939, EPI_ISL_921941, EPI_ISL_921943, EPI_ISL_921944, EPI_ISL_921945, EPI_ISL_921946, EPI_ISL_921949, EPI_ISL_921950, EPI_ISL_921951, EPI_ISL_921954, EPI_ISL_921955, EPI_ISL_921956, EPI_ISL_921957, EPI_ISL_921958, EPI_ISL_921959, EPI_ISL_921960, EPI_ISL_921961, EPI_ISL_921962, EPI_ISL_921963, EPI_ISL_921964, EPI_ISL_921965, EPI_ISL_921967, EPI_ISL_921970, EPI_ISL_921971, EPI_ISL_921972, EPI_ISL_921973, EPI_ISL_921974, EPI_ISL_921975, EPI_ISL_921976, EPI_ISL_921977, EPI_ISL_921979, EPI_ISL_921980			
see above	Quadram Institute Bioscience	COVID-19 Genomics UK (COG-UK) Consortium	Dave J. Baker, Gemma L. Kay, Alp Aydin, Thanh Le-Viet, Steven Rudder, Ana P. Tedim, Anastasia Kolyva, Maria Diaz, Leonardo de Oliveira Martins, Nabil-Fareed Alikhan, Lizzie Meadows, Rachael Stanley, Ngozi Elumogo, Muhammed Yasir, Nicholas M. Thomson, Alexander J Trotter, Rachel Gilroy, Samuel Bloomfield, Claire Stuart, Andrew Bell, Reenesh Prakash, Samir Dervisevic, Alison E. Mather, John Wain, Mark Webber, Andrew J. Page, Justin O'Grady
EPI_ISL_921981, EPI_ISL_921982, EPI_ISL_921983, EPI_ISL_921984, EPI_ISL_921985, EPI_ISL_921986, EPI_ISL_921987, EPI_ISL_921988, EPI_ISL_921989, EPI_ISL_921990, EPI_ISL_921991, EPI_ISL_921992, EPI_ISL_921993, EPI_ISL_921994, EPI_ISL_921995, EPI_ISL_921996, EPI_ISL_921997, EPI_ISL_921998			
see above	Queens Medical Centre, Clinical Microbiology Department / DeepSeq Nottingham	COVID-19 Genomics UK (COG-UK) Consortium	Gemma Clark, Wendy Smith, Manjinder Khakh, Vicki M Fleming, Michelle M Lister, Hannah Howson-Wells, Jonathan Ball, Patrick McClure, Joseph Chappell, Theocharis Tsoleridis, Nadine Holmes, Matthew Carlisle, Christopher Moore, Fei Sang, Johnny Debebe, Victoria Wright, Matthew Loose
EPI_ISL_922164, EPI_ISL_922165, EPI_ISL_922166, EPI_ISL_922167, EPI_ISL_922168	Lincolnshire Hospitals and DeepSeq Nottingham	COVID-19 Genomics UK (COG-UK) Consortium	Nichola Duckworth, Tim Sloan, Sarah Walsh, Jonathan Ball, Patrick McClure, Joeseph Chappell, Nadine Holmes, Matthew Carlisle, Christopher Moore, Fei Sang, Johnny Debebe, Victoria Wright, Matthew Loose
EPI_ISL_922545, EPI_ISL_922557, EPI_ISL_922562, EPI_ISL_922569, EPI_ISL_922578, EPI_ISL_922579, EPI_ISL_922583, EPI_ISL_922584, EPI_ISL_922612, EPI_ISL_922613, EPI_ISL_922614, EPI_ISL_922615, EPI_ISL_922617, EPI_ISL_922619, EPI_ISL_922620, EPI_ISL_922621, EPI_ISL_922622, EPI_ISL_922623, EPI_ISL_922624, EPI_ISL_922625, EPI_ISL_922630, EPI_ISL_922631, EPI_ISL_922632, EPI_ISL_922635, EPI_ISL_922636, EPI_ISL_922736, EPI_ISL_922742, EPI_ISL_922769, EPI_ISL_922778, EPI_ISL_922795, EPI_ISL_922799, EPI_ISL_922804, EPI_ISL_922808, EPI_ISL_922826, EPI_ISL_922827, EPI_ISL_922828, EPI_ISL_922829, EPI_ISL_922830, EPI_ISL_922831, EPI_ISL_922832, EPI_ISL_922833, EPI_ISL_922834, EPI_ISL_922839, EPI_ISL_922840, EPI_ISL_922842, EPI_ISL_922843, EPI_ISL_922844, EPI_ISL_922845, EPI_ISL_922847, EPI_ISL_922848, EPI_ISL_922850, EPI_ISL_922851, EPI_ISL_922854, EPI_ISL_922857, EPI_ISL_922859, EPI_ISL_922860, EPI_ISL_922861, EPI_ISL_922863, EPI_ISL_922864, EPI_ISL_922865, EPI_ISL_922866, EPI_ISL_922867, EPI_ISL_922868, EPI_ISL_922869, EPI_ISL_922870, EPI_ISL_922871, EPI_ISL_922872, EPI_ISL_922873, EPI_ISL_922874, EPI_ISL_922876, EPI_ISL_922877, EPI_ISL_922878, EPI_ISL_922879, EPI_ISL_922880, EPI_ISL_922881, EPI_ISL_922885, EPI_ISL_922886, EPI_ISL_922888, EPI_ISL_922889, EPI_ISL_922890, EPI_ISL_922896, EPI_ISL_922897, EPI_ISL_922898, EPI_ISL_922899, EPI_ISL_922900, EPI_ISL_922916, EPI_ISL_922917, EPI_ISL_922918, EPI_ISL_922919, EPI_ISL_922920, EPI_ISL_922921, EPI_ISL_922922, EPI_ISL_922936, EPI_ISL_922937, EPI_ISL_922939, EPI_ISL_922962, EPI_ISL_922963, EPI_ISL_922971, EPI_ISL_923048, EPI_ISL_923052, EPI_ISL_923053, EPI_ISL_923054, EPI_ISL_923055, EPI_ISL_923056, EPI_ISL_923057, EPI_ISL_923058, EPI_ISL_923059, EPI_ISL_923060, EPI_ISL_923061, EPI_ISL_923062, EPI_ISL_923063, EPI_ISL_923064, EPI_ISL_923065, EPI_ISL_923066, EPI_ISL_923067, EPI_ISL_923068, EPI_ISL_923069, EPI_ISL_923070, EPI_ISL_923071, EPI_ISL_923073, EPI_ISL_923074, EPI_ISL_923075, EPI_ISL_923076, EPI_ISL_923084, EPI_ISL_923088, EPI_ISL_923089, EPI_ISL_923091, EPI_ISL_923092, EPI_ISL_923093, EPI_ISL_923095, EPI_ISL_923096, EPI_ISL_923097, EPI_ISL_923098, EPI_ISL_923099, EPI_ISL_923100, EPI_ISL_923103, EPI_ISL_923104, EPI_ISL_923105, EPI_ISL_923106, EPI_ISL_923107, EPI_ISL_923108, EPI_ISL_923109, EPI_ISL_923111, EPI_ISL_923112, EPI_ISL_923115, EPI_ISL_923116, EPI_ISL_923118, EPI_ISL_923121, EPI_ISL_923123, EPI_ISL_923125, EPI_ISL_923126, EPI_ISL_923127, EPI_ISL_923129, EPI_ISL_923130, EPI_ISL_923131, EPI_ISL_923133, EPI_ISL_923134, EPI_ISL_923135, EPI_ISL_923137, EPI_ISL_923138, EPI_ISL_923139, EPI_ISL_923140, EPI_ISL_923141, EPI_ISL_923144, EPI_ISL_923145, EPI_ISL_923148, EPI_ISL_923149, EPI_ISL_923150, EPI_ISL_923151, EPI_ISL_923152, EPI_ISL_923154, EPI_ISL_923157, EPI_ISL_923161, EPI_ISL_923163, EPI_ISL_923164, EPI_ISL_923165, EPI_ISL_923166, EPI_ISL_923167			
see above	Wales Specialist Virology Centre Sequencing lab: Pathogen Genomics Unit	Public Health Wales Microbiology Cardiff Wales Specialist Virology Centre	Catherine Moore, Johnathan Evans, Laura Gifford, Malorie Perry, Simon Cottrell, Angela Marchbank, Alec Birchley, Alexander Adams, Amy Gaskin, Bree Gatica-Wilcox, Jason Coombes, Joel Southgate, Lauren Gilbert, Lee Graham, Nicole Pacchiarini, Sara Kumziene-Summerhayes, Sarah Taylor, Sophie Jones, Sara Rey, Matthew Bull, Joanne Watkins, Sally Corden, Tom Connor
EPI_ISL_923320, EPI_ISL_923321, EPI_ISL_923322, EPI_ISL_923323, EPI_ISL_923324, EPI_ISL_923325, EPI_ISL_923326, EPI_ISL_923327, EPI_ISL_923329, EPI_ISL_923331, EPI_ISL_923332, EPI_ISL_923333, EPI_ISL_923335, EPI_ISL_923336, EPI_ISL_923337, EPI_ISL_923338, EPI_ISL_923339, EPI_ISL_923340, EPI_ISL_923341, EPI_ISL_923342, EPI_ISL_923343, EPI_ISL_923344, EPI_ISL_923346, EPI_ISL_923347, EPI_ISL_923348, EPI_ISL_923350, EPI_ISL_923352, EPI_ISL_923353, EPI_ISL_923354, EPI_ISL_923436, EPI_ISL_923598			
see above	Centre for Enzyme Innovation, University of Portsmouth / Translational Research Laboratory, Portsmouth Hospitals NHS Trust	COVID-19 Genomics UK (COG-UK) Consortium	Angela Beckett,Salman Goudarzi,Christopher Fearn,Kate Cook,Katie Loveson,Sharon Glaysher,Scott Elliott,Samuel Robson
EPI_ISL_924098, EPI_ISL_924102, EPI_ISL_924106, EPI_ISL_924114, EPI_ISL_924123, EPI_ISL_924159, EPI_ISL_924169, EPI_ISL_924170, EPI_ISL_924174, EPI_ISL_924189, EPI_ISL_924204, EPI_ISL_924206, EPI_ISL_924207, EPI_ISL_924208, EPI_ISL_924253, EPI_ISL_924255, EPI_ISL_924256, EPI_ISL_924266, EPI_ISL_924284, EPI_ISL_924291, EPI_ISL_924299, EPI_ISL_924307, EPI_ISL_924310, EPI_ISL_924311, EPI_ISL_924312, EPI_ISL_924314, EPI_ISL_924337, EPI_ISL_924343, EPI_ISL_924345, EPI_ISL_924348, EPI_ISL_924358, EPI_ISL_924360, EPI_ISL_924363, EPI_ISL_924364, EPI_ISL_924380, EPI_ISL_924386, EPI_ISL_924388, EPI_ISL_924394, EPI_ISL_924413			

see above	Virology Department, Sheffield Teaching Hospitals NHS Foundation Trust/Department of Infection, Immunity and Cardiovascular Disease, The Medical School, University of Sheffield	COVID-19 Genomics UK (COG-UK) Consortium	Thushan de Silva, Matthew Parker, Nikki Smith, Adri Angyal, Rebecca Brown, Luke Green, Rachel Tucker, Paul Parsons, Danielle Groves, Katie Johnson, Laura Carrilero, Alex Keeley, Dave Partridge, Matthew Wyles, Benjamin Lindsey, Mehmet Yavuz, Mohammad Raza, Cariad Evans
EPI_ISL_924652, EPI_ISL_924654, EPI_ISL_924655, EPI_ISL_924656, EPI_ISL_924658, EPI_ISL_924659, EPI_ISL_924660, EPI_ISL_924662, EPI_ISL_924663, EPI_ISL_924665, EPI_ISL_924670, EPI_ISL_924673, EPI_ISL_924678, EPI_ISL_924679, EPI_ISL_924680, EPI_ISL_924681, EPI_ISL_924682, EPI_ISL_924686, EPI_ISL_924687, EPI_ISL_924703, EPI_ISL_924710, EPI_ISL_924711, EPI_ISL_924829, EPI_ISL_924831, EPI_ISL_924833, EPI_ISL_924835, EPI_ISL_924837, EPI_ISL_924840, EPI_ISL_924843, EPI_ISL_924844, EPI_ISL_924847, EPI_ISL_924852, EPI_ISL_924854, EPI_ISL_924856, EPI_ISL_924861, EPI_ISL_924862, EPI_ISL_924866, EPI_ISL_924868, EPI_ISL_924870, EPI_ISL_924872, EPI_ISL_924874, EPI_ISL_924875, EPI_ISL_924878, EPI_ISL_924881, EPI_ISL_924885, EPI_ISL_924888, EPI_ISL_924889			
see above	Bioinformatics and Biostatistics Lab, Advanced Sequencing Facility	COVID-19 Genomics UK (COG-UK) Consortium	Aengus Stewart,Jerome Nicod,Chelsea Sawyer,Laura Cubitt,Harshil Patel,Margaret Crawford
EPI_ISL_925173	Laboratorio de Referencia Nacional de Enteropatógenos. Instituto Nacional de Salud del Perú	Laboratorio de Referencia Nacional de Enteropatógenos. Instituto Nacional de Salud del Perú	Ronnie Gavilan Chavez, Junior Caro Castro, Willi Quino Sifuentes, Veronica Hurtado Vela, Iris Silva Molina, Fiorella Orellana Peralta,
EPI_ISL_925917, EPI_ISL_925919, EPI_ISL_925920, EPI_ISL_925921, EPI_ISL_925922, EPI_ISL_925925, EPI_ISL_925926, EPI_ISL_925927, EPI_ISL_925928, EPI_ISL_925930, EPI_ISL_925931, EPI_ISL_925932, EPI_ISL_925934, EPI_ISL_925935, EPI_ISL_925936, EPI_ISL_925938, EPI_ISL_925941, EPI_ISL_925943, EPI_ISL_925945, EPI_ISL_925946, EPI_ISL_925947, EPI_ISL_925948, EPI_ISL_925951, EPI_ISL_925954, EPI_ISL_925956, EPI_ISL_925957, EPI_ISL_925959, EPI_ISL_925960, EPI_ISL_925962, EPI_ISL_925963, EPI_ISL_925964, EPI_ISL_925966, EPI_ISL_925972, EPI_ISL_925973, EPI_ISL_925975, EPI_ISL_925977, EPI_ISL_925978, EPI_ISL_925980, EPI_ISL_925981, EPI_ISL_925982, EPI_ISL_925984, EPI_ISL_925986, EPI_ISL_925989, EPI_ISL_925991, EPI_ISL_925993, EPI_ISL_925994, EPI_ISL_925995, EPI_ISL_925996, EPI_ISL_925997, EPI_ISL_925998, EPI_ISL_925999, EPI_ISL_926001, EPI_ISL_926004, EPI_ISL_926006, EPI_ISL_926009, EPI_ISL_926013, EPI_ISL_926014, EPI_ISL_926016, EPI_ISL_926019, EPI_ISL_926022, EPI_ISL_926023, EPI_ISL_926026, EPI_ISL_926027, EPI_ISL_926028, EPI_ISL_926032, EPI_ISL_926033, EPI_ISL_926034, EPI_ISL_926036, EPI_ISL_926039, EPI_ISL_926040, EPI_ISL_926042, EPI_ISL_926049, EPI_ISL_926052, EPI_ISL_926054, EPI_ISL_926056, EPI_ISL_926057, EPI_ISL_926058, EPI_ISL_926060, EPI_ISL_926061, EPI_ISL_926062, EPI_ISL_926066, EPI_ISL_926067, EPI_ISL_926068, EPI_ISL_926069, EPI_ISL_926072, EPI_ISL_926074, EPI_ISL_926076, EPI_ISL_926077, EPI_ISL_926078, EPI_ISL_926080, EPI_ISL_926081, EPI_ISL_926084, EPI_ISL_926085, EPI_ISL_926087, EPI_ISL_926089, EPI_ISL_926091, EPI_ISL_926092, EPI_ISL_926094, EPI_ISL_926097, EPI_ISL_926098, EPI_ISL_926100, EPI_ISL_926101, EPI_ISL_926102, EPI_ISL_926105, EPI_ISL_926106, EPI_ISL_926108, EPI_ISL_926109, EPI_ISL_926110, EPI_ISL_926111, EPI_ISL_926112, EPI_ISL_926115, EPI_ISL_926116, EPI_ISL_926119, EPI_ISL_926121, EPI_ISL_926123, EPI_ISL_926125, EPI_ISL_926127, EPI_ISL_926130, EPI_ISL_926131, EPI_ISL_926132, EPI_ISL_926133, EPI_ISL_926135, EPI_ISL_926136, EPI_ISL_926138, EPI_ISL_926141, EPI_ISL_926143, EPI_ISL_926145, EPI_ISL_926146, EPI_ISL_926148, EPI_ISL_926149, EPI_ISL_926152, EPI_ISL_926154, EPI_ISL_926155, EPI_ISL_926159, EPI_ISL_926160, EPI_ISL_926161, EPI_ISL_926165, EPI_ISL_926167, EPI_ISL_926168, EPI_ISL_926170, EPI_ISL_926171, EPI_ISL_926172, EPI_ISL_926173, EPI_ISL_926175, EPI_ISL_926176, EPI_ISL_926179, EPI_ISL_926180, EPI_ISL_926181, EPI_ISL_926182, EPI_ISL_926184, EPI_ISL_926185, EPI_ISL_926186, EPI_ISL_926188, EPI_ISL_926189, EPI_ISL_926190, EPI_ISL_926191, EPI_ISL_926194, EPI_ISL_926195, EPI_ISL_926196, EPI_ISL_926197, EPI_ISL_926198, EPI_ISL_926200, EPI_ISL_926202, EPI_ISL_926203, EPI_ISL_926204, EPI_ISL_926205, EPI_ISL_926206, EPI_ISL_926207, EPI_ISL_926208, EPI_ISL_926212, EPI_ISL_926213, EPI_ISL_926214, EPI_ISL_926215, EPI_ISL_926217, EPI_ISL_926219, EPI_ISL_926220, EPI_ISL_926223, EPI_ISL_926228, EPI_ISL_926229, EPI_ISL_926230, EPI_ISL_926231, EPI_ISL_926232, EPI_ISL_926234, EPI_ISL_926236, EPI_ISL_926239, EPI_ISL_926240, EPI_ISL_926241, EPI_ISL_926242, EPI_ISL_926244, EPI_ISL_926246, EPI_ISL_926248, EPI_ISL_926249, EPI_ISL_926250, EPI_ISL_926251, EPI_ISL_926253, EPI_ISL_926254, EPI_ISL_926256, EPI_ISL_926258, EPI_ISL_926260, EPI_ISL_926262, EPI_ISL_926263, EPI_ISL_926264, EPI_ISL_926266, EPI_ISL_926267, EPI_ISL_926268, EPI_ISL_926269, EPI_ISL_926271, EPI_ISL_926273, EPI_ISL_926276, EPI_ISL_926277, EPI_ISL_926278, EPI_ISL_926279, EPI_ISL_926280, EPI_ISL_926284, EPI_ISL_926285, EPI_ISL_926287, EPI_ISL_926288, EPI_ISL_926290, EPI_ISL_926293, EPI_ISL_926294, EPI_ISL_926296, EPI_ISL_926298, EPI_ISL_926300, EPI_ISL_926301, EPI_ISL_926302, EPI_ISL_926305, EPI_ISL_926306, EPI_ISL_926307, EPI_ISL_926309, EPI_ISL_926310, EPI_ISL_926312, EPI_ISL_926314, EPI_ISL_926317, EPI_ISL_926318, EPI_ISL_926319, EPI_ISL_926322, EPI_ISL_926325, EPI_ISL_926326, EPI_ISL_926329, EPI_ISL_926331, EPI_ISL_926332, EPI_ISL_926333, EPI_ISL_926335, EPI_ISL_926336, EPI_ISL_926338, EPI_ISL_926340, EPI_ISL_926341, EPI_ISL_926343, EPI_ISL_926344, EPI_ISL_926349, EPI_ISL_926351, EPI_ISL_926352, EPI_ISL_926353, EPI_ISL_926354, EPI_ISL_926355, EPI_ISL_926356, EPI_ISL_926358, EPI_ISL_926362, EPI_ISL_926363, EPI_ISL_926366, EPI_ISL_926368, EPI_ISL_926369, EPI_ISL_926370, EPI_ISL_926371, EPI_ISL_926373, EPI_ISL_926375, EPI_ISL_926376, EPI_ISL_926377, EPI_ISL_926378, EPI_ISL_926379, EPI_ISL_926382, EPI_ISL_926383, EPI_ISL_926384, EPI_ISL_926387, EPI_ISL_926391, EPI_ISL_926392, EPI_ISL_926393, EPI_ISL_926400, EPI_ISL_926401, EPI_ISL_926403, EPI_ISL_926405, EPI_ISL_926408, EPI_ISL_926409, EPI_ISL_926411, EPI_ISL_926412, EPI_ISL_926413, EPI_ISL_926414, EPI_ISL_926415, EPI_ISL_926417, EPI_ISL_926418, EPI_ISL_926421, EPI_ISL_926424, EPI_ISL_926425, EPI_ISL_926426, EPI_ISL_926427, EPI_ISL_926429, EPI_ISL_926430, EPI_ISL_926431, EPI_ISL_926433, EPI_ISL_926434, EPI_ISL_926443, EPI_ISL_926444, EPI_ISL_926445, EPI_ISL_926447, EPI_ISL_926448, EPI_ISL_926449, EPI_ISL_926450, EPI_ISL_926453, EPI_ISL_926454, EPI_ISL_926456, EPI_ISL_926457, EPI_ISL_926458, EPI_ISL_926460, EPI_ISL_926462, EPI_ISL_926463, EPI_ISL_926464, EPI_ISL_926467, EPI_ISL_926468, EPI_ISL_926470, EPI_ISL_926471, EPI_ISL_926472, EPI_ISL_926473, EPI_ISL_926474, EPI_ISL_926475, EPI_ISL_926477, EPI_ISL_926480, EPI_ISL_926481, EPI_ISL_926486, EPI_ISL_926488, EPI_ISL_926490, EPI_ISL_926493, EPI_ISL_926495, EPI_ISL_926496, EPI_ISL_926497, EPI_ISL_926498, EPI_ISL_926500, EPI_ISL_926501, EPI_ISL_926502, EPI_ISL_926503, EPI_ISL_926506, EPI_ISL_926507, EPI_ISL_926508, EPI_ISL_926509, EPI_ISL_926510, EPI_ISL_926511, EPI_ISL_926513, EPI_ISL_926514, EPI_ISL_926516, EPI_ISL_926518, EPI_ISL_926519, EPI_ISL_926523, EPI_ISL_926524, EPI_ISL_926526, EPI_ISL_926527, EPI_ISL_926529, EPI_ISL_926530, EPI_ISL_926531, EPI_ISL_926533, EPI_ISL_926534, EPI_ISL_926540, EPI_ISL_926542, EPI_ISL_926543, EPI_ISL_926544, EPI_ISL_926545, EPI_ISL_926548, EPI_ISL_926550, EPI_ISL_926554, EPI_ISL_926556, EPI_ISL_926557, EPI_ISL_926561, EPI_ISL_926562, EPI_ISL_926563, EPI_ISL_926565, EPI_ISL_926568, EPI_ISL_926571, EPI_ISL_926572, EPI_ISL_926574, EPI_ISL_926577, EPI_ISL_926578, EPI_ISL_926579, EPI_ISL_926580, EPI_ISL_926581, EPI_ISL_926582, EPI_ISL_926583, EPI_ISL_926584, EPI_ISL_926585, EPI_ISL_926589, EPI_ISL_926590, EPI_ISL_926592, EPI_ISL_926595, EPI_ISL_926597, EPI_ISL_926599, EPI_ISL_926600, EPI_ISL_926602, EPI_ISL_926604, EPI_ISL_926606, EPI_ISL_926608, EPI_ISL_926610, EPI_ISL_926611, EPI_ISL_926612, EPI_ISL_926613, EPI_ISL_926614, EPI_ISL_926618, EPI_ISL_926623, EPI_ISL_926624, EPI_ISL_926625, EPI_ISL_926626, EPI_ISL_926629, EPI_ISL_926630, EPI_ISL_926634, EPI_ISL_926635, EPI_ISL_926636, EPI_ISL_926637, EPI_ISL_926638, EPI_ISL_926639, EPI_ISL_926640, EPI_ISL_926641, EPI_ISL_926644, EPI_ISL_926645, EPI_ISL_926646, EPI_ISL_926647, EPI_ISL_926650, EPI_ISL_926652, EPI_ISL_926653, EPI_ISL_926654, EPI_ISL_926657, EPI_ISL_926658, EPI_ISL_926659, EPI_ISL_926660, EPI_ISL_926662, EPI_ISL_926664, EPI_ISL_926665, EPI_ISL_926669, EPI_ISL_926670, EPI_ISL_926672, EPI_ISL_926673, EPI_ISL_926675, EPI_ISL_926677, EPI_ISL_926678, EPI_ISL_926680, EPI_ISL_926682, EPI_ISL_926683, EPI_ISL_926685, EPI_ISL_926686, EPI_ISL_926688, EPI_ISL_926689, EPI_ISL_926690, EPI_ISL_926691, EPI_ISL_926694, EPI_ISL_926695, EPI_ISL_926696, EPI_ISL_926699, EPI_ISL_926700, EPI_ISL_926701, EPI_ISL_926702, EPI_ISL_926703, EPI_ISL_926704, EPI_ISL_926705, EPI_ISL_926706, EPI_ISL_926707, EPI_ISL_926709, EPI_ISL_926711, EPI_ISL_926712, EPI_ISL_926715, EPI_ISL_926718, EPI_ISL_926719, EPI_ISL_926720, EPI_ISL_926723, EPI_ISL_926724, EPI_ISL_926725, EPI_ISL_926726, EPI_ISL_926728, EPI_ISL_926729, EPI_ISL_926731, EPI_ISL_926732, EPI_ISL_926733, EPI_ISL_926734, EPI_ISL_926736, EPI_ISL_926737, EPI_ISL_926738, EPI_ISL_926739, EPI_ISL_926740, EPI_ISL_926741, EPI_ISL_926742, EPI_ISL_926743, EPI_ISL_926744, EPI_ISL_926745, EPI_ISL_926746, EPI_ISL_926747, EPI_ISL_926748, EPI_ISL_926749, EPI_ISL_926750, EPI_ISL_926753, EPI_ISL_926754, EPI_ISL_926755, EPI_ISL_926756, EPI_ISL_926759, EPI_ISL_926761, EPI_ISL_926762, EPI_ISL_926764, EPI_ISL_926766, EPI_ISL_926769, EPI_ISL_926771, EPI_ISL_926772, EPI_ISL_926774, EPI_ISL_926777, EPI_ISL_926779, EPI_ISL_926780, EPI_ISL_926781, EPI_ISL_926782, EPI_ISL_926783, EPI_ISL_926785, EPI_ISL_926788, EPI_ISL_926790, EPI_ISL_926792, EPI_ISL_926796, EPI_ISL_926798, EPI_ISL_926802, EPI_ISL_926803, EPI_ISL_926804, EPI_ISL_926807, EPI_ISL_926808, EPI_ISL_926812, EPI_ISL_926814, EPI_ISL_926815, EPI_ISL_926816, EPI_ISL_926819, EPI_ISL_926822, EPI_ISL_926823, EPI_ISL_926824, EPI_ISL_926828, EPI_ISL_926829, EPI_ISL_926831, EPI_ISL_926832, EPI_ISL_926834, EPI_ISL_926835, EPI_ISL_926837, EPI_ISL_926839, EPI_ISL_926840, EPI_ISL_926841, EPI_ISL_926842, EPI_ISL_926843, EPI_ISL_926845, EPI_ISL_926846, EPI_ISL_926847, EPI_ISL_926849, EPI_ISL_926850, EPI_ISL_926854, EPI_ISL_926856, EPI_ISL_926857, EPI_ISL_926858, EPI_ISL_926860, EPI_ISL_926861, EPI_ISL_926863, EPI_ISL_926864, EPI_ISL_926867, EPI_ISL_926868, EPI_ISL_926869, EPI_ISL_926871, EPI_ISL_926874, EPI_ISL_926875, EPI_ISL_926876, EPI_ISL_926877, EPI_ISL_926879, EPI_ISL_926883, EPI_ISL_926884, EPI_ISL_926886, EPI_ISL_926887, EPI_ISL_926888, EPI_ISL_926889, EPI_ISL_926890, EPI_ISL_926891, EPI_ISL_926892, EPI_ISL_926894, EPI_ISL_926895, EPI_ISL_926896, EPI_ISL_926897, EPI_ISL_926898, EPI_ISL_926901, EPI_ISL_926904, EPI_ISL_926905, EPI_ISL_926906, EPI_ISL_926907, EPI_ISL_926908, EPI_ISL_926910, EPI_ISL_926912, EPI_ISL_926913, EPI_ISL_926918, EPI_ISL_926919, EPI_ISL_926920, EPI_ISL_926921, EPI_ISL_926922, EPI_ISL_926924, EPI_ISL_926925, EPI_ISL_926926, EPI_ISL_926928, EPI_ISL_926929, EPI_ISL_926930, EPI_ISL_926935, EPI_ISL_926938, EPI_ISL_926939, EPI_ISL_926943, EPI_ISL_926944, EPI_ISL_926946, EPI_ISL_926947, EPI_ISL_926948, EPI_ISL_926950, EPI_ISL_926951, EPI_ISL_926952, EPI_ISL_926953, EPI_ISL_926954, EPI_ISL_926956, EPI_ISL_926957, EPI_ISL_926958, EPI_ISL_926961, EPI_ISL_926963, EPI_ISL_926966, EPI_ISL_926969, EPI_ISL_926971, EPI_ISL_926974, EPI_ISL_926975, EPI_ISL_926977, EPI_ISL_926978, EPI_ISL_926983, EPI_ISL_926984, EPI_ISL_926985, EPI_ISL_926986, EPI_ISL_926988, EPI_ISL_926991, EPI_ISL_926992, EPI_ISL_926993, EPI_ISL_926994, EPI_ISL_926996, EPI_ISL_926997, EPI_ISL_926998, EPI_ISL_927000, EPI_ISL_927001, EPI_ISL_927005, EPI_ISL_927006, EPI_ISL_927007, EPI_ISL_927008, EPI_ISL_927009, EPI_ISL_927010, EPI_ISL_927012, EPI_ISL_927013, EPI_ISL_927015, EPI_ISL_927016, EPI_ISL_927017, EPI_ISL_927019, EPI_ISL_927021, EPI_ISL_927022, EPI_ISL_927023, EPI_ISL_927026, EPI_ISL_927027, EPI_ISL_927029, EPI_ISL_927030, EPI_ISL_927031, EPI_ISL_927033, EPI_ISL_927034, EPI_ISL_927035, EPI_ISL_927037, EPI_ISL_927038, EPI_ISL_927040, EPI_ISL_927041, EPI_ISL_927042, EPI_ISL_927043, EPI_ISL_927044, EPI_ISL_927045, EPI_ISL_927046, EPI_ISL_927048, EPI_ISL_927049, EPI_ISL_927050, EPI_ISL_927051, EPI_ISL_927052, EPI_ISL_927054, EPI_ISL_927055, EPI_ISL_927059, EPI_ISL_927062, EPI_ISL_927064, EPI_ISL_927065, EPI_ISL_927067, EPI_ISL_927068, EPI_ISL_927071, EPI_ISL_927073, EPI_ISL_927074, EPI_ISL_927076, EPI_ISL_927077, EPI_ISL_927078, EPI_ISL_927080, EPI_ISL_927083, EPI_ISL_927085, EPI_ISL_927086, EPI_ISL_927087, EPI_ISL_927088, EPI_ISL_927090, EPI_ISL_927093, EPI_ISL_927094, EPI_ISL_927096, EPI_ISL_927098, EPI_ISL_927102, EPI_ISL_927104, EPI_ISL_927105, EPI_ISL_927108, EPI_ISL_927109, EPI_ISL_927110, EPI_ISL_927111, EPI_ISL_927112, EPI_ISL_927113, EPI_ISL_927115, EPI_ISL_927116, EPI_ISL_927120, EPI_ISL_927121, EPI_ISL_927124, EPI_ISL_927126, EPI_ISL_927129, EPI_ISL_927130, EPI_ISL_927131, EPI_ISL_927135, EPI_ISL_927136, EPI_ISL_927137, EPI_ISL_927138, EPI_ISL_927139, EPI_ISL_927142, EPI_ISL_927143, EPI_ISL_927146, EPI_ISL_927147, EPI_ISL_927150, EPI_ISL_927152, EPI_ISL_927154, EPI_ISL_927155, EPI_ISL_927156, EPI_ISL_927158, EPI_ISL_927159, EPI_ISL_927163, EPI_ISL_927165, EPI_ISL_927166, EPI_ISL_927169, EPI_ISL_927170, EPI_ISL_927171, EPI_ISL_927172, EPI_ISL_927173, EPI_ISL_927175, EPI_ISL_927178, EPI_ISL_927179, EPI_ISL_927180, EPI_ISL_927183, EPI_ISL_927187, EPI_ISL_927188, EPI_ISL_927191, EPI_ISL_927193, EPI_ISL_927195, EPI_ISL_927198, EPI_ISL_927201, EPI_ISL_927202, EPI_ISL_927204, EPI_ISL_927206, EPI_ISL_927208, EPI_ISL_927210, EPI_ISL_927212, EPI_ISL_927214, EPI_ISL_927217, EPI_ISL_927218, EPI_ISL_927219, EPI_ISL_927220, EPI_ISL_927221, EPI_ISL_927225, EPI_ISL_927226, EPI_ISL_927227, EPI_ISL_927229, EPI_ISL_927230, EPI_ISL_927231, EPI_ISL_927232, EPI_ISL_927233, EPI_ISL_927234, EPI_ISL_927235, EPI_ISL_927237, EPI_ISL_927238, EPI_ISL_927242, EPI_ISL_927244, EPI_ISL_927245, EPI_ISL_927250, EPI_ISL_927251, EPI_ISL_927253, EPI_ISL_927254, EPI_ISL_927256, EPI_ISL_927260, EPI_ISL_927264, EPI_ISL_927265, EPI_ISL_927267, EPI_ISL_927268, EPI_ISL_927269, EPI_ISL_927271, EPI_ISL_927272, EPI_ISL_927273, EPI_ISL_927275, EPI_ISL_927279, EPI_ISL_927280, EPI_ISL_927281, EPI_ISL_927282, EPI_ISL_927285, EPI_ISL_927286, EPI_ISL_927289, EPI_ISL_927290, EPI_ISL_927292, EPI_ISL_927294, EPI_ISL_927296, EPI_ISL_927297, EPI_ISL_927303, EPI_ISL_927304, EPI_ISL_927305, EPI_ISL_927307, EPI_ISL_927308, EPI_ISL_927310, EPI_ISL_927312, EPI_ISL_927313, EPI_ISL_927315, EPI_ISL_927316, EPI_ISL_927317, EPI_ISL_927318, EPI_ISL_927322, EPI_ISL_927323, EPI_ISL_927324, EPI_ISL_927326, EPI_ISL_927328, EPI_ISL_927329, EPI_ISL_927330, EPI_ISL_927331, EPI_ISL_927332, EPI_ISL_927333, EPI_ISL_927334, EPI_ISL_927335, EPI_ISL_927336, EPI_ISL_927339, EPI_ISL_927340, EPI_ISL_927343, EPI_ISL_927344, EPI_ISL_927349, EPI_ISL_927350, EPI_ISL_927354, EPI_ISL_927355, EPI_ISL_927357, EPI_ISL_927358, EPI_ISL_927359, EPI_ISL_927360, EPI_ISL_927361, EPI_ISL_927362, EPI_ISL_927363, EPI_ISL_927365, EPI_ISL_927368, EPI_ISL_927369, EPI_ISL_927372, EPI_ISL_927373, EPI_ISL_927374, EPI_ISL_927376, EPI_ISL_927377, EPI_ISL_927378, EPI_ISL_927379, EPI_ISL_927380, EPI_ISL_927381, EPI_ISL_927382, EPI_ISL_927383, EPI_ISL_927386, EPI_ISL_927387, EPI_ISL_927388, EPI_ISL_927390, EPI_ISL_927391, EPI_ISL_927392, EPI_ISL_927393, EPI_ISL_927394, EPI_ISL_927395, EPI_ISL_927396, EPI_ISL_927401, EPI_ISL_927403, EPI_ISL_927404, EPI_ISL_927405, EPI_ISL_927406, EPI_ISL_927407, EPI_ISL_927409, EPI_ISL_927410, EPI_ISL_927412, EPI_ISL_927413, EPI_ISL_927414, EPI_ISL_927415, EPI_ISL_927416, EPI_ISL_927417, EPI_ISL_927419, EPI_ISL_927421, EPI_ISL_927426, EPI_ISL_927427, EPI_ISL_927429, EPI_ISL_927430, EPI_ISL_927434, EPI_ISL_927435, EPI_ISL_927436, EPI_ISL_927437, EPI_ISL_927438, EPI_ISL_927442, EPI_ISL_927443, EPI_ISL_927445, EPI_ISL_927446, EPI_ISL_927448, EPI_ISL_927449, EPI_ISL_927450, EPI_ISL_927451, EPI_ISL_927452, EPI_ISL_927453, EPI_ISL_927454, EPI_ISL_927457, EPI_ISL_927458, EPI_ISL_927462, EPI_ISL_927466, EPI_ISL_927467, EPI_ISL_927468, EPI_ISL_927469, EPI_ISL_927471, EPI_ISL_927477, EPI_ISL_927478, EPI_ISL_927481, EPI_ISL_927482, EPI_ISL_927487, EPI_ISL_927488, EPI_ISL_927490, EPI_ISL_927494, EPI_ISL_927495, EPI_ISL_927496, EPI_ISL_927497, EPI_ISL_927499, EPI_ISL_927500, EPI_ISL_927503, EPI_ISL_927506, EPI_ISL_927509, EPI_ISL_927511, EPI_ISL_927512, EPI_ISL_927513, EPI_ISL_927514, EPI_ISL_927515, EPI_ISL_927516, EPI_ISL_927517, EPI_ISL_927518, EPI_ISL_927520, EPI_ISL_927521, EPI_ISL_927523, EPI_ISL_927524, EPI_ISL_927527, EPI_ISL_927528, EPI_ISL_927529, EPI_ISL_927530, EPI_ISL_927531, EPI_ISL_927532, EPI_ISL_927533, EPI_ISL_927535, EPI_ISL_927537, EPI_ISL_927539, EPI_ISL_927540, EPI_ISL_927541, EPI_ISL_927542, EPI_ISL_927545, EPI_ISL_927547, EPI_ISL_927548, EPI_ISL_927549, EPI_ISL_927552, EPI_ISL_927556, EPI_ISL_927557, EPI_ISL_927560, EPI_ISL_927561, EPI_ISL_927562, EPI_ISL_927563, EPI_ISL_927564, EPI_ISL_927567, EPI_ISL_927572, EPI_ISL_927574, EPI_ISL_927575, EPI_ISL_927576, EPI_ISL_927579, EPI_ISL_927581, EPI_ISL_927582, EPI_ISL_927583, EPI_ISL_927584, EPI_ISL_927585, EPI_ISL_927586, EPI_ISL_927588, EPI_ISL_927590, EPI_ISL_927591, EPI_ISL_927592, EPI_ISL_927594, EPI_ISL_927597, EPI_ISL_927604, EPI_ISL_927605, EPI_ISL_927606, EPI_ISL_927607, EPI_ISL_927609, EPI_ISL_927611, EPI_ISL_927618, EPI_ISL_927619, EPI_ISL_927620, EPI_ISL_927625, EPI_ISL_927629, EPI_ISL_927631, EPI_ISL_927632, EPI_ISL_927635, EPI_ISL_927638, EPI_ISL_927640, EPI_ISL_927642, EPI_ISL_927645, EPI_ISL_927646, EPI_ISL_927647, EPI_ISL_927649, EPI_ISL_927650, EPI_ISL_927654, EPI_ISL_927655, EPI_ISL_927656, EPI_ISL_927661, EPI_ISL_927663, EPI_ISL_927664, EPI_ISL_927666, EPI_ISL_927667, EPI_ISL_927670, EPI_ISL_927672, EPI_ISL_927676, EPI_ISL_927677, EPI_ISL_927678, EPI_ISL_927680, EPI_ISL_927681, EPI_ISL_927684, EPI_ISL_927686, EPI_ISL_927687, EPI_ISL_927688, EPI_ISL_927690, EPI_ISL_927691, EPI_ISL_927692, EPI_ISL_927693, EPI_ISL_927694, EPI_ISL_927696, EPI_ISL_927697, EPI_ISL_927699, EPI_ISL_927700, EPI_ISL_927701, EPI_ISL_927702, EPI_ISL_927704, EPI_ISL_927710, EPI_ISL_927712, EPI_ISL_927713, EPI_ISL_927714, EPI_ISL_927715, EPI_ISL_927717, EPI_ISL_927718, EPI_ISL_927719, EPI_ISL_927720, EPI_ISL_927722, EPI_ISL_927724, EPI_ISL_927725, EPI_ISL_927728, EPI_ISL_927729, EPI_ISL_927731, EPI_ISL_927734, EPI_ISL_927735, EPI_ISL_927736, EPI_ISL_927737, EPI_ISL_927738, EPI_ISL_927741, EPI_ISL_927742, EPI_ISL_927743, EPI_ISL_927745, EPI_ISL_927746, EPI_ISL_927747, EPI_ISL_927749, EPI_ISL_927750, EPI_ISL_927753, EPI_ISL_927754, EPI_ISL_927757, EPI_ISL_927759, EPI_ISL_927760, EPI_ISL_927761, EPI_ISL_927762, EPI_ISL_927764, EPI_ISL_927765, EPI_ISL_927766, EPI_ISL_927767, EPI_ISL_927768, EPI_ISL_927772, EPI_ISL_927773, EPI_ISL_927775, EPI_ISL_927776, EPI_ISL_927777, EPI_ISL_927779, EPI_ISL_927780, EPI_ISL_927783, EPI_ISL_927785, EPI_ISL_927786, EPI_ISL_927790, EPI_ISL_927791, EPI_ISL_927793, EPI_ISL_927795, EPI_ISL_927796, EPI_ISL_927798, EPI_ISL_927799, EPI_ISL_927800, EPI_ISL_927801, EPI_ISL_927802, EPI_ISL_927803, EPI_ISL_927804, EPI_ISL_927805, EPI_ISL_927806, EPI_ISL_927807, EPI_ISL_927809, EPI_ISL_927810, EPI_ISL_927811, EPI_ISL_927812, EPI_ISL_927814, EPI_ISL_927815, EPI_ISL_927816, EPI_ISL_927817, EPI_ISL_927819, EPI_ISL_927821, EPI_ISL_927823, EPI_ISL_927825, EPI_ISL_927827, EPI_ISL_927828, EPI_ISL_927829, EPI_ISL_927834, EPI_ISL_927835, EPI_ISL_927836, EPI_ISL_927837, EPI_ISL_927838, EPI_ISL_927841, EPI_ISL_927842, EPI_ISL_927844, EPI_ISL_927848, EPI_ISL_927850, EPI_ISL_927851, EPI_ISL_927852, EPI_ISL_927853, EPI_ISL_927854, EPI_ISL_927857, EPI_ISL_927865, EPI_ISL_927867, EPI_ISL_927870, EPI_ISL_927874, EPI_ISL_927875, EPI_ISL_927884, EPI_ISL_927885, EPI_ISL_927886, EPI_ISL_927889, EPI_ISL_927890, EPI_ISL_927892, EPI_ISL_927893, EPI_ISL_927894, EPI_ISL_927898, EPI_ISL_927899, EPI_ISL_927900, EPI_ISL_927903, EPI_ISL_927906, EPI_ISL_927908, EPI_ISL_927909, EPI_ISL_927910, EPI_ISL_927912, EPI_ISL_927913, EPI_ISL_927914, EPI_ISL_927915, EPI_ISL_927916, EPI_ISL_927917, EPI_ISL_927920, EPI_ISL_927921, EPI_ISL_927922, EPI_ISL_927923, EPI_ISL_927924, EPI_ISL_927929, EPI_ISL_927931, EPI_ISL_927935, EPI_ISL_927936, EPI_ISL_927937, EPI_ISL_927938, EPI_ISL_927939, EPI_ISL_927940, EPI_ISL_927941, EPI_ISL_927942, EPI_ISL_927944, EPI_ISL_927947, EPI_ISL_927948, EPI_ISL_927949, EPI_ISL_927950, EPI_ISL_927951, EPI_ISL_927954, EPI_ISL_927960, EPI_ISL_927963, EPI_ISL_927965, EPI_ISL_927966, EPI_ISL_927970, EPI_ISL_927971, EPI_ISL_927973, EPI_ISL_927974, EPI_ISL_927975, EPI_ISL_927977, EPI_ISL_927983, EPI_ISL_927985, EPI_ISL_927986, EPI_ISL_927989, EPI_ISL_927990, EPI_ISL_927992, EPI_ISL_927993, EPI_ISL_927994, EPI_ISL_927995, EPI_ISL_927997, EPI_ISL_927999, EPI_ISL_928000, EPI_ISL_928002, EPI_ISL_928004, EPI_ISL_928007, EPI_ISL_928008, EPI_ISL_928010, EPI_ISL_928011, EPI_ISL_928013, EPI_ISL_928015, EPI_ISL_928016, EPI_ISL_928017, EPI_ISL_928018, EPI_ISL_928019, EPI_ISL_928021, EPI_ISL_928023, EPI_ISL_928024, EPI_ISL_928027, EPI_ISL_928028, EPI_ISL_928029, EPI_ISL_928030, EPI_ISL_928031, EPI_ISL_928033, EPI_ISL_928034, EPI_ISL_928035, EPI_ISL_928036, EPI_ISL_928037, EPI_ISL_928039, EPI_ISL_928040, EPI_ISL_928041, EPI_ISL_928042, EPI_ISL_928045, EPI_ISL_928046, EPI_ISL_928050, EPI_ISL_928052, EPI_ISL_928053, EPI_ISL_928054, EPI_ISL_928055, EPI_ISL_928056, EPI_ISL_928057, EPI_ISL_928058, EPI_ISL_928060, EPI_ISL_928065, EPI_ISL_928069, EPI_ISL_928075, EPI_ISL_928077, EPI_ISL_928078, EPI_ISL_928079, EPI_ISL_928082, EPI_ISL_928083, EPI_ISL_928084, EPI_ISL_928086, EPI_ISL_928088, EPI_ISL_928090, EPI_ISL_928091, EPI_ISL_928093, EPI_ISL_928094, EPI_ISL_928095, EPI_ISL_928098, EPI_ISL_928099, EPI_ISL_928101, EPI_ISL_928103, EPI_ISL_928104, EPI_ISL_928107, EPI_ISL_928108, EPI_ISL_928110, EPI_ISL_928114, EPI_ISL_928115, EPI_ISL_928116, EPI_ISL_928117, EPI_ISL_928119, EPI_ISL_928122, EPI_ISL_928124, EPI_ISL_928126, EPI_ISL_928127, EPI_ISL_928128, EPI_ISL_928129, EPI_ISL_928130, EPI_ISL_928132, EPI_ISL_928133, EPI_ISL_928134, EPI_ISL_928138, EPI_ISL_928139, EPI_ISL_928140, EPI_ISL_928142, EPI_ISL_928143, EPI_ISL_928144, EPI_ISL_928145, EPI_ISL_928146, EPI_ISL_928151, EPI_ISL_928152, EPI_ISL_928153, EPI_ISL_928156, EPI_ISL_928158, EPI_ISL_928159, EPI_ISL_928160, EPI_ISL_928161, EPI_ISL_928162, EPI_ISL_928165, EPI_ISL_928168, EPI_ISL_928169, EPI_ISL_928172, EPI_ISL_928173, EPI_ISL_928174, EPI_ISL_928176, EPI_ISL_928177, EPI_ISL_928178, EPI_ISL_928180, EPI_ISL_928181, EPI_ISL_928182, EPI_ISL_928183, EPI_ISL_928184, EPI_ISL_928186, EPI_ISL_928187, EPI_ISL_928188, EPI_ISL_928189, EPI_ISL_928193, EPI_ISL_928194, EPI_ISL_928195, EPI_ISL_928196, EPI_ISL_928198, EPI_ISL_928199, EPI_ISL_928201, EPI_ISL_928203, EPI_ISL_928204, EPI_ISL_928205, EPI_ISL_928206, EPI_ISL_928207, EPI_ISL_928209,			

EPI_ISL_928215, EPI_ISL_928216, EPI_ISL_928219, EPI_ISL_928220, EPI_ISL_928222, EPI_ISL_928223, EPI_ISL_928226, EPI_ISL_928227, EPI_ISL_928228, EPI_ISL_928232, EPI_ISL_928234, EPI_ISL_928235, EPI_ISL_928238, EPI_ISL_928242, EPI_ISL_928243, EPI_ISL_928244, EPI_ISL_928245, EPI_ISL_928246, EPI_ISL_928253, EPI_ISL_928254, EPI_ISL_928255, EPI_ISL_928256, EPI_ISL_928257, EPI_ISL_928260, EPI_ISL_928265, EPI_ISL_928267, EPI_ISL_928268, EPI_ISL_928269, EPI_ISL_928270, EPI_ISL_928271, EPI_ISL_928275, EPI_ISL_928277, EPI_ISL_928278, EPI_ISL_928279, EPI_ISL_928280, EPI_ISL_928283, EPI_ISL_928284, EPI_ISL_928286, EPI_ISL_928287, EPI_ISL_928288, EPI_ISL_928289, EPI_ISL_928290, EPI_ISL_928291, EPI_ISL_928292, EPI_ISL_928293, EPI_ISL_928294, EPI_ISL_928295, EPI_ISL_928296, EPI_ISL_928300, EPI_ISL_928301, EPI_ISL_928302, EPI_ISL_928303, EPI_ISL_928304, EPI_ISL_928306, EPI_ISL_928309, EPI_ISL_928310, EPI_ISL_928311, EPI_ISL_928312, EPI_ISL_928313, EPI_ISL_928314, EPI_ISL_928317, EPI_ISL_928318, EPI_ISL_928319, EPI_ISL_928321, EPI_ISL_928324, EPI_ISL_928325, EPI_ISL_928326, EPI_ISL_928328, EPI_ISL_928329, EPI_ISL_928330, EPI_ISL_928331, EPI_ISL_928334, EPI_ISL_928336, EPI_ISL_928337, EPI_ISL_928338, EPI_ISL_928339, EPI_ISL_928341, EPI_ISL_928342, EPI_ISL_928346, EPI_ISL_928347, EPI_ISL_928348, EPI_ISL_928349, EPI_ISL_928350, EPI_ISL_928351, EPI_ISL_928352, EPI_ISL_928353, EPI_ISL_928354, EPI_ISL_928357, EPI_ISL_928358, EPI_ISL_928360, EPI_ISL_928364, EPI_ISL_928366, EPI_ISL_928368, EPI_ISL_928369, EPI_ISL_928370, EPI_ISL_928371, EPI_ISL_928373, EPI_ISL_928374, EPI_ISL_928375, EPI_ISL_928376, EPI_ISL_928377, EPI_ISL_928378, EPI_ISL_928379, EPI_ISL_928381, EPI_ISL_928382, EPI_ISL_928384, EPI_ISL_928385, EPI_ISL_928387, EPI_ISL_928390, EPI_ISL_928391, EPI_ISL_928393, EPI_ISL_928394, EPI_ISL_928396, EPI_ISL_928398, EPI_ISL_928399, EPI_ISL_928400, EPI_ISL_928401, EPI_ISL_928402, EPI_ISL_928405, EPI_ISL_928407, EPI_ISL_928408, EPI_ISL_928410, EPI_ISL_928411, EPI_ISL_928414, EPI_ISL_928416, EPI_ISL_928418, EPI_ISL_928419, EPI_ISL_928421, EPI_ISL_928423, EPI_ISL_928429, EPI_ISL_928433, EPI_ISL_928435, EPI_ISL_928436, EPI_ISL_928437, EPI_ISL_928438, EPI_ISL_928440, EPI_ISL_928448, EPI_ISL_928449, EPI_ISL_928451, EPI_ISL_928452, EPI_ISL_928453, EPI_ISL_928457, EPI_ISL_928458, EPI_ISL_928460, EPI_ISL_928461, EPI_ISL_928463, EPI_ISL_928465, EPI_ISL_928469, EPI_ISL_928471, EPI_ISL_928472, EPI_ISL_928473, EPI_ISL_928474, EPI_ISL_928475, EPI_ISL_928476, EPI_ISL_928477, EPI_ISL_928478, EPI_ISL_928479, EPI_ISL_928482, EPI_ISL_928484, EPI_ISL_928486, EPI_ISL_928487, EPI_ISL_928488, EPI_ISL_928492, EPI_ISL_928496, EPI_ISL_928497, EPI_ISL_928498, EPI_ISL_928500, EPI_ISL_928501, EPI_ISL_928502, EPI_ISL_928505, EPI_ISL_928506, EPI_ISL_928507, EPI_ISL_928508, EPI_ISL_928510, EPI_ISL_928511, EPI_ISL_928512, EPI_ISL_928515, EPI_ISL_928516, EPI_ISL_928517, EPI_ISL_928518, EPI_ISL_928519, EPI_ISL_928520, EPI_ISL_928521, EPI_ISL_928526, EPI_ISL_928530, EPI_ISL_928531, EPI_ISL_928533, EPI_ISL_928534, EPI_ISL_928535, EPI_ISL_928536, EPI_ISL_928538, EPI_ISL_928540, EPI_ISL_928542, EPI_ISL_928543, EPI_ISL_928544, EPI_ISL_928545, EPI_ISL_928547, EPI_ISL_928549, EPI_ISL_928551, EPI_ISL_928554, EPI_ISL_928555, EPI_ISL_928556, EPI_ISL_928558, EPI_ISL_928559, EPI_ISL_928562, EPI_ISL_928564, EPI_ISL_928566, EPI_ISL_928567, EPI_ISL_928571, EPI_ISL_928572, EPI_ISL_928573, EPI_ISL_928574, EPI_ISL_928575, EPI_ISL_928576, EPI_ISL_928577, EPI_ISL_928578, EPI_ISL_928579, EPI_ISL_928582, EPI_ISL_928583, EPI_ISL_928584, EPI_ISL_928589, EPI_ISL_928590, EPI_ISL_928591, EPI_ISL_928592, EPI_ISL_928593, EPI_ISL_928594, EPI_ISL_928596, EPI_ISL_928597, EPI_ISL_928598, EPI_ISL_928599, EPI_ISL_928602, EPI_ISL_928603, EPI_ISL_928605, EPI_ISL_928607, EPI_ISL_928608, EPI_ISL_928610, EPI_ISL_928611, EPI_ISL_928612, EPI_ISL_928613, EPI_ISL_928615, EPI_ISL_928616, EPI_ISL_928618, EPI_ISL_928619, EPI_ISL_928620, EPI_ISL_928622, EPI_ISL_928627, EPI_ISL_928632, EPI_ISL_928634, EPI_ISL_928635, EPI_ISL_928637, EPI_ISL_928639, EPI_ISL_928640, EPI_ISL_928641, EPI_ISL_928642, EPI_ISL_928643, EPI_ISL_928644, EPI_ISL_928645, EPI_ISL_928647, EPI_ISL_928649, EPI_ISL_928656, EPI_ISL_928658, EPI_ISL_928659, EPI_ISL_928662, EPI_ISL_928663, EPI_ISL_928666, EPI_ISL_928667, EPI_ISL_928668, EPI_ISL_928669, EPI_ISL_928671, EPI_ISL_928672, EPI_ISL_928673, EPI_ISL_928680, EPI_ISL_928681, EPI_ISL_928684, EPI_ISL_928685, EPI_ISL_928686, EPI_ISL_928691, EPI_ISL_928692, EPI_ISL_928693, EPI_ISL_928694, EPI_ISL_928695, EPI_ISL_928697, EPI_ISL_928698, EPI_ISL_928701, EPI_ISL_928702, EPI_ISL_928703, EPI_ISL_928704, EPI_ISL_928707, EPI_ISL_928709, EPI_ISL_928710, EPI_ISL_928712, EPI_ISL_928713, EPI_ISL_928715, EPI_ISL_928721, EPI_ISL_928724, EPI_ISL_928725, EPI_ISL_928726, EPI_ISL_928729, EPI_ISL_928731, EPI_ISL_928732, EPI_ISL_928733, EPI_ISL_928734, EPI_ISL_928737, EPI_ISL_928739, EPI_ISL_928740, EPI_ISL_928741, EPI_ISL_928742, EPI_ISL_928744, EPI_ISL_928746, EPI_ISL_928747, EPI_ISL_928748, EPI_ISL_928749, EPI_ISL_928752, EPI_ISL_928753, EPI_ISL_928756, EPI_ISL_928758, EPI_ISL_928759, EPI_ISL_928760, EPI_ISL_928761, EPI_ISL_928762, EPI_ISL_928763, EPI_ISL_928764, EPI_ISL_928765, EPI_ISL_928766, EPI_ISL_928767, EPI_ISL_928770, EPI_ISL_928771, EPI_ISL_928773, EPI_ISL_928775, EPI_ISL_928776, EPI_ISL_928779, EPI_ISL_928781, EPI_ISL_928782, EPI_ISL_928783, EPI_ISL_928784, EPI_ISL_928785, EPI_ISL_928787, EPI_ISL_928790, EPI_ISL_928792, EPI_ISL_928793, EPI_ISL_928795, EPI_ISL_928798, EPI_ISL_928799, EPI_ISL_928800, EPI_ISL_928801, EPI_ISL_928802, EPI_ISL_928803, EPI_ISL_928805, EPI_ISL_928806, EPI_ISL_928807, EPI_ISL_928809, EPI_ISL_928810, EPI_ISL_928811, EPI_ISL_928813, EPI_ISL_928815, EPI_ISL_928818, EPI_ISL_928819, EPI_ISL_928824, EPI_ISL_928825, EPI_ISL_928826, EPI_ISL_928827, EPI_ISL_928828, EPI_ISL_928829, EPI_ISL_928830, EPI_ISL_928831, EPI_ISL_928832, EPI_ISL_928835, EPI_ISL_928836, EPI_ISL_928837, EPI_ISL_928838, EPI_ISL_928839, EPI_ISL_928840, EPI_ISL_928841, EPI_ISL_928844, EPI_ISL_928845, EPI_ISL_928846, EPI_ISL_928847, EPI_ISL_928848, EPI_ISL_928851, EPI_ISL_928852, EPI_ISL_928854, EPI_ISL_928855, EPI_ISL_928856, EPI_ISL_928857, EPI_ISL_928858, EPI_ISL_928862, EPI_ISL_928865, EPI_ISL_928866, EPI_ISL_928868, EPI_ISL_928870, EPI_ISL_928871, EPI_ISL_928873, EPI_ISL_928874, EPI_ISL_928875, EPI_ISL_928877, EPI_ISL_928879, EPI_ISL_928880, EPI_ISL_928887, EPI_ISL_928889, EPI_ISL_928890, EPI_ISL_928891, EPI_ISL_928896, EPI_ISL_928897, EPI_ISL_928898, EPI_ISL_928900, EPI_ISL_928901, EPI_ISL_928902, EPI_ISL_928903, EPI_ISL_928904, EPI_ISL_928906, EPI_ISL_928907, EPI_ISL_928908, EPI_ISL_928910, EPI_ISL_928911, EPI_ISL_928912, EPI_ISL_928913, EPI_ISL_928914, EPI_ISL_928917, EPI_ISL_928920, EPI_ISL_928921, EPI_ISL_928922, EPI_ISL_928923, EPI_ISL_928925, EPI_ISL_928927, EPI_ISL_928928, EPI_ISL_928929, EPI_ISL_928930, EPI_ISL_928931, EPI_ISL_928933, EPI_ISL_928936, EPI_ISL_928937, EPI_ISL_928938, EPI_ISL_928941, EPI_ISL_928945, EPI_ISL_928947, EPI_ISL_928948, EPI_ISL_928949, EPI_ISL_928950, EPI_ISL_928952, EPI_ISL_928953, EPI_ISL_928955, EPI_ISL_928956, EPI_ISL_928957, EPI_ISL_928958, EPI_ISL_928959, EPI_ISL_928960, EPI_ISL_928962, EPI_ISL_928963, EPI_ISL_928964, EPI_ISL_928968, EPI_ISL_928969, EPI_ISL_928970, EPI_ISL_928971, EPI_ISL_928972, EPI_ISL_928973, EPI_ISL_928974, EPI_ISL_928975, EPI_ISL_928977, EPI_ISL_928979, EPI_ISL_928981, EPI_ISL_928982, EPI_ISL_928986, EPI_ISL_928990, EPI_ISL_928991, EPI_ISL_928992, EPI_ISL_928993, EPI_ISL_928996, EPI_ISL_928997, EPI_ISL_928998, EPI_ISL_928999, EPI_ISL_929000, EPI_ISL_929001, EPI_ISL_929002, EPI_ISL_929003, EPI_ISL_929004, EPI_ISL_929007, EPI_ISL_929008, EPI_ISL_929009, EPI_ISL_929012, EPI_ISL_929013, EPI_ISL_929014, EPI_ISL_929016, EPI_ISL_929017, EPI_ISL_929019, EPI_ISL_929021, EPI_ISL_929023, EPI_ISL_929024, EPI_ISL_929025, EPI_ISL_929028, EPI_ISL_929029, EPI_ISL_929031, EPI_ISL_929032, EPI_ISL_929033, EPI_ISL_929036, EPI_ISL_929037, EPI_ISL_929038, EPI_ISL_929041, EPI_ISL_929042, EPI_ISL_929045, EPI_ISL_929048, EPI_ISL_929049, EPI_ISL_929050, EPI_ISL_929051, EPI_ISL_929052, EPI_ISL_929053, EPI_ISL_929054, EPI_ISL_929059, EPI_ISL_929060, EPI_ISL_929065, EPI_ISL_929067, EPI_ISL_929068, EPI_ISL_929070, EPI_ISL_929071, EPI_ISL_929072, EPI_ISL_929073, EPI_ISL_929074, EPI_ISL_929077, EPI_ISL_929078, EPI_ISL_929080, EPI_ISL_929081, EPI_ISL_929084, EPI_ISL_929087, EPI_ISL_929089, EPI_ISL_929091, EPI_ISL_929094, EPI_ISL_929095, EPI_ISL_929098, EPI_ISL_929100, EPI_ISL_929101, EPI_ISL_929102, EPI_ISL_929103, EPI_ISL_929104, EPI_ISL_929105, EPI_ISL_929106, EPI_ISL_929107, EPI_ISL_929108, EPI_ISL_929109, EPI_ISL_929110, EPI_ISL_929111, EPI_ISL_929113, EPI_ISL_929114, EPI_ISL_929115, EPI_ISL_929117, EPI_ISL_929118, EPI_ISL_929121, EPI_ISL_929123, EPI_ISL_929125, EPI_ISL_929126, EPI_ISL_929127, EPI_ISL_929131, EPI_ISL_929133, EPI_ISL_929134, EPI_ISL_929135, EPI_ISL_929137, EPI_ISL_929138, EPI_ISL_929139, EPI_ISL_929143, EPI_ISL_929144, EPI_ISL_929148, EPI_ISL_929150, EPI_ISL_929151, EPI_ISL_929152, EPI_ISL_929154, EPI_ISL_929155, EPI_ISL_929160, EPI_ISL_929161, EPI_ISL_929163, EPI_ISL_929164, EPI_ISL_929167, EPI_ISL_929172, EPI_ISL_929174, EPI_ISL_929177, EPI_ISL_929178, EPI_ISL_929179, EPI_ISL_929181, EPI_ISL_929183, EPI_ISL_929186, EPI_ISL_929187, EPI_ISL_929189, EPI_ISL_929190, EPI_ISL_929192, EPI_ISL_929193, EPI_ISL_929195, EPI_ISL_929204, EPI_ISL_929206, EPI_ISL_929208, EPI_ISL_929209, EPI_ISL_929210, EPI_ISL_929211, EPI_ISL_929212, EPI_ISL_929216, EPI_ISL_929217, EPI_ISL_929218, EPI_ISL_929220, EPI_ISL_929221, EPI_ISL_929225, EPI_ISL_929232, EPI_ISL_929233, EPI_ISL_929234, EPI_ISL_929236, EPI_ISL_929237, EPI_ISL_929238, EPI_ISL_929239, EPI_ISL_929240, EPI_ISL_929242, EPI_ISL_929244, EPI_ISL_929245, EPI_ISL_929246, EPI_ISL_929248, EPI_ISL_929249, EPI_ISL_929251, EPI_ISL_929252, EPI_ISL_929255, EPI_ISL_929256, EPI_ISL_929257, EPI_ISL_929259, EPI_ISL_929260, EPI_ISL_929263, EPI_ISL_929264, EPI_ISL_929266, EPI_ISL_929267, EPI_ISL_929271, EPI_ISL_929272, EPI_ISL_929273, EPI_ISL_929274, EPI_ISL_929275, EPI_ISL_929276, EPI_ISL_929277, EPI_ISL_929278, EPI_ISL_929283, EPI_ISL_929285, EPI_ISL_929287, EPI_ISL_929288, EPI_ISL_929289, EPI_ISL_929290, EPI_ISL_929293, EPI_ISL_929294, EPI_ISL_929295, EPI_ISL_929296, EPI_ISL_929298, EPI_ISL_929300, EPI_ISL_929301, EPI_ISL_929302, EPI_ISL_929303, EPI_ISL_929304, EPI_ISL_929305, EPI_ISL_929306, EPI_ISL_929310, EPI_ISL_929311, EPI_ISL_929312, EPI_ISL_929315, EPI_ISL_929316, EPI_ISL_929320, EPI_ISL_929321, EPI_ISL_929324, EPI_ISL_929325, EPI_ISL_929327, EPI_ISL_929328, EPI_ISL_929330, EPI_ISL_929333, EPI_ISL_929335, EPI_ISL_929336, EPI_ISL_929337, EPI_ISL_929341, EPI_ISL_929342, EPI_ISL_929343, EPI_ISL_929345, EPI_ISL_929346, EPI_ISL_929347, EPI_ISL_929348, EPI_ISL_929350, EPI_ISL_929351, EPI_ISL_929352, EPI_ISL_929354, EPI_ISL_929358, EPI_ISL_929359, EPI_ISL_929361, EPI_ISL_929363, EPI_ISL_929364, EPI_ISL_929365, EPI_ISL_929367, EPI_ISL_929369, EPI_ISL_929373, EPI_ISL_929374, EPI_ISL_929375, EPI_ISL_929376, EPI_ISL_929377, EPI_ISL_929378, EPI_ISL_929379, EPI_ISL_929380, EPI_ISL_929381, EPI_ISL_929382, EPI_ISL_929384, EPI_ISL_929386, EPI_ISL_929392, EPI_ISL_929393, EPI_ISL_929394, EPI_ISL_929395, EPI_ISL_929396, EPI_ISL_929397, EPI_ISL_929399, EPI_ISL_929401, EPI_ISL_929402, EPI_ISL_929404, EPI_ISL_929405, EPI_ISL_929406, EPI_ISL_929407, EPI_ISL_929408, EPI_ISL_929410, EPI_ISL_929411, EPI_ISL_929412, EPI_ISL_929413, EPI_ISL_929414, EPI_ISL_929416, EPI_ISL_929417, EPI_ISL_929419, EPI_ISL_929420, EPI_ISL_929423, EPI_ISL_929426, EPI_ISL_929427, EPI_ISL_929430, EPI_ISL_929431, EPI_ISL_929432, EPI_ISL_929434, EPI_ISL_929436, EPI_ISL_929437, EPI_ISL_929439, EPI_ISL_929440, EPI_ISL_929441, EPI_ISL_929442, EPI_ISL_929443, EPI_ISL_929445, EPI_ISL_929447, EPI_ISL_929448, EPI_ISL_929453, EPI_ISL_929454, EPI_ISL_929455, EPI_ISL_929457, EPI_ISL_929459, EPI_ISL_929460, EPI_ISL_929465, EPI_ISL_929470, EPI_ISL_929471, EPI_ISL_929472, EPI_ISL_929473, EPI_ISL_929474, EPI_ISL_929475, EPI_ISL_929478, EPI_ISL_929479, EPI_ISL_929480, EPI_ISL_929481, EPI_ISL_929482, EPI_ISL_929483, EPI_ISL_929484, EPI_ISL_929485, EPI_ISL_929487, EPI_ISL_929489, EPI_ISL_929490, EPI_ISL_929491, EPI_ISL_929492, EPI_ISL_929493, EPI_ISL_929496, EPI_ISL_929498, EPI_ISL_929499, EPI_ISL_929500, EPI_ISL_929502, EPI_ISL_929503, EPI_ISL_929504, EPI_ISL_929505, EPI_ISL_929506, EPI_ISL_929507, EPI_ISL_929508, EPI_ISL_929509, EPI_ISL_929510, EPI_ISL_929511, EPI_ISL_929512, EPI_ISL_929514, EPI_ISL_929516, EPI_ISL_929517, EPI_ISL_929520, EPI_ISL_929521, EPI_ISL_929522, EPI_ISL_929525, EPI_ISL_929528, EPI_ISL_929529, EPI_ISL_929532, EPI_ISL_929536, EPI_ISL_929537, EPI_ISL_929539, EPI_ISL_929541, EPI_ISL_929542, EPI_ISL_929543, EPI_ISL_929544, EPI_ISL_929545, EPI_ISL_929548, EPI_ISL_929549, EPI_ISL_929550, EPI_ISL_929551, EPI_ISL_929553, EPI_ISL_929554, EPI_ISL_929555, EPI_ISL_929556, EPI_ISL_929557, EPI_ISL_929558, EPI_ISL_929562, EPI_ISL_929563, EPI_ISL_929564, EPI_ISL_929566, EPI_ISL_929567, EPI_ISL_929569, EPI_ISL_929571, EPI_ISL_929573, EPI_ISL_929574, EPI_ISL_929576, EPI_ISL_929577, EPI_ISL_929580, EPI_ISL_929581, EPI_ISL_929582, EPI_ISL_929584, EPI_ISL_929585, EPI_ISL_929586, EPI_ISL_929587, EPI_ISL_929590, EPI_ISL_929591, EPI_ISL_929592, EPI_ISL_929593, EPI_ISL_929594, EPI_ISL_929596, EPI_ISL_929597, EPI_ISL_929599, EPI_ISL_929604, EPI_ISL_929606, EPI_ISL_929615, EPI_ISL_929616, EPI_ISL_929620, EPI_ISL_929621, EPI_ISL_929622, EPI_ISL_929625, EPI_ISL_929626, EPI_ISL_929629, EPI_ISL_929630, EPI_ISL_929632, EPI_ISL_929633, EPI_ISL_929635, EPI_ISL_929636, EPI_ISL_929640, EPI_ISL_929641, EPI_ISL_929642, EPI_ISL_929644, EPI_ISL_929646, EPI_ISL_929649, EPI_ISL_929650, EPI_ISL_929652, EPI_ISL_929654, EPI_ISL_929656, EPI_ISL_929663, EPI_ISL_929664, EPI_ISL_929665, EPI_ISL_929666, EPI_ISL_929667, EPI_ISL_929668, EPI_ISL_929670, EPI_ISL_929671, EPI_ISL_929675, EPI_ISL_929679, EPI_ISL_929680, EPI_ISL_929681, EPI_ISL_929682, EPI_ISL_929684, EPI_ISL_929690, EPI_ISL_929691, EPI_ISL_929692, EPI_ISL_929696, EPI_ISL_929697, EPI_ISL_929698, EPI_ISL_929699, EPI_ISL_929700, EPI_ISL_929703, EPI_ISL_929704, EPI_ISL_929705, EPI_ISL_929707, EPI_ISL_929708, EPI_ISL_929710, EPI_ISL_929713, EPI_ISL_929714, EPI_ISL_929715, EPI_ISL_929717, EPI_ISL_929718, EPI_ISL_929719, EPI_ISL_929721, EPI_ISL_929724, EPI_ISL_929727, EPI_ISL_929728, EPI_ISL_929729, EPI_ISL_929731, EPI_ISL_929732, EPI_ISL_929733, EPI_ISL_929736, EPI_ISL_929739, EPI_ISL_929742, EPI_ISL_929743, EPI_ISL_929747, EPI_ISL_929751, EPI_ISL_929752, EPI_ISL_929753, EPI_ISL_929754, EPI_ISL_929759, EPI_ISL_929760, EPI_ISL_929761, EPI_ISL_929764, EPI_ISL_929766, EPI_ISL_929767, EPI_ISL_929768, EPI_ISL_929769, EPI_ISL_929774, EPI_ISL_929775, EPI_ISL_929777, EPI_ISL_929778, EPI_ISL_929779, EPI_ISL_929785, EPI_ISL_929786, EPI_ISL_929787, EPI_ISL_929789, EPI_ISL_929791, EPI_ISL_929792, EPI_ISL_929793, EPI_ISL_929795, EPI_ISL_929798, EPI_ISL_929799, EPI_ISL_929801, EPI_ISL_929804, EPI_ISL_929805, EPI_ISL_929806, EPI_ISL_929809, EPI_ISL_929812, EPI_ISL_929813, EPI_ISL_929814, EPI_ISL_929815, EPI_ISL_929817, EPI_ISL_929818, EPI_ISL_929819, EPI_ISL_929820, EPI_ISL_929821, EPI_ISL_929822, EPI_ISL_929824, EPI_ISL_929825, EPI_ISL_929826, EPI_ISL_929828, EPI_ISL_929831, EPI_ISL_929834, EPI_ISL_929835, EPI_ISL_929836, EPI_ISL_929838, EPI_ISL_929839, EPI_ISL_929841, EPI_ISL_929842, EPI_ISL_929843, EPI_ISL_929844, EPI_ISL_929846, EPI_ISL_929847, EPI_ISL_929849, EPI_ISL_929850, EPI_ISL_929851, EPI_ISL_929852, EPI_ISL_929853, EPI_ISL_929855, EPI_ISL_929858, EPI_ISL_929859, EPI_ISL_929862, EPI_ISL_929863, EPI_ISL_929865, EPI_ISL_929869, EPI_ISL_929871, EPI_ISL_929874, EPI_ISL_929875, EPI_ISL_929876, EPI_ISL_929877, EPI_ISL_929878, EPI_ISL_929879, EPI_ISL_929883, EPI_ISL_929884, EPI_ISL_929885, EPI_ISL_929888, EPI_ISL_929889, EPI_ISL_929891, EPI_ISL_929893, EPI_ISL_929895, EPI_ISL_929896, EPI_ISL_929897, EPI_ISL_929898, EPI_ISL_929901, EPI_ISL_929902, EPI_ISL_929903, EPI_ISL_929904, EPI_ISL_929905, EPI_ISL_929906, EPI_ISL_929909, EPI_ISL_929912, EPI_ISL_929915, EPI_ISL_929918, EPI_ISL_929920, EPI_ISL_929922, EPI_ISL_929924, EPI_ISL_929925, EPI_ISL_929927, EPI_ISL_929930, EPI_ISL_929932, EPI_ISL_929934, EPI_ISL_929935, EPI_ISL_929938, EPI_ISL_929943, EPI_ISL_929946, EPI_ISL_929947, EPI_ISL_929949, EPI_ISL_929950, EPI_ISL_929952, EPI_ISL_929953, EPI_ISL_929955, EPI_ISL_929959, EPI_ISL_929961, EPI_ISL_929962, EPI_ISL_929963, EPI_ISL_929965, EPI_ISL_929967, EPI_ISL_929968, EPI_ISL_929969, EPI_ISL_929970, EPI_ISL_929971, EPI_ISL_929973, EPI_ISL_929974, EPI_ISL_929975, EPI_ISL_929977, EPI_ISL_929978, EPI_ISL_929979, EPI_ISL_929981, EPI_ISL_929982, EPI_ISL_929983, EPI_ISL_929984, EPI_ISL_929985, EPI_ISL_929986, EPI_ISL_929989, EPI_ISL_929990, EPI_ISL_929991, EPI_ISL_929992, EPI_ISL_929996, EPI_ISL_929997, EPI_ISL_929999, EPI_ISL_930000, EPI_ISL_930002, EPI_ISL_930005, EPI_ISL_930009, EPI_ISL_930012, EPI_ISL_930013, EPI_ISL_930014, EPI_ISL_930015, EPI_ISL_930018, EPI_ISL_930020, EPI_ISL_930022, EPI_ISL_930023, EPI_ISL_930024, EPI_ISL_930025, EPI_ISL_930026, EPI_ISL_930028, EPI_ISL_930032, EPI_ISL_930033, EPI_ISL_930036, EPI_ISL_930038, EPI_ISL_930039, EPI_ISL_930042, EPI_ISL_930046, EPI_ISL_930047, EPI_ISL_930048, EPI_ISL_930051, EPI_ISL_930052, EPI_ISL_930055, EPI_ISL_930056, EPI_ISL_930057, EPI_ISL_930060, EPI_ISL_930061, EPI_ISL_930066, EPI_ISL_930067, EPI_ISL_930070, EPI_ISL_930071, EPI_ISL_930072, EPI_ISL_930073, EPI_ISL_930076, EPI_ISL_930077, EPI_ISL_930078, EPI_ISL_930084, EPI_ISL_930085, EPI_ISL_930086, EPI_ISL_930087, EPI_ISL_930089, EPI_ISL_930091, EPI_ISL_930092, EPI_ISL_930094, EPI_ISL_930097, EPI_ISL_930098, EPI_ISL_930099, EPI_ISL_930101, EPI_ISL_930102, EPI_ISL_930103, EPI_ISL_930106, EPI_ISL_930113, EPI_ISL_930114, EPI_ISL_930116, EPI_ISL_930118, EPI_ISL_930119, EPI_ISL_930120, EPI_ISL_930121, EPI_ISL_930122, EPI_ISL_930124, EPI_ISL_930125, EPI_ISL_930127, EPI_ISL_930130, EPI_ISL_930131, EPI_ISL_930132, EPI_ISL_930133, EPI_ISL_930136, EPI_ISL_930139, EPI_ISL_930141, EPI_ISL_930142, EPI_ISL_930144, EPI_ISL_930145, EPI_ISL_930146, EPI_ISL_930147, EPI_ISL_930148, EPI_ISL_930149, EPI_ISL_930150, EPI_ISL_930152, EPI_ISL_930155, EPI_ISL_930159, EPI_ISL_930163, EPI_ISL_930164, EPI_ISL_930165, EPI_ISL_930167, EPI_ISL_930168, EPI_ISL_930169, EPI_ISL_930171, EPI_ISL_930175, EPI_ISL_930176, EPI_ISL_930177, EPI_ISL_930179, EPI_ISL_930182, EPI_ISL_930183, EPI_ISL_930190, EPI_ISL_930191, EPI_ISL_930192, EPI_ISL_930195, EPI_ISL_930196, EPI_ISL_930197, EPI_ISL_930198, EPI_ISL_930199, EPI_ISL_930201, EPI_ISL_930203, EPI_ISL_930204, EPI_ISL_930205, EPI_ISL_930207, EPI_ISL_930209, EPI_ISL_930213, EPI_ISL_930214, EPI_ISL_930217, EPI_ISL_930218, EPI_ISL_930226, EPI_ISL_930229, EPI_ISL_930230, EPI_ISL_930231, EPI_ISL_930232, EPI_ISL_930234, EPI_ISL_930236, EPI_ISL_930240, EPI_ISL_930241, EPI_ISL_930242, EPI_ISL_930243, EPI_ISL_930245, EPI_ISL_930246, EPI_ISL_930248, EPI_ISL_930250, EPI_ISL_930251, EPI_ISL_930256, EPI_ISL_930260, EPI_ISL_930261, EPI_ISL_930262, EPI_ISL_930264, EPI_ISL_930266, EPI_ISL_930267, EPI_ISL_930269, EPI_ISL_930271, EPI_ISL_930276, EPI_ISL_930277, EPI_ISL_930278, EPI_ISL_930279, EPI_ISL_930281, EPI_ISL_930282, EPI_ISL_930283, EPI_ISL_930287, EPI_ISL_930291, EPI_ISL_930293, EPI_ISL_930295, EPI_ISL_930297, EPI_ISL_930298, EPI_ISL_930301, EPI_ISL_930302, EPI_ISL_930304, EPI_ISL_930305, EPI_ISL_930306, EPI_ISL_930308, EPI_ISL_930309, EPI_ISL_930312, EPI_ISL_930313, EPI_ISL_930316, EPI_ISL_930317, EPI_ISL_930318, EPI_ISL_930323, EPI_ISL_930325, EPI_ISL_930326, EPI_ISL_930328, EPI_ISL_930329, EPI_ISL_930330, EPI_ISL_930331, EPI_ISL_930332, EPI_ISL_930336, EPI_ISL_930339, EPI_ISL_930340, EPI_ISL_930344, EPI_ISL_930346, EPI_ISL_930349, EPI_ISL_930350, EPI_ISL_930352, EPI_ISL_930354, EPI_ISL_930355, EPI_ISL_930357, EPI_ISL_930358, EPI_ISL_930359, EPI_ISL_930361, EPI_ISL_930362, EPI_ISL_930363, EPI_ISL_930364, EPI_ISL_930365, EPI_ISL_930366, EPI_ISL_930369, EPI_ISL_930370, EPI_ISL_930373, EPI_ISL_930374, EPI_ISL_930377, EPI_ISL_930378, EPI_ISL_930379, EPI_ISL_930380, EPI_ISL_930381, EPI_ISL_930382, EPI_ISL_930383, EPI_ISL_930384, EPI_ISL_930386, EPI_ISL_930388, EPI_ISL_930389, EPI_ISL_930393, EPI_ISL_930395, EPI_ISL_930397, EPI_ISL_930398, EPI_ISL_930400, EPI_ISL_930401, EPI_ISL_930402, EPI_ISL_930405, EPI_ISL_930408, EPI_ISL_930411, EPI_ISL_930413, EPI_ISL_930415, EPI_ISL_930416, EPI_ISL_930417, EPI_ISL_930418, EPI_ISL_930420, EPI_ISL_930424, EPI_ISL_930425, EPI_ISL_930426, EPI_ISL_930427, EPI_ISL_930428, EPI_ISL_930429, EPI_ISL_930435, EPI_ISL_930436, EPI_ISL_930437, EPI_ISL_930438, EPI_ISL_930439, EPI_ISL_930440, EPI_ISL_930441, EPI_ISL_930445, EPI_ISL_930446, EPI_ISL_930447, EPI_ISL_930448, EPI_ISL_930451, EPI_ISL_930456, EPI_ISL_930459, EPI_ISL_930462, EPI_ISL_930463, EPI_ISL_930466, EPI_ISL_930468, EPI_ISL_930469, EPI_ISL_930470, EPI_ISL_930471, EPI_ISL_930472, EPI_ISL_930473, EPI_ISL_930474, EPI_ISL_930475, EPI_ISL_930477, EPI_ISL_930479, EPI_ISL_930480, EPI_ISL_930481, EPI_ISL_930482, EPI_ISL_930483, EPI_ISL_930484, EPI_ISL_930487, EPI_ISL_930489, EPI_ISL_930492, EPI_ISL_930493, EPI_ISL_930495, EPI_ISL_930498, EPI_ISL_930500, EPI_ISL_930501, EPI_ISL_930502, EPI_ISL_930507, EPI_ISL_930508, EPI_ISL_930512, EPI_ISL_930515, EPI_ISL_930517, EPI_ISL_930520, EPI_ISL_930521, EPI_ISL_930525, EPI_ISL_930528, EPI_ISL_930530, EPI_ISL_930531, EPI_ISL_930532, EPI_ISL_930533, EPI_ISL_930534, EPI_ISL_930535, EPI_ISL_930536, EPI_ISL_930537, EPI_ISL_930538, EPI_ISL_930539, EPI_ISL_930540, EPI_ISL_930541, EPI_ISL_930542, EPI_ISL_930543, EPI_ISL_930544, EPI_ISL_930545, EPI_ISL_930548, EPI_ISL_930549, EPI_ISL_930550, EPI_ISL_930551, EPI_ISL_930555, EPI_ISL_930556, EPI_ISL_930558, EPI_ISL_930560, EPI_ISL_930561, EPI_ISL_930564, EPI_ISL_930565

see above	Department of Virus and Microbiological Special Diagnostics, Statens Serum Institut, Copenhagen, Denmark	Aalborg University	Danish Covid-19 Genome Consortium

EPI_ISL_930579, EPI_ISL_930585, EPI_ISL_930586, EPI_ISL_930587, EPI_ISL_930589, EPI_ISL_930590, EPI_ISL_930591, EPI_ISL_930592, EPI_ISL_930593, EPI_ISL_930598, EPI_ISL_930599, EPI_ISL_930600, EPI_ISL_930601, EPI_ISL_930602

see above	Synlab	GIGA Medical Genomics	Keith Durkin, Maria Artesi, Sébastien Bontems, Raphaël Boreux, Bouchra Boujemla, Cécile Meex, Pierrette Melin, Marie-Pierre Hayette, Vincent Bours
EPI_ISL_930788, EPI_ISL_930789, EPI_ISL_930791, EPI_ISL_930833, EPI_ISL_930845, EPI_ISL_930847, EPI_ISL_930848, EPI_ISL_930851	Monterey County Public Health Laboratory	Monterey County Public Health Laboratory	C. Anaya et al.,
EPI_ISL_930852	Monterey County Public Health Laboratory	Monterey County Public Health Laboratory	C. Anaya et al.

EPI_ISL_931455, EPI_ISL_931456, EPI_ISL_931457, EPI_ISL_931458, EPI_ISL_931459, EPI_ISL_931487	Maryland Public Health Laboratory (MD PHL)	Maryland Public Health Laboratory (MD PHL)	Maryland Department of Health Laboratories Administration
EPI_ISL_932257, EPI_ISL_932263, EPI_ISL_932276, EPI_ISL_932294, EPI_ISL_932319, EPI_ISL_932329, EPI_ISL_932358, EPI_ISL_932366, EPI_ISL_932372, EPI_ISL_932380, EPI_ISL_932391, EPI_ISL_932395, EPI_ISL_932403, EPI_ISL_932417, EPI_ISL_932456, EPI_ISL_932505, EPI_ISL_932507, EPI_ISL_932542, EPI_ISL_932563, EPI_ISL_932575, EPI_ISL_932627, EPI_ISL_932631, EPI_ISL_932756, EPI_ISL_932793, EPI_ISL_932874, EPI_ISL_932886, EPI_ISL_932895, EPI_ISL_932913, EPI_ISL_932974, EPI_ISL_933022, EPI_ISL_933061			
see above	Lighthouse Lab in Milton Keynes	Wellcome Sanger Institute for the COVID-19 Genomics UK (COG-UK) Consortium	The Lighthouse Lab in Milton Keynes and Alex Alderton, Roberto Amato, Sonia Goncalves, Ewan Harrison, David K. Jackson, Ian Johnston, Dominic Kwiatkowski, Cordelia Langford, John Sillitoe on behalf of the Wellcome Sanger Institute COVID-19 Surveillance Team
EPI_ISL_933113, EPI_ISL_933123, EPI_ISL_933124, EPI_ISL_933127, EPI_ISL_933130, EPI_ISL_933134, EPI_ISL_933136, EPI_ISL_933141, EPI_ISL_933143, EPI_ISL_933149, EPI_ISL_933150, EPI_ISL_933154, EPI_ISL_933158, EPI_ISL_933166, EPI_ISL_933167, EPI_ISL_933170, EPI_ISL_933171, EPI_ISL_933181, EPI_ISL_933187, EPI_ISL_933188, EPI_ISL_933196, EPI_ISL_933199, EPI_ISL_933200, EPI_ISL_933207, EPI_ISL_933213, EPI_ISL_933214, EPI_ISL_933215, EPI_ISL_933221, EPI_ISL_933226, EPI_ISL_933227, EPI_ISL_933228, EPI_ISL_933233, EPI_ISL_933239, EPI_ISL_933243, EPI_ISL_933256, EPI_ISL_933259, EPI_ISL_933265, EPI_ISL_933266, EPI_ISL_933271, EPI_ISL_933272, EPI_ISL_933273, EPI_ISL_933277, EPI_ISL_933282, EPI_ISL_933283, EPI_ISL_933289, EPI_ISL_933293, EPI_ISL_933298, EPI_ISL_933303, EPI_ISL_933321, EPI_ISL_933325, EPI_ISL_933326, EPI_ISL_933336, EPI_ISL_933340, EPI_ISL_933341, EPI_ISL_933343, EPI_ISL_933344, EPI_ISL_933345, EPI_ISL_933346, EPI_ISL_933348, EPI_ISL_933350, EPI_ISL_933354, EPI_ISL_933358, EPI_ISL_933359, EPI_ISL_933364, EPI_ISL_933371, EPI_ISL_933375, EPI_ISL_933382, EPI_ISL_933386, EPI_ISL_933387, EPI_ISL_933388, EPI_ISL_933393, EPI_ISL_933396, EPI_ISL_933397, EPI_ISL_933400, EPI_ISL_933401, EPI_ISL_933410, EPI_ISL_933412, EPI_ISL_933414, EPI_ISL_933417			
see above	Lighthouse Lab in Cambridge	Wellcome Sanger Institute for the COVID-19 Genomics UK (COG-UK) Consortium	Rob Howes, The Lighthouse Lab in Cambridge and Alex Alderton, Roberto Amato, Sonia Goncalves, Ewan Harrison, David K. Jackson, Ian Johnston, Dominic Kwiatkowski, Cordelia Langford, John Sillitoe on behalf of the Wellcome Sanger Institute COVID-19 Surveillance Team
EPI_ISL_933730, EPI_ISL_933731, EPI_ISL_933733	Servicio de Microbiología Hospital Ramón y Cajal	Servicio de Microbiología Hospital Ramón y Cajal	José M Gonzalez-Alba, Concepción Rodríguez, Melanie Abreu, Laura Martínez, Val F Lanza, Luz Leticia Olavarrieta, Rafael Cantón, JC Galán
EPI_ISL_933736, EPI_ISL_933737, EPI_ISL_933738, EPI_ISL_933762, EPI_ISL_933763	DPHL	Delaware Public Health Lab	Gregory Hovan
EPI_ISL_933767	Servicio de Microbiología Hospital Ramón y Cajal	Servicio de Microbiología Hospital Ramón y Cajal	José M Gonzalez-Alba, Concepción Rodríguez, Melanie Abreu, Laura Martínez, Val F Lanza, Luz Leticia Olavarrieta, Rafael Cantón, JC Galán
EPI_ISL_933790	PathWest Laboratory Medicine WA	PathWest Laboratory Medicine WA Microbial Surveillance Unit	PathWest Laboratory Medicine WA Microbial Surveillance Unit
EPI_ISL_934308, EPI_ISL_934309, EPI_ISL_934310, EPI_ISL_934311, EPI_ISL_934312, EPI_ISL_934313	Vilnius university hospital Santaros Klinikos, Center of Laboratory Medicine	Vilnius university hospital Santaros Klinikos, Center of Laboratory Medicine	Ingrida Olendraite, Daniel Naumovas, Rimvydas Norvilas, Dovile Ezerskyte, Justinas Slikas, Gytis Dudas
EPI_ISL_934382, EPI_ISL_934383, EPI_ISL_934387	Klinisk mikrobiologi	The Public Health Agency of Sweden	Anna-Malin Linde, Maria Lind Karlberg, Carlo Berg, Oskar Karlsson Lindsjo, Sofia Stamouli, Reza Advani, Mattias Haukland, Petra Holmstrom, Noura Walai, Petra Edquist, Mia Brytting, Anna Risberg, Karin Tegmark-Wisell
EPI_ISL_934391, EPI_ISL_934392, EPI_ISL_934393, EPI_ISL_934394, EPI_ISL_934396	Klinisk Mikrobiologi	The Public Health Agency of Sweden	Anna-Malin Linde, Maria Lind Karlberg, Carlo Berg, Oskar Karlsson Lindsjo, Sofia Stamouli, Reza Advani, Mattias Haukland, Petra Holmstrom, Noura Walai, Petra Edquist, Mia Brytting, Anna Risberg, Karin Tegmark-Wisell
EPI_ISL_934979, EPI_ISL_934985, EPI_ISL_934988, EPI_ISL_934992	ADMED Microbiologie	Genomics and Transcriptomics, Philip Morris International	Reto Lienhard, Marie-Lise Tritten, Emmanuel Guedj, Nicolas Sierro, Rémi Dulize, David Bornand, Mehdi Auberson, Maxime Berthouzoz, Nikolai Ivanov, Manuel Peitsch
EPI_ISL_935048, EPI_ISL_935049, EPI_ISL_935050, EPI_ISL_935051, EPI_ISL_935052, EPI_ISL_935053, EPI_ISL_935054, EPI_ISL_935055, EPI_ISL_935056, EPI_ISL_935057, EPI_ISL_935058, EPI_ISL_935059, EPI_ISL_935060, EPI_ISL_935061, EPI_ISL_935062, EPI_ISL_935063, EPI_ISL_935064, EPI_ISL_935065, EPI_ISL_935066, EPI_ISL_935067, EPI_ISL_935068, EPI_ISL_935069			
see above	Ministry of Health Turkey	Ministry of Health Turkey	Fatma Bayrakdar, Yasemin Cogun, Süleyman Yalcin, Aye Baak Alta, Gülay Korukluolu
EPI_ISL_935187, EPI_ISL_935188, EPI_ISL_935189, EPI_ISL_935190, EPI_ISL_935191, EPI_ISL_935192, EPI_ISL_935193, EPI_ISL_935194, EPI_ISL_935198, EPI_ISL_935201, EPI_ISL_935227, EPI_ISL_935228, EPI_ISL_935229, EPI_ISL_935230, EPI_ISL_935231, EPI_ISL_935232, EPI_ISL_935233, EPI_ISL_935234, EPI_ISL_935235, EPI_ISL_935236, EPI_ISL_935244, EPI_ISL_935245, EPI_ISL_935246, EPI_ISL_935247, EPI_ISL_935248, EPI_ISL_935249, EPI_ISL_935250, EPI_ISL_935251, EPI_ISL_935252, EPI_ISL_935253, EPI_ISL_935254, EPI_ISL_935255, EPI_ISL_935256, EPI_ISL_935257, EPI_ISL_935258, EPI_ISL_935259, EPI_ISL_935260, EPI_ISL_935261, EPI_ISL_935262, EPI_ISL_935263, EPI_ISL_935281, EPI_ISL_935282, EPI_ISL_935283, EPI_ISL_935326, EPI_ISL_935329			
see above	KU Leuven, Rega Institute, Clinical and Epidemiological Virology	KU Leuven, Rega Institute, Clinical and Epidemiological Virology	Tony Wawina-Bokalanga, Bert Vanmechelen, Joan Marti-Carerras, Piet Maes
EPI_ISL_935533	Labo Analyses Med	National Reference Center for Viruses of Respiratory Infections, Institut Pasteur, Paris	Marion Barbet, Sylvie Behillil, Méline Bizard, Angela Brisebarre, Camille Capel, Etienne Simon-Lorière, Vincent Enouf, Maud Vanpeene, Sylvie van der Werf,Jacques Alexandra
EPI_ISL_935534, EPI_ISL_935535	Labo Analyses Med	National Reference Center for Viruses of Respiratory Infections, Institut Pasteur, Paris	Marion Barbet, Sylvie Behillil, Méline Bizard, Angela Brisebarre, Camille Capel, Etienne Simon-Lorière, Vincent Enouf, Maud Vanpeene, Sylvie van der Werf,Garel FréDéRick
EPI_ISL_935536	Labo Analyses Med	National Reference Center for Viruses of Respiratory Infections, Institut Pasteur, Paris	Marion Barbet, Sylvie Behillil, Méline Bizard, Angela Brisebarre, Camille Capel, Etienne Simon-Lorière, Vincent Enouf, Maud Vanpeene, Sylvie van der Werf,Wittersheim Pascal
EPI_ISL_935546	Labo Analyses Med	National Reference Center for Viruses of Respiratory Infections, Institut Pasteur, Paris	Marion Barbet, Sylvie Behillil, Méline Bizard, Angela Brisebarre, Camille Capel, Etienne Simon-Lorière, Vincent Enouf, Maud Vanpeene, Sylvie van der Werf,Besson J
EPI_ISL_935549	Labo Analyses Med	National Reference Center for Viruses of Respiratory Infections, Institut Pasteur, Paris	Marion Barbet, Sylvie Behillil, Méline Bizard, Angela Brisebarre, Camille Capel, Etienne Simon-Lorière, Vincent Enouf, Maud Vanpeene, Sylvie van der Werf
EPI_ISL_935558, EPI_ISL_935559	Hopital	National Reference Center for Viruses of Respiratory Infections, Institut Pasteur, Paris	Marion Barbet, Sylvie Behillil, Méline Bizard, Angela Brisebarre, Camille Capel, Etienne Simon-Lorière, Vincent Enouf, Maud Vanpeene, Sylvie van der Werf
EPI_ISL_935561	Hopital	National Reference Center for Viruses of Respiratory Infections, Institut Pasteur, Paris	Marion Barbet, Sylvie Behillil, Méline Bizard, Angela Brisebarre, Camille Capel, Etienne Simon-Lorière, Vincent Enouf, Maud Vanpeene, Sylvie van der Werf,Gaschet Anne
EPI_ISL_935563	Labo Analyses Med	National Reference Center for Viruses of Respiratory Infections, Institut Pasteur, Paris	Marion Barbet, Sylvie Behillil, Méline Bizard, Angela Brisebarre, Camille Capel, Etienne Simon-Lorière, Vincent Enouf, Maud Vanpeene, Sylvie van der Werf
EPI_ISL_935569	Hopital	National Reference Center for Viruses of Respiratory Infections, Institut Pasteur, Paris	Marion Barbet, Sylvie Behillil, Méline Bizard, Angela Brisebarre, Camille Capel, Etienne Simon-Lorière, Vincent Enouf, Maud Vanpeene, Sylvie van der Werf,Bret Laurent
EPI_ISL_935576	Labo Analyses Med	National Reference Center for Viruses of Respiratory Infections, Institut Pasteur, Paris	Marion Barbet, Sylvie Behillil, Méline Bizard, Angela Brisebarre, Camille Capel, Etienne Simon-Lorière, Vincent Enouf, Maud Vanpeene, Sylvie van der Werf,Breton-Cazaux JéRôMe
EPI_ISL_935577, EPI_ISL_935578, EPI_ISL_935579, EPI_ISL_935580	Hopital	National Reference Center for Viruses of Respiratory Infections, Institut Pasteur, Paris	Marion Barbet, Sylvie Behillil, Méline Bizard, Angela Brisebarre, Camille Capel, Etienne Simon-Lorière, Vincent Enouf, Maud Vanpeene, Sylvie van der Werf,Brichler SéGolèNe
EPI_ISL_935586	Hopital	National Reference Center for Viruses of Respiratory Infections, Institut Pasteur, Paris	Marion Barbet, Sylvie Behillil, Méline Bizard, Angela Brisebarre, Camille Capel, Etienne Simon-Lorière, Vincent Enouf, Maud Vanpeene, Sylvie van der Werf,Fourgeaud Jacques
EPI_ISL_935590	Labo Analyses Med	National Reference Center for Viruses of Respiratory Infections, Institut Pasteur, Paris	Marion Barbet, Sylvie Behillil, Méline Bizard, Angela Brisebarre, Camille Capel, Etienne Simon-Lorière, Vincent Enouf, Maud Vanpeene, Sylvie van der Werf,Amzalag Jonas
EPI_ISL_935591, EPI_ISL_935592, EPI_ISL_935593, EPI_ISL_935594, EPI_ISL_935599, EPI_ISL_935604	Labo Analyses med	National Reference Center for Viruses of Respiratory Infections, Institut Pasteur, Paris	Marion Barbet, Sylvie Behillil, Méline Bizard, Angela Brisebarre, Camille Capel, Etienne Simon-Lorière, Vincent Enouf, Maud Vanpeene, Sylvie van der Werf,Amzalag Jonas
EPI_ISL_935605, EPI_ISL_935606	Labo Analyses Med	National Reference Center for Viruses of Respiratory Infections, Institut Pasteur, Paris	Marion Barbet, Sylvie Behillil, Méline Bizard, Angela Brisebarre, Camille Capel, Etienne Simon-Lorière, Vincent Enouf, Maud Vanpeene, Sylvie van der Werf,Amzalag Jonas
EPI_ISL_935607, EPI_ISL_935608, EPI_ISL_935609, EPI_ISL_935610, EPI_ISL_935611, EPI_ISL_935612, EPI_ISL_935613, EPI_ISL_935614, EPI_ISL_935615, EPI_ISL_935616, EPI_ISL_935617, EPI_ISL_935618, EPI_ISL_935619, EPI_ISL_935620, EPI_ISL_935629, EPI_ISL_935630, EPI_ISL_935631, EPI_ISL_935632,			

EPI_ISL_935633, EPI_ISL_935634, EPI_ISL_935635, EPI_ISL_935636			
see above	Labo Analyses med	National Reference Center for Viruses of Respiratory Infections, Institut Pasteur, Paris	Marion Barbet, Sylvie Behillil, Méline Bizard, Angela Brisebarre, Camille Capel, Etienne Simon-Lorière, Vincent Enouf, Maud Vanpeene, Sylvie van der Werf,Amzalag Jonas
EPI_ISL_935637	Labo Analyses Med	National Reference Center for Viruses of Respiratory Infections, Institut Pasteur, Paris	Marion Barbet, Sylvie Behillil, Méline Bizard, Angela Brisebarre, Camille Capel, Etienne Simon-Lorière, Vincent Enouf, Maud Vanpeene, Sylvie van der Werf,Jacques Alexandra
EPI_ISL_935639	Hopital	National Reference Center for Viruses of Respiratory Infections, Institut Pasteur, Paris	Marion Barbet, Sylvie Behillil, Méline Bizard, Angela Brisebarre, Camille Capel, Etienne Simon-Lorière, Vincent Enouf, Maud Vanpeene, Sylvie van der Werf,Breuil J.
EPI_ISL_935642, EPI_ISL_935645, EPI_ISL_935646, EPI_ISL_935647, EPI_ISL_935648, EPI_ISL_935649	Labo Analyses Med	National Reference Center for Viruses of Respiratory Infections, Institut Pasteur, Paris	Marion Barbet, Sylvie Behillil, Méline Bizard, Angela Brisebarre, Camille Capel, Etienne Simon-Lorière, Vincent Enouf, Maud Vanpeene, Sylvie van der Werf
EPI_ISL_936252, EPI_ISL_936254, EPI_ISL_936255, EPI_ISL_936256, EPI_ISL_936257, EPI_ISL_936262, EPI_ISL_936263, EPI_ISL_936268, EPI_ISL_936270, EPI_ISL_936274, EPI_ISL_936276, EPI_ISL_936277, EPI_ISL_936278, EPI_ISL_936280, EPI_ISL_936283, EPI_ISL_936284, EPI_ISL_936291, EPI_ISL_936294, EPI_ISL_936295, EPI_ISL_936298			
see above	MONTEFIORE MEDICAL CENTER LABORATORIES	Wadsworth Center, New York State Department of Health	Kirsten St. George, Daryl M. Lamson, Alexis Russel, Matthew Shudt, Melissa A Leisner, Jonathan Plitnick, Navjot Singh, John Kelly, Erasmus Schneider, Erica Lasek-Nesselquist
EPI_ISL_936478, EPI_ISL_936480, EPI_ISL_936481	DPH, Massachusetts State Public Health Lab	DPH, Massachusetts State Public Health Lab	Lang,A.S., Fink,T., Gallagher,G.R., Smole,S.C.
EPI_ISL_937126, EPI_ISL_937127, EPI_ISL_937128	DOHMH Riverside	New York City Public Health Laboratory	Jade Wang, et al.
EPI_ISL_937129, EPI_ISL_937130, EPI_ISL_937131	DOHMH Morrisania	New York City Public Health Laboratory	Jade Wang, et al.
EPI_ISL_937132, EPI_ISL_937133	DOHMH Central Harlem	New York City Public Health Laboratory	Jade Wang, et al.
EPI_ISL_937134, EPI_ISL_937135	DOHMH Fort Greene	New York City Public Health Laboratory	Jade Wang, et al.
EPI_ISL_937136, EPI_ISL_937137	DOHMH Chelsea	New York City Public Health Laboratory	Jade Wang, et al.
EPI_ISL_937138	DOHMH Crown Heights	New York City Public Health Laboratory	Jade Wang, et al.
EPI_ISL_937139, EPI_ISL_937140, EPI_ISL_937141, EPI_ISL_937142, EPI_ISL_937143, EPI_ISL_937144	DOHMH Corona	New York City Public Health Laboratory	Jade Wang, et al.
EPI_ISL_937145, EPI_ISL_937146, EPI_ISL_937147, EPI_ISL_937148, EPI_ISL_937149, EPI_ISL_937150, EPI_ISL_937151	DOHMH Jamaica	New York City Public Health Laboratory	Jade Wang, et al.
EPI_ISL_937152, EPI_ISL_937153	DOHMH PHL	New York City Public Health Laboratory	Jade Wang, et al.
EPI_ISL_937167	DOHMH Central Harlem	New York City Public Health Laboratory	Jade Wang, et al.
EPI_ISL_937168	DOHMH Crown Heights	New York City Public Health Laboratory	Jade Wang, et al.
EPI_ISL_937188	OCME Office Of Chief Medical Examiner	New York City Public Health Laboratory	Jade Wang, et al.
EPI_ISL_937191	Department of Homeless Services	New York City Public Health Laboratory	Jade Wang, et al.
EPI_ISL_937193, EPI_ISL_937194	OCME Office Of Chief Medical Examiner	New York City Public Health Laboratory	Jade Wang, et al.
EPI_ISL_937383, EPI_ISL_937384, EPI_ISL_937388, EPI_ISL_937389, EPI_ISL_937390, EPI_ISL_937391, EPI_ISL_937414, EPI_ISL_937415, EPI_ISL_937416, EPI_ISL_937417, EPI_ISL_937418, EPI_ISL_937419, EPI_ISL_937420, EPI_ISL_937428, EPI_ISL_937429, EPI_ISL_937430, EPI_ISL_937431, EPI_ISL_937432, EPI_ISL_937433, EPI_ISL_937434, EPI_ISL_937438, EPI_ISL_937439, EPI_ISL_937440, EPI_ISL_937441, EPI_ISL_937442, EPI_ISL_937443, EPI_ISL_937444, EPI_ISL_937449, EPI_ISL_937450, EPI_ISL_937451, EPI_ISL_937452, EPI_ISL_937453, EPI_ISL_937454, EPI_ISL_937455, EPI_ISL_937456			
see above	Maine Health and Environmental Testing Laboratory (Maine HETL)	Tewhey Lab, The Jackson Laboratory	Matluk,N., Dewey,H., Iosue,F., Barter,M., Lynch,R., Munger,H. and Tewhey,R.
EPI_ISL_938057, EPI_ISL_938120, EPI_ISL_938151	Lighthouse Lab in Milton Keynes	Wellcome Sanger Institute for the COVID-19 Genomics UK (COG-UK) Consortium	The Lighthouse Lab in Milton Keynes and Alex Alderton, Roberto Amato, Sonia Goncalves, Ewan Harrison, David K. Jackson, Ian Johnston, Dominic Kwiatkowski, Cordelia Langford, John Sillitoe on behalf of the Wellcome Sanger Institute COVID-19 Surveillance Team
EPI_ISL_939630, EPI_ISL_939631, EPI_ISL_939632, EPI_ISL_939633, EPI_ISL_939634, EPI_ISL_939635, EPI_ISL_939637, EPI_ISL_939638, EPI_ISL_939639	MEPHI, Aix Marseille University	MEPHI, Aix Marseille University	Anthony LEVASSEUR
EPI_ISL_940069, EPI_ISL_940070	University Hospitals of Geneva, Laboratory of Virology	HUG, Laboratory of Virology and the Health2030 Genome Center	Samuel Cordey, Ana Rita Goncalves, Laurent Kaiser, Lorenzo Cerutti, Henri Pegeot, Melyssa Elies, Deborah Penet, Keith Harshman, Ioannis Xenarios, Emmanouil Dermitzakis
EPI_ISL_940564	Servizo de Microbioloxía. Complexo Hospitalario Universitario de Santiago de Compostela	Servizo de Microbioloxía. Complexo Hospitalario Universitario de Santiago de Compostela	Antonio Aguilera, Gema Barbeito, Amparo Coira, Rocio Trastoy, María Luisa Pérez del Molino
EPI_ISL_940565	University of Bari Biomedical Sciences and Human Oncology	University of Bari Biomedical Sciences and Human Oncology	Chironna M., Sallustio A., Loconsole D., Accogli M.
EPI_ISL_940609	Hospital e Maternidade Celso Pierro	Instituto Adolfo Lutz, Interdiciplinary Procedures Center, Strategic Laboratory	Claudio Tavares Sacchi, Claudia Regina Gonçalves, Erica Valessa Ramos Gomes, Karoline Rodrigues Campos
EPI_ISL_940613, EPI_ISL_940614, EPI_ISL_940615, EPI_ISL_940616, EPI_ISL_940617, EPI_ISL_940618	LACEN-PI DR. Costa Alvarenga	Instituto Adolfo Lutz, Interdiciplinary Procedures Center, Strategic Laboratory	Claudio Tavares Sacchi, Claudia Regina Gonçalves, Erica Valessa Ramos Gomes, Karoline Rodrigues Campos
EPI_ISL_940625	Hospital Sao Joaquim - Beneficiencia Portuguesa	Instituto Adolfo Lutz, Interdiciplinary Procedures Center, Strategic Laboratory	Claudio Tavares Sacchi, Claudia Regina Gonçalves, Erica Valessa Ramos Gomes, Karoline Rodrigues Campos
EPI_ISL_940631, EPI_ISL_940632	University of Bari Biomedical Sciences and Human Oncology	University of Bari Biomedical Sciences and Human Oncology	Chironna M., Sallustio A., Loconsole D., Accogli M.
EPI_ISL_940644, EPI_ISL_940645, EPI_ISL_940646, EPI_ISL_940647, EPI_ISL_940648, EPI_ISL_940649, EPI_ISL_940650, EPI_ISL_940651, EPI_ISL_940652, EPI_ISL_940653	Ministry of Health Turkey	Ministry of Health Turkey	Fatma Bayrakdar, Yasemin Cogun, Süleyman Yalcin, Aye Baak Alta, Gülay Korukluolu
EPI_ISL_940683	Servizo de Microbioloxía. Complexo Hospitalario Universitario de Santiago de Compostela	Servizo de Microbioloxía. Complexo Hospitalario Universitario de Santiago de Compostela	Antonio Aguilera, Gema Barbeito, Amparo Coira, Rocio Trastoy, María Luisa Pérez del Molino
EPI_ISL_940712	Ministry of Health Turkey	Ministry of Health Turkey	Fatma Bayrakdar, Yasemin Cogun, Süleyman Yalcin, Aye Baak Alta, Gülay Korukluolu
EPI_ISL_940757	GZA Sint-Augustinus Hospital	UAntwerp, Laboratory of Medical Microbiology, Campus Drie	Basil Britto Xavier, Jasmine Coppens, Marie Le Mercier, Christine Lammens, Veerle Matheeussen, Herman Goossens

		Eiken S6.26, Universiteitsplein 1, 2610, Wilrijk, Belgium	
EPI_ISL_940762	Botswana Harvard HIV Reference Laboratory	Botswana Harvard HIV Reference Laboratory	Sikhulile Moyo, Wonderful Choga, Dorcas Maruapula, Botshelo Radibe, Boitumelo Zuze, David Lawrence, Roger Shapiro, Shahin Lockman, Mosepele Mosepele, Joseph, Makhema, Simani Gaseitsiwe
EPI_ISL_940763	ZNA Middelheim	UAntwerp, Laboratory of Medical Microbiology, Campus Drie Eiken S6.26, Universiteitsplein 1, 2610, Wilrijk, Belgium	Basil Britto Xavier, Jasmine Coppens, Marie Le Mercier, Christine Lammens, Veerle Matheeussen, Herman Goossens
EPI_ISL_940784, EPI_ISL_940786, EPI_ISL_940788, EPI_ISL_940789, EPI_ISL_940790, EPI_ISL_940791	City of Milwaukee Health Department Laboratory	City of Milwaukee Health Department Laboratory	Sanjib Bhattacharyya
EPI_ISL_940843, EPI_ISL_940847, EPI_ISL_940849	Platform BIS UZA/UAntwerpen	UAntwerp, Laboratory of Medical Microbiology	Basil Britto Xavier, Jasmine Coppens, Marie Le Mercier, Christine Lammens, Veerle Matheeussen, Herman Goossens
EPI_ISL_940891	Vaccines and Infectious Diseases Analytics Research Unit (VIDA)	KRISP, KZN Research Innovation and Sequencing Platform	Baillie Vicky, du Plessis Jeanine, Giandhari Jennifer, Pillay Sureshnee, Naidoo Yeshnee, Tegally Houriiyah, de Oliveira Tulio, Madhi Shabir
EPI_ISL_940893	Platform BIS UZA/UAntwerpen	UAntwerp, Laboratory of Medical Microbiology, Campus Drie Eiken S6.26, Universiteitsplein 1, 2610, Wilrijk, Belgium	Basil Britto Xavier, Jasmine Coppens, Marie Le Mercier, Christine Lammens, Veerle Matheeussen, Herman Goossens
EPI_ISL_940996	Sentinelles Idf	National Reference Center for Viruses of Respiratory Infections, Institut Pasteur, Paris	Marion Barbet, Sylvie Behillil, Méline Bizard, Angela Brisebarre, Camille Capel, Etienne Simon-Lorière, Vincent Enouf, Maud Vanpeene, Sylvie van der Werf, Garrigues Anne
EPI_ISL_941026	Labo Analyses Med	National Reference Center for Viruses of Respiratory Infections, Institut Pasteur, Paris	Marion Barbet, Sylvie Behillil, Méline Bizard, Angela Brisebarre, Camille Capel, Etienne Simon-Lorière, Vincent Enouf, Maud Vanpeene, Sylvie van der Werf, Griscelli Franck
EPI_ISL_941027	Hopital	National Reference Center for Viruses of Respiratory Infections, Institut Pasteur, Paris	Marion Barbet, Sylvie Behillil, Méline Bizard, Angela Brisebarre, Camille Capel, Etienne Simon-Lorière, Vincent Enouf, Maud Vanpeene, Sylvie van der Werf, Marque Juillet StéPhanie
EPI_ISL_941035, EPI_ISL_941036, EPI_ISL_941037, EPI_ISL_941040, EPI_ISL_941041, EPI_ISL_941058, EPI_ISL_941063, EPI_ISL_941065, EPI_ISL_941071	Labo Analyses Med	National Reference Center for Viruses of Respiratory Infections, Institut Pasteur, Paris	Marion Barbet, Sylvie Behillil, Méline Bizard, Angela Brisebarre, Camille Capel, Etienne Simon-Lorière, Vincent Enouf, Maud Vanpeene, Sylvie van der Werf, Merah Kader
EPI_ISL_941296, EPI_ISL_941297	Nigeria Centre for Disease Control (NCDC)	African Centre of Excellence for Genomics of Infectious Diseases (ACEGID), Redeemer's University	Oluniyi P.E. et al
EPI_ISL_941437, EPI_ISL_941438, EPI_ISL_941439, EPI_ISL_941440, EPI_ISL_941441, EPI_ISL_941610, EPI_ISL_941611	Instituto Nacional de Saude (INSA)	Instituto Nacional de Saude (INSA)	Borges et al
EPI_ISL_941842, EPI_ISL_941845, EPI_ISL_941849, EPI_ISL_941850, EPI_ISL_941852, EPI_ISL_941853, EPI_ISL_941854, EPI_ISL_941855, EPI_ISL_941856, EPI_ISL_941857, EPI_ISL_941858, EPI_ISL_941859, EPI_ISL_941867			
see above	Instituto Nacional de Saude (INSA) and Instituto Gulbenkian de Ciencia (IGC)	Instituto Nacional de Saude (INSA) and Instituto Gulbenkian de Ciencia (IGC)	Borges et al
EPI_ISL_942376, EPI_ISL_942406	Viollier AG	University Hospital Basel, Clinical Bacteriology	Tim Roloff, Madlen Stange, Helena MB Seth-Smith, Alfredo Mari, Karoline Leuzinger, Julia Bielicki, Christiane Beckmann, Manuel Battegay, Hans Hirsch, Adrian Egli
EPI_ISL_942515	University Hospital Basel, Clinical Virology	University Hospital Basel, Clinical Bacteriology	Tim Roloff, Madlen Stange, Helena MB Seth-Smith, Alfredo Mari, Karoline Leuzinger, Julia Bielicki, Manuel Battegay, Hans Hirsch, Adrian Egli
EPI_ISL_942851, EPI_ISL_942852, EPI_ISL_942853, EPI_ISL_942854, EPI_ISL_942855, EPI_ISL_942856, EPI_ISL_942857, EPI_ISL_942858, EPI_ISL_942859, EPI_ISL_942860, EPI_ISL_942861			
see above	Gundersen Molecular Diagnostics Laboratory	Kabara Cancer Research Institute	Craig S. Richmond, Paraic A. Kenny
EPI_ISL_942927	LESP Nuevo Leon/Grupo de Diagnostico ARIES	Instituto de Diagnostico y Referencia Epidemiologicos (INDRE)	Claudia Wong-Arambula, Abril Rodriguez-Maldonado, Fabiola Garces-Ayala, Natividad Cruz-Ortiz, Tatiana Nunez-Garcia, Gisela Barrera-Badillo, Lucia Hernandez-Rivas, Irma Lopez-Martinez, Ernesto Ramirez-Gonzalez.
EPI_ISL_942946	Gundersen Molecular Diagnostics Laboratory	Kabara Cancer Research Institute	Craig S. Richmond, Paraic A. Kenny
EPI_ISL_942972, EPI_ISL_942973, EPI_ISL_942974	Servizo de Microbioloxía. Complexo Hospitalario Universitario de Santiago de Compostela	Servizo de Microbioloxía. Complexo Hospitalario Universitario de Santiago de Compostela	Antonio Aguilera, Gema Barbeito, Amparo Coira, José Costa, Rocio Trastoy, María Luisa Pérez del Molino.
EPI_ISL_942998, EPI_ISL_943027, EPI_ISL_943042, EPI_ISL_943043, EPI_ISL_943048, EPI_ISL_943060, EPI_ISL_943061, EPI_ISL_943062, EPI_ISL_943081, EPI_ISL_943083, EPI_ISL_943140, EPI_ISL_943146, EPI_ISL_943168, EPI_ISL_943169, EPI_ISL_943170, EPI_ISL_943171, EPI_ISL_943172, EPI_ISL_943175, EPI_ISL_943178, EPI_ISL_943179, EPI_ISL_943180, EPI_ISL_943237, EPI_ISL_943238, EPI_ISL_943239, EPI_ISL_943259, EPI_ISL_943261, EPI_ISL_943273, EPI_ISL_943285, EPI_ISL_943311, EPI_ISL_943312, EPI_ISL_943315, EPI_ISL_943323, EPI_ISL_943324, EPI_ISL_943325, EPI_ISL_943352, EPI_ISL_943378, EPI_ISL_943385, EPI_ISL_943388, EPI_ISL_943389, EPI_ISL_943391, EPI_ISL_943392, EPI_ISL_943440, EPI_ISL_943470, EPI_ISL_943471, EPI_ISL_943472, EPI_ISL_943476, EPI_ISL_943487, EPI_ISL_943488, EPI_ISL_943517, EPI_ISL_943546			
see above	Dutch COVID-19 response team	National Institute for Public Health and the Environment (RIVM)	Adam Meijer, Harry Vennema, Dirk Eggink, Jeroen Cremer, Sharon van den Brink, Bas van der Veer, AnneMarie van den Brandt, Florian Zwagemaker, Dennis Schmitz, Chantal Reusken, on behalf of the national COVID-19 response team
EPI_ISL_943549	National Institute of Laboratory Medicine and Referral Center	Genomic Research Lab, BCSIR	Md. Murshed Hasan Sarkar, Mohammad Samir Uzzaman,Eshrar Osman, Md. Ahashan Habib,Shahina Akter, Tanjina Akhtar Banu,Abu Sayeed Mohammad Mahmud,Barna Goswami,Iffat Jahan, Md. Saddam Hossain, Tasnim Nafisa, Md. Maruf Ahmed Molla, Mahmuda Yeasmin, Asish Kumar Ghosh, Arifa Akram, A. K. M. Shamsuzzaman,Md. Salim Khan
EPI_ISL_943554	National Institute of Laboratory Medicine and Referral Center	Genomic Research Lab, BCSIR	Barna Goswami,Mohammad Samir Uzzaman, Eshrar Osman, Md. Ahashan Habib,Shahina Akter, Tanjina Akhter Banu, Abu Sayeed Mohammad Mahmud, Md. Murshed Hasan Sarkar,Iffat Jahan, Md. Saddam Hossain, Tasnim Nafisa, Md. Maruf Ahmed Molla, Mahmuda Yeasmin, Asish Kumar Ghosh,Arifa Akram, A. K. M. Shamsuzzaman, Md. Salim Khan
EPI_ISL_943556, EPI_ISL_943558	Servizo de Microbioloxía. Complexo Hospitalario Universitario de Santiago de Compostela	Servizo de Microbioloxía. Complexo Hospitalario Universitario de Santiago de Compostela	Antonio Aguilera, Gema Barbeito, Amparo Coira, José Costa, Rocio Trastoy, María Luisa Pérez del Molino.
EPI_ISL_943559	National Institute of Laboratory Medicine and Referral Center	Genomic Research Lab, BCSIR	Shahina Akter, Mohammad Samir Uzzaman, Eshrar Osman, Md. Ahashan Habib,Tanjina Akhtar Banu, Abu Sayeed Mohammad Mahmud, Md. Murshed Hasan Sarkar, Barna Goswami, Iffat Jahan, Md. Saddam Hossain, Tasnim Nafisa, Md. Maruf Ahmed Molla, Mahmuda Yeasmin, Asish Kumar Ghosh, Arifa Akram, A. K. M. Shamsuzzaman, Md. Salim Khan
EPI_ISL_943560, EPI_ISL_943562	Servizo de Microbioloxía. Complexo Hospitalario Universitario de Santiago de Compostela	Servizo de Microbioloxía. Complexo Hospitalario Universitario de Santiago de Compostela	Antonio Aguilera, Gema Barbeito, Amparo Coira, José Costa, Rocio Trastoy, María Luisa Pérez del Molino.
EPI_ISL_943563	National Institute of Laboratory Medicine and Referral Center	Genomic Research Lab, BCSIR	Md. Saddam Hossain,Mohammad Samir Uzzaman, Eshrar Osman, Md. Ahashan Habib,Shahina Akter,Tanjina Akhter Banu, Abu Sayeed Mohammad Mahmud, Md. Murshed Hasan Sarkar, Barna Goswami, Iffat Jahan, Tasnim Nafisa, Md. Maruf Ahmed Molla, Mahmuda Yeasmin, Asish Kumar Ghosh, Arifa Akram, A. K. M. Shamsuzzaman, Md. Salim Khan
EPI_ISL_943572	National Institute of Laboratory Medicine and Referral Center	Genomic Research Lab, BCSIR	Md. Maruf Ahmed Molla,Mohammad Samir Uzzaman, Eshrar Osman, Md. Ahashan Habib, Shahina Akter,Tanjina Akhtar Banu, Abu Sayeed Mohammad Mahmud, Md. Murshed Hasan Sarkar, Barna Goswami, Iffat Jahan, Md. Saddam Hossain, Tasnim Nafisa,Mahmuda Yeasmin, Asish Kumar Ghosh,Arifa Akram, A. K. M. Shamsuzzaman,Md. Salim Khan
EPI_ISL_943814, EPI_ISL_943815, EPI_ISL_943816, EPI_ISL_943817, EPI_ISL_943930, EPI_ISL_943934, EPI_ISL_943937	Utah Public Health Laboratory	Utah Public Health Laboratory	Erin L. Young, Kelly F. Oakeson, Tara Gallagher

EPI_ISL_943990	LACEN do Estado de Goias	Instituto Adolfo Lutz, Interdiciplinary Procedures Center, Strategic Laboratory	Claudio Tavares Sacchi, Claudia Regina Gonçalves, Erica Valessa Ramos Gomes, Karoline Rodrigues Campos
EPI_ISL_944590	Botswana Harvard HIV Reference Laboratory	Botswana Harvard HIV Reference Laboratory	Sikhulile Moyo, Wonderful T. Choga, Dorcas Maruapula, Botshelo Radibe, Boitumelo Zuze, David Lawrence, Roger Shapiro, Shahin Lockman, Mosepele Mosepele, Joseph Makhema, Simani Gaseitsiwe
EPI_ISL_944740, EPI_ISL_944741, EPI_ISL_944742, EPI_ISL_944743	unknown	Public Health Virology-Forensic and Scientific Services (PHV-FSS)	Son Nguyen et al.
EPI_ISL_944782	Botswana Harvard HIV Reference Laboratory	Botswana Harvard HIV Reference Laboratory	Sikhulile Moyo, Wonderful T. Choga, Dorcas Maruapula, Botshelo Radibe, Boitumelo Zuze, David Lawrence, Roger Shapiro, Shahin Lockman, Mosepele Mosepele, Joseph Makhema, Simani Gaseitsiwe
EPI_ISL_948973, EPI_ISL_948975, EPI_ISL_948978, EPI_ISL_948981, EPI_ISL_948984, EPI_ISL_948989, EPI_ISL_948991, EPI_ISL_948994, EPI_ISL_948997, EPI_ISL_949000, EPI_ISL_949003, EPI_ISL_949005, EPI_ISL_949008, EPI_ISL_949011, EPI_ISL_949013, EPI_ISL_949016, EPI_ISL_949019, EPI_ISL_949022, EPI_ISL_949024, EPI_ISL_949069, EPI_ISL_949070, EPI_ISL_949095, EPI_ISL_949096, EPI_ISL_949097, EPI_ISL_949098, EPI_ISL_949099, EPI_ISL_949100, EPI_ISL_949101, EPI_ISL_949102, EPI_ISL_949103, EPI_ISL_949104, EPI_ISL_949105			
see above	Jessa	Jessa	Jessa_cmdLab
EPI_ISL_949182	University of Bari Biomedical Sciences and Human Oncology	University of Bari Biomedical Sciences and Human Oncology	Chironna M., Sallustio A., Loconsole D., Accogli M.
EPI_ISL_949254	Jessa	Jessa	Jessa_cmdLab
EPI_ISL_949408, EPI_ISL_949426	University of Birmingham	COVID-19 Genomics UK (COG-UK) Consortium	Institute of Microbiology, University of Birmingham: Claire McMurray, Joanne Stockton, Samuel Nicholls, Radoslaw Poplawski, Will Rowe, Josh Quick, Nicholas Loman. University of Birmingham Testing Laboratory: Celina M Whalley, Andrew Bosworth, Charlotte Poxon, Kasun Wanigasooriya, Oliver Pickles, Mike Kidd, Alex Richter, Andrew D Beggs PHE Heartlands Lab: Husam Osman, Andrew Bosworth. Queen Elizabeth Hospital: Anna Casey
EPI_ISL_949428, EPI_ISL_949442, EPI_ISL_949443, EPI_ISL_949444, EPI_ISL_949448, EPI_ISL_949478, EPI_ISL_949480, EPI_ISL_949481, EPI_ISL_949482, EPI_ISL_949483, EPI_ISL_949484, EPI_ISL_949486, EPI_ISL_949488, EPI_ISL_949489, EPI_ISL_949491, EPI_ISL_949493, EPI_ISL_949495, EPI_ISL_949497, EPI_ISL_949499, EPI_ISL_949501, EPI_ISL_949503, EPI_ISL_949505, EPI_ISL_949507, EPI_ISL_949509, EPI_ISL_949511, EPI_ISL_949513, EPI_ISL_949515, EPI_ISL_949517			
see above	Department of Pathology, University of Cambridge	COVID-19 Genomics UK (COG-UK) Consortium	Aminu S. Jahun, Yasmin Chaudhry, Iliana Georgana, Myra Hosmillo, Rhys Izu, Martin D. Curran, Surendra Parmar, Ian Goodfellow
EPI_ISL_949612	West of Scotland Specialist Virology Centre, NHSGGC / MRC-University of Glasgow Centre for Virus Research	COVID-19 Genomics UK (COG-UK) Consortium	Ana da Silva Filipe, Natasha Johnson, Kathy Smollett, Daniel Mair, Stephen Carmichael, Alice Broos, Lily Tong, Jenna Nichols, Kyriaki Nomikou; Sarah McDonald; Richard Orton, Joseph Hughes, Sreenu Vattipally, David L Robertson; Alasdair MacLean, Rory Gunson; Sharif Shaaban, Matthew Holden; Rachel Blacow, Guy Mollett, Kathy Li, James Shepherd, Antonia Ho, Emma Thomson
EPI_ISL_949639	Virology Department, Royal Infirmary of Edinburgh, NHS Lothian / School of Biological Sciences, University of Edinburgh / Institute of Genetics and Molecular Medicine, University of Edinburgh	COVID-19 Genomics UK (COG-UK) Consortium	McHugh M, Dewar R, Rooke S, Gallagher M, Balcaza C, O'Toole Á, Scher E, Hill V, McCrone JT, Colquhoun R, Yu X, Jackson B, Rambaut A, Williams TC, Templeton K
EPI_ISL_949791, EPI_ISL_949979	University College London, Great Ormond Street Hospital for Children NHS Foundation Trust, Imperial College Healthcare NHS Trust	COVID-19 Genomics UK (COG-UK) Consortium	Sergi Castellano, Rachel Williams, Mark Kristiansen, Paola Resende Silva, Sunando Roy, Tony Brooks, Helena Tutill, Paola Niola, Patricia Dyal, Charlotte Williams, Leysa Forrest, Yasmin Panchbhaya, Jacqueline Findlay, Samuel Weeks, Julianne Brown, Kathryn Harris, Paul Randell, James Price, Alison Holmes, Judith Breuer
EPI_ISL_950339, EPI_ISL_950340, EPI_ISL_950343, EPI_ISL_950344, EPI_ISL_950345	Northumbria University / South Tees Hospitals NHS Foundation Trust / North Cumbria Integrated Care NHS Foundation Trust / North Tees and Hartlepool NHS Foundation Trust / Newcastle Hospitals NHS Foundation Trust	COVID-19 Genomics UK (COG-UK) Consortium	Darren L Smith,Andrew Nelson,Matthew Bashton,Greg R Young,Joshua Loh,John Allan,Mohammad A Tariq,Giles S Holt,Gary Black,Wen C Yew,Lynn Dover,Paul Baker,Steve Liggett,Sarah Essex,Jane Greenaway,Debra Padgett,Clive Graham,Garren Scott,Edward Barton,Emma Swindells,Brendan Payne,Jennifer Collins,Yusri Taha,Gary Eltringham
EPI_ISL_950563, EPI_ISL_950564, EPI_ISL_950565, EPI_ISL_950567, EPI_ISL_950568, EPI_ISL_950569, EPI_ISL_950570, EPI_ISL_950571	Quadram Institute Bioscience	COVID-19 Genomics UK (COG-UK) Consortium	Dave J. Baker, Gemma L. Kay, Alp Aydin, Thanh Le-Viet, Steven Rudder, Ana P. Tedim, Anastasia Kolyva, Maria Diaz, Leonardo de Oliveira Martins, Nabil-Fareed Alikhan, Lizzie Meadows, Rachael Stanley, Ngozi Elumogo, Muhammed Yasir, Nicholas M. Thomson, Alexander J Trotter, Rachel Gilroy, Samuel Bloomfield, Claire Stuart, Andrew Bell, Reenesh Prakash, Samir Dervisevic, Alison E. Mather, John Wain, Mark Webber, Andrew J. Page, Justin O'Grady
EPI_ISL_950716	Lincolnshire Hospitals and DeepSeq Nottingham	COVID-19 Genomics UK (COG-UK) Consortium	Nichola Duckworth, Tim Sloan, Sarah Walsh, Jonathan Ball, Patrick McClure, Joeseph Chappell, Nadine Holmes, Matthew Carlisle, Christopher Moore, Fei Sang, Johnny Debebe, Victoria Wright, Matthew Loose
EPI_ISL_951360, EPI_ISL_951369, EPI_ISL_951370, EPI_ISL_951371, EPI_ISL_951466, EPI_ISL_951468, EPI_ISL_951470, EPI_ISL_951471, EPI_ISL_951476, EPI_ISL_951479, EPI_ISL_951480, EPI_ISL_951481, EPI_ISL_951482, EPI_ISL_951484, EPI_ISL_951485, EPI_ISL_951489			
see above	Oxford Viromics, NDM, University of Oxford; Oxford University Hospitals; Basingstoke and North Hampshire Hospital	COVID-19 Genomics UK (COG-UK) Consortium	Tanya Golubchik, David Bonsall, George Macintyre, Amy Trebes, Mariateresa de Cesare, Catrin Moore, Alex Mobbs, Anita Justice, Robert Shaw, Monique Andersson, Timothy Peto, Emma Wise, Nathan Moore, Jessica Lynch, Nick Cortes, Matilde Mori, Stephen Kidd, David Buck, John Todd, Christophe Fraser
EPI_ISL_951590, EPI_ISL_951591, EPI_ISL_951592, EPI_ISL_951593, EPI_ISL_951632, EPI_ISL_951633, EPI_ISL_951634, EPI_ISL_951635, EPI_ISL_951637, EPI_ISL_951648, EPI_ISL_951740, EPI_ISL_951756, EPI_ISL_952187, EPI_ISL_952188, EPI_ISL_952189, EPI_ISL_952190			
see above	Originating lab: Wales Specialist Virology Centre Sequencing lab: Pathogen Genomics Unit	Public Health Wales Microbiology Cardiff Wales Specialist Virology Centre	Catherine Moore, Johnathan Evans, Laura Gifford, Malorie Perry, Simon Cottrell, Angela Marchbank, Alec Birchley, Alexander Adams, Amy Gaskin, Bree Gatica-Wilcox, Jason Coombes, Joel Southgate, Lauren Gilbert, Lee Graham, Nicole Pacchiarini, Sara Kumziene-Summerhayes, Sarah Taylor, Sophie Jones, Sara Rey, Matthew Bull, Joanne Watkins, Sally Corden, Tom Connor
EPI_ISL_952405, EPI_ISL_952410, EPI_ISL_952412, EPI_ISL_952416, EPI_ISL_952432, EPI_ISL_952489, EPI_ISL_952490, EPI_ISL_952521, EPI_ISL_952522, EPI_ISL_952528, EPI_ISL_952529, EPI_ISL_952538, EPI_ISL_952673, EPI_ISL_952675, EPI_ISL_952677, EPI_ISL_952680, EPI_ISL_952689, EPI_ISL_952820, EPI_ISL_952830, EPI_ISL_952831, EPI_ISL_952832, EPI_ISL_952842, EPI_ISL_952844, EPI_ISL_952845, EPI_ISL_952846, EPI_ISL_952847, EPI_ISL_952848, EPI_ISL_952849, EPI_ISL_952850, EPI_ISL_952852, EPI_ISL_952853			
see above	Centre for Enzyme Innovation, University of Portsmouth / Translational Research Laboratory, Portsmouth Hospitals NHS Trust	COVID-19 Genomics UK (COG-UK) Consortium	Angela Beckett,Salman Goudarzi,Christopher Fearn,Kate Cook,Katie Loveson,Sharon Glaysher,Scott Elliott,Samuel Robson
EPI_ISL_953504, EPI_ISL_953515, EPI_ISL_953523, EPI_ISL_953524, EPI_ISL_953525, EPI_ISL_953599, EPI_ISL_953600, EPI_ISL_953601, EPI_ISL_953626, EPI_ISL_953652, EPI_ISL_953662, EPI_ISL_953671, EPI_ISL_953672, EPI_ISL_953673, EPI_ISL_953674, EPI_ISL_953675, EPI_ISL_953676, EPI_ISL_953677, EPI_ISL_953678, EPI_ISL_953679, EPI_ISL_953680, EPI_ISL_953681, EPI_ISL_953682, EPI_ISL_953683, EPI_ISL_953684, EPI_ISL_953685, EPI_ISL_953686, EPI_ISL_953687, EPI_ISL_953688, EPI_ISL_953689, EPI_ISL_953690, EPI_ISL_953691, EPI_ISL_953692, EPI_ISL_953693, EPI_ISL_953694, EPI_ISL_953695, EPI_ISL_953696, EPI_ISL_953697, EPI_ISL_953698, EPI_ISL_953699, EPI_ISL_953700, EPI_ISL_953701, EPI_ISL_953702, EPI_ISL_953703, EPI_ISL_953704, EPI_ISL_953705, EPI_ISL_953706			
see above	University Hospitals of Geneva, Laboratory of Virology	HUG, Laboratory of Virology and the Health2030 Genome Center	Samuel Cordey, Ana Rita Goncalves, Laurent Kaiser, Lorenzo Cerutti, Henri Pegeot, Melyssa Elies, Deborah Penet, Keith Harshman, Ioannis Xenarios, Emmanouil Dermitzakis
EPI_ISL_953946, EPI_ISL_953947, EPI_ISL_953948	Hopital	National Reference Center for Viruses of Respiratory Infections, Institut Pasteur, Paris	Marion Barbet, Sylvie Behillil, Méline Bizard, Angela Brisebarre, Camille Capel, Etienne Simon-Lorière, Vincent Enouf, Maud Vanpeene, Sylvie van der Werf,Cady Anne
EPI_ISL_953986	Labo Analyses Med	National Reference Center for Viruses of Respiratory Infections, Institut Pasteur, Paris	Marion Barbet, Sylvie Behillil, Méline Bizard, Angela Brisebarre, Camille Capel, Etienne Simon-Lorière, Vincent Enouf, Maud Vanpeene, Sylvie van der Werf
EPI_ISL_953987	Hopital	National Reference Center for Viruses of Respiratory Infections, Institut Pasteur, Paris	Marion Barbet, Sylvie Behillil, Méline Bizard, Angela Brisebarre, Camille Capel, Etienne Simon-Lorière, Vincent Enouf, Maud Vanpeene, Sylvie van der Werf,Lagathu GisèLe
EPI_ISL_953988	Labo Analyses Med	National Reference Center for Viruses of Respiratory Infections, Institut Pasteur, Paris	Marion Barbet, Sylvie Behillil, Méline Bizard, Angela Brisebarre, Camille Capel, Etienne Simon-Lorière, Vincent Enouf, Maud Vanpeene, Sylvie van der Werf,Selas Labomaine
EPI_ISL_953989	Hopital	National Reference Center for Viruses of Respiratory Infections, Institut Pasteur, Paris	Marion Barbet, Sylvie Behillil, Méline Bizard, Angela Brisebarre, Camille Capel, Etienne Simon-Lorière, Vincent Enouf, Maud Vanpeene, Sylvie van der Werf,Lesimple BéAtrice
EPI_ISL_953990	Hopital	National Reference Center for Viruses of Respiratory Infections, Institut Pasteur, Paris	Marion Barbet, Sylvie Behillil, Méline Bizard, Angela Brisebarre, Camille Capel, Etienne Simon-Lorière, Vincent Enouf, Maud Vanpeene, Sylvie van der Werf,Eloy Clarence
EPI_ISL_954020	Hopital	National Reference Center for Viruses of Respiratory Infections, Institut Pasteur, Paris	Marion Barbet, Sylvie Behillil, Méline Bizard, Angela Brisebarre, Camille Capel, Etienne Simon-Lorière, Vincent Enouf, Maud Vanpeene, Sylvie van der Werf,Breuil J.
EPI_ISL_954021	hopital	National Reference Center for Viruses of Respiratory Infections, Institut Pasteur, Paris	Marion Barbet, Sylvie Behillil, Méline Bizard, Angela Brisebarre, Camille Capel, Etienne Simon-Lorière, Vincent Enouf, Maud Vanpeene, Sylvie van der Werf,Isabelle Joly

EPI_ISL_954091	Labo Analyses Med	National Reference Center for Viruses of Respiratory Infections, Institut Pasteur, Paris	Marion Barbet, Sylvie Behillil, Méline Bizard, Angela Brisebarre, Camille Capel, Etienne Simon-Lorière, Vincent Enouf, Maud Vanpeene, Sylvie van der Werf,Besson J
EPI_ISL_954092	Labo Analyses Med	National Reference Center for Viruses of Respiratory Infections, Institut Pasteur, Paris	Marion Barbet, Sylvie Behillil, Méline Bizard, Angela Brisebarre, Camille Capel, Etienne Simon-Lorière, Vincent Enouf, Maud Vanpeene, Sylvie van der Werf,Dlab Dieppe
EPI_ISL_954093	Labo Analyses Med	National Reference Center for Viruses of Respiratory Infections, Institut Pasteur, Paris	Marion Barbet, Sylvie Behillil, Méline Bizard, Angela Brisebarre, Camille Capel, Etienne Simon-Lorière, Vincent Enouf, Maud Vanpeene, Sylvie van der Werf,Holstein Anne
EPI_ISL_954094, EPI_ISL_954095, EPI_ISL_954096, EPI_ISL_954097, EPI_ISL_954098, EPI_ISL_954099, EPI_ISL_954100, EPI_ISL_954101, EPI_ISL_954102	Outre Mer	National Reference Center for Viruses of Respiratory Infections, Institut Pasteur, Paris	Marion Barbet, Sylvie Behillil, Méline Bizard, Angela Brisebarre, Camille Capel, Etienne Simon-Lorière, Vincent Enouf, Maud Vanpeene, Sylvie van der Werf,Rousset Dominique
EPI_ISL_954103, EPI_ISL_954104	Hopital	National Reference Center for Viruses of Respiratory Infections, Institut Pasteur, Paris	Marion Barbet, Sylvie Behillil, Méline Bizard, Angela Brisebarre, Camille Capel, Etienne Simon-Lorière, Vincent Enouf, Maud Vanpeene, Sylvie van der Werf,Raulin Olivia
EPI_ISL_954105	Labo Analyses Med	National Reference Center for Viruses of Respiratory Infections, Institut Pasteur, Paris	Marion Barbet, Sylvie Behillil, Méline Bizard, Angela Brisebarre, Camille Capel, Etienne Simon-Lorière, Vincent Enouf, Maud Vanpeene, Sylvie van der Werf,Takoudju Eve-Marie
EPI_ISL_954106, EPI_ISL_954107	Hopital	National Reference Center for Viruses of Respiratory Infections, Institut Pasteur, Paris	Marion Barbet, Sylvie Behillil, Méline Bizard, Angela Brisebarre, Camille Capel, Etienne Simon-Lorière, Vincent Enouf, Maud Vanpeene, Sylvie van der Werf,Lesimple BéAtrice
EPI_ISL_954108, EPI_ISL_954109, EPI_ISL_954110, EPI_ISL_954111	Outre Mer	National Reference Center for Viruses of Respiratory Infections, Institut Pasteur, Paris	Marion Barbet, Sylvie Behillil, Méline Bizard, Angela Brisebarre, Camille Capel, Etienne Simon-Lorière, Vincent Enouf, Maud Vanpeene, Sylvie van der Werf,Rousset Dominique
EPI_ISL_954142, EPI_ISL_954156, EPI_ISL_954157, EPI_ISL_954158, EPI_ISL_954159, EPI_ISL_954160, EPI_ISL_954161, EPI_ISL_954162, EPI_ISL_954163, EPI_ISL_954164, EPI_ISL_954173, EPI_ISL_954174, EPI_ISL_954182			
see above	Hospital Universitari Vall d'Hebron - Vall d'Hebron Institut de Rercerca	Hospital Universitari Vall d'Hebron	Cristina Andrés, Maria Piñana, Josep F Abril, Damir Garcia-Cehic, Ariadna Rando, Juliana Esperalba, Maria Gema Codina, Carla Castillo, Maria Carmen Martin, Tomàs Pumarola, Josep Quer, Andrés Antón
EPI_ISL_954753	City of Milwaukee Health Department Laboratory	City of Milwaukee Health Department Laboratory	Sanjib Bhattacharyya
EPI_ISL_954783	Hospital Universitario Virgen de la Arrixaca	Instituto de Salud Carlos III	Iglesias-Caballero, M. Camarero, S. Sandonís,V. Vázquez, S. Pozo, F. Casas, I. Jiménez, P. Zaballos, A. Monzón, S. Varona, S. Cuesta, I. Moreno, L.
EPI_ISL_954785	Complejo Asistencial Universitario de Salamanca	Instituto de Salud Carlos III	Iglesias-Caballero, M. Camarero, S. Sandonís,V. Vázquez, S. Pozo, F. Casas, I. Jiménez, P. Zaballos, A. Monzón, S. Varona, S. Cuesta, I. Ávila,A.
EPI_ISL_954797, EPI_ISL_954801, EPI_ISL_954803	Hospital Los Arcos	Instituto de Salud Carlos III	Iglesias-Caballero, M. Camarero, S. Sandonís,V. Vázquez, S. Pozo, F. Casas, I. Jiménez, P. Zaballos, A. Monzón, S. Varona, S. Cuesta, I. Camara, M.
EPI_ISL_954821, EPI_ISL_954828, EPI_ISL_954847, EPI_ISL_954855, EPI_ISL_954863, EPI_ISL_954868, EPI_ISL_954873, EPI_ISL_954875, EPI_ISL_954999, EPI_ISL_955000, EPI_ISL_955001, EPI_ISL_955002, EPI_ISL_955003, EPI_ISL_955004, EPI_ISL_955005, EPI_ISL_955006, EPI_ISL_955007, EPI_ISL_955008, EPI_ISL_955009, EPI_ISL_955010, EPI_ISL_955011, EPI_ISL_955012, EPI_ISL_955013, EPI_ISL_955014, EPI_ISL_955015, EPI_ISL_955016, EPI_ISL_955017, EPI_ISL_955018, EPI_ISL_955019, EPI_ISL_955020, EPI_ISL_955021, EPI_ISL_955022, EPI_ISL_955023, EPI_ISL_955024, EPI_ISL_955025, EPI_ISL_955026, EPI_ISL_955027, EPI_ISL_955028, EPI_ISL_955029, EPI_ISL_955030, EPI_ISL_955031, EPI_ISL_955032, EPI_ISL_955033, EPI_ISL_955034, EPI_ISL_955035, EPI_ISL_955036, EPI_ISL_955037, EPI_ISL_955038, EPI_ISL_955039, EPI_ISL_955040			
see above	Colorado Department of Public Health and Environment	Colorado Department of Puplic Health and Environment	Laura Bankers, Molly C. Hetherington-Rauth, Diana Ir, Shannon Ely, Shannon R. Matzinger, Sarah Elizabeth Totten, Emily A. Travanty
EPI_ISL_955064, EPI_ISL_955075, EPI_ISL_955100	Hopital	National Reference Center for Viruses of Respiratory Infections, Institut Pasteur, Paris	Marion Barbet, Sylvie Behillil, Méline Bizard, Angela Brisebarre, Camille Capel, Etienne Simon-Lorière, Vincent Enouf, Maud Vanpeene, Sylvie van der Werf,Guinoiseau Thibault
EPI_ISL_955103	National Institute of Laboratory Medicine and Referral Center	Genomic Research Lab, BCSIR	Md. Ahashan Habib,Mohammad Samir Uzzaman, Eshrar Osman, Shahina Akter,Tanjina Akhtar Banu, Abu Sayeed Mohammad Mahmud, Md. Murshed Hasan Sarkar, Barna Goswami, Iffat Jahan, Md. Saddam Hossain, Tasnim Nafisa, Md. Maruf Ahmed Molla, Mahmuda Yeasmin, Asish Kumar Ghosh, Arifa Akram, A. K. M. Shamsuzzaman, Md. Salim Khan
EPI_ISL_955120, EPI_ISL_955121, EPI_ISL_955122, EPI_ISL_955123, EPI_ISL_955124, EPI_ISL_955125	Maryland Public Health Laboratory	Maryland Public Health Laboratory	Maryland Department of Health Laboratories Administration
EPI_ISL_955143, EPI_ISL_955145	Platform BIS UZA/UAntwerpen	UAntwerp, Laboratory of Medical Microbiology	Basil Britto Xavier, Jasmine Coppens, Marie Le Mercier, Christine Lammens, Veerle Matheeussen, Herman Goossens
EPI_ISL_955176	Hopital	National Reference Center for Viruses of Respiratory Infections, Institut Pasteur, Paris	Marion Barbet, Sylvie Behillil, Méline Bizard, Angela Brisebarre, Camille Capel, Etienne Simon-Lorière, Vincent Enouf, Maud Vanpeene, Sylvie van der Werf,Brichler SéGolèNe
EPI_ISL_955177	Hopital	National Reference Center for Viruses of Respiratory Infections, Institut Pasteur, Paris	Marion Barbet, Sylvie Behillil, Méline Bizard, Angela Brisebarre, Camille Capel, Etienne Simon-Lorière, Vincent Enouf, Maud Vanpeene, Sylvie van der Werf,Fourgeaud Jacques
EPI_ISL_955178, EPI_ISL_955179	Labo Analyses med	National Reference Center for Viruses of Respiratory Infections, Institut Pasteur, Paris	Marion Barbet, Sylvie Behillil, Méline Bizard, Angela Brisebarre, Camille Capel, Etienne Simon-Lorière, Vincent Enouf, Maud Vanpeene, Sylvie van der Werf,Amzalag Jonas
EPI_ISL_955180, EPI_ISL_955181	Labo Analyses Med	National Reference Center for Viruses of Respiratory Infections, Institut Pasteur, Paris	Marion Barbet, Sylvie Behillil, Méline Bizard, Angela Brisebarre, Camille Capel, Etienne Simon-Lorière, Vincent Enouf, Maud Vanpeene, Sylvie van der Werf,Amzalag Jonas
EPI_ISL_955183	Labo Analyses Med	National Reference Center for Viruses of Respiratory Infections, Institut Pasteur, Paris	Marion Barbet, Sylvie Behillil, Méline Bizard, Angela Brisebarre, Camille Capel, Etienne Simon-Lorière, Vincent Enouf, Maud Vanpeene, Sylvie van der Werf,Le Berre (D) David
EPI_ISL_955224, EPI_ISL_955225	Indiana Animal Disease Diagnostic Laboratory	Carpi Laboratory - Purdue University	Jack Dorman, Ilinca I Ciubotariu, Lev Gorenstein, Abebe A Fola, G Kenitra Hendrix, Rebecca P Wilkes, Giovanna Carpi
EPI_ISL_955782, EPI_ISL_955785, EPI_ISL_955787, EPI_ISL_955794, EPI_ISL_955795, EPI_ISL_955796, EPI_ISL_955797, EPI_ISL_955798, EPI_ISL_955799, EPI_ISL_955800, EPI_ISL_955801, EPI_ISL_955803, EPI_ISL_955804, EPI_ISL_955805, EPI_ISL_955806, EPI_ISL_955807, EPI_ISL_955808, EPI_ISL_955809, EPI_ISL_955812, EPI_ISL_955813, EPI_ISL_955814, EPI_ISL_955837, EPI_ISL_955838, EPI_ISL_955839, EPI_ISL_955845, EPI_ISL_955846, EPI_ISL_955847, EPI_ISL_955848, EPI_ISL_955849, EPI_ISL_955850, EPI_ISL_955851			
see above	University of Michigan Clinical Microbiology Laboratory	Lauring Lab, University of Michigan, Department of Microbiology and Immunology	Valesano
EPI_ISL_956284, EPI_ISL_956285, EPI_ISL_956286, EPI_ISL_956294	Instituto Nacional de Salud- Dirección de Redes de Laboratorios de Salud Pública	Instituto Nacional de Salud- Dirección de Investigación en Salud Pública	Katherine Laiton-Donato, Diego A. Álvarez-Díaz, Carlos Franco-Muñoz, Mauricio Pacheco-Montealegre, Hector Alejandro Ruiz-Moreno, Maria T. Herrera-Sepúlveda, Diego Andrés Prada, Jhonnatan Reales-González, Sheryll Corchuelo, Julian Naizaque, Gerardo Santamaría, Magdalena Wiesner, Martha Lucia Ospina Martinez, Marcela Mercado-Reyes
EPI_ISL_956329	Laboratory Medicine	Department of Laboratory Medicine, Lin-Kou Chang Gung Memorial Hospital, Taoyuan, Taiwan	Kuo-Chien Tsao, Yu-Nong Gong, Shu-Li Yang, Yi-Chun Liu, Chung-Guei Huang, Mei-Jen Hsiao, Po-Wei Huang, Cheng-Ta Yang, Cheng-Hsun Chiu, Peng-Nien Huang, Kuo-Ming Lee, Guang-Wu Chen, Shin-Ru Shih
EPI_ISL_956333	Utah Public Health Laboratory, Utah Public Health Laboratory Infectious Disease submission group	Utah Public Health Laboratory, Utah Public Health Laboratory Infectious Disease submission group	Gallagher,T., Young,E.L., Oakeson,K.F.
EPI_ISL_959309	National Institute of Laboratory Medicine and Referral Center	Genomic Research Lab, BCSIR	Md. Maruf Ahmed Molla, Mohammad Samir Uzzaman, Eshrar Osman, Md. Ahashan Habib, Shahina Akter,Tanjina Akhtar Banu, Abu Sayeed Mohammad Mahmud, Md. Murshed Hasan Sarkar, Barna Goswami, Iffat Jahan, Md. Saddam Hossain, Tasnim Nafisa, Mahmuda Yeasmin, Asish Kumar Ghosh, Arifa Akram, A. K. M. Shamsuzzaman, Md. Salim Khan
EPI_ISL_959311, EPI_ISL_959312, EPI_ISL_959313, EPI_ISL_959316, EPI_ISL_959317, EPI_ISL_959318, EPI_ISL_959319, EPI_ISL_959320, EPI_ISL_959321, EPI_ISL_959322, EPI_ISL_959323, EPI_ISL_959324, EPI_ISL_959325, EPI_ISL_959326, EPI_ISL_959327, EPI_ISL_959328, EPI_ISL_959329, EPI_ISL_959330, EPI_ISL_959331, EPI_ISL_959332, EPI_ISL_959334			
see above	Servicio de Microbiología, Laboratori Clínic Metropolitana Nord. Hospital Universitari Germans Trias i Pujol. Institut d'Investigació en Ciències de la Salut Germans Trias i Pujol (IGTP)	IrsiCaixa - Can Ruti CovidSeq	Marc Noguera-Julian, Mariona Parera, Maria Casadellà, Pilar Armengol, Francesc Catala-Moll, Roger Paredes, Bonaventura Clotet Elisa Martró, Verónica Saludes, Anna Not, Ana Pérez, Montserrat Giménez, Ignacio Blanco, Cristina Casañ, Antoni E. Bordoy, Adrián Antuori

EPI_ISL_959371	National Institute of Laboratory Medicine and Referral Center	Genomic Research Lab, BCSIR	Shahina Akter, Mohammad Samir Uzzaman, Eshrar Osman, Md. Ahashan Habib, Tanjina Akhtar Banu, Abu Sayeed Mohammad Mahmud, Md. Murshed Hasan Sarkar, Barna Goswami, Iffat Jahan, Md. Saddam Hossain, Tasnim Nafisa, Md. Maruf Ahmed Molla, Mahmuda Yeasmin, Asish Kumar Ghosh, Arifa Akram, A. K. M. Shamsuzzaman, Md. Salim Khan
EPI_ISL_959372	National Institute of Laboratory Medicine and Referral Center	Genomic Research Lab, BCSIR	Tanjina Akhtar Banu, Mohammad Samir Uzzaman, Eshrar Osman, Md. Ahashan Habib, Shahina Akter, Abu Sayeed Mohammad Mahmud, Md. Murshed Hasan Sarkar, Barna Goswami, Iffat Jahan, Md. Saddam Hossain, Tasnim Nafisa, Md. Maruf Ahmed Molla, Mahmuda Yeasmin, Asish Kumar Ghosh, Arifa Akram, A. K. M. Shamsuzzaman, Md. Salim Khan
EPI_ISL_959424, EPI_ISL_959427, EPI_ISL_959428, EPI_ISL_959430, EPI_ISL_959431, EPI_ISL_959439, EPI_ISL_959440, EPI_ISL_959446, EPI_ISL_959463, EPI_ISL_959464, EPI_ISL_959465, EPI_ISL_959466, EPI_ISL_959467			
see above	Servicio de Microbiología, Hospital Universitario Son Espases	SeqCOVID-SPAIN consortium/IBV(CSIC)	Carla López-Causapé, Jordi Reina, Antonio Oliver and SeqCOVID-SPAIN consortium
EPI_ISL_959538	National Institute of Laboratory Medicine and Referral Center	Genomic Research Lab, BCSIR	Barna Goswami, Mohammad Samir Uzzaman, Eshrar Osman, Md. Ahashan Habib, Shahina Akter, Tanjina Akhtar Banu, Abu Sayeed Mohammad Mahmud, Md. Murshed Hasan Sarkar, Iffat Jahan, Md. Saddam Hossain, Tasnim Nafisa, Md. Maruf Ahmed Molla, Mahmuda Yeasmin, Asish Kumar Ghosh, Arifa Akram, A. K. M. Shamsuzzaman, Md. Salim Khan
EPI_ISL_959539, EPI_ISL_959540	University of Michigan Clinical Microbiology Laboratory	Lauring Lab, University of Michigan, Department of Microbiology and Immunology	Valesano
EPI_ISL_959545	National Institute of Laboratory Medicine and Referral Center	Genomic Research Lab, BCSIR	Md. Saddam Hossain, Mohammad Samir Uzzaman, Eshrar Osman, Md. Ahashan Habib, Shahina Akter, Tanjina Akhtar Banu, Abu Sayeed Mohammad Mahmud, Md. Murshed Hasan Sarkar, Barna Goswami, Iffat Jahan, Tasnim Nafisa, Md. Maruf Ahmed Molla, Mahmuda Yeasmin, Asish Kumar Ghosh, Arifa Akram, A. K. M.Shamsuzzaman, Md. Salim Khan
EPI_ISL_959567, EPI_ISL_959568	Vivalia - Clinique Saint-Joseph	GIGA Medical Genomics	Keith Durkin, Maria Artesi, Sébastien Bontems, Raphaël Boreux, Bouchra Boujemla, Cécile Meex, Pierrette Melin, Marie-Pierre Hayette, Vincent Bours
EPI_ISL_959569, EPI_ISL_959570, EPI_ISL_959571, EPI_ISL_959572, EPI_ISL_959573, EPI_ISL_959574, EPI_ISL_959575, EPI_ISL_959576, EPI_ISL_959577, EPI_ISL_959578, EPI_ISL_959579, EPI_ISL_959580, EPI_ISL_959581, EPI_ISL_959582, EPI_ISL_959583, EPI_ISL_959584			
see above	Labo Luc Olivier	GIGA Medical Genomics	Keith Durkin, Maria Artesi, Sébastien Bontems, Raphaël Boreux, Bouchra Boujemla, Cécile Meex, Pierrette Melin, Marie-Pierre Hayette, Vincent Bours
EPI_ISL_959774, EPI_ISL_959775, EPI_ISL_959776, EPI_ISL_959777, EPI_ISL_959778, EPI_ISL_959779, EPI_ISL_959780, EPI_ISL_959781, EPI_ISL_959782, EPI_ISL_959783, EPI_ISL_959784, EPI_ISL_959785, EPI_ISL_959786, EPI_ISL_959787, EPI_ISL_959788, EPI_ISL_959789, EPI_ISL_959790, EPI_ISL_959791, EPI_ISL_959792, EPI_ISL_959793, EPI_ISL_959794, EPI_ISL_959795, EPI_ISL_959796, EPI_ISL_959797, EPI_ISL_959798, EPI_ISL_959799, EPI_ISL_959800, EPI_ISL_959801, EPI_ISL_959802, EPI_ISL_959803, EPI_ISL_959804, EPI_ISL_959805, EPI_ISL_959806, EPI_ISL_959807, EPI_ISL_959808, EPI_ISL_959809, EPI_ISL_959810, EPI_ISL_959811, EPI_ISL_959812, EPI_ISL_959813, EPI_ISL_959814, EPI_ISL_959815, EPI_ISL_959816, EPI_ISL_959817, EPI_ISL_959818, EPI_ISL_959819, EPI_ISL_959820, EPI_ISL_959821, EPI_ISL_959822, EPI_ISL_959823, EPI_ISL_959824, EPI_ISL_959825, EPI_ISL_959826, EPI_ISL_959827, EPI_ISL_959828, EPI_ISL_959829, EPI_ISL_959830, EPI_ISL_959831, EPI_ISL_959832, EPI_ISL_959833, EPI_ISL_959834, EPI_ISL_959835, EPI_ISL_959836, EPI_ISL_959837, EPI_ISL_959838, EPI_ISL_959854, EPI_ISL_959873			
see above	National Virus Reference Laboratory	National Virus Reference Laboratory	Michael Carr, Gabriel Gonzalez, Jonathan Dean, Cillian F De Gascun
EPI_ISL_960312	Botswana Harvard HIV Reference Laboratory	Botswana Harvard HIV Reference Laboratory	Sikhulile Moyo, Wonderful T. Choga, Dorcas Maruapula, Botshelo Radibe, Boitumelo Zuze, David Lawrence, Roger Shapiro, Shahin Lockman, Mosepele Mosepele, Joseph Makhema, Simani Gaseitsiwe
EPI_ISL_960422, EPI_ISL_960427, EPI_ISL_960429	The National Institute of Public Health	State Veterinary Institute Prague	Nagy,A;Vecerova,J;Cernikova,L;Stara,M;Jirincova,H;Trnka,D
EPI_ISL_960855, EPI_ISL_960860, EPI_ISL_960862, EPI_ISL_960869, EPI_ISL_960870, EPI_ISL_960871, EPI_ISL_960880	Institute of Medical Microbiology and Hospital Hygiene	Institute of Medical Microbiology and Hospital Hygiene	Prof. Dr. Achim Kaasch, Aljoscha Tersteegen
EPI_ISL_961052, EPI_ISL_961053, EPI_ISL_961054	SIESP DIPARTIMENTO DI PREVENZIONE CHIETI	Istituto Zooprofilattico Sperimentale dell'Abruzzo e Molise "G. Caporale"	Lorusso A, Marcacci M, Di Domenico M, Ancora M, Curini V, Mangone I, Rinaldi A, Scialabba S, Di Pasquale A, Cammà C, Puglia I, Calistri P, Savini G
EPI_ISL_961055, EPI_ISL_961056	SIESP CHIETI - DRIVE IN ORTONA	Istituto Zooprofilattico Sperimentale dell'Abruzzo e Molise "G. Caporale"	Lorusso A, Marcacci M, Di Domenico M, Ancora M, Curini V, Mangone I, Rinaldi A, Scialabba S, Di Pasquale A, Cammà C, Puglia I, Calistri P, Savini G
EPI_ISL_961057, EPI_ISL_961058, EPI_ISL_961059, EPI_ISL_961060	SIESP CHIETI - DRIVE IN CHIETI	Istituto Zooprofilattico Sperimentale dell'Abruzzo e Molise "G. Caporale"	Lorusso A, Marcacci M, Di Domenico M, Ancora M, Curini V, Mangone I, Rinaldi A, Scialabba S, Di Pasquale A, Cammà C, Puglia I, Calistri P, Savini G
EPI_ISL_961061	SIESP CHIETI - DRIVE IN ORTONA	Istituto Zooprofilattico Sperimentale dell'Abruzzo e Molise "G. Caporale"	Lorusso A, Marcacci M, Di Domenico M, Ancora M, Curini V, Mangone I, Rinaldi A, Scialabba S, Di Pasquale A, Cammà C, Puglia I, Calistri P, Savini G
EPI_ISL_961062	SIESP CHIETI - DRIVE IN CHIETI	Istituto Zooprofilattico Sperimentale dell'Abruzzo e Molise "G. Caporale"	Lorusso A, Marcacci M, Di Domenico M, Ancora M, Curini V, Mangone I, Rinaldi A, Scialabba S, Di Pasquale A, Cammà C, Puglia I, Calistri P, Savini G
EPI_ISL_961063	SIESP DIPARTIMENTO DI PREVENZIONE CHIETI	Istituto Zooprofilattico Sperimentale dell'Abruzzo e Molise "G. Caporale"	Lorusso A, Marcacci M, Di Domenico M, Ancora M, Curini V, Mangone I, Rinaldi A, Scialabba S, Di Pasquale A, Cammà C, Puglia I, Calistri P, Savini G
EPI_ISL_961064, EPI_ISL_961065	SIESP CHIETI - DRIVE IN ORTONA	Istituto Zooprofilattico Sperimentale dell'Abruzzo e Molise "G. Caporale"	Lorusso A, Marcacci M, Di Domenico M, Ancora M, Curini V, Mangone I, Rinaldi A, Scialabba S, Di Pasquale A, Cammà C, Puglia I, Calistri P, Savini G
EPI_ISL_961084	SIESP TERAMO	Istituto Zooprofilattico Sperimentale dell'Abruzzo e Molise "G. Caporale"	Lorusso A, Marcacci M, Di Domenico M, Ancora M, Curini V, Mangone I, Rinaldi A, Scialabba S, Di Pasquale A, Cammà C, Puglia I, Calistri P, Savini G
EPI_ISL_961085	SIESP CHIETI - DRIVE IN ORTONA	Istituto Zooprofilattico Sperimentale dell'Abruzzo e Molise "G. Caporale"	Lorusso A, Marcacci M, Di Domenico M, Ancora M, Curini V, Mangone I, Rinaldi A, Scialabba S, Di Pasquale A, Cammà C, Puglia I, Calistri P, Savini G
EPI_ISL_961086	SIESP CHIETI - DRIVE IN CHIETI	Istituto Zooprofilattico Sperimentale dell'Abruzzo e Molise "G. Caporale"	Lorusso A, Marcacci M, Di Domenico M, Ancora M, Curini V, Mangone I, Rinaldi A, Scialabba S, Di Pasquale A, Cammà C, Puglia I, Calistri P, Savini G
EPI_ISL_961087, EPI_ISL_961088, EPI_ISL_961089	SIESP DIPARTIMENTO DI PREVENZIONE CHIETI	Istituto Zooprofilattico Sperimentale dell'Abruzzo e Molise "G. Caporale"	Lorusso A, Marcacci M, Di Domenico M, Ancora M, Curini V, Mangone I, Rinaldi A, Scialabba S, Di Pasquale A, Cammà C, Puglia I, Calistri P, Savini G
EPI_ISL_961090, EPI_ISL_961091, EPI_ISL_961092	SIESP CHIETI - DRIVE IN CHIETI	Istituto Zooprofilattico Sperimentale dell'Abruzzo e Molise "G. Caporale"	Lorusso A, Marcacci M, Di Domenico M, Ancora M, Curini V, Mangone I, Rinaldi A, Scialabba S, Di Pasquale A, Cammà C, Puglia I, Calistri P, Savini G
EPI_ISL_961093	Ospedale SS Annunziata	Istituto Zooprofilattico Sperimentale dell'Abruzzo e Molise "G. Caporale"	Lorusso A, Marcacci M, Di Domenico M, Ancora M, Curini V, Mangone I, Rinaldi A, Scialabba S, Di Pasquale A, Cammà C, Puglia I, Calistri P, Savini G
EPI_ISL_961094, EPI_ISL_961095	DIP. PREV. AVEZZANO SERVIZIO DI IGIENE EPIDEMIOLOGIAE SANITA' PUBBLICA	Istituto Zooprofilattico Sperimentale dell'Abruzzo e Molise "G. Caporale"	Lorusso A, Marcacci M, Di Domenico M, Ancora M, Curini V, Mangone I, Rinaldi A, Scialabba S, Di Pasquale A, Cammà C, Puglia I, Calistri P, Savini G
EPI_ISL_961171, EPI_ISL_961175	Texas Department of State Health Services	Texas Department of State Health Services	Bonnie Oh, Anita Pokharel, James Daniel Bonser, Myong Koag, Chung Wang, Rachel Lee, Grace Kubin, Rashmi Tuladhar, Mayela Pedrueza, Maliha Rahman, Jenny Zhang
EPI_ISL_961215, EPI_ISL_961217, EPI_ISL_961220, EPI_ISL_961221, EPI_ISL_961222, EPI_ISL_961224, EPI_ISL_961227, EPI_ISL_961234, EPI_ISL_961238	Hospital General Universitario de Alicante - Instituto de Investigación Sanitaria y Biomédica de Alicante	SeqCOVID-SPAIN consortium/IBV(CSIC)	Maripaz Ventero Martín, Carmen Molina Pardines and SeqCOVID-SPAIN consortium
EPI_ISL_961368, EPI_ISL_961369, EPI_ISL_961371, EPI_ISL_961372, EPI_ISL_961373, EPI_ISL_961374, EPI_ISL_961375, EPI_ISL_961376, EPI_ISL_961377, EPI_ISL_961378, EPI_ISL_961382, EPI_ISL_961383, EPI_ISL_961384, EPI_ISL_961385, EPI_ISL_961386, EPI_ISL_961387, EPI_ISL_961388, EPI_ISL_961389, EPI_ISL_961390, EPI_ISL_961391, EPI_ISL_961392, EPI_ISL_961393, EPI_ISL_961394, EPI_ISL_961395, EPI_ISL_961396, EPI_ISL_961397, EPI_ISL_961398, EPI_ISL_961399, EPI_ISL_961400, EPI_ISL_961401, EPI_ISL_961402, EPI_ISL_961403, EPI_ISL_961404, EPI_ISL_961405, EPI_ISL_961406, EPI_ISL_961407, EPI_ISL_961408, EPI_ISL_961409, EPI_ISL_961410, EPI_ISL_961411, EPI_ISL_961412, EPI_ISL_961413, EPI_ISL_961414, EPI_ISL_961415, EPI_ISL_961416, EPI_ISL_961417, EPI_ISL_961418, EPI_ISL_961419, EPI_ISL_961420, EPI_ISL_961421, EPI_ISL_961422, EPI_ISL_961423, EPI_ISL_961424, EPI_ISL_961425			
see above	Toronto Invasive Bacterial Diseases Network	McMaster University	Allison McGeer, Patryk Aftanas, Hooman Derakhshani, Angel Li, Kuganya Nirmalarajah, Emily Panousis, Ahmed Draia, Jalees Nasir, Michael Surette,

			Samira Mubareka, Andrew G. McArthur
EPI_ISL_961467, EPI_ISL_961468, EPI_ISL_961469	LESP Nuevo Leon/Grupo de Diagnostico ARIES	Instituto de Diagnostico y Referencia Epidemiologicos (INDRE)	Claudia Wong-Arambula, Abril Rodriguez-Maldonado, Fabiola Garces-Ayala, Natividad Cruz-Ortiz, Tatiana Nunez-Garcia, Gisela Barrera-Badillo, Lucia Hernandez-Rivas, Irma Lopez-Martinez, Ernesto Ramirez-Gonzalez.
EPI_ISL_961472, EPI_ISL_961504, EPI_ISL_961505, EPI_ISL_961517, EPI_ISL_961523, EPI_ISL_961539, EPI_ISL_961540, EPI_ISL_961541, EPI_ISL_961542, EPI_ISL_961543	Michigan Department of Health and Human Services, Bureau of Laboratories	Michigan Department of Health and Human Services, Bureau of Laboratories	Blankenship HM, Riner D, Soehnlen MK
EPI_ISL_961581, EPI_ISL_961582, EPI_ISL_961588, EPI_ISL_961589, EPI_ISL_961590, EPI_ISL_961620, EPI_ISL_961621, EPI_ISL_961622, EPI_ISL_961623, EPI_ISL_961624, EPI_ISL_961625, EPI_ISL_961626, EPI_ISL_961627, EPI_ISL_961628, EPI_ISL_961629			
see above	Hôpital Georges L. Dumont	National Microbiology Laboratory (NML)	Anna Majer, Shari Tyson, Grace Seo, Philip Mabon, Elsie Grudeski, Rhiannon Huzarewich, Russell Mandes, Anneliese Landgraff, Jennifer Tanner, Natalie Knox, Morag Graham, Gary Van Domselaar, Richard Garceau, Guillaume Desnoyers, Nathalie Bastien, Yan Li, Timothy Booth, Darian Hole, Madison Chapel, Kirsten Biggar, CanCOGeN's metadata curation team, Public Health Agency of Canada CanCOGeN team
EPI_ISL_961802, EPI_ISL_961804	E. Gulbja laboratorija	Latvian Biomedical Research and Study Centre	Janis Pjalkovskis, Nikita Zrelovs, Monta Ustinova, Ivars Silamikelis, Liga Birzniece, Kaspars Megnis, Vita Rovite, Lauma Freimane, Laila Silamikele, Laura Ansone, Davids Fridmanis, Mikus Gavars, Dmitrijs Perminovs, Jurijs Perevoscikovs, Uga Dumpis, Janis Klovins
EPI_ISL_961806	Latvijas Infektologijas Centrs	Latvian Biomedical Research and Study Centre	Janis Pjalkovskis, Nikita Zrelovs, Monta Ustinova, Ivars Silamikelis, Liga Birzniece, Kaspars Megnis, Vita Rovite, Lauma Freimane, Laila Silamikele, Laura Ansone, Davids Fridmanis,Reinis Zeltmatis, Diana Dusacka, Jurijs Perevoscikovs, Uga Dumpis, Janis Klovins
EPI_ISL_961853, EPI_ISL_961854, EPI_ISL_961855, EPI_ISL_961856, EPI_ISL_961857, EPI_ISL_961858, EPI_ISL_961859, EPI_ISL_961860, EPI_ISL_961861	E. Gulbja laboratorija	Latvian Biomedical Research and Study Centre	Janis Pjalkovskis, Nikita Zrelovs, Monta Ustinova, Ivars Silamikelis, Liga Birzniece, Kaspars Megnis, Vita Rovite, Lauma Freimane, Laila Silamikele, Laura Ansone, Davids Fridmanis, Mikus Gavars, Dmitrijs Perminovs, Jurijs Perevoscikovs, Uga Dumpis, Janis Klovins
EPI_ISL_961862, EPI_ISL_961863	Latvijas Infektologijas Centrs	Latvian Biomedical Research and Study Centre	Janis Pjalkovskis, Nikita Zrelovs, Monta Ustinova, Ivars Silamikelis, Liga Birzniece, Kaspars Megnis, Vita Rovite, Lauma Freimane, Laila Silamikele, Laura Ansone, Davids Fridmanis,Reinis Zeltmatis, Diana Dusacka, Jurijs Perevoscikovs, Uga Dumpis, Janis Klovins
EPI_ISL_961864, EPI_ISL_961865	E. Gulbja laboratorija	Latvian Biomedical Research and Study Centre	Janis Pjalkovskis, Nikita Zrelovs, Monta Ustinova, Ivars Silamikelis, Liga Birzniece, Kaspars Megnis, Vita Rovite, Lauma Freimane, Laila Silamikele, Laura Ansone, Davids Fridmanis, Mikus Gavars, Dmitrijs Perminovs, Jurijs Perevoscikovs, Uga Dumpis, Janis Klovins
EPI_ISL_961866	Latvijas Infektologijas Centrs	Latvian Biomedical Research and Study Centre	Janis Pjalkovskis, Nikita Zrelovs, Monta Ustinova, Ivars Silamikelis, Liga Birzniece, Kaspars Megnis, Vita Rovite, Lauma Freimane, Laila Silamikele, Laura Ansone, Davids Fridmanis,Reinis Zeltmatis, Diana Dusacka, Jurijs Perevoscikovs, Uga Dumpis, Janis Klovins
EPI_ISL_961867, EPI_ISL_961868	E. Gulbja laboratorija	Latvian Biomedical Research and Study Centre	Janis Pjalkovskis, Nikita Zrelovs, Monta Ustinova, Ivars Silamikelis, Liga Birzniece, Kaspars Megnis, Vita Rovite, Lauma Freimane, Laila Silamikele, Laura Ansone, Davids Fridmanis, Mikus Gavars, Dmitrijs Perminovs, Jurijs Perevoscikovs, Uga Dumpis, Janis Klovins
EPI_ISL_961869, EPI_ISL_961870	Latvijas Infektologijas Centrs	Latvian Biomedical Research and Study Centre	Janis Pjalkovskis, Nikita Zrelovs, Monta Ustinova, Ivars Silamikelis, Liga Birzniece, Kaspars Megnis, Vita Rovite, Lauma Freimane, Laila Silamikele, Laura Ansone, Davids Fridmanis,Reinis Zeltmatis, Diana Dusacka, Jurijs Perevoscikovs, Uga Dumpis, Janis Klovins
EPI_ISL_961871, EPI_ISL_961872	E. Gulbja laboratorija	Latvian Biomedical Research and Study Centre	Janis Pjalkovskis, Nikita Zrelovs, Monta Ustinova, Ivars Silamikelis, Liga Birzniece, Kaspars Megnis, Vita Rovite, Lauma Freimane, Laila Silamikele, Laura Ansone, Davids Fridmanis, Mikus Gavars, Dmitrijs Perminovs, Jurijs Perevoscikovs, Uga Dumpis, Janis Klovins
EPI_ISL_962183	Toronto Invasive Bacterial Diseases Network	McMaster University	Allison McGeer, Patryk Aftanas, Hooman Derakhshani, Angel Li, Kuganya Nirmalarajah, Emily Panousis, Ahmed Draia, Jalees Nasir, Michael Surette, Samira Mubareka, Andrew G. McArthur
EPI_ISL_962205	Institute for Medical Research, Infectious Disease Research Centre, National Institutes of Health, Ministry of Health Malaysia	Institute for Medical Research, Infectious Disease Research Centre, National Institutes of Health, Ministry of Health Malaysia	Suppiah J, Kamel K, Azizan MA, Thayan R
EPI_ISL_962264, EPI_ISL_962265, EPI_ISL_962266, EPI_ISL_962267, EPI_ISL_962268, EPI_ISL_962269	Seattle Flu Study	Seattle Flu Study	Deborah A. Nickerson, Chris D. Frazar, Jover Lee, Benjamin Pelle, Erica Ryke, Matthew Richardson, Amanda Adler, Elisabeth Brandstetter, Peter D. Han, Kairsten Fay, Misja Ilcisin, Kirsten Lacombe, Thomas R. Sibley, Melissa Truong, Caitlin R. Wolf, Michael Boeckh, Janet A. Englund, Michael Famulare, Barry R. Lutz, Mark J. Rieder, Lea M. Starita, Matthew Thompson, Jay Shendure, Trevor Bedford, Helen Y. Chu
EPI_ISL_962285, EPI_ISL_962286, EPI_ISL_962288, EPI_ISL_962289, EPI_ISL_962290, EPI_ISL_962292, EPI_ISL_962293, EPI_ISL_962294, EPI_ISL_962295, EPI_ISL_962296, EPI_ISL_962297, EPI_ISL_962298, EPI_ISL_962299			
see above	Seattle Flu Study	Seattle Flu Study	Deborah A. Nickerson, Chris D. Frazar, Jover Lee, Benjamin Pelle, Erica Ryke, Matthew Richardson, Amanda Adler, Elisabeth Brandstetter, Peter D. Han, Kairsten Fay, Misja Ilcisin, Kirsten Lacombe, Thomas R. Sibley, Melissa Truong, Caitlin R. Wolf, Karen Cowgill, Stephanie Schrag, Jeff Duchin, Michael Boeckh, Janet A. Englund, Michael Famulare, Barry R. Lutz, Mark J. Rieder, Lea M. Starita, Matthew Thompson, Helen Y. Chu, Trevor Bedford, Jay Shendure
EPI_ISL_962305, EPI_ISL_962306, EPI_ISL_962307, EPI_ISL_962308, EPI_ISL_962309, EPI_ISL_962310, EPI_ISL_962311, EPI_ISL_962312, EPI_ISL_962313, EPI_ISL_962314, EPI_ISL_962315, EPI_ISL_962316, EPI_ISL_962317, EPI_ISL_962318, EPI_ISL_962319, EPI_ISL_962320, EPI_ISL_962321, EPI_ISL_962322, EPI_ISL_962323, EPI_ISL_962324, EPI_ISL_962325, EPI_ISL_962326, EPI_ISL_962327, EPI_ISL_962328, EPI_ISL_962329, EPI_ISL_962330, EPI_ISL_962331, EPI_ISL_962332, EPI_ISL_962333, EPI_ISL_962334, EPI_ISL_962335, EPI_ISL_962336, EPI_ISL_962337, EPI_ISL_962338, EPI_ISL_962339, EPI_ISL_962340, EPI_ISL_962341, EPI_ISL_962342, EPI_ISL_962343, EPI_ISL_962344, EPI_ISL_962345, EPI_ISL_962346, EPI_ISL_962347, EPI_ISL_962348, EPI_ISL_962349, EPI_ISL_962350, EPI_ISL_962351, EPI_ISL_962352, EPI_ISL_962353, EPI_ISL_962354, EPI_ISL_962355, EPI_ISL_962356, EPI_ISL_962357, EPI_ISL_962358, EPI_ISL_962359, EPI_ISL_962360, EPI_ISL_962361, EPI_ISL_962362, EPI_ISL_962363, EPI_ISL_962364, EPI_ISL_962365, EPI_ISL_962366, EPI_ISL_962367			
see above	Altius Institute for Biomedical Sciences	Seattle Flu Study	Deborah A. Nickerson, Chris D. Frazar, Jover Lee, Benjamin Pelle, Erica Ryke, Matthew Richardson, Amanda Adler, Elisabeth Brandstetter, Peter D. Han, Kairsten Fay, Misja Ilcisin, Kirsten Lacombe, Thomas R. Sibley, Melissa Truong, Caitlin R. Wolf, Ryan Alexander, Daniel Bates, Rebecca Bruders, Stephanie DeBaun, Clem Green, Muhammad Halimun, Jessica Halow, Kneshay Harper, Matt Hartman, Andrew Meuser, Alex Nguyen, Truong Nguyen, Sofia Olsson, Sadie Patraw, Hannah Petersen, Tobias Ragoczy, Joshua Richards, Jacob Rodriguez, John Stamatoyannopoulos, Julia Wald, Olivia Waltner, Michael Boeckh, Janet A. Englund, Michael Famulare, Barry R. Lutz, Mark J. Rieder, Lea M. Starita, Matthew Thompson, Helen Y. Chu, Jay Shendure, Trevor Bedford
EPI_ISL_962369	Seattle Flu Study	Seattle Flu Study	Deborah A. Nickerson, Chris D. Frazar, Jover Lee, Benjamin Pelle, Erica Ryke, Matthew Richardson, Amanda Adler, Elisabeth Brandstetter, Peter D. Han, Kairsten Fay, Misja Ilcisin, Kirsten Lacombe, Thomas R. Sibley, Melissa Truong, Caitlin R. Wolf, Karen Cowgill, Stephanie Schrag, Jeff Duchin, Michael Boeckh, Janet A. Englund, Michael Famulare, Barry R. Lutz, Mark J. Rieder, Lea M. Starita, Matthew Thompson, Helen Y. Chu, Trevor Bedford, Jay Shendure
EPI_ISL_962403, EPI_ISL_962404, EPI_ISL_962405, EPI_ISL_962406, EPI_ISL_962407, EPI_ISL_962408, EPI_ISL_962409, EPI_ISL_962410, EPI_ISL_962411, EPI_ISL_962412, EPI_ISL_962413, EPI_ISL_962414, EPI_ISL_962415, EPI_ISL_962416, EPI_ISL_962418, EPI_ISL_962419, EPI_ISL_962420			
see above	Washington State Department of Health	Seattle Flu Study	Deborah A. Nickerson, Chris D. Frazar, Jover Lee, Benjamin Pelle, Erica Ryke, Matthew Richardson, Amanda Adler, Elisabeth Brandstetter, Peter D. Han, Kairsten Fay, Misja Ilcisin, Kirsten Lacombe, Thomas R. Sibley, Melissa Truong, Caitlin R. Wolf, Romesh Gautom, Geoff Melly, Brian Hiatt, Philip Dykema, Scott Lindquist, Michael Boeckh, Janet A. Englund, Michael Famulare, Barry R. Lutz, Mark J. Rieder, Lea M. Starita, Matthew Thompson, Helen Y. Chu, Jay Shendure, Trevor Bedford
EPI_ISL_962521	UCLA Clinical Micro Lab	Los Angeles County PHL	P. Hemarajata et al.
EPI_ISL_962527	Institute for Medical Research, Infectious Disease Research Centre, National Institutes of Health, Ministry of Health Malaysia	Institute for Medical Research, Infectious Disease Research Centre, National Institutes of Health, Ministry of Health Malaysia	Suppiah J, Kamel K, Azizan MA, Thayan R
EPI_ISL_962618, EPI_ISL_962629, EPI_ISL_962641	Scripps Medical Laboratory	Andersen lab at Scripps Research	SEARCH Alliance San Diego with Michael Quigley, Ellen Stefanski, Ian Mchardy
EPI_ISL_962814	Robert Garry lab	Andersen lab at Scripps Research	Allison Smither, Gilberto Sabino-Santos, Patricia Snarski, Lilia Melnik, Antoinette Bell, Kaylynn Genemaras, Arnaud Drouin, Dahlene Fusco, Robert Garry with SEARCH Alliance San Diego

EPI_ISL_962827	Microbiological Diagnostic Unit - Public Health Laboratory (MDU-PHL)	MDU-PHL	Seemann T., Sait, M.L., Sherry, N.L.
EPI_ISL_962847	MD Laboratories	Los Angeles County PHL	P. Hemarajata et al.
EPI_ISL_962897	Akershus University Hospital, Department for Microbiology and Infectious Disease Control	Norwegian Institute of Public Health, Department of Virology	Kathrine Stene-Johansen, Kamilla Heddeland Instefjord, Hilde Elshaug, Ignacio Garcia Llorente, Serina B Engebretsen, Atiya R Ali,Marie Paulsen Madsen, Rasmus Riis Kopperud, Hilde Vollan, Karoline Bragstad, Olav Hungnes
EPI_ISL_962901, EPI_ISL_962902, EPI_ISL_962903	Furst Medical Laboratory	Norwegian Institute of Public Health, Department of Virology	Kathrine Stene-Johansen, Kamilla Heddeland Instefjord, Hilde Elshaug, Ignacio Garcia Llorente, Serina B Engebretsen, Atiya R Ali,Marie Paulsen Madsen, Rasmus Riis Kopperud, Hilde Vollan, Karoline Bragstad, Olav Hungnes
EPI_ISL_962908	Medical Microbiology Unit, Department for Laboratory Medicine, Drammen Hospital, Vestre Viken Health Trust,	Norwegian Institute of Public Health, Department of Virology	Kathrine Stene-Johansen, Kamilla Heddeland Instefjord, Hilde Elshaug, Ignacio Garcia Llorente, Serina B Engebretsen, Atiya R Ali,Marie Paulsen Madsen, Rasmus Riis Kopperud, Hilde Vollan, Karoline Bragstad, Olav Hungnes
EPI_ISL_962928	Servicio de Microbiología, Laboratori Clínic Metropolitana Nord. Hospital Universitari Germans Trias i Pujol. Institut d'Investigació en Ciències de la Salut Germans Trias i Pujol (IGTP)	SeqCOVID-SPAIN consortium/IBV(CSIC)	Elisa Martró, Antoni E. Bordoy, Anna Not, Adrián Antuori, Anabel Fernández, Nona Romaní, Verónica Saludes, Cristina Casañ and SeqCOVID-SPAIN consortium
EPI_ISL_962947, EPI_ISL_962948, EPI_ISL_962949, EPI_ISL_962950, EPI_ISL_962951, EPI_ISL_962952, EPI_ISL_962953, EPI_ISL_962956, EPI_ISL_962957	Hospital Universitario de Gran Canaria Dr. Negrín	SeqCOVID-SPAIN consortium/IBV(CSIC)	M. Carmen Pérez González, Francisco J. Chamizo López, Ana Bordes Benítez and SeqCOVID-SPAIN consortium
EPI_ISL_964295	Oslo University Hospital, Department of Medical Microbiology	Norwegian Institute of Public Health, Department of Virology	Kathrine Stene-Johansen, Kamilla Heddeland Instefjord, Hilde Elshaug, Ignacio Garcia Llorente, Serina B Engebretsen, Atiya R Ali, Marie Paulsen Madsen, Rasmus Riis Kopperud, Hilde Vollan, Karoline Bragstad, Olav Hungnes
EPI_ISL_964880	National Institute of Laboratory Medicine and Referral Center	Sher-e-Bangla Nagar, Agargaon, Dhaka-1207, Bangladesh.	Shahina Akter, Mohammad Samir Uzzaman, Eshrar Osman, Md. Ahashan Habib, Tanjina Akhtar Banu, Abu Sayeed Mohammad Mahmud, Md. Murshed Hasan Sarkar, Barna Goswami, Iffat Jahan, Md. Saddam Hossain, Tasnim Nafisa, Md. Maruf Ahmed Molla, Mahmuda Yeasmin, Asish Kumar Ghosh, Arifa Akram, A. K. M.Shamsuzzaman, Md. Salim Khan
EPI_ISL_964886	National Institute of Laboratory Medicine and Referral Center	Genomic Research Lab, BCSIR	Tasnim Nafisa, Mohammad Samir Uzzaman, Eshrar Osman, Md. Ahashan Habib, Shahina Akter,Tanjina Akhtar Banu, Abu Sayeed Mohammad Mahmud, Md. Murshed Hasan Sarkar, Barna Goswami, Iffat Jahan, Md. Saddam Hossain, Md. Maruf Ahmed Molla, Mahmuda Yeasmin, Asish Kumar Ghosh, Arifa Akram, A. K. M.Shamsuzzaman, Md. Salim Khan
EPI_ISL_964950	Laboratory of Virology and Molecular Diagnostics	Institute of Public Health of Republic of North Macedonia Laboratory of Virology and Molecular Diagnostics	Kuzmanovska M., Boshevska G.
EPI_ISL_964958	Akershus University Hospital, Department for Microbiology and Infectious Disease Control	Norwegian Institute of Public Health, Department of Virology	Kathrine Stene-Johansen, Kamilla Heddeland Instefjord, Hilde Elshaug, Ignacio Garcia Llorente, Serina B Engebretsen, Atiya R Ali,Marie Paulsen Madsen, Rasmus Riis Kopperud, Hilde Vollan, Karoline Bragstad, Olav Hungnes
EPI_ISL_964961	Dept. of Medical Microbiology, Stavanger University Hospital, Helse Stavanger HF	Norwegian Institute of Public Health, Department of Virology	Kathrine Stene-Johansen, Kamilla Heddeland Instefjord, Hilde Elshaug, Ignacio Garcia Llorente, Serina B Engebretsen, Atiya R Ali,Marie Paulsen Madsen, Rasmus Riis Kopperud, Hilde Vollan, Karoline Bragstad, Olav Hungnes
EPI_ISL_964969	University Hospital of Northern Norway, Department for Microbiology and Infectious Disease Control	Norwegian Institute of Public Health, Department of Virology	Kathrine Stene-Johansen, Kamilla Heddeland Instefjord, Hilde Elshaug, Ignacio Garcia Llorente, Serina B Engebretsen, Atiya R Ali,Marie Paulsen Madsen, Rasmus Riis Kopperud, Hilde Vollan, Karoline Bragstad, Olav Hungnes
EPI_ISL_964973	Ostfold Hospital Trust - Kalnes, Centre for Laboratory Medicine, Section for gene technology and infection serology	Norwegian Institute of Public Health, Department of Virology	Kathrine Stene-Johansen, Kamilla Heddeland Instefjord, Hilde Elshaug, Ignacio Garcia Llorente, Serina B Engebretsen, Atiya R Ali,Marie Paulsen Madsen, Rasmus Riis Kopperud, Hilde Vollan, Karoline Bragstad, Olav Hungnes
EPI_ISL_964981, EPI_ISL_964982	Department of Medical Microbiology - section Molde, Molde Hospital	Norwegian Institute of Public Health, Department of Virology	Kathrine Stene-Johansen, Kamilla Heddeland Instefjord, Hilde Elshaug, Ignacio Garcia Llorente, Serina B Engebretsen, Atiya R Ali,Marie Paulsen Madsen, Rasmus Riis Kopperud, Hilde Vollan, Karoline Bragstad, Olav Hungnes
EPI_ISL_965005, EPI_ISL_965006, EPI_ISL_965007	Dept. of Medical Microbiology, Stavanger University Hospital, Helse Stavanger HF	Norwegian Institute of Public Health, Department of Virology	Kathrine Stene-Johansen, Kamilla Heddeland Instefjord, Hilde Elshaug, Ignacio Garcia Llorente, Serina B Engebretsen, Atiya R Ali, Marie Paulsen Madsen, Rasmus Riis Kopperud, Hilde Vollan, Karoline Bragstad, Olav Hungnes
EPI_ISL_965015	Medical Microbiology Unit, Department for Laboratory Medicine, Drammen Hospital, Vestre Viken Health Trust,	Norwegian Institute of Public Health, Department of Virology	Kathrine Stene-Johansen, Kamilla Heddeland Instefjord, Hilde Elshaug, Ignacio Garcia Llorente, Serina B Engebretsen, Atiya R Ali, Marie Paulsen Madsen, Rasmus Riis Kopperud, Hilde Vollan, Karoline Bragstad, Olav Hungnes
EPI_ISL_965020	Florida Bureau of Public Health Laboratories	Florida Bureau of Public Health Laboratories	Sarah Schmedes, Jason Blanton
EPI_ISL_965086, EPI_ISL_965087, EPI_ISL_965088, EPI_ISL_965089, EPI_ISL_965090, EPI_ISL_965091, EPI_ISL_965092, EPI_ISL_965093, EPI_ISL_965094, EPI_ISL_965095, EPI_ISL_965096, EPI_ISL_965097, EPI_ISL_965098, EPI_ISL_965099, EPI_ISL_965100			
see above	Wyoming Public Health Laboratory	Wyoming Public Health Laboratory	Noah Hull, Taylor Fearing, Lynette Gumbleton, Channing Weber, Ashley Norberg, Bailey Bowcutt, and Wanda Manley
EPI_ISL_965128	INMI Lazzaro Spallanzani IRCCS	INMI Lazzaro Spallanzani IRCCS	M. Rueca, E Giombini, B Bartolini, O Butera, C.E.M Gruber, F Messina, A Di Caro, MR Capobianchi
EPI_ISL_965140	UOC "Microbiologia e Virologia" AOU "Policlinico Umberto I", Roma; Dipartimento di Medicina Molecolare Sapienza Università di Roma	INMI Lazzaro Spallanzani IRCCS	F Messina, C.E.M Gruber, B Bartolini, E Giombini, M Rueca, O Butera, G Antonelli, O Turriziani, MR Capobianchi, A Di Caro
EPI_ISL_965700	Dutch COVID-19 response team	Medical Microbiology, Maastricht University Medical Centre	Jozef Dingemans*, Brian van der Veer*, Erik Beuken, Carmen Reumkens, Lieke van Alphen, Christian Hoebe, Paul Savelkoul
EPI_ISL_965909	Botswana Harvard HIV Reference Laboratory	Botswana Harvard HIV Reference Laboratory	Sikhulile Moyo, Wonderful T. Choga, Dorcas Maruapula, Botshelo Radibe, Boitumelo Zuze, David Lawrence, Roger Shapiro, Shahin Lockman, Mosepele Mosepele, Joseph Makhema, Simani Gaseitsiwe
EPI_ISL_965914	Hospital General Universitario de Ciudad Real	Instituto de Salud Carlos III	Vázquez, S. Iglesias-Caballero, M. Sandonís,V. Camarero, S. Pozo, F. Casas, I. Jiménez, P. Zaballos, A. Monzón, S. Varona, S. Cuesta, I. Illescas, S.
EPI_ISL_965920, EPI_ISL_965924	Servicio Murciano de Salud	Instituto de Salud Carlos III	Vázquez, S. Iglesias-Caballero, M. Sandonís,V. Camarero, S. Pozo, F. Casas, I. Jiménez, P. Zaballos, A. Monzón, S. Varona, S. Cuesta, I. Blázquez, A.
EPI_ISL_965926	Hospital General Universitario de Ciudad Real	Instituto de Salud Carlos III	Sandonís,V. Vázquez, S. Iglesias-Caballero, M. Camarero, S. Pozo, F. Casas, I. Jiménez, P. Zaballos, A. Monzón, S. Varona, S. Cuesta, I. Illescas, S.
EPI_ISL_965946	Hospital General Universitario de Ciudad Real	Instituto de Salud Carlos III	Iglesias-Caballero, M. Sandonís,V. Vázquez, S. Camarero, S. Pozo, F. Casas, I. Jiménez, P. Zaballos, A. Monzón, S. Varona, S. Cuesta, I. Illescas, S.
EPI_ISL_965954	Servicio Murciano de Salud	Instituto de Salud Carlos III	Iglesias-Caballero, M. Sandonís,V. Vázquez, S. Camarero, S. Pozo, F. Casas, I. Jiménez, P. Zaballos, A. Monzón, S. Varona, S. Cuesta, I. Blázquez, A.
EPI_ISL_966497, EPI_ISL_966498, EPI_ISL_966499, EPI_ISL_966553, EPI_ISL_966554, EPI_ISL_966556, EPI_ISL_966557, EPI_ISL_966558, EPI_ISL_966559, EPI_ISL_966564, EPI_ISL_966566, EPI_ISL_966567, EPI_ISL_966568, EPI_ISL_966569, EPI_ISL_966570, EPI_ISL_966571, EPI_ISL_966572, EPI_ISL_966573, EPI_ISL_966574, EPI_ISL_966575, EPI_ISL_966576, EPI_ISL_966592, EPI_ISL_966593, EPI_ISL_966594, EPI_ISL_966595, EPI_ISL_966596, EPI_ISL_966597, EPI_ISL_966598, EPI_ISL_966599, EPI_ISL_966602, EPI_ISL_966608, EPI_ISL_966618, EPI_ISL_966752, EPI_ISL_966753, EPI_ISL_966754, EPI_ISL_966755, EPI_ISL_967182, EPI_ISL_967425, EPI_ISL_967426, EPI_ISL_967427, EPI_ISL_967428, EPI_ISL_967429, EPI_ISL_967430, EPI_ISL_967456, EPI_ISL_967457, EPI_ISL_967458, EPI_ISL_967459, EPI_ISL_967460, EPI_ISL_967461, EPI_ISL_967462, EPI_ISL_967463, EPI_ISL_967464, EPI_ISL_967465, EPI_ISL_967466, EPI_ISL_967467, EPI_ISL_967468, EPI_ISL_967469, EPI_ISL_967470, EPI_ISL_967471, EPI_ISL_967472, EPI_ISL_967473, EPI_ISL_967474, EPI_ISL_967475, EPI_ISL_967476, EPI_ISL_967477, EPI_ISL_967478, EPI_ISL_967479, EPI_ISL_967480, EPI_ISL_967481, EPI_ISL_967482, EPI_ISL_967483, EPI_ISL_967484, EPI_ISL_967485, EPI_ISL_967486, EPI_ISL_967487, EPI_ISL_967488, EPI_ISL_967489, EPI_ISL_967490, EPI_ISL_967491, EPI_ISL_967492, EPI_ISL_967493, EPI_ISL_967494, EPI_ISL_967495, EPI_ISL_967496, EPI_ISL_967497, EPI_ISL_967498, EPI_ISL_967499, EPI_ISL_967500, EPI_ISL_967501, EPI_ISL_967502, EPI_ISL_967503, EPI_ISL_967504, EPI_ISL_967505, EPI_ISL_967506, EPI_ISL_967507, EPI_ISL_967508, EPI_ISL_967509, EPI_ISL_967510, EPI_ISL_967511, EPI_ISL_967512, EPI_ISL_967513, EPI_ISL_967514, EPI_ISL_967515, EPI_ISL_967516, EPI_ISL_967517, EPI_ISL_967518, EPI_ISL_967519, EPI_ISL_967520, EPI_ISL_967521, EPI_ISL_967522, EPI_ISL_967524, EPI_ISL_967531, EPI_ISL_967537, EPI_ISL_967547, EPI_ISL_967554, EPI_ISL_967567, EPI_ISL_967576, EPI_ISL_967583, EPI_ISL_967590, EPI_ISL_967599, EPI_ISL_967609, EPI_ISL_967617, EPI_ISL_967629, EPI_ISL_967642, EPI_ISL_967655, EPI_ISL_967662, EPI_ISL_967671, EPI_ISL_967678, EPI_ISL_967684, EPI_ISL_967693, EPI_ISL_967700, EPI_ISL_967710, EPI_ISL_967719, EPI_ISL_967726, EPI_ISL_967736, EPI_ISL_967748, EPI_ISL_967753			
see above	Helix/Illumina	Respiratory Viruses Branch, Division of Viral Diseases, Centers for Disease Control and Prevention	Peter W. Cook,Dakota Howard,Dhwani Batra,Ben L. Rambo-Martin,Eileen de Feo,Jan Antico,Christine Tran,Matthew Tolentino,Shannon Wickline,Kim Gietzen,Brad Sickler,Jingtao Liu,Eric Allen,Phil Febbo,Summer Galloway,Nicole L. Washington,Simon White,Geraint Levan,Kelly Schiabor Barrett,Elizabeth Cirulli,Alexandre Bolze,Ary Ascencio,Charlotte Rivera-Garcia,Ryan Cho,Jason Nguyen,Sherry Wang,Jimmy Ramirez,Tyler Cassens,Efren Sandoval,Magnus Isaksson,William Lee,David Becker,Marc Laurent,James Lu,Clinton R. Paden,Suxiang Tong,Duncan MacCannell,
EPI_ISL_967755	State Laboratories Division, Hawaii State Department of Health	State Laboratories Division, Hawaii State Department of Health	Pamela O'Brien, Drew Kuwazaki, Ayana Garnet, Razvan Sultana, Edward Desmond

EPI_ISL_967761, EPI_ISL_967767	Helix/Illumina	Respiratory Viruses Branch, Division of Viral Diseases, Centers for Disease Control and Prevention	Peter W. Cook,Dakota Howard,Dhwani Batra,Ben L. Rambo-Martin,Eileen de Feo,Jan Antico,Christine Tran,Matthew Tolentino,Shannon Wickline,Kim Gietzen,Brad Sickler,Jingtao Liu,Eric Allen,Phil Febbo,Summer Galloway,Nicole L. Washington,Simon White,Geraint Levan,Kelly Schiabor Barrett,Elizabeth Cirulli,Alexandre Bolze,Ary Ascencio,Charlotte Rivera-Garcia,Ryan Cho,Jason Nguyen,Sherry Wang,Jimmy Ramirez,Tyler Cassens,Efren Sandoval,Magnus Isaksson,William Lee,David Becker,Marc Laurent,James Lu,Clinton R. Paden,Suxiang Tong,Duncan MacCannell,
EPI_ISL_967768, EPI_ISL_967769	State Laboratories Division, Hawaii State Department of Health	State Laboratories Division, Hawaii State Department of Health	Pamela O'Brien, Drew Kuwazaki, Ayana Garnet, Razvan Sultana, Edward Desmond
EPI_ISL_967772, EPI_ISL_967773, EPI_ISL_967774, EPI_ISL_967775, EPI_ISL_967776, EPI_ISL_967777, EPI_ISL_967778, EPI_ISL_967779, EPI_ISL_967780	Helix/Illumina	Respiratory Viruses Branch, Division of Viral Diseases, Centers for Disease Control and Prevention	Peter W. Cook,Dakota Howard,Dhwani Batra,Ben L. Rambo-Martin,Eileen de Feo,Jan Antico,Christine Tran,Matthew Tolentino,Shannon Wickline,Kim Gietzen,Brad Sickler,Jingtao Liu,Eric Allen,Phil Febbo,Summer Galloway,Nicole L. Washington,Simon White,Geraint Levan,Kelly Schiabor Barrett,Elizabeth Cirulli,Alexandre Bolze,Ary Ascencio,Charlotte Rivera-Garcia,Ryan Cho,Jason Nguyen,Sherry Wang,Jimmy Ramirez,Tyler Cassens,Efren Sandoval,Magnus Isaksson,William Lee,David Becker,Marc Laurent,James Lu,Clinton R. Paden,Suxiang Tong,Duncan MacCannell,
EPI_ISL_968069, EPI_ISL_968070, EPI_ISL_968071, EPI_ISL_968072, EPI_ISL_968073, EPI_ISL_968074, EPI_ISL_968075, EPI_ISL_968076	Monterey County Public Health Laboratory	Monterey County Public Health Laboratory	Monterey County Public Health Laboratory
EPI_ISL_968252	Botswana Harvard HIV Reference Laboratory	Botswana Harvard HIV Reference Laboratory	Sikhulile Moyo, Dorcas Maruapula, Wonderful Choga, Botshelo Radibe, Boitumelo Zuze, David Lawrence, Roger Shapiro, Shahin Lockman, Mosepele Mosepele, Joseph Makhema, Simani Gaseitsiwe
EPI_ISL_969001, EPI_ISL_969002, EPI_ISL_969023, EPI_ISL_969052	KEMRI-Wellcome Trust Research Programme/KEMRI-CGMR-C Kilifi	KEMRI-Wellcome Trust Research Programme/KEMRI-CGMR-C Kilifi	Githinji et al
EPI_ISL_970827, EPI_ISL_970849, EPI_ISL_970867, EPI_ISL_970907, EPI_ISL_970961, EPI_ISL_970966, EPI_ISL_971266, EPI_ISL_971346, EPI_ISL_971349, EPI_ISL_971391, EPI_ISL_971436, EPI_ISL_971473, EPI_ISL_971478, EPI_ISL_971560, EPI_ISL_971566, EPI_ISL_971613, EPI_ISL_971690, EPI_ISL_971719, EPI_ISL_971839, EPI_ISL_972072, EPI_ISL_972137, EPI_ISL_972139, EPI_ISL_972202, EPI_ISL_972225, EPI_ISL_972232, EPI_ISL_972251, EPI_ISL_972273, EPI_ISL_972275, EPI_ISL_972297, EPI_ISL_972352, EPI_ISL_972363, EPI_ISL_972369, EPI_ISL_972371, EPI_ISL_972418, EPI_ISL_972456, EPI_ISL_972514, EPI_ISL_972575, EPI_ISL_972608, EPI_ISL_972618, EPI_ISL_972722, EPI_ISL_972750, EPI_ISL_972761, EPI_ISL_972778, EPI_ISL_972793, EPI_ISL_972835, EPI_ISL_972874, EPI_ISL_972884, EPI_ISL_973028, EPI_ISL_973035, EPI_ISL_973078, EPI_ISL_973128, EPI_ISL_973131, EPI_ISL_973263, EPI_ISL_973271, EPI_ISL_973310, EPI_ISL_973325, EPI_ISL_973390, EPI_ISL_973492, EPI_ISL_973523, EPI_ISL_973568, EPI_ISL_973591, EPI_ISL_973672, EPI_ISL_973804			
see above	Department of Virus and Microbiological Special Diagnostics, Statens Serum Institut, Copenhagen, Denmark	Aalborg University	Danish Covid-19 Genome Consortium
EPI_ISL_977065, EPI_ISL_977066, EPI_ISL_977067, EPI_ISL_977069, EPI_ISL_977070, EPI_ISL_977071, EPI_ISL_977072, EPI_ISL_977076, EPI_ISL_977077	Rhode Island Department of Health	Infectious Disease Program, Broad Institute of Harvard and MIT	Lemieux,J.E., Siddle,K.J., Huard,R., King,E., Azevedo,K., Miller,A., Adams,G., Gladden-Young,A., Lagerborg,K., Rudy,M., DeRuff,K., Carter,A., Normandin,E., Bauer,M., Reilly,S., Tomkins-Tinch,C., Loreth,C., Chaluvadi,S., Birren,B.W., Gallagher,G., Smole,S., Park,D.J., MacInnis,B.L., and Sabeti,P.C.
EPI_ISL_977133, EPI_ISL_977134, EPI_ISL_977135, EPI_ISL_977136, EPI_ISL_977137	Flow Health	Infectious Disease Program, Broad Institute of Harvard and MIT	Lemieux,J.E., Siddle,K.J., Adams,G., Gladden-Young,A., Lagerborg,K., Rudy,M., DeRuff,K., Carter,A., Normandin,E., Bauer,M., Reilly,S., Tomkins-Tinch,C., Loreth,C., Chaluvadi,S., Birren,B.W., Gallagher,G., Smole,S., Park,D.J., MacInnis,B.L., and Sabeti,P.C.
EPI_ISL_977270, EPI_ISL_977334, EPI_ISL_977350, EPI_ISL_977351, EPI_ISL_977468	University of Zambia, School of Veterinary Medicine	UNZAVET and PATH	Mulenga Mwenda-Chimfwembe, Ngonda Saasa, Daniel Bridges
EPI_ISL_977538	Caribbean Public Health Agency	Carrington Lab, Department of PreClinical Sciences	Nikita S. D. Sahadeo, Arianne Brown-Jordan, Vernie Ramkissoon, Sarah Hill, Naresh Nandram, Dr. Sharon Belmar-George Avery Hinds, Jerome Foster, Stanley Giddings, Karla Georges, Marsha Ivey, Rahul Naidu, Risha Singh, SueMin Nathaniel, Rajini Haraksingh, Jaya Jayaraman, Chinna Chinnadurai, Adesh Ramsubhag, Nuno Faria, Oliver Pybus, Christopher Oura, Gabriel Escobar, Christine V. F. Carrington
EPI_ISL_977539	Caribbean Public Health Agency	Carrington Lab, Department of PreClinical Sciences	Nikita S. D. Sahadeo, Arianne Brown-Jordan, Vernie Ramkissoon, Sarah Hill, Naresh Nandram, Avery Hinds, Dr. Sharon Belmar-George, Jerome Foster, Stanley Giddings, Karla Georges, Marsha Ivey, Rahul Naidu, Risha Singh, SueMin Nathaniel, Rajini Haraksingh, Jaya Jayaraman, Chinna Chinnadurai, Adesh Ramsubhag, Nuno Faria, Oliver Pybus, Christopher Oura, Gabriel Escobar, Christine V. F. Carrington
EPI_ISL_978158, EPI_ISL_978159	Chiu Laboratory, University of California, San Francisco	Chiu Laboratory, University of California, San Francisco	Charles Chiu, Xianding (Wayne) Deng, Candace Wang, Venice Servellita, Jill Hacker, Debra Wadford
EPI_ISL_978226	Virginia Division of Consolidated Laboratory Services	Virginia Division of Consolidated Laboratory Services	Virginia DCLS
EPI_ISL_978274, EPI_ISL_978275, EPI_ISL_978276, EPI_ISL_978277, EPI_ISL_978278, EPI_ISL_978279, EPI_ISL_978350, EPI_ISL_978351, EPI_ISL_978352	Texas Department of State Health Services	Texas Department of State Health Services	Bonnie Oh, Anita Pokharel, James Daniel Bonser, Myong Koag, Chung Wang, Rachel Lee, Grace Kubin, Rashmi Tuladhar, Mayela Pedrueza, Maliha Rahman, Jenny Zhang
EPI_ISL_978383, EPI_ISL_978384, EPI_ISL_978385, EPI_ISL_978386, EPI_ISL_978387, EPI_ISL_978388, EPI_ISL_978389, EPI_ISL_978390, EPI_ISL_978446, EPI_ISL_978447, EPI_ISL_978448, EPI_ISL_978449, EPI_ISL_978450, EPI_ISL_978451, EPI_ISL_978452, EPI_ISL_978453, EPI_ISL_978454, EPI_ISL_978455, EPI_ISL_978456, EPI_ISL_978457, EPI_ISL_978458, EPI_ISL_978459, EPI_ISL_978460, EPI_ISL_978461, EPI_ISL_978468, EPI_ISL_978469			
see above	Arizona State Public Health Laboratory	Arizona State Public Health Laboratory	Trung Huynh, Jessica Escobar, Katherine Fullerton, Nobuko Fukushima, Stacy White, Linda Getsinger, Victor Waddell
EPI_ISL_978880, EPI_ISL_978881, EPI_ISL_978882, EPI_ISL_978883, EPI_ISL_978884, EPI_ISL_978885, EPI_ISL_978932, EPI_ISL_978934, EPI_ISL_978937	Centre for Dengue Research and AICBU, Department of Immunology and Molecular Medicine	Centre for Dengue Research and AICBU, Department of Immunology and Molecular Medicine	Chandima Jeewandara, Deshni Jayathilaka, Dinuka Ariyaratne, Tibutius Thanesh Pramanayagam, Diyanath Ranasinghe, Laksiri Gomes, Gathsaurie Neelika Malavige
EPI_ISL_979133, EPI_ISL_979134, EPI_ISL_979143, EPI_ISL_979144	Santa Clara County Public Health Laboratory	Chan-Zuckerberg Biohub	CZB Cliahub Consortium
EPI_ISL_979252	Institute of Microbiology and Immunology, Faculty of Medicine, University of Ljubljana	Institute of Microbiology and Immunology, Faculty of Medicine, University of Ljubljana	Samo Zakotnik, Tomaž Mark Zorec, Matic Brvar, Doroteja Vlaj, Patricija Pozvek,Špela Pleh, Miša Korva, Mario Poljak, Tatjana Avši - Županc
EPI_ISL_979312, EPI_ISL_979324	Cadham Provincial laboratory	National Microbiology Laboratory (NML)	Anna Majer, Shari Tyson, Grace Seo, Philip Mabon, Elsie Grudeski, Rhiannon Huzarewich, Russell Mandes, Anneliese Landgraff, Jennifer Tanner, Natalie Knox, Morag Graham, Gary Van Domselaar, Paul Van Caeseele, Jared Bullard, David Alexander, Kerry Dust, Nathalie Bastien, Yan Li, Timothy Booth, Darian Hole, Madison Chapel, Kirsten Biggar, CanCOGeN's metadata curation team, Public Health Agency of Canada CanCOGeN team
EPI_ISL_979372	The Jackson Laboratory	The Jackson Laboratory	Lloyd M, Sanderson B, Srivastava A, Maurya R, Renzette N, Omerza G, Kelly K, Li L, Wei C L, Adams M
EPI_ISL_979638, EPI_ISL_979639, EPI_ISL_979640, EPI_ISL_979641, EPI_ISL_979642	Santa Clara County Public Health Laboratory	Chan-Zuckerberg Biohub	CZB Cliahub Consortium
EPI_ISL_980907, EPI_ISL_980908, EPI_ISL_980914, EPI_ISL_980940	Innovative Genomics Institute, UC Berkeley	Innovative Genomics Institute, UC Berkeley	Stacia Wyman, Haridha Shivram, Phil Frankino, Liana Lareau
EPI_ISL_981059, EPI_ISL_981065, EPI_ISL_981066, EPI_ISL_981067, EPI_ISL_981072	Johns Hopkins Hospital Department of Pathology	Johns Hopkins Hospital Department of Pathology	C. Paul Morris, Chun Huai Luo, Adannaya Amadi, Matthew Schwartz, Nicholas Gallagher, Heba H. Mostafa
EPI_ISL_981270, EPI_ISL_981272,	Hospital Universitari de Bellvitge	Hospital Universitari Vall d'Hebron	Cristina Andrés, Maria Piñana, Josep F Abril, Damir Garcia-Cehic, Ariadna Rando, Juliana Esperalba, Maria Gema Codina, Carla Castillo, Maria Carmen

EPI_ISL_981295			Martin, Tomàs Pumarola, Josep Quer, Andrés Antón
EPI_ISL_981298, EPI_ISL_981299, EPI_ISL_981301, EPI_ISL_981302, EPI_ISL_981303	Hospital Universitari Vall d'Hebron	Hospital Universitari Vall d'Hebron	Cristina Andrés, Maria Piñana, Josep F Abril, Damir Garcia-Cehic, Ariadna Rando, Juliana Esperalba, Maria Gema Codina, Carla Castillo, Maria Carmen Martin, Tomàs Pumarola, Josep Quer, Andrés Antón
EPI_ISL_981373	AZ Klina	AZ Klina	Dr. C. Vael
EPI_ISL_981384, EPI_ISL_981386	Botswana Harvard HIV Reference Laboratory	Botswana Harvard HIV Reference Laboratory	Sikhulile Moyo, Dorcas Maruapula, Wonderful Choga, Botshelo Radibe, Boitumelo Zuze, David Lawrence, Roger Shapiro, Shahin Lockman, Mosepele Mosepele, Joseph Makhema, Simani Gaseitsiwe
EPI_ISL_981387	IAL Regional de Bauru	Instituto Adolfo Lutz, Interdiciplinary Procedures Center, Strategic Laboratory	Claudio Tavares Sacchi, Claudia Regina Gonçalves, Erica Valessa Ramos Gomes, Karoline Rodrigues Campos
EPI_ISL_981548	Botswana Harvard HIV Reference Laboratory	Botswana Harvard HIV Reference Laboratory	Sikhulile Moyo, Dorcas Maruapula, Wonderful T. Choga, Botshelo Radibe, Boitumelo Zuze, David Lawrence, Roger Shapiro, Shahin Lockman, Mosepele Mosepele, Joseph Makhema, Simani Gaseitsiwe
EPI_ISL_981971, EPI_ISL_981972, EPI_ISL_981973	Microbiology Service, Hospital Universitario Clinico San Cecilio, Granada	Microbiology Service, Hospital Universitario Clinico San Cecilio, Granada	Adolfo de Salazar, Natalia Chueca, Laura Viñuela, Ana Fuentes, Federico García
EPI_ISL_982048, EPI_ISL_982057, EPI_ISL_982097	TGen North	TGen North	"Jolene Bowers, Megan Folkerts, Chris French, Hayley Yaglom, Ashlyn Pfeiffer, Darrin Lemmer, Dave Engelthaler, The Arizona COVID Genomics Union (ACGU)"
EPI_ISL_982244, EPI_ISL_982273, EPI_ISL_982285	Lab voor klinische biologie	Lab voor klinische biologie	Hannelore Hamerlinck, Marija Janevska, Bruno Verhasselt
EPI_ISL_982395	M Health Fairview	Minnesota Department of Health, Public Health Laboratory	Alexandra Lorentz, Jacob Garfin, Matt Plumb, and Xiong Wang
EPI_ISL_982505	National Institute of Laboratory Medicine and Referral Center	Genomic Research Lab, BCSIR	Iffat Jahan, Mohammad Samir Uzzaman, Eshrar Osman, Md. Ahashan Habib, Shahina Akter, Tanjina Akhtar Banu, Abu Sayeed Mohammad Mahmud, Md. Murshed Hasan Sarkar, Barna Goswami, Md. Saddam Hossain, Tasnim Nafisa, Md. Maruf Ahmed Molla, Mahmuda Yeasmin, Asish Kumar Ghosh, Arifa Akram, A. K. M.Shamsuzzaman, Md. Salim Khan
EPI_ISL_982781, EPI_ISL_982782, EPI_ISL_982784, EPI_ISL_982785, EPI_ISL_982786, EPI_ISL_982788, EPI_ISL_982789, EPI_ISL_982790, EPI_ISL_982791, EPI_ISL_982792, EPI_ISL_982793, EPI_ISL_982794, EPI_ISL_982795, EPI_ISL_982796, EPI_ISL_982797, EPI_ISL_982798, EPI_ISL_982799, EPI_ISL_982800, EPI_ISL_982801, EPI_ISL_982802, EPI_ISL_982803, EPI_ISL_982804, EPI_ISL_982805, EPI_ISL_982806, EPI_ISL_982807, EPI_ISL_982808, EPI_ISL_982809, EPI_ISL_982810, EPI_ISL_982812, EPI_ISL_982813, EPI_ISL_982814, EPI_ISL_982816, EPI_ISL_982819, EPI_ISL_982820, EPI_ISL_982821, EPI_ISL_982822, EPI_ISL_982823, EPI_ISL_982824, EPI_ISL_982825, EPI_ISL_982826, EPI_ISL_982827, EPI_ISL_982828, EPI_ISL_982829, EPI_ISL_982830, EPI_ISL_982832, EPI_ISL_982833, EPI_ISL_982834, EPI_ISL_982835, EPI_ISL_982836, EPI_ISL_982837, EPI_ISL_982838, EPI_ISL_982840			
see above	University Health Network/Mount Sinai Hospital Department of Microbiology	Ontario Institute for Cancer Research	Marie-Ming Aynaud, Javier Hernandez, Seda Barutcu, Kin Chan, Jessica Bourke, Marc Mazzulli, Tony Mazzulli, Laurence Pelletier, Jeff Wrana, Aimee Paterson, Angel Liu, Allison McGeer, Patryk Aftanas, Kuganya Nirmalarajah, Samira Mubareka, Ilinca Lungu, Cassandra Bergwerff, Lubaina Kothari, Bernard Lam, Paul Krzyzanowski, Michael Laszloffy, Lawrence E. Heisler, Richard de Borja, Jared T. Simpson
EPI_ISL_982841	National Institute of Laboratory Medicine and Referral Center	Genomic Research Lab, BCSIR	Md. Saddam Hossain, Mohammad Samir Uzzaman, Eshrar Osman, Md. Ahashan Habib, Shahina Akter, Tanjina Akhtar Banu, Abu Sayeed Mohammad Mahmud, Md. Murshed Hasan Sarkar, Barna Goswami, Iffat Jahan, Tasnim Nafisa, Md. Maruf Ahmed Molla, Mahmuda Yeasmin, Asish Kumar Ghosh, Arifa Akram, A. K. M.Shamsuzzaman, Md. Salim Khan
EPI_ISL_982885, EPI_ISL_982886, EPI_ISL_982892, EPI_ISL_982893, EPI_ISL_982924, EPI_ISL_982925	MEPHI Aix Marseille University (AMU)	MEPHI Aix Marseille University (AMU)	Anthony LEVASSEUR
EPI_ISL_982937, EPI_ISL_982965, EPI_ISL_982968, EPI_ISL_982971, EPI_ISL_982975, EPI_ISL_982989, EPI_ISL_982997, EPI_ISL_982998, EPI_ISL_983003, EPI_ISL_983004, EPI_ISL_983006, EPI_ISL_983007, EPI_ISL_983009, EPI_ISL_983010, EPI_ISL_983012, EPI_ISL_983013, EPI_ISL_983014, EPI_ISL_983015, EPI_ISL_983016, EPI_ISL_983017, EPI_ISL_983018, EPI_ISL_983019, EPI_ISL_983020, EPI_ISL_983023, EPI_ISL_983025, EPI_ISL_983026, EPI_ISL_983029, EPI_ISL_983033, EPI_ISL_983034, EPI_ISL_983035, EPI_ISL_983036, EPI_ISL_983037, EPI_ISL_983038, EPI_ISL_983040, EPI_ISL_983041, EPI_ISL_983042, EPI_ISL_983043, EPI_ISL_983046, EPI_ISL_983047, EPI_ISL_983048, EPI_ISL_983050, EPI_ISL_983052, EPI_ISL_983056, EPI_ISL_983057, EPI_ISL_983058, EPI_ISL_983059, EPI_ISL_983060, EPI_ISL_983062			
see above	University Health Network/Mount Sinai Hospital Department of Microbiology	Ontario Institute for Cancer Research	Marie-Ming Aynaud, Javier Hernandez, Seda Barutcu, Kin Chan, Jessica Bourke, Marc Mazzulli, Tony Mazzulli, Laurence Pelletier, Jeff Wrana, Aimee Paterson, Angel Liu, Allison McGeer, Patryk Aftanas, Kuganya Nirmalarajah, Samira Mubareka, Ilinca Lungu, Cassandra Bergwerff, Lubaina Kothari, Bernard Lam, Paul Krzyzanowski, Michael Laszloffy, Lawrence E. Heisler, Richard de Borja, Jared T. Simpson
EPI_ISL_983082	Kentucky State Public Health Lab	Kentucky State Public Health Lab	Stephanie Lunn, Karim George, Joshua Tobias, William Grooms, Vaneet Arora, Matthew Johnson, Rachel Zinner, Rhonda Lucas
EPI_ISL_983089, EPI_ISL_983090, EPI_ISL_983091	Gravity Diagnostics	Kentucky State Public Health Lab	Stephanie Lunn, Karim George, Joshua Tobias, William Grooms, Vaneet Arora, Matthew Johnson, Rachel Zinner, Rhonda Lucas
EPI_ISL_983092, EPI_ISL_983095	Kentucky State Public Health Lab	Kentucky State Public Health Lab	Stephanie Lunn, Karim George, Joshua Tobias, William Grooms, Vaneet Arora, Matthew Johnson, Rachel Zinner, Rhonda Lucas
EPI_ISL_983171, EPI_ISL_983172, EPI_ISL_983173, EPI_ISL_983174, EPI_ISL_983175, EPI_ISL_983176, EPI_ISL_983177, EPI_ISL_983178, EPI_ISL_983179, EPI_ISL_983180, EPI_ISL_983181, EPI_ISL_983182, EPI_ISL_983183, EPI_ISL_983184, EPI_ISL_983185, EPI_ISL_983186, EPI_ISL_983187, EPI_ISL_983188, EPI_ISL_983189, EPI_ISL_983190, EPI_ISL_983191, EPI_ISL_983192, EPI_ISL_983193, EPI_ISL_983194, EPI_ISL_983195, EPI_ISL_983196, EPI_ISL_983197, EPI_ISL_983198, EPI_ISL_983199, EPI_ISL_983200, EPI_ISL_983201, EPI_ISL_983202, EPI_ISL_983203, EPI_ISL_983204, EPI_ISL_983205, EPI_ISL_983206, EPI_ISL_983207, EPI_ISL_983208, EPI_ISL_983209, EPI_ISL_983210, EPI_ISL_983211, EPI_ISL_983212, EPI_ISL_983213, EPI_ISL_983214, EPI_ISL_983215, EPI_ISL_983216, EPI_ISL_983217, EPI_ISL_983218, EPI_ISL_983219, EPI_ISL_983220, EPI_ISL_983221, EPI_ISL_983222, EPI_ISL_983223, EPI_ISL_983224, EPI_ISL_983225, EPI_ISL_983226, EPI_ISL_983227, EPI_ISL_983228, EPI_ISL_983229, EPI_ISL_983230, EPI_ISL_983231, EPI_ISL_983232, EPI_ISL_983233, EPI_ISL_983234, EPI_ISL_983235, EPI_ISL_983236, EPI_ISL_983237, EPI_ISL_983238, EPI_ISL_983239			
see above	University Health Network/Mount Sinai Hospital Department of Microbiology	Ontario Institute for Cancer Research	Marie-Ming Aynaud, Javier Hernandez, Seda Barutcu, Kin Chan, Jessica Bourke, Marc Mazzulli, Tony Mazzulli, Laurence Pelletier, Jeff Wrana, Aimee Paterson, Angel Liu, Allison McGeer, Patryk Aftanas, Kuganya Nirmalarajah, Samira Mubareka, Ilinca Lungu, Cassandra Bergwerff, Lubaina Kothari, Bernard Lam, Paul Krzyzanowski, Michael Laszloffy, Lawrence E. Heisler, Richard de Borja, Jared T. Simpson
EPI_ISL_983372, EPI_ISL_983373	Utah Public Health Laboratory	Utah Public Health Laboratory	Erin L. Young, Kelly F. Oakeson, Tara Gallagher
EPI_ISL_983500	National Institute of Laboratory Medicine and Referral Center	Genomic Research Lab, BCSIR	Iffat Jahan, Mohammad Samir Uzzaman, Eshrar Osman, Md. Ahashan Habib, Shahina Akter, Tanjina Akhtar Banu, Abu Sayeed Mohammad Mahmud, Md. Murshed Hasan Sarkar, Barna Goswami, Md. Saddam Hossain, Tasnim Nafisa, Md. Maruf Ahmed Molla, Mahmuda Yeasmin, Asish Kumar Ghosh, Arifa Akram, A. K. M. Shamsuzzaman, Md. Salim Khan
EPI_ISL_983614, EPI_ISL_983615	Texas Department of State Health Services	Texas Department of State Health Services	Bonnie Oh, Anita Pokharel, James Daniel Bonser, Myong Koag, Chung Wang, Rachel Lee, Grace Kubin, Rashmi Tuladhar, Mayela Pedrueza, Maliha Rahman, Jenny Zhang
EPI_ISL_983799, EPI_ISL_983800	Colorado Department of Public Health and Environment	Colorado Department of Puplic Health and Environment	Laura Bankers, Molly C. Hetherington-Rauth, Diana Ir, Shannon Ely, Shannon R. Matzinger, Sarah Elizabeth Totten, Emily A. Travanty
