# Supplementary material for: Detection and characterization of the SARS-CoV-2 lineage B.1.526 in New York
Source: Nat Commun. 2021 Aug 9;12:4886. doi: 10.1038/s41467-021-25168-4 (PMC8352861; doi:10.1038/s41467-021-25168-4)
Supplement: Supplementary file 8 — Supplementary Data 4 [file 41467_2021_25168_MOESM8_ESM.zip › GISAID_acknowledements_tables/gisaid_hcov-19_acknowledgement_table_2021_02_13_010-11.pdf]

We gratefully acknowledge the following Authors from the Originating laboratories responsible for obtaining the specimens, as well as the Submitting laboratories where the genome data were generated and shared via GISAID, on which this research is based.

All Submitters of data may be contacted directly via www.gisaid.org

Authors are sorted alphabetically.

Accession ID	Originating Laboratory	Submitting Laboratory	Authors
EPI_ISL_812359	Area of Virology, Serology and Virology Division (SAViD), New South Wales Health Pathology Randwick	Virology Research Laboratory; Area of Virology, Serology and Virology Division (SAViD), New South Wales Health Pathology Randwick	Foster, C.; Au, J.; Ruiz Silva, M.; Deveson, I.; Bull, R.; Van Hal, S.; Rawlinson, W.
EPI_ISL_831663	Institute of Virology, Biomedical Research Center of the Slovak Academy of Sciences, Bratislava	Faculty of Natural Sciences, Comenius University, Bratislava	Kristína Boršová, Viktória abanová, Broa Brejová, Viktória Hodorová, Sabina Fumaová Havlíková, Juraj Kopáek, Martina Liková, ubomíra Lukáiková, Martina Neboháová, Monika Sláviková, Tomáš Vina, Boris Klempa, Jozef Nosek
EPI_ISL_831664, EPI_ISL_831665	Institute of Virology, Biomedical Research Center of the Slovak Academy of Sciences, Bratislava	Faculty of Natural Sciences, Comenius University, Bratislava	Broa Brejová, Viktória abanová, Kristína Boršová, Viktória Hodorová, Sabina Fumaová Havlíková, Juraj Kopáek, Martina Liková, ubomíra Lukáiková, Martina Neboháová, Monika Sláviková, Tomáš Vina, Jozef Nosek, Boris Klempa
EPI_ISL_831666	Institute of Virology, Biomedical Research Center of the Slovak Academy of Sciences, Bratislava	Faculty of Natural Sciences, Comenius University, Bratislava	Viktória abanová, Kristína Boršová, Broa Brejová, Viktória Hodorová, Sabina Fumaová Havlíková, Juraj Kopáek, Martina Liková, ubomíra Lukáiková, Martina Neboháová, Monika Sláviková, Tomáš Vina, Jozef Nosek, Boris Klempa
EPI_ISL_833373, EPI_ISL_833374, EPI_ISL_833375, EPI_ISL_833376, EPI_ISL_833377, EPI_ISL_833379, EPI_ISL_833380, EPI_ISL_833381, EPI_ISL_833382, EPI_ISL_833383, EPI_ISL_833384, EPI_ISL_833385, EPI_ISL_833386			
see above	National Public Health Laboratory, National Centre for Infectious Diseases	National Public Health Laboratory, National Centre for Infectious Diseases	Tze Minn Mak, Sophie Octavia, Zhenyang Zhou, Lin Cui, Raymond Tzer Pin Lin
EPI_ISL_833432	Torrance Memorial Medical Center Microbiology	Los Angeles County PHL	P. Hemarajata et al.
EPI_ISL_837058, EPI_ISL_837063, EPI_ISL_837065, EPI_ISL_837073, EPI_ISL_837087, EPI_ISL_837183, EPI_ISL_837196, EPI_ISL_837197, EPI_ISL_837245, EPI_ISL_837246	Respiratory Virus Unit, National Infection Service, Public Health England	COVID-19 Genomics UK (COG-UK) Consortium	PHE Covid Sequencing Team
EPI_ISL_838015, EPI_ISL_838017, EPI_ISL_838018, EPI_ISL_838021, EPI_ISL_838022, EPI_ISL_838025, EPI_ISL_838035, EPI_ISL_838038, EPI_ISL_838048	Department of Pathology, University of Cambridge	COVID-19 Genomics UK (COG-UK) Consortium	Aminu S. Jahun, Yasmin Chaudhry, Grant Hall, Iliana Georgana, Myra Hosmillo, Martin D. Curran, Malte Pinckert, Surendra Parmar, Ian Goodfellow
EPI_ISL_839006, EPI_ISL_839020	University College London, Great Ormond Street Hospital for Children NHS Foundation Trust, Imperial College Healthcare NHS Trust	COVID-19 Genomics UK (COG-UK) Consortium	Sergi Castellano, Rachel Williams, Mark Kristiansen, Paola Resende Silva, Sunando Roy, Tony Brooks, Helena Tutill, Paola Niola, Patricia Dyal, Charlotte Williams, Leysa Forrest, Yasmin Panchbhaya, Jacqueline Findlay, Samuel Weeks, Julianne Brown, Kathryn Harris, Paul Randell, James Price, Alison Holmes, Judith Breuer
EPI_ISL_845617	KU Leuven, Rega Institute, Clinical and Epidemiological Virology	KU Leuven, Rega Institute, Clinical and Epidemiological Virology	Tony Wawina-Bokalanga, Bert Vanmechelen, Joan Marti-Carerras, Piet Maes
EPI_ISL_845655	Laboratorio de Salud Pública - Secretaría Distrital de Salud	Instituto Nacional de Salud - Dirección de Investigación en Salud Pública	Katherine Laiton-Donato, Diego A. Álvarez-Díaz, Carlos Franco-Muñoz, Mauricio Pacheco-Montealegre, Maria T. Herrera-Sepúlveda, Jonathan Reales, Sheryll Corchuelo, Julian Naizaque, Gerardo Santamaría, Paola Muñoz-Laiton, Diego Andrés Prada, Magdalena Wiesner, Martha Lucia Ospina Martinez, Marcela Mercado-Reyes
EPI_ISL_845806	Sydney South West Pathology Service (SSWPS) - Royal Prince Alfred Hospital - NSW Health Pathology	NSW Health Pathology - Institute of Clinical Pathology and Medical Research; Westmead Hospital; University of Sydney	CIDM-PH et al.
EPI_ISL_846553, EPI_ISL_846554, EPI_ISL_846555, EPI_ISL_846556, EPI_ISL_846557, EPI_ISL_846568, EPI_ISL_846569, EPI_ISL_846570, EPI_ISL_846571, EPI_ISL_846572, EPI_ISL_846573, EPI_ISL_846574, EPI_ISL_846578, EPI_ISL_846579, EPI_ISL_846580			
see above	Lab voor klinische biologie	Lab voor klinische biologie	Hannelore Hamerlinck, Bruno Verhasselt
EPI_ISL_848200, EPI_ISL_848201, EPI_ISL_848202	Charité Universitätsmedizin Berlin, Institute of Virology, Charitéplatz 1, 10117 Berlin, Germany	Charité Universitätsmedizin Berlin, Institute of Virology, Charitéplatz 1, 10117 Berlin, Germany	Victor M Corman, Julia Schneider, Jörn Beheim-Schwarzbach, Tobias Bleicker, Julia Tesch, Barbara Mühlemann, Talitha Veith, Terry Jones, Christian Drosten
EPI_ISL_849761	unknown	PHV-FSS	Son Nguyen et al.
EPI_ISL_852968, EPI_ISL_853006, EPI_ISL_853013, EPI_ISL_853014, EPI_ISL_853015	Hospital General Universitario Gregorio Marañón	SeqCOVID-SPAIN consortium/IBV(CSIC)	Darío García de Viedma, Laura Pérez-Lago, Pedro J Sola-Campoy, Sergio Buenestado-Serrano, Marta Herranz, Victor Manuel de la Cueva, Julia Suárez, Pilar Catalán, Patricia Muñoz and SeqCOVID-SPAIN consortium
EPI_ISL_854594	Faroese National Reference Laboratory for Fish and Animal Diseases	Faroese National Reference Laboratory for Fish and Animal Diseases	Maria Marjunardóttir Dahl, Petra Elisabeth Petersen, Arnfinnur Kallsberg Junior, Debes Hammershaimb Christiansen
EPI_ISL_854754, EPI_ISL_854758	Microbiological Diagnostic Unit - Public Health Laboratory (MDU-PHL)	MDU-PHL	Seemann T., Sait, M.L., Sherry, N.L.
EPI_ISL_854759, EPI_ISL_854762	Victorian Infectious Diseases Reference Laboratory (VIDRL)	VIDRL and MDU-PHL	Caly L., Seemann T., Sait, M.L., Druce J., Sherry, N.L.
EPI_ISL_855603	Respiratory Virus Unit, National Infection Service, Public Health England	COVID-19 Genomics UK (COG-UK) Consortium	PHE Covid Sequencing Team
EPI_ISL_856707, EPI_ISL_856708, EPI_ISL_856709, EPI_ISL_856710	MFD Veselbas centrs "Dziedniecba"	Latvian Biomedical Research and Study Centre	Ivars Silamielis, Kaspars Megnis, Monta Ustinova, Jnis Pjalkovskis, ikita Zrelovs, Vita Rovte, Iveta Bere, Marija Donaka, Uga Dumpis, Jnis Kloviš
EPI_ISL_856726, EPI_ISL_856727, EPI_ISL_856728, EPI_ISL_856729, EPI_ISL_856749, EPI_ISL_856750, EPI_ISL_856751, EPI_ISL_856752, EPI_ISL_856753, EPI_ISL_856754	Department of Clinical Microbiology	GIGA Medical Genomics	Keith Durkin, Maria Artesi, Sébastien Bontems, Raphaël Boreux, Bouchra Boujemla, Cécile Meex, Pierrette Melin, Marie-Pierre Hayette, Vincent Bours
EPI_ISL_857309	Medlab Pathology	NSW Health Pathology - Institute of Clinical Pathology and Medical Research; Westmead Hospital; University of Sydney	CIDM-PH et al.
EPI_ISL_857348	Caribbean Public Health Agency	Carrington Lab, Department of PreClinical Sciences, Faculty of Medical Sciences, The University of the West Indies	Nikita S. D. Sahadeo, Arianne Brown-Jordan, Vernie Ramkissoon, Sarah Hill, Vernie Ramkissoon, Roshan Parasram, Naresh Nandram, Avery Hinds, Jerome Foster, Stanley Giddings, Karla Georges, Marsha Ivey, Rahul Naidu, Risha Singh, SueMin Nathaniel, Rajini Haraksingh, Jaya Jayaraman, Chinna Chinnadurai, Adesh Ramsubhag, Nuno Faria, Oliver Pybus, Christopher Oura, Gabriel Escobar, Christine V. F. Carrington
EPI_ISL_857396, EPI_ISL_857397, EPI_ISL_857398, EPI_ISL_857399, EPI_ISL_857400, EPI_ISL_857401, EPI_ISL_857407, EPI_ISL_857410, EPI_ISL_857412, EPI_ISL_857413, EPI_ISL_857415, EPI_ISL_857416, EPI_ISL_857417, EPI_ISL_857418, EPI_ISL_857419, EPI_ISL_857420, EPI_ISL_857421, EPI_ISL_857422, EPI_ISL_857423, EPI_ISL_857424, EPI_ISL_857425, EPI_ISL_857426, EPI_ISL_857427, EPI_ISL_857428, EPI_ISL_857429, EPI_ISL_857430, EPI_ISL_857431, EPI_ISL_857432, EPI_ISL_857433, EPI_ISL_857434, EPI_ISL_857435, EPI_ISL_857436, EPI_ISL_857437, EPI_ISL_857438, EPI_ISL_857439, EPI_ISL_857440,			

EPI_ISL_857441, EPI_ISL_857442, EPI_ISL_857443, EPI_ISL_857447			
see above	Maine HETL	Tewhey Lab, The Jackson Laboratory	Matluk,N., Dewey,H., Iosue,F., Barter,M., Lynch,R., Munger,H. and Tewhey,R.
EPI_ISL_857468	PathWest Laboratory Medicine WA	PathWest Laboratory Medicine WA Microbial Surveillance Unit	PathWest Laboratory Medicine WA Microbial Surveillance Unit
EPI_ISL_857485	National Public Health Laboratory, National Centre for Infectious Diseases	National Public Health Laboratory, National Centre for Infectious Diseases	Tze Minn Mak, Sophie Octavia, Zhenyang Zhou, Lin Cui, Raymond Tzer Pin Lin
EPI_ISL_857486	PathWest Laboratory Medicine WA	PathWest Laboratory Medicine WA Microbial Surveillance Unit	PathWest Laboratory Medicine WA Microbial Surveillance Unit
EPI_ISL_857855	Lighthouse Lab in Cambridge	Wellcome Sanger Institute for the COVID-19 Genomics UK (COG-UK) Consortium	Rob Howes, The Lighthouse Lab in Cambridge and Alex Alderton, Roberto Amato, Sonia Goncalves, Ewan Harrison, David K. Jackson, Ian Johnston, Dominic Kwiatkowski, Cordelia Langford, John Sillitoe on behalf of the Wellcome Sanger Institute COVID-19 Surveillance Team
EPI_ISL_860262	Department of Medical Microbiology - section Molde, Molde Hospital	Norwegian Institute of Public Health, Department of Virology	Kathrine Stene-Johansen, Kamilla Heddeland Instefjord, Hilde Elshaug, Atiya R Ali,Marie Paulsen Madsen, Rasmus Riis Kopperud, Hilde Vollan, Karoline Bragstad, Olav Hungnes
EPI_ISL_860278	Haukeland University Hospital, Dept. of Microbiology	Norwegian Institute of Public Health, Department of Virology	Kathrine Stene-Johansen, Kamilla Heddeland Instefjord, Hilde Elshaug, Atiya R Ali,Marie Paulsen Madsen, Rasmus Riis Kopperud, Hilde Vollan, Karoline Bragstad, Olav Hungnes
EPI_ISL_860686, EPI_ISL_860695, EPI_ISL_860709	Respiratory Virus Unit, National Infection Service, Public Health England	COVID-19 Genomics UK (COG-UK) Consortium	PHE Covid Sequencing Team
EPI_ISL_860721, EPI_ISL_860722, EPI_ISL_860723, EPI_ISL_860726	Institute for Infectious Diseases, University of Bern, Switzerland	Institute for Infectious Diseases, University of Bern, Switzerland	Michel C Koch, Christian Baumann, Miguel A Terrazos Miani, Cora Sägesser, Pascal Bittel, Stephen L Leib, Peter Keller, Franziska Suter-Riniker, Alban Ramette
EPI_ISL_860954, EPI_ISL_860959, EPI_ISL_860961, EPI_ISL_860963, EPI_ISL_860966, EPI_ISL_860967, EPI_ISL_860968, EPI_ISL_860975, EPI_ISL_860983, EPI_ISL_860986, EPI_ISL_860990, EPI_ISL_860991, EPI_ISL_860993, EPI_ISL_861001, EPI_ISL_861005, EPI_ISL_861006, EPI_ISL_861007, EPI_ISL_861009, EPI_ISL_861012, EPI_ISL_861015, EPI_ISL_861034, EPI_ISL_861038, EPI_ISL_861046, EPI_ISL_861049, EPI_ISL_861053, EPI_ISL_861057, EPI_ISL_861066, EPI_ISL_861069, EPI_ISL_861071, EPI_ISL_861072, EPI_ISL_861073, EPI_ISL_861084, EPI_ISL_861092, EPI_ISL_861093, EPI_ISL_861094			
see above	Johns Hopkins Hospital Department of Pathology	Johns Hopkins Hospital Department of Pathology	C. Paul Morris, Chun Huai Luo, Adannaya Amadi, Nicholas Gallagher, Heba H. Mostafa
EPI_ISL_861447, EPI_ISL_861448, EPI_ISL_861452, EPI_ISL_861453, EPI_ISL_861455	Clinical Molecular Microbiology Laboratory, UNC Hospitals	Jeremy Wang	Jeremy Wang, Alexander Rubinsteyn, Colleen Rice, Jason Smedberg, Melissa Miller, Corbin Jones, Robert Hagan
EPI_ISL_861507	Cerballiance Provence	CERBA LAB	Roig JC; Delaunay E; Prots L.
EPI_ISL_861710	Laboratory for Respiratory Viruses, Cantacuzino National Military-Medical Institute for Research and Development	Cantacuzino Institute Virology	Mihaela Lazar, Luiza Ustea, Nicoleta Parashiv, Tim Durfee
EPI_ISL_861711, EPI_ISL_861712, EPI_ISL_861713	Laboratory for Respiratory Viruses, Cantacuzino National Military-Medical Institute for Research and Development	Cantacuzino Institute Virology	Mihaela Lazar, Luiza Ustea, Nicoleta Paraschiv, Tim Durfee
EPI_ISL_861735, EPI_ISL_861738, EPI_ISL_861741	Tempus	Grubaugh Lab - Yale School of Public Health	Tara Alpert, Joseph Fauver, Anderson Brito, Mallery Breban, Anne Wyllie, Chantal Vogels, Mary Petrone, Annie Watkins, Chaney Kalinich, Isabel Ott, Nathan Grubaugh
EPI_ISL_861745	Yale Pathology Lab	Grubaugh Lab - Yale School of Public Health	Tara Alpert, Joseph Fauver, Chen Liu, Pei Hui, Jianhui Wang, Susan Bell and Han Zhou, Anderson Brito, Mallery Breban, Anne Wyllie, Chantal Vogels, Mary Petrone, Chaney Kalinich, Isabel Ott, Arnau Casanovas, Catherine Muenker, Adam Moore, Alice Lu, Maria Tokuyama, Patrick Wong, Peiwen Lu, Saad Omer, Richard Martinello, Allison Nelson, Shelli Farhadian, Akiko Iwasaki, Charlese Dela Cruz, Albert Ko, Nathan Grubaugh
EPI_ISL_861757, EPI_ISL_861759, EPI_ISL_861760	Tempus	Grubaugh Lab - Yale School of Public Health	Tara Alpert, Joseph Fauver, Anderson Brito, Mallery Breban, Anne Wyllie, Chantal Vogels, Mary Petrone, Annie Watkins, Chaney Kalinich, Isabel Ott, Nathan Grubaugh
EPI_ISL_861768	Yale Pathology Lab	Grubaugh Lab - Yale School of Public Health	Tara Alpert, Joseph Fauver, Chen Liu, Pei Hui, Jianhui Wang, Susan Bell and Han Zhou, Anderson Brito, Mallery Breban, Anne Wyllie, Chantal Vogels, Mary Petrone, Chaney Kalinich, Isabel Ott, Arnau Casanovas, Catherine Muenker, Adam Moore, Alice Lu, Maria Tokuyama, Patrick Wong, Peiwen Lu, Saad Omer, Richard Martinello, Allison Nelson, Shelli Farhadian, Akiko Iwasaki, Charlese Dela Cruz, Albert Ko, Nathan Grubaugh
EPI_ISL_861769	Tempus	Grubaugh Lab - Yale School of Public Health	Tara Alpert, Joseph Fauver, Anderson Brito, Mallery Breban, Anne Wyllie, Chantal Vogels, Mary Petrone, Annie Watkins, Chaney Kalinich, Isabel Ott, Nathan Grubaugh
EPI_ISL_861770	Yale Pathology Lab	Grubaugh Lab - Yale School of Public Health	Tara Alpert, Joseph Fauver, Chen Liu, Pei Hui, Jianhui Wang, Susan Bell and Han Zhou, Anderson Brito, Mallery Breban, Anne Wyllie, Chantal Vogels, Mary Petrone, Chaney Kalinich, Isabel Ott, Arnau Casanovas, Catherine Muenker, Adam Moore, Alice Lu, Maria Tokuyama, Patrick Wong, Peiwen Lu, Saad Omer, Richard Martinello, Allison Nelson, Shelli Farhadian, Akiko Iwasaki, Charlese Dela Cruz, Albert Ko, Nathan Grubaugh
EPI_ISL_862124, EPI_ISL_862136, EPI_ISL_862139, EPI_ISL_862141, EPI_ISL_862144, EPI_ISL_862146, EPI_ISL_862151, EPI_ISL_862152, EPI_ISL_862164, EPI_ISL_862165, EPI_ISL_862167, EPI_ISL_862168, EPI_ISL_862169, EPI_ISL_862171, EPI_ISL_862176, EPI_ISL_862177, EPI_ISL_862178			
see above	Charité Universitätsmedizin Berlin, Institut fur Virologie/Labor Berlin	Charité Universitätsmedizin Berlin, Institut für Virologie	Victor M Corman, Barbara Mühlemann, Jörn Beheim-Schwarzbach, Tobias Bleicker, Julia Tesch, Talitha Veith, Julia Schneider, Terry Jones, Christian Drosten
EPI_ISL_862193	Respiratory Virus Unit, National Infection Service, Public Health England	COVID-19 Genomics UK (COG-UK) Consortium	PHE Covid Sequencing Team
EPI_ISL_862718, EPI_ISL_862719	Centre for Dengue Research and AICBU, Department of Immunology and Molecular Medicine	Centre for Dengue Research and AICBU, Department of Immunology and Molecular Medicine	Chandima Jeewandara, Deshni Jayathilaka, Dinuka Ariyaratne, Diyanath Ranasinghe, Laksiri Gomes, Gathsaurie Neelika Malavige
EPI_ISL_862723	San Diego County Public Health Laboratory	Andersen lab at Scripps Research	SEARCH Alliance San Diego with Tracy Basler, Jovan Shephard, Brett Austin
EPI_ISL_862725	Centre for Dengue Research and AICBU, Department of Immunology and Molecular Medicine	Centre for Dengue Research and AICBU, Department of Immunology and Molecular Medicine	Chandima Jeewandara, Deshni Jayathilaka, Dinuka Ariyaratne, Tibutius Thanesh Pramanayagam, Diyanath Ranasinghe, Laksiri Gomes, Gathsaurie Neelika Malavige
EPI_ISL_862833	National Institute of Infectious Diseases-Prof. Dr. Matei Bals Molecular Diagnostics Laboratory	National Institute of Infectious Diseases-Prof. Dr. Matei Bals Molecular Diagnostics Laboratory	Leontina Banica, Marius Surleac, Corina Casangiu, Petre Milu, Andreea Tudor, Simona Paraschiv, Dan Otelea
EPI_ISL_863245, EPI_ISL_863246, EPI_ISL_863247, EPI_ISL_863248, EPI_ISL_863249, EPI_ISL_863250	Lighthouse Lab in Glasgow	Wellcome Sanger Institute for the COVID-19 Genomics UK (COG-UK) Consortium	Harper VanSteenhouse, Yumi Kasai, David Gray, Carol Clugston, Anna Dominiczak and Alex Alderton, Roberto Amato, Sonia Goncalves, Ewan Harrison, David K. Jackson, Ian Johnston, Dominic Kwiatkowski, Cordelia Langford, John Sillitoe on behalf of the Wellcome Sanger Institute COVID-19 Surveillance Team
EPI_ISL_863251	Lighthouse Lab in Alderley Park	Wellcome Sanger Institute for the COVID-19 Genomics UK (COG-UK) Consortium	Jacquelyn Wynn, Mairead Hyland, The Lighthouse Lab in Alderley Park and Alex Alderton, Roberto Amato, Sonia Goncalves, Ewan Harrison, David K. Jackson, Ian Johnston, Dominic Kwiatkowski, Cordelia Langford, John Sillitoe on behalf of the Wellcome Sanger Institute COVID-19 Surveillance Team
EPI_ISL_863252, EPI_ISL_863253	Lighthouse Lab in Glasgow	Wellcome Sanger Institute for the COVID-19 Genomics UK (COG-UK) Consortium	Harper VanSteenhouse, Yumi Kasai, David Gray, Carol Clugston, Anna Dominiczak and Alex Alderton, Roberto Amato, Sonia Goncalves, Ewan Harrison, David K. Jackson, Ian Johnston, Dominic Kwiatkowski, Cordelia Langford, John Sillitoe on behalf of the Wellcome Sanger Institute COVID-19 Surveillance Team
EPI_ISL_863254	Lighthouse Lab in Alderley Park	Wellcome Sanger Institute for the COVID-19 Genomics UK (COG-UK) Consortium	Jacquelyn Wynn, Mairead Hyland, The Lighthouse Lab in Alderley Park and Alex Alderton, Roberto Amato, Sonia Goncalves, Ewan Harrison, David K. Jackson, Ian Johnston, Dominic Kwiatkowski, Cordelia Langford, John Sillitoe on behalf of the Wellcome Sanger Institute COVID-19 Surveillance Team
EPI_ISL_863255, EPI_ISL_863256, EPI_ISL_863257	Lighthouse Lab in Glasgow	Wellcome Sanger Institute for the COVID-19 Genomics UK (COG-UK) Consortium	Harper VanSteenhouse, Yumi Kasai, David Gray, Carol Clugston, Anna Dominiczak and Alex Alderton, Roberto Amato, Sonia Goncalves, Ewan Harrison, David K. Jackson, Ian Johnston, Dominic Kwiatkowski, Cordelia Langford, John Sillitoe on behalf of the Wellcome Sanger Institute COVID-19 Surveillance Team
EPI_ISL_863258	Lighthouse Lab in Alderley Park	Wellcome Sanger Institute for the COVID-19 Genomics UK (COG-UK) Consortium	Jacquelyn Wynn, Mairead Hyland, The Lighthouse Lab in Alderley Park and Alex Alderton, Roberto Amato, Sonia Goncalves, Ewan Harrison, David K. Jackson, Ian Johnston, Dominic Kwiatkowski, Cordelia Langford, John Sillitoe on behalf of the Wellcome Sanger Institute COVID-19 Surveillance Team

EPI_ISL_863259, EPI_ISL_863260, EPI_ISL_863261, EPI_ISL_863262, EPI_ISL_863263, EPI_ISL_863264, EPI_ISL_863265, EPI_ISL_863266, EPI_ISL_863267	Lighthouse Lab in Glasgow	Wellcome Sanger Institute for the COVID-19 Genomics UK (COG-UK) Consortium	Harper VanSteenhouse, Yumi Kasai, David Gray, Carol Clugston, Anna Dominiczak and Alex Alderton, Roberto Amato, Sonia Goncalves, Ewan Harrison, David K. Jackson, Ian Johnston, Dominic Kwiatkowski, Cordelia Langford, John Sillitoe on behalf of the Wellcome Sanger Institute COVID-19 Surveillance Team
EPI_ISL_863268	Lighthouse Lab in Alderley Park	Wellcome Sanger Institute for the COVID-19 Genomics UK (COG-UK) Consortium	Jacquelyn Wynn, Mairead Hyland, The Lighthouse Lab in Alderley Park and Alex Alderton, Roberto Amato, Sonia Goncalves, Ewan Harrison, David K. Jackson, Ian Johnston, Dominic Kwiatkowski, Cordelia Langford, John Sillitoe on behalf of the Wellcome Sanger Institute COVID-19 Surveillance Team
EPI_ISL_863269	Lighthouse Lab in Glasgow	Wellcome Sanger Institute for the COVID-19 Genomics UK (COG-UK) Consortium	Harper VanSteenhouse, Yumi Kasai, David Gray, Carol Clugston, Anna Dominiczak and Alex Alderton, Roberto Amato, Sonia Goncalves, Ewan Harrison, David K. Jackson, Ian Johnston, Dominic Kwiatkowski, Cordelia Langford, John Sillitoe on behalf of the Wellcome Sanger Institute COVID-19 Surveillance Team
EPI_ISL_863270, EPI_ISL_863271, EPI_ISL_863272	Lighthouse Lab in Alderley Park	Wellcome Sanger Institute for the COVID-19 Genomics UK (COG-UK) Consortium	Jacquelyn Wynn, Mairead Hyland, The Lighthouse Lab in Alderley Park and Alex Alderton, Roberto Amato, Sonia Goncalves, Ewan Harrison, David K. Jackson, Ian Johnston, Dominic Kwiatkowski, Cordelia Langford, John Sillitoe on behalf of the Wellcome Sanger Institute COVID-19 Surveillance Team
EPI_ISL_863273	Lighthouse Lab in Glasgow	Wellcome Sanger Institute for the COVID-19 Genomics UK (COG-UK) Consortium	Harper VanSteenhouse, Yumi Kasai, David Gray, Carol Clugston, Anna Dominiczak and Alex Alderton, Roberto Amato, Sonia Goncalves, Ewan Harrison, David K. Jackson, Ian Johnston, Dominic Kwiatkowski, Cordelia Langford, John Sillitoe on behalf of the Wellcome Sanger Institute COVID-19 Surveillance Team
EPI_ISL_863274, EPI_ISL_863275	Lighthouse Lab in Alderley Park	Wellcome Sanger Institute for the COVID-19 Genomics UK (COG-UK) Consortium	Jacquelyn Wynn, Mairead Hyland, The Lighthouse Lab in Alderley Park and Alex Alderton, Roberto Amato, Sonia Goncalves, Ewan Harrison, David K. Jackson, Ian Johnston, Dominic Kwiatkowski, Cordelia Langford, John Sillitoe on behalf of the Wellcome Sanger Institute COVID-19 Surveillance Team
EPI_ISL_863276, EPI_ISL_863277, EPI_ISL_863278, EPI_ISL_863279	Lighthouse Lab in Glasgow	Wellcome Sanger Institute for the COVID-19 Genomics UK (COG-UK) Consortium	Harper VanSteenhouse, Yumi Kasai, David Gray, Carol Clugston, Anna Dominiczak and Alex Alderton, Roberto Amato, Sonia Goncalves, Ewan Harrison, David K. Jackson, Ian Johnston, Dominic Kwiatkowski, Cordelia Langford, John Sillitoe on behalf of the Wellcome Sanger Institute COVID-19 Surveillance Team
EPI_ISL_863280, EPI_ISL_863281	Lighthouse Lab in Alderley Park	Wellcome Sanger Institute for the COVID-19 Genomics UK (COG-UK) Consortium	Jacquelyn Wynn, Mairead Hyland, The Lighthouse Lab in Alderley Park and Alex Alderton, Roberto Amato, Sonia Goncalves, Ewan Harrison, David K. Jackson, Ian Johnston, Dominic Kwiatkowski, Cordelia Langford, John Sillitoe on behalf of the Wellcome Sanger Institute COVID-19 Surveillance Team
EPI_ISL_863282, EPI_ISL_863283, EPI_ISL_863284, EPI_ISL_863285, EPI_ISL_863286, EPI_ISL_863287, EPI_ISL_863288	Lighthouse Lab in Glasgow	Wellcome Sanger Institute for the COVID-19 Genomics UK (COG-UK) Consortium	Harper VanSteenhouse, Yumi Kasai, David Gray, Carol Clugston, Anna Dominiczak and Alex Alderton, Roberto Amato, Sonia Goncalves, Ewan Harrison, David K. Jackson, Ian Johnston, Dominic Kwiatkowski, Cordelia Langford, John Sillitoe on behalf of the Wellcome Sanger Institute COVID-19 Surveillance Team
EPI_ISL_863289, EPI_ISL_863290	Lighthouse Lab in Alderley Park	Wellcome Sanger Institute for the COVID-19 Genomics UK (COG-UK) Consortium	Jacquelyn Wynn, Mairead Hyland, The Lighthouse Lab in Alderley Park and Alex Alderton, Roberto Amato, Sonia Goncalves, Ewan Harrison, David K. Jackson, Ian Johnston, Dominic Kwiatkowski, Cordelia Langford, John Sillitoe on behalf of the Wellcome Sanger Institute COVID-19 Surveillance Team
EPI_ISL_863291, EPI_ISL_863292, EPI_ISL_863293, EPI_ISL_863294, EPI_ISL_863295, EPI_ISL_863296, EPI_ISL_863297, EPI_ISL_863298	Lighthouse Lab in Glasgow	Wellcome Sanger Institute for the COVID-19 Genomics UK (COG-UK) Consortium	Harper VanSteenhouse, Yumi Kasai, David Gray, Carol Clugston, Anna Dominiczak and Alex Alderton, Roberto Amato, Sonia Goncalves, Ewan Harrison, David K. Jackson, Ian Johnston, Dominic Kwiatkowski, Cordelia Langford, John Sillitoe on behalf of the Wellcome Sanger Institute COVID-19 Surveillance Team
EPI_ISL_863299	Lighthouse Lab in Alderley Park	Wellcome Sanger Institute for the COVID-19 Genomics UK (COG-UK) Consortium	Jacquelyn Wynn, Mairead Hyland, The Lighthouse Lab in Alderley Park and Alex Alderton, Roberto Amato, Sonia Goncalves, Ewan Harrison, David K. Jackson, Ian Johnston, Dominic Kwiatkowski, Cordelia Langford, John Sillitoe on behalf of the Wellcome Sanger Institute COVID-19 Surveillance Team
EPI_ISL_863300, EPI_ISL_863301	Lighthouse Lab in Glasgow	Wellcome Sanger Institute for the COVID-19 Genomics UK (COG-UK) Consortium	Harper VanSteenhouse, Yumi Kasai, David Gray, Carol Clugston, Anna Dominiczak and Alex Alderton, Roberto Amato, Sonia Goncalves, Ewan Harrison, David K. Jackson, Ian Johnston, Dominic Kwiatkowski, Cordelia Langford, John Sillitoe on behalf of the Wellcome Sanger Institute COVID-19 Surveillance Team
EPI_ISL_863302	Lighthouse Lab in Alderley Park	Wellcome Sanger Institute for the COVID-19 Genomics UK (COG-UK) Consortium	Jacquelyn Wynn, Mairead Hyland, The Lighthouse Lab in Alderley Park and Alex Alderton, Roberto Amato, Sonia Goncalves, Ewan Harrison, David K. Jackson, Ian Johnston, Dominic Kwiatkowski, Cordelia Langford, John Sillitoe on behalf of the Wellcome Sanger Institute COVID-19 Surveillance Team
EPI_ISL_863303, EPI_ISL_863304, EPI_ISL_863305	Lighthouse Lab in Glasgow	Wellcome Sanger Institute for the COVID-19 Genomics UK (COG-UK) Consortium	Harper VanSteenhouse, Yumi Kasai, David Gray, Carol Clugston, Anna Dominiczak and Alex Alderton, Roberto Amato, Sonia Goncalves, Ewan Harrison, David K. Jackson, Ian Johnston, Dominic Kwiatkowski, Cordelia Langford, John Sillitoe on behalf of the Wellcome Sanger Institute COVID-19 Surveillance Team
EPI_ISL_863306	Lighthouse Lab in Alderley Park	Wellcome Sanger Institute for the COVID-19 Genomics UK (COG-UK) Consortium	Jacquelyn Wynn, Mairead Hyland, The Lighthouse Lab in Alderley Park and Alex Alderton, Roberto Amato, Sonia Goncalves, Ewan Harrison, David K. Jackson, Ian Johnston, Dominic Kwiatkowski, Cordelia Langford, John Sillitoe on behalf of the Wellcome Sanger Institute COVID-19 Surveillance Team
EPI_ISL_863307, EPI_ISL_863308, EPI_ISL_863309	Lighthouse Lab in Glasgow	Wellcome Sanger Institute for the COVID-19 Genomics UK (COG-UK) Consortium	Harper VanSteenhouse, Yumi Kasai, David Gray, Carol Clugston, Anna Dominiczak and Alex Alderton, Roberto Amato, Sonia Goncalves, Ewan Harrison, David K. Jackson, Ian Johnston, Dominic Kwiatkowski, Cordelia Langford, John Sillitoe on behalf of the Wellcome Sanger Institute COVID-19 Surveillance Team
EPI_ISL_863310, EPI_ISL_863311	Lighthouse Lab in Alderley Park	Wellcome Sanger Institute for the COVID-19 Genomics UK (COG-UK) Consortium	Jacquelyn Wynn, Mairead Hyland, The Lighthouse Lab in Alderley Park and Alex Alderton, Roberto Amato, Sonia Goncalves, Ewan Harrison, David K. Jackson, Ian Johnston, Dominic Kwiatkowski, Cordelia Langford, John Sillitoe on behalf of the Wellcome Sanger Institute COVID-19 Surveillance Team
EPI_ISL_863312, EPI_ISL_863313, EPI_ISL_863314, EPI_ISL_863315, EPI_ISL_863316, EPI_ISL_863317	Lighthouse Lab in Glasgow	Wellcome Sanger Institute for the COVID-19 Genomics UK (COG-UK) Consortium	Harper VanSteenhouse, Yumi Kasai, David Gray, Carol Clugston, Anna Dominiczak and Alex Alderton, Roberto Amato, Sonia Goncalves, Ewan Harrison, David K. Jackson, Ian Johnston, Dominic Kwiatkowski, Cordelia Langford, John Sillitoe on behalf of the Wellcome Sanger Institute COVID-19 Surveillance Team
EPI_ISL_863318, EPI_ISL_863319	Lighthouse Lab in Alderley Park	Wellcome Sanger Institute for the COVID-19 Genomics UK (COG-UK) Consortium	Jacquelyn Wynn, Mairead Hyland, The Lighthouse Lab in Alderley Park and Alex Alderton, Roberto Amato, Sonia Goncalves, Ewan Harrison, David K. Jackson, Ian Johnston, Dominic Kwiatkowski, Cordelia Langford, John Sillitoe on behalf of the Wellcome Sanger Institute COVID-19 Surveillance Team
EPI_ISL_863320, EPI_ISL_863321, EPI_ISL_863322, EPI_ISL_863323, EPI_ISL_863324, EPI_ISL_863325	Lighthouse Lab in Glasgow	Wellcome Sanger Institute for the COVID-19 Genomics UK (COG-UK) Consortium	Harper VanSteenhouse, Yumi Kasai, David Gray, Carol Clugston, Anna Dominiczak and Alex Alderton, Roberto Amato, Sonia Goncalves, Ewan Harrison, David K. Jackson, Ian Johnston, Dominic Kwiatkowski, Cordelia Langford, John Sillitoe on behalf of the Wellcome Sanger Institute COVID-19 Surveillance Team
EPI_ISL_863326	Lighthouse Lab in Alderley Park	Wellcome Sanger Institute for the COVID-19 Genomics UK (COG-UK) Consortium	Jacquelyn Wynn, Mairead Hyland, The Lighthouse Lab in Alderley Park and Alex Alderton, Roberto Amato, Sonia Goncalves, Ewan Harrison, David K. Jackson, Ian Johnston, Dominic Kwiatkowski, Cordelia Langford, John Sillitoe on behalf of the Wellcome Sanger Institute COVID-19 Surveillance Team
EPI_ISL_863327, EPI_ISL_863328, EPI_ISL_863329, EPI_ISL_863330, EPI_ISL_863331, EPI_ISL_863332, EPI_ISL_863333, EPI_ISL_863335, EPI_ISL_863336, EPI_ISL_863337	Lighthouse Lab in Glasgow	Wellcome Sanger Institute for the COVID-19 Genomics UK (COG-UK) Consortium	Harper VanSteenhouse, Yumi Kasai, David Gray, Carol Clugston, Anna Dominiczak and Alex Alderton, Roberto Amato, Sonia Goncalves, Ewan Harrison, David K. Jackson, Ian Johnston, Dominic Kwiatkowski, Cordelia Langford, John Sillitoe on behalf of the Wellcome Sanger Institute COVID-19 Surveillance Team
EPI_ISL_863338	Lighthouse Lab in Alderley Park	Wellcome Sanger Institute for the COVID-19 Genomics UK (COG-UK) Consortium	Jacquelyn Wynn, Mairead Hyland, The Lighthouse Lab in Alderley Park and Alex Alderton, Roberto Amato, Sonia Goncalves, Ewan Harrison, David K. Jackson, Ian Johnston, Dominic Kwiatkowski, Cordelia Langford, John Sillitoe on behalf of the Wellcome Sanger Institute COVID-19 Surveillance Team
EPI_ISL_863339, EPI_ISL_863340, EPI_ISL_863341	Lighthouse Lab in Glasgow	Wellcome Sanger Institute for the COVID-19 Genomics UK (COG-UK) Consortium	Harper VanSteenhouse, Yumi Kasai, David Gray, Carol Clugston, Anna Dominiczak and Alex Alderton, Roberto Amato, Sonia Goncalves, Ewan Harrison, David K. Jackson, Ian Johnston, Dominic Kwiatkowski, Cordelia Langford, John Sillitoe on behalf of the Wellcome Sanger Institute COVID-19 Surveillance Team
EPI_ISL_863342, EPI_ISL_863343	Lighthouse Lab in Alderley Park	Wellcome Sanger Institute for the COVID-19 Genomics UK (COG-UK) Consortium	Jacquelyn Wynn, Mairead Hyland, The Lighthouse Lab in Alderley Park and Alex Alderton, Roberto Amato, Sonia Goncalves, Ewan Harrison, David K. Jackson, Ian Johnston, Dominic Kwiatkowski, Cordelia Langford, John Sillitoe on behalf of the Wellcome Sanger Institute COVID-19 Surveillance Team
EPI_ISL_863344, EPI_ISL_863345	Lighthouse Lab in Glasgow	Wellcome Sanger Institute for the COVID-19 Genomics UK (COG-UK) Consortium	Harper VanSteenhouse, Yumi Kasai, David Gray, Carol Clugston, Anna Dominiczak and Alex Alderton, Roberto Amato, Sonia Goncalves, Ewan Harrison, David K. Jackson, Ian Johnston, Dominic Kwiatkowski, Cordelia Langford, John Sillitoe on behalf of the Wellcome Sanger Institute COVID-19 Surveillance Team

EPI_ISL_863346	Lighthouse Lab in Alderley Park	Wellcome Sanger Institute for the COVID-19 Genomics UK (COG-UK) Consortium	Jacquelyn Wynn, Mairead Hyland, The Lighthouse Lab in Alderley Park and Alex Alderton, Roberto Amato, Sonia Goncalves, Ewan Harrison, David K. Jackson, Ian Johnston, Dominic Kwiatkowski, Cordelia Langford, John Sillitoe on behalf of the Wellcome Sanger Institute COVID-19 Surveillance Team
EPI_ISL_863347, EPI_ISL_863348, EPI_ISL_863349, EPI_ISL_863350, EPI_ISL_863351, EPI_ISL_863352, EPI_ISL_863353, EPI_ISL_863354, EPI_ISL_863355	Lighthouse Lab in Glasgow	Wellcome Sanger Institute for the COVID-19 Genomics UK (COG-UK) Consortium	Harper VanSteenhouse, Yumi Kasai, David Gray, Carol Clugston, Anna Dominiczak and Alex Alderton, Roberto Amato, Sonia Goncalves, Ewan Harrison, David K. Jackson, Ian Johnston, Dominic Kwiatkowski, Cordelia Langford, John Sillitoe on behalf of the Wellcome Sanger Institute COVID-19 Surveillance Team
EPI_ISL_863356, EPI_ISL_863357	Lighthouse Lab in Alderley Park	Wellcome Sanger Institute for the COVID-19 Genomics UK (COG-UK) Consortium	Jacquelyn Wynn, Mairead Hyland, The Lighthouse Lab in Alderley Park and Alex Alderton, Roberto Amato, Sonia Goncalves, Ewan Harrison, David K. Jackson, Ian Johnston, Dominic Kwiatkowski, Cordelia Langford, John Sillitoe on behalf of the Wellcome Sanger Institute COVID-19 Surveillance Team
EPI_ISL_863358, EPI_ISL_863359	Lighthouse Lab in Glasgow	Wellcome Sanger Institute for the COVID-19 Genomics UK (COG-UK) Consortium	Harper VanSteenhouse, Yumi Kasai, David Gray, Carol Clugston, Anna Dominiczak and Alex Alderton, Roberto Amato, Sonia Goncalves, Ewan Harrison, David K. Jackson, Ian Johnston, Dominic Kwiatkowski, Cordelia Langford, John Sillitoe on behalf of the Wellcome Sanger Institute COVID-19 Surveillance Team
EPI_ISL_863360	Lighthouse Lab in Alderley Park	Wellcome Sanger Institute for the COVID-19 Genomics UK (COG-UK) Consortium	Jacquelyn Wynn, Mairead Hyland, The Lighthouse Lab in Alderley Park and Alex Alderton, Roberto Amato, Sonia Goncalves, Ewan Harrison, David K. Jackson, Ian Johnston, Dominic Kwiatkowski, Cordelia Langford, John Sillitoe on behalf of the Wellcome Sanger Institute COVID-19 Surveillance Team
EPI_ISL_863361	Lighthouse Lab in Glasgow	Wellcome Sanger Institute for the COVID-19 Genomics UK (COG-UK) Consortium	Harper VanSteenhouse, Yumi Kasai, David Gray, Carol Clugston, Anna Dominiczak and Alex Alderton, Roberto Amato, Sonia Goncalves, Ewan Harrison, David K. Jackson, Ian Johnston, Dominic Kwiatkowski, Cordelia Langford, John Sillitoe on behalf of the Wellcome Sanger Institute COVID-19 Surveillance Team
EPI_ISL_863362, EPI_ISL_863363, EPI_ISL_863364	Lighthouse Lab in Alderley Park	Wellcome Sanger Institute for the COVID-19 Genomics UK (COG-UK) Consortium	Jacquelyn Wynn, Mairead Hyland, The Lighthouse Lab in Alderley Park and Alex Alderton, Roberto Amato, Sonia Goncalves, Ewan Harrison, David K. Jackson, Ian Johnston, Dominic Kwiatkowski, Cordelia Langford, John Sillitoe on behalf of the Wellcome Sanger Institute COVID-19 Surveillance Team
EPI_ISL_863365, EPI_ISL_863366, EPI_ISL_863367, EPI_ISL_863368, EPI_ISL_863369, EPI_ISL_863370, EPI_ISL_863371, EPI_ISL_863372, EPI_ISL_863373, EPI_ISL_863374, EPI_ISL_863375, EPI_ISL_863376			
see above	Lighthouse Lab in Glasgow	Wellcome Sanger Institute for the COVID-19 Genomics UK (COG-UK) Consortium	Harper VanSteenhouse, Yumi Kasai, David Gray, Carol Clugston, Anna Dominiczak and Alex Alderton, Roberto Amato, Sonia Goncalves, Ewan Harrison, David K. Jackson, Ian Johnston, Dominic Kwiatkowski, Cordelia Langford, John Sillitoe on behalf of the Wellcome Sanger Institute COVID-19 Surveillance Team
EPI_ISL_863377	Lighthouse Lab in Alderley Park	Wellcome Sanger Institute for the COVID-19 Genomics UK (COG-UK) Consortium	Jacquelyn Wynn, Mairead Hyland, The Lighthouse Lab in Alderley Park and Alex Alderton, Roberto Amato, Sonia Goncalves, Ewan Harrison, David K. Jackson, Ian Johnston, Dominic Kwiatkowski, Cordelia Langford, John Sillitoe on behalf of the Wellcome Sanger Institute COVID-19 Surveillance Team
EPI_ISL_863378	Lighthouse Lab in Glasgow	Wellcome Sanger Institute for the COVID-19 Genomics UK (COG-UK) Consortium	Harper VanSteenhouse, Yumi Kasai, David Gray, Carol Clugston, Anna Dominiczak and Alex Alderton, Roberto Amato, Sonia Goncalves, Ewan Harrison, David K. Jackson, Ian Johnston, Dominic Kwiatkowski, Cordelia Langford, John Sillitoe on behalf of the Wellcome Sanger Institute COVID-19 Surveillance Team
EPI_ISL_863379	Lighthouse Lab in Alderley Park	Wellcome Sanger Institute for the COVID-19 Genomics UK (COG-UK) Consortium	Jacquelyn Wynn, Mairead Hyland, The Lighthouse Lab in Alderley Park and Alex Alderton, Roberto Amato, Sonia Goncalves, Ewan Harrison, David K. Jackson, Ian Johnston, Dominic Kwiatkowski, Cordelia Langford, John Sillitoe on behalf of the Wellcome Sanger Institute COVID-19 Surveillance Team
EPI_ISL_863380, EPI_ISL_863381, EPI_ISL_863382	Lighthouse Lab in Glasgow	Wellcome Sanger Institute for the COVID-19 Genomics UK (COG-UK) Consortium	Harper VanSteenhouse, Yumi Kasai, David Gray, Carol Clugston, Anna Dominiczak and Alex Alderton, Roberto Amato, Sonia Goncalves, Ewan Harrison, David K. Jackson, Ian Johnston, Dominic Kwiatkowski, Cordelia Langford, John Sillitoe on behalf of the Wellcome Sanger Institute COVID-19 Surveillance Team
EPI_ISL_863383	Lighthouse Lab in Alderley Park	Wellcome Sanger Institute for the COVID-19 Genomics UK (COG-UK) Consortium	Jacquelyn Wynn, Mairead Hyland, The Lighthouse Lab in Alderley Park and Alex Alderton, Roberto Amato, Sonia Goncalves, Ewan Harrison, David K. Jackson, Ian Johnston, Dominic Kwiatkowski, Cordelia Langford, John Sillitoe on behalf of the Wellcome Sanger Institute COVID-19 Surveillance Team
EPI_ISL_863384, EPI_ISL_863385, EPI_ISL_863386	Lighthouse Lab in Glasgow	Wellcome Sanger Institute for the COVID-19 Genomics UK (COG-UK) Consortium	Harper VanSteenhouse, Yumi Kasai, David Gray, Carol Clugston, Anna Dominiczak and Alex Alderton, Roberto Amato, Sonia Goncalves, Ewan Harrison, David K. Jackson, Ian Johnston, Dominic Kwiatkowski, Cordelia Langford, John Sillitoe on behalf of the Wellcome Sanger Institute COVID-19 Surveillance Team
EPI_ISL_863387	Lighthouse Lab in Alderley Park	Wellcome Sanger Institute for the COVID-19 Genomics UK (COG-UK) Consortium	Jacquelyn Wynn, Mairead Hyland, The Lighthouse Lab in Alderley Park and Alex Alderton, Roberto Amato, Sonia Goncalves, Ewan Harrison, David K. Jackson, Ian Johnston, Dominic Kwiatkowski, Cordelia Langford, John Sillitoe on behalf of the Wellcome Sanger Institute COVID-19 Surveillance Team
EPI_ISL_863388, EPI_ISL_863389, EPI_ISL_863390, EPI_ISL_863391, EPI_ISL_863392, EPI_ISL_863393, EPI_ISL_863394	Lighthouse Lab in Glasgow	Wellcome Sanger Institute for the COVID-19 Genomics UK (COG-UK) Consortium	Harper VanSteenhouse, Yumi Kasai, David Gray, Carol Clugston, Anna Dominiczak and Alex Alderton, Roberto Amato, Sonia Goncalves, Ewan Harrison, David K. Jackson, Ian Johnston, Dominic Kwiatkowski, Cordelia Langford, John Sillitoe on behalf of the Wellcome Sanger Institute COVID-19 Surveillance Team
EPI_ISL_863395, EPI_ISL_863397	Lighthouse Lab in Alderley Park	Wellcome Sanger Institute for the COVID-19 Genomics UK (COG-UK) Consortium	Jacquelyn Wynn, Mairead Hyland, The Lighthouse Lab in Alderley Park and Alex Alderton, Roberto Amato, Sonia Goncalves, Ewan Harrison, David K. Jackson, Ian Johnston, Dominic Kwiatkowski, Cordelia Langford, John Sillitoe on behalf of the Wellcome Sanger Institute COVID-19 Surveillance Team
EPI_ISL_863398, EPI_ISL_863399, EPI_ISL_863400, EPI_ISL_863402	Lighthouse Lab in Glasgow	Wellcome Sanger Institute for the COVID-19 Genomics UK (COG-UK) Consortium	Harper VanSteenhouse, Yumi Kasai, David Gray, Carol Clugston, Anna Dominiczak and Alex Alderton, Roberto Amato, Sonia Goncalves, Ewan Harrison, David K. Jackson, Ian Johnston, Dominic Kwiatkowski, Cordelia Langford, John Sillitoe on behalf of the Wellcome Sanger Institute COVID-19 Surveillance Team
EPI_ISL_863404	Lighthouse Lab in Alderley Park	Wellcome Sanger Institute for the COVID-19 Genomics UK (COG-UK) Consortium	Jacquelyn Wynn, Mairead Hyland, The Lighthouse Lab in Alderley Park and Alex Alderton, Roberto Amato, Sonia Goncalves, Ewan Harrison, David K. Jackson, Ian Johnston, Dominic Kwiatkowski, Cordelia Langford, John Sillitoe on behalf of the Wellcome Sanger Institute COVID-19 Surveillance Team
EPI_ISL_863405	Lighthouse Lab in Glasgow	Wellcome Sanger Institute for the COVID-19 Genomics UK (COG-UK) Consortium	Harper VanSteenhouse, Yumi Kasai, David Gray, Carol Clugston, Anna Dominiczak and Alex Alderton, Roberto Amato, Sonia Goncalves, Ewan Harrison, David K. Jackson, Ian Johnston, Dominic Kwiatkowski, Cordelia Langford, John Sillitoe on behalf of the Wellcome Sanger Institute COVID-19 Surveillance Team
EPI_ISL_863406	Lighthouse Lab in Alderley Park	Wellcome Sanger Institute for the COVID-19 Genomics UK (COG-UK) Consortium	Jacquelyn Wynn, Mairead Hyland, The Lighthouse Lab in Alderley Park and Alex Alderton, Roberto Amato, Sonia Goncalves, Ewan Harrison, David K. Jackson, Ian Johnston, Dominic Kwiatkowski, Cordelia Langford, John Sillitoe on behalf of the Wellcome Sanger Institute COVID-19 Surveillance Team
EPI_ISL_863407	Lighthouse Lab in Glasgow	Wellcome Sanger Institute for the COVID-19 Genomics UK (COG-UK) Consortium	Harper VanSteenhouse, Yumi Kasai, David Gray, Carol Clugston, Anna Dominiczak and Alex Alderton, Roberto Amato, Sonia Goncalves, Ewan Harrison, David K. Jackson, Ian Johnston, Dominic Kwiatkowski, Cordelia Langford, John Sillitoe on behalf of the Wellcome Sanger Institute COVID-19 Surveillance Team
EPI_ISL_863408	Lighthouse Lab in Alderley Park	Wellcome Sanger Institute for the COVID-19 Genomics UK (COG-UK) Consortium	Jacquelyn Wynn, Mairead Hyland, The Lighthouse Lab in Alderley Park and Alex Alderton, Roberto Amato, Sonia Goncalves, Ewan Harrison, David K. Jackson, Ian Johnston, Dominic Kwiatkowski, Cordelia Langford, John Sillitoe on behalf of the Wellcome Sanger Institute COVID-19 Surveillance Team
EPI_ISL_863409, EPI_ISL_863410	Lighthouse Lab in Glasgow	Wellcome Sanger Institute for the COVID-19 Genomics UK (COG-UK) Consortium	Harper VanSteenhouse, Yumi Kasai, David Gray, Carol Clugston, Anna Dominiczak and Alex Alderton, Roberto Amato, Sonia Goncalves, Ewan Harrison, David K. Jackson, Ian Johnston, Dominic Kwiatkowski, Cordelia Langford, John Sillitoe on behalf of the Wellcome Sanger Institute COVID-19 Surveillance Team
EPI_ISL_863411	Lighthouse Lab in Alderley Park	Wellcome Sanger Institute for the COVID-19 Genomics UK (COG-UK) Consortium	Jacquelyn Wynn, Mairead Hyland, The Lighthouse Lab in Alderley Park and Alex Alderton, Roberto Amato, Sonia Goncalves, Ewan Harrison, David K. Jackson, Ian Johnston, Dominic Kwiatkowski, Cordelia Langford, John Sillitoe on behalf of the Wellcome Sanger Institute COVID-19 Surveillance Team
EPI_ISL_863412, EPI_ISL_863413	Lighthouse Lab in Glasgow	Wellcome Sanger Institute for the COVID-19 Genomics UK (COG-UK) Consortium	Harper VanSteenhouse, Yumi Kasai, David Gray, Carol Clugston, Anna Dominiczak and Alex Alderton, Roberto Amato, Sonia Goncalves, Ewan Harrison, David K. Jackson, Ian Johnston, Dominic Kwiatkowski, Cordelia Langford, John Sillitoe on behalf of the Wellcome Sanger Institute COVID-19 Surveillance Team
EPI_ISL_863414	Lighthouse Lab in Alderley Park	Wellcome Sanger Institute for the COVID-19 Genomics UK (COG-UK) Consortium	Jacquelyn Wynn, Mairead Hyland, The Lighthouse Lab in Alderley Park and Alex Alderton, Roberto Amato, Sonia Goncalves, Ewan Harrison, David K. Jackson, Ian Johnston, Dominic Kwiatkowski, Cordelia Langford, John Sillitoe on behalf of the Wellcome Sanger Institute COVID-19 Surveillance Team
EPI_ISL_863415	Lighthouse Lab in Glasgow	Wellcome Sanger Institute for the COVID-19 Genomics UK (COG-UK) Consortium	Harper VanSteenhouse, Yumi Kasai, David Gray, Carol Clugston, Anna Dominiczak and Alex Alderton, Roberto Amato, Sonia Goncalves, Ewan Harrison, David K. Jackson, Ian Johnston, Dominic Kwiatkowski, Cordelia Langford, John Sillitoe on behalf of the Wellcome Sanger Institute COVID-19 Surveillance

			Team
EPI_ISL_863416	Lighthouse Lab in Alderley Park	Wellcome Sanger Institute for the COVID-19 Genomics UK (COG-UK) Consortium	Jacquelyn Wynn, Mairead Hyland, The Lighthouse Lab in Alderley Park and Alex Alderton, Roberto Amato, Sonia Goncalves, Ewan Harrison, David K. Jackson, Ian Johnston, Dominic Kwiatkowski, Cordelia Langford, John Sillitoe on behalf of the Wellcome Sanger Institute COVID-19 Surveillance Team
EPI_ISL_863417, EPI_ISL_863418, EPI_ISL_863419	Lighthouse Lab in Glasgow	Wellcome Sanger Institute for the COVID-19 Genomics UK (COG-UK) Consortium	Harper VanSteenhouse, Yumi Kasai, David Gray, Carol Clugston, Anna Dominiczak and Alex Alderton, Roberto Amato, Sonia Goncalves, Ewan Harrison, David K. Jackson, Ian Johnston, Dominic Kwiatkowski, Cordelia Langford, John Sillitoe on behalf of the Wellcome Sanger Institute COVID-19 Surveillance Team
EPI_ISL_863420, EPI_ISL_863421	Lighthouse Lab in Alderley Park	Wellcome Sanger Institute for the COVID-19 Genomics UK (COG-UK) Consortium	Jacquelyn Wynn, Mairead Hyland, The Lighthouse Lab in Alderley Park and Alex Alderton, Roberto Amato, Sonia Goncalves, Ewan Harrison, David K. Jackson, Ian Johnston, Dominic Kwiatkowski, Cordelia Langford, John Sillitoe on behalf of the Wellcome Sanger Institute COVID-19 Surveillance Team
EPI_ISL_863422	Lighthouse Lab in Glasgow	Wellcome Sanger Institute for the COVID-19 Genomics UK (COG-UK) Consortium	Harper VanSteenhouse, Yumi Kasai, David Gray, Carol Clugston, Anna Dominiczak and Alex Alderton, Roberto Amato, Sonia Goncalves, Ewan Harrison, David K. Jackson, Ian Johnston, Dominic Kwiatkowski, Cordelia Langford, John Sillitoe on behalf of the Wellcome Sanger Institute COVID-19 Surveillance Team
EPI_ISL_863423	Lighthouse Lab in Alderley Park	Wellcome Sanger Institute for the COVID-19 Genomics UK (COG-UK) Consortium	Jacquelyn Wynn, Mairead Hyland, The Lighthouse Lab in Alderley Park and Alex Alderton, Roberto Amato, Sonia Goncalves, Ewan Harrison, David K. Jackson, Ian Johnston, Dominic Kwiatkowski, Cordelia Langford, John Sillitoe on behalf of the Wellcome Sanger Institute COVID-19 Surveillance Team
EPI_ISL_863425	Lighthouse Lab in Glasgow	Wellcome Sanger Institute for the COVID-19 Genomics UK (COG-UK) Consortium	Harper VanSteenhouse, Yumi Kasai, David Gray, Carol Clugston, Anna Dominiczak and Alex Alderton, Roberto Amato, Sonia Goncalves, Ewan Harrison, David K. Jackson, Ian Johnston, Dominic Kwiatkowski, Cordelia Langford, John Sillitoe on behalf of the Wellcome Sanger Institute COVID-19 Surveillance Team
EPI_ISL_863426	Lighthouse Lab in Alderley Park	Wellcome Sanger Institute for the COVID-19 Genomics UK (COG-UK) Consortium	Jacquelyn Wynn, Mairead Hyland, The Lighthouse Lab in Alderley Park and Alex Alderton, Roberto Amato, Sonia Goncalves, Ewan Harrison, David K. Jackson, Ian Johnston, Dominic Kwiatkowski, Cordelia Langford, John Sillitoe on behalf of the Wellcome Sanger Institute COVID-19 Surveillance Team
EPI_ISL_863427, EPI_ISL_863428, EPI_ISL_863429, EPI_ISL_863430, EPI_ISL_863433	Lighthouse Lab in Glasgow	Wellcome Sanger Institute for the COVID-19 Genomics UK (COG-UK) Consortium	Harper VanSteenhouse, Yumi Kasai, David Gray, Carol Clugston, Anna Dominiczak and Alex Alderton, Roberto Amato, Sonia Goncalves, Ewan Harrison, David K. Jackson, Ian Johnston, Dominic Kwiatkowski, Cordelia Langford, John Sillitoe on behalf of the Wellcome Sanger Institute COVID-19 Surveillance Team
EPI_ISL_863434	Lighthouse Lab in Alderley Park	Wellcome Sanger Institute for the COVID-19 Genomics UK (COG-UK) Consortium	Jacquelyn Wynn, Mairead Hyland, The Lighthouse Lab in Alderley Park and Alex Alderton, Roberto Amato, Sonia Goncalves, Ewan Harrison, David K. Jackson, Ian Johnston, Dominic Kwiatkowski, Cordelia Langford, John Sillitoe on behalf of the Wellcome Sanger Institute COVID-19 Surveillance Team
EPI_ISL_863435, EPI_ISL_863436	Lighthouse Lab in Glasgow	Wellcome Sanger Institute for the COVID-19 Genomics UK (COG-UK) Consortium	Harper VanSteenhouse, Yumi Kasai, David Gray, Carol Clugston, Anna Dominiczak and Alex Alderton, Roberto Amato, Sonia Goncalves, Ewan Harrison, David K. Jackson, Ian Johnston, Dominic Kwiatkowski, Cordelia Langford, John Sillitoe on behalf of the Wellcome Sanger Institute COVID-19 Surveillance Team
EPI_ISL_863437	Lighthouse Lab in Alderley Park	Wellcome Sanger Institute for the COVID-19 Genomics UK (COG-UK) Consortium	Jacquelyn Wynn, Mairead Hyland, The Lighthouse Lab in Alderley Park and Alex Alderton, Roberto Amato, Sonia Goncalves, Ewan Harrison, David K. Jackson, Ian Johnston, Dominic Kwiatkowski, Cordelia Langford, John Sillitoe on behalf of the Wellcome Sanger Institute COVID-19 Surveillance Team
EPI_ISL_863438, EPI_ISL_863439, EPI_ISL_863440, EPI_ISL_863441, EPI_ISL_863442	Lighthouse Lab in Glasgow	Wellcome Sanger Institute for the COVID-19 Genomics UK (COG-UK) Consortium	Harper VanSteenhouse, Yumi Kasai, David Gray, Carol Clugston, Anna Dominiczak and Alex Alderton, Roberto Amato, Sonia Goncalves, Ewan Harrison, David K. Jackson, Ian Johnston, Dominic Kwiatkowski, Cordelia Langford, John Sillitoe on behalf of the Wellcome Sanger Institute COVID-19 Surveillance Team
EPI_ISL_863443	Lighthouse Lab in Alderley Park	Wellcome Sanger Institute for the COVID-19 Genomics UK (COG-UK) Consortium	Jacquelyn Wynn, Mairead Hyland, The Lighthouse Lab in Alderley Park and Alex Alderton, Roberto Amato, Sonia Goncalves, Ewan Harrison, David K. Jackson, Ian Johnston, Dominic Kwiatkowski, Cordelia Langford, John Sillitoe on behalf of the Wellcome Sanger Institute COVID-19 Surveillance Team
EPI_ISL_863444, EPI_ISL_863445	Lighthouse Lab in Glasgow	Wellcome Sanger Institute for the COVID-19 Genomics UK (COG-UK) Consortium	Harper VanSteenhouse, Yumi Kasai, David Gray, Carol Clugston, Anna Dominiczak and Alex Alderton, Roberto Amato, Sonia Goncalves, Ewan Harrison, David K. Jackson, Ian Johnston, Dominic Kwiatkowski, Cordelia Langford, John Sillitoe on behalf of the Wellcome Sanger Institute COVID-19 Surveillance Team
EPI_ISL_863446, EPI_ISL_863447, EPI_ISL_863448	Lighthouse Lab in Alderley Park	Wellcome Sanger Institute for the COVID-19 Genomics UK (COG-UK) Consortium	Jacquelyn Wynn, Mairead Hyland, The Lighthouse Lab in Alderley Park and Alex Alderton, Roberto Amato, Sonia Goncalves, Ewan Harrison, David K. Jackson, Ian Johnston, Dominic Kwiatkowski, Cordelia Langford, John Sillitoe on behalf of the Wellcome Sanger Institute COVID-19 Surveillance Team
EPI_ISL_863449, EPI_ISL_863450, EPI_ISL_863451, EPI_ISL_863452, EPI_ISL_863453	Lighthouse Lab in Glasgow	Wellcome Sanger Institute for the COVID-19 Genomics UK (COG-UK) Consortium	Harper VanSteenhouse, Yumi Kasai, David Gray, Carol Clugston, Anna Dominiczak and Alex Alderton, Roberto Amato, Sonia Goncalves, Ewan Harrison, David K. Jackson, Ian Johnston, Dominic Kwiatkowski, Cordelia Langford, John Sillitoe on behalf of the Wellcome Sanger Institute COVID-19 Surveillance Team
EPI_ISL_863454	Lighthouse Lab in Alderley Park	Wellcome Sanger Institute for the COVID-19 Genomics UK (COG-UK) Consortium	Jacquelyn Wynn, Mairead Hyland, The Lighthouse Lab in Alderley Park and Alex Alderton, Roberto Amato, Sonia Goncalves, Ewan Harrison, David K. Jackson, Ian Johnston, Dominic Kwiatkowski, Cordelia Langford, John Sillitoe on behalf of the Wellcome Sanger Institute COVID-19 Surveillance Team
EPI_ISL_863455, EPI_ISL_863456, EPI_ISL_863457, EPI_ISL_863458, EPI_ISL_863459, EPI_ISL_863460	Lighthouse Lab in Glasgow	Wellcome Sanger Institute for the COVID-19 Genomics UK (COG-UK) Consortium	Harper VanSteenhouse, Yumi Kasai, David Gray, Carol Clugston, Anna Dominiczak and Alex Alderton, Roberto Amato, Sonia Goncalves, Ewan Harrison, David K. Jackson, Ian Johnston, Dominic Kwiatkowski, Cordelia Langford, John Sillitoe on behalf of the Wellcome Sanger Institute COVID-19 Surveillance Team
EPI_ISL_863461	Lighthouse Lab in Alderley Park	Wellcome Sanger Institute for the COVID-19 Genomics UK (COG-UK) Consortium	Jacquelyn Wynn, Mairead Hyland, The Lighthouse Lab in Alderley Park and Alex Alderton, Roberto Amato, Sonia Goncalves, Ewan Harrison, David K. Jackson, Ian Johnston, Dominic Kwiatkowski, Cordelia Langford, John Sillitoe on behalf of the Wellcome Sanger Institute COVID-19 Surveillance Team
EPI_ISL_863462, EPI_ISL_863463, EPI_ISL_863464, EPI_ISL_863465, EPI_ISL_863466, EPI_ISL_863468, EPI_ISL_863469	Lighthouse Lab in Glasgow	Wellcome Sanger Institute for the COVID-19 Genomics UK (COG-UK) Consortium	Harper VanSteenhouse, Yumi Kasai, David Gray, Carol Clugston, Anna Dominiczak and Alex Alderton, Roberto Amato, Sonia Goncalves, Ewan Harrison, David K. Jackson, Ian Johnston, Dominic Kwiatkowski, Cordelia Langford, John Sillitoe on behalf of the Wellcome Sanger Institute COVID-19 Surveillance Team
EPI_ISL_863470	Lighthouse Lab in Alderley Park	Wellcome Sanger Institute for the COVID-19 Genomics UK (COG-UK) Consortium	Jacquelyn Wynn, Mairead Hyland, The Lighthouse Lab in Alderley Park and Alex Alderton, Roberto Amato, Sonia Goncalves, Ewan Harrison, David K. Jackson, Ian Johnston, Dominic Kwiatkowski, Cordelia Langford, John Sillitoe on behalf of the Wellcome Sanger Institute COVID-19 Surveillance Team
EPI_ISL_863471, EPI_ISL_863472, EPI_ISL_863473, EPI_ISL_863474, EPI_ISL_863475, EPI_ISL_863476	Lighthouse Lab in Glasgow	Wellcome Sanger Institute for the COVID-19 Genomics UK (COG-UK) Consortium	Harper VanSteenhouse, Yumi Kasai, David Gray, Carol Clugston, Anna Dominiczak and Alex Alderton, Roberto Amato, Sonia Goncalves, Ewan Harrison, David K. Jackson, Ian Johnston, Dominic Kwiatkowski, Cordelia Langford, John Sillitoe on behalf of the Wellcome Sanger Institute COVID-19 Surveillance Team
EPI_ISL_863477	Lighthouse Lab in Alderley Park	Wellcome Sanger Institute for the COVID-19 Genomics UK (COG-UK) Consortium	Jacquelyn Wynn, Mairead Hyland, The Lighthouse Lab in Alderley Park and Alex Alderton, Roberto Amato, Sonia Goncalves, Ewan Harrison, David K. Jackson, Ian Johnston, Dominic Kwiatkowski, Cordelia Langford, John Sillitoe on behalf of the Wellcome Sanger Institute COVID-19 Surveillance Team
EPI_ISL_863478	Lighthouse Lab in Glasgow	Wellcome Sanger Institute for the COVID-19 Genomics UK (COG-UK) Consortium	Harper VanSteenhouse, Yumi Kasai, David Gray, Carol Clugston, Anna Dominiczak and Alex Alderton, Roberto Amato, Sonia Goncalves, Ewan Harrison, David K. Jackson, Ian Johnston, Dominic Kwiatkowski, Cordelia Langford, John Sillitoe on behalf of the Wellcome Sanger Institute COVID-19 Surveillance Team
EPI_ISL_863479	Lighthouse Lab in Alderley Park	Wellcome Sanger Institute for the COVID-19 Genomics UK (COG-UK) Consortium	Jacquelyn Wynn, Mairead Hyland, The Lighthouse Lab in Alderley Park and Alex Alderton, Roberto Amato, Sonia Goncalves, Ewan Harrison, David K. Jackson, Ian Johnston, Dominic Kwiatkowski, Cordelia Langford, John Sillitoe on behalf of the Wellcome Sanger Institute COVID-19 Surveillance Team
EPI_ISL_863480, EPI_ISL_863481, EPI_ISL_863482, EPI_ISL_863483	Lighthouse Lab in Glasgow	Wellcome Sanger Institute for the COVID-19 Genomics UK (COG-UK) Consortium	Harper VanSteenhouse, Yumi Kasai, David Gray, Carol Clugston, Anna Dominiczak and Alex Alderton, Roberto Amato, Sonia Goncalves, Ewan Harrison, David K. Jackson, Ian Johnston, Dominic Kwiatkowski, Cordelia Langford, John Sillitoe on behalf of the Wellcome Sanger Institute COVID-19 Surveillance Team
EPI_ISL_863484	Lighthouse Lab in Alderley Park	Wellcome Sanger Institute for the COVID-19 Genomics UK (COG-UK) Consortium	Jacquelyn Wynn, Mairead Hyland, The Lighthouse Lab in Alderley Park and Alex Alderton, Roberto Amato, Sonia Goncalves, Ewan Harrison, David K. Jackson, Ian Johnston, Dominic Kwiatkowski, Cordelia Langford, John Sillitoe on behalf of the Wellcome Sanger Institute COVID-19 Surveillance Team
EPI_ISL_863485, EPI_ISL_863486, EPI_ISL_863487, EPI_ISL_863488, EPI_ISL_863489, EPI_ISL_863490	Lighthouse Lab in Glasgow	Wellcome Sanger Institute for the COVID-19 Genomics UK (COG-UK) Consortium	Harper VanSteenhouse, Yumi Kasai, David Gray, Carol Clugston, Anna Dominiczak and Alex Alderton, Roberto Amato, Sonia Goncalves, Ewan Harrison, David K. Jackson, Ian Johnston, Dominic Kwiatkowski, Cordelia Langford, John Sillitoe on behalf of the Wellcome Sanger Institute COVID-19 Surveillance Team
EPI_ISL_863491	Lighthouse Lab in Alderley Park	Wellcome Sanger Institute for the COVID-19 Genomics UK	Jacquelyn Wynn, Mairead Hyland, The Lighthouse Lab in Alderley Park and Alex Alderton, Roberto Amato, Sonia Goncalves, Ewan Harrison, David K.

		(COG-UK) Consortium	Jackson, Ian Johnston, Dominic Kwiatkowski, Cordelia Langford, John Sillitoe on behalf of the Wellcome Sanger Institute COVID-19 Surveillance Team
EPI_ISL_863492, EPI_ISL_863493, EPI_ISL_863494, EPI_ISL_863495, EPI_ISL_863496, EPI_ISL_863497, EPI_ISL_863498, EPI_ISL_863499, EPI_ISL_863500, EPI_ISL_863501, EPI_ISL_863502			
see above	Lighthouse Lab in Glasgow	Wellcome Sanger Institute for the COVID-19 Genomics UK (COG-UK) Consortium	Harper VanSteenhouse, Yumi Kasai, David Gray, Carol Clugston, Anna Dominiczak and Alex Alderton, Roberto Amato, Sonia Goncalves, Ewan Harrison, David K. Jackson, Ian Johnston, Dominic Kwiatkowski, Cordelia Langford, John Sillitoe on behalf of the Wellcome Sanger Institute COVID-19 Surveillance Team
EPI_ISL_863503, EPI_ISL_863504	Lighthouse Lab in Alderley Park	Wellcome Sanger Institute for the COVID-19 Genomics UK (COG-UK) Consortium	Jacquelyn Wynn, Mairead Hyland, The Lighthouse Lab in Alderley Park and Alex Alderton, Roberto Amato, Sonia Goncalves, Ewan Harrison, David K. Jackson, Ian Johnston, Dominic Kwiatkowski, Cordelia Langford, John Sillitoe on behalf of the Wellcome Sanger Institute COVID-19 Surveillance Team
EPI_ISL_863505	Lighthouse Lab in Glasgow	Wellcome Sanger Institute for the COVID-19 Genomics UK (COG-UK) Consortium	Harper VanSteenhouse, Yumi Kasai, David Gray, Carol Clugston, Anna Dominiczak and Alex Alderton, Roberto Amato, Sonia Goncalves, Ewan Harrison, David K. Jackson, Ian Johnston, Dominic Kwiatkowski, Cordelia Langford, John Sillitoe on behalf of the Wellcome Sanger Institute COVID-19 Surveillance Team
EPI_ISL_863506	Lighthouse Lab in Alderley Park	Wellcome Sanger Institute for the COVID-19 Genomics UK (COG-UK) Consortium	Jacquelyn Wynn, Mairead Hyland, The Lighthouse Lab in Alderley Park and Alex Alderton, Roberto Amato, Sonia Goncalves, Ewan Harrison, David K. Jackson, Ian Johnston, Dominic Kwiatkowski, Cordelia Langford, John Sillitoe on behalf of the Wellcome Sanger Institute COVID-19 Surveillance Team
EPI_ISL_863507, EPI_ISL_863508, EPI_ISL_863509, EPI_ISL_863510, EPI_ISL_863511, EPI_ISL_863512, EPI_ISL_863513	Lighthouse Lab in Glasgow	Wellcome Sanger Institute for the COVID-19 Genomics UK (COG-UK) Consortium	Harper VanSteenhouse, Yumi Kasai, David Gray, Carol Clugston, Anna Dominiczak and Alex Alderton, Roberto Amato, Sonia Goncalves, Ewan Harrison, David K. Jackson, Ian Johnston, Dominic Kwiatkowski, Cordelia Langford, John Sillitoe on behalf of the Wellcome Sanger Institute COVID-19 Surveillance Team
EPI_ISL_863515, EPI_ISL_863516	Lighthouse Lab in Alderley Park	Wellcome Sanger Institute for the COVID-19 Genomics UK (COG-UK) Consortium	Jacquelyn Wynn, Mairead Hyland, The Lighthouse Lab in Alderley Park and Alex Alderton, Roberto Amato, Sonia Goncalves, Ewan Harrison, David K. Jackson, Ian Johnston, Dominic Kwiatkowski, Cordelia Langford, John Sillitoe on behalf of the Wellcome Sanger Institute COVID-19 Surveillance Team
EPI_ISL_863517, EPI_ISL_863518	Lighthouse Lab in Glasgow	Wellcome Sanger Institute for the COVID-19 Genomics UK (COG-UK) Consortium	Harper VanSteenhouse, Yumi Kasai, David Gray, Carol Clugston, Anna Dominiczak and Alex Alderton, Roberto Amato, Sonia Goncalves, Ewan Harrison, David K. Jackson, Ian Johnston, Dominic Kwiatkowski, Cordelia Langford, John Sillitoe on behalf of the Wellcome Sanger Institute COVID-19 Surveillance Team
EPI_ISL_863519, EPI_ISL_863520	Lighthouse Lab in Alderley Park	Wellcome Sanger Institute for the COVID-19 Genomics UK (COG-UK) Consortium	Jacquelyn Wynn, Mairead Hyland, The Lighthouse Lab in Alderley Park and Alex Alderton, Roberto Amato, Sonia Goncalves, Ewan Harrison, David K. Jackson, Ian Johnston, Dominic Kwiatkowski, Cordelia Langford, John Sillitoe on behalf of the Wellcome Sanger Institute COVID-19 Surveillance Team
EPI_ISL_863521, EPI_ISL_863522, EPI_ISL_863523	Lighthouse Lab in Glasgow	Wellcome Sanger Institute for the COVID-19 Genomics UK (COG-UK) Consortium	Harper VanSteenhouse, Yumi Kasai, David Gray, Carol Clugston, Anna Dominiczak and Alex Alderton, Roberto Amato, Sonia Goncalves, Ewan Harrison, David K. Jackson, Ian Johnston, Dominic Kwiatkowski, Cordelia Langford, John Sillitoe on behalf of the Wellcome Sanger Institute COVID-19 Surveillance Team
EPI_ISL_863524	Lighthouse Lab in Alderley Park	Wellcome Sanger Institute for the COVID-19 Genomics UK (COG-UK) Consortium	Jacquelyn Wynn, Mairead Hyland, The Lighthouse Lab in Alderley Park and Alex Alderton, Roberto Amato, Sonia Goncalves, Ewan Harrison, David K. Jackson, Ian Johnston, Dominic Kwiatkowski, Cordelia Langford, John Sillitoe on behalf of the Wellcome Sanger Institute COVID-19 Surveillance Team
EPI_ISL_863525, EPI_ISL_863526	Lighthouse Lab in Glasgow	Wellcome Sanger Institute for the COVID-19 Genomics UK (COG-UK) Consortium	Harper VanSteenhouse, Yumi Kasai, David Gray, Carol Clugston, Anna Dominiczak and Alex Alderton, Roberto Amato, Sonia Goncalves, Ewan Harrison, David K. Jackson, Ian Johnston, Dominic Kwiatkowski, Cordelia Langford, John Sillitoe on behalf of the Wellcome Sanger Institute COVID-19 Surveillance Team
EPI_ISL_863527	Lighthouse Lab in Alderley Park	Wellcome Sanger Institute for the COVID-19 Genomics UK (COG-UK) Consortium	Jacquelyn Wynn, Mairead Hyland, The Lighthouse Lab in Alderley Park and Alex Alderton, Roberto Amato, Sonia Goncalves, Ewan Harrison, David K. Jackson, Ian Johnston, Dominic Kwiatkowski, Cordelia Langford, John Sillitoe on behalf of the Wellcome Sanger Institute COVID-19 Surveillance Team
EPI_ISL_863528, EPI_ISL_863530	Lighthouse Lab in Glasgow	Wellcome Sanger Institute for the COVID-19 Genomics UK (COG-UK) Consortium	Harper VanSteenhouse, Yumi Kasai, David Gray, Carol Clugston, Anna Dominiczak and Alex Alderton, Roberto Amato, Sonia Goncalves, Ewan Harrison, David K. Jackson, Ian Johnston, Dominic Kwiatkowski, Cordelia Langford, John Sillitoe on behalf of the Wellcome Sanger Institute COVID-19 Surveillance Team
EPI_ISL_863869, EPI_ISL_863870, EPI_ISL_863871, EPI_ISL_863872, EPI_ISL_863874, EPI_ISL_863875, EPI_ISL_863876, EPI_ISL_863877, EPI_ISL_863878, EPI_ISL_863879, EPI_ISL_863880, EPI_ISL_863881, EPI_ISL_863882, EPI_ISL_863883, EPI_ISL_863884, EPI_ISL_863885, EPI_ISL_863886, EPI_ISL_863887, EPI_ISL_863888, EPI_ISL_863889, EPI_ISL_863890, EPI_ISL_863891, EPI_ISL_863892, EPI_ISL_863893, EPI_ISL_863894, EPI_ISL_863895, EPI_ISL_863896, EPI_ISL_863897, EPI_ISL_863898, EPI_ISL_863900, EPI_ISL_863902, EPI_ISL_863903, EPI_ISL_863904, EPI_ISL_863905, EPI_ISL_863906, EPI_ISL_863907, EPI_ISL_863908, EPI_ISL_863909, EPI_ISL_863910, EPI_ISL_863911, EPI_ISL_863914, EPI_ISL_863915, EPI_ISL_863916, EPI_ISL_863917, EPI_ISL_863918, EPI_ISL_863919, EPI_ISL_863920, EPI_ISL_863923, EPI_ISL_863924, EPI_ISL_863925, EPI_ISL_863926, EPI_ISL_863927, EPI_ISL_863928, EPI_ISL_863929, EPI_ISL_863930, EPI_ISL_863931, EPI_ISL_863932, EPI_ISL_863933, EPI_ISL_863934, EPI_ISL_863935, EPI_ISL_863936, EPI_ISL_863937, EPI_ISL_863938, EPI_ISL_863939, EPI_ISL_863940, EPI_ISL_863941, EPI_ISL_863942, EPI_ISL_863943, EPI_ISL_863944, EPI_ISL_863945, EPI_ISL_863947, EPI_ISL_863948, EPI_ISL_863949, EPI_ISL_863950, EPI_ISL_863951, EPI_ISL_863952, EPI_ISL_863953, EPI_ISL_863954, EPI_ISL_863955, EPI_ISL_863957, EPI_ISL_863958, EPI_ISL_863959, EPI_ISL_863960, EPI_ISL_863961, EPI_ISL_863962, EPI_ISL_863963, EPI_ISL_863964, EPI_ISL_863965, EPI_ISL_863966, EPI_ISL_863967, EPI_ISL_863968, EPI_ISL_863970, EPI_ISL_863971, EPI_ISL_863972, EPI_ISL_863973, EPI_ISL_863974, EPI_ISL_863975, EPI_ISL_863976, EPI_ISL_863977, EPI_ISL_863978, EPI_ISL_863979, EPI_ISL_863980, EPI_ISL_863981, EPI_ISL_863982, EPI_ISL_863983, EPI_ISL_863984, EPI_ISL_863985, EPI_ISL_863986, EPI_ISL_863987, EPI_ISL_863988, EPI_ISL_863989, EPI_ISL_863990, EPI_ISL_863991, EPI_ISL_863992, EPI_ISL_863993, EPI_ISL_863994, EPI_ISL_863995, EPI_ISL_863997, EPI_ISL_863998, EPI_ISL_863999, EPI_ISL_864000, EPI_ISL_864001, EPI_ISL_864002, EPI_ISL_864003, EPI_ISL_864006, EPI_ISL_864007, EPI_ISL_864008, EPI_ISL_864010, EPI_ISL_864011, EPI_ISL_864012, EPI_ISL_864013, EPI_ISL_864014, EPI_ISL_864015, EPI_ISL_864016, EPI_ISL_864017, EPI_ISL_864018, EPI_ISL_864019, EPI_ISL_864020, EPI_ISL_864021, EPI_ISL_864022, EPI_ISL_864023, EPI_ISL_864024, EPI_ISL_864025, EPI_ISL_864027, EPI_ISL_864028, EPI_ISL_864029, EPI_ISL_864030, EPI_ISL_864031, EPI_ISL_864032, EPI_ISL_864033, EPI_ISL_864034, EPI_ISL_864035, EPI_ISL_864037, EPI_ISL_864038, EPI_ISL_864040, EPI_ISL_864041, EPI_ISL_864042, EPI_ISL_864044, EPI_ISL_864045, EPI_ISL_864046, EPI_ISL_864047, EPI_ISL_864048, EPI_ISL_864049, EPI_ISL_864050, EPI_ISL_864051, EPI_ISL_864052, EPI_ISL_864053, EPI_ISL_864054, EPI_ISL_864055, EPI_ISL_864056, EPI_ISL_864057, EPI_ISL_864058, EPI_ISL_864059, EPI_ISL_864060, EPI_ISL_864061, EPI_ISL_864062, EPI_ISL_864063, EPI_ISL_864065, EPI_ISL_864066, EPI_ISL_864067, EPI_ISL_864068, EPI_ISL_864069, EPI_ISL_864070, EPI_ISL_864071, EPI_ISL_864072, EPI_ISL_864073, EPI_ISL_864074, EPI_ISL_864075, EPI_ISL_864076, EPI_ISL_864077, EPI_ISL_864078, EPI_ISL_864079, EPI_ISL_864080, EPI_ISL_864081, EPI_ISL_864082, EPI_ISL_864083, EPI_ISL_864084, EPI_ISL_864085, EPI_ISL_864086, EPI_ISL_864087, EPI_ISL_864088, EPI_ISL_864089, EPI_ISL_864090, EPI_ISL_864091, EPI_ISL_864092, EPI_ISL_864093, EPI_ISL_864095, EPI_ISL_864096, EPI_ISL_864097, EPI_ISL_864099, EPI_ISL_864100, EPI_ISL_864101, EPI_ISL_864102, EPI_ISL_864103, EPI_ISL_864104, EPI_ISL_864105, EPI_ISL_864106, EPI_ISL_864107, EPI_ISL_864108, EPI_ISL_864109, EPI_ISL_864111, EPI_ISL_864112, EPI_ISL_864113, EPI_ISL_864114, EPI_ISL_864115, EPI_ISL_864116, EPI_ISL_864117, EPI_ISL_864118, EPI_ISL_864119, EPI_ISL_864120, EPI_ISL_864121, EPI_ISL_864122, EPI_ISL_864123, EPI_ISL_864125, EPI_ISL_864126, EPI_ISL_864127, EPI_ISL_864128, EPI_ISL_864129, EPI_ISL_864130, EPI_ISL_864131, EPI_ISL_864132, EPI_ISL_864134, EPI_ISL_864135, EPI_ISL_864136, EPI_ISL_864137, EPI_ISL_864138, EPI_ISL_864139, EPI_ISL_864140, EPI_ISL_864141, EPI_ISL_864143, EPI_ISL_864144, EPI_ISL_864146, EPI_ISL_864147, EPI_ISL_864148, EPI_ISL_864149, EPI_ISL_864150, EPI_ISL_864151, EPI_ISL_864152, EPI_ISL_864153, EPI_ISL_864154, EPI_ISL_864155, EPI_ISL_864156, EPI_ISL_864157, EPI_ISL_864158, EPI_ISL_864159, EPI_ISL_864160, EPI_ISL_864161, EPI_ISL_864162, EPI_ISL_864163, EPI_ISL_864164, EPI_ISL_864166, EPI_ISL_864167, EPI_ISL_864168, EPI_ISL_864170, EPI_ISL_864171, EPI_ISL_864172, EPI_ISL_864174, EPI_ISL_864177, EPI_ISL_864179, EPI_ISL_864180, EPI_ISL_864181, EPI_ISL_864182, EPI_ISL_864183, EPI_ISL_864184, EPI_ISL_864185, EPI_ISL_864186, EPI_ISL_864187, EPI_ISL_864188, EPI_ISL_864189, EPI_ISL_864190, EPI_ISL_864192, EPI_ISL_864194, EPI_ISL_864195, EPI_ISL_864196, EPI_ISL_864197, EPI_ISL_864198, EPI_ISL_864199, EPI_ISL_864200, EPI_ISL_864201, EPI_ISL_864202			
see above	Lighthouse Lab in Alderley Park	Wellcome Sanger Institute for the COVID-19 Genomics UK (COG-UK) Consortium	Jacquelyn Wynn, Mairead Hyland, The Lighthouse Lab in Alderley Park and Alex Alderton, Roberto Amato, Sonia Goncalves, Ewan Harrison, David K. Jackson, Ian Johnston, Dominic Kwiatkowski, Cordelia Langford, John Sillitoe on behalf of the Wellcome Sanger Institute COVID-19 Surveillance Team
EPI_ISL_864581, EPI_ISL_864584, EPI_ISL_864586, EPI_ISL_864588, EPI_ISL_864589, EPI_ISL_864590, EPI_ISL_864591, EPI_ISL_864592, EPI_ISL_864593	CHU Purpan - Laboratoire de Virologie - Institut Fédératif de Biologie	CHU Purpan - Laboratoire de Virologie - Institut Fédératif de Biologie	Latour J., Ranger N., Dubois M., Carcenac R., Harter A., Boyer P., Tremeaux P., Izopet J.
EPI_ISL_864598, EPI_ISL_864599, EPI_ISL_864608, EPI_ISL_864609, EPI_ISL_864610, EPI_ISL_864611, EPI_ISL_864619, EPI_ISL_864620, EPI_ISL_864624, EPI_ISL_864630, EPI_ISL_864635, EPI_ISL_864707, EPI_ISL_864708, EPI_ISL_864709, EPI_ISL_864710, EPI_ISL_864711, EPI_ISL_864712, EPI_ISL_864713, EPI_ISL_864714, EPI_ISL_864715, EPI_ISL_864728, EPI_ISL_864729, EPI_ISL_864730, EPI_ISL_864731, EPI_ISL_864732, EPI_ISL_864733			
see above	University Hospitals of Geneva, Laboratory of Virology	HUG, Laboratory of Virology and the Health2030 Genome Center	Samuel Cordey, Ana Rita Goncalves, Laurent Kaiser, Lorenzo Cerutti, Henri Pegeot, Melyssa Elies, Deborah Penet, Keith Harshman, Ioannis Xenarios, Emmanouil Dermitzakis
EPI_ISL_864896, EPI_ISL_864897, EPI_ISL_864898, EPI_ISL_864899, EPI_ISL_864900, EPI_ISL_864901, EPI_ISL_864902, EPI_ISL_864903, EPI_ISL_864904, EPI_ISL_864905, EPI_ISL_864906, EPI_ISL_864907, EPI_ISL_864908, EPI_ISL_864909, EPI_ISL_864910, EPI_ISL_864911, EPI_ISL_864912, EPI_ISL_864913, EPI_ISL_864914, EPI_ISL_864915, EPI_ISL_864916, EPI_ISL_864919, EPI_ISL_864920, EPI_ISL_864921, EPI_ISL_864922, EPI_ISL_864923, EPI_ISL_864924, EPI_ISL_864933, EPI_ISL_864935, EPI_ISL_864937, EPI_ISL_864938, EPI_ISL_864939, EPI_ISL_864940, EPI_ISL_864941, EPI_ISL_864942, EPI_ISL_864943, EPI_ISL_864944, EPI_ISL_864949, EPI_ISL_864950, EPI_ISL_864951, EPI_ISL_864952, EPI_ISL_864954, EPI_ISL_864957			
see above	Department of Pathology, University of Cambridge	COVID-19 Genomics UK (COG-UK) Consortium	Aminu S. Jahun, Yasmin Chaudhry, Grant Hall, Iliana Georgana, Myra Hosmillo, Martin D. Curran, Malte Pinckert, Surendra Parmar, Ian Goodfellow
EPI_ISL_865092, EPI_ISL_865093, EPI_ISL_865105, EPI_ISL_865106, EPI_ISL_865107, EPI_ISL_865108, EPI_ISL_865109, EPI_ISL_865110, EPI_ISL_865136, EPI_ISL_865138, EPI_ISL_865139, EPI_ISL_865141, EPI_ISL_865144			
see above	Virology Department, Royal Infirmary of Edinburgh, NHS Lothian / School of Biological Sciences, University of Edinburgh / Institute of Genetics and Molecular Medicine, University of Edinburgh	COVID-19 Genomics UK (COG-UK) Consortium	McHugh M, Dewar R, Rooke S, Gallagher M, Balcaza C, O'Toole Á, Scher E, Hill V, McCrone JT, Colquhoun R, Yu X, Jackson B, Rambaut A, Williams TC, Templeton K
EPI_ISL_865472, EPI_ISL_865474, EPI_ISL_865475, EPI_ISL_865476	Liverpool Clinical Laboratories	COVID-19 Genomics UK (COG-UK) Consortium	Sam Haldenby, Anita Lucaci, Steve Paterson, Julian Hiscox, Alistair Darby, M Almsaud, A Alrezaihi, Muhannad Alruwaili, Stuart D Armstrong, Jones Benjamin, Eleanor G Bentley, Anu Chawla, Jordan J Clark, Angela Cowell, Richard Eccles, Isabel García-Dorival, Matthew Gemmell, Alessandro Gerada,

			PKF Gilmore, Richard Gregory, Ximeng Han, Catherine Hartley, Margaret Hughes, Miren Iturriza-Gomara, James Johnson, L Luu, Jenifer Manson, Charlotte Nelson, Elaine O'Toole, Cassie Olateju, Rebekah Penrice-Randal , Lucille Rainbow, N.P Randle, Trevor Ian Robinson, Parul Sharma, Ghada T Shawli, James P Stewart, Neil Swainston, Ecaterina Vamos, Joanne Watts, Mark Whitehead
EPI_ISL_865485, EPI_ISL_865486, EPI_ISL_865487, EPI_ISL_865585, EPI_ISL_865586, EPI_ISL_865587, EPI_ISL_865588, EPI_ISL_865589, EPI_ISL_865590, EPI_ISL_865591, EPI_ISL_865592, EPI_ISL_865593, EPI_ISL_865594, EPI_ISL_865595, EPI_ISL_865596, EPI_ISL_865599, EPI_ISL_865602, EPI_ISL_865603, EPI_ISL_865604, EPI_ISL_865848, EPI_ISL_865849, EPI_ISL_865850, EPI_ISL_865851, EPI_ISL_865852, EPI_ISL_865864, EPI_ISL_865867, EPI_ISL_865868, EPI_ISL_865869, EPI_ISL_865870, EPI_ISL_865871, EPI_ISL_865872, EPI_ISL_865873, EPI_ISL_865874, EPI_ISL_865875, EPI_ISL_865876, EPI_ISL_865877, EPI_ISL_865878, EPI_ISL_865879, EPI_ISL_865880, EPI_ISL_865881, EPI_ISL_865883, EPI_ISL_865891, EPI_ISL_865895, EPI_ISL_865897, EPI_ISL_865905, EPI_ISL_865906, EPI_ISL_865909, EPI_ISL_865910, EPI_ISL_865911, EPI_ISL_865913, EPI_ISL_865914, EPI_ISL_865915, EPI_ISL_865916, EPI_ISL_865918, EPI_ISL_865919, EPI_ISL_865921, EPI_ISL_865922, EPI_ISL_865923, EPI_ISL_865924, EPI_ISL_865925, EPI_ISL_865929, EPI_ISL_865930, EPI_ISL_865931, EPI_ISL_865932, EPI_ISL_865934, EPI_ISL_865940, EPI_ISL_865941, EPI_ISL_865942, EPI_ISL_865943, EPI_ISL_865944, EPI_ISL_865945, EPI_ISL_865949, EPI_ISL_865950, EPI_ISL_865952, EPI_ISL_865953, EPI_ISL_865954, EPI_ISL_865955, EPI_ISL_865956, EPI_ISL_865957, EPI_ISL_865958, EPI_ISL_865959, EPI_ISL_865960, EPI_ISL_865961, EPI_ISL_865962, EPI_ISL_865963, EPI_ISL_865964, EPI_ISL_865965, EPI_ISL_865966, EPI_ISL_865968, EPI_ISL_866000, EPI_ISL_866012			
see above	University College London, Great Ormond Street Hospital for Children NHS Foundation Trust, Imperial College Healthcare NHS Trust	COVID-19 Genomics UK (COG-UK) Consortium	Sergi Castellano, Rachel Williams, Mark Kristiansen, Paola Resende Silva, Sunando Roy, Tony Brooks, Helena Tutill, Paola Niola, Patricia Dyal, Charlotte Williams, Leysa Forrest, Yasmin Panchbhaya, Jacqueline Findlay, Samuel Weeks, Julianne Brown, Kathryn Harris, Paul Randell, James Price, Alison Holmes, Judith Breuer
EPI_ISL_866150, EPI_ISL_866153, EPI_ISL_866155, EPI_ISL_866156, EPI_ISL_866158, EPI_ISL_866159, EPI_ISL_866161, EPI_ISL_866163, EPI_ISL_866165, EPI_ISL_866166, EPI_ISL_866167, EPI_ISL_866169, EPI_ISL_866170, EPI_ISL_866172, EPI_ISL_866173, EPI_ISL_866175, EPI_ISL_866176, EPI_ISL_866177, EPI_ISL_866181, EPI_ISL_866182, EPI_ISL_866184, EPI_ISL_866185			
see above	University College London Hospital	COVID-19 Genomics UK (COG-UK) Consortium	Judith Heaney, Matthew Byott, Catherine Houlihan, Dan Frampton, Stuart Kirk, Moira Spyer and Eleni Nastouli
EPI_ISL_866198, EPI_ISL_866201, EPI_ISL_866215, EPI_ISL_866217, EPI_ISL_866219, EPI_ISL_866221, EPI_ISL_866231, EPI_ISL_866262, EPI_ISL_866263, EPI_ISL_866264, EPI_ISL_866265, EPI_ISL_866266, EPI_ISL_866267, EPI_ISL_866268, EPI_ISL_866269, EPI_ISL_866270, EPI_ISL_866271, EPI_ISL_866272, EPI_ISL_866273, EPI_ISL_866274, EPI_ISL_866275, EPI_ISL_866276, EPI_ISL_866277, EPI_ISL_866278, EPI_ISL_866279, EPI_ISL_866280, EPI_ISL_866281, EPI_ISL_866282, EPI_ISL_866283, EPI_ISL_866284, EPI_ISL_866285, EPI_ISL_866286, EPI_ISL_866287, EPI_ISL_866288, EPI_ISL_866294, EPI_ISL_866295, EPI_ISL_866296, EPI_ISL_866297, EPI_ISL_866299, EPI_ISL_866300, EPI_ISL_866308, EPI_ISL_866309, EPI_ISL_866310, EPI_ISL_866313, EPI_ISL_866314, EPI_ISL_866315, EPI_ISL_866316, EPI_ISL_866317, EPI_ISL_866318, EPI_ISL_866319			
see above	University College London, Great Ormond Street Hospital for Children NHS Foundation Trust, Imperial College Healthcare NHS Trust	COVID-19 Genomics UK (COG-UK) Consortium	Sergi Castellano, Rachel Williams, Mark Kristiansen, Paola Resende Silva, Sunando Roy, Tony Brooks, Helena Tutill, Paola Niola, Patricia Dyal, Charlotte Williams, Leysa Forrest, Yasmin Panchbhaya, Jacqueline Findlay, Samuel Weeks, Julianne Brown, Kathryn Harris, Paul Randell, James Price, Alison Holmes, Judith Breuer
EPI_ISL_866541, EPI_ISL_866548, EPI_ISL_866556, EPI_ISL_866566, EPI_ISL_866569, EPI_ISL_866748, EPI_ISL_866749, EPI_ISL_866750, EPI_ISL_866751, EPI_ISL_866752, EPI_ISL_866753, EPI_ISL_866754, EPI_ISL_866755, EPI_ISL_866756, EPI_ISL_866757, EPI_ISL_866760, EPI_ISL_866763, EPI_ISL_866764, EPI_ISL_866767, EPI_ISL_866770, EPI_ISL_866774, EPI_ISL_866775, EPI_ISL_866781, EPI_ISL_866788, EPI_ISL_866789, EPI_ISL_866801, EPI_ISL_866835, EPI_ISL_866836, EPI_ISL_866837, EPI_ISL_866838, EPI_ISL_866839, EPI_ISL_866840, EPI_ISL_866841, EPI_ISL_866842, EPI_ISL_866843, EPI_ISL_866844, EPI_ISL_866845, EPI_ISL_866846, EPI_ISL_866847, EPI_ISL_866848, EPI_ISL_866849, EPI_ISL_866850, EPI_ISL_866851, EPI_ISL_866852, EPI_ISL_866853, EPI_ISL_866854, EPI_ISL_866855, EPI_ISL_866856, EPI_ISL_866857, EPI_ISL_866858, EPI_ISL_866859, EPI_ISL_866860, EPI_ISL_866861, EPI_ISL_866862, EPI_ISL_866863, EPI_ISL_866864, EPI_ISL_866865, EPI_ISL_866866, EPI_ISL_866867, EPI_ISL_866868, EPI_ISL_866869, EPI_ISL_866871, EPI_ISL_866872, EPI_ISL_866873, EPI_ISL_866874, EPI_ISL_866875, EPI_ISL_866876, EPI_ISL_866877, EPI_ISL_866878, EPI_ISL_866879, EPI_ISL_866880, EPI_ISL_866881, EPI_ISL_866882, EPI_ISL_866883, EPI_ISL_866884, EPI_ISL_866885, EPI_ISL_866886, EPI_ISL_866887, EPI_ISL_866888			
see above	Quadram Institute Bioscience	COVID-19 Genomics UK (COG-UK) Consortium	Dave J. Baker, Gemma L. Kay, Alp Aydin, Thanh Le-Viet, Steven Rudder, Ana P. Tedim, Anastasia Kolyva, Maria Diaz, Leonardo de Oliveira Martins, Nabil-Fareed Alikhan, Lizzie Meadows, Rachael Stanley, Ngozi Elumogo, Muhammed Yasir, Nicholas M. Thomson, Alexander J Trotter, Rachel Gilroy, Samuel Bloomfield, Claire Stuart, Andrew Bell, Reenesh Prakash, Samir Dervisevic, Alison E. Mather, John Wain, Mark Webber, Andrew J. Page, Justin O'Grady
EPI_ISL_866921, EPI_ISL_866922, EPI_ISL_866923, EPI_ISL_866924, EPI_ISL_866925, EPI_ISL_866926, EPI_ISL_866927, EPI_ISL_866928, EPI_ISL_866929, EPI_ISL_866930, EPI_ISL_866931, EPI_ISL_866932, EPI_ISL_866933, EPI_ISL_866934, EPI_ISL_866935, EPI_ISL_866936, EPI_ISL_866937, EPI_ISL_866938, EPI_ISL_866939, EPI_ISL_866940, EPI_ISL_866941, EPI_ISL_866942, EPI_ISL_866971, EPI_ISL_866972, EPI_ISL_866973, EPI_ISL_866974, EPI_ISL_866975, EPI_ISL_866976, EPI_ISL_866977, EPI_ISL_866978, EPI_ISL_866979, EPI_ISL_866980, EPI_ISL_866981, EPI_ISL_866982, EPI_ISL_866983, EPI_ISL_866984, EPI_ISL_866986, EPI_ISL_866987, EPI_ISL_866988, EPI_ISL_866989			
see above	Queens Medical Centre, Clinical Microbiology Department / DeepSeq Nottingham	COVID-19 Genomics UK (COG-UK) Consortium	Gemma Clark, Wendy Smith, Manjinder Khakh, Vicki M Fleming, Michelle M Lister, Hannah Howson-Wells, Jonathan Ball, Patrick McClure, Joseph Chappell, Theocharis Tsoleridis, Nadine Holmes, Matthew Carlisle, Christopher Moore, Fei Sang, Johnny Debebe, Victoria Wright, Matthew Loose
EPI_ISL_867234, EPI_ISL_867235, EPI_ISL_867236, EPI_ISL_867259, EPI_ISL_867260, EPI_ISL_867262, EPI_ISL_867263, EPI_ISL_867264, EPI_ISL_867265, EPI_ISL_867266, EPI_ISL_867267, EPI_ISL_867268, EPI_ISL_867407, EPI_ISL_867408, EPI_ISL_867409, EPI_ISL_867410, EPI_ISL_867411, EPI_ISL_867412, EPI_ISL_867413, EPI_ISL_867414, EPI_ISL_867415, EPI_ISL_867420, EPI_ISL_867421, EPI_ISL_867422, EPI_ISL_867423, EPI_ISL_867425, EPI_ISL_867426, EPI_ISL_867428, EPI_ISL_867436, EPI_ISL_867444, EPI_ISL_867445, EPI_ISL_867446, EPI_ISL_867447, EPI_ISL_867448, EPI_ISL_867449, EPI_ISL_867452, EPI_ISL_867453, EPI_ISL_867454, EPI_ISL_867455, EPI_ISL_867460, EPI_ISL_867462, EPI_ISL_867463, EPI_ISL_867464, EPI_ISL_867465, EPI_ISL_867466, EPI_ISL_867467, EPI_ISL_867468, EPI_ISL_867469, EPI_ISL_867470, EPI_ISL_867471, EPI_ISL_867472, EPI_ISL_867473, EPI_ISL_867474, EPI_ISL_867475, EPI_ISL_867476, EPI_ISL_867477, EPI_ISL_867478, EPI_ISL_867479, EPI_ISL_867480, EPI_ISL_867481, EPI_ISL_867482, EPI_ISL_867483, EPI_ISL_867484, EPI_ISL_867485, EPI_ISL_867487, EPI_ISL_867488, EPI_ISL_867489, EPI_ISL_867495, EPI_ISL_867496, EPI_ISL_867502, EPI_ISL_867549, EPI_ISL_867576, EPI_ISL_867577, EPI_ISL_867585, EPI_ISL_867591, EPI_ISL_867592, EPI_ISL_867594, EPI_ISL_867595, EPI_ISL_867596, EPI_ISL_867597, EPI_ISL_867599, EPI_ISL_867600, EPI_ISL_867601, EPI_ISL_867602, EPI_ISL_867605, EPI_ISL_867607, EPI_ISL_867608, EPI_ISL_867610, EPI_ISL_867611, EPI_ISL_867613, EPI_ISL_867616, EPI_ISL_867617, EPI_ISL_867618, EPI_ISL_867620, EPI_ISL_867621, EPI_ISL_867623, EPI_ISL_867624, EPI_ISL_867625, EPI_ISL_867628, EPI_ISL_867629, EPI_ISL_867630, EPI_ISL_867631, EPI_ISL_867633, EPI_ISL_867634, EPI_ISL_867635, EPI_ISL_867636, EPI_ISL_867637, EPI_ISL_867639, EPI_ISL_867640, EPI_ISL_867641, EPI_ISL_867642, EPI_ISL_867643, EPI_ISL_867644, EPI_ISL_867647, EPI_ISL_867649, EPI_ISL_867650, EPI_ISL_867651, EPI_ISL_867652, EPI_ISL_867653, EPI_ISL_867654, EPI_ISL_867655, EPI_ISL_867656, EPI_ISL_867657, EPI_ISL_867658, EPI_ISL_867659, EPI_ISL_867660, EPI_ISL_867661, EPI_ISL_867662, EPI_ISL_867663, EPI_ISL_867664, EPI_ISL_867665, EPI_ISL_867667, EPI_ISL_867668, EPI_ISL_867670, EPI_ISL_867671, EPI_ISL_867672, EPI_ISL_867673, EPI_ISL_867674, EPI_ISL_867675, EPI_ISL_867676, EPI_ISL_867677, EPI_ISL_867678, EPI_ISL_867679, EPI_ISL_867680, EPI_ISL_867681, EPI_ISL_867682, EPI_ISL_867683, EPI_ISL_867684, EPI_ISL_867686, EPI_ISL_867688, EPI_ISL_867689, EPI_ISL_867690, EPI_ISL_867692, EPI_ISL_867696, EPI_ISL_867697, EPI_ISL_867698, EPI_ISL_867699, EPI_ISL_867700, EPI_ISL_867701, EPI_ISL_867727, EPI_ISL_867732, EPI_ISL_867733, EPI_ISL_867734, EPI_ISL_867735, EPI_ISL_867909, EPI_ISL_867917, EPI_ISL_867922, EPI_ISL_867923, EPI_ISL_867924, EPI_ISL_867926, EPI_ISL_867927, EPI_ISL_867928, EPI_ISL_867929			
see above	Originating lab: Wales Specialist Virology Centre Sequencing lab: Pathogen Genomics Unit	Public Health Wales Microbiology Cardiff Wales Specialist Virology Centre	Catherine Moore, Johnathan Evans, Laura Gifford, Malorie Perry, Simon Cottrell, Angela Marchbank, Alec Birchley, Alexander Adams, Amy Gaskin, Bree Gatica-Wilcox, Jason Coombes, Joel Southgate, Lauren Gilbert, Lee Graham, Nicole Pacchiarini, Sara Kumziene-Summerhayes, Sarah Taylor, Sophie Jones, Sara Rey, Matthew Bull, Joanne Watkins, Sally Corden, Tom Connor
EPI_ISL_868034, EPI_ISL_868102, EPI_ISL_868155, EPI_ISL_868156, EPI_ISL_868157, EPI_ISL_868158, EPI_ISL_868159, EPI_ISL_868160, EPI_ISL_868175, EPI_ISL_868176, EPI_ISL_868180, EPI_ISL_868195, EPI_ISL_868205, EPI_ISL_868206, EPI_ISL_868207, EPI_ISL_868208, EPI_ISL_868209, EPI_ISL_868210, EPI_ISL_868212, EPI_ISL_868213, EPI_ISL_868214, EPI_ISL_868215, EPI_ISL_868216, EPI_ISL_868219, EPI_ISL_868220, EPI_ISL_868221, EPI_ISL_868222, EPI_ISL_868224, EPI_ISL_868225, EPI_ISL_868238			
see above	Centre for Enzyme Innovation, University of Portsmouth / Translational Research Laboratory, Portsmouth Hospitals NHS Trust	COVID-19 Genomics UK (COG-UK) Consortium	Angela Beckett,Yann Bourgeois,Garry Scarlett,Sharon Glaysher,Scott Elliott,Kelly Bicknell,Robert Impey,Allyson Lloyd,Sarah Wyllie,Ethan Butcher,Anoop Chauhan,Samuel Robson
EPI_ISL_868361, EPI_ISL_868362, EPI_ISL_868365, EPI_ISL_868367, EPI_ISL_868368, EPI_ISL_868378, EPI_ISL_868379, EPI_ISL_868389, EPI_ISL_868391, EPI_ISL_868398, EPI_ISL_868407, EPI_ISL_868424, EPI_ISL_868425, EPI_ISL_868440, EPI_ISL_868451, EPI_ISL_868476, EPI_ISL_868482, EPI_ISL_868484, EPI_ISL_868492, EPI_ISL_868496, EPI_ISL_868500, EPI_ISL_868523, EPI_ISL_868527, EPI_ISL_868536, EPI_ISL_868578, EPI_ISL_868579, EPI_ISL_868602, EPI_ISL_868618, EPI_ISL_868626, EPI_ISL_868632, EPI_ISL_868639, EPI_ISL_868642, EPI_ISL_868661, EPI_ISL_868662, EPI_ISL_868679, EPI_ISL_868690, EPI_ISL_868696, EPI_ISL_868698, EPI_ISL_868703			
see above	Virology Department, Sheffield Teaching Hospitals NHS Foundation Trust/Department of Infection, Immunity and Cardiovascular Disease, The Medical School, University of Sheffield	COVID-19 Genomics UK (COG-UK) Consortium	Thushan de Silva, Matthew Parker, Nikki Smith, Adri Angyal, Rebecca Brown, Luke Green, Rachel Tucker, Paul Parsons, Danielle Groves, Katie Johnson, Laura Carrilero, Alex Keeley, Dave Partridge, Matthew Wyles, Benjamin Lindsey, Mehmet Yavuz, Mohammad Raza, Cariad Evans
EPI_ISL_869082	A. Krumbholz, Labor Dr. Krause und Kollegen MVZ GmbH, Kiel	Charité Universitätsmedizin Berlin, Institut für Virologie	Victor M Corman, Barbara Mühlemann, Jörn Beheim-Schwarzbach, Tobias Bleicker, Julia Tesch, Talitha Veith, Julia Schneider, Terry Jones, Christian Drosten
EPI_ISL_869085	Charité Universitätsmedizin Berlin, Institut für Virologie/Labor Berlin	Charité Universitätsmedizin Berlin, Institut für Virologie	Victor M Corman, Barbara Mühlemann, Jörn Beheim-Schwarzbach, Tobias Bleicker, Julia Tesch, Talitha Veith, Julia Schneider, Terry Jones, Christian Drosten
EPI_ISL_869086, EPI_ISL_869087, EPI_ISL_869088, EPI_ISL_869089, EPI_ISL_869090, EPI_ISL_869091, EPI_ISL_869092, EPI_ISL_869093, EPI_ISL_869094, EPI_ISL_869095, EPI_ISL_869096, EPI_ISL_869097, EPI_ISL_869098, EPI_ISL_869099, EPI_ISL_869100, EPI_ISL_869101			
see above	A. Krumbholz, Labor Dr. Krause und Kollegen MVZ GmbH, Kiel	Charité Universitätsmedizin Berlin, Institut für Virologie	Victor M Corman, Barbara Mühlemann, Jörn Beheim-Schwarzbach, Tobias Bleicker, Julia Tesch, Talitha Veith, Julia Schneider, Terry Jones, Christian Drosten
EPI_ISL_869131, EPI_ISL_869134, EPI_ISL_869136, EPI_ISL_869143	Charité Universitätsmedizin Berlin, Institut für Virologie/Labor Berlin	Charité Universitätsmedizin Berlin, Institut für Virologie	Victor M Corman, Barbara Mühlemann, Jörn Beheim-Schwarzbach, Tobias Bleicker, Julia Tesch, Talitha Veith, Julia Schneider, Terry Jones, Christian Drosten
EPI_ISL_869241	Suceava County Emergency Hospital	"Stefan cel Mare" University Metagenomics Laboratory	Lobiuc Andrei, Puscaselu Roxana
EPI_ISL_872038, EPI_ISL_872039, EPI_ISL_872042, EPI_ISL_872043, EPI_ISL_872049, EPI_ISL_872050	Department of Clinical Microbiology	GIGA Medical Genomics	Keith Durkin, Maria Artesi, Sébastien Bontems, Raphaël Boreux, Bouchra Boujemla, Cécile Meex, Pierrette Melin, Marie-Pierre Hayette, Vincent Bours
EPI_ISL_872067, EPI_ISL_872068	AZ Vesale (Tongres)	GIGA Medical Genomics	Keith Durkin, Maria Artesi, Sébastien Bontems, Raphaël Boreux, Bouchra Boujemla, Cécile Meex, Pierrette Melin, Marie-Pierre Hayette, Vincent Bours
EPI_ISL_872069, EPI_ISL_872070,	Department of Clinical Microbiology	GIGA Medical Genomics	Keith Durkin, Maria Artesi, Sébastien Bontems, Raphaël Boreux, Bouchra Boujemla, Cécile Meex, Pierrette Melin, Marie-Pierre Hayette, Vincent Bours

EPI_ISL_872071, EPI_ISL_872072			
EPI_ISL_872152	CHR Citadelle	GIGA Medical Genomics	Keith Durkin, Maria Artesi, Sébastien Bontems, Raphaël Boreux, Bouchra Boujemla, Cécile Meex, Pierrette Melin, Marie-Pierre Hayette, Vincent Bours
EPI_ISL_872176, EPI_ISL_872177, EPI_ISL_872180, EPI_ISL_872181	Wyoming Public Health Laboratory	Wyoming Public Health Laboratory	Noah Hull, Taylor Fearing, Lynette Gumbleton, Channing Weber, Ashley Norberg, Bailey Bowcutt, and Wanda Manley
EPI_ISL_872211	Hopital	National Reference Center for Viruses of Respiratory Infections, Institut Pasteur, Paris	Marion Barbet, Sylvie Behillil, Méline Bizard, Angela Brisebarre, Camille Capel, Etienne Simon-Lorière, Vincent Enouf, Maud Vanpeene, Sylvie van der Werf,Guigon AuréLie
EPI_ISL_872220	Hopital	National Reference Center for Viruses of Respiratory Infections, Institut Pasteur, Paris	Marion Barbet, Sylvie Behillil, Méline Bizard, Angela Brisebarre, Camille Capel, Etienne Simon-Lorière, Vincent Enouf, Maud Vanpeene, Sylvie van der Werf,Brichler Ségolène
EPI_ISL_872262	Hopital	National Reference Center for Viruses of Respiratory Infections, Institut Pasteur, Paris	Marion Barbet, Sylvie Behillil, Méline Bizard, Angela Brisebarre, Camille Capel, Etienne Simon-Lorière, Vincent Enouf, Maud Vanpeene, Sylvie van der Werf,Florin CéCile
EPI_ISL_872269	Hopital	National Reference Center for Viruses of Respiratory Infections, Institut Pasteur, Paris	Marion Barbet, Sylvie Behillil, Méline Bizard, Angela Brisebarre, Camille Capel, Etienne Simon-Lorière, Vincent Enouf, Maud Vanpeene, Sylvie van der Werf,Foissaud Vincent
EPI_ISL_872284	Labo Analyses Med	National Reference Center for Viruses of Respiratory Infections, Institut Pasteur, Paris	Marion Barbet, Sylvie Behillil, Méline Bizard, Angela Brisebarre, Camille Capel, Etienne Simon-Lorière, Vincent Enouf, Maud Vanpeene, Sylvie van der Werf
EPI_ISL_872302, EPI_ISL_872303, EPI_ISL_872304, EPI_ISL_872305	Hopital	National Reference Center for Viruses of Respiratory Infections, Institut Pasteur, Paris	Marion Barbet, Sylvie Behillil, Méline Bizard, Angela Brisebarre, Camille Capel, Etienne Simon-Lorière, Vincent Enouf, Maud Vanpeene, Sylvie van der Werf,Florin CéCile
EPI_ISL_872306, EPI_ISL_872307	Hopital	National Reference Center for Viruses of Respiratory Infections, Institut Pasteur, Paris	Marion Barbet, Sylvie Behillil, Méline Bizard, Angela Brisebarre, Camille Capel, Etienne Simon-Lorière, Vincent Enouf, Maud Vanpeene, Sylvie van der Werf,Castelain Sandrine
EPI_ISL_872324	Labo Analyses Med	National Reference Center for Viruses of Respiratory Infections, Institut Pasteur, Paris	Marion Barbet, Sylvie Behillil, Méline Bizard, Angela Brisebarre, Camille Capel, Etienne Simon-Lorière, Vincent Enouf, Maud Vanpeene, Sylvie van der Werf
EPI_ISL_872394	Centre for Dengue Research and AICBU, Department of Immunology and Molecular Medicine	Centre for Dengue Research and AICBU, Department of Immunology and Molecular Medicine	Chandima Jeewandara, Deshni Jayathilaka, Dinuka Ariyaratne, Tibutius Thanesh Pramanayagam, Diyanath Ranasinghe, Laksiri Gomes, Gathsaurie Neelika Malavige
EPI_ISL_872513, EPI_ISL_872514, EPI_ISL_872515, EPI_ISL_872516, EPI_ISL_872517, EPI_ISL_872518, EPI_ISL_872519, EPI_ISL_872520, EPI_ISL_872521, EPI_ISL_872522, EPI_ISL_872523, EPI_ISL_872524, EPI_ISL_872525, EPI_ISL_872526, EPI_ISL_872527, EPI_ISL_872528, EPI_ISL_872529, EPI_ISL_872530, EPI_ISL_872531, EPI_ISL_872532, EPI_ISL_872533, EPI_ISL_872534, EPI_ISL_872535, EPI_ISL_872536, EPI_ISL_872537, EPI_ISL_872538, EPI_ISL_872539, EPI_ISL_872540, EPI_ISL_872541, EPI_ISL_872542, EPI_ISL_872543, EPI_ISL_872544, EPI_ISL_872545, EPI_ISL_872546, EPI_ISL_872547, EPI_ISL_872548, EPI_ISL_872549, EPI_ISL_872550, EPI_ISL_872551, EPI_ISL_872552, EPI_ISL_872553, EPI_ISL_872554, EPI_ISL_872555, EPI_ISL_872556, EPI_ISL_872562, EPI_ISL_872563			
see above	New Mexico Department of Health Scientific Laboratory	Center for Global Health, University of New Mexico Health Sciences Center	Daryl Domman, Kurt Schwalm, Twila Kunde, Joseph Hicks, Anastacia Griego, Michael Edwards, Darrell Dinwiddie
EPI_ISL_872588	Sydney South West Pathology Service (SSWPS) - Royal Prince Alfred Hospital - NSW Health Pathology	NSW Health Pathology - Institute of Clinical Pathology and Medical Research; Westmead Hospital; University of Sydney	CIDM-PH et al.
EPI_ISL_872778, EPI_ISL_872779, EPI_ISL_872780, EPI_ISL_872782, EPI_ISL_872783, EPI_ISL_872784, EPI_ISL_872785, EPI_ISL_872786, EPI_ISL_872787, EPI_ISL_872788, EPI_ISL_872789, EPI_ISL_872790, EPI_ISL_872791, EPI_ISL_872793, EPI_ISL_872796, EPI_ISL_872800, EPI_ISL_872801, EPI_ISL_872805, EPI_ISL_872806, EPI_ISL_872807, EPI_ISL_872810, EPI_ISL_872812, EPI_ISL_872814, EPI_ISL_872816, EPI_ISL_872817, EPI_ISL_872820, EPI_ISL_872822, EPI_ISL_872823, EPI_ISL_872824, EPI_ISL_872828, EPI_ISL_872829, EPI_ISL_872833, EPI_ISL_872835, EPI_ISL_872837, EPI_ISL_872839, EPI_ISL_872840, EPI_ISL_872841, EPI_ISL_872842, EPI_ISL_872843, EPI_ISL_872845, EPI_ISL_872847, EPI_ISL_872854, EPI_ISL_872856, EPI_ISL_872858, EPI_ISL_872862, EPI_ISL_872864, EPI_ISL_872867, EPI_ISL_872869, EPI_ISL_872870, EPI_ISL_872872, EPI_ISL_872874, EPI_ISL_872880, EPI_ISL_872882, EPI_ISL_872886, EPI_ISL_872888, EPI_ISL_872890, EPI_ISL_872891, EPI_ISL_872893, EPI_ISL_872894			
see above	University of Wisconsin-Madison AIDS Vaccine Research Laboratories	University of Wisconsin-Madison AIDS Vaccine Research Laboratories	Gage Moreno, Katarina Braun, et al. AIDS Vaccine Research Laboratories
EPI_ISL_873158, EPI_ISL_873160	University of Michigan Clinical Microbiology Laboratory	Lauring Lab, University of Michigan, Department of Microbiology and Immunology	Valesano
EPI_ISL_873281	Lighthouse Lab in Milton Keynes	Wellcome Sanger Institute for the COVID-19 Genomics UK (COG-UK) Consortium	The Lighthouse Lab in Milton Keynes and Alex Alderton, Roberto Amato, Sonia Goncalves, Ewan Harrison, David K. Jackson, Ian Johnston, Dominic Kwiatkowski, Cordelia Langford, John Sillitoe on behalf of the Wellcome Sanger Institute COVID-19 Surveillance Team
EPI_ISL_873292	Lighthouse Lab in Cambridge	Wellcome Sanger Institute for the COVID-19 Genomics UK (COG-UK) Consortium	Rob Howes, The Lighthouse Lab in Cambridge and Alex Alderton, Roberto Amato, Sonia Goncalves, Ewan Harrison, David K. Jackson, Ian Johnston, Dominic Kwiatkowski, Cordelia Langford, John Sillitoe on behalf of the Wellcome Sanger Institute COVID-19 Surveillance Team
EPI_ISL_873294	Lighthouse Lab in Milton Keynes	Wellcome Sanger Institute for the COVID-19 Genomics UK (COG-UK) Consortium	The Lighthouse Lab in Milton Keynes and Alex Alderton, Roberto Amato, Sonia Goncalves, Ewan Harrison, David K. Jackson, Ian Johnston, Dominic Kwiatkowski, Cordelia Langford, John Sillitoe on behalf of the Wellcome Sanger Institute COVID-19 Surveillance Team
EPI_ISL_873297	Lighthouse Lab in Cambridge	Wellcome Sanger Institute for the COVID-19 Genomics UK (COG-UK) Consortium	Rob Howes, The Lighthouse Lab in Cambridge and Alex Alderton, Roberto Amato, Sonia Goncalves, Ewan Harrison, David K. Jackson, Ian Johnston, Dominic Kwiatkowski, Cordelia Langford, John Sillitoe on behalf of the Wellcome Sanger Institute COVID-19 Surveillance Team
EPI_ISL_873298	Lighthouse Lab in Milton Keynes	Wellcome Sanger Institute for the COVID-19 Genomics UK (COG-UK) Consortium	The Lighthouse Lab in Milton Keynes and Alex Alderton, Roberto Amato, Sonia Goncalves, Ewan Harrison, David K. Jackson, Ian Johnston, Dominic Kwiatkowski, Cordelia Langford, John Sillitoe on behalf of the Wellcome Sanger Institute COVID-19 Surveillance Team
EPI_ISL_873302, EPI_ISL_873304, EPI_ISL_873306, EPI_ISL_873310	Lighthouse Lab in Cambridge	Wellcome Sanger Institute for the COVID-19 Genomics UK (COG-UK) Consortium	Rob Howes, The Lighthouse Lab in Cambridge and Alex Alderton, Roberto Amato, Sonia Goncalves, Ewan Harrison, David K. Jackson, Ian Johnston, Dominic Kwiatkowski, Cordelia Langford, John Sillitoe on behalf of the Wellcome Sanger Institute COVID-19 Surveillance Team
EPI_ISL_873311	Lighthouse Lab in Milton Keynes	Wellcome Sanger Institute for the COVID-19 Genomics UK (COG-UK) Consortium	The Lighthouse Lab in Milton Keynes and Alex Alderton, Roberto Amato, Sonia Goncalves, Ewan Harrison, David K. Jackson, Ian Johnston, Dominic Kwiatkowski, Cordelia Langford, John Sillitoe on behalf of the Wellcome Sanger Institute COVID-19 Surveillance Team
EPI_ISL_873313, EPI_ISL_873316, EPI_ISL_873318, EPI_ISL_873320	Lighthouse Lab in Cambridge	Wellcome Sanger Institute for the COVID-19 Genomics UK (COG-UK) Consortium	Rob Howes, The Lighthouse Lab in Cambridge and Alex Alderton, Roberto Amato, Sonia Goncalves, Ewan Harrison, David K. Jackson, Ian Johnston, Dominic Kwiatkowski, Cordelia Langford, John Sillitoe on behalf of the Wellcome Sanger Institute COVID-19 Surveillance Team
EPI_ISL_873321	Lighthouse Lab in Milton Keynes	Wellcome Sanger Institute for the COVID-19 Genomics UK (COG-UK) Consortium	The Lighthouse Lab in Milton Keynes and Alex Alderton, Roberto Amato, Sonia Goncalves, Ewan Harrison, David K. Jackson, Ian Johnston, Dominic Kwiatkowski, Cordelia Langford, John Sillitoe on behalf of the Wellcome Sanger Institute COVID-19 Surveillance Team
EPI_ISL_873324, EPI_ISL_873325, EPI_ISL_873326	Lighthouse Lab in Cambridge	Wellcome Sanger Institute for the COVID-19 Genomics UK (COG-UK) Consortium	Rob Howes, The Lighthouse Lab in Cambridge and Alex Alderton, Roberto Amato, Sonia Goncalves, Ewan Harrison, David K. Jackson, Ian Johnston, Dominic Kwiatkowski, Cordelia Langford, John Sillitoe on behalf of the Wellcome Sanger Institute COVID-19 Surveillance Team
EPI_ISL_873330	Lighthouse Lab in Milton Keynes	Wellcome Sanger Institute for the COVID-19 Genomics UK (COG-UK) Consortium	The Lighthouse Lab in Milton Keynes and Alex Alderton, Roberto Amato, Sonia Goncalves, Ewan Harrison, David K. Jackson, Ian Johnston, Dominic Kwiatkowski, Cordelia Langford, John Sillitoe on behalf of the Wellcome Sanger Institute COVID-19 Surveillance Team
EPI_ISL_873334	Lighthouse Lab in Cambridge	Wellcome Sanger Institute for the COVID-19 Genomics UK (COG-UK) Consortium	Rob Howes, The Lighthouse Lab in Cambridge and Alex Alderton, Roberto Amato, Sonia Goncalves, Ewan Harrison, David K. Jackson, Ian Johnston, Dominic Kwiatkowski, Cordelia Langford, John Sillitoe on behalf of the Wellcome Sanger Institute COVID-19 Surveillance Team
EPI_ISL_873336	Lighthouse Lab in Milton Keynes	Wellcome Sanger Institute for the COVID-19 Genomics UK (COG-UK) Consortium	The Lighthouse Lab in Milton Keynes and Alex Alderton, Roberto Amato, Sonia Goncalves, Ewan Harrison, David K. Jackson, Ian Johnston, Dominic Kwiatkowski, Cordelia Langford, John Sillitoe on behalf of the Wellcome Sanger Institute COVID-19 Surveillance Team
EPI_ISL_873337, EPI_ISL_873339, EPI_ISL_873341, EPI_ISL_873342, EPI_ISL_873343, EPI_ISL_873350	Lighthouse Lab in Cambridge	Wellcome Sanger Institute for the COVID-19 Genomics UK (COG-UK) Consortium	Rob Howes, The Lighthouse Lab in Cambridge and Alex Alderton, Roberto Amato, Sonia Goncalves, Ewan Harrison, David K. Jackson, Ian Johnston, Dominic Kwiatkowski, Cordelia Langford, John Sillitoe on behalf of the Wellcome Sanger Institute COVID-19 Surveillance Team
EPI_ISL_873354	Lighthouse Lab in Milton Keynes	Wellcome Sanger Institute for the COVID-19 Genomics UK (COG-UK) Consortium	The Lighthouse Lab in Milton Keynes and Alex Alderton, Roberto Amato, Sonia Goncalves, Ewan Harrison, David K. Jackson, Ian Johnston, Dominic Kwiatkowski, Cordelia Langford, John Sillitoe on behalf of the Wellcome Sanger Institute COVID-19 Surveillance Team
EPI_ISL_873357, EPI_ISL_873359	Lighthouse Lab in Cambridge	Wellcome Sanger Institute for the COVID-19 Genomics UK (COG-UK) Consortium	Rob Howes, The Lighthouse Lab in Cambridge and Alex Alderton, Roberto Amato, Sonia Goncalves, Ewan Harrison, David K. Jackson, Ian Johnston, Dominic Kwiatkowski, Cordelia Langford, John Sillitoe on behalf of the Wellcome Sanger Institute COVID-19 Surveillance Team
EPI_ISL_873361	Lighthouse Lab in Milton Keynes	Wellcome Sanger Institute for the COVID-19 Genomics UK	The Lighthouse Lab in Milton Keynes and Alex Alderton, Roberto Amato, Sonia Goncalves, Ewan Harrison, David K. Jackson, Ian Johnston, Dominic

		(COG-UK) Consortium	Kwiatkowski, Cordelia Langford, John Sillitoe on behalf of the Wellcome Sanger Institute COVID-19 Surveillance Team
EPI_ISL_873362, EPI_ISL_873364, EPI_ISL_873374	Lighthouse Lab in Cambridge	Wellcome Sanger Institute for the COVID-19 Genomics UK (COG-UK) Consortium	Rob Howes, The Lighthouse Lab in Cambridge and Alex Alderton, Roberto Amato, Sonia Goncalves, Ewan Harrison, David K. Jackson, Ian Johnston, Dominic Kwiatkowski, Cordelia Langford, John Sillitoe on behalf of the Wellcome Sanger Institute COVID-19 Surveillance Team
EPI_ISL_873376, EPI_ISL_873378, EPI_ISL_873386, EPI_ISL_873389, EPI_ISL_873396, EPI_ISL_873411	Lighthouse Lab in Milton Keynes	Wellcome Sanger Institute for the COVID-19 Genomics UK (COG-UK) Consortium	The Lighthouse Lab in Milton Keynes and Alex Alderton, Roberto Amato, Sonia Goncalves, Ewan Harrison, David K. Jackson, Ian Johnston, Dominic Kwiatkowski, Cordelia Langford, John Sillitoe on behalf of the Wellcome Sanger Institute COVID-19 Surveillance Team
EPI_ISL_873412	Lighthouse Lab in Cambridge	Wellcome Sanger Institute for the COVID-19 Genomics UK (COG-UK) Consortium	Rob Howes, The Lighthouse Lab in Cambridge and Alex Alderton, Roberto Amato, Sonia Goncalves, Ewan Harrison, David K. Jackson, Ian Johnston, Dominic Kwiatkowski, Cordelia Langford, John Sillitoe on behalf of the Wellcome Sanger Institute COVID-19 Surveillance Team
EPI_ISL_873413, EPI_ISL_873414, EPI_ISL_873415	Lighthouse Lab in Milton Keynes	Wellcome Sanger Institute for the COVID-19 Genomics UK (COG-UK) Consortium	The Lighthouse Lab in Milton Keynes and Alex Alderton, Roberto Amato, Sonia Goncalves, Ewan Harrison, David K. Jackson, Ian Johnston, Dominic Kwiatkowski, Cordelia Langford, John Sillitoe on behalf of the Wellcome Sanger Institute COVID-19 Surveillance Team
EPI_ISL_873416	Lighthouse Lab in Cambridge	Wellcome Sanger Institute for the COVID-19 Genomics UK (COG-UK) Consortium	Rob Howes, The Lighthouse Lab in Cambridge and Alex Alderton, Roberto Amato, Sonia Goncalves, Ewan Harrison, David K. Jackson, Ian Johnston, Dominic Kwiatkowski, Cordelia Langford, John Sillitoe on behalf of the Wellcome Sanger Institute COVID-19 Surveillance Team
EPI_ISL_873420	Lighthouse Lab in Milton Keynes	Wellcome Sanger Institute for the COVID-19 Genomics UK (COG-UK) Consortium	The Lighthouse Lab in Milton Keynes and Alex Alderton, Roberto Amato, Sonia Goncalves, Ewan Harrison, David K. Jackson, Ian Johnston, Dominic Kwiatkowski, Cordelia Langford, John Sillitoe on behalf of the Wellcome Sanger Institute COVID-19 Surveillance Team
EPI_ISL_873422	Lighthouse Lab in Cambridge	Wellcome Sanger Institute for the COVID-19 Genomics UK (COG-UK) Consortium	Rob Howes, The Lighthouse Lab in Cambridge and Alex Alderton, Roberto Amato, Sonia Goncalves, Ewan Harrison, David K. Jackson, Ian Johnston, Dominic Kwiatkowski, Cordelia Langford, John Sillitoe on behalf of the Wellcome Sanger Institute COVID-19 Surveillance Team
EPI_ISL_873429, EPI_ISL_873430	Lighthouse Lab in Milton Keynes	Wellcome Sanger Institute for the COVID-19 Genomics UK (COG-UK) Consortium	The Lighthouse Lab in Milton Keynes and Alex Alderton, Roberto Amato, Sonia Goncalves, Ewan Harrison, David K. Jackson, Ian Johnston, Dominic Kwiatkowski, Cordelia Langford, John Sillitoe on behalf of the Wellcome Sanger Institute COVID-19 Surveillance Team
EPI_ISL_873431, EPI_ISL_873433	Lighthouse Lab in Cambridge	Wellcome Sanger Institute for the COVID-19 Genomics UK (COG-UK) Consortium	Rob Howes, The Lighthouse Lab in Cambridge and Alex Alderton, Roberto Amato, Sonia Goncalves, Ewan Harrison, David K. Jackson, Ian Johnston, Dominic Kwiatkowski, Cordelia Langford, John Sillitoe on behalf of the Wellcome Sanger Institute COVID-19 Surveillance Team
EPI_ISL_873435, EPI_ISL_873438, EPI_ISL_873439	Lighthouse Lab in Milton Keynes	Wellcome Sanger Institute for the COVID-19 Genomics UK (COG-UK) Consortium	The Lighthouse Lab in Milton Keynes and Alex Alderton, Roberto Amato, Sonia Goncalves, Ewan Harrison, David K. Jackson, Ian Johnston, Dominic Kwiatkowski, Cordelia Langford, John Sillitoe on behalf of the Wellcome Sanger Institute COVID-19 Surveillance Team
EPI_ISL_873443	Lighthouse Lab in Cambridge	Wellcome Sanger Institute for the COVID-19 Genomics UK (COG-UK) Consortium	Rob Howes, The Lighthouse Lab in Cambridge and Alex Alderton, Roberto Amato, Sonia Goncalves, Ewan Harrison, David K. Jackson, Ian Johnston, Dominic Kwiatkowski, Cordelia Langford, John Sillitoe on behalf of the Wellcome Sanger Institute COVID-19 Surveillance Team
EPI_ISL_873444, EPI_ISL_873445, EPI_ISL_873450	Lighthouse Lab in Milton Keynes	Wellcome Sanger Institute for the COVID-19 Genomics UK (COG-UK) Consortium	The Lighthouse Lab in Milton Keynes and Alex Alderton, Roberto Amato, Sonia Goncalves, Ewan Harrison, David K. Jackson, Ian Johnston, Dominic Kwiatkowski, Cordelia Langford, John Sillitoe on behalf of the Wellcome Sanger Institute COVID-19 Surveillance Team
EPI_ISL_873454	Lighthouse Lab in Cambridge	Wellcome Sanger Institute for the COVID-19 Genomics UK (COG-UK) Consortium	Rob Howes, The Lighthouse Lab in Cambridge and Alex Alderton, Roberto Amato, Sonia Goncalves, Ewan Harrison, David K. Jackson, Ian Johnston, Dominic Kwiatkowski, Cordelia Langford, John Sillitoe on behalf of the Wellcome Sanger Institute COVID-19 Surveillance Team
EPI_ISL_873455	Lighthouse Lab in Milton Keynes	Wellcome Sanger Institute for the COVID-19 Genomics UK (COG-UK) Consortium	The Lighthouse Lab in Milton Keynes and Alex Alderton, Roberto Amato, Sonia Goncalves, Ewan Harrison, David K. Jackson, Ian Johnston, Dominic Kwiatkowski, Cordelia Langford, John Sillitoe on behalf of the Wellcome Sanger Institute COVID-19 Surveillance Team
EPI_ISL_873457, EPI_ISL_873459	Lighthouse Lab in Cambridge	Wellcome Sanger Institute for the COVID-19 Genomics UK (COG-UK) Consortium	Rob Howes, The Lighthouse Lab in Cambridge and Alex Alderton, Roberto Amato, Sonia Goncalves, Ewan Harrison, David K. Jackson, Ian Johnston, Dominic Kwiatkowski, Cordelia Langford, John Sillitoe on behalf of the Wellcome Sanger Institute COVID-19 Surveillance Team
EPI_ISL_873462, EPI_ISL_873466, EPI_ISL_873473	Lighthouse Lab in Milton Keynes	Wellcome Sanger Institute for the COVID-19 Genomics UK (COG-UK) Consortium	The Lighthouse Lab in Milton Keynes and Alex Alderton, Roberto Amato, Sonia Goncalves, Ewan Harrison, David K. Jackson, Ian Johnston, Dominic Kwiatkowski, Cordelia Langford, John Sillitoe on behalf of the Wellcome Sanger Institute COVID-19 Surveillance Team
EPI_ISL_873476, EPI_ISL_873481	Lighthouse Lab in Cambridge	Wellcome Sanger Institute for the COVID-19 Genomics UK (COG-UK) Consortium	Rob Howes, The Lighthouse Lab in Cambridge and Alex Alderton, Roberto Amato, Sonia Goncalves, Ewan Harrison, David K. Jackson, Ian Johnston, Dominic Kwiatkowski, Cordelia Langford, John Sillitoe on behalf of the Wellcome Sanger Institute COVID-19 Surveillance Team
EPI_ISL_873487, EPI_ISL_873493	Lighthouse Lab in Milton Keynes	Wellcome Sanger Institute for the COVID-19 Genomics UK (COG-UK) Consortium	The Lighthouse Lab in Milton Keynes and Alex Alderton, Roberto Amato, Sonia Goncalves, Ewan Harrison, David K. Jackson, Ian Johnston, Dominic Kwiatkowski, Cordelia Langford, John Sillitoe on behalf of the Wellcome Sanger Institute COVID-19 Surveillance Team
EPI_ISL_873494, EPI_ISL_873495	Lighthouse Lab in Cambridge	Wellcome Sanger Institute for the COVID-19 Genomics UK (COG-UK) Consortium	Rob Howes, The Lighthouse Lab in Cambridge and Alex Alderton, Roberto Amato, Sonia Goncalves, Ewan Harrison, David K. Jackson, Ian Johnston, Dominic Kwiatkowski, Cordelia Langford, John Sillitoe on behalf of the Wellcome Sanger Institute COVID-19 Surveillance Team
EPI_ISL_873496	Lighthouse Lab in Milton Keynes	Wellcome Sanger Institute for the COVID-19 Genomics UK (COG-UK) Consortium	The Lighthouse Lab in Milton Keynes and Alex Alderton, Roberto Amato, Sonia Goncalves, Ewan Harrison, David K. Jackson, Ian Johnston, Dominic Kwiatkowski, Cordelia Langford, John Sillitoe on behalf of the Wellcome Sanger Institute COVID-19 Surveillance Team
EPI_ISL_873497, EPI_ISL_873499, EPI_ISL_873500	Lighthouse Lab in Cambridge	Wellcome Sanger Institute for the COVID-19 Genomics UK (COG-UK) Consortium	Rob Howes, The Lighthouse Lab in Cambridge and Alex Alderton, Roberto Amato, Sonia Goncalves, Ewan Harrison, David K. Jackson, Ian Johnston, Dominic Kwiatkowski, Cordelia Langford, John Sillitoe on behalf of the Wellcome Sanger Institute COVID-19 Surveillance Team
EPI_ISL_873503, EPI_ISL_873504, EPI_ISL_873509	Lighthouse Lab in Milton Keynes	Wellcome Sanger Institute for the COVID-19 Genomics UK (COG-UK) Consortium	The Lighthouse Lab in Milton Keynes and Alex Alderton, Roberto Amato, Sonia Goncalves, Ewan Harrison, David K. Jackson, Ian Johnston, Dominic Kwiatkowski, Cordelia Langford, John Sillitoe on behalf of the Wellcome Sanger Institute COVID-19 Surveillance Team
EPI_ISL_873510	Lighthouse Lab in Cambridge	Wellcome Sanger Institute for the COVID-19 Genomics UK (COG-UK) Consortium	Rob Howes, The Lighthouse Lab in Cambridge and Alex Alderton, Roberto Amato, Sonia Goncalves, Ewan Harrison, David K. Jackson, Ian Johnston, Dominic Kwiatkowski, Cordelia Langford, John Sillitoe on behalf of the Wellcome Sanger Institute COVID-19 Surveillance Team
EPI_ISL_873515, EPI_ISL_873520, EPI_ISL_873531	Lighthouse Lab in Milton Keynes	Wellcome Sanger Institute for the COVID-19 Genomics UK (COG-UK) Consortium	The Lighthouse Lab in Milton Keynes and Alex Alderton, Roberto Amato, Sonia Goncalves, Ewan Harrison, David K. Jackson, Ian Johnston, Dominic Kwiatkowski, Cordelia Langford, John Sillitoe on behalf of the Wellcome Sanger Institute COVID-19 Surveillance Team
EPI_ISL_873532	Lighthouse Lab in Cambridge	Wellcome Sanger Institute for the COVID-19 Genomics UK (COG-UK) Consortium	Rob Howes, The Lighthouse Lab in Cambridge and Alex Alderton, Roberto Amato, Sonia Goncalves, Ewan Harrison, David K. Jackson, Ian Johnston, Dominic Kwiatkowski, Cordelia Langford, John Sillitoe on behalf of the Wellcome Sanger Institute COVID-19 Surveillance Team
EPI_ISL_873551	Lighthouse Lab in Milton Keynes	Wellcome Sanger Institute for the COVID-19 Genomics UK (COG-UK) Consortium	The Lighthouse Lab in Milton Keynes and Alex Alderton, Roberto Amato, Sonia Goncalves, Ewan Harrison, David K. Jackson, Ian Johnston, Dominic Kwiatkowski, Cordelia Langford, John Sillitoe on behalf of the Wellcome Sanger Institute COVID-19 Surveillance Team
EPI_ISL_873552, EPI_ISL_873566, EPI_ISL_873571, EPI_ISL_873573, EPI_ISL_873576	Lighthouse Lab in Cambridge	Wellcome Sanger Institute for the COVID-19 Genomics UK (COG-UK) Consortium	Rob Howes, The Lighthouse Lab in Cambridge and Alex Alderton, Roberto Amato, Sonia Goncalves, Ewan Harrison, David K. Jackson, Ian Johnston, Dominic Kwiatkowski, Cordelia Langford, John Sillitoe on behalf of the Wellcome Sanger Institute COVID-19 Surveillance Team
EPI_ISL_873585, EPI_ISL_873596, EPI_ISL_873600, EPI_ISL_873603, EPI_ISL_873604	Lighthouse Lab in Milton Keynes	Wellcome Sanger Institute for the COVID-19 Genomics UK (COG-UK) Consortium	The Lighthouse Lab in Milton Keynes and Alex Alderton, Roberto Amato, Sonia Goncalves, Ewan Harrison, David K. Jackson, Ian Johnston, Dominic Kwiatkowski, Cordelia Langford, John Sillitoe on behalf of the Wellcome Sanger Institute COVID-19 Surveillance Team
EPI_ISL_873605	Lighthouse Lab in Cambridge	Wellcome Sanger Institute for the COVID-19 Genomics UK (COG-UK) Consortium	Rob Howes, The Lighthouse Lab in Cambridge and Alex Alderton, Roberto Amato, Sonia Goncalves, Ewan Harrison, David K. Jackson, Ian Johnston, Dominic Kwiatkowski, Cordelia Langford, John Sillitoe on behalf of the Wellcome Sanger Institute COVID-19 Surveillance Team
EPI_ISL_873607	Lighthouse Lab in Milton Keynes	Wellcome Sanger Institute for the COVID-19 Genomics UK (COG-UK) Consortium	The Lighthouse Lab in Milton Keynes and Alex Alderton, Roberto Amato, Sonia Goncalves, Ewan Harrison, David K. Jackson, Ian Johnston, Dominic Kwiatkowski, Cordelia Langford, John Sillitoe on behalf of the Wellcome Sanger Institute COVID-19 Surveillance Team
EPI_ISL_873614, EPI_ISL_873621, EPI_ISL_873629, EPI_ISL_873632, EPI_ISL_873633, EPI_ISL_873634, EPI_ISL_873635, EPI_ISL_873636, EPI_ISL_873638, EPI_ISL_873639, EPI_ISL_873640, EPI_ISL_873642, EPI_ISL_873643, EPI_ISL_873644			
see above	Lighthouse Lab in Cambridge	Wellcome Sanger Institute for the COVID-19 Genomics UK (COG-UK) Consortium	Rob Howes, The Lighthouse Lab in Cambridge and Alex Alderton, Roberto Amato, Sonia Goncalves, Ewan Harrison, David K. Jackson, Ian Johnston, Dominic Kwiatkowski, Cordelia Langford, John Sillitoe on behalf of the Wellcome Sanger Institute COVID-19 Surveillance Team
EPI_ISL_873645	Lighthouse Lab in Milton Keynes	Wellcome Sanger Institute for the COVID-19 Genomics UK (COG-UK) Consortium	The Lighthouse Lab in Milton Keynes and Alex Alderton, Roberto Amato, Sonia Goncalves, Ewan Harrison, David K. Jackson, Ian Johnston, Dominic Kwiatkowski, Cordelia Langford, John Sillitoe on behalf of the Wellcome Sanger Institute COVID-19 Surveillance Team
EPI_ISL_873646, EPI_ISL_873647, EPI_ISL_873648, EPI_ISL_873649,	Lighthouse Lab in Cambridge	Wellcome Sanger Institute for the COVID-19 Genomics UK (COG-UK) Consortium	Rob Howes, The Lighthouse Lab in Cambridge and Alex Alderton, Roberto Amato, Sonia Goncalves, Ewan Harrison, David K. Jackson, Ian Johnston, Dominic Kwiatkowski, Cordelia Langford, John Sillitoe on behalf of the Wellcome Sanger Institute COVID-19 Surveillance Team

EPI_ISL_873650, EPI_ISL_873651			
EPI_ISL_873652	Lighthouse Lab in Milton Keynes	Wellcome Sanger Institute for the COVID-19 Genomics UK (COG-UK) Consortium	The Lighthouse Lab in Milton Keynes and Alex Alderton, Roberto Amato, Sonia Goncalves, Ewan Harrison, David K. Jackson, Ian Johnston, Dominic Kwiatkowski, Cordelia Langford, John Sillitoe on behalf of the Wellcome Sanger Institute COVID-19 Surveillance Team
EPI_ISL_873653, EPI_ISL_873655, EPI_ISL_873658, EPI_ISL_873659, EPI_ISL_873661, EPI_ISL_873662, EPI_ISL_873663, EPI_ISL_873664, EPI_ISL_873665, EPI_ISL_873666, EPI_ISL_873667, EPI_ISL_873668, EPI_ISL_873670, EPI_ISL_873671, EPI_ISL_873673, EPI_ISL_873675, EPI_ISL_873676, EPI_ISL_873677, EPI_ISL_873678			
see above	Lighthouse Lab in Cambridge	Wellcome Sanger Institute for the COVID-19 Genomics UK (COG-UK) Consortium	Rob Howes, The Lighthouse Lab in Cambridge and Alex Alderton, Roberto Amato, Sonia Goncalves, Ewan Harrison, David K. Jackson, Ian Johnston, Dominic Kwiatkowski, Cordelia Langford, John Sillitoe on behalf of the Wellcome Sanger Institute COVID-19 Surveillance Team
EPI_ISL_873679	Lighthouse Lab in Milton Keynes	Wellcome Sanger Institute for the COVID-19 Genomics UK (COG-UK) Consortium	The Lighthouse Lab in Milton Keynes and Alex Alderton, Roberto Amato, Sonia Goncalves, Ewan Harrison, David K. Jackson, Ian Johnston, Dominic Kwiatkowski, Cordelia Langford, John Sillitoe on behalf of the Wellcome Sanger Institute COVID-19 Surveillance Team
EPI_ISL_873680, EPI_ISL_873681, EPI_ISL_873682, EPI_ISL_873683	Lighthouse Lab in Cambridge	Wellcome Sanger Institute for the COVID-19 Genomics UK (COG-UK) Consortium	Rob Howes, The Lighthouse Lab in Cambridge and Alex Alderton, Roberto Amato, Sonia Goncalves, Ewan Harrison, David K. Jackson, Ian Johnston, Dominic Kwiatkowski, Cordelia Langford, John Sillitoe on behalf of the Wellcome Sanger Institute COVID-19 Surveillance Team
EPI_ISL_873684	Lighthouse Lab in Milton Keynes	Wellcome Sanger Institute for the COVID-19 Genomics UK (COG-UK) Consortium	The Lighthouse Lab in Milton Keynes and Alex Alderton, Roberto Amato, Sonia Goncalves, Ewan Harrison, David K. Jackson, Ian Johnston, Dominic Kwiatkowski, Cordelia Langford, John Sillitoe on behalf of the Wellcome Sanger Institute COVID-19 Surveillance Team
EPI_ISL_873685, EPI_ISL_873686, EPI_ISL_873687, EPI_ISL_873688, EPI_ISL_873689, EPI_ISL_873690, EPI_ISL_873691, EPI_ISL_873692, EPI_ISL_873693, EPI_ISL_873694, EPI_ISL_873695, EPI_ISL_873696, EPI_ISL_873697, EPI_ISL_873698, EPI_ISL_873699, EPI_ISL_873700, EPI_ISL_873701, EPI_ISL_873703, EPI_ISL_873705, EPI_ISL_873706, EPI_ISL_873707, EPI_ISL_873708, EPI_ISL_873709, EPI_ISL_873710, EPI_ISL_873711, EPI_ISL_873712, EPI_ISL_873713, EPI_ISL_873714, EPI_ISL_873715			
see above	Lighthouse Lab in Cambridge	Wellcome Sanger Institute for the COVID-19 Genomics UK (COG-UK) Consortium	Rob Howes, The Lighthouse Lab in Cambridge and Alex Alderton, Roberto Amato, Sonia Goncalves, Ewan Harrison, David K. Jackson, Ian Johnston, Dominic Kwiatkowski, Cordelia Langford, John Sillitoe on behalf of the Wellcome Sanger Institute COVID-19 Surveillance Team
EPI_ISL_873716	Lighthouse Lab in Milton Keynes	Wellcome Sanger Institute for the COVID-19 Genomics UK (COG-UK) Consortium	The Lighthouse Lab in Milton Keynes and Alex Alderton, Roberto Amato, Sonia Goncalves, Ewan Harrison, David K. Jackson, Ian Johnston, Dominic Kwiatkowski, Cordelia Langford, John Sillitoe on behalf of the Wellcome Sanger Institute COVID-19 Surveillance Team
EPI_ISL_873717, EPI_ISL_873718, EPI_ISL_873719, EPI_ISL_873720, EPI_ISL_873721, EPI_ISL_873722, EPI_ISL_873723, EPI_ISL_873727, EPI_ISL_873728, EPI_ISL_873731, EPI_ISL_873732, EPI_ISL_873733, EPI_ISL_873735, EPI_ISL_873736, EPI_ISL_873738, EPI_ISL_873739, EPI_ISL_873740, EPI_ISL_873742, EPI_ISL_873745, EPI_ISL_873747			
see above	Lighthouse Lab in Cambridge	Wellcome Sanger Institute for the COVID-19 Genomics UK (COG-UK) Consortium	Rob Howes, The Lighthouse Lab in Cambridge and Alex Alderton, Roberto Amato, Sonia Goncalves, Ewan Harrison, David K. Jackson, Ian Johnston, Dominic Kwiatkowski, Cordelia Langford, John Sillitoe on behalf of the Wellcome Sanger Institute COVID-19 Surveillance Team
EPI_ISL_873748	Lighthouse Lab in Milton Keynes	Wellcome Sanger Institute for the COVID-19 Genomics UK (COG-UK) Consortium	The Lighthouse Lab in Milton Keynes and Alex Alderton, Roberto Amato, Sonia Goncalves, Ewan Harrison, David K. Jackson, Ian Johnston, Dominic Kwiatkowski, Cordelia Langford, John Sillitoe on behalf of the Wellcome Sanger Institute COVID-19 Surveillance Team
EPI_ISL_873751, EPI_ISL_873753, EPI_ISL_873754	Lighthouse Lab in Cambridge	Wellcome Sanger Institute for the COVID-19 Genomics UK (COG-UK) Consortium	Rob Howes, The Lighthouse Lab in Cambridge and Alex Alderton, Roberto Amato, Sonia Goncalves, Ewan Harrison, David K. Jackson, Ian Johnston, Dominic Kwiatkowski, Cordelia Langford, John Sillitoe on behalf of the Wellcome Sanger Institute COVID-19 Surveillance Team
EPI_ISL_873755	Lighthouse Lab in Milton Keynes	Wellcome Sanger Institute for the COVID-19 Genomics UK (COG-UK) Consortium	The Lighthouse Lab in Milton Keynes and Alex Alderton, Roberto Amato, Sonia Goncalves, Ewan Harrison, David K. Jackson, Ian Johnston, Dominic Kwiatkowski, Cordelia Langford, John Sillitoe on behalf of the Wellcome Sanger Institute COVID-19 Surveillance Team
EPI_ISL_873756, EPI_ISL_873757, EPI_ISL_873758, EPI_ISL_873759, EPI_ISL_873761, EPI_ISL_873762, EPI_ISL_873763, EPI_ISL_873765, EPI_ISL_873766, EPI_ISL_873767, EPI_ISL_873768, EPI_ISL_873770, EPI_ISL_873771, EPI_ISL_873772, EPI_ISL_873773, EPI_ISL_873774, EPI_ISL_873775, EPI_ISL_873776, EPI_ISL_873777, EPI_ISL_873778, EPI_ISL_873779, EPI_ISL_873780, EPI_ISL_873781, EPI_ISL_873782			
see above	Lighthouse Lab in Cambridge	Wellcome Sanger Institute for the COVID-19 Genomics UK (COG-UK) Consortium	Rob Howes, The Lighthouse Lab in Cambridge and Alex Alderton, Roberto Amato, Sonia Goncalves, Ewan Harrison, David K. Jackson, Ian Johnston, Dominic Kwiatkowski, Cordelia Langford, John Sillitoe on behalf of the Wellcome Sanger Institute COVID-19 Surveillance Team
EPI_ISL_873783	Lighthouse Lab in Milton Keynes	Wellcome Sanger Institute for the COVID-19 Genomics UK (COG-UK) Consortium	The Lighthouse Lab in Milton Keynes and Alex Alderton, Roberto Amato, Sonia Goncalves, Ewan Harrison, David K. Jackson, Ian Johnston, Dominic Kwiatkowski, Cordelia Langford, John Sillitoe on behalf of the Wellcome Sanger Institute COVID-19 Surveillance Team
EPI_ISL_873784, EPI_ISL_873785, EPI_ISL_873787, EPI_ISL_873788, EPI_ISL_873789, EPI_ISL_873790, EPI_ISL_873791, EPI_ISL_873792, EPI_ISL_873793, EPI_ISL_873795, EPI_ISL_873796			
see above	Lighthouse Lab in Cambridge	Wellcome Sanger Institute for the COVID-19 Genomics UK (COG-UK) Consortium	Rob Howes, The Lighthouse Lab in Cambridge and Alex Alderton, Roberto Amato, Sonia Goncalves, Ewan Harrison, David K. Jackson, Ian Johnston, Dominic Kwiatkowski, Cordelia Langford, John Sillitoe on behalf of the Wellcome Sanger Institute COVID-19 Surveillance Team
EPI_ISL_873797	Lighthouse Lab in Milton Keynes	Wellcome Sanger Institute for the COVID-19 Genomics UK (COG-UK) Consortium	The Lighthouse Lab in Milton Keynes and Alex Alderton, Roberto Amato, Sonia Goncalves, Ewan Harrison, David K. Jackson, Ian Johnston, Dominic Kwiatkowski, Cordelia Langford, John Sillitoe on behalf of the Wellcome Sanger Institute COVID-19 Surveillance Team
EPI_ISL_873798, EPI_ISL_873799, EPI_ISL_873800, EPI_ISL_873801, EPI_ISL_873802, EPI_ISL_873803, EPI_ISL_873804, EPI_ISL_873805, EPI_ISL_873807, EPI_ISL_873808, EPI_ISL_873810, EPI_ISL_873811, EPI_ISL_873813, EPI_ISL_873815, EPI_ISL_873816, EPI_ISL_873818, EPI_ISL_873819, EPI_ISL_873821			
see above	Lighthouse Lab in Cambridge	Wellcome Sanger Institute for the COVID-19 Genomics UK (COG-UK) Consortium	Rob Howes, The Lighthouse Lab in Cambridge and Alex Alderton, Roberto Amato, Sonia Goncalves, Ewan Harrison, David K. Jackson, Ian Johnston, Dominic Kwiatkowski, Cordelia Langford, John Sillitoe on behalf of the Wellcome Sanger Institute COVID-19 Surveillance Team
EPI_ISL_873822	Lighthouse Lab in Milton Keynes	Wellcome Sanger Institute for the COVID-19 Genomics UK (COG-UK) Consortium	The Lighthouse Lab in Milton Keynes and Alex Alderton, Roberto Amato, Sonia Goncalves, Ewan Harrison, David K. Jackson, Ian Johnston, Dominic Kwiatkowski, Cordelia Langford, John Sillitoe on behalf of the Wellcome Sanger Institute COVID-19 Surveillance Team
EPI_ISL_873823, EPI_ISL_873824, EPI_ISL_873826	Lighthouse Lab in Cambridge	Wellcome Sanger Institute for the COVID-19 Genomics UK (COG-UK) Consortium	Rob Howes, The Lighthouse Lab in Cambridge and Alex Alderton, Roberto Amato, Sonia Goncalves, Ewan Harrison, David K. Jackson, Ian Johnston, Dominic Kwiatkowski, Cordelia Langford, John Sillitoe on behalf of the Wellcome Sanger Institute COVID-19 Surveillance Team
EPI_ISL_873827	Lighthouse Lab in Milton Keynes	Wellcome Sanger Institute for the COVID-19 Genomics UK (COG-UK) Consortium	The Lighthouse Lab in Milton Keynes and Alex Alderton, Roberto Amato, Sonia Goncalves, Ewan Harrison, David K. Jackson, Ian Johnston, Dominic Kwiatkowski, Cordelia Langford, John Sillitoe on behalf of the Wellcome Sanger Institute COVID-19 Surveillance Team
EPI_ISL_873828	Lighthouse Lab in Cambridge	Wellcome Sanger Institute for the COVID-19 Genomics UK (COG-UK) Consortium	Rob Howes, The Lighthouse Lab in Cambridge and Alex Alderton, Roberto Amato, Sonia Goncalves, Ewan Harrison, David K. Jackson, Ian Johnston, Dominic Kwiatkowski, Cordelia Langford, John Sillitoe on behalf of the Wellcome Sanger Institute COVID-19 Surveillance Team
EPI_ISL_873829	Lighthouse Lab in Milton Keynes	Wellcome Sanger Institute for the COVID-19 Genomics UK (COG-UK) Consortium	The Lighthouse Lab in Milton Keynes and Alex Alderton, Roberto Amato, Sonia Goncalves, Ewan Harrison, David K. Jackson, Ian Johnston, Dominic Kwiatkowski, Cordelia Langford, John Sillitoe on behalf of the Wellcome Sanger Institute COVID-19 Surveillance Team
EPI_ISL_873830, EPI_ISL_873831, EPI_ISL_873832, EPI_ISL_873835, EPI_ISL_873836, EPI_ISL_873837, EPI_ISL_873838	Lighthouse Lab in Cambridge	Wellcome Sanger Institute for the COVID-19 Genomics UK (COG-UK) Consortium	Rob Howes, The Lighthouse Lab in Cambridge and Alex Alderton, Roberto Amato, Sonia Goncalves, Ewan Harrison, David K. Jackson, Ian Johnston, Dominic Kwiatkowski, Cordelia Langford, John Sillitoe on behalf of the Wellcome Sanger Institute COVID-19 Surveillance Team
EPI_ISL_873840	Lighthouse Lab in Milton Keynes	Wellcome Sanger Institute for the COVID-19 Genomics UK (COG-UK) Consortium	The Lighthouse Lab in Milton Keynes and Alex Alderton, Roberto Amato, Sonia Goncalves, Ewan Harrison, David K. Jackson, Ian Johnston, Dominic Kwiatkowski, Cordelia Langford, John Sillitoe on behalf of the Wellcome Sanger Institute COVID-19 Surveillance Team
EPI_ISL_873841	Lighthouse Lab in Cambridge	Wellcome Sanger Institute for the COVID-19 Genomics UK (COG-UK) Consortium	Rob Howes, The Lighthouse Lab in Cambridge and Alex Alderton, Roberto Amato, Sonia Goncalves, Ewan Harrison, David K. Jackson, Ian Johnston, Dominic Kwiatkowski, Cordelia Langford, John Sillitoe on behalf of the Wellcome Sanger Institute COVID-19 Surveillance Team
EPI_ISL_873842	Lighthouse Lab in Milton Keynes	Wellcome Sanger Institute for the COVID-19 Genomics UK (COG-UK) Consortium	The Lighthouse Lab in Milton Keynes and Alex Alderton, Roberto Amato, Sonia Goncalves, Ewan Harrison, David K. Jackson, Ian Johnston, Dominic Kwiatkowski, Cordelia Langford, John Sillitoe on behalf of the Wellcome Sanger Institute COVID-19 Surveillance Team
EPI_ISL_873843, EPI_ISL_873844, EPI_ISL_873845, EPI_ISL_873846	Lighthouse Lab in Cambridge	Wellcome Sanger Institute for the COVID-19 Genomics UK (COG-UK) Consortium	Rob Howes, The Lighthouse Lab in Cambridge and Alex Alderton, Roberto Amato, Sonia Goncalves, Ewan Harrison, David K. Jackson, Ian Johnston, Dominic Kwiatkowski, Cordelia Langford, John Sillitoe on behalf of the Wellcome Sanger Institute COVID-19 Surveillance Team
EPI_ISL_873848	Lighthouse Lab in Milton Keynes	Wellcome Sanger Institute for the COVID-19 Genomics UK (COG-UK) Consortium	The Lighthouse Lab in Milton Keynes and Alex Alderton, Roberto Amato, Sonia Goncalves, Ewan Harrison, David K. Jackson, Ian Johnston, Dominic Kwiatkowski, Cordelia Langford, John Sillitoe on behalf of the Wellcome Sanger Institute COVID-19 Surveillance Team
EPI_ISL_873849, EPI_ISL_873851, EPI_ISL_873852	Lighthouse Lab in Cambridge	Wellcome Sanger Institute for the COVID-19 Genomics UK (COG-UK) Consortium	Rob Howes, The Lighthouse Lab in Cambridge and Alex Alderton, Roberto Amato, Sonia Goncalves, Ewan Harrison, David K. Jackson, Ian Johnston, Dominic Kwiatkowski, Cordelia Langford, John Sillitoe on behalf of the Wellcome Sanger Institute COVID-19 Surveillance Team
EPI_ISL_873853	Lighthouse Lab in Milton Keynes	Wellcome Sanger Institute for the COVID-19 Genomics UK	The Lighthouse Lab in Milton Keynes and Alex Alderton, Roberto Amato, Sonia Goncalves, Ewan Harrison, David K. Jackson, Ian Johnston, Dominic

		(COG-UK) Consortium	Kwiatkowski, Cordelia Langford, John Sillitoe on behalf of the Wellcome Sanger Institute COVID-19 Surveillance Team
EPI_ISL_873854, EPI_ISL_873855, EPI_ISL_873856, EPI_ISL_873858	Lighthouse Lab in Cambridge	Wellcome Sanger Institute for the COVID-19 Genomics UK (COG-UK) Consortium	Rob Howes, The Lighthouse Lab in Cambridge and Alex Alderton, Roberto Amato, Sonia Goncalves, Ewan Harrison, David K. Jackson, Ian Johnston, Dominic Kwiatkowski, Cordelia Langford, John Sillitoe on behalf of the Wellcome Sanger Institute COVID-19 Surveillance Team
EPI_ISL_873860	Lighthouse Lab in Milton Keynes	Wellcome Sanger Institute for the COVID-19 Genomics UK (COG-UK) Consortium	The Lighthouse Lab in Milton Keynes and Alex Alderton, Roberto Amato, Sonia Goncalves, Ewan Harrison, David K. Jackson, Ian Johnston, Dominic Kwiatkowski, Cordelia Langford, John Sillitoe on behalf of the Wellcome Sanger Institute COVID-19 Surveillance Team
EPI_ISL_873861	Lighthouse Lab in Cambridge	Wellcome Sanger Institute for the COVID-19 Genomics UK (COG-UK) Consortium	Rob Howes, The Lighthouse Lab in Cambridge and Alex Alderton, Roberto Amato, Sonia Goncalves, Ewan Harrison, David K. Jackson, Ian Johnston, Dominic Kwiatkowski, Cordelia Langford, John Sillitoe on behalf of the Wellcome Sanger Institute COVID-19 Surveillance Team
EPI_ISL_873862	Lighthouse Lab in Milton Keynes	Wellcome Sanger Institute for the COVID-19 Genomics UK (COG-UK) Consortium	The Lighthouse Lab in Milton Keynes and Alex Alderton, Roberto Amato, Sonia Goncalves, Ewan Harrison, David K. Jackson, Ian Johnston, Dominic Kwiatkowski, Cordelia Langford, John Sillitoe on behalf of the Wellcome Sanger Institute COVID-19 Surveillance Team
EPI_ISL_873863, EPI_ISL_873864, EPI_ISL_873866, EPI_ISL_873867, EPI_ISL_873868, EPI_ISL_873869, EPI_ISL_873871, EPI_ISL_873872, EPI_ISL_873874, EPI_ISL_873877, EPI_ISL_873878, EPI_ISL_873879			
see above	Lighthouse Lab in Cambridge	Wellcome Sanger Institute for the COVID-19 Genomics UK (COG-UK) Consortium	Rob Howes, The Lighthouse Lab in Cambridge and Alex Alderton, Roberto Amato, Sonia Goncalves, Ewan Harrison, David K. Jackson, Ian Johnston, Dominic Kwiatkowski, Cordelia Langford, John Sillitoe on behalf of the Wellcome Sanger Institute COVID-19 Surveillance Team
EPI_ISL_873881	Lighthouse Lab in Milton Keynes	Wellcome Sanger Institute for the COVID-19 Genomics UK (COG-UK) Consortium	The Lighthouse Lab in Milton Keynes and Alex Alderton, Roberto Amato, Sonia Goncalves, Ewan Harrison, David K. Jackson, Ian Johnston, Dominic Kwiatkowski, Cordelia Langford, John Sillitoe on behalf of the Wellcome Sanger Institute COVID-19 Surveillance Team
EPI_ISL_873883, EPI_ISL_873885	Lighthouse Lab in Cambridge	Wellcome Sanger Institute for the COVID-19 Genomics UK (COG-UK) Consortium	Rob Howes, The Lighthouse Lab in Cambridge and Alex Alderton, Roberto Amato, Sonia Goncalves, Ewan Harrison, David K. Jackson, Ian Johnston, Dominic Kwiatkowski, Cordelia Langford, John Sillitoe on behalf of the Wellcome Sanger Institute COVID-19 Surveillance Team
EPI_ISL_873886	Lighthouse Lab in Milton Keynes	Wellcome Sanger Institute for the COVID-19 Genomics UK (COG-UK) Consortium	The Lighthouse Lab in Milton Keynes and Alex Alderton, Roberto Amato, Sonia Goncalves, Ewan Harrison, David K. Jackson, Ian Johnston, Dominic Kwiatkowski, Cordelia Langford, John Sillitoe on behalf of the Wellcome Sanger Institute COVID-19 Surveillance Team
EPI_ISL_873888, EPI_ISL_873889, EPI_ISL_873890, EPI_ISL_873891, EPI_ISL_873892, EPI_ISL_873893, EPI_ISL_873894, EPI_ISL_873895, EPI_ISL_873896, EPI_ISL_873897, EPI_ISL_873898, EPI_ISL_873899, EPI_ISL_873901, EPI_ISL_873902, EPI_ISL_873906, EPI_ISL_873907, EPI_ISL_873908, EPI_ISL_873909, EPI_ISL_873911, EPI_ISL_873912, EPI_ISL_873914, EPI_ISL_873915, EPI_ISL_873917, EPI_ISL_873918, EPI_ISL_873919, EPI_ISL_873920			
see above	Lighthouse Lab in Cambridge	Wellcome Sanger Institute for the COVID-19 Genomics UK (COG-UK) Consortium	Rob Howes, The Lighthouse Lab in Cambridge and Alex Alderton, Roberto Amato, Sonia Goncalves, Ewan Harrison, David K. Jackson, Ian Johnston, Dominic Kwiatkowski, Cordelia Langford, John Sillitoe on behalf of the Wellcome Sanger Institute COVID-19 Surveillance Team
EPI_ISL_873921	Lighthouse Lab in Milton Keynes	Wellcome Sanger Institute for the COVID-19 Genomics UK (COG-UK) Consortium	The Lighthouse Lab in Milton Keynes and Alex Alderton, Roberto Amato, Sonia Goncalves, Ewan Harrison, David K. Jackson, Ian Johnston, Dominic Kwiatkowski, Cordelia Langford, John Sillitoe on behalf of the Wellcome Sanger Institute COVID-19 Surveillance Team
EPI_ISL_873922	Lighthouse Lab in Cambridge	Wellcome Sanger Institute for the COVID-19 Genomics UK (COG-UK) Consortium	Rob Howes, The Lighthouse Lab in Cambridge and Alex Alderton, Roberto Amato, Sonia Goncalves, Ewan Harrison, David K. Jackson, Ian Johnston, Dominic Kwiatkowski, Cordelia Langford, John Sillitoe on behalf of the Wellcome Sanger Institute COVID-19 Surveillance Team
EPI_ISL_873926	Lighthouse Lab in Milton Keynes	Wellcome Sanger Institute for the COVID-19 Genomics UK (COG-UK) Consortium	The Lighthouse Lab in Milton Keynes and Alex Alderton, Roberto Amato, Sonia Goncalves, Ewan Harrison, David K. Jackson, Ian Johnston, Dominic Kwiatkowski, Cordelia Langford, John Sillitoe on behalf of the Wellcome Sanger Institute COVID-19 Surveillance Team
EPI_ISL_873927, EPI_ISL_873929, EPI_ISL_873930, EPI_ISL_873931, EPI_ISL_873932, EPI_ISL_873933, EPI_ISL_873936, EPI_ISL_873938, EPI_ISL_873939, EPI_ISL_873940, EPI_ISL_873941			
see above	Lighthouse Lab in Cambridge	Wellcome Sanger Institute for the COVID-19 Genomics UK (COG-UK) Consortium	Rob Howes, The Lighthouse Lab in Cambridge and Alex Alderton, Roberto Amato, Sonia Goncalves, Ewan Harrison, David K. Jackson, Ian Johnston, Dominic Kwiatkowski, Cordelia Langford, John Sillitoe on behalf of the Wellcome Sanger Institute COVID-19 Surveillance Team
EPI_ISL_873942	Lighthouse Lab in Milton Keynes	Wellcome Sanger Institute for the COVID-19 Genomics UK (COG-UK) Consortium	The Lighthouse Lab in Milton Keynes and Alex Alderton, Roberto Amato, Sonia Goncalves, Ewan Harrison, David K. Jackson, Ian Johnston, Dominic Kwiatkowski, Cordelia Langford, John Sillitoe on behalf of the Wellcome Sanger Institute COVID-19 Surveillance Team
EPI_ISL_873944, EPI_ISL_873945, EPI_ISL_873946, EPI_ISL_873949	Lighthouse Lab in Cambridge	Wellcome Sanger Institute for the COVID-19 Genomics UK (COG-UK) Consortium	Rob Howes, The Lighthouse Lab in Cambridge and Alex Alderton, Roberto Amato, Sonia Goncalves, Ewan Harrison, David K. Jackson, Ian Johnston, Dominic Kwiatkowski, Cordelia Langford, John Sillitoe on behalf of the Wellcome Sanger Institute COVID-19 Surveillance Team
EPI_ISL_873950	Lighthouse Lab in Milton Keynes	Wellcome Sanger Institute for the COVID-19 Genomics UK (COG-UK) Consortium	The Lighthouse Lab in Milton Keynes and Alex Alderton, Roberto Amato, Sonia Goncalves, Ewan Harrison, David K. Jackson, Ian Johnston, Dominic Kwiatkowski, Cordelia Langford, John Sillitoe on behalf of the Wellcome Sanger Institute COVID-19 Surveillance Team
EPI_ISL_873951, EPI_ISL_873952, EPI_ISL_873953, EPI_ISL_873955	Lighthouse Lab in Cambridge	Wellcome Sanger Institute for the COVID-19 Genomics UK (COG-UK) Consortium	Rob Howes, The Lighthouse Lab in Cambridge and Alex Alderton, Roberto Amato, Sonia Goncalves, Ewan Harrison, David K. Jackson, Ian Johnston, Dominic Kwiatkowski, Cordelia Langford, John Sillitoe on behalf of the Wellcome Sanger Institute COVID-19 Surveillance Team
EPI_ISL_873956	Lighthouse Lab in Milton Keynes	Wellcome Sanger Institute for the COVID-19 Genomics UK (COG-UK) Consortium	The Lighthouse Lab in Milton Keynes and Alex Alderton, Roberto Amato, Sonia Goncalves, Ewan Harrison, David K. Jackson, Ian Johnston, Dominic Kwiatkowski, Cordelia Langford, John Sillitoe on behalf of the Wellcome Sanger Institute COVID-19 Surveillance Team
EPI_ISL_873957, EPI_ISL_873958, EPI_ISL_873959	Lighthouse Lab in Cambridge	Wellcome Sanger Institute for the COVID-19 Genomics UK (COG-UK) Consortium	Rob Howes, The Lighthouse Lab in Cambridge and Alex Alderton, Roberto Amato, Sonia Goncalves, Ewan Harrison, David K. Jackson, Ian Johnston, Dominic Kwiatkowski, Cordelia Langford, John Sillitoe on behalf of the Wellcome Sanger Institute COVID-19 Surveillance Team
EPI_ISL_873964, EPI_ISL_873965, EPI_ISL_873974, EPI_ISL_873976, EPI_ISL_873984, EPI_ISL_873987, EPI_ISL_873990, EPI_ISL_873999, EPI_ISL_874004, EPI_ISL_874007, EPI_ISL_874009, EPI_ISL_874010, EPI_ISL_874012, EPI_ISL_874014, EPI_ISL_874017, EPI_ISL_874021, EPI_ISL_874022, EPI_ISL_874032, EPI_ISL_874037, EPI_ISL_874038, EPI_ISL_874039, EPI_ISL_874047, EPI_ISL_874055, EPI_ISL_874061, EPI_ISL_874062, EPI_ISL_874064, EPI_ISL_874071, EPI_ISL_874076, EPI_ISL_874085, EPI_ISL_874090, EPI_ISL_874092, EPI_ISL_874096, EPI_ISL_874097, EPI_ISL_874115, EPI_ISL_874118, EPI_ISL_874130, EPI_ISL_874141, EPI_ISL_874143, EPI_ISL_874148, EPI_ISL_874153, EPI_ISL_874154, EPI_ISL_874157, EPI_ISL_874160, EPI_ISL_874163, EPI_ISL_874164, EPI_ISL_874165, EPI_ISL_874169, EPI_ISL_874180, EPI_ISL_874188, EPI_ISL_874191, EPI_ISL_874193, EPI_ISL_874195, EPI_ISL_874197, EPI_ISL_874198, EPI_ISL_874199, EPI_ISL_874202, EPI_ISL_874204, EPI_ISL_874207, EPI_ISL_874209, EPI_ISL_874211, EPI_ISL_874215, EPI_ISL_874238, EPI_ISL_874240, EPI_ISL_874246, EPI_ISL_874248, EPI_ISL_874254, EPI_ISL_874258, EPI_ISL_874263, EPI_ISL_874266, EPI_ISL_874268, EPI_ISL_874275, EPI_ISL_874278, EPI_ISL_874283, EPI_ISL_874290, EPI_ISL_874293, EPI_ISL_874297, EPI_ISL_874301, EPI_ISL_874311, EPI_ISL_874312, EPI_ISL_874313, EPI_ISL_874314, EPI_ISL_874316, EPI_ISL_874317, EPI_ISL_874318, EPI_ISL_874319, EPI_ISL_874320, EPI_ISL_874321, EPI_ISL_874322, EPI_ISL_874323, EPI_ISL_874324, EPI_ISL_874325, EPI_ISL_874326, EPI_ISL_874327, EPI_ISL_874328, EPI_ISL_874329, EPI_ISL_874330, EPI_ISL_874331, EPI_ISL_874332, EPI_ISL_874333, EPI_ISL_874334, EPI_ISL_874335, EPI_ISL_874336, EPI_ISL_874337, EPI_ISL_874338, EPI_ISL_874339, EPI_ISL_874340, EPI_ISL_874341, EPI_ISL_874342, EPI_ISL_874343, EPI_ISL_874344, EPI_ISL_874345, EPI_ISL_874346, EPI_ISL_874347, EPI_ISL_874348, EPI_ISL_874349, EPI_ISL_874350, EPI_ISL_874351, EPI_ISL_874352, EPI_ISL_874353, EPI_ISL_874354, EPI_ISL_874355, EPI_ISL_874356, EPI_ISL_874357, EPI_ISL_874358, EPI_ISL_874359, EPI_ISL_874360, EPI_ISL_874361, EPI_ISL_874362, EPI_ISL_874363, EPI_ISL_874364, EPI_ISL_874365, EPI_ISL_874366, EPI_ISL_874367, EPI_ISL_874368, EPI_ISL_874369, EPI_ISL_874370, EPI_ISL_874371, EPI_ISL_874372, EPI_ISL_874373, EPI_ISL_874374, EPI_ISL_874375, EPI_ISL_874376, EPI_ISL_874377, EPI_ISL_874378, EPI_ISL_874380, EPI_ISL_874381, EPI_ISL_874382, EPI_ISL_874383, EPI_ISL_874384, EPI_ISL_874385, EPI_ISL_874386, EPI_ISL_874387, EPI_ISL_874388, EPI_ISL_874389, EPI_ISL_874390, EPI_ISL_874391, EPI_ISL_874392, EPI_ISL_874393, EPI_ISL_874394, EPI_ISL_874395, EPI_ISL_874396, EPI_ISL_874397, EPI_ISL_874398, EPI_ISL_874399, EPI_ISL_874400, EPI_ISL_874402, EPI_ISL_874403, EPI_ISL_874405, EPI_ISL_874406, EPI_ISL_874407, EPI_ISL_874408, EPI_ISL_874409, EPI_ISL_874410, EPI_ISL_874411, EPI_ISL_874412, EPI_ISL_874413, EPI_ISL_874414, EPI_ISL_874415, EPI_ISL_874416, EPI_ISL_874417, EPI_ISL_874418, EPI_ISL_874419, EPI_ISL_874420, EPI_ISL_874421, EPI_ISL_874422, EPI_ISL_874423, EPI_ISL_874424, EPI_ISL_874425, EPI_ISL_874426, EPI_ISL_874427, EPI_ISL_874428, EPI_ISL_874429, EPI_ISL_874430, EPI_ISL_874431, EPI_ISL_874432, EPI_ISL_874433, EPI_ISL_874434, EPI_ISL_874435, EPI_ISL_874436, EPI_ISL_874437, EPI_ISL_874438, EPI_ISL_874439, EPI_ISL_874440, EPI_ISL_874441, EPI_ISL_874442, EPI_ISL_874444, EPI_ISL_874445, EPI_ISL_874446, EPI_ISL_874447, EPI_ISL_874449, EPI_ISL_874450, EPI_ISL_874451, EPI_ISL_874452, EPI_ISL_874453, EPI_ISL_874454, EPI_ISL_874455, EPI_ISL_874456, EPI_ISL_874457, EPI_ISL_874458, EPI_ISL_874459, EPI_ISL_874460, EPI_ISL_874461, EPI_ISL_874462, EPI_ISL_874463, EPI_ISL_874464, EPI_ISL_874465, EPI_ISL_874466, EPI_ISL_874467, EPI_ISL_874468, EPI_ISL_874470, EPI_ISL_874471, EPI_ISL_874472, EPI_ISL_874473, EPI_ISL_874474, EPI_ISL_874475, EPI_ISL_874476, EPI_ISL_874477, EPI_ISL_874478, EPI_ISL_874479, EPI_ISL_874480, EPI_ISL_874481, EPI_ISL_874483, EPI_ISL_874484, EPI_ISL_874485, EPI_ISL_874486, EPI_ISL_874487, EPI_ISL_874488, EPI_ISL_874489, EPI_ISL_874490, EPI_ISL_874491, EPI_ISL_874492, EPI_ISL_874493, EPI_ISL_874494, EPI_ISL_874495, EPI_ISL_874496, EPI_ISL_874497, EPI_ISL_874498, EPI_ISL_874500, EPI_ISL_874501, EPI_ISL_874503, EPI_ISL_874504, EPI_ISL_874505, EPI_ISL_874506, EPI_ISL_874507, EPI_ISL_874508, EPI_ISL_874509, EPI_ISL_874510, EPI_ISL_874511, EPI_ISL_874512, EPI_ISL_874513, EPI_ISL_874514, EPI_ISL_874515, EPI_ISL_874516, EPI_ISL_874517, EPI_ISL_874518, EPI_ISL_874519, EPI_ISL_874520, EPI_ISL_874521, EPI_ISL_874522, EPI_ISL_874524, EPI_ISL_874525, EPI_ISL_874526, EPI_ISL_874529, EPI_ISL_874531, EPI_ISL_874532, EPI_ISL_874533, EPI_ISL_874534, EPI_ISL_874535, EPI_ISL_874536, EPI_ISL_874537, EPI_ISL_874538, EPI_ISL_874539, EPI_ISL_874540, EPI_ISL_874541, EPI_ISL_874542, EPI_ISL_874544, EPI_ISL_874545, EPI_ISL_874546, EPI_ISL_874547, EPI_ISL_874548, EPI_ISL_874549, EPI_ISL_874550, EPI_ISL_874551, EPI_ISL_874552, EPI_ISL_874553, EPI_ISL_874554, EPI_ISL_874555, EPI_ISL_874556, EPI_ISL_874557, EPI_ISL_874559, EPI_ISL_874560, EPI_ISL_874561, EPI_ISL_874562, EPI_ISL_874563, EPI_ISL_874564, EPI_ISL_874565, EPI_ISL_874566, EPI_ISL_874567, EPI_ISL_874568, EPI_ISL_874569, EPI_ISL_874570, EPI_ISL_874571, EPI_ISL_874572, EPI_ISL_874573, EPI_ISL_874574, EPI_ISL_874575, EPI_ISL_874576, EPI_ISL_874577, EPI_ISL_874578, EPI_ISL_874579, EPI_ISL_874580, EPI_ISL_874581, EPI_ISL_874582, EPI_ISL_874583, EPI_ISL_874584, EPI_ISL_874585, EPI_ISL_874586, EPI_ISL_874587, EPI_ISL_874588, EPI_ISL_874589, EPI_ISL_874590, EPI_ISL_874591, EPI_ISL_874592, EPI_ISL_874593, EPI_ISL_874594, EPI_ISL_874595, EPI_ISL_874596, EPI_ISL_874597, EPI_ISL_874598, EPI_ISL_874599, EPI_ISL_874600, EPI_ISL_874601, EPI_ISL_874602, EPI_ISL_874603, EPI_ISL_874604, EPI_ISL_874605, EPI_ISL_874606, EPI_ISL_874607, EPI_ISL_874608, EPI_ISL_874609, EPI_ISL_874610, EPI_ISL_874611, EPI_ISL_874612, EPI_ISL_874613, EPI_ISL_874614, EPI_ISL_874615, EPI_ISL_874616, EPI_ISL_874617, EPI_ISL_874618, EPI_ISL_874619, EPI_ISL_874620, EPI_ISL_874621, EPI_ISL_874622, EPI_ISL_874623, EPI_ISL_874625, EPI_ISL_874626, EPI_ISL_874627, EPI_ISL_874628, EPI_ISL_874629, EPI_ISL_874630, EPI_ISL_874631, EPI_ISL_874632, EPI_ISL_874633, EPI_ISL_874634, EPI_ISL_874635, EPI_ISL_874636, EPI_ISL_874637, EPI_ISL_874638, EPI_ISL_874639, EPI_ISL_874640, EPI_ISL_874641, EPI_ISL_874642, EPI_ISL_874643, EPI_ISL_874644, EPI_ISL_874645, EPI_ISL_874646, EPI_ISL_874647, EPI_ISL_874648, EPI_ISL_874649, EPI_ISL_874650, EPI_ISL_874651, EPI_ISL_874652, EPI_ISL_874653, EPI_ISL_874654, EPI_ISL_874655			
see above	Lighthouse Lab in Alderley Park	Wellcome Sanger Institute for the COVID-19 Genomics UK (COG-UK) Consortium	Jacquelyn Wynn, Mairead Hyland, The Lighthouse Lab in Alderley Park and Alex Alderton, Roberto Amato, Sonia Goncalves, Ewan Harrison, David K. Jackson, Ian Johnston, Dominic Kwiatkowski, Cordelia Langford, John Sillitoe on behalf of the Wellcome Sanger Institute COVID-19 Surveillance Team
EPI_ISL_874656	Lighthouse Lab in Glasgow	Wellcome Sanger Institute for the COVID-19 Genomics UK (COG-UK) Consortium	Harper VanSteenhouse, Yumi Kasai, David Gray, Carol Clugston, Anna Dominiczak and Alex Alderton, Roberto Amato, Sonia Goncalves, Ewan Harrison, David K. Jackson, Ian Johnston, Dominic Kwiatkowski, Cordelia Langford, John Sillitoe on behalf of the Wellcome Sanger Institute COVID-19 Surveillance Team
EPI_ISL_874657, EPI_ISL_874658, EPI_ISL_874659, EPI_ISL_874660, EPI_ISL_874661, EPI_ISL_874662, EPI_ISL_874663, EPI_ISL_874664, EPI_ISL_874665, EPI_ISL_874666, EPI_ISL_874667, EPI_ISL_874668, EPI_ISL_874669, EPI_ISL_874670, EPI_ISL_874671, EPI_ISL_874672, EPI_ISL_874673, EPI_ISL_874674, EPI_ISL_874675			
see above	Lighthouse Lab in Alderley Park	Wellcome Sanger Institute for the COVID-19 Genomics UK	Jacquelyn Wynn, Mairead Hyland, The Lighthouse Lab in Alderley Park and Alex Alderton, Roberto Amato, Sonia Goncalves, Ewan Harrison, David K.

		(COG-UK) Consortium	Jackson, Ian Johnston, Dominic Kwiatkowski, Cordelia Langford, John Sillitoe on behalf of the Wellcome Sanger Institute COVID-19 Surveillance Team
EPI_ISL_874676	Lighthouse Lab in Glasgow	Wellcome Sanger Institute for the COVID-19 Genomics UK (COG-UK) Consortium	Harper VanSteenhouse, Yumi Kasai, David Gray, Carol Clugston, Anna Dominiczak and Alex Alderton, Roberto Amato, Sonia Goncalves, Ewan Harrison, David K. Jackson, Ian Johnston, Dominic Kwiatkowski, Cordelia Langford, John Sillitoe on behalf of the Wellcome Sanger Institute COVID-19 Surveillance Team
EPI_ISL_874677, EPI_ISL_874678, EPI_ISL_874679, EPI_ISL_874680, EPI_ISL_874681, EPI_ISL_874682, EPI_ISL_874683, EPI_ISL_874684, EPI_ISL_874685, EPI_ISL_874686, EPI_ISL_874687, EPI_ISL_874688, EPI_ISL_874689, EPI_ISL_874690, EPI_ISL_874691, EPI_ISL_874692, EPI_ISL_874693, EPI_ISL_874694, EPI_ISL_874695, EPI_ISL_874696, EPI_ISL_874697, EPI_ISL_874698, EPI_ISL_874699, EPI_ISL_874700, EPI_ISL_874701, EPI_ISL_874702, EPI_ISL_874703, EPI_ISL_874704, EPI_ISL_874705, EPI_ISL_874706, EPI_ISL_874707, EPI_ISL_874708, EPI_ISL_874709, EPI_ISL_874710, EPI_ISL_874711, EPI_ISL_874712, EPI_ISL_874713, EPI_ISL_874714, EPI_ISL_874715, EPI_ISL_874716, EPI_ISL_874717, EPI_ISL_874718, EPI_ISL_874719, EPI_ISL_874720, EPI_ISL_874721, EPI_ISL_874722, EPI_ISL_874723, EPI_ISL_874724, EPI_ISL_874725, EPI_ISL_874726, EPI_ISL_874727, EPI_ISL_874728, EPI_ISL_874729, EPI_ISL_874730, EPI_ISL_874731, EPI_ISL_874732			
see above	Lighthouse Lab in Alderley Park	Wellcome Sanger Institute for the COVID-19 Genomics UK (COG-UK) Consortium	Jacquelyn Wynn, Mairead Hyland, The Lighthouse Lab in Alderley Park and Alex Alderton, Roberto Amato, Sonia Goncalves, Ewan Harrison, David K. Jackson, Ian Johnston, Dominic Kwiatkowski, Cordelia Langford, John Sillitoe on behalf of the Wellcome Sanger Institute COVID-19 Surveillance Team
EPI_ISL_874733	Lighthouse Lab in Glasgow	Wellcome Sanger Institute for the COVID-19 Genomics UK (COG-UK) Consortium	Harper VanSteenhouse, Yumi Kasai, David Gray, Carol Clugston, Anna Dominiczak and Alex Alderton, Roberto Amato, Sonia Goncalves, Ewan Harrison, David K. Jackson, Ian Johnston, Dominic Kwiatkowski, Cordelia Langford, John Sillitoe on behalf of the Wellcome Sanger Institute COVID-19 Surveillance Team
EPI_ISL_874734, EPI_ISL_874735	Lighthouse Lab in Alderley Park	Wellcome Sanger Institute for the COVID-19 Genomics UK (COG-UK) Consortium	Jacquelyn Wynn, Mairead Hyland, The Lighthouse Lab in Alderley Park and Alex Alderton, Roberto Amato, Sonia Goncalves, Ewan Harrison, David K. Jackson, Ian Johnston, Dominic Kwiatkowski, Cordelia Langford, John Sillitoe on behalf of the Wellcome Sanger Institute COVID-19 Surveillance Team
EPI_ISL_874736, EPI_ISL_874737	Lighthouse Lab in Glasgow	Wellcome Sanger Institute for the COVID-19 Genomics UK (COG-UK) Consortium	Harper VanSteenhouse, Yumi Kasai, David Gray, Carol Clugston, Anna Dominiczak and Alex Alderton, Roberto Amato, Sonia Goncalves, Ewan Harrison, David K. Jackson, Ian Johnston, Dominic Kwiatkowski, Cordelia Langford, John Sillitoe on behalf of the Wellcome Sanger Institute COVID-19 Surveillance Team
EPI_ISL_874738	Lighthouse Lab in Alderley Park	Wellcome Sanger Institute for the COVID-19 Genomics UK (COG-UK) Consortium	Jacquelyn Wynn, Mairead Hyland, The Lighthouse Lab in Alderley Park and Alex Alderton, Roberto Amato, Sonia Goncalves, Ewan Harrison, David K. Jackson, Ian Johnston, Dominic Kwiatkowski, Cordelia Langford, John Sillitoe on behalf of the Wellcome Sanger Institute COVID-19 Surveillance Team
EPI_ISL_874739	Lighthouse Lab in Glasgow	Wellcome Sanger Institute for the COVID-19 Genomics UK (COG-UK) Consortium	Harper VanSteenhouse, Yumi Kasai, David Gray, Carol Clugston, Anna Dominiczak and Alex Alderton, Roberto Amato, Sonia Goncalves, Ewan Harrison, David K. Jackson, Ian Johnston, Dominic Kwiatkowski, Cordelia Langford, John Sillitoe on behalf of the Wellcome Sanger Institute COVID-19 Surveillance Team
EPI_ISL_874740, EPI_ISL_874741, EPI_ISL_874742, EPI_ISL_874743, EPI_ISL_874744, EPI_ISL_874745, EPI_ISL_874746, EPI_ISL_874747, EPI_ISL_874748, EPI_ISL_874749, EPI_ISL_874750, EPI_ISL_874751, EPI_ISL_874752			
see above	Lighthouse Lab in Alderley Park	Wellcome Sanger Institute for the COVID-19 Genomics UK (COG-UK) Consortium	Jacquelyn Wynn, Mairead Hyland, The Lighthouse Lab in Alderley Park and Alex Alderton, Roberto Amato, Sonia Goncalves, Ewan Harrison, David K. Jackson, Ian Johnston, Dominic Kwiatkowski, Cordelia Langford, John Sillitoe on behalf of the Wellcome Sanger Institute COVID-19 Surveillance Team
EPI_ISL_874753	Lighthouse Lab in Glasgow	Wellcome Sanger Institute for the COVID-19 Genomics UK (COG-UK) Consortium	Harper VanSteenhouse, Yumi Kasai, David Gray, Carol Clugston, Anna Dominiczak and Alex Alderton, Roberto Amato, Sonia Goncalves, Ewan Harrison, David K. Jackson, Ian Johnston, Dominic Kwiatkowski, Cordelia Langford, John Sillitoe on behalf of the Wellcome Sanger Institute COVID-19 Surveillance Team
EPI_ISL_874754, EPI_ISL_874755, EPI_ISL_874756, EPI_ISL_874757, EPI_ISL_874758, EPI_ISL_874760, EPI_ISL_874761, EPI_ISL_874762, EPI_ISL_874763, EPI_ISL_874764, EPI_ISL_874765, EPI_ISL_874766, EPI_ISL_874767, EPI_ISL_874768, EPI_ISL_874769, EPI_ISL_874770, EPI_ISL_874771, EPI_ISL_874772, EPI_ISL_874773, EPI_ISL_874774			
see above	Lighthouse Lab in Alderley Park	Wellcome Sanger Institute for the COVID-19 Genomics UK (COG-UK) Consortium	Jacquelyn Wynn, Mairead Hyland, The Lighthouse Lab in Alderley Park and Alex Alderton, Roberto Amato, Sonia Goncalves, Ewan Harrison, David K. Jackson, Ian Johnston, Dominic Kwiatkowski, Cordelia Langford, John Sillitoe on behalf of the Wellcome Sanger Institute COVID-19 Surveillance Team
EPI_ISL_874775	Lighthouse Lab in Glasgow	Wellcome Sanger Institute for the COVID-19 Genomics UK (COG-UK) Consortium	Harper VanSteenhouse, Yumi Kasai, David Gray, Carol Clugston, Anna Dominiczak and Alex Alderton, Roberto Amato, Sonia Goncalves, Ewan Harrison, David K. Jackson, Ian Johnston, Dominic Kwiatkowski, Cordelia Langford, John Sillitoe on behalf of the Wellcome Sanger Institute COVID-19 Surveillance Team
EPI_ISL_874776, EPI_ISL_874777, EPI_ISL_874778, EPI_ISL_874779, EPI_ISL_874780, EPI_ISL_874781, EPI_ISL_874782, EPI_ISL_874783, EPI_ISL_874784, EPI_ISL_874785, EPI_ISL_874786, EPI_ISL_874787, EPI_ISL_874788, EPI_ISL_874789, EPI_ISL_874790, EPI_ISL_874791, EPI_ISL_874792, EPI_ISL_874793, EPI_ISL_874794, EPI_ISL_874795, EPI_ISL_874796, EPI_ISL_874797			
see above	Lighthouse Lab in Alderley Park	Wellcome Sanger Institute for the COVID-19 Genomics UK (COG-UK) Consortium	Jacquelyn Wynn, Mairead Hyland, The Lighthouse Lab in Alderley Park and Alex Alderton, Roberto Amato, Sonia Goncalves, Ewan Harrison, David K. Jackson, Ian Johnston, Dominic Kwiatkowski, Cordelia Langford, John Sillitoe on behalf of the Wellcome Sanger Institute COVID-19 Surveillance Team
EPI_ISL_874798	Lighthouse Lab in Glasgow	Wellcome Sanger Institute for the COVID-19 Genomics UK (COG-UK) Consortium	Harper VanSteenhouse, Yumi Kasai, David Gray, Carol Clugston, Anna Dominiczak and Alex Alderton, Roberto Amato, Sonia Goncalves, Ewan Harrison, David K. Jackson, Ian Johnston, Dominic Kwiatkowski, Cordelia Langford, John Sillitoe on behalf of the Wellcome Sanger Institute COVID-19 Surveillance Team
EPI_ISL_874799, EPI_ISL_874800, EPI_ISL_874801, EPI_ISL_874802, EPI_ISL_874803, EPI_ISL_874804, EPI_ISL_874805	Lighthouse Lab in Alderley Park	Wellcome Sanger Institute for the COVID-19 Genomics UK (COG-UK) Consortium	Jacquelyn Wynn, Mairead Hyland, The Lighthouse Lab in Alderley Park and Alex Alderton, Roberto Amato, Sonia Goncalves, Ewan Harrison, David K. Jackson, Ian Johnston, Dominic Kwiatkowski, Cordelia Langford, John Sillitoe on behalf of the Wellcome Sanger Institute COVID-19 Surveillance Team
EPI_ISL_874806	Lighthouse Lab in Glasgow	Wellcome Sanger Institute for the COVID-19 Genomics UK (COG-UK) Consortium	Harper VanSteenhouse, Yumi Kasai, David Gray, Carol Clugston, Anna Dominiczak and Alex Alderton, Roberto Amato, Sonia Goncalves, Ewan Harrison, David K. Jackson, Ian Johnston, Dominic Kwiatkowski, Cordelia Langford, John Sillitoe on behalf of the Wellcome Sanger Institute COVID-19 Surveillance Team
EPI_ISL_874807	Lighthouse Lab in Alderley Park	Wellcome Sanger Institute for the COVID-19 Genomics UK (COG-UK) Consortium	Jacquelyn Wynn, Mairead Hyland, The Lighthouse Lab in Alderley Park and Alex Alderton, Roberto Amato, Sonia Goncalves, Ewan Harrison, David K. Jackson, Ian Johnston, Dominic Kwiatkowski, Cordelia Langford, John Sillitoe on behalf of the Wellcome Sanger Institute COVID-19 Surveillance Team
EPI_ISL_874808	Lighthouse Lab in Glasgow	Wellcome Sanger Institute for the COVID-19 Genomics UK (COG-UK) Consortium	Harper VanSteenhouse, Yumi Kasai, David Gray, Carol Clugston, Anna Dominiczak and Alex Alderton, Roberto Amato, Sonia Goncalves, Ewan Harrison, David K. Jackson, Ian Johnston, Dominic Kwiatkowski, Cordelia Langford, John Sillitoe on behalf of the Wellcome Sanger Institute COVID-19 Surveillance Team
EPI_ISL_874809, EPI_ISL_874810, EPI_ISL_874811, EPI_ISL_874812, EPI_ISL_874813, EPI_ISL_874814, EPI_ISL_874815, EPI_ISL_874817, EPI_ISL_874818, EPI_ISL_874819, EPI_ISL_874820, EPI_ISL_874821, EPI_ISL_874822, EPI_ISL_874823, EPI_ISL_874824, EPI_ISL_874825, EPI_ISL_874826, EPI_ISL_874827, EPI_ISL_874828, EPI_ISL_874829, EPI_ISL_874830, EPI_ISL_874831, EPI_ISL_874832, EPI_ISL_874833, EPI_ISL_874834, EPI_ISL_874835, EPI_ISL_874836, EPI_ISL_874837, EPI_ISL_874838, EPI_ISL_874839, EPI_ISL_874840, EPI_ISL_874841, EPI_ISL_874842, EPI_ISL_874843, EPI_ISL_874844, EPI_ISL_874845, EPI_ISL_874846, EPI_ISL_874847, EPI_ISL_874848, EPI_ISL_874849, EPI_ISL_874850, EPI_ISL_874851, EPI_ISL_874852, EPI_ISL_874854, EPI_ISL_874855, EPI_ISL_874856, EPI_ISL_874857, EPI_ISL_874858, EPI_ISL_874859, EPI_ISL_874860, EPI_ISL_874861, EPI_ISL_874862			
see above	Lighthouse Lab in Alderley Park	Wellcome Sanger Institute for the COVID-19 Genomics UK (COG-UK) Consortium	Jacquelyn Wynn, Mairead Hyland, The Lighthouse Lab in Alderley Park and Alex Alderton, Roberto Amato, Sonia Goncalves, Ewan Harrison, David K. Jackson, Ian Johnston, Dominic Kwiatkowski, Cordelia Langford, John Sillitoe on behalf of the Wellcome Sanger Institute COVID-19 Surveillance Team
EPI_ISL_874863	Lighthouse Lab in Glasgow	Wellcome Sanger Institute for the COVID-19 Genomics UK (COG-UK) Consortium	Harper VanSteenhouse, Yumi Kasai, David Gray, Carol Clugston, Anna Dominiczak and Alex Alderton, Roberto Amato, Sonia Goncalves, Ewan Harrison, David K. Jackson, Ian Johnston, Dominic Kwiatkowski, Cordelia Langford, John Sillitoe on behalf of the Wellcome Sanger Institute COVID-19 Surveillance Team
EPI_ISL_874864, EPI_ISL_874865, EPI_ISL_874866, EPI_ISL_874867, EPI_ISL_874868, EPI_ISL_874869, EPI_ISL_874870, EPI_ISL_874871, EPI_ISL_874872, EPI_ISL_874873, EPI_ISL_874874, EPI_ISL_874875, EPI_ISL_874876, EPI_ISL_874877, EPI_ISL_874878, EPI_ISL_874879, EPI_ISL_874880, EPI_ISL_874881, EPI_ISL_874882, EPI_ISL_874883, EPI_ISL_874884, EPI_ISL_874885, EPI_ISL_874886, EPI_ISL_874887, EPI_ISL_874888, EPI_ISL_874889, EPI_ISL_874890, EPI_ISL_874891, EPI_ISL_874892, EPI_ISL_874893, EPI_ISL_874894, EPI_ISL_874895			
see above	Lighthouse Lab in Alderley Park	Wellcome Sanger Institute for the COVID-19 Genomics UK (COG-UK) Consortium	Jacquelyn Wynn, Mairead Hyland, The Lighthouse Lab in Alderley Park and Alex Alderton, Roberto Amato, Sonia Goncalves, Ewan Harrison, David K. Jackson, Ian Johnston, Dominic Kwiatkowski, Cordelia Langford, John Sillitoe on behalf of the Wellcome Sanger Institute COVID-19 Surveillance Team
EPI_ISL_874896	Lighthouse Lab in Glasgow	Wellcome Sanger Institute for the COVID-19 Genomics UK (COG-UK) Consortium	Harper VanSteenhouse, Yumi Kasai, David Gray, Carol Clugston, Anna Dominiczak and Alex Alderton, Roberto Amato, Sonia Goncalves, Ewan Harrison, David K. Jackson, Ian Johnston, Dominic Kwiatkowski, Cordelia Langford, John Sillitoe on behalf of the Wellcome Sanger Institute COVID-19 Surveillance Team
EPI_ISL_874897	Lighthouse Lab in Alderley Park	Wellcome Sanger Institute for the COVID-19 Genomics UK (COG-UK) Consortium	Jacquelyn Wynn, Mairead Hyland, The Lighthouse Lab in Alderley Park and Alex Alderton, Roberto Amato, Sonia Goncalves, Ewan Harrison, David K. Jackson, Ian Johnston, Dominic Kwiatkowski, Cordelia Langford, John Sillitoe on behalf of the Wellcome Sanger Institute COVID-19 Surveillance Team
EPI_ISL_874898	Lighthouse Lab in Glasgow	Wellcome Sanger Institute for the COVID-19 Genomics UK	Harper VanSteenhouse, Yumi Kasai, David Gray, Carol Clugston, Anna Dominiczak and Alex Alderton, Roberto Amato, Sonia Goncalves, Ewan Harrison,

		(COG-UK) Consortium	David K. Jackson, Ian Johnston, Dominic Kwiatkowski, Cordelia Langford, John Sillitoe on behalf of the Wellcome Sanger Institute COVID-19 Surveillance Team
EPI_ISL_874899, EPI_ISL_874900, EPI_ISL_874901, EPI_ISL_874902, EPI_ISL_874903, EPI_ISL_874904	Lighthouse Lab in Alderley Park	Wellcome Sanger Institute for the COVID-19 Genomics UK (COG-UK) Consortium	Jacquelyn Wynn, Mairead Hyland, The Lighthouse Lab in Alderley Park and Alex Alderton, Roberto Amato, Sonia Goncalves, Ewan Harrison, David K. Jackson, Ian Johnston, Dominic Kwiatkowski, Cordelia Langford, John Sillitoe on behalf of the Wellcome Sanger Institute COVID-19 Surveillance Team
EPI_ISL_874905	Lighthouse Lab in Glasgow	Wellcome Sanger Institute for the COVID-19 Genomics UK (COG-UK) Consortium	Harper VanSteenhouse, Yumi Kasai, David Gray, Carol Clugston, Anna Dominiczak and Alex Alderton, Roberto Amato, Sonia Goncalves, Ewan Harrison, David K. Jackson, Ian Johnston, Dominic Kwiatkowski, Cordelia Langford, John Sillitoe on behalf of the Wellcome Sanger Institute COVID-19 Surveillance Team
EPI_ISL_874906, EPI_ISL_874907, EPI_ISL_874908, EPI_ISL_874909, EPI_ISL_874910, EPI_ISL_874911, EPI_ISL_874912, EPI_ISL_874913, EPI_ISL_874914, EPI_ISL_874915, EPI_ISL_874916, EPI_ISL_874917, EPI_ISL_874918, EPI_ISL_874919, EPI_ISL_874920, EPI_ISL_874921			
see above	Lighthouse Lab in Alderley Park	Wellcome Sanger Institute for the COVID-19 Genomics UK (COG-UK) Consortium	Jacquelyn Wynn, Mairead Hyland, The Lighthouse Lab in Alderley Park and Alex Alderton, Roberto Amato, Sonia Goncalves, Ewan Harrison, David K. Jackson, Ian Johnston, Dominic Kwiatkowski, Cordelia Langford, John Sillitoe on behalf of the Wellcome Sanger Institute COVID-19 Surveillance Team
EPI_ISL_874922	Lighthouse Lab in Glasgow	Wellcome Sanger Institute for the COVID-19 Genomics UK (COG-UK) Consortium	Harper VanSteenhouse, Yumi Kasai, David Gray, Carol Clugston, Anna Dominiczak and Alex Alderton, Roberto Amato, Sonia Goncalves, Ewan Harrison, David K. Jackson, Ian Johnston, Dominic Kwiatkowski, Cordelia Langford, John Sillitoe on behalf of the Wellcome Sanger Institute COVID-19 Surveillance Team
EPI_ISL_874923, EPI_ISL_874924, EPI_ISL_874925, EPI_ISL_874926, EPI_ISL_874927, EPI_ISL_874928, EPI_ISL_874929, EPI_ISL_874930, EPI_ISL_874931, EPI_ISL_874932, EPI_ISL_874933, EPI_ISL_874934			
see above	Lighthouse Lab in Alderley Park	Wellcome Sanger Institute for the COVID-19 Genomics UK (COG-UK) Consortium	Jacquelyn Wynn, Mairead Hyland, The Lighthouse Lab in Alderley Park and Alex Alderton, Roberto Amato, Sonia Goncalves, Ewan Harrison, David K. Jackson, Ian Johnston, Dominic Kwiatkowski, Cordelia Langford, John Sillitoe on behalf of the Wellcome Sanger Institute COVID-19 Surveillance Team
EPI_ISL_874935	Lighthouse Lab in Glasgow	Wellcome Sanger Institute for the COVID-19 Genomics UK (COG-UK) Consortium	Harper VanSteenhouse, Yumi Kasai, David Gray, Carol Clugston, Anna Dominiczak and Alex Alderton, Roberto Amato, Sonia Goncalves, Ewan Harrison, David K. Jackson, Ian Johnston, Dominic Kwiatkowski, Cordelia Langford, John Sillitoe on behalf of the Wellcome Sanger Institute COVID-19 Surveillance Team
EPI_ISL_874936, EPI_ISL_874937, EPI_ISL_874939, EPI_ISL_874940, EPI_ISL_874941, EPI_ISL_874942	Lighthouse Lab in Alderley Park	Wellcome Sanger Institute for the COVID-19 Genomics UK (COG-UK) Consortium	Jacquelyn Wynn, Mairead Hyland, The Lighthouse Lab in Alderley Park and Alex Alderton, Roberto Amato, Sonia Goncalves, Ewan Harrison, David K. Jackson, Ian Johnston, Dominic Kwiatkowski, Cordelia Langford, John Sillitoe on behalf of the Wellcome Sanger Institute COVID-19 Surveillance Team
EPI_ISL_874943	Lighthouse Lab in Glasgow	Wellcome Sanger Institute for the COVID-19 Genomics UK (COG-UK) Consortium	Harper VanSteenhouse, Yumi Kasai, David Gray, Carol Clugston, Anna Dominiczak and Alex Alderton, Roberto Amato, Sonia Goncalves, Ewan Harrison, David K. Jackson, Ian Johnston, Dominic Kwiatkowski, Cordelia Langford, John Sillitoe on behalf of the Wellcome Sanger Institute COVID-19 Surveillance Team
EPI_ISL_874944, EPI_ISL_874945, EPI_ISL_874946, EPI_ISL_874947, EPI_ISL_874948, EPI_ISL_874949	Lighthouse Lab in Alderley Park	Wellcome Sanger Institute for the COVID-19 Genomics UK (COG-UK) Consortium	Jacquelyn Wynn, Mairead Hyland, The Lighthouse Lab in Alderley Park and Alex Alderton, Roberto Amato, Sonia Goncalves, Ewan Harrison, David K. Jackson, Ian Johnston, Dominic Kwiatkowski, Cordelia Langford, John Sillitoe on behalf of the Wellcome Sanger Institute COVID-19 Surveillance Team
EPI_ISL_874950	Lighthouse Lab in Glasgow	Wellcome Sanger Institute for the COVID-19 Genomics UK (COG-UK) Consortium	Harper VanSteenhouse, Yumi Kasai, David Gray, Carol Clugston, Anna Dominiczak and Alex Alderton, Roberto Amato, Sonia Goncalves, Ewan Harrison, David K. Jackson, Ian Johnston, Dominic Kwiatkowski, Cordelia Langford, John Sillitoe on behalf of the Wellcome Sanger Institute COVID-19 Surveillance Team
EPI_ISL_874951, EPI_ISL_874952, EPI_ISL_874953, EPI_ISL_874954, EPI_ISL_874955, EPI_ISL_874957, EPI_ISL_874958, EPI_ISL_874959, EPI_ISL_874960, EPI_ISL_874961, EPI_ISL_874962, EPI_ISL_874963, EPI_ISL_874964			
see above	Lighthouse Lab in Alderley Park	Wellcome Sanger Institute for the COVID-19 Genomics UK (COG-UK) Consortium	Jacquelyn Wynn, Mairead Hyland, The Lighthouse Lab in Alderley Park and Alex Alderton, Roberto Amato, Sonia Goncalves, Ewan Harrison, David K. Jackson, Ian Johnston, Dominic Kwiatkowski, Cordelia Langford, John Sillitoe on behalf of the Wellcome Sanger Institute COVID-19 Surveillance Team
EPI_ISL_874965, EPI_ISL_874967	Lighthouse Lab in Glasgow	Wellcome Sanger Institute for the COVID-19 Genomics UK (COG-UK) Consortium	Harper VanSteenhouse, Yumi Kasai, David Gray, Carol Clugston, Anna Dominiczak and Alex Alderton, Roberto Amato, Sonia Goncalves, Ewan Harrison, David K. Jackson, Ian Johnston, Dominic Kwiatkowski, Cordelia Langford, John Sillitoe on behalf of the Wellcome Sanger Institute COVID-19 Surveillance Team
EPI_ISL_874968, EPI_ISL_874969, EPI_ISL_874970, EPI_ISL_874971, EPI_ISL_874972, EPI_ISL_874973, EPI_ISL_874974, EPI_ISL_874975, EPI_ISL_874976, EPI_ISL_874977, EPI_ISL_874978, EPI_ISL_874979, EPI_ISL_874980, EPI_ISL_874981, EPI_ISL_874982, EPI_ISL_874983, EPI_ISL_874984, EPI_ISL_874985, EPI_ISL_874986, EPI_ISL_874987, EPI_ISL_874988, EPI_ISL_874989			
see above	Lighthouse Lab in Alderley Park	Wellcome Sanger Institute for the COVID-19 Genomics UK (COG-UK) Consortium	Jacquelyn Wynn, Mairead Hyland, The Lighthouse Lab in Alderley Park and Alex Alderton, Roberto Amato, Sonia Goncalves, Ewan Harrison, David K. Jackson, Ian Johnston, Dominic Kwiatkowski, Cordelia Langford, John Sillitoe on behalf of the Wellcome Sanger Institute COVID-19 Surveillance Team
EPI_ISL_875353, EPI_ISL_875354, EPI_ISL_875355, EPI_ISL_875356, EPI_ISL_875357, EPI_ISL_875358, EPI_ISL_875359, EPI_ISL_875360, EPI_ISL_875361, EPI_ISL_875362, EPI_ISL_875363, EPI_ISL_875364, EPI_ISL_875365, EPI_ISL_875366, EPI_ISL_875367, EPI_ISL_875368, EPI_ISL_875369, EPI_ISL_875370, EPI_ISL_875371, EPI_ISL_875372			
see above	National Virus Reference Laboratory	National Virus Reference Laboratory	Michael Carr, Gabriel Gonzalez, Jonathan Dean, Cillian F De Gascun
EPI_ISL_875539	Institute of Virology, Biomedical Research Center of the Slovak Academy of Sciences, Bratislava	Faculty of Natural Sciences, Comenius University, Bratislava	Kristína Boršová, Viktória abanová, Broa Brejová, Viktória Hodorová, Sabina Fumaová Havlíková, Juraj Kopáek, Martina Liková, ubomíra Lukáiková, Martina Neboháová, Monika Sláviková, Tomáš Vina, Boris Klempa, Jozef Nosek
EPI_ISL_875558	Microbiologia e Virologia	Istituto Zooprofilattico Sperimentale delle Venezie	Adelaide Milani, Alessia Schivo, Annalisa Salviato, Erika Giorgia Quaranta, Ambra Pastori, Bianca Zecchin, Alice Fusaro, Isabella Monne, Calogero Terregino, Antonia Ricci
EPI_ISL_875666, EPI_ISL_875667, EPI_ISL_875668	CHU Purpan - Laboratoire de Virologie - Institut Fédératif de Biologie	CHU Purpan - Laboratoire de Virologie - Institut Fédératif de Biologie	Latour J., Ranger N., Dubois M., Carcenac R., Harter A., Boyer P., Tremeaux P., Izopet J.
EPI_ISL_876065, EPI_ISL_876087, EPI_ISL_876095, EPI_ISL_876099, EPI_ISL_876124, EPI_ISL_876202, EPI_ISL_876215, EPI_ISL_876234, EPI_ISL_876235, EPI_ISL_876236, EPI_ISL_876237, EPI_ISL_876238			
see above	Massachusetts State Public Health Laboratory	Massachusetts State Public Health Laboratory	Andrew Lang, Timelia Fink, Glen Gallagher, Sandra Smole
EPI_ISL_877044, EPI_ISL_877045, EPI_ISL_877051, EPI_ISL_877052, EPI_ISL_877053, EPI_ISL_877054, EPI_ISL_877055, EPI_ISL_877056, EPI_ISL_877057, EPI_ISL_877058	Quest Diagnostics	Quest Diagnostics	Rosenthal,S.H., Gerasimova,A., Kagan,R.M., Anderson, B., Hua, M., Liu Y., Bernstein, L.E., Livingston, K.E., Perez, A., Shalhout, D.F., Shlyakhter, I.A., Owen, R., Tanpaiboon, P., Lacbawan, F.
EPI_ISL_877133, EPI_ISL_877134, EPI_ISL_877135, EPI_ISL_877136, EPI_ISL_877137, EPI_ISL_877138, EPI_ISL_877139, EPI_ISL_877140, EPI_ISL_877141, EPI_ISL_877142, EPI_ISL_877143, EPI_ISL_877144, EPI_ISL_877145, EPI_ISL_877153			
see above	Univeristy of New Mexico Hospital	Center for Global Health, University of New Mexico Health Sciences Center	Daryl Domman, Kurt Schwalm, Justin Bacca, Jon Femling, Darrell Dinwiddie
EPI_ISL_877219	Canterbury Health Laboratories	Institute of Environmental Science and Research (ESR)	Xiaoyun Ren, Matt Storey, Nikki Freed, Muhammad Faisal, Jing Wang, Hermes Perez, Anja Werno, Antje van der Linden, Arlo Upton, Chris Mansell, David Hammer, Dragana Drinkovic, Gary McAuliffe, Hana Sofia Andersson, James Ussher, Jill Sherwood, Josh Freeman, Julia Howard, Juliet Elvy, Mary DeAlmeida, Matt Blakiston, Matthew Rogers, Max Bloomfield, Michael Addidle, Michelle Balm, Sally Roberts, Sarah Jefferies, Sharmini Muttaiyah, Susan Morpeth, Susan Taylor, Timothy Blackmore, Vani Sathyendran, Veronica Playle, Virginia Hope, Erasmus Smit, Lauren Jelly, Olin Silander, Joep de Ligt
EPI_ISL_877221, EPI_ISL_877222, EPI_ISL_877223, EPI_ISL_877224, EPI_ISL_877225, EPI_ISL_877226	LabPLUS	Institute of Environmental Science and Research (ESR)	Xiaoyun Ren, Matt Storey, Nikki Freed, Muhammad Faisal, Jing Wang, Hermes Perez, Anja Werno, Antje van der Linden, Arlo Upton, Chris Mansell, David Hammer, Dragana Drinkovic, Gary McAuliffe, Hana Sofia Andersson, James Ussher, Jill Sherwood, Josh Freeman, Julia Howard, Juliet Elvy, Mary DeAlmeida, Matt Blakiston, Matthew Rogers, Max Bloomfield, Michael Addidle, Michelle Balm, Sally Roberts, Sarah Jefferies, Sharmini Muttaiyah, Susan Morpeth, Susan Taylor, Timothy Blackmore, Vani Sathyendran, Veronica Playle, Virginia Hope, Erasmus Smit, Lauren Jelly, Olin Silander, Joep de Ligt
EPI_ISL_877551	Institute of Microbiology, Universidad San Francisco de Quito	Institute of Microbiology, Universidad San Francisco de Quito	Belén Prado-Vivar, Sully Márquez, Juan José Guadalupe, Monica Becerra-Wong, Bernardo Gutiérrez, Marcos DiStefano, Verónica Barragán, Patricio Rojas-Silva, Gabriel Trueba, Michelle Grunauer, Paúl Cárdenas
EPI_ISL_877771, EPI_ISL_877807, EPI_ISL_877821, EPI_ISL_877828, EPI_ISL_877844, EPI_ISL_877903, EPI_ISL_877940, EPI_ISL_877972, EPI_ISL_878089, EPI_ISL_878118, EPI_ISL_878124, EPI_ISL_878126, EPI_ISL_878163, EPI_ISL_878173, EPI_ISL_878176, EPI_ISL_878179, EPI_ISL_878192, EPI_ISL_878208,			

EPI_ISL_878249, EPI_ISL_878252, EPI_ISL_878265, EPI_ISL_878277, EPI_ISL_878285, EPI_ISL_878323, EPI_ISL_878337, EPI_ISL_878339, EPI_ISL_878420, EPI_ISL_878422, EPI_ISL_878449, EPI_ISL_878454, EPI_ISL_878456, EPI_ISL_878458, EPI_ISL_878466, EPI_ISL_878481, EPI_ISL_878484, EPI_ISL_878486, EPI_ISL_878505, EPI_ISL_878507, EPI_ISL_878512, EPI_ISL_878530, EPI_ISL_878542, EPI_ISL_878556, EPI_ISL_878570, EPI_ISL_878572, EPI_ISL_878586, EPI_ISL_878598, EPI_ISL_878609, EPI_ISL_878614, EPI_ISL_878618, EPI_ISL_878652, EPI_ISL_878654, EPI_ISL_878681, EPI_ISL_878683, EPI_ISL_878688, EPI_ISL_878689, EPI_ISL_878701, EPI_ISL_878704, EPI_ISL_878706, EPI_ISL_878712, EPI_ISL_878730			
see above	Lighthouse Lab in Alderley Park	Wellcome Sanger Institute for the COVID-19 Genomics UK (COG-UK) Consortium	Jacquelyn Wynn, Mairead Hyland, The Lighthouse Lab in Alderley Park and Alex Alderton, Roberto Amato, Sonia Goncalves, Ewan Harrison, David K. Jackson, Ian Johnston, Dominic Kwiatkowski, Cordelia Langford, John Sillitoe on behalf of the Wellcome Sanger Institute COVID-19 Surveillance Team
EPI_ISL_878751, EPI_ISL_878756, EPI_ISL_878759, EPI_ISL_878772, EPI_ISL_878775, EPI_ISL_878778, EPI_ISL_878780, EPI_ISL_878782, EPI_ISL_878792, EPI_ISL_878793, EPI_ISL_878798, EPI_ISL_878800, EPI_ISL_878806, EPI_ISL_878807, EPI_ISL_878814			
see above	Lighthouse Lab in Milton Keynes	Wellcome Sanger Institute for the COVID-19 Genomics UK (COG-UK) Consortium	The Lighthouse Lab in Milton Keynes and Alex Alderton, Roberto Amato, Sonia Goncalves, Ewan Harrison, David K. Jackson, Ian Johnston, Dominic Kwiatkowski, Cordelia Langford, John Sillitoe on behalf of the Wellcome Sanger Institute COVID-19 Surveillance Team
EPI_ISL_878819	Lighthouse Lab in Alderley Park	Wellcome Sanger Institute for the COVID-19 Genomics UK (COG-UK) Consortium	Jacquelyn Wynn, Mairead Hyland, The Lighthouse Lab in Alderley Park and Alex Alderton, Roberto Amato, Sonia Goncalves, Ewan Harrison, David K. Jackson, Ian Johnston, Dominic Kwiatkowski, Cordelia Langford, John Sillitoe on behalf of the Wellcome Sanger Institute COVID-19 Surveillance Team
EPI_ISL_878824, EPI_ISL_878842, EPI_ISL_878845, EPI_ISL_878846, EPI_ISL_878848, EPI_ISL_878851, EPI_ISL_878852, EPI_ISL_878854, EPI_ISL_878858, EPI_ISL_878860, EPI_ISL_878861, EPI_ISL_878874			
see above	Lighthouse Lab in Milton Keynes	Wellcome Sanger Institute for the COVID-19 Genomics UK (COG-UK) Consortium	The Lighthouse Lab in Milton Keynes and Alex Alderton, Roberto Amato, Sonia Goncalves, Ewan Harrison, David K. Jackson, Ian Johnston, Dominic Kwiatkowski, Cordelia Langford, John Sillitoe on behalf of the Wellcome Sanger Institute COVID-19 Surveillance Team
EPI_ISL_878876	Lighthouse Lab in Alderley Park	Wellcome Sanger Institute for the COVID-19 Genomics UK (COG-UK) Consortium	Jacquelyn Wynn, Mairead Hyland, The Lighthouse Lab in Alderley Park and Alex Alderton, Roberto Amato, Sonia Goncalves, Ewan Harrison, David K. Jackson, Ian Johnston, Dominic Kwiatkowski, Cordelia Langford, John Sillitoe on behalf of the Wellcome Sanger Institute COVID-19 Surveillance Team
EPI_ISL_878879, EPI_ISL_878880, EPI_ISL_878882, EPI_ISL_878884, EPI_ISL_878892, EPI_ISL_878896, EPI_ISL_878902, EPI_ISL_878906	Lighthouse Lab in Milton Keynes	Wellcome Sanger Institute for the COVID-19 Genomics UK (COG-UK) Consortium	The Lighthouse Lab in Milton Keynes and Alex Alderton, Roberto Amato, Sonia Goncalves, Ewan Harrison, David K. Jackson, Ian Johnston, Dominic Kwiatkowski, Cordelia Langford, John Sillitoe on behalf of the Wellcome Sanger Institute COVID-19 Surveillance Team
EPI_ISL_878913	Lighthouse Lab in Alderley Park	Wellcome Sanger Institute for the COVID-19 Genomics UK (COG-UK) Consortium	Jacquelyn Wynn, Mairead Hyland, The Lighthouse Lab in Alderley Park and Alex Alderton, Roberto Amato, Sonia Goncalves, Ewan Harrison, David K. Jackson, Ian Johnston, Dominic Kwiatkowski, Cordelia Langford, John Sillitoe on behalf of the Wellcome Sanger Institute COVID-19 Surveillance Team
EPI_ISL_878918, EPI_ISL_878919, EPI_ISL_878934, EPI_ISL_878941, EPI_ISL_878942, EPI_ISL_878943, EPI_ISL_878944, EPI_ISL_878945, EPI_ISL_878951, EPI_ISL_878952, EPI_ISL_878960, EPI_ISL_878962, EPI_ISL_878967, EPI_ISL_878971, EPI_ISL_878972, EPI_ISL_878982, EPI_ISL_878990			
see above	Lighthouse Lab in Milton Keynes	Wellcome Sanger Institute for the COVID-19 Genomics UK (COG-UK) Consortium	The Lighthouse Lab in Milton Keynes and Alex Alderton, Roberto Amato, Sonia Goncalves, Ewan Harrison, David K. Jackson, Ian Johnston, Dominic Kwiatkowski, Cordelia Langford, John Sillitoe on behalf of the Wellcome Sanger Institute COVID-19 Surveillance Team
EPI_ISL_878993	Lighthouse Lab in Alderley Park	Wellcome Sanger Institute for the COVID-19 Genomics UK (COG-UK) Consortium	Jacquelyn Wynn, Mairead Hyland, The Lighthouse Lab in Alderley Park and Alex Alderton, Roberto Amato, Sonia Goncalves, Ewan Harrison, David K. Jackson, Ian Johnston, Dominic Kwiatkowski, Cordelia Langford, John Sillitoe on behalf of the Wellcome Sanger Institute COVID-19 Surveillance Team
EPI_ISL_879001, EPI_ISL_879009, EPI_ISL_879013, EPI_ISL_879014, EPI_ISL_879016, EPI_ISL_879019, EPI_ISL_879021, EPI_ISL_879023, EPI_ISL_879025, EPI_ISL_879028, EPI_ISL_879032, EPI_ISL_879034, EPI_ISL_879037, EPI_ISL_879038			
see above	Lighthouse Lab in Milton Keynes	Wellcome Sanger Institute for the COVID-19 Genomics UK (COG-UK) Consortium	The Lighthouse Lab in Milton Keynes and Alex Alderton, Roberto Amato, Sonia Goncalves, Ewan Harrison, David K. Jackson, Ian Johnston, Dominic Kwiatkowski, Cordelia Langford, John Sillitoe on behalf of the Wellcome Sanger Institute COVID-19 Surveillance Team
EPI_ISL_879040	Lighthouse Lab in Alderley Park	Wellcome Sanger Institute for the COVID-19 Genomics UK (COG-UK) Consortium	Jacquelyn Wynn, Mairead Hyland, The Lighthouse Lab in Alderley Park and Alex Alderton, Roberto Amato, Sonia Goncalves, Ewan Harrison, David K. Jackson, Ian Johnston, Dominic Kwiatkowski, Cordelia Langford, John Sillitoe on behalf of the Wellcome Sanger Institute COVID-19 Surveillance Team
EPI_ISL_879042, EPI_ISL_879043, EPI_ISL_879044, EPI_ISL_879047, EPI_ISL_879049, EPI_ISL_879050, EPI_ISL_879052, EPI_ISL_879055	Lighthouse Lab in Milton Keynes	Wellcome Sanger Institute for the COVID-19 Genomics UK (COG-UK) Consortium	The Lighthouse Lab in Milton Keynes and Alex Alderton, Roberto Amato, Sonia Goncalves, Ewan Harrison, David K. Jackson, Ian Johnston, Dominic Kwiatkowski, Cordelia Langford, John Sillitoe on behalf of the Wellcome Sanger Institute COVID-19 Surveillance Team
EPI_ISL_879056	Lighthouse Lab in Alderley Park	Wellcome Sanger Institute for the COVID-19 Genomics UK (COG-UK) Consortium	Jacquelyn Wynn, Mairead Hyland, The Lighthouse Lab in Alderley Park and Alex Alderton, Roberto Amato, Sonia Goncalves, Ewan Harrison, David K. Jackson, Ian Johnston, Dominic Kwiatkowski, Cordelia Langford, John Sillitoe on behalf of the Wellcome Sanger Institute COVID-19 Surveillance Team
EPI_ISL_879057, EPI_ISL_879063, EPI_ISL_879064, EPI_ISL_879065	Lighthouse Lab in Milton Keynes	Wellcome Sanger Institute for the COVID-19 Genomics UK (COG-UK) Consortium	The Lighthouse Lab in Milton Keynes and Alex Alderton, Roberto Amato, Sonia Goncalves, Ewan Harrison, David K. Jackson, Ian Johnston, Dominic Kwiatkowski, Cordelia Langford, John Sillitoe on behalf of the Wellcome Sanger Institute COVID-19 Surveillance Team
EPI_ISL_879073	Lighthouse Lab in Alderley Park	Wellcome Sanger Institute for the COVID-19 Genomics UK (COG-UK) Consortium	Jacquelyn Wynn, Mairead Hyland, The Lighthouse Lab in Alderley Park and Alex Alderton, Roberto Amato, Sonia Goncalves, Ewan Harrison, David K. Jackson, Ian Johnston, Dominic Kwiatkowski, Cordelia Langford, John Sillitoe on behalf of the Wellcome Sanger Institute COVID-19 Surveillance Team
EPI_ISL_879075, EPI_ISL_879086, EPI_ISL_879087, EPI_ISL_879089, EPI_ISL_879092, EPI_ISL_879093, EPI_ISL_879094, EPI_ISL_879096	Lighthouse Lab in Milton Keynes	Wellcome Sanger Institute for the COVID-19 Genomics UK (COG-UK) Consortium	The Lighthouse Lab in Milton Keynes and Alex Alderton, Roberto Amato, Sonia Goncalves, Ewan Harrison, David K. Jackson, Ian Johnston, Dominic Kwiatkowski, Cordelia Langford, John Sillitoe on behalf of the Wellcome Sanger Institute COVID-19 Surveillance Team
EPI_ISL_879116, EPI_ISL_879117, EPI_ISL_879120, EPI_ISL_879138, EPI_ISL_879154, EPI_ISL_879185, EPI_ISL_879193, EPI_ISL_879220, EPI_ISL_879235, EPI_ISL_879253, EPI_ISL_879278, EPI_ISL_879290, EPI_ISL_879314, EPI_ISL_879322, EPI_ISL_879331, EPI_ISL_879338, EPI_ISL_879344, EPI_ISL_879363, EPI_ISL_879377			
see above	Lighthouse Lab in Alderley Park	Wellcome Sanger Institute for the COVID-19 Genomics UK (COG-UK) Consortium	Jacquelyn Wynn, Mairead Hyland, The Lighthouse Lab in Alderley Park and Alex Alderton, Roberto Amato, Sonia Goncalves, Ewan Harrison, David K. Jackson, Ian Johnston, Dominic Kwiatkowski, Cordelia Langford, John Sillitoe on behalf of the Wellcome Sanger Institute COVID-19 Surveillance Team
EPI_ISL_879378, EPI_ISL_879379, EPI_ISL_879380	Lighthouse Lab in Milton Keynes	Wellcome Sanger Institute for the COVID-19 Genomics UK (COG-UK) Consortium	The Lighthouse Lab in Milton Keynes and Alex Alderton, Roberto Amato, Sonia Goncalves, Ewan Harrison, David K. Jackson, Ian Johnston, Dominic Kwiatkowski, Cordelia Langford, John Sillitoe on behalf of the Wellcome Sanger Institute COVID-19 Surveillance Team
EPI_ISL_879384	Lighthouse Lab in Alderley Park	Wellcome Sanger Institute for the COVID-19 Genomics UK (COG-UK) Consortium	Jacquelyn Wynn, Mairead Hyland, The Lighthouse Lab in Alderley Park and Alex Alderton, Roberto Amato, Sonia Goncalves, Ewan Harrison, David K. Jackson, Ian Johnston, Dominic Kwiatkowski, Cordelia Langford, John Sillitoe on behalf of the Wellcome Sanger Institute COVID-19 Surveillance Team
EPI_ISL_879388, EPI_ISL_879395, EPI_ISL_879396, EPI_ISL_879397, EPI_ISL_879399, EPI_ISL_879404, EPI_ISL_879409, EPI_ISL_879412, EPI_ISL_879413, EPI_ISL_879424, EPI_ISL_879425, EPI_ISL_879426, EPI_ISL_879429			
see above	Lighthouse Lab in Milton Keynes	Wellcome Sanger Institute for the COVID-19 Genomics UK (COG-UK) Consortium	The Lighthouse Lab in Milton Keynes and Alex Alderton, Roberto Amato, Sonia Goncalves, Ewan Harrison, David K. Jackson, Ian Johnston, Dominic Kwiatkowski, Cordelia Langford, John Sillitoe on behalf of the Wellcome Sanger Institute COVID-19 Surveillance Team
EPI_ISL_879434	Lighthouse Lab in Alderley Park	Wellcome Sanger Institute for the COVID-19 Genomics UK (COG-UK) Consortium	Jacquelyn Wynn, Mairead Hyland, The Lighthouse Lab in Alderley Park and Alex Alderton, Roberto Amato, Sonia Goncalves, Ewan Harrison, David K. Jackson, Ian Johnston, Dominic Kwiatkowski, Cordelia Langford, John Sillitoe on behalf of the Wellcome Sanger Institute COVID-19 Surveillance Team
EPI_ISL_879442, EPI_ISL_879448, EPI_ISL_879449, EPI_ISL_879450	Lighthouse Lab in Milton Keynes	Wellcome Sanger Institute for the COVID-19 Genomics UK (COG-UK) Consortium	The Lighthouse Lab in Milton Keynes and Alex Alderton, Roberto Amato, Sonia Goncalves, Ewan Harrison, David K. Jackson, Ian Johnston, Dominic Kwiatkowski, Cordelia Langford, John Sillitoe on behalf of the Wellcome Sanger Institute COVID-19 Surveillance Team
EPI_ISL_879452	Lighthouse Lab in Alderley Park	Wellcome Sanger Institute for the COVID-19 Genomics UK (COG-UK) Consortium	Jacquelyn Wynn, Mairead Hyland, The Lighthouse Lab in Alderley Park and Alex Alderton, Roberto Amato, Sonia Goncalves, Ewan Harrison, David K. Jackson, Ian Johnston, Dominic Kwiatkowski, Cordelia Langford, John Sillitoe on behalf of the Wellcome Sanger Institute COVID-19 Surveillance Team
EPI_ISL_879455, EPI_ISL_879457, EPI_ISL_879458, EPI_ISL_879459, EPI_ISL_879461	Lighthouse Lab in Milton Keynes	Wellcome Sanger Institute for the COVID-19 Genomics UK (COG-UK) Consortium	The Lighthouse Lab in Milton Keynes and Alex Alderton, Roberto Amato, Sonia Goncalves, Ewan Harrison, David K. Jackson, Ian Johnston, Dominic Kwiatkowski, Cordelia Langford, John Sillitoe on behalf of the Wellcome Sanger Institute COVID-19 Surveillance Team
EPI_ISL_879462	Lighthouse Lab in Alderley Park	Wellcome Sanger Institute for the COVID-19 Genomics UK (COG-UK) Consortium	Jacquelyn Wynn, Mairead Hyland, The Lighthouse Lab in Alderley Park and Alex Alderton, Roberto Amato, Sonia Goncalves, Ewan Harrison, David K. Jackson, Ian Johnston, Dominic Kwiatkowski, Cordelia Langford, John Sillitoe on behalf of the Wellcome Sanger Institute COVID-19 Surveillance Team
EPI_ISL_879464	Lighthouse Lab in Milton Keynes	Wellcome Sanger Institute for the COVID-19 Genomics UK (COG-UK) Consortium	The Lighthouse Lab in Milton Keynes and Alex Alderton, Roberto Amato, Sonia Goncalves, Ewan Harrison, David K. Jackson, Ian Johnston, Dominic Kwiatkowski, Cordelia Langford, John Sillitoe on behalf of the Wellcome Sanger Institute COVID-19 Surveillance Team
EPI_ISL_879466	Lighthouse Lab in Alderley Park	Wellcome Sanger Institute for the COVID-19 Genomics UK (COG-UK) Consortium	Jacquelyn Wynn, Mairead Hyland, The Lighthouse Lab in Alderley Park and Alex Alderton, Roberto Amato, Sonia Goncalves, Ewan Harrison, David K. Jackson, Ian Johnston, Dominic Kwiatkowski, Cordelia Langford, John Sillitoe on behalf of the Wellcome Sanger Institute COVID-19 Surveillance Team

EPI_ISL_879467, EPI_ISL_879471	Lighthouse Lab in Milton Keynes	Wellcome Sanger Institute for the COVID-19 Genomics UK (COG-UK) Consortium	The Lighthouse Lab in Milton Keynes and Alex Alderton, Roberto Amato, Sonia Goncalves, Ewan Harrison, David K. Jackson, Ian Johnston, Dominic Kwiatkowski, Cordelia Langford, John Sillitoe on behalf of the Wellcome Sanger Institute COVID-19 Surveillance Team
EPI_ISL_879473, EPI_ISL_879475	Lighthouse Lab in Alderley Park	Wellcome Sanger Institute for the COVID-19 Genomics UK (COG-UK) Consortium	Jacquelyn Wynn, Mairead Hyland, The Lighthouse Lab in Alderley Park and Alex Alderton, Roberto Amato, Sonia Goncalves, Ewan Harrison, David K. Jackson, Ian Johnston, Dominic Kwiatkowski, Cordelia Langford, John Sillitoe on behalf of the Wellcome Sanger Institute COVID-19 Surveillance Team
EPI_ISL_879477, EPI_ISL_879479	Lighthouse Lab in Milton Keynes	Wellcome Sanger Institute for the COVID-19 Genomics UK (COG-UK) Consortium	The Lighthouse Lab in Milton Keynes and Alex Alderton, Roberto Amato, Sonia Goncalves, Ewan Harrison, David K. Jackson, Ian Johnston, Dominic Kwiatkowski, Cordelia Langford, John Sillitoe on behalf of the Wellcome Sanger Institute COVID-19 Surveillance Team
EPI_ISL_879486, EPI_ISL_879487	Lighthouse Lab in Alderley Park	Wellcome Sanger Institute for the COVID-19 Genomics UK (COG-UK) Consortium	Jacquelyn Wynn, Mairead Hyland, The Lighthouse Lab in Alderley Park and Alex Alderton, Roberto Amato, Sonia Goncalves, Ewan Harrison, David K. Jackson, Ian Johnston, Dominic Kwiatkowski, Cordelia Langford, John Sillitoe on behalf of the Wellcome Sanger Institute COVID-19 Surveillance Team
EPI_ISL_879492, EPI_ISL_879495, EPI_ISL_879499, EPI_ISL_879502	Lighthouse Lab in Milton Keynes	Wellcome Sanger Institute for the COVID-19 Genomics UK (COG-UK) Consortium	The Lighthouse Lab in Milton Keynes and Alex Alderton, Roberto Amato, Sonia Goncalves, Ewan Harrison, David K. Jackson, Ian Johnston, Dominic Kwiatkowski, Cordelia Langford, John Sillitoe on behalf of the Wellcome Sanger Institute COVID-19 Surveillance Team
EPI_ISL_879504, EPI_ISL_879510	Lighthouse Lab in Alderley Park	Wellcome Sanger Institute for the COVID-19 Genomics UK (COG-UK) Consortium	Jacquelyn Wynn, Mairead Hyland, The Lighthouse Lab in Alderley Park and Alex Alderton, Roberto Amato, Sonia Goncalves, Ewan Harrison, David K. Jackson, Ian Johnston, Dominic Kwiatkowski, Cordelia Langford, John Sillitoe on behalf of the Wellcome Sanger Institute COVID-19 Surveillance Team
EPI_ISL_879514	Lighthouse Lab in Milton Keynes	Wellcome Sanger Institute for the COVID-19 Genomics UK (COG-UK) Consortium	The Lighthouse Lab in Milton Keynes and Alex Alderton, Roberto Amato, Sonia Goncalves, Ewan Harrison, David K. Jackson, Ian Johnston, Dominic Kwiatkowski, Cordelia Langford, John Sillitoe on behalf of the Wellcome Sanger Institute COVID-19 Surveillance Team
EPI_ISL_879519, EPI_ISL_879520	Lighthouse Lab in Alderley Park	Wellcome Sanger Institute for the COVID-19 Genomics UK (COG-UK) Consortium	Jacquelyn Wynn, Mairead Hyland, The Lighthouse Lab in Alderley Park and Alex Alderton, Roberto Amato, Sonia Goncalves, Ewan Harrison, David K. Jackson, Ian Johnston, Dominic Kwiatkowski, Cordelia Langford, John Sillitoe on behalf of the Wellcome Sanger Institute COVID-19 Surveillance Team
EPI_ISL_879526, EPI_ISL_879532, EPI_ISL_879538, EPI_ISL_879539, EPI_ISL_879540	Lighthouse Lab in Milton Keynes	Wellcome Sanger Institute for the COVID-19 Genomics UK (COG-UK) Consortium	The Lighthouse Lab in Milton Keynes and Alex Alderton, Roberto Amato, Sonia Goncalves, Ewan Harrison, David K. Jackson, Ian Johnston, Dominic Kwiatkowski, Cordelia Langford, John Sillitoe on behalf of the Wellcome Sanger Institute COVID-19 Surveillance Team
EPI_ISL_879546, EPI_ISL_879547	Lighthouse Lab in Alderley Park	Wellcome Sanger Institute for the COVID-19 Genomics UK (COG-UK) Consortium	Jacquelyn Wynn, Mairead Hyland, The Lighthouse Lab in Alderley Park and Alex Alderton, Roberto Amato, Sonia Goncalves, Ewan Harrison, David K. Jackson, Ian Johnston, Dominic Kwiatkowski, Cordelia Langford, John Sillitoe on behalf of the Wellcome Sanger Institute COVID-19 Surveillance Team
EPI_ISL_879548, EPI_ISL_879549	Lighthouse Lab in Milton Keynes	Wellcome Sanger Institute for the COVID-19 Genomics UK (COG-UK) Consortium	The Lighthouse Lab in Milton Keynes and Alex Alderton, Roberto Amato, Sonia Goncalves, Ewan Harrison, David K. Jackson, Ian Johnston, Dominic Kwiatkowski, Cordelia Langford, John Sillitoe on behalf of the Wellcome Sanger Institute COVID-19 Surveillance Team
EPI_ISL_879550	Lighthouse Lab in Alderley Park	Wellcome Sanger Institute for the COVID-19 Genomics UK (COG-UK) Consortium	Jacquelyn Wynn, Mairead Hyland, The Lighthouse Lab in Alderley Park and Alex Alderton, Roberto Amato, Sonia Goncalves, Ewan Harrison, David K. Jackson, Ian Johnston, Dominic Kwiatkowski, Cordelia Langford, John Sillitoe on behalf of the Wellcome Sanger Institute COVID-19 Surveillance Team
EPI_ISL_879552, EPI_ISL_879553, EPI_ISL_879554	Lighthouse Lab in Milton Keynes	Wellcome Sanger Institute for the COVID-19 Genomics UK (COG-UK) Consortium	The Lighthouse Lab in Milton Keynes and Alex Alderton, Roberto Amato, Sonia Goncalves, Ewan Harrison, David K. Jackson, Ian Johnston, Dominic Kwiatkowski, Cordelia Langford, John Sillitoe on behalf of the Wellcome Sanger Institute COVID-19 Surveillance Team
EPI_ISL_879556	Lighthouse Lab in Alderley Park	Wellcome Sanger Institute for the COVID-19 Genomics UK (COG-UK) Consortium	Jacquelyn Wynn, Mairead Hyland, The Lighthouse Lab in Alderley Park and Alex Alderton, Roberto Amato, Sonia Goncalves, Ewan Harrison, David K. Jackson, Ian Johnston, Dominic Kwiatkowski, Cordelia Langford, John Sillitoe on behalf of the Wellcome Sanger Institute COVID-19 Surveillance Team
EPI_ISL_879560, EPI_ISL_879561	Lighthouse Lab in Milton Keynes	Wellcome Sanger Institute for the COVID-19 Genomics UK (COG-UK) Consortium	The Lighthouse Lab in Milton Keynes and Alex Alderton, Roberto Amato, Sonia Goncalves, Ewan Harrison, David K. Jackson, Ian Johnston, Dominic Kwiatkowski, Cordelia Langford, John Sillitoe on behalf of the Wellcome Sanger Institute COVID-19 Surveillance Team
EPI_ISL_879565	Lighthouse Lab in Alderley Park	Wellcome Sanger Institute for the COVID-19 Genomics UK (COG-UK) Consortium	Jacquelyn Wynn, Mairead Hyland, The Lighthouse Lab in Alderley Park and Alex Alderton, Roberto Amato, Sonia Goncalves, Ewan Harrison, David K. Jackson, Ian Johnston, Dominic Kwiatkowski, Cordelia Langford, John Sillitoe on behalf of the Wellcome Sanger Institute COVID-19 Surveillance Team
EPI_ISL_879566, EPI_ISL_879571, EPI_ISL_879572, EPI_ISL_879575, EPI_ISL_879576, EPI_ISL_879581, EPI_ISL_879585, EPI_ISL_879586, EPI_ISL_879591, EPI_ISL_879592, EPI_ISL_879594, EPI_ISL_879597, EPI_ISL_879600, EPI_ISL_879601, EPI_ISL_879608, EPI_ISL_879611, EPI_ISL_879613			
see above	Lighthouse Lab in Milton Keynes	Wellcome Sanger Institute for the COVID-19 Genomics UK (COG-UK) Consortium	The Lighthouse Lab in Milton Keynes and Alex Alderton, Roberto Amato, Sonia Goncalves, Ewan Harrison, David K. Jackson, Ian Johnston, Dominic Kwiatkowski, Cordelia Langford, John Sillitoe on behalf of the Wellcome Sanger Institute COVID-19 Surveillance Team
EPI_ISL_879617	Lighthouse Lab in Alderley Park	Wellcome Sanger Institute for the COVID-19 Genomics UK (COG-UK) Consortium	Jacquelyn Wynn, Mairead Hyland, The Lighthouse Lab in Alderley Park and Alex Alderton, Roberto Amato, Sonia Goncalves, Ewan Harrison, David K. Jackson, Ian Johnston, Dominic Kwiatkowski, Cordelia Langford, John Sillitoe on behalf of the Wellcome Sanger Institute COVID-19 Surveillance Team
EPI_ISL_879618, EPI_ISL_879621, EPI_ISL_879623, EPI_ISL_879628	Lighthouse Lab in Milton Keynes	Wellcome Sanger Institute for the COVID-19 Genomics UK (COG-UK) Consortium	The Lighthouse Lab in Milton Keynes and Alex Alderton, Roberto Amato, Sonia Goncalves, Ewan Harrison, David K. Jackson, Ian Johnston, Dominic Kwiatkowski, Cordelia Langford, John Sillitoe on behalf of the Wellcome Sanger Institute COVID-19 Surveillance Team
EPI_ISL_879629, EPI_ISL_879631	Lighthouse Lab in Alderley Park	Wellcome Sanger Institute for the COVID-19 Genomics UK (COG-UK) Consortium	Jacquelyn Wynn, Mairead Hyland, The Lighthouse Lab in Alderley Park and Alex Alderton, Roberto Amato, Sonia Goncalves, Ewan Harrison, David K. Jackson, Ian Johnston, Dominic Kwiatkowski, Cordelia Langford, John Sillitoe on behalf of the Wellcome Sanger Institute COVID-19 Surveillance Team
EPI_ISL_879642, EPI_ISL_879643	Lighthouse Lab in Milton Keynes	Wellcome Sanger Institute for the COVID-19 Genomics UK (COG-UK) Consortium	The Lighthouse Lab in Milton Keynes and Alex Alderton, Roberto Amato, Sonia Goncalves, Ewan Harrison, David K. Jackson, Ian Johnston, Dominic Kwiatkowski, Cordelia Langford, John Sillitoe on behalf of the Wellcome Sanger Institute COVID-19 Surveillance Team
EPI_ISL_879646	Lighthouse Lab in Alderley Park	Wellcome Sanger Institute for the COVID-19 Genomics UK (COG-UK) Consortium	Jacquelyn Wynn, Mairead Hyland, The Lighthouse Lab in Alderley Park and Alex Alderton, Roberto Amato, Sonia Goncalves, Ewan Harrison, David K. Jackson, Ian Johnston, Dominic Kwiatkowski, Cordelia Langford, John Sillitoe on behalf of the Wellcome Sanger Institute COVID-19 Surveillance Team
EPI_ISL_879653, EPI_ISL_879654, EPI_ISL_879655, EPI_ISL_879656, EPI_ISL_879663, EPI_ISL_879664, EPI_ISL_879675, EPI_ISL_879678, EPI_ISL_879679, EPI_ISL_879681, EPI_ISL_879686, EPI_ISL_879689			
see above	Lighthouse Lab in Milton Keynes	Wellcome Sanger Institute for the COVID-19 Genomics UK (COG-UK) Consortium	The Lighthouse Lab in Milton Keynes and Alex Alderton, Roberto Amato, Sonia Goncalves, Ewan Harrison, David K. Jackson, Ian Johnston, Dominic Kwiatkowski, Cordelia Langford, John Sillitoe on behalf of the Wellcome Sanger Institute COVID-19 Surveillance Team
EPI_ISL_879692, EPI_ISL_879693, EPI_ISL_879700	Lighthouse Lab in Alderley Park	Wellcome Sanger Institute for the COVID-19 Genomics UK (COG-UK) Consortium	Jacquelyn Wynn, Mairead Hyland, The Lighthouse Lab in Alderley Park and Alex Alderton, Roberto Amato, Sonia Goncalves, Ewan Harrison, David K. Jackson, Ian Johnston, Dominic Kwiatkowski, Cordelia Langford, John Sillitoe on behalf of the Wellcome Sanger Institute COVID-19 Surveillance Team
EPI_ISL_879703, EPI_ISL_879707, EPI_ISL_879710, EPI_ISL_879711, EPI_ISL_879712, EPI_ISL_879713	Lighthouse Lab in Milton Keynes	Wellcome Sanger Institute for the COVID-19 Genomics UK (COG-UK) Consortium	The Lighthouse Lab in Milton Keynes and Alex Alderton, Roberto Amato, Sonia Goncalves, Ewan Harrison, David K. Jackson, Ian Johnston, Dominic Kwiatkowski, Cordelia Langford, John Sillitoe on behalf of the Wellcome Sanger Institute COVID-19 Surveillance Team
EPI_ISL_879714	Lighthouse Lab in Alderley Park	Wellcome Sanger Institute for the COVID-19 Genomics UK (COG-UK) Consortium	Jacquelyn Wynn, Mairead Hyland, The Lighthouse Lab in Alderley Park and Alex Alderton, Roberto Amato, Sonia Goncalves, Ewan Harrison, David K. Jackson, Ian Johnston, Dominic Kwiatkowski, Cordelia Langford, John Sillitoe on behalf of the Wellcome Sanger Institute COVID-19 Surveillance Team
EPI_ISL_879715, EPI_ISL_879717	Lighthouse Lab in Milton Keynes	Wellcome Sanger Institute for the COVID-19 Genomics UK (COG-UK) Consortium	The Lighthouse Lab in Milton Keynes and Alex Alderton, Roberto Amato, Sonia Goncalves, Ewan Harrison, David K. Jackson, Ian Johnston, Dominic Kwiatkowski, Cordelia Langford, John Sillitoe on behalf of the Wellcome Sanger Institute COVID-19 Surveillance Team
EPI_ISL_879719	Lighthouse Lab in Alderley Park	Wellcome Sanger Institute for the COVID-19 Genomics UK (COG-UK) Consortium	Jacquelyn Wynn, Mairead Hyland, The Lighthouse Lab in Alderley Park and Alex Alderton, Roberto Amato, Sonia Goncalves, Ewan Harrison, David K. Jackson, Ian Johnston, Dominic Kwiatkowski, Cordelia Langford, John Sillitoe on behalf of the Wellcome Sanger Institute COVID-19 Surveillance Team
EPI_ISL_879720	Lighthouse Lab in Cambridge	Wellcome Sanger Institute for the COVID-19 Genomics UK (COG-UK) Consortium	Rob Howes, The Lighthouse Lab in Cambridge and Alex Alderton, Roberto Amato, Sonia Goncalves, Ewan Harrison, David K. Jackson, Ian Johnston, Dominic Kwiatkowski, Cordelia Langford, John Sillitoe on behalf of the Wellcome Sanger Institute COVID-19 Surveillance Team
EPI_ISL_879721	Lighthouse Lab in Alderley Park	Wellcome Sanger Institute for the COVID-19 Genomics UK (COG-UK) Consortium	Jacquelyn Wynn, Mairead Hyland, The Lighthouse Lab in Alderley Park and Alex Alderton, Roberto Amato, Sonia Goncalves, Ewan Harrison, David K. Jackson, Ian Johnston, Dominic Kwiatkowski, Cordelia Langford, John Sillitoe on behalf of the Wellcome Sanger Institute COVID-19 Surveillance Team
EPI_ISL_879723, EPI_ISL_879724, EPI_ISL_879725	Lighthouse Lab in Cambridge	Wellcome Sanger Institute for the COVID-19 Genomics UK (COG-UK) Consortium	Rob Howes, The Lighthouse Lab in Cambridge and Alex Alderton, Roberto Amato, Sonia Goncalves, Ewan Harrison, David K. Jackson, Ian Johnston, Dominic Kwiatkowski, Cordelia Langford, John Sillitoe on behalf of the Wellcome Sanger Institute COVID-19 Surveillance Team
EPI_ISL_879726	Lighthouse Lab in Alderley Park	Wellcome Sanger Institute for the COVID-19 Genomics UK (COG-UK) Consortium	Jacquelyn Wynn, Mairead Hyland, The Lighthouse Lab in Alderley Park and Alex Alderton, Roberto Amato, Sonia Goncalves, Ewan Harrison, David K. Jackson, Ian Johnston, Dominic Kwiatkowski, Cordelia Langford, John Sillitoe on behalf of the Wellcome Sanger Institute COVID-19 Surveillance Team
EPI_ISL_879727	Lighthouse Lab in Cambridge	Wellcome Sanger Institute for the COVID-19 Genomics UK (COG-UK) Consortium	Rob Howes, The Lighthouse Lab in Cambridge and Alex Alderton, Roberto Amato, Sonia Goncalves, Ewan Harrison, David K. Jackson, Ian Johnston, Dominic Kwiatkowski, Cordelia Langford, John Sillitoe on behalf of the Wellcome Sanger Institute COVID-19 Surveillance Team

EPI_ISL_879728	Lighthouse Lab in Alderley Park	Wellcome Sanger Institute for the COVID-19 Genomics UK (COG-UK) Consortium	Jacquelyn Wynn, Mairead Hyland, The Lighthouse Lab in Alderley Park and Alex Alderton, Roberto Amato, Sonia Goncalves, Ewan Harrison, David K. Jackson, Ian Johnston, Dominic Kwiatkowski, Cordelia Langford, John Sillitoe on behalf of the Wellcome Sanger Institute COVID-19 Surveillance Team
EPI_ISL_879729, EPI_ISL_879730, EPI_ISL_879731, EPI_ISL_879732, EPI_ISL_879734	Lighthouse Lab in Cambridge	Wellcome Sanger Institute for the COVID-19 Genomics UK (COG-UK) Consortium	Rob Howes, The Lighthouse Lab in Cambridge and Alex Alderton, Roberto Amato, Sonia Goncalves, Ewan Harrison, David K. Jackson, Ian Johnston, Dominic Kwiatkowski, Cordelia Langford, John Sillitoe on behalf of the Wellcome Sanger Institute COVID-19 Surveillance Team
EPI_ISL_879736, EPI_ISL_879737	Lighthouse Lab in Alderley Park	Wellcome Sanger Institute for the COVID-19 Genomics UK (COG-UK) Consortium	Jacquelyn Wynn, Mairead Hyland, The Lighthouse Lab in Alderley Park and Alex Alderton, Roberto Amato, Sonia Goncalves, Ewan Harrison, David K. Jackson, Ian Johnston, Dominic Kwiatkowski, Cordelia Langford, John Sillitoe on behalf of the Wellcome Sanger Institute COVID-19 Surveillance Team
EPI_ISL_879738, EPI_ISL_879739	Lighthouse Lab in Cambridge	Wellcome Sanger Institute for the COVID-19 Genomics UK (COG-UK) Consortium	Rob Howes, The Lighthouse Lab in Cambridge and Alex Alderton, Roberto Amato, Sonia Goncalves, Ewan Harrison, David K. Jackson, Ian Johnston, Dominic Kwiatkowski, Cordelia Langford, John Sillitoe on behalf of the Wellcome Sanger Institute COVID-19 Surveillance Team
EPI_ISL_879740	Lighthouse Lab in Alderley Park	Wellcome Sanger Institute for the COVID-19 Genomics UK (COG-UK) Consortium	Jacquelyn Wynn, Mairead Hyland, The Lighthouse Lab in Alderley Park and Alex Alderton, Roberto Amato, Sonia Goncalves, Ewan Harrison, David K. Jackson, Ian Johnston, Dominic Kwiatkowski, Cordelia Langford, John Sillitoe on behalf of the Wellcome Sanger Institute COVID-19 Surveillance Team
EPI_ISL_879741	Lighthouse Lab in Cambridge	Wellcome Sanger Institute for the COVID-19 Genomics UK (COG-UK) Consortium	Rob Howes, The Lighthouse Lab in Cambridge and Alex Alderton, Roberto Amato, Sonia Goncalves, Ewan Harrison, David K. Jackson, Ian Johnston, Dominic Kwiatkowski, Cordelia Langford, John Sillitoe on behalf of the Wellcome Sanger Institute COVID-19 Surveillance Team
EPI_ISL_879742, EPI_ISL_879744, EPI_ISL_879747, EPI_ISL_879748	Lighthouse Lab in Alderley Park	Wellcome Sanger Institute for the COVID-19 Genomics UK (COG-UK) Consortium	Jacquelyn Wynn, Mairead Hyland, The Lighthouse Lab in Alderley Park and Alex Alderton, Roberto Amato, Sonia Goncalves, Ewan Harrison, David K. Jackson, Ian Johnston, Dominic Kwiatkowski, Cordelia Langford, John Sillitoe on behalf of the Wellcome Sanger Institute COVID-19 Surveillance Team
EPI_ISL_879749	Lighthouse Lab in Cambridge	Wellcome Sanger Institute for the COVID-19 Genomics UK (COG-UK) Consortium	Rob Howes, The Lighthouse Lab in Cambridge and Alex Alderton, Roberto Amato, Sonia Goncalves, Ewan Harrison, David K. Jackson, Ian Johnston, Dominic Kwiatkowski, Cordelia Langford, John Sillitoe on behalf of the Wellcome Sanger Institute COVID-19 Surveillance Team
EPI_ISL_879750, EPI_ISL_879751, EPI_ISL_879752, EPI_ISL_879753	Lighthouse Lab in Alderley Park	Wellcome Sanger Institute for the COVID-19 Genomics UK (COG-UK) Consortium	Jacquelyn Wynn, Mairead Hyland, The Lighthouse Lab in Alderley Park and Alex Alderton, Roberto Amato, Sonia Goncalves, Ewan Harrison, David K. Jackson, Ian Johnston, Dominic Kwiatkowski, Cordelia Langford, John Sillitoe on behalf of the Wellcome Sanger Institute COVID-19 Surveillance Team
EPI_ISL_879754	Lighthouse Lab in Cambridge	Wellcome Sanger Institute for the COVID-19 Genomics UK (COG-UK) Consortium	Rob Howes, The Lighthouse Lab in Cambridge and Alex Alderton, Roberto Amato, Sonia Goncalves, Ewan Harrison, David K. Jackson, Ian Johnston, Dominic Kwiatkowski, Cordelia Langford, John Sillitoe on behalf of the Wellcome Sanger Institute COVID-19 Surveillance Team
EPI_ISL_879755, EPI_ISL_879756, EPI_ISL_879757, EPI_ISL_879758, EPI_ISL_879759, EPI_ISL_879760, EPI_ISL_879761, EPI_ISL_879762	Lighthouse Lab in Alderley Park	Wellcome Sanger Institute for the COVID-19 Genomics UK (COG-UK) Consortium	Jacquelyn Wynn, Mairead Hyland, The Lighthouse Lab in Alderley Park and Alex Alderton, Roberto Amato, Sonia Goncalves, Ewan Harrison, David K. Jackson, Ian Johnston, Dominic Kwiatkowski, Cordelia Langford, John Sillitoe on behalf of the Wellcome Sanger Institute COVID-19 Surveillance Team
EPI_ISL_879763, EPI_ISL_879764	Lighthouse Lab in Cambridge	Wellcome Sanger Institute for the COVID-19 Genomics UK (COG-UK) Consortium	Rob Howes, The Lighthouse Lab in Cambridge and Alex Alderton, Roberto Amato, Sonia Goncalves, Ewan Harrison, David K. Jackson, Ian Johnston, Dominic Kwiatkowski, Cordelia Langford, John Sillitoe on behalf of the Wellcome Sanger Institute COVID-19 Surveillance Team
EPI_ISL_879765, EPI_ISL_879766, EPI_ISL_879767	Lighthouse Lab in Alderley Park	Wellcome Sanger Institute for the COVID-19 Genomics UK (COG-UK) Consortium	Jacquelyn Wynn, Mairead Hyland, The Lighthouse Lab in Alderley Park and Alex Alderton, Roberto Amato, Sonia Goncalves, Ewan Harrison, David K. Jackson, Ian Johnston, Dominic Kwiatkowski, Cordelia Langford, John Sillitoe on behalf of the Wellcome Sanger Institute COVID-19 Surveillance Team
EPI_ISL_879768, EPI_ISL_879769, EPI_ISL_879771, EPI_ISL_879772, EPI_ISL_879773, EPI_ISL_879774	Lighthouse Lab in Cambridge	Wellcome Sanger Institute for the COVID-19 Genomics UK (COG-UK) Consortium	Rob Howes, The Lighthouse Lab in Cambridge and Alex Alderton, Roberto Amato, Sonia Goncalves, Ewan Harrison, David K. Jackson, Ian Johnston, Dominic Kwiatkowski, Cordelia Langford, John Sillitoe on behalf of the Wellcome Sanger Institute COVID-19 Surveillance Team
EPI_ISL_879775	Lighthouse Lab in Alderley Park	Wellcome Sanger Institute for the COVID-19 Genomics UK (COG-UK) Consortium	Jacquelyn Wynn, Mairead Hyland, The Lighthouse Lab in Alderley Park and Alex Alderton, Roberto Amato, Sonia Goncalves, Ewan Harrison, David K. Jackson, Ian Johnston, Dominic Kwiatkowski, Cordelia Langford, John Sillitoe on behalf of the Wellcome Sanger Institute COVID-19 Surveillance Team
EPI_ISL_879776, EPI_ISL_879777	Lighthouse Lab in Cambridge	Wellcome Sanger Institute for the COVID-19 Genomics UK (COG-UK) Consortium	Rob Howes, The Lighthouse Lab in Cambridge and Alex Alderton, Roberto Amato, Sonia Goncalves, Ewan Harrison, David K. Jackson, Ian Johnston, Dominic Kwiatkowski, Cordelia Langford, John Sillitoe on behalf of the Wellcome Sanger Institute COVID-19 Surveillance Team
EPI_ISL_879778	Lighthouse Lab in Alderley Park	Wellcome Sanger Institute for the COVID-19 Genomics UK (COG-UK) Consortium	Jacquelyn Wynn, Mairead Hyland, The Lighthouse Lab in Alderley Park and Alex Alderton, Roberto Amato, Sonia Goncalves, Ewan Harrison, David K. Jackson, Ian Johnston, Dominic Kwiatkowski, Cordelia Langford, John Sillitoe on behalf of the Wellcome Sanger Institute COVID-19 Surveillance Team
EPI_ISL_879779	Lighthouse Lab in Cambridge	Wellcome Sanger Institute for the COVID-19 Genomics UK (COG-UK) Consortium	Rob Howes, The Lighthouse Lab in Cambridge and Alex Alderton, Roberto Amato, Sonia Goncalves, Ewan Harrison, David K. Jackson, Ian Johnston, Dominic Kwiatkowski, Cordelia Langford, John Sillitoe on behalf of the Wellcome Sanger Institute COVID-19 Surveillance Team
EPI_ISL_879780	Lighthouse Lab in Alderley Park	Wellcome Sanger Institute for the COVID-19 Genomics UK (COG-UK) Consortium	Jacquelyn Wynn, Mairead Hyland, The Lighthouse Lab in Alderley Park and Alex Alderton, Roberto Amato, Sonia Goncalves, Ewan Harrison, David K. Jackson, Ian Johnston, Dominic Kwiatkowski, Cordelia Langford, John Sillitoe on behalf of the Wellcome Sanger Institute COVID-19 Surveillance Team
EPI_ISL_879781	Lighthouse Lab in Cambridge	Wellcome Sanger Institute for the COVID-19 Genomics UK (COG-UK) Consortium	Rob Howes, The Lighthouse Lab in Cambridge and Alex Alderton, Roberto Amato, Sonia Goncalves, Ewan Harrison, David K. Jackson, Ian Johnston, Dominic Kwiatkowski, Cordelia Langford, John Sillitoe on behalf of the Wellcome Sanger Institute COVID-19 Surveillance Team
EPI_ISL_879783, EPI_ISL_879784, EPI_ISL_879785, EPI_ISL_879787	Lighthouse Lab in Alderley Park	Wellcome Sanger Institute for the COVID-19 Genomics UK (COG-UK) Consortium	Jacquelyn Wynn, Mairead Hyland, The Lighthouse Lab in Alderley Park and Alex Alderton, Roberto Amato, Sonia Goncalves, Ewan Harrison, David K. Jackson, Ian Johnston, Dominic Kwiatkowski, Cordelia Langford, John Sillitoe on behalf of the Wellcome Sanger Institute COVID-19 Surveillance Team
EPI_ISL_879788	Lighthouse Lab in Cambridge	Wellcome Sanger Institute for the COVID-19 Genomics UK (COG-UK) Consortium	Rob Howes, The Lighthouse Lab in Cambridge and Alex Alderton, Roberto Amato, Sonia Goncalves, Ewan Harrison, David K. Jackson, Ian Johnston, Dominic Kwiatkowski, Cordelia Langford, John Sillitoe on behalf of the Wellcome Sanger Institute COVID-19 Surveillance Team
EPI_ISL_879789	Lighthouse Lab in Alderley Park	Wellcome Sanger Institute for the COVID-19 Genomics UK (COG-UK) Consortium	Jacquelyn Wynn, Mairead Hyland, The Lighthouse Lab in Alderley Park and Alex Alderton, Roberto Amato, Sonia Goncalves, Ewan Harrison, David K. Jackson, Ian Johnston, Dominic Kwiatkowski, Cordelia Langford, John Sillitoe on behalf of the Wellcome Sanger Institute COVID-19 Surveillance Team
EPI_ISL_879790	Lighthouse Lab in Cambridge	Wellcome Sanger Institute for the COVID-19 Genomics UK (COG-UK) Consortium	Rob Howes, The Lighthouse Lab in Cambridge and Alex Alderton, Roberto Amato, Sonia Goncalves, Ewan Harrison, David K. Jackson, Ian Johnston, Dominic Kwiatkowski, Cordelia Langford, John Sillitoe on behalf of the Wellcome Sanger Institute COVID-19 Surveillance Team
EPI_ISL_879791, EPI_ISL_879792, EPI_ISL_879793, EPI_ISL_879794	Lighthouse Lab in Alderley Park	Wellcome Sanger Institute for the COVID-19 Genomics UK (COG-UK) Consortium	Jacquelyn Wynn, Mairead Hyland, The Lighthouse Lab in Alderley Park and Alex Alderton, Roberto Amato, Sonia Goncalves, Ewan Harrison, David K. Jackson, Ian Johnston, Dominic Kwiatkowski, Cordelia Langford, John Sillitoe on behalf of the Wellcome Sanger Institute COVID-19 Surveillance Team
EPI_ISL_879795, EPI_ISL_879797, EPI_ISL_879798	Lighthouse Lab in Cambridge	Wellcome Sanger Institute for the COVID-19 Genomics UK (COG-UK) Consortium	Rob Howes, The Lighthouse Lab in Cambridge and Alex Alderton, Roberto Amato, Sonia Goncalves, Ewan Harrison, David K. Jackson, Ian Johnston, Dominic Kwiatkowski, Cordelia Langford, John Sillitoe on behalf of the Wellcome Sanger Institute COVID-19 Surveillance Team
EPI_ISL_879800, EPI_ISL_879801, EPI_ISL_879802, EPI_ISL_879803	Lighthouse Lab in Alderley Park	Wellcome Sanger Institute for the COVID-19 Genomics UK (COG-UK) Consortium	Jacquelyn Wynn, Mairead Hyland, The Lighthouse Lab in Alderley Park and Alex Alderton, Roberto Amato, Sonia Goncalves, Ewan Harrison, David K. Jackson, Ian Johnston, Dominic Kwiatkowski, Cordelia Langford, John Sillitoe on behalf of the Wellcome Sanger Institute COVID-19 Surveillance Team
EPI_ISL_879806	Lighthouse Lab in Cambridge	Wellcome Sanger Institute for the COVID-19 Genomics UK (COG-UK) Consortium	Rob Howes, The Lighthouse Lab in Cambridge and Alex Alderton, Roberto Amato, Sonia Goncalves, Ewan Harrison, David K. Jackson, Ian Johnston, Dominic Kwiatkowski, Cordelia Langford, John Sillitoe on behalf of the Wellcome Sanger Institute COVID-19 Surveillance Team
EPI_ISL_879808	Lighthouse Lab in Alderley Park	Wellcome Sanger Institute for the COVID-19 Genomics UK (COG-UK) Consortium	Jacquelyn Wynn, Mairead Hyland, The Lighthouse Lab in Alderley Park and Alex Alderton, Roberto Amato, Sonia Goncalves, Ewan Harrison, David K. Jackson, Ian Johnston, Dominic Kwiatkowski, Cordelia Langford, John Sillitoe on behalf of the Wellcome Sanger Institute COVID-19 Surveillance Team
EPI_ISL_879809	Lighthouse Lab in Cambridge	Wellcome Sanger Institute for the COVID-19 Genomics UK (COG-UK) Consortium	Rob Howes, The Lighthouse Lab in Cambridge and Alex Alderton, Roberto Amato, Sonia Goncalves, Ewan Harrison, David K. Jackson, Ian Johnston, Dominic Kwiatkowski, Cordelia Langford, John Sillitoe on behalf of the Wellcome Sanger Institute COVID-19 Surveillance Team
EPI_ISL_879810, EPI_ISL_879811, EPI_ISL_879813, EPI_ISL_879814	Lighthouse Lab in Alderley Park	Wellcome Sanger Institute for the COVID-19 Genomics UK (COG-UK) Consortium	Jacquelyn Wynn, Mairead Hyland, The Lighthouse Lab in Alderley Park and Alex Alderton, Roberto Amato, Sonia Goncalves, Ewan Harrison, David K. Jackson, Ian Johnston, Dominic Kwiatkowski, Cordelia Langford, John Sillitoe on behalf of the Wellcome Sanger Institute COVID-19 Surveillance Team
EPI_ISL_879816	Lighthouse Lab in Cambridge	Wellcome Sanger Institute for the COVID-19 Genomics UK (COG-UK) Consortium	Rob Howes, The Lighthouse Lab in Cambridge and Alex Alderton, Roberto Amato, Sonia Goncalves, Ewan Harrison, David K. Jackson, Ian Johnston, Dominic Kwiatkowski, Cordelia Langford, John Sillitoe on behalf of the Wellcome Sanger Institute COVID-19 Surveillance Team
EPI_ISL_879817	Lighthouse Lab in Alderley Park	Wellcome Sanger Institute for the COVID-19 Genomics UK (COG-UK) Consortium	Jacquelyn Wynn, Mairead Hyland, The Lighthouse Lab in Alderley Park and Alex Alderton, Roberto Amato, Sonia Goncalves, Ewan Harrison, David K. Jackson, Ian Johnston, Dominic Kwiatkowski, Cordelia Langford, John Sillitoe on behalf of the Wellcome Sanger Institute COVID-19 Surveillance Team
EPI_ISL_879818	Lighthouse Lab in Cambridge	Wellcome Sanger Institute for the COVID-19 Genomics UK	Rob Howes, The Lighthouse Lab in Cambridge and Alex Alderton, Roberto Amato, Sonia Goncalves, Ewan Harrison, David K. Jackson, Ian Johnston,

		(COG-UK) Consortium	Dominic Kwiatkowski, Cordelia Langford, John Sillitoe on behalf of the Wellcome Sanger Institute COVID-19 Surveillance Team
EPI_ISL_879819, EPI_ISL_879821, EPI_ISL_879822	Lighthouse Lab in Alderley Park	Wellcome Sanger Institute for the COVID-19 Genomics UK (COG-UK) Consortium	Jacquelyn Wynn, Mairead Hyland, The Lighthouse Lab in Alderley Park and Alex Alderton, Roberto Amato, Sonia Goncalves, Ewan Harrison, David K. Jackson, Ian Johnston, Dominic Kwiatkowski, Cordelia Langford, John Sillitoe on behalf of the Wellcome Sanger Institute COVID-19 Surveillance Team
EPI_ISL_879823	Lighthouse Lab in Cambridge	Wellcome Sanger Institute for the COVID-19 Genomics UK (COG-UK) Consortium	Rob Howes, The Lighthouse Lab in Cambridge and Alex Alderton, Roberto Amato, Sonia Goncalves, Ewan Harrison, David K. Jackson, Ian Johnston, Dominic Kwiatkowski, Cordelia Langford, John Sillitoe on behalf of the Wellcome Sanger Institute COVID-19 Surveillance Team
EPI_ISL_879825, EPI_ISL_879826, EPI_ISL_879827	Lighthouse Lab in Alderley Park	Wellcome Sanger Institute for the COVID-19 Genomics UK (COG-UK) Consortium	Jacquelyn Wynn, Mairead Hyland, The Lighthouse Lab in Alderley Park and Alex Alderton, Roberto Amato, Sonia Goncalves, Ewan Harrison, David K. Jackson, Ian Johnston, Dominic Kwiatkowski, Cordelia Langford, John Sillitoe on behalf of the Wellcome Sanger Institute COVID-19 Surveillance Team
EPI_ISL_879828	Lighthouse Lab in Cambridge	Wellcome Sanger Institute for the COVID-19 Genomics UK (COG-UK) Consortium	Rob Howes, The Lighthouse Lab in Cambridge and Alex Alderton, Roberto Amato, Sonia Goncalves, Ewan Harrison, David K. Jackson, Ian Johnston, Dominic Kwiatkowski, Cordelia Langford, John Sillitoe on behalf of the Wellcome Sanger Institute COVID-19 Surveillance Team
EPI_ISL_879829, EPI_ISL_879831	Lighthouse Lab in Alderley Park	Wellcome Sanger Institute for the COVID-19 Genomics UK (COG-UK) Consortium	Jacquelyn Wynn, Mairead Hyland, The Lighthouse Lab in Alderley Park and Alex Alderton, Roberto Amato, Sonia Goncalves, Ewan Harrison, David K. Jackson, Ian Johnston, Dominic Kwiatkowski, Cordelia Langford, John Sillitoe on behalf of the Wellcome Sanger Institute COVID-19 Surveillance Team
EPI_ISL_879832, EPI_ISL_879833, EPI_ISL_879834, EPI_ISL_879835	Lighthouse Lab in Cambridge	Wellcome Sanger Institute for the COVID-19 Genomics UK (COG-UK) Consortium	Rob Howes, The Lighthouse Lab in Cambridge and Alex Alderton, Roberto Amato, Sonia Goncalves, Ewan Harrison, David K. Jackson, Ian Johnston, Dominic Kwiatkowski, Cordelia Langford, John Sillitoe on behalf of the Wellcome Sanger Institute COVID-19 Surveillance Team
EPI_ISL_879836, EPI_ISL_879837, EPI_ISL_879838	Lighthouse Lab in Alderley Park	Wellcome Sanger Institute for the COVID-19 Genomics UK (COG-UK) Consortium	Jacquelyn Wynn, Mairead Hyland, The Lighthouse Lab in Alderley Park and Alex Alderton, Roberto Amato, Sonia Goncalves, Ewan Harrison, David K. Jackson, Ian Johnston, Dominic Kwiatkowski, Cordelia Langford, John Sillitoe on behalf of the Wellcome Sanger Institute COVID-19 Surveillance Team
EPI_ISL_879839	Lighthouse Lab in Cambridge	Wellcome Sanger Institute for the COVID-19 Genomics UK (COG-UK) Consortium	Rob Howes, The Lighthouse Lab in Cambridge and Alex Alderton, Roberto Amato, Sonia Goncalves, Ewan Harrison, David K. Jackson, Ian Johnston, Dominic Kwiatkowski, Cordelia Langford, John Sillitoe on behalf of the Wellcome Sanger Institute COVID-19 Surveillance Team
EPI_ISL_879841, EPI_ISL_879842, EPI_ISL_879843, EPI_ISL_879844	Lighthouse Lab in Alderley Park	Wellcome Sanger Institute for the COVID-19 Genomics UK (COG-UK) Consortium	Jacquelyn Wynn, Mairead Hyland, The Lighthouse Lab in Alderley Park and Alex Alderton, Roberto Amato, Sonia Goncalves, Ewan Harrison, David K. Jackson, Ian Johnston, Dominic Kwiatkowski, Cordelia Langford, John Sillitoe on behalf of the Wellcome Sanger Institute COVID-19 Surveillance Team
EPI_ISL_879845	Lighthouse Lab in Cambridge	Wellcome Sanger Institute for the COVID-19 Genomics UK (COG-UK) Consortium	Rob Howes, The Lighthouse Lab in Cambridge and Alex Alderton, Roberto Amato, Sonia Goncalves, Ewan Harrison, David K. Jackson, Ian Johnston, Dominic Kwiatkowski, Cordelia Langford, John Sillitoe on behalf of the Wellcome Sanger Institute COVID-19 Surveillance Team
EPI_ISL_879846	Lighthouse Lab in Alderley Park	Wellcome Sanger Institute for the COVID-19 Genomics UK (COG-UK) Consortium	Jacquelyn Wynn, Mairead Hyland, The Lighthouse Lab in Alderley Park and Alex Alderton, Roberto Amato, Sonia Goncalves, Ewan Harrison, David K. Jackson, Ian Johnston, Dominic Kwiatkowski, Cordelia Langford, John Sillitoe on behalf of the Wellcome Sanger Institute COVID-19 Surveillance Team
EPI_ISL_879848	Lighthouse Lab in Cambridge	Wellcome Sanger Institute for the COVID-19 Genomics UK (COG-UK) Consortium	Rob Howes, The Lighthouse Lab in Cambridge and Alex Alderton, Roberto Amato, Sonia Goncalves, Ewan Harrison, David K. Jackson, Ian Johnston, Dominic Kwiatkowski, Cordelia Langford, John Sillitoe on behalf of the Wellcome Sanger Institute COVID-19 Surveillance Team
EPI_ISL_879849	Lighthouse Lab in Alderley Park	Wellcome Sanger Institute for the COVID-19 Genomics UK (COG-UK) Consortium	Jacquelyn Wynn, Mairead Hyland, The Lighthouse Lab in Alderley Park and Alex Alderton, Roberto Amato, Sonia Goncalves, Ewan Harrison, David K. Jackson, Ian Johnston, Dominic Kwiatkowski, Cordelia Langford, John Sillitoe on behalf of the Wellcome Sanger Institute COVID-19 Surveillance Team
EPI_ISL_879850	Lighthouse Lab in Cambridge	Wellcome Sanger Institute for the COVID-19 Genomics UK (COG-UK) Consortium	Rob Howes, The Lighthouse Lab in Cambridge and Alex Alderton, Roberto Amato, Sonia Goncalves, Ewan Harrison, David K. Jackson, Ian Johnston, Dominic Kwiatkowski, Cordelia Langford, John Sillitoe on behalf of the Wellcome Sanger Institute COVID-19 Surveillance Team
EPI_ISL_879851	Lighthouse Lab in Alderley Park	Wellcome Sanger Institute for the COVID-19 Genomics UK (COG-UK) Consortium	Jacquelyn Wynn, Mairead Hyland, The Lighthouse Lab in Alderley Park and Alex Alderton, Roberto Amato, Sonia Goncalves, Ewan Harrison, David K. Jackson, Ian Johnston, Dominic Kwiatkowski, Cordelia Langford, John Sillitoe on behalf of the Wellcome Sanger Institute COVID-19 Surveillance Team
EPI_ISL_879852	Lighthouse Lab in Cambridge	Wellcome Sanger Institute for the COVID-19 Genomics UK (COG-UK) Consortium	Rob Howes, The Lighthouse Lab in Cambridge and Alex Alderton, Roberto Amato, Sonia Goncalves, Ewan Harrison, David K. Jackson, Ian Johnston, Dominic Kwiatkowski, Cordelia Langford, John Sillitoe on behalf of the Wellcome Sanger Institute COVID-19 Surveillance Team
EPI_ISL_879854, EPI_ISL_879855, EPI_ISL_879856, EPI_ISL_879858, EPI_ISL_879859	Lighthouse Lab in Alderley Park	Wellcome Sanger Institute for the COVID-19 Genomics UK (COG-UK) Consortium	Jacquelyn Wynn, Mairead Hyland, The Lighthouse Lab in Alderley Park and Alex Alderton, Roberto Amato, Sonia Goncalves, Ewan Harrison, David K. Jackson, Ian Johnston, Dominic Kwiatkowski, Cordelia Langford, John Sillitoe on behalf of the Wellcome Sanger Institute COVID-19 Surveillance Team
EPI_ISL_879860, EPI_ISL_879861, EPI_ISL_879862	Lighthouse Lab in Cambridge	Wellcome Sanger Institute for the COVID-19 Genomics UK (COG-UK) Consortium	Rob Howes, The Lighthouse Lab in Cambridge and Alex Alderton, Roberto Amato, Sonia Goncalves, Ewan Harrison, David K. Jackson, Ian Johnston, Dominic Kwiatkowski, Cordelia Langford, John Sillitoe on behalf of the Wellcome Sanger Institute COVID-19 Surveillance Team
EPI_ISL_879863	Lighthouse Lab in Alderley Park	Wellcome Sanger Institute for the COVID-19 Genomics UK (COG-UK) Consortium	Jacquelyn Wynn, Mairead Hyland, The Lighthouse Lab in Alderley Park and Alex Alderton, Roberto Amato, Sonia Goncalves, Ewan Harrison, David K. Jackson, Ian Johnston, Dominic Kwiatkowski, Cordelia Langford, John Sillitoe on behalf of the Wellcome Sanger Institute COVID-19 Surveillance Team
EPI_ISL_879864	Lighthouse Lab in Cambridge	Wellcome Sanger Institute for the COVID-19 Genomics UK (COG-UK) Consortium	Rob Howes, The Lighthouse Lab in Cambridge and Alex Alderton, Roberto Amato, Sonia Goncalves, Ewan Harrison, David K. Jackson, Ian Johnston, Dominic Kwiatkowski, Cordelia Langford, John Sillitoe on behalf of the Wellcome Sanger Institute COVID-19 Surveillance Team
EPI_ISL_879865, EPI_ISL_879866	Lighthouse Lab in Alderley Park	Wellcome Sanger Institute for the COVID-19 Genomics UK (COG-UK) Consortium	Jacquelyn Wynn, Mairead Hyland, The Lighthouse Lab in Alderley Park and Alex Alderton, Roberto Amato, Sonia Goncalves, Ewan Harrison, David K. Jackson, Ian Johnston, Dominic Kwiatkowski, Cordelia Langford, John Sillitoe on behalf of the Wellcome Sanger Institute COVID-19 Surveillance Team
EPI_ISL_879867	Lighthouse Lab in Cambridge	Wellcome Sanger Institute for the COVID-19 Genomics UK (COG-UK) Consortium	Rob Howes, The Lighthouse Lab in Cambridge and Alex Alderton, Roberto Amato, Sonia Goncalves, Ewan Harrison, David K. Jackson, Ian Johnston, Dominic Kwiatkowski, Cordelia Langford, John Sillitoe on behalf of the Wellcome Sanger Institute COVID-19 Surveillance Team
EPI_ISL_879868, EPI_ISL_879869, EPI_ISL_879870, EPI_ISL_879871, EPI_ISL_879872, EPI_ISL_879873	Lighthouse Lab in Alderley Park	Wellcome Sanger Institute for the COVID-19 Genomics UK (COG-UK) Consortium	Jacquelyn Wynn, Mairead Hyland, The Lighthouse Lab in Alderley Park and Alex Alderton, Roberto Amato, Sonia Goncalves, Ewan Harrison, David K. Jackson, Ian Johnston, Dominic Kwiatkowski, Cordelia Langford, John Sillitoe on behalf of the Wellcome Sanger Institute COVID-19 Surveillance Team
EPI_ISL_879874	Lighthouse Lab in Cambridge	Wellcome Sanger Institute for the COVID-19 Genomics UK (COG-UK) Consortium	Rob Howes, The Lighthouse Lab in Cambridge and Alex Alderton, Roberto Amato, Sonia Goncalves, Ewan Harrison, David K. Jackson, Ian Johnston, Dominic Kwiatkowski, Cordelia Langford, John Sillitoe on behalf of the Wellcome Sanger Institute COVID-19 Surveillance Team
EPI_ISL_879875	Lighthouse Lab in Alderley Park	Wellcome Sanger Institute for the COVID-19 Genomics UK (COG-UK) Consortium	Jacquelyn Wynn, Mairead Hyland, The Lighthouse Lab in Alderley Park and Alex Alderton, Roberto Amato, Sonia Goncalves, Ewan Harrison, David K. Jackson, Ian Johnston, Dominic Kwiatkowski, Cordelia Langford, John Sillitoe on behalf of the Wellcome Sanger Institute COVID-19 Surveillance Team
EPI_ISL_879876, EPI_ISL_879877	Lighthouse Lab in Cambridge	Wellcome Sanger Institute for the COVID-19 Genomics UK (COG-UK) Consortium	Rob Howes, The Lighthouse Lab in Cambridge and Alex Alderton, Roberto Amato, Sonia Goncalves, Ewan Harrison, David K. Jackson, Ian Johnston, Dominic Kwiatkowski, Cordelia Langford, John Sillitoe on behalf of the Wellcome Sanger Institute COVID-19 Surveillance Team
EPI_ISL_879878, EPI_ISL_879879	Lighthouse Lab in Alderley Park	Wellcome Sanger Institute for the COVID-19 Genomics UK (COG-UK) Consortium	Jacquelyn Wynn, Mairead Hyland, The Lighthouse Lab in Alderley Park and Alex Alderton, Roberto Amato, Sonia Goncalves, Ewan Harrison, David K. Jackson, Ian Johnston, Dominic Kwiatkowski, Cordelia Langford, John Sillitoe on behalf of the Wellcome Sanger Institute COVID-19 Surveillance Team
EPI_ISL_879880	Lighthouse Lab in Cambridge	Wellcome Sanger Institute for the COVID-19 Genomics UK (COG-UK) Consortium	Rob Howes, The Lighthouse Lab in Cambridge and Alex Alderton, Roberto Amato, Sonia Goncalves, Ewan Harrison, David K. Jackson, Ian Johnston, Dominic Kwiatkowski, Cordelia Langford, John Sillitoe on behalf of the Wellcome Sanger Institute COVID-19 Surveillance Team
EPI_ISL_879882, EPI_ISL_879884, EPI_ISL_879885, EPI_ISL_879886, EPI_ISL_879887, EPI_ISL_879888	Lighthouse Lab in Alderley Park	Wellcome Sanger Institute for the COVID-19 Genomics UK (COG-UK) Consortium	Jacquelyn Wynn, Mairead Hyland, The Lighthouse Lab in Alderley Park and Alex Alderton, Roberto Amato, Sonia Goncalves, Ewan Harrison, David K. Jackson, Ian Johnston, Dominic Kwiatkowski, Cordelia Langford, John Sillitoe on behalf of the Wellcome Sanger Institute COVID-19 Surveillance Team
EPI_ISL_879889, EPI_ISL_879890, EPI_ISL_879891, EPI_ISL_879892	Lighthouse Lab in Cambridge	Wellcome Sanger Institute for the COVID-19 Genomics UK (COG-UK) Consortium	Rob Howes, The Lighthouse Lab in Cambridge and Alex Alderton, Roberto Amato, Sonia Goncalves, Ewan Harrison, David K. Jackson, Ian Johnston, Dominic Kwiatkowski, Cordelia Langford, John Sillitoe on behalf of the Wellcome Sanger Institute COVID-19 Surveillance Team
EPI_ISL_879894, EPI_ISL_879895	Lighthouse Lab in Alderley Park	Wellcome Sanger Institute for the COVID-19 Genomics UK (COG-UK) Consortium	Jacquelyn Wynn, Mairead Hyland, The Lighthouse Lab in Alderley Park and Alex Alderton, Roberto Amato, Sonia Goncalves, Ewan Harrison, David K. Jackson, Ian Johnston, Dominic Kwiatkowski, Cordelia Langford, John Sillitoe on behalf of the Wellcome Sanger Institute COVID-19 Surveillance Team
EPI_ISL_879896	Lighthouse Lab in Cambridge	Wellcome Sanger Institute for the COVID-19 Genomics UK (COG-UK) Consortium	Rob Howes, The Lighthouse Lab in Cambridge and Alex Alderton, Roberto Amato, Sonia Goncalves, Ewan Harrison, David K. Jackson, Ian Johnston, Dominic Kwiatkowski, Cordelia Langford, John Sillitoe on behalf of the Wellcome Sanger Institute COVID-19 Surveillance Team
EPI_ISL_879897, EPI_ISL_879898, EPI_ISL_879901, EPI_ISL_879903	Lighthouse Lab in Alderley Park	Wellcome Sanger Institute for the COVID-19 Genomics UK (COG-UK) Consortium	Jacquelyn Wynn, Mairead Hyland, The Lighthouse Lab in Alderley Park and Alex Alderton, Roberto Amato, Sonia Goncalves, Ewan Harrison, David K. Jackson, Ian Johnston, Dominic Kwiatkowski, Cordelia Langford, John Sillitoe on behalf of the Wellcome Sanger Institute COVID-19 Surveillance Team
EPI_ISL_879904	Lighthouse Lab in Cambridge	Wellcome Sanger Institute for the COVID-19 Genomics UK	Rob Howes, The Lighthouse Lab in Cambridge and Alex Alderton, Roberto Amato, Sonia Goncalves, Ewan Harrison, David K. Jackson, Ian Johnston,

		(COG-UK) Consortium	Dominic Kwiatkowski, Cordelia Langford, John Sillitoe on behalf of the Wellcome Sanger Institute COVID-19 Surveillance Team
EPI_ISL_879905, EPI_ISL_879906, EPI_ISL_879907	Lighthouse Lab in Alderley Park	Wellcome Sanger Institute for the COVID-19 Genomics UK (COG-UK) Consortium	Jacquelyn Wynn, Mairead Hyland, The Lighthouse Lab in Alderley Park and Alex Alderton, Roberto Amato, Sonia Goncalves, Ewan Harrison, David K. Jackson, Ian Johnston, Dominic Kwiatkowski, Cordelia Langford, John Sillitoe on behalf of the Wellcome Sanger Institute COVID-19 Surveillance Team
EPI_ISL_879908, EPI_ISL_879909, EPI_ISL_879911	Lighthouse Lab in Cambridge	Wellcome Sanger Institute for the COVID-19 Genomics UK (COG-UK) Consortium	Rob Howes, The Lighthouse Lab in Cambridge and Alex Alderton, Roberto Amato, Sonia Goncalves, Ewan Harrison, David K. Jackson, Ian Johnston, Dominic Kwiatkowski, Cordelia Langford, John Sillitoe on behalf of the Wellcome Sanger Institute COVID-19 Surveillance Team
EPI_ISL_879912, EPI_ISL_879913, EPI_ISL_879914, EPI_ISL_879915, EPI_ISL_879916, EPI_ISL_879918, EPI_ISL_879919	Lighthouse Lab in Alderley Park	Wellcome Sanger Institute for the COVID-19 Genomics UK (COG-UK) Consortium	Jacquelyn Wynn, Mairead Hyland, The Lighthouse Lab in Alderley Park and Alex Alderton, Roberto Amato, Sonia Goncalves, Ewan Harrison, David K. Jackson, Ian Johnston, Dominic Kwiatkowski, Cordelia Langford, John Sillitoe on behalf of the Wellcome Sanger Institute COVID-19 Surveillance Team
EPI_ISL_879920, EPI_ISL_879921	Lighthouse Lab in Cambridge	Wellcome Sanger Institute for the COVID-19 Genomics UK (COG-UK) Consortium	Rob Howes, The Lighthouse Lab in Cambridge and Alex Alderton, Roberto Amato, Sonia Goncalves, Ewan Harrison, David K. Jackson, Ian Johnston, Dominic Kwiatkowski, Cordelia Langford, John Sillitoe on behalf of the Wellcome Sanger Institute COVID-19 Surveillance Team
EPI_ISL_879922, EPI_ISL_879923, EPI_ISL_879925, EPI_ISL_879927	Lighthouse Lab in Alderley Park	Wellcome Sanger Institute for the COVID-19 Genomics UK (COG-UK) Consortium	Jacquelyn Wynn, Mairead Hyland, The Lighthouse Lab in Alderley Park and Alex Alderton, Roberto Amato, Sonia Goncalves, Ewan Harrison, David K. Jackson, Ian Johnston, Dominic Kwiatkowski, Cordelia Langford, John Sillitoe on behalf of the Wellcome Sanger Institute COVID-19 Surveillance Team
EPI_ISL_879928, EPI_ISL_879930	Lighthouse Lab in Cambridge	Wellcome Sanger Institute for the COVID-19 Genomics UK (COG-UK) Consortium	Rob Howes, The Lighthouse Lab in Cambridge and Alex Alderton, Roberto Amato, Sonia Goncalves, Ewan Harrison, David K. Jackson, Ian Johnston, Dominic Kwiatkowski, Cordelia Langford, John Sillitoe on behalf of the Wellcome Sanger Institute COVID-19 Surveillance Team
EPI_ISL_879931, EPI_ISL_879933	Lighthouse Lab in Alderley Park	Wellcome Sanger Institute for the COVID-19 Genomics UK (COG-UK) Consortium	Jacquelyn Wynn, Mairead Hyland, The Lighthouse Lab in Alderley Park and Alex Alderton, Roberto Amato, Sonia Goncalves, Ewan Harrison, David K. Jackson, Ian Johnston, Dominic Kwiatkowski, Cordelia Langford, John Sillitoe on behalf of the Wellcome Sanger Institute COVID-19 Surveillance Team
EPI_ISL_879935	Lighthouse Lab in Cambridge	Wellcome Sanger Institute for the COVID-19 Genomics UK (COG-UK) Consortium	Rob Howes, The Lighthouse Lab in Cambridge and Alex Alderton, Roberto Amato, Sonia Goncalves, Ewan Harrison, David K. Jackson, Ian Johnston, Dominic Kwiatkowski, Cordelia Langford, John Sillitoe on behalf of the Wellcome Sanger Institute COVID-19 Surveillance Team
EPI_ISL_879936, EPI_ISL_879938	Lighthouse Lab in Alderley Park	Wellcome Sanger Institute for the COVID-19 Genomics UK (COG-UK) Consortium	Jacquelyn Wynn, Mairead Hyland, The Lighthouse Lab in Alderley Park and Alex Alderton, Roberto Amato, Sonia Goncalves, Ewan Harrison, David K. Jackson, Ian Johnston, Dominic Kwiatkowski, Cordelia Langford, John Sillitoe on behalf of the Wellcome Sanger Institute COVID-19 Surveillance Team
EPI_ISL_879940, EPI_ISL_879941, EPI_ISL_879944, EPI_ISL_879946	Lighthouse Lab in Cambridge	Wellcome Sanger Institute for the COVID-19 Genomics UK (COG-UK) Consortium	Rob Howes, The Lighthouse Lab in Cambridge and Alex Alderton, Roberto Amato, Sonia Goncalves, Ewan Harrison, David K. Jackson, Ian Johnston, Dominic Kwiatkowski, Cordelia Langford, John Sillitoe on behalf of the Wellcome Sanger Institute COVID-19 Surveillance Team
EPI_ISL_879948, EPI_ISL_879949	Lighthouse Lab in Alderley Park	Wellcome Sanger Institute for the COVID-19 Genomics UK (COG-UK) Consortium	Jacquelyn Wynn, Mairead Hyland, The Lighthouse Lab in Alderley Park and Alex Alderton, Roberto Amato, Sonia Goncalves, Ewan Harrison, David K. Jackson, Ian Johnston, Dominic Kwiatkowski, Cordelia Langford, John Sillitoe on behalf of the Wellcome Sanger Institute COVID-19 Surveillance Team
EPI_ISL_879954	Lighthouse Lab in Cambridge	Wellcome Sanger Institute for the COVID-19 Genomics UK (COG-UK) Consortium	Rob Howes, The Lighthouse Lab in Cambridge and Alex Alderton, Roberto Amato, Sonia Goncalves, Ewan Harrison, David K. Jackson, Ian Johnston, Dominic Kwiatkowski, Cordelia Langford, John Sillitoe on behalf of the Wellcome Sanger Institute COVID-19 Surveillance Team
EPI_ISL_879955, EPI_ISL_879957	Lighthouse Lab in Alderley Park	Wellcome Sanger Institute for the COVID-19 Genomics UK (COG-UK) Consortium	Jacquelyn Wynn, Mairead Hyland, The Lighthouse Lab in Alderley Park and Alex Alderton, Roberto Amato, Sonia Goncalves, Ewan Harrison, David K. Jackson, Ian Johnston, Dominic Kwiatkowski, Cordelia Langford, John Sillitoe on behalf of the Wellcome Sanger Institute COVID-19 Surveillance Team
EPI_ISL_879958, EPI_ISL_879960, EPI_ISL_879962, EPI_ISL_879963	Lighthouse Lab in Cambridge	Wellcome Sanger Institute for the COVID-19 Genomics UK (COG-UK) Consortium	Rob Howes, The Lighthouse Lab in Cambridge and Alex Alderton, Roberto Amato, Sonia Goncalves, Ewan Harrison, David K. Jackson, Ian Johnston, Dominic Kwiatkowski, Cordelia Langford, John Sillitoe on behalf of the Wellcome Sanger Institute COVID-19 Surveillance Team
EPI_ISL_879965, EPI_ISL_879966, EPI_ISL_879968, EPI_ISL_879970, EPI_ISL_879971	Lighthouse Lab in Alderley Park	Wellcome Sanger Institute for the COVID-19 Genomics UK (COG-UK) Consortium	Jacquelyn Wynn, Mairead Hyland, The Lighthouse Lab in Alderley Park and Alex Alderton, Roberto Amato, Sonia Goncalves, Ewan Harrison, David K. Jackson, Ian Johnston, Dominic Kwiatkowski, Cordelia Langford, John Sillitoe on behalf of the Wellcome Sanger Institute COVID-19 Surveillance Team
EPI_ISL_879973	Lighthouse Lab in Cambridge	Wellcome Sanger Institute for the COVID-19 Genomics UK (COG-UK) Consortium	Rob Howes, The Lighthouse Lab in Cambridge and Alex Alderton, Roberto Amato, Sonia Goncalves, Ewan Harrison, David K. Jackson, Ian Johnston, Dominic Kwiatkowski, Cordelia Langford, John Sillitoe on behalf of the Wellcome Sanger Institute COVID-19 Surveillance Team
EPI_ISL_879974, EPI_ISL_879976, EPI_ISL_879978, EPI_ISL_879979, EPI_ISL_879981, EPI_ISL_879984, EPI_ISL_879986, EPI_ISL_879988	Lighthouse Lab in Alderley Park	Wellcome Sanger Institute for the COVID-19 Genomics UK (COG-UK) Consortium	Jacquelyn Wynn, Mairead Hyland, The Lighthouse Lab in Alderley Park and Alex Alderton, Roberto Amato, Sonia Goncalves, Ewan Harrison, David K. Jackson, Ian Johnston, Dominic Kwiatkowski, Cordelia Langford, John Sillitoe on behalf of the Wellcome Sanger Institute COVID-19 Surveillance Team
EPI_ISL_879989	Lighthouse Lab in Cambridge	Wellcome Sanger Institute for the COVID-19 Genomics UK (COG-UK) Consortium	Rob Howes, The Lighthouse Lab in Cambridge and Alex Alderton, Roberto Amato, Sonia Goncalves, Ewan Harrison, David K. Jackson, Ian Johnston, Dominic Kwiatkowski, Cordelia Langford, John Sillitoe on behalf of the Wellcome Sanger Institute COVID-19 Surveillance Team
EPI_ISL_879991	Lighthouse Lab in Alderley Park	Wellcome Sanger Institute for the COVID-19 Genomics UK (COG-UK) Consortium	Jacquelyn Wynn, Mairead Hyland, The Lighthouse Lab in Alderley Park and Alex Alderton, Roberto Amato, Sonia Goncalves, Ewan Harrison, David K. Jackson, Ian Johnston, Dominic Kwiatkowski, Cordelia Langford, John Sillitoe on behalf of the Wellcome Sanger Institute COVID-19 Surveillance Team
EPI_ISL_879994, EPI_ISL_879996	Lighthouse Lab in Cambridge	Wellcome Sanger Institute for the COVID-19 Genomics UK (COG-UK) Consortium	Rob Howes, The Lighthouse Lab in Cambridge and Alex Alderton, Roberto Amato, Sonia Goncalves, Ewan Harrison, David K. Jackson, Ian Johnston, Dominic Kwiatkowski, Cordelia Langford, John Sillitoe on behalf of the Wellcome Sanger Institute COVID-19 Surveillance Team
EPI_ISL_879997, EPI_ISL_879999, EPI_ISL_880002	Lighthouse Lab in Alderley Park	Wellcome Sanger Institute for the COVID-19 Genomics UK (COG-UK) Consortium	Jacquelyn Wynn, Mairead Hyland, The Lighthouse Lab in Alderley Park and Alex Alderton, Roberto Amato, Sonia Goncalves, Ewan Harrison, David K. Jackson, Ian Johnston, Dominic Kwiatkowski, Cordelia Langford, John Sillitoe on behalf of the Wellcome Sanger Institute COVID-19 Surveillance Team
EPI_ISL_880004, EPI_ISL_880005	Lighthouse Lab in Cambridge	Wellcome Sanger Institute for the COVID-19 Genomics UK (COG-UK) Consortium	Rob Howes, The Lighthouse Lab in Cambridge and Alex Alderton, Roberto Amato, Sonia Goncalves, Ewan Harrison, David K. Jackson, Ian Johnston, Dominic Kwiatkowski, Cordelia Langford, John Sillitoe on behalf of the Wellcome Sanger Institute COVID-19 Surveillance Team
EPI_ISL_880007, EPI_ISL_880008, EPI_ISL_880010	Lighthouse Lab in Alderley Park	Wellcome Sanger Institute for the COVID-19 Genomics UK (COG-UK) Consortium	Jacquelyn Wynn, Mairead Hyland, The Lighthouse Lab in Alderley Park and Alex Alderton, Roberto Amato, Sonia Goncalves, Ewan Harrison, David K. Jackson, Ian Johnston, Dominic Kwiatkowski, Cordelia Langford, John Sillitoe on behalf of the Wellcome Sanger Institute COVID-19 Surveillance Team
EPI_ISL_880012, EPI_ISL_880013, EPI_ISL_880015	Lighthouse Lab in Cambridge	Wellcome Sanger Institute for the COVID-19 Genomics UK (COG-UK) Consortium	Rob Howes, The Lighthouse Lab in Cambridge and Alex Alderton, Roberto Amato, Sonia Goncalves, Ewan Harrison, David K. Jackson, Ian Johnston, Dominic Kwiatkowski, Cordelia Langford, John Sillitoe on behalf of the Wellcome Sanger Institute COVID-19 Surveillance Team
EPI_ISL_880016, EPI_ISL_880018	Lighthouse Lab in Alderley Park	Wellcome Sanger Institute for the COVID-19 Genomics UK (COG-UK) Consortium	Jacquelyn Wynn, Mairead Hyland, The Lighthouse Lab in Alderley Park and Alex Alderton, Roberto Amato, Sonia Goncalves, Ewan Harrison, David K. Jackson, Ian Johnston, Dominic Kwiatkowski, Cordelia Langford, John Sillitoe on behalf of the Wellcome Sanger Institute COVID-19 Surveillance Team
EPI_ISL_880020	Lighthouse Lab in Cambridge	Wellcome Sanger Institute for the COVID-19 Genomics UK (COG-UK) Consortium	Rob Howes, The Lighthouse Lab in Cambridge and Alex Alderton, Roberto Amato, Sonia Goncalves, Ewan Harrison, David K. Jackson, Ian Johnston, Dominic Kwiatkowski, Cordelia Langford, John Sillitoe on behalf of the Wellcome Sanger Institute COVID-19 Surveillance Team
EPI_ISL_880021, EPI_ISL_880023	Lighthouse Lab in Alderley Park	Wellcome Sanger Institute for the COVID-19 Genomics UK (COG-UK) Consortium	Jacquelyn Wynn, Mairead Hyland, The Lighthouse Lab in Alderley Park and Alex Alderton, Roberto Amato, Sonia Goncalves, Ewan Harrison, David K. Jackson, Ian Johnston, Dominic Kwiatkowski, Cordelia Langford, John Sillitoe on behalf of the Wellcome Sanger Institute COVID-19 Surveillance Team
EPI_ISL_880025	Lighthouse Lab in Cambridge	Wellcome Sanger Institute for the COVID-19 Genomics UK (COG-UK) Consortium	Rob Howes, The Lighthouse Lab in Cambridge and Alex Alderton, Roberto Amato, Sonia Goncalves, Ewan Harrison, David K. Jackson, Ian Johnston, Dominic Kwiatkowski, Cordelia Langford, John Sillitoe on behalf of the Wellcome Sanger Institute COVID-19 Surveillance Team
EPI_ISL_880028, EPI_ISL_880030, EPI_ISL_880031	Lighthouse Lab in Alderley Park	Wellcome Sanger Institute for the COVID-19 Genomics UK (COG-UK) Consortium	Jacquelyn Wynn, Mairead Hyland, The Lighthouse Lab in Alderley Park and Alex Alderton, Roberto Amato, Sonia Goncalves, Ewan Harrison, David K. Jackson, Ian Johnston, Dominic Kwiatkowski, Cordelia Langford, John Sillitoe on behalf of the Wellcome Sanger Institute COVID-19 Surveillance Team
EPI_ISL_880033, EPI_ISL_880035	Lighthouse Lab in Cambridge	Wellcome Sanger Institute for the COVID-19 Genomics UK (COG-UK) Consortium	Rob Howes, The Lighthouse Lab in Cambridge and Alex Alderton, Roberto Amato, Sonia Goncalves, Ewan Harrison, David K. Jackson, Ian Johnston, Dominic Kwiatkowski, Cordelia Langford, John Sillitoe on behalf of the Wellcome Sanger Institute COVID-19 Surveillance Team
EPI_ISL_880036	Lighthouse Lab in Alderley Park	Wellcome Sanger Institute for the COVID-19 Genomics UK (COG-UK) Consortium	Jacquelyn Wynn, Mairead Hyland, The Lighthouse Lab in Alderley Park and Alex Alderton, Roberto Amato, Sonia Goncalves, Ewan Harrison, David K. Jackson, Ian Johnston, Dominic Kwiatkowski, Cordelia Langford, John Sillitoe on behalf of the Wellcome Sanger Institute COVID-19 Surveillance Team
EPI_ISL_880039	Lighthouse Lab in Cambridge	Wellcome Sanger Institute for the COVID-19 Genomics UK (COG-UK) Consortium	Rob Howes, The Lighthouse Lab in Cambridge and Alex Alderton, Roberto Amato, Sonia Goncalves, Ewan Harrison, David K. Jackson, Ian Johnston, Dominic Kwiatkowski, Cordelia Langford, John Sillitoe on behalf of the Wellcome Sanger Institute COVID-19 Surveillance Team
EPI_ISL_880041, EPI_ISL_880043	Lighthouse Lab in Alderley Park	Wellcome Sanger Institute for the COVID-19 Genomics UK	Jacquelyn Wynn, Mairead Hyland, The Lighthouse Lab in Alderley Park and Alex Alderton, Roberto Amato, Sonia Goncalves, Ewan Harrison, David K.

		(COG-UK) Consortium	Jackson, Ian Johnston, Dominic Kwiatkowski, Cordelia Langford, John Sillitoe on behalf of the Wellcome Sanger Institute COVID-19 Surveillance Team
EPI_ISL_880044, EPI_ISL_880046, EPI_ISL_880047, EPI_ISL_880049	Lighthouse Lab in Cambridge	Wellcome Sanger Institute for the COVID-19 Genomics UK (COG-UK) Consortium	Rob Howes, The Lighthouse Lab in Cambridge and Alex Alderton, Roberto Amato, Sonia Goncalves, Ewan Harrison, David K. Jackson, Ian Johnston, Dominic Kwiatkowski, Cordelia Langford, John Sillitoe on behalf of the Wellcome Sanger Institute COVID-19 Surveillance Team
EPI_ISL_880052	Lighthouse Lab in Alderley Park	Wellcome Sanger Institute for the COVID-19 Genomics UK (COG-UK) Consortium	Jacquelyn Wynn, Mairead Hyland, The Lighthouse Lab in Alderley Park and Alex Alderton, Roberto Amato, Sonia Goncalves, Ewan Harrison, David K. Jackson, Ian Johnston, Dominic Kwiatkowski, Cordelia Langford, John Sillitoe on behalf of the Wellcome Sanger Institute COVID-19 Surveillance Team
EPI_ISL_880054	Lighthouse Lab in Cambridge	Wellcome Sanger Institute for the COVID-19 Genomics UK (COG-UK) Consortium	Rob Howes, The Lighthouse Lab in Cambridge and Alex Alderton, Roberto Amato, Sonia Goncalves, Ewan Harrison, David K. Jackson, Ian Johnston, Dominic Kwiatkowski, Cordelia Langford, John Sillitoe on behalf of the Wellcome Sanger Institute COVID-19 Surveillance Team
EPI_ISL_880055, EPI_ISL_880057	Lighthouse Lab in Alderley Park	Wellcome Sanger Institute for the COVID-19 Genomics UK (COG-UK) Consortium	Jacquelyn Wynn, Mairead Hyland, The Lighthouse Lab in Alderley Park and Alex Alderton, Roberto Amato, Sonia Goncalves, Ewan Harrison, David K. Jackson, Ian Johnston, Dominic Kwiatkowski, Cordelia Langford, John Sillitoe on behalf of the Wellcome Sanger Institute COVID-19 Surveillance Team
EPI_ISL_880059, EPI_ISL_880060	Lighthouse Lab in Cambridge	Wellcome Sanger Institute for the COVID-19 Genomics UK (COG-UK) Consortium	Rob Howes, The Lighthouse Lab in Cambridge and Alex Alderton, Roberto Amato, Sonia Goncalves, Ewan Harrison, David K. Jackson, Ian Johnston, Dominic Kwiatkowski, Cordelia Langford, John Sillitoe on behalf of the Wellcome Sanger Institute COVID-19 Surveillance Team
EPI_ISL_880062, EPI_ISL_880064	Lighthouse Lab in Alderley Park	Wellcome Sanger Institute for the COVID-19 Genomics UK (COG-UK) Consortium	Jacquelyn Wynn, Mairead Hyland, The Lighthouse Lab in Alderley Park and Alex Alderton, Roberto Amato, Sonia Goncalves, Ewan Harrison, David K. Jackson, Ian Johnston, Dominic Kwiatkowski, Cordelia Langford, John Sillitoe on behalf of the Wellcome Sanger Institute COVID-19 Surveillance Team
EPI_ISL_880065	Lighthouse Lab in Cambridge	Wellcome Sanger Institute for the COVID-19 Genomics UK (COG-UK) Consortium	Rob Howes, The Lighthouse Lab in Cambridge and Alex Alderton, Roberto Amato, Sonia Goncalves, Ewan Harrison, David K. Jackson, Ian Johnston, Dominic Kwiatkowski, Cordelia Langford, John Sillitoe on behalf of the Wellcome Sanger Institute COVID-19 Surveillance Team
EPI_ISL_880067	Lighthouse Lab in Alderley Park	Wellcome Sanger Institute for the COVID-19 Genomics UK (COG-UK) Consortium	Jacquelyn Wynn, Mairead Hyland, The Lighthouse Lab in Alderley Park and Alex Alderton, Roberto Amato, Sonia Goncalves, Ewan Harrison, David K. Jackson, Ian Johnston, Dominic Kwiatkowski, Cordelia Langford, John Sillitoe on behalf of the Wellcome Sanger Institute COVID-19 Surveillance Team
EPI_ISL_880068, EPI_ISL_880070	Lighthouse Lab in Cambridge	Wellcome Sanger Institute for the COVID-19 Genomics UK (COG-UK) Consortium	Rob Howes, The Lighthouse Lab in Cambridge and Alex Alderton, Roberto Amato, Sonia Goncalves, Ewan Harrison, David K. Jackson, Ian Johnston, Dominic Kwiatkowski, Cordelia Langford, John Sillitoe on behalf of the Wellcome Sanger Institute COVID-19 Surveillance Team
EPI_ISL_880072	Lighthouse Lab in Alderley Park	Wellcome Sanger Institute for the COVID-19 Genomics UK (COG-UK) Consortium	Jacquelyn Wynn, Mairead Hyland, The Lighthouse Lab in Alderley Park and Alex Alderton, Roberto Amato, Sonia Goncalves, Ewan Harrison, David K. Jackson, Ian Johnston, Dominic Kwiatkowski, Cordelia Langford, John Sillitoe on behalf of the Wellcome Sanger Institute COVID-19 Surveillance Team
EPI_ISL_880075, EPI_ISL_880076, EPI_ISL_880078	Lighthouse Lab in Cambridge	Wellcome Sanger Institute for the COVID-19 Genomics UK (COG-UK) Consortium	Rob Howes, The Lighthouse Lab in Cambridge and Alex Alderton, Roberto Amato, Sonia Goncalves, Ewan Harrison, David K. Jackson, Ian Johnston, Dominic Kwiatkowski, Cordelia Langford, John Sillitoe on behalf of the Wellcome Sanger Institute COVID-19 Surveillance Team
EPI_ISL_880083, EPI_ISL_880084, EPI_ISL_880086, EPI_ISL_880088, EPI_ISL_880089, EPI_ISL_880093, EPI_ISL_880094, EPI_ISL_880096, EPI_ISL_880097, EPI_ISL_880099, EPI_ISL_880102, EPI_ISL_880104, EPI_ISL_880105, EPI_ISL_880107, EPI_ISL_880108, EPI_ISL_880110, EPI_ISL_880115, EPI_ISL_880116, EPI_ISL_880118, EPI_ISL_880121, EPI_ISL_880127, EPI_ISL_880129, EPI_ISL_880131, EPI_ISL_880132, EPI_ISL_880134, EPI_ISL_880137, EPI_ISL_880139, EPI_ISL_880210, EPI_ISL_880211, EPI_ISL_880213, EPI_ISL_880214, EPI_ISL_880217, EPI_ISL_880219, EPI_ISL_880221, EPI_ISL_880224, EPI_ISL_880226, EPI_ISL_880227, EPI_ISL_880229, EPI_ISL_880230, EPI_ISL_880232, EPI_ISL_880234, EPI_ISL_880235, EPI_ISL_880237, EPI_ISL_880238, EPI_ISL_880240, EPI_ISL_880242, EPI_ISL_880243, EPI_ISL_880245, EPI_ISL_880247, EPI_ISL_880248, EPI_ISL_880250, EPI_ISL_880252, EPI_ISL_880254, EPI_ISL_880255, EPI_ISL_880257, EPI_ISL_880262, EPI_ISL_880267, EPI_ISL_880269, EPI_ISL_880270, EPI_ISL_880272, EPI_ISL_880273, EPI_ISL_880275, EPI_ISL_880276, EPI_ISL_880278, EPI_ISL_880280, EPI_ISL_880281, EPI_ISL_880283, EPI_ISL_880285, EPI_ISL_880289, EPI_ISL_880293, EPI_ISL_880295, EPI_ISL_880296, EPI_ISL_880299, EPI_ISL_880302, EPI_ISL_880304, EPI_ISL_880307, EPI_ISL_880309, EPI_ISL_880311, EPI_ISL_880312, EPI_ISL_880314, EPI_ISL_880315, EPI_ISL_880317, EPI_ISL_880318, EPI_ISL_880320, EPI_ISL_880322, EPI_ISL_880323, EPI_ISL_880325, EPI_ISL_880327, EPI_ISL_880328, EPI_ISL_880330, EPI_ISL_880331, EPI_ISL_880333, EPI_ISL_880334, EPI_ISL_880336, EPI_ISL_880338, EPI_ISL_880339, EPI_ISL_880341, EPI_ISL_880342, EPI_ISL_880344, EPI_ISL_880345, EPI_ISL_880346, EPI_ISL_880347, EPI_ISL_880349, EPI_ISL_880350, EPI_ISL_880351, EPI_ISL_880353, EPI_ISL_880354, EPI_ISL_880355, EPI_ISL_880357, EPI_ISL_880358, EPI_ISL_880359, EPI_ISL_880360, EPI_ISL_880362, EPI_ISL_880363, EPI_ISL_880364, EPI_ISL_880365, EPI_ISL_880367, EPI_ISL_880368, EPI_ISL_880369, EPI_ISL_880370, EPI_ISL_880371, EPI_ISL_880372, EPI_ISL_880373, EPI_ISL_880374, EPI_ISL_880377, EPI_ISL_880378, EPI_ISL_880380, EPI_ISL_880381, EPI_ISL_880382, EPI_ISL_880383, EPI_ISL_880384, EPI_ISL_880385, EPI_ISL_880386, EPI_ISL_880388, EPI_ISL_880389, EPI_ISL_880391, EPI_ISL_880393, EPI_ISL_880394, EPI_ISL_880395, EPI_ISL_880396, EPI_ISL_880397, EPI_ISL_880398, EPI_ISL_880399, EPI_ISL_880401, EPI_ISL_880403, EPI_ISL_880404, EPI_ISL_880405, EPI_ISL_880406, EPI_ISL_880407, EPI_ISL_880408, EPI_ISL_880409, EPI_ISL_880410, EPI_ISL_880411, EPI_ISL_880412, EPI_ISL_880413, EPI_ISL_880414, EPI_ISL_880415, EPI_ISL_880416, EPI_ISL_880419, EPI_ISL_880420, EPI_ISL_880421, EPI_ISL_880422, EPI_ISL_880423, EPI_ISL_880424, EPI_ISL_880425, EPI_ISL_880428, EPI_ISL_880429, EPI_ISL_880430, EPI_ISL_880431, EPI_ISL_880432, EPI_ISL_880434, EPI_ISL_880437, EPI_ISL_880438, EPI_ISL_880439, EPI_ISL_880440, EPI_ISL_880441, EPI_ISL_880442, EPI_ISL_880444, EPI_ISL_880445, EPI_ISL_880447, EPI_ISL_880448, EPI_ISL_880449, EPI_ISL_880450, EPI_ISL_880451, EPI_ISL_880452, EPI_ISL_880453, EPI_ISL_880454, EPI_ISL_880455, EPI_ISL_880457, EPI_ISL_880459, EPI_ISL_880460, EPI_ISL_880461, EPI_ISL_880462, EPI_ISL_880463, EPI_ISL_880465, EPI_ISL_880466, EPI_ISL_880468, EPI_ISL_880469, EPI_ISL_880470, EPI_ISL_880471, EPI_ISL_880472, EPI_ISL_880473, EPI_ISL_880474, EPI_ISL_880476, EPI_ISL_880478, EPI_ISL_880479, EPI_ISL_880480, EPI_ISL_880481, EPI_ISL_880483, EPI_ISL_880484, EPI_ISL_880485, EPI_ISL_880486, EPI_ISL_880487, EPI_ISL_880488, EPI_ISL_880489, EPI_ISL_880490, EPI_ISL_880491, EPI_ISL_880492, EPI_ISL_880494, EPI_ISL_880495, EPI_ISL_880496, EPI_ISL_880497, EPI_ISL_880499, EPI_ISL_880500, EPI_ISL_880501, EPI_ISL_880503, EPI_ISL_880504, EPI_ISL_880505, EPI_ISL_880506, EPI_ISL_880507, EPI_ISL_880510, EPI_ISL_880511, EPI_ISL_880512, EPI_ISL_880513, EPI_ISL_880514, EPI_ISL_880515, EPI_ISL_880516, EPI_ISL_880517, EPI_ISL_880520, EPI_ISL_880521, EPI_ISL_880522, EPI_ISL_880523, EPI_ISL_880524, EPI_ISL_880525, EPI_ISL_880526, EPI_ISL_880527, EPI_ISL_880528, EPI_ISL_880530, EPI_ISL_880532, EPI_ISL_880533, EPI_ISL_880534, EPI_ISL_880535, EPI_ISL_880536, EPI_ISL_880537, EPI_ISL_880538, EPI_ISL_880539, EPI_ISL_880540, EPI_ISL_880541, EPI_ISL_880542, EPI_ISL_880543, EPI_ISL_880544, EPI_ISL_880545, EPI_ISL_880546, EPI_ISL_880547, EPI_ISL_880548, EPI_ISL_880549, EPI_ISL_880550, EPI_ISL_880551, EPI_ISL_880552, EPI_ISL_880553, EPI_ISL_880554, EPI_ISL_880555, EPI_ISL_880556, EPI_ISL_880557, EPI_ISL_880558, EPI_ISL_880559, EPI_ISL_880560, EPI_ISL_880561, EPI_ISL_880562, EPI_ISL_880563, EPI_ISL_880564, EPI_ISL_880565, EPI_ISL_880566, EPI_ISL_880567, EPI_ISL_880568, EPI_ISL_880569, EPI_ISL_880570, EPI_ISL_880571, EPI_ISL_880572, EPI_ISL_880573, EPI_ISL_880574, EPI_ISL_880576, EPI_ISL_880577, EPI_ISL_880578, EPI_ISL_880579, EPI_ISL_880580, EPI_ISL_880581, EPI_ISL_880582			
see above	Lighthouse Lab in Alderley Park	Wellcome Sanger Institute for the COVID-19 Genomics UK (COG-UK) Consortium	Jacquelyn Wynn, Mairead Hyland, The Lighthouse Lab in Alderley Park and Alex Alderton, Roberto Amato, Sonia Goncalves, Ewan Harrison, David K. Jackson, Ian Johnston, Dominic Kwiatkowski, Cordelia Langford, John Sillitoe on behalf of the Wellcome Sanger Institute COVID-19 Surveillance Team
EPI_ISL_880584, EPI_ISL_880585, EPI_ISL_880587, EPI_ISL_880588, EPI_ISL_880589, EPI_ISL_880592, EPI_ISL_880593, EPI_ISL_880594, EPI_ISL_880597, EPI_ISL_880598, EPI_ISL_880600, EPI_ISL_880603, EPI_ISL_880604, EPI_ISL_880605, EPI_ISL_880607, EPI_ISL_880612, EPI_ISL_880614, EPI_ISL_880617, EPI_ISL_880621, EPI_ISL_880622, EPI_ISL_880624, EPI_ISL_880625, EPI_ISL_880628, EPI_ISL_880630, EPI_ISL_880631, EPI_ISL_880632, EPI_ISL_880633, EPI_ISL_880634, EPI_ISL_880635, EPI_ISL_880636, EPI_ISL_880638, EPI_ISL_880640, EPI_ISL_880643, EPI_ISL_880644, EPI_ISL_880645, EPI_ISL_880646, EPI_ISL_880648, EPI_ISL_880649, EPI_ISL_880650, EPI_ISL_880652, EPI_ISL_880656, EPI_ISL_880658, EPI_ISL_880659, EPI_ISL_880661, EPI_ISL_880663, EPI_ISL_880664, EPI_ISL_880666, EPI_ISL_880670, EPI_ISL_880671, EPI_ISL_880676, EPI_ISL_880678, EPI_ISL_880680, EPI_ISL_880681, EPI_ISL_880683, EPI_ISL_880684, EPI_ISL_880685, EPI_ISL_880686, EPI_ISL_880687, EPI_ISL_880688, EPI_ISL_880689, EPI_ISL_880690, EPI_ISL_880691, EPI_ISL_880692, EPI_ISL_880693, EPI_ISL_880694, EPI_ISL_880696, EPI_ISL_880697, EPI_ISL_880698, EPI_ISL_880699, EPI_ISL_880700, EPI_ISL_880702, EPI_ISL_880703, EPI_ISL_880704, EPI_ISL_880707, EPI_ISL_880711, EPI_ISL_880712, EPI_ISL_880714, EPI_ISL_880715, EPI_ISL_880716, EPI_ISL_880719, EPI_ISL_880720, EPI_ISL_880723, EPI_ISL_880728, EPI_ISL_880730, EPI_ISL_880731, EPI_ISL_880732, EPI_ISL_880733, EPI_ISL_880734, EPI_ISL_880735, EPI_ISL_880741, EPI_ISL_880742, EPI_ISL_880743, EPI_ISL_880744, EPI_ISL_880747, EPI_ISL_880748, EPI_ISL_880749, EPI_ISL_880750, EPI_ISL_880754, EPI_ISL_880756, EPI_ISL_880757, EPI_ISL_880758, EPI_ISL_880760, EPI_ISL_880761, EPI_ISL_880764, EPI_ISL_880765, EPI_ISL_880767, EPI_ISL_880768, EPI_ISL_880770, EPI_ISL_880771, EPI_ISL_880772, EPI_ISL_880773, EPI_ISL_880777, EPI_ISL_880778, EPI_ISL_880780, EPI_ISL_880782, EPI_ISL_880783, EPI_ISL_880784, EPI_ISL_880787, EPI_ISL_880788, EPI_ISL_880790, EPI_ISL_880793, EPI_ISL_880794, EPI_ISL_880795, EPI_ISL_880796, EPI_ISL_880801, EPI_ISL_880802, EPI_ISL_880804, EPI_ISL_880805, EPI_ISL_880806, EPI_ISL_880807, EPI_ISL_880808, EPI_ISL_880810, EPI_ISL_880811, EPI_ISL_880814, EPI_ISL_880815, EPI_ISL_880816, EPI_ISL_880820, EPI_ISL_880821, EPI_ISL_880823, EPI_ISL_880825, EPI_ISL_880827, EPI_ISL_880828, EPI_ISL_880832, EPI_ISL_880834, EPI_ISL_880837, EPI_ISL_880842, EPI_ISL_880844, EPI_ISL_880845, EPI_ISL_880846, EPI_ISL_880848, EPI_ISL_880849, EPI_ISL_880850, EPI_ISL_880851, EPI_ISL_880852, EPI_ISL_880855, EPI_ISL_880859, EPI_ISL_880860, EPI_ISL_880861, EPI_ISL_880863, EPI_ISL_880864, EPI_ISL_880865, EPI_ISL_880867, EPI_ISL_880869, EPI_ISL_880871, EPI_ISL_880872, EPI_ISL_880873, EPI_ISL_880874, EPI_ISL_880875, EPI_ISL_880876, EPI_ISL_880877, EPI_ISL_880880, EPI_ISL_880881, EPI_ISL_880882, EPI_ISL_880885, EPI_ISL_880887, EPI_ISL_880888, EPI_ISL_880890, EPI_ISL_880891, EPI_ISL_880892, EPI_ISL_880893, EPI_ISL_880896, EPI_ISL_880897, EPI_ISL_880899, EPI_ISL_880902, EPI_ISL_880904, EPI_ISL_880905, EPI_ISL_880906, EPI_ISL_880907, EPI_ISL_880912, EPI_ISL_880913, EPI_ISL_880917, EPI_ISL_880918, EPI_ISL_880919, EPI_ISL_880920, EPI_ISL_880922, EPI_ISL_880923, EPI_ISL_880924, EPI_ISL_880926, EPI_ISL_880927, EPI_ISL_880928, EPI_ISL_880929, EPI_ISL_880930, EPI_ISL_880931, EPI_ISL_880932			
see above	Lighthouse Lab in Milton Keynes	Wellcome Sanger Institute for the COVID-19 Genomics UK (COG-UK) Consortium	The Lighthouse Lab in Milton Keynes and Alex Alderton, Roberto Amato, Sonia Goncalves, Ewan Harrison, David K. Jackson, Ian Johnston, Dominic Kwiatkowski, Cordelia Langford, John Sillitoe on behalf of the Wellcome Sanger Institute COVID-19 Surveillance Team
EPI_ISL_880936, EPI_ISL_880937, EPI_ISL_880938, EPI_ISL_880939, EPI_ISL_880940, EPI_ISL_880941, EPI_ISL_880942, EPI_ISL_880943, EPI_ISL_880944, EPI_ISL_880945, EPI_ISL_880946, EPI_ISL_880947, EPI_ISL_880948, EPI_ISL_880949, EPI_ISL_880950, EPI_ISL_880951, EPI_ISL_880952, EPI_ISL_880953, EPI_ISL_880954, EPI_ISL_880955, EPI_ISL_880956, EPI_ISL_880957, EPI_ISL_880958, EPI_ISL_880959, EPI_ISL_880960, EPI_ISL_880961, EPI_ISL_880962, EPI_ISL_880963, EPI_ISL_880964, EPI_ISL_880965, EPI_ISL_880966, EPI_ISL_880967, EPI_ISL_880968, EPI_ISL_880969, EPI_ISL_880970, EPI_ISL_880971, EPI_ISL_880972, EPI_ISL_880973, EPI_ISL_880974, EPI_ISL_880975, EPI_ISL_880976, EPI_ISL_880977, EPI_ISL_880978, EPI_ISL_880979, EPI_ISL_880980, EPI_ISL_880981, EPI_ISL_880982, EPI_ISL_880983, EPI_ISL_880984, EPI_ISL_880985, EPI_ISL_880986, EPI_ISL_880987, EPI_ISL_880988, EPI_ISL_880989, EPI_ISL_880990, EPI_ISL_880991, EPI_ISL_880992, EPI_ISL_880993, EPI_ISL_880994, EPI_ISL_880995, EPI_ISL_880996, EPI_ISL_880997, EPI_ISL_880998, EPI_ISL_880999, EPI_ISL_881000, EPI_ISL_881001, EPI_ISL_881002, EPI_ISL_881003, EPI_ISL_881004, EPI_ISL_881005, EPI_ISL_881006, EPI_ISL_881007, EPI_ISL_881008, EPI_ISL_881009, EPI_ISL_881010, EPI_ISL_881011, EPI_ISL_881012, EPI_ISL_881013, EPI_ISL_881014, EPI_ISL_881015, EPI_ISL_881016, EPI_ISL_881017, EPI_ISL_881018, EPI_ISL_881019, EPI_ISL_881020, EPI_ISL_881021, EPI_ISL_881022, EPI_ISL_881023, EPI_ISL_881024, EPI_ISL_881025, EPI_ISL_881026, EPI_ISL_881027, EPI_ISL_881028, EPI_ISL_881029, EPI_ISL_881030, EPI_ISL_881031, EPI_ISL_881032, EPI_ISL_881033, EPI_ISL_881034, EPI_ISL_881035, EPI_ISL_881036, EPI_ISL_881037, EPI_ISL_881038, EPI_ISL_881039, EPI_ISL_881040, EPI_ISL_881041, EPI_ISL_881042, EPI_ISL_881043, EPI_ISL_881044, EPI_ISL_881045, EPI_ISL_881046, EPI_ISL_881047, EPI_ISL_881048, EPI_ISL_881049, EPI_ISL_881050, EPI_ISL_881051, EPI_ISL_881052, EPI_ISL_881053, EPI_ISL_881054, EPI_ISL_881055, EPI_ISL_881056, EPI_ISL_881057, EPI_ISL_881058, EPI_ISL_881059, EPI_ISL_881060, EPI_ISL_881061, EPI_ISL_881062, EPI_ISL_881063, EPI_ISL_881064, EPI_ISL_881065, EPI_ISL_881066, EPI_ISL_881067, EPI_ISL_881068, EPI_ISL_881069, EPI_ISL_881070, EPI_ISL_881071, EPI_ISL_881072, EPI_ISL_881073, EPI_ISL_881074, EPI_ISL_881075, EPI_ISL_881076, EPI_ISL_881077, EPI_ISL_881078, EPI_ISL_881079, EPI_ISL_881080, EPI_ISL_881081, EPI_ISL_881082, EPI_ISL_881083, EPI_ISL_881084, EPI_ISL_881085, EPI_ISL_881086, EPI_ISL_881087, EPI_ISL_881088, EPI_ISL_881089, EPI_ISL_881090, EPI_ISL_881091, EPI_ISL_881092, EPI_ISL_881093, EPI_ISL_881094, EPI_ISL_881095, EPI_ISL_881096, EPI_ISL_881097, EPI_ISL_881098, EPI_ISL_881099, EPI_ISL_881100, EPI_ISL_881101, EPI_ISL_881102, EPI_ISL_881103, EPI_ISL_881104, EPI_ISL_881105, EPI_ISL_881106, EPI_ISL_881107, EPI_ISL_881108, EPI_ISL_881109, EPI_ISL_881110, EPI_ISL_881111, EPI_ISL_881112, EPI_ISL_881113, EPI_ISL_881114, EPI_ISL_881115, EPI_ISL_881116, EPI_ISL_881117, EPI_ISL_881118, EPI_ISL_881119, EPI_ISL_881120, EPI_ISL_881121, EPI_ISL_881122, EPI_ISL_881123, EPI_ISL_881124, EPI_ISL_881125, EPI_ISL_881126, EPI_ISL_881127, EPI_ISL_881128, EPI_ISL_881129, EPI_ISL_881130, EPI_ISL_881131, EPI_ISL_881132, EPI_ISL_881133, EPI_ISL_881134, EPI_ISL_881135, EPI_ISL_881136, EPI_ISL_881137, EPI_ISL_881138, EPI_ISL_881139, EPI_ISL_881140, EPI_ISL_881141, EPI_ISL_881142, EPI_ISL_881143, EPI_ISL_881144, EPI_ISL_881145, EPI_ISL_881146, EPI_ISL_881147, EPI_ISL_881148, EPI_ISL_881149, EPI_ISL_881150, EPI_ISL_881151, EPI_ISL_881152, EPI_ISL_881153, EPI_ISL_881154, EPI_ISL_881155, EPI_ISL_881156, EPI_ISL_881157, EPI_ISL_881158, EPI_ISL_881159, EPI_ISL_881160, EPI_ISL_881161, EPI_ISL_881162, EPI_ISL_881163, EPI_ISL_881164, EPI_ISL_881165, EPI_ISL_881166, EPI_ISL_881167, EPI_ISL_881168, EPI_ISL_881169, EPI_ISL_881170, EPI_ISL_881171, EPI_ISL_881172, EPI_ISL_881173, EPI_ISL_881174, EPI_ISL_881175, EPI_ISL_881176, EPI_ISL_881177, EPI_ISL_881178, EPI_ISL_881179, EPI_ISL_881180, EPI_ISL_881181, EPI_ISL_881182, EPI_ISL_881183, EPI_ISL_881184, EPI_ISL_881185, EPI_ISL_881186, EPI_ISL_881187, EPI_ISL_881188, EPI_ISL_881189, EPI_ISL_881190, EPI_ISL_881191, EPI_ISL_881192, EPI_ISL_881193, EPI_ISL_881194, EPI_ISL_881195, EPI_ISL_881196, EPI_ISL_881197, EPI_ISL_881198, EPI_ISL_881199, EPI_ISL_881200, EPI_ISL_881201, EPI_ISL_881202, EPI_ISL_881203, EPI_ISL_881204, EPI_ISL_881205, EPI_ISL_881206, EPI_ISL_881207, EPI_ISL_881208, EPI_ISL_881209, EPI_ISL_881210, EPI_ISL_881211, EPI_ISL_881212, EPI_ISL_881213, EPI_ISL_881214, EPI_ISL_881215, EPI_ISL_881216, EPI_ISL_881217, EPI_ISL_881218, EPI_ISL_881219, EPI_ISL_881220, EPI_ISL_881221, EPI_ISL_881222, EPI_ISL_881223, EPI_ISL_881224, EPI_ISL_881225, EPI_ISL_881226, EPI_ISL_881227, EPI_ISL_881228, EPI_ISL_881229, EPI_ISL_881230, EPI_ISL_881231, EPI_ISL_881232, EPI_ISL_881233, EPI_ISL_881234, EPI_ISL_881235, EPI_ISL_881236, EPI_ISL_881237, EPI_ISL_881238, EPI_ISL_881239, EPI_ISL_881240, EPI_ISL_881241, EPI_ISL_881242, EPI_ISL_881243, EPI_ISL_881244, EPI_ISL_881245, EPI_ISL_881246, EPI_ISL_881247, EPI_ISL_881248, EPI_ISL_881249, EPI_ISL_881250, EPI_ISL_881251, EPI_ISL_881252, EPI_ISL_881253, EPI_ISL_881254, EPI_ISL_881255, EPI_ISL_881256, EPI_ISL_881257, EPI_ISL_881258, EPI_ISL_881259, EPI_ISL_881260, EPI_ISL_881261, EPI_ISL_881262, EPI_ISL_881263, EPI_ISL_881264, EPI_ISL_881265, EPI_ISL_881266, EPI_ISL_881267, EPI_ISL_881268, EPI_ISL_881269, EPI_ISL_881270, EPI_ISL_881271, EPI_ISL_881272, EPI_ISL_881273, EPI_ISL_881274, EPI_ISL_881275, EPI_ISL_881276, EPI_ISL_881278, EPI_ISL_881279, EPI_ISL_881280, EPI_ISL_881282, EPI_ISL_881284, EPI_ISL_881285, EPI_ISL_881286, EPI_ISL_881287, EPI_ISL_881288, EPI_ISL_881292, EPI_ISL_881294, EPI_ISL_881295, EPI_ISL_881296, EPI_ISL_881297, EPI_ISL_881299, EPI_ISL_881300, EPI_ISL_881301, EPI_ISL_881302, EPI_ISL_881303, EPI_ISL_881304, EPI_ISL_881305, EPI_ISL_881308, EPI_ISL_881311, EPI_ISL_881312, EPI_ISL_881313, EPI_ISL_881314, EPI_ISL_881316, EPI_ISL_881317, EPI_ISL_881319, EPI_ISL_881320, EPI_ISL_881321, EPI_ISL_881322, EPI_ISL_881323, EPI_ISL_881324, EPI_ISL_881325, EPI_ISL_881327, EPI_ISL_881328, EPI_ISL_881329, EPI_ISL_881330, EPI_ISL_881331, EPI_ISL_881332, EPI_ISL_881333, EPI_ISL_881334, EPI_ISL_881335, EPI_ISL_881336, EPI_ISL_881337, EPI_ISL_881338, EPI_ISL_881339, EPI_ISL_881340, EPI_ISL_881342, EPI_ISL_881343, EPI_ISL_881344, EPI_ISL_881345, EPI_ISL_881346, EPI_ISL_881347, EPI_ISL_881348, EPI_ISL_881349, EPI_ISL_881350, EPI_ISL_881351, EPI_ISL_881352, EPI_ISL_881353, EPI_ISL_881354, EPI_ISL_881355, EPI_ISL_881356, EPI_ISL_881357, EPI_ISL_881358, EPI_ISL_881360, EPI_ISL_881361, EPI_ISL_881362, EPI_ISL_881363, EPI_ISL_881364, EPI_ISL_881365, EPI_ISL_881366,			

EPI_ISL_881367, EPI_ISL_881368, EPI_ISL_881369, EPI_ISL_881370, EPI_ISL_881371, EPI_ISL_881372, EPI_ISL_881373, EPI_ISL_881374, EPI_ISL_881375, EPI_ISL_881376, EPI_ISL_881377, EPI_ISL_881378, EPI_ISL_881379, EPI_ISL_881380, EPI_ISL_881381, EPI_ISL_881382, EPI_ISL_881383, EPI_ISL_881384, EPI_ISL_881385, EPI_ISL_881387, EPI_ISL_881389, EPI_ISL_881390, EPI_ISL_881391, EPI_ISL_881392, EPI_ISL_881393, EPI_ISL_881394, EPI_ISL_881395, EPI_ISL_881396, EPI_ISL_881397, EPI_ISL_881398, EPI_ISL_881399, EPI_ISL_881400, EPI_ISL_881401, EPI_ISL_881402, EPI_ISL_881403, EPI_ISL_881404, EPI_ISL_881405, EPI_ISL_881406, EPI_ISL_881407, EPI_ISL_881408, EPI_ISL_881409, EPI_ISL_881410, EPI_ISL_881411, EPI_ISL_881412, EPI_ISL_881413, EPI_ISL_881415, EPI_ISL_881416, EPI_ISL_881417, EPI_ISL_881418, EPI_ISL_881419, EPI_ISL_881420, EPI_ISL_881421, EPI_ISL_881422, EPI_ISL_881423, EPI_ISL_881424, EPI_ISL_881425, EPI_ISL_881426, EPI_ISL_881427, EPI_ISL_881428, EPI_ISL_881430, EPI_ISL_881431, EPI_ISL_881432, EPI_ISL_881433, EPI_ISL_881434, EPI_ISL_881435, EPI_ISL_881436, EPI_ISL_881437, EPI_ISL_881438, EPI_ISL_881439, EPI_ISL_881440, EPI_ISL_881441, EPI_ISL_881442, EPI_ISL_881443, EPI_ISL_881444, EPI_ISL_881445, EPI_ISL_881446, EPI_ISL_881447, EPI_ISL_881448, EPI_ISL_881449, EPI_ISL_881450, EPI_ISL_881451, EPI_ISL_881452, EPI_ISL_881453, EPI_ISL_881454, EPI_ISL_881456, EPI_ISL_881457, EPI_ISL_881458, EPI_ISL_881459, EPI_ISL_881460, EPI_ISL_881462, EPI_ISL_881463, EPI_ISL_881464, EPI_ISL_881465, EPI_ISL_881466, EPI_ISL_881467, EPI_ISL_881468, EPI_ISL_881469, EPI_ISL_881470, EPI_ISL_881471, EPI_ISL_881472, EPI_ISL_881473, EPI_ISL_881474, EPI_ISL_881475, EPI_ISL_881476, EPI_ISL_881477, EPI_ISL_881478, EPI_ISL_881479, EPI_ISL_881480, EPI_ISL_881481, EPI_ISL_881482, EPI_ISL_881483, EPI_ISL_881484, EPI_ISL_881485, EPI_ISL_881486, EPI_ISL_881488, EPI_ISL_881489, EPI_ISL_881490, EPI_ISL_881491, EPI_ISL_881492, EPI_ISL_881493, EPI_ISL_881496, EPI_ISL_881497, EPI_ISL_881498, EPI_ISL_881499, EPI_ISL_881500, EPI_ISL_881501, EPI_ISL_881502, EPI_ISL_881503, EPI_ISL_881504, EPI_ISL_881505, EPI_ISL_881506, EPI_ISL_881507, EPI_ISL_881508, EPI_ISL_881510, EPI_ISL_881511, EPI_ISL_881512, EPI_ISL_881513, EPI_ISL_881514, EPI_ISL_881515, EPI_ISL_881516, EPI_ISL_881517, EPI_ISL_881519, EPI_ISL_881520, EPI_ISL_881523, EPI_ISL_881524, EPI_ISL_881525, EPI_ISL_881526, EPI_ISL_881527, EPI_ISL_881528, EPI_ISL_881529, EPI_ISL_881530, EPI_ISL_881532, EPI_ISL_881533, EPI_ISL_881534, EPI_ISL_881535, EPI_ISL_881536, EPI_ISL_881537, EPI_ISL_881538, EPI_ISL_881539, EPI_ISL_881541, EPI_ISL_881542, EPI_ISL_881544, EPI_ISL_881545, EPI_ISL_881547, EPI_ISL_881548, EPI_ISL_881549, EPI_ISL_881551, EPI_ISL_881552, EPI_ISL_881553, EPI_ISL_881554, EPI_ISL_881555, EPI_ISL_881556, EPI_ISL_881557, EPI_ISL_881558, EPI_ISL_881559, EPI_ISL_881560, EPI_ISL_881561, EPI_ISL_881562, EPI_ISL_881563, EPI_ISL_881564, EPI_ISL_881565, EPI_ISL_881566, EPI_ISL_881568, EPI_ISL_881569, EPI_ISL_881572, EPI_ISL_881573, EPI_ISL_881574, EPI_ISL_881575, EPI_ISL_881577, EPI_ISL_881578, EPI_ISL_881579, EPI_ISL_881581, EPI_ISL_881583, EPI_ISL_881584, EPI_ISL_881585, EPI_ISL_881587, EPI_ISL_881589, EPI_ISL_881590, EPI_ISL_881591, EPI_ISL_881592, EPI_ISL_881593, EPI_ISL_881594, EPI_ISL_881595, EPI_ISL_881596, EPI_ISL_881597, EPI_ISL_881599, EPI_ISL_881601, EPI_ISL_881602, EPI_ISL_881603, EPI_ISL_881604, EPI_ISL_881605, EPI_ISL_881606, EPI_ISL_881608, EPI_ISL_881609, EPI_ISL_881610, EPI_ISL_881611, EPI_ISL_881613, EPI_ISL_881614, EPI_ISL_881615, EPI_ISL_881616, EPI_ISL_881617			
see above	Lighthouse Lab in Alderley Park	Wellcome Sanger Institute for the COVID-19 Genomics UK (COG-UK) Consortium	Jacquelyn Wynn, Mairead Hyland, The Lighthouse Lab in Alderley Park and Alex Alderton, Roberto Amato, Sonia Goncalves, Ewan Harrison, David K. Jackson, Ian Johnston, Dominic Kwiatkowski, Cordelia Langford, John Sillitoe on behalf of the Wellcome Sanger Institute COVID-19 Surveillance Team
EPI_ISL_881620, EPI_ISL_881622	Lighthouse Lab in Cambridge	Wellcome Sanger Institute for the COVID-19 Genomics UK (COG-UK) Consortium	Rob Howes, The Lighthouse Lab in Cambridge and Alex Alderton, Roberto Amato, Sonia Goncalves, Ewan Harrison, David K. Jackson, Ian Johnston, Dominic Kwiatkowski, Cordelia Langford, John Sillitoe on behalf of the Wellcome Sanger Institute COVID-19 Surveillance Team
EPI_ISL_882300, EPI_ISL_882605, EPI_ISL_882606, EPI_ISL_882607, EPI_ISL_882608	Lighthouse Lab in Alderley Park	Wellcome Sanger Institute for the COVID-19 Genomics UK (COG-UK) Consortium	Jacquelyn Wynn, Mairead Hyland, The Lighthouse Lab in Alderley Park and Alex Alderton, Roberto Amato, Sonia Goncalves, Ewan Harrison, David K. Jackson, Ian Johnston, Dominic Kwiatkowski, Cordelia Langford, John Sillitoe on behalf of the Wellcome Sanger Institute COVID-19 Surveillance Team
EPI_ISL_882609	Lighthouse Lab in Glasgow	Wellcome Sanger Institute for the COVID-19 Genomics UK (COG-UK) Consortium	Harper VanSteenhouse, Yumi Kasai, David Gray, Carol Clugston, Anna Dominiczak and Alex Alderton, Roberto Amato, Sonia Goncalves, Ewan Harrison, David K. Jackson, Ian Johnston, Dominic Kwiatkowski, Cordelia Langford, John Sillitoe on behalf of the Wellcome Sanger Institute COVID-19 Surveillance Team
EPI_ISL_882670	Hospital Samaritano Paulista	Instituto Adolfo Lutz, Interdiciplinary Procedures Center, Strategic Laboratory	Claudio Tavares Sacchi, Claudia Regina Gonçalves, Erica Valessa Ramos Gomes, Karoline Rodrigues Campos
EPI_ISL_882671, EPI_ISL_882672	Hospital Municipal Dr. Guido Guida	Instituto Adolfo Lutz, Interdiciplinary Procedures Center, Strategic Laboratory	Claudio Tavares Sacchi, Claudia Regina Gonçalves, Erica Valessa Ramos Gomes, Karoline Rodrigues Campos
EPI_ISL_882776	Institute for Urban Disease Control and Prevention	COVID-19 Network Investigations (CONI) Alliance	Kamolthip Atsawawaranunt, Elizabeth Batty, Wasun Chantratita, Thanat Chookajorn, Stefan Fernandez, Angkana Huang, Anthony R. Jones, Khajohn Joonsalak, Chonticha Klungtong, Theerarat Kochakarn, Prayuth Kaewmalang, Amornmas Kongklieng, Namfon Kotanan, Krittikorn Kumpornsin, Duangkamon Loesbanluechai, Wudtichai Manasatienkij, Anek Mungaomklang, Bhakbhoom Panthan, Pukkaporn Parnwijitkul, Ekawat Pasomsub, Vichan Pawun, Kingkan Rakmanee, Insee Sensorn, Janjira Thaipadungpanit, Arporn Wangwiwatsin, Treewat Watthanachockchai
EPI_ISL_882922	Sant'Eugenio/CTO ASL Roma 2	INMI Lazzaro Spallanzani IRCCS	E Giombini, M Rueca, B Bartolini, O Butera, C.E.M Gruber, F Messina, F Bondanini, GC Cocciolillo, C Disegni, MR Capobianchi, A Di Caro
EPI_ISL_882933	Sant'Eugenio/CTO ASL Roma 2	INMI Lazzaro Spallanzani IRCCS	E Giombini, M. Rueca, B Bartolini, O Butera, C.E.M Gruber, F Bondanini, GC Cocciolillo, C Disegni, F Messina, MR Capobianchi, A Di Caro
EPI_ISL_882934	Sant'Eugenio/CTO ASL Roma 2	INMI Lazzaro Spallanzani IRCCS	B Bartolini, O Butera, C.E.M Gruber, M Rueca, F Messina, E Giombini, F Bondanini, GC Cocciolillo, C Disegni, MR Capobianchi, A Di Caro
EPI_ISL_882935	Sant'Eugenio/CTO ASL Roma 2	INMI Lazzaro Spallanzani IRCCS	F Messina, O Butera, E Giombini, M Rueca, B Bartolini, C.E.M Gruber, F Bondanini, C Disegni, GC Cocciolillo, MR Capobianchi, A Di Caro
EPI_ISL_882936	Sant'Eugenio/CTO ASL Roma 2	INMI Lazzaro Spallanzani IRCCS	E Giombini, M Rueca, B Bartolini, O Butera, C.E.M Gruber, F Messina, F Bondanini, GC Cocciolillo, C Disegni, MR Capobianchi, A Di Caro
EPI_ISL_882937	Azienda Ospedaliera San Giovanni Addolorata	INMI Lazzaro Spallanzani IRCCS	F Messina, O Butera, E Giombini, M Rueca, B Bartolini, C.E.M Gruber, M Gaudio, PM Placanica, MR Capobianchi, A Di Caro
EPI_ISL_882938	Azienda Ospedaliera San Giovanni Addolorata	INMI Lazzaro Spallanzani IRCCS	C.E.M Gruber, B Bartolini, E Giombini, M Rueca, O Butera, F Messina, PM Placanica, M Gaudio, A Di Caro, MR Capobianchi
EPI_ISL_882939	Azienda Ospedaliera San Giovanni Addolorata	INMI Lazzaro Spallanzani IRCCS	B Bartolini, O Butera, C.E.M Gruber, M Rueca, F Messina, E Giombini, M Gaudio, PM Placanica, MR Capobianchi, A Di Caro
EPI_ISL_882940	Ospedale San Camillo De Lellis Rieti	INMI Lazzaro Spallanzani IRCCS	M Rueca, E Giombini, C.E.M Gruber, B Bartolini, O Butera, S Venarubea, A De Luca, L Casertano, F Messina, A Di Caro, MR Capobianchi
EPI_ISL_882941	Ospedale San Camillo De Lellis Rieti	INMI Lazzaro Spallanzani IRCCS	C.E.M Gruber, B Bartolini, E Giombini, M Rueca, O Butera, F Messina, S Venarubea, L Casertano, A De Luca, A Di Caro, MR Capobianchi
EPI_ISL_882942	Ospedale San Camillo De Lellis Rieti	INMI Lazzaro Spallanzani IRCCS	M Rueca, E Giombini, C.E.M Gruber, B Bartolini, O Butera, F Messina, S Venarubea, A De Luca, L Casertano, A Di Caro, MR Capobianchi
EPI_ISL_882967, EPI_ISL_882968, EPI_ISL_882969, EPI_ISL_882970, EPI_ISL_882971, EPI_ISL_882972, EPI_ISL_882973, EPI_ISL_882974, EPI_ISL_882975, EPI_ISL_882976, EPI_ISL_882977, EPI_ISL_882978			
see above	UCLA Clinical Micro Lab	Los Angeles County PHL	P. Hemarajata et al.
EPI_ISL_882999, EPI_ISL_883000, EPI_ISL_883001, EPI_ISL_883002, EPI_ISL_883018, EPI_ISL_883019, EPI_ISL_883020, EPI_ISL_883021, EPI_ISL_883022, EPI_ISL_883023, EPI_ISL_883024, EPI_ISL_883027, EPI_ISL_883028, EPI_ISL_883029, EPI_ISL_883030, EPI_ISL_883031, EPI_ISL_883032			
see above	Maryland Public Health Laboratory	Maryland Public Health Laboratory	Maryland Department of Health Laboratories Administration
EPI_ISL_883309	DOHMH Central Harlem	New York City Public Health Laboratory	Jade Wang, et al.
EPI_ISL_883310	DOHMH Jamaica	New York City Public Health Laboratory	Jade Wang, et al.
EPI_ISL_883312, EPI_ISL_883313	DOHMH Corona	New York City Public Health Laboratory	Jade Wang, et al.
EPI_ISL_883314	DOHMH Jamaica	New York City Public Health Laboratory	Jade Wang, et al.
EPI_ISL_883315, EPI_ISL_883316	OCME Office Of Chief Medical Examiner	New York City Public Health Laboratory	Jade Wang, et al.
EPI_ISL_883318, EPI_ISL_883319, EPI_ISL_883320, EPI_ISL_883321, EPI_ISL_883322, EPI_ISL_883323	DOHMH Jamaica	New York City Public Health Laboratory	Jade Wang, et al.
EPI_ISL_883326	Department of Homeless Services	New York City Public Health Laboratory	Jade Wang, et al.
EPI_ISL_883327	DOHMH Central Harlem	New York City Public Health Laboratory	Jade Wang, et al.
EPI_ISL_883328	OCME Office Of Chief Medical Examiner	New York City Public Health Laboratory	Jade Wang, et al.
EPI_ISL_883329	DOHMH Central Harlem	New York City Public Health Laboratory	Jade Wang, et al.
EPI_ISL_883333, EPI_ISL_883334	DOHMH Riverside	New York City Public Health Laboratory	Jade Wang, et al.
EPI_ISL_883335	DOHMH Chelsea	New York City Public Health Laboratory	Jade Wang, et al.
EPI_ISL_883336	OCME Office Of Chief Medical Examiner	New York City Public Health Laboratory	Jade Wang, et al.
EPI_ISL_883338	DOHMH Morrisania	New York City Public Health Laboratory	Jade Wang, et al.
EPI_ISL_883339	DOHMH Chelsea	New York City Public Health Laboratory	Jade Wang, et al.
EPI_ISL_883340, EPI_ISL_883341, EPI_ISL_883342, EPI_ISL_883343, EPI_ISL_883346	DOHMH PHL	New York City Public Health Laboratory	Jade Wang, et al.
EPI_ISL_883348, EPI_ISL_883354, EPI_ISL_883355, EPI_ISL_883356	DOHMH Corona	New York City Public Health Laboratory	Jade Wang, et al.

EPI_ISL_883357, EPI_ISL_883358, EPI_ISL_883359, EPI_ISL_883360	DOHMH Crown Heights	New York City Public Health Laboratory	Jade Wang, et al.
EPI_ISL_883361, EPI_ISL_883362, EPI_ISL_883363	DOHMH PHL	New York City Public Health Laboratory	Jade Wang, et al.
EPI_ISL_883364	DOHMH Crown Heights	New York City Public Health Laboratory	Jade Wang, et al.
EPI_ISL_883365, EPI_ISL_883366, EPI_ISL_883367	DOHMH Morrisania	New York City Public Health Laboratory	Jade Wang, et al.
EPI_ISL_883368, EPI_ISL_883369	DOHMH Chelsea	New York City Public Health Laboratory	Jade Wang, et al.
EPI_ISL_883370	DOHMH Crown Heights	New York City Public Health Laboratory	Jade Wang, et al.
EPI_ISL_883371, EPI_ISL_883372	DOHMH Central Harlem	New York City Public Health Laboratory	Jade Wang, et al.
EPI_ISL_883373, EPI_ISL_883374, EPI_ISL_883375	DOHMH Jamaica	New York City Public Health Laboratory	Jade Wang, et al.
EPI_ISL_883376, EPI_ISL_883377, EPI_ISL_883378, EPI_ISL_883379	DOHMH Central Harlem	New York City Public Health Laboratory	Jade Wang, et al.
EPI_ISL_883380, EPI_ISL_883381, EPI_ISL_883382	DOHMH PHL	New York City Public Health Laboratory	Jade Wang, et al.
EPI_ISL_883390, EPI_ISL_883391, EPI_ISL_883392, EPI_ISL_883393, EPI_ISL_883394	OCME Office Of Chief Medical Examiner	New York City Public Health Laboratory	Jade Wang, et al.
EPI_ISL_883395, EPI_ISL_883396	Department of Homeless Services	New York City Public Health Laboratory	Jade Wang, et al.
EPI_ISL_883397, EPI_ISL_883398, EPI_ISL_883399, EPI_ISL_883400, EPI_ISL_883401, EPI_ISL_883402	OCME Office Of Chief Medical Examiner	New York City Public Health Laboratory	Jade Wang, et al.
EPI_ISL_883409	DOHMH Central Harlem	New York City Public Health Laboratory	Jade Wang, et al.
EPI_ISL_883423	DOHMH Jamaica	New York City Public Health Laboratory	Jade Wang, et al.
EPI_ISL_883425	BOSTON HEART DIAGNOSTICS CORP	Wadsworth Center, New York State Department of Health	Kirsten St. George, Daryl M. Lamson, Alexis Russel, Matthew Shudt, Melissa A Leisner, Jonathan Plitnick, Navjot Singh, John Kelly, Erasmus Schneider, Erica Lasek-Nesselquist
EPI_ISL_883436, EPI_ISL_883437, EPI_ISL_883438, EPI_ISL_883440, EPI_ISL_883441, EPI_ISL_883442, EPI_ISL_883443, EPI_ISL_883444, EPI_ISL_883473	ADIRONDACK MEDICAL CENTER	Wadsworth Center, New York State Department of Health	Kirsten St. George, Daryl M. Lamson, Alexis Russel, Matthew Shudt, Melissa A Leisner, Jonathan Plitnick, Navjot Singh, John Kelly, Erasmus Schneider, Erica Lasek-Nesselquist
EPI_ISL_884021, EPI_ISL_884022, EPI_ISL_884023, EPI_ISL_884024, EPI_ISL_884025, EPI_ISL_884026, EPI_ISL_884027, EPI_ISL_884028, EPI_ISL_884029, EPI_ISL_884030, EPI_ISL_884031			
see above	ALBANY MEDICAL CENTER HOSPITAL CLINICAL LABORATORIES	Wadsworth Center, New York State Department of Health	Kirsten St. George, Daryl M. Lamson, Alexis Russel, Matthew Shudt, Melissa A Leisner, Jonathan Plitnick, Navjot Singh, John Kelly, Erasmus Schneider, Erica Lasek-Nesselquist
EPI_ISL_884032, EPI_ISL_884033, EPI_ISL_884034, EPI_ISL_884035, EPI_ISL_884036, EPI_ISL_884037, EPI_ISL_884038, EPI_ISL_884039, EPI_ISL_884040, EPI_ISL_884041	Wadsworth Center, New York State Department of Health	Wadsworth Center, New York State Department of Health	Kirsten St. George, Daryl M. Lamson, Alexis Russel, Matthew Shudt, Melissa A Leisner, Jonathan Plitnick, Navjot Singh, John Kelly, Erasmus Schneider, Erica Lasek-Nesselquist
EPI_ISL_884042, EPI_ISL_884043, EPI_ISL_884044, EPI_ISL_884045, EPI_ISL_884046	ALBANY MEDICAL CENTER HOSPITAL CLINICAL LABORATORIES	Wadsworth Center, New York State Department of Health	Kirsten St. George, Daryl M. Lamson, Alexis Russel, Matthew Shudt, Melissa A Leisner, Jonathan Plitnick, Navjot Singh, John Kelly, Erasmus Schneider, Erica Lasek-Nesselquist
EPI_ISL_884047	Wadsworth Center, New York State Department of Health	Wadsworth Center, New York State Department of Health	Kirsten St. George, Daryl M. Lamson, Alexis Russel, Matthew Shudt, Melissa A Leisner, Jonathan Plitnick, Navjot Singh, John Kelly, Erasmus Schneider, Erica Lasek-Nesselquist
EPI_ISL_884048, EPI_ISL_884049, EPI_ISL_884050, EPI_ISL_884051, EPI_ISL_884052, EPI_ISL_884053	ALBANY MEDICAL CENTER HOSPITAL CLINICAL LABORATORIES	Wadsworth Center, New York State Department of Health	Kirsten St. George, Daryl M. Lamson, Alexis Russel, Matthew Shudt, Melissa A Leisner, Jonathan Plitnick, Navjot Singh, John Kelly, Erasmus Schneider, Erica Lasek-Nesselquist
EPI_ISL_884054	TEMPUS LABS INC	Wadsworth Center, New York State Department of Health	Kirsten St. George, Daryl M. Lamson, Alexis Russel, Matthew Shudt, Melissa A Leisner, Jonathan Plitnick, Navjot Singh, John Kelly, Erasmus Schneider, Erica Lasek-Nesselquist
EPI_ISL_884206, EPI_ISL_884212, EPI_ISL_884213	Wyoming Public Health Laboratory	Wyoming Public Health Laboratory	Noah Hull, Taylor Fearing, Lynette Gumbleton, Channing Weber, Ashley Norberg, Bailey Bowcutt, and Wanda Manley
EPI_ISL_884227, EPI_ISL_884228, EPI_ISL_884229, EPI_ISL_884230	Kansas Health and Environmental Lab	Kansas Health and Environmental Lab	Mike Grose, Paige Drury, Carissa Robertson, Ben Olsen, and Phil Adam
EPI_ISL_884256, EPI_ISL_884257, EPI_ISL_884258, EPI_ISL_884259	Maryland Public Health Laboratory	Maryland Public Health Laboratory	Maryland Department of Health Laboratories Administration
EPI_ISL_884590, EPI_ISL_884591, EPI_ISL_884592, EPI_ISL_884593, EPI_ISL_884594, EPI_ISL_884595, EPI_ISL_884596, EPI_ISL_884597, EPI_ISL_884598, EPI_ISL_884599, EPI_ISL_884600, EPI_ISL_884601, EPI_ISL_884602, EPI_ISL_884603, EPI_ISL_884604, EPI_ISL_884605, EPI_ISL_884606, EPI_ISL_884607, EPI_ISL_884608, EPI_ISL_884609, EPI_ISL_884610, EPI_ISL_884611, EPI_ISL_884612, EPI_ISL_884613, EPI_ISL_884614, EPI_ISL_884615, EPI_ISL_884616, EPI_ISL_884617, EPI_ISL_884618, EPI_ISL_884619, EPI_ISL_884620, EPI_ISL_884621, EPI_ISL_884622, EPI_ISL_884623, EPI_ISL_884624, EPI_ISL_884625, EPI_ISL_884626, EPI_ISL_884627, EPI_ISL_884628, EPI_ISL_884629, EPI_ISL_884630, EPI_ISL_884631, EPI_ISL_884632, EPI_ISL_884633, EPI_ISL_884634, EPI_ISL_884635, EPI_ISL_884636, EPI_ISL_884637, EPI_ISL_884638, EPI_ISL_884639, EPI_ISL_884640, EPI_ISL_884641, EPI_ISL_884642, EPI_ISL_884643, EPI_ISL_884644, EPI_ISL_884645, EPI_ISL_884646, EPI_ISL_884647, EPI_ISL_884648, EPI_ISL_884649, EPI_ISL_884650, EPI_ISL_884651, EPI_ISL_884652, EPI_ISL_884653, EPI_ISL_884654, EPI_ISL_884655, EPI_ISL_884656, EPI_ISL_884657, EPI_ISL_884658, EPI_ISL_884659, EPI_ISL_884660, EPI_ISL_884661, EPI_ISL_884662, EPI_ISL_884663, EPI_ISL_884664, EPI_ISL_884665, EPI_ISL_884666, EPI_ISL_884667, EPI_ISL_884668, EPI_ISL_884669, EPI_ISL_884670, EPI_ISL_884671, EPI_ISL_884672, EPI_ISL_884673, EPI_ISL_884674, EPI_ISL_884675, EPI_ISL_884676, EPI_ISL_884677, EPI_ISL_884678, EPI_ISL_884679, EPI_ISL_884680, EPI_ISL_884681, EPI_ISL_884682, EPI_ISL_884683, EPI_ISL_884684, EPI_ISL_884685, EPI_ISL_884686, EPI_ISL_884687, EPI_ISL_884688, EPI_ISL_884689, EPI_ISL_884690, EPI_ISL_884691, EPI_ISL_884692, EPI_ISL_884693, EPI_ISL_884694, EPI_ISL_884695, EPI_ISL_884696, EPI_ISL_884697, EPI_ISL_884698, EPI_ISL_884699, EPI_ISL_884700, EPI_ISL_884701, EPI_ISL_884702, EPI_ISL_884703, EPI_ISL_884704, EPI_ISL_884705, EPI_ISL_884706, EPI_ISL_884707, EPI_ISL_884708, EPI_ISL_884709, EPI_ISL_884710, EPI_ISL_884711, EPI_ISL_884712, EPI_ISL_884713, EPI_ISL_884714, EPI_ISL_884715, EPI_ISL_884716, EPI_ISL_884717, EPI_ISL_884718, EPI_ISL_884719, EPI_ISL_884720, EPI_ISL_884721, EPI_ISL_884722, EPI_ISL_884723, EPI_ISL_884724, EPI_ISL_884725, EPI_ISL_884726, EPI_ISL_884727, EPI_ISL_884728			
see above	Respiratory Viruses Branch, Centers for Disease Control and Prevention	Respiratory Viruses Branch, Centers for Disease Control and Prevention	Cook,P.W., Batra,D., Rambo-Martin,B.L., de Feo,E., Antico,J., Tran,C., Tolentino,M., Wickline,S., Gietzen,K., Sickler,B., Liu,J., Allen,E., Febbo,P., Galloway,S., Washington,N.L., White,S., Levan,G., Barret,K.S., Cirulli,E., Bolze,A., Ascencio,A., Rivera-Garcia,C., Cho,R., Nguyen,J., Wang,S., Ramirez,J., Cassens,T., Sandoval,E., Isaksson,M., Lee,W., Becker,D., Laurent,M., Lu,J., Paden,C.R., Tong,S., MacCannell,D.
EPI_ISL_885146	Genomic Laboratory (GLAB) (Conjoint lab of Health Directorate of Istanbul and Istanbul Technical University)	Genomic Laboratory (GLAB), Istanbul Technical University	Ilker Karacan, Tugba Kizilboga Akgun, Payam Zolfagharian, Nisan Denizce Can, Pari Sharifli, Levent Doganay, Gizem Dinler Doganay
EPI_ISL_885183, EPI_ISL_885204, EPI_ISL_885211, EPI_ISL_885244, EPI_ISL_885291, EPI_ISL_885321, EPI_ISL_885349, EPI_ISL_885359, EPI_ISL_885392, EPI_ISL_885411, EPI_ISL_885434, EPI_ISL_885503, EPI_ISL_885580, EPI_ISL_885589, EPI_ISL_885813, EPI_ISL_885880, EPI_ISL_885889, EPI_ISL_885916, EPI_ISL_886016			
see above	Lighthouse Lab in Alderley Park	Wellcome Sanger Institute for the COVID-19 Genomics UK	Jacquelyn Wynn, Mairead Hyland, The Lighthouse Lab in Alderley Park and Alex Alderton, Roberto Amato, Sonia Goncalves, Ewan Harrison, David K.

		(COG-UK) Consortium	Jackson, Ian Johnston, Dominic Kwiatkowski, Cordelia Langford, John Sillitoe on behalf of the Wellcome Sanger Institute COVID-19 Surveillance Team
EPI_ISL_886169, EPI_ISL_886172, EPI_ISL_886214, EPI_ISL_886246, EPI_ISL_886247, EPI_ISL_886248, EPI_ISL_886270, EPI_ISL_886318, EPI_ISL_886322, EPI_ISL_886347, EPI_ISL_886351, EPI_ISL_886362, EPI_ISL_886364, EPI_ISL_886365, EPI_ISL_886424, EPI_ISL_886442, EPI_ISL_886452, EPI_ISL_886465, EPI_ISL_886471, EPI_ISL_886494, EPI_ISL_886522, EPI_ISL_886545, EPI_ISL_886546, EPI_ISL_886554, EPI_ISL_886580, EPI_ISL_886583, EPI_ISL_886592, EPI_ISL_886616, EPI_ISL_886633, EPI_ISL_886636, EPI_ISL_886650, EPI_ISL_886662, EPI_ISL_886666, EPI_ISL_886701, EPI_ISL_886720, EPI_ISL_886735, EPI_ISL_886761, EPI_ISL_886790, EPI_ISL_886808, EPI_ISL_886837, EPI_ISL_886853, EPI_ISL_886860, EPI_ISL_886866, EPI_ISL_886870, EPI_ISL_886895, EPI_ISL_886898, EPI_ISL_886907, EPI_ISL_886963, EPI_ISL_886965, EPI_ISL_886975, EPI_ISL_886990, EPI_ISL_886997, EPI_ISL_887006, EPI_ISL_887067, EPI_ISL_887087, EPI_ISL_887091			
see above	Labcorp	Genomics and Discovery, Respiratory Viruses Branch, Division of Viral Diseases, Centers for Disease Control and Prevention	Peter W. Cook,Dhwani Batra,Ben L. Rambo-Martin,Summer Galloway,Brian Krueger,Minoo Agarwal,Eyad Almasri,Debbie Boles,Ayla Burns,Nuthawin Charoensri,Oren Cohen,Susan Countryman,Mary Ann Cristobal,Bobbi Croy,Suzanne Dale,Hrushikesh Deshmukh,Amanda Douglas,Vincent Drouillon,Marcia Eisenberg,Howard Engler,Rama Ghatti,Prashant Gupta,Susan Hicks,Jake Humphrey,Lax Iyer,Manoj Jain,Mohan Kolli,Tim Kuphal,Stanley Letovsky,Michael Levandoski,Craig Lukasik,Jonathan Meltzer,Brian Norvell,Mindy Nye,Scott Parker,Christos Petropoulos,John Pruitt,Steven Ragan,Scott Ryan,Mike Sapeta,Jana Schroth,Suresh Babu Selvaraju,Goran Stevovic,Amanda Suchanek,Andrea Throop,Lyndon Tilson,Thomas Urban,Joe Voshell,Kimberly Wagner,Jonathan Williams,Mary Williamson,Qian Zeng,Tricia Zwiefelhofer,Clinton R. Paden,Suxiang Tong,Duncan MacCannell,
EPI_ISL_887136, EPI_ISL_887137, EPI_ISL_887144	Institute of Medical Microbiology and Hospital Hygiene	Institute of Medical Microbiology and Hospital Hygiene	Prof. Dr. Achim Kaasch, Aljoscha Tersteegen
EPI_ISL_887508, EPI_ISL_887509, EPI_ISL_887510, EPI_ISL_887511, EPI_ISL_887513, EPI_ISL_887522, EPI_ISL_887523, EPI_ISL_887529, EPI_ISL_887530, EPI_ISL_887532, EPI_ISL_887533, EPI_ISL_887538, EPI_ISL_887544, EPI_ISL_887550, EPI_ISL_887552, EPI_ISL_887558, EPI_ISL_887559, EPI_ISL_887560, EPI_ISL_887563, EPI_ISL_887567, EPI_ISL_887568, EPI_ISL_887577, EPI_ISL_887580, EPI_ISL_887581, EPI_ISL_887582, EPI_ISL_887583			
see above	Johns Hopkins Hospital Department of Pathology	Johns Hopkins Hospital Department of Pathology	C. Paul Morris, Chun Huai Luo, Adannaya Amadi, Matthew Schwartz, Nicholas Gallagher, Heba H. Mostafa
EPI_ISL_888599, EPI_ISL_888600, EPI_ISL_888602, EPI_ISL_888603, EPI_ISL_888604, EPI_ISL_888605, EPI_ISL_888606, EPI_ISL_888607, EPI_ISL_888608, EPI_ISL_888609, EPI_ISL_888610, EPI_ISL_888611, EPI_ISL_888612, EPI_ISL_888613, EPI_ISL_888614, EPI_ISL_888615, EPI_ISL_888616, EPI_ISL_888617, EPI_ISL_888618, EPI_ISL_888619, EPI_ISL_888620, EPI_ISL_888621, EPI_ISL_888622			
see above	Wyoming Public Health Laboratory	Wyoming Public Health Laboratory	Noah Hull, Taylor Fearing, Lynette Gumbleton, Channing Weber, Ashley Norberg, Bailey Bowcutt, and Wanda Manley
EPI_ISL_888667, EPI_ISL_888668, EPI_ISL_888669, EPI_ISL_888670	University of Michigan Clinical Microbiology Laboratory	Lauring Lab, University of Michigan, Department of Microbiology and Immunology	Valesano
EPI_ISL_888810, EPI_ISL_888811, EPI_ISL_888812, EPI_ISL_888813, EPI_ISL_888814, EPI_ISL_888815, EPI_ISL_888816, EPI_ISL_888817	Univeristy of New Mexico Hospital	Center for Global Health, University of New Mexico Health Sciences Center	Daryl Domman, Kurt Schwalm, Justin Bacca, Jon Femling, Darrell Dinwiddie
EPI_ISL_888830, EPI_ISL_888831	National Virus Reference Laboratory	National Virus Reference Laboratory	Michael Carr, Gabriel Gonzalez, Jonathan Dean, Cillian F De Gascun
EPI_ISL_888851, EPI_ISL_888856, EPI_ISL_888903	Michigan Department of Health and Human Services, Bureau of Laboratories	Michigan Department of Health and Human Services, Bureau of Laboratories	Blankenship HM, Riner D, Soehnlen MK
EPI_ISL_888908, EPI_ISL_888910, EPI_ISL_888911, EPI_ISL_888914, EPI_ISL_888934, EPI_ISL_888936, EPI_ISL_888941, EPI_ISL_888942, EPI_ISL_888943, EPI_ISL_888944, EPI_ISL_888949, EPI_ISL_888959			
see above	Wyoming Public Health Laboratory	Wyoming Public Health Laboratory	Noah Hull, Taylor Fearing, Lynette Gumbleton, Channing Weber, Ashley Norberg, Bailey Bowcutt, and Wanda Manley
EPI_ISL_889703, EPI_ISL_889704, EPI_ISL_889705, EPI_ISL_889706, EPI_ISL_889707, EPI_ISL_889708, EPI_ISL_889709, EPI_ISL_889710, EPI_ISL_889711, EPI_ISL_889712, EPI_ISL_889714, EPI_ISL_889715, EPI_ISL_889716, EPI_ISL_889717, EPI_ISL_889718, EPI_ISL_889719, EPI_ISL_889720, EPI_ISL_889721, EPI_ISL_889722, EPI_ISL_889723, EPI_ISL_889724, EPI_ISL_889725, EPI_ISL_889726, EPI_ISL_889727, EPI_ISL_889728, EPI_ISL_889729, EPI_ISL_889730, EPI_ISL_889731, EPI_ISL_889732, EPI_ISL_889733, EPI_ISL_889734, EPI_ISL_889735, EPI_ISL_889736, EPI_ISL_889737, EPI_ISL_889738, EPI_ISL_889739, EPI_ISL_889740, EPI_ISL_889741, EPI_ISL_889742, EPI_ISL_889745			
see above	LSUHS Emerging Viral Threat Laboratory	Microbial Genome Sequencing Center	Jeremy P. Kamil, Jennifer L. Carroll, Camille F. Abshire, Maarten Van Diest, Mohammed N.A. Siddiquey, Andrew D. Yurochko, Martin J. Sapp, Rona S. Scott, Christopher G. Kevil, Daniel J. Snyder, Vaughn S. Cooper, John A. Vanchiere
EPI_ISL_890232	School of Pharmacy, Shenandoah University	School of Pharmacy, Shenandoah University	Adams,S.M., Harralson,A.F., Kidd,R.S., Sawyer,G.W.
EPI_ISL_890365, EPI_ISL_890366	LSUHS Emerging Viral Threat Laboratory	Microbial Genome Sequencing Center	Jeremy P. Kamil, Jennifer L. Carroll, Camille F. Abshire, Maarten Van Diest, Mohammed N.A. Siddiquey, Andrew D. Yurochko, Martin J. Sapp, Rona S. Scott, Christopher G. Kevil, Daniel J. Snyder, Vaughn S. Cooper, John A. Vanchiere
EPI_ISL_890985, EPI_ISL_890986, EPI_ISL_890987, EPI_ISL_890988	Seattle Flu Study	Seattle Flu Study	Deborah A. Nickerson, Chris D. Frazar, Jover Lee, Benjamin Pelle, Erica Ryke, Matthew Richardson, Amanda Adler, Elisabeth Brandstetter, Peter D. Han, Kairsten Fay, Misja Ilcisin, Kirsten Lacombe, Thomas R. Sibley, Melissa Truong, Caitlin R. Wolf, Michael Boeckh, Janet A. Englund, Michael Famulare, Barry R. Lutz, Mark J. Rieder, Lea M. Starita, Matthew Thompson, Jay Shendure, Trevor Bedford, Helen Y. Chu
EPI_ISL_890989	Seattle Flu Study	Seattle Flu Study	Deborah A. Nickerson, Chris D. Frazar, Jover Lee, Benjamin Pelle, Erica Ryke, Matthew Richardson, Amanda Adler, Elisabeth Brandstetter, Peter D. Han, Kairsten Fay, Misja Ilcisin, Kirsten Lacombe, Thomas R. Sibley, Melissa Truong, Caitlin R. Wolf, Karen Cowgill, Stephanie Schrag, Jeff Duchin, Michael Boeckh, Janet A. Englund, Michael Famulare, Barry R. Lutz, Mark J. Rieder, Lea M. Starita, Matthew Thompson, Helen Y. Chu, Trevor Bedford, Jay Shendure
EPI_ISL_890991, EPI_ISL_890992, EPI_ISL_890994, EPI_ISL_890995, EPI_ISL_890996, EPI_ISL_890997	Seattle Flu Study	Seattle Flu Study	Deborah A. Nickerson, Chris D. Frazar, Jover Lee, Benjamin Pelle, Erica Ryke, Matthew Richardson, Amanda Adler, Elisabeth Brandstetter, Peter D. Han, Kairsten Fay, Misja Ilcisin, Kirsten Lacombe, Thomas R. Sibley, Melissa Truong, Caitlin R. Wolf, Michael Boeckh, Janet A. Englund, Michael Famulare, Barry R. Lutz, Mark J. Rieder, Lea M. Starita, Matthew Thompson, Jay Shendure, Trevor Bedford, Helen Y. Chu
EPI_ISL_891058	Seattle Flu Study	Seattle Flu Study	Deborah A. Nickerson, Chris D. Frazar, Jover Lee, Benjamin Pelle, Erica Ryke, Matthew Richardson, Amanda Adler, Elisabeth Brandstetter, Peter D. Han, Kairsten Fay, Misja Ilcisin, Kirsten Lacombe, Thomas R. Sibley, Melissa Truong, Caitlin R. Wolf, Karen Cowgill, Stephanie Schrag, Jeff Duchin, Michael Boeckh, Janet A. Englund, Michael Famulare, Barry R. Lutz, Mark J. Rieder, Lea M. Starita, Matthew Thompson, Helen Y. Chu, Trevor Bedford, Jay Shendure
EPI_ISL_891060, EPI_ISL_891061, EPI_ISL_891062, EPI_ISL_891063	Seattle Flu Study	Seattle Flu Study	Deborah A. Nickerson, Chris D. Frazar, Jover Lee, Benjamin Pelle, Erica Ryke, Matthew Richardson, Amanda Adler, Elisabeth Brandstetter, Peter D. Han, Kairsten Fay, Misja Ilcisin, Kirsten Lacombe, Thomas R. Sibley, Melissa Truong, Caitlin R. Wolf, Michael Boeckh, Janet A. Englund, Michael Famulare, Barry R. Lutz, Mark J. Rieder, Lea M. Starita, Matthew Thompson, Jay Shendure, Trevor Bedford, Helen Y. Chu
EPI_ISL_891064, EPI_ISL_891065, EPI_ISL_891066, EPI_ISL_891067, EPI_ISL_891068, EPI_ISL_891069	Seattle Flu Study	Seattle Flu Study	Deborah A. Nickerson, Chris D. Frazar, Jover Lee, Benjamin Pelle, Erica Ryke, Matthew Richardson, Amanda Adler, Elisabeth Brandstetter, Peter D. Han, Kairsten Fay, Misja Ilcisin, Kirsten Lacombe, Thomas R. Sibley, Melissa Truong, Caitlin R. Wolf, Karen Cowgill, Stephanie Schrag, Jeff Duchin, Michael Boeckh, Janet A. Englund, Michael Famulare, Barry R. Lutz, Mark J. Rieder, Lea M. Starita, Matthew Thompson, Helen Y. Chu, Trevor Bedford, Jay Shendure
EPI_ISL_891144	Indiana Animal Disease Diagnostic Laboratory	Carpi Laboratory - Purdue University	Jack Dorman, Ilinca I Ciubotariu, Lev Gorenstein, Abebe A Fola, G Kenitra Hendrix, Rebecca P Wilkes, Giovanna Carpi
EPI_ISL_891146, EPI_ISL_891147, EPI_ISL_891148, EPI_ISL_891149, EPI_ISL_891150	University of Wisconsin-Madison AIDS Vaccine Research Laboratories	University of Wisconsin-Madison AIDS Vaccine Research Laboratories	Gage Moreno, Katarina Braun, et al. AIDS Vaccine Research Laboratories
EPI_ISL_891206	DPH, Massachusetts State Public Health Lab	DPH, Massachusetts State Public Health Lab	Lang,A.S., Fink,T., Gallagher,G.R., Smole,S.C.
EPI_ISL_891252	Singapore General Hospital	Department of Microbiology	Nurdyana Abdul Rahman, Kun Lee Lim, Chenhao Li, Sui Sin Goh, Kenneth Xin Long Chan, Kian Sing Chan, Lynette Oon, Kern Rei Chng, Niranjan Nagarajan, Karrie Ko
EPI_ISL_891903, EPI_ISL_891909, EPI_ISL_891918, EPI_ISL_891928, EPI_ISL_891945, EPI_ISL_891958, EPI_ISL_891967, EPI_ISL_891972, EPI_ISL_891973, EPI_ISL_891974, EPI_ISL_891975, EPI_ISL_891976, EPI_ISL_891977, EPI_ISL_891978, EPI_ISL_891979, EPI_ISL_891980, EPI_ISL_891981, EPI_ISL_891982, EPI_ISL_891983, EPI_ISL_891984, EPI_ISL_891985, EPI_ISL_891986, EPI_ISL_891987, EPI_ISL_891988, EPI_ISL_891989, EPI_ISL_891990, EPI_ISL_891991, EPI_ISL_891992, EPI_ISL_891993, EPI_ISL_891994, EPI_ISL_891995, EPI_ISL_891996, EPI_ISL_891997, EPI_ISL_891998, EPI_ISL_891999, EPI_ISL_892000, EPI_ISL_892001, EPI_ISL_892003, EPI_ISL_892004, EPI_ISL_892007, EPI_ISL_892008, EPI_ISL_892013, EPI_ISL_892014, EPI_ISL_892015, EPI_ISL_892016, EPI_ISL_892017, EPI_ISL_892018, EPI_ISL_892019, EPI_ISL_892020, EPI_ISL_892021, EPI_ISL_892022, EPI_ISL_892023, EPI_ISL_892024, EPI_ISL_892025, EPI_ISL_892026, EPI_ISL_892027, EPI_ISL_892028, EPI_ISL_892029, EPI_ISL_892030, EPI_ISL_892031, EPI_ISL_892032, EPI_ISL_892033, EPI_ISL_892034, EPI_ISL_892035, EPI_ISL_892036, EPI_ISL_892037, EPI_ISL_892038, EPI_ISL_892039, EPI_ISL_892040, EPI_ISL_892041, EPI_ISL_892042, EPI_ISL_892043, EPI_ISL_892044, EPI_ISL_892045, EPI_ISL_892046, EPI_ISL_892047, EPI_ISL_892048, EPI_ISL_892049, EPI_ISL_892056, EPI_ISL_892127, EPI_ISL_892128, EPI_ISL_892129, EPI_ISL_892130, EPI_ISL_892131, EPI_ISL_892132, EPI_ISL_892133, EPI_ISL_892134, EPI_ISL_892135, EPI_ISL_892136, EPI_ISL_892137,			

EPI_ISL_892138, EPI_ISL_892139, EPI_ISL_892140, EPI_ISL_892141, EPI_ISL_892142, EPI_ISL_892143, EPI_ISL_892144, EPI_ISL_892145, EPI_ISL_892146, EPI_ISL_892147, EPI_ISL_892148, EPI_ISL_892149, EPI_ISL_892150, EPI_ISL_892151, EPI_ISL_892152, EPI_ISL_892153, EPI_ISL_892154, EPI_ISL_892155, EPI_ISL_892156, EPI_ISL_892157, EPI_ISL_892158, EPI_ISL_892159, EPI_ISL_892160, EPI_ISL_892161, EPI_ISL_892162, EPI_ISL_892163, EPI_ISL_892164, EPI_ISL_892165, EPI_ISL_892166, EPI_ISL_892167, EPI_ISL_892168, EPI_ISL_892169, EPI_ISL_892170, EPI_ISL_892171, EPI_ISL_892172, EPI_ISL_892173, EPI_ISL_892174, EPI_ISL_892175, EPI_ISL_892176, EPI_ISL_892177, EPI_ISL_892178, EPI_ISL_892180, EPI_ISL_892181, EPI_ISL_892182, EPI_ISL_892183, EPI_ISL_892184, EPI_ISL_892185, EPI_ISL_892186, EPI_ISL_892187, EPI_ISL_892188, EPI_ISL_892198, EPI_ISL_892199, EPI_ISL_892200			
see above	Lighthouse Lab in Alderley Park	Wellcome Sanger Institute for the COVID-19 Genomics UK (COG-UK) Consortium	Jacquelyn Wynn, Mairead Hyland, The Lighthouse Lab in Alderley Park and Alex Alderton, Roberto Amato, Sonia Goncalves, Ewan Harrison, David K. Jackson, Ian Johnston, Dominic Kwiatkowski, Cordelia Langford, John Sillitoe on behalf of the Wellcome Sanger Institute COVID-19 Surveillance Team
EPI_ISL_892201	Lighthouse Lab in Glasgow	Wellcome Sanger Institute for the COVID-19 Genomics UK (COG-UK) Consortium	Harper VanSteenhouse, Yumi Kasai, David Gray, Carol Clugston, Anna Dominiczak and Alex Alderton, Roberto Amato, Sonia Goncalves, Ewan Harrison, David K. Jackson, Ian Johnston, Dominic Kwiatkowski, Cordelia Langford, John Sillitoe on behalf of the Wellcome Sanger Institute COVID-19 Surveillance Team
EPI_ISL_892202, EPI_ISL_892203, EPI_ISL_892204, EPI_ISL_892205	Lighthouse Lab in Alderley Park	Wellcome Sanger Institute for the COVID-19 Genomics UK (COG-UK) Consortium	Jacquelyn Wynn, Mairead Hyland, The Lighthouse Lab in Alderley Park and Alex Alderton, Roberto Amato, Sonia Goncalves, Ewan Harrison, David K. Jackson, Ian Johnston, Dominic Kwiatkowski, Cordelia Langford, John Sillitoe on behalf of the Wellcome Sanger Institute COVID-19 Surveillance Team
EPI_ISL_893781	Institute of Virology, Medical Center, University of Freiburg, Freiburg, Germany	Institute of Virology, Clinial Virus Genomics, Medical Center, University of Freiburg, Freiburg, Germany	Jonas Fuchs, Lisa Kern, Sandra Reuter, Hajo Grundmann, Marcus Panning
EPI_ISL_894220, EPI_ISL_894221, EPI_ISL_894222, EPI_ISL_894227, EPI_ISL_894228, EPI_ISL_894229, EPI_ISL_894230, EPI_ISL_894231	CH de Mayotte - Laboratoire de Biologie	National Reference Center for Viruses of Respiratory Infections, Institut Pasteur, Paris	Marion Barbet, Sylvie Behillil, Méline Bizard, Angela Brisebarre, Camille Capel, Etienne Simon-Lorière, Vincent Enouf, Maud Vanpeene, Sylvie van der Werf,Combe Patrice
EPI_ISL_894255, EPI_ISL_894256, EPI_ISL_894257, EPI_ISL_894258, EPI_ISL_894259, EPI_ISL_894260, EPI_ISL_894261, EPI_ISL_894262, EPI_ISL_894263, EPI_ISL_894264, EPI_ISL_894265, EPI_ISL_894266, EPI_ISL_894267, EPI_ISL_894268, EPI_ISL_894269, EPI_ISL_894270, EPI_ISL_894271, EPI_ISL_894272, EPI_ISL_894273, EPI_ISL_894274, EPI_ISL_894275, EPI_ISL_894276			
see above	Ministry of Health Turkey	Ministry of Health Turkey	Fatma Bayrakdar, Yasemin Cogun, Süleyman Yalcin, Aye Baak Alta, Gülay Korukluolu
EPI_ISL_896070	Rothen Medizinische Laboratorien AG	University Hospital Basel, Clinical Bacteriology	Tim Roloff, Madlen Stange, Helena MB Seth-Smith, Alfredo Mari, Karoline Leuzinger, Julia Bielicki, Ingrid Steffen, Manuel Battegay, Hans Hirsch, Adrian Egli
EPI_ISL_896211, EPI_ISL_896213	MEPHI, Aix Marseille University	MEPHI, Aix Marseille University	Anthony LEVASSEUR
EPI_ISL_896219, EPI_ISL_896221, EPI_ISL_896222, EPI_ISL_896223, EPI_ISL_896225, EPI_ISL_896230, EPI_ISL_896235, EPI_ISL_896247, EPI_ISL_896252, EPI_ISL_896253, EPI_ISL_896256, EPI_ISL_896257, EPI_ISL_896258, EPI_ISL_896259, EPI_ISL_896260, EPI_ISL_896261, EPI_ISL_896262, EPI_ISL_896263, EPI_ISL_896264, EPI_ISL_896265, EPI_ISL_896266, EPI_ISL_896267, EPI_ISL_896268, EPI_ISL_896269, EPI_ISL_896270, EPI_ISL_896271, EPI_ISL_896272, EPI_ISL_896273, EPI_ISL_896274, EPI_ISL_896275, EPI_ISL_896276, EPI_ISL_896277, EPI_ISL_896279, EPI_ISL_896280, EPI_ISL_896283, EPI_ISL_896284, EPI_ISL_896285, EPI_ISL_896287, EPI_ISL_896288, EPI_ISL_896292, EPI_ISL_896293			
see above	SUNY UPSTATE MEDICAL UNIVERSITY	Wadsworth Center, New York State Department of Health	Kirsten St. George, Daryl M. Lamson, Alexis Russel, Matthew Shudt, Melissa A Leisner, Jonathan Plitnick, Navjot Singh, John Kelly, Erasmus Schneider, Erica Lasek-Nesselquist
EPI_ISL_896380	Suceava County Emergency Hospital	"Stefan cel Mare" University Metagenomics Lab	Lobiuc Andrei, Gheorghita Roxana
EPI_ISL_896413	URMC LABS	Wadsworth Center, New York State Department of Health	Kirsten St. George, Daryl M. Lamson, Alexis Russel, Matthew Shudt, Melissa A Leisner, Jonathan Plitnick, Navjot Singh, John Kelly, Erasmus Schneider, Erica Lasek-Nesselquist
EPI_ISL_896451, EPI_ISL_896452, EPI_ISL_896453, EPI_ISL_896455, EPI_ISL_896457, EPI_ISL_896461, EPI_ISL_896462, EPI_ISL_896463, EPI_ISL_896464, EPI_ISL_896465, EPI_ISL_896469, EPI_ISL_896470, EPI_ISL_896471, EPI_ISL_896472, EPI_ISL_896475			
see above	KU Leuven, Rega Institute, Clinical and Epidemiological Virology	KU Leuven, Rega Institute, Clinical and Epidemiological Virology	Tony Wawina-Bokalanga, Bert Vanmechelen, Joan Marti-Carerras, Piet Maes
EPI_ISL_897598, EPI_ISL_897603, EPI_ISL_897604, EPI_ISL_897611, EPI_ISL_897616, EPI_ISL_897617, EPI_ISL_897618, EPI_ISL_897621, EPI_ISL_897622, EPI_ISL_897623, EPI_ISL_897624, EPI_ISL_897625, EPI_ISL_897670, EPI_ISL_897674, EPI_ISL_897677, EPI_ISL_897680, EPI_ISL_897681, EPI_ISL_897682, EPI_ISL_897685, EPI_ISL_897688, EPI_ISL_897689, EPI_ISL_897692, EPI_ISL_897693, EPI_ISL_897694, EPI_ISL_897697, EPI_ISL_897699, EPI_ISL_897700, EPI_ISL_897704, EPI_ISL_897705, EPI_ISL_897708, EPI_ISL_897711, EPI_ISL_897713, EPI_ISL_897714, EPI_ISL_897716, EPI_ISL_897721, EPI_ISL_897722, EPI_ISL_897723, EPI_ISL_897724, EPI_ISL_897725, EPI_ISL_897726, EPI_ISL_897727, EPI_ISL_897728, EPI_ISL_897729, EPI_ISL_897730, EPI_ISL_897731, EPI_ISL_897732, EPI_ISL_897733, EPI_ISL_897735, EPI_ISL_897736, EPI_ISL_897737, EPI_ISL_897738, EPI_ISL_897739, EPI_ISL_897740, EPI_ISL_897741, EPI_ISL_897742, EPI_ISL_897743, EPI_ISL_897744, EPI_ISL_897745, EPI_ISL_897746, EPI_ISL_897747, EPI_ISL_897748, EPI_ISL_897749, EPI_ISL_897750, EPI_ISL_897751, EPI_ISL_897752, EPI_ISL_897753, EPI_ISL_897754, EPI_ISL_897755, EPI_ISL_897756, EPI_ISL_897757, EPI_ISL_897758, EPI_ISL_897759, EPI_ISL_897760, EPI_ISL_897761, EPI_ISL_897762, EPI_ISL_897763, EPI_ISL_897764, EPI_ISL_897765, EPI_ISL_897766, EPI_ISL_897767, EPI_ISL_897768, EPI_ISL_897769, EPI_ISL_897770, EPI_ISL_897903, EPI_ISL_897904, EPI_ISL_897906, EPI_ISL_897907, EPI_ISL_897908, EPI_ISL_897909, EPI_ISL_897910, EPI_ISL_897911, EPI_ISL_897912, EPI_ISL_897913, EPI_ISL_897914, EPI_ISL_897915, EPI_ISL_897916, EPI_ISL_897917, EPI_ISL_897918, EPI_ISL_897919, EPI_ISL_897942, EPI_ISL_897943, EPI_ISL_897944, EPI_ISL_897945, EPI_ISL_897946, EPI_ISL_897947, EPI_ISL_897948, EPI_ISL_897949, EPI_ISL_897950, EPI_ISL_897951, EPI_ISL_897952, EPI_ISL_897954, EPI_ISL_897955, EPI_ISL_897956, EPI_ISL_897957, EPI_ISL_897958, EPI_ISL_897959, EPI_ISL_897960, EPI_ISL_897961, EPI_ISL_897962, EPI_ISL_897963, EPI_ISL_897964, EPI_ISL_897965, EPI_ISL_897980, EPI_ISL_897981, EPI_ISL_897982, EPI_ISL_897983, EPI_ISL_897984, EPI_ISL_897985			
see above	University Hospitals of Geneva, Laboratory of Virology	HUG, Laboratory of Virology and the Health2030 Genome Center	Samuel Cordey, Ana Rita Goncalves, Laurent Kaiser, Lorenzo Cerutti, Henri Pegeot, Melyssa Elies, Deborah Penet, Keith Harshman, Ioannis Xenarios, Emmanouil Dermitzakis
EPI_ISL_897986, EPI_ISL_897989, EPI_ISL_897990, EPI_ISL_897993, EPI_ISL_897996, EPI_ISL_897998, EPI_ISL_898000, EPI_ISL_898001, EPI_ISL_898002, EPI_ISL_898004, EPI_ISL_898005, EPI_ISL_898007, EPI_ISL_898015, EPI_ISL_898016, EPI_ISL_898023			
see above	KU Leuven, Rega Institute, Clinical and Epidemiological Virology	KU Leuven, Rega Institute, Clinical and Epidemiological Virology	Tony Wawina-Bokalanga, Bert Vanmechelen, Joan Marti-Carerras, Piet Maes
EPI_ISL_898993, EPI_ISL_898994, EPI_ISL_898995, EPI_ISL_898996	Viollier AG	Department of Biosystems Science and Engineering, ETH Zürich	Christian Beisel, Sarah Nadeau, Chaoran Chen, Ivan Topolsky, Philipp Jablonski, Lara Fuhrmann, David Dreifuss, Katharina Jahn, Tobias Schär, Ina Nissen, Natascha Santacroce, Elodie Burcklen, Christiane Beckmann, Maurice Redondo, Olivier Kobel, Christoph Noppen, Sophie Seidel, Noemie Santamaria de Souza, Niko Beerenwinkel, Tanja Stadler
EPI_ISL_898998, EPI_ISL_899000, EPI_ISL_899005, EPI_ISL_899007, EPI_ISL_899011	Viollier AG	Department of Biosystems Science and Engineering, ETH Zürich	Chaoran Chen, Sarah Nadeau, Ivan Topolsky, Emmanouil Dermitzakis, Keith Harshman, Ioannis Xenarios, Henri Pegeot, Lorenzo Cerutti, Deborah Penet, Philipp Jablonski, Lara Fuhrmann, David Dreifuss, Katharina Jahn, Christiane Beckmann, Maurice Redondo, Olivier Kobel, Christoph Noppen, Sophie Seidel, Noemie Santamaria de Souza, Niko Beerenwinkel, Tanja Stadler
EPI_ISL_899023	Viollier AG	Department of Biosystems Science and Engineering, ETH Zürich	Christian Beisel, Sarah Nadeau, Chaoran Chen, Ivan Topolsky, Philipp Jablonski, Lara Fuhrmann, David Dreifuss, Katharina Jahn, Tobias Schär, Ina Nissen, Natascha Santacroce, Elodie Burcklen, Christiane Beckmann, Maurice Redondo, Olivier Kobel, Christoph Noppen, Sophie Seidel, Noemie Santamaria de Souza, Niko Beerenwinkel, Tanja Stadler
EPI_ISL_899026, EPI_ISL_899028, EPI_ISL_899029, EPI_ISL_899030	Viollier AG	Department of Biosystems Science and Engineering, ETH Zürich	Chaoran Chen, Sarah Nadeau, Ivan Topolsky, Emmanouil Dermitzakis, Keith Harshman, Ioannis Xenarios, Henri Pegeot, Lorenzo Cerutti, Deborah Penet, Philipp Jablonski, Lara Fuhrmann, David Dreifuss, Katharina Jahn, Christiane Beckmann, Maurice Redondo, Olivier Kobel, Christoph Noppen, Sophie Seidel, Noemie Santamaria de Souza, Niko Beerenwinkel, Tanja Stadler
EPI_ISL_899031	Viollier AG	Department of Biosystems Science and Engineering, ETH Zürich	Christian Beisel, Sarah Nadeau, Chaoran Chen, Ivan Topolsky, Philipp Jablonski, Lara Fuhrmann, David Dreifuss, Katharina Jahn, Tobias Schär, Ina Nissen, Natascha Santacroce, Elodie Burcklen, Christiane Beckmann, Maurice Redondo, Olivier Kobel, Christoph Noppen, Sophie Seidel, Noemie Santamaria de Souza, Niko Beerenwinkel, Tanja Stadler
EPI_ISL_899032, EPI_ISL_899034, EPI_ISL_899036, EPI_ISL_899038, EPI_ISL_899041, EPI_ISL_899042, EPI_ISL_899044	Viollier AG	Department of Biosystems Science and Engineering, ETH Zürich	Chaoran Chen, Sarah Nadeau, Ivan Topolsky, Emmanouil Dermitzakis, Keith Harshman, Ioannis Xenarios, Henri Pegeot, Lorenzo Cerutti, Deborah Penet, Philipp Jablonski, Lara Fuhrmann, David Dreifuss, Katharina Jahn, Christiane Beckmann, Maurice Redondo, Olivier Kobel, Christoph Noppen, Sophie Seidel, Noemie Santamaria de Souza, Niko Beerenwinkel, Tanja Stadler
EPI_ISL_899052	Viollier AG	Department of Biosystems Science and Engineering, ETH Zürich	Christian Beisel, Sarah Nadeau, Chaoran Chen, Ivan Topolsky, Philipp Jablonski, Lara Fuhrmann, David Dreifuss, Katharina Jahn, Tobias Schär, Ina Nissen, Natascha Santacroce, Elodie Burcklen, Christiane Beckmann, Maurice Redondo, Olivier Kobel, Christoph Noppen, Sophie Seidel, Noemie Santamaria de Souza, Niko Beerenwinkel, Tanja Stadler
EPI_ISL_899054, EPI_ISL_899057, EPI_ISL_899058, EPI_ISL_899059	Viollier AG	Department of Biosystems Science and Engineering, ETH Zürich	Chaoran Chen, Sarah Nadeau, Ivan Topolsky, Emmanouil Dermitzakis, Keith Harshman, Ioannis Xenarios, Henri Pegeot, Lorenzo Cerutti, Deborah Penet, Philipp Jablonski, Lara Fuhrmann, David Dreifuss, Katharina Jahn, Christiane Beckmann, Maurice Redondo, Olivier Kobel, Christoph Noppen, Sophie Seidel, Noemie Santamaria de Souza, Niko Beerenwinkel, Tanja Stadler
EPI_ISL_899069	Viollier AG	Department of Biosystems Science and Engineering, ETH Zürich	Christian Beisel, Sarah Nadeau, Chaoran Chen, Ivan Topolsky, Philipp Jablonski, Lara Fuhrmann, David Dreifuss, Katharina Jahn, Tobias Schär, Ina Nissen, Natascha Santacroce, Elodie Burcklen, Christiane Beckmann, Maurice Redondo, Olivier Kobel, Christoph Noppen, Sophie Seidel, Noemie

			Santamaria de Souza, Niko Beerenwinkel, Tanja Stadler
EPI_ISL_899071, EPI_ISL_899074, EPI_ISL_899075, EPI_ISL_899078, EPI_ISL_899081, EPI_ISL_899082	Viollier AG	Department of Biosystems Science and Engineering, ETH Zürich	Chaoran Chen, Sarah Nadeau, Ivan Topolsky, Emmanouil Dermitzakis, Keith Harshman, Ioannis Xenarios, Henri Pegeot, Lorenzo Cerutti, Deborah Penet, Philipp Jablonski, Lara Fuhrmann, David Dreifuss, Katharina Jahn, Christiane Beckmann, Maurice Redondo, Olivier Kobel, Christoph Noppen, Sophie Seidel, Noemie Santamaria de Souza, Niko Beerenwinkel, Tanja Stadler
EPI_ISL_899090	Viollier AG	Department of Biosystems Science and Engineering, ETH Zürich	Christian Beisel, Sarah Nadeau, Chaoran Chen, Ivan Topolsky, Philipp Jablonski, Lara Fuhrmann, David Dreifuss, Katharina Jahn, Tobias Schär, Ina Nissen, Natascha Santacroce, Elodie Burcklen, Christiane Beckmann, Maurice Redondo, Olivier Kobel, Christoph Noppen, Sophie Seidel, Noemie Santamaria de Souza, Niko Beerenwinkel, Tanja Stadler
EPI_ISL_899094, EPI_ISL_899106	Viollier AG	Department of Biosystems Science and Engineering, ETH Zürich	Chaoran Chen, Sarah Nadeau, Ivan Topolsky, Emmanouil Dermitzakis, Keith Harshman, Ioannis Xenarios, Henri Pegeot, Lorenzo Cerutti, Deborah Penet, Philipp Jablonski, Lara Fuhrmann, David Dreifuss, Katharina Jahn, Christiane Beckmann, Maurice Redondo, Olivier Kobel, Christoph Noppen, Sophie Seidel, Noemie Santamaria de Souza, Niko Beerenwinkel, Tanja Stadler
EPI_ISL_899123	Viollier AG	Department of Biosystems Science and Engineering, ETH Zürich	Christian Beisel, Sarah Nadeau, Chaoran Chen, Ivan Topolsky, Philipp Jablonski, Lara Fuhrmann, David Dreifuss, Katharina Jahn, Tobias Schär, Ina Nissen, Natascha Santacroce, Elodie Burcklen, Christiane Beckmann, Maurice Redondo, Olivier Kobel, Christoph Noppen, Sophie Seidel, Noemie Santamaria de Souza, Niko Beerenwinkel, Tanja Stadler
EPI_ISL_899126, EPI_ISL_899132, EPI_ISL_899133	Viollier AG	Department of Biosystems Science and Engineering, ETH Zürich	Chaoran Chen, Sarah Nadeau, Ivan Topolsky, Emmanouil Dermitzakis, Keith Harshman, Ioannis Xenarios, Henri Pegeot, Lorenzo Cerutti, Deborah Penet, Philipp Jablonski, Lara Fuhrmann, David Dreifuss, Katharina Jahn, Christiane Beckmann, Maurice Redondo, Olivier Kobel, Christoph Noppen, Sophie Seidel, Noemie Santamaria de Souza, Niko Beerenwinkel, Tanja Stadler
EPI_ISL_899137, EPI_ISL_899138, EPI_ISL_899151, EPI_ISL_899152, EPI_ISL_899167	Viollier AG	Department of Biosystems Science and Engineering, ETH Zürich	Christian Beisel, Sarah Nadeau, Chaoran Chen, Ivan Topolsky, Philipp Jablonski, Lara Fuhrmann, David Dreifuss, Katharina Jahn, Tobias Schär, Ina Nissen, Natascha Santacroce, Elodie Burcklen, Christiane Beckmann, Maurice Redondo, Olivier Kobel, Christoph Noppen, Sophie Seidel, Noemie Santamaria de Souza, Niko Beerenwinkel, Tanja Stadler
EPI_ISL_899169	Viollier AG	Department of Biosystems Science and Engineering, ETH Zürich	Chaoran Chen, Sarah Nadeau, Ivan Topolsky, Emmanouil Dermitzakis, Keith Harshman, Ioannis Xenarios, Henri Pegeot, Lorenzo Cerutti, Deborah Penet, Philipp Jablonski, Lara Fuhrmann, David Dreifuss, Katharina Jahn, Christiane Beckmann, Maurice Redondo, Olivier Kobel, Christoph Noppen, Sophie Seidel, Noemie Santamaria de Souza, Niko Beerenwinkel, Tanja Stadler
EPI_ISL_899176, EPI_ISL_899242, EPI_ISL_899244, EPI_ISL_899245, EPI_ISL_899247, EPI_ISL_899248, EPI_ISL_899249	Viollier AG	Department of Biosystems Science and Engineering, ETH Zürich	Christian Beisel, Sarah Nadeau, Chaoran Chen, Ivan Topolsky, Philipp Jablonski, Lara Fuhrmann, David Dreifuss, Katharina Jahn, Tobias Schär, Ina Nissen, Natascha Santacroce, Elodie Burcklen, Christiane Beckmann, Maurice Redondo, Olivier Kobel, Christoph Noppen, Sophie Seidel, Noemie Santamaria de Souza, Niko Beerenwinkel, Tanja Stadler
EPI_ISL_899268, EPI_ISL_899269, EPI_ISL_899270, EPI_ISL_899271, EPI_ISL_899272, EPI_ISL_899273, EPI_ISL_899274, EPI_ISL_899275, EPI_ISL_899276, EPI_ISL_899277	Viollier AG	Department of Biosystems Science and Engineering, ETH Zürich	Chaoran Chen, Sarah Nadeau, Ivan Topolsky, Emmanouil Dermitzakis, Keith Harshman, Ioannis Xenarios, Henri Pegeot, Lorenzo Cerutti, Deborah Penet, Philipp Jablonski, Lara Fuhrmann, David Dreifuss, Katharina Jahn, Christiane Beckmann, Maurice Redondo, Olivier Kobel, Christoph Noppen, Sophie Seidel, Noemie Santamaria de Souza, Niko Beerenwinkel, Tanja Stadler
EPI_ISL_899342, EPI_ISL_899343, EPI_ISL_899344, EPI_ISL_899345, EPI_ISL_899350, EPI_ISL_899351, EPI_ISL_899352, EPI_ISL_899353	Viollier AG	Department of Biosystems Science and Engineering, ETH Zürich	Christian Beisel, Sarah Nadeau, Chaoran Chen, Ivan Topolsky, Philipp Jablonski, Lara Fuhrmann, David Dreifuss, Katharina Jahn, Tobias Schär, Ina Nissen, Natascha Santacroce, Elodie Burcklen, Christiane Beckmann, Maurice Redondo, Olivier Kobel, Christoph Noppen, Sophie Seidel, Noemie Santamaria de Souza, Niko Beerenwinkel, Tanja Stadler
EPI_ISL_899373, EPI_ISL_899374, EPI_ISL_899375, EPI_ISL_899376, EPI_ISL_899377, EPI_ISL_899378, EPI_ISL_899379, EPI_ISL_899380, EPI_ISL_899381, EPI_ISL_899382, EPI_ISL_899383, EPI_ISL_899384			
see above	Viollier AG	Department of Biosystems Science and Engineering, ETH Zürich	Chaoran Chen, Sarah Nadeau, Ivan Topolsky, Emmanouil Dermitzakis, Keith Harshman, Ioannis Xenarios, Henri Pegeot, Lorenzo Cerutti, Deborah Penet, Philipp Jablonski, Lara Fuhrmann, David Dreifuss, Katharina Jahn, Christiane Beckmann, Maurice Redondo, Olivier Kobel, Christoph Noppen, Sophie Seidel, Noemie Santamaria de Souza, Niko Beerenwinkel, Tanja Stadler
EPI_ISL_899458, EPI_ISL_899459, EPI_ISL_899460, EPI_ISL_899461, EPI_ISL_899462, EPI_ISL_899463, EPI_ISL_899464, EPI_ISL_899465, EPI_ISL_899466, EPI_ISL_899467, EPI_ISL_899468, EPI_ISL_899469, EPI_ISL_899470, EPI_ISL_899471, EPI_ISL_899472, EPI_ISL_899473, EPI_ISL_899474, EPI_ISL_899478			
see above	Viollier AG	Department of Biosystems Science and Engineering, ETH Zürich	Christian Beisel, Sarah Nadeau, Chaoran Chen, Ivan Topolsky, Philipp Jablonski, Lara Fuhrmann, David Dreifuss, Katharina Jahn, Tobias Schär, Ina Nissen, Natascha Santacroce, Elodie Burcklen, Christiane Beckmann, Maurice Redondo, Olivier Kobel, Christoph Noppen, Sophie Seidel, Noemie Santamaria de Souza, Niko Beerenwinkel, Tanja Stadler
EPI_ISL_899509, EPI_ISL_899510, EPI_ISL_899511, EPI_ISL_899512, EPI_ISL_899513, EPI_ISL_899514	Viollier AG	Department of Biosystems Science and Engineering, ETH Zürich	Chaoran Chen, Sarah Nadeau, Ivan Topolsky, Emmanouil Dermitzakis, Keith Harshman, Ioannis Xenarios, Henri Pegeot, Lorenzo Cerutti, Deborah Penet, Philipp Jablonski, Lara Fuhrmann, David Dreifuss, Katharina Jahn, Christiane Beckmann, Maurice Redondo, Olivier Kobel, Christoph Noppen, Sophie Seidel, Noemie Santamaria de Souza, Niko Beerenwinkel, Tanja Stadler
EPI_ISL_899531	Viollier AG	Department of Biosystems Science and Engineering, ETH Zürich	Christian Beisel, Sarah Nadeau, Chaoran Chen, Ivan Topolsky, Philipp Jablonski, Lara Fuhrmann, David Dreifuss, Katharina Jahn, Tobias Schär, Ina Nissen, Natascha Santacroce, Elodie Burcklen, Christiane Beckmann, Maurice Redondo, Olivier Kobel, Christoph Noppen, Sophie Seidel, Noemie Santamaria de Souza, Niko Beerenwinkel, Tanja Stadler
EPI_ISL_899535, EPI_ISL_899536	Viollier AG	Department of Biosystems Science and Engineering, ETH Zürich	Chaoran Chen, Sarah Nadeau, Ivan Topolsky, Emmanouil Dermitzakis, Keith Harshman, Ioannis Xenarios, Henri Pegeot, Lorenzo Cerutti, Deborah Penet, Philipp Jablonski, Lara Fuhrmann, David Dreifuss, Katharina Jahn, Christiane Beckmann, Maurice Redondo, Olivier Kobel, Christoph Noppen, Sophie Seidel, Noemie Santamaria de Souza, Niko Beerenwinkel, Tanja Stadler
EPI_ISL_899542, EPI_ISL_899543, EPI_ISL_899545	Viollier AG	Department of Biosystems Science and Engineering, ETH Zürich	Christian Beisel, Sarah Nadeau, Chaoran Chen, Ivan Topolsky, Philipp Jablonski, Lara Fuhrmann, David Dreifuss, Katharina Jahn, Tobias Schär, Ina Nissen, Natascha Santacroce, Elodie Burcklen, Christiane Beckmann, Maurice Redondo, Olivier Kobel, Christoph Noppen, Sophie Seidel, Noemie Santamaria de Souza, Niko Beerenwinkel, Tanja Stadler
EPI_ISL_899546	Viollier AG	Department of Biosystems Science and Engineering, ETH Zürich	Chaoran Chen, Sarah Nadeau, Ivan Topolsky, Emmanouil Dermitzakis, Keith Harshman, Ioannis Xenarios, Henri Pegeot, Lorenzo Cerutti, Deborah Penet, Philipp Jablonski, Lara Fuhrmann, David Dreifuss, Katharina Jahn, Christiane Beckmann, Maurice Redondo, Olivier Kobel, Christoph Noppen, Sophie Seidel, Noemie Santamaria de Souza, Niko Beerenwinkel, Tanja Stadler
EPI_ISL_899554	Viollier AG	Department of Biosystems Science and Engineering, ETH Zürich	Christian Beisel, Sarah Nadeau, Chaoran Chen, Ivan Topolsky, Philipp Jablonski, Lara Fuhrmann, David Dreifuss, Katharina Jahn, Tobias Schär, Ina Nissen, Natascha Santacroce, Elodie Burcklen, Christiane Beckmann, Maurice Redondo, Olivier Kobel, Christoph Noppen, Sophie Seidel, Noemie Santamaria de Souza, Niko Beerenwinkel, Tanja Stadler
EPI_ISL_899557, EPI_ISL_899558, EPI_ISL_899561	Viollier AG	Department of Biosystems Science and Engineering, ETH Zürich	Chaoran Chen, Sarah Nadeau, Ivan Topolsky, Emmanouil Dermitzakis, Keith Harshman, Ioannis Xenarios, Henri Pegeot, Lorenzo Cerutti, Deborah Penet, Philipp Jablonski, Lara Fuhrmann, David Dreifuss, Katharina Jahn, Christiane Beckmann, Maurice Redondo, Olivier Kobel, Christoph Noppen, Sophie Seidel, Noemie Santamaria de Souza, Niko Beerenwinkel, Tanja Stadler
EPI_ISL_899571, EPI_ISL_899572, EPI_ISL_899573	Viollier AG	Department of Biosystems Science and Engineering, ETH Zürich	Christian Beisel, Sarah Nadeau, Chaoran Chen, Ivan Topolsky, Philipp Jablonski, Lara Fuhrmann, David Dreifuss, Katharina Jahn, Tobias Schär, Ina Nissen, Natascha Santacroce, Elodie Burcklen, Christiane Beckmann, Maurice Redondo, Olivier Kobel, Christoph Noppen, Sophie Seidel, Noemie Santamaria de Souza, Niko Beerenwinkel, Tanja Stadler
EPI_ISL_899574, EPI_ISL_899575	Viollier AG	Department of Biosystems Science and Engineering, ETH Zürich	Chaoran Chen, Sarah Nadeau, Ivan Topolsky, Emmanouil Dermitzakis, Keith Harshman, Ioannis Xenarios, Henri Pegeot, Lorenzo Cerutti, Deborah Penet, Philipp Jablonski, Lara Fuhrmann, David Dreifuss, Katharina Jahn, Christiane Beckmann, Maurice Redondo, Olivier Kobel, Christoph Noppen, Sophie Seidel, Noemie Santamaria de Souza, Niko Beerenwinkel, Tanja Stadler
EPI_ISL_899583, EPI_ISL_899584, EPI_ISL_899585, EPI_ISL_899586, EPI_ISL_899587, EPI_ISL_899588, EPI_ISL_899591, EPI_ISL_899592	Viollier AG	Department of Biosystems Science and Engineering, ETH Zürich	Christian Beisel, Sarah Nadeau, Chaoran Chen, Ivan Topolsky, Philipp Jablonski, Lara Fuhrmann, David Dreifuss, Katharina Jahn, Tobias Schär, Ina Nissen, Natascha Santacroce, Elodie Burcklen, Christiane Beckmann, Maurice Redondo, Olivier Kobel, Christoph Noppen, Sophie Seidel, Noemie Santamaria de Souza, Niko Beerenwinkel, Tanja Stadler
EPI_ISL_899596	Viollier AG	Department of Biosystems Science and Engineering, ETH	Chaoran Chen, Sarah Nadeau, Ivan Topolsky, Emmanouil Dermitzakis, Keith Harshman, Ioannis Xenarios, Henri Pegeot, Lorenzo Cerutti, Deborah Penet,

		Zürich	Philipp Jablonski, Lara Fuhrmann, David Dreifuss, Katharina Jahn, Christiane Beckmann, Maurice Redondo, Olivier Kobel, Christoph Noppen, Sophie Seidel, Noemie Santamaria de Souza, Niko Beerenwinkel, Tanja Stadler
EPI_ISL_899600, EPI_ISL_899601, EPI_ISL_899606, EPI_ISL_899609, EPI_ISL_899610	Viollier AG	Department of Biosystems Science and Engineering, ETH Zürich	Christian Beisel, Sarah Nadeau, Chaoran Chen, Ivan Topolsky, Philipp Jablonski, Lara Fuhrmann, David Dreifuss, Katharina Jahn, Tobias Schär, Ina Nissen, Natascha Santacroce, Elodie Burcklen, Christiane Beckmann, Maurice Redondo, Olivier Kobel, Christoph Noppen, Sophie Seidel, Noemie Santamaria de Souza, Niko Beerenwinkel, Tanja Stadler
EPI_ISL_899612, EPI_ISL_899613	Viollier AG	Department of Biosystems Science and Engineering, ETH Zürich	Chaoran Chen, Sarah Nadeau, Ivan Topolsky, Emmanouil Dermitzakis, Keith Harshman, Ioannis Xenarios, Henri Pegeot, Lorenzo Cerutti, Deborah Penet, Philipp Jablonski, Lara Fuhrmann, David Dreifuss, Katharina Jahn, Christiane Beckmann, Maurice Redondo, Olivier Kobel, Christoph Noppen, Sophie Seidel, Noemie Santamaria de Souza, Niko Beerenwinkel, Tanja Stadler
EPI_ISL_899640	Viollier AG	Department of Biosystems Science and Engineering, ETH Zürich	Christian Beisel, Sarah Nadeau, Chaoran Chen, Ivan Topolsky, Philipp Jablonski, Lara Fuhrmann, David Dreifuss, Katharina Jahn, Tobias Schär, Ina Nissen, Natascha Santacroce, Elodie Burcklen, Christiane Beckmann, Maurice Redondo, Olivier Kobel, Christoph Noppen, Sophie Seidel, Noemie Santamaria de Souza, Niko Beerenwinkel, Tanja Stadler
EPI_ISL_899646	Viollier AG	Department of Biosystems Science and Engineering, ETH Zürich	Chaoran Chen, Sarah Nadeau, Ivan Topolsky, Emmanouil Dermitzakis, Keith Harshman, Ioannis Xenarios, Henri Pegeot, Lorenzo Cerutti, Deborah Penet, Philipp Jablonski, Lara Fuhrmann, David Dreifuss, Katharina Jahn, Christiane Beckmann, Maurice Redondo, Olivier Kobel, Christoph Noppen, Sophie Seidel, Noemie Santamaria de Souza, Niko Beerenwinkel, Tanja Stadler
EPI_ISL_899721, EPI_ISL_899725, EPI_ISL_899726, EPI_ISL_899727, EPI_ISL_899728, EPI_ISL_899729, EPI_ISL_899730, EPI_ISL_899731, EPI_ISL_899732, EPI_ISL_899733, EPI_ISL_899734, EPI_ISL_899735, EPI_ISL_899736, EPI_ISL_899738			
see above	Viollier AG	Department of Biosystems Science and Engineering, ETH Zürich	Christian Beisel, Sarah Nadeau, Chaoran Chen, Ivan Topolsky, Philipp Jablonski, Lara Fuhrmann, David Dreifuss, Katharina Jahn, Tobias Schär, Ina Nissen, Natascha Santacroce, Elodie Burcklen, Christiane Beckmann, Maurice Redondo, Olivier Kobel, Christoph Noppen, Sophie Seidel, Noemie Santamaria de Souza, Niko Beerenwinkel, Tanja Stadler
EPI_ISL_899756, EPI_ISL_899757, EPI_ISL_899758	Viollier AG	Department of Biosystems Science and Engineering, ETH Zürich	Chaoran Chen, Sarah Nadeau, Ivan Topolsky, Emmanouil Dermitzakis, Keith Harshman, Ioannis Xenarios, Henri Pegeot, Lorenzo Cerutti, Deborah Penet, Philipp Jablonski, Lara Fuhrmann, David Dreifuss, Katharina Jahn, Christiane Beckmann, Maurice Redondo, Olivier Kobel, Christoph Noppen, Sophie Seidel, Noemie Santamaria de Souza, Niko Beerenwinkel, Tanja Stadler
EPI_ISL_899787, EPI_ISL_899788, EPI_ISL_899789, EPI_ISL_899790, EPI_ISL_899791, EPI_ISL_899793	Viollier AG	Department of Biosystems Science and Engineering, ETH Zürich	Christian Beisel, Sarah Nadeau, Chaoran Chen, Ivan Topolsky, Philipp Jablonski, Lara Fuhrmann, David Dreifuss, Katharina Jahn, Tobias Schär, Ina Nissen, Natascha Santacroce, Elodie Burcklen, Christiane Beckmann, Maurice Redondo, Olivier Kobel, Christoph Noppen, Sophie Seidel, Noemie Santamaria de Souza, Niko Beerenwinkel, Tanja Stadler
EPI_ISL_899805, EPI_ISL_899806, EPI_ISL_899807, EPI_ISL_899808, EPI_ISL_899809	Viollier AG	Department of Biosystems Science and Engineering, ETH Zürich	Chaoran Chen, Sarah Nadeau, Ivan Topolsky, Emmanouil Dermitzakis, Keith Harshman, Ioannis Xenarios, Henri Pegeot, Lorenzo Cerutti, Deborah Penet, Philipp Jablonski, Lara Fuhrmann, David Dreifuss, Katharina Jahn, Christiane Beckmann, Maurice Redondo, Olivier Kobel, Christoph Noppen, Sophie Seidel, Noemie Santamaria de Souza, Niko Beerenwinkel, Tanja Stadler
EPI_ISL_899843, EPI_ISL_899845	Viollier AG	Department of Biosystems Science and Engineering, ETH Zürich	Christian Beisel, Sarah Nadeau, Chaoran Chen, Ivan Topolsky, Philipp Jablonski, Lara Fuhrmann, David Dreifuss, Katharina Jahn, Tobias Schär, Ina Nissen, Natascha Santacroce, Elodie Burcklen, Christiane Beckmann, Maurice Redondo, Olivier Kobel, Christoph Noppen, Sophie Seidel, Noemie Santamaria de Souza, Niko Beerenwinkel, Tanja Stadler
EPI_ISL_899847	Viollier AG	Department of Biosystems Science and Engineering, ETH Zürich	Chaoran Chen, Sarah Nadeau, Ivan Topolsky, Emmanouil Dermitzakis, Keith Harshman, Ioannis Xenarios, Henri Pegeot, Lorenzo Cerutti, Deborah Penet, Philipp Jablonski, Lara Fuhrmann, David Dreifuss, Katharina Jahn, Christiane Beckmann, Maurice Redondo, Olivier Kobel, Christoph Noppen, Sophie Seidel, Noemie Santamaria de Souza, Niko Beerenwinkel, Tanja Stadler
EPI_ISL_899850	Viollier AG	Department of Biosystems Science and Engineering, ETH Zürich	Christian Beisel, Sarah Nadeau, Chaoran Chen, Ivan Topolsky, Philipp Jablonski, Lara Fuhrmann, David Dreifuss, Katharina Jahn, Tobias Schär, Ina Nissen, Natascha Santacroce, Elodie Burcklen, Christiane Beckmann, Maurice Redondo, Olivier Kobel, Christoph Noppen, Sophie Seidel, Noemie Santamaria de Souza, Niko Beerenwinkel, Tanja Stadler
EPI_ISL_899851, EPI_ISL_899852, EPI_ISL_899853	Viollier AG	Department of Biosystems Science and Engineering, ETH Zürich	Chaoran Chen, Sarah Nadeau, Ivan Topolsky, Emmanouil Dermitzakis, Keith Harshman, Ioannis Xenarios, Henri Pegeot, Lorenzo Cerutti, Deborah Penet, Philipp Jablonski, Lara Fuhrmann, David Dreifuss, Katharina Jahn, Christiane Beckmann, Maurice Redondo, Olivier Kobel, Christoph Noppen, Sophie Seidel, Noemie Santamaria de Souza, Niko Beerenwinkel, Tanja Stadler
EPI_ISL_899854, EPI_ISL_899855, EPI_ISL_899856, EPI_ISL_899857, EPI_ISL_899858, EPI_ISL_899859, EPI_ISL_899860, EPI_ISL_899861, EPI_ISL_899864, EPI_ISL_899865, EPI_ISL_899866, EPI_ISL_899867, EPI_ISL_899868, EPI_ISL_899869, EPI_ISL_899870, EPI_ISL_899871, EPI_ISL_899872, EPI_ISL_899873, EPI_ISL_899885			
see above	Viollier AG	Department of Biosystems Science and Engineering, ETH Zürich	Christian Beisel, Sarah Nadeau, Chaoran Chen, Ivan Topolsky, Philipp Jablonski, Lara Fuhrmann, David Dreifuss, Katharina Jahn, Tobias Schär, Ina Nissen, Natascha Santacroce, Elodie Burcklen, Christiane Beckmann, Maurice Redondo, Olivier Kobel, Christoph Noppen, Sophie Seidel, Noemie Santamaria de Souza, Niko Beerenwinkel, Tanja Stadler
EPI_ISL_899889, EPI_ISL_899890, EPI_ISL_899891, EPI_ISL_899892, EPI_ISL_899893, EPI_ISL_899894, EPI_ISL_899895, EPI_ISL_899896, EPI_ISL_899897, EPI_ISL_899898, EPI_ISL_899899, EPI_ISL_899900, EPI_ISL_899901, EPI_ISL_899902, EPI_ISL_899903, EPI_ISL_899904, EPI_ISL_899905, EPI_ISL_899906			
see above	Viollier AG	Department of Biosystems Science and Engineering, ETH Zürich	Chaoran Chen, Sarah Nadeau, Ivan Topolsky, Emmanouil Dermitzakis, Keith Harshman, Ioannis Xenarios, Henri Pegeot, Lorenzo Cerutti, Deborah Penet, Philipp Jablonski, Lara Fuhrmann, David Dreifuss, Katharina Jahn, Christiane Beckmann, Maurice Redondo, Olivier Kobel, Christoph Noppen, Sophie Seidel, Noemie Santamaria de Souza, Niko Beerenwinkel, Tanja Stadler
EPI_ISL_899913, EPI_ISL_899914, EPI_ISL_899916	Viollier AG	Department of Biosystems Science and Engineering, ETH Zürich	Christian Beisel, Sarah Nadeau, Chaoran Chen, Ivan Topolsky, Philipp Jablonski, Lara Fuhrmann, David Dreifuss, Katharina Jahn, Tobias Schär, Ina Nissen, Natascha Santacroce, Elodie Burcklen, Christiane Beckmann, Maurice Redondo, Olivier Kobel, Christoph Noppen, Sophie Seidel, Noemie Santamaria de Souza, Niko Beerenwinkel, Tanja Stadler
EPI_ISL_899919, EPI_ISL_899920, EPI_ISL_899922	Viollier AG	Department of Biosystems Science and Engineering, ETH Zürich	Chaoran Chen, Sarah Nadeau, Ivan Topolsky, Emmanouil Dermitzakis, Keith Harshman, Ioannis Xenarios, Henri Pegeot, Lorenzo Cerutti, Deborah Penet, Philipp Jablonski, Lara Fuhrmann, David Dreifuss, Katharina Jahn, Christiane Beckmann, Maurice Redondo, Olivier Kobel, Christoph Noppen, Sophie Seidel, Noemie Santamaria de Souza, Niko Beerenwinkel, Tanja Stadler
EPI_ISL_900008, EPI_ISL_900009, EPI_ISL_900013, EPI_ISL_900014, EPI_ISL_900015, EPI_ISL_900019, EPI_ISL_900020, EPI_ISL_900021, EPI_ISL_900022	Viollier AG	Department of Biosystems Science and Engineering, ETH Zürich	Christian Beisel, Sarah Nadeau, Chaoran Chen, Ivan Topolsky, Philipp Jablonski, Lara Fuhrmann, David Dreifuss, Katharina Jahn, Tobias Schär, Ina Nissen, Natascha Santacroce, Elodie Burcklen, Christiane Beckmann, Maurice Redondo, Olivier Kobel, Christoph Noppen, Sophie Seidel, Noemie Santamaria de Souza, Niko Beerenwinkel, Tanja Stadler
EPI_ISL_900037, EPI_ISL_900038, EPI_ISL_900039, EPI_ISL_900040, EPI_ISL_900041, EPI_ISL_900042, EPI_ISL_900043, EPI_ISL_900044, EPI_ISL_900045, EPI_ISL_900046, EPI_ISL_900047, EPI_ISL_900048, EPI_ISL_900049, EPI_ISL_900050, EPI_ISL_900051, EPI_ISL_900052, EPI_ISL_900053			
see above	Viollier AG	Department of Biosystems Science and Engineering, ETH Zürich	Chaoran Chen, Sarah Nadeau, Ivan Topolsky, Emmanouil Dermitzakis, Keith Harshman, Ioannis Xenarios, Henri Pegeot, Lorenzo Cerutti, Deborah Penet, Philipp Jablonski, Lara Fuhrmann, David Dreifuss, Katharina Jahn, Christiane Beckmann, Maurice Redondo, Olivier Kobel, Christoph Noppen, Sophie Seidel, Noemie Santamaria de Souza, Niko Beerenwinkel, Tanja Stadler
EPI_ISL_900492, EPI_ISL_900496, EPI_ISL_900501	Althaia. Xarxa Assistencial Universitària de Manresa	IrsiCaixa - Can Ruti CovidSeq	Fundació irsiCaixa. Hospital Universitari Germans Trias i Pujol(HUGTiP), 2a planta, maternal Ctra Canyet s/n, Badalona Gloria Trujillo, Rafel Perez Vidal, Jaume Trape Pujol, Carolina Gonzalez Fernandez, Roger Paredes, Eulalia Grau, Bonaventura Clotet
EPI_ISL_902750	Ospedale "F. Spaziani" Frosinone	INMI Lazzaro Spallanzani IRCCS	F Messina, C.E.M Gruber, B Bartolini, E Giombini, M Rueca, O Butera, C. Gargiulo, C Sias, A Di Caro, MR Capobianchi
EPI_ISL_902751	Ospedale Sandro Pertini ASL Roma2	INMI Lazzaro Spallanzani IRCCS	B Bartolini, O Butera, C.E.M Gruber, M Rueca, F Messina, E Giombini, G Cappiello, MC Cava, MR Capobianchi, A Di Caro
EPI_ISL_902752	Ospedale Sandro Pertini ASL Roma2	INMI Lazzaro Spallanzani IRCCS	O Butera, F Messina, C.E.M Gruber, B Bartolini, E Giombini, M Rueca, S Romano, MC Cava, A Di Caro, MR Capobianchi
EPI_ISL_902753	Azienda Ospedaliera San Camillo Forlanini	INMI Lazzaro Spallanzani IRCCS	C.E.M Gruber, B Bartolini, E Giombini, M Rueca, O Butera, F Messina, D.Gallone, G Parisi, A Di Caro, MR Capobianchi
EPI_ISL_902757	San Camillo De Lellis Rieti Hospital	INMI Lazzaro Spallanzani IRCCS	C.E.M Gruber, B Bartolini, E Giombini, M Rueca, O Butera, F Messina, S Venarubea, A De Luca, A Di Caro, MR Capobianchi
EPI_ISL_902927, EPI_ISL_902928, EPI_ISL_902929, EPI_ISL_902930, EPI_ISL_902931, EPI_ISL_902932, EPI_ISL_902935, EPI_ISL_902936, EPI_ISL_902937, EPI_ISL_902938, EPI_ISL_902939, EPI_ISL_902940, EPI_ISL_902941, EPI_ISL_902943, EPI_ISL_902944, EPI_ISL_902945, EPI_ISL_902946, EPI_ISL_902947			
see above	Maryland Public Health Laboratory	Maryland Public Health Laboratory	Maryland Department of Health Laboratories Administration

EPI_ISL_903348, EPI_ISL_903358	Wyoming Public Health Laboratory	Wyoming Public Health Laboratory	Noah Hull, Taylor Fearing, Lynette Gumbleton, Channing Weber, Ashley Norberg, Bailey Bowcutt, and Wanda Manley
EPI_ISL_903371	Los Angeles County PHL	Los Angeles County PHL	P. Hemarajata et al.
EPI_ISL_904118	New Mexico Department of Health Scientific Laboratory	New Mexico Department of Health Scientific Laboratory	Ellie Johnson, Anastacia Griego-Fisher, D'eldra Malone
EPI_ISL_904123, EPI_ISL_904124	National Institute of Public Health - National Institute of Hygiene	National Institute of Public Health - National Institute of Hygiene	Wokowicz Tomasz, Zacharczuk Katarzyna
EPI_ISL_904135, EPI_ISL_904212, EPI_ISL_904213, EPI_ISL_904214, EPI_ISL_904215, EPI_ISL_904216, EPI_ISL_904247, EPI_ISL_904314, EPI_ISL_904374, EPI_ISL_904378, EPI_ISL_904379, EPI_ISL_904380, EPI_ISL_904588, EPI_ISL_904589, EPI_ISL_904590, EPI_ISL_904591, EPI_ISL_904592, EPI_ISL_904593, EPI_ISL_904594, EPI_ISL_904595, EPI_ISL_904596, EPI_ISL_904597, EPI_ISL_904598, EPI_ISL_904599, EPI_ISL_904600, EPI_ISL_904601, EPI_ISL_904602			
see above	Dutch COVID-19 response team	Erasmus Medical Center	Bas Oude Munnink, Reina Sikkema, David Nieuwenhuijse, Irina Chestakova, Anne van der Linden, Marjan Boter, Emmanuelle Munger, Corine GeurtsvanKessel, Annemiek van der Eijk, Richard Molenkamp, Marion Koopmans, on behalf of the Dutch national COVID-19 response team.
EPI_ISL_904660, EPI_ISL_904682, EPI_ISL_904683, EPI_ISL_904684, EPI_ISL_904686, EPI_ISL_904687, EPI_ISL_904688, EPI_ISL_904691, EPI_ISL_904692, EPI_ISL_904693, EPI_ISL_904694, EPI_ISL_904697, EPI_ISL_904698, EPI_ISL_904699, EPI_ISL_904710, EPI_ISL_904714, EPI_ISL_904719, EPI_ISL_904720, EPI_ISL_904722, EPI_ISL_904724, EPI_ISL_904730, EPI_ISL_904731, EPI_ISL_904732, EPI_ISL_904740, EPI_ISL_904741, EPI_ISL_904744, EPI_ISL_904752, EPI_ISL_904753, EPI_ISL_904754, EPI_ISL_904770, EPI_ISL_904771, EPI_ISL_904778, EPI_ISL_904779, EPI_ISL_904782, EPI_ISL_904783, EPI_ISL_904787, EPI_ISL_904788, EPI_ISL_904791, EPI_ISL_904792, EPI_ISL_904794, EPI_ISL_904799, EPI_ISL_904804, EPI_ISL_904805, EPI_ISL_904806, EPI_ISL_904807, EPI_ISL_904810, EPI_ISL_904814, EPI_ISL_904815, EPI_ISL_904816, EPI_ISL_904817, EPI_ISL_904818, EPI_ISL_904819, EPI_ISL_904820, EPI_ISL_904821, EPI_ISL_904822, EPI_ISL_904823, EPI_ISL_904824, EPI_ISL_904825, EPI_ISL_904828, EPI_ISL_904829, EPI_ISL_904833, EPI_ISL_904834, EPI_ISL_904835, EPI_ISL_904841, EPI_ISL_904843, EPI_ISL_904844, EPI_ISL_904845, EPI_ISL_904851, EPI_ISL_904853, EPI_ISL_904854, EPI_ISL_904855, EPI_ISL_904856, EPI_ISL_904857, EPI_ISL_904858, EPI_ISL_904859, EPI_ISL_904866, EPI_ISL_904867, EPI_ISL_904869, EPI_ISL_904870, EPI_ISL_904875, EPI_ISL_904876, EPI_ISL_904877, EPI_ISL_904879, EPI_ISL_904880, EPI_ISL_904894, EPI_ISL_904906, EPI_ISL_904907, EPI_ISL_904908, EPI_ISL_904909, EPI_ISL_904910, EPI_ISL_904911, EPI_ISL_904912, EPI_ISL_904913, EPI_ISL_904925, EPI_ISL_904926, EPI_ISL_904958, EPI_ISL_904959, EPI_ISL_904960, EPI_ISL_904961, EPI_ISL_904962, EPI_ISL_904963, EPI_ISL_904964, EPI_ISL_904965, EPI_ISL_904966, EPI_ISL_904967, EPI_ISL_904968, EPI_ISL_904969, EPI_ISL_904970, EPI_ISL_904971, EPI_ISL_904976, EPI_ISL_904977, EPI_ISL_904978, EPI_ISL_904979, EPI_ISL_904980, EPI_ISL_904981, EPI_ISL_904995, EPI_ISL_905017, EPI_ISL_905031, EPI_ISL_905032, EPI_ISL_905057, EPI_ISL_905058, EPI_ISL_905062, EPI_ISL_905064, EPI_ISL_905065, EPI_ISL_905068, EPI_ISL_905112, EPI_ISL_905114, EPI_ISL_905115, EPI_ISL_905117, EPI_ISL_905171, EPI_ISL_905177, EPI_ISL_905191, EPI_ISL_905194, EPI_ISL_905239, EPI_ISL_905240, EPI_ISL_905241, EPI_ISL_905242, EPI_ISL_905251, EPI_ISL_905252, EPI_ISL_905253, EPI_ISL_905255, EPI_ISL_905256, EPI_ISL_905257, EPI_ISL_905258, EPI_ISL_905259, EPI_ISL_905306, EPI_ISL_905309, EPI_ISL_905310, EPI_ISL_905608, EPI_ISL_905611, EPI_ISL_905612, EPI_ISL_905613, EPI_ISL_905614, EPI_ISL_905660, EPI_ISL_905694, EPI_ISL_905695, EPI_ISL_905696, EPI_ISL_905721, EPI_ISL_905722, EPI_ISL_905726, EPI_ISL_905727, EPI_ISL_905730			
see above	Dutch COVID-19 response team	National Institute for Public Health and the Environment (RIVM)	Adam Meijer, Harry Vennema, Dirk Eggink, Jeroen Cremer, Sharon van den Brink, Bas van der Veer, AnneMarie van den Brandt, Florian Zwagemaker, Dennis Schmitz, Chantal Reusken, on behalf of the national COVID-19 response team
EPI_ISL_905739, EPI_ISL_905748, EPI_ISL_905749, EPI_ISL_905750, EPI_ISL_905751, EPI_ISL_905754, EPI_ISL_905755	National Institute of Public Health - National Institute of Hygiene	National Institute of Public Health - National Institute of Hygiene	Wokowicz Tomasz, Zacharczuk Katarzyna
EPI_ISL_906149, EPI_ISL_906151, EPI_ISL_906152, EPI_ISL_906154, EPI_ISL_906155, EPI_ISL_906156, EPI_ISL_906157, EPI_ISL_906160, EPI_ISL_906162, EPI_ISL_906163, EPI_ISL_906165, EPI_ISL_906166, EPI_ISL_906168, EPI_ISL_906170, EPI_ISL_906171, EPI_ISL_906173, EPI_ISL_906175, EPI_ISL_906177, EPI_ISL_906179, EPI_ISL_906180, EPI_ISL_906183, EPI_ISL_906184, EPI_ISL_906186, EPI_ISL_906188			
see above	University of Wisconsin-Madison AIDS Vaccine Research Laboratories	University of Wisconsin-Madison AIDS Vaccine Research Laboratories	Gage Moreno, Katarina Braun, et al. AIDS Vaccine Research Laboratories
EPI_ISL_906282, EPI_ISL_906283	Nigeria Centre for Disease Control (NCDC)	African Centre of Excellence for Genomics of Infectious Diseases (ACEGID), Redeemer's University	Oluniyi P.E. et al
EPI_ISL_906530	Laboratorio de salud publica de Bogota	Instituto Nacional de Salud- Dirección de Investigación en Salud Pública, Universidad de los Andes- Applied genomics research group, Vicerrectoria de Investigación y Creación, Universidad de los Andes- Systems and Computing Engineering Department	Katherine Laiton-Donato, Diego A. Álvarez-Díaz, Carlos Franco-Muñoz, Mauricio Pacheco-Montealegre, Héctor Alejandro Ruiz-Moreno, Maria T. Herrera-Sepúlveda, Diego Andrés Prada, Jhonnatan Reales-González, Sheryll Corchuelo, Julian Naizaque, Gerardo Santamaría Jorge Duitama, Laura Natalia Gonzalez, Jorge Ivan Diaz, Silvia Restrepo-Restrepo, Magdalena Wiesner, Martha Lucia Ospina Martinez, Marcela Mercado-Reyes
EPI_ISL_906531	Laboratorio de salud publica del Valle del Cauca	Instituto Nacional de Salud- Dirección de Investigación en Salud Pública, Universidad de los Andes- Applied genomics research group, Vicerrectoria de Investigación y Creación, Universidad de los Andes- Systems and Computing Engineering Department	Katherine Laiton-Donato, Diego A. Álvarez-Díaz, Carlos Franco-Muñoz, Mauricio Pacheco-Montealegre, Héctor Alejandro Ruiz-Moreno, Maria T. Herrera-Sepúlveda, Diego Andrés Prada, Jhonnatan Reales-González, Sheryll Corchuelo, Julian Naizaque, Gerardo Santamaría Jorge Duitama, Laura Natalia Gonzalez, Jorge Ivan Diaz, Silvia Restrepo-Restrepo, Magdalena Wiesner, Martha Lucia Ospina Martinez, Marcela Mercado-Reyes
EPI_ISL_906532	Hospital San Jose de Maicao	Instituto Nacional de Salud- Dirección de Investigación en Salud Pública, Universidad de los Andes- Applied genomics research group, Vicerrectoria de Investigación y Creación, Universidad de los Andes- Systems and Computing Engineering Department	Katherine Laiton-Donato, Diego A. Álvarez-Díaz, Carlos Franco-Muñoz, Mauricio Pacheco-Montealegre, Héctor Alejandro Ruiz-Moreno, Maria T. Herrera-Sepúlveda, Diego Andrés Prada, Jhonnatan Reales-González, Sheryll Corchuelo, Julian Naizaque, Gerardo Santamaría Jorge Duitama, Laura Natalia Gonzalez, Jorge Ivan Diaz, Silvia Restrepo-Restrepo, Magdalena Wiesner, Martha Lucia Ospina Martinez, Marcela Mercado-Reyes
EPI_ISL_906552	Laboratorio Angel Diagnostica	Instituto Nacional de Salud- Dirección de Investigación en Salud Pública, Universidad de los Andes- Applied genomics research group, Vicerrectoria de Investigación y Creación, Universidad de los Andes- Systems and Computing Engineering Department	Katherine Laiton-Donato, Diego A. Álvarez-Díaz, Carlos Franco-Muñoz, Mauricio Pacheco-Montealegre, Héctor Alejandro Ruiz-Moreno, Maria T. Herrera-Sepúlveda, Diego Andrés Prada, Jhonnatan Reales-González, Sheryll Corchuelo, Julian Naizaque, Gerardo Santamaría Jorge Duitama, Laura Natalia Gonzalez, Jorge Ivan Diaz, Silvia Restrepo-Restrepo, Magdalena Wiesner, Martha Lucia Ospina Martinez, Marcela Mercado-Reyes
EPI_ISL_906556	DIRECCION DEPARTAMENTAL DE SALUD DE GUAJIRA	Instituto Nacional de Salud- Dirección de Investigación en Salud Pública, Universidad de los Andes- Applied genomics research group, Vicerrectoria de Investigación y Creación, Universidad de los Andes- Systems and Computing Engineering Department	Katherine Laiton-Donato, Diego A. Álvarez-Díaz, Carlos Franco-Muñoz, Mauricio Pacheco-Montealegre, Héctor Alejandro Ruiz-Moreno, Maria T. Herrera-Sepúlveda, Diego Andrés Prada, Jhonnatan Reales-González, Sheryll Corchuelo, Julian Naizaque, Gerardo Santamaría Jorge Duitama, Laura Natalia Gonzalez, Jorge Ivan Diaz, Silvia Restrepo-Restrepo, Magdalena Wiesner, Martha Lucia Ospina Martinez, Marcela Mercado-Reyes
EPI_ISL_906557	Laboratorio de Salud Publica de Amazonas	Instituto Nacional de Salud- Dirección de Investigación en Salud Pública, Universidad de los Andes- Applied genomics research group, Vicerrectoria de Investigación y Creación, Universidad de los Andes- Systems and Computing Engineering Department	Katherine Laiton-Donato, Diego A. Álvarez-Díaz, Carlos Franco-Muñoz, Mauricio Pacheco-Montealegre, Héctor Alejandro Ruiz-Moreno, Maria T. Herrera-Sepúlveda, Diego Andrés Prada, Jhonnatan Reales-González, Sheryll Corchuelo, Julian Naizaque, Gerardo Santamaría Jorge Duitama, Laura Natalia Gonzalez, Jorge Ivan Diaz, Silvia Restrepo-Restrepo, Magdalena Wiesner, Martha Lucia Ospina Martinez, Marcela Mercado-Reyes
EPI_ISL_906603, EPI_ISL_906604, EPI_ISL_906605, EPI_ISL_906606, EPI_ISL_906607, EPI_ISL_906608, EPI_ISL_906609, EPI_ISL_906610, EPI_ISL_906611, EPI_ISL_906612, EPI_ISL_906613, EPI_ISL_906614, EPI_ISL_906615, EPI_ISL_906616, EPI_ISL_906617, EPI_ISL_906618, EPI_ISL_906619, EPI_ISL_906620, EPI_ISL_906621, EPI_ISL_906622, EPI_ISL_906623, EPI_ISL_906624, EPI_ISL_906625, EPI_ISL_906626, EPI_ISL_906627, EPI_ISL_906628, EPI_ISL_906629, EPI_ISL_906630, EPI_ISL_906631, EPI_ISL_906632, EPI_ISL_906633, EPI_ISL_906634, EPI_ISL_906635, EPI_ISL_906636, EPI_ISL_906637, EPI_ISL_906638, EPI_ISL_906639, EPI_ISL_906640, EPI_ISL_906641, EPI_ISL_906642, EPI_ISL_906643, EPI_ISL_906644, EPI_ISL_906645, EPI_ISL_906646, EPI_ISL_906647, EPI_ISL_906648, EPI_ISL_906649, EPI_ISL_906650, EPI_ISL_906651			
see above	Maine Health and Environmental Testing Laboratory (Maine HETL)	Tewhey Lab, The Jackson Laboratory	Matluk,N., Dewey,H., Iosue,F., Barter,M., Lynch,R., Munger,H. and Tewhey,R.
EPI_ISL_906720, EPI_ISL_906727, EPI_ISL_906730, EPI_ISL_906736	Laboratory of Molecular Biology, Diagnostyka sp. z o.o.	Laboratory of Recombinant Vaccines	Lukasz Rabalski, Maciej Kosinski, Anna Piotrowska-Mietelska,Izabela Szczygielska, Boguslaw Szewczyk, Krystyna Bienkowska-Szewczyk
EPI_ISL_906793, EPI_ISL_906796	Innlandet Hospital Trust, Division Lillehammer, Department for Medical Microbiology	Norwegian Institute of Public Health, Department of Virology	Kathrine Stene-Johansen, Kamilla Heddeland Instefjord, Hilde Elshaug, Atiya R Ali,Marie Paulsen Madsen, Rasmus Riis Kopperud, Hilde Vollan, Karoline Bragstad, Olav Hungnes
EPI_ISL_906808, EPI_ISL_906809, EPI_ISL_906810, EPI_ISL_906811	University Hospital of Northern Norway, Department for Microbiology and Infectious Disease Control	Norwegian Institute of Public Health, Department of Virology	Kathrine Stene-Johansen, Kamilla Heddeland Instefjord, Hilde Elshaug, Atiya R Ali,Marie Paulsen Madsen, Rasmus Riis Kopperud, Hilde Vollan, Karoline Bragstad, Olav Hungnes
EPI_ISL_906812	Innlandet Hospital Trust, Division Lillehammer, Department for Medical Microbiology	Norwegian Institute of Public Health, Department of Virology	Kathrine Stene-Johansen, Kamilla Heddeland Instefjord, Hilde Elshaug, Atiya R Ali,Marie Paulsen Madsen, Rasmus Riis Kopperud, Hilde Vollan, Karoline Bragstad, Olav Hungnes
EPI_ISL_906817, EPI_ISL_906819, EPI_ISL_906820	Akershus University Hospital, Department for Microbiology and Infectious Disease Control	Norwegian Institute of Public Health, Department of Virology	Kathrine Stene-Johansen, Kamilla Heddeland Instefjord, Hilde Elshaug, Atiya R Ali,Marie Paulsen Madsen, Rasmus Riis Kopperud, Hilde Vollan, Karoline Bragstad, Olav Hungnes

EPI_ISL_906821	Oslo University Hospital, Department of Medical Microbiology	Norwegian Institute of Public Health, Department of Virology	Kathrine Stene-Johansen, Kamilla Heddeland Instefjord, Hilde Elshaug, Atiya R Ali,Marie Paulsen Madsen, Rasmus Riis Kopperud, Hilde Vollan, Karoline Bragstad, Olav Hungnes
EPI_ISL_906825	Department of Medical Microbiology - section Molde, Molde Hospital	Norwegian Institute of Public Health, Department of Virology	Kathrine Stene-Johansen, Kamilla Heddeland Instefjord, Hilde Elshaug, Atiya R Ali,Marie Paulsen Madsen, Rasmus Riis Kopperud, Hilde Vollan, Karoline Bragstad, Olav Hungnes
EPI_ISL_906916, EPI_ISL_906917	Bureau of Public Health Laboratories, Florida Department of Health (BPHL, FLDOH)	Bureau of Public Health Laboratories, Florida Department of Health (BPHL, FLDOH)	Schmedes,S., Blanton,J.
EPI_ISL_908934, EPI_ISL_908966, EPI_ISL_908969, EPI_ISL_908971, EPI_ISL_908972, EPI_ISL_908973, EPI_ISL_908975, EPI_ISL_908980, EPI_ISL_908981, EPI_ISL_909000, EPI_ISL_909001, EPI_ISL_909033, EPI_ISL_909045, EPI_ISL_909074, EPI_ISL_909098, EPI_ISL_909112, EPI_ISL_909115, EPI_ISL_909140, EPI_ISL_909142, EPI_ISL_909151, EPI_ISL_909164, EPI_ISL_909168, EPI_ISL_909175, EPI_ISL_909188, EPI_ISL_909190, EPI_ISL_909225, EPI_ISL_909239, EPI_ISL_909249, EPI_ISL_909253			
see above	Lighthouse Lab in Milton Keynes	Wellcome Sanger Institute for the COVID-19 Genomics UK (COG-UK) Consortium	The Lighthouse Lab in Milton Keynes and Alex Alderton, Roberto Amato, Sonia Goncalves, Ewan Harrison, David K. Jackson, Ian Johnston, Dominic Kwiatkowski, Cordelia Langford, John Sillitoe on behalf of the Wellcome Sanger Institute COVID-19 Surveillance Team
EPI_ISL_909657, EPI_ISL_909659	Labo Analyses med	National Reference Center for Viruses of Respiratory Infections, Institut Pasteur, Paris	Marion Barbet, Sylvie Behillil, Méline Bizard, Angela Brisebarre, Camille Capel, Etienne Simon-Lorière, Vincent Enouf, Maud Vanpeene, Sylvie van der Werf,Amzalag Jonas
EPI_ISL_909666	Hopital Avicenne. Laboratoire central de bactériologie virologie hygiène	National Reference Center for Viruses of Respiratory Infections, Institut Pasteur, Paris	Marion Barbet, Sylvie Behillil, Méline Bizard, Angela Brisebarre, Camille Capel, Etienne Simon-Lorière, Vincent Enouf, Maud Vanpeene, Sylvie van der Werf,Brichler Ségolène
EPI_ISL_909667, EPI_ISL_909668	Labo Analyses Med	National Reference Center for Viruses of Respiratory Infections, Institut Pasteur, Paris	Marion Barbet, Sylvie Behillil, Méline Bizard, Angela Brisebarre, Camille Capel, Etienne Simon-Lorière, Vincent Enouf, Maud Vanpeene, Sylvie van der Werf,Le Vicky
EPI_ISL_909674	CH de Mayotte - Laboratoire de Biologie	National Reference Center for Viruses of Respiratory Infections, Institut Pasteur, Paris	Marion Barbet, Sylvie Behillil, Méline Bizard, Angela Brisebarre, Camille Capel, Etienne Simon-Lorière, Vincent Enouf, Maud Vanpeene, Sylvie van der Werf,Combe Patrice
EPI_ISL_909682, EPI_ISL_909683	Hopital Avicenne. Laboratoire central de bactériologie virologie hygiène	National Reference Center for Viruses of Respiratory Infections, Institut Pasteur, Paris	Marion Barbet, Sylvie Behillil, Méline Bizard, Angela Brisebarre, Camille Capel, Etienne Simon-Lorière, Vincent Enouf, Maud Vanpeene, Sylvie van der Werf,Brichler Ségolène
EPI_ISL_909687, EPI_ISL_909697	CH de Mayotte - Laboratoire de Biologie	National Reference Center for Viruses of Respiratory Infections, Institut Pasteur, Paris	Marion Barbet, Sylvie Behillil, Méline Bizard, Angela Brisebarre, Camille Capel, Etienne Simon-Lorière, Vincent Enouf, Maud Vanpeene, Sylvie van der Werf,Combe Patrice
EPI_ISL_909702	CHU de Nantes - Hôtel Dieu. Laboratoire de Virologie	National Reference Center for Viruses of Respiratory Infections, Institut Pasteur, Paris	Marion Barbet, Sylvie Behillil, Méline Bizard, Angela Brisebarre, Camille Capel, Etienne Simon-Lorière, Vincent Enouf, Maud Vanpeene, Sylvie van der Werf,Bressollette CéLine
EPI_ISL_909707	Labo Analyses Med	National Reference Center for Viruses of Respiratory Infections, Institut Pasteur, Paris	Marion Barbet, Sylvie Behillil, Méline Bizard, Angela Brisebarre, Camille Capel, Etienne Simon-Lorière, Vincent Enouf, Maud Vanpeene, Sylvie van der Werf
EPI_ISL_909708, EPI_ISL_909709, EPI_ISL_909714, EPI_ISL_909723	CH de Mayotte - Laboratoire de Biologie	National Reference Center for Viruses of Respiratory Infections, Institut Pasteur, Paris	Marion Barbet, Sylvie Behillil, Méline Bizard, Angela Brisebarre, Camille Capel, Etienne Simon-Lorière, Vincent Enouf, Maud Vanpeene, Sylvie van der Werf,Combe Patrice
EPI_ISL_909724, EPI_ISL_909725, EPI_ISL_909726, EPI_ISL_909727, EPI_ISL_909729, EPI_ISL_909730, EPI_ISL_909731	Labo Analyses Med	National Reference Center for Viruses of Respiratory Infections, Institut Pasteur, Paris	Marion Barbet, Sylvie Behillil, Méline Bizard, Angela Brisebarre, Camille Capel, Etienne Simon-Lorière, Vincent Enouf, Maud Vanpeene, Sylvie van der Werf,Nizard Patrice
EPI_ISL_909733, EPI_ISL_909734, EPI_ISL_909735	Labo Analyses Med	National Reference Center for Viruses of Respiratory Infections, Institut Pasteur, Paris	Marion Barbet, Sylvie Behillil, Méline Bizard, Angela Brisebarre, Camille Capel, Etienne Simon-Lorière, Vincent Enouf, Maud Vanpeene, Sylvie van der Werf
EPI_ISL_909747, EPI_ISL_909752, EPI_ISL_909753, EPI_ISL_909754, EPI_ISL_909758, EPI_ISL_909759, EPI_ISL_909760, EPI_ISL_909771	Charité Universitätsmedizin Berlin, Institut für Virologie/Labor Berlin	Charité Universitätsmedizin Berlin, Institut für Virologie	Victor M Corman, Barbara Mühlemann, Jörn Beheim-Schwarzbach, Tobias Bleicker, Julia Tesch, Talitha Veith, Julia Schneider, Terry Jones, Christian Drosten
EPI_ISL_909775, EPI_ISL_909816, EPI_ISL_909817, EPI_ISL_909818, EPI_ISL_909827, EPI_ISL_909828, EPI_ISL_909829, EPI_ISL_909830, EPI_ISL_909831, EPI_ISL_909833, EPI_ISL_909834, EPI_ISL_909841, EPI_ISL_909844, EPI_ISL_909847, EPI_ISL_909851, EPI_ISL_909854, EPI_ISL_909855, EPI_ISL_909856, EPI_ISL_909857, EPI_ISL_909859, EPI_ISL_909860, EPI_ISL_909861, EPI_ISL_909862, EPI_ISL_909864, EPI_ISL_909865, EPI_ISL_909866, EPI_ISL_909867, EPI_ISL_909869, EPI_ISL_909870, EPI_ISL_909871, EPI_ISL_909872, EPI_ISL_909875, EPI_ISL_909903, EPI_ISL_909905, EPI_ISL_909908, EPI_ISL_909909, EPI_ISL_909910, EPI_ISL_909911, EPI_ISL_909912, EPI_ISL_909914, EPI_ISL_909918, EPI_ISL_909920, EPI_ISL_909921, EPI_ISL_909923, EPI_ISL_909924, EPI_ISL_909929, EPI_ISL_909933, EPI_ISL_909936, EPI_ISL_909938, EPI_ISL_909940, EPI_ISL_909942, EPI_ISL_909943, EPI_ISL_909944			
see above	National Virus Reference Laboratory	National Virus Reference Laboratory	Michael Carr, Gabriel Gonzalez, Jonathan Dean, Cillian F De Gascun
EPI_ISL_911134, EPI_ISL_911136, EPI_ISL_911137, EPI_ISL_911138, EPI_ISL_911139, EPI_ISL_911140, EPI_ISL_911141, EPI_ISL_911142, EPI_ISL_911143, EPI_ISL_911144, EPI_ISL_911145, EPI_ISL_911146, EPI_ISL_911147, EPI_ISL_911148, EPI_ISL_911149, EPI_ISL_911150, EPI_ISL_911151, EPI_ISL_911152, EPI_ISL_911153, EPI_ISL_911154, EPI_ISL_911155, EPI_ISL_911156, EPI_ISL_911157, EPI_ISL_911158, EPI_ISL_911159, EPI_ISL_911160, EPI_ISL_911161, EPI_ISL_911162, EPI_ISL_911163, EPI_ISL_911164, EPI_ISL_911165, EPI_ISL_911166, EPI_ISL_911232, EPI_ISL_911234			
see above	Laboratoire national de sante, Microbiology, Virology	Laboratoire national de sante, Microbiology, Microbial Genomics Platform	Anke Wienecke-Baldacchino, Catherine Ragimbeau,Jessica Tapp, Fatu Djabi, Lise Pignon, Raoul Salmon, Tamir Abdelrahman
EPI_ISL_911242, EPI_ISL_911243, EPI_ISL_911244, EPI_ISL_911246	National Virus Reference Laboratory	National Virus Reference Laboratory	Michael Carr, Gabriel Gonzalez, Jonathan Dean, Cillian F De Gascun
EPI_ISL_911295, EPI_ISL_911305, EPI_ISL_911315, EPI_ISL_911325, EPI_ISL_911332, EPI_ISL_911340, EPI_ISL_911349	Servicio de Microbiología, Hospital Universitario Son Espases	SeqCOVID-SPAIN consortium/IBV(CSIC)	Carla López-Causapé, Jordi Reina, Antonio Oliver and SeqCOVID-SPAIN consortium
EPI_ISL_911383, EPI_ISL_911384, EPI_ISL_911385	University of Michigan Clinical Microbiology Laboratory	Lauring Lab, University of Michigan, Department of Microbiology and Immunology	Valesano
EPI_ISL_911580	Clinical Molecular Microbiology Laboratory, UNC Hospitals	Jeremy Wang	Jeremy Wang, Alexander Rubinsteyn, Colleen Rice, Jason Smedberg, Melissa Miller, Corbin Jones, Robert Hagan
EPI_ISL_911746, EPI_ISL_911747	Toronto Invasive Bacterial Diseases Network	McMaster University	Allison McGeer, Patryk Aftanas, Hooman Derakhshani, Angel Li, Kuganya Nirmalarajah, Emily Panousis, Ahmed Draia, Jalees Nasir, Michael Surette, Samira Mubareka, Andrew G. McArthur
EPI_ISL_911767, EPI_ISL_911774, EPI_ISL_911792, EPI_ISL_911793, EPI_ISL_911796, EPI_ISL_911797, EPI_ISL_911798, EPI_ISL_911902, EPI_ISL_911903, EPI_ISL_911907, EPI_ISL_911908, EPI_ISL_911909, EPI_ISL_911910			
see above	Johns Hopkins Hospital Department of Pathology	Johns Hopkins Hospital Department of Pathology	C. Paul Morris, Chun Huai Luo, Adannaya Amadi, Matthew Schwartz, Nicholas Gallagher, Heba H. Mostafa
EPI_ISL_911918, EPI_ISL_911919, EPI_ISL_911920, EPI_ISL_911921, EPI_ISL_911922	SRL (Sonic Reference Laboratory - Sonic Healthcare)	Pathogen Discovery, Respiratory Viruses Branch, Division of Viral Diseases, Centers for Disease Control and Prevention	Ying Tao, Yan Li, Jing Zhang, Krista Queen, Anna Uehara, Peter Cook, Clinton R. Paden, Haibin Wang, Suxiang Tong
EPI_ISL_911952, EPI_ISL_911953, EPI_ISL_911954, EPI_ISL_911955, EPI_ISL_911956, EPI_ISL_911957, EPI_ISL_911958, EPI_ISL_911959, EPI_ISL_911960, EPI_ISL_911961, EPI_ISL_911962, EPI_ISL_911963, EPI_ISL_911964, EPI_ISL_911965			
see above	Wyoming Public Health Laboratory	Wyoming Public Health Laboratory	Noah Hull, Taylor Fearing, Lynette Gumbleton, Channing Weber, Ashley Norberg, Bailey Bowcutt, and Wanda Manley
EPI_ISL_912094, EPI_ISL_912096, EPI_ISL_912097, EPI_ISL_912098, EPI_ISL_912099, EPI_ISL_912100, EPI_ISL_912101, EPI_ISL_912102, EPI_ISL_912103, EPI_ISL_912104, EPI_ISL_912105, EPI_ISL_912106, EPI_ISL_912107, EPI_ISL_912108			
see above	Washington State Department of Health	Seattle Flu Study	Deborah A. Nickerson, Chris D. Frazar, Jover Lee, Benjamin Pelle, Erica Ryke, Matthew Richardson, Amanda Adler, Elisabeth Brandstetter, Peter D. Han, Kairsten Fay, Misja Ilcisin, Kirsten Lacombe, Thomas R. Sibley, Melissa Truong, Caitlin R. Wolf, Romesh Gautom, Geoff Melly, Brian Hiatt, Philip Dykema, Scott Lindquist, Michael Boeckh, Janet A. Englund, Michael Famulare, Barry R. Lutz, Mark J. Rieder, Lea M. Starita, Matthew Thompson, Helen Y. Chu, Jay Shendure, Trevor Bedford
EPI_ISL_912227, EPI_ISL_912228, EPI_ISL_912229, EPI_ISL_912230, EPI_ISL_912231, EPI_ISL_912232, EPI_ISL_912233, EPI_ISL_912234, EPI_ISL_912235, EPI_ISL_912247, EPI_ISL_912249			
see above	Charité Universitätsmedizin Berlin, Institut für Virologie/Labor	Charité Universitätsmedizin Berlin, Institut für Virologie	Victor M Corman, Barbara Mühlemann, Jörn Beheim-Schwarzbach, Tobias Bleicker, Julia Tesch, Talitha Veith, Julia Schneider, Terry Jones, Christian

	Berlin		Drosten
EPI_ISL_912274, EPI_ISL_912275, EPI_ISL_912277, EPI_ISL_912278, EPI_ISL_912281, EPI_ISL_912284, EPI_ISL_912286, EPI_ISL_912290, EPI_ISL_912303, EPI_ISL_912310, EPI_ISL_912314			
see above	Hospital General Universitario Gregorio Marañón	SeqCOVID-SPAIN consortium / IBV (CSIC)	Darío García de Viedma, Laura Pérez-Lago, Pedro J Sola-Campoy, Sergio Buenestado-Serrano, Marta Herranz, Victor Manuel de la Cueva, Julia Suárez, Pilar Catalán, Patricia Muñoz and SeqCOVID-SPAIN consortium
EPI_ISL_912316	Hospital General Universitario Gregorio Marañón	SeqCOVID-SPAIN consortium / IBV (CSIC)	Sergio Buenestado Serrano, Pedro J. Sola Campoy, Laura Pérez-Lago, Pilar Catalán, Arturo fraile Torres, Andrés Von Wernitz, Carmen del Arco, Patricia Muñoz, Laura Cardeñoso, Darío García de Viedma and SeqCOVID-SPAIN consortium
EPI_ISL_912320, EPI_ISL_912323, EPI_ISL_912328, EPI_ISL_912329	Hospital General Universitario Gregorio Marañón	SeqCOVID-SPAIN consortium / IBV (CSIC)	Darío García de Viedma, Laura Pérez-Lago, Pedro J Sola-Campoy, Sergio Buenestado-Serrano, Marta Herranz, Victor Manuel de la Cueva, Julia Suárez, Pilar Catalán, Patricia Muñoz and SeqCOVID-SPAIN consortium
EPI_ISL_912337, EPI_ISL_912338	Hospital General Universitario Gregorio Marañón	SeqCOVID-SPAIN consortium / IBV (CSIC)	Sergio Buenestado Serrano, Pedro J. Sola Campoy, Laura Pérez-Lago, Pilar Catalán, Arturo fraile Torres, Andrés Von Wernitz, Carmen del Arco, Patricia Muñoz, Laura Cardeñoso, Darío García de Viedma and SeqCOVID-SPAIN consortium
EPI_ISL_912340, EPI_ISL_912343, EPI_ISL_912344, EPI_ISL_912348, EPI_ISL_912350	Hospital General Universitario Gregorio Marañón	SeqCOVID-SPAIN consortium / IBV (CSIC)	Darío García de Viedma, Laura Pérez-Lago, Pedro J Sola-Campoy, Sergio Buenestado-Serrano, Marta Herranz, Victor Manuel de la Cueva, Julia Suárez, Pilar Catalán, Patricia Muñoz and SeqCOVID-SPAIN consortium
EPI_ISL_912402	CH de Mayotte - Laboratoire de Biologie	National Reference Center for Viruses of Respiratory Infections, Institut Pasteur, Paris	Marion Barbet, Sylvie Behillil, Méline Bizard, Angela Brisebarre, Camille Capel, Etienne Simon-Lorière, Vincent Enouf, Maud Vanpeene, Sylvie van der Werf,Combe Patrice
EPI_ISL_912424, EPI_ISL_912428, EPI_ISL_912430, EPI_ISL_912431, EPI_ISL_912432, EPI_ISL_912433, EPI_ISL_912434, EPI_ISL_912435, EPI_ISL_912436, EPI_ISL_912437, EPI_ISL_912438, EPI_ISL_912439, EPI_ISL_912440, EPI_ISL_912441, EPI_ISL_912442, EPI_ISL_912443, EPI_ISL_912444, EPI_ISL_912445, EPI_ISL_912446, EPI_ISL_912447, EPI_ISL_912448			
see above	KU Leuven, Rega Institute, Clinical and Epidemiological Virology	KU Leuven, Rega Institute, Clinical and Epidemiological Virology	Tony Wawina-Bokalanga, Bert Vanmechelen, Joan Marti-Carerras, Piet Maes
EPI_ISL_912554, EPI_ISL_912555, EPI_ISL_912556	C.H.R. d'Orléans - Hôpital de la Source	National Reference Center for Viruses of Respiratory Infections, Institut Pasteur, Paris	Marion Barbet, Sylvie Behillil, Méline Bizard, Angela Brisebarre, Camille Capel, Etienne Simon-Lorière, Vincent Enouf, Maud Vanpeene, Sylvie van der Werf,Guinard Jérome
EPI_ISL_912559	Hopital Avicenne Laboratoire central de bactériologie virologie hygiène	National Reference Center for Viruses of Respiratory Infections, Institut Pasteur, Paris	Marion Barbet, Sylvie Behillil, Méline Bizard, Angela Brisebarre, Camille Capel, Etienne Simon-Lorière, Vincent Enouf, Maud Vanpeene, Sylvie van der Werf,Brichler Ségolène
EPI_ISL_912563, EPI_ISL_912564	Laboratoire Central - American Hospital of Paris	National Reference Center for Viruses of Respiratory Infections, Institut Pasteur, Paris	Marion Barbet, Sylvie Behillil, Méline Bizard, Angela Brisebarre, Camille Capel, Etienne Simon-Lorière, Vincent Enouf, Maud Vanpeene, Sylvie van der Werf,Ramirez José-Manuel
EPI_ISL_912566	Hopital	National Reference Center for Viruses of Respiratory Infections, Institut Pasteur, Paris	Marion Barbet, Sylvie Behillil, Méline Bizard, Angela Brisebarre, Camille Capel, Etienne Simon-Lorière, Vincent Enouf, Maud Vanpeene, Sylvie van der Werf
EPI_ISL_912569	Labo Analyses Med	National Reference Center for Viruses of Respiratory Infections, Institut Pasteur, Paris	Marion Barbet, Sylvie Behillil, Méline Bizard, Angela Brisebarre, Camille Capel, Etienne Simon-Lorière, Vincent Enouf, Maud Vanpeene, Sylvie van der Werf
EPI_ISL_912573	C.H.R. d'Orléans - Hôpital de la Source	National Reference Center for Viruses of Respiratory Infections, Institut Pasteur, Paris	Marion Barbet, Sylvie Behillil, Méline Bizard, Angela Brisebarre, Camille Capel, Etienne Simon-Lorière, Vincent Enouf, Maud Vanpeene, Sylvie van der Werf,Guinard Jérome
EPI_ISL_912574	Laboratoire Central - American Hospital of Paris	National Reference Center for Viruses of Respiratory Infections, Institut Pasteur, Paris	Marion Barbet, Sylvie Behillil, Méline Bizard, Angela Brisebarre, Camille Capel, Etienne Simon-Lorière, Vincent Enouf, Maud Vanpeene, Sylvie van der Werf,Ramirez José-Manuel
EPI_ISL_912575, EPI_ISL_912576	Laboratoire de Virologie du CHU de Lille Bâtiment Paul Boulanger	National Reference Center for Viruses of Respiratory Infections, Institut Pasteur, Paris	Marion Barbet, Sylvie Behillil, Méline Bizard, Angela Brisebarre, Camille Capel, Etienne Simon-Lorière, Vincent Enouf, Maud Vanpeene, Sylvie van der Werf,Guigon Aurélie
EPI_ISL_912582, EPI_ISL_912583	CENTRE HOSPITALIER LEON BINET	National Reference Center for Viruses of Respiratory Infections, Institut Pasteur, Paris	Marion Barbet, Sylvie Behillil, Méline Bizard, Angela Brisebarre, Camille Capel, Etienne Simon-Lorière, Vincent Enouf, Maud Vanpeene, Sylvie van der Werf,Meziane Ilham
EPI_ISL_912599	HOPITAL SIMONE VEIL	National Reference Center for Viruses of Respiratory Infections, Institut Pasteur, Paris	Marion Barbet, Sylvie Behillil, Méline Bizard, Angela Brisebarre, Camille Capel, Etienne Simon-Lorière, Vincent Enouf, Maud Vanpeene, Sylvie van der Werf,Moreau Farida
EPI_ISL_912600	Labo Analyses Med	National Reference Center for Viruses of Respiratory Infections, Institut Pasteur, Paris	Marion Barbet, Sylvie Behillil, Méline Bizard, Angela Brisebarre, Camille Capel, Etienne Simon-Lorière, Vincent Enouf, Maud Vanpeene, Sylvie van der Werf,Gaumery Bertrand
EPI_ISL_912602	Laboratoire de Virologie du CHU de Lille Bâtiment Paul Boulanger	National Reference Center for Viruses of Respiratory Infections, Institut Pasteur, Paris	Marion Barbet, Sylvie Behillil, Méline Bizard, Angela Brisebarre, Camille Capel, Etienne Simon-Lorière, Vincent Enouf, Maud Vanpeene, Sylvie van der Werf,Guigon Aurélie
EPI_ISL_912604	Labo Analyses Med	National Reference Center for Viruses of Respiratory Infections, Institut Pasteur, Paris	Marion Barbet, Sylvie Behillil, Méline Bizard, Angela Brisebarre, Camille Capel, Etienne Simon-Lorière, Vincent Enouf, Maud Vanpeene, Sylvie van der Werf
EPI_ISL_912607, EPI_ISL_912608, EPI_ISL_912609, EPI_ISL_912610, EPI_ISL_912611	Labo Analyses Med	National Reference Center for Viruses of Respiratory Infections, Institut Pasteur, Paris	Marion Barbet, Sylvie Behillil, Méline Bizard, Angela Brisebarre, Camille Capel, Etienne Simon-Lorière, Vincent Enouf, Maud Vanpeene, Sylvie van der Werf,Holstein Anne
EPI_ISL_912612, EPI_ISL_912613	Centre Hospitalier de Compiègne Laboratoire Compiègne	National Reference Center for Viruses of Respiratory Infections, Institut Pasteur, Paris	Marion Barbet, Sylvie Behillil, Méline Bizard, Angela Brisebarre, Camille Capel, Etienne Simon-Lorière, Vincent Enouf, Maud Vanpeene, Sylvie van der Werf,Romand Sylvie
EPI_ISL_912622	Labo Analyses Med	National Reference Center for Viruses of Respiratory Infections, Institut Pasteur, Paris	Marion Barbet, Sylvie Behillil, Méline Bizard, Angela Brisebarre, Camille Capel, Etienne Simon-Lorière, Vincent Enouf, Maud Vanpeene, Sylvie van der Werf
EPI_ISL_912642	Hôpital Henri Mondor	Department of Virology, Henri Mondor University Hospital, Assistance Publique Hôpitaux de Paris, Université Paris-Est Créteil, INSERM U955	Christophe Rodriguez, Slim Fourati, Vanessa Demontant, Guillaume Gricourt, Melissa N'Debi, Alexandre Soulier, Elisabeth Trawinski, Jean-Michel Pawlotsky
EPI_ISL_912660	Hôpital Pitié-Salpêtrière	Department of Virology, Henri Mondor University Hospital, Assistance Publique Hôpitaux de Paris, Université Paris-Est Créteil, INSERM U955	Christophe Rodriguez, Slim Fourati, Vanessa Demontant, Guillaume Gricourt, Melissa N'Debi, Alexandre Soulier, Elisabeth Trawinski, Jean-Michel Pawlotsky
EPI_ISL_912695, EPI_ISL_912713, EPI_ISL_912734	Hôpital Henri Mondor	Department of Virology, Henri Mondor University Hospital, Assistance Publique Hôpitaux de Paris, Université Paris-Est Créteil, INSERM U955	Christophe Rodriguez, Slim Fourati, Vanessa Demontant, Guillaume Gricourt, Melissa N'Debi, Alexandre Soulier, Elisabeth Trawinski, Jean-Michel Pawlotsky
EPI_ISL_912791, EPI_ISL_912792	Hôpital Pitié-Salpêtrière	Department of Virology, Henri Mondor University Hospital, Assistance Publique Hôpitaux de Paris, Université Paris-Est Créteil, INSERM U955	Christophe Rodriguez, Slim Fourati, Vanessa Demontant, Guillaume Gricourt, Melissa N'Debi, Alexandre Soulier, Elisabeth Trawinski, Jean-Michel Pawlotsky
EPI_ISL_912812, EPI_ISL_912814, EPI_ISL_912818, EPI_ISL_912821, EPI_ISL_912822, EPI_ISL_912823, EPI_ISL_912827, EPI_ISL_912828, EPI_ISL_912830, EPI_ISL_912837, EPI_ISL_912838, EPI_ISL_912840, EPI_ISL_912841, EPI_ISL_912844, EPI_ISL_912845, EPI_ISL_912851, EPI_ISL_912852, EPI_ISL_912853, EPI_ISL_912854, EPI_ISL_912855, EPI_ISL_912856, EPI_ISL_912857, EPI_ISL_912858, EPI_ISL_912859, EPI_ISL_912860, EPI_ISL_912861, EPI_ISL_912862, EPI_ISL_912899, EPI_ISL_912908			
see above	Hôpital Henri Mondor	Department of Virology, Henri Mondor University Hospital, Assistance Publique Hôpitaux de Paris, Université Paris-Est Créteil, INSERM U955	Christophe Rodriguez, Slim Fourati, Vanessa Demontant, Guillaume Gricourt, Melissa N'Debi, Alexandre Soulier, Elisabeth Trawinski, Jean-Michel Pawlotsky
EPI_ISL_912912, EPI_ISL_912913, EPI_ISL_912914, EPI_ISL_912915, EPI_ISL_912917, EPI_ISL_912918	Hôpital Pitié-Salpêtrière	Department of Virology, Henri Mondor University Hospital, Assistance Publique Hôpitaux de Paris, Université Paris-Est Créteil, INSERM U955	Christophe Rodriguez, Slim Fourati, Vanessa Demontant, Guillaume Gricourt, Melissa N'Debi, Alexandre Soulier, Elisabeth Trawinski, Jean-Michel Pawlotsky

EPI_ISL_912953, EPI_ISL_912954, EPI_ISL_912955, EPI_ISL_912956, EPI_ISL_912957	Hôpital Henri Mondor	Department of Virology, Henri Mondor University Hospital, Assistance Publique Hôpitaux de Paris, Université Paris-Est Créteil, INSERM U955	Christophe Rodriguez, Slim Fourati, Vanessa Demontant, Guillaume Gricourt, Melissa N'Debi, Alexandre Soulier, Elisabeth Trawinski, Jean-Michel Pawlotsky
EPI_ISL_913028, EPI_ISL_913030, EPI_ISL_913031, EPI_ISL_913036, EPI_ISL_913037	Hospital los Arcos	Instituto de Salud Carlos III	Iglesias-Caballero, M. Camarero, S. Sandonís,V. Vázquez, S. Pozo, F. Casas, I. Jiménez, P. Zaballos, A. Monzón, S. Varona, S. Cuesta, I. Cámara, M.
EPI_ISL_913057	Hospital San Pedro de Alcántara	Instituto de Salud Carlos III	Iglesias-Caballero, M. Camarero, S. Sandonís,V. Vázquez, S. Pozo, F. Casas, I. Jiménez, P. Zaballos, A. Monzón, S. Varona, S. Cuesta, I. Rodríguez, G.
EPI_ISL_913061, EPI_ISL_913062	Hospital Nuestra Señora de Sonsoles	Instituto de Salud Carlos III	Iglesias-Caballero, M. Camarero, S. Sandonís,V. Vázquez, S. Pozo, F. Casas, I. Jiménez, P. Zaballos, A. Monzón, S. Varona, S. Cuesta, I. Sánchez, R.
EPI_ISL_913114	CHU Purpan - Laboratoire de Virologie - Institut Fédératif de Biologie	CHU Purpan - Laboratoire de Virologie - Institut Fédératif de Biologie	Latour J., Ranger N., Dubois M., Carcenac R., Harter A., Boyer P., Tremeaux P., Izopet J.
EPI_ISL_913116, EPI_ISL_913118, EPI_ISL_913119, EPI_ISL_913120, EPI_ISL_913126, EPI_ISL_913128, EPI_ISL_913129, EPI_ISL_913131, EPI_ISL_913133, EPI_ISL_913137, EPI_ISL_913138, EPI_ISL_913139, EPI_ISL_913141, EPI_ISL_913142, EPI_ISL_913144, EPI_ISL_913150, EPI_ISL_913174, EPI_ISL_913177, EPI_ISL_913178, EPI_ISL_913180, EPI_ISL_913182, EPI_ISL_913183, EPI_ISL_913184, EPI_ISL_913185, EPI_ISL_913186, EPI_ISL_913187, EPI_ISL_913188, EPI_ISL_913189, EPI_ISL_913190, EPI_ISL_913191, EPI_ISL_913192, EPI_ISL_913193, EPI_ISL_913194, EPI_ISL_913195, EPI_ISL_913196, EPI_ISL_913197, EPI_ISL_913198, EPI_ISL_913199, EPI_ISL_913200			
see above	University of Michigan Clinical Microbiology Laboratory	Lauring Lab, University of Michigan, Department of Microbiology and Immunology	Valesano
EPI_ISL_913266	Unilabs AB Skovde	The Public Health Agency of Sweden	Anna-Malin Linde, Maria Lind Karlberg, Carlo Berg, Oskar Karlsson Lindsjo, Sofia Stamouli, Reza Advani, Mattias Haukland, Petra Holmstrom, Noura Walai, Petra Edquist, Mia Brytting, Anna Risberg, Karin Tegmark-Wisell
EPI_ISL_913373	Klinisk mikrobiologi	The Public Health Agency of Sweden	Anna-Malin Linde, Maria Lind Karlberg, Carlo Berg, Oskar Karlsson Lindsjo, Sofia Stamouli, Reza Advani, Mattias Haukland, Petra Holmstrom, Noura Walai, Petra Edquist, Mia Brytting, Anna Risberg, Karin Tegmark-Wisell
EPI_ISL_913394, EPI_ISL_913396, EPI_ISL_913401, EPI_ISL_913404, EPI_ISL_913405, EPI_ISL_913406, EPI_ISL_913409, EPI_ISL_913411, EPI_ISL_913420	Massachusetts State Public Health Laboratory	Massachusetts State Public Health Laboratory	Andrew Lang, Timelia Fink, Glen Gallagher, Sandra Smole
EPI_ISL_913455	Unilabs AB Skovde	The Public Health Agency of Sweden	Anna-Malin Linde, Maria Lind Karlberg, Carlo Berg, Oskar Karlsson Lindsjo, Sofia Stamouli, Reza Advani, Mattias Haukland, Petra Holmstrom, Noura Walai, Petra Edquist, Mia Brytting, Anna Risberg, Karin Tegmark-Wisell
EPI_ISL_913456	Synlab Medilab, Mikrobiologi	The Public Health Agency of Sweden	Anna-Malin Linde, Maria Lind Karlberg, Carlo Berg, Oskar Karlsson Lindsjo, Sofia Stamouli, Reza Advani, Mattias Haukland, Petra Holmstrom, Noura Walai, Petra Edquist, Mia Brytting, Anna Risberg, Karin Tegmark-Wisell
EPI_ISL_913470	Klinisk mikrobiologi	The Public Health Agency of Sweden	Anna-Malin Linde, Maria Lind Karlberg, Carlo Berg, Oskar Karlsson Lindsjo, Sofia Stamouli, Reza Advani, Mattias Haukland, Petra Holmstrom, Noura Walai, Petra Edquist, Mia Brytting, Anna Risberg, Karin Tegmark-Wisell
EPI_ISL_913514, EPI_ISL_913515, EPI_ISL_913516	University of Michigan Clinical Microbiology Laboratory	Lauring Lab, University of Michigan, Department of Microbiology and Immunology	Valesano
EPI_ISL_913584	M Health Fairview	Minnesota Department of Health, Public Health Laboratory	Alexandra Lorentz, Jacob Garfin, Matt Plumb, and Xiong Wang
EPI_ISL_913586, EPI_ISL_913588, EPI_ISL_913589, EPI_ISL_913590, EPI_ISL_913591	Vault Health	Minnesota Department of Health, Public Health Laboratory	Alexandra Lorentz, Jacob Garfin, Matt Plumb, and Xiong Wang
EPI_ISL_913616	Michigan Department of Health and Human Services, Bureau of Laboratories	Michigan Department of Health and Human Services, Bureau of Laboratories	Blankenship HM, Riner D, Soehnlen MK
EPI_ISL_913667	LA Office of Public Health Laboratories	Centers for Disease Control and Prevention Division of Viral Diseases, Pathogen Discovery	Ying Tao, Yan Li, Jing Zhang, Krista Queen, Anna Uehara, Peter Cook, Clinton R. Paden, Haibin Wang, Suxiang Tong
EPI_ISL_913748, EPI_ISL_913749, EPI_ISL_913750, EPI_ISL_913751, EPI_ISL_913752, EPI_ISL_913753, EPI_ISL_913754, EPI_ISL_913755, EPI_ISL_913756, EPI_ISL_913758, EPI_ISL_913759, EPI_ISL_913760, EPI_ISL_913761, EPI_ISL_913762, EPI_ISL_913763, EPI_ISL_913764, EPI_ISL_913765, EPI_ISL_913768, EPI_ISL_913794, EPI_ISL_913796, EPI_ISL_913797, EPI_ISL_913799, EPI_ISL_913800, EPI_ISL_913810, EPI_ISL_913812, EPI_ISL_913813, EPI_ISL_913814, EPI_ISL_913816, EPI_ISL_913817, EPI_ISL_913819, EPI_ISL_913820, EPI_ISL_913821, EPI_ISL_913823, EPI_ISL_913824, EPI_ISL_913826, EPI_ISL_913827, EPI_ISL_913828, EPI_ISL_913830, EPI_ISL_913831, EPI_ISL_913833, EPI_ISL_913834, EPI_ISL_913835, EPI_ISL_913837, EPI_ISL_913838, EPI_ISL_913840, EPI_ISL_913841, EPI_ISL_913843, EPI_ISL_913844, EPI_ISL_913845, EPI_ISL_913847, EPI_ISL_913848, EPI_ISL_913850, EPI_ISL_913851, EPI_ISL_913853, EPI_ISL_913854, EPI_ISL_913855, EPI_ISL_913857, EPI_ISL_913858, EPI_ISL_913867, EPI_ISL_913868, EPI_ISL_913869, EPI_ISL_913871, EPI_ISL_913872, EPI_ISL_913874, EPI_ISL_913875, EPI_ISL_913876, EPI_ISL_913878			
see above	KU Leuven, Rega Institute, Clinical and Epidemiological Virology	KU Leuven, Rega Institute, Clinical and Epidemiological Virology	Tony Wawina-Bokalanga, Bert Vanmechelen, Joan Marti-Carerras, Piet Maes
EPI_ISL_913985, EPI_ISL_913986, EPI_ISL_913987, EPI_ISL_913988, EPI_ISL_913989	TX DSHS, Lab Services Section MC 1947	Pathogen Discovery, Respiratory Viruses Branch, Division of Viral Diseases, Centers for Disease Control and Prevention	Ying Tao, Yan Li, Jing Zhang, Krista Queen, Anna Uehara, Peter Cook, Clinton R. Paden, Haibin Wang, Suxiang Tong
EPI_ISL_913992, EPI_ISL_913993, EPI_ISL_913994	TN Division of Laboratory Services	Pathogen Discovery, Respiratory Viruses Branch, Division of Viral Diseases, Centers for Disease Control and Prevention	Ying Tao, Yan Li, Jing Zhang, Krista Queen, Anna Uehara, Peter Cook, Clinton R. Paden, Haibin Wang, Suxiang Tong
EPI_ISL_914027, EPI_ISL_914028, EPI_ISL_914029	IL Department of Public Health Chicago Laboratory	Pathogen Discovery, Respiratory Viruses Branch, Division of Viral Diseases, Centers for Disease Control and Prevention	Ying Tao, Yan Li, Jing Zhang, Krista Queen, Anna Uehara, Peter Cook, Clinton R. Paden, Haibin Wang, Suxiang Tong
EPI_ISL_914738, EPI_ISL_914742, EPI_ISL_914752, EPI_ISL_914754, EPI_ISL_914772	Wyoming Public Health Laboratory	Wyoming Public Health Laboratory	Noah Hull, Taylor Fearing, Lynette Gumbleton, Channing Weber, Ashley Norberg, Bailey Bowcutt, and Wanda Manley
EPI_ISL_914832	LABORATORIO CLINICO LABIN	Inciensa, Instituto Costarricense de Investigación y Enseñanza en Nutrición y Salud	Francisco Duarte, Hebleen Porras, Claudio Soto-Garita, Estela Cordero, Adriana Godínez, Melany Calderón & Pei Ling Chan Ma
EPI_ISL_914835	AREA DE SALUD PARAISO-CERVANTES	Inciensa, Instituto Costarricense de Investigación y Enseñanza en Nutrición y Salud	Francisco Duarte, Hebleen Porras, Claudio Soto-Garita, Estela Cordero, Adriana Godínez, Melany Calderón & Mariel López
EPI_ISL_914836	HOSPITAL MEXICO	Inciensa, Instituto Costarricense de Investigación y Enseñanza en Nutrición y Salud	Francisco Duarte, Hebleen Porras, Claudio Soto-Garita, Estela Cordero, Adriana Godínez, Melany Calderón & Mariel López
EPI_ISL_914837, EPI_ISL_914838, EPI_ISL_914839	HOSPITAL MEXICO	Inciensa, Instituto Costarricense de Investigación y Enseñanza en Nutrición y Salud	Francisco Duarte, Hebleen Porras, Claudio Soto-Garita, Estela Cordero, Adriana Godínez, Melany Calderón & Teresita Somogyi
EPI_ISL_914866, EPI_ISL_914867	Kansas Health and Environmental Lab	Kansas Health and Environmental Lab	Mike Grose, Paige Drury, Carissa Robertson, Ben Olsen, and Phil Adam
EPI_ISL_914890, EPI_ISL_914891, EPI_ISL_914892, EPI_ISL_914893, EPI_ISL_914894, EPI_ISL_914895, EPI_ISL_914896, EPI_ISL_914897, EPI_ISL_914898, EPI_ISL_914899, EPI_ISL_914900, EPI_ISL_914901, EPI_ISL_914902, EPI_ISL_914903, EPI_ISL_914904, EPI_ISL_914905, EPI_ISL_914906, EPI_ISL_914907, EPI_ISL_914908, EPI_ISL_914909, EPI_ISL_914910, EPI_ISL_914911, EPI_ISL_914912, EPI_ISL_914913, EPI_ISL_914915, EPI_ISL_914916, EPI_ISL_914917, EPI_ISL_914918, EPI_ISL_914919, EPI_ISL_914920, EPI_ISL_914921, EPI_ISL_914922, EPI_ISL_914923, EPI_ISL_914924, EPI_ISL_914925, EPI_ISL_914926, EPI_ISL_914927, EPI_ISL_914928, EPI_ISL_914929, EPI_ISL_914930, EPI_ISL_914931, EPI_ISL_914932, EPI_ISL_914933, EPI_ISL_914934, EPI_ISL_914935, EPI_ISL_914936, EPI_ISL_914937, EPI_ISL_914938, EPI_ISL_914939, EPI_ISL_914940, EPI_ISL_914941, EPI_ISL_914942, EPI_ISL_914943, EPI_ISL_914945, EPI_ISL_914946, EPI_ISL_914947, EPI_ISL_914948, EPI_ISL_914949, EPI_ISL_914950, EPI_ISL_914951, EPI_ISL_914952, EPI_ISL_914953, EPI_ISL_914954, EPI_ISL_914955, EPI_ISL_914956, EPI_ISL_914957, EPI_ISL_914958, EPI_ISL_914959, EPI_ISL_914960, EPI_ISL_914961, EPI_ISL_914962, EPI_ISL_914963, EPI_ISL_914964, EPI_ISL_914965, EPI_ISL_914966, EPI_ISL_914967, EPI_ISL_914968, EPI_ISL_914969, EPI_ISL_915195, EPI_ISL_915196, EPI_ISL_915197, EPI_ISL_915199, EPI_ISL_915200, EPI_ISL_915201, EPI_ISL_915203, EPI_ISL_915204, EPI_ISL_915205, EPI_ISL_915208, EPI_ISL_915209, EPI_ISL_915210, EPI_ISL_915214, EPI_ISL_915216, EPI_ISL_915217, EPI_ISL_915218, EPI_ISL_915219, EPI_ISL_915220, EPI_ISL_915221, EPI_ISL_915222, EPI_ISL_915223, EPI_ISL_915225, EPI_ISL_915229, EPI_ISL_915233, EPI_ISL_915240, EPI_ISL_915241, EPI_ISL_915242, EPI_ISL_915243, EPI_ISL_915244, EPI_ISL_915245,			

EPI_ISL_915246, EPI_ISL_915247, EPI_ISL_915248, EPI_ISL_915249, EPI_ISL_915250, EPI_ISL_915251, EPI_ISL_915252, EPI_ISL_915253, EPI_ISL_915254, EPI_ISL_915255, EPI_ISL_915256, EPI_ISL_915257, EPI_ISL_915258, EPI_ISL_915259, EPI_ISL_915260, EPI_ISL_915261, EPI_ISL_915262, EPI_ISL_915263, EPI_ISL_915265, EPI_ISL_915266, EPI_ISL_915275, EPI_ISL_915277, EPI_ISL_915290, EPI_ISL_915291, EPI_ISL_915292, EPI_ISL_915293, EPI_ISL_915294, EPI_ISL_915295, EPI_ISL_915296, EPI_ISL_915297, EPI_ISL_915298, EPI_ISL_915299, EPI_ISL_915300, EPI_ISL_915301, EPI_ISL_915303, EPI_ISL_915304, EPI_ISL_915305, EPI_ISL_915307, EPI_ISL_915309, EPI_ISL_915315, EPI_ISL_915317, EPI_ISL_915327, EPI_ISL_915342, EPI_ISL_915343, EPI_ISL_915345, EPI_ISL_915350, EPI_ISL_915352, EPI_ISL_915354			
see above	Quest Diagnostics	Quest Diagnostics	Rosenthal,S.H., Gerasimova,A., Kagan,R.M., Anderson, B., Hua, M., Liu Y., Bernstein, L.E., Livingston, K.E., Perez, A., Shalhout, D.F., Shlyakhter, I.A., Owen, R., Tanpaiboon, P., Lacbawan, F.
EPI_ISL_916622, EPI_ISL_916623, EPI_ISL_916624	Lighthouse Lab in Alderley Park	Wellcome Sanger Institute for the COVID-19 Genomics UK (COG-UK) Consortium	Jacquelyn Wynn, Mairead Hyland, The Lighthouse Lab in Alderley Park and Alex Alderton, Roberto Amato, Sonia Goncalves, Ewan Harrison, David K. Jackson, Ian Johnston, Dominic Kwiatkowski, Cordelia Langford, John Sillitoe on behalf of the Wellcome Sanger Institute COVID-19 Surveillance Team
EPI_ISL_916625, EPI_ISL_916626	Lighthouse Lab in Glasgow	Wellcome Sanger Institute for the COVID-19 Genomics UK (COG-UK) Consortium	Harper VanSteenhouse, Yumi Kasai, David Gray, Carol Clugston, Anna Dominiczak and Alex Alderton, Roberto Amato, Sonia Goncalves, Ewan Harrison, David K. Jackson, Ian Johnston, Dominic Kwiatkowski, Cordelia Langford, John Sillitoe on behalf of the Wellcome Sanger Institute COVID-19 Surveillance Team
EPI_ISL_916627, EPI_ISL_916628, EPI_ISL_916629, EPI_ISL_916631, EPI_ISL_916632, EPI_ISL_916634, EPI_ISL_916635, EPI_ISL_916636, EPI_ISL_916638, EPI_ISL_916639, EPI_ISL_916640, EPI_ISL_916641, EPI_ISL_916642, EPI_ISL_916643, EPI_ISL_916644			
see above	Lighthouse Lab in Alderley Park	Wellcome Sanger Institute for the COVID-19 Genomics UK (COG-UK) Consortium	Jacquelyn Wynn, Mairead Hyland, The Lighthouse Lab in Alderley Park and Alex Alderton, Roberto Amato, Sonia Goncalves, Ewan Harrison, David K. Jackson, Ian Johnston, Dominic Kwiatkowski, Cordelia Langford, John Sillitoe on behalf of the Wellcome Sanger Institute COVID-19 Surveillance Team
EPI_ISL_916645	Lighthouse Lab in Glasgow	Wellcome Sanger Institute for the COVID-19 Genomics UK (COG-UK) Consortium	Harper VanSteenhouse, Yumi Kasai, David Gray, Carol Clugston, Anna Dominiczak and Alex Alderton, Roberto Amato, Sonia Goncalves, Ewan Harrison, David K. Jackson, Ian Johnston, Dominic Kwiatkowski, Cordelia Langford, John Sillitoe on behalf of the Wellcome Sanger Institute COVID-19 Surveillance Team
EPI_ISL_916646, EPI_ISL_916647	Lighthouse Lab in Alderley Park	Wellcome Sanger Institute for the COVID-19 Genomics UK (COG-UK) Consortium	Jacquelyn Wynn, Mairead Hyland, The Lighthouse Lab in Alderley Park and Alex Alderton, Roberto Amato, Sonia Goncalves, Ewan Harrison, David K. Jackson, Ian Johnston, Dominic Kwiatkowski, Cordelia Langford, John Sillitoe on behalf of the Wellcome Sanger Institute COVID-19 Surveillance Team
EPI_ISL_916648	Lighthouse Lab in Glasgow	Wellcome Sanger Institute for the COVID-19 Genomics UK (COG-UK) Consortium	Harper VanSteenhouse, Yumi Kasai, David Gray, Carol Clugston, Anna Dominiczak and Alex Alderton, Roberto Amato, Sonia Goncalves, Ewan Harrison, David K. Jackson, Ian Johnston, Dominic Kwiatkowski, Cordelia Langford, John Sillitoe on behalf of the Wellcome Sanger Institute COVID-19 Surveillance Team
EPI_ISL_916650, EPI_ISL_916652, EPI_ISL_916653, EPI_ISL_916654, EPI_ISL_916655, EPI_ISL_916656, EPI_ISL_916657, EPI_ISL_916658, EPI_ISL_916659, EPI_ISL_916660	Lighthouse Lab in Alderley Park	Wellcome Sanger Institute for the COVID-19 Genomics UK (COG-UK) Consortium	Jacquelyn Wynn, Mairead Hyland, The Lighthouse Lab in Alderley Park and Alex Alderton, Roberto Amato, Sonia Goncalves, Ewan Harrison, David K. Jackson, Ian Johnston, Dominic Kwiatkowski, Cordelia Langford, John Sillitoe on behalf of the Wellcome Sanger Institute COVID-19 Surveillance Team
EPI_ISL_916662	Lighthouse Lab in Glasgow	Wellcome Sanger Institute for the COVID-19 Genomics UK (COG-UK) Consortium	Harper VanSteenhouse, Yumi Kasai, David Gray, Carol Clugston, Anna Dominiczak and Alex Alderton, Roberto Amato, Sonia Goncalves, Ewan Harrison, David K. Jackson, Ian Johnston, Dominic Kwiatkowski, Cordelia Langford, John Sillitoe on behalf of the Wellcome Sanger Institute COVID-19 Surveillance Team
EPI_ISL_916663, EPI_ISL_916664, EPI_ISL_916665, EPI_ISL_916666, EPI_ISL_916667	Lighthouse Lab in Alderley Park	Wellcome Sanger Institute for the COVID-19 Genomics UK (COG-UK) Consortium	Jacquelyn Wynn, Mairead Hyland, The Lighthouse Lab in Alderley Park and Alex Alderton, Roberto Amato, Sonia Goncalves, Ewan Harrison, David K. Jackson, Ian Johnston, Dominic Kwiatkowski, Cordelia Langford, John Sillitoe on behalf of the Wellcome Sanger Institute COVID-19 Surveillance Team
EPI_ISL_916668	Lighthouse Lab in Glasgow	Wellcome Sanger Institute for the COVID-19 Genomics UK (COG-UK) Consortium	Harper VanSteenhouse, Yumi Kasai, David Gray, Carol Clugston, Anna Dominiczak and Alex Alderton, Roberto Amato, Sonia Goncalves, Ewan Harrison, David K. Jackson, Ian Johnston, Dominic Kwiatkowski, Cordelia Langford, John Sillitoe on behalf of the Wellcome Sanger Institute COVID-19 Surveillance Team
EPI_ISL_916669, EPI_ISL_916670, EPI_ISL_916671, EPI_ISL_916672, EPI_ISL_916673, EPI_ISL_916674, EPI_ISL_916676, EPI_ISL_916677, EPI_ISL_916678, EPI_ISL_916679, EPI_ISL_916680, EPI_ISL_916681, EPI_ISL_916683			
see above	Lighthouse Lab in Alderley Park	Wellcome Sanger Institute for the COVID-19 Genomics UK (COG-UK) Consortium	Jacquelyn Wynn, Mairead Hyland, The Lighthouse Lab in Alderley Park and Alex Alderton, Roberto Amato, Sonia Goncalves, Ewan Harrison, David K. Jackson, Ian Johnston, Dominic Kwiatkowski, Cordelia Langford, John Sillitoe on behalf of the Wellcome Sanger Institute COVID-19 Surveillance Team
EPI_ISL_916684	Lighthouse Lab in Glasgow	Wellcome Sanger Institute for the COVID-19 Genomics UK (COG-UK) Consortium	Harper VanSteenhouse, Yumi Kasai, David Gray, Carol Clugston, Anna Dominiczak and Alex Alderton, Roberto Amato, Sonia Goncalves, Ewan Harrison, David K. Jackson, Ian Johnston, Dominic Kwiatkowski, Cordelia Langford, John Sillitoe on behalf of the Wellcome Sanger Institute COVID-19 Surveillance Team
EPI_ISL_916685, EPI_ISL_916686, EPI_ISL_916687, EPI_ISL_916689, EPI_ISL_916690, EPI_ISL_916691, EPI_ISL_916692, EPI_ISL_916693, EPI_ISL_916695, EPI_ISL_916697, EPI_ISL_916698, EPI_ISL_916699, EPI_ISL_916700, EPI_ISL_916701, EPI_ISL_916702, EPI_ISL_916703, EPI_ISL_916706, EPI_ISL_916707, EPI_ISL_916708, EPI_ISL_916709, EPI_ISL_916710			
see above	Lighthouse Lab in Alderley Park	Wellcome Sanger Institute for the COVID-19 Genomics UK (COG-UK) Consortium	Jacquelyn Wynn, Mairead Hyland, The Lighthouse Lab in Alderley Park and Alex Alderton, Roberto Amato, Sonia Goncalves, Ewan Harrison, David K. Jackson, Ian Johnston, Dominic Kwiatkowski, Cordelia Langford, John Sillitoe on behalf of the Wellcome Sanger Institute COVID-19 Surveillance Team
EPI_ISL_916711	Lighthouse Lab in Glasgow	Wellcome Sanger Institute for the COVID-19 Genomics UK (COG-UK) Consortium	Harper VanSteenhouse, Yumi Kasai, David Gray, Carol Clugston, Anna Dominiczak and Alex Alderton, Roberto Amato, Sonia Goncalves, Ewan Harrison, David K. Jackson, Ian Johnston, Dominic Kwiatkowski, Cordelia Langford, John Sillitoe on behalf of the Wellcome Sanger Institute COVID-19 Surveillance Team
EPI_ISL_916712, EPI_ISL_916714, EPI_ISL_916715	Lighthouse Lab in Alderley Park	Wellcome Sanger Institute for the COVID-19 Genomics UK (COG-UK) Consortium	Jacquelyn Wynn, Mairead Hyland, The Lighthouse Lab in Alderley Park and Alex Alderton, Roberto Amato, Sonia Goncalves, Ewan Harrison, David K. Jackson, Ian Johnston, Dominic Kwiatkowski, Cordelia Langford, John Sillitoe on behalf of the Wellcome Sanger Institute COVID-19 Surveillance Team
EPI_ISL_916716	Lighthouse Lab in Glasgow	Wellcome Sanger Institute for the COVID-19 Genomics UK (COG-UK) Consortium	Harper VanSteenhouse, Yumi Kasai, David Gray, Carol Clugston, Anna Dominiczak and Alex Alderton, Roberto Amato, Sonia Goncalves, Ewan Harrison, David K. Jackson, Ian Johnston, Dominic Kwiatkowski, Cordelia Langford, John Sillitoe on behalf of the Wellcome Sanger Institute COVID-19 Surveillance Team
EPI_ISL_916717, EPI_ISL_916718, EPI_ISL_916719, EPI_ISL_916720, EPI_ISL_916721, EPI_ISL_916722, EPI_ISL_916723, EPI_ISL_916724, EPI_ISL_916725, EPI_ISL_916726, EPI_ISL_916727, EPI_ISL_916729, EPI_ISL_916731, EPI_ISL_916733, EPI_ISL_916734, EPI_ISL_916735			
see above	Lighthouse Lab in Alderley Park	Wellcome Sanger Institute for the COVID-19 Genomics UK (COG-UK) Consortium	Jacquelyn Wynn, Mairead Hyland, The Lighthouse Lab in Alderley Park and Alex Alderton, Roberto Amato, Sonia Goncalves, Ewan Harrison, David K. Jackson, Ian Johnston, Dominic Kwiatkowski, Cordelia Langford, John Sillitoe on behalf of the Wellcome Sanger Institute COVID-19 Surveillance Team
EPI_ISL_916736	Lighthouse Lab in Glasgow	Wellcome Sanger Institute for the COVID-19 Genomics UK (COG-UK) Consortium	Harper VanSteenhouse, Yumi Kasai, David Gray, Carol Clugston, Anna Dominiczak and Alex Alderton, Roberto Amato, Sonia Goncalves, Ewan Harrison, David K. Jackson, Ian Johnston, Dominic Kwiatkowski, Cordelia Langford, John Sillitoe on behalf of the Wellcome Sanger Institute COVID-19 Surveillance Team
EPI_ISL_916737, EPI_ISL_916739, EPI_ISL_916740, EPI_ISL_916741, EPI_ISL_916742, EPI_ISL_916743, EPI_ISL_916744, EPI_ISL_916745, EPI_ISL_916747, EPI_ISL_916748, EPI_ISL_916749, EPI_ISL_916750, EPI_ISL_916751, EPI_ISL_916752, EPI_ISL_916753, EPI_ISL_916754, EPI_ISL_916755, EPI_ISL_916756, EPI_ISL_916757			
see above	Lighthouse Lab in Alderley Park	Wellcome Sanger Institute for the COVID-19 Genomics UK (COG-UK) Consortium	Jacquelyn Wynn, Mairead Hyland, The Lighthouse Lab in Alderley Park and Alex Alderton, Roberto Amato, Sonia Goncalves, Ewan Harrison, David K. Jackson, Ian Johnston, Dominic Kwiatkowski, Cordelia Langford, John Sillitoe on behalf of the Wellcome Sanger Institute COVID-19 Surveillance Team
EPI_ISL_916758	Lighthouse Lab in Glasgow	Wellcome Sanger Institute for the COVID-19 Genomics UK (COG-UK) Consortium	Harper VanSteenhouse, Yumi Kasai, David Gray, Carol Clugston, Anna Dominiczak and Alex Alderton, Roberto Amato, Sonia Goncalves, Ewan Harrison, David K. Jackson, Ian Johnston, Dominic Kwiatkowski, Cordelia Langford, John Sillitoe on behalf of the Wellcome Sanger Institute COVID-19 Surveillance Team
EPI_ISL_916759, EPI_ISL_916760	Lighthouse Lab in Alderley Park	Wellcome Sanger Institute for the COVID-19 Genomics UK (COG-UK) Consortium	Jacquelyn Wynn, Mairead Hyland, The Lighthouse Lab in Alderley Park and Alex Alderton, Roberto Amato, Sonia Goncalves, Ewan Harrison, David K. Jackson, Ian Johnston, Dominic Kwiatkowski, Cordelia Langford, John Sillitoe on behalf of the Wellcome Sanger Institute COVID-19 Surveillance Team
EPI_ISL_916761	Lighthouse Lab in Glasgow	Wellcome Sanger Institute for the COVID-19 Genomics UK (COG-UK) Consortium	Harper VanSteenhouse, Yumi Kasai, David Gray, Carol Clugston, Anna Dominiczak and Alex Alderton, Roberto Amato, Sonia Goncalves, Ewan Harrison, David K. Jackson, Ian Johnston, Dominic Kwiatkowski, Cordelia Langford, John Sillitoe on behalf of the Wellcome Sanger Institute COVID-19 Surveillance Team
EPI_ISL_916762, EPI_ISL_916763, EPI_ISL_916764, EPI_ISL_916765, EPI_ISL_916767, EPI_ISL_916768, EPI_ISL_916769, EPI_ISL_916770, EPI_ISL_916772, EPI_ISL_916773, EPI_ISL_916774, EPI_ISL_916776, EPI_ISL_916777, EPI_ISL_916778, EPI_ISL_916780, EPI_ISL_916781, EPI_ISL_916783, EPI_ISL_916785, EPI_ISL_916786, EPI_ISL_916787, EPI_ISL_916788, EPI_ISL_916789, EPI_ISL_916790, EPI_ISL_916791, EPI_ISL_916792			

see above	Lighthouse Lab in Alderley Park	Wellcome Sanger Institute for the COVID-19 Genomics UK (COG-UK) Consortium	Jacquelyn Wynn, Mairead Hyland, The Lighthouse Lab in Alderley Park and Alex Alderton, Roberto Amato, Sonia Goncalves, Ewan Harrison, David K. Jackson, Ian Johnston, Dominic Kwiatkowski, Cordelia Langford, John Sillitoe on behalf of the Wellcome Sanger Institute COVID-19 Surveillance Team
EPI_ISL_916793	Lighthouse Lab in Glasgow	Wellcome Sanger Institute for the COVID-19 Genomics UK (COG-UK) Consortium	Harper VanSteenhouse, Yumi Kasai, David Gray, Carol Clugston, Anna Dominiczak and Alex Alderton, Roberto Amato, Sonia Goncalves, Ewan Harrison, David K. Jackson, Ian Johnston, Dominic Kwiatkowski, Cordelia Langford, John Sillitoe on behalf of the Wellcome Sanger Institute COVID-19 Surveillance Team
EPI_ISL_916794, EPI_ISL_916795, EPI_ISL_916796, EPI_ISL_916797, EPI_ISL_916798, EPI_ISL_916799, EPI_ISL_916801, EPI_ISL_916803, EPI_ISL_916804, EPI_ISL_916805, EPI_ISL_916806, EPI_ISL_916807, EPI_ISL_916808, EPI_ISL_916811, EPI_ISL_916813, EPI_ISL_916814, EPI_ISL_916816, EPI_ISL_916817, EPI_ISL_916818, EPI_ISL_916819			
see above	Lighthouse Lab in Alderley Park	Wellcome Sanger Institute for the COVID-19 Genomics UK (COG-UK) Consortium	Jacquelyn Wynn, Mairead Hyland, The Lighthouse Lab in Alderley Park and Alex Alderton, Roberto Amato, Sonia Goncalves, Ewan Harrison, David K. Jackson, Ian Johnston, Dominic Kwiatkowski, Cordelia Langford, John Sillitoe on behalf of the Wellcome Sanger Institute COVID-19 Surveillance Team
EPI_ISL_916820	Lighthouse Lab in Glasgow	Wellcome Sanger Institute for the COVID-19 Genomics UK (COG-UK) Consortium	Harper VanSteenhouse, Yumi Kasai, David Gray, Carol Clugston, Anna Dominiczak and Alex Alderton, Roberto Amato, Sonia Goncalves, Ewan Harrison, David K. Jackson, Ian Johnston, Dominic Kwiatkowski, Cordelia Langford, John Sillitoe on behalf of the Wellcome Sanger Institute COVID-19 Surveillance Team
EPI_ISL_916822, EPI_ISL_916823, EPI_ISL_916824, EPI_ISL_916826, EPI_ISL_916827, EPI_ISL_916828, EPI_ISL_916829, EPI_ISL_916830, EPI_ISL_916831, EPI_ISL_916832, EPI_ISL_916835, EPI_ISL_916836, EPI_ISL_916837, EPI_ISL_916839			
see above	Lighthouse Lab in Alderley Park	Wellcome Sanger Institute for the COVID-19 Genomics UK (COG-UK) Consortium	Jacquelyn Wynn, Mairead Hyland, The Lighthouse Lab in Alderley Park and Alex Alderton, Roberto Amato, Sonia Goncalves, Ewan Harrison, David K. Jackson, Ian Johnston, Dominic Kwiatkowski, Cordelia Langford, John Sillitoe on behalf of the Wellcome Sanger Institute COVID-19 Surveillance Team
EPI_ISL_916840	Lighthouse Lab in Glasgow	Wellcome Sanger Institute for the COVID-19 Genomics UK (COG-UK) Consortium	Harper VanSteenhouse, Yumi Kasai, David Gray, Carol Clugston, Anna Dominiczak and Alex Alderton, Roberto Amato, Sonia Goncalves, Ewan Harrison, David K. Jackson, Ian Johnston, Dominic Kwiatkowski, Cordelia Langford, John Sillitoe on behalf of the Wellcome Sanger Institute COVID-19 Surveillance Team
EPI_ISL_916841	Lighthouse Lab in Alderley Park	Wellcome Sanger Institute for the COVID-19 Genomics UK (COG-UK) Consortium	Jacquelyn Wynn, Mairead Hyland, The Lighthouse Lab in Alderley Park and Alex Alderton, Roberto Amato, Sonia Goncalves, Ewan Harrison, David K. Jackson, Ian Johnston, Dominic Kwiatkowski, Cordelia Langford, John Sillitoe on behalf of the Wellcome Sanger Institute COVID-19 Surveillance Team
EPI_ISL_916842	Lighthouse Lab in Glasgow	Wellcome Sanger Institute for the COVID-19 Genomics UK (COG-UK) Consortium	Harper VanSteenhouse, Yumi Kasai, David Gray, Carol Clugston, Anna Dominiczak and Alex Alderton, Roberto Amato, Sonia Goncalves, Ewan Harrison, David K. Jackson, Ian Johnston, Dominic Kwiatkowski, Cordelia Langford, John Sillitoe on behalf of the Wellcome Sanger Institute COVID-19 Surveillance Team
EPI_ISL_916843, EPI_ISL_916844, EPI_ISL_916846, EPI_ISL_916848, EPI_ISL_916851, EPI_ISL_916852, EPI_ISL_916853, EPI_ISL_916854	Lighthouse Lab in Alderley Park	Wellcome Sanger Institute for the COVID-19 Genomics UK (COG-UK) Consortium	Jacquelyn Wynn, Mairead Hyland, The Lighthouse Lab in Alderley Park and Alex Alderton, Roberto Amato, Sonia Goncalves, Ewan Harrison, David K. Jackson, Ian Johnston, Dominic Kwiatkowski, Cordelia Langford, John Sillitoe on behalf of the Wellcome Sanger Institute COVID-19 Surveillance Team
EPI_ISL_916855	Lighthouse Lab in Glasgow	Wellcome Sanger Institute for the COVID-19 Genomics UK (COG-UK) Consortium	Harper VanSteenhouse, Yumi Kasai, David Gray, Carol Clugston, Anna Dominiczak and Alex Alderton, Roberto Amato, Sonia Goncalves, Ewan Harrison, David K. Jackson, Ian Johnston, Dominic Kwiatkowski, Cordelia Langford, John Sillitoe on behalf of the Wellcome Sanger Institute COVID-19 Surveillance Team
EPI_ISL_916856, EPI_ISL_916857, EPI_ISL_916858, EPI_ISL_916859, EPI_ISL_916860, EPI_ISL_916861, EPI_ISL_916862, EPI_ISL_916863, EPI_ISL_916864, EPI_ISL_916865, EPI_ISL_916866, EPI_ISL_916867, EPI_ISL_916868			
see above	Lighthouse Lab in Alderley Park	Wellcome Sanger Institute for the COVID-19 Genomics UK (COG-UK) Consortium	Jacquelyn Wynn, Mairead Hyland, The Lighthouse Lab in Alderley Park and Alex Alderton, Roberto Amato, Sonia Goncalves, Ewan Harrison, David K. Jackson, Ian Johnston, Dominic Kwiatkowski, Cordelia Langford, John Sillitoe on behalf of the Wellcome Sanger Institute COVID-19 Surveillance Team
EPI_ISL_916869	Lighthouse Lab in Glasgow	Wellcome Sanger Institute for the COVID-19 Genomics UK (COG-UK) Consortium	Harper VanSteenhouse, Yumi Kasai, David Gray, Carol Clugston, Anna Dominiczak and Alex Alderton, Roberto Amato, Sonia Goncalves, Ewan Harrison, David K. Jackson, Ian Johnston, Dominic Kwiatkowski, Cordelia Langford, John Sillitoe on behalf of the Wellcome Sanger Institute COVID-19 Surveillance Team
EPI_ISL_916870, EPI_ISL_916871, EPI_ISL_916872, EPI_ISL_916873, EPI_ISL_916874, EPI_ISL_916875, EPI_ISL_916876, EPI_ISL_916877, EPI_ISL_916878	Lighthouse Lab in Alderley Park	Wellcome Sanger Institute for the COVID-19 Genomics UK (COG-UK) Consortium	Jacquelyn Wynn, Mairead Hyland, The Lighthouse Lab in Alderley Park and Alex Alderton, Roberto Amato, Sonia Goncalves, Ewan Harrison, David K. Jackson, Ian Johnston, Dominic Kwiatkowski, Cordelia Langford, John Sillitoe on behalf of the Wellcome Sanger Institute COVID-19 Surveillance Team
EPI_ISL_916879	Lighthouse Lab in Glasgow	Wellcome Sanger Institute for the COVID-19 Genomics UK (COG-UK) Consortium	Harper VanSteenhouse, Yumi Kasai, David Gray, Carol Clugston, Anna Dominiczak and Alex Alderton, Roberto Amato, Sonia Goncalves, Ewan Harrison, David K. Jackson, Ian Johnston, Dominic Kwiatkowski, Cordelia Langford, John Sillitoe on behalf of the Wellcome Sanger Institute COVID-19 Surveillance Team
EPI_ISL_916880, EPI_ISL_916881, EPI_ISL_916883, EPI_ISL_916884, EPI_ISL_916885, EPI_ISL_916886, EPI_ISL_916888, EPI_ISL_916889, EPI_ISL_916890	Lighthouse Lab in Alderley Park	Wellcome Sanger Institute for the COVID-19 Genomics UK (COG-UK) Consortium	Jacquelyn Wynn, Mairead Hyland, The Lighthouse Lab in Alderley Park and Alex Alderton, Roberto Amato, Sonia Goncalves, Ewan Harrison, David K. Jackson, Ian Johnston, Dominic Kwiatkowski, Cordelia Langford, John Sillitoe on behalf of the Wellcome Sanger Institute COVID-19 Surveillance Team
EPI_ISL_916891	Lighthouse Lab in Glasgow	Wellcome Sanger Institute for the COVID-19 Genomics UK (COG-UK) Consortium	Harper VanSteenhouse, Yumi Kasai, David Gray, Carol Clugston, Anna Dominiczak and Alex Alderton, Roberto Amato, Sonia Goncalves, Ewan Harrison, David K. Jackson, Ian Johnston, Dominic Kwiatkowski, Cordelia Langford, John Sillitoe on behalf of the Wellcome Sanger Institute COVID-19 Surveillance Team
EPI_ISL_916892, EPI_ISL_916893	Lighthouse Lab in Alderley Park	Wellcome Sanger Institute for the COVID-19 Genomics UK (COG-UK) Consortium	Jacquelyn Wynn, Mairead Hyland, The Lighthouse Lab in Alderley Park and Alex Alderton, Roberto Amato, Sonia Goncalves, Ewan Harrison, David K. Jackson, Ian Johnston, Dominic Kwiatkowski, Cordelia Langford, John Sillitoe on behalf of the Wellcome Sanger Institute COVID-19 Surveillance Team
EPI_ISL_916894	Lighthouse Lab in Glasgow	Wellcome Sanger Institute for the COVID-19 Genomics UK (COG-UK) Consortium	Harper VanSteenhouse, Yumi Kasai, David Gray, Carol Clugston, Anna Dominiczak and Alex Alderton, Roberto Amato, Sonia Goncalves, Ewan Harrison, David K. Jackson, Ian Johnston, Dominic Kwiatkowski, Cordelia Langford, John Sillitoe on behalf of the Wellcome Sanger Institute COVID-19 Surveillance Team
EPI_ISL_916895, EPI_ISL_916897	Lighthouse Lab in Alderley Park	Wellcome Sanger Institute for the COVID-19 Genomics UK (COG-UK) Consortium	Jacquelyn Wynn, Mairead Hyland, The Lighthouse Lab in Alderley Park and Alex Alderton, Roberto Amato, Sonia Goncalves, Ewan Harrison, David K. Jackson, Ian Johnston, Dominic Kwiatkowski, Cordelia Langford, John Sillitoe on behalf of the Wellcome Sanger Institute COVID-19 Surveillance Team
EPI_ISL_916899, EPI_ISL_916900	Lighthouse Lab in Glasgow	Wellcome Sanger Institute for the COVID-19 Genomics UK (COG-UK) Consortium	Harper VanSteenhouse, Yumi Kasai, David Gray, Carol Clugston, Anna Dominiczak and Alex Alderton, Roberto Amato, Sonia Goncalves, Ewan Harrison, David K. Jackson, Ian Johnston, Dominic Kwiatkowski, Cordelia Langford, John Sillitoe on behalf of the Wellcome Sanger Institute COVID-19 Surveillance Team
EPI_ISL_916901, EPI_ISL_916902, EPI_ISL_916903, EPI_ISL_916904, EPI_ISL_916906, EPI_ISL_916910, EPI_ISL_916913, EPI_ISL_916914, EPI_ISL_916915, EPI_ISL_916917, EPI_ISL_916920, EPI_ISL_916921, EPI_ISL_916922, EPI_ISL_916924, EPI_ISL_916925, EPI_ISL_916926, EPI_ISL_916927, EPI_ISL_916928, EPI_ISL_916929, EPI_ISL_916930, EPI_ISL_916931, EPI_ISL_916932, EPI_ISL_916933, EPI_ISL_916935, EPI_ISL_916936, EPI_ISL_916937, EPI_ISL_916938, EPI_ISL_916939, EPI_ISL_916941			
see above	Lighthouse Lab in Alderley Park	Wellcome Sanger Institute for the COVID-19 Genomics UK (COG-UK) Consortium	Jacquelyn Wynn, Mairead Hyland, The Lighthouse Lab in Alderley Park and Alex Alderton, Roberto Amato, Sonia Goncalves, Ewan Harrison, David K. Jackson, Ian Johnston, Dominic Kwiatkowski, Cordelia Langford, John Sillitoe on behalf of the Wellcome Sanger Institute COVID-19 Surveillance Team
EPI_ISL_916942	Lighthouse Lab in Glasgow	Wellcome Sanger Institute for the COVID-19 Genomics UK (COG-UK) Consortium	Harper VanSteenhouse, Yumi Kasai, David Gray, Carol Clugston, Anna Dominiczak and Alex Alderton, Roberto Amato, Sonia Goncalves, Ewan Harrison, David K. Jackson, Ian Johnston, Dominic Kwiatkowski, Cordelia Langford, John Sillitoe on behalf of the Wellcome Sanger Institute COVID-19 Surveillance Team
EPI_ISL_916943, EPI_ISL_916944, EPI_ISL_916947	Lighthouse Lab in Alderley Park	Wellcome Sanger Institute for the COVID-19 Genomics UK (COG-UK) Consortium	Jacquelyn Wynn, Mairead Hyland, The Lighthouse Lab in Alderley Park and Alex Alderton, Roberto Amato, Sonia Goncalves, Ewan Harrison, David K. Jackson, Ian Johnston, Dominic Kwiatkowski, Cordelia Langford, John Sillitoe on behalf of the Wellcome Sanger Institute COVID-19 Surveillance Team
EPI_ISL_916951	Lighthouse Lab in Glasgow	Wellcome Sanger Institute for the COVID-19 Genomics UK (COG-UK) Consortium	Harper VanSteenhouse, Yumi Kasai, David Gray, Carol Clugston, Anna Dominiczak and Alex Alderton, Roberto Amato, Sonia Goncalves, Ewan Harrison, David K. Jackson, Ian Johnston, Dominic Kwiatkowski, Cordelia Langford, John Sillitoe on behalf of the Wellcome Sanger Institute COVID-19 Surveillance Team

EPI_ISL_916952, EPI_ISL_916955, EPI_ISL_916957, EPI_ISL_916958, EPI_ISL_916959, EPI_ISL_916960, EPI_ISL_916962, EPI_ISL_916963, EPI_ISL_916964, EPI_ISL_916965, EPI_ISL_916966			
see above	Lighthouse Lab in Alderley Park	Wellcome Sanger Institute for the COVID-19 Genomics UK (COG-UK) Consortium	Jacquelyn Wynn, Mairead Hyland, The Lighthouse Lab in Alderley Park and Alex Alderton, Roberto Amato, Sonia Goncalves, Ewan Harrison, David K. Jackson, Ian Johnston, Dominic Kwiatkowski, Cordelia Langford, John Sillitoe on behalf of the Wellcome Sanger Institute COVID-19 Surveillance Team
EPI_ISL_916967	Lighthouse Lab in Glasgow	Wellcome Sanger Institute for the COVID-19 Genomics UK (COG-UK) Consortium	Harper VanSteenhouse, Yumi Kasai, David Gray, Carol Clugston, Anna Dominiczak and Alex Alderton, Roberto Amato, Sonia Goncalves, Ewan Harrison, David K. Jackson, Ian Johnston, Dominic Kwiatkowski, Cordelia Langford, John Sillitoe on behalf of the Wellcome Sanger Institute COVID-19 Surveillance Team
EPI_ISL_916969, EPI_ISL_916971, EPI_ISL_916972, EPI_ISL_916973, EPI_ISL_916975, EPI_ISL_916978	Lighthouse Lab in Alderley Park	Wellcome Sanger Institute for the COVID-19 Genomics UK (COG-UK) Consortium	Jacquelyn Wynn, Mairead Hyland, The Lighthouse Lab in Alderley Park and Alex Alderton, Roberto Amato, Sonia Goncalves, Ewan Harrison, David K. Jackson, Ian Johnston, Dominic Kwiatkowski, Cordelia Langford, John Sillitoe on behalf of the Wellcome Sanger Institute COVID-19 Surveillance Team
EPI_ISL_916979	Lighthouse Lab in Glasgow	Wellcome Sanger Institute for the COVID-19 Genomics UK (COG-UK) Consortium	Harper VanSteenhouse, Yumi Kasai, David Gray, Carol Clugston, Anna Dominiczak and Alex Alderton, Roberto Amato, Sonia Goncalves, Ewan Harrison, David K. Jackson, Ian Johnston, Dominic Kwiatkowski, Cordelia Langford, John Sillitoe on behalf of the Wellcome Sanger Institute COVID-19 Surveillance Team
EPI_ISL_916980, EPI_ISL_916982, EPI_ISL_916983, EPI_ISL_916986, EPI_ISL_916988, EPI_ISL_916989, EPI_ISL_916990, EPI_ISL_916992, EPI_ISL_916993, EPI_ISL_916994, EPI_ISL_916995, EPI_ISL_916996, EPI_ISL_916997			
see above	Lighthouse Lab in Alderley Park	Wellcome Sanger Institute for the COVID-19 Genomics UK (COG-UK) Consortium	Jacquelyn Wynn, Mairead Hyland, The Lighthouse Lab in Alderley Park and Alex Alderton, Roberto Amato, Sonia Goncalves, Ewan Harrison, David K. Jackson, Ian Johnston, Dominic Kwiatkowski, Cordelia Langford, John Sillitoe on behalf of the Wellcome Sanger Institute COVID-19 Surveillance Team
EPI_ISL_916998	Lighthouse Lab in Glasgow	Wellcome Sanger Institute for the COVID-19 Genomics UK (COG-UK) Consortium	Harper VanSteenhouse, Yumi Kasai, David Gray, Carol Clugston, Anna Dominiczak and Alex Alderton, Roberto Amato, Sonia Goncalves, Ewan Harrison, David K. Jackson, Ian Johnston, Dominic Kwiatkowski, Cordelia Langford, John Sillitoe on behalf of the Wellcome Sanger Institute COVID-19 Surveillance Team
EPI_ISL_917000, EPI_ISL_917001	Lighthouse Lab in Alderley Park	Wellcome Sanger Institute for the COVID-19 Genomics UK (COG-UK) Consortium	Jacquelyn Wynn, Mairead Hyland, The Lighthouse Lab in Alderley Park and Alex Alderton, Roberto Amato, Sonia Goncalves, Ewan Harrison, David K. Jackson, Ian Johnston, Dominic Kwiatkowski, Cordelia Langford, John Sillitoe on behalf of the Wellcome Sanger Institute COVID-19 Surveillance Team
EPI_ISL_917002	Lighthouse Lab in Glasgow	Wellcome Sanger Institute for the COVID-19 Genomics UK (COG-UK) Consortium	Harper VanSteenhouse, Yumi Kasai, David Gray, Carol Clugston, Anna Dominiczak and Alex Alderton, Roberto Amato, Sonia Goncalves, Ewan Harrison, David K. Jackson, Ian Johnston, Dominic Kwiatkowski, Cordelia Langford, John Sillitoe on behalf of the Wellcome Sanger Institute COVID-19 Surveillance Team
EPI_ISL_917003, EPI_ISL_917004, EPI_ISL_917005, EPI_ISL_917006, EPI_ISL_917007, EPI_ISL_917008, EPI_ISL_917010, EPI_ISL_917015, EPI_ISL_917016, EPI_ISL_917017, EPI_ISL_917018, EPI_ISL_917020, EPI_ISL_917023, EPI_ISL_917027, EPI_ISL_917028, EPI_ISL_917030, EPI_ISL_917033, EPI_ISL_917034, EPI_ISL_917035, EPI_ISL_917037, EPI_ISL_917039, EPI_ISL_917040, EPI_ISL_917041, EPI_ISL_917043, EPI_ISL_917044, EPI_ISL_917045, EPI_ISL_917046, EPI_ISL_917048			
see above	Lighthouse Lab in Alderley Park	Wellcome Sanger Institute for the COVID-19 Genomics UK (COG-UK) Consortium	Jacquelyn Wynn, Mairead Hyland, The Lighthouse Lab in Alderley Park and Alex Alderton, Roberto Amato, Sonia Goncalves, Ewan Harrison, David K. Jackson, Ian Johnston, Dominic Kwiatkowski, Cordelia Langford, John Sillitoe on behalf of the Wellcome Sanger Institute COVID-19 Surveillance Team
EPI_ISL_917049	Lighthouse Lab in Glasgow	Wellcome Sanger Institute for the COVID-19 Genomics UK (COG-UK) Consortium	Harper VanSteenhouse, Yumi Kasai, David Gray, Carol Clugston, Anna Dominiczak and Alex Alderton, Roberto Amato, Sonia Goncalves, Ewan Harrison, David K. Jackson, Ian Johnston, Dominic Kwiatkowski, Cordelia Langford, John Sillitoe on behalf of the Wellcome Sanger Institute COVID-19 Surveillance Team
EPI_ISL_917050, EPI_ISL_917051, EPI_ISL_917052	Lighthouse Lab in Alderley Park	Wellcome Sanger Institute for the COVID-19 Genomics UK (COG-UK) Consortium	Jacquelyn Wynn, Mairead Hyland, The Lighthouse Lab in Alderley Park and Alex Alderton, Roberto Amato, Sonia Goncalves, Ewan Harrison, David K. Jackson, Ian Johnston, Dominic Kwiatkowski, Cordelia Langford, John Sillitoe on behalf of the Wellcome Sanger Institute COVID-19 Surveillance Team
EPI_ISL_917053	Lighthouse Lab in Glasgow	Wellcome Sanger Institute for the COVID-19 Genomics UK (COG-UK) Consortium	Harper VanSteenhouse, Yumi Kasai, David Gray, Carol Clugston, Anna Dominiczak and Alex Alderton, Roberto Amato, Sonia Goncalves, Ewan Harrison, David K. Jackson, Ian Johnston, Dominic Kwiatkowski, Cordelia Langford, John Sillitoe on behalf of the Wellcome Sanger Institute COVID-19 Surveillance Team
EPI_ISL_917056, EPI_ISL_917057, EPI_ISL_917058, EPI_ISL_917059, EPI_ISL_917061, EPI_ISL_917062, EPI_ISL_917064, EPI_ISL_917065, EPI_ISL_917066, EPI_ISL_917068, EPI_ISL_917069, EPI_ISL_917072, EPI_ISL_917073			
see above	Lighthouse Lab in Alderley Park	Wellcome Sanger Institute for the COVID-19 Genomics UK (COG-UK) Consortium	Jacquelyn Wynn, Mairead Hyland, The Lighthouse Lab in Alderley Park and Alex Alderton, Roberto Amato, Sonia Goncalves, Ewan Harrison, David K. Jackson, Ian Johnston, Dominic Kwiatkowski, Cordelia Langford, John Sillitoe on behalf of the Wellcome Sanger Institute COVID-19 Surveillance Team
EPI_ISL_917076	Lighthouse Lab in Glasgow	Wellcome Sanger Institute for the COVID-19 Genomics UK (COG-UK) Consortium	Harper VanSteenhouse, Yumi Kasai, David Gray, Carol Clugston, Anna Dominiczak and Alex Alderton, Roberto Amato, Sonia Goncalves, Ewan Harrison, David K. Jackson, Ian Johnston, Dominic Kwiatkowski, Cordelia Langford, John Sillitoe on behalf of the Wellcome Sanger Institute COVID-19 Surveillance Team
EPI_ISL_917078, EPI_ISL_917080, EPI_ISL_917084, EPI_ISL_917085, EPI_ISL_917087, EPI_ISL_917089, EPI_ISL_917090, EPI_ISL_917092, EPI_ISL_917094, EPI_ISL_917097, EPI_ISL_917098, EPI_ISL_917100, EPI_ISL_917101, EPI_ISL_917102, EPI_ISL_917103			
see above	Lighthouse Lab in Alderley Park	Wellcome Sanger Institute for the COVID-19 Genomics UK (COG-UK) Consortium	Jacquelyn Wynn, Mairead Hyland, The Lighthouse Lab in Alderley Park and Alex Alderton, Roberto Amato, Sonia Goncalves, Ewan Harrison, David K. Jackson, Ian Johnston, Dominic Kwiatkowski, Cordelia Langford, John Sillitoe on behalf of the Wellcome Sanger Institute COVID-19 Surveillance Team
EPI_ISL_917104	Lighthouse Lab in Glasgow	Wellcome Sanger Institute for the COVID-19 Genomics UK (COG-UK) Consortium	Harper VanSteenhouse, Yumi Kasai, David Gray, Carol Clugston, Anna Dominiczak and Alex Alderton, Roberto Amato, Sonia Goncalves, Ewan Harrison, David K. Jackson, Ian Johnston, Dominic Kwiatkowski, Cordelia Langford, John Sillitoe on behalf of the Wellcome Sanger Institute COVID-19 Surveillance Team
EPI_ISL_917106, EPI_ISL_917108, EPI_ISL_917109, EPI_ISL_917111	Lighthouse Lab in Alderley Park	Wellcome Sanger Institute for the COVID-19 Genomics UK (COG-UK) Consortium	Jacquelyn Wynn, Mairead Hyland, The Lighthouse Lab in Alderley Park and Alex Alderton, Roberto Amato, Sonia Goncalves, Ewan Harrison, David K. Jackson, Ian Johnston, Dominic Kwiatkowski, Cordelia Langford, John Sillitoe on behalf of the Wellcome Sanger Institute COVID-19 Surveillance Team
EPI_ISL_917112	Lighthouse Lab in Glasgow	Wellcome Sanger Institute for the COVID-19 Genomics UK (COG-UK) Consortium	Harper VanSteenhouse, Yumi Kasai, David Gray, Carol Clugston, Anna Dominiczak and Alex Alderton, Roberto Amato, Sonia Goncalves, Ewan Harrison, David K. Jackson, Ian Johnston, Dominic Kwiatkowski, Cordelia Langford, John Sillitoe on behalf of the Wellcome Sanger Institute COVID-19 Surveillance Team
EPI_ISL_917113	Lighthouse Lab in Alderley Park	Wellcome Sanger Institute for the COVID-19 Genomics UK (COG-UK) Consortium	Jacquelyn Wynn, Mairead Hyland, The Lighthouse Lab in Alderley Park and Alex Alderton, Roberto Amato, Sonia Goncalves, Ewan Harrison, David K. Jackson, Ian Johnston, Dominic Kwiatkowski, Cordelia Langford, John Sillitoe on behalf of the Wellcome Sanger Institute COVID-19 Surveillance Team
EPI_ISL_917114	Lighthouse Lab in Glasgow	Wellcome Sanger Institute for the COVID-19 Genomics UK (COG-UK) Consortium	Harper VanSteenhouse, Yumi Kasai, David Gray, Carol Clugston, Anna Dominiczak and Alex Alderton, Roberto Amato, Sonia Goncalves, Ewan Harrison, David K. Jackson, Ian Johnston, Dominic Kwiatkowski, Cordelia Langford, John Sillitoe on behalf of the Wellcome Sanger Institute COVID-19 Surveillance Team
EPI_ISL_917115, EPI_ISL_917117, EPI_ISL_917118, EPI_ISL_917119, EPI_ISL_917122, EPI_ISL_917123, EPI_ISL_917124, EPI_ISL_917126, EPI_ISL_917127, EPI_ISL_917130, EPI_ISL_917131, EPI_ISL_917132, EPI_ISL_917134, EPI_ISL_917135, EPI_ISL_917136, EPI_ISL_917137, EPI_ISL_917138, EPI_ISL_917140, EPI_ISL_917142			
see above	Lighthouse Lab in Alderley Park	Wellcome Sanger Institute for the COVID-19 Genomics UK (COG-UK) Consortium	Jacquelyn Wynn, Mairead Hyland, The Lighthouse Lab in Alderley Park and Alex Alderton, Roberto Amato, Sonia Goncalves, Ewan Harrison, David K. Jackson, Ian Johnston, Dominic Kwiatkowski, Cordelia Langford, John Sillitoe on behalf of the Wellcome Sanger Institute COVID-19 Surveillance Team
EPI_ISL_917144	Lighthouse Lab in Glasgow	Wellcome Sanger Institute for the COVID-19 Genomics UK (COG-UK) Consortium	Harper VanSteenhouse, Yumi Kasai, David Gray, Carol Clugston, Anna Dominiczak and Alex Alderton, Roberto Amato, Sonia Goncalves, Ewan Harrison, David K. Jackson, Ian Johnston, Dominic Kwiatkowski, Cordelia Langford, John Sillitoe on behalf of the Wellcome Sanger Institute COVID-19 Surveillance Team
EPI_ISL_917146, EPI_ISL_917147, EPI_ISL_917150, EPI_ISL_917152, EPI_ISL_917153, EPI_ISL_917154, EPI_ISL_917155, EPI_ISL_917157, EPI_ISL_917158	Lighthouse Lab in Alderley Park	Wellcome Sanger Institute for the COVID-19 Genomics UK (COG-UK) Consortium	Jacquelyn Wynn, Mairead Hyland, The Lighthouse Lab in Alderley Park and Alex Alderton, Roberto Amato, Sonia Goncalves, Ewan Harrison, David K. Jackson, Ian Johnston, Dominic Kwiatkowski, Cordelia Langford, John Sillitoe on behalf of the Wellcome Sanger Institute COVID-19 Surveillance Team
EPI_ISL_917161	Lighthouse Lab in Glasgow	Wellcome Sanger Institute for the COVID-19 Genomics UK (COG-UK) Consortium	Harper VanSteenhouse, Yumi Kasai, David Gray, Carol Clugston, Anna Dominiczak and Alex Alderton, Roberto Amato, Sonia Goncalves, Ewan Harrison, David K. Jackson, Ian Johnston, Dominic Kwiatkowski, Cordelia Langford, John Sillitoe on behalf of the Wellcome Sanger Institute COVID-19 Surveillance Team

EPI_ISL_917162, EPI_ISL_917164, EPI_ISL_917165	Lighthouse Lab in Alderley Park	Wellcome Sanger Institute for the COVID-19 Genomics UK (COG-UK) Consortium	Jacquelyn Wynn, Mairead Hyland, The Lighthouse Lab in Alderley Park and Alex Alderton, Roberto Amato, Sonia Goncalves, Ewan Harrison, David K. Jackson, Ian Johnston, Dominic Kwiatkowski, Cordelia Langford, John Sillitoe on behalf of the Wellcome Sanger Institute COVID-19 Surveillance Team
EPI_ISL_917166	Lighthouse Lab in Glasgow	Wellcome Sanger Institute for the COVID-19 Genomics UK (COG-UK) Consortium	Harper VanSteenhouse, Yumi Kasai, David Gray, Carol Clugston, Anna Dominiczak and Alex Alderton, Roberto Amato, Sonia Goncalves, Ewan Harrison, David K. Jackson, Ian Johnston, Dominic Kwiatkowski, Cordelia Langford, John Sillitoe on behalf of the Wellcome Sanger Institute COVID-19 Surveillance Team
EPI_ISL_917167, EPI_ISL_918061	Lighthouse Lab in Alderley Park	Wellcome Sanger Institute for the COVID-19 Genomics UK (COG-UK) Consortium	Jacquelyn Wynn, Mairead Hyland, The Lighthouse Lab in Alderley Park and Alex Alderton, Roberto Amato, Sonia Goncalves, Ewan Harrison, David K. Jackson, Ian Johnston, Dominic Kwiatkowski, Cordelia Langford, John Sillitoe on behalf of the Wellcome Sanger Institute COVID-19 Surveillance Team
EPI_ISL_918062, EPI_ISL_918063, EPI_ISL_918064, EPI_ISL_918065, EPI_ISL_918066, EPI_ISL_918067, EPI_ISL_918068, EPI_ISL_918069, EPI_ISL_918070, EPI_ISL_918071, EPI_ISL_918072, EPI_ISL_918073, EPI_ISL_918074, EPI_ISL_918075, EPI_ISL_918076, EPI_ISL_918077, EPI_ISL_918078, EPI_ISL_918079, EPI_ISL_918080, EPI_ISL_918081, EPI_ISL_918082, EPI_ISL_918083, EPI_ISL_918084, EPI_ISL_918085, EPI_ISL_918086, EPI_ISL_918087, EPI_ISL_918088, EPI_ISL_918089, EPI_ISL_918090, EPI_ISL_918091, EPI_ISL_918092, EPI_ISL_918093, EPI_ISL_918094, EPI_ISL_918095, EPI_ISL_918096, EPI_ISL_918097, EPI_ISL_918098, EPI_ISL_918099, EPI_ISL_918100, EPI_ISL_918101, EPI_ISL_918102, EPI_ISL_918103, EPI_ISL_918104, EPI_ISL_918105, EPI_ISL_918106, EPI_ISL_918107, EPI_ISL_918108, EPI_ISL_918109, EPI_ISL_918110, EPI_ISL_918111, EPI_ISL_918112, EPI_ISL_918113, EPI_ISL_918114, EPI_ISL_918115, EPI_ISL_918116, EPI_ISL_918117, EPI_ISL_918118, EPI_ISL_918119, EPI_ISL_918120, EPI_ISL_918121, EPI_ISL_918122, EPI_ISL_918123, EPI_ISL_918124			
see above	Lighthouse Lab in Glasgow	Wellcome Sanger Institute for the COVID-19 Genomics UK (COG-UK) Consortium	Harper VanSteenhouse, Yumi Kasai, David Gray, Carol Clugston, Anna Dominiczak and Alex Alderton, Roberto Amato, Sonia Goncalves, Ewan Harrison, David K. Jackson, Ian Johnston, Dominic Kwiatkowski, Cordelia Langford, John Sillitoe on behalf of the Wellcome Sanger Institute COVID-19 Surveillance Team
EPI_ISL_918165	UMR190-Unité des virus emergents	UMR190-Unité des virus emergents	Cecile Baronti
EPI_ISL_918179, EPI_ISL_918182, EPI_ISL_918188, EPI_ISL_918190, EPI_ISL_918195, EPI_ISL_918196, EPI_ISL_918198, EPI_ISL_918199, EPI_ISL_918204, EPI_ISL_918226, EPI_ISL_918231, EPI_ISL_918232, EPI_ISL_918235, EPI_ISL_918236, EPI_ISL_918238, EPI_ISL_918245, EPI_ISL_918246, EPI_ISL_918247, EPI_ISL_918252, EPI_ISL_918253, EPI_ISL_918260			
see above	Innovative Genomics Institute, UC Berkeley	Innovative Genomics Institute, UC Berkeley	Stacia Wyman, Haridha Shivram, Phil Frankino, Liana Lareau, Shana McDevitt, Justin Choi
EPI_ISL_918288	Hospital Universitari Vall d'Hebron - Vall d'Hebron Institut de Rercerca	Hospital Universitari Vall d'Hebron	Cristina Andrés, Maria Piñana, Josep F Abril, Damir Garcia-Cehic, Ariadna Rando, Juliana Esperalba, Maria Gema Codina, Carla Castillo, Maria Carmen Martin, Tomàs Pumarola, Josep Quer, Andrés Antón
EPI_ISL_918543, EPI_ISL_918544	LACEN - Laboratório Central de Saúde Pública do Ceara	Evandro Chagas Institute	Santos, M.C.; Silva, A.M.; Junior, W.D.C.; Barbagelata, L.S.; Ferreira, J.A.; Sousa, E.M.A.; da Silva, P.S.; Pinheiro, K.C.; L.C.; Sousa Junior, E.C.
EPI_ISL_918764, EPI_ISL_918765, EPI_ISL_918766, EPI_ISL_918767, EPI_ISL_918768, EPI_ISL_918769, EPI_ISL_918770, EPI_ISL_918771, EPI_ISL_918772, EPI_ISL_918773, EPI_ISL_918774, EPI_ISL_918776, EPI_ISL_918777, EPI_ISL_918778, EPI_ISL_918780, EPI_ISL_918781, EPI_ISL_918782, EPI_ISL_918783, EPI_ISL_918784, EPI_ISL_918785, EPI_ISL_918786, EPI_ISL_918787, EPI_ISL_918788, EPI_ISL_918789, EPI_ISL_918790, EPI_ISL_918791, EPI_ISL_918792, EPI_ISL_918793, EPI_ISL_918794, EPI_ISL_918795, EPI_ISL_918796, EPI_ISL_918797, EPI_ISL_918798, EPI_ISL_918799, EPI_ISL_918800, EPI_ISL_918801, EPI_ISL_918802, EPI_ISL_918803, EPI_ISL_918804, EPI_ISL_918805, EPI_ISL_918806, EPI_ISL_918807, EPI_ISL_918808, EPI_ISL_918809, EPI_ISL_918810, EPI_ISL_918811, EPI_ISL_918812, EPI_ISL_918813, EPI_ISL_918814, EPI_ISL_918815, EPI_ISL_918816, EPI_ISL_918817, EPI_ISL_918818, EPI_ISL_918819, EPI_ISL_918820, EPI_ISL_918821, EPI_ISL_918822, EPI_ISL_918823, EPI_ISL_918824, EPI_ISL_918825, EPI_ISL_918826, EPI_ISL_918827, EPI_ISL_918842, EPI_ISL_918843, EPI_ISL_918844, EPI_ISL_918845, EPI_ISL_918856, EPI_ISL_918869, EPI_ISL_918874, EPI_ISL_918875, EPI_ISL_918876, EPI_ISL_918877, EPI_ISL_918878, EPI_ISL_918882, EPI_ISL_918912, EPI_ISL_918913, EPI_ISL_918977, EPI_ISL_918978, EPI_ISL_918979, EPI_ISL_918980, EPI_ISL_918981, EPI_ISL_918982, EPI_ISL_918983			
see above	University of Birmingham	COVID-19 Genomics UK (COG-UK) Consortium	Institute of Microbiology, University of Birmingham: Claire McMurray, Joanne Stockton, Samuel Nicholls, Radoslaw Poplawski, Will Rowe, Josh Quick, Nicholas Loman. University of Birmingham Testing Laboratory: Celina M Whalley, Andrew Bosworth, Charlotte Poxon, Kasun Wanigasooriya, Oliver Pickles, Mike Kidd, Alex Richter, Andrew D Beggs PHE Heartlands Lab: Husam Osman, Andrew Bosworth. Queen Elizabeth Hospital: Anna Casey
EPI_ISL_919057, EPI_ISL_919059, EPI_ISL_919060, EPI_ISL_919061, EPI_ISL_919064, EPI_ISL_919065, EPI_ISL_919066, EPI_ISL_919068, EPI_ISL_919069, EPI_ISL_919072, EPI_ISL_919076, EPI_ISL_919078, EPI_ISL_919083, EPI_ISL_919085, EPI_ISL_919086, EPI_ISL_919088, EPI_ISL_919090, EPI_ISL_919094, EPI_ISL_919096, EPI_ISL_919098, EPI_ISL_919100			
see above	Department of Pathology, University of Cambridge	COVID-19 Genomics UK (COG-UK) Consortium	Aminu S. Jahun, Yasmin Chaudhry, Iliana Georgana, Myra Hosmillo, Rhys Izu, Martin D. Curran, Surendra Parmar, Ian Goodfellow
EPI_ISL_919198, EPI_ISL_919199, EPI_ISL_919200, EPI_ISL_919201, EPI_ISL_919202, EPI_ISL_919203, EPI_ISL_919204	West of Scotland Specialist Virology Centre, NHSGGC / MRC-University of Glasgow Centre for Virus Research	COVID-19 Genomics UK (COG-UK) Consortium	Ana da Silva Filipe, Natasha Johnson, Kathy Smollett, Daniel Mair, Stephen Carmichael, Alice Broos, Lily Tong, Jenna Nichols, Kyriaki Nomikou; Sarah McDonald; Richard Orton, Joseph Hughes, Sreenu Vattipally, David L Robertson; Alasdair MacLean, Rory Gunson; Sharif Shaaban, Matthew Holden; Rachel Blacow, Guy Mollett, Kathy Li, James Shepherd, Antonia Ho, Emma Thomson
EPI_ISL_919339, EPI_ISL_919340, EPI_ISL_919341	Virology Department, Royal Infirmary of Edinburgh, NHS Lothian / School of Biological Sciences, University of Edinburgh / Institute of Genetics and Molecular Medicine, University of Edinburgh	COVID-19 Genomics UK (COG-UK) Consortium	McHugh M, Dewar R, Rooke S, Gallagher M, Balcaza C, O'Toole Á, Scher E, Hill V, McCrone JT, Colquhoun R, Yu X, Jackson B, Rambaut A, Williams TC, Templeton K
EPI_ISL_919479, EPI_ISL_919480, EPI_ISL_919481, EPI_ISL_919482, EPI_ISL_919483, EPI_ISL_919484, EPI_ISL_919485, EPI_ISL_919486, EPI_ISL_919487, EPI_ISL_919516, EPI_ISL_919524, EPI_ISL_919526, EPI_ISL_919527, EPI_ISL_919528, EPI_ISL_919539, EPI_ISL_919540, EPI_ISL_919546, EPI_ISL_919553, EPI_ISL_919555, EPI_ISL_919560, EPI_ISL_919566, EPI_ISL_919567, EPI_ISL_919569, EPI_ISL_919580, EPI_ISL_919583, EPI_ISL_919584, EPI_ISL_919586, EPI_ISL_919592, EPI_ISL_919596, EPI_ISL_919597, EPI_ISL_919598, EPI_ISL_919599, EPI_ISL_919600, EPI_ISL_919601, EPI_ISL_919602, EPI_ISL_919632, EPI_ISL_919634, EPI_ISL_919637, EPI_ISL_919641, EPI_ISL_919642, EPI_ISL_919643, EPI_ISL_919645, EPI_ISL_919646, EPI_ISL_919651, EPI_ISL_919660			
see above	Liverpool Clinical Laboratories	COVID-19 Genomics UK (COG-UK) Consortium	Sam Haldenby, Anita Lucaci, Steve Paterson, Julian Hiscox, Alistair Darby, M Almsaud, A Alrezaihi, Muhannad Alruwaili, Stuart D Armstrong, Jones Benjamin, Eleanor G Bentley, Anu Chawla, Jordan J Clark, Angela Cowell, Richard Eccles, Isabel García-Dorival, Matthew Gemmell, Alessandro Gerada, PKF Gilmore, Richard Gregory, Ximeng Han, Catherine Hartley, Margaret Hughes, Miren Iturriza-Gomara, James Johnson, L Luu, Jenifer Manson, Charlotte Nelson, Elaine O'Toole, Cassie Olateju, Rebekah Penrice-Randal , Lucille Rainbow, N.P Randle, Trevor Ian Robinson, Parul Sharma, Ghada T Shawli, James P Stewart, Neil Swainston, Ecaterina Vamos, Joanne Watts, Mark Whitehead
EPI_ISL_919853	Barts Health NHS Trust	COVID-19 Genomics UK (COG-UK) Consortium	CUTINO-MOGUEL, Maria-Teresa; HARRINGTON, David; OWOYEMI, Dola; KULASEGARAN-SHYLINI, Raghavendran; BROAD, Claire; KELE, Beatrix
EPI_ISL_920030, EPI_ISL_920031, EPI_ISL_920032, EPI_ISL_920033, EPI_ISL_920035, EPI_ISL_920036, EPI_ISL_920037, EPI_ISL_920038, EPI_ISL_920039, EPI_ISL_920043, EPI_ISL_920044, EPI_ISL_920045			
see above	University College London, Great Ormond Street Hospital for Children NHS Foundation Trust, Imperial College Healthcare NHS Trust	COVID-19 Genomics UK (COG-UK) Consortium	Sergi Castellano, Rachel Williams, Mark Kristiansen, Paola Resende Silva, Sunando Roy, Tony Brooks, Helena Tutill, Paola Niola, Patricia Dyal, Charlotte Williams, Leysa Forrest, Yasmin Panchbhaya, Jacqueline Findlay, Samuel Weeks, Julianne Brown, Kathryn Harris, Paul Randell, James Price, Alison Holmes, Judith Breuer
EPI_ISL_920174, EPI_ISL_920175, EPI_ISL_920177, EPI_ISL_920180, EPI_ISL_920181, EPI_ISL_920182, EPI_ISL_920183, EPI_ISL_920184, EPI_ISL_920186, EPI_ISL_920190, EPI_ISL_920194, EPI_ISL_920196, EPI_ISL_920197, EPI_ISL_920198, EPI_ISL_920200, EPI_ISL_920201, EPI_ISL_920202, EPI_ISL_920203, EPI_ISL_920205, EPI_ISL_920206, EPI_ISL_920207, EPI_ISL_920208, EPI_ISL_920210, EPI_ISL_920211, EPI_ISL_920212, EPI_ISL_920215, EPI_ISL_920217, EPI_ISL_920219, EPI_ISL_920222, EPI_ISL_920224, EPI_ISL_920225, EPI_ISL_920226, EPI_ISL_920227, EPI_ISL_920228, EPI_ISL_920229, EPI_ISL_920230, EPI_ISL_920232, EPI_ISL_920233, EPI_ISL_920237, EPI_ISL_920238, EPI_ISL_920245, EPI_ISL_920246, EPI_ISL_920253, EPI_ISL_920254, EPI_ISL_920263, EPI_ISL_920264, EPI_ISL_920265, EPI_ISL_920273, EPI_ISL_920274, EPI_ISL_920275, EPI_ISL_920285, EPI_ISL_920286, EPI_ISL_920294, EPI_ISL_920295, EPI_ISL_920296, EPI_ISL_920305, EPI_ISL_920306, EPI_ISL_920315, EPI_ISL_920360			
see above	University College London Hospital	COVID-19 Genomics UK (COG-UK) Consortium	Judith Heaney, Matthew Byott, Catherine Houlihan, Dan Frampton, Stuart Kirk, Moira Spyer and Eleni Nastouli
EPI_ISL_920882, EPI_ISL_920883, EPI_ISL_920884, EPI_ISL_920885, EPI_ISL_920886, EPI_ISL_920887, EPI_ISL_920888, EPI_ISL_920889, EPI_ISL_920890, EPI_ISL_920891, EPI_ISL_920892			
see above	University College London, Great Ormond Street Hospital for Children NHS Foundation Trust, Imperial College Healthcare NHS Trust	COVID-19 Genomics UK (COG-UK) Consortium	Sergi Castellano, Rachel Williams, Mark Kristiansen, Paola Resende Silva, Sunando Roy, Tony Brooks, Helena Tutill, Paola Niola, Patricia Dyal, Charlotte Williams, Leysa Forrest, Yasmin Panchbhaya, Jacqueline Findlay, Samuel Weeks, Julianne Brown, Kathryn Harris, Paul Randell, James Price, Alison Holmes, Judith Breuer
EPI_ISL_920901, EPI_ISL_920902, EPI_ISL_920903, EPI_ISL_920904, EPI_ISL_920905, EPI_ISL_920906, EPI_ISL_920907, EPI_ISL_920908, EPI_ISL_920909, EPI_ISL_920911, EPI_ISL_920912, EPI_ISL_920913, EPI_ISL_920914, EPI_ISL_920915, EPI_ISL_920916, EPI_ISL_920917, EPI_ISL_920918, EPI_ISL_920919, EPI_ISL_920920, EPI_ISL_920921, EPI_ISL_920922, EPI_ISL_920923, EPI_ISL_920925, EPI_ISL_920926, EPI_ISL_920927, EPI_ISL_920928, EPI_ISL_920929, EPI_ISL_920930, EPI_ISL_920931, EPI_ISL_920932, EPI_ISL_920933, EPI_ISL_920935, EPI_ISL_920936, EPI_ISL_920937, EPI_ISL_920938, EPI_ISL_920939, EPI_ISL_920940, EPI_ISL_920941, EPI_ISL_920942, EPI_ISL_920944, EPI_ISL_920945, EPI_ISL_920946, EPI_ISL_920947, EPI_ISL_920948, EPI_ISL_920949, EPI_ISL_920950, EPI_ISL_920951, EPI_ISL_920952, EPI_ISL_920953, EPI_ISL_920954, EPI_ISL_920955, EPI_ISL_920956, EPI_ISL_920957, EPI_ISL_920958, EPI_ISL_920959, EPI_ISL_920960, EPI_ISL_920961, EPI_ISL_920962, EPI_ISL_920963, EPI_ISL_920964, EPI_ISL_920965, EPI_ISL_920966, EPI_ISL_920967, EPI_ISL_920968, EPI_ISL_920969, EPI_ISL_920970, EPI_ISL_920971, EPI_ISL_920972, EPI_ISL_920973, EPI_ISL_920974			
see above	Department of Pathology, University of Cambridge	COVID-19 Genomics UK (COG-UK) Consortium	Aminu S. Jahun, Yasmin Chaudhry, Iliana Georgana, Myra Hosmillo, Rhys Izu, Martin D. Curran, Surendra Parmar, Ian Goodfellow
EPI_ISL_920976, EPI_ISL_920977, EPI_ISL_920978, EPI_ISL_920979, EPI_ISL_920981, EPI_ISL_920982, EPI_ISL_920983, EPI_ISL_920984, EPI_ISL_920985, EPI_ISL_920986, EPI_ISL_920987, EPI_ISL_920988, EPI_ISL_920989, EPI_ISL_920990, EPI_ISL_920991, EPI_ISL_920992, EPI_ISL_920993, EPI_ISL_920994, EPI_ISL_920995, EPI_ISL_921011, EPI_ISL_921012, EPI_ISL_921013, EPI_ISL_921014, EPI_ISL_921015, EPI_ISL_921016, EPI_ISL_921017			
see above	Regional Virus Laboratory, Belfast Health and Social Care Trust	COVID-19 Genomics UK (COG-UK) Consortium	Conall McCaughey, James McKenna, Tanya Curran, Susan Feeney, Alison Watt, Ciara Cox, Mairead Connor, Zoltan Molnar, David Simpson, Derek Fairley
EPI_ISL_921428, EPI_ISL_921486, EPI_ISL_921487, EPI_ISL_921502, EPI_ISL_921521, EPI_ISL_921523, EPI_ISL_921524, EPI_ISL_921525, EPI_ISL_921526, EPI_ISL_921527, EPI_ISL_921528, EPI_ISL_921529, EPI_ISL_921531, EPI_ISL_921532, EPI_ISL_921533, EPI_ISL_921534, EPI_ISL_921535, EPI_ISL_921536, EPI_ISL_921537, EPI_ISL_921538, EPI_ISL_921539, EPI_ISL_921540, EPI_ISL_921542, EPI_ISL_921543, EPI_ISL_921544, EPI_ISL_921545, EPI_ISL_921547, EPI_ISL_921548, EPI_ISL_921549, EPI_ISL_921550, EPI_ISL_921551, EPI_ISL_921552, EPI_ISL_921553, EPI_ISL_921554, EPI_ISL_921555, EPI_ISL_921556,			

EPI_ISL_921557, EPI_ISL_921559, EPI_ISL_921562, EPI_ISL_921567, EPI_ISL_921568, EPI_ISL_921570, EPI_ISL_921571, EPI_ISL_921573, EPI_ISL_921574			
see above	Northumbria University / South Tees Hospitals NHS Foundation Trust / North Cumbria Integrated Care NHS Foundation Trust / North Tees and Hartlepool NHS Foundation Trust / Newcastle Hospitals NHS Foundation Trust	COVID-19 Genomics UK (COG-UK) Consortium	Darren L Smith,Andrew Nelson,Matthew Bashton,Greg R Young,Joshua Loh,John Allan,Mohammad A Tariq,Giles S Holt,Gary Black,Wen C Yew,Lynn Dover,Paul Baker,Steve Liggett,Sarah Essex,Jane Greenaway,Debra Padgett,Clive Graham,Garren Scott,Edward Barton,Emma Swindells,Brendan Payne,Jennifer Collins,Yusri Taha,Gary Eltringham
EPI_ISL_922113, EPI_ISL_922114, EPI_ISL_922117, EPI_ISL_922118, EPI_ISL_922119, EPI_ISL_922120, EPI_ISL_922121, EPI_ISL_922122, EPI_ISL_922123, EPI_ISL_922124, EPI_ISL_922125, EPI_ISL_922126, EPI_ISL_922127, EPI_ISL_922128, EPI_ISL_922129, EPI_ISL_922130, EPI_ISL_922131, EPI_ISL_922132, EPI_ISL_922133, EPI_ISL_922134			
see above	Lincolnshire Hospitals and DeepSeq Nottingham	COVID-19 Genomics UK (COG-UK) Consortium	Nichola Duckworth, Tim Sloan, Sarah Walsh, Jonathan Ball, Patrick McClure, Joeseph Chappell, Nadine Holmes, Matthew Carlisle, Christopher Moore, Fei Sang, Johnny Debebe, Victoria Wright, Matthew Loose
EPI_ISL_922231, EPI_ISL_922232, EPI_ISL_922233, EPI_ISL_922234, EPI_ISL_922238, EPI_ISL_922239, EPI_ISL_922240	Oxford Viromics, NDM, University of Oxford; Oxford University Hospitals; Basingstoke and North Hampshire Hospital	COVID-19 Genomics UK (COG-UK) Consortium	Tanya Golubchik, David Bonsall, George Macintyre, Amy Trebes, Mariateresa de Cesare, Catrin Moore, Alex Mobbs, Anita Justice, Robert Shaw, Monique Andersson, Timothy Peto, Emma Wise, Nathan Moore, Jessica Lynch, Nick Cortes, Matilde Mori, Stephen Kidd, David Buck, John Todd, Christophe Fraser
EPI_ISL_922360, EPI_ISL_922362, EPI_ISL_922363, EPI_ISL_922365, EPI_ISL_922384, EPI_ISL_922392, EPI_ISL_922395, EPI_ISL_922396, EPI_ISL_922398, EPI_ISL_922399, EPI_ISL_922401, EPI_ISL_922402, EPI_ISL_922403, EPI_ISL_922404, EPI_ISL_922406, EPI_ISL_922407, EPI_ISL_922408, EPI_ISL_922409, EPI_ISL_922410, EPI_ISL_922411, EPI_ISL_922413, EPI_ISL_922415, EPI_ISL_922416, EPI_ISL_922417, EPI_ISL_922418, EPI_ISL_922419, EPI_ISL_922420, EPI_ISL_922421, EPI_ISL_922423, EPI_ISL_922425, EPI_ISL_922428, EPI_ISL_922430, EPI_ISL_922432, EPI_ISL_922433, EPI_ISL_922434, EPI_ISL_922435, EPI_ISL_922440, EPI_ISL_922442, EPI_ISL_922444, EPI_ISL_922445, EPI_ISL_922446, EPI_ISL_922448, EPI_ISL_922452, EPI_ISL_922455, EPI_ISL_922457, EPI_ISL_922458, EPI_ISL_922459, EPI_ISL_922460, EPI_ISL_922462, EPI_ISL_922463, EPI_ISL_922464, EPI_ISL_922465, EPI_ISL_922466, EPI_ISL_922467, EPI_ISL_922468, EPI_ISL_922469, EPI_ISL_922470, EPI_ISL_922473, EPI_ISL_922474, EPI_ISL_922481, EPI_ISL_922553, EPI_ISL_922563, EPI_ISL_922565, EPI_ISL_922566, EPI_ISL_922567, EPI_ISL_922568, EPI_ISL_922570, EPI_ISL_922571, EPI_ISL_922572, EPI_ISL_922574, EPI_ISL_922580, EPI_ISL_922581, EPI_ISL_922582, EPI_ISL_922590, EPI_ISL_922591, EPI_ISL_922592, EPI_ISL_922593, EPI_ISL_922594, EPI_ISL_922595, EPI_ISL_922596, EPI_ISL_922597, EPI_ISL_922598, EPI_ISL_922599, EPI_ISL_922601, EPI_ISL_922633, EPI_ISL_922634, EPI_ISL_922643, EPI_ISL_922645, EPI_ISL_922646, EPI_ISL_922647, EPI_ISL_922648, EPI_ISL_922649, EPI_ISL_922650, EPI_ISL_922651, EPI_ISL_922652, EPI_ISL_922653, EPI_ISL_922654, EPI_ISL_922655, EPI_ISL_922659, EPI_ISL_922660, EPI_ISL_922663, EPI_ISL_922664, EPI_ISL_922665, EPI_ISL_922666, EPI_ISL_922667, EPI_ISL_922668, EPI_ISL_922669, EPI_ISL_922672, EPI_ISL_922673, EPI_ISL_922674, EPI_ISL_922675, EPI_ISL_922676, EPI_ISL_922677, EPI_ISL_922678, EPI_ISL_922679, EPI_ISL_922680, EPI_ISL_922681, EPI_ISL_922682, EPI_ISL_922683, EPI_ISL_922684, EPI_ISL_922685, EPI_ISL_922686, EPI_ISL_922687, EPI_ISL_922688, EPI_ISL_922689, EPI_ISL_922690, EPI_ISL_922693, EPI_ISL_922695, EPI_ISL_922696, EPI_ISL_922698, EPI_ISL_922701, EPI_ISL_922702, EPI_ISL_922705, EPI_ISL_922706, EPI_ISL_922707, EPI_ISL_922710, EPI_ISL_922895, EPI_ISL_922903, EPI_ISL_922904, EPI_ISL_922905, EPI_ISL_922913, EPI_ISL_922923, EPI_ISL_922925, EPI_ISL_922926, EPI_ISL_922927, EPI_ISL_922928, EPI_ISL_922929, EPI_ISL_922930, EPI_ISL_922931, EPI_ISL_922965, EPI_ISL_922970, EPI_ISL_922974, EPI_ISL_922975, EPI_ISL_922976, EPI_ISL_922977, EPI_ISL_922978, EPI_ISL_922979, EPI_ISL_922980, EPI_ISL_922981, EPI_ISL_922982, EPI_ISL_922983, EPI_ISL_922984, EPI_ISL_922985, EPI_ISL_922986, EPI_ISL_922987, EPI_ISL_922988, EPI_ISL_922989, EPI_ISL_922990, EPI_ISL_922991, EPI_ISL_922992, EPI_ISL_922993, EPI_ISL_922994, EPI_ISL_922995, EPI_ISL_922996, EPI_ISL_922997, EPI_ISL_922998, EPI_ISL_922999, EPI_ISL_923000, EPI_ISL_923001, EPI_ISL_923002, EPI_ISL_923003, EPI_ISL_923004, EPI_ISL_923005, EPI_ISL_923006, EPI_ISL_923007, EPI_ISL_923008, EPI_ISL_923009, EPI_ISL_923010, EPI_ISL_923011, EPI_ISL_923013, EPI_ISL_923014, EPI_ISL_923015			
see above	Wales Specialist Virology Centre Sequencing lab: Pathogen Genomics Unit	Public Health Wales Microbiology Cardiff Wales Specialist Virology Centre	Catherine Moore, Johnathan Evans, Laura Gifford, Malorie Perry, Simon Cottrell, Angela Marchbank, Alec Birchley, Alexander Adams, Amy Gaskin, Bree Gatica-Wilcox, Jason Coombes, Joel Southgate, Lauren Gilbert, Lee Graham, Nicole Pacchiarini, Sara Kumziene-Summerhayes, Sarah Taylor, Sophie Jones, Sara Rey, Matthew Bull, Joanne Watkins, Sally Corden, Tom Connor
EPI_ISL_923276, EPI_ISL_923280, EPI_ISL_923281, EPI_ISL_923287, EPI_ISL_923290, EPI_ISL_923291, EPI_ISL_923292, EPI_ISL_923294, EPI_ISL_923298, EPI_ISL_923302, EPI_ISL_923303, EPI_ISL_923304, EPI_ISL_923307, EPI_ISL_923308, EPI_ISL_923309, EPI_ISL_923310, EPI_ISL_923311, EPI_ISL_923312, EPI_ISL_923313, EPI_ISL_923314, EPI_ISL_923317, EPI_ISL_923355, EPI_ISL_923356, EPI_ISL_923359, EPI_ISL_923361, EPI_ISL_923364, EPI_ISL_923365, EPI_ISL_923371, EPI_ISL_923414, EPI_ISL_923416, EPI_ISL_923419, EPI_ISL_923438, EPI_ISL_923589, EPI_ISL_923590, EPI_ISL_923594, EPI_ISL_923628, EPI_ISL_923660, EPI_ISL_923664, EPI_ISL_923665, EPI_ISL_923666, EPI_ISL_923667, EPI_ISL_923668, EPI_ISL_923669, EPI_ISL_923670, EPI_ISL_923672, EPI_ISL_923673, EPI_ISL_923674, EPI_ISL_923675, EPI_ISL_923676, EPI_ISL_923677, EPI_ISL_923681, EPI_ISL_923683			
see above	Centre for Enzyme Innovation, University of Portsmouth / Translational Research Laboratory, Portsmouth Hospitals NHS Trust	COVID-19 Genomics UK (COG-UK) Consortium	Angela Beckett,Salman Goudarzi,Christopher Fearn,Kate Cook,Katie Loveson,Sharon Glaysher,Scott Elliott,Samuel Robson
EPI_ISL_923685, EPI_ISL_923692, EPI_ISL_923695, EPI_ISL_923712, EPI_ISL_923713, EPI_ISL_923714, EPI_ISL_923715, EPI_ISL_923726, EPI_ISL_923738, EPI_ISL_923742, EPI_ISL_923748, EPI_ISL_923752, EPI_ISL_923767, EPI_ISL_923768, EPI_ISL_923776, EPI_ISL_923778, EPI_ISL_923798, EPI_ISL_923804, EPI_ISL_923810, EPI_ISL_923864, EPI_ISL_923916, EPI_ISL_923924, EPI_ISL_923925, EPI_ISL_923939, EPI_ISL_923950, EPI_ISL_923951, EPI_ISL_923952, EPI_ISL_923959, EPI_ISL_923962, EPI_ISL_923982, EPI_ISL_923983, EPI_ISL_923990, EPI_ISL_923996, EPI_ISL_924001, EPI_ISL_924009, EPI_ISL_924014, EPI_ISL_924026, EPI_ISL_924038, EPI_ISL_924041, EPI_ISL_924043, EPI_ISL_924047, EPI_ISL_924048, EPI_ISL_924051, EPI_ISL_924053, EPI_ISL_924067, EPI_ISL_924070, EPI_ISL_924076			
see above	Department of Pathology, University of Cambridge	COVID-19 Genomics UK (COG-UK) Consortium	Aminu S. Jahun, Yasmin Chaudhry, Iliana Georgana, Myra Hosmillo, Rhys Izu, Martin D. Curran, Surendra Parmar, Ian Goodfellow
EPI_ISL_924083, EPI_ISL_924145, EPI_ISL_924150, EPI_ISL_924177, EPI_ISL_924181, EPI_ISL_924220, EPI_ISL_924224, EPI_ISL_924230, EPI_ISL_924233, EPI_ISL_924239, EPI_ISL_924242, EPI_ISL_924244, EPI_ISL_924247, EPI_ISL_924278, EPI_ISL_924303, EPI_ISL_924325, EPI_ISL_924326, EPI_ISL_924399			
see above	Virology Department, Sheffield Teaching Hospitals NHS Foundation Trust/Department of Infection, Immunity and Cardiovascular Disease, The Medical School, University of Sheffield	COVID-19 Genomics UK (COG-UK) Consortium	Thushan de Silva, Matthew Parker, Nikki Smith, Adri Angyal, Rebecca Brown, Luke Green, Rachel Tucker, Paul Parsons, Danielle Groves, Katie Johnson, Laura Carrilero, Alex Keeley, Dave Partridge, Matthew Wyles, Benjamin Lindsey, Mehmet Yavuz, Mohammad Raza, Cariad Evans
EPI_ISL_924796, EPI_ISL_924801, EPI_ISL_924802, EPI_ISL_924803, EPI_ISL_924804, EPI_ISL_924806, EPI_ISL_924810, EPI_ISL_924811, EPI_ISL_924812, EPI_ISL_924813, EPI_ISL_924814, EPI_ISL_924815, EPI_ISL_924817, EPI_ISL_924820, EPI_ISL_924821, EPI_ISL_924822, EPI_ISL_924823, EPI_ISL_924824, EPI_ISL_924825, EPI_ISL_924826, EPI_ISL_924830, EPI_ISL_924834, EPI_ISL_924841, EPI_ISL_924842, EPI_ISL_924845, EPI_ISL_924857, EPI_ISL_924859, EPI_ISL_924948, EPI_ISL_924949, EPI_ISL_924952, EPI_ISL_924953, EPI_ISL_924954, EPI_ISL_924956, EPI_ISL_924957, EPI_ISL_924958, EPI_ISL_924959, EPI_ISL_924960, EPI_ISL_924961, EPI_ISL_924962, EPI_ISL_924963, EPI_ISL_924964, EPI_ISL_924965, EPI_ISL_924966, EPI_ISL_924967, EPI_ISL_924968, EPI_ISL_924969, EPI_ISL_924970, EPI_ISL_924971, EPI_ISL_924972, EPI_ISL_924973, EPI_ISL_924974, EPI_ISL_924976, EPI_ISL_924977, EPI_ISL_924978, EPI_ISL_924979, EPI_ISL_924981, EPI_ISL_924982, EPI_ISL_924983, EPI_ISL_924984, EPI_ISL_924985, EPI_ISL_924986, EPI_ISL_924988, EPI_ISL_924989, EPI_ISL_924990, EPI_ISL_924991, EPI_ISL_924992, EPI_ISL_924993, EPI_ISL_924994, EPI_ISL_924995, EPI_ISL_924996, EPI_ISL_924997, EPI_ISL_924998, EPI_ISL_924999, EPI_ISL_925000, EPI_ISL_925001, EPI_ISL_925002, EPI_ISL_925003, EPI_ISL_925004, EPI_ISL_925005, EPI_ISL_925006, EPI_ISL_925007, EPI_ISL_925008, EPI_ISL_925009, EPI_ISL_925010, EPI_ISL_925011, EPI_ISL_925012, EPI_ISL_925013			
see above	Bioinformatics and Biostatistics Lab, Advanced Sequencing Facility	COVID-19 Genomics UK (COG-UK) Consortium	Aengus Stewart,Jerome Nicod,Chelsea Sawyer,Laura Cubitt,Harshil Patel,Margaret Crawford
EPI_ISL_925078, EPI_ISL_925079, EPI_ISL_925080	TXDSHS	TXDSHS	Bonnie Oh, Anita Pokharel, James Daniel Bonser, Myong Koag, Chung Wang, Rachel Lee, Grace Kubin, Rashmi Tuladhar, Mayela Pedrueza, Maliha Rahman, Jenny Zhang
EPI_ISL_925137, EPI_ISL_925138, EPI_ISL_925139, EPI_ISL_925140	Quest Diagnostics	Quest Diagnostics	Rosenthal,S.H., Gerasimova,A., Kagan,R.M., Anderson, B., Hua, M., Liu Y., Bernstein, L.E., Livingston, K.E., Perez, A., Shalhout, D.F., Shlyakhter, I.A., Owen, R., Tanpaiboon, P., Lacbawan, F.
EPI_ISL_925216, EPI_ISL_925217, EPI_ISL_925218, EPI_ISL_925219, EPI_ISL_925220, EPI_ISL_925221, EPI_ISL_925222, EPI_ISL_925223, EPI_ISL_925226, EPI_ISL_925228, EPI_ISL_925229, EPI_ISL_925230, EPI_ISL_925231, EPI_ISL_925232, EPI_ISL_925233, EPI_ISL_925234, EPI_ISL_925235, EPI_ISL_925236, EPI_ISL_925237, EPI_ISL_925238, EPI_ISL_925239, EPI_ISL_925240, EPI_ISL_925241, EPI_ISL_925242, EPI_ISL_925243, EPI_ISL_925244, EPI_ISL_925245, EPI_ISL_925246, EPI_ISL_925247, EPI_ISL_925248, EPI_ISL_925249, EPI_ISL_925250, EPI_ISL_925251, EPI_ISL_925252, EPI_ISL_925254, EPI_ISL_925255, EPI_ISL_925256, EPI_ISL_925257			
see above	New Mexico Department of Health Scientific Laboratory	New Mexico Department of Health Scientific Laboratory	Ellie Johnson, Anastacia Griego-Fisher, D'eldra Malone, Jennifer Benoit
EPI_ISL_925266, EPI_ISL_925268, EPI_ISL_925270, EPI_ISL_925271, EPI_ISL_925274, EPI_ISL_925276, EPI_ISL_925288, EPI_ISL_925292, EPI_ISL_925297, EPI_ISL_925298, EPI_ISL_925299			
see above	Wyoming Public Health Laboratory	Wyoming Public Health Laboratory	Noah Hull, Taylor Fearing, Lynette Gumbleton, Channing Weber, Ashley Norberg, Bailey Bowcutt, and Wanda Manley
EPI_ISL_925402, EPI_ISL_925403, EPI_ISL_925404, EPI_ISL_925405	Department of Clinical Microbiology	GIGA Medical Genomics	Keith Durkin, Maria Artesi, Sébastien Bontems, Raphaël Boreux, Bouchra Boujemla, Cécile Meex, Pierrette Melin, Marie-Pierre Hayette, Vincent Bours
EPI_ISL_925888, EPI_ISL_925889, EPI_ISL_925890, EPI_ISL_925891, EPI_ISL_925892, EPI_ISL_925909, EPI_ISL_925910	Nucleic Acid Testing, National Reference Laboratory	GIGA Medical Genomics	Yvan Butera, Keith Durkin, Maria Artesi, Bouchra Boujemla, Robert Rutayisire, Patrick Tuyisenge, Esperence Umumararungu, Sébastien Bontems, Marie-Pierre Hayette, Nathalie Renotte, Swaibu Gatare, Jacob Souopgui, Sabin Nsanzimana, Vincent Bours, Léon Mutesa
EPI_ISL_930784	Hematology Laboratory, Section of Molecular Diagnostics, University Clinical Centre, Medical University of Gdansk	Laboratory of Recombinant Vaccines	Lukasz Rabalski, Maciej Kosinski, Maciej Grzybek, Adam Sodol, Aneta Szulc, Krzysztof Lewandowski, Ewa Milosz, Marlena Robakowska, Boguslaw Szewczyk, Krystyna Bienkowska-Szewczyk
EPI_ISL_930785	Laboratory of Molecular Biology, Diagnostyka sp. z o.o.	Laboratory of Recombinant Vaccines	Lukasz Rabalski, Maciej Kosinski, Anna Piotrowska-Mietelska,Izabela Szczygielska, Boguslaw Szewczyk, Krystyna Bienkowska-Szewczyk
EPI_ISL_931584, EPI_ISL_933718, EPI_ISL_933721, EPI_ISL_933734, EPI_ISL_933740	Servicio de Microbiología Hospital Ramón y Cajal	Servicio de Microbiología Hospital Ramón y Cajal	José M Gonzalez-Alba, Concepción Rodríguez, Melanie Abreu, Laura Martínez, Val F Lanza, Luz Leticia Olavarrieta, Rafael Cantón, JC Galán
EPI_ISL_933760	DPHL	Delaware Public Health Lab	Gregory Hovan
EPI_ISL_933768, EPI_ISL_933770	Servicio de Microbiología Hospital Ramón y Cajal	Servicio de Microbiología Hospital Ramón y Cajal	José M Gonzalez-Alba, Concepción Rodríguez, Melanie Abreu, Laura Martínez, Val F Lanza, Luz Leticia Olavarrieta, Rafael Cantón, JC Galán

EPI_ISL_933791	PathWest Laboratory Medicine WA	PathWest Laboratory Medicine WA Microbial Surveillance Unit	PathWest Laboratory Medicine WA Microbial Surveillance Unit
EPI_ISL_934234, EPI_ISL_934235, EPI_ISL_934236, EPI_ISL_934237, EPI_ISL_934239, EPI_ISL_934240, EPI_ISL_934242, EPI_ISL_934243, EPI_ISL_934244, EPI_ISL_934245, EPI_ISL_934246, EPI_ISL_934247, EPI_ISL_934248, EPI_ISL_934249, EPI_ISL_934250, EPI_ISL_934251, EPI_ISL_934252, EPI_ISL_934253, EPI_ISL_934254, EPI_ISL_934255, EPI_ISL_934256, EPI_ISL_934257, EPI_ISL_934258, EPI_ISL_934259, EPI_ISL_934260, EPI_ISL_934261, EPI_ISL_934262, EPI_ISL_934263, EPI_ISL_934264, EPI_ISL_934265, EPI_ISL_934266, EPI_ISL_934267, EPI_ISL_934268, EPI_ISL_934269, EPI_ISL_934270, EPI_ISL_934271, EPI_ISL_934272, EPI_ISL_934273, EPI_ISL_934274, EPI_ISL_934275, EPI_ISL_934276, EPI_ISL_934277, EPI_ISL_934278, EPI_ISL_934279			
see above	Vilnius university hospital Santaros Klinikos, Center of Laboratory Medicine	Vilnius university hospital Santaros Klinikos, Center of Laboratory Medicine	Ingrida Olendraite, Daniel Naumovas, Rimvydas Norvilas, Dovile Ezerskyte, Justinas Slikas, Gytis Dudas
EPI_ISL_934315	Narhalsan Fjallbacka VC	The Public Health Agency of Sweden	Anna-Malin Linde, Maria Lind Karlberg, Carlo Berg, Oskar Karlsson Lindsjo, Sofia Stamouli, Reza Advani, Mattias Haukland, Petra Holmstrom, Noura Walai, Petra Edquist, Mia Brytting, Anna Risberg, Karin Tegmark-Wisell
EPI_ISL_934375, EPI_ISL_934376, EPI_ISL_934379, EPI_ISL_934380	Klinisk mikrobiologi	The Public Health Agency of Sweden	Anna-Malin Linde, Maria Lind Karlberg, Carlo Berg, Oskar Karlsson Lindsjo, Sofia Stamouli, Reza Advani, Mattias Haukland, Petra Holmstrom, Noura Walai, Petra Edquist, Mia Brytting, Anna Risberg, Karin Tegmark-Wisell
EPI_ISL_934399, EPI_ISL_934401, EPI_ISL_934402, EPI_ISL_934403, EPI_ISL_934404	Synlab Medilab, Mikrobiologi	The Public Health Agency of Sweden	Anna-Malin Linde, Maria Lind Karlberg, Carlo Berg, Oskar Karlsson Lindsjo, Sofia Stamouli, Reza Advani, Mattias Haukland, Petra Holmstrom, Noura Walai, Petra Edquist, Mia Brytting, Anna Risberg, Karin Tegmark-Wisell
EPI_ISL_934408, EPI_ISL_934409	Klinisk Mikrobiologi	The Public Health Agency of Sweden	Anna-Malin Linde, Maria Lind Karlberg, Carlo Berg, Oskar Karlsson Lindsjo, Sofia Stamouli, Reza Advani, Mattias Haukland, Petra Holmstrom, Noura Walai, Petra Edquist, Mia Brytting, Anna Risberg, Karin Tegmark-Wisell
EPI_ISL_934410, EPI_ISL_934411, EPI_ISL_934412	Synlab Medilab, Mikrobiologi	The Public Health Agency of Sweden	Anna-Malin Linde, Maria Lind Karlberg, Carlo Berg, Oskar Karlsson Lindsjo, Sofia Stamouli, Reza Advani, Mattias Haukland, Petra Holmstrom, Noura Walai, Petra Edquist, Mia Brytting, Anna Risberg, Karin Tegmark-Wisell
EPI_ISL_934548, EPI_ISL_934549, EPI_ISL_934550, EPI_ISL_934551, EPI_ISL_934552, EPI_ISL_934553, EPI_ISL_934554, EPI_ISL_934555, EPI_ISL_934556, EPI_ISL_934557, EPI_ISL_934558, EPI_ISL_934559, EPI_ISL_934560, EPI_ISL_934561, EPI_ISL_934562, EPI_ISL_934563, EPI_ISL_934564, EPI_ISL_934565, EPI_ISL_934566, EPI_ISL_934567, EPI_ISL_934568			
see above	Department of Laboratory Medicine, Division of Clinical Virology, University of Medicine, Vienna	Bergthaler laboratory, CeMM Research Center for Molecular Medicine of the Austrian Academy of Sciences	Lukas Endler, Anna Schedl, Thomas Penz, Benedikt Agerer, Maelle Le Moing, Michael Schuster, Bekir Erguner, Jan Laine, Martin Senekowitsch, Christoph Bock, Andreas Bergthaler
EPI_ISL_934651	Institute for Water Quality and Resource Management, Technical University Vienna	Bergthaler laboratory, CeMM Research Center for Molecular Medicine of the Austrian Academy of Sciences	Lukas Endler, Anna Schedl, Thomas Penz, Benedikt Agerer, Maelle Le Moing, Michael Schuster, Bekir Erguner, Jan Laine, Martin Senekowitsch, Christoph Bock, Andreas Bergthaler
EPI_ISL_934652, EPI_ISL_934653, EPI_ISL_934654	Klinikum Wels-Grieskirchen	Bergthaler laboratory, CeMM Research Center for Molecular Medicine of the Austrian Academy of Sciences	Lukas Endler, Anna Schedl, Thomas Penz, Benedikt Agerer, Maelle Le Moing, Michael Schuster, Bekir Erguner, Jan Laine, Martin Senekowitsch, Christoph Bock, Andreas Bergthaler
EPI_ISL_934682, EPI_ISL_934683, EPI_ISL_934684, EPI_ISL_934685, EPI_ISL_934686, EPI_ISL_934687, EPI_ISL_934688, EPI_ISL_934689, EPI_ISL_934690, EPI_ISL_934691, EPI_ISL_934692, EPI_ISL_934693, EPI_ISL_934694, EPI_ISL_934695			
see above	Department of Laboratory Medicine, Division of Clinical Virology, University of Medicine, Vienna	Bergthaler laboratory, CeMM Research Center for Molecular Medicine of the Austrian Academy of Sciences	Lukas Endler, Anna Schedl, Thomas Penz, Benedikt Agerer, Maelle Le Moing, Michael Schuster, Bekir Erguner, Jan Laine, Martin Senekowitsch, Christoph Bock, Andreas Bergthaler
EPI_ISL_934980, EPI_ISL_934990, EPI_ISL_934997, EPI_ISL_935005, EPI_ISL_935006, EPI_ISL_935007, EPI_ISL_935008, EPI_ISL_935009, EPI_ISL_935010	ADMED Microbiologie	Genomics and Transcriptomics, Philip Morris International	Reto Lienhard, Marie-Lise Tritten, Emmanuel Guedj, Nicolas Sierro, Rémi Dulize, David Bornand, Mehdi Auberson, Maxime Berthouzoz, Nikolai Ivanov, Manuel Peitsch
EPI_ISL_935195, EPI_ISL_935212, EPI_ISL_935213, EPI_ISL_935222, EPI_ISL_935237, EPI_ISL_935238, EPI_ISL_935239	KU Leuven, Rega Institute, Clinical and Epidemiological Virology	KU Leuven, Rega Institute, Clinical and Epidemiological Virology	Tony Wawina-Bokalanga, Bert Vanmechelen, Joan Marti-Carerras, Piet Maes
EPI_ISL_935527, EPI_ISL_935528, EPI_ISL_935529, EPI_ISL_935530, EPI_ISL_935531	Labo Analyses Med	National Reference Center for Viruses of Respiratory Infections, Institut Pasteur, Paris	Marion Barbet, Sylvie Behillil, Méline Bizard, Angela Brisebarre, Camille Capel, Etienne Simon-Lorière, Vincent Enouf, Maud Vanpeene, Sylvie van der Werf,Merah Kader
EPI_ISL_935537	Labo Analyses Med	National Reference Center for Viruses of Respiratory Infections, Institut Pasteur, Paris	Marion Barbet, Sylvie Behillil, Méline Bizard, Angela Brisebarre, Camille Capel, Etienne Simon-Lorière, Vincent Enouf, Maud Vanpeene, Sylvie van der Werf,Bonnaudet GéRaldine
EPI_ISL_935547	Labo Analyses Med	National Reference Center for Viruses of Respiratory Infections, Institut Pasteur, Paris	Marion Barbet, Sylvie Behillil, Méline Bizard, Angela Brisebarre, Camille Capel, Etienne Simon-Lorière, Vincent Enouf, Maud Vanpeene, Sylvie van der Werf,Besson J
EPI_ISL_935548	Labo Analyses Med	National Reference Center for Viruses of Respiratory Infections, Institut Pasteur, Paris	Marion Barbet, Sylvie Behillil, Méline Bizard, Angela Brisebarre, Camille Capel, Etienne Simon-Lorière, Vincent Enouf, Maud Vanpeene, Sylvie van der Werf,Pasquier
EPI_ISL_935551, EPI_ISL_935552, EPI_ISL_935553, EPI_ISL_935554, EPI_ISL_935555	Hopital	National Reference Center for Viruses of Respiratory Infections, Institut Pasteur, Paris	Marion Barbet, Sylvie Behillil, Méline Bizard, Angela Brisebarre, Camille Capel, Etienne Simon-Lorière, Vincent Enouf, Maud Vanpeene, Sylvie van der Werf,Goudeau Alain
EPI_ISL_935567, EPI_ISL_935568	Hopital	National Reference Center for Viruses of Respiratory Infections, Institut Pasteur, Paris	Marion Barbet, Sylvie Behillil, Méline Bizard, Angela Brisebarre, Camille Capel, Etienne Simon-Lorière, Vincent Enouf, Maud Vanpeene, Sylvie van der Werf,Bret Laurent
EPI_ISL_935581	Hopital	National Reference Center for Viruses of Respiratory Infections, Institut Pasteur, Paris	Marion Barbet, Sylvie Behillil, Méline Bizard, Angela Brisebarre, Camille Capel, Etienne Simon-Lorière, Vincent Enouf, Maud Vanpeene, Sylvie van der Werf,Brichler SéGolèNe
EPI_ISL_935644	Labo Analyses Med	National Reference Center for Viruses of Respiratory Infections, Institut Pasteur, Paris	Marion Barbet, Sylvie Behillil, Méline Bizard, Angela Brisebarre, Camille Capel, Etienne Simon-Lorière, Vincent Enouf, Maud Vanpeene, Sylvie van der Werf
EPI_ISL_936005	SUNY UPSTATE MEDICAL UNIVERSITY	Wadsworth Center, New York State Department of Health	Kirsten St. George, Daryl M. Lamson, Alexis Russel, Matthew Shudt, Melissa A Leisner, Jonathan Plitnick, Navjot Singh, John Kelly, Erasmus Schneider, Erica Lasek-Nesselquist
EPI_ISL_936026, EPI_ISL_936034, EPI_ISL_936035	THE MARY IMOGENE BASSETT HOSPITAL	Wadsworth Center, New York State Department of Health	Kirsten St. George, Daryl M. Lamson, Alexis Russel, Matthew Shudt, Melissa A Leisner, Jonathan Plitnick, Navjot Singh, John Kelly, Erasmus Schneider, Erica Lasek-Nesselquist
EPI_ISL_936237, EPI_ISL_936238, EPI_ISL_936239, EPI_ISL_936240, EPI_ISL_936241	Wadsworth Center, New York State Department of Health	Wadsworth Center, New York State Department of Health	Kirsten St. George, Daryl M. Lamson, Alexis Russel, Matthew Shudt, Melissa A Leisner, Jonathan Plitnick, Navjot Singh, John Kelly, Erasmus Schneider, Erica Lasek-Nesselquist
EPI_ISL_936249, EPI_ISL_936260, EPI_ISL_936261, EPI_ISL_936264, EPI_ISL_936266, EPI_ISL_936267, EPI_ISL_936269, EPI_ISL_936272, EPI_ISL_936273, EPI_ISL_936279, EPI_ISL_936285, EPI_ISL_936288, EPI_ISL_936289, EPI_ISL_936292, EPI_ISL_936296			
see above	MONTEFIORE MEDICAL CENTER LABORATORIES	Wadsworth Center, New York State Department of Health	Kirsten St. George, Daryl M. Lamson, Alexis Russel, Matthew Shudt, Melissa A Leisner, Jonathan Plitnick, Navjot Singh, John Kelly, Erasmus Schneider, Erica Lasek-Nesselquist
EPI_ISL_936464, EPI_ISL_936465, EPI_ISL_936468, EPI_ISL_936479	DPH, Massachusetts State Public Health Lab	DPH, Massachusetts State Public Health Lab	Lang,A.S., Fink,T., Gallagher,G.R., Smole,S.C.
EPI_ISL_937122, EPI_ISL_937123, EPI_ISL_937124, EPI_ISL_937125	Quest Diagnostics	Quest Diagnostics	Rosenthal,S.H., Gerasimova,A., Kagan,R.M., Anderson, B., Hua, M., Liu Y., Bernstein, L.E., Livingston, K.E., Perez, A., Shalhout, D.F., Shlyakhter, I.A., Owen, R., Tanpaiboon, P., Lacbawan, F.
EPI_ISL_937362, EPI_ISL_937364, EPI_ISL_937365, EPI_ISL_937366, EPI_ISL_937367, EPI_ISL_937368, EPI_ISL_937369, EPI_ISL_937370, EPI_ISL_937371, EPI_ISL_937372, EPI_ISL_937374, EPI_ISL_937375, EPI_ISL_937378, EPI_ISL_937380			
see above	Utah Public Health Laboratory	Utah Public Health Laboratory	Erin L. Young, Kelly F. Oakeson, Tara Gallagher

EPI_ISL_937408	Maine Health and Environmental Testing Laboratory (Maine HETL)	Tewhey Lab, The Jackson Laboratory	Matluk,N., Dewey,H., Iosue,F., Barter,M., Lynch,R., Munger,H. and Tewhey,R.
EPI_ISL_940068, EPI_ISL_940071, EPI_ISL_940072, EPI_ISL_940073	University Hospitals of Geneva, Laboratory of Virology	HUG, Laboratory of Virology and the Health2030 Genome Center	Samuel Cordey, Ana Rita Goncalves, Laurent Kaiser, Lorenzo Cerutti, Henri Pegeot, Melyssa Elies, Deborah Penet, Keith Harshman, Ioannis Xenarios, Emmanouil Dermitzakis
EPI_ISL_940157	Laboratory of Virology and Molecular Diagnostics	Institute of Public Health of Republic of North Macedonia Laboratory of Virology and Molecular Diagnostics	Maja Kuzmanovska, Golubinka Bosehvska, Elizabeta Janchevska
EPI_ISL_940620, EPI_ISL_940621	Hospital Sao Joaquim - Beneficiencia Portuguesa	Instituto Adolfo Lutz, Interdiciplinary Procedures Center, Strategic Laboratory	Claudio Tavares Sacchi, Claudia Regina Gonçalves, Erica Valessa Ramos Gomes, Karoline Rodrigues Campos
EPI_ISL_940732	City of Milwaukee Health Department Laboratory	City of Milwaukee Health Department Laboratory	Sanjib Bhattacharyya
EPI_ISL_940749	University of Bari Biomedical Sciences and Human Oncology	University of Bari Biomedical Sciences and Human Oncology	Chironna M., Sallustio A., Loconsole D., Accogli M.
EPI_ISL_940868, EPI_ISL_940871, EPI_ISL_940875, EPI_ISL_940888, EPI_ISL_940889	Vaccines and Infectious Diseases Analytics Research Unit (VIDA)	KRISP, KZN Research Innovation and Sequencing Platform	Baillie Vicky, du Plessis Jeanine, Giandhari Jennifer, Pillay Sureshnee, Naidoo Yeshnee, Tegally Houriiyah, de Oliveira Tulio, Madhi Shabir
EPI_ISL_940999	Labo Analyses Med	National Reference Center for Viruses of Respiratory Infections, Institut Pasteur, Paris	Marion Barbet, Sylvie Behillil, Méline Bizard, Angela Brisebarre, Camille Capel, Etienne Simon-Lorière, Vincent Enouf, Maud Vanpeene, Sylvie van der Werf, Gestin (B) Brieuc
EPI_ISL_941000	Hopital	National Reference Center for Viruses of Respiratory Infections, Institut Pasteur, Paris	Marion Barbet, Sylvie Behillil, Méline Bizard, Angela Brisebarre, Camille Capel, Etienne Simon-Lorière, Vincent Enouf, Maud Vanpeene, Sylvie van der Werf, Cron Sophie
EPI_ISL_941079, EPI_ISL_941080, EPI_ISL_941082, EPI_ISL_941084, EPI_ISL_941086, EPI_ISL_941087, EPI_ISL_941088, EPI_ISL_941091, EPI_ISL_941093, EPI_ISL_941095, EPI_ISL_941096, EPI_ISL_941097, EPI_ISL_941100, EPI_ISL_941101, EPI_ISL_941103			
see above	Labo Analyses Med	National Reference Center for Viruses of Respiratory Infections, Institut Pasteur, Paris	Marion Barbet, Sylvie Behillil, Méline Bizard, Angela Brisebarre, Camille Capel, Etienne Simon-Lorière, Vincent Enouf, Maud Vanpeene, Sylvie van der Werf, Merah Kader
EPI_ISL_941165, EPI_ISL_941166, EPI_ISL_941167, EPI_ISL_941168, EPI_ISL_941169, EPI_ISL_941170, EPI_ISL_941171	Hospital Universitari i Politècnic La Fe de València	SeqCOVID-SPAIN consortium/IBV(CSIC)	María Dolores Gómez Ruiz, Eva González Barberá, Ana Gil Brusola, Salvador Giner Almaraz, José Luis López Hontangas and SeqCOVID-SPAIN consortium
EPI_ISL_941229, EPI_ISL_941230, EPI_ISL_941231, EPI_ISL_941232	Laboratorio de Microbiología. Hospital General Universitario de Elda, Alicante	SeqCOVID-SPAIN consortium/IBV(CSIC)	Mª Isabel Gascón Ros, Cristina Torregrosa Hetland, Eva Pastor Boix, Paloma Cascales Ramos and SeqCOVID-SPAIN consortium
EPI_ISL_941293	Nigeria Centre for Disease Control (NCDC)	African Centre of Excellence for Genomics of Infectious Diseases (ACEGID), Redeemer's University	Oluniyi P.E. et al
EPI_ISL_941338	INMI Lazzaro Spallanzani IRCCS	INMI Lazzaro Spallanzani IRCCS	Emanuela Giombini, Ornella Butera, Cesare E.M. Gruber, Martina Rueca, Barbara Bartolini, Francesco Messina, Giandomenico Russo, Daniele Castiglia , Stefania Madonna, Antonino Di Caro, Maria R. Capobianchi
EPI_ISL_941346, EPI_ISL_941347, EPI_ISL_941348, EPI_ISL_941349, EPI_ISL_941350, EPI_ISL_941351, EPI_ISL_941352, EPI_ISL_941353, EPI_ISL_941354, EPI_ISL_941355, EPI_ISL_941356, EPI_ISL_941357, EPI_ISL_941358, EPI_ISL_941359, EPI_ISL_941389, EPI_ISL_941390, EPI_ISL_941391, EPI_ISL_941394, EPI_ISL_941395, EPI_ISL_941396, EPI_ISL_941397, EPI_ISL_941398, EPI_ISL_941399, EPI_ISL_941400, EPI_ISL_941401, EPI_ISL_941403, EPI_ISL_941404, EPI_ISL_941411, EPI_ISL_941412, EPI_ISL_941415, EPI_ISL_941416, EPI_ISL_941417, EPI_ISL_941418, EPI_ISL_941419, EPI_ISL_941420, EPI_ISL_941421, EPI_ISL_941422, EPI_ISL_941428, EPI_ISL_941429, EPI_ISL_941433, EPI_ISL_941434, EPI_ISL_941435, EPI_ISL_941436, EPI_ISL_941462, EPI_ISL_941463, EPI_ISL_941464, EPI_ISL_941465, EPI_ISL_941466, EPI_ISL_941467, EPI_ISL_941468, EPI_ISL_941469, EPI_ISL_941473, EPI_ISL_941474, EPI_ISL_941475, EPI_ISL_941476, EPI_ISL_941477, EPI_ISL_941478, EPI_ISL_941479, EPI_ISL_941480, EPI_ISL_941481, EPI_ISL_941482, EPI_ISL_941483, EPI_ISL_941484, EPI_ISL_941485, EPI_ISL_941508, EPI_ISL_941510, EPI_ISL_941511, EPI_ISL_941512, EPI_ISL_941513, EPI_ISL_941515, EPI_ISL_941517, EPI_ISL_941520, EPI_ISL_941521, EPI_ISL_941522, EPI_ISL_941527, EPI_ISL_941528, EPI_ISL_941529, EPI_ISL_941530, EPI_ISL_941531, EPI_ISL_941532, EPI_ISL_941533, EPI_ISL_941534, EPI_ISL_941535, EPI_ISL_941539, EPI_ISL_941540, EPI_ISL_941541, EPI_ISL_941542, EPI_ISL_941547, EPI_ISL_941548, EPI_ISL_941549, EPI_ISL_941550, EPI_ISL_941551, EPI_ISL_941552, EPI_ISL_941574, EPI_ISL_941575, EPI_ISL_941576, EPI_ISL_941577, EPI_ISL_941578, EPI_ISL_941579, EPI_ISL_941580, EPI_ISL_941581, EPI_ISL_941582, EPI_ISL_941583, EPI_ISL_941584, EPI_ISL_941585, EPI_ISL_941586, EPI_ISL_941587, EPI_ISL_941588, EPI_ISL_941589, EPI_ISL_941590, EPI_ISL_941591, EPI_ISL_941592, EPI_ISL_941593, EPI_ISL_941594, EPI_ISL_941595, EPI_ISL_941603, EPI_ISL_941633, EPI_ISL_941636, EPI_ISL_941637, EPI_ISL_941638, EPI_ISL_941640, EPI_ISL_941641, EPI_ISL_941642			
see above	Instituto Nacional de Saude (INSA)	Instituto Nacional de Saude (INSA)	Borges et al
EPI_ISL_941650, EPI_ISL_941651, EPI_ISL_941652, EPI_ISL_941663, EPI_ISL_941667, EPI_ISL_941670, EPI_ISL_941671, EPI_ISL_941672, EPI_ISL_941673, EPI_ISL_941674, EPI_ISL_941675, EPI_ISL_941676, EPI_ISL_941677, EPI_ISL_941678, EPI_ISL_941680, EPI_ISL_941681, EPI_ISL_941682, EPI_ISL_941683, EPI_ISL_941685, EPI_ISL_941686, EPI_ISL_941687, EPI_ISL_941691, EPI_ISL_941692, EPI_ISL_941693, EPI_ISL_941694, EPI_ISL_941695, EPI_ISL_941704, EPI_ISL_941705, EPI_ISL_941707, EPI_ISL_941712, EPI_ISL_941713, EPI_ISL_941714, EPI_ISL_941715, EPI_ISL_941716, EPI_ISL_941717, EPI_ISL_941718, EPI_ISL_941719, EPI_ISL_941733, EPI_ISL_941734, EPI_ISL_941735, EPI_ISL_941736, EPI_ISL_941737, EPI_ISL_941738, EPI_ISL_941739, EPI_ISL_941740, EPI_ISL_941741, EPI_ISL_941742, EPI_ISL_941743, EPI_ISL_941744, EPI_ISL_941745, EPI_ISL_941746, EPI_ISL_941747, EPI_ISL_941748, EPI_ISL_941749, EPI_ISL_941750, EPI_ISL_941751, EPI_ISL_941752, EPI_ISL_941753, EPI_ISL_941754, EPI_ISL_941755, EPI_ISL_941759, EPI_ISL_941760, EPI_ISL_941761, EPI_ISL_941767, EPI_ISL_941768, EPI_ISL_941769, EPI_ISL_941770, EPI_ISL_941776, EPI_ISL_941777, EPI_ISL_941778, EPI_ISL_941779, EPI_ISL_941780, EPI_ISL_941781, EPI_ISL_941782, EPI_ISL_941783, EPI_ISL_941784, EPI_ISL_941785, EPI_ISL_941786, EPI_ISL_941787, EPI_ISL_941788, EPI_ISL_941800, EPI_ISL_941801, EPI_ISL_941802, EPI_ISL_941809, EPI_ISL_941810, EPI_ISL_941811, EPI_ISL_941812, EPI_ISL_941813, EPI_ISL_941819, EPI_ISL_941828, EPI_ISL_941829, EPI_ISL_941830, EPI_ISL_941831, EPI_ISL_941837, EPI_ISL_941838, EPI_ISL_941839, EPI_ISL_941840, EPI_ISL_941841, EPI_ISL_941846, EPI_ISL_941860, EPI_ISL_941862, EPI_ISL_941863, EPI_ISL_941864, EPI_ISL_941865, EPI_ISL_941866, EPI_ISL_941868, EPI_ISL_941869, EPI_ISL_941870, EPI_ISL_941871, EPI_ISL_941874, EPI_ISL_941875, EPI_ISL_941878, EPI_ISL_941883, EPI_ISL_941884, EPI_ISL_941890, EPI_ISL_941891, EPI_ISL_941892, EPI_ISL_941893, EPI_ISL_941894, EPI_ISL_941895, EPI_ISL_941896, EPI_ISL_941897			
see above	Instituto Nacional de Saude (INSA) and Instituto Gulbenkian de Ciencia (IGC)	Instituto Nacional de Saude (INSA) and Instituto Gulbenkian de Ciencia (IGC)	Borges et al
EPI_ISL_942822, EPI_ISL_942823, EPI_ISL_942824, EPI_ISL_942825, EPI_ISL_942826, EPI_ISL_942827, EPI_ISL_942828, EPI_ISL_942829, EPI_ISL_942830, EPI_ISL_942831, EPI_ISL_942832, EPI_ISL_942833, EPI_ISL_942834			
see above	Gundersen Molecular Diagnostics Laboratory	Kabara Cancer Research Institute	Craig S. Richmond, Paraic A. Kenny
EPI_ISL_942928	Instituto de Diagnostico y Referencia Epidemiologicos INDRE_RNLSP	Instituto de Diagnostico y Referencia Epidemiologicos (INDRE)	Claudia Wong-Arambula, Abril Rodriguez-Maldonado, Fabiola Garces-Ayala, Natividad Cruz-Ortiz, Tatiana Nunez-Garcia, Gisela Barrera-Badillo, Lucia Hernandez-Rivas, Irma Lopez-Martinez, Ernesto Ramirez-Gonzalez.
EPI_ISL_943025, EPI_ISL_943053, EPI_ISL_943065, EPI_ISL_943075, EPI_ISL_943076, EPI_ISL_943077, EPI_ISL_943078, EPI_ISL_943086, EPI_ISL_943087, EPI_ISL_943088, EPI_ISL_943089, EPI_ISL_943090, EPI_ISL_943091, EPI_ISL_943117, EPI_ISL_943174, EPI_ISL_943275, EPI_ISL_943280, EPI_ISL_943284, EPI_ISL_943287, EPI_ISL_943288, EPI_ISL_943331, EPI_ISL_943335, EPI_ISL_943336, EPI_ISL_943341, EPI_ISL_943346, EPI_ISL_943353, EPI_ISL_943371, EPI_ISL_943381, EPI_ISL_943507, EPI_ISL_943523			
see above	Dutch COVID-19 response team	National Institute for Public Health and the Environment (RIVM)	Adam Meijer, Harry Vennema, Dirk Eggink, Jeroen Cremer, Sharon van den Brink, Bas van der Veer, AnneMarie van den Brandt, Florian Zwagemaker, Dennis Schmitz, Chantal Reusken, on behalf of the national COVID-19 response team
EPI_ISL_943805, EPI_ISL_943809	Servizo de Microbioloxía. Complexo Hospitalario Universitario de Santiago de Compostela	Servizo de Microbioloxía. Complexo Hospitalario Universitario de Santiago de Compostela	Antonio Aguilera, Gema Barbeito, Amparo Coira, José Costa, Rocio Trastoy, María Luisa Pérez del Molino.
EPI_ISL_943810, EPI_ISL_943811, EPI_ISL_943841, EPI_ISL_943842, EPI_ISL_943843, EPI_ISL_943844, EPI_ISL_943845, EPI_ISL_943846, EPI_ISL_943847, EPI_ISL_943868, EPI_ISL_943917, EPI_ISL_943918, EPI_ISL_943919, EPI_ISL_943920, EPI_ISL_943921, EPI_ISL_943925, EPI_ISL_943926, EPI_ISL_943935, EPI_ISL_943945, EPI_ISL_943948, EPI_ISL_943953, EPI_ISL_943954, EPI_ISL_943959, EPI_ISL_943960, EPI_ISL_943961, EPI_ISL_943963, EPI_ISL_943965			
see above	Utah Public Health Laboratory	Utah Public Health Laboratory	Erin L. Young, Kelly F. Oakeson, Tara Gallagher
EPI_ISL_944189, EPI_ISL_944190, EPI_ISL_944191, EPI_ISL_944192, EPI_ISL_944194, EPI_ISL_944195, EPI_ISL_944199, EPI_ISL_944200, EPI_ISL_944201, EPI_ISL_944206, EPI_ISL_944207, EPI_ISL_944214, EPI_ISL_944215, EPI_ISL_944217, EPI_ISL_944218, EPI_ISL_944220, EPI_ISL_944221, EPI_ISL_944224, EPI_ISL_944228, EPI_ISL_944230, EPI_ISL_944231, EPI_ISL_944242, EPI_ISL_944247, EPI_ISL_944248, EPI_ISL_944251, EPI_ISL_944252, EPI_ISL_944253, EPI_ISL_944254, EPI_ISL_944255, EPI_ISL_944339, EPI_ISL_944340, EPI_ISL_944341, EPI_ISL_944342, EPI_ISL_944343, EPI_ISL_944344, EPI_ISL_944345, EPI_ISL_944346, EPI_ISL_944347, EPI_ISL_944348, EPI_ISL_944349, EPI_ISL_944350, EPI_ISL_944351, EPI_ISL_944352, EPI_ISL_944353, EPI_ISL_944354, EPI_ISL_944355, EPI_ISL_944356, EPI_ISL_944357, EPI_ISL_944358, EPI_ISL_944359, EPI_ISL_944360, EPI_ISL_944361, EPI_ISL_944362, EPI_ISL_944363, EPI_ISL_944364, EPI_ISL_944365, EPI_ISL_944367, EPI_ISL_944368, EPI_ISL_944372, EPI_ISL_944375, EPI_ISL_944376, EPI_ISL_944377, EPI_ISL_944384, EPI_ISL_944385, EPI_ISL_944386, EPI_ISL_944390, EPI_ISL_944391, EPI_ISL_944392, EPI_ISL_944393, EPI_ISL_944394, EPI_ISL_944395, EPI_ISL_944397, EPI_ISL_944401, EPI_ISL_944402, EPI_ISL_944407, EPI_ISL_944408, EPI_ISL_944409, EPI_ISL_944410, EPI_ISL_944420, EPI_ISL_944425, EPI_ISL_944426, EPI_ISL_944427, EPI_ISL_944428, EPI_ISL_944516, EPI_ISL_944517, EPI_ISL_944518, EPI_ISL_944519, EPI_ISL_944520, EPI_ISL_944521, EPI_ISL_944522, EPI_ISL_944523, EPI_ISL_944524, EPI_ISL_944525, EPI_ISL_944526, EPI_ISL_944527, EPI_ISL_944528, EPI_ISL_944529, EPI_ISL_944530, EPI_ISL_944531, EPI_ISL_944532, EPI_ISL_944533, EPI_ISL_944534, EPI_ISL_944535, EPI_ISL_944536, EPI_ISL_944537, EPI_ISL_944538, EPI_ISL_944539, EPI_ISL_944540, EPI_ISL_944541, EPI_ISL_944542, EPI_ISL_944543, EPI_ISL_944544, EPI_ISL_944545, EPI_ISL_944546, EPI_ISL_944547, EPI_ISL_944548, EPI_ISL_944549, EPI_ISL_944550, EPI_ISL_944551, EPI_ISL_944552, EPI_ISL_944553, EPI_ISL_944554, EPI_ISL_944555, EPI_ISL_944556, EPI_ISL_944557, EPI_ISL_944558, EPI_ISL_944559, EPI_ISL_944560, EPI_ISL_944561, EPI_ISL_944562, EPI_ISL_944563, EPI_ISL_944564, EPI_ISL_944565			
see above	Israel Central Virology laboratory	Israel National Consortium for SARS-CoV-2 sequencing	Neta Zuckerman, Efrat Dahan Bucris, Michal Mandelboim, Dana Bar-Ilan, Oran Erster, Tzvia Mann, Omer Murik, David A. Zeevi, Assaf Rokney, Joseph Jaffe, Eva Nachum, Maya Davidovich Cohen, Ephraim Fass, Gal Zizelski Valenci, Mor Rubinstein, Efrat Rorman, Israel Nissan, Efrat Glick-Saar, Omri Nayshool, Gideon Rechavi, Ella Mendelson, Orna Mor
EPI_ISL_944617, EPI_ISL_944618, EPI_ISL_944620, EPI_ISL_944621,	Instituto Nacional de Medicina Genomica	Instituto Nacional de Medicina Genomica	Hidalgo-Miranda A, Mendoza-Vargas A, Reyes-Grajeda JP, Cisneros-Villanueva M, Cedro-Tanda A,Peñaloza-Figueroa F, Herrera-Montalvo LA

EPI_ISL_944622, EPI_ISL_944626, EPI_ISL_944629, EPI_ISL_944630, EPI_ISL_944632			
EPI_ISL_944672, EPI_ISL_944674, EPI_ISL_944676, EPI_ISL_944682, EPI_ISL_944685, EPI_ISL_944686, EPI_ISL_944690, EPI_ISL_944697, EPI_ISL_944698, EPI_ISL_944706, EPI_ISL_944708, EPI_ISL_944714, EPI_ISL_944734			
see above	Department of Biochemistry, Cell and Molecular Biology, West African Centre for Cell Biology of Infectious Pathogens (WACCBIP), University of Ghana	Department of Biochemistry, Cell and Molecular Biology, West African Centre for Cell Biology of Infectious Pathogens (WACCBIP), University of Ghana	Morang'a,C.M., Ngoi,J.M., Quansah,E.B., Saiid,S., Amuzu,D.S., Asante,I., Bonney,J.H., Bonney,E., Odoom,J.K., Ndam,N.T., Tei-Maya,F., Adusei-Poku,M., Ofori-Boadu,L., Ampofo,W.K., Amenga-Etego,L.N., Quashie,P., Bediako,Y., Awandare,G.A.
EPI_ISL_944738	unknown	Public Health Virology-Forensic and Scientific Services (PHV-FSS)	Son Nguyen et al.
EPI_ISL_949094	Jessa	Jessa	Jessa_cmdLab
EPI_ISL_949318	Department of Pathology, University of Cambridge	COVID-19 Genomics UK (COG-UK) Consortium	Aminu S. Jahun, Yasmin Chaudhry, Iliana Georgana, Myra Hosmillo, Rhys Izu, Martin D. Curran, Surendra Parmar, Ian Goodfellow
EPI_ISL_949640, EPI_ISL_949643, EPI_ISL_949644	Virology Department, Royal Infirmary of Edinburgh, NHS Lothian / School of Biological Sciences, University of Edinburgh / Institute of Genetics and Molecular Medicine, University of Edinburgh	COVID-19 Genomics UK (COG-UK) Consortium	McHugh M, Dewar R, Rooke S, Gallagher M, Balcaza C, O'Toole Á, Scher E, Hill V, McCrone JT, Colquhoun R, Yu X, Jackson B, Rambaut A, Williams TC, Templeton K
EPI_ISL_949749, EPI_ISL_949777, EPI_ISL_949778, EPI_ISL_949780, EPI_ISL_949783	Barts Health NHS Trust	COVID-19 Genomics UK (COG-UK) Consortium	CUTINO-MOGUEL, Maria-Teresa; HARRINGTON, David; OWOYEMI, Dola; KULASEGARAN-SHYLINI, Raghavendran; BROAD, Claire; KELE, Beatrix
EPI_ISL_950211, EPI_ISL_950212, EPI_ISL_950213, EPI_ISL_950214, EPI_ISL_950215, EPI_ISL_950216, EPI_ISL_950217, EPI_ISL_950218, EPI_ISL_950219, EPI_ISL_950220, EPI_ISL_950221, EPI_ISL_950229, EPI_ISL_950230, EPI_ISL_950231			
see above	University College London, Great Ormond Street Hospital for Children NHS Foundation Trust, Imperial College Healthcare NHS Trust	COVID-19 Genomics UK (COG-UK) Consortium	Sergi Castellano, Rachel Williams, Mark Kristiansen, Paola Resende Silva, Sunando Roy, Tony Brooks, Helena Tutill, Paola Niola, Patricia Dyal, Charlotte Williams, Leysa Forrest, Yasmin Panchbhaya, Jacqueline Findlay, Samuel Weeks, Julianne Brown, Kathryn Harris, Paul Randell, James Price, Alison Holmes, Judith Breuer
EPI_ISL_950304, EPI_ISL_950306, EPI_ISL_950307, EPI_ISL_950308, EPI_ISL_950309, EPI_ISL_950310, EPI_ISL_950311, EPI_ISL_950312, EPI_ISL_950317, EPI_ISL_950324, EPI_ISL_950325, EPI_ISL_950330, EPI_ISL_950331, EPI_ISL_950332, EPI_ISL_950333, EPI_ISL_950335, EPI_ISL_950336			
see above	Northumbria University / South Tees Hospitals NHS Foundation Trust / North Cumbria Integrated Care NHS Foundation Trust / North Tees and Hartlepool NHS Foundation Trust / Newcastle Hospitals NHS Foundation Trust	COVID-19 Genomics UK (COG-UK) Consortium	Darren L Smith,Andrew Nelson,Matthew Bashton,Greg R Young,Joshua Loh,John Allan,Mohammad A Tariq,Giles S Holt,Gary Black,Wen C Yew,Lynn Dover,Paul Baker,Steve Liggett,Sarah Essex,Jane Greenaway,Debra Padgett,Clive Graham,Garren Scott,Edward Barton,Emma Swindells,Brendan Payne,Jennifer Collins,Yusri Taha,Gary Eltringham
EPI_ISL_950714	Lincolnshire Hospitals and DeepSeq Nottingham	COVID-19 Genomics UK (COG-UK) Consortium	Nichola Duckworth, Tim Sloan, Sarah Walsh, Jonathan Ball, Patrick McClure, Joeseph Chappell, Nadine Holmes, Matthew Carlisle, Christopher Moore, Fei Sang, Johnny Debebe, Victoria Wright, Matthew Loose
EPI_ISL_951305, EPI_ISL_951311, EPI_ISL_951313, EPI_ISL_951318, EPI_ISL_951374, EPI_ISL_951375, EPI_ISL_951378, EPI_ISL_951379, EPI_ISL_951380, EPI_ISL_951383, EPI_ISL_951387, EPI_ISL_951388, EPI_ISL_951416, EPI_ISL_951419, EPI_ISL_951420, EPI_ISL_951421, EPI_ISL_951423, EPI_ISL_951424, EPI_ISL_951425, EPI_ISL_951428, EPI_ISL_951435, EPI_ISL_951436, EPI_ISL_951437, EPI_ISL_951438, EPI_ISL_951442, EPI_ISL_951444, EPI_ISL_951445, EPI_ISL_951446, EPI_ISL_951447, EPI_ISL_951450, EPI_ISL_951451, EPI_ISL_951452, EPI_ISL_951453, EPI_ISL_951456, EPI_ISL_951457			
see above	Oxford Viromics, NDM, University of Oxford; Oxford University Hospitals; Basingstoke and North Hampshire Hospital	COVID-19 Genomics UK (COG-UK) Consortium	Tanya Golubchik, David Bonsall, George Macintyre, Amy Trebes, Mariateresa de Cesare, Catrin Moore, Alex Mobbs, Anita Justice, Robert Shaw, Monique Andersson, Timothy Peto, Emma Wise, Nathan Moore, Jessica Lynch, Nick Cortes, Matilde Mori, Stephen Kidd, David Buck, John Todd, Christophe Fraser
EPI_ISL_951629, EPI_ISL_951816	Originating lab: Wales Specialist Virology Centre Sequencing lab: Pathogen Genomics Unit	Public Health Wales Microbiology Cardiff Wales Specialist Virology Centre	Catherine Moore, Johnathan Evans, Laura Gifford, Malorie Perry, Simon Cottrell, Angela Marchbank, Alec Birchley, Alexander Adams, Amy Gaskin, Bree Gatica-Wilcox, Jason Coombes, Joel Southgate, Lauren Gilbert, Lee Graham, Nicole Pacchiarini, Sara Kumziene-Summerhayes, Sarah Taylor, Sophie Jones, Sara Rey, Matthew Bull, Joanne Watkins, Sally Corden, Tom Connor
EPI_ISL_952377, EPI_ISL_952379, EPI_ISL_952382, EPI_ISL_952383, EPI_ISL_952385, EPI_ISL_952392, EPI_ISL_952394, EPI_ISL_952397, EPI_ISL_952398, EPI_ISL_952409, EPI_ISL_952411, EPI_ISL_952414, EPI_ISL_952418, EPI_ISL_952422, EPI_ISL_952424, EPI_ISL_952427, EPI_ISL_952429, EPI_ISL_952433, EPI_ISL_952471, EPI_ISL_952472, EPI_ISL_952473, EPI_ISL_952474, EPI_ISL_952475, EPI_ISL_952476, EPI_ISL_952526, EPI_ISL_952569, EPI_ISL_952570, EPI_ISL_952571, EPI_ISL_952572, EPI_ISL_952573, EPI_ISL_952574, EPI_ISL_952575, EPI_ISL_952576, EPI_ISL_952578, EPI_ISL_952580, EPI_ISL_952581, EPI_ISL_952582, EPI_ISL_952583, EPI_ISL_952584, EPI_ISL_952585, EPI_ISL_952586, EPI_ISL_952587, EPI_ISL_952588, EPI_ISL_952589, EPI_ISL_952590, EPI_ISL_952591, EPI_ISL_952592, EPI_ISL_952594, EPI_ISL_952595, EPI_ISL_952597, EPI_ISL_952598, EPI_ISL_952599, EPI_ISL_952600, EPI_ISL_952778, EPI_ISL_952779, EPI_ISL_952780, EPI_ISL_952789, EPI_ISL_952793, EPI_ISL_952794, EPI_ISL_952795, EPI_ISL_952798, EPI_ISL_952799, EPI_ISL_952804			
see above	Centre for Enzyme Innovation, University of Portsmouth / Translational Research Laboratory, Portsmouth Hospitals NHS Trust	COVID-19 Genomics UK (COG-UK) Consortium	Angela Beckett,Salman Goudarzi,Christopher Fearn,Kate Cook,Katie Loveson,Sharon Glaysher,Scott Elliott,Samuel Robson
EPI_ISL_952899, EPI_ISL_952908, EPI_ISL_952909, EPI_ISL_952912	Department of Pathology, University of Cambridge	COVID-19 Genomics UK (COG-UK) Consortium	Aminu S. Jahun, Yasmin Chaudhry, Iliana Georgana, Myra Hosmillo, Rhys Izu, Martin D. Curran, Surendra Parmar, Ian Goodfellow
EPI_ISL_953024, EPI_ISL_953025, EPI_ISL_953026, EPI_ISL_953027, EPI_ISL_953028, EPI_ISL_953029, EPI_ISL_953030, EPI_ISL_953031, EPI_ISL_953032, EPI_ISL_953033, EPI_ISL_953034, EPI_ISL_953035, EPI_ISL_953036, EPI_ISL_953037, EPI_ISL_953038, EPI_ISL_953039, EPI_ISL_953040, EPI_ISL_953041, EPI_ISL_953042, EPI_ISL_953043, EPI_ISL_953044, EPI_ISL_953045, EPI_ISL_953046, EPI_ISL_953047, EPI_ISL_953048, EPI_ISL_953049, EPI_ISL_953050, EPI_ISL_953051, EPI_ISL_953052, EPI_ISL_953053, EPI_ISL_953054, EPI_ISL_953055, EPI_ISL_953056, EPI_ISL_953057, EPI_ISL_953058, EPI_ISL_953059, EPI_ISL_953060, EPI_ISL_953061, EPI_ISL_953062, EPI_ISL_953063, EPI_ISL_953064, EPI_ISL_953066, EPI_ISL_953067, EPI_ISL_953068, EPI_ISL_953069, EPI_ISL_953070, EPI_ISL_953071, EPI_ISL_953072, EPI_ISL_953073, EPI_ISL_953074, EPI_ISL_953075			
see above	Bioinformatics and Biostatistics Lab, Advanced Sequencing Facility	COVID-19 Genomics UK (COG-UK) Consortium	Aengus Stewart,Jerome Nicod,Chelsea Sawyer,Laura Cubitt,Harshil Patel,Margaret Crawford
EPI_ISL_953522, EPI_ISL_953666, EPI_ISL_953667	University Hospitals of Geneva, Laboratory of Virology	HUG, Laboratory of Virology and the Health2030 Genome Center	Samuel Cordey, Ana Rita Goncalves, Laurent Kaiser, Lorenzo Cerutti, Henri Pegeot, Melyssa Elies, Deborah Penet, Keith Harshman, Ioannis Xenarios, Emmanouil Dermitzakis
EPI_ISL_953940, EPI_ISL_953941	Hopital	National Reference Center for Viruses of Respiratory Infections, Institut Pasteur, Paris	Marion Barbet, Sylvie Behillil, Méline Bizard, Angela Brisebarre, Camille Capel, Etienne Simon-Lorière, Vincent Enouf, Maud Vanpeene, Sylvie van der Werf,Lagathu GisèLe
EPI_ISL_953954	Outre Mer	National Reference Center for Viruses of Respiratory Infections, Institut Pasteur, Paris	Marion Barbet, Sylvie Behillil, Méline Bizard, Angela Brisebarre, Camille Capel, Etienne Simon-Lorière, Vincent Enouf, Maud Vanpeene, Sylvie van der Werf,Rousset Dominique
EPI_ISL_953994	Labo Analyses Med	National Reference Center for Viruses of Respiratory Infections, Institut Pasteur, Paris	Marion Barbet, Sylvie Behillil, Méline Bizard, Angela Brisebarre, Camille Capel, Etienne Simon-Lorière, Vincent Enouf, Maud Vanpeene, Sylvie van der Werf,Amzalag Jonas
EPI_ISL_953995, EPI_ISL_953996	Hopital	National Reference Center for Viruses of Respiratory Infections, Institut Pasteur, Paris	Marion Barbet, Sylvie Behillil, Méline Bizard, Angela Brisebarre, Camille Capel, Etienne Simon-Lorière, Vincent Enouf, Maud Vanpeene, Sylvie van der Werf,Lagathu GisèLe
EPI_ISL_954007, EPI_ISL_954022, EPI_ISL_954023, EPI_ISL_954024, EPI_ISL_954025, EPI_ISL_954031	Labo Analyses Med	National Reference Center for Viruses of Respiratory Infections, Institut Pasteur, Paris	Marion Barbet, Sylvie Behillil, Méline Bizard, Angela Brisebarre, Camille Capel, Etienne Simon-Lorière, Vincent Enouf, Maud Vanpeene, Sylvie van der Werf,Amzalag Jonas
EPI_ISL_954035	Outre Mer	National Reference Center for Viruses of Respiratory Infections, Institut Pasteur, Paris	Marion Barbet, Sylvie Behillil, Méline Bizard, Angela Brisebarre, Camille Capel, Etienne Simon-Lorière, Vincent Enouf, Maud Vanpeene, Sylvie van der Werf,Rousset Dominique
EPI_ISL_954119	Labo Analyses Med	National Reference Center for Viruses of Respiratory Infections, Institut Pasteur, Paris	Marion Barbet, Sylvie Behillil, Méline Bizard, Angela Brisebarre, Camille Capel, Etienne Simon-Lorière, Vincent Enouf, Maud Vanpeene, Sylvie van der Werf,Takoudju Eve-Marie
EPI_ISL_954120	Hopital	National Reference Center for Viruses of Respiratory Infections, Institut Pasteur, Paris	Marion Barbet, Sylvie Behillil, Méline Bizard, Angela Brisebarre, Camille Capel, Etienne Simon-Lorière, Vincent Enouf, Maud Vanpeene, Sylvie van der Werf,Lagathu GisèLe
EPI_ISL_954121	Labo Analyses Med	National Reference Center for Viruses of Respiratory Infections, Institut Pasteur, Paris	Marion Barbet, Sylvie Behillil, Méline Bizard, Angela Brisebarre, Camille Capel, Etienne Simon-Lorière, Vincent Enouf, Maud Vanpeene, Sylvie van der Werf,Takoudju Eve-Marie
EPI_ISL_954122	Hopital	National Reference Center for Viruses of Respiratory	Marion Barbet, Sylvie Behillil, Méline Bizard, Angela Brisebarre, Camille Capel, Etienne Simon-Lorière, Vincent Enouf, Maud Vanpeene, Sylvie van der

		Infections, Institut Pasteur, Paris	Werf,Lagathu GisèLe
EPI_ISL_954123, EPI_ISL_954124	Labo Analyses Med	National Reference Center for Viruses of Respiratory Infections, Institut Pasteur, Paris	Marion Barbet, Sylvie Behillil, Méline Bizard, Angela Brisebarre, Camille Capel, Etienne Simon-Lorière, Vincent Enouf, Maud Vanpeene, Sylvie van der Werf,Takoudju Eve-Marie
EPI_ISL_954125	Outre Mer	National Reference Center for Viruses of Respiratory Infections, Institut Pasteur, Paris	Marion Barbet, Sylvie Behillil, Méline Bizard, Angela Brisebarre, Camille Capel, Etienne Simon-Lorière, Vincent Enouf, Maud Vanpeene, Sylvie van der Werf,Rousset Dominique
EPI_ISL_954165, EPI_ISL_954166, EPI_ISL_954167, EPI_ISL_954168, EPI_ISL_954169, EPI_ISL_954170, EPI_ISL_954171, EPI_ISL_954175, EPI_ISL_954176, EPI_ISL_954177, EPI_ISL_954178			
see above	Hospital Universitari Vall d'Hebron - Vall d'Hebron Institut de Rercerca	Hospital Universitari Vall d'Hebron	Cristina Andrés, Maria Piñana, Josep F Abril, Damir Garcia-Cehic, Ariadna Rando, Juliana Esperalba, Maria Gema Codina, Carla Castillo, Maria Carmen Martin, Tomàs Pumarola, Josep Quer, Andrés Antón
EPI_ISL_954750	Diagen	Diagen	Koliada O
EPI_ISL_954754	City of Milwaukee Health Department Laboratory	City of Milwaukee Health Department Laboratory	Sanjib Bhattacharyya
EPI_ISL_954780	COMPLEJO HOSPITALARIO XERAL-CALDE	Instituto de Salud Carlos III	Iglesias-Caballero, M. Camarero, S. Sandonís,V. Vázquez, S. Pozo, F. Casas, I. Jiménez, P. Zaballos, A. Monzón, S. Varona, S. Cuesta, I. Alonso, P.
EPI_ISL_954786	Complejo Hospitalario de Navarra	Instituto de Salud Carlos III	Iglesias-Caballero, M. Camarero, S. Sandonís,V. Vázquez, S. Pozo, F. Casas, I. Jiménez, P. Zaballos, A. Monzón, S. Varona, S. Cuesta, I. Ezpeleta,C.
EPI_ISL_954798	Hospital Comarcal de Melilla	Instituto de Salud Carlos III	Iglesias-Caballero, M. Camarero, S. Sandonís,V. Vázquez, S. Pozo, F. Casas, I. Jiménez, P. Zaballos, A. Monzón, S. Varona, S. Cuesta, I. Roman, S.
EPI_ISL_954806	Gerencia de Asistencia Sanitaria de Soria	Instituto de Salud Carlos III	Iglesias-Caballero, M. Camarero, S. Sandonís,V. Vázquez, S. Pozo, F. Casas, I. Jiménez, P. Zaballos, A. Monzón, S. Varona, S. Cuesta, I. Aldea, C.
EPI_ISL_954807, EPI_ISL_954808	Complejo Hospitalario Universitario La Coruña	Instituto de Salud Carlos III	Iglesias-Caballero, M. Camarero, S. Sandonís,V. Vázquez, S. Pozo, F. Casas, I. Jiménez, P. Zaballos, A. Monzón, S. Varona, S. Cuesta, I. Peña, F.
EPI_ISL_954811, EPI_ISL_954813	HOSPITAL UNIVERSITARIO DE GUADALAJARA	Instituto de Salud Carlos III	Iglesias-Caballero, M. Camarero, S. Sandonís,V. Vázquez, S. Pozo, F. Casas, I. Jiménez, P. Zaballos, A. Monzón, S. Varona, S. Cuesta, I. González, A.
EPI_ISL_954818, EPI_ISL_954830, EPI_ISL_954840, EPI_ISL_954845, EPI_ISL_954854, EPI_ISL_954879, EPI_ISL_954936, EPI_ISL_954937, EPI_ISL_954938, EPI_ISL_954939, EPI_ISL_954940, EPI_ISL_954941, EPI_ISL_954942			
see above	Colorado Department of Public Health and Environment	Colorado Department of Puplic Health and Environment	Laura Bankers, Molly C. Hetherington-Rauth, Diana Ir, Shannon Ely, Shannon R. Matzinger, Sarah Elizabeth Totten, Emily A. Travanty
EPI_ISL_954943	Laboratory of Virology and Molecular Diagnostics	Institute of Public Health of Republic of North Macedonia Laboratory of Virology and Molecular Diagnostics	M. Kuzmanovska, G. Boshevska
EPI_ISL_954944, EPI_ISL_954945, EPI_ISL_954946, EPI_ISL_954947, EPI_ISL_954948, EPI_ISL_954949, EPI_ISL_954950	Colorado Department of Public Health and Environment	Colorado Department of Puplic Health and Environment	Laura Bankers, Molly C. Hetherington-Rauth, Diana Ir, Shannon Ely, Shannon R. Matzinger, Sarah Elizabeth Totten, Emily A. Travanty
EPI_ISL_955129	Innovative Genomics Institute, UC Berkeley	Innovative Genomics Institute, UC Berkeley	Stacia Wyman, Haridha Shivram, Phil Frankino, Liana Lareau, Shana McDevitt, Justin Choi
EPI_ISL_955153, EPI_ISL_955154	Genomic Laboratory (GLAB) (Conjoint lab of Health Directorate of Istanbul and Istanbul Technical University)	Genomic Laboratory (GLAB), Istanbul Technical University	Ilker Karacan, Tugba Kizilboga Akgun, Payam Zolfagharian, Nisan Denizce Can, Pari Sharifli, Levent Doganay, Gizem Dinler Doganay
EPI_ISL_955182	Labo Analyses Med	National Reference Center for Viruses of Respiratory Infections, Institut Pasteur, Paris	Marion Barbet, Sylvie Behillil, Méline Bizard, Angela Brisebarre, Camille Capel, Etienne Simon-Lorière, Vincent Enouf, Maud Vanpeene, Sylvie van der Werf
EPI_ISL_955212, EPI_ISL_955216, EPI_ISL_955217, EPI_ISL_955226	Indiana Animal Disease Diagnostic Laboratory	Carpi Laboratory - Purdue University	Jack Dorman, Ilinca I Ciubotariu, Lev Gorenstein, Abebe A Fola, G Kenitra Hendrix, Rebecca P Wilkes, Giovanna Carpi
EPI_ISL_955230	Mary Eliza Mahoney HC	NJ Public Health and Environmental Laboratories	Lindsey Bodnar, Shiv Verma, Dana Woell, Byeong Jeong
EPI_ISL_955323	American Esoteric Laboratory	Pathogen Discovery, Respiratory Viruses Branch, Division of Viral Diseases, Centers for Disease Control and Prevention	Ying Tao, Jing Zhang, Yan Li, Krista Queen, Anna Uehara, Peter Cook, Clinton R. Paden, Haibin Wang, Suxiang Tong
EPI_ISL_955426	Alameda County Public Health Lab	Chan-Zuckerberg Biohub	CZB Cliahub Consortium
EPI_ISL_955779, EPI_ISL_955781, EPI_ISL_955786, EPI_ISL_955789, EPI_ISL_955790	University of Michigan Clinical Microbiology Laboratory	Lauring Lab, University of Michigan, Department of Microbiology and Immunology	Valesano
EPI_ISL_956282, EPI_ISL_956288, EPI_ISL_956289, EPI_ISL_956290	Instituto Nacional de Salud- Dirección de Redes de Laboratorios de Salud Pública	Instituto Nacional de Salud- Dirección de Investigación en Salud Pública	Katherine Laiton-Donato, Diego A. Álvarez-Díaz, Carlos Franco-Muñoz, Mauricio Pacheco-Montealegre, Hector Alejandro Ruiz-Moreno, Maria T. Herrera-Sepúlveda, Diego Andrés Prada, Jhonnatan Reales-González, Sheryll Corchuelo, Julian Naizaque, Gerardo Santamaría, Magdalena Wiesner, Martha Lucia Ospina Martinez, Marcela Mercado-Reyes
EPI_ISL_956298	Laboratorio de salud publica del Valle del Cauca	Instituto Nacional de Salud- Dirección de Investigación en Salud Pública	Katherine Laiton-Donato, Diego A. Álvarez-Díaz, Carlos Franco-Muñoz, Mauricio Pacheco-Montealegre, Hector Alejandro Ruiz-Moreno, Maria T. Herrera-Sepúlveda, Diego Andrés Prada, Jhonnatan Reales-González, Sheryll Corchuelo, Julian Naizaque, Gerardo Santamaría, Magdalena Wiesner, Martha Lucia Ospina Martinez, Marcela Mercado-Reyes
EPI_ISL_956413, EPI_ISL_956416, EPI_ISL_956420, EPI_ISL_959287	Laboratory of Virology and Molecular Diagnostics	Institute of Public Health of Republic of North Macedonia Laboratory of Virology and Molecular Diagnostics	Maja Kuzmanovska, Golubinka Boshevska
EPI_ISL_959290, EPI_ISL_959375, EPI_ISL_959376	Laboratory of Virology and Molecular Diagnostics	Institute of Public Health of Republic of North Macedonia Laboratory of Virology and Molecular Diagnostics	Kuzmanovska M., Boshevska G.
EPI_ISL_959426	Servicio de Microbiología, Hospital Universitario Son Espases	SeqCOVID-SPAIN consortium/IBV(CSIC)	Carla López-Causapé, Jordi Reina, Antonio Oliver and SeqCOVID-SPAIN consortium
EPI_ISL_959544	Laboratory of Virology and Molecular Diagnostics	Institute of Public Health of Republic of North Macedonia Laboratory of Virology and Molecular Diagnostics	Kuzmanovska M., Boshevska G.
EPI_ISL_959555, EPI_ISL_959556, EPI_ISL_959557, EPI_ISL_959558	Synlab	GIGA Medical Genomics	Keith Durkin, Maria Artesi, Sébastien Bontems, Raphaël Boreux, Bouchra Boujemla, Cécile Meex, Pierrette Melin, Marie-Pierre Hayette, Vincent Bours
EPI_ISL_959559, EPI_ISL_959560	Vivalia - Clinique Saint-Joseph	GIGA Medical Genomics	Keith Durkin, Maria Artesi, Sébastien Bontems, Raphaël Boreux, Bouchra Boujemla, Cécile Meex, Pierrette Melin, Marie-Pierre Hayette, Vincent Bours
EPI_ISL_959561	University of Liège COVID-19 testing center	GIGA Medical Genomics	Keith Durkin, Maria Artesi, Sébastien Bontems, Raphaël Boreux, Bouchra Boujemla, Cécile Meex, Pierrette Melin, Marie-Pierre Hayette, Vincent Bours
EPI_ISL_959562	Vivalia - Clinique Saint-Joseph	GIGA Medical Genomics	Keith Durkin, Maria Artesi, Sébastien Bontems, Raphaël Boreux, Bouchra Boujemla, Cécile Meex, Pierrette Melin, Marie-Pierre Hayette, Vincent Bours
EPI_ISL_959840, EPI_ISL_959841, EPI_ISL_959842, EPI_ISL_959843, EPI_ISL_959844, EPI_ISL_959845, EPI_ISL_959846, EPI_ISL_959847, EPI_ISL_959848	National Virus Reference Laboratory	National Virus Reference Laboratory	Michael Carr, Gabriel Gonzalez, Jonathan Dean, Cillian F De Gascun
EPI_ISL_960223	FidaLab	Seattle Flu Study	Deborah A. Nickerson, Chris D. Frazar, Jover Lee, Benjamin Pelle, Erica Ryke, Matthew Richardson, Amanda Adler, Elisabeth Brandstetter, Peter D. Han, Kairsten Fay, Misja Ilcisin, Kirsten Lacombe, Thomas R. Sibley, Melissa Truong, Caitlin R. Wolf, Romesh Gautom, Geoff Melly, Brian Hiatt, Philip Dykema, Scott Lindquist, Michael Boeckh, Janet A. Englund, Michael Famulare, Barry R. Lutz, Mark J. Rieder, Lea M. Starita, Matthew Thompson, Helen Y. Chu, Jay Shendure, Trevor Bedford
EPI_ISL_960415	The National Institute of Public Health	State Veterinary Institute Prague	Nagy,A;Vecerova,J;Cernikova,L;Stara,M;Jirincova,H;Trnka,D
EPI_ISL_960461, EPI_ISL_960573	Istituto Zooprofilattico Sperimentale del Mezzogiorno	TIGEM	Patrizia Annunziata, Andrea Ballabio, Valentina Bouche, Davide Cacchiarelli, Pellegrino Cerino, Chiara Colantuono, Maria Concetta Cuomo, Denise Di Concilio, Lucio Di Filippo, Antonio Grimaldi, Antonio Limone, Anna Manfredi, Francesco Panariello, Biancamaria Pierri, Marcello Salvi

EPI_ISL_960989, EPI_ISL_960990, EPI_ISL_960992, EPI_ISL_960993, EPI_ISL_960995, EPI_ISL_960997, EPI_ISL_960998, EPI_ISL_961001, EPI_ISL_961003, EPI_ISL_961004, EPI_ISL_961005, EPI_ISL_961006, EPI_ISL_961007, EPI_ISL_961008			
see above	AIID	Irish Coronavirus Sequencing Consortium-Teagasc Grange	Matthew McCabe, Aljandro Abner Garcia Leon, Fiona Crispie, Calum Walsh, Michael Carr, John Kenny, Paul Cotter, Patrick Mallon, Gabriel Gonzalez
EPI_ISL_961466	Laboratorios Lister	Instituto de Diagnostico y Referencia Epidemiologicos (INDRE)	Claudia Wong-Arambula, Abril Rodriguez-Maldonado, Fabiola Garces-Ayala, Natividad Cruz-Ortiz, Tatiana Nunez-Garcia, Gisela Barrera-Badillo, Lucia Hernandez-Rivas, Irma Lopez-Martinez, Ernesto Ramirez-Gonzalez.
EPI_ISL_961597, EPI_ISL_961609, EPI_ISL_961637, EPI_ISL_961638, EPI_ISL_961639, EPI_ISL_961640, EPI_ISL_961641	Hôpital Georges L. Dumont	National Microbiology Laboratory (NML)	Anna Majer, Shari Tyson, Grace Seo, Philip Mabon, Elsie Grudeski, Rhiannon Huzarewich, Russell Mandes, Anneliese Landgraff, Jennifer Tanner, Natalie Knox, Morag Graham, Gary Van Domselaar, Richard Garceau, Guillaume Desnoyers, Nathalie Bastien, Yan Li, Timothy Booth, Darian Hole, Madison Chapel, Kirsten Biggar, CanCOGeN's metadata curation team, Public Health Agency of Canada CanCOGeN team
EPI_ISL_961875	E. Gulbja laboratorija	Latvian Biomedical Research and Study Centre	Janis Pjalkovskis, Nikita Zrelovs, Monta Ustinova, Ivars Silamikelis, Liga Birzniece, Kaspars Megnis, Vita Rovite, Lauma Freimane, Laila Silamikele, Laura Ansone, Davids Fridmanis, Mikus Gavars, Dmitrijs Perminovs, Jurijs Perevoscikovs, Uga Dumpis, Janis Klovins
EPI_ISL_961876	Latvijas Infektologijas Centrs	Latvian Biomedical Research and Study Centre	Janis Pjalkovskis, Nikita Zrelovs, Monta Ustinova, Ivars Silamikelis, Liga Birzniece, Kaspars Megnis, Vita Rovite, Lauma Freimane, Laila Silamikele, Laura Ansone, Davids Fridmanis,Reinis Zeltmatis, Diana Dusacka, Jurijs Perevoscikovs, Uga Dumpis, Janis Klovins
EPI_ISL_961899, EPI_ISL_961926, EPI_ISL_961934, EPI_ISL_961941, EPI_ISL_961942, EPI_ISL_961949, EPI_ISL_961950, EPI_ISL_961957, EPI_ISL_961974, EPI_ISL_961981, EPI_ISL_961982, EPI_ISL_961991, EPI_ISL_962014, EPI_ISL_962174, EPI_ISL_962175			
see above	Illinois Department of Public Health	Gagnon Lab, Southern Illinois University	Keith Gagnon
EPI_ISL_962368	Seattle Flu Study	Seattle Flu Study	Deborah A. Nickerson, Chris D. Frazar, Jover Lee, Benjamin Pelle, Erica Ryke, Matthew Richardson, Amanda Adler, Elisabeth Brandstetter, Peter D. Han, Kairsten Fay, Misja Ilcisin, Kirsten Lacombe, Thomas R. Sibley, Melissa Truong, Caitlin R. Wolf, Karen Cowgill, Stephanie Schrag, Jeff Duchin, Michael Boeckh, Janet A. Englund, Michael Famulare, Barry R. Lutz, Mark J. Rieder, Lea M. Starita, Matthew Thompson, Helen Y. Chu, Trevor Bedford, Jay Shendure
EPI_ISL_962379, EPI_ISL_962380, EPI_ISL_962381, EPI_ISL_962382, EPI_ISL_962383, EPI_ISL_962384, EPI_ISL_962385, EPI_ISL_962386, EPI_ISL_962387, EPI_ISL_962388	Washington State Department of Health	Seattle Flu Study	Deborah A. Nickerson, Chris D. Frazar, Jover Lee, Benjamin Pelle, Erica Ryke, Matthew Richardson, Amanda Adler, Elisabeth Brandstetter, Peter D. Han, Kairsten Fay, Misja Ilcisin, Kirsten Lacombe, Thomas R. Sibley, Melissa Truong, Caitlin R. Wolf, Romesh Gautom, Geoff Melly, Brian Hiatt, Philip Dykema, Scott Lindquist, Michael Boeckh, Janet A. Englund, Michael Famulare, Barry R. Lutz, Mark J. Rieder, Lea M. Starita, Matthew Thompson, Helen Y. Chu, Jay Shendure, Trevor Bedford
EPI_ISL_962512	UCLA Clinical Micro Lab	Los Angeles County PHL	P. Hemarajata et al.
EPI_ISL_962602, EPI_ISL_962603, EPI_ISL_962604, EPI_ISL_962605, EPI_ISL_962606, EPI_ISL_962607, EPI_ISL_962608	Sharp HealthCare Laboratory	Andersen lab at Scripps Research	SEARCH Alliance San Diego with Aaron Harding, Jacquelyn Berumen, Cathy Woerle, Liam McGinnis, Art Mendoza, Omid Bakhtar
EPI_ISL_962619, EPI_ISL_962620, EPI_ISL_962624, EPI_ISL_962626, EPI_ISL_962627, EPI_ISL_962632, EPI_ISL_962633, EPI_ISL_962636, EPI_ISL_962643, EPI_ISL_962645, EPI_ISL_962648, EPI_ISL_962649			
see above	Scripps Medical Laboratory	Andersen lab at Scripps Research	SEARCH Alliance San Diego with Michael Quigley, Ellen Stefanski, Ian Mchardy
EPI_ISL_962650, EPI_ISL_962651, EPI_ISL_962652, EPI_ISL_962653, EPI_ISL_962654, EPI_ISL_962655, EPI_ISL_962656, EPI_ISL_962657, EPI_ISL_962658, EPI_ISL_962659, EPI_ISL_962660, EPI_ISL_962661, EPI_ISL_962662, EPI_ISL_962663, EPI_ISL_962664, EPI_ISL_962665, EPI_ISL_962666, EPI_ISL_962667, EPI_ISL_962668, EPI_ISL_962669, EPI_ISL_962670, EPI_ISL_962671, EPI_ISL_962672, EPI_ISL_962673, EPI_ISL_962674, EPI_ISL_962675, EPI_ISL_962676, EPI_ISL_962677, EPI_ISL_962678, EPI_ISL_962679, EPI_ISL_962680, EPI_ISL_962681, EPI_ISL_962682, EPI_ISL_962683, EPI_ISL_962684, EPI_ISL_962685, EPI_ISL_962686, EPI_ISL_962687, EPI_ISL_962688, EPI_ISL_962689, EPI_ISL_962690, EPI_ISL_962691, EPI_ISL_962692, EPI_ISL_962693, EPI_ISL_962694, EPI_ISL_962695, EPI_ISL_962696, EPI_ISL_962697, EPI_ISL_962698, EPI_ISL_962699, EPI_ISL_962700, EPI_ISL_962701, EPI_ISL_962702, EPI_ISL_962703, EPI_ISL_962704, EPI_ISL_962705, EPI_ISL_962706, EPI_ISL_962707, EPI_ISL_962708, EPI_ISL_962709, EPI_ISL_962719, EPI_ISL_962727, EPI_ISL_962730, EPI_ISL_962739, EPI_ISL_962744, EPI_ISL_962745, EPI_ISL_962754, EPI_ISL_962756, EPI_ISL_962758, EPI_ISL_962759, EPI_ISL_962765			
see above	Sharp HealthCare Laboratory	Andersen lab at Scripps Research	SEARCH Alliance San Diego with Aaron Harding, Jacquelyn Berumen, Cathy Woerle, Liam McGinnis, Art Mendoza, Omid Bakhtar
EPI_ISL_962896	Norwegian Institute of Public Health, Department of Virology	Norwegian Institute of Public Health, Department of Virology	Kathrine Stene-Johansen, Kamilla Heddeland Instefjord, Hilde Elshaug, Ignacio Garcia Llorente, Serina B Engebretsen, Atiya R Ali,Marie Paulsen Madsen, Rasmus Riis Kopperud, Hilde Vollan, Karoline Bragstad, Olav Hungnes
EPI_ISL_962906	Ostfold Hospital Trust - Kalnes, Centre for Laboratory Medicine, Section for gene technology and infection serology	Norwegian Institute of Public Health, Department of Virology	Kathrine Stene-Johansen, Kamilla Heddeland Instefjord, Hilde Elshaug, Ignacio Garcia Llorente, Serina B Engebretsen, Atiya R Ali,Marie Paulsen Madsen, Rasmus Riis Kopperud, Hilde Vollan, Karoline Bragstad, Olav Hungnes
EPI_ISL_962945, EPI_ISL_962946	Hospital Universitario de Gran Canaria Dr. Negrín	SeqCOVID-SPAIN consortium/IBV(CSIC)	M. Carmen Pérez González, Francisco J. Chamizo López, Ana Bordes Benítez and SeqCOVID-SPAIN consortium
EPI_ISL_963321	Lighthouse Lab in Alderley Park	Wellcome Sanger Institute for the COVID-19 Genomics UK (COG-UK) Consortium	Jacquelyn Wynn, Mairead Hyland, The Lighthouse Lab in Alderley Park and Alex Alderton, Roberto Amato, Sonia Goncalves, Ewan Harrison, David K. Jackson, Ian Johnston, Dominic Kwiatkowski, Cordelia Langford, John Sillitoe on behalf of the Wellcome Sanger Institute COVID-19 Surveillance Team
EPI_ISL_964257	Akershus University Hospital, Department for Microbiology and Infectious Disease Control	Norwegian Institute of Public Health, Department of Virology	Kathrine Stene-Johansen, Kamilla Heddeland Instefjord, Hilde Elshaug, Ignacio Garcia Llorente, Serina B Engebretsen, Atiya R Ali, Marie Paulsen Madsen, Rasmus Riis Kopperud, Hilde Vollan, Karoline Bragstad, Olav Hungnes
EPI_ISL_964918, EPI_ISL_964921, EPI_ISL_964932, EPI_ISL_964933, EPI_ISL_964940, EPI_ISL_964941	Instituto Nacional de Saude (INS), Mozambique	KRISP, KZN Research Innovation and Sequencing Platform	Nalia Ismael, Nadia Sitoe, Paulo Arnaldo, Nedio Mabunda, Giandhari J, Pillay S, Emmanuel S, Tegally H, Wilkinson E, de Oliveira T
EPI_ISL_964952	Ostfold Hospital Trust - Kalnes, Centre for Laboratory Medicine, Section for gene technology and infection serology	Norwegian Institute of Public Health, Department of Virology	Kathrine Stene-Johansen, Kamilla Heddeland Instefjord, Hilde Elshaug, Ignacio Garcia Llorente, Serina B Engebretsen, Atiya R Ali,Marie Paulsen Madsen, Rasmus Riis Kopperud, Hilde Vollan, Karoline Bragstad, Olav Hungnes
EPI_ISL_964953, EPI_ISL_964954	Akershus University Hospital, Department for Microbiology and Infectious Disease Control	Norwegian Institute of Public Health, Department of Virology	Kathrine Stene-Johansen, Kamilla Heddeland Instefjord, Hilde Elshaug, Ignacio Garcia Llorente, Serina B Engebretsen, Atiya R Ali,Marie Paulsen Madsen, Rasmus Riis Kopperud, Hilde Vollan, Karoline Bragstad, Olav Hungnes
EPI_ISL_964955, EPI_ISL_964960	Oslo University Hospital, Department of Medical Microbiology	Norwegian Institute of Public Health, Department of Virology	Kathrine Stene-Johansen, Kamilla Heddeland Instefjord, Hilde Elshaug, Ignacio Garcia Llorente, Serina B Engebretsen, Atiya R Ali,Marie Paulsen Madsen, Rasmus Riis Kopperud, Hilde Vollan, Karoline Bragstad, Olav Hungnes
EPI_ISL_964974	Ostfold Hospital Trust - Kalnes, Centre for Laboratory Medicine, Section for gene technology and infection serology	Norwegian Institute of Public Health, Department of Virology	Kathrine Stene-Johansen, Kamilla Heddeland Instefjord, Hilde Elshaug, Ignacio Garcia Llorente, Serina B Engebretsen, Atiya R Ali,Marie Paulsen Madsen, Rasmus Riis Kopperud, Hilde Vollan, Karoline Bragstad, Olav Hungnes
EPI_ISL_964980	Oslo University Hospital, Department of Medical Microbiology	Norwegian Institute of Public Health, Department of Virology	Kathrine Stene-Johansen, Kamilla Heddeland Instefjord, Hilde Elshaug, Ignacio Garcia Llorente, Serina B Engebretsen, Atiya R Ali,Marie Paulsen Madsen, Rasmus Riis Kopperud, Hilde Vollan, Karoline Bragstad, Olav Hungnes
EPI_ISL_965009, EPI_ISL_965010	Nordland Hospital - Bodo, Laboratory Department, Molecular Biology Unit	Norwegian Institute of Public Health, Department of Virology	Kathrine Stene-Johansen, Kamilla Heddeland Instefjord, Hilde Elshaug, Ignacio Garcia Llorente, Serina B Engebretsen, Atiya R Ali, Marie Paulsen Madsen, Rasmus Riis Kopperud, Hilde Vollan, Karoline Bragstad, Olav Hungnes
EPI_ISL_965133	Azienda Ospedaliera San Camillo Forlanini	INMI Lazzaro Spallanzani IRCCS	B Bartolini, O Butera, C.E.M Gruber, M Rueca, F Messina, E Giombini, F Basile, A D'Agostino, G Parisi, MR Capobianchi, A Di Caro
EPI_ISL_965134	San Gallicano Dermatological Institute I.F.O.	INMI Lazzaro Spallanzani IRCCS	M Rueca, O Butera, F Messina, CEM Gruber, B Bartolini, E Giombini, F Pimpinelli, F Ensoli, A Mastrofrancesco, A Di Caro, MR Capobianchi
EPI_ISL_965135	Presidio Ospedaliero G. B. Grassi ASL RM3	INMI Lazzaro Spallanzani IRCCS	F Messina, C.E.M Gruber, B Bartolini, E Giombini, M Rueca, O Butera, F Tabacco, E Ristori, A Di Caro, MR Capobianchi
EPI_ISL_965139	Fondazione Policlinico Universitario "A. Gemelli" IRCCS	INMI Lazzaro Spallanzani IRCCS	B Bartolini, O Butera, C.E.M Gruber, M Rueca, F Messina, E Giombini, P Cattani,M Sanguinetti, MR Capobianchi, A Di Caro
EPI_ISL_965559, EPI_ISL_965780	Dutch COVID-19 response team	Medical Microbiology, Maastricht University Medical Centre	Jozef Dingemans*, Brian van der Veer*, Erik Beuken, Carmen Reumkens, Lieke van Alphen, Christian Hoebe, Paul Savelkoul
EPI_ISL_965840, EPI_ISL_965841, EPI_ISL_965842, EPI_ISL_965843, EPI_ISL_965844, EPI_ISL_965845, EPI_ISL_965846, EPI_ISL_965853, EPI_ISL_965854	GA Department of Public Health	GA Department of Public Health	Stacy Reeves, Jonathan Edwards, Cynthia Dixey, Tonia Parrott
EPI_ISL_965934	Hospital General Universitario de Ciudad Real	Instituto de Salud Carlos III	Sandonís,V. Vázquez, S. Iglesias-Caballero, M. Camarero, S. Pozo, F. Casas, I. Jiménez, P. Zaballos, A. Monzón, S. Varona, S. Cuesta, I. Illescas, S.

EPI_ISL_965938	Consejería de Sanidad y Asuntos Sociales de Castilla La Mancha	Instituto de Salud Carlos III	Sandonís,V. Vázquez, S. Iglesias-Caballero, M. Camarero, S. Pozo, F. Casas, I. Jiménez, P. Zaballos, A. Monzón, S. Varona, S. Cuesta, I. Gutiérrez, G.
EPI_ISL_965959	Hospital General Universitario de Ciudad Real	Instituto de Salud Carlos III	Iglesias-Caballero, M. Sandonís,V. Vázquez, S. Camarero, S. Pozo, F. Casas, I. Jiménez, P. Zaballos, A. Monzón, S. Varona, S. Cuesta, I. Illescas, S.
EPI_ISL_965974	Vancouver Clinic	Seattle Flu Study	Deborah A. Nickerson, Chris D. Frazar, Jover Lee, Benjamin Pelle, Erica Ryke, Matthew Richardson, Amanda Adler, Elisabeth Brandstetter, Peter D. Han, Kairsten Fay, Misja Ilcisin, Kirsten Lacombe, Thomas R. Sibley, Melissa Truong, Caitlin R. Wolf, Romesh Gautom, Geoff Melly, Brian Hiatt, Philip Dykema, Scott Lindquist, Michael Boeckh, Janet A. Englund, Michael Famulare, Barry R. Lutz, Mark J. Rieder, Lea M. Starita, Matthew Thompson, Helen Y. Chu, Jay Shendure, Trevor Bedford
EPI_ISL_966312	Toronto Invasive Bacterial Diseases Network	McMaster University	Allison McGeer, Patryk Aftanas, Hooman Derakhshani, Angel Li, Kuganya Nirmalarajah, Emily Panousis, Ahmed Draia, Jalees Nasir, Michael Surette, Samira Mubareka, Andrew G. McArthur
EPI_ISL_966532, EPI_ISL_966534, EPI_ISL_966535, EPI_ISL_966536, EPI_ISL_966537, EPI_ISL_966538, EPI_ISL_966539, EPI_ISL_966540, EPI_ISL_966541, EPI_ISL_966542, EPI_ISL_966543, EPI_ISL_966544, EPI_ISL_966545, EPI_ISL_966546, EPI_ISL_967169, EPI_ISL_967171, EPI_ISL_967174, EPI_ISL_967175, EPI_ISL_967179, EPI_ISL_967180, EPI_ISL_967181, EPI_ISL_967183, EPI_ISL_967184, EPI_ISL_967192, EPI_ISL_967193, EPI_ISL_967194, EPI_ISL_967195, EPI_ISL_967196, EPI_ISL_967197, EPI_ISL_967198, EPI_ISL_967199, EPI_ISL_967200, EPI_ISL_967201, EPI_ISL_967213, EPI_ISL_967214, EPI_ISL_967218, EPI_ISL_967243, EPI_ISL_967244, EPI_ISL_967245, EPI_ISL_967246, EPI_ISL_967247, EPI_ISL_967248, EPI_ISL_967249, EPI_ISL_967250, EPI_ISL_967251, EPI_ISL_967252, EPI_ISL_967253, EPI_ISL_967254, EPI_ISL_967255, EPI_ISL_967256, EPI_ISL_967257, EPI_ISL_967258, EPI_ISL_967259, EPI_ISL_967260, EPI_ISL_967261, EPI_ISL_967262, EPI_ISL_967263, EPI_ISL_967264, EPI_ISL_967265, EPI_ISL_967266, EPI_ISL_967267, EPI_ISL_967268, EPI_ISL_967269, EPI_ISL_967270, EPI_ISL_967271, EPI_ISL_967272, EPI_ISL_967273, EPI_ISL_967274, EPI_ISL_967275, EPI_ISL_967276, EPI_ISL_967277, EPI_ISL_967278, EPI_ISL_967279, EPI_ISL_967280, EPI_ISL_967281, EPI_ISL_967282, EPI_ISL_967283, EPI_ISL_967284, EPI_ISL_967285, EPI_ISL_967286, EPI_ISL_967287, EPI_ISL_967288, EPI_ISL_967289, EPI_ISL_967290, EPI_ISL_967291, EPI_ISL_967292, EPI_ISL_967293, EPI_ISL_967294, EPI_ISL_967295, EPI_ISL_967296, EPI_ISL_967297, EPI_ISL_967298, EPI_ISL_967299, EPI_ISL_967300, EPI_ISL_967301, EPI_ISL_967302, EPI_ISL_967303, EPI_ISL_967304, EPI_ISL_967305, EPI_ISL_967306, EPI_ISL_967307, EPI_ISL_967308, EPI_ISL_967309, EPI_ISL_967310, EPI_ISL_967311, EPI_ISL_967312, EPI_ISL_967313, EPI_ISL_967314, EPI_ISL_967315, EPI_ISL_967316, EPI_ISL_967317, EPI_ISL_967318, EPI_ISL_967319, EPI_ISL_967320, EPI_ISL_967321, EPI_ISL_967322, EPI_ISL_967323, EPI_ISL_967324, EPI_ISL_967325, EPI_ISL_967326			
see above	Helix/Illumina	Respiratory Viruses Branch, Division of Viral Diseases, Centers for Disease Control and Prevention	Peter W. Cook,Dakota Howard,Dhwani Batra,Ben L. Rambo-Martin,Eileen de Feo,Jan Antico,Christine Tran,Matthew Tolentino,Shannon Wickline,Kim Gietzen,Brad Sickler,Jingtao Liu,Eric Allen,Phil Febbo,Summer Galloway,Nicole L. Washington,Simon White,Geraint Levan,Kelly Schiabor Barrett,Elizabeth Cirulli,Alexandre Bolze,Ary Ascencio,Charlotte Rivera-Garcia,Ryan Cho,Jason Nguyen,Sherry Wang,Jimmy Ramirez,Tyler Cassens,Efren Sandoval,Magnus Isaksson,William Lee,David Becker,Marc Laurent,James Lu,Clinton R. Paden,Suxiang Tong,Duncan MacCannell,
EPI_ISL_967697, EPI_ISL_967702, EPI_ISL_967703, EPI_ISL_967745	State Laboratories Division, Hawaii State Department of Health	State Laboratories Division, Hawaii State Department of Health	Pamela O'Brien, Drew Kuwazaki, Ayana Garnet, Razvan Sultana, Edward Desmond
EPI_ISL_967843, EPI_ISL_967844, EPI_ISL_967845, EPI_ISL_967846, EPI_ISL_967847, EPI_ISL_967848, EPI_ISL_967849, EPI_ISL_967850, EPI_ISL_967851, EPI_ISL_967852, EPI_ISL_967853, EPI_ISL_967854, EPI_ISL_967855, EPI_ISL_967856, EPI_ISL_967857, EPI_ISL_967858, EPI_ISL_967859, EPI_ISL_967860, EPI_ISL_967861, EPI_ISL_967862, EPI_ISL_967863, EPI_ISL_967864, EPI_ISL_967865, EPI_ISL_967866, EPI_ISL_967867, EPI_ISL_967868, EPI_ISL_967869, EPI_ISL_967870, EPI_ISL_967871, EPI_ISL_967872, EPI_ISL_967873, EPI_ISL_967874, EPI_ISL_967875, EPI_ISL_967876, EPI_ISL_967877, EPI_ISL_967878, EPI_ISL_967879, EPI_ISL_967880, EPI_ISL_967881			
see above	Helix/Illumina	Respiratory Viruses Branch, Division of Viral Diseases, Centers for Disease Control and Prevention	Peter W. Cook,Dakota Howard,Dhwani Batra,Ben L. Rambo-Martin,Eileen de Feo,Jan Antico,Christine Tran,Matthew Tolentino,Shannon Wickline,Kim Gietzen,Brad Sickler,Jingtao Liu,Eric Allen,Phil Febbo,Summer Galloway,Nicole L. Washington,Simon White,Geraint Levan,Kelly Schiabor Barrett,Elizabeth Cirulli,Alexandre Bolze,Ary Ascencio,Charlotte Rivera-Garcia,Ryan Cho,Jason Nguyen,Sherry Wang,Jimmy Ramirez,Tyler Cassens,Efren Sandoval,Magnus Isaksson,William Lee,David Becker,Marc Laurent,James Lu,Clinton R. Paden,Suxiang Tong,Duncan MacCannell,
EPI_ISL_967885, EPI_ISL_967886, EPI_ISL_967888, EPI_ISL_967889	TGen North	TGen North	"Jolene Bowers, Megan Folkerts, Chris French, Hayley Yaglom, Ashlyn Pfeiffer, Darrin Lemmer, Dave Engelthaler, The Arizona COVID Genomics Union (ACGU)"
EPI_ISL_967890, EPI_ISL_967893	TGen North	Sonora Quest Laboratories	"Jolene Bowers, Megan Folkerts, Chris French, Hayley Yaglom, Ashlyn Pfeiffer, Darrin Lemmer, Dave Engelthaler, The Arizona COVID Genomics Union (ACGU)"
EPI_ISL_967894	TGen North	TGen North	"Jolene Bowers, Megan Folkerts, Chris French, Hayley Yaglom, Ashlyn Pfeiffer, Darrin Lemmer, Dave Engelthaler, The Arizona COVID Genomics Union (ACGU)"
EPI_ISL_967895, EPI_ISL_967896, EPI_ISL_967898	TGen North	Sonora Quest Laboratories	"Jolene Bowers, Megan Folkerts, Chris French, Hayley Yaglom, Ashlyn Pfeiffer, Darrin Lemmer, Dave Engelthaler, The Arizona COVID Genomics Union (ACGU)"
EPI_ISL_967902	TGen North	TGen North	"Jolene Bowers, Megan Folkerts, Chris French, Hayley Yaglom, Ashlyn Pfeiffer, Darrin Lemmer, Dave Engelthaler, The Arizona COVID Genomics Union (ACGU)"
EPI_ISL_967904	TGen North	Sonora Quest Laboratories	"Jolene Bowers, Megan Folkerts, Chris French, Hayley Yaglom, Ashlyn Pfeiffer, Darrin Lemmer, Dave Engelthaler, The Arizona COVID Genomics Union (ACGU)"
EPI_ISL_967905, EPI_ISL_967908	TGen North	TGen North	"Jolene Bowers, Megan Folkerts, Chris French, Hayley Yaglom, Ashlyn Pfeiffer, Darrin Lemmer, Dave Engelthaler, The Arizona COVID Genomics Union (ACGU)"
EPI_ISL_967909	TGen North	Sonora Quest Laboratories	"Jolene Bowers, Megan Folkerts, Chris French, Hayley Yaglom, Ashlyn Pfeiffer, Darrin Lemmer, Dave Engelthaler, The Arizona COVID Genomics Union (ACGU)"
EPI_ISL_967910, EPI_ISL_967911, EPI_ISL_967912, EPI_ISL_967914	TGen North	TGen North	"Jolene Bowers, Megan Folkerts, Chris French, Hayley Yaglom, Ashlyn Pfeiffer, Darrin Lemmer, Dave Engelthaler, The Arizona COVID Genomics Union (ACGU)"
EPI_ISL_967917, EPI_ISL_967918	TGen North	Sonora Quest Laboratories	"Jolene Bowers, Megan Folkerts, Chris French, Hayley Yaglom, Ashlyn Pfeiffer, Darrin Lemmer, Dave Engelthaler, The Arizona COVID Genomics Union (ACGU)"
EPI_ISL_967919, EPI_ISL_967921	TGen North	TGen North	"Jolene Bowers, Megan Folkerts, Chris French, Hayley Yaglom, Ashlyn Pfeiffer, Darrin Lemmer, Dave Engelthaler, The Arizona COVID Genomics Union (ACGU)"
EPI_ISL_967922	TGen North	Sonora Quest Laboratories	"Jolene Bowers, Megan Folkerts, Chris French, Hayley Yaglom, Ashlyn Pfeiffer, Darrin Lemmer, Dave Engelthaler, The Arizona COVID Genomics Union (ACGU)"
EPI_ISL_967923	TGen North	TGen North	"Jolene Bowers, Megan Folkerts, Chris French, Hayley Yaglom, Ashlyn Pfeiffer, Darrin Lemmer, Dave Engelthaler, The Arizona COVID Genomics Union (ACGU)"
EPI_ISL_967924	TGen North	Sonora Quest Laboratories	"Jolene Bowers, Megan Folkerts, Chris French, Hayley Yaglom, Ashlyn Pfeiffer, Darrin Lemmer, Dave Engelthaler, The Arizona COVID Genomics Union (ACGU)"
EPI_ISL_967925	TGen North	TGen North	"Jolene Bowers, Megan Folkerts, Chris French, Hayley Yaglom, Ashlyn Pfeiffer, Darrin Lemmer, Dave Engelthaler, The Arizona COVID Genomics Union (ACGU)"
EPI_ISL_967927	TGen North	Sonora Quest Laboratories	"Jolene Bowers, Megan Folkerts, Chris French, Hayley Yaglom, Ashlyn Pfeiffer, Darrin Lemmer, Dave Engelthaler, The Arizona COVID Genomics Union (ACGU)"
EPI_ISL_967930, EPI_ISL_967931, EPI_ISL_967935, EPI_ISL_967937, EPI_ISL_967939, EPI_ISL_967941, EPI_ISL_967944, EPI_ISL_967945, EPI_ISL_967947, EPI_ISL_967948, EPI_ISL_967949, EPI_ISL_967950, EPI_ISL_967952, EPI_ISL_967953, EPI_ISL_967956, EPI_ISL_967959, EPI_ISL_967960, EPI_ISL_967961			
see above	TGen North	TGen North	"Jolene Bowers, Megan Folkerts, Chris French, Hayley Yaglom, Ashlyn Pfeiffer, Darrin Lemmer, Dave Engelthaler, The Arizona COVID Genomics Union (ACGU)"
EPI_ISL_967962, EPI_ISL_967963	TGen North	Sonora Quest Laboratories	"Jolene Bowers, Megan Folkerts, Chris French, Hayley Yaglom, Ashlyn Pfeiffer, Darrin Lemmer, Dave Engelthaler, The Arizona COVID Genomics Union (ACGU)"
EPI_ISL_967970, EPI_ISL_967971, EPI_ISL_967973, EPI_ISL_967975	TGen North	TGen North	"Jolene Bowers, Megan Folkerts, Chris French, Hayley Yaglom, Ashlyn Pfeiffer, Darrin Lemmer, Dave Engelthaler, The Arizona COVID Genomics Union (ACGU)"
EPI_ISL_967985	TGen North	Sonora Quest Laboratories	"Jolene Bowers, Megan Folkerts, Chris French, Hayley Yaglom, Ashlyn Pfeiffer, Darrin Lemmer, Dave Engelthaler, The Arizona COVID Genomics Union (ACGU)"
EPI_ISL_967986, EPI_ISL_967987, EPI_ISL_967988	TGen North	TGen North	"Jolene Bowers, Megan Folkerts, Chris French, Hayley Yaglom, Ashlyn Pfeiffer, Darrin Lemmer, Dave Engelthaler, The Arizona COVID Genomics Union (ACGU)"

EPI_ISL_967991	TGen North	Sonora Quest Laboratories	"Jolene Bowers, Megan Folkerts, Chris French, Hayley Yaglom, Ashlyn Pfeiffer, Darrin Lemmer, Dave Engelthaler, The Arizona COVID Genomics Union (ACGU)"
EPI_ISL_967992	TGen North	TGen North	"Jolene Bowers, Megan Folkerts, Chris French, Hayley Yaglom, Ashlyn Pfeiffer, Darrin Lemmer, Dave Engelthaler, The Arizona COVID Genomics Union (ACGU)"
EPI_ISL_967995	TGen North	Sonora Quest Laboratories	"Jolene Bowers, Megan Folkerts, Chris French, Hayley Yaglom, Ashlyn Pfeiffer, Darrin Lemmer, Dave Engelthaler, The Arizona COVID Genomics Union (ACGU)"
EPI_ISL_967997, EPI_ISL_967999, EPI_ISL_968003, EPI_ISL_968005, EPI_ISL_968007	TGen North	TGen North	"Jolene Bowers, Megan Folkerts, Chris French, Hayley Yaglom, Ashlyn Pfeiffer, Darrin Lemmer, Dave Engelthaler, The Arizona COVID Genomics Union (ACGU)"
EPI_ISL_968008	TGen North	Sonora Quest Laboratories	"Jolene Bowers, Megan Folkerts, Chris French, Hayley Yaglom, Ashlyn Pfeiffer, Darrin Lemmer, Dave Engelthaler, The Arizona COVID Genomics Union (ACGU)"
EPI_ISL_968009, EPI_ISL_968011, EPI_ISL_968012, EPI_ISL_968013, EPI_ISL_968015, EPI_ISL_968016	TGen North	TGen North	"Jolene Bowers, Megan Folkerts, Chris French, Hayley Yaglom, Ashlyn Pfeiffer, Darrin Lemmer, Dave Engelthaler, The Arizona COVID Genomics Union (ACGU)"
EPI_ISL_968025	TGen North	Sonora Quest Laboratories	"Jolene Bowers, Megan Folkerts, Chris French, Hayley Yaglom, Ashlyn Pfeiffer, Darrin Lemmer, Dave Engelthaler, The Arizona COVID Genomics Union (ACGU)"
EPI_ISL_968032, EPI_ISL_968034	TGen North	TGen North	"Jolene Bowers, Megan Folkerts, Chris French, Hayley Yaglom, Ashlyn Pfeiffer, Darrin Lemmer, Dave Engelthaler, The Arizona COVID Genomics Union (ACGU)"
EPI_ISL_968035	TGen North	Sonora Quest Laboratories	"Jolene Bowers, Megan Folkerts, Chris French, Hayley Yaglom, Ashlyn Pfeiffer, Darrin Lemmer, Dave Engelthaler, The Arizona COVID Genomics Union (ACGU)"
EPI_ISL_968037, EPI_ISL_968038	TGen North	TGen North	"Jolene Bowers, Megan Folkerts, Chris French, Hayley Yaglom, Ashlyn Pfeiffer, Darrin Lemmer, Dave Engelthaler, The Arizona COVID Genomics Union (ACGU)"
EPI_ISL_968039	TGen North	Sonora Quest Laboratories	"Jolene Bowers, Megan Folkerts, Chris French, Hayley Yaglom, Ashlyn Pfeiffer, Darrin Lemmer, Dave Engelthaler, The Arizona COVID Genomics Union (ACGU)"
EPI_ISL_968041	TGen North	TGen North	"Jolene Bowers, Megan Folkerts, Chris French, Hayley Yaglom, Ashlyn Pfeiffer, Darrin Lemmer, Dave Engelthaler, The Arizona COVID Genomics Union (ACGU)"
EPI_ISL_968043	TGen North	Sonora Quest Laboratories	"Jolene Bowers, Megan Folkerts, Chris French, Hayley Yaglom, Ashlyn Pfeiffer, Darrin Lemmer, Dave Engelthaler, The Arizona COVID Genomics Union (ACGU)"
EPI_ISL_968044, EPI_ISL_968045	TGen North	TGen North	"Jolene Bowers, Megan Folkerts, Chris French, Hayley Yaglom, Ashlyn Pfeiffer, Darrin Lemmer, Dave Engelthaler, The Arizona COVID Genomics Union (ACGU)"
EPI_ISL_968046, EPI_ISL_968049	TGen North	Sonora Quest Laboratories	"Jolene Bowers, Megan Folkerts, Chris French, Hayley Yaglom, Ashlyn Pfeiffer, Darrin Lemmer, Dave Engelthaler, The Arizona COVID Genomics Union (ACGU)"
EPI_ISL_968050, EPI_ISL_968051, EPI_ISL_968053	TGen North	TGen North	"Jolene Bowers, Megan Folkerts, Chris French, Hayley Yaglom, Ashlyn Pfeiffer, Darrin Lemmer, Dave Engelthaler, The Arizona COVID Genomics Union (ACGU)"
EPI_ISL_968058, EPI_ISL_968059, EPI_ISL_968061	TGen North	Sonora Quest Laboratories	"Jolene Bowers, Megan Folkerts, Chris French, Hayley Yaglom, Ashlyn Pfeiffer, Darrin Lemmer, Dave Engelthaler, The Arizona COVID Genomics Union (ACGU)"
EPI_ISL_968064	TGen North	TGen North	"Jolene Bowers, Megan Folkerts, Chris French, Hayley Yaglom, Ashlyn Pfeiffer, Darrin Lemmer, Dave Engelthaler, The Arizona COVID Genomics Union (ACGU)"
EPI_ISL_968066	TGen North	Sonora Quest Laboratories	"Jolene Bowers, Megan Folkerts, Chris French, Hayley Yaglom, Ashlyn Pfeiffer, Darrin Lemmer, Dave Engelthaler, The Arizona COVID Genomics Union (ACGU)"
EPI_ISL_968067, EPI_ISL_968068	TGen North	TGen North	"Jolene Bowers, Megan Folkerts, Chris French, Hayley Yaglom, Ashlyn Pfeiffer, Darrin Lemmer, Dave Engelthaler, The Arizona COVID Genomics Union (ACGU)"
EPI_ISL_968079	Monterey County Public Health Laboratory	Monterey County Public Health Laboratory	Monterey County Public Health Laboratory
EPI_ISL_977035, EPI_ISL_977036, EPI_ISL_977037, EPI_ISL_977038, EPI_ISL_977039, EPI_ISL_977040, EPI_ISL_977041, EPI_ISL_977042, EPI_ISL_977043, EPI_ISL_977044, EPI_ISL_977045, EPI_ISL_977046, EPI_ISL_977047, EPI_ISL_977048, EPI_ISL_977049, EPI_ISL_977050, EPI_ISL_977051, EPI_ISL_977052, EPI_ISL_977053, EPI_ISL_977054, EPI_ISL_977055, EPI_ISL_977056, EPI_ISL_977057, EPI_ISL_977058, EPI_ISL_977059, EPI_ISL_977060, EPI_ISL_977061, EPI_ISL_977062, EPI_ISL_977063, EPI_ISL_977064, EPI_ISL_977073, EPI_ISL_977074, EPI_ISL_977075			
see above	Rhode Island Department of Health	Infectious Disease Program, Broad Institute of Harvard and MIT	Lemieux,J.E., Siddle,K.J., Huard,R., King,E., Azevedo,K., Miller,A., Adams,G., Gladden-Young,A., Lagerborg,K., Rudy,M., DeRuff,K., Carter,A., Normandin,E., Bauer,M., Reilly,S., Tomkins-Tinch,C., Loreth,C., Chaluvadi,S., Birren,B.W., Gallagher,G., Smole,S., Park,D.J., MacInnis,B.L., and Sabeti,P.C.
EPI_ISL_977101, EPI_ISL_977112, EPI_ISL_977113, EPI_ISL_977114, EPI_ISL_977118, EPI_ISL_977119	Massachusetts General Hospital	Infectious Disease Program, Broad Institute of Harvard and MIT	Lemieux,J.E., Siddle,K.J., Shaw,B., Adams,G., Pierce,V., Turbett,S., Anahtar,M., Branda,J., Slater,D., Harris,J., Lin,A.E., Gladden-Young,A., Lagerborg,K., Rudy,M., DeRuff,K., Carter,A., Normandin,E., Bauer,M., Reilly,S., Tomkins-Tinch,C., Loreth,C., Chaluvadi,S., Neumann,A., Cusick,C., Chapman,S.B., Gnirke,A., Flowers,K., Cerrato,F., Birren,B.W., Gallagher,G., Smole,S., Park,D.J., MacInnis,B.L., Ryan,E., LaRocque,R., Rosenberg,E. and Sabeti,P.C.
EPI_ISL_977121, EPI_ISL_977122, EPI_ISL_977123, EPI_ISL_977124, EPI_ISL_977125, EPI_ISL_977126, EPI_ISL_977127, EPI_ISL_977128, EPI_ISL_977129	Flow Health	Infectious Disease Program, Broad Institute of Harvard and MIT	Lemieux,J.E., Siddle,K.J., Adams,G., Gladden-Young,A., Lagerborg,K., Rudy,M., DeRuff,K., Carter,A., Normandin,E., Bauer,M., Reilly,S., Tomkins-Tinch,C., Loreth,C., Chaluvadi,S., Birren,B.W., Gallagher,G., Smole,S., Park,D.J., MacInnis,B.L., and Sabeti,P.C.
EPI_ISL_977160, EPI_ISL_977246	Microbiologia e Virologia	Istituto Zooprofilattico Sperimentale delle Venezie	Adelaide Milani, Alessia Schivo, Annalisa Salviato, Erika Giorgia Quaranta, Ambra Pastori, Bianca Zecchin, Alice Fusaro, Isabella Monne, Calogero Terregino, Antonia Ricci
EPI_ISL_977348, EPI_ISL_977349	University of Zambia, School of Veterinary Medicine	UNZAVET and PATH	Mulenga Mwenda-Chimfwembe, Ngonda Saasa, Daniel Bridges
EPI_ISL_977540, EPI_ISL_977550, EPI_ISL_977553, EPI_ISL_977554	Nigeria Centre of Disease Control (NCDC)	African Centre of Excellence for Genomics of Infectious Diseases (ACEGID), Redeemer's University	Olawoye I. B. et al
EPI_ISL_977581, EPI_ISL_977584, EPI_ISL_977594	Caribbean Public Health Agency	Carrington Lab, Department of PreClinical Sciences	Nikita S. D. Sahadeo, Arianne Brown-Jordan, Vernie Ramkissoon, Sarah Hill, Naresh Nandram, Avery Hinds, Dr. Sharon Belmar-George, Jerome Foster, Stanley Giddings, Karla Georges, Marsha Ivey, Rahul Naidu, Risha Singh, SueMin Nathaniel, Rajini Haraksingh, Jaya Jayaraman, Chinna Chinnadurai, Adesh Ramsubhag, Nuno Faria, Oliver Pybus, Christopher Oura, Gabriel Escobar, Christine V. F. Carrington
EPI_ISL_977940, EPI_ISL_977941, EPI_ISL_977942, EPI_ISL_977943, EPI_ISL_977944, EPI_ISL_977945, EPI_ISL_977946, EPI_ISL_977947, EPI_ISL_977948, EPI_ISL_977949, EPI_ISL_977950, EPI_ISL_977951, EPI_ISL_977952, EPI_ISL_977953, EPI_ISL_977954, EPI_ISL_977955, EPI_ISL_977956, EPI_ISL_977957, EPI_ISL_977958, EPI_ISL_977959, EPI_ISL_977960, EPI_ISL_977961, EPI_ISL_977980			
see above	Chiu Laboratory, University of California, San Francisco	Chiu Laboratory, University of California, San Francisco	Charles Chiu, Xianding (Wayne) Deng, Candace Wang, Venice Servellita, Jill Hacker, Debra Wadford
EPI_ISL_978219, EPI_ISL_978220, EPI_ISL_978221	Virginia Division of Consolidated Laboratory Services	Virginia Division of Consolidated Laboratory Services	Virginia DCLS
EPI_ISL_978282, EPI_ISL_978283, EPI_ISL_978284, EPI_ISL_978285, EPI_ISL_978286, EPI_ISL_978287, EPI_ISL_978288, EPI_ISL_978289, EPI_ISL_978290, EPI_ISL_978291, EPI_ISL_978292, EPI_ISL_978293			
see above	Texas Department of State Health Services	Texas Department of State Health Services	Bonnie Oh, Anita Pokharel, James Daniel Bonser, Myong Koag, Chung Wang, Rachel Lee, Grace Kubin, Rashmi Tuladhar, Mayela Pedrueza, Maliha

			Rahman, Jenny Zhang
EPI_ISL_978353, EPI_ISL_978354, EPI_ISL_978355, EPI_ISL_978358, EPI_ISL_978359, EPI_ISL_978360, EPI_ISL_978361, EPI_ISL_978362, EPI_ISL_978396	Arizona State Public Health Laboratory	Arizona State Public Health Laboratory	Trung Huynh, Jessica Escobar, Katherine Fullerton, Nobuko Fukushima, Stacy White, Linda Getsinger, Victor Waddell
EPI_ISL_978811, EPI_ISL_978926, EPI_ISL_978927, EPI_ISL_978928	Centre for Dengue Research and AICBU, Department of Immunology and Molecular Medicine	Centre for Dengue Research and AICBU, Department of Immunology and Molecular Medicine	Chandima Jeewandara, Deshni Jayathilaka, Dinuka Ariyaratne, Tibutius Thanesh Pramanayagam, Diyanath Ranasinghe, Laksiri Gomes, Gathsaurie Neelika Malavige
EPI_ISL_979056, EPI_ISL_979057, EPI_ISL_979058, EPI_ISL_979059, EPI_ISL_979061, EPI_ISL_979062, EPI_ISL_979063, EPI_ISL_979064, EPI_ISL_979065, EPI_ISL_979066, EPI_ISL_979067, EPI_ISL_979068, EPI_ISL_979069, EPI_ISL_979070, EPI_ISL_979071, EPI_ISL_979072, EPI_ISL_979073, EPI_ISL_979074, EPI_ISL_979075, EPI_ISL_979076, EPI_ISL_979077, EPI_ISL_979078, EPI_ISL_979079, EPI_ISL_979080, EPI_ISL_979081, EPI_ISL_979082, EPI_ISL_979083, EPI_ISL_979099, EPI_ISL_979100, EPI_ISL_979101, EPI_ISL_979102, EPI_ISL_979108, EPI_ISL_979109, EPI_ISL_979111, EPI_ISL_979113, EPI_ISL_979114, EPI_ISL_979123, EPI_ISL_979127, EPI_ISL_979128			
see above	Santa Clara County Public Health Laboratory	Chan-Zuckerberg Biohub	CZB Cliahub Consortium
EPI_ISL_979160, EPI_ISL_979161	County of San Luis Obispo Public Health Laboratory	Chan-Zuckerberg Biohub	CZB Cliahub Consortium
EPI_ISL_979260, EPI_ISL_979261, EPI_ISL_979262, EPI_ISL_979263, EPI_ISL_979264, EPI_ISL_979265, EPI_ISL_979266, EPI_ISL_979267	Institute of Microbiology and Immunology, Faculty of Medicine, University of Ljubljana	Institute of Microbiology and Immunology, Faculty of Medicine, University of Ljubljana	Samo Zakotnik, Tomaž Mark Zorec, Matic Brvar, Doroteja Vlaj, Patricija Pozvek,Špela Pleh, Miša Korva, Mario Poljak, Tatjana Avši - Županc
EPI_ISL_979321, EPI_ISL_979323	Cadham Provincial laboratory	National Microbiology Laboratory (NML)	Anna Majer, Shari Tyson, Grace Seo, Philip Mabon, Elsie Grudeski, Rhiannon Huzarewich, Russell Mandes, Anneliese Landgraff, Jennifer Tanner, Natalie Knox, Morag Graham, Gary Van Domselaar, Paul Van Caeseele, Jared Bullard, David Alexander, Kerry Dust, Nathalie Bastien, Yan Li, Timothy Booth, Darian Hole, Madison Chapel, Kirsten Biggar, CanCOGeN's metadata curation team, Public Health Agency of Canada CanCOGeN team
EPI_ISL_979368, EPI_ISL_979370, EPI_ISL_979371, EPI_ISL_979373, EPI_ISL_979374, EPI_ISL_979375, EPI_ISL_979377	The Jackson Laboratory	The Jackson Laboratory	Lloyd M, Sanderson B, Srivastava A, Maurya R, Renzette N, Omerza G, Kelly K, Li L, Wei C L, Adams M
EPI_ISL_980845, EPI_ISL_980852, EPI_ISL_980854, EPI_ISL_980855, EPI_ISL_980859, EPI_ISL_980897, EPI_ISL_980898, EPI_ISL_980899, EPI_ISL_980902, EPI_ISL_980916, EPI_ISL_980920, EPI_ISL_980921, EPI_ISL_980922, EPI_ISL_980935, EPI_ISL_980936, EPI_ISL_980937, EPI_ISL_980977, EPI_ISL_980978, EPI_ISL_980979, EPI_ISL_980980, EPI_ISL_980981			
see above	Innovative Genomics Institute, UC Berkeley	Innovative Genomics Institute, UC Berkeley	Stacia Wyman, Haridha Shivram, Phil Frankino, Liana Lareau
EPI_ISL_981071	Johns Hopkins Hospital Department of Pathology	Johns Hopkins Hospital Department of Pathology	C. Paul Morris, Chun Huai Luo, Adannaya Amadi, Matthew Schwartz, Nicholas Gallagher, Heba H. Mostafa
EPI_ISL_981271, EPI_ISL_981273, EPI_ISL_981274, EPI_ISL_981275, EPI_ISL_981276, EPI_ISL_981277, EPI_ISL_981278, EPI_ISL_981279, EPI_ISL_981280, EPI_ISL_981281, EPI_ISL_981282, EPI_ISL_981283, EPI_ISL_981284, EPI_ISL_981285, EPI_ISL_981286, EPI_ISL_981287, EPI_ISL_981288, EPI_ISL_981289, EPI_ISL_981290, EPI_ISL_981291, EPI_ISL_981292, EPI_ISL_981293, EPI_ISL_981294, EPI_ISL_981296			
see above	Hospital Universitari de Bellvitge	Hospital Universitari Vall d'Hebron	Cristina Andrés, Maria Piñana, Josep F Abril, Damir Garcia-Cehic, Ariadna Rando, Juliana Esperalba, Maria Gema Codina, Carla Castillo, Maria Carmen Martin, Tomàs Pumarola, Josep Quer, Andrés Antón
EPI_ISL_981297, EPI_ISL_981300, EPI_ISL_981304	Hospital Universitari Vall d'Hebron	Hospital Universitari Vall d'Hebron	Cristina Andrés, Maria Piñana, Josep F Abril, Damir Garcia-Cehic, Ariadna Rando, Juliana Esperalba, Maria Gema Codina, Carla Castillo, Maria Carmen Martin, Tomàs Pumarola, Josep Quer, Andrés Antón
EPI_ISL_981964, EPI_ISL_981965, EPI_ISL_981966, EPI_ISL_981969	Microbiology Service, Hospital Universitario Clinico San Cecilio, Granada	Microbiology Service, Hospital Universitario Clinico San Cecilio, Granada	Adolfo de Salazar, Natalia Chueca, Laura Viñuela, Ana Fuentes, Federico García
EPI_ISL_981981, EPI_ISL_982001	TGen North	TGen North	"Jolene Bowers, Megan Folkerts, Chris French, Hayley Yaglom, Ashlyn Pfeiffer, Darrin Lemmer, Dave Engelthaler, The Arizona COVID Genomics Union (ACGU)"
EPI_ISL_982045, EPI_ISL_982064, EPI_ISL_982067	TGen North	Sonora Quest Laboratories	"Jolene Bowers, Megan Folkerts, Chris French, Hayley Yaglom, Ashlyn Pfeiffer, Darrin Lemmer, Dave Engelthaler, The Arizona COVID Genomics Union (ACGU)"
EPI_ISL_982241, EPI_ISL_982245, EPI_ISL_982248, EPI_ISL_982283	Lab voor klinische biologie	Lab voor klinische biologie	Hannelore Hamerlinck, Marija Janevska, Bruno Verhasselt
EPI_ISL_982344, EPI_ISL_982346, EPI_ISL_982347, EPI_ISL_982389, EPI_ISL_982390, EPI_ISL_982391, EPI_ISL_982394, EPI_ISL_982397, EPI_ISL_982398, EPI_ISL_982400, EPI_ISL_982401, EPI_ISL_982402, EPI_ISL_982406, EPI_ISL_982407, EPI_ISL_982408, EPI_ISL_982409, EPI_ISL_982410, EPI_ISL_982411, EPI_ISL_982412, EPI_ISL_982413, EPI_ISL_982414, EPI_ISL_982415, EPI_ISL_982416, EPI_ISL_982417, EPI_ISL_982418, EPI_ISL_982419			
see above	M Health Fairview	Minnesota Department of Health, Public Health Laboratory	Alexandra Lorentz, Jacob Garfin, Matt Plumb, and Xiong Wang
EPI_ISL_982502, EPI_ISL_982503, EPI_ISL_982504	ADIRONDACK MEDICAL CENTER	Wadsworth Center, New York State Department of Health	Kirsten St. George, Daryl M. Lamson, Alexis Russel, Matthew Shudt, Melissa A Leisner, Jonathan Plitnick, Navjot Singh, John Kelly, Erasmus Schneider, Erica Lasek-Nesselquist
EPI_ISL_982529, EPI_ISL_982535, EPI_ISL_982537, EPI_ISL_982545, EPI_ISL_982547, EPI_ISL_982548, EPI_ISL_982550, EPI_ISL_982551, EPI_ISL_982552, EPI_ISL_982555, EPI_ISL_982557, EPI_ISL_982558, EPI_ISL_982561, EPI_ISL_982567, EPI_ISL_982573, EPI_ISL_982576, EPI_ISL_982583, EPI_ISL_982589, EPI_ISL_982596, EPI_ISL_982603, EPI_ISL_982609, EPI_ISL_982615, EPI_ISL_982624, EPI_ISL_982635, EPI_ISL_982637, EPI_ISL_982641, EPI_ISL_982642, EPI_ISL_982647, EPI_ISL_982664, EPI_ISL_982665, EPI_ISL_982666, EPI_ISL_982667, EPI_ISL_982668, EPI_ISL_982670, EPI_ISL_982674, EPI_ISL_982684, EPI_ISL_982693, EPI_ISL_982695, EPI_ISL_982707, EPI_ISL_982708, EPI_ISL_982709, EPI_ISL_982720, EPI_ISL_982721, EPI_ISL_982722, EPI_ISL_982723, EPI_ISL_982724, EPI_ISL_982725, EPI_ISL_982726, EPI_ISL_982727, EPI_ISL_982732, EPI_ISL_982733, EPI_ISL_982738, EPI_ISL_982742, EPI_ISL_982746, EPI_ISL_982758, EPI_ISL_982760			
see above	US Air Force School of Aerospace Medicine	US Air Force School of Aerospace Medicine	Anthony Fries, Jennifer Meyer, William Gruner, William Buggele, Amanda Javorina, Sarah Purves, Clarise Starr, Elizabeth Macias
EPI_ISL_983063, EPI_ISL_983086, EPI_ISL_983087	Gravity Diagnostics	Kentucky State Public Health Lab	Stephanie Lunn, Karim George, Joshua Tobias, William Grooms, Vaneet Arora, Matthew Johnson, Rachel Zinner, Rhonda Lucas
EPI_ISL_983118	SUNY UPSTATE MEDICAL UNIVERSITY	Wadsworth Center, New York State Department of Health	Kirsten St. George, Daryl M. Lamson, Alexis Russel, Matthew Shudt, Melissa A Leisner, Jonathan Plitnick, Navjot Singh, John Kelly, Erasmus Schneider, Erica Lasek-Nesselquist
EPI_ISL_983130, EPI_ISL_983131	THE MARY IMOGENE BASSETT HOSPITAL	Wadsworth Center, New York State Department of Health	Kirsten St. George, Daryl M. Lamson, Alexis Russel, Matthew Shudt, Melissa A Leisner, Jonathan Plitnick, Navjot Singh, John Kelly, Erasmus Schneider, Erica Lasek-Nesselquist
EPI_ISL_983321	Eurofins Genoma Group s.r.l.	INMI Lazzaro Spallanzani IRCCS	O Butera, F Messina, CEM Gruber, B Bartolini, E Giombini, M Rueca, F Spinella, F Fiorentino, MR Capobianchi, A Di Caro
EPI_ISL_983616, EPI_ISL_983617	Texas Department of State Health Services	Texas Department of State Health Services	Bonnie Oh, Anita Pokharel, James Daniel Bonser, Myong Koag, Chung Wang, Rachel Lee, Grace Kubin, Rashmi Tuladhar, Mayela Pedrueza, Maliha Rahman, Jenny Zhang
EPI_ISL_983698	Vault Health	Minnesota Department of Health, Public Health Laboratory	Alexandra Lorentz, Jacob Garfin, Matt Plumb, and Xiong Wang
EPI_ISL_983710, EPI_ISL_983717, EPI_ISL_983801, EPI_ISL_983802, EPI_ISL_983803, EPI_ISL_983804	Colorado Department of Public Health and Environment	Colorado Department of Puplic Health and Environment	Laura Bankers, Molly C. Hetherington-Rauth, Diana Ir, Shannon Ely, Shannon R. Matzinger, Sarah Elizabeth Totten, Emily A. Travanty
