# Supplementary material for: Detection and characterization of the SARS-CoV-2 lineage B.1.526 in New York
Source: Nat Commun. 2021 Aug 9;12:4886. doi: 10.1038/s41467-021-25168-4 (PMC8352861; doi:10.1038/s41467-021-25168-4)
Supplement: Supplementary file 8 — Supplementary Data 4 [file 41467_2021_25168_MOESM8_ESM.zip › GISAID_acknowledements_tables/gisaid_hcov-19_acknowledgement_table_2021_02_13_010-10.pdf]

We gratefully acknowledge the following Authors from the Originating laboratories responsible for obtaining the specimens, as well as the Submitting laboratories where the genome data were generated and shared via GISAID, on which this research is based.

All Submitters of data may be contacted directly via www.gisaid.org

Authors are sorted alphabetically.

Accession ID	Originating Laboratory	Submitting Laboratory	Authors
EPI_ISL_803109	Pathology West - NSW Health Pathology	NSW Health Pathology - Institute of Clinical Pathology and Medical Research; Westmead Hospital; University of Sydney	CIDM-PH et al.
EPI_ISL_825550, EPI_ISL_825551, EPI_ISL_825573, EPI_ISL_825574	Respiratory Virus Unit, National Infection Service, Public Health England	COVID-19 Genomics UK (COG-UK) Consortium	PHE Covid Sequencing Team
EPI_ISL_830726	Wadsworth Center, New York State Department of Health	Wadsworth Center, New York State Department of Health	Kirsten St. George, Daryl M. Lamson, Alexis Russel, Matthew Shudt, Melissa A Leisner, Jonathan Plitnick, Navjot Singh, John Kelly, Erasmus Schneider, Erica Lasek-Nesselquist
EPI_ISL_831669	Institute of Virology, Biomedical Research Center of the Slovak Academy of Sciences, Bratislava	Faculty of Natural Sciences, Comenius University, Bratislava	Kristína Boršová, Viktória abanová, Broa Brejová, Viktória Hodorová, Sabina Fumaová Havlíková, Juraj Kopáek, Martina Liková, ubomíra Lukáiková, Martina Neboháová, Monika Sláviková, Tomáš Vina, Boris Klempa, Jozef Nosek
EPI_ISL_831670	Institute of Virology, Biomedical Research Center of the Slovak Academy of Sciences, Bratislava	Faculty of Natural Sciences, Comenius University, Bratislava	Viktória abanová, Kristína Boršová, Broa Brejová, Viktória Hodorová, Sabina Fumaová Havlíková, Juraj Kopáek, Martina Liková, ubomíra Lukáiková, Martina Neboháová, Monika Sláviková, Tomáš Vina, Jozef Nosek, Boris Klempa
EPI_ISL_831671	Institute of Virology, Biomedical Research Center of the Slovak Academy of Sciences, Bratislava	Faculty of Natural Sciences, Comenius University, Bratislava	Broa Brejová, Viktória abanová, Kristína Boršová, Viktória Hodorová, Sabina Fumaová Havlíková, Juraj Kopáek, Martina Liková, ubomíra Lukáiková, Martina Neboháová, Monika Sláviková, Tomáš Vina, Jozef Nosek, Boris Klempa
EPI_ISL_831673	Institute of Virology, Biomedical Research Center of the Slovak Academy of Sciences, Bratislava	Faculty of Natural Sciences, Comenius University, Bratislava	Kristína Boršová, Viktória abanová, Broa Brejová, Viktória Hodorová, Sabina Fumaová Havlíková, Juraj Kopáek, Martina Liková, ubomíra Lukáiková, Martina Neboháová, Monika Sláviková, Tomáš Vina, Boris Klempa, Jozef Nosek
EPI_ISL_832242	Department of Clinical Microbiology	GIGA Medical Genomics	Keith Durkin, Maria Artesi, Sébastien Bontems, Raphaël Boreux, Bouchra Boujemla, Cécile Meex, Pierrette Melin, Marie-Pierre Hayette, Vincent Bours
EPI_ISL_833378, EPI_ISL_833387	National Public Health Laboratory, National Centre for Infectious Diseases	National Public Health Laboratory, National Centre for Infectious Diseases	Tze Minn Mak, Sophie Octavia, Zhenyang Zhou, Lin Cui, Raymond Tzer Pin Lin
EPI_ISL_833434, EPI_ISL_833481, EPI_ISL_833483, EPI_ISL_833484, EPI_ISL_833486	UZA/UAntwerpen (BIS lab), University Hospital Antwerp, Edegem, Belgium	Laboratory of Medical Microbiology, University of Antwerp, Campus Drie Eiken, S6.26, Universiteitsplein 1, 2610, Wilrijk, Antwerp, Belgium	Basil Britto Xavier, Jasmine Coppens, Christine Lammens, Veerle Matheeussen, Herman Goossens
EPI_ISL_837057, EPI_ISL_837182	Respiratory Virus Unit, National Infection Service, Public Health England	COVID-19 Genomics UK (COG-UK) Consortium	PHE Covid Sequencing Team
EPI_ISL_837973, EPI_ISL_837975, EPI_ISL_837978, EPI_ISL_837984, EPI_ISL_837985, EPI_ISL_837990, EPI_ISL_838010, EPI_ISL_838014, EPI_ISL_838019, EPI_ISL_838020, EPI_ISL_838023, EPI_ISL_838024, EPI_ISL_838027, EPI_ISL_838028, EPI_ISL_838029, EPI_ISL_838030, EPI_ISL_838031, EPI_ISL_838033, EPI_ISL_838036, EPI_ISL_838039, EPI_ISL_838040, EPI_ISL_838041, EPI_ISL_838042, EPI_ISL_838043, EPI_ISL_838044, EPI_ISL_838045, EPI_ISL_838046, EPI_ISL_838047			
see above	Department of Pathology, University of Cambridge	COVID-19 Genomics UK (COG-UK) Consortium	Aminu S. Jahun, Yasmin Chaudhry, Grant Hall, Iliana Georgana, Myra Hosmillo, Martin D. Curran, Malte Pinckert, Surendra Parmar, Ian Goodfellow
EPI_ISL_838997, EPI_ISL_838998, EPI_ISL_839014, EPI_ISL_839016, EPI_ISL_839017, EPI_ISL_839018, EPI_ISL_839019, EPI_ISL_839021, EPI_ISL_839022, EPI_ISL_839023, EPI_ISL_839024, EPI_ISL_839025			
see above	University College London, Great Ormond Street Hospital for Children NHS Foundation Trust, Imperial College Healthcare NHS Trust	COVID-19 Genomics UK (COG-UK) Consortium	Sergi Castellano, Rachel Williams, Mark Kristiansen, Paola Resende Silva, Sunando Roy, Tony Brooks, Helena Tutill, Paola Niola, Patricia Dyal, Charlotte Williams, Leysa Forrest, Yasmin Panchbhaya, Jacqueline Findlay, Samuel Weeks, Julianne Brown, Kathryn Harris, Paul Randell, James Price, Alison Holmes, Judith Breuer
EPI_ISL_840061	Queens Medical Centre, Clinical Microbiology Department / DeepSeq Nottingham	COVID-19 Genomics UK (COG-UK) Consortium	Gemma Clark, Wendy Smith, Manjinder Khakh, Vicki M Fleming, Michelle M Lister, Hannah Howson-Wells, Jonathan Ball, Patrick McClure, Joseph Chappell, Theocharis Tsoleridis, Nadine Holmes, Matthew Carlisle, Christopher Moore, Fei Sang, Johnny Debebe, Victoria Wright, Matthew Loose
EPI_ISL_841526, EPI_ISL_841543, EPI_ISL_841544, EPI_ISL_841545, EPI_ISL_841546, EPI_ISL_841552, EPI_ISL_841555, EPI_ISL_841556, EPI_ISL_841741, EPI_ISL_841742, EPI_ISL_841743, EPI_ISL_841744, EPI_ISL_841745, EPI_ISL_841746, EPI_ISL_841747, EPI_ISL_841748, EPI_ISL_841749, EPI_ISL_841750, EPI_ISL_841751, EPI_ISL_841752, EPI_ISL_841753			
see above	Originating lab: Wales Specialist Virology Centre Sequencing lab: Pathogen Genomics Unit	Public Health Wales Microbiology Cardiff Wales Specialist Virology Centre	Catherine Moore, Johnathan Evans, Laura Gifford, Malorie Perry, Simon Cottrell, Angela Marchbank, Alec Birchley, Alexander Adams, Amy Gaskin, Bree Gatica-Wilcox, Jason Coombes, Joel Southgate, Lauren Gilbert, Lee Graham, Nicole Pacchiarini, Sara Kumziene-Summerhayes, Sarah Taylor, Sophie Jones, Sara Rey, Matthew Bull, Joanne Watkins, Sally Corden, Tom Connor
EPI_ISL_842201, EPI_ISL_842210	Virology Department, Sheffield Teaching Hospitals NHS Foundation Trust/Department of Infection, Immunity and Cardiovascular Disease, The Medical School, University of Sheffield	COVID-19 Genomics UK (COG-UK) Consortium	Thushan de Silva, Matthew Parker, Nikki Smith, Adri Angyal, Rebecca Brown, Luke Green, Rachel Tucker, Paul Parsons, Danielle Groves, Katie Johnson, Laura Carrilero, Alex Keeley, Dave Partridge, Matthew Wyles, Benjamin Lindsey, Mehmet Yavuz, Mohammad Raza, Cariad Evans
EPI_ISL_843188	Maryland Public Health Laboratory	Maryland Public Health Laboratory	Maryland Department of Health Laboratories Administration
EPI_ISL_844543, EPI_ISL_844545, EPI_ISL_844552, EPI_ISL_844553, EPI_ISL_844556, EPI_ISL_844557, EPI_ISL_844558, EPI_ISL_844559, EPI_ISL_844560, EPI_ISL_844561, EPI_ISL_844562, EPI_ISL_844563, EPI_ISL_844564, EPI_ISL_844565, EPI_ISL_844566, EPI_ISL_844567, EPI_ISL_844568, EPI_ISL_844569, EPI_ISL_844570, EPI_ISL_844571, EPI_ISL_844572, EPI_ISL_844573, EPI_ISL_844574, EPI_ISL_844575, EPI_ISL_844576, EPI_ISL_844577, EPI_ISL_844635, EPI_ISL_844636, EPI_ISL_844637, EPI_ISL_844638, EPI_ISL_844639, EPI_ISL_844653, EPI_ISL_844654, EPI_ISL_844655, EPI_ISL_844666, EPI_ISL_844667, EPI_ISL_844669, EPI_ISL_844670, EPI_ISL_844676, EPI_ISL_844680, EPI_ISL_844681, EPI_ISL_844693, EPI_ISL_844694, EPI_ISL_844702, EPI_ISL_844706, EPI_ISL_844707, EPI_ISL_844708, EPI_ISL_844716, EPI_ISL_844717, EPI_ISL_844718, EPI_ISL_844730, EPI_ISL_844731, EPI_ISL_844732, EPI_ISL_844733, EPI_ISL_844734, EPI_ISL_844735, EPI_ISL_844736, EPI_ISL_844737, EPI_ISL_844738, EPI_ISL_844747, EPI_ISL_844758, EPI_ISL_844759, EPI_ISL_844763, EPI_ISL_844766, EPI_ISL_844767, EPI_ISL_844768, EPI_ISL_844788, EPI_ISL_844805, EPI_ISL_844807, EPI_ISL_844808, EPI_ISL_844818, EPI_ISL_844821, EPI_ISL_844824, EPI_ISL_844834, EPI_ISL_844836, EPI_ISL_844837, EPI_ISL_844842, EPI_ISL_844843, EPI_ISL_844844, EPI_ISL_844845, EPI_ISL_844846, EPI_ISL_844847, EPI_ISL_844848, EPI_ISL_844849, EPI_ISL_844860, EPI_ISL_844861, EPI_ISL_844862, EPI_ISL_844863, EPI_ISL_844864, EPI_ISL_844865, EPI_ISL_844911, EPI_ISL_844912, EPI_ISL_844931, EPI_ISL_844932, EPI_ISL_844948, EPI_ISL_844951, EPI_ISL_844953, EPI_ISL_844954, EPI_ISL_844955, EPI_ISL_844956, EPI_ISL_844957, EPI_ISL_844958, EPI_ISL_844978, EPI_ISL_844981, EPI_ISL_844988, EPI_ISL_844991, EPI_ISL_844992, EPI_ISL_844993, EPI_ISL_844998, EPI_ISL_844999, EPI_ISL_845000, EPI_ISL_845001, EPI_ISL_845002, EPI_ISL_845003, EPI_ISL_845004, EPI_ISL_845005, EPI_ISL_845006, EPI_ISL_845007, EPI_ISL_845008, EPI_ISL_845009, EPI_ISL_845010, EPI_ISL_845011, EPI_ISL_845012, EPI_ISL_845013, EPI_ISL_845014, EPI_ISL_845015, EPI_ISL_845016, EPI_ISL_845017, EPI_ISL_845018, EPI_ISL_845019, EPI_ISL_845020, EPI_ISL_845021, EPI_ISL_845022, EPI_ISL_845023, EPI_ISL_845024, EPI_ISL_845025, EPI_ISL_845026, EPI_ISL_845027, EPI_ISL_845028, EPI_ISL_845029, EPI_ISL_845030, EPI_ISL_845031, EPI_ISL_845032, EPI_ISL_845033, EPI_ISL_845034, EPI_ISL_845035, EPI_ISL_845036, EPI_ISL_845037, EPI_ISL_845038, EPI_ISL_845039, EPI_ISL_845040, EPI_ISL_845041, EPI_ISL_845042, EPI_ISL_845043, EPI_ISL_845044, EPI_ISL_845045, EPI_ISL_845046, EPI_ISL_845047, EPI_ISL_845048, EPI_ISL_845049, EPI_ISL_845050, EPI_ISL_845051, EPI_ISL_845052, EPI_ISL_845053, EPI_ISL_845054, EPI_ISL_845055, EPI_ISL_845056, EPI_ISL_845057, EPI_ISL_845058, EPI_ISL_845059, EPI_ISL_845060, EPI_ISL_845061, EPI_ISL_845062, EPI_ISL_845063, EPI_ISL_845064, EPI_ISL_845065, EPI_ISL_845066, EPI_ISL_845067, EPI_ISL_845068, EPI_ISL_845069, EPI_ISL_845070, EPI_ISL_845071, EPI_ISL_845072, EPI_ISL_845073, EPI_ISL_845074, EPI_ISL_845075, EPI_ISL_845076, EPI_ISL_845077, EPI_ISL_845078, EPI_ISL_845079, EPI_ISL_845080, EPI_ISL_845275, EPI_ISL_845276, EPI_ISL_845277, EPI_ISL_845278, EPI_ISL_845279, EPI_ISL_845280, EPI_ISL_845377, EPI_ISL_845378, EPI_ISL_845379, EPI_ISL_845380, EPI_ISL_845381, EPI_ISL_845382, EPI_ISL_845383, EPI_ISL_845384, EPI_ISL_845385, EPI_ISL_845386, EPI_ISL_845387, EPI_ISL_845388, EPI_ISL_845389, EPI_ISL_845390, EPI_ISL_845391, EPI_ISL_845392, EPI_ISL_845393, EPI_ISL_845394, EPI_ISL_845395, EPI_ISL_845396, EPI_ISL_845397, EPI_ISL_845398, EPI_ISL_845399, EPI_ISL_845498, EPI_ISL_845499, EPI_ISL_845500			
see above	Department of Virus and Microbiological Special Diagnostics, Statens Serum Institut, Copenhagen, Denmark	Albertsen Lab, Department of Chemistry and Bioscience, Aalborg University, Denmark	Danish Covid-19 Genome Consortium
EPI_ISL_846544, EPI_ISL_846550, EPI_ISL_846551, EPI_ISL_846558, EPI_ISL_846561, EPI_ISL_846562, EPI_ISL_846563, EPI_ISL_846564, EPI_ISL_846565, EPI_ISL_846566, EPI_ISL_846567, EPI_ISL_846577			
see above	Lab voor klinische biologie	Lab voor klinische biologie	Hannelore Hamerlinck, Bruno Verhasselt
EPI_ISL_846775, EPI_ISL_846776, EPI_ISL_846777, EPI_ISL_846778, EPI_ISL_846780, EPI_ISL_846782, EPI_ISL_846783, EPI_ISL_846784, EPI_ISL_846785, EPI_ISL_846786, EPI_ISL_846787, EPI_ISL_846788, EPI_ISL_846789, EPI_ISL_846790, EPI_ISL_846791, EPI_ISL_846792, EPI_ISL_846793, EPI_ISL_846794, EPI_ISL_846795, EPI_ISL_846796, EPI_ISL_846797, EPI_ISL_846798, EPI_ISL_846799, EPI_ISL_846800, EPI_ISL_846801, EPI_ISL_846802, EPI_ISL_846803, EPI_ISL_846804, EPI_ISL_846805, EPI_ISL_846806, EPI_ISL_846807, EPI_ISL_846808, EPI_ISL_846809, EPI_ISL_846810, EPI_ISL_846811, EPI_ISL_846821, EPI_ISL_846822, EPI_ISL_846823, EPI_ISL_846824, EPI_ISL_846825, EPI_ISL_846826, EPI_ISL_846827, EPI_ISL_846828, EPI_ISL_846829, EPI_ISL_846830, EPI_ISL_846831, EPI_ISL_846832, EPI_ISL_846833, EPI_ISL_846834, EPI_ISL_846835, EPI_ISL_846836, EPI_ISL_846837, EPI_ISL_846838, EPI_ISL_846839, EPI_ISL_846840, EPI_ISL_846841, EPI_ISL_846842, EPI_ISL_846843, EPI_ISL_846844, EPI_ISL_846845, EPI_ISL_846846, EPI_ISL_846847, EPI_ISL_846848, EPI_ISL_846849, EPI_ISL_846850, EPI_ISL_846854, EPI_ISL_846855, EPI_ISL_846856, EPI_ISL_846857, EPI_ISL_846858, EPI_ISL_846859, EPI_ISL_846862, EPI_ISL_846863, EPI_ISL_846864, EPI_ISL_846865, EPI_ISL_846867, EPI_ISL_846868, EPI_ISL_846869, EPI_ISL_846870, EPI_ISL_846871, EPI_ISL_846872, EPI_ISL_846873, EPI_ISL_846874, EPI_ISL_846875, EPI_ISL_846876, EPI_ISL_846878, EPI_ISL_846879, EPI_ISL_846880, EPI_ISL_846881, EPI_ISL_846882,			

EPI_ISL_846883, EPI_ISL_846885, EPI_ISL_846887, EPI_ISL_846890, EPI_ISL_846891, EPI_ISL_846892, EPI_ISL_846896, EPI_ISL_846897, EPI_ISL_846899, EPI_ISL_846900, EPI_ISL_846901, EPI_ISL_846902, EPI_ISL_846903, EPI_ISL_846905, EPI_ISL_846906, EPI_ISL_846907, EPI_ISL_846908, EPI_ISL_846909, EPI_ISL_846911, EPI_ISL_846912, EPI_ISL_846914, EPI_ISL_846915, EPI_ISL_846916, EPI_ISL_846917, EPI_ISL_846918, EPI_ISL_846919, EPI_ISL_846920, EPI_ISL_846921, EPI_ISL_846922, EPI_ISL_846923, EPI_ISL_846924, EPI_ISL_846925, EPI_ISL_846926, EPI_ISL_846927, EPI_ISL_846928, EPI_ISL_846929, EPI_ISL_846930, EPI_ISL_846931, EPI_ISL_846932, EPI_ISL_846933, EPI_ISL_846934, EPI_ISL_846935, EPI_ISL_846936, EPI_ISL_846937, EPI_ISL_846938, EPI_ISL_846939, EPI_ISL_846940, EPI_ISL_846941, EPI_ISL_846942, EPI_ISL_846943, EPI_ISL_846944, EPI_ISL_846945, EPI_ISL_846946, EPI_ISL_846947, EPI_ISL_846948, EPI_ISL_846949, EPI_ISL_846950, EPI_ISL_846951, EPI_ISL_846952, EPI_ISL_846953, EPI_ISL_846954, EPI_ISL_846955, EPI_ISL_846956, EPI_ISL_846957, EPI_ISL_846958, EPI_ISL_846959, EPI_ISL_846960, EPI_ISL_846961, EPI_ISL_846962, EPI_ISL_846963, EPI_ISL_846964, EPI_ISL_846965, EPI_ISL_846966, EPI_ISL_846967, EPI_ISL_846968, EPI_ISL_846969, EPI_ISL_846970, EPI_ISL_846971, EPI_ISL_846972, EPI_ISL_846973, EPI_ISL_846974, EPI_ISL_846975, EPI_ISL_846976, EPI_ISL_846977, EPI_ISL_846978, EPI_ISL_846979, EPI_ISL_846980, EPI_ISL_846981, EPI_ISL_846982, EPI_ISL_846983, EPI_ISL_846984, EPI_ISL_846985, EPI_ISL_846986, EPI_ISL_846987, EPI_ISL_846988, EPI_ISL_846989, EPI_ISL_846990, EPI_ISL_846991, EPI_ISL_846992, EPI_ISL_846993, EPI_ISL_846994, EPI_ISL_846995, EPI_ISL_846996, EPI_ISL_846997, EPI_ISL_846998, EPI_ISL_846999, EPI_ISL_847000, EPI_ISL_847001, EPI_ISL_847002, EPI_ISL_847003, EPI_ISL_847004, EPI_ISL_847005, EPI_ISL_847006, EPI_ISL_847007, EPI_ISL_847008, EPI_ISL_847009, EPI_ISL_847010, EPI_ISL_847011, EPI_ISL_847012, EPI_ISL_847013, EPI_ISL_847014, EPI_ISL_847015, EPI_ISL_847016, EPI_ISL_847017, EPI_ISL_847018, EPI_ISL_847019, EPI_ISL_847020, EPI_ISL_847021, EPI_ISL_847022, EPI_ISL_847023, EPI_ISL_847024, EPI_ISL_847025, EPI_ISL_847026, EPI_ISL_847027, EPI_ISL_847028, EPI_ISL_847029, EPI_ISL_847030, EPI_ISL_847031, EPI_ISL_847032, EPI_ISL_847033, EPI_ISL_847034, EPI_ISL_847035, EPI_ISL_847036, EPI_ISL_847037, EPI_ISL_847038, EPI_ISL_847039, EPI_ISL_847040, EPI_ISL_847041, EPI_ISL_847042, EPI_ISL_847043, EPI_ISL_847044, EPI_ISL_847045, EPI_ISL_847046, EPI_ISL_847047, EPI_ISL_847048, EPI_ISL_847049, EPI_ISL_847050, EPI_ISL_847051, EPI_ISL_847052, EPI_ISL_847053, EPI_ISL_847054, EPI_ISL_847055, EPI_ISL_847056, EPI_ISL_847057, EPI_ISL_847058, EPI_ISL_847059, EPI_ISL_847060, EPI_ISL_847061, EPI_ISL_847062, EPI_ISL_847063, EPI_ISL_847064, EPI_ISL_847065, EPI_ISL_847066, EPI_ISL_847067, EPI_ISL_847068, EPI_ISL_847069, EPI_ISL_847070, EPI_ISL_847071, EPI_ISL_847072, EPI_ISL_847073, EPI_ISL_847074, EPI_ISL_847075, EPI_ISL_847076, EPI_ISL_847077, EPI_ISL_847078, EPI_ISL_847079, EPI_ISL_847080, EPI_ISL_847081, EPI_ISL_847082, EPI_ISL_847083, EPI_ISL_847084, EPI_ISL_847085, EPI_ISL_847086, EPI_ISL_847087, EPI_ISL_847088, EPI_ISL_847089, EPI_ISL_847090, EPI_ISL_847091, EPI_ISL_847092, EPI_ISL_847093, EPI_ISL_847094, EPI_ISL_847095, EPI_ISL_847096, EPI_ISL_847097, EPI_ISL_847098, EPI_ISL_847099, EPI_ISL_847100, EPI_ISL_847101, EPI_ISL_847102, EPI_ISL_847103, EPI_ISL_847104, EPI_ISL_847105, EPI_ISL_847106, EPI_ISL_847107, EPI_ISL_847108, EPI_ISL_847109, EPI_ISL_847110, EPI_ISL_847111, EPI_ISL_847112, EPI_ISL_847113, EPI_ISL_847114, EPI_ISL_847115, EPI_ISL_847116, EPI_ISL_847117, EPI_ISL_847118, EPI_ISL_847119, EPI_ISL_847120, EPI_ISL_847121, EPI_ISL_847122, EPI_ISL_847123, EPI_ISL_847124, EPI_ISL_847180, EPI_ISL_847181, EPI_ISL_847182, EPI_ISL_847183, EPI_ISL_847184, EPI_ISL_847185, EPI_ISL_847186, EPI_ISL_847187, EPI_ISL_847188, EPI_ISL_847189, EPI_ISL_847190, EPI_ISL_847191, EPI_ISL_847192, EPI_ISL_847193, EPI_ISL_847194, EPI_ISL_847195, EPI_ISL_847196, EPI_ISL_847197, EPI_ISL_847198, EPI_ISL_847206, EPI_ISL_847207, EPI_ISL_847208, EPI_ISL_847209, EPI_ISL_847210, EPI_ISL_847211, EPI_ISL_847212, EPI_ISL_847213, EPI_ISL_847214, EPI_ISL_847215, EPI_ISL_847216, EPI_ISL_847217, EPI_ISL_847218, EPI_ISL_847219, EPI_ISL_847220, EPI_ISL_847221, EPI_ISL_847222, EPI_ISL_847223, EPI_ISL_847224, EPI_ISL_847225, EPI_ISL_847226, EPI_ISL_847227, EPI_ISL_847228, EPI_ISL_847229, EPI_ISL_847230, EPI_ISL_847231, EPI_ISL_847232, EPI_ISL_847238, EPI_ISL_847239, EPI_ISL_847240, EPI_ISL_847241, EPI_ISL_847242, EPI_ISL_847243, EPI_ISL_847250, EPI_ISL_847251, EPI_ISL_847257, EPI_ISL_847258, EPI_ISL_847259, EPI_ISL_847261, EPI_ISL_847265, EPI_ISL_847267, EPI_ISL_847268, EPI_ISL_847269, EPI_ISL_847271, EPI_ISL_847272, EPI_ISL_847273, EPI_ISL_847275, EPI_ISL_847276, EPI_ISL_847277, EPI_ISL_847278, EPI_ISL_847279, EPI_ISL_847281, EPI_ISL_847282, EPI_ISL_847283, EPI_ISL_847284, EPI_ISL_847285, EPI_ISL_847286, EPI_ISL_847287, EPI_ISL_847288, EPI_ISL_847289, EPI_ISL_847290, EPI_ISL_847291, EPI_ISL_847292, EPI_ISL_847293, EPI_ISL_847294, EPI_ISL_847295, EPI_ISL_847296, EPI_ISL_847297, EPI_ISL_847298, EPI_ISL_847299, EPI_ISL_847300, EPI_ISL_847301, EPI_ISL_847302, EPI_ISL_847303, EPI_ISL_847304, EPI_ISL_847305, EPI_ISL_847306, EPI_ISL_847307, EPI_ISL_847308, EPI_ISL_847309, EPI_ISL_847310, EPI_ISL_847311, EPI_ISL_847312, EPI_ISL_847313, EPI_ISL_847314, EPI_ISL_847315, EPI_ISL_847316, EPI_ISL_847317, EPI_ISL_847318, EPI_ISL_847319, EPI_ISL_847321, EPI_ISL_847322, EPI_ISL_847323, EPI_ISL_847324, EPI_ISL_847325, EPI_ISL_847326, EPI_ISL_847327, EPI_ISL_847328, EPI_ISL_847329, EPI_ISL_847330, EPI_ISL_847331, EPI_ISL_847333, EPI_ISL_847334, EPI_ISL_847336, EPI_ISL_847338, EPI_ISL_847339, EPI_ISL_847340, EPI_ISL_847341, EPI_ISL_847342, EPI_ISL_847343, EPI_ISL_847344, EPI_ISL_847345, EPI_ISL_847346, EPI_ISL_847347, EPI_ISL_847350, EPI_ISL_847354, EPI_ISL_847355, EPI_ISL_847356, EPI_ISL_847357, EPI_ISL_847359, EPI_ISL_847360, EPI_ISL_847361, EPI_ISL_847362, EPI_ISL_847363, EPI_ISL_847364, EPI_ISL_847365, EPI_ISL_847367, EPI_ISL_847371, EPI_ISL_847372, EPI_ISL_847373, EPI_ISL_847374, EPI_ISL_847375, EPI_ISL_847376, EPI_ISL_847378, EPI_ISL_847379, EPI_ISL_847380, EPI_ISL_847381, EPI_ISL_847382, EPI_ISL_847383, EPI_ISL_847384, EPI_ISL_847385, EPI_ISL_847386, EPI_ISL_847387, EPI_ISL_847388, EPI_ISL_847389, EPI_ISL_847390, EPI_ISL_847391, EPI_ISL_847392, EPI_ISL_847393, EPI_ISL_847394, EPI_ISL_847395, EPI_ISL_847396, EPI_ISL_847397, EPI_ISL_847398, EPI_ISL_847399, EPI_ISL_847400, EPI_ISL_847401, EPI_ISL_847402, EPI_ISL_847403, EPI_ISL_847404, EPI_ISL_847405, EPI_ISL_847406, EPI_ISL_847407, EPI_ISL_847408, EPI_ISL_847409, EPI_ISL_847410, EPI_ISL_847411, EPI_ISL_847412, EPI_ISL_847413, EPI_ISL_847414, EPI_ISL_847415, EPI_ISL_847416, EPI_ISL_847417, EPI_ISL_847418, EPI_ISL_847419, EPI_ISL_847420, EPI_ISL_847421, EPI_ISL_847422, EPI_ISL_847423, EPI_ISL_847424, EPI_ISL_847425, EPI_ISL_847426, EPI_ISL_847427, EPI_ISL_847428, EPI_ISL_847429, EPI_ISL_847430, EPI_ISL_847431, EPI_ISL_847432, EPI_ISL_847433, EPI_ISL_847434, EPI_ISL_847435, EPI_ISL_847436, EPI_ISL_847437, EPI_ISL_847438, EPI_ISL_847439, EPI_ISL_847440, EPI_ISL_847441, EPI_ISL_847442, EPI_ISL_847443, EPI_ISL_847444, EPI_ISL_847445, EPI_ISL_847446, EPI_ISL_847447, EPI_ISL_847448, EPI_ISL_847449, EPI_ISL_847450, EPI_ISL_847451, EPI_ISL_847452, EPI_ISL_847453, EPI_ISL_847454, EPI_ISL_847455, EPI_ISL_847456, EPI_ISL_847457, EPI_ISL_847458, EPI_ISL_847459, EPI_ISL_847460, EPI_ISL_847461, EPI_ISL_847462, EPI_ISL_847463, EPI_ISL_847464, EPI_ISL_847465, EPI_ISL_847466, EPI_ISL_847467, EPI_ISL_847468, EPI_ISL_847469, EPI_ISL_847470, EPI_ISL_847471, EPI_ISL_847472, EPI_ISL_847473, EPI_ISL_847474, EPI_ISL_847475, EPI_ISL_847476, EPI_ISL_847477, EPI_ISL_847478, EPI_ISL_847479, EPI_ISL_847480, EPI_ISL_847481, EPI_ISL_847482, EPI_ISL_847483, EPI_ISL_847484, EPI_ISL_847485			
see above	Department of Virus and Microbiological Special Diagnostics, Statens Serum Institut, Copenhagen, Denmark	Albertsen Lab, Department of Chemistry and Bioscience, Aalborg University, Denmark	Danish Covid-19 Genome Consortium
EPI_ISL_847564	California Department of Public Health	Chiu Laboratory, University of California, San Francisco	Charles Chiu, Xianding (Wayne) Deng, Candace Wang, Brian Bushnell, Scot Federman, Jill Hacker, Debra Wadford
EPI_ISL_849758, EPI_ISL_849759, EPI_ISL_849760	unknown	PHV-FSS	Son Nguyen et al.
EPI_ISL_851101, EPI_ISL_851126, EPI_ISL_851142, EPI_ISL_851180, EPI_ISL_851187, EPI_ISL_851190, EPI_ISL_851196, EPI_ISL_851217, EPI_ISL_851229, EPI_ISL_851236, EPI_ISL_851241, EPI_ISL_851258, EPI_ISL_851329, EPI_ISL_851365, EPI_ISL_851374, EPI_ISL_851674			
see above	Lighthouse Lab in Glasgow	Wellcome Sanger Institute for the COVID-19 Genomics UK (COG-UK) Consortium	Harper VanSteenhouse, Yumi Kasai, David Gray, Carol Clugston, Anna Dominiczak and Alex Alderton, Roberto Amato, Sonia Goncalves, Ewan Harrison, David K. Jackson, Ian Johnston, Dominic Kwiatkowski, Cordelia Langford, John Sillitoe on behalf of the Wellcome Sanger Institute COVID-19 Surveillance Team
EPI_ISL_852949, EPI_ISL_852951, EPI_ISL_852956, EPI_ISL_852957, EPI_ISL_852959, EPI_ISL_852960, EPI_ISL_852965, EPI_ISL_852966, EPI_ISL_852967, EPI_ISL_852969, EPI_ISL_852970, EPI_ISL_852971, EPI_ISL_852972, EPI_ISL_852973, EPI_ISL_852975, EPI_ISL_852976, EPI_ISL_852977, EPI_ISL_852978, EPI_ISL_852979, EPI_ISL_852980, EPI_ISL_852981, EPI_ISL_852982, EPI_ISL_852984, EPI_ISL_852985, EPI_ISL_852986, EPI_ISL_852987, EPI_ISL_853017, EPI_ISL_853030, EPI_ISL_853031			
see above	Hospital General Universitario Gregorio Marañón	SeqCOVID-SPAIN consortium/IBV(CSIC)	Darío García de Viedma, Laura Pérez-Lago, Pedro J Sola-Campoy, Sergio Buenestado-Serrano, Marta Herranz, Victor Manuel de la Cueva, Julia Suárez, Pilar Catalán, Patricia Muñoz and SeqCOVID-SPAIN consortium
EPI_ISL_853721, EPI_ISL_853741, EPI_ISL_853746, EPI_ISL_853766, EPI_ISL_853807, EPI_ISL_853809, EPI_ISL_853810, EPI_ISL_854176, EPI_ISL_854177, EPI_ISL_854178, EPI_ISL_854180, EPI_ISL_854181, EPI_ISL_854182, EPI_ISL_854183, EPI_ISL_854235			
see above	Austrian Agency for Health and Food Safety (AGES)	Bergthaler laboratory, CeMM Research Center for Molecular Medicine of the Austrian Academy of Sciences	Lukas Endler, Alexandra Popa, Benedikt Agerer, Jakob-Wendelin Genger, Alexander Lercher, Anna Schedl, Thomas Penz, Michael Schuster, Jan Laine, Martin Senekowitsch, Christoph Bock, Andreas Bergthaler
EPI_ISL_855344	SA Pathology	SA Pathology	Lex Leong, Chuan Kok Lim, Mark Turra, Karin Kassahn, Ivan Bastian, Geoff Higgins
EPI_ISL_855605	Respiratory Virus Unit, National Infection Service, Public Health England	COVID-19 Genomics UK (COG-UK) Consortium	PHE Covid Sequencing Team
EPI_ISL_855615, EPI_ISL_855616, EPI_ISL_855617, EPI_ISL_855618, EPI_ISL_855619, EPI_ISL_855620, EPI_ISL_855621, EPI_ISL_855622, EPI_ISL_855623, EPI_ISL_855624, EPI_ISL_855625, EPI_ISL_855626, EPI_ISL_855627, EPI_ISL_855628, EPI_ISL_855629, EPI_ISL_855630, EPI_ISL_855631, EPI_ISL_855632, EPI_ISL_855633, EPI_ISL_855634, EPI_ISL_855635, EPI_ISL_855636, EPI_ISL_855637, EPI_ISL_855638, EPI_ISL_855639, EPI_ISL_855640, EPI_ISL_855641, EPI_ISL_855642, EPI_ISL_855643, EPI_ISL_855644, EPI_ISL_855645, EPI_ISL_855646, EPI_ISL_855647, EPI_ISL_855648, EPI_ISL_855649, EPI_ISL_855650, EPI_ISL_855651, EPI_ISL_855652, EPI_ISL_855653, EPI_ISL_855654, EPI_ISL_855655, EPI_ISL_855656, EPI_ISL_855657, EPI_ISL_855658, EPI_ISL_855659, EPI_ISL_855660, EPI_ISL_855661, EPI_ISL_855662, EPI_ISL_855663, EPI_ISL_855664, EPI_ISL_855665, EPI_ISL_855666, EPI_ISL_855667, EPI_ISL_855668, EPI_ISL_855669, EPI_ISL_855670, EPI_ISL_855671, EPI_ISL_855672, EPI_ISL_855673, EPI_ISL_855674, EPI_ISL_855675, EPI_ISL_855676, EPI_ISL_855677, EPI_ISL_855678, EPI_ISL_855679, EPI_ISL_855680, EPI_ISL_855681, EPI_ISL_855682, EPI_ISL_855683, EPI_ISL_855684, EPI_ISL_855685, EPI_ISL_855686, EPI_ISL_855687, EPI_ISL_855688, EPI_ISL_855689, EPI_ISL_855690, EPI_ISL_855691, EPI_ISL_855692, EPI_ISL_855693, EPI_ISL_855694, EPI_ISL_855695, EPI_ISL_855696, EPI_ISL_855697, EPI_ISL_855698, EPI_ISL_855699, EPI_ISL_855700, EPI_ISL_855701, EPI_ISL_855702, EPI_ISL_855703, EPI_ISL_855704, EPI_ISL_855705, EPI_ISL_855706, EPI_ISL_855707, EPI_ISL_855708, EPI_ISL_855709, EPI_ISL_855710, EPI_ISL_855711, EPI_ISL_855712, EPI_ISL_855713, EPI_ISL_855714, EPI_ISL_855715, EPI_ISL_855716, EPI_ISL_855717, EPI_ISL_855718, EPI_ISL_855719, EPI_ISL_855720, EPI_ISL_855721, EPI_ISL_855722, EPI_ISL_855723, EPI_ISL_855724, EPI_ISL_855725, EPI_ISL_855726, EPI_ISL_855727, EPI_ISL_855728, EPI_ISL_855729, EPI_ISL_855730, EPI_ISL_855731, EPI_ISL_855732, EPI_ISL_855733, EPI_ISL_855734, EPI_ISL_855735, EPI_ISL_855736, EPI_ISL_855737, EPI_ISL_855738, EPI_ISL_855739, EPI_ISL_855740, EPI_ISL_855741, EPI_ISL_855742, EPI_ISL_855743, EPI_ISL_855744, EPI_ISL_855745, EPI_ISL_855746, EPI_ISL_855747, EPI_ISL_855748, EPI_ISL_855749, EPI_ISL_855750, EPI_ISL_855751, EPI_ISL_855752, EPI_ISL_855753, EPI_ISL_855754, EPI_ISL_855755, EPI_ISL_855756, EPI_ISL_855757, EPI_ISL_855758, EPI_ISL_855759, EPI_ISL_855760, EPI_ISL_855761, EPI_ISL_855762, EPI_ISL_855763, EPI_ISL_855764, EPI_ISL_855765, EPI_ISL_855766, EPI_ISL_855767, EPI_ISL_855768, EPI_ISL_855769, EPI_ISL_855770, EPI_ISL_855771, EPI_ISL_855772, EPI_ISL_855773, EPI_ISL_855774, EPI_ISL_855775, EPI_ISL_855776, EPI_ISL_855777, EPI_ISL_855778, EPI_ISL_855779, EPI_ISL_855780, EPI_ISL_855781, EPI_ISL_855782, EPI_ISL_855783, EPI_ISL_855784, EPI_ISL_855785, EPI_ISL_855786, EPI_ISL_855787, EPI_ISL_855788, EPI_ISL_855789, EPI_ISL_855790, EPI_ISL_855791, EPI_ISL_855792, EPI_ISL_855793, EPI_ISL_855794, EPI_ISL_855795, EPI_ISL_855796, EPI_ISL_855797, EPI_ISL_855798, EPI_ISL_855799, EPI_ISL_855800, EPI_ISL_855801, EPI_ISL_855802, EPI_ISL_855803, EPI_ISL_855804, EPI_ISL_855805, EPI_ISL_855806, EPI_ISL_855807, EPI_ISL_855808, EPI_ISL_855809, EPI_ISL_855810, EPI_ISL_855811, EPI_ISL_855812, EPI_ISL_855813, EPI_ISL_855814, EPI_ISL_855815, EPI_ISL_855816, EPI_ISL_855817, EPI_ISL_855818, EPI_ISL_855819, EPI_ISL_855820, EPI_ISL_855821, EPI_ISL_855822, EPI_ISL_855823, EPI_ISL_855824, EPI_ISL_855825, EPI_ISL_855826, EPI_ISL_855827, EPI_ISL_855828, EPI_ISL_855829, EPI_ISL_855830, EPI_ISL_855831, EPI_ISL_855832, EPI_ISL_855833, EPI_ISL_855834, EPI_ISL_855835, EPI_ISL_855836, EPI_ISL_855837, EPI_ISL_855838, EPI_ISL_855839, EPI_ISL_855840, EPI_ISL_855841, EPI_ISL_855842, EPI_ISL_855843, EPI_ISL_855844, EPI_ISL_855845, EPI_ISL_855846, EPI_ISL_855847, EPI_ISL_855848, EPI_ISL_855849, EPI_ISL_855850, EPI_ISL_855851, EPI_ISL_855852, EPI_ISL_855853, EPI_ISL_855854, EPI_ISL_855855, EPI_ISL_855856, EPI_ISL_855857, EPI_ISL_855858, EPI_ISL_855859, EPI_ISL_855860, EPI_ISL_855861, EPI_ISL_855862, EPI_ISL_855863, EPI_ISL_855864, EPI_ISL_855865, EPI_ISL_855866, EPI_ISL_855867, EPI_ISL_855868, EPI_ISL_855869, EPI_ISL_855870, EPI_ISL_855871, EPI_ISL_855872, EPI_ISL_855873, EPI_ISL_855874, EPI_ISL_855875, EPI_ISL_855876, EPI_ISL_855877, EPI_ISL_855878, EPI_ISL_855879, EPI_ISL_855880, EPI_ISL_855881, EPI_ISL_855882, EPI_ISL_855883, EPI_ISL_855884, EPI_ISL_855885, EPI_ISL_855886, EPI_ISL_855887, EPI_ISL_855888, EPI_ISL_855889, EPI_ISL_855890, EPI_ISL_855891, EPI_ISL_855892, EPI_ISL_855893, EPI_ISL_855894, EPI_ISL_855895, EPI_ISL_855896, EPI_ISL_855897, EPI_ISL_855898, EPI_ISL_855899, EPI_ISL_855900, EPI_ISL_855901, EPI_ISL_855903, EPI_ISL_855905, EPI_ISL_855907, EPI_ISL_855909, EPI_ISL_855911, EPI_ISL_855913, EPI_ISL_855915, EPI_ISL_855918, EPI_ISL_855920, EPI_ISL_855922, EPI_ISL_855924, EPI_ISL_855926, EPI_ISL_855929, EPI_ISL_855931, EPI_ISL_855933, EPI_ISL_855935, EPI_ISL_855937, EPI_ISL_855939, EPI_ISL_855941, EPI_ISL_855943, EPI_ISL_855946, EPI_ISL_855948, EPI_ISL_855950, EPI_ISL_855952, EPI_ISL_855954, EPI_ISL_855956, EPI_ISL_855958, EPI_ISL_855961, EPI_ISL_855963, EPI_ISL_855965, EPI_ISL_855967, EPI_ISL_855969, EPI_ISL_855970, EPI_ISL_855971, EPI_ISL_855972, EPI_ISL_855973, EPI_ISL_855974, EPI_ISL_855975, EPI_ISL_855976, EPI_ISL_855977, EPI_ISL_855978, EPI_ISL_855979, EPI_ISL_855980, EPI_ISL_855981, EPI_ISL_855982, EPI_ISL_855983, EPI_ISL_855984, EPI_ISL_855985, EPI_ISL_855986, EPI_ISL_855987, EPI_ISL_855988, EPI_ISL_855989, EPI_ISL_855990, EPI_ISL_855991, EPI_ISL_855992, EPI_ISL_855993, EPI_ISL_855994, EPI_ISL_855995, EPI_ISL_855996, EPI_ISL_855997, EPI_ISL_855998, EPI_ISL_855999, EPI_ISL_856000, EPI_ISL_856001, EPI_ISL_856002, EPI_ISL_856003, EPI_ISL_856004, EPI_ISL_856005, EPI_ISL_856006, EPI_ISL_856007, EPI_ISL_856008, EPI_ISL_856009, EPI_ISL_856010, EPI_ISL_856011, EPI_ISL_856012, EPI_ISL_856013, EPI_ISL_856014, EPI_ISL_856015, EPI_ISL_856016, EPI_ISL_856017, EPI_ISL_856018, EPI_ISL_856019, EPI_ISL_856020, EPI_ISL_856021, EPI_ISL_856022, EPI_ISL_856023, EPI_ISL_856024, EPI_ISL_856025, EPI_ISL_856026, EPI_ISL_856027, EPI_ISL_856028, EPI_ISL_856029, EPI_ISL_856030, EPI_ISL_856031, EPI_ISL_856032, EPI_ISL_856033, EPI_ISL_856034, EPI_ISL_856035, EPI_ISL_856036, EPI_ISL_856037, EPI_ISL_856038, EPI_ISL_856039, EPI_ISL_856040, EPI_ISL_856041, EPI_ISL_856042, EPI_ISL_856043, EPI_ISL_856044, EPI_ISL_856045, EPI_ISL_856046, EPI_ISL_856047, EPI_ISL_856048, EPI_ISL_856049, EPI_ISL_856050, EPI_ISL_856051, EPI_ISL_856052, EPI_ISL_856053, EPI_ISL_856054, EPI_ISL_856055, EPI_ISL_856056, EPI_ISL_856057, EPI_ISL_856058, EPI_ISL_856059, EPI_ISL_856060, EPI_ISL_856061, EPI_ISL_856062, EPI_ISL_856063, EPI_ISL_856064, EPI_ISL_856065, EPI_ISL_856066, EPI_ISL_856067, EPI_ISL_856068, EPI_ISL_856069, EPI_ISL_856070, EPI_ISL_856071, EPI_ISL_856072, EPI_ISL_856073, EPI_ISL_856074, EPI_ISL_856075, EPI_ISL_856076, EPI_ISL_856077, EPI_ISL_856078, EPI_ISL_856079, EPI_ISL_856080, EPI_ISL_856081, EPI_ISL_856082, EPI_ISL_856083, EPI_ISL_856084, EPI_ISL_856085, EPI_ISL_856086, EPI_ISL_856087, EPI_ISL_856088, EPI_ISL_856089, EPI_ISL_856090, EPI_ISL_856091, EPI_ISL_856092, EPI_ISL_856093, EPI_ISL_856094, EPI_ISL_856095, EPI_ISL_856096, EPI_ISL_856097, EPI_ISL_856098, EPI_ISL_856099, EPI_ISL_856100, EPI_ISL_856101, EPI_ISL_856102, EPI_ISL_856103, EPI_ISL_856104, EPI_ISL_856105, EPI_ISL_856106, EPI_ISL_856107, EPI_ISL_856108, EPI_ISL_856109, EPI_ISL_856110, EPI_ISL_856111, EPI_ISL_856112, EPI_ISL_856113, EPI_ISL_856114, EPI_ISL_856115, EPI_ISL_856116, EPI_ISL_856117, EPI_ISL_856118, EPI_ISL_856119, EPI_ISL_856120, EPI_ISL_856121, EPI_ISL_856122, EPI_ISL_856123, EPI_ISL_856124, EPI_ISL_856125, EPI_ISL_856126, EPI_ISL_856127, EPI_ISL_856128, EPI_ISL_856129, EPI_ISL_856130, EPI_ISL_856131, EPI_ISL_856132, EPI_ISL_856133, EPI_ISL_856134, EPI_ISL_856135, EPI_ISL_856136, EPI_ISL_856137, EPI_ISL_856138, EPI_ISL_856139, EPI_ISL_856140, EPI_ISL_856141, EPI_ISL_856142, EPI_ISL_856143, EPI_ISL_856144, EPI_ISL_856145, EPI_ISL_856146, EPI_ISL_856147, EPI_ISL_856148, EPI_ISL_856149, EPI_ISL_856150, EPI_ISL_856151, EPI_ISL_856152, EPI_ISL_856153, EPI_ISL_856154, EPI_ISL_856155, EPI_ISL_856156, EPI_ISL_856157, EPI_ISL_856158, EPI_ISL_856159, EPI_ISL_856160, EPI_ISL_856161, EPI_ISL_856162, EPI_ISL_856163, EPI_ISL_856164, EPI_ISL_856165, EPI_ISL_856166, EPI_ISL_856167, EPI_ISL_856168, EPI_ISL_856169, EPI_ISL_856170, EPI_ISL_856171, EPI_ISL_856172, EPI_ISL_856173, EPI_ISL_856174, EPI_ISL_856175, EPI_ISL_856176, EPI_ISL_856177, EPI_ISL_856178, EPI_ISL_856179, EPI_ISL_856180, EPI_ISL_856181, EPI_ISL_856182, EPI_ISL_856183, EPI_ISL_856184, EPI_ISL_856185, EPI_ISL_856186, EPI_ISL_856187, EPI_ISL_856188, EPI_ISL_856189, EPI_ISL_856190, EPI_ISL_856191, EPI_ISL_856192, EPI_ISL_856193, EPI_ISL_856194, EPI_ISL_856195, EPI_ISL_856196, EPI_ISL_856197, EPI_ISL_856198, EPI_ISL_856199, EPI_ISL_856200, EPI_ISL_856201, EPI_ISL_856202, EPI_ISL_856203, EPI_ISL_856204, EPI_ISL_856205, EPI_ISL_856206, EPI_ISL_856207, EPI_ISL_856208, EPI_ISL_856209, EPI_ISL_856210, EPI_ISL_856211, EPI_ISL_856212, EPI_ISL_856213, EPI_ISL_856214, EPI_ISL_856215, EPI_ISL_856216, EPI_ISL_856217, EPI_ISL_856218, EPI_ISL_856219, EPI_ISL_856220, EPI_ISL_856221, EPI_ISL_856222, EPI_ISL_856223, EPI_ISL_856224, EPI_ISL_856225, EPI_ISL_856226, EPI_ISL_856227, EPI_ISL_856228, EPI_ISL_856229, EPI_ISL_856230, EPI_ISL_856231, EPI_ISL_856232, EPI_ISL_856233, EPI_ISL_856234, EPI_ISL_856235, EPI_ISL_856236, EPI_ISL_856237, EPI_ISL_856238, EPI_ISL_856239, EPI_ISL_856240, EPI_ISL_856241, EPI_ISL_856242, EPI_ISL_856243, EPI_ISL_856244, EPI_ISL_856245, EPI_ISL_856246, EPI_ISL_856247, EPI_ISL_856248, EPI_ISL_856249, EPI_ISL_856250, EPI_ISL_856251, EPI_ISL_856252, EPI_ISL_856253, EPI_ISL_856254, EPI_ISL_856255, EPI_ISL_856256, EPI_ISL_856257, EPI_ISL_856258, EPI_ISL_856259, EPI_ISL_856260, EPI_ISL_856261, EPI_ISL_856262,			

EPI_ISL_856263, EPI_ISL_856264, EPI_ISL_856265, EPI_ISL_856266, EPI_ISL_856267, EPI_ISL_856268, EPI_ISL_856269, EPI_ISL_856270, EPI_ISL_856271, EPI_ISL_856272, EPI_ISL_856273, EPI_ISL_856274, EPI_ISL_856275, EPI_ISL_856276, EPI_ISL_856277, EPI_ISL_856278, EPI_ISL_856279, EPI_ISL_856280, EPI_ISL_856281, EPI_ISL_856282, EPI_ISL_856283, EPI_ISL_856284, EPI_ISL_856285, EPI_ISL_856286, EPI_ISL_856287, EPI_ISL_856288, EPI_ISL_856289, EPI_ISL_856290, EPI_ISL_856291			
see above	Department of Virus and Microbiological Special Diagnostics, Statens Serum Institut, Copenhagen, Denmark	Aalborg University	Danish Covid-19 Genome Consortium
EPI_ISL_856723, EPI_ISL_856724, EPI_ISL_856725, EPI_ISL_856732, EPI_ISL_856733, EPI_ISL_856734, EPI_ISL_856735, EPI_ISL_856736, EPI_ISL_856737, EPI_ISL_856738, EPI_ISL_856739, EPI_ISL_856740, EPI_ISL_856741, EPI_ISL_856742, EPI_ISL_856743, EPI_ISL_856744, EPI_ISL_856745, EPI_ISL_856746, EPI_ISL_856747, EPI_ISL_856748			
see above	Department of Clinical Microbiology	GIGA Medical Genomics	Keith Durkin, Maria Artesi, Sébastien Bontems, Raphaël Boreux, Bouchra Boujemla, Cécile Meex, Pierrette Melin, Marie-Pierre Hayette, Vincent Bours
EPI_ISL_856972	The Institute of Tropical Medicine in Antwerp, Belgium	UAntwerp, Laboratory of Medical Microbiology, Campus Drie Eiken S6.26, Universiteitsplein 1, 2610, Wilrijk, Antwerp, Belgium	Basil Britto Xavier, Jasmine Coppens, Christine Lammens, Veerle Matheeussen, Herman Goossens
EPI_ISL_857158	DOHMH Corona	New York City Public Health Laboratory	Jade Wang, et al.
EPI_ISL_857159, EPI_ISL_857160	DOHMH Morrisania	New York City Public Health Laboratory	Jade Wang, et al.
EPI_ISL_857161, EPI_ISL_857162	DOHMH PHL	New York City Public Health Laboratory	Jade Wang, et al.
EPI_ISL_857163	DOHMH Central Harlem	New York City Public Health Laboratory	Jade Wang, et al.
EPI_ISL_857164	DOHMH Morrisania	New York City Public Health Laboratory	Jade Wang, et al.
EPI_ISL_857165	DOHMH Corona	New York City Public Health Laboratory	Jade Wang, et al.
EPI_ISL_857166, EPI_ISL_857167	DOHMH Fort Greene	New York City Public Health Laboratory	Jade Wang, et al.
EPI_ISL_857168	DOHMH Corona	New York City Public Health Laboratory	Jade Wang, et al.
EPI_ISL_857169, EPI_ISL_857170, EPI_ISL_857171	DOHMH Morrisania	New York City Public Health Laboratory	Jade Wang, et al.
EPI_ISL_857172, EPI_ISL_857173	DOHMH Crown Heights	New York City Public Health Laboratory	Jade Wang, et al.
EPI_ISL_857174, EPI_ISL_857175, EPI_ISL_857176, EPI_ISL_857177	DOHMH Jamaica	New York City Public Health Laboratory	Jade Wang, et al.
EPI_ISL_857178	DOHMH Riverside	New York City Public Health Laboratory	Jade Wang, et al.
EPI_ISL_857179	DOHMH Jamaica	New York City Public Health Laboratory	Jade Wang, et al.
EPI_ISL_857180, EPI_ISL_857181, EPI_ISL_857182, EPI_ISL_857183	DOHMH Central Harlem	New York City Public Health Laboratory	Jade Wang, et al.
EPI_ISL_857184	DOHMH Riverside	New York City Public Health Laboratory	Jade Wang, et al.
EPI_ISL_857185	DOHMH PHL	New York City Public Health Laboratory	Jade Wang, et al.
EPI_ISL_857395	S.M.S.Medical College, Jaipur, Rajasthan	S.M.S.Medical College, Jaipur, Rajasthan	Dr. Bharti Malhotra, Dr. Swati Gautam
EPI_ISL_857444, EPI_ISL_857446	Maine HETL	Tewhey Lab, The Jackson Laboratory	Matluk,N., Dewey,H., Iosue,F., Barter,M., Lynch,R., Munger,H. and Tewhey,R.
EPI_ISL_857466, EPI_ISL_857467	PathWest Laboratory Medicine WA	PathWest Laboratory Medicine WA Microbial Surveillance Unit	PathWest Laboratory Medicine WA Microbial Surveillance Unit
EPI_ISL_857543, EPI_ISL_857554, EPI_ISL_857559, EPI_ISL_857564, EPI_ISL_857566, EPI_ISL_857572, EPI_ISL_857581, EPI_ISL_857587, EPI_ISL_857593, EPI_ISL_857595, EPI_ISL_857596, EPI_ISL_857600, EPI_ISL_857602, EPI_ISL_857605, EPI_ISL_857607, EPI_ISL_857611, EPI_ISL_857613, EPI_ISL_857614, EPI_ISL_857616, EPI_ISL_857619, EPI_ISL_857621, EPI_ISL_857622, EPI_ISL_857625, EPI_ISL_857626, EPI_ISL_857631, EPI_ISL_857632, EPI_ISL_857633, EPI_ISL_857635, EPI_ISL_857638, EPI_ISL_857641, EPI_ISL_857646, EPI_ISL_857649, EPI_ISL_857653, EPI_ISL_857659, EPI_ISL_857660, EPI_ISL_857662, EPI_ISL_857675, EPI_ISL_857677, EPI_ISL_857679, EPI_ISL_857687, EPI_ISL_857691, EPI_ISL_857700, EPI_ISL_857705, EPI_ISL_857709, EPI_ISL_857717, EPI_ISL_857719, EPI_ISL_857720, EPI_ISL_857723, EPI_ISL_857728, EPI_ISL_857731, EPI_ISL_857734, EPI_ISL_857736, EPI_ISL_857738, EPI_ISL_857739, EPI_ISL_857740, EPI_ISL_857743, EPI_ISL_857744, EPI_ISL_857749, EPI_ISL_857751, EPI_ISL_857754, EPI_ISL_857756, EPI_ISL_857763, EPI_ISL_857769, EPI_ISL_857773, EPI_ISL_857776, EPI_ISL_857778, EPI_ISL_857780, EPI_ISL_857782, EPI_ISL_857783, EPI_ISL_857786, EPI_ISL_857787, EPI_ISL_857792, EPI_ISL_857794, EPI_ISL_857795, EPI_ISL_857798, EPI_ISL_857799, EPI_ISL_857803, EPI_ISL_857811, EPI_ISL_857820, EPI_ISL_857825, EPI_ISL_857829, EPI_ISL_857835, EPI_ISL_857839, EPI_ISL_857840, EPI_ISL_857845, EPI_ISL_857846, EPI_ISL_857847, EPI_ISL_857853, EPI_ISL_857857, EPI_ISL_857863, EPI_ISL_857866			
see above	Lighthouse Lab in Cambridge	Wellcome Sanger Institute for the COVID-19 Genomics UK (COG-UK) Consortium	Rob Howes, The Lighthouse Lab in Cambridge and Alex Alderton, Roberto Amato, Sonia Goncalves, Ewan Harrison, David K. Jackson, Ian Johnston, Dominic Kwiatkowski, Cordelia Langford, John Sillitoe on behalf of the Wellcome Sanger Institute COVID-19 Surveillance Team
EPI_ISL_857892, EPI_ISL_857893, EPI_ISL_857895, EPI_ISL_857896, EPI_ISL_857897, EPI_ISL_857898, EPI_ISL_857899, EPI_ISL_857900, EPI_ISL_857901, EPI_ISL_857902, EPI_ISL_857903, EPI_ISL_857904, EPI_ISL_857905, EPI_ISL_857906, EPI_ISL_857907, EPI_ISL_857908, EPI_ISL_857909, EPI_ISL_857910			
see above	Lighthouse Lab in Glasgow	Wellcome Sanger Institute for the COVID-19 Genomics UK (COG-UK) Consortium	Harper VanSteenhouse, Yumi Kasai, David Gray, Carol Clugston, Anna Dominiczak and Alex Alderton, Roberto Amato, Sonia Goncalves, Ewan Harrison, David K. Jackson, Ian Johnston, Dominic Kwiatkowski, Cordelia Langford, John Sillitoe on behalf of the Wellcome Sanger Institute COVID-19 Surveillance Team
EPI_ISL_860280, EPI_ISL_860282, EPI_ISL_860283	Nordland Hospital - Bodo, Laboratory Department, Molecular Biology Unit	Norwegian Institute of Public Health, Department of Virology	Kathrine Stene-Johansen, Kamilla Heddeland Instefjord, Hilde Elshaug, Atiya R Ali,Marie Paulsen Madsen, Rasmus Riis Kopperud, Hilde Vollan, Karoline Bragstad, Olav Hungnes
EPI_ISL_860720	Institute for Infectious Diseases, University of Bern, Switzerland	Institute for Infectious Diseases, University of Bern, Switzerland	Michel C Koch, Christian Baumann, Miguel A Terrazos Miani, Cora Sägesser, Pascal Bittel, Stephen L Leib, Peter Keller, Franziska Suter-Riniker, Alban Ramette
EPI_ISL_860785, EPI_ISL_860789, EPI_ISL_860790	Charité Universitätsmedizin Berlin, Institute of Virology, Charitéplatz 1, 10117 Berlin, Germany	Charité Universitätsmedizin Berlin, Institute of Virology, Charitéplatz 1, 10117 Berlin, Germany	Victor M Corman, Julia Schneider, Jörn Beheim-Schwarzbach, Tobias Bleicker, Julia Tesch, Barbara Mühlemann, Talitha Veith, Terry Jones, Christian Drosten
EPI_ISL_860850, EPI_ISL_860851	HIA PERCY, Biologie médicale	National Reference Center for Viruses of Respiratory Infections, Institut Pasteur, Paris	Marion Barbet, Sylvie Behillil, Méline Bizard, Angela Brisebarre, Camille Capel, Etienne Simon-Lorière, Vincent Enouf, Maud Vanpeene, Sylvie van der Werf,Foissaud Vincent
EPI_ISL_861004, EPI_ISL_861014, EPI_ISL_861027, EPI_ISL_861043, EPI_ISL_861048, EPI_ISL_861052, EPI_ISL_861082, EPI_ISL_861104	Johns Hopkins Hospital Department of Pathology	Johns Hopkins Hospital Department of Pathology	C. Paul Morris, Chun Huai Luo, Adannaya Amadi, Nicholas Gallagher, Heba H. Mostafa
EPI_ISL_861183, EPI_ISL_861184, EPI_ISL_861199, EPI_ISL_861200, EPI_ISL_861201, EPI_ISL_861202, EPI_ISL_861203, EPI_ISL_861204, EPI_ISL_861206, EPI_ISL_861207, EPI_ISL_861208, EPI_ISL_861209			
see above	URMC LABS	Wadsworth Center, New York State Department of Health	Kirsten St. George, Daryl M. Lamson, Alexis Russel, Matthew Shudt, Melissa A Leisner, Jonathan Plitnick, Navjot Singh, John Kelly, Erasmus Schneider, Erica Lasek-Nesselquist
EPI_ISL_861332	DOHMH Central Harlem	New York City Public Health Laboratory	Jade Wang, et al.
EPI_ISL_861446	Clinical Molecular Microbiology Laboratory, UNC Hospitals	Jeremy Wang	Jeremy Wang, Alexander Rubinsteyn, Colleen Rice, Jason Smedberg, Melissa Miller, Corbin Jones, Robert Hagan
EPI_ISL_861749	Tempus	Grubaugh Lab - Yale School of Public Health	Tara Alpert, Joseph Fauver, Anderson Brito, Mallery Breban, Anne Wyllie, Chantal Vogels, Mary Petrone, Annie Watkins, Chaney Kalinich, Isabel Ott, Nathan Grubaugh
EPI_ISL_862038	Johns Hopkins Hospital Department of Pathology	Johns Hopkins Hospital Department of Pathology	C. Paul Morris, Chun Huai Luo, Adannaya Amadi, Matthew Schwartz, Nicholas Gallagher, Heba H. Mostafa
EPI_ISL_862129, EPI_ISL_862130, EPI_ISL_862134, EPI_ISL_862135, EPI_ISL_862140, EPI_ISL_862142, EPI_ISL_862147, EPI_ISL_862150, EPI_ISL_862163, EPI_ISL_862166, EPI_ISL_862170, EPI_ISL_862179, EPI_ISL_862180, EPI_ISL_862181, EPI_ISL_862183, EPI_ISL_862187, EPI_ISL_862659, EPI_ISL_862661, EPI_ISL_862662, EPI_ISL_862663, EPI_ISL_862664			
see above	Charité Universitätsmedizin Berlin, Institut fur Virologie/Labor	Charité Universitätsmedizin Berlin, Institut für Virologie	Victor M Corman, Barbara Mühlemann, Jörn Beheim-Schwarzbach, Tobias Bleicker, Julia Tesch, Talitha Veith, Julia Schneider, Terry Jones, Christian

	Berlin		Drosten
EPI_ISL_862670, EPI_ISL_862671, EPI_ISL_862672, EPI_ISL_862673, EPI_ISL_862674, EPI_ISL_862675, EPI_ISL_862677, EPI_ISL_862678, EPI_ISL_862679, EPI_ISL_862680, EPI_ISL_862681, EPI_ISL_862682, EPI_ISL_862683, EPI_ISL_862684, EPI_ISL_862685, EPI_ISL_862686, EPI_ISL_862687, EPI_ISL_862688, EPI_ISL_862689, EPI_ISL_862690, EPI_ISL_862691, EPI_ISL_862692, EPI_ISL_862693, EPI_ISL_862694, EPI_ISL_862695, EPI_ISL_862696, EPI_ISL_862697, EPI_ISL_862698, EPI_ISL_862699, EPI_ISL_862700, EPI_ISL_862701, EPI_ISL_862702, EPI_ISL_862703, EPI_ISL_862704, EPI_ISL_862705, EPI_ISL_862706, EPI_ISL_862707, EPI_ISL_862708, EPI_ISL_862709, EPI_ISL_862713			
see above	New Mexico Department of Health Scientific Laboratory	New Mexico Department of Health Scientific Laboratory	D'eldra Malone, Ellie Johnson, Anastacia Griego-Fisher
EPI_ISL_862889, EPI_ISL_862890, EPI_ISL_862891, EPI_ISL_862892, EPI_ISL_862893, EPI_ISL_862894, EPI_ISL_862895, EPI_ISL_862896, EPI_ISL_862897, EPI_ISL_862898, EPI_ISL_862899, EPI_ISL_862901, EPI_ISL_862902, EPI_ISL_862903, EPI_ISL_862904, EPI_ISL_862905, EPI_ISL_862906, EPI_ISL_862907, EPI_ISL_862908, EPI_ISL_862909, EPI_ISL_862910, EPI_ISL_862911, EPI_ISL_862912, EPI_ISL_862913, EPI_ISL_862914, EPI_ISL_862916, EPI_ISL_862917, EPI_ISL_862918, EPI_ISL_862919, EPI_ISL_862920, EPI_ISL_862921, EPI_ISL_862922, EPI_ISL_862923, EPI_ISL_862924, EPI_ISL_862925, EPI_ISL_862926, EPI_ISL_862927, EPI_ISL_862928, EPI_ISL_862929, EPI_ISL_862930, EPI_ISL_862931, EPI_ISL_862932, EPI_ISL_862933, EPI_ISL_862934, EPI_ISL_862935, EPI_ISL_862936, EPI_ISL_862937, EPI_ISL_862939, EPI_ISL_862940, EPI_ISL_862941, EPI_ISL_862942, EPI_ISL_862943, EPI_ISL_862944, EPI_ISL_862945, EPI_ISL_862946, EPI_ISL_862947, EPI_ISL_862949, EPI_ISL_862950, EPI_ISL_862951, EPI_ISL_862952, EPI_ISL_862953, EPI_ISL_862954, EPI_ISL_862955, EPI_ISL_862956, EPI_ISL_862957, EPI_ISL_862958, EPI_ISL_862959, EPI_ISL_862960, EPI_ISL_862961, EPI_ISL_862962, EPI_ISL_862963, EPI_ISL_862964, EPI_ISL_862965, EPI_ISL_862966, EPI_ISL_862967, EPI_ISL_862968, EPI_ISL_862969, EPI_ISL_862970, EPI_ISL_862971, EPI_ISL_862972, EPI_ISL_862973, EPI_ISL_862974, EPI_ISL_862975, EPI_ISL_862976, EPI_ISL_862977, EPI_ISL_862978, EPI_ISL_862979, EPI_ISL_862980, EPI_ISL_862981, EPI_ISL_862982, EPI_ISL_862984, EPI_ISL_862985, EPI_ISL_862986, EPI_ISL_862987, EPI_ISL_862988, EPI_ISL_862989, EPI_ISL_862991, EPI_ISL_862992, EPI_ISL_862993, EPI_ISL_862994, EPI_ISL_862995, EPI_ISL_862996, EPI_ISL_862997, EPI_ISL_862998, EPI_ISL_862999, EPI_ISL_863000, EPI_ISL_863001, EPI_ISL_863002, EPI_ISL_863003, EPI_ISL_863004, EPI_ISL_863005, EPI_ISL_863006, EPI_ISL_863007, EPI_ISL_863008, EPI_ISL_863009, EPI_ISL_863010, EPI_ISL_863011, EPI_ISL_863012, EPI_ISL_863014, EPI_ISL_863015, EPI_ISL_863016, EPI_ISL_863017, EPI_ISL_863018, EPI_ISL_863019, EPI_ISL_863020, EPI_ISL_863021, EPI_ISL_863022, EPI_ISL_863024, EPI_ISL_863025, EPI_ISL_863026, EPI_ISL_863027, EPI_ISL_863028, EPI_ISL_863029, EPI_ISL_863030, EPI_ISL_863031, EPI_ISL_863032, EPI_ISL_863033, EPI_ISL_863034, EPI_ISL_863035, EPI_ISL_863036, EPI_ISL_863037, EPI_ISL_863038, EPI_ISL_863039, EPI_ISL_863040, EPI_ISL_863041, EPI_ISL_863042, EPI_ISL_863043, EPI_ISL_863044, EPI_ISL_863045, EPI_ISL_863047, EPI_ISL_863048, EPI_ISL_863049, EPI_ISL_863050, EPI_ISL_863051, EPI_ISL_863052, EPI_ISL_863053, EPI_ISL_863054, EPI_ISL_863055, EPI_ISL_863056, EPI_ISL_863057, EPI_ISL_863059, EPI_ISL_863060, EPI_ISL_863062, EPI_ISL_863063, EPI_ISL_863064, EPI_ISL_863065, EPI_ISL_863066, EPI_ISL_863067, EPI_ISL_863068, EPI_ISL_863069, EPI_ISL_863070, EPI_ISL_863071, EPI_ISL_863072, EPI_ISL_863073, EPI_ISL_863075, EPI_ISL_863076, EPI_ISL_863077, EPI_ISL_863078, EPI_ISL_863079, EPI_ISL_863080, EPI_ISL_863081, EPI_ISL_863082, EPI_ISL_863083, EPI_ISL_863085, EPI_ISL_863086, EPI_ISL_863087, EPI_ISL_863088, EPI_ISL_863090, EPI_ISL_863092, EPI_ISL_863093, EPI_ISL_863094, EPI_ISL_863095, EPI_ISL_863096, EPI_ISL_863097, EPI_ISL_863098, EPI_ISL_863099, EPI_ISL_863100, EPI_ISL_863102, EPI_ISL_863104, EPI_ISL_863105, EPI_ISL_863107, EPI_ISL_863108, EPI_ISL_863109, EPI_ISL_863110, EPI_ISL_863111, EPI_ISL_863113, EPI_ISL_863114, EPI_ISL_863115, EPI_ISL_863116, EPI_ISL_863117, EPI_ISL_863118, EPI_ISL_863119, EPI_ISL_863120, EPI_ISL_863121, EPI_ISL_863123, EPI_ISL_863124, EPI_ISL_863125, EPI_ISL_863127, EPI_ISL_863128, EPI_ISL_863129, EPI_ISL_863130, EPI_ISL_863131, EPI_ISL_863132, EPI_ISL_863133, EPI_ISL_863134, EPI_ISL_863135, EPI_ISL_863139, EPI_ISL_863141, EPI_ISL_863142, EPI_ISL_863143, EPI_ISL_863144, EPI_ISL_863145, EPI_ISL_863146, EPI_ISL_863147, EPI_ISL_863148, EPI_ISL_863149, EPI_ISL_863150, EPI_ISL_863152, EPI_ISL_863153, EPI_ISL_863154, EPI_ISL_863155, EPI_ISL_863157, EPI_ISL_863158, EPI_ISL_863159, EPI_ISL_863160, EPI_ISL_863161, EPI_ISL_863163, EPI_ISL_863164, EPI_ISL_863165, EPI_ISL_863166, EPI_ISL_863167, EPI_ISL_863168, EPI_ISL_863169, EPI_ISL_863170, EPI_ISL_863171, EPI_ISL_863172, EPI_ISL_863173, EPI_ISL_863174, EPI_ISL_863175, EPI_ISL_863176, EPI_ISL_863177, EPI_ISL_863178, EPI_ISL_863181, EPI_ISL_863182, EPI_ISL_863183, EPI_ISL_863184, EPI_ISL_863185, EPI_ISL_863186, EPI_ISL_863187, EPI_ISL_863189, EPI_ISL_863190, EPI_ISL_863191, EPI_ISL_863194, EPI_ISL_863195, EPI_ISL_863196, EPI_ISL_863197, EPI_ISL_863198, EPI_ISL_863199, EPI_ISL_863200, EPI_ISL_863201, EPI_ISL_863202, EPI_ISL_863203, EPI_ISL_863204, EPI_ISL_863205, EPI_ISL_863206, EPI_ISL_863207, EPI_ISL_863208, EPI_ISL_863209, EPI_ISL_863211, EPI_ISL_863212, EPI_ISL_863213, EPI_ISL_863214, EPI_ISL_863215, EPI_ISL_863216, EPI_ISL_863217, EPI_ISL_863219, EPI_ISL_863220, EPI_ISL_863221, EPI_ISL_863222, EPI_ISL_863223, EPI_ISL_863224, EPI_ISL_863225, EPI_ISL_863226, EPI_ISL_863227, EPI_ISL_863228, EPI_ISL_863229, EPI_ISL_863230, EPI_ISL_863231, EPI_ISL_863232, EPI_ISL_863233, EPI_ISL_863234, EPI_ISL_863235, EPI_ISL_863236, EPI_ISL_863237, EPI_ISL_863239, EPI_ISL_863240, EPI_ISL_863241, EPI_ISL_863242, EPI_ISL_863243, EPI_ISL_863244, EPI_ISL_863334, EPI_ISL_863396, EPI_ISL_863401, EPI_ISL_863403, EPI_ISL_863424			
see above	Lighthouse Lab in Alderley Park	Wellcome Sanger Institute for the COVID-19 Genomics UK (COG-UK) Consortium	Jacquelyn Wynn, Mairead Hyland, The Lighthouse Lab in Alderley Park and Alex Alderton, Roberto Amato, Sonia Goncalves, Ewan Harrison, David K. Jackson, Ian Johnston, Dominic Kwiatkowski, Cordelia Langford, John Sillitoe on behalf of the Wellcome Sanger Institute COVID-19 Surveillance Team
EPI_ISL_863431	Lighthouse Lab in Glasgow	Wellcome Sanger Institute for the COVID-19 Genomics UK (COG-UK) Consortium	Harper VanSteenhouse, Yumi Kasai, David Gray, Carol Clugston, Anna Dominiczak and Alex Alderton, Roberto Amato, Sonia Goncalves, Ewan Harrison, David K. Jackson, Ian Johnston, Dominic Kwiatkowski, Cordelia Langford, John Sillitoe on behalf of the Wellcome Sanger Institute COVID-19 Surveillance Team
EPI_ISL_863432, EPI_ISL_863467, EPI_ISL_863514, EPI_ISL_863529	Lighthouse Lab in Alderley Park	Wellcome Sanger Institute for the COVID-19 Genomics UK (COG-UK) Consortium	Jacquelyn Wynn, Mairead Hyland, The Lighthouse Lab in Alderley Park and Alex Alderton, Roberto Amato, Sonia Goncalves, Ewan Harrison, David K. Jackson, Ian Johnston, Dominic Kwiatkowski, Cordelia Langford, John Sillitoe on behalf of the Wellcome Sanger Institute COVID-19 Surveillance Team
EPI_ISL_863531, EPI_ISL_863533, EPI_ISL_863539, EPI_ISL_863540, EPI_ISL_863542, EPI_ISL_863544, EPI_ISL_863546, EPI_ISL_863549, EPI_ISL_863551, EPI_ISL_863552, EPI_ISL_863555, EPI_ISL_863556, EPI_ISL_863557, EPI_ISL_863558, EPI_ISL_863560, EPI_ISL_863565, EPI_ISL_863566, EPI_ISL_863570, EPI_ISL_863572, EPI_ISL_863574, EPI_ISL_863576, EPI_ISL_863578, EPI_ISL_863579, EPI_ISL_863581, EPI_ISL_863582, EPI_ISL_863583, EPI_ISL_863584, EPI_ISL_863586, EPI_ISL_863591, EPI_ISL_863592, EPI_ISL_863593, EPI_ISL_863594, EPI_ISL_863595, EPI_ISL_863603, EPI_ISL_863604, EPI_ISL_863608, EPI_ISL_863609, EPI_ISL_863610, EPI_ISL_863612, EPI_ISL_863614, EPI_ISL_863616, EPI_ISL_863618, EPI_ISL_863619, EPI_ISL_863621, EPI_ISL_863625, EPI_ISL_863626, EPI_ISL_863627, EPI_ISL_863629, EPI_ISL_863630, EPI_ISL_863636, EPI_ISL_863640, EPI_ISL_863641, EPI_ISL_863643, EPI_ISL_863644, EPI_ISL_863648, EPI_ISL_863652, EPI_ISL_863654, EPI_ISL_863656, EPI_ISL_863658, EPI_ISL_863659, EPI_ISL_863661, EPI_ISL_863662, EPI_ISL_863664, EPI_ISL_863665, EPI_ISL_863667, EPI_ISL_863668, EPI_ISL_863671, EPI_ISL_863672, EPI_ISL_863673, EPI_ISL_863674, EPI_ISL_863675, EPI_ISL_863676, EPI_ISL_863680, EPI_ISL_863682, EPI_ISL_863685, EPI_ISL_863687, EPI_ISL_863688, EPI_ISL_863689, EPI_ISL_863690, EPI_ISL_863691, EPI_ISL_863692, EPI_ISL_863694, EPI_ISL_863695, EPI_ISL_863697, EPI_ISL_863698, EPI_ISL_863701, EPI_ISL_863702, EPI_ISL_863704, EPI_ISL_863706, EPI_ISL_863708, EPI_ISL_863710, EPI_ISL_863712, EPI_ISL_863713, EPI_ISL_863715, EPI_ISL_863720, EPI_ISL_863722, EPI_ISL_863727, EPI_ISL_863728, EPI_ISL_863730, EPI_ISL_863731, EPI_ISL_863736, EPI_ISL_863738, EPI_ISL_863740, EPI_ISL_863743, EPI_ISL_863744, EPI_ISL_863748, EPI_ISL_863749, EPI_ISL_863750, EPI_ISL_863751, EPI_ISL_863752, EPI_ISL_863754, EPI_ISL_863755, EPI_ISL_863756, EPI_ISL_863757, EPI_ISL_863758, EPI_ISL_863760, EPI_ISL_863762, EPI_ISL_863763, EPI_ISL_863764, EPI_ISL_863765, EPI_ISL_863767, EPI_ISL_863769, EPI_ISL_863770, EPI_ISL_863771, EPI_ISL_863772, EPI_ISL_863774, EPI_ISL_863777, EPI_ISL_863778, EPI_ISL_863779, EPI_ISL_863780, EPI_ISL_863782, EPI_ISL_863786, EPI_ISL_863787, EPI_ISL_863790, EPI_ISL_863791, EPI_ISL_863794, EPI_ISL_863798, EPI_ISL_863799, EPI_ISL_863800, EPI_ISL_863801, EPI_ISL_863813, EPI_ISL_863816, EPI_ISL_863818, EPI_ISL_863819, EPI_ISL_863820, EPI_ISL_863821, EPI_ISL_863822, EPI_ISL_863823, EPI_ISL_863824, EPI_ISL_863826, EPI_ISL_863827, EPI_ISL_863828, EPI_ISL_863831, EPI_ISL_863832, EPI_ISL_863836, EPI_ISL_863837, EPI_ISL_863839, EPI_ISL_863840, EPI_ISL_863843, EPI_ISL_863844, EPI_ISL_863846, EPI_ISL_863849, EPI_ISL_863852, EPI_ISL_863854, EPI_ISL_863861, EPI_ISL_863862, EPI_ISL_863864, EPI_ISL_863866, EPI_ISL_863868			
see above	Lighthouse Lab in Milton Keynes	Wellcome Sanger Institute for the COVID-19 Genomics UK (COG-UK) Consortium	The Lighthouse Lab in Milton Keynes and Alex Alderton, Roberto Amato, Sonia Goncalves, Ewan Harrison, David K. Jackson, Ian Johnston, Dominic Kwiatkowski, Cordelia Langford, John Sillitoe on behalf of the Wellcome Sanger Institute COVID-19 Surveillance Team
EPI_ISL_863873, EPI_ISL_863946, EPI_ISL_864004, EPI_ISL_864064, EPI_ISL_864094, EPI_ISL_864142, EPI_ISL_864145, EPI_ISL_864178	Lighthouse Lab in Alderley Park	Wellcome Sanger Institute for the COVID-19 Genomics UK (COG-UK) Consortium	Jacquelyn Wynn, Mairead Hyland, The Lighthouse Lab in Alderley Park and Alex Alderton, Roberto Amato, Sonia Goncalves, Ewan Harrison, David K. Jackson, Ian Johnston, Dominic Kwiatkowski, Cordelia Langford, John Sillitoe on behalf of the Wellcome Sanger Institute COVID-19 Surveillance Team
EPI_ISL_864595, EPI_ISL_864607, EPI_ISL_864612, EPI_ISL_864616, EPI_ISL_864617, EPI_ISL_864618, EPI_ISL_864625, EPI_ISL_864628, EPI_ISL_864629, EPI_ISL_864634, EPI_ISL_864689, EPI_ISL_864690, EPI_ISL_864691, EPI_ISL_864692, EPI_ISL_864693, EPI_ISL_864694, EPI_ISL_864695, EPI_ISL_864696, EPI_ISL_864697, EPI_ISL_864698, EPI_ISL_864699, EPI_ISL_864700, EPI_ISL_864701, EPI_ISL_864702, EPI_ISL_864703, EPI_ISL_864704, EPI_ISL_864705, EPI_ISL_864706, EPI_ISL_864716, EPI_ISL_864725, EPI_ISL_864726, EPI_ISL_864727			
see above	University Hospitals of Geneva, Laboratory of Virology	HUG, Laboratory of Virology and the Health2030 Genome Center	Samuel Cordey, Ana Rita Goncalves, Laurent Kaiser, Lorenzo Cerutti, Henri Pegeot, Melyssa Elies, Deborah Penet, Keith Harshman, Ioannis Xenarios, Emmanouil Dermitzakis
EPI_ISL_864926	Department of Pathology, University of Cambridge	COVID-19 Genomics UK (COG-UK) Consortium	Aminu S. Jahun, Yasmin Chaudhry, Grant Hall, Iliana Georgana, Myra Hosmillo, Martin D. Curran, Malte Pinckert, Surendra Parmar, Ian Goodfellow
EPI_ISL_865035, EPI_ISL_865036	West of Scotland Specialist Virology Centre, NHSGGC / MRC-University of Glasgow Centre for Virus Research	COVID-19 Genomics UK (COG-UK) Consortium	Ana da Silva Filipe, Natasha Johnson, Kathy Smollett, Daniel Mair, Stephen Carmichael, Alice Broos, Lily Tong, Jenna Nichols, Kyriaki Nomikou; Sarah McDonald; Richard Orton, Joseph Hughes, Sreenu Vattipally, David L Robertson; Alasdair MacLean, Rory Gunson; Sharif Shaaban, Matthew Holden; Rachel Blacow, Guy Mollett, Kathy Li, James Shepherd, Antonia Ho, Emma Thomson
EPI_ISL_865068	Lighthouse Lab in Glasgow / MRC-University of Glasgow Centre for Virus Research	COVID-19 Genomics UK (COG-UK) Consortium	Ana da Silva Filipe, Natasha Johnson, Kathy Smollett, Daniel Mair, Stephen Carmichael, Alice Broos, Lily Tong, Jenna Nichols, Kyriaki Nomikou; Sarah McDonald; Harper VanSteenhouse, Yumi Kasai, David Gray, Carol Clugston, Anna Dominiczak; Alasdair MacLean, Rory Gunson; Richard Orton, Joseph Hughes, Sreenu Vattipally, David L Robertson; Sharif Shaaban, Matthew Holden; Kathy Li, James Shepherd, Antonia Ho, Emma Thomson
EPI_ISL_865140	Virology Department, Royal Infirmary of Edinburgh, NHS Lothian / School of Biological Sciences, University of Edinburgh / Institute of Genetics and Molecular Medicine, University of Edinburgh	COVID-19 Genomics UK (COG-UK) Consortium	McHugh M, Dewar R, Rooke S, Gallagher M, Balcaza C, O'Toole Á, Scher E, Hill V, McCrone JT, Colquhoun R, Yu X, Jackson B, Rambaut A, Williams TC, Templeton K
EPI_ISL_865343, EPI_ISL_865344, EPI_ISL_865345, EPI_ISL_865348, EPI_ISL_865349, EPI_ISL_865351, EPI_ISL_865353, EPI_ISL_865449	Liverpool Clinical Laboratories	COVID-19 Genomics UK (COG-UK) Consortium	Sam Haldenby, Anita Lucaci, Steve Paterson, Julian Hiscox, Alistair Darby, M Almsaud, A Alrezaihi, Muhannad Alruwaili, Stuart D Armstrong, Jones Benjamin, Eleanor G Bentley, Anu Chawla, Jordan J Clark, Angela Cowell, Richard Eccles, Isabel García-Dorival, Matthew Gemmell, Alessandro Gerada, PKF Gilmore, Richard Gregory, Ximeng Han, Catherine Hartley, Margaret Hughes, Miren Iturriza-Gomara, James Johnson, L Luu, Jenifer Manson, Charlotte Nelson, Elaine O'Toole, Cassie Olateju, Rebekah Penrice-Randal , Lucille Rainbow, N.P Randle, Trevor Ian Robinson, Parul Sharma, Ghada T Shawli, James P Stewart, Neil Swainston, Ecaterina Vamos, Joanne Watts, Mark Whitehead
EPI_ISL_865847	University College London, Great Ormond Street Hospital for Children NHS Foundation Trust, Imperial College Healthcare NHS Trust	COVID-19 Genomics UK (COG-UK) Consortium	Sergi Castellano, Rachel Williams, Mark Kristiansen, Paola Resende Silva, Sunando Roy, Tony Brooks, Helena Tutill, Paola Niola, Patricia Dyal, Charlotte Williams, Leysa Forrest, Yasmin Panchbhaya, Jacqueline Findlay, Samuel Weeks, Julianne Brown, Kathryn Harris, Paul Randell, James Price, Alison Holmes, Judith Breuer
EPI_ISL_866103, EPI_ISL_866104, EPI_ISL_866105, EPI_ISL_866117, EPI_ISL_866120, EPI_ISL_866125, EPI_ISL_866126, EPI_ISL_866134, EPI_ISL_866142, EPI_ISL_866143, EPI_ISL_866146, EPI_ISL_866152, EPI_ISL_866154, EPI_ISL_866160, EPI_ISL_866171, EPI_ISL_866174, EPI_ISL_866178, EPI_ISL_866186			
see above	University College London Hospital	COVID-19 Genomics UK (COG-UK) Consortium	Judith Heaney, Matthew Byott, Catherine Houlihan, Dan Frampton, Stuart Kirk, Moira Spyer and Eleni Nastouli
EPI_ISL_866195, EPI_ISL_866197, EPI_ISL_866199, EPI_ISL_866200, EPI_ISL_866203, EPI_ISL_866204, EPI_ISL_866205, EPI_ISL_866214, EPI_ISL_866216, EPI_ISL_866218, EPI_ISL_866220, EPI_ISL_866224, EPI_ISL_866226, EPI_ISL_866227, EPI_ISL_866233, EPI_ISL_866234, EPI_ISL_866245, EPI_ISL_866246, EPI_ISL_866248, EPI_ISL_866249, EPI_ISL_866252, EPI_ISL_866254			
see above	University College London, Great Ormond Street Hospital for Children NHS Foundation Trust, Imperial College Healthcare NHS Trust	COVID-19 Genomics UK (COG-UK) Consortium	Sergi Castellano, Rachel Williams, Mark Kristiansen, Paola Resende Silva, Sunando Roy, Tony Brooks, Helena Tutill, Paola Niola, Patricia Dyal, Charlotte Williams, Leysa Forrest, Yasmin Panchbhaya, Jacqueline Findlay, Samuel Weeks, Julianne Brown, Kathryn Harris, Paul Randell, James Price, Alison Holmes, Judith Breuer

EPI_ISL_866542, EPI_ISL_866543, EPI_ISL_866544, EPI_ISL_866546, EPI_ISL_866547, EPI_ISL_866549, EPI_ISL_866550, EPI_ISL_866551, EPI_ISL_866552, EPI_ISL_866553, EPI_ISL_866554, EPI_ISL_866555, EPI_ISL_866557, EPI_ISL_866558, EPI_ISL_866559, EPI_ISL_866561, EPI_ISL_866562, EPI_ISL_866563, EPI_ISL_866564, EPI_ISL_866565, EPI_ISL_866567, EPI_ISL_866568, EPI_ISL_866570, EPI_ISL_866571, EPI_ISL_866572, EPI_ISL_866574, EPI_ISL_866575, EPI_ISL_866576, EPI_ISL_866603, EPI_ISL_866604, EPI_ISL_866605, EPI_ISL_866606, EPI_ISL_866607, EPI_ISL_866608, EPI_ISL_866609, EPI_ISL_866610, EPI_ISL_866611, EPI_ISL_866612, EPI_ISL_866613, EPI_ISL_866614, EPI_ISL_866615, EPI_ISL_866616, EPI_ISL_866617, EPI_ISL_866620, EPI_ISL_866623, EPI_ISL_866624, EPI_ISL_866625, EPI_ISL_866626, EPI_ISL_866630, EPI_ISL_866632, EPI_ISL_866633, EPI_ISL_866634, EPI_ISL_866636, EPI_ISL_866637, EPI_ISL_866638, EPI_ISL_866639, EPI_ISL_866640, EPI_ISL_866641, EPI_ISL_866644, EPI_ISL_866645, EPI_ISL_866646, EPI_ISL_866647, EPI_ISL_866648, EPI_ISL_866649, EPI_ISL_866650, EPI_ISL_866739, EPI_ISL_866758, EPI_ISL_866759, EPI_ISL_866761, EPI_ISL_866762, EPI_ISL_866765, EPI_ISL_866766, EPI_ISL_866768, EPI_ISL_866769, EPI_ISL_866771, EPI_ISL_866772, EPI_ISL_866773, EPI_ISL_866776, EPI_ISL_866777, EPI_ISL_866778, EPI_ISL_866779, EPI_ISL_866780, EPI_ISL_866783, EPI_ISL_866784, EPI_ISL_866785, EPI_ISL_866786, EPI_ISL_866787, EPI_ISL_866790, EPI_ISL_866791, EPI_ISL_866792, EPI_ISL_866794, EPI_ISL_866795, EPI_ISL_866796, EPI_ISL_866797, EPI_ISL_866798, EPI_ISL_866799, EPI_ISL_866800			
see above	Quadram Institute Bioscience	COVID-19 Genomics UK (COG-UK) Consortium	Dave J. Baker, Gemma L. Kay, Alp Aydin, Thanh Le-Viet, Steven Rudder, Ana P. Tedim, Anastasia Kolyva, Maria Diaz, Leonardo de Oliveira Martins, Nabil-Fareed Alikhan, Lizzie Meadows, Rachael Stanley, Ngozi Elumogo, Muhammed Yasir, Nicholas M. Thomson, Alexander J Trotter, Rachel Gilroy, Samuel Bloomfield, Claire Stuart, Andrew Bell, Reenesh Prakash, Samir Dervisevic, Alison E. Mather, John Wain, Mark Webber, Andrew J. Page, Justin O'Grady
EPI_ISL_867192, EPI_ISL_867193, EPI_ISL_867194, EPI_ISL_867195, EPI_ISL_867202, EPI_ISL_867215, EPI_ISL_867227, EPI_ISL_867228, EPI_ISL_867229, EPI_ISL_867230, EPI_ISL_867231, EPI_ISL_867232, EPI_ISL_867233, EPI_ISL_867252, EPI_ISL_867253, EPI_ISL_867254, EPI_ISL_867255, EPI_ISL_867256, EPI_ISL_867257, EPI_ISL_867258, EPI_ISL_867261, EPI_ISL_867269, EPI_ISL_867298, EPI_ISL_867299, EPI_ISL_867300, EPI_ISL_867301, EPI_ISL_867353, EPI_ISL_867358, EPI_ISL_867360, EPI_ISL_867362, EPI_ISL_867364, EPI_ISL_867365, EPI_ISL_867366, EPI_ISL_867367, EPI_ISL_867369, EPI_ISL_867370, EPI_ISL_867372, EPI_ISL_867373, EPI_ISL_867374, EPI_ISL_867375, EPI_ISL_867377, EPI_ISL_867378, EPI_ISL_867379, EPI_ISL_867381, EPI_ISL_867382, EPI_ISL_867383, EPI_ISL_867384, EPI_ISL_867385, EPI_ISL_867386, EPI_ISL_867387, EPI_ISL_867388, EPI_ISL_867390, EPI_ISL_867391, EPI_ISL_867392, EPI_ISL_867393, EPI_ISL_867394, EPI_ISL_867395, EPI_ISL_867396, EPI_ISL_867397, EPI_ISL_867398, EPI_ISL_867399, EPI_ISL_867400, EPI_ISL_867401, EPI_ISL_867403, EPI_ISL_867404, EPI_ISL_867405, EPI_ISL_867419, EPI_ISL_867424, EPI_ISL_867430, EPI_ISL_867450, EPI_ISL_867451, EPI_ISL_867459, EPI_ISL_867491, EPI_ISL_867492, EPI_ISL_867494, EPI_ISL_867525, EPI_ISL_867536, EPI_ISL_867538, EPI_ISL_867539, EPI_ISL_867540, EPI_ISL_867541, EPI_ISL_867556, EPI_ISL_867557, EPI_ISL_867561, EPI_ISL_867562, EPI_ISL_867567, EPI_ISL_867572, EPI_ISL_867573, EPI_ISL_867574, EPI_ISL_867575, EPI_ISL_867579, EPI_ISL_867582, EPI_ISL_867584, EPI_ISL_867586, EPI_ISL_867587, EPI_ISL_867588, EPI_ISL_867589, EPI_ISL_867590, EPI_ISL_867704, EPI_ISL_867705, EPI_ISL_867706, EPI_ISL_867716, EPI_ISL_867726, EPI_ISL_867728, EPI_ISL_867737, EPI_ISL_867738, EPI_ISL_867739, EPI_ISL_867740, EPI_ISL_867741, EPI_ISL_867819, EPI_ISL_867834, EPI_ISL_867890, EPI_ISL_867899, EPI_ISL_867900, EPI_ISL_867901, EPI_ISL_867904, EPI_ISL_867906, EPI_ISL_867911, EPI_ISL_867912, EPI_ISL_867913, EPI_ISL_867914, EPI_ISL_867915, EPI_ISL_867916, EPI_ISL_867918, EPI_ISL_867921			
see above	Originating lab: Wales Specialist Virology Centre Sequencing lab: Pathogen Genomics Unit	Public Health Wales Microbiology Cardiff Wales Specialist Virology Centre	Catherine Moore, Johnathan Evans, Laura Gifford, Malorie Perry, Simon Cottrell, Angela Marchbank, Alec Birchley, Alexander Adams, Amy Gaskin, Bree Gatica-Wilcox, Jason Coombes, Joel Southgate, Lauren Gilbert, Lee Graham, Nicole Pacchiarini, Sara Kumziene-Summerhayes, Sarah Taylor, Sophie Jones, Sara Rey, Matthew Bull, Joanne Watkins, Sally Corden, Tom Connor
EPI_ISL_868141, EPI_ISL_868148, EPI_ISL_868150, EPI_ISL_868152, EPI_ISL_868153, EPI_ISL_868154, EPI_ISL_868177, EPI_ISL_868178, EPI_ISL_868179, EPI_ISL_868203, EPI_ISL_868204			
see above	Centre for Enzyme Innovation, University of Portsmouth / Translational Research Laboratory, Portsmouth Hospitals NHS Trust	COVID-19 Genomics UK (COG-UK) Consortium	Angela Beckett,Yann Bourgeois,Garry Scarlett,Sharon Glaysher,Scott Elliott,Kelly Bicknell,Robert Impey,Allyson Lloyd,Sarah Wyllie,Ethan Butcher,Anoop Chauhan,Samuel Robson
EPI_ISL_868358, EPI_ISL_868364, EPI_ISL_868386, EPI_ISL_868399, EPI_ISL_868413, EPI_ISL_868428, EPI_ISL_868430, EPI_ISL_868432, EPI_ISL_868434, EPI_ISL_868445, EPI_ISL_868446, EPI_ISL_868448, EPI_ISL_868459, EPI_ISL_868467, EPI_ISL_868471, EPI_ISL_868487, EPI_ISL_868503, EPI_ISL_868522, EPI_ISL_868535, EPI_ISL_868547, EPI_ISL_868552, EPI_ISL_868556, EPI_ISL_868557, EPI_ISL_868573, EPI_ISL_868593, EPI_ISL_868621, EPI_ISL_868631, EPI_ISL_868648, EPI_ISL_868656, EPI_ISL_868660, EPI_ISL_868677, EPI_ISL_868684, EPI_ISL_868693, EPI_ISL_868700, EPI_ISL_868715			
see above	Virology Department, Sheffield Teaching Hospitals NHS Foundation Trust/Department of Infection, Immunity and Cardiovascular Disease, The Medical School, University of Sheffield	COVID-19 Genomics UK (COG-UK) Consortium	Thushan de Silva, Matthew Parker, Nikki Smith, Adri Angyal, Rebecca Brown, Luke Green, Rachel Tucker, Paul Parsons, Danielle Groves, Katie Johnson, Laura Carrilero, Alex Keeley, Dave Partridge, Matthew Wyles, Benjamin Lindsey, Mehmet Yavuz, Mohammad Raza, Cariad Evans
EPI_ISL_869132	Charité Universitätsmedizin Berlin, Institut für Virologie/Labor Berlin	Charité Universitätsmedizin Berlin, Institut für Virologie	Victor M Corman, Barbara Mühlemann, Jörn Beheim-Schwarzbach, Tobias Bleicker, Julia Tesch, Talitha Veith, Julia Schneider, Terry Jones, Christian Drosten
EPI_ISL_869644, EPI_ISL_869645, EPI_ISL_869646, EPI_ISL_869647, EPI_ISL_869648, EPI_ISL_869649, EPI_ISL_869650, EPI_ISL_869651, EPI_ISL_869652, EPI_ISL_869653, EPI_ISL_869654, EPI_ISL_869655, EPI_ISL_869656, EPI_ISL_869657, EPI_ISL_869658, EPI_ISL_869659, EPI_ISL_869660, EPI_ISL_869661, EPI_ISL_869662, EPI_ISL_869663, EPI_ISL_869664, EPI_ISL_869665, EPI_ISL_869666, EPI_ISL_869667, EPI_ISL_869668, EPI_ISL_869669, EPI_ISL_869670, EPI_ISL_869671, EPI_ISL_869672, EPI_ISL_869673, EPI_ISL_869674, EPI_ISL_869675, EPI_ISL_869676, EPI_ISL_869677, EPI_ISL_869678, EPI_ISL_869679, EPI_ISL_869680, EPI_ISL_869681, EPI_ISL_869682, EPI_ISL_869683, EPI_ISL_869684, EPI_ISL_869685, EPI_ISL_869686, EPI_ISL_869687, EPI_ISL_869688, EPI_ISL_869689, EPI_ISL_869690, EPI_ISL_869691, EPI_ISL_869692, EPI_ISL_869693, EPI_ISL_869694, EPI_ISL_869695, EPI_ISL_869696, EPI_ISL_869697, EPI_ISL_869698, EPI_ISL_869699, EPI_ISL_869700, EPI_ISL_869701, EPI_ISL_869702, EPI_ISL_869703, EPI_ISL_869704, EPI_ISL_869705, EPI_ISL_869706, EPI_ISL_869707, EPI_ISL_869708, EPI_ISL_869709, EPI_ISL_869710, EPI_ISL_869711, EPI_ISL_869712, EPI_ISL_869713, EPI_ISL_869714, EPI_ISL_869715, EPI_ISL_869716, EPI_ISL_869717, EPI_ISL_869718, EPI_ISL_869719, EPI_ISL_869720, EPI_ISL_869721, EPI_ISL_869722, EPI_ISL_869723, EPI_ISL_869724, EPI_ISL_869725, EPI_ISL_869726, EPI_ISL_869727, EPI_ISL_869728, EPI_ISL_869729, EPI_ISL_869730, EPI_ISL_869731, EPI_ISL_869732, EPI_ISL_869733, EPI_ISL_869734, EPI_ISL_869735, EPI_ISL_869736, EPI_ISL_869737, EPI_ISL_869738, EPI_ISL_869739, EPI_ISL_869740, EPI_ISL_869741, EPI_ISL_869742, EPI_ISL_869743, EPI_ISL_869744, EPI_ISL_869745, EPI_ISL_869746, EPI_ISL_869747, EPI_ISL_869748, EPI_ISL_869749, EPI_ISL_869750, EPI_ISL_869751, EPI_ISL_869752, EPI_ISL_869753, EPI_ISL_869754, EPI_ISL_869755, EPI_ISL_869756, EPI_ISL_869757, EPI_ISL_869758, EPI_ISL_869759, EPI_ISL_869760, EPI_ISL_869761, EPI_ISL_869762, EPI_ISL_869763, EPI_ISL_869764, EPI_ISL_869765, EPI_ISL_869766, EPI_ISL_869767, EPI_ISL_869768, EPI_ISL_869769, EPI_ISL_869770, EPI_ISL_869771, EPI_ISL_869772, EPI_ISL_869773, EPI_ISL_869774, EPI_ISL_869775, EPI_ISL_869776, EPI_ISL_869777, EPI_ISL_869778, EPI_ISL_869779, EPI_ISL_869780, EPI_ISL_869781, EPI_ISL_869782, EPI_ISL_869783, EPI_ISL_869784, EPI_ISL_869785, EPI_ISL_869786, EPI_ISL_869787, EPI_ISL_869788, EPI_ISL_869789, EPI_ISL_869790, EPI_ISL_869791, EPI_ISL_869792, EPI_ISL_869793, EPI_ISL_869794, EPI_ISL_869795, EPI_ISL_869796, EPI_ISL_869797, EPI_ISL_869798, EPI_ISL_869799, EPI_ISL_869800, EPI_ISL_869801, EPI_ISL_869802, EPI_ISL_869803, EPI_ISL_869804, EPI_ISL_869805, EPI_ISL_869806, EPI_ISL_869807, EPI_ISL_869808, EPI_ISL_869809, EPI_ISL_869810, EPI_ISL_869811, EPI_ISL_869812, EPI_ISL_869813, EPI_ISL_869814, EPI_ISL_869815, EPI_ISL_869816, EPI_ISL_869817, EPI_ISL_869818, EPI_ISL_869819, EPI_ISL_869820, EPI_ISL_869821, EPI_ISL_869822, EPI_ISL_869823, EPI_ISL_869824, EPI_ISL_869825, EPI_ISL_869826, EPI_ISL_869827, EPI_ISL_869828, EPI_ISL_869829, EPI_ISL_869830, EPI_ISL_869831, EPI_ISL_869832, EPI_ISL_869833, EPI_ISL_869834, EPI_ISL_869835, EPI_ISL_869836, EPI_ISL_869837, EPI_ISL_869838, EPI_ISL_869839, EPI_ISL_869840, EPI_ISL_869841, EPI_ISL_869842, EPI_ISL_869843, EPI_ISL_869844, EPI_ISL_869845, EPI_ISL_869846, EPI_ISL_869847, EPI_ISL_869848, EPI_ISL_869849, EPI_ISL_869850, EPI_ISL_869851, EPI_ISL_869852, EPI_ISL_869853, EPI_ISL_869854, EPI_ISL_869855, EPI_ISL_869856, EPI_ISL_869857, EPI_ISL_869858, EPI_ISL_869859, EPI_ISL_869860, EPI_ISL_869861, EPI_ISL_869862, EPI_ISL_869863, EPI_ISL_869864, EPI_ISL_869865, EPI_ISL_869866, EPI_ISL_869867, EPI_ISL_869868, EPI_ISL_869869, EPI_ISL_869870, EPI_ISL_869871, EPI_ISL_869872, EPI_ISL_869873, EPI_ISL_869874, EPI_ISL_869875, EPI_ISL_869876, EPI_ISL_869877, EPI_ISL_869878, EPI_ISL_869879, EPI_ISL_869880, EPI_ISL_869881, EPI_ISL_869882, EPI_ISL_869883, EPI_ISL_869884, EPI_ISL_869885, EPI_ISL_869886, EPI_ISL_869887, EPI_ISL_869888, EPI_ISL_869889, EPI_ISL_869890, EPI_ISL_869891, EPI_ISL_869892, EPI_ISL_869893, EPI_ISL_869894, EPI_ISL_869895, EPI_ISL_869896, EPI_ISL_869897, EPI_ISL_869898, EPI_ISL_869899, EPI_ISL_869900, EPI_ISL_869901, EPI_ISL_869902, EPI_ISL_869903, EPI_ISL_869904, EPI_ISL_869905, EPI_ISL_869906, EPI_ISL_869907, EPI_ISL_869908, EPI_ISL_869909, EPI_ISL_869910, EPI_ISL_869911, EPI_ISL_869912, EPI_ISL_869913, EPI_ISL_869914, EPI_ISL_869915, EPI_ISL_869916, EPI_ISL_869917, EPI_ISL_869918, EPI_ISL_869919, EPI_ISL_869920, EPI_ISL_869921, EPI_ISL_869922, EPI_ISL_869923, EPI_ISL_869924, EPI_ISL_869925, EPI_ISL_869926, EPI_ISL_869927, EPI_ISL_869928, EPI_ISL_869929, EPI_ISL_869930, EPI_ISL_869931, EPI_ISL_869932, EPI_ISL_869933, EPI_ISL_869934, EPI_ISL_869935, EPI_ISL_869936, EPI_ISL_869937, EPI_ISL_869938, EPI_ISL_869939, EPI_ISL_869940, EPI_ISL_869941, EPI_ISL_869942, EPI_ISL_869943, EPI_ISL_869944, EPI_ISL_869945, EPI_ISL_869946, EPI_ISL_869947, EPI_ISL_869948, EPI_ISL_869949, EPI_ISL_869950, EPI_ISL_869951, EPI_ISL_869952, EPI_ISL_869953, EPI_ISL_869954, EPI_ISL_869955, EPI_ISL_869956, EPI_ISL_869957, EPI_ISL_869958, EPI_ISL_869959, EPI_ISL_869960, EPI_ISL_869961, EPI_ISL_869962, EPI_ISL_869963, EPI_ISL_869964, EPI_ISL_869965, EPI_ISL_869966, EPI_ISL_869967, EPI_ISL_869968, EPI_ISL_869969, EPI_ISL_869970, EPI_ISL_869971, EPI_ISL_869972, EPI_ISL_869973, EPI_ISL_869974, EPI_ISL_869975, EPI_ISL_869976, EPI_ISL_869977, EPI_ISL_869978, EPI_ISL_869979, EPI_ISL_869980, EPI_ISL_869981, EPI_ISL_869982, EPI_ISL_869983, EPI_ISL_869984, EPI_ISL_869985, EPI_ISL_869986, EPI_ISL_869987, EPI_ISL_869988, EPI_ISL_869989, EPI_ISL_869990, EPI_ISL_869991, EPI_ISL_869992, EPI_ISL_869993, EPI_ISL_869994, EPI_ISL_869995, EPI_ISL_869996, EPI_ISL_869997, EPI_ISL_869998, EPI_ISL_869999, EPI_ISL_870000, EPI_ISL_870001, EPI_ISL_870002, EPI_ISL_870003, EPI_ISL_870004, EPI_ISL_870005, EPI_ISL_870006, EPI_ISL_870007, EPI_ISL_870008, EPI_ISL_870009, EPI_ISL_870010, EPI_ISL_870011, EPI_ISL_870012, EPI_ISL_870013, EPI_ISL_870014, EPI_ISL_870015, EPI_ISL_870016, EPI_ISL_870017, EPI_ISL_870018, EPI_ISL_870019, EPI_ISL_870020, EPI_ISL_870021, EPI_ISL_870022, EPI_ISL_870023, EPI_ISL_870024, EPI_ISL_870025, EPI_ISL_870026, EPI_ISL_870027, EPI_ISL_870028, EPI_ISL_870029, EPI_ISL_870030, EPI_ISL_870031, EPI_ISL_870032, EPI_ISL_870033, EPI_ISL_870034, EPI_ISL_870035, EPI_ISL_870036, EPI_ISL_870037, EPI_ISL_870038, EPI_ISL_870039, EPI_ISL_870040, EPI_ISL_870041, EPI_ISL_870042, EPI_ISL_870043, EPI_ISL_870044, EPI_ISL_870045, EPI_ISL_870046, EPI_ISL_870047, EPI_ISL_870048, EPI_ISL_870049, EPI_ISL_870050, EPI_ISL_870051, EPI_ISL_870052, EPI_ISL_870053, EPI_ISL_870054, EPI_ISL_870055, EPI_ISL_870056, EPI_ISL_870057, EPI_ISL_870058, EPI_ISL_870059, EPI_ISL_870060, EPI_ISL_870061, EPI_ISL_870062, EPI_ISL_870063, EPI_ISL_870064, EPI_ISL_870066, EPI_ISL_870067, EPI_ISL_870068, EPI_ISL_870069, EPI_ISL_870070, EPI_ISL_870071, EPI_ISL_870072, EPI_ISL_870073, EPI_ISL_870074, EPI_ISL_870075, EPI_ISL_870076, EPI_ISL_870077, EPI_ISL_870078, EPI_ISL_870079, EPI_ISL_870080, EPI_ISL_870081, EPI_ISL_870082, EPI_ISL_870083, EPI_ISL_870084, EPI_ISL_870085, EPI_ISL_870086, EPI_ISL_870087, EPI_ISL_870088, EPI_ISL_870089, EPI_ISL_870090, EPI_ISL_870091, EPI_ISL_870092, EPI_ISL_870093, EPI_ISL_870094, EPI_ISL_870095, EPI_ISL_870096, EPI_ISL_870097, EPI_ISL_870098, EPI_ISL_870099, EPI_ISL_870100, EPI_ISL_870101, EPI_ISL_870102, EPI_ISL_870103, EPI_ISL_870104, EPI_ISL_870105, EPI_ISL_870106, EPI_ISL_870107, EPI_ISL_870108, EPI_ISL_870109, EPI_ISL_870110, EPI_ISL_870111, EPI_ISL_870112, EPI_ISL_870113, EPI_ISL_870114, EPI_ISL_870115, EPI_ISL_870116, EPI_ISL_870117, EPI_ISL_870118, EPI_ISL_870119, EPI_ISL_870120, EPI_ISL_870121, EPI_ISL_870122, EPI_ISL_870123, EPI_ISL_870124, EPI_ISL_870125, EPI_ISL_870126, EPI_ISL_870127, EPI_ISL_870128, EPI_ISL_870129, EPI_ISL_870130, EPI_ISL_870131, EPI_ISL_870132, EPI_ISL_870133, EPI_ISL_870134, EPI_ISL_870135, EPI_ISL_870136, EPI_ISL_870137, EPI_ISL_870138, EPI_ISL_870139, EPI_ISL_870140, EPI_ISL_870141, EPI_ISL_870142, EPI_ISL_870143, EPI_ISL_870144, EPI_ISL_870145, EPI_ISL_870146, EPI_ISL_870147, EPI_ISL_870148, EPI_ISL_870149, EPI_ISL_870150, EPI_ISL_870151, EPI_ISL_870152, EPI_ISL_870153, EPI_ISL_870154, EPI_ISL_870155, EPI_ISL_870156, EPI_ISL_870157, EPI_ISL_870158, EPI_ISL_870159, EPI_ISL_870160, EPI_ISL_870161, EPI_ISL_870162, EPI_ISL_870163, EPI_ISL_870164, EPI_ISL_870165, EPI_ISL_870166, EPI_ISL_870167, EPI_ISL_870168, EPI_ISL_870169, EPI_ISL_870170, EPI_ISL_870171, EPI_ISL_870172, EPI_ISL_870173, EPI_ISL_870174, EPI_ISL_870175, EPI_ISL_870176, EPI_ISL_870177, EPI_ISL_870178, EPI_ISL_870179, EPI_ISL_870180, EPI_ISL_870181, EPI_ISL_870182, EPI_ISL_870183, EPI_ISL_870184, EPI_ISL_870185, EPI_ISL_870186, EPI_ISL_870187, EPI_ISL_870188, EPI_ISL_870189, EPI_ISL_870190, EPI_ISL_870191, EPI_ISL_870192, EPI_ISL_870193, EPI_ISL_870194, EPI_ISL_870195, EPI_ISL_870196, EPI_ISL_870197, EPI_ISL_870198, EPI_ISL_870199, EPI_ISL_870200, EPI_ISL_870201, EPI_ISL_870202, EPI_ISL_870203, EPI_ISL_870204, EPI_ISL_870205, EPI_ISL_870206, EPI_ISL_870207, EPI_ISL_870208, EPI_ISL_870209, EPI_ISL_870210, EPI_ISL_870211, EPI_ISL_870212, EPI_ISL_870213, EPI_ISL_870214, EPI_ISL_870215, EPI_ISL_870216, EPI_ISL_870217, EPI_ISL_870218, EPI_ISL_870219, EPI_ISL_870220, EPI_ISL_870221, EPI_ISL_870222, EPI_ISL_870223, EPI_ISL_870224, EPI_ISL_870225, EPI_ISL_870226, EPI_ISL_870227, EPI_ISL_870228, EPI_ISL_870229, EPI_ISL_870230, EPI_ISL_870231, EPI_ISL_870232, EPI_ISL_870233, EPI_ISL_870234, EPI_ISL_870235, EPI_ISL_870236, EPI_ISL_870237, EPI_ISL_870238, EPI_ISL_870239, EPI_ISL_870240, EPI_ISL_870241, EPI_ISL_870242, EPI_ISL_870243, EPI_ISL_870244, EPI_ISL_870245, EPI_ISL_870246, EPI_ISL_870247, EPI_ISL_870248, EPI_ISL_870249, EPI_ISL_870250, EPI_ISL_870251, EPI_ISL_870252, EPI_ISL_870253, EPI_ISL_870254, EPI_ISL_870255, EPI_ISL_870256, EPI_ISL_870257, EPI_ISL_870258, EPI_ISL_870259, EPI_ISL_870260, EPI_ISL_870261, EPI_ISL_870262, EPI_ISL_870263, EPI_ISL_870264, EPI_ISL_870265, EPI_ISL_870266, EPI_ISL_870267, EPI_ISL_870268, EPI_ISL_870269, EPI_ISL_870270, EPI_ISL_870271, EPI_ISL_870272, EPI_ISL_870273, EPI_ISL_870274, EPI_ISL_870275, EPI_ISL_870276, EPI_ISL_870277, EPI_ISL_870278, EPI_ISL_870279, EPI_ISL_870280, EPI_ISL_870281, EPI_ISL_870282, EPI_ISL_870283, EPI_ISL_870284, EPI_ISL_870285, EPI_ISL_870286, EPI_ISL_870287, EPI_ISL_870288, EPI_ISL_870289, EPI_ISL_870290, EPI_ISL_870291, EPI_ISL_870292, EPI_ISL_870293, EPI_ISL_870294, EPI_ISL_870295, EPI_ISL_870296, EPI_ISL_870297, EPI_ISL_870298, EPI_ISL_870299, EPI_ISL_870300, EPI_ISL_870301, EPI_ISL_870302, EPI_ISL_870303, EPI_ISL_870304, EPI_ISL_870305, EPI_ISL_870306, EPI_ISL_870307, EPI_ISL_870308, EPI_ISL_870309, EPI_ISL_870310, EPI_ISL_870311, EPI_ISL_870312, EPI_ISL_870313, EPI_ISL_870314, EPI_ISL_870316, EPI_ISL_870318, EPI_ISL_870320			
see above	Department of Virus and Microbiological Special Diagnostics, Statens Serum Institut, Copenhagen, Denmark	Aalborg University	Danish Covid-19 Genome Consortium
EPI_ISL_870321	University of Michigan Clinical Microbiology Laboratory	Lauring Lab, University of Michigan, Department of Microbiology and Immunology	Valesano
EPI_ISL_870323, EPI_ISL_870324, EPI_ISL_870325, EPI_ISL_870326, EPI_ISL_870327, EPI_ISL_870328, EPI_ISL_870329, EPI_ISL_870330, EPI_ISL_870331, EPI_ISL_870332, EPI_ISL_870333, EPI_ISL_870334, EPI_ISL_870335, EPI_ISL_870336, EPI_ISL_870337, EPI_ISL_870338, EPI_ISL_870339, EPI_ISL_870340, EPI_ISL_870341, EPI_ISL_870342, EPI_ISL_870343, EPI_ISL_870344, EPI_ISL_870345, EPI_ISL_870346, EPI_ISL_870347, EPI_ISL_870348, EPI_ISL_870349, EPI_ISL_870350, EPI_ISL_870351, EPI_ISL_870352, EPI_ISL_870353, EPI_ISL_870354, EPI_ISL_870355, EPI_ISL_870356, EPI_ISL_870357, EPI_ISL_870358, EPI_ISL_870359, EPI_ISL_870360, EPI_ISL_870361, EPI_ISL_870362, EPI_ISL_870363, EPI_ISL_870364, EPI_ISL_870365, EPI_ISL_870366, EPI_ISL_870367, EPI_ISL_870368, EPI_ISL_870369, EPI_ISL_870370, EPI_ISL_870371, EPI_ISL_870372, EPI_ISL_870373, EPI_ISL_870374, EPI_ISL_870375, EPI_ISL_870376, EPI_ISL_870377, EPI_ISL_870378, EPI_ISL_870379, EPI_ISL_870380, EPI_ISL_870381, EPI_ISL_870382, EPI_ISL_870383, EPI_ISL_870384, EPI_ISL_870385, EPI_ISL_870386, EPI_ISL_870387, EPI_ISL_870388, EPI_ISL_870389, EPI_ISL_870390, EPI_ISL_870391, EPI_ISL_870392, EPI_ISL_870393, EPI_ISL_870394, EPI_ISL_870395, EPI_ISL_870396, EPI_ISL_870397, EPI_ISL_870398, EPI_ISL_870399, EPI_ISL_870400, EPI_ISL_870401, EPI_ISL_870402, EPI_ISL_870403, EPI_ISL_870404, EPI_ISL_870405, EPI_ISL_870406, EPI_ISL_870407, EPI_ISL_870408, EPI_ISL_870409, EPI_ISL_870410, EPI_ISL_870411, EPI_ISL_870412, EPI_ISL_870413, EPI_ISL_870414, EPI_ISL_870415, EPI_ISL_870416, EPI_ISL_870417, EPI_ISL_870418, EPI_ISL_870419, EPI_ISL_870420, EPI_ISL_870421, EPI_ISL_870422, EPI_ISL_870423, EPI_ISL_870424, EPI_ISL_870425, EPI_ISL_870426, EPI_ISL_870427, EPI_ISL_870428, EPI_ISL_870429, EPI_ISL_870430, EPI_ISL_870431, EPI_ISL_870432, EPI_ISL_870433, EPI_ISL_870434, EPI_ISL_870435, EPI_ISL_870436, EPI_ISL_870437, EPI_ISL_870438, EPI_ISL_870439, EPI_ISL_870440, EPI_ISL_870441, EPI_ISL_870442, EPI_ISL_870443, EPI_ISL_870444, EPI_ISL_870445, EPI_ISL_870446, EPI_ISL_870447, EPI_ISL_870448,			

EPI_ISL_870449, EPI_ISL_870450, EPI_ISL_870451, EPI_ISL_870452, EPI_ISL_870453, EPI_ISL_870454, EPI_ISL_870455, EPI_ISL_870456, EPI_ISL_870457, EPI_ISL_870458, EPI_ISL_870459, EPI_ISL_870460, EPI_ISL_870461, EPI_ISL_870462, EPI_ISL_870463, EPI_ISL_870464, EPI_ISL_870465, EPI_ISL_870466, EPI_ISL_870467, EPI_ISL_870468, EPI_ISL_870469, EPI_ISL_870470, EPI_ISL_870471, EPI_ISL_870472, EPI_ISL_870473, EPI_ISL_870474, EPI_ISL_870475, EPI_ISL_870476, EPI_ISL_870477, EPI_ISL_870478, EPI_ISL_870479, EPI_ISL_870480, EPI_ISL_870481, EPI_ISL_870482, EPI_ISL_870483, EPI_ISL_870484, EPI_ISL_870485, EPI_ISL_870486, EPI_ISL_870487, EPI_ISL_870488, EPI_ISL_870489, EPI_ISL_870490, EPI_ISL_870491, EPI_ISL_870492, EPI_ISL_870493, EPI_ISL_870494, EPI_ISL_870495, EPI_ISL_870496, EPI_ISL_870497, EPI_ISL_870498, EPI_ISL_870499, EPI_ISL_870500, EPI_ISL_870501, EPI_ISL_870502, EPI_ISL_870503, EPI_ISL_870504, EPI_ISL_870505, EPI_ISL_870506, EPI_ISL_870507, EPI_ISL_870508, EPI_ISL_870509, EPI_ISL_870510, EPI_ISL_870511, EPI_ISL_870512, EPI_ISL_870513, EPI_ISL_870514, EPI_ISL_870515, EPI_ISL_870516, EPI_ISL_870517, EPI_ISL_870518, EPI_ISL_870519, EPI_ISL_870520, EPI_ISL_870521, EPI_ISL_870522, EPI_ISL_870523, EPI_ISL_870524, EPI_ISL_870525, EPI_ISL_870526, EPI_ISL_870527, EPI_ISL_870528, EPI_ISL_870529, EPI_ISL_870530, EPI_ISL_870531, EPI_ISL_870532, EPI_ISL_870533, EPI_ISL_870534, EPI_ISL_870535, EPI_ISL_870536, EPI_ISL_870537, EPI_ISL_870538, EPI_ISL_870539, EPI_ISL_870540, EPI_ISL_870541, EPI_ISL_870542, EPI_ISL_870543, EPI_ISL_870544, EPI_ISL_870545, EPI_ISL_870546, EPI_ISL_870547, EPI_ISL_870548, EPI_ISL_870549, EPI_ISL_870550, EPI_ISL_870551, EPI_ISL_870552, EPI_ISL_870553, EPI_ISL_870554, EPI_ISL_870555, EPI_ISL_870556, EPI_ISL_870557, EPI_ISL_870558, EPI_ISL_870559, EPI_ISL_870560, EPI_ISL_870561, EPI_ISL_870562, EPI_ISL_870563, EPI_ISL_870564, EPI_ISL_870565, EPI_ISL_870566, EPI_ISL_870567, EPI_ISL_870568, EPI_ISL_870569, EPI_ISL_870570, EPI_ISL_870571, EPI_ISL_870572, EPI_ISL_870573, EPI_ISL_870574, EPI_ISL_870575, EPI_ISL_870576, EPI_ISL_870577, EPI_ISL_870578, EPI_ISL_870579, EPI_ISL_870580, EPI_ISL_870581, EPI_ISL_870582, EPI_ISL_870583, EPI_ISL_870584, EPI_ISL_870585, EPI_ISL_870586, EPI_ISL_870587, EPI_ISL_870588, EPI_ISL_870589, EPI_ISL_870590, EPI_ISL_870591, EPI_ISL_870592, EPI_ISL_870593, EPI_ISL_870594, EPI_ISL_870595, EPI_ISL_870596, EPI_ISL_870597, EPI_ISL_870598, EPI_ISL_870599, EPI_ISL_870600, EPI_ISL_870601, EPI_ISL_870602, EPI_ISL_870603, EPI_ISL_870604, EPI_ISL_870605, EPI_ISL_870606, EPI_ISL_870607, EPI_ISL_870608, EPI_ISL_870609, EPI_ISL_870610, EPI_ISL_870611, EPI_ISL_870612, EPI_ISL_870613, EPI_ISL_870614, EPI_ISL_870615, EPI_ISL_870616, EPI_ISL_870617, EPI_ISL_870618, EPI_ISL_870619, EPI_ISL_870620, EPI_ISL_870621, EPI_ISL_870622, EPI_ISL_870623, EPI_ISL_870624, EPI_ISL_870625, EPI_ISL_870626, EPI_ISL_870627, EPI_ISL_870628, EPI_ISL_870629, EPI_ISL_870630, EPI_ISL_870631, EPI_ISL_870632, EPI_ISL_870633, EPI_ISL_870634, EPI_ISL_870635, EPI_ISL_870636, EPI_ISL_870637, EPI_ISL_870638, EPI_ISL_870639, EPI_ISL_870640, EPI_ISL_870641, EPI_ISL_870642, EPI_ISL_870643, EPI_ISL_870644, EPI_ISL_870645, EPI_ISL_870646, EPI_ISL_870647, EPI_ISL_870648, EPI_ISL_870649, EPI_ISL_870650, EPI_ISL_870651, EPI_ISL_870652, EPI_ISL_870653, EPI_ISL_870654, EPI_ISL_870655, EPI_ISL_870656, EPI_ISL_870657, EPI_ISL_870658, EPI_ISL_870659, EPI_ISL_870660, EPI_ISL_870661, EPI_ISL_870662, EPI_ISL_870663, EPI_ISL_870664, EPI_ISL_870665, EPI_ISL_870666, EPI_ISL_870667, EPI_ISL_870668, EPI_ISL_870669, EPI_ISL_870670, EPI_ISL_870671, EPI_ISL_870672, EPI_ISL_870673, EPI_ISL_870674, EPI_ISL_870675, EPI_ISL_870676, EPI_ISL_870677, EPI_ISL_870678, EPI_ISL_870679, EPI_ISL_870680, EPI_ISL_870681, EPI_ISL_870682, EPI_ISL_870683, EPI_ISL_870684, EPI_ISL_870685, EPI_ISL_870686, EPI_ISL_870687, EPI_ISL_870688, EPI_ISL_870689, EPI_ISL_870690, EPI_ISL_870691, EPI_ISL_870692, EPI_ISL_870693, EPI_ISL_870694, EPI_ISL_870695, EPI_ISL_870696, EPI_ISL_870697, EPI_ISL_870698, EPI_ISL_870699, EPI_ISL_870700, EPI_ISL_870701, EPI_ISL_870702, EPI_ISL_870703, EPI_ISL_870704, EPI_ISL_870705, EPI_ISL_870706, EPI_ISL_870707, EPI_ISL_870708, EPI_ISL_870709, EPI_ISL_870710, EPI_ISL_870711, EPI_ISL_870712, EPI_ISL_870713, EPI_ISL_870714, EPI_ISL_870715, EPI_ISL_870716, EPI_ISL_870717, EPI_ISL_870718, EPI_ISL_870719, EPI_ISL_870720, EPI_ISL_870721, EPI_ISL_870722, EPI_ISL_870723, EPI_ISL_870724, EPI_ISL_870725, EPI_ISL_870726, EPI_ISL_870727, EPI_ISL_870728, EPI_ISL_870729, EPI_ISL_870730, EPI_ISL_870731, EPI_ISL_870732, EPI_ISL_870733, EPI_ISL_870734, EPI_ISL_870735, EPI_ISL_870736, EPI_ISL_870737, EPI_ISL_870738, EPI_ISL_870739, EPI_ISL_870740, EPI_ISL_870741, EPI_ISL_870742, EPI_ISL_870743, EPI_ISL_870744, EPI_ISL_870745, EPI_ISL_870746, EPI_ISL_870747, EPI_ISL_870748, EPI_ISL_870749, EPI_ISL_870750, EPI_ISL_870751, EPI_ISL_870752, EPI_ISL_870753, EPI_ISL_870754, EPI_ISL_870755, EPI_ISL_870756, EPI_ISL_870757, EPI_ISL_870758, EPI_ISL_870759, EPI_ISL_870760, EPI_ISL_870761, EPI_ISL_870762, EPI_ISL_870763, EPI_ISL_870764, EPI_ISL_870765, EPI_ISL_870766, EPI_ISL_870767, EPI_ISL_870768, EPI_ISL_870769, EPI_ISL_870770, EPI_ISL_870771, EPI_ISL_870772, EPI_ISL_870773, EPI_ISL_870774, EPI_ISL_870775, EPI_ISL_870776, EPI_ISL_870777, EPI_ISL_870778, EPI_ISL_870779, EPI_ISL_870780, EPI_ISL_870781, EPI_ISL_870782, EPI_ISL_870783, EPI_ISL_870784, EPI_ISL_870785, EPI_ISL_870786, EPI_ISL_870787, EPI_ISL_870788, EPI_ISL_870789, EPI_ISL_870790, EPI_ISL_870791, EPI_ISL_870792, EPI_ISL_870793, EPI_ISL_870794, EPI_ISL_870795, EPI_ISL_870796, EPI_ISL_870797, EPI_ISL_870798, EPI_ISL_870799, EPI_ISL_870800, EPI_ISL_870801, EPI_ISL_870802, EPI_ISL_870803, EPI_ISL_870804, EPI_ISL_870805, EPI_ISL_870806, EPI_ISL_870807, EPI_ISL_870808, EPI_ISL_870809, EPI_ISL_870810, EPI_ISL_870811, EPI_ISL_870812, EPI_ISL_870813, EPI_ISL_870814, EPI_ISL_870815, EPI_ISL_870816, EPI_ISL_870817, EPI_ISL_870818, EPI_ISL_870819, EPI_ISL_870820, EPI_ISL_870821, EPI_ISL_870822, EPI_ISL_870823, EPI_ISL_870824, EPI_ISL_870825, EPI_ISL_870826, EPI_ISL_870827, EPI_ISL_870828, EPI_ISL_870829, EPI_ISL_870830, EPI_ISL_870831, EPI_ISL_870832, EPI_ISL_870833, EPI_ISL_870834, EPI_ISL_870835, EPI_ISL_870836, EPI_ISL_870837, EPI_ISL_870838, EPI_ISL_870839, EPI_ISL_870840, EPI_ISL_870841, EPI_ISL_870842, EPI_ISL_870843, EPI_ISL_870844, EPI_ISL_870845, EPI_ISL_870846, EPI_ISL_870847, EPI_ISL_870848, EPI_ISL_870849, EPI_ISL_870850, EPI_ISL_870851, EPI_ISL_870852, EPI_ISL_870853, EPI_ISL_870854, EPI_ISL_870855, EPI_ISL_870856, EPI_ISL_870857, EPI_ISL_870858, EPI_ISL_870859, EPI_ISL_870860, EPI_ISL_870861, EPI_ISL_870862, EPI_ISL_870863, EPI_ISL_870864, EPI_ISL_870865, EPI_ISL_870866, EPI_ISL_870867, EPI_ISL_870868, EPI_ISL_870869, EPI_ISL_870870, EPI_ISL_870871, EPI_ISL_870872, EPI_ISL_870873, EPI_ISL_870874, EPI_ISL_870875, EPI_ISL_870876, EPI_ISL_870877, EPI_ISL_870878, EPI_ISL_870879, EPI_ISL_870880, EPI_ISL_870881, EPI_ISL_870882, EPI_ISL_870883, EPI_ISL_870884, EPI_ISL_870885, EPI_ISL_870886, EPI_ISL_870887, EPI_ISL_870888, EPI_ISL_870889, EPI_ISL_870890, EPI_ISL_870891, EPI_ISL_870892, EPI_ISL_870893, EPI_ISL_870894, EPI_ISL_870895, EPI_ISL_870896, EPI_ISL_870897, EPI_ISL_870898, EPI_ISL_870899, EPI_ISL_870900, EPI_ISL_870901, EPI_ISL_870902, EPI_ISL_870903, EPI_ISL_870904, EPI_ISL_870905, EPI_ISL_870906, EPI_ISL_870907, EPI_ISL_870908, EPI_ISL_870909, EPI_ISL_870910, EPI_ISL_870911, EPI_ISL_870912, EPI_ISL_870913, EPI_ISL_870914, EPI_ISL_870915, EPI_ISL_870916, EPI_ISL_870917, EPI_ISL_870918, EPI_ISL_870919, EPI_ISL_870920, EPI_ISL_870921, EPI_ISL_870922, EPI_ISL_870923, EPI_ISL_870924, EPI_ISL_870925, EPI_ISL_870926, EPI_ISL_870927, EPI_ISL_870928, EPI_ISL_870929, EPI_ISL_870930, EPI_ISL_870931, EPI_ISL_870932, EPI_ISL_870933, EPI_ISL_870934, EPI_ISL_870935, EPI_ISL_870936, EPI_ISL_870937, EPI_ISL_870938, EPI_ISL_870939, EPI_ISL_870940, EPI_ISL_870941, EPI_ISL_870942, EPI_ISL_870943, EPI_ISL_870944, EPI_ISL_870945, EPI_ISL_870946, EPI_ISL_870947, EPI_ISL_870948, EPI_ISL_870949, EPI_ISL_870950, EPI_ISL_870951, EPI_ISL_870952, EPI_ISL_870953, EPI_ISL_870954, EPI_ISL_870955, EPI_ISL_870956, EPI_ISL_870957, EPI_ISL_870958, EPI_ISL_870959, EPI_ISL_870960, EPI_ISL_870961, EPI_ISL_870962, EPI_ISL_870963, EPI_ISL_870964, EPI_ISL_870965, EPI_ISL_870966, EPI_ISL_870967, EPI_ISL_870968, EPI_ISL_870969, EPI_ISL_870970, EPI_ISL_870971, EPI_ISL_870972, EPI_ISL_870973, EPI_ISL_870974, EPI_ISL_870975, EPI_ISL_870976, EPI_ISL_870977, EPI_ISL_870978, EPI_ISL_870979, EPI_ISL_870980, EPI_ISL_870981, EPI_ISL_870982, EPI_ISL_870983, EPI_ISL_870984, EPI_ISL_870985, EPI_ISL_870986, EPI_ISL_870987, EPI_ISL_870988, EPI_ISL_870989, EPI_ISL_870990, EPI_ISL_870991, EPI_ISL_870992, EPI_ISL_870993, EPI_ISL_870994, EPI_ISL_870995, EPI_ISL_870996, EPI_ISL_870997, EPI_ISL_870998, EPI_ISL_870999, EPI_ISL_871000, EPI_ISL_871001, EPI_ISL_871002, EPI_ISL_871003, EPI_ISL_871004, EPI_ISL_871005, EPI_ISL_871006, EPI_ISL_871007, EPI_ISL_871008, EPI_ISL_871009, EPI_ISL_871010, EPI_ISL_871011, EPI_ISL_871012, EPI_ISL_871013, EPI_ISL_871014, EPI_ISL_871015, EPI_ISL_871016, EPI_ISL_871017, EPI_ISL_871018, EPI_ISL_871019, EPI_ISL_871020, EPI_ISL_871021, EPI_ISL_871022, EPI_ISL_871023, EPI_ISL_871024, EPI_ISL_871025, EPI_ISL_871026, EPI_ISL_871027, EPI_ISL_871028, EPI_ISL_871029, EPI_ISL_871030, EPI_ISL_871031, EPI_ISL_871032, EPI_ISL_871033, EPI_ISL_871034, EPI_ISL_871035, EPI_ISL_871036, EPI_ISL_871037, EPI_ISL_871038, EPI_ISL_871039, EPI_ISL_871040, EPI_ISL_871041, EPI_ISL_871042, EPI_ISL_871043, EPI_ISL_871044, EPI_ISL_871045, EPI_ISL_871046, EPI_ISL_871047, EPI_ISL_871048, EPI_ISL_871049, EPI_ISL_871050, EPI_ISL_871051, EPI_ISL_871052, EPI_ISL_871053, EPI_ISL_871054, EPI_ISL_871055, EPI_ISL_871056, EPI_ISL_871057, EPI_ISL_871058, EPI_ISL_871059, EPI_ISL_871060, EPI_ISL_871061, EPI_ISL_871062, EPI_ISL_871063, EPI_ISL_871064, EPI_ISL_871065, EPI_ISL_871066, EPI_ISL_871067, EPI_ISL_871068, EPI_ISL_871069, EPI_ISL_871070, EPI_ISL_871071, EPI_ISL_871072, EPI_ISL_871073, EPI_ISL_871074, EPI_ISL_871075, EPI_ISL_871076, EPI_ISL_871077, EPI_ISL_871078, EPI_ISL_871079, EPI_ISL_871080, EPI_ISL_871081, EPI_ISL_871082, EPI_ISL_871083, EPI_ISL_871084, EPI_ISL_871085, EPI_ISL_871086, EPI_ISL_871087, EPI_ISL_871088, EPI_ISL_871089, EPI_ISL_871090, EPI_ISL_871091, EPI_ISL_871092, EPI_ISL_871093, EPI_ISL_871094, EPI_ISL_871095, EPI_ISL_871096, EPI_ISL_871097, EPI_ISL_871098, EPI_ISL_871099, EPI_ISL_871100, EPI_ISL_871101, EPI_ISL_871102, EPI_ISL_871103, EPI_ISL_871104, EPI_ISL_871105, EPI_ISL_871106, EPI_ISL_871107, EPI_ISL_871108, EPI_ISL_871109, EPI_ISL_871110, EPI_ISL_871111, EPI_ISL_871112, EPI_ISL_871113, EPI_ISL_871114, EPI_ISL_871115, EPI_ISL_871116, EPI_ISL_871117, EPI_ISL_871118, EPI_ISL_871119, EPI_ISL_871120, EPI_ISL_871121, EPI_ISL_871122, EPI_ISL_871123, EPI_ISL_871124, EPI_ISL_871125, EPI_ISL_871126, EPI_ISL_871127, EPI_ISL_871128, EPI_ISL_871129, EPI_ISL_871130, EPI_ISL_871131, EPI_ISL_871132			
see above	Department of Virus and Microbiological Special Diagnostics, Statens Serum Institut, Copenhagen, Denmark	Aalborg University	Danish Covid-19 Genome Consortium
EPI_ISL_871820	Jessa	Jessa	Brigitte Maes, Bert Cruys
EPI_ISL_872065, EPI_ISL_872066	AZ Vesale (Tongres)	GIGA Medical Genomics	Keith Durkin, Maria Artesi, Sébastien Bontems, Raphaël Boreux, Bouchra Boujemla, Cécile Meex, Pierrette Melin, Marie-Pierre Hayette, Vincent Bours
EPI_ISL_872197, EPI_ISL_872200, EPI_ISL_872202	hopital	National Reference Center for Viruses of Respiratory Infections, Institut Pasteur, Paris	Marion Barbet, Sylvie Behillil, Méline Bizard, Angela Brisebarre, Camille Capel, Etienne Simon-Lorière, Vincent Enouf, Maud Vanpeene, Sylvie van der Werf,Combe Patrice
EPI_ISL_872207	Labo Analyses Med	National Reference Center for Viruses of Respiratory Infections, Institut Pasteur, Paris	Marion Barbet, Sylvie Behillil, Méline Bizard, Angela Brisebarre, Camille Capel, Etienne Simon-Lorière, Vincent Enouf, Maud Vanpeene, Sylvie van der Werf
EPI_ISL_872210	Hopital	National Reference Center for Viruses of Respiratory Infections, Institut Pasteur, Paris	Marion Barbet, Sylvie Behillil, Méline Bizard, Angela Brisebarre, Camille Capel, Etienne Simon-Lorière, Vincent Enouf, Maud Vanpeene, Sylvie van der Werf,Guigon AuréLie
EPI_ISL_872222, EPI_ISL_872223, EPI_ISL_872224, EPI_ISL_872225, EPI_ISL_872226	hopital	National Reference Center for Viruses of Respiratory Infections, Institut Pasteur, Paris	Marion Barbet, Sylvie Behillil, Méline Bizard, Angela Brisebarre, Camille Capel, Etienne Simon-Lorière, Vincent Enouf, Maud Vanpeene, Sylvie van der Werf,Combe Patrice
EPI_ISL_872227	Hopital	National Reference Center for Viruses of Respiratory Infections, Institut Pasteur, Paris	Marion Barbet, Sylvie Behillil, Méline Bizard, Angela Brisebarre, Camille Capel, Etienne Simon-Lorière, Vincent Enouf, Maud Vanpeene, Sylvie van der Werf
EPI_ISL_872232	hopital	National Reference Center for Viruses of Respiratory Infections, Institut Pasteur, Paris	Marion Barbet, Sylvie Behillil, Méline Bizard, Angela Brisebarre, Camille Capel, Etienne Simon-Lorière, Vincent Enouf, Maud Vanpeene, Sylvie van der Werf,Combe Patrice
EPI_ISL_872235	Labo Analyses Med	National Reference Center for Viruses of Respiratory Infections, Institut Pasteur, Paris	Marion Barbet, Sylvie Behillil, Méline Bizard, Angela Brisebarre, Camille Capel, Etienne Simon-Lorière, Vincent Enouf, Maud Vanpeene, Sylvie van der Werf
EPI_ISL_872237	Hopital	National Reference Center for Viruses of Respiratory Infections, Institut Pasteur, Paris	Marion Barbet, Sylvie Behillil, Méline Bizard, Angela Brisebarre, Camille Capel, Etienne Simon-Lorière, Vincent Enouf, Maud Vanpeene, Sylvie van der Werf,Florin CéCile
EPI_ISL_872242, EPI_ISL_872243, EPI_ISL_872244, EPI_ISL_872245, EPI_ISL_872246, EPI_ISL_872247, EPI_ISL_872248	hopital	National Reference Center for Viruses of Respiratory Infections, Institut Pasteur, Paris	Marion Barbet, Sylvie Behillil, Méline Bizard, Angela Brisebarre, Camille Capel, Etienne Simon-Lorière, Vincent Enouf, Maud Vanpeene, Sylvie van der Werf,Combe Patrice
EPI_ISL_872260	Labo Analyses Med	National Reference Center for Viruses of Respiratory Infections, Institut Pasteur, Paris	Marion Barbet, Sylvie Behillil, Méline Bizard, Angela Brisebarre, Camille Capel, Etienne Simon-Lorière, Vincent Enouf, Maud Vanpeene, Sylvie van der Werf
EPI_ISL_872263	Hopital	National Reference Center for Viruses of Respiratory Infections, Institut Pasteur, Paris	Marion Barbet, Sylvie Behillil, Méline Bizard, Angela Brisebarre, Camille Capel, Etienne Simon-Lorière, Vincent Enouf, Maud Vanpeene, Sylvie van der Werf,Florin CéCile
EPI_ISL_872271, EPI_ISL_872272, EPI_ISL_872273	hopital	National Reference Center for Viruses of Respiratory Infections, Institut Pasteur, Paris	Marion Barbet, Sylvie Behillil, Méline Bizard, Angela Brisebarre, Camille Capel, Etienne Simon-Lorière, Vincent Enouf, Maud Vanpeene, Sylvie van der Werf,Combe Patrice
EPI_ISL_872285, EPI_ISL_872286, EPI_ISL_872287, EPI_ISL_872288, EPI_ISL_872289	Labo Analyses Med	National Reference Center for Viruses of Respiratory Infections, Institut Pasteur, Paris	Marion Barbet, Sylvie Behillil, Méline Bizard, Angela Brisebarre, Camille Capel, Etienne Simon-Lorière, Vincent Enouf, Maud Vanpeene, Sylvie van der Werf
EPI_ISL_872295, EPI_ISL_872296, EPI_ISL_872297	Hopital	National Reference Center for Viruses of Respiratory Infections, Institut Pasteur, Paris	Marion Barbet, Sylvie Behillil, Méline Bizard, Angela Brisebarre, Camille Capel, Etienne Simon-Lorière, Vincent Enouf, Maud Vanpeene, Sylvie van der Werf,Guigon AuréLie
EPI_ISL_872299, EPI_ISL_872301	Hopital	National Reference Center for Viruses of Respiratory Infections, Institut Pasteur, Paris	Marion Barbet, Sylvie Behillil, Méline Bizard, Angela Brisebarre, Camille Capel, Etienne Simon-Lorière, Vincent Enouf, Maud Vanpeene, Sylvie van der Werf,Florin CéCile
EPI_ISL_872308	Hopital	National Reference Center for Viruses of Respiratory Infections, Institut Pasteur, Paris	Marion Barbet, Sylvie Behillil, Méline Bizard, Angela Brisebarre, Camille Capel, Etienne Simon-Lorière, Vincent Enouf, Maud Vanpeene, Sylvie van der Werf,Brichler Ségolène

EPI_ISL_872321, EPI_ISL_872322	Labo Analyses Med	National Reference Center for Viruses of Respiratory Infections, Institut Pasteur, Paris	Marion Barbet, Sylvie Behillil, Méline Bizard, Angela Brisebarre, Camille Capel, Etienne Simon-Lorière, Vincent Enouf, Maud Vanpeene, Sylvie van der Werf
EPI_ISL_872325	Hopital	National Reference Center for Viruses of Respiratory Infections, Institut Pasteur, Paris	Marion Barbet, Sylvie Behillil, Méline Bizard, Angela Brisebarre, Camille Capel, Etienne Simon-Lorière, Vincent Enouf, Maud Vanpeene, Sylvie van der Werf,Brichler Ségolène
EPI_ISL_872330, EPI_ISL_872331, EPI_ISL_872332, EPI_ISL_872333, EPI_ISL_872334, EPI_ISL_872335, EPI_ISL_872336, EPI_ISL_872337, EPI_ISL_872338	hopital	National Reference Center for Viruses of Respiratory Infections, Institut Pasteur, Paris	Marion Barbet, Sylvie Behillil, Méline Bizard, Angela Brisebarre, Camille Capel, Etienne Simon-Lorière, Vincent Enouf, Maud Vanpeene, Sylvie van der Werf,Combe Patrice
EPI_ISL_872478	Centre for Dengue Research and AICBU, Department of Immunology and Molecular Medicine	Centre for Dengue Research and AICBU, Department of Immunology and Molecular Medicine	Chandima Jeewandara, Deshni Jayathilaka, Dinuka Ariyaratne, Tibutius Thanesh Pramanayagam, Diyanath Ranasinghe, Laksiri Gomes, Gathsaurie Neelika Malavige
EPI_ISL_872494, EPI_ISL_872495, EPI_ISL_872496, EPI_ISL_872497, EPI_ISL_872498, EPI_ISL_872499, EPI_ISL_872500, EPI_ISL_872501, EPI_ISL_872502, EPI_ISL_872503, EPI_ISL_872504, EPI_ISL_872505, EPI_ISL_872506, EPI_ISL_872507, EPI_ISL_872508, EPI_ISL_872509, EPI_ISL_872510			
see above	New Mexico Department of Health Scientific Laboratory	Center for Global Health, University of New Mexico Health Sciences Center	Daryl Domman, Kurt Schwalm, Twila Kunde, Joseph Hicks, Anastacia Griego, Michael Edwards, Darrell Dinwiddie
EPI_ISL_872781, EPI_ISL_872792, EPI_ISL_872794, EPI_ISL_872795, EPI_ISL_872797, EPI_ISL_872798, EPI_ISL_872799, EPI_ISL_872802, EPI_ISL_872803, EPI_ISL_872804, EPI_ISL_872808, EPI_ISL_872809, EPI_ISL_872811, EPI_ISL_872813, EPI_ISL_872815, EPI_ISL_872818, EPI_ISL_872819, EPI_ISL_872821			
see above	University of Wisconsin-Madison AIDS Vaccine Research Laboratories	University of Wisconsin-Madison AIDS Vaccine Research Laboratories	Gage Moreno, Katarina Braun, et al. AIDS Vaccine Research Laboratories
EPI_ISL_873157, EPI_ISL_873162	University of Michigan Clinical Microbiology Laboratory	Lauring Lab, University of Michigan, Department of Microbiology and Immunology	Valesano
EPI_ISL_873258, EPI_ISL_873261, EPI_ISL_873263, EPI_ISL_873264, EPI_ISL_873265, EPI_ISL_873266, EPI_ISL_873267, EPI_ISL_873268, EPI_ISL_873269	M Health Fairview	Minnesota Department of Health, Public Health Laboratory	Alexandra Lorentz, Jacob Garfin, Matt Plumb, and Xiong Wang
EPI_ISL_873279	Lighthouse Lab in Milton Keynes	Wellcome Sanger Institute for the COVID-19 Genomics UK (COG-UK) Consortium	The Lighthouse Lab in Milton Keynes and Alex Alderton, Roberto Amato, Sonia Goncalves, Ewan Harrison, David K. Jackson, Ian Johnston, Dominic Kwiatkowski, Cordelia Langford, John Sillitoe on behalf of the Wellcome Sanger Institute COVID-19 Surveillance Team
EPI_ISL_873282	Lighthouse Lab in Cambridge	Wellcome Sanger Institute for the COVID-19 Genomics UK (COG-UK) Consortium	Rob Howes, The Lighthouse Lab in Cambridge and Alex Alderton, Roberto Amato, Sonia Goncalves, Ewan Harrison, David K. Jackson, Ian Johnston, Dominic Kwiatkowski, Cordelia Langford, John Sillitoe on behalf of the Wellcome Sanger Institute COVID-19 Surveillance Team
EPI_ISL_873284, EPI_ISL_873285, EPI_ISL_873287, EPI_ISL_873290, EPI_ISL_873293	Lighthouse Lab in Milton Keynes	Wellcome Sanger Institute for the COVID-19 Genomics UK (COG-UK) Consortium	The Lighthouse Lab in Milton Keynes and Alex Alderton, Roberto Amato, Sonia Goncalves, Ewan Harrison, David K. Jackson, Ian Johnston, Dominic Kwiatkowski, Cordelia Langford, John Sillitoe on behalf of the Wellcome Sanger Institute COVID-19 Surveillance Team
EPI_ISL_873295	Lighthouse Lab in Cambridge	Wellcome Sanger Institute for the COVID-19 Genomics UK (COG-UK) Consortium	Rob Howes, The Lighthouse Lab in Cambridge and Alex Alderton, Roberto Amato, Sonia Goncalves, Ewan Harrison, David K. Jackson, Ian Johnston, Dominic Kwiatkowski, Cordelia Langford, John Sillitoe on behalf of the Wellcome Sanger Institute COVID-19 Surveillance Team
EPI_ISL_873296, EPI_ISL_873299, EPI_ISL_873300, EPI_ISL_873301, EPI_ISL_873303, EPI_ISL_873305, EPI_ISL_873307, EPI_ISL_873314, EPI_ISL_873315, EPI_ISL_873317, EPI_ISL_873319, EPI_ISL_873322, EPI_ISL_873323, EPI_ISL_873327, EPI_ISL_873328, EPI_ISL_873329, EPI_ISL_873332, EPI_ISL_873333, EPI_ISL_873335, EPI_ISL_873338, EPI_ISL_873340, EPI_ISL_873344, EPI_ISL_873346, EPI_ISL_873349, EPI_ISL_873351, EPI_ISL_873353, EPI_ISL_873355, EPI_ISL_873356, EPI_ISL_873358, EPI_ISL_873363			
see above	Lighthouse Lab in Milton Keynes	Wellcome Sanger Institute for the COVID-19 Genomics UK (COG-UK) Consortium	The Lighthouse Lab in Milton Keynes and Alex Alderton, Roberto Amato, Sonia Goncalves, Ewan Harrison, David K. Jackson, Ian Johnston, Dominic Kwiatkowski, Cordelia Langford, John Sillitoe on behalf of the Wellcome Sanger Institute COVID-19 Surveillance Team
EPI_ISL_873365	Lighthouse Lab in Cambridge	Wellcome Sanger Institute for the COVID-19 Genomics UK (COG-UK) Consortium	Rob Howes, The Lighthouse Lab in Cambridge and Alex Alderton, Roberto Amato, Sonia Goncalves, Ewan Harrison, David K. Jackson, Ian Johnston, Dominic Kwiatkowski, Cordelia Langford, John Sillitoe on behalf of the Wellcome Sanger Institute COVID-19 Surveillance Team
EPI_ISL_873366, EPI_ISL_873367	Lighthouse Lab in Milton Keynes	Wellcome Sanger Institute for the COVID-19 Genomics UK (COG-UK) Consortium	The Lighthouse Lab in Milton Keynes and Alex Alderton, Roberto Amato, Sonia Goncalves, Ewan Harrison, David K. Jackson, Ian Johnston, Dominic Kwiatkowski, Cordelia Langford, John Sillitoe on behalf of the Wellcome Sanger Institute COVID-19 Surveillance Team
EPI_ISL_873368	Lighthouse Lab in Cambridge	Wellcome Sanger Institute for the COVID-19 Genomics UK (COG-UK) Consortium	Rob Howes, The Lighthouse Lab in Cambridge and Alex Alderton, Roberto Amato, Sonia Goncalves, Ewan Harrison, David K. Jackson, Ian Johnston, Dominic Kwiatkowski, Cordelia Langford, John Sillitoe on behalf of the Wellcome Sanger Institute COVID-19 Surveillance Team
EPI_ISL_873373, EPI_ISL_873375, EPI_ISL_873380, EPI_ISL_873384, EPI_ISL_873387, EPI_ISL_873391, EPI_ISL_873392, EPI_ISL_873393, EPI_ISL_873394, EPI_ISL_873397, EPI_ISL_873398, EPI_ISL_873400, EPI_ISL_873402, EPI_ISL_873404, EPI_ISL_873405, EPI_ISL_873406, EPI_ISL_873408, EPI_ISL_873410			
see above	Lighthouse Lab in Milton Keynes	Wellcome Sanger Institute for the COVID-19 Genomics UK (COG-UK) Consortium	The Lighthouse Lab in Milton Keynes and Alex Alderton, Roberto Amato, Sonia Goncalves, Ewan Harrison, David K. Jackson, Ian Johnston, Dominic Kwiatkowski, Cordelia Langford, John Sillitoe on behalf of the Wellcome Sanger Institute COVID-19 Surveillance Team
EPI_ISL_873418	Lighthouse Lab in Cambridge	Wellcome Sanger Institute for the COVID-19 Genomics UK (COG-UK) Consortium	Rob Howes, The Lighthouse Lab in Cambridge and Alex Alderton, Roberto Amato, Sonia Goncalves, Ewan Harrison, David K. Jackson, Ian Johnston, Dominic Kwiatkowski, Cordelia Langford, John Sillitoe on behalf of the Wellcome Sanger Institute COVID-19 Surveillance Team
EPI_ISL_873421, EPI_ISL_873423, EPI_ISL_873424	Lighthouse Lab in Milton Keynes	Wellcome Sanger Institute for the COVID-19 Genomics UK (COG-UK) Consortium	The Lighthouse Lab in Milton Keynes and Alex Alderton, Roberto Amato, Sonia Goncalves, Ewan Harrison, David K. Jackson, Ian Johnston, Dominic Kwiatkowski, Cordelia Langford, John Sillitoe on behalf of the Wellcome Sanger Institute COVID-19 Surveillance Team
EPI_ISL_873432	Lighthouse Lab in Cambridge	Wellcome Sanger Institute for the COVID-19 Genomics UK (COG-UK) Consortium	Rob Howes, The Lighthouse Lab in Cambridge and Alex Alderton, Roberto Amato, Sonia Goncalves, Ewan Harrison, David K. Jackson, Ian Johnston, Dominic Kwiatkowski, Cordelia Langford, John Sillitoe on behalf of the Wellcome Sanger Institute COVID-19 Surveillance Team
EPI_ISL_873434, EPI_ISL_873436, EPI_ISL_873437, EPI_ISL_873440, EPI_ISL_873441, EPI_ISL_873442, EPI_ISL_873446, EPI_ISL_873448, EPI_ISL_873449, EPI_ISL_873453, EPI_ISL_873456, EPI_ISL_873460, EPI_ISL_873461, EPI_ISL_873464, EPI_ISL_873465, EPI_ISL_873467, EPI_ISL_873470, EPI_ISL_873471, EPI_ISL_873472, EPI_ISL_873474, EPI_ISL_873475, EPI_ISL_873477, EPI_ISL_873478, EPI_ISL_873479, EPI_ISL_873480, EPI_ISL_873484			
see above	Lighthouse Lab in Milton Keynes	Wellcome Sanger Institute for the COVID-19 Genomics UK (COG-UK) Consortium	The Lighthouse Lab in Milton Keynes and Alex Alderton, Roberto Amato, Sonia Goncalves, Ewan Harrison, David K. Jackson, Ian Johnston, Dominic Kwiatkowski, Cordelia Langford, John Sillitoe on behalf of the Wellcome Sanger Institute COVID-19 Surveillance Team
EPI_ISL_873489	Lighthouse Lab in Cambridge	Wellcome Sanger Institute for the COVID-19 Genomics UK (COG-UK) Consortium	Rob Howes, The Lighthouse Lab in Cambridge and Alex Alderton, Roberto Amato, Sonia Goncalves, Ewan Harrison, David K. Jackson, Ian Johnston, Dominic Kwiatkowski, Cordelia Langford, John Sillitoe on behalf of the Wellcome Sanger Institute COVID-19 Surveillance Team
EPI_ISL_873491, EPI_ISL_873498, EPI_ISL_873502, EPI_ISL_873506, EPI_ISL_873507, EPI_ISL_873508, EPI_ISL_873511, EPI_ISL_873512	Lighthouse Lab in Milton Keynes	Wellcome Sanger Institute for the COVID-19 Genomics UK (COG-UK) Consortium	The Lighthouse Lab in Milton Keynes and Alex Alderton, Roberto Amato, Sonia Goncalves, Ewan Harrison, David K. Jackson, Ian Johnston, Dominic Kwiatkowski, Cordelia Langford, John Sillitoe on behalf of the Wellcome Sanger Institute COVID-19 Surveillance Team
EPI_ISL_873513	Lighthouse Lab in Cambridge	Wellcome Sanger Institute for the COVID-19 Genomics UK (COG-UK) Consortium	Rob Howes, The Lighthouse Lab in Cambridge and Alex Alderton, Roberto Amato, Sonia Goncalves, Ewan Harrison, David K. Jackson, Ian Johnston, Dominic Kwiatkowski, Cordelia Langford, John Sillitoe on behalf of the Wellcome Sanger Institute COVID-19 Surveillance Team
EPI_ISL_873514, EPI_ISL_873516, EPI_ISL_873519, EPI_ISL_873522, EPI_ISL_873523, EPI_ISL_873524, EPI_ISL_873525, EPI_ISL_873526, EPI_ISL_873529, EPI_ISL_873530, EPI_ISL_873533, EPI_ISL_873534, EPI_ISL_873539, EPI_ISL_873540, EPI_ISL_873541, EPI_ISL_873544, EPI_ISL_873545, EPI_ISL_873548, EPI_ISL_873550, EPI_ISL_873553, EPI_ISL_873555, EPI_ISL_873556, EPI_ISL_873558, EPI_ISL_873559, EPI_ISL_873560			
see above	Lighthouse Lab in Milton Keynes	Wellcome Sanger Institute for the COVID-19 Genomics UK (COG-UK) Consortium	The Lighthouse Lab in Milton Keynes and Alex Alderton, Roberto Amato, Sonia Goncalves, Ewan Harrison, David K. Jackson, Ian Johnston, Dominic Kwiatkowski, Cordelia Langford, John Sillitoe on behalf of the Wellcome Sanger Institute COVID-19 Surveillance Team
EPI_ISL_873561	Lighthouse Lab in Cambridge	Wellcome Sanger Institute for the COVID-19 Genomics UK (COG-UK) Consortium	Rob Howes, The Lighthouse Lab in Cambridge and Alex Alderton, Roberto Amato, Sonia Goncalves, Ewan Harrison, David K. Jackson, Ian Johnston, Dominic Kwiatkowski, Cordelia Langford, John Sillitoe on behalf of the Wellcome Sanger Institute COVID-19 Surveillance Team
EPI_ISL_873564, EPI_ISL_873567, EPI_ISL_873568, EPI_ISL_873569, EPI_ISL_873572, EPI_ISL_873578, EPI_ISL_873579, EPI_ISL_873580, EPI_ISL_873581, EPI_ISL_873582, EPI_ISL_873584, EPI_ISL_873586, EPI_ISL_873587, EPI_ISL_873588, EPI_ISL_873592, EPI_ISL_873593, EPI_ISL_873594, EPI_ISL_873597,			

EPI_ISL_873598, EPI_ISL_873601, EPI_ISL_873606, EPI_ISL_873608, EPI_ISL_873609, EPI_ISL_873610			
see above	Lighthouse Lab in Milton Keynes	Wellcome Sanger Institute for the COVID-19 Genomics UK (COG-UK) Consortium	The Lighthouse Lab in Milton Keynes and Alex Alderton, Roberto Amato, Sonia Goncalves, Ewan Harrison, David K. Jackson, Ian Johnston, Dominic Kwiatkowski, Cordelia Langford, John Sillitoe on behalf of the Wellcome Sanger Institute COVID-19 Surveillance Team
EPI_ISL_873611	Lighthouse Lab in Cambridge	Wellcome Sanger Institute for the COVID-19 Genomics UK (COG-UK) Consortium	Rob Howes, The Lighthouse Lab in Cambridge and Alex Alderton, Roberto Amato, Sonia Goncalves, Ewan Harrison, David K. Jackson, Ian Johnston, Dominic Kwiatkowski, Cordelia Langford, John Sillitoe on behalf of the Wellcome Sanger Institute COVID-19 Surveillance Team
EPI_ISL_873612	Lighthouse Lab in Milton Keynes	Wellcome Sanger Institute for the COVID-19 Genomics UK (COG-UK) Consortium	The Lighthouse Lab in Milton Keynes and Alex Alderton, Roberto Amato, Sonia Goncalves, Ewan Harrison, David K. Jackson, Ian Johnston, Dominic Kwiatkowski, Cordelia Langford, John Sillitoe on behalf of the Wellcome Sanger Institute COVID-19 Surveillance Team
EPI_ISL_873613	Lighthouse Lab in Cambridge	Wellcome Sanger Institute for the COVID-19 Genomics UK (COG-UK) Consortium	Rob Howes, The Lighthouse Lab in Cambridge and Alex Alderton, Roberto Amato, Sonia Goncalves, Ewan Harrison, David K. Jackson, Ian Johnston, Dominic Kwiatkowski, Cordelia Langford, John Sillitoe on behalf of the Wellcome Sanger Institute COVID-19 Surveillance Team
EPI_ISL_873615, EPI_ISL_873616, EPI_ISL_873617, EPI_ISL_873619, EPI_ISL_873620, EPI_ISL_873622, EPI_ISL_873626, EPI_ISL_873628, EPI_ISL_873631, EPI_ISL_873637, EPI_ISL_873641, EPI_ISL_873654, EPI_ISL_873657, EPI_ISL_873660, EPI_ISL_873669, EPI_ISL_873704, EPI_ISL_873725, EPI_ISL_873729, EPI_ISL_873734, EPI_ISL_873741, EPI_ISL_873743, EPI_ISL_873744, EPI_ISL_873749			
see above	Lighthouse Lab in Milton Keynes	Wellcome Sanger Institute for the COVID-19 Genomics UK (COG-UK) Consortium	The Lighthouse Lab in Milton Keynes and Alex Alderton, Roberto Amato, Sonia Goncalves, Ewan Harrison, David K. Jackson, Ian Johnston, Dominic Kwiatkowski, Cordelia Langford, John Sillitoe on behalf of the Wellcome Sanger Institute COVID-19 Surveillance Team
EPI_ISL_873750, EPI_ISL_873752	Lighthouse Lab in Cambridge	Wellcome Sanger Institute for the COVID-19 Genomics UK (COG-UK) Consortium	Rob Howes, The Lighthouse Lab in Cambridge and Alex Alderton, Roberto Amato, Sonia Goncalves, Ewan Harrison, David K. Jackson, Ian Johnston, Dominic Kwiatkowski, Cordelia Langford, John Sillitoe on behalf of the Wellcome Sanger Institute COVID-19 Surveillance Team
EPI_ISL_873760, EPI_ISL_873764, EPI_ISL_873794, EPI_ISL_873806, EPI_ISL_873809, EPI_ISL_873812, EPI_ISL_873817	Lighthouse Lab in Milton Keynes	Wellcome Sanger Institute for the COVID-19 Genomics UK (COG-UK) Consortium	The Lighthouse Lab in Milton Keynes and Alex Alderton, Roberto Amato, Sonia Goncalves, Ewan Harrison, David K. Jackson, Ian Johnston, Dominic Kwiatkowski, Cordelia Langford, John Sillitoe on behalf of the Wellcome Sanger Institute COVID-19 Surveillance Team
EPI_ISL_873825	Lighthouse Lab in Cambridge	Wellcome Sanger Institute for the COVID-19 Genomics UK (COG-UK) Consortium	Rob Howes, The Lighthouse Lab in Cambridge and Alex Alderton, Roberto Amato, Sonia Goncalves, Ewan Harrison, David K. Jackson, Ian Johnston, Dominic Kwiatkowski, Cordelia Langford, John Sillitoe on behalf of the Wellcome Sanger Institute COVID-19 Surveillance Team
EPI_ISL_873833, EPI_ISL_873834, EPI_ISL_873847, EPI_ISL_873857, EPI_ISL_873859, EPI_ISL_873870, EPI_ISL_873873, EPI_ISL_873880, EPI_ISL_873884, EPI_ISL_873887, EPI_ISL_873900, EPI_ISL_873905, EPI_ISL_873910, EPI_ISL_873913, EPI_ISL_873916, EPI_ISL_873923, EPI_ISL_873924, EPI_ISL_873928, EPI_ISL_873934, EPI_ISL_873937, EPI_ISL_873948, EPI_ISL_873960			
see above	Lighthouse Lab in Milton Keynes	Wellcome Sanger Institute for the COVID-19 Genomics UK (COG-UK) Consortium	The Lighthouse Lab in Milton Keynes and Alex Alderton, Roberto Amato, Sonia Goncalves, Ewan Harrison, David K. Jackson, Ian Johnston, Dominic Kwiatkowski, Cordelia Langford, John Sillitoe on behalf of the Wellcome Sanger Institute COVID-19 Surveillance Team
EPI_ISL_873961, EPI_ISL_873963, EPI_ISL_873966, EPI_ISL_873967, EPI_ISL_873968, EPI_ISL_873969, EPI_ISL_873970, EPI_ISL_873971, EPI_ISL_873972, EPI_ISL_873973, EPI_ISL_873975, EPI_ISL_873977, EPI_ISL_873978, EPI_ISL_873979, EPI_ISL_873980, EPI_ISL_873983, EPI_ISL_873985, EPI_ISL_873988, EPI_ISL_873989, EPI_ISL_873991, EPI_ISL_873992, EPI_ISL_873993, EPI_ISL_873994, EPI_ISL_873995, EPI_ISL_873996, EPI_ISL_873997, EPI_ISL_873998, EPI_ISL_874000, EPI_ISL_874001, EPI_ISL_874002, EPI_ISL_874003, EPI_ISL_874005, EPI_ISL_874006, EPI_ISL_874008, EPI_ISL_874013, EPI_ISL_874015, EPI_ISL_874016, EPI_ISL_874018, EPI_ISL_874019, EPI_ISL_874020, EPI_ISL_874023, EPI_ISL_874025, EPI_ISL_874026, EPI_ISL_874027, EPI_ISL_874028, EPI_ISL_874029, EPI_ISL_874030, EPI_ISL_874031, EPI_ISL_874033, EPI_ISL_874034, EPI_ISL_874035, EPI_ISL_874036, EPI_ISL_874040, EPI_ISL_874041, EPI_ISL_874042, EPI_ISL_874043, EPI_ISL_874044, EPI_ISL_874045, EPI_ISL_874046, EPI_ISL_874048, EPI_ISL_874049, EPI_ISL_874050, EPI_ISL_874051, EPI_ISL_874052, EPI_ISL_874053, EPI_ISL_874054, EPI_ISL_874056, EPI_ISL_874057, EPI_ISL_874058, EPI_ISL_874059, EPI_ISL_874060, EPI_ISL_874063, EPI_ISL_874065, EPI_ISL_874066, EPI_ISL_874067, EPI_ISL_874068, EPI_ISL_874069, EPI_ISL_874072, EPI_ISL_874073, EPI_ISL_874074, EPI_ISL_874075, EPI_ISL_874077, EPI_ISL_874079, EPI_ISL_874083, EPI_ISL_874084, EPI_ISL_874086, EPI_ISL_874087, EPI_ISL_874088, EPI_ISL_874089, EPI_ISL_874091, EPI_ISL_874094, EPI_ISL_874095, EPI_ISL_874099, EPI_ISL_874100, EPI_ISL_874101, EPI_ISL_874102, EPI_ISL_874103, EPI_ISL_874104, EPI_ISL_874105, EPI_ISL_874106, EPI_ISL_874107, EPI_ISL_874108, EPI_ISL_874110, EPI_ISL_874111, EPI_ISL_874112, EPI_ISL_874113, EPI_ISL_874114, EPI_ISL_874116, EPI_ISL_874117, EPI_ISL_874119, EPI_ISL_874120, EPI_ISL_874122, EPI_ISL_874123, EPI_ISL_874124, EPI_ISL_874125, EPI_ISL_874126, EPI_ISL_874127, EPI_ISL_874128, EPI_ISL_874129, EPI_ISL_874131, EPI_ISL_874132, EPI_ISL_874133, EPI_ISL_874134, EPI_ISL_874135, EPI_ISL_874136, EPI_ISL_874137, EPI_ISL_874138, EPI_ISL_874139, EPI_ISL_874140, EPI_ISL_874142, EPI_ISL_874144, EPI_ISL_874146, EPI_ISL_874147, EPI_ISL_874150, EPI_ISL_874151, EPI_ISL_874152, EPI_ISL_874156, EPI_ISL_874158, EPI_ISL_874161, EPI_ISL_874162, EPI_ISL_874166, EPI_ISL_874167, EPI_ISL_874168, EPI_ISL_874171, EPI_ISL_874172, EPI_ISL_874173, EPI_ISL_874174, EPI_ISL_874175, EPI_ISL_874176, EPI_ISL_874177, EPI_ISL_874178, EPI_ISL_874179, EPI_ISL_874182, EPI_ISL_874183, EPI_ISL_874184, EPI_ISL_874186, EPI_ISL_874187, EPI_ISL_874189, EPI_ISL_874190, EPI_ISL_874192, EPI_ISL_874194, EPI_ISL_874200, EPI_ISL_874201, EPI_ISL_874205, EPI_ISL_874206, EPI_ISL_874208, EPI_ISL_874212, EPI_ISL_874213, EPI_ISL_874214, EPI_ISL_874216, EPI_ISL_874217, EPI_ISL_874219, EPI_ISL_874221, EPI_ISL_874222, EPI_ISL_874223, EPI_ISL_874224, EPI_ISL_874226, EPI_ISL_874228, EPI_ISL_874229, EPI_ISL_874230, EPI_ISL_874231, EPI_ISL_874232, EPI_ISL_874233, EPI_ISL_874234, EPI_ISL_874235, EPI_ISL_874236, EPI_ISL_874237, EPI_ISL_874239, EPI_ISL_874241, EPI_ISL_874242, EPI_ISL_874244, EPI_ISL_874247, EPI_ISL_874250, EPI_ISL_874251, EPI_ISL_874253, EPI_ISL_874255, EPI_ISL_874256, EPI_ISL_874260, EPI_ISL_874261, EPI_ISL_874262, EPI_ISL_874264, EPI_ISL_874265, EPI_ISL_874267, EPI_ISL_874269, EPI_ISL_874270, EPI_ISL_874271, EPI_ISL_874272, EPI_ISL_874274, EPI_ISL_874276, EPI_ISL_874277, EPI_ISL_874279, EPI_ISL_874280, EPI_ISL_874282, EPI_ISL_874285, EPI_ISL_874286, EPI_ISL_874288, EPI_ISL_874289, EPI_ISL_874292, EPI_ISL_874294, EPI_ISL_874295, EPI_ISL_874296, EPI_ISL_874298, EPI_ISL_874299, EPI_ISL_874300, EPI_ISL_874302, EPI_ISL_874303, EPI_ISL_874304, EPI_ISL_874305, EPI_ISL_874306, EPI_ISL_874307, EPI_ISL_874308, EPI_ISL_874309, EPI_ISL_874315, EPI_ISL_874379, EPI_ISL_874401, EPI_ISL_874404, EPI_ISL_874443, EPI_ISL_874448, EPI_ISL_874469, EPI_ISL_874482, EPI_ISL_874499, EPI_ISL_874523, EPI_ISL_874527, EPI_ISL_874528, EPI_ISL_874543, EPI_ISL_874558, EPI_ISL_874624, EPI_ISL_874759, EPI_ISL_874816, EPI_ISL_874938, EPI_ISL_874956			
see above	Lighthouse Lab in Alderley Park	Wellcome Sanger Institute for the COVID-19 Genomics UK (COG-UK) Consortium	Jacquelyn Wynn, Mairead Hyland, The Lighthouse Lab in Alderley Park and Alex Alderton, Roberto Amato, Sonia Goncalves, Ewan Harrison, David K. Jackson, Ian Johnston, Dominic Kwiatkowski, Cordelia Langford, John Sillitoe on behalf of the Wellcome Sanger Institute COVID-19 Surveillance Team
EPI_ISL_875373, EPI_ISL_875374, EPI_ISL_875375, EPI_ISL_875376, EPI_ISL_875377, EPI_ISL_875378, EPI_ISL_875379, EPI_ISL_875380, EPI_ISL_875381, EPI_ISL_875382, EPI_ISL_875383, EPI_ISL_875384, EPI_ISL_875385, EPI_ISL_875386, EPI_ISL_875387, EPI_ISL_875388, EPI_ISL_875389, EPI_ISL_875390, EPI_ISL_875391, EPI_ISL_875392, EPI_ISL_875393, EPI_ISL_875394, EPI_ISL_875395, EPI_ISL_875396, EPI_ISL_875397, EPI_ISL_875398, EPI_ISL_875399, EPI_ISL_875400, EPI_ISL_875401, EPI_ISL_875402, EPI_ISL_875403, EPI_ISL_875404, EPI_ISL_875405, EPI_ISL_875406, EPI_ISL_875407, EPI_ISL_875408, EPI_ISL_875409			
see above	National Virus Reference Laboratory	National Virus Reference Laboratory	Michael Carr, Gabriel Gonzalez, Jonathan Dean, Cillian F De Gascun
EPI_ISL_875557	ULSS 2 Treviso	Istituto Zooprofilattico Sperimentale delle Venezie	Adelaide Milani, Alessia Schivo, Annalisa Salviato, Erika Giorgia Quaranta, Ambra Pastori, Bianca Zecchin, Alice Fusaro, Isabella Monne, Calogero Terregino, Antonia Ricci
EPI_ISL_875664, EPI_ISL_875665	University of Michigan Clinical Microbiology Laboratory	Lauring Lab, University of Michigan, Department of Microbiology and Immunology	Valesano
EPI_ISL_876050, EPI_ISL_876070, EPI_ISL_876080, EPI_ISL_876096, EPI_ISL_876162, EPI_ISL_876190, EPI_ISL_876239, EPI_ISL_876240, EPI_ISL_876241, EPI_ISL_876242, EPI_ISL_876243			
see above	Massachusetts State Public Health Laboratory	Massachusetts State Public Health Laboratory	Andrew Lang, Timelia Fink, Glen Gallagher, Sandra Smole
EPI_ISL_876595	TN Division of Laboratory Services	Pathogen Discovery, Respiratory Viruses Branch, Division of Viral Diseases, Centers for Disease Control and Prevention	Ying Tao, Yan Li, Jing Zhang, Krista Queen, Anna Uehara, Peter Cook, Clinton R. Paden, Haibin Wang, Suxiang Tong
EPI_ISL_876741	Istituto Zooprofilattico Sperimentale della Puglia e della Basilicata	Istituto Zooprofilattico Sperimentale della Puglia e della Basilicata	Parisi A., Bianco A., Capozzi L., Del Sambro L., Manzulli V, Rondinone V., Pace L., Cipolletta D., Galante D.
EPI_ISL_877236, EPI_ISL_877329, EPI_ISL_877330	University College Sedaya International (UCSI University)	Institute for Medical Research, Infectious Disease Research Centre, National Institutes of Health, Ministry of Health Malaysia	Suppiah J, Kamel K, Azizan MA, Sekaran SD, Thayan R
EPI_ISL_877913, EPI_ISL_877955	Lighthouse Lab in Alderley Park	Wellcome Sanger Institute for the COVID-19 Genomics UK (COG-UK) Consortium	Jacquelyn Wynn, Mairead Hyland, The Lighthouse Lab in Alderley Park and Alex Alderton, Roberto Amato, Sonia Goncalves, Ewan Harrison, David K. Jackson, Ian Johnston, Dominic Kwiatkowski, Cordelia Langford, John Sillitoe on behalf of the Wellcome Sanger Institute COVID-19 Surveillance Team
EPI_ISL_878769	Lighthouse Lab in Milton Keynes	Wellcome Sanger Institute for the COVID-19 Genomics UK (COG-UK) Consortium	The Lighthouse Lab in Milton Keynes and Alex Alderton, Roberto Amato, Sonia Goncalves, Ewan Harrison, David K. Jackson, Ian Johnston, Dominic Kwiatkowski, Cordelia Langford, John Sillitoe on behalf of the Wellcome Sanger Institute COVID-19 Surveillance Team
EPI_ISL_878844, EPI_ISL_879274	Lighthouse Lab in Alderley Park	Wellcome Sanger Institute for the COVID-19 Genomics UK (COG-UK) Consortium	Jacquelyn Wynn, Mairead Hyland, The Lighthouse Lab in Alderley Park and Alex Alderton, Roberto Amato, Sonia Goncalves, Ewan Harrison, David K. Jackson, Ian Johnston, Dominic Kwiatkowski, Cordelia Langford, John Sillitoe on behalf of the Wellcome Sanger Institute COVID-19 Surveillance Team
EPI_ISL_879820, EPI_ISL_880038	Lighthouse Lab in Cambridge	Wellcome Sanger Institute for the COVID-19 Genomics UK (COG-UK) Consortium	Rob Howes, The Lighthouse Lab in Cambridge and Alex Alderton, Roberto Amato, Sonia Goncalves, Ewan Harrison, David K. Jackson, Ian Johnston, Dominic Kwiatkowski, Cordelia Langford, John Sillitoe on behalf of the Wellcome Sanger Institute COVID-19 Surveillance Team
EPI_ISL_880586, EPI_ISL_880590, EPI_ISL_880591, EPI_ISL_880595, EPI_ISL_880596, EPI_ISL_880599, EPI_ISL_880602, EPI_ISL_880606, EPI_ISL_880608, EPI_ISL_880609, EPI_ISL_880610, EPI_ISL_880611, EPI_ISL_880613, EPI_ISL_880615, EPI_ISL_880616, EPI_ISL_880618, EPI_ISL_880619, EPI_ISL_880623, EPI_ISL_880626, EPI_ISL_880627, EPI_ISL_880639, EPI_ISL_880641, EPI_ISL_880642, EPI_ISL_880647, EPI_ISL_880651, EPI_ISL_880653, EPI_ISL_880654, EPI_ISL_880655, EPI_ISL_880657, EPI_ISL_880660, EPI_ISL_880662, EPI_ISL_880665, EPI_ISL_880667, EPI_ISL_880668, EPI_ISL_880672, EPI_ISL_880673, EPI_ISL_880675, EPI_ISL_880677, EPI_ISL_880679, EPI_ISL_880695, EPI_ISL_880701, EPI_ISL_880705, EPI_ISL_880706, EPI_ISL_880708, EPI_ISL_880713, EPI_ISL_880722, EPI_ISL_880724, EPI_ISL_880725, EPI_ISL_880726, EPI_ISL_880727, EPI_ISL_880729, EPI_ISL_880736, EPI_ISL_880737, EPI_ISL_880738, EPI_ISL_880739, EPI_ISL_880740, EPI_ISL_880745, EPI_ISL_880751, EPI_ISL_880752, EPI_ISL_880755, EPI_ISL_880759, EPI_ISL_880762, EPI_ISL_880766, EPI_ISL_880769, EPI_ISL_880781, EPI_ISL_880785, EPI_ISL_880786, EPI_ISL_880791, EPI_ISL_880792, EPI_ISL_880797, EPI_ISL_880798, EPI_ISL_880800, EPI_ISL_880803, EPI_ISL_880809, EPI_ISL_880813, EPI_ISL_880817, EPI_ISL_880818, EPI_ISL_880819, EPI_ISL_880822, EPI_ISL_880824, EPI_ISL_880826, EPI_ISL_880829, EPI_ISL_880830, EPI_ISL_880833, EPI_ISL_880836, EPI_ISL_880838, EPI_ISL_880839, EPI_ISL_880840, EPI_ISL_880841, EPI_ISL_880843, EPI_ISL_880847, EPI_ISL_880854, EPI_ISL_880856, EPI_ISL_880857, EPI_ISL_880858, EPI_ISL_880862, EPI_ISL_880866, EPI_ISL_880870, EPI_ISL_880878, EPI_ISL_880883, EPI_ISL_880884, EPI_ISL_880886, EPI_ISL_880889, EPI_ISL_880894, EPI_ISL_880895, EPI_ISL_880898, EPI_ISL_880900, EPI_ISL_880901, EPI_ISL_880903, EPI_ISL_880908, EPI_ISL_880909, EPI_ISL_880910, EPI_ISL_880911, EPI_ISL_880914, EPI_ISL_880916, EPI_ISL_880921, EPI_ISL_880925, EPI_ISL_880933, EPI_ISL_880934, EPI_ISL_880935			

see above	Lighthouse Lab in Milton Keynes	Wellcome Sanger Institute for the COVID-19 Genomics UK (COG-UK) Consortium	The Lighthouse Lab in Milton Keynes and Alex Alderton, Roberto Amato, Sonia Goncalves, Ewan Harrison, David K. Jackson, Ian Johnston, Dominic Kwiatkowski, Cordelia Langford, John Sillitoe on behalf of the Wellcome Sanger Institute COVID-19 Surveillance Team
EPI_ISL_881277, EPI_ISL_881281, EPI_ISL_881283, EPI_ISL_881289, EPI_ISL_881290, EPI_ISL_881291, EPI_ISL_881293, EPI_ISL_881298, EPI_ISL_881306, EPI_ISL_881307, EPI_ISL_881309, EPI_ISL_881310, EPI_ISL_881315, EPI_ISL_881341, EPI_ISL_881359, EPI_ISL_881386, EPI_ISL_881388, EPI_ISL_881414, EPI_ISL_881429, EPI_ISL_881455, EPI_ISL_881461, EPI_ISL_881487, EPI_ISL_881494, EPI_ISL_881495, EPI_ISL_881509, EPI_ISL_881518, EPI_ISL_881521, EPI_ISL_881522, EPI_ISL_881531, EPI_ISL_881540, EPI_ISL_881543, EPI_ISL_881546, EPI_ISL_881550, EPI_ISL_881567, EPI_ISL_881570, EPI_ISL_881571, EPI_ISL_881576, EPI_ISL_881580, EPI_ISL_881582, EPI_ISL_881586, EPI_ISL_881588, EPI_ISL_881598, EPI_ISL_881600, EPI_ISL_881607, EPI_ISL_881612			
see above	Lighthouse Lab in Alderley Park	Wellcome Sanger Institute for the COVID-19 Genomics UK (COG-UK) Consortium	Jacquelyn Wynn, Mairead Hyland, The Lighthouse Lab in Alderley Park and Alex Alderton, Roberto Amato, Sonia Goncalves, Ewan Harrison, David K. Jackson, Ian Johnston, Dominic Kwiatkowski, Cordelia Langford, John Sillitoe on behalf of the Wellcome Sanger Institute COVID-19 Surveillance Team
EPI_ISL_881619	Lighthouse Lab in Cambridge	Wellcome Sanger Institute for the COVID-19 Genomics UK (COG-UK) Consortium	Rob Howes, The Lighthouse Lab in Cambridge and Alex Alderton, Roberto Amato, Sonia Goncalves, Ewan Harrison, David K. Jackson, Ian Johnston, Dominic Kwiatkowski, Cordelia Langford, John Sillitoe on behalf of the Wellcome Sanger Institute COVID-19 Surveillance Team
EPI_ISL_882299	Lighthouse Lab in Alderley Park	Wellcome Sanger Institute for the COVID-19 Genomics UK (COG-UK) Consortium	Jacquelyn Wynn, Mairead Hyland, The Lighthouse Lab in Alderley Park and Alex Alderton, Roberto Amato, Sonia Goncalves, Ewan Harrison, David K. Jackson, Ian Johnston, Dominic Kwiatkowski, Cordelia Langford, John Sillitoe on behalf of the Wellcome Sanger Institute COVID-19 Surveillance Team
EPI_ISL_882669	UPA Vila Santa Catarina	Instituto Adolfo Lutz, Interdiciplinary Procedures Center, Strategic Laboratory	Claudio Tavares Sacchi, Claudia Regina Gonçalves, Erica Valessa Ramos Gomes, Karoline Rodrigues Campos
EPI_ISL_882782	Institute for Urban Disease Control and Prevention	COVID-19 Network Investigations (CONI) Alliance	Kamolthip Atsawawaranunt, Elizabeth Batty, Wasun Chantratita, Thanat Chookajorn, Stefan Fernandez, Angkana Huang, Anthony R. Jones, Khajohn Joonsalak, Chonticha Klungtong, Theerarat Kochakarn, Prayuth Kaewmalang, Amornmas Kongklieng, Namfon Kotanan, Krittikorn Kumpornsin, Duangkamon Loesbanluechai, Wudtichai Manasatienkij, Anek Mungaomklang, Bhakbhoom Panthan, Pukkaporn Parnwijitkul, Ekawat Pasomsub, Vichan Pawun, Kingkan Rakmanee, Insee Sensorn, Janjira Thaipadungpanit, Arporn Wangwiwatsin, Treewat Watthanachockchai
EPI_ISL_882979, EPI_ISL_882980, EPI_ISL_882981, EPI_ISL_882982, EPI_ISL_882983, EPI_ISL_882984	UCLA Clinical Micro Lab	Los Angeles County PHL	P. Hemarajata et al.
EPI_ISL_882985	MD Laboratories	Los Angeles County PHL	P. Hemarajata et al.
EPI_ISL_883004, EPI_ISL_883005, EPI_ISL_883011, EPI_ISL_883015, EPI_ISL_883025, EPI_ISL_883026	Maryland Public Health Laboratory	Maryland Public Health Laboratory	Maryland Department of Health Laboratories Administration
EPI_ISL_883426	NORTHWELL HEALTH LABORATORIES	Wadsworth Center, New York State Department of Health	Kirsten St. George, Daryl M. Lamson, Alexis Russel, Matthew Shudt, Melissa A Leisner, Jonathan Plitnick, Navjot Singh, John Kelly, Erasmus Schneider, Erica Lasek-Nesselquist
EPI_ISL_883429	BOSTON HEART DIAGNOSTICS CORP	Wadsworth Center, New York State Department of Health	Kirsten St. George, Daryl M. Lamson, Alexis Russel, Matthew Shudt, Melissa A Leisner, Jonathan Plitnick, Navjot Singh, John Kelly, Erasmus Schneider, Erica Lasek-Nesselquist
EPI_ISL_883435	NORTHWELL HEALTH LABORATORIES	Wadsworth Center, New York State Department of Health	Kirsten St. George, Daryl M. Lamson, Alexis Russel, Matthew Shudt, Melissa A Leisner, Jonathan Plitnick, Navjot Singh, John Kelly, Erasmus Schneider, Erica Lasek-Nesselquist
EPI_ISL_883439	ADIRONDACK MEDICAL CENTER	Wadsworth Center, New York State Department of Health	Kirsten St. George, Daryl M. Lamson, Alexis Russel, Matthew Shudt, Melissa A Leisner, Jonathan Plitnick, Navjot Singh, John Kelly, Erasmus Schneider, Erica Lasek-Nesselquist
EPI_ISL_883480, EPI_ISL_883482, EPI_ISL_883487, EPI_ISL_883488, EPI_ISL_883489, EPI_ISL_883490, EPI_ISL_883492, EPI_ISL_883493, EPI_ISL_883495, EPI_ISL_883496, EPI_ISL_883497			
see above	NORTHWELL HEALTH LABORATORIES	Wadsworth Center, New York State Department of Health	Kirsten St. George, Daryl M. Lamson, Alexis Russel, Matthew Shudt, Melissa A Leisner, Jonathan Plitnick, Navjot Singh, John Kelly, Erasmus Schneider, Erica Lasek-Nesselquist
EPI_ISL_883960	Labo Analyses Med	National Reference Center for Viruses of Respiratory Infections, Institut Pasteur, Paris	Marion Barbet, Sylvie Behillil, Méline Bizard, Angela Brisebarre, Camille Capel, Etienne Simon-Lorière, Vincent Enouf, Maud Vanpeene, Sylvie van der Werf,Jacques Alexandra
EPI_ISL_884055, EPI_ISL_884056, EPI_ISL_884057, EPI_ISL_884058	Wadsworth Center, New York State Department of Health	Wadsworth Center, New York State Department of Health	Kirsten St. George, Daryl M. Lamson, Alexis Russel, Matthew Shudt, Melissa A Leisner, Jonathan Plitnick, Navjot Singh, John Kelly, Erasmus Schneider, Erica Lasek-Nesselquist
EPI_ISL_884059, EPI_ISL_884060, EPI_ISL_884061, EPI_ISL_884062, EPI_ISL_884063, EPI_ISL_884064	ALBANY MEDICAL CENTER HOSPITAL CLINICAL LABORATORIES	Wadsworth Center, New York State Department of Health	Kirsten St. George, Daryl M. Lamson, Alexis Russel, Matthew Shudt, Melissa A Leisner, Jonathan Plitnick, Navjot Singh, John Kelly, Erasmus Schneider, Erica Lasek-Nesselquist
EPI_ISL_884204, EPI_ISL_884205, EPI_ISL_884207, EPI_ISL_884210	Wyoming Public Health Laboratory	Wyoming Public Health Laboratory	Noah Hull, Taylor Fearing, Lynette Gumbleton, Channing Weber, Ashley Norberg, Bailey Bowcutt, and Wanda Manley
EPI_ISL_884231, EPI_ISL_884232, EPI_ISL_884233, EPI_ISL_884234, EPI_ISL_884235, EPI_ISL_884236, EPI_ISL_884237, EPI_ISL_884238, EPI_ISL_884239, EPI_ISL_884240, EPI_ISL_884241			
see above	Kansas Health and Environmental Lab	Kansas Health and Environmental Lab	Mike Grose, Paige Drury, Carissa Robertson, Ben Olsen, and Phil Adam
EPI_ISL_884255	Maryland Public Health Laboratory	Maryland Public Health Laboratory	Maryland Department of Health Laboratories Administration
EPI_ISL_884729, EPI_ISL_884730, EPI_ISL_884731, EPI_ISL_884732, EPI_ISL_884733, EPI_ISL_884734, EPI_ISL_884735, EPI_ISL_884736, EPI_ISL_884737, EPI_ISL_884738, EPI_ISL_884739, EPI_ISL_884740, EPI_ISL_884741, EPI_ISL_884742, EPI_ISL_884743, EPI_ISL_884744, EPI_ISL_884745, EPI_ISL_884746, EPI_ISL_884747, EPI_ISL_884748, EPI_ISL_884749, EPI_ISL_884750, EPI_ISL_884751, EPI_ISL_884752, EPI_ISL_884753, EPI_ISL_884754, EPI_ISL_884755, EPI_ISL_884756, EPI_ISL_884757, EPI_ISL_884758, EPI_ISL_884759, EPI_ISL_884760, EPI_ISL_884761, EPI_ISL_884762, EPI_ISL_884763, EPI_ISL_884764, EPI_ISL_884765, EPI_ISL_884766, EPI_ISL_884767, EPI_ISL_884768, EPI_ISL_884769, EPI_ISL_884770, EPI_ISL_884771, EPI_ISL_884772, EPI_ISL_884773, EPI_ISL_884774, EPI_ISL_884775, EPI_ISL_884776, EPI_ISL_884777, EPI_ISL_884778, EPI_ISL_884779, EPI_ISL_884780, EPI_ISL_884781, EPI_ISL_884782, EPI_ISL_884783, EPI_ISL_884784, EPI_ISL_884785, EPI_ISL_884786, EPI_ISL_884787, EPI_ISL_884788			
see above	Respiratory Viruses Branch, Centers for Disease Control and Prevention	Respiratory Viruses Branch, Centers for Disease Control and Prevention	Cook,P.W., Batra,D., Rambo-Martin,B.L., de Feo,E., Antico,J., Tran,C., Tolentino,M., Wickline,S., Gietzen,K., Sickler,B., Liu,J., Allen,E., Febbo,P., Galloway,S., Washington,N.L., White,S., Levan,G., Barret,K.S., Cirulli,E., Bolze,A., Ascencio,A., Rivera-Garcia,C., Cho,R., Nguyen,J., Wang,S., Ramirez,J., Cassens,T., Sandoval,E., Isaksson,M., Lee,W., Becker,D., Laurent,M., Lu,J., Paden,C.R., Tong,S., MacCannell,D.
EPI_ISL_885240, EPI_ISL_885327, EPI_ISL_885409, EPI_ISL_885514	Lighthouse Lab in Alderley Park	Wellcome Sanger Institute for the COVID-19 Genomics UK (COG-UK) Consortium	Jacquelyn Wynn, Mairead Hyland, The Lighthouse Lab in Alderley Park and Alex Alderton, Roberto Amato, Sonia Goncalves, Ewan Harrison, David K. Jackson, Ian Johnston, Dominic Kwiatkowski, Cordelia Langford, John Sillitoe on behalf of the Wellcome Sanger Institute COVID-19 Surveillance Team
EPI_ISL_886185, EPI_ISL_886186, EPI_ISL_886194, EPI_ISL_886195, EPI_ISL_886213, EPI_ISL_886215, EPI_ISL_886219, EPI_ISL_886221, EPI_ISL_886227, EPI_ISL_886228, EPI_ISL_886235, EPI_ISL_886238, EPI_ISL_886240, EPI_ISL_886249, EPI_ISL_886252, EPI_ISL_886253, EPI_ISL_886255, EPI_ISL_886261, EPI_ISL_886266, EPI_ISL_886274, EPI_ISL_886283, EPI_ISL_886285, EPI_ISL_886286, EPI_ISL_886288, EPI_ISL_886292, EPI_ISL_886294, EPI_ISL_886295, EPI_ISL_886305, EPI_ISL_886306, EPI_ISL_886308, EPI_ISL_886310, EPI_ISL_886312, EPI_ISL_886324, EPI_ISL_886344, EPI_ISL_886359, EPI_ISL_886360, EPI_ISL_886366, EPI_ISL_886380, EPI_ISL_886384, EPI_ISL_886392, EPI_ISL_886393, EPI_ISL_886401, EPI_ISL_886404, EPI_ISL_886405, EPI_ISL_886407, EPI_ISL_886412, EPI_ISL_886414, EPI_ISL_886420, EPI_ISL_886437, EPI_ISL_886460, EPI_ISL_886470, EPI_ISL_886474, EPI_ISL_886476, EPI_ISL_886505, EPI_ISL_886506, EPI_ISL_886523, EPI_ISL_886529, EPI_ISL_886531, EPI_ISL_886562, EPI_ISL_886565, EPI_ISL_886566, EPI_ISL_886567, EPI_ISL_886570, EPI_ISL_886595, EPI_ISL_886617, EPI_ISL_886626, EPI_ISL_886634, EPI_ISL_886635, EPI_ISL_886647, EPI_ISL_886661, EPI_ISL_886671, EPI_ISL_886677, EPI_ISL_886689, EPI_ISL_886690, EPI_ISL_886697, EPI_ISL_886700, EPI_ISL_886703, EPI_ISL_886705, EPI_ISL_886723, EPI_ISL_886734, EPI_ISL_886736, EPI_ISL_886748, EPI_ISL_886750, EPI_ISL_886753, EPI_ISL_886758, EPI_ISL_886759, EPI_ISL_886765, EPI_ISL_886775, EPI_ISL_886776, EPI_ISL_886784, EPI_ISL_886789, EPI_ISL_886799, EPI_ISL_886814, EPI_ISL_886816, EPI_ISL_886817, EPI_ISL_886819, EPI_ISL_886821, EPI_ISL_886822, EPI_ISL_886827, EPI_ISL_886830, EPI_ISL_886833, EPI_ISL_886834, EPI_ISL_886838, EPI_ISL_886839, EPI_ISL_886841, EPI_ISL_886854, EPI_ISL_886855, EPI_ISL_886857, EPI_ISL_886858, EPI_ISL_886864, EPI_ISL_886871, EPI_ISL_886880, EPI_ISL_886886, EPI_ISL_886890, EPI_ISL_886897, EPI_ISL_886900, EPI_ISL_886918, EPI_ISL_886927, EPI_ISL_886933, EPI_ISL_886944, EPI_ISL_886946, EPI_ISL_886967, EPI_ISL_886968, EPI_ISL_886973, EPI_ISL_886980, EPI_ISL_886991, EPI_ISL_886992, EPI_ISL_886993, EPI_ISL_886996, EPI_ISL_887007, EPI_ISL_887011, EPI_ISL_887013, EPI_ISL_887016, EPI_ISL_887022, EPI_ISL_887023, EPI_ISL_887026, EPI_ISL_887046, EPI_ISL_887053, EPI_ISL_887085, EPI_ISL_887086, EPI_ISL_887088, EPI_ISL_887093			
see above	Labcorp	Genomics and Discovery, Respiratory Viruses Branch, Division of Viral Diseases, Centers for Disease Control and Prevention	Peter W. Cook,Dhwani Batra,Ben L. Rambo-Martin,Summer Galloway,Brian Krueger,Minoo Agarwal,Eyad Almasri,Debbie Boles,Ayla Burns,Nuthawin Charoensri,Oren Cohen,Susan Countryman,Mary Ann Cristobal,Bobbi Croy,Suzanne Dale,Hrushikesh Deshmukh,Amanda Douglas,Vincent Drouillon,Marcia Eisenberg,Howard Engler,Rama Ghatti,Prashant Gupta,Susan Hicks,Jake Humphrey,Lax Iyer,Manoj Jain,Mohan Kolli,Tim Kuphal,Stanley Letovsky,Michael Levandoski,Craig Lukasik,Jonathan Meltzer,Brian Norvell,Mindy Nye,Scott Parker,Christos Petropoulos,John Pruitt,Steven Ragan,Scott Ryan,Mike Sapeta,Jana Schroth,Suresh Babu Selvaraju,Goran Stevovic,Amanda Suchanek,Andrea Throop,Lyndon Tilson,Thomas Urban,Joe Voshell,Kimberly Wagner,Jonathan Williams,Mary Williamson,Qian Zeng,Tricia Zwiefelhofer,Clinton R. Paden,Suxiang Tong,Duncan MacCannell,
EPI_ISL_887121, EPI_ISL_887130, EPI_ISL_887131, EPI_ISL_887141	Institute of Medical Microbiology and Hospital Hygiene	Institute of Medical Microbiology and Hospital Hygiene	Prof. Dr. Achim Kaasch, Aljoscha Tersteegen
EPI_ISL_888597, EPI_ISL_888598,	Wyoming Public Health Laboratory	Wyoming Public Health Laboratory	Noah Hull, Taylor Fearing, Lynette Gumbleton, Channing Weber, Ashley Norberg, Bailey Bowcutt, and Wanda Manley

EPI_ISL_888601			
EPI_ISL_888803, EPI_ISL_888804, EPI_ISL_888805, EPI_ISL_888806, EPI_ISL_888807, EPI_ISL_888808, EPI_ISL_888809	Univeristy of New Mexico Hospital	Center for Global Health, University of New Mexico Health Sciences Center	Daryl Domman, Kurt Schwalm, Justin Bacca, Jon Femling, Darrell Dinwiddie
EPI_ISL_888855, EPI_ISL_888897, EPI_ISL_888898, EPI_ISL_888899, EPI_ISL_888900, EPI_ISL_888902, EPI_ISL_888904, EPI_ISL_888905	Michigan Department of Health and Human Services, Bureau of Laboratories	Michigan Department of Health and Human Services, Bureau of Laboratories	Blankenship HM, Riner D, Soehnlen MK
EPI_ISL_889005	Eijkman Institute for Molecular Biology, Ministry of Research and Technology/National Agency for Research and Innovation	Eijkman Institute for Molecular Biology, Ministry of Research and Technology/National Agency for Research and Innovation	Willy Agustine, Edison Johar, Hidayat Trimarsanto, Iskandar Adnan, Lydia V. Panggalo, Sukma Oktavianthi, Frilasita A Yudhaputri, Safarina G Malik, Khin Saw Myint, Amin Soebandrio
EPI_ISL_889006	RSU Harapan Bunda	Eijkman Institute for Molecular Biology, Ministry of Research and Technology/National Agency for Research and Innovation	Willy Agustine, Edison Johar, Hidayat Trimarsanto, Iskandar Adnan, Lydia V. Panggalo, Sukma Oktavianthi, Frilasita A Yudhaputri, Safarina G Malik, Khin Saw Myint, Amin Soebandrio
EPI_ISL_889007	RSUD Cileungsi	Eijkman Institute for Molecular Biology, Ministry of Research and Technology/National Agency for Research and Innovation	Willy Agustine, Edison Johar, Hidayat Trimarsanto, Iskandar Adnan, Lydia V. Panggalo, Sukma Oktavianthi, Frilasita A Yudhaputri, Safarina G Malik, Khin Saw Myint, Amin Soebandrio
EPI_ISL_889686, EPI_ISL_889687, EPI_ISL_889688, EPI_ISL_889689, EPI_ISL_889690, EPI_ISL_889691, EPI_ISL_889692, EPI_ISL_889693, EPI_ISL_889694, EPI_ISL_889695, EPI_ISL_889696, EPI_ISL_889697, EPI_ISL_889698, EPI_ISL_889699, EPI_ISL_889700, EPI_ISL_889701, EPI_ISL_889702, EPI_ISL_889713			
see above	LSUHS Emerging Viral Threat Laboratory	Microbial Genome Sequencing Center	Jeremy P. Kamil, Jennifer L. Carroll, Camille F. Abshire, Maarten Van Diest, Mohammed N.A. Siddiquey, Andrew D. Yurochko, Martin J. Sapp, Rona S. Scott, Christopher G. Kevil, Daniel J. Snyder, Vaughn S. Cooper, John A. Vanchiere
EPI_ISL_890358	Hospital	National Reference Center for Viruses of Respiratory Infections, Institut Pasteur, Paris	Marion Barbet, Sylvie Behillil, Méline Bizard, Angela Brisebarre, Camille Capel, Etienne Simon-Lorière, Vincent Enouf, Maud Vanpeene, Sylvie van der Werf,Combe Patrice
EPI_ISL_890967, EPI_ISL_890969	Seattle Flu Study	Seattle Flu Study	Deborah A. Nickerson, Chris D. Frazar, Jover Lee, Benjamin Pelle, Erica Ryke, Matthew Richardson, Amanda Adler, Elisabeth Brandstetter, Peter D. Han, Kairsten Fay, Misja Ilcisin, Kirsten Lacombe, Thomas R. Sibley, Melissa Truong, Caitlin R. Wolf, Michael Boeckh, Janet A. Englund, Michael Famulare, Barry R. Lutz, Mark J. Rieder, Lea M. Starita, Matthew Thompson, Jay Shendure, Trevor Bedford, Helen Y. Chu
EPI_ISL_890970, EPI_ISL_890971, EPI_ISL_890972, EPI_ISL_890973, EPI_ISL_890974, EPI_ISL_890975, EPI_ISL_890976, EPI_ISL_890977, EPI_ISL_890978, EPI_ISL_890979, EPI_ISL_890980, EPI_ISL_890981, EPI_ISL_890982, EPI_ISL_890983, EPI_ISL_890984, EPI_ISL_890990, EPI_ISL_890993			
see above	Seattle Flu Study	Seattle Flu Study	Deborah A. Nickerson, Chris D. Frazar, Jover Lee, Benjamin Pelle, Erica Ryke, Matthew Richardson, Amanda Adler, Elisabeth Brandstetter, Peter D. Han, Kairsten Fay, Misja Ilcisin, Kirsten Lacombe, Thomas R. Sibley, Melissa Truong, Caitlin R. Wolf, Karen Cowgill, Stephanie Schrag, Jeff Duchin, Michael Boeckh, Janet A. Englund, Michael Famulare, Barry R. Lutz, Mark J. Rieder, Lea M. Starita, Matthew Thompson, Helen Y. Chu, Trevor Bedford, Jay Shendure
EPI_ISL_891145	University of Wisconsin-Madison AIDS Vaccine Research Laboratories	University of Wisconsin-Madison AIDS Vaccine Research Laboratories	Gage Moreno, Katarina Braun, et al. AIDS Vaccine Research Laboratories
EPI_ISL_891204	DPH, Massachusetts State Public Health Lab	DPH, Massachusetts State Public Health Lab	Lang,A.S., Fink,T., Gallagher,G.R., Smole,S.C.
EPI_ISL_892050, EPI_ISL_892051, EPI_ISL_892054, EPI_ISL_892057, EPI_ISL_892058, EPI_ISL_892126, EPI_ISL_892179, EPI_ISL_892189, EPI_ISL_892190, EPI_ISL_892191, EPI_ISL_892192, EPI_ISL_892193, EPI_ISL_892194, EPI_ISL_892195, EPI_ISL_892196, EPI_ISL_892197			
see above	Lighthouse Lab in Alderley Park	Wellcome Sanger Institute for the COVID-19 Genomics UK (COG-UK) Consortium	Jacquelyn Wynn, Mairead Hyland, The Lighthouse Lab in Alderley Park and Alex Alderton, Roberto Amato, Sonia Goncalves, Ewan Harrison, David K. Jackson, Ian Johnston, Dominic Kwiatkowski, Cordelia Langford, John Sillitoe on behalf of the Wellcome Sanger Institute COVID-19 Surveillance Team
EPI_ISL_892250, EPI_ISL_892252, EPI_ISL_892253, EPI_ISL_892254, EPI_ISL_892255, EPI_ISL_892256, EPI_ISL_892257, EPI_ISL_892258, EPI_ISL_892259, EPI_ISL_892260, EPI_ISL_892261, EPI_ISL_892262			
see above	MD Laboratories	Los Angeles County PHL	P. Hemarajata et al.
EPI_ISL_894189, EPI_ISL_894191, EPI_ISL_894198, EPI_ISL_894210	KU Leuven, Rega Institute, Clinical and Epidemiological Virology	KU Leuven, Rega Institute, Clinical and Epidemiological Virology	Tony Wawina-Bokalanga, Bert Vanmechelen, Joan Marti-Carerras, Piet Maes
EPI_ISL_894224, EPI_ISL_894225, EPI_ISL_894226, EPI_ISL_894236	CH de Mayotte - Laboratoire de Biologie	National Reference Center for Viruses of Respiratory Infections, Institut Pasteur, Paris	Marion Barbet, Sylvie Behillil, Méline Bizard, Angela Brisebarre, Camille Capel, Etienne Simon-Lorière, Vincent Enouf, Maud Vanpeene, Sylvie van der Werf,Combe Patrice
EPI_ISL_894248, EPI_ISL_894249, EPI_ISL_894250, EPI_ISL_894251, EPI_ISL_894252, EPI_ISL_894253, EPI_ISL_894254	Ministry of Health Turkey	Ministry of Health Turkey	Fatma Bayrakdar, Yasemin Cogun, Süleyman Yalcin, Aye Baak Alta, Gülay Korukluolu
EPI_ISL_896086, EPI_ISL_896100, EPI_ISL_896111	Viollier AG	University Hospital Basel, Clinical Bacteriology	Tim Roloff, Madlen Stange, Helena MB Seth-Smith, Alfredo Mari, Karoline Leuzinger, Julia Bielicki, Christiane Beckmann, Manuel Battegay, Hans Hirsch, Adrian Egli
EPI_ISL_896169, EPI_ISL_896212, EPI_ISL_896214, EPI_ISL_896215	MEPHI, Aix Marseille University	MEPHI, Aix Marseille University	Anthony LEVASSEUR
EPI_ISL_896220, EPI_ISL_896228, EPI_ISL_896248, EPI_ISL_896249, EPI_ISL_896250, EPI_ISL_896251, EPI_ISL_896254, EPI_ISL_896255, EPI_ISL_896290	SUNY UPSTATE MEDICAL UNIVERSITY	Wadsworth Center, New York State Department of Health	Kirsten St. George, Daryl M. Lamson, Alexis Russel, Matthew Shudt, Melissa A Leisner, Jonathan Plitnick, Navjot Singh, John Kelly, Erasmus Schneider, Erica Lasek-Nesselquist
EPI_ISL_896385, EPI_ISL_896387, EPI_ISL_896388, EPI_ISL_896396, EPI_ISL_896397, EPI_ISL_896398, EPI_ISL_896399, EPI_ISL_896400, EPI_ISL_896401, EPI_ISL_896402, EPI_ISL_896403, EPI_ISL_896404, EPI_ISL_896405, EPI_ISL_896406, EPI_ISL_896407, EPI_ISL_896408, EPI_ISL_896409, EPI_ISL_896410, EPI_ISL_896411, EPI_ISL_896412, EPI_ISL_896414, EPI_ISL_896415, EPI_ISL_896416, EPI_ISL_896419, EPI_ISL_896420			
see above	URMC LABS	Wadsworth Center, New York State Department of Health	Kirsten St. George, Daryl M. Lamson, Alexis Russel, Matthew Shudt, Melissa A Leisner, Jonathan Plitnick, Navjot Singh, John Kelly, Erasmus Schneider, Erica Lasek-Nesselquist
EPI_ISL_896456, EPI_ISL_896474, EPI_ISL_896493, EPI_ISL_896494	KU Leuven, Rega Institute, Clinical and Epidemiological Virology	KU Leuven, Rega Institute, Clinical and Epidemiological Virology	Tony Wawina-Bokalanga, Bert Vanmechelen, Joan Marti-Carerras, Piet Maes
EPI_ISL_897605, EPI_ISL_897673, EPI_ISL_897676, EPI_ISL_897734, EPI_ISL_897934, EPI_ISL_897935, EPI_ISL_897936, EPI_ISL_897938, EPI_ISL_897939, EPI_ISL_897940, EPI_ISL_897941			
see above	University Hospitals of Geneva, Laboratory of Virology	HUG, Laboratory of Virology and the Health2030 Genome Center	Samuel Cordey, Ana Rita Goncalves, Laurent Kaiser, Lorenzo Cerutti, Henri Pegeot, Melyssa Elies, Deborah Penet, Keith Harshman, Ioannis Xenarios, Emmanouil Dermitzakis
EPI_ISL_897988, EPI_ISL_897991, EPI_ISL_897992, EPI_ISL_897994, EPI_ISL_897995, EPI_ISL_897997, EPI_ISL_897999, EPI_ISL_898003, EPI_ISL_898006, EPI_ISL_898017, EPI_ISL_898018, EPI_ISL_898019, EPI_ISL_898020, EPI_ISL_898021, EPI_ISL_898022, EPI_ISL_898032			
see above	KU Leuven, Rega Institute, Clinical and Epidemiological Virology	KU Leuven, Rega Institute, Clinical and Epidemiological Virology	Tony Wawina-Bokalanga, Bert Vanmechelen, Joan Marti-Carerras, Piet Maes
EPI_ISL_898997, EPI_ISL_898999, EPI_ISL_899002, EPI_ISL_899004	Viollier AG	Department of Biosystems Science and Engineering, ETH Zürich	Chaoran Chen, Sarah Nadeau, Ivan Topolsky, Emmanouil Dermitzakis, Keith Harshman, Ioannis Xenarios, Henri Pegeot, Lorenzo Cerutti, Deborah Penet, Philipp Jablonski, Lara Fuhrmann, David Dreifuss, Katharina Jahn, Christiane Beckmann, Maurice Redondo, Olivier Kobel, Christoph Noppen, Sophie Seidel, Noemie Santamaria de Souza, Niko Beerenwinkel, Tanja Stadler
EPI_ISL_899009	Viollier AG	Department of Biosystems Science and Engineering, ETH Zürich	Christian Beisel, Sarah Nadeau, Chaoran Chen, Ivan Topolsky, Philipp Jablonski, Lara Fuhrmann, David Dreifuss, Katharina Jahn, Tobias Schär, Ina Nissen, Natascha Santacroce, Elodie Burcklen, Christiane Beckmann, Maurice Redondo, Olivier Kobel, Christoph Noppen, Sophie Seidel, Noemie Santamaria de Souza, Niko Beerenwinkel, Tanja Stadler

EPI_ISL_899010, EPI_ISL_899016, EPI_ISL_899022, EPI_ISL_899024, EPI_ISL_899025, EPI_ISL_899035, EPI_ISL_899039, EPI_ISL_899043, EPI_ISL_899053, EPI_ISL_899056, EPI_ISL_899060, EPI_ISL_899064			
see above	Viollier AG	Department of Biosystems Science and Engineering, ETH Zürich	Chaoran Chen, Sarah Nadeau, Ivan Topolsky, Emmanouil Dermitzakis, Keith Harshman, Ioannis Xenarios, Henri Pegeot, Lorenzo Cerutti, Deborah Penet, Philipp Jablonski, Lara Fuhrmann, David Dreifuss, Katharina Jahn, Christiane Beckmann, Maurice Redondo, Olivier Kobel, Christoph Noppen, Sophie Seidel, Noemie Santamaria de Souza, Niko Beerenwinkel, Tanja Stadler
EPI_ISL_899070	Viollier AG	Department of Biosystems Science and Engineering, ETH Zürich	Christian Beisel, Sarah Nadeau, Chaoran Chen, Ivan Topolsky, Philipp Jablonski, Lara Fuhrmann, David Dreifuss, Katharina Jahn, Tobias Schär, Ina Nissen, Natascha Santacroce, Elodie Burcklen, Christiane Beckmann, Maurice Redondo, Olivier Kobel, Christoph Noppen, Sophie Seidel, Noemie Santamaria de Souza, Niko Beerenwinkel, Tanja Stadler
EPI_ISL_899073, EPI_ISL_899076, EPI_ISL_899079	Viollier AG	Department of Biosystems Science and Engineering, ETH Zürich	Chaoran Chen, Sarah Nadeau, Ivan Topolsky, Emmanouil Dermitzakis, Keith Harshman, Ioannis Xenarios, Henri Pegeot, Lorenzo Cerutti, Deborah Penet, Philipp Jablonski, Lara Fuhrmann, David Dreifuss, Katharina Jahn, Christiane Beckmann, Maurice Redondo, Olivier Kobel, Christoph Noppen, Sophie Seidel, Noemie Santamaria de Souza, Niko Beerenwinkel, Tanja Stadler
EPI_ISL_899080	Viollier AG	Department of Biosystems Science and Engineering, ETH Zürich	Christian Beisel, Sarah Nadeau, Chaoran Chen, Ivan Topolsky, Philipp Jablonski, Lara Fuhrmann, David Dreifuss, Katharina Jahn, Tobias Schär, Ina Nissen, Natascha Santacroce, Elodie Burcklen, Christiane Beckmann, Maurice Redondo, Olivier Kobel, Christoph Noppen, Sophie Seidel, Noemie Santamaria de Souza, Niko Beerenwinkel, Tanja Stadler
EPI_ISL_899084, EPI_ISL_899086, EPI_ISL_899087, EPI_ISL_899093	Viollier AG	Department of Biosystems Science and Engineering, ETH Zürich	Chaoran Chen, Sarah Nadeau, Ivan Topolsky, Emmanouil Dermitzakis, Keith Harshman, Ioannis Xenarios, Henri Pegeot, Lorenzo Cerutti, Deborah Penet, Philipp Jablonski, Lara Fuhrmann, David Dreifuss, Katharina Jahn, Christiane Beckmann, Maurice Redondo, Olivier Kobel, Christoph Noppen, Sophie Seidel, Noemie Santamaria de Souza, Niko Beerenwinkel, Tanja Stadler
EPI_ISL_899096	Viollier AG	Department of Biosystems Science and Engineering, ETH Zürich	Christian Beisel, Sarah Nadeau, Chaoran Chen, Ivan Topolsky, Philipp Jablonski, Lara Fuhrmann, David Dreifuss, Katharina Jahn, Tobias Schär, Ina Nissen, Natascha Santacroce, Elodie Burcklen, Christiane Beckmann, Maurice Redondo, Olivier Kobel, Christoph Noppen, Sophie Seidel, Noemie Santamaria de Souza, Niko Beerenwinkel, Tanja Stadler
EPI_ISL_899098, EPI_ISL_899100	Viollier AG	Department of Biosystems Science and Engineering, ETH Zürich	Chaoran Chen, Sarah Nadeau, Ivan Topolsky, Emmanouil Dermitzakis, Keith Harshman, Ioannis Xenarios, Henri Pegeot, Lorenzo Cerutti, Deborah Penet, Philipp Jablonski, Lara Fuhrmann, David Dreifuss, Katharina Jahn, Christiane Beckmann, Maurice Redondo, Olivier Kobel, Christoph Noppen, Sophie Seidel, Noemie Santamaria de Souza, Niko Beerenwinkel, Tanja Stadler
EPI_ISL_899117	Viollier AG	Department of Biosystems Science and Engineering, ETH Zürich	Christian Beisel, Sarah Nadeau, Chaoran Chen, Ivan Topolsky, Philipp Jablonski, Lara Fuhrmann, David Dreifuss, Katharina Jahn, Tobias Schär, Ina Nissen, Natascha Santacroce, Elodie Burcklen, Christiane Beckmann, Maurice Redondo, Olivier Kobel, Christoph Noppen, Sophie Seidel, Noemie Santamaria de Souza, Niko Beerenwinkel, Tanja Stadler
EPI_ISL_899124	Viollier AG	Department of Biosystems Science and Engineering, ETH Zürich	Chaoran Chen, Sarah Nadeau, Ivan Topolsky, Emmanouil Dermitzakis, Keith Harshman, Ioannis Xenarios, Henri Pegeot, Lorenzo Cerutti, Deborah Penet, Philipp Jablonski, Lara Fuhrmann, David Dreifuss, Katharina Jahn, Christiane Beckmann, Maurice Redondo, Olivier Kobel, Christoph Noppen, Sophie Seidel, Noemie Santamaria de Souza, Niko Beerenwinkel, Tanja Stadler
EPI_ISL_899125	Viollier AG	Department of Biosystems Science and Engineering, ETH Zürich	Christian Beisel, Sarah Nadeau, Chaoran Chen, Ivan Topolsky, Philipp Jablonski, Lara Fuhrmann, David Dreifuss, Katharina Jahn, Tobias Schär, Ina Nissen, Natascha Santacroce, Elodie Burcklen, Christiane Beckmann, Maurice Redondo, Olivier Kobel, Christoph Noppen, Sophie Seidel, Noemie Santamaria de Souza, Niko Beerenwinkel, Tanja Stadler
EPI_ISL_899153	Viollier AG	Department of Biosystems Science and Engineering, ETH Zürich	Chaoran Chen, Sarah Nadeau, Ivan Topolsky, Emmanouil Dermitzakis, Keith Harshman, Ioannis Xenarios, Henri Pegeot, Lorenzo Cerutti, Deborah Penet, Philipp Jablonski, Lara Fuhrmann, David Dreifuss, Katharina Jahn, Christiane Beckmann, Maurice Redondo, Olivier Kobel, Christoph Noppen, Sophie Seidel, Noemie Santamaria de Souza, Niko Beerenwinkel, Tanja Stadler
EPI_ISL_899154	Viollier AG	Department of Biosystems Science and Engineering, ETH Zürich	Christian Beisel, Sarah Nadeau, Chaoran Chen, Ivan Topolsky, Philipp Jablonski, Lara Fuhrmann, David Dreifuss, Katharina Jahn, Tobias Schär, Ina Nissen, Natascha Santacroce, Elodie Burcklen, Christiane Beckmann, Maurice Redondo, Olivier Kobel, Christoph Noppen, Sophie Seidel, Noemie Santamaria de Souza, Niko Beerenwinkel, Tanja Stadler
EPI_ISL_899159, EPI_ISL_899160, EPI_ISL_899164	Viollier AG	Department of Biosystems Science and Engineering, ETH Zürich	Chaoran Chen, Sarah Nadeau, Ivan Topolsky, Emmanouil Dermitzakis, Keith Harshman, Ioannis Xenarios, Henri Pegeot, Lorenzo Cerutti, Deborah Penet, Philipp Jablonski, Lara Fuhrmann, David Dreifuss, Katharina Jahn, Christiane Beckmann, Maurice Redondo, Olivier Kobel, Christoph Noppen, Sophie Seidel, Noemie Santamaria de Souza, Niko Beerenwinkel, Tanja Stadler
EPI_ISL_899241, EPI_ISL_899243, EPI_ISL_899246	Viollier AG	Department of Biosystems Science and Engineering, ETH Zürich	Christian Beisel, Sarah Nadeau, Chaoran Chen, Ivan Topolsky, Philipp Jablonski, Lara Fuhrmann, David Dreifuss, Katharina Jahn, Tobias Schär, Ina Nissen, Natascha Santacroce, Elodie Burcklen, Christiane Beckmann, Maurice Redondo, Olivier Kobel, Christoph Noppen, Sophie Seidel, Noemie Santamaria de Souza, Niko Beerenwinkel, Tanja Stadler
EPI_ISL_899261, EPI_ISL_899262, EPI_ISL_899263, EPI_ISL_899264, EPI_ISL_899265, EPI_ISL_899266, EPI_ISL_899267	Viollier AG	Department of Biosystems Science and Engineering, ETH Zürich	Chaoran Chen, Sarah Nadeau, Ivan Topolsky, Emmanouil Dermitzakis, Keith Harshman, Ioannis Xenarios, Henri Pegeot, Lorenzo Cerutti, Deborah Penet, Philipp Jablonski, Lara Fuhrmann, David Dreifuss, Katharina Jahn, Christiane Beckmann, Maurice Redondo, Olivier Kobel, Christoph Noppen, Sophie Seidel, Noemie Santamaria de Souza, Niko Beerenwinkel, Tanja Stadler
EPI_ISL_899340, EPI_ISL_899341, EPI_ISL_899346, EPI_ISL_899347, EPI_ISL_899348, EPI_ISL_899349	Viollier AG	Department of Biosystems Science and Engineering, ETH Zürich	Christian Beisel, Sarah Nadeau, Chaoran Chen, Ivan Topolsky, Philipp Jablonski, Lara Fuhrmann, David Dreifuss, Katharina Jahn, Tobias Schär, Ina Nissen, Natascha Santacroce, Elodie Burcklen, Christiane Beckmann, Maurice Redondo, Olivier Kobel, Christoph Noppen, Sophie Seidel, Noemie Santamaria de Souza, Niko Beerenwinkel, Tanja Stadler
EPI_ISL_899360, EPI_ISL_899370, EPI_ISL_899371, EPI_ISL_899372	Viollier AG	Department of Biosystems Science and Engineering, ETH Zürich	Chaoran Chen, Sarah Nadeau, Ivan Topolsky, Emmanouil Dermitzakis, Keith Harshman, Ioannis Xenarios, Henri Pegeot, Lorenzo Cerutti, Deborah Penet, Philipp Jablonski, Lara Fuhrmann, David Dreifuss, Katharina Jahn, Christiane Beckmann, Maurice Redondo, Olivier Kobel, Christoph Noppen, Sophie Seidel, Noemie Santamaria de Souza, Niko Beerenwinkel, Tanja Stadler
EPI_ISL_899456, EPI_ISL_899457, EPI_ISL_899475, EPI_ISL_899476, EPI_ISL_899477	Viollier AG	Department of Biosystems Science and Engineering, ETH Zürich	Christian Beisel, Sarah Nadeau, Chaoran Chen, Ivan Topolsky, Philipp Jablonski, Lara Fuhrmann, David Dreifuss, Katharina Jahn, Tobias Schär, Ina Nissen, Natascha Santacroce, Elodie Burcklen, Christiane Beckmann, Maurice Redondo, Olivier Kobel, Christoph Noppen, Sophie Seidel, Noemie Santamaria de Souza, Niko Beerenwinkel, Tanja Stadler
EPI_ISL_899498, EPI_ISL_899499, EPI_ISL_899500, EPI_ISL_899501, EPI_ISL_899502, EPI_ISL_899503, EPI_ISL_899504, EPI_ISL_899505, EPI_ISL_899506, EPI_ISL_899507, EPI_ISL_899508			
see above	Viollier AG	Department of Biosystems Science and Engineering, ETH Zürich	Chaoran Chen, Sarah Nadeau, Ivan Topolsky, Emmanouil Dermitzakis, Keith Harshman, Ioannis Xenarios, Henri Pegeot, Lorenzo Cerutti, Deborah Penet, Philipp Jablonski, Lara Fuhrmann, David Dreifuss, Katharina Jahn, Christiane Beckmann, Maurice Redondo, Olivier Kobel, Christoph Noppen, Sophie Seidel, Noemie Santamaria de Souza, Niko Beerenwinkel, Tanja Stadler
EPI_ISL_899530	Viollier AG	Department of Biosystems Science and Engineering, ETH Zürich	Christian Beisel, Sarah Nadeau, Chaoran Chen, Ivan Topolsky, Philipp Jablonski, Lara Fuhrmann, David Dreifuss, Katharina Jahn, Tobias Schär, Ina Nissen, Natascha Santacroce, Elodie Burcklen, Christiane Beckmann, Maurice Redondo, Olivier Kobel, Christoph Noppen, Sophie Seidel, Noemie Santamaria de Souza, Niko Beerenwinkel, Tanja Stadler
EPI_ISL_899532, EPI_ISL_899533, EPI_ISL_899534	Viollier AG	Department of Biosystems Science and Engineering, ETH Zürich	Chaoran Chen, Sarah Nadeau, Ivan Topolsky, Emmanouil Dermitzakis, Keith Harshman, Ioannis Xenarios, Henri Pegeot, Lorenzo Cerutti, Deborah Penet, Philipp Jablonski, Lara Fuhrmann, David Dreifuss, Katharina Jahn, Christiane Beckmann, Maurice Redondo, Olivier Kobel, Christoph Noppen, Sophie Seidel, Noemie Santamaria de Souza, Niko Beerenwinkel, Tanja Stadler
EPI_ISL_899538, EPI_ISL_899544	Viollier AG	Department of Biosystems Science and Engineering, ETH Zürich	Christian Beisel, Sarah Nadeau, Chaoran Chen, Ivan Topolsky, Philipp Jablonski, Lara Fuhrmann, David Dreifuss, Katharina Jahn, Tobias Schär, Ina Nissen, Natascha Santacroce, Elodie Burcklen, Christiane Beckmann, Maurice Redondo, Olivier Kobel, Christoph Noppen, Sophie Seidel, Noemie Santamaria de Souza, Niko Beerenwinkel, Tanja Stadler
EPI_ISL_899560	Viollier AG	Department of Biosystems Science and Engineering, ETH Zürich	Chaoran Chen, Sarah Nadeau, Ivan Topolsky, Emmanouil Dermitzakis, Keith Harshman, Ioannis Xenarios, Henri Pegeot, Lorenzo Cerutti, Deborah Penet, Philipp Jablonski, Lara Fuhrmann, David Dreifuss, Katharina Jahn, Christiane Beckmann, Maurice Redondo, Olivier Kobel, Christoph Noppen, Sophie Seidel, Noemie Santamaria de Souza, Niko Beerenwinkel, Tanja Stadler
EPI_ISL_899589, EPI_ISL_899590	Viollier AG	Department of Biosystems Science and Engineering, ETH Zürich	Christian Beisel, Sarah Nadeau, Chaoran Chen, Ivan Topolsky, Philipp Jablonski, Lara Fuhrmann, David Dreifuss, Katharina Jahn, Tobias Schär, Ina Nissen, Natascha Santacroce, Elodie Burcklen, Christiane Beckmann, Maurice Redondo, Olivier Kobel, Christoph Noppen, Sophie Seidel, Noemie Santamaria de Souza, Niko Beerenwinkel, Tanja Stadler

EPI_ISL_899593, EPI_ISL_899594, EPI_ISL_899595	Viollier AG	Department of Biosystems Science and Engineering, ETH Zürich	Chaoran Chen, Sarah Nadeau, Ivan Topolsky, Emmanouil Dermitzakis, Keith Harshman, Ioannis Xenarios, Henri Pegeot, Lorenzo Cerutti, Deborah Penet, Philipp Jablonski, Lara Fuhrmann, David Dreifuss, Katharina Jahn, Christiane Beckmann, Maurice Redondo, Olivier Kobel, Christoph Noppen, Sophie Seidel, Noemie Santamaria de Souza, Niko Beerenwinkel, Tanja Stadler
EPI_ISL_899599, EPI_ISL_899602, EPI_ISL_899603, EPI_ISL_899604, EPI_ISL_899605, EPI_ISL_899607, EPI_ISL_899608	Viollier AG	Department of Biosystems Science and Engineering, ETH Zürich	Christian Beisel, Sarah Nadeau, Chaoran Chen, Ivan Topolsky, Philipp Jablonski, Lara Fuhrmann, David Dreifuss, Katharina Jahn, Tobias Schär, Ina Nissen, Natascha Santacroce, Elodie Burcklen, Christiane Beckmann, Maurice Redondo, Olivier Kobel, Christoph Noppen, Sophie Seidel, Noemie Santamaria de Souza, Niko Beerenwinkel, Tanja Stadler
EPI_ISL_899611, EPI_ISL_899643, EPI_ISL_899644, EPI_ISL_899645	Viollier AG	Department of Biosystems Science and Engineering, ETH Zürich	Chaoran Chen, Sarah Nadeau, Ivan Topolsky, Emmanouil Dermitzakis, Keith Harshman, Ioannis Xenarios, Henri Pegeot, Lorenzo Cerutti, Deborah Penet, Philipp Jablonski, Lara Fuhrmann, David Dreifuss, Katharina Jahn, Christiane Beckmann, Maurice Redondo, Olivier Kobel, Christoph Noppen, Sophie Seidel, Noemie Santamaria de Souza, Niko Beerenwinkel, Tanja Stadler
EPI_ISL_899722, EPI_ISL_899723, EPI_ISL_899724, EPI_ISL_899737	Viollier AG	Department of Biosystems Science and Engineering, ETH Zürich	Christian Beisel, Sarah Nadeau, Chaoran Chen, Ivan Topolsky, Philipp Jablonski, Lara Fuhrmann, David Dreifuss, Katharina Jahn, Tobias Schär, Ina Nissen, Natascha Santacroce, Elodie Burcklen, Christiane Beckmann, Maurice Redondo, Olivier Kobel, Christoph Noppen, Sophie Seidel, Noemie Santamaria de Souza, Niko Beerenwinkel, Tanja Stadler
EPI_ISL_899750, EPI_ISL_899751, EPI_ISL_899752, EPI_ISL_899753, EPI_ISL_899754, EPI_ISL_899755	Viollier AG	Department of Biosystems Science and Engineering, ETH Zürich	Chaoran Chen, Sarah Nadeau, Ivan Topolsky, Emmanouil Dermitzakis, Keith Harshman, Ioannis Xenarios, Henri Pegeot, Lorenzo Cerutti, Deborah Penet, Philipp Jablonski, Lara Fuhrmann, David Dreifuss, Katharina Jahn, Christiane Beckmann, Maurice Redondo, Olivier Kobel, Christoph Noppen, Sophie Seidel, Noemie Santamaria de Souza, Niko Beerenwinkel, Tanja Stadler
EPI_ISL_899792	Viollier AG	Department of Biosystems Science and Engineering, ETH Zürich	Christian Beisel, Sarah Nadeau, Chaoran Chen, Ivan Topolsky, Philipp Jablonski, Lara Fuhrmann, David Dreifuss, Katharina Jahn, Tobias Schär, Ina Nissen, Natascha Santacroce, Elodie Burcklen, Christiane Beckmann, Maurice Redondo, Olivier Kobel, Christoph Noppen, Sophie Seidel, Noemie Santamaria de Souza, Niko Beerenwinkel, Tanja Stadler
EPI_ISL_899800, EPI_ISL_899801, EPI_ISL_899802, EPI_ISL_899803, EPI_ISL_899804	Viollier AG	Department of Biosystems Science and Engineering, ETH Zürich	Chaoran Chen, Sarah Nadeau, Ivan Topolsky, Emmanouil Dermitzakis, Keith Harshman, Ioannis Xenarios, Henri Pegeot, Lorenzo Cerutti, Deborah Penet, Philipp Jablonski, Lara Fuhrmann, David Dreifuss, Katharina Jahn, Christiane Beckmann, Maurice Redondo, Olivier Kobel, Christoph Noppen, Sophie Seidel, Noemie Santamaria de Souza, Niko Beerenwinkel, Tanja Stadler
EPI_ISL_899844, EPI_ISL_899848, EPI_ISL_899849, EPI_ISL_899874, EPI_ISL_899912, EPI_ISL_899915	Viollier AG	Department of Biosystems Science and Engineering, ETH Zürich	Christian Beisel, Sarah Nadeau, Chaoran Chen, Ivan Topolsky, Philipp Jablonski, Lara Fuhrmann, David Dreifuss, Katharina Jahn, Tobias Schär, Ina Nissen, Natascha Santacroce, Elodie Burcklen, Christiane Beckmann, Maurice Redondo, Olivier Kobel, Christoph Noppen, Sophie Seidel, Noemie Santamaria de Souza, Niko Beerenwinkel, Tanja Stadler
EPI_ISL_899917, EPI_ISL_899918	Viollier AG	Department of Biosystems Science and Engineering, ETH Zürich	Chaoran Chen, Sarah Nadeau, Ivan Topolsky, Emmanouil Dermitzakis, Keith Harshman, Ioannis Xenarios, Henri Pegeot, Lorenzo Cerutti, Deborah Penet, Philipp Jablonski, Lara Fuhrmann, David Dreifuss, Katharina Jahn, Christiane Beckmann, Maurice Redondo, Olivier Kobel, Christoph Noppen, Sophie Seidel, Noemie Santamaria de Souza, Niko Beerenwinkel, Tanja Stadler
EPI_ISL_900010, EPI_ISL_900011, EPI_ISL_900012, EPI_ISL_900016, EPI_ISL_900017, EPI_ISL_900018	Viollier AG	Department of Biosystems Science and Engineering, ETH Zürich	Christian Beisel, Sarah Nadeau, Chaoran Chen, Ivan Topolsky, Philipp Jablonski, Lara Fuhrmann, David Dreifuss, Katharina Jahn, Tobias Schär, Ina Nissen, Natascha Santacroce, Elodie Burcklen, Christiane Beckmann, Maurice Redondo, Olivier Kobel, Christoph Noppen, Sophie Seidel, Noemie Santamaria de Souza, Niko Beerenwinkel, Tanja Stadler
EPI_ISL_900030, EPI_ISL_900031, EPI_ISL_900032, EPI_ISL_900033, EPI_ISL_900034, EPI_ISL_900035, EPI_ISL_900036	Viollier AG	Department of Biosystems Science and Engineering, ETH Zürich	Chaoran Chen, Sarah Nadeau, Ivan Topolsky, Emmanouil Dermitzakis, Keith Harshman, Ioannis Xenarios, Henri Pegeot, Lorenzo Cerutti, Deborah Penet, Philipp Jablonski, Lara Fuhrmann, David Dreifuss, Katharina Jahn, Christiane Beckmann, Maurice Redondo, Olivier Kobel, Christoph Noppen, Sophie Seidel, Noemie Santamaria de Souza, Niko Beerenwinkel, Tanja Stadler
EPI_ISL_900061	MEPHI, Aix Marseille University	MEPHI, Aix Marseille University	Anthony LEVASSEUR
EPI_ISL_900499	Althaia. Xarxa Assistencial Universitària de Manresa	IrsiCaixa - Can Ruti CovidSeq	Fundació irsiCaixa. Hospital Universitari Germans Trias i Pujol(HUGTiP), 2a planta, maternal Ctra Canyet s/n, Badalona Gloria Trujillo, Rafel Perez Vidal, Jaume Trape Pujol, Carolina Gonzalez Fernandez, Roger Paredes, Eulalia Grau, Bonaventura Clotet
EPI_ISL_902916	Department of Virology and Immunology, University of Helsinki and Helsinki University Hospital, Huslab Finland	Department of Virology, Faculty of Medicine, University of Helsinki, Helsinki, Finland	Teemu Smura, Ravi Kant, Phuoc Truong, Hussein Alburkat, Hannimari Kallio-Kokko, Jenni Virtanen, Maija Suvanto, Essi Korhonen, Sari Hannula, Harri Kangas, Hanna Liimatainen, Satu Kurkela, Hanna Jarva, Maija Lappalainen, Pekka Ellonen, Olli Vapalahti
EPI_ISL_902948	Maryland Public Health Laboratory	Maryland Public Health Laboratory	Maryland Department of Health Laboratories Administration
EPI_ISL_903347	Ministry of Health Turkey	Ministry of Health Turkey	Fatma Bayrakdar, Yasemin Cogun, Süleyman Yalcin, Aye Baak Alta, Gülay Korukluolu
EPI_ISL_903372	Los Angeles County PHL	Los Angeles County PHL	P. Hemarajata et al.
EPI_ISL_904068, EPI_ISL_904069, EPI_ISL_904070	New Mexico Department of Health Scientific Laboratory	New Mexico Department of Health Scientific Laboratory	Ellie Johnson, Anastacia Griego-Fisher, D'eldra Malone
EPI_ISL_904139, EPI_ISL_904156, EPI_ISL_904163, EPI_ISL_904207, EPI_ISL_904208, EPI_ISL_904209, EPI_ISL_904210, EPI_ISL_904211, EPI_ISL_904249, EPI_ISL_904275, EPI_ISL_904276, EPI_ISL_904277, EPI_ISL_904319, EPI_ISL_904369, EPI_ISL_904579, EPI_ISL_904580, EPI_ISL_904581, EPI_ISL_904582, EPI_ISL_904583, EPI_ISL_904584, EPI_ISL_904585, EPI_ISL_904586, EPI_ISL_904587			
see above	Dutch COVID-19 response team	Erasmus Medical Center	Bas Oude Munnink, Reina Sikkema, David Nieuwenhuijse, Irina Chestakova, Anne van der Linden, Marjan Boter, Emmanuelle Munger, Corine GeurtsvanKessel, Annemiek van der Eijk, Richard Molenkamp, Marion Koopmans, on behalf of the Dutch national COVID-19 response team.
EPI_ISL_904659, EPI_ISL_904673, EPI_ISL_904690, EPI_ISL_904696, EPI_ISL_904728, EPI_ISL_904729, EPI_ISL_904769, EPI_ISL_904786, EPI_ISL_904789, EPI_ISL_904801, EPI_ISL_904802, EPI_ISL_904803, EPI_ISL_904812, EPI_ISL_904826, EPI_ISL_904827, EPI_ISL_904832, EPI_ISL_904840, EPI_ISL_904852, EPI_ISL_904873, EPI_ISL_904874, EPI_ISL_904878, EPI_ISL_904882, EPI_ISL_904883, EPI_ISL_905048, EPI_ISL_905051, EPI_ISL_905052, EPI_ISL_905053, EPI_ISL_905054, EPI_ISL_905055, EPI_ISL_905056, EPI_ISL_905061, EPI_ISL_905063, EPI_ISL_905066, EPI_ISL_905067, EPI_ISL_905076, EPI_ISL_905118, EPI_ISL_905119, EPI_ISL_905176, EPI_ISL_905178, EPI_ISL_905180, EPI_ISL_905192, EPI_ISL_905193, EPI_ISL_905197, EPI_ISL_905204, EPI_ISL_905245, EPI_ISL_905246, EPI_ISL_905247, EPI_ISL_905248, EPI_ISL_905249, EPI_ISL_905250, EPI_ISL_905262, EPI_ISL_905267, EPI_ISL_905281, EPI_ISL_905299, EPI_ISL_905300, EPI_ISL_905301, EPI_ISL_905302, EPI_ISL_905303, EPI_ISL_905304, EPI_ISL_905305, EPI_ISL_905307, EPI_ISL_905308, EPI_ISL_905316, EPI_ISL_905317, EPI_ISL_905318, EPI_ISL_905325, EPI_ISL_905326, EPI_ISL_905548, EPI_ISL_905549, EPI_ISL_905550			
see above	Dutch COVID-19 response team	National Institute for Public Health and the Environment (RIVM)	Adam Meijer, Harry Vennema, Dirk Eggink, Jeroen Cremer, Sharon van den Brink, Bas van der Veer, AnneMarie van den Brandt, Florian Zwagemaker, Dennis Schmitz, Chantal Reusken, on behalf of the national COVID-19 response team
EPI_ISL_905741, EPI_ISL_905742, EPI_ISL_905743, EPI_ISL_905744, EPI_ISL_905745, EPI_ISL_905746, EPI_ISL_905747	National Institute of Public Health - National Institute of Hygiene	National Institute of Public Health - National Institute of Hygiene	Wokowicz Tomasz, Zacharczuk Katarzyna
EPI_ISL_906045	OHSU Lab Services Molecular Microbiology Lab	Oregon SARS-CoV-2 Genome Sequencing Center	Brendan L. O'Connell, Sally Grindstaff, Kayla Carter, Ruth V. Nichols, Alec J. Hirsch, Donna Hansel, Guang Fan, Xuan, Qin, Daniel N. Streblow, William B. Messer, Andrew C. Adey, Benjamin N. Bimber, Brian J. O'Roak
EPI_ISL_906279, EPI_ISL_906280, EPI_ISL_906281, EPI_ISL_906284	Nigeria Centre for Disease Control (NCDC)	African Centre of Excellence for Genomics of Infectious Diseases (ACEGID), Redeemer's University	Oluniyi P.E. et al
EPI_ISL_906306	Gorgas memorial Institute For Health Studies	Gorgas memorial Institute For Health Studies	Diaz Y, Franco D, Moreno B, Moreno A, Gondola Y, Saenz L, Abrego L, Chavarria O, Pitti Y, Castillo M, Lopez-Verguez S, Martinez Alexander A.
EPI_ISL_906559	Laboratorio de Salud Publica de Amazonas	Instituto Nacional de Salud- Dirección de Investigación en Salud Pública, Universidad de los Andes- Applied genomics research group, Vicerrectoria de Investigación y Creación, Universidad de los Andes- Systems and Computing Engineering Department	Katherine Laiton-Donato, Diego A. Álvarez-Díaz, Carlos Franco-Muñoz, Mauricio Pacheco-Montealegre, Héctor Alejandro Ruiz-Moreno, Maria T. Herrera-Sepúlveda, Diego Andrés Prada, Jhonnatan Reales-González, Sheryll Corchuelo, Julian Naizaque, Gerardo Santamaría Jorge Duitama, Laura Natalia Gonzalez, Jorge Ivan Diaz, Silvia Restrepo-Restrepo, Magdalena Wiesner, Martha Lucia Ospina Martinez, Marcela Mercado-Reyes
EPI_ISL_906590, EPI_ISL_906591, EPI_ISL_906592, EPI_ISL_906593, EPI_ISL_906594, EPI_ISL_906595, EPI_ISL_906596, EPI_ISL_906597, EPI_ISL_906598, EPI_ISL_906599, EPI_ISL_906600, EPI_ISL_906601, EPI_ISL_906602			
see above	Maine Health and Environmental Testing Laboratory (Maine HETL)	Tewhey Lab, The Jackson Laboratory	Matluk,N., Dewey,H., Iosue,F., Barter,M., Lynch,R., Munger,H. and Tewhey,R.
EPI_ISL_906806, EPI_ISL_906807	University Hospital of Northern Norway, Department for	Norwegian Institute of Public Health, Department of Virology	Kathrine Stene-Johansen, Kamilla Heddeland Instefjord, Hilde Elshaug, Atiya R Ali,Marie Paulsen Madsen, Rasmus Riis Kopperud, Hilde Vollan, Karoline

	Microbiology and Infectious Disease Control		Bragstad, Olav Hungnes
EPI_ISL_906818	Akershus University Hospital, Department for Microbiology and Infectious Disease Control	Norwegian Institute of Public Health, Department of Virology	Kathrine Stene-Johansen, Kamilla Heddeland Instefjord, Hilde Elshaug, Atiya R Ali,Marie Paulsen Madsen, Rasmus Riis Kopperud, Hilde Vollan, Karoline Bragstad, Olav Hungnes
EPI_ISL_906910, EPI_ISL_906911, EPI_ISL_906912, EPI_ISL_906913, EPI_ISL_906914	Bureau of Public Health Laboratories, Florida Department of Health (BPHL, FLDOH)	Bureau of Public Health Laboratories, Florida Department of Health (BPHL, FLDOH)	Schmedes,S., Blanton,J.
EPI_ISL_907210, EPI_ISL_907750	Lighthouse Lab in Glasgow	Wellcome Sanger Institute for the COVID-19 Genomics UK (COG-UK) Consortium	Harper VanSteenhouse, Yumi Kasai, David Gray, Carol Clugston, Anna Dominiczak and Alex Alderton, Roberto Amato, Sonia Goncalves, Ewan Harrison, David K. Jackson, Ian Johnston, Dominic Kwiatkowski, Cordelia Langford, John Sillitoe on behalf of the Wellcome Sanger Institute COVID-19 Surveillance Team
EPI_ISL_909665	Labo Analyses Med	National Reference Center for Viruses of Respiratory Infections, Institut Pasteur, Paris	Marion Barbet, Sylvie Behillil, Méline Bizard, Angela Brisebarre, Camille Capel, Etienne Simon-Lorière, Vincent Enouf, Maud Vanpeene, Sylvie van der Werf,Le Vicky
EPI_ISL_909835, EPI_ISL_909836, EPI_ISL_909837, EPI_ISL_909838, EPI_ISL_909839, EPI_ISL_909840, EPI_ISL_909842, EPI_ISL_909843, EPI_ISL_909845, EPI_ISL_909846, EPI_ISL_909848, EPI_ISL_909852, EPI_ISL_909853, EPI_ISL_909858, EPI_ISL_909863, EPI_ISL_909868, EPI_ISL_909873, EPI_ISL_909874, EPI_ISL_909897, EPI_ISL_909904, EPI_ISL_909906, EPI_ISL_909907, EPI_ISL_909913, EPI_ISL_909915, EPI_ISL_909916, EPI_ISL_909919, EPI_ISL_909928, EPI_ISL_909931, EPI_ISL_909937, EPI_ISL_909939			
see above	National Virus Reference Laboratory	National Virus Reference Laboratory	Michael Carr, Gabriel Gonzalez, Jonathan Dean, Cillian F De Gascun
EPI_ISL_910329, EPI_ISL_910334	ALEA dr Kandic	Alea Genetic Centre	Rijad Konjhodzic, Dino Pecar, Lana Salihefendic
EPI_ISL_910954, EPI_ISL_910955, EPI_ISL_910956, EPI_ISL_911125, EPI_ISL_911126, EPI_ISL_911127, EPI_ISL_911128, EPI_ISL_911129, EPI_ISL_911130, EPI_ISL_911131, EPI_ISL_911132, EPI_ISL_911133, EPI_ISL_911135, EPI_ISL_911168			
see above	Laboratoire national de sante, Microbiology, Virology	Laboratoire national de sante, Microbiology, Microbial Genomics Platform	Anke Wienecke-Baldacchino, Catherine Ragimbeau,Jessica Tapp, Fatu Djabi, Lise Pignon, Raoul Salmon, Tamir Abdelrahman
EPI_ISL_911245, EPI_ISL_911247	National Virus Reference Laboratory	National Virus Reference Laboratory	Michael Carr, Gabriel Gonzalez, Jonathan Dean, Cillian F De Gascun
EPI_ISL_911289, EPI_ISL_911342, EPI_ISL_911350	Servicio de Microbiología, Hospital Universitario Son Espases	SeqCOVID-SPAIN consortium/IBV(CSIC)	Carla López-Causapé, Jordi Reina, Antonio Oliver and SeqCOVID-SPAIN consortium
EPI_ISL_911931, EPI_ISL_911932, EPI_ISL_911933, EPI_ISL_911934, EPI_ISL_911935, EPI_ISL_911936, EPI_ISL_911937, EPI_ISL_911938, EPI_ISL_911939, EPI_ISL_911940, EPI_ISL_911941, EPI_ISL_911942, EPI_ISL_911943, EPI_ISL_911944, EPI_ISL_911945, EPI_ISL_911946, EPI_ISL_911947, EPI_ISL_911948, EPI_ISL_911949, EPI_ISL_911950, EPI_ISL_911951			
see above	Wyoming Public Health Laboratory	Wyoming Public Health Laboratory	Noah Hull, Taylor Fearing, Lynette Gumbleton, Channing Weber, Ashley Norberg, Bailey Bowcutt, and Wanda Manley
EPI_ISL_912006, EPI_ISL_912007, EPI_ISL_912008, EPI_ISL_912009, EPI_ISL_912010, EPI_ISL_912011, EPI_ISL_912012, EPI_ISL_912013, EPI_ISL_912014, EPI_ISL_912015, EPI_ISL_912016, EPI_ISL_912017, EPI_ISL_912018, EPI_ISL_912019, EPI_ISL_912020, EPI_ISL_912021, EPI_ISL_912022, EPI_ISL_912023, EPI_ISL_912024, EPI_ISL_912025, EPI_ISL_912026, EPI_ISL_912027, EPI_ISL_912028, EPI_ISL_912029, EPI_ISL_912030, EPI_ISL_912031, EPI_ISL_912032, EPI_ISL_912033, EPI_ISL_912034			
see above	Altius Institute for Biomedical Sciences	Seattle Flu Study	Deborah A. Nickerson, Chris D. Frazar, Jover Lee, Benjamin Pelle, Erica Ryke, Matthew Richardson, Amanda Adler, Elisabeth Brandstetter, Peter D. Han, Kairsten Fay, Misja Ilcisin, Kirsten Lacombe, Thomas R. Sibley, Melissa Truong, Caitlin R. Wolf, Ryan Alexander, Daniel Bates, Rebecca Bruders, Stephanie DeBaun, Clem Green, Muhammad Halimun, Jessica Halow, Kneshay Harper, Matt Hartman, Andrew Meuser, Alex Nguyen, Truong Nguyen, Sofia Olsson, Sadie Patraw, Hannah Petersen, Tobias Ragoczy, Joshua Richards, Jacob Rodriguez, John Stamatoyannopoulos, Julia Wald, Olivia Waltner, Michael Boeckh, Janet A. Englund, Michael Famulare, Barry R. Lutz, Mark J. Rieder, Lea M. Starita, Matthew Thompson, Helen Y. Chu, Jay Shendure, Trevor Bedford
EPI_ISL_912079, EPI_ISL_912080, EPI_ISL_912081, EPI_ISL_912082, EPI_ISL_912083, EPI_ISL_912084, EPI_ISL_912085, EPI_ISL_912086, EPI_ISL_912087, EPI_ISL_912088, EPI_ISL_912089, EPI_ISL_912090, EPI_ISL_912091, EPI_ISL_912092, EPI_ISL_912093, EPI_ISL_912095			
see above	Washington State Department of Health	Seattle Flu Study	Deborah A. Nickerson, Chris D. Frazar, Jover Lee, Benjamin Pelle, Erica Ryke, Matthew Richardson, Amanda Adler, Elisabeth Brandstetter, Peter D. Han, Kairsten Fay, Misja Ilcisin, Kirsten Lacombe, Thomas R. Sibley, Melissa Truong, Caitlin R. Wolf, Romesh Gautom, Geoff Melly, Brian Hiatt, Philip Dykema, Scott Lindquist, Michael Boeckh, Janet A. Englund, Michael Famulare, Barry R. Lutz, Mark J. Rieder, Lea M. Starita, Matthew Thompson, Helen Y. Chu, Jay Shendure, Trevor Bedford
EPI_ISL_912166	Natual State Genomics	Grubaugh Lab - Yale School of Public Health	Tara Alpert, Joseph Fauver, Anderson Brito, Mallery Breban, Anne Wyllie, Chantal Vogels, Mary Petrone, Anne Watkins, Chaney Kalinich, Isabel Ott, Nathan Grubaugh
EPI_ISL_912248, EPI_ISL_912255	Charité Universitätsmedizin Berlin, Institut für Virologie/Labor Berlin	Charité Universitätsmedizin Berlin, Institut für Virologie	Victor M Corman, Barbara Mühlemann, Jörn Beheim-Schwarzbach, Tobias Bleicker, Julia Tesch, Talitha Veith, Julia Schneider, Terry Jones, Christian Drosten
EPI_ISL_912413, EPI_ISL_912416	KU Leuven, Rega Institute, Clinical and Epidemiological Virology	KU Leuven, Rega Institute, Clinical and Epidemiological Virology	Tony Wawina-Bokalanga, Bert Vanmechelen, Joan Marti-Carerras, Piet Maes
EPI_ISL_912539, EPI_ISL_912580, EPI_ISL_912581	CENTRE HOSPITALIER LEON BINET	National Reference Center for Viruses of Respiratory Infections, Institut Pasteur, Paris	Marion Barbet, Sylvie Behillil, Méline Bizard, Angela Brisebarre, Camille Capel, Etienne Simon-Lorière, Vincent Enouf, Maud Vanpeene, Sylvie van der Werf,Meziane Ilham
EPI_ISL_912617, EPI_ISL_912618, EPI_ISL_912619, EPI_ISL_912620	Labo Analyses Med	National Reference Center for Viruses of Respiratory Infections, Institut Pasteur, Paris	Marion Barbet, Sylvie Behillil, Méline Bizard, Angela Brisebarre, Camille Capel, Etienne Simon-Lorière, Vincent Enouf, Maud Vanpeene, Sylvie van der Werf,Le Vicky
EPI_ISL_912643	Hôpital Pitié-Salpêtrière	Department of Virology, Henri Mondor University Hospital, Assistance Publique Hôpitaux de Paris, Université Paris-Est Créteil, INSERM U955	Christophe Rodriguez, Slim Fourati, Vanessa Demontant, Guillaume Gricourt, Melissa N'Debi, Alexandre Soulier, Elisabeth Trawinski, Jean-Michel Pawlotsky
EPI_ISL_912648, EPI_ISL_912672, EPI_ISL_912748, EPI_ISL_912749	Hôpital Henri Mondor	Department of Virology, Henri Mondor University Hospital, Assistance Publique Hôpitaux de Paris, Université Paris-Est Créteil, INSERM U955	Christophe Rodriguez, Slim Fourati, Vanessa Demontant, Guillaume Gricourt, Melissa N'Debi, Alexandre Soulier, Elisabeth Trawinski, Jean-Michel Pawlotsky
EPI_ISL_912789, EPI_ISL_912790, EPI_ISL_912916	Hôpital Pitié-Salpêtrière	Department of Virology, Henri Mondor University Hospital, Assistance Publique Hôpitaux de Paris, Université Paris-Est Créteil, INSERM U955	Christophe Rodriguez, Slim Fourati, Vanessa Demontant, Guillaume Gricourt, Melissa N'Debi, Alexandre Soulier, Elisabeth Trawinski, Jean-Michel Pawlotsky
EPI_ISL_912958, EPI_ISL_912959	Hôpital Henri Mondor	Department of Virology, Henri Mondor University Hospital, Assistance Publique Hôpitaux de Paris, Université Paris-Est Créteil, INSERM U955	Christophe Rodriguez, Slim Fourati, Vanessa Demontant, Guillaume Gricourt, Melissa N'Debi, Alexandre Soulier, Elisabeth Trawinski, Jean-Michel Pawlotsky
EPI_ISL_913032	Hospital San Pedro de Alcántara	Instituto de Salud Carlos III	Iglesias-Caballero, M. Camarero, S. Sandonís,V. Vázquez, S. Pozo, F. Casas, I. Jiménez, P. Zaballos, A. Monzón, S. Varona, S. Cuesta, I. Rodríguez, G.
EPI_ISL_913121, EPI_ISL_913125, EPI_ISL_913136, EPI_ISL_913155, EPI_ISL_913162, EPI_ISL_913163, EPI_ISL_913168, EPI_ISL_913169, EPI_ISL_913171, EPI_ISL_913172, EPI_ISL_913173, EPI_ISL_913176, EPI_ISL_913179, EPI_ISL_913181			
see above	University of Michigan Clinical Microbiology Laboratory	Lauring Lab, University of Michigan, Department of Microbiology and Immunology	Valesano
EPI_ISL_913412, EPI_ISL_913421, EPI_ISL_913429, EPI_ISL_913431	Massachusetts State Public Health Laboratory	Massachusetts State Public Health Laboratory	Andrew Lang, Timelia Fink, Glen Gallagher, Sandra Smole
EPI_ISL_913469	Klinisk mikrobiologi	The Public Health Agency of Sweden	Anna-Malin Linde, Maria Lind Karlberg, Carlo Berg, Oskar Karlsson Lindsjo, Sofia Stamouli, Reza Advani, Mattias Haukland, Petra Holmstrom, Noura Walai, Petra Edquist, Mia Brytting, Anna Risberg, Karin Tegmark-Wisell
EPI_ISL_913587	Vault Health	Minnesota Department of Health, Public Health Laboratory	Alexandra Lorentz, Jacob Garfin, Matt Plumb, and Xiong Wang
EPI_ISL_913757, EPI_ISL_913803, EPI_ISL_913804, EPI_ISL_913806, EPI_ISL_913807, EPI_ISL_913809, EPI_ISL_913860, EPI_ISL_913861, EPI_ISL_913863, EPI_ISL_913864, EPI_ISL_913865			
see above	KU Leuven, Rega Institute, Clinical and Epidemiological Virology	KU Leuven, Rega Institute, Clinical and Epidemiological Virology	Tony Wawina-Bokalanga, Bert Vanmechelen, Joan Marti-Carerras, Piet Maes

EPI_ISL_914025, EPI_ISL_914026	IL Department of Public Health Chicago Laboratory	Pathogen Discovery, Respiratory Viruses Branch, Division of Viral Diseases, Centers for Disease Control and Prevention	Ying Tao, Yan Li, Jing Zhang, Krista Queen, Anna Uehara, Peter Cook, Clinton R. Paden, Haibin Wang, Suxiang Tong
EPI_ISL_914039	United States Air Force School of Aerospace Medicine	U.S. Air Force School of Aerospace Medicine	Anthony Fries, Jennifer Meyer, William Gruner, William Buggele, Amanda Javorina, Sarah Purves, Clarise Starr, Elizabeth Macias
EPI_ISL_914829	TAMIZAJE COMUNITARIO - PASO CANOAS	Inciensa, Instituto Costarricense de Investigación y Enseñanza en Nutrición y Salud	Francisco Duarte, Hebleen Porras, Claudio Soto-Garita, Estela Cordero, Adriana Godínez, Melany Calderón & Mariel López
EPI_ISL_915427	Bundeswehr Institute of Microbiology	Bundeswehr Institute of Microbiology	Markus Antwerpen, Alexandra Rehn, Mathias Walter, Malena Bestehorn-Willmann, Sabine Zange, Enrico Georgi, Roman Wölfel
EPI_ISL_916630, EPI_ISL_916637, EPI_ISL_916651, EPI_ISL_916732, EPI_ISL_916746, EPI_ISL_916766, EPI_ISL_916775, EPI_ISL_916779, EPI_ISL_916849, EPI_ISL_916919, EPI_ISL_916934, EPI_ISL_916950, EPI_ISL_917011, EPI_ISL_917060, EPI_ISL_917095, EPI_ISL_917121, EPI_ISL_917792			
see above	Lighthouse Lab in Alderley Park	Wellcome Sanger Institute for the COVID-19 Genomics UK (COG-UK) Consortium	Jacquelyn Wynn, Mairead Hyland, The Lighthouse Lab in Alderley Park and Alex Alderton, Roberto Amato, Sonia Goncalves, Ewan Harrison, David K. Jackson, Ian Johnston, Dominic Kwiatkowski, Cordelia Langford, John Sillitoe on behalf of the Wellcome Sanger Institute COVID-19 Surveillance Team
EPI_ISL_918203, EPI_ISL_918229, EPI_ISL_918234, EPI_ISL_918256, EPI_ISL_918257, EPI_ISL_918258, EPI_ISL_918262	Innovative Genomics Institute, UC Berkeley	Innovative Genomics Institute, UC Berkeley	Stacia Wyman, Haridha Shivram, Phil Frankino, Liana Lareau, Shana McDevitt, Justin Choi
EPI_ISL_918282, EPI_ISL_918283, EPI_ISL_918284, EPI_ISL_918285, EPI_ISL_918286, EPI_ISL_918287	Hospital Universitari Vall d'Hebron - Vall d'Hebron Institut de Rercerca	Hospital Universitari Vall d'Hebron	Cristina Andrés, Maria Piñana, Josep F Abril, Damir Garcia-Cehic, Ariadna Rando, Juliana Esperalba, Maria Gema Codina, Carla Castillo, Maria Carmen Martin, Tomàs Pumarola, Josep Quer, Andrés Antón
EPI_ISL_918407	ALEA Dr Kandic	Alea Genetic Centre	Rijad Konjhodzic, Dino Pecar, Lana Salihefendic
EPI_ISL_918550	LACEN - Laboratório Central de Saúde Pública do Para	Evandro Chagas Institute	Santos, M.C.; Silva, A.M.; Junior, W.D.C.; Barbagelata, L.S.; Ferreira, J.A.; Sousa, E.M.A.; da Silva, P.S.; Pinheiro, K.C.; L.C.; Sousa Junior, E.C.
EPI_ISL_918859, EPI_ISL_918862, EPI_ISL_918863, EPI_ISL_918864, EPI_ISL_918866, EPI_ISL_918867, EPI_ISL_918868, EPI_ISL_918870, EPI_ISL_918871, EPI_ISL_918872, EPI_ISL_918873, EPI_ISL_918879, EPI_ISL_918880, EPI_ISL_918881, EPI_ISL_918914, EPI_ISL_918915, EPI_ISL_918935			
see above	University of Birmingham	COVID-19 Genomics UK (COG-UK) Consortium	Institute of Microbiology, University of Birmingham: Claire McMurray, Joanne Stockton, Samuel Nicholls, Radoslaw Poplawski, Will Rowe, Josh Quick, Nicholas Loman. University of Birmingham Testing Laboratory: Celina M Whalley, Andrew Bosworth, Charlotte Poxon, Kasun Wanigasooriya, Oliver Pickles, Mike Kidd, Alex Richter, Andrew D Beggs PHE Heartlands Lab: Husam Osman, Andrew Bosworth. Queen Elizabeth Hospital: Anna Casey
EPI_ISL_919011, EPI_ISL_919012, EPI_ISL_919017, EPI_ISL_919037, EPI_ISL_919039, EPI_ISL_919041, EPI_ISL_919043, EPI_ISL_919045, EPI_ISL_919047, EPI_ISL_919049, EPI_ISL_919051, EPI_ISL_919089, EPI_ISL_919105			
see above	Department of Pathology, University of Cambridge	COVID-19 Genomics UK (COG-UK) Consortium	Aminu S. Jahun, Yasmin Chaudhry, Iliana Georgana, Myra Hosmillo, Rhys Izu, Martin D. Curran, Surendra Parmar, Ian Goodfellow
EPI_ISL_919197	West of Scotland Specialist Virology Centre, NHSGGC / MRC-University of Glasgow Centre for Virus Research	COVID-19 Genomics UK (COG-UK) Consortium	Ana da Silva Filipe, Natasha Johnson, Kathy Smollett, Daniel Mair, Stephen Carmichael, Alice Broos, Lily Tong, Jenna Nichols, Kyriaki Nomikou; Sarah McDonald; Richard Orton, Joseph Hughes, Sreenu Vattipally, David L Robertson; Alasdair MacLean, Rory Gunson; Sharif Shaaban, Matthew Holden; Rachel Blacow, Guy Mollett, Kathy Li, James Shepherd, Antonia Ho, Emma Thomson
EPI_ISL_919338	Virology Department, Royal Infirmary of Edinburgh, NHS Lothian / School of Biological Sciences, University of Edinburgh / Institute of Genetics and Molecular Medicine, University of Edinburgh	COVID-19 Genomics UK (COG-UK) Consortium	McHugh M, Dewar R, Rooke S, Gallagher M, Balcaza C, O'Toole Á, Scher E, Hill V, McCrone JT, Colquhoun R, Yu X, Jackson B, Rambaut A, Williams TC, Templeton K
EPI_ISL_919475, EPI_ISL_919478, EPI_ISL_919515, EPI_ISL_919517, EPI_ISL_919591, EPI_ISL_919644	Liverpool Clinical Laboratories	COVID-19 Genomics UK (COG-UK) Consortium	Sam Haldenby, Anita Lucaci, Steve Paterson, Julian Hiscox, Alistair Darby, M Almsaud, A Alrezaihi, Muhannad Alruwaili, Stuart D Armstrong, Jones Benjamin, Eleanor G Bentley, Anu Chawla, Jordan J Clark, Angela Cowell, Richard Eccles, Isabel García-Dorival, Matthew Gemmell, Alessandro Gerada, PKF Gilmore, Richard Gregory, Ximeng Han, Catherine Hartley, Margaret Hughes, Miren Iturriza-Gomara, James Johnson, L Luu, Jenifer Manson, Charlotte Nelson, Elaine O'Toole, Cassie Olateju, Rebekah Penrice-Randal , Lucille Rainbow, N.P Randle, Trevor Ian Robinson, Parul Sharma, Ghada T Shawli, James P Stewart, Neil Swainston, Ecaterina Vamos, Joanne Watts, Mark Whitehead
EPI_ISL_919852	Barts Health NHS Trust	COVID-19 Genomics UK (COG-UK) Consortium	CUTINO-MOGUEL, Maria-Teresa; HARRINGTON, David; OWOYEMI, Dola; KULASEGARAN-SHYLINI, Raghavendran; BROAD, Claire; KELE, Beatrix
EPI_ISL_920012, EPI_ISL_920013, EPI_ISL_920015, EPI_ISL_920016, EPI_ISL_920017, EPI_ISL_920018, EPI_ISL_920019, EPI_ISL_920020, EPI_ISL_920021, EPI_ISL_920022, EPI_ISL_920023, EPI_ISL_920024, EPI_ISL_920025, EPI_ISL_920027, EPI_ISL_920028, EPI_ISL_920029, EPI_ISL_920034			
see above	University College London, Great Ormond Street Hospital for Children NHS Foundation Trust, Imperial College Healthcare NHS Trust	COVID-19 Genomics UK (COG-UK) Consortium	Sergi Castellano, Rachel Williams, Mark Kristiansen, Paola Resende Silva, Sunando Roy, Tony Brooks, Helena Tutill, Paola Niola, Patricia Dyal, Charlotte Williams, Leysa Forrest, Yasmin Panchbhaya, Jacqueline Findlay, Samuel Weeks, Julianne Brown, Kathryn Harris, Paul Randell, James Price, Alison Holmes, Judith Breuer
EPI_ISL_920188, EPI_ISL_920193, EPI_ISL_920299, EPI_ISL_920309, EPI_ISL_920318, EPI_ISL_920327, EPI_ISL_920328, EPI_ISL_920330, EPI_ISL_920337, EPI_ISL_920338, EPI_ISL_920339, EPI_ISL_920353, EPI_ISL_920362, EPI_ISL_920363, EPI_ISL_920371, EPI_ISL_920466, EPI_ISL_920501			
see above	University College London Hospital	COVID-19 Genomics UK (COG-UK) Consortium	Judith Heaney, Matthew Byott, Catherine Houlihan, Dan Frampton, Stuart Kirk, Moira Spyer and Eleni Nastouli
EPI_ISL_920867, EPI_ISL_920868, EPI_ISL_920869, EPI_ISL_920870, EPI_ISL_920871, EPI_ISL_920872, EPI_ISL_920873, EPI_ISL_920874, EPI_ISL_920875, EPI_ISL_920876, EPI_ISL_920877, EPI_ISL_920878, EPI_ISL_920879, EPI_ISL_920880, EPI_ISL_920881			
see above	University College London, Great Ormond Street Hospital for Children NHS Foundation Trust, Imperial College Healthcare NHS Trust	COVID-19 Genomics UK (COG-UK) Consortium	Sergi Castellano, Rachel Williams, Mark Kristiansen, Paola Resende Silva, Sunando Roy, Tony Brooks, Helena Tutill, Paola Niola, Patricia Dyal, Charlotte Williams, Leysa Forrest, Yasmin Panchbhaya, Jacqueline Findlay, Samuel Weeks, Julianne Brown, Kathryn Harris, Paul Randell, James Price, Alison Holmes, Judith Breuer
EPI_ISL_920894, EPI_ISL_920895, EPI_ISL_920896, EPI_ISL_920897, EPI_ISL_920898, EPI_ISL_920899	Department of Pathology, University of Cambridge	COVID-19 Genomics UK (COG-UK) Consortium	Aminu S. Jahun, Yasmin Chaudhry, Iliana Georgana, Myra Hosmillo, Rhys Izu, Martin D. Curran, Surendra Parmar, Ian Goodfellow
EPI_ISL_921007	Regional Virus Laboratory, Belfast Health and Social Care Trust	COVID-19 Genomics UK (COG-UK) Consortium	Conall McCaughey, James McKenna, Tanya Curran, Susan Feeney, Alison Watt, Ciara Cox, Mairead Connor, Zoltan Molnar, David Simpson, Derek Fairley
EPI_ISL_921508, EPI_ISL_921509, EPI_ISL_921510, EPI_ISL_921511, EPI_ISL_921512, EPI_ISL_921513, EPI_ISL_921514, EPI_ISL_921516, EPI_ISL_921518, EPI_ISL_921520	Northumbria University / South Tees Hospitals NHS Foundation Trust / North Cumbria Integrated Care NHS Foundation Trust / North Tees and Hartlepool NHS Foundation Trust / Newcastle Hospitals NHS Foundation Trust	COVID-19 Genomics UK (COG-UK) Consortium	Darren L Smith,Andrew Nelson,Matthew Bashton,Greg R Young,Joshua Loh,John Allan,Mohammad A Tariq,Giles S Holt,Gary Black,Wen C Yew,Lynn Dover,Paul Baker,Steve Liggett,Sarah Essex,Jane Greenaway,Debra Padgett,Clive Graham,Garren Scott,Edward Barton,Emma Swindells,Brendan Payne,Jennifer Collins,Yusri Taha,Gary Eltringham
EPI_ISL_922101, EPI_ISL_922105, EPI_ISL_922106, EPI_ISL_922108, EPI_ISL_922109, EPI_ISL_922110, EPI_ISL_922111, EPI_ISL_922112, EPI_ISL_922115, EPI_ISL_922116	Lincolnshire Hospitals and DeepSeq Nottingham	COVID-19 Genomics UK (COG-UK) Consortium	Nichola Duckworth, Tim Sloan, Sarah Walsh, Jonathan Ball, Patrick McClure, Joeseph Chappell, Nadine Holmes, Matthew Carlisle, Christopher Moore, Fei Sang, Johnny Debebe, Victoria Wright, Matthew Loose
EPI_ISL_922206, EPI_ISL_922207, EPI_ISL_922211, EPI_ISL_922223, EPI_ISL_922349	Oxford Viromics, NDM, University of Oxford; Oxford University Hospitals; Basingstoke and North Hampshire Hospital	COVID-19 Genomics UK (COG-UK) Consortium	Tanya Golubchik, David Bonsall, George Macintyre, Amy Trebes, Mariateresa de Cesare, Catrin Moore, Alex Mobbs, Anita Justice, Robert Shaw, Monique Andersson, Timothy Peto, Emma Wise, Nathan Moore, Jessica Lynch, Nick Cortes, Matilde Mori, Stephen Kidd, David Buck, John Todd, Christophe Fraser
EPI_ISL_922361, EPI_ISL_922364, EPI_ISL_922387, EPI_ISL_922388, EPI_ISL_922393, EPI_ISL_922397, EPI_ISL_922588, EPI_ISL_922589, EPI_ISL_922644	Wales Specialist Virology Centre Sequencing lab: Pathogen Genomics Unit	Public Health Wales Microbiology Cardiff Wales Specialist Virology Centre	Catherine Moore, Johnathan Evans, Laura Gifford, Malorie Perry, Simon Cottrell, Angela Marchbank, Alec Birchley, Alexander Adams, Amy Gaskin, Bree Gatica-Wilcox, Jason Coombes, Joel Southgate, Lauren Gilbert, Lee Graham, Nicole Pacchiarini, Sara Kumziene-Summerhayes, Sarah Taylor, Sophie Jones, Sara Rey, Matthew Bull, Joanne Watkins, Sally Corden, Tom Connor
EPI_ISL_923279, EPI_ISL_923284, EPI_ISL_923293, EPI_ISL_923301, EPI_ISL_923305, EPI_ISL_923306, EPI_ISL_923605, EPI_ISL_923611, EPI_ISL_923613, EPI_ISL_923671, EPI_ISL_923678, EPI_ISL_923679			
see above	Centre for Enzyme Innovation, University of Portsmouth /	COVID-19 Genomics UK (COG-UK) Consortium	Angela Beckett,Salman Goudarzi,Christopher Fearn,Kate Cook,Katie Loveson,Sharon Glaysher,Scott Elliott,Samuel Robson

	Translational Research Laboratory, Portsmouth Hospitals NHS Trust		
EPI_ISL_924091, EPI_ISL_924182	Virology Department, Sheffield Teaching Hospitals NHS Foundation Trust/Department of Infection, Immunity and Cardiovascular Disease, The Medical School, University of Sheffield	COVID-19 Genomics UK (COG-UK) Consortium	Thushan de Silva, Matthew Parker, Nikki Smith, Adri Angyal, Rebecca Brown, Luke Green, Rachel Tucker, Paul Parsons, Danielle Groves, Katie Johnson, Laura Carrilero, Alex Keeley, Dave Partridge, Matthew Wyles, Benjamin Lindsey, Mehmet Yavuz, Mohammad Raza, Cariad Evans
EPI_ISL_924809, EPI_ISL_924818, EPI_ISL_924832, EPI_ISL_924836, EPI_ISL_924846, EPI_ISL_924860	Bioinformatics and Biostatistics Lab, Advanced Sequencing Facility	COVID-19 Genomics UK (COG-UK) Consortium	Aengus Stewart,Jerome Nicod,Chelsea Sawyer,Laura Cubitt,Harshil Patel,Margaret Crawford
EPI_ISL_925048, EPI_ISL_925049, EPI_ISL_925050, EPI_ISL_925051, EPI_ISL_925052, EPI_ISL_925053, EPI_ISL_925054, EPI_ISL_925055, EPI_ISL_925056, EPI_ISL_925057, EPI_ISL_925058, EPI_ISL_925059, EPI_ISL_925075, EPI_ISL_925076, EPI_ISL_925077			
see above	TXDSHS	TXDSHS	Bonnie Oh, Anita Pokharel, James Daniel Bonser, Myong Koag, Chung Wang, Rachel Lee, Grace Kubin, Rashmi Tuladhar, Mayela Pedrueza, Maliha Rahman, Jenny Zhang
EPI_ISL_925209, EPI_ISL_925210, EPI_ISL_925211, EPI_ISL_925212, EPI_ISL_925213, EPI_ISL_925214, EPI_ISL_925215, EPI_ISL_925224, EPI_ISL_925225, EPI_ISL_925227, EPI_ISL_925253			
see above	New Mexico Department of Health Scientific Laboratory	New Mexico Department of Health Scientific Laboratory	Ellie Johnson, Anastacia Griego-Fisher, D'eldra Malone, Jennifer Benoit
EPI_ISL_925258, EPI_ISL_925261, EPI_ISL_925262, EPI_ISL_925277, EPI_ISL_925282, EPI_ISL_925283, EPI_ISL_925306	Wyoming Public Health Laboratory	Wyoming Public Health Laboratory	Noah Hull, Taylor Fearing, Lynette Gumbleton, Channing Weber, Ashley Norberg, Bailey Bowcutt, and Wanda Manley
EPI_ISL_925401	Department of Clinical Microbiology	GIGA Medical Genomics	Keith Durkin, Maria Artesi, Sébastien Bontems, Raphaël Boreux, Bouchra Boujemla, Cécile Meex, Pierrette Melin, Marie-Pierre Hayette, Vincent Bours
EPI_ISL_925882, EPI_ISL_925884, EPI_ISL_925885, EPI_ISL_925886	Nucleic Acid Testing, National Reference Laboratory	GIGA Medical Genomics	Yvan Butera, Keith Durkin, Maria Artesi, Bouchra Boujemla, Robert Rutayisire, Patrick Tuyisenge, Esperence Umumararungu, Sébastien Bontems, Marie-Pierre Hayette, Nathalie Renotte, Swaibu Gatare, Jacob Souopgui, Sabin Nsanzimana, Vincent Bours, Léon Mutesa
EPI_ISL_925950, EPI_ISL_925961, EPI_ISL_925967, EPI_ISL_925974, EPI_ISL_925976, EPI_ISL_926000, EPI_ISL_926008, EPI_ISL_926021, EPI_ISL_926035, EPI_ISL_926038, EPI_ISL_926047, EPI_ISL_926051, EPI_ISL_926063, EPI_ISL_926064, EPI_ISL_926065, EPI_ISL_926096, EPI_ISL_926103, EPI_ISL_926114, EPI_ISL_926122, EPI_ISL_926139, EPI_ISL_926144, EPI_ISL_926150, EPI_ISL_926169, EPI_ISL_926221, EPI_ISL_926226, EPI_ISL_926281, EPI_ISL_926282, EPI_ISL_926299, EPI_ISL_926313, EPI_ISL_926359, EPI_ISL_926380, EPI_ISL_926385, EPI_ISL_926394, EPI_ISL_926395, EPI_ISL_926419, EPI_ISL_926428, EPI_ISL_926436, EPI_ISL_926439, EPI_ISL_926466, EPI_ISL_926482, EPI_ISL_926491, EPI_ISL_926522, EPI_ISL_926532, EPI_ISL_926553, EPI_ISL_926559, EPI_ISL_926569, EPI_ISL_926570, EPI_ISL_926591, EPI_ISL_926598, EPI_ISL_926605, EPI_ISL_926619, EPI_ISL_926621, EPI_ISL_926633, EPI_ISL_926651, EPI_ISL_926663, EPI_ISL_926692, EPI_ISL_926713, EPI_ISL_926714, EPI_ISL_926730, EPI_ISL_926784, EPI_ISL_926786, EPI_ISL_926789, EPI_ISL_926827, EPI_ISL_926830, EPI_ISL_926893, EPI_ISL_926902, EPI_ISL_926927, EPI_ISL_926933, EPI_ISL_926945, EPI_ISL_926979, EPI_ISL_927053, EPI_ISL_927057, EPI_ISL_927058, EPI_ISL_927061, EPI_ISL_927063, EPI_ISL_927107, EPI_ISL_927128, EPI_ISL_927160, EPI_ISL_927176, EPI_ISL_927185, EPI_ISL_927190, EPI_ISL_927197, EPI_ISL_927200, EPI_ISL_927205, EPI_ISL_927207, EPI_ISL_927209, EPI_ISL_927224, EPI_ISL_927246, EPI_ISL_927257, EPI_ISL_927258, EPI_ISL_927259, EPI_ISL_927274, EPI_ISL_927287, EPI_ISL_927291, EPI_ISL_927301, EPI_ISL_927306, EPI_ISL_927309, EPI_ISL_927320, EPI_ISL_927347, EPI_ISL_927367, EPI_ISL_927370, EPI_ISL_927400, EPI_ISL_927418, EPI_ISL_927423, EPI_ISL_927425, EPI_ISL_927463, EPI_ISL_927465, EPI_ISL_927475, EPI_ISL_927485, EPI_ISL_927486, EPI_ISL_927489, EPI_ISL_927492, EPI_ISL_927507, EPI_ISL_927508, EPI_ISL_927510, EPI_ISL_927526, EPI_ISL_927538, EPI_ISL_927551, EPI_ISL_927553, EPI_ISL_927559, EPI_ISL_927570, EPI_ISL_927573, EPI_ISL_927596, EPI_ISL_927613, EPI_ISL_927622, EPI_ISL_927630, EPI_ISL_927659, EPI_ISL_927674, EPI_ISL_927675, EPI_ISL_927682, EPI_ISL_927706, EPI_ISL_927732, EPI_ISL_927739, EPI_ISL_927752, EPI_ISL_927755, EPI_ISL_927770, EPI_ISL_927782, EPI_ISL_927784, EPI_ISL_927797, EPI_ISL_927813, EPI_ISL_927826, EPI_ISL_927846, EPI_ISL_927847, EPI_ISL_927883, EPI_ISL_927901, EPI_ISL_927902, EPI_ISL_927907, EPI_ISL_927911, EPI_ISL_927934, EPI_ISL_927961, EPI_ISL_927962, EPI_ISL_927968, EPI_ISL_927991, EPI_ISL_928001, EPI_ISL_928006, EPI_ISL_928009, EPI_ISL_928014, EPI_ISL_928020, EPI_ISL_928059, EPI_ISL_928063, EPI_ISL_928097, EPI_ISL_928111, EPI_ISL_928120, EPI_ISL_928121, EPI_ISL_928123, EPI_ISL_928147, EPI_ISL_928164, EPI_ISL_928190, EPI_ISL_928202, EPI_ISL_928214, EPI_ISL_928233, EPI_ISL_928241, EPI_ISL_928248, EPI_ISL_928252, EPI_ISL_928264, EPI_ISL_928266, EPI_ISL_928272, EPI_ISL_928276, EPI_ISL_928281, EPI_ISL_928308, EPI_ISL_928323, EPI_ISL_928332, EPI_ISL_928345, EPI_ISL_928359, EPI_ISL_928361, EPI_ISL_928406, EPI_ISL_928409, EPI_ISL_928413, EPI_ISL_928415, EPI_ISL_928447, EPI_ISL_928450, EPI_ISL_928485, EPI_ISL_928489, EPI_ISL_928495, EPI_ISL_928514, EPI_ISL_928541, EPI_ISL_928552, EPI_ISL_928553, EPI_ISL_928557, EPI_ISL_928570, EPI_ISL_928601, EPI_ISL_928604, EPI_ISL_928623, EPI_ISL_928624, EPI_ISL_928626, EPI_ISL_928633, EPI_ISL_928653, EPI_ISL_928654, EPI_ISL_928661, EPI_ISL_928674, EPI_ISL_928677, EPI_ISL_928679, EPI_ISL_928700, EPI_ISL_928717, EPI_ISL_928728, EPI_ISL_928736, EPI_ISL_928738, EPI_ISL_928745, EPI_ISL_928754, EPI_ISL_928774, EPI_ISL_928789, EPI_ISL_928822, EPI_ISL_928882, EPI_ISL_928883, EPI_ISL_928893, EPI_ISL_928905, EPI_ISL_928961, EPI_ISL_929011, EPI_ISL_929043, EPI_ISL_929044, EPI_ISL_929079, EPI_ISL_929085, EPI_ISL_929092, EPI_ISL_929099, EPI_ISL_929122, EPI_ISL_929128, EPI_ISL_929129, EPI_ISL_929130, EPI_ISL_929132, EPI_ISL_929146, EPI_ISL_929149, EPI_ISL_929156, EPI_ISL_929170, EPI_ISL_929176, EPI_ISL_929184, EPI_ISL_929191, EPI_ISL_929196, EPI_ISL_929201, EPI_ISL_929231, EPI_ISL_929247, EPI_ISL_929254, EPI_ISL_929258, EPI_ISL_929282, EPI_ISL_929284, EPI_ISL_929297, EPI_ISL_929313, EPI_ISL_929318, EPI_ISL_929322, EPI_ISL_929329, EPI_ISL_929344, EPI_ISL_929385, EPI_ISL_929398, EPI_ISL_929400, EPI_ISL_929403, EPI_ISL_929421, EPI_ISL_929424, EPI_ISL_929429, EPI_ISL_929456, EPI_ISL_929526, EPI_ISL_929530, EPI_ISL_929561, EPI_ISL_929565, EPI_ISL_929572, EPI_ISL_929589, EPI_ISL_929595, EPI_ISL_929602, EPI_ISL_929611, EPI_ISL_929613, EPI_ISL_929634, EPI_ISL_929648, EPI_ISL_929669, EPI_ISL_929674, EPI_ISL_929716, EPI_ISL_929737, EPI_ISL_929749, EPI_ISL_929765, EPI_ISL_929776, EPI_ISL_929780, EPI_ISL_929784, EPI_ISL_929794, EPI_ISL_929800, EPI_ISL_929837, EPI_ISL_929848, EPI_ISL_929857, EPI_ISL_929867, EPI_ISL_929892, EPI_ISL_929894, EPI_ISL_929914, EPI_ISL_929933, EPI_ISL_929941, EPI_ISL_929942, EPI_ISL_929948, EPI_ISL_930006, EPI_ISL_930035, EPI_ISL_930037, EPI_ISL_930041, EPI_ISL_930050, EPI_ISL_930069, EPI_ISL_930095, EPI_ISL_930108, EPI_ISL_930110, EPI_ISL_930143, EPI_ISL_930156, EPI_ISL_930162, EPI_ISL_930193, EPI_ISL_930194, EPI_ISL_930216, EPI_ISL_930219, EPI_ISL_930225, EPI_ISL_930228, EPI_ISL_930233, EPI_ISL_930237, EPI_ISL_930244, EPI_ISL_930294, EPI_ISL_930299, EPI_ISL_930310, EPI_ISL_930311, EPI_ISL_930337, EPI_ISL_930345, EPI_ISL_930348, EPI_ISL_930351, EPI_ISL_930353, EPI_ISL_930367, EPI_ISL_930391, EPI_ISL_930404, EPI_ISL_930406, EPI_ISL_930433, EPI_ISL_930443, EPI_ISL_930449, EPI_ISL_930467, EPI_ISL_930490, EPI_ISL_930491, EPI_ISL_930505, EPI_ISL_930506, EPI_ISL_930514, EPI_ISL_930529, EPI_ISL_930553, EPI_ISL_930557			
see above	Department of Virus and Microbiological Special Diagnostics, Statens Serum Institut, Copenhagen, Denmark	Aalborg University	Danish Covid-19 Genome Consortium
EPI_ISL_930656, EPI_ISL_930657, EPI_ISL_930658, EPI_ISL_930659, EPI_ISL_930660, EPI_ISL_930664	Arizona State Public Health Laboratory	Arizona State Public Health Laboratory	Trung Huynh, Jessica Escobar, Katherine Fullerton, Nobuko Fukushima, Stacy White, Linda Getsinger, Victor Waddell
EPI_ISL_933761	DPHL	Delaware Public Health Lab	Gregory Hovan
EPI_ISL_934241	Vilnius university hospital Santaros Klinikos, Center of Laboratory Medicine	Vilnius university hospital Santaros Klinikos, Center of Laboratory Medicine	Ingrida Olendraite, Daniel Naumovas, Rimvydas Norvilas, Dovile Ezerskyte, Justinas Slikas, Gytis Dudas
EPI_ISL_934378	Klinisk mikrobiologi	The Public Health Agency of Sweden	Anna-Malin Linde, Maria Lind Karlberg, Carlo Berg, Oskar Karlsson Lindsjo, Sofia Stamouli, Reza Advani, Mattias Haukland, Petra Holmstrom, Noura Walai, Petra Edquist, Mia Brytting, Anna Risberg, Karin Tegmark-Wisell
EPI_ISL_934452, EPI_ISL_934453, EPI_ISL_934454, EPI_ISL_934457, EPI_ISL_934459, EPI_ISL_934465, EPI_ISL_934466, EPI_ISL_934467, EPI_ISL_934468, EPI_ISL_934469, EPI_ISL_934470, EPI_ISL_934471, EPI_ISL_934472, EPI_ISL_934479, EPI_ISL_934481, EPI_ISL_934482, EPI_ISL_934483, EPI_ISL_934485, EPI_ISL_934487, EPI_ISL_934490, EPI_ISL_934491, EPI_ISL_934492, EPI_ISL_934495, EPI_ISL_934539			
see above	Austrian Agency for Health and Food Safety (AGES)	Bergthaler laboratory, CeMM Research Center for Molecular Medicine of the Austrian Academy of Sciences	Lukas Endler, Anna Schedl, Thomas Penz, Benedikt Agerer, Maelle Le Moing, Michael Schuster, Bekir Erguner, Jan Laine, Martin Senekowitsch, Christoph Bock, Andreas Bergthaler
EPI_ISL_934589, EPI_ISL_934590, EPI_ISL_934591, EPI_ISL_934592	Institute of Legal Medicine, Medical University of Innsbruck	Bergthaler laboratory, CeMM Research Center for Molecular Medicine of the Austrian Academy of Sciences	Lukas Endler, Anna Schedl, Thomas Penz, Benedikt Agerer, Maelle Le Moing, Michael Schuster, Bekir Erguner, Jan Laine, Martin Senekowitsch, Christoph Bock, Andreas Bergthaler
EPI_ISL_934696	Institute for Water Quality and Resource Management, Technical University Vienna	Bergthaler laboratory, CeMM Research Center for Molecular Medicine of the Austrian Academy of Sciences	Lukas Endler, Anna Schedl, Thomas Penz, Benedikt Agerer, Maelle Le Moing, Michael Schuster, Bekir Erguner, Jan Laine, Martin Senekowitsch, Christoph Bock, Andreas Bergthaler
EPI_ISL_934986, EPI_ISL_934989, EPI_ISL_934995, EPI_ISL_934996, EPI_ISL_935000, EPI_ISL_935001, EPI_ISL_935002, EPI_ISL_935003, EPI_ISL_935004	ADMED Microbiologie	Genomics and Transcriptomics, Philip Morris International	Reto Lienhard, Marie-Lise Tritten, Emmanuel Guedj, Nicolas Sierro, Rémi Dulize, David Bornand, Mehdi Auberson, Maxime Berthouzoz, Nikolai Ivanov, Manuel Peitsch
EPI_ISL_935199, EPI_ISL_935210, EPI_ISL_935225, EPI_ISL_935274, EPI_ISL_935275, EPI_ISL_935276, EPI_ISL_935277, EPI_ISL_935278, EPI_ISL_935279, EPI_ISL_935280, EPI_ISL_935284, EPI_ISL_935285, EPI_ISL_935286, EPI_ISL_935287, EPI_ISL_935288, EPI_ISL_935289, EPI_ISL_935290, EPI_ISL_935291, EPI_ISL_935292, EPI_ISL_935293, EPI_ISL_935294, EPI_ISL_935295, EPI_ISL_935296, EPI_ISL_935297, EPI_ISL_935298, EPI_ISL_935299, EPI_ISL_935300			
see above	KU Leuven, Rega Institute, Clinical and Epidemiological Virology	KU Leuven, Rega Institute, Clinical and Epidemiological Virology	Tony Wawina-Bokalanga, Bert Vanmechelen, Joan Marti-Carerras, Piet Maes
EPI_ISL_935532	Labo Analyses Med	National Reference Center for Viruses of Respiratory Infections, Institut Pasteur, Paris	Marion Barbet, Sylvie Behillil, Méline Bizard, Angela Brisebarre, Camille Capel, Etienne Simon-Lorière, Vincent Enouf, Maud Vanpeene, Sylvie van der Werf,Merah Kader
EPI_ISL_935562	Hopital	National Reference Center for Viruses of Respiratory Infections, Institut Pasteur, Paris	Marion Barbet, Sylvie Behillil, Méline Bizard, Angela Brisebarre, Camille Capel, Etienne Simon-Lorière, Vincent Enouf, Maud Vanpeene, Sylvie van der Werf,Fiette
EPI_ISL_936003, EPI_ISL_936004, EPI_ISL_936009, EPI_ISL_936010, EPI_ISL_936022	SUNY UPSTATE MEDICAL UNIVERSITY	Wadsworth Center, New York State Department of Health	Kirsten St. George, Daryl M. Lamson, Alexis Russel, Matthew Shudt, Melissa A Leisner, Jonathan Plitnick, Navjot Singh, John Kelly, Erasmus Schneider, Erica Lasek-Nesselquist

EPI_ISL_936214, EPI_ISL_936215, EPI_ISL_936216, EPI_ISL_936217, EPI_ISL_936218, EPI_ISL_936219, EPI_ISL_936220, EPI_ISL_936221, EPI_ISL_936222, EPI_ISL_936223, EPI_ISL_936224, EPI_ISL_936225, EPI_ISL_936226, EPI_ISL_936227, EPI_ISL_936228, EPI_ISL_936229, EPI_ISL_936230, EPI_ISL_936231, EPI_ISL_936232, EPI_ISL_936233, EPI_ISL_936234, EPI_ISL_936235, EPI_ISL_936236			
see above	Wadsworth Center, New York State Department of Health	Wadsworth Center, New York State Department of Health	Kirsten St. George, Daryl M. Lamson, Alexis Russel, Matthew Shudt, Melissa A Leisner, Jonathan Plitnick, Navjot Singh, John Kelly, Erasmus Schneider, Erica Lasek-Nesselquist
EPI_ISL_936476	DPH, Massachusetts State Public Health Lab	DPH, Massachusetts State Public Health Lab	Lang,A.S., Fink,T., Gallagher,G.R., Smole,S.C.
EPI_ISL_937356, EPI_ISL_937357, EPI_ISL_937358, EPI_ISL_937359, EPI_ISL_937360, EPI_ISL_937361, EPI_ISL_937363, EPI_ISL_937373, EPI_ISL_937376, EPI_ISL_937377, EPI_ISL_937379, EPI_ISL_937381, EPI_ISL_937382			
see above	Utah Public Health Laboratory	Utah Public Health Laboratory	Erin L. Young, Kelly F. Oakeson, Tara Gallagher
EPI_ISL_937435, EPI_ISL_937436, EPI_ISL_937445, EPI_ISL_937446, EPI_ISL_937447, EPI_ISL_937448, EPI_ISL_937457, EPI_ISL_937458	Maine Health and Environmental Testing Laboratory (Maine HETL)	Tewhey Lab, The Jackson Laboratory	Matluk,N., Dewey,H., Iosue,F., Barter,M., Lynch,R., Munger,H. and Tewhey,R.
EPI_ISL_937818	Lighthouse Lab in Alderley Park	Wellcome Sanger Institute for the COVID-19 Genomics UK (COG-UK) Consortium	Jacquelyn Wynn, Mairead Hyland, The Lighthouse Lab in Alderley Park and Alex Alderton, Roberto Amato, Sonia Goncalves, Ewan Harrison, David K. Jackson, Ian Johnston, Dominic Kwiatkowski, Cordelia Langford, John Sillitoe on behalf of the Wellcome Sanger Institute COVID-19 Surveillance Team
EPI_ISL_940146	Laboratory of Virology and Molecular Diagnostics	Institute of Public Health of Republic of North Macedonia Laboratory of Virology and Molecular Diagnostics	Maja Kuzmanovska, Golubinka Boshevska, Elizabeta Janchevska
EPI_ISL_940728, EPI_ISL_940729, EPI_ISL_940730, EPI_ISL_940731	City of Milwaukee Health Department Laboratory	City of Milwaukee Health Department Laboratory	Sanjib Bhattacharyya
EPI_ISL_941083, EPI_ISL_941085, EPI_ISL_941090, EPI_ISL_941098, EPI_ISL_941099, EPI_ISL_941102	Labo Analyses Med	National Reference Center for Viruses of Respiratory Infections, Institut Pasteur, Paris	Marion Barbet, Sylvie Behillil, Méline Bizard, Angela Brisebarre, Camille Capel, Etienne Simon-Lorière, Vincent Enouf, Maud Vanpeene, Sylvie van der Werf, Merah Kader
EPI_ISL_941341, EPI_ISL_941342, EPI_ISL_941343, EPI_ISL_941344, EPI_ISL_941345, EPI_ISL_941382, EPI_ISL_941383, EPI_ISL_941384, EPI_ISL_941387, EPI_ISL_941470, EPI_ISL_941471, EPI_ISL_941472, EPI_ISL_941537, EPI_ISL_941538, EPI_ISL_941543, EPI_ISL_941544, EPI_ISL_941545, EPI_ISL_941546, EPI_ISL_941571, EPI_ISL_941572, EPI_ISL_941573			
see above	Instituto Nacional de Saude (INSA)	Instituto Nacional de Saude (INSA)	Borges et al
EPI_ISL_941647, EPI_ISL_941648, EPI_ISL_941649, EPI_ISL_941659, EPI_ISL_941662, EPI_ISL_941664, EPI_ISL_941665, EPI_ISL_941666, EPI_ISL_941688, EPI_ISL_941689, EPI_ISL_941690, EPI_ISL_941702, EPI_ISL_941703, EPI_ISL_941709, EPI_ISL_941710, EPI_ISL_941711, EPI_ISL_941726, EPI_ISL_941727, EPI_ISL_941728, EPI_ISL_941729, EPI_ISL_941730, EPI_ISL_941731, EPI_ISL_941732, EPI_ISL_941762, EPI_ISL_941765, EPI_ISL_941766, EPI_ISL_941772, EPI_ISL_941773, EPI_ISL_941774, EPI_ISL_941775, EPI_ISL_941797, EPI_ISL_941798, EPI_ISL_941799, EPI_ISL_941808, EPI_ISL_941818, EPI_ISL_941820, EPI_ISL_941821, EPI_ISL_941822, EPI_ISL_941823, EPI_ISL_941824, EPI_ISL_941825, EPI_ISL_941826, EPI_ISL_941827, EPI_ISL_941835, EPI_ISL_941836, EPI_ISL_941873, EPI_ISL_941877, EPI_ISL_941881, EPI_ISL_941882			
see above	Instituto Nacional de Saude (INSA) and Instituto Gulbenkian de Ciencia (IGC)	Instituto Nacional de Saude (INSA) and Instituto Gulbenkian de Ciencia (IGC)	Borges et al
EPI_ISL_942819, EPI_ISL_942820, EPI_ISL_942821	Gundersen Molecular Diagnostics Laboratory	Kabara Cancer Research Institute	Craig S. Richmond, Paraic A. Kenny
EPI_ISL_943011, EPI_ISL_943300	Dutch COVID-19 response team	National Institute for Public Health and the Environment (RIVM)	Adam Meijer, Harry Vennema, Dirk Eggink, Jeroen Cremer, Sharon van den Brink, Bas van der Veer, AnneMarie van den Brandt, Florian Zwagemaker, Dennis Schmitz, Chantal Reusken, on behalf of the national COVID-19 response team
EPI_ISL_943927, EPI_ISL_943928, EPI_ISL_943933, EPI_ISL_943962	Utah Public Health Laboratory	Utah Public Health Laboratory	Erin L. Young, Kelly F. Oakeson, Tara Gallagher
EPI_ISL_944203, EPI_ISL_944209, EPI_ISL_944210, EPI_ISL_944211, EPI_ISL_944212, EPI_ISL_944219, EPI_ISL_944223, EPI_ISL_944227, EPI_ISL_944322, EPI_ISL_944323, EPI_ISL_944324, EPI_ISL_944325, EPI_ISL_944326, EPI_ISL_944327, EPI_ISL_944328, EPI_ISL_944329, EPI_ISL_944330, EPI_ISL_944331, EPI_ISL_944332, EPI_ISL_944333, EPI_ISL_944334, EPI_ISL_944335, EPI_ISL_944336, EPI_ISL_944337, EPI_ISL_944338, EPI_ISL_944369, EPI_ISL_944370, EPI_ISL_944374, EPI_ISL_944398, EPI_ISL_944404, EPI_ISL_944405, EPI_ISL_944406, EPI_ISL_944411, EPI_ISL_944417, EPI_ISL_944418, EPI_ISL_944419, EPI_ISL_944423, EPI_ISL_944424, EPI_ISL_944430, EPI_ISL_944464, EPI_ISL_944465, EPI_ISL_944466, EPI_ISL_944467, EPI_ISL_944468, EPI_ISL_944469, EPI_ISL_944470, EPI_ISL_944471, EPI_ISL_944472, EPI_ISL_944473, EPI_ISL_944474, EPI_ISL_944475, EPI_ISL_944476, EPI_ISL_944477, EPI_ISL_944478, EPI_ISL_944479, EPI_ISL_944480, EPI_ISL_944481, EPI_ISL_944482, EPI_ISL_944483, EPI_ISL_944484, EPI_ISL_944485, EPI_ISL_944486, EPI_ISL_944487, EPI_ISL_944488, EPI_ISL_944489, EPI_ISL_944490, EPI_ISL_944491, EPI_ISL_944492, EPI_ISL_944493, EPI_ISL_944494, EPI_ISL_944495, EPI_ISL_944496, EPI_ISL_944497, EPI_ISL_944498, EPI_ISL_944499, EPI_ISL_944500, EPI_ISL_944501, EPI_ISL_944502, EPI_ISL_944503, EPI_ISL_944504, EPI_ISL_944505, EPI_ISL_944506, EPI_ISL_944507, EPI_ISL_944508, EPI_ISL_944509, EPI_ISL_944510, EPI_ISL_944511, EPI_ISL_944512, EPI_ISL_944513, EPI_ISL_944514, EPI_ISL_944515			
see above	Israel Central Virology laboratory	Israel National Consortium for SARS-CoV-2 sequencing	Neta Zuckerman, Efrat Dahan Bucris, Michal Mandelboim, Dana Bar-Ilan, Oran Erster, Tzvia Mann, Omer Murik, David A. Zeevi, Assaf Rokney, Joseph Jaffe, Eva Nachum, Maya Davidovich Cohen, Ephraim Fass, Gal Zizelski Valenci, Mor Rubinstein, Efrat Rorman, Israel Nissan, Efrat Glick-Saar, Omri Nayshool, Gideon Rechavi, Ella Mendelson, Orna Mor
EPI_ISL_944611, EPI_ISL_944612, EPI_ISL_944613, EPI_ISL_944615, EPI_ISL_944616, EPI_ISL_944619, EPI_ISL_944627, EPI_ISL_944628, EPI_ISL_944631, EPI_ISL_944633	Instituto Nacional de Medicina Genomica	Instituto Nacional de Medicina Genomica	Hidalgo-Miranda A, Mendoza-Vargas A, Reyes-Grajeda JP, Cisneros-Villanueva M, Cedro-Tanda A,Peñaloza-Figueroa F, Herrera-Montalvo LA
EPI_ISL_944660, EPI_ISL_944722	Department of Biochemistry, Cell and Molecular Biology, West African Centre for Cell Biology of Infectious Pathogens (WACCBIP), University of Ghana	Department of Biochemistry, Cell and Molecular Biology, West African Centre for Cell Biology of Infectious Pathogens (WACCBIP), University of Ghana	Morang'a,C.M., Ngoi,J.M., Quansah,E.B., Saiid,S., Amuzu,D.S., Asante,I., Bonney,J.H., Bonney,E., Odoom,J.K., Ndam,N.T., Tei-Maya,F., Adusei-Poku,M., Ofori-Boadu,L., Ampofo,W.K., Amenga-Etego,L.N., Quashie,P., Bediako,Y., Awandare,G.A.
EPI_ISL_946701, EPI_ISL_946780	Lighthouse Lab in Alderley Park	Wellcome Sanger Institute for the COVID-19 Genomics UK (COG-UK) Consortium	Jacquelyn Wynn, Mairead Hyland, The Lighthouse Lab in Alderley Park and Alex Alderton, Roberto Amato, Sonia Goncalves, Ewan Harrison, David K. Jackson, Ian Johnston, Dominic Kwiatkowski, Cordelia Langford, John Sillitoe on behalf of the Wellcome Sanger Institute COVID-19 Surveillance Team
EPI_ISL_949093	Jessa	Jessa	Jessa_cmdLab
EPI_ISL_949750, EPI_ISL_949768, EPI_ISL_949776, EPI_ISL_949779, EPI_ISL_949782	Barts Health NHS Trust	COVID-19 Genomics UK (COG-UK) Consortium	CUTINO-MOGUEL, Maria-Teresa; HARRINGTON, David; OWOYEMI, Dola; KULASEGARAN-SHYLINI, Raghavendran; BROAD, Claire; KELE, Beatrix
EPI_ISL_949790, EPI_ISL_950162, EPI_ISL_950163, EPI_ISL_950164	University College London, Great Ormond Street Hospital for Children NHS Foundation Trust, Imperial College Healthcare NHS Trust	COVID-19 Genomics UK (COG-UK) Consortium	Sergi Castellano, Rachel Williams, Mark Kristiansen, Paola Resende Silva, Sunando Roy, Tony Brooks, Helena Tutill, Paola Niola, Patricia Dyal, Charlotte Williams, Leysa Forrest, Yasmin Panchbhaya, Jacqueline Findlay, Samuel Weeks, Julianne Brown, Kathryn Harris, Paul Randell, James Price, Alison Holmes, Judith Breuer
EPI_ISL_950280, EPI_ISL_950282, EPI_ISL_950283, EPI_ISL_950284, EPI_ISL_950285, EPI_ISL_950289, EPI_ISL_950290, EPI_ISL_950291, EPI_ISL_950292	Northumbria University / South Tees Hospitals NHS Foundation Trust / North Cumbria Integrated Care NHS Foundation Trust / North Tees and Hartlepool NHS Foundation Trust / Newcastle Hospitals NHS Foundation Trust	COVID-19 Genomics UK (COG-UK) Consortium	Darren L Smith,Andrew Nelson,Matthew Bashton,Greg R Young,Joshua Loh,John Allan,Mohammad A Tariq,Giles S Holt,Gary Black,Wen C Yew,Lynn Dover,Paul Baker,Steve Liggett,Sarah Essex,Jane Greenaway,Debra Padgett,Clive Graham,Garren Scott,Edward Barton,Emma Swindells,Brendan Payne,Jennifer Collins,Yusri Taha,Gary Eltringham
EPI_ISL_951274, EPI_ISL_951290, EPI_ISL_951291, EPI_ISL_951293, EPI_ISL_951297, EPI_ISL_951304, EPI_ISL_951307, EPI_ISL_951317, EPI_ISL_951362, EPI_ISL_951363, EPI_ISL_951376, EPI_ISL_951377, EPI_ISL_951412, EPI_ISL_951413, EPI_ISL_951415, EPI_ISL_951417, EPI_ISL_951439			
see above	Oxford Viromics, NDM, University of Oxford; Oxford University Hospitals; Basingstoke and North Hampshire Hospital	COVID-19 Genomics UK (COG-UK) Consortium	Tanya Golubchik, David Bonsall, George Macintyre, Amy Trebes, Mariateresa de Cesare, Catrin Moore, Alex Mobbs, Anita Justice, Robert Shaw, Monique Andersson, Timothy Peto, Emma Wise, Nathan Moore, Jessica Lynch, Nick Cortes, Matilde Mori, Stephen Kidd, David Buck, John Todd, Christophe Fraser
EPI_ISL_952421, EPI_ISL_952434, EPI_ISL_952559, EPI_ISL_952561, EPI_ISL_952563, EPI_ISL_952565, EPI_ISL_952566, EPI_ISL_952567, EPI_ISL_952568, EPI_ISL_952777, EPI_ISL_952790, EPI_ISL_952792, EPI_ISL_952796, EPI_ISL_952797, EPI_ISL_952801, EPI_ISL_952803			
see above	Centre for Enzyme Innovation, University of Portsmouth / Translational Research Laboratory, Portsmouth Hospitals NHS Trust	COVID-19 Genomics UK (COG-UK) Consortium	Angela Beckett,Salman Goudarzi,Christopher Fearn,Kate Cook,Katie Loveson,Sharon Glaysher,Scott Elliott,Samuel Robson

EPI_ISL_953493	University Hospitals of Geneva, Laboratory of Virology	HUG, Laboratory of Virology and the Health2030 Genome Center	Samuel Cordey, Ana Rita Goncalves, Laurent Kaiser, Lorenzo Cerutti, Henri Pegeot, Melyssa Elies, Deborah Penet, Keith Harshman, Ioannis Xenarios, Emmanouil Dermitzakis
EPI_ISL_953952	Outre Mer	National Reference Center for Viruses of Respiratory Infections, Institut Pasteur, Paris	Marion Barbet, Sylvie Behillil, Méline Bizard, Angela Brisebarre, Camille Capel, Etienne Simon-Lorière, Vincent Enouf, Maud Vanpeene, Sylvie van der Werf,Rousset Dominique
EPI_ISL_954126	Hopital	National Reference Center for Viruses of Respiratory Infections, Institut Pasteur, Paris	Marion Barbet, Sylvie Behillil, Méline Bizard, Angela Brisebarre, Camille Capel, Etienne Simon-Lorière, Vincent Enouf, Maud Vanpeene, Sylvie van der Werf,Lesimple BéAtrice
EPI_ISL_954127	Labo Analyses Med	National Reference Center for Viruses of Respiratory Infections, Institut Pasteur, Paris	Marion Barbet, Sylvie Behillil, Méline Bizard, Angela Brisebarre, Camille Capel, Etienne Simon-Lorière, Vincent Enouf, Maud Vanpeene, Sylvie van der Werf,Takoudju Eve-Marie
EPI_ISL_954784	Complejo Hospitalario de Navarra	Instituto de Salud Carlos III	Iglesias-Caballero, M. Camarero, S. Sandonís,V. Vázquez, S. Pozo, F. Casas, I. Jiménez, P. Zaballos, A. Monzón, S. Varona, S. Cuesta, I. Ezpeleta,C.
EPI_ISL_954788	Servicio Murciano de Salud	Instituto de Salud Carlos III	Iglesias-Caballero, M. Camarero, S. Sandonís,V. Vázquez, S. Pozo, F. Casas, I. Jiménez, P. Zaballos, A. Monzón, S. Varona, S. Cuesta, I. Blázquez, A.
EPI_ISL_954842, EPI_ISL_954931, EPI_ISL_954932, EPI_ISL_954933, EPI_ISL_954934, EPI_ISL_954935	Colorado Department of Public Health and Environment	Colorado Department of Puplic Health and Environment	Laura Bankers, Molly C. Hetherington-Rauth, Diana Ir, Shannon Ely, Shannon R. Matzinger, Sarah Elizabeth Totten, Emily A. Travanty
EPI_ISL_955227, EPI_ISL_955228	Indiana Animal Disease Diagnostic Laboratory	Carpi Laboratory - Purdue University	Jack Dorman, Ilinca I Ciubotariu, Lev Gorenstein, Abebe A Fola, G Kenitra Hendrix, Rebecca P Wilkes, Giovanna Carpi
EPI_ISL_956299	Laboratorio de salud publica Arauca	Instituto Nacional de Salud- Dirección de Investigación en Salud Pública	Katherine Laiton-Donato, Diego A. Álvarez-Díaz, Carlos Franco-Muñoz, Mauricio Pacheco-Montealegre, Hector Alejandro Ruiz-Moreno, Maria T. Herrera-Sepúlveda, Diego Andrés Prada, Jhonnatan Reales-González, Sheryll Corchuelo, Julian Naizaque, Gerardo Santamaría, Magdalena Wiesner, Martha Lucia Ospina Martinez, Marcela Mercado-Reyes
EPI_ISL_956314	Paru Hospital	Institute of Tropical Disease, Universitas Airlangga	Rima R Prasetya, Krisnoadi Rahardjo, Aldise M Nastri, Jezzy R Dewantari, Dyah Retno, Gatot Soegiarto, Laksmi Wulandari, Resti Yudhawati, Yasuko Mori, Soetjipto, Kazufumi Shimizu, Maria I Lusida
EPI_ISL_956335	Utah Public Health Laboratory, Utah Public Health Laboratory Infectious Disease submission group	Utah Public Health Laboratory, Utah Public Health Laboratory Infectious Disease submission group	Gallagher,T., Young,E.L., Oakeson,K.F.
EPI_ISL_959273	Lighthouse Lab in Milton Keynes	Wellcome Sanger Institute for the COVID-19 Genomics UK (COG-UK) Consortium	The Lighthouse Lab in Milton Keynes and Alex Alderton, Roberto Amato, Sonia Goncalves, Ewan Harrison, David K. Jackson, Ian Johnston, Dominic Kwiatkowski, Cordelia Langford, John Sillitoe on behalf of the Wellcome Sanger Institute COVID-19 Surveillance Team
EPI_ISL_959286	General Hospital - Strumica	Research Center for Genetic Engineering and Biotechnology "Georgi D. Efremov" , Macedonian Academy of Sciences and Arts	Aleksandar J. Dimovski, Dijana Plasheska-Karanfilska, Predrag Noveski, Gjorgji Bozinovski, Milena Jakimovska
EPI_ISL_959288	General Hospital - Shtip	Research Center for Genetic Engineering and Biotechnology "Georgi D. Efremov" , Macedonian Academy of Sciences and Arts	Aleksandar J. Dimovski, Dijana Plasheska-Karanfilska, Predrag Noveski, Gjorgji Bozinovski, Milena Jakimovska
EPI_ISL_959289, EPI_ISL_959291	General Hospital - Ohrid	Research Center for Genetic Engineering and Biotechnology "Georgi D. Efremov" , Macedonian Academy of Sciences and Arts	Aleksandar J. Dimovski, Dijana Plasheska-Karanfilska, Predrag Noveski, Gjorgji Bozinovski, Milena Jakimovska
EPI_ISL_959850	National Virus Reference Laboratory	National Virus Reference Laboratory	Michael Carr, Gabriel Gonzalez, Jonathan Dean, Cillian F De Gascun
EPI_ISL_960405, EPI_ISL_960431, EPI_ISL_960440	The National Institute of Public Health	State Veterinary Institute Prague	Nagy,A;Vecerova,J;Cernikova,L;Stara,M;Jirincova,H;Trnka,D
EPI_ISL_960470, EPI_ISL_960572	Istituto Zooprofilattico Sperimentale del Mezzogiorno	TIGEM	Patrizia Annunziata, Andrea Ballabio, Valentina Bouche, Davide Cacchiarelli, Pellegrino Cerino, Chiara Colantuono, Maria Concetta Cuomo, Denise Di Concilio, Lucio Di Filippo, Antonio Grimaldi, Antonio Limone, Anna Manfredi, Francesco Panariello, Biancamaria Pierri, Marcello Salvi
EPI_ISL_960877, EPI_ISL_960878, EPI_ISL_960879	Institute of Medical Microbiology and Hospital Hygiene	Institute of Medical Microbiology and Hospital Hygiene	Prof. Dr. Achim Kaasch, Aljoscha Tersteegen
EPI_ISL_960996	AIID	Irish Coronavirus Sequencing Consortium-Teagasc Grange	Matthew McCabe, Aljandro Abner Garcia Leon, Fiona Crispie, Calum Walsh, Michael Carr, John Kenny, Paul Cotter, Patrick Mallon, Gabriel Gonzalez
EPI_ISL_961129, EPI_ISL_961130	Delaware Public Health Laboratory	Delaware Public Health Lab	Gregory Hovan
EPI_ISL_961381	Toronto Invasive Bacterial Diseases Network	McMaster University	Allison McGeer, Patryk Aftanas, Hooman Derakhshani, Angel Li, Kuganya Nirmalarajah, Emily Panousis, Ahmed Draia, Jalees Nasir, Michael Surette, Samira Mubareka, Andrew G. McArthur
EPI_ISL_961642	Hôpital Georges L. Dumont	National Microbiology Laboratory (NML)	Anna Majer, Shari Tyson, Grace Seo, Philip Mabon, Elsie Grudeski, Rhiannon Huzarewich, Russell Mandes, Anneliese Landgraff, Jennifer Tanner, Natalie Knox, Morag Graham, Gary Van Domselaar, Richard Garceau, Guillaume Desnoyers, Nathalie Bastien, Yan Li, Timothy Booth, Darian Hole, Madison Chapel, Kirsten Biggar, CanCOGeN's metadata curation team, Public Health Agency of Canada CanCOGeN team
EPI_ISL_961877, EPI_ISL_961878, EPI_ISL_961879, EPI_ISL_961880	E. Gulbja laboratorija	Latvian Biomedical Research and Study Centre	Janis Pjalkovskis, Nikita Zrelovs, Monta Ustinova, Ivars Silamikelis, Liga Birzniece, Kaspars Megnis, Vita Rovite, Lauma Freimane, Laila Silamikele, Laura Ansone, Davids Fridmanis, Mikus Gavars, Dmitrijs Perminovs, Jurijs Perevoscikovs, Uga Dumpis, Janis Klovins
EPI_ISL_961905, EPI_ISL_961906, EPI_ISL_961907, EPI_ISL_961933, EPI_ISL_961940, EPI_ISL_961944, EPI_ISL_961970, EPI_ISL_962010, EPI_ISL_962011, EPI_ISL_962171, EPI_ISL_962173			
see above	Illinois Department of Public Health	Gagnon Lab, Southern Illinois University	Keith Gagnon
EPI_ISL_962182	Ohio Department of Health Laboratory	Ohio Department of Health Laboratory	Holmes, Jennifer; Eric Brandt, Keoni Omura, Glen McGillivary, Caitlin McDonnell, Jade Mowery, Stephanie Mccracken, Tyler Payne, Kirtana Ramadugu, Erica Leasure, Brent Lee, Kelsey Florek, Heather Blankenship, Quanta Brown, and Tammy Bannerman
EPI_ISL_962206	Institute for Medical Research, Infectious Disease Research Centre, National Institutes of Health, Ministry of Health Malaysia	Institute for Medical Research, Infectious Disease Research Centre, National Institutes of Health, Ministry of Health Malaysia	Suppiah J, Kamel K, Azizan MA, Thayan R
EPI_ISL_962515	MD Laboratories	Los Angeles County PHL	P. Hemarajata et al.
EPI_ISL_962909, EPI_ISL_962910	Norwegian Institute of Public Health, Department of Virology	Norwegian Institute of Public Health, Department of Virology	Kathrine Stene-Johansen, Kamilla Heddeland Instefjord, Hilde Elshaug, Ignacio Garcia Llorente, Serina B Engebretsen, Atiya R Ali,Marie Paulsen Madsen, Rasmus Riis Kopperud, Hilde Vollan, Karoline Bragstad, Olav Hungnes
EPI_ISL_962924, EPI_ISL_962925, EPI_ISL_962926, EPI_ISL_962927	Servicio de Microbiología, Laboratori Clínic Metropolitana Nord. Hospital Universitari Germans Trias i Pujol. Institut d'Investigació en Ciències de la Salut Germans Trias i Pujol (IGTP)	SeqCOVID-SPAIN consortium/IBV(CSIC)	Elisa Martró, Antoni E. Bordoy, Anna Not, Adrián Antuori, Anabel Fernández, Nona Romaní, Verónica Saludes, Cristina Casañ and SeqCOVID-SPAIN consortium
EPI_ISL_962939	Hospital Universitario de Gran Canaria Dr. Negrín	SeqCOVID-SPAIN consortium/IBV(CSIC)	M. Carmen Pérez González, Francisco J. Chamizo López, Ana Bordes Benítez and SeqCOVID-SPAIN consortium
EPI_ISL_963061, EPI_ISL_963296, EPI_ISL_963955	Lighthouse Lab in Alderley Park	Wellcome Sanger Institute for the COVID-19 Genomics UK (COG-UK) Consortium	Jacquelyn Wynn, Mairead Hyland, The Lighthouse Lab in Alderley Park and Alex Alderton, Roberto Amato, Sonia Goncalves, Ewan Harrison, David K. Jackson, Ian Johnston, Dominic Kwiatkowski, Cordelia Langford, John Sillitoe on behalf of the Wellcome Sanger Institute COVID-19 Surveillance Team
EPI_ISL_964278	Oslo University Hospital, Department of Medical Microbiology	Norwegian Institute of Public Health, Department of Virology	Kathrine Stene-Johansen, Kamilla Heddeland Instefjord, Hilde Elshaug, Ignacio Garcia Llorente, Serina B Engebretsen, Atiya R Ali, Marie Paulsen Madsen, Rasmus Riis Kopperud, Hilde Vollan, Karoline Bragstad, Olav Hungnes
EPI_ISL_964882	General Hospital "Abdulah Nakas" Sarajevo	Alea Genetic Centre	Lana Salihefendic, Dino Pecar, Adis Kandic, Sead Jazic, Rijad Konjhodzic
EPI_ISL_965008	Nordland Hospital - Bodo, Laboratory Department, Molecular Biology Unit	Norwegian Institute of Public Health, Department of Virology	Kathrine Stene-Johansen, Kamilla Heddeland Instefjord, Hilde Elshaug, Ignacio Garcia Llorente, Serina B Engebretsen, Atiya R Ali, Marie Paulsen Madsen, Rasmus Riis Kopperud, Hilde Vollan, Karoline Bragstad, Olav Hungnes

EPI_ISL_965113, EPI_ISL_965121	Ohio Department of Health Laboratory	Ohio Department of Health Laboratory	Holmes, Jennifer; Eric Brandt, Keoni Omura, Glen McGillivary, Caitlin McDonnell, Jade Mowery, Stephanie Mccracken, Tyler Payne, Kirtana Ramadugu, Erica Leasure, Brent Lee, Kelsey Florek, Heather Blankenship, Quanta Brown, and Tammy Bannerman
EPI_ISL_965129	Sant'Eugenio/CTO ASL Roma 2.	INMI Lazzaro Spallanzani IRCCS	F Messina, C.E.M Gruber, B Bartolini, E Giombini, M Rueca, O Butera, F Bondanini, GC Cocciolillo, C Disegni, A Di Caro, MR Capobianchi
EPI_ISL_965130	Ospedale Sandro Pertini ASL Roma2	INMI Lazzaro Spallanzani IRCCS	B Bartolini, O Butera, C.E.M Gruber, M Rueca, F Messina, E Giombini, R Longo, V Michela, MR Capobianchi, A Di Caro
EPI_ISL_965132	Azienda Ospedaliera San Giovanni Addolorata	INMI Lazzaro Spallanzani IRCCS	CEM Gruber, B Bartolini, E Giombini, M Rueca, O Butera, F Messina, M Gaudio, PM Placanica, A Di Caro, MR Capobianchi
EPI_ISL_965137	Fondazione Policlinico Universitario "A. Gemelli" IRCCS	INMI Lazzaro Spallanzani IRCCS	E Giombini, M. Rueca, B Bartolini, O Butera, C.E.M Gruber, F Messina, P Cattani,M Sanguinetti, A Di Caro, MR Capobianchi
EPI_ISL_965143, EPI_ISL_965149, EPI_ISL_965301, EPI_ISL_965302, EPI_ISL_965813	Ohio Department of Health Laboratory	Ohio Department of Health Laboratory	Holmes, Jennifer; Eric Brandt, Keoni Omura, Glen McGillivary, Caitlin McDonnell, Jade Mowery, Stephanie Mccracken, Tyler Payne, Kirtana Ramadugu, Erica Leasure, Brent Lee, Kelsey Florek, Heather Blankenship, Quanta Brown, and Tammy Bannerman
EPI_ISL_965890, EPI_ISL_965891, EPI_ISL_965892, EPI_ISL_965893, EPI_ISL_965894	Massachusetts State Public Health Laboratory	Massachusetts State Public Health Laboratory	Andrew Lang, Timelia Fink, Glen Gallagher, Sandra Smole
EPI_ISL_965952	Consejería de Sanidad y Asuntos Sociales de Castilla La Mancha	Instituto de Salud Carlos III	Iglesias-Caballero, M. Sandonís,V. Vázquez, S. Camarero, S. Pozo, F. Casas, I. Jiménez, P. Zaballos, A. Monzón, S. Varona, S. Cuesta, I. Gutiérrez, G.
EPI_ISL_966547, EPI_ISL_966548, EPI_ISL_967186, EPI_ISL_967187, EPI_ISL_967188, EPI_ISL_967189, EPI_ISL_967190, EPI_ISL_967191, EPI_ISL_967216, EPI_ISL_967221, EPI_ISL_967222, EPI_ISL_967223, EPI_ISL_967224, EPI_ISL_967225, EPI_ISL_967226, EPI_ISL_967227, EPI_ISL_967228, EPI_ISL_967229, EPI_ISL_967230, EPI_ISL_967231, EPI_ISL_967232, EPI_ISL_967233, EPI_ISL_967234, EPI_ISL_967235, EPI_ISL_967236, EPI_ISL_967237, EPI_ISL_967238, EPI_ISL_967239, EPI_ISL_967240, EPI_ISL_967241, EPI_ISL_967242			
see above	Helix/Illumina	Respiratory Viruses Branch, Division of Viral Diseases, Centers for Disease Control and Prevention	Peter W. Cook,Dakota Howard,Dhwani Batra,Ben L. Rambo-Martin,Eileen de Feo,Jan Antico,Christine Tran,Matthew Tolentino,Shannon Wickline,Kim Gietzen,Brad Sickler,Jingtao Liu,Eric Allen,Phil Febbo,Summer Galloway,Nicole L. Washington,Simon White,Geraint Levan,Kelly Schiabor Barrett,Elizabeth Cirulli,Alexandre Bolze,Ary Ascencio,Charlotte Rivera-Garcia,Ryan Cho,Jason Nguyen,Sherry Wang,Jimmy Ramirez,Tyler Cassens,Efren Sandoval,Magnus Isaksson,William Lee,David Becker,Marc Laurent,James Lu,Clinton R. Paden,Suxiang Tong,Duncan MacCannell,
EPI_ISL_967683, EPI_ISL_967713, EPI_ISL_967714, EPI_ISL_967744	State Laboratories Division, Hawaii State Department of Health	State Laboratories Division, Hawaii State Department of Health	Pamela O'Brien, Drew Kuwazaki, Ayana Garnet, Razvan Sultana, Edward Desmond
EPI_ISL_967897, EPI_ISL_967913	TGen North	TGen North	"Jolene Bowers, Megan Folkerts, Chris French, Hayley Yaglom, Ashlyn Pfeiffer, Darrin Lemmer, Dave Engelthaler, The Arizona COVID Genomics Union (ACGU)"
EPI_ISL_967916	TGen North	Sonora Quest Laboratories	"Jolene Bowers, Megan Folkerts, Chris French, Hayley Yaglom, Ashlyn Pfeiffer, Darrin Lemmer, Dave Engelthaler, The Arizona COVID Genomics Union (ACGU)"
EPI_ISL_967928, EPI_ISL_967936	TGen North	TGen North	"Jolene Bowers, Megan Folkerts, Chris French, Hayley Yaglom, Ashlyn Pfeiffer, Darrin Lemmer, Dave Engelthaler, The Arizona COVID Genomics Union (ACGU)"
EPI_ISL_967957, EPI_ISL_967964	TGen North	Sonora Quest Laboratories	"Jolene Bowers, Megan Folkerts, Chris French, Hayley Yaglom, Ashlyn Pfeiffer, Darrin Lemmer, Dave Engelthaler, The Arizona COVID Genomics Union (ACGU)"
EPI_ISL_967966, EPI_ISL_967976, EPI_ISL_967977, EPI_ISL_967982, EPI_ISL_967983, EPI_ISL_967990, EPI_ISL_968002, EPI_ISL_968017, EPI_ISL_968020	TGen North	TGen North	"Jolene Bowers, Megan Folkerts, Chris French, Hayley Yaglom, Ashlyn Pfeiffer, Darrin Lemmer, Dave Engelthaler, The Arizona COVID Genomics Union (ACGU)"
EPI_ISL_968060	TGen North	Sonora Quest Laboratories	"Jolene Bowers, Megan Folkerts, Chris French, Hayley Yaglom, Ashlyn Pfeiffer, Darrin Lemmer, Dave Engelthaler, The Arizona COVID Genomics Union (ACGU)"
EPI_ISL_968063	TGen North	TGen North	"Jolene Bowers, Megan Folkerts, Chris French, Hayley Yaglom, Ashlyn Pfeiffer, Darrin Lemmer, Dave Engelthaler, The Arizona COVID Genomics Union (ACGU)"
EPI_ISL_970829, EPI_ISL_970846, EPI_ISL_970936, EPI_ISL_970992, EPI_ISL_971028, EPI_ISL_971030, EPI_ISL_971065, EPI_ISL_971133, EPI_ISL_971145, EPI_ISL_971155, EPI_ISL_971519, EPI_ISL_971586, EPI_ISL_971610, EPI_ISL_971695, EPI_ISL_971699, EPI_ISL_971791, EPI_ISL_971793, EPI_ISL_971911, EPI_ISL_971919, EPI_ISL_971949, EPI_ISL_971977, EPI_ISL_972044, EPI_ISL_972055, EPI_ISL_972091, EPI_ISL_972094, EPI_ISL_972098, EPI_ISL_972109, EPI_ISL_972178, EPI_ISL_972216, EPI_ISL_972218, EPI_ISL_972221, EPI_ISL_972226, EPI_ISL_972240, EPI_ISL_972270, EPI_ISL_972277, EPI_ISL_972290, EPI_ISL_972291, EPI_ISL_972353, EPI_ISL_972382, EPI_ISL_972392, EPI_ISL_972399, EPI_ISL_972413, EPI_ISL_972442, EPI_ISL_972484, EPI_ISL_972496, EPI_ISL_972502, EPI_ISL_972522, EPI_ISL_972554, EPI_ISL_972557, EPI_ISL_972607, EPI_ISL_972633, EPI_ISL_972635, EPI_ISL_972671, EPI_ISL_972688, EPI_ISL_972709, EPI_ISL_972734, EPI_ISL_972875, EPI_ISL_972885, EPI_ISL_972899, EPI_ISL_972913, EPI_ISL_972933, EPI_ISL_972944, EPI_ISL_972950, EPI_ISL_972969, EPI_ISL_973077, EPI_ISL_973138, EPI_ISL_973157, EPI_ISL_973175, EPI_ISL_973180, EPI_ISL_973197, EPI_ISL_973227, EPI_ISL_973239, EPI_ISL_973265, EPI_ISL_973298, EPI_ISL_973339, EPI_ISL_973350, EPI_ISL_973351, EPI_ISL_973379, EPI_ISL_973389, EPI_ISL_973397, EPI_ISL_973407, EPI_ISL_973420, EPI_ISL_973438, EPI_ISL_973535, EPI_ISL_973614, EPI_ISL_973666, EPI_ISL_973676, EPI_ISL_973752			
see above	Department of Virus and Microbiological Special Diagnostics, Statens Serum Institut, Copenhagen, Denmark	Aalborg University	Danish Covid-19 Genome Consortium
EPI_ISL_974150	Laboratorio de Virología / Hospital Universitario Central de Asturias (HUCA)	Laboratorio de Virología / Hospital Universitario Central de Asturias (HUCA)	Castelló C, Gómez de Oña J, Boga JA, Rojo S, Alvarez-Arguelles ME, Abreu F, Costales I, Sandoval M, Perez-Martínez Z, Martín-Rodríguez G, Coto E, Melón S
EPI_ISL_977117	Massachusetts General Hospital	Infectious Disease Program, Broad Institute of Harvard and MIT	Lemieux,J.E., Siddle,K.J., Shaw,B., Adams,G., Pierce,V., Turbett,S., Anahtar,M., Branda,J., Slater,D., Harris,J., Lin,A.E., Gladden-Young,A., Lagerborg,K., Rudy,M., DeRuff,K., Carter,A., Normandin,E., Bauer,M., Reilly,S., Tomkins-Tinch,C., Loreth,C., Chaluvadi,S., Neumann,A., Cusick,C., Chapman,S.B., Gnirke,A., Flowers,K., Cerrato,F., Birren,B.W., Gallagher,G., Smole,S., Park,D.J., MacInnis,B.L., Ryan,E., LaRocque,R., Rosenberg,E. and Sabeti,P.C.
EPI_ISL_977120	Flow Health	Infectious Disease Program, Broad Institute of Harvard and MIT	Lemieux,J.E., Siddle,K.J., Adams,G., Gladden-Young,A., Lagerborg,K., Rudy,M., DeRuff,K., Carter,A., Normandin,E., Bauer,M., Reilly,S., Tomkins-Tinch,C., Loreth,C., Chaluvadi,S., Birren,B.W., Gallagher,G., Smole,S., Park,D.J., MacInnis,B.L., and Sabeti,P.C.
EPI_ISL_977278, EPI_ISL_977292, EPI_ISL_977346	University of Zambia, School of Veterinary Medicine	UNZAVET and PATH	Mulenga Mwenda-Chimfwembe, Ngonda Saasa, Daniel Bridges
EPI_ISL_977543, EPI_ISL_977551, EPI_ISL_977557, EPI_ISL_977558, EPI_ISL_977559	Nigeria Centre of Disease Control (NCDC)	African Centre of Excellence for Genomics of Infectious Diseases (ACEGID), Redeemer's University	Olawoye I. B. et al
EPI_ISL_977664	Hopital	National Reference Center for Viruses of Respiratory Infections, Institut Pasteur, Paris	Marion Barbet, Sylvie Behillil, Méline Bizard, Angela Brisebarre, Camille Capel, Etienne Simon-Lorière, Vincent Enouf, Maud Vanpeene, Sylvie van der Werf,Combe Patrice
EPI_ISL_978294, EPI_ISL_978295, EPI_ISL_978296, EPI_ISL_978297, EPI_ISL_978298, EPI_ISL_978299, EPI_ISL_978347, EPI_ISL_978348, EPI_ISL_978349	Texas Department of State Health Services	Texas Department of State Health Services	Bonnie Oh, Anita Pokharel, James Daniel Bonser, Myong Koag, Chung Wang, Rachel Lee, Grace Kubin, Rashmi Tuladhar, Mayela Pedrueza, Maliha Rahman, Jenny Zhang
EPI_ISL_978356, EPI_ISL_978363, EPI_ISL_978364, EPI_ISL_978365	Arizona State Public Health Laboratory	Arizona State Public Health Laboratory	Trung Huynh, Jessica Escobar, Katherine Fullerton, Nobuko Fukushima, Stacy White, Linda Getsinger, Victor Waddell
EPI_ISL_978622, EPI_ISL_978623, EPI_ISL_978624	Helix/Illumina	Respiratory Viruses Branch, Division of Viral Diseases, Centers for Disease Control and Prevention	Peter W. Cook,Dakota Howard,Dhwani Batra,Ben L. Rambo-Martin,Eileen de Feo,Jan Antico,Christine Tran,Matthew Tolentino,Shannon Wickline,Kim Gietzen,Brad Sickler,Jingtao Liu,Eric Allen,Phil Febbo,Summer Galloway,Nicole L. Washington,Simon White,Geraint Levan,Kelly Schiabor Barrett,Elizabeth Cirulli,Alexandre Bolze,Ary Ascencio,Charlotte Rivera-Garcia,Ryan Cho,Jason Nguyen,Sherry Wang,Jimmy Ramirez,Tyler Cassens,Efren Sandoval,Magnus Isaksson,William Lee,David Becker,Marc Laurent,James Lu,Clinton R. Paden,Suxiang Tong,Duncan MacCannell,
EPI_ISL_979084, EPI_ISL_979086, EPI_ISL_979087, EPI_ISL_979088,	Santa Clara County Public Health Laboratory	Chan-Zuckerberg Biohub	CZB Cliahub Consortium

EPI_ISL_979090, EPI_ISL_979091			
EPI_ISL_979254, EPI_ISL_979255, EPI_ISL_979256, EPI_ISL_979257, EPI_ISL_979258, EPI_ISL_979259	Institute of Microbiology and Immunology, Faculty of Medicine, University of Ljubljana	Institute of Microbiology and Immunology, Faculty of Medicine, University of Ljubljana	Samo Zakotnik, Tomaž Mark Zorec, Matic Brvar, Doroteja Vlaj, Patricija Pozvek,Špela Pleh, Miša Korva, Mario Poljak, Tatjana Avši - Županc
EPI_ISL_979376	The Jackson Laboratory	The Jackson Laboratory	Lloyd M, Sanderson B, Srivastava A, Maurya R, Renzette N, Omerza G, Kelly K, Li L, Wei C L, Adams M
EPI_ISL_981063, EPI_ISL_981064	Johns Hopkins Hospital Department of Pathology	Johns Hopkins Hospital Department of Pathology	C. Paul Morris, Chun Huai Luo, Adannaya Amadi, Matthew Schwartz, Nicholas Gallagher, Heba H. Mostafa
EPI_ISL_981202, EPI_ISL_981305, EPI_ISL_981306, EPI_ISL_981307, EPI_ISL_981368, EPI_ISL_981369	Ohio Department of Health Laboratory	Ohio Department of Health Laboratory	Holmes, Jennifer; Eric Brandt, Keoni Omura, Glen McGillivary, Caitlin McDonnell, Jade Mowery, Stephanie Mccracken, Tyler Payne, Kirtana Ramadugu, Erica Leasure, Brent Lee, Kelsey Florek, Heather Blankenship, Quanta Brown, and Tammy Bannerman
EPI_ISL_981961, EPI_ISL_981962, EPI_ISL_981963	Microbiology Service, Hospital Universitario Clinico San Cecilio, Granada	Microbiology Service, Hospital Universitario Clinico San Cecilio, Granada	Adolfo de Salazar, Natalia Chueca, Laura Viñuela, Ana Fuentes, Federico García
EPI_ISL_981979, EPI_ISL_982024, EPI_ISL_982082, EPI_ISL_982084, EPI_ISL_982094, EPI_ISL_982099, EPI_ISL_982104	TGen North	Sonora Quest Laboratories	"Jolene Bowers, Megan Folkerts, Chris French, Hayley Yaglom, Ashlyn Pfeiffer, Darrin Lemmer, Dave Engelthaler, The Arizona COVID Genomics Union (ACGU)"
EPI_ISL_982247	Lab voor klinische biologie	Lab voor klinische biologie	Hannelore Hamerlinck, Marija Janevska, Bruno Verhasselt
EPI_ISL_982349, EPI_ISL_982352, EPI_ISL_982393, EPI_ISL_982396, EPI_ISL_982399, EPI_ISL_982403, EPI_ISL_982404, EPI_ISL_982405	M Health Fairview	Minnesota Department of Health, Public Health Laboratory	Alexandra Lorentz, Jacob Garfin, Matt Plumb, and Xiong Wang
EPI_ISL_982499, EPI_ISL_982500, EPI_ISL_982501	ADIRONDACK MEDICAL CENTER	Wadsworth Center, New York State Department of Health	Kirsten St. George, Daryl M. Lamson, Alexis Russel, Matthew Shudt, Melissa A Leisner, Jonathan Plitnick, Navjot Singh, John Kelly, Erasmus Schneider, Erica Lasek-Nesselquist
EPI_ISL_982544, EPI_ISL_982546, EPI_ISL_982560, EPI_ISL_982563, EPI_ISL_982568, EPI_ISL_982591, EPI_ISL_982617, EPI_ISL_982626, EPI_ISL_982652, EPI_ISL_982662, EPI_ISL_982663, EPI_ISL_982677, EPI_ISL_982683, EPI_ISL_982731			
see above	US Air Force School of Aerospace Medicine	US Air Force School of Aerospace Medicine	Anthony Fries, Jennifer Meyer, William Gruner, William Buggele, Amanda Javorina, Sarah Purves, Clarise Starr, Elizabeth Macias
EPI_ISL_983116, EPI_ISL_983117	SUNY UPSTATE MEDICAL UNIVERSITY	Wadsworth Center, New York State Department of Health	Kirsten St. George, Daryl M. Lamson, Alexis Russel, Matthew Shudt, Melissa A Leisner, Jonathan Plitnick, Navjot Singh, John Kelly, Erasmus Schneider, Erica Lasek-Nesselquist
EPI_ISL_983126, EPI_ISL_983127, EPI_ISL_983128	THE MARY IMOGENE BASSETT HOSPITAL	Wadsworth Center, New York State Department of Health	Kirsten St. George, Daryl M. Lamson, Alexis Russel, Matthew Shudt, Melissa A Leisner, Jonathan Plitnick, Navjot Singh, John Kelly, Erasmus Schneider, Erica Lasek-Nesselquist
EPI_ISL_983322	ASL LATINA PRESIDIO OSPEDALIERO NORD	INMI Lazzaro Spallanzani IRCCS	E Giombini, M. Rueca, B Bartolini, O Butera, C.E.M Gruber, F Messina, A Lucci, S Pignalosa, A Di Caro, MR Capobianchi
EPI_ISL_983618, EPI_ISL_983619, EPI_ISL_983620, EPI_ISL_983621, EPI_ISL_983622, EPI_ISL_983623	Texas Department of State Health Services	Texas Department of State Health Services	Bonnie Oh, Anita Pokharel, James Daniel Bonser, Myong Koag, Chung Wang, Rachel Lee, Grace Kubin, Rashmi Tuladhar, Mayela Pedrueza, Maliha Rahman, Jenny Zhang
EPI_ISL_983713, EPI_ISL_983730, EPI_ISL_983805, EPI_ISL_983806, EPI_ISL_983807, EPI_ISL_983808, EPI_ISL_983809, EPI_ISL_983810	Colorado Department of Public Health and Environment	Colorado Department of Puplic Health and Environment	Laura Bankers, Molly C. Hetherington-Rauth, Diana Ir, Shannon Ely, Shannon R. Matzinger, Sarah Elizabeth Totten, Emily A. Travanty
