# Supplementary material for: Detection and characterization of the SARS-CoV-2 lineage B.1.526 in New York
Source: Nat Commun. 2021 Aug 9;12:4886. doi: 10.1038/s41467-021-25168-4 (PMC8352861; doi:10.1038/s41467-021-25168-4)
Supplement: Supplementary file 8 — Supplementary Data 4 [file 41467_2021_25168_MOESM8_ESM.zip › GISAID_acknowledements_tables/gisaid_hcov-19_acknowledgement_table_2021_02_13_01-3.pdf]

We gratefully acknowledge the following Authors from the Originating laboratories responsible for obtaining the specimens, as well as the Submitting laboratories where the genome data were generated and shared via GISAID, on which this research is based.

All Submitters of data may be contacted directly via www.gisaid.org

Authors are sorted alphabetically.

Accession ID	Originating Laboratory	Submitting Laboratory	Authors
EPI_ISL_936486	Medicine and Surgery, University of Insubria	Medicine and Surgery, University of Insubria	Novazzi,F., Genoni,A., Baj,A., Spezia,P.G., Focosi,D., Zago,C.,Colombo,A., Cassani,G., Pasciuta,R., Tamborini,A., Rossi,A.,Prestia,M., Capuano,R. and Maggi,F.
EPI_ISL_949900, EPI_ISL_949902	University College London, Great Ormond Street Hospital for Children NHS Foundation Trust, Imperial College Healthcare NHS Trust	COVID-19 Genomics UK (COG-UK) Consortium	Sergi Castellano, Rachel Williams, Mark Kristiansen, Paola Resende Silva, Sunando Roy, Tony Brooks, Helena Tutill, Paola Niola, Patricia Dyal, Charlotte Williams, Leysa Forrest, Yasmin Panchbhaya, Jacqueline Findlay, Samuel Weeks, Julianne Brown, Kathryn Harris, Paul Randell, James Price, Alison Holmes, Judith Breuer
EPI_ISL_950723, EPI_ISL_950724, EPI_ISL_950725, EPI_ISL_950726, EPI_ISL_950727, EPI_ISL_950728	Queens Medical Centre, Clinical Microbiology Department / DeepSeq Nottingham	COVID-19 Genomics UK (COG-UK) Consortium	Gemma Clark, Wendy Smith, Manjinder Khakh, Vicki M Fleming, Michelle M Lister, Hannah Howson-Wells, Jonathan Ball, Patrick McClure, Joseph Chappell, Theocharis Tsoleridis, Nadine Holmes, Matthew Carlisle, Christopher Moore, Fei Sang, Johnny Debebe, Victoria Wright, Matthew Loose
EPI_ISL_952956, EPI_ISL_952960, EPI_ISL_952961, EPI_ISL_952962, EPI_ISL_952963, EPI_ISL_952966, EPI_ISL_952967, EPI_ISL_952971, EPI_ISL_952973, EPI_ISL_952974, EPI_ISL_952981, EPI_ISL_952987, EPI_ISL_952989, EPI_ISL_952992, EPI_ISL_952993, EPI_ISL_952996, EPI_ISL_953001, EPI_ISL_953005, EPI_ISL_953007, EPI_ISL_953008, EPI_ISL_953011, EPI_ISL_953018, EPI_ISL_953019, EPI_ISL_953023			
see above	Virology Department, Sheffield Teaching Hospitals NHS Foundation Trust/Department of Infection, Immunity and Cardiovascular Disease, The Medical School, University of Sheffield	COVID-19 Genomics UK (COG-UK) Consortium	Thushan de Silva, Matthew Parker, Nikki Smith, Adri Angyal, Rebecca Brown, Luke Green, Rachel Tucker, Paul Parsons, Danielle Groves, Katie Johnson, Laura Carrilero, Alex Keeley, Dave Partridge, Matthew Wyles, Benjamin Lindsey, Mehmet Yavuz, Mohammad Raza, Cariad Evans
EPI_ISL_953377, EPI_ISL_953378, EPI_ISL_953379, EPI_ISL_953380, EPI_ISL_953381, EPI_ISL_953382, EPI_ISL_953383, EPI_ISL_953384	Jessa	Jessa	Jessa_cmdLab
EPI_ISL_953385, EPI_ISL_953386, EPI_ISL_953388, EPI_ISL_953390, EPI_ISL_953394	National Public Health Laboratory, National Centre for Infectious Diseases	National Public Health Laboratory, National Centre for Infectious Diseases	Tze Minn Mak, Zhenyang Zhou, Lin Cui, Raymond Tzer Pin Lin
EPI_ISL_960176, EPI_ISL_960177, EPI_ISL_960178, EPI_ISL_960179, EPI_ISL_960180, EPI_ISL_960181, EPI_ISL_960182, EPI_ISL_960183, EPI_ISL_960184, EPI_ISL_960185, EPI_ISL_960186, EPI_ISL_960187			
see above	Jessa	Jessa	Jessa_cmdLab
EPI_ISL_960835, EPI_ISL_960843, EPI_ISL_960846, EPI_ISL_960849, EPI_ISL_960851, EPI_ISL_960852, EPI_ISL_960884, EPI_ISL_960885, EPI_ISL_960887, EPI_ISL_960891, EPI_ISL_960892, EPI_ISL_960894, EPI_ISL_960895, EPI_ISL_960896, EPI_ISL_960897, EPI_ISL_960898			
see above	Landesamt für Verbraucherschutz Sachsen Anhalt, Magdeburg	Institute of Medical Microbiology and Hospital Hygiene	Prof. Dr. Achim Kaasch, Aljoscha Tersteegen
EPI_ISL_960900, EPI_ISL_960901	Institute of Medical Microbiology and Hospital Hygiene	Institute of Medical Microbiology and Hospital Hygiene	Prof. Dr. Achim Kaasch, Aljoscha Tersteegen
EPI_ISL_960935, EPI_ISL_960936, EPI_ISL_960937, EPI_ISL_960938, EPI_ISL_960939, EPI_ISL_960940	Landesamt für Verbraucherschutz Sachsen Anhalt, Magdeburg	Institute of Medical Microbiology and Hospital Hygiene	Prof. Dr. Achim Kaasch, Aljoscha Tersteegen
EPI_ISL_961181, EPI_ISL_961182, EPI_ISL_961183, EPI_ISL_961184, EPI_ISL_961185, EPI_ISL_961186, EPI_ISL_961187, EPI_ISL_961188, EPI_ISL_961189, EPI_ISL_961190, EPI_ISL_961191, EPI_ISL_961192, EPI_ISL_961193, EPI_ISL_961194, EPI_ISL_961195, EPI_ISL_961196, EPI_ISL_961197, EPI_ISL_961198, EPI_ISL_961199, EPI_ISL_961200, EPI_ISL_961201, EPI_ISL_961202, EPI_ISL_961203			
see above	AZDelta	AZDelta	Geert Martens; Dieter De Smet
EPI_ISL_961673	SIESP CHIETI DRIVE IN LANCIANO	Istituto Zooprofilattico Sperimentale dell'Abruzzo e Molise "G. Caporale"	Lorusso A, Marcacci M, Di Domenico M, Ancora M, Curini V, Mangone I, Rinaldi A, Scialabba S, Di Pasquale A, Cammà C, Puglia I, Calistri P, Savini G
EPI_ISL_961674, EPI_ISL_961675, EPI_ISL_961676, EPI_ISL_961677	SIESP CHIETI - DRIVE IN CHIETI	Istituto Zooprofilattico Sperimentale dell'Abruzzo e Molise "G. Caporale"	Lorusso A, Marcacci M, Di Domenico M, Ancora M, Curini V, Mangone I, Rinaldi A, Scialabba S, Di Pasquale A, Cammà C, Puglia I, Calistri P, Savini G
EPI_ISL_961678	SIESP TERAMO	Istituto Zooprofilattico Sperimentale dell'Abruzzo e Molise "G. Caporale"	Lorusso A, Marcacci M, Di Domenico M, Ancora M, Curini V, Mangone I, Rinaldi A, Scialabba S, Di Pasquale A, Cammà C, Puglia I, Calistri P, Savini G
EPI_ISL_961679, EPI_ISL_961680, EPI_ISL_961681, EPI_ISL_961682	SIESP CHIETI - DRIVE IN CHIETI	Istituto Zooprofilattico Sperimentale dell'Abruzzo e Molise "G. Caporale"	Lorusso A, Marcacci M, Di Domenico M, Ancora M, Curini V, Mangone I, Rinaldi A, Scialabba S, Di Pasquale A, Cammà C, Puglia I, Calistri P, Savini G
EPI_ISL_961683	SIESP TERAMO	Istituto Zooprofilattico Sperimentale dell'Abruzzo e Molise "G. Caporale"	Lorusso A, Marcacci M, Di Domenico M, Ancora M, Curini V, Mangone I, Rinaldi A, Scialabba S, Di Pasquale A, Cammà C, Puglia I, Calistri P, Savini G
EPI_ISL_961684	SIESP CHIETI - DRIVE IN CHIETI	Istituto Zooprofilattico Sperimentale dell'Abruzzo e Molise "G. Caporale"	Lorusso A, Marcacci M, Di Domenico M, Ancora M, Curini V, Mangone I, Rinaldi A, Scialabba S, Di Pasquale A, Cammà C, Puglia I, Calistri P, Savini G
EPI_ISL_961685	SIESP DIPARTIMENTO DI PREVENZIONE CHIETI	Istituto Zooprofilattico Sperimentale dell'Abruzzo e Molise "G. Caporale"	Lorusso A, Marcacci M, Di Domenico M, Ancora M, Curini V, Mangone I, Rinaldi A, Scialabba S, Di Pasquale A, Cammà C, Puglia I, Calistri P, Savini G
EPI_ISL_961686, EPI_ISL_961687	SIESP CHIETI - DRIVE IN ORTONA	Istituto Zooprofilattico Sperimentale dell'Abruzzo e Molise "G. Caporale"	Lorusso A, Marcacci M, Di Domenico M, Ancora M, Curini V, Mangone I, Rinaldi A, Scialabba S, Di Pasquale A, Cammà C, Puglia I, Calistri P, Savini G
EPI_ISL_961688	SIESP TERAMO	Istituto Zooprofilattico Sperimentale dell'Abruzzo e Molise "G. Caporale"	Lorusso A, Marcacci M, Di Domenico M, Ancora M, Curini V, Mangone I, Rinaldi A, Scialabba S, Di Pasquale A, Cammà C, Puglia I, Calistri P, Savini G
EPI_ISL_961689, EPI_ISL_961690	SIESP CHIETI - DRIVE IN CHIETI	Istituto Zooprofilattico Sperimentale dell'Abruzzo e Molise "G. Caporale"	Lorusso A, Marcacci M, Di Domenico M, Ancora M, Curini V, Mangone I, Rinaldi A, Scialabba S, Di Pasquale A, Cammà C, Puglia I, Calistri P, Savini G
EPI_ISL_961691, EPI_ISL_961692, EPI_ISL_961693	SIESP CHIETI - DRIVE IN ORTONA	Istituto Zooprofilattico Sperimentale dell'Abruzzo e Molise "G. Caporale"	Lorusso A, Marcacci M, Di Domenico M, Ancora M, Curini V, Mangone I, Rinaldi A, Scialabba S, Di Pasquale A, Cammà C, Puglia I, Calistri P, Savini G
EPI_ISL_961694	SIESP CHIETI - DRIVE IN CHIETI	Istituto Zooprofilattico Sperimentale dell'Abruzzo e Molise "G. Caporale"	Lorusso A, Marcacci M, Di Domenico M, Ancora M, Curini V, Mangone I, Rinaldi A, Scialabba S, Di Pasquale A, Cammà C, Puglia I, Calistri P, Savini G
EPI_ISL_961695	SIESP DIPARTIMENTO DI PREVENZIONE CHIETI	Istituto Zooprofilattico Sperimentale dell'Abruzzo e Molise "G. Caporale"	Lorusso A, Marcacci M, Di Domenico M, Ancora M, Curini V, Mangone I, Rinaldi A, Scialabba S, Di Pasquale A, Cammà C, Puglia I, Calistri P, Savini G
EPI_ISL_961696	SIESP SULMONA	Istituto Zooprofilattico Sperimentale dell'Abruzzo e Molise "G. Caporale"	Lorusso A, Marcacci M, Di Domenico M, Ancora M, Curini V, Mangone I, Rinaldi A, Scialabba S, Di Pasquale A, Cammà C, Puglia I, Calistri P, Savini G

EPI_ISL_961697, EPI_ISL_961698	SIESP CHIETI - DRIVE IN CHIETI	Istituto Zooprofilattico Sperimentale dell'Abruzzo e Molise "G. Caporale"	Lorusso A, Marcacci M, Di Domenico M, Ancora M, Curini V, Mangone I, Rinaldi A, Scialabba S, Di Pasquale A, Cammà C, Puglia I, Calistri P, Savini G
EPI_ISL_961699	SIESP CHIETI - DRIVE IN ORTONA	Istituto Zooprofilattico Sperimentale dell'Abruzzo e Molise "G. Caporale"	Lorusso A, Marcacci M, Di Domenico M, Ancora M, Curini V, Mangone I, Rinaldi A, Scialabba S, Di Pasquale A, Cammà C, Puglia I, Calistri P, Savini G
EPI_ISL_961700	SIESP CHIETI - DRIVE IN CHIETI	Istituto Zooprofilattico Sperimentale dell'Abruzzo e Molise "G. Caporale"	Lorusso A, Marcacci M, Di Domenico M, Ancora M, Curini V, Mangone I, Rinaldi A, Scialabba S, Di Pasquale A, Cammà C, Puglia I, Calistri P, Savini G
EPI_ISL_961701	Ospedale Civile Teramo	Istituto Zooprofilattico Sperimentale dell'Abruzzo e Molise "G. Caporale"	Lorusso A, Marcacci M, Di Domenico M, Ancora M, Curini V, Mangone I, Rinaldi A, Scialabba S, Di Pasquale A, Cammà C, Puglia I, Calistri P, Savini G
EPI_ISL_961702	SIESP CHIETI - DRIVE IN CHIETI	Istituto Zooprofilattico Sperimentale dell'Abruzzo e Molise "G. Caporale"	Lorusso A, Marcacci M, Di Domenico M, Ancora M, Curini V, Mangone I, Rinaldi A, Scialabba S, Di Pasquale A, Cammà C, Puglia I, Calistri P, Savini G
EPI_ISL_961703	SIESP DIPARTIMENTO DI PREVENZIONE CHIETI	Istituto Zooprofilattico Sperimentale dell'Abruzzo e Molise "G. Caporale"	Lorusso A, Marcacci M, Di Domenico M, Ancora M, Curini V, Mangone I, Rinaldi A, Scialabba S, Di Pasquale A, Cammà C, Puglia I, Calistri P, Savini G
EPI_ISL_961704	SIESP SULMONA	Istituto Zooprofilattico Sperimentale dell'Abruzzo e Molise "G. Caporale"	Lorusso A, Marcacci M, Di Domenico M, Ancora M, Curini V, Mangone I, Rinaldi A, Scialabba S, Di Pasquale A, Cammà C, Puglia I, Calistri P, Savini G
EPI_ISL_961705	Ospedale San Salvatore UOC Malattie Infettive	Istituto Zooprofilattico Sperimentale dell'Abruzzo e Molise "G. Caporale"	Lorusso A, Marcacci M, Di Domenico M, Ancora M, Curini V, Mangone I, Rinaldi A, Scialabba S, Di Pasquale A, Cammà C, Puglia I, Calistri P, Savini G
EPI_ISL_961706	SIESP CHIETI - DRIVE IN CHIETI	Istituto Zooprofilattico Sperimentale dell'Abruzzo e Molise "G. Caporale"	Lorusso A, Marcacci M, Di Domenico M, Ancora M, Curini V, Mangone I, Rinaldi A, Scialabba S, Di Pasquale A, Cammà C, Puglia I, Calistri P, Savini G
EPI_ISL_961707, EPI_ISL_961708	SIESP DIPARTIMENTO DI PREVENZIONE CHIETI	Istituto Zooprofilattico Sperimentale dell'Abruzzo e Molise "G. Caporale"	Lorusso A, Marcacci M, Di Domenico M, Ancora M, Curini V, Mangone I, Rinaldi A, Scialabba S, Di Pasquale A, Cammà C, Puglia I, Calistri P, Savini G
EPI_ISL_961709, EPI_ISL_961710, EPI_ISL_961711	SIESP CHIETI - DRIVE IN CHIETI	Istituto Zooprofilattico Sperimentale dell'Abruzzo e Molise "G. Caporale"	Lorusso A, Marcacci M, Di Domenico M, Ancora M, Curini V, Mangone I, Rinaldi A, Scialabba S, Di Pasquale A, Cammà C, Puglia I, Calistri P, Savini G
EPI_ISL_961712, EPI_ISL_961713	SIESP CHIETI DRIVE IN LANCIANO	Istituto Zooprofilattico Sperimentale dell'Abruzzo e Molise "G. Caporale"	Lorusso A, Marcacci M, Di Domenico M, Ancora M, Curini V, Mangone I, Rinaldi A, Scialabba S, Di Pasquale A, Cammà C, Puglia I, Calistri P, Savini G
EPI_ISL_961714	SIESP CHIETI - DRIVE IN CHIETI	Istituto Zooprofilattico Sperimentale dell'Abruzzo e Molise "G. Caporale"	Lorusso A, Marcacci M, Di Domenico M, Ancora M, Curini V, Mangone I, Rinaldi A, Scialabba S, Di Pasquale A, Cammà C, Puglia I, Calistri P, Savini G
EPI_ISL_961715, EPI_ISL_961716	SIESP CHIETI - DRIVE IN ORTONA	Istituto Zooprofilattico Sperimentale dell'Abruzzo e Molise "G. Caporale"	Lorusso A, Marcacci M, Di Domenico M, Ancora M, Curini V, Mangone I, Rinaldi A, Scialabba S, Di Pasquale A, Cammà C, Puglia I, Calistri P, Savini G
EPI_ISL_961717	SIESP CHIETI - DRIVE IN CHIETI	Istituto Zooprofilattico Sperimentale dell'Abruzzo e Molise "G. Caporale"	Lorusso A, Marcacci M, Di Domenico M, Ancora M, Curini V, Mangone I, Rinaldi A, Scialabba S, Di Pasquale A, Cammà C, Puglia I, Calistri P, Savini G
EPI_ISL_961718, EPI_ISL_961719, EPI_ISL_961720, EPI_ISL_961721, EPI_ISL_961722	SIESP CHIETI - DRIVE IN ORTONA	Istituto Zooprofilattico Sperimentale dell'Abruzzo e Molise "G. Caporale"	Lorusso A, Marcacci M, Di Domenico M, Ancora M, Curini V, Mangone I, Rinaldi A, Scialabba S, Di Pasquale A, Cammà C, Puglia I, Calistri P, Savini G
EPI_ISL_961723	SIESP CHIETI DRIVE IN LANCIANO	Istituto Zooprofilattico Sperimentale dell'Abruzzo e Molise "G. Caporale"	Lorusso A, Marcacci M, Di Domenico M, Ancora M, Curini V, Mangone I, Rinaldi A, Scialabba S, Di Pasquale A, Cammà C, Puglia I, Calistri P, Savini G
EPI_ISL_961724	SIESP CHIETI - DRIVE IN ORTONA	Istituto Zooprofilattico Sperimentale dell'Abruzzo e Molise "G. Caporale"	Lorusso A, Marcacci M, Di Domenico M, Ancora M, Curini V, Mangone I, Rinaldi A, Scialabba S, Di Pasquale A, Cammà C, Puglia I, Calistri P, Savini G
EPI_ISL_961725	SIESP CHIETI DRIVE IN GISSI	Istituto Zooprofilattico Sperimentale dell'Abruzzo e Molise "G. Caporale"	Lorusso A, Marcacci M, Di Domenico M, Ancora M, Curini V, Mangone I, Rinaldi A, Scialabba S, Di Pasquale A, Cammà C, Puglia I, Calistri P, Savini G
EPI_ISL_961726, EPI_ISL_961727	SIESP CHIETI - DRIVE IN CHIETI	Istituto Zooprofilattico Sperimentale dell'Abruzzo e Molise "G. Caporale"	Lorusso A, Marcacci M, Di Domenico M, Ancora M, Curini V, Mangone I, Rinaldi A, Scialabba S, Di Pasquale A, Cammà C, Puglia I, Calistri P, Savini G
EPI_ISL_961728	SIESP DIPARTIMENTO DI PREVENZIONE CHIETI	Istituto Zooprofilattico Sperimentale dell'Abruzzo e Molise "G. Caporale"	Lorusso A, Marcacci M, Di Domenico M, Ancora M, Curini V, Mangone I, Rinaldi A, Scialabba S, Di Pasquale A, Cammà C, Puglia I, Calistri P, Savini G
EPI_ISL_961729	SIESP CHIETI - DRIVE IN CHIETI	Istituto Zooprofilattico Sperimentale dell'Abruzzo e Molise "G. Caporale"	Lorusso A, Marcacci M, Di Domenico M, Ancora M, Curini V, Mangone I, Rinaldi A, Scialabba S, Di Pasquale A, Cammà C, Puglia I, Calistri P, Savini G
EPI_ISL_961730	Dipartimento Prevenzione Avezzano-Servizio Igiene epidemiologia Sanità Pubblica	Istituto Zooprofilattico Sperimentale dell'Abruzzo e Molise "G. Caporale"	Lorusso A, Marcacci M, Di Domenico M, Ancora M, Curini V, Mangone I, Rinaldi A, Scialabba S, Di Pasquale A, Cammà C, Puglia I, Calistri P, Savini G
EPI_ISL_961731, EPI_ISL_961732	SIESP CHIETI - DRIVE IN CHIETI	Istituto Zooprofilattico Sperimentale dell'Abruzzo e Molise "G. Caporale"	Lorusso A, Marcacci M, Di Domenico M, Ancora M, Curini V, Mangone I, Rinaldi A, Scialabba S, Di Pasquale A, Cammà C, Puglia I, Calistri P, Savini G
EPI_ISL_961733	SIESP CHIETI - DRIVE IN ORTONA	Istituto Zooprofilattico Sperimentale dell'Abruzzo e Molise "G. Caporale"	Lorusso A, Marcacci M, Di Domenico M, Ancora M, Curini V, Mangone I, Rinaldi A, Scialabba S, Di Pasquale A, Cammà C, Puglia I, Calistri P, Savini G
EPI_ISL_961734	Dipartimento Prevenzione Avezzano-Servizio Igiene epidemiologia Sanità Pubblica	Istituto Zooprofilattico Sperimentale dell'Abruzzo e Molise "G. Caporale"	Lorusso A, Marcacci M, Di Domenico M, Ancora M, Curini V, Mangone I, Rinaldi A, Scialabba S, Di Pasquale A, Cammà C, Puglia I, Calistri P, Savini G
EPI_ISL_961735, EPI_ISL_961736	SIESP CHIETI - DRIVE IN CHIETI	Istituto Zooprofilattico Sperimentale dell'Abruzzo e Molise "G. Caporale"	Lorusso A, Marcacci M, Di Domenico M, Ancora M, Curini V, Mangone I, Rinaldi A, Scialabba S, Di Pasquale A, Cammà C, Puglia I, Calistri P, Savini G
EPI_ISL_961737	SIESP CHIETI- DRIVE IN VASTO	Istituto Zooprofilattico Sperimentale dell'Abruzzo e Molise "G. Caporale"	Lorusso A, Marcacci M, Di Domenico M, Ancora M, Curini V, Mangone I, Rinaldi A, Scialabba S, Di Pasquale A, Cammà C, Puglia I, Calistri P, Savini G
EPI_ISL_961738, EPI_ISL_961739	SIESP CHIETI DRIVE IN LANCIANO	Istituto Zooprofilattico Sperimentale dell'Abruzzo e Molise "G. Caporale"	Lorusso A, Marcacci M, Di Domenico M, Ancora M, Curini V, Mangone I, Rinaldi A, Scialabba S, Di Pasquale A, Cammà C, Puglia I, Calistri P, Savini G
EPI_ISL_961740	SIESP CHIETI - DRIVE IN CHIETI	Istituto Zooprofilattico Sperimentale dell'Abruzzo e Molise "G. Caporale"	Lorusso A, Marcacci M, Di Domenico M, Ancora M, Curini V, Mangone I, Rinaldi A, Scialabba S, Di Pasquale A, Cammà C, Puglia I, Calistri P, Savini G
EPI_ISL_961741	SIESP CHIETI- DRIVE IN VASTO	Istituto Zooprofilattico Sperimentale dell'Abruzzo e Molise "G. Caporale"	Lorusso A, Marcacci M, Di Domenico M, Ancora M, Curini V, Mangone I, Rinaldi A, Scialabba S, Di Pasquale A, Cammà C, Puglia I, Calistri P, Savini G
EPI_ISL_961742	SIESP CHIETI - DRIVE IN CHIETI	Istituto Zooprofilattico Sperimentale dell'Abruzzo e Molise "G. Caporale"	Lorusso A, Marcacci M, Di Domenico M, Ancora M, Curini V, Mangone I, Rinaldi A, Scialabba S, Di Pasquale A, Cammà C, Puglia I, Calistri P, Savini G
EPI_ISL_961743	SIESP CHIETI DRIVE IN GISSI	Istituto Zooprofilattico Sperimentale dell'Abruzzo e Molise "G. Caporale"	Lorusso A, Marcacci M, Di Domenico M, Ancora M, Curini V, Mangone I, Rinaldi A, Scialabba S, Di Pasquale A, Cammà C, Puglia I, Calistri P, Savini G
EPI_ISL_961744	SIESP CHIETI - DRIVE IN ORTONA	Istituto Zooprofilattico Sperimentale dell'Abruzzo e Molise "G. Caporale"	Lorusso A, Marcacci M, Di Domenico M, Ancora M, Curini V, Mangone I, Rinaldi A, Scialabba S, Di Pasquale A, Cammà C, Puglia I, Calistri P, Savini G
EPI_ISL_961745	SIESP CHIETI DRIVE IN GISSI	Istituto Zooprofilattico Sperimentale dell'Abruzzo e Molise "G. Caporale"	Lorusso A, Marcacci M, Di Domenico M, Ancora M, Curini V, Mangone I, Rinaldi A, Scialabba S, Di Pasquale A, Cammà C, Puglia I, Calistri P, Savini G

EPI_ISL_961746	SIESP CHIETI - DRIVE IN CHIETI	Istituto Zooprofilattico Sperimentale dell'Abruzzo e Molise "G. Caporale"	Lorusso A, Marcacci M, Di Domenico M, Ancora M, Curini V, Mangone I, Rinaldi A, Scialabba S, Di Pasquale A, Cammà C, Puglia I, Calistri P, Savini G
EPI_ISL_961748	SIESP CHIETI DRIVE IN LANCIANO	Istituto Zooprofilattico Sperimentale dell'Abruzzo e Molise "G. Caporale"	Lorusso A, Marcacci M, Di Domenico M, Ancora M, Curini V, Mangone I, Rinaldi A, Scialabba S, Di Pasquale A, Cammà C, Puglia I, Calistri P, Savini G
EPI_ISL_961749	SIESP DIPARTIMENTO DI PREVENZIONE CHIETI	Istituto Zooprofilattico Sperimentale dell'Abruzzo e Molise "G. Caporale"	Lorusso A, Marcacci M, Di Domenico M, Ancora M, Curini V, Mangone I, Rinaldi A, Scialabba S, Di Pasquale A, Cammà C, Puglia I, Calistri P, Savini G
EPI_ISL_961750	Ospedale Cvile Atri Med. Interna	Istituto Zooprofilattico Sperimentale dell'Abruzzo e Molise "G. Caporale"	Lorusso A, Marcacci M, Di Domenico M, Ancora M, Curini V, Mangone I, Rinaldi A, Scialabba S, Di Pasquale A, Cammà C, Puglia I, Calistri P, Savini G
EPI_ISL_961751	SIESP DIPARTIMENTO DI PREVENZIONE CHIETI	Istituto Zooprofilattico Sperimentale dell'Abruzzo e Molise "G. Caporale"	Lorusso A, Marcacci M, Di Domenico M, Ancora M, Curini V, Mangone I, Rinaldi A, Scialabba S, Di Pasquale A, Cammà C, Puglia I, Calistri P, Savini G
EPI_ISL_962180	unknown	Public Health Virology-Forensic and Scientific Services (PHV-FSS)	Son Nguyen et al.
EPI_ISL_965025	S.C. Microbiologia e Virologia Laboratorio Virologia -Speciale Centro Influenza - AOU di Sassari - Viale san Pietro 43/B Palazzo Infettivologia	Laboratorio specialistico UOC Ematologia - Ospedale "San Francesco" - ATS-ASSL Nuoro	Piras Giovanna, Malune Paolo, Asproni Rosanna, Monne Maria Itria, Palmas Angelo Domenico Serra Caterina, Rimini Elena, Rubino Salvatore
EPI_ISL_965028, EPI_ISL_965114	Laboratorio Biologia Molecolare Sars Cov2 - UOC Laboratorio Analisi - Servizio Medicina di Laboratorio, Ospedale "San Francesco" - ATS-ASSL Nuoro	Laboratorio specialistico UOC Ematologia - Ospedale "San Francesco" - ATS-ASSL Nuoro	Piras Giovanna, Asproni Rosanna, Malune Paolo, Fiamma Maura, Monne Maria Itria, Palmas Angelo Domenico, Lo Maglio Iana, Mameli Giuseppe
EPI_ISL_965223	SIESP SULMONA	Istituto Zooprofilattico Sperimentale dell'Abruzzo e Molise "G. Caporale"	Lorusso A, Marcacci M, Di Domenico M, Ancora M, Curini V, Mangone I, Rinaldi A, Scialabba S, Di Pasquale A, Cammà C, Puglia I, Calistri P, Savini G
EPI_ISL_965225, EPI_ISL_965226	USCA SULMONA	Istituto Zooprofilattico Sperimentale dell'Abruzzo e Molise "G. Caporale"	Lorusso A, Marcacci M, Di Domenico M, Ancora M, Curini V, Mangone I, Rinaldi A, Scialabba S, Di Pasquale A, Cammà C, Puglia I, Calistri P, Savini G
EPI_ISL_965227, EPI_ISL_965228, EPI_ISL_965229	SIESP CHIETI - DRIVE IN CHIETI	Istituto Zooprofilattico Sperimentale dell'Abruzzo e Molise "G. Caporale"	Lorusso A, Marcacci M, Di Domenico M, Ancora M, Curini V, Mangone I, Rinaldi A, Scialabba S, Di Pasquale A, Cammà C, Puglia I, Calistri P, Savini G
EPI_ISL_965230	SIESP TERAMO DIP PREV	Istituto Zooprofilattico Sperimentale dell'Abruzzo e Molise "G. Caporale"	Lorusso A, Marcacci M, Di Domenico M, Ancora M, Curini V, Mangone I, Rinaldi A, Scialabba S, Di Pasquale A, Cammà C, Puglia I, Calistri P, Savini G
EPI_ISL_965231	Ospedale San Salvatore UOC MED INT.	Istituto Zooprofilattico Sperimentale dell'Abruzzo e Molise "G. Caporale"	Lorusso A, Marcacci M, Di Domenico M, Ancora M, Curini V, Mangone I, Rinaldi A, Scialabba S, Di Pasquale A, Cammà C, Puglia I, Calistri P, Savini G
EPI_ISL_965232, EPI_ISL_965233	SIESP CHIETI - DRIVE IN CHIETI	Istituto Zooprofilattico Sperimentale dell'Abruzzo e Molise "G. Caporale"	Lorusso A, Marcacci M, Di Domenico M, Ancora M, Curini V, Mangone I, Rinaldi A, Scialabba S, Di Pasquale A, Cammà C, Puglia I, Calistri P, Savini G
EPI_ISL_965234	SIESP SULMONA	Istituto Zooprofilattico Sperimentale dell'Abruzzo e Molise "G. Caporale"	Lorusso A, Marcacci M, Di Domenico M, Ancora M, Curini V, Mangone I, Rinaldi A, Scialabba S, Di Pasquale A, Cammà C, Puglia I, Calistri P, Savini G
EPI_ISL_965235	SIESP TERAMO DIP PREV	Istituto Zooprofilattico Sperimentale dell'Abruzzo e Molise "G. Caporale"	Lorusso A, Marcacci M, Di Domenico M, Ancora M, Curini V, Mangone I, Rinaldi A, Scialabba S, Di Pasquale A, Cammà C, Puglia I, Calistri P, Savini G
EPI_ISL_965236, EPI_ISL_965237, EPI_ISL_965238, EPI_ISL_965239	SIESP CHIETI - DRIVE IN CHIETI	Istituto Zooprofilattico Sperimentale dell'Abruzzo e Molise "G. Caporale"	Lorusso A, Marcacci M, Di Domenico M, Ancora M, Curini V, Mangone I, Rinaldi A, Scialabba S, Di Pasquale A, Cammà C, Puglia I, Calistri P, Savini G
EPI_ISL_965240	SIESP CHIETI	Istituto Zooprofilattico Sperimentale dell'Abruzzo e Molise "G. Caporale"	Lorusso A, Marcacci M, Di Domenico M, Ancora M, Curini V, Mangone I, Rinaldi A, Scialabba S, Di Pasquale A, Cammà C, Puglia I, Calistri P, Savini G
EPI_ISL_965241, EPI_ISL_965242	USCA SULMONA	Istituto Zooprofilattico Sperimentale dell'Abruzzo e Molise "G. Caporale"	Lorusso A, Marcacci M, Di Domenico M, Ancora M, Curini V, Mangone I, Rinaldi A, Scialabba S, Di Pasquale A, Cammà C, Puglia I, Calistri P, Savini G
EPI_ISL_965243, EPI_ISL_965244	SIESP CHIETI-DRIVE IN ORTONA	Istituto Zooprofilattico Sperimentale dell'Abruzzo e Molise "G. Caporale"	Lorusso A, Marcacci M, Di Domenico M, Ancora M, Curini V, Mangone I, Rinaldi A, Scialabba S, Di Pasquale A, Cammà C, Puglia I, Calistri P, Savini G
EPI_ISL_965253	SIESP CHIETI	Istituto Zooprofilattico Sperimentale dell'Abruzzo e Molise "G. Caporale"	Lorusso A, Marcacci M, Di Domenico M, Ancora M, Curini V, Mangone I, Rinaldi A, Scialabba S, Di Pasquale A, Cammà C, Puglia I, Calistri P, Savini G
EPI_ISL_965254, EPI_ISL_965255	SIESP CHIETI - DRIVE IN CHIETI	Istituto Zooprofilattico Sperimentale dell'Abruzzo e Molise "G. Caporale"	Lorusso A, Marcacci M, Di Domenico M, Ancora M, Curini V, Mangone I, Rinaldi A, Scialabba S, Di Pasquale A, Cammà C, Puglia I, Calistri P, Savini G
EPI_ISL_965256	SIESP CHIETI-DRIVE IN VASTO	Istituto Zooprofilattico Sperimentale dell'Abruzzo e Molise "G. Caporale"	Lorusso A, Marcacci M, Di Domenico M, Ancora M, Curini V, Mangone I, Rinaldi A, Scialabba S, Di Pasquale A, Cammà C, Puglia I, Calistri P, Savini G
EPI_ISL_965257	SIESP CHIETI-DRIVE IN GISSI	Istituto Zooprofilattico Sperimentale dell'Abruzzo e Molise "G. Caporale"	Lorusso A, Marcacci M, Di Domenico M, Ancora M, Curini V, Mangone I, Rinaldi A, Scialabba S, Di Pasquale A, Cammà C, Puglia I, Calistri P, Savini G
EPI_ISL_965258, EPI_ISL_965259	SIESP CHIETI-DRIVE IN VASTO	Istituto Zooprofilattico Sperimentale dell'Abruzzo e Molise "G. Caporale"	Lorusso A, Marcacci M, Di Domenico M, Ancora M, Curini V, Mangone I, Rinaldi A, Scialabba S, Di Pasquale A, Cammà C, Puglia I, Calistri P, Savini G
EPI_ISL_965260	SIESP CHIETI-DRIVE IN GISSI	Istituto Zooprofilattico Sperimentale dell'Abruzzo e Molise "G. Caporale"	Lorusso A, Marcacci M, Di Domenico M, Ancora M, Curini V, Mangone I, Rinaldi A, Scialabba S, Di Pasquale A, Cammà C, Puglia I, Calistri P, Savini G
EPI_ISL_965261	SIESP CHIETI	Istituto Zooprofilattico Sperimentale dell'Abruzzo e Molise "G. Caporale"	Lorusso A, Marcacci M, Di Domenico M, Ancora M, Curini V, Mangone I, Rinaldi A, Scialabba S, Di Pasquale A, Cammà C, Puglia I, Calistri P, Savini G
EPI_ISL_965262	SIESP CHIETI, DISTRETTO SAN CHIETI	Istituto Zooprofilattico Sperimentale dell'Abruzzo e Molise "G. Caporale"	Lorusso A, Marcacci M, Di Domenico M, Ancora M, Curini V, Mangone I, Rinaldi A, Scialabba S, Di Pasquale A, Cammà C, Puglia I, Calistri P, Savini G
EPI_ISL_965263	SIESP CHIETI-DRIVE IN ORTONA	Istituto Zooprofilattico Sperimentale dell'Abruzzo e Molise "G. Caporale"	Lorusso A, Marcacci M, Di Domenico M, Ancora M, Curini V, Mangone I, Rinaldi A, Scialabba S, Di Pasquale A, Cammà C, Puglia I, Calistri P, Savini G
EPI_ISL_965265	SIESP CHIETI - DRIVE IN CHIETI	Istituto Zooprofilattico Sperimentale dell'Abruzzo e Molise "G. Caporale"	Lorusso A, Marcacci M, Di Domenico M, Ancora M, Curini V, Mangone I, Rinaldi A, Scialabba S, Di Pasquale A, Cammà C, Puglia I, Calistri P, Savini G
EPI_ISL_965267	SIESP CHIETI, DISTRETTON SAN CHIETI	Istituto Zooprofilattico Sperimentale dell'Abruzzo e Molise "G. Caporale"	Lorusso A, Marcacci M, Di Domenico M, Ancora M, Curini V, Mangone I, Rinaldi A, Scialabba S, Di Pasquale A, Cammà C, Puglia I, Calistri P, Savini G
EPI_ISL_965268, EPI_ISL_965269	SIESP CHIETI, DISTRETTO SAN CHIETI	Istituto Zooprofilattico Sperimentale dell'Abruzzo e Molise "G. Caporale"	Lorusso A, Marcacci M, Di Domenico M, Ancora M, Curini V, Mangone I, Rinaldi A, Scialabba S, Di Pasquale A, Cammà C, Puglia I, Calistri P, Savini G
EPI_ISL_965270	SIESP CHIETI	Istituto Zooprofilattico Sperimentale dell'Abruzzo e Molise "G. Caporale"	Lorusso A, Marcacci M, Di Domenico M, Ancora M, Curini V, Mangone I, Rinaldi A, Scialabba S, Di Pasquale A, Cammà C, Puglia I, Calistri P, Savini G
EPI_ISL_965271	SIESP CHIETI, DISTRETTO SAN CHIETI	Istituto Zooprofilattico Sperimentale dell'Abruzzo e Molise "G. Caporale"	Lorusso A, Marcacci M, Di Domenico M, Ancora M, Curini V, Mangone I, Rinaldi A, Scialabba S, Di Pasquale A, Cammà C, Puglia I, Calistri P, Savini G

EPI_ISL_965272	SIESP CHIETI	Istituto Zooprofilattico Sperimentale dell'Abruzzo e Molise "G. Caporale"	Lorusso A, Marcacci M, Di Domenico M, Ancora M, Curini V, Mangone I, Rinaldi A, Scialabba S, Di Pasquale A, Cammà C, Puglia I, Calistri P, Savini G
EPI_ISL_965273, EPI_ISL_965274, EPI_ISL_965275	SIESP CHIETI, DISTRETTO SAN CHIETI	Istituto Zooprofilattico Sperimentale dell'Abruzzo e Molise "G. Caporale"	Lorusso A, Marcacci M, Di Domenico M, Ancora M, Curini V, Mangone I, Rinaldi A, Scialabba S, Di Pasquale A, Cammà C, Puglia I, Calistri P, Savini G
EPI_ISL_965278, EPI_ISL_965279, EPI_ISL_965280, EPI_ISL_965281, EPI_ISL_965282, EPI_ISL_965283, EPI_ISL_965284, EPI_ISL_965285, EPI_ISL_965286, EPI_ISL_965287, EPI_ISL_965288, EPI_ISL_965289, EPI_ISL_965290, EPI_ISL_965291, EPI_ISL_965292, EPI_ISL_965293, EPI_ISL_965294, EPI_ISL_965295, EPI_ISL_965296, EPI_ISL_965297, EPI_ISL_965298, EPI_ISL_965299, EPI_ISL_965300			
see above	P.O.CARDARELLI	Istituto Zooprofilattico Sperimentale dell'Abruzzo e Molise "G. Caporale"	Scutellà M, Niro G, Lorusso A, Marcacci M, Di Domenico M, Ancora M, Curini V, Mangone I, Rinaldi A, Scialabba S, Di Pasquale A, Cammà C, Puglia I, Calistri P, Savini G
EPI_ISL_966245	NYU Langone Health	Departments of Pathology and Medicine, New York University School of Medicine	Adriana Heguy, Dacia Dimartino, Emily Guzman, Christian Marier, Peter Meyn, Sitharam Ramaswami, Gael Westby, Paul Zappile, Yutong Zhang, Paolo Cotzia, Guiqing Wang
EPI_ISL_966346, EPI_ISL_966347, EPI_ISL_966348, EPI_ISL_966349, EPI_ISL_966350, EPI_ISL_966351, EPI_ISL_966352, EPI_ISL_966353, EPI_ISL_966354	Kansas Health and Environmental Lab	Kansas Health and Environmental Lab	Mike Grose, Paige Drury, Carissa Robertson, Ben Olsen, and Phil Adam
EPI_ISL_966807, EPI_ISL_966808, EPI_ISL_966809, EPI_ISL_966810, EPI_ISL_966811, EPI_ISL_966812, EPI_ISL_966813, EPI_ISL_966814, EPI_ISL_966815, EPI_ISL_966816, EPI_ISL_966817, EPI_ISL_966818, EPI_ISL_966819, EPI_ISL_966820, EPI_ISL_966821, EPI_ISL_966822, EPI_ISL_966823, EPI_ISL_966824, EPI_ISL_966825, EPI_ISL_966826, EPI_ISL_966827, EPI_ISL_966828, EPI_ISL_966829, EPI_ISL_966830, EPI_ISL_966831, EPI_ISL_966832, EPI_ISL_966833, EPI_ISL_966834, EPI_ISL_966835, EPI_ISL_966836, EPI_ISL_966837, EPI_ISL_966838, EPI_ISL_966839, EPI_ISL_966840, EPI_ISL_966841, EPI_ISL_966842, EPI_ISL_966843, EPI_ISL_966844, EPI_ISL_966845, EPI_ISL_966846, EPI_ISL_966847, EPI_ISL_966848, EPI_ISL_966849, EPI_ISL_966850, EPI_ISL_966851			
see above	Maine HETL	Tewhey Lab, The Jackson Laboratory	Matluk,N., Dewey,H., Iosue,F., Barter,M., Lynch,R., Munger,H. and Tewhey,R.
EPI_ISL_967882, EPI_ISL_967883	Arizona State Public Health Laboratory	Arizona State Public Health Laboratory	Trung Huynh, Jessica Escobar, Katherine Fullerton, Nobuko Fukushima, Stacy White, Linda Getsinger, Victor Waddell
EPI_ISL_968212	PHV-FSS	PHV-FSS	Son Nguyen et al.
EPI_ISL_969131	Laboratorio Microbiologia e Virologia P.O. Cotugno A.O. dei Colli	Laboratorio Microbiologia e Virologia P.O. Cotugno A.O. dei Colli	Luigi Atripaldi, Claudia Tiberio, Anna Perfetti,
EPI_ISL_969227	Laboratorio Microbiologia e Virologia P.O. Cotugno A.O. dei Colli	Laboratorio Microbiologia e Virologia P.O. Cotugno A.O. dei Colli	Luigi Atripaldi, Claudia Tiberio, Anna Perfetti
EPI_ISL_969297	Laboratorio Microbiologia e Virologia, P.O. Cotugno, A.O. dei Colli	Laboratorio Microbiologia e Virologia, P.O. Cotugno, A.O. dei Colli	Luigi Atripaldi, Claudia Tiberio, Anna Perfetti
EPI_ISL_969456	Lighthouse Lab in Milton Keynes	Wellcome Sanger Institute for the COVID-19 Genomics UK (COG-UK) Consortium	The Lighthouse Lab in Milton Keynes and Alex Alderton, Roberto Amato, Sonia Goncalves, Ewan Harrison, David K. Jackson, Ian Johnston, Dominic Kwiatkowski, Cordelia Langford, John Sillitoe on behalf of the Wellcome Sanger Institute COVID-19 Surveillance Team
EPI_ISL_969884	Laboratorio Microbiologia e Virologia P.O. Cotugno A.O. dei Colli	Laboratorio Microbiologia e Virologia P.O. Cotugno A.O. dei Colli	Luigi Atripaldi,Claudia Tiberio, Anna Perfetti
EPI_ISL_970647	Laboratorio Microbiologia e Virologia P.O. Cotugno A.O. dei Colli	Laboratorio Microbiologia e Virologia P.O. Cotugno A.O. dei Colli	Luigi Atripaldi, Claudia Tiberio,Anna Perfetti
EPI_ISL_974745	Laboratorio Microbiologia e Virologia P.O. Cotugno A.O. dei Colli	Laboratorio Microbiologia e Virologia P.O. Cotugno A.O. dei Colli	Luigi Atripaldi, Claudia Tiberio, Anna Perfetti,
EPI_ISL_977495, EPI_ISL_977496, EPI_ISL_977497, EPI_ISL_977498	University of Bari Biomedical Sciences and Human Oncology	University of Bari Biomedical Sciences and Human Oncology	Chironna M., Sallustio A., Loconsole D., Accogli M.
EPI_ISL_977568, EPI_ISL_977569, EPI_ISL_977570, EPI_ISL_977571, EPI_ISL_977572, EPI_ISL_977573, EPI_ISL_977574	University of Michigan Clinical Microbiology Laboratory	Lauring Lab, University of Michigan, Department of Microbiology and Immunology	Valesano
EPI_ISL_977598	Laboratorio Microbiologia e Virologia P.O. Cotugno A.O. dei Colli	Laboratorio Microbiologia e Virologia P.O. Cotugno A.O. dei Colli	Luigi Atripaldi, Claudia Tiberio, Anna Perfetti
EPI_ISL_977601, EPI_ISL_977603	Laboratorio Microbiologia e Virologia P.O. Cotugno A.O. dei Colli	Laboratorio Microbiologia e Virologia P.O. Cotugno A.O. dei Colli	Luigi Atripaldi, Claudia Tiberio, Anna Perfetti,
EPI_ISL_977604, EPI_ISL_977605, EPI_ISL_977606, EPI_ISL_977607, EPI_ISL_977608, EPI_ISL_977609, EPI_ISL_977610, EPI_ISL_977611	SC (UCO) Igiene e Sanità Pubblica (funzione integrata con SC Microbiologia e Virologia) e Laboratory of Molecular Virology of the International Centre for Genetic Engineering and Biotechnology (ICGEB)	ARGO Laboratorio Genomica ed Epigenomica	Licastro D, Dal Monego S, Degasperi M, Marcello A, D'Agaro P, De Rosa R
EPI_ISL_977612, EPI_ISL_977616, EPI_ISL_977617, EPI_ISL_977618, EPI_ISL_977619	SC (UCO) Igiene e Sanità Pubblica (funzione integrata con SC Microbiologia e Virologia) e Laboratory of Molecular Virology of the International Centre for Genetic Engineering and Biotechnology (ICGEB)	ARGO Laboratorio Genomica ed Epigenomica	Licastro D, Dal Monego S, Degasperi M, Marcello A, D'Agaro P, Pipan C
EPI_ISL_977621	SC (UCO) Igiene e Sanità Pubblica (funzione integrata con SC Microbiologia e Virologia) e Laboratory of Molecular Virology of the International Centre for Genetic Engineering and Biotechnology (ICGEB)	ARGO Laboratorio Genomica ed Epigenomica	Licastro D, Dal Monego S, Degasperi M, Marcello A, D'Agaro P
EPI_ISL_977622, EPI_ISL_977623, EPI_ISL_977624, EPI_ISL_977625, EPI_ISL_977626, EPI_ISL_977627, EPI_ISL_977628, EPI_ISL_977629, EPI_ISL_977630, EPI_ISL_977631, EPI_ISL_977632, EPI_ISL_977633, EPI_ISL_977634, EPI_ISL_977635, EPI_ISL_977636, EPI_ISL_977637, EPI_ISL_977638, EPI_ISL_977639, EPI_ISL_977640			
see above	SC (UCO) Igiene e Sanità Pubblica (funzione integrata con SC Microbiologia e Virologia) e Laboratory of Molecular Virology of the International Centre for Genetic Engineering and Biotechnology (ICGEB)	ARGO Laboratorio Genomica ed Epigenomica	Licastro D, Dal Monego S, Degasperi M, Marcello A, Segat L, Piscianz E, D'Agaro P
EPI_ISL_977641, EPI_ISL_977642, EPI_ISL_977643, EPI_ISL_977644, EPI_ISL_977645, EPI_ISL_977646, EPI_ISL_977647, EPI_ISL_977648, EPI_ISL_977649, EPI_ISL_977650	SC (UCO) Igiene e Sanità Pubblica (funzione integrata con SC Microbiologia e Virologia) e Laboratory of Molecular Virology of the International Centre for Genetic Engineering and Biotechnology (ICGEB)	ARGO Laboratorio Genomica ed Epigenomica	Licastro D, Dal Monego S, Degasperi M, Marcello A, D'Agaro P, Lombardo F
EPI_ISL_977651	Laboratorio Microbiologia e Virologia P.O. Cotugno A.O. dei Colli	Laboratorio Microbiologia e Virologia P.O. Cotugno A.O. dei Colli	Luigi Atripaldi, Claudia Tiberio, Anna Perfetti,
EPI_ISL_978788, EPI_ISL_978789, EPI_ISL_978790, EPI_ISL_978791, EPI_ISL_978792, EPI_ISL_978793, EPI_ISL_978794, EPI_ISL_978795, EPI_ISL_978796, EPI_ISL_978797, EPI_ISL_978798, EPI_ISL_978799, EPI_ISL_978800, EPI_ISL_978801, EPI_ISL_978802, EPI_ISL_978803, EPI_ISL_978804, EPI_ISL_978805, EPI_ISL_978806, EPI_ISL_978807, EPI_ISL_978808, EPI_ISL_978809, EPI_ISL_978810, EPI_ISL_978812, EPI_ISL_978813, EPI_ISL_978814, EPI_ISL_978815, EPI_ISL_978816, EPI_ISL_978817, EPI_ISL_978818, EPI_ISL_978819, EPI_ISL_978820, EPI_ISL_978821, EPI_ISL_978822, EPI_ISL_978823, EPI_ISL_978824, EPI_ISL_978825, EPI_ISL_978826, EPI_ISL_978827, EPI_ISL_978828, EPI_ISL_978829, EPI_ISL_978830, EPI_ISL_978831, EPI_ISL_978832, EPI_ISL_978833, EPI_ISL_978834, EPI_ISL_978835, EPI_ISL_978836, EPI_ISL_978837, EPI_ISL_978838, EPI_ISL_978839, EPI_ISL_978840, EPI_ISL_978841, EPI_ISL_978842,			

EPI_ISL_978843, EPI_ISL_978844, EPI_ISL_978845, EPI_ISL_978846, EPI_ISL_978847, EPI_ISL_978848, EPI_ISL_978849, EPI_ISL_978850, EPI_ISL_978851, EPI_ISL_978852, EPI_ISL_978853, EPI_ISL_978854, EPI_ISL_978855, EPI_ISL_978856, EPI_ISL_978857, EPI_ISL_978858, EPI_ISL_978859, EPI_ISL_978860, EPI_ISL_978861, EPI_ISL_978862, EPI_ISL_978863, EPI_ISL_978864, EPI_ISL_978865, EPI_ISL_978866, EPI_ISL_978868, EPI_ISL_978869, EPI_ISL_978870, EPI_ISL_978871, EPI_ISL_978872, EPI_ISL_978873, EPI_ISL_978874, EPI_ISL_978875, EPI_ISL_978876, EPI_ISL_978877, EPI_ISL_978878, EPI_ISL_978879			
see above	AZDelta	AZDelta	Geert Martens; Dieter De Smet
EPI_ISL_979360, EPI_ISL_979361	Microbiological Diagnostic Unit - Public Health Laboratory (MDU-PHL)	MDU-PHL	Seemann T., Sait, M.L., Sherry, N.L.
EPI_ISL_979362, EPI_ISL_979363, EPI_ISL_979364	Victorian Infectious Diseases Reference Laboratory (VIDRL)	VIDRL and MDU-PHL	Caly L., Seemann T., Sait, M.L., Druce J., Sherry, N.L.
EPI_ISL_979968, EPI_ISL_979969	National Institute of Infectious Diseases-Prof. Dr. Matei Bals Molecular Diagnostics Laboratory	National Institute of Infectious Diseases-Prof. Dr. Matei Bals Molecular Diagnostics Laboratory	Leontina Banica, Marius Surleac, Corina Casangiu, Petre Milu, Andreea Tudor, Simona Paraschiv, Dan Otelea
EPI_ISL_980235, EPI_ISL_980236, EPI_ISL_980237, EPI_ISL_980238, EPI_ISL_980239, EPI_ISL_980240, EPI_ISL_980241, EPI_ISL_980242, EPI_ISL_980243, EPI_ISL_980244, EPI_ISL_980245, EPI_ISL_980246, EPI_ISL_980247, EPI_ISL_980248, EPI_ISL_980249, EPI_ISL_980250, EPI_ISL_980251, EPI_ISL_980252, EPI_ISL_980253, EPI_ISL_980254, EPI_ISL_980255, EPI_ISL_980256, EPI_ISL_980257, EPI_ISL_980258, EPI_ISL_980259, EPI_ISL_980260, EPI_ISL_980261, EPI_ISL_980262, EPI_ISL_980263, EPI_ISL_980264, EPI_ISL_980265, EPI_ISL_980266, EPI_ISL_980267, EPI_ISL_980268, EPI_ISL_980269, EPI_ISL_980270, EPI_ISL_980271, EPI_ISL_980272, EPI_ISL_980273, EPI_ISL_980274, EPI_ISL_980275, EPI_ISL_980276, EPI_ISL_980277, EPI_ISL_980278, EPI_ISL_980279, EPI_ISL_980281, EPI_ISL_980282, EPI_ISL_980283, EPI_ISL_980284, EPI_ISL_980285, EPI_ISL_980286, EPI_ISL_980287, EPI_ISL_980288, EPI_ISL_980289, EPI_ISL_980290, EPI_ISL_980291, EPI_ISL_980292, EPI_ISL_980293, EPI_ISL_980294, EPI_ISL_980295, EPI_ISL_980296, EPI_ISL_980297, EPI_ISL_980298, EPI_ISL_980299, EPI_ISL_980300, EPI_ISL_980301, EPI_ISL_980302, EPI_ISL_980303, EPI_ISL_980304, EPI_ISL_980305, EPI_ISL_980306, EPI_ISL_980307, EPI_ISL_980308, EPI_ISL_980309, EPI_ISL_980310, EPI_ISL_980311, EPI_ISL_980312, EPI_ISL_980313, EPI_ISL_980314, EPI_ISL_980315, EPI_ISL_980316, EPI_ISL_980317, EPI_ISL_980318, EPI_ISL_980319, EPI_ISL_980320, EPI_ISL_980321, EPI_ISL_980322, EPI_ISL_980323, EPI_ISL_980324, EPI_ISL_980325, EPI_ISL_980326, EPI_ISL_980327, EPI_ISL_980328, EPI_ISL_980329, EPI_ISL_980330, EPI_ISL_980331, EPI_ISL_980332, EPI_ISL_980333, EPI_ISL_980334, EPI_ISL_980335, EPI_ISL_980336, EPI_ISL_980337, EPI_ISL_980338, EPI_ISL_980339, EPI_ISL_980340, EPI_ISL_980341, EPI_ISL_980342, EPI_ISL_980343, EPI_ISL_980344, EPI_ISL_980345, EPI_ISL_980346, EPI_ISL_980347, EPI_ISL_980348, EPI_ISL_980349, EPI_ISL_980350, EPI_ISL_980351, EPI_ISL_980352, EPI_ISL_980353, EPI_ISL_980354, EPI_ISL_980355, EPI_ISL_980357, EPI_ISL_980358, EPI_ISL_980359, EPI_ISL_980360, EPI_ISL_980361, EPI_ISL_980362, EPI_ISL_980363, EPI_ISL_980364, EPI_ISL_980365, EPI_ISL_980366, EPI_ISL_980367, EPI_ISL_980368, EPI_ISL_980369, EPI_ISL_980370, EPI_ISL_980371, EPI_ISL_980372, EPI_ISL_980373, EPI_ISL_980374, EPI_ISL_980375, EPI_ISL_980376, EPI_ISL_980377, EPI_ISL_980378, EPI_ISL_980379, EPI_ISL_980380, EPI_ISL_980381, EPI_ISL_980382, EPI_ISL_980383, EPI_ISL_980384, EPI_ISL_980385, EPI_ISL_980386, EPI_ISL_980387, EPI_ISL_980388, EPI_ISL_980389, EPI_ISL_980390, EPI_ISL_980391, EPI_ISL_980392, EPI_ISL_980393, EPI_ISL_980394, EPI_ISL_980395, EPI_ISL_980396, EPI_ISL_980397, EPI_ISL_980398, EPI_ISL_980399, EPI_ISL_980400, EPI_ISL_980401, EPI_ISL_980402, EPI_ISL_980403, EPI_ISL_980404, EPI_ISL_980405, EPI_ISL_980406, EPI_ISL_980407, EPI_ISL_980408, EPI_ISL_980409, EPI_ISL_980410, EPI_ISL_980411, EPI_ISL_980412, EPI_ISL_980413, EPI_ISL_980414, EPI_ISL_980415, EPI_ISL_980417, EPI_ISL_980418, EPI_ISL_980420, EPI_ISL_980421, EPI_ISL_980422, EPI_ISL_980423, EPI_ISL_980424, EPI_ISL_980425, EPI_ISL_980426, EPI_ISL_980427, EPI_ISL_980428, EPI_ISL_980429, EPI_ISL_980430, EPI_ISL_980431, EPI_ISL_980432, EPI_ISL_980434, EPI_ISL_980435, EPI_ISL_980436, EPI_ISL_980437, EPI_ISL_980438, EPI_ISL_980439, EPI_ISL_980440, EPI_ISL_980441, EPI_ISL_980442, EPI_ISL_980443, EPI_ISL_980444, EPI_ISL_980445, EPI_ISL_980446, EPI_ISL_980447, EPI_ISL_980448, EPI_ISL_980450, EPI_ISL_980451, EPI_ISL_980452, EPI_ISL_980453, EPI_ISL_980454, EPI_ISL_980455, EPI_ISL_980456, EPI_ISL_980457, EPI_ISL_980458, EPI_ISL_980459, EPI_ISL_980460, EPI_ISL_980461, EPI_ISL_980462, EPI_ISL_980463, EPI_ISL_980464, EPI_ISL_980465, EPI_ISL_980466, EPI_ISL_980467, EPI_ISL_980468, EPI_ISL_980469, EPI_ISL_980470, EPI_ISL_980471, EPI_ISL_980472, EPI_ISL_980473, EPI_ISL_980474, EPI_ISL_980475, EPI_ISL_980476, EPI_ISL_980477, EPI_ISL_980478, EPI_ISL_980479, EPI_ISL_980480, EPI_ISL_980481, EPI_ISL_980482, EPI_ISL_980483, EPI_ISL_980484, EPI_ISL_980485, EPI_ISL_980486, EPI_ISL_980487, EPI_ISL_980488, EPI_ISL_980489, EPI_ISL_980490, EPI_ISL_980491, EPI_ISL_980492, EPI_ISL_980493, EPI_ISL_980494, EPI_ISL_980495, EPI_ISL_980496, EPI_ISL_980497, EPI_ISL_980498, EPI_ISL_980499, EPI_ISL_980500, EPI_ISL_980501, EPI_ISL_980502, EPI_ISL_980503, EPI_ISL_980504, EPI_ISL_980505, EPI_ISL_980506, EPI_ISL_980507, EPI_ISL_980508, EPI_ISL_980509, EPI_ISL_980510, EPI_ISL_980511, EPI_ISL_980513, EPI_ISL_980514, EPI_ISL_980515, EPI_ISL_980516, EPI_ISL_980517, EPI_ISL_980518, EPI_ISL_980519, EPI_ISL_980520, EPI_ISL_980521, EPI_ISL_980522, EPI_ISL_980523, EPI_ISL_980524, EPI_ISL_980525, EPI_ISL_980526, EPI_ISL_980527, EPI_ISL_980528, EPI_ISL_980529, EPI_ISL_980530, EPI_ISL_980531, EPI_ISL_980532, EPI_ISL_980533, EPI_ISL_980534, EPI_ISL_980535, EPI_ISL_980536, EPI_ISL_980537, EPI_ISL_980538, EPI_ISL_980539, EPI_ISL_980540, EPI_ISL_980541, EPI_ISL_980542, EPI_ISL_980543, EPI_ISL_980544, EPI_ISL_980545, EPI_ISL_980546, EPI_ISL_980547, EPI_ISL_980548, EPI_ISL_980549, EPI_ISL_980550			
see above	Lighthouse Lab in Cambridge	Wellcome Sanger Institute for the COVID-19 Genomics UK (COG-UK) Consortium	Rob Howes, The Lighthouse Lab in Cambridge and Alex Alderton, Roberto Amato, Sonia Goncalves, Ewan Harrison, David K. Jackson, Ian Johnston, Dominic Kwiatkowski, Cordelia Langford, John Sillitoe on behalf of the Wellcome Sanger Institute COVID-19 Surveillance Team
EPI_ISL_981004	Thai Red Cross Emerging Infectious Diseases Health Science Centre, Chulalongkorn Hospital, Faculty of Medicine, Chulalongkorn University	Thai Red Cross Emerging Infectious Diseases Center and Faculty of Medicine, Chulalongkorn University	Rome Buathong, Wichai Thanasopon, Sopon Iamsirithaworn, Opass Putcharoen,Sininat Petcharat, Yutthana Joyjinda, Weenassarin Ampoot, Apaporn Rodpan, Thiravat Hemachudha, Supaporn Wacharapluesadee
EPI_ISL_981007, EPI_ISL_981008, EPI_ISL_981009, EPI_ISL_981010, EPI_ISL_981011, EPI_ISL_981012, EPI_ISL_981013, EPI_ISL_981014, EPI_ISL_981015, EPI_ISL_981016, EPI_ISL_981017, EPI_ISL_981018, EPI_ISL_981019, EPI_ISL_981020, EPI_ISL_981021, EPI_ISL_981022, EPI_ISL_981023, EPI_ISL_981024, EPI_ISL_981025, EPI_ISL_981026, EPI_ISL_981027, EPI_ISL_981028, EPI_ISL_981029			
see above	National Public Health Laboratory, National Centre for Infectious Diseases	National Public Health Laboratory, National Centre for Infectious Diseases	Tze Minn Mak, Zhenyang Zhou, Lin Cui, Raymond Tzer Pin Lin
EPI_ISL_981077, EPI_ISL_981088, EPI_ISL_981093, EPI_ISL_981094, EPI_ISL_981095, EPI_ISL_981097, EPI_ISL_981100, EPI_ISL_981114, EPI_ISL_981115, EPI_ISL_981116, EPI_ISL_981117, EPI_ISL_981120, EPI_ISL_981121, EPI_ISL_981128, EPI_ISL_981129, EPI_ISL_981130, EPI_ISL_981131, EPI_ISL_981132, EPI_ISL_981135, EPI_ISL_981158, EPI_ISL_981159, EPI_ISL_981160, EPI_ISL_981161, EPI_ISL_981162, EPI_ISL_981163, EPI_ISL_981164, EPI_ISL_981165, EPI_ISL_981166, EPI_ISL_981167, EPI_ISL_981168, EPI_ISL_981169, EPI_ISL_981170, EPI_ISL_981171, EPI_ISL_981172, EPI_ISL_981173, EPI_ISL_981175, EPI_ISL_981191, EPI_ISL_981192, EPI_ISL_981193, EPI_ISL_981194, EPI_ISL_981195, EPI_ISL_981196, EPI_ISL_981197, EPI_ISL_981198, EPI_ISL_981199, EPI_ISL_981200, EPI_ISL_981201			
see above	Johns Hopkins Hospital Department of Pathology	Johns Hopkins Hospital Department of Pathology	C. Paul Morris, Chun Huai Luo, Adannaya Amadi, Matthew Schwartz, Nicholas Gallagher, Heba H. Mostafa
EPI_ISL_981372	Laborarory for Respiratory Viruses, "Cantacuzino" National Military-Medical Institute for Resararch and Development	Cantacuzino Institute Virology	Luiza Ustea, Nicoleta Paraschiv, Catalina Pascu, Mihaela Lazar
EPI_ISL_981374	Laboratory for Respiratory Viruses, Cantacuzino National Military-Medical Institute for Research and Development	Cantacuzino Institute Virology	Luiza Ustea, Nicoleta Paraschiv, Catalina Pascu, Mihaela Lazar
EPI_ISL_981551, EPI_ISL_981557, EPI_ISL_981611, EPI_ISL_981612, EPI_ISL_981613, EPI_ISL_981614, EPI_ISL_981615, EPI_ISL_981616, EPI_ISL_981617, EPI_ISL_981618, EPI_ISL_981619, EPI_ISL_981620, EPI_ISL_981621, EPI_ISL_981622, EPI_ISL_981623, EPI_ISL_981624, EPI_ISL_981625, EPI_ISL_981626, EPI_ISL_981627, EPI_ISL_981628, EPI_ISL_981629, EPI_ISL_981630, EPI_ISL_981631, EPI_ISL_981632, EPI_ISL_981633, EPI_ISL_981634, EPI_ISL_981635, EPI_ISL_981636, EPI_ISL_981637, EPI_ISL_981638, EPI_ISL_981639, EPI_ISL_981640, EPI_ISL_981641, EPI_ISL_981644, EPI_ISL_981645, EPI_ISL_981646, EPI_ISL_981647, EPI_ISL_981648, EPI_ISL_981649, EPI_ISL_981690, EPI_ISL_981691, EPI_ISL_981692, EPI_ISL_981693, EPI_ISL_981694, EPI_ISL_981695, EPI_ISL_981705, EPI_ISL_981710, EPI_ISL_981711, EPI_ISL_981712, EPI_ISL_981718, EPI_ISL_981720, EPI_ISL_981808, EPI_ISL_981809, EPI_ISL_981810, EPI_ISL_981811, EPI_ISL_981812, EPI_ISL_981814, EPI_ISL_981815, EPI_ISL_981816, EPI_ISL_981817, EPI_ISL_981818, EPI_ISL_981819, EPI_ISL_981820, EPI_ISL_981821, EPI_ISL_981822, EPI_ISL_981823, EPI_ISL_981824, EPI_ISL_981825, EPI_ISL_981826, EPI_ISL_981836			
see above	University Hospitals of Geneva, Laboratory of Virology	HUG, Laboratory of Virology and the Health2030 Genome Center	Samuel Cordey, Ana Rita Goncalves, Laurent Kaiser, Lorenzo Cerutti, Henri Pegeot, Melyssa Elies, Deborah Penet, Keith Harshman, Ioannis Xenarios, Emmanouil Dermitzakis
EPI_ISL_983328	Azienda Ospedaliera San Camillo Forlanini	INMI Lazzaro Spallanzani IRCCS	E Giombini, M. Rueca, B Bartolini, O Butera, C.E.M Gruber, F Messina, G Parisi, ML Guarino, A Di Caro, MR Capobianchi
EPI_ISL_983329	Istituto Zooprofilattico Sperimentale Lazio e Toscana "M. Aleandri"	INMI Lazzaro Spallanzani IRCCS	CEM Gruber, B Bartolini, E Giombini, M Rueca, O Butera, F Messina, MT Scicluna, G Manna, A Cersini, A Di Caro, MR Capobianchi
EPI_ISL_983632, EPI_ISL_983633, EPI_ISL_983634, EPI_ISL_983635, EPI_ISL_983636, EPI_ISL_983637, EPI_ISL_983638, EPI_ISL_983639	University of Michigan Clinical Microbiology Laboratory	Lauring Lab, University of Michigan, Department of Microbiology and Immunology	Valesano
EPI_ISL_983736, EPI_ISL_983737	Colorado Department of Public Health and Environment	Colorado Department of Puplic Health and Environment	Laura Bankers, Molly C. Hetherington-Rauth, Diana Ir, Shannon Ely, Shannon R. Matzinger, Sarah Elizabeth Totten, Emily A. Travanty
