# Supplementary material for: Detection and characterization of the SARS-CoV-2 lineage B.1.526 in New York
Source: Nat Commun. 2021 Aug 9;12:4886. doi: 10.1038/s41467-021-25168-4 (PMC8352861; doi:10.1038/s41467-021-25168-4)
Supplement: Supplementary file 8 — Supplementary Data 4 [file 41467_2021_25168_MOESM8_ESM.zip › GISAID_acknowledements_tables/gisaid_hcov-19_acknowledgement_table_2021_02_13_010-8.pdf]

We gratefully acknowledge the following Authors from the Originating laboratories responsible for obtaining the specimens, as well as the Submitting laboratories where the genome data were generated and shared via GISAID, on which this research is based.

All Submitters of data may be contacted directly via www.gisaid.org

Authors are sorted alphabetically.

Accession ID	Originating Laboratory	Submitting Laboratory	Authors
EPI_ISL_791422, EPI_ISL_791423, EPI_ISL_791425, EPI_ISL_791429	Johns Hopkins Hospital Department of Pathology	Johns Hopkins Hospital Department of Pathology	C. Paul Morris, Chun Huai Luo, Adannaya Amadi, Nicholas Gallagher, Heba H. Mostafa
EPI_ISL_792090	Toronto Invasive Bacterial Diseases Network	McMaster University	Allison McGeer, Patryk Aftanas, Hooman Derakhshani, Angel Li, Kuganya Nirmalarajah, Emily Panousis, Ahmed Draia, Jalees Nasir, Michael Surette, Samira Mubareka, Andrew G. McArthur
EPI_ISL_794309, EPI_ISL_794310, EPI_ISL_794311, EPI_ISL_794312, EPI_ISL_794313, EPI_ISL_794314, EPI_ISL_794315, EPI_ISL_794316, EPI_ISL_794317, EPI_ISL_794318, EPI_ISL_794319, EPI_ISL_794320			
see above	Wadsworth Center, New York State Department.of Health	Wadsworth Center, New York State Department.of Health	Kirsten St. George, Daryl M. Lamson, Alexis Russel, Matthew Shudt, Melissa A Leisner, Jonathan Plitnick, Navjot Singh, John Kelly, Sara Griesemer, Erasmus Schneider, Erica Lasek-Nesselquist
EPI_ISL_794640	USC Clinical Lab	Los Angeles County PHL	P. Hemarajata et al.
EPI_ISL_794641, EPI_ISL_794642, EPI_ISL_794643, EPI_ISL_794644, EPI_ISL_794645, EPI_ISL_794646, EPI_ISL_794647, EPI_ISL_794648	UCLA Clinical Micro Lab	Los Angeles County PHL	P. Hemarajata et al.
EPI_ISL_794666, EPI_ISL_794667	SYNLAB REGIONAL NOROCCIDENTE	Instituto Nacional de Salud - Dirección de Investigación en Salud Pública	Katherine Laiton-Donato, Diego A. Álvarez-Díaz, Carlos Franco-Muñoz, Mauricio Pacheco-Montealegre, Jonathan Reales, Sheryl Corchuelo, Maria T. Herrera, Julian Naizaque, Gerardo Santamaría, Paola Muñoz-Laiton, Diego Andrés Prada, Magdalena Wiesner, Martha Lucia Ospina Martinez, Marcela Mercado-Reyes
EPI_ISL_794670	UCLA Clinical Micro Lab	Los Angeles County PHL	P. Hemarajata et al.
EPI_ISL_794671	Area of Virology, Serology and Virology Division (SAViD), New South Wales Health Pathology Randwick	Area of Virology, Serology and Virology Division (SAViD), New South Wales Health Pathology Randwick	Rawlinson, W., Deveson, I., Van Haal, S., Bull, R.
EPI_ISL_794735, EPI_ISL_794736, EPI_ISL_794737, EPI_ISL_794738	1-Laboratory of Microbiology, National Reference Lab, Charles Nicolle Hospital; 2-University of Tunis ElManar, Faculty of Medicine of Tunis, LR99ES09, Tunis, Tunisia	1-Clinical and Experimental Pharmacology Lab, LR16SP02, National Center of Pharmacovigilance, University of Tunis El Manar, Tunis, Tunisia. 2-Neurodegenerative diseases and psychiatric troubles, LR18SP03, Razi Hospital, University of Tunis El Manar, Tunis, Tunisia. 3- Ministry of Health, National Observatory of New and Emerging Diseases, 1006, Tunis, Tunisia	Ilhem Boutiba-Ben Boubaker, Sameh Trabelsi, Nissaf Ben Alaya, Maher Kharrat, Alia BenKahla, Jalila Ben Khelil, Salma Abid, Sana Ferjani, Mouna Ben Sassi, Mouna Safer, Zaineb Hamzaoui, Guedi Ali Barreh, Habiba Ben Romdhane, Souissi Amira, Sarra Chamman, Hanen El Jebari, Asma Ferjani, Gaies Emna, Riadh Daghfous, Riadh Gouider.
EPI_ISL_803098	Institute of Microbiology, Universidad San Francisco de Quito	Institute of Microbiology, Universidad San Francisco de Quito	Sully Márquez, Belén Prado-Vivar, Juan José Guadalupe, Monica Becerra-Wong, Bernardo Gutiérrez, Eulalia Pazmiño, Katalina Pacheco, Verónica Barragán, Patricio Rojas-Silva, Gabriel Trueba, Michelle Grunauer, Paúl Cárdenas
EPI_ISL_803120, EPI_ISL_803430	Laboratory of Microbiology, National Reference Lab, Charles Nicolle Hospital; 2-University of Tunis ElManar, Faculty of Medicine of Tunis, LR99ES09, Tunis, Tunisia	Clinical and Experimental Pharmacology Lab, LR16SP02, National Center of Pharmacovigilance, University of Tunis El Manar, Tunis, Tunisia. 2-Neurodegenerative diseases and psychiatric troubles, LR18SP03, Razi Hospital, University of Tunis El Manar, Tunis, Tunisia. 3- Ministry of Health, National Observatory of New and Emerging Diseases, 1006, Tunis, Tunisia	Ilhem Boutiba-Ben Boubaker, Sameh Trabelsi, Nissaf Ben Alaya, Maher Kharrat, Alia BenKahla, Jalila Ben Khelil, Salma Abid, Sana Ferjani, Mouna Ben Sassi, Mouna Safer, Zaineb Hamzaoui, Habiba Ben Romdhane, Souissi Amira, Sarra Chamman, Hanen El Jebari, Ahmed Fakhfakh, Gaies Emna, Riadh Daghfous, Riadh Gouider.
EPI_ISL_803962, EPI_ISL_803963, EPI_ISL_803964, EPI_ISL_803965, EPI_ISL_803999, EPI_ISL_804006	National Public Health Laboratory, National Centre for Infectious Diseases	National Public Health Laboratory, National Centre for Infectious Diseases	Tze Minn Mak, Sophie Octavia, Zhenyang Zhou, Lin Cui, Raymond Tzer Pin Lin
EPI_ISL_804259, EPI_ISL_804369	Respiratory Virus Unit, National Infection Service, Public Health England	COVID-19 Genomics UK (COG-UK) Consortium	PHE Covid Sequencing Team
EPI_ISL_804947	Wadsworth Center, New York State Department.of Health	Wadsworth Center, New York State Department.of Health	Kirsten St. George, Daryl M. Lamson, Alexis Russel, Matthew Shudt, Melissa A Leisner, Jonathan Plitnick, Navjot Singh, John Kelly, Sara Griesemer, Erasmus Schneider, Erica Lasek-Nesselquist
EPI_ISL_806544	INSPI Instituto Nacional de Investigación en Salud Pública	Av. Julián Coronel 905 entre Esmeraldas y José Mascote Av. Juan Tanca Marengo No. 100 y Av. de las Américas	Leandro Patiño, Doménica de Mora, Maritza Olmedo, Andrés Carrazco, Orson Mestanza, Mary Regato, Melissa Zambrano, Manuel González, Alfredo Bruno, Alberto Orlando.
EPI_ISL_806729	Sydney South West Pathology Service (SSWPS) - Royal Prince Alfred Hospital - NSW Health Pathology	NSW Health Pathology - Institute of Clinical Pathology and Medical Research; Westmead Hospital; University of Sydney	CIDM-PH et al.
EPI_ISL_806808	Gundersen Molecular Diagnostics Laboratory	Kabara Cancer Research Institute	Craig S. Richmond, Paraic A. Kenny
EPI_ISL_807148, EPI_ISL_807149	Department of Virology	Department of Virology	Massab Umair, Aamer Ikram, Muhammad Salman, Sana Tamim, Nazish Badar, Adnan Khurshid, Salmaan Sharif, Zaira Rehman, Abdul Ahad, Hamza Ahmad, Samee Ullah
EPI_ISL_810967	PathWest Laboratory Medicine WA	PathWest Laboratory Medicine WA Microbial Surveillance Unit	PathWest Laboratory Medicine WA Microbial Surveillance Unit
EPI_ISL_811127	Cantonal Hospital Winterthur	Institute of Medical Virology, University of Zurich	Stefan Schmutz, Maryam Zaheri, Verena Kufner, Annette Audigé, Maria Grünberg, Kevin Steiner, Jon Huder, Cyril Shah, Riccarda Capaul, Guido Bloemberg, Jürg Böni, Michael Huber, Alexandra Trkola, Alexander Wepf, Urs Karrer
EPI_ISL_811147	WHO National Influenza Centre Russian Federation	WHO National Influenza Centre Russian Federation	Andrey Komissarov, Artem Fadeev, Maxim Burlakov, Anna Ivanova, Kseniya Komissarova, Dmitry Bazhenov, Daria Danilenko, Ksenia Safina, Elena Nabieva, Georgii Bazykin, Dmitry Lioznov
EPI_ISL_812122	Department of Clinical Microbiology	GIGA Medical Genomics	Keith Durkin, Maria Artesi, Sébastien Bontems, Raphaël Boreux, Bouchra Boujemla, Cécile Meex, Pierrette Melin, Marie-Pierre Hayette, Vincent Bours
EPI_ISL_812356, EPI_ISL_812357	SA Pathology	SA Pathology	Lex Leong, Julien Soubrier, Chuan Kok Lim, Song Gao, Mark Turra, Karin Kassahn, Ivan Bastian, Geoff Higgins
EPI_ISL_812440	Victorian Infectious Diseases Reference Laboratory (VIDRL)	VIDRL and MDU-PHL	Caly L., Seemann T., Sait, M.L., Druce J., Sherry, N.L.
EPI_ISL_812778	Ministry of Health Turkey	Ministry of Health Turkey	Fatma Bayrakdar, Yasemin Cogun, Süleyman Yalcin, Aye Baak Alta, Gülay Korukluolu
EPI_ISL_816258, EPI_ISL_816328, EPI_ISL_816363, EPI_ISL_816371, EPI_ISL_816373, EPI_ISL_816377, EPI_ISL_816413, EPI_ISL_816424, EPI_ISL_816451, EPI_ISL_816479, EPI_ISL_816506, EPI_ISL_816519, EPI_ISL_816614, EPI_ISL_816653			
see above	Virology Department, Sheffield Teaching Hospitals NHS Foundation Trust/Department of Infection, Immunity and Cardiovascular Disease, The Medical School, University of Sheffield	COVID-19 Genomics UK (COG-UK) Consortium	Thushan de Silva, Matthew Parker, Nikki Smith, Adri Angyal, Rebecca Brown, Luke Green, Rachel Tucker, Paul Parsons, Danielle Groves, Katie Johnson, Laura Carrilero, Alex Keeley, Dave Partridge, Matthew Wyles, Benjamin Lindsey, Mehmet Yavuz, Mohammad Raza, Cariad Evans

EPI_ISL_819125	Wyoming Public Health Laboratory	Wyoming Public Health Laboratory	Noah Hull, Taylor Fearing, Lynette Gumbleton, Channing Weber, Ashley Norberg, Bailey Bowcutt, and Wanda Manley
EPI_ISL_819134, EPI_ISL_819139, EPI_ISL_819141, EPI_ISL_819145, EPI_ISL_819147, EPI_ISL_819151, EPI_ISL_819152, EPI_ISL_819153, EPI_ISL_819154, EPI_ISL_819155, EPI_ISL_819158, EPI_ISL_819159, EPI_ISL_819160, EPI_ISL_819161, EPI_ISL_819162, EPI_ISL_819164, EPI_ISL_819165, EPI_ISL_819177			
see above	Servicio de Microbiología, Hospital Universitario Son Espases	SeqCOVID-SPAIN consortium/IBV(CSIC)	Carla López-Causapé, Jordi Reina, Antonio Oliver and SeqCOVID-SPAIN consortium
EPI_ISL_819204, EPI_ISL_819206, EPI_ISL_819208, EPI_ISL_819211, EPI_ISL_819212, EPI_ISL_819214, EPI_ISL_819224, EPI_ISL_819225, EPI_ISL_819226, EPI_ISL_819227, EPI_ISL_819228, EPI_ISL_819229, EPI_ISL_819230, EPI_ISL_819231, EPI_ISL_819232, EPI_ISL_819233, EPI_ISL_819234, EPI_ISL_819235, EPI_ISL_819240, EPI_ISL_819246, EPI_ISL_819247, EPI_ISL_819248, EPI_ISL_819249, EPI_ISL_819250, EPI_ISL_819251, EPI_ISL_819252, EPI_ISL_819253, EPI_ISL_819254, EPI_ISL_819255, EPI_ISL_819256, EPI_ISL_819257, EPI_ISL_819258, EPI_ISL_819259, EPI_ISL_819260, EPI_ISL_819261, EPI_ISL_819262, EPI_ISL_819263, EPI_ISL_819264, EPI_ISL_819265, EPI_ISL_819266			
see above	Wyoming Public Health Laboratory	Wyoming Public Health Laboratory	Noah Hull, Taylor Fearing, Lynette Gumbleton, Channing Weber, Ashley Norberg, Bailey Bowcutt, and Wanda Manley
EPI_ISL_820596, EPI_ISL_820598, EPI_ISL_820601, EPI_ISL_820603, EPI_ISL_820605, EPI_ISL_820607, EPI_ISL_820610	Queens Medical Centre, Clinical Microbiology Department / DeepSeq Nottingham	COVID-19 Genomics UK (COG-UK) Consortium	Gemma Clark, Wendy Smith, Manjinder Khakh, Vicki M Fleming, Michelle M Lister, Hannah Howson-Wells, Jonathan Ball, Patrick McClure, Joseph Chappell, Theocharis Tsoleridis, Nadine Holmes, Matthew Carlisle, Christopher Moore, Fei Sang, Johnny Debebe, Victoria Wright, Matthew Loose
EPI_ISL_821088, EPI_ISL_821139	Lighthouse Lab in Alderley Park	Wellcome Sanger Institute for the COVID-19 Genomics UK (COG-UK) Consortium	Jacquelyn Wynn, Mairead Hyland, The Lighthouse Lab in Alderley Park and Alex Alderton, Roberto Amato, Sonia Goncalves, Ewan Harrison, David K. Jackson, Ian Johnston, Dominic Kwiatkowski, Cordelia Langford, John Sillitoe on behalf of the Wellcome Sanger Institute COVID-19 Surveillance Team
EPI_ISL_823989, EPI_ISL_824261, EPI_ISL_824262, EPI_ISL_824263, EPI_ISL_824279, EPI_ISL_824280	Dutch COVID-19 response team	National Institute for Public Health and the Environment (RIVM)	Adam Meijer, Harry Vennema, Jeroen Cremer, Sharon van den Brink, Bas van der Veer, AnneMarie van den Brandt, Florian Zwagemaker, Dennis Schmitz, Chantal Reusken, on behalf of the national COVID-19 response team
EPI_ISL_824292	Institute of Microbiology, Universidad San Francisco de Quito	Institute of Microbiology, Universidad San Francisco de Quito	Belén Prado-Vivar, Sully Márquez, Juan José Guadalupe, Monica Becerra-Wong, Bernardo Gutiérrez, Tania Guayasamin, Patricio Reyes, Verónica Barragán, Patricio Rojas-Silva, Gabriel Trueba, Michelle Grunauer, Paúl Cárdenas
EPI_ISL_824293, EPI_ISL_824294	Institute of Microbiology, Universidad San Francisco de Quito	Institute of Microbiology, Universidad San Francisco de Quito	Belén Prado-Vivar, Sully Márquez, Juan José Guadalupe, Monica Becerra-Wong, Bernardo Gutiérrez, Eulalia Pazmiño, Katalina Pacheco, Verónica Barragán, Patricio Rojas-Silva, Gabriel Trueba, Michelle Grunauer, Paúl Cárdenas
EPI_ISL_824852, EPI_ISL_824853, EPI_ISL_824854, EPI_ISL_824855, EPI_ISL_824856, EPI_ISL_824857, EPI_ISL_824858, EPI_ISL_824859, EPI_ISL_824860, EPI_ISL_824861, EPI_ISL_824862, EPI_ISL_824863			
see above	Department of Clinical Microbiology	GIGA Medical Genomics	Keith Durkin, Maria Artesi, Sébastien Bontems, Raphaël Boreux, Bouchra Boujemla, Cécile Meex, Pierrette Melin, Marie-Pierre Hayette, Vincent Bours
EPI_ISL_824966, EPI_ISL_824967, EPI_ISL_824968, EPI_ISL_824969, EPI_ISL_824970, EPI_ISL_824971, EPI_ISL_824972, EPI_ISL_824973, EPI_ISL_824978, EPI_ISL_824979	Maryland Public Health Laboratory	Maryland Public Health Laboratory	Maryland Department of Health Laboratories Administration
EPI_ISL_824985, EPI_ISL_824986	Charité Universitätsmedizin Berlin, Institute of Virology, Charitéplatz 1, 10117 Berlin, Germany	Charité Universitätsmedizin Berlin, Institute of Virology, Charitéplatz 1, 10117 Berlin, Germany	Victor M Corman, Jörn Beheim-Schwarzbach, Tobias Bleicker, Julia Tesch, Barbara Mühlemann, Talitha Veith, Julia Schneider, Terry Jones, Christian Drosten
EPI_ISL_824995	Arizona State Public Health Laboratory	Arizona State Public Health Laboratory	Trung Huynh, Jessica Escobar, Katherine Fullerton, Nobuko Fukushima, Stacy White, Linda Getsinger, Victor Waddell
EPI_ISL_825013	Johns Hopkins Pathology	Johns Hopkins Hospital Department of Pathology	C. Paul Morris, Chun Huai Luo, Adannaya Amadi, Nicholas Gallagher, Heba H. Mostafa
EPI_ISL_825069, EPI_ISL_825074, EPI_ISL_825076, EPI_ISL_825080	National Public Health Laboratory, National Centre for Infectious Diseases	National Public Health Laboratory, National Centre for Infectious Diseases	Tze Minn Mak, Sophie Octavia, Zhenyang Zhou, Lin Cui, Raymond Tzer Pin Lin
EPI_ISL_826269	University of Debrecen, Department of Medical Microbiology	National Laboratory of Virology, Szentágothai Research Centre	Endre Gábor Tóth, Balázs Somogyi, Brigitta Zana, Eszter Csoma, Ferenc Jakab, Gábor Kemenesi
EPI_ISL_826304, EPI_ISL_826306	Wyoming Public Health Laboratory	Wyoming Public Health Laboratory	Noah Hull, Taylor Fearing, Lynette Gumbleton, Channing Weber, Ashley Norberg, Bailey Bowcutt, and Wanda Manley
EPI_ISL_826461	Montana Public Health Laboratory	Wyoming Public Health Laboratory	Noah Hull, Joy Ritter, Taylor Fearing, Lynette Gumbleton, Channing Weber, Ashley Norberg, Bailey Bowcutt, Wanda Manley, Deborah Gibson
EPI_ISL_826896, EPI_ISL_826918, EPI_ISL_827031, EPI_ISL_827120, EPI_ISL_827307, EPI_ISL_827345, EPI_ISL_827358, EPI_ISL_827416, EPI_ISL_827689, EPI_ISL_827690, EPI_ISL_827757, EPI_ISL_827780, EPI_ISL_827871, EPI_ISL_827951, EPI_ISL_827992, EPI_ISL_828262, EPI_ISL_828497, EPI_ISL_829977, EPI_ISL_829982			
see above	deCODE genetics	deCODE genetics	Daniel F Gudbjartsson; Agnar Helgason; Hakon Jonsson; Olafur T Magnusson; Pall Melsted; Gudmundur L Norddahl; Jona Saemundsdottir; Asgeir Sigurdsson; Patrick Sulem; Arna B Agustsdottir; Hannes Eggertsson; Berglind Eiriksdottir; Run Fridriksdottir; Elisabet E Gardarsdottir; Gudmundur Georgsson; Olafia S Gretarsdottir; Kjartan R Gudmundsson; Thora R Gunnarsdottir; Arnaldur Gylfason; Hilma Holm; Brynjar O Jensson; Aslaug Jonasdottir; Kamilla S Josefsdottir; Thordur Kristjansson; Droplaug N Magnusdottir; Solvi Rognvaldsson; Louise le Roux; Gudrun Sigmundsdottir; Gardar Sveinbjornsson; Kristin E Sveinsdottir; Maney Sveinsdottir; Emil A Thorarensen; Bjarni Thorbjornsson; Gisli Masson; Ingileif Jonsdottir; Alma Moller; Thorolfur Gudnason; Karl G Kristinsson; Unnur Thorsteinsdottir; Kari Stefansson
EPI_ISL_830216, EPI_ISL_830218, EPI_ISL_830220, EPI_ISL_830221, EPI_ISL_830263, EPI_ISL_830265, EPI_ISL_830267, EPI_ISL_830269, EPI_ISL_830270, EPI_ISL_830272, EPI_ISL_830274, EPI_ISL_830276, EPI_ISL_830278, EPI_ISL_830280, EPI_ISL_830282, EPI_ISL_830284, EPI_ISL_830286, EPI_ISL_830288, EPI_ISL_830289, EPI_ISL_830291, EPI_ISL_830293, EPI_ISL_830294, EPI_ISL_830296, EPI_ISL_830298, EPI_ISL_830300, EPI_ISL_830302, EPI_ISL_830304, EPI_ISL_830306, EPI_ISL_830308, EPI_ISL_830310, EPI_ISL_830312, EPI_ISL_830313, EPI_ISL_830315, EPI_ISL_830317, EPI_ISL_830319, EPI_ISL_830321, EPI_ISL_830323, EPI_ISL_830325, EPI_ISL_830326, EPI_ISL_830329, EPI_ISL_830331, EPI_ISL_830333, EPI_ISL_830334, EPI_ISL_830335, EPI_ISL_830337, EPI_ISL_830338, EPI_ISL_830340, EPI_ISL_830342, EPI_ISL_830344, EPI_ISL_830346, EPI_ISL_830348, EPI_ISL_830349			
see above	Wadsworth Center, New York State Department of Health	Wadsworth Center, New York State Department of Health	Kirsten St. George, Daryl M. Lamson, Alexis Russel, Matthew Shudt, Melissa A Leisner, Jonathan Plitnick, Navjot Singh, John Kelly, Erasmus Schneider, Erica Lasek-Nesselquist
EPI_ISL_830682	SUNY UPSTATE MEDICAL UNIVERSITY	Wadsworth Center, New York State Department of Health	Kirsten St. George, Daryl M. Lamson, Alexis Russel, Matthew Shudt, Melissa A Leisner, Jonathan Plitnick, Navjot Singh, John Kelly, Erasmus Schneider, Erica Lasek-Nesselquist
EPI_ISL_830704, EPI_ISL_830705, EPI_ISL_830706	ALBANY MEDICAL CENTER HOSPITAL CLINICAL LABORATORIES	Wadsworth Center, New York State Department of Health	Kirsten St. George, Daryl M. Lamson, Alexis Russel, Matthew Shudt, Melissa A Leisner, Jonathan Plitnick, Navjot Singh, John Kelly, Erasmus Schneider, Erica Lasek-Nesselquist
EPI_ISL_831366, EPI_ISL_831367	Clinical Molecular Microbiology Laboratory, UNC Hospitals	Jeremy Wang	Jeremy Wang, Alexander Rubinsteyn, Colleen Rice, Jason Smedberg, Melissa Miller, Corbin Jones, Robert Hagan
EPI_ISL_831479, EPI_ISL_831480, EPI_ISL_831481, EPI_ISL_831483, EPI_ISL_831486, EPI_ISL_831487, EPI_ISL_831496, EPI_ISL_831511, EPI_ISL_831521, EPI_ISL_831527, EPI_ISL_831529, EPI_ISL_831530, EPI_ISL_831533, EPI_ISL_831534, EPI_ISL_831536, EPI_ISL_831538, EPI_ISL_831539, EPI_ISL_831540, EPI_ISL_831542, EPI_ISL_831543, EPI_ISL_831544, EPI_ISL_831545, EPI_ISL_831546, EPI_ISL_831548, EPI_ISL_831550, EPI_ISL_831623, EPI_ISL_831624, EPI_ISL_831625, EPI_ISL_831626, EPI_ISL_831627, EPI_ISL_831628, EPI_ISL_831629, EPI_ISL_831630, EPI_ISL_831631, EPI_ISL_831632, EPI_ISL_831633, EPI_ISL_831634, EPI_ISL_831635, EPI_ISL_831636, EPI_ISL_831637, EPI_ISL_831638, EPI_ISL_831639, EPI_ISL_831640, EPI_ISL_831641, EPI_ISL_831642, EPI_ISL_831643			
see above	University of Wisconsin-Madison AIDS Vaccine Research Laboratories	University of Wisconsin-Madison AIDS Vaccine Research Laboratories	Gage Moreno, Katarina Braun, et al. AIDS Vaccine Research Laboratories
EPI_ISL_831667	Institute of Virology, Biomedical Research Center of the Slovak Academy of Sciences, Bratislava	Faculty of Natural Sciences, Comenius University, Bratislava	Viktória abanová, Kristína Boršová, Broa Brejová, Viktória Hodorová, Sabina Fumaová Havlíková, Juraj Kopáek, Martina Liková, ubomíra Lukáiková, Martina Neboháová, Monika Sláviková, Tomáš Vina, Jozef Nosek, Boris Klempa
EPI_ISL_831668	Institute of Virology, Biomedical Research Center of the Slovak Academy of Sciences, Bratislava	Faculty of Natural Sciences, Comenius University, Bratislava	Kristína Boršová, Viktória abanová, Broa Brejová, Viktória Hodorová, Sabina Fumaová Havlíková, Juraj Kopáek, Martina Liková, ubomíra Lukáiková, Martina Neboháová, Monika Sláviková, Tomáš Vina, Boris Klempa, Jozef Nosek
EPI_ISL_831672	Institute of Virology, Biomedical Research Center of the Slovak Academy of Sciences, Bratislava	Faculty of Natural Sciences, Comenius University, Bratislava	Viktória abanová, Kristína Boršová, Broa Brejová, Viktória Hodorová, Sabina Fumaová Havlíková, Juraj Kopáek, Martina Liková, ubomíra Lukáiková, Martina Neboháová, Monika Sláviková, Tomáš Vina, Jozef Nosek, Boris Klempa
EPI_ISL_831906, EPI_ISL_831907, EPI_ISL_831911, EPI_ISL_831928, EPI_ISL_831929, EPI_ISL_831930	New Mexico Department of Health Scientific Laboratory	New Mexico Department of Health Scientific Laboratory	Ellie Johnson, Anastacia Griego-Fisher, D'eldra Malone
EPI_ISL_831941, EPI_ISL_831942	Klinisk mikrobiologi	The Public Health Agency of Sweden	Department of Microbiology, The Public Health Agency of Sweden

EPI_ISL_832022, EPI_ISL_832065, EPI_ISL_832096, EPI_ISL_832097, EPI_ISL_832098, EPI_ISL_832100	Santa Clara County Public Health Laboratory	Santa Clara County Public Health Laboratory	Santa Clara County Public Health Department
EPI_ISL_832122, EPI_ISL_832123	Clinical Molecular Microbiology Laboratory, UNC Hospitals	Jeremy Wang	Jeremy Wang, Alexander Rubinsteyn, Colleen Rice, Jason Smedberg, Melissa Miller, Corbin Jones, Robert Hagan
EPI_ISL_832210, EPI_ISL_832211, EPI_ISL_832212, EPI_ISL_832213, EPI_ISL_832214, EPI_ISL_832215, EPI_ISL_832216, EPI_ISL_832217, EPI_ISL_832218, EPI_ISL_832219, EPI_ISL_832220, EPI_ISL_832221, EPI_ISL_832222, EPI_ISL_832223, EPI_ISL_832224, EPI_ISL_832225, EPI_ISL_832226, EPI_ISL_832227, EPI_ISL_832228, EPI_ISL_832229, EPI_ISL_832230, EPI_ISL_832231			
see above	Department of Clinical Microbiology	GIGA Medical Genomics	Keith Durkin, Maria Artesi, Sébastien Bontems, Raphaël Boreux, Bouchra Boujemla, Cécile Meex, Pierrette Melin, Marie-Pierre Hayette, Vincent Bours
EPI_ISL_832378	Ohio State	James Molecular Lab	Huolin Tu, Matthew R Avenarius, Laura Kubatko, Matthew Hunt, Xiaokang Pan, Peng Ru, Jason Garee, Keelie Thomas, Peter Mohler, Preeti Pancholi, Dan Jones
EPI_ISL_832968, EPI_ISL_832969, EPI_ISL_832970, EPI_ISL_832971, EPI_ISL_832972, EPI_ISL_832973, EPI_ISL_832974, EPI_ISL_832975, EPI_ISL_832976, EPI_ISL_832977, EPI_ISL_832986, EPI_ISL_832987, EPI_ISL_832988, EPI_ISL_832989, EPI_ISL_832990, EPI_ISL_832991, EPI_ISL_832992, EPI_ISL_832993, EPI_ISL_832994, EPI_ISL_832995, EPI_ISL_832996, EPI_ISL_832997, EPI_ISL_832998, EPI_ISL_832999, EPI_ISL_833000, EPI_ISL_833001, EPI_ISL_833002, EPI_ISL_833003, EPI_ISL_833004, EPI_ISL_833005, EPI_ISL_833006, EPI_ISL_833007, EPI_ISL_833008, EPI_ISL_833009, EPI_ISL_833010, EPI_ISL_833011, EPI_ISL_833012, EPI_ISL_833013, EPI_ISL_833014, EPI_ISL_833015, EPI_ISL_833016, EPI_ISL_833017, EPI_ISL_833018, EPI_ISL_833019, EPI_ISL_833020, EPI_ISL_833021, EPI_ISL_833022, EPI_ISL_833023, EPI_ISL_833024, EPI_ISL_833025, EPI_ISL_833026, EPI_ISL_833027, EPI_ISL_833029, EPI_ISL_833030, EPI_ISL_833031, EPI_ISL_833032, EPI_ISL_833033, EPI_ISL_833034, EPI_ISL_833035, EPI_ISL_833036, EPI_ISL_833037			
see above	Maine HETL	Tewhey Lab, The Jackson Laboratory	Matluk,N., Dewey,H., Iosue,F., Barter,M., Lynch,R., Munger,H. and Tewhey,R.
EPI_ISL_833147	Genomic Laboratory (GLAB) (Conjoint lab of Health Directorate of Istanbul and Istanbul Technical University)	Genomic Laboratory (GLAB), Istanbul Technical University	Ilker Karacan, Tugba Kizilboga Akgun, Payam Zolfagharian, Nisan Denizce Can, Pari Sharifli, Levent Doganay, Gizem Dinler Doganay
EPI_ISL_833165, EPI_ISL_833166	Hospital Samaritano	Instituto Adolfo Lutz, Interdiciplinary Procedures Center, Strategic Laboratory	Claudio Tavares Sacchi, Claudia Regina Gonçalves, Erica Valessa Ramos Gomes, Karoline Rodrigues Campos
EPI_ISL_833185, EPI_ISL_833189, EPI_ISL_833190	Department of Clinical Microbiology	GIGA Medical Genomics	Keith Durkin, Maria Artesi, Sébastien Bontems, Raphaël Boreux, Bouchra Boujemla, Cécile Meex, Pierrette Melin, Marie-Pierre Hayette, Vincent Bours
EPI_ISL_833401, EPI_ISL_833402, EPI_ISL_833403	St. Francis Medical Center	Los Angeles County PHL	P. Hemarajata et al.
EPI_ISL_833405, EPI_ISL_833406, EPI_ISL_833407, EPI_ISL_833408, EPI_ISL_833409, EPI_ISL_833410, EPI_ISL_833411, EPI_ISL_833412	Beverly Hospital	Los Angeles County PHL	P. Hemarajata et al.
EPI_ISL_833413	City of Hope Comprehensive Cancer Center	Los Angeles County PHL	P. Hemarajata et al.
EPI_ISL_833417, EPI_ISL_833419, EPI_ISL_833420, EPI_ISL_833421, EPI_ISL_833422, EPI_ISL_833423, EPI_ISL_833424, EPI_ISL_833425, EPI_ISL_833426, EPI_ISL_833427	MD Laboratories	Los Angeles County PHL	P. Hemarajata et al.
EPI_ISL_833428, EPI_ISL_833429, EPI_ISL_833430, EPI_ISL_833431	UCLA Clinical Micro Lab	Los Angeles County PHL	P. Hemarajata et al.
EPI_ISL_833489	St. Francis Medical Center	Los Angeles County PHL	P. Hemarajata et al.
EPI_ISL_833586, EPI_ISL_833590, EPI_ISL_833593, EPI_ISL_833596, EPI_ISL_833597, EPI_ISL_833599, EPI_ISL_833602, EPI_ISL_833604, EPI_ISL_833606, EPI_ISL_833607	Lighthouse Lab in Milton Keynes	Wellcome Sanger Institute for the COVID-19 Genomics UK (COG-UK) Consortium	The Lighthouse Lab in Milton Keynes and Alex Alderton, Roberto Amato, Sonia Goncalves, Ewan Harrison, David K. Jackson, Ian Johnston, Dominic Kwiatkowski, Cordelia Langford, John Sillitoe on behalf of the Wellcome Sanger Institute COVID-19 Surveillance Team
EPI_ISL_833609, EPI_ISL_833610	Lighthouse Lab in Cambridge	Wellcome Sanger Institute for the COVID-19 Genomics UK (COG-UK) Consortium	Rob Howes, The Lighthouse Lab in Cambridge and Alex Alderton, Roberto Amato, Sonia Goncalves, Ewan Harrison, David K. Jackson, Ian Johnston, Dominic Kwiatkowski, Cordelia Langford, John Sillitoe on behalf of the Wellcome Sanger Institute COVID-19 Surveillance Team
EPI_ISL_833611, EPI_ISL_833612, EPI_ISL_833614, EPI_ISL_833615	Lighthouse Lab in Milton Keynes	Wellcome Sanger Institute for the COVID-19 Genomics UK (COG-UK) Consortium	The Lighthouse Lab in Milton Keynes and Alex Alderton, Roberto Amato, Sonia Goncalves, Ewan Harrison, David K. Jackson, Ian Johnston, Dominic Kwiatkowski, Cordelia Langford, John Sillitoe on behalf of the Wellcome Sanger Institute COVID-19 Surveillance Team
EPI_ISL_833616	Lighthouse Lab in Cambridge	Wellcome Sanger Institute for the COVID-19 Genomics UK (COG-UK) Consortium	Rob Howes, The Lighthouse Lab in Cambridge and Alex Alderton, Roberto Amato, Sonia Goncalves, Ewan Harrison, David K. Jackson, Ian Johnston, Dominic Kwiatkowski, Cordelia Langford, John Sillitoe on behalf of the Wellcome Sanger Institute COVID-19 Surveillance Team
EPI_ISL_833617, EPI_ISL_833618, EPI_ISL_833619, EPI_ISL_833620, EPI_ISL_833621	Lighthouse Lab in Milton Keynes	Wellcome Sanger Institute for the COVID-19 Genomics UK (COG-UK) Consortium	The Lighthouse Lab in Milton Keynes and Alex Alderton, Roberto Amato, Sonia Goncalves, Ewan Harrison, David K. Jackson, Ian Johnston, Dominic Kwiatkowski, Cordelia Langford, John Sillitoe on behalf of the Wellcome Sanger Institute COVID-19 Surveillance Team
EPI_ISL_833626	Lighthouse Lab in Cambridge	Wellcome Sanger Institute for the COVID-19 Genomics UK (COG-UK) Consortium	Rob Howes, The Lighthouse Lab in Cambridge and Alex Alderton, Roberto Amato, Sonia Goncalves, Ewan Harrison, David K. Jackson, Ian Johnston, Dominic Kwiatkowski, Cordelia Langford, John Sillitoe on behalf of the Wellcome Sanger Institute COVID-19 Surveillance Team
EPI_ISL_833629	Lighthouse Lab in Milton Keynes	Wellcome Sanger Institute for the COVID-19 Genomics UK (COG-UK) Consortium	The Lighthouse Lab in Milton Keynes and Alex Alderton, Roberto Amato, Sonia Goncalves, Ewan Harrison, David K. Jackson, Ian Johnston, Dominic Kwiatkowski, Cordelia Langford, John Sillitoe on behalf of the Wellcome Sanger Institute COVID-19 Surveillance Team
EPI_ISL_833630, EPI_ISL_833631	Lighthouse Lab in Cambridge	Wellcome Sanger Institute for the COVID-19 Genomics UK (COG-UK) Consortium	Rob Howes, The Lighthouse Lab in Cambridge and Alex Alderton, Roberto Amato, Sonia Goncalves, Ewan Harrison, David K. Jackson, Ian Johnston, Dominic Kwiatkowski, Cordelia Langford, John Sillitoe on behalf of the Wellcome Sanger Institute COVID-19 Surveillance Team
EPI_ISL_833632, EPI_ISL_833634	Lighthouse Lab in Milton Keynes	Wellcome Sanger Institute for the COVID-19 Genomics UK (COG-UK) Consortium	The Lighthouse Lab in Milton Keynes and Alex Alderton, Roberto Amato, Sonia Goncalves, Ewan Harrison, David K. Jackson, Ian Johnston, Dominic Kwiatkowski, Cordelia Langford, John Sillitoe on behalf of the Wellcome Sanger Institute COVID-19 Surveillance Team
EPI_ISL_833635, EPI_ISL_833636, EPI_ISL_833640	Lighthouse Lab in Cambridge	Wellcome Sanger Institute for the COVID-19 Genomics UK (COG-UK) Consortium	Rob Howes, The Lighthouse Lab in Cambridge and Alex Alderton, Roberto Amato, Sonia Goncalves, Ewan Harrison, David K. Jackson, Ian Johnston, Dominic Kwiatkowski, Cordelia Langford, John Sillitoe on behalf of the Wellcome Sanger Institute COVID-19 Surveillance Team
EPI_ISL_833643	Lighthouse Lab in Milton Keynes	Wellcome Sanger Institute for the COVID-19 Genomics UK (COG-UK) Consortium	The Lighthouse Lab in Milton Keynes and Alex Alderton, Roberto Amato, Sonia Goncalves, Ewan Harrison, David K. Jackson, Ian Johnston, Dominic Kwiatkowski, Cordelia Langford, John Sillitoe on behalf of the Wellcome Sanger Institute COVID-19 Surveillance Team
EPI_ISL_833646	Lighthouse Lab in Cambridge	Wellcome Sanger Institute for the COVID-19 Genomics UK (COG-UK) Consortium	Rob Howes, The Lighthouse Lab in Cambridge and Alex Alderton, Roberto Amato, Sonia Goncalves, Ewan Harrison, David K. Jackson, Ian Johnston, Dominic Kwiatkowski, Cordelia Langford, John Sillitoe on behalf of the Wellcome Sanger Institute COVID-19 Surveillance Team
EPI_ISL_833647, EPI_ISL_833648	Lighthouse Lab in Milton Keynes	Wellcome Sanger Institute for the COVID-19 Genomics UK (COG-UK) Consortium	The Lighthouse Lab in Milton Keynes and Alex Alderton, Roberto Amato, Sonia Goncalves, Ewan Harrison, David K. Jackson, Ian Johnston, Dominic Kwiatkowski, Cordelia Langford, John Sillitoe on behalf of the Wellcome Sanger Institute COVID-19 Surveillance Team
EPI_ISL_833650, EPI_ISL_833651	Lighthouse Lab in Cambridge	Wellcome Sanger Institute for the COVID-19 Genomics UK (COG-UK) Consortium	Rob Howes, The Lighthouse Lab in Cambridge and Alex Alderton, Roberto Amato, Sonia Goncalves, Ewan Harrison, David K. Jackson, Ian Johnston, Dominic Kwiatkowski, Cordelia Langford, John Sillitoe on behalf of the Wellcome Sanger Institute COVID-19 Surveillance Team
EPI_ISL_833652	Lighthouse Lab in Milton Keynes	Wellcome Sanger Institute for the COVID-19 Genomics UK (COG-UK) Consortium	The Lighthouse Lab in Milton Keynes and Alex Alderton, Roberto Amato, Sonia Goncalves, Ewan Harrison, David K. Jackson, Ian Johnston, Dominic Kwiatkowski, Cordelia Langford, John Sillitoe on behalf of the Wellcome Sanger Institute COVID-19 Surveillance Team
EPI_ISL_833654	Lighthouse Lab in Cambridge	Wellcome Sanger Institute for the COVID-19 Genomics UK (COG-UK) Consortium	Rob Howes, The Lighthouse Lab in Cambridge and Alex Alderton, Roberto Amato, Sonia Goncalves, Ewan Harrison, David K. Jackson, Ian Johnston, Dominic Kwiatkowski, Cordelia Langford, John Sillitoe on behalf of the Wellcome Sanger Institute COVID-19 Surveillance Team
EPI_ISL_833656, EPI_ISL_833658	Lighthouse Lab in Milton Keynes	Wellcome Sanger Institute for the COVID-19 Genomics UK	The Lighthouse Lab in Milton Keynes and Alex Alderton, Roberto Amato, Sonia Goncalves, Ewan Harrison, David K. Jackson, Ian Johnston, Dominic

		(COG-UK) Consortium	Kwiatkowski, Cordelia Langford, John Sillitoe on behalf of the Wellcome Sanger Institute COVID-19 Surveillance Team
EPI_ISL_833662, EPI_ISL_833665	Lighthouse Lab in Cambridge	Wellcome Sanger Institute for the COVID-19 Genomics UK (COG-UK) Consortium	Rob Howes, The Lighthouse Lab in Cambridge and Alex Alderton, Roberto Amato, Sonia Goncalves, Ewan Harrison, David K. Jackson, Ian Johnston, Dominic Kwiatkowski, Cordelia Langford, John Sillitoe on behalf of the Wellcome Sanger Institute COVID-19 Surveillance Team
EPI_ISL_833667	Lighthouse Lab in Milton Keynes	Wellcome Sanger Institute for the COVID-19 Genomics UK (COG-UK) Consortium	The Lighthouse Lab in Milton Keynes and Alex Alderton, Roberto Amato, Sonia Goncalves, Ewan Harrison, David K. Jackson, Ian Johnston, Dominic Kwiatkowski, Cordelia Langford, John Sillitoe on behalf of the Wellcome Sanger Institute COVID-19 Surveillance Team
EPI_ISL_833668, EPI_ISL_833670, EPI_ISL_833671, EPI_ISL_833673	Lighthouse Lab in Cambridge	Wellcome Sanger Institute for the COVID-19 Genomics UK (COG-UK) Consortium	Rob Howes, The Lighthouse Lab in Cambridge and Alex Alderton, Roberto Amato, Sonia Goncalves, Ewan Harrison, David K. Jackson, Ian Johnston, Dominic Kwiatkowski, Cordelia Langford, John Sillitoe on behalf of the Wellcome Sanger Institute COVID-19 Surveillance Team
EPI_ISL_833676, EPI_ISL_833678	Lighthouse Lab in Milton Keynes	Wellcome Sanger Institute for the COVID-19 Genomics UK (COG-UK) Consortium	The Lighthouse Lab in Milton Keynes and Alex Alderton, Roberto Amato, Sonia Goncalves, Ewan Harrison, David K. Jackson, Ian Johnston, Dominic Kwiatkowski, Cordelia Langford, John Sillitoe on behalf of the Wellcome Sanger Institute COVID-19 Surveillance Team
EPI_ISL_833679, EPI_ISL_833680, EPI_ISL_833683, EPI_ISL_833684	Lighthouse Lab in Cambridge	Wellcome Sanger Institute for the COVID-19 Genomics UK (COG-UK) Consortium	Rob Howes, The Lighthouse Lab in Cambridge and Alex Alderton, Roberto Amato, Sonia Goncalves, Ewan Harrison, David K. Jackson, Ian Johnston, Dominic Kwiatkowski, Cordelia Langford, John Sillitoe on behalf of the Wellcome Sanger Institute COVID-19 Surveillance Team
EPI_ISL_833685	Lighthouse Lab in Milton Keynes	Wellcome Sanger Institute for the COVID-19 Genomics UK (COG-UK) Consortium	The Lighthouse Lab in Milton Keynes and Alex Alderton, Roberto Amato, Sonia Goncalves, Ewan Harrison, David K. Jackson, Ian Johnston, Dominic Kwiatkowski, Cordelia Langford, John Sillitoe on behalf of the Wellcome Sanger Institute COVID-19 Surveillance Team
EPI_ISL_833686, EPI_ISL_833687, EPI_ISL_833689, EPI_ISL_833691	Lighthouse Lab in Cambridge	Wellcome Sanger Institute for the COVID-19 Genomics UK (COG-UK) Consortium	Rob Howes, The Lighthouse Lab in Cambridge and Alex Alderton, Roberto Amato, Sonia Goncalves, Ewan Harrison, David K. Jackson, Ian Johnston, Dominic Kwiatkowski, Cordelia Langford, John Sillitoe on behalf of the Wellcome Sanger Institute COVID-19 Surveillance Team
EPI_ISL_833693	Lighthouse Lab in Milton Keynes	Wellcome Sanger Institute for the COVID-19 Genomics UK (COG-UK) Consortium	The Lighthouse Lab in Milton Keynes and Alex Alderton, Roberto Amato, Sonia Goncalves, Ewan Harrison, David K. Jackson, Ian Johnston, Dominic Kwiatkowski, Cordelia Langford, John Sillitoe on behalf of the Wellcome Sanger Institute COVID-19 Surveillance Team
EPI_ISL_833695, EPI_ISL_833696	Lighthouse Lab in Cambridge	Wellcome Sanger Institute for the COVID-19 Genomics UK (COG-UK) Consortium	Rob Howes, The Lighthouse Lab in Cambridge and Alex Alderton, Roberto Amato, Sonia Goncalves, Ewan Harrison, David K. Jackson, Ian Johnston, Dominic Kwiatkowski, Cordelia Langford, John Sillitoe on behalf of the Wellcome Sanger Institute COVID-19 Surveillance Team
EPI_ISL_833700	Lighthouse Lab in Milton Keynes	Wellcome Sanger Institute for the COVID-19 Genomics UK (COG-UK) Consortium	The Lighthouse Lab in Milton Keynes and Alex Alderton, Roberto Amato, Sonia Goncalves, Ewan Harrison, David K. Jackson, Ian Johnston, Dominic Kwiatkowski, Cordelia Langford, John Sillitoe on behalf of the Wellcome Sanger Institute COVID-19 Surveillance Team
EPI_ISL_833702, EPI_ISL_833703, EPI_ISL_833704, EPI_ISL_833705, EPI_ISL_833706, EPI_ISL_833707, EPI_ISL_833708, EPI_ISL_833709, EPI_ISL_833710	Lighthouse Lab in Cambridge	Wellcome Sanger Institute for the COVID-19 Genomics UK (COG-UK) Consortium	Rob Howes, The Lighthouse Lab in Cambridge and Alex Alderton, Roberto Amato, Sonia Goncalves, Ewan Harrison, David K. Jackson, Ian Johnston, Dominic Kwiatkowski, Cordelia Langford, John Sillitoe on behalf of the Wellcome Sanger Institute COVID-19 Surveillance Team
EPI_ISL_833712	Lighthouse Lab in Milton Keynes	Wellcome Sanger Institute for the COVID-19 Genomics UK (COG-UK) Consortium	The Lighthouse Lab in Milton Keynes and Alex Alderton, Roberto Amato, Sonia Goncalves, Ewan Harrison, David K. Jackson, Ian Johnston, Dominic Kwiatkowski, Cordelia Langford, John Sillitoe on behalf of the Wellcome Sanger Institute COVID-19 Surveillance Team
EPI_ISL_833714	Lighthouse Lab in Cambridge	Wellcome Sanger Institute for the COVID-19 Genomics UK (COG-UK) Consortium	Rob Howes, The Lighthouse Lab in Cambridge and Alex Alderton, Roberto Amato, Sonia Goncalves, Ewan Harrison, David K. Jackson, Ian Johnston, Dominic Kwiatkowski, Cordelia Langford, John Sillitoe on behalf of the Wellcome Sanger Institute COVID-19 Surveillance Team
EPI_ISL_833716, EPI_ISL_833719, EPI_ISL_833721	Lighthouse Lab in Milton Keynes	Wellcome Sanger Institute for the COVID-19 Genomics UK (COG-UK) Consortium	The Lighthouse Lab in Milton Keynes and Alex Alderton, Roberto Amato, Sonia Goncalves, Ewan Harrison, David K. Jackson, Ian Johnston, Dominic Kwiatkowski, Cordelia Langford, John Sillitoe on behalf of the Wellcome Sanger Institute COVID-19 Surveillance Team
EPI_ISL_833724	Lighthouse Lab in Cambridge	Wellcome Sanger Institute for the COVID-19 Genomics UK (COG-UK) Consortium	Rob Howes, The Lighthouse Lab in Cambridge and Alex Alderton, Roberto Amato, Sonia Goncalves, Ewan Harrison, David K. Jackson, Ian Johnston, Dominic Kwiatkowski, Cordelia Langford, John Sillitoe on behalf of the Wellcome Sanger Institute COVID-19 Surveillance Team
EPI_ISL_833725, EPI_ISL_833726	Lighthouse Lab in Milton Keynes	Wellcome Sanger Institute for the COVID-19 Genomics UK (COG-UK) Consortium	The Lighthouse Lab in Milton Keynes and Alex Alderton, Roberto Amato, Sonia Goncalves, Ewan Harrison, David K. Jackson, Ian Johnston, Dominic Kwiatkowski, Cordelia Langford, John Sillitoe on behalf of the Wellcome Sanger Institute COVID-19 Surveillance Team
EPI_ISL_833727, EPI_ISL_833728, EPI_ISL_833733	Lighthouse Lab in Cambridge	Wellcome Sanger Institute for the COVID-19 Genomics UK (COG-UK) Consortium	Rob Howes, The Lighthouse Lab in Cambridge and Alex Alderton, Roberto Amato, Sonia Goncalves, Ewan Harrison, David K. Jackson, Ian Johnston, Dominic Kwiatkowski, Cordelia Langford, John Sillitoe on behalf of the Wellcome Sanger Institute COVID-19 Surveillance Team
EPI_ISL_833734, EPI_ISL_833735, EPI_ISL_833736, EPI_ISL_833738	Lighthouse Lab in Milton Keynes	Wellcome Sanger Institute for the COVID-19 Genomics UK (COG-UK) Consortium	The Lighthouse Lab in Milton Keynes and Alex Alderton, Roberto Amato, Sonia Goncalves, Ewan Harrison, David K. Jackson, Ian Johnston, Dominic Kwiatkowski, Cordelia Langford, John Sillitoe on behalf of the Wellcome Sanger Institute COVID-19 Surveillance Team
EPI_ISL_833741, EPI_ISL_833742	Lighthouse Lab in Cambridge	Wellcome Sanger Institute for the COVID-19 Genomics UK (COG-UK) Consortium	Rob Howes, The Lighthouse Lab in Cambridge and Alex Alderton, Roberto Amato, Sonia Goncalves, Ewan Harrison, David K. Jackson, Ian Johnston, Dominic Kwiatkowski, Cordelia Langford, John Sillitoe on behalf of the Wellcome Sanger Institute COVID-19 Surveillance Team
EPI_ISL_833743	Lighthouse Lab in Milton Keynes	Wellcome Sanger Institute for the COVID-19 Genomics UK (COG-UK) Consortium	The Lighthouse Lab in Milton Keynes and Alex Alderton, Roberto Amato, Sonia Goncalves, Ewan Harrison, David K. Jackson, Ian Johnston, Dominic Kwiatkowski, Cordelia Langford, John Sillitoe on behalf of the Wellcome Sanger Institute COVID-19 Surveillance Team
EPI_ISL_833746, EPI_ISL_833748, EPI_ISL_833749, EPI_ISL_833750, EPI_ISL_833753	Lighthouse Lab in Cambridge	Wellcome Sanger Institute for the COVID-19 Genomics UK (COG-UK) Consortium	Rob Howes, The Lighthouse Lab in Cambridge and Alex Alderton, Roberto Amato, Sonia Goncalves, Ewan Harrison, David K. Jackson, Ian Johnston, Dominic Kwiatkowski, Cordelia Langford, John Sillitoe on behalf of the Wellcome Sanger Institute COVID-19 Surveillance Team
EPI_ISL_833755, EPI_ISL_833756	Lighthouse Lab in Milton Keynes	Wellcome Sanger Institute for the COVID-19 Genomics UK (COG-UK) Consortium	The Lighthouse Lab in Milton Keynes and Alex Alderton, Roberto Amato, Sonia Goncalves, Ewan Harrison, David K. Jackson, Ian Johnston, Dominic Kwiatkowski, Cordelia Langford, John Sillitoe on behalf of the Wellcome Sanger Institute COVID-19 Surveillance Team
EPI_ISL_833758	Lighthouse Lab in Cambridge	Wellcome Sanger Institute for the COVID-19 Genomics UK (COG-UK) Consortium	Rob Howes, The Lighthouse Lab in Cambridge and Alex Alderton, Roberto Amato, Sonia Goncalves, Ewan Harrison, David K. Jackson, Ian Johnston, Dominic Kwiatkowski, Cordelia Langford, John Sillitoe on behalf of the Wellcome Sanger Institute COVID-19 Surveillance Team
EPI_ISL_833759	Lighthouse Lab in Milton Keynes	Wellcome Sanger Institute for the COVID-19 Genomics UK (COG-UK) Consortium	The Lighthouse Lab in Milton Keynes and Alex Alderton, Roberto Amato, Sonia Goncalves, Ewan Harrison, David K. Jackson, Ian Johnston, Dominic Kwiatkowski, Cordelia Langford, John Sillitoe on behalf of the Wellcome Sanger Institute COVID-19 Surveillance Team
EPI_ISL_833762	Lighthouse Lab in Cambridge	Wellcome Sanger Institute for the COVID-19 Genomics UK (COG-UK) Consortium	Rob Howes, The Lighthouse Lab in Cambridge and Alex Alderton, Roberto Amato, Sonia Goncalves, Ewan Harrison, David K. Jackson, Ian Johnston, Dominic Kwiatkowski, Cordelia Langford, John Sillitoe on behalf of the Wellcome Sanger Institute COVID-19 Surveillance Team
EPI_ISL_833766, EPI_ISL_833767, EPI_ISL_833768	Lighthouse Lab in Milton Keynes	Wellcome Sanger Institute for the COVID-19 Genomics UK (COG-UK) Consortium	The Lighthouse Lab in Milton Keynes and Alex Alderton, Roberto Amato, Sonia Goncalves, Ewan Harrison, David K. Jackson, Ian Johnston, Dominic Kwiatkowski, Cordelia Langford, John Sillitoe on behalf of the Wellcome Sanger Institute COVID-19 Surveillance Team
EPI_ISL_833770, EPI_ISL_833771, EPI_ISL_833773, EPI_ISL_833774, EPI_ISL_833778	Lighthouse Lab in Cambridge	Wellcome Sanger Institute for the COVID-19 Genomics UK (COG-UK) Consortium	Rob Howes, The Lighthouse Lab in Cambridge and Alex Alderton, Roberto Amato, Sonia Goncalves, Ewan Harrison, David K. Jackson, Ian Johnston, Dominic Kwiatkowski, Cordelia Langford, John Sillitoe on behalf of the Wellcome Sanger Institute COVID-19 Surveillance Team
EPI_ISL_833779, EPI_ISL_833780	Lighthouse Lab in Milton Keynes	Wellcome Sanger Institute for the COVID-19 Genomics UK (COG-UK) Consortium	The Lighthouse Lab in Milton Keynes and Alex Alderton, Roberto Amato, Sonia Goncalves, Ewan Harrison, David K. Jackson, Ian Johnston, Dominic Kwiatkowski, Cordelia Langford, John Sillitoe on behalf of the Wellcome Sanger Institute COVID-19 Surveillance Team
EPI_ISL_833784	Lighthouse Lab in Cambridge	Wellcome Sanger Institute for the COVID-19 Genomics UK (COG-UK) Consortium	Rob Howes, The Lighthouse Lab in Cambridge and Alex Alderton, Roberto Amato, Sonia Goncalves, Ewan Harrison, David K. Jackson, Ian Johnston, Dominic Kwiatkowski, Cordelia Langford, John Sillitoe on behalf of the Wellcome Sanger Institute COVID-19 Surveillance Team
EPI_ISL_833789, EPI_ISL_833791	Lighthouse Lab in Milton Keynes	Wellcome Sanger Institute for the COVID-19 Genomics UK (COG-UK) Consortium	The Lighthouse Lab in Milton Keynes and Alex Alderton, Roberto Amato, Sonia Goncalves, Ewan Harrison, David K. Jackson, Ian Johnston, Dominic Kwiatkowski, Cordelia Langford, John Sillitoe on behalf of the Wellcome Sanger Institute COVID-19 Surveillance Team
EPI_ISL_833792, EPI_ISL_833794	Lighthouse Lab in Cambridge	Wellcome Sanger Institute for the COVID-19 Genomics UK (COG-UK) Consortium	Rob Howes, The Lighthouse Lab in Cambridge and Alex Alderton, Roberto Amato, Sonia Goncalves, Ewan Harrison, David K. Jackson, Ian Johnston, Dominic Kwiatkowski, Cordelia Langford, John Sillitoe on behalf of the Wellcome Sanger Institute COVID-19 Surveillance Team
EPI_ISL_833795, EPI_ISL_833796, EPI_ISL_833797	Lighthouse Lab in Milton Keynes	Wellcome Sanger Institute for the COVID-19 Genomics UK (COG-UK) Consortium	The Lighthouse Lab in Milton Keynes and Alex Alderton, Roberto Amato, Sonia Goncalves, Ewan Harrison, David K. Jackson, Ian Johnston, Dominic Kwiatkowski, Cordelia Langford, John Sillitoe on behalf of the Wellcome Sanger Institute COVID-19 Surveillance Team
EPI_ISL_833800	Lighthouse Lab in Cambridge	Wellcome Sanger Institute for the COVID-19 Genomics UK	Rob Howes, The Lighthouse Lab in Cambridge and Alex Alderton, Roberto Amato, Sonia Goncalves, Ewan Harrison, David K. Jackson, Ian Johnston,

		(COG-UK) Consortium	Dominic Kwiatkowski, Cordelia Langford, John Sillitoe on behalf of the Wellcome Sanger Institute COVID-19 Surveillance Team
EPI_ISL_833801, EPI_ISL_833803, EPI_ISL_833804	Lighthouse Lab in Milton Keynes	Wellcome Sanger Institute for the COVID-19 Genomics UK (COG-UK) Consortium	The Lighthouse Lab in Milton Keynes and Alex Alderton, Roberto Amato, Sonia Goncalves, Ewan Harrison, David K. Jackson, Ian Johnston, Dominic Kwiatkowski, Cordelia Langford, John Sillitoe on behalf of the Wellcome Sanger Institute COVID-19 Surveillance Team
EPI_ISL_833806, EPI_ISL_833807	Lighthouse Lab in Cambridge	Wellcome Sanger Institute for the COVID-19 Genomics UK (COG-UK) Consortium	Rob Howes, The Lighthouse Lab in Cambridge and Alex Alderton, Roberto Amato, Sonia Goncalves, Ewan Harrison, David K. Jackson, Ian Johnston, Dominic Kwiatkowski, Cordelia Langford, John Sillitoe on behalf of the Wellcome Sanger Institute COVID-19 Surveillance Team
EPI_ISL_833809	Lighthouse Lab in Milton Keynes	Wellcome Sanger Institute for the COVID-19 Genomics UK (COG-UK) Consortium	The Lighthouse Lab in Milton Keynes and Alex Alderton, Roberto Amato, Sonia Goncalves, Ewan Harrison, David K. Jackson, Ian Johnston, Dominic Kwiatkowski, Cordelia Langford, John Sillitoe on behalf of the Wellcome Sanger Institute COVID-19 Surveillance Team
EPI_ISL_833810, EPI_ISL_833812, EPI_ISL_833813	Lighthouse Lab in Cambridge	Wellcome Sanger Institute for the COVID-19 Genomics UK (COG-UK) Consortium	Rob Howes, The Lighthouse Lab in Cambridge and Alex Alderton, Roberto Amato, Sonia Goncalves, Ewan Harrison, David K. Jackson, Ian Johnston, Dominic Kwiatkowski, Cordelia Langford, John Sillitoe on behalf of the Wellcome Sanger Institute COVID-19 Surveillance Team
EPI_ISL_833814	Lighthouse Lab in Milton Keynes	Wellcome Sanger Institute for the COVID-19 Genomics UK (COG-UK) Consortium	The Lighthouse Lab in Milton Keynes and Alex Alderton, Roberto Amato, Sonia Goncalves, Ewan Harrison, David K. Jackson, Ian Johnston, Dominic Kwiatkowski, Cordelia Langford, John Sillitoe on behalf of the Wellcome Sanger Institute COVID-19 Surveillance Team
EPI_ISL_833815, EPI_ISL_833816	Lighthouse Lab in Cambridge	Wellcome Sanger Institute for the COVID-19 Genomics UK (COG-UK) Consortium	Rob Howes, The Lighthouse Lab in Cambridge and Alex Alderton, Roberto Amato, Sonia Goncalves, Ewan Harrison, David K. Jackson, Ian Johnston, Dominic Kwiatkowski, Cordelia Langford, John Sillitoe on behalf of the Wellcome Sanger Institute COVID-19 Surveillance Team
EPI_ISL_833817, EPI_ISL_833818, EPI_ISL_833821, EPI_ISL_833823	Lighthouse Lab in Milton Keynes	Wellcome Sanger Institute for the COVID-19 Genomics UK (COG-UK) Consortium	The Lighthouse Lab in Milton Keynes and Alex Alderton, Roberto Amato, Sonia Goncalves, Ewan Harrison, David K. Jackson, Ian Johnston, Dominic Kwiatkowski, Cordelia Langford, John Sillitoe on behalf of the Wellcome Sanger Institute COVID-19 Surveillance Team
EPI_ISL_833829	Lighthouse Lab in Cambridge	Wellcome Sanger Institute for the COVID-19 Genomics UK (COG-UK) Consortium	Rob Howes, The Lighthouse Lab in Cambridge and Alex Alderton, Roberto Amato, Sonia Goncalves, Ewan Harrison, David K. Jackson, Ian Johnston, Dominic Kwiatkowski, Cordelia Langford, John Sillitoe on behalf of the Wellcome Sanger Institute COVID-19 Surveillance Team
EPI_ISL_833830	Lighthouse Lab in Milton Keynes	Wellcome Sanger Institute for the COVID-19 Genomics UK (COG-UK) Consortium	The Lighthouse Lab in Milton Keynes and Alex Alderton, Roberto Amato, Sonia Goncalves, Ewan Harrison, David K. Jackson, Ian Johnston, Dominic Kwiatkowski, Cordelia Langford, John Sillitoe on behalf of the Wellcome Sanger Institute COVID-19 Surveillance Team
EPI_ISL_833831, EPI_ISL_833832, EPI_ISL_833833	Lighthouse Lab in Cambridge	Wellcome Sanger Institute for the COVID-19 Genomics UK (COG-UK) Consortium	Rob Howes, The Lighthouse Lab in Cambridge and Alex Alderton, Roberto Amato, Sonia Goncalves, Ewan Harrison, David K. Jackson, Ian Johnston, Dominic Kwiatkowski, Cordelia Langford, John Sillitoe on behalf of the Wellcome Sanger Institute COVID-19 Surveillance Team
EPI_ISL_833834, EPI_ISL_833835	Lighthouse Lab in Milton Keynes	Wellcome Sanger Institute for the COVID-19 Genomics UK (COG-UK) Consortium	The Lighthouse Lab in Milton Keynes and Alex Alderton, Roberto Amato, Sonia Goncalves, Ewan Harrison, David K. Jackson, Ian Johnston, Dominic Kwiatkowski, Cordelia Langford, John Sillitoe on behalf of the Wellcome Sanger Institute COVID-19 Surveillance Team
EPI_ISL_833840	Lighthouse Lab in Cambridge	Wellcome Sanger Institute for the COVID-19 Genomics UK (COG-UK) Consortium	Rob Howes, The Lighthouse Lab in Cambridge and Alex Alderton, Roberto Amato, Sonia Goncalves, Ewan Harrison, David K. Jackson, Ian Johnston, Dominic Kwiatkowski, Cordelia Langford, John Sillitoe on behalf of the Wellcome Sanger Institute COVID-19 Surveillance Team
EPI_ISL_833841	Lighthouse Lab in Milton Keynes	Wellcome Sanger Institute for the COVID-19 Genomics UK (COG-UK) Consortium	The Lighthouse Lab in Milton Keynes and Alex Alderton, Roberto Amato, Sonia Goncalves, Ewan Harrison, David K. Jackson, Ian Johnston, Dominic Kwiatkowski, Cordelia Langford, John Sillitoe on behalf of the Wellcome Sanger Institute COVID-19 Surveillance Team
EPI_ISL_833842, EPI_ISL_833844	Lighthouse Lab in Cambridge	Wellcome Sanger Institute for the COVID-19 Genomics UK (COG-UK) Consortium	Rob Howes, The Lighthouse Lab in Cambridge and Alex Alderton, Roberto Amato, Sonia Goncalves, Ewan Harrison, David K. Jackson, Ian Johnston, Dominic Kwiatkowski, Cordelia Langford, John Sillitoe on behalf of the Wellcome Sanger Institute COVID-19 Surveillance Team
EPI_ISL_833845, EPI_ISL_833846, EPI_ISL_833848	Lighthouse Lab in Milton Keynes	Wellcome Sanger Institute for the COVID-19 Genomics UK (COG-UK) Consortium	The Lighthouse Lab in Milton Keynes and Alex Alderton, Roberto Amato, Sonia Goncalves, Ewan Harrison, David K. Jackson, Ian Johnston, Dominic Kwiatkowski, Cordelia Langford, John Sillitoe on behalf of the Wellcome Sanger Institute COVID-19 Surveillance Team
EPI_ISL_833849, EPI_ISL_833852, EPI_ISL_833853, EPI_ISL_833854, EPI_ISL_833855	Lighthouse Lab in Cambridge	Wellcome Sanger Institute for the COVID-19 Genomics UK (COG-UK) Consortium	Rob Howes, The Lighthouse Lab in Cambridge and Alex Alderton, Roberto Amato, Sonia Goncalves, Ewan Harrison, David K. Jackson, Ian Johnston, Dominic Kwiatkowski, Cordelia Langford, John Sillitoe on behalf of the Wellcome Sanger Institute COVID-19 Surveillance Team
EPI_ISL_833856	Lighthouse Lab in Milton Keynes	Wellcome Sanger Institute for the COVID-19 Genomics UK (COG-UK) Consortium	The Lighthouse Lab in Milton Keynes and Alex Alderton, Roberto Amato, Sonia Goncalves, Ewan Harrison, David K. Jackson, Ian Johnston, Dominic Kwiatkowski, Cordelia Langford, John Sillitoe on behalf of the Wellcome Sanger Institute COVID-19 Surveillance Team
EPI_ISL_833859, EPI_ISL_833860	Lighthouse Lab in Cambridge	Wellcome Sanger Institute for the COVID-19 Genomics UK (COG-UK) Consortium	Rob Howes, The Lighthouse Lab in Cambridge and Alex Alderton, Roberto Amato, Sonia Goncalves, Ewan Harrison, David K. Jackson, Ian Johnston, Dominic Kwiatkowski, Cordelia Langford, John Sillitoe on behalf of the Wellcome Sanger Institute COVID-19 Surveillance Team
EPI_ISL_833861	Lighthouse Lab in Milton Keynes	Wellcome Sanger Institute for the COVID-19 Genomics UK (COG-UK) Consortium	The Lighthouse Lab in Milton Keynes and Alex Alderton, Roberto Amato, Sonia Goncalves, Ewan Harrison, David K. Jackson, Ian Johnston, Dominic Kwiatkowski, Cordelia Langford, John Sillitoe on behalf of the Wellcome Sanger Institute COVID-19 Surveillance Team
EPI_ISL_833862, EPI_ISL_833863, EPI_ISL_833864	Lighthouse Lab in Cambridge	Wellcome Sanger Institute for the COVID-19 Genomics UK (COG-UK) Consortium	Rob Howes, The Lighthouse Lab in Cambridge and Alex Alderton, Roberto Amato, Sonia Goncalves, Ewan Harrison, David K. Jackson, Ian Johnston, Dominic Kwiatkowski, Cordelia Langford, John Sillitoe on behalf of the Wellcome Sanger Institute COVID-19 Surveillance Team
EPI_ISL_833865	Lighthouse Lab in Milton Keynes	Wellcome Sanger Institute for the COVID-19 Genomics UK (COG-UK) Consortium	The Lighthouse Lab in Milton Keynes and Alex Alderton, Roberto Amato, Sonia Goncalves, Ewan Harrison, David K. Jackson, Ian Johnston, Dominic Kwiatkowski, Cordelia Langford, John Sillitoe on behalf of the Wellcome Sanger Institute COVID-19 Surveillance Team
EPI_ISL_833866	Lighthouse Lab in Cambridge	Wellcome Sanger Institute for the COVID-19 Genomics UK (COG-UK) Consortium	Rob Howes, The Lighthouse Lab in Cambridge and Alex Alderton, Roberto Amato, Sonia Goncalves, Ewan Harrison, David K. Jackson, Ian Johnston, Dominic Kwiatkowski, Cordelia Langford, John Sillitoe on behalf of the Wellcome Sanger Institute COVID-19 Surveillance Team
EPI_ISL_833868, EPI_ISL_833872	Lighthouse Lab in Milton Keynes	Wellcome Sanger Institute for the COVID-19 Genomics UK (COG-UK) Consortium	The Lighthouse Lab in Milton Keynes and Alex Alderton, Roberto Amato, Sonia Goncalves, Ewan Harrison, David K. Jackson, Ian Johnston, Dominic Kwiatkowski, Cordelia Langford, John Sillitoe on behalf of the Wellcome Sanger Institute COVID-19 Surveillance Team
EPI_ISL_833876, EPI_ISL_833877	Lighthouse Lab in Cambridge	Wellcome Sanger Institute for the COVID-19 Genomics UK (COG-UK) Consortium	Rob Howes, The Lighthouse Lab in Cambridge and Alex Alderton, Roberto Amato, Sonia Goncalves, Ewan Harrison, David K. Jackson, Ian Johnston, Dominic Kwiatkowski, Cordelia Langford, John Sillitoe on behalf of the Wellcome Sanger Institute COVID-19 Surveillance Team
EPI_ISL_833879, EPI_ISL_833881	Lighthouse Lab in Milton Keynes	Wellcome Sanger Institute for the COVID-19 Genomics UK (COG-UK) Consortium	The Lighthouse Lab in Milton Keynes and Alex Alderton, Roberto Amato, Sonia Goncalves, Ewan Harrison, David K. Jackson, Ian Johnston, Dominic Kwiatkowski, Cordelia Langford, John Sillitoe on behalf of the Wellcome Sanger Institute COVID-19 Surveillance Team
EPI_ISL_833882	Lighthouse Lab in Cambridge	Wellcome Sanger Institute for the COVID-19 Genomics UK (COG-UK) Consortium	Rob Howes, The Lighthouse Lab in Cambridge and Alex Alderton, Roberto Amato, Sonia Goncalves, Ewan Harrison, David K. Jackson, Ian Johnston, Dominic Kwiatkowski, Cordelia Langford, John Sillitoe on behalf of the Wellcome Sanger Institute COVID-19 Surveillance Team
EPI_ISL_833884	Lighthouse Lab in Milton Keynes	Wellcome Sanger Institute for the COVID-19 Genomics UK (COG-UK) Consortium	The Lighthouse Lab in Milton Keynes and Alex Alderton, Roberto Amato, Sonia Goncalves, Ewan Harrison, David K. Jackson, Ian Johnston, Dominic Kwiatkowski, Cordelia Langford, John Sillitoe on behalf of the Wellcome Sanger Institute COVID-19 Surveillance Team
EPI_ISL_833886, EPI_ISL_833888	Lighthouse Lab in Cambridge	Wellcome Sanger Institute for the COVID-19 Genomics UK (COG-UK) Consortium	Rob Howes, The Lighthouse Lab in Cambridge and Alex Alderton, Roberto Amato, Sonia Goncalves, Ewan Harrison, David K. Jackson, Ian Johnston, Dominic Kwiatkowski, Cordelia Langford, John Sillitoe on behalf of the Wellcome Sanger Institute COVID-19 Surveillance Team
EPI_ISL_833889, EPI_ISL_833890	Lighthouse Lab in Milton Keynes	Wellcome Sanger Institute for the COVID-19 Genomics UK (COG-UK) Consortium	The Lighthouse Lab in Milton Keynes and Alex Alderton, Roberto Amato, Sonia Goncalves, Ewan Harrison, David K. Jackson, Ian Johnston, Dominic Kwiatkowski, Cordelia Langford, John Sillitoe on behalf of the Wellcome Sanger Institute COVID-19 Surveillance Team
EPI_ISL_833892	Lighthouse Lab in Cambridge	Wellcome Sanger Institute for the COVID-19 Genomics UK (COG-UK) Consortium	Rob Howes, The Lighthouse Lab in Cambridge and Alex Alderton, Roberto Amato, Sonia Goncalves, Ewan Harrison, David K. Jackson, Ian Johnston, Dominic Kwiatkowski, Cordelia Langford, John Sillitoe on behalf of the Wellcome Sanger Institute COVID-19 Surveillance Team
EPI_ISL_833894, EPI_ISL_833895	Lighthouse Lab in Milton Keynes	Wellcome Sanger Institute for the COVID-19 Genomics UK (COG-UK) Consortium	The Lighthouse Lab in Milton Keynes and Alex Alderton, Roberto Amato, Sonia Goncalves, Ewan Harrison, David K. Jackson, Ian Johnston, Dominic Kwiatkowski, Cordelia Langford, John Sillitoe on behalf of the Wellcome Sanger Institute COVID-19 Surveillance Team
EPI_ISL_833896, EPI_ISL_833898	Lighthouse Lab in Cambridge	Wellcome Sanger Institute for the COVID-19 Genomics UK (COG-UK) Consortium	Rob Howes, The Lighthouse Lab in Cambridge and Alex Alderton, Roberto Amato, Sonia Goncalves, Ewan Harrison, David K. Jackson, Ian Johnston, Dominic Kwiatkowski, Cordelia Langford, John Sillitoe on behalf of the Wellcome Sanger Institute COVID-19 Surveillance Team
EPI_ISL_833901	Lighthouse Lab in Milton Keynes	Wellcome Sanger Institute for the COVID-19 Genomics UK (COG-UK) Consortium	The Lighthouse Lab in Milton Keynes and Alex Alderton, Roberto Amato, Sonia Goncalves, Ewan Harrison, David K. Jackson, Ian Johnston, Dominic Kwiatkowski, Cordelia Langford, John Sillitoe on behalf of the Wellcome Sanger Institute COVID-19 Surveillance Team
EPI_ISL_833903, EPI_ISL_833907	Lighthouse Lab in Cambridge	Wellcome Sanger Institute for the COVID-19 Genomics UK (COG-UK) Consortium	Rob Howes, The Lighthouse Lab in Cambridge and Alex Alderton, Roberto Amato, Sonia Goncalves, Ewan Harrison, David K. Jackson, Ian Johnston, Dominic Kwiatkowski, Cordelia Langford, John Sillitoe on behalf of the Wellcome Sanger Institute COVID-19 Surveillance Team

EPI_ISL_833908	Lighthouse Lab in Milton Keynes	Wellcome Sanger Institute for the COVID-19 Genomics UK (COG-UK) Consortium	The Lighthouse Lab in Milton Keynes and Alex Alderton, Roberto Amato, Sonia Goncalves, Ewan Harrison, David K. Jackson, Ian Johnston, Dominic Kwiatkowski, Cordelia Langford, John Sillitoe on behalf of the Wellcome Sanger Institute COVID-19 Surveillance Team
EPI_ISL_833909, EPI_ISL_833910, EPI_ISL_833912	Lighthouse Lab in Cambridge	Wellcome Sanger Institute for the COVID-19 Genomics UK (COG-UK) Consortium	Rob Howes, The Lighthouse Lab in Cambridge and Alex Alderton, Roberto Amato, Sonia Goncalves, Ewan Harrison, David K. Jackson, Ian Johnston, Dominic Kwiatkowski, Cordelia Langford, John Sillitoe on behalf of the Wellcome Sanger Institute COVID-19 Surveillance Team
EPI_ISL_833916	Lighthouse Lab in Milton Keynes	Wellcome Sanger Institute for the COVID-19 Genomics UK (COG-UK) Consortium	The Lighthouse Lab in Milton Keynes and Alex Alderton, Roberto Amato, Sonia Goncalves, Ewan Harrison, David K. Jackson, Ian Johnston, Dominic Kwiatkowski, Cordelia Langford, John Sillitoe on behalf of the Wellcome Sanger Institute COVID-19 Surveillance Team
EPI_ISL_833917, EPI_ISL_833918, EPI_ISL_833919	Lighthouse Lab in Cambridge	Wellcome Sanger Institute for the COVID-19 Genomics UK (COG-UK) Consortium	Rob Howes, The Lighthouse Lab in Cambridge and Alex Alderton, Roberto Amato, Sonia Goncalves, Ewan Harrison, David K. Jackson, Ian Johnston, Dominic Kwiatkowski, Cordelia Langford, John Sillitoe on behalf of the Wellcome Sanger Institute COVID-19 Surveillance Team
EPI_ISL_833922, EPI_ISL_833924, EPI_ISL_833930, EPI_ISL_833931	Lighthouse Lab in Milton Keynes	Wellcome Sanger Institute for the COVID-19 Genomics UK (COG-UK) Consortium	The Lighthouse Lab in Milton Keynes and Alex Alderton, Roberto Amato, Sonia Goncalves, Ewan Harrison, David K. Jackson, Ian Johnston, Dominic Kwiatkowski, Cordelia Langford, John Sillitoe on behalf of the Wellcome Sanger Institute COVID-19 Surveillance Team
EPI_ISL_833932	Lighthouse Lab in Cambridge	Wellcome Sanger Institute for the COVID-19 Genomics UK (COG-UK) Consortium	Rob Howes, The Lighthouse Lab in Cambridge and Alex Alderton, Roberto Amato, Sonia Goncalves, Ewan Harrison, David K. Jackson, Ian Johnston, Dominic Kwiatkowski, Cordelia Langford, John Sillitoe on behalf of the Wellcome Sanger Institute COVID-19 Surveillance Team
EPI_ISL_833933, EPI_ISL_833934, EPI_ISL_833935, EPI_ISL_833936, EPI_ISL_833937, EPI_ISL_833938, EPI_ISL_833939, EPI_ISL_833940, EPI_ISL_833941, EPI_ISL_833942, EPI_ISL_833943, EPI_ISL_833945, EPI_ISL_833946, EPI_ISL_833947, EPI_ISL_833948, EPI_ISL_833949, EPI_ISL_833950, EPI_ISL_833951, EPI_ISL_833952, EPI_ISL_833953, EPI_ISL_833954, EPI_ISL_833955, EPI_ISL_833957, EPI_ISL_833958, EPI_ISL_833959, EPI_ISL_833960, EPI_ISL_833961, EPI_ISL_833962, EPI_ISL_833963, EPI_ISL_833964, EPI_ISL_833965, EPI_ISL_833967, EPI_ISL_833969, EPI_ISL_833970, EPI_ISL_833971, EPI_ISL_833972, EPI_ISL_833973, EPI_ISL_833974, EPI_ISL_833975, EPI_ISL_833976, EPI_ISL_833977, EPI_ISL_833978, EPI_ISL_833979, EPI_ISL_833980, EPI_ISL_833981, EPI_ISL_833982, EPI_ISL_833983, EPI_ISL_833984, EPI_ISL_833985, EPI_ISL_833986, EPI_ISL_833987, EPI_ISL_833988, EPI_ISL_833989, EPI_ISL_833990, EPI_ISL_833991, EPI_ISL_833992, EPI_ISL_833993, EPI_ISL_833994, EPI_ISL_833996, EPI_ISL_833998, EPI_ISL_833999, EPI_ISL_834000, EPI_ISL_834001, EPI_ISL_834002, EPI_ISL_834003, EPI_ISL_834004, EPI_ISL_834005, EPI_ISL_834006, EPI_ISL_834007, EPI_ISL_834008, EPI_ISL_834010, EPI_ISL_834011, EPI_ISL_834012, EPI_ISL_834013, EPI_ISL_834014, EPI_ISL_834015, EPI_ISL_834016, EPI_ISL_834017, EPI_ISL_834018, EPI_ISL_834019, EPI_ISL_834020, EPI_ISL_834021, EPI_ISL_834023, EPI_ISL_834024, EPI_ISL_834025, EPI_ISL_834026, EPI_ISL_834027, EPI_ISL_834028, EPI_ISL_834030, EPI_ISL_834031, EPI_ISL_834032, EPI_ISL_834033, EPI_ISL_834034, EPI_ISL_834035, EPI_ISL_834036, EPI_ISL_834037, EPI_ISL_834038, EPI_ISL_834039, EPI_ISL_834040, EPI_ISL_834041, EPI_ISL_834043, EPI_ISL_834044, EPI_ISL_834045, EPI_ISL_834046, EPI_ISL_834047, EPI_ISL_834048, EPI_ISL_834049, EPI_ISL_834050, EPI_ISL_834051, EPI_ISL_834052, EPI_ISL_834053, EPI_ISL_834054, EPI_ISL_834055, EPI_ISL_834056, EPI_ISL_834057, EPI_ISL_834058, EPI_ISL_834059, EPI_ISL_834060, EPI_ISL_834061, EPI_ISL_834062, EPI_ISL_834063, EPI_ISL_834064, EPI_ISL_834065, EPI_ISL_834066, EPI_ISL_834067, EPI_ISL_834068, EPI_ISL_834070, EPI_ISL_834071, EPI_ISL_834072, EPI_ISL_834073, EPI_ISL_834074, EPI_ISL_834075, EPI_ISL_834076, EPI_ISL_834077, EPI_ISL_834078, EPI_ISL_834079, EPI_ISL_834080, EPI_ISL_834081, EPI_ISL_834083, EPI_ISL_834084, EPI_ISL_834085, EPI_ISL_834086, EPI_ISL_834087, EPI_ISL_834088, EPI_ISL_834089, EPI_ISL_834090, EPI_ISL_834091, EPI_ISL_834092, EPI_ISL_834093, EPI_ISL_834094, EPI_ISL_834095, EPI_ISL_834096, EPI_ISL_834097, EPI_ISL_834098, EPI_ISL_834099, EPI_ISL_834100, EPI_ISL_834101, EPI_ISL_834102, EPI_ISL_834103, EPI_ISL_834105, EPI_ISL_834106, EPI_ISL_834107, EPI_ISL_834108, EPI_ISL_834109, EPI_ISL_834110, EPI_ISL_834111, EPI_ISL_834112, EPI_ISL_834113, EPI_ISL_834114, EPI_ISL_834115, EPI_ISL_834116, EPI_ISL_834117, EPI_ISL_834118, EPI_ISL_834119, EPI_ISL_834120, EPI_ISL_834121, EPI_ISL_834122, EPI_ISL_834123, EPI_ISL_834124, EPI_ISL_834125, EPI_ISL_834126, EPI_ISL_834127, EPI_ISL_834128, EPI_ISL_834129, EPI_ISL_834130, EPI_ISL_834131, EPI_ISL_834132, EPI_ISL_834133, EPI_ISL_834135, EPI_ISL_834136, EPI_ISL_834137, EPI_ISL_834138, EPI_ISL_834139, EPI_ISL_834140, EPI_ISL_834141, EPI_ISL_834142, EPI_ISL_834143, EPI_ISL_834145, EPI_ISL_834147, EPI_ISL_834148, EPI_ISL_834149, EPI_ISL_834150, EPI_ISL_834151, EPI_ISL_834152, EPI_ISL_834153, EPI_ISL_834154, EPI_ISL_834155, EPI_ISL_834156, EPI_ISL_834157, EPI_ISL_834158, EPI_ISL_834159, EPI_ISL_834160, EPI_ISL_834161, EPI_ISL_834162, EPI_ISL_834163, EPI_ISL_834164, EPI_ISL_834165, EPI_ISL_834166, EPI_ISL_834167, EPI_ISL_834168, EPI_ISL_834169, EPI_ISL_834170, EPI_ISL_834171, EPI_ISL_834172, EPI_ISL_834173, EPI_ISL_834174, EPI_ISL_834175, EPI_ISL_834176, EPI_ISL_834177, EPI_ISL_834178, EPI_ISL_834179, EPI_ISL_834180, EPI_ISL_834181, EPI_ISL_834182, EPI_ISL_834183, EPI_ISL_834184, EPI_ISL_834185, EPI_ISL_834186, EPI_ISL_834187, EPI_ISL_834188, EPI_ISL_834189, EPI_ISL_834190, EPI_ISL_834191, EPI_ISL_834192, EPI_ISL_834193, EPI_ISL_834194, EPI_ISL_834195, EPI_ISL_834196, EPI_ISL_834198, EPI_ISL_834199, EPI_ISL_834200, EPI_ISL_834201, EPI_ISL_834202, EPI_ISL_834203, EPI_ISL_834204, EPI_ISL_834205, EPI_ISL_834206, EPI_ISL_834207, EPI_ISL_834208, EPI_ISL_834209			
see above	Lighthouse Lab in Alderley Park	Wellcome Sanger Institute for the COVID-19 Genomics UK (COG-UK) Consortium	Jacquelyn Wynn, Mairead Hyland, The Lighthouse Lab in Alderley Park and Alex Alderton, Roberto Amato, Sonia Goncalves, Ewan Harrison, David K. Jackson, Ian Johnston, Dominic Kwiatkowski, Cordelia Langford, John Sillitoe on behalf of the Wellcome Sanger Institute COVID-19 Surveillance Team
EPI_ISL_834553, EPI_ISL_834554, EPI_ISL_834555, EPI_ISL_834556, EPI_ISL_834557, EPI_ISL_834559, EPI_ISL_834560, EPI_ISL_834563, EPI_ISL_834564, EPI_ISL_834565, EPI_ISL_834566, EPI_ISL_834567, EPI_ISL_834569, EPI_ISL_834570, EPI_ISL_834571, EPI_ISL_834573, EPI_ISL_834574, EPI_ISL_834575, EPI_ISL_834576, EPI_ISL_834577, EPI_ISL_834580, EPI_ISL_834582, EPI_ISL_834585, EPI_ISL_834586, EPI_ISL_834587, EPI_ISL_834589, EPI_ISL_834590, EPI_ISL_834591, EPI_ISL_834594, EPI_ISL_834595, EPI_ISL_834596, EPI_ISL_834597, EPI_ISL_834598, EPI_ISL_834601, EPI_ISL_834602, EPI_ISL_834605, EPI_ISL_834606, EPI_ISL_834607, EPI_ISL_834609, EPI_ISL_834610, EPI_ISL_834611, EPI_ISL_834613, EPI_ISL_834614, EPI_ISL_834615, EPI_ISL_834616, EPI_ISL_834617, EPI_ISL_834618, EPI_ISL_834620, EPI_ISL_834621, EPI_ISL_834622, EPI_ISL_834624, EPI_ISL_834625, EPI_ISL_834626, EPI_ISL_834627, EPI_ISL_834628, EPI_ISL_834630, EPI_ISL_834631, EPI_ISL_834633, EPI_ISL_834634, EPI_ISL_834635, EPI_ISL_834636, EPI_ISL_834637, EPI_ISL_834638, EPI_ISL_834641, EPI_ISL_834643, EPI_ISL_834647, EPI_ISL_834649, EPI_ISL_834651, EPI_ISL_834652, EPI_ISL_834653, EPI_ISL_834654, EPI_ISL_834655, EPI_ISL_834656, EPI_ISL_834657, EPI_ISL_834659, EPI_ISL_834660, EPI_ISL_834661, EPI_ISL_834662, EPI_ISL_834663, EPI_ISL_834664, EPI_ISL_834665, EPI_ISL_834666, EPI_ISL_834667, EPI_ISL_834668, EPI_ISL_834669, EPI_ISL_834671, EPI_ISL_834674, EPI_ISL_834675, EPI_ISL_834676, EPI_ISL_834677, EPI_ISL_834678, EPI_ISL_834679, EPI_ISL_834680, EPI_ISL_834681, EPI_ISL_834682, EPI_ISL_834684, EPI_ISL_834685, EPI_ISL_834688, EPI_ISL_834693, EPI_ISL_834694, EPI_ISL_834696, EPI_ISL_834697, EPI_ISL_834698, EPI_ISL_834700, EPI_ISL_834701, EPI_ISL_834702, EPI_ISL_834704, EPI_ISL_834705, EPI_ISL_834706, EPI_ISL_834707, EPI_ISL_834709, EPI_ISL_834711, EPI_ISL_834712, EPI_ISL_834713, EPI_ISL_834715, EPI_ISL_834716, EPI_ISL_834719, EPI_ISL_834720, EPI_ISL_834721, EPI_ISL_834722, EPI_ISL_834723, EPI_ISL_834724, EPI_ISL_834725, EPI_ISL_834727, EPI_ISL_834728, EPI_ISL_834730, EPI_ISL_834731, EPI_ISL_834732, EPI_ISL_834735, EPI_ISL_834736, EPI_ISL_834737, EPI_ISL_834739, EPI_ISL_834740, EPI_ISL_834742, EPI_ISL_834743, EPI_ISL_834744, EPI_ISL_834745, EPI_ISL_834747, EPI_ISL_834748, EPI_ISL_834749, EPI_ISL_834750, EPI_ISL_834752, EPI_ISL_834753, EPI_ISL_834755, EPI_ISL_834758, EPI_ISL_834759, EPI_ISL_834762, EPI_ISL_834763, EPI_ISL_834766, EPI_ISL_834768, EPI_ISL_834769, EPI_ISL_834770, EPI_ISL_834771, EPI_ISL_834772, EPI_ISL_834773, EPI_ISL_834774, EPI_ISL_834775, EPI_ISL_834776, EPI_ISL_834777, EPI_ISL_834778, EPI_ISL_834779, EPI_ISL_834780, EPI_ISL_834781, EPI_ISL_834784, EPI_ISL_834785, EPI_ISL_834786, EPI_ISL_834787, EPI_ISL_834788, EPI_ISL_834789, EPI_ISL_834790, EPI_ISL_834792, EPI_ISL_834794, EPI_ISL_834795, EPI_ISL_834796, EPI_ISL_834797			
see above	Lighthouse Lab in Glasgow	Wellcome Sanger Institute for the COVID-19 Genomics UK (COG-UK) Consortium	Harper VanSteenhouse, Yumi Kasai, David Gray, Carol Clugston, Anna Dominiczak and Alex Alderton, Roberto Amato, Sonia Goncalves, Ewan Harrison, David K. Jackson, Ian Johnston, Dominic Kwiatkowski, Cordelia Langford, John Sillitoe on behalf of the Wellcome Sanger Institute COVID-19 Surveillance Team
EPI_ISL_834801, EPI_ISL_834868, EPI_ISL_834968, EPI_ISL_834976, EPI_ISL_834985, EPI_ISL_835010, EPI_ISL_835016, EPI_ISL_835065, EPI_ISL_835068, EPI_ISL_835108, EPI_ISL_835110, EPI_ISL_835121			
see above	Lighthouse Lab in Alderley Park	Wellcome Sanger Institute for the COVID-19 Genomics UK (COG-UK) Consortium	Jacquelyn Wynn, Mairead Hyland, The Lighthouse Lab in Alderley Park and Alex Alderton, Roberto Amato, Sonia Goncalves, Ewan Harrison, David K. Jackson, Ian Johnston, Dominic Kwiatkowski, Cordelia Langford, John Sillitoe on behalf of the Wellcome Sanger Institute COVID-19 Surveillance Team
EPI_ISL_835143, EPI_ISL_835144, EPI_ISL_835146, EPI_ISL_835147, EPI_ISL_835148, EPI_ISL_835150, EPI_ISL_835151, EPI_ISL_835152, EPI_ISL_835156, EPI_ISL_835157, EPI_ISL_835158, EPI_ISL_835159, EPI_ISL_835160, EPI_ISL_835161, EPI_ISL_835162, EPI_ISL_835163, EPI_ISL_835164, EPI_ISL_835165, EPI_ISL_835166, EPI_ISL_835167, EPI_ISL_835168, EPI_ISL_835169, EPI_ISL_835170, EPI_ISL_835171, EPI_ISL_835172, EPI_ISL_835173, EPI_ISL_835175, EPI_ISL_835176, EPI_ISL_835178, EPI_ISL_835180, EPI_ISL_835182, EPI_ISL_835183, EPI_ISL_835184, EPI_ISL_835185, EPI_ISL_835186, EPI_ISL_835187, EPI_ISL_835188, EPI_ISL_835190, EPI_ISL_835191, EPI_ISL_835193, EPI_ISL_835194, EPI_ISL_835195, EPI_ISL_835196, EPI_ISL_835197, EPI_ISL_835199, EPI_ISL_835201, EPI_ISL_835203, EPI_ISL_835204, EPI_ISL_835206, EPI_ISL_835207, EPI_ISL_835208, EPI_ISL_835209, EPI_ISL_835210, EPI_ISL_835211, EPI_ISL_835213, EPI_ISL_835215, EPI_ISL_835216, EPI_ISL_835217, EPI_ISL_835218, EPI_ISL_835220, EPI_ISL_835221, EPI_ISL_835224, EPI_ISL_835225, EPI_ISL_835226, EPI_ISL_835227, EPI_ISL_835228, EPI_ISL_835229, EPI_ISL_835230, EPI_ISL_835232, EPI_ISL_835233, EPI_ISL_835234, EPI_ISL_835236, EPI_ISL_835237, EPI_ISL_835238, EPI_ISL_835239, EPI_ISL_835240, EPI_ISL_835242, EPI_ISL_835244, EPI_ISL_835245, EPI_ISL_835248, EPI_ISL_835249, EPI_ISL_835250, EPI_ISL_835251, EPI_ISL_835252, EPI_ISL_835254, EPI_ISL_835255, EPI_ISL_835256, EPI_ISL_835258, EPI_ISL_835262, EPI_ISL_835263, EPI_ISL_835264, EPI_ISL_835265, EPI_ISL_835266, EPI_ISL_835267, EPI_ISL_835269, EPI_ISL_835270, EPI_ISL_835271, EPI_ISL_835272, EPI_ISL_835273, EPI_ISL_835274, EPI_ISL_835275, EPI_ISL_835276, EPI_ISL_835277, EPI_ISL_835278, EPI_ISL_835279, EPI_ISL_835280, EPI_ISL_835281, EPI_ISL_835283, EPI_ISL_835284, EPI_ISL_835285, EPI_ISL_835286, EPI_ISL_835287, EPI_ISL_835288, EPI_ISL_835290, EPI_ISL_835291, EPI_ISL_835292, EPI_ISL_835293, EPI_ISL_835294, EPI_ISL_835295, EPI_ISL_835296, EPI_ISL_835297, EPI_ISL_835299, EPI_ISL_835301, EPI_ISL_835302, EPI_ISL_835304, EPI_ISL_835305, EPI_ISL_835306, EPI_ISL_835308, EPI_ISL_835309, EPI_ISL_835311, EPI_ISL_835312, EPI_ISL_835314, EPI_ISL_835315, EPI_ISL_835317, EPI_ISL_835318, EPI_ISL_835322, EPI_ISL_835323, EPI_ISL_835324, EPI_ISL_835325, EPI_ISL_835326, EPI_ISL_835327, EPI_ISL_835328, EPI_ISL_835329, EPI_ISL_835331, EPI_ISL_835332, EPI_ISL_835335, EPI_ISL_835336, EPI_ISL_835337, EPI_ISL_835340, EPI_ISL_835341, EPI_ISL_835342, EPI_ISL_835343, EPI_ISL_835344, EPI_ISL_835345, EPI_ISL_835346, EPI_ISL_835347, EPI_ISL_835348, EPI_ISL_835349, EPI_ISL_835350, EPI_ISL_835351, EPI_ISL_835352, EPI_ISL_835353, EPI_ISL_835355, EPI_ISL_835356, EPI_ISL_835358, EPI_ISL_835360, EPI_ISL_835363, EPI_ISL_835364, EPI_ISL_835367, EPI_ISL_835370, EPI_ISL_835371, EPI_ISL_835372, EPI_ISL_835373, EPI_ISL_835374, EPI_ISL_835376, EPI_ISL_835377, EPI_ISL_835379, EPI_ISL_835380, EPI_ISL_835381, EPI_ISL_835382, EPI_ISL_835383, EPI_ISL_835386, EPI_ISL_835387, EPI_ISL_835388, EPI_ISL_835391, EPI_ISL_835392, EPI_ISL_835393, EPI_ISL_835394, EPI_ISL_835395, EPI_ISL_835396, EPI_ISL_835397, EPI_ISL_835398, EPI_ISL_835399, EPI_ISL_835401, EPI_ISL_835402, EPI_ISL_835404, EPI_ISL_835405, EPI_ISL_835406, EPI_ISL_835407, EPI_ISL_835408, EPI_ISL_835411, EPI_ISL_835412, EPI_ISL_835413, EPI_ISL_835414, EPI_ISL_835417, EPI_ISL_835418, EPI_ISL_835419, EPI_ISL_835422, EPI_ISL_835423, EPI_ISL_835424, EPI_ISL_835425			
see above	Lighthouse Lab in Glasgow	Wellcome Sanger Institute for the COVID-19 Genomics UK (COG-UK) Consortium	Harper VanSteenhouse, Yumi Kasai, David Gray, Carol Clugston, Anna Dominiczak and Alex Alderton, Roberto Amato, Sonia Goncalves, Ewan Harrison, David K. Jackson, Ian Johnston, Dominic Kwiatkowski, Cordelia Langford, John Sillitoe on behalf of the Wellcome Sanger Institute COVID-19 Surveillance Team
EPI_ISL_836423, EPI_ISL_836424, EPI_ISL_836431, EPI_ISL_836432, EPI_ISL_836439, EPI_ISL_836444, EPI_ISL_836445, EPI_ISL_836451, EPI_ISL_836466, EPI_ISL_836471, EPI_ISL_836482, EPI_ISL_836483, EPI_ISL_836491, EPI_ISL_836494, EPI_ISL_836496, EPI_ISL_836500, EPI_ISL_836501, EPI_ISL_836504, EPI_ISL_836506, EPI_ISL_836517, EPI_ISL_836527, EPI_ISL_836528, EPI_ISL_836530, EPI_ISL_836536, EPI_ISL_836548, EPI_ISL_836556, EPI_ISL_836562, EPI_ISL_836567, EPI_ISL_836572, EPI_ISL_836576, EPI_ISL_836585, EPI_ISL_836592, EPI_ISL_836595, EPI_ISL_836596, EPI_ISL_836597, EPI_ISL_836603, EPI_ISL_836611, EPI_ISL_836617, EPI_ISL_836620, EPI_ISL_836623, EPI_ISL_836626, EPI_ISL_836627, EPI_ISL_836636, EPI_ISL_836644, EPI_ISL_836645, EPI_ISL_836651, EPI_ISL_836652, EPI_ISL_836655, EPI_ISL_836656, EPI_ISL_836657, EPI_ISL_836665, EPI_ISL_836679, EPI_ISL_836681, EPI_ISL_836682, EPI_ISL_836683, EPI_ISL_836686, EPI_ISL_836688, EPI_ISL_836689, EPI_ISL_836695, EPI_ISL_836697, EPI_ISL_836712, EPI_ISL_836713, EPI_ISL_836722, EPI_ISL_836725			
see above	Lighthouse Lab in Cambridge	Wellcome Sanger Institute for the COVID-19 Genomics UK (COG-UK) Consortium	Rob Howes, The Lighthouse Lab in Cambridge and Alex Alderton, Roberto Amato, Sonia Goncalves, Ewan Harrison, David K. Jackson, Ian Johnston, Dominic Kwiatkowski, Cordelia Langford, John Sillitoe on behalf of the Wellcome Sanger Institute COVID-19 Surveillance Team
EPI_ISL_836751, EPI_ISL_836759, EPI_ISL_836770, EPI_ISL_836778, EPI_ISL_836784, EPI_ISL_836820, EPI_ISL_836825, EPI_ISL_836827, EPI_ISL_836829, EPI_ISL_836831, EPI_ISL_836834, EPI_ISL_836847, EPI_ISL_836849, EPI_ISL_836850, EPI_ISL_836857, EPI_ISL_836864, EPI_ISL_836870, EPI_ISL_836893, EPI_ISL_836895, EPI_ISL_836898, EPI_ISL_836900, EPI_ISL_836902, EPI_ISL_836908, EPI_ISL_836924, EPI_ISL_836930, EPI_ISL_836931, EPI_ISL_836942, EPI_ISL_836944, EPI_ISL_836949, EPI_ISL_836954, EPI_ISL_836956, EPI_ISL_836957, EPI_ISL_836969, EPI_ISL_836971, EPI_ISL_836973, EPI_ISL_836974			
see above	Lighthouse Lab in Glasgow	Wellcome Sanger Institute for the COVID-19 Genomics UK (COG-UK) Consortium	Harper VanSteenhouse, Yumi Kasai, David Gray, Carol Clugston, Anna Dominiczak and Alex Alderton, Roberto Amato, Sonia Goncalves, Ewan Harrison, David K. Jackson, Ian Johnston, Dominic Kwiatkowski, Cordelia Langford, John Sillitoe on behalf of the Wellcome Sanger Institute COVID-19 Surveillance Team
EPI_ISL_837055, EPI_ISL_837176, EPI_ISL_837177, EPI_ISL_837178, EPI_ISL_837179, EPI_ISL_837180	Respiratory Virus Unit, National Infection Service, Public Health England	COVID-19 Genomics UK (COG-UK) Consortium	PHE Covid Sequencing Team
EPI_ISL_837847, EPI_ISL_837848, EPI_ISL_837937, EPI_ISL_837938, EPI_ISL_837939, EPI_ISL_837941, EPI_ISL_837944, EPI_ISL_837945, EPI_ISL_837946, EPI_ISL_837947, EPI_ISL_837948, EPI_ISL_837949, EPI_ISL_837950, EPI_ISL_837951, EPI_ISL_837952, EPI_ISL_837953, EPI_ISL_837954, EPI_ISL_837956, EPI_ISL_837958, EPI_ISL_837960, EPI_ISL_837961, EPI_ISL_837962, EPI_ISL_837963, EPI_ISL_837964, EPI_ISL_837965, EPI_ISL_837966, EPI_ISL_837967, EPI_ISL_837968, EPI_ISL_837969, EPI_ISL_837970			
see above	Department of Pathology, University of Cambridge	COVID-19 Genomics UK (COG-UK) Consortium	Aminu S. Jahun, Yasmin Chaudhry, Grant Hall, Iliana Georgana, Myra Hosmillo, Martin D. Curran, Malte Pinckert, Surendra Parmar, Ian Goodfellow

EPI_ISL_838290, EPI_ISL_838291, EPI_ISL_838293, EPI_ISL_838294, EPI_ISL_838295, EPI_ISL_838296, EPI_ISL_838297, EPI_ISL_838298, EPI_ISL_838299, EPI_ISL_838300, EPI_ISL_838301, EPI_ISL_838302, EPI_ISL_838303, EPI_ISL_838304, EPI_ISL_838305, EPI_ISL_838306, EPI_ISL_838307, EPI_ISL_838308, EPI_ISL_838309, EPI_ISL_838310, EPI_ISL_838311, EPI_ISL_838312			
see above	Virology Department, Royal Infirmary of Edinburgh, NHS Lothian / School of Biological Sciences, University of Edinburgh / Institute of Genetics and Molecular Medicine, University of Edinburgh	COVID-19 Genomics UK (COG-UK) Consortium	McHugh M, Dewar R, Rooke S, Gallagher M, Balcaza C, O'Toole Á, Scher E, Hill V, McCrone JT, Colquhoun R, Yu X, Jackson B, Rambaut A, Williams TC, Templeton K
EPI_ISL_838341	University of Exeter	COVID-19 Genomics UK (COG-UK) Consortium	Ben Temperton,Aaron Jeffries,Michelle Michelsen,Joanna Warwick-Dugdale,Audrey Farbos,Robyn Manley,Stephen Michell,Jane Masoli
EPI_ISL_838895, EPI_ISL_838896, EPI_ISL_838897, EPI_ISL_838899, EPI_ISL_838900, EPI_ISL_838901, EPI_ISL_838902, EPI_ISL_838903, EPI_ISL_838904, EPI_ISL_838905, EPI_ISL_838909, EPI_ISL_838910, EPI_ISL_838911, EPI_ISL_838912, EPI_ISL_838913, EPI_ISL_838914, EPI_ISL_838915, EPI_ISL_838917, EPI_ISL_838919, EPI_ISL_838920, EPI_ISL_838926, EPI_ISL_838927, EPI_ISL_838928, EPI_ISL_838929, EPI_ISL_838930, EPI_ISL_838931, EPI_ISL_838932, EPI_ISL_838933, EPI_ISL_838934, EPI_ISL_838935, EPI_ISL_838936, EPI_ISL_838937, EPI_ISL_838938, EPI_ISL_838939, EPI_ISL_838940, EPI_ISL_838941, EPI_ISL_838942, EPI_ISL_838943, EPI_ISL_838944, EPI_ISL_838945, EPI_ISL_838946, EPI_ISL_838947, EPI_ISL_838949, EPI_ISL_838950, EPI_ISL_838951, EPI_ISL_838952, EPI_ISL_838953, EPI_ISL_838954, EPI_ISL_838955, EPI_ISL_838956, EPI_ISL_838957, EPI_ISL_838958, EPI_ISL_838959, EPI_ISL_838969, EPI_ISL_838988, EPI_ISL_838989, EPI_ISL_838990, EPI_ISL_839013, EPI_ISL_839015, EPI_ISL_839026, EPI_ISL_839027, EPI_ISL_839028, EPI_ISL_839029, EPI_ISL_839030, EPI_ISL_839031, EPI_ISL_839032, EPI_ISL_839033, EPI_ISL_839034, EPI_ISL_839035, EPI_ISL_839036, EPI_ISL_839037, EPI_ISL_839038, EPI_ISL_839039, EPI_ISL_839128, EPI_ISL_839133, EPI_ISL_839134, EPI_ISL_839135, EPI_ISL_839136, EPI_ISL_839137, EPI_ISL_839138, EPI_ISL_839139, EPI_ISL_839140, EPI_ISL_839141, EPI_ISL_839142, EPI_ISL_839143, EPI_ISL_839144, EPI_ISL_839145, EPI_ISL_839146, EPI_ISL_839147, EPI_ISL_839148, EPI_ISL_839149, EPI_ISL_839150, EPI_ISL_839151, EPI_ISL_839152, EPI_ISL_839153, EPI_ISL_839154, EPI_ISL_839155, EPI_ISL_839157, EPI_ISL_839158, EPI_ISL_839159, EPI_ISL_839160, EPI_ISL_839161, EPI_ISL_839162, EPI_ISL_839163, EPI_ISL_839165, EPI_ISL_839166, EPI_ISL_839167, EPI_ISL_839168, EPI_ISL_839169, EPI_ISL_839170, EPI_ISL_839171, EPI_ISL_839173, EPI_ISL_839174, EPI_ISL_839175, EPI_ISL_839188, EPI_ISL_839190, EPI_ISL_839191, EPI_ISL_839193, EPI_ISL_839194, EPI_ISL_839196, EPI_ISL_839197, EPI_ISL_839200, EPI_ISL_839212, EPI_ISL_839250, EPI_ISL_839251, EPI_ISL_839252, EPI_ISL_839253, EPI_ISL_839254, EPI_ISL_839255, EPI_ISL_839256, EPI_ISL_839257, EPI_ISL_839258, EPI_ISL_839260, EPI_ISL_839261, EPI_ISL_839262, EPI_ISL_839264, EPI_ISL_839266, EPI_ISL_839267, EPI_ISL_839268, EPI_ISL_839269, EPI_ISL_839270, EPI_ISL_839271, EPI_ISL_839272, EPI_ISL_839273, EPI_ISL_839274, EPI_ISL_839275, EPI_ISL_839276			
see above	University College London, Great Ormond Street Hospital for Children NHS Foundation Trust, Imperial College Healthcare NHS Trust	COVID-19 Genomics UK (COG-UK) Consortium	Sergi Castellano, Rachel Williams, Mark Kristiansen, Paola Resende Silva, Sunando Roy, Tony Brooks, Helena Tutill, Paola Niola, Patricia Dyal, Charlotte Williams, Leysa Forrest, Yasmin Panchbhaya, Jacqueline Findlay, Samuel Weeks, Julianne Brown, Kathryn Harris, Paul Randell, James Price, Alison Holmes, Judith Breuer
EPI_ISL_839855, EPI_ISL_839856, EPI_ISL_839857, EPI_ISL_839858, EPI_ISL_839859, EPI_ISL_839860, EPI_ISL_839861, EPI_ISL_839862, EPI_ISL_839863, EPI_ISL_839864, EPI_ISL_839865, EPI_ISL_839867, EPI_ISL_839868, EPI_ISL_839869, EPI_ISL_839870, EPI_ISL_839871, EPI_ISL_839872, EPI_ISL_839873, EPI_ISL_839874, EPI_ISL_839875, EPI_ISL_839876, EPI_ISL_839877, EPI_ISL_839878, EPI_ISL_839879, EPI_ISL_839880, EPI_ISL_839881, EPI_ISL_839882, EPI_ISL_839883, EPI_ISL_839884, EPI_ISL_839885, EPI_ISL_839886, EPI_ISL_839887, EPI_ISL_839888, EPI_ISL_839889, EPI_ISL_839890, EPI_ISL_839891, EPI_ISL_839892, EPI_ISL_839893, EPI_ISL_839894, EPI_ISL_839895, EPI_ISL_839896, EPI_ISL_839897, EPI_ISL_839898, EPI_ISL_839900, EPI_ISL_839939, EPI_ISL_839940, EPI_ISL_839941, EPI_ISL_839942, EPI_ISL_839943, EPI_ISL_839944, EPI_ISL_839945, EPI_ISL_839946, EPI_ISL_839947, EPI_ISL_839948, EPI_ISL_839949, EPI_ISL_839950, EPI_ISL_839951, EPI_ISL_839952, EPI_ISL_839953, EPI_ISL_839954, EPI_ISL_839955, EPI_ISL_839956, EPI_ISL_839957, EPI_ISL_839958, EPI_ISL_839959, EPI_ISL_839960, EPI_ISL_839961, EPI_ISL_839962, EPI_ISL_839963, EPI_ISL_839964, EPI_ISL_839965, EPI_ISL_839966, EPI_ISL_839967, EPI_ISL_839968, EPI_ISL_839969, EPI_ISL_839970, EPI_ISL_839971, EPI_ISL_839972, EPI_ISL_839973			
see above	Quadram Institute Bioscience	COVID-19 Genomics UK (COG-UK) Consortium	Dave J. Baker, Gemma L. Kay, Alp Aydin, Thanh Le-Viet, Steven Rudder, Ana P. Tedim, Anastasia Kolyva, Maria Diaz, Leonardo de Oliveira Martins, Nabil-Fareed Alikhan, Lizzie Meadows, Rachael Stanley, Ngozi Elumogo, Muhammed Yasir, Nicholas M. Thomson, Alexander J Trotter, Rachel Gilroy, Samuel Bloomfield, Claire Stuart, Andrew Bell, Reenesh Prakash, Samir Dervisevic, Alison E. Mather, John Wain, Mark Webber, Andrew J. Page, Justin O'Grady
EPI_ISL_839998, EPI_ISL_839999, EPI_ISL_840000, EPI_ISL_840001, EPI_ISL_840002, EPI_ISL_840003, EPI_ISL_840004, EPI_ISL_840005, EPI_ISL_840006, EPI_ISL_840007, EPI_ISL_840008, EPI_ISL_840009, EPI_ISL_840010, EPI_ISL_840011, EPI_ISL_840012, EPI_ISL_840013, EPI_ISL_840014, EPI_ISL_840015, EPI_ISL_840016, EPI_ISL_840051, EPI_ISL_840052			
see above	Queens Medical Centre, Clinical Microbiology Department / DeepSeq Nottingham	COVID-19 Genomics UK (COG-UK) Consortium	Gemma Clark, Wendy Smith, Manjinder Khakh, Vicki M Fleming, Michelle M Lister, Hannah Howson-Wells, Jonathan Ball, Patrick McClure, Joseph Chappell, Theocharis Tsoleridis, Nadine Holmes, Matthew Carlisle, Christopher Moore, Fei Sang, Johnny Debebe, Victoria Wright, Matthew Loose
EPI_ISL_840139, EPI_ISL_840145	Lincolnshire Hospitals and DeepSeq Nottingham	COVID-19 Genomics UK (COG-UK) Consortium	Nichola Duckworth, Tim Sloan, Sarah Walsh, Jonathan Ball, Patrick McClure, Joeseph Chappell, Nadine Holmes, Matthew Carlisle, Christopher Moore, Fei Sang, Johnny Debebe, Victoria Wright, Matthew Loose
EPI_ISL_840861, EPI_ISL_840862, EPI_ISL_840863, EPI_ISL_840864, EPI_ISL_840865, EPI_ISL_840866, EPI_ISL_840867, EPI_ISL_840869, EPI_ISL_840870, EPI_ISL_840871, EPI_ISL_840872, EPI_ISL_840874, EPI_ISL_840875, EPI_ISL_840876, EPI_ISL_840877, EPI_ISL_840878, EPI_ISL_840880, EPI_ISL_840882, EPI_ISL_840883, EPI_ISL_840884, EPI_ISL_841008, EPI_ISL_841010, EPI_ISL_841012, EPI_ISL_841013, EPI_ISL_841014, EPI_ISL_841015, EPI_ISL_841016, EPI_ISL_841017, EPI_ISL_841018, EPI_ISL_841019, EPI_ISL_841020, EPI_ISL_841021, EPI_ISL_841027, EPI_ISL_841029, EPI_ISL_841030, EPI_ISL_841031, EPI_ISL_841036, EPI_ISL_841037, EPI_ISL_841038, EPI_ISL_841039, EPI_ISL_841045, EPI_ISL_841046, EPI_ISL_841047, EPI_ISL_841049, EPI_ISL_841050, EPI_ISL_841053, EPI_ISL_841054, EPI_ISL_841055, EPI_ISL_841056, EPI_ISL_841067, EPI_ISL_841068, EPI_ISL_841069, EPI_ISL_841070, EPI_ISL_841071, EPI_ISL_841072, EPI_ISL_841073, EPI_ISL_841074, EPI_ISL_841075, EPI_ISL_841076, EPI_ISL_841077, EPI_ISL_841078, EPI_ISL_841079, EPI_ISL_841080, EPI_ISL_841108, EPI_ISL_841147, EPI_ISL_841148, EPI_ISL_841149, EPI_ISL_841150, EPI_ISL_841151, EPI_ISL_841152, EPI_ISL_841153, EPI_ISL_841154, EPI_ISL_841155, EPI_ISL_841182, EPI_ISL_841191, EPI_ISL_841192, EPI_ISL_841194, EPI_ISL_841196, EPI_ISL_841197, EPI_ISL_841200, EPI_ISL_841201, EPI_ISL_841202, EPI_ISL_841203, EPI_ISL_841205, EPI_ISL_841206, EPI_ISL_841207, EPI_ISL_841209, EPI_ISL_841210, EPI_ISL_841213, EPI_ISL_841216, EPI_ISL_841217, EPI_ISL_841220, EPI_ISL_841221, EPI_ISL_841222, EPI_ISL_841223, EPI_ISL_841224, EPI_ISL_841226, EPI_ISL_841227, EPI_ISL_841229, EPI_ISL_841230, EPI_ISL_841231, EPI_ISL_841232, EPI_ISL_841233, EPI_ISL_841234, EPI_ISL_841235, EPI_ISL_841236, EPI_ISL_841237, EPI_ISL_841238, EPI_ISL_841240, EPI_ISL_841242, EPI_ISL_841244, EPI_ISL_841245, EPI_ISL_841246, EPI_ISL_841247, EPI_ISL_841248, EPI_ISL_841250, EPI_ISL_841251, EPI_ISL_841252, EPI_ISL_841253, EPI_ISL_841255, EPI_ISL_841256, EPI_ISL_841257, EPI_ISL_841258, EPI_ISL_841259, EPI_ISL_841261, EPI_ISL_841262, EPI_ISL_841263, EPI_ISL_841264, EPI_ISL_841265, EPI_ISL_841266, EPI_ISL_841267, EPI_ISL_841268, EPI_ISL_841269, EPI_ISL_841270, EPI_ISL_841271, EPI_ISL_841273, EPI_ISL_841275, EPI_ISL_841276, EPI_ISL_841277, EPI_ISL_841278, EPI_ISL_841280, EPI_ISL_841281, EPI_ISL_841283, EPI_ISL_841284, EPI_ISL_841287, EPI_ISL_841288, EPI_ISL_841290, EPI_ISL_841291, EPI_ISL_841292, EPI_ISL_841293, EPI_ISL_841294, EPI_ISL_841295, EPI_ISL_841296, EPI_ISL_841297, EPI_ISL_841298, EPI_ISL_841299, EPI_ISL_841300, EPI_ISL_841314, EPI_ISL_841315			
see above	Wales Specialist Virology Centre Sequencing lab: Pathogen Genomics Unit	Public Health Wales Microbiology Cardiff Wales Specialist Virology Centre	Catherine Moore, Johnathan Evans, Laura Gifford, Malorie Perry, Simon Cottrell, Angela Marchbank, Alec Birchley, Alexander Adams, Amy Gaskin, Bree Gatica-Wilcox, Jason Coombes, Joel Southgate, Lauren Gilbert, Lee Graham, Nicole Pacchiarini, Sara Kumziene-Summerhayes, Sarah Taylor, Sophie Jones, Sara Rey, Matthew Bull, Joanne Watkins, Sally Corden, Tom Connor
EPI_ISL_841317, EPI_ISL_841318, EPI_ISL_841319, EPI_ISL_841320, EPI_ISL_841322, EPI_ISL_841323, EPI_ISL_841324, EPI_ISL_841327, EPI_ISL_841335, EPI_ISL_841336, EPI_ISL_841337, EPI_ISL_841338, EPI_ISL_841339, EPI_ISL_841341, EPI_ISL_841342, EPI_ISL_841343, EPI_ISL_841344, EPI_ISL_841345, EPI_ISL_841346, EPI_ISL_841347, EPI_ISL_841348, EPI_ISL_841353, EPI_ISL_841354, EPI_ISL_841358, EPI_ISL_841378, EPI_ISL_841379, EPI_ISL_841380, EPI_ISL_841381, EPI_ISL_841382, EPI_ISL_841383, EPI_ISL_841384, EPI_ISL_841385, EPI_ISL_841386, EPI_ISL_841391, EPI_ISL_841392, EPI_ISL_841393, EPI_ISL_841394, EPI_ISL_841395, EPI_ISL_841396, EPI_ISL_841397, EPI_ISL_841399, EPI_ISL_841400, EPI_ISL_841401, EPI_ISL_841402, EPI_ISL_841403, EPI_ISL_841404, EPI_ISL_841405, EPI_ISL_841406, EPI_ISL_841413, EPI_ISL_841414, EPI_ISL_841416, EPI_ISL_841417, EPI_ISL_841418, EPI_ISL_841419, EPI_ISL_841420, EPI_ISL_841421, EPI_ISL_841422, EPI_ISL_841423, EPI_ISL_841424, EPI_ISL_841425, EPI_ISL_841428, EPI_ISL_841429, EPI_ISL_841431, EPI_ISL_841432, EPI_ISL_841434, EPI_ISL_841435, EPI_ISL_841436, EPI_ISL_841437, EPI_ISL_841440, EPI_ISL_841443, EPI_ISL_841444, EPI_ISL_841445, EPI_ISL_841446, EPI_ISL_841448, EPI_ISL_841451, EPI_ISL_841453, EPI_ISL_841454, EPI_ISL_841455, EPI_ISL_841456, EPI_ISL_841458, EPI_ISL_841460, EPI_ISL_841461, EPI_ISL_841462, EPI_ISL_841464, EPI_ISL_841465, EPI_ISL_841467, EPI_ISL_841468, EPI_ISL_841469, EPI_ISL_841470, EPI_ISL_841472, EPI_ISL_841473, EPI_ISL_841474, EPI_ISL_841475, EPI_ISL_841476, EPI_ISL_841477, EPI_ISL_841478, EPI_ISL_841480, EPI_ISL_841481, EPI_ISL_841502, EPI_ISL_841513, EPI_ISL_841514, EPI_ISL_841515, EPI_ISL_841516, EPI_ISL_841517, EPI_ISL_841518, EPI_ISL_841519, EPI_ISL_841520, EPI_ISL_841548, EPI_ISL_841594, EPI_ISL_841595, EPI_ISL_841597, EPI_ISL_841598, EPI_ISL_841599, EPI_ISL_841600, EPI_ISL_841601, EPI_ISL_841602, EPI_ISL_841603, EPI_ISL_841604, EPI_ISL_841605, EPI_ISL_841606, EPI_ISL_841607, EPI_ISL_841613, EPI_ISL_841615, EPI_ISL_841616, EPI_ISL_841621, EPI_ISL_841624, EPI_ISL_841628, EPI_ISL_841629, EPI_ISL_841631, EPI_ISL_841632, EPI_ISL_841642, EPI_ISL_841644, EPI_ISL_841645			
see above	Originating lab: Wales Specialist Virology Centre Sequencing lab: Pathogen Genomics Unit	Public Health Wales Microbiology Cardiff Wales Specialist Virology Centre	Catherine Moore, Johnathan Evans, Laura Gifford, Malorie Perry, Simon Cottrell, Angela Marchbank, Alec Birchley, Alexander Adams, Amy Gaskin, Bree Gatica-Wilcox, Jason Coombes, Joel Southgate, Lauren Gilbert, Lee Graham, Nicole Pacchiarini, Sara Kumziene-Summerhayes, Sarah Taylor, Sophie Jones, Sara Rey, Matthew Bull, Joanne Watkins, Sally Corden, Tom Connor
EPI_ISL_842202, EPI_ISL_842203, EPI_ISL_842205, EPI_ISL_842206, EPI_ISL_842219, EPI_ISL_842220, EPI_ISL_842227, EPI_ISL_842231, EPI_ISL_842237, EPI_ISL_842240, EPI_ISL_842244, EPI_ISL_842245, EPI_ISL_842253, EPI_ISL_842257, EPI_ISL_842260, EPI_ISL_842262, EPI_ISL_842268, EPI_ISL_842269, EPI_ISL_842279, EPI_ISL_842284, EPI_ISL_842286, EPI_ISL_842290, EPI_ISL_842298, EPI_ISL_842299, EPI_ISL_842307, EPI_ISL_842308, EPI_ISL_842311, EPI_ISL_842326, EPI_ISL_842332, EPI_ISL_842344, EPI_ISL_842345, EPI_ISL_842346			
see above	Virology Department, Sheffield Teaching Hospitals NHS Foundation Trust/Department of Infection, Immunity and Cardiovascular Disease, The Medical School, University of Sheffield	COVID-19 Genomics UK (COG-UK) Consortium	Thushan de Silva, Matthew Parker, Nikki Smith, Adri Angyal, Rebecca Brown, Luke Green, Rachel Tucker, Paul Parsons, Danielle Groves, Katie Johnson, Laura Carrilero, Alex Keeley, Dave Partridge, Matthew Wyles, Benjamin Lindsey, Mehmet Yavuz, Mohammad Raza, Cariad Evans
EPI_ISL_842634, EPI_ISL_842635, EPI_ISL_842636	Medical Microbiology Unit, Department for Laboratory Medicine, Drammen Hospital, Vestre Viken Health Trust,	Norwegian Institute of Public Health, Department of Virology	Kathrine Stene-Johansen, Kamilla Heddeland Instefjord, Hilde Elshaug, Atiya R Ali,Marie Paulsen Madsen, Rasmus Riis Kopperud, Hilde Vollan, Karoline Bragstad, Olav Hungnes
EPI_ISL_842639	Oslo University Hospital, Department of Medical Microbiology	Norwegian Institute of Public Health, Department of Virology	Kathrine Stene-Johansen, Kamilla Heddeland Instefjord, Hilde Elshaug, Atiya R Ali,Marie Paulsen Madsen, Rasmus Riis Kopperud, Hilde Vollan, Karoline Bragstad, Olav Hungnes
EPI_ISL_842644	Furst Medical Laboratory	Norwegian Institute of Public Health, Department of Virology	Kathrine Stene-Johansen, Kamilla Heddeland Instefjord, Hilde Elshaug, Atiya R Ali,Marie Paulsen Madsen, Rasmus Riis Kopperud, Hilde Vollan, Karoline Bragstad, Olav Hungnes
EPI_ISL_843170, EPI_ISL_843171, EPI_ISL_843172, EPI_ISL_843173, EPI_ISL_843174, EPI_ISL_843175, EPI_ISL_843176, EPI_ISL_843177, EPI_ISL_843178, EPI_ISL_843179, EPI_ISL_843180, EPI_ISL_843181, EPI_ISL_843182, EPI_ISL_843183			
see above	Maryland Public Health Laboratory	Maryland Public Health Laboratory	Maryland Department of Health Laboratories Administration
EPI_ISL_843195, EPI_ISL_843197, EPI_ISL_843198	Canterbury Health Laboratories	Institute of Environmental Science and Research (ESR)	Xiaoyun Ren, Matt Storey, Nikki Freed, Muhammad Faisal, Jing Wang, Hermes Perez, Anja Werno, Antje van der Linden, Arlo Upton, Chris Mansell, David Hammer, Dragana Drinkovic, Gary McAuliffe, Hana Sofia Andersson, James Ussher, Jill Sherwood, Josh Freeman, Julia Howard, Juliet Elvy, Mary DeAlmeida, Matt Blakiston, Matthew Rogers, Max Bloomfield, Michael Addidle, Michelle Balm, Sally Roberts, Sarah Jefferies, Sharmini Muttaiyah, Susan Morpeth, Susan Taylor, Timothy Blackmore, Vani Sathyendran, Veronica Playle, Virginia Hope, Erasmus Smit, Lauren Jelly, Olin Silander, Joep de Ligt
EPI_ISL_845575, EPI_ISL_845586,	KU Leuven, Rega Institute, Clinical and Epidemiological	KU Leuven, Rega Institute, Clinical and Epidemiological	Tony Wawina-Bokalanga, Bert Vanmechelen, Joan Marti-Carreras, Piet Maes

EPI_ISL_845612, EPI_ISL_845613, EPI_ISL_845616	Virology	Virology	
EPI_ISL_845752	Toronto Invasive Bacterial Diseases Network	McMaster University	Allison McGeer, Patryk Aftanas, Hooman Derakhshani, Angel Li, Kuganya Nirmalarajah, Emily Panousis, Ahmed Draia, Jalees Nasir, Michael Surette, Samira Mubareka, Andrew G. McArthur
EPI_ISL_845761, EPI_ISL_845762, EPI_ISL_845763, EPI_ISL_845764, EPI_ISL_845765, EPI_ISL_845766, EPI_ISL_845767, EPI_ISL_845768, EPI_ISL_845769, EPI_ISL_845772, EPI_ISL_845773, EPI_ISL_845774, EPI_ISL_845775, EPI_ISL_845776, EPI_ISL_845777, EPI_ISL_845778, EPI_ISL_845779, EPI_ISL_845780, EPI_ISL_845781, EPI_ISL_845782			
see above	Emory Molecular Diagnostics Laboratory, Emory Healthcare	Piantadosi Lab, Emory Department of Pathology	Ahmed Babiker, Anne Piantadosi
EPI_ISL_845893, EPI_ISL_845896, EPI_ISL_845973, EPI_ISL_846007, EPI_ISL_846022, EPI_ISL_846023, EPI_ISL_846052, EPI_ISL_846059, EPI_ISL_846070, EPI_ISL_846084, EPI_ISL_846139, EPI_ISL_846147, EPI_ISL_846152, EPI_ISL_846168			
see above	Lighthouse Lab in Cambridge	Wellcome Sanger Institute for the COVID-19 Genomics UK (COG-UK) Consortium	Rob Howes, The Lighthouse Lab in Cambridge and Alex Alderton, Roberto Amato, Sonia Goncalves, Ewan Harrison, David K. Jackson, Ian Johnston, Dominic Kwiatkowski, Cordelia Langford, John Sillitoe on behalf of the Wellcome Sanger Institute COVID-19 Surveillance Team
EPI_ISL_846283, EPI_ISL_846287, EPI_ISL_846299, EPI_ISL_846315, EPI_ISL_846319, EPI_ISL_846321, EPI_ISL_846354, EPI_ISL_846399, EPI_ISL_846400, EPI_ISL_846421, EPI_ISL_846429, EPI_ISL_846431, EPI_ISL_846443, EPI_ISL_846491			
see above	Lighthouse Lab in Alderley Park	Wellcome Sanger Institute for the COVID-19 Genomics UK (COG-UK) Consortium	Jacquelyn Wynn, Mairead Hyland, The Lighthouse Lab in Alderley Park and Alex Alderton, Roberto Amato, Sonia Goncalves, Ewan Harrison, David K. Jackson, Ian Johnston, Dominic Kwiatkowski, Cordelia Langford, John Sillitoe on behalf of the Wellcome Sanger Institute COVID-19 Surveillance Team
EPI_ISL_846509	Lighthouse Lab in Cambridge	Wellcome Sanger Institute for the COVID-19 Genomics UK (COG-UK) Consortium	Rob Howes, The Lighthouse Lab in Cambridge and Alex Alderton, Roberto Amato, Sonia Goncalves, Ewan Harrison, David K. Jackson, Ian Johnston, Dominic Kwiatkowski, Cordelia Langford, John Sillitoe on behalf of the Wellcome Sanger Institute COVID-19 Surveillance Team
EPI_ISL_846510, EPI_ISL_846511, EPI_ISL_846513, EPI_ISL_846514, EPI_ISL_846515, EPI_ISL_846516, EPI_ISL_846517, EPI_ISL_846518, EPI_ISL_846519, EPI_ISL_846520, EPI_ISL_846521, EPI_ISL_846522			
see above	Lighthouse Lab in Alderley Park	Wellcome Sanger Institute for the COVID-19 Genomics UK (COG-UK) Consortium	Jacquelyn Wynn, Mairead Hyland, The Lighthouse Lab in Alderley Park and Alex Alderton, Roberto Amato, Sonia Goncalves, Ewan Harrison, David K. Jackson, Ian Johnston, Dominic Kwiatkowski, Cordelia Langford, John Sillitoe on behalf of the Wellcome Sanger Institute COVID-19 Surveillance Team
EPI_ISL_846534, EPI_ISL_846538, EPI_ISL_846539	Lighthouse Lab in Glasgow	Wellcome Sanger Institute for the COVID-19 Genomics UK (COG-UK) Consortium	Harper VanSteenhouse, Yumi Kasai, David Gray, Carol Clugston, Anna Dominiczak and Alex Alderton, Roberto Amato, Sonia Goncalves, Ewan Harrison, David K. Jackson, Ian Johnston, Dominic Kwiatkowski, Cordelia Langford, John Sillitoe on behalf of the Wellcome Sanger Institute COVID-19 Surveillance Team
EPI_ISL_846540	National Laboratory for Health, Environment and Food	National Laboratory for Health, Environment and Food	Aleksander Mahnic, Sandra Janezic, Maja Rupnik
EPI_ISL_846545, EPI_ISL_846546	Lab voor klinische biologie	Lab voor klinische biologie	Hannelore Hamerlinck, Bruno Verhasselt
EPI_ISL_846598	Respiratory Virus Unit, National Infection Service, Public Health England	COVID-19 Genomics UK (COG-UK) Consortium	PHE Covid Sequencing Team
EPI_ISL_846644	Hopital	National Reference Center for Viruses of Respiratory Infections, Institut Pasteur, Paris	Marion Barbet, Sylvie Behillil, Méline Bizard, Angela Brisebarre, Camille Capel, Etienne Simon-Lorière, Vincent Enouf, Maud Vanpeene, Sylvie van der Werf
EPI_ISL_846648	Labo Analyses Med	National Reference Center for Viruses of Respiratory Infections, Institut Pasteur, Paris	Marion Barbet, Sylvie Behillil, Méline Bizard, Angela Brisebarre, Camille Capel, Etienne Simon-Lorière, Vincent Enouf, Maud Vanpeene, Sylvie van der Werf
EPI_ISL_847835	Oak Ridge National Laboratory	Grubaugh Lab - Yale School of Public Health	Tara Alpert, Joseph Fauver, Anderson Brito, Mallery Breban, Anne Wyllie, Chantal Vogels, Mary Petrone, Chaney Kalinich, Isabel Ott, Nathan Grubaugh
EPI_ISL_847987, EPI_ISL_848050, EPI_ISL_848055	Michigan Department of Health and Human Services, Bureau of Laboratories	Michigan Department of Health and Human Services, Bureau of Laboratories	Blankenship HM, Riner D, Soehnlen MK
EPI_ISL_848065, EPI_ISL_848066, EPI_ISL_848067, EPI_ISL_848068	CHU Purpan - Laboratoire de Virologie - Institut Fédératif de Biologie	CHU Purpan - Laboratoire de Virologie - Institut Fédératif de Biologie	Latour J., Ranger N., Dubois M., Carcenac R., Harter A., Boyer P., Tremeaux P., Izopet J.
EPI_ISL_848069	Tulane Medical Center	Centers for Disease Control and Prevention Division of Viral Diseases, Pathogen Discovery	Ying Tao, Yan Li, Jing Zhang, Krista Queen, Anna Uehara, Peter Cook, Clinton R. Paden, Haibin Wang, Suxiang Tong
EPI_ISL_848630, EPI_ISL_848631, EPI_ISL_848632	Montana Public Health Laboratory	Wyoming Public Health Laboratory	Noah Hull, Joy Ritter, Taylor Fearing, Lynette Gumbleton, Channing Weber, Ashley Norberg, Bailey Bowcutt, Wanda Manley, Deborah Gibson
EPI_ISL_849753, EPI_ISL_849754, EPI_ISL_849755	unknown	PHV-FSS	Son Nguyen et al.
EPI_ISL_849906, EPI_ISL_849907, EPI_ISL_849908, EPI_ISL_849909, EPI_ISL_849910, EPI_ISL_849911, EPI_ISL_849912, EPI_ISL_849921	Utah Public Health Laboratory	Utah Public Health Laboratory	Erin L. Young, Kelly F. Oakeson, Tara Gallagher
EPI_ISL_849923	UCSF Clinical Microbiology Laboratory	Chan-Zuckerberg Biohub	CZB Cliahub Consortium
EPI_ISL_850677, EPI_ISL_850679, EPI_ISL_850684, EPI_ISL_850685, EPI_ISL_850686, EPI_ISL_850688	The National Institute of Public Health	State Veterinary Institute Prague	Nagy,A;Jirincova,H;Trnka,D;Vecerova,J
EPI_ISL_850689, EPI_ISL_850690, EPI_ISL_850691, EPI_ISL_850692, EPI_ISL_850693, EPI_ISL_850694, EPI_ISL_850695, EPI_ISL_850696, EPI_ISL_850697, EPI_ISL_850698, EPI_ISL_850699, EPI_ISL_850700, EPI_ISL_850701, EPI_ISL_850702, EPI_ISL_850703, EPI_ISL_850704, EPI_ISL_850705, EPI_ISL_850706, EPI_ISL_850707, EPI_ISL_850708, EPI_ISL_850709, EPI_ISL_850710, EPI_ISL_850711, EPI_ISL_850712, EPI_ISL_850713, EPI_ISL_850714, EPI_ISL_850715, EPI_ISL_850716, EPI_ISL_850717, EPI_ISL_850718, EPI_ISL_850719, EPI_ISL_850720, EPI_ISL_850721, EPI_ISL_850722, EPI_ISL_850723, EPI_ISL_850724, EPI_ISL_850725, EPI_ISL_850726, EPI_ISL_850727, EPI_ISL_850728, EPI_ISL_850729, EPI_ISL_850730, EPI_ISL_850731, EPI_ISL_850732, EPI_ISL_850733, EPI_ISL_850734, EPI_ISL_850735, EPI_ISL_850736, EPI_ISL_850737, EPI_ISL_850738, EPI_ISL_850739, EPI_ISL_850740, EPI_ISL_850741, EPI_ISL_850742, EPI_ISL_850743, EPI_ISL_850744, EPI_ISL_850745, EPI_ISL_850746, EPI_ISL_850747, EPI_ISL_850748, EPI_ISL_850749, EPI_ISL_850750, EPI_ISL_850751, EPI_ISL_850752, EPI_ISL_850753, EPI_ISL_850754, EPI_ISL_850755, EPI_ISL_850756, EPI_ISL_850757, EPI_ISL_850758, EPI_ISL_850759, EPI_ISL_850760, EPI_ISL_850761, EPI_ISL_850762, EPI_ISL_850763, EPI_ISL_850764, EPI_ISL_850765, EPI_ISL_850766, EPI_ISL_850767, EPI_ISL_850768, EPI_ISL_850769, EPI_ISL_850770, EPI_ISL_850771, EPI_ISL_850772, EPI_ISL_850773, EPI_ISL_850774, EPI_ISL_850775, EPI_ISL_850776, EPI_ISL_850777, EPI_ISL_850778, EPI_ISL_850779, EPI_ISL_850780, EPI_ISL_850781, EPI_ISL_850782, EPI_ISL_850783, EPI_ISL_850784, EPI_ISL_850785, EPI_ISL_850786, EPI_ISL_850787, EPI_ISL_850788, EPI_ISL_850789, EPI_ISL_850790, EPI_ISL_850791, EPI_ISL_850792, EPI_ISL_850793, EPI_ISL_850794, EPI_ISL_850795, EPI_ISL_850796, EPI_ISL_850797, EPI_ISL_850798, EPI_ISL_850799, EPI_ISL_850800, EPI_ISL_850801, EPI_ISL_850802, EPI_ISL_850803, EPI_ISL_850804, EPI_ISL_850805, EPI_ISL_850806, EPI_ISL_850807, EPI_ISL_850808, EPI_ISL_850809, EPI_ISL_850810, EPI_ISL_850811, EPI_ISL_850812, EPI_ISL_850813, EPI_ISL_850814, EPI_ISL_850815, EPI_ISL_850816			
see above	Helix / Illumina	Genomics and Discovery, Respiratory Viruses Branch, Division of Viral Diseases, Centers for Disease Control and Prevention	Peter W. Cook, Dhwani Batra, Ben L. Rambo-Martin Eileen de Feo, Jan Antico, Christine Tran, Matthew Tolentino, Shannon Wickline, Kim Gietzen, Brad Sickler, Jingtao Liu, Eric Allen, Phil Febbo, Summer Galloway, Nicole L. Washington, Simon White, Geraint Levan, Kelly Schiabor Barrett, Elizabeth Cirulli, Alexandre Bolze, Ary Ascencio, Charlotte Rivera-Garcia, Ryan Cho, Jason Nguyen, Sherry Wang, Jimmy Ramirez, Tyler Cassens, Efren Sandoval, Magnus Isaksson, William Lee, David Becker, Marc Laurent, James Lu, Clinton R. Paden, Suxiang Tong, Duncan MacCannell
EPI_ISL_851062, EPI_ISL_851063, EPI_ISL_851079, EPI_ISL_851083, EPI_ISL_851110, EPI_ISL_851115, EPI_ISL_851133, EPI_ISL_851143, EPI_ISL_851150, EPI_ISL_851166, EPI_ISL_851170, EPI_ISL_851175, EPI_ISL_851189, EPI_ISL_851193, EPI_ISL_851194, EPI_ISL_851202, EPI_ISL_851208, EPI_ISL_851213, EPI_ISL_851214, EPI_ISL_851251, EPI_ISL_851254, EPI_ISL_851269, EPI_ISL_851273, EPI_ISL_851287, EPI_ISL_851298, EPI_ISL_851332, EPI_ISL_851338, EPI_ISL_851342, EPI_ISL_851343, EPI_ISL_851353, EPI_ISL_851354, EPI_ISL_851370, EPI_ISL_851377			
see above	Lighthouse Lab in Cambridge	Wellcome Sanger Institute for the COVID-19 Genomics UK (COG-UK) Consortium	Rob Howes, The Lighthouse Lab in Cambridge and Alex Alderton, Roberto Amato, Sonia Goncalves, Ewan Harrison, David K. Jackson, Ian Johnston, Dominic Kwiatkowski, Cordelia Langford, John Sillitoe on behalf of the Wellcome Sanger Institute COVID-19 Surveillance Team
EPI_ISL_851390, EPI_ISL_851395, EPI_ISL_851413, EPI_ISL_851423, EPI_ISL_851436, EPI_ISL_851448, EPI_ISL_851454, EPI_ISL_851455, EPI_ISL_851461, EPI_ISL_851467, EPI_ISL_851484, EPI_ISL_851514, EPI_ISL_851559, EPI_ISL_851567, EPI_ISL_851572, EPI_ISL_851582, EPI_ISL_851590, EPI_ISL_851594, EPI_ISL_851598, EPI_ISL_851601, EPI_ISL_851614, EPI_ISL_851617, EPI_ISL_851629, EPI_ISL_851633, EPI_ISL_851635, EPI_ISL_851646, EPI_ISL_851648, EPI_ISL_851657, EPI_ISL_851664			
see above	Lighthouse Lab in Glasgow	Wellcome Sanger Institute for the COVID-19 Genomics UK (COG-UK) Consortium	Harper VanSteenhouse, Yumi Kasai, David Gray, Carol Clugston, Anna Dominiczak and Alex Alderton, Roberto Amato, Sonia Goncalves, Ewan Harrison, David K. Jackson, Ian Johnston, Dominic Kwiatkowski, Cordelia Langford, John Sillitoe on behalf of the Wellcome Sanger Institute COVID-19 Surveillance Team
EPI_ISL_851750, EPI_ISL_851875	Lighthouse Lab in Alderley Park	Wellcome Sanger Institute for the COVID-19 Genomics UK (COG-UK) Consortium	Jacquelyn Wynn, Mairead Hyland, The Lighthouse Lab in Alderley Park and Alex Alderton, Roberto Amato, Sonia Goncalves, Ewan Harrison, David K. Jackson, Ian Johnston, Dominic Kwiatkowski, Cordelia Langford, John Sillitoe on behalf of the Wellcome Sanger Institute COVID-19 Surveillance Team

EPI_ISL_851954	Lighthouse Lab in Glasgow	Wellcome Sanger Institute for the COVID-19 Genomics UK (COG-UK) Consortium	Harper VanSteenhouse, Yumi Kasai, David Gray, Carol Clugston, Anna Dominiczak and Alex Alderton, Roberto Amato, Sonia Goncalves, Ewan Harrison, David K. Jackson, Ian Johnston, Dominic Kwiatkowski, Cordelia Langford, John Sillitoe on behalf of the Wellcome Sanger Institute COVID-19 Surveillance Team
EPI_ISL_851963	Lighthouse Lab in Milton Keynes	Wellcome Sanger Institute for the COVID-19 Genomics UK (COG-UK) Consortium	The Lighthouse Lab in Milton Keynes and Alex Alderton, Roberto Amato, Sonia Goncalves, Ewan Harrison, David K. Jackson, Ian Johnston, Dominic Kwiatkowski, Cordelia Langford, John Sillitoe on behalf of the Wellcome Sanger Institute COVID-19 Surveillance Team
EPI_ISL_851964	Lighthouse Lab in Alderley Park	Wellcome Sanger Institute for the COVID-19 Genomics UK (COG-UK) Consortium	Jacquelyn Wynn, Mairead Hyland, The Lighthouse Lab in Alderley Park and Alex Alderton, Roberto Amato, Sonia Goncalves, Ewan Harrison, David K. Jackson, Ian Johnston, Dominic Kwiatkowski, Cordelia Langford, John Sillitoe on behalf of the Wellcome Sanger Institute COVID-19 Surveillance Team
EPI_ISL_851968, EPI_ISL_851972, EPI_ISL_851974, EPI_ISL_851976, EPI_ISL_851979, EPI_ISL_851985, EPI_ISL_851993, EPI_ISL_852002, EPI_ISL_852009	Lighthouse Lab in Milton Keynes	Wellcome Sanger Institute for the COVID-19 Genomics UK (COG-UK) Consortium	The Lighthouse Lab in Milton Keynes and Alex Alderton, Roberto Amato, Sonia Goncalves, Ewan Harrison, David K. Jackson, Ian Johnston, Dominic Kwiatkowski, Cordelia Langford, John Sillitoe on behalf of the Wellcome Sanger Institute COVID-19 Surveillance Team
EPI_ISL_852025	Lighthouse Lab in Alderley Park	Wellcome Sanger Institute for the COVID-19 Genomics UK (COG-UK) Consortium	Jacquelyn Wynn, Mairead Hyland, The Lighthouse Lab in Alderley Park and Alex Alderton, Roberto Amato, Sonia Goncalves, Ewan Harrison, David K. Jackson, Ian Johnston, Dominic Kwiatkowski, Cordelia Langford, John Sillitoe on behalf of the Wellcome Sanger Institute COVID-19 Surveillance Team
EPI_ISL_852029, EPI_ISL_852042, EPI_ISL_852062, EPI_ISL_852077, EPI_ISL_852086, EPI_ISL_852093, EPI_ISL_852100, EPI_ISL_852114, EPI_ISL_852118, EPI_ISL_852124, EPI_ISL_852127, EPI_ISL_852132, EPI_ISL_852135, EPI_ISL_852165, EPI_ISL_852174			
see above	Lighthouse Lab in Milton Keynes	Wellcome Sanger Institute for the COVID-19 Genomics UK (COG-UK) Consortium	The Lighthouse Lab in Milton Keynes and Alex Alderton, Roberto Amato, Sonia Goncalves, Ewan Harrison, David K. Jackson, Ian Johnston, Dominic Kwiatkowski, Cordelia Langford, John Sillitoe on behalf of the Wellcome Sanger Institute COVID-19 Surveillance Team
EPI_ISL_852183	Lighthouse Lab in Alderley Park	Wellcome Sanger Institute for the COVID-19 Genomics UK (COG-UK) Consortium	Jacquelyn Wynn, Mairead Hyland, The Lighthouse Lab in Alderley Park and Alex Alderton, Roberto Amato, Sonia Goncalves, Ewan Harrison, David K. Jackson, Ian Johnston, Dominic Kwiatkowski, Cordelia Langford, John Sillitoe on behalf of the Wellcome Sanger Institute COVID-19 Surveillance Team
EPI_ISL_852184, EPI_ISL_852190, EPI_ISL_852203, EPI_ISL_852206, EPI_ISL_852213	Lighthouse Lab in Milton Keynes	Wellcome Sanger Institute for the COVID-19 Genomics UK (COG-UK) Consortium	The Lighthouse Lab in Milton Keynes and Alex Alderton, Roberto Amato, Sonia Goncalves, Ewan Harrison, David K. Jackson, Ian Johnston, Dominic Kwiatkowski, Cordelia Langford, John Sillitoe on behalf of the Wellcome Sanger Institute COVID-19 Surveillance Team
EPI_ISL_852214	Lighthouse Lab in Alderley Park	Wellcome Sanger Institute for the COVID-19 Genomics UK (COG-UK) Consortium	Jacquelyn Wynn, Mairead Hyland, The Lighthouse Lab in Alderley Park and Alex Alderton, Roberto Amato, Sonia Goncalves, Ewan Harrison, David K. Jackson, Ian Johnston, Dominic Kwiatkowski, Cordelia Langford, John Sillitoe on behalf of the Wellcome Sanger Institute COVID-19 Surveillance Team
EPI_ISL_852218, EPI_ISL_852234, EPI_ISL_852236	Lighthouse Lab in Milton Keynes	Wellcome Sanger Institute for the COVID-19 Genomics UK (COG-UK) Consortium	The Lighthouse Lab in Milton Keynes and Alex Alderton, Roberto Amato, Sonia Goncalves, Ewan Harrison, David K. Jackson, Ian Johnston, Dominic Kwiatkowski, Cordelia Langford, John Sillitoe on behalf of the Wellcome Sanger Institute COVID-19 Surveillance Team
EPI_ISL_852256, EPI_ISL_852257, EPI_ISL_852258, EPI_ISL_852259, EPI_ISL_852260, EPI_ISL_852261, EPI_ISL_852262, EPI_ISL_852263, EPI_ISL_852264, EPI_ISL_852265, EPI_ISL_852266, EPI_ISL_852267, EPI_ISL_852269, EPI_ISL_852270, EPI_ISL_852271, EPI_ISL_852272, EPI_ISL_852273, EPI_ISL_852274, EPI_ISL_852275, EPI_ISL_852276, EPI_ISL_852277, EPI_ISL_852278, EPI_ISL_852279, EPI_ISL_852280, EPI_ISL_852281, EPI_ISL_852282, EPI_ISL_852283, EPI_ISL_852284, EPI_ISL_852285, EPI_ISL_852287, EPI_ISL_852288, EPI_ISL_852289, EPI_ISL_852290, EPI_ISL_852291, EPI_ISL_852292, EPI_ISL_852293, EPI_ISL_852294, EPI_ISL_852295, EPI_ISL_852296, EPI_ISL_852297, EPI_ISL_852298, EPI_ISL_852299, EPI_ISL_852300, EPI_ISL_852301, EPI_ISL_852302, EPI_ISL_852304, EPI_ISL_852305, EPI_ISL_852306, EPI_ISL_852307, EPI_ISL_852308, EPI_ISL_852309, EPI_ISL_852310, EPI_ISL_852311, EPI_ISL_852312, EPI_ISL_852313, EPI_ISL_852314, EPI_ISL_852315, EPI_ISL_852316, EPI_ISL_852317, EPI_ISL_852318, EPI_ISL_852320, EPI_ISL_852321, EPI_ISL_852322, EPI_ISL_852323, EPI_ISL_852324, EPI_ISL_852325, EPI_ISL_852326, EPI_ISL_852327, EPI_ISL_852328, EPI_ISL_852329, EPI_ISL_852330, EPI_ISL_852331, EPI_ISL_852332, EPI_ISL_852333, EPI_ISL_852334, EPI_ISL_852335, EPI_ISL_852336, EPI_ISL_852337, EPI_ISL_852339, EPI_ISL_852340, EPI_ISL_852341, EPI_ISL_852342, EPI_ISL_852343, EPI_ISL_852344, EPI_ISL_852345, EPI_ISL_852346, EPI_ISL_852347, EPI_ISL_852348, EPI_ISL_852349, EPI_ISL_852350, EPI_ISL_852351, EPI_ISL_852352, EPI_ISL_852353, EPI_ISL_852354, EPI_ISL_852355, EPI_ISL_852356, EPI_ISL_852357, EPI_ISL_852358, EPI_ISL_852359, EPI_ISL_852360, EPI_ISL_852362, EPI_ISL_852363, EPI_ISL_852364, EPI_ISL_852365, EPI_ISL_852366, EPI_ISL_852367, EPI_ISL_852368, EPI_ISL_852369, EPI_ISL_852370, EPI_ISL_852371, EPI_ISL_852372, EPI_ISL_852373, EPI_ISL_852374, EPI_ISL_852376, EPI_ISL_852377, EPI_ISL_852378, EPI_ISL_852379, EPI_ISL_852380, EPI_ISL_852381, EPI_ISL_852382, EPI_ISL_852384, EPI_ISL_852385, EPI_ISL_852386, EPI_ISL_852387, EPI_ISL_852388, EPI_ISL_852390, EPI_ISL_852391, EPI_ISL_852392, EPI_ISL_852393, EPI_ISL_852394, EPI_ISL_852395, EPI_ISL_852396, EPI_ISL_852397, EPI_ISL_852398, EPI_ISL_852399, EPI_ISL_852400, EPI_ISL_852401, EPI_ISL_852402, EPI_ISL_852403, EPI_ISL_852404, EPI_ISL_852405, EPI_ISL_852408, EPI_ISL_852409, EPI_ISL_852410, EPI_ISL_852411, EPI_ISL_852412, EPI_ISL_852415, EPI_ISL_852417, EPI_ISL_852418, EPI_ISL_852419, EPI_ISL_852420, EPI_ISL_852421, EPI_ISL_852422, EPI_ISL_852423, EPI_ISL_852424, EPI_ISL_852425, EPI_ISL_852426, EPI_ISL_852427, EPI_ISL_852428, EPI_ISL_852429, EPI_ISL_852430, EPI_ISL_852431, EPI_ISL_852432, EPI_ISL_852433, EPI_ISL_852434, EPI_ISL_852435, EPI_ISL_852436, EPI_ISL_852437, EPI_ISL_852439, EPI_ISL_852440, EPI_ISL_852441, EPI_ISL_852442, EPI_ISL_852443, EPI_ISL_852444, EPI_ISL_852445, EPI_ISL_852446, EPI_ISL_852447, EPI_ISL_852449, EPI_ISL_852450, EPI_ISL_852451, EPI_ISL_852452, EPI_ISL_852453, EPI_ISL_852454, EPI_ISL_852455, EPI_ISL_852456, EPI_ISL_852457, EPI_ISL_852458, EPI_ISL_852460, EPI_ISL_852461, EPI_ISL_852462, EPI_ISL_852463, EPI_ISL_852464, EPI_ISL_852465, EPI_ISL_852466, EPI_ISL_852467, EPI_ISL_852468, EPI_ISL_852469, EPI_ISL_852470, EPI_ISL_852471, EPI_ISL_852472, EPI_ISL_852473, EPI_ISL_852474, EPI_ISL_852475, EPI_ISL_852476, EPI_ISL_852477, EPI_ISL_852478, EPI_ISL_852479, EPI_ISL_852480, EPI_ISL_852481, EPI_ISL_852482, EPI_ISL_852483, EPI_ISL_852484, EPI_ISL_852485, EPI_ISL_852486, EPI_ISL_852487, EPI_ISL_852488, EPI_ISL_852489, EPI_ISL_852490, EPI_ISL_852491, EPI_ISL_852492, EPI_ISL_852493, EPI_ISL_852494, EPI_ISL_852495, EPI_ISL_852496, EPI_ISL_852497, EPI_ISL_852498, EPI_ISL_852499, EPI_ISL_852501, EPI_ISL_852502, EPI_ISL_852503, EPI_ISL_852504, EPI_ISL_852505, EPI_ISL_852506, EPI_ISL_852507, EPI_ISL_852508, EPI_ISL_852509, EPI_ISL_852510, EPI_ISL_852511, EPI_ISL_852512, EPI_ISL_852513, EPI_ISL_852514, EPI_ISL_852515, EPI_ISL_852516, EPI_ISL_852518, EPI_ISL_852519, EPI_ISL_852520, EPI_ISL_852521, EPI_ISL_852522, EPI_ISL_852523, EPI_ISL_852525, EPI_ISL_852526, EPI_ISL_852528, EPI_ISL_852529, EPI_ISL_852530, EPI_ISL_852531, EPI_ISL_852532, EPI_ISL_852533, EPI_ISL_852534, EPI_ISL_852537, EPI_ISL_852538, EPI_ISL_852539, EPI_ISL_852540, EPI_ISL_852541, EPI_ISL_852542, EPI_ISL_852543, EPI_ISL_852545, EPI_ISL_852546, EPI_ISL_852547, EPI_ISL_852548, EPI_ISL_852550, EPI_ISL_852551, EPI_ISL_852552, EPI_ISL_852553, EPI_ISL_852554, EPI_ISL_852555, EPI_ISL_852556, EPI_ISL_852557, EPI_ISL_852558, EPI_ISL_852559, EPI_ISL_852560, EPI_ISL_852561, EPI_ISL_852562, EPI_ISL_852563			
see above	Lighthouse Lab in Glasgow	Wellcome Sanger Institute for the COVID-19 Genomics UK (COG-UK) Consortium	Harper VanSteenhouse, Yumi Kasai, David Gray, Carol Clugston, Anna Dominiczak and Alex Alderton, Roberto Amato, Sonia Goncalves, Ewan Harrison, David K. Jackson, Ian Johnston, Dominic Kwiatkowski, Cordelia Langford, John Sillitoe on behalf of the Wellcome Sanger Institute COVID-19 Surveillance Team
EPI_ISL_852861	Florida Bureau of Public Health Laboratories	Florida Bureau of Public Health Laboratories	Sarah Schmedes, Jason Blanton
EPI_ISL_852947, EPI_ISL_852948, EPI_ISL_852950, EPI_ISL_852954, EPI_ISL_852964, EPI_ISL_853003, EPI_ISL_853009, EPI_ISL_853010, EPI_ISL_853011, EPI_ISL_853012, EPI_ISL_853024, EPI_ISL_853025, EPI_ISL_853026, EPI_ISL_853027, EPI_ISL_853028, EPI_ISL_853029, EPI_ISL_853032			
see above	Hospital General Universitario Gregorio Marañón	SeqCOVID-SPAIN consortium/IBV(CSIC)	Darío García de Viedma, Laura Pérez-Lago, Pedro J Sola-Campoy, Sergio Buenestado-Serrano, Marta Herranz, Victor Manuel de la Cueva, Julia Suárez, Pilar Catalán, Patricia Muñoz and SeqCOVID-SPAIN consortium
EPI_ISL_853397, EPI_ISL_853398, EPI_ISL_853399	Charité Universitätsmedizin Berlin, Institut für Virologie/Labor Berlin	Charité Universitätsmedizin Berlin, Institut für Virologie	Victor M Corman, Julia Schneider, Barbara Mühlemann, Jörn Beheim-Schwarzbach, Talitha Veith, Julia Tesch, Tobias Bleicker, Terry Jones, Christian Drosten
EPI_ISL_853649	THE MARY IMOGENE BASSETT HOSPITAL	Wadsworth Center, New York State Department of Health	Kirsten St. George, Daryl M. Lamson, Alexis Russel, Matthew Shudt, Melissa A Leisner, Jonathan Plitnick, Navjot Singh, John Kelly, Erasmus Schneider, Erica Lasek-Nesselquist
EPI_ISL_853650, EPI_ISL_853651	NORTHWELL HEALTH LABORATORIES	Wadsworth Center, New York State Department of Health	Kirsten St. George, Daryl M. Lamson, Alexis Russel, Matthew Shudt, Melissa A Leisner, Jonathan Plitnick, Navjot Singh, John Kelly, Erasmus Schneider, Erica Lasek-Nesselquist
EPI_ISL_853652	THE MARY IMOGENE BASSETT HOSPITAL	Wadsworth Center, New York State Department of Health	Kirsten St. George, Daryl M. Lamson, Alexis Russel, Matthew Shudt, Melissa A Leisner, Jonathan Plitnick, Navjot Singh, John Kelly, Erasmus Schneider, Erica Lasek-Nesselquist
EPI_ISL_853653, EPI_ISL_853654, EPI_ISL_853655, EPI_ISL_853656, EPI_ISL_853657, EPI_ISL_853658, EPI_ISL_853659, EPI_ISL_853660, EPI_ISL_853661, EPI_ISL_853662, EPI_ISL_853663, EPI_ISL_853664, EPI_ISL_853665, EPI_ISL_853666			
see above	NORTHWELL HEALTH LABORATORIES	Wadsworth Center, New York State Department of Health	Kirsten St. George, Daryl M. Lamson, Alexis Russel, Matthew Shudt, Melissa A Leisner, Jonathan Plitnick, Navjot Singh, John Kelly, Erasmus Schneider, Erica Lasek-Nesselquist
EPI_ISL_853667	THE MARY IMOGENE BASSETT HOSPITAL	Wadsworth Center, New York State Department of Health	Kirsten St. George, Daryl M. Lamson, Alexis Russel, Matthew Shudt, Melissa A Leisner, Jonathan Plitnick, Navjot Singh, John Kelly, Erasmus Schneider, Erica Lasek-Nesselquist
EPI_ISL_853713, EPI_ISL_853714, EPI_ISL_853716, EPI_ISL_853717	NORTHWELL HEALTH LABORATORIES	Wadsworth Center, New York State Department of Health	Kirsten St. George, Daryl M. Lamson, Alexis Russel, Matthew Shudt, Melissa A Leisner, Jonathan Plitnick, Navjot Singh, John Kelly, Erasmus Schneider, Erica Lasek-Nesselquist
EPI_ISL_854305	ESOTERIX GENETIC LABORATORIES LLC	Wadsworth Center, New York State Department of Health	Kirsten St. George, Daryl M. Lamson, Alexis Russel, Matthew Shudt, Melissa A Leisner, Jonathan Plitnick, Navjot Singh, John Kelly, Erasmus Schneider, Erica Lasek-Nesselquist
EPI_ISL_854307, EPI_ISL_854308, EPI_ISL_854309, EPI_ISL_854310, EPI_ISL_854311, EPI_ISL_854312, EPI_ISL_854313, EPI_ISL_854314, EPI_ISL_854315, EPI_ISL_854316, EPI_ISL_854317, EPI_ISL_854318, EPI_ISL_854319, EPI_ISL_854320			
see above	URMC LABS	Wadsworth Center, New York State Department of Health	Kirsten St. George, Daryl M. Lamson, Alexis Russel, Matthew Shudt, Melissa A Leisner, Jonathan Plitnick, Navjot Singh, John Kelly, Erasmus Schneider, Erica Lasek-Nesselquist
EPI_ISL_854368	Wadsworth Center, New York State Department of Health	Wadsworth Center, New York State Department of Health	Kirsten St. George, Daryl M. Lamson, Alexis Russel, Matthew Shudt, Melissa A Leisner, Jonathan Plitnick, Navjot Singh, John Kelly, Erasmus Schneider, Erica Lasek-Nesselquist
EPI_ISL_854381, EPI_ISL_854382, EPI_ISL_854383, EPI_ISL_854384, EPI_ISL_854385, EPI_ISL_854386, EPI_ISL_854387, EPI_ISL_854388, EPI_ISL_854389, EPI_ISL_854390, EPI_ISL_854391, EPI_ISL_854392, EPI_ISL_854393, EPI_ISL_854394, EPI_ISL_854395, EPI_ISL_854396, EPI_ISL_854397, EPI_ISL_854398			
see above	WESTCHESTER MEDICAL CENTER	Wadsworth Center, New York State Department of Health	Kirsten St. George, Daryl M. Lamson, Alexis Russel, Matthew Shudt, Melissa A Leisner, Jonathan Plitnick, Navjot Singh, John Kelly, Erasmus Schneider,

			Erica Lasek-Nesselquist
EPI_ISL_854399	URMC LABS	Wadsworth Center, New York State Department of Health	Kirsten St. George, Daryl M. Lamson, Alexis Russel, Matthew Shudt, Melissa A Leisner, Jonathan Plitnick, Navjot Singh, John Kelly, Erasmus Schneider, Erica Lasek-Nesselquist
EPI_ISL_854400, EPI_ISL_854401, EPI_ISL_854402, EPI_ISL_854403	WESTCHESTER MEDICAL CENTER	Wadsworth Center, New York State Department of Health	Kirsten St. George, Daryl M. Lamson, Alexis Russel, Matthew Shudt, Melissa A Leisner, Jonathan Plitnick, Navjot Singh, John Kelly, Erasmus Schneider, Erica Lasek-Nesselquist
EPI_ISL_854446	BOSTON HEART DIAGNOSTICS CORP	Wadsworth Center, New York State Department of Health	Kirsten St. George, Daryl M. Lamson, Alexis Russel, Matthew Shudt, Melissa A Leisner, Jonathan Plitnick, Navjot Singh, John Kelly, Erasmus Schneider, Erica Lasek-Nesselquist
EPI_ISL_854447, EPI_ISL_854448	TEMPUS LABS INC	Wadsworth Center, New York State Department of Health	Kirsten St. George, Daryl M. Lamson, Alexis Russel, Matthew Shudt, Melissa A Leisner, Jonathan Plitnick, Navjot Singh, John Kelly, Erasmus Schneider, Erica Lasek-Nesselquist
EPI_ISL_854449	WESTCHESTER MEDICAL CENTER	Wadsworth Center, New York State Department of Health	Kirsten St. George, Daryl M. Lamson, Alexis Russel, Matthew Shudt, Melissa A Leisner, Jonathan Plitnick, Navjot Singh, John Kelly, Erasmus Schneider, Erica Lasek-Nesselquist
EPI_ISL_854460	Northwestern Memorial Hospital	Ozer Lab	Ramon Lorenzo-Redondo, Lacy M. Simons, Lawrence J. Jennings, Michael G. Ison, Judd F. Hultquist, Egon A. Ozer
EPI_ISL_854745	Royal Darwin Hospital Pathology	MDU-PHL	Meumann, E., Caly L., Seemann T., Sait, M.L., Druce J., Sherry, N.L.
EPI_ISL_854810, EPI_ISL_854813, EPI_ISL_854816, EPI_ISL_854817, EPI_ISL_854818, EPI_ISL_854820, EPI_ISL_854824, EPI_ISL_854828, EPI_ISL_854829, EPI_ISL_854830, EPI_ISL_854831, EPI_ISL_854832, EPI_ISL_854833, EPI_ISL_854834, EPI_ISL_854842, EPI_ISL_854844, EPI_ISL_854845, EPI_ISL_854846, EPI_ISL_854847, EPI_ISL_854848, EPI_ISL_854849, EPI_ISL_854851, EPI_ISL_854852, EPI_ISL_854853, EPI_ISL_854854, EPI_ISL_854855, EPI_ISL_854856, EPI_ISL_854857, EPI_ISL_854858, EPI_ISL_854859, EPI_ISL_854860, EPI_ISL_854861, EPI_ISL_854863, EPI_ISL_854864, EPI_ISL_854865, EPI_ISL_854866, EPI_ISL_854868, EPI_ISL_854869, EPI_ISL_854870, EPI_ISL_854871, EPI_ISL_854872, EPI_ISL_854873, EPI_ISL_854874, EPI_ISL_854875, EPI_ISL_854876, EPI_ISL_854877, EPI_ISL_854878, EPI_ISL_854879, EPI_ISL_854880, EPI_ISL_854881, EPI_ISL_854882, EPI_ISL_854883, EPI_ISL_854884, EPI_ISL_854886, EPI_ISL_854887, EPI_ISL_854888, EPI_ISL_854889, EPI_ISL_854891, EPI_ISL_854893, EPI_ISL_854894, EPI_ISL_854895, EPI_ISL_854896, EPI_ISL_854897, EPI_ISL_854899, EPI_ISL_854900, EPI_ISL_854901, EPI_ISL_854902, EPI_ISL_854903, EPI_ISL_854904, EPI_ISL_854905, EPI_ISL_854906, EPI_ISL_854907, EPI_ISL_854908, EPI_ISL_854909, EPI_ISL_854910, EPI_ISL_854911, EPI_ISL_854912, EPI_ISL_854913, EPI_ISL_854914, EPI_ISL_854915, EPI_ISL_854916, EPI_ISL_854917, EPI_ISL_854918, EPI_ISL_854919, EPI_ISL_854920, EPI_ISL_854921, EPI_ISL_854922, EPI_ISL_854923, EPI_ISL_854924, EPI_ISL_854925, EPI_ISL_854926, EPI_ISL_854927, EPI_ISL_854928, EPI_ISL_854929, EPI_ISL_854930, EPI_ISL_854931, EPI_ISL_854932, EPI_ISL_854933, EPI_ISL_854934, EPI_ISL_854935, EPI_ISL_854936, EPI_ISL_854937, EPI_ISL_854938, EPI_ISL_854939, EPI_ISL_854940, EPI_ISL_854941, EPI_ISL_854942, EPI_ISL_854944, EPI_ISL_854945, EPI_ISL_854946, EPI_ISL_854947, EPI_ISL_854948, EPI_ISL_854949, EPI_ISL_854950, EPI_ISL_854951, EPI_ISL_854952, EPI_ISL_854953, EPI_ISL_854954, EPI_ISL_854955, EPI_ISL_854956, EPI_ISL_854957, EPI_ISL_854958, EPI_ISL_854959, EPI_ISL_854960, EPI_ISL_854961, EPI_ISL_854962, EPI_ISL_854966, EPI_ISL_854968, EPI_ISL_854969, EPI_ISL_854970, EPI_ISL_854971, EPI_ISL_854972, EPI_ISL_854973, EPI_ISL_854974, EPI_ISL_854975, EPI_ISL_854976, EPI_ISL_855015, EPI_ISL_855018, EPI_ISL_855020, EPI_ISL_855021, EPI_ISL_855024, EPI_ISL_855025, EPI_ISL_855026, EPI_ISL_855028, EPI_ISL_855029, EPI_ISL_855032, EPI_ISL_855036, EPI_ISL_855040, EPI_ISL_855042, EPI_ISL_855043, EPI_ISL_855044, EPI_ISL_855045, EPI_ISL_855048, EPI_ISL_855051, EPI_ISL_855056, EPI_ISL_855064, EPI_ISL_855065, EPI_ISL_855066, EPI_ISL_855067, EPI_ISL_855068, EPI_ISL_855069, EPI_ISL_855071, EPI_ISL_855072, EPI_ISL_855073, EPI_ISL_855074, EPI_ISL_855075, EPI_ISL_855081, EPI_ISL_855082, EPI_ISL_855083, EPI_ISL_855084, EPI_ISL_855085, EPI_ISL_855086, EPI_ISL_855087, EPI_ISL_855088, EPI_ISL_855089, EPI_ISL_855090, EPI_ISL_855091, EPI_ISL_855092, EPI_ISL_855093, EPI_ISL_855094, EPI_ISL_855095, EPI_ISL_855096, EPI_ISL_855097, EPI_ISL_855098, EPI_ISL_855099, EPI_ISL_855100, EPI_ISL_855101, EPI_ISL_855102, EPI_ISL_855103, EPI_ISL_855104, EPI_ISL_855105, EPI_ISL_855106, EPI_ISL_855107, EPI_ISL_855108, EPI_ISL_855109, EPI_ISL_855110, EPI_ISL_855111, EPI_ISL_855112, EPI_ISL_855113, EPI_ISL_855114, EPI_ISL_855116, EPI_ISL_855117, EPI_ISL_855118, EPI_ISL_855119, EPI_ISL_855120, EPI_ISL_855121, EPI_ISL_855122, EPI_ISL_855123, EPI_ISL_855124, EPI_ISL_855125, EPI_ISL_855126, EPI_ISL_855127, EPI_ISL_855128, EPI_ISL_855130, EPI_ISL_855131, EPI_ISL_855132, EPI_ISL_855133, EPI_ISL_855134, EPI_ISL_855135, EPI_ISL_855136, EPI_ISL_855137, EPI_ISL_855138, EPI_ISL_855139, EPI_ISL_855140, EPI_ISL_855141, EPI_ISL_855142, EPI_ISL_855143, EPI_ISL_855144, EPI_ISL_855145, EPI_ISL_855146, EPI_ISL_855147, EPI_ISL_855148, EPI_ISL_855149, EPI_ISL_855150, EPI_ISL_855151, EPI_ISL_855152, EPI_ISL_855153, EPI_ISL_855155, EPI_ISL_855156, EPI_ISL_855157, EPI_ISL_855158, EPI_ISL_855160, EPI_ISL_855161, EPI_ISL_855162, EPI_ISL_855163, EPI_ISL_855165, EPI_ISL_855166, EPI_ISL_855167, EPI_ISL_855168, EPI_ISL_855169, EPI_ISL_855170, EPI_ISL_855171, EPI_ISL_855172, EPI_ISL_855173, EPI_ISL_855174, EPI_ISL_855175, EPI_ISL_855176, EPI_ISL_855177, EPI_ISL_855178, EPI_ISL_855179, EPI_ISL_855180, EPI_ISL_855181, EPI_ISL_855182, EPI_ISL_855183, EPI_ISL_855185			
see above	Quest Diagnostics	Quest Diagnostics	Rosenthal,S.H., Gerasimova,A., Kagan,R.M., Anderson, B., Hua, M., Liu Y., Bernstein, L.E., Livingston, K.E., Perez, A., Shalhout, D.F., Shlyakhter, I.A., Owen, R., Tanpaiboon, P., Lacbawan, F.
EPI_ISL_855358	Hospital	National Reference Center for Viruses of Respiratory Infections, Institut Pasteur, Paris	Marion Barbet, Sylvie Behillil, Méline Bizard, Angela Brisebarre, Camille Capel, Etienne Simon-Lorière, Vincent Enouf, Maud Vanpeene, Sylvie van der Werf,Griscelli Franck
EPI_ISL_855359	Hospital	National Reference Center for Viruses of Respiratory Infections, Institut Pasteur, Paris	Marion Barbet, Sylvie Behillil, Méline Bizard, Angela Brisebarre, Camille Capel, Etienne Simon-Lorière, Vincent Enouf, Maud Vanpeene, Sylvie van der Werf,Fourgeaud Jacques
EPI_ISL_855360, EPI_ISL_855361, EPI_ISL_855362, EPI_ISL_855363, EPI_ISL_855364, EPI_ISL_855365, EPI_ISL_855366, EPI_ISL_855367, EPI_ISL_855368, EPI_ISL_855369, EPI_ISL_855370, EPI_ISL_855371, EPI_ISL_855372, EPI_ISL_855373, EPI_ISL_855374, EPI_ISL_855375, EPI_ISL_855376, EPI_ISL_855377, EPI_ISL_855378, EPI_ISL_855379, EPI_ISL_855380			
see above	Hospital	National Reference Center for Viruses of Respiratory Infections, Institut Pasteur, Paris	Marion Barbet, Sylvie Behillil, Méline Bizard, Angela Brisebarre, Camille Capel, Etienne Simon-Lorière, Vincent Enouf, Maud Vanpeene, Sylvie van der Werf,Combe Patrice
EPI_ISL_855381	Labo Analyses Med	National Reference Center for Viruses of Respiratory Infections, Institut Pasteur, Paris	Marion Barbet, Sylvie Behillil, Méline Bizard, Angela Brisebarre, Camille Capel, Etienne Simon-Lorière, Vincent Enouf, Maud Vanpeene, Sylvie van der Werf
EPI_ISL_855385	Hospital	National Reference Center for Viruses of Respiratory Infections, Institut Pasteur, Paris	Marion Barbet, Sylvie Behillil, Méline Bizard, Angela Brisebarre, Camille Capel, Etienne Simon-Lorière, Vincent Enouf, Maud Vanpeene, Sylvie van der Werf
EPI_ISL_855551	Cerballiance Wilson	CERBA LAB	Roquebert B; Merah K; Olivi M; Herhira S; Lecorche E; Trombert S; Verdurme L; Malek R; Zimmer S; Costa JM; Haïm-Boukobza S
EPI_ISL_855574, EPI_ISL_855575, EPI_ISL_855577, EPI_ISL_855578, EPI_ISL_855579, EPI_ISL_855581, EPI_ISL_855582, EPI_ISL_855584, EPI_ISL_855589, EPI_ISL_855590, EPI_ISL_855591, EPI_ISL_855592, EPI_ISL_855593, EPI_ISL_855594, EPI_ISL_855595, EPI_ISL_855596, EPI_ISL_855597, EPI_ISL_855598, EPI_ISL_855599, EPI_ISL_855600, EPI_ISL_855601, EPI_ISL_855602, EPI_ISL_855606, EPI_ISL_855607, EPI_ISL_855608, EPI_ISL_855609			
see above	Respiratory Virus Unit, National Infection Service, Public Health England	COVID-19 Genomics UK (COG-UK) Consortium	PHE Covid Sequencing Team
EPI_ISL_855904, EPI_ISL_855930	Lab voor klinische biologie	Onderzoeksgroep Virologie	Laurens Lambrechts, Nick Vereecke, Marthe Pauwels, Bruno Verhasselt, Linos Vandekerckhove, Hans Nauwynck, Sebastiaan Theuns
EPI_ISL_855936, EPI_ISL_855938, EPI_ISL_855944, EPI_ISL_855945, EPI_ISL_855947	Lab voor klinische biologie	Onderzoeksgroep Virologie	Nick Vereecke, Laurens Lambrechts, Marthe Pauwels, Bruno Verhasselt, Linos Vandekerckhove, Hans Nauwynck, Sebastiaan Theuns
EPI_ISL_856703, EPI_ISL_856704, EPI_ISL_856705, EPI_ISL_856706	Respiratory Virus Unit, National Infection Service, Public Health England	COVID-19 Genomics UK (COG-UK) Consortium	PHE Covid Sequencing Team
EPI_ISL_856711	Wyoming Public Health Laboratory	Wyoming Public Health Laboratory	Noah Hull, Kevin Libuit, Joel Sevinsky, Taylor Fearing, Lynette Gumbleton, Channing Weber, Ashley Norberg, Bailey Bowcutt, and Wanda Manley
EPI_ISL_856913, EPI_ISL_856914, EPI_ISL_856915, EPI_ISL_856916, EPI_ISL_856917, EPI_ISL_856918, EPI_ISL_856919, EPI_ISL_856932, EPI_ISL_856933, EPI_ISL_856935, EPI_ISL_856936, EPI_ISL_856937, EPI_ISL_856945, EPI_ISL_856946, EPI_ISL_856947, EPI_ISL_856948, EPI_ISL_856949, EPI_ISL_856950, EPI_ISL_856951, EPI_ISL_856952, EPI_ISL_856953, EPI_ISL_856954, EPI_ISL_856955, EPI_ISL_856956, EPI_ISL_856957, EPI_ISL_856958, EPI_ISL_856959, EPI_ISL_856960			
see above	Wyoming Public Health Laboratory	Wyoming Public Health Laboratory	Noah Hull, Taylor Fearing, Lynette Gumbleton, Channing Weber, Ashley Norberg, Bailey Bowcutt, and Wanda Manley
EPI_ISL_857042	Platform BIS UZA/UAntwerpen, University Hospital Antwerp, Edegem, Belgium	UAntwerp, Laboratory of Medical Microbiology, Campus Drie Eiken S6.26, Universiteitsplein 1, 2610, Wilrijk, Antwerp, Belgium	Basil Britto Xavier, Jasmine Coppens, Christine Lammens, Veerle Matheeussen, Herman Goossens
EPI_ISL_857043, EPI_ISL_857044, EPI_ISL_857045	Colorado Department of Public Health and Environment	Colorado Department of Puplic Health and Environment	Laura Bankers, Molly C. Hetherington-Rauth, Diana Ir, Shannon Ely, Shannon R. Matzinger, Sarah Elizabeth Totten, Emily A. Travanty
EPI_ISL_857053, EPI_ISL_857054	Platform BIS UZA/UAntwerpen, University Hospital Antwerp, Edegem, Belgium	UAntwerp, Laboratory of Medical Microbiology, Campus Drie Eiken S6.26, Universiteitsplein 1, 2610, Wilrijk, Antwerp, Belgium	Basil Britto Xavier, Jasmine Coppens, Christine Lammens, Veerle Matheeussen, Herman Goossens
EPI_ISL_857067	DOHMH Corona	New York City Public Health Laboratory	Jade Wang, et al.
EPI_ISL_857068	DOHMH PHL	New York City Public Health Laboratory	Jade Wang, et al.
EPI_ISL_857069	DOHMH Jamaica	New York City Public Health Laboratory	Jade Wang, et al.
EPI_ISL_857070	DOHMH PHL	New York City Public Health Laboratory	Jade Wang, et al.
EPI_ISL_857071	DOHMH Morrisania	New York City Public Health Laboratory	Jade Wang, et al.
EPI_ISL_857072	DOHMH PHL	New York City Public Health Laboratory	Jade Wang, et al.

EPI_ISL_857073	DOHMH Corona	New York City Public Health Laboratory	Jade Wang, et al.
EPI_ISL_857074	DOHMH Morrisania	New York City Public Health Laboratory	Jade Wang, et al.
EPI_ISL_857075	DOHMH Jamaica	New York City Public Health Laboratory	Jade Wang, et al.
EPI_ISL_857076	DOHMH Chelsea	New York City Public Health Laboratory	Jade Wang, et al.
EPI_ISL_857077, EPI_ISL_857078, EPI_ISL_857079, EPI_ISL_857080	DOHMH Central Harlem	New York City Public Health Laboratory	Jade Wang, et al.
EPI_ISL_857081	DOHMH Morrisania	New York City Public Health Laboratory	Jade Wang, et al.
EPI_ISL_857082, EPI_ISL_857083, EPI_ISL_857084	DOHMH Corona	New York City Public Health Laboratory	Jade Wang, et al.
EPI_ISL_857085, EPI_ISL_857086	DOHMH Central Harlem	New York City Public Health Laboratory	Jade Wang, et al.
EPI_ISL_857087, EPI_ISL_857088	DOHMH PHL	New York City Public Health Laboratory	Jade Wang, et al.
EPI_ISL_857089	DOHMH Crown Heights	New York City Public Health Laboratory	Jade Wang, et al.
EPI_ISL_857090	DOHMH Riverside	New York City Public Health Laboratory	Jade Wang, et al.
EPI_ISL_857091	DOHMH Jamaica	New York City Public Health Laboratory	Jade Wang, et al.
EPI_ISL_857092	DOHMH Riverside	New York City Public Health Laboratory	Jade Wang, et al.
EPI_ISL_857093	DOHMH Morrisania	New York City Public Health Laboratory	Jade Wang, et al.
EPI_ISL_857094	DOHMH Central Harlem	New York City Public Health Laboratory	Jade Wang, et al.
EPI_ISL_857095	DOHMH Fort Greene	New York City Public Health Laboratory	Jade Wang, et al.
EPI_ISL_857096	DOHMH Jamaica	New York City Public Health Laboratory	Jade Wang, et al.
EPI_ISL_857097	DOHMH Crown Heights	New York City Public Health Laboratory	Jade Wang, et al.
EPI_ISL_857098, EPI_ISL_857099	DOHMH Morrisania	New York City Public Health Laboratory	Jade Wang, et al.
EPI_ISL_857100	DOHMH Central Harlem	New York City Public Health Laboratory	Jade Wang, et al.
EPI_ISL_857101, EPI_ISL_857102, EPI_ISL_857103, EPI_ISL_857104, EPI_ISL_857105	DOHMH Corona	New York City Public Health Laboratory	Jade Wang, et al.
EPI_ISL_857106, EPI_ISL_857107, EPI_ISL_857108, EPI_ISL_857109, EPI_ISL_857110	DOHMH Chelsea	New York City Public Health Laboratory	Jade Wang, et al.
EPI_ISL_857111	DOHMH Riverside	New York City Public Health Laboratory	Jade Wang, et al.
EPI_ISL_857112, EPI_ISL_857113, EPI_ISL_857114, EPI_ISL_857115	DOHMH Fort Greene	New York City Public Health Laboratory	Jade Wang, et al.
EPI_ISL_857116	DOHMH Central Harlem	New York City Public Health Laboratory	Jade Wang, et al.
EPI_ISL_857117	DOHMH Chelsea	New York City Public Health Laboratory	Jade Wang, et al.
EPI_ISL_857118	DOHMH Central Harlem	New York City Public Health Laboratory	Jade Wang, et al.
EPI_ISL_857119	DOHMH Morrisania	New York City Public Health Laboratory	Jade Wang, et al.
EPI_ISL_857120	DOHMH Central Harlem	New York City Public Health Laboratory	Jade Wang, et al.
EPI_ISL_857121	DOHMH PHL	New York City Public Health Laboratory	Jade Wang, et al.
EPI_ISL_857122, EPI_ISL_857123, EPI_ISL_857124, EPI_ISL_857125	DOHMH Morrisania	New York City Public Health Laboratory	Jade Wang, et al.
EPI_ISL_857126	DOHMH Central Harlem	New York City Public Health Laboratory	Jade Wang, et al.
EPI_ISL_857127, EPI_ISL_857128, EPI_ISL_857129	DOHMH PHL	New York City Public Health Laboratory	Jade Wang, et al.
EPI_ISL_857130, EPI_ISL_857131	DOHMH Chelsea	New York City Public Health Laboratory	Jade Wang, et al.
EPI_ISL_857132, EPI_ISL_857133	DOHMH Central Harlem	New York City Public Health Laboratory	Jade Wang, et al.
EPI_ISL_857349, EPI_ISL_857350, EPI_ISL_857351, EPI_ISL_857352, EPI_ISL_857353, EPI_ISL_857354, EPI_ISL_857355, EPI_ISL_857356, EPI_ISL_857357, EPI_ISL_857358, EPI_ISL_857359, EPI_ISL_857360, EPI_ISL_857361, EPI_ISL_857362, EPI_ISL_857363, EPI_ISL_857364, EPI_ISL_857365			
see above	Texas Department of State Health Services (TXDSHS)	Texas Department of State Health Services (TXDSHS)	Anita Pokharel, Bonnie Oh, James Daniel Bonser, Rashmi Tuladhar, Mayela Pedrueza, Jenny Zhang, Maliha Rahman, Myong Koag, Chung Wang, Rachel Lee, Grace Kubin
EPI_ISL_857402, EPI_ISL_857454, EPI_ISL_857456, EPI_ISL_857458, EPI_ISL_857459	Maine HETL	Tewhey Lab, The Jackson Laboratory	Matluk,N., Dewey,H., Iosue,F., Barter,M., Lynch,R., Munger,H. and Tewhey,R.
EPI_ISL_857478	National Public Health Laboratory, National Centre for Infectious Diseases	National Public Health Laboratory, National Centre for Infectious Diseases	Tze Minn Mak, Sophie Octavia, Zhenyang Zhou, Lin Cui, Raymond Tzer Pin Lin
EPI_ISL_857546, EPI_ISL_857548, EPI_ISL_857550, EPI_ISL_857553, EPI_ISL_857562, EPI_ISL_857571, EPI_ISL_857584, EPI_ISL_857599, EPI_ISL_857603, EPI_ISL_857604, EPI_ISL_857606, EPI_ISL_857618, EPI_ISL_857642, EPI_ISL_857645, EPI_ISL_857654, EPI_ISL_857656, EPI_ISL_857669, EPI_ISL_857680, EPI_ISL_857682, EPI_ISL_857685, EPI_ISL_857699, EPI_ISL_857701, EPI_ISL_857715, EPI_ISL_857733, EPI_ISL_857752, EPI_ISL_857759, EPI_ISL_857781, EPI_ISL_857790, EPI_ISL_857802, EPI_ISL_857818, EPI_ISL_857819, EPI_ISL_857821, EPI_ISL_857823, EPI_ISL_857852			
see above	Lighthouse Lab in Cambridge	Wellcome Sanger Institute for the COVID-19 Genomics UK (COG-UK) Consortium	Rob Howes, The Lighthouse Lab in Cambridge and Alex Alderton, Roberto Amato, Sonia Goncalves, Ewan Harrison, David K. Jackson, Ian Johnston, Dominic Kwiatkowski, Cordelia Langford, John Sillitoe on behalf of the Wellcome Sanger Institute COVID-19 Surveillance Team
EPI_ISL_857875, EPI_ISL_857879, EPI_ISL_857880, EPI_ISL_857882, EPI_ISL_857890, EPI_ISL_857960, EPI_ISL_857965, EPI_ISL_857966, EPI_ISL_857984, EPI_ISL_858000	Lighthouse Lab in Alderley Park	Wellcome Sanger Institute for the COVID-19 Genomics UK (COG-UK) Consortium	Jacquelyn Wynn, Mairead Hyland, The Lighthouse Lab in Alderley Park and Alex Alderton, Roberto Amato, Sonia Goncalves, Ewan Harrison, David K. Jackson, Ian Johnston, Dominic Kwiatkowski, Cordelia Langford, John Sillitoe on behalf of the Wellcome Sanger Institute COVID-19 Surveillance Team
EPI_ISL_858012, EPI_ISL_858013, EPI_ISL_858014, EPI_ISL_858015, EPI_ISL_858016, EPI_ISL_858017, EPI_ISL_858018, EPI_ISL_858019, EPI_ISL_858020, EPI_ISL_858021, EPI_ISL_858023, EPI_ISL_858024, EPI_ISL_858026, EPI_ISL_858027, EPI_ISL_858028, EPI_ISL_858029, EPI_ISL_858030, EPI_ISL_858031, EPI_ISL_858032, EPI_ISL_858033, EPI_ISL_858034, EPI_ISL_858036, EPI_ISL_858037, EPI_ISL_858038, EPI_ISL_858041, EPI_ISL_858042, EPI_ISL_858044, EPI_ISL_858045, EPI_ISL_858046, EPI_ISL_858047, EPI_ISL_858048, EPI_ISL_858049, EPI_ISL_858050, EPI_ISL_858051, EPI_ISL_858052, EPI_ISL_858053, EPI_ISL_858055, EPI_ISL_858056, EPI_ISL_858057, EPI_ISL_858058, EPI_ISL_858059, EPI_ISL_858060, EPI_ISL_858061, EPI_ISL_858062, EPI_ISL_858063			
see above	Lighthouse Lab in Glasgow	Wellcome Sanger Institute for the COVID-19 Genomics UK (COG-UK) Consortium	Harper VanSteenhouse, Yumi Kasai, David Gray, Carol Clugston, Anna Dominiczak and Alex Alderton, Roberto Amato, Sonia Goncalves, Ewan Harrison, David K. Jackson, Ian Johnston, Dominic Kwiatkowski, Cordelia Langford, John Sillitoe on behalf of the Wellcome Sanger Institute COVID-19 Surveillance

			Team
EPI_ISL_858064, EPI_ISL_858065, EPI_ISL_858066, EPI_ISL_858067, EPI_ISL_858068, EPI_ISL_858069, EPI_ISL_858070, EPI_ISL_858071, EPI_ISL_858072, EPI_ISL_858073, EPI_ISL_858074, EPI_ISL_858075, EPI_ISL_858076, EPI_ISL_858078, EPI_ISL_858079, EPI_ISL_858080, EPI_ISL_858081, EPI_ISL_858082, EPI_ISL_858083, EPI_ISL_858084, EPI_ISL_858087, EPI_ISL_858088, EPI_ISL_858089, EPI_ISL_858090, EPI_ISL_858091, EPI_ISL_858092, EPI_ISL_858093, EPI_ISL_858094, EPI_ISL_858095, EPI_ISL_858096, EPI_ISL_858097, EPI_ISL_858098, EPI_ISL_858099, EPI_ISL_858100, EPI_ISL_858102, EPI_ISL_858103, EPI_ISL_858104, EPI_ISL_858105, EPI_ISL_858106, EPI_ISL_858107, EPI_ISL_858108, EPI_ISL_858109, EPI_ISL_858110, EPI_ISL_858111, EPI_ISL_858112, EPI_ISL_858113, EPI_ISL_858114, EPI_ISL_858115, EPI_ISL_858116, EPI_ISL_858117, EPI_ISL_858118, EPI_ISL_858119, EPI_ISL_858120, EPI_ISL_858121, EPI_ISL_858123, EPI_ISL_858124, EPI_ISL_858125, EPI_ISL_858126, EPI_ISL_858127, EPI_ISL_858128, EPI_ISL_858129, EPI_ISL_858130, EPI_ISL_858131, EPI_ISL_858132, EPI_ISL_858133, EPI_ISL_858134, EPI_ISL_858135, EPI_ISL_858137, EPI_ISL_858138, EPI_ISL_858139, EPI_ISL_858140, EPI_ISL_858141			
see above	Lighthouse Lab in Alderley Park	Wellcome Sanger Institute for the COVID-19 Genomics UK (COG-UK) Consortium	Jacquelyn Wynn, Mairead Hyland, The Lighthouse Lab in Alderley Park and Alex Alderton, Roberto Amato, Sonia Goncalves, Ewan Harrison, David K. Jackson, Ian Johnston, Dominic Kwiatkowski, Cordelia Langford, John Sillitoe on behalf of the Wellcome Sanger Institute COVID-19 Surveillance Team
EPI_ISL_858158, EPI_ISL_858159, EPI_ISL_858161, EPI_ISL_858162, EPI_ISL_858163, EPI_ISL_858164, EPI_ISL_858165, EPI_ISL_858166, EPI_ISL_858167, EPI_ISL_858168, EPI_ISL_858169, EPI_ISL_858170, EPI_ISL_858171, EPI_ISL_858172, EPI_ISL_858173, EPI_ISL_858174, EPI_ISL_858175, EPI_ISL_858176, EPI_ISL_858177, EPI_ISL_858178, EPI_ISL_858179, EPI_ISL_858180, EPI_ISL_858181, EPI_ISL_858182, EPI_ISL_858183, EPI_ISL_858184, EPI_ISL_858185, EPI_ISL_858186, EPI_ISL_858187, EPI_ISL_858188, EPI_ISL_858189, EPI_ISL_858190, EPI_ISL_858191, EPI_ISL_858192, EPI_ISL_858193, EPI_ISL_858194, EPI_ISL_858195, EPI_ISL_858196, EPI_ISL_858197, EPI_ISL_858198, EPI_ISL_858199, EPI_ISL_858200, EPI_ISL_858201, EPI_ISL_858202, EPI_ISL_858203, EPI_ISL_858204, EPI_ISL_858205, EPI_ISL_858206, EPI_ISL_858223, EPI_ISL_858224, EPI_ISL_858225, EPI_ISL_858226, EPI_ISL_858227, EPI_ISL_858228, EPI_ISL_858229, EPI_ISL_858230, EPI_ISL_858231, EPI_ISL_858232, EPI_ISL_858233, EPI_ISL_858234, EPI_ISL_858235, EPI_ISL_858236, EPI_ISL_858237, EPI_ISL_858238, EPI_ISL_858239, EPI_ISL_858240, EPI_ISL_858242, EPI_ISL_858243, EPI_ISL_858244, EPI_ISL_858245, EPI_ISL_858247, EPI_ISL_858248, EPI_ISL_858249, EPI_ISL_858250, EPI_ISL_858251, EPI_ISL_858252, EPI_ISL_858253, EPI_ISL_858254, EPI_ISL_858255, EPI_ISL_858256, EPI_ISL_858257, EPI_ISL_858258, EPI_ISL_858259, EPI_ISL_858260, EPI_ISL_858261, EPI_ISL_858262, EPI_ISL_858264, EPI_ISL_858265, EPI_ISL_858266, EPI_ISL_858271, EPI_ISL_858273, EPI_ISL_858275, EPI_ISL_858276, EPI_ISL_858277, EPI_ISL_858279, EPI_ISL_858280, EPI_ISL_858281, EPI_ISL_858282, EPI_ISL_858283, EPI_ISL_858284, EPI_ISL_858286, EPI_ISL_858288, EPI_ISL_858289, EPI_ISL_858291, EPI_ISL_858292, EPI_ISL_858293, EPI_ISL_858294, EPI_ISL_858295, EPI_ISL_858297, EPI_ISL_858298, EPI_ISL_858299, EPI_ISL_858300, EPI_ISL_858301, EPI_ISL_858302, EPI_ISL_858304, EPI_ISL_858305, EPI_ISL_858314, EPI_ISL_858315, EPI_ISL_858316, EPI_ISL_858317, EPI_ISL_858319, EPI_ISL_858320, EPI_ISL_858321, EPI_ISL_858322, EPI_ISL_858323, EPI_ISL_858327, EPI_ISL_858331, EPI_ISL_858333, EPI_ISL_858335, EPI_ISL_858336, EPI_ISL_858337, EPI_ISL_858338, EPI_ISL_858340, EPI_ISL_858342, EPI_ISL_858344, EPI_ISL_858345, EPI_ISL_858346, EPI_ISL_858347, EPI_ISL_858348, EPI_ISL_858349, EPI_ISL_858352, EPI_ISL_858353, EPI_ISL_858355, EPI_ISL_858356, EPI_ISL_858358, EPI_ISL_858359, EPI_ISL_858360, EPI_ISL_858361, EPI_ISL_858362, EPI_ISL_858364, EPI_ISL_858365, EPI_ISL_858366, EPI_ISL_858367, EPI_ISL_858369, EPI_ISL_858372, EPI_ISL_858373, EPI_ISL_858374, EPI_ISL_858376, EPI_ISL_858377, EPI_ISL_858379, EPI_ISL_858380, EPI_ISL_858384, EPI_ISL_858390, EPI_ISL_858396, EPI_ISL_858401, EPI_ISL_858402, EPI_ISL_858404, EPI_ISL_858405, EPI_ISL_858406, EPI_ISL_858407			
see above	Lighthouse Lab in Glasgow	Wellcome Sanger Institute for the COVID-19 Genomics UK (COG-UK) Consortium	Harper VanSteenhouse, Yumi Kasai, David Gray, Carol Clugston, Anna Dominiczak and Alex Alderton, Roberto Amato, Sonia Goncalves, Ewan Harrison, David K. Jackson, Ian Johnston, Dominic Kwiatkowski, Cordelia Langford, John Sillitoe on behalf of the Wellcome Sanger Institute COVID-19 Surveillance Team
EPI_ISL_860198	Railway Hospital	Department of Neurovirology, National Institute of Mental Health and Neurosciences (NIMHANS)	Chitra Pattabiraman, Pramada Prasad, Anson Kunjumon George, Risha Rasheed, Darshan Sreenivas, Nakka Vijay Kiran Reddy, Anita S Desai, V Ravi
EPI_ISL_860240, EPI_ISL_860244, EPI_ISL_860245, EPI_ISL_860246, EPI_ISL_860251, EPI_ISL_860253, EPI_ISL_860255	University Hospitals of Geneva, Laboratory of Virology	HUG, Laboratory of Virology and Universitätsspital Basel	Samuel Cordey, Ana Rita Goncalves, Laurent Kaiser, Tim Roloff, Madlen Stange, Helena MB Seth-Smith, Alfredo Mari, Karoline Leuzinger, Julia Bielicki, Manuel Battegay, Hans Hirsch, Adrian Egli
EPI_ISL_860271, EPI_ISL_860273	Ostfold Hospital Trust - Kalnes, Centre for Laboratory Medicine, Section for gene technology and infection serology	Norwegian Institute of Public Health, Department of Virology	Kathrine Stene-Johansen, Kamilla Heddeland Instefjord, Hilde Elshaug, Atiya R Ali,Marie Paulsen Madsen, Rasmus Riis Kopperud, Hilde Vollan, Karoline Bragstad, Olav Hungnes
EPI_ISL_860274	Foerde Hospital, Department of Microbiology	Norwegian Institute of Public Health, Department of Virology	Kathrine Stene-Johansen, Kamilla Heddeland Instefjord, Hilde Elshaug, Atiya R Ali,Marie Paulsen Madsen, Rasmus Riis Kopperud, Hilde Vollan, Karoline Bragstad, Olav Hungnes
EPI_ISL_860279	Ostfold Hospital Trust - Kalnes, Centre for Laboratory Medicine, Section for gene technology and infection serology	Norwegian Institute of Public Health, Department of Virology	Kathrine Stene-Johansen, Kamilla Heddeland Instefjord, Hilde Elshaug, Atiya R Ali,Marie Paulsen Madsen, Rasmus Riis Kopperud, Hilde Vollan, Karoline Bragstad, Olav Hungnes
EPI_ISL_860294	Norwegian Institute of Public Health, Department of Virology	Norwegian Institute of Public Health, Department of Virology	Kathrine Stene-Johansen, Kamilla Heddeland Instefjord, Hilde Elshaug, Atiya R Ali,Marie Paulsen Madsen, Rasmus Riis Kopperud, Hilde Vollan, Karoline Bragstad, Olav Hungnes
EPI_ISL_860313, EPI_ISL_860314, EPI_ISL_860315	Unit 17: Influenza & Other Respiratory Viruses, German National Influenza Center	Project group Epidemiology of Highly Pathogenic Microorganisms, Robert Koch-Institute	Andreas Sachse, Grit Schubert, Essia Belarbi, Sébastien Calvignac-Spencer, Thorsten Wolff, Ralf Dürrwald, Djin-Ye Oh, Marianne Wedde
EPI_ISL_860317	Instituto Adolfo Lutz - Regional de Sorocaba	Instituto Adolfo Lutz, Interdiciplinary Procedures Center, Strategic Laboratory	Claudio Tavares Sacchi, Claudia Regina Gonçalves, Erica Valessa Ramos Gomes, Karoline Rodrigues Campos
EPI_ISL_860553	Ampath-Netcare	KRISP, KZn Research Innovation and Sequencing Platform	Giandhari J, Pillay S, Lessells R, Mdlalose K, York D, Khan S, Tegally H, Wilkinson E, de Oliveira T
EPI_ISL_860554, EPI_ISL_860555, EPI_ISL_860556, EPI_ISL_860557, EPI_ISL_860558	NHLS-IALCH	KRISP, KZn Research Innovation and Sequencing Platform	Giandhari J, Pillay S, Lessells R, Mdlalose K, York D, Khan S, Tegally H, Wilkinson E, de Oliveira T
EPI_ISL_860633	Instituto Adolfo Lutz - Regional de Sorocaba	Instituto Adolfo Lutz, Interdiciplinary Procedures Center, Strategic Laboratory	Claudio Tavares Sacchi, Claudia Regina Gonçalves, Erica Valessa Ramos Gomes, Karoline Rodrigues Campos
EPI_ISL_860671, EPI_ISL_860674, EPI_ISL_860675, EPI_ISL_860676, EPI_ISL_860680, EPI_ISL_860681, EPI_ISL_860682, EPI_ISL_860683, EPI_ISL_860684	Respiratory Virus Unit, National Infection Service, Public Health England	COVID-19 Genomics UK (COG-UK) Consortium	PHE Covid Sequencing Team
EPI_ISL_860719	Institute for Infectious Diseases, University of Bern, Switzerland	Institute for Infectious Diseases, University of Bern, Switzerland	Michel C Koch, Christian Baumann, Miguel A Terrazos Miani, Cora Sägesser, Pascal Bittel, Stephen L Leib, Peter Keller, Franziska Suter-Riniker, Alban Ramette
EPI_ISL_860784, EPI_ISL_860786, EPI_ISL_860788	Charité Universitätsmedizin Berlin, Institute of Virology, Charitéplatz 1, 10117 Berlin, Germany	Charité Universitätsmedizin Berlin, Institute of Virology, Charitéplatz 1, 10117 Berlin, Germany	Victor M Corman, Julia Schneider, Jörn Beheim-Schwarzbach, Tobias Bleicker, Julia Tesch, Barbara Mühlemann, Talitha Veith, Terry Jones, Christian Drosten
EPI_ISL_860821	CHU de Nantes - Hôtel Dieu - Labo. Virologie	National Reference Center for Viruses of Respiratory Infections, Institut Pasteur, Paris	Marion Barbet, Sylvie Behillil, Méline Bizard, Angela Brisebarre, Camille Capel, Etienne Simon-Lorière, Vincent Enouf, Maud Vanpeene, Sylvie van der Werf,Andre-Garnier Elisabeth
EPI_ISL_860822	CHU Tours - Virologie	National Reference Center for Viruses of Respiratory Infections, Institut Pasteur, Paris	Marion Barbet, Sylvie Behillil, Méline Bizard, Angela Brisebarre, Camille Capel, Etienne Simon-Lorière, Vincent Enouf, Maud Vanpeene, Sylvie van der Werf,Gaudy Graffin Catherine
EPI_ISL_860824	Labo Analyses Med	National Reference Center for Viruses of Respiratory Infections, Institut Pasteur, Paris	Marion Barbet, Sylvie Behillil, Méline Bizard, Angela Brisebarre, Camille Capel, Etienne Simon-Lorière, Vincent Enouf, Maud Vanpeene, Sylvie van der Werf,Amzalag Jonas
EPI_ISL_860826	Labo Analyses med	National Reference Center for Viruses of Respiratory Infections, Institut Pasteur, Paris	Marion Barbet, Sylvie Behillil, Méline Bizard, Angela Brisebarre, Camille Capel, Etienne Simon-Lorière, Vincent Enouf, Maud Vanpeene, Sylvie van der Werf,Amzalag Jonas
EPI_ISL_860828	CHU de Nantes - Hôtel Dieu - Labo. Virologie	National Reference Center for Viruses of Respiratory Infections, Institut Pasteur, Paris	Marion Barbet, Sylvie Behillil, Méline Bizard, Angela Brisebarre, Camille Capel, Etienne Simon-Lorière, Vincent Enouf, Maud Vanpeene, Sylvie van der Werf,Andre-Garnier Elisabeth
EPI_ISL_860831, EPI_ISL_860833, EPI_ISL_860836, EPI_ISL_860838, EPI_ISL_860840	Labo Analyses Med	National Reference Center for Viruses of Respiratory Infections, Institut Pasteur, Paris	Marion Barbet, Sylvie Behillil, Méline Bizard, Angela Brisebarre, Camille Capel, Etienne Simon-Lorière, Vincent Enouf, Maud Vanpeene, Sylvie van der Werf,Amzalag Jonas
EPI_ISL_860841	Labo Analyses Med	National Reference Center for Viruses of Respiratory Infections, Institut Pasteur, Paris	Marion Barbet, Sylvie Behillil, Méline Bizard, Angela Brisebarre, Camille Capel, Etienne Simon-Lorière, Vincent Enouf, Maud Vanpeene, Sylvie van der Werf,
EPI_ISL_860842, EPI_ISL_860843, EPI_ISL_860844	Labo Analyses med	National Reference Center for Viruses of Respiratory Infections, Institut Pasteur, Paris	Marion Barbet, Sylvie Behillil, Méline Bizard, Angela Brisebarre, Camille Capel, Etienne Simon-Lorière, Vincent Enouf, Maud Vanpeene, Sylvie van der Werf,Amzalag Jonas
EPI_ISL_860849	HIA BÉGIN Service de Biologie	National Reference Center for Viruses of Respiratory Infections, Institut Pasteur, Paris	Marion Barbet, Sylvie Behillil, Méline Bizard, Angela Brisebarre, Camille Capel, Etienne Simon-Lorière, Vincent Enouf, Maud Vanpeene, Sylvie van der Werf,Merens Audrey
EPI_ISL_860856	Labo Analyses Med	National Reference Center for Viruses of Respiratory	Marion Barbet, Sylvie Behillil, Méline Bizard, Angela Brisebarre, Camille Capel, Etienne Simon-Lorière, Vincent Enouf, Maud Vanpeene, Sylvie van der

		Infections, Institut Pasteur, Paris	Werf,Amzalag Jonas
EPI_ISL_860860, EPI_ISL_860861, EPI_ISL_860862	Labo Analyses med	National Reference Center for Viruses of Respiratory Infections, Institut Pasteur, Paris	Marion Barbet, Sylvie Behillil, Méline Bizard, Angela Brisebarre, Camille Capel, Etienne Simon-Lorière, Vincent Enouf, Maud Vanpeene, Sylvie van der Werf,Amzalag Jonas
EPI_ISL_860863, EPI_ISL_860865	CHU de Nantes - Hôtel Dieu - Labo. Virologie	National Reference Center for Viruses of Respiratory Infections, Institut Pasteur, Paris	Marion Barbet, Sylvie Behillil, Méline Bizard, Angela Brisebarre, Camille Capel, Etienne Simon-Lorière, Vincent Enouf, Maud Vanpeene, Sylvie van der Werf,Andre-Garnier Elisabeth
EPI_ISL_860866	Labo Analyses Med	National Reference Center for Viruses of Respiratory Infections, Institut Pasteur, Paris	Marion Barbet, Sylvie Behillil, Méline Bizard, Angela Brisebarre, Camille Capel, Etienne Simon-Lorière, Vincent Enouf, Maud Vanpeene, Sylvie van der Werf,Lefaure Brieuc
EPI_ISL_860867	CHU Tours - Virologie	National Reference Center for Viruses of Respiratory Infections, Institut Pasteur, Paris	Marion Barbet, Sylvie Behillil, Méline Bizard, Angela Brisebarre, Camille Capel, Etienne Simon-Lorière, Vincent Enouf, Maud Vanpeene, Sylvie van der Werf,Gaudy Graffin Catherine
EPI_ISL_860868	CHU de Nantes - Hôtel Dieu - Labo. Virologie	National Reference Center for Viruses of Respiratory Infections, Institut Pasteur, Paris	Marion Barbet, Sylvie Behillil, Méline Bizard, Angela Brisebarre, Camille Capel, Etienne Simon-Lorière, Vincent Enouf, Maud Vanpeene, Sylvie van der Werf,Andre-Garnier Elisabeth
EPI_ISL_860872, EPI_ISL_860873, EPI_ISL_860874, EPI_ISL_860875, EPI_ISL_860876, EPI_ISL_860877, EPI_ISL_860878, EPI_ISL_860886, EPI_ISL_860887	Labo Analyses Med	National Reference Center for Viruses of Respiratory Infections, Institut Pasteur, Paris	Marion Barbet, Sylvie Behillil, Méline Bizard, Angela Brisebarre, Camille Capel, Etienne Simon-Lorière, Vincent Enouf, Maud Vanpeene, Sylvie van der Werf,Lefaure Brieuc
EPI_ISL_860889	Labo Analyses Med	National Reference Center for Viruses of Respiratory Infections, Institut Pasteur, Paris	Marion Barbet, Sylvie Behillil, Méline Bizard, Angela Brisebarre, Camille Capel, Etienne Simon-Lorière, Vincent Enouf, Maud Vanpeene, Sylvie van der Werf,Amzalag Jonas
EPI_ISL_860891, EPI_ISL_860893, EPI_ISL_860894, EPI_ISL_860896	CHU Tours - Virologie	National Reference Center for Viruses of Respiratory Infections, Institut Pasteur, Paris	Marion Barbet, Sylvie Behillil, Méline Bizard, Angela Brisebarre, Camille Capel, Etienne Simon-Lorière, Vincent Enouf, Maud Vanpeene, Sylvie van der Werf,Gaudy Graffin Catherine
EPI_ISL_860905	CHU Angers - Dpt des Agents Infectieux	National Reference Center for Viruses of Respiratory Infections, Institut Pasteur, Paris	Marion Barbet, Sylvie Behillil, Méline Bizard, Angela Brisebarre, Camille Capel, Etienne Simon-Lorière, Vincent Enouf, Maud Vanpeene, Sylvie van der Werf,Ducancelle Alexandra
EPI_ISL_860907	CHU Tours - Virologie	National Reference Center for Viruses of Respiratory Infections, Institut Pasteur, Paris	Marion Barbet, Sylvie Behillil, Méline Bizard, Angela Brisebarre, Camille Capel, Etienne Simon-Lorière, Vincent Enouf, Maud Vanpeene, Sylvie van der Werf,Gaudy Graffin Catherine
EPI_ISL_860911, EPI_ISL_860912	CHU Angers - Dpt des Agents Infectieux	National Reference Center for Viruses of Respiratory Infections, Institut Pasteur, Paris	Marion Barbet, Sylvie Behillil, Méline Bizard, Angela Brisebarre, Camille Capel, Etienne Simon-Lorière, Vincent Enouf, Maud Vanpeene, Sylvie van der Werf,Ducancelle Alexandra
EPI_ISL_860913, EPI_ISL_860918, EPI_ISL_860919, EPI_ISL_860920, EPI_ISL_860921, EPI_ISL_860926, EPI_ISL_860927, EPI_ISL_860928, EPI_ISL_860929, EPI_ISL_860930	CHU de Nantes - Hôtel Dieu - Labo. Virologie	National Reference Center for Viruses of Respiratory Infections, Institut Pasteur, Paris	Marion Barbet, Sylvie Behillil, Méline Bizard, Angela Brisebarre, Camille Capel, Etienne Simon-Lorière, Vincent Enouf, Maud Vanpeene, Sylvie van der Werf,Andre-Garnier Elisabeth
EPI_ISL_860952, EPI_ISL_860953, EPI_ISL_860965, EPI_ISL_860977, EPI_ISL_860984, EPI_ISL_860988, EPI_ISL_860999, EPI_ISL_861002, EPI_ISL_861008, EPI_ISL_861016, EPI_ISL_861030, EPI_ISL_861041, EPI_ISL_861042, EPI_ISL_861044, EPI_ISL_861047, EPI_ISL_861050, EPI_ISL_861074, EPI_ISL_861076			
see above	Johns Hopkins Hospital Department of Pathology	Johns Hopkins Hospital Department of Pathology	C. Paul Morris, Chun Huai Luo, Adannaya Amadi, Nicholas Gallagher, Heba H. Mostafa
EPI_ISL_861235	CHU Tours - Virologie	National Reference Center for Viruses of Respiratory Infections, Institut Pasteur, Paris	Marion Barbet, Sylvie Behillil, Méline Bizard, Angela Brisebarre, Camille Capel, Etienne Simon-Lorière, Vincent Enouf, Maud Vanpeene, Sylvie van der Werf,Gaudy Graffin Catherine
EPI_ISL_861236	CHU Angers - Dpt des Agents Infectieux	National Reference Center for Viruses of Respiratory Infections, Institut Pasteur, Paris	Marion Barbet, Sylvie Behillil, Méline Bizard, Angela Brisebarre, Camille Capel, Etienne Simon-Lorière, Vincent Enouf, Maud Vanpeene, Sylvie van der Werf,Ducancelle Alexandra
EPI_ISL_861239	Labo Analyses med	National Reference Center for Viruses of Respiratory Infections, Institut Pasteur, Paris	Marion Barbet, Sylvie Behillil, Méline Bizard, Angela Brisebarre, Camille Capel, Etienne Simon-Lorière, Vincent Enouf, Maud Vanpeene, Sylvie van der Werf,Amzalag Jonas
EPI_ISL_861333, EPI_ISL_861337, EPI_ISL_861340, EPI_ISL_861348, EPI_ISL_861355, EPI_ISL_861356, EPI_ISL_861373, EPI_ISL_861374, EPI_ISL_861375, EPI_ISL_861376, EPI_ISL_861377, EPI_ISL_861378, EPI_ISL_861379, EPI_ISL_861380, EPI_ISL_861382, EPI_ISL_861383, EPI_ISL_861384, EPI_ISL_861385, EPI_ISL_861386, EPI_ISL_861387, EPI_ISL_861389, EPI_ISL_861392, EPI_ISL_861393, EPI_ISL_861394, EPI_ISL_861395, EPI_ISL_861396, EPI_ISL_861397, EPI_ISL_861399, EPI_ISL_861400, EPI_ISL_861401, EPI_ISL_861402, EPI_ISL_861403, EPI_ISL_861404, EPI_ISL_861405, EPI_ISL_861406, EPI_ISL_861407, EPI_ISL_861408, EPI_ISL_861409, EPI_ISL_861410, EPI_ISL_861411, EPI_ISL_861412			
see above	WESTCHESTER MEDICAL CENTER	Wadsworth Center, New York State Department of Health	Kirsten St. George, Daryl M. Lamson, Alexis Russel, Matthew Shudt, Melissa A Leisner, Jonathan Plitnick, Navjot Singh, John Kelly, Erasmus Schneider, Erica Lasek-Nesselquist
EPI_ISL_861417	NORTH SHORE UNIVERSITY HOSPITAL	Wadsworth Center, New York State Department of Health	Kirsten St. George, Daryl M. Lamson, Alexis Russel, Matthew Shudt, Melissa A Leisner, Jonathan Plitnick, Navjot Singh, John Kelly, Erasmus Schneider, Erica Lasek-Nesselquist
EPI_ISL_861428, EPI_ISL_861430, EPI_ISL_861432, EPI_ISL_861476, EPI_ISL_861477, EPI_ISL_861478, EPI_ISL_861479, EPI_ISL_861482, EPI_ISL_861483, EPI_ISL_861484, EPI_ISL_861485, EPI_ISL_861486			
see above	Wyoming Public Health Laboratory	Wyoming Public Health Laboratory	Noah Hull, Taylor Fearing, Lynette Gumbleton, Channing Weber, Ashley Norberg, Bailey Bowcutt, and Wanda Manley
EPI_ISL_861496	Cerballiance Côte d'Azur	CERBA LAB	Bausset O; Juvet O; Prots L.
EPI_ISL_861503	Cerballiance Alpes Durance	CERBA LAB	Dyen P; Prots L.
EPI_ISL_861505	Cerballiance Provence	CERBA LAB	Delaunay E; Roig JC; Prots L.
EPI_ISL_861506	Cerballiance Provence	CERBA LAB	Roig JC; Delaunay E; Prots L.
EPI_ISL_861508	Cerballiance Haut de France	CERBA LAB	Felloni C; Mainardi A; Lahmidi S.
EPI_ISL_861535, EPI_ISL_861541, EPI_ISL_861542, EPI_ISL_861543, EPI_ISL_861544, EPI_ISL_861545, EPI_ISL_861546, EPI_ISL_861547, EPI_ISL_861548, EPI_ISL_861549, EPI_ISL_861551, EPI_ISL_861558, EPI_ISL_861559, EPI_ISL_861565, EPI_ISL_861566, EPI_ISL_861578, EPI_ISL_861580, EPI_ISL_861592, EPI_ISL_861593, EPI_ISL_861594, EPI_ISL_861595, EPI_ISL_861596, EPI_ISL_861597, EPI_ISL_861598, EPI_ISL_861599, EPI_ISL_861600, EPI_ISL_861601, EPI_ISL_861602, EPI_ISL_861603, EPI_ISL_861604, EPI_ISL_861605, EPI_ISL_861606, EPI_ISL_861607, EPI_ISL_861608, EPI_ISL_861609, EPI_ISL_861610, EPI_ISL_861611, EPI_ISL_861612, EPI_ISL_861613, EPI_ISL_861614, EPI_ISL_861615, EPI_ISL_861616, EPI_ISL_861617, EPI_ISL_861618, EPI_ISL_861619, EPI_ISL_861620, EPI_ISL_861621, EPI_ISL_861622, EPI_ISL_861623			
see above	Instituto Nacional de Saude (INSA)	Instituto Nacional de Saude (INSA)	Borges et al
EPI_ISL_861687, EPI_ISL_861688, EPI_ISL_861691	Los Angeles County PHL	Los Angeles County PHL	P. Hemarajata et al.
EPI_ISL_861700, EPI_ISL_861701, EPI_ISL_861702, EPI_ISL_861703	MD Laboratories	Los Angeles County PHL	P. Hemarajata et al.
EPI_ISL_861704, EPI_ISL_861705, EPI_ISL_861706	UCLA Clinical Micro Lab	Los Angeles County PHL	P. Hemarajata et al.
EPI_ISL_861707, EPI_ISL_861708, EPI_ISL_861709	Instituto Nacional de Saude (INSA)	Instituto Nacional de Saude (INSA)	Borges et al
EPI_ISL_861714	Cerba	CERBA LAB	Roquebert B; Costa JM; Hedbaut E; Trombert S; Lecorche E; Verdurme L; Malek Ramdane, Olivi M; Haïm-Boukobza S.
EPI_ISL_861715	CERBA	CERBA LAB	Roquebert B; Costa JM; Hedbaut E; Trombert S; Lecorche E; Verdurme L; Malek Ramdane, Olivi M; Haïm-Boukobza S.
EPI_ISL_861727	CERBA	CERBA LAB	Roquebert B; Costa JM; Hedbaut E; Trombert S; Lecorche E; Verdurme L; Malek Ramdane; Olivi M; Haïm-Boukobza S.
EPI_ISL_861734	Yale Pathology Lab	Grubaugh Lab - Yale School of Public Health	Tara Alpert, Joseph Fauver, Chen Liu, Pei Hui, Jianhui Wang, Susan Bell and Han Zhou, Anderson Brito, Mallery Breban, Anne Wyllie, Chantal Vogels,

			Mary Petrone, Chaney Kalinich, Isabel Ott, Arnau Casanovas, Catherine Muenker, Adam Moore, Alice Lu, Maria Tokuyama, Patrick Wong, Peiwen Lu, Saad Omer, Richard Martinello, Allison Nelson, Shelli Farhadian, Akiko Iwasaki, Charlese Dela Cruz, Albert Ko, Nathan Grubaugh
EPI_ISL_861739, EPI_ISL_861740, EPI_ISL_861743, EPI_ISL_861747	Tempus	Grubaugh Lab - Yale School of Public Health	Tara Alpert, Joseph Fauver, Anderson Brito, Mallery Breban, Anne Wyllie, Chantal Vogels, Mary Petrone, Annie Watkins, Chaney Kalinich, Isabel Ott, Nathan Grubaugh
EPI_ISL_861752, EPI_ISL_861753	Yale Clinical Virology Lab	Grubaugh Lab - Yale School of Public Health	Tara Alpert, Joseph Fauver, Anderson Brito, Mallery Breban, Anne Wyllie, Chantal Vogels, Mary Petrone, Chaney Kalinich, Isabel Ott, Nathan Grubaugh
EPI_ISL_861762, EPI_ISL_861763	Tempus	Grubaugh Lab - Yale School of Public Health	Tara Alpert, Joseph Fauver, Anderson Brito, Mallery Breban, Anne Wyllie, Chantal Vogels, Mary Petrone, Annie Watkins, Chaney Kalinich, Isabel Ott, Nathan Grubaugh
EPI_ISL_861764	Yale Pathology Lab	Grubaugh Lab - Yale School of Public Health	Tara Alpert, Joseph Fauver, Chen Liu, Pei Hui, Jianhui Wang, Susan Bell and Han Zhou, Anderson Brito, Mallery Breban, Anne Wyllie, Chantal Vogels, Mary Petrone, Chaney Kalinich, Isabel Ott, Arnau Casanovas, Catherine Muenker, Adam Moore, Alice Lu, Maria Tokuyama, Patrick Wong, Peiwen Lu, Saad Omer, Richard Martinello, Allison Nelson, Shelli Farhadian, Akiko Iwasaki, Charlese Dela Cruz, Albert Ko, Nathan Grubaugh
EPI_ISL_861765	Tempus	Grubaugh Lab - Yale School of Public Health	Tara Alpert, Joseph Fauver, Anderson Brito, Mallery Breban, Anne Wyllie, Chantal Vogels, Mary Petrone, Annie Watkins, Chaney Kalinich, Isabel Ott, Nathan Grubaugh
EPI_ISL_862043, EPI_ISL_862044, EPI_ISL_862045	CERBA	CERBA LAB	Roquebert B; Costa JM; Hedbaut E; Trombert S; Lecorche E; Verdurme L; Malek Ramdane, Olivi M; Haïm-Boukobza S.
EPI_ISL_862046, EPI_ISL_862047	Cerballiance Val de Loire	CERBA LAB	Abs G.
EPI_ISL_862121	MD Laboratories	Los Angeles County PHL	P. Hemarajata et al.
EPI_ISL_862122, EPI_ISL_862123	UCLA Clinical Micro Lab	Los Angeles County PHL	P. Hemarajata et al.
EPI_ISL_862131, EPI_ISL_862148, EPI_ISL_862154, EPI_ISL_862157, EPI_ISL_862158, EPI_ISL_862172, EPI_ISL_862173, EPI_ISL_862174, EPI_ISL_862182, EPI_ISL_862184, EPI_ISL_862185			
see above	Charité Universitätsmedizin Berlin, Institut fur Virologie/Labor Berlin	Charité Universitätsmedizin Berlin, Institut für Virologie	Victor M Corman, Barbara Mühlemann, Jörn Beheim-Schwarzbach, Tobias Bleicker, Julia Tesch, Talitha Veith, Julia Schneider, Terry Jones, Christian Drosten
EPI_ISL_862676	New Mexico Department of Health Scientific Laboratory	New Mexico Department of Health Scientific Laboratory	D'eldra Malone, Ellie Johnson, Anastacia Griego-Fisher
EPI_ISL_862730, EPI_ISL_862731, EPI_ISL_862733, EPI_ISL_862737, EPI_ISL_862745, EPI_ISL_862752, EPI_ISL_862772, EPI_ISL_862774, EPI_ISL_862779	Utah Public Health Laboratory, Utah Public Health Laboratory Infectious Disease submission group	Utah Public Health Laboratory, Utah Public Health Laboratory Infectious Disease submission group	Young,E.L., Oakeson,K.F., Gallagher,T.
EPI_ISL_863809	Lighthouse Lab in Milton Keynes	Wellcome Sanger Institute for the COVID-19 Genomics UK (COG-UK) Consortium	The Lighthouse Lab in Milton Keynes and Alex Alderton, Roberto Amato, Sonia Goncalves, Ewan Harrison, David K. Jackson, Ian Johnston, Dominic Kwiatkowski, Cordelia Langford, John Sillitoe on behalf of the Wellcome Sanger Institute COVID-19 Surveillance Team
EPI_ISL_864039	Lighthouse Lab in Alderley Park	Wellcome Sanger Institute for the COVID-19 Genomics UK (COG-UK) Consortium	Jacquelyn Wynn, Mairead Hyland, The Lighthouse Lab in Alderley Park and Alex Alderton, Roberto Amato, Sonia Goncalves, Ewan Harrison, David K. Jackson, Ian Johnston, Dominic Kwiatkowski, Cordelia Langford, John Sillitoe on behalf of the Wellcome Sanger Institute COVID-19 Surveillance Team
EPI_ISL_864204, EPI_ISL_864211, EPI_ISL_864213, EPI_ISL_864216, EPI_ISL_864217, EPI_ISL_864221, EPI_ISL_864227, EPI_ISL_864228, EPI_ISL_864230, EPI_ISL_864239, EPI_ISL_864244, EPI_ISL_864255, EPI_ISL_864262, EPI_ISL_864266, EPI_ISL_864274, EPI_ISL_864276, EPI_ISL_864281, EPI_ISL_864288, EPI_ISL_864291, EPI_ISL_864297, EPI_ISL_864301, EPI_ISL_864305, EPI_ISL_864309, EPI_ISL_864311, EPI_ISL_864313, EPI_ISL_864317, EPI_ISL_864321, EPI_ISL_864324, EPI_ISL_864328, EPI_ISL_864336, EPI_ISL_864352, EPI_ISL_864354, EPI_ISL_864356, EPI_ISL_864361, EPI_ISL_864365, EPI_ISL_864370, EPI_ISL_864383, EPI_ISL_864384, EPI_ISL_864390, EPI_ISL_864396, EPI_ISL_864399, EPI_ISL_864400, EPI_ISL_864402, EPI_ISL_864403, EPI_ISL_864409, EPI_ISL_864411, EPI_ISL_864414, EPI_ISL_864422, EPI_ISL_864436, EPI_ISL_864439, EPI_ISL_864440, EPI_ISL_864443, EPI_ISL_864445, EPI_ISL_864447, EPI_ISL_864452, EPI_ISL_864461, EPI_ISL_864465, EPI_ISL_864467, EPI_ISL_864469, EPI_ISL_864472, EPI_ISL_864475, EPI_ISL_864478, EPI_ISL_864483, EPI_ISL_864484, EPI_ISL_864485, EPI_ISL_864487, EPI_ISL_864493, EPI_ISL_864501, EPI_ISL_864513, EPI_ISL_864515, EPI_ISL_864516, EPI_ISL_864520, EPI_ISL_864527, EPI_ISL_864528, EPI_ISL_864529, EPI_ISL_864534, EPI_ISL_864539, EPI_ISL_864540, EPI_ISL_864542			
see above	Lighthouse Lab in Milton Keynes	Wellcome Sanger Institute for the COVID-19 Genomics UK (COG-UK) Consortium	The Lighthouse Lab in Milton Keynes and Alex Alderton, Roberto Amato, Sonia Goncalves, Ewan Harrison, David K. Jackson, Ian Johnston, Dominic Kwiatkowski, Cordelia Langford, John Sillitoe on behalf of the Wellcome Sanger Institute COVID-19 Surveillance Team
EPI_ISL_864582	CHU Purpan - Laboratoire de Virologie - Institut Fédératif de Biologie	CHU Purpan - Laboratoire de Virologie - Institut Fédératif de Biologie	Latour J., Ranger N., Dubois M., Carcenac R., Harter A., Boyer P., Tremeaux P., Izopet J.
EPI_ISL_864719, EPI_ISL_864720, EPI_ISL_864734, EPI_ISL_864737	University Hospitals of Geneva, Laboratory of Virology	HUG, Laboratory of Virology and the Health2030 Genome Center	Samuel Cordey, Ana Rita Goncalves, Laurent Kaiser, Lorenzo Cerutti, Henri Pegeot, Melyssa Elies, Deborah Penet, Keith Harshman, Ioannis Xenarios, Emmanouil Dermitzakis
EPI_ISL_864754, EPI_ISL_864760, EPI_ISL_864797, EPI_ISL_864799, EPI_ISL_864803, EPI_ISL_864807, EPI_ISL_864809, EPI_ISL_864810, EPI_ISL_864811, EPI_ISL_864813, EPI_ISL_864814, EPI_ISL_864815, EPI_ISL_864818, EPI_ISL_864829, EPI_ISL_864846, EPI_ISL_864875, EPI_ISL_864883, EPI_ISL_864889, EPI_ISL_864893			
see above	Department of Pathology, University of Cambridge	COVID-19 Genomics UK (COG-UK) Consortium	Aminu S. Jahun, Yasmin Chaudhry, Grant Hall, Iliana Georgana, Myra Hosmillo, Martin D. Curran, Malte Pinckert, Surendra Parmar, Ian Goodfellow
EPI_ISL_865012	Lighthouse Lab in Glasgow / MRC-University of Glasgow Centre for Virus Research	COVID-19 Genomics UK (COG-UK) Consortium	Ana da Silva Filipe, Natasha Johnson, Kathy Smollett, Daniel Mair, Stephen Carmichael, Alice Broos, Lily Tong, Jenna Nichols, Kyriaki Nomikou; Sarah McDonald; Harper VanSteenhouse, Yumi Kasai, David Gray, Carol Clugston, Anna Dominiczak; Alasdair MacLean, Rory Gunson; Richard Orton, Joseph Hughes, Sreenu Vattipally, David L Robertson; Sharif Shaaban, Matthew Holden; Kathy Li, James Shepherd, Antonia Ho, Emma Thomson
EPI_ISL_865014, EPI_ISL_865015, EPI_ISL_865016, EPI_ISL_865017, EPI_ISL_865018, EPI_ISL_865020, EPI_ISL_865041, EPI_ISL_865042, EPI_ISL_865043, EPI_ISL_865044, EPI_ISL_865045, EPI_ISL_865046, EPI_ISL_865047, EPI_ISL_865048, EPI_ISL_865049, EPI_ISL_865050, EPI_ISL_865051, EPI_ISL_865052, EPI_ISL_865054, EPI_ISL_865055, EPI_ISL_865066, EPI_ISL_865067			
see above	West of Scotland Specialist Virology Centre, NHSGGC / MRC-University of Glasgow Centre for Virus Research	COVID-19 Genomics UK (COG-UK) Consortium	Ana da Silva Filipe, Natasha Johnson, Kathy Smollett, Daniel Mair, Stephen Carmichael, Alice Broos, Lily Tong, Jenna Nichols, Kyriaki Nomikou; Sarah McDonald; Richard Orton, Joseph Hughes, Sreenu Vattipally, David L Robertson; Alasdair MacLean, Rory Gunson; Sharif Shaaban, Matthew Holden; Rachel Blacow, Guy Mollett, Kathy Li, James Shepherd, Antonia Ho, Emma Thomson
EPI_ISL_865074, EPI_ISL_865075, EPI_ISL_865076, EPI_ISL_865077, EPI_ISL_865089, EPI_ISL_865115, EPI_ISL_865117, EPI_ISL_865143, EPI_ISL_865149, EPI_ISL_865150, EPI_ISL_865151, EPI_ISL_865152			
see above	Virology Department, Royal Infirmary of Edinburgh, NHS Lothian / School of Biological Sciences, University of Edinburgh / Institute of Genetics and Molecular Medicine, University of Edinburgh	COVID-19 Genomics UK (COG-UK) Consortium	McHugh M, Dewar R, Rooke S, Gallagher M, Balcaza C, O'Toole Á, Scher E, Hill V, McCrone JT, Colquhoun R, Yu X, Jackson B, Rambaut A, Williams TC, Templeton K
EPI_ISL_865203, EPI_ISL_865204, EPI_ISL_865205, EPI_ISL_865206, EPI_ISL_865270, EPI_ISL_865271, EPI_ISL_865322, EPI_ISL_865324, EPI_ISL_865331, EPI_ISL_865332, EPI_ISL_865333, EPI_ISL_865336, EPI_ISL_865338, EPI_ISL_865339, EPI_ISL_865340, EPI_ISL_865479, EPI_ISL_865480, EPI_ISL_865484			
see above	Liverpool Clinical Laboratories	COVID-19 Genomics UK (COG-UK) Consortium	Sam Haldenby, Anita Lucaci, Steve Paterson, Julian Hiscox, Alistair Darby, M Almsaud, A Alrezaihi, Muhannad Alruwaili, Stuart D Armstrong, Jones Benjamin, Eleanor G Bentley, Anu Chawla, Jordan J Clark, Angela Cowell, Richard Eccles, Isabel García-Dorival, Matthew Gemmell, Alessandro Gerada, PKF Gilmore, Richard Gregory, Ximeng Han, Catherine Hartley, Margaret Hughes, Miren Iturriza-Gomara, James Johnson, L Luu, Jenifer Manson, Charlotte Nelson, Elaine O'Toole, Cassie Olateju, Rebekah Penrice-Randal , Lucille Rainbow, N.P Randle, Trevor Ian Robinson, Parul Sharma, Ghada T Shawli, James P Stewart, Neil Swainston, Ecaterina Vamos, Joanne Watts, Mark Whitehead
EPI_ISL_866077, EPI_ISL_866078, EPI_ISL_866079, EPI_ISL_866080, EPI_ISL_866109, EPI_ISL_866110, EPI_ISL_866111	University College London Hospital	COVID-19 Genomics UK (COG-UK) Consortium	Judith Heaney, Matthew Byott, Catherine Houlihan, Dan Frampton, Stuart Kirk, Moira Spyer and Eleni Nastouli
EPI_ISL_866191, EPI_ISL_866192, EPI_ISL_866193	University College London, Great Ormond Street Hospital for Children NHS Foundation Trust, Imperial College Healthcare NHS Trust	COVID-19 Genomics UK (COG-UK) Consortium	Sergi Castellano, Rachel Williams, Mark Kristiansen, Paola Resende Silva, Sunando Roy, Tony Brooks, Helena Tutill, Paola Niola, Patricia Dyal, Charlotte Williams, Leysa Forrest, Yasmin Panchbhaya, Jacqueline Findlay, Samuel Weeks, Julianne Brown, Kathryn Harris, Paul Randell, James Price, Alison Holmes, Judith Breuer
EPI_ISL_866354, EPI_ISL_866355, EPI_ISL_866357, EPI_ISL_866358, EPI_ISL_866359, EPI_ISL_866362, EPI_ISL_866366, EPI_ISL_866367, EPI_ISL_866373, EPI_ISL_866374, EPI_ISL_866375, EPI_ISL_866376, EPI_ISL_866377, EPI_ISL_866378, EPI_ISL_866379, EPI_ISL_866380, EPI_ISL_866381, EPI_ISL_866382, EPI_ISL_866384, EPI_ISL_866385, EPI_ISL_866386, EPI_ISL_866387, EPI_ISL_866388, EPI_ISL_866395, EPI_ISL_866396, EPI_ISL_866397, EPI_ISL_866398, EPI_ISL_866399			

see above	Regional Virus Laboratory, Belfast Health and Social Care Trust	COVID-19 Genomics UK (COG-UK) Consortium	Conall McCaughey, James McKenna, Tanya Curran, Susan Feeney, Alison Watt, Ciara Cox, Mairead Connor, Zoltan Molnar, David Simpson, Derek Fairley
EPI_ISL_866662, EPI_ISL_866675, EPI_ISL_866676, EPI_ISL_866678, EPI_ISL_866680, EPI_ISL_866685, EPI_ISL_866687, EPI_ISL_866690, EPI_ISL_866693, EPI_ISL_866694	Quadram Institute Bioscience	COVID-19 Genomics UK (COG-UK) Consortium	Dave J. Baker, Gemma L. Kay, Alp Aydin, Thanh Le-Viet, Steven Rudder, Ana P. Tedim, Anastasia Kolyva, Maria Diaz, Leonardo de Oliveira Martins, Nabil-Fareed Alikhan, Lizzie Meadows, Rachael Stanley, Ngozi Elumogo, Muhammed Yasir, Nicholas M. Thomson, Alexander J Trotter, Rachel Gilroy, Samuel Bloomfield, Claire Stuart, Andrew Bell, Reenesh Prakash, Samir Dervisevic, Alison E. Mather, John Wain, Mark Webber, Andrew J. Page, Justin O'Grady
EPI_ISL_866907, EPI_ISL_866908	Queens Medical Centre, Clinical Microbiology Department / DeepSeq Nottingham	COVID-19 Genomics UK (COG-UK) Consortium	Gemma Clark, Wendy Smith, Manjinder Khakh, Vicki M Fleming, Michelle M Lister, Hannah Howson-Wells, Jonathan Ball, Patrick McClure, Joseph Chappell, Theocharis Tsoleridis, Nadine Holmes, Matthew Carlisle, Christopher Moore, Fei Sang, Johnny Debebe, Victoria Wright, Matthew Loose
EPI_ISL_867034	Lincolnshire Hospitals and DeepSeq Nottingham	COVID-19 Genomics UK (COG-UK) Consortium	Nichola Duckworth, Tim Sloan, Sarah Walsh, Jonathan Ball, Patrick McClure, Joeseph Chappell, Nadine Holmes, Matthew Carlisle, Christopher Moore, Fei Sang, Johnny Debebe, Victoria Wright, Matthew Loose
EPI_ISL_867164, EPI_ISL_867165, EPI_ISL_867167	Oxford Viromics, NDM, University of Oxford; Oxford University Hospitals; Basingstoke and North Hampshire Hospital	COVID-19 Genomics UK (COG-UK) Consortium	Tanya Golubchik, David Bonsall, George Macintyre, Amy Trebes, Mariateresa de Cesare, Catrin Moore, Alex Mobbs, Anita Justice, Robert Shaw, Monique Andersson, Timothy Peto, Emma Wise, Nathan Moore, Jessica Lynch, Nick Cortes, Matilde Mori, Stephen Kidd, David Buck, John Todd, Christophe Fraser
EPI_ISL_867208, EPI_ISL_867209, EPI_ISL_867210, EPI_ISL_867211, EPI_ISL_867219, EPI_ISL_867220, EPI_ISL_867313, EPI_ISL_867314, EPI_ISL_867315, EPI_ISL_867416, EPI_ISL_867456, EPI_ISL_867503, EPI_ISL_867511, EPI_ISL_867512, EPI_ISL_867515, EPI_ISL_867751, EPI_ISL_867758, EPI_ISL_867759, EPI_ISL_867762, EPI_ISL_867763, EPI_ISL_867764, EPI_ISL_867766, EPI_ISL_867768, EPI_ISL_867771, EPI_ISL_867773, EPI_ISL_867776, EPI_ISL_867779			
see above	Originating lab: Wales Specialist Virology Centre Sequencing lab: Pathogen Genomics Unit	Public Health Wales Microbiology Cardiff Wales Specialist Virology Centre	Catherine Moore, Johnathan Evans, Laura Gifford, Malorie Perry, Simon Cottrell, Angela Marchbank, Alec Birchley, Alexander Adams, Amy Gaskin, Bree Gatica-Wilcox, Jason Coombes, Joel Southgate, Lauren Gilbert, Lee Graham, Nicole Pacchiarini, Sara Kumziene-Summerhayes, Sarah Taylor, Sophie Jones, Sara Rey, Matthew Bull, Joanne Watkins, Sally Corden, Tom Connor
EPI_ISL_868001, EPI_ISL_868008, EPI_ISL_868309, EPI_ISL_868310, EPI_ISL_868322, EPI_ISL_868323, EPI_ISL_868324, EPI_ISL_868325, EPI_ISL_868329, EPI_ISL_868330, EPI_ISL_868331, EPI_ISL_868339, EPI_ISL_868340, EPI_ISL_868341, EPI_ISL_868342, EPI_ISL_868343, EPI_ISL_868344, EPI_ISL_868345, EPI_ISL_868346, EPI_ISL_868347, EPI_ISL_868348, EPI_ISL_868353			
see above	Centre for Enzyme Innovation, University of Portsmouth / Translational Research Laboratory, Portsmouth Hospitals NHS Trust	COVID-19 Genomics UK (COG-UK) Consortium	Angela Beckett,Yann Bourgeois,Garry Scarlett,Sharon Glaysher,Scott Elliott,Kelly Bicknell,Robert Impey,Allyson Lloyd,Sarah Wyllie,Ethan Butcher,Anoop Chauhan,Samuel Robson
EPI_ISL_868370, EPI_ISL_868390, EPI_ISL_868392, EPI_ISL_868400, EPI_ISL_868402, EPI_ISL_868406, EPI_ISL_868416, EPI_ISL_868429, EPI_ISL_868431, EPI_ISL_868444, EPI_ISL_868450, EPI_ISL_868453, EPI_ISL_868454, EPI_ISL_868455, EPI_ISL_868468, EPI_ISL_868470, EPI_ISL_868474, EPI_ISL_868478, EPI_ISL_868481, EPI_ISL_868491, EPI_ISL_868498, EPI_ISL_868499, EPI_ISL_868502, EPI_ISL_868507, EPI_ISL_868515, EPI_ISL_868516, EPI_ISL_868518, EPI_ISL_868525, EPI_ISL_868531, EPI_ISL_868537, EPI_ISL_868542, EPI_ISL_868543, EPI_ISL_868545, EPI_ISL_868561, EPI_ISL_868565, EPI_ISL_868572, EPI_ISL_868590, EPI_ISL_868598, EPI_ISL_868601, EPI_ISL_868603, EPI_ISL_868608, EPI_ISL_868609, EPI_ISL_868612, EPI_ISL_868613, EPI_ISL_868620, EPI_ISL_868622, EPI_ISL_868625, EPI_ISL_868627, EPI_ISL_868630, EPI_ISL_868644, EPI_ISL_868654, EPI_ISL_868659, EPI_ISL_868664, EPI_ISL_868665, EPI_ISL_868675, EPI_ISL_868678, EPI_ISL_868680, EPI_ISL_868683, EPI_ISL_868699			
see above	Virology Department, Sheffield Teaching Hospitals NHS Foundation Trust/Department of Infection, Immunity and Cardiovascular Disease, The Medical School, University of Sheffield	COVID-19 Genomics UK (COG-UK) Consortium	Thushan de Silva, Matthew Parker, Nikki Smith, Adri Angyal, Rebecca Brown, Luke Green, Rachel Tucker, Paul Parsons, Danielle Groves, Katie Johnson, Laura Carrilero, Alex Keeley, Dave Partridge, Matthew Wyles, Benjamin Lindsey, Mehmet Yavuz, Mohammad Raza, Cariad Evans
EPI_ISL_869167, EPI_ISL_869168, EPI_ISL_869170, EPI_ISL_869171, EPI_ISL_869174, EPI_ISL_869175, EPI_ISL_869176, EPI_ISL_869180, EPI_ISL_869181, EPI_ISL_869184, EPI_ISL_869185, EPI_ISL_869186, EPI_ISL_869187, EPI_ISL_869188, EPI_ISL_869189, EPI_ISL_869190, EPI_ISL_869191, EPI_ISL_869192, EPI_ISL_869193, EPI_ISL_869194, EPI_ISL_869195, EPI_ISL_869196, EPI_ISL_869197, EPI_ISL_869198, EPI_ISL_869199, EPI_ISL_869200, EPI_ISL_869201, EPI_ISL_869202, EPI_ISL_869203, EPI_ISL_869204, EPI_ISL_869205, EPI_ISL_869206, EPI_ISL_869207, EPI_ISL_869208, EPI_ISL_869209, EPI_ISL_869210, EPI_ISL_869211, EPI_ISL_869212, EPI_ISL_869213, EPI_ISL_869214, EPI_ISL_869215, EPI_ISL_869216, EPI_ISL_869225, EPI_ISL_869227			
see above	New Mexico Department of Health Scientific Laboratory	Center for Global Health, University of New Mexico Health Sciences Center	Daryl Domman, Kurt Schwalm, Twila Kunde, Joseph Hicks, Anastacia Griego, Michael Edwards, Darrell Dinwiddie
EPI_ISL_871789	Cerballiance Normandie	CERBA LAB	LAFOREST D, BRIAND G, LEPIGEON, technicien V; COUDRAY J; MARTINS-AMARAL F; COULON C, THOMAS Johan, VITEL R, HAMEL A.
EPI_ISL_871827, EPI_ISL_871828, EPI_ISL_871829, EPI_ISL_871830, EPI_ISL_871831	Jessa	Jessa	Brigitte Maes, Bert Cruys
EPI_ISL_871844, EPI_ISL_871845, EPI_ISL_871846, EPI_ISL_871848, EPI_ISL_871849, EPI_ISL_871870, EPI_ISL_871875	Wyoming Public Health Laboratory	Wyoming Public Health Laboratory	Noah Hull, Taylor Fearing, Lynette Gumbleton, Channing Weber, Ashley Norberg, Bailey Bowcutt, and Wanda Manley
EPI_ISL_871876	Cerballiance Côte d'Azur	CERBA LAB	Prots L; Barrieu-Moussat S; Roquebert B; Costa JM; Hedbaut E; Trombert S; Lecorche E; Verdurme L; Malek Ramdane, Olivi M; Haïm-Boukobza S.
EPI_ISL_871879, EPI_ISL_871880, EPI_ISL_871881, EPI_ISL_871882, EPI_ISL_871883, EPI_ISL_871884, EPI_ISL_871885, EPI_ISL_871886, EPI_ISL_871887, EPI_ISL_871888, EPI_ISL_871889, EPI_ISL_871892, EPI_ISL_871894, EPI_ISL_871895, EPI_ISL_871897, EPI_ISL_871898, EPI_ISL_871899, EPI_ISL_871900, EPI_ISL_871901, EPI_ISL_871902, EPI_ISL_871903, EPI_ISL_871904, EPI_ISL_871905, EPI_ISL_871906, EPI_ISL_871907			
see above	Hospital Universitario Marqués de Valdecilla - IDIVAL (Santander, Cantabria)	SeqCOVID-SPAIN consortium/IBV(CSIC)	Mónica Gozalo Margüello, María Eliecer Cano García, Jose Manuel Méndez Legaza, Daniel Pablo Marcos, Jesús Rodríguez Rodríguez, María Siller Ruiz and SeqCOVID-SPAIN consortium
EPI_ISL_871913, EPI_ISL_871938, EPI_ISL_871942, EPI_ISL_871943, EPI_ISL_871944, EPI_ISL_871945, EPI_ISL_871946, EPI_ISL_871947, EPI_ISL_871948, EPI_ISL_871949, EPI_ISL_871950, EPI_ISL_871951, EPI_ISL_871952, EPI_ISL_871953, EPI_ISL_871955, EPI_ISL_871957, EPI_ISL_871958			
see above	Servicio de Microbiología Clínica (Complejo Hospitalario de Navarra, Pamplona), Instituto de Investigación Sanitaria de Navarra (IdiSNA)	SeqCOVID-SPAIN consortium/IBV(CSIC)	Carmen Ezpeleta Baquedano, Ana Navascués, Ana Miqueleiz and SeqCOVID-SPAIN consortium
EPI_ISL_871999, EPI_ISL_872001, EPI_ISL_872094	Wyoming Public Health Laboratory	Wyoming Public Health Laboratory	Noah Hull, Taylor Fearing, Lynette Gumbleton, Channing Weber, Ashley Norberg, Bailey Bowcutt, and Wanda Manley
EPI_ISL_872104	Chu Tivoli	GIGA Medical Genomics	Keith Durkin, Maria Artesi, Sébastien Bontems, Raphaël Boreux, Bouchra Boujemla, Cécile Meex, Pierrette Melin, Marie-Pierre Hayette, Vincent Bours
EPI_ISL_872198	Hopital	National Reference Center for Viruses of Respiratory Infections, Institut Pasteur, Paris	Marion Barbet, Sylvie Behillil, Méline Bizard, Angela Brisebarre, Camille Capel, Etienne Simon-Lorière, Vincent Enouf, Maud Vanpeene, Sylvie van der Werf
EPI_ISL_872201, EPI_ISL_872205	Labo Analyses Med	National Reference Center for Viruses of Respiratory Infections, Institut Pasteur, Paris	Marion Barbet, Sylvie Behillil, Méline Bizard, Angela Brisebarre, Camille Capel, Etienne Simon-Lorière, Vincent Enouf, Maud Vanpeene, Sylvie van der Werf,Girard Sophie
EPI_ISL_872206	Hopital	National Reference Center for Viruses of Respiratory Infections, Institut Pasteur, Paris	Marion Barbet, Sylvie Behillil, Méline Bizard, Angela Brisebarre, Camille Capel, Etienne Simon-Lorière, Vincent Enouf, Maud Vanpeene, Sylvie van der Werf,Morvan Odile
EPI_ISL_872208	Labo Analyses Med	National Reference Center for Viruses of Respiratory Infections, Institut Pasteur, Paris	Marion Barbet, Sylvie Behillil, Méline Bizard, Angela Brisebarre, Camille Capel, Etienne Simon-Lorière, Vincent Enouf, Maud Vanpeene, Sylvie van der Werf
EPI_ISL_872212	Labo analyses med	National Reference Center for Viruses of Respiratory Infections, Institut Pasteur, Paris	Marion Barbet, Sylvie Behillil, Méline Bizard, Angela Brisebarre, Camille Capel, Etienne Simon-Lorière, Vincent Enouf, Maud Vanpeene, Sylvie van der Werf,Amzalag Jonas
EPI_ISL_872213, EPI_ISL_872214, EPI_ISL_872215, EPI_ISL_872216, EPI_ISL_872217, EPI_ISL_872218, EPI_ISL_872219, EPI_ISL_872233	Hopital	National Reference Center for Viruses of Respiratory Infections, Institut Pasteur, Paris	Marion Barbet, Sylvie Behillil, Méline Bizard, Angela Brisebarre, Camille Capel, Etienne Simon-Lorière, Vincent Enouf, Maud Vanpeene, Sylvie van der Werf,Fourgeaud Jacques
EPI_ISL_872234	Labo Analyses Med	National Reference Center for Viruses of Respiratory Infections, Institut Pasteur, Paris	Marion Barbet, Sylvie Behillil, Méline Bizard, Angela Brisebarre, Camille Capel, Etienne Simon-Lorière, Vincent Enouf, Maud Vanpeene, Sylvie van der Werf,Girard Sophie
EPI_ISL_872236	Hopital	National Reference Center for Viruses of Respiratory	Marion Barbet, Sylvie Behillil, Méline Bizard, Angela Brisebarre, Camille Capel, Etienne Simon-Lorière, Vincent Enouf, Maud Vanpeene, Sylvie van der

		Infections, Institut Pasteur, Paris	Werf,Guigon AuréLie
EPI_ISL_872238	Labo analyses med	National Reference Center for Viruses of Respiratory Infections, Institut Pasteur, Paris	Marion Barbet, Sylvie Behillil, Méline Bizard, Angela Brisebarre, Camille Capel, Etienne Simon-Lorière, Vincent Enouf, Maud Vanpeene, Sylvie van der Werf,Amzalag Jonas
EPI_ISL_872254	hopital	National Reference Center for Viruses of Respiratory Infections, Institut Pasteur, Paris	Marion Barbet, Sylvie Behillil, Méline Bizard, Angela Brisebarre, Camille Capel, Etienne Simon-Lorière, Vincent Enouf, Maud Vanpeene, Sylvie van der Werf,Ducancelle Alexandra
EPI_ISL_872256	Labo Analyses Med	National Reference Center for Viruses of Respiratory Infections, Institut Pasteur, Paris	Marion Barbet, Sylvie Behillil, Méline Bizard, Angela Brisebarre, Camille Capel, Etienne Simon-Lorière, Vincent Enouf, Maud Vanpeene, Sylvie van der Werf
EPI_ISL_872264, EPI_ISL_872265	Labo analyses med	National Reference Center for Viruses of Respiratory Infections, Institut Pasteur, Paris	Marion Barbet, Sylvie Behillil, Méline Bizard, Angela Brisebarre, Camille Capel, Etienne Simon-Lorière, Vincent Enouf, Maud Vanpeene, Sylvie van der Werf,Amzalag Jonas
EPI_ISL_872266, EPI_ISL_872267, EPI_ISL_872268, EPI_ISL_872270	Hopital	National Reference Center for Viruses of Respiratory Infections, Institut Pasteur, Paris	Marion Barbet, Sylvie Behillil, Méline Bizard, Angela Brisebarre, Camille Capel, Etienne Simon-Lorière, Vincent Enouf, Maud Vanpeene, Sylvie van der Werf,Fourgeaud Jacques
EPI_ISL_872274	Hopital	National Reference Center for Viruses of Respiratory Infections, Institut Pasteur, Paris	Marion Barbet, Sylvie Behillil, Méline Bizard, Angela Brisebarre, Camille Capel, Etienne Simon-Lorière, Vincent Enouf, Maud Vanpeene, Sylvie van der Werf,Irimia Alix
EPI_ISL_872278	Hopital	National Reference Center for Viruses of Respiratory Infections, Institut Pasteur, Paris	Marion Barbet, Sylvie Behillil, Méline Bizard, Angela Brisebarre, Camille Capel, Etienne Simon-Lorière, Vincent Enouf, Maud Vanpeene, Sylvie van der Werf,Ramanantsoa CéLine
EPI_ISL_872279	Labo Analyses Med	National Reference Center for Viruses of Respiratory Infections, Institut Pasteur, Paris	Marion Barbet, Sylvie Behillil, Méline Bizard, Angela Brisebarre, Camille Capel, Etienne Simon-Lorière, Vincent Enouf, Maud Vanpeene, Sylvie van der Werf,Bour Jean Baptiste
EPI_ISL_872291, EPI_ISL_872292	Hopital	National Reference Center for Viruses of Respiratory Infections, Institut Pasteur, Paris	Marion Barbet, Sylvie Behillil, Méline Bizard, Angela Brisebarre, Camille Capel, Etienne Simon-Lorière, Vincent Enouf, Maud Vanpeene, Sylvie van der Werf,Guigon AuréLie
EPI_ISL_872309, EPI_ISL_872310, EPI_ISL_872311, EPI_ISL_872312, EPI_ISL_872314	Labo analyses med	National Reference Center for Viruses of Respiratory Infections, Institut Pasteur, Paris	Marion Barbet, Sylvie Behillil, Méline Bizard, Angela Brisebarre, Camille Capel, Etienne Simon-Lorière, Vincent Enouf, Maud Vanpeene, Sylvie van der Werf,Amzalag Jonas
EPI_ISL_872315, EPI_ISL_872316, EPI_ISL_872317, EPI_ISL_872318, EPI_ISL_872319	Hopital	National Reference Center for Viruses of Respiratory Infections, Institut Pasteur, Paris	Marion Barbet, Sylvie Behillil, Méline Bizard, Angela Brisebarre, Camille Capel, Etienne Simon-Lorière, Vincent Enouf, Maud Vanpeene, Sylvie van der Werf,Fourgeaud Jacques
EPI_ISL_872339, EPI_ISL_872340	Hopital	National Reference Center for Viruses of Respiratory Infections, Institut Pasteur, Paris	Marion Barbet, Sylvie Behillil, Méline Bizard, Angela Brisebarre, Camille Capel, Etienne Simon-Lorière, Vincent Enouf, Maud Vanpeene, Sylvie van der Werf,Ducancelle Alexandra
EPI_ISL_872347	Hopital	National Reference Center for Viruses of Respiratory Infections, Institut Pasteur, Paris	Marion Barbet, Sylvie Behillil, Méline Bizard, Angela Brisebarre, Camille Capel, Etienne Simon-Lorière, Vincent Enouf, Maud Vanpeene, Sylvie van der Werf,Irimia Alix
EPI_ISL_872512	New Mexico Department of Health Scientific Laboratory	Center for Global Health, University of New Mexico Health Sciences Center	Daryl Domman, Kurt Schwalm, Twila Kunde, Joseph Hicks, Anastacia Griego, Michael Edwards, Darrell Dinwiddie
EPI_ISL_872576	Laverty Pathology	NSW Health Pathology - Institute of Clinical Pathology and Medical Research; Westmead Hospital; University of Sydney	CIDM-PH et al.
EPI_ISL_872578	Sydney South West Pathology Service (SSWPS) - Liverpool Hospital - NSW Health Pathology	NSW Health Pathology - Institute of Clinical Pathology and Medical Research; Westmead Hospital; University of Sydney	CIDM-PH et al.
EPI_ISL_872825, EPI_ISL_872826, EPI_ISL_872827, EPI_ISL_872830, EPI_ISL_872831, EPI_ISL_872832, EPI_ISL_872834, EPI_ISL_872836, EPI_ISL_872838, EPI_ISL_872844, EPI_ISL_872846, EPI_ISL_872848, EPI_ISL_872855, EPI_ISL_872857, EPI_ISL_872859, EPI_ISL_872860, EPI_ISL_872861, EPI_ISL_872863, EPI_ISL_872865, EPI_ISL_872866, EPI_ISL_872868, EPI_ISL_872871, EPI_ISL_872873, EPI_ISL_872875, EPI_ISL_872876, EPI_ISL_872877, EPI_ISL_872878, EPI_ISL_872879, EPI_ISL_872881, EPI_ISL_872883, EPI_ISL_872884, EPI_ISL_872885, EPI_ISL_872887, EPI_ISL_872889, EPI_ISL_872892, EPI_ISL_872895, EPI_ISL_872896			
see above	University of Wisconsin-Madison AIDS Vaccine Research Laboratories	University of Wisconsin-Madison AIDS Vaccine Research Laboratories	Gage Moreno, Katarina Braun, et al. AIDS Vaccine Research Laboratories
EPI_ISL_873055, EPI_ISL_873057, EPI_ISL_873062, EPI_ISL_873063, EPI_ISL_873065, EPI_ISL_873066, EPI_ISL_873067, EPI_ISL_873068, EPI_ISL_873069, EPI_ISL_873070, EPI_ISL_873071, EPI_ISL_873072, EPI_ISL_873073, EPI_ISL_873074, EPI_ISL_873075, EPI_ISL_873076, EPI_ISL_873077, EPI_ISL_873078, EPI_ISL_873079, EPI_ISL_873080, EPI_ISL_873081, EPI_ISL_873082, EPI_ISL_873083, EPI_ISL_873084, EPI_ISL_873085, EPI_ISL_873086, EPI_ISL_873087, EPI_ISL_873088, EPI_ISL_873089, EPI_ISL_873090, EPI_ISL_873091, EPI_ISL_873092, EPI_ISL_873093, EPI_ISL_873094, EPI_ISL_873095, EPI_ISL_873096, EPI_ISL_873097, EPI_ISL_873098, EPI_ISL_873099, EPI_ISL_873100, EPI_ISL_873101, EPI_ISL_873102, EPI_ISL_873103, EPI_ISL_873104, EPI_ISL_873105, EPI_ISL_873106, EPI_ISL_873107, EPI_ISL_873108, EPI_ISL_873109, EPI_ISL_873111, EPI_ISL_873113, EPI_ISL_873116, EPI_ISL_873117, EPI_ISL_873119, EPI_ISL_873120, EPI_ISL_873122, EPI_ISL_873156, EPI_ISL_873159			
see above	University of Michigan Clinical Microbiology Laboratory	Lauring Lab, University of Michigan, Department of Microbiology and Immunology	Valesano
EPI_ISL_873165	Medical Laboratory Sciences, Arab American University	Medical Laboratory Sciences, Arab American University	Dumaidi,k., Al-Jawabreh,A., Ereqat,S., Al-Jawabreh,H., Nasereddin,A.
EPI_ISL_873176, EPI_ISL_873183	Microbiology Division, South Carolina Department of Health and Environmental Control (SC DHEC)	Microbiology Division, South Carolina Department of Health and Environmental Control (SC DHEC)	Flores,H., Freeman,J.
EPI_ISL_873195, EPI_ISL_873196, EPI_ISL_873198, EPI_ISL_873199, EPI_ISL_873200, EPI_ISL_873202, EPI_ISL_873203, EPI_ISL_873206	North Dakota Department of Health, Public Health Laboratory	North Dakota Department of Health, Public Health Laboratory	Lisa Wingerter
EPI_ISL_873238, EPI_ISL_873241, EPI_ISL_873243, EPI_ISL_873245, EPI_ISL_873246, EPI_ISL_873249, EPI_ISL_873250, EPI_ISL_873252, EPI_ISL_873254	M Health Fairview	Minnesota Department of Health, Public Health Laboratory	Alexandra Lorentz, Jacob Garfin, Matt Plumb, and Xiong Wang
EPI_ISL_873273, EPI_ISL_873274, EPI_ISL_873275	Vault Health	Minnesota Department of Health, Public Health Laboratory	Alexandra Lorentz, Jacob Garfin, Matt Plumb, and Xiong Wang
EPI_ISL_873277	Environmental and Global Health, University of Florida	Environmental and Global Health, University of Florida	Iovine,N.M., Waltzek,T.B., Subramaniam,K., Loeb,J.C.,Stephenson,C.J., Lessard,K., Reeves,M., Wilkerson,G., Morris,J.G. and Lednicky,J.A.
EPI_ISL_873624, EPI_ISL_873746	Lighthouse Lab in Cambridge	Wellcome Sanger Institute for the COVID-19 Genomics UK (COG-UK) Consortium	Rob Howes, The Lighthouse Lab in Cambridge and Alex Alderton, Roberto Amato, Sonia Goncalves, Ewan Harrison, David K. Jackson, Ian Johnston, Dominic Kwiatkowski, Cordelia Langford, John Sillitoe on behalf of the Wellcome Sanger Institute COVID-19 Surveillance Team
EPI_ISL_874170, EPI_ISL_874281	Lighthouse Lab in Alderley Park	Wellcome Sanger Institute for the COVID-19 Genomics UK (COG-UK) Consortium	Jacquelyn Wynn, Mairead Hyland, The Lighthouse Lab in Alderley Park and Alex Alderton, Roberto Amato, Sonia Goncalves, Ewan Harrison, David K. Jackson, Ian Johnston, Dominic Kwiatkowski, Cordelia Langford, John Sillitoe on behalf of the Wellcome Sanger Institute COVID-19 Surveillance Team
EPI_ISL_874990, EPI_ISL_874991, EPI_ISL_874992, EPI_ISL_874993, EPI_ISL_874994, EPI_ISL_874995, EPI_ISL_874996, EPI_ISL_874997, EPI_ISL_874998, EPI_ISL_874999, EPI_ISL_875000, EPI_ISL_875001, EPI_ISL_875002, EPI_ISL_875003, EPI_ISL_875004, EPI_ISL_875005, EPI_ISL_875006, EPI_ISL_875007, EPI_ISL_875008, EPI_ISL_875009, EPI_ISL_875010, EPI_ISL_875011, EPI_ISL_875012, EPI_ISL_875013, EPI_ISL_875014, EPI_ISL_875015, EPI_ISL_875016, EPI_ISL_875017, EPI_ISL_875018, EPI_ISL_875019, EPI_ISL_875020, EPI_ISL_875021, EPI_ISL_875022, EPI_ISL_875023, EPI_ISL_875024, EPI_ISL_875025, EPI_ISL_875026, EPI_ISL_875027, EPI_ISL_875028, EPI_ISL_875029, EPI_ISL_875030, EPI_ISL_875031, EPI_ISL_875032, EPI_ISL_875033, EPI_ISL_875034, EPI_ISL_875035, EPI_ISL_875036, EPI_ISL_875037, EPI_ISL_875038, EPI_ISL_875039, EPI_ISL_875040, EPI_ISL_875041, EPI_ISL_875042, EPI_ISL_875043, EPI_ISL_875044, EPI_ISL_875045, EPI_ISL_875046, EPI_ISL_875047, EPI_ISL_875048, EPI_ISL_875049, EPI_ISL_875050, EPI_ISL_875051, EPI_ISL_875052, EPI_ISL_875053, EPI_ISL_875054, EPI_ISL_875055, EPI_ISL_875056, EPI_ISL_875057, EPI_ISL_875058, EPI_ISL_875059, EPI_ISL_875060, EPI_ISL_875061, EPI_ISL_875062, EPI_ISL_875063, EPI_ISL_875064, EPI_ISL_875065, EPI_ISL_875066, EPI_ISL_875067, EPI_ISL_875068, EPI_ISL_875069, EPI_ISL_875070, EPI_ISL_875071, EPI_ISL_875072, EPI_ISL_875073, EPI_ISL_875074, EPI_ISL_875075, EPI_ISL_875076, EPI_ISL_875077, EPI_ISL_875078, EPI_ISL_875079, EPI_ISL_875080, EPI_ISL_875081, EPI_ISL_875082, EPI_ISL_875083, EPI_ISL_875084, EPI_ISL_875085, EPI_ISL_875086, EPI_ISL_875087, EPI_ISL_875088, EPI_ISL_875089, EPI_ISL_875090, EPI_ISL_875091, EPI_ISL_875092, EPI_ISL_875093, EPI_ISL_875094, EPI_ISL_875095, EPI_ISL_875096, EPI_ISL_875097, EPI_ISL_875098, EPI_ISL_875099, EPI_ISL_875100, EPI_ISL_875101, EPI_ISL_875102, EPI_ISL_875103, EPI_ISL_875104, EPI_ISL_875105, EPI_ISL_875106, EPI_ISL_875107, EPI_ISL_875108, EPI_ISL_875109, EPI_ISL_875110, EPI_ISL_875111, EPI_ISL_875112, EPI_ISL_875113, EPI_ISL_875114, EPI_ISL_875115, EPI_ISL_875116, EPI_ISL_875117, EPI_ISL_875118, EPI_ISL_875119, EPI_ISL_875120, EPI_ISL_875121, EPI_ISL_875122, EPI_ISL_875123, EPI_ISL_875124, EPI_ISL_875125, EPI_ISL_875126, EPI_ISL_875127, EPI_ISL_875128, EPI_ISL_875129, EPI_ISL_875130, EPI_ISL_875131, EPI_ISL_875132, EPI_ISL_875133,			

EPI_ISL_875134, EPI_ISL_875135, EPI_ISL_875136, EPI_ISL_875137, EPI_ISL_875138, EPI_ISL_875139, EPI_ISL_875140, EPI_ISL_875141, EPI_ISL_875142, EPI_ISL_875143, EPI_ISL_875144, EPI_ISL_875145, EPI_ISL_875146, EPI_ISL_875147, EPI_ISL_875148, EPI_ISL_875149, EPI_ISL_875150, EPI_ISL_875151, EPI_ISL_875152, EPI_ISL_875153, EPI_ISL_875154, EPI_ISL_875155, EPI_ISL_875156, EPI_ISL_875157, EPI_ISL_875158, EPI_ISL_875159, EPI_ISL_875160, EPI_ISL_875161, EPI_ISL_875162, EPI_ISL_875163, EPI_ISL_875164, EPI_ISL_875165, EPI_ISL_875166, EPI_ISL_875167, EPI_ISL_875168, EPI_ISL_875169, EPI_ISL_875170, EPI_ISL_875171, EPI_ISL_875172, EPI_ISL_875173, EPI_ISL_875174, EPI_ISL_875175, EPI_ISL_875176, EPI_ISL_875177, EPI_ISL_875178, EPI_ISL_875179, EPI_ISL_875180, EPI_ISL_875181, EPI_ISL_875182, EPI_ISL_875183, EPI_ISL_875184, EPI_ISL_875185, EPI_ISL_875186, EPI_ISL_875187, EPI_ISL_875188, EPI_ISL_875189, EPI_ISL_875190, EPI_ISL_875191, EPI_ISL_875192, EPI_ISL_875193, EPI_ISL_875194, EPI_ISL_875195, EPI_ISL_875196, EPI_ISL_875197, EPI_ISL_875198, EPI_ISL_875199, EPI_ISL_875200, EPI_ISL_875201, EPI_ISL_875202, EPI_ISL_875203, EPI_ISL_875204, EPI_ISL_875205, EPI_ISL_875206, EPI_ISL_875207, EPI_ISL_875208, EPI_ISL_875209, EPI_ISL_875210, EPI_ISL_875211, EPI_ISL_875212, EPI_ISL_875213, EPI_ISL_875214, EPI_ISL_875215, EPI_ISL_875216, EPI_ISL_875217, EPI_ISL_875218, EPI_ISL_875219, EPI_ISL_875220, EPI_ISL_875221, EPI_ISL_875222, EPI_ISL_875223, EPI_ISL_875224, EPI_ISL_875225, EPI_ISL_875226, EPI_ISL_875227, EPI_ISL_875228, EPI_ISL_875229, EPI_ISL_875230, EPI_ISL_875231, EPI_ISL_875232, EPI_ISL_875233, EPI_ISL_875234, EPI_ISL_875235, EPI_ISL_875236, EPI_ISL_875237, EPI_ISL_875238, EPI_ISL_875239, EPI_ISL_875240, EPI_ISL_875241, EPI_ISL_875242, EPI_ISL_875243, EPI_ISL_875244, EPI_ISL_875245, EPI_ISL_875246, EPI_ISL_875247, EPI_ISL_875248, EPI_ISL_875249, EPI_ISL_875250, EPI_ISL_875251, EPI_ISL_875252, EPI_ISL_875253, EPI_ISL_875254, EPI_ISL_875255, EPI_ISL_875256, EPI_ISL_875257, EPI_ISL_875258, EPI_ISL_875259, EPI_ISL_875260, EPI_ISL_875261, EPI_ISL_875262, EPI_ISL_875263, EPI_ISL_875264, EPI_ISL_875265, EPI_ISL_875266, EPI_ISL_875267, EPI_ISL_875268, EPI_ISL_875269, EPI_ISL_875270, EPI_ISL_875271, EPI_ISL_875272, EPI_ISL_875273, EPI_ISL_875274, EPI_ISL_875275, EPI_ISL_875276, EPI_ISL_875277, EPI_ISL_875278, EPI_ISL_875279, EPI_ISL_875280, EPI_ISL_875281, EPI_ISL_875282, EPI_ISL_875283, EPI_ISL_875284, EPI_ISL_875285, EPI_ISL_875286, EPI_ISL_875287, EPI_ISL_875288, EPI_ISL_875289, EPI_ISL_875290, EPI_ISL_875291, EPI_ISL_875292, EPI_ISL_875293, EPI_ISL_875294, EPI_ISL_875295, EPI_ISL_875296, EPI_ISL_875297, EPI_ISL_875298, EPI_ISL_875299, EPI_ISL_875300, EPI_ISL_875301, EPI_ISL_875302, EPI_ISL_875303, EPI_ISL_875304, EPI_ISL_875305, EPI_ISL_875306, EPI_ISL_875307, EPI_ISL_875308, EPI_ISL_875309, EPI_ISL_875310, EPI_ISL_875311, EPI_ISL_875312, EPI_ISL_875313, EPI_ISL_875314, EPI_ISL_875315, EPI_ISL_875316, EPI_ISL_875317, EPI_ISL_875318, EPI_ISL_875319, EPI_ISL_875320, EPI_ISL_875321, EPI_ISL_875322, EPI_ISL_875323, EPI_ISL_875324, EPI_ISL_875325, EPI_ISL_875326, EPI_ISL_875327, EPI_ISL_875328, EPI_ISL_875329, EPI_ISL_875330, EPI_ISL_875331, EPI_ISL_875332, EPI_ISL_875333, EPI_ISL_875334, EPI_ISL_875335, EPI_ISL_875336, EPI_ISL_875337, EPI_ISL_875338, EPI_ISL_875339, EPI_ISL_875340, EPI_ISL_875341			
see above	Lighthouse Lab in Milton Keynes	Wellcome Sanger Institute for the COVID-19 Genomics UK (COG-UK) Consortium	The Lighthouse Lab in Milton Keynes and Alex Alderton, Roberto Amato, Sonia Goncalves, Ewan Harrison, David K. Jackson, Ian Johnston, Dominic Kwiatkowski, Cordelia Langford, John Sillitoe on behalf of the Wellcome Sanger Institute COVID-19 Surveillance Team
EPI_ISL_875346	National Institute of Infectious Diseases-Prof. Dr. Matei Bals Molecular Diagnostics Laboratory	National Institute of Infectious Diseases-Prof. Dr. Matei Bals Molecular Diagnostics Laboratory	Leontina Banica, Marius Surleac, Carmen Casangiu, Petre Milu, Andreea Tudor, Simona Paraschiv, Dan Otelea
EPI_ISL_875460, EPI_ISL_875461, EPI_ISL_875462, EPI_ISL_875463, EPI_ISL_875464, EPI_ISL_875465, EPI_ISL_875466, EPI_ISL_875467, EPI_ISL_875468, EPI_ISL_875469, EPI_ISL_875470, EPI_ISL_875471, EPI_ISL_875472, EPI_ISL_875473, EPI_ISL_875474, EPI_ISL_875475, EPI_ISL_875476, EPI_ISL_875477, EPI_ISL_875478, EPI_ISL_875479, EPI_ISL_875480, EPI_ISL_875481, EPI_ISL_875482, EPI_ISL_875483, EPI_ISL_875484, EPI_ISL_875485, EPI_ISL_875486, EPI_ISL_875487, EPI_ISL_875488, EPI_ISL_875489, EPI_ISL_875490, EPI_ISL_875491, EPI_ISL_875492, EPI_ISL_875493, EPI_ISL_875494, EPI_ISL_875495, EPI_ISL_875496, EPI_ISL_875497, EPI_ISL_875498, EPI_ISL_875499, EPI_ISL_875500, EPI_ISL_875501, EPI_ISL_875502, EPI_ISL_875503, EPI_ISL_875504, EPI_ISL_875505, EPI_ISL_875506, EPI_ISL_875507, EPI_ISL_875508, EPI_ISL_875509, EPI_ISL_875510			
see above	National Virus Reference Laboratory	National Virus Reference Laboratory	Michael Carr, Gabriel Gonzalez, Jonathan Dean, Cillian F De Gascun
EPI_ISL_875518	Institute of Virology, Biomedical Research Center of the Slovak Academy of Sciences, Bratislava	Faculty of Natural Sciences, Comenius University, Bratislava	Broa Brejová, Viktória abanová, Kristína Boršová, Viktória Hodorová, Sabina Fumaová Havlíková, Juraj Kopáek, Martina Liková, ubomíra Lukáiková, Martina Neboháová, Monika Sláviková, Tomáš Vina, Jozef Nosek, Boris Klempa
EPI_ISL_875520	Institute of Virology, Biomedical Research Center of the Slovak Academy of Sciences, Bratislava	Faculty of Natural Sciences, Comenius University, Bratislava	Kristína Boršová, Viktória abanová, Broa Brejová, Viktória Hodorová, Sabina Fumaová Havlíková, Juraj Kopáek, Martina Liková, ubomíra Lukáiková, Martina Neboháová, Monika Sláviková, Tomáš Vina, Boris Klempa, Jozef Nosek
EPI_ISL_875521, EPI_ISL_875522	Institute of Virology, Biomedical Research Center of the Slovak Academy of Sciences, Bratislava	Faculty of Natural Sciences, Comenius University, Bratislava	Viktória abanová, Kristína Boršová, Broa Brejová, Viktória Hodorová, Sabina Fumaová Havlíková, Juraj Kopáek, Martina Liková, ubomíra Lukáiková, Martina Neboháová, Monika Sláviková, Tomáš Vina, Jozef Nosek, Boris Klempa
EPI_ISL_875523	Institute of Virology, Biomedical Research Center of the Slovak Academy of Sciences, Bratislava	Faculty of Natural Sciences, Comenius University, Bratislava	Kristína Boršová, Viktória abanová, Broa Brejová, Viktória Hodorová, Sabina Fumaová Havlíková, Juraj Kopáek, Martina Liková, ubomíra Lukáiková, Martina Neboháová, Monika Sláviková, Tomáš Vina, Boris Klempa, Jozef Nosek
EPI_ISL_875524	Institute of Virology, Biomedical Research Center of the Slovak Academy of Sciences, Bratislava	Faculty of Natural Sciences, Comenius University, Bratislava	Viktória abanová, Kristína Boršová, Broa Brejová, Viktória Hodorová, Sabina Fumaová Havlíková, Juraj Kopáek, Martina Liková, ubomíra Lukáiková, Martina Neboháová, Monika Sláviková, Tomáš Vina, Jozef Nosek, Boris Klempa
EPI_ISL_875525	Institute of Virology, Biomedical Research Center of the Slovak Academy of Sciences, Bratislava	Faculty of Natural Sciences, Comenius University, Bratislava	Kristína Boršová, Viktória abanová, Broa Brejová, Viktória Hodorová, Sabina Fumaová Havlíková, Juraj Kopáek, Martina Liková, ubomíra Lukáiková, Martina Neboháová, Monika Sláviková, Tomáš Vina, Boris Klempa, Jozef Nosek
EPI_ISL_875526, EPI_ISL_875527	Institute of Virology, Biomedical Research Center of the Slovak Academy of Sciences, Bratislava	Faculty of Natural Sciences, Comenius University, Bratislava	Broa Brejová, Viktória abanová, Kristína Boršová, Viktória Hodorová, Sabina Fumaová Havlíková, Juraj Kopáek, Martina Liková, ubomíra Lukáiková, Martina Neboháová, Monika Sláviková, Tomáš Vina, Jozef Nosek, Boris Klempa
EPI_ISL_875528	Institute of Virology, Biomedical Research Center of the Slovak Academy of Sciences, Bratislava	Faculty of Natural Sciences, Comenius University, Bratislava	Kristína Boršová, Viktória abanová, Broa Brejová, Viktória Hodorová, Sabina Fumaová Havlíková, Juraj Kopáek, Martina Liková, ubomíra Lukáiková, Martina Neboháová, Monika Sláviková, Tomáš Vina, Boris Klempa, Jozef Nosek
EPI_ISL_875530	Institute of Virology, Biomedical Research Center of the Slovak Academy of Sciences, Bratislava	Faculty of Natural Sciences, Comenius University, Bratislava	Viktória abanová, Kristína Boršová, Broa Brejová, Viktória Hodorová, Sabina Fumaová Havlíková, Juraj Kopáek, Martina Liková, ubomíra Lukáiková, Martina Neboháová, Monika Sláviková, Tomáš Vina, Jozef Nosek, Boris Klempa
EPI_ISL_875660, EPI_ISL_875661	University of Michigan Clinical Microbiology Laboratory	Lauring Lab, University of Michigan, Department of Microbiology and Immunology	Valesano
EPI_ISL_875686	National Virus Reference Laboratory	National Virus Reference Laboratory	Michael Carr, Gabriel Gonzalez, Jonathan Dean, Cillian F De Gascun
EPI_ISL_876052, EPI_ISL_876053, EPI_ISL_876056, EPI_ISL_876059, EPI_ISL_876060, EPI_ISL_876062, EPI_ISL_876081, EPI_ISL_876127, EPI_ISL_876128, EPI_ISL_876131, EPI_ISL_876137, EPI_ISL_876146, EPI_ISL_876147, EPI_ISL_876149, EPI_ISL_876163, EPI_ISL_876172, EPI_ISL_876173, EPI_ISL_876178, EPI_ISL_876180, EPI_ISL_876184, EPI_ISL_876186, EPI_ISL_876196, EPI_ISL_876198, EPI_ISL_876200, EPI_ISL_876203, EPI_ISL_876208, EPI_ISL_876245, EPI_ISL_876246, EPI_ISL_876247, EPI_ISL_876248, EPI_ISL_876249, EPI_ISL_876250, EPI_ISL_876251			
see above	Massachusetts State Public Health Laboratory	Massachusetts State Public Health Laboratory	Andrew Lang, Timelia Fink, Glen Gallagher, Sandra Smole
EPI_ISL_876588	Blackhawk Genomics	Pathogen Discovery, Respiratory Viruses Branch, Division of Viral Diseases, Centers for Disease Control and Prevention	Ying Tao, Yan Li, Jing Zhang, Krista Queen, Anna Uehara, Peter Cook, Clinton R. Paden, Haibin Wang, Suxiang Tong
EPI_ISL_876594, EPI_ISL_876596	TN Division of Laboratory Services	Pathogen Discovery, Respiratory Viruses Branch, Division of Viral Diseases, Centers for Disease Control and Prevention	Ying Tao, Yan Li, Jing Zhang, Krista Queen, Anna Uehara, Peter Cook, Clinton R. Paden, Haibin Wang, Suxiang Tong
EPI_ISL_876625, EPI_ISL_876626, EPI_ISL_876628, EPI_ISL_876629, EPI_ISL_876630, EPI_ISL_876632, EPI_ISL_876633, EPI_ISL_876634, EPI_ISL_876635, EPI_ISL_876636, EPI_ISL_876637, EPI_ISL_876638, EPI_ISL_876639, EPI_ISL_876640, EPI_ISL_876641, EPI_ISL_876643, EPI_ISL_876644, EPI_ISL_876645, EPI_ISL_876646, EPI_ISL_876647, EPI_ISL_876648, EPI_ISL_876649, EPI_ISL_876650, EPI_ISL_876651, EPI_ISL_876652, EPI_ISL_876653, EPI_ISL_876654, EPI_ISL_876655, EPI_ISL_876656, EPI_ISL_876657, EPI_ISL_876658, EPI_ISL_876660, EPI_ISL_876661, EPI_ISL_876662, EPI_ISL_876663, EPI_ISL_876664, EPI_ISL_876665, EPI_ISL_876666, EPI_ISL_876668, EPI_ISL_876670, EPI_ISL_876671, EPI_ISL_876672, EPI_ISL_876673, EPI_ISL_876674, EPI_ISL_876675, EPI_ISL_876676, EPI_ISL_876677, EPI_ISL_876678, EPI_ISL_876680, EPI_ISL_876681, EPI_ISL_876682, EPI_ISL_876683, EPI_ISL_876684, EPI_ISL_876685, EPI_ISL_876686, EPI_ISL_876687, EPI_ISL_876688, EPI_ISL_876689, EPI_ISL_876691, EPI_ISL_876692, EPI_ISL_876693, EPI_ISL_876695, EPI_ISL_876696, EPI_ISL_876698, EPI_ISL_876699, EPI_ISL_876700, EPI_ISL_876701, EPI_ISL_876702, EPI_ISL_876703, EPI_ISL_876704, EPI_ISL_876705, EPI_ISL_876706, EPI_ISL_876708, EPI_ISL_876709, EPI_ISL_876711, EPI_ISL_876712, EPI_ISL_876713, EPI_ISL_876714, EPI_ISL_876715, EPI_ISL_876716, EPI_ISL_876718, EPI_ISL_876719, EPI_ISL_876720, EPI_ISL_876721, EPI_ISL_876722, EPI_ISL_876723, EPI_ISL_876724, EPI_ISL_876725, EPI_ISL_876726, EPI_ISL_876727, EPI_ISL_876728, EPI_ISL_876729, EPI_ISL_876730, EPI_ISL_876731, EPI_ISL_876732, EPI_ISL_876733, EPI_ISL_876734, EPI_ISL_876735, EPI_ISL_876736			
see above	Helix/Illumina	Genomics and Discovery, Respiratory Viruses Branch, Division of Viral Diseases, Centers for Disease Control and Prevention	Peter W. Cook,Dhwani Batra,Ben L. Rambo-Martin,Eileen de Feo,Jan Antico,Christine Tran,Matthew Tolentino,Shannon Wickline,Kim Gietzen,Brad Sickler,Jingtao Liu,Eric Allen,Phil Febbo,Summer Galloway,Nicole L. Washington,Simon White,Geraint Levan,Kelly Schiabor Barrett,Elizabeth Cirulli,Alexandre Bolze,Ary Ascencio,Charlotte Rivera-Garcia,Ryan Cho,Jason Nguyen,Sherry Wang,Jimmy Ramirez,Tyler Cassens,Efren Sandoval,Magnus Isaksson,William Lee,David Becker,Marc Laurent,James Lu,Clinton R. Paden,Suxiang Tong,Duncan MacCannell,
EPI_ISL_877187, EPI_ISL_877189, EPI_ISL_877190, EPI_ISL_877191, EPI_ISL_877192, EPI_ISL_877193, EPI_ISL_877194, EPI_ISL_877195, EPI_ISL_877196, EPI_ISL_877197, EPI_ISL_877198, EPI_ISL_877199, EPI_ISL_877200, EPI_ISL_877201, EPI_ISL_877202, EPI_ISL_877203, EPI_ISL_877204			
see above	Quest Diagnostics	Quest Diagnostics	Rosenthal,S.H., Gerasimova,A., Kagan,R.M., Anderson, B., Hua, M., Liu Y., Bernstein, L.E., Livingston, K.E., Perez, A., Shalhout, D.F., Shlyakhter, I.A., Owen, R., Tanpaiboon, P., Lacbawan, F.
EPI_ISL_877205, EPI_ISL_877206, EPI_ISL_877207, EPI_ISL_877208, EPI_ISL_877209	LabPLUS	Institute of Environmental Science and Research (ESR)	Xiaoyun Ren, Matt Storey, Nikki Freed, Muhammad Faisal, Jing Wang, Hermes Perez, Anja Werno, Antje van der Linden, Arlo Upton, Chris Mansell, David Hammer, Dragana Drinkovic, Gary McAuliffe, Hana Sofia Andersson, James Ussher, Jill Sherwood, Josh Freeman, Julia Howard, Juliet Elvy, Mary DeAlmeida, Matt Blakiston, Matthew Rogers, Max Bloomfield, Michael Addidle, Michelle Balm, Sally Roberts, Sarah Jefferies, Sharmini Muttaiyah, Susan Morpeth, Susan Taylor, Timothy Blackmore, Vani Sathyendran, Veronica Playle, Virginia Hope, Erasmus Smit, Lauren Jelly, Olin Silander, Joep de Ligt
EPI_ISL_877214	Middlemore Hospital	Institute of Environmental Science and Research (ESR)	Xiaoyun Ren, Matt Storey, Nikki Freed, Muhammad Faisal, Jing Wang, Hermes Perez, Anja Werno, Antje van der Linden, Arlo Upton, Chris Mansell, David Hammer, Dragana Drinkovic, Gary McAuliffe, Hana Sofia Andersson, James Ussher, Jill Sherwood, Josh Freeman, Julia Howard, Juliet Elvy, Mary DeAlmeida, Matt Blakiston, Matthew Rogers, Max Bloomfield, Michael Addidle, Michelle Balm, Sally Roberts, Sarah Jefferies, Sharmini Muttaiyah, Susan Morpeth, Susan Taylor, Timothy Blackmore, Vani Sathyendran, Veronica Playle, Virginia Hope, Erasmus Smit, Lauren Jelly, Olin Silander, Joep de Ligt
EPI_ISL_877453	Institute of Microbiology and Immunology, Faculty of Medicine, University of Ljubljana	Institute of Microbiology and Immunology, Faculty of Medicine, University of Ljubljana	Samo Zakotnik, Tomaž Mark Zorec, Matic Brvar, Miša Korva, Mario Poljak, Tatjana Avši - Županc
EPI_ISL_878477, EPI_ISL_878480, EPI_ISL_878483, EPI_ISL_878485, EPI_ISL_878488, EPI_ISL_878491, EPI_ISL_878492, EPI_ISL_878503, EPI_ISL_878506, EPI_ISL_878509, EPI_ISL_878511, EPI_ISL_878514, EPI_ISL_878516, EPI_ISL_878519, EPI_ISL_878522, EPI_ISL_878524, EPI_ISL_878526			
see above	Biolab Diagnostic Laboratories	Andersen lab at Scripps Research	Issa Abu-Dayyeh, Ahmad Tibi, Lama Hussein, Lina Mohammad, Zein Naber, Amid Abdelnour with SEARCH Alliance San Diego

EPI_ISL_880037, EPI_ISL_880045, EPI_ISL_880048, EPI_ISL_880050, EPI_ISL_880053, EPI_ISL_880056, EPI_ISL_880061, EPI_ISL_880063, EPI_ISL_880071, EPI_ISL_880074, EPI_ISL_880079, EPI_ISL_880082, EPI_ISL_880090, EPI_ISL_880092, EPI_ISL_880095, EPI_ISL_880101, EPI_ISL_880103, EPI_ISL_880106, EPI_ISL_880117, EPI_ISL_880119, EPI_ISL_880122, EPI_ISL_880125, EPI_ISL_880128, EPI_ISL_880130, EPI_ISL_880133, EPI_ISL_880135, EPI_ISL_880138, EPI_ISL_880140, EPI_ISL_880141, EPI_ISL_880143, EPI_ISL_880144, EPI_ISL_880146, EPI_ISL_880147, EPI_ISL_880149, EPI_ISL_880150, EPI_ISL_880151, EPI_ISL_880152, EPI_ISL_880156, EPI_ISL_880157, EPI_ISL_880159, EPI_ISL_880161, EPI_ISL_880162, EPI_ISL_880164, EPI_ISL_880165, EPI_ISL_880168, EPI_ISL_880169			
see above	Sharp HealthCare Laboratory	Andersen lab at Scripps Research	SEARCH Alliance San Diego with Aaron Harding, Jacquelyn Berumen, Cathy Woerle, Liam McGinnis, Art Mendoza, Omid Bakhtar
EPI_ISL_880182, EPI_ISL_880183, EPI_ISL_880192	Rady's Childrens Hospital	Andersen lab at Scripps Research	SEARCH Alliance San Diego with Nanda Radamchar, David Dimmock, Linda Luo, Christina Clarke, Kathryn Bouic, Teresa Mueller, Denise Malicki
EPI_ISL_880201, EPI_ISL_880202, EPI_ISL_880203, EPI_ISL_880204, EPI_ISL_880205, EPI_ISL_880206, EPI_ISL_880207, EPI_ISL_880208, EPI_ISL_880209, EPI_ISL_880212, EPI_ISL_880215, EPI_ISL_880218, EPI_ISL_880220, EPI_ISL_880223, EPI_ISL_880225, EPI_ISL_880228, EPI_ISL_880231, EPI_ISL_880233, EPI_ISL_880236, EPI_ISL_880239, EPI_ISL_880241, EPI_ISL_880244, EPI_ISL_880246, EPI_ISL_880249, EPI_ISL_880251, EPI_ISL_880253, EPI_ISL_880256, EPI_ISL_880259, EPI_ISL_880261, EPI_ISL_880263, EPI_ISL_880266, EPI_ISL_880268, EPI_ISL_880271, EPI_ISL_880274, EPI_ISL_880277, EPI_ISL_880279, EPI_ISL_880282, EPI_ISL_880284, EPI_ISL_880287, EPI_ISL_880290, EPI_ISL_880292, EPI_ISL_880294, EPI_ISL_880297, EPI_ISL_880300, EPI_ISL_880303, EPI_ISL_880305, EPI_ISL_880308, EPI_ISL_880310, EPI_ISL_880313, EPI_ISL_880316, EPI_ISL_880319, EPI_ISL_880321, EPI_ISL_880324, EPI_ISL_880326, EPI_ISL_880329			
see above	Scripps Medical Laboratory	Andersen lab at Scripps Research	SEARCH Alliance San Diego with Michael Quigley, Ellen Stefanski, Ian Mchardy
EPI_ISL_882610	Lighthouse Lab in Milton Keynes	Wellcome Sanger Institute for the COVID-19 Genomics UK (COG-UK) Consortium	The Lighthouse Lab in Milton Keynes and Alex Alderton, Roberto Amato, Sonia Goncalves, Ewan Harrison, David K. Jackson, Ian Johnston, Dominic Kwiatkowski, Cordelia Langford, John Sillitoe on behalf of the Wellcome Sanger Institute COVID-19 Surveillance Team
EPI_ISL_882616, EPI_ISL_882618, EPI_ISL_882628, EPI_ISL_882629	Hospital Ramón y Cajal	Hospital Ramón y Cajal	José M Gonzalez-Alba, Concepción Rodríguez, Melanie Abreu, Laura Martínez, Val F Lanza, Luz Leticia Olavarrieta, Rafael Cantón, JC Galán
EPI_ISL_882642, EPI_ISL_882643, EPI_ISL_882644	Azerbaijan National Hematology Center Division of Medical Genetics	Azerbaijan National Hematology Center Division of Medical Genetics	Aghayev Agha Rza
EPI_ISL_882646	Medical Research Center, Faculty of Medicine, Syarif Hidayatullah State Islamic University Jakarta	Medical Research Center, Faculty of Medicine, Syarif Hidayatullah State Islamic University Jakarta	Chris Adhiyanto, Laifa Hendarmin, Zeti Harriyati, Endah Wulandari, Flori Ratna Sari, Fika Ekayanti, Hari Hendarto
EPI_ISL_882656	Medical Research Center, Faculty of Medicine, Syarif Hidayatullah State Islamic University Jakarta	Medical Research Center, Faculty of Medicine, Syarif Hidayatullah State Islamic University Jakarta	Erike A Suwarsono, Chris Adhiyanto, Laifa Hendarmin, Zeti Harriyati, Flori Ratna Sari, Endah Wulandari, Fika Ekayanti
EPI_ISL_882665	Unidade de Pronto Atendimento Dra Zilda Arns	Instituto Adolfo Lutz, Interdiciplinary Procedures Center, Strategic Laboratory	Claudio Tavares Sacchi, Claudia Regina Gonçalves, Erica Valessa Ramos Gomes, Karoline Rodrigues Campos
EPI_ISL_882773, EPI_ISL_882774, EPI_ISL_882775, EPI_ISL_882777, EPI_ISL_882778	Institute for Urban Disease Control and Prevention	COVID-19 Network Investigations (CONI) Alliance	Kamolthip Atsawawaranunt, Elizabeth Batty, Wasun Chantratita, Thanat Chookajorn, Stefan Fernandez, Angkana Huang, Anthony R. Jones, Khajohn Joonsalak, Chonticha Klungtong, Theerarat Kochakarn, Prayuth Kaewmalang, Amornmas Kongklieng, Namfon Kotanan, Krittikorn Kumpornsin, Duangkamon Loesbanluechai, Wudtichai Manasatienkij, Anek Mungaomklang, Bhakbhoom Panthan, Pukkaporn Parnwijitkul, Ekawat Pasomsub, Vichan Pawun, Kingkan Rakmanee, Insee Sensorn, Janjira Thaipadungpanit, Arporn Wangwiwatsin, Treewat Watthanachockchai
EPI_ISL_882780	Ramathibodi Chakri Naruebodindra Hospital	COVID-19 Network Investigations (CONI) Alliance	Elizabeth Batty, Wasun Chantratita, Thanat Chookajorn, Stefan Fernandez, Angkana Huang, Anthony R. Jones, Khajohn Joonsalak, Chonticha Klungtong, Theerarat Kochakarn, Namfon Kotanan, Krittikorn Kumpornsin, Wudtichai Manasatienkij, Bhakbhoom Panthan, Pukkaporn Parnwijitkul, Ekawat Pasomsub, Kingkan Rakmanee, Insee Sensorn, Janjira Thaipadungpanit, Arporn Wangwiwatsin,Treewat Watthanachockchai
EPI_ISL_882783, EPI_ISL_882784, EPI_ISL_882785, EPI_ISL_882786, EPI_ISL_882787, EPI_ISL_882788, EPI_ISL_882789, EPI_ISL_882790, EPI_ISL_882791	Institute for Urban Disease Control and Prevention	COVID-19 Network Investigations (CONI) Alliance	Kamolthip Atsawawaranunt, Elizabeth Batty, Wasun Chantratita, Thanat Chookajorn, Stefan Fernandez, Angkana Huang, Anthony R. Jones, Khajohn Joonsalak, Chonticha Klungtong, Theerarat Kochakarn, Prayuth Kaewmalang, Amornmas Kongklieng, Namfon Kotanan, Krittikorn Kumpornsin, Duangkamon Loesbanluechai, Wudtichai Manasatienkij, Anek Mungaomklang, Bhakbhoom Panthan, Pukkaporn Parnwijitkul, Ekawat Pasomsub, Vichan Pawun, Kingkan Rakmanee, Insee Sensorn, Janjira Thaipadungpanit, Arporn Wangwiwatsin, Treewat Watthanachockchai
EPI_ISL_882805	MSHS Clinical Microbiology Laboratories	MSHS Pathogen Surveillance Program	Ana S. Gonzalez-Reiche, Hala Alshammary, Mitchell J. Sullivan, Brianne Ciferri, Ajay Obla, Angela Amoako, Mahmoud Awawda, Elena Hirsch, Ashley S. Salimbangon, Levy Sominsky, Katherine Beach, Kayla Russo, Charles Gleason, Shelcie Fabre, Giulio Kleiner, Zenab Khan, Bremy Alburquerque, Adriana van de Guchte, Komal Srivastava, Matthew M. Hernandez, Jayeeta Dutta, Denise Jurczyszak, Emily Ferreri, Rachel Chernet, Nancy Francoeur, Betsaida Salom Melo, Irina Oussenko, Gintaras Deikus, Juan Soto, Shwetha Hara Sridhar, Ying-Chih Wang, Kathryn Twyman, Andrew Kasarskis, Deena R. Altman, Robert Sebra, Adolfo García-Sastre, Marta Luksza, Gopi Patel, Sarah Schaefer, Melissa Gitman, Michael D. Nowak, Alberto Paniz-Mondolfi, Emilia Mia Sordillo, Viviana Simon, Harm van Bakel
EPI_ISL_882919	INMI Lazzaro Spallanzani IRCCS	INMI Lazzaro Spallanzani IRCCS	C.E.M Gruber, B Bartolini, E Giombini, M Rueca, O Butera, F Messina, A Di Caro, MR Capobianchi
EPI_ISL_882944, EPI_ISL_882950, EPI_ISL_882951, EPI_ISL_882952, EPI_ISL_882953	The National Institute of Public Health	State Veterinary Institute Prague	Nagy,A;Jirincova,H;Vecerova,JTrnka,D
EPI_ISL_882987	MD Laboratories	Los Angeles County PHL	P. Hemarajata et al.
EPI_ISL_883035	Maryland Public Health Laboratory	Maryland Public Health Laboratory	Maryland Department of Health Laboratories Administration
EPI_ISL_883293	SIESP CHIETI-DRIVE IN LANCIANO	Istituto Zooprofilattico Sperimentale dell'Abruzzo e Molise "G. Caporale"	Lorusso A, Marcacci M, Di Domenico M, Ancora M, Curini V, Mangone I, Rinaldi A, Scialabba S, Di Pasquale A, Cammà C, Puglia I, Calistri P, Savini G
EPI_ISL_883294, EPI_ISL_883295, EPI_ISL_883301, EPI_ISL_883302	RP Guardiagrele-Ospedale di Comunità	Istituto Zooprofilattico Sperimentale dell'Abruzzo e Molise "G. Caporale"	Lorusso A, Marcacci M, Di Domenico M, Ancora M, Curini V, Mangone I, Rinaldi A, Scialabba S, Di Pasquale A, Cammà C, Puglia I, Calistri P, Savini G
EPI_ISL_883303, EPI_ISL_883304, EPI_ISL_883305	SIESP DIPARTIMENTO DI PREVENZIONE CHIE	Istituto Zooprofilattico Sperimentale dell'Abruzzo e Molise "G. Caporale"	Lorusso A, Marcacci M, Di Domenico M, Ancora M, Curini V, Mangone I, Rinaldi A, Scialabba S, Di Pasquale A, Cammà C, Puglia I, Calistri P, Savini G
EPI_ISL_883427, EPI_ISL_883452, EPI_ISL_883453, EPI_ISL_883456, EPI_ISL_883458, EPI_ISL_883459, EPI_ISL_883460, EPI_ISL_883461, EPI_ISL_883462, EPI_ISL_883463, EPI_ISL_883464, EPI_ISL_883465, EPI_ISL_883466, EPI_ISL_883467, EPI_ISL_883468, EPI_ISL_883469, EPI_ISL_883470, EPI_ISL_883481			
see above	NORTHWELL HEALTH LABORATORIES	Wadsworth Center, New York State Department of Health	Kirsten St. George, Daryl M. Lamson, Alexis Russel, Matthew Shudt, Melissa A Leisner, Jonathan Plitnick, Navjot Singh, John Kelly, Erasmus Schneider, Erica Lasek-Nesselquist
EPI_ISL_883957	Medical Research Center, Faculty of Medicine, Syarif Hidayatullah State Islamic University Jakarta	Medical Research Center, Faculty of Medicine, Syarif Hidayatullah State Islamic University Jakarta	Erike A Suwarsono, Chris Adhiyanto, Laifa Hendarmin, Zeti Harriyati, Endah Wulandari, Flori Ratna Sari, Fika Ekayanti, Hari Hendarto
EPI_ISL_883961	Labo Analyses Med	National Reference Center for Viruses of Respiratory Infections, Institut Pasteur, Paris	Marion Barbet, Sylvie Behillil, Méline Bizard, Angela Brisebarre, Camille Capel, Etienne Simon-Lorière, Vincent Enouf, Maud Vanpeene, Sylvie van der Werf,Jacques Alexandra
EPI_ISL_883962, EPI_ISL_883963	Labo Analyses Med	National Reference Center for Viruses of Respiratory Infections, Institut Pasteur, Paris	Marion Barbet, Sylvie Behillil, Méline Bizard, Angela Brisebarre, Camille Capel, Etienne Simon-Lorière, Vincent Enouf, Maud Vanpeene, Sylvie van der Werf,Amzalag Jonas
EPI_ISL_883964, EPI_ISL_883967, EPI_ISL_883968, EPI_ISL_883969, EPI_ISL_883970	Labo Analyses med	National Reference Center for Viruses of Respiratory Infections, Institut Pasteur, Paris	Marion Barbet, Sylvie Behillil, Méline Bizard, Angela Brisebarre, Camille Capel, Etienne Simon-Lorière, Vincent Enouf, Maud Vanpeene, Sylvie van der Werf,Amzalag Jonas
EPI_ISL_883972, EPI_ISL_883973, EPI_ISL_883974, EPI_ISL_883975, EPI_ISL_883976, EPI_ISL_883979, EPI_ISL_883980, EPI_ISL_883981, EPI_ISL_883985	Hopital	National Reference Center for Viruses of Respiratory Infections, Institut Pasteur, Paris	Marion Barbet, Sylvie Behillil, Méline Bizard, Angela Brisebarre, Camille Capel, Etienne Simon-Lorière, Vincent Enouf, Maud Vanpeene, Sylvie van der Werf,Fourgeaud Jacques
EPI_ISL_883986, EPI_ISL_883987, EPI_ISL_883988, EPI_ISL_883989	Hopital	National Reference Center for Viruses of Respiratory Infections, Institut Pasteur, Paris	Marion Barbet, Sylvie Behillil, Méline Bizard, Angela Brisebarre, Camille Capel, Etienne Simon-Lorière, Vincent Enouf, Maud Vanpeene, Sylvie van der Werf
EPI_ISL_883990, EPI_ISL_883991,	hopital	National Reference Center for Viruses of Respiratory	Marion Barbet, Sylvie Behillil, Méline Bizard, Angela Brisebarre, Camille Capel, Etienne Simon-Lorière, Vincent Enouf, Maud Vanpeene, Sylvie van der

EPI_ISL_883992, EPI_ISL_883993, EPI_ISL_883994, EPI_ISL_883995, EPI_ISL_883996		Infections, Institut Pasteur, Paris	Werf,Andre-Garnier Elisabeth
EPI_ISL_884001, EPI_ISL_884003	Labo Analyses Med	National Reference Center for Viruses of Respiratory Infections, Institut Pasteur, Paris	Marion Barbet, Sylvie Behillil, Méline Bizard, Angela Brisebarre, Camille Capel, Etienne Simon-Lorière, Vincent Enouf, Maud Vanpeene, Sylvie van der Werf,Rousset Dominique
EPI_ISL_884080	Wadsworth Center, New York State Department of Health	Wadsworth Center, New York State Department of Health	Kirsten St. George, Daryl M. Lamson, Alexis Russel, Matthew Shudt, Melissa A Leisner, Jonathan Plitnick, Navjot Singh, John Kelly, Erasmus Schneider, Erica Lasek-Nesselquist
EPI_ISL_884081, EPI_ISL_884082, EPI_ISL_884083, EPI_ISL_884084, EPI_ISL_884085	ALBANY MEDICAL CENTER HOSPITAL CLINICAL LABORATORIES	Wadsworth Center, New York State Department of Health	Kirsten St. George, Daryl M. Lamson, Alexis Russel, Matthew Shudt, Melissa A Leisner, Jonathan Plitnick, Navjot Singh, John Kelly, Erasmus Schneider, Erica Lasek-Nesselquist
EPI_ISL_884086	Wadsworth Center, New York State Department of Health	Wadsworth Center, New York State Department of Health	Kirsten St. George, Daryl M. Lamson, Alexis Russel, Matthew Shudt, Melissa A Leisner, Jonathan Plitnick, Navjot Singh, John Kelly, Erasmus Schneider, Erica Lasek-Nesselquist
EPI_ISL_884089, EPI_ISL_884090, EPI_ISL_884091, EPI_ISL_884092, EPI_ISL_884093, EPI_ISL_884094, EPI_ISL_884095, EPI_ISL_884096, EPI_ISL_884097, EPI_ISL_884098, EPI_ISL_884099, EPI_ISL_884100, EPI_ISL_884101, EPI_ISL_884102, EPI_ISL_884103, EPI_ISL_884104, EPI_ISL_884105, EPI_ISL_884106, EPI_ISL_884107, EPI_ISL_884108, EPI_ISL_884109, EPI_ISL_884110, EPI_ISL_884111, EPI_ISL_884112, EPI_ISL_884113, EPI_ISL_884114, EPI_ISL_884115, EPI_ISL_884116, EPI_ISL_884117, EPI_ISL_884118, EPI_ISL_884119, EPI_ISL_884120, EPI_ISL_884121, EPI_ISL_884122, EPI_ISL_884123, EPI_ISL_884124, EPI_ISL_884125, EPI_ISL_884126, EPI_ISL_884127, EPI_ISL_884128, EPI_ISL_884129, EPI_ISL_884130, EPI_ISL_884131, EPI_ISL_884132, EPI_ISL_884133, EPI_ISL_884134, EPI_ISL_884135, EPI_ISL_884136, EPI_ISL_884137, EPI_ISL_884138, EPI_ISL_884139, EPI_ISL_884140, EPI_ISL_884141, EPI_ISL_884142, EPI_ISL_884143, EPI_ISL_884144, EPI_ISL_884145, EPI_ISL_884146, EPI_ISL_884147, EPI_ISL_884148, EPI_ISL_884149, EPI_ISL_884150, EPI_ISL_884151, EPI_ISL_884152, EPI_ISL_884153, EPI_ISL_884154, EPI_ISL_884155, EPI_ISL_884156, EPI_ISL_884157, EPI_ISL_884158, EPI_ISL_884159, EPI_ISL_884160, EPI_ISL_884161, EPI_ISL_884162, EPI_ISL_884163, EPI_ISL_884164, EPI_ISL_884165, EPI_ISL_884166, EPI_ISL_884167, EPI_ISL_884168, EPI_ISL_884169, EPI_ISL_884170, EPI_ISL_884171, EPI_ISL_884172, EPI_ISL_884173, EPI_ISL_884174, EPI_ISL_884175, EPI_ISL_884176, EPI_ISL_884177, EPI_ISL_884178, EPI_ISL_884179, EPI_ISL_884180, EPI_ISL_884181			
see above	Eurofins Diatherix	Hudsonalpha Genome Sequencing Center	Jane Grimwood, Melissa Williams, Lori H. Handley, Joshua Stough, Leslie Malone, Stefan Brzezinski, Ada Stewart, Teresa Jones, Jenell Webber, John Lovell, Jennifer Cart, and Jeremy Schmutz
EPI_ISL_884248	Kansas Health and Environmental Lab	Kansas Health and Environmental Lab	Mike Grose, Paige Drury, Carissa Robertson, Ben Olsen, and Phil Adam
EPI_ISL_884296, EPI_ISL_884297, EPI_ISL_884298, EPI_ISL_884299, EPI_ISL_884300, EPI_ISL_884301, EPI_ISL_884302	Santa Clara County Public Health Laboratory	Santa Clara County Public Health Laboratory	Santa Clara County Public Health Department
EPI_ISL_886159, EPI_ISL_886165, EPI_ISL_886178, EPI_ISL_886179, EPI_ISL_886182, EPI_ISL_886192, EPI_ISL_886202, EPI_ISL_886210, EPI_ISL_886216, EPI_ISL_886225, EPI_ISL_886226, EPI_ISL_886260, EPI_ISL_886262, EPI_ISL_886273, EPI_ISL_886275, EPI_ISL_886284, EPI_ISL_886287, EPI_ISL_886314, EPI_ISL_886319, EPI_ISL_886327, EPI_ISL_886343, EPI_ISL_886356, EPI_ISL_886386, EPI_ISL_886387, EPI_ISL_886396, EPI_ISL_886427, EPI_ISL_886439, EPI_ISL_886440, EPI_ISL_886457, EPI_ISL_886461, EPI_ISL_886475, EPI_ISL_886478, EPI_ISL_886502, EPI_ISL_886509, EPI_ISL_886516, EPI_ISL_886521, EPI_ISL_886532, EPI_ISL_886533, EPI_ISL_886537, EPI_ISL_886538, EPI_ISL_886539, EPI_ISL_886540, EPI_ISL_886541, EPI_ISL_886542, EPI_ISL_886552, EPI_ISL_886555, EPI_ISL_886579, EPI_ISL_886582, EPI_ISL_886590, EPI_ISL_886603, EPI_ISL_886613, EPI_ISL_886614, EPI_ISL_886624, EPI_ISL_886628, EPI_ISL_886640, EPI_ISL_886652, EPI_ISL_886674, EPI_ISL_886676, EPI_ISL_886678, EPI_ISL_886682, EPI_ISL_886683, EPI_ISL_886687, EPI_ISL_886695, EPI_ISL_886709, EPI_ISL_886713, EPI_ISL_886718, EPI_ISL_886737, EPI_ISL_886740, EPI_ISL_886741, EPI_ISL_886768, EPI_ISL_886781, EPI_ISL_886800, EPI_ISL_886820, EPI_ISL_886823, EPI_ISL_886859, EPI_ISL_886865, EPI_ISL_886879, EPI_ISL_886881, EPI_ISL_886882, EPI_ISL_886884, EPI_ISL_886887, EPI_ISL_886892, EPI_ISL_886903, EPI_ISL_886908, EPI_ISL_886911, EPI_ISL_886912, EPI_ISL_886917, EPI_ISL_886923, EPI_ISL_886932, EPI_ISL_886949, EPI_ISL_886954, EPI_ISL_886955, EPI_ISL_886956, EPI_ISL_887001, EPI_ISL_887008, EPI_ISL_887010, EPI_ISL_887019, EPI_ISL_887029, EPI_ISL_887034, EPI_ISL_887038, EPI_ISL_887042, EPI_ISL_887045, EPI_ISL_887058, EPI_ISL_887071, EPI_ISL_887077, EPI_ISL_887080, EPI_ISL_887083, EPI_ISL_887084			
see above	Labcorp	Genomics and Discovery, Respiratory Viruses Branch, Division of Viral Diseases, Centers for Disease Control and Prevention	Peter W. Cook,Dhwani Batra,Ben L. Rambo-Martin,Summer Galloway,Brian Krueger,Minoo Agarwal,Eyad Almasri,Debbie Boles,Ayla Burns,Nuthawin Charoensri,Oren Cohen,Susan Countryman,Mary Ann Cristobal,Bobbi Croy,Suzanne Dale,Hrushikesh Deshmukh,Amanda Douglas,Vincent Drouillon,Marcia Eisenberg,Howard Engler,Rama Ghatti,Prashant Gupta,Susan Hicks,Jake Humphrey,Lax Iyer,Manoj Jain,Mohan Kolli,Tim Kuphal,Stanley Letovsky,Michael Levandoski,Craig Lukasik,Jonathan Meltzer,Brian Norvell,Mindy Nye,Scott Parker,Christos Petropoulos,John Pruitt,Steven Ragan,Scott Ryan,Mike Sapeta,Jana Schroth,Suresh Babu Selvaraju,Goran Stevovic,Amanda Suchanek,Andrea Throop,Lyndon Tilson,Thomas Urban,Joe Voshell,Kimberly Wagner,Jonathan Williams,Mary Williamson,Qian Zeng,Tricia Zwiefelhofer,Clinton R. Paden,Suxiang Tong,Duncan MacCannell,
EPI_ISL_887105	Institute for Urban Disease Control and Prevention	COVID-19 Network Investigations (CONI) Alliance	Kamolthip Atsawawaranunt, Elizabeth Batty, Wasun Chantratita, Thanat Chookajorn, Stefan Fernandez, Angkana Huang, Anthony R. Jones, Khajohn Joonsalak, Chonticha Klungtong, Theerarat Kochakarn, Prayuth Kaewmalang, Amornmas Kongklieng, Namfon Kotanan, Krittikorn Kumpornsin, Duangkamon Loesbanluechai, Wudtichai Manasatienkij, Anek Mungaomklang, Bhakbhoom Panthan, Pukkaporn Parnwijitkul, Ekawat Pasomsub, Vichan Pawun, Kingkan Rakmanee, Insee Sensorn, Janjira Thaipadungpanit, Arporn Wangwiwatsin, Treewat Watthanachockchai
EPI_ISL_887117, EPI_ISL_887123, EPI_ISL_887129, EPI_ISL_887134	Institute of Medical Microbiology and Hospital Hygiene	Institute of Medical Microbiology and Hospital Hygiene	Prof. Dr. Achim Kaasch, Aljoscha Tersteegen
EPI_ISL_887419, EPI_ISL_887422, EPI_ISL_887423, EPI_ISL_887427, EPI_ISL_887430, EPI_ISL_887432, EPI_ISL_887433, EPI_ISL_887448, EPI_ISL_887449, EPI_ISL_887450, EPI_ISL_887451, EPI_ISL_887452, EPI_ISL_887453, EPI_ISL_887454, EPI_ISL_887504, EPI_ISL_887505			
see above	Instituto Nacional de Saude (INS), Mozambique	KRISP, KZN Research Innovation and Sequencing Platform	Nalia Ismael, Nadia Sitoe, Paulo Arnaldo, Nedio Mabunda, Giandhari J, Pillay S, Tegally H, Wilkinson E, de Oliveira T
EPI_ISL_887514, EPI_ISL_887515, EPI_ISL_887519, EPI_ISL_887520, EPI_ISL_887521, EPI_ISL_887525, EPI_ISL_887526, EPI_ISL_887527, EPI_ISL_887537, EPI_ISL_887561, EPI_ISL_887562, EPI_ISL_887564, EPI_ISL_887565, EPI_ISL_887566, EPI_ISL_887584, EPI_ISL_887585, EPI_ISL_887586			
see above	Johns Hopkins Hospital Department of Pathology	Johns Hopkins Hospital Department of Pathology	C. Paul Morris, Chun Huai Luo, Adannaya Amadi, Matthew Schwartz, Nicholas Gallagher, Heba H. Mostafa
EPI_ISL_887606, EPI_ISL_887723, EPI_ISL_887965, EPI_ISL_888062	Labcorp	Genomics and Discovery, Respiratory Viruses Branch, Division of Viral Diseases, Centers for Disease Control and Prevention	Peter W. Cook,Dhwani Batra,Ben L. Rambo-Martin,Summer Galloway,Brian Krueger,Minoo Agarwal,Eyad Almasri,Debbie Boles,Ayla Burns,Nuthawin Charoensri,Oren Cohen,Susan Countryman,Mary Ann Cristobal,Bobbi Croy,Suzanne Dale,Hrushikesh Deshmukh,Amanda Douglas,Vincent Drouillon,Marcia Eisenberg,Howard Engler,Rama Ghatti,Prashant Gupta,Susan Hicks,Jake Humphrey,Lax Iyer,Manoj Jain,Mohan Kolli,Tim Kuphal,Stanley Letovsky,Michael Levandoski,Craig Lukasik,Jonathan Meltzer,Brian Norvell,Mindy Nye,Scott Parker,Christos Petropoulos,John Pruitt,Steven Ragan,Scott Ryan,Mike Sapeta,Jana Schroth,Suresh Babu Selvaraju,Goran Stevovic,Amanda Suchanek,Andrea Throop,Lyndon Tilson,Thomas Urban,Joe Voshell,Kimberly Wagner,Jonathan Williams,Mary Williamson,Qian Zeng,Tricia Zwiefelhofer,Clinton R. Paden,Suxiang Tong,Duncan MacCannell,
EPI_ISL_888628, EPI_ISL_888629, EPI_ISL_888630, EPI_ISL_888631, EPI_ISL_888632, EPI_ISL_888633, EPI_ISL_888634, EPI_ISL_888635, EPI_ISL_888636, EPI_ISL_888637, EPI_ISL_888638, EPI_ISL_888639, EPI_ISL_888640, EPI_ISL_888641, EPI_ISL_888642, EPI_ISL_888643, EPI_ISL_888649, EPI_ISL_888650			
see above	Univeristy of New Mexico Hospital	Center for Global Health, University of New Mexico Health Sciences Center	Daryl Domman, Kurt Schwalm, Justin Bacca, Jon Femling, Darrell Dinwiddie
EPI_ISL_888854, EPI_ISL_888870, EPI_ISL_888871, EPI_ISL_888879, EPI_ISL_888880, EPI_ISL_888894, EPI_ISL_888895	Michigan Department of Health and Human Services, Bureau of Laboratories	Michigan Department of Health and Human Services, Bureau of Laboratories	Blankenship HM, Riner D, Soehnlen MK
EPI_ISL_888979	RS Bhakti Mulia	Eijkman Institute for Molecular Biology, Ministry of Research and Technology/National Agency for Research and Innovation	Willy Agustine, Edison Johar, Hidayat Trimarsanto, Iskandar Adnan, Lydia V. Panggalo, Sukma Oktavianthi, Frilasita A Yudhaputri, Safarina G Malik, Khin Saw Myint, Amin Soebandrio
EPI_ISL_889000	RSUD Cileungsi	Eijkman Institute for Molecular Biology, Ministry of Research and Technology/National Agency for Research and Innovation	Sukma Oktavianthi, Willy Agustine, Edison Johar, Hidayat Trimarsanto, Iskandar Adnan, Lydia V. Panggalo, Frilasita A Yudhaputri, Safarina G Malik, Khin Saw Myint, Amin Soebandrio
EPI_ISL_889001	RSU Azra	Eijkman Institute for Molecular Biology, Ministry of Research and Technology/National Agency for Research and Innovation	Sukma Oktavianthi, Willy Agustine, Edison Johar, Hidayat Trimarsanto, Iskandar Adnan, Lydia V. Panggalo, Frilasita A Yudhaputri, Safarina G Malik, Khin Saw Myint, Amin Soebandrio
EPI_ISL_889002	RSU Sumber Waras	Eijkman Institute for Molecular Biology, Ministry of Research and Technology/National Agency for Research and Innovation	Sukma Oktavianthi, Willy Agustine, Edison Johar, Hidayat Trimarsanto, Iskandar Adnan, Lydia V. Panggalo, Frilasita A Yudhaputri, Safarina G Malik, Khin Saw Myint, Amin Soebandrio
EPI_ISL_889607, EPI_ISL_889608, EPI_ISL_889609, EPI_ISL_889610, EPI_ISL_889611, EPI_ISL_889612, EPI_ISL_889613, EPI_ISL_889614, EPI_ISL_889615, EPI_ISL_889616, EPI_ISL_889617, EPI_ISL_889618, EPI_ISL_889619, EPI_ISL_889620, EPI_ISL_889621, EPI_ISL_889622, EPI_ISL_889623, EPI_ISL_889624, EPI_ISL_889625, EPI_ISL_889626, EPI_ISL_889627, EPI_ISL_889628, EPI_ISL_889629, EPI_ISL_889630, EPI_ISL_889631, EPI_ISL_889632, EPI_ISL_889633, EPI_ISL_889634, EPI_ISL_889635, EPI_ISL_889636, EPI_ISL_889637, EPI_ISL_889638, EPI_ISL_889639, EPI_ISL_889640, EPI_ISL_889641, EPI_ISL_889642, EPI_ISL_889643, EPI_ISL_889644, EPI_ISL_889645, EPI_ISL_889646, EPI_ISL_889647, EPI_ISL_889648, EPI_ISL_889649, EPI_ISL_889650, EPI_ISL_889651, EPI_ISL_889652, EPI_ISL_889653, EPI_ISL_889654, EPI_ISL_889655, EPI_ISL_889656, EPI_ISL_889657, EPI_ISL_889658, EPI_ISL_889659, EPI_ISL_889660, EPI_ISL_889661, EPI_ISL_889662, EPI_ISL_889663, EPI_ISL_889664, EPI_ISL_889665, EPI_ISL_889666, EPI_ISL_889667, EPI_ISL_889668, EPI_ISL_889669, EPI_ISL_889670			
see above	LSUHS Emerging Viral Threat Laboratory	Microbial Genome Sequencing Center	Jeremy P. Kamil, Jennifer L. Carroll, Camille F. Abshire, Maarten Van Diest, Mohammed N.A. Siddiquey, Andrew D. Yurochko, Martin J. Sapp, Rona S. Scott, Christopher G. Kevil, Daniel J. Snyder, Vaughn S. Cooper, John A. Vanchiere

EPI_ISL_890235	Respiratory Viruses Branch, Centers for Disease Control and Prevention	Centers for Disease Control and Prevention	Tao,Y., Li,Y., Zhang,J., Queen,K., Uehara,A., Cook,P., Paden,C.R., Wang,H. and Tong,S.
EPI_ISL_890237	Virology, International Centre for Diarrhoeal Disease Research, Bangladesh (ICDDR,B)	International Centre for Diarrhoeal Disease Research (ICDDR,B)	Hossain,M.E., Rahman,M.M., Sumiya,M.K., Alam,M.S., Karim,M.Y.,Hoque,A.F., Rahman,M.Z. and Rahman,M.
EPI_ISL_890282, EPI_ISL_890286, EPI_ISL_890290, EPI_ISL_890293, EPI_ISL_890296, EPI_ISL_890300, EPI_ISL_890308, EPI_ISL_890311, EPI_ISL_890313, EPI_ISL_890314, EPI_ISL_890316, EPI_ISL_890317, EPI_ISL_890319, EPI_ISL_890321, EPI_ISL_890323, EPI_ISL_890326, EPI_ISL_890330, EPI_ISL_890334, EPI_ISL_890338, EPI_ISL_890347, EPI_ISL_890351			
see above	KU Leuven, Rega Institute, Clinical and Epidemiological Virology	KU Leuven, Rega Institute, Clinical and Epidemiological Virology	Tony Wawina-Bokalanga, Bert Vanmechelen, Joan Marti-Carerras, Piet Maes
EPI_ISL_890354	Labo Analyses Med	National Reference Center for Viruses of Respiratory Infections, Institut Pasteur, Paris	Marion Barbet, Sylvie Behillil, Méline Bizard, Angela Brisebarre, Camille Capel, Etienne Simon-Lorière, Vincent Enouf, Maud Vanpeene, Sylvie van der Werf,Coignard Catherine
EPI_ISL_890359	Hospital	National Reference Center for Viruses of Respiratory Infections, Institut Pasteur, Paris	Marion Barbet, Sylvie Behillil, Méline Bizard, Angela Brisebarre, Camille Capel, Etienne Simon-Lorière, Vincent Enouf, Maud Vanpeene, Sylvie van der Werf,Guigon AuréLie
EPI_ISL_890363, EPI_ISL_890364	LSUHS Emerging Viral Threat Laboratory	Microbial Genome Sequencing Center	Jeremy P. Kamil, Jennifer L. Carroll, Camille F. Abshire, Maarten Van Diest, Mohammed N.A. Siddiquey, Andrew D. Yurochko, Martin J. Sapp, Rona S. Scott, Christopher G. Kevil, Daniel J. Snyder, Vaughn S. Cooper, John A. Vanchiere
EPI_ISL_891139, EPI_ISL_891140	The Jackson Laboratory	The Jackson Laboratory	Lloyd M, Sanderson B, Srivastava A, Maurya R, Renzette N, Omerza G, Kelly K, Li L, Wei C L, Adams M
EPI_ISL_891200, EPI_ISL_891202, EPI_ISL_891203	DPH, Massachusetts State Public Health Lab	DPH, Massachusetts State Public Health Lab	Lang,A.S., Fink,T., Gallagher,G.R., Smole,S.C.
EPI_ISL_891212	Population Medicine and Diagnostic Sciences, Cornell University	Population Medicine and Diagnostic Sciences, Cornell University	Cardia Caserta,L., Mitchel,P.K., Plachorzyk,E. and Diel,D.G.
EPI_ISL_892207, EPI_ISL_892208, EPI_ISL_892209	Lighthouse Lab in Alderley Park	Wellcome Sanger Institute for the COVID-19 Genomics UK (COG-UK) Consortium	Jacquelyn Wynn, Mairead Hyland, The Lighthouse Lab in Alderley Park and Alex Alderton, Roberto Amato, Sonia Goncalves, Ewan Harrison, David K. Jackson, Ian Johnston, Dominic Kwiatkowski, Cordelia Langford, John Sillitoe on behalf of the Wellcome Sanger Institute COVID-19 Surveillance Team
EPI_ISL_892210	Lighthouse Lab in Glasgow	Wellcome Sanger Institute for the COVID-19 Genomics UK (COG-UK) Consortium	Harper VanSteenhouse, Yumi Kasai, David Gray, Carol Clugston, Anna Dominiczak and Alex Alderton, Roberto Amato, Sonia Goncalves, Ewan Harrison, David K. Jackson, Ian Johnston, Dominic Kwiatkowski, Cordelia Langford, John Sillitoe on behalf of the Wellcome Sanger Institute COVID-19 Surveillance Team
EPI_ISL_892266	MD Laboratories	Los Angeles County PHL	P. Hemarajata et al.
EPI_ISL_892267, EPI_ISL_892268	UCLA Clinical Micro Lab	Los Angeles County PHL	P. Hemarajata et al.
EPI_ISL_893750, EPI_ISL_893752, EPI_ISL_893755, EPI_ISL_893757, EPI_ISL_893759, EPI_ISL_893761, EPI_ISL_893778	Institute of Virology, Medical Center, University of Freiburg, Freiburg, Germany	Institute of Virology, Clinial Virus Genomics, Medical Center, University of Freiburg, Freiburg, Germany	Jonas Fuchs, Lisa Kern, Sandra Reuter, Hajo Grundmann, Marcus Panning
EPI_ISL_894176, EPI_ISL_894178, EPI_ISL_894179, EPI_ISL_894180, EPI_ISL_894181, EPI_ISL_894185, EPI_ISL_894186, EPI_ISL_894192, EPI_ISL_894193, EPI_ISL_894196, EPI_ISL_894197, EPI_ISL_894209, EPI_ISL_894211, EPI_ISL_894212, EPI_ISL_894213, EPI_ISL_894216			
see above	KU Leuven, Rega Institute, Clinical and Epidemiological Virology	KU Leuven, Rega Institute, Clinical and Epidemiological Virology	Tony Wawina-Bokalanga, Bert Vanmechelen, Joan Marti-Carerras, Piet Maes
EPI_ISL_894219	Hopital	National Reference Center for Viruses of Respiratory Infections, Institut Pasteur, Paris	Marion Barbet, Sylvie Behillil, Méline Bizard, Angela Brisebarre, Camille Capel, Etienne Simon-Lorière, Vincent Enouf, Maud Vanpeene, Sylvie van der Werf,Guigon AuréLie
EPI_ISL_894223, EPI_ISL_894232, EPI_ISL_894244, EPI_ISL_894245	Labo Analyses med	National Reference Center for Viruses of Respiratory Infections, Institut Pasteur, Paris	Marion Barbet, Sylvie Behillil, Méline Bizard, Angela Brisebarre, Camille Capel, Etienne Simon-Lorière, Vincent Enouf, Maud Vanpeene, Sylvie van der Werf,Amzalag Jonas
EPI_ISL_894246, EPI_ISL_894247	Ministry of Health Turkey	Ministry of Health Turkey	Fatma Bayrakdar, Yasemin Cogun, Süleyman Yalcin, Aye Baak Alta, Gülay Korukluolu
EPI_ISL_896071, EPI_ISL_896084	Labormedizinisches Zentrum Dr Risch	University Hospital Basel, Clinical Bacteriology	Tim Roloff, Madlen Stange, Helena MB Seth-Smith, Alfredo Mari, Karoline Leuzinger, Julia Bielicki, Nadia Wohlwend,Martin Risch, Lorenz Risch, Manuel Battegay, Hans Hirsch, Adrian Egli
EPI_ISL_896089	Rothen Medizinische Laboratorien AG	University Hospital Basel, Clinical Bacteriology	Tim Roloff, Madlen Stange, Helena MB Seth-Smith, Alfredo Mari, Karoline Leuzinger, Julia Bielicki, Ingrid Steffen, Manuel Battegay, Hans Hirsch, Adrian Egli
EPI_ISL_896090	Labormedizinisches Zentrum Dr Risch	University Hospital Basel, Clinical Bacteriology	Tim Roloff, Madlen Stange, Helena MB Seth-Smith, Alfredo Mari, Karoline Leuzinger, Julia Bielicki, Nadia Wohlwend,Martin Risch, Lorenz Risch, Manuel Battegay, Hans Hirsch, Adrian Egli
EPI_ISL_896091	Kantonsspital Aarau, Institut für Labormedizin	University Hospital Basel, Clinical Bacteriology	Tim Roloff, Madlen Stange, Helena MB Seth-Smith, Alfredo Mari, Karoline Leuzinger, Julia Bielicki, Michael Oberle, Manuel Battegay, Hans Hirsch, Adrian Egli
EPI_ISL_896112	Labormedizinisches Zentrum Dr Risch	University Hospital Basel, Clinical Bacteriology	Tim Roloff, Madlen Stange, Helena MB Seth-Smith, Alfredo Mari, Karoline Leuzinger, Julia Bielicki, Nadia Wohlwend,Martin Risch, Lorenz Risch, Manuel Battegay, Hans Hirsch, Adrian Egli
EPI_ISL_896113	Viollier AG	University Hospital Basel, Clinical Bacteriology	Tim Roloff, Madlen Stange, Helena MB Seth-Smith, Alfredo Mari, Karoline Leuzinger, Julia Bielicki, Christiane Beckmann, Manuel Battegay, Hans Hirsch, Adrian Egli
EPI_ISL_896210	MEPHI, Aix Marseille University	MEPHI, Aix Marseille University	Anthony LEVASSEUR
EPI_ISL_896296	New York Presbyterian Hospital	Wadsworth Center, New York State Department of Health	Kirsten St. George, Daryl M. Lamson, Alexis Russel, Matthew Shudt, Melissa A Leisner, Jonathan Plitnick, Navjot Singh, John Kelly, Erasmus Schneider, Erica Lasek-Nesselquist
EPI_ISL_896297	BIO-REFERENCE LABORATORIES	Wadsworth Center, New York State Department of Health	Kirsten St. George, Daryl M. Lamson, Alexis Russel, Matthew Shudt, Melissa A Leisner, Jonathan Plitnick, Navjot Singh, John Kelly, Erasmus Schneider, Erica Lasek-Nesselquist
EPI_ISL_896302, EPI_ISL_896303, EPI_ISL_896305, EPI_ISL_896306, EPI_ISL_896366, EPI_ISL_896367, EPI_ISL_896368, EPI_ISL_896369, EPI_ISL_896370, EPI_ISL_896371, EPI_ISL_896372			
see above	New York Presbyterian Hospital	Wadsworth Center, New York State Department of Health	Kirsten St. George, Daryl M. Lamson, Alexis Russel, Matthew Shudt, Melissa A Leisner, Jonathan Plitnick, Navjot Singh, John Kelly, Erasmus Schneider, Erica Lasek-Nesselquist
EPI_ISL_896487	KU Leuven, Rega Institute, Clinical and Epidemiological Virology	KU Leuven, Rega Institute, Clinical and Epidemiological Virology	Tony Wawina-Bokalanga, Bert Vanmechelen, Joan Marti-Carerras, Piet Maes
EPI_ISL_896504, EPI_ISL_896505, EPI_ISL_896506, EPI_ISL_896507, EPI_ISL_896525, EPI_ISL_896526, EPI_ISL_896527, EPI_ISL_896528, EPI_ISL_896529, EPI_ISL_896531, EPI_ISL_896532, EPI_ISL_896534, EPI_ISL_896537, EPI_ISL_896538, EPI_ISL_896539, EPI_ISL_896540, EPI_ISL_896549, EPI_ISL_896550, EPI_ISL_896551, EPI_ISL_896553, EPI_ISL_896554, EPI_ISL_896556, EPI_ISL_896557, EPI_ISL_896558, EPI_ISL_896559, EPI_ISL_896560, EPI_ISL_896561			
see above	New York Presbyterian Hospital	Wadsworth Center, New York State Department of Health	Kirsten St. George, Daryl M. Lamson, Alexis Russel, Matthew Shudt, Melissa A Leisner, Jonathan Plitnick, Navjot Singh, John Kelly, Erasmus Schneider, Erica Lasek-Nesselquist
EPI_ISL_897987, EPI_ISL_898008, EPI_ISL_898029	KU Leuven, Rega Institute, Clinical and Epidemiological Virology	KU Leuven, Rega Institute, Clinical and Epidemiological Virology	Tony Wawina-Bokalanga, Bert Vanmechelen, Joan Marti-Carerras, Piet Maes
EPI_ISL_899063, EPI_ISL_899065, EPI_ISL_899097, EPI_ISL_899102, EPI_ISL_899136, EPI_ISL_899139, EPI_ISL_899147, EPI_ISL_899148, EPI_ISL_899149, EPI_ISL_899150, EPI_ISL_899157, EPI_ISL_899163, EPI_ISL_899178, EPI_ISL_899180, EPI_ISL_899181, EPI_ISL_899236, EPI_ISL_899238, EPI_ISL_899239, EPI_ISL_899240, EPI_ISL_899325, EPI_ISL_899326, EPI_ISL_899327, EPI_ISL_899328, EPI_ISL_899329, EPI_ISL_899330, EPI_ISL_899331, EPI_ISL_899332, EPI_ISL_899333, EPI_ISL_899334, EPI_ISL_899335, EPI_ISL_899336, EPI_ISL_899337, EPI_ISL_899338, EPI_ISL_899339, EPI_ISL_899442, EPI_ISL_899443, EPI_ISL_899444, EPI_ISL_899445, EPI_ISL_899446, EPI_ISL_899447, EPI_ISL_899448, EPI_ISL_899449, EPI_ISL_899450, EPI_ISL_899451, EPI_ISL_899452, EPI_ISL_899453, EPI_ISL_899454, EPI_ISL_899455, EPI_ISL_899529, EPI_ISL_899539, EPI_ISL_899540, EPI_ISL_899541, EPI_ISL_899552, EPI_ISL_899553, EPI_ISL_899569, EPI_ISL_899570, EPI_ISL_899700, EPI_ISL_899701, EPI_ISL_899702, EPI_ISL_899703, EPI_ISL_899704, EPI_ISL_899705, EPI_ISL_899706, EPI_ISL_899707, EPI_ISL_899708, EPI_ISL_899709, EPI_ISL_899710, EPI_ISL_899712, EPI_ISL_899713, EPI_ISL_899714, EPI_ISL_899715, EPI_ISL_899716,			

EPI_ISL_899717, EPI_ISL_899718, EPI_ISL_899719, EPI_ISL_899720, EPI_ISL_899840, EPI_ISL_899841, EPI_ISL_899842, EPI_ISL_899884, EPI_ISL_899907, EPI_ISL_899908, EPI_ISL_899909, EPI_ISL_899910, EPI_ISL_899911, EPI_ISL_899988, EPI_ISL_899989, EPI_ISL_899990, EPI_ISL_899991, EPI_ISL_899992, EPI_ISL_899993, EPI_ISL_899994, EPI_ISL_899995, EPI_ISL_899996, EPI_ISL_899997, EPI_ISL_899998, EPI_ISL_899999, EPI_ISL_900000, EPI_ISL_900001, EPI_ISL_900002, EPI_ISL_900003, EPI_ISL_900004, EPI_ISL_900005, EPI_ISL_900006, EPI_ISL_900007			
see above	Viollier AG	Department of Biosystems Science and Engineering, ETH Zürich	Christian Beisel, Sarah Nadeau, Chaoran Chen, Ivan Topolsky, Philipp Jablonski, Lara Fuhrmann, David Dreifuss, Katharina Jahn, Tobias Schär, Ina Nissen, Natascha Santacroce, Elodie Burcklen, Christiane Beckmann, Maurice Redondo, Olivier Kobel, Christoph Noppen, Sophie Seidel, Noemie Santamaria de Souza, Niko Beerenwinkel, Tanja Stadler
EPI_ISL_900023	Viollier AG	Department of Biosystems Science and Engineering, ETH Zürich	Chaoran Chen, Sarah Nadeau, Ivan Topolsky, Emmanouil Dermitzakis, Keith Harshman, Ioannis Xenarios, Henri Pegeot, Lorenzo Cerutti, Deborah Penet, Philipp Jablonski, Lara Fuhrmann, David Dreifuss, Katharina Jahn, Christiane Beckmann, Maurice Redondo, Olivier Kobel, Christoph Noppen, Sophie Seidel, Noemie Santamaria de Souza, Niko Beerenwinkel, Tanja Stadler
EPI_ISL_900568	Synlab	CNR Virus des Infections Respiratoires - France SUD	Antonin Bal, Gregory Destras, Gwendolyne Burfin, Hadrien Règue, Quentin Semanas, Martine Valette, Bruno Lina, Sylvie Larrat, Laurence Josset
EPI_ISL_902736	Dirección Departamental de Salud de Pública Leticia	Instituto Nacional de Salud- Dirección de Investigación en Salud Pública, Universidad de los Andes- Applied genomics research group, Vicerrectoria de Investigación y Creación, Universidad de los Andes- Systems and Computing Engineering Department	Katherine Laiton-Donato, Diego A. Álvarez-Díaz, Carlos Franco-Muñoz, Mauricio Pacheco-Montealegre, Maria T. Herrera-Sepúlveda, Diego Andrés Prada, Jorge Duitama, Laura Natalia Gonzalez, Jorge Ivan Diaz, Silvia Restrepo-Restrepo, Magdalena Wiesner, Martha Lucia Ospina Martinez, Marcela Mercado-Reyes
EPI_ISL_902889, EPI_ISL_902890, EPI_ISL_902891, EPI_ISL_902892, EPI_ISL_902893	Department of Virology and Immunology, University of Helsinki and Helsinki University Hospital, Huslab Finland	Department of Virology, Faculty of Medicine, University of Helsinki, Helsinki, Finland	Teemu Smura, Ravi Kant, Phuoc Truong, Hussein Alburkat, Hannimari Kallio-Kokko, Jenni Virtanen, Maija Suvanto, Essi Korhonen, Sari Hannula, Harri Kangas, Hanna Liimatainen, Satu Kurkela, Hanna Jarva, Maija Lappalainen, Pekka Ellonen, Olli Vapalahti
EPI_ISL_903066, EPI_ISL_903067, EPI_ISL_903068, EPI_ISL_903069	Seattle Flu Study	Seattle Flu Study	Deborah A. Nickerson, Chris D. Frazar, Jover Lee, Benjamin Pelle, Erica Ryke, Matthew Richardson, Amanda Adler, Elisabeth Brandstetter, Peter D. Han, Kairsten Fay, Misja Ilcisin, Kirsten Lacombe, Thomas R. Sibley, Melissa Truong, Caitlin R. Wolf, Michael Boeckh, Janet A. Englund, Michael Famulare, Barry R. Lutz, Mark J. Rieder, Lea M. Starita, Matthew Thompson, Jay Shendure, Trevor Bedford, Helen Y. Chu
EPI_ISL_903073	Seattle Flu Study	Seattle Flu Study	Deborah A. Nickerson, Chris D. Frazar, Jover Lee, Benjamin Pelle, Erica Ryke, Matthew Richardson, Amanda Adler, Elisabeth Brandstetter, Peter D. Han, Kairsten Fay, Misja Ilcisin, Kirsten Lacombe, Thomas R. Sibley, Melissa Truong, Caitlin R. Wolf, Karen Cowgill, Stephanie Schrag, Jeff Duchin, Michael Boeckh, Janet A. Englund, Michael Famulare, Barry R. Lutz, Mark J. Rieder, Lea M. Starita, Matthew Thompson, Helen Y. Chu, Trevor Bedford, Jay Shendure
EPI_ISL_903074, EPI_ISL_903075, EPI_ISL_903076, EPI_ISL_903077	Seattle Flu Study	Seattle Flu Study	Deborah A. Nickerson, Chris D. Frazar, Jover Lee, Benjamin Pelle, Erica Ryke, Matthew Richardson, Amanda Adler, Elisabeth Brandstetter, Peter D. Han, Kairsten Fay, Misja Ilcisin, Kirsten Lacombe, Thomas R. Sibley, Melissa Truong, Caitlin R. Wolf, Michael Boeckh, Janet A. Englund, Michael Famulare, Barry R. Lutz, Mark J. Rieder, Lea M. Starita, Matthew Thompson, Jay Shendure, Trevor Bedford, Helen Y. Chu
EPI_ISL_903078, EPI_ISL_903079, EPI_ISL_903080, EPI_ISL_903104	Seattle Flu Study	Seattle Flu Study	Deborah A. Nickerson, Chris D. Frazar, Jover Lee, Benjamin Pelle, Erica Ryke, Matthew Richardson, Amanda Adler, Elisabeth Brandstetter, Peter D. Han, Kairsten Fay, Misja Ilcisin, Kirsten Lacombe, Thomas R. Sibley, Melissa Truong, Caitlin R. Wolf, Karen Cowgill, Stephanie Schrag, Jeff Duchin, Michael Boeckh, Janet A. Englund, Michael Famulare, Barry R. Lutz, Mark J. Rieder, Lea M. Starita, Matthew Thompson, Helen Y. Chu, Trevor Bedford, Jay Shendure
EPI_ISL_903151, EPI_ISL_903153, EPI_ISL_903154, EPI_ISL_903155, EPI_ISL_903156, EPI_ISL_903157, EPI_ISL_903158, EPI_ISL_903159, EPI_ISL_903160, EPI_ISL_903161, EPI_ISL_903162, EPI_ISL_903164, EPI_ISL_903170, EPI_ISL_903171, EPI_ISL_903172, EPI_ISL_903173, EPI_ISL_903174, EPI_ISL_903175, EPI_ISL_903176, EPI_ISL_903177, EPI_ISL_903178, EPI_ISL_903179			
see above	Washington State Department of Health	Seattle Flu Study	Deborah A. Nickerson, Chris D. Frazar, Jover Lee, Benjamin Pelle, Erica Ryke, Matthew Richardson, Amanda Adler, Elisabeth Brandstetter, Peter D. Han, Kairsten Fay, Misja Ilcisin, Kirsten Lacombe, Thomas R. Sibley, Melissa Truong, Caitlin R. Wolf, Romesh Gautom, Geoff Melly, Brian Hiatt, Philip Dykema, Scott Lindquist, Michael Boeckh, Janet A. Englund, Michael Famulare, Barry R. Lutz, Mark J. Rieder, Lea M. Starita, Matthew Thompson, Helen Y. Chu, Jay Shendure, Trevor Bedford
EPI_ISL_903185, EPI_ISL_903186, EPI_ISL_903187, EPI_ISL_903188, EPI_ISL_903189, EPI_ISL_903190, EPI_ISL_903191, EPI_ISL_903192, EPI_ISL_903193, EPI_ISL_903194, EPI_ISL_903195, EPI_ISL_903196, EPI_ISL_903197, EPI_ISL_903198, EPI_ISL_903199, EPI_ISL_903200, EPI_ISL_903201, EPI_ISL_903202, EPI_ISL_903203, EPI_ISL_903205			
see above	Seattle Flu Study	Seattle Flu Study	Deborah A. Nickerson, Chris D. Frazar, Jover Lee, Benjamin Pelle, Erica Ryke, Matthew Richardson, Amanda Adler, Elisabeth Brandstetter, Peter D. Han, Kairsten Fay, Misja Ilcisin, Kirsten Lacombe, Thomas R. Sibley, Melissa Truong, Caitlin R. Wolf, Karen Cowgill, Stephanie Schrag, Jeff Duchin, Michael Boeckh, Janet A. Englund, Michael Famulare, Barry R. Lutz, Mark J. Rieder, Lea M. Starita, Matthew Thompson, Helen Y. Chu, Trevor Bedford, Jay Shendure
EPI_ISL_903361, EPI_ISL_903362, EPI_ISL_903363	Arizona State University	Arizona State University	Emily A. Kaelin, LaRinda A. Holland, Peter T. Skidmore, Nicholas J. Mellor, Kristina Buss, Joy M. Blain, Valerie Harris, Joshua LaBaer, Vel Murugan, Efrem S. Lim
EPI_ISL_903394, EPI_ISL_903398, EPI_ISL_903400, EPI_ISL_903406, EPI_ISL_903417, EPI_ISL_903421, EPI_ISL_903423, EPI_ISL_903425, EPI_ISL_903437, EPI_ISL_903444, EPI_ISL_903459, EPI_ISL_903467, EPI_ISL_903473, EPI_ISL_903478, EPI_ISL_903483, EPI_ISL_903498, EPI_ISL_903504, EPI_ISL_903526, EPI_ISL_903527, EPI_ISL_903528, EPI_ISL_903529, EPI_ISL_903530, EPI_ISL_903531, EPI_ISL_903532, EPI_ISL_903533, EPI_ISL_903534, EPI_ISL_903535, EPI_ISL_903536, EPI_ISL_903537, EPI_ISL_903538, EPI_ISL_903539, EPI_ISL_903540, EPI_ISL_903541, EPI_ISL_903542, EPI_ISL_903543, EPI_ISL_903544, EPI_ISL_903545, EPI_ISL_903546, EPI_ISL_903547, EPI_ISL_903548, EPI_ISL_903549, EPI_ISL_903550, EPI_ISL_903551, EPI_ISL_903553, EPI_ISL_903558, EPI_ISL_903563, EPI_ISL_903564			
see above	Quest Diagnostics	Quest Diagnostics	Rosenthal,S.H., Gerasimova,A., Kagan,R.M., Anderson, B., Hua, M., Liu Y., Bernstein, L.E., Livingston, K.E., Perez, A., Shalhout, D.F., Shlyakhter, I.A., Owen, R., Tanpaiboon, P., Lacbawan, F.
EPI_ISL_904058, EPI_ISL_904059, EPI_ISL_904060, EPI_ISL_904061, EPI_ISL_904062, EPI_ISL_904063	New Mexico Department of Health Scientific Laboratory	New Mexico Department of Health Scientific Laboratory	Ellie Johnson, Anastacia Griego-Fisher, D'eldra Malone
EPI_ISL_904122	National Institute of Public Health - National Institute of Hygiene	National Institute of Public Health - National Institute of Hygiene	Wokowicz Tomasz, Zacharczuk Katarzyna
EPI_ISL_904125	Laboratorium Analityczne Szpital Specjalistyczny w Chorzowie	National Institute of Public Health - National Institute of Hygiene	Wokowicz Tomasz, Zacharczuk Katarzyna
EPI_ISL_904128, EPI_ISL_904130, EPI_ISL_904131, EPI_ISL_904132, EPI_ISL_904133, EPI_ISL_904134	Quest Diagnostics	Quest Diagnostics	Rosenthal,S.H., Gerasimova,A., Kagan,R.M., Anderson, B., Hua, M., Liu Y., Bernstein, L.E., Livingston, K.E., Perez, A., Shalhout, D.F., Shlyakhter, I.A., Owen, R., Tanpaiboon, P., Lacbawan, F.
EPI_ISL_904144, EPI_ISL_904161, EPI_ISL_904201, EPI_ISL_904202, EPI_ISL_904203, EPI_ISL_904219, EPI_ISL_904220, EPI_ISL_904237, EPI_ISL_904246, EPI_ISL_904271, EPI_ISL_904272, EPI_ISL_904305, EPI_ISL_904306, EPI_ISL_904307, EPI_ISL_904308, EPI_ISL_904309, EPI_ISL_904310, EPI_ISL_904318, EPI_ISL_904366, EPI_ISL_904367, EPI_ISL_904548, EPI_ISL_904549, EPI_ISL_904550, EPI_ISL_904551, EPI_ISL_904552, EPI_ISL_904553, EPI_ISL_904554, EPI_ISL_904555, EPI_ISL_904556, EPI_ISL_904557, EPI_ISL_904558, EPI_ISL_904559, EPI_ISL_904560, EPI_ISL_904561, EPI_ISL_904562, EPI_ISL_904563, EPI_ISL_904564			
see above	Dutch COVID-19 response team	Erasmus Medical Center	Bas Oude Munnink, Reina Sikkema, David Nieuwenhuijse, Irina Chestakova, Anne van der Linden, Marjan Boter, Emmanuelle Munger, Corine GeurtsvanKessel, Annemiek van der Eijk, Richard Molenkamp, Marion Koopmans, on behalf of the Dutch national COVID-19 response team.
EPI_ISL_904631, EPI_ISL_904634, EPI_ISL_904636, EPI_ISL_904637, EPI_ISL_904638, EPI_ISL_904640, EPI_ISL_904641, EPI_ISL_904642, EPI_ISL_904647, EPI_ISL_904650, EPI_ISL_904651, EPI_ISL_904652, EPI_ISL_904654, EPI_ISL_904656, EPI_ISL_904657			
see above	Servicio de Microbiología, Laboratori Clínic Metropolitana Nord. Hospital Universitari Germans Trias i Pujol. Institut d'Investigació en Ciències de la Salut Germans Trias i Pujol (IGTP)	IrsiCaixa - Can Ruti CovidSeq	Marc Noguera-Julian, Mariona Parera, Maria Casadellà, Pilar Armengol, Francesc Catala-Moll, Roger Paredes, Bonaventura Clotet Elisa Martró, Verónica Saludes, Anna Not, Ana Pérez, Montserrat Giménez, Ignacio Blanco, Cristina Casañ, Antoni E. Bordoy, Adrián Antuori
EPI_ISL_904747, EPI_ISL_904781, EPI_ISL_904892, EPI_ISL_904975, EPI_ISL_904983, EPI_ISL_904987, EPI_ISL_904989, EPI_ISL_905000, EPI_ISL_905003, EPI_ISL_905007, EPI_ISL_905029, EPI_ISL_905035, EPI_ISL_905036, EPI_ISL_905037, EPI_ISL_905038, EPI_ISL_905039, EPI_ISL_905040, EPI_ISL_905041, EPI_ISL_905042, EPI_ISL_905043, EPI_ISL_905044, EPI_ISL_905045, EPI_ISL_905046, EPI_ISL_905047, EPI_ISL_905072, EPI_ISL_905073, EPI_ISL_905074, EPI_ISL_905075, EPI_ISL_905107, EPI_ISL_905108, EPI_ISL_905109, EPI_ISL_905111, EPI_ISL_905120, EPI_ISL_905121, EPI_ISL_905175, EPI_ISL_905190, EPI_ISL_905202, EPI_ISL_905203, EPI_ISL_905263, EPI_ISL_905269, EPI_ISL_905270, EPI_ISL_905271, EPI_ISL_905272, EPI_ISL_905273, EPI_ISL_905277, EPI_ISL_905278, EPI_ISL_905279, EPI_ISL_905289, EPI_ISL_905290, EPI_ISL_905349, EPI_ISL_905363, EPI_ISL_905364, EPI_ISL_905365, EPI_ISL_905366, EPI_ISL_905367, EPI_ISL_905368, EPI_ISL_905369, EPI_ISL_905387, EPI_ISL_905389, EPI_ISL_905390, EPI_ISL_905392, EPI_ISL_905405, EPI_ISL_905406, EPI_ISL_905407, EPI_ISL_905408, EPI_ISL_905409, EPI_ISL_905410, EPI_ISL_905411, EPI_ISL_905412, EPI_ISL_905413, EPI_ISL_905415, EPI_ISL_905416, EPI_ISL_905417, EPI_ISL_905425, EPI_ISL_905426, EPI_ISL_905427, EPI_ISL_905450, EPI_ISL_905451, EPI_ISL_905452, EPI_ISL_905453, EPI_ISL_905454, EPI_ISL_905455, EPI_ISL_905456, EPI_ISL_905457, EPI_ISL_905458, EPI_ISL_905474, EPI_ISL_905475, EPI_ISL_905476, EPI_ISL_905477, EPI_ISL_905478,			

EPI_ISL_905479, EPI_ISL_905480, EPI_ISL_905482, EPI_ISL_905485, EPI_ISL_905511, EPI_ISL_905512, EPI_ISL_905517, EPI_ISL_905518, EPI_ISL_905523, EPI_ISL_905525, EPI_ISL_905526, EPI_ISL_905527, EPI_ISL_905528, EPI_ISL_905529, EPI_ISL_905530, EPI_ISL_905531, EPI_ISL_905545, EPI_ISL_905546, EPI_ISL_905547, EPI_ISL_905569, EPI_ISL_905570, EPI_ISL_905571, EPI_ISL_905572, EPI_ISL_905573, EPI_ISL_905574, EPI_ISL_905576, EPI_ISL_905582, EPI_ISL_905585, EPI_ISL_905587, EPI_ISL_905588, EPI_ISL_905597, EPI_ISL_905598, EPI_ISL_905599, EPI_ISL_905600, EPI_ISL_905601, EPI_ISL_905602, EPI_ISL_905609, EPI_ISL_905615, EPI_ISL_905616, EPI_ISL_905622, EPI_ISL_905629, EPI_ISL_905630, EPI_ISL_905631, EPI_ISL_905632, EPI_ISL_905633, EPI_ISL_905634, EPI_ISL_905635, EPI_ISL_905636, EPI_ISL_905643, EPI_ISL_905644, EPI_ISL_905645, EPI_ISL_905646, EPI_ISL_905664, EPI_ISL_905665, EPI_ISL_905666, EPI_ISL_905676, EPI_ISL_905681, EPI_ISL_905682, EPI_ISL_905683, EPI_ISL_905684, EPI_ISL_905697, EPI_ISL_905698, EPI_ISL_905699, EPI_ISL_905719, EPI_ISL_905720			
see above	Dutch COVID-19 response team	National Institute for Public Health and the Environment (RIVM)	Adam Meijer, Harry Vennema, Dirk Eggink, Jeroen Cremer, Sharon van den Brink, Bas van der Veer, AnneMarie van den Brandt, Florian Zwagemaker, Dennis Schmitz, Chantal Reusken, on behalf of the national COVID-19 response team
EPI_ISL_905732, EPI_ISL_905733, EPI_ISL_905734, EPI_ISL_905735, EPI_ISL_905736, EPI_ISL_905737, EPI_ISL_905738	National Institute of Public Health - National Institute of Hygiene	National Institute of Public Health - National Institute of Hygiene	Wokowicz Tomasz, Zacharczuk Katarzyna
EPI_ISL_905927, EPI_ISL_905928, EPI_ISL_905929, EPI_ISL_905930, EPI_ISL_905931, EPI_ISL_905932, EPI_ISL_905933, EPI_ISL_905934, EPI_ISL_905935, EPI_ISL_905936, EPI_ISL_905937, EPI_ISL_905938, EPI_ISL_905939, EPI_ISL_905940, EPI_ISL_905941, EPI_ISL_905942, EPI_ISL_905943, EPI_ISL_905944, EPI_ISL_905945, EPI_ISL_905946, EPI_ISL_905947, EPI_ISL_905948, EPI_ISL_905949, EPI_ISL_905950, EPI_ISL_905951, EPI_ISL_905952, EPI_ISL_905953, EPI_ISL_905954, EPI_ISL_905955, EPI_ISL_905956, EPI_ISL_905957, EPI_ISL_905958, EPI_ISL_905959, EPI_ISL_905960, EPI_ISL_905961, EPI_ISL_905962, EPI_ISL_905963, EPI_ISL_905964, EPI_ISL_905965, EPI_ISL_905966, EPI_ISL_905967, EPI_ISL_905968, EPI_ISL_905969, EPI_ISL_905970, EPI_ISL_905971, EPI_ISL_905972, EPI_ISL_905973, EPI_ISL_905974, EPI_ISL_905975, EPI_ISL_905976, EPI_ISL_905977, EPI_ISL_905978, EPI_ISL_905979, EPI_ISL_905980, EPI_ISL_905981, EPI_ISL_905982, EPI_ISL_905983, EPI_ISL_905984, EPI_ISL_905985, EPI_ISL_905986, EPI_ISL_905987, EPI_ISL_905988, EPI_ISL_905989, EPI_ISL_905990, EPI_ISL_905991, EPI_ISL_905992, EPI_ISL_905993, EPI_ISL_905994, EPI_ISL_905995, EPI_ISL_905996, EPI_ISL_905997, EPI_ISL_905998, EPI_ISL_905999, EPI_ISL_906000, EPI_ISL_906001, EPI_ISL_906002, EPI_ISL_906003, EPI_ISL_906004, EPI_ISL_906005, EPI_ISL_906006, EPI_ISL_906007, EPI_ISL_906008, EPI_ISL_906009, EPI_ISL_906010, EPI_ISL_906011, EPI_ISL_906012, EPI_ISL_906013, EPI_ISL_906014, EPI_ISL_906015, EPI_ISL_906016, EPI_ISL_906017, EPI_ISL_906018, EPI_ISL_906019, EPI_ISL_906020, EPI_ISL_906021, EPI_ISL_906022, EPI_ISL_906023, EPI_ISL_906024, EPI_ISL_906025, EPI_ISL_906026, EPI_ISL_906027, EPI_ISL_906028, EPI_ISL_906029, EPI_ISL_906030, EPI_ISL_906031, EPI_ISL_906032, EPI_ISL_906033, EPI_ISL_906034, EPI_ISL_906035, EPI_ISL_906036, EPI_ISL_906037, EPI_ISL_906038, EPI_ISL_906039, EPI_ISL_906040, EPI_ISL_906041, EPI_ISL_906042, EPI_ISL_906043, EPI_ISL_906044, EPI_ISL_906046			
see above	OHSU Lab Services Molecular Microbiology Lab	Oregon SARS-CoV-2 Genome Sequencing Center	Brendan L. O'Connell, Sally Grindstaff, Kayla Carter, Ruth V. Nichols, Alec J. Hirsch, Donna Hansel, Guang Fan, Xuan, Qin, Daniel N. Streblow, William B. Messer, Andrew C. Adey, Benjamin N. Bimber, Brian J. O'Roak
EPI_ISL_906086	Child Health Research Foundation	Child Health Research Foundation	Senjuti Saha, Syed Muktadir Al Sium, Sharmistha Goswami, Afroza Akter Tanni, Arif Mohammad Tanmoy, Roly Malaker, Md Hafizur Rahman, Samir K Saha
EPI_ISL_906145	Laboratorio de Salud Publica de Amazonas	Instituto Nacional de Salud- Dirección de Investigación en Salud Pública, Universidad de los Andes- Applied genomics research group, Vicerrectoria de Investigación y Creación, Universidad de los Andes- Systems and Computing Engineering Department	Katherine Laiton-Donato, Diego A. Álvarez-Díaz, Carlos Franco-Muñoz, Mauricio Pacheco-Montealegre, Héctor Alejandro Ruiz-Moreno, Maria T. Herrera-Sepúlveda, Diego Andrés Prada, Jhonnatan Reales-González, Sheryll Corchuelo, Julian Naizaque, Gerardo Santamaría Jorge Duitama, Laura Natalia Gonzalez, Jorge Ivan Diaz, Silvia Restrepo-Restrepo, Magdalena Wiesner, Martha Lucia Ospina Martinez, Marcela Mercado-Reyes
EPI_ISL_906147, EPI_ISL_906148, EPI_ISL_906150, EPI_ISL_906153, EPI_ISL_906158, EPI_ISL_906159, EPI_ISL_906161, EPI_ISL_906164, EPI_ISL_906167, EPI_ISL_906169, EPI_ISL_906172, EPI_ISL_906174, EPI_ISL_906176, EPI_ISL_906178, EPI_ISL_906181, EPI_ISL_906182, EPI_ISL_906185, EPI_ISL_906187			
see above	University of Wisconsin-Madison AIDS Vaccine Research Laboratories	University of Wisconsin-Madison AIDS Vaccine Research Laboratories	Gage Moreno, Katarina Braun, et al. AIDS Vaccine Research Laboratories
EPI_ISL_906277, EPI_ISL_906278	Nigeria Centre for Disease Control (NCDC)	African Centre of Excellence for Genomics of Infectious Diseases (ACEGID), Redeemer's University	Oluniyi P.E. et al
EPI_ISL_906315, EPI_ISL_906339, EPI_ISL_906342, EPI_ISL_906365, EPI_ISL_906369, EPI_ISL_906389, EPI_ISL_906394, EPI_ISL_906396, EPI_ISL_906403, EPI_ISL_906424, EPI_ISL_906425, EPI_ISL_906426, EPI_ISL_906427, EPI_ISL_906428, EPI_ISL_906429, EPI_ISL_906430, EPI_ISL_906431, EPI_ISL_906432, EPI_ISL_906434, EPI_ISL_906435, EPI_ISL_906437, EPI_ISL_906438, EPI_ISL_906439, EPI_ISL_906440, EPI_ISL_906441, EPI_ISL_906442, EPI_ISL_906443, EPI_ISL_906444, EPI_ISL_906445, EPI_ISL_906446, EPI_ISL_906447, EPI_ISL_906449, EPI_ISL_906450, EPI_ISL_906451, EPI_ISL_906452, EPI_ISL_906453, EPI_ISL_906459, EPI_ISL_906460, EPI_ISL_906464			
see above	Quest Diagnostics	Quest Diagnostics	Rosenthal,S.H., Gerasimova,A., Kagan,R.M., Anderson, B., Hua, M., Liu Y., Bernstein, L.E., Livingston, K.E., Perez, A., Shalhout, D.F., Shlyakhter, I.A., Owen, R., Tanpaiboon, P., Lacbawan, F.
EPI_ISL_906529	Direccion de partamental de salud del Amazonas	Instituto Nacional de Salud- Dirección de Investigación en Salud Pública, Universidad de los Andes- Applied genomics research group, Vicerrectoria de Investigación y Creación, Universidad de los Andes- Systems and Computing Engineering Department	Katherine Laiton-Donato, Diego A. Álvarez-Díaz, Carlos Franco-Muñoz, Mauricio Pacheco-Montealegre, Héctor Alejandro Ruiz-Moreno, Maria T. Herrera-Sepúlveda, Diego Andrés Prada, Jhonnatan Reales-González, Sheryll Corchuelo, Julian Naizaque, Gerardo Santamaría Jorge Duitama, Laura Natalia Gonzalez, Jorge Ivan Diaz, Silvia Restrepo-Restrepo, Magdalena Wiesner, Martha Lucia Ospina Martinez, Marcela Mercado-Reyes
EPI_ISL_906535, EPI_ISL_906537	HOSPITAL DEPARTAMENTAL DE VILLAVICENCIO E.S.E.	Instituto Nacional de Salud- Dirección de Investigación en Salud Pública, Universidad de los Andes- Applied genomics research group, Vicerrectoria de Investigación y Creación, Universidad de los Andes- Systems and Computing Engineering Department	Katherine Laiton-Donato, Diego A. Álvarez-Díaz, Carlos Franco-Muñoz, Mauricio Pacheco-Montealegre, Héctor Alejandro Ruiz-Moreno, Maria T. Herrera-Sepúlveda, Diego Andrés Prada, Jhonnatan Reales-González, Sheryll Corchuelo, Julian Naizaque, Gerardo Santamaría Jorge Duitama, Laura Natalia Gonzalez, Jorge Ivan Diaz, Silvia Restrepo-Restrepo, Magdalena Wiesner, Martha Lucia Ospina Martinez, Marcela Mercado-Reyes
EPI_ISL_906550, EPI_ISL_906551	DIRECCION DEPARTAMENTAL DE SALUD DE AMAZONAS	Instituto Nacional de Salud- Dirección de Investigación en Salud Pública, Universidad de los Andes- Applied genomics research group, Vicerrectoria de Investigación y Creación, Universidad de los Andes- Systems and Computing Engineering Department	Katherine Laiton-Donato, Diego A. Álvarez-Díaz, Carlos Franco-Muñoz, Mauricio Pacheco-Montealegre, Héctor Alejandro Ruiz-Moreno, Maria T. Herrera-Sepúlveda, Diego Andrés Prada, Jhonnatan Reales-González, Sheryll Corchuelo, Julian Naizaque, Gerardo Santamaría Jorge Duitama, Laura Natalia Gonzalez, Jorge Ivan Diaz, Silvia Restrepo-Restrepo, Magdalena Wiesner, Martha Lucia Ospina Martinez, Marcela Mercado-Reyes
EPI_ISL_906716, EPI_ISL_906718, EPI_ISL_906719, EPI_ISL_906722, EPI_ISL_906732, EPI_ISL_906734, EPI_ISL_906737, EPI_ISL_906739, EPI_ISL_906741, EPI_ISL_906742, EPI_ISL_906745, EPI_ISL_906747, EPI_ISL_906750, EPI_ISL_906751, EPI_ISL_906753, EPI_ISL_906754			
see above	Hematology Laboratory, Section of Molecular Diagnostics, University Clinical Centre, Medical University of Gdansk	Laboratory of Recombinant Vaccines	Lukasz Rabalski, Maciej Kosinski, Maciej Grzybek, Adam Sodol, Aneta Szulc, Krzysztof Lewandowski, Ewa Milosz, Marlena Robakowska, Boguslaw Szewczyk, Krystyna Bienkowska-Szewczyk
EPI_ISL_906798, EPI_ISL_906799, EPI_ISL_906801, EPI_ISL_906803	Nordland Hospital - Bodo, Laboratory Department, Molecular Biology Unit	Norwegian Institute of Public Health, Department of Virology	Kathrine Stene-Johansen, Kamilla Heddeland Instefjord, Hilde Elshaug, Atiya R Ali,Marie Paulsen Madsen, Rasmus Riis Kopperud, Hilde Vollan, Karoline Bragstad, Olav Hungnes
EPI_ISL_906822, EPI_ISL_906823	Department of Medical Microbiology - section Molde, Molde Hospital	Norwegian Institute of Public Health, Department of Virology	Kathrine Stene-Johansen, Kamilla Heddeland Instefjord, Hilde Elshaug, Atiya R Ali,Marie Paulsen Madsen, Rasmus Riis Kopperud, Hilde Vollan, Karoline Bragstad, Olav Hungnes
EPI_ISL_906826, EPI_ISL_906827, EPI_ISL_906828, EPI_ISL_906829	Akershus University Hospital, Department for Microbiology and Infectious Disease Control	Norwegian Institute of Public Health, Department of Virology	Kathrine Stene-Johansen, Kamilla Heddeland Instefjord, Hilde Elshaug, Atiya R Ali,Marie Paulsen Madsen, Rasmus Riis Kopperud, Hilde Vollan, Karoline Bragstad, Olav Hungnes
EPI_ISL_906884, EPI_ISL_906885, EPI_ISL_906886, EPI_ISL_906887, EPI_ISL_906888, EPI_ISL_906889, EPI_ISL_906890, EPI_ISL_906891, EPI_ISL_906892, EPI_ISL_906893, EPI_ISL_906894, EPI_ISL_906895, EPI_ISL_906896, EPI_ISL_906897, EPI_ISL_906898, EPI_ISL_906899, EPI_ISL_906900, EPI_ISL_906901, EPI_ISL_906902, EPI_ISL_906903, EPI_ISL_906904			
see above	Bureau of Public Health Laboratories, Florida Department of Health (BPHL, FLDOH)	Bureau of Public Health Laboratories, Florida Department of Health (BPHL, FLDOH)	Schmedes,S., Blanton,J.
EPI_ISL_906924, EPI_ISL_906930, EPI_ISL_906954, EPI_ISL_906964, EPI_ISL_906978, EPI_ISL_907064, EPI_ISL_907066, EPI_ISL_907068, EPI_ISL_907070, EPI_ISL_907074	Infectious Diseases, Quest Diagnostics	Infectious Diseases, Quest Diagnostics	Rosenthal,S.H., Gerasimova,A., Kagan,R.M., Anderson,B., Bernstein,L.E., Livingston,K.E., Hua,M., Liu,Y., Shalhout,D.F., Owen,R., Lacbawan,F.
EPI_ISL_909946, EPI_ISL_909947	CUSL/UCLouvain COVID testing federal platform	UCLouvain/IREC/MBLG	Jean Ruelle, Lysa Pinsmaye, Benoit Kabamba Mukadi
EPI_ISL_910029, EPI_ISL_910335	Laboratory for Respiratory Viruses, Cantacuzino National Military-Medical Institute for Research and Development	Cantacuzino Institute Virology	Luiza Ustea, Nicoleta Paraschiv, Mihaela Lazar
EPI_ISL_910902, EPI_ISL_910903, EPI_ISL_910904, EPI_ISL_910905, EPI_ISL_910906, EPI_ISL_910907, EPI_ISL_910908, EPI_ISL_910909, EPI_ISL_910910, EPI_ISL_910911, EPI_ISL_910912, EPI_ISL_910913, EPI_ISL_910914, EPI_ISL_910915, EPI_ISL_910916, EPI_ISL_910917, EPI_ISL_910918, EPI_ISL_910919, EPI_ISL_910920, EPI_ISL_910921, EPI_ISL_910922, EPI_ISL_910923, EPI_ISL_910924, EPI_ISL_910925, EPI_ISL_910926, EPI_ISL_910927, EPI_ISL_910928, EPI_ISL_910929, EPI_ISL_910930, EPI_ISL_910931, EPI_ISL_910932, EPI_ISL_910933, EPI_ISL_910934, EPI_ISL_910935, EPI_ISL_910936, EPI_ISL_910937, EPI_ISL_910938, EPI_ISL_910939, EPI_ISL_910940, EPI_ISL_910942			

see above	Laboratoire national de sante, Microbiology, Virology	Laboratoire national de sante, Microbiology, Microbial Genomics Platform	Anke Wienecke-Baldacchino, Catherine Ragimbeau,Jessica Tapp, Fatu Djabi, Lise Pignon, Raoul Salmon, Tamir Abdelrahman
EPI_ISL_911292, EPI_ISL_911302, EPI_ISL_911311, EPI_ISL_911321, EPI_ISL_911329, EPI_ISL_911346	Servicio de Microbiología, Hospital Universitario Son Espases	SeqCOVID-SPAIN consortium/IBV(CSIC)	Carla López-Causapé, Jordi Reina, Antonio Oliver and SeqCOVID-SPAIN consortium
EPI_ISL_911518, EPI_ISL_911519, EPI_ISL_911520, EPI_ISL_911521, EPI_ISL_911522, EPI_ISL_911523	Florida Bureau of Public Health Laboratories	Florida Bureau of Public Health Laboratories	Sarah Schmedes, Jason Blanton
EPI_ISL_911526, EPI_ISL_911527	Microbiology and Virology Unit, Florence Careggi University Hospital	Microbiology and Virology Unit, Florence Careggi University Hospital	Vincenzo Di Pilato, Marco Coppi, Fabio Morecchiato, Noemi Aiezza, Ilaria Baccani, Alberto Antonelli, Emanuele Gori, Gian Maria Rossolini
EPI_ISL_911635, EPI_ISL_911636, EPI_ISL_911637, EPI_ISL_911638, EPI_ISL_911641, EPI_ISL_911642, EPI_ISL_911643, EPI_ISL_911644, EPI_ISL_911645, EPI_ISL_911646, EPI_ISL_911647, EPI_ISL_911648, EPI_ISL_911649, EPI_ISL_911670, EPI_ISL_911671, EPI_ISL_911672, EPI_ISL_911673			
see above	Texas Department of State Health Services (TXDSHS)	Texas Department of State Health Services (TXDSHS)	Bonnie Oh, Anita Pokharel, James Daniel Bonser, Myong Koag, Chung Wang, Rachel Lee, Grace Kubin, Rashmi Tuladhar, Mayela Pedrueza, Maliha Rahman, Jenny Zhang
EPI_ISL_911789, EPI_ISL_911824, EPI_ISL_911825	Johns Hopkins Hospital Department of Pathology	Johns Hopkins Hospital Department of Pathology	C. Paul Morris, Chun Huai Luo, Adannaya Amadi, Matthew Schwartz, Nicholas Gallagher, Heba H. Mostafa
EPI_ISL_912035, EPI_ISL_912036, EPI_ISL_912037, EPI_ISL_912038, EPI_ISL_912039, EPI_ISL_912040, EPI_ISL_912041, EPI_ISL_912042, EPI_ISL_912043, EPI_ISL_912044, EPI_ISL_912045, EPI_ISL_912046			
see above	Washington State Department of Health	Seattle Flu Study	Deborah A. Nickerson, Chris D. Frazar, Jover Lee, Benjamin Pelle, Erica Ryke, Matthew Richardson, Amanda Adler, Elisabeth Brandstetter, Peter D. Han, Kairsten Fay, Misja Ilcisin, Kirsten Lacombe, Thomas R. Sibley, Melissa Truong, Caitlin R. Wolf, Romesh Gautom, Geoff Melly, Brian Hiatt, Philip Dykema, Scott Lindquist, Michael Boeckh, Janet A. Englund, Michael Famulare, Barry R. Lutz, Mark J. Rieder, Lea M. Starita, Matthew Thompson, Helen Y. Chu, Jay Shendure, Trevor Bedford
EPI_ISL_912162	Baptist Medical Center	Grubaugh Lab - Yale School of Public Health	Tara Alpert, Joseph Fauver, Anderson Brito, Mallery Breban, Anne Wyllie, Chantal Vogels, Mary Petrone, Annie Watkins, Chaney Kalinich, Isabel Ott, Nathan Grubaugh
EPI_ISL_912169	East Gene	Grubaugh Lab - Yale School of Public Health	Tara Alpert, Joseph Fauver, Anderson Brito, Mallery Breban, Anne Wyllie, Chantal Vogels, Mary Petrone, Annie Watkins, Chaney Kalinich, Isabel Ott, Nathan Grubaugh
EPI_ISL_912185	Connecticut Department of Health	Grubaugh Lab - Yale School of Public Health	Tara Alpert, Joseph Fauver, Anderson Brito, Mallery Breban, Anne Wyllie, Chantal Vogels, Mary Petrone, Annie Watkins, Chaney Kalinich, Isabel Ott, Nathan Grubaugh
EPI_ISL_912280	Hospital General Universitario Gregorio Marañón	SeqCOVID-SPAIN consortium / IBV (CSIC)	Darío García de Viedma, Laura Pérez-Lago, Pedro J Sola-Campoy, Sergio Buenestado-Serrano, Marta Herranz, Victor Manuel de la Cueva, Julia Suárez, Pilar Catalán, Patricia Muñoz and SeqCOVID-SPAIN consortium
EPI_ISL_912305, EPI_ISL_912309, EPI_ISL_912312	Hospital General Universitario Gregorio Marañón	SeqCOVID-SPAIN consortium / IBV (CSIC)	Sergio Buenestado Serrano, Pedro J. Sola Campoy, Laura Pérez-Lago, Pilar Catalán, Arturo fraile Torres, Andrés Von Wernitz, Carmen del Arco, Patricia Muñoz, Laura Cardeñoso, Darío García de Viedma and SeqCOVID-SPAIN consortium
EPI_ISL_912317, EPI_ISL_912319	Hospital General Universitario Gregorio Marañón	SeqCOVID-SPAIN consortium / IBV (CSIC)	Darío García de Viedma, Laura Pérez-Lago, Pedro J Sola-Campoy, Sergio Buenestado-Serrano, Marta Herranz, Victor Manuel de la Cueva, Julia Suárez, Pilar Catalán, Patricia Muñoz and SeqCOVID-SPAIN consortium
EPI_ISL_912332	Hospital General Universitario Gregorio Marañón	SeqCOVID-SPAIN consortium / IBV (CSIC)	Sergio Buenestado Serrano, Pedro J. Sola Campoy, Laura Pérez-Lago, Pilar Catalán, Arturo fraile Torres, Andrés Von Wernitz, Carmen del Arco, Patricia Muñoz, Laura Cardeñoso, Darío García de Viedma and SeqCOVID-SPAIN consortium
EPI_ISL_912333, EPI_ISL_912335	Hospital General Universitario Gregorio Marañón	SeqCOVID-SPAIN consortium / IBV (CSIC)	Darío García de Viedma, Laura Pérez-Lago, Pedro J Sola-Campoy, Sergio Buenestado-Serrano, Marta Herranz, Victor Manuel de la Cueva, Julia Suárez, Pilar Catalán, Patricia Muñoz and SeqCOVID-SPAIN consortium
EPI_ISL_912379, EPI_ISL_912380, EPI_ISL_912386, EPI_ISL_912387, EPI_ISL_912388, EPI_ISL_912389, EPI_ISL_912390	Fondation Congolaise pour la recherche medicale (FCRM), Francine Ntoumi	NGS Competence Center Tuebingen, Institut für Medizinische Mikrobiologie und Hygiene, Universitaetsklinikum Tübingen	Angel Angelov
EPI_ISL_912404, EPI_ISL_912420, EPI_ISL_912449, EPI_ISL_912450, EPI_ISL_912451, EPI_ISL_912452, EPI_ISL_912455	KU Leuven, Rega Institute, Clinical and Epidemiological Virology	KU Leuven, Rega Institute, Clinical and Epidemiological Virology	Tony Wawina-Bokalanga, Bert Vanmechelen, Joan Marti-Carerras, Piet Maes
EPI_ISL_912521, EPI_ISL_912524	NHLS Universitas Academic	UFS Virology	PA Bester, MM Nyaga, P Nthiga, MT Mogotsi, D Goedhals, T de Oliveira
EPI_ISL_912597	Labo Analyses Med	National Reference Center for Viruses of Respiratory Infections, Institut Pasteur, Paris	Marion Barbet, Sylvie Behillil, Méline Bizard, Angela Brisebarre, Camille Capel, Etienne Simon-Lorière, Vincent Enouf, Maud Vanpeene, Sylvie van der Werf,Le Vicky
EPI_ISL_912647, EPI_ISL_912652, EPI_ISL_912680, EPI_ISL_912688, EPI_ISL_912689, EPI_ISL_912693, EPI_ISL_912701, EPI_ISL_912705, EPI_ISL_912708, EPI_ISL_912714, EPI_ISL_912715, EPI_ISL_912725, EPI_ISL_912726, EPI_ISL_912735, EPI_ISL_912736, EPI_ISL_912737, EPI_ISL_912738, EPI_ISL_912739, EPI_ISL_912740, EPI_ISL_912741, EPI_ISL_912850			
see above	Hôpital Henri Mondor	Department of Virology, Henri Mondor University Hospital, Assistance Publique Hôpitaux de Paris, Université Paris-Est Créteil, INSERM U955	Christophe Rodriguez, Slim Fourati, Vanessa Demontant, Guillaume Gricourt, Melissa N'Debi, Alexandre Soulier, Elisabeth Trawinski, Jean-Michel Pawlotsky
EPI_ISL_912919, EPI_ISL_912920	Hôpital Pitié-Salpêtrière	Department of Virology, Henri Mondor University Hospital, Assistance Publique Hôpitaux de Paris, Université Paris-Est Créteil, INSERM U955	Christophe Rodriguez, Slim Fourati, Vanessa Demontant, Guillaume Gricourt, Melissa N'Debi, Alexandre Soulier, Elisabeth Trawinski, Jean-Michel Pawlotsky
EPI_ISL_913020	Hospital Universitario de Ceuta	Instituto de Salud Carlos III	Iglesias-Caballero, M. Camarero, S. Sandonís,V. Vázquez, S. Pozo, F. Casas, I. Jiménez, P. Zaballos, A. Monzón, S. Varona, S. Cuesta, I. López, J.
EPI_ISL_913038	Complejo Asistencial Universitario de Burgos	Instituto de Salud Carlos III	Iglesias-Caballero, M. Camarero, S. Sandonís,V. Vázquez, S. Pozo, F. Casas, I. Jiménez, P. Zaballos, A. Monzón, S. Varona, S. Cuesta, I. Megías, G.
EPI_ISL_913042, EPI_ISL_913043, EPI_ISL_913046	Hospital Comarcal Sierrallana	Instituto de Salud Carlos III	Iglesias-Caballero, M. Camarero, S. Sandonís,V. Vázquez, S. Pozo, F. Casas, I. Jiménez, P. Zaballos, A. Monzón, S. Varona, S. Cuesta, I. De Benito, I.
EPI_ISL_913054, EPI_ISL_913055, EPI_ISL_913056, EPI_ISL_913058	Hospital Universitario de Ceuta	Instituto de Salud Carlos III	Iglesias-Caballero, M. Camarero, S. Sandonís,V. Vázquez, S. Pozo, F. Casas, I. Jiménez, P. Zaballos, A. Monzón, S. Varona, S. Cuesta, I. López, J.
EPI_ISL_913077, EPI_ISL_913078	Center for Virology	Center for Virology	Jeremy V. Camp, Irene Goerzer, Monika Redlberger-Fritz, Stephan W. Aberle
EPI_ISL_913127, EPI_ISL_913134	University of Michigan Clinical Microbiology Laboratory	Lauring Lab, University of Michigan, Department of Microbiology and Immunology	Valesano
EPI_ISL_913259	Klinisk mikrobiologi	The Public Health Agency of Sweden	Anna-Malin Linde, Maria Lind Karlberg, Carlo Berg, Oskar Karlsson Lindsjo, Sofia Stamouli, Reza Advani, Mattias Haukland, Petra Holmstrom, Noura Walai, Petra Edquist, Mia Brytting, Anna Risberg, Karin Tegmark-Wisell
EPI_ISL_913263, EPI_ISL_913267	The Public Health Agency of Sweden	The Public Health Agency of Sweden	Anna-Malin Linde, Maria Lind Karlberg, Carlo Berg, Oskar Karlsson Lindsjo, Sofia Stamouli, Reza Advani, Mattias Haukland, Petra Holmstrom, Noura Walai, Petra Edquist, Mia Brytting, Anna Risberg, Karin Tegmark-Wisell
EPI_ISL_913268, EPI_ISL_913272, EPI_ISL_913300	Klinisk mikrobiologi	The Public Health Agency of Sweden	Anna-Malin Linde, Maria Lind Karlberg, Carlo Berg, Oskar Karlsson Lindsjo, Sofia Stamouli, Reza Advani, Mattias Haukland, Petra Holmstrom, Noura Walai, Petra Edquist, Mia Brytting, Anna Risberg, Karin Tegmark-Wisell
EPI_ISL_913370	Klinisk Mikrobiologi	The Public Health Agency of Sweden	Anna-Malin Linde, Maria Lind Karlberg, Carlo Berg, Oskar Karlsson Lindsjo, Sofia Stamouli, Reza Advani, Mattias Haukland, Petra Holmstrom, Noura

			Walai, Petra Edquist, Mia Brytting, Anna Risberg, Karin Tegmark-Wisell
EPI_ISL_913371, EPI_ISL_913372, EPI_ISL_913374	Klinisk mikrobiologi	The Public Health Agency of Sweden	Anna-Malin Linde, Maria Lind Karlberg, Carlo Berg, Oskar Karlsson Lindsjo, Sofia Stamouli, Reza Advani, Mattias Haukland, Petra Holmstrom, Noura Walai, Petra Edquist, Mia Brytting, Anna Risberg, Karin Tegmark-Wisell
EPI_ISL_913375, EPI_ISL_913376, EPI_ISL_913377	The Public Health Agency of Sweden	The Public Health Agency of Sweden	Anna-Malin Linde, Maria Lind Karlberg, Carlo Berg, Oskar Karlsson Lindsjo, Sofia Stamouli, Reza Advani, Mattias Haukland, Petra Holmstrom, Noura Walai, Petra Edquist, Mia Brytting, Anna Risberg, Karin Tegmark-Wisell
EPI_ISL_913399, EPI_ISL_913417, EPI_ISL_913430	Massachusetts State Public Health Laboratory	Massachusetts State Public Health Laboratory	Andrew Lang, Timelia Fink, Glen Gallagher, Sandra Smole
EPI_ISL_913451, EPI_ISL_913452	The Public Health Agency of Sweden	The Public Health Agency of Sweden	Anna-Malin Linde, Maria Lind Karlberg, Carlo Berg, Oskar Karlsson Lindsjo, Sofia Stamouli, Reza Advani, Mattias Haukland, Petra Holmstrom, Noura Walai, Petra Edquist, Mia Brytting, Anna Risberg, Karin Tegmark-Wisell
EPI_ISL_913466	Synlab Medilab, Mikrobiologi	The Public Health Agency of Sweden	Anna-Malin Linde, Maria Lind Karlberg, Carlo Berg, Oskar Karlsson Lindsjo, Sofia Stamouli, Reza Advani, Mattias Haukland, Petra Holmstrom, Noura Walai, Petra Edquist, Mia Brytting, Anna Risberg, Karin Tegmark-Wisell
EPI_ISL_913471, EPI_ISL_913493	Klinisk mikrobiologi	The Public Health Agency of Sweden	Anna-Malin Linde, Maria Lind Karlberg, Carlo Berg, Oskar Karlsson Lindsjo, Sofia Stamouli, Reza Advani, Mattias Haukland, Petra Holmstrom, Noura Walai, Petra Edquist, Mia Brytting, Anna Risberg, Karin Tegmark-Wisell
EPI_ISL_913567, EPI_ISL_913569, EPI_ISL_913570, EPI_ISL_913571, EPI_ISL_913572, EPI_ISL_913573, EPI_ISL_913574, EPI_ISL_913575, EPI_ISL_913576, EPI_ISL_913577, EPI_ISL_913578, EPI_ISL_913579, EPI_ISL_913580, EPI_ISL_913581, EPI_ISL_913582, EPI_ISL_913583, EPI_ISL_913585			
see above	M Health Fairview	Minnesota Department of Health, Public Health Laboratory	Alexandra Lorentz, Jacob Garfin, Matt Plumb, and Xiong Wang
EPI_ISL_913651, EPI_ISL_913652, EPI_ISL_913653, EPI_ISL_913654	Michigan Department of Health and Human Services, Bureau of Laboratories	Michigan Department of Health and Human Services, Bureau of Laboratories	Blankenship HM, Riner D, Soehnlen MK
EPI_ISL_913720, EPI_ISL_913721, EPI_ISL_913722, EPI_ISL_913723, EPI_ISL_913724, EPI_ISL_913725	Minnesota Department of Health, Public Health Laboratory	Minnesota Department of Health, Public Health Laboratory	Alexandra Lorentz, Jacob Garfin, Matt Plumb, and Xiong Wang
EPI_ISL_913727, EPI_ISL_913728, EPI_ISL_913729, EPI_ISL_913730, EPI_ISL_913731	M Health Fairview	Minnesota Department of Health, Public Health Laboratory	Alexandra Lorentz, Jacob Garfin, Matt Plumb, and Xiong Wang
EPI_ISL_913737, EPI_ISL_913738, EPI_ISL_913739	Minnesota Department of Health, Public Health Laboratory	Minnesota Department of Health, Public Health Laboratory	Alexandra Lorentz, Jacob Garfin, Matt Plumb, and Xiong Wang
EPI_ISL_913771, EPI_ISL_913774, EPI_ISL_913782, EPI_ISL_913788, EPI_ISL_913795, EPI_ISL_913798, EPI_ISL_913805, EPI_ISL_913815, EPI_ISL_913829, EPI_ISL_913836, EPI_ISL_913849, EPI_ISL_913852, EPI_ISL_913856, EPI_ISL_913873, EPI_ISL_913879, EPI_ISL_913881, EPI_ISL_913885, EPI_ISL_913886, EPI_ISL_913890, EPI_ISL_913894, EPI_ISL_913895, EPI_ISL_913896, EPI_ISL_913899, EPI_ISL_913900, EPI_ISL_913901, EPI_ISL_913907, EPI_ISL_913908			
see above	TGen North	TGen North	"Jolene Bowers, Megan Folkerts, Chris French, Hayley Yaglom, Ashlyn Pfeiffer, Darrin Lemmer, Dave Engelthaler, The Arizona COVID Genomics Union (ACGU)"
EPI_ISL_914000, EPI_ISL_914001	Quest Diagnostics Tucker GA	Pathogen Discovery, Respiratory Viruses Branch, Division of Viral Diseases, Centers for Disease Control and Prevention	Ying Tao, Yan Li, Jing Zhang, Krista Queen, Anna Uehara, Peter Cook, Clinton R. Paden, Haibin Wang, Suxiang Tong
EPI_ISL_914013, EPI_ISL_914014	IL Department of Public Health Chicago Laboratory	Pathogen Discovery, Respiratory Viruses Branch, Division of Viral Diseases, Centers for Disease Control and Prevention	Ying Tao, Yan Li, Jing Zhang, Krista Queen, Anna Uehara, Peter Cook, Clinton R. Paden, Haibin Wang, Suxiang Tong
EPI_ISL_914641, EPI_ISL_914645	Santa Clara County Public Health Laboratory	Santa Clara County Public Health Laboratory	Santa Clara County Public Health Department
EPI_ISL_914825	AREA DE SALUD MORAVIA	Inciensa, Instituto Costarricense de Investigación y Enseñanza en Nutrición y Salud	Francisco Duarte, Hebleen Porras, Claudio Soto-Garita, Estela Cordero, Adriana Godínez, Melany Calderón & Mariel López
EPI_ISL_914826	HLE - MANOS ABIERTAS	Inciensa, Instituto Costarricense de Investigación y Enseñanza en Nutrición y Salud	Francisco Duarte, Hebleen Porras, Claudio Soto-Garita, Estela Cordero, Adriana Godínez, Melany Calderón & Mariel López
EPI_ISL_914827	HLE - ASOCIACION DE ALBERGUE PARA ANCIANOS DE GOLFITO	Inciensa, Instituto Costarricense de Investigación y Enseñanza en Nutrición y Salud	Francisco Duarte, Hebleen Porras, Claudio Soto-Garita, Estela Cordero, Adriana Godínez, Melany Calderón & Mariel López
EPI_ISL_914830	AREA DE SALUD CARRILLO	Inciensa, Instituto Costarricense de Investigación y Enseñanza en Nutrición y Salud	Francisco Duarte, Hebleen Porras, Claudio Soto-Garita, Estela Cordero, Adriana Godínez, Melany Calderón & Adriana Bermúdez-Espinoza
EPI_ISL_914970, EPI_ISL_915192	MRCG at LSHTM Genomics lab	MRCG at LSHTM Genomics lab	Abdul Karim sesay, Abdoulie Kanteh, Jarra Manneh, Mariama Kujabi, Bakary Sanyang
EPI_ISL_915326, EPI_ISL_915333, EPI_ISL_915334, EPI_ISL_915341	Quest Diagnostics	Quest Diagnostics	Rosenthal,S.H., Gerasimova,A., Kagan,R.M., Anderson, B., Hua, M., Liu Y., Bernstein, L.E., Livingston, K.E., Perez, A., Shalhout, D.F., Shlyakhter, I.A., Owen, R., Tanpaiboon, P., Lacbawan, F.
EPI_ISL_915434	MD Laboratories	Los Angeles County PHL	P. Hemarajata et al.
EPI_ISL_917941	Los Angeles County PHL	Los Angeles County PHL	P. Hemarajata et al.
EPI_ISL_918176	Department of Infectious Diseases and Immunology, National Hospital Organization Nagoya Medical Center	Clinical Research Center, National Hospital Organization Nagoya Medical Center	Yoshihiro Nakata, Hirotaka Ode, Mai Kubota, Masakazu Matsuda, Kazuhiro Matsuoka, Miho Nakasuji, Mikiko Mori, Mayumi Imahashi, Yoshiyuki Yokomaku, Yasumasa Iwatani
EPI_ISL_918177, EPI_ISL_918180, EPI_ISL_918183, EPI_ISL_918185, EPI_ISL_918187, EPI_ISL_918200, EPI_ISL_918237, EPI_ISL_918243, EPI_ISL_918249, EPI_ISL_918251, EPI_ISL_918254, EPI_ISL_918255			
see above	Innovative Genomics Institute, UC Berkeley	Innovative Genomics Institute, UC Berkeley	Stacia Wyman, Haridha Shivram, Phil Frankino, Liana Lareau, Shana McDevitt, Justin Choi
EPI_ISL_918263	SIESP DIPARTIMENTO DI PREVENZIONE CHIE	Istituto Zooprofilattico Sperimentale dell'Abruzzo e Molise "G. Caporale"	Lorusso A, Marcacci M, Di Domenico M, Ancora M, Curini V, Mangone I, Rinaldi A, Scialabba S, Di Pasquale A, Cammà C, Puglia I, Calistri P, Savini G
EPI_ISL_918266	SIESP CHIETI-Drive in Lanciano	Istituto Zooprofilattico Sperimentale dell'Abruzzo e Molise "G. Caporale"	Lorusso A, Marcacci M, Di Domenico M, Ancora M, Curini V, Mangone I, Rinaldi A, Scialabba S, Di Pasquale A, Cammà C, Puglia I, Calistri P, Savini G
EPI_ISL_918267	SIESP CHIETI - DRIVE IN CHIETI	Istituto Zooprofilattico Sperimentale dell'Abruzzo e Molise "G. Caporale"	Lorusso A, Marcacci M, Di Domenico M, Ancora M, Curini V, Mangone I, Rinaldi A, Scialabba S, Di Pasquale A, Cammà C, Puglia I, Calistri P, Savini G
EPI_ISL_918268	SIESP CHIETI - DRIVE IN ORTONA	Istituto Zooprofilattico Sperimentale dell'Abruzzo e Molise "G. Caporale"	Lorusso A, Marcacci M, Di Domenico M, Ancora M, Curini V, Mangone I, Rinaldi A, Scialabba S, Di Pasquale A, Cammà C, Puglia I, Calistri P, Savini G
EPI_ISL_918271	SIESP DIPARTIMENTO DI PREVENZIONE CHIETI	Istituto Zooprofilattico Sperimentale dell'Abruzzo e Molise "G. Caporale"	Lorusso A, Marcacci M, Di Domenico M, Ancora M, Curini V, Mangone I, Rinaldi A, Scialabba S, Di Pasquale A, Cammà C, Puglia I, Calistri P, Savini G
EPI_ISL_918277, EPI_ISL_918278, EPI_ISL_918279	Hospital Universitari Vall d'Hebron - Vall d'Hebron Institut de Rercerca	Hospital Universitari Vall d'Hebron	Cristina Andrés, Maria Piñana, Josep F Abril, Damir Garcia-Cehic, Ariadna Rando, Juliana Esperalba, Maria Gema Codina, Carla Castillo, Maria Carmen Martin, Tomàs Pumarola, Josep Quer, Andrés Antón
EPI_ISL_918489	CUSL/UCLouvain COVID testing federal platform	UCLouvain/IREC/MBLG	Jean Ruelle, Lysa Pinsmaye, Benoit Kabamba Mukadi
EPI_ISL_918507, EPI_ISL_918508, EPI_ISL_918509, EPI_ISL_918510, EPI_ISL_918535	LACEN - Laboratório Central de Saúde Pública do Amazonas	Evandro Chagas Institute	Santos, M.C.; Silva, A.M.; Junior, W.D.C.; Barbagelata, L.S.; Ferreira, J.A.; Sousa, E.M.A.; da Silva, P.S.; Pinheiro, K.C.; L.C.; Sousa Junior, E.C.
EPI_ISL_918537, EPI_ISL_918538	LACEN - Laboratório Central de Saúde Pública do Ceara	Evandro Chagas Institute	Santos, M.C.; Silva, A.M.; Junior, W.D.C.; Barbagelata, L.S.; Ferreira, J.A.; Sousa, E.M.A.; da Silva, P.S.; Pinheiro, K.C.; L.C.; Sousa Junior, E.C.
EPI_ISL_918545, EPI_ISL_918546,	LACEN - Laboratório Central de Saúde Pública do Para	Evandro Chagas Institute	Santos, M.C.; Silva, A.M.; Junior, W.D.C.; Barbagelata, L.S.; Ferreira, J.A.; Sousa, E.M.A.; da Silva, P.S.; Pinheiro, K.C.; L.C.; Sousa Junior, E.C.

EPI_ISL_918547, EPI_ISL_918548, EPI_ISL_918549			
EPI_ISL_918697, EPI_ISL_918698, EPI_ISL_918699, EPI_ISL_918700, EPI_ISL_918702, EPI_ISL_918703, EPI_ISL_918704, EPI_ISL_918705, EPI_ISL_918706, EPI_ISL_918707, EPI_ISL_918708, EPI_ISL_918709, EPI_ISL_918710, EPI_ISL_918712, EPI_ISL_918713, EPI_ISL_918714, EPI_ISL_918715, EPI_ISL_918716, EPI_ISL_918717, EPI_ISL_918718, EPI_ISL_918719, EPI_ISL_918720, EPI_ISL_918722, EPI_ISL_918723, EPI_ISL_918724, EPI_ISL_918725, EPI_ISL_918726, EPI_ISL_918727, EPI_ISL_918728, EPI_ISL_918729, EPI_ISL_918730, EPI_ISL_918731, EPI_ISL_918732, EPI_ISL_918735, EPI_ISL_918736, EPI_ISL_918737, EPI_ISL_918740, EPI_ISL_918904, EPI_ISL_918905			
see above	University of Birmingham	COVID-19 Genomics UK (COG-UK) Consortium	Institute of Microbiology, University of Birmingham: Claire McMurray, Joanne Stockton, Samuel Nicholls, Radoslaw Poplawski, Will Rowe, Josh Quick, Nicholas Loman. University of Birmingham Testing Laboratory: Celina M Whalley, Andrew Bosworth, Charlotte Poxon, Kasun Wanigasooriya, Oliver Pickles, Mike Kidd, Alex Richter, Andrew D Beggs PHE Heartlands Lab: Husam Osman, Andrew Bosworth. Queen Elizabeth Hospital: Anna Casey
EPI_ISL_919071, EPI_ISL_919073, EPI_ISL_919077, EPI_ISL_919081, EPI_ISL_919104	Department of Pathology, University of Cambridge	COVID-19 Genomics UK (COG-UK) Consortium	Aminu S. Jahun, Yasmin Chaudhry, Iliana Georgana, Myra Hosmillo, Rhys Izu, Martin D. Curran, Surendra Parmar, Ian Goodfellow
EPI_ISL_919196	West of Scotland Specialist Virology Centre, NHSGGC / MRC-University of Glasgow Centre for Virus Research	COVID-19 Genomics UK (COG-UK) Consortium	Ana da Silva Filipe, Natasha Johnson, Kathy Smollett, Daniel Mair, Stephen Carmichael, Alice Broos, Lily Tong, Jenna Nichols, Kyriaki Nomikou; Sarah McDonald; Richard Orton, Joseph Hughes, Sreenu Vattipally, David L Robertson; Alasdair MacLean, Rory Gunson; Sharif Shaaban, Matthew Holden; Rachel Blacow, Guy Mollett, Kathy Li, James Shepherd, Antonia Ho, Emma Thomson
EPI_ISL_919460, EPI_ISL_919462, EPI_ISL_919463, EPI_ISL_919465, EPI_ISL_919594, EPI_ISL_919595	Liverpool Clinical Laboratories	COVID-19 Genomics UK (COG-UK) Consortium	Sam Haldenby, Anita Lucaci, Steve Paterson, Julian Hiscox, Alistair Darby, M Almsaud, A Alrezaihi, Muhannad Alruwaili, Stuart D Armstrong, Jones Benjamin, Eleanor G Bentley, Anu Chawla, Jordan J Clark, Angela Cowell, Richard Eccles, Isabel García-Dorival, Matthew Gemmell, Alessandro Gerada, PKF Gilmore, Richard Gregory, Ximeng Han, Catherine Hartley, Margaret Hughes, Miren Iturriza-Gomara, James Johnson, L Luu, Jenifer Manson, Charlotte Nelson, Elaine O'Toole, Cassie Olateju, Rebekah Penrice-Randal , Lucille Rainbow, N.P Randle, Trevor Ian Robinson, Parul Sharma, Ghada T Shawli, James P Stewart, Neil Swainston, Ecaterina Vamos, Joanne Watts, Mark Whitehead
EPI_ISL_919779, EPI_ISL_919780, EPI_ISL_919797, EPI_ISL_919803, EPI_ISL_919804, EPI_ISL_919826, EPI_ISL_919827, EPI_ISL_919828, EPI_ISL_919829, EPI_ISL_919830, EPI_ISL_919831, EPI_ISL_919832, EPI_ISL_919833, EPI_ISL_919834, EPI_ISL_919835, EPI_ISL_919836, EPI_ISL_919837, EPI_ISL_919838, EPI_ISL_919839, EPI_ISL_919840, EPI_ISL_919841, EPI_ISL_919845, EPI_ISL_919846, EPI_ISL_919847, EPI_ISL_919848, EPI_ISL_919849, EPI_ISL_919850, EPI_ISL_919851			
see above	Barts Health NHS Trust	COVID-19 Genomics UK (COG-UK) Consortium	CUTINO-MOGUEL, Maria-Teresa; HARRINGTON, David; OWOYEMI, Dola; KULASEGARAN-SHYLINI, Raghavendran; BROAD, Claire; KELE, Beatrix
EPI_ISL_919982, EPI_ISL_919983, EPI_ISL_919984, EPI_ISL_919986, EPI_ISL_919988, EPI_ISL_919989, EPI_ISL_919990, EPI_ISL_919991, EPI_ISL_919992, EPI_ISL_919993, EPI_ISL_919994, EPI_ISL_919995, EPI_ISL_919996, EPI_ISL_919997, EPI_ISL_919998, EPI_ISL_919999, EPI_ISL_920041, EPI_ISL_920048			
see above	University College London, Great Ormond Street Hospital for Children NHS Foundation Trust, Imperial College Healthcare NHS Trust	COVID-19 Genomics UK (COG-UK) Consortium	Sergi Castellano, Rachel Williams, Mark Kristiansen, Paola Resende Silva, Sunando Roy, Tony Brooks, Helena Tutill, Paola Niola, Patricia Dyal, Charlotte Williams, Leysa Forrest, Yasmin Panchbhaya, Jacqueline Findlay, Samuel Weeks, Julianne Brown, Kathryn Harris, Paul Randell, James Price, Alison Holmes, Judith Breuer
EPI_ISL_920179, EPI_ISL_920288, EPI_ISL_920297, EPI_ISL_920311, EPI_ISL_920689	University College London Hospital	COVID-19 Genomics UK (COG-UK) Consortium	Judith Heaney, Matthew Byott, Catherine Houlihan, Dan Frampton, Stuart Kirk, Moira Spyer and Eleni Nastouli
EPI_ISL_920839, EPI_ISL_920850, EPI_ISL_920852, EPI_ISL_920853, EPI_ISL_920856, EPI_ISL_920857, EPI_ISL_920858, EPI_ISL_920859	University College London, Great Ormond Street Hospital for Children NHS Foundation Trust, Imperial College Healthcare NHS Trust	COVID-19 Genomics UK (COG-UK) Consortium	Sergi Castellano, Rachel Williams, Mark Kristiansen, Paola Resende Silva, Sunando Roy, Tony Brooks, Helena Tutill, Paola Niola, Patricia Dyal, Charlotte Williams, Leysa Forrest, Yasmin Panchbhaya, Jacqueline Findlay, Samuel Weeks, Julianne Brown, Kathryn Harris, Paul Randell, James Price, Alison Holmes, Judith Breuer
EPI_ISL_920980, EPI_ISL_921019, EPI_ISL_921026, EPI_ISL_921126, EPI_ISL_921127, EPI_ISL_921129, EPI_ISL_921130	Regional Virus Laboratory, Belfast Health and Social Care Trust	COVID-19 Genomics UK (COG-UK) Consortium	Conall McCaughey, James McKenna, Tanya Curran, Susan Feeney, Alison Watt, Ciara Cox, Mairead Connor, Zoltan Molnar, David Simpson, Derek Fairley
EPI_ISL_921300, EPI_ISL_921302, EPI_ISL_921307, EPI_ISL_921308, EPI_ISL_921309, EPI_ISL_921310, EPI_ISL_921311, EPI_ISL_921312, EPI_ISL_921315, EPI_ISL_921319, EPI_ISL_921320, EPI_ISL_921452, EPI_ISL_921453, EPI_ISL_921454, EPI_ISL_921455, EPI_ISL_921457, EPI_ISL_921458, EPI_ISL_921459, EPI_ISL_921462, EPI_ISL_921464, EPI_ISL_921465, EPI_ISL_921468, EPI_ISL_921469, EPI_ISL_921470, EPI_ISL_921471, EPI_ISL_921472, EPI_ISL_921473, EPI_ISL_921474, EPI_ISL_921475, EPI_ISL_921476, EPI_ISL_921477, EPI_ISL_921478, EPI_ISL_921479, EPI_ISL_921480, EPI_ISL_921481, EPI_ISL_921482, EPI_ISL_921483, EPI_ISL_921484, EPI_ISL_921494			
see above	Northumbria University / South Tees Hospitals NHS Foundation Trust / North Cumbria Integrated Care NHS Foundation Trust / North Tees and Hartlepool NHS Foundation Trust / Newcastle Hospitals NHS Foundation Trust	COVID-19 Genomics UK (COG-UK) Consortium	Darren L Smith,Andrew Nelson,Matthew Bashton,Greg R Young,Joshua Loh,John Allan,Mohammad A Tariq,Giles S Holt,Gary Black,Wen C Yew,Lynn Dover,Paul Baker,Steve Liggett,Sarah Essex,Jane Greenaway,Debra Padgett,Clive Graham,Garren Scott,Edward Barton,Emma Swindells,Brendan Payne,Jennifer Collins,Yusri Taha,Gary Eltringham
EPI_ISL_922041, EPI_ISL_922042, EPI_ISL_922043, EPI_ISL_922044, EPI_ISL_922045, EPI_ISL_922046, EPI_ISL_922047, EPI_ISL_922048, EPI_ISL_922049, EPI_ISL_922050, EPI_ISL_922051, EPI_ISL_922052, EPI_ISL_922053, EPI_ISL_922054, EPI_ISL_922055, EPI_ISL_922056, EPI_ISL_922057, EPI_ISL_922058, EPI_ISL_922059, EPI_ISL_922060, EPI_ISL_922061, EPI_ISL_922062, EPI_ISL_922063, EPI_ISL_922064, EPI_ISL_922065, EPI_ISL_922066, EPI_ISL_922067, EPI_ISL_922068, EPI_ISL_922069, EPI_ISL_922070, EPI_ISL_922073			
see above	Lincolnshire Hospitals and DeepSeq Nottingham	COVID-19 Genomics UK (COG-UK) Consortium	Nichola Duckworth, Tim Sloan, Sarah Walsh, Jonathan Ball, Patrick McClure, Joeseph Chappell, Nadine Holmes, Matthew Carlisle, Christopher Moore, Fei Sang, Johnny Debebe, Victoria Wright, Matthew Loose
EPI_ISL_922174, EPI_ISL_922177, EPI_ISL_922182, EPI_ISL_922184, EPI_ISL_922185, EPI_ISL_922189, EPI_ISL_922190, EPI_ISL_922191, EPI_ISL_922194, EPI_ISL_922197, EPI_ISL_922198, EPI_ISL_922199, EPI_ISL_922200, EPI_ISL_922201, EPI_ISL_922203, EPI_ISL_922208, EPI_ISL_922209, EPI_ISL_922244, EPI_ISL_922246, EPI_ISL_922252, EPI_ISL_922254, EPI_ISL_922258, EPI_ISL_922284, EPI_ISL_922295, EPI_ISL_922323, EPI_ISL_922324, EPI_ISL_922325, EPI_ISL_922326, EPI_ISL_922328, EPI_ISL_922329, EPI_ISL_922330, EPI_ISL_922331, EPI_ISL_922332, EPI_ISL_922333, EPI_ISL_922334, EPI_ISL_922335, EPI_ISL_922338, EPI_ISL_922342, EPI_ISL_922343, EPI_ISL_922345, EPI_ISL_922346, EPI_ISL_922347, EPI_ISL_922348			
see above	Oxford Viromics, NDM, University of Oxford; Oxford University Hospitals; Basingstoke and North Hampshire Hospital	COVID-19 Genomics UK (COG-UK) Consortium	Tanya Golubchik, David Bonsall, George Macintyre, Amy Trebes, Mariateresa de Cesare, Catrin Moore, Alex Mobbs, Anita Justice, Robert Shaw, Monique Andersson, Timothy Peto, Emma Wise, Nathan Moore, Jessica Lynch, Nick Cortes, Matilde Mori, Stephen Kidd, David Buck, John Todd, Christophe Fraser
EPI_ISL_923256, EPI_ISL_923257, EPI_ISL_923260, EPI_ISL_923261, EPI_ISL_923263, EPI_ISL_923266, EPI_ISL_923275, EPI_ISL_923296, EPI_ISL_923300, EPI_ISL_923372, EPI_ISL_923374, EPI_ISL_923377, EPI_ISL_923379, EPI_ISL_923380, EPI_ISL_923381, EPI_ISL_923382, EPI_ISL_923383, EPI_ISL_923384, EPI_ISL_923385, EPI_ISL_923386, EPI_ISL_923387, EPI_ISL_923388, EPI_ISL_923430, EPI_ISL_923443, EPI_ISL_923663			
see above	Centre for Enzyme Innovation, University of Portsmouth / Translational Research Laboratory, Portsmouth Hospitals NHS Trust	COVID-19 Genomics UK (COG-UK) Consortium	Angela Beckett,Salman Goudarzi,Christopher Fearn,Kate Cook,Katie Loveson,Sharon Glaysher,Scott Elliott,Samuel Robson
EPI_ISL_924285, EPI_ISL_924369, EPI_ISL_924383	Virology Department, Sheffield Teaching Hospitals NHS Foundation Trust/Department of Infection, Immunity and Cardiovascular Disease, The Medical School, University of Sheffield	COVID-19 Genomics UK (COG-UK) Consortium	Thushan de Silva, Matthew Parker, Nikki Smith, Adri Angyal, Rebecca Brown, Luke Green, Rachel Tucker, Paul Parsons, Danielle Groves, Katie Johnson, Laura Carrilero, Alex Keeley, Dave Partridge, Matthew Wyles, Benjamin Lindsey, Mehmet Yavuz, Mohammad Raza, Cariad Evans
EPI_ISL_924623, EPI_ISL_924625, EPI_ISL_924626, EPI_ISL_924627, EPI_ISL_924628, EPI_ISL_924629, EPI_ISL_924631, EPI_ISL_924632, EPI_ISL_924633, EPI_ISL_924635, EPI_ISL_924636, EPI_ISL_924637, EPI_ISL_924638, EPI_ISL_924639, EPI_ISL_924640, EPI_ISL_924641, EPI_ISL_924642, EPI_ISL_924644, EPI_ISL_924645, EPI_ISL_924646, EPI_ISL_924647, EPI_ISL_924648, EPI_ISL_924649, EPI_ISL_924650, EPI_ISL_924651, EPI_ISL_924723, EPI_ISL_924724, EPI_ISL_924725, EPI_ISL_924726, EPI_ISL_924727, EPI_ISL_924728, EPI_ISL_924730, EPI_ISL_924732, EPI_ISL_924733, EPI_ISL_924734, EPI_ISL_924735, EPI_ISL_924736, EPI_ISL_924737, EPI_ISL_924739, EPI_ISL_924740, EPI_ISL_924741, EPI_ISL_924742, EPI_ISL_924743, EPI_ISL_924744, EPI_ISL_924745, EPI_ISL_924747, EPI_ISL_924748, EPI_ISL_924750, EPI_ISL_924752, EPI_ISL_924753, EPI_ISL_924754, EPI_ISL_924755, EPI_ISL_924756, EPI_ISL_924757, EPI_ISL_924758, EPI_ISL_924759, EPI_ISL_924760, EPI_ISL_924761, EPI_ISL_924762, EPI_ISL_924763, EPI_ISL_924765, EPI_ISL_924766, EPI_ISL_924768, EPI_ISL_924769, EPI_ISL_924771, EPI_ISL_924772, EPI_ISL_924773, EPI_ISL_924775, EPI_ISL_924776, EPI_ISL_924777, EPI_ISL_924778, EPI_ISL_924779, EPI_ISL_924780, EPI_ISL_924781, EPI_ISL_924782, EPI_ISL_924897			
see above	Bioinformatics and Biostatistics Lab, Advanced Sequencing Facility	COVID-19 Genomics UK (COG-UK) Consortium	Aengus Stewart,Jerome Nicod,Chelsea Sawyer,Laura Cubitt,Harshil Patel,Margaret Crawford
EPI_ISL_925065, EPI_ISL_925066, EPI_ISL_925067, EPI_ISL_925068, EPI_ISL_925069, EPI_ISL_925070, EPI_ISL_925071, EPI_ISL_925072, EPI_ISL_925073, EPI_ISL_925074	TXDSHS	TXDSHS	Bonnie Oh, Anita Pokharel, James Daniel Bonser, Myong Koag, Chung Wang, Rachel Lee, Grace Kubin, Rashmi Tuladhar, Mayela Pedrueza, Maliha Rahman, Jenny Zhang
EPI_ISL_925290, EPI_ISL_925304	Wyoming Public Health Laboratory	Wyoming Public Health Laboratory	Noah Hull, Taylor Fearing, Lynette Gumbleton, Channing Weber, Ashley Norberg, Bailey Bowcutt, and Wanda Manley

EPI_ISL_925509, EPI_ISL_925510, EPI_ISL_925511, EPI_ISL_925512, EPI_ISL_925522, EPI_ISL_925523, EPI_ISL_925525, EPI_ISL_925526, EPI_ISL_925534, EPI_ISL_925824, EPI_ISL_925825, EPI_ISL_925826, EPI_ISL_925827, EPI_ISL_925828, EPI_ISL_925829, EPI_ISL_925830, EPI_ISL_925831, EPI_ISL_925833, EPI_ISL_925834, EPI_ISL_925835, EPI_ISL_925836, EPI_ISL_925837, EPI_ISL_925838, EPI_ISL_925839, EPI_ISL_925840, EPI_ISL_925841, EPI_ISL_925842, EPI_ISL_925843, EPI_ISL_925844, EPI_ISL_925845			
see above	Arizona State Public Health Laboratory	Arizona State Public Health Laboratory	Trung Huynh, Jessica Escobar, Katherine Fullerton, Nobuko Fukushima, Stacy White, Linda Getsinger, Victor Waddell
EPI_ISL_925887, EPI_ISL_925908, EPI_ISL_925912	Nucleic Acid Testing, National Reference Laboratory	GIGA Medical Genomics	Yvan Butera, Keith Durkin, Maria Artesi, Bouchra Boujemla, Robert Rutayisire, Patrick Tuyisenge, Esperence Umumararungu, Sébastien Bontems, Marie-Pierre Hayette, Nathalie Renotte, Swaibu Gatare, Jacob Souopgui, Sabin Nsanzimana, Vincent Bours, Léon Mutesa
EPI_ISL_930636, EPI_ISL_930649, EPI_ISL_930650, EPI_ISL_930661	Arizona State Public Health Laboratory	Arizona State Public Health Laboratory	Trung Huynh, Jessica Escobar, Katherine Fullerton, Nobuko Fukushima, Stacy White, Linda Getsinger, Victor Waddell
EPI_ISL_931489, EPI_ISL_931496, EPI_ISL_931497, EPI_ISL_931498, EPI_ISL_931499, EPI_ISL_931500	Maryland Public Health Laboratory (MD PHL)	Maryland Public Health Laboratory (MD PHL)	Maryland Department of Health Laboratories Administration
EPI_ISL_931818	Lighthouse Lab in Alderley Park	Wellcome Sanger Institute for the COVID-19 Genomics UK (COG-UK) Consortium	Jacquelyn Wynn, Mairead Hyland, The Lighthouse Lab in Alderley Park and Alex Alderton, Roberto Amato, Sonia Goncalves, Ewan Harrison, David K. Jackson, Ian Johnston, Dominic Kwiatkowski, Cordelia Langford, John Sillitoe on behalf of the Wellcome Sanger Institute COVID-19 Surveillance Team
EPI_ISL_933661, EPI_ISL_933662, EPI_ISL_933680, EPI_ISL_933681, EPI_ISL_933682, EPI_ISL_933683, EPI_ISL_933684, EPI_ISL_933685, EPI_ISL_933686, EPI_ISL_933687, EPI_ISL_933688, EPI_ISL_933689, EPI_ISL_933704			
see above	Instituto de Diagnostico y Referencia Epidemiologicos INDRE_RNLSP	Instituto de Diagnostico y Referencia Epidemiologicos (INDRE)	Claudia Wong-Arambula, Abril Rodriguez-Maldonado, Fabiola Garces-Ayala, Adnan Araiza-Rodriguez, David Fragoso-Fonseca, Sergio Rangel-Guerrero, Mayra Jimenez-Morales, Nancy Munoz-Hernandez, Natividad Cruz-Ortiz, Tatiana Nunez-Garcia, Gisela Barrera-Badillo, Lucia Hernandez-Rivas, Irma Lopez-Martinez, Ernesto Ramirez-Gonzalez.
EPI_ISL_934182, EPI_ISL_934183, EPI_ISL_934184, EPI_ISL_934185, EPI_ISL_934186, EPI_ISL_934195, EPI_ISL_934196, EPI_ISL_934197, EPI_ISL_934198, EPI_ISL_934199, EPI_ISL_934200, EPI_ISL_934201, EPI_ISL_934229, EPI_ISL_934230, EPI_ISL_934231, EPI_ISL_934232, EPI_ISL_934233			
see above	Vilnius university hospital Santaros Klinikos, Center of Laboratory Medicine	Vilnius university hospital Santaros Klinikos, Center of Laboratory Medicine	Ingrida Olendraite, Daniel Naumovas, Rimvydas Norvilas, Dovile Ezerskyte, Justinas Slikas, Gytis Dudas
EPI_ISL_934343, EPI_ISL_934344, EPI_ISL_934355, EPI_ISL_934356, EPI_ISL_934357, EPI_ISL_934360	Klinisk mikrobiologi	The Public Health Agency of Sweden	Anna-Malin Linde, Maria Lind Karlberg, Carlo Berg, Oskar Karlsson Lindsjo, Sofia Stamouli, Reza Advani, Mattias Haukland, Petra Holmstrom, Noura Walai, Petra Edquist, Mia Brytting, Anna Risberg, Karin Tegmark-Wisell
EPI_ISL_934366, EPI_ISL_934367, EPI_ISL_934368, EPI_ISL_934369	Synlab Medilab, Mikrobiologi	The Public Health Agency of Sweden	Anna-Malin Linde, Maria Lind Karlberg, Carlo Berg, Oskar Karlsson Lindsjo, Sofia Stamouli, Reza Advani, Mattias Haukland, Petra Holmstrom, Noura Walai, Petra Edquist, Mia Brytting, Anna Risberg, Karin Tegmark-Wisell
EPI_ISL_934431, EPI_ISL_934432, EPI_ISL_934433	ILV Kärnten	Bergthaler laboratory, CeMM Research Center for Molecular Medicine of the Austrian Academy of Sciences	Lukas Endler, Anna Schedl, Thomas Penz, Benedikt Agerer, Maelle Le Moing, Michael Schuster, Bekir Erguner, Jan Laine, Martin Senekowitsch, Christoph Bock, Andreas Bergthaler
EPI_ISL_934456, EPI_ISL_934460, EPI_ISL_934486, EPI_ISL_934510, EPI_ISL_934511, EPI_ISL_934512, EPI_ISL_934513, EPI_ISL_934514, EPI_ISL_934515, EPI_ISL_934516, EPI_ISL_934517, EPI_ISL_934518, EPI_ISL_934519, EPI_ISL_934520, EPI_ISL_934521, EPI_ISL_934522, EPI_ISL_934523, EPI_ISL_934524, EPI_ISL_934525, EPI_ISL_934526, EPI_ISL_934527, EPI_ISL_934528, EPI_ISL_934529, EPI_ISL_934530, EPI_ISL_934531, EPI_ISL_934532, EPI_ISL_934533, EPI_ISL_934534, EPI_ISL_934535, EPI_ISL_934536, EPI_ISL_934537, EPI_ISL_934538			
see above	Austrian Agency for Health and Food Safety (AGES)	Bergthaler laboratory, CeMM Research Center for Molecular Medicine of the Austrian Academy of Sciences	Lukas Endler, Anna Schedl, Thomas Penz, Benedikt Agerer, Maelle Le Moing, Michael Schuster, Bekir Erguner, Jan Laine, Martin Senekowitsch, Christoph Bock, Andreas Bergthaler
EPI_ISL_934634, EPI_ISL_934641	Department of Microbiology, University Innsbruck	Bergthaler laboratory, CeMM Research Center for Molecular Medicine of the Austrian Academy of Sciences	Lukas Endler, Anna Schedl, Thomas Penz, Benedikt Agerer, Maelle Le Moing, Michael Schuster, Bekir Erguner, Jan Laine, Martin Senekowitsch, Christoph Bock, Andreas Bergthaler
EPI_ISL_935026	Biolab Diagnostic Laboratories	Princess Haya Biotechnology Center, Jordan University of Science and Technology	Saied Jaradat, Hazem Haddad, Areej Alquran, Maha Karam, Shereen Issa,Suha Hasan, Amid Abdelnour, Issa Abu-Dayyeh, Mustafa Ababneh, Moh''D Al-Zghoul, Mohammad Alboom
EPI_ISL_935038, EPI_ISL_935039, EPI_ISL_935041	Biolab Diagnostic Laboratories	Princess Haya Biotechnology Center, Jordan University of Science and Technology	Saied Jaradat, Hazem Haddad, Areej Alquran, Maha Karam, Shereen Issa,Suha Hasan, Amid Abdelnour, Issa Abu-Dayyeh
EPI_ISL_935043	Biolab Diagnostic Laboratories	Princess Haya Biotechnology Center/ Jordan University of Science & Technology	Saied Jaradat, Hazem Haddad, Areej Alquran, Maha Karam, Shereen Issa,Suha Hasan, Amid Abdelnour, Issa Abu-Dayyeh
EPI_ISL_935046	Biolab Diagnostic Laboratories	Princess Haya Biotechnology Center/ Jordan University of Science & Technology	Saied Jaradat, Hazem Haddad, Areej Alquran, Maha Karam, Shereen Issa,Suha Hasan, Amid Abdelnour, Issa Abu-Dayyeh, Mustafa Ababneh, Moh''D Al-Zghoul, Mohammad Alboom
EPI_ISL_935157	SIESP CHIETI-DRIVE IN ORTONA	Istituto Zooprofilattico Sperimentale dell'Abruzzo e Molise "G. Caporale"	Lorusso A, Marcacci M, Di Domenico M, Ancora M, Curini V, Mangone I, Rinaldi A, Scialabba S, Di Pasquale A, Cammà C, Puglia I, Calistri P, Savini G
EPI_ISL_935158	SIESP CHIETI	Istituto Zooprofilattico Sperimentale dell'Abruzzo e Molise "G. Caporale"	Lorusso A, Marcacci M, Di Domenico M, Ancora M, Curini V, Mangone I, Rinaldi A, Scialabba S, Di Pasquale A, Cammà C, Puglia I, Calistri P, Savini G
EPI_ISL_935226	KU Leuven, Rega Institute, Clinical and Epidemiological Virology	KU Leuven, Rega Institute, Clinical and Epidemiological Virology	Tony Wawina-Bokalanga, Bert Vanmechelen, Joan Marti-Carerras, Piet Maes
EPI_ISL_935362, EPI_ISL_935365, EPI_ISL_935366	Florida Bureau of Public Health Laboratories	Florida Bureau of Public Health Laboratories	Sarah Schmedes, Jason Blanton
EPI_ISL_935974, EPI_ISL_935976, EPI_ISL_935977, EPI_ISL_935978, EPI_ISL_935979, EPI_ISL_935980, EPI_ISL_935981	GLENS FALLS HOSPITAL LABORATORY	Wadsworth Center, New York State Department of Health	Kirsten St. George, Daryl M. Lamson, Alexis Russel, Matthew Shudt, Melissa A Leisner, Jonathan Plitnick, Navjot Singh, John Kelly, Erasmus Schneider, Erica Lasek-Nesselquist
EPI_ISL_935993, EPI_ISL_935994, EPI_ISL_935995, EPI_ISL_935996, EPI_ISL_935997, EPI_ISL_935998, EPI_ISL_935999, EPI_ISL_936000, EPI_ISL_936001	SUNY UPSTATE MEDICAL UNIVERSITY	Wadsworth Center, New York State Department of Health	Kirsten St. George, Daryl M. Lamson, Alexis Russel, Matthew Shudt, Melissa A Leisner, Jonathan Plitnick, Navjot Singh, John Kelly, Erasmus Schneider, Erica Lasek-Nesselquist
EPI_ISL_936023, EPI_ISL_936024, EPI_ISL_936029, EPI_ISL_936030, EPI_ISL_936032, EPI_ISL_936033	THE MARY IMOGENE BASSETT HOSPITAL	Wadsworth Center, New York State Department of Health	Kirsten St. George, Daryl M. Lamson, Alexis Russel, Matthew Shudt, Melissa A Leisner, Jonathan Plitnick, Navjot Singh, John Kelly, Erasmus Schneider, Erica Lasek-Nesselquist
EPI_ISL_936046, EPI_ISL_936047, EPI_ISL_936049, EPI_ISL_936052, EPI_ISL_936053, EPI_ISL_936054, EPI_ISL_936056	ADIRONDACK MEDICAL CENTER	Wadsworth Center, New York State Department of Health	Kirsten St. George, Daryl M. Lamson, Alexis Russel, Matthew Shudt, Melissa A Leisner, Jonathan Plitnick, Navjot Singh, John Kelly, Erasmus Schneider, Erica Lasek-Nesselquist
EPI_ISL_936062, EPI_ISL_936098, EPI_ISL_936099, EPI_ISL_936100, EPI_ISL_936102	KALEIDA CENTER FOR LABORATORY MEDICINE	Wadsworth Center, New York State Department of Health	Kirsten St. George, Daryl M. Lamson, Alexis Russel, Matthew Shudt, Melissa A Leisner, Jonathan Plitnick, Navjot Singh, John Kelly, Erasmus Schneider, Erica Lasek-Nesselquist
EPI_ISL_936139	THE MARY IMOGENE BASSETT HOSPITAL	Wadsworth Center, New York State Department of Health	Kirsten St. George, Daryl M. Lamson, Alexis Russel, Matthew Shudt, Melissa A Leisner, Jonathan Plitnick, Navjot Singh, John Kelly, Erasmus Schneider, Erica Lasek-Nesselquist
EPI_ISL_936143	SUNY UPSTATE MEDICAL UNIVERSITY	Wadsworth Center, New York State Department of Health	Kirsten St. George, Daryl M. Lamson, Alexis Russel, Matthew Shudt, Melissa A Leisner, Jonathan Plitnick, Navjot Singh, John Kelly, Erasmus Schneider, Erica Lasek-Nesselquist
EPI_ISL_936145	New York Presbyterian Hospital	Wadsworth Center, New York State Department of Health	Kirsten St. George, Daryl M. Lamson, Alexis Russel, Matthew Shudt, Melissa A Leisner, Jonathan Plitnick, Navjot Singh, John Kelly, Erasmus Schneider,

			Erica Lasek-Nesselquist
EPI_ISL_936212, EPI_ISL_936213	Wadsworth Center, New York State Department of Health	Wadsworth Center, New York State Department of Health	Kirsten St. George, Daryl M. Lamson, Alexis Russel, Matthew Shudt, Melissa A Leisner, Jonathan Plitnick, Navjot Singh, John Kelly, Erasmus Schneider, Erica Lasek-Nesselquist
EPI_ISL_936387, EPI_ISL_936389, EPI_ISL_936393, EPI_ISL_936394, EPI_ISL_936399, EPI_ISL_936403, EPI_ISL_936404, EPI_ISL_936409, EPI_ISL_936411, EPI_ISL_936412, EPI_ISL_936413, EPI_ISL_936416, EPI_ISL_936417, EPI_ISL_936418, EPI_ISL_936422, EPI_ISL_936425, EPI_ISL_936427, EPI_ISL_936428, EPI_ISL_936429, EPI_ISL_936430, EPI_ISL_936434, EPI_ISL_936438, EPI_ISL_936440, EPI_ISL_936441, EPI_ISL_936443, EPI_ISL_936444, EPI_ISL_936446, EPI_ISL_936448, EPI_ISL_936452, EPI_ISL_936454, EPI_ISL_936455, EPI_ISL_936457, EPI_ISL_936461, EPI_ISL_936462			
see above	TGen North	TGen North	Jolene Bowers, Megan Folkerts, Chris French, Hayley Yaglom, Ashlyn Pfeiffer, Darrin Lemmer, Dave Engelthaler, The Arizona COVID Genomics Union (ACGU)
EPI_ISL_936469	DPH, Massachusetts State Public Health Lab	DPH, Massachusetts State Public Health Lab	Lang,A.S., Fink,T., Gallagher,G.R., Smole,S.C.
EPI_ISL_936888, EPI_ISL_936890, EPI_ISL_936891, EPI_ISL_936892, EPI_ISL_936894, EPI_ISL_936905, EPI_ISL_936906, EPI_ISL_936907, EPI_ISL_936908, EPI_ISL_936909, EPI_ISL_936910, EPI_ISL_936911, EPI_ISL_936912, EPI_ISL_936913, EPI_ISL_936914, EPI_ISL_936915, EPI_ISL_936916, EPI_ISL_936917, EPI_ISL_936918, EPI_ISL_936923, EPI_ISL_936924, EPI_ISL_936925, EPI_ISL_936926, EPI_ISL_936927, EPI_ISL_936928, EPI_ISL_936929, EPI_ISL_936930, EPI_ISL_936931, EPI_ISL_936932, EPI_ISL_936933, EPI_ISL_936934, EPI_ISL_936935, EPI_ISL_936936, EPI_ISL_936937, EPI_ISL_936938, EPI_ISL_936939, EPI_ISL_936940, EPI_ISL_936941, EPI_ISL_936942, EPI_ISL_936943, EPI_ISL_936944, EPI_ISL_936945, EPI_ISL_936946, EPI_ISL_936947, EPI_ISL_936948, EPI_ISL_936949, EPI_ISL_936950, EPI_ISL_936951, EPI_ISL_936952, EPI_ISL_936953, EPI_ISL_936954, EPI_ISL_936955, EPI_ISL_936956, EPI_ISL_936957, EPI_ISL_936958, EPI_ISL_936959, EPI_ISL_936960, EPI_ISL_936961, EPI_ISL_936962, EPI_ISL_936963, EPI_ISL_936964, EPI_ISL_936965, EPI_ISL_936966			
see above	Northwestern Memorial Hospital	Ozer Lab	Ramon Lorenzo-Redondo, Lacy M. Simons, Chad J. Achenbach, Lawrence J. Jennings, Michael G. Ison, Judd F. Hultquist, Egon A. Ozer
EPI_ISL_937259	NYC HH Elmhurst Hospital Medical Center	New York City Public Health Laboratory	Jade Wang, et al.
EPI_ISL_940723, EPI_ISL_940724, EPI_ISL_940725, EPI_ISL_940726, EPI_ISL_940734	City of Milwaukee Health Department Laboratory	City of Milwaukee Health Department Laboratory	Sanjib Bhattacharyya
EPI_ISL_940852, EPI_ISL_940856, EPI_ISL_940862, EPI_ISL_940865, EPI_ISL_940866, EPI_ISL_940867, EPI_ISL_940869, EPI_ISL_940870, EPI_ISL_940874, EPI_ISL_940876, EPI_ISL_940886, EPI_ISL_940887			
see above	Vaccines and Infectious Diseases Analytics Research Unit (VIDA)	KRISP, KZN Research Innovation and Sequencing Platform	Baillie Vicky, du Plessis Jeanine, Giandhari Jennifer, Pillay Sureshnee, Naidoo Yeshnee, Tegally Houriiyah, de Oliveira Tulio, Madhi Shabir
EPI_ISL_941005, EPI_ISL_941006, EPI_ISL_941009, EPI_ISL_941010, EPI_ISL_941011, EPI_ISL_941012, EPI_ISL_941014	Labo Analyses Med	National Reference Center for Viruses of Respiratory Infections, Institut Pasteur, Paris	Marion Barbet, Sylvie Behillil, Méline Bizard, Angela Brisebarre, Camille Capel, Etienne Simon-Lorière, Vincent Enouf, Maud Vanpeene, Sylvie van der Werf, Amzalag Jonas
EPI_ISL_941016, EPI_ISL_941019, EPI_ISL_941021, EPI_ISL_941022	Hopital	National Reference Center for Viruses of Respiratory Infections, Institut Pasteur, Paris	Marion Barbet, Sylvie Behillil, Méline Bizard, Angela Brisebarre, Camille Capel, Etienne Simon-Lorière, Vincent Enouf, Maud Vanpeene, Sylvie van der Werf, Bour Jean Baptiste
EPI_ISL_941089, EPI_ISL_941092, EPI_ISL_941094	Labo Analyses Med	National Reference Center for Viruses of Respiratory Infections, Institut Pasteur, Paris	Marion Barbet, Sylvie Behillil, Méline Bizard, Angela Brisebarre, Camille Capel, Etienne Simon-Lorière, Vincent Enouf, Maud Vanpeene, Sylvie van der Werf, Merah Kader
EPI_ISL_941164	Servicio de Microbiología. Hospital Arnau de Vilanova	SeqCOVID-SPAIN consortium/IBV(CSIC)	Victoria Domínguez, Rocío Falcón, Amparo Farga and SeqCOVID-SPAIN consortium
EPI_ISL_941220, EPI_ISL_941221, EPI_ISL_941222, EPI_ISL_941224	Laboratorio de Microbiología. Hospital General Universitario de Elda, Alicante	SeqCOVID-SPAIN consortium/IBV(CSIC)	Mª Isabel Gascón Ros, Cristina Torregrosa Hetland, Eva Pastor Boix, Paloma Cascales Ramos and SeqCOVID-SPAIN consortium
EPI_ISL_941375, EPI_ISL_941451, EPI_ISL_941452, EPI_ISL_941453, EPI_ISL_941454, EPI_ISL_941629, EPI_ISL_941630	Instituto Nacional de Saude (INSA)	Instituto Nacional de Saude (INSA)	Borges et al
EPI_ISL_941901	Virginia DCLS	Virginia DCLS	Virginia DCLS
EPI_ISL_942795, EPI_ISL_942796, EPI_ISL_942797, EPI_ISL_942798, EPI_ISL_942799, EPI_ISL_942800, EPI_ISL_942801, EPI_ISL_942802, EPI_ISL_942803, EPI_ISL_942804, EPI_ISL_942805, EPI_ISL_942806, EPI_ISL_942807, EPI_ISL_942808, EPI_ISL_942944			
see above	Gundersen Molecular Diagnostics Laboratory	Kabara Cancer Research Institute	Craig S. Richmond, Paraic A. Kenny
EPI_ISL_942996, EPI_ISL_943010, EPI_ISL_943013, EPI_ISL_943014, EPI_ISL_943019, EPI_ISL_943067, EPI_ISL_943148, EPI_ISL_943149, EPI_ISL_943185, EPI_ISL_943186, EPI_ISL_943187, EPI_ISL_943188, EPI_ISL_943189, EPI_ISL_943190, EPI_ISL_943191, EPI_ISL_943192, EPI_ISL_943292, EPI_ISL_943337, EPI_ISL_943358, EPI_ISL_943380, EPI_ISL_943398, EPI_ISL_943399, EPI_ISL_943400, EPI_ISL_943401, EPI_ISL_943402, EPI_ISL_943403, EPI_ISL_943404, EPI_ISL_943437, EPI_ISL_943497, EPI_ISL_943508, EPI_ISL_943511, EPI_ISL_943512			
see above	Dutch COVID-19 response team	National Institute for Public Health and the Environment (RIVM)	Adam Meijer, Harry Vennema, Dirk Eggink, Jeroen Cremer, Sharon van den Brink, Bas van der Veer, AnneMarie van den Brandt, Florian Zwagemaker, Dennis Schmitz, Chantal Reusken, on behalf of the national COVID-19 response team
EPI_ISL_943548	National Institute of Health Research and Development	National Institute of Health Research and Development	Subangkit, Hana Apsari Pawestri, Kartika Dewi Puspa, Arie Ardiansyah Nugraha, Hartanti Dian Ikawati, Krisna Nur Andriana Pangesti, Yuni Rukminiati, Ririn Ramadhany, Agustiningsih, Kindi Adam, Holy Arif Wibowo, Triyani Soekarso, Ni Ketut Susilarini, Nurika Hariastuti, Ully Alfi Nikmah, Reni Herman, Nike Susanti, Herna, Tati Febriyanti, Natalie Laurencia Kipuw, Fauzul Muna, Irene Lorinda Indalao, Nelly Puspandari, Vivi Setiawaty
EPI_ISL_943824, EPI_ISL_943825, EPI_ISL_943826, EPI_ISL_943827, EPI_ISL_943828, EPI_ISL_943829, EPI_ISL_943830, EPI_ISL_943836, EPI_ISL_943950	Utah Public Health Laboratory	Utah Public Health Laboratory	Erin L. Young, Kelly F. Oakeson, Tara Gallagher
EPI_ISL_944130, EPI_ISL_944132, EPI_ISL_944134, EPI_ISL_944136, EPI_ISL_944138, EPI_ISL_944139, EPI_ISL_944144, EPI_ISL_944146, EPI_ISL_944147, EPI_ISL_944150, EPI_ISL_944160, EPI_ISL_944161, EPI_ISL_944165, EPI_ISL_944172, EPI_ISL_944177			
see above	National Health Laboratory Service, South Africa	KRISP, KZN Research Innovation and Sequencing Platform	Laguda-Akingba O, Giandhari J, Pillay S, Lessells R, Mdlalose K, York D, Khan S, Emmanuel SJ, Tegally H, Wilkinson E, de Oliveira T
EPI_ISL_944234, EPI_ISL_944235, EPI_ISL_944246, EPI_ISL_944249, EPI_ISL_944250, EPI_ISL_944279, EPI_ISL_944280, EPI_ISL_944281, EPI_ISL_944282, EPI_ISL_944283, EPI_ISL_944284, EPI_ISL_944285, EPI_ISL_944286, EPI_ISL_944287, EPI_ISL_944288, EPI_ISL_944289, EPI_ISL_944290, EPI_ISL_944291, EPI_ISL_944292, EPI_ISL_944293, EPI_ISL_944294, EPI_ISL_944295, EPI_ISL_944296, EPI_ISL_944297, EPI_ISL_944298, EPI_ISL_944299, EPI_ISL_944300, EPI_ISL_944301, EPI_ISL_944302, EPI_ISL_944303, EPI_ISL_944304, EPI_ISL_944305, EPI_ISL_944306, EPI_ISL_944307, EPI_ISL_944308, EPI_ISL_944309, EPI_ISL_944310, EPI_ISL_944311, EPI_ISL_944312, EPI_ISL_944313, EPI_ISL_944314, EPI_ISL_944315, EPI_ISL_944316, EPI_ISL_944317, EPI_ISL_944318, EPI_ISL_944319, EPI_ISL_944320, EPI_ISL_944321, EPI_ISL_944396, EPI_ISL_944399, EPI_ISL_944400, EPI_ISL_944416			
see above	Israel Central Virology laboratory	Israel National Consortium for SARS-CoV-2 sequencing	Neta Zuckerman, Efrat Dahan Bucris, Michal Mandelboim, Dana Bar-Ilan, Oran Erster, Tzvia Mann, Omer Murik, David A. Zeevi, Assaf Rokney, Joseph Jaffe, Eva Nachum, Maya Davidovich Cohen, Ephraim Fass, Gal Zizelski Valenci, Mor Rubinstein, Efrat Rorman, Israel Nissan, Efrat Glick-Saar, Omri Nayshool, Gideon Rechavi, Ella Mendelson, Orna Mor
EPI_ISL_944644	Public Health Virology-Forensic and Scientific Services (PHV-FSS)	Public Health Virology-Forensic and Scientific Services (PHV-FSS)	Alyssa T. Pyke et al.
EPI_ISL_944646, EPI_ISL_944647, EPI_ISL_944648, EPI_ISL_944649, EPI_ISL_944650, EPI_ISL_944651, EPI_ISL_944653, EPI_ISL_944654, EPI_ISL_944655, EPI_ISL_944658, EPI_ISL_944659, EPI_ISL_944661, EPI_ISL_944662, EPI_ISL_944663, EPI_ISL_944664, EPI_ISL_944665, EPI_ISL_944666, EPI_ISL_944667, EPI_ISL_944668, EPI_ISL_944670, EPI_ISL_944671, EPI_ISL_944675, EPI_ISL_944677, EPI_ISL_944678, EPI_ISL_944684, EPI_ISL_944689, EPI_ISL_944692, EPI_ISL_944694, EPI_ISL_944695, EPI_ISL_944696, EPI_ISL_944700, EPI_ISL_944701, EPI_ISL_944702, EPI_ISL_944703, EPI_ISL_944704, EPI_ISL_944705, EPI_ISL_944709, EPI_ISL_944710, EPI_ISL_944712, EPI_ISL_944715, EPI_ISL_944716, EPI_ISL_944717, EPI_ISL_944718, EPI_ISL_944720, EPI_ISL_944721, EPI_ISL_944723, EPI_ISL_944724, EPI_ISL_944725, EPI_ISL_944728, EPI_ISL_944729, EPI_ISL_944730, EPI_ISL_944731, EPI_ISL_944732, EPI_ISL_944733, EPI_ISL_944735			
see above	Department of Biochemistry, Cell and Molecular Biology, West African Centre for Cell Biology of Infectious Pathogens (WACCBIP), University of Ghana	Department of Biochemistry, Cell and Molecular Biology, West African Centre for Cell Biology of Infectious Pathogens (WACCBIP), University of Ghana	Morang'a,C.M., Ngoi,J.M., Quansah,E.B., Saiid,S., Amuzu,D.S., Asante,I., Bonney,J.H., Bonney,E., Odoom,J.K., Ndam,N.T., Tei-Maya,F., Adusei-Poku,M., Ofori-Boadu,L., Ampofo,W.K., Amenga-Etego,L.N., Quashie,P., Bediako,Y., Awandare,G.A.
EPI_ISL_945050, EPI_ISL_945053, EPI_ISL_945055, EPI_ISL_945058, EPI_ISL_945059, EPI_ISL_945060, EPI_ISL_945062	Lighthouse Lab in Milton Keynes	Wellcome Sanger Institute for the COVID-19 Genomics UK (COG-UK) Consortium	The Lighthouse Lab in Milton Keynes and Alex Alderton, Roberto Amato, Sonia Goncalves, Ewan Harrison, David K. Jackson, Ian Johnston, Dominic Kwiatkowski, Cordelia Langford, John Sillitoe on behalf of the Wellcome Sanger Institute COVID-19 Surveillance Team
EPI_ISL_945063	Lighthouse Lab in Cambridge	Wellcome Sanger Institute for the COVID-19 Genomics UK	Rob Howes, The Lighthouse Lab in Cambridge and Alex Alderton, Roberto Amato, Sonia Goncalves, Ewan Harrison, David K. Jackson, Ian Johnston,

		(COG-UK) Consortium	Dominic Kwiatkowski, Cordelia Langford, John Sillitoe on behalf of the Wellcome Sanger Institute COVID-19 Surveillance Team
EPI_ISL_945066	Lighthouse Lab in Milton Keynes	Wellcome Sanger Institute for the COVID-19 Genomics UK (COG-UK) Consortium	The Lighthouse Lab in Milton Keynes and Alex Alderton, Roberto Amato, Sonia Goncalves, Ewan Harrison, David K. Jackson, Ian Johnston, Dominic Kwiatkowski, Cordelia Langford, John Sillitoe on behalf of the Wellcome Sanger Institute COVID-19 Surveillance Team
EPI_ISL_945067	Lighthouse Lab in Cambridge	Wellcome Sanger Institute for the COVID-19 Genomics UK (COG-UK) Consortium	Rob Howes, The Lighthouse Lab in Cambridge and Alex Alderton, Roberto Amato, Sonia Goncalves, Ewan Harrison, David K. Jackson, Ian Johnston, Dominic Kwiatkowski, Cordelia Langford, John Sillitoe on behalf of the Wellcome Sanger Institute COVID-19 Surveillance Team
EPI_ISL_945069	Lighthouse Lab in Milton Keynes	Wellcome Sanger Institute for the COVID-19 Genomics UK (COG-UK) Consortium	The Lighthouse Lab in Milton Keynes and Alex Alderton, Roberto Amato, Sonia Goncalves, Ewan Harrison, David K. Jackson, Ian Johnston, Dominic Kwiatkowski, Cordelia Langford, John Sillitoe on behalf of the Wellcome Sanger Institute COVID-19 Surveillance Team
EPI_ISL_945076	Lighthouse Lab in Cambridge	Wellcome Sanger Institute for the COVID-19 Genomics UK (COG-UK) Consortium	Rob Howes, The Lighthouse Lab in Cambridge and Alex Alderton, Roberto Amato, Sonia Goncalves, Ewan Harrison, David K. Jackson, Ian Johnston, Dominic Kwiatkowski, Cordelia Langford, John Sillitoe on behalf of the Wellcome Sanger Institute COVID-19 Surveillance Team
EPI_ISL_945080, EPI_ISL_945082	Lighthouse Lab in Milton Keynes	Wellcome Sanger Institute for the COVID-19 Genomics UK (COG-UK) Consortium	The Lighthouse Lab in Milton Keynes and Alex Alderton, Roberto Amato, Sonia Goncalves, Ewan Harrison, David K. Jackson, Ian Johnston, Dominic Kwiatkowski, Cordelia Langford, John Sillitoe on behalf of the Wellcome Sanger Institute COVID-19 Surveillance Team
EPI_ISL_945083	Lighthouse Lab in Cambridge	Wellcome Sanger Institute for the COVID-19 Genomics UK (COG-UK) Consortium	Rob Howes, The Lighthouse Lab in Cambridge and Alex Alderton, Roberto Amato, Sonia Goncalves, Ewan Harrison, David K. Jackson, Ian Johnston, Dominic Kwiatkowski, Cordelia Langford, John Sillitoe on behalf of the Wellcome Sanger Institute COVID-19 Surveillance Team
EPI_ISL_945087, EPI_ISL_945090, EPI_ISL_945091, EPI_ISL_945092, EPI_ISL_945093, EPI_ISL_945094, EPI_ISL_945096	Lighthouse Lab in Milton Keynes	Wellcome Sanger Institute for the COVID-19 Genomics UK (COG-UK) Consortium	The Lighthouse Lab in Milton Keynes and Alex Alderton, Roberto Amato, Sonia Goncalves, Ewan Harrison, David K. Jackson, Ian Johnston, Dominic Kwiatkowski, Cordelia Langford, John Sillitoe on behalf of the Wellcome Sanger Institute COVID-19 Surveillance Team
EPI_ISL_945100	Lighthouse Lab in Cambridge	Wellcome Sanger Institute for the COVID-19 Genomics UK (COG-UK) Consortium	Rob Howes, The Lighthouse Lab in Cambridge and Alex Alderton, Roberto Amato, Sonia Goncalves, Ewan Harrison, David K. Jackson, Ian Johnston, Dominic Kwiatkowski, Cordelia Langford, John Sillitoe on behalf of the Wellcome Sanger Institute COVID-19 Surveillance Team
EPI_ISL_945101	Lighthouse Lab in Milton Keynes	Wellcome Sanger Institute for the COVID-19 Genomics UK (COG-UK) Consortium	The Lighthouse Lab in Milton Keynes and Alex Alderton, Roberto Amato, Sonia Goncalves, Ewan Harrison, David K. Jackson, Ian Johnston, Dominic Kwiatkowski, Cordelia Langford, John Sillitoe on behalf of the Wellcome Sanger Institute COVID-19 Surveillance Team
EPI_ISL_945103, EPI_ISL_945106	Lighthouse Lab in Cambridge	Wellcome Sanger Institute for the COVID-19 Genomics UK (COG-UK) Consortium	Rob Howes, The Lighthouse Lab in Cambridge and Alex Alderton, Roberto Amato, Sonia Goncalves, Ewan Harrison, David K. Jackson, Ian Johnston, Dominic Kwiatkowski, Cordelia Langford, John Sillitoe on behalf of the Wellcome Sanger Institute COVID-19 Surveillance Team
EPI_ISL_945108, EPI_ISL_945112	Lighthouse Lab in Milton Keynes	Wellcome Sanger Institute for the COVID-19 Genomics UK (COG-UK) Consortium	The Lighthouse Lab in Milton Keynes and Alex Alderton, Roberto Amato, Sonia Goncalves, Ewan Harrison, David K. Jackson, Ian Johnston, Dominic Kwiatkowski, Cordelia Langford, John Sillitoe on behalf of the Wellcome Sanger Institute COVID-19 Surveillance Team
EPI_ISL_945116	Lighthouse Lab in Cambridge	Wellcome Sanger Institute for the COVID-19 Genomics UK (COG-UK) Consortium	Rob Howes, The Lighthouse Lab in Cambridge and Alex Alderton, Roberto Amato, Sonia Goncalves, Ewan Harrison, David K. Jackson, Ian Johnston, Dominic Kwiatkowski, Cordelia Langford, John Sillitoe on behalf of the Wellcome Sanger Institute COVID-19 Surveillance Team
EPI_ISL_945118, EPI_ISL_945120, EPI_ISL_945121, EPI_ISL_945124, EPI_ISL_945128, EPI_ISL_945129	Lighthouse Lab in Milton Keynes	Wellcome Sanger Institute for the COVID-19 Genomics UK (COG-UK) Consortium	The Lighthouse Lab in Milton Keynes and Alex Alderton, Roberto Amato, Sonia Goncalves, Ewan Harrison, David K. Jackson, Ian Johnston, Dominic Kwiatkowski, Cordelia Langford, John Sillitoe on behalf of the Wellcome Sanger Institute COVID-19 Surveillance Team
EPI_ISL_945131	Lighthouse Lab in Cambridge	Wellcome Sanger Institute for the COVID-19 Genomics UK (COG-UK) Consortium	Rob Howes, The Lighthouse Lab in Cambridge and Alex Alderton, Roberto Amato, Sonia Goncalves, Ewan Harrison, David K. Jackson, Ian Johnston, Dominic Kwiatkowski, Cordelia Langford, John Sillitoe on behalf of the Wellcome Sanger Institute COVID-19 Surveillance Team
EPI_ISL_945133, EPI_ISL_945134	Lighthouse Lab in Milton Keynes	Wellcome Sanger Institute for the COVID-19 Genomics UK (COG-UK) Consortium	The Lighthouse Lab in Milton Keynes and Alex Alderton, Roberto Amato, Sonia Goncalves, Ewan Harrison, David K. Jackson, Ian Johnston, Dominic Kwiatkowski, Cordelia Langford, John Sillitoe on behalf of the Wellcome Sanger Institute COVID-19 Surveillance Team
EPI_ISL_945139	Lighthouse Lab in Cambridge	Wellcome Sanger Institute for the COVID-19 Genomics UK (COG-UK) Consortium	Rob Howes, The Lighthouse Lab in Cambridge and Alex Alderton, Roberto Amato, Sonia Goncalves, Ewan Harrison, David K. Jackson, Ian Johnston, Dominic Kwiatkowski, Cordelia Langford, John Sillitoe on behalf of the Wellcome Sanger Institute COVID-19 Surveillance Team
EPI_ISL_945141, EPI_ISL_945145	Lighthouse Lab in Milton Keynes	Wellcome Sanger Institute for the COVID-19 Genomics UK (COG-UK) Consortium	The Lighthouse Lab in Milton Keynes and Alex Alderton, Roberto Amato, Sonia Goncalves, Ewan Harrison, David K. Jackson, Ian Johnston, Dominic Kwiatkowski, Cordelia Langford, John Sillitoe on behalf of the Wellcome Sanger Institute COVID-19 Surveillance Team
EPI_ISL_945148	Lighthouse Lab in Cambridge	Wellcome Sanger Institute for the COVID-19 Genomics UK (COG-UK) Consortium	Rob Howes, The Lighthouse Lab in Cambridge and Alex Alderton, Roberto Amato, Sonia Goncalves, Ewan Harrison, David K. Jackson, Ian Johnston, Dominic Kwiatkowski, Cordelia Langford, John Sillitoe on behalf of the Wellcome Sanger Institute COVID-19 Surveillance Team
EPI_ISL_945149, EPI_ISL_945150, EPI_ISL_945153, EPI_ISL_945156	Lighthouse Lab in Milton Keynes	Wellcome Sanger Institute for the COVID-19 Genomics UK (COG-UK) Consortium	The Lighthouse Lab in Milton Keynes and Alex Alderton, Roberto Amato, Sonia Goncalves, Ewan Harrison, David K. Jackson, Ian Johnston, Dominic Kwiatkowski, Cordelia Langford, John Sillitoe on behalf of the Wellcome Sanger Institute COVID-19 Surveillance Team
EPI_ISL_945157	Lighthouse Lab in Cambridge	Wellcome Sanger Institute for the COVID-19 Genomics UK (COG-UK) Consortium	Rob Howes, The Lighthouse Lab in Cambridge and Alex Alderton, Roberto Amato, Sonia Goncalves, Ewan Harrison, David K. Jackson, Ian Johnston, Dominic Kwiatkowski, Cordelia Langford, John Sillitoe on behalf of the Wellcome Sanger Institute COVID-19 Surveillance Team
EPI_ISL_945161, EPI_ISL_945164	Lighthouse Lab in Milton Keynes	Wellcome Sanger Institute for the COVID-19 Genomics UK (COG-UK) Consortium	The Lighthouse Lab in Milton Keynes and Alex Alderton, Roberto Amato, Sonia Goncalves, Ewan Harrison, David K. Jackson, Ian Johnston, Dominic Kwiatkowski, Cordelia Langford, John Sillitoe on behalf of the Wellcome Sanger Institute COVID-19 Surveillance Team
EPI_ISL_945166	Lighthouse Lab in Cambridge	Wellcome Sanger Institute for the COVID-19 Genomics UK (COG-UK) Consortium	Rob Howes, The Lighthouse Lab in Cambridge and Alex Alderton, Roberto Amato, Sonia Goncalves, Ewan Harrison, David K. Jackson, Ian Johnston, Dominic Kwiatkowski, Cordelia Langford, John Sillitoe on behalf of the Wellcome Sanger Institute COVID-19 Surveillance Team
EPI_ISL_945172, EPI_ISL_945175	Lighthouse Lab in Milton Keynes	Wellcome Sanger Institute for the COVID-19 Genomics UK (COG-UK) Consortium	The Lighthouse Lab in Milton Keynes and Alex Alderton, Roberto Amato, Sonia Goncalves, Ewan Harrison, David K. Jackson, Ian Johnston, Dominic Kwiatkowski, Cordelia Langford, John Sillitoe on behalf of the Wellcome Sanger Institute COVID-19 Surveillance Team
EPI_ISL_945176	Lighthouse Lab in Cambridge	Wellcome Sanger Institute for the COVID-19 Genomics UK (COG-UK) Consortium	Rob Howes, The Lighthouse Lab in Cambridge and Alex Alderton, Roberto Amato, Sonia Goncalves, Ewan Harrison, David K. Jackson, Ian Johnston, Dominic Kwiatkowski, Cordelia Langford, John Sillitoe on behalf of the Wellcome Sanger Institute COVID-19 Surveillance Team
EPI_ISL_945177, EPI_ISL_945178, EPI_ISL_945179, EPI_ISL_945183, EPI_ISL_945185	Lighthouse Lab in Milton Keynes	Wellcome Sanger Institute for the COVID-19 Genomics UK (COG-UK) Consortium	The Lighthouse Lab in Milton Keynes and Alex Alderton, Roberto Amato, Sonia Goncalves, Ewan Harrison, David K. Jackson, Ian Johnston, Dominic Kwiatkowski, Cordelia Langford, John Sillitoe on behalf of the Wellcome Sanger Institute COVID-19 Surveillance Team
EPI_ISL_945186	Lighthouse Lab in Cambridge	Wellcome Sanger Institute for the COVID-19 Genomics UK (COG-UK) Consortium	Rob Howes, The Lighthouse Lab in Cambridge and Alex Alderton, Roberto Amato, Sonia Goncalves, Ewan Harrison, David K. Jackson, Ian Johnston, Dominic Kwiatkowski, Cordelia Langford, John Sillitoe on behalf of the Wellcome Sanger Institute COVID-19 Surveillance Team
EPI_ISL_945189	Lighthouse Lab in Milton Keynes	Wellcome Sanger Institute for the COVID-19 Genomics UK (COG-UK) Consortium	The Lighthouse Lab in Milton Keynes and Alex Alderton, Roberto Amato, Sonia Goncalves, Ewan Harrison, David K. Jackson, Ian Johnston, Dominic Kwiatkowski, Cordelia Langford, John Sillitoe on behalf of the Wellcome Sanger Institute COVID-19 Surveillance Team
EPI_ISL_945191, EPI_ISL_945192	Lighthouse Lab in Cambridge	Wellcome Sanger Institute for the COVID-19 Genomics UK (COG-UK) Consortium	Rob Howes, The Lighthouse Lab in Cambridge and Alex Alderton, Roberto Amato, Sonia Goncalves, Ewan Harrison, David K. Jackson, Ian Johnston, Dominic Kwiatkowski, Cordelia Langford, John Sillitoe on behalf of the Wellcome Sanger Institute COVID-19 Surveillance Team
EPI_ISL_945193, EPI_ISL_945198	Lighthouse Lab in Milton Keynes	Wellcome Sanger Institute for the COVID-19 Genomics UK (COG-UK) Consortium	The Lighthouse Lab in Milton Keynes and Alex Alderton, Roberto Amato, Sonia Goncalves, Ewan Harrison, David K. Jackson, Ian Johnston, Dominic Kwiatkowski, Cordelia Langford, John Sillitoe on behalf of the Wellcome Sanger Institute COVID-19 Surveillance Team
EPI_ISL_945199, EPI_ISL_945203	Lighthouse Lab in Cambridge	Wellcome Sanger Institute for the COVID-19 Genomics UK (COG-UK) Consortium	Rob Howes, The Lighthouse Lab in Cambridge and Alex Alderton, Roberto Amato, Sonia Goncalves, Ewan Harrison, David K. Jackson, Ian Johnston, Dominic Kwiatkowski, Cordelia Langford, John Sillitoe on behalf of the Wellcome Sanger Institute COVID-19 Surveillance Team
EPI_ISL_945205, EPI_ISL_945210, EPI_ISL_945218, EPI_ISL_945220	Lighthouse Lab in Milton Keynes	Wellcome Sanger Institute for the COVID-19 Genomics UK (COG-UK) Consortium	The Lighthouse Lab in Milton Keynes and Alex Alderton, Roberto Amato, Sonia Goncalves, Ewan Harrison, David K. Jackson, Ian Johnston, Dominic Kwiatkowski, Cordelia Langford, John Sillitoe on behalf of the Wellcome Sanger Institute COVID-19 Surveillance Team
EPI_ISL_945222	Lighthouse Lab in Cambridge	Wellcome Sanger Institute for the COVID-19 Genomics UK (COG-UK) Consortium	Rob Howes, The Lighthouse Lab in Cambridge and Alex Alderton, Roberto Amato, Sonia Goncalves, Ewan Harrison, David K. Jackson, Ian Johnston, Dominic Kwiatkowski, Cordelia Langford, John Sillitoe on behalf of the Wellcome Sanger Institute COVID-19 Surveillance Team
EPI_ISL_945228, EPI_ISL_945230	Lighthouse Lab in Milton Keynes	Wellcome Sanger Institute for the COVID-19 Genomics UK (COG-UK) Consortium	The Lighthouse Lab in Milton Keynes and Alex Alderton, Roberto Amato, Sonia Goncalves, Ewan Harrison, David K. Jackson, Ian Johnston, Dominic Kwiatkowski, Cordelia Langford, John Sillitoe on behalf of the Wellcome Sanger Institute COVID-19 Surveillance Team

EPI_ISL_945232, EPI_ISL_945237	Lighthouse Lab in Cambridge	Wellcome Sanger Institute for the COVID-19 Genomics UK (COG-UK) Consortium	Rob Howes, The Lighthouse Lab in Cambridge and Alex Alderton, Roberto Amato, Sonia Goncalves, Ewan Harrison, David K. Jackson, Ian Johnston, Dominic Kwiatkowski, Cordelia Langford, John Sillitoe on behalf of the Wellcome Sanger Institute COVID-19 Surveillance Team
EPI_ISL_945239, EPI_ISL_945246, EPI_ISL_945251, EPI_ISL_945253, EPI_ISL_945256	Lighthouse Lab in Milton Keynes	Wellcome Sanger Institute for the COVID-19 Genomics UK (COG-UK) Consortium	The Lighthouse Lab in Milton Keynes and Alex Alderton, Roberto Amato, Sonia Goncalves, Ewan Harrison, David K. Jackson, Ian Johnston, Dominic Kwiatkowski, Cordelia Langford, John Sillitoe on behalf of the Wellcome Sanger Institute COVID-19 Surveillance Team
EPI_ISL_945257	Lighthouse Lab in Cambridge	Wellcome Sanger Institute for the COVID-19 Genomics UK (COG-UK) Consortium	Rob Howes, The Lighthouse Lab in Cambridge and Alex Alderton, Roberto Amato, Sonia Goncalves, Ewan Harrison, David K. Jackson, Ian Johnston, Dominic Kwiatkowski, Cordelia Langford, John Sillitoe on behalf of the Wellcome Sanger Institute COVID-19 Surveillance Team
EPI_ISL_945258	Lighthouse Lab in Milton Keynes	Wellcome Sanger Institute for the COVID-19 Genomics UK (COG-UK) Consortium	The Lighthouse Lab in Milton Keynes and Alex Alderton, Roberto Amato, Sonia Goncalves, Ewan Harrison, David K. Jackson, Ian Johnston, Dominic Kwiatkowski, Cordelia Langford, John Sillitoe on behalf of the Wellcome Sanger Institute COVID-19 Surveillance Team
EPI_ISL_945261	Lighthouse Lab in Cambridge	Wellcome Sanger Institute for the COVID-19 Genomics UK (COG-UK) Consortium	Rob Howes, The Lighthouse Lab in Cambridge and Alex Alderton, Roberto Amato, Sonia Goncalves, Ewan Harrison, David K. Jackson, Ian Johnston, Dominic Kwiatkowski, Cordelia Langford, John Sillitoe on behalf of the Wellcome Sanger Institute COVID-19 Surveillance Team
EPI_ISL_945266	Lighthouse Lab in Milton Keynes	Wellcome Sanger Institute for the COVID-19 Genomics UK (COG-UK) Consortium	The Lighthouse Lab in Milton Keynes and Alex Alderton, Roberto Amato, Sonia Goncalves, Ewan Harrison, David K. Jackson, Ian Johnston, Dominic Kwiatkowski, Cordelia Langford, John Sillitoe on behalf of the Wellcome Sanger Institute COVID-19 Surveillance Team
EPI_ISL_945268, EPI_ISL_945271	Lighthouse Lab in Cambridge	Wellcome Sanger Institute for the COVID-19 Genomics UK (COG-UK) Consortium	Rob Howes, The Lighthouse Lab in Cambridge and Alex Alderton, Roberto Amato, Sonia Goncalves, Ewan Harrison, David K. Jackson, Ian Johnston, Dominic Kwiatkowski, Cordelia Langford, John Sillitoe on behalf of the Wellcome Sanger Institute COVID-19 Surveillance Team
EPI_ISL_945276, EPI_ISL_945277, EPI_ISL_945278, EPI_ISL_945287, EPI_ISL_945292	Lighthouse Lab in Milton Keynes	Wellcome Sanger Institute for the COVID-19 Genomics UK (COG-UK) Consortium	The Lighthouse Lab in Milton Keynes and Alex Alderton, Roberto Amato, Sonia Goncalves, Ewan Harrison, David K. Jackson, Ian Johnston, Dominic Kwiatkowski, Cordelia Langford, John Sillitoe on behalf of the Wellcome Sanger Institute COVID-19 Surveillance Team
EPI_ISL_945309	Lighthouse Lab in Cambridge	Wellcome Sanger Institute for the COVID-19 Genomics UK (COG-UK) Consortium	Rob Howes, The Lighthouse Lab in Cambridge and Alex Alderton, Roberto Amato, Sonia Goncalves, Ewan Harrison, David K. Jackson, Ian Johnston, Dominic Kwiatkowski, Cordelia Langford, John Sillitoe on behalf of the Wellcome Sanger Institute COVID-19 Surveillance Team
EPI_ISL_945313, EPI_ISL_945314, EPI_ISL_945315	Lighthouse Lab in Milton Keynes	Wellcome Sanger Institute for the COVID-19 Genomics UK (COG-UK) Consortium	The Lighthouse Lab in Milton Keynes and Alex Alderton, Roberto Amato, Sonia Goncalves, Ewan Harrison, David K. Jackson, Ian Johnston, Dominic Kwiatkowski, Cordelia Langford, John Sillitoe on behalf of the Wellcome Sanger Institute COVID-19 Surveillance Team
EPI_ISL_945321	Lighthouse Lab in Cambridge	Wellcome Sanger Institute for the COVID-19 Genomics UK (COG-UK) Consortium	Rob Howes, The Lighthouse Lab in Cambridge and Alex Alderton, Roberto Amato, Sonia Goncalves, Ewan Harrison, David K. Jackson, Ian Johnston, Dominic Kwiatkowski, Cordelia Langford, John Sillitoe on behalf of the Wellcome Sanger Institute COVID-19 Surveillance Team
EPI_ISL_945322, EPI_ISL_945325, EPI_ISL_945329, EPI_ISL_945331, EPI_ISL_945333, EPI_ISL_945336	Lighthouse Lab in Milton Keynes	Wellcome Sanger Institute for the COVID-19 Genomics UK (COG-UK) Consortium	The Lighthouse Lab in Milton Keynes and Alex Alderton, Roberto Amato, Sonia Goncalves, Ewan Harrison, David K. Jackson, Ian Johnston, Dominic Kwiatkowski, Cordelia Langford, John Sillitoe on behalf of the Wellcome Sanger Institute COVID-19 Surveillance Team
EPI_ISL_945341	Lighthouse Lab in Cambridge	Wellcome Sanger Institute for the COVID-19 Genomics UK (COG-UK) Consortium	Rob Howes, The Lighthouse Lab in Cambridge and Alex Alderton, Roberto Amato, Sonia Goncalves, Ewan Harrison, David K. Jackson, Ian Johnston, Dominic Kwiatkowski, Cordelia Langford, John Sillitoe on behalf of the Wellcome Sanger Institute COVID-19 Surveillance Team
EPI_ISL_945344	Lighthouse Lab in Milton Keynes	Wellcome Sanger Institute for the COVID-19 Genomics UK (COG-UK) Consortium	The Lighthouse Lab in Milton Keynes and Alex Alderton, Roberto Amato, Sonia Goncalves, Ewan Harrison, David K. Jackson, Ian Johnston, Dominic Kwiatkowski, Cordelia Langford, John Sillitoe on behalf of the Wellcome Sanger Institute COVID-19 Surveillance Team
EPI_ISL_945346	Lighthouse Lab in Cambridge	Wellcome Sanger Institute for the COVID-19 Genomics UK (COG-UK) Consortium	Rob Howes, The Lighthouse Lab in Cambridge and Alex Alderton, Roberto Amato, Sonia Goncalves, Ewan Harrison, David K. Jackson, Ian Johnston, Dominic Kwiatkowski, Cordelia Langford, John Sillitoe on behalf of the Wellcome Sanger Institute COVID-19 Surveillance Team
EPI_ISL_945352	Lighthouse Lab in Milton Keynes	Wellcome Sanger Institute for the COVID-19 Genomics UK (COG-UK) Consortium	The Lighthouse Lab in Milton Keynes and Alex Alderton, Roberto Amato, Sonia Goncalves, Ewan Harrison, David K. Jackson, Ian Johnston, Dominic Kwiatkowski, Cordelia Langford, John Sillitoe on behalf of the Wellcome Sanger Institute COVID-19 Surveillance Team
EPI_ISL_945356	Lighthouse Lab in Cambridge	Wellcome Sanger Institute for the COVID-19 Genomics UK (COG-UK) Consortium	Rob Howes, The Lighthouse Lab in Cambridge and Alex Alderton, Roberto Amato, Sonia Goncalves, Ewan Harrison, David K. Jackson, Ian Johnston, Dominic Kwiatkowski, Cordelia Langford, John Sillitoe on behalf of the Wellcome Sanger Institute COVID-19 Surveillance Team
EPI_ISL_945362	Lighthouse Lab in Milton Keynes	Wellcome Sanger Institute for the COVID-19 Genomics UK (COG-UK) Consortium	The Lighthouse Lab in Milton Keynes and Alex Alderton, Roberto Amato, Sonia Goncalves, Ewan Harrison, David K. Jackson, Ian Johnston, Dominic Kwiatkowski, Cordelia Langford, John Sillitoe on behalf of the Wellcome Sanger Institute COVID-19 Surveillance Team
EPI_ISL_945365	Lighthouse Lab in Cambridge	Wellcome Sanger Institute for the COVID-19 Genomics UK (COG-UK) Consortium	Rob Howes, The Lighthouse Lab in Cambridge and Alex Alderton, Roberto Amato, Sonia Goncalves, Ewan Harrison, David K. Jackson, Ian Johnston, Dominic Kwiatkowski, Cordelia Langford, John Sillitoe on behalf of the Wellcome Sanger Institute COVID-19 Surveillance Team
EPI_ISL_945368, EPI_ISL_945372, EPI_ISL_945373	Lighthouse Lab in Milton Keynes	Wellcome Sanger Institute for the COVID-19 Genomics UK (COG-UK) Consortium	The Lighthouse Lab in Milton Keynes and Alex Alderton, Roberto Amato, Sonia Goncalves, Ewan Harrison, David K. Jackson, Ian Johnston, Dominic Kwiatkowski, Cordelia Langford, John Sillitoe on behalf of the Wellcome Sanger Institute COVID-19 Surveillance Team
EPI_ISL_946746	Lighthouse Lab in Alderley Park	Wellcome Sanger Institute for the COVID-19 Genomics UK (COG-UK) Consortium	Jacquelyn Wynn, Mairead Hyland, The Lighthouse Lab in Alderley Park and Alex Alderton, Roberto Amato, Sonia Goncalves, Ewan Harrison, David K. Jackson, Ian Johnston, Dominic Kwiatkowski, Cordelia Langford, John Sillitoe on behalf of the Wellcome Sanger Institute COVID-19 Surveillance Team
EPI_ISL_947322	RS Mitra Keluarga Kelapa Gading	Eijkman Institute for Molecular Biology, Ministry of Research and Technology/National Agency for Research and Innovation	Sukma Oktavianthi, Willy Agustine, Edison Johar, Hidayat Trimarsanto, Iskandar Adnan, Lydia V. Panggalo, Frilasita A Yudhaputri, Safarina G Malik, Khin Saw Myint, Amin Soebandrio
EPI_ISL_947324	RSU Medirossa	Eijkman Institute for Molecular Biology, Ministry of Research and Technology/National Agency for Research and Innovation	Sukma Oktavianthi, Willy Agustine, Edison Johar, Hidayat Trimarsanto, Iskandar Adnan, Lydia V. Panggalo, Frilasita A Yudhaputri, Safarina G Malik, Khin Saw Myint, Amin Soebandrio
EPI_ISL_947325	RSIA PKU Muhammadiyah	Eijkman Institute for Molecular Biology, Ministry of Research and Technology/National Agency for Research and Innovation	Sukma Oktavianthi, Willy Agustine, Edison Johar, Hidayat Trimarsanto, Iskandar Adnan, Lydia V. Panggalo, Frilasita A Yudhaputri, Safarina G Malik, Khin Saw Myint, Amin Soebandrio
EPI_ISL_947327	RS Mitra Keluarga Gading Serpong	Eijkman Institute for Molecular Biology, Ministry of Research and Technology/National Agency for Research and Innovation	Willy Agustine, Edison Johar, Hidayat Trimarsanto, Iskandar Adnan, Lydia V. Panggalo, Sukma Oktavianthi, Frilasita A Yudhaputri, Safarina G Malik, Khin Saw Myint, Amin Soebandrio
EPI_ISL_949183	National Institute of Health Research and Development	National Institute of Health Research and Development	Kindi Adam; Holy Arif Wibowo; Ririn Ramadhany; Yuni Rukminiati; Agustiningsih; Hana Apsari Pawestri; Subangkit; Kartika Dewi Puspa; Arie Ardiansyah Nugraha; Hartanti Dian Ikawati; Krisna Nur Andriana Pangesti; Triyani Soekarso; Ni Ketut Susilarini; Nur Ika Hariastuti; Uly Alfi Nikmah; Mursinah; Asri Febriyani; Reni Herman; Nike Susanti; Herna; Tati Febriyanti; Natalie Laurencia Kipuw; Fauzul Muna; Irene Lorinda Indalao; Aulia Rizki; Nelly Puspandari; Vivi Setiawaty
EPI_ISL_949411	University of Birmingham	COVID-19 Genomics UK (COG-UK) Consortium	Institute of Microbiology, University of Birmingham: Claire McMurray, Joanne Stockton, Samuel Nicholls, Radoslaw Poplawski, Will Rowe, Josh Quick, Nicholas Loman. University of Birmingham Testing Laboratory: Celina M Whalley, Andrew Bosworth, Charlotte Poxon, Kasun Wanigasooriya, Oliver Pickles, Mike Kidd, Alex Richter, Andrew D Beggs PHE Heartlands Lab: Husam Osman, Andrew Bosworth. Queen Elizabeth Hospital: Anna Casey
EPI_ISL_949754, EPI_ISL_949756, EPI_ISL_949764, EPI_ISL_949769, EPI_ISL_949771, EPI_ISL_949773, EPI_ISL_949775, EPI_ISL_949781	Barts Health NHS Trust	COVID-19 Genomics UK (COG-UK) Consortium	CUTINO-MOGUEL, Maria-Teresa; HARRINGTON, David; OWOYEMI, Dola; KULASEGARAN-SHYLINI, Raghavendran; BROAD, Claire; KELE, Beatrix
EPI_ISL_949788, EPI_ISL_949927, EPI_ISL_950068, EPI_ISL_950073, EPI_ISL_950076, EPI_ISL_950081, EPI_ISL_950087, EPI_ISL_950089, EPI_ISL_950090, EPI_ISL_950091, EPI_ISL_950093, EPI_ISL_950095, EPI_ISL_950096, EPI_ISL_950099, EPI_ISL_950103, EPI_ISL_950104, EPI_ISL_950106, EPI_ISL_950107, EPI_ISL_950108, EPI_ISL_950109, EPI_ISL_950113, EPI_ISL_950116, EPI_ISL_950117, EPI_ISL_950118, EPI_ISL_950119, EPI_ISL_950120, EPI_ISL_950122, EPI_ISL_950123, EPI_ISL_950124, EPI_ISL_950125, EPI_ISL_950128, EPI_ISL_950129, EPI_ISL_950130, EPI_ISL_950133, EPI_ISL_950136, EPI_ISL_950142, EPI_ISL_950147, EPI_ISL_950148, EPI_ISL_950152, EPI_ISL_950154			
see above	University College London, Great Ormond Street Hospital for Children NHS Foundation Trust, Imperial College Healthcare NHS Trust	COVID-19 Genomics UK (COG-UK) Consortium	Sergi Castellano, Rachel Williams, Mark Kristiansen, Paola Resende Silva, Sunando Roy, Tony Brooks, Helena Tutill, Paola Niola, Patricia Dyal, Charlotte Williams, Leysa Forrest, Yasmin Panchbhaya, Jacqueline Findlay, Samuel Weeks, Julianne Brown, Kathryn Harris, Paul Randell, James Price, Alison Holmes, Judith Breuer
EPI_ISL_950268, EPI_ISL_950270	Northumbria University / South Tees Hospitals NHS Foundation Trust / North Cumbria Integrated Care NHS Foundation Trust / North Tees and Hartlepool NHS	COVID-19 Genomics UK (COG-UK) Consortium	Darren L Smith,Andrew Nelson,Matthew Bashton,Greg R Young,Joshua Loh,John Allan,Mohammad A Tariq,Giles S Holt,Gary Black,Wen C Yew,Lynn Dover,Paul Baker,Steve Liggett,Sarah Essex,Jane Greenaway,Debra Padgett,Clive Graham,Garren Scott,Edward Barton,Emma Swindells,Brendan Payne,Jennifer Collins,Yusri Taha,Gary Eltringham

	Foundation Trust / Newcastle Hospitals NHS Foundation Trust		
EPI_ISL_950712	Lincolnshire Hospitals and DeepSeq Nottingham	COVID-19 Genomics UK (COG-UK) Consortium	Nichola Duckworth, Tim Sloan, Sarah Walsh, Jonathan Ball, Patrick McClure, Joeseph Chappell, Nadine Holmes, Matthew Carlisle, Christopher Moore, Fei Sang, Johnny Debebe, Victoria Wright, Matthew Loose
EPI_ISL_951125, EPI_ISL_951141, EPI_ISL_951152, EPI_ISL_951154, EPI_ISL_951158, EPI_ISL_951160, EPI_ISL_951172, EPI_ISL_951174, EPI_ISL_951175, EPI_ISL_951176, EPI_ISL_951177, EPI_ISL_951180, EPI_ISL_951181, EPI_ISL_951182, EPI_ISL_951184, EPI_ISL_951186, EPI_ISL_951189, EPI_ISL_951193, EPI_ISL_951195, EPI_ISL_951196, EPI_ISL_951197, EPI_ISL_951199, EPI_ISL_951201, EPI_ISL_951202, EPI_ISL_951204, EPI_ISL_951205, EPI_ISL_951206, EPI_ISL_951207, EPI_ISL_951209, EPI_ISL_951210, EPI_ISL_951211, EPI_ISL_951212, EPI_ISL_951213, EPI_ISL_951215, EPI_ISL_951216, EPI_ISL_951218, EPI_ISL_951220, EPI_ISL_951221, EPI_ISL_951223, EPI_ISL_951224, EPI_ISL_951225, EPI_ISL_951226, EPI_ISL_951227, EPI_ISL_951228, EPI_ISL_951233, EPI_ISL_951234, EPI_ISL_951235, EPI_ISL_951237, EPI_ISL_951238, EPI_ISL_951241, EPI_ISL_951242, EPI_ISL_951264, EPI_ISL_951267, EPI_ISL_951301, EPI_ISL_951320, EPI_ISL_951323, EPI_ISL_951324, EPI_ISL_951326, EPI_ISL_951327, EPI_ISL_951328, EPI_ISL_951329, EPI_ISL_951331, EPI_ISL_951333, EPI_ISL_951334, EPI_ISL_951335, EPI_ISL_951336, EPI_ISL_951337, EPI_ISL_951339, EPI_ISL_951340, EPI_ISL_951341, EPI_ISL_951342, EPI_ISL_951344, EPI_ISL_951345, EPI_ISL_951346, EPI_ISL_951347, EPI_ISL_951348, EPI_ISL_951349, EPI_ISL_951350, EPI_ISL_951351, EPI_ISL_951352, EPI_ISL_951353, EPI_ISL_951354, EPI_ISL_951382, EPI_ISL_951397, EPI_ISL_951398, EPI_ISL_951400, EPI_ISL_951401, EPI_ISL_951402, EPI_ISL_951404, EPI_ISL_951405, EPI_ISL_951410			
see above	Oxford Viromics, NDM, University of Oxford; Oxford University Hospitals; Basingstoke and North Hampshire Hospital	COVID-19 Genomics UK (COG-UK) Consortium	Tanya Golubchik, David Bonsall, George Macintyre, Amy Trebes, Mariateresa de Cesare, Catrin Moore, Alex Mobbs, Anita Justice, Robert Shaw, Monique Andersson, Timothy Peto, Emma Wise, Nathan Moore, Jessica Lynch, Nick Cortes, Matilde Mori, Stephen Kidd, David Buck, John Todd, Christophe Fraser
EPI_ISL_952401, EPI_ISL_952407, EPI_ISL_952408, EPI_ISL_952413, EPI_ISL_952415, EPI_ISL_952785, EPI_ISL_952813, EPI_ISL_952814, EPI_ISL_952815	Centre for Enzyme Innovation, University of Portsmouth / Translational Research Laboratory, Portsmouth Hospitals NHS Trust	COVID-19 Genomics UK (COG-UK) Consortium	Angela Beckett,Salman Goudarzi,Christopher Fearn,Kate Cook,Katie Loveson,Sharon Glaysher,Scott Elliott,Samuel Robson
EPI_ISL_953178, EPI_ISL_953210, EPI_ISL_953214, EPI_ISL_953242, EPI_ISL_953260, EPI_ISL_953273, EPI_ISL_953274, EPI_ISL_953278	Bioinformatics and Biostatistics Lab, Advanced Sequencing Facility	COVID-19 Genomics UK (COG-UK) Consortium	Aengus Stewart,Jerome Nicod,Chelsea Sawyer,Laura Cubitt,Harshil Patel,Margaret Crawford
EPI_ISL_953997	Hopital	National Reference Center for Viruses of Respiratory Infections, Institut Pasteur, Paris	Marion Barbet, Sylvie Behillil, Méline Bizard, Angela Brisebarre, Camille Capel, Etienne Simon-Lorière, Vincent Enouf, Maud Vanpeene, Sylvie van der Werf,Lagathu GisèLe
EPI_ISL_954220, EPI_ISL_954221, EPI_ISL_954222	1.AO Universitaria 'S. Giovanni di Dio e Ruggi D'Aragona, Scuola Medica Salernitana' Hospital / 2.UOC di Virologia e Microbiologia, Università della Campania 'L. Vanvitelli' / 3.AO Universitaria 'Federico II' Napoli Hospital / 4.AORN 'San Giuseppe Moscati' Avellino Hospital / 5.AO 'San Pio - presidio G. Rummo' Benevento Hospital / 6.AO 'Sant'Anna e San Sebastiano' Caserta Hospital / 7.PO 'Maria Santissima Addolorata' Eboli Hospital / 8.Biogem Istituto di Ricerche Genetiche	1. Genome Research Center for Health (CRGS) / 2. Laboratory of Molecular Medicine and Genomics(LMMGe) / 3. Center for Research in Pure and Applied Mathematics (CRMPA)	Giorgio Giurato, Francesca Rizzo, Alessandro Weisz, Gianluigi Franci, Giovanni Nassa, Pasquale Pagliano, Roberta Tarallo, Elena Alexandrova, Ylenia D'Agostino, Carlo Ferravante, Jessica Lamberti, Viola Melone, Domenico Memoli, Valeria Mirici Cappa, Domenico Palumbo, Giovanni Pecoraro, Assunta Sellitto, Oriana Strianese, Ilaria Terenzi, Giuseppe Fenza, Aniello Gentile, Antonello Saccomanno, Sonia Amabile, Teresa Rocco, Annamaria Salvati, Emilia Vaccaro, Massimiliano Galdiero, Michele Cennamo, Giuseppe Portella, Maria Grazia Foti, Mariarosaria Ingino, Maria Landi, Maurizio Fumi, Vincenzo Rocco, Rita Greco, Vittoria Letizia, Arnolfo Petruzziello, Maddalena Schioppa, Gregorio Goffredi, Francesca Marciano, Michele Caraglia, Alessia Cossu, Marianna Scrima, Edmondo Adorisio, Morena D'Avenia, Michela Iacobellis, Rosanna Piluscio, Giorgio Dirani, Vittorio Sambri, Simona Semprini, Silvia Zanoli, Francesco Curcio, Stefania Marzinotto, Andreina Baj, Fausto Sessa.
EPI_ISL_954831, EPI_ISL_954844, EPI_ISL_954848, EPI_ISL_954852, EPI_ISL_954856, EPI_ISL_954857, EPI_ISL_954862, EPI_ISL_954864, EPI_ISL_954886, EPI_ISL_954902, EPI_ISL_954903, EPI_ISL_954904, EPI_ISL_954905, EPI_ISL_954906, EPI_ISL_954907, EPI_ISL_954908, EPI_ISL_954909, EPI_ISL_954910, EPI_ISL_954911, EPI_ISL_954912, EPI_ISL_954913, EPI_ISL_954914, EPI_ISL_954915, EPI_ISL_954916, EPI_ISL_954917, EPI_ISL_954918			
see above	Colorado Department of Public Health and Environment	Colorado Department of Puplic Health and Environment	Laura Bankers, Molly C. Hetherington-Rauth, Diana Ir, Shannon Ely, Shannon R. Matzinger, Sarah Elizabeth Totten, Emily A. Travanty
EPI_ISL_955127	Innovative Genomics Institute, UC Berkeley	Innovative Genomics Institute, UC Berkeley	Stacia Wyman, Haridha Shivram, Phil Frankino, Liana Lareau, Shana McDevitt, Justin Choi
EPI_ISL_955184	University of Sarajevo, Veterinary Faculty, Laboratory for Molecular Diagnostic and Research Laboratory	University of Sarajevo, Veterinary Faculty, Laboratory for Molecular Diagnostic and Research Laboratory	Goleti Š., Goleti T., Softi A., Ali-Šeho A., Nicevi M., Hodži A., Terzi I., Šabi E., Jaži A.
EPI_ISL_955185	University of Sarajevo, Veterinary Faculty, Laboratory for Molecular Diagnostic and Research Laboratory	University of Sarajevo, Veterinary Faculty, Laboratory for Molecular Diagnostic and Research Laboratory	Goleti Š., Goleti T., Ali-Šeho A., Softi A., Terzi I., Šabi E., Jaži A., Nicevi M., Hodži A.
EPI_ISL_955211	University of Sarajevo, Veterinary Faculty, Laboratory for Molecular Diagnostic and Research Laboratory	University of Sarajevo, Veterinary Faculty, Laboratory for Molecular Diagnostic and Research Laboratory	Goleti Š., Goleti T., Ali-Šeho A., Softi A., Jaži A., Terzi I., Šabi E., Nicevi M., Hodži A.
EPI_ISL_955274, EPI_ISL_955285	American Esoteric Laboratory	Pathogen Discovery, Respiratory Viruses Branch, Division of Viral Diseases, Centers for Disease Control and Prevention	Ying Tao, Jing Zhang, Yan Li, Krista Queen, Anna Uehara, Peter Cook, Clinton R. Paden, Haibin Wang, Suxiang Tong
EPI_ISL_955311	TX DSHS, Lab Services Section MC 1947	Pathogen Discovery, Respiratory Viruses Branch, Division of Viral Diseases, Centers for Disease Control and Prevention	Ying Tao, Jing Zhang, Yan Li, Krista Queen, Anna Uehara, Peter Cook, Clinton R. Paden, Haibin Wang, Suxiang Tong
EPI_ISL_955324, EPI_ISL_955325, EPI_ISL_955326, EPI_ISL_955327	American Esoteric Laboratory	Pathogen Discovery, Respiratory Viruses Branch, Division of Viral Diseases, Centers for Disease Control and Prevention	Ying Tao, Jing Zhang, Yan Li, Krista Queen, Anna Uehara, Peter Cook, Clinton R. Paden, Haibin Wang, Suxiang Tong
EPI_ISL_955330, EPI_ISL_955335	GA Department of Public Health Laboratory	Pathogen Discovery, Respiratory Viruses Branch, Division of Viral Diseases, Centers for Disease Control and Prevention	Ying Tao, Jing Zhang, Yan Li, Krista Queen, Anna Uehara, Peter Cook, Clinton R. Paden, Haibin Wang, Suxiang Tong
EPI_ISL_955411, EPI_ISL_955417, EPI_ISL_955418, EPI_ISL_955419, EPI_ISL_955420, EPI_ISL_955421, EPI_ISL_955422, EPI_ISL_955423, EPI_ISL_955424, EPI_ISL_955425	Alameda County Public Health Lab	Chan-Zuckerberg Biohub	CZB Cliahub Consortium
EPI_ISL_955447, EPI_ISL_955452, EPI_ISL_955453, EPI_ISL_955464, EPI_ISL_955465, EPI_ISL_955466, EPI_ISL_955467, EPI_ISL_955470, EPI_ISL_955471, EPI_ISL_955472, EPI_ISL_955473, EPI_ISL_955474, EPI_ISL_955475, EPI_ISL_955476, EPI_ISL_955477, EPI_ISL_955478, EPI_ISL_955479, EPI_ISL_955480, EPI_ISL_955481, EPI_ISL_955482, EPI_ISL_955483, EPI_ISL_955484, EPI_ISL_955485, EPI_ISL_955486, EPI_ISL_955487, EPI_ISL_955488, EPI_ISL_955489, EPI_ISL_955490, EPI_ISL_955491, EPI_ISL_955492, EPI_ISL_955493, EPI_ISL_955494, EPI_ISL_955495			
see above	Orange County Public Health Lab	Chan-Zuckerberg Biohub	CZB Cliahub Consortium
EPI_ISL_955777	Humboldt County Public Health Laboratory	Chan-Zuckerberg Biohub	CZB Cliahub Consortium
EPI_ISL_956300	Laboratorio de salud publica Arauca	Instituto Nacional de Salud- Dirección de Investigación en Salud Pública	Katherine Laiton-Donato, Diego A. Álvarez-Díaz, Carlos Franco-Muñoz, Mauricio Pacheco-Montealegre, Hector Alejandro Ruiz-Moreno, Maria T. Herrera-Sepúlveda, Diego Andrés Prada, Jhonnatan Reales-González, Sheryll Corchuelo, Julian Naizaque, Gerardo Santamaría, Magdalena Wiesner, Martha Lucia Ospina Martinez, Marcela Mercado-Reyes
EPI_ISL_956316	Institute of Tropical Disease	Institute of Tropical Disease, Universitas Airlangga	Krisnoadi Rahardjo, Aldise M Nastri, Jezzy R Dewantari, Rima R Prasetya, Gatot Soegiarto, Laksmi Wulandari, Resti Yudhawati, Yasuko Mori, Soetjipto, Kazufumi Shimizu, Maria I Lusida
EPI_ISL_956421, EPI_ISL_956668	General Hospital - Kumanovo	Research Center for Genetic Engineering and Biotechnology "Georgi D. Efremov" , Macedonian Academy of Sciences and Arts	Aleksandar J. Dimovski, Dijana Plasheska-Karanfilska, Predrag Noveski, Gjorgji Bozinovski, Milena Jakimovska
EPI_ISL_957423	General Hospital - Bitola	Research Center for Genetic Engineering and Biotechnology "Georgi D. Efremov" , Macedonian Academy of Sciences and Arts	Aleksandar J. Dimovski, Dijana Plasheska-Karanfilska, Predrag Noveski, Gjorgji Bozinovski, Milena Jakimovska
EPI_ISL_957966	General Hospital - Tetovo	Research Center for Genetic Engineering and Biotechnology "Georgi D. Efremov" , Macedonian Academy of Sciences and Arts	Aleksandar J. Dimovski, Dijana Plasheska-Karanfilska, Predrag Noveski, Gjorgji Bozinovski, Milena Jakimovska
EPI_ISL_958564, EPI_ISL_958565	Lighthouse Lab in Milton Keynes	Wellcome Sanger Institute for the COVID-19 Genomics UK (COG-UK) Consortium	The Lighthouse Lab in Milton Keynes and Alex Alderton, Roberto Amato, Sonia Goncalves, Ewan Harrison, David K. Jackson, Ian Johnston, Dominic Kwiatkowski, Cordelia Langford, John Sillitoe on behalf of the Wellcome Sanger Institute COVID-19 Surveillance Team

EPI_ISL_958751	General Hospital - Bitola	Research Center for Genetic Engineering and Biotechnology "Georgi D. Efremov" , Macedonian Academy of Sciences and Arts	Aleksandar J. Dimovski, Dijana Plasheska-Karanfilska, Predrag Noveski, Gjorgji Bozinovski, Milena Jakimovska
EPI_ISL_959851	National Virus Reference Laboratory	National Virus Reference Laboratory	Michael Carr, Gabriel Gonzalez, Jonathan Dean, Cillian F De Gascun
EPI_ISL_960313	University of Wisconsin-Madison AIDS Vaccine Research Laboratories	University of Wisconsin-Madison AIDS Vaccine Research Laboratories	Gage Moreno, Katarina Braun, et al. AIDS Vaccine Research Laboratories
EPI_ISL_960409, EPI_ISL_960411, EPI_ISL_960416, EPI_ISL_960426, EPI_ISL_960432	The National Institute of Public Health	State Veterinary Institute Prague	Nagy,A;Vecerova,J;Cernikova,L;Stara,M;Jirincova,H;Trnka,D
EPI_ISL_960465	Istituto Zooprofilattico Sperimentale del Mezzogiorno	TIGEM	Patrizia Annunziata, Andrea Ballabio, Valentina Bouche, Davide Cacchiarelli, Pellegrino Cerino, Chiara Colantuono, Maria Concetta Cuomo, Denise Di Concilio, Lucio Di Filippo, Antonio Grimaldi, Antonio Limone, Anna Manfredi, Francesco Panariello, Biancamaria Pierri, Marcello Salvi
EPI_ISL_960853, EPI_ISL_960868	Institute of Medical Microbiology and Hospital Hygiene	Institute of Medical Microbiology and Hospital Hygiene	Prof. Dr. Achim Kaasch, Aljoscha Tersteegen
EPI_ISL_961127, EPI_ISL_961128	Delaware Public Health Laboratory	Delaware Public Health Lab	Gregory Hovan
EPI_ISL_961223, EPI_ISL_961230	Hospital General Universitario de Alicante - Instituto de Investigación Sanitaria y Biomédica de Alicante	SeqCOVID-SPAIN consortium/IBV(CSIC)	Maripaz Ventero Martín, Carmen Molina Pardines and SeqCOVID-SPAIN consortium
EPI_ISL_961470, EPI_ISL_961476, EPI_ISL_961493, EPI_ISL_961496, EPI_ISL_961498, EPI_ISL_961510, EPI_ISL_961512, EPI_ISL_961519, EPI_ISL_961525, EPI_ISL_961548, EPI_ISL_961549, EPI_ISL_961550, EPI_ISL_961551			
see above	Michigan Department of Health and Human Services, Bureau of Laboratories	Michigan Department of Health and Human Services, Bureau of Laboratories	Blankenship HM, Riner D, Soehnlen MK
EPI_ISL_961560, EPI_ISL_961599, EPI_ISL_961600, EPI_ISL_961601, EPI_ISL_961602, EPI_ISL_961603, EPI_ISL_961649, EPI_ISL_961650, EPI_ISL_961651	Hôpital Georges L. Dumont	National Microbiology Laboratory (NML)	Anna Majer, Shari Tyson, Grace Seo, Philip Mabon, Elsie Grudeski, Rhiannon Huzarewich, Russell Mandes, Anneliese Landgraff, Jennifer Tanner, Natalie Knox, Morag Graham, Gary Van Domselaar, Richard Garceau, Guillaume Desnoyers, Nathalie Bastien, Yan Li, Timothy Booth, Darian Hole, Madison Chapel, Kirsten Biggar, CanCOGeN's metadata curation team, Public Health Agency of Canada CanCOGeN team
EPI_ISL_961803	E. Gulbja laboratorija	Latvian Biomedical Research and Study Centre	Janis Pjalkovskis, Nikita Zrelovs, Monta Ustinova, Ivars Silamikelis, Liga Birzniece, Kaspars Megnis, Vita Rovite, Lauma Freimane, Laila Silamikele, Laura Ansone, Davids Fridmanis, Mikus Gavars, Dmitrijs Perminovs, Jurijs Perevoscikovs, Uga Dumpis, Janis Klovins
EPI_ISL_961888, EPI_ISL_961889, EPI_ISL_961896, EPI_ISL_961900, EPI_ISL_961918, EPI_ISL_961968, EPI_ISL_961985, EPI_ISL_961990, EPI_ISL_961996, EPI_ISL_962138, EPI_ISL_962139, EPI_ISL_962141, EPI_ISL_962142, EPI_ISL_962143, EPI_ISL_962144, EPI_ISL_962145, EPI_ISL_962146, EPI_ISL_962147, EPI_ISL_962148, EPI_ISL_962149, EPI_ISL_962150, EPI_ISL_962151, EPI_ISL_962152, EPI_ISL_962153, EPI_ISL_962160			
see above	Illinois Department of Public Health	Gagnon Lab, Southern Illinois University	Keith Gagnon
EPI_ISL_962510	UCLA Clinical Micro Lab	Los Angeles County PHL	P. Hemarajata et al.
EPI_ISL_962585, EPI_ISL_962586, EPI_ISL_962587, EPI_ISL_962588, EPI_ISL_962589, EPI_ISL_962590, EPI_ISL_962591, EPI_ISL_962592, EPI_ISL_962593, EPI_ISL_962594, EPI_ISL_962595, EPI_ISL_962596, EPI_ISL_962597, EPI_ISL_962598, EPI_ISL_962599, EPI_ISL_962600, EPI_ISL_962601			
see above	Scripps Medical Laboratory	Andersen lab at Scripps Research	SEARCH Alliance San Diego with Michael Quigley, Ellen Stefanski, Ian Mchardy
EPI_ISL_962904, EPI_ISL_962905	Ostfold Hospital Trust - Kalnes, Centre for Laboratory Medicine, Section for gene technology and infection serology	Norwegian Institute of Public Health, Department of Virology	Kathrine Stene-Johansen, Kamilla Heddeland Instefjord, Hilde Elshaug, Ignacio Garcia Llorente, Serina B Engebretsen, Atiya R Ali,Marie Paulsen Madsen, Rasmus Riis Kopperud, Hilde Vollan, Karoline Bragstad, Olav Hungnes
EPI_ISL_962919, EPI_ISL_962920, EPI_ISL_962921	Servicio de Microbiología, Laboratori Clínic Metropolitana Nord. Hospital Universitari Germans Trias i Pujol. Institut d'Investigació en Ciències de la Salut Germans Trias i Pujol (IGTP)	SeqCOVID-SPAIN consortium/IBV(CSIC)	Elisa Martró, Antoni E. Bordoy, Anna Not, Adrián Antuori, Anabel Fernández, Nona Romaní, Verónica Saludes, Cristina Casañ and SeqCOVID-SPAIN consortium
EPI_ISL_962935	Hospital Universitario de Gran Canaria Dr. Negrín	SeqCOVID-SPAIN consortium/IBV(CSIC)	M. Carmen Pérez González, Francisco J. Chamizo López, Ana Bordes Benítez and SeqCOVID-SPAIN consortium
EPI_ISL_963106, EPI_ISL_963980, EPI_ISL_964107, EPI_ISL_964219, EPI_ISL_964234	Lighthouse Lab in Alderley Park	Wellcome Sanger Institute for the COVID-19 Genomics UK (COG-UK) Consortium	Jacquelyn Wynn, Mairead Hyland, The Lighthouse Lab in Alderley Park and Alex Alderton, Roberto Amato, Sonia Goncalves, Ewan Harrison, David K. Jackson, Ian Johnston, Dominic Kwiatkowski, Cordelia Langford, John Sillitoe on behalf of the Wellcome Sanger Institute COVID-19 Surveillance Team
EPI_ISL_964281	Oslo University Hospital, Department of Medical Microbiology	Norwegian Institute of Public Health, Department of Virology	Kathrine Stene-Johansen, Kamilla Heddeland Instefjord, Hilde Elshaug, Ignacio Garcia Llorente, Serina B Engebretsen, Atiya R Ali, Marie Paulsen Madsen, Rasmus Riis Kopperud, Hilde Vollan, Karoline Bragstad, Olav Hungnes
EPI_ISL_964303	Akershus University Hospital, Department for Microbiology and Infectious Disease Control	Norwegian Institute of Public Health, Department of Virology	Kathrine Stene-Johansen, Kamilla Heddeland Instefjord, Hilde Elshaug, Ignacio Garcia Llorente, Serina B Engebretsen, Atiya R Ali, Marie Paulsen Madsen, Rasmus Riis Kopperud, Hilde Vollan, Karoline Bragstad, Olav Hungnes
EPI_ISL_965191, EPI_ISL_965192, EPI_ISL_965193, EPI_ISL_965194, EPI_ISL_965195, EPI_ISL_965196, EPI_ISL_965197	Virginia Division of Consolidated Laboratory Services	Virginia Division of Consolidated Laboratory Services	Virginia DCLS
EPI_ISL_965579, EPI_ISL_965585, EPI_ISL_965614, EPI_ISL_965615, EPI_ISL_965625, EPI_ISL_965632, EPI_ISL_965640, EPI_ISL_965668, EPI_ISL_965676, EPI_ISL_965686, EPI_ISL_965752, EPI_ISL_965765, EPI_ISL_965795, EPI_ISL_965810, EPI_ISL_965811			
see above	Dutch COVID-19 response team	Medical Microbiology, Maastricht University Medical Centre	Jozef Dingemans*, Brian van der Veer*, Erik Beuken, Carmen Reumkens, Lieke van Alphen, Christian Hoebe, Paul Savelkoul
EPI_ISL_966777, EPI_ISL_966778, EPI_ISL_966779, EPI_ISL_966780, EPI_ISL_966781	Maine HETL	Tewhey Lab, The Jackson Laboratory	Matluk,N., Dewey,H., Iosue,F., Barter,M., Lynch,R., Munger,H. and Tewhey,R.
EPI_ISL_967589, EPI_ISL_967638, EPI_ISL_967653, EPI_ISL_967654, EPI_ISL_967666, EPI_ISL_967667, EPI_ISL_967668, EPI_ISL_967669, EPI_ISL_967670, EPI_ISL_967673, EPI_ISL_967674, EPI_ISL_967682, EPI_ISL_967694, EPI_ISL_967695, EPI_ISL_967709, EPI_ISL_967711, EPI_ISL_967712, EPI_ISL_967734, EPI_ISL_967735, EPI_ISL_967737, EPI_ISL_967738, EPI_ISL_967739, EPI_ISL_967740, EPI_ISL_967741, EPI_ISL_967742, EPI_ISL_967763, EPI_ISL_967765			
see above	State Laboratories Division, Hawaii State Department of Health	State Laboratories Division, Hawaii State Department of Health	Pamela O'Brien, Drew Kuwazaki, Ayana Garnet, Razvan Sultana, Edward Desmond
EPI_ISL_968860, EPI_ISL_968861, EPI_ISL_968863, EPI_ISL_968864, EPI_ISL_968865, EPI_ISL_968868, EPI_ISL_968869, EPI_ISL_968871, EPI_ISL_968873, EPI_ISL_968877	KEMRI-Wellcome Trust Research Programme/KEMRI-CGMR-C Kilifi	KEMRI-Wellcome Trust Research Programme/KEMRI-CGMR-C Kilifi	Githinji et al
EPI_ISL_976945, EPI_ISL_976953, EPI_ISL_976961, EPI_ISL_976969, EPI_ISL_976977, EPI_ISL_976982, EPI_ISL_976986, EPI_ISL_976994, EPI_ISL_977001, EPI_ISL_977008, EPI_ISL_977014			
see above	Broad Institute Clinical Research Sequencing Platform	Infectious Disease Program, Broad Institute of Harvard and MIT	Lemieux,J.E., Siddle,K.J., Adams,G., Gladden-Young,A., Lagerborg,K., Rudy,M., DeRuff,K., Carter,A., Normandin,E., Bauer,M., Reilly,S., Tomkins-Tinch,C., Loreth,C., Chaluvadi,S., Birren,B.W., Gallagher,G., Smole,S., Park,D.J., MacInnis,B.L., and Sabeti,P.C.
EPI_ISL_977130	Flow Health	Infectious Disease Program, Broad Institute of Harvard and MIT	Lemieux,J.E., Siddle,K.J., Adams,G., Gladden-Young,A., Lagerborg,K., Rudy,M., DeRuff,K., Carter,A., Normandin,E., Bauer,M., Reilly,S., Tomkins-Tinch,C., Loreth,C., Chaluvadi,S., Birren,B.W., Gallagher,G., Smole,S., Park,D.J., MacInnis,B.L., and Sabeti,P.C.
EPI_ISL_977158	ULSS 03 Venezia	Istituto Zooprofilattico Sperimentale delle Venezie	Adelaide Milani, Alessia Schivo, Annalisa Salviato, Erika Giorgia Quaranta, Ambra Pastori, Bianca Zecchin, Alice Fusaro, Isabella Monne, Calogero Terregino, Antonia Ricci
EPI_ISL_977544, EPI_ISL_977549,	Nigeria Centre of Disease Control (NCDC)	African Centre of Excellence for Genomics of Infectious	Olawoye I. B. et al

EPI_ISL_977555, EPI_ISL_977556, EPI_ISL_977560, EPI_ISL_977561, EPI_ISL_977562, EPI_ISL_977564		Diseases (ACEGID), Redeemer's University	
EPI_ISL_977661, EPI_ISL_977662, EPI_ISL_977663	Labo analyses med	National Reference Center for Viruses of Respiratory Infections, Institut Pasteur, Paris	Marion Barbet, Sylvie Behillil, Méline Bizard, Angela Brisebarre, Camille Capel, Etienne Simon-Lorière, Vincent Enouf, Maud Vanpeene, Sylvie van der Werf,Amzalag Jonas
EPI_ISL_978132, EPI_ISL_978133, EPI_ISL_978134, EPI_ISL_978135, EPI_ISL_978136, EPI_ISL_978137, EPI_ISL_978138, EPI_ISL_978139, EPI_ISL_978140, EPI_ISL_978141, EPI_ISL_978142, EPI_ISL_978143, EPI_ISL_978144, EPI_ISL_978145, EPI_ISL_978146, EPI_ISL_978147, EPI_ISL_978148, EPI_ISL_978149, EPI_ISL_978150, EPI_ISL_978151			
see above	Chiu Laboratory, University of California, San Francisco	Chiu Laboratory, University of California, San Francisco	Charles Chiu, Xianding (Wayne) Deng, Candace Wang, Venice Servellita, Jill Hacker, Debra Wadford
EPI_ISL_978371, EPI_ISL_978372, EPI_ISL_978397, EPI_ISL_978398, EPI_ISL_978399, EPI_ISL_978400, EPI_ISL_978401, EPI_ISL_978402, EPI_ISL_978403, EPI_ISL_978404, EPI_ISL_978405, EPI_ISL_978406, EPI_ISL_978407, EPI_ISL_978408			
see above	Arizona State Public Health Laboratory	Arizona State Public Health Laboratory	Trung Huynh, Jessica Escobar, Katherine Fullerton, Nobuko Fukushima, Stacy White, Linda Getsinger, Victor Waddell
EPI_ISL_978741, EPI_ISL_978742, EPI_ISL_978743, EPI_ISL_978744, EPI_ISL_978745, EPI_ISL_978746, EPI_ISL_978747, EPI_ISL_978748, EPI_ISL_978749, EPI_ISL_978750, EPI_ISL_978751, EPI_ISL_978752, EPI_ISL_978753, EPI_ISL_978754, EPI_ISL_978755, EPI_ISL_978756, EPI_ISL_978757, EPI_ISL_978758, EPI_ISL_978759, EPI_ISL_978760, EPI_ISL_978761, EPI_ISL_978762, EPI_ISL_978763, EPI_ISL_978764, EPI_ISL_978765, EPI_ISL_978766, EPI_ISL_978767, EPI_ISL_978768, EPI_ISL_978769, EPI_ISL_978770, EPI_ISL_978771, EPI_ISL_978772, EPI_ISL_978773, EPI_ISL_978774, EPI_ISL_978775, EPI_ISL_978776, EPI_ISL_978777, EPI_ISL_978778, EPI_ISL_978779, EPI_ISL_978780, EPI_ISL_978781, EPI_ISL_978782, EPI_ISL_978783, EPI_ISL_978784, EPI_ISL_978785, EPI_ISL_978786, EPI_ISL_978787			
see above	Helix/Illumina	Respiratory Viruses Branch, Division of Viral Diseases, Centers for Disease Control and Prevention	Peter W. Cook,Dakota Howard,Dhwani Batra,Ben L. Rambo-Martin,Eileen de Feo,Jan Antico,Christine Tran,Matthew Tolentino,Shannon Wickline,Kim Gietzen,Brad Sickler,Jingtao Liu,Eric Allen,Phil Febbo,Summer Galloway,Nicole L. Washington,Simon White,Geraint Levan,Kelly Schiabor Barrett,Elizabeth Cirulli,Alexandre Bolze,Ary Ascencio,Charlotte Rivera-Garcia,Ryan Cho,Jason Nguyen,Sherry Wang,Jimmy Ramirez,Tyler Cassens,Efren Sandoval,Magnus Isaksson,William Lee,David Becker,Marc Laurent,James Lu,Clinton R. Paden,Suxiang Tong,Duncan MacCannell,
EPI_ISL_978924	Centre for Dengue Research and AICBU, Department of Immunology and Molecular Medicine	Centre for Dengue Research and AICBU, Department of Immunology and Molecular Medicine	Chandima Jeewandara, Deshni Jayathilaka, Dinuka Ariyaratne, Tibutius Thanesh Pramanayagam, Diyanath Ranasinghe, Laksiri Gomes, Gathsaurie Neelika Malavige
EPI_ISL_978931, EPI_ISL_978933, EPI_ISL_978935, EPI_ISL_978936	Caribbean Public Health Agency	Carrington Lab, Department of PreClinical Sciences, Building 36, First Floor Biochemistry Unit, Faculty of Medical Sciences, The University of the West Indies	Nikita S. D. Sahadeo, Arianne Brown-Jordan, Vernie Ramkissoon, Sarah Hill, Naresh Nandram, Avery Hinds, Irad Potter, Jerome Foster, Stanley Giddings, Karla Georges, Marsha Ivey, Rahul Naidu, Risha Singh, SueMin Nathaniel, Rajini Haraksingh, Jaya Jayaraman, Chinna Chinnadurai, Adesh Ramsubhag, Nuno Faria, Oliver Pybus, Christopher Oura, Gabriel Escobar, Christine V. F. Carrington
EPI_ISL_978962, EPI_ISL_978963, EPI_ISL_978964, EPI_ISL_978965, EPI_ISL_978966	Chiu Laboratory, University of California, San Francisco	Chiu Laboratory, University of California, San Francisco	Charles Chiu, Xianding (Wayne) Deng, Candace Wang, Venice Servellita, Jill Hacker, Debra Wadford
EPI_ISL_979137	Santa Clara County Public Health Laboratory	Chan-Zuckerberg Biohub	CZB Cliahub Consortium
EPI_ISL_979159	County of San Luis Obispo Public Health Laboratory	Chan-Zuckerberg Biohub	CZB Cliahub Consortium
EPI_ISL_979454, EPI_ISL_979456	University of Iowa Hospitals and Clinics	State Hygienic Laboratory at the University of Iowa	Valerie Reeb, Wes Hottel, Alankar Kampoowale
EPI_ISL_981136	Johns Hopkins Hospital Department of Pathology	Johns Hopkins Hospital Department of Pathology	C. Paul Morris, Chun Huai Luo, Adannaya Amadi, Matthew Schwartz, Nicholas Gallagher, Heba H. Mostafa
EPI_ISL_981370	AZ Klina	AZ Klina	Dr. C. Vael
EPI_ISL_981946	Microbiology Service, Hospital Universitario Clinico San Cecilio, Granada	Microbiology Service, Hospital Universitario Clinico San Cecilio, Granada	Adolfo de Salazar, Natalia Chueca, Laura Viñuela, Ana Fuentes, Federico García
EPI_ISL_981976, EPI_ISL_981980, EPI_ISL_981984, EPI_ISL_981986, EPI_ISL_981991, EPI_ISL_981993, EPI_ISL_981994	TGen North	Sonora Quest Laboratories	"Jolene Bowers, Megan Folkerts, Chris French, Hayley Yaglom, Ashlyn Pfeiffer, Darrin Lemmer, Dave Engelthaler, The Arizona COVID Genomics Union (ACGU)"
EPI_ISL_982008	TGen North	TGen North	"Jolene Bowers, Megan Folkerts, Chris French, Hayley Yaglom, Ashlyn Pfeiffer, Darrin Lemmer, Dave Engelthaler, The Arizona COVID Genomics Union (ACGU)"
EPI_ISL_982009	TGen North	Sonora Quest Laboratories	"Jolene Bowers, Megan Folkerts, Chris French, Hayley Yaglom, Ashlyn Pfeiffer, Darrin Lemmer, Dave Engelthaler, The Arizona COVID Genomics Union (ACGU)"
EPI_ISL_982010, EPI_ISL_982020	TGen North	TGen North	"Jolene Bowers, Megan Folkerts, Chris French, Hayley Yaglom, Ashlyn Pfeiffer, Darrin Lemmer, Dave Engelthaler, The Arizona COVID Genomics Union (ACGU)"
EPI_ISL_982025, EPI_ISL_982028, EPI_ISL_982030, EPI_ISL_982031, EPI_ISL_982033, EPI_ISL_982036, EPI_ISL_982037, EPI_ISL_982039, EPI_ISL_982040, EPI_ISL_982042, EPI_ISL_982051, EPI_ISL_982054, EPI_ISL_982058, EPI_ISL_982062, EPI_ISL_982069, EPI_ISL_982076, EPI_ISL_982091, EPI_ISL_982095, EPI_ISL_982096, EPI_ISL_982098, EPI_ISL_982101			
see above	TGen North	Sonora Quest Laboratories	"Jolene Bowers, Megan Folkerts, Chris French, Hayley Yaglom, Ashlyn Pfeiffer, Darrin Lemmer, Dave Engelthaler, The Arizona COVID Genomics Union (ACGU)"
EPI_ISL_982105	TGen North	TGen North	"Jolene Bowers, Megan Folkerts, Chris French, Hayley Yaglom, Ashlyn Pfeiffer, Darrin Lemmer, Dave Engelthaler, The Arizona COVID Genomics Union (ACGU)"
EPI_ISL_982240, EPI_ISL_982242	Lab voor klinische biologie	Lab voor klinische biologie	Hannelore Hamerlinck, Marija Janevska, Bruno Verhasselt
EPI_ISL_982425, EPI_ISL_982436, EPI_ISL_982441, EPI_ISL_982449, EPI_ISL_982450, EPI_ISL_982453, EPI_ISL_982457, EPI_ISL_982460, EPI_ISL_982463, EPI_ISL_982464, EPI_ISL_982466, EPI_ISL_982471, EPI_ISL_982473, EPI_ISL_982476, EPI_ISL_982477, EPI_ISL_982479, EPI_ISL_982490, EPI_ISL_982493, EPI_ISL_982494			
see above	MONTEFIORE MEDICAL CENTER LABORATORIES	Wadsworth Center, New York State Department of Health	Kirsten St. George, Daryl M. Lamson, Alexis Russel, Matthew Shudt, Melissa A Leisner, Jonathan Plitnick, Navjot Singh, John Kelly, Erasmus Schneider, Erica Lasek-Nesselquist
EPI_ISL_982527, EPI_ISL_982528, EPI_ISL_982531, EPI_ISL_982532, EPI_ISL_982533, EPI_ISL_982536, EPI_ISL_982543, EPI_ISL_982549, EPI_ISL_982554, EPI_ISL_982556, EPI_ISL_982559, EPI_ISL_982562, EPI_ISL_982564, EPI_ISL_982565, EPI_ISL_982570, EPI_ISL_982572, EPI_ISL_982577, EPI_ISL_982580, EPI_ISL_982581, EPI_ISL_982582, EPI_ISL_982584, EPI_ISL_982587, EPI_ISL_982588, EPI_ISL_982590, EPI_ISL_982595, EPI_ISL_982598, EPI_ISL_982599, EPI_ISL_982604, EPI_ISL_982607, EPI_ISL_982610, EPI_ISL_982612, EPI_ISL_982614, EPI_ISL_982618, EPI_ISL_982619, EPI_ISL_982620, EPI_ISL_982621, EPI_ISL_982622, EPI_ISL_982631, EPI_ISL_982632, EPI_ISL_982633, EPI_ISL_982636, EPI_ISL_982638, EPI_ISL_982639, EPI_ISL_982640, EPI_ISL_982644, EPI_ISL_982645, EPI_ISL_982646, EPI_ISL_982648, EPI_ISL_982649, EPI_ISL_982651, EPI_ISL_982654, EPI_ISL_982655, EPI_ISL_982656, EPI_ISL_982657, EPI_ISL_982658, EPI_ISL_982659, EPI_ISL_982660, EPI_ISL_982671, EPI_ISL_982681, EPI_ISL_982682, EPI_ISL_982685, EPI_ISL_982686, EPI_ISL_982687, EPI_ISL_982688, EPI_ISL_982689, EPI_ISL_982690, EPI_ISL_982694, EPI_ISL_982702, EPI_ISL_982703, EPI_ISL_982704, EPI_ISL_982705, EPI_ISL_982710, EPI_ISL_982711, EPI_ISL_982712, EPI_ISL_982713, EPI_ISL_982714, EPI_ISL_982715, EPI_ISL_982716, EPI_ISL_982717, EPI_ISL_982718, EPI_ISL_982729, EPI_ISL_982737, EPI_ISL_982740, EPI_ISL_982743, EPI_ISL_982744, EPI_ISL_982745, EPI_ISL_982751, EPI_ISL_982752, EPI_ISL_982753, EPI_ISL_982754, EPI_ISL_982755, EPI_ISL_982756, EPI_ISL_982757			
see above	US Air Force School of Aerospace Medicine	US Air Force School of Aerospace Medicine	Anthony Fries, Jennifer Meyer, William Gruner, William Buggele, Amanda Javorina, Sarah Purves, Clarise Starr, Elizabeth Macias
EPI_ISL_983066, EPI_ISL_983067, EPI_ISL_983068, EPI_ISL_983069, EPI_ISL_983070, EPI_ISL_983071, EPI_ISL_983072	Gravity Diagnostics	Kentucky State Public Health Lab	Stephanie Lunn, Karim George, Joshua Tobias, William Grooms, Vaneet Arora, Matthew Johnson, Rachel Zinner, Rhonda Lucas
EPI_ISL_983073, EPI_ISL_983074	Kentucky State Public Health Lab	Kentucky State Public Health Lab	Stephanie Lunn, Karim George, Joshua Tobias, William Grooms, Vaneet Arora, Matthew Johnson, Rachel Zinner, Rhonda Lucas
EPI_ISL_983075, EPI_ISL_983076, EPI_ISL_983077, EPI_ISL_983078, EPI_ISL_983079, EPI_ISL_983080	Gravity Diagnostics	Kentucky State Public Health Lab	Stephanie Lunn, Karim George, Joshua Tobias, William Grooms, Vaneet Arora, Matthew Johnson, Rachel Zinner, Rhonda Lucas
EPI_ISL_983100, EPI_ISL_983107, EPI_ISL_983109, EPI_ISL_983110, EPI_ISL_983114, EPI_ISL_983136, EPI_ISL_983139, EPI_ISL_983143, EPI_ISL_983145, EPI_ISL_983148, EPI_ISL_983151, EPI_ISL_983153, EPI_ISL_983155, EPI_ISL_983158, EPI_ISL_983159, EPI_ISL_983160, EPI_ISL_983161, EPI_ISL_983165			
see above	MONTEFIORE MEDICAL CENTER LABORATORIES	Wadsworth Center, New York State Department of Health	Kirsten St. George, Daryl M. Lamson, Alexis Russel, Matthew Shudt, Melissa A Leisner, Jonathan Plitnick, Navjot Singh, John Kelly, Erasmus Schneider, Erica Lasek-Nesselquist

EPI_ISL_983240, EPI_ISL_983241, EPI_ISL_983242	KALEIDA CENTER FOR LABORATORY MEDICINE	Wadsworth Center, New York State Department of Health	Kirsten St. George, Daryl M. Lamson, Alexis Russel, Matthew Shudt, Melissa A Leisner, Jonathan Plitnick, Navjot Singh, John Kelly, Erasmus Schneider, Erica Lasek-Nesselquist
EPI_ISL_983243	SUNY UPSTATE MEDICAL UNIVERSITY	Wadsworth Center, New York State Department of Health	Kirsten St. George, Daryl M. Lamson, Alexis Russel, Matthew Shudt, Melissa A Leisner, Jonathan Plitnick, Navjot Singh, John Kelly, Erasmus Schneider, Erica Lasek-Nesselquist
EPI_ISL_983244, EPI_ISL_983246	KALEIDA CENTER FOR LABORATORY MEDICINE	Wadsworth Center, New York State Department of Health	Kirsten St. George, Daryl M. Lamson, Alexis Russel, Matthew Shudt, Melissa A Leisner, Jonathan Plitnick, Navjot Singh, John Kelly, Erasmus Schneider, Erica Lasek-Nesselquist
EPI_ISL_983249	SUNY UPSTATE MEDICAL UNIVERSITY	Wadsworth Center, New York State Department of Health	Kirsten St. George, Daryl M. Lamson, Alexis Russel, Matthew Shudt, Melissa A Leisner, Jonathan Plitnick, Navjot Singh, John Kelly, Erasmus Schneider, Erica Lasek-Nesselquist
EPI_ISL_983250, EPI_ISL_983263, EPI_ISL_983264, EPI_ISL_983265, EPI_ISL_983266, EPI_ISL_983267, EPI_ISL_983268, EPI_ISL_983269, EPI_ISL_983270, EPI_ISL_983271, EPI_ISL_983272, EPI_ISL_983273, EPI_ISL_983274, EPI_ISL_983275, EPI_ISL_983276, EPI_ISL_983277, EPI_ISL_983278, EPI_ISL_983279, EPI_ISL_983280, EPI_ISL_983281, EPI_ISL_983282			
see above	KALEIDA CENTER FOR LABORATORY MEDICINE	Wadsworth Center, New York State Department of Health	Kirsten St. George, Daryl M. Lamson, Alexis Russel, Matthew Shudt, Melissa A Leisner, Jonathan Plitnick, Navjot Singh, John Kelly, Erasmus Schneider, Erica Lasek-Nesselquist
EPI_ISL_983283	SUNY UPSTATE MEDICAL UNIVERSITY	Wadsworth Center, New York State Department of Health	Kirsten St. George, Daryl M. Lamson, Alexis Russel, Matthew Shudt, Melissa A Leisner, Jonathan Plitnick, Navjot Singh, John Kelly, Erasmus Schneider, Erica Lasek-Nesselquist
EPI_ISL_983284, EPI_ISL_983285, EPI_ISL_983286, EPI_ISL_983287, EPI_ISL_983288	KALEIDA CENTER FOR LABORATORY MEDICINE	Wadsworth Center, New York State Department of Health	Kirsten St. George, Daryl M. Lamson, Alexis Russel, Matthew Shudt, Melissa A Leisner, Jonathan Plitnick, Navjot Singh, John Kelly, Erasmus Schneider, Erica Lasek-Nesselquist
EPI_ISL_983289, EPI_ISL_983290, EPI_ISL_983291, EPI_ISL_983292, EPI_ISL_983293, EPI_ISL_983294, EPI_ISL_983295	SUNY UPSTATE MEDICAL UNIVERSITY	Wadsworth Center, New York State Department of Health	Kirsten St. George, Daryl M. Lamson, Alexis Russel, Matthew Shudt, Melissa A Leisner, Jonathan Plitnick, Navjot Singh, John Kelly, Erasmus Schneider, Erica Lasek-Nesselquist
EPI_ISL_983324	INMI Lazzaro Spallanzani IRCCS	INMI Lazzaro Spallanzani IRCCS	B Bartolini, O Butera, C.E.M Gruber, M Rueca, F Messina, E Giombini, MR Capobianchi, A Di Caro
EPI_ISL_983382, EPI_ISL_983383, EPI_ISL_983384, EPI_ISL_983385, EPI_ISL_983386, EPI_ISL_983387, EPI_ISL_983388, EPI_ISL_983389, EPI_ISL_983390, EPI_ISL_983391, EPI_ISL_983392, EPI_ISL_983393, EPI_ISL_983394, EPI_ISL_983395, EPI_ISL_983396, EPI_ISL_983397, EPI_ISL_983398, EPI_ISL_983399, EPI_ISL_983400, EPI_ISL_983401, EPI_ISL_983402, EPI_ISL_983403, EPI_ISL_983404, EPI_ISL_983405, EPI_ISL_983406, EPI_ISL_983407			
see above	Utah Public Health Laboratory	Utah Public Health Laboratory	Erin L. Young, Kelly F. Oakeson, Tara Gallagher
EPI_ISL_983720, EPI_ISL_983735, EPI_ISL_983813, EPI_ISL_983814, EPI_ISL_983815, EPI_ISL_983816, EPI_ISL_983817, EPI_ISL_983818, EPI_ISL_983819, EPI_ISL_983820, EPI_ISL_983821, EPI_ISL_983822, EPI_ISL_983823, EPI_ISL_983824, EPI_ISL_983825			
see above	Colorado Department of Public Health and Environment	Colorado Department of Puplic Health and Environment	Laura Bankers, Molly C. Hetherington-Rauth, Diana Ir, Shannon Ely, Shannon R. Matzinger, Sarah Elizabeth Totten, Emily A. Travanty
