# Supplementary material for: Detection and characterization of the SARS-CoV-2 lineage B.1.526 in New York
Source: Nat Commun. 2021 Aug 9;12:4886. doi: 10.1038/s41467-021-25168-4 (PMC8352861; doi:10.1038/s41467-021-25168-4)
Supplement: Supplementary file 8 — Supplementary Data 4 [file 41467_2021_25168_MOESM8_ESM.zip › GISAID_acknowledements_tables/gisaid_hcov-19_acknowledgement_table_2021_02_13_010-9.pdf]

We gratefully acknowledge the following Authors from the Originating laboratories responsible for obtaining the specimens, as well as the Submitting laboratories where the genome data were generated and shared via GISAID, on which this research is based.

All Submitters of data may be contacted directly via www.gisaid.org

Authors are sorted alphabetically.

Accession ID	Originating Laboratory	Submitting Laboratory	Authors
EPI_ISL_803108	Pathology West - NSW Health Pathology	NSW Health Pathology - Institute of Clinical Pathology and Medical Research; Westmead Hospital; University of Sydney	CIDM-PH et al.
EPI_ISL_803110, EPI_ISL_803111, EPI_ISL_803112, EPI_ISL_803113, EPI_ISL_803114	Sydney South West Pathology Service (SSWPS) - Royal Prince Alfred Hospital - NSW Health Pathology	NSW Health Pathology - Institute of Clinical Pathology and Medical Research; Westmead Hospital; University of Sydney	CIDM-PH et al.
EPI_ISL_803119, EPI_ISL_803851	Laboratory of Microbiology, National Reference Lab, Charles Nicolle Hospital; 2-University of Tunis ElManar, Faculty of Medicine of Tunis, LR99ES09, Tunis, Tunisia	Clinical and Experimental Pharmacology Lab, LR16SP02, National Center of Pharmacovigilance, University of Tunis El Manar, Tunis, Tunisia. 2-Neurodegenerative diseases and psychiatric troubles, LR18SP03, Razi Hospital, University of Tunis El Manar, Tunis, Tunisia. 3- Ministry of Health, National Observatory of New and Emerging Diseases, 1006, Tunis, Tunisia	Ilhem Boutiba-Ben Boubaker, Sameh Trabelsi, Nissaf Ben Alaya, Maher Kharrat, Alia BenKahla, Jalila Ben Khelil, Salma Abid, Sana Ferjani, Mouna Ben Sassi, Mouna Safer, Zaineb Hamzaoui, Habiba Ben Romdhane, Souissi Amira, Sarra Chamman, Hanen El Jebari, Ahmed Fakhfakh, Gaies Emna, Riadh Daghfous, Riadh Gouider.
EPI_ISL_810968, EPI_ISL_810969	PathWest Laboratory Medicine WA	PathWest Laboratory Medicine WA Microbial Surveillance Unit	PathWest Laboratory Medicine WA Microbial Surveillance Unit
EPI_ISL_811123, EPI_ISL_811124, EPI_ISL_811125, EPI_ISL_811126, EPI_ISL_811150	Respiratory Virus Unit, National Infection Service, Public Health England	COVID-19 Genomics UK (COG-UK) Consortium	PHE Covid Sequencing Team
EPI_ISL_812256	University Hospital Zürich	Institute of Medical Virology, University of Zurich	Stefan Schmutz, Maryam Zaheri, Verena Kufner, Annette Audigé, Maria Grünberg, Kevin Steiner, Jon Huder, Cyril Shah, Riccarda Capaul, Guido Bloemberg, Jürg Böni, Michael Huber, Alexandra Trkola
EPI_ISL_812346	Oklahoma Animal Disease Diagnostic Laboratory	Oklahoma Animal Disease Diagnostic Laboratory	Sai Narayanan, Girish Patil, Sunil More, Jeremiah Saliki, Akhilesh Ramachandran
EPI_ISL_812347	Area of Virology, Serology and Virology Division (SAViD), New South Wales Health Pathology Randwick	Virology Research Laboratory; Area of Virology, Serology and Virology Division (SAViD), New South Wales Health Pathology Randwick	Foster, C.; Au, J.; Ruiz Silva, M.; Deveson, I.; Bull, R.; Van Hal, S.; Rawlinson, W.
EPI_ISL_812358	SA Pathology	SA Pathology	Lex Leong, Julien Soubrier, Chuan Kok Lim, Song Gao, Mark Turra, Karin Kassahn, Ivan Bastian, Geoff Higgins
EPI_ISL_812441, EPI_ISL_812442	Victorian Infectious Diseases Reference Laboratory (VIDRL)	VIDRL and MDU-PHL	Caly L., Seemann T., Sait, M.L., Druce J., Sherry, N.L.
EPI_ISL_812521	Pathology North - Royal North Shore Hospital - NSW Health Pathology	NSW Health Pathology - Institute of Clinical Pathology and Medical Research; Westmead Hospital; University of Sydney	CIDM-PH et al.
EPI_ISL_825016	Sydney South West Pathology Service (SSWPS) - Royal Prince Alfred Hospital - NSW Health Pathology	NSW Health Pathology - Institute of Clinical Pathology and Medical Research; Westmead Hospital; University of Sydney	CIDM-PH et al.
EPI_ISL_825064, EPI_ISL_825065, EPI_ISL_825066, EPI_ISL_825067, EPI_ISL_825068, EPI_ISL_825070, EPI_ISL_825071, EPI_ISL_825072, EPI_ISL_825073, EPI_ISL_825075, EPI_ISL_825077, EPI_ISL_825078, EPI_ISL_825079, EPI_ISL_825081, EPI_ISL_825082, EPI_ISL_825083			
see above	National Public Health Laboratory, National Centre for Infectious Diseases	National Public Health Laboratory, National Centre for Infectious Diseases	Tze Minn Mak, Sophie Octavia, Zhenyang Zhou, Lin Cui, Raymond Tzer Pin Lin
EPI_ISL_825491, EPI_ISL_825616	Respiratory Virus Unit, National Infection Service, Public Health England	COVID-19 Genomics UK (COG-UK) Consortium	PHE Covid Sequencing Team
EPI_ISL_826270, EPI_ISL_826271, EPI_ISL_826272, EPI_ISL_826273	University of Debrecen, Department of Medical Microbiology	National Laboratory of Virology, Szentágothai Research Centre	Endre Gábor Tóth, Balázs Somogyi, Brigitta Zana, Eszter Csoma, Ferenc Jakab, Gábor Kemenesi
EPI_ISL_826282	Respiratory Virus Unit, National Infection Service, Public Health England	COVID-19 Genomics UK (COG-UK) Consortium	PHE Covid Sequencing Team
EPI_ISL_826297, EPI_ISL_826298, EPI_ISL_826299, EPI_ISL_826300, EPI_ISL_826301, EPI_ISL_826302, EPI_ISL_826303, EPI_ISL_826305, EPI_ISL_826307, EPI_ISL_826308, EPI_ISL_826309, EPI_ISL_826310, EPI_ISL_826311, EPI_ISL_826312, EPI_ISL_826313, EPI_ISL_826314, EPI_ISL_826315, EPI_ISL_826316, EPI_ISL_826317, EPI_ISL_826318			
see above	Wyoming Public Health Laboratory	Wyoming Public Health Laboratory	Noah Hull, Taylor Fearing, Lynette Gumbleton, Channing Weber, Ashley Norberg, Bailey Bowcutt, and Wanda Manley
EPI_ISL_830643, EPI_ISL_830644, EPI_ISL_830645, EPI_ISL_830646, EPI_ISL_830647	Wadsworth Center, New York State Department of Health	Wadsworth Center, New York State Department of Health	Kirsten St. George, Daryl M. Lamson, Alexis Russel, Matthew Shudt, Melissa A Leisner, Jonathan Plitnick, Navjot Singh, John Kelly, Erasmus Schneider, Erica Lasek-Nesselquist
EPI_ISL_831361, EPI_ISL_831362, EPI_ISL_831363, EPI_ISL_831364, EPI_ISL_831365, EPI_ISL_831368, EPI_ISL_831369	Clinical Molecular Microbiology Laboratory, UNC Hospitals	Jeremy Wang	Jeremy Wang, Alexander Rubinsteyn, Colleen Rice, Jason Smedberg, Melissa Miller, Corbin Jones, Robert Hagan
EPI_ISL_831476, EPI_ISL_831478, EPI_ISL_831484, EPI_ISL_831489, EPI_ISL_831495, EPI_ISL_831504, EPI_ISL_831505, EPI_ISL_831506, EPI_ISL_831507, EPI_ISL_831508, EPI_ISL_831509, EPI_ISL_831510, EPI_ISL_831512, EPI_ISL_831513, EPI_ISL_831514, EPI_ISL_831515, EPI_ISL_831516, EPI_ISL_831517, EPI_ISL_831518, EPI_ISL_831519, EPI_ISL_831520, EPI_ISL_831522, EPI_ISL_831523, EPI_ISL_831524, EPI_ISL_831525, EPI_ISL_831526, EPI_ISL_831528, EPI_ISL_831531, EPI_ISL_831532, EPI_ISL_831535, EPI_ISL_831537, EPI_ISL_831541, EPI_ISL_831547, EPI_ISL_831549			
see above	University of Wisconsin-Madison AIDS Vaccine Research Laboratories	University of Wisconsin-Madison AIDS Vaccine Research Laboratories	Gage Moreno, Katarina Braun, et al. AIDS Vaccine Research Laboratories
EPI_ISL_831674	Institute of Virology, Biomedical Research Center of the Slovak Academy of Sciences, Bratislava	Faculty of Natural Sciences, Comenius University, Bratislava	Kristína Boršová, Viktória abanová, Broa Brejová, Viktória Hodorová, Sabina Fumaová Havlíková, Juraj Kopáek, Martina Liková, ubomíra Lukáiková, Martina Neboháová, Monika Sláviková, Tomáš Vina, Boris Klempa, Jozef Nosek
EPI_ISL_831675	Institute of Virology, Biomedical Research Center of the Slovak Academy of Sciences, Bratislava	Faculty of Natural Sciences, Comenius University, Bratislava	Viktória abanová, Kristína Boršová, Broa Brejová, Viktória Hodorová, Sabina Fumaová Havlíková, Juraj Kopáek, Martina Liková, ubomíra Lukáiková, Martina Neboháová, Monika Sláviková, Tomáš Vina, Jozef Nosek, Boris Klempa
EPI_ISL_831676	Institute of Virology, Biomedical Research Center of the Slovak Academy of Sciences, Bratislava	Faculty of Natural Sciences, Comenius University, Bratislava	Broa Brejová, Viktória abanová, Kristína Boršová, Viktória Hodorová, Sabina Fumaová Havlíková, Juraj Kopáek, Martina Liková, ubomíra Lukáiková, Martina Neboháová, Monika Sláviková, Tomáš Vina, Jozef Nosek, Boris Klempa
EPI_ISL_831900, EPI_ISL_831901, EPI_ISL_831903, EPI_ISL_831905, EPI_ISL_831908, EPI_ISL_831910, EPI_ISL_831920, EPI_ISL_831921, EPI_ISL_831922, EPI_ISL_831923, EPI_ISL_831924, EPI_ISL_831925, EPI_ISL_831926, EPI_ISL_831927, EPI_ISL_831931, EPI_ISL_831932, EPI_ISL_831933, EPI_ISL_831934, EPI_ISL_831935, EPI_ISL_831936, EPI_ISL_831937			
see above	New Mexico Department of Health Scientific Laboratory	New Mexico Department of Health Scientific Laboratory	Ellie Johnson, Anastacia Griego-Fisher, D'eldra Malone
EPI_ISL_832015, EPI_ISL_832016	University of Wisconsin-Madison AIDS Vaccine Research	University of Wisconsin-Madison AIDS Vaccine Research	Gage Moreno, Katarina Braun, et al. AIDS Vaccine Research Laboratories

	Laboratories	Laboratories	
EPI_ISL_832125, EPI_ISL_832126	Clinical Molecular Microbiology Laboratory, UNC Hospitals	Jeremy Wang	Jeremy Wang, Alexander Rubinsteyn, Colleen Rice, Jason Smedberg, Melissa Miller, Corbin Jones, Robert Hagan
EPI_ISL_832127	Michigan Department of Health and Human Services, Bureau of Laboratories	Michigan Department of Health and Human Services, Bureau of Laboratories	Blankenship HM, Riner D, Soehnlen MK
EPI_ISL_832203, EPI_ISL_832204	Northwest Pathology	UW Virology Lab	Pavitra Roychoudhury, Hong Xie, Lasata Shrestha, Michelle Lin, Meei-Li Huang, Keith R Jerome, Alexander Greninger
EPI_ISL_832232, EPI_ISL_832233, EPI_ISL_832234, EPI_ISL_832235, EPI_ISL_832236, EPI_ISL_832237, EPI_ISL_832238, EPI_ISL_832239, EPI_ISL_832240, EPI_ISL_832241	Department of Clinical Microbiology	GIGA Medical Genomics	Keith Durkin, Maria Artesi, Sébastien Bontems, Raphaël Boreux, Bouchra Boujemla, Cécile Meex, Pierrette Melin, Marie-Pierre Hayette, Vincent Bours
EPI_ISL_833028	Maine HETL	Tewhey Lab, The Jackson Laboratory	Matluk,N., Dewey,H., Iosue,F., Barter,M., Lynch,R., Munger,H. and Tewhey,R.
EPI_ISL_833148	Genomic Laboratory (GLAB) (Conjoint lab of Health Directorate of Istanbul and Istanbul Technical University)	Genomic Laboratory (GLAB), Istanbul Technical University	Ilker Karacan, Tugba Kizilboga Akgun, Payam Zolfagharian, Nisan Denizce Can, Pari Sharifli, Levent Doganay, Gizem Dinler Doganay
EPI_ISL_833249	Division of Emerging Infectious Diseases, Bureau of Infectious Diseases Diagnosis Control, Korea Disease Control and Prevention Agency	Division of Emerging Infectious Diseases, Bureau of Infectious Diseases Diagnosis Control, Korea Disease Control and Prevention Agency	Ae Kyung Park, Il-Hwan Kim, Heui Man Kim, Jeong-Min Kim, Namjoo Lee, Chaeyoung Lee, Sang Hee Woo, Eun-Jin Kim
EPI_ISL_833404	St. Francis Medical Center	Los Angeles County PHL	P. Hemarajata et al.
EPI_ISL_833418, EPI_ISL_833490, EPI_ISL_833491	MD Laboratories	Los Angeles County PHL	P. Hemarajata et al.
EPI_ISL_833578	National Reference Laboratory for COVID-19, Pasteur Institute of Iran	National Reference Laboratory for COVID-19, Pasteur Institute of Iran	Zahra Ahmadi, Marzieh Sadjadi, Tahmineh Jalali, Mohammad Hassan Pouriayevali, Mahsa Tavakoli, Zahra Fereydouni, Setareh Kashanian, Sanam Azad-Manjiri, Tahereh Mohammadi, Kayhan Azadmanesh, Zabihollah Shoja, Parastoo Yekta, Farideh Niknam, Hessam Nemati, Ahmad Ghasemi, Sahar Khakifirouz, Sepideh Gerdooei,Maryam Rostamtabar,Sana Eybpoosh,Mohammad Mehdi Mortazavipour, Mohamad Sadegh Shams Nosrati, Zeynab VeisiZadeh, Mostafa Salehi-Vaziri
EPI_ISL_833585, EPI_ISL_833587, EPI_ISL_833588, EPI_ISL_833589, EPI_ISL_833591, EPI_ISL_833592, EPI_ISL_833594, EPI_ISL_833595, EPI_ISL_833598, EPI_ISL_833600, EPI_ISL_833601, EPI_ISL_833603, EPI_ISL_833605, EPI_ISL_833608, EPI_ISL_833613, EPI_ISL_833622, EPI_ISL_833623, EPI_ISL_833624, EPI_ISL_833625, EPI_ISL_833627, EPI_ISL_833628, EPI_ISL_833633, EPI_ISL_833637, EPI_ISL_833638, EPI_ISL_833639, EPI_ISL_833641, EPI_ISL_833642, EPI_ISL_833644, EPI_ISL_833645, EPI_ISL_833649, EPI_ISL_833653, EPI_ISL_833655, EPI_ISL_833657, EPI_ISL_833659, EPI_ISL_833660, EPI_ISL_833661, EPI_ISL_833663, EPI_ISL_833664, EPI_ISL_833666, EPI_ISL_833669, EPI_ISL_833672, EPI_ISL_833674, EPI_ISL_833675, EPI_ISL_833677, EPI_ISL_833681, EPI_ISL_833682, EPI_ISL_833688, EPI_ISL_833690, EPI_ISL_833692, EPI_ISL_833694, EPI_ISL_833697, EPI_ISL_833698, EPI_ISL_833699, EPI_ISL_833701, EPI_ISL_833711, EPI_ISL_833713, EPI_ISL_833715, EPI_ISL_833717, EPI_ISL_833718, EPI_ISL_833720, EPI_ISL_833722, EPI_ISL_833723, EPI_ISL_833729, EPI_ISL_833730, EPI_ISL_833731, EPI_ISL_833732, EPI_ISL_833737, EPI_ISL_833739, EPI_ISL_833740, EPI_ISL_833744, EPI_ISL_833745, EPI_ISL_833747, EPI_ISL_833751, EPI_ISL_833752, EPI_ISL_833754, EPI_ISL_833757, EPI_ISL_833760, EPI_ISL_833761, EPI_ISL_833763, EPI_ISL_833764, EPI_ISL_833765, EPI_ISL_833769, EPI_ISL_833772, EPI_ISL_833775, EPI_ISL_833776, EPI_ISL_833777, EPI_ISL_833781, EPI_ISL_833782, EPI_ISL_833783, EPI_ISL_833785, EPI_ISL_833786, EPI_ISL_833787, EPI_ISL_833788, EPI_ISL_833790, EPI_ISL_833793, EPI_ISL_833798, EPI_ISL_833799, EPI_ISL_833802, EPI_ISL_833805, EPI_ISL_833808, EPI_ISL_833811, EPI_ISL_833819, EPI_ISL_833820, EPI_ISL_833822, EPI_ISL_833824, EPI_ISL_833825, EPI_ISL_833826, EPI_ISL_833827, EPI_ISL_833828, EPI_ISL_833836, EPI_ISL_833837, EPI_ISL_833838, EPI_ISL_833839, EPI_ISL_833843, EPI_ISL_833847, EPI_ISL_833850, EPI_ISL_833851, EPI_ISL_833857, EPI_ISL_833858, EPI_ISL_833867, EPI_ISL_833869, EPI_ISL_833870, EPI_ISL_833871, EPI_ISL_833873, EPI_ISL_833874, EPI_ISL_833875, EPI_ISL_833878, EPI_ISL_833880, EPI_ISL_833883, EPI_ISL_833885, EPI_ISL_833887, EPI_ISL_833891, EPI_ISL_833893, EPI_ISL_833897, EPI_ISL_833899, EPI_ISL_833900, EPI_ISL_833902, EPI_ISL_833904, EPI_ISL_833905, EPI_ISL_833906, EPI_ISL_833911, EPI_ISL_833913, EPI_ISL_833914, EPI_ISL_833915, EPI_ISL_833920, EPI_ISL_833921, EPI_ISL_833923, EPI_ISL_833925, EPI_ISL_833926, EPI_ISL_833927, EPI_ISL_833928, EPI_ISL_833929			
see above	Lighthouse Lab in Cambridge	Wellcome Sanger Institute for the COVID-19 Genomics UK (COG-UK) Consortium	Rob Howes, The Lighthouse Lab in Cambridge and Alex Alderton, Roberto Amato, Sonia Goncalves, Ewan Harrison, David K. Jackson, Ian Johnston, Dominic Kwiatkowski, Cordelia Langford, John Sillitoe on behalf of the Wellcome Sanger Institute COVID-19 Surveillance Team
EPI_ISL_835041	Lighthouse Lab in Alderley Park	Wellcome Sanger Institute for the COVID-19 Genomics UK (COG-UK) Consortium	Jacquelyn Wynn, Mairead Hyland, The Lighthouse Lab in Alderley Park and Alex Alderton, Roberto Amato, Sonia Goncalves, Ewan Harrison, David K. Jackson, Ian Johnston, Dominic Kwiatkowski, Cordelia Langford, John Sillitoe on behalf of the Wellcome Sanger Institute COVID-19 Surveillance Team
EPI_ISL_837056, EPI_ISL_837059, EPI_ISL_837060, EPI_ISL_837069, EPI_ISL_837181, EPI_ISL_837243, EPI_ISL_837244	Respiratory Virus Unit, National Infection Service, Public Health England	COVID-19 Genomics UK (COG-UK) Consortium	PHE Covid Sequencing Team
EPI_ISL_837387	National Virus Reference Laboratory	National Virus Reference Laboratory	Michael Carr, Gabriel Gonzalez, Jonathan Dean, Cillian F De Gascun
EPI_ISL_837957, EPI_ISL_837959, EPI_ISL_837974, EPI_ISL_837976, EPI_ISL_837977, EPI_ISL_837979, EPI_ISL_837980, EPI_ISL_837982, EPI_ISL_837983, EPI_ISL_837986, EPI_ISL_837987, EPI_ISL_837988, EPI_ISL_837991, EPI_ISL_837993, EPI_ISL_837997, EPI_ISL_838000, EPI_ISL_838001, EPI_ISL_838003, EPI_ISL_838004, EPI_ISL_838005, EPI_ISL_838006, EPI_ISL_838007, EPI_ISL_838008, EPI_ISL_838009, EPI_ISL_838011, EPI_ISL_838012, EPI_ISL_838013, EPI_ISL_838016, EPI_ISL_838026, EPI_ISL_838032, EPI_ISL_838049, EPI_ISL_838050, EPI_ISL_838051, EPI_ISL_838052, EPI_ISL_838053, EPI_ISL_838054			
see above	Department of Pathology, University of Cambridge	COVID-19 Genomics UK (COG-UK) Consortium	Aminu S. Jahun, Yasmin Chaudhry, Grant Hall, Iliana Georgana, Myra Hosmillo, Martin D. Curran, Malte Pinckert, Surendra Parmar, Ian Goodfellow
EPI_ISL_838315	University of Exeter	COVID-19 Genomics UK (COG-UK) Consortium	Ben Temperton,Aaron Jeffries,Michelle Michelsen,Joanna Warwick-Dugdale,Audrey Farbos,Robyn Manley,Stephen Michell,Jane Masoli
EPI_ISL_838960, EPI_ISL_838961, EPI_ISL_838962, EPI_ISL_838963, EPI_ISL_838964, EPI_ISL_838965, EPI_ISL_838966, EPI_ISL_838967, EPI_ISL_838968, EPI_ISL_838970, EPI_ISL_838971, EPI_ISL_838972, EPI_ISL_838973, EPI_ISL_838974, EPI_ISL_838975, EPI_ISL_838976, EPI_ISL_838977, EPI_ISL_838978, EPI_ISL_838979, EPI_ISL_838980, EPI_ISL_838982, EPI_ISL_838983, EPI_ISL_838984, EPI_ISL_838985, EPI_ISL_838986, EPI_ISL_838987, EPI_ISL_838991, EPI_ISL_838992, EPI_ISL_838993, EPI_ISL_838994, EPI_ISL_838995, EPI_ISL_838996, EPI_ISL_838999, EPI_ISL_839000, EPI_ISL_839001, EPI_ISL_839002, EPI_ISL_839003, EPI_ISL_839004, EPI_ISL_839005, EPI_ISL_839007, EPI_ISL_839008, EPI_ISL_839009, EPI_ISL_839010, EPI_ISL_839011, EPI_ISL_839012, EPI_ISL_839126, EPI_ISL_839127, EPI_ISL_839129, EPI_ISL_839130, EPI_ISL_839131, EPI_ISL_839132, EPI_ISL_839233, EPI_ISL_839234, EPI_ISL_839235, EPI_ISL_839236, EPI_ISL_839237, EPI_ISL_839238, EPI_ISL_839239, EPI_ISL_839240, EPI_ISL_839241, EPI_ISL_839242, EPI_ISL_839243, EPI_ISL_839244, EPI_ISL_839245, EPI_ISL_839246, EPI_ISL_839247, EPI_ISL_839248, EPI_ISL_839249, EPI_ISL_839259, EPI_ISL_839263, EPI_ISL_839277, EPI_ISL_839278, EPI_ISL_839279, EPI_ISL_839280, EPI_ISL_839281, EPI_ISL_839282, EPI_ISL_839283, EPI_ISL_839284, EPI_ISL_839285, EPI_ISL_839286, EPI_ISL_839287, EPI_ISL_839288, EPI_ISL_839289, EPI_ISL_839290, EPI_ISL_839291, EPI_ISL_839292, EPI_ISL_839293, EPI_ISL_839294, EPI_ISL_839295, EPI_ISL_839296, EPI_ISL_839297, EPI_ISL_839298, EPI_ISL_839299, EPI_ISL_839300, EPI_ISL_839301, EPI_ISL_839302, EPI_ISL_839303, EPI_ISL_839304			
see above	University College London, Great Ormond Street Hospital for Children NHS Foundation Trust, Imperial College Healthcare NHS Trust	COVID-19 Genomics UK (COG-UK) Consortium	Sergi Castellano, Rachel Williams, Mark Kristiansen, Paola Resende Silva, Sunando Roy, Tony Brooks, Helena Tutill, Paola Niola, Patricia Dyal, Charlotte Williams, Leysa Forrest, Yasmin Panchbhaya, Jacqueline Findlay, Samuel Weeks, Julianne Brown, Kathryn Harris, Paul Randell, James Price, Alison Holmes, Judith Breuer
EPI_ISL_840053, EPI_ISL_840054, EPI_ISL_840055, EPI_ISL_840056, EPI_ISL_840057, EPI_ISL_840058, EPI_ISL_840059, EPI_ISL_840060	Queens Medical Centre, Clinical Microbiology Department / DeepSeq Nottingham	COVID-19 Genomics UK (COG-UK) Consortium	Gemma Clark, Wendy Smith, Manjinder Khakh, Vicki M Fleming, Michelle M Lister, Hannah Howson-Wells, Jonathan Ball, Patrick McClure, Joseph Chappell, Theocharis Tsoleridis, Nadine Holmes, Matthew Carlisle, Christopher Moore, Fei Sang, Johnny Debebe, Victoria Wright, Matthew Loose
EPI_ISL_841326, EPI_ISL_841328, EPI_ISL_841330, EPI_ISL_841331, EPI_ISL_841340, EPI_ISL_841349, EPI_ISL_841350, EPI_ISL_841351, EPI_ISL_841352, EPI_ISL_841426, EPI_ISL_841427, EPI_ISL_841430, EPI_ISL_841457, EPI_ISL_841484, EPI_ISL_841487, EPI_ISL_841488, EPI_ISL_841489, EPI_ISL_841492, EPI_ISL_841493, EPI_ISL_841494, EPI_ISL_841495, EPI_ISL_841496, EPI_ISL_841497, EPI_ISL_841523, EPI_ISL_841524, EPI_ISL_841525, EPI_ISL_841527, EPI_ISL_841528, EPI_ISL_841530, EPI_ISL_841532, EPI_ISL_841533, EPI_ISL_841534, EPI_ISL_841535, EPI_ISL_841536, EPI_ISL_841537, EPI_ISL_841538, EPI_ISL_841539, EPI_ISL_841540, EPI_ISL_841541, EPI_ISL_841542, EPI_ISL_841547, EPI_ISL_841549, EPI_ISL_841550, EPI_ISL_841551, EPI_ISL_841553, EPI_ISL_841554, EPI_ISL_841557, EPI_ISL_841558, EPI_ISL_841614, EPI_ISL_841625, EPI_ISL_841659, EPI_ISL_841661, EPI_ISL_841665, EPI_ISL_841667, EPI_ISL_841669, EPI_ISL_841670, EPI_ISL_841673, EPI_ISL_841676, EPI_ISL_841677, EPI_ISL_841679, EPI_ISL_841684, EPI_ISL_841685, EPI_ISL_841686, EPI_ISL_841687, EPI_ISL_841688, EPI_ISL_841689, EPI_ISL_841690, EPI_ISL_841694, EPI_ISL_841695, EPI_ISL_841700, EPI_ISL_841702, EPI_ISL_841704, EPI_ISL_841705, EPI_ISL_841706, EPI_ISL_841707, EPI_ISL_841709, EPI_ISL_841710, EPI_ISL_841711, EPI_ISL_841712, EPI_ISL_841713, EPI_ISL_841714, EPI_ISL_841715, EPI_ISL_841716, EPI_ISL_841717, EPI_ISL_841718, EPI_ISL_841719, EPI_ISL_841720, EPI_ISL_841721, EPI_ISL_841722, EPI_ISL_841723, EPI_ISL_841724, EPI_ISL_841725, EPI_ISL_841726, EPI_ISL_841727, EPI_ISL_841728, EPI_ISL_841729, EPI_ISL_841730, EPI_ISL_841733, EPI_ISL_841734, EPI_ISL_841735, EPI_ISL_841736, EPI_ISL_841738, EPI_ISL_841739, EPI_ISL_841740			
see above	Originating lab: Wales Specialist Virology Centre Sequencing lab: Pathogen Genomics Unit	Public Health Wales Microbiology Cardiff Wales Specialist Virology Centre	Catherine Moore, Johnathan Evans, Laura Gifford, Malorie Perry, Simon Cottrell, Angela Marchbank, Alec Birchley, Alexander Adams, Amy Gaskin, Bree Gatica-Wilcox, Jason Coombes, Joel Southgate, Lauren Gilbert, Lee Graham, Nicole Pacchiarini, Sara Kumziene-Summerhayes, Sarah Taylor, Sophie Jones, Sara Rey, Matthew Bull, Joanne Watkins, Sally Corden, Tom Connor
EPI_ISL_841921	Centre for Enzyme Innovation, University of Portsmouth / Translational Research Laboratory, Portsmouth Hospitals NHS Trust	COVID-19 Genomics UK (COG-UK) Consortium	Angela Beckett,Yann Bourgeois,Garry Scarlett,Sharon Glaysher,Scott Elliott,Kelly Bicknell,Robert Impey,Allyson Lloyd,Sarah Wyllie,Ethan Butcher,Anoop Chauhan,Samuel Robson
EPI_ISL_842207, EPI_ISL_842213, EPI_ISL_842222, EPI_ISL_842223, EPI_ISL_842225, EPI_ISL_842229, EPI_ISL_842234, EPI_ISL_842242, EPI_ISL_842243, EPI_ISL_842247, EPI_ISL_842249, EPI_ISL_842252, EPI_ISL_842259, EPI_ISL_842267, EPI_ISL_842271, EPI_ISL_842275, EPI_ISL_842276, EPI_ISL_842277, EPI_ISL_842296, EPI_ISL_842302, EPI_ISL_842309, EPI_ISL_842310, EPI_ISL_842314, EPI_ISL_842320, EPI_ISL_842321, EPI_ISL_842323, EPI_ISL_842328, EPI_ISL_842331, EPI_ISL_842333, EPI_ISL_842336, EPI_ISL_842337, EPI_ISL_842343			
see above	Virology Department, Sheffield Teaching Hospitals NHS Foundation Trust/Department of Infection, Immunity and	COVID-19 Genomics UK (COG-UK) Consortium	Thushan de Silva, Matthew Parker, Nikki Smith, Adri Angyal, Rebecca Brown, Luke Green, Rachel Tucker, Paul Parsons, Danielle Groves, Katie Johnson, Laura Carrilero, Alex Keeley, Dave Partridge, Matthew Wyles, Benjamin Lindsey, Mehmet Yavuz, Mohammad Raza, Cariad Evans

	Cardiovascular Disease, The Medical School, University of Sheffield		
EPI_ISL_842637	Medical Microbiology Unit, Department for Laboratory Medicine, Drammen Hospital, Vestre Viken Health Trust,	Norwegian Institute of Public Health, Department of Virology	Kathrine Stene-Johansen, Kamilla Heddeland Instefjord, Hilde Elshaug, Atiya R Ali,Marie Paulsen Madsen, Rasmus Riis Kopperud, Hilde Vollan, Karoline Bragstad, Olav Hungnes
EPI_ISL_842642	Norwegian Institute of Public Health, Department of Virology	Norwegian Institute of Public Health, Department of Virology	Kathrine Stene-Johansen, Kamilla Heddeland Instefjord, Hilde Elshaug, Atiya R Ali,Marie Paulsen Madsen, Rasmus Riis Kopperud, Hilde Vollan, Karoline Bragstad, Olav Hungnes
EPI_ISL_843184, EPI_ISL_843185, EPI_ISL_843186, EPI_ISL_843187	Maryland Public Health Laboratory	Maryland Public Health Laboratory	Maryland Department of Health Laboratories Administration
EPI_ISL_843194, EPI_ISL_843199, EPI_ISL_843200	Canterbury Health Laboratories	Institute of Environmental Science and Research (ESR)	Xiaoyun Ren, Matt Storey, Nikki Freed, Muhammad Faisal, Jing Wang, Hermes Perez, Anja Werno, Antje van der Linden, Arlo Upton, Chris Mansell, David Hammer, Dragana Drinkovic, Gary McAuliffe, Hana Sofia Andersson, James Ussher, Jill Sherwood, Josh Freeman, Julia Howard, Juliet Elvy, Mary DeAlmeida, Matt Blakiston, Matthew Rogers, Max Bloomfield, Michael Addidle, Michelle Balm, Sally Roberts, Sarah Jefferies, Sharmini Muttaiyah, Susan Morpeth, Susan Taylor, Timothy Blackmore, Vani Sathyendran, Veronica Playle, Virginia Hope, Erasmus Smit, Lauren Jelly, Olin Silander, Joep de Ligt
EPI_ISL_845638, EPI_ISL_845639, EPI_ISL_845640, EPI_ISL_845646, EPI_ISL_845647, EPI_ISL_845654	Laboratorio de Salud Pública - Secretaría Distrital de Salud	Instituto Nacional de Salud - Dirección de Investigación en Salud Pública	Katherine Laiton-Donato, Diego A. Álvarez-Díaz, Carlos Franco-Muñoz, Mauricio Pacheco-Montealegre, Maria T. Herrera-Sepúlveda, Jonathan Reales, Sheryll Corchuelo, Julian Naizaque, Gerardo Santamaría, Paola Muñoz-Laiton, Diego Andrés Prada, Magdalena Wiesner, Martha Lucia Ospina Martinez, Marcela Mercado-Reyes
EPI_ISL_845753	Toronto Invasive Bacterial Diseases Network	McMaster University	Allison McGeer, Patryk Aftanas, Hooman Derakhshani, Angel Li, Kuganya Nirmalarajah, Emily Panousis, Ahmed Draia, Jalees Nasir, Michael Surette, Samira Mubareka, Andrew G. McArthur
EPI_ISL_845797	South Eastern Area Laboratory Services (SEALS)	NSW Health Pathology - Institute of Clinical Pathology and Medical Research; Westmead Hospital; University of Sydney	CIDM-PH et al.
EPI_ISL_845798, EPI_ISL_845799	Sydney South West Pathology Service (SSWPS) - Royal Prince Alfred Hospital - NSW Health Pathology	NSW Health Pathology - Institute of Clinical Pathology and Medical Research; Westmead Hospital; University of Sydney	CIDM-PH et al.
EPI_ISL_845894, EPI_ISL_845895, EPI_ISL_845897, EPI_ISL_845898, EPI_ISL_845899, EPI_ISL_845900, EPI_ISL_845901, EPI_ISL_845902, EPI_ISL_845903, EPI_ISL_845904, EPI_ISL_845905, EPI_ISL_845906, EPI_ISL_845907, EPI_ISL_845908, EPI_ISL_845909, EPI_ISL_845910, EPI_ISL_845911, EPI_ISL_845912, EPI_ISL_845913, EPI_ISL_845914, EPI_ISL_845915, EPI_ISL_845916, EPI_ISL_845917, EPI_ISL_845918, EPI_ISL_845919, EPI_ISL_845920, EPI_ISL_845921, EPI_ISL_845922, EPI_ISL_845923, EPI_ISL_845924, EPI_ISL_845925, EPI_ISL_845926, EPI_ISL_845927, EPI_ISL_845928, EPI_ISL_845929, EPI_ISL_845930, EPI_ISL_845931, EPI_ISL_845932, EPI_ISL_845933, EPI_ISL_845934, EPI_ISL_845935, EPI_ISL_845936, EPI_ISL_845937, EPI_ISL_845938, EPI_ISL_845939, EPI_ISL_845940, EPI_ISL_845941, EPI_ISL_845942, EPI_ISL_845943, EPI_ISL_845944, EPI_ISL_845945, EPI_ISL_845946, EPI_ISL_845947, EPI_ISL_845948, EPI_ISL_845949, EPI_ISL_845950, EPI_ISL_845951, EPI_ISL_845952, EPI_ISL_845953, EPI_ISL_845954, EPI_ISL_845955, EPI_ISL_845956, EPI_ISL_845957, EPI_ISL_845958, EPI_ISL_845959, EPI_ISL_845960, EPI_ISL_845961, EPI_ISL_845962, EPI_ISL_845963, EPI_ISL_845964, EPI_ISL_845965, EPI_ISL_845966, EPI_ISL_845967, EPI_ISL_845968, EPI_ISL_845969, EPI_ISL_845970, EPI_ISL_845971, EPI_ISL_845972, EPI_ISL_845974, EPI_ISL_845975, EPI_ISL_845976, EPI_ISL_845977, EPI_ISL_845978, EPI_ISL_845979, EPI_ISL_845980, EPI_ISL_845981, EPI_ISL_845982, EPI_ISL_845983, EPI_ISL_845984, EPI_ISL_845985, EPI_ISL_845986, EPI_ISL_845987, EPI_ISL_845988, EPI_ISL_845989, EPI_ISL_845990, EPI_ISL_845991, EPI_ISL_845992, EPI_ISL_845993, EPI_ISL_845994, EPI_ISL_845995, EPI_ISL_845996, EPI_ISL_845997, EPI_ISL_845998, EPI_ISL_845999, EPI_ISL_846000, EPI_ISL_846001, EPI_ISL_846002, EPI_ISL_846003, EPI_ISL_846004, EPI_ISL_846005, EPI_ISL_846006, EPI_ISL_846008, EPI_ISL_846009, EPI_ISL_846010, EPI_ISL_846011, EPI_ISL_846012, EPI_ISL_846013, EPI_ISL_846014, EPI_ISL_846015, EPI_ISL_846016, EPI_ISL_846017, EPI_ISL_846018, EPI_ISL_846019, EPI_ISL_846020, EPI_ISL_846021, EPI_ISL_846024, EPI_ISL_846025, EPI_ISL_846026, EPI_ISL_846027, EPI_ISL_846028, EPI_ISL_846029, EPI_ISL_846030, EPI_ISL_846031, EPI_ISL_846032, EPI_ISL_846033, EPI_ISL_846034, EPI_ISL_846035, EPI_ISL_846036, EPI_ISL_846037, EPI_ISL_846038, EPI_ISL_846039, EPI_ISL_846040, EPI_ISL_846041, EPI_ISL_846042, EPI_ISL_846043, EPI_ISL_846044, EPI_ISL_846045, EPI_ISL_846046, EPI_ISL_846047, EPI_ISL_846048, EPI_ISL_846049, EPI_ISL_846050, EPI_ISL_846051, EPI_ISL_846053, EPI_ISL_846054, EPI_ISL_846055, EPI_ISL_846056, EPI_ISL_846057, EPI_ISL_846058, EPI_ISL_846060, EPI_ISL_846061, EPI_ISL_846062, EPI_ISL_846063, EPI_ISL_846064, EPI_ISL_846065, EPI_ISL_846066, EPI_ISL_846067, EPI_ISL_846068, EPI_ISL_846069, EPI_ISL_846071, EPI_ISL_846072, EPI_ISL_846073, EPI_ISL_846074, EPI_ISL_846075, EPI_ISL_846076, EPI_ISL_846077, EPI_ISL_846078, EPI_ISL_846079, EPI_ISL_846080, EPI_ISL_846081, EPI_ISL_846082, EPI_ISL_846083, EPI_ISL_846085, EPI_ISL_846086, EPI_ISL_846087, EPI_ISL_846088, EPI_ISL_846089, EPI_ISL_846090, EPI_ISL_846091, EPI_ISL_846092, EPI_ISL_846093, EPI_ISL_846094, EPI_ISL_846095, EPI_ISL_846096, EPI_ISL_846097, EPI_ISL_846098, EPI_ISL_846099, EPI_ISL_846100, EPI_ISL_846101, EPI_ISL_846102, EPI_ISL_846103, EPI_ISL_846104, EPI_ISL_846105, EPI_ISL_846106, EPI_ISL_846107, EPI_ISL_846108, EPI_ISL_846109, EPI_ISL_846110, EPI_ISL_846111, EPI_ISL_846112, EPI_ISL_846113, EPI_ISL_846114, EPI_ISL_846115, EPI_ISL_846116, EPI_ISL_846117, EPI_ISL_846118, EPI_ISL_846119, EPI_ISL_846120, EPI_ISL_846121, EPI_ISL_846122, EPI_ISL_846123, EPI_ISL_846124, EPI_ISL_846125, EPI_ISL_846126, EPI_ISL_846127, EPI_ISL_846128, EPI_ISL_846129, EPI_ISL_846130, EPI_ISL_846131, EPI_ISL_846132, EPI_ISL_846133, EPI_ISL_846134, EPI_ISL_846135, EPI_ISL_846136, EPI_ISL_846137, EPI_ISL_846138, EPI_ISL_846140, EPI_ISL_846141, EPI_ISL_846142, EPI_ISL_846143, EPI_ISL_846144, EPI_ISL_846145, EPI_ISL_846146, EPI_ISL_846148, EPI_ISL_846149, EPI_ISL_846150, EPI_ISL_846151, EPI_ISL_846153, EPI_ISL_846154, EPI_ISL_846155, EPI_ISL_846156, EPI_ISL_846157, EPI_ISL_846158, EPI_ISL_846159, EPI_ISL_846160, EPI_ISL_846161, EPI_ISL_846162, EPI_ISL_846163, EPI_ISL_846164, EPI_ISL_846165, EPI_ISL_846166, EPI_ISL_846167, EPI_ISL_846169, EPI_ISL_846170, EPI_ISL_846171, EPI_ISL_846172, EPI_ISL_846173, EPI_ISL_846174, EPI_ISL_846175, EPI_ISL_846176, EPI_ISL_846177, EPI_ISL_846178, EPI_ISL_846179, EPI_ISL_846180, EPI_ISL_846181, EPI_ISL_846182, EPI_ISL_846183, EPI_ISL_846184, EPI_ISL_846185, EPI_ISL_846186, EPI_ISL_846187, EPI_ISL_846188, EPI_ISL_846189, EPI_ISL_846190, EPI_ISL_846191, EPI_ISL_846192, EPI_ISL_846193, EPI_ISL_846194, EPI_ISL_846195, EPI_ISL_846196, EPI_ISL_846197, EPI_ISL_846198, EPI_ISL_846199			
see above	Lighthouse Lab in Cambridge	Wellcome Sanger Institute for the COVID-19 Genomics UK (COG-UK) Consortium	Rob Howes, The Lighthouse Lab in Cambridge and Alex Alderton, Roberto Amato, Sonia Goncalves, Ewan Harrison, David K. Jackson, Ian Johnston, Dominic Kwiatkowski, Cordelia Langford, John Sillitoe on behalf of the Wellcome Sanger Institute COVID-19 Surveillance Team
EPI_ISL_846200, EPI_ISL_846201, EPI_ISL_846202, EPI_ISL_846203, EPI_ISL_846204, EPI_ISL_846205, EPI_ISL_846206, EPI_ISL_846207, EPI_ISL_846208, EPI_ISL_846209, EPI_ISL_846210, EPI_ISL_846211, EPI_ISL_846212, EPI_ISL_846213, EPI_ISL_846214, EPI_ISL_846215, EPI_ISL_846216, EPI_ISL_846217, EPI_ISL_846218, EPI_ISL_846219, EPI_ISL_846220, EPI_ISL_846221, EPI_ISL_846222, EPI_ISL_846223, EPI_ISL_846224, EPI_ISL_846225, EPI_ISL_846226, EPI_ISL_846227, EPI_ISL_846228, EPI_ISL_846229, EPI_ISL_846230, EPI_ISL_846231, EPI_ISL_846232, EPI_ISL_846233, EPI_ISL_846234, EPI_ISL_846235, EPI_ISL_846236, EPI_ISL_846237, EPI_ISL_846238, EPI_ISL_846239, EPI_ISL_846240, EPI_ISL_846241, EPI_ISL_846242, EPI_ISL_846243, EPI_ISL_846244, EPI_ISL_846245, EPI_ISL_846246, EPI_ISL_846247, EPI_ISL_846248, EPI_ISL_846249, EPI_ISL_846250, EPI_ISL_846251, EPI_ISL_846252, EPI_ISL_846253, EPI_ISL_846254, EPI_ISL_846255, EPI_ISL_846256, EPI_ISL_846257, EPI_ISL_846258, EPI_ISL_846259, EPI_ISL_846260, EPI_ISL_846261, EPI_ISL_846262, EPI_ISL_846263, EPI_ISL_846264, EPI_ISL_846265, EPI_ISL_846266, EPI_ISL_846267, EPI_ISL_846268, EPI_ISL_846269, EPI_ISL_846270, EPI_ISL_846271, EPI_ISL_846272, EPI_ISL_846273, EPI_ISL_846274, EPI_ISL_846275, EPI_ISL_846276, EPI_ISL_846277, EPI_ISL_846278, EPI_ISL_846279, EPI_ISL_846280, EPI_ISL_846281, EPI_ISL_846282, EPI_ISL_846284, EPI_ISL_846285, EPI_ISL_846286, EPI_ISL_846288, EPI_ISL_846289, EPI_ISL_846290, EPI_ISL_846291, EPI_ISL_846292, EPI_ISL_846293, EPI_ISL_846294, EPI_ISL_846295, EPI_ISL_846296, EPI_ISL_846297, EPI_ISL_846298, EPI_ISL_846300, EPI_ISL_846301, EPI_ISL_846302, EPI_ISL_846303, EPI_ISL_846304, EPI_ISL_846305, EPI_ISL_846306, EPI_ISL_846307, EPI_ISL_846308, EPI_ISL_846309, EPI_ISL_846310, EPI_ISL_846311, EPI_ISL_846312, EPI_ISL_846313, EPI_ISL_846314, EPI_ISL_846316, EPI_ISL_846317, EPI_ISL_846318, EPI_ISL_846320, EPI_ISL_846322, EPI_ISL_846323, EPI_ISL_846324, EPI_ISL_846325, EPI_ISL_846326, EPI_ISL_846327, EPI_ISL_846328, EPI_ISL_846329, EPI_ISL_846330, EPI_ISL_846331, EPI_ISL_846332, EPI_ISL_846333, EPI_ISL_846334, EPI_ISL_846335, EPI_ISL_846336, EPI_ISL_846337, EPI_ISL_846338, EPI_ISL_846339, EPI_ISL_846340, EPI_ISL_846341, EPI_ISL_846342, EPI_ISL_846343, EPI_ISL_846344, EPI_ISL_846345, EPI_ISL_846346, EPI_ISL_846347, EPI_ISL_846348, EPI_ISL_846349, EPI_ISL_846350, EPI_ISL_846351, EPI_ISL_846352, EPI_ISL_846353, EPI_ISL_846355, EPI_ISL_846356, EPI_ISL_846357, EPI_ISL_846358, EPI_ISL_846359, EPI_ISL_846360, EPI_ISL_846361, EPI_ISL_846362, EPI_ISL_846363, EPI_ISL_846364, EPI_ISL_846365, EPI_ISL_846366, EPI_ISL_846367, EPI_ISL_846368, EPI_ISL_846369, EPI_ISL_846370, EPI_ISL_846371, EPI_ISL_846372, EPI_ISL_846373, EPI_ISL_846374, EPI_ISL_846375, EPI_ISL_846376, EPI_ISL_846377, EPI_ISL_846378, EPI_ISL_846379, EPI_ISL_846380, EPI_ISL_846381, EPI_ISL_846382, EPI_ISL_846383, EPI_ISL_846384, EPI_ISL_846385, EPI_ISL_846386, EPI_ISL_846387, EPI_ISL_846388, EPI_ISL_846389, EPI_ISL_846390, EPI_ISL_846391, EPI_ISL_846392, EPI_ISL_846393, EPI_ISL_846394, EPI_ISL_846395, EPI_ISL_846396, EPI_ISL_846397, EPI_ISL_846398, EPI_ISL_846401, EPI_ISL_846402, EPI_ISL_846403, EPI_ISL_846404, EPI_ISL_846405, EPI_ISL_846406, EPI_ISL_846407, EPI_ISL_846408, EPI_ISL_846409, EPI_ISL_846410, EPI_ISL_846411, EPI_ISL_846412, EPI_ISL_846413, EPI_ISL_846414, EPI_ISL_846415, EPI_ISL_846416, EPI_ISL_846417, EPI_ISL_846418, EPI_ISL_846419, EPI_ISL_846420, EPI_ISL_846422, EPI_ISL_846423, EPI_ISL_846424, EPI_ISL_846425, EPI_ISL_846426, EPI_ISL_846427, EPI_ISL_846428, EPI_ISL_846430, EPI_ISL_846432, EPI_ISL_846433, EPI_ISL_846434, EPI_ISL_846435, EPI_ISL_846436, EPI_ISL_846437, EPI_ISL_846438, EPI_ISL_846439, EPI_ISL_846440, EPI_ISL_846441, EPI_ISL_846442, EPI_ISL_846444, EPI_ISL_846445, EPI_ISL_846446, EPI_ISL_846447, EPI_ISL_846448, EPI_ISL_846449, EPI_ISL_846450, EPI_ISL_846451, EPI_ISL_846452, EPI_ISL_846453, EPI_ISL_846454, EPI_ISL_846455, EPI_ISL_846456, EPI_ISL_846457, EPI_ISL_846458, EPI_ISL_846459, EPI_ISL_846460, EPI_ISL_846461, EPI_ISL_846462, EPI_ISL_846463, EPI_ISL_846464, EPI_ISL_846465, EPI_ISL_846466, EPI_ISL_846467, EPI_ISL_846468, EPI_ISL_846469, EPI_ISL_846470, EPI_ISL_846471, EPI_ISL_846472, EPI_ISL_846473, EPI_ISL_846474, EPI_ISL_846475, EPI_ISL_846476, EPI_ISL_846477, EPI_ISL_846478, EPI_ISL_846479, EPI_ISL_846480, EPI_ISL_846481, EPI_ISL_846482, EPI_ISL_846483, EPI_ISL_846484, EPI_ISL_846485, EPI_ISL_846486, EPI_ISL_846487, EPI_ISL_846488, EPI_ISL_846489, EPI_ISL_846490, EPI_ISL_846492, EPI_ISL_846493, EPI_ISL_846494, EPI_ISL_846495, EPI_ISL_846496, EPI_ISL_846497, EPI_ISL_846498, EPI_ISL_846499, EPI_ISL_846500, EPI_ISL_846501, EPI_ISL_846502, EPI_ISL_846503, EPI_ISL_846504, EPI_ISL_846505, EPI_ISL_846506, EPI_ISL_846507, EPI_ISL_846508, EPI_ISL_846512			
see above	Lighthouse Lab in Alderley Park	Wellcome Sanger Institute for the COVID-19 Genomics UK (COG-UK) Consortium	Jacquelyn Wynn, Mairead Hyland, The Lighthouse Lab in Alderley Park and Alex Alderton, Roberto Amato, Sonia Goncalves, Ewan Harrison, David K. Jackson, Ian Johnston, Dominic Kwiatkowski, Cordelia Langford, John Sillitoe on behalf of the Wellcome Sanger Institute COVID-19 Surveillance Team
EPI_ISL_846542, EPI_ISL_846547, EPI_ISL_846548, EPI_ISL_846549, EPI_ISL_846559, EPI_ISL_846560, EPI_ISL_846575, EPI_ISL_846576	Lab voor klinische biologie	Lab voor klinische biologie	Hannelore Hamerlinck, Bruno Verhasselt
EPI_ISL_846597	Respiratory Virus Unit, National Infection Service, Public Health England	COVID-19 Genomics UK (COG-UK) Consortium	PHE Covid Sequencing Team
EPI_ISL_847589	California Department of Public Health	Chiu Laboratory, University of California, San Francisco	Charles Chiu, Xianding (Wayne) Deng, Candace Wang, Brian Bushnell, Scot Federman, Jill Hacker, Debra Wadford
EPI_ISL_848192	National Laboratory for Health, Environment and Food	National Laboratory for Health, Environment and Food	Aleksander Mahnic, Sandra Janezic, Maja Rupnik
EPI_ISL_849736, EPI_ISL_849737, EPI_ISL_849738, EPI_ISL_849739, EPI_ISL_849740, EPI_ISL_849741, EPI_ISL_849742, EPI_ISL_849743	Special Operations Medical Research Division, Defence Services Medical Research Centre	Special Operations Medical Research Division, Defence Services Medical Research Centre	Oo,K.Z., Aung,N.M., Win,K.K., Aung,P.K., Htun,Z.W., Zaw,T., Myint,K., Lwin,K.K.
EPI_ISL_849756, EPI_ISL_849757	unknown	PHV-FSS	Son Nguyen et al.
EPI_ISL_849763	Molecular Biology, IZS Sicilia	Molecular Biology, IZS Sicilia	Reale,S., Seidita,G., Di Gaudio,F., Scibetta,S. and Vitale,F.
EPI_ISL_849913, EPI_ISL_849914, EPI_ISL_849915, EPI_ISL_849916, EPI_ISL_849917, EPI_ISL_849918, EPI_ISL_849919, EPI_ISL_849920	Utah Public Health Laboratory	Utah Public Health Laboratory	Erin L. Young, Kelly F. Oakeson, Tara Gallagher

EPI_ISL_850511, EPI_ISL_850512, EPI_ISL_850513, EPI_ISL_850515	Hospital Universitari de Bellvitge	Hospital Universitari Vall d'Hebron - Vall d'Hebron Institut de Rercerca	Cristina Andrés, Maria Piñana, Josep F Abril, Damir Garcia-Cehic, Ariadna Rando, Juliana Esperalba, Maria Gema Codina, Carla Castillo, Maria Carmen Martin, Tomàs Pumarola, Josep Quer, Andrés Antón
EPI_ISL_850516	Hospital Universitari Vall d'Hebron - Vall d'Hebron Institut de Rercerca	Hospital Universitari Vall d'Hebron - Vall d'Hebron Institut de Rercerca	Cristina Andrés, Maria Piñana, Josep F Abril, Damir Garcia-Cehic, Ariadna Rando, Juliana Esperalba, Maria Gema Codina, Carla Castillo, Maria Carmen Martin, Tomàs Pumarola, Josep Quer, Andrés Antón
EPI_ISL_851064, EPI_ISL_851065, EPI_ISL_851066, EPI_ISL_851067, EPI_ISL_851068, EPI_ISL_851069	Lighthouse Lab in Cambridge	Wellcome Sanger Institute for the COVID-19 Genomics UK (COG-UK) Consortium	Rob Howes, The Lighthouse Lab in Cambridge and Alex Alderton, Roberto Amato, Sonia Goncalves, Ewan Harrison, David K. Jackson, Ian Johnston, Dominic Kwiatkowski, Cordelia Langford, John Sillitoe on behalf of the Wellcome Sanger Institute COVID-19 Surveillance Team
EPI_ISL_851070	Lighthouse Lab in Glasgow	Wellcome Sanger Institute for the COVID-19 Genomics UK (COG-UK) Consortium	Harper VanSteenhouse, Yumi Kasai, David Gray, Carol Clugston, Anna Dominiczak and Alex Alderton, Roberto Amato, Sonia Goncalves, Ewan Harrison, David K. Jackson, Ian Johnston, Dominic Kwiatkowski, Cordelia Langford, John Sillitoe on behalf of the Wellcome Sanger Institute COVID-19 Surveillance Team
EPI_ISL_851071, EPI_ISL_851072, EPI_ISL_851073, EPI_ISL_851074, EPI_ISL_851075, EPI_ISL_851076	Lighthouse Lab in Cambridge	Wellcome Sanger Institute for the COVID-19 Genomics UK (COG-UK) Consortium	Rob Howes, The Lighthouse Lab in Cambridge and Alex Alderton, Roberto Amato, Sonia Goncalves, Ewan Harrison, David K. Jackson, Ian Johnston, Dominic Kwiatkowski, Cordelia Langford, John Sillitoe on behalf of the Wellcome Sanger Institute COVID-19 Surveillance Team
EPI_ISL_851077	Lighthouse Lab in Glasgow	Wellcome Sanger Institute for the COVID-19 Genomics UK (COG-UK) Consortium	Harper VanSteenhouse, Yumi Kasai, David Gray, Carol Clugston, Anna Dominiczak and Alex Alderton, Roberto Amato, Sonia Goncalves, Ewan Harrison, David K. Jackson, Ian Johnston, Dominic Kwiatkowski, Cordelia Langford, John Sillitoe on behalf of the Wellcome Sanger Institute COVID-19 Surveillance Team
EPI_ISL_851078, EPI_ISL_851080	Lighthouse Lab in Cambridge	Wellcome Sanger Institute for the COVID-19 Genomics UK (COG-UK) Consortium	Rob Howes, The Lighthouse Lab in Cambridge and Alex Alderton, Roberto Amato, Sonia Goncalves, Ewan Harrison, David K. Jackson, Ian Johnston, Dominic Kwiatkowski, Cordelia Langford, John Sillitoe on behalf of the Wellcome Sanger Institute COVID-19 Surveillance Team
EPI_ISL_851081, EPI_ISL_851082	Lighthouse Lab in Glasgow	Wellcome Sanger Institute for the COVID-19 Genomics UK (COG-UK) Consortium	Harper VanSteenhouse, Yumi Kasai, David Gray, Carol Clugston, Anna Dominiczak and Alex Alderton, Roberto Amato, Sonia Goncalves, Ewan Harrison, David K. Jackson, Ian Johnston, Dominic Kwiatkowski, Cordelia Langford, John Sillitoe on behalf of the Wellcome Sanger Institute COVID-19 Surveillance Team
EPI_ISL_851084	Lighthouse Lab in Cambridge	Wellcome Sanger Institute for the COVID-19 Genomics UK (COG-UK) Consortium	Rob Howes, The Lighthouse Lab in Cambridge and Alex Alderton, Roberto Amato, Sonia Goncalves, Ewan Harrison, David K. Jackson, Ian Johnston, Dominic Kwiatkowski, Cordelia Langford, John Sillitoe on behalf of the Wellcome Sanger Institute COVID-19 Surveillance Team
EPI_ISL_851085	Lighthouse Lab in Glasgow	Wellcome Sanger Institute for the COVID-19 Genomics UK (COG-UK) Consortium	Harper VanSteenhouse, Yumi Kasai, David Gray, Carol Clugston, Anna Dominiczak and Alex Alderton, Roberto Amato, Sonia Goncalves, Ewan Harrison, David K. Jackson, Ian Johnston, Dominic Kwiatkowski, Cordelia Langford, John Sillitoe on behalf of the Wellcome Sanger Institute COVID-19 Surveillance Team
EPI_ISL_851086, EPI_ISL_851087, EPI_ISL_851088, EPI_ISL_851089, EPI_ISL_851090, EPI_ISL_851091, EPI_ISL_851092, EPI_ISL_851093	Lighthouse Lab in Cambridge	Wellcome Sanger Institute for the COVID-19 Genomics UK (COG-UK) Consortium	Rob Howes, The Lighthouse Lab in Cambridge and Alex Alderton, Roberto Amato, Sonia Goncalves, Ewan Harrison, David K. Jackson, Ian Johnston, Dominic Kwiatkowski, Cordelia Langford, John Sillitoe on behalf of the Wellcome Sanger Institute COVID-19 Surveillance Team
EPI_ISL_851094, EPI_ISL_851095	Lighthouse Lab in Glasgow	Wellcome Sanger Institute for the COVID-19 Genomics UK (COG-UK) Consortium	Harper VanSteenhouse, Yumi Kasai, David Gray, Carol Clugston, Anna Dominiczak and Alex Alderton, Roberto Amato, Sonia Goncalves, Ewan Harrison, David K. Jackson, Ian Johnston, Dominic Kwiatkowski, Cordelia Langford, John Sillitoe on behalf of the Wellcome Sanger Institute COVID-19 Surveillance Team
EPI_ISL_851096, EPI_ISL_851097, EPI_ISL_851098, EPI_ISL_851099, EPI_ISL_851100	Lighthouse Lab in Cambridge	Wellcome Sanger Institute for the COVID-19 Genomics UK (COG-UK) Consortium	Rob Howes, The Lighthouse Lab in Cambridge and Alex Alderton, Roberto Amato, Sonia Goncalves, Ewan Harrison, David K. Jackson, Ian Johnston, Dominic Kwiatkowski, Cordelia Langford, John Sillitoe on behalf of the Wellcome Sanger Institute COVID-19 Surveillance Team
EPI_ISL_851102	Lighthouse Lab in Glasgow	Wellcome Sanger Institute for the COVID-19 Genomics UK (COG-UK) Consortium	Harper VanSteenhouse, Yumi Kasai, David Gray, Carol Clugston, Anna Dominiczak and Alex Alderton, Roberto Amato, Sonia Goncalves, Ewan Harrison, David K. Jackson, Ian Johnston, Dominic Kwiatkowski, Cordelia Langford, John Sillitoe on behalf of the Wellcome Sanger Institute COVID-19 Surveillance Team
EPI_ISL_851103	Lighthouse Lab in Cambridge	Wellcome Sanger Institute for the COVID-19 Genomics UK (COG-UK) Consortium	Rob Howes, The Lighthouse Lab in Cambridge and Alex Alderton, Roberto Amato, Sonia Goncalves, Ewan Harrison, David K. Jackson, Ian Johnston, Dominic Kwiatkowski, Cordelia Langford, John Sillitoe on behalf of the Wellcome Sanger Institute COVID-19 Surveillance Team
EPI_ISL_851104	Lighthouse Lab in Glasgow	Wellcome Sanger Institute for the COVID-19 Genomics UK (COG-UK) Consortium	Harper VanSteenhouse, Yumi Kasai, David Gray, Carol Clugston, Anna Dominiczak and Alex Alderton, Roberto Amato, Sonia Goncalves, Ewan Harrison, David K. Jackson, Ian Johnston, Dominic Kwiatkowski, Cordelia Langford, John Sillitoe on behalf of the Wellcome Sanger Institute COVID-19 Surveillance Team
EPI_ISL_851105, EPI_ISL_851106	Lighthouse Lab in Cambridge	Wellcome Sanger Institute for the COVID-19 Genomics UK (COG-UK) Consortium	Rob Howes, The Lighthouse Lab in Cambridge and Alex Alderton, Roberto Amato, Sonia Goncalves, Ewan Harrison, David K. Jackson, Ian Johnston, Dominic Kwiatkowski, Cordelia Langford, John Sillitoe on behalf of the Wellcome Sanger Institute COVID-19 Surveillance Team
EPI_ISL_851107	Lighthouse Lab in Glasgow	Wellcome Sanger Institute for the COVID-19 Genomics UK (COG-UK) Consortium	Harper VanSteenhouse, Yumi Kasai, David Gray, Carol Clugston, Anna Dominiczak and Alex Alderton, Roberto Amato, Sonia Goncalves, Ewan Harrison, David K. Jackson, Ian Johnston, Dominic Kwiatkowski, Cordelia Langford, John Sillitoe on behalf of the Wellcome Sanger Institute COVID-19 Surveillance Team
EPI_ISL_851108, EPI_ISL_851109, EPI_ISL_851111, EPI_ISL_851112, EPI_ISL_851113, EPI_ISL_851114	Lighthouse Lab in Cambridge	Wellcome Sanger Institute for the COVID-19 Genomics UK (COG-UK) Consortium	Rob Howes, The Lighthouse Lab in Cambridge and Alex Alderton, Roberto Amato, Sonia Goncalves, Ewan Harrison, David K. Jackson, Ian Johnston, Dominic Kwiatkowski, Cordelia Langford, John Sillitoe on behalf of the Wellcome Sanger Institute COVID-19 Surveillance Team
EPI_ISL_851116	Lighthouse Lab in Glasgow	Wellcome Sanger Institute for the COVID-19 Genomics UK (COG-UK) Consortium	Harper VanSteenhouse, Yumi Kasai, David Gray, Carol Clugston, Anna Dominiczak and Alex Alderton, Roberto Amato, Sonia Goncalves, Ewan Harrison, David K. Jackson, Ian Johnston, Dominic Kwiatkowski, Cordelia Langford, John Sillitoe on behalf of the Wellcome Sanger Institute COVID-19 Surveillance Team
EPI_ISL_851117, EPI_ISL_851118	Lighthouse Lab in Cambridge	Wellcome Sanger Institute for the COVID-19 Genomics UK (COG-UK) Consortium	Rob Howes, The Lighthouse Lab in Cambridge and Alex Alderton, Roberto Amato, Sonia Goncalves, Ewan Harrison, David K. Jackson, Ian Johnston, Dominic Kwiatkowski, Cordelia Langford, John Sillitoe on behalf of the Wellcome Sanger Institute COVID-19 Surveillance Team
EPI_ISL_851119	Lighthouse Lab in Glasgow	Wellcome Sanger Institute for the COVID-19 Genomics UK (COG-UK) Consortium	Harper VanSteenhouse, Yumi Kasai, David Gray, Carol Clugston, Anna Dominiczak and Alex Alderton, Roberto Amato, Sonia Goncalves, Ewan Harrison, David K. Jackson, Ian Johnston, Dominic Kwiatkowski, Cordelia Langford, John Sillitoe on behalf of the Wellcome Sanger Institute COVID-19 Surveillance Team
EPI_ISL_851120, EPI_ISL_851121, EPI_ISL_851122, EPI_ISL_851123	Lighthouse Lab in Cambridge	Wellcome Sanger Institute for the COVID-19 Genomics UK (COG-UK) Consortium	Rob Howes, The Lighthouse Lab in Cambridge and Alex Alderton, Roberto Amato, Sonia Goncalves, Ewan Harrison, David K. Jackson, Ian Johnston, Dominic Kwiatkowski, Cordelia Langford, John Sillitoe on behalf of the Wellcome Sanger Institute COVID-19 Surveillance Team
EPI_ISL_851124, EPI_ISL_851125	Lighthouse Lab in Glasgow	Wellcome Sanger Institute for the COVID-19 Genomics UK (COG-UK) Consortium	Harper VanSteenhouse, Yumi Kasai, David Gray, Carol Clugston, Anna Dominiczak and Alex Alderton, Roberto Amato, Sonia Goncalves, Ewan Harrison, David K. Jackson, Ian Johnston, Dominic Kwiatkowski, Cordelia Langford, John Sillitoe on behalf of the Wellcome Sanger Institute COVID-19 Surveillance Team
EPI_ISL_851127, EPI_ISL_851128, EPI_ISL_851129	Lighthouse Lab in Cambridge	Wellcome Sanger Institute for the COVID-19 Genomics UK (COG-UK) Consortium	Rob Howes, The Lighthouse Lab in Cambridge and Alex Alderton, Roberto Amato, Sonia Goncalves, Ewan Harrison, David K. Jackson, Ian Johnston, Dominic Kwiatkowski, Cordelia Langford, John Sillitoe on behalf of the Wellcome Sanger Institute COVID-19 Surveillance Team
EPI_ISL_851130	Lighthouse Lab in Glasgow	Wellcome Sanger Institute for the COVID-19 Genomics UK (COG-UK) Consortium	Harper VanSteenhouse, Yumi Kasai, David Gray, Carol Clugston, Anna Dominiczak and Alex Alderton, Roberto Amato, Sonia Goncalves, Ewan Harrison, David K. Jackson, Ian Johnston, Dominic Kwiatkowski, Cordelia Langford, John Sillitoe on behalf of the Wellcome Sanger Institute COVID-19 Surveillance Team
EPI_ISL_851131, EPI_ISL_851132	Lighthouse Lab in Cambridge	Wellcome Sanger Institute for the COVID-19 Genomics UK (COG-UK) Consortium	Rob Howes, The Lighthouse Lab in Cambridge and Alex Alderton, Roberto Amato, Sonia Goncalves, Ewan Harrison, David K. Jackson, Ian Johnston, Dominic Kwiatkowski, Cordelia Langford, John Sillitoe on behalf of the Wellcome Sanger Institute COVID-19 Surveillance Team
EPI_ISL_851134, EPI_ISL_851135	Lighthouse Lab in Glasgow	Wellcome Sanger Institute for the COVID-19 Genomics UK (COG-UK) Consortium	Harper VanSteenhouse, Yumi Kasai, David Gray, Carol Clugston, Anna Dominiczak and Alex Alderton, Roberto Amato, Sonia Goncalves, Ewan Harrison, David K. Jackson, Ian Johnston, Dominic Kwiatkowski, Cordelia Langford, John Sillitoe on behalf of the Wellcome Sanger Institute COVID-19 Surveillance

			Team
EPI_ISL_851136	Lighthouse Lab in Cambridge	Wellcome Sanger Institute for the COVID-19 Genomics UK (COG-UK) Consortium	Rob Howes, The Lighthouse Lab in Cambridge and Alex Alderton, Roberto Amato, Sonia Goncalves, Ewan Harrison, David K. Jackson, Ian Johnston, Dominic Kwiatkowski, Cordelia Langford, John Sillitoe on behalf of the Wellcome Sanger Institute COVID-19 Surveillance Team
EPI_ISL_851137	Lighthouse Lab in Glasgow	Wellcome Sanger Institute for the COVID-19 Genomics UK (COG-UK) Consortium	Harper VanSteenhouse, Yumi Kasai, David Gray, Carol Clugston, Anna Dominiczak and Alex Alderton, Roberto Amato, Sonia Goncalves, Ewan Harrison, David K. Jackson, Ian Johnston, Dominic Kwiatkowski, Cordelia Langford, John Sillitoe on behalf of the Wellcome Sanger Institute COVID-19 Surveillance Team
EPI_ISL_851138, EPI_ISL_851139, EPI_ISL_851140, EPI_ISL_851141, EPI_ISL_851144, EPI_ISL_851145	Lighthouse Lab in Cambridge	Wellcome Sanger Institute for the COVID-19 Genomics UK (COG-UK) Consortium	Rob Howes, The Lighthouse Lab in Cambridge and Alex Alderton, Roberto Amato, Sonia Goncalves, Ewan Harrison, David K. Jackson, Ian Johnston, Dominic Kwiatkowski, Cordelia Langford, John Sillitoe on behalf of the Wellcome Sanger Institute COVID-19 Surveillance Team
EPI_ISL_851146, EPI_ISL_851147, EPI_ISL_851148	Lighthouse Lab in Glasgow	Wellcome Sanger Institute for the COVID-19 Genomics UK (COG-UK) Consortium	Harper VanSteenhouse, Yumi Kasai, David Gray, Carol Clugston, Anna Dominiczak and Alex Alderton, Roberto Amato, Sonia Goncalves, Ewan Harrison, David K. Jackson, Ian Johnston, Dominic Kwiatkowski, Cordelia Langford, John Sillitoe on behalf of the Wellcome Sanger Institute COVID-19 Surveillance Team
EPI_ISL_851149, EPI_ISL_851151, EPI_ISL_851152, EPI_ISL_851153	Lighthouse Lab in Cambridge	Wellcome Sanger Institute for the COVID-19 Genomics UK (COG-UK) Consortium	Rob Howes, The Lighthouse Lab in Cambridge and Alex Alderton, Roberto Amato, Sonia Goncalves, Ewan Harrison, David K. Jackson, Ian Johnston, Dominic Kwiatkowski, Cordelia Langford, John Sillitoe on behalf of the Wellcome Sanger Institute COVID-19 Surveillance Team
EPI_ISL_851154	Lighthouse Lab in Glasgow	Wellcome Sanger Institute for the COVID-19 Genomics UK (COG-UK) Consortium	Harper VanSteenhouse, Yumi Kasai, David Gray, Carol Clugston, Anna Dominiczak and Alex Alderton, Roberto Amato, Sonia Goncalves, Ewan Harrison, David K. Jackson, Ian Johnston, Dominic Kwiatkowski, Cordelia Langford, John Sillitoe on behalf of the Wellcome Sanger Institute COVID-19 Surveillance Team
EPI_ISL_851155, EPI_ISL_851156, EPI_ISL_851157, EPI_ISL_851158, EPI_ISL_851159, EPI_ISL_851160, EPI_ISL_851161, EPI_ISL_851162, EPI_ISL_851163, EPI_ISL_851164, EPI_ISL_851165			
see above	Lighthouse Lab in Cambridge	Wellcome Sanger Institute for the COVID-19 Genomics UK (COG-UK) Consortium	Rob Howes, The Lighthouse Lab in Cambridge and Alex Alderton, Roberto Amato, Sonia Goncalves, Ewan Harrison, David K. Jackson, Ian Johnston, Dominic Kwiatkowski, Cordelia Langford, John Sillitoe on behalf of the Wellcome Sanger Institute COVID-19 Surveillance Team
EPI_ISL_851167	Lighthouse Lab in Glasgow	Wellcome Sanger Institute for the COVID-19 Genomics UK (COG-UK) Consortium	Harper VanSteenhouse, Yumi Kasai, David Gray, Carol Clugston, Anna Dominiczak and Alex Alderton, Roberto Amato, Sonia Goncalves, Ewan Harrison, David K. Jackson, Ian Johnston, Dominic Kwiatkowski, Cordelia Langford, John Sillitoe on behalf of the Wellcome Sanger Institute COVID-19 Surveillance Team
EPI_ISL_851168, EPI_ISL_851169, EPI_ISL_851171, EPI_ISL_851172, EPI_ISL_851173, EPI_ISL_851174, EPI_ISL_851176, EPI_ISL_851177	Lighthouse Lab in Cambridge	Wellcome Sanger Institute for the COVID-19 Genomics UK (COG-UK) Consortium	Rob Howes, The Lighthouse Lab in Cambridge and Alex Alderton, Roberto Amato, Sonia Goncalves, Ewan Harrison, David K. Jackson, Ian Johnston, Dominic Kwiatkowski, Cordelia Langford, John Sillitoe on behalf of the Wellcome Sanger Institute COVID-19 Surveillance Team
EPI_ISL_851178	Lighthouse Lab in Glasgow	Wellcome Sanger Institute for the COVID-19 Genomics UK (COG-UK) Consortium	Harper VanSteenhouse, Yumi Kasai, David Gray, Carol Clugston, Anna Dominiczak and Alex Alderton, Roberto Amato, Sonia Goncalves, Ewan Harrison, David K. Jackson, Ian Johnston, Dominic Kwiatkowski, Cordelia Langford, John Sillitoe on behalf of the Wellcome Sanger Institute COVID-19 Surveillance Team
EPI_ISL_851179, EPI_ISL_851181	Lighthouse Lab in Cambridge	Wellcome Sanger Institute for the COVID-19 Genomics UK (COG-UK) Consortium	Rob Howes, The Lighthouse Lab in Cambridge and Alex Alderton, Roberto Amato, Sonia Goncalves, Ewan Harrison, David K. Jackson, Ian Johnston, Dominic Kwiatkowski, Cordelia Langford, John Sillitoe on behalf of the Wellcome Sanger Institute COVID-19 Surveillance Team
EPI_ISL_851182	Lighthouse Lab in Glasgow	Wellcome Sanger Institute for the COVID-19 Genomics UK (COG-UK) Consortium	Harper VanSteenhouse, Yumi Kasai, David Gray, Carol Clugston, Anna Dominiczak and Alex Alderton, Roberto Amato, Sonia Goncalves, Ewan Harrison, David K. Jackson, Ian Johnston, Dominic Kwiatkowski, Cordelia Langford, John Sillitoe on behalf of the Wellcome Sanger Institute COVID-19 Surveillance Team
EPI_ISL_851183, EPI_ISL_851184, EPI_ISL_851185, EPI_ISL_851186, EPI_ISL_851188, EPI_ISL_851191, EPI_ISL_851192, EPI_ISL_851195, EPI_ISL_851197, EPI_ISL_851198, EPI_ISL_851199, EPI_ISL_851200, EPI_ISL_851201, EPI_ISL_851203, EPI_ISL_851204, EPI_ISL_851205			
see above	Lighthouse Lab in Cambridge	Wellcome Sanger Institute for the COVID-19 Genomics UK (COG-UK) Consortium	Rob Howes, The Lighthouse Lab in Cambridge and Alex Alderton, Roberto Amato, Sonia Goncalves, Ewan Harrison, David K. Jackson, Ian Johnston, Dominic Kwiatkowski, Cordelia Langford, John Sillitoe on behalf of the Wellcome Sanger Institute COVID-19 Surveillance Team
EPI_ISL_851206, EPI_ISL_851207	Lighthouse Lab in Glasgow	Wellcome Sanger Institute for the COVID-19 Genomics UK (COG-UK) Consortium	Harper VanSteenhouse, Yumi Kasai, David Gray, Carol Clugston, Anna Dominiczak and Alex Alderton, Roberto Amato, Sonia Goncalves, Ewan Harrison, David K. Jackson, Ian Johnston, Dominic Kwiatkowski, Cordelia Langford, John Sillitoe on behalf of the Wellcome Sanger Institute COVID-19 Surveillance Team
EPI_ISL_851209, EPI_ISL_851210, EPI_ISL_851211, EPI_ISL_851212	Lighthouse Lab in Cambridge	Wellcome Sanger Institute for the COVID-19 Genomics UK (COG-UK) Consortium	Rob Howes, The Lighthouse Lab in Cambridge and Alex Alderton, Roberto Amato, Sonia Goncalves, Ewan Harrison, David K. Jackson, Ian Johnston, Dominic Kwiatkowski, Cordelia Langford, John Sillitoe on behalf of the Wellcome Sanger Institute COVID-19 Surveillance Team
EPI_ISL_851215	Lighthouse Lab in Glasgow	Wellcome Sanger Institute for the COVID-19 Genomics UK (COG-UK) Consortium	Harper VanSteenhouse, Yumi Kasai, David Gray, Carol Clugston, Anna Dominiczak and Alex Alderton, Roberto Amato, Sonia Goncalves, Ewan Harrison, David K. Jackson, Ian Johnston, Dominic Kwiatkowski, Cordelia Langford, John Sillitoe on behalf of the Wellcome Sanger Institute COVID-19 Surveillance Team
EPI_ISL_851216	Lighthouse Lab in Cambridge	Wellcome Sanger Institute for the COVID-19 Genomics UK (COG-UK) Consortium	Rob Howes, The Lighthouse Lab in Cambridge and Alex Alderton, Roberto Amato, Sonia Goncalves, Ewan Harrison, David K. Jackson, Ian Johnston, Dominic Kwiatkowski, Cordelia Langford, John Sillitoe on behalf of the Wellcome Sanger Institute COVID-19 Surveillance Team
EPI_ISL_851218	Lighthouse Lab in Glasgow	Wellcome Sanger Institute for the COVID-19 Genomics UK (COG-UK) Consortium	Harper VanSteenhouse, Yumi Kasai, David Gray, Carol Clugston, Anna Dominiczak and Alex Alderton, Roberto Amato, Sonia Goncalves, Ewan Harrison, David K. Jackson, Ian Johnston, Dominic Kwiatkowski, Cordelia Langford, John Sillitoe on behalf of the Wellcome Sanger Institute COVID-19 Surveillance Team
EPI_ISL_851219, EPI_ISL_851220, EPI_ISL_851221, EPI_ISL_851222, EPI_ISL_851223, EPI_ISL_851224	Lighthouse Lab in Cambridge	Wellcome Sanger Institute for the COVID-19 Genomics UK (COG-UK) Consortium	Rob Howes, The Lighthouse Lab in Cambridge and Alex Alderton, Roberto Amato, Sonia Goncalves, Ewan Harrison, David K. Jackson, Ian Johnston, Dominic Kwiatkowski, Cordelia Langford, John Sillitoe on behalf of the Wellcome Sanger Institute COVID-19 Surveillance Team
EPI_ISL_851225	Lighthouse Lab in Glasgow	Wellcome Sanger Institute for the COVID-19 Genomics UK (COG-UK) Consortium	Harper VanSteenhouse, Yumi Kasai, David Gray, Carol Clugston, Anna Dominiczak and Alex Alderton, Roberto Amato, Sonia Goncalves, Ewan Harrison, David K. Jackson, Ian Johnston, Dominic Kwiatkowski, Cordelia Langford, John Sillitoe on behalf of the Wellcome Sanger Institute COVID-19 Surveillance Team
EPI_ISL_851226, EPI_ISL_851227, EPI_ISL_851228, EPI_ISL_851230, EPI_ISL_851231, EPI_ISL_851232, EPI_ISL_851233, EPI_ISL_851234, EPI_ISL_851235	Lighthouse Lab in Cambridge	Wellcome Sanger Institute for the COVID-19 Genomics UK (COG-UK) Consortium	Rob Howes, The Lighthouse Lab in Cambridge and Alex Alderton, Roberto Amato, Sonia Goncalves, Ewan Harrison, David K. Jackson, Ian Johnston, Dominic Kwiatkowski, Cordelia Langford, John Sillitoe on behalf of the Wellcome Sanger Institute COVID-19 Surveillance Team
EPI_ISL_851237	Lighthouse Lab in Glasgow	Wellcome Sanger Institute for the COVID-19 Genomics UK (COG-UK) Consortium	Harper VanSteenhouse, Yumi Kasai, David Gray, Carol Clugston, Anna Dominiczak and Alex Alderton, Roberto Amato, Sonia Goncalves, Ewan Harrison, David K. Jackson, Ian Johnston, Dominic Kwiatkowski, Cordelia Langford, John Sillitoe on behalf of the Wellcome Sanger Institute COVID-19 Surveillance Team
EPI_ISL_851238, EPI_ISL_851239, EPI_ISL_851240, EPI_ISL_851242, EPI_ISL_851243, EPI_ISL_851244, EPI_ISL_851245	Lighthouse Lab in Cambridge	Wellcome Sanger Institute for the COVID-19 Genomics UK (COG-UK) Consortium	Rob Howes, The Lighthouse Lab in Cambridge and Alex Alderton, Roberto Amato, Sonia Goncalves, Ewan Harrison, David K. Jackson, Ian Johnston, Dominic Kwiatkowski, Cordelia Langford, John Sillitoe on behalf of the Wellcome Sanger Institute COVID-19 Surveillance Team
EPI_ISL_851246	Lighthouse Lab in Glasgow	Wellcome Sanger Institute for the COVID-19 Genomics UK (COG-UK) Consortium	Harper VanSteenhouse, Yumi Kasai, David Gray, Carol Clugston, Anna Dominiczak and Alex Alderton, Roberto Amato, Sonia Goncalves, Ewan Harrison, David K. Jackson, Ian Johnston, Dominic Kwiatkowski, Cordelia Langford, John Sillitoe on behalf of the Wellcome Sanger Institute COVID-19 Surveillance Team
EPI_ISL_851247, EPI_ISL_851248, EPI_ISL_851249, EPI_ISL_851250	Lighthouse Lab in Cambridge	Wellcome Sanger Institute for the COVID-19 Genomics UK (COG-UK) Consortium	Rob Howes, The Lighthouse Lab in Cambridge and Alex Alderton, Roberto Amato, Sonia Goncalves, Ewan Harrison, David K. Jackson, Ian Johnston, Dominic Kwiatkowski, Cordelia Langford, John Sillitoe on behalf of the Wellcome Sanger Institute COVID-19 Surveillance Team

EPI_ISL_851252, EPI_ISL_851253	Lighthouse Lab in Glasgow	Wellcome Sanger Institute for the COVID-19 Genomics UK (COG-UK) Consortium	Harper VanSteenhouse, Yumi Kasai, David Gray, Carol Clugston, Anna Dominiczak and Alex Alderton, Roberto Amato, Sonia Goncalves, Ewan Harrison, David K. Jackson, Ian Johnston, Dominic Kwiatkowski, Cordelia Langford, John Sillitoe on behalf of the Wellcome Sanger Institute COVID-19 Surveillance Team
EPI_ISL_851255, EPI_ISL_851256	Lighthouse Lab in Cambridge	Wellcome Sanger Institute for the COVID-19 Genomics UK (COG-UK) Consortium	Rob Howes, The Lighthouse Lab in Cambridge and Alex Alderton, Roberto Amato, Sonia Goncalves, Ewan Harrison, David K. Jackson, Ian Johnston, Dominic Kwiatkowski, Cordelia Langford, John Sillitoe on behalf of the Wellcome Sanger Institute COVID-19 Surveillance Team
EPI_ISL_851257, EPI_ISL_851259	Lighthouse Lab in Glasgow	Wellcome Sanger Institute for the COVID-19 Genomics UK (COG-UK) Consortium	Harper VanSteenhouse, Yumi Kasai, David Gray, Carol Clugston, Anna Dominiczak and Alex Alderton, Roberto Amato, Sonia Goncalves, Ewan Harrison, David K. Jackson, Ian Johnston, Dominic Kwiatkowski, Cordelia Langford, John Sillitoe on behalf of the Wellcome Sanger Institute COVID-19 Surveillance Team
EPI_ISL_851260, EPI_ISL_851261, EPI_ISL_851262, EPI_ISL_851263	Lighthouse Lab in Cambridge	Wellcome Sanger Institute for the COVID-19 Genomics UK (COG-UK) Consortium	Rob Howes, The Lighthouse Lab in Cambridge and Alex Alderton, Roberto Amato, Sonia Goncalves, Ewan Harrison, David K. Jackson, Ian Johnston, Dominic Kwiatkowski, Cordelia Langford, John Sillitoe on behalf of the Wellcome Sanger Institute COVID-19 Surveillance Team
EPI_ISL_851264	Lighthouse Lab in Glasgow	Wellcome Sanger Institute for the COVID-19 Genomics UK (COG-UK) Consortium	Harper VanSteenhouse, Yumi Kasai, David Gray, Carol Clugston, Anna Dominiczak and Alex Alderton, Roberto Amato, Sonia Goncalves, Ewan Harrison, David K. Jackson, Ian Johnston, Dominic Kwiatkowski, Cordelia Langford, John Sillitoe on behalf of the Wellcome Sanger Institute COVID-19 Surveillance Team
EPI_ISL_851265, EPI_ISL_851266, EPI_ISL_851267, EPI_ISL_851268	Lighthouse Lab in Cambridge	Wellcome Sanger Institute for the COVID-19 Genomics UK (COG-UK) Consortium	Rob Howes, The Lighthouse Lab in Cambridge and Alex Alderton, Roberto Amato, Sonia Goncalves, Ewan Harrison, David K. Jackson, Ian Johnston, Dominic Kwiatkowski, Cordelia Langford, John Sillitoe on behalf of the Wellcome Sanger Institute COVID-19 Surveillance Team
EPI_ISL_851270	Lighthouse Lab in Glasgow	Wellcome Sanger Institute for the COVID-19 Genomics UK (COG-UK) Consortium	Harper VanSteenhouse, Yumi Kasai, David Gray, Carol Clugston, Anna Dominiczak and Alex Alderton, Roberto Amato, Sonia Goncalves, Ewan Harrison, David K. Jackson, Ian Johnston, Dominic Kwiatkowski, Cordelia Langford, John Sillitoe on behalf of the Wellcome Sanger Institute COVID-19 Surveillance Team
EPI_ISL_851271, EPI_ISL_851272, EPI_ISL_851274, EPI_ISL_851275	Lighthouse Lab in Cambridge	Wellcome Sanger Institute for the COVID-19 Genomics UK (COG-UK) Consortium	Rob Howes, The Lighthouse Lab in Cambridge and Alex Alderton, Roberto Amato, Sonia Goncalves, Ewan Harrison, David K. Jackson, Ian Johnston, Dominic Kwiatkowski, Cordelia Langford, John Sillitoe on behalf of the Wellcome Sanger Institute COVID-19 Surveillance Team
EPI_ISL_851276	Lighthouse Lab in Glasgow	Wellcome Sanger Institute for the COVID-19 Genomics UK (COG-UK) Consortium	Harper VanSteenhouse, Yumi Kasai, David Gray, Carol Clugston, Anna Dominiczak and Alex Alderton, Roberto Amato, Sonia Goncalves, Ewan Harrison, David K. Jackson, Ian Johnston, Dominic Kwiatkowski, Cordelia Langford, John Sillitoe on behalf of the Wellcome Sanger Institute COVID-19 Surveillance Team
EPI_ISL_851277, EPI_ISL_851278, EPI_ISL_851279, EPI_ISL_851280, EPI_ISL_851281, EPI_ISL_851282, EPI_ISL_851283, EPI_ISL_851284	Lighthouse Lab in Cambridge	Wellcome Sanger Institute for the COVID-19 Genomics UK (COG-UK) Consortium	Rob Howes, The Lighthouse Lab in Cambridge and Alex Alderton, Roberto Amato, Sonia Goncalves, Ewan Harrison, David K. Jackson, Ian Johnston, Dominic Kwiatkowski, Cordelia Langford, John Sillitoe on behalf of the Wellcome Sanger Institute COVID-19 Surveillance Team
EPI_ISL_851285	Lighthouse Lab in Glasgow	Wellcome Sanger Institute for the COVID-19 Genomics UK (COG-UK) Consortium	Harper VanSteenhouse, Yumi Kasai, David Gray, Carol Clugston, Anna Dominiczak and Alex Alderton, Roberto Amato, Sonia Goncalves, Ewan Harrison, David K. Jackson, Ian Johnston, Dominic Kwiatkowski, Cordelia Langford, John Sillitoe on behalf of the Wellcome Sanger Institute COVID-19 Surveillance Team
EPI_ISL_851286, EPI_ISL_851288, EPI_ISL_851289, EPI_ISL_851290, EPI_ISL_851291, EPI_ISL_851292, EPI_ISL_851293, EPI_ISL_851294, EPI_ISL_851295	Lighthouse Lab in Cambridge	Wellcome Sanger Institute for the COVID-19 Genomics UK (COG-UK) Consortium	Rob Howes, The Lighthouse Lab in Cambridge and Alex Alderton, Roberto Amato, Sonia Goncalves, Ewan Harrison, David K. Jackson, Ian Johnston, Dominic Kwiatkowski, Cordelia Langford, John Sillitoe on behalf of the Wellcome Sanger Institute COVID-19 Surveillance Team
EPI_ISL_851296	Lighthouse Lab in Glasgow	Wellcome Sanger Institute for the COVID-19 Genomics UK (COG-UK) Consortium	Harper VanSteenhouse, Yumi Kasai, David Gray, Carol Clugston, Anna Dominiczak and Alex Alderton, Roberto Amato, Sonia Goncalves, Ewan Harrison, David K. Jackson, Ian Johnston, Dominic Kwiatkowski, Cordelia Langford, John Sillitoe on behalf of the Wellcome Sanger Institute COVID-19 Surveillance Team
EPI_ISL_851297, EPI_ISL_851299, EPI_ISL_851300	Lighthouse Lab in Cambridge	Wellcome Sanger Institute for the COVID-19 Genomics UK (COG-UK) Consortium	Rob Howes, The Lighthouse Lab in Cambridge and Alex Alderton, Roberto Amato, Sonia Goncalves, Ewan Harrison, David K. Jackson, Ian Johnston, Dominic Kwiatkowski, Cordelia Langford, John Sillitoe on behalf of the Wellcome Sanger Institute COVID-19 Surveillance Team
EPI_ISL_851301	Lighthouse Lab in Glasgow	Wellcome Sanger Institute for the COVID-19 Genomics UK (COG-UK) Consortium	Harper VanSteenhouse, Yumi Kasai, David Gray, Carol Clugston, Anna Dominiczak and Alex Alderton, Roberto Amato, Sonia Goncalves, Ewan Harrison, David K. Jackson, Ian Johnston, Dominic Kwiatkowski, Cordelia Langford, John Sillitoe on behalf of the Wellcome Sanger Institute COVID-19 Surveillance Team
EPI_ISL_851302, EPI_ISL_851303, EPI_ISL_851304	Lighthouse Lab in Cambridge	Wellcome Sanger Institute for the COVID-19 Genomics UK (COG-UK) Consortium	Rob Howes, The Lighthouse Lab in Cambridge and Alex Alderton, Roberto Amato, Sonia Goncalves, Ewan Harrison, David K. Jackson, Ian Johnston, Dominic Kwiatkowski, Cordelia Langford, John Sillitoe on behalf of the Wellcome Sanger Institute COVID-19 Surveillance Team
EPI_ISL_851305, EPI_ISL_851306, EPI_ISL_851307	Lighthouse Lab in Glasgow	Wellcome Sanger Institute for the COVID-19 Genomics UK (COG-UK) Consortium	Harper VanSteenhouse, Yumi Kasai, David Gray, Carol Clugston, Anna Dominiczak and Alex Alderton, Roberto Amato, Sonia Goncalves, Ewan Harrison, David K. Jackson, Ian Johnston, Dominic Kwiatkowski, Cordelia Langford, John Sillitoe on behalf of the Wellcome Sanger Institute COVID-19 Surveillance Team
EPI_ISL_851308, EPI_ISL_851309	Lighthouse Lab in Cambridge	Wellcome Sanger Institute for the COVID-19 Genomics UK (COG-UK) Consortium	Rob Howes, The Lighthouse Lab in Cambridge and Alex Alderton, Roberto Amato, Sonia Goncalves, Ewan Harrison, David K. Jackson, Ian Johnston, Dominic Kwiatkowski, Cordelia Langford, John Sillitoe on behalf of the Wellcome Sanger Institute COVID-19 Surveillance Team
EPI_ISL_851310	Lighthouse Lab in Glasgow	Wellcome Sanger Institute for the COVID-19 Genomics UK (COG-UK) Consortium	Harper VanSteenhouse, Yumi Kasai, David Gray, Carol Clugston, Anna Dominiczak and Alex Alderton, Roberto Amato, Sonia Goncalves, Ewan Harrison, David K. Jackson, Ian Johnston, Dominic Kwiatkowski, Cordelia Langford, John Sillitoe on behalf of the Wellcome Sanger Institute COVID-19 Surveillance

EPI_ISL_851311, EPI_ISL_851312, EPI_ISL_851313, EPI_ISL_851314, EPI_ISL_851315, EPI_ISL_851316, EPI_ISL_851317, EPI_ISL_851318, EPI_ISL_851319, EPI_ISL_851320, EPI_ISL_851321, EPI_ISL_851322, EPI_ISL_851323, EPI_ISL_851324

see above	Lighthouse Lab in Cambridge	Wellcome Sanger Institute for the COVID-19 Genomics UK (COG-UK) Consortium	Rob Howes, The Lighthouse Lab in Cambridge and Alex Alderton, Roberto Amato, Sonia Goncalves, Ewan Harrison, David K. Jackson, Ian Johnston, Dominic Kwiatkowski, Cordelia Langford, John Sillitoe on behalf of the Wellcome Sanger Institute COVID-19 Surveillance Team
EPI_ISL_851325	Lighthouse Lab in Glasgow	Wellcome Sanger Institute for the COVID-19 Genomics UK (COG-UK) Consortium	Harper VanSteenhouse, Yumi Kasai, David Gray, Carol Clugston, Anna Dominiczak and Alex Alderton, Roberto Amato, Sonia Goncalves, Ewan Harrison, David K. Jackson, Ian Johnston, Dominic Kwiatkowski, Cordelia Langford, John Sillitoe on behalf of the Wellcome Sanger Institute COVID-19 Surveillance Team
EPI_ISL_851326	Lighthouse Lab in Cambridge	Wellcome Sanger Institute for the COVID-19 Genomics UK (COG-UK) Consortium	Rob Howes, The Lighthouse Lab in Cambridge and Alex Alderton, Roberto Amato, Sonia Goncalves, Ewan Harrison, David K. Jackson, Ian Johnston, Dominic Kwiatkowski, Cordelia Langford, John Sillitoe on behalf of the Wellcome Sanger Institute COVID-19 Surveillance Team
EPI_ISL_851327	Lighthouse Lab in Glasgow	Wellcome Sanger Institute for the COVID-19 Genomics UK (COG-UK) Consortium	Harper VanSteenhouse, Yumi Kasai, David Gray, Carol Clugston, Anna Dominiczak and Alex Alderton, Roberto Amato, Sonia Goncalves, Ewan Harrison, David K. Jackson, Ian Johnston, Dominic Kwiatkowski, Cordelia Langford, John Sillitoe on behalf of the Wellcome Sanger Institute COVID-19 Surveillance Team
EPI_ISL_851328, EPI_ISL_851330	Lighthouse Lab in Cambridge	Wellcome Sanger Institute for the COVID-19 Genomics UK (COG-UK) Consortium	Rob Howes, The Lighthouse Lab in Cambridge and Alex Alderton, Roberto Amato, Sonia Goncalves, Ewan Harrison, David K. Jackson, Ian Johnston, Dominic Kwiatkowski, Cordelia Langford, John Sillitoe on behalf of the Wellcome Sanger Institute COVID-19 Surveillance Team
EPI_ISL_851331	Lighthouse Lab in Glasgow	Wellcome Sanger Institute for the COVID-19 Genomics UK (COG-UK) Consortium	Harper VanSteenhouse, Yumi Kasai, David Gray, Carol Clugston, Anna Dominiczak and Alex Alderton, Roberto Amato, Sonia Goncalves, Ewan Harrison, David K. Jackson, Ian Johnston, Dominic Kwiatkowski, Cordelia Langford, John Sillitoe on behalf of the Wellcome Sanger Institute COVID-19 Surveillance Team
EPI_ISL_851333, EPI_ISL_851334	Lighthouse Lab in Cambridge	Wellcome Sanger Institute for the COVID-19 Genomics UK (COG-UK) Consortium	Rob Howes, The Lighthouse Lab in Cambridge and Alex Alderton, Roberto Amato, Sonia Goncalves, Ewan Harrison, David K. Jackson, Ian Johnston, Dominic Kwiatkowski, Cordelia Langford, John Sillitoe on behalf of the Wellcome Sanger Institute COVID-19 Surveillance Team
EPI_ISL_851335	Lighthouse Lab in Glasgow	Wellcome Sanger Institute for the COVID-19 Genomics UK (COG-UK) Consortium	Harper VanSteenhouse, Yumi Kasai, David Gray, Carol Clugston, Anna Dominiczak and Alex Alderton, Roberto Amato, Sonia Goncalves, Ewan Harrison, David K. Jackson, Ian Johnston, Dominic Kwiatkowski, Cordelia Langford, John Sillitoe on behalf of the Wellcome Sanger Institute COVID-19 Surveillance Team

EPI_ISL_851336, EPI_ISL_851337, EPI_ISL_851339, EPI_ISL_851340	Lighthouse Lab in Cambridge	Wellcome Sanger Institute for the COVID-19 Genomics UK (COG-UK) Consortium	Rob Howes, The Lighthouse Lab in Cambridge and Alex Alderton, Roberto Amato, Sonia Goncalves, Ewan Harrison, David K. Jackson, Ian Johnston, Dominic Kwiatkowski, Cordelia Langford, John Sillitoe on behalf of the Wellcome Sanger Institute COVID-19 Surveillance Team
EPI_ISL_851341	Lighthouse Lab in Glasgow	Wellcome Sanger Institute for the COVID-19 Genomics UK (COG-UK) Consortium	Harper VanSteenhouse, Yumi Kasai, David Gray, Carol Clugston, Anna Dominiczak and Alex Alderton, Roberto Amato, Sonia Goncalves, Ewan Harrison, David K. Jackson, Ian Johnston, Dominic Kwiatkowski, Cordelia Langford, John Sillitoe on behalf of the Wellcome Sanger Institute COVID-19 Surveillance Team
EPI_ISL_851344, EPI_ISL_851345	Lighthouse Lab in Cambridge	Wellcome Sanger Institute for the COVID-19 Genomics UK (COG-UK) Consortium	Rob Howes, The Lighthouse Lab in Cambridge and Alex Alderton, Roberto Amato, Sonia Goncalves, Ewan Harrison, David K. Jackson, Ian Johnston, Dominic Kwiatkowski, Cordelia Langford, John Sillitoe on behalf of the Wellcome Sanger Institute COVID-19 Surveillance Team
EPI_ISL_851346	Lighthouse Lab in Glasgow	Wellcome Sanger Institute for the COVID-19 Genomics UK (COG-UK) Consortium	Harper VanSteenhouse, Yumi Kasai, David Gray, Carol Clugston, Anna Dominiczak and Alex Alderton, Roberto Amato, Sonia Goncalves, Ewan Harrison, David K. Jackson, Ian Johnston, Dominic Kwiatkowski, Cordelia Langford, John Sillitoe on behalf of the Wellcome Sanger Institute COVID-19 Surveillance Team
EPI_ISL_851347, EPI_ISL_851348, EPI_ISL_851349, EPI_ISL_851350	Lighthouse Lab in Cambridge	Wellcome Sanger Institute for the COVID-19 Genomics UK (COG-UK) Consortium	Rob Howes, The Lighthouse Lab in Cambridge and Alex Alderton, Roberto Amato, Sonia Goncalves, Ewan Harrison, David K. Jackson, Ian Johnston, Dominic Kwiatkowski, Cordelia Langford, John Sillitoe on behalf of the Wellcome Sanger Institute COVID-19 Surveillance Team
EPI_ISL_851351	Lighthouse Lab in Glasgow	Wellcome Sanger Institute for the COVID-19 Genomics UK (COG-UK) Consortium	Harper VanSteenhouse, Yumi Kasai, David Gray, Carol Clugston, Anna Dominiczak and Alex Alderton, Roberto Amato, Sonia Goncalves, Ewan Harrison, David K. Jackson, Ian Johnston, Dominic Kwiatkowski, Cordelia Langford, John Sillitoe on behalf of the Wellcome Sanger Institute COVID-19 Surveillance Team
EPI_ISL_851352, EPI_ISL_851355, EPI_ISL_851356, EPI_ISL_851357	Lighthouse Lab in Cambridge	Wellcome Sanger Institute for the COVID-19 Genomics UK (COG-UK) Consortium	Rob Howes, The Lighthouse Lab in Cambridge and Alex Alderton, Roberto Amato, Sonia Goncalves, Ewan Harrison, David K. Jackson, Ian Johnston, Dominic Kwiatkowski, Cordelia Langford, John Sillitoe on behalf of the Wellcome Sanger Institute COVID-19 Surveillance Team
EPI_ISL_851358	Lighthouse Lab in Glasgow	Wellcome Sanger Institute for the COVID-19 Genomics UK (COG-UK) Consortium	Harper VanSteenhouse, Yumi Kasai, David Gray, Carol Clugston, Anna Dominiczak and Alex Alderton, Roberto Amato, Sonia Goncalves, Ewan Harrison, David K. Jackson, Ian Johnston, Dominic Kwiatkowski, Cordelia Langford, John Sillitoe on behalf of the Wellcome Sanger Institute COVID-19 Surveillance Team
EPI_ISL_851359, EPI_ISL_851360, EPI_ISL_851361, EPI_ISL_851362, EPI_ISL_851363, EPI_ISL_851364, EPI_ISL_851366	Lighthouse Lab in Cambridge	Wellcome Sanger Institute for the COVID-19 Genomics UK (COG-UK) Consortium	Rob Howes, The Lighthouse Lab in Cambridge and Alex Alderton, Roberto Amato, Sonia Goncalves, Ewan Harrison, David K. Jackson, Ian Johnston, Dominic Kwiatkowski, Cordelia Langford, John Sillitoe on behalf of the Wellcome Sanger Institute COVID-19 Surveillance Team
EPI_ISL_851367	Lighthouse Lab in Glasgow	Wellcome Sanger Institute for the COVID-19 Genomics UK (COG-UK) Consortium	Harper VanSteenhouse, Yumi Kasai, David Gray, Carol Clugston, Anna Dominiczak and Alex Alderton, Roberto Amato, Sonia Goncalves, Ewan Harrison, David K. Jackson, Ian Johnston, Dominic Kwiatkowski, Cordelia Langford, John Sillitoe on behalf of the Wellcome Sanger Institute COVID-19 Surveillance Team
EPI_ISL_851368, EPI_ISL_851369	Lighthouse Lab in Cambridge	Wellcome Sanger Institute for the COVID-19 Genomics UK (COG-UK) Consortium	Rob Howes, The Lighthouse Lab in Cambridge and Alex Alderton, Roberto Amato, Sonia Goncalves, Ewan Harrison, David K. Jackson, Ian Johnston, Dominic Kwiatkowski, Cordelia Langford, John Sillitoe on behalf of the Wellcome Sanger Institute COVID-19 Surveillance Team
EPI_ISL_851371	Lighthouse Lab in Glasgow	Wellcome Sanger Institute for the COVID-19 Genomics UK (COG-UK) Consortium	Harper VanSteenhouse, Yumi Kasai, David Gray, Carol Clugston, Anna Dominiczak and Alex Alderton, Roberto Amato, Sonia Goncalves, Ewan Harrison, David K. Jackson, Ian Johnston, Dominic Kwiatkowski, Cordelia Langford, John Sillitoe on behalf of the Wellcome Sanger Institute COVID-19 Surveillance Team
EPI_ISL_851372, EPI_ISL_851373, EPI_ISL_851375, EPI_ISL_851376, EPI_ISL_851378, EPI_ISL_851379	Lighthouse Lab in Cambridge	Wellcome Sanger Institute for the COVID-19 Genomics UK (COG-UK) Consortium	Rob Howes, The Lighthouse Lab in Cambridge and Alex Alderton, Roberto Amato, Sonia Goncalves, Ewan Harrison, David K. Jackson, Ian Johnston, Dominic Kwiatkowski, Cordelia Langford, John Sillitoe on behalf of the Wellcome Sanger Institute COVID-19 Surveillance Team
EPI_ISL_851380	Lighthouse Lab in Glasgow	Wellcome Sanger Institute for the COVID-19 Genomics UK (COG-UK) Consortium	Harper VanSteenhouse, Yumi Kasai, David Gray, Carol Clugston, Anna Dominiczak and Alex Alderton, Roberto Amato, Sonia Goncalves, Ewan Harrison, David K. Jackson, Ian Johnston, Dominic Kwiatkowski, Cordelia Langford, John Sillitoe on behalf of the Wellcome Sanger Institute COVID-19 Surveillance Team
EPI_ISL_851381	Lighthouse Lab in Cambridge	Wellcome Sanger Institute for the COVID-19 Genomics UK (COG-UK) Consortium	Rob Howes, The Lighthouse Lab in Cambridge and Alex Alderton, Roberto Amato, Sonia Goncalves, Ewan Harrison, David K. Jackson, Ian Johnston, Dominic Kwiatkowski, Cordelia Langford, John Sillitoe on behalf of the Wellcome Sanger Institute COVID-19 Surveillance Team
EPI_ISL_851382, EPI_ISL_851383, EPI_ISL_851384, EPI_ISL_851385, EPI_ISL_851386, EPI_ISL_851387, EPI_ISL_851388, EPI_ISL_851389, EPI_ISL_851391, EPI_ISL_851392, EPI_ISL_851393, EPI_ISL_851394, EPI_ISL_851396, EPI_ISL_851397, EPI_ISL_851398, EPI_ISL_851399, EPI_ISL_851400, EPI_ISL_851401, EPI_ISL_851402, EPI_ISL_851403, EPI_ISL_851404, EPI_ISL_851405, EPI_ISL_851406, EPI_ISL_851407, EPI_ISL_851408, EPI_ISL_851409, EPI_ISL_851410, EPI_ISL_851411, EPI_ISL_851412, EPI_ISL_851414, EPI_ISL_851415, EPI_ISL_851416, EPI_ISL_851417, EPI_ISL_851418, EPI_ISL_851419, EPI_ISL_851420, EPI_ISL_851421, EPI_ISL_851422, EPI_ISL_851424, EPI_ISL_851425, EPI_ISL_851426, EPI_ISL_851427, EPI_ISL_851428, EPI_ISL_851429, EPI_ISL_851430, EPI_ISL_851431, EPI_ISL_851432, EPI_ISL_851433, EPI_ISL_851434, EPI_ISL_851435, EPI_ISL_851437, EPI_ISL_851438, EPI_ISL_851439, EPI_ISL_851440, EPI_ISL_851441, EPI_ISL_851442, EPI_ISL_851443, EPI_ISL_851444, EPI_ISL_851445, EPI_ISL_851446, EPI_ISL_851447, EPI_ISL_851449, EPI_ISL_851450, EPI_ISL_851451, EPI_ISL_851452, EPI_ISL_851453, EPI_ISL_851456, EPI_ISL_851457, EPI_ISL_851458, EPI_ISL_851459, EPI_ISL_851460, EPI_ISL_851462, EPI_ISL_851463, EPI_ISL_851464, EPI_ISL_851465, EPI_ISL_851466, EPI_ISL_851468, EPI_ISL_851469, EPI_ISL_851470, EPI_ISL_851471, EPI_ISL_851472, EPI_ISL_851473, EPI_ISL_851474, EPI_ISL_851475, EPI_ISL_851476, EPI_ISL_851477, EPI_ISL_851478, EPI_ISL_851479, EPI_ISL_851480, EPI_ISL_851481, EPI_ISL_851482, EPI_ISL_851483, EPI_ISL_851485, EPI_ISL_851486, EPI_ISL_851487, EPI_ISL_851488, EPI_ISL_851489, EPI_ISL_851490, EPI_ISL_851491, EPI_ISL_851492, EPI_ISL_851493, EPI_ISL_851494, EPI_ISL_851495, EPI_ISL_851496, EPI_ISL_851497, EPI_ISL_851498, EPI_ISL_851499, EPI_ISL_851500, EPI_ISL_851501, EPI_ISL_851502, EPI_ISL_851503, EPI_ISL_851504, EPI_ISL_851505, EPI_ISL_851506, EPI_ISL_851507, EPI_ISL_851508, EPI_ISL_851509, EPI_ISL_851510, EPI_ISL_851511, EPI_ISL_851512, EPI_ISL_851513, EPI_ISL_851515, EPI_ISL_851516, EPI_ISL_851517, EPI_ISL_851518, EPI_ISL_851519, EPI_ISL_851520, EPI_ISL_851521, EPI_ISL_851522, EPI_ISL_851523, EPI_ISL_851524, EPI_ISL_851525, EPI_ISL_851526, EPI_ISL_851527, EPI_ISL_851528, EPI_ISL_851529, EPI_ISL_851530, EPI_ISL_851531, EPI_ISL_851532, EPI_ISL_851533, EPI_ISL_851534, EPI_ISL_851535, EPI_ISL_851536, EPI_ISL_851537, EPI_ISL_851538, EPI_ISL_851539, EPI_ISL_851540, EPI_ISL_851541, EPI_ISL_851542, EPI_ISL_851543, EPI_ISL_851544, EPI_ISL_851545, EPI_ISL_851546, EPI_ISL_851547, EPI_ISL_851548, EPI_ISL_851549, EPI_ISL_851550, EPI_ISL_851551, EPI_ISL_851552, EPI_ISL_851553, EPI_ISL_851554, EPI_ISL_851555, EPI_ISL_851556, EPI_ISL_851557, EPI_ISL_851558, EPI_ISL_851560, EPI_ISL_851561, EPI_ISL_851562, EPI_ISL_851563, EPI_ISL_851564, EPI_ISL_851565, EPI_ISL_851566, EPI_ISL_851568, EPI_ISL_851569, EPI_ISL_851570, EPI_ISL_851571, EPI_ISL_851573, EPI_ISL_851574, EPI_ISL_851575, EPI_ISL_851576, EPI_ISL_851577, EPI_ISL_851578, EPI_ISL_851579, EPI_ISL_851580, EPI_ISL_851581, EPI_ISL_851583, EPI_ISL_851584, EPI_ISL_851585, EPI_ISL_851586, EPI_ISL_851587, EPI_ISL_851588, EPI_ISL_851589, EPI_ISL_851591, EPI_ISL_851592, EPI_ISL_851593, EPI_ISL_851595, EPI_ISL_851596, EPI_ISL_851597, EPI_ISL_851599, EPI_ISL_851600, EPI_ISL_851602, EPI_ISL_851603, EPI_ISL_851604, EPI_ISL_851605, EPI_ISL_851606, EPI_ISL_851607, EPI_ISL_851608, EPI_ISL_851609, EPI_ISL_851610, EPI_ISL_851611, EPI_ISL_851612, EPI_ISL_851613, EPI_ISL_851615, EPI_ISL_851616, EPI_ISL_851618, EPI_ISL_851619, EPI_ISL_851620, EPI_ISL_851621, EPI_ISL_851622, EPI_ISL_851623, EPI_ISL_851624, EPI_ISL_851625, EPI_ISL_851626, EPI_ISL_851627, EPI_ISL_851628, EPI_ISL_851630, EPI_ISL_851631, EPI_ISL_851632, EPI_ISL_851634, EPI_ISL_851636, EPI_ISL_851637, EPI_ISL_851638, EPI_ISL_851639, EPI_ISL_851640, EPI_ISL_851641, EPI_ISL_851642, EPI_ISL_851643, EPI_ISL_851644, EPI_ISL_851645, EPI_ISL_851647, EPI_ISL_851649, EPI_ISL_851650, EPI_ISL_851651, EPI_ISL_851652, EPI_ISL_851653, EPI_ISL_851654, EPI_ISL_851655, EPI_ISL_851656, EPI_ISL_851658, EPI_ISL_851659, EPI_ISL_851660, EPI_ISL_851661, EPI_ISL_851662, EPI_ISL_851663, EPI_ISL_851665, EPI_ISL_851666, EPI_ISL_851667, EPI_ISL_851668, EPI_ISL_851669, EPI_ISL_851670, EPI_ISL_851671, EPI_ISL_851672			
see above	Lighthouse Lab in Glasgow	Wellcome Sanger Institute for the COVID-19 Genomics UK (COG-UK) Consortium	Harper VanSteenhouse, Yumi Kasai, David Gray, Carol Clugston, Anna Dominiczak and Alex Alderton, Roberto Amato, Sonia Goncalves, Ewan Harrison, David K. Jackson, Ian Johnston, Dominic Kwiatkowski, Cordelia Langford, John Sillitoe on behalf of the Wellcome Sanger Institute COVID-19 Surveillance Team
EPI_ISL_851673, EPI_ISL_851675	Lighthouse Lab in Alderley Park	Wellcome Sanger Institute for the COVID-19 Genomics UK (COG-UK) Consortium	Jacquelyn Wynn, Mairead Hyland, The Lighthouse Lab in Alderley Park and Alex Alderton, Roberto Amato, Sonia Goncalves, Ewan Harrison, David K. Jackson, Ian Johnston, Dominic Kwiatkowski, Cordelia Langford, John Sillitoe on behalf of the Wellcome Sanger Institute COVID-19 Surveillance Team
EPI_ISL_851676	Lighthouse Lab in Glasgow	Wellcome Sanger Institute for the COVID-19 Genomics UK (COG-UK) Consortium	Harper VanSteenhouse, Yumi Kasai, David Gray, Carol Clugston, Anna Dominiczak and Alex Alderton, Roberto Amato, Sonia Goncalves, Ewan Harrison, David K. Jackson, Ian Johnston, Dominic Kwiatkowski, Cordelia Langford, John Sillitoe on behalf of the Wellcome Sanger Institute COVID-19 Surveillance Team
EPI_ISL_851677, EPI_ISL_851678, EPI_ISL_851679, EPI_ISL_851680	Lighthouse Lab in Alderley Park	Wellcome Sanger Institute for the COVID-19 Genomics UK (COG-UK) Consortium	Jacquelyn Wynn, Mairead Hyland, The Lighthouse Lab in Alderley Park and Alex Alderton, Roberto Amato, Sonia Goncalves, Ewan Harrison, David K. Jackson, Ian Johnston, Dominic Kwiatkowski, Cordelia Langford, John Sillitoe on behalf of the Wellcome Sanger Institute COVID-19 Surveillance Team
EPI_ISL_851681	Lighthouse Lab in Glasgow	Wellcome Sanger Institute for the COVID-19 Genomics UK (COG-UK) Consortium	Harper VanSteenhouse, Yumi Kasai, David Gray, Carol Clugston, Anna Dominiczak and Alex Alderton, Roberto Amato, Sonia Goncalves, Ewan Harrison, David K. Jackson, Ian Johnston, Dominic Kwiatkowski, Cordelia Langford, John Sillitoe on behalf of the Wellcome Sanger Institute COVID-19 Surveillance Team
EPI_ISL_851682, EPI_ISL_851683, EPI_ISL_851684	Lighthouse Lab in Alderley Park	Wellcome Sanger Institute for the COVID-19 Genomics UK (COG-UK) Consortium	Jacquelyn Wynn, Mairead Hyland, The Lighthouse Lab in Alderley Park and Alex Alderton, Roberto Amato, Sonia Goncalves, Ewan Harrison, David K. Jackson, Ian Johnston, Dominic Kwiatkowski, Cordelia Langford, John Sillitoe on behalf of the Wellcome Sanger Institute COVID-19 Surveillance Team
EPI_ISL_851685, EPI_ISL_851686, EPI_ISL_851687	Lighthouse Lab in Glasgow	Wellcome Sanger Institute for the COVID-19 Genomics UK (COG-UK) Consortium	Harper VanSteenhouse, Yumi Kasai, David Gray, Carol Clugston, Anna Dominiczak and Alex Alderton, Roberto Amato, Sonia Goncalves, Ewan Harrison, David K. Jackson, Ian Johnston, Dominic Kwiatkowski, Cordelia Langford, John Sillitoe on behalf of the Wellcome Sanger Institute COVID-19 Surveillance Team
EPI_ISL_851688	Lighthouse Lab in Alderley Park	Wellcome Sanger Institute for the COVID-19 Genomics UK (COG-UK) Consortium	Jacquelyn Wynn, Mairead Hyland, The Lighthouse Lab in Alderley Park and Alex Alderton, Roberto Amato, Sonia Goncalves, Ewan Harrison, David K. Jackson, Ian Johnston, Dominic Kwiatkowski, Cordelia Langford, John Sillitoe on behalf of the Wellcome Sanger Institute COVID-19 Surveillance Team

EPI_ISL_851689, EPI_ISL_851690	Lighthouse Lab in Glasgow	Wellcome Sanger Institute for the COVID-19 Genomics UK (COG-UK) Consortium	Harper VanSteenhouse, Yumi Kasai, David Gray, Carol Clugston, Anna Dominiczak and Alex Alderton, Roberto Amato, Sonia Goncalves, Ewan Harrison, David K. Jackson, Ian Johnston, Dominic Kwiatkowski, Cordelia Langford, John Sillitoe on behalf of the Wellcome Sanger Institute COVID-19 Surveillance Team
EPI_ISL_851691, EPI_ISL_851692	Lighthouse Lab in Alderley Park	Wellcome Sanger Institute for the COVID-19 Genomics UK (COG-UK) Consortium	Jacquelyn Wynn, Mairead Hyland, The Lighthouse Lab in Alderley Park and Alex Alderton, Roberto Amato, Sonia Goncalves, Ewan Harrison, David K. Jackson, Ian Johnston, Dominic Kwiatkowski, Cordelia Langford, John Sillitoe on behalf of the Wellcome Sanger Institute COVID-19 Surveillance Team
EPI_ISL_851693	Lighthouse Lab in Glasgow	Wellcome Sanger Institute for the COVID-19 Genomics UK (COG-UK) Consortium	Harper VanSteenhouse, Yumi Kasai, David Gray, Carol Clugston, Anna Dominiczak and Alex Alderton, Roberto Amato, Sonia Goncalves, Ewan Harrison, David K. Jackson, Ian Johnston, Dominic Kwiatkowski, Cordelia Langford, John Sillitoe on behalf of the Wellcome Sanger Institute COVID-19 Surveillance Team
EPI_ISL_851694, EPI_ISL_851695, EPI_ISL_851696, EPI_ISL_851697, EPI_ISL_851698, EPI_ISL_851699, EPI_ISL_851700	Lighthouse Lab in Alderley Park	Wellcome Sanger Institute for the COVID-19 Genomics UK (COG-UK) Consortium	Jacquelyn Wynn, Mairead Hyland, The Lighthouse Lab in Alderley Park and Alex Alderton, Roberto Amato, Sonia Goncalves, Ewan Harrison, David K. Jackson, Ian Johnston, Dominic Kwiatkowski, Cordelia Langford, John Sillitoe on behalf of the Wellcome Sanger Institute COVID-19 Surveillance Team
EPI_ISL_851701	Lighthouse Lab in Glasgow	Wellcome Sanger Institute for the COVID-19 Genomics UK (COG-UK) Consortium	Harper VanSteenhouse, Yumi Kasai, David Gray, Carol Clugston, Anna Dominiczak and Alex Alderton, Roberto Amato, Sonia Goncalves, Ewan Harrison, David K. Jackson, Ian Johnston, Dominic Kwiatkowski, Cordelia Langford, John Sillitoe on behalf of the Wellcome Sanger Institute COVID-19 Surveillance Team
EPI_ISL_851702, EPI_ISL_851703, EPI_ISL_851704, EPI_ISL_851705, EPI_ISL_851706, EPI_ISL_851707, EPI_ISL_851708, EPI_ISL_851709, EPI_ISL_851710, EPI_ISL_851711, EPI_ISL_851712, EPI_ISL_851713			
see above	Lighthouse Lab in Alderley Park	Wellcome Sanger Institute for the COVID-19 Genomics UK (COG-UK) Consortium	Jacquelyn Wynn, Mairead Hyland, The Lighthouse Lab in Alderley Park and Alex Alderton, Roberto Amato, Sonia Goncalves, Ewan Harrison, David K. Jackson, Ian Johnston, Dominic Kwiatkowski, Cordelia Langford, John Sillitoe on behalf of the Wellcome Sanger Institute COVID-19 Surveillance Team
EPI_ISL_851714	Lighthouse Lab in Glasgow	Wellcome Sanger Institute for the COVID-19 Genomics UK (COG-UK) Consortium	Harper VanSteenhouse, Yumi Kasai, David Gray, Carol Clugston, Anna Dominiczak and Alex Alderton, Roberto Amato, Sonia Goncalves, Ewan Harrison, David K. Jackson, Ian Johnston, Dominic Kwiatkowski, Cordelia Langford, John Sillitoe on behalf of the Wellcome Sanger Institute COVID-19 Surveillance Team
EPI_ISL_851715, EPI_ISL_851716, EPI_ISL_851717, EPI_ISL_851718, EPI_ISL_851719	Lighthouse Lab in Alderley Park	Wellcome Sanger Institute for the COVID-19 Genomics UK (COG-UK) Consortium	Jacquelyn Wynn, Mairead Hyland, The Lighthouse Lab in Alderley Park and Alex Alderton, Roberto Amato, Sonia Goncalves, Ewan Harrison, David K. Jackson, Ian Johnston, Dominic Kwiatkowski, Cordelia Langford, John Sillitoe on behalf of the Wellcome Sanger Institute COVID-19 Surveillance Team
EPI_ISL_851720	Lighthouse Lab in Glasgow	Wellcome Sanger Institute for the COVID-19 Genomics UK (COG-UK) Consortium	Harper VanSteenhouse, Yumi Kasai, David Gray, Carol Clugston, Anna Dominiczak and Alex Alderton, Roberto Amato, Sonia Goncalves, Ewan Harrison, David K. Jackson, Ian Johnston, Dominic Kwiatkowski, Cordelia Langford, John Sillitoe on behalf of the Wellcome Sanger Institute COVID-19 Surveillance Team
EPI_ISL_851721, EPI_ISL_851722, EPI_ISL_851723, EPI_ISL_851724	Lighthouse Lab in Alderley Park	Wellcome Sanger Institute for the COVID-19 Genomics UK (COG-UK) Consortium	Jacquelyn Wynn, Mairead Hyland, The Lighthouse Lab in Alderley Park and Alex Alderton, Roberto Amato, Sonia Goncalves, Ewan Harrison, David K. Jackson, Ian Johnston, Dominic Kwiatkowski, Cordelia Langford, John Sillitoe on behalf of the Wellcome Sanger Institute COVID-19 Surveillance Team
EPI_ISL_851725	Lighthouse Lab in Glasgow	Wellcome Sanger Institute for the COVID-19 Genomics UK (COG-UK) Consortium	Harper VanSteenhouse, Yumi Kasai, David Gray, Carol Clugston, Anna Dominiczak and Alex Alderton, Roberto Amato, Sonia Goncalves, Ewan Harrison, David K. Jackson, Ian Johnston, Dominic Kwiatkowski, Cordelia Langford, John Sillitoe on behalf of the Wellcome Sanger Institute COVID-19 Surveillance Team
EPI_ISL_851726	Lighthouse Lab in Alderley Park	Wellcome Sanger Institute for the COVID-19 Genomics UK (COG-UK) Consortium	Jacquelyn Wynn, Mairead Hyland, The Lighthouse Lab in Alderley Park and Alex Alderton, Roberto Amato, Sonia Goncalves, Ewan Harrison, David K. Jackson, Ian Johnston, Dominic Kwiatkowski, Cordelia Langford, John Sillitoe on behalf of the Wellcome Sanger Institute COVID-19 Surveillance Team
EPI_ISL_851727	Lighthouse Lab in Glasgow	Wellcome Sanger Institute for the COVID-19 Genomics UK (COG-UK) Consortium	Harper VanSteenhouse, Yumi Kasai, David Gray, Carol Clugston, Anna Dominiczak and Alex Alderton, Roberto Amato, Sonia Goncalves, Ewan Harrison, David K. Jackson, Ian Johnston, Dominic Kwiatkowski, Cordelia Langford, John Sillitoe on behalf of the Wellcome Sanger Institute COVID-19 Surveillance Team
EPI_ISL_851728	Lighthouse Lab in Alderley Park	Wellcome Sanger Institute for the COVID-19 Genomics UK (COG-UK) Consortium	Jacquelyn Wynn, Mairead Hyland, The Lighthouse Lab in Alderley Park and Alex Alderton, Roberto Amato, Sonia Goncalves, Ewan Harrison, David K. Jackson, Ian Johnston, Dominic Kwiatkowski, Cordelia Langford, John Sillitoe on behalf of the Wellcome Sanger Institute COVID-19 Surveillance Team
EPI_ISL_851729	Lighthouse Lab in Glasgow	Wellcome Sanger Institute for the COVID-19 Genomics UK (COG-UK) Consortium	Harper VanSteenhouse, Yumi Kasai, David Gray, Carol Clugston, Anna Dominiczak and Alex Alderton, Roberto Amato, Sonia Goncalves, Ewan Harrison, David K. Jackson, Ian Johnston, Dominic Kwiatkowski, Cordelia Langford, John Sillitoe on behalf of the Wellcome Sanger Institute COVID-19 Surveillance Team
EPI_ISL_851730, EPI_ISL_851731, EPI_ISL_851732, EPI_ISL_851733	Lighthouse Lab in Alderley Park	Wellcome Sanger Institute for the COVID-19 Genomics UK (COG-UK) Consortium	Jacquelyn Wynn, Mairead Hyland, The Lighthouse Lab in Alderley Park and Alex Alderton, Roberto Amato, Sonia Goncalves, Ewan Harrison, David K. Jackson, Ian Johnston, Dominic Kwiatkowski, Cordelia Langford, John Sillitoe on behalf of the Wellcome Sanger Institute COVID-19 Surveillance Team
EPI_ISL_851734	Lighthouse Lab in Glasgow	Wellcome Sanger Institute for the COVID-19 Genomics UK (COG-UK) Consortium	Harper VanSteenhouse, Yumi Kasai, David Gray, Carol Clugston, Anna Dominiczak and Alex Alderton, Roberto Amato, Sonia Goncalves, Ewan Harrison, David K. Jackson, Ian Johnston, Dominic Kwiatkowski, Cordelia Langford, John Sillitoe on behalf of the Wellcome Sanger Institute COVID-19 Surveillance Team
EPI_ISL_851735, EPI_ISL_851736, EPI_ISL_851737	Lighthouse Lab in Alderley Park	Wellcome Sanger Institute for the COVID-19 Genomics UK (COG-UK) Consortium	Jacquelyn Wynn, Mairead Hyland, The Lighthouse Lab in Alderley Park and Alex Alderton, Roberto Amato, Sonia Goncalves, Ewan Harrison, David K. Jackson, Ian Johnston, Dominic Kwiatkowski, Cordelia Langford, John Sillitoe on behalf of the Wellcome Sanger Institute COVID-19 Surveillance Team
EPI_ISL_851738	Lighthouse Lab in Glasgow	Wellcome Sanger Institute for the COVID-19 Genomics UK (COG-UK) Consortium	Harper VanSteenhouse, Yumi Kasai, David Gray, Carol Clugston, Anna Dominiczak and Alex Alderton, Roberto Amato, Sonia Goncalves, Ewan Harrison, David K. Jackson, Ian Johnston, Dominic Kwiatkowski, Cordelia Langford, John Sillitoe on behalf of the Wellcome Sanger Institute COVID-19 Surveillance Team
EPI_ISL_851739, EPI_ISL_851740, EPI_ISL_851741, EPI_ISL_851742	Lighthouse Lab in Alderley Park	Wellcome Sanger Institute for the COVID-19 Genomics UK (COG-UK) Consortium	Jacquelyn Wynn, Mairead Hyland, The Lighthouse Lab in Alderley Park and Alex Alderton, Roberto Amato, Sonia Goncalves, Ewan Harrison, David K. Jackson, Ian Johnston, Dominic Kwiatkowski, Cordelia Langford, John Sillitoe on behalf of the Wellcome Sanger Institute COVID-19 Surveillance Team
EPI_ISL_851743	Lighthouse Lab in Glasgow	Wellcome Sanger Institute for the COVID-19 Genomics UK (COG-UK) Consortium	Harper VanSteenhouse, Yumi Kasai, David Gray, Carol Clugston, Anna Dominiczak and Alex Alderton, Roberto Amato, Sonia Goncalves, Ewan Harrison, David K. Jackson, Ian Johnston, Dominic Kwiatkowski, Cordelia Langford, John Sillitoe on behalf of the Wellcome Sanger Institute COVID-19 Surveillance Team
EPI_ISL_851744, EPI_ISL_851745, EPI_ISL_851746	Lighthouse Lab in Alderley Park	Wellcome Sanger Institute for the COVID-19 Genomics UK (COG-UK) Consortium	Jacquelyn Wynn, Mairead Hyland, The Lighthouse Lab in Alderley Park and Alex Alderton, Roberto Amato, Sonia Goncalves, Ewan Harrison, David K. Jackson, Ian Johnston, Dominic Kwiatkowski, Cordelia Langford, John Sillitoe on behalf of the Wellcome Sanger Institute COVID-19 Surveillance Team
EPI_ISL_851747	Lighthouse Lab in Glasgow	Wellcome Sanger Institute for the COVID-19 Genomics UK (COG-UK) Consortium	Harper VanSteenhouse, Yumi Kasai, David Gray, Carol Clugston, Anna Dominiczak and Alex Alderton, Roberto Amato, Sonia Goncalves, Ewan Harrison, David K. Jackson, Ian Johnston, Dominic Kwiatkowski, Cordelia Langford, John Sillitoe on behalf of the Wellcome Sanger Institute COVID-19 Surveillance Team
EPI_ISL_851748	Lighthouse Lab in Alderley Park	Wellcome Sanger Institute for the COVID-19 Genomics UK (COG-UK) Consortium	Jacquelyn Wynn, Mairead Hyland, The Lighthouse Lab in Alderley Park and Alex Alderton, Roberto Amato, Sonia Goncalves, Ewan Harrison, David K. Jackson, Ian Johnston, Dominic Kwiatkowski, Cordelia Langford, John Sillitoe on behalf of the Wellcome Sanger Institute COVID-19 Surveillance Team
EPI_ISL_851749	Lighthouse Lab in Glasgow	Wellcome Sanger Institute for the COVID-19 Genomics UK (COG-UK) Consortium	Harper VanSteenhouse, Yumi Kasai, David Gray, Carol Clugston, Anna Dominiczak and Alex Alderton, Roberto Amato, Sonia Goncalves, Ewan Harrison, David K. Jackson, Ian Johnston, Dominic Kwiatkowski, Cordelia Langford, John Sillitoe on behalf of the Wellcome Sanger Institute COVID-19 Surveillance Team
EPI_ISL_851751, EPI_ISL_851752, EPI_ISL_851753	Lighthouse Lab in Alderley Park	Wellcome Sanger Institute for the COVID-19 Genomics UK (COG-UK) Consortium	Jacquelyn Wynn, Mairead Hyland, The Lighthouse Lab in Alderley Park and Alex Alderton, Roberto Amato, Sonia Goncalves, Ewan Harrison, David K. Jackson, Ian Johnston, Dominic Kwiatkowski, Cordelia Langford, John Sillitoe on behalf of the Wellcome Sanger Institute COVID-19 Surveillance Team
EPI_ISL_851754	Lighthouse Lab in Glasgow	Wellcome Sanger Institute for the COVID-19 Genomics UK (COG-UK) Consortium	Harper VanSteenhouse, Yumi Kasai, David Gray, Carol Clugston, Anna Dominiczak and Alex Alderton, Roberto Amato, Sonia Goncalves, Ewan Harrison, David K. Jackson, Ian Johnston, Dominic Kwiatkowski, Cordelia Langford, John Sillitoe on behalf of the Wellcome Sanger Institute COVID-19 Surveillance Team
EPI_ISL_851755, EPI_ISL_851756	Lighthouse Lab in Alderley Park	Wellcome Sanger Institute for the COVID-19 Genomics UK	Jacquelyn Wynn, Mairead Hyland, The Lighthouse Lab in Alderley Park and Alex Alderton, Roberto Amato, Sonia Goncalves, Ewan Harrison, David K.

		(COG-UK) Consortium	Jackson, Ian Johnston, Dominic Kwiatkowski, Cordelia Langford, John Sillitoe on behalf of the Wellcome Sanger Institute COVID-19 Surveillance Team
EPI_ISL_851757	Lighthouse Lab in Glasgow	Wellcome Sanger Institute for the COVID-19 Genomics UK (COG-UK) Consortium	Harper VanSteenhouse, Yumi Kasai, David Gray, Carol Clugston, Anna Dominiczak and Alex Alderton, Roberto Amato, Sonia Goncalves, Ewan Harrison, David K. Jackson, Ian Johnston, Dominic Kwiatkowski, Cordelia Langford, John Sillitoe on behalf of the Wellcome Sanger Institute COVID-19 Surveillance Team
EPI_ISL_851758, EPI_ISL_851759	Lighthouse Lab in Alderley Park	Wellcome Sanger Institute for the COVID-19 Genomics UK (COG-UK) Consortium	Jacquelyn Wynn, Mairead Hyland, The Lighthouse Lab in Alderley Park and Alex Alderton, Roberto Amato, Sonia Goncalves, Ewan Harrison, David K. Jackson, Ian Johnston, Dominic Kwiatkowski, Cordelia Langford, John Sillitoe on behalf of the Wellcome Sanger Institute COVID-19 Surveillance Team
EPI_ISL_851760	Lighthouse Lab in Glasgow	Wellcome Sanger Institute for the COVID-19 Genomics UK (COG-UK) Consortium	Harper VanSteenhouse, Yumi Kasai, David Gray, Carol Clugston, Anna Dominiczak and Alex Alderton, Roberto Amato, Sonia Goncalves, Ewan Harrison, David K. Jackson, Ian Johnston, Dominic Kwiatkowski, Cordelia Langford, John Sillitoe on behalf of the Wellcome Sanger Institute COVID-19 Surveillance Team
EPI_ISL_851761, EPI_ISL_851762	Lighthouse Lab in Alderley Park	Wellcome Sanger Institute for the COVID-19 Genomics UK (COG-UK) Consortium	Jacquelyn Wynn, Mairead Hyland, The Lighthouse Lab in Alderley Park and Alex Alderton, Roberto Amato, Sonia Goncalves, Ewan Harrison, David K. Jackson, Ian Johnston, Dominic Kwiatkowski, Cordelia Langford, John Sillitoe on behalf of the Wellcome Sanger Institute COVID-19 Surveillance Team
EPI_ISL_851763	Lighthouse Lab in Glasgow	Wellcome Sanger Institute for the COVID-19 Genomics UK (COG-UK) Consortium	Harper VanSteenhouse, Yumi Kasai, David Gray, Carol Clugston, Anna Dominiczak and Alex Alderton, Roberto Amato, Sonia Goncalves, Ewan Harrison, David K. Jackson, Ian Johnston, Dominic Kwiatkowski, Cordelia Langford, John Sillitoe on behalf of the Wellcome Sanger Institute COVID-19 Surveillance Team
EPI_ISL_851764, EPI_ISL_851765, EPI_ISL_851766	Lighthouse Lab in Alderley Park	Wellcome Sanger Institute for the COVID-19 Genomics UK (COG-UK) Consortium	Jacquelyn Wynn, Mairead Hyland, The Lighthouse Lab in Alderley Park and Alex Alderton, Roberto Amato, Sonia Goncalves, Ewan Harrison, David K. Jackson, Ian Johnston, Dominic Kwiatkowski, Cordelia Langford, John Sillitoe on behalf of the Wellcome Sanger Institute COVID-19 Surveillance Team
EPI_ISL_851767	Lighthouse Lab in Glasgow	Wellcome Sanger Institute for the COVID-19 Genomics UK (COG-UK) Consortium	Harper VanSteenhouse, Yumi Kasai, David Gray, Carol Clugston, Anna Dominiczak and Alex Alderton, Roberto Amato, Sonia Goncalves, Ewan Harrison, David K. Jackson, Ian Johnston, Dominic Kwiatkowski, Cordelia Langford, John Sillitoe on behalf of the Wellcome Sanger Institute COVID-19 Surveillance Team
EPI_ISL_851768, EPI_ISL_851769	Lighthouse Lab in Alderley Park	Wellcome Sanger Institute for the COVID-19 Genomics UK (COG-UK) Consortium	Jacquelyn Wynn, Mairead Hyland, The Lighthouse Lab in Alderley Park and Alex Alderton, Roberto Amato, Sonia Goncalves, Ewan Harrison, David K. Jackson, Ian Johnston, Dominic Kwiatkowski, Cordelia Langford, John Sillitoe on behalf of the Wellcome Sanger Institute COVID-19 Surveillance Team
EPI_ISL_851770	Lighthouse Lab in Glasgow	Wellcome Sanger Institute for the COVID-19 Genomics UK (COG-UK) Consortium	Harper VanSteenhouse, Yumi Kasai, David Gray, Carol Clugston, Anna Dominiczak and Alex Alderton, Roberto Amato, Sonia Goncalves, Ewan Harrison, David K. Jackson, Ian Johnston, Dominic Kwiatkowski, Cordelia Langford, John Sillitoe on behalf of the Wellcome Sanger Institute COVID-19 Surveillance Team
EPI_ISL_851771	Lighthouse Lab in Alderley Park	Wellcome Sanger Institute for the COVID-19 Genomics UK (COG-UK) Consortium	Jacquelyn Wynn, Mairead Hyland, The Lighthouse Lab in Alderley Park and Alex Alderton, Roberto Amato, Sonia Goncalves, Ewan Harrison, David K. Jackson, Ian Johnston, Dominic Kwiatkowski, Cordelia Langford, John Sillitoe on behalf of the Wellcome Sanger Institute COVID-19 Surveillance Team
EPI_ISL_851772	Lighthouse Lab in Glasgow	Wellcome Sanger Institute for the COVID-19 Genomics UK (COG-UK) Consortium	Harper VanSteenhouse, Yumi Kasai, David Gray, Carol Clugston, Anna Dominiczak and Alex Alderton, Roberto Amato, Sonia Goncalves, Ewan Harrison, David K. Jackson, Ian Johnston, Dominic Kwiatkowski, Cordelia Langford, John Sillitoe on behalf of the Wellcome Sanger Institute COVID-19 Surveillance Team
EPI_ISL_851773	Lighthouse Lab in Alderley Park	Wellcome Sanger Institute for the COVID-19 Genomics UK (COG-UK) Consortium	Jacquelyn Wynn, Mairead Hyland, The Lighthouse Lab in Alderley Park and Alex Alderton, Roberto Amato, Sonia Goncalves, Ewan Harrison, David K. Jackson, Ian Johnston, Dominic Kwiatkowski, Cordelia Langford, John Sillitoe on behalf of the Wellcome Sanger Institute COVID-19 Surveillance Team
EPI_ISL_851774	Lighthouse Lab in Glasgow	Wellcome Sanger Institute for the COVID-19 Genomics UK (COG-UK) Consortium	Harper VanSteenhouse, Yumi Kasai, David Gray, Carol Clugston, Anna Dominiczak and Alex Alderton, Roberto Amato, Sonia Goncalves, Ewan Harrison, David K. Jackson, Ian Johnston, Dominic Kwiatkowski, Cordelia Langford, John Sillitoe on behalf of the Wellcome Sanger Institute COVID-19 Surveillance Team
EPI_ISL_851775, EPI_ISL_851776, EPI_ISL_851777, EPI_ISL_851778, EPI_ISL_851779	Lighthouse Lab in Alderley Park	Wellcome Sanger Institute for the COVID-19 Genomics UK (COG-UK) Consortium	Jacquelyn Wynn, Mairead Hyland, The Lighthouse Lab in Alderley Park and Alex Alderton, Roberto Amato, Sonia Goncalves, Ewan Harrison, David K. Jackson, Ian Johnston, Dominic Kwiatkowski, Cordelia Langford, John Sillitoe on behalf of the Wellcome Sanger Institute COVID-19 Surveillance Team
EPI_ISL_851780, EPI_ISL_851781	Lighthouse Lab in Glasgow	Wellcome Sanger Institute for the COVID-19 Genomics UK (COG-UK) Consortium	Harper VanSteenhouse, Yumi Kasai, David Gray, Carol Clugston, Anna Dominiczak and Alex Alderton, Roberto Amato, Sonia Goncalves, Ewan Harrison, David K. Jackson, Ian Johnston, Dominic Kwiatkowski, Cordelia Langford, John Sillitoe on behalf of the Wellcome Sanger Institute COVID-19 Surveillance Team
EPI_ISL_851782, EPI_ISL_851783, EPI_ISL_851784	Lighthouse Lab in Alderley Park	Wellcome Sanger Institute for the COVID-19 Genomics UK (COG-UK) Consortium	Jacquelyn Wynn, Mairead Hyland, The Lighthouse Lab in Alderley Park and Alex Alderton, Roberto Amato, Sonia Goncalves, Ewan Harrison, David K. Jackson, Ian Johnston, Dominic Kwiatkowski, Cordelia Langford, John Sillitoe on behalf of the Wellcome Sanger Institute COVID-19 Surveillance Team
EPI_ISL_851785	Lighthouse Lab in Glasgow	Wellcome Sanger Institute for the COVID-19 Genomics UK (COG-UK) Consortium	Harper VanSteenhouse, Yumi Kasai, David Gray, Carol Clugston, Anna Dominiczak and Alex Alderton, Roberto Amato, Sonia Goncalves, Ewan Harrison, David K. Jackson, Ian Johnston, Dominic Kwiatkowski, Cordelia Langford, John Sillitoe on behalf of the Wellcome Sanger Institute COVID-19 Surveillance Team
EPI_ISL_851786, EPI_ISL_851787, EPI_ISL_851788, EPI_ISL_851789, EPI_ISL_851790, EPI_ISL_851791	Lighthouse Lab in Alderley Park	Wellcome Sanger Institute for the COVID-19 Genomics UK (COG-UK) Consortium	Jacquelyn Wynn, Mairead Hyland, The Lighthouse Lab in Alderley Park and Alex Alderton, Roberto Amato, Sonia Goncalves, Ewan Harrison, David K. Jackson, Ian Johnston, Dominic Kwiatkowski, Cordelia Langford, John Sillitoe on behalf of the Wellcome Sanger Institute COVID-19 Surveillance Team
EPI_ISL_851792	Lighthouse Lab in Glasgow	Wellcome Sanger Institute for the COVID-19 Genomics UK (COG-UK) Consortium	Harper VanSteenhouse, Yumi Kasai, David Gray, Carol Clugston, Anna Dominiczak and Alex Alderton, Roberto Amato, Sonia Goncalves, Ewan Harrison, David K. Jackson, Ian Johnston, Dominic Kwiatkowski, Cordelia Langford, John Sillitoe on behalf of the Wellcome Sanger Institute COVID-19 Surveillance Team
EPI_ISL_851793	Lighthouse Lab in Alderley Park	Wellcome Sanger Institute for the COVID-19 Genomics UK (COG-UK) Consortium	Jacquelyn Wynn, Mairead Hyland, The Lighthouse Lab in Alderley Park and Alex Alderton, Roberto Amato, Sonia Goncalves, Ewan Harrison, David K. Jackson, Ian Johnston, Dominic Kwiatkowski, Cordelia Langford, John Sillitoe on behalf of the Wellcome Sanger Institute COVID-19 Surveillance Team
EPI_ISL_851794	Lighthouse Lab in Glasgow	Wellcome Sanger Institute for the COVID-19 Genomics UK (COG-UK) Consortium	Harper VanSteenhouse, Yumi Kasai, David Gray, Carol Clugston, Anna Dominiczak and Alex Alderton, Roberto Amato, Sonia Goncalves, Ewan Harrison, David K. Jackson, Ian Johnston, Dominic Kwiatkowski, Cordelia Langford, John Sillitoe on behalf of the Wellcome Sanger Institute COVID-19 Surveillance Team
EPI_ISL_851795	Lighthouse Lab in Alderley Park	Wellcome Sanger Institute for the COVID-19 Genomics UK (COG-UK) Consortium	Jacquelyn Wynn, Mairead Hyland, The Lighthouse Lab in Alderley Park and Alex Alderton, Roberto Amato, Sonia Goncalves, Ewan Harrison, David K. Jackson, Ian Johnston, Dominic Kwiatkowski, Cordelia Langford, John Sillitoe on behalf of the Wellcome Sanger Institute COVID-19 Surveillance Team
EPI_ISL_851796, EPI_ISL_851797, EPI_ISL_851798, EPI_ISL_851799	Lighthouse Lab in Glasgow	Wellcome Sanger Institute for the COVID-19 Genomics UK (COG-UK) Consortium	Harper VanSteenhouse, Yumi Kasai, David Gray, Carol Clugston, Anna Dominiczak and Alex Alderton, Roberto Amato, Sonia Goncalves, Ewan Harrison, David K. Jackson, Ian Johnston, Dominic Kwiatkowski, Cordelia Langford, John Sillitoe on behalf of the Wellcome Sanger Institute COVID-19 Surveillance Team
EPI_ISL_851800, EPI_ISL_851801, EPI_ISL_851802	Lighthouse Lab in Alderley Park	Wellcome Sanger Institute for the COVID-19 Genomics UK (COG-UK) Consortium	Jacquelyn Wynn, Mairead Hyland, The Lighthouse Lab in Alderley Park and Alex Alderton, Roberto Amato, Sonia Goncalves, Ewan Harrison, David K. Jackson, Ian Johnston, Dominic Kwiatkowski, Cordelia Langford, John Sillitoe on behalf of the Wellcome Sanger Institute COVID-19 Surveillance Team
EPI_ISL_851803	Lighthouse Lab in Glasgow	Wellcome Sanger Institute for the COVID-19 Genomics UK (COG-UK) Consortium	Harper VanSteenhouse, Yumi Kasai, David Gray, Carol Clugston, Anna Dominiczak and Alex Alderton, Roberto Amato, Sonia Goncalves, Ewan Harrison, David K. Jackson, Ian Johnston, Dominic Kwiatkowski, Cordelia Langford, John Sillitoe on behalf of the Wellcome Sanger Institute COVID-19 Surveillance Team
EPI_ISL_851804	Lighthouse Lab in Alderley Park	Wellcome Sanger Institute for the COVID-19 Genomics UK (COG-UK) Consortium	Jacquelyn Wynn, Mairead Hyland, The Lighthouse Lab in Alderley Park and Alex Alderton, Roberto Amato, Sonia Goncalves, Ewan Harrison, David K. Jackson, Ian Johnston, Dominic Kwiatkowski, Cordelia Langford, John Sillitoe on behalf of the Wellcome Sanger Institute COVID-19 Surveillance Team
EPI_ISL_851805	Lighthouse Lab in Glasgow	Wellcome Sanger Institute for the COVID-19 Genomics UK (COG-UK) Consortium	Harper VanSteenhouse, Yumi Kasai, David Gray, Carol Clugston, Anna Dominiczak and Alex Alderton, Roberto Amato, Sonia Goncalves, Ewan Harrison, David K. Jackson, Ian Johnston, Dominic Kwiatkowski, Cordelia Langford, John Sillitoe on behalf of the Wellcome Sanger Institute COVID-19 Surveillance Team
EPI_ISL_851806	Lighthouse Lab in Alderley Park	Wellcome Sanger Institute for the COVID-19 Genomics UK (COG-UK) Consortium	Jacquelyn Wynn, Mairead Hyland, The Lighthouse Lab in Alderley Park and Alex Alderton, Roberto Amato, Sonia Goncalves, Ewan Harrison, David K. Jackson, Ian Johnston, Dominic Kwiatkowski, Cordelia Langford, John Sillitoe on behalf of the Wellcome Sanger Institute COVID-19 Surveillance Team

EPI_ISL_851807	Lighthouse Lab in Glasgow	Wellcome Sanger Institute for the COVID-19 Genomics UK (COG-UK) Consortium	Harper VanSteenhouse, Yumi Kasai, David Gray, Carol Clugston, Anna Dominiczak and Alex Alderton, Roberto Amato, Sonia Goncalves, Ewan Harrison, David K. Jackson, Ian Johnston, Dominic Kwiatkowski, Cordelia Langford, John Sillitoe on behalf of the Wellcome Sanger Institute COVID-19 Surveillance Team
EPI_ISL_851808, EPI_ISL_851809, EPI_ISL_851810, EPI_ISL_851811	Lighthouse Lab in Alderley Park	Wellcome Sanger Institute for the COVID-19 Genomics UK (COG-UK) Consortium	Jacquelyn Wynn, Mairead Hyland, The Lighthouse Lab in Alderley Park and Alex Alderton, Roberto Amato, Sonia Goncalves, Ewan Harrison, David K. Jackson, Ian Johnston, Dominic Kwiatkowski, Cordelia Langford, John Sillitoe on behalf of the Wellcome Sanger Institute COVID-19 Surveillance Team
EPI_ISL_851812	Lighthouse Lab in Glasgow	Wellcome Sanger Institute for the COVID-19 Genomics UK (COG-UK) Consortium	Harper VanSteenhouse, Yumi Kasai, David Gray, Carol Clugston, Anna Dominiczak and Alex Alderton, Roberto Amato, Sonia Goncalves, Ewan Harrison, David K. Jackson, Ian Johnston, Dominic Kwiatkowski, Cordelia Langford, John Sillitoe on behalf of the Wellcome Sanger Institute COVID-19 Surveillance Team
EPI_ISL_851813	Lighthouse Lab in Alderley Park	Wellcome Sanger Institute for the COVID-19 Genomics UK (COG-UK) Consortium	Jacquelyn Wynn, Mairead Hyland, The Lighthouse Lab in Alderley Park and Alex Alderton, Roberto Amato, Sonia Goncalves, Ewan Harrison, David K. Jackson, Ian Johnston, Dominic Kwiatkowski, Cordelia Langford, John Sillitoe on behalf of the Wellcome Sanger Institute COVID-19 Surveillance Team
EPI_ISL_851814	Lighthouse Lab in Glasgow	Wellcome Sanger Institute for the COVID-19 Genomics UK (COG-UK) Consortium	Harper VanSteenhouse, Yumi Kasai, David Gray, Carol Clugston, Anna Dominiczak and Alex Alderton, Roberto Amato, Sonia Goncalves, Ewan Harrison, David K. Jackson, Ian Johnston, Dominic Kwiatkowski, Cordelia Langford, John Sillitoe on behalf of the Wellcome Sanger Institute COVID-19 Surveillance Team
EPI_ISL_851815, EPI_ISL_851816, EPI_ISL_851817, EPI_ISL_851818, EPI_ISL_851819, EPI_ISL_851820, EPI_ISL_851821, EPI_ISL_851822, EPI_ISL_851823, EPI_ISL_851824, EPI_ISL_851825, EPI_ISL_851826, EPI_ISL_851827, EPI_ISL_851828			
see above	Lighthouse Lab in Alderley Park	Wellcome Sanger Institute for the COVID-19 Genomics UK (COG-UK) Consortium	Jacquelyn Wynn, Mairead Hyland, The Lighthouse Lab in Alderley Park and Alex Alderton, Roberto Amato, Sonia Goncalves, Ewan Harrison, David K. Jackson, Ian Johnston, Dominic Kwiatkowski, Cordelia Langford, John Sillitoe on behalf of the Wellcome Sanger Institute COVID-19 Surveillance Team
EPI_ISL_851829	Lighthouse Lab in Glasgow	Wellcome Sanger Institute for the COVID-19 Genomics UK (COG-UK) Consortium	Harper VanSteenhouse, Yumi Kasai, David Gray, Carol Clugston, Anna Dominiczak and Alex Alderton, Roberto Amato, Sonia Goncalves, Ewan Harrison, David K. Jackson, Ian Johnston, Dominic Kwiatkowski, Cordelia Langford, John Sillitoe on behalf of the Wellcome Sanger Institute COVID-19 Surveillance Team
EPI_ISL_851830, EPI_ISL_851831, EPI_ISL_851832, EPI_ISL_851833, EPI_ISL_851834, EPI_ISL_851835, EPI_ISL_851836	Lighthouse Lab in Alderley Park	Wellcome Sanger Institute for the COVID-19 Genomics UK (COG-UK) Consortium	Jacquelyn Wynn, Mairead Hyland, The Lighthouse Lab in Alderley Park and Alex Alderton, Roberto Amato, Sonia Goncalves, Ewan Harrison, David K. Jackson, Ian Johnston, Dominic Kwiatkowski, Cordelia Langford, John Sillitoe on behalf of the Wellcome Sanger Institute COVID-19 Surveillance Team
EPI_ISL_851837	Lighthouse Lab in Glasgow	Wellcome Sanger Institute for the COVID-19 Genomics UK (COG-UK) Consortium	Harper VanSteenhouse, Yumi Kasai, David Gray, Carol Clugston, Anna Dominiczak and Alex Alderton, Roberto Amato, Sonia Goncalves, Ewan Harrison, David K. Jackson, Ian Johnston, Dominic Kwiatkowski, Cordelia Langford, John Sillitoe on behalf of the Wellcome Sanger Institute COVID-19 Surveillance Team
EPI_ISL_851838, EPI_ISL_851839	Lighthouse Lab in Alderley Park	Wellcome Sanger Institute for the COVID-19 Genomics UK (COG-UK) Consortium	Jacquelyn Wynn, Mairead Hyland, The Lighthouse Lab in Alderley Park and Alex Alderton, Roberto Amato, Sonia Goncalves, Ewan Harrison, David K. Jackson, Ian Johnston, Dominic Kwiatkowski, Cordelia Langford, John Sillitoe on behalf of the Wellcome Sanger Institute COVID-19 Surveillance Team
EPI_ISL_851840	Lighthouse Lab in Glasgow	Wellcome Sanger Institute for the COVID-19 Genomics UK (COG-UK) Consortium	Harper VanSteenhouse, Yumi Kasai, David Gray, Carol Clugston, Anna Dominiczak and Alex Alderton, Roberto Amato, Sonia Goncalves, Ewan Harrison, David K. Jackson, Ian Johnston, Dominic Kwiatkowski, Cordelia Langford, John Sillitoe on behalf of the Wellcome Sanger Institute COVID-19 Surveillance Team
EPI_ISL_851841, EPI_ISL_851842, EPI_ISL_851843, EPI_ISL_851844, EPI_ISL_851845	Lighthouse Lab in Alderley Park	Wellcome Sanger Institute for the COVID-19 Genomics UK (COG-UK) Consortium	Jacquelyn Wynn, Mairead Hyland, The Lighthouse Lab in Alderley Park and Alex Alderton, Roberto Amato, Sonia Goncalves, Ewan Harrison, David K. Jackson, Ian Johnston, Dominic Kwiatkowski, Cordelia Langford, John Sillitoe on behalf of the Wellcome Sanger Institute COVID-19 Surveillance Team
EPI_ISL_851846, EPI_ISL_851847	Lighthouse Lab in Glasgow	Wellcome Sanger Institute for the COVID-19 Genomics UK (COG-UK) Consortium	Harper VanSteenhouse, Yumi Kasai, David Gray, Carol Clugston, Anna Dominiczak and Alex Alderton, Roberto Amato, Sonia Goncalves, Ewan Harrison, David K. Jackson, Ian Johnston, Dominic Kwiatkowski, Cordelia Langford, John Sillitoe on behalf of the Wellcome Sanger Institute COVID-19 Surveillance Team
EPI_ISL_851848, EPI_ISL_851849, EPI_ISL_851850	Lighthouse Lab in Alderley Park	Wellcome Sanger Institute for the COVID-19 Genomics UK (COG-UK) Consortium	Jacquelyn Wynn, Mairead Hyland, The Lighthouse Lab in Alderley Park and Alex Alderton, Roberto Amato, Sonia Goncalves, Ewan Harrison, David K. Jackson, Ian Johnston, Dominic Kwiatkowski, Cordelia Langford, John Sillitoe on behalf of the Wellcome Sanger Institute COVID-19 Surveillance Team
EPI_ISL_851851	Lighthouse Lab in Glasgow	Wellcome Sanger Institute for the COVID-19 Genomics UK (COG-UK) Consortium	Harper VanSteenhouse, Yumi Kasai, David Gray, Carol Clugston, Anna Dominiczak and Alex Alderton, Roberto Amato, Sonia Goncalves, Ewan Harrison, David K. Jackson, Ian Johnston, Dominic Kwiatkowski, Cordelia Langford, John Sillitoe on behalf of the Wellcome Sanger Institute COVID-19 Surveillance Team
EPI_ISL_851852, EPI_ISL_851853, EPI_ISL_851854, EPI_ISL_851855	Lighthouse Lab in Alderley Park	Wellcome Sanger Institute for the COVID-19 Genomics UK (COG-UK) Consortium	Jacquelyn Wynn, Mairead Hyland, The Lighthouse Lab in Alderley Park and Alex Alderton, Roberto Amato, Sonia Goncalves, Ewan Harrison, David K. Jackson, Ian Johnston, Dominic Kwiatkowski, Cordelia Langford, John Sillitoe on behalf of the Wellcome Sanger Institute COVID-19 Surveillance Team
EPI_ISL_851856, EPI_ISL_851857	Lighthouse Lab in Glasgow	Wellcome Sanger Institute for the COVID-19 Genomics UK (COG-UK) Consortium	Harper VanSteenhouse, Yumi Kasai, David Gray, Carol Clugston, Anna Dominiczak and Alex Alderton, Roberto Amato, Sonia Goncalves, Ewan Harrison, David K. Jackson, Ian Johnston, Dominic Kwiatkowski, Cordelia Langford, John Sillitoe on behalf of the Wellcome Sanger Institute COVID-19 Surveillance Team
EPI_ISL_851858	Lighthouse Lab in Alderley Park	Wellcome Sanger Institute for the COVID-19 Genomics UK (COG-UK) Consortium	Jacquelyn Wynn, Mairead Hyland, The Lighthouse Lab in Alderley Park and Alex Alderton, Roberto Amato, Sonia Goncalves, Ewan Harrison, David K. Jackson, Ian Johnston, Dominic Kwiatkowski, Cordelia Langford, John Sillitoe on behalf of the Wellcome Sanger Institute COVID-19 Surveillance Team
EPI_ISL_851859	Lighthouse Lab in Glasgow	Wellcome Sanger Institute for the COVID-19 Genomics UK (COG-UK) Consortium	Harper VanSteenhouse, Yumi Kasai, David Gray, Carol Clugston, Anna Dominiczak and Alex Alderton, Roberto Amato, Sonia Goncalves, Ewan Harrison, David K. Jackson, Ian Johnston, Dominic Kwiatkowski, Cordelia Langford, John Sillitoe on behalf of the Wellcome Sanger Institute COVID-19 Surveillance Team
EPI_ISL_851860, EPI_ISL_851861, EPI_ISL_851862	Lighthouse Lab in Alderley Park	Wellcome Sanger Institute for the COVID-19 Genomics UK (COG-UK) Consortium	Jacquelyn Wynn, Mairead Hyland, The Lighthouse Lab in Alderley Park and Alex Alderton, Roberto Amato, Sonia Goncalves, Ewan Harrison, David K. Jackson, Ian Johnston, Dominic Kwiatkowski, Cordelia Langford, John Sillitoe on behalf of the Wellcome Sanger Institute COVID-19 Surveillance Team
EPI_ISL_851863, EPI_ISL_851864, EPI_ISL_851865, EPI_ISL_851866	Lighthouse Lab in Glasgow	Wellcome Sanger Institute for the COVID-19 Genomics UK (COG-UK) Consortium	Harper VanSteenhouse, Yumi Kasai, David Gray, Carol Clugston, Anna Dominiczak and Alex Alderton, Roberto Amato, Sonia Goncalves, Ewan Harrison, David K. Jackson, Ian Johnston, Dominic Kwiatkowski, Cordelia Langford, John Sillitoe on behalf of the Wellcome Sanger Institute COVID-19 Surveillance Team
EPI_ISL_851867, EPI_ISL_851868, EPI_ISL_851869	Lighthouse Lab in Alderley Park	Wellcome Sanger Institute for the COVID-19 Genomics UK (COG-UK) Consortium	Jacquelyn Wynn, Mairead Hyland, The Lighthouse Lab in Alderley Park and Alex Alderton, Roberto Amato, Sonia Goncalves, Ewan Harrison, David K. Jackson, Ian Johnston, Dominic Kwiatkowski, Cordelia Langford, John Sillitoe on behalf of the Wellcome Sanger Institute COVID-19 Surveillance Team
EPI_ISL_851870, EPI_ISL_851871	Lighthouse Lab in Glasgow	Wellcome Sanger Institute for the COVID-19 Genomics UK (COG-UK) Consortium	Harper VanSteenhouse, Yumi Kasai, David Gray, Carol Clugston, Anna Dominiczak and Alex Alderton, Roberto Amato, Sonia Goncalves, Ewan Harrison, David K. Jackson, Ian Johnston, Dominic Kwiatkowski, Cordelia Langford, John Sillitoe on behalf of the Wellcome Sanger Institute COVID-19 Surveillance Team
EPI_ISL_851872, EPI_ISL_851873, EPI_ISL_851874	Lighthouse Lab in Alderley Park	Wellcome Sanger Institute for the COVID-19 Genomics UK (COG-UK) Consortium	Jacquelyn Wynn, Mairead Hyland, The Lighthouse Lab in Alderley Park and Alex Alderton, Roberto Amato, Sonia Goncalves, Ewan Harrison, David K. Jackson, Ian Johnston, Dominic Kwiatkowski, Cordelia Langford, John Sillitoe on behalf of the Wellcome Sanger Institute COVID-19 Surveillance Team
EPI_ISL_851876, EPI_ISL_851877	Lighthouse Lab in Glasgow	Wellcome Sanger Institute for the COVID-19 Genomics UK (COG-UK) Consortium	Harper VanSteenhouse, Yumi Kasai, David Gray, Carol Clugston, Anna Dominiczak and Alex Alderton, Roberto Amato, Sonia Goncalves, Ewan Harrison, David K. Jackson, Ian Johnston, Dominic Kwiatkowski, Cordelia Langford, John Sillitoe on behalf of the Wellcome Sanger Institute COVID-19 Surveillance Team
EPI_ISL_851878, EPI_ISL_851879, EPI_ISL_851880, EPI_ISL_851881, EPI_ISL_851882, EPI_ISL_851883, EPI_ISL_851884	Lighthouse Lab in Alderley Park	Wellcome Sanger Institute for the COVID-19 Genomics UK (COG-UK) Consortium	Jacquelyn Wynn, Mairead Hyland, The Lighthouse Lab in Alderley Park and Alex Alderton, Roberto Amato, Sonia Goncalves, Ewan Harrison, David K. Jackson, Ian Johnston, Dominic Kwiatkowski, Cordelia Langford, John Sillitoe on behalf of the Wellcome Sanger Institute COVID-19 Surveillance Team
EPI_ISL_851885	Lighthouse Lab in Glasgow	Wellcome Sanger Institute for the COVID-19 Genomics UK (COG-UK) Consortium	Harper VanSteenhouse, Yumi Kasai, David Gray, Carol Clugston, Anna Dominiczak and Alex Alderton, Roberto Amato, Sonia Goncalves, Ewan Harrison, David K. Jackson, Ian Johnston, Dominic Kwiatkowski, Cordelia Langford, John Sillitoe on behalf of the Wellcome Sanger Institute COVID-19 Surveillance Team

EPI_ISL_851886, EPI_ISL_851887	Lighthouse Lab in Alderley Park	Wellcome Sanger Institute for the COVID-19 Genomics UK (COG-UK) Consortium	Jacquelyn Wynn, Mairead Hyland, The Lighthouse Lab in Alderley Park and Alex Alderton, Roberto Amato, Sonia Goncalves, Ewan Harrison, David K. Jackson, Ian Johnston, Dominic Kwiatkowski, Cordelia Langford, John Sillitoe on behalf of the Wellcome Sanger Institute COVID-19 Surveillance Team
EPI_ISL_851888	Lighthouse Lab in Glasgow	Wellcome Sanger Institute for the COVID-19 Genomics UK (COG-UK) Consortium	Harper VanSteenhouse, Yumi Kasai, David Gray, Carol Clugston, Anna Dominiczak and Alex Alderton, Roberto Amato, Sonia Goncalves, Ewan Harrison, David K. Jackson, Ian Johnston, Dominic Kwiatkowski, Cordelia Langford, John Sillitoe on behalf of the Wellcome Sanger Institute COVID-19 Surveillance Team
EPI_ISL_851889, EPI_ISL_851890	Lighthouse Lab in Alderley Park	Wellcome Sanger Institute for the COVID-19 Genomics UK (COG-UK) Consortium	Jacquelyn Wynn, Mairead Hyland, The Lighthouse Lab in Alderley Park and Alex Alderton, Roberto Amato, Sonia Goncalves, Ewan Harrison, David K. Jackson, Ian Johnston, Dominic Kwiatkowski, Cordelia Langford, John Sillitoe on behalf of the Wellcome Sanger Institute COVID-19 Surveillance Team
EPI_ISL_851891, EPI_ISL_851892	Lighthouse Lab in Glasgow	Wellcome Sanger Institute for the COVID-19 Genomics UK (COG-UK) Consortium	Harper VanSteenhouse, Yumi Kasai, David Gray, Carol Clugston, Anna Dominiczak and Alex Alderton, Roberto Amato, Sonia Goncalves, Ewan Harrison, David K. Jackson, Ian Johnston, Dominic Kwiatkowski, Cordelia Langford, John Sillitoe on behalf of the Wellcome Sanger Institute COVID-19 Surveillance Team
EPI_ISL_851893, EPI_ISL_851894	Lighthouse Lab in Alderley Park	Wellcome Sanger Institute for the COVID-19 Genomics UK (COG-UK) Consortium	Jacquelyn Wynn, Mairead Hyland, The Lighthouse Lab in Alderley Park and Alex Alderton, Roberto Amato, Sonia Goncalves, Ewan Harrison, David K. Jackson, Ian Johnston, Dominic Kwiatkowski, Cordelia Langford, John Sillitoe on behalf of the Wellcome Sanger Institute COVID-19 Surveillance Team
EPI_ISL_851895	Lighthouse Lab in Glasgow	Wellcome Sanger Institute for the COVID-19 Genomics UK (COG-UK) Consortium	Harper VanSteenhouse, Yumi Kasai, David Gray, Carol Clugston, Anna Dominiczak and Alex Alderton, Roberto Amato, Sonia Goncalves, Ewan Harrison, David K. Jackson, Ian Johnston, Dominic Kwiatkowski, Cordelia Langford, John Sillitoe on behalf of the Wellcome Sanger Institute COVID-19 Surveillance Team
EPI_ISL_851896, EPI_ISL_851897	Lighthouse Lab in Alderley Park	Wellcome Sanger Institute for the COVID-19 Genomics UK (COG-UK) Consortium	Jacquelyn Wynn, Mairead Hyland, The Lighthouse Lab in Alderley Park and Alex Alderton, Roberto Amato, Sonia Goncalves, Ewan Harrison, David K. Jackson, Ian Johnston, Dominic Kwiatkowski, Cordelia Langford, John Sillitoe on behalf of the Wellcome Sanger Institute COVID-19 Surveillance Team
EPI_ISL_851898	Lighthouse Lab in Glasgow	Wellcome Sanger Institute for the COVID-19 Genomics UK (COG-UK) Consortium	Harper VanSteenhouse, Yumi Kasai, David Gray, Carol Clugston, Anna Dominiczak and Alex Alderton, Roberto Amato, Sonia Goncalves, Ewan Harrison, David K. Jackson, Ian Johnston, Dominic Kwiatkowski, Cordelia Langford, John Sillitoe on behalf of the Wellcome Sanger Institute COVID-19 Surveillance Team
EPI_ISL_851899, EPI_ISL_851900	Lighthouse Lab in Alderley Park	Wellcome Sanger Institute for the COVID-19 Genomics UK (COG-UK) Consortium	Jacquelyn Wynn, Mairead Hyland, The Lighthouse Lab in Alderley Park and Alex Alderton, Roberto Amato, Sonia Goncalves, Ewan Harrison, David K. Jackson, Ian Johnston, Dominic Kwiatkowski, Cordelia Langford, John Sillitoe on behalf of the Wellcome Sanger Institute COVID-19 Surveillance Team
EPI_ISL_851901	Lighthouse Lab in Glasgow	Wellcome Sanger Institute for the COVID-19 Genomics UK (COG-UK) Consortium	Harper VanSteenhouse, Yumi Kasai, David Gray, Carol Clugston, Anna Dominiczak and Alex Alderton, Roberto Amato, Sonia Goncalves, Ewan Harrison, David K. Jackson, Ian Johnston, Dominic Kwiatkowski, Cordelia Langford, John Sillitoe on behalf of the Wellcome Sanger Institute COVID-19 Surveillance Team
EPI_ISL_851902, EPI_ISL_851903, EPI_ISL_851904	Lighthouse Lab in Alderley Park	Wellcome Sanger Institute for the COVID-19 Genomics UK (COG-UK) Consortium	Jacquelyn Wynn, Mairead Hyland, The Lighthouse Lab in Alderley Park and Alex Alderton, Roberto Amato, Sonia Goncalves, Ewan Harrison, David K. Jackson, Ian Johnston, Dominic Kwiatkowski, Cordelia Langford, John Sillitoe on behalf of the Wellcome Sanger Institute COVID-19 Surveillance Team
EPI_ISL_851905, EPI_ISL_851906	Lighthouse Lab in Glasgow	Wellcome Sanger Institute for the COVID-19 Genomics UK (COG-UK) Consortium	Harper VanSteenhouse, Yumi Kasai, David Gray, Carol Clugston, Anna Dominiczak and Alex Alderton, Roberto Amato, Sonia Goncalves, Ewan Harrison, David K. Jackson, Ian Johnston, Dominic Kwiatkowski, Cordelia Langford, John Sillitoe on behalf of the Wellcome Sanger Institute COVID-19 Surveillance Team
EPI_ISL_851907, EPI_ISL_851908, EPI_ISL_851909, EPI_ISL_851910, EPI_ISL_851911, EPI_ISL_851912, EPI_ISL_851913, EPI_ISL_851914, EPI_ISL_851915	Lighthouse Lab in Alderley Park	Wellcome Sanger Institute for the COVID-19 Genomics UK (COG-UK) Consortium	Jacquelyn Wynn, Mairead Hyland, The Lighthouse Lab in Alderley Park and Alex Alderton, Roberto Amato, Sonia Goncalves, Ewan Harrison, David K. Jackson, Ian Johnston, Dominic Kwiatkowski, Cordelia Langford, John Sillitoe on behalf of the Wellcome Sanger Institute COVID-19 Surveillance Team
EPI_ISL_851916	Lighthouse Lab in Glasgow	Wellcome Sanger Institute for the COVID-19 Genomics UK (COG-UK) Consortium	Harper VanSteenhouse, Yumi Kasai, David Gray, Carol Clugston, Anna Dominiczak and Alex Alderton, Roberto Amato, Sonia Goncalves, Ewan Harrison, David K. Jackson, Ian Johnston, Dominic Kwiatkowski, Cordelia Langford, John Sillitoe on behalf of the Wellcome Sanger Institute COVID-19 Surveillance Team
EPI_ISL_851917, EPI_ISL_851918, EPI_ISL_851919	Lighthouse Lab in Alderley Park	Wellcome Sanger Institute for the COVID-19 Genomics UK (COG-UK) Consortium	Jacquelyn Wynn, Mairead Hyland, The Lighthouse Lab in Alderley Park and Alex Alderton, Roberto Amato, Sonia Goncalves, Ewan Harrison, David K. Jackson, Ian Johnston, Dominic Kwiatkowski, Cordelia Langford, John Sillitoe on behalf of the Wellcome Sanger Institute COVID-19 Surveillance Team
EPI_ISL_851920	Lighthouse Lab in Glasgow	Wellcome Sanger Institute for the COVID-19 Genomics UK (COG-UK) Consortium	Harper VanSteenhouse, Yumi Kasai, David Gray, Carol Clugston, Anna Dominiczak and Alex Alderton, Roberto Amato, Sonia Goncalves, Ewan Harrison, David K. Jackson, Ian Johnston, Dominic Kwiatkowski, Cordelia Langford, John Sillitoe on behalf of the Wellcome Sanger Institute COVID-19 Surveillance Team
EPI_ISL_851921	Lighthouse Lab in Alderley Park	Wellcome Sanger Institute for the COVID-19 Genomics UK (COG-UK) Consortium	Jacquelyn Wynn, Mairead Hyland, The Lighthouse Lab in Alderley Park and Alex Alderton, Roberto Amato, Sonia Goncalves, Ewan Harrison, David K. Jackson, Ian Johnston, Dominic Kwiatkowski, Cordelia Langford, John Sillitoe on behalf of the Wellcome Sanger Institute COVID-19 Surveillance Team
EPI_ISL_851922	Lighthouse Lab in Glasgow	Wellcome Sanger Institute for the COVID-19 Genomics UK (COG-UK) Consortium	Harper VanSteenhouse, Yumi Kasai, David Gray, Carol Clugston, Anna Dominiczak and Alex Alderton, Roberto Amato, Sonia Goncalves, Ewan Harrison, David K. Jackson, Ian Johnston, Dominic Kwiatkowski, Cordelia Langford, John Sillitoe on behalf of the Wellcome Sanger Institute COVID-19 Surveillance Team
EPI_ISL_851923, EPI_ISL_851924, EPI_ISL_851925, EPI_ISL_851926, EPI_ISL_851927, EPI_ISL_851928	Lighthouse Lab in Alderley Park	Wellcome Sanger Institute for the COVID-19 Genomics UK (COG-UK) Consortium	Jacquelyn Wynn, Mairead Hyland, The Lighthouse Lab in Alderley Park and Alex Alderton, Roberto Amato, Sonia Goncalves, Ewan Harrison, David K. Jackson, Ian Johnston, Dominic Kwiatkowski, Cordelia Langford, John Sillitoe on behalf of the Wellcome Sanger Institute COVID-19 Surveillance Team
EPI_ISL_851929	Lighthouse Lab in Glasgow	Wellcome Sanger Institute for the COVID-19 Genomics UK (COG-UK) Consortium	Harper VanSteenhouse, Yumi Kasai, David Gray, Carol Clugston, Anna Dominiczak and Alex Alderton, Roberto Amato, Sonia Goncalves, Ewan Harrison, David K. Jackson, Ian Johnston, Dominic Kwiatkowski, Cordelia Langford, John Sillitoe on behalf of the Wellcome Sanger Institute COVID-19 Surveillance Team
EPI_ISL_851930, EPI_ISL_851931, EPI_ISL_851932, EPI_ISL_851933	Lighthouse Lab in Alderley Park	Wellcome Sanger Institute for the COVID-19 Genomics UK (COG-UK) Consortium	Jacquelyn Wynn, Mairead Hyland, The Lighthouse Lab in Alderley Park and Alex Alderton, Roberto Amato, Sonia Goncalves, Ewan Harrison, David K. Jackson, Ian Johnston, Dominic Kwiatkowski, Cordelia Langford, John Sillitoe on behalf of the Wellcome Sanger Institute COVID-19 Surveillance Team
EPI_ISL_851934	Lighthouse Lab in Glasgow	Wellcome Sanger Institute for the COVID-19 Genomics UK (COG-UK) Consortium	Harper VanSteenhouse, Yumi Kasai, David Gray, Carol Clugston, Anna Dominiczak and Alex Alderton, Roberto Amato, Sonia Goncalves, Ewan Harrison, David K. Jackson, Ian Johnston, Dominic Kwiatkowski, Cordelia Langford, John Sillitoe on behalf of the Wellcome Sanger Institute COVID-19 Surveillance Team
EPI_ISL_851935	Lighthouse Lab in Alderley Park	Wellcome Sanger Institute for the COVID-19 Genomics UK (COG-UK) Consortium	Jacquelyn Wynn, Mairead Hyland, The Lighthouse Lab in Alderley Park and Alex Alderton, Roberto Amato, Sonia Goncalves, Ewan Harrison, David K. Jackson, Ian Johnston, Dominic Kwiatkowski, Cordelia Langford, John Sillitoe on behalf of the Wellcome Sanger Institute COVID-19 Surveillance Team
EPI_ISL_851936	Lighthouse Lab in Glasgow	Wellcome Sanger Institute for the COVID-19 Genomics UK (COG-UK) Consortium	Harper VanSteenhouse, Yumi Kasai, David Gray, Carol Clugston, Anna Dominiczak and Alex Alderton, Roberto Amato, Sonia Goncalves, Ewan Harrison, David K. Jackson, Ian Johnston, Dominic Kwiatkowski, Cordelia Langford, John Sillitoe on behalf of the Wellcome Sanger Institute COVID-19 Surveillance Team
EPI_ISL_851937, EPI_ISL_851938, EPI_ISL_851939	Lighthouse Lab in Alderley Park	Wellcome Sanger Institute for the COVID-19 Genomics UK (COG-UK) Consortium	Jacquelyn Wynn, Mairead Hyland, The Lighthouse Lab in Alderley Park and Alex Alderton, Roberto Amato, Sonia Goncalves, Ewan Harrison, David K. Jackson, Ian Johnston, Dominic Kwiatkowski, Cordelia Langford, John Sillitoe on behalf of the Wellcome Sanger Institute COVID-19 Surveillance Team
EPI_ISL_851940	Lighthouse Lab in Glasgow	Wellcome Sanger Institute for the COVID-19 Genomics UK (COG-UK) Consortium	Harper VanSteenhouse, Yumi Kasai, David Gray, Carol Clugston, Anna Dominiczak and Alex Alderton, Roberto Amato, Sonia Goncalves, Ewan Harrison, David K. Jackson, Ian Johnston, Dominic Kwiatkowski, Cordelia Langford, John Sillitoe on behalf of the Wellcome Sanger Institute COVID-19 Surveillance Team
EPI_ISL_851941, EPI_ISL_851942, EPI_ISL_851943, EPI_ISL_851944, EPI_ISL_851945, EPI_ISL_851946, EPI_ISL_851947, EPI_ISL_851948, EPI_ISL_851949	Lighthouse Lab in Alderley Park	Wellcome Sanger Institute for the COVID-19 Genomics UK (COG-UK) Consortium	Jacquelyn Wynn, Mairead Hyland, The Lighthouse Lab in Alderley Park and Alex Alderton, Roberto Amato, Sonia Goncalves, Ewan Harrison, David K. Jackson, Ian Johnston, Dominic Kwiatkowski, Cordelia Langford, John Sillitoe on behalf of the Wellcome Sanger Institute COVID-19 Surveillance Team

EPI_ISL_851950	Lighthouse Lab in Glasgow	Wellcome Sanger Institute for the COVID-19 Genomics UK (COG-UK) Consortium	Harper VanSteenhouse, Yumi Kasai, David Gray, Carol Clugston, Anna Dominiczak and Alex Alderton, Roberto Amato, Sonia Goncalves, Ewan Harrison, David K. Jackson, Ian Johnston, Dominic Kwiatkowski, Cordelia Langford, John Sillitoe on behalf of the Wellcome Sanger Institute COVID-19 Surveillance Team
EPI_ISL_851951, EPI_ISL_851952, EPI_ISL_851953	Lighthouse Lab in Alderley Park	Wellcome Sanger Institute for the COVID-19 Genomics UK (COG-UK) Consortium	Jacquelyn Wynn, Mairead Hyland, The Lighthouse Lab in Alderley Park and Alex Alderton, Roberto Amato, Sonia Goncalves, Ewan Harrison, David K. Jackson, Ian Johnston, Dominic Kwiatkowski, Cordelia Langford, John Sillitoe on behalf of the Wellcome Sanger Institute COVID-19 Surveillance Team
EPI_ISL_851955, EPI_ISL_851956	Lighthouse Lab in Glasgow	Wellcome Sanger Institute for the COVID-19 Genomics UK (COG-UK) Consortium	Harper VanSteenhouse, Yumi Kasai, David Gray, Carol Clugston, Anna Dominiczak and Alex Alderton, Roberto Amato, Sonia Goncalves, Ewan Harrison, David K. Jackson, Ian Johnston, Dominic Kwiatkowski, Cordelia Langford, John Sillitoe on behalf of the Wellcome Sanger Institute COVID-19 Surveillance Team
EPI_ISL_851957, EPI_ISL_851958, EPI_ISL_851959, EPI_ISL_851960, EPI_ISL_851961, EPI_ISL_851962, EPI_ISL_851965, EPI_ISL_851966, EPI_ISL_851967, EPI_ISL_851969, EPI_ISL_851970, EPI_ISL_851971, EPI_ISL_851973, EPI_ISL_851975, EPI_ISL_851977, EPI_ISL_851978, EPI_ISL_851980, EPI_ISL_851981, EPI_ISL_851982, EPI_ISL_851983, EPI_ISL_851984, EPI_ISL_851986, EPI_ISL_851987, EPI_ISL_851988, EPI_ISL_851990, EPI_ISL_851991, EPI_ISL_851992, EPI_ISL_851994, EPI_ISL_851995, EPI_ISL_851996, EPI_ISL_851997, EPI_ISL_851998, EPI_ISL_851999, EPI_ISL_852000, EPI_ISL_852001, EPI_ISL_852003, EPI_ISL_852004, EPI_ISL_852005, EPI_ISL_852006, EPI_ISL_852007, EPI_ISL_852008, EPI_ISL_852010, EPI_ISL_852011, EPI_ISL_852012, EPI_ISL_852013, EPI_ISL_852015, EPI_ISL_852016, EPI_ISL_852018, EPI_ISL_852019, EPI_ISL_852020, EPI_ISL_852021, EPI_ISL_852022, EPI_ISL_852023, EPI_ISL_852024, EPI_ISL_852026, EPI_ISL_852027, EPI_ISL_852028, EPI_ISL_852030, EPI_ISL_852031, EPI_ISL_852032, EPI_ISL_852033, EPI_ISL_852034, EPI_ISL_852035, EPI_ISL_852036, EPI_ISL_852037, EPI_ISL_852038, EPI_ISL_852039, EPI_ISL_852040, EPI_ISL_852041, EPI_ISL_852043, EPI_ISL_852044, EPI_ISL_852045, EPI_ISL_852046, EPI_ISL_852048, EPI_ISL_852049, EPI_ISL_852050, EPI_ISL_852051, EPI_ISL_852052, EPI_ISL_852053, EPI_ISL_852054, EPI_ISL_852055, EPI_ISL_852056, EPI_ISL_852057, EPI_ISL_852058, EPI_ISL_852059, EPI_ISL_852060, EPI_ISL_852061, EPI_ISL_852063, EPI_ISL_852064, EPI_ISL_852065, EPI_ISL_852066, EPI_ISL_852067, EPI_ISL_852068, EPI_ISL_852069, EPI_ISL_852070, EPI_ISL_852071, EPI_ISL_852072, EPI_ISL_852073, EPI_ISL_852075, EPI_ISL_852076, EPI_ISL_852078, EPI_ISL_852079, EPI_ISL_852080, EPI_ISL_852081, EPI_ISL_852082, EPI_ISL_852084, EPI_ISL_852085, EPI_ISL_852087, EPI_ISL_852088, EPI_ISL_852089, EPI_ISL_852090			
see above	Lighthouse Lab in Milton Keynes	Wellcome Sanger Institute for the COVID-19 Genomics UK (COG-UK) Consortium	The Lighthouse Lab in Milton Keynes and Alex Alderton, Roberto Amato, Sonia Goncalves, Ewan Harrison, David K. Jackson, Ian Johnston, Dominic Kwiatkowski, Cordelia Langford, John Sillitoe on behalf of the Wellcome Sanger Institute COVID-19 Surveillance Team
EPI_ISL_852091	Lighthouse Lab in Alderley Park	Wellcome Sanger Institute for the COVID-19 Genomics UK (COG-UK) Consortium	Jacquelyn Wynn, Mairead Hyland, The Lighthouse Lab in Alderley Park and Alex Alderton, Roberto Amato, Sonia Goncalves, Ewan Harrison, David K. Jackson, Ian Johnston, Dominic Kwiatkowski, Cordelia Langford, John Sillitoe on behalf of the Wellcome Sanger Institute COVID-19 Surveillance Team
EPI_ISL_852092, EPI_ISL_852094, EPI_ISL_852095, EPI_ISL_852096, EPI_ISL_852097, EPI_ISL_852098, EPI_ISL_852099, EPI_ISL_852101, EPI_ISL_852102, EPI_ISL_852103, EPI_ISL_852104, EPI_ISL_852105, EPI_ISL_852106			
see above	Lighthouse Lab in Milton Keynes	Wellcome Sanger Institute for the COVID-19 Genomics UK (COG-UK) Consortium	The Lighthouse Lab in Milton Keynes and Alex Alderton, Roberto Amato, Sonia Goncalves, Ewan Harrison, David K. Jackson, Ian Johnston, Dominic Kwiatkowski, Cordelia Langford, John Sillitoe on behalf of the Wellcome Sanger Institute COVID-19 Surveillance Team
EPI_ISL_852107	Lighthouse Lab in Alderley Park	Wellcome Sanger Institute for the COVID-19 Genomics UK (COG-UK) Consortium	Jacquelyn Wynn, Mairead Hyland, The Lighthouse Lab in Alderley Park and Alex Alderton, Roberto Amato, Sonia Goncalves, Ewan Harrison, David K. Jackson, Ian Johnston, Dominic Kwiatkowski, Cordelia Langford, John Sillitoe on behalf of the Wellcome Sanger Institute COVID-19 Surveillance Team
EPI_ISL_852108, EPI_ISL_852109, EPI_ISL_852110, EPI_ISL_852111, EPI_ISL_852112, EPI_ISL_852113, EPI_ISL_852115, EPI_ISL_852116, EPI_ISL_852117, EPI_ISL_852119, EPI_ISL_852120, EPI_ISL_852121, EPI_ISL_852122, EPI_ISL_852123, EPI_ISL_852125, EPI_ISL_852126, EPI_ISL_852128, EPI_ISL_852129, EPI_ISL_852130, EPI_ISL_852131, EPI_ISL_852133, EPI_ISL_852134, EPI_ISL_852136, EPI_ISL_852137, EPI_ISL_852138, EPI_ISL_852139, EPI_ISL_852140, EPI_ISL_852141, EPI_ISL_852142, EPI_ISL_852143, EPI_ISL_852144, EPI_ISL_852145, EPI_ISL_852146, EPI_ISL_852147, EPI_ISL_852148, EPI_ISL_852149, EPI_ISL_852150, EPI_ISL_852151, EPI_ISL_852152, EPI_ISL_852153, EPI_ISL_852154, EPI_ISL_852155, EPI_ISL_852156, EPI_ISL_852157, EPI_ISL_852158, EPI_ISL_852159, EPI_ISL_852160, EPI_ISL_852161, EPI_ISL_852162, EPI_ISL_852163, EPI_ISL_852164, EPI_ISL_852166, EPI_ISL_852167, EPI_ISL_852168, EPI_ISL_852169, EPI_ISL_852170, EPI_ISL_852171, EPI_ISL_852172, EPI_ISL_852173, EPI_ISL_852175, EPI_ISL_852176, EPI_ISL_852177, EPI_ISL_852178, EPI_ISL_852179, EPI_ISL_852180, EPI_ISL_852181, EPI_ISL_852182, EPI_ISL_852185, EPI_ISL_852186, EPI_ISL_852187, EPI_ISL_852188, EPI_ISL_852189, EPI_ISL_852192, EPI_ISL_852193, EPI_ISL_852194, EPI_ISL_852195, EPI_ISL_852196, EPI_ISL_852197, EPI_ISL_852198, EPI_ISL_852199, EPI_ISL_852200, EPI_ISL_852201, EPI_ISL_852202, EPI_ISL_852204, EPI_ISL_852205, EPI_ISL_852207, EPI_ISL_852208, EPI_ISL_852209, EPI_ISL_852210, EPI_ISL_852211, EPI_ISL_852212, EPI_ISL_852215, EPI_ISL_852216, EPI_ISL_852217, EPI_ISL_852219, EPI_ISL_852220, EPI_ISL_852221, EPI_ISL_852222, EPI_ISL_852223, EPI_ISL_852224, EPI_ISL_852226, EPI_ISL_852227, EPI_ISL_852228, EPI_ISL_852229, EPI_ISL_852230, EPI_ISL_852231, EPI_ISL_852232, EPI_ISL_852233, EPI_ISL_852235, EPI_ISL_852237, EPI_ISL_852238, EPI_ISL_852239, EPI_ISL_852240, EPI_ISL_852241, EPI_ISL_852242, EPI_ISL_852243, EPI_ISL_852244, EPI_ISL_852245, EPI_ISL_852246, EPI_ISL_852247, EPI_ISL_852248, EPI_ISL_852249, EPI_ISL_852250, EPI_ISL_852251, EPI_ISL_852252, EPI_ISL_852253, EPI_ISL_852254, EPI_ISL_852255			
see above	Lighthouse Lab in Milton Keynes	Wellcome Sanger Institute for the COVID-19 Genomics UK (COG-UK) Consortium	The Lighthouse Lab in Milton Keynes and Alex Alderton, Roberto Amato, Sonia Goncalves, Ewan Harrison, David K. Jackson, Ian Johnston, Dominic Kwiatkowski, Cordelia Langford, John Sillitoe on behalf of the Wellcome Sanger Institute COVID-19 Surveillance Team
EPI_ISL_852268, EPI_ISL_852286, EPI_ISL_852303, EPI_ISL_852319, EPI_ISL_852338, EPI_ISL_852361, EPI_ISL_852375, EPI_ISL_852389, EPI_ISL_852406, EPI_ISL_852407, EPI_ISL_852413, EPI_ISL_852414, EPI_ISL_852416, EPI_ISL_852438, EPI_ISL_852448, EPI_ISL_852459, EPI_ISL_852500, EPI_ISL_852517, EPI_ISL_852524, EPI_ISL_852527, EPI_ISL_852535, EPI_ISL_852536, EPI_ISL_852544, EPI_ISL_852549			
see above	Lighthouse Lab in Glasgow	Wellcome Sanger Institute for the COVID-19 Genomics UK (COG-UK) Consortium	Harper VanSteenhouse, Yumi Kasai, David Gray, Carol Clugston, Anna Dominiczak and Alex Alderton, Roberto Amato, Sonia Goncalves, Ewan Harrison, David K. Jackson, Ian Johnston, Dominic Kwiatkowski, Cordelia Langford, John Sillitoe on behalf of the Wellcome Sanger Institute COVID-19 Surveillance Team
EPI_ISL_852952, EPI_ISL_852955, EPI_ISL_852962, EPI_ISL_852963, EPI_ISL_852992, EPI_ISL_852993, EPI_ISL_852994, EPI_ISL_852995, EPI_ISL_852996, EPI_ISL_852997, EPI_ISL_852999, EPI_ISL_853001, EPI_ISL_853007, EPI_ISL_853008, EPI_ISL_853018, EPI_ISL_853019, EPI_ISL_853020, EPI_ISL_853021, EPI_ISL_853022, EPI_ISL_853023			
see above	Hospital General Universitario Gregorio Marañón	SeqCOVID-SPAIN consortium/IBV(CSIC)	Darío García de Viedma, Laura Pérez-Lago, Pedro J Sola-Campoy, Sergio Buenestado-Serrano, Marta Herranz, Victor Manuel de la Cueva, Julia Suárez, Pilar Catalán, Patricia Muñoz and SeqCOVID-SPAIN consortium
EPI_ISL_853394, EPI_ISL_853395, EPI_ISL_853396	Charité Universitätsmedizin Berlin, Institut für Virologie/Labor Berlin	Charité Universitätsmedizin Berlin, Institut für Virologie	Victor M Corman, Julia Schneider, Barbara Mühlemann, Jörn Beheim-Schwarzbach, Talitha Veith, Julia Tesch, Tobias Bleicker, Terry Jones, Christian Drosten
EPI_ISL_853757, EPI_ISL_853808, EPI_ISL_853811, EPI_ISL_853812, EPI_ISL_853813, EPI_ISL_854187, EPI_ISL_854188, EPI_ISL_854189, EPI_ISL_854190, EPI_ISL_854191, EPI_ISL_854192, EPI_ISL_854193, EPI_ISL_854194, EPI_ISL_854195, EPI_ISL_854196, EPI_ISL_854197, EPI_ISL_854198, EPI_ISL_854199, EPI_ISL_854200, EPI_ISL_854201, EPI_ISL_854202, EPI_ISL_854203, EPI_ISL_854204, EPI_ISL_854205, EPI_ISL_854206, EPI_ISL_854207, EPI_ISL_854208			
see above	Austrian Agency for Health and Food Safety (AGES)	Bergthaler laboratory, CeMM Research Center for Molecular Medicine of the Austrian Academy of Sciences	Lukas Endler, Alexandra Popa, Benedikt Agerer, Jakob-Wendelin Genger, Alexander Lercher, Anna Schedl, Thomas Penz, Michael Schuster, Jan Laine, Martin Senekowitsch, Christoph Bock, Andreas Bergthaler
EPI_ISL_854374, EPI_ISL_854375, EPI_ISL_854376, EPI_ISL_854377, EPI_ISL_854378, EPI_ISL_854379, EPI_ISL_854380, EPI_ISL_854451	WESTCHESTER MEDICAL CENTER	Wadsworth Center, New York State Department of Health	Kirsten St. George, Daryl M. Lamson, Alexis Russel, Matthew Shudt, Melissa A Leisner, Jonathan Plitnick, Navjot Singh, John Kelly, Erasmus Schneider, Erica Lasek-Nesselquist
EPI_ISL_854750	Microbiological Diagnostic Unit - Public Health Laboratory (MDU-PHL)	MDU-PHL	Seemann T., Sait, M.L., Sherry, N.L.
EPI_ISL_854751, EPI_ISL_854752, EPI_ISL_854753	Victorian Infectious Diseases Reference Laboratory (VIDRL)	VIDRL and MDU-PHL	Caly L., Seemann T., Sait, M.L., Druce J., Sherry, N.L.
EPI_ISL_854755, EPI_ISL_854756	Microbiological Diagnostic Unit - Public Health Laboratory (MDU-PHL)	MDU-PHL	Seemann T., Sait, M.L., Sherry, N.L.
EPI_ISL_854757	Victorian Infectious Diseases Reference Laboratory (VIDRL)	VIDRL and MDU-PHL	Caly L., Seemann T., Sait, M.L., Druce J., Sherry, N.L.
EPI_ISL_854811, EPI_ISL_854812, EPI_ISL_854814, EPI_ISL_854815, EPI_ISL_854819, EPI_ISL_854821, EPI_ISL_854822, EPI_ISL_854823, EPI_ISL_854825, EPI_ISL_854826, EPI_ISL_854827, EPI_ISL_854835, EPI_ISL_854836, EPI_ISL_854837, EPI_ISL_854838, EPI_ISL_854839, EPI_ISL_854840, EPI_ISL_854841, EPI_ISL_855014, EPI_ISL_855017, EPI_ISL_855022, EPI_ISL_855035, EPI_ISL_855055, EPI_ISL_855058, EPI_ISL_855070, EPI_ISL_855076, EPI_ISL_855077, EPI_ISL_855078, EPI_ISL_855079, EPI_ISL_855080, EPI_ISL_855154, EPI_ISL_855159, EPI_ISL_855184			
see above	Quest Diagnostics	Quest Diagnostics	Rosenthal,S.H., Gerasimova,A., Kagan,R.M., Anderson, B., Hua, M., Liu Y., Bernstein, L.E., Livingston, K.E., Perez, A., Shalhout, D.F., Shlyakhter, I.A., Owen, R., Tanpaiboon, P., Lacbawan, F.
EPI_ISL_855357	Labo Analyses Med	National Reference Center for Viruses of Respiratory Infections, Institut Pasteur, Paris	Marion Barbet, Sylvie Behillil, Méline Bizard, Angela Brisebarre, Camille Capel, Etienne Simon-Lorière, Vincent Enouf, Maud Vanpeene, Sylvie van der Werf,Harich
EPI_ISL_856678	Charité Universitätsmedizin Berlin, Institute of Virology, Charitéplatz 1, 10117 Berlin, Germany	Charité Universitätsmedizin Berlin, Institute of Virology, Charitéplatz 1, 10117 Berlin, Germany	Victor M Corman, Julia Schneider, Jörn Beheim-Schwarzbach, Tobias Bleicker, Julia Tesch, Barbara Mühlemann, Talitha Veith, Terry Jones, Christian Drosten
EPI_ISL_856722, EPI_ISL_856730, EPI_ISL_856731	Department of Clinical Microbiology	GIGA Medical Genomics	Keith Durkin, Maria Artesi, Sébastien Bontems, Raphaël Boreux, Bouchra Boujemla, Cécile Meex, Pierrette Melin, Marie-Pierre Hayette, Vincent Bours
EPI_ISL_856912, EPI_ISL_856920, EPI_ISL_856921, EPI_ISL_856922, EPI_ISL_856923, EPI_ISL_856924, EPI_ISL_856925, EPI_ISL_856926, EPI_ISL_856927, EPI_ISL_856928, EPI_ISL_856929			
see above	Wyoming Public Health Laboratory	Wyoming Public Health Laboratory	Noah Hull, Taylor Fearing, Lynette Gumbleton, Channing Weber, Ashley Norberg, Bailey Bowcutt, and Wanda Manley
EPI_ISL_857041	Platform BIS UZA/UAntwerpen, University Hospital Antwerp,	UAntwerp, Laboratory of Medical Microbiology, Campus Drie	Basil Britto Xavier, Jasmine Coppens, Christine Lammens, Veerle Matheeussen, Herman Goossens

	Edegem, Belgium	Eiken S6.26, Universiteitsplein 1, 2610, Wilrijk, Antwerp, Belgium	
EPI_ISL_857134	DOHMH PHL	New York City Public Health Laboratory	Jade Wang, et al.
EPI_ISL_857135	DOHMH Corona	New York City Public Health Laboratory	Jade Wang, et al.
EPI_ISL_857136, EPI_ISL_857137, EPI_ISL_857138, EPI_ISL_857139	DOHMH Chelsea	New York City Public Health Laboratory	Jade Wang, et al.
EPI_ISL_857140, EPI_ISL_857141	DOHMH Morrisania	New York City Public Health Laboratory	Jade Wang, et al.
EPI_ISL_857142	DOHMH Central Harlem	New York City Public Health Laboratory	Jade Wang, et al.
EPI_ISL_857143, EPI_ISL_857144	DOHMH Riverside	New York City Public Health Laboratory	Jade Wang, et al.
EPI_ISL_857145, EPI_ISL_857146, EPI_ISL_857147	DOHMH Corona	New York City Public Health Laboratory	Jade Wang, et al.
EPI_ISL_857148	DOHMH Fort Greene	New York City Public Health Laboratory	Jade Wang, et al.
EPI_ISL_857149, EPI_ISL_857150, EPI_ISL_857151, EPI_ISL_857152	DOHMH Jamaica	New York City Public Health Laboratory	Jade Wang, et al.
EPI_ISL_857153	DOHMH Corona	New York City Public Health Laboratory	Jade Wang, et al.
EPI_ISL_857154, EPI_ISL_857155	DOHMH Jamaica	New York City Public Health Laboratory	Jade Wang, et al.
EPI_ISL_857156	DOHMH Corona	New York City Public Health Laboratory	Jade Wang, et al.
EPI_ISL_857157	DOHMH PHL	New York City Public Health Laboratory	Jade Wang, et al.
EPI_ISL_857308	South Eastern Area Laboratory Services (SEALS)	NSW Health Pathology - Institute of Clinical Pathology and Medical Research; Westmead Hospital; University of Sydney	CIDM-PH et al.
EPI_ISL_857390, EPI_ISL_857391, EPI_ISL_857392, EPI_ISL_857393, EPI_ISL_857394	Texas Department of State Health Services (TXDSHS)	Texas Department of State Health Services (TXDSHS)	Anita Pokharel, Bonnie Oh, James Daniel Bonser, Rashmi Tuladhar, Mayela Pedrueza, Jenny Zhang, Maliha Rahman, Myong Koag, Chung Wang, Rachel Lee, Grace Kubin
EPI_ISL_857403, EPI_ISL_857404, EPI_ISL_857405, EPI_ISL_857406, EPI_ISL_857445, EPI_ISL_857448, EPI_ISL_857449, EPI_ISL_857450, EPI_ISL_857451, EPI_ISL_857452, EPI_ISL_857453, EPI_ISL_857455, EPI_ISL_857457, EPI_ISL_857460, EPI_ISL_857461, EPI_ISL_857462, EPI_ISL_857463, EPI_ISL_857464, EPI_ISL_857465			
see above	Maine HETL	Tewhey Lab, The Jackson Laboratory	Matluk,N., Dewey,H., Iosue,F., Barter,M., Lynch,R., Munger,H. and Tewhey,R.
EPI_ISL_857542, EPI_ISL_857544, EPI_ISL_857545, EPI_ISL_857547, EPI_ISL_857549, EPI_ISL_857551, EPI_ISL_857552, EPI_ISL_857555, EPI_ISL_857556, EPI_ISL_857557, EPI_ISL_857558, EPI_ISL_857560, EPI_ISL_857561, EPI_ISL_857563, EPI_ISL_857565, EPI_ISL_857567, EPI_ISL_857568, EPI_ISL_857569, EPI_ISL_857570, EPI_ISL_857573, EPI_ISL_857574, EPI_ISL_857575, EPI_ISL_857576, EPI_ISL_857577, EPI_ISL_857578, EPI_ISL_857579, EPI_ISL_857580, EPI_ISL_857582, EPI_ISL_857583, EPI_ISL_857585, EPI_ISL_857586, EPI_ISL_857588, EPI_ISL_857589, EPI_ISL_857590, EPI_ISL_857591, EPI_ISL_857592, EPI_ISL_857594, EPI_ISL_857597, EPI_ISL_857598, EPI_ISL_857601, EPI_ISL_857608, EPI_ISL_857609, EPI_ISL_857610, EPI_ISL_857612, EPI_ISL_857615, EPI_ISL_857617, EPI_ISL_857620, EPI_ISL_857623, EPI_ISL_857624, EPI_ISL_857627, EPI_ISL_857628, EPI_ISL_857629, EPI_ISL_857630, EPI_ISL_857634, EPI_ISL_857636, EPI_ISL_857637, EPI_ISL_857639, EPI_ISL_857640, EPI_ISL_857643, EPI_ISL_857644, EPI_ISL_857647, EPI_ISL_857648, EPI_ISL_857650, EPI_ISL_857651, EPI_ISL_857652, EPI_ISL_857655, EPI_ISL_857657, EPI_ISL_857658, EPI_ISL_857661, EPI_ISL_857663, EPI_ISL_857664, EPI_ISL_857665, EPI_ISL_857666, EPI_ISL_857667, EPI_ISL_857668, EPI_ISL_857670, EPI_ISL_857671, EPI_ISL_857672, EPI_ISL_857673, EPI_ISL_857674, EPI_ISL_857676, EPI_ISL_857678, EPI_ISL_857681, EPI_ISL_857683, EPI_ISL_857684, EPI_ISL_857686, EPI_ISL_857688, EPI_ISL_857689, EPI_ISL_857690, EPI_ISL_857692, EPI_ISL_857693, EPI_ISL_857694, EPI_ISL_857695, EPI_ISL_857696, EPI_ISL_857697, EPI_ISL_857698, EPI_ISL_857702, EPI_ISL_857703, EPI_ISL_857704, EPI_ISL_857706, EPI_ISL_857707, EPI_ISL_857708, EPI_ISL_857710, EPI_ISL_857711, EPI_ISL_857712, EPI_ISL_857713, EPI_ISL_857714, EPI_ISL_857716, EPI_ISL_857718, EPI_ISL_857721, EPI_ISL_857722, EPI_ISL_857724, EPI_ISL_857725, EPI_ISL_857726, EPI_ISL_857727, EPI_ISL_857729, EPI_ISL_857730, EPI_ISL_857732, EPI_ISL_857735, EPI_ISL_857737, EPI_ISL_857741, EPI_ISL_857742, EPI_ISL_857745, EPI_ISL_857746, EPI_ISL_857747, EPI_ISL_857748, EPI_ISL_857750, EPI_ISL_857753, EPI_ISL_857755, EPI_ISL_857757, EPI_ISL_857758, EPI_ISL_857760, EPI_ISL_857761, EPI_ISL_857762, EPI_ISL_857764, EPI_ISL_857765, EPI_ISL_857766, EPI_ISL_857767, EPI_ISL_857768, EPI_ISL_857770, EPI_ISL_857771, EPI_ISL_857772, EPI_ISL_857774, EPI_ISL_857775, EPI_ISL_857777, EPI_ISL_857779, EPI_ISL_857784, EPI_ISL_857785, EPI_ISL_857788, EPI_ISL_857789, EPI_ISL_857791, EPI_ISL_857793, EPI_ISL_857796, EPI_ISL_857797, EPI_ISL_857800, EPI_ISL_857801, EPI_ISL_857804, EPI_ISL_857805, EPI_ISL_857806, EPI_ISL_857807, EPI_ISL_857808, EPI_ISL_857809, EPI_ISL_857810, EPI_ISL_857812, EPI_ISL_857813, EPI_ISL_857814, EPI_ISL_857815, EPI_ISL_857816, EPI_ISL_857817, EPI_ISL_857822, EPI_ISL_857824, EPI_ISL_857826, EPI_ISL_857827, EPI_ISL_857828, EPI_ISL_857830, EPI_ISL_857831, EPI_ISL_857832, EPI_ISL_857833, EPI_ISL_857834, EPI_ISL_857836, EPI_ISL_857837, EPI_ISL_857838, EPI_ISL_857841, EPI_ISL_857842, EPI_ISL_857843, EPI_ISL_857844, EPI_ISL_857848, EPI_ISL_857849, EPI_ISL_857850, EPI_ISL_857851, EPI_ISL_857854, EPI_ISL_857856, EPI_ISL_857858, EPI_ISL_857859, EPI_ISL_857860, EPI_ISL_857861, EPI_ISL_857862, EPI_ISL_857864, EPI_ISL_857865, EPI_ISL_857867, EPI_ISL_857868, EPI_ISL_857869			
see above	Lighthouse Lab in Cambridge	Wellcome Sanger Institute for the COVID-19 Genomics UK (COG-UK) Consortium	Rob Howes, The Lighthouse Lab in Cambridge and Alex Alderton, Roberto Amato, Sonia Goncalves, Ewan Harrison, David K. Jackson, Ian Johnston, Dominic Kwiatkowski, Cordelia Langford, John Sillitoe on behalf of the Wellcome Sanger Institute COVID-19 Surveillance Team
EPI_ISL_857870, EPI_ISL_857871, EPI_ISL_857872, EPI_ISL_857873	Lighthouse Lab in Glasgow	Wellcome Sanger Institute for the COVID-19 Genomics UK (COG-UK) Consortium	Harper VanSteenhouse, Yumi Kasai, David Gray, Carol Clugston, Anna Dominiczak and Alex Alderton, Roberto Amato, Sonia Goncalves, Ewan Harrison, David K. Jackson, Ian Johnston, Dominic Kwiatkowski, Cordelia Langford, John Sillitoe on behalf of the Wellcome Sanger Institute COVID-19 Surveillance Team
EPI_ISL_857874	Lighthouse Lab in Alderley Park	Wellcome Sanger Institute for the COVID-19 Genomics UK (COG-UK) Consortium	Jacquelyn Wynn, Mairead Hyland, The Lighthouse Lab in Alderley Park and Alex Alderton, Roberto Amato, Sonia Goncalves, Ewan Harrison, David K. Jackson, Ian Johnston, Dominic Kwiatkowski, Cordelia Langford, John Sillitoe on behalf of the Wellcome Sanger Institute COVID-19 Surveillance Team
EPI_ISL_857876	Lighthouse Lab in Glasgow	Wellcome Sanger Institute for the COVID-19 Genomics UK (COG-UK) Consortium	Harper VanSteenhouse, Yumi Kasai, David Gray, Carol Clugston, Anna Dominiczak and Alex Alderton, Roberto Amato, Sonia Goncalves, Ewan Harrison, David K. Jackson, Ian Johnston, Dominic Kwiatkowski, Cordelia Langford, John Sillitoe on behalf of the Wellcome Sanger Institute COVID-19 Surveillance Team
EPI_ISL_857877	Lighthouse Lab in Alderley Park	Wellcome Sanger Institute for the COVID-19 Genomics UK (COG-UK) Consortium	Jacquelyn Wynn, Mairead Hyland, The Lighthouse Lab in Alderley Park and Alex Alderton, Roberto Amato, Sonia Goncalves, Ewan Harrison, David K. Jackson, Ian Johnston, Dominic Kwiatkowski, Cordelia Langford, John Sillitoe on behalf of the Wellcome Sanger Institute COVID-19 Surveillance Team
EPI_ISL_857878, EPI_ISL_857881	Lighthouse Lab in Glasgow	Wellcome Sanger Institute for the COVID-19 Genomics UK (COG-UK) Consortium	Harper VanSteenhouse, Yumi Kasai, David Gray, Carol Clugston, Anna Dominiczak and Alex Alderton, Roberto Amato, Sonia Goncalves, Ewan Harrison, David K. Jackson, Ian Johnston, Dominic Kwiatkowski, Cordelia Langford, John Sillitoe on behalf of the Wellcome Sanger Institute COVID-19 Surveillance Team
EPI_ISL_857883, EPI_ISL_857884	Lighthouse Lab in Alderley Park	Wellcome Sanger Institute for the COVID-19 Genomics UK (COG-UK) Consortium	Jacquelyn Wynn, Mairead Hyland, The Lighthouse Lab in Alderley Park and Alex Alderton, Roberto Amato, Sonia Goncalves, Ewan Harrison, David K. Jackson, Ian Johnston, Dominic Kwiatkowski, Cordelia Langford, John Sillitoe on behalf of the Wellcome Sanger Institute COVID-19 Surveillance Team
EPI_ISL_857885	Lighthouse Lab in Glasgow	Wellcome Sanger Institute for the COVID-19 Genomics UK (COG-UK) Consortium	Harper VanSteenhouse, Yumi Kasai, David Gray, Carol Clugston, Anna Dominiczak and Alex Alderton, Roberto Amato, Sonia Goncalves, Ewan Harrison, David K. Jackson, Ian Johnston, Dominic Kwiatkowski, Cordelia Langford, John Sillitoe on behalf of the Wellcome Sanger Institute COVID-19 Surveillance Team
EPI_ISL_857886	Lighthouse Lab in Alderley Park	Wellcome Sanger Institute for the COVID-19 Genomics UK (COG-UK) Consortium	Jacquelyn Wynn, Mairead Hyland, The Lighthouse Lab in Alderley Park and Alex Alderton, Roberto Amato, Sonia Goncalves, Ewan Harrison, David K. Jackson, Ian Johnston, Dominic Kwiatkowski, Cordelia Langford, John Sillitoe on behalf of the Wellcome Sanger Institute COVID-19 Surveillance Team
EPI_ISL_857887, EPI_ISL_857888, EPI_ISL_857889, EPI_ISL_857894, EPI_ISL_857911, EPI_ISL_857912, EPI_ISL_857913, EPI_ISL_857914, EPI_ISL_857915, EPI_ISL_857916, EPI_ISL_857917, EPI_ISL_857918, EPI_ISL_857919, EPI_ISL_857920, EPI_ISL_857921, EPI_ISL_857922, EPI_ISL_857923, EPI_ISL_857924, EPI_ISL_857925, EPI_ISL_857926, EPI_ISL_857927, EPI_ISL_857928, EPI_ISL_857929, EPI_ISL_857930, EPI_ISL_857931, EPI_ISL_857932, EPI_ISL_857933, EPI_ISL_857934, EPI_ISL_857935, EPI_ISL_857936, EPI_ISL_857937, EPI_ISL_857938, EPI_ISL_857939, EPI_ISL_857940, EPI_ISL_857941, EPI_ISL_857942, EPI_ISL_857943, EPI_ISL_857944, EPI_ISL_857945, EPI_ISL_857946, EPI_ISL_857947, EPI_ISL_857948, EPI_ISL_857949, EPI_ISL_857950, EPI_ISL_857951, EPI_ISL_857952, EPI_ISL_857953, EPI_ISL_857954, EPI_ISL_857955, EPI_ISL_857956, EPI_ISL_857957, EPI_ISL_857958, EPI_ISL_857959			
see above	Lighthouse Lab in Glasgow	Wellcome Sanger Institute for the COVID-19 Genomics UK (COG-UK) Consortium	Harper VanSteenhouse, Yumi Kasai, David Gray, Carol Clugston, Anna Dominiczak and Alex Alderton, Roberto Amato, Sonia Goncalves, Ewan Harrison, David K. Jackson, Ian Johnston, Dominic Kwiatkowski, Cordelia Langford, John Sillitoe on behalf of the Wellcome Sanger Institute COVID-19 Surveillance Team
EPI_ISL_857961, EPI_ISL_857962, EPI_ISL_857963, EPI_ISL_857964, EPI_ISL_857967, EPI_ISL_857968, EPI_ISL_857969, EPI_ISL_857970, EPI_ISL_857971, EPI_ISL_857972, EPI_ISL_857973, EPI_ISL_857974, EPI_ISL_857975, EPI_ISL_857976, EPI_ISL_857977, EPI_ISL_857978, EPI_ISL_857979, EPI_ISL_857980, EPI_ISL_857981, EPI_ISL_857982, EPI_ISL_857983, EPI_ISL_857985, EPI_ISL_857986, EPI_ISL_857987, EPI_ISL_857988, EPI_ISL_857989, EPI_ISL_857990, EPI_ISL_857991, EPI_ISL_857992, EPI_ISL_857993, EPI_ISL_857994, EPI_ISL_857995, EPI_ISL_857996, EPI_ISL_857997, EPI_ISL_857998, EPI_ISL_857999, EPI_ISL_858001, EPI_ISL_858002, EPI_ISL_858003, EPI_ISL_858004, EPI_ISL_858005, EPI_ISL_858006, EPI_ISL_858007, EPI_ISL_858008, EPI_ISL_858009, EPI_ISL_858010			
see above	Lighthouse Lab in Alderley Park	Wellcome Sanger Institute for the COVID-19 Genomics UK (COG-UK) Consortium	Jacquelyn Wynn, Mairead Hyland, The Lighthouse Lab in Alderley Park and Alex Alderton, Roberto Amato, Sonia Goncalves, Ewan Harrison, David K. Jackson, Ian Johnston, Dominic Kwiatkowski, Cordelia Langford, John Sillitoe on behalf of the Wellcome Sanger Institute COVID-19 Surveillance Team
EPI_ISL_858011, EPI_ISL_858022,	Lighthouse Lab in Glasgow	Wellcome Sanger Institute for the COVID-19 Genomics UK	Harper VanSteenhouse, Yumi Kasai, David Gray, Carol Clugston, Anna Dominiczak and Alex Alderton, Roberto Amato, Sonia Goncalves, Ewan Harrison,

EPI_ISL_858025, EPI_ISL_858035, EPI_ISL_858039, EPI_ISL_858040, EPI_ISL_858043, EPI_ISL_858054		(COG-UK) Consortium	David K. Jackson, Ian Johnston, Dominic Kwiatkowski, Cordelia Langford, John Sillitoe on behalf of the Wellcome Sanger Institute COVID-19 Surveillance Team
EPI_ISL_860260	Akershus University Hospital, Department for Microbiology and Infectious Disease Control	Norwegian Institute of Public Health, Department of Virology	Kathrine Stene-Johansen, Kamilla Heddeland Instefjord, Hilde Elshaug, Atiya R Ali,Marie Paulsen Madsen, Rasmus Riis Kopperud, Hilde Vollan, Karoline Bragstad, Olav Hungnes
EPI_ISL_860263	Oslo University Hospital, Department of Medical Microbiology	Norwegian Institute of Public Health, Department of Virology	Kathrine Stene-Johansen, Kamilla Heddeland Instefjord, Hilde Elshaug, Atiya R Ali,Marie Paulsen Madsen, Rasmus Riis Kopperud, Hilde Vollan, Karoline Bragstad, Olav Hungnes
EPI_ISL_860268	Vestfold Hospital, Toensberg Department of Microbiology	Norwegian Institute of Public Health, Department of Virology	Kathrine Stene-Johansen, Kamilla Heddeland Instefjord, Hilde Elshaug, Atiya R Ali,Marie Paulsen Madsen, Rasmus Riis Kopperud, Hilde Vollan, Karoline Bragstad, Olav Hungnes
EPI_ISL_860269	Akershus University Hospital, Department for Microbiology and Infectious Disease Control	Norwegian Institute of Public Health, Department of Virology	Kathrine Stene-Johansen, Kamilla Heddeland Instefjord, Hilde Elshaug, Atiya R Ali,Marie Paulsen Madsen, Rasmus Riis Kopperud, Hilde Vollan, Karoline Bragstad, Olav Hungnes
EPI_ISL_860281	Furst Medical Laboratory	Norwegian Institute of Public Health, Department of Virology	Kathrine Stene-Johansen, Kamilla Heddeland Instefjord, Hilde Elshaug, Atiya R Ali,Marie Paulsen Madsen, Rasmus Riis Kopperud, Hilde Vollan, Karoline Bragstad, Olav Hungnes
EPI_ISL_860688	Respiratory Virus Unit, National Infection Service, Public Health England	COVID-19 Genomics UK (COG-UK) Consortium	PHE Covid Sequencing Team
EPI_ISL_860716	Cerballiance Côte d'Azur	CERBA LAB	Roquebert B; Prots.L; Barrieu-Moussat S;Haïm-Boukobza S.
EPI_ISL_860724, EPI_ISL_860725	Institute for Infectious Diseases, University of Bern, Switzerland	Institute for Infectious Diseases, University of Bern, Switzerland	Michel C Koch, Christian Baumann, Miguel A Terrazos Miani, Cora Sägesser, Pascal Bittel, Stephen L Leib, Peter Keller, Franziska Suter-Riniker, Alban Ramette
EPI_ISL_860787	Charité Universitätsmedizin Berlin, Institute of Virology, Charitéplatz 1, 10117 Berlin, Germany	Charité Universitätsmedizin Berlin, Institute of Virology, Charitéplatz 1, 10117 Berlin, Germany	Victor M Corman, Julia Schneider, Jörn Beheim-Schwarzbach, Tobias Bleicker, Julia Tesch, Barbara Mühlemann, Talitha Veith, Terry Jones, Christian Drosten
EPI_ISL_860820, EPI_ISL_860823, EPI_ISL_860825, EPI_ISL_860827	CHU Tours - Virologie	National Reference Center for Viruses of Respiratory Infections, Institut Pasteur, Paris	Marion Barbet, Sylvie Behillil, Méline Bizard, Angela Brisebarre, Camille Capel, Etienne Simon-Lorière, Vincent Enouf, Maud Vanpeene, Sylvie van der Werf,Gaudy Graffin Catherine
EPI_ISL_860829, EPI_ISL_860830	Labo Analyses Med	National Reference Center for Viruses of Respiratory Infections, Institut Pasteur, Paris	Marion Barbet, Sylvie Behillil, Méline Bizard, Angela Brisebarre, Camille Capel, Etienne Simon-Lorière, Vincent Enouf, Maud Vanpeene, Sylvie van der Werf,Amzalag Jonas
EPI_ISL_860832	CHU Tours - Virologie	National Reference Center for Viruses of Respiratory Infections, Institut Pasteur, Paris	Marion Barbet, Sylvie Behillil, Méline Bizard, Angela Brisebarre, Camille Capel, Etienne Simon-Lorière, Vincent Enouf, Maud Vanpeene, Sylvie van der Werf,Gaudy Graffin Catherine
EPI_ISL_860834, EPI_ISL_860835, EPI_ISL_860837, EPI_ISL_860839	Labo Analyses Med	National Reference Center for Viruses of Respiratory Infections, Institut Pasteur, Paris	Marion Barbet, Sylvie Behillil, Méline Bizard, Angela Brisebarre, Camille Capel, Etienne Simon-Lorière, Vincent Enouf, Maud Vanpeene, Sylvie van der Werf,Amzalag Jonas
EPI_ISL_860845	Labo Analyses med	National Reference Center for Viruses of Respiratory Infections, Institut Pasteur, Paris	Marion Barbet, Sylvie Behillil, Méline Bizard, Angela Brisebarre, Camille Capel, Etienne Simon-Lorière, Vincent Enouf, Maud Vanpeene, Sylvie van der Werf,Amzalag Jonas
EPI_ISL_860847	Centre Hospitalier Compiègne-Noyon - Laboratoire	National Reference Center for Viruses of Respiratory Infections, Institut Pasteur, Paris	Marion Barbet, Sylvie Behillil, Méline Bizard, Angela Brisebarre, Camille Capel, Etienne Simon-Lorière, Vincent Enouf, Maud Vanpeene, Sylvie van der Werf,Foissaud Vincent
EPI_ISL_860848	CH Dunkerque Laboratoire de Biologie	National Reference Center for Viruses of Respiratory Infections, Institut Pasteur, Paris	Marion Barbet, Sylvie Behillil, Méline Bizard, Angela Brisebarre, Camille Capel, Etienne Simon-Lorière, Vincent Enouf, Maud Vanpeene, Sylvie van der Werf,Joly Isabelle
EPI_ISL_860852, EPI_ISL_860853, EPI_ISL_860854, EPI_ISL_860855, EPI_ISL_860857, EPI_ISL_860858, EPI_ISL_860859	Labo Analyses Med	National Reference Center for Viruses of Respiratory Infections, Institut Pasteur, Paris	Marion Barbet, Sylvie Behillil, Méline Bizard, Angela Brisebarre, Camille Capel, Etienne Simon-Lorière, Vincent Enouf, Maud Vanpeene, Sylvie van der Werf,Amzalag Jonas
EPI_ISL_860890	CHU de Nantes - Hôtel Dieu - Labo. Virologie	National Reference Center for Viruses of Respiratory Infections, Institut Pasteur, Paris	Marion Barbet, Sylvie Behillil, Méline Bizard, Angela Brisebarre, Camille Capel, Etienne Simon-Lorière, Vincent Enouf, Maud Vanpeene, Sylvie van der Werf,Andre-Garnier Elisabeth
EPI_ISL_860892, EPI_ISL_860895, EPI_ISL_860897	CHU Tours - Virologie	National Reference Center for Viruses of Respiratory Infections, Institut Pasteur, Paris	Marion Barbet, Sylvie Behillil, Méline Bizard, Angela Brisebarre, Camille Capel, Etienne Simon-Lorière, Vincent Enouf, Maud Vanpeene, Sylvie van der Werf,Gaudy Graffin Catherine
EPI_ISL_860900, EPI_ISL_860901, EPI_ISL_860902, EPI_ISL_860903, EPI_ISL_860904	CH du MANS - Lab. Bio. Moléculaire	National Reference Center for Viruses of Respiratory Infections, Institut Pasteur, Paris	Marion Barbet, Sylvie Behillil, Méline Bizard, Angela Brisebarre, Camille Capel, Etienne Simon-Lorière, Vincent Enouf, Maud Vanpeene, Sylvie van der Werf,Ramanantsoa CéLine
EPI_ISL_860906	CHU Angers - Dpt des Agents Infectieux	National Reference Center for Viruses of Respiratory Infections, Institut Pasteur, Paris	Marion Barbet, Sylvie Behillil, Méline Bizard, Angela Brisebarre, Camille Capel, Etienne Simon-Lorière, Vincent Enouf, Maud Vanpeene, Sylvie van der Werf,Ducancelle Alexandra
EPI_ISL_860908, EPI_ISL_860909	CHU Tours - Virologie	National Reference Center for Viruses of Respiratory Infections, Institut Pasteur, Paris	Marion Barbet, Sylvie Behillil, Méline Bizard, Angela Brisebarre, Camille Capel, Etienne Simon-Lorière, Vincent Enouf, Maud Vanpeene, Sylvie van der Werf,Gaudy Graffin Catherine
EPI_ISL_860910	CH du MANS - Lab. Bio. Moléculaire	National Reference Center for Viruses of Respiratory Infections, Institut Pasteur, Paris	Marion Barbet, Sylvie Behillil, Méline Bizard, Angela Brisebarre, Camille Capel, Etienne Simon-Lorière, Vincent Enouf, Maud Vanpeene, Sylvie van der Werf,Ramanantsoa CéLine
EPI_ISL_860914, EPI_ISL_860915, EPI_ISL_860916, EPI_ISL_860917, EPI_ISL_860922, EPI_ISL_860923, EPI_ISL_860924, EPI_ISL_860925	CHU de Nantes - Hôtel Dieu - Labo. Virologie	National Reference Center for Viruses of Respiratory Infections, Institut Pasteur, Paris	Marion Barbet, Sylvie Behillil, Méline Bizard, Angela Brisebarre, Camille Capel, Etienne Simon-Lorière, Vincent Enouf, Maud Vanpeene, Sylvie van der Werf,Andre-Garnier Elisabeth
EPI_ISL_860931	Labo Analyses Med	National Reference Center for Viruses of Respiratory Infections, Institut Pasteur, Paris	Marion Barbet, Sylvie Behillil, Méline Bizard, Angela Brisebarre, Camille Capel, Etienne Simon-Lorière, Vincent Enouf, Maud Vanpeene, Sylvie van der Werf
EPI_ISL_860956, EPI_ISL_860970, EPI_ISL_860972, EPI_ISL_860973, EPI_ISL_860974, EPI_ISL_860978, EPI_ISL_860980, EPI_ISL_860982, EPI_ISL_860985, EPI_ISL_860994, EPI_ISL_860997, EPI_ISL_861000, EPI_ISL_861017, EPI_ISL_861019, EPI_ISL_861020, EPI_ISL_861022, EPI_ISL_861024, EPI_ISL_861029, EPI_ISL_861032, EPI_ISL_861033, EPI_ISL_861039, EPI_ISL_861040, EPI_ISL_861045, EPI_ISL_861051, EPI_ISL_861058, EPI_ISL_861064, EPI_ISL_861065, EPI_ISL_861078, EPI_ISL_861081, EPI_ISL_861083, EPI_ISL_861086, EPI_ISL_861088, EPI_ISL_861100			
see above	Johns Hopkins Hospital Department of Pathology	Johns Hopkins Hospital Department of Pathology	C. Paul Morris, Chun Huai Luo, Adannaya Amadi, Nicholas Gallagher, Heba H. Mostafa
EPI_ISL_861205	URMC LABS	Wadsworth Center, New York State Department of Health	Kirsten St. George, Daryl M. Lamson, Alexis Russel, Matthew Shudt, Melissa A Leisner, Jonathan Plitnick, Navjot Singh, John Kelly, Erasmus Schneider, Erica Lasek-Nesselquist
EPI_ISL_861234, EPI_ISL_861237	Labo Analyses Med	National Reference Center for Viruses of Respiratory Infections, Institut Pasteur, Paris	Marion Barbet, Sylvie Behillil, Méline Bizard, Angela Brisebarre, Camille Capel, Etienne Simon-Lorière, Vincent Enouf, Maud Vanpeene, Sylvie van der Werf
EPI_ISL_861238	CHU Tours - Virologie	National Reference Center for Viruses of Respiratory Infections, Institut Pasteur, Paris	Marion Barbet, Sylvie Behillil, Méline Bizard, Angela Brisebarre, Camille Capel, Etienne Simon-Lorière, Vincent Enouf, Maud Vanpeene, Sylvie van der Werf,Gaudy Graffin Catherine
EPI_ISL_861334, EPI_ISL_861335, EPI_ISL_861336, EPI_ISL_861338, EPI_ISL_861339, EPI_ISL_861341, EPI_ISL_861342, EPI_ISL_861343, EPI_ISL_861344, EPI_ISL_861345, EPI_ISL_861346, EPI_ISL_861347, EPI_ISL_861349, EPI_ISL_861350, EPI_ISL_861351, EPI_ISL_861352, EPI_ISL_861353, EPI_ISL_861354, EPI_ISL_861357, EPI_ISL_861358, EPI_ISL_861359			
see above	WESTCHESTER MEDICAL CENTER	Wadsworth Center, New York State Department of Health	Kirsten St. George, Daryl M. Lamson, Alexis Russel, Matthew Shudt, Melissa A Leisner, Jonathan Plitnick, Navjot Singh, John Kelly, Erasmus Schneider, Erica Lasek-Nesselquist
EPI_ISL_861422, EPI_ISL_861423,	Oklahoma Animal Disease Diagnostic Laboratory	Oklahoma Animal Disease Diagnostic Laboratory	Sai Narayanan, Girish Patil, Sunil More, Jeremiah Saliki, Akhilesh Ramachandran

EPI_ISL_861424, EPI_ISL_861425, EPI_ISL_861426			
EPI_ISL_861445	Clinical Molecular Microbiology Laboratory, UNC Hospitals	Jeremy Wang	Jeremy Wang, Alexander Rubinsteyn, Colleen Rice, Jason Smedberg, Melissa Miller, Corbin Jones, Robert Hagan
EPI_ISL_861474, EPI_ISL_861475	Gundersen Molecular Diagnostics Laboratory	Kabara Cancer Research Institute	Craig S. Richmond, Paraic A. Kenny
EPI_ISL_861480	Wyoming Public Health Laboratory	Wyoming Public Health Laboratory	Noah Hull, Taylor Fearing, Lynette Gumbleton, Channing Weber, Ashley Norberg, Bailey Bowcutt, and Wanda Manley
EPI_ISL_861504	Cerballiance Provence	CERBA LAB	Delaunay E; Roig JC; Prots L.
EPI_ISL_861529, EPI_ISL_861553, EPI_ISL_861554, EPI_ISL_861555, EPI_ISL_861560, EPI_ISL_861561, EPI_ISL_861562, EPI_ISL_861563, EPI_ISL_861564, EPI_ISL_861576, EPI_ISL_861579, EPI_ISL_861624			
see above	Instituto Nacional de Saude (INSA)	Instituto Nacional de Saude (INSA)	Borges et al
EPI_ISL_861716, EPI_ISL_861728	CERBA	CERBA LAB	Roquebert B; Costa JM; Hedbaut E; Trombert S; Lecorche E; Verdurme L; Malek Ramdane; Olivi M; Haïm-Boukobza S.
EPI_ISL_861732, EPI_ISL_861733, EPI_ISL_861748, EPI_ISL_861755	Tempus	Grubaugh Lab - Yale School of Public Health	Tara Alpert, Joseph Fauver, Anderson Brito, Mallery Breban, Anne Wyllie, Chantal Vogels, Mary Petrone, Annie Watkins, Chaney Kalinich, Isabel Ott, Nathan Grubaugh
EPI_ISL_861758	Murphy Medical Associates	Grubaugh Lab - Yale School of Public Health	Tara Alpert, Joseph Fauver, Anderson Brito, Mallery Breban, Anne Wyllie, Chantal Vogels, Mary Petrone, Annie Watkins, Chaney Kalinich, Isabel Ott, Nathan Grubaugh
EPI_ISL_861761, EPI_ISL_861766	Tempus	Grubaugh Lab - Yale School of Public Health	Tara Alpert, Joseph Fauver, Anderson Brito, Mallery Breban, Anne Wyllie, Chantal Vogels, Mary Petrone, Annie Watkins, Chaney Kalinich, Isabel Ott, Nathan Grubaugh
EPI_ISL_862042	CERBA	CERBA LAB	Roquebert B; Costa JM; Hedbaut E; Trombert S; Lecorche E; Verdurme L; Malek Ramdane, Olivi M; Haïm-Boukobza S.
EPI_ISL_862126, EPI_ISL_862143, EPI_ISL_862149, EPI_ISL_862175, EPI_ISL_862186	Charité Universitätsmedizin Berlin, Institut fur Virologie/Labor Berlin	Charité Universitätsmedizin Berlin, Institut für Virologie	Victor M Corman, Barbara Mühlemann, Jörn Beheim-Schwarzbach, Tobias Bleicker, Julia Tesch, Talitha Veith, Julia Schneider, Terry Jones, Christian Drosten
EPI_ISL_862669, EPI_ISL_862710, EPI_ISL_862711, EPI_ISL_862712	New Mexico Department of Health Scientific Laboratory	New Mexico Department of Health Scientific Laboratory	D'eldra Malone, Ellie Johnson, Anastacia Griego-Fisher
EPI_ISL_862722	San Diego County Public Health Laboratory	Andersen lab at Scripps Research	SEARCH Alliance San Diego with Tracy Basler, Jovan Shephard, Brett Austin
EPI_ISL_862729, EPI_ISL_862732, EPI_ISL_862734	Utah Public Health Laboratory, Utah Public Health Laboratory Infectious Disease submission group	Utah Public Health Laboratory, Utah Public Health Laboratory Infectious Disease submission group	Young,E.L., Oakeson,K.F., Gallagher,T.
EPI_ISL_862886, EPI_ISL_862887, EPI_ISL_862888, EPI_ISL_862900, EPI_ISL_862915, EPI_ISL_862938, EPI_ISL_862948, EPI_ISL_862983, EPI_ISL_862990, EPI_ISL_863013, EPI_ISL_863023, EPI_ISL_863046, EPI_ISL_863058, EPI_ISL_863061, EPI_ISL_863074, EPI_ISL_863084, EPI_ISL_863089, EPI_ISL_863091, EPI_ISL_863101, EPI_ISL_863103, EPI_ISL_863106, EPI_ISL_863112, EPI_ISL_863122, EPI_ISL_863126, EPI_ISL_863136, EPI_ISL_863137, EPI_ISL_863138, EPI_ISL_863140, EPI_ISL_863151, EPI_ISL_863156, EPI_ISL_863162, EPI_ISL_863179, EPI_ISL_863180, EPI_ISL_863188, EPI_ISL_863192, EPI_ISL_863193, EPI_ISL_863210, EPI_ISL_863218, EPI_ISL_863238			
see above	Lighthouse Lab in Alderley Park	Wellcome Sanger Institute for the COVID-19 Genomics UK (COG-UK) Consortium	Jacquelyn Wynn, Mairead Hyland, The Lighthouse Lab in Alderley Park and Alex Alderton, Roberto Amato, Sonia Goncalves, Ewan Harrison, David K. Jackson, Ian Johnston, Dominic Kwiatkowski, Cordelia Langford, John Sillitoe on behalf of the Wellcome Sanger Institute COVID-19 Surveillance Team
EPI_ISL_863537, EPI_ISL_863543, EPI_ISL_863547, EPI_ISL_863553, EPI_ISL_863561, EPI_ISL_863562, EPI_ISL_863567, EPI_ISL_863577, EPI_ISL_863580, EPI_ISL_863587, EPI_ISL_863588, EPI_ISL_863589, EPI_ISL_863599, EPI_ISL_863601, EPI_ISL_863605, EPI_ISL_863606, EPI_ISL_863622, EPI_ISL_863623, EPI_ISL_863628, EPI_ISL_863633, EPI_ISL_863647, EPI_ISL_863649, EPI_ISL_863650, EPI_ISL_863655, EPI_ISL_863663, EPI_ISL_863666, EPI_ISL_863670, EPI_ISL_863683, EPI_ISL_863684, EPI_ISL_863686, EPI_ISL_863693, EPI_ISL_863703, EPI_ISL_863714, EPI_ISL_863716, EPI_ISL_863717, EPI_ISL_863718, EPI_ISL_863719, EPI_ISL_863724, EPI_ISL_863732, EPI_ISL_863735, EPI_ISL_863737, EPI_ISL_863739, EPI_ISL_863745, EPI_ISL_863753, EPI_ISL_863768, EPI_ISL_863775, EPI_ISL_863783, EPI_ISL_863805, EPI_ISL_863807, EPI_ISL_863808, EPI_ISL_863814, EPI_ISL_863815, EPI_ISL_863817, EPI_ISL_863825, EPI_ISL_863829, EPI_ISL_863833, EPI_ISL_863834, EPI_ISL_863835, EPI_ISL_863855, EPI_ISL_863857, EPI_ISL_863859, EPI_ISL_863867, EPI_ISL_863899, EPI_ISL_863901, EPI_ISL_863912, EPI_ISL_863913, EPI_ISL_863921, EPI_ISL_863922, EPI_ISL_863956, EPI_ISL_863969, EPI_ISL_863996, EPI_ISL_864005, EPI_ISL_864009, EPI_ISL_864026, EPI_ISL_864036, EPI_ISL_864043, EPI_ISL_864098			
see above	Lighthouse Lab in Milton Keynes	Wellcome Sanger Institute for the COVID-19 Genomics UK (COG-UK) Consortium	The Lighthouse Lab in Milton Keynes and Alex Alderton, Roberto Amato, Sonia Goncalves, Ewan Harrison, David K. Jackson, Ian Johnston, Dominic Kwiatkowski, Cordelia Langford, John Sillitoe on behalf of the Wellcome Sanger Institute COVID-19 Surveillance Team
EPI_ISL_864110	Lighthouse Lab in Alderley Park	Wellcome Sanger Institute for the COVID-19 Genomics UK (COG-UK) Consortium	Jacquelyn Wynn, Mairead Hyland, The Lighthouse Lab in Alderley Park and Alex Alderton, Roberto Amato, Sonia Goncalves, Ewan Harrison, David K. Jackson, Ian Johnston, Dominic Kwiatkowski, Cordelia Langford, John Sillitoe on behalf of the Wellcome Sanger Institute COVID-19 Surveillance Team
EPI_ISL_864124, EPI_ISL_864133, EPI_ISL_864165, EPI_ISL_864169, EPI_ISL_864173, EPI_ISL_864175, EPI_ISL_864176, EPI_ISL_864191, EPI_ISL_864193, EPI_ISL_864203, EPI_ISL_864205, EPI_ISL_864206, EPI_ISL_864207, EPI_ISL_864208, EPI_ISL_864209, EPI_ISL_864210, EPI_ISL_864212, EPI_ISL_864214, EPI_ISL_864215, EPI_ISL_864218, EPI_ISL_864219, EPI_ISL_864220, EPI_ISL_864222, EPI_ISL_864223, EPI_ISL_864224, EPI_ISL_864225			
see above	Lighthouse Lab in Milton Keynes	Wellcome Sanger Institute for the COVID-19 Genomics UK (COG-UK) Consortium	The Lighthouse Lab in Milton Keynes and Alex Alderton, Roberto Amato, Sonia Goncalves, Ewan Harrison, David K. Jackson, Ian Johnston, Dominic Kwiatkowski, Cordelia Langford, John Sillitoe on behalf of the Wellcome Sanger Institute COVID-19 Surveillance Team
EPI_ISL_864226	Lighthouse Lab in Alderley Park	Wellcome Sanger Institute for the COVID-19 Genomics UK (COG-UK) Consortium	Jacquelyn Wynn, Mairead Hyland, The Lighthouse Lab in Alderley Park and Alex Alderton, Roberto Amato, Sonia Goncalves, Ewan Harrison, David K. Jackson, Ian Johnston, Dominic Kwiatkowski, Cordelia Langford, John Sillitoe on behalf of the Wellcome Sanger Institute COVID-19 Surveillance Team
EPI_ISL_864229, EPI_ISL_864231, EPI_ISL_864232, EPI_ISL_864233, EPI_ISL_864234, EPI_ISL_864235, EPI_ISL_864236, EPI_ISL_864237, EPI_ISL_864238, EPI_ISL_864240, EPI_ISL_864241, EPI_ISL_864242, EPI_ISL_864243, EPI_ISL_864245, EPI_ISL_864246, EPI_ISL_864247, EPI_ISL_864248, EPI_ISL_864249, EPI_ISL_864250, EPI_ISL_864251, EPI_ISL_864252, EPI_ISL_864253, EPI_ISL_864254, EPI_ISL_864256, EPI_ISL_864257, EPI_ISL_864258, EPI_ISL_864259, EPI_ISL_864260, EPI_ISL_864261, EPI_ISL_864263, EPI_ISL_864264, EPI_ISL_864265			
see above	Lighthouse Lab in Milton Keynes	Wellcome Sanger Institute for the COVID-19 Genomics UK (COG-UK) Consortium	The Lighthouse Lab in Milton Keynes and Alex Alderton, Roberto Amato, Sonia Goncalves, Ewan Harrison, David K. Jackson, Ian Johnston, Dominic Kwiatkowski, Cordelia Langford, John Sillitoe on behalf of the Wellcome Sanger Institute COVID-19 Surveillance Team
EPI_ISL_864267	Lighthouse Lab in Alderley Park	Wellcome Sanger Institute for the COVID-19 Genomics UK (COG-UK) Consortium	Jacquelyn Wynn, Mairead Hyland, The Lighthouse Lab in Alderley Park and Alex Alderton, Roberto Amato, Sonia Goncalves, Ewan Harrison, David K. Jackson, Ian Johnston, Dominic Kwiatkowski, Cordelia Langford, John Sillitoe on behalf of the Wellcome Sanger Institute COVID-19 Surveillance Team
EPI_ISL_864268, EPI_ISL_864269, EPI_ISL_864270, EPI_ISL_864271, EPI_ISL_864272, EPI_ISL_864273, EPI_ISL_864275, EPI_ISL_864277, EPI_ISL_864278, EPI_ISL_864279, EPI_ISL_864280, EPI_ISL_864282, EPI_ISL_864283, EPI_ISL_864284, EPI_ISL_864285, EPI_ISL_864286, EPI_ISL_864287, EPI_ISL_864289, EPI_ISL_864290, EPI_ISL_864292, EPI_ISL_864293, EPI_ISL_864294, EPI_ISL_864295, EPI_ISL_864296, EPI_ISL_864298, EPI_ISL_864299, EPI_ISL_864300, EPI_ISL_864302, EPI_ISL_864303, EPI_ISL_864304, EPI_ISL_864306, EPI_ISL_864307			
see above	Lighthouse Lab in Milton Keynes	Wellcome Sanger Institute for the COVID-19 Genomics UK (COG-UK) Consortium	The Lighthouse Lab in Milton Keynes and Alex Alderton, Roberto Amato, Sonia Goncalves, Ewan Harrison, David K. Jackson, Ian Johnston, Dominic Kwiatkowski, Cordelia Langford, John Sillitoe on behalf of the Wellcome Sanger Institute COVID-19 Surveillance Team
EPI_ISL_864308	Lighthouse Lab in Alderley Park	Wellcome Sanger Institute for the COVID-19 Genomics UK (COG-UK) Consortium	Jacquelyn Wynn, Mairead Hyland, The Lighthouse Lab in Alderley Park and Alex Alderton, Roberto Amato, Sonia Goncalves, Ewan Harrison, David K. Jackson, Ian Johnston, Dominic Kwiatkowski, Cordelia Langford, John Sillitoe on behalf of the Wellcome Sanger Institute COVID-19 Surveillance Team
EPI_ISL_864310, EPI_ISL_864312, EPI_ISL_864314, EPI_ISL_864315, EPI_ISL_864316, EPI_ISL_864318, EPI_ISL_864319, EPI_ISL_864320, EPI_ISL_864322, EPI_ISL_864323, EPI_ISL_864325, EPI_ISL_864326, EPI_ISL_864327, EPI_ISL_864329, EPI_ISL_864330, EPI_ISL_864331, EPI_ISL_864332, EPI_ISL_864333, EPI_ISL_864334, EPI_ISL_864335, EPI_ISL_864337, EPI_ISL_864338, EPI_ISL_864339, EPI_ISL_864340, EPI_ISL_864341, EPI_ISL_864342, EPI_ISL_864343, EPI_ISL_864344, EPI_ISL_864345, EPI_ISL_864346, EPI_ISL_864347, EPI_ISL_864348, EPI_ISL_864349, EPI_ISL_864350, EPI_ISL_864351, EPI_ISL_864353, EPI_ISL_864355, EPI_ISL_864357, EPI_ISL_864358, EPI_ISL_864359, EPI_ISL_864360, EPI_ISL_864362, EPI_ISL_864363, EPI_ISL_864364, EPI_ISL_864366, EPI_ISL_864367, EPI_ISL_864369, EPI_ISL_864371, EPI_ISL_864372, EPI_ISL_864373, EPI_ISL_864374, EPI_ISL_864375, EPI_ISL_864376, EPI_ISL_864377, EPI_ISL_864378, EPI_ISL_864379, EPI_ISL_864380, EPI_ISL_864381, EPI_ISL_864382, EPI_ISL_864385, EPI_ISL_864386, EPI_ISL_864387, EPI_ISL_864388, EPI_ISL_864389, EPI_ISL_864391, EPI_ISL_864392, EPI_ISL_864393, EPI_ISL_864394, EPI_ISL_864395, EPI_ISL_864397, EPI_ISL_864398, EPI_ISL_864401, EPI_ISL_864404, EPI_ISL_864405, EPI_ISL_864406, EPI_ISL_864407, EPI_ISL_864408, EPI_ISL_864410, EPI_ISL_864412, EPI_ISL_864413, EPI_ISL_864415, EPI_ISL_864416, EPI_ISL_864417, EPI_ISL_864418, EPI_ISL_864419, EPI_ISL_864420, EPI_ISL_864421, EPI_ISL_864423, EPI_ISL_864424, EPI_ISL_864425, EPI_ISL_864426, EPI_ISL_864427, EPI_ISL_864428, EPI_ISL_864429, EPI_ISL_864430, EPI_ISL_864431, EPI_ISL_864432, EPI_ISL_864433, EPI_ISL_864434, EPI_ISL_864435, EPI_ISL_864437, EPI_ISL_864438, EPI_ISL_864441, EPI_ISL_864442, EPI_ISL_864444, EPI_ISL_864446, EPI_ISL_864448, EPI_ISL_864449, EPI_ISL_864450, EPI_ISL_864451, EPI_ISL_864453, EPI_ISL_864454, EPI_ISL_864455, EPI_ISL_864456, EPI_ISL_864457, EPI_ISL_864458, EPI_ISL_864459, EPI_ISL_864460, EPI_ISL_864462, EPI_ISL_864463, EPI_ISL_864464, EPI_ISL_864466, EPI_ISL_864468, EPI_ISL_864470, EPI_ISL_864471, EPI_ISL_864473, EPI_ISL_864474, EPI_ISL_864476, EPI_ISL_864477, EPI_ISL_864479, EPI_ISL_864480, EPI_ISL_864481, EPI_ISL_864482, EPI_ISL_864486, EPI_ISL_864488, EPI_ISL_864489, EPI_ISL_864490, EPI_ISL_864491, EPI_ISL_864492, EPI_ISL_864494, EPI_ISL_864495, EPI_ISL_864496, EPI_ISL_864497, EPI_ISL_864498, EPI_ISL_864499, EPI_ISL_864500, EPI_ISL_864502, EPI_ISL_864503, EPI_ISL_864504, EPI_ISL_864505, EPI_ISL_864506, EPI_ISL_864507, EPI_ISL_864508, EPI_ISL_864509, EPI_ISL_864510, EPI_ISL_864511, EPI_ISL_864512, EPI_ISL_864514, EPI_ISL_864517, EPI_ISL_864518, EPI_ISL_864519, EPI_ISL_864521, EPI_ISL_864522, EPI_ISL_864523, EPI_ISL_864524, EPI_ISL_864525, EPI_ISL_864526, EPI_ISL_864530, EPI_ISL_864531, EPI_ISL_864532, EPI_ISL_864533, EPI_ISL_864535, EPI_ISL_864536, EPI_ISL_864537, EPI_ISL_864538, EPI_ISL_864541			
see above	Lighthouse Lab in Milton Keynes	Wellcome Sanger Institute for the COVID-19 Genomics UK (COG-UK) Consortium	The Lighthouse Lab in Milton Keynes and Alex Alderton, Roberto Amato, Sonia Goncalves, Ewan Harrison, David K. Jackson, Ian Johnston, Dominic Kwiatkowski, Cordelia Langford, John Sillitoe on behalf of the Wellcome Sanger Institute COVID-19 Surveillance Team
EPI_ISL_864594, EPI_ISL_864596, EPI_ISL_864597, EPI_ISL_864600, EPI_ISL_864601, EPI_ISL_864602, EPI_ISL_864603, EPI_ISL_864604, EPI_ISL_864605, EPI_ISL_864606, EPI_ISL_864613, EPI_ISL_864614, EPI_ISL_864615, EPI_ISL_864621, EPI_ISL_864622, EPI_ISL_864623, EPI_ISL_864626, EPI_ISL_864627, EPI_ISL_864631, EPI_ISL_864632, EPI_ISL_864633, EPI_ISL_864636, EPI_ISL_864637, EPI_ISL_864638, EPI_ISL_864639, EPI_ISL_864640, EPI_ISL_864641, EPI_ISL_864642, EPI_ISL_864643, EPI_ISL_864644, EPI_ISL_864645, EPI_ISL_864646, EPI_ISL_864647, EPI_ISL_864648, EPI_ISL_864649, EPI_ISL_864650,			

EPI_ISL_864651, EPI_ISL_864652, EPI_ISL_864653, EPI_ISL_864654, EPI_ISL_864655, EPI_ISL_864656, EPI_ISL_864657, EPI_ISL_864658, EPI_ISL_864659, EPI_ISL_864660, EPI_ISL_864661, EPI_ISL_864662, EPI_ISL_864663, EPI_ISL_864664, EPI_ISL_864665, EPI_ISL_864666, EPI_ISL_864667, EPI_ISL_864668, EPI_ISL_864669, EPI_ISL_864670, EPI_ISL_864671, EPI_ISL_864672, EPI_ISL_864673, EPI_ISL_864674, EPI_ISL_864675, EPI_ISL_864676, EPI_ISL_864677, EPI_ISL_864678, EPI_ISL_864679, EPI_ISL_864680, EPI_ISL_864681, EPI_ISL_864682, EPI_ISL_864683, EPI_ISL_864684, EPI_ISL_864685, EPI_ISL_864686, EPI_ISL_864687, EPI_ISL_864688, EPI_ISL_864717, EPI_ISL_864718, EPI_ISL_864721, EPI_ISL_864722, EPI_ISL_864723, EPI_ISL_864724, EPI_ISL_864735, EPI_ISL_864736			
see above	University Hospitals of Geneva, Laboratory of Virology	HUG, Laboratory of Virology and the Health2030 Genome Center	Samuel Cordey, Ana Rita Goncalves, Laurent Kaiser, Lorenzo Cerutti, Henri Pegeot, Melyssa Elies, Deborah Penet, Keith Harshman, Ioannis Xenarios, Emmanouil Dermitzakis
EPI_ISL_865019, EPI_ISL_865021, EPI_ISL_865022, EPI_ISL_865023, EPI_ISL_865024, EPI_ISL_865025, EPI_ISL_865026, EPI_ISL_865027, EPI_ISL_865028, EPI_ISL_865029, EPI_ISL_865030, EPI_ISL_865031, EPI_ISL_865032, EPI_ISL_865033, EPI_ISL_865034, EPI_ISL_865037, EPI_ISL_865038, EPI_ISL_865039, EPI_ISL_865040, EPI_ISL_865053, EPI_ISL_865056, EPI_ISL_865057, EPI_ISL_865058, EPI_ISL_865059, EPI_ISL_865060, EPI_ISL_865061, EPI_ISL_865062, EPI_ISL_865063, EPI_ISL_865064, EPI_ISL_865065			
see above	West of Scotland Specialist Virology Centre, NHSGGC / MRC-University of Glasgow Centre for Virus Research	COVID-19 Genomics UK (COG-UK) Consortium	Ana da Silva Filipe, Natasha Johnson, Kathy Smollett, Daniel Mair, Stephen Carmichael, Alice Broos, Lily Tong, Jenna Nichols, Kyriaki Nomikou; Sarah McDonald; Richard Orton, Joseph Hughes, Sreenu Vattipally, David L Robertson; Alasdair MacLean, Rory Gunson; Sharif Shaaban, Matthew Holden; Rachel Blacow, Guy Mollett, Kathy Li, James Shepherd, Antonia Ho, Emma Thomson
EPI_ISL_865111, EPI_ISL_865112, EPI_ISL_865113, EPI_ISL_865114, EPI_ISL_865116, EPI_ISL_865118, EPI_ISL_865119, EPI_ISL_865120, EPI_ISL_865121, EPI_ISL_865122, EPI_ISL_865123, EPI_ISL_865124, EPI_ISL_865125, EPI_ISL_865126, EPI_ISL_865127, EPI_ISL_865128, EPI_ISL_865129, EPI_ISL_865130, EPI_ISL_865132, EPI_ISL_865133, EPI_ISL_865134, EPI_ISL_865135, EPI_ISL_865145			
see above	Virology Department, Royal Infirmary of Edinburgh, NHS Lothian / School of Biological Sciences, University of Edinburgh / Institute of Genetics and Molecular Medicine, University of Edinburgh	COVID-19 Genomics UK (COG-UK) Consortium	McHugh M, Dewar R, Rooke S, Gallagher M, Balcaza C, O'Toole Á, Scher E, Hill V, McCrone JT, Colquhoun R, Yu X, Jackson B, Rambaut A, Williams TC, Templeton K
EPI_ISL_865272, EPI_ISL_865273, EPI_ISL_865274, EPI_ISL_865275, EPI_ISL_865276, EPI_ISL_865277, EPI_ISL_865280, EPI_ISL_865281, EPI_ISL_865282, EPI_ISL_865284, EPI_ISL_865285, EPI_ISL_865286, EPI_ISL_865287, EPI_ISL_865288, EPI_ISL_865289, EPI_ISL_865290, EPI_ISL_865291, EPI_ISL_865292, EPI_ISL_865293, EPI_ISL_865294, EPI_ISL_865295, EPI_ISL_865296, EPI_ISL_865297, EPI_ISL_865298, EPI_ISL_865300, EPI_ISL_865301, EPI_ISL_865302, EPI_ISL_865303, EPI_ISL_865304, EPI_ISL_865305, EPI_ISL_865306, EPI_ISL_865307, EPI_ISL_865308, EPI_ISL_865309, EPI_ISL_865310, EPI_ISL_865311, EPI_ISL_865312, EPI_ISL_865313, EPI_ISL_865314, EPI_ISL_865315, EPI_ISL_865317, EPI_ISL_865318, EPI_ISL_865319, EPI_ISL_865320, EPI_ISL_865321, EPI_ISL_865326, EPI_ISL_865327, EPI_ISL_865328, EPI_ISL_865341, EPI_ISL_865342, EPI_ISL_865346, EPI_ISL_865347, EPI_ISL_865350			
see above	Liverpool Clinical Laboratories	COVID-19 Genomics UK (COG-UK) Consortium	Sam Haldenby, Anita Lucaci, Steve Paterson, Julian Hiscox, Alistair Darby, M Almsaud, A Alrezaihi, Muhannad Alruwaili, Stuart D Armstrong, Jones Benjamin, Eleanor G Bentley, Anu Chawla, Jordan J Clark, Angela Cowell, Richard Eccles, Isabel García-Dorival, Matthew Gemmell, Alessandro Gerada, PKF Gilmore, Richard Gregory, Ximeng Han, Catherine Hartley, Margaret Hughes, Miren Iturriza-Gomara, James Johnson, L Luu, Jenifer Manson, Charlotte Nelson, Elaine O'Toole, Cassie Olateju, Rebekah Penrice-Randal , Lucille Rainbow, N.P Randle, Trevor Ian Robinson, Parul Sharma, Ghada T Shawli, James P Stewart, Neil Swainston, Ecaterina Vamos, Joanne Watts, Mark Whitehead
EPI_ISL_866082, EPI_ISL_866083, EPI_ISL_866085, EPI_ISL_866086, EPI_ISL_866087, EPI_ISL_866088, EPI_ISL_866089, EPI_ISL_866090, EPI_ISL_866091, EPI_ISL_866092, EPI_ISL_866093, EPI_ISL_866094, EPI_ISL_866095, EPI_ISL_866096, EPI_ISL_866097, EPI_ISL_866098, EPI_ISL_866099, EPI_ISL_866100, EPI_ISL_866101, EPI_ISL_866102, EPI_ISL_866112, EPI_ISL_866113, EPI_ISL_866114			
see above	University College London Hospital	COVID-19 Genomics UK (COG-UK) Consortium	Judith Heaney, Matthew Byott, Catherine Houlihan, Dan Frampton, Stuart Kirk, Moira Spyer and Eleni Nastouli
EPI_ISL_866194, EPI_ISL_866196, EPI_ISL_866202, EPI_ISL_866206, EPI_ISL_866207, EPI_ISL_866208, EPI_ISL_866209, EPI_ISL_866210, EPI_ISL_866211, EPI_ISL_866212, EPI_ISL_866213, EPI_ISL_866222, EPI_ISL_866223, EPI_ISL_866225, EPI_ISL_866228, EPI_ISL_866229, EPI_ISL_866230, EPI_ISL_866232, EPI_ISL_866235, EPI_ISL_866236, EPI_ISL_866237, EPI_ISL_866238, EPI_ISL_866239, EPI_ISL_866240, EPI_ISL_866241, EPI_ISL_866242, EPI_ISL_866243, EPI_ISL_866244, EPI_ISL_866247, EPI_ISL_866250, EPI_ISL_866253, EPI_ISL_866255, EPI_ISL_866256, EPI_ISL_866257, EPI_ISL_866258, EPI_ISL_866259, EPI_ISL_866260, EPI_ISL_866261			
see above	University College London, Great Ormond Street Hospital for Children NHS Foundation Trust, Imperial College Healthcare NHS Trust	COVID-19 Genomics UK (COG-UK) Consortium	Sergi Castellano, Rachel Williams, Mark Kristiansen, Paola Resende Silva, Sunando Roy, Tony Brooks, Helena Tutill, Paola Niola, Patricia Dyal, Charlotte Williams, Leysa Forrest, Yasmin Panchbhaya, Jacqueline Findlay, Samuel Weeks, Julianne Brown, Kathryn Harris, Paul Randell, James Price, Alison Holmes, Judith Breuer
EPI_ISL_866393, EPI_ISL_866394	Regional Virus Laboratory, Belfast Health and Social Care Trust	COVID-19 Genomics UK (COG-UK) Consortium	Conall McCaughey, James McKenna, Tanya Curran, Susan Feeney, Alison Watt, Ciara Cox, Mairead Connor, Zoltan Molnar, David Simpson, Derek Fairley
EPI_ISL_866545, EPI_ISL_866573, EPI_ISL_866618, EPI_ISL_866621, EPI_ISL_866622, EPI_ISL_866627, EPI_ISL_866628, EPI_ISL_866629, EPI_ISL_866631, EPI_ISL_866635, EPI_ISL_866642, EPI_ISL_866643, EPI_ISL_866651, EPI_ISL_866652, EPI_ISL_866653, EPI_ISL_866654, EPI_ISL_866655, EPI_ISL_866656, EPI_ISL_866657, EPI_ISL_866658, EPI_ISL_866659, EPI_ISL_866660, EPI_ISL_866661, EPI_ISL_866663, EPI_ISL_866664, EPI_ISL_866665, EPI_ISL_866666, EPI_ISL_866667, EPI_ISL_866668, EPI_ISL_866669, EPI_ISL_866670, EPI_ISL_866671, EPI_ISL_866672, EPI_ISL_866673, EPI_ISL_866674, EPI_ISL_866677, EPI_ISL_866679, EPI_ISL_866681, EPI_ISL_866682, EPI_ISL_866683, EPI_ISL_866684, EPI_ISL_866686, EPI_ISL_866688, EPI_ISL_866689, EPI_ISL_866691, EPI_ISL_866692, EPI_ISL_866695, EPI_ISL_866696, EPI_ISL_866697, EPI_ISL_866698, EPI_ISL_866699, EPI_ISL_866700, EPI_ISL_866701, EPI_ISL_866702, EPI_ISL_866703, EPI_ISL_866704, EPI_ISL_866705, EPI_ISL_866706, EPI_ISL_866707, EPI_ISL_866708, EPI_ISL_866709, EPI_ISL_866710, EPI_ISL_866711, EPI_ISL_866712, EPI_ISL_866713, EPI_ISL_866714, EPI_ISL_866715, EPI_ISL_866716, EPI_ISL_866717, EPI_ISL_866718, EPI_ISL_866719, EPI_ISL_866720, EPI_ISL_866721, EPI_ISL_866722, EPI_ISL_866723, EPI_ISL_866724, EPI_ISL_866725, EPI_ISL_866726, EPI_ISL_866727, EPI_ISL_866728, EPI_ISL_866729, EPI_ISL_866730, EPI_ISL_866731, EPI_ISL_866732, EPI_ISL_866733, EPI_ISL_866734, EPI_ISL_866735, EPI_ISL_866736, EPI_ISL_866737, EPI_ISL_866738, EPI_ISL_866740, EPI_ISL_866741, EPI_ISL_866742, EPI_ISL_866744, EPI_ISL_866745, EPI_ISL_866746, EPI_ISL_866747, EPI_ISL_866831, EPI_ISL_866832, EPI_ISL_866833, EPI_ISL_866834			
see above	Quadram Institute Bioscience	COVID-19 Genomics UK (COG-UK) Consortium	Dave J. Baker, Gemma L. Kay, Alp Aydin, Thanh Le-Viet, Steven Rudder, Ana P. Tedim, Anastasia Kolyva, Maria Diaz, Leonardo de Oliveira Martins, Nabil-Fareed Alikhan, Lizzie Meadows, Rachael Stanley, Ngozi Elumogo, Muhammed Yasir, Nicholas M. Thomson, Alexander J Trotter, Rachel Gilroy, Samuel Bloomfield, Claire Stuart, Andrew Bell, Reenesh Prakash, Samir Dervisevic, Alison E. Mather, John Wain, Mark Webber, Andrew J. Page, Justin O'Grady
EPI_ISL_866909, EPI_ISL_866910, EPI_ISL_866911, EPI_ISL_866912, EPI_ISL_866913, EPI_ISL_866914, EPI_ISL_866915, EPI_ISL_866916, EPI_ISL_866917, EPI_ISL_866918, EPI_ISL_866919, EPI_ISL_866920			
see above	Queens Medical Centre, Clinical Microbiology Department / DeepSeq Nottingham	COVID-19 Genomics UK (COG-UK) Consortium	Gemma Clark, Wendy Smith, Manjinder Khakh, Vicki M Fleming, Michelle M Lister, Hannah Howson-Wells, Jonathan Ball, Patrick McClure, Joseph Chappell, Theocharis Tsoleridis, Nadine Holmes, Matthew Carlisle, Christopher Moore, Fei Sang, Johnny Debebe, Victoria Wright, Matthew Loose
EPI_ISL_867190, EPI_ISL_867191, EPI_ISL_867196, EPI_ISL_867197, EPI_ISL_867212, EPI_ISL_867213, EPI_ISL_867214, EPI_ISL_867221, EPI_ISL_867222, EPI_ISL_867223, EPI_ISL_867224, EPI_ISL_867225, EPI_ISL_867226, EPI_ISL_867237, EPI_ISL_867238, EPI_ISL_867239, EPI_ISL_867240, EPI_ISL_867241, EPI_ISL_867242, EPI_ISL_867243, EPI_ISL_867244, EPI_ISL_867245, EPI_ISL_867246, EPI_ISL_867247, EPI_ISL_867248, EPI_ISL_867249, EPI_ISL_867250, EPI_ISL_867251, EPI_ISL_867270, EPI_ISL_867305, EPI_ISL_867306, EPI_ISL_867307, EPI_ISL_867308, EPI_ISL_867309, EPI_ISL_867310, EPI_ISL_867311, EPI_ISL_867312, EPI_ISL_867317, EPI_ISL_867318, EPI_ISL_867319, EPI_ISL_867320, EPI_ISL_867321, EPI_ISL_867322, EPI_ISL_867323, EPI_ISL_867324, EPI_ISL_867325, EPI_ISL_867326, EPI_ISL_867327, EPI_ISL_867328, EPI_ISL_867329, EPI_ISL_867330, EPI_ISL_867331, EPI_ISL_867332, EPI_ISL_867333, EPI_ISL_867334, EPI_ISL_867335, EPI_ISL_867336, EPI_ISL_867337, EPI_ISL_867338, EPI_ISL_867339, EPI_ISL_867340, EPI_ISL_867341, EPI_ISL_867342, EPI_ISL_867343, EPI_ISL_867344, EPI_ISL_867345, EPI_ISL_867346, EPI_ISL_867348, EPI_ISL_867349, EPI_ISL_867350, EPI_ISL_867351, EPI_ISL_867352, EPI_ISL_867354, EPI_ISL_867355, EPI_ISL_867357, EPI_ISL_867359, EPI_ISL_867361, EPI_ISL_867368, EPI_ISL_867371, EPI_ISL_867417, EPI_ISL_867429, EPI_ISL_867457, EPI_ISL_867458, EPI_ISL_867497, EPI_ISL_867498, EPI_ISL_867506, EPI_ISL_867509, EPI_ISL_867510, EPI_ISL_867518, EPI_ISL_867519, EPI_ISL_867520, EPI_ISL_867521, EPI_ISL_867522, EPI_ISL_867523, EPI_ISL_867542, EPI_ISL_867543, EPI_ISL_867547, EPI_ISL_867550, EPI_ISL_867552, EPI_ISL_867553, EPI_ISL_867708, EPI_ISL_867717, EPI_ISL_867718, EPI_ISL_867719, EPI_ISL_867720, EPI_ISL_867721, EPI_ISL_867722, EPI_ISL_867723, EPI_ISL_867724, EPI_ISL_867725, EPI_ISL_867730, EPI_ISL_867731, EPI_ISL_867795, EPI_ISL_867796, EPI_ISL_867797, EPI_ISL_867800, EPI_ISL_867801, EPI_ISL_867802, EPI_ISL_867804, EPI_ISL_867805, EPI_ISL_867806, EPI_ISL_867807, EPI_ISL_867808, EPI_ISL_867809, EPI_ISL_867810, EPI_ISL_867811, EPI_ISL_867812, EPI_ISL_867815, EPI_ISL_867816, EPI_ISL_867821, EPI_ISL_867823, EPI_ISL_867824, EPI_ISL_867825, EPI_ISL_867828, EPI_ISL_867830, EPI_ISL_867832, EPI_ISL_867833, EPI_ISL_867837, EPI_ISL_867838, EPI_ISL_867839, EPI_ISL_867841, EPI_ISL_867842, EPI_ISL_867843, EPI_ISL_867844, EPI_ISL_867845, EPI_ISL_867847, EPI_ISL_867848, EPI_ISL_867849, EPI_ISL_867850, EPI_ISL_867851, EPI_ISL_867853, EPI_ISL_867855, EPI_ISL_867856, EPI_ISL_867859, EPI_ISL_867860, EPI_ISL_867861, EPI_ISL_867862, EPI_ISL_867863, EPI_ISL_867868, EPI_ISL_867873, EPI_ISL_867875, EPI_ISL_867876, EPI_ISL_867878, EPI_ISL_867879, EPI_ISL_867880, EPI_ISL_867881, EPI_ISL_867883, EPI_ISL_867884, EPI_ISL_867885, EPI_ISL_867889, EPI_ISL_867893, EPI_ISL_867895			
see above	Originating lab: Wales Specialist Virology Centre Sequencing lab: Pathogen Genomics Unit	Public Health Wales Microbiology Cardiff Wales Specialist Virology Centre	Catherine Moore, Johnathan Evans, Laura Gifford, Malorie Perry, Simon Cottrell, Angela Marchbank, Alec Birchley, Alexander Adams, Amy Gaskin, Bree Gatica-Wilcox, Jason Coombes, Joel Southgate, Lauren Gilbert, Lee Graham, Nicole Pacchiarini, Sara Kumziene-Summerhayes, Sarah Taylor, Sophie Jones, Sara Rey, Matthew Bull, Joanne Watkins, Sally Corden, Tom Connor
EPI_ISL_868142, EPI_ISL_868143, EPI_ISL_868144, EPI_ISL_868145, EPI_ISL_868146, EPI_ISL_868147, EPI_ISL_868161, EPI_ISL_868162, EPI_ISL_868163, EPI_ISL_868164, EPI_ISL_868165, EPI_ISL_868166, EPI_ISL_868167, EPI_ISL_868168, EPI_ISL_868169, EPI_ISL_868170, EPI_ISL_868171, EPI_ISL_868172, EPI_ISL_868173, EPI_ISL_868174, EPI_ISL_868181, EPI_ISL_868182, EPI_ISL_868183, EPI_ISL_868184, EPI_ISL_868185, EPI_ISL_868186, EPI_ISL_868187, EPI_ISL_868188, EPI_ISL_868189, EPI_ISL_868190, EPI_ISL_868191, EPI_ISL_868192, EPI_ISL_868193, EPI_ISL_868194, EPI_ISL_868196, EPI_ISL_868197, EPI_ISL_868198, EPI_ISL_868199, EPI_ISL_868200, EPI_ISL_868201, EPI_ISL_868217, EPI_ISL_868218, EPI_ISL_868332, EPI_ISL_868333, EPI_ISL_868334, EPI_ISL_868335, EPI_ISL_868336, EPI_ISL_868337, EPI_ISL_868338, EPI_ISL_868354, EPI_ISL_868355			
see above	Centre for Enzyme Innovation, University of Portsmouth / Translational Research Laboratory, Portsmouth Hospitals NHS Trust	COVID-19 Genomics UK (COG-UK) Consortium	Angela Beckett,Yann Bourgeois,Garry Scarlett,Sharon Glaysher,Scott Elliott,Kelly Bicknell,Robert Impey,Allyson Lloyd,Sarah Wyllie,Ethan Butcher,Anoop Chauhan,Samuel Robson
EPI_ISL_868373, EPI_ISL_868381, EPI_ISL_868382, EPI_ISL_868388, EPI_ISL_868395, EPI_ISL_868401, EPI_ISL_868408, EPI_ISL_868409, EPI_ISL_868415, EPI_ISL_868442, EPI_ISL_868464, EPI_ISL_868465, EPI_ISL_868469, EPI_ISL_868480, EPI_ISL_868486, EPI_ISL_868495, EPI_ISL_868508, EPI_ISL_868513, EPI_ISL_868521, EPI_ISL_868524, EPI_ISL_868533, EPI_ISL_868541, EPI_ISL_868560, EPI_ISL_868564, EPI_ISL_868566, EPI_ISL_868588, EPI_ISL_868594, EPI_ISL_868597, EPI_ISL_868599, EPI_ISL_868638, EPI_ISL_868643, EPI_ISL_868651, EPI_ISL_868655, EPI_ISL_868674, EPI_ISL_868681, EPI_ISL_868688, EPI_ISL_868692, EPI_ISL_868701			
see above	Virology Department, Sheffield Teaching Hospitals NHS Foundation Trust/Department of Infection, Immunity and Cardiovascular Disease, The Medical School, University of Sheffield	COVID-19 Genomics UK (COG-UK) Consortium	Thushan de Silva, Matthew Parker, Nikki Smith, Adri Angyal, Rebecca Brown, Luke Green, Rachel Tucker, Paul Parsons, Danielle Groves, Katie Johnson, Laura Carrilero, Alex Keeley, Dave Partridge, Matthew Wyles, Benjamin Lindsey, Mehmet Yavuz, Mohammad Raza, Cariad Evans
EPI_ISL_869169, EPI_ISL_869172, EPI_ISL_869173, EPI_ISL_869177, EPI_ISL_869178, EPI_ISL_869179, EPI_ISL_869217, EPI_ISL_869218, EPI_ISL_869219, EPI_ISL_869220, EPI_ISL_869221, EPI_ISL_869222, EPI_ISL_869223, EPI_ISL_869224, EPI_ISL_869226, EPI_ISL_869228, EPI_ISL_869229, EPI_ISL_869230, EPI_ISL_869231, EPI_ISL_869232, EPI_ISL_869233, EPI_ISL_869234, EPI_ISL_869235, EPI_ISL_869236, EPI_ISL_869237, EPI_ISL_869238, EPI_ISL_869239			

see above	New Mexico Department of Health Scientific Laboratory	Center for Global Health, University of New Mexico Health Sciences Center	Daryl Domman, Kurt Schwalm, Twila Kunde, Joseph Hicks, Anastacia Griego, Michael Edwards, Darrell Dinwiddie
EPI_ISL_871792	Cerballiance Normandie	CERBA LAB	LAFOREST D, BRIAND G, LEPIGEON, technicien V; COUDRAY J; MARTINS-AMARAL F; COULON C, THOMAS Johan, VITEL R, HAMEL A.
EPI_ISL_871821, EPI_ISL_871822, EPI_ISL_871823, EPI_ISL_871824, EPI_ISL_871825, EPI_ISL_871826	Jessa	Jessa	Brigitte Maes, Bert Cruys
EPI_ISL_871840, EPI_ISL_871847, EPI_ISL_871869, EPI_ISL_871873, EPI_ISL_871874, EPI_ISL_871877, EPI_ISL_871878	Wyoming Public Health Laboratory	Wyoming Public Health Laboratory	Noah Hull, Taylor Fearing, Lynette Gumbleton, Channing Weber, Ashley Norberg, Bailey Bowcutt, and Wanda Manley
EPI_ISL_872199	Hopital	National Reference Center for Viruses of Respiratory Infections, Institut Pasteur, Paris	Marion Barbet, Sylvie Behillil, Méline Bizard, Angela Brisebarre, Camille Capel, Etienne Simon-Lorière, Vincent Enouf, Maud Vanpeene, Sylvie van der Werf,Ramanantsoa CéLine
EPI_ISL_872221	hopital	National Reference Center for Viruses of Respiratory Infections, Institut Pasteur, Paris	Marion Barbet, Sylvie Behillil, Méline Bizard, Angela Brisebarre, Camille Capel, Etienne Simon-Lorière, Vincent Enouf, Maud Vanpeene, Sylvie van der Werf,Combe Patrice
EPI_ISL_872228	Hopital	National Reference Center for Viruses of Respiratory Infections, Institut Pasteur, Paris	Marion Barbet, Sylvie Behillil, Méline Bizard, Angela Brisebarre, Camille Capel, Etienne Simon-Lorière, Vincent Enouf, Maud Vanpeene, Sylvie van der Werf,Irimia Alix
EPI_ISL_872229, EPI_ISL_872230	Hopital	National Reference Center for Viruses of Respiratory Infections, Institut Pasteur, Paris	Marion Barbet, Sylvie Behillil, Méline Bizard, Angela Brisebarre, Camille Capel, Etienne Simon-Lorière, Vincent Enouf, Maud Vanpeene, Sylvie van der Werf,Ramanantsoa CéLine
EPI_ISL_872231	Hopital	National Reference Center for Viruses of Respiratory Infections, Institut Pasteur, Paris	Marion Barbet, Sylvie Behillil, Méline Bizard, Angela Brisebarre, Camille Capel, Etienne Simon-Lorière, Vincent Enouf, Maud Vanpeene, Sylvie van der Werf,Florin CéCile
EPI_ISL_872240, EPI_ISL_872241	hopital	National Reference Center for Viruses of Respiratory Infections, Institut Pasteur, Paris	Marion Barbet, Sylvie Behillil, Méline Bizard, Angela Brisebarre, Camille Capel, Etienne Simon-Lorière, Vincent Enouf, Maud Vanpeene, Sylvie van der Werf,Combe Patrice
EPI_ISL_872249, EPI_ISL_872250	Hopital	National Reference Center for Viruses of Respiratory Infections, Institut Pasteur, Paris	Marion Barbet, Sylvie Behillil, Méline Bizard, Angela Brisebarre, Camille Capel, Etienne Simon-Lorière, Vincent Enouf, Maud Vanpeene, Sylvie van der Werf,Ducancelle Alexandra
EPI_ISL_872251	Hopital	National Reference Center for Viruses of Respiratory Infections, Institut Pasteur, Paris	Marion Barbet, Sylvie Behillil, Méline Bizard, Angela Brisebarre, Camille Capel, Etienne Simon-Lorière, Vincent Enouf, Maud Vanpeene, Sylvie van der Werf,Irimia Alix
EPI_ISL_872252	Hopital	National Reference Center for Viruses of Respiratory Infections, Institut Pasteur, Paris	Marion Barbet, Sylvie Behillil, Méline Bizard, Angela Brisebarre, Camille Capel, Etienne Simon-Lorière, Vincent Enouf, Maud Vanpeene, Sylvie van der Werf,Ramanantsoa CéLine
EPI_ISL_872253	Hopital	National Reference Center for Viruses of Respiratory Infections, Institut Pasteur, Paris	Marion Barbet, Sylvie Behillil, Méline Bizard, Angela Brisebarre, Camille Capel, Etienne Simon-Lorière, Vincent Enouf, Maud Vanpeene, Sylvie van der Werf,Irimia Alix
EPI_ISL_872255	Hopital	National Reference Center for Viruses of Respiratory Infections, Institut Pasteur, Paris	Marion Barbet, Sylvie Behillil, Méline Bizard, Angela Brisebarre, Camille Capel, Etienne Simon-Lorière, Vincent Enouf, Maud Vanpeene, Sylvie van der Werf,Philippi Jean-FrançOis
EPI_ISL_872257	Labo Analyses Med	National Reference Center for Viruses of Respiratory Infections, Institut Pasteur, Paris	Marion Barbet, Sylvie Behillil, Méline Bizard, Angela Brisebarre, Camille Capel, Etienne Simon-Lorière, Vincent Enouf, Maud Vanpeene, Sylvie van der Werf,Bour Jean Baptiste
EPI_ISL_872261	Hopital	National Reference Center for Viruses of Respiratory Infections, Institut Pasteur, Paris	Marion Barbet, Sylvie Behillil, Méline Bizard, Angela Brisebarre, Camille Capel, Etienne Simon-Lorière, Vincent Enouf, Maud Vanpeene, Sylvie van der Werf,Guigon AuréLie
EPI_ISL_872275, EPI_ISL_872276, EPI_ISL_872277	Hopital	National Reference Center for Viruses of Respiratory Infections, Institut Pasteur, Paris	Marion Barbet, Sylvie Behillil, Méline Bizard, Angela Brisebarre, Camille Capel, Etienne Simon-Lorière, Vincent Enouf, Maud Vanpeene, Sylvie van der Werf,Ramanantsoa CéLine
EPI_ISL_872280	Labo Analyses Med	National Reference Center for Viruses of Respiratory Infections, Institut Pasteur, Paris	Marion Barbet, Sylvie Behillil, Méline Bizard, Angela Brisebarre, Camille Capel, Etienne Simon-Lorière, Vincent Enouf, Maud Vanpeene, Sylvie van der Werf,Bour Jean Baptiste
EPI_ISL_872283	Labo Analyses Med	National Reference Center for Viruses of Respiratory Infections, Institut Pasteur, Paris	Marion Barbet, Sylvie Behillil, Méline Bizard, Angela Brisebarre, Camille Capel, Etienne Simon-Lorière, Vincent Enouf, Maud Vanpeene, Sylvie van der Werf,Girard Sophie
EPI_ISL_872293, EPI_ISL_872294	Hopital	National Reference Center for Viruses of Respiratory Infections, Institut Pasteur, Paris	Marion Barbet, Sylvie Behillil, Méline Bizard, Angela Brisebarre, Camille Capel, Etienne Simon-Lorière, Vincent Enouf, Maud Vanpeene, Sylvie van der Werf,Guigon AuréLie
EPI_ISL_872300	Hopital	National Reference Center for Viruses of Respiratory Infections, Institut Pasteur, Paris	Marion Barbet, Sylvie Behillil, Méline Bizard, Angela Brisebarre, Camille Capel, Etienne Simon-Lorière, Vincent Enouf, Maud Vanpeene, Sylvie van der Werf,Florin CéCile
EPI_ISL_872320	Labo Analyses Med	National Reference Center for Viruses of Respiratory Infections, Institut Pasteur, Paris	Marion Barbet, Sylvie Behillil, Méline Bizard, Angela Brisebarre, Camille Capel, Etienne Simon-Lorière, Vincent Enouf, Maud Vanpeene, Sylvie van der Werf
EPI_ISL_872326, EPI_ISL_872327, EPI_ISL_872328, EPI_ISL_872329	hopital	National Reference Center for Viruses of Respiratory Infections, Institut Pasteur, Paris	Marion Barbet, Sylvie Behillil, Méline Bizard, Angela Brisebarre, Camille Capel, Etienne Simon-Lorière, Vincent Enouf, Maud Vanpeene, Sylvie van der Werf,Combe Patrice
EPI_ISL_872341, EPI_ISL_872342	Hopital	National Reference Center for Viruses of Respiratory Infections, Institut Pasteur, Paris	Marion Barbet, Sylvie Behillil, Méline Bizard, Angela Brisebarre, Camille Capel, Etienne Simon-Lorière, Vincent Enouf, Maud Vanpeene, Sylvie van der Werf,Ducancelle Alexandra
EPI_ISL_872343	Hopital	National Reference Center for Viruses of Respiratory Infections, Institut Pasteur, Paris	Marion Barbet, Sylvie Behillil, Méline Bizard, Angela Brisebarre, Camille Capel, Etienne Simon-Lorière, Vincent Enouf, Maud Vanpeene, Sylvie van der Werf,Irimia Alix
EPI_ISL_872344, EPI_ISL_872345, EPI_ISL_872346	Hopital	National Reference Center for Viruses of Respiratory Infections, Institut Pasteur, Paris	Marion Barbet, Sylvie Behillil, Méline Bizard, Angela Brisebarre, Camille Capel, Etienne Simon-Lorière, Vincent Enouf, Maud Vanpeene, Sylvie van der Werf,Ramanantsoa CéLine
EPI_ISL_872348	Hopital	National Reference Center for Viruses of Respiratory Infections, Institut Pasteur, Paris	Marion Barbet, Sylvie Behillil, Méline Bizard, Angela Brisebarre, Camille Capel, Etienne Simon-Lorière, Vincent Enouf, Maud Vanpeene, Sylvie van der Werf,Irimia Alix
EPI_ISL_872479, EPI_ISL_872480, EPI_ISL_872481, EPI_ISL_872482, EPI_ISL_872483, EPI_ISL_872484, EPI_ISL_872485, EPI_ISL_872486, EPI_ISL_872487, EPI_ISL_872488, EPI_ISL_872489, EPI_ISL_872490, EPI_ISL_872491, EPI_ISL_872492, EPI_ISL_872493, EPI_ISL_872511			
see above	New Mexico Department of Health Scientific Laboratory	Center for Global Health, University of New Mexico Health Sciences Center	Daryl Domman, Kurt Schwalm, Twila Kunde, Joseph Hicks, Anastacia Griego, Michael Edwards, Darrell Dinwiddie
EPI_ISL_872579	Sydney South West Pathology Service (SSWPS) - Royal Prince Alfred Hospital - NSW Health Pathology	NSW Health Pathology - Institute of Clinical Pathology and Medical Research; Westmead Hospital; University of Sydney	CIDM-PH et al.
EPI_ISL_872580	Histopath	NSW Health Pathology - Institute of Clinical Pathology and Medical Research; Westmead Hospital; University of Sydney	CIDM-PH et al.
EPI_ISL_872581	St Vincent's Pathology (SydPath)	NSW Health Pathology - Institute of Clinical Pathology and Medical Research; Westmead Hospital; University of Sydney	CIDM-PH et al.
EPI_ISL_872582	South Eastern Area Laboratory Services (SEALS)	NSW Health Pathology - Institute of Clinical Pathology and Medical Research; Westmead Hospital; University of Sydney	CIDM-PH et al.
EPI_ISL_872849, EPI_ISL_872850,	University of Wisconsin-Madison AIDS Vaccine Research	University of Wisconsin-Madison AIDS Vaccine Research	Gage Moreno, Katarina Braun, et al. AIDS Vaccine Research Laboratories

EPI_ISL_872851, EPI_ISL_872852, EPI_ISL_872853	Laboratories	Laboratories	
EPI_ISL_873040	WHO National Influenza Centre Russian Federation	WHO National Influenza Centre Russian Federation	Andrey Komissarov, Artem Fadeev, Anna Ivanova, Kseniya Komissarova, Dmitry Bazhenov, Mikhail Bakaev, Daria Danilenko, Ksenia Safina, Elena Nabieva, Georgii Bazykin, Dmitry Lioznov
EPI_ISL_873110, EPI_ISL_873112, EPI_ISL_873114, EPI_ISL_873115, EPI_ISL_873118, EPI_ISL_873121, EPI_ISL_873123	University of Michigan Clinical Microbiology Laboratory	Lauring Lab, University of Michigan, Department of Microbiology and Immunology	Valesano
EPI_ISL_873166, EPI_ISL_873167, EPI_ISL_873168, EPI_ISL_873169, EPI_ISL_873170, EPI_ISL_873171, EPI_ISL_873172, EPI_ISL_873173, EPI_ISL_873174, EPI_ISL_873175, EPI_ISL_873177, EPI_ISL_873178, EPI_ISL_873179, EPI_ISL_873180, EPI_ISL_873181, EPI_ISL_873182			
see above	Microbiology Division, South Carolina Department of Health and Environmental Control (SC DHEC)	Microbiology Division, South Carolina Department of Health and Environmental Control (SC DHEC)	Flores,H., Freeman,J.
EPI_ISL_873235, EPI_ISL_873236, EPI_ISL_873237, EPI_ISL_873239, EPI_ISL_873240, EPI_ISL_873242, EPI_ISL_873244, EPI_ISL_873247, EPI_ISL_873248, EPI_ISL_873251, EPI_ISL_873253, EPI_ISL_873255, EPI_ISL_873256, EPI_ISL_873257, EPI_ISL_873259, EPI_ISL_873260, EPI_ISL_873262			
see above	M Health Fairview	Minnesota Department of Health, Public Health Laboratory	Alexandra Lorentz, Jacob Garfin, Matt Plumb, and Xiong Wang
EPI_ISL_873272	Vault Health	Minnesota Department of Health, Public Health Laboratory	Alexandra Lorentz, Jacob Garfin, Matt Plumb, and Xiong Wang
EPI_ISL_873276	Hennepin County Medical Center	Minnesota Department of Health, Public Health Laboratory	Alexandra Lorentz, Jacob Garfin, Matt Plumb, and Xiong Wang
EPI_ISL_873280, EPI_ISL_873288, EPI_ISL_873289, EPI_ISL_873308, EPI_ISL_873309, EPI_ISL_873312, EPI_ISL_873331, EPI_ISL_873345, EPI_ISL_873352, EPI_ISL_873360, EPI_ISL_873372, EPI_ISL_873377, EPI_ISL_873379, EPI_ISL_873382, EPI_ISL_873383, EPI_ISL_873385, EPI_ISL_873390, EPI_ISL_873395, EPI_ISL_873401, EPI_ISL_873403, EPI_ISL_873407, EPI_ISL_873409, EPI_ISL_873417, EPI_ISL_873419, EPI_ISL_873425, EPI_ISL_873427, EPI_ISL_873447, EPI_ISL_873452, EPI_ISL_873458, EPI_ISL_873463, EPI_ISL_873468, EPI_ISL_873469, EPI_ISL_873483, EPI_ISL_873485, EPI_ISL_873486, EPI_ISL_873488, EPI_ISL_873490, EPI_ISL_873505, EPI_ISL_873517, EPI_ISL_873521, EPI_ISL_873527, EPI_ISL_873528, EPI_ISL_873535, EPI_ISL_873536, EPI_ISL_873538, EPI_ISL_873542, EPI_ISL_873543, EPI_ISL_873546, EPI_ISL_873547, EPI_ISL_873549, EPI_ISL_873554, EPI_ISL_873557, EPI_ISL_873562, EPI_ISL_873563, EPI_ISL_873565, EPI_ISL_873570, EPI_ISL_873575, EPI_ISL_873577, EPI_ISL_873583, EPI_ISL_873589, EPI_ISL_873590, EPI_ISL_873591, EPI_ISL_873595, EPI_ISL_873599, EPI_ISL_873602, EPI_ISL_873618, EPI_ISL_873623, EPI_ISL_873627, EPI_ISL_873656, EPI_ISL_873672, EPI_ISL_873724, EPI_ISL_873726, EPI_ISL_873730, EPI_ISL_873769, EPI_ISL_873786, EPI_ISL_873850, EPI_ISL_873865, EPI_ISL_873875			
see above	Lighthouse Lab in Milton Keynes	Wellcome Sanger Institute for the COVID-19 Genomics UK (COG-UK) Consortium	The Lighthouse Lab in Milton Keynes and Alex Alderton, Roberto Amato, Sonia Goncalves, Ewan Harrison, David K. Jackson, Ian Johnston, Dominic Kwiatkowski, Cordelia Langford, John Sillitoe on behalf of the Wellcome Sanger Institute COVID-19 Surveillance Team
EPI_ISL_873882	Lighthouse Lab in Cambridge	Wellcome Sanger Institute for the COVID-19 Genomics UK (COG-UK) Consortium	Rob Howes, The Lighthouse Lab in Cambridge and Alex Alderton, Roberto Amato, Sonia Goncalves, Ewan Harrison, David K. Jackson, Ian Johnston, Dominic Kwiatkowski, Cordelia Langford, John Sillitoe on behalf of the Wellcome Sanger Institute COVID-19 Surveillance Team
EPI_ISL_873904, EPI_ISL_873925, EPI_ISL_873935, EPI_ISL_873943, EPI_ISL_873947, EPI_ISL_873954	Lighthouse Lab in Milton Keynes	Wellcome Sanger Institute for the COVID-19 Genomics UK (COG-UK) Consortium	The Lighthouse Lab in Milton Keynes and Alex Alderton, Roberto Amato, Sonia Goncalves, Ewan Harrison, David K. Jackson, Ian Johnston, Dominic Kwiatkowski, Cordelia Langford, John Sillitoe on behalf of the Wellcome Sanger Institute COVID-19 Surveillance Team
EPI_ISL_873962, EPI_ISL_873981, EPI_ISL_873982, EPI_ISL_873986, EPI_ISL_874011, EPI_ISL_874024, EPI_ISL_874070, EPI_ISL_874078, EPI_ISL_874080, EPI_ISL_874081, EPI_ISL_874082, EPI_ISL_874093, EPI_ISL_874098, EPI_ISL_874109, EPI_ISL_874121, EPI_ISL_874145, EPI_ISL_874149, EPI_ISL_874155, EPI_ISL_874159, EPI_ISL_874181, EPI_ISL_874185, EPI_ISL_874196, EPI_ISL_874203, EPI_ISL_874210, EPI_ISL_874218, EPI_ISL_874220, EPI_ISL_874225, EPI_ISL_874227, EPI_ISL_874243, EPI_ISL_874245, EPI_ISL_874249, EPI_ISL_874252, EPI_ISL_874257, EPI_ISL_874259, EPI_ISL_874273, EPI_ISL_874284, EPI_ISL_874287, EPI_ISL_874291, EPI_ISL_874310, EPI_ISL_874502, EPI_ISL_874530, EPI_ISL_874853, EPI_ISL_874966			
see above	Lighthouse Lab in Alderley Park	Wellcome Sanger Institute for the COVID-19 Genomics UK (COG-UK) Consortium	Jacquelyn Wynn, Mairead Hyland, The Lighthouse Lab in Alderley Park and Alex Alderton, Roberto Amato, Sonia Goncalves, Ewan Harrison, David K. Jackson, Ian Johnston, Dominic Kwiatkowski, Cordelia Langford, John Sillitoe on behalf of the Wellcome Sanger Institute COVID-19 Surveillance Team
EPI_ISL_875342	Pakistan Institute of Medical Sciences	Department of Virology	Massab Umair, Aamer Ikram, Muhammad Salman, Sana Tamim, Nazish Badar, Adnan Khurshid, Zaira Rahman, Abdul Ahad, Hamza Ahmad
EPI_ISL_875343, EPI_ISL_875345	Pakistan Institute of Medical Sciences	Department of Virology	Massab Umair, Aamer Ikram, Muhammad Salman, Nazish Badar, Sana Tamim, Adnan Khurshid, Zaira Rahman, Abdul Ahad, Hamza Ahmad
EPI_ISL_875410, EPI_ISL_875411, EPI_ISL_875412, EPI_ISL_875413, EPI_ISL_875414, EPI_ISL_875415, EPI_ISL_875416, EPI_ISL_875417, EPI_ISL_875418, EPI_ISL_875419, EPI_ISL_875420, EPI_ISL_875421, EPI_ISL_875422, EPI_ISL_875423, EPI_ISL_875424, EPI_ISL_875425, EPI_ISL_875426, EPI_ISL_875427, EPI_ISL_875428, EPI_ISL_875429, EPI_ISL_875430, EPI_ISL_875431, EPI_ISL_875432, EPI_ISL_875433, EPI_ISL_875434, EPI_ISL_875435, EPI_ISL_875436, EPI_ISL_875437, EPI_ISL_875438, EPI_ISL_875439, EPI_ISL_875440, EPI_ISL_875441, EPI_ISL_875442, EPI_ISL_875443, EPI_ISL_875444, EPI_ISL_875445, EPI_ISL_875446, EPI_ISL_875447, EPI_ISL_875448, EPI_ISL_875449, EPI_ISL_875450, EPI_ISL_875451, EPI_ISL_875452, EPI_ISL_875453, EPI_ISL_875454, EPI_ISL_875455, EPI_ISL_875456, EPI_ISL_875457, EPI_ISL_875458, EPI_ISL_875459			
see above	National Virus Reference Laboratory	National Virus Reference Laboratory	Michael Carr, Gabriel Gonzalez, Jonathan Dean, Cillian F De Gascun
EPI_ISL_875519	Institute of Virology, Biomedical Research Center of the Slovak Academy of Sciences, Bratislava	Faculty of Natural Sciences, Comenius University, Bratislava	Broa Brejová, Viktória abanová, Kristína Boršová, Viktória Hodorová, Sabina Fumaová Havlíková, Juraj Kopáek, Martina Liková, ubomíra Lukáiková, Martina Neboháová, Monika Sláviková, Tomáš Vina, Jozef Nosek, Boris Klempa
EPI_ISL_875529	Institute of Virology, Biomedical Research Center of the Slovak Academy of Sciences, Bratislava	Faculty of Natural Sciences, Comenius University, Bratislava	Kristína Boršová, Viktória abanová, Broa Brejová, Viktória Hodorová, Sabina Fumaová Havlíková, Juraj Kopáek, Martina Liková, ubomíra Lukáiková, Martina Neboháová, Monika Sláviková, Tomáš Vina, Boris Klempa, Jozef Nosek
EPI_ISL_875532	Institute of Virology, Biomedical Research Center of the Slovak Academy of Sciences, Bratislava	Faculty of Natural Sciences, Comenius University, Bratislava	Broa Brejová, Viktória abanová, Kristína Boršová, Viktória Hodorová, Sabina Fumaová Havlíková, Juraj Kopáek, Martina Liková, ubomíra Lukáiková, Martina Neboháová, Monika Sláviková, Tomáš Vina, Jozef Nosek, Boris Klempa
EPI_ISL_875662, EPI_ISL_875663	University of Michigan Clinical Microbiology Laboratory	Lauring Lab, University of Michigan, Department of Microbiology and Immunology	Valesano
EPI_ISL_875671	CHU Purpan - Laboratoire de Virologie - Institut Fédératif de Biologie	CHU Purpan - Laboratoire de Virologie - Institut Fédératif de Biologie	Latour J., Ranger N., Dubois M., Carcenac R., Harter A., Boyer P., Tremeaux P., Izopet J.
EPI_ISL_875685	National Virus Reference Laboratory	National Virus Reference Laboratory	Michael Carr, Gabriel Gonzalez, Jonathan Dean, Cillian F De Gascun
EPI_ISL_876063, EPI_ISL_876077, EPI_ISL_876111, EPI_ISL_876144, EPI_ISL_876155, EPI_ISL_876244	Massachusetts State Public Health Laboratory	Massachusetts State Public Health Laboratory	Andrew Lang, Timelia Fink, Glen Gallagher, Sandra Smole
EPI_ISL_876589	Blackhawk Genomics	Pathogen Discovery, Respiratory Viruses Branch, Division of Viral Diseases, Centers for Disease Control and Prevention	Ying Tao, Yan Li, Jing Zhang, Krista Queen, Anna Uehara, Peter Cook, Clinton R. Paden, Haibin Wang, Suxiang Tong
EPI_ISL_877210, EPI_ISL_877211, EPI_ISL_877212, EPI_ISL_877213	LabPLUS	Institute of Environmental Science and Research (ESR)	Xiaoyun Ren, Matt Storey, Nikki Freed, Muhammad Faisal, Jing Wang, Hermes Perez, Anja Werno, Antje van der Linden, Arlo Upton, Chris Mansell, David Hammer, Dragana Drinkovic, Gary McAuliffe, Hana Sofia Andersson, James Ussher, Jill Sherwood, Josh Freeman, Julia Howard, Juliet Elvy, Mary DeAlmeida, Matt Blakiston, Matthew Rogers, Max Bloomfield, Michael Addidle, Michelle Balm, Sally Roberts, Sarah Jefferies, Sharmini Muttaiyah, Susan Morpeth, Susan Taylor, Timothy Blackmore, Vani Sathyendran, Veronica Playle, Virginia Hope, Erasmus Smit, Lauren Jelly, Olin Silander, Joep de Ligt
EPI_ISL_877215, EPI_ISL_877216, EPI_ISL_877217, EPI_ISL_877218	Middlemore Hospital	Institute of Environmental Science and Research (ESR)	Xiaoyun Ren, Matt Storey, Nikki Freed, Muhammad Faisal, Jing Wang, Hermes Perez, Anja Werno, Antje van der Linden, Arlo Upton, Chris Mansell, David Hammer, Dragana Drinkovic, Gary McAuliffe, Hana Sofia Andersson, James Ussher, Jill Sherwood, Josh Freeman, Julia Howard, Juliet Elvy, Mary DeAlmeida, Matt Blakiston, Matthew Rogers, Max Bloomfield, Michael Addidle, Michelle Balm, Sally Roberts, Sarah Jefferies, Sharmini Muttaiyah, Susan Morpeth, Susan Taylor, Timothy Blackmore, Vani Sathyendran, Veronica Playle, Virginia Hope, Erasmus Smit, Lauren Jelly, Olin Silander, Joep de Ligt
EPI_ISL_877227	Thai Red Cross Emerging Infectious Diseases Health Science Centre, Chulalongkorn Hospital, Faculty of Medicine, Chulalongkorn University	Thai Red Cross Emerging Infectious Diseases Center and Faculty of Medicine, Chulalongkorn University	Krirk Asavametha, Panpit Suwangool, Sininat Petcharat, Yutthana Joyjinda, Weenassarin Ampoot, Apaporn Rodpan, Opass Putcharoen, Thiravat Hemachudha, Supaporn Wacharapluesadee
EPI_ISL_877229, EPI_ISL_877230, EPI_ISL_877231, EPI_ISL_877232, EPI_ISL_877233, EPI_ISL_877234, EPI_ISL_877235	University College Sedaya International (UCSI University)	Institute for Medical Research, Infectious Disease Research Centre, National Institutes of Health, Ministry of Health Malaysia	Suppiah J, Kamel K, Azizan MA, Sekaran SD, Thayan R
EPI_ISL_878833	Lighthouse Lab in Milton Keynes	Wellcome Sanger Institute for the COVID-19 Genomics UK (COG-UK) Consortium	The Lighthouse Lab in Milton Keynes and Alex Alderton, Roberto Amato, Sonia Goncalves, Ewan Harrison, David K. Jackson, Ian Johnston, Dominic Kwiatkowski, Cordelia Langford, John Sillitoe on behalf of the Wellcome Sanger Institute COVID-19 Surveillance Team
EPI_ISL_880040, EPI_ISL_880042, EPI_ISL_880058, EPI_ISL_880066, EPI_ISL_880069, EPI_ISL_880077, EPI_ISL_880085, EPI_ISL_880087, EPI_ISL_880098, EPI_ISL_880109, EPI_ISL_880111, EPI_ISL_880114, EPI_ISL_880142, EPI_ISL_880145, EPI_ISL_880148, EPI_ISL_880153, EPI_ISL_880154, EPI_ISL_880155,			

EPI_ISL_880158, EPI_ISL_880160, EPI_ISL_880163, EPI_ISL_880166, EPI_ISL_880167, EPI_ISL_880170			
see above	Sharp HealthCare Laboratory	Andersen lab at Scripps Research	SEARCH Alliance San Diego with Aaron Harding, Jacquelyn Berumen, Cathy Woerle, Liam McGinnis, Art Mendoza, Omid Bakhtar
EPI_ISL_880179	Rady's Childrens Hospital	Andersen lab at Scripps Research	SEARCH Alliance San Diego with Nanda Radamchar, David Dimmock, Linda Luo, Christina Clarke, Kathryn Bouic, Teresa Mueller, Denise Malicki
EPI_ISL_880601, EPI_ISL_880620, EPI_ISL_880629, EPI_ISL_880637, EPI_ISL_880669, EPI_ISL_880674, EPI_ISL_880682, EPI_ISL_880709, EPI_ISL_880710, EPI_ISL_880717, EPI_ISL_880718, EPI_ISL_880721, EPI_ISL_880746, EPI_ISL_880753, EPI_ISL_880763, EPI_ISL_880774, EPI_ISL_880775, EPI_ISL_880776, EPI_ISL_880779, EPI_ISL_880789, EPI_ISL_880799, EPI_ISL_880812, EPI_ISL_880831, EPI_ISL_880835, EPI_ISL_880853, EPI_ISL_880868, EPI_ISL_880879, EPI_ISL_880915			
see above	Lighthouse Lab in Milton Keynes	Wellcome Sanger Institute for the COVID-19 Genomics UK (COG-UK) Consortium	The Lighthouse Lab in Milton Keynes and Alex Alderton, Roberto Amato, Sonia Goncalves, Ewan Harrison, David K. Jackson, Ian Johnston, Dominic Kwiatkowski, Cordelia Langford, John Sillitoe on behalf of the Wellcome Sanger Institute COVID-19 Surveillance Team
EPI_ISL_881318, EPI_ISL_881326	Lighthouse Lab in Alderley Park	Wellcome Sanger Institute for the COVID-19 Genomics UK (COG-UK) Consortium	Jacquelyn Wynn, Mairead Hyland, The Lighthouse Lab in Alderley Park and Alex Alderton, Roberto Amato, Sonia Goncalves, Ewan Harrison, David K. Jackson, Ian Johnston, Dominic Kwiatkowski, Cordelia Langford, John Sillitoe on behalf of the Wellcome Sanger Institute COVID-19 Surveillance Team
EPI_ISL_881618, EPI_ISL_881621	Lighthouse Lab in Milton Keynes	Wellcome Sanger Institute for the COVID-19 Genomics UK (COG-UK) Consortium	The Lighthouse Lab in Milton Keynes and Alex Alderton, Roberto Amato, Sonia Goncalves, Ewan Harrison, David K. Jackson, Ian Johnston, Dominic Kwiatkowski, Cordelia Langford, John Sillitoe on behalf of the Wellcome Sanger Institute COVID-19 Surveillance Team
EPI_ISL_881623, EPI_ISL_881624, EPI_ISL_881625, EPI_ISL_881626, EPI_ISL_881627, EPI_ISL_881628, EPI_ISL_881629, EPI_ISL_881630, EPI_ISL_881631, EPI_ISL_881632, EPI_ISL_881633, EPI_ISL_881634, EPI_ISL_881635, EPI_ISL_881636, EPI_ISL_881637, EPI_ISL_881638, EPI_ISL_881639, EPI_ISL_881640, EPI_ISL_881641, EPI_ISL_881642, EPI_ISL_881643, EPI_ISL_881644, EPI_ISL_881645, EPI_ISL_881646, EPI_ISL_881647, EPI_ISL_881648, EPI_ISL_881649, EPI_ISL_881650, EPI_ISL_881651, EPI_ISL_881652, EPI_ISL_881653, EPI_ISL_881654, EPI_ISL_881655, EPI_ISL_881656, EPI_ISL_881657, EPI_ISL_881658, EPI_ISL_881659, EPI_ISL_881660, EPI_ISL_881661, EPI_ISL_881662, EPI_ISL_881663, EPI_ISL_881664, EPI_ISL_881665, EPI_ISL_881666, EPI_ISL_881667, EPI_ISL_881668, EPI_ISL_881669, EPI_ISL_881670, EPI_ISL_881671, EPI_ISL_881672, EPI_ISL_881673, EPI_ISL_881674, EPI_ISL_881675, EPI_ISL_881676, EPI_ISL_881677, EPI_ISL_881678, EPI_ISL_881679, EPI_ISL_881680, EPI_ISL_881681, EPI_ISL_881682, EPI_ISL_881683, EPI_ISL_881684, EPI_ISL_881685, EPI_ISL_881686, EPI_ISL_881687, EPI_ISL_881688, EPI_ISL_881689, EPI_ISL_881690, EPI_ISL_881691, EPI_ISL_881692, EPI_ISL_881693, EPI_ISL_881694, EPI_ISL_881695, EPI_ISL_881696, EPI_ISL_881697, EPI_ISL_881698, EPI_ISL_881699, EPI_ISL_881700, EPI_ISL_881701, EPI_ISL_881702, EPI_ISL_881703, EPI_ISL_881704, EPI_ISL_881705, EPI_ISL_881706, EPI_ISL_881707, EPI_ISL_881708, EPI_ISL_881709, EPI_ISL_881710, EPI_ISL_881711, EPI_ISL_881712, EPI_ISL_881713, EPI_ISL_881714, EPI_ISL_881715, EPI_ISL_881716, EPI_ISL_881717, EPI_ISL_881718, EPI_ISL_881719, EPI_ISL_881720, EPI_ISL_881721, EPI_ISL_881722, EPI_ISL_881723, EPI_ISL_881724, EPI_ISL_881725, EPI_ISL_881726, EPI_ISL_881727, EPI_ISL_881728, EPI_ISL_881729, EPI_ISL_881730, EPI_ISL_881731, EPI_ISL_881732, EPI_ISL_881733, EPI_ISL_881734, EPI_ISL_881735, EPI_ISL_881736, EPI_ISL_881737, EPI_ISL_881738, EPI_ISL_881739, EPI_ISL_881740, EPI_ISL_881741, EPI_ISL_881742, EPI_ISL_881743, EPI_ISL_881744, EPI_ISL_881745, EPI_ISL_881746, EPI_ISL_881747, EPI_ISL_881748, EPI_ISL_881749, EPI_ISL_881750, EPI_ISL_881751, EPI_ISL_881752, EPI_ISL_881753, EPI_ISL_881754, EPI_ISL_881755, EPI_ISL_881756, EPI_ISL_881757, EPI_ISL_881758, EPI_ISL_881759, EPI_ISL_881760, EPI_ISL_881761, EPI_ISL_881762, EPI_ISL_881763, EPI_ISL_881764, EPI_ISL_881765, EPI_ISL_881766, EPI_ISL_881767, EPI_ISL_881768, EPI_ISL_881769, EPI_ISL_881770, EPI_ISL_881771, EPI_ISL_881772, EPI_ISL_881773, EPI_ISL_881774, EPI_ISL_881775, EPI_ISL_881776, EPI_ISL_881777, EPI_ISL_881778, EPI_ISL_881779, EPI_ISL_881780, EPI_ISL_881781, EPI_ISL_881782, EPI_ISL_881783, EPI_ISL_881784, EPI_ISL_881785, EPI_ISL_881786, EPI_ISL_881787, EPI_ISL_881788, EPI_ISL_881789, EPI_ISL_881790, EPI_ISL_881791, EPI_ISL_881792, EPI_ISL_881793, EPI_ISL_881794, EPI_ISL_881795, EPI_ISL_881796, EPI_ISL_881797, EPI_ISL_881798, EPI_ISL_881799, EPI_ISL_881800, EPI_ISL_881801, EPI_ISL_881802, EPI_ISL_881803, EPI_ISL_881804, EPI_ISL_881805, EPI_ISL_881806, EPI_ISL_881807, EPI_ISL_881808, EPI_ISL_881809, EPI_ISL_881810, EPI_ISL_881811, EPI_ISL_881812, EPI_ISL_881813, EPI_ISL_881814, EPI_ISL_881815, EPI_ISL_881816, EPI_ISL_881817, EPI_ISL_881818, EPI_ISL_881819, EPI_ISL_881820, EPI_ISL_881821, EPI_ISL_881822, EPI_ISL_881823, EPI_ISL_881824, EPI_ISL_881825, EPI_ISL_881826, EPI_ISL_881827, EPI_ISL_881828, EPI_ISL_881829, EPI_ISL_881830, EPI_ISL_881831, EPI_ISL_881832, EPI_ISL_881833, EPI_ISL_881834, EPI_ISL_881835, EPI_ISL_881836, EPI_ISL_881837, EPI_ISL_881838, EPI_ISL_881839, EPI_ISL_881840, EPI_ISL_881841, EPI_ISL_881842, EPI_ISL_881843, EPI_ISL_881844, EPI_ISL_881845, EPI_ISL_881846, EPI_ISL_881847, EPI_ISL_881848, EPI_ISL_881849, EPI_ISL_881850, EPI_ISL_881851, EPI_ISL_881852, EPI_ISL_881853, EPI_ISL_881854, EPI_ISL_881855, EPI_ISL_881856, EPI_ISL_881857, EPI_ISL_881858, EPI_ISL_881859, EPI_ISL_881860, EPI_ISL_881861, EPI_ISL_881862, EPI_ISL_881863, EPI_ISL_881864, EPI_ISL_881865, EPI_ISL_881866, EPI_ISL_881867, EPI_ISL_881868, EPI_ISL_881869, EPI_ISL_881870, EPI_ISL_881871, EPI_ISL_881872, EPI_ISL_881873, EPI_ISL_881874, EPI_ISL_881875, EPI_ISL_881876, EPI_ISL_881877, EPI_ISL_881878, EPI_ISL_881879, EPI_ISL_881880, EPI_ISL_881881, EPI_ISL_881882, EPI_ISL_881883, EPI_ISL_881884, EPI_ISL_881885, EPI_ISL_881886, EPI_ISL_881887, EPI_ISL_881888, EPI_ISL_881889, EPI_ISL_881890, EPI_ISL_881891, EPI_ISL_881892, EPI_ISL_881893, EPI_ISL_881894, EPI_ISL_881895, EPI_ISL_881896, EPI_ISL_881897, EPI_ISL_881898, EPI_ISL_881899, EPI_ISL_881900, EPI_ISL_881901, EPI_ISL_881902, EPI_ISL_881903, EPI_ISL_881904, EPI_ISL_881905, EPI_ISL_881906, EPI_ISL_881907, EPI_ISL_881908, EPI_ISL_881909, EPI_ISL_881910, EPI_ISL_881911, EPI_ISL_881912, EPI_ISL_881913, EPI_ISL_881914, EPI_ISL_881915, EPI_ISL_881916, EPI_ISL_881917, EPI_ISL_881918, EPI_ISL_881919, EPI_ISL_881920, EPI_ISL_881921, EPI_ISL_881922, EPI_ISL_881923, EPI_ISL_881924, EPI_ISL_881925, EPI_ISL_881926, EPI_ISL_881927, EPI_ISL_881928, EPI_ISL_881929, EPI_ISL_881930, EPI_ISL_881931, EPI_ISL_881932, EPI_ISL_881933, EPI_ISL_881934, EPI_ISL_881935, EPI_ISL_881936, EPI_ISL_881937, EPI_ISL_881938, EPI_ISL_881939, EPI_ISL_881940, EPI_ISL_881941, EPI_ISL_881942, EPI_ISL_881943, EPI_ISL_881944, EPI_ISL_881945, EPI_ISL_881946, EPI_ISL_881947, EPI_ISL_881948, EPI_ISL_881949, EPI_ISL_881950, EPI_ISL_881951, EPI_ISL_881952, EPI_ISL_881953, EPI_ISL_881954, EPI_ISL_881955, EPI_ISL_881956, EPI_ISL_881957, EPI_ISL_881958, EPI_ISL_881959, EPI_ISL_881960, EPI_ISL_881961, EPI_ISL_881962, EPI_ISL_881963, EPI_ISL_881964, EPI_ISL_881965, EPI_ISL_881966, EPI_ISL_881967, EPI_ISL_881968, EPI_ISL_881969, EPI_ISL_881970, EPI_ISL_881971, EPI_ISL_881972, EPI_ISL_881973, EPI_ISL_881974, EPI_ISL_881975, EPI_ISL_881976, EPI_ISL_881977, EPI_ISL_881978, EPI_ISL_881979, EPI_ISL_881980, EPI_ISL_881981, EPI_ISL_881982, EPI_ISL_881983, EPI_ISL_881984, EPI_ISL_881985, EPI_ISL_881986, EPI_ISL_881987, EPI_ISL_881988, EPI_ISL_881989, EPI_ISL_881990, EPI_ISL_881991, EPI_ISL_881992, EPI_ISL_881993, EPI_ISL_881994, EPI_ISL_881995, EPI_ISL_881996, EPI_ISL_881997, EPI_ISL_881998, EPI_ISL_881999, EPI_ISL_882000, EPI_ISL_882001, EPI_ISL_882002, EPI_ISL_882003, EPI_ISL_882004, EPI_ISL_882005, EPI_ISL_882006, EPI_ISL_882007, EPI_ISL_882008, EPI_ISL_882009, EPI_ISL_882010, EPI_ISL_882011, EPI_ISL_882012, EPI_ISL_882013, EPI_ISL_882014, EPI_ISL_882015, EPI_ISL_882016, EPI_ISL_882017, EPI_ISL_882018, EPI_ISL_882019, EPI_ISL_882020, EPI_ISL_882021, EPI_ISL_882022, EPI_ISL_882023, EPI_ISL_882024, EPI_ISL_882025, EPI_ISL_882026, EPI_ISL_882027, EPI_ISL_882028, EPI_ISL_882029, EPI_ISL_882030, EPI_ISL_882031, EPI_ISL_882032, EPI_ISL_882033, EPI_ISL_882034, EPI_ISL_882035, EPI_ISL_882036, EPI_ISL_882037, EPI_ISL_882038, EPI_ISL_882039, EPI_ISL_882040, EPI_ISL_882041, EPI_ISL_882042, EPI_ISL_882043, EPI_ISL_882044, EPI_ISL_882045, EPI_ISL_882046, EPI_ISL_882047, EPI_ISL_882048, EPI_ISL_882049, EPI_ISL_882050, EPI_ISL_882051, EPI_ISL_882052, EPI_ISL_882053, EPI_ISL_882054, EPI_ISL_882055, EPI_ISL_882056, EPI_ISL_882057, EPI_ISL_882058, EPI_ISL_882059, EPI_ISL_882060, EPI_ISL_882061, EPI_ISL_882062, EPI_ISL_882063, EPI_ISL_882064, EPI_ISL_882065, EPI_ISL_882066, EPI_ISL_882067, EPI_ISL_882068, EPI_ISL_882069, EPI_ISL_882070, EPI_ISL_882071, EPI_ISL_882072, EPI_ISL_882073, EPI_ISL_882074, EPI_ISL_882075, EPI_ISL_882076, EPI_ISL_882077, EPI_ISL_882078, EPI_ISL_882079, EPI_ISL_882080, EPI_ISL_882081, EPI_ISL_882082, EPI_ISL_882083, EPI_ISL_882084, EPI_ISL_882085, EPI_ISL_882086, EPI_ISL_882087, EPI_ISL_882088, EPI_ISL_882089, EPI_ISL_882090, EPI_ISL_882091, EPI_ISL_882092, EPI_ISL_882093, EPI_ISL_882094, EPI_ISL_882095, EPI_ISL_882096, EPI_ISL_882097, EPI_ISL_882098, EPI_ISL_882099, EPI_ISL_882100, EPI_ISL_882101, EPI_ISL_882102, EPI_ISL_882103, EPI_ISL_882104, EPI_ISL_882105, EPI_ISL_882106, EPI_ISL_882107, EPI_ISL_882108, EPI_ISL_882109, EPI_ISL_882110, EPI_ISL_882111, EPI_ISL_882112, EPI_ISL_882113, EPI_ISL_882114, EPI_ISL_882115, EPI_ISL_882116, EPI_ISL_882117, EPI_ISL_882118, EPI_ISL_882119, EPI_ISL_882120, EPI_ISL_882121, EPI_ISL_882122, EPI_ISL_882123, EPI_ISL_882124, EPI_ISL_882125, EPI_ISL_882126, EPI_ISL_882127, EPI_ISL_882128, EPI_ISL_882129, EPI_ISL_882130, EPI_ISL_882131, EPI_ISL_882132, EPI_ISL_882133, EPI_ISL_882134, EPI_ISL_882135, EPI_ISL_882136, EPI_ISL_882137, EPI_ISL_882138, EPI_ISL_882139, EPI_ISL_882140, EPI_ISL_882141, EPI_ISL_882142, EPI_ISL_882143, EPI_ISL_882144, EPI_ISL_882145, EPI_ISL_882146, EPI_ISL_882147, EPI_ISL_882148, EPI_ISL_882149, EPI_ISL_882150, EPI_ISL_882151, EPI_ISL_882152, EPI_ISL_882153, EPI_ISL_882154, EPI_ISL_882155, EPI_ISL_882156, EPI_ISL_882157, EPI_ISL_882158, EPI_ISL_882159, EPI_ISL_882160, EPI_ISL_882161, EPI_ISL_882162, EPI_ISL_882163, EPI_ISL_882164, EPI_ISL_882165, EPI_ISL_882166, EPI_ISL_882167, EPI_ISL_882168, EPI_ISL_882169, EPI_ISL_882170, EPI_ISL_882171, EPI_ISL_882172, EPI_ISL_882173, EPI_ISL_882174, EPI_ISL_882175, EPI_ISL_882176, EPI_ISL_882177, EPI_ISL_882178, EPI_ISL_882179, EPI_ISL_882180, EPI_ISL_882181, EPI_ISL_882182, EPI_ISL_882183, EPI_ISL_882184, EPI_ISL_882185, EPI_ISL_882186, EPI_ISL_882187, EPI_ISL_882188, EPI_ISL_882189, EPI_ISL_882190, EPI_ISL_882191, EPI_ISL_882192, EPI_ISL_882193, EPI_ISL_882194, EPI_ISL_882195, EPI_ISL_882196, EPI_ISL_882197, EPI_ISL_882198, EPI_ISL_882199, EPI_ISL_882200, EPI_ISL_882201, EPI_ISL_882202, EPI_ISL_882203, EPI_ISL_882204, EPI_ISL_882205, EPI_ISL_882206, EPI_ISL_882207, EPI_ISL_882208, EPI_ISL_882209, EPI_ISL_882210, EPI_ISL_882211, EPI_ISL_882212, EPI_ISL_882213, EPI_ISL_882214, EPI_ISL_882215, EPI_ISL_882216, EPI_ISL_882217, EPI_ISL_882218, EPI_ISL_882219, EPI_ISL_882220, EPI_ISL_882221, EPI_ISL_882222, EPI_ISL_882223, EPI_ISL_882224, EPI_ISL_882225, EPI_ISL_882226, EPI_ISL_882227, EPI_ISL_882228, EPI_ISL_882229, EPI_ISL_882230, EPI_ISL_882231, EPI_ISL_882232, EPI_ISL_882233, EPI_ISL_882234, EPI_ISL_882235, EPI_ISL_882236, EPI_ISL_882237, EPI_ISL_882238, EPI_ISL_882239, EPI_ISL_882240, EPI_ISL_882241, EPI_ISL_882242, EPI_ISL_882243, EPI_ISL_882244, EPI_ISL_882245, EPI_ISL_882246, EPI_ISL_882247, EPI_ISL_882248, EPI_ISL_882249, EPI_ISL_882250, EPI_ISL_882251, EPI_ISL_882252, EPI_ISL_882253, EPI_ISL_882254, EPI_ISL_882255, EPI_ISL_882256, EPI_ISL_882257, EPI_ISL_882258, EPI_ISL_882259, EPI_ISL_882260, EPI_ISL_882261, EPI_ISL_882262, EPI_ISL_882263, EPI_ISL_882264, EPI_ISL_882265, EPI_ISL_882266, EPI_ISL_882267, EPI_ISL_882268, EPI_ISL_882269, EPI_ISL_882270, EPI_ISL_882271, EPI_ISL_882272, EPI_ISL_882273, EPI_ISL_882274, EPI_ISL_882275, EPI_ISL_882276, EPI_ISL_882277, EPI_ISL_882278, EPI_ISL_882279, EPI_ISL_882280, EPI_ISL_882281, EPI_ISL_882282, EPI_ISL_882283, EPI_ISL_882284, EPI_ISL_882285, EPI_ISL_882286, EPI_ISL_882287, EPI_ISL_882288, EPI_ISL_882289, EPI_ISL_882290, EPI_ISL_882291, EPI_ISL_882292, EPI_ISL_882293, EPI_ISL_882294, EPI_ISL_882295, EPI_ISL_882296, EPI_ISL_882297, EPI_ISL_882298			
see above	Lighthouse Lab in Alderley Park	Wellcome Sanger Institute for the COVID-19 Genomics UK (COG-UK) Consortium	Jacquelyn Wynn, Mairead Hyland, The Lighthouse Lab in Alderley Park and Alex Alderton, Roberto Amato, Sonia Goncalves, Ewan Harrison, David K. Jackson, Ian Johnston, Dominic Kwiatkowski, Cordelia Langford, John Sillitoe on behalf of the Wellcome Sanger Institute COVID-19 Surveillance Team
EPI_ISL_882620, EPI_ISL_882624, EPI_ISL_882625, EPI_ISL_882627, EPI_ISL_882631, EPI_ISL_882632	Hospital Ramón y Cajal	Hospital Ramón y Cajal	José M Gonzalez-Alba, Concepción Rodríguez, Melanie Abreu, Laura Martínez, Val F Lanza, Luz Leticia Olavarrieta, Rafael Cantón, JC Galán
EPI_ISL_882666	UMS de Juquitiba	Instituto Adolfo Lutz, Interdiciplinary Procedures Center, Strategic Laboratory	Claudio Tavares Sacchi, Claudia Regina Gonçalves, Erica Valessa Ramos Gomes, Karoline Rodrigues Campos
EPI_ISL_882667	Hospital de Clinicas Caieiras	Instituto Adolfo Lutz, Interdiciplinary Procedures Center, Strategic Laboratory	Claudio Tavares Sacchi, Claudia Regina Gonçalves, Erica Valessa Ramos Gomes, Karoline Rodrigues Campos
EPI_ISL_882668	UMS de Juquitiba	Instituto Adolfo Lutz, Interdiciplinary Procedures Center, Strategic Laboratory	Claudio Tavares Sacchi, Claudia Regina Gonçalves, Erica Valessa Ramos Gomes, Karoline Rodrigues Campos
EPI_ISL_882963, EPI_ISL_882964, EPI_ISL_882965, EPI_ISL_882966, EPI_ISL_882986, EPI_ISL_882988, EPI_ISL_882989, EPI_ISL_882990, EPI_ISL_882991, EPI_ISL_882992, EPI_ISL_882993, EPI_ISL_882994, EPI_ISL_882995, EPI_ISL_882996			
see above	MD Laboratories	Los Angeles County PHL	P. Hemarajata et al.
EPI_ISL_883012, EPI_ISL_883013, EPI_ISL_883014, EPI_ISL_883016, EPI_ISL_883017	Maryland Public Health Laboratory	Maryland Public Health Laboratory	Maryland Department of Health Laboratories Administration
EPI_ISL_883428, EPI_ISL_883431, EPI_ISL_883432, EPI_ISL_883433, EPI_ISL_883434, EPI_ISL_883445, EPI_ISL_883446, EPI_ISL_883447, EPI_ISL_883471, EPI_ISL_883472, EPI_ISL_883474, EPI_ISL_883475, EPI_ISL_883476, EPI_ISL_883477, EPI_ISL_883478, EPI_ISL_883479, EPI_ISL_883483, EPI_ISL_883484, EPI_ISL_883485, EPI_ISL_883486, EPI_ISL_883491, EPI_ISL_883494, EPI_ISL_883498			
see above	NORTHWELL HEALTH LABORATORIES	Wadsworth Center, New York State Department of Health	Kirsten St. George, Daryl M. Lamson, Alexis Russel, Matthew Shudt, Melissa A Leisner, Jonathan Plitnick, Navjot Singh, John Kelly, Erasmus Schneider, Erica Lasek-Nesselquist
EPI_ISL_883959	Labo Analyses Med	National Reference Center for Viruses of Respiratory Infections, Institut Pasteur, Paris	Marion Barbet, Sylvie Behillil, Méline Bizard, Angela Brisebarre, Camille Capel, Etienne Simon-Lorière, Vincent Enouf, Maud Vanpeene, Sylvie van der Werf
EPI_ISL_883965, EPI_ISL_883966, EPI_ISL_883971	Labo Analyses med	National Reference Center for Viruses of Respiratory Infections, Institut Pasteur, Paris	Marion Barbet, Sylvie Behillil, Méline Bizard, Angela Brisebarre, Camille Capel, Etienne Simon-Lorière, Vincent Enouf, Maud Vanpeene, Sylvie van der Werf,Amzalag Jonas
EPI_ISL_883977, EPI_ISL_883978, EPI_ISL_883982, EPI_ISL_883983, EPI_ISL_883984	Hopital	National Reference Center for Viruses of Respiratory Infections, Institut Pasteur, Paris	Marion Barbet, Sylvie Behillil, Méline Bizard, Angela Brisebarre, Camille Capel, Etienne Simon-Lorière, Vincent Enouf, Maud Vanpeene, Sylvie van der Werf,Fourgeaud Jacques
EPI_ISL_883997	Labo Analyses Med	National Reference Center for Viruses of Respiratory	Marion Barbet, Sylvie Behillil, Méline Bizard, Angela Brisebarre, Camille Capel, Etienne Simon-Lorière, Vincent Enouf, Maud Vanpeene, Sylvie van der

		Infections, Institut Pasteur, Paris	Werf,Selas Labomaine
EPI_ISL_884004, EPI_ISL_884005	Labo Analyses Med	National Reference Center for Viruses of Respiratory Infections, Institut Pasteur, Paris	Marion Barbet, Sylvie Behillil, Méline Bizard, Angela Brisebarre, Camille Capel, Etienne Simon-Lorière, Vincent Enouf, Maud Vanpeene, Sylvie van der Werf,Rousset Dominique
EPI_ISL_884006, EPI_ISL_884007	hopital	National Reference Center for Viruses of Respiratory Infections, Institut Pasteur, Paris	Marion Barbet, Sylvie Behillil, Méline Bizard, Angela Brisebarre, Camille Capel, Etienne Simon-Lorière, Vincent Enouf, Maud Vanpeene, Sylvie van der Werf,Combe Patrice
EPI_ISL_884065, EPI_ISL_884066, EPI_ISL_884067, EPI_ISL_884068, EPI_ISL_884069, EPI_ISL_884070, EPI_ISL_884071, EPI_ISL_884072, EPI_ISL_884073, EPI_ISL_884074, EPI_ISL_884075, EPI_ISL_884076, EPI_ISL_884077, EPI_ISL_884078, EPI_ISL_884079			
see above	ALBANY MEDICAL CENTER HOSPITAL CLINICAL LABORATORIES	Wadsworth Center, New York State Department of Health	Kirsten St. George, Daryl M. Lamson, Alexis Russel, Matthew Shudt, Melissa A Leisner, Jonathan Plitnick, Navjot Singh, John Kelly, Erasmus Schneider, Erica Lasek-Nesselquist
EPI_ISL_884223, EPI_ISL_884224, EPI_ISL_884225, EPI_ISL_884226, EPI_ISL_884242, EPI_ISL_884243, EPI_ISL_884244, EPI_ISL_884245, EPI_ISL_884246, EPI_ISL_884247	Kansas Health and Environmental Lab	Kansas Health and Environmental Lab	Mike Grose, Paige Drury, Carissa Robertson, Ben Olsen, and Phil Adam
EPI_ISL_884789, EPI_ISL_884790, EPI_ISL_884791, EPI_ISL_884792, EPI_ISL_884793, EPI_ISL_884794, EPI_ISL_884795, EPI_ISL_884796, EPI_ISL_884797, EPI_ISL_884798, EPI_ISL_884799, EPI_ISL_884800, EPI_ISL_884801, EPI_ISL_884802, EPI_ISL_884803, EPI_ISL_884804, EPI_ISL_884805, EPI_ISL_884806, EPI_ISL_884807, EPI_ISL_884808, EPI_ISL_884809, EPI_ISL_884810, EPI_ISL_884811, EPI_ISL_884812, EPI_ISL_884813, EPI_ISL_884814, EPI_ISL_884815, EPI_ISL_884816, EPI_ISL_884817, EPI_ISL_884818, EPI_ISL_884819, EPI_ISL_884820, EPI_ISL_884821, EPI_ISL_884822, EPI_ISL_884823, EPI_ISL_884824, EPI_ISL_884825			
see above	Respiratory Viruses Branch, Centers for Disease Control and Prevention	Respiratory Viruses Branch, Centers for Disease Control and Prevention	Cook,P.W., Batra,D., Rambo-Martin,B.L., de Feo,E., Antico,J., Tran,C., Tolentino,M., Wickline,S., Gietzen,K., Sickler,B., Liu,J., Allen,E., Febbo,P., Galloway,S., Washington,N.L., White,S., Levan,G., Barret,K.S., Cirulli,E., Bolze,A., Ascencio,A., Rivera-Garcia,C., Cho,R., Nguyen,J., Wang,S., Ramirez,J., Cassens,T., Sandoval,E., Isaksson,M., Lee,W., Becker,D., Laurent,M., Lu,J., Paden,C.R., Tong,S., MacCannell,D.
EPI_ISL_886158, EPI_ISL_886160, EPI_ISL_886174, EPI_ISL_886176, EPI_ISL_886177, EPI_ISL_886180, EPI_ISL_886181, EPI_ISL_886184, EPI_ISL_886188, EPI_ISL_886189, EPI_ISL_886191, EPI_ISL_886199, EPI_ISL_886200, EPI_ISL_886201, EPI_ISL_886204, EPI_ISL_886205, EPI_ISL_886207, EPI_ISL_886212, EPI_ISL_886218, EPI_ISL_886220, EPI_ISL_886223, EPI_ISL_886224, EPI_ISL_886233, EPI_ISL_886237, EPI_ISL_886241, EPI_ISL_886243, EPI_ISL_886244, EPI_ISL_886245, EPI_ISL_886254, EPI_ISL_886259, EPI_ISL_886263, EPI_ISL_886265, EPI_ISL_886267, EPI_ISL_886269, EPI_ISL_886271, EPI_ISL_886276, EPI_ISL_886280, EPI_ISL_886281, EPI_ISL_886289, EPI_ISL_886293, EPI_ISL_886297, EPI_ISL_886298, EPI_ISL_886302, EPI_ISL_886303, EPI_ISL_886321, EPI_ISL_886330, EPI_ISL_886331, EPI_ISL_886333, EPI_ISL_886336, EPI_ISL_886337, EPI_ISL_886338, EPI_ISL_886339, EPI_ISL_886341, EPI_ISL_886342, EPI_ISL_886345, EPI_ISL_886348, EPI_ISL_886350, EPI_ISL_886352, EPI_ISL_886353, EPI_ISL_886354, EPI_ISL_886355, EPI_ISL_886358, EPI_ISL_886363, EPI_ISL_886367, EPI_ISL_886369, EPI_ISL_886370, EPI_ISL_886371, EPI_ISL_886372, EPI_ISL_886374, EPI_ISL_886375, EPI_ISL_886376, EPI_ISL_886378, EPI_ISL_886385, EPI_ISL_886388, EPI_ISL_886391, EPI_ISL_886402, EPI_ISL_886403, EPI_ISL_886406, EPI_ISL_886409, EPI_ISL_886411, EPI_ISL_886416, EPI_ISL_886418, EPI_ISL_886423, EPI_ISL_886426, EPI_ISL_886430, EPI_ISL_886431, EPI_ISL_886432, EPI_ISL_886433, EPI_ISL_886434, EPI_ISL_886435, EPI_ISL_886436, EPI_ISL_886438, EPI_ISL_886443, EPI_ISL_886445, EPI_ISL_886447, EPI_ISL_886448, EPI_ISL_886451, EPI_ISL_886463, EPI_ISL_886464, EPI_ISL_886466, EPI_ISL_886467, EPI_ISL_886469, EPI_ISL_886473, EPI_ISL_886479, EPI_ISL_886482, EPI_ISL_886483, EPI_ISL_886487, EPI_ISL_886488, EPI_ISL_886489, EPI_ISL_886495, EPI_ISL_886496, EPI_ISL_886498, EPI_ISL_886499, EPI_ISL_886504, EPI_ISL_886510, EPI_ISL_886511, EPI_ISL_886514, EPI_ISL_886515, EPI_ISL_886518, EPI_ISL_886519, EPI_ISL_886520, EPI_ISL_886525, EPI_ISL_886528, EPI_ISL_886530, EPI_ISL_886534, EPI_ISL_886535, EPI_ISL_886547, EPI_ISL_886553, EPI_ISL_886557, EPI_ISL_886558, EPI_ISL_886564, EPI_ISL_886568, EPI_ISL_886569, EPI_ISL_886571, EPI_ISL_886572, EPI_ISL_886574, EPI_ISL_886575, EPI_ISL_886577, EPI_ISL_886584, EPI_ISL_886587, EPI_ISL_886588, EPI_ISL_886591, EPI_ISL_886594, EPI_ISL_886597, EPI_ISL_886599, EPI_ISL_886602, EPI_ISL_886606, EPI_ISL_886608, EPI_ISL_886609, EPI_ISL_886618, EPI_ISL_886620, EPI_ISL_886622, EPI_ISL_886625, EPI_ISL_886630, EPI_ISL_886632, EPI_ISL_886637, EPI_ISL_886638, EPI_ISL_886645, EPI_ISL_886646, EPI_ISL_886649, EPI_ISL_886653, EPI_ISL_886655, EPI_ISL_886656, EPI_ISL_886657, EPI_ISL_886659, EPI_ISL_886663, EPI_ISL_886668, EPI_ISL_886669, EPI_ISL_886672, EPI_ISL_886684, EPI_ISL_886685, EPI_ISL_886688, EPI_ISL_886699, EPI_ISL_886706, EPI_ISL_886707, EPI_ISL_886710, EPI_ISL_886711, EPI_ISL_886716, EPI_ISL_886719, EPI_ISL_886721, EPI_ISL_886725, EPI_ISL_886726, EPI_ISL_886728, EPI_ISL_886729, EPI_ISL_886730, EPI_ISL_886743, EPI_ISL_886745, EPI_ISL_886746, EPI_ISL_886747, EPI_ISL_886749, EPI_ISL_886751, EPI_ISL_886757, EPI_ISL_886764, EPI_ISL_886767, EPI_ISL_886771, EPI_ISL_886773, EPI_ISL_886774, EPI_ISL_886777, EPI_ISL_886778, EPI_ISL_886779, EPI_ISL_886783, EPI_ISL_886788, EPI_ISL_886794, EPI_ISL_886796, EPI_ISL_886802, EPI_ISL_886807, EPI_ISL_886813, EPI_ISL_886818, EPI_ISL_886824, EPI_ISL_886828, EPI_ISL_886829, EPI_ISL_886835, EPI_ISL_886836, EPI_ISL_886840, EPI_ISL_886842, EPI_ISL_886847, EPI_ISL_886848, EPI_ISL_886849, EPI_ISL_886850, EPI_ISL_886851, EPI_ISL_886852, EPI_ISL_886856, EPI_ISL_886861, EPI_ISL_886867, EPI_ISL_886869, EPI_ISL_886873, EPI_ISL_886874, EPI_ISL_886875, EPI_ISL_886876, EPI_ISL_886883, EPI_ISL_886885, EPI_ISL_886888, EPI_ISL_886894, EPI_ISL_886896, EPI_ISL_886901, EPI_ISL_886902, EPI_ISL_886904, EPI_ISL_886905, EPI_ISL_886906, EPI_ISL_886910, EPI_ISL_886914, EPI_ISL_886915, EPI_ISL_886926, EPI_ISL_886934, EPI_ISL_886935, EPI_ISL_886936, EPI_ISL_886940, EPI_ISL_886941, EPI_ISL_886948, EPI_ISL_886950, EPI_ISL_886952, EPI_ISL_886953, EPI_ISL_886957, EPI_ISL_886958, EPI_ISL_886959, EPI_ISL_886961, EPI_ISL_886964, EPI_ISL_886966, EPI_ISL_886969, EPI_ISL_886970, EPI_ISL_886971, EPI_ISL_886972, EPI_ISL_886974, EPI_ISL_886976, EPI_ISL_886978, EPI_ISL_886981, EPI_ISL_886983, EPI_ISL_886986, EPI_ISL_886988, EPI_ISL_887012, EPI_ISL_887017, EPI_ISL_887018, EPI_ISL_887020, EPI_ISL_887025, EPI_ISL_887028, EPI_ISL_887032, EPI_ISL_887033, EPI_ISL_887037, EPI_ISL_887041, EPI_ISL_887043, EPI_ISL_887048, EPI_ISL_887049, EPI_ISL_887050, EPI_ISL_887052, EPI_ISL_887061, EPI_ISL_887073, EPI_ISL_887082, EPI_ISL_887089, EPI_ISL_887094, EPI_ISL_887095, EPI_ISL_887097, EPI_ISL_887098, EPI_ISL_887099			
see above	Labcorp	Genomics and Discovery, Respiratory Viruses Branch, Division of Viral Diseases, Centers for Disease Control and Prevention	Peter W. Cook,Dhwani Batra,Ben L. Rambo-Martin,Summer Galloway,Brian Krueger,Minoo Agarwal,Eyad Almasri,Debbie Boles,Ayla Burns,Nuthawin Charoensri,Oren Cohen,Susan Countryman,Mary Ann Cristobal,Bobbi Croy,Suzanne Dale,Hrushikesh Deshmukh,Amanda Douglas,Vincent Drouillon,Marcia Eisenberg,Howard Engler,Rama Ghatti,Prashant Gupta,Susan Hicks,Jake Humphrey,Lax Iyer,Manoj Jain,Mohan Kolli,Tim Kuphal,Stanley Letovsky,Michael Levandoski,Craig Lukasik,Jonathan Meltzer,Brian Norvell,Mindy Nye,Scott Parker,Christos Petropoulos,John Pruitt,Steven Ragan,Scott Ryan,Mike Sapeta,Jana Schroth,Suresh Babu Selvaraju,Goran Stevovic,Amanda Suchanek,Andrea Throop,Lyndon Tilson,Thomas Urban,Joe Voshell,Kimberly Wagner,Jonathan Williams,Mary Williamson,Qian Zeng,Tricia Zwiefelhofer,Clinton R. Paden,Suxiang Tong,Duncan MacCannell,
EPI_ISL_887133, EPI_ISL_887140	Institute of Medical Microbiology and Hospital Hygiene	Institute of Medical Microbiology and Hospital Hygiene	Prof. Dr. Achim Kaasch, Aljoscha Tersteegen
EPI_ISL_888644, EPI_ISL_888645, EPI_ISL_888646, EPI_ISL_888647, EPI_ISL_888648, EPI_ISL_888651, EPI_ISL_888791, EPI_ISL_888792, EPI_ISL_888793, EPI_ISL_888794, EPI_ISL_888795, EPI_ISL_888796, EPI_ISL_888797, EPI_ISL_888798, EPI_ISL_888799, EPI_ISL_888800, EPI_ISL_888801, EPI_ISL_888802			
see above	Univeristy of New Mexico Hospital	Center for Global Health, University of New Mexico Health Sciences Center	Daryl Domman, Kurt Schwalm, Justin Bacca, Jon Femling, Darrell Dinwiddie
EPI_ISL_888848, EPI_ISL_888849, EPI_ISL_888872, EPI_ISL_888896, EPI_ISL_888901	Michigan Department of Health and Human Services, Bureau of Laboratories	Michigan Department of Health and Human Services, Bureau of Laboratories	Blankenship HM, Riner D, Soehnlen MK
EPI_ISL_889671, EPI_ISL_889672, EPI_ISL_889673, EPI_ISL_889674, EPI_ISL_889675, EPI_ISL_889676, EPI_ISL_889677, EPI_ISL_889678, EPI_ISL_889679, EPI_ISL_889680, EPI_ISL_889681, EPI_ISL_889682, EPI_ISL_889683, EPI_ISL_889684, EPI_ISL_889685			
see above	LSUHS Emerging Viral Threat Laboratory	Microbial Genome Sequencing Center	Jeremy P. Kamil, Jennifer L. Carroll, Camille F. Abshire, Maarten Van Diest, Mohammed N.A. Siddiquey, Andrew D. Yurochko, Martin J. Sapp, Rona S. Scott, Christopher G. Kevil, Daniel J. Snyder, Vaughn S. Cooper, John A. Vanchiere
EPI_ISL_890195, EPI_ISL_890196, EPI_ISL_890197, EPI_ISL_890198, EPI_ISL_890199, EPI_ISL_890200, EPI_ISL_890201, EPI_ISL_890202, EPI_ISL_890203, EPI_ISL_890204, EPI_ISL_890205, EPI_ISL_890206, EPI_ISL_890207, EPI_ISL_890208, EPI_ISL_890209, EPI_ISL_890210, EPI_ISL_890211, EPI_ISL_890212, EPI_ISL_890213, EPI_ISL_890214, EPI_ISL_890215, EPI_ISL_890216, EPI_ISL_890217, EPI_ISL_890218, EPI_ISL_890219, EPI_ISL_890220, EPI_ISL_890221, EPI_ISL_890222, EPI_ISL_890223, EPI_ISL_890224, EPI_ISL_890225, EPI_ISL_890226, EPI_ISL_890227, EPI_ISL_890228, EPI_ISL_890229, EPI_ISL_890230, EPI_ISL_890231			
see above	Cancer Biology Department, National Cancer Institute	Cancer Biology Department, National Cancer Institute	Zekri,A.N., Sedawy,M.G., Ahmed,O.S., Hafez,M.M., Soliman,H.K., Bahnassy,A.A., Elhosieny,F.W., Gad,A.E., Hamdy,M.S., Soliman,M.S., Soliman,L., Abouelhoda,M.
EPI_ISL_890344, EPI_ISL_890348	KU Leuven, Rega Institute, Clinical and Epidemiological Virology	KU Leuven, Rega Institute, Clinical and Epidemiological Virology	Tony Wawina-Bokalanga, Bert Vanmechelen, Joan Marti-Carerras, Piet Maes
EPI_ISL_890355, EPI_ISL_890356, EPI_ISL_890357	Hospital	National Reference Center for Viruses of Respiratory Infections, Institut Pasteur, Paris	Marion Barbet, Sylvie Behillil, Méline Bizard, Angela Brisebarre, Camille Capel, Etienne Simon-Lorière, Vincent Enouf, Maud Vanpeene, Sylvie van der Werf,Combe Patrice
EPI_ISL_890960, EPI_ISL_890961, EPI_ISL_890962, EPI_ISL_890963, EPI_ISL_890964, EPI_ISL_890965, EPI_ISL_890966, EPI_ISL_890968	Seattle Flu Study	Seattle Flu Study	Deborah A. Nickerson, Chris D. Frazar, Jover Lee, Benjamin Pelle, Erica Ryke, Matthew Richardson, Amanda Adler, Elisabeth Brandstetter, Peter D. Han, Kairsten Fay, Misja Ilcisin, Kirsten Lacombe, Thomas R. Sibley, Melissa Truong, Caitlin R. Wolf, Karen Cowgill, Stephanie Schrag, Jeff Duchin, Michael Boeckh, Janet A. Englund, Michael Famulare, Barry R. Lutz, Mark J. Rieder, Lea M. Starita, Matthew Thompson, Helen Y. Chu, Trevor Bedford, Jay Shendure
EPI_ISL_891059	FidaLab	Seattle Flu Study	Deborah A. Nickerson, Chris D. Frazar, Jover Lee, Benjamin Pelle, Erica Ryke, Matthew Richardson, Amanda Adler, Elisabeth Brandstetter, Peter D. Han, Kairsten Fay, Misja Ilcisin, Kirsten Lacombe, Thomas R. Sibley, Melissa Truong, Caitlin R. Wolf, Romesh Gautom, Geoff Melly, Brian Hiatt, Philip Dykema, Scott Lindquist, Michael Boeckh, Janet A. Englund, Michael Famulare, Barry R. Lutz, Mark J. Rieder, Lea M. Starita, Matthew Thompson, Helen Y. Chu, Jay Shendure, Trevor Bedford
EPI_ISL_891084, EPI_ISL_891085, EPI_ISL_891086, EPI_ISL_891087, EPI_ISL_891088, EPI_ISL_891089, EPI_ISL_891090, EPI_ISL_891091	Seattle Flu Study	Seattle Flu Study	Deborah A. Nickerson, Chris D. Frazar, Jover Lee, Benjamin Pelle, Erica Ryke, Matthew Richardson, Amanda Adler, Elisabeth Brandstetter, Peter D. Han, Kairsten Fay, Misja Ilcisin, Kirsten Lacombe, Thomas R. Sibley, Melissa Truong, Caitlin R. Wolf, Karen Cowgill, Stephanie Schrag, Jeff Duchin, Michael Boeckh, Janet A. Englund, Michael Famulare, Barry R. Lutz, Mark J. Rieder, Lea M. Starita, Matthew Thompson, Helen Y. Chu, Trevor Bedford, Jay Shendure
EPI_ISL_891094, EPI_ISL_891095, EPI_ISL_891096, EPI_ISL_891097, EPI_ISL_891098, EPI_ISL_891099, EPI_ISL_891100, EPI_ISL_891101, EPI_ISL_891102, EPI_ISL_891103, EPI_ISL_891104, EPI_ISL_891105, EPI_ISL_891106, EPI_ISL_891107, EPI_ISL_891108, EPI_ISL_891109, EPI_ISL_891110, EPI_ISL_891111, EPI_ISL_891112, EPI_ISL_891113, EPI_ISL_891114, EPI_ISL_891115, EPI_ISL_891116, EPI_ISL_891117, EPI_ISL_891118, EPI_ISL_891119, EPI_ISL_891120, EPI_ISL_891121, EPI_ISL_891122, EPI_ISL_891123, EPI_ISL_891124, EPI_ISL_891125, EPI_ISL_891126, EPI_ISL_891127, EPI_ISL_891128, EPI_ISL_891129, EPI_ISL_891130, EPI_ISL_891131, EPI_ISL_891132, EPI_ISL_891133, EPI_ISL_891134, EPI_ISL_891135			

see above	Altius Institute for Biomedical Sciences	Seattle Flu Study	Deborah A. Nickerson, Chris D. Frazar, Jover Lee, Benjamin Pelle, Erica Ryke, Matthew Richardson, Amanda Adler, Elisabeth Brandstetter, Peter D. Han, Kairsten Fay, Misja Ilcisin, Kirsten Lacombe, Thomas R. Sibley, Melissa Truong, Caitlin R. Wolf, Ryan Alexander, Daniel Bates, Rebecca Bruders, Stephanie DeBaun, Clem Green, Muhammad Halimun, Kneshay Harper, Matt Hartman, Andrew Meuser, Alex Nguyen, Truong Nguyen, Sofia Olsson, Sadie Patraw, Hannah Petersen, Tobias Ragoczy, Joshua Richards, Jacob Rodriguez, John Stamatoyannopoulos, Julia Wald, Olivia Waltner, Michael Boeckh, Janet A. Englund, Michael Famulare, Barry R. Lutz, Mark J. Rieder, Lea M. Starita, Matthew Thompson, Helen Y. Chu, Jay Shendure, Trevor Bedford
EPI_ISL_891141	Indiana Animal Disease Diagnostic Laboratory	Carpi Laboratory - Purdue University	Jack Dorman, Ilinca I Ciubotariu, Lev Gorenstein, Abebe A Fola, G Kenitra Hendrix, Rebecca P Wilkes, Giovanna Carpi
EPI_ISL_892052, EPI_ISL_892053, EPI_ISL_892055, EPI_ISL_892067, EPI_ISL_892068, EPI_ISL_892069, EPI_ISL_892070, EPI_ISL_892071, EPI_ISL_892072, EPI_ISL_892073, EPI_ISL_892074, EPI_ISL_892075, EPI_ISL_892076, EPI_ISL_892077, EPI_ISL_892078, EPI_ISL_892079, EPI_ISL_892080, EPI_ISL_892081, EPI_ISL_892082, EPI_ISL_892083, EPI_ISL_892084, EPI_ISL_892085, EPI_ISL_892086, EPI_ISL_892087, EPI_ISL_892088, EPI_ISL_892089, EPI_ISL_892090, EPI_ISL_892091, EPI_ISL_892092, EPI_ISL_892093, EPI_ISL_892094, EPI_ISL_892095, EPI_ISL_892096, EPI_ISL_892097, EPI_ISL_892098, EPI_ISL_892099, EPI_ISL_892100, EPI_ISL_892101, EPI_ISL_892102, EPI_ISL_892103, EPI_ISL_892104, EPI_ISL_892105, EPI_ISL_892106, EPI_ISL_892107, EPI_ISL_892108, EPI_ISL_892109, EPI_ISL_892110, EPI_ISL_892111, EPI_ISL_892112, EPI_ISL_892113, EPI_ISL_892114, EPI_ISL_892115, EPI_ISL_892116, EPI_ISL_892117, EPI_ISL_892118, EPI_ISL_892119, EPI_ISL_892120, EPI_ISL_892121, EPI_ISL_892122, EPI_ISL_892123, EPI_ISL_892124, EPI_ISL_892125, EPI_ISL_892206			
see above	Lighthouse Lab in Alderley Park	Wellcome Sanger Institute for the COVID-19 Genomics UK (COG-UK) Consortium	Jacquelyn Wynn, Mairead Hyland, The Lighthouse Lab in Alderley Park and Alex Alderton, Roberto Amato, Sonia Goncalves, Ewan Harrison, David K. Jackson, Ian Johnston, Dominic Kwiatkowski, Cordelia Langford, John Sillitoe on behalf of the Wellcome Sanger Institute COVID-19 Surveillance Team
EPI_ISL_892249, EPI_ISL_892251, EPI_ISL_892263, EPI_ISL_892264, EPI_ISL_892265	MD Laboratories	Los Angeles County PHL	P. Hemarajata et al.
EPI_ISL_893214	Indiana Animal Disease Diagnostic Laboratory	Carpi Laboratory - Purdue University	Jack Dorman, Ilinca I Ciubotariu, Lev Gorenstein, Abebe A Fola, G Kenitra Hendrix, Rebecca P Wilkes, Giovanna Carpi
EPI_ISL_893764, EPI_ISL_893766	Institute of Virology, Medical Center, University of Freiburg, Freiburg, Germany	Institute of Virology, Clinial Virus Genomics, Medical Center, University of Freiburg, Freiburg, Germany	Jonas Fuchs, Lisa Kern, Sandra Reuter, Hajo Grundmann, Marcus Panning
EPI_ISL_894175, EPI_ISL_894177, EPI_ISL_894182, EPI_ISL_894184, EPI_ISL_894188, EPI_ISL_894190, EPI_ISL_894194, EPI_ISL_894195, EPI_ISL_894199, EPI_ISL_894200, EPI_ISL_894201, EPI_ISL_894202, EPI_ISL_894203, EPI_ISL_894204, EPI_ISL_894205, EPI_ISL_894206, EPI_ISL_894207, EPI_ISL_894208, EPI_ISL_894214, EPI_ISL_894215, EPI_ISL_894217			
see above	KU Leuven, Rega Institute, Clinical and Epidemiological Virology	KU Leuven, Rega Institute, Clinical and Epidemiological Virology	Tony Wawina-Bokalanga, Bert Vanmechelen, Joan Marti-Carerras, Piet Maes
EPI_ISL_896069	Labormedizinisches Zentrum Dr Risch	University Hospital Basel, Clinical Bacteriology	Tim Roloff, Madlen Stange, Helena MB Seth-Smith, Alfredo Mari, Karoline Leuzinger, Julia Bielicki, Nadia Wohlwend,Martin Risch, Lorenz Risch, Manuel Battegay, Hans Hirsch, Adrian Egli
EPI_ISL_896085	Viollier AG	University Hospital Basel, Clinical Bacteriology	Tim Roloff, Madlen Stange, Helena MB Seth-Smith, Alfredo Mari, Karoline Leuzinger, Julia Bielicki, Christiane Beckmann, Manuel Battegay, Hans Hirsch, Adrian Egli
EPI_ISL_896099	Labormedizinisches Zentrum Dr Risch	University Hospital Basel, Clinical Bacteriology	Tim Roloff, Madlen Stange, Helena MB Seth-Smith, Alfredo Mari, Karoline Leuzinger, Julia Bielicki, Nadia Wohlwend,Martin Risch, Lorenz Risch, Manuel Battegay, Hans Hirsch, Adrian Egli
EPI_ISL_896149, EPI_ISL_896158	MEPHI, Aix Marseille University	MEPHI, Aix Marseille University	Anthony LEVASSEUR
EPI_ISL_896216, EPI_ISL_896227, EPI_ISL_896234, EPI_ISL_896244, EPI_ISL_896245, EPI_ISL_896246	Columbia University Irving Medical Center	Wadsworth Center, New York State Department of Health	Kirsten St. George, Daryl M. Lamson, Alexis Russel, Matthew Shudt, Melissa A Leisner, Jonathan Plitnick, Navjot Singh, John Kelly, Erasmus Schneider, Erica Lasek-Nesselquist
EPI_ISL_896300	BIO-REFERENCE LABORATORIES	Wadsworth Center, New York State Department of Health	Kirsten St. George, Daryl M. Lamson, Alexis Russel, Matthew Shudt, Melissa A Leisner, Jonathan Plitnick, Navjot Singh, John Kelly, Erasmus Schneider, Erica Lasek-Nesselquist
EPI_ISL_896390, EPI_ISL_896417, EPI_ISL_896418, EPI_ISL_896421	URMC LABS	Wadsworth Center, New York State Department of Health	Kirsten St. George, Daryl M. Lamson, Alexis Russel, Matthew Shudt, Melissa A Leisner, Jonathan Plitnick, Navjot Singh, John Kelly, Erasmus Schneider, Erica Lasek-Nesselquist
EPI_ISL_896488	KU Leuven, Rega Institute, Clinical and Epidemiological Virology	KU Leuven, Rega Institute, Clinical and Epidemiological Virology	Tony Wawina-Bokalanga, Bert Vanmechelen, Joan Marti-Carerras, Piet Maes
EPI_ISL_897687, EPI_ISL_897937	University Hospitals of Geneva, Laboratory of Virology	HUG, Laboratory of Virology and the Health2030 Genome Center	Samuel Cordey, Ana Rita Goncalves, Laurent Kaiser, Lorenzo Cerutti, Henri Pegeot, Melyssa Elies, Deborah Penet, Keith Harshman, Ioannis Xenarios, Emmanouil Dermitzakis
EPI_ISL_898009, EPI_ISL_898024, EPI_ISL_898030, EPI_ISL_898031	KU Leuven, Rega Institute, Clinical and Epidemiological Virology	KU Leuven, Rega Institute, Clinical and Epidemiological Virology	Tony Wawina-Bokalanga, Bert Vanmechelen, Joan Marti-Carerras, Piet Maes
EPI_ISL_899001, EPI_ISL_899012, EPI_ISL_899013, EPI_ISL_899014, EPI_ISL_899018, EPI_ISL_899037, EPI_ISL_899040, EPI_ISL_899055, EPI_ISL_899061, EPI_ISL_899072, EPI_ISL_899077, EPI_ISL_899083, EPI_ISL_899089, EPI_ISL_899099, EPI_ISL_899103, EPI_ISL_899104, EPI_ISL_899158, EPI_ISL_899174, EPI_ISL_899250, EPI_ISL_899251, EPI_ISL_899252, EPI_ISL_899253, EPI_ISL_899254, EPI_ISL_899255, EPI_ISL_899256, EPI_ISL_899257, EPI_ISL_899258, EPI_ISL_899259, EPI_ISL_899260, EPI_ISL_899354, EPI_ISL_899355, EPI_ISL_899356, EPI_ISL_899357, EPI_ISL_899358, EPI_ISL_899359, EPI_ISL_899361, EPI_ISL_899362, EPI_ISL_899363, EPI_ISL_899364, EPI_ISL_899365, EPI_ISL_899366, EPI_ISL_899367, EPI_ISL_899368, EPI_ISL_899369, EPI_ISL_899479, EPI_ISL_899480, EPI_ISL_899481, EPI_ISL_899482, EPI_ISL_899483, EPI_ISL_899484, EPI_ISL_899485, EPI_ISL_899486, EPI_ISL_899487, EPI_ISL_899488, EPI_ISL_899489, EPI_ISL_899490, EPI_ISL_899491, EPI_ISL_899492, EPI_ISL_899493, EPI_ISL_899494, EPI_ISL_899495, EPI_ISL_899496, EPI_ISL_899497, EPI_ISL_899555, EPI_ISL_899556, EPI_ISL_899641, EPI_ISL_899642, EPI_ISL_899739, EPI_ISL_899740, EPI_ISL_899741, EPI_ISL_899742, EPI_ISL_899743, EPI_ISL_899744, EPI_ISL_899745, EPI_ISL_899746, EPI_ISL_899747, EPI_ISL_899748, EPI_ISL_899749, EPI_ISL_899794, EPI_ISL_899795, EPI_ISL_899796, EPI_ISL_899797, EPI_ISL_899798, EPI_ISL_899799, EPI_ISL_899846, EPI_ISL_899886, EPI_ISL_899887, EPI_ISL_899888, EPI_ISL_900024, EPI_ISL_900025, EPI_ISL_900026, EPI_ISL_900027, EPI_ISL_900028, EPI_ISL_900029			
see above	Viollier AG	Department of Biosystems Science and Engineering, ETH Zürich	Chaoran Chen, Sarah Nadeau, Ivan Topolsky, Emmanouil Dermitzakis, Keith Harshman, Ioannis Xenarios, Henri Pegeot, Lorenzo Cerutti, Deborah Penet, Philipp Jablonski, Lara Fuhrmann, David Dreifuss, Katharina Jahn, Christiane Beckmann, Maurice Redondo, Olivier Kobel, Christoph Noppen, Sophie Seidel, Noemie Santamaria de Souza, Niko Beerenwinkel, Tanja Stadler
EPI_ISL_902894, EPI_ISL_902895, EPI_ISL_902896, EPI_ISL_902897, EPI_ISL_902898, EPI_ISL_902899, EPI_ISL_902900, EPI_ISL_902901, EPI_ISL_902902, EPI_ISL_902903, EPI_ISL_902904, EPI_ISL_902905, EPI_ISL_902906, EPI_ISL_902907, EPI_ISL_902908, EPI_ISL_902909, EPI_ISL_902910, EPI_ISL_902911, EPI_ISL_902912, EPI_ISL_902913, EPI_ISL_902914, EPI_ISL_902915			
see above	Department of Virology and Immunology, University of Helsinki and Helsinki University Hospital, Huslab Finland	Department of Virology, Faculty of Medicine, University of Helsinki, Helsinki, Finland	Teemu Smura, Ravi Kant, Phuoc Truong, Hussein Alburkat, Hannimari Kallio-Kokko, Jenni Virtanen, Maija Suvanto, Essi Korhonen, Sari Hannula, Harri Kangas, Hanna Liimatainen, Satu Kurkela, Hanna Jarva, Maija Lappalainen, Pekka Ellonen, Olli Vapalahti
EPI_ISL_902925	MD Laboratories	Los Angeles County PHL	P. Hemarajata et al.
EPI_ISL_902926	SC (UCO) Igiene e Sanità Pubblica (funzione integrata con SC Microbiologia e Virologia) e Laboratory of Molecular Virology of the International Centre for Genetic Engineering and Biotechnology (ICGEB)	ARGO Laboratorio Genomica ed Epigenomica	Licastro D, Dal Monego S, Degasperi M, Marcello A, D'Agaro P
EPI_ISL_903081, EPI_ISL_903084	Seattle Flu Study	Seattle Flu Study	Deborah A. Nickerson, Chris D. Frazar, Jover Lee, Benjamin Pelle, Erica Ryke, Matthew Richardson, Amanda Adler, Elisabeth Brandstetter, Peter D. Han, Kairsten Fay, Misja Ilcisin, Kirsten Lacombe, Thomas R. Sibley, Melissa Truong, Caitlin R. Wolf, Michael Boeckh, Janet A. Englund, Michael Famulare, Barry R. Lutz, Mark J. Rieder, Lea M. Starita, Matthew Thompson, Jay Shendure, Trevor Bedford, Helen Y. Chu
EPI_ISL_903085, EPI_ISL_903086, EPI_ISL_903204, EPI_ISL_903206, EPI_ISL_903207	Seattle Flu Study	Seattle Flu Study	Deborah A. Nickerson, Chris D. Frazar, Jover Lee, Benjamin Pelle, Erica Ryke, Matthew Richardson, Amanda Adler, Elisabeth Brandstetter, Peter D. Han, Kairsten Fay, Misja Ilcisin, Kirsten Lacombe, Thomas R. Sibley, Melissa Truong, Caitlin R. Wolf, Karen Cowgill, Stephanie Schrag, Jeff Duchin, Michael Boeckh, Janet A. Englund, Michael Famulare, Barry R. Lutz, Mark J. Rieder, Lea M. Starita, Matthew Thompson, Helen Y. Chu, Trevor Bedford, Jay Shendure
EPI_ISL_903357	Wyoming Public Health Laboratory	Wyoming Public Health Laboratory	Noah Hull, Taylor Fearing, Lynette Gumbleton, Channing Weber, Ashley Norberg, Bailey Bowcutt, and Wanda Manley
EPI_ISL_903373, EPI_ISL_903374	MD Laboratories	Los Angeles County PHL	P. Hemarajata et al.
EPI_ISL_903390, EPI_ISL_903391, EPI_ISL_903392, EPI_ISL_903393, EPI_ISL_903395, EPI_ISL_903396, EPI_ISL_903397, EPI_ISL_903399, EPI_ISL_903401, EPI_ISL_903402, EPI_ISL_903403, EPI_ISL_903404, EPI_ISL_903405, EPI_ISL_903407, EPI_ISL_903408, EPI_ISL_903409, EPI_ISL_903410, EPI_ISL_903411, EPI_ISL_903412, EPI_ISL_903413, EPI_ISL_903414, EPI_ISL_903415, EPI_ISL_903416, EPI_ISL_903418, EPI_ISL_903419, EPI_ISL_903420, EPI_ISL_903422, EPI_ISL_903424, EPI_ISL_903426, EPI_ISL_903427, EPI_ISL_903428, EPI_ISL_903429, EPI_ISL_903430, EPI_ISL_903431, EPI_ISL_903432, EPI_ISL_903433, EPI_ISL_903434, EPI_ISL_903435, EPI_ISL_903436, EPI_ISL_903438, EPI_ISL_903439, EPI_ISL_903440, EPI_ISL_903441, EPI_ISL_903442, EPI_ISL_903443, EPI_ISL_903445, EPI_ISL_903446, EPI_ISL_903447, EPI_ISL_903448, EPI_ISL_903449, EPI_ISL_903450, EPI_ISL_903451, EPI_ISL_903452, EPI_ISL_903453, EPI_ISL_903454, EPI_ISL_903455, EPI_ISL_903456, EPI_ISL_903457, EPI_ISL_903458, EPI_ISL_903460, EPI_ISL_903461, EPI_ISL_903462, EPI_ISL_903463, EPI_ISL_903464, EPI_ISL_903465, EPI_ISL_903466, EPI_ISL_903468, EPI_ISL_903469, EPI_ISL_903470, EPI_ISL_903471, EPI_ISL_903472, EPI_ISL_903474,			

EPI_ISL_903475, EPI_ISL_903476, EPI_ISL_903477, EPI_ISL_903479, EPI_ISL_903480, EPI_ISL_903481, EPI_ISL_903482, EPI_ISL_903484, EPI_ISL_903485, EPI_ISL_903486, EPI_ISL_903487, EPI_ISL_903488, EPI_ISL_903489, EPI_ISL_903490, EPI_ISL_903491, EPI_ISL_903492, EPI_ISL_903493, EPI_ISL_903494, EPI_ISL_903495, EPI_ISL_903496, EPI_ISL_903497, EPI_ISL_903499, EPI_ISL_903500, EPI_ISL_903501, EPI_ISL_903502, EPI_ISL_903503, EPI_ISL_903505, EPI_ISL_903506, EPI_ISL_903507, EPI_ISL_903508, EPI_ISL_903509, EPI_ISL_903510, EPI_ISL_903511, EPI_ISL_903512, EPI_ISL_903513, EPI_ISL_903514, EPI_ISL_903515, EPI_ISL_903516, EPI_ISL_903517, EPI_ISL_903518, EPI_ISL_903519, EPI_ISL_903520, EPI_ISL_903521, EPI_ISL_903522, EPI_ISL_903523, EPI_ISL_903524, EPI_ISL_903525, EPI_ISL_903552, EPI_ISL_903554, EPI_ISL_903555, EPI_ISL_903556, EPI_ISL_903557, EPI_ISL_903559, EPI_ISL_903560, EPI_ISL_903561, EPI_ISL_903562			
see above	Quest Diagnostics	Quest Diagnostics	Rosenthal,S.H., Gerasimova,A., Kagan,R.M., Anderson, B., Hua, M., Liu Y., Bernstein, L.E., Livingston, K.E., Perez, A., Shalhout, D.F., Shlyakhter, I.A., Owen, R., Tanpaiboon, P., Lacbawan, F.
EPI_ISL_903979	Institute of Virology, Biomedical Research Center of the Slovak Academy of Sciences, Bratislava	Faculty of Natural Sciences, Comenius University, Bratislava	Viktória abanová, Kristína Boršová, Broa Brejová, Viktória Hodorová, Sabina Fumaová Havlíková, Juraj Kopáek, Martina Liková, ubomíra Lukáiková, Martina Neboháová, Monika Sláviková, Tomáš Vina, Jozef Nosek, Boris Klempa
EPI_ISL_904064, EPI_ISL_904065, EPI_ISL_904066, EPI_ISL_904067	New Mexico Department of Health Scientific Laboratory	New Mexico Department of Health Scientific Laboratory	Ellie Johnson, Anastacia Griego-Fisher, D'eldra Malone
EPI_ISL_904120, EPI_ISL_904121	LACEN - Laboratório Central de Saúde Pública do Pará	Evandro Chagas Institute	Santos, M.C.; Silva, A.M.; Junior, W.D.C.; Barbagelata, L.S.; Ferreira, J.A.; Sousa, E.M.A.; da Silva, P.S.; Pinheiro, K.C.; L.C.; Sousa Junior, E.C.
EPI_ISL_904127, EPI_ISL_904129	Quest Diagnostics	Quest Diagnostics	Rosenthal,S.H., Gerasimova,A., Kagan,R.M., Anderson, B., Hua, M., Liu Y., Bernstein, L.E., Livingston, K.E., Perez, A., Shalhout, D.F., Shlyakhter, I.A., Owen, R., Tanpaiboon, P., Lacbawan, F.
EPI_ISL_904138, EPI_ISL_904141, EPI_ISL_904142, EPI_ISL_904152, EPI_ISL_904157, EPI_ISL_904173, EPI_ISL_904184, EPI_ISL_904204, EPI_ISL_904205, EPI_ISL_904206, EPI_ISL_904232, EPI_ISL_904255, EPI_ISL_904273, EPI_ISL_904274, EPI_ISL_904311, EPI_ISL_904312, EPI_ISL_904313, EPI_ISL_904368, EPI_ISL_904377, EPI_ISL_904565, EPI_ISL_904566, EPI_ISL_904567, EPI_ISL_904568, EPI_ISL_904569, EPI_ISL_904570, EPI_ISL_904571, EPI_ISL_904572, EPI_ISL_904573, EPI_ISL_904574, EPI_ISL_904575, EPI_ISL_904576, EPI_ISL_904577, EPI_ISL_904578			
see above	Dutch COVID-19 response team	Erasmus Medical Center	Bas Oude Munnink, Reina Sikkema, David Nieuwenhuijse, Irina Chestakova, Anne van der Linden, Marjan Boter, Emmanuelle Munger, Corine GeurtsvanKessel, Annemiek van der Eijk, Richard Molenkamp, Marion Koopmans, on behalf of the Dutch national COVID-19 response team.
EPI_ISL_904627	Servicio de Microbiología, Laboratori Clínic Metropolitana Nord. Hospital Universitari Germans Trias i Pujol. Institut d'Investigació en Ciències de la Salut Germans Trias i Pujol (IGTP)	IrsiCaixa - Can Ruti CovidSeq	Marc Noguera-Julian, Mariona Parera, Maria Casadellà, Pilar Armengol, Francesc Catala-Moll, Roger Paredes, Bonaventura Clotet Elisa Martró, Verónica Saludes, Anna Not, Ana Pérez, Montserrat Giménez, Ignacio Blanco, Cristina Casañ, Antoni E. Bordoy, Adrián Antuori
EPI_ISL_904665, EPI_ISL_904668, EPI_ISL_904678, EPI_ISL_904715, EPI_ISL_904723, EPI_ISL_904725, EPI_ISL_904726, EPI_ISL_904727, EPI_ISL_904764, EPI_ISL_904800, EPI_ISL_904872, EPI_ISL_904974, EPI_ISL_904986, EPI_ISL_904988, EPI_ISL_904990, EPI_ISL_904997, EPI_ISL_904998, EPI_ISL_904999, EPI_ISL_905001, EPI_ISL_905002, EPI_ISL_905050, EPI_ISL_905059, EPI_ISL_905060, EPI_ISL_905071, EPI_ISL_905110, EPI_ISL_905113, EPI_ISL_905116, EPI_ISL_905122, EPI_ISL_905185, EPI_ISL_905186, EPI_ISL_905188, EPI_ISL_905195, EPI_ISL_905196, EPI_ISL_905243, EPI_ISL_905244, EPI_ISL_905254, EPI_ISL_905260, EPI_ISL_905261, EPI_ISL_905264, EPI_ISL_905265, EPI_ISL_905266, EPI_ISL_905274, EPI_ISL_905280, EPI_ISL_905283, EPI_ISL_905291, EPI_ISL_905312, EPI_ISL_905313, EPI_ISL_905314, EPI_ISL_905315, EPI_ISL_905321, EPI_ISL_905322, EPI_ISL_905323, EPI_ISL_905324, EPI_ISL_905327, EPI_ISL_905328, EPI_ISL_905329, EPI_ISL_905333, EPI_ISL_905334, EPI_ISL_905335, EPI_ISL_905336, EPI_ISL_905337, EPI_ISL_905345, EPI_ISL_905350, EPI_ISL_905355, EPI_ISL_905356, EPI_ISL_905357, EPI_ISL_905358, EPI_ISL_905359, EPI_ISL_905577, EPI_ISL_905578, EPI_ISL_905579, EPI_ISL_905580, EPI_ISL_905581, EPI_ISL_905583, EPI_ISL_905584, EPI_ISL_905586, EPI_ISL_905589, EPI_ISL_905590, EPI_ISL_905591, EPI_ISL_905617, EPI_ISL_905618, EPI_ISL_905619, EPI_ISL_905620, EPI_ISL_905621, EPI_ISL_905623, EPI_ISL_905624, EPI_ISL_905625, EPI_ISL_905626, EPI_ISL_905627, EPI_ISL_905628, EPI_ISL_905637, EPI_ISL_905638, EPI_ISL_905639, EPI_ISL_905640, EPI_ISL_905667, EPI_ISL_905668, EPI_ISL_905669, EPI_ISL_905670, EPI_ISL_905677, EPI_ISL_905678, EPI_ISL_905679, EPI_ISL_905680, EPI_ISL_905700, EPI_ISL_905701, EPI_ISL_905702, EPI_ISL_905703, EPI_ISL_905704, EPI_ISL_905705, EPI_ISL_905706, EPI_ISL_905707, EPI_ISL_905708, EPI_ISL_905716, EPI_ISL_905717, EPI_ISL_905723, EPI_ISL_905724			
see above	Dutch COVID-19 response team	National Institute for Public Health and the Environment (RIVM)	Adam Meijer, Harry Vennema, Dirk Eggink, Jeroen Cremer, Sharon van den Brink, Bas van der Veer, AnneMarie van den Brandt, Florian Zwagemaker, Dennis Schmitz, Chantal Reusken, on behalf of the national COVID-19 response team
EPI_ISL_905765, EPI_ISL_905766, EPI_ISL_905767, EPI_ISL_905768, EPI_ISL_905769, EPI_ISL_905770, EPI_ISL_905771, EPI_ISL_905772	MD Laboratories	Los Angeles County PHL	P. Hemarajata et al.
EPI_ISL_906047, EPI_ISL_906048	MD Laboratories	Los Angeles County Public Health Laboratory	P. Hemarajata et al.
EPI_ISL_906144	Laboratorio de Salud Publica de Amazonas	Instituto Nacional de Salud- Dirección de Investigación en Salud Pública, Universidad de los Andes- Applied genomics research group, Vicerrectoria de Investigación y Creación, Universidad de los Andes- Systems and Computing Engineering Department	Katherine Laiton-Donato, Diego A. Álvarez-Díaz, Carlos Franco-Muñoz, Mauricio Pacheco-Montealegre, Héctor Alejandro Ruiz-Moreno, Maria T. Herrera-Sepúlveda, Diego Andrés Prada, Jhonnatan Reales-González, Sheryll Corchuelo, Julian Naizaque, Gerardo Santamaría Jorge Duitama, Laura Natalia Gonzalez, Jorge Ivan Diaz, Silvia Restrepo-Restrepo, Magdalena Wiesner, Martha Lucia Ospina Martinez, Marcela Mercado-Reyes
EPI_ISL_906307, EPI_ISL_906308, EPI_ISL_906309, EPI_ISL_906310, EPI_ISL_906311, EPI_ISL_906312, EPI_ISL_906313, EPI_ISL_906314, EPI_ISL_906316, EPI_ISL_906317, EPI_ISL_906318, EPI_ISL_906319, EPI_ISL_906320, EPI_ISL_906321, EPI_ISL_906322, EPI_ISL_906323, EPI_ISL_906324, EPI_ISL_906325, EPI_ISL_906326, EPI_ISL_906327, EPI_ISL_906328, EPI_ISL_906329, EPI_ISL_906330, EPI_ISL_906331, EPI_ISL_906332, EPI_ISL_906333, EPI_ISL_906334, EPI_ISL_906335, EPI_ISL_906336, EPI_ISL_906337, EPI_ISL_906338, EPI_ISL_906340, EPI_ISL_906341, EPI_ISL_906343, EPI_ISL_906344, EPI_ISL_906345, EPI_ISL_906346, EPI_ISL_906347, EPI_ISL_906348, EPI_ISL_906349, EPI_ISL_906350, EPI_ISL_906351, EPI_ISL_906352, EPI_ISL_906353, EPI_ISL_906354, EPI_ISL_906355, EPI_ISL_906356, EPI_ISL_906357, EPI_ISL_906358, EPI_ISL_906359, EPI_ISL_906360, EPI_ISL_906361, EPI_ISL_906362, EPI_ISL_906363, EPI_ISL_906364, EPI_ISL_906366, EPI_ISL_906367, EPI_ISL_906368, EPI_ISL_906370, EPI_ISL_906371, EPI_ISL_906372, EPI_ISL_906373, EPI_ISL_906374, EPI_ISL_906375, EPI_ISL_906376, EPI_ISL_906377, EPI_ISL_906378, EPI_ISL_906379, EPI_ISL_906380, EPI_ISL_906381, EPI_ISL_906382, EPI_ISL_906383, EPI_ISL_906384, EPI_ISL_906385, EPI_ISL_906386, EPI_ISL_906387, EPI_ISL_906388, EPI_ISL_906390, EPI_ISL_906391, EPI_ISL_906392, EPI_ISL_906393, EPI_ISL_906395, EPI_ISL_906397, EPI_ISL_906398, EPI_ISL_906399, EPI_ISL_906400, EPI_ISL_906401, EPI_ISL_906402, EPI_ISL_906404, EPI_ISL_906405, EPI_ISL_906406, EPI_ISL_906407, EPI_ISL_906408, EPI_ISL_906409, EPI_ISL_906410, EPI_ISL_906411, EPI_ISL_906412, EPI_ISL_906413, EPI_ISL_906414, EPI_ISL_906415, EPI_ISL_906416, EPI_ISL_906417, EPI_ISL_906418, EPI_ISL_906419, EPI_ISL_906420, EPI_ISL_906421, EPI_ISL_906422, EPI_ISL_906423, EPI_ISL_906433, EPI_ISL_906436, EPI_ISL_906448, EPI_ISL_906454, EPI_ISL_906455, EPI_ISL_906456, EPI_ISL_906457, EPI_ISL_906458, EPI_ISL_906461, EPI_ISL_906462, EPI_ISL_906463			
see above	Quest Diagnostics	Quest Diagnostics	Rosenthal,S.H., Gerasimova,A., Kagan,R.M., Anderson, B., Hua, M., Liu Y., Bernstein, L.E., Livingston, K.E., Perez, A., Shalhout, D.F., Shlyakhter, I.A., Owen, R., Tanpaiboon, P., Lacbawan, F.
EPI_ISL_906534, EPI_ISL_906560, EPI_ISL_906561	Laboratorio de Salud Publica de Amazonas	Instituto Nacional de Salud- Dirección de Investigación en Salud Pública, Universidad de los Andes- Applied genomics research group, Vicerrectoria de Investigación y Creación, Universidad de los Andes- Systems and Computing Engineering Department	Katherine Laiton-Donato, Diego A. Álvarez-Díaz, Carlos Franco-Muñoz, Mauricio Pacheco-Montealegre, Héctor Alejandro Ruiz-Moreno, Maria T. Herrera-Sepúlveda, Diego Andrés Prada, Jhonnatan Reales-González, Sheryll Corchuelo, Julian Naizaque, Gerardo Santamaría Jorge Duitama, Laura Natalia Gonzalez, Jorge Ivan Diaz, Silvia Restrepo-Restrepo, Magdalena Wiesner, Martha Lucia Ospina Martinez, Marcela Mercado-Reyes
EPI_ISL_906575, EPI_ISL_906576, EPI_ISL_906577, EPI_ISL_906578, EPI_ISL_906579, EPI_ISL_906580, EPI_ISL_906581, EPI_ISL_906582, EPI_ISL_906583, EPI_ISL_906584, EPI_ISL_906585, EPI_ISL_906586, EPI_ISL_906587, EPI_ISL_906588, EPI_ISL_906589			
see above	Maine Health and Environmental Testing Laboratory (Maine HETL)	Tewhey Lab, The Jackson Laboratory	Matluk,N., Dewey,H., Iosue,F., Barter,M., Lynch,R., Munger,H. and Tewhey,R.
EPI_ISL_906715, EPI_ISL_906717, EPI_ISL_906721, EPI_ISL_906723, EPI_ISL_906724, EPI_ISL_906729, EPI_ISL_906731, EPI_ISL_906733, EPI_ISL_906735, EPI_ISL_906738, EPI_ISL_906748, EPI_ISL_906749, EPI_ISL_906756			
see above	Hematology Laboratory, Section of Molecular Diagnostics, University Clinical Centre, Medical University of Gdansk	Laboratory of Recombinant Vaccines	Lukasz Rabalski, Maciej Kosinski, Maciej Grzybek, Adam Sodol, Aneta Szulc, Krzysztof Lewandowski, Ewa Milosz, Marlena Robakowska, Boguslaw Szewczyk, Krystyna Bienkowska-Szewczyk
EPI_ISL_906779	New Mexico Department of Health Scientific Laboratory	New Mexico Department of Health Scientific Laboratory	Ellie Johnson, Anastacia Griego-Fisher, D'eldra Malone
EPI_ISL_906805	Vestfold Hospital, Toensberg Department of Microbiology	Norwegian Institute of Public Health, Department of Virology	Kathrine Stene-Johansen, Kamilla Heddeland Instefjord, Hilde Elshaug, Atiya R Ali,Marie Paulsen Madsen, Rasmus Riis Kopperud, Hilde Vollan, Karoline Bragstad, Olav Hungnes
EPI_ISL_906905, EPI_ISL_906906, EPI_ISL_906907, EPI_ISL_906908, EPI_ISL_906909, EPI_ISL_906915, EPI_ISL_906919	Bureau of Public Health Laboratories, Florida Department of Health (BPHL, FLDOH)	Bureau of Public Health Laboratories, Florida Department of Health (BPHL, FLDOH)	Schmedes,S., Blanton,J.
EPI_ISL_906920, EPI_ISL_906921, EPI_ISL_906922, EPI_ISL_906923, EPI_ISL_906925, EPI_ISL_906926, EPI_ISL_906927, EPI_ISL_906928, EPI_ISL_906929, EPI_ISL_906931, EPI_ISL_906932, EPI_ISL_906933, EPI_ISL_906934, EPI_ISL_906935, EPI_ISL_906936, EPI_ISL_906937, EPI_ISL_906938, EPI_ISL_906939, EPI_ISL_906940, EPI_ISL_906941, EPI_ISL_906942, EPI_ISL_906943, EPI_ISL_906944, EPI_ISL_906945, EPI_ISL_906946, EPI_ISL_906947, EPI_ISL_906948, EPI_ISL_906949, EPI_ISL_906950, EPI_ISL_906951, EPI_ISL_906952, EPI_ISL_906953, EPI_ISL_906955, EPI_ISL_906956, EPI_ISL_906957, EPI_ISL_906958, EPI_ISL_906959, EPI_ISL_906960, EPI_ISL_906961, EPI_ISL_906962, EPI_ISL_906963, EPI_ISL_906965, EPI_ISL_906966, EPI_ISL_906967, EPI_ISL_906968, EPI_ISL_906969, EPI_ISL_906970, EPI_ISL_906971, EPI_ISL_906972, EPI_ISL_906973, EPI_ISL_906974, EPI_ISL_906975, EPI_ISL_906976, EPI_ISL_906977, EPI_ISL_906979, EPI_ISL_906980, EPI_ISL_906981, EPI_ISL_906982, EPI_ISL_906983, EPI_ISL_906984, EPI_ISL_906985, EPI_ISL_906986, EPI_ISL_906987, EPI_ISL_906988, EPI_ISL_906989, EPI_ISL_906990, EPI_ISL_906991, EPI_ISL_906992, EPI_ISL_906993, EPI_ISL_906994, EPI_ISL_906995, EPI_ISL_906996, EPI_ISL_906997, EPI_ISL_906998, EPI_ISL_906999, EPI_ISL_907000, EPI_ISL_907001, EPI_ISL_907002, EPI_ISL_907003, EPI_ISL_907004, EPI_ISL_907005, EPI_ISL_907006, EPI_ISL_907007, EPI_ISL_907008, EPI_ISL_907009, EPI_ISL_907010, EPI_ISL_907011, EPI_ISL_907012, EPI_ISL_907013, EPI_ISL_907014, EPI_ISL_907015, EPI_ISL_907016, EPI_ISL_907017, EPI_ISL_907018, EPI_ISL_907019, EPI_ISL_907020, EPI_ISL_907021, EPI_ISL_907022, EPI_ISL_907023, EPI_ISL_907024, EPI_ISL_907025, EPI_ISL_907026, EPI_ISL_907027, EPI_ISL_907028, EPI_ISL_907029, EPI_ISL_907030, EPI_ISL_907031, EPI_ISL_907032,			

EPI_ISL_907033, EPI_ISL_907034, EPI_ISL_907035, EPI_ISL_907036, EPI_ISL_907037, EPI_ISL_907038, EPI_ISL_907039, EPI_ISL_907040, EPI_ISL_907041, EPI_ISL_907042, EPI_ISL_907043, EPI_ISL_907044, EPI_ISL_907045, EPI_ISL_907046, EPI_ISL_907047, EPI_ISL_907048, EPI_ISL_907049, EPI_ISL_907050, EPI_ISL_907051, EPI_ISL_907052, EPI_ISL_907053, EPI_ISL_907054, EPI_ISL_907055, EPI_ISL_907056, EPI_ISL_907057, EPI_ISL_907058, EPI_ISL_907059, EPI_ISL_907060, EPI_ISL_907061, EPI_ISL_907062, EPI_ISL_907063, EPI_ISL_907065, EPI_ISL_907067, EPI_ISL_907069, EPI_ISL_907071, EPI_ISL_907072, EPI_ISL_907073			
see above	Infectious Diseases, Quest Diagnostics	Infectious Diseases, Quest Diagnostics	Rosenthal,S.H., Gerasimova,A., Kagan,R.M., Anderson,B., Bernstein,L.E., Livingston,K.E., Hua,M., Liu,Y., Shalhout,D.F., Owen,R., Lacbawan,F.
EPI_ISL_909595, EPI_ISL_909596, EPI_ISL_909597, EPI_ISL_909598, EPI_ISL_909599, EPI_ISL_909600, EPI_ISL_909601, EPI_ISL_909602, EPI_ISL_909603, EPI_ISL_909604, EPI_ISL_909605, EPI_ISL_909606, EPI_ISL_909607, EPI_ISL_909608, EPI_ISL_909609, EPI_ISL_909610, EPI_ISL_909611, EPI_ISL_909612, EPI_ISL_909613, EPI_ISL_909614, EPI_ISL_909615, EPI_ISL_909616, EPI_ISL_909617, EPI_ISL_909618, EPI_ISL_909619, EPI_ISL_909620, EPI_ISL_909621, EPI_ISL_909622, EPI_ISL_909623, EPI_ISL_909624, EPI_ISL_909625, EPI_ISL_909626, EPI_ISL_909627, EPI_ISL_909628, EPI_ISL_909629, EPI_ISL_909630, EPI_ISL_909631, EPI_ISL_909632, EPI_ISL_909633, EPI_ISL_909634, EPI_ISL_909635, EPI_ISL_909636, EPI_ISL_909637, EPI_ISL_909638, EPI_ISL_909639, EPI_ISL_909640, EPI_ISL_909641, EPI_ISL_909642, EPI_ISL_909643, EPI_ISL_909644, EPI_ISL_909645, EPI_ISL_909646, EPI_ISL_909647, EPI_ISL_909648, EPI_ISL_909649, EPI_ISL_909650, EPI_ISL_909651			
see above	Lighthouse Lab in Alderley Park	Wellcome Sanger Institute for the COVID-19 Genomics UK (COG-UK) Consortium	Jacquelyn Wynn, Mairead Hyland, The Lighthouse Lab in Alderley Park and Alex Alderton, Roberto Amato, Sonia Goncalves, Ewan Harrison, David K. Jackson, Ian Johnston, Dominic Kwiatkowski, Cordelia Langford, John Sillitoe on behalf of the Wellcome Sanger Institute COVID-19 Surveillance Team
EPI_ISL_909656, EPI_ISL_909660, EPI_ISL_909661, EPI_ISL_909662	Labo Analyses med	National Reference Center for Viruses of Respiratory Infections, Institut Pasteur, Paris	Marion Barbet, Sylvie Behillil, Méline Bizard, Angela Brisebarre, Camille Capel, Etienne Simon-Lorière, Vincent Enouf, Maud Vanpeene, Sylvie van der Werf,Amzalag Jonas
EPI_ISL_909675, EPI_ISL_909676, EPI_ISL_909677, EPI_ISL_909678, EPI_ISL_909679, EPI_ISL_909680, EPI_ISL_909681	Labo Analyses Med	National Reference Center for Viruses of Respiratory Infections, Institut Pasteur, Paris	Marion Barbet, Sylvie Behillil, Méline Bizard, Angela Brisebarre, Camille Capel, Etienne Simon-Lorière, Vincent Enouf, Maud Vanpeene, Sylvie van der Werf,Le Vicky
EPI_ISL_909949, EPI_ISL_909950	CUSL/UCLouvain COVID testing federal platform	UCLouvain/IREC/MBLG	Jean Ruelle, Lysa Pinsmaye, Benoit Kabamba Mukadi
EPI_ISL_910941, EPI_ISL_910943, EPI_ISL_910944, EPI_ISL_910945, EPI_ISL_910946, EPI_ISL_910947, EPI_ISL_910948, EPI_ISL_910949, EPI_ISL_910950, EPI_ISL_910951, EPI_ISL_910952, EPI_ISL_910953, EPI_ISL_911104, EPI_ISL_911105, EPI_ISL_911106, EPI_ISL_911107, EPI_ISL_911108, EPI_ISL_911109, EPI_ISL_911110, EPI_ISL_911111, EPI_ISL_911112, EPI_ISL_911113, EPI_ISL_911114, EPI_ISL_911115, EPI_ISL_911116, EPI_ISL_911117, EPI_ISL_911118, EPI_ISL_911119, EPI_ISL_911120, EPI_ISL_911121, EPI_ISL_911122, EPI_ISL_911123, EPI_ISL_911124, EPI_ISL_911167, EPI_ISL_911169, EPI_ISL_911170, EPI_ISL_911171, EPI_ISL_911172, EPI_ISL_911173			
see above	Laboratoire national de sante, Microbiology, Virology	Laboratoire national de sante, Microbiology, Microbial Genomics Platform	Anke Wienecke-Baldacchino, Catherine Ragimbeau,Jessica Tapp, Fatu Djabi, Lise Pignon, Raoul Salmon, Tamir Abdelrahman
EPI_ISL_911297, EPI_ISL_911307, EPI_ISL_911316, EPI_ISL_911327, EPI_ISL_911334, EPI_ISL_911337	Servicio de Microbiología, Hospital Universitario Son Espases	SeqCOVID-SPAIN consortium/IBV(CSIC)	Carla López-Causapé, Jordi Reina, Antonio Oliver and SeqCOVID-SPAIN consortium
EPI_ISL_911634	Texas Department of State Health Services (Texas Department of State Health Services (TXDSHS))	Texas Department of State Health Services (TXDSHS)	Bonnie Oh, Anita Pokharel, James Daniel Bonser, Myong Koag, Chung Wang, Rachel Lee, Grace Kubin, Rashmi Tuladhar, Mayela Pedrueza, Maliha Rahman, Jenny Zhang
EPI_ISL_911639, EPI_ISL_911640	Texas Department of State Health Services (TXDSHS)	Texas Department of State Health Services (TXDSHS)	Bonnie Oh, Anita Pokharel, James Daniel Bonser, Myong Koag, Chung Wang, Rachel Lee, Grace Kubin, Rashmi Tuladhar, Mayela Pedrueza, Maliha Rahman, Jenny Zhang
EPI_ISL_911704, EPI_ISL_911705, EPI_ISL_911706	Quest Diagnostics	Quest Diagnostics	Rosenthal,S.H., Gerasimova,A., Kagan,R.M., Anderson, B., Hua, M., Liu Y., Bernstein, L.E., Livingston, K.E., Perez, A., Shalhout, D.F., Shlyakhter, I.A., Owen, R., Tanpaiboon, P., Lacbawan, F.
EPI_ISL_911794	Johns Hopkins Hospital Department of Pathology	Johns Hopkins Hospital Department of Pathology	C. Paul Morris, Chun Huai Luo, Adannaya Amadi, Matthew Schwartz, Nicholas Gallagher, Heba H. Mostafa
EPI_ISL_911929, EPI_ISL_911930	Wyoming Public Health Laboratory	Wyoming Public Health Laboratory	Noah Hull, Taylor Fearing, Lynette Gumbleton, Channing Weber, Ashley Norberg, Bailey Bowcutt, and Wanda Manley
EPI_ISL_911972, EPI_ISL_911973, EPI_ISL_911974, EPI_ISL_911975, EPI_ISL_911976, EPI_ISL_911977, EPI_ISL_911978, EPI_ISL_911979, EPI_ISL_911980, EPI_ISL_911981, EPI_ISL_911982, EPI_ISL_911983, EPI_ISL_911984, EPI_ISL_911985, EPI_ISL_911986, EPI_ISL_911987, EPI_ISL_911988, EPI_ISL_911989, EPI_ISL_911990, EPI_ISL_911991, EPI_ISL_911992, EPI_ISL_911993, EPI_ISL_911994, EPI_ISL_911995, EPI_ISL_911996, EPI_ISL_911997, EPI_ISL_911998, EPI_ISL_911999, EPI_ISL_912000, EPI_ISL_912001, EPI_ISL_912002, EPI_ISL_912003, EPI_ISL_912004, EPI_ISL_912005			
see above	Altius Institute for Biomedical Sciences	Seattle Flu Study	Deborah A. Nickerson, Chris D. Frazar, Jover Lee, Benjamin Pelle, Erica Ryke, Matthew Richardson, Amanda Adler, Elisabeth Brandstetter, Peter D. Han, Kairsten Fay, Misja Ilcisin, Kirsten Lacombe, Thomas R. Sibley, Melissa Truong, Caitlin R. Wolf, Ryan Alexander, Daniel Bates, Rebecca Bruders, Stephanie DeBaun, Clem Green, Muhammad Halimun, Jessica Halow, Kneshay Harper, Matt Hartman, Andrew Meuser, Alex Nguyen, Truong Nguyen, Sofia Olsson, Sadie Patraw, Hannah Petersen, Tobias Ragoczy, Joshua Richards, Jacob Rodriguez, John Stamatoyannopoulos, Julia Wald, Olivia Waltner, Michael Boeckh, Janet A. Englund, Michael Famulare, Barry R. Lutz, Mark J. Rieder, Lea M. Starita, Matthew Thompson, Helen Y. Chu, Jay Shendure, Trevor Bedford
EPI_ISL_912047, EPI_ISL_912048, EPI_ISL_912049, EPI_ISL_912050, EPI_ISL_912051, EPI_ISL_912052, EPI_ISL_912053, EPI_ISL_912054, EPI_ISL_912055, EPI_ISL_912056, EPI_ISL_912057, EPI_ISL_912058, EPI_ISL_912059, EPI_ISL_912060, EPI_ISL_912061, EPI_ISL_912062, EPI_ISL_912063, EPI_ISL_912064, EPI_ISL_912065, EPI_ISL_912066, EPI_ISL_912067, EPI_ISL_912068, EPI_ISL_912069, EPI_ISL_912070, EPI_ISL_912071, EPI_ISL_912072, EPI_ISL_912073, EPI_ISL_912074, EPI_ISL_912075, EPI_ISL_912076, EPI_ISL_912077, EPI_ISL_912078			
see above	Washington State Department of Health	Seattle Flu Study	Deborah A. Nickerson, Chris D. Frazar, Jover Lee, Benjamin Pelle, Erica Ryke, Matthew Richardson, Amanda Adler, Elisabeth Brandstetter, Peter D. Han, Kairsten Fay, Misja Ilcisin, Kirsten Lacombe, Thomas R. Sibley, Melissa Truong, Caitlin R. Wolf, Romesh Gautom, Geoff Melly, Brian Hiatt, Philip Dykema, Scott Lindquist, Michael Boeckh, Janet A. Englund, Michael Famulare, Barry R. Lutz, Mark J. Rieder, Lea M. Starita, Matthew Thompson, Helen Y. Chu, Jay Shendure, Trevor Bedford
EPI_ISL_912195	Respiratory Viruses Branch, Centers for Disease Control and Prevention	Respiratory Viruses Branch, Centers for Disease Control and Prevention	Cook,P.W., Batra,D., Rambo-Martin,B.L., Galloway,S., Krueger,B.,Agarwal,M., Almasri,E., Boles,D., Burns,A., Charoensri,N.,Cohen,O., Countryman,S., Cristobal,M.A., Croy,B., Dale,S.,Deshmukh,H., Douglas,A., Drouillon,V., Eisenberg,M., Engler,H.,Ghatti,R., Gupta,P., Hicks,S., Humphrey,J., Iyer,L., Jain,M.,Kolli,M., Kuphal,T., Letovsky,S., Levandoski,M., Lukasik,C.,Meltzer,J., Norvell,B., Nye,M., Parker,S., Petropoulos,C.,Pruitt,J., Ragan,S., Ryan,S., Sapeta,M., Schroth,J.,Selvaraju,S.B., Stevovic,G., Suchanek,A., Throop,A., Tilson,L.,Urban,T., Voshell,J., Wagner,K., Williams,J., Williamson,M.,Zeng,Q., Zwiefelhofer,T., Paden,C.R., Tong,S. and MacCannell,D.
EPI_ISL_912226	Lighthouse Lab in Alderley Park	Wellcome Sanger Institute for the COVID-19 Genomics UK (COG-UK) Consortium	Jacquelyn Wynn, Mairead Hyland, The Lighthouse Lab in Alderley Park and Alex Alderton, Roberto Amato, Sonia Goncalves, Ewan Harrison, David K. Jackson, Ian Johnston, Dominic Kwiatkowski, Cordelia Langford, John Sillitoe on behalf of the Wellcome Sanger Institute COVID-19 Surveillance Team (http://www.sanger.ac.uk/covid-team)
EPI_ISL_912256	Charité Universitätsmedizin Berlin, Institut für Virologie/Labor Berlin	Charité Universitätsmedizin Berlin, Institut für Virologie	Victor M Corman, Barbara Mühlemann, Jörn Beheim-Schwarzbach, Tobias Bleicker, Julia Tesch, Talitha Veith, Julia Schneider, Terry Jones, Christian Drosten
EPI_ISL_912401	PathCare	National Health Laboratory Service (NHLS), Tygerberg	Susan Engelbrecht, Jean Maritz, Bronwyn Kleinhans, Houriiyah Tegally, Eduan Wilkindon, Gert van Zyl, Wolfgang Preiser, Tulio de Oliveira
EPI_ISL_912414, EPI_ISL_912415, EPI_ISL_912423, EPI_ISL_912425	KU Leuven, Rega Institute, Clinical and Epidemiological Virology	KU Leuven, Rega Institute, Clinical and Epidemiological Virology	Tony Wawina-Bokalanga, Bert Vanmechelen, Joan Marti-Carerras, Piet Maes
EPI_ISL_912547, EPI_ISL_912548	Labo Analyses Med	National Reference Center for Viruses of Respiratory Infections, Institut Pasteur, Paris	Marion Barbet, Sylvie Behillil, Méline Bizard, Angela Brisebarre, Camille Capel, Etienne Simon-Lorière, Vincent Enouf, Maud Vanpeene, Sylvie van der Werf,Le Vicky
EPI_ISL_912560	HOPITAL SIMONE VEIL	National Reference Center for Viruses of Respiratory Infections, Institut Pasteur, Paris	Marion Barbet, Sylvie Behillil, Méline Bizard, Angela Brisebarre, Camille Capel, Etienne Simon-Lorière, Vincent Enouf, Maud Vanpeene, Sylvie van der Werf,Moreau Farida
EPI_ISL_912584, EPI_ISL_912585, EPI_ISL_912586, EPI_ISL_912587, EPI_ISL_912588, EPI_ISL_912589, EPI_ISL_912590, EPI_ISL_912591, EPI_ISL_912592, EPI_ISL_912593, EPI_ISL_912594, EPI_ISL_912595, EPI_ISL_912596			
see above	Labo Analyses Med	National Reference Center for Viruses of Respiratory Infections, Institut Pasteur, Paris	Marion Barbet, Sylvie Behillil, Méline Bizard, Angela Brisebarre, Camille Capel, Etienne Simon-Lorière, Vincent Enouf, Maud Vanpeene, Sylvie van der Werf,Le Vicky
EPI_ISL_912635, EPI_ISL_912742, EPI_ISL_912743, EPI_ISL_912744, EPI_ISL_912745, EPI_ISL_912746, EPI_ISL_912747	Hôpital Henri Mondor	Department of Virology, Henri Mondor University Hospital, Assistance Publique Hôpitaux de Paris, Université Paris-Est Créteil, INSERM U955	Christophe Rodriguez, Slim Fourati, Vanessa Demontant, Guillaume Gricourt, Melissa N'Debi, Alexandre Soulier, Elisabeth Trawinski, Jean-Michel Pawlotsky
EPI_ISL_912909, EPI_ISL_912910, EPI_ISL_912911, EPI_ISL_912923,	Hôpital Pitié-Salpêtrière	Department of Virology, Henri Mondor University Hospital, Assistance Publique Hôpitaux de Paris, Université Paris-Est	Christophe Rodriguez, Slim Fourati, Vanessa Demontant, Guillaume Gricourt, Melissa N'Debi, Alexandre Soulier, Elisabeth Trawinski, Jean-Michel Pawlotsky

EPI_ISL_912926, EPI_ISL_912934		Créteil, INSERM U955	
EPI_ISL_913039	Complejo Hospitalario Xeral-Calde	Instituto de Salud Carlos III	Iglesias-Caballero, M. Camarero, S. Sandonís,V. Vázquez, S. Pozo, F. Casas, I. Jiménez, P. Zaballos, A. Monzón, S. Varona, S. Cuesta, I. Alonso, P.
EPI_ISL_913060	Hospital Universitario Virgen de la Arrixaca	Instituto de Salud Carlos III	Iglesias-Caballero, M. Camarero, S. Sandonís,V. Vázquez, S. Pozo, F. Casas, I. Jiménez, P. Zaballos, A. Monzón, S. Varona, S. Cuesta, I. Moreno, A.
EPI_ISL_913130, EPI_ISL_913135, EPI_ISL_913143, EPI_ISL_913146, EPI_ISL_913149, EPI_ISL_913158, EPI_ISL_913159, EPI_ISL_913161, EPI_ISL_913164, EPI_ISL_913165, EPI_ISL_913166, EPI_ISL_913167, EPI_ISL_913170, EPI_ISL_913175			
see above	University of Michigan Clinical Microbiology Laboratory	Lauring Lab, University of Michigan, Department of Microbiology and Immunology	Valesano
EPI_ISL_913258, EPI_ISL_913260, EPI_ISL_913261, EPI_ISL_913262, EPI_ISL_913264, EPI_ISL_913265, EPI_ISL_913270, EPI_ISL_913271, EPI_ISL_913273, EPI_ISL_913307, EPI_ISL_913308, EPI_ISL_913359, EPI_ISL_913360, EPI_ISL_913361, EPI_ISL_913362			
see above	Klinisk mikrobiologi	The Public Health Agency of Sweden	Anna-Malin Linde, Maria Lind Karlberg, Carlo Berg, Oskar Karlsson Lindsjo, Sofia Stamouli, Reza Advani, Mattias Haukland, Petra Holmstrom, Noura Walai, Petra Edquist, Mia Brytting, Anna Risberg, Karin Tegmark-Wisell
EPI_ISL_913428	Massachusetts State Public Health Laboratory	Massachusetts State Public Health Laboratory	Andrew Lang, Timelia Fink, Glen Gallagher, Sandra Smole
EPI_ISL_913453	Klinisk mikrobiologi	The Public Health Agency of Sweden	Anna-Malin Linde, Maria Lind Karlberg, Carlo Berg, Oskar Karlsson Lindsjo, Sofia Stamouli, Reza Advani, Mattias Haukland, Petra Holmstrom, Noura Walai, Petra Edquist, Mia Brytting, Anna Risberg, Karin Tegmark-Wisell
EPI_ISL_913454	Dynamic Code AB	The Public Health Agency of Sweden	Anna-Malin Linde, Maria Lind Karlberg, Carlo Berg, Oskar Karlsson Lindsjo, Sofia Stamouli, Reza Advani, Mattias Haukland, Petra Holmstrom, Noura Walai, Petra Edquist, Mia Brytting, Anna Risberg, Karin Tegmark-Wisell
EPI_ISL_913467	Synlab Medilab, Mikrobiologi	The Public Health Agency of Sweden	Anna-Malin Linde, Maria Lind Karlberg, Carlo Berg, Oskar Karlsson Lindsjo, Sofia Stamouli, Reza Advani, Mattias Haukland, Petra Holmstrom, Noura Walai, Petra Edquist, Mia Brytting, Anna Risberg, Karin Tegmark-Wisell
EPI_ISL_913468, EPI_ISL_913494	The Public Health Agency of Sweden	The Public Health Agency of Sweden	Anna-Malin Linde, Maria Lind Karlberg, Carlo Berg, Oskar Karlsson Lindsjo, Sofia Stamouli, Reza Advani, Mattias Haukland, Petra Holmstrom, Noura Walai, Petra Edquist, Mia Brytting, Anna Risberg, Karin Tegmark-Wisell
EPI_ISL_913746, EPI_ISL_913747	DC Public Health Lab/ Dept. of Forensic Sciences	DC Public Health Lab/ Dept. of Forensic Sciences	Scott Nguyen, Elizabeth Zelaya, Connie Maza, Monica Mann, Brittany Hamilton, David Payne, Jocelyn Hauser
EPI_ISL_913769, EPI_ISL_913770, EPI_ISL_913772, EPI_ISL_913773, EPI_ISL_913775, EPI_ISL_913776, EPI_ISL_913777, EPI_ISL_913779, EPI_ISL_913780, EPI_ISL_913783, EPI_ISL_913784, EPI_ISL_913786, EPI_ISL_913787, EPI_ISL_913789, EPI_ISL_913790, EPI_ISL_913792, EPI_ISL_913793			
see above	KU Leuven, Rega Institute, Clinical and Epidemiological Virology	KU Leuven, Rega Institute, Clinical and Epidemiological Virology	Tony Wawina-Bokalanga, Bert Vanmechelen, Joan Marti-Carerras, Piet Maes
EPI_ISL_914002	Quest Diagnostics Tucker GA	Pathogen Discovery, Respiratory Viruses Branch, Division of Viral Diseases, Centers for Disease Control and Prevention	Ying Tao, Yan Li, Jing Zhang, Krista Queen, Anna Uehara, Peter Cook, Clinton R. Paden, Haibin Wang, Suxiang Tong
EPI_ISL_914015, EPI_ISL_914016, EPI_ISL_914017, EPI_ISL_914018, EPI_ISL_914019, EPI_ISL_914020, EPI_ISL_914021, EPI_ISL_914022, EPI_ISL_914023, EPI_ISL_914024	IL Department of Public Health Chicago Laboratory	Pathogen Discovery, Respiratory Viruses Branch, Division of Viral Diseases, Centers for Disease Control and Prevention	Ying Tao, Yan Li, Jing Zhang, Krista Queen, Anna Uehara, Peter Cook, Clinton R. Paden, Haibin Wang, Suxiang Tong
EPI_ISL_914828	AREA DE SALUD ALAJUELA SUR	Inciensa, Instituto Costarricense de Investigación y Enseñanza en Nutrición y Salud	Francisco Duarte, Hebleen Porras, Claudio Soto-Garita, Estela Cordero, Adriana Godínez, Melany Calderón & Mariel López
EPI_ISL_914831	AREA DE SALUD CARRILLO	Inciensa, Instituto Costarricense de Investigación y Enseñanza en Nutrición y Salud	Francisco Duarte, Hebleen Porras, Claudio Soto-Garita, Estela Cordero, Adriana Godínez, Melany Calderón & Adriana Bermúdez-Espinoza
EPI_ISL_914833	LABORATORIO CLINICO LABIN	Inciensa, Instituto Costarricense de Investigación y Enseñanza en Nutrición y Salud	Francisco Duarte, Hebleen Porras, Claudio Soto-Garita, Estela Cordero, Adriana Godínez, Melany Calderón & Pei Ling Chan Ma
EPI_ISL_914834	HOSPITAL METROPOLITANO	Inciensa, Instituto Costarricense de Investigación y Enseñanza en Nutrición y Salud	Francisco Duarte, Hebleen Porras, Claudio Soto-Garita, Estela Cordero, Adriana Godínez, Melany Calderón & Margarita Lee-Lui
EPI_ISL_915198, EPI_ISL_915202, EPI_ISL_915235, EPI_ISL_915236, EPI_ISL_915237, EPI_ISL_915238, EPI_ISL_915239, EPI_ISL_915284, EPI_ISL_915285, EPI_ISL_915286, EPI_ISL_915287, EPI_ISL_915288, EPI_ISL_915289, EPI_ISL_915323, EPI_ISL_915325			
see above	Quest Diagnostics	Quest Diagnostics	Rosenthal,S.H., Gerasimova,A., Kagan,R.M., Anderson, B., Hua, M., Liu Y., Bernstein, L.E., Livingston, K.E., Perez, A., Shalhout, D.F., Shlyakhter, I.A., Owen, R., Tanpaiboon, P., Lacbawan, F.
EPI_ISL_915435, EPI_ISL_915436, EPI_ISL_915437, EPI_ISL_917944, EPI_ISL_917945, EPI_ISL_917946, EPI_ISL_917947, EPI_ISL_917948, EPI_ISL_917949, EPI_ISL_917950	MD Laboratories	Los Angeles County PHL	P. Hemarajata et al.
EPI_ISL_918181, EPI_ISL_918201, EPI_ISL_918259	Innovative Genomics Institute, UC Berkeley	Innovative Genomics Institute, UC Berkeley	Stacia Wyman, Haridha Shivram, Phil Frankino, Liana Lareau, Shana McDevitt, Justin Choi
EPI_ISL_918264	SIESP CHIETI-DRIVE IN CHIETI	Istituto Zooprofilattico Sperimentale dell'Abruzzo e Molise "G. Caporale"	Lorusso A, Marcacci M, Di Domenico M, Ancora M, Curini V, Mangone I, Rinaldi A, Scialabba S, Di Pasquale A, Cammà C, Puglia I, Calistri P, Savini G
EPI_ISL_918265	OSPEDALE ATRI	Istituto Zooprofilattico Sperimentale dell'Abruzzo e Molise "G. Caporale"	Lorusso A, Marcacci M, Di Domenico M, Ancora M, Curini V, Mangone I, Rinaldi A, Scialabba S, Di Pasquale A, Cammà C, Puglia I, Calistri P, Savini G
EPI_ISL_918270	SIESP CHIETI - DRIVE IN LANCIANO	Istituto Zooprofilattico Sperimentale dell'Abruzzo e Molise "G. Caporale"	Lorusso A, Marcacci M, Di Domenico M, Ancora M, Curini V, Mangone I, Rinaldi A, Scialabba S, Di Pasquale A, Cammà C, Puglia I, Calistri P, Savini G
EPI_ISL_918280, EPI_ISL_918281	Hospital Universitari Vall d'Hebron - Vall d'Hebron Institut de Rercerca	Hospital Universitari Vall d'Hebron	Cristina Andrés, Maria Piñana, Josep F Abril, Damir Garcia-Cehic, Ariadna Rando, Juliana Esperalba, Maria Gema Codina, Carla Castillo, Maria Carmen Martin, Tomàs Pumarola, Josep Quer, Andrés Antón
EPI_ISL_918356	Institute of Virology, Medical Center, University of Freiburg, Freiburg, Germany	Institute of Virology, Clinial Virus Genomics, Medical Center, University of Freiburg, Freiburg, Germany	Jonas Fuchs, Lisa Kern, Sandra Reuter, Hajo Grundmann, Marcus Panning
EPI_ISL_918490	CUSL/UCLouvain COVID testing federal platform	UCLouvain/IREC/MBLG	Jean Ruelle, Lysa Pinsmaye, Benoit Kabamba Mukadi
EPI_ISL_918539	LACEN - Laboratório Central de Saúde Pública do Ceara	Evandro Chagas Institute	Santos, M.C.; Silva, A.M.; Junior, W.D.C.; Barbagelata, L.S.; Ferreira, J.A.; Sousa, E.M.A.; da Silva, P.S.; Pinheiro, K.C.; L.C.; Sousa Junior, E.C.
EPI_ISL_918560, EPI_ISL_918561	LACEN - Laboratório Central de Saúde Pública do Amapa	Evandro Chagas Institute	Santos, M.C.; Silva, A.M.; Junior, W.D.C.; Barbagelata, L.S.; Ferreira, J.A.; Sousa, E.M.A.; da Silva, P.S.; Pinheiro, K.C.; L.C.; Sousa Junior, E.C.
EPI_ISL_918686, EPI_ISL_918687, EPI_ISL_918688, EPI_ISL_918689, EPI_ISL_918690, EPI_ISL_918691, EPI_ISL_918692, EPI_ISL_918693, EPI_ISL_918694, EPI_ISL_918695, EPI_ISL_918696, EPI_ISL_918701, EPI_ISL_918733, EPI_ISL_918734, EPI_ISL_918860, EPI_ISL_918861, EPI_ISL_918884, EPI_ISL_918885, EPI_ISL_918886, EPI_ISL_918887, EPI_ISL_918888, EPI_ISL_918889, EPI_ISL_918890, EPI_ISL_918891, EPI_ISL_918892, EPI_ISL_918893, EPI_ISL_918894, EPI_ISL_918895, EPI_ISL_918896, EPI_ISL_918897, EPI_ISL_918898, EPI_ISL_918899, EPI_ISL_918916, EPI_ISL_918917, EPI_ISL_918918, EPI_ISL_918919, EPI_ISL_918920, EPI_ISL_918921, EPI_ISL_918922, EPI_ISL_918923, EPI_ISL_918924, EPI_ISL_918925, EPI_ISL_918926, EPI_ISL_918927, EPI_ISL_918928, EPI_ISL_918929, EPI_ISL_918930, EPI_ISL_918931, EPI_ISL_918932, EPI_ISL_918933, EPI_ISL_918934, EPI_ISL_918936, EPI_ISL_918937, EPI_ISL_918938, EPI_ISL_918939, EPI_ISL_918973, EPI_ISL_918974, EPI_ISL_918975, EPI_ISL_918976			
see above	University of Birmingham	COVID-19 Genomics UK (COG-UK) Consortium	Institute of Microbiology, University of Birmingham: Claire McMurray, Joanne Stockton, Samuel Nicholls, Radoslaw Poplawski, Will Rowe, Josh Quick, Nicholas Loman. University of Birmingham Testing Laboratory: Celina M Whalley, Andrew Bosworth, Charlotte Poxon, Kasun Wanigasooriya, Oliver Pickles, Mike Kidd, Alex Richter, Andrew D Beggs PHE Heartlands Lab: Husam Osman, Andrew Bosworth. Queen Elizabeth Hospital: Anna Casey
EPI_ISL_919029, EPI_ISL_919044, EPI_ISL_919046, EPI_ISL_919048, EPI_ISL_919050, EPI_ISL_919052,	Department of Pathology, University of Cambridge	COVID-19 Genomics UK (COG-UK) Consortium	Aminu S. Jahun, Yasmin Chaudhry, Iliana Georgana, Myra Hosmillo, Rhys Izu, Martin D. Curran, Surendra Parmar, Ian Goodfellow

EPI_ISL_919056			
EPI_ISL_919190, EPI_ISL_919191, EPI_ISL_919192, EPI_ISL_919193, EPI_ISL_919194, EPI_ISL_919195	West of Scotland Specialist Virology Centre, NHSGGC / MRC-University of Glasgow Centre for Virus Research	COVID-19 Genomics UK (COG-UK) Consortium	Ana da Silva Filipe, Natasha Johnson, Kathy Smollett, Daniel Mair, Stephen Carmichael, Alice Broos, Lily Tong, Jenna Nichols, Kyriaki Nomikou; Sarah McDonald; Richard Orton, Joseph Hughes, Sreenu Vattipally, David L Robertson; Alasdair MacLean, Rory Gunson; Sharif Shaaban, Matthew Holden; Rachel Blacow, Guy Mollett, Kathy Li, James Shepherd, Antonia Ho, Emma Thomson
EPI_ISL_919337	Virology Department, Royal Infirmary of Edinburgh, NHS Lothian / School of Biological Sciences, University of Edinburgh / Institute of Genetics and Molecular Medicine, University of Edinburgh	COVID-19 Genomics UK (COG-UK) Consortium	McHugh M, Dewar R, Rooke S, Gallagher M, Balcaza C, O'Toole Á, Scher E, Hill V, McCrone JT, Colquhoun R, Yu X, Jackson B, Rambaut A, Williams TC, Templeton K
EPI_ISL_919467, EPI_ISL_919468, EPI_ISL_919469, EPI_ISL_919470, EPI_ISL_919471, EPI_ISL_919472, EPI_ISL_919473, EPI_ISL_919474, EPI_ISL_919593	Liverpool Clinical Laboratories	COVID-19 Genomics UK (COG-UK) Consortium	Sam Haldenby, Anita Lucaci, Steve Paterson, Julian Hiscox, Alistair Darby, M Almsaud, A Alrezaihi, Muhannad Alruwaili, Stuart D Armstrong, Jones Benjamin, Eleanor G Bentley, Anu Chawla, Jordan J Clark, Angela Cowell, Richard Eccles, Isabel García-Dorival, Matthew Gemmell, Alessandro Gerada, PKF Gilmore, Richard Gregory, Ximeng Han, Catherine Hartley, Margaret Hughes, Miren Iturriza-Gomara, James Johnson, L Luu, Jenifer Manson, Charlotte Nelson, Elaine O'Toole, Cassie Olateju, Rebekah Penrice-Randal , Lucille Rainbow, N.P Randle, Trevor Ian Robinson, Parul Sharma, Ghada T Shawli, James P Stewart, Neil Swainston, Ecaterina Vamos, Joanne Watts, Mark Whitehead
EPI_ISL_920000, EPI_ISL_920001, EPI_ISL_920002, EPI_ISL_920003, EPI_ISL_920004, EPI_ISL_920005, EPI_ISL_920006, EPI_ISL_920007, EPI_ISL_920008, EPI_ISL_920009, EPI_ISL_920010, EPI_ISL_920011, EPI_ISL_920014, EPI_ISL_920042, EPI_ISL_920050			
see above	University College London, Great Ormond Street Hospital for Children NHS Foundation Trust, Imperial College Healthcare NHS Trust	COVID-19 Genomics UK (COG-UK) Consortium	Sergi Castellano, Rachel Williams, Mark Kristiansen, Paola Resende Silva, Sunando Roy, Tony Brooks, Helena Tutill, Paola Niola, Patricia Dyal, Charlotte Williams, Leysa Forrest, Yasmin Panchbhaya, Jacqueline Findlay, Samuel Weeks, Julianne Brown, Kathryn Harris, Paul Randell, James Price, Alison Holmes, Judith Breuer
EPI_ISL_920191, EPI_ISL_920241, EPI_ISL_920298, EPI_ISL_920310, EPI_ISL_920329, EPI_ISL_920345, EPI_ISL_920357, EPI_ISL_920458, EPI_ISL_920467	University College London Hospital	COVID-19 Genomics UK (COG-UK) Consortium	Judith Heaney, Matthew Byott, Catherine Houlihan, Dan Frampton, Stuart Kirk, Moira Spyer and Eleni Nastouli
EPI_ISL_920851, EPI_ISL_920860, EPI_ISL_920861, EPI_ISL_920862, EPI_ISL_920863, EPI_ISL_920864, EPI_ISL_920865, EPI_ISL_920866	University College London, Great Ormond Street Hospital for Children NHS Foundation Trust, Imperial College Healthcare NHS Trust	COVID-19 Genomics UK (COG-UK) Consortium	Sergi Castellano, Rachel Williams, Mark Kristiansen, Paola Resende Silva, Sunando Roy, Tony Brooks, Helena Tutill, Paola Niola, Patricia Dyal, Charlotte Williams, Leysa Forrest, Yasmin Panchbhaya, Jacqueline Findlay, Samuel Weeks, Julianne Brown, Kathryn Harris, Paul Randell, James Price, Alison Holmes, Judith Breuer
EPI_ISL_920900	Department of Pathology, University of Cambridge	COVID-19 Genomics UK (COG-UK) Consortium	Aminu S. Jahun, Yasmin Chaudhry, Iliana Georgana, Myra Hosmillo, Rhys Izu, Martin D. Curran, Surendra Parmar, Ian Goodfellow
EPI_ISL_920996, EPI_ISL_920997, EPI_ISL_920998, EPI_ISL_920999, EPI_ISL_921000, EPI_ISL_921001, EPI_ISL_921002, EPI_ISL_921003, EPI_ISL_921004, EPI_ISL_921005, EPI_ISL_921006, EPI_ISL_921008, EPI_ISL_921009, EPI_ISL_921010, EPI_ISL_921018			
see above	Regional Virus Laboratory, Belfast Health and Social Care Trust	COVID-19 Genomics UK (COG-UK) Consortium	Conall McCaughey, James McKenna, Tanya Curran, Susan Feeney, Alison Watt, Ciara Cox, Mairead Connor, Zoltan Molnar, David Simpson, Derek Fairley
EPI_ISL_921491, EPI_ISL_921492, EPI_ISL_921495, EPI_ISL_921496, EPI_ISL_921497, EPI_ISL_921498, EPI_ISL_921499, EPI_ISL_921500, EPI_ISL_921506, EPI_ISL_921515, EPI_ISL_921517			
see above	Northumbria University / South Tees Hospitals NHS Foundation Trust / North Cumbria Integrated Care NHS Foundation Trust / North Tees and Hartlepool NHS Foundation Trust / Newcastle Hospitals NHS Foundation Trust	COVID-19 Genomics UK (COG-UK) Consortium	Darren L Smith,Andrew Nelson,Matthew Bashton,Greg R Young,Joshua Loh,John Allan,Mohammad A Tariq,Giles S Holt,Gary Black,Wen C Yew,Lynn Dover,Paul Baker,Steve Liggett,Sarah Essex,Jane Greenaway,Debra Padgett,Clive Graham,Garren Scott,Edward Barton,Emma Swindells,Brendan Payne,Jennifer Collins,Yusri Taha,Gary Eltringham
EPI_ISL_922071, EPI_ISL_922072, EPI_ISL_922074, EPI_ISL_922075	Lincolnshire Hospitals and DeepSeq Nottingham	COVID-19 Genomics UK (COG-UK) Consortium	Nichola Duckworth, Tim Sloan, Sarah Walsh, Jonathan Ball, Patrick McClure, Joeseph Chappell, Nadine Holmes, Matthew Carlisle, Christopher Moore, Fei Sang, Johnny Debebe, Victoria Wright, Matthew Loose
EPI_ISL_922076	General Hospital #Abdulah Nakas" Sarajevo	Alea Genetic Centre	Rijad Konjhodzic, Dino Pecar, Lana Salihefendic, Sead Jazic
EPI_ISL_922077, EPI_ISL_922078, EPI_ISL_922079, EPI_ISL_922080, EPI_ISL_922081, EPI_ISL_922082, EPI_ISL_922083, EPI_ISL_922084, EPI_ISL_922085, EPI_ISL_922086, EPI_ISL_922087, EPI_ISL_922088, EPI_ISL_922089, EPI_ISL_922090, EPI_ISL_922091, EPI_ISL_922092, EPI_ISL_922093, EPI_ISL_922094, EPI_ISL_922095, EPI_ISL_922096, EPI_ISL_922097, EPI_ISL_922098, EPI_ISL_922099, EPI_ISL_922100, EPI_ISL_922102, EPI_ISL_922103, EPI_ISL_922104, EPI_ISL_922107			
see above	Lincolnshire Hospitals and DeepSeq Nottingham	COVID-19 Genomics UK (COG-UK) Consortium	Nichola Duckworth, Tim Sloan, Sarah Walsh, Jonathan Ball, Patrick McClure, Joeseph Chappell, Nadine Holmes, Matthew Carlisle, Christopher Moore, Fei Sang, Johnny Debebe, Victoria Wright, Matthew Loose
EPI_ISL_922212, EPI_ISL_922213, EPI_ISL_922214, EPI_ISL_922215, EPI_ISL_922216, EPI_ISL_922217, EPI_ISL_922218, EPI_ISL_922219, EPI_ISL_922220, EPI_ISL_922224, EPI_ISL_922225, EPI_ISL_922226, EPI_ISL_922227, EPI_ISL_922228, EPI_ISL_922229, EPI_ISL_922230, EPI_ISL_922237, EPI_ISL_922259, EPI_ISL_922294			
see above	Oxford Viromics, NDM, University of Oxford; Oxford University Hospitals; Basingstoke and North Hampshire Hospital	COVID-19 Genomics UK (COG-UK) Consortium	Tanya Golubchik, David Bonsall, George Macintyre, Amy Trebes, Mariateresa de Cesare, Catrin Moore, Alex Mobbs, Anita Justice, Robert Shaw, Monique Andersson, Timothy Peto, Emma Wise, Nathan Moore, Jessica Lynch, Nick Cortes, Matilde Mori, Stephen Kidd, David Buck, John Todd, Christophe Fraser
EPI_ISL_922380, EPI_ISL_922558	Wales Specialist Virology Centre Sequencing lab: Pathogen Genomics Unit	Public Health Wales Microbiology Cardiff Wales Specialist Virology Centre	Catherine Moore, Johnathan Evans, Laura Gifford, Malorie Perry, Simon Cottrell, Angela Marchbank, Alec Birchley, Alexander Adams, Amy Gaskin, Bree Gatica-Wilcox, Jason Coombes, Joel Southgate, Lauren Gilbert, Lee Graham, Nicole Pacchiarini, Sara Kumziene-Summerhayes, Sarah Taylor, Sophie Jones, Sara Rey, Matthew Bull, Joanne Watkins, Sally Corden, Tom Connor
EPI_ISL_923262, EPI_ISL_923373, EPI_ISL_923375, EPI_ISL_923376, EPI_ISL_923378, EPI_ISL_923390, EPI_ISL_923392, EPI_ISL_923393, EPI_ISL_923394, EPI_ISL_923395, EPI_ISL_923396, EPI_ISL_923397, EPI_ISL_923398, EPI_ISL_923399, EPI_ISL_923401, EPI_ISL_923402, EPI_ISL_923403, EPI_ISL_923404, EPI_ISL_923405, EPI_ISL_923406, EPI_ISL_923407, EPI_ISL_923408, EPI_ISL_923409, EPI_ISL_923410, EPI_ISL_923411, EPI_ISL_923412, EPI_ISL_923413, EPI_ISL_923539, EPI_ISL_923593			
see above	Centre for Enzyme Innovation, University of Portsmouth / Translational Research Laboratory, Portsmouth Hospitals NHS Trust	COVID-19 Genomics UK (COG-UK) Consortium	Angela Beckett,Salman Goudarzi,Christopher Fearn,Kate Cook,Katie Loveson,Sharon Glaysher,Scott Elliott,Samuel Robson
EPI_ISL_924270	Virology Department, Sheffield Teaching Hospitals NHS Foundation Trust/Department of Infection, Immunity and Cardiovascular Disease, The Medical School, University of Sheffield	COVID-19 Genomics UK (COG-UK) Consortium	Thushan de Silva, Matthew Parker, Nikki Smith, Adri Angyal, Rebecca Brown, Luke Green, Rachel Tucker, Paul Parsons, Danielle Groves, Katie Johnson, Laura Carrilero, Alex Keeley, Dave Partridge, Matthew Wyles, Benjamin Lindsey, Mehmet Yavuz, Mohammad Raza, Cariad Evans
EPI_ISL_924729, EPI_ISL_924731, EPI_ISL_924746, EPI_ISL_924749, EPI_ISL_924764, EPI_ISL_924770, EPI_ISL_924774, EPI_ISL_924794, EPI_ISL_924795, EPI_ISL_924797, EPI_ISL_924798, EPI_ISL_924800, EPI_ISL_924805, EPI_ISL_924807, EPI_ISL_924808, EPI_ISL_924816, EPI_ISL_924819, EPI_ISL_924827, EPI_ISL_924828, EPI_ISL_924838, EPI_ISL_924839, EPI_ISL_924848, EPI_ISL_924849, EPI_ISL_924850, EPI_ISL_924851, EPI_ISL_924855, EPI_ISL_924863, EPI_ISL_924864, EPI_ISL_924865, EPI_ISL_924867, EPI_ISL_924871, EPI_ISL_924877, EPI_ISL_924879, EPI_ISL_924880, EPI_ISL_924883, EPI_ISL_924886, EPI_ISL_924887, EPI_ISL_924890, EPI_ISL_924891, EPI_ISL_924892, EPI_ISL_924895, EPI_ISL_924899, EPI_ISL_924901, EPI_ISL_924902, EPI_ISL_924904, EPI_ISL_924905, EPI_ISL_924907, EPI_ISL_924908, EPI_ISL_924909, EPI_ISL_924910, EPI_ISL_924911, EPI_ISL_924912, EPI_ISL_924913, EPI_ISL_924914, EPI_ISL_924915, EPI_ISL_924916, EPI_ISL_924917, EPI_ISL_924918, EPI_ISL_924919, EPI_ISL_924921, EPI_ISL_924922, EPI_ISL_924923, EPI_ISL_924924, EPI_ISL_924925, EPI_ISL_924926, EPI_ISL_924927, EPI_ISL_924928, EPI_ISL_924929, EPI_ISL_924930, EPI_ISL_924931, EPI_ISL_924933, EPI_ISL_924935, EPI_ISL_924936, EPI_ISL_924937, EPI_ISL_924938, EPI_ISL_924939, EPI_ISL_924940, EPI_ISL_924941, EPI_ISL_924942, EPI_ISL_924943, EPI_ISL_924944, EPI_ISL_924945, EPI_ISL_924946, EPI_ISL_924947, EPI_ISL_924950, EPI_ISL_924975, EPI_ISL_924980			
see above	Bioinformatics and Biostatistics Lab, Advanced Sequencing Facility	COVID-19 Genomics UK (COG-UK) Consortium	Aengus Stewart,Jerome Nicod,Chelsea Sawyer,Laura Cubitt,Harshil Patel,Margaret Crawford
EPI_ISL_925263, EPI_ISL_925284, EPI_ISL_925294, EPI_ISL_925302, EPI_ISL_925305	Wyoming Public Health Laboratory	Wyoming Public Health Laboratory	Noah Hull, Taylor Fearing, Lynette Gumbleton, Channing Weber, Ashley Norberg, Bailey Bowcutt, and Wanda Manley
EPI_ISL_925399, EPI_ISL_925400	Department of Clinical Microbiology	GIGA Medical Genomics	Keith Durkin, Maria Artesi, Sébastien Bontems, Raphaël Boreux, Bouchra Boujemla, Cécile Meex, Pierrette Melin, Marie-Pierre Hayette, Vincent Bours
EPI_ISL_925914	Nucleic Acid Testing, National Reference Laboratory	GIGA Medical Genomics	Yvan Butera, Keith Durkin, Maria Artesi, Bouchra Boujemla, Robert Rutayisire, Patrick Tuyisenge, Esperence Umumararungu, Sébastien Bontems, Marie-Pierre Hayette, Nathalie Renotte, Swaibu Gatare, Jacob Souopgui, Sabin Nsanzimana, Vincent Bours, Léon Mutesa
EPI_ISL_930635, EPI_ISL_930637, EPI_ISL_930638, EPI_ISL_930639, EPI_ISL_930640, EPI_ISL_930641, EPI_ISL_930642, EPI_ISL_930643, EPI_ISL_930644, EPI_ISL_930645, EPI_ISL_930646, EPI_ISL_930647, EPI_ISL_930648, EPI_ISL_930651, EPI_ISL_930652, EPI_ISL_930653, EPI_ISL_930654, EPI_ISL_930655,			

EPI_ISL_930662, EPI_ISL_930663			
see above	Arizona State Public Health Laboratory	Arizona State Public Health Laboratory	Trung Huynh, Jessica Escobar, Katherine Fullerton, Nobuko Fukushima, Stacy White, Linda Getsinger, Victor Waddell
EPI_ISL_933663, EPI_ISL_933690, EPI_ISL_933691, EPI_ISL_933692, EPI_ISL_933705	Instituto de Diagnostico y Referencia Epidemiologicos INDRE_RNLSP	Instituto de Diagnostico y Referencia Epidemiologicos (INDRE)	Claudia Wong-Arambula, Abril Rodriguez-Maldonado, Fabiola Garces-Ayala, Adnan Araiza-Rodriguez, David Fragoso-Fonseca, Sergio Rangel-Guerrero, Mayra Jimenez-Morales, Nancy Munoz-Hernandez, Natividad Cruz-Ortiz, Tatiana Nunez-Garcia, Gisela Barrera-Badillo, Lucia Hernandez-Rivas, Irma Lopez-Martinez, Ernesto Ramirez-Gonzalez.
EPI_ISL_934238	Vilnius university hospital Santaros Klinikos, Center of Laboratory Medicine	Vilnius university hospital Santaros Klinikos, Center of Laboratory Medicine	Ingrida Olendraite, Daniel Naumovas, Rimvydas Norvilas, Dovile Ezerskyte, Justinas Slikas, Gytis Dudas
EPI_ISL_934324, EPI_ISL_934340, EPI_ISL_934350, EPI_ISL_934351, EPI_ISL_934352, EPI_ISL_934353	Klinisk mikrobiologi	The Public Health Agency of Sweden	Anna-Malin Linde, Maria Lind Karlberg, Carlo Berg, Oskar Karlsson Lindsjo, Sofia Stamouli, Reza Advani, Mattias Haukland, Petra Holmstrom, Noura Walai, Petra Edquist, Mia Brytting, Anna Risberg, Karin Tegmark-Wisell
EPI_ISL_934370, EPI_ISL_934371, EPI_ISL_934372, EPI_ISL_934373, EPI_ISL_934374	Synlab Medilab, Mikrobiologi	The Public Health Agency of Sweden	Anna-Malin Linde, Maria Lind Karlberg, Carlo Berg, Oskar Karlsson Lindsjo, Sofia Stamouli, Reza Advani, Mattias Haukland, Petra Holmstrom, Noura Walai, Petra Edquist, Mia Brytting, Anna Risberg, Karin Tegmark-Wisell
EPI_ISL_934437, EPI_ISL_934438, EPI_ISL_934439, EPI_ISL_934440, EPI_ISL_934441, EPI_ISL_934442, EPI_ISL_934443, EPI_ISL_934444, EPI_ISL_934445, EPI_ISL_934446, EPI_ISL_934447, EPI_ISL_934448, EPI_ISL_934449, EPI_ISL_934450, EPI_ISL_934451, EPI_ISL_934455, EPI_ISL_934458, EPI_ISL_934461, EPI_ISL_934462, EPI_ISL_934463, EPI_ISL_934464, EPI_ISL_934473, EPI_ISL_934474, EPI_ISL_934475, EPI_ISL_934476, EPI_ISL_934477, EPI_ISL_934478, EPI_ISL_934480, EPI_ISL_934484, EPI_ISL_934488, EPI_ISL_934489, EPI_ISL_934493, EPI_ISL_934494, EPI_ISL_934503, EPI_ISL_934504, EPI_ISL_934505, EPI_ISL_934506, EPI_ISL_934507, EPI_ISL_934508, EPI_ISL_934509			
see above	Austrian Agency for Health and Food Safety (AGES)	Bergthaler laboratory, CeMM Research Center for Molecular Medicine of the Austrian Academy of Sciences	Lukas Endler, Anna Schedl, Thomas Penz, Benedikt Agerer, Maelle Le Moing, Michael Schuster, Bekir Erguner, Jan Laine, Martin Senekowitsch, Christoph Bock, Andreas Bergthaler
EPI_ISL_934635, EPI_ISL_934645, EPI_ISL_934648, EPI_ISL_934649	Department of Microbiology, University Innsbruck	Bergthaler laboratory, CeMM Research Center for Molecular Medicine of the Austrian Academy of Sciences	Lukas Endler, Anna Schedl, Thomas Penz, Benedikt Agerer, Maelle Le Moing, Michael Schuster, Bekir Erguner, Jan Laine, Martin Senekowitsch, Christoph Bock, Andreas Bergthaler
EPI_ISL_935156, EPI_ISL_935159	SIESP CHIETI	Istituto Zooprofilattico Sperimentale dell'Abruzzo e Molise "G. Caporale"	Lorusso A, Marcacci M, Di Domenico M, Ancora M, Curini V, Mangone I, Rinaldi A, Scialabba S, Di Pasquale A, Cammà C, Puglia I, Calistri P, Savini G
EPI_ISL_935160	SIESP CHIETI-DRIVE IN ORTONA	Istituto Zooprofilattico Sperimentale dell'Abruzzo e Molise "G. Caporale"	Lorusso A, Marcacci M, Di Domenico M, Ancora M, Curini V, Mangone I, Rinaldi A, Scialabba S, Di Pasquale A, Cammà C, Puglia I, Calistri P, Savini G
EPI_ISL_935161, EPI_ISL_935162	SIESP CHIETI	Istituto Zooprofilattico Sperimentale dell'Abruzzo e Molise "G. Caporale"	Lorusso A, Marcacci M, Di Domenico M, Ancora M, Curini V, Mangone I, Rinaldi A, Scialabba S, Di Pasquale A, Cammà C, Puglia I, Calistri P, Savini G
EPI_ISL_935163, EPI_ISL_935164	SIESP CHIETI-DRIVE IN ORTONA	Istituto Zooprofilattico Sperimentale dell'Abruzzo e Molise "G. Caporale"	Lorusso A, Marcacci M, Di Domenico M, Ancora M, Curini V, Mangone I, Rinaldi A, Scialabba S, Di Pasquale A, Cammà C, Puglia I, Calistri P, Savini G
EPI_ISL_935165, EPI_ISL_935166, EPI_ISL_935167, EPI_ISL_935168, EPI_ISL_935169	SIESP CHIETI	Istituto Zooprofilattico Sperimentale dell'Abruzzo e Molise "G. Caporale"	Lorusso A, Marcacci M, Di Domenico M, Ancora M, Curini V, Mangone I, Rinaldi A, Scialabba S, Di Pasquale A, Cammà C, Puglia I, Calistri P, Savini G
EPI_ISL_935206, EPI_ISL_935208, EPI_ISL_935221, EPI_ISL_935264, EPI_ISL_935265, EPI_ISL_935266, EPI_ISL_935267, EPI_ISL_935268, EPI_ISL_935269, EPI_ISL_935270, EPI_ISL_935271, EPI_ISL_935272, EPI_ISL_935273, EPI_ISL_935301, EPI_ISL_935302, EPI_ISL_935303			
see above	KU Leuven, Rega Institute, Clinical and Epidemiological Virology	KU Leuven, Rega Institute, Clinical and Epidemiological Virology	Tony Wawina-Bokalanga, Bert Vanmechelen, Joan Marti-Carerras, Piet Maes
EPI_ISL_935971, EPI_ISL_935972	SIESP CHIETI	Istituto Zooprofilattico Sperimentale dell'Abruzzo e Molise "G. Caporale"	Lorusso A, Marcacci M, Di Domenico M, Ancora M, Curini V, Mangone I, Rinaldi A, Scialabba S, Di Pasquale A, Cammà C, Puglia I, Calistri P, Savini G
EPI_ISL_935973, EPI_ISL_935975, EPI_ISL_935991	GLENS FALLS HOSPITAL LABORATORY	Wadsworth Center, New York State Department of Health	Kirsten St. George, Daryl M. Lamson, Alexis Russel, Matthew Shudt, Melissa A Leisner, Jonathan Plitnick, Navjot Singh, John Kelly, Erasmus Schneider, Erica Lasek-Nesselquist
EPI_ISL_936002, EPI_ISL_936006, EPI_ISL_936007, EPI_ISL_936008, EPI_ISL_936011, EPI_ISL_936012, EPI_ISL_936013, EPI_ISL_936014, EPI_ISL_936015, EPI_ISL_936016, EPI_ISL_936017, EPI_ISL_936018, EPI_ISL_936019, EPI_ISL_936020, EPI_ISL_936021			
see above	SUNY UPSTATE MEDICAL UNIVERSITY	Wadsworth Center, New York State Department of Health	Kirsten St. George, Daryl M. Lamson, Alexis Russel, Matthew Shudt, Melissa A Leisner, Jonathan Plitnick, Navjot Singh, John Kelly, Erasmus Schneider, Erica Lasek-Nesselquist
EPI_ISL_936025, EPI_ISL_936027, EPI_ISL_936028, EPI_ISL_936031	THE MARY IMOGENE BASSETT HOSPITAL	Wadsworth Center, New York State Department of Health	Kirsten St. George, Daryl M. Lamson, Alexis Russel, Matthew Shudt, Melissa A Leisner, Jonathan Plitnick, Navjot Singh, John Kelly, Erasmus Schneider, Erica Lasek-Nesselquist
EPI_ISL_936050, EPI_ISL_936051, EPI_ISL_936055, EPI_ISL_936057, EPI_ISL_936058, EPI_ISL_936059	ADIRONDACK MEDICAL CENTER	Wadsworth Center, New York State Department of Health	Kirsten St. George, Daryl M. Lamson, Alexis Russel, Matthew Shudt, Melissa A Leisner, Jonathan Plitnick, Navjot Singh, John Kelly, Erasmus Schneider, Erica Lasek-Nesselquist
EPI_ISL_936138, EPI_ISL_936140, EPI_ISL_936141	THE MARY IMOGENE BASSETT HOSPITAL	Wadsworth Center, New York State Department of Health	Kirsten St. George, Daryl M. Lamson, Alexis Russel, Matthew Shudt, Melissa A Leisner, Jonathan Plitnick, Navjot Singh, John Kelly, Erasmus Schneider, Erica Lasek-Nesselquist
EPI_ISL_936146	New York Presbyterian Hospital	Wadsworth Center, New York State Department of Health	Kirsten St. George, Daryl M. Lamson, Alexis Russel, Matthew Shudt, Melissa A Leisner, Jonathan Plitnick, Navjot Singh, John Kelly, Erasmus Schneider, Erica Lasek-Nesselquist
EPI_ISL_936153	THE MARY IMOGENE BASSETT HOSPITAL	Wadsworth Center, New York State Department of Health	Kirsten St. George, Daryl M. Lamson, Alexis Russel, Matthew Shudt, Melissa A Leisner, Jonathan Plitnick, Navjot Singh, John Kelly, Erasmus Schneider, Erica Lasek-Nesselquist
EPI_ISL_936388, EPI_ISL_936398	TGen North	TGen North	Jolene Bowers, Megan Folkerts, Chris French, Hayley Yaglom, Ashlyn Pfeiffer, Darrin Lemmer, Dave Engelthaler, The Arizona COVID Genomics Union (ACGU)
EPI_ISL_936466	DPH, Massachusetts State Public Health Lab	DPH, Massachusetts State Public Health Lab	Lang,A.S., Fink,T., Gallagher,G.R., Smole,S.C.
EPI_ISL_936967, EPI_ISL_936968, EPI_ISL_936969, EPI_ISL_936970, EPI_ISL_936971, EPI_ISL_936972, EPI_ISL_936973, EPI_ISL_936974, EPI_ISL_936975, EPI_ISL_936976, EPI_ISL_936977, EPI_ISL_936978, EPI_ISL_936979, EPI_ISL_936980, EPI_ISL_936981, EPI_ISL_936982, EPI_ISL_936983, EPI_ISL_936984, EPI_ISL_936985, EPI_ISL_936986, EPI_ISL_936987, EPI_ISL_936988, EPI_ISL_936989, EPI_ISL_936990, EPI_ISL_936991, EPI_ISL_936992, EPI_ISL_936993, EPI_ISL_936994, EPI_ISL_936995, EPI_ISL_936996, EPI_ISL_936997, EPI_ISL_936998, EPI_ISL_936999, EPI_ISL_937000, EPI_ISL_937001, EPI_ISL_937002, EPI_ISL_937003, EPI_ISL_937004, EPI_ISL_937005, EPI_ISL_937006, EPI_ISL_937007, EPI_ISL_937008, EPI_ISL_937009, EPI_ISL_937010, EPI_ISL_937011, EPI_ISL_937012, EPI_ISL_937013, EPI_ISL_937014, EPI_ISL_937016, EPI_ISL_937017, EPI_ISL_937018, EPI_ISL_937019, EPI_ISL_937020, EPI_ISL_937021, EPI_ISL_937022, EPI_ISL_937023			
see above	Northwestern Memorial Hospital	Ozer Lab	Ramon Lorenzo-Redondo, Lacy M. Simons, Chad J. Achenbach, Lawrence J. Jennings, Michael G. Ison, Judd F. Hultquist, Egon A. Ozer
EPI_ISL_937437	Maine Health and Environmental Testing Laboratory (Maine HETL)	Tewhey Lab, The Jackson Laboratory	Matluk,N., Dewey,H., Iosue,F., Barter,M., Lynch,R., Munger,H. and Tewhey,R.
EPI_ISL_939628	Laboratory of Virology and Molecular Diagnostics	Institute of Public Health of Republic of North Macedonia Laboratory of Virology and Molecular Diagnostics	Maja Kuzmanovska. Golubinka Boshevska, Elizabeta Janchevska
EPI_ISL_939644, EPI_ISL_939649	Laboratory of Virology and Molecular Diagnostics	Institute of Public Health of Republic of North Macedonia Laboratory of Virology and Molecular Diagnostics	Maja Kuzmanovska, Golubinka Boshevska, Elizabeta Janchevska
EPI_ISL_939650	Laboratory of Virology and Molecular Diagnostics	Institute of Public Health of Republic of North Macedonia Laboratory of Virology and Molecular Diagnostics	Maja Kuzmanovska,Golubinka Boshevska, Elizabeta Janchevska
EPI_ISL_939651	MD Laboratories	Los Angeles County PHL	P. Hemarajata et al.
EPI_ISL_939652	Los Angeles County PHL	Los Angeles County PHL	P. Hemarajata et al.

EPI_ISL_939653	MD Laboratories	Los Angeles County PHL	P. Hemarajata et al.
EPI_ISL_940623	Hospital Sao Joaquim - Beneficiencia Portuguesa	Instituto Adolfo Lutz, Interdiciplinary Procedures Center, Strategic Laboratory	Claudio Tavares Sacchi, Claudia Regina Gonçalves, Erica Valessa Ramos Gomes, Karoline Rodrigues Campos
EPI_ISL_940727	City of Milwaukee Health Department Laboratory	City of Milwaukee Health Department Laboratory	Sanjib Bhattacharyya
EPI_ISL_940766	Laboratory of Virology and Molecular Diagnostics	Institute of Public Health of Republic of North Macedonia Laboratory of Virology and Molecular Diagnostics	Maja Kuzmanovska, Golubinka Boshevska, Elizabeta Janchevska
EPI_ISL_940767	Heilig Hart Lier	UAntwerp, Laboratory of Medical Microbiology, Campus Drie Eiken S6.26, Universiteitsplein 1, 2610, Wilrijk, Belgium	Basil Britto Xavier, Jasmine Coppens, Marie Le Mercier, Christine Lammens, Veerle Matheeussen, Herman Goossens
EPI_ISL_940864	Vaccines and Infectious Diseases Analytics Research Unit (VIDA)	KRISP, KZN Research Innovation and Sequencing Platform	Baillie Vicky, du Plessis Jeanine, Giandhari Jennifer, Pillay Sureshnee, Naidoo Yeshnee, Tegally Houriiyah, de Oliveira Tulio, Madhi Shabir
EPI_ISL_940995	Hopital	National Reference Center for Viruses of Respiratory Infections, Institut Pasteur, Paris	Marion Barbet, Sylvie Behillil, Méline Bizard, Angela Brisebarre, Camille Capel, Etienne Simon-Lorière, Vincent Enouf, Maud Vanpeene, Sylvie van der Werf
EPI_ISL_940997, EPI_ISL_940998	Labo Analyses Med	National Reference Center for Viruses of Respiratory Infections, Institut Pasteur, Paris	Marion Barbet, Sylvie Behillil, Méline Bizard, Angela Brisebarre, Camille Capel, Etienne Simon-Lorière, Vincent Enouf, Maud Vanpeene, Sylvie van der Werf, Gestin (B) Brieuc
EPI_ISL_941004, EPI_ISL_941007, EPI_ISL_941008, EPI_ISL_941013	Labo Analyses Med	National Reference Center for Viruses of Respiratory Infections, Institut Pasteur, Paris	Marion Barbet, Sylvie Behillil, Méline Bizard, Angela Brisebarre, Camille Capel, Etienne Simon-Lorière, Vincent Enouf, Maud Vanpeene, Sylvie van der Werf, Amzalag Jonas
EPI_ISL_941081	Labo Analyses Med	National Reference Center for Viruses of Respiratory Infections, Institut Pasteur, Paris	Marion Barbet, Sylvie Behillil, Méline Bizard, Angela Brisebarre, Camille Capel, Etienne Simon-Lorière, Vincent Enouf, Maud Vanpeene, Sylvie van der Werf, Merah Kader
EPI_ISL_941163	Laboratory of Virology and Molecular Diagnostics	Institute of Public Health of Republic of North Macedonia Laboratory of Virology and Molecular Diagnostics	Maja Kuzmanovska
EPI_ISL_941228	Laboratorio de Microbiología. Hospital General Universitario de Elda, Alicante	SeqCOVID-SPAIN consortium/IBV(CSIC)	Mª Isabel Gascón Ros, Cristina Torregrosa Hetland, Eva Pastor Boix, Paloma Cascales Ramos and SeqCOVID-SPAIN consortium
EPI_ISL_941336	Ospedale San Camillo De Lellis di Rieti	INMI Lazzaro Spallanzani IRCCS	Cesare E.M. Gruber, Francesco Messina, Martina Rueca, Emanuela Giombini, Barbara Bartolini, Ornella Butera, Stefano Venarubea, Luca Casertano, Antonino Di Caro, Maria R. Capobianchi
EPI_ISL_941337	Ospedale San Camillo De Lellis di Rieti	INMI Lazzaro Spallanzani IRCCS	Ornella Butera, Barbara Bartolini, Cesare E.M. Gruber, Martina Rueca, Francesco Messina, Emanuela Giombini, Stefano Venarubea, Assunta De Luca, Maria R. Capobianchi, Antonino Di Caro
EPI_ISL_941376, EPI_ISL_941377, EPI_ISL_941378, EPI_ISL_941379, EPI_ISL_941380, EPI_ISL_941381, EPI_ISL_941385, EPI_ISL_941386, EPI_ISL_941388, EPI_ISL_941414, EPI_ISL_941426, EPI_ISL_941427, EPI_ISL_941455, EPI_ISL_941456, EPI_ISL_941457, EPI_ISL_941458, EPI_ISL_941459, EPI_ISL_941460, EPI_ISL_941461, EPI_ISL_941631, EPI_ISL_941632, EPI_ISL_941634			
see above	Instituto Nacional de Saude (INSA)	Instituto Nacional de Saude (INSA)	Borges et al
EPI_ISL_941706, EPI_ISL_941771, EPI_ISL_941796	Instituto Nacional de Saude (INSA) and Instituto Gulbenkian de Ciencia (IGC)	Instituto Nacional de Saude (INSA) and Instituto Gulbenkian de Ciencia (IGC)	Borges et al
EPI_ISL_942166	Wisconsin State Laboratory of Hygiene Communicable Disease Division	Wisconsin State Laboratory of Hygiene Communicable Disease Division	Kelsey R. Florek, Abigail C. Shockey
EPI_ISL_942809, EPI_ISL_942810, EPI_ISL_942811, EPI_ISL_942812, EPI_ISL_942813, EPI_ISL_942814, EPI_ISL_942815, EPI_ISL_942816, EPI_ISL_942817, EPI_ISL_942818	Gundersen Molecular Diagnostics Laboratory	Kabara Cancer Research Institute	Craig S. Richmond, Paraic A. Kenny
EPI_ISL_942916, EPI_ISL_942917, EPI_ISL_942918, EPI_ISL_942919, EPI_ISL_942920, EPI_ISL_942921, EPI_ISL_942922, EPI_ISL_942923, EPI_ISL_942924, EPI_ISL_942925, EPI_ISL_942926			
see above	Houston Health Dept.	Houston Health Dept.	Ryker Penn, Pamela Brown, Adolpho Lara
EPI_ISL_942945	Gundersen Molecular Diagnostics Laboratory	Kabara Cancer Research Institute	Craig S. Richmond, Paraic A. Kenny
EPI_ISL_942977, EPI_ISL_943012, EPI_ISL_943017, EPI_ISL_943018, EPI_ISL_943020, EPI_ISL_943021, EPI_ISL_943066, EPI_ISL_943144, EPI_ISL_943145, EPI_ISL_943150, EPI_ISL_943151, EPI_ISL_943152, EPI_ISL_943193, EPI_ISL_943194, EPI_ISL_943195, EPI_ISL_943196, EPI_ISL_943197, EPI_ISL_943198, EPI_ISL_943199, EPI_ISL_943243, EPI_ISL_943244, EPI_ISL_943266, EPI_ISL_943269, EPI_ISL_943296, EPI_ISL_943317, EPI_ISL_943360, EPI_ISL_943361, EPI_ISL_943376, EPI_ISL_943377, EPI_ISL_943405, EPI_ISL_943406, EPI_ISL_943407, EPI_ISL_943435, EPI_ISL_943449, EPI_ISL_943461, EPI_ISL_943462, EPI_ISL_943463, EPI_ISL_943464, EPI_ISL_943479, EPI_ISL_943480, EPI_ISL_943496, EPI_ISL_943506, EPI_ISL_943513			
see above	Dutch COVID-19 response team	National Institute for Public Health and the Environment (RIVM)	Adam Meijer, Harry Vennema, Dirk Eggink, Jeroen Cremer, Sharon van den Brink, Bas van der Veer, AnneMarie van den Brandt, Florian Zwagemaker, Dennis Schmitz, Chantal Reusken, on behalf of the national COVID-19 response team
EPI_ISL_943831, EPI_ISL_943832, EPI_ISL_943833, EPI_ISL_943834, EPI_ISL_943835, EPI_ISL_943940	Utah Public Health Laboratory	Utah Public Health Laboratory	Erin L. Young, Kelly F. Oakeson, Tara Gallagher
EPI_ISL_944623, EPI_ISL_944624, EPI_ISL_944625	Instituto Nacional de Medicina Genomica	Instituto Nacional de Medicina Genomica	Hidalgo-Miranda A, Mendoza-Vargas A, Reyes-Grajeda JP, Cisneros-Villanueva M, Cedro-Tanda A,Peñaloza-Figueroa F, Herrera-Montalvo LA
EPI_ISL_944656, EPI_ISL_944657, EPI_ISL_944669, EPI_ISL_944679, EPI_ISL_944680, EPI_ISL_944681, EPI_ISL_944687, EPI_ISL_944688, EPI_ISL_944693, EPI_ISL_944699, EPI_ISL_944707, EPI_ISL_944711			
see above	Department of Biochemistry, Cell and Molecular Biology, West African Centre for Cell Biology of Infectious Pathogens (WACCBIP), University of Ghana	Department of Biochemistry, Cell and Molecular Biology, West African Centre for Cell Biology of Infectious Pathogens (WACCBIP), University of Ghana	Morang'a,C.M., Ngoi,J.M., Quansah,E.B., Saiid,S., Amuzu,D.S., Asante,I., Bonney,J.H., Bonney,E., Odoom,J.K., Ndam,N.T., Tei-Maya,F., Adusei-Poku,M., Ofori-Boadu,L., Ampofo,W.K., Amenga-Etego,L.N., Quashie,P., Bediako,Y., Awandare,G.A.
EPI_ISL_944787	Laboratory of Virology and Molecular Diagnostics	Institute of Public Health of Republic of North Macedonia Laboratory of Virology and Molecular Diagnostics	Maja Kuzmanovska, Golubinka Boshevska, Elizabeta Janchevska
EPI_ISL_945049, EPI_ISL_945056, EPI_ISL_945061, EPI_ISL_945072, EPI_ISL_945084, EPI_ISL_945095, EPI_ISL_945114, EPI_ISL_945115, EPI_ISL_945122, EPI_ISL_945125, EPI_ISL_945130, EPI_ISL_945142, EPI_ISL_945143, EPI_ISL_945146, EPI_ISL_945147, EPI_ISL_945152, EPI_ISL_945155, EPI_ISL_945163, EPI_ISL_945169, EPI_ISL_945181, EPI_ISL_945190, EPI_ISL_945197, EPI_ISL_945207, EPI_ISL_945208, EPI_ISL_945212, EPI_ISL_945217, EPI_ISL_945221, EPI_ISL_945236, EPI_ISL_945238, EPI_ISL_945241, EPI_ISL_945243, EPI_ISL_945244, EPI_ISL_945248, EPI_ISL_945250, EPI_ISL_945254, EPI_ISL_945275, EPI_ISL_945279, EPI_ISL_945285, EPI_ISL_945288, EPI_ISL_945297, EPI_ISL_945304, EPI_ISL_945311, EPI_ISL_945319, EPI_ISL_945324, EPI_ISL_945332, EPI_ISL_945334, EPI_ISL_945337, EPI_ISL_945345, EPI_ISL_945347, EPI_ISL_945363, EPI_ISL_945364, EPI_ISL_945366			
see above	Lighthouse Lab in Cambridge	Wellcome Sanger Institute for the COVID-19 Genomics UK (COG-UK) Consortium	Rob Howes, The Lighthouse Lab in Cambridge and Alex Alderton, Roberto Amato, Sonia Goncalves, Ewan Harrison, David K. Jackson, Ian Johnston, Dominic Kwiatkowski, Cordelia Langford, John Sillitoe on behalf of the Wellcome Sanger Institute COVID-19 Surveillance Team
EPI_ISL_945547, EPI_ISL_946913	Laboratory of Virology and Molecular Diagnostics	Institute of Public Health of Republic of North Macedonia Laboratory of Virology and Molecular Diagnostics	Maja Kuzmanovska, Golubinka Boshevska, Elizabeta Janchevska
EPI_ISL_947328	RS Mitra Keluarga Depok	Eijkman Institute for Molecular Biology, Ministry of Research and Technology/National Agency for Research and Innovation	Willy Agustine, Edison Johar, Hidayat Trimarsanto, Iskandar Adnan, Lydia V. Panggalo, Sukma Oktavianthi, Frilasita A Yudhaputri, Safarina G Malik, Khin Saw Myint, Amin Soebandrio
EPI_ISL_947329, EPI_ISL_947330	RSU Bunda Mulia	Eijkman Institute for Molecular Biology, Ministry of Research and Technology/National Agency for Research and Innovation	Willy Agustine, Edison Johar, Hidayat Trimarsanto, Iskandar Adnan, Lydia V. Panggalo, Sukma Oktavianthi, Frilasita A Yudhaputri, Safarina G Malik, Khin Saw Myint, Amin Soebandrio
EPI_ISL_949124	SC (UCO) Igiene e Sanità Pubblica (funzione integrata con SC Microbiologia e Virologia) e Laboratory of Molecular Virology of the International Centre for Genetic Engineering	ARGO Laboratorio Genomica ed Epigenomica	Licastro D, Dal Monego S, Degasperi M, Marcello A, D'Agaro P

	and Biotechnology (ICGEB)		
EPI_ISL_949192	National Institute of Health Research and Development	National Institute of Health Research and Development	Ririn Ramadhany; Yuni Rukminiati; Agustiningsih; Kindi Adam; Holy Arif Wibowo; Hana Apsari Pawestri; Subangkit; Kartika Dewi Puspa; Arie Ardiansyah Nugraha; Hartanti Dian Ikawati; Krisna Nur Andriana Pangesti; Ni Ketut Susilarini; Nur Ika Hariastuti; Uly Alfi Nikmah; Mursinah; Asri Febriyani; Reni Herman; Nike Susanti; Herna; Tati Febriyanti; Natalie Laurentia Kipuw; Fauzul Muna; Irene Lorinda Indalao; Aulia Rizki; Nelly Puspandari; Vivi Setiawaty
EPI_ISL_949757, EPI_ISL_949759, EPI_ISL_949760, EPI_ISL_949762, EPI_ISL_949763, EPI_ISL_949765, EPI_ISL_949766, EPI_ISL_949772, EPI_ISL_949774	Barts Health NHS Trust	COVID-19 Genomics UK (COG-UK) Consortium	CUTINO-MOGUEL, Maria-Teresa; HARRINGTON, David; OWOYEMI, Dola; KULASEGARAN-SHYLINI, Raghavendran; BROAD, Claire; KELE, Beatrix
EPI_ISL_949789, EPI_ISL_950069, EPI_ISL_950070, EPI_ISL_950072, EPI_ISL_950074, EPI_ISL_950075, EPI_ISL_950077, EPI_ISL_950079, EPI_ISL_950080, EPI_ISL_950083, EPI_ISL_950084, EPI_ISL_950085, EPI_ISL_950097, EPI_ISL_950098, EPI_ISL_950100, EPI_ISL_950102, EPI_ISL_950110, EPI_ISL_950112, EPI_ISL_950115, EPI_ISL_950126, EPI_ISL_950150, EPI_ISL_950169			
see above	University College London, Great Ormond Street Hospital for Children NHS Foundation Trust, Imperial College Healthcare NHS Trust	COVID-19 Genomics UK (COG-UK) Consortium	Sergi Castellano, Rachel Williams, Mark Kristiansen, Paola Resende Silva, Sunando Roy, Tony Brooks, Helena Tutill, Paola Niola, Patricia Dyal, Charlotte Williams, Leysa Forrest, Yasmin Panchbhaya, Jacqueline Findlay, Samuel Weeks, Julianne Brown, Kathryn Harris, Paul Randell, James Price, Alison Holmes, Judith Breuer
EPI_ISL_950272	Northumbria University / South Tees Hospitals NHS Foundation Trust / North Cumbria Integrated Care NHS Foundation Trust / North Tees and Hartlepool NHS Foundation Trust / Newcastle Hospitals NHS Foundation Trust	COVID-19 Genomics UK (COG-UK) Consortium	Darren L Smith,Andrew Nelson,Matthew Bashton,Greg R Young,Joshua Loh,John Allan,Mohammad A Tariq,Giles S Holt,Gary Black,Wen C Yew,Lynn Dover,Paul Baker,Steve Liggett,Sarah Essex,Jane Greenaway,Debra Padgett,Clive Graham,Garren Scott,Edward Barton,Emma Swindells,Brendan Payne,Jennifer Collins,Yusri Taha,Gary Eltringham
EPI_ISL_950713	Lincolnshire Hospitals and DeepSeq Nottingham	COVID-19 Genomics UK (COG-UK) Consortium	Nichola Duckworth, Tim Sloan, Sarah Walsh, Jonathan Ball, Patrick McClure, Joeseph Chappell, Nadine Holmes, Matthew Carlisle, Christopher Moore, Fei Sang, Johnny Debebe, Victoria Wright, Matthew Loose
EPI_ISL_951231, EPI_ISL_951232, EPI_ISL_951239, EPI_ISL_951245, EPI_ISL_951246, EPI_ISL_951247, EPI_ISL_951248, EPI_ISL_951252, EPI_ISL_951254, EPI_ISL_951255, EPI_ISL_951256, EPI_ISL_951258, EPI_ISL_951265, EPI_ISL_951268, EPI_ISL_951269, EPI_ISL_951270, EPI_ISL_951271, EPI_ISL_951272, EPI_ISL_951273, EPI_ISL_951275, EPI_ISL_951276, EPI_ISL_951277, EPI_ISL_951281, EPI_ISL_951282, EPI_ISL_951283, EPI_ISL_951286, EPI_ISL_951287, EPI_ISL_951288, EPI_ISL_951289, EPI_ISL_951294, EPI_ISL_951299, EPI_ISL_951300, EPI_ISL_951308, EPI_ISL_951309, EPI_ISL_951316, EPI_ISL_951356, EPI_ISL_951364, EPI_ISL_951366, EPI_ISL_951367, EPI_ISL_951389, EPI_ISL_951391, EPI_ISL_951392, EPI_ISL_951393, EPI_ISL_951395, EPI_ISL_951396, EPI_ISL_951406, EPI_ISL_951407, EPI_ISL_951408, EPI_ISL_951409, EPI_ISL_951414, EPI_ISL_951426, EPI_ISL_951427, EPI_ISL_951429			
see above	Oxford Viromics, NDM, University of Oxford; Oxford University Hospitals; Basingstoke and North Hampshire Hospital	COVID-19 Genomics UK (COG-UK) Consortium	Tanya Golubchik, David Bonsall, George Macintyre, Amy Trebes, Mariateresa de Cesare, Catrin Moore, Alex Mobbs, Anita Justice, Robert Shaw, Monique Andersson, Timothy Peto, Emma Wise, Nathan Moore, Jessica Lynch, Nick Cortes, Matilde Mori, Stephen Kidd, David Buck, John Todd, Christophe Fraser
EPI_ISL_952381, EPI_ISL_952384, EPI_ISL_952388, EPI_ISL_952396, EPI_ISL_952400, EPI_ISL_952402, EPI_ISL_952406, EPI_ISL_952419, EPI_ISL_952425, EPI_ISL_952426, EPI_ISL_952781, EPI_ISL_952782, EPI_ISL_952784, EPI_ISL_952786, EPI_ISL_952787, EPI_ISL_952788, EPI_ISL_952791, EPI_ISL_952800, EPI_ISL_952802, EPI_ISL_952805, EPI_ISL_952806, EPI_ISL_952807, EPI_ISL_952808, EPI_ISL_952809, EPI_ISL_952810, EPI_ISL_952811, EPI_ISL_952812, EPI_ISL_952819			
see above	Centre for Enzyme Innovation, University of Portsmouth / Translational Research Laboratory, Portsmouth Hospitals NHS Trust	COVID-19 Genomics UK (COG-UK) Consortium	Angela Beckett,Salman Goudarzi,Christopher Fearn,Kate Cook,Katie Loveson,Sharon Glaysher,Scott Elliott,Samuel Robson
EPI_ISL_953177, EPI_ISL_953180, EPI_ISL_953181, EPI_ISL_953182, EPI_ISL_953185, EPI_ISL_953187, EPI_ISL_953193, EPI_ISL_953199, EPI_ISL_953205, EPI_ISL_953207, EPI_ISL_953216, EPI_ISL_953217, EPI_ISL_953219, EPI_ISL_953222, EPI_ISL_953226, EPI_ISL_953230, EPI_ISL_953231, EPI_ISL_953232, EPI_ISL_953233, EPI_ISL_953236, EPI_ISL_953238, EPI_ISL_953239			
see above	Bioinformatics and Biostatistics Lab, Advanced Sequencing Facility	COVID-19 Genomics UK (COG-UK) Consortium	Aengus Stewart,Jerome Nicod,Chelsea Sawyer,Laura Cubitt,Harshil Patel,Margaret Crawford
EPI_ISL_953521	University Hospitals of Geneva, Laboratory of Virology	HUG, Laboratory of Virology and the Health2030 Genome Center	Samuel Cordey, Ana Rita Goncalves, Laurent Kaiser, Lorenzo Cerutti, Henri Pegeot, Melyssa Elies, Deborah Penet, Keith Harshman, Ioannis Xenarios, Emmanouil Dermitzakis
EPI_ISL_954128	Labo Analyses Med	National Reference Center for Viruses of Respiratory Infections, Institut Pasteur, Paris	Marion Barbet, Sylvie Behillil, Méline Bizard, Angela Brisebarre, Camille Capel, Etienne Simon-Lorière, Vincent Enouf, Maud Vanpeene, Sylvie van der Werf,Takoudju Eve-Marie
EPI_ISL_954223, EPI_ISL_954224, EPI_ISL_954225	1.AO Universitaria 'S. Giovanni di Dio e Ruggi D'Aragona, Scuola Medica Salernitana' Hospital / 2.UOC di Virologia e Microbiologia, Università della Campania 'L. Vanvitelli' / 3.AO Universitaria 'Federico II' Napoli Hospital / 4.AORN 'San Giuseppe Moscati' Avellino Hospital / 5.AO 'San Pio - presidio G. Rummo' Benevento Hospital / 6.AO 'Sant'Anna e San Sebastiano' Caserta Hospital / 7.PO 'Maria Santissima Addolorata' Eboli Hospital / 8.Biogem Istituto di Ricerche Genetiche	1. Genome Research Center for Health (CRGS) / 2. Laboratory of Molecular Medicine and Genomics(LMMGe) / 3. Center for Research in Pure and Applied Mathematics (CRMPA)	Giorgio Giurato, Francesca Rizzo, Alessandro Weisz, Gianluigi Franci, Giovanni Nassa, Pasquale Pagliano, Roberta Tarallo, Elena Alexandrova, Ylenia D'Agostino, Carlo Ferravante, Jessica Lamberti, Viola Melone, Domenico Memoli, Valeria Mirici Cappa, Domenico Palumbo, Giovanni Pecoraro, Assunta Sellitto, Oriana Strianese, Ilaria Terenzi, Giuseppe Fenza, Aniello Gentile, Antonello Saccomanno, Sonia Amabile, Teresa Rocco, Annamaria Salvati, Emilia Vaccaro, Massimiliano Galdiero, Michele Cennamo, Giuseppe Portella, Maria Grazia Foti, Mariarosaria Ingino, Maria Landi, Maurizio Fumi, Vincenzo Rocco, Rita Greco, Vittoria Letizia, Arnolfo Petruzziello, Maddalena Schioppa, Gregorio Goffredi, Francesca Marciano, Michele Caraglia, Alessia Cossu, Marianna Scrima, Edmondo Adorisio, Morena D'Avenia, Michela Iacobellis, Rosanna Piluscio, Giorgio Dirani, Vittorio Sambri, Simona Semprini, Silvia Zanoli, Francesco Curcio, Stefania Marzinotto, Andreina Baj, Fausto Sessa.
EPI_ISL_954781	Hospital General Universitario de Ciudad Real	Instituto de Salud Carlos III	Iglesias-Caballero, M. Camarero, S. Sandonís,V. Vázquez, S. Pozo, F. Casas, I. Jiménez, P. Zaballos, A. Monzón, S. Varona, S. Cuesta, I. Illescas, S.
EPI_ISL_954793	Hospital Universitario Virgen de la Arrixaca	Instituto de Salud Carlos III	Iglesias-Caballero, M. Camarero, S. Sandonís,V. Vázquez, S. Pozo, F. Casas, I. Jiménez, P. Zaballos, A. Monzón, S. Varona, S. Cuesta, I. Moreno, L.
EPI_ISL_954796	HOSPITAL COMARCAL DE MELILLA	Instituto de Salud Carlos III	Iglesias-Caballero, M. Camarero, S. Sandonís,V. Vázquez, S. Pozo, F. Casas, I. Jiménez, P. Zaballos, A. Monzón, S. Varona, S. Cuesta, I. Illescas, S.
EPI_ISL_954804	Complejo Hospitalario de Navarra	Instituto de Salud Carlos III	Iglesias-Caballero, M. Camarero, S. Sandonís,V. Vázquez, S. Pozo, F. Casas, I. Jiménez, P. Zaballos, A. Monzón, S. Varona, S. Cuesta, I. Ezpeleta,C.
EPI_ISL_954858, EPI_ISL_954876, EPI_ISL_954919, EPI_ISL_954920, EPI_ISL_954921, EPI_ISL_954922, EPI_ISL_954923, EPI_ISL_954924, EPI_ISL_954925, EPI_ISL_954926, EPI_ISL_954927, EPI_ISL_954928, EPI_ISL_954929, EPI_ISL_954930			
see above	Colorado Department of Public Health and Environment	Colorado Department of Puplic Health and Environment	Laura Bankers, Molly C. Hetherington-Rauth, Diana Ir, Shannon Ely, Shannon R. Matzinger, Sarah Elizabeth Totten, Emily A. Travanty
EPI_ISL_955277, EPI_ISL_955319, EPI_ISL_955329, EPI_ISL_955334	GA Department of Public Health Laboratory	Pathogen Discovery, Respiratory Viruses Branch, Division of Viral Diseases, Centers for Disease Control and Prevention	Ying Tao, Jing Zhang, Yan Li, Krista Queen, Anna Uehara, Peter Cook, Clinton R. Paden, Haibin Wang, Suxiang Tong
EPI_ISL_956304, EPI_ISL_956305	ESE INSTITUTO NACIONAL DE CANCEROLOGIA	Instituto Nacional de Salud- Dirección de Investigación en Salud Pública	Katherine Laiton-Donato, Diego A. Álvarez-Díaz, Carlos Franco-Muñoz, Mauricio Pacheco-Montealegre, Hector Alejandro Ruiz-Moreno, Maria T. Herrera-Sepúlveda, Diego Andrés Prada, Jhonnatan Reales-González, Sheryll Corchuelo, Julian Naizaque, Gerardo Santamaría, Magdalena Wiesner, Martha Lucia Ospina Martinez, Marcela Mercado-Reyes
EPI_ISL_956313	Siti Khodijah Hospital	Institute of Tropical Disease, Universitas Airlangga	Jezzy R Dewantari, Rima R Prasetya, Krisnoadi Rahardjo, Aldise M Nastri, Muhammad Hamdan, Gatot Soegiarto, Laksmi Wulandari, Resti Yudhawati, Yasuko Mori, Soetjipto, Kazufumi Shimizu, Maria I Lusida
EPI_ISL_959274	General Hospital - Tetovo	Research Center for Genetic Engineering and Biotechnology "Georgi D. Efremov" , Macedonian Academy of Sciences and Arts	Aleksandar J. Dimovski, Dijana Plasheska-Karanfilska, Predrag Noveski, Gjorgji Bozinovski, Milena Jakimovska
EPI_ISL_959285	General Hospital - Prilep	Research Center for Genetic Engineering and Biotechnology "Georgi D. Efremov" , Macedonian Academy of Sciences and Arts	Aleksandar J. Dimovski, Dijana Plasheska-Karanfilska, Predrag Noveski, Gjorgji Bozinovski, Milena Jakimovska
EPI_ISL_959551, EPI_ISL_959552	CHC Liège	GIGA Medical Genomics	Keith Durkin, Maria Artesi, Sébastien Bontems, Raphaël Boreux, Bouchra Boujemla, Cécile Meex, Pierrette Melin, Marie-Pierre Hayette, Vincent Bours
EPI_ISL_959553	CHR Namur	GIGA Medical Genomics	Keith Durkin, Maria Artesi, Sébastien Bontems, Raphaël Boreux, Bouchra Boujemla, Cécile Meex, Pierrette Melin, Marie-Pierre Hayette, Vincent Bours
EPI_ISL_959554	Vivalia - Clinique Saint-Joseph	GIGA Medical Genomics	Keith Durkin, Maria Artesi, Sébastien Bontems, Raphaël Boreux, Bouchra Boujemla, Cécile Meex, Pierrette Melin, Marie-Pierre Hayette, Vincent Bours
EPI_ISL_959650	General Hospital "Abdulah Nakas" Sarajevo	Alea Genetic Centre	Dino Pecar, Lana Salihefendic, Sead Jazic, Rijad Konjhodzic
EPI_ISL_959855, EPI_ISL_959858	National Virus Reference Laboratory	National Virus Reference Laboratory	Michael Carr, Gabriel Gonzalez, Jonathan Dean, Cillian F De Gascun

EPI_ISL_960324	University of Wisconsin-Madison AIDS Vaccine Research Laboratories	University of Wisconsin-Madison AIDS Vaccine Research Laboratories	Gage Moreno, Katarina Braun, et al. AIDS Vaccine Research Laboratories
EPI_ISL_960404, EPI_ISL_960407, EPI_ISL_960413, EPI_ISL_960419, EPI_ISL_960430, EPI_ISL_960436	The National Institute of Public Health	State Veterinary Institute Prague	Nagy,A;Vecerova,J;Cernikova,L;Stara,M;Jirincova,H;Trnka,D
EPI_ISL_960834, EPI_ISL_960876	Institute of Medical Microbiology and Hospital Hygiene	Institute of Medical Microbiology and Hospital Hygiene	Prof. Dr. Achim Kaasch, Aljoscha Tersteegen
EPI_ISL_961105, EPI_ISL_961106, EPI_ISL_961107, EPI_ISL_961108, EPI_ISL_961109, EPI_ISL_961110, EPI_ISL_961111, EPI_ISL_961112, EPI_ISL_961113, EPI_ISL_961114, EPI_ISL_961115, EPI_ISL_961116, EPI_ISL_961117, EPI_ISL_961118, EPI_ISL_961119, EPI_ISL_961120, EPI_ISL_961121, EPI_ISL_961122, EPI_ISL_961123, EPI_ISL_961124, EPI_ISL_961125, EPI_ISL_961126, EPI_ISL_961131			
see above	Delaware Public Health Laboratory	Delaware Public Health Lab	Gregory Hovan
EPI_ISL_961475, EPI_ISL_961487, EPI_ISL_961500, EPI_ISL_961501, EPI_ISL_961507, EPI_ISL_961515, EPI_ISL_961516, EPI_ISL_961547	Michigan Department of Health and Human Services, Bureau of Laboratories	Michigan Department of Health and Human Services, Bureau of Laboratories	Blankenship HM, Riner D, Soehnlen MK
EPI_ISL_961556, EPI_ISL_961557, EPI_ISL_961558, EPI_ISL_961559, EPI_ISL_961598, EPI_ISL_961643, EPI_ISL_961644, EPI_ISL_961645, EPI_ISL_961646, EPI_ISL_961647, EPI_ISL_961648			
see above	Hôpital Georges L. Dumont	National Microbiology Laboratory (NML)	Anna Majer, Shari Tyson, Grace Seo, Philip Mabon, Elsie Grudeski, Rhiannon Huzarewich, Russell Mandes, Anneliese Landgraff, Jennifer Tanner, Natalie Knox, Morag Graham, Gary Van Domselaar, Richard Garceau, Guillaume Desnoyers, Nathalie Bastien, Yan Li, Timothy Booth, Darian Hole, Madison Chapel, Kirsten Biggar, CanCOGeN's metadata curation team, Public Health Agency of Canada CanCOGeN team
EPI_ISL_961881, EPI_ISL_961882	E. Gulbja laboratorija	Latvian Biomedical Research and Study Centre	Janis Pjalkovskis, Nikita Zrelovs, Monta Ustinova, Ivars Silamikelis, Liga Birzniece, Kaspars Megnis, Vita Rovite, Lauma Freimane, Laila Silamikele, Laura Ansone, Davids Fridmanis, Mikus Gavars, Dmitrijs Perminovs, Jurijs Perevoscikovs, Uga Dumpis, Janis Klovins
EPI_ISL_961897, EPI_ISL_961920, EPI_ISL_961925, EPI_ISL_961930, EPI_ISL_961931, EPI_ISL_961932, EPI_ISL_961935, EPI_ISL_961938, EPI_ISL_961960, EPI_ISL_961962, EPI_ISL_961969, EPI_ISL_961971, EPI_ISL_962154, EPI_ISL_962161, EPI_ISL_962162, EPI_ISL_962163, EPI_ISL_962164, EPI_ISL_962165, EPI_ISL_962166, EPI_ISL_962167, EPI_ISL_962168, EPI_ISL_962169, EPI_ISL_962170, EPI_ISL_962172			
see above	Illinois Department of Public Health	Gagnon Lab, Southern Illinois University	Keith Gagnon
EPI_ISL_962511	UCLA Clinical Micro Lab	Los Angeles County PHL	P. Hemarajata et al.
EPI_ISL_962516	MD Laboratories	Los Angeles County PHL	P. Hemarajata et al.
EPI_ISL_962812	Robert Garry lab	Andersen lab at Scripps Research	Allison Smither, Gilberto Sabino-Santos, Patricia Snarski, Lilia Melnik, Antoinette Bell, Kaylynn Genemaras, Arnaud Drouin, Dahlene Fusco, Robert Garry with SEARCH Alliance San Diego
EPI_ISL_962825	Victorian Infectious Diseases Reference Laboratory (VIDRL)	VIDRL and MDU-PHL	Caly L., Seemann T., Sait, M.L., Druce J., Sherry, N.L.
EPI_ISL_962889	Laboratory of Virology and Molecular Diagnostics	Institute of Public Health of Republic of North Macedonia Laboratory of Virology and Molecular Diagnostics	Kuzmanovska M., Boshevska G.
EPI_ISL_962915, EPI_ISL_962917, EPI_ISL_962922, EPI_ISL_962923	Servicio de Microbiología, Laboratori Clínic Metropolitana Nord. Hospital Universitari Germans Trias i Pujol. Institut d'Investigació en Ciències de la Salut Germans Trias i Pujol (IGTP)	SeqCOVID-SPAIN consortium/IBV(CSIC)	Elisa Martró, Antoni E. Bordoy, Anna Not, Adrián Antuori, Anabel Fernández, Nona Romaní, Verónica Saludes, Cristina Casañ and SeqCOVID-SPAIN consortium
EPI_ISL_962934	Hospital Universitario de Gran Canaria Dr. Negrín	SeqCOVID-SPAIN consortium/IBV(CSIC)	M. Carmen Pérez González, Francisco J. Chamizo López, Ana Bordes Benítez and SeqCOVID-SPAIN consortium
EPI_ISL_964292	Oslo University Hospital, Department of Medical Microbiology	Norwegian Institute of Public Health, Department of Virology	Kathrine Stene-Johansen, Kamilla Heddeland Instefjord, Hilde Elshaug, Ignacio Garcia Llorente, Serina B Engebretsen, Atiya R Ali, Marie Paulsen Madsen, Rasmus Riis Kopperud, Hilde Vollan, Karoline Bragstad, Olav Hungnes
EPI_ISL_964924	Instituto Nacional de Saude (INS), Mozambique	KRISP, KZN Research Innovation and Sequencing Platform	Nalia Ismael, Nadia Sitoe, Paulo Arnaldo, Nedio Mabunda, Giandhari J, Pillay S, Emmanuel S, Tegally H, Wilkinson E, de Oliveira T
EPI_ISL_964951	Haukeland University Hospital, Dept. of Microbiology	Norwegian Institute of Public Health, Department of Virology	Kathrine Stene-Johansen, Kamilla Heddeland Instefjord, Hilde Elshaug, Ignacio Garcia Llorente, Serina B Engebretsen, Atiya R Ali,Marie Paulsen Madsen, Rasmus Riis Kopperud, Hilde Vollan, Karoline Bragstad, Olav Hungnes
EPI_ISL_965131	Azienda Ospedaliera San Giovanni Addolorata	INMI Lazzaro Spallanzani IRCCS	O Butera, F Messina, CEM Gruber, B Bartolini, E Giombini, M Rueca, M Gaudio, PM Placanica, MR Capobianchi, A Di Caro
EPI_ISL_965136	Azienda Sanitaria Locale Roma 5	INMI Lazzaro Spallanzani IRCCS	O Butera, F Messina, CEM Gruber, B Bartolini, E Giombini, M Rueca, D Di Fusco, D Cerini, MR Capobianchi, A Di Caro
EPI_ISL_965657, EPI_ISL_965690, EPI_ISL_965763, EPI_ISL_965793, EPI_ISL_965806	Dutch COVID-19 response team	Medical Microbiology, Maastricht University Medical Centre	Jozef Dingemans*, Brian van der Veer*, Erik Beuken, Carmen Reumkens, Lieke van Alphen, Christian Hoebe, Paul Savelkoul
EPI_ISL_966782	Maine HETL	Tewhey Lab, The Jackson Laboratory	Matluk,N., Dewey,H., Iosue,F., Barter,M., Lynch,R., Munger,H. and Tewhey,R.
EPI_ISL_967220	Helix/Illumina	Respiratory Viruses Branch, Division of Viral Diseases, Centers for Disease Control and Prevention	Peter W. Cook,Dakota Howard,Dhwani Batra,Ben L. Rambo-Martin,Eileen de Feo,Jan Antico,Christine Tran,Matthew Tolentino,Shannon Wickline,Kim Gietzen,Brad Sickler,Jingtao Liu,Eric Allen,Phil Febbo,Summer Galloway,Nicole L. Washington,Simon White,Geraint Levan,Kelly Schiabor Barrett,Elizabeth Cirulli,Alexandre Bolze,Ary Ascencio,Charlotte Rivera-Garcia,Ryan Cho,Jason Nguyen,Sherry Wang,Jimmy Ramirez,Tyler Cassens,Efren Sandoval,Magnus Isaksson,William Lee,David Becker,Marc Laurent,James Lu,Clinton R. Paden,Suxiang Tong,Duncan MacCannell,
EPI_ISL_967696, EPI_ISL_967743	State Laboratories Division, Hawaii State Department of Health	State Laboratories Division, Hawaii State Department of Health	Pamela O'Brien, Drew Kuwazaki, Ayana Garnet, Razvan Sultana, Edward Desmond
EPI_ISL_967901, EPI_ISL_967906, EPI_ISL_967938, EPI_ISL_967955, EPI_ISL_967974, EPI_ISL_968001, EPI_ISL_968055	TGen North	TGen North	"Jolene Bowers, Megan Folkerts, Chris French, Hayley Yaglom, Ashlyn Pfeiffer, Darrin Lemmer, Dave Engelthaler, The Arizona COVID Genomics Union (ACGU)"
EPI_ISL_970179	Hospital San Agustin, Avilés	Laboratorio de Virología HUCA	Castelló C, Gómez de Oña J, Boga JA, Rojo S, Alvarez-Arguelles ME, Abreu F, Costales I, Sandoval M, Perez-Martínez Z, Martín-Rodríguez G, Coto E, Melón S
EPI_ISL_976941, EPI_ISL_976942, EPI_ISL_976943, EPI_ISL_976944, EPI_ISL_976946, EPI_ISL_976947, EPI_ISL_976948, EPI_ISL_976949, EPI_ISL_976950, EPI_ISL_976951, EPI_ISL_976952, EPI_ISL_976954, EPI_ISL_976955, EPI_ISL_976956, EPI_ISL_976957, EPI_ISL_976958, EPI_ISL_976959, EPI_ISL_976960, EPI_ISL_976962, EPI_ISL_976963, EPI_ISL_976964, EPI_ISL_976965, EPI_ISL_976966, EPI_ISL_976967, EPI_ISL_976968, EPI_ISL_976970, EPI_ISL_976971, EPI_ISL_976972, EPI_ISL_976973, EPI_ISL_976974, EPI_ISL_976975, EPI_ISL_976976, EPI_ISL_976978, EPI_ISL_976979, EPI_ISL_976980, EPI_ISL_976983, EPI_ISL_976984, EPI_ISL_976985, EPI_ISL_976987, EPI_ISL_976988, EPI_ISL_976989, EPI_ISL_976990, EPI_ISL_976991, EPI_ISL_976992, EPI_ISL_976993, EPI_ISL_976995, EPI_ISL_976996, EPI_ISL_976997, EPI_ISL_976998, EPI_ISL_976999, EPI_ISL_977000, EPI_ISL_977002, EPI_ISL_977003, EPI_ISL_977004, EPI_ISL_977005, EPI_ISL_977006, EPI_ISL_977007, EPI_ISL_977009, EPI_ISL_977010, EPI_ISL_977011, EPI_ISL_977012, EPI_ISL_977013, EPI_ISL_977015, EPI_ISL_977016, EPI_ISL_977017, EPI_ISL_977018, EPI_ISL_977019, EPI_ISL_977020, EPI_ISL_977021, EPI_ISL_977022, EPI_ISL_977023			
see above	Broad Institute Clinical Research Sequencing Platform	Infectious Disease Program, Broad Institute of Harvard and MIT	Lemieux,J.E., Siddle,K.J., Adams,G., Gladden-Young,A., Lagerborg,K., Rudy,M., DeRuff,K., Carter,A., Normandin,E., Bauer,M., Reilly,S., Tomkins-Tinch,C., Loreth,C., Chaluvadi,S., Birren,B.W., Gallagher,G., Smole,S., Park,D.J., MacInnis,B.L., and Sabeti,P.C.
EPI_ISL_977083	Massachusetts General Hospital	Infectious Disease Program, Broad Institute of Harvard and MIT	Lemieux,J.E., Siddle,K.J., Shaw,B., Adams,G., Pierce,V., Turbett,S., Anahtar,M., Branda,J., Slater,D., Harris,J., Lin,A.E., Gladden-Young,A., Lagerborg,K., Rudy,M., DeRuff,K., Carter,A., Normandin,E., Bauer,M., Reilly,S., Tomkins-Tinch,C., Loreth,C., Chaluvadi,S., Neumann,A., Cusick,C., Chapman,S.B., Gnirke,A., Flowers,K., Cerrato,F., Birren,B.W., Gallagher,G., Smole,S., Park,D.J., MacInnis,B.L., Ryan,E., LaRocque,R., Rosenberg,E. and Sabeti,P.C.
EPI_ISL_977155, EPI_ISL_977157	ULSS 03 Venezia	Istituto Zooprofilattico Sperimentale delle Venezie	Adelaide Milani, Alessia Schivo, Annalisa Salviato, Erika Giorgia Quaranta, Ambra Pastori, Bianca Zecchin, Alice Fusaro, Isabella Monne, Calogero Terregino, Antonia Ricci
EPI_ISL_977347	University of Zambia, School of Veterinary Medicine	UNZAVET and PATH	Mulenga Mwenda-Chimfwembe, Ngonda Saasa, Daniel Bridges
EPI_ISL_977541, EPI_ISL_977542, EPI_ISL_977545, EPI_ISL_977546,	Nigeria Centre of Disease Control (NCDC)	African Centre of Excellence for Genomics of Infectious Diseases (ACEGID), Redeemer's University	Olawoye I. B. et al

EPI_ISL_977547, EPI_ISL_977548, EPI_ISL_977552, EPI_ISL_977563, EPI_ISL_977565			
EPI_ISL_977652	Michigan Department of Health and Human Services, Bureau of Laboratories	Michigan Department of Health and Human Services, Bureau of Laboratories	Blankenship HM, Riner D, Soehnlen MK
EPI_ISL_977962, EPI_ISL_977963, EPI_ISL_977964, EPI_ISL_977965, EPI_ISL_977966, EPI_ISL_977967, EPI_ISL_977968, EPI_ISL_977969, EPI_ISL_977970, EPI_ISL_977971, EPI_ISL_977972, EPI_ISL_977973, EPI_ISL_977974, EPI_ISL_977975, EPI_ISL_977976, EPI_ISL_977977, EPI_ISL_977978			
see above	Chiu Laboratory, University of California, San Francisco	Chiu Laboratory, University of California, San Francisco	Charles Chiu, Xianding (Wayne) Deng, Candace Wang, Venice Servellita, Jill Hacker, Debra Wadford
EPI_ISL_978357, EPI_ISL_978366, EPI_ISL_978367, EPI_ISL_978368, EPI_ISL_978369, EPI_ISL_978370	Arizona State Public Health Laboratory	Arizona State Public Health Laboratory	Trung Huynh, Jessica Escobar, Katherine Fullerton, Nobuko Fukushima, Stacy White, Linda Getsinger, Victor Waddell
EPI_ISL_978625, EPI_ISL_978626, EPI_ISL_978627, EPI_ISL_978628, EPI_ISL_978629, EPI_ISL_978630, EPI_ISL_978631, EPI_ISL_978632, EPI_ISL_978633, EPI_ISL_978634, EPI_ISL_978635, EPI_ISL_978636, EPI_ISL_978637, EPI_ISL_978638, EPI_ISL_978639, EPI_ISL_978640, EPI_ISL_978641, EPI_ISL_978642, EPI_ISL_978643, EPI_ISL_978644, EPI_ISL_978645, EPI_ISL_978646, EPI_ISL_978647, EPI_ISL_978648, EPI_ISL_978649, EPI_ISL_978650, EPI_ISL_978651, EPI_ISL_978652, EPI_ISL_978653, EPI_ISL_978654, EPI_ISL_978655, EPI_ISL_978656, EPI_ISL_978657, EPI_ISL_978658, EPI_ISL_978659, EPI_ISL_978660, EPI_ISL_978661, EPI_ISL_978662, EPI_ISL_978663, EPI_ISL_978664, EPI_ISL_978665, EPI_ISL_978666, EPI_ISL_978667, EPI_ISL_978668, EPI_ISL_978669, EPI_ISL_978670, EPI_ISL_978671, EPI_ISL_978672, EPI_ISL_978673, EPI_ISL_978674, EPI_ISL_978675, EPI_ISL_978676, EPI_ISL_978677, EPI_ISL_978678, EPI_ISL_978679, EPI_ISL_978680, EPI_ISL_978681, EPI_ISL_978682, EPI_ISL_978683, EPI_ISL_978684, EPI_ISL_978685, EPI_ISL_978686, EPI_ISL_978687, EPI_ISL_978688, EPI_ISL_978689, EPI_ISL_978690, EPI_ISL_978691, EPI_ISL_978692, EPI_ISL_978693, EPI_ISL_978694, EPI_ISL_978695, EPI_ISL_978696, EPI_ISL_978697, EPI_ISL_978698, EPI_ISL_978699, EPI_ISL_978700, EPI_ISL_978701, EPI_ISL_978702, EPI_ISL_978703, EPI_ISL_978704, EPI_ISL_978705, EPI_ISL_978706, EPI_ISL_978707, EPI_ISL_978708, EPI_ISL_978709, EPI_ISL_978710, EPI_ISL_978711, EPI_ISL_978712, EPI_ISL_978713, EPI_ISL_978714, EPI_ISL_978715, EPI_ISL_978716, EPI_ISL_978717, EPI_ISL_978718, EPI_ISL_978719, EPI_ISL_978720, EPI_ISL_978721, EPI_ISL_978722, EPI_ISL_978723, EPI_ISL_978724, EPI_ISL_978725, EPI_ISL_978726, EPI_ISL_978727, EPI_ISL_978728, EPI_ISL_978729, EPI_ISL_978730, EPI_ISL_978731, EPI_ISL_978732, EPI_ISL_978733, EPI_ISL_978734, EPI_ISL_978735, EPI_ISL_978736, EPI_ISL_978737, EPI_ISL_978738, EPI_ISL_978739, EPI_ISL_978740			
see above	Helix/Illumina	Respiratory Viruses Branch, Division of Viral Diseases, Centers for Disease Control and Prevention	Peter W. Cook,Dakota Howard,Dhwani Batra,Ben L. Rambo-Martin,Eileen de Feo,Jan Antico,Christine Tran,Matthew Tolentino,Shannon Wickline,Kim Gietzen,Brad Sickler,Jingtao Liu,Eric Allen,Phil Febbo,Summer Galloway,Nicole L. Washington,Simon White,Geraint Levan,Kelly Schiabor Barrett,Elizabeth Cirulli,Alexandre Bolze,Ary Ascencio,Charlotte Rivera-Garcia,Ryan Cho,Jason Nguyen,Sherry Wang,Jimmy Ramirez,Tyler Cassens,Efren Sandoval,Magnus Isaksson,William Lee,David Becker,Marc Laurent,James Lu,Clinton R. Paden,Suxiang Tong,Duncan MacCannell,
EPI_ISL_978973	Chiu Laboratory, University of California, San Francisco	Chiu Laboratory, University of California, San Francisco	Charles Chiu, Xianding (Wayne) Deng, Candace Wang, Venice Servellita, Jill Hacker, Debra Wadford
EPI_ISL_979085, EPI_ISL_979089, EPI_ISL_979092, EPI_ISL_979093, EPI_ISL_979094, EPI_ISL_979095, EPI_ISL_979096, EPI_ISL_979097, EPI_ISL_979098	Santa Clara County Public Health Laboratory	Chan-Zuckerberg Biohub	CZB Cliahub Consortium
EPI_ISL_979343, EPI_ISL_979344, EPI_ISL_979345, EPI_ISL_979346, EPI_ISL_979347, EPI_ISL_979348, EPI_ISL_979349, EPI_ISL_979350, EPI_ISL_979351, EPI_ISL_979352	Laboratorio Estatal de Salud Pública de Nuevo León	Laboratorio de Infectología Molecular, Departamento de Bioquímica y Medicina Molecular,Facultad de Medicina - Universidad Autónoma de Nuevo León	Kame A. Galán-Huerta, María F. Herrera-Saldivar, Natalia Martínez-Acuña, Sonia A. Lozano-Sepúlveda, Daniel Arellanos-Soto, Ana M. Rivas-Estilla, Samuel Buentello-Wong, Else del Carmen García-García, Gloria A. Jasso-de-la-Peña, Roberto Montes-de-Oca, Consuelo Treviño-Garza, Manuel E. de-la-O-Cavazos
EPI_ISL_979379, EPI_ISL_979380, EPI_ISL_979381, EPI_ISL_979382, EPI_ISL_979383, EPI_ISL_979384, EPI_ISL_979385, EPI_ISL_979386, EPI_ISL_979387, EPI_ISL_979388, EPI_ISL_979389, EPI_ISL_979390, EPI_ISL_979391, EPI_ISL_979392, EPI_ISL_979393, EPI_ISL_979394, EPI_ISL_979395, EPI_ISL_979396, EPI_ISL_979397, EPI_ISL_979398, EPI_ISL_979399, EPI_ISL_979400, EPI_ISL_979401, EPI_ISL_979402, EPI_ISL_979403, EPI_ISL_979404, EPI_ISL_979405, EPI_ISL_979406, EPI_ISL_979407, EPI_ISL_979408, EPI_ISL_979409			
see above	Houston Health Dept.	Houston Health Dept.	Ryker Penn, Pamela Brown, Adolpho Lara
EPI_ISL_981947, EPI_ISL_981948, EPI_ISL_981949, EPI_ISL_981950, EPI_ISL_981951, EPI_ISL_981952	Microbiology Service, Hospital Universitario Clinico San Cecilio, Granada	Microbiology Service, Hospital Universitario Clinico San Cecilio, Granada	Adolfo de Salazar, Natalia Chueca, Laura Viñuela, Ana Fuentes, Federico García
EPI_ISL_981983, EPI_ISL_981989, EPI_ISL_981992, EPI_ISL_981995, EPI_ISL_982000, EPI_ISL_982013, EPI_ISL_982014, EPI_ISL_982015, EPI_ISL_982017, EPI_ISL_982018, EPI_ISL_982032, EPI_ISL_982035, EPI_ISL_982041, EPI_ISL_982046, EPI_ISL_982049, EPI_ISL_982050, EPI_ISL_982060, EPI_ISL_982061, EPI_ISL_982066, EPI_ISL_982073, EPI_ISL_982074, EPI_ISL_982077, EPI_ISL_982079, EPI_ISL_982083, EPI_ISL_982088, EPI_ISL_982089, EPI_ISL_982090, EPI_ISL_982092, EPI_ISL_982100, EPI_ISL_982108, EPI_ISL_982112			
see above	TGen North	Sonora Quest Laboratories	"Jolene Bowers, Megan Folkerts, Chris French, Hayley Yaglom, Ashlyn Pfeiffer, Darrin Lemmer, Dave Engelthaler, The Arizona COVID Genomics Union (ACGU)"
EPI_ISL_982232	MEPHI, Aix Marseille University	MEPHI, Aix Marseille University	Anthony LEVASSEUR
EPI_ISL_982246, EPI_ISL_982249, EPI_ISL_982250	Lab voor klinische biologie	Lab voor klinische biologie	Hannelore Hamerlinck, Marija Janevska, Bruno Verhasselt
EPI_ISL_982345, EPI_ISL_982353, EPI_ISL_982354, EPI_ISL_982385, EPI_ISL_982386, EPI_ISL_982387, EPI_ISL_982388, EPI_ISL_982392	M Health Fairview	Minnesota Department of Health, Public Health Laboratory	Alexandra Lorentz, Jacob Garfin, Matt Plumb, and Xiong Wang
EPI_ISL_982539	Landstuhl Regional Medical Center	US Air Force School of Aerospace Medicine	Anthony Fries, Jennifer Meyer, William Gruner, William Buggele, Amanda Javorina, Sarah Purves, Fritz Castillo, Cole Anderson, Clarise Starr, Elizabeth Macias
EPI_ISL_982540, EPI_ISL_982569, EPI_ISL_982578, EPI_ISL_982597, EPI_ISL_982600, EPI_ISL_982623	US Air Force School of Aerospace Medicine	US Air Force School of Aerospace Medicine	Anthony Fries, Jennifer Meyer, William Gruner, William Buggele, Amanda Javorina, Sarah Purves, Clarise Starr, Elizabeth Macias
EPI_ISL_982627, EPI_ISL_982628, EPI_ISL_982629, EPI_ISL_982630	Landstuhl Regional Medical Center	US Air Force School of Aerospace Medicine	Anthony Fries, Jennifer Meyer, William Gruner, William Buggele, Amanda Javorina, Sarah Purves, Fritz Castillo, Cole Anderson, Clarise Starr, Elizabeth Macias
EPI_ISL_982634, EPI_ISL_982661, EPI_ISL_982672, EPI_ISL_982673, EPI_ISL_982676, EPI_ISL_982691, EPI_ISL_982692, EPI_ISL_982706, EPI_ISL_982719	US Air Force School of Aerospace Medicine	US Air Force School of Aerospace Medicine	Anthony Fries, Jennifer Meyer, William Gruner, William Buggele, Amanda Javorina, Sarah Purves, Clarise Starr, Elizabeth Macias
EPI_ISL_983064, EPI_ISL_983065	Gravity Diagnostics	Kentucky State Public Health Lab	Stephanie Lunn, Karim George, Joshua Tobias, William Grooms, Vaneet Arora, Matthew Johnson, Rachel Zinner, Rhonda Lucas
EPI_ISL_983119, EPI_ISL_983120, EPI_ISL_983121, EPI_ISL_983122, EPI_ISL_983123, EPI_ISL_983124, EPI_ISL_983125, EPI_ISL_983129	THE MARY IMOGENE BASSETT HOSPITAL	Wadsworth Center, New York State Department of Health	Kirsten St. George, Daryl M. Lamson, Alexis Russel, Matthew Shudt, Melissa A Leisner, Jonathan Plitnick, Navjot Singh, John Kelly, Erasmus Schneider, Erica Lasek-Nesselquist
EPI_ISL_983251, EPI_ISL_983252, EPI_ISL_983253, EPI_ISL_983254, EPI_ISL_983255, EPI_ISL_983256, EPI_ISL_983257, EPI_ISL_983258, EPI_ISL_983259, EPI_ISL_983260, EPI_ISL_983261, EPI_ISL_983262			
see above	KALEIDA CENTER FOR LABORATORY MEDICINE	Wadsworth Center, New York State Department of Health	Kirsten St. George, Daryl M. Lamson, Alexis Russel, Matthew Shudt, Melissa A Leisner, Jonathan Plitnick, Navjot Singh, John Kelly, Erasmus Schneider, Erica Lasek-Nesselquist
EPI_ISL_983711, EPI_ISL_983811, EPI_ISL_983812	Colorado Department of Public Health and Environment	Colorado Department of Puplic Health and Environment	Laura Bankers, Molly C. Hetherington-Rauth, Diana Ir, Shannon Ely, Shannon R. Matzinger, Sarah Elizabeth Totten, Emily A. Travanty
