# Supplementary material for: Detection and characterization of the SARS-CoV-2 lineage B.1.526 in New York
Source: Nat Commun. 2021 Aug 9;12:4886. doi: 10.1038/s41467-021-25168-4 (PMC8352861; doi:10.1038/s41467-021-25168-4)
Supplement: Supplementary file 8 — Supplementary Data 4 [file 41467_2021_25168_MOESM8_ESM.zip › GISAID_acknowledements_tables/gisaid_hcov-19_acknowledgement_table_2021_02_13_01-2.pdf]

We gratefully acknowledge the following Authors from the Originating laboratories responsible for obtaining the specimens, as well as the Submitting laboratories where the genome data were generated and shared via GISAID, on which this research is based.

All Submitters of data may be contacted directly via www.gisaid.org

Authors are sorted alphabetically.

Accession ID	Originating Laboratory	Submitting Laboratory	Authors
EPI_ISL_906273, EPI_ISL_906274	University of Wisconsin-Madison AIDS Vaccine Research Laboratories	University of Wisconsin-Madison AIDS Vaccine Research Laboratories	Gage Moreno, Katarina Braun, et al. AIDS Vaccine Research Laboratories
EPI_ISL_907237	Lighthouse Lab in Glasgow	Wellcome Sanger Institute for the COVID-19 Genomics UK (COG-UK) Consortium	Harper VanSteenhouse, Yumi Kasai, David Gray, Carol Clugston, Anna Dominiczak and Alex Alderton, Roberto Amato, Sonia Goncalves, Ewan Harrison, David K. Jackson, Ian Johnston, Dominic Kwiatkowski, Cordelia Langford, John Sillitoe on behalf of the Wellcome Sanger Institute COVID-19 Surveillance Team
EPI_ISL_911372, EPI_ISL_911374, EPI_ISL_911375, EPI_ISL_911378, EPI_ISL_911379, EPI_ISL_911380	University of Michigan Clinical Microbiology Laboratory	Lauring Lab, University of Michigan, Department of Microbiology and Immunology	Valesano
EPI_ISL_918430	Bundeswehrkrankenhaus Westerstede	Bundeswehr Institute of Microbiology	Markus Antwerpen, Klaus Peter Ebert, Alexandra Rehn, Mathias Walter, Malena Bestehorn-Willmann, Sabine Zange, Enrico Georgi, Roman Wölfel
EPI_ISL_918431, EPI_ISL_918432, EPI_ISL_918433	Bundeswehrkrankenhaus Berlin	Bundeswehr Institute of Microbiology	Markus Antwerpen, Martin Müller, Alexandra Rehn, Mathias Walter, Malena Bestehorn-Willmann, Sabine Zange, Enrico Georgi, Roman Wölfel
EPI_ISL_918483	Laboratory of Microbiology, ASST Settelaghi, Varese, Italy	Laboratory of Microbiology, ASST Settelaghi, Varese, Italy	Novazzi,F., Genoni,A., Baj,A., Focosi,D., Spezia,P.G., Zago,C., Colombo,A., Cassani,G., Pasciuta,R., Tamborini,A., Rossi,A., Prestia,M., Capuano,R., Maggi,F.
EPI_ISL_924084, EPI_ISL_924101, EPI_ISL_924104, EPI_ISL_924108, EPI_ISL_924119, EPI_ISL_924122, EPI_ISL_924128, EPI_ISL_924148, EPI_ISL_924164, EPI_ISL_924166, EPI_ISL_924176, EPI_ISL_924196, EPI_ISL_924197, EPI_ISL_924203, EPI_ISL_924215, EPI_ISL_924225, EPI_ISL_924231, EPI_ISL_924240, EPI_ISL_924245, EPI_ISL_924252, EPI_ISL_924265, EPI_ISL_924274, EPI_ISL_924281, EPI_ISL_924290, EPI_ISL_924296, EPI_ISL_924302, EPI_ISL_924328, EPI_ISL_924332, EPI_ISL_924334, EPI_ISL_924351, EPI_ISL_924376, EPI_ISL_924384, EPI_ISL_924389, EPI_ISL_924392, EPI_ISL_924397, EPI_ISL_924406			
see above	Virology Department, Sheffield Teaching Hospitals NHS Foundation Trust/Department of Infection, Immunity and Cardiovascular Disease, The Medical School, University of Sheffield	COVID-19 Genomics UK (COG-UK) Consortium	Thushan de Silva, Matthew Parker, Nikki Smith, Adri Angyal, Rebecca Brown, Luke Green, Rachel Tucker, Paul Parsons, Danielle Groves, Katie Johnson, Laura Carrilero, Alex Keeley, Dave Partridge, Matthew Wyles, Benjamin Lindsey, Mehmet Yavuz, Mohammad Raza, Cariad Evans
EPI_ISL_933798, EPI_ISL_933799, EPI_ISL_933800, EPI_ISL_933801	PathWest Laboratory Medicine WA	PathWest Laboratory Medicine WA Microbial Surveillance Unit	PathWest Laboratory Medicine WA Microbial Surveillance Unit
EPI_ISL_934971, EPI_ISL_934972	Thai Red Cross Emerging Infectious Diseases Health Science Centre, Chulalongkorn Hospital, Faculty of Medicine, Chulalongkorn University	Thai Red Cross Emerging Infectious Diseases Center and Faculty of Medicine, Chulalongkorn University	Rome Buathong, Sopon Iamsirithaworn, Sininat Petcharat, Yutthana Joyjinda, Weenassarin Ampoot, Apaporn Rodpan, Opass Putcharoen, Thiravat Hemachudha, Supaporn Wacharapluesadee
EPI_ISL_935017, EPI_ISL_935021	Laboratory for Respiratory Viruses, Cantacuzino National Military-Medical Institute for Research and Development	Cantacuzino Institute Virology	Luiza Ustea, Nicoleta Paraschiv, Mihaela Lazar
EPI_ISL_935102	Ministry of Health Turkey	Ministry of Health Turkey	Fatma Bayrakdar, Yasemin Cogun, Süleyman Yalcin, Aye Baak Alta, Gülay Korukluolu
EPI_ISL_935181, EPI_ISL_935182, EPI_ISL_935183, EPI_ISL_935184, EPI_ISL_935185, EPI_ISL_935186	Wyoming Public Health Laboratory	Wyoming Public Health Laboratory	Noah Hull, Taylor Fearing, Lynette Gumbleton, Channing Weber, Ashley Norberg, Bailey Bowcutt, and Wanda Manley
EPI_ISL_937468, EPI_ISL_937469, EPI_ISL_937470, EPI_ISL_937471, EPI_ISL_937472, EPI_ISL_937473, EPI_ISL_937474, EPI_ISL_937475, EPI_ISL_937476, EPI_ISL_937477, EPI_ISL_937478, EPI_ISL_937479, EPI_ISL_937480, EPI_ISL_937481, EPI_ISL_937482, EPI_ISL_937483, EPI_ISL_937484, EPI_ISL_937485, EPI_ISL_937486, EPI_ISL_937487, EPI_ISL_937488, EPI_ISL_937489, EPI_ISL_937490, EPI_ISL_937506, EPI_ISL_937508, EPI_ISL_937509, EPI_ISL_937511			
see above	Maine Health and Environmental Testing Laboratory (Maine HETL)	Tewhey Lab, The Jackson Laboratory	Matluk,N., Dewey,H., Iosue,F., Barter,M., Lynch,R., Munger,H. and Tewhey,R.
EPI_ISL_937515, EPI_ISL_937516, EPI_ISL_937517, EPI_ISL_937518, EPI_ISL_937519, EPI_ISL_937520, EPI_ISL_937521, EPI_ISL_937522, EPI_ISL_937523, EPI_ISL_937524, EPI_ISL_937525, EPI_ISL_937526, EPI_ISL_937527, EPI_ISL_937528, EPI_ISL_937529, EPI_ISL_937530			
see above	National Public Health Laboratory, National Centre for Infectious Diseases	National Public Health Laboratory, National Centre for Infectious Diseases	Tze Minn Mak, Zhenyang Zhou, Lin Cui, Raymond Tzer Pin Lin
EPI_ISL_939647, EPI_ISL_940144	Laboratory for Respiratory Viruses, Cantacuzino National Military-Medical Institute for Research and Development	Cantacuzino Institute Virology	Luiza Ustea, Nicoleta Paraschiv, Mihaela Lazar
EPI_ISL_940577, EPI_ISL_940578, EPI_ISL_940579	Jessa	Jessa	Jessa_cmdLab
EPI_ISL_940741	LHUB-ULB	UAntwerp, Laboratory of Medical Microbiology, Campus Drie Eiken S6.26, Universiteitsplein 1, 2610, Wilrijk, Belgium	Basil Britto Xavier, Jasmine Coppens, Marie Le Mercier, Christine Lammens, Veerle Matheeussen, Herman Goossens
EPI_ISL_941335	New Mexico Department of Health Scientific Laboratory	New Mexico Department of Health Scientific Laboratory	Ellie Johnson, Anastacia Griego-Fisher, D'eldra Malone, Jennifer Benoit
EPI_ISL_942354, EPI_ISL_942366, EPI_ISL_942369, EPI_ISL_942370, EPI_ISL_942371, EPI_ISL_942372, EPI_ISL_942373	MD PHL	MD PHL	Maryland Department of Health Laboratories Administration
EPI_ISL_942894	Gundersen Molecular Diagnostics Laboratory	Kabara Cancer Research Institute	Craig S. Richmond, Paraic A. Kenny
EPI_ISL_942895	South Eastern Area Laboratory Services (SEALS)	NSW Health Pathology - Institute of Clinical Pathology and Medical Research; Westmead Hospital; University of Sydney	CIDM-PH et al.
EPI_ISL_942935, EPI_ISL_942936, EPI_ISL_942937, EPI_ISL_942938, EPI_ISL_942939	Wyoming Public Health Laboratory	Wyoming Public Health Laboratory	Noah Hull, Taylor Fearing, Lynette Gumbleton, Channing Weber, Ashley Norberg, Bailey Bowcutt, and Wanda Manley
EPI_ISL_943570	Laboratorio de Referencia Nacional de Virus Respiratorio. Instituto Nacional de Salud Perú	Laboratorio de Referencia Nacional de Biotecnología y Biología Molecular. Instituto Nacional de Salud Perú	Carlos Padilla Rojas, Karolyn Vega Chozo, Luis Barcena, Priscila Lope Pari, Omar Caceres Rey, Marco Galarza Perez, Maribel Huaringa Nuñez, Johanna Balbuena Torrez, Henri Bailon Calderon, Nancy Rojas Serrano
EPI_ISL_944748	unknown	Public Health Virology-Forensic and Scientific Services (PHV-FSS)	Son Nguyen et al.
EPI_ISL_944785	Laboratory of Microbiology and Virology, Ospedale Amedeo di Savoia, ASL "Città di Torino"	U.O. Genomics, S.S. Genetics and Advanced Omics Techniques, Istituto Zooprofilattico Sperimentale del Piemonte Liguria e Valle d'Aosta	Chiara Beltramo, Francesco Cerutti, Valeria Ghisetti, Elena Bozzetta, Pier Luigi Acutis, Simone Peletto

EPI_ISL_944792, EPI_ISL_944793, EPI_ISL_944794, EPI_ISL_944795, EPI_ISL_944796, EPI_ISL_944797, EPI_ISL_944798, EPI_ISL_944800, EPI_ISL_944801, EPI_ISL_944802, EPI_ISL_944803, EPI_ISL_944804, EPI_ISL_944806, EPI_ISL_944807, EPI_ISL_944808, EPI_ISL_944809, EPI_ISL_944810, EPI_ISL_944811, EPI_ISL_944812, EPI_ISL_944813, EPI_ISL_944814, EPI_ISL_944815, EPI_ISL_944816, EPI_ISL_944817, EPI_ISL_944818, EPI_ISL_944819, EPI_ISL_944820, EPI_ISL_944821, EPI_ISL_944822, EPI_ISL_944823, EPI_ISL_944825, EPI_ISL_944826, EPI_ISL_944827, EPI_ISL_944828, EPI_ISL_944829, EPI_ISL_944830, EPI_ISL_944831, EPI_ISL_944832, EPI_ISL_944833, EPI_ISL_944834, EPI_ISL_944835, EPI_ISL_944836, EPI_ISL_944837, EPI_ISL_944838, EPI_ISL_944839, EPI_ISL_944840, EPI_ISL_944841, EPI_ISL_944842, EPI_ISL_944843, EPI_ISL_944845, EPI_ISL_944846, EPI_ISL_944847, EPI_ISL_944848, EPI_ISL_944849, EPI_ISL_944850, EPI_ISL_944851, EPI_ISL_944852, EPI_ISL_944853, EPI_ISL_944854, EPI_ISL_944855, EPI_ISL_944856, EPI_ISL_944857, EPI_ISL_944858, EPI_ISL_944859, EPI_ISL_944860, EPI_ISL_944861, EPI_ISL_944862, EPI_ISL_944864, EPI_ISL_944865, EPI_ISL_944866, EPI_ISL_944867, EPI_ISL_944868, EPI_ISL_944869, EPI_ISL_944870, EPI_ISL_944871, EPI_ISL_944872, EPI_ISL_944873, EPI_ISL_944874, EPI_ISL_944875, EPI_ISL_944876, EPI_ISL_944877, EPI_ISL_944878, EPI_ISL_944879, EPI_ISL_944880, EPI_ISL_944881, EPI_ISL_944882, EPI_ISL_944884, EPI_ISL_944885, EPI_ISL_944886, EPI_ISL_944887, EPI_ISL_944888, EPI_ISL_944889, EPI_ISL_944890, EPI_ISL_944891, EPI_ISL_944892, EPI_ISL_944893, EPI_ISL_944894, EPI_ISL_944895, EPI_ISL_944896, EPI_ISL_944897, EPI_ISL_944898, EPI_ISL_944899, EPI_ISL_944900, EPI_ISL_944901, EPI_ISL_944902, EPI_ISL_944903, EPI_ISL_944904, EPI_ISL_944905, EPI_ISL_944906, EPI_ISL_944907, EPI_ISL_944909, EPI_ISL_944910, EPI_ISL_944911, EPI_ISL_944912, EPI_ISL_944913, EPI_ISL_944915, EPI_ISL_944916, EPI_ISL_944917, EPI_ISL_944918, EPI_ISL_944919, EPI_ISL_944920, EPI_ISL_944921, EPI_ISL_944922, EPI_ISL_944924, EPI_ISL_944925, EPI_ISL_944926, EPI_ISL_944927, EPI_ISL_944928, EPI_ISL_944929, EPI_ISL_944930, EPI_ISL_944931, EPI_ISL_944932, EPI_ISL_944933, EPI_ISL_944934, EPI_ISL_944935, EPI_ISL_944936, EPI_ISL_944937, EPI_ISL_944938, EPI_ISL_944939, EPI_ISL_944940, EPI_ISL_944941, EPI_ISL_944942, EPI_ISL_944943, EPI_ISL_944944, EPI_ISL_944945, EPI_ISL_944946, EPI_ISL_944947, EPI_ISL_944948, EPI_ISL_944949, EPI_ISL_944950, EPI_ISL_944951, EPI_ISL_944952, EPI_ISL_944954, EPI_ISL_944955, EPI_ISL_944956, EPI_ISL_944957, EPI_ISL_944958, EPI_ISL_944959, EPI_ISL_944960, EPI_ISL_944961, EPI_ISL_944962, EPI_ISL_944963, EPI_ISL_944964, EPI_ISL_944965, EPI_ISL_944966, EPI_ISL_944967, EPI_ISL_944968, EPI_ISL_944969, EPI_ISL_944970, EPI_ISL_944971, EPI_ISL_944972, EPI_ISL_944973, EPI_ISL_944974, EPI_ISL_944975, EPI_ISL_944977, EPI_ISL_944979, EPI_ISL_944980, EPI_ISL_944981, EPI_ISL_944982, EPI_ISL_944983, EPI_ISL_944984, EPI_ISL_944985, EPI_ISL_944986, EPI_ISL_944987, EPI_ISL_944988, EPI_ISL_944989, EPI_ISL_944990, EPI_ISL_944991, EPI_ISL_944992, EPI_ISL_944993, EPI_ISL_944994, EPI_ISL_944995, EPI_ISL_944996, EPI_ISL_944997, EPI_ISL_944998, EPI_ISL_944999, EPI_ISL_945000, EPI_ISL_945001, EPI_ISL_945002, EPI_ISL_945003, EPI_ISL_945004, EPI_ISL_945005, EPI_ISL_945006, EPI_ISL_945007, EPI_ISL_945008, EPI_ISL_945009, EPI_ISL_945010, EPI_ISL_945011, EPI_ISL_945012, EPI_ISL_945013, EPI_ISL_945014, EPI_ISL_945015, EPI_ISL_945016, EPI_ISL_945017, EPI_ISL_945018, EPI_ISL_945019, EPI_ISL_945020, EPI_ISL_945021, EPI_ISL_945022, EPI_ISL_945024, EPI_ISL_945025, EPI_ISL_945026, EPI_ISL_945027, EPI_ISL_945028, EPI_ISL_945029, EPI_ISL_945030, EPI_ISL_945031, EPI_ISL_945033, EPI_ISL_945035, EPI_ISL_945036, EPI_ISL_945037, EPI_ISL_945038, EPI_ISL_945039, EPI_ISL_945040, EPI_ISL_945041, EPI_ISL_945042, EPI_ISL_945043, EPI_ISL_945044, EPI_ISL_945045, EPI_ISL_945046, EPI_ISL_945047, EPI_ISL_945048			
see above	Lighthouse Lab in Glasgow	Wellcome Sanger Institute for the COVID-19 Genomics UK (COG-UK) Consortium	Harper VanSteenhouse, Yumi Kasai, David Gray, Carol Clugston, Anna Dominiczak and Alex Alderton, Roberto Amato, Sonia Goncalves, Ewan Harrison, David K. Jackson, Ian Johnston, Dominic Kwiatkowski, Cordelia Langford, John Sillitoe on behalf of the Wellcome Sanger Institute COVID-19 Surveillance Team
EPI_ISL_945374, EPI_ISL_945375, EPI_ISL_945376, EPI_ISL_945379, EPI_ISL_945386, EPI_ISL_945387, EPI_ISL_945389, EPI_ISL_945402, EPI_ISL_945408, EPI_ISL_945409, EPI_ISL_945419, EPI_ISL_945420, EPI_ISL_945422, EPI_ISL_945423, EPI_ISL_945433, EPI_ISL_945444, EPI_ISL_945446, EPI_ISL_945449, EPI_ISL_945458, EPI_ISL_945459, EPI_ISL_945466, EPI_ISL_945467, EPI_ISL_945468, EPI_ISL_945469, EPI_ISL_945475, EPI_ISL_945480, EPI_ISL_945485, EPI_ISL_945490, EPI_ISL_945494, EPI_ISL_945495, EPI_ISL_945498, EPI_ISL_945502, EPI_ISL_945506, EPI_ISL_945508, EPI_ISL_945514, EPI_ISL_945518, EPI_ISL_945522, EPI_ISL_945525, EPI_ISL_945529, EPI_ISL_945538, EPI_ISL_945539, EPI_ISL_945543, EPI_ISL_945545, EPI_ISL_945546, EPI_ISL_945548, EPI_ISL_945549, EPI_ISL_945555, EPI_ISL_945561, EPI_ISL_945563, EPI_ISL_945569, EPI_ISL_945579, EPI_ISL_945582, EPI_ISL_945587, EPI_ISL_945589, EPI_ISL_945591, EPI_ISL_945597, EPI_ISL_945619, EPI_ISL_945621, EPI_ISL_945627, EPI_ISL_945635, EPI_ISL_945636, EPI_ISL_945648, EPI_ISL_945650, EPI_ISL_945656, EPI_ISL_945664, EPI_ISL_945668, EPI_ISL_945672, EPI_ISL_945674, EPI_ISL_945677, EPI_ISL_945678, EPI_ISL_945681, EPI_ISL_945682, EPI_ISL_945692, EPI_ISL_945696, EPI_ISL_945697, EPI_ISL_945698, EPI_ISL_945701, EPI_ISL_945704			
see above	Lighthouse Lab in Alderley Park	Wellcome Sanger Institute for the COVID-19 Genomics UK (COG-UK) Consortium	Jacquelyn Wynn, Mairead Hyland, The Lighthouse Lab in Alderley Park and Alex Alderton, Roberto Amato, Sonia Goncalves, Ewan Harrison, David K. Jackson, Ian Johnston, Dominic Kwiatkowski, Cordelia Langford, John Sillitoe on behalf of the Wellcome Sanger Institute COVID-19 Surveillance Team
EPI_ISL_945716, EPI_ISL_945718, EPI_ISL_945722, EPI_ISL_945725, EPI_ISL_945726, EPI_ISL_945728, EPI_ISL_945729, EPI_ISL_945730, EPI_ISL_945732, EPI_ISL_945733, EPI_ISL_945735, EPI_ISL_945736, EPI_ISL_945737, EPI_ISL_945738, EPI_ISL_945741, EPI_ISL_945742, EPI_ISL_945743, EPI_ISL_945744, EPI_ISL_945747, EPI_ISL_945753, EPI_ISL_945755, EPI_ISL_945757, EPI_ISL_945758, EPI_ISL_945759, EPI_ISL_945762, EPI_ISL_945763, EPI_ISL_945768, EPI_ISL_945771, EPI_ISL_945772, EPI_ISL_945776, EPI_ISL_945777, EPI_ISL_945781, EPI_ISL_945782, EPI_ISL_945788, EPI_ISL_945792, EPI_ISL_945795, EPI_ISL_945796, EPI_ISL_945799, EPI_ISL_945800, EPI_ISL_945802, EPI_ISL_945803, EPI_ISL_945804, EPI_ISL_945806, EPI_ISL_945807, EPI_ISL_945808, EPI_ISL_945809, EPI_ISL_945812, EPI_ISL_945813, EPI_ISL_945815, EPI_ISL_945816, EPI_ISL_945817, EPI_ISL_945818, EPI_ISL_945821, EPI_ISL_945822, EPI_ISL_945824, EPI_ISL_945826, EPI_ISL_945828, EPI_ISL_945831, EPI_ISL_945835, EPI_ISL_945836, EPI_ISL_945839, EPI_ISL_945841, EPI_ISL_945843, EPI_ISL_945844, EPI_ISL_945846, EPI_ISL_945848, EPI_ISL_945849, EPI_ISL_945850, EPI_ISL_945851, EPI_ISL_945853, EPI_ISL_945860, EPI_ISL_945862, EPI_ISL_945863, EPI_ISL_945865, EPI_ISL_945867, EPI_ISL_945868, EPI_ISL_945869, EPI_ISL_945870, EPI_ISL_945871, EPI_ISL_945872, EPI_ISL_945873, EPI_ISL_945874, EPI_ISL_945877, EPI_ISL_945878, EPI_ISL_945879, EPI_ISL_945882, EPI_ISL_945884, EPI_ISL_945885, EPI_ISL_945889, EPI_ISL_945895, EPI_ISL_945898, EPI_ISL_945899, EPI_ISL_945900, EPI_ISL_945901, EPI_ISL_945904, EPI_ISL_945906, EPI_ISL_945907, EPI_ISL_945908, EPI_ISL_945909, EPI_ISL_945910, EPI_ISL_945911, EPI_ISL_945914, EPI_ISL_945916, EPI_ISL_945917, EPI_ISL_945920, EPI_ISL_945921, EPI_ISL_945926, EPI_ISL_945933, EPI_ISL_945935, EPI_ISL_945938, EPI_ISL_945939, EPI_ISL_945940, EPI_ISL_945944, EPI_ISL_945945, EPI_ISL_945950, EPI_ISL_945951, EPI_ISL_945954, EPI_ISL_945956, EPI_ISL_945958, EPI_ISL_945960, EPI_ISL_945961, EPI_ISL_945962, EPI_ISL_945963, EPI_ISL_945965, EPI_ISL_945967, EPI_ISL_945969, EPI_ISL_945970, EPI_ISL_945971, EPI_ISL_945972, EPI_ISL_945976, EPI_ISL_945979, EPI_ISL_945980, EPI_ISL_945983, EPI_ISL_945984, EPI_ISL_945988, EPI_ISL_945990, EPI_ISL_945993, EPI_ISL_945994, EPI_ISL_945997, EPI_ISL_945998, EPI_ISL_945999, EPI_ISL_946000, EPI_ISL_946002, EPI_ISL_946005, EPI_ISL_946006, EPI_ISL_946007, EPI_ISL_946008, EPI_ISL_946009, EPI_ISL_946010, EPI_ISL_946011, EPI_ISL_946014, EPI_ISL_946021, EPI_ISL_946023, EPI_ISL_946026, EPI_ISL_946028, EPI_ISL_946030, EPI_ISL_946035, EPI_ISL_946037, EPI_ISL_946041, EPI_ISL_946048, EPI_ISL_946049, EPI_ISL_946052, EPI_ISL_946055, EPI_ISL_946057, EPI_ISL_946058, EPI_ISL_946059, EPI_ISL_946062, EPI_ISL_946066, EPI_ISL_946067, EPI_ISL_946068, EPI_ISL_946069, EPI_ISL_946070, EPI_ISL_946071, EPI_ISL_946072, EPI_ISL_946073, EPI_ISL_946076, EPI_ISL_946080, EPI_ISL_946086, EPI_ISL_946087, EPI_ISL_946088, EPI_ISL_946090, EPI_ISL_946093, EPI_ISL_946096, EPI_ISL_946106, EPI_ISL_946109, EPI_ISL_946110, EPI_ISL_946112, EPI_ISL_946113, EPI_ISL_946115, EPI_ISL_946123, EPI_ISL_946126, EPI_ISL_946127, EPI_ISL_946129, EPI_ISL_946135, EPI_ISL_946137, EPI_ISL_946145, EPI_ISL_946147, EPI_ISL_946149, EPI_ISL_946150, EPI_ISL_946157, EPI_ISL_946160, EPI_ISL_946162, EPI_ISL_946169, EPI_ISL_946173, EPI_ISL_946174, EPI_ISL_946176, EPI_ISL_946177, EPI_ISL_946180, EPI_ISL_946181, EPI_ISL_946182, EPI_ISL_946188, EPI_ISL_946190, EPI_ISL_946192, EPI_ISL_946196, EPI_ISL_946199, EPI_ISL_946202, EPI_ISL_946206, EPI_ISL_946208, EPI_ISL_946210, EPI_ISL_946211, EPI_ISL_946213, EPI_ISL_946215, EPI_ISL_946218, EPI_ISL_946220, EPI_ISL_946221, EPI_ISL_946224, EPI_ISL_946225, EPI_ISL_946227, EPI_ISL_946229, EPI_ISL_946231, EPI_ISL_946232, EPI_ISL_946233, EPI_ISL_946234, EPI_ISL_946243, EPI_ISL_946250, EPI_ISL_946253, EPI_ISL_946254, EPI_ISL_946260, EPI_ISL_946264, EPI_ISL_946270, EPI_ISL_946271, EPI_ISL_946274, EPI_ISL_946281, EPI_ISL_946284, EPI_ISL_946285, EPI_ISL_946286, EPI_ISL_946292, EPI_ISL_946298, EPI_ISL_946301, EPI_ISL_946302, EPI_ISL_946304, EPI_ISL_946318, EPI_ISL_946319, EPI_ISL_946326, EPI_ISL_946328, EPI_ISL_946333, EPI_ISL_946335, EPI_ISL_946338, EPI_ISL_946339, EPI_ISL_946351, EPI_ISL_946356, EPI_ISL_946357, EPI_ISL_946360, EPI_ISL_946361, EPI_ISL_946365, EPI_ISL_946366, EPI_ISL_946367, EPI_ISL_946371, EPI_ISL_946374, EPI_ISL_946375, EPI_ISL_946378, EPI_ISL_946379, EPI_ISL_946380, EPI_ISL_946385, EPI_ISL_946391, EPI_ISL_946397, EPI_ISL_946400, EPI_ISL_946405, EPI_ISL_946407, EPI_ISL_946410, EPI_ISL_946411			
see above	Lighthouse Lab in Glasgow	Wellcome Sanger Institute for the COVID-19 Genomics UK (COG-UK) Consortium	Harper VanSteenhouse, Yumi Kasai, David Gray, Carol Clugston, Anna Dominiczak and Alex Alderton, Roberto Amato, Sonia Goncalves, Ewan Harrison, David K. Jackson, Ian Johnston, Dominic Kwiatkowski, Cordelia Langford, John Sillitoe on behalf of the Wellcome Sanger Institute COVID-19 Surveillance Team
EPI_ISL_949405	University of Birmingham	COVID-19 Genomics UK (COG-UK) Consortium	Institute of Microbiology, University of Birmingham: Claire McMurray, Joanne Stockton, Samuel Nicholls, Radoslaw Poplawski, Will Rowe, Josh Quick, Nicholas Loman. University of Birmingham Testing Laboratory: Celina M Whalley, Andrew Bosworth, Charlotte Poxon, Kasun Wanigasooriya, Oliver Pickles, Mike Kidd, Alex Richter, Andrew D Beggs PHE Heartlands Lab: Husam Osman, Andrew Bosworth. Queen Elizabeth Hospital: Anna Casey
EPI_ISL_949543, EPI_ISL_949545, EPI_ISL_949547, EPI_ISL_949551, EPI_ISL_949552, EPI_ISL_949557, EPI_ISL_949558, EPI_ISL_949559, EPI_ISL_949560, EPI_ISL_949561, EPI_ISL_949562, EPI_ISL_949563, EPI_ISL_949564, EPI_ISL_949565, EPI_ISL_949566, EPI_ISL_949567, EPI_ISL_949568, EPI_ISL_949569, EPI_ISL_949570, EPI_ISL_949571, EPI_ISL_949572, EPI_ISL_949573, EPI_ISL_949574, EPI_ISL_949575, EPI_ISL_949576, EPI_ISL_949577, EPI_ISL_949578, EPI_ISL_949579, EPI_ISL_949580, EPI_ISL_949582, EPI_ISL_949583, EPI_ISL_949584, EPI_ISL_949586, EPI_ISL_949587, EPI_ISL_949588, EPI_ISL_949589, EPI_ISL_949590, EPI_ISL_949591, EPI_ISL_949592, EPI_ISL_949593, EPI_ISL_949594, EPI_ISL_949595, EPI_ISL_949596, EPI_ISL_949597, EPI_ISL_949598, EPI_ISL_949599, EPI_ISL_949600, EPI_ISL_949601, EPI_ISL_949602, EPI_ISL_949603, EPI_ISL_949604, EPI_ISL_949605, EPI_ISL_949606, EPI_ISL_949607			
see above	Department of Pathology, University of Cambridge	COVID-19 Genomics UK (COG-UK) Consortium	Aminu S. Jahun, Yasmin Chaudhry, Iliana Georgana, Myra Hosmillo, Rhys Izu, Martin D. Curran, Surendra Parmar, Ian Goodfellow
EPI_ISL_949613, EPI_ISL_949616, EPI_ISL_949617, EPI_ISL_949619, EPI_ISL_949621, EPI_ISL_949623, EPI_ISL_949625, EPI_ISL_949627, EPI_ISL_949629	West of Scotland Specialist Virology Centre, NHSGGC / MRC-University of Glasgow Centre for Virus Research	COVID-19 Genomics UK (COG-UK) Consortium	Ana da Silva Filipe, Natasha Johnson, Kathy Smollett, Daniel Mair, Stephen Carmichael, Alice Broos, Lily Tong, Jenna Nichols, Kyriaki Nomikou; Sarah McDonald; Richard Orton, Joseph Hughes, Sreenu Vattipally, David L Robertson; Alasdair MacLean, Rory Gunson; Sharif Shaaban, Matthew Holden; Rachel Blacow, Guy Mollett, Kathy Li, James Shepherd, Antonia Ho, Emma Thomson
EPI_ISL_949662, EPI_ISL_949663, EPI_ISL_949664, EPI_ISL_949666, EPI_ISL_949668, EPI_ISL_949669, EPI_ISL_949670, EPI_ISL_949708, EPI_ISL_949709, EPI_ISL_949710, EPI_ISL_949711, EPI_ISL_949712, EPI_ISL_949713, EPI_ISL_949714, EPI_ISL_949715, EPI_ISL_949716, EPI_ISL_949717, EPI_ISL_949718, EPI_ISL_949719, EPI_ISL_949720, EPI_ISL_949721, EPI_ISL_949722, EPI_ISL_949723, EPI_ISL_949724, EPI_ISL_949725, EPI_ISL_949726, EPI_ISL_949727, EPI_ISL_949728, EPI_ISL_949729, EPI_ISL_949730, EPI_ISL_949731, EPI_ISL_949732, EPI_ISL_949733, EPI_ISL_949734, EPI_ISL_949735, EPI_ISL_949736, EPI_ISL_949737, EPI_ISL_949738, EPI_ISL_949739, EPI_ISL_949740, EPI_ISL_949741, EPI_ISL_949742, EPI_ISL_949743, EPI_ISL_949744, EPI_ISL_949745, EPI_ISL_949746			
see above	Liverpool Clinical Laboratories	COVID-19 Genomics UK (COG-UK) Consortium	Sam Haldenby, Anita Lucaci, Steve Paterson, Julian Hiscox, Alistair Darby, M Almsaud, A Alrezaihi, Muhannad Alruwaili, Stuart D Armstrong, Jones Benjamin, Eleanor G Bentley, Anu Chawla, Jordan J Clark, Angela Cowell, Richard Eccles, Isabel García-Dorival, Matthew Gemmell, Alessandro Gerada, PKF Gilmore, Richard Gregory, Ximeng Han, Catherine Hartley, Margaret Hughes, Miren Iturriza-Gomara, James Johnson, L Luu, Jenifer Manson, Charlotte Nelson, Elaine O'Toole, Cassie Olateju, Rebekah Penrice-Randal , Lucille Rainbow, N.P Randle, Trevor Ian Robinson, Parul Sharma, Ghada T Shawli, James P Stewart, Neil Swainston, Ecaterina Vamos, Joanne Watts, Mark Whitehead
EPI_ISL_949795, EPI_ISL_949796, EPI_ISL_949797, EPI_ISL_949798, EPI_ISL_949799, EPI_ISL_949800, EPI_ISL_949801, EPI_ISL_949803, EPI_ISL_949804, EPI_ISL_949805, EPI_ISL_949806, EPI_ISL_949807, EPI_ISL_949808, EPI_ISL_949809, EPI_ISL_949810, EPI_ISL_949811, EPI_ISL_949812, EPI_ISL_949813, EPI_ISL_949814, EPI_ISL_949815, EPI_ISL_949816, EPI_ISL_949817, EPI_ISL_949818, EPI_ISL_949819, EPI_ISL_949820, EPI_ISL_949821, EPI_ISL_949822, EPI_ISL_949823, EPI_ISL_949824, EPI_ISL_949825, EPI_ISL_949826, EPI_ISL_949827, EPI_ISL_949828, EPI_ISL_949829, EPI_ISL_949830, EPI_ISL_949831, EPI_ISL_949832, EPI_ISL_949833, EPI_ISL_949834, EPI_ISL_949835, EPI_ISL_949836, EPI_ISL_949837, EPI_ISL_949838, EPI_ISL_949839, EPI_ISL_949840, EPI_ISL_949841, EPI_ISL_949842, EPI_ISL_949843, EPI_ISL_949844, EPI_ISL_949845, EPI_ISL_949846, EPI_ISL_949847, EPI_ISL_949848, EPI_ISL_949850, EPI_ISL_949851, EPI_ISL_949852, EPI_ISL_949853, EPI_ISL_949855, EPI_ISL_949856, EPI_ISL_949858, EPI_ISL_949859, EPI_ISL_949860, EPI_ISL_949861, EPI_ISL_949862, EPI_ISL_949863, EPI_ISL_949864, EPI_ISL_949865, EPI_ISL_949866, EPI_ISL_949867, EPI_ISL_949868, EPI_ISL_949869, EPI_ISL_949871, EPI_ISL_949872, EPI_ISL_949873, EPI_ISL_949874, EPI_ISL_949875, EPI_ISL_949876, EPI_ISL_949877, EPI_ISL_949878, EPI_ISL_949879, EPI_ISL_949880, EPI_ISL_949881, EPI_ISL_949883, EPI_ISL_949884, EPI_ISL_949885, EPI_ISL_949886, EPI_ISL_949887, EPI_ISL_949888, EPI_ISL_949889, EPI_ISL_949890, EPI_ISL_949891, EPI_ISL_949892, EPI_ISL_949893, EPI_ISL_949895, EPI_ISL_949896, EPI_ISL_949897, EPI_ISL_949898, EPI_ISL_949901, EPI_ISL_949903, EPI_ISL_949904, EPI_ISL_949915, EPI_ISL_949916, EPI_ISL_949918, EPI_ISL_949920, EPI_ISL_949921, EPI_ISL_949922, EPI_ISL_949923, EPI_ISL_949926, EPI_ISL_949928, EPI_ISL_949929, EPI_ISL_949930, EPI_ISL_949932, EPI_ISL_949933, EPI_ISL_949934, EPI_ISL_949935, EPI_ISL_949936, EPI_ISL_949937, EPI_ISL_949938, EPI_ISL_949939, EPI_ISL_949940, EPI_ISL_949941, EPI_ISL_949942, EPI_ISL_949943, EPI_ISL_949944, EPI_ISL_949945, EPI_ISL_949947, EPI_ISL_949949, EPI_ISL_949950, EPI_ISL_949951, EPI_ISL_949952, EPI_ISL_949953, EPI_ISL_949954, EPI_ISL_949955, EPI_ISL_949956, EPI_ISL_949957, EPI_ISL_949958, EPI_ISL_949961, EPI_ISL_949962, EPI_ISL_949964, EPI_ISL_949965, EPI_ISL_949966, EPI_ISL_949967, EPI_ISL_949968, EPI_ISL_949969, EPI_ISL_949970, EPI_ISL_949971, EPI_ISL_949972, EPI_ISL_949973, EPI_ISL_949974, EPI_ISL_950173, EPI_ISL_950177, EPI_ISL_950195, EPI_ISL_950198, EPI_ISL_950200, EPI_ISL_950201, EPI_ISL_950202, EPI_ISL_950203, EPI_ISL_950204, EPI_ISL_950205, EPI_ISL_950206, EPI_ISL_950207, EPI_ISL_950208, EPI_ISL_950209, EPI_ISL_950210			
see above	University College London, Great Ormond Street Hospital for Children NHS Foundation Trust, Imperial College Healthcare NHS Trust	COVID-19 Genomics UK (COG-UK) Consortium	Sergi Castellano, Rachel Williams, Mark Kristiansen, Paola Resende Silva, Sunando Roy, Tony Brooks, Helena Tutill, Paola Niola, Patricia Dyal, Charlotte Williams, Leysa Forrest, Yasmin Panchbhaya, Jacqueline Findlay, Samuel Weeks, Julianne Brown, Kathryn Harris, Paul Randell, James Price, Alison Holmes, Judith Breuer
EPI_ISL_950402, EPI_ISL_950404, EPI_ISL_950405, EPI_ISL_950407, EPI_ISL_950408, EPI_ISL_950409, EPI_ISL_950411, EPI_ISL_950412, EPI_ISL_950413, EPI_ISL_950415, EPI_ISL_950424, EPI_ISL_950428, EPI_ISL_950431, EPI_ISL_950433, EPI_ISL_950434			
see above	Northumbria University / South Tees Hospitals NHS Foundation Trust / North Cumbria Integrated Care NHS	COVID-19 Genomics UK (COG-UK) Consortium	Darren L Smith,Andrew Nelson,Matthew Bashton,Greg R Young,Joshua Loh,John Allan,Mohammad A Tariq,Giles S Holt,Gary Black,Wen C Yew,Lynn Dover,Paul Baker,Steve Liggett,Sarah Essex,Jane Greenaway,Debra Padgett,Clive Graham,Garren Scott,Edward Barton,Emma Swindells,Brendan

	Foundation Trust / North Tees and Hartlepool NHS Foundation Trust / Newcastle Hospitals NHS Foundation Trust		Payne,Jennifer Collins,Yusri Taha,Gary Eltringham
EPI_ISL_950646, EPI_ISL_950647, EPI_ISL_950648, EPI_ISL_950649, EPI_ISL_950650, EPI_ISL_950651, EPI_ISL_950652, EPI_ISL_950653, EPI_ISL_950654, EPI_ISL_950655, EPI_ISL_950656, EPI_ISL_950657, EPI_ISL_950658, EPI_ISL_950659, EPI_ISL_950660, EPI_ISL_950661, EPI_ISL_950662, EPI_ISL_950663, EPI_ISL_950665, EPI_ISL_950666, EPI_ISL_950667, EPI_ISL_950668, EPI_ISL_950669, EPI_ISL_950670, EPI_ISL_950671, EPI_ISL_950672, EPI_ISL_950673, EPI_ISL_950674, EPI_ISL_950675, EPI_ISL_950676, EPI_ISL_950677, EPI_ISL_950678, EPI_ISL_950679, EPI_ISL_950680, EPI_ISL_950681, EPI_ISL_950682, EPI_ISL_950683, EPI_ISL_950684, EPI_ISL_950685, EPI_ISL_950686, EPI_ISL_950687, EPI_ISL_950688, EPI_ISL_950689, EPI_ISL_950690, EPI_ISL_950691, EPI_ISL_950692, EPI_ISL_950693, EPI_ISL_950694, EPI_ISL_950695, EPI_ISL_950696, EPI_ISL_950697, EPI_ISL_950717, EPI_ISL_950718, EPI_ISL_950719, EPI_ISL_950720, EPI_ISL_950721, EPI_ISL_950722, EPI_ISL_950762			
see above	Queens Medical Centre, Clinical Microbiology Department / DeepSeq Nottingham	COVID-19 Genomics UK (COG-UK) Consortium	Gemma Clark, Wendy Smith, Manjinder Khakh, Vicki M Fleming, Michelle M Lister, Hannah Howson-Wells, Jonathan Ball, Patrick McClure, Joseph Chappell, Theocharis Tsoleridis, Nadine Holmes, Matthew Carlisle, Christopher Moore, Fei Sang, Johnny Debebe, Victoria Wright, Matthew Loose
EPI_ISL_951783, EPI_ISL_951971, EPI_ISL_951975, EPI_ISL_951976, EPI_ISL_951977, EPI_ISL_951978, EPI_ISL_951979, EPI_ISL_951980, EPI_ISL_951981, EPI_ISL_951982, EPI_ISL_951983, EPI_ISL_951984, EPI_ISL_951985, EPI_ISL_951986, EPI_ISL_952021, EPI_ISL_952023, EPI_ISL_952028, EPI_ISL_952044, EPI_ISL_952046, EPI_ISL_952047, EPI_ISL_952048, EPI_ISL_952049, EPI_ISL_952050, EPI_ISL_952052, EPI_ISL_952053, EPI_ISL_952054, EPI_ISL_952055, EPI_ISL_952056, EPI_ISL_952057, EPI_ISL_952058, EPI_ISL_952060, EPI_ISL_952251, EPI_ISL_952252, EPI_ISL_952253, EPI_ISL_952255, EPI_ISL_952256, EPI_ISL_952257, EPI_ISL_952258, EPI_ISL_952259, EPI_ISL_952261, EPI_ISL_952262, EPI_ISL_952263, EPI_ISL_952264, EPI_ISL_952266, EPI_ISL_952267, EPI_ISL_952268, EPI_ISL_952269, EPI_ISL_952270, EPI_ISL_952271, EPI_ISL_952272, EPI_ISL_952273, EPI_ISL_952274, EPI_ISL_952275, EPI_ISL_952276, EPI_ISL_952277, EPI_ISL_952278, EPI_ISL_952279, EPI_ISL_952280, EPI_ISL_952281, EPI_ISL_952282, EPI_ISL_952283, EPI_ISL_952284, EPI_ISL_952285, EPI_ISL_952286, EPI_ISL_952287, EPI_ISL_952288, EPI_ISL_952289, EPI_ISL_952290, EPI_ISL_952291, EPI_ISL_952292, EPI_ISL_952293, EPI_ISL_952294, EPI_ISL_952295, EPI_ISL_952296, EPI_ISL_952297, EPI_ISL_952299, EPI_ISL_952300, EPI_ISL_952301, EPI_ISL_952302, EPI_ISL_952303, EPI_ISL_952304, EPI_ISL_952305, EPI_ISL_952306, EPI_ISL_952307, EPI_ISL_952308, EPI_ISL_952309, EPI_ISL_952310, EPI_ISL_952311, EPI_ISL_952312, EPI_ISL_952313, EPI_ISL_952314, EPI_ISL_952315, EPI_ISL_952316, EPI_ISL_952317, EPI_ISL_952318, EPI_ISL_952319, EPI_ISL_952320, EPI_ISL_952321, EPI_ISL_952322, EPI_ISL_952323, EPI_ISL_952324, EPI_ISL_952325, EPI_ISL_952326, EPI_ISL_952327, EPI_ISL_952328, EPI_ISL_952329, EPI_ISL_952330, EPI_ISL_952331, EPI_ISL_952332, EPI_ISL_952333, EPI_ISL_952334, EPI_ISL_952335, EPI_ISL_952337, EPI_ISL_952338, EPI_ISL_952339, EPI_ISL_952340, EPI_ISL_952341, EPI_ISL_952342, EPI_ISL_952343, EPI_ISL_952344, EPI_ISL_952346, EPI_ISL_952347, EPI_ISL_952348, EPI_ISL_952349, EPI_ISL_952350, EPI_ISL_952351, EPI_ISL_952352, EPI_ISL_952353, EPI_ISL_952354, EPI_ISL_952355, EPI_ISL_952356, EPI_ISL_952357, EPI_ISL_952358, EPI_ISL_952359, EPI_ISL_952360, EPI_ISL_952361, EPI_ISL_952362, EPI_ISL_952363, EPI_ISL_952364, EPI_ISL_952366, EPI_ISL_952367, EPI_ISL_952368, EPI_ISL_952370, EPI_ISL_952371, EPI_ISL_952372, EPI_ISL_952373, EPI_ISL_952374, EPI_ISL_952375, EPI_ISL_952376			
see above	Originating lab: Wales Specialist Virology Centre Sequencing lab: Pathogen Genomics Unit	Public Health Wales Microbiology Cardiff Wales Specialist Virology Centre	Catherine Moore, Johnathan Evans, Laura Gifford, Malorie Perry, Simon Cottrell, Angela Marchbank, Alec Birchley, Alexander Adams, Amy Gaskin, Bree Gatica-Wilcox, Jason Coombes, Joel Southgate, Lauren Gilbert, Lee Graham, Nicole Pacchiarini, Sara Kumziene-Summerhayes, Sarah Taylor, Sophie Jones, Sara Rey, Matthew Bull, Joanne Watkins, Sally Corden, Tom Connor
EPI_ISL_952486, EPI_ISL_952487, EPI_ISL_952488	Centre for Enzyme Innovation, University of Portsmouth / Translational Research Laboratory, Portsmouth Hospitals NHS Trust	COVID-19 Genomics UK (COG-UK) Consortium	Angela Beckett,Salman Goudarzi,Christopher Fearn,Kate Cook,Katie Loveson,Sharon Glaysher,Scott Elliott,Samuel Robson
EPI_ISL_952954, EPI_ISL_952955, EPI_ISL_952957, EPI_ISL_952958, EPI_ISL_952959, EPI_ISL_952964, EPI_ISL_952965, EPI_ISL_952968, EPI_ISL_952970, EPI_ISL_952972, EPI_ISL_952975, EPI_ISL_952976, EPI_ISL_952977, EPI_ISL_952979, EPI_ISL_952980, EPI_ISL_952982, EPI_ISL_952984, EPI_ISL_952985, EPI_ISL_952986, EPI_ISL_952988, EPI_ISL_952990, EPI_ISL_952991, EPI_ISL_952994, EPI_ISL_952995, EPI_ISL_952997, EPI_ISL_952998, EPI_ISL_952999, EPI_ISL_953000, EPI_ISL_953002, EPI_ISL_953003, EPI_ISL_953004, EPI_ISL_953006, EPI_ISL_953009, EPI_ISL_953010, EPI_ISL_953012, EPI_ISL_953013, EPI_ISL_953015, EPI_ISL_953016, EPI_ISL_953017, EPI_ISL_953020, EPI_ISL_953021, EPI_ISL_953022			
see above	Virology Department, Sheffield Teaching Hospitals NHS Foundation Trust/Department of Infection, Immunity and Cardiovascular Disease, The Medical School, University of Sheffield	COVID-19 Genomics UK (COG-UK) Consortium	Thushan de Silva, Matthew Parker, Nikki Smith, Adri Angyal, Rebecca Brown, Luke Green, Rachel Tucker, Paul Parsons, Danielle Groves, Katie Johnson, Laura Carrilero, Alex Keeley, Dave Partridge, Matthew Wyles, Benjamin Lindsey, Mehmet Yavuz, Mohammad Raza, Cariad Evans
EPI_ISL_953240, EPI_ISL_953245, EPI_ISL_953248, EPI_ISL_953249, EPI_ISL_953250, EPI_ISL_953252, EPI_ISL_953253, EPI_ISL_953255, EPI_ISL_953256, EPI_ISL_953257, EPI_ISL_953258, EPI_ISL_953264, EPI_ISL_953265, EPI_ISL_953266, EPI_ISL_953268, EPI_ISL_953269, EPI_ISL_953270, EPI_ISL_953271, EPI_ISL_953275, EPI_ISL_953276, EPI_ISL_953277, EPI_ISL_953279, EPI_ISL_953280, EPI_ISL_953282, EPI_ISL_953283, EPI_ISL_953284, EPI_ISL_953285			
see above	Bioinformatics and Biostatistics Lab, Advanced Sequencing Facility	COVID-19 Genomics UK (COG-UK) Consortium	Aengus Stewart,Jerome Nicod,Chelsea Sawyer,Laura Cubitt,Harshil Patel,Margaret Crawford
EPI_ISL_953376	Jessa	Jessa	Jessa_cmdLab
EPI_ISL_953387, EPI_ISL_953389, EPI_ISL_953391, EPI_ISL_953392, EPI_ISL_953393, EPI_ISL_953395, EPI_ISL_953396, EPI_ISL_953397, EPI_ISL_953398, EPI_ISL_953399, EPI_ISL_953400, EPI_ISL_953401			
see above	National Public Health Laboratory, National Centre for Infectious Diseases	National Public Health Laboratory, National Centre for Infectious Diseases	Tze Minn Mak, Zhenyang Zhou, Lin Cui, Raymond Tzer Pin Lin
EPI_ISL_953428, EPI_ISL_953429, EPI_ISL_953430, EPI_ISL_953431, EPI_ISL_953432, EPI_ISL_953433, EPI_ISL_953434, EPI_ISL_953435, EPI_ISL_953436, EPI_ISL_953437, EPI_ISL_953438, EPI_ISL_953439, EPI_ISL_953440, EPI_ISL_953441, EPI_ISL_953442, EPI_ISL_953443, EPI_ISL_953444, EPI_ISL_953445, EPI_ISL_953446, EPI_ISL_953447, EPI_ISL_953448, EPI_ISL_953449, EPI_ISL_953450, EPI_ISL_953451, EPI_ISL_953453, EPI_ISL_953454, EPI_ISL_953455, EPI_ISL_953456, EPI_ISL_953457, EPI_ISL_953458, EPI_ISL_953459, EPI_ISL_953460, EPI_ISL_953461, EPI_ISL_953462, EPI_ISL_953463, EPI_ISL_953464, EPI_ISL_953465, EPI_ISL_953466, EPI_ISL_953467, EPI_ISL_953468, EPI_ISL_953469, EPI_ISL_953470, EPI_ISL_953471, EPI_ISL_953472, EPI_ISL_953473, EPI_ISL_953474, EPI_ISL_953475, EPI_ISL_953476, EPI_ISL_953477, EPI_ISL_953478, EPI_ISL_953479, EPI_ISL_953480, EPI_ISL_953481, EPI_ISL_953482, EPI_ISL_953483, EPI_ISL_953484, EPI_ISL_953485, EPI_ISL_953486, EPI_ISL_953487, EPI_ISL_953488			
see above	Jessa	Jessa	Jessa_cmdLab
EPI_ISL_953499, EPI_ISL_953502, EPI_ISL_953503, EPI_ISL_953588, EPI_ISL_953589, EPI_ISL_953590, EPI_ISL_953591, EPI_ISL_953592, EPI_ISL_953593, EPI_ISL_953594, EPI_ISL_953595, EPI_ISL_953596, EPI_ISL_953597, EPI_ISL_953598, EPI_ISL_953621, EPI_ISL_953622, EPI_ISL_953623, EPI_ISL_953624, EPI_ISL_953625, EPI_ISL_953649, EPI_ISL_953650, EPI_ISL_953651, EPI_ISL_953922, EPI_ISL_953923, EPI_ISL_953924, EPI_ISL_953925, EPI_ISL_953926, EPI_ISL_953927, EPI_ISL_953928, EPI_ISL_953929, EPI_ISL_953930, EPI_ISL_953931, EPI_ISL_953932, EPI_ISL_953933, EPI_ISL_953934, EPI_ISL_953935, EPI_ISL_953936, EPI_ISL_953937			
see above	University Hospitals of Geneva, Laboratory of Virology	HUG, Laboratory of Virology and the Health2030 Genome Center	Samuel Cordey, Ana Rita Goncalves, Laurent Kaiser, Lorenzo Cerutti, Henri Pegeot, Melyssa Elies, Deborah Penet, Keith Harshman, Ioannis Xenarios, Emmanouil Dermitzakis
EPI_ISL_954755, EPI_ISL_954756, EPI_ISL_954757, EPI_ISL_954758, EPI_ISL_954759, EPI_ISL_954760, EPI_ISL_954761, EPI_ISL_954762, EPI_ISL_954763, EPI_ISL_954764, EPI_ISL_954765, EPI_ISL_954766, EPI_ISL_954767, EPI_ISL_954768, EPI_ISL_954769, EPI_ISL_954770, EPI_ISL_954771, EPI_ISL_954772, EPI_ISL_954773, EPI_ISL_954774, EPI_ISL_954775, EPI_ISL_954777, EPI_ISL_954778, EPI_ISL_954779			
see above	AZDelta	AZDelta	Geert Martens; Dieter De Smet
EPI_ISL_955104, EPI_ISL_955105, EPI_ISL_955106, EPI_ISL_955107, EPI_ISL_955108, EPI_ISL_955109, EPI_ISL_955110, EPI_ISL_955111	Maryland Public Health Laboratory	Maryland Public Health Laboratory	Maryland Department of Health Laboratories Administration
EPI_ISL_955218, EPI_ISL_955220, EPI_ISL_955221	Indiana Animal Disease Diagnostic Laboratory	Carpi Laboratory - Purdue University	Jack Dorman, Ilinca I Ciubotariu, Lev Gorenstein, Abebe A Fola, G Kenitra Hendrix, Rebecca P Wilkes, Giovanna Carpi
EPI_ISL_955290, EPI_ISL_955292	TMC/Pathology	Pathogen Discovery, Respiratory Viruses Branch, Division of Viral Diseases, Centers for Disease Control and Prevention	Ying Tao, Jing Zhang, Yan Li, Krista Queen, Anna Uehara, Peter Cook, Clinton R. Paden, Haibin Wang, Suxiang Tong
EPI_ISL_955308	OK Public Health Laboratory, Oklahoma State DOH	Pathogen Discovery, Respiratory Viruses Branch, Division of Viral Diseases, Centers for Disease Control and Prevention	Ying Tao, Jing Zhang, Yan Li, Krista Queen, Anna Uehara, Peter Cook, Clinton R. Paden, Haibin Wang, Suxiang Tong
EPI_ISL_955831, EPI_ISL_955832, EPI_ISL_955833, EPI_ISL_955834, EPI_ISL_955835, EPI_ISL_955836, EPI_ISL_955906, EPI_ISL_955907, EPI_ISL_955908, EPI_ISL_955909, EPI_ISL_955910, EPI_ISL_955911, EPI_ISL_955912, EPI_ISL_955913, EPI_ISL_955914, EPI_ISL_955915, EPI_ISL_955916, EPI_ISL_955917, EPI_ISL_955922, EPI_ISL_955923, EPI_ISL_955924, EPI_ISL_955925, EPI_ISL_955926, EPI_ISL_955927, EPI_ISL_955928, EPI_ISL_955929, EPI_ISL_955930, EPI_ISL_955931, EPI_ISL_955932, EPI_ISL_955933, EPI_ISL_955934, EPI_ISL_955935, EPI_ISL_955936, EPI_ISL_955937, EPI_ISL_955938			
see above	University of Michigan Clinical Microbiology Laboratory	Lauring Lab, University of Michigan, Department of Microbiology and Immunology	Valesano
EPI_ISL_956272	Thai Red Cross Emerging Infectious Diseases Health Science Centre, Chulalongkorn Hospital, Faculty of Medicine, Chulalongkorn University	Thai Red Cross Emerging Infectious Diseases Center and Faculty of Medicine, Chulalongkorn University	Rome Buathong, Sopon Iamsirithaworn, Sininat Petcharat, Yutthana Joyjinda, Weenassarin Ampoot, Apaporn Rodpan, Opass Putcharoen, Thiravat Hemachudha, Supaporn Wacharapluesadee
EPI_ISL_956296	Instituto Nacional de Salud- Dirección de Redes de Laboratorios de Salud Pública	Instituto Nacional de Salud- Dirección de Investigación en Salud Pública	Katherine Laiton-Donato, Diego A. Álvarez-Díaz, Carlos Franco-Muñoz, Mauricio Pacheco-Montealegre, Hector Alejandro Ruiz-Moreno, Maria T. Herrera-Sepúlveda, Diego Andrés Prada, Jhonnatan Reales-González, Sheryll Corchuelo, Julian Naizaque, Gerardo Santamaría, Magdalena Wiesner, Martha Lucia Ospina Martinez, Marcela Mercado-Reyes
EPI_ISL_956422, EPI_ISL_956423, EPI_ISL_956424, EPI_ISL_956425, EPI_ISL_956426, EPI_ISL_956427, EPI_ISL_956428, EPI_ISL_956429, EPI_ISL_956430, EPI_ISL_956431, EPI_ISL_956432, EPI_ISL_956433, EPI_ISL_956434, EPI_ISL_956435, EPI_ISL_956436, EPI_ISL_956437, EPI_ISL_956438, EPI_ISL_956439, EPI_ISL_956440, EPI_ISL_956441, EPI_ISL_956442, EPI_ISL_956443, EPI_ISL_956444, EPI_ISL_956445, EPI_ISL_956447, EPI_ISL_956448, EPI_ISL_956449, EPI_ISL_956450, EPI_ISL_956451, EPI_ISL_956452, EPI_ISL_956453, EPI_ISL_956454, EPI_ISL_956455, EPI_ISL_956456, EPI_ISL_956457, EPI_ISL_956458, EPI_ISL_956459, EPI_ISL_956460, EPI_ISL_956461, EPI_ISL_956462, EPI_ISL_956463, EPI_ISL_956464, EPI_ISL_956465, EPI_ISL_956466, EPI_ISL_956467, EPI_ISL_956468, EPI_ISL_956469, EPI_ISL_956470, EPI_ISL_956471, EPI_ISL_956472, EPI_ISL_956473, EPI_ISL_956474, EPI_ISL_956475, EPI_ISL_956476,			

EPI_ISL_956477, EPI_ISL_956478, EPI_ISL_956479, EPI_ISL_956480, EPI_ISL_956481, EPI_ISL_956482, EPI_ISL_956483, EPI_ISL_956484, EPI_ISL_956485, EPI_ISL_956486, EPI_ISL_956488, EPI_ISL_956489, EPI_ISL_956490, EPI_ISL_956491, EPI_ISL_956492, EPI_ISL_956493, EPI_ISL_956494, EPI_ISL_956495, EPI_ISL_956496, EPI_ISL_956497, EPI_ISL_956498, EPI_ISL_956499, EPI_ISL_956500, EPI_ISL_956501, EPI_ISL_956502, EPI_ISL_956503, EPI_ISL_956504, EPI_ISL_956505, EPI_ISL_956506, EPI_ISL_956507, EPI_ISL_956509, EPI_ISL_956510, EPI_ISL_956511, EPI_ISL_956512, EPI_ISL_956513, EPI_ISL_956514, EPI_ISL_956515, EPI_ISL_956516, EPI_ISL_956517, EPI_ISL_956518, EPI_ISL_956519, EPI_ISL_956520, EPI_ISL_956521, EPI_ISL_956522, EPI_ISL_956523, EPI_ISL_956524, EPI_ISL_956525, EPI_ISL_956526, EPI_ISL_956527, EPI_ISL_956528, EPI_ISL_956529, EPI_ISL_956530, EPI_ISL_956531, EPI_ISL_956532, EPI_ISL_956533, EPI_ISL_956534, EPI_ISL_956535, EPI_ISL_956536, EPI_ISL_956537, EPI_ISL_956538, EPI_ISL_956539, EPI_ISL_956540, EPI_ISL_956541, EPI_ISL_956542, EPI_ISL_956543, EPI_ISL_956544, EPI_ISL_956545, EPI_ISL_956546, EPI_ISL_956547, EPI_ISL_956548, EPI_ISL_956549, EPI_ISL_956550, EPI_ISL_956551, EPI_ISL_956552, EPI_ISL_956553, EPI_ISL_956554, EPI_ISL_956555, EPI_ISL_956556, EPI_ISL_956557, EPI_ISL_956558, EPI_ISL_956559, EPI_ISL_956560, EPI_ISL_956561, EPI_ISL_956563, EPI_ISL_956564, EPI_ISL_956565, EPI_ISL_956566, EPI_ISL_956567, EPI_ISL_956568, EPI_ISL_956569, EPI_ISL_956570, EPI_ISL_956571, EPI_ISL_956572, EPI_ISL_956573, EPI_ISL_956574, EPI_ISL_956575, EPI_ISL_956577, EPI_ISL_956578, EPI_ISL_956579, EPI_ISL_956580, EPI_ISL_956581, EPI_ISL_956582, EPI_ISL_956583, EPI_ISL_956584, EPI_ISL_956586, EPI_ISL_956588, EPI_ISL_956589, EPI_ISL_956590, EPI_ISL_956591, EPI_ISL_956593, EPI_ISL_956594, EPI_ISL_956595, EPI_ISL_956596, EPI_ISL_956597, EPI_ISL_956598, EPI_ISL_956599, EPI_ISL_956600, EPI_ISL_956603, EPI_ISL_956604, EPI_ISL_956605, EPI_ISL_956607, EPI_ISL_956608, EPI_ISL_956609, EPI_ISL_956610, EPI_ISL_956611, EPI_ISL_956612, EPI_ISL_956613, EPI_ISL_956614, EPI_ISL_956615, EPI_ISL_956616, EPI_ISL_956617, EPI_ISL_956619, EPI_ISL_956620, EPI_ISL_956621, EPI_ISL_956622, EPI_ISL_956623, EPI_ISL_956624, EPI_ISL_956627, EPI_ISL_956628, EPI_ISL_956629, EPI_ISL_956630, EPI_ISL_956632, EPI_ISL_956633, EPI_ISL_956634, EPI_ISL_956635, EPI_ISL_956637, EPI_ISL_956638, EPI_ISL_956639, EPI_ISL_956640, EPI_ISL_956641, EPI_ISL_956642, EPI_ISL_956643, EPI_ISL_956644, EPI_ISL_956645, EPI_ISL_956646, EPI_ISL_956647, EPI_ISL_956648, EPI_ISL_956649, EPI_ISL_956650, EPI_ISL_956652, EPI_ISL_956653, EPI_ISL_956654, EPI_ISL_956655, EPI_ISL_956656, EPI_ISL_956657, EPI_ISL_956658, EPI_ISL_956659, EPI_ISL_956660, EPI_ISL_956661, EPI_ISL_956662, EPI_ISL_956663, EPI_ISL_956664, EPI_ISL_956665, EPI_ISL_956666, EPI_ISL_956667, EPI_ISL_956669, EPI_ISL_956670, EPI_ISL_956671, EPI_ISL_956672, EPI_ISL_956673, EPI_ISL_956674, EPI_ISL_956675, EPI_ISL_956676, EPI_ISL_956677, EPI_ISL_956678, EPI_ISL_956679, EPI_ISL_956680, EPI_ISL_956681, EPI_ISL_956682, EPI_ISL_956683, EPI_ISL_956684, EPI_ISL_956685, EPI_ISL_956686, EPI_ISL_956687, EPI_ISL_956688, EPI_ISL_956689, EPI_ISL_956692, EPI_ISL_956693, EPI_ISL_956695, EPI_ISL_956696, EPI_ISL_956697, EPI_ISL_956698, EPI_ISL_956700, EPI_ISL_956701, EPI_ISL_956702, EPI_ISL_956703, EPI_ISL_956704, EPI_ISL_956705, EPI_ISL_956706, EPI_ISL_956707, EPI_ISL_956709, EPI_ISL_956710, EPI_ISL_956711, EPI_ISL_956712, EPI_ISL_956713, EPI_ISL_956714, EPI_ISL_956715, EPI_ISL_956716, EPI_ISL_956717, EPI_ISL_956718, EPI_ISL_956719, EPI_ISL_956720, EPI_ISL_956721, EPI_ISL_956722, EPI_ISL_956723, EPI_ISL_956724, EPI_ISL_956725, EPI_ISL_956726, EPI_ISL_956727, EPI_ISL_956728, EPI_ISL_956730, EPI_ISL_956731, EPI_ISL_956732, EPI_ISL_956733, EPI_ISL_956734, EPI_ISL_956735, EPI_ISL_956736, EPI_ISL_956737, EPI_ISL_956738, EPI_ISL_956739, EPI_ISL_956740, EPI_ISL_956741, EPI_ISL_956742, EPI_ISL_956743, EPI_ISL_956744, EPI_ISL_956745, EPI_ISL_956746, EPI_ISL_956747, EPI_ISL_956748, EPI_ISL_956749, EPI_ISL_956750, EPI_ISL_956751, EPI_ISL_956752, EPI_ISL_956753, EPI_ISL_956754, EPI_ISL_956755, EPI_ISL_956756, EPI_ISL_956757, EPI_ISL_956758, EPI_ISL_956759, EPI_ISL_956760, EPI_ISL_956761, EPI_ISL_956762, EPI_ISL_956763, EPI_ISL_956764			
see above	Lighthouse Lab in Alderley Park	Wellcome Sanger Institute for the COVID-19 Genomics UK (COG-UK) Consortium	Jacquelyn Wynn, Mairead Hyland, The Lighthouse Lab in Alderley Park and Alex Alderton, Roberto Amato, Sonia Goncalves, Ewan Harrison, David K. Jackson, Ian Johnston, Dominic Kwiatkowski, Cordelia Langford, John Sillitoe on behalf of the Wellcome Sanger Institute COVID-19 Surveillance Team
EPI_ISL_956765, EPI_ISL_956766, EPI_ISL_956767, EPI_ISL_956768, EPI_ISL_956769, EPI_ISL_956770, EPI_ISL_956771, EPI_ISL_956772, EPI_ISL_956773, EPI_ISL_956774, EPI_ISL_956775, EPI_ISL_956776, EPI_ISL_956777, EPI_ISL_956778, EPI_ISL_956779, EPI_ISL_956780, EPI_ISL_956781, EPI_ISL_956782, EPI_ISL_956783, EPI_ISL_956784, EPI_ISL_956785, EPI_ISL_956786, EPI_ISL_956787, EPI_ISL_956788, EPI_ISL_956789, EPI_ISL_956790, EPI_ISL_956791, EPI_ISL_956792, EPI_ISL_956793, EPI_ISL_956794, EPI_ISL_956795, EPI_ISL_956796, EPI_ISL_956797, EPI_ISL_956798, EPI_ISL_956799, EPI_ISL_956800, EPI_ISL_956801, EPI_ISL_956802, EPI_ISL_956803, EPI_ISL_956804, EPI_ISL_956805, EPI_ISL_956806, EPI_ISL_956807, EPI_ISL_956808, EPI_ISL_956809, EPI_ISL_956810, EPI_ISL_956811, EPI_ISL_956812, EPI_ISL_956813, EPI_ISL_956814, EPI_ISL_956815, EPI_ISL_956816, EPI_ISL_956817, EPI_ISL_956818, EPI_ISL_956819, EPI_ISL_956820, EPI_ISL_956821, EPI_ISL_956822, EPI_ISL_956823, EPI_ISL_956824, EPI_ISL_956825, EPI_ISL_956826, EPI_ISL_956827, EPI_ISL_956828, EPI_ISL_956829, EPI_ISL_956830, EPI_ISL_956831, EPI_ISL_956832, EPI_ISL_956833, EPI_ISL_956834, EPI_ISL_956835, EPI_ISL_956836, EPI_ISL_956837, EPI_ISL_956838, EPI_ISL_956839, EPI_ISL_956840, EPI_ISL_956841, EPI_ISL_956842, EPI_ISL_956843, EPI_ISL_956844, EPI_ISL_956845, EPI_ISL_956846, EPI_ISL_956847, EPI_ISL_956848, EPI_ISL_956849, EPI_ISL_956850, EPI_ISL_956851, EPI_ISL_956852, EPI_ISL_956853, EPI_ISL_956854, EPI_ISL_956855, EPI_ISL_956856, EPI_ISL_956857, EPI_ISL_956858, EPI_ISL_956859, EPI_ISL_956860, EPI_ISL_956861, EPI_ISL_956862, EPI_ISL_956863, EPI_ISL_956864, EPI_ISL_956865, EPI_ISL_956866, EPI_ISL_956867, EPI_ISL_956868, EPI_ISL_956869, EPI_ISL_956870, EPI_ISL_956871, EPI_ISL_956872, EPI_ISL_956873, EPI_ISL_956874, EPI_ISL_956875, EPI_ISL_956876, EPI_ISL_956877, EPI_ISL_956878, EPI_ISL_956879, EPI_ISL_956880, EPI_ISL_956881, EPI_ISL_956882, EPI_ISL_956883, EPI_ISL_956884, EPI_ISL_956885, EPI_ISL_956886, EPI_ISL_956887, EPI_ISL_956888, EPI_ISL_956889, EPI_ISL_956890, EPI_ISL_956891, EPI_ISL_956892, EPI_ISL_956893, EPI_ISL_956894, EPI_ISL_956895, EPI_ISL_956896, EPI_ISL_956897, EPI_ISL_956898, EPI_ISL_956899, EPI_ISL_956900, EPI_ISL_956901, EPI_ISL_956902, EPI_ISL_956903, EPI_ISL_956904, EPI_ISL_956905, EPI_ISL_956906, EPI_ISL_956907, EPI_ISL_956908, EPI_ISL_956909, EPI_ISL_956910, EPI_ISL_956911, EPI_ISL_956912, EPI_ISL_956913, EPI_ISL_956914, EPI_ISL_956915, EPI_ISL_956916, EPI_ISL_956917, EPI_ISL_956918, EPI_ISL_956919, EPI_ISL_956920, EPI_ISL_956921, EPI_ISL_956922, EPI_ISL_956923, EPI_ISL_956924, EPI_ISL_956925, EPI_ISL_956926, EPI_ISL_956927, EPI_ISL_956928, EPI_ISL_956929, EPI_ISL_956930, EPI_ISL_956931, EPI_ISL_956932, EPI_ISL_956933, EPI_ISL_956934, EPI_ISL_956935, EPI_ISL_956936, EPI_ISL_956937, EPI_ISL_956938, EPI_ISL_956939, EPI_ISL_956940, EPI_ISL_956941, EPI_ISL_956942, EPI_ISL_956943, EPI_ISL_956944, EPI_ISL_956945, EPI_ISL_956946, EPI_ISL_956947, EPI_ISL_956948, EPI_ISL_956949, EPI_ISL_956950, EPI_ISL_956951, EPI_ISL_956952, EPI_ISL_956953, EPI_ISL_956954, EPI_ISL_956955, EPI_ISL_956956, EPI_ISL_956957, EPI_ISL_956958, EPI_ISL_956959, EPI_ISL_956960, EPI_ISL_956961, EPI_ISL_956962, EPI_ISL_956963, EPI_ISL_956964, EPI_ISL_956965, EPI_ISL_956966, EPI_ISL_956967, EPI_ISL_956968, EPI_ISL_956969, EPI_ISL_956970, EPI_ISL_956971, EPI_ISL_956972, EPI_ISL_956973, EPI_ISL_956974, EPI_ISL_956975, EPI_ISL_956976, EPI_ISL_956977, EPI_ISL_956978, EPI_ISL_956979, EPI_ISL_956980, EPI_ISL_956981, EPI_ISL_956982, EPI_ISL_956983, EPI_ISL_956984, EPI_ISL_956985, EPI_ISL_956986, EPI_ISL_956987, EPI_ISL_956988, EPI_ISL_956989, EPI_ISL_956990, EPI_ISL_956991, EPI_ISL_956992, EPI_ISL_956993, EPI_ISL_956994, EPI_ISL_956995, EPI_ISL_956996, EPI_ISL_956997, EPI_ISL_956998, EPI_ISL_956999, EPI_ISL_957000, EPI_ISL_957001, EPI_ISL_957002, EPI_ISL_957003, EPI_ISL_957004, EPI_ISL_957005, EPI_ISL_957006, EPI_ISL_957007, EPI_ISL_957008, EPI_ISL_957009, EPI_ISL_957010, EPI_ISL_957011, EPI_ISL_957012, EPI_ISL_957013, EPI_ISL_957014, EPI_ISL_957015, EPI_ISL_957016, EPI_ISL_957017, EPI_ISL_957018, EPI_ISL_957019, EPI_ISL_957020, EPI_ISL_957021, EPI_ISL_957022, EPI_ISL_957023, EPI_ISL_957024, EPI_ISL_957025, EPI_ISL_957026, EPI_ISL_957027, EPI_ISL_957028, EPI_ISL_957029, EPI_ISL_957030, EPI_ISL_957031, EPI_ISL_957032, EPI_ISL_957033, EPI_ISL_957034, EPI_ISL_957035, EPI_ISL_957036, EPI_ISL_957037, EPI_ISL_957038, EPI_ISL_957039, EPI_ISL_957040, EPI_ISL_957041, EPI_ISL_957042, EPI_ISL_957043, EPI_ISL_957044, EPI_ISL_957045, EPI_ISL_957046, EPI_ISL_957047, EPI_ISL_957048, EPI_ISL_957049, EPI_ISL_957050, EPI_ISL_957051, EPI_ISL_957052, EPI_ISL_957053			
see above	Lighthouse Lab in Milton Keynes	Wellcome Sanger Institute for the COVID-19 Genomics UK (COG-UK) Consortium	The Lighthouse Lab in Milton Keynes and Alex Alderton, Roberto Amato, Sonia Goncalves, Ewan Harrison, David K. Jackson, Ian Johnston, Dominic Kwiatkowski, Cordelia Langford, John Sillitoe on behalf of the Wellcome Sanger Institute COVID-19 Surveillance Team
EPI_ISL_957054, EPI_ISL_957055	Lighthouse Lab in Glasgow	Wellcome Sanger Institute for the COVID-19 Genomics UK (COG-UK) Consortium	Harper VanSteenhouse, Yumi Kasai, David Gray, Carol Clugston, Anna Dominiczak and Alex Alderton, Roberto Amato, Sonia Goncalves, Ewan Harrison, David K. Jackson, Ian Johnston, Dominic Kwiatkowski, Cordelia Langford, John Sillitoe on behalf of the Wellcome Sanger Institute COVID-19 Surveillance Team
EPI_ISL_957056	Lighthouse Lab in Milton Keynes	Wellcome Sanger Institute for the COVID-19 Genomics UK (COG-UK) Consortium	The Lighthouse Lab in Milton Keynes and Alex Alderton, Roberto Amato, Sonia Goncalves, Ewan Harrison, David K. Jackson, Ian Johnston, Dominic Kwiatkowski, Cordelia Langford, John Sillitoe on behalf of the Wellcome Sanger Institute COVID-19 Surveillance Team
EPI_ISL_957057	Lighthouse Lab in Glasgow	Wellcome Sanger Institute for the COVID-19 Genomics UK (COG-UK) Consortium	Harper VanSteenhouse, Yumi Kasai, David Gray, Carol Clugston, Anna Dominiczak and Alex Alderton, Roberto Amato, Sonia Goncalves, Ewan Harrison, David K. Jackson, Ian Johnston, Dominic Kwiatkowski, Cordelia Langford, John Sillitoe on behalf of the Wellcome Sanger Institute COVID-19 Surveillance Team
EPI_ISL_957058, EPI_ISL_957059	Lighthouse Lab in Milton Keynes	Wellcome Sanger Institute for the COVID-19 Genomics UK (COG-UK) Consortium	The Lighthouse Lab in Milton Keynes and Alex Alderton, Roberto Amato, Sonia Goncalves, Ewan Harrison, David K. Jackson, Ian Johnston, Dominic Kwiatkowski, Cordelia Langford, John Sillitoe on behalf of the Wellcome Sanger Institute COVID-19 Surveillance Team
EPI_ISL_957060, EPI_ISL_957061	Lighthouse Lab in Glasgow	Wellcome Sanger Institute for the COVID-19 Genomics UK (COG-UK) Consortium	Harper VanSteenhouse, Yumi Kasai, David Gray, Carol Clugston, Anna Dominiczak and Alex Alderton, Roberto Amato, Sonia Goncalves, Ewan Harrison, David K. Jackson, Ian Johnston, Dominic Kwiatkowski, Cordelia Langford, John Sillitoe on behalf of the Wellcome Sanger Institute COVID-19 Surveillance Team
EPI_ISL_957062, EPI_ISL_957063, EPI_ISL_957064	Lighthouse Lab in Milton Keynes	Wellcome Sanger Institute for the COVID-19 Genomics UK (COG-UK) Consortium	The Lighthouse Lab in Milton Keynes and Alex Alderton, Roberto Amato, Sonia Goncalves, Ewan Harrison, David K. Jackson, Ian Johnston, Dominic Kwiatkowski, Cordelia Langford, John Sillitoe on behalf of the Wellcome Sanger Institute COVID-19 Surveillance Team
EPI_ISL_957065	Lighthouse Lab in Glasgow	Wellcome Sanger Institute for the COVID-19 Genomics UK (COG-UK) Consortium	Harper VanSteenhouse, Yumi Kasai, David Gray, Carol Clugston, Anna Dominiczak and Alex Alderton, Roberto Amato, Sonia Goncalves, Ewan Harrison, David K. Jackson, Ian Johnston, Dominic Kwiatkowski, Cordelia Langford, John Sillitoe on behalf of the Wellcome Sanger Institute COVID-19 Surveillance Team
EPI_ISL_957066	Lighthouse Lab in Milton Keynes	Wellcome Sanger Institute for the COVID-19 Genomics UK (COG-UK) Consortium	The Lighthouse Lab in Milton Keynes and Alex Alderton, Roberto Amato, Sonia Goncalves, Ewan Harrison, David K. Jackson, Ian Johnston, Dominic Kwiatkowski, Cordelia Langford, John Sillitoe on behalf of the Wellcome Sanger Institute COVID-19 Surveillance Team
EPI_ISL_957067, EPI_ISL_957068	Lighthouse Lab in Glasgow	Wellcome Sanger Institute for the COVID-19 Genomics UK (COG-UK) Consortium	Harper VanSteenhouse, Yumi Kasai, David Gray, Carol Clugston, Anna Dominiczak and Alex Alderton, Roberto Amato, Sonia Goncalves, Ewan Harrison, David K. Jackson, Ian Johnston, Dominic Kwiatkowski, Cordelia Langford, John Sillitoe on behalf of the Wellcome Sanger Institute COVID-19 Surveillance Team
EPI_ISL_957069, EPI_ISL_957070	Lighthouse Lab in Milton Keynes	Wellcome Sanger Institute for the COVID-19 Genomics UK (COG-UK) Consortium	The Lighthouse Lab in Milton Keynes and Alex Alderton, Roberto Amato, Sonia Goncalves, Ewan Harrison, David K. Jackson, Ian Johnston, Dominic Kwiatkowski, Cordelia Langford, John Sillitoe on behalf of the Wellcome Sanger Institute COVID-19 Surveillance Team
EPI_ISL_957071, EPI_ISL_957072, EPI_ISL_957073	Lighthouse Lab in Glasgow	Wellcome Sanger Institute for the COVID-19 Genomics UK (COG-UK) Consortium	Harper VanSteenhouse, Yumi Kasai, David Gray, Carol Clugston, Anna Dominiczak and Alex Alderton, Roberto Amato, Sonia Goncalves, Ewan Harrison, David K. Jackson, Ian Johnston, Dominic Kwiatkowski, Cordelia Langford, John Sillitoe on behalf of the Wellcome Sanger Institute COVID-19 Surveillance Team
EPI_ISL_957074	Lighthouse Lab in Milton Keynes	Wellcome Sanger Institute for the COVID-19 Genomics UK (COG-UK) Consortium	The Lighthouse Lab in Milton Keynes and Alex Alderton, Roberto Amato, Sonia Goncalves, Ewan Harrison, David K. Jackson, Ian Johnston, Dominic Kwiatkowski, Cordelia Langford, John Sillitoe on behalf of the Wellcome Sanger Institute COVID-19 Surveillance Team
EPI_ISL_957075	Lighthouse Lab in Glasgow	Wellcome Sanger Institute for the COVID-19 Genomics UK (COG-UK) Consortium	Harper VanSteenhouse, Yumi Kasai, David Gray, Carol Clugston, Anna Dominiczak and Alex Alderton, Roberto Amato, Sonia Goncalves, Ewan Harrison, David K. Jackson, Ian Johnston, Dominic Kwiatkowski, Cordelia Langford, John Sillitoe on behalf of the Wellcome Sanger Institute COVID-19 Surveillance Team
EPI_ISL_957076	Lighthouse Lab in Milton Keynes	Wellcome Sanger Institute for the COVID-19 Genomics UK (COG-UK) Consortium	The Lighthouse Lab in Milton Keynes and Alex Alderton, Roberto Amato, Sonia Goncalves, Ewan Harrison, David K. Jackson, Ian Johnston, Dominic Kwiatkowski, Cordelia Langford, John Sillitoe on behalf of the Wellcome Sanger Institute COVID-19 Surveillance Team
EPI_ISL_957077, EPI_ISL_957078, EPI_ISL_957079, EPI_ISL_957080, EPI_ISL_957081, EPI_ISL_957082, EPI_ISL_957083, EPI_ISL_957084	Lighthouse Lab in Glasgow	Wellcome Sanger Institute for the COVID-19 Genomics UK (COG-UK) Consortium	Harper VanSteenhouse, Yumi Kasai, David Gray, Carol Clugston, Anna Dominiczak and Alex Alderton, Roberto Amato, Sonia Goncalves, Ewan Harrison, David K. Jackson, Ian Johnston, Dominic Kwiatkowski, Cordelia Langford, John Sillitoe on behalf of the Wellcome Sanger Institute COVID-19 Surveillance Team
EPI_ISL_957085	Lighthouse Lab in Milton Keynes	Wellcome Sanger Institute for the COVID-19 Genomics UK (COG-UK) Consortium	The Lighthouse Lab in Milton Keynes and Alex Alderton, Roberto Amato, Sonia Goncalves, Ewan Harrison, David K. Jackson, Ian Johnston, Dominic Kwiatkowski, Cordelia Langford, John Sillitoe on behalf of the Wellcome Sanger Institute COVID-19 Surveillance Team

EPI_ISL_957086	Lighthouse Lab in Glasgow	Wellcome Sanger Institute for the COVID-19 Genomics UK (COG-UK) Consortium	Harper VanSteenhouse, Yumi Kasai, David Gray, Carol Clugston, Anna Dominiczak and Alex Alderton, Roberto Amato, Sonia Goncalves, Ewan Harrison, David K. Jackson, Ian Johnston, Dominic Kwiatkowski, Cordelia Langford, John Sillitoe on behalf of the Wellcome Sanger Institute COVID-19 Surveillance Team
EPI_ISL_957087, EPI_ISL_957088	Lighthouse Lab in Milton Keynes	Wellcome Sanger Institute for the COVID-19 Genomics UK (COG-UK) Consortium	The Lighthouse Lab in Milton Keynes and Alex Alderton, Roberto Amato, Sonia Goncalves, Ewan Harrison, David K. Jackson, Ian Johnston, Dominic Kwiatkowski, Cordelia Langford, John Sillitoe on behalf of the Wellcome Sanger Institute COVID-19 Surveillance Team
EPI_ISL_957089, EPI_ISL_957090, EPI_ISL_957091, EPI_ISL_957092, EPI_ISL_957093, EPI_ISL_957094, EPI_ISL_957095	Lighthouse Lab in Glasgow	Wellcome Sanger Institute for the COVID-19 Genomics UK (COG-UK) Consortium	Harper VanSteenhouse, Yumi Kasai, David Gray, Carol Clugston, Anna Dominiczak and Alex Alderton, Roberto Amato, Sonia Goncalves, Ewan Harrison, David K. Jackson, Ian Johnston, Dominic Kwiatkowski, Cordelia Langford, John Sillitoe on behalf of the Wellcome Sanger Institute COVID-19 Surveillance Team
EPI_ISL_957096	Lighthouse Lab in Milton Keynes	Wellcome Sanger Institute for the COVID-19 Genomics UK (COG-UK) Consortium	The Lighthouse Lab in Milton Keynes and Alex Alderton, Roberto Amato, Sonia Goncalves, Ewan Harrison, David K. Jackson, Ian Johnston, Dominic Kwiatkowski, Cordelia Langford, John Sillitoe on behalf of the Wellcome Sanger Institute COVID-19 Surveillance Team
EPI_ISL_957097	Lighthouse Lab in Glasgow	Wellcome Sanger Institute for the COVID-19 Genomics UK (COG-UK) Consortium	Harper VanSteenhouse, Yumi Kasai, David Gray, Carol Clugston, Anna Dominiczak and Alex Alderton, Roberto Amato, Sonia Goncalves, Ewan Harrison, David K. Jackson, Ian Johnston, Dominic Kwiatkowski, Cordelia Langford, John Sillitoe on behalf of the Wellcome Sanger Institute COVID-19 Surveillance Team
EPI_ISL_957098	Lighthouse Lab in Milton Keynes	Wellcome Sanger Institute for the COVID-19 Genomics UK (COG-UK) Consortium	The Lighthouse Lab in Milton Keynes and Alex Alderton, Roberto Amato, Sonia Goncalves, Ewan Harrison, David K. Jackson, Ian Johnston, Dominic Kwiatkowski, Cordelia Langford, John Sillitoe on behalf of the Wellcome Sanger Institute COVID-19 Surveillance Team
EPI_ISL_957099	Lighthouse Lab in Glasgow	Wellcome Sanger Institute for the COVID-19 Genomics UK (COG-UK) Consortium	Harper VanSteenhouse, Yumi Kasai, David Gray, Carol Clugston, Anna Dominiczak and Alex Alderton, Roberto Amato, Sonia Goncalves, Ewan Harrison, David K. Jackson, Ian Johnston, Dominic Kwiatkowski, Cordelia Langford, John Sillitoe on behalf of the Wellcome Sanger Institute COVID-19 Surveillance Team
EPI_ISL_957100	Lighthouse Lab in Milton Keynes	Wellcome Sanger Institute for the COVID-19 Genomics UK (COG-UK) Consortium	The Lighthouse Lab in Milton Keynes and Alex Alderton, Roberto Amato, Sonia Goncalves, Ewan Harrison, David K. Jackson, Ian Johnston, Dominic Kwiatkowski, Cordelia Langford, John Sillitoe on behalf of the Wellcome Sanger Institute COVID-19 Surveillance Team
EPI_ISL_957102, EPI_ISL_957103, EPI_ISL_957104, EPI_ISL_957105	Lighthouse Lab in Glasgow	Wellcome Sanger Institute for the COVID-19 Genomics UK (COG-UK) Consortium	Harper VanSteenhouse, Yumi Kasai, David Gray, Carol Clugston, Anna Dominiczak and Alex Alderton, Roberto Amato, Sonia Goncalves, Ewan Harrison, David K. Jackson, Ian Johnston, Dominic Kwiatkowski, Cordelia Langford, John Sillitoe on behalf of the Wellcome Sanger Institute COVID-19 Surveillance Team
EPI_ISL_957106	Lighthouse Lab in Milton Keynes	Wellcome Sanger Institute for the COVID-19 Genomics UK (COG-UK) Consortium	The Lighthouse Lab in Milton Keynes and Alex Alderton, Roberto Amato, Sonia Goncalves, Ewan Harrison, David K. Jackson, Ian Johnston, Dominic Kwiatkowski, Cordelia Langford, John Sillitoe on behalf of the Wellcome Sanger Institute COVID-19 Surveillance Team
EPI_ISL_957107	Lighthouse Lab in Glasgow	Wellcome Sanger Institute for the COVID-19 Genomics UK (COG-UK) Consortium	Harper VanSteenhouse, Yumi Kasai, David Gray, Carol Clugston, Anna Dominiczak and Alex Alderton, Roberto Amato, Sonia Goncalves, Ewan Harrison, David K. Jackson, Ian Johnston, Dominic Kwiatkowski, Cordelia Langford, John Sillitoe on behalf of the Wellcome Sanger Institute COVID-19 Surveillance Team
EPI_ISL_957108, EPI_ISL_957109	Lighthouse Lab in Milton Keynes	Wellcome Sanger Institute for the COVID-19 Genomics UK (COG-UK) Consortium	The Lighthouse Lab in Milton Keynes and Alex Alderton, Roberto Amato, Sonia Goncalves, Ewan Harrison, David K. Jackson, Ian Johnston, Dominic Kwiatkowski, Cordelia Langford, John Sillitoe on behalf of the Wellcome Sanger Institute COVID-19 Surveillance Team
EPI_ISL_957110	Lighthouse Lab in Glasgow	Wellcome Sanger Institute for the COVID-19 Genomics UK (COG-UK) Consortium	Harper VanSteenhouse, Yumi Kasai, David Gray, Carol Clugston, Anna Dominiczak and Alex Alderton, Roberto Amato, Sonia Goncalves, Ewan Harrison, David K. Jackson, Ian Johnston, Dominic Kwiatkowski, Cordelia Langford, John Sillitoe on behalf of the Wellcome Sanger Institute COVID-19 Surveillance Team
EPI_ISL_957111, EPI_ISL_957112, EPI_ISL_957113	Lighthouse Lab in Milton Keynes	Wellcome Sanger Institute for the COVID-19 Genomics UK (COG-UK) Consortium	The Lighthouse Lab in Milton Keynes and Alex Alderton, Roberto Amato, Sonia Goncalves, Ewan Harrison, David K. Jackson, Ian Johnston, Dominic Kwiatkowski, Cordelia Langford, John Sillitoe on behalf of the Wellcome Sanger Institute COVID-19 Surveillance Team
EPI_ISL_957114, EPI_ISL_957115	Lighthouse Lab in Glasgow	Wellcome Sanger Institute for the COVID-19 Genomics UK (COG-UK) Consortium	Harper VanSteenhouse, Yumi Kasai, David Gray, Carol Clugston, Anna Dominiczak and Alex Alderton, Roberto Amato, Sonia Goncalves, Ewan Harrison, David K. Jackson, Ian Johnston, Dominic Kwiatkowski, Cordelia Langford, John Sillitoe on behalf of the Wellcome Sanger Institute COVID-19 Surveillance Team
EPI_ISL_957116	Lighthouse Lab in Milton Keynes	Wellcome Sanger Institute for the COVID-19 Genomics UK (COG-UK) Consortium	The Lighthouse Lab in Milton Keynes and Alex Alderton, Roberto Amato, Sonia Goncalves, Ewan Harrison, David K. Jackson, Ian Johnston, Dominic Kwiatkowski, Cordelia Langford, John Sillitoe on behalf of the Wellcome Sanger Institute COVID-19 Surveillance Team
EPI_ISL_957117, EPI_ISL_957118	Lighthouse Lab in Glasgow	Wellcome Sanger Institute for the COVID-19 Genomics UK (COG-UK) Consortium	Harper VanSteenhouse, Yumi Kasai, David Gray, Carol Clugston, Anna Dominiczak and Alex Alderton, Roberto Amato, Sonia Goncalves, Ewan Harrison, David K. Jackson, Ian Johnston, Dominic Kwiatkowski, Cordelia Langford, John Sillitoe on behalf of the Wellcome Sanger Institute COVID-19 Surveillance Team
EPI_ISL_957119, EPI_ISL_957120	Lighthouse Lab in Milton Keynes	Wellcome Sanger Institute for the COVID-19 Genomics UK (COG-UK) Consortium	The Lighthouse Lab in Milton Keynes and Alex Alderton, Roberto Amato, Sonia Goncalves, Ewan Harrison, David K. Jackson, Ian Johnston, Dominic Kwiatkowski, Cordelia Langford, John Sillitoe on behalf of the Wellcome Sanger Institute COVID-19 Surveillance Team
EPI_ISL_957121, EPI_ISL_957122	Lighthouse Lab in Glasgow	Wellcome Sanger Institute for the COVID-19 Genomics UK (COG-UK) Consortium	Harper VanSteenhouse, Yumi Kasai, David Gray, Carol Clugston, Anna Dominiczak and Alex Alderton, Roberto Amato, Sonia Goncalves, Ewan Harrison, David K. Jackson, Ian Johnston, Dominic Kwiatkowski, Cordelia Langford, John Sillitoe on behalf of the Wellcome Sanger Institute COVID-19 Surveillance Team
EPI_ISL_957123	Lighthouse Lab in Milton Keynes	Wellcome Sanger Institute for the COVID-19 Genomics UK (COG-UK) Consortium	The Lighthouse Lab in Milton Keynes and Alex Alderton, Roberto Amato, Sonia Goncalves, Ewan Harrison, David K. Jackson, Ian Johnston, Dominic Kwiatkowski, Cordelia Langford, John Sillitoe on behalf of the Wellcome Sanger Institute COVID-19 Surveillance Team
EPI_ISL_957124	Lighthouse Lab in Glasgow	Wellcome Sanger Institute for the COVID-19 Genomics UK (COG-UK) Consortium	Harper VanSteenhouse, Yumi Kasai, David Gray, Carol Clugston, Anna Dominiczak and Alex Alderton, Roberto Amato, Sonia Goncalves, Ewan Harrison, David K. Jackson, Ian Johnston, Dominic Kwiatkowski, Cordelia Langford, John Sillitoe on behalf of the Wellcome Sanger Institute COVID-19 Surveillance Team
EPI_ISL_957125, EPI_ISL_957126, EPI_ISL_957127, EPI_ISL_957128, EPI_ISL_957129	Lighthouse Lab in Milton Keynes	Wellcome Sanger Institute for the COVID-19 Genomics UK (COG-UK) Consortium	The Lighthouse Lab in Milton Keynes and Alex Alderton, Roberto Amato, Sonia Goncalves, Ewan Harrison, David K. Jackson, Ian Johnston, Dominic Kwiatkowski, Cordelia Langford, John Sillitoe on behalf of the Wellcome Sanger Institute COVID-19 Surveillance Team
EPI_ISL_957130	Lighthouse Lab in Glasgow	Wellcome Sanger Institute for the COVID-19 Genomics UK (COG-UK) Consortium	Harper VanSteenhouse, Yumi Kasai, David Gray, Carol Clugston, Anna Dominiczak and Alex Alderton, Roberto Amato, Sonia Goncalves, Ewan Harrison, David K. Jackson, Ian Johnston, Dominic Kwiatkowski, Cordelia Langford, John Sillitoe on behalf of the Wellcome Sanger Institute COVID-19 Surveillance Team
EPI_ISL_957131, EPI_ISL_957132	Lighthouse Lab in Milton Keynes	Wellcome Sanger Institute for the COVID-19 Genomics UK (COG-UK) Consortium	The Lighthouse Lab in Milton Keynes and Alex Alderton, Roberto Amato, Sonia Goncalves, Ewan Harrison, David K. Jackson, Ian Johnston, Dominic Kwiatkowski, Cordelia Langford, John Sillitoe on behalf of the Wellcome Sanger Institute COVID-19 Surveillance Team
EPI_ISL_957133	Lighthouse Lab in Glasgow	Wellcome Sanger Institute for the COVID-19 Genomics UK (COG-UK) Consortium	Harper VanSteenhouse, Yumi Kasai, David Gray, Carol Clugston, Anna Dominiczak and Alex Alderton, Roberto Amato, Sonia Goncalves, Ewan Harrison, David K. Jackson, Ian Johnston, Dominic Kwiatkowski, Cordelia Langford, John Sillitoe on behalf of the Wellcome Sanger Institute COVID-19 Surveillance Team
EPI_ISL_957134, EPI_ISL_957135, EPI_ISL_957136, EPI_ISL_957137, EPI_ISL_957138, EPI_ISL_957139	Lighthouse Lab in Milton Keynes	Wellcome Sanger Institute for the COVID-19 Genomics UK (COG-UK) Consortium	The Lighthouse Lab in Milton Keynes and Alex Alderton, Roberto Amato, Sonia Goncalves, Ewan Harrison, David K. Jackson, Ian Johnston, Dominic Kwiatkowski, Cordelia Langford, John Sillitoe on behalf of the Wellcome Sanger Institute COVID-19 Surveillance Team
EPI_ISL_957140, EPI_ISL_957141	Lighthouse Lab in Glasgow	Wellcome Sanger Institute for the COVID-19 Genomics UK (COG-UK) Consortium	Harper VanSteenhouse, Yumi Kasai, David Gray, Carol Clugston, Anna Dominiczak and Alex Alderton, Roberto Amato, Sonia Goncalves, Ewan Harrison, David K. Jackson, Ian Johnston, Dominic Kwiatkowski, Cordelia Langford, John Sillitoe on behalf of the Wellcome Sanger Institute COVID-19 Surveillance Team
EPI_ISL_957142, EPI_ISL_957143, EPI_ISL_957144, EPI_ISL_957145, EPI_ISL_957146, EPI_ISL_957147,	Lighthouse Lab in Milton Keynes	Wellcome Sanger Institute for the COVID-19 Genomics UK (COG-UK) Consortium	The Lighthouse Lab in Milton Keynes and Alex Alderton, Roberto Amato, Sonia Goncalves, Ewan Harrison, David K. Jackson, Ian Johnston, Dominic Kwiatkowski, Cordelia Langford, John Sillitoe on behalf of the Wellcome Sanger Institute COVID-19 Surveillance Team

EPI_ISL_957148			
EPI_ISL_957149, EPI_ISL_957150, EPI_ISL_957151	Lighthouse Lab in Glasgow	Wellcome Sanger Institute for the COVID-19 Genomics UK (COG-UK) Consortium	Harper VanSteenhouse, Yumi Kasai, David Gray, Carol Clugston, Anna Dominiczak and Alex Alderton, Roberto Amato, Sonia Goncalves, Ewan Harrison, David K. Jackson, Ian Johnston, Dominic Kwiatkowski, Cordelia Langford, John Sillitoe on behalf of the Wellcome Sanger Institute COVID-19 Surveillance Team
EPI_ISL_957152, EPI_ISL_957153	Lighthouse Lab in Milton Keynes	Wellcome Sanger Institute for the COVID-19 Genomics UK (COG-UK) Consortium	The Lighthouse Lab in Milton Keynes and Alex Alderton, Roberto Amato, Sonia Goncalves, Ewan Harrison, David K. Jackson, Ian Johnston, Dominic Kwiatkowski, Cordelia Langford, John Sillitoe on behalf of the Wellcome Sanger Institute COVID-19 Surveillance Team
EPI_ISL_957154, EPI_ISL_957155	Lighthouse Lab in Glasgow	Wellcome Sanger Institute for the COVID-19 Genomics UK (COG-UK) Consortium	Harper VanSteenhouse, Yumi Kasai, David Gray, Carol Clugston, Anna Dominiczak and Alex Alderton, Roberto Amato, Sonia Goncalves, Ewan Harrison, David K. Jackson, Ian Johnston, Dominic Kwiatkowski, Cordelia Langford, John Sillitoe on behalf of the Wellcome Sanger Institute COVID-19 Surveillance Team
EPI_ISL_957156	Lighthouse Lab in Milton Keynes	Wellcome Sanger Institute for the COVID-19 Genomics UK (COG-UK) Consortium	The Lighthouse Lab in Milton Keynes and Alex Alderton, Roberto Amato, Sonia Goncalves, Ewan Harrison, David K. Jackson, Ian Johnston, Dominic Kwiatkowski, Cordelia Langford, John Sillitoe on behalf of the Wellcome Sanger Institute COVID-19 Surveillance Team
EPI_ISL_957157, EPI_ISL_957158	Lighthouse Lab in Glasgow	Wellcome Sanger Institute for the COVID-19 Genomics UK (COG-UK) Consortium	Harper VanSteenhouse, Yumi Kasai, David Gray, Carol Clugston, Anna Dominiczak and Alex Alderton, Roberto Amato, Sonia Goncalves, Ewan Harrison, David K. Jackson, Ian Johnston, Dominic Kwiatkowski, Cordelia Langford, John Sillitoe on behalf of the Wellcome Sanger Institute COVID-19 Surveillance Team
EPI_ISL_957159	Lighthouse Lab in Milton Keynes	Wellcome Sanger Institute for the COVID-19 Genomics UK (COG-UK) Consortium	The Lighthouse Lab in Milton Keynes and Alex Alderton, Roberto Amato, Sonia Goncalves, Ewan Harrison, David K. Jackson, Ian Johnston, Dominic Kwiatkowski, Cordelia Langford, John Sillitoe on behalf of the Wellcome Sanger Institute COVID-19 Surveillance Team
EPI_ISL_957160	Lighthouse Lab in Glasgow	Wellcome Sanger Institute for the COVID-19 Genomics UK (COG-UK) Consortium	Harper VanSteenhouse, Yumi Kasai, David Gray, Carol Clugston, Anna Dominiczak and Alex Alderton, Roberto Amato, Sonia Goncalves, Ewan Harrison, David K. Jackson, Ian Johnston, Dominic Kwiatkowski, Cordelia Langford, John Sillitoe on behalf of the Wellcome Sanger Institute COVID-19 Surveillance Team
EPI_ISL_957161	Lighthouse Lab in Milton Keynes	Wellcome Sanger Institute for the COVID-19 Genomics UK (COG-UK) Consortium	The Lighthouse Lab in Milton Keynes and Alex Alderton, Roberto Amato, Sonia Goncalves, Ewan Harrison, David K. Jackson, Ian Johnston, Dominic Kwiatkowski, Cordelia Langford, John Sillitoe on behalf of the Wellcome Sanger Institute COVID-19 Surveillance Team
EPI_ISL_957162	Lighthouse Lab in Glasgow	Wellcome Sanger Institute for the COVID-19 Genomics UK (COG-UK) Consortium	Harper VanSteenhouse, Yumi Kasai, David Gray, Carol Clugston, Anna Dominiczak and Alex Alderton, Roberto Amato, Sonia Goncalves, Ewan Harrison, David K. Jackson, Ian Johnston, Dominic Kwiatkowski, Cordelia Langford, John Sillitoe on behalf of the Wellcome Sanger Institute COVID-19 Surveillance Team
EPI_ISL_957163, EPI_ISL_957164	Lighthouse Lab in Milton Keynes	Wellcome Sanger Institute for the COVID-19 Genomics UK (COG-UK) Consortium	The Lighthouse Lab in Milton Keynes and Alex Alderton, Roberto Amato, Sonia Goncalves, Ewan Harrison, David K. Jackson, Ian Johnston, Dominic Kwiatkowski, Cordelia Langford, John Sillitoe on behalf of the Wellcome Sanger Institute COVID-19 Surveillance Team
EPI_ISL_957165, EPI_ISL_957166	Lighthouse Lab in Glasgow	Wellcome Sanger Institute for the COVID-19 Genomics UK (COG-UK) Consortium	Harper VanSteenhouse, Yumi Kasai, David Gray, Carol Clugston, Anna Dominiczak and Alex Alderton, Roberto Amato, Sonia Goncalves, Ewan Harrison, David K. Jackson, Ian Johnston, Dominic Kwiatkowski, Cordelia Langford, John Sillitoe on behalf of the Wellcome Sanger Institute COVID-19 Surveillance Team
EPI_ISL_957167	Lighthouse Lab in Milton Keynes	Wellcome Sanger Institute for the COVID-19 Genomics UK (COG-UK) Consortium	The Lighthouse Lab in Milton Keynes and Alex Alderton, Roberto Amato, Sonia Goncalves, Ewan Harrison, David K. Jackson, Ian Johnston, Dominic Kwiatkowski, Cordelia Langford, John Sillitoe on behalf of the Wellcome Sanger Institute COVID-19 Surveillance Team
EPI_ISL_957168	Lighthouse Lab in Glasgow	Wellcome Sanger Institute for the COVID-19 Genomics UK (COG-UK) Consortium	Harper VanSteenhouse, Yumi Kasai, David Gray, Carol Clugston, Anna Dominiczak and Alex Alderton, Roberto Amato, Sonia Goncalves, Ewan Harrison, David K. Jackson, Ian Johnston, Dominic Kwiatkowski, Cordelia Langford, John Sillitoe on behalf of the Wellcome Sanger Institute COVID-19 Surveillance Team
EPI_ISL_957169	Lighthouse Lab in Milton Keynes	Wellcome Sanger Institute for the COVID-19 Genomics UK (COG-UK) Consortium	The Lighthouse Lab in Milton Keynes and Alex Alderton, Roberto Amato, Sonia Goncalves, Ewan Harrison, David K. Jackson, Ian Johnston, Dominic Kwiatkowski, Cordelia Langford, John Sillitoe on behalf of the Wellcome Sanger Institute COVID-19 Surveillance Team
EPI_ISL_957170, EPI_ISL_957171, EPI_ISL_957172	Lighthouse Lab in Glasgow	Wellcome Sanger Institute for the COVID-19 Genomics UK (COG-UK) Consortium	Harper VanSteenhouse, Yumi Kasai, David Gray, Carol Clugston, Anna Dominiczak and Alex Alderton, Roberto Amato, Sonia Goncalves, Ewan Harrison, David K. Jackson, Ian Johnston, Dominic Kwiatkowski, Cordelia Langford, John Sillitoe on behalf of the Wellcome Sanger Institute COVID-19 Surveillance Team
EPI_ISL_957173, EPI_ISL_957174, EPI_ISL_957175	Lighthouse Lab in Milton Keynes	Wellcome Sanger Institute for the COVID-19 Genomics UK (COG-UK) Consortium	The Lighthouse Lab in Milton Keynes and Alex Alderton, Roberto Amato, Sonia Goncalves, Ewan Harrison, David K. Jackson, Ian Johnston, Dominic Kwiatkowski, Cordelia Langford, John Sillitoe on behalf of the Wellcome Sanger Institute COVID-19 Surveillance Team
EPI_ISL_957176, EPI_ISL_957177	Lighthouse Lab in Glasgow	Wellcome Sanger Institute for the COVID-19 Genomics UK (COG-UK) Consortium	Harper VanSteenhouse, Yumi Kasai, David Gray, Carol Clugston, Anna Dominiczak and Alex Alderton, Roberto Amato, Sonia Goncalves, Ewan Harrison, David K. Jackson, Ian Johnston, Dominic Kwiatkowski, Cordelia Langford, John Sillitoe on behalf of the Wellcome Sanger Institute COVID-19 Surveillance Team
EPI_ISL_957178, EPI_ISL_957179	Lighthouse Lab in Milton Keynes	Wellcome Sanger Institute for the COVID-19 Genomics UK (COG-UK) Consortium	The Lighthouse Lab in Milton Keynes and Alex Alderton, Roberto Amato, Sonia Goncalves, Ewan Harrison, David K. Jackson, Ian Johnston, Dominic Kwiatkowski, Cordelia Langford, John Sillitoe on behalf of the Wellcome Sanger Institute COVID-19 Surveillance Team
EPI_ISL_957180	Lighthouse Lab in Glasgow	Wellcome Sanger Institute for the COVID-19 Genomics UK (COG-UK) Consortium	Harper VanSteenhouse, Yumi Kasai, David Gray, Carol Clugston, Anna Dominiczak and Alex Alderton, Roberto Amato, Sonia Goncalves, Ewan Harrison, David K. Jackson, Ian Johnston, Dominic Kwiatkowski, Cordelia Langford, John Sillitoe on behalf of the Wellcome Sanger Institute COVID-19 Surveillance Team
EPI_ISL_957181, EPI_ISL_957182	Lighthouse Lab in Milton Keynes	Wellcome Sanger Institute for the COVID-19 Genomics UK (COG-UK) Consortium	The Lighthouse Lab in Milton Keynes and Alex Alderton, Roberto Amato, Sonia Goncalves, Ewan Harrison, David K. Jackson, Ian Johnston, Dominic Kwiatkowski, Cordelia Langford, John Sillitoe on behalf of the Wellcome Sanger Institute COVID-19 Surveillance Team
EPI_ISL_957183	Lighthouse Lab in Glasgow	Wellcome Sanger Institute for the COVID-19 Genomics UK (COG-UK) Consortium	Harper VanSteenhouse, Yumi Kasai, David Gray, Carol Clugston, Anna Dominiczak and Alex Alderton, Roberto Amato, Sonia Goncalves, Ewan Harrison, David K. Jackson, Ian Johnston, Dominic Kwiatkowski, Cordelia Langford, John Sillitoe on behalf of the Wellcome Sanger Institute COVID-19 Surveillance Team
EPI_ISL_957184, EPI_ISL_957185, EPI_ISL_957186	Lighthouse Lab in Milton Keynes	Wellcome Sanger Institute for the COVID-19 Genomics UK (COG-UK) Consortium	The Lighthouse Lab in Milton Keynes and Alex Alderton, Roberto Amato, Sonia Goncalves, Ewan Harrison, David K. Jackson, Ian Johnston, Dominic Kwiatkowski, Cordelia Langford, John Sillitoe on behalf of the Wellcome Sanger Institute COVID-19 Surveillance Team
EPI_ISL_957187, EPI_ISL_957188	Lighthouse Lab in Glasgow	Wellcome Sanger Institute for the COVID-19 Genomics UK (COG-UK) Consortium	Harper VanSteenhouse, Yumi Kasai, David Gray, Carol Clugston, Anna Dominiczak and Alex Alderton, Roberto Amato, Sonia Goncalves, Ewan Harrison, David K. Jackson, Ian Johnston, Dominic Kwiatkowski, Cordelia Langford, John Sillitoe on behalf of the Wellcome Sanger Institute COVID-19 Surveillance Team
EPI_ISL_957189	Lighthouse Lab in Milton Keynes	Wellcome Sanger Institute for the COVID-19 Genomics UK (COG-UK) Consortium	The Lighthouse Lab in Milton Keynes and Alex Alderton, Roberto Amato, Sonia Goncalves, Ewan Harrison, David K. Jackson, Ian Johnston, Dominic Kwiatkowski, Cordelia Langford, John Sillitoe on behalf of the Wellcome Sanger Institute COVID-19 Surveillance Team
EPI_ISL_957190, EPI_ISL_957191	Lighthouse Lab in Glasgow	Wellcome Sanger Institute for the COVID-19 Genomics UK (COG-UK) Consortium	Harper VanSteenhouse, Yumi Kasai, David Gray, Carol Clugston, Anna Dominiczak and Alex Alderton, Roberto Amato, Sonia Goncalves, Ewan Harrison, David K. Jackson, Ian Johnston, Dominic Kwiatkowski, Cordelia Langford, John Sillitoe on behalf of the Wellcome Sanger Institute COVID-19 Surveillance Team
EPI_ISL_957192, EPI_ISL_957193	Lighthouse Lab in Milton Keynes	Wellcome Sanger Institute for the COVID-19 Genomics UK (COG-UK) Consortium	The Lighthouse Lab in Milton Keynes and Alex Alderton, Roberto Amato, Sonia Goncalves, Ewan Harrison, David K. Jackson, Ian Johnston, Dominic Kwiatkowski, Cordelia Langford, John Sillitoe on behalf of the Wellcome Sanger Institute COVID-19 Surveillance Team
EPI_ISL_957194	Lighthouse Lab in Glasgow	Wellcome Sanger Institute for the COVID-19 Genomics UK (COG-UK) Consortium	Harper VanSteenhouse, Yumi Kasai, David Gray, Carol Clugston, Anna Dominiczak and Alex Alderton, Roberto Amato, Sonia Goncalves, Ewan Harrison, David K. Jackson, Ian Johnston, Dominic Kwiatkowski, Cordelia Langford, John Sillitoe on behalf of the Wellcome Sanger Institute COVID-19 Surveillance Team
EPI_ISL_957195, EPI_ISL_957196, EPI_ISL_957197	Lighthouse Lab in Milton Keynes	Wellcome Sanger Institute for the COVID-19 Genomics UK (COG-UK) Consortium	The Lighthouse Lab in Milton Keynes and Alex Alderton, Roberto Amato, Sonia Goncalves, Ewan Harrison, David K. Jackson, Ian Johnston, Dominic Kwiatkowski, Cordelia Langford, John Sillitoe on behalf of the Wellcome Sanger Institute COVID-19 Surveillance Team
EPI_ISL_957198	Lighthouse Lab in Glasgow	Wellcome Sanger Institute for the COVID-19 Genomics UK (COG-UK) Consortium	Harper VanSteenhouse, Yumi Kasai, David Gray, Carol Clugston, Anna Dominiczak and Alex Alderton, Roberto Amato, Sonia Goncalves, Ewan Harrison, David K. Jackson, Ian Johnston, Dominic Kwiatkowski, Cordelia Langford, John Sillitoe on behalf of the Wellcome Sanger Institute COVID-19 Surveillance

			Team
EPI_ISL_957199	Lighthouse Lab in Milton Keynes	Wellcome Sanger Institute for the COVID-19 Genomics UK (COG-UK) Consortium	The Lighthouse Lab in Milton Keynes and Alex Alderton, Roberto Amato, Sonia Goncalves, Ewan Harrison, David K. Jackson, Ian Johnston, Dominic Kwiatkowski, Cordelia Langford, John Sillitoe on behalf of the Wellcome Sanger Institute COVID-19 Surveillance Team
EPI_ISL_957200, EPI_ISL_957201	Lighthouse Lab in Glasgow	Wellcome Sanger Institute for the COVID-19 Genomics UK (COG-UK) Consortium	Harper VanSteenhouse, Yumi Kasai, David Gray, Carol Clugston, Anna Dominiczak and Alex Alderton, Roberto Amato, Sonia Goncalves, Ewan Harrison, David K. Jackson, Ian Johnston, Dominic Kwiatkowski, Cordelia Langford, John Sillitoe on behalf of the Wellcome Sanger Institute COVID-19 Surveillance Team
EPI_ISL_957202, EPI_ISL_957203, EPI_ISL_957204	Lighthouse Lab in Milton Keynes	Wellcome Sanger Institute for the COVID-19 Genomics UK (COG-UK) Consortium	The Lighthouse Lab in Milton Keynes and Alex Alderton, Roberto Amato, Sonia Goncalves, Ewan Harrison, David K. Jackson, Ian Johnston, Dominic Kwiatkowski, Cordelia Langford, John Sillitoe on behalf of the Wellcome Sanger Institute COVID-19 Surveillance Team
EPI_ISL_957205, EPI_ISL_957206	Lighthouse Lab in Glasgow	Wellcome Sanger Institute for the COVID-19 Genomics UK (COG-UK) Consortium	Harper VanSteenhouse, Yumi Kasai, David Gray, Carol Clugston, Anna Dominiczak and Alex Alderton, Roberto Amato, Sonia Goncalves, Ewan Harrison, David K. Jackson, Ian Johnston, Dominic Kwiatkowski, Cordelia Langford, John Sillitoe on behalf of the Wellcome Sanger Institute COVID-19 Surveillance Team
EPI_ISL_957207	Lighthouse Lab in Milton Keynes	Wellcome Sanger Institute for the COVID-19 Genomics UK (COG-UK) Consortium	The Lighthouse Lab in Milton Keynes and Alex Alderton, Roberto Amato, Sonia Goncalves, Ewan Harrison, David K. Jackson, Ian Johnston, Dominic Kwiatkowski, Cordelia Langford, John Sillitoe on behalf of the Wellcome Sanger Institute COVID-19 Surveillance Team
EPI_ISL_957208	Lighthouse Lab in Glasgow	Wellcome Sanger Institute for the COVID-19 Genomics UK (COG-UK) Consortium	Harper VanSteenhouse, Yumi Kasai, David Gray, Carol Clugston, Anna Dominiczak and Alex Alderton, Roberto Amato, Sonia Goncalves, Ewan Harrison, David K. Jackson, Ian Johnston, Dominic Kwiatkowski, Cordelia Langford, John Sillitoe on behalf of the Wellcome Sanger Institute COVID-19 Surveillance Team
EPI_ISL_957209	Lighthouse Lab in Milton Keynes	Wellcome Sanger Institute for the COVID-19 Genomics UK (COG-UK) Consortium	The Lighthouse Lab in Milton Keynes and Alex Alderton, Roberto Amato, Sonia Goncalves, Ewan Harrison, David K. Jackson, Ian Johnston, Dominic Kwiatkowski, Cordelia Langford, John Sillitoe on behalf of the Wellcome Sanger Institute COVID-19 Surveillance Team
EPI_ISL_957210, EPI_ISL_957211, EPI_ISL_957212	Lighthouse Lab in Glasgow	Wellcome Sanger Institute for the COVID-19 Genomics UK (COG-UK) Consortium	Harper VanSteenhouse, Yumi Kasai, David Gray, Carol Clugston, Anna Dominiczak and Alex Alderton, Roberto Amato, Sonia Goncalves, Ewan Harrison, David K. Jackson, Ian Johnston, Dominic Kwiatkowski, Cordelia Langford, John Sillitoe on behalf of the Wellcome Sanger Institute COVID-19 Surveillance Team
EPI_ISL_957213, EPI_ISL_957214	Lighthouse Lab in Milton Keynes	Wellcome Sanger Institute for the COVID-19 Genomics UK (COG-UK) Consortium	The Lighthouse Lab in Milton Keynes and Alex Alderton, Roberto Amato, Sonia Goncalves, Ewan Harrison, David K. Jackson, Ian Johnston, Dominic Kwiatkowski, Cordelia Langford, John Sillitoe on behalf of the Wellcome Sanger Institute COVID-19 Surveillance Team
EPI_ISL_957215, EPI_ISL_957216	Lighthouse Lab in Glasgow	Wellcome Sanger Institute for the COVID-19 Genomics UK (COG-UK) Consortium	Harper VanSteenhouse, Yumi Kasai, David Gray, Carol Clugston, Anna Dominiczak and Alex Alderton, Roberto Amato, Sonia Goncalves, Ewan Harrison, David K. Jackson, Ian Johnston, Dominic Kwiatkowski, Cordelia Langford, John Sillitoe on behalf of the Wellcome Sanger Institute COVID-19 Surveillance Team
EPI_ISL_957217, EPI_ISL_957218	Lighthouse Lab in Milton Keynes	Wellcome Sanger Institute for the COVID-19 Genomics UK (COG-UK) Consortium	The Lighthouse Lab in Milton Keynes and Alex Alderton, Roberto Amato, Sonia Goncalves, Ewan Harrison, David K. Jackson, Ian Johnston, Dominic Kwiatkowski, Cordelia Langford, John Sillitoe on behalf of the Wellcome Sanger Institute COVID-19 Surveillance Team
EPI_ISL_957219	Lighthouse Lab in Glasgow	Wellcome Sanger Institute for the COVID-19 Genomics UK (COG-UK) Consortium	Harper VanSteenhouse, Yumi Kasai, David Gray, Carol Clugston, Anna Dominiczak and Alex Alderton, Roberto Amato, Sonia Goncalves, Ewan Harrison, David K. Jackson, Ian Johnston, Dominic Kwiatkowski, Cordelia Langford, John Sillitoe on behalf of the Wellcome Sanger Institute COVID-19 Surveillance Team
EPI_ISL_957220, EPI_ISL_957221, EPI_ISL_957222	Lighthouse Lab in Milton Keynes	Wellcome Sanger Institute for the COVID-19 Genomics UK (COG-UK) Consortium	The Lighthouse Lab in Milton Keynes and Alex Alderton, Roberto Amato, Sonia Goncalves, Ewan Harrison, David K. Jackson, Ian Johnston, Dominic Kwiatkowski, Cordelia Langford, John Sillitoe on behalf of the Wellcome Sanger Institute COVID-19 Surveillance Team
EPI_ISL_957223, EPI_ISL_957224, EPI_ISL_957225	Lighthouse Lab in Glasgow	Wellcome Sanger Institute for the COVID-19 Genomics UK (COG-UK) Consortium	Harper VanSteenhouse, Yumi Kasai, David Gray, Carol Clugston, Anna Dominiczak and Alex Alderton, Roberto Amato, Sonia Goncalves, Ewan Harrison, David K. Jackson, Ian Johnston, Dominic Kwiatkowski, Cordelia Langford, John Sillitoe on behalf of the Wellcome Sanger Institute COVID-19 Surveillance Team
EPI_ISL_957226	Lighthouse Lab in Milton Keynes	Wellcome Sanger Institute for the COVID-19 Genomics UK (COG-UK) Consortium	The Lighthouse Lab in Milton Keynes and Alex Alderton, Roberto Amato, Sonia Goncalves, Ewan Harrison, David K. Jackson, Ian Johnston, Dominic Kwiatkowski, Cordelia Langford, John Sillitoe on behalf of the Wellcome Sanger Institute COVID-19 Surveillance Team
EPI_ISL_957227	Lighthouse Lab in Glasgow	Wellcome Sanger Institute for the COVID-19 Genomics UK (COG-UK) Consortium	Harper VanSteenhouse, Yumi Kasai, David Gray, Carol Clugston, Anna Dominiczak and Alex Alderton, Roberto Amato, Sonia Goncalves, Ewan Harrison, David K. Jackson, Ian Johnston, Dominic Kwiatkowski, Cordelia Langford, John Sillitoe on behalf of the Wellcome Sanger Institute COVID-19 Surveillance Team
EPI_ISL_957228	Lighthouse Lab in Milton Keynes	Wellcome Sanger Institute for the COVID-19 Genomics UK (COG-UK) Consortium	The Lighthouse Lab in Milton Keynes and Alex Alderton, Roberto Amato, Sonia Goncalves, Ewan Harrison, David K. Jackson, Ian Johnston, Dominic Kwiatkowski, Cordelia Langford, John Sillitoe on behalf of the Wellcome Sanger Institute COVID-19 Surveillance Team
EPI_ISL_957229	Lighthouse Lab in Glasgow	Wellcome Sanger Institute for the COVID-19 Genomics UK (COG-UK) Consortium	Harper VanSteenhouse, Yumi Kasai, David Gray, Carol Clugston, Anna Dominiczak and Alex Alderton, Roberto Amato, Sonia Goncalves, Ewan Harrison, David K. Jackson, Ian Johnston, Dominic Kwiatkowski, Cordelia Langford, John Sillitoe on behalf of the Wellcome Sanger Institute COVID-19 Surveillance Team
EPI_ISL_957230	Lighthouse Lab in Milton Keynes	Wellcome Sanger Institute for the COVID-19 Genomics UK (COG-UK) Consortium	The Lighthouse Lab in Milton Keynes and Alex Alderton, Roberto Amato, Sonia Goncalves, Ewan Harrison, David K. Jackson, Ian Johnston, Dominic Kwiatkowski, Cordelia Langford, John Sillitoe on behalf of the Wellcome Sanger Institute COVID-19 Surveillance Team
EPI_ISL_957231	Lighthouse Lab in Glasgow	Wellcome Sanger Institute for the COVID-19 Genomics UK (COG-UK) Consortium	Harper VanSteenhouse, Yumi Kasai, David Gray, Carol Clugston, Anna Dominiczak and Alex Alderton, Roberto Amato, Sonia Goncalves, Ewan Harrison, David K. Jackson, Ian Johnston, Dominic Kwiatkowski, Cordelia Langford, John Sillitoe on behalf of the Wellcome Sanger Institute COVID-19 Surveillance Team
EPI_ISL_957232, EPI_ISL_957233, EPI_ISL_957234, EPI_ISL_957235, EPI_ISL_957236	Lighthouse Lab in Milton Keynes	Wellcome Sanger Institute for the COVID-19 Genomics UK (COG-UK) Consortium	The Lighthouse Lab in Milton Keynes and Alex Alderton, Roberto Amato, Sonia Goncalves, Ewan Harrison, David K. Jackson, Ian Johnston, Dominic Kwiatkowski, Cordelia Langford, John Sillitoe on behalf of the Wellcome Sanger Institute COVID-19 Surveillance Team
EPI_ISL_957237	Lighthouse Lab in Glasgow	Wellcome Sanger Institute for the COVID-19 Genomics UK (COG-UK) Consortium	Harper VanSteenhouse, Yumi Kasai, David Gray, Carol Clugston, Anna Dominiczak and Alex Alderton, Roberto Amato, Sonia Goncalves, Ewan Harrison, David K. Jackson, Ian Johnston, Dominic Kwiatkowski, Cordelia Langford, John Sillitoe on behalf of the Wellcome Sanger Institute COVID-19 Surveillance Team
EPI_ISL_957238	Lighthouse Lab in Milton Keynes	Wellcome Sanger Institute for the COVID-19 Genomics UK (COG-UK) Consortium	The Lighthouse Lab in Milton Keynes and Alex Alderton, Roberto Amato, Sonia Goncalves, Ewan Harrison, David K. Jackson, Ian Johnston, Dominic Kwiatkowski, Cordelia Langford, John Sillitoe on behalf of the Wellcome Sanger Institute COVID-19 Surveillance Team
EPI_ISL_957239	Lighthouse Lab in Glasgow	Wellcome Sanger Institute for the COVID-19 Genomics UK (COG-UK) Consortium	Harper VanSteenhouse, Yumi Kasai, David Gray, Carol Clugston, Anna Dominiczak and Alex Alderton, Roberto Amato, Sonia Goncalves, Ewan Harrison, David K. Jackson, Ian Johnston, Dominic Kwiatkowski, Cordelia Langford, John Sillitoe on behalf of the Wellcome Sanger Institute COVID-19 Surveillance Team
EPI_ISL_957240, EPI_ISL_957241	Lighthouse Lab in Milton Keynes	Wellcome Sanger Institute for the COVID-19 Genomics UK (COG-UK) Consortium	The Lighthouse Lab in Milton Keynes and Alex Alderton, Roberto Amato, Sonia Goncalves, Ewan Harrison, David K. Jackson, Ian Johnston, Dominic Kwiatkowski, Cordelia Langford, John Sillitoe on behalf of the Wellcome Sanger Institute COVID-19 Surveillance Team
EPI_ISL_957242, EPI_ISL_957243	Lighthouse Lab in Glasgow	Wellcome Sanger Institute for the COVID-19 Genomics UK (COG-UK) Consortium	Harper VanSteenhouse, Yumi Kasai, David Gray, Carol Clugston, Anna Dominiczak and Alex Alderton, Roberto Amato, Sonia Goncalves, Ewan Harrison, David K. Jackson, Ian Johnston, Dominic Kwiatkowski, Cordelia Langford, John Sillitoe on behalf of the Wellcome Sanger Institute COVID-19 Surveillance Team
EPI_ISL_957244	Lighthouse Lab in Milton Keynes	Wellcome Sanger Institute for the COVID-19 Genomics UK (COG-UK) Consortium	The Lighthouse Lab in Milton Keynes and Alex Alderton, Roberto Amato, Sonia Goncalves, Ewan Harrison, David K. Jackson, Ian Johnston, Dominic Kwiatkowski, Cordelia Langford, John Sillitoe on behalf of the Wellcome Sanger Institute COVID-19 Surveillance Team
EPI_ISL_957245, EPI_ISL_957246	Lighthouse Lab in Glasgow	Wellcome Sanger Institute for the COVID-19 Genomics UK (COG-UK) Consortium	Harper VanSteenhouse, Yumi Kasai, David Gray, Carol Clugston, Anna Dominiczak and Alex Alderton, Roberto Amato, Sonia Goncalves, Ewan Harrison, David K. Jackson, Ian Johnston, Dominic Kwiatkowski, Cordelia Langford, John Sillitoe on behalf of the Wellcome Sanger Institute COVID-19 Surveillance Team
EPI_ISL_957247	Lighthouse Lab in Milton Keynes	Wellcome Sanger Institute for the COVID-19 Genomics UK	The Lighthouse Lab in Milton Keynes and Alex Alderton, Roberto Amato, Sonia Goncalves, Ewan Harrison, David K. Jackson, Ian Johnston, Dominic

		(COG-UK) Consortium	Kwiatkowski, Cordelia Langford, John Sillitoe on behalf of the Wellcome Sanger Institute COVID-19 Surveillance Team
EPI_ISL_957248	Lighthouse Lab in Glasgow	Wellcome Sanger Institute for the COVID-19 Genomics UK (COG-UK) Consortium	Harper VanSteenhouse, Yumi Kasai, David Gray, Carol Clugston, Anna Dominiczak and Alex Alderton, Roberto Amato, Sonia Goncalves, Ewan Harrison, David K. Jackson, Ian Johnston, Dominic Kwiatkowski, Cordelia Langford, John Sillitoe on behalf of the Wellcome Sanger Institute COVID-19 Surveillance Team
EPI_ISL_957249	Lighthouse Lab in Milton Keynes	Wellcome Sanger Institute for the COVID-19 Genomics UK (COG-UK) Consortium	The Lighthouse Lab in Milton Keynes and Alex Alderton, Roberto Amato, Sonia Goncalves, Ewan Harrison, David K. Jackson, Ian Johnston, Dominic Kwiatkowski, Cordelia Langford, John Sillitoe on behalf of the Wellcome Sanger Institute COVID-19 Surveillance Team
EPI_ISL_957250	Lighthouse Lab in Glasgow	Wellcome Sanger Institute for the COVID-19 Genomics UK (COG-UK) Consortium	Harper VanSteenhouse, Yumi Kasai, David Gray, Carol Clugston, Anna Dominiczak and Alex Alderton, Roberto Amato, Sonia Goncalves, Ewan Harrison, David K. Jackson, Ian Johnston, Dominic Kwiatkowski, Cordelia Langford, John Sillitoe on behalf of the Wellcome Sanger Institute COVID-19 Surveillance Team
EPI_ISL_957251, EPI_ISL_957252	Lighthouse Lab in Milton Keynes	Wellcome Sanger Institute for the COVID-19 Genomics UK (COG-UK) Consortium	The Lighthouse Lab in Milton Keynes and Alex Alderton, Roberto Amato, Sonia Goncalves, Ewan Harrison, David K. Jackson, Ian Johnston, Dominic Kwiatkowski, Cordelia Langford, John Sillitoe on behalf of the Wellcome Sanger Institute COVID-19 Surveillance Team
EPI_ISL_957253, EPI_ISL_957254, EPI_ISL_957255, EPI_ISL_957256, EPI_ISL_957257	Lighthouse Lab in Glasgow	Wellcome Sanger Institute for the COVID-19 Genomics UK (COG-UK) Consortium	Harper VanSteenhouse, Yumi Kasai, David Gray, Carol Clugston, Anna Dominiczak and Alex Alderton, Roberto Amato, Sonia Goncalves, Ewan Harrison, David K. Jackson, Ian Johnston, Dominic Kwiatkowski, Cordelia Langford, John Sillitoe on behalf of the Wellcome Sanger Institute COVID-19 Surveillance Team
EPI_ISL_957258	Lighthouse Lab in Milton Keynes	Wellcome Sanger Institute for the COVID-19 Genomics UK (COG-UK) Consortium	The Lighthouse Lab in Milton Keynes and Alex Alderton, Roberto Amato, Sonia Goncalves, Ewan Harrison, David K. Jackson, Ian Johnston, Dominic Kwiatkowski, Cordelia Langford, John Sillitoe on behalf of the Wellcome Sanger Institute COVID-19 Surveillance Team
EPI_ISL_957259, EPI_ISL_957260	Lighthouse Lab in Glasgow	Wellcome Sanger Institute for the COVID-19 Genomics UK (COG-UK) Consortium	Harper VanSteenhouse, Yumi Kasai, David Gray, Carol Clugston, Anna Dominiczak and Alex Alderton, Roberto Amato, Sonia Goncalves, Ewan Harrison, David K. Jackson, Ian Johnston, Dominic Kwiatkowski, Cordelia Langford, John Sillitoe on behalf of the Wellcome Sanger Institute COVID-19 Surveillance Team
EPI_ISL_957261	Lighthouse Lab in Milton Keynes	Wellcome Sanger Institute for the COVID-19 Genomics UK (COG-UK) Consortium	The Lighthouse Lab in Milton Keynes and Alex Alderton, Roberto Amato, Sonia Goncalves, Ewan Harrison, David K. Jackson, Ian Johnston, Dominic Kwiatkowski, Cordelia Langford, John Sillitoe on behalf of the Wellcome Sanger Institute COVID-19 Surveillance Team
EPI_ISL_957262, EPI_ISL_957263	Lighthouse Lab in Glasgow	Wellcome Sanger Institute for the COVID-19 Genomics UK (COG-UK) Consortium	Harper VanSteenhouse, Yumi Kasai, David Gray, Carol Clugston, Anna Dominiczak and Alex Alderton, Roberto Amato, Sonia Goncalves, Ewan Harrison, David K. Jackson, Ian Johnston, Dominic Kwiatkowski, Cordelia Langford, John Sillitoe on behalf of the Wellcome Sanger Institute COVID-19 Surveillance Team
EPI_ISL_957264, EPI_ISL_957265	Lighthouse Lab in Milton Keynes	Wellcome Sanger Institute for the COVID-19 Genomics UK (COG-UK) Consortium	The Lighthouse Lab in Milton Keynes and Alex Alderton, Roberto Amato, Sonia Goncalves, Ewan Harrison, David K. Jackson, Ian Johnston, Dominic Kwiatkowski, Cordelia Langford, John Sillitoe on behalf of the Wellcome Sanger Institute COVID-19 Surveillance Team
EPI_ISL_957266	Lighthouse Lab in Glasgow	Wellcome Sanger Institute for the COVID-19 Genomics UK (COG-UK) Consortium	Harper VanSteenhouse, Yumi Kasai, David Gray, Carol Clugston, Anna Dominiczak and Alex Alderton, Roberto Amato, Sonia Goncalves, Ewan Harrison, David K. Jackson, Ian Johnston, Dominic Kwiatkowski, Cordelia Langford, John Sillitoe on behalf of the Wellcome Sanger Institute COVID-19 Surveillance Team
EPI_ISL_957267	Lighthouse Lab in Milton Keynes	Wellcome Sanger Institute for the COVID-19 Genomics UK (COG-UK) Consortium	The Lighthouse Lab in Milton Keynes and Alex Alderton, Roberto Amato, Sonia Goncalves, Ewan Harrison, David K. Jackson, Ian Johnston, Dominic Kwiatkowski, Cordelia Langford, John Sillitoe on behalf of the Wellcome Sanger Institute COVID-19 Surveillance Team
EPI_ISL_957268	Lighthouse Lab in Glasgow	Wellcome Sanger Institute for the COVID-19 Genomics UK (COG-UK) Consortium	Harper VanSteenhouse, Yumi Kasai, David Gray, Carol Clugston, Anna Dominiczak and Alex Alderton, Roberto Amato, Sonia Goncalves, Ewan Harrison, David K. Jackson, Ian Johnston, Dominic Kwiatkowski, Cordelia Langford, John Sillitoe on behalf of the Wellcome Sanger Institute COVID-19 Surveillance Team
EPI_ISL_957269	Lighthouse Lab in Milton Keynes	Wellcome Sanger Institute for the COVID-19 Genomics UK (COG-UK) Consortium	The Lighthouse Lab in Milton Keynes and Alex Alderton, Roberto Amato, Sonia Goncalves, Ewan Harrison, David K. Jackson, Ian Johnston, Dominic Kwiatkowski, Cordelia Langford, John Sillitoe on behalf of the Wellcome Sanger Institute COVID-19 Surveillance Team
EPI_ISL_957270, EPI_ISL_957271, EPI_ISL_957272	Lighthouse Lab in Glasgow	Wellcome Sanger Institute for the COVID-19 Genomics UK (COG-UK) Consortium	Harper VanSteenhouse, Yumi Kasai, David Gray, Carol Clugston, Anna Dominiczak and Alex Alderton, Roberto Amato, Sonia Goncalves, Ewan Harrison, David K. Jackson, Ian Johnston, Dominic Kwiatkowski, Cordelia Langford, John Sillitoe on behalf of the Wellcome Sanger Institute COVID-19 Surveillance Team
EPI_ISL_957273	Lighthouse Lab in Milton Keynes	Wellcome Sanger Institute for the COVID-19 Genomics UK (COG-UK) Consortium	The Lighthouse Lab in Milton Keynes and Alex Alderton, Roberto Amato, Sonia Goncalves, Ewan Harrison, David K. Jackson, Ian Johnston, Dominic Kwiatkowski, Cordelia Langford, John Sillitoe on behalf of the Wellcome Sanger Institute COVID-19 Surveillance Team
EPI_ISL_957274, EPI_ISL_957275, EPI_ISL_957276	Lighthouse Lab in Glasgow	Wellcome Sanger Institute for the COVID-19 Genomics UK (COG-UK) Consortium	Harper VanSteenhouse, Yumi Kasai, David Gray, Carol Clugston, Anna Dominiczak and Alex Alderton, Roberto Amato, Sonia Goncalves, Ewan Harrison, David K. Jackson, Ian Johnston, Dominic Kwiatkowski, Cordelia Langford, John Sillitoe on behalf of the Wellcome Sanger Institute COVID-19 Surveillance Team
EPI_ISL_957277, EPI_ISL_957278	Lighthouse Lab in Milton Keynes	Wellcome Sanger Institute for the COVID-19 Genomics UK (COG-UK) Consortium	The Lighthouse Lab in Milton Keynes and Alex Alderton, Roberto Amato, Sonia Goncalves, Ewan Harrison, David K. Jackson, Ian Johnston, Dominic Kwiatkowski, Cordelia Langford, John Sillitoe on behalf of the Wellcome Sanger Institute COVID-19 Surveillance Team
EPI_ISL_957279	Lighthouse Lab in Glasgow	Wellcome Sanger Institute for the COVID-19 Genomics UK (COG-UK) Consortium	Harper VanSteenhouse, Yumi Kasai, David Gray, Carol Clugston, Anna Dominiczak and Alex Alderton, Roberto Amato, Sonia Goncalves, Ewan Harrison, David K. Jackson, Ian Johnston, Dominic Kwiatkowski, Cordelia Langford, John Sillitoe on behalf of the Wellcome Sanger Institute COVID-19 Surveillance Team
EPI_ISL_957280, EPI_ISL_957281, EPI_ISL_957282	Lighthouse Lab in Milton Keynes	Wellcome Sanger Institute for the COVID-19 Genomics UK (COG-UK) Consortium	The Lighthouse Lab in Milton Keynes and Alex Alderton, Roberto Amato, Sonia Goncalves, Ewan Harrison, David K. Jackson, Ian Johnston, Dominic Kwiatkowski, Cordelia Langford, John Sillitoe on behalf of the Wellcome Sanger Institute COVID-19 Surveillance Team
EPI_ISL_957283	Lighthouse Lab in Glasgow	Wellcome Sanger Institute for the COVID-19 Genomics UK (COG-UK) Consortium	Harper VanSteenhouse, Yumi Kasai, David Gray, Carol Clugston, Anna Dominiczak and Alex Alderton, Roberto Amato, Sonia Goncalves, Ewan Harrison, David K. Jackson, Ian Johnston, Dominic Kwiatkowski, Cordelia Langford, John Sillitoe on behalf of the Wellcome Sanger Institute COVID-19 Surveillance Team
EPI_ISL_957284	Lighthouse Lab in Milton Keynes	Wellcome Sanger Institute for the COVID-19 Genomics UK (COG-UK) Consortium	The Lighthouse Lab in Milton Keynes and Alex Alderton, Roberto Amato, Sonia Goncalves, Ewan Harrison, David K. Jackson, Ian Johnston, Dominic Kwiatkowski, Cordelia Langford, John Sillitoe on behalf of the Wellcome Sanger Institute COVID-19 Surveillance Team
EPI_ISL_957285, EPI_ISL_957286, EPI_ISL_957287	Lighthouse Lab in Glasgow	Wellcome Sanger Institute for the COVID-19 Genomics UK (COG-UK) Consortium	Harper VanSteenhouse, Yumi Kasai, David Gray, Carol Clugston, Anna Dominiczak and Alex Alderton, Roberto Amato, Sonia Goncalves, Ewan Harrison, David K. Jackson, Ian Johnston, Dominic Kwiatkowski, Cordelia Langford, John Sillitoe on behalf of the Wellcome Sanger Institute COVID-19 Surveillance Team
EPI_ISL_957288	Lighthouse Lab in Milton Keynes	Wellcome Sanger Institute for the COVID-19 Genomics UK (COG-UK) Consortium	The Lighthouse Lab in Milton Keynes and Alex Alderton, Roberto Amato, Sonia Goncalves, Ewan Harrison, David K. Jackson, Ian Johnston, Dominic Kwiatkowski, Cordelia Langford, John Sillitoe on behalf of the Wellcome Sanger Institute COVID-19 Surveillance Team
EPI_ISL_957289	Lighthouse Lab in Glasgow	Wellcome Sanger Institute for the COVID-19 Genomics UK (COG-UK) Consortium	Harper VanSteenhouse, Yumi Kasai, David Gray, Carol Clugston, Anna Dominiczak and Alex Alderton, Roberto Amato, Sonia Goncalves, Ewan Harrison, David K. Jackson, Ian Johnston, Dominic Kwiatkowski, Cordelia Langford, John Sillitoe on behalf of the Wellcome Sanger Institute COVID-19 Surveillance Team
EPI_ISL_957290	Lighthouse Lab in Milton Keynes	Wellcome Sanger Institute for the COVID-19 Genomics UK (COG-UK) Consortium	The Lighthouse Lab in Milton Keynes and Alex Alderton, Roberto Amato, Sonia Goncalves, Ewan Harrison, David K. Jackson, Ian Johnston, Dominic Kwiatkowski, Cordelia Langford, John Sillitoe on behalf of the Wellcome Sanger Institute COVID-19 Surveillance Team
EPI_ISL_957291, EPI_ISL_957292	Lighthouse Lab in Glasgow	Wellcome Sanger Institute for the COVID-19 Genomics UK (COG-UK) Consortium	Harper VanSteenhouse, Yumi Kasai, David Gray, Carol Clugston, Anna Dominiczak and Alex Alderton, Roberto Amato, Sonia Goncalves, Ewan Harrison, David K. Jackson, Ian Johnston, Dominic Kwiatkowski, Cordelia Langford, John Sillitoe on behalf of the Wellcome Sanger Institute COVID-19 Surveillance Team
EPI_ISL_957293	Lighthouse Lab in Milton Keynes	Wellcome Sanger Institute for the COVID-19 Genomics UK (COG-UK) Consortium	The Lighthouse Lab in Milton Keynes and Alex Alderton, Roberto Amato, Sonia Goncalves, Ewan Harrison, David K. Jackson, Ian Johnston, Dominic Kwiatkowski, Cordelia Langford, John Sillitoe on behalf of the Wellcome Sanger Institute COVID-19 Surveillance Team
EPI_ISL_957294	Lighthouse Lab in Glasgow	Wellcome Sanger Institute for the COVID-19 Genomics UK (COG-UK) Consortium	Harper VanSteenhouse, Yumi Kasai, David Gray, Carol Clugston, Anna Dominiczak and Alex Alderton, Roberto Amato, Sonia Goncalves, Ewan Harrison, David K. Jackson, Ian Johnston, Dominic Kwiatkowski, Cordelia Langford, John Sillitoe on behalf of the Wellcome Sanger Institute COVID-19 Surveillance

			Team
EPI_ISL_957295, EPI_ISL_957296	Lighthouse Lab in Milton Keynes	Wellcome Sanger Institute for the COVID-19 Genomics UK (COG-UK) Consortium	The Lighthouse Lab in Milton Keynes and Alex Alderton, Roberto Amato, Sonia Goncalves, Ewan Harrison, David K. Jackson, Ian Johnston, Dominic Kwiatkowski, Cordelia Langford, John Sillitoe on behalf of the Wellcome Sanger Institute COVID-19 Surveillance Team
EPI_ISL_957297	Lighthouse Lab in Glasgow	Wellcome Sanger Institute for the COVID-19 Genomics UK (COG-UK) Consortium	Harper VanSteenhouse, Yumi Kasai, David Gray, Carol Clugston, Anna Dominiczak and Alex Alderton, Roberto Amato, Sonia Goncalves, Ewan Harrison, David K. Jackson, Ian Johnston, Dominic Kwiatkowski, Cordelia Langford, John Sillitoe on behalf of the Wellcome Sanger Institute COVID-19 Surveillance Team
EPI_ISL_957298	Lighthouse Lab in Milton Keynes	Wellcome Sanger Institute for the COVID-19 Genomics UK (COG-UK) Consortium	The Lighthouse Lab in Milton Keynes and Alex Alderton, Roberto Amato, Sonia Goncalves, Ewan Harrison, David K. Jackson, Ian Johnston, Dominic Kwiatkowski, Cordelia Langford, John Sillitoe on behalf of the Wellcome Sanger Institute COVID-19 Surveillance Team
EPI_ISL_957299	Lighthouse Lab in Glasgow	Wellcome Sanger Institute for the COVID-19 Genomics UK (COG-UK) Consortium	Harper VanSteenhouse, Yumi Kasai, David Gray, Carol Clugston, Anna Dominiczak and Alex Alderton, Roberto Amato, Sonia Goncalves, Ewan Harrison, David K. Jackson, Ian Johnston, Dominic Kwiatkowski, Cordelia Langford, John Sillitoe on behalf of the Wellcome Sanger Institute COVID-19 Surveillance Team
EPI_ISL_957300, EPI_ISL_957301	Lighthouse Lab in Milton Keynes	Wellcome Sanger Institute for the COVID-19 Genomics UK (COG-UK) Consortium	The Lighthouse Lab in Milton Keynes and Alex Alderton, Roberto Amato, Sonia Goncalves, Ewan Harrison, David K. Jackson, Ian Johnston, Dominic Kwiatkowski, Cordelia Langford, John Sillitoe on behalf of the Wellcome Sanger Institute COVID-19 Surveillance Team
EPI_ISL_957302	Lighthouse Lab in Glasgow	Wellcome Sanger Institute for the COVID-19 Genomics UK (COG-UK) Consortium	Harper VanSteenhouse, Yumi Kasai, David Gray, Carol Clugston, Anna Dominiczak and Alex Alderton, Roberto Amato, Sonia Goncalves, Ewan Harrison, David K. Jackson, Ian Johnston, Dominic Kwiatkowski, Cordelia Langford, John Sillitoe on behalf of the Wellcome Sanger Institute COVID-19 Surveillance Team
EPI_ISL_957303	Lighthouse Lab in Milton Keynes	Wellcome Sanger Institute for the COVID-19 Genomics UK (COG-UK) Consortium	The Lighthouse Lab in Milton Keynes and Alex Alderton, Roberto Amato, Sonia Goncalves, Ewan Harrison, David K. Jackson, Ian Johnston, Dominic Kwiatkowski, Cordelia Langford, John Sillitoe on behalf of the Wellcome Sanger Institute COVID-19 Surveillance Team
EPI_ISL_957304	Lighthouse Lab in Glasgow	Wellcome Sanger Institute for the COVID-19 Genomics UK (COG-UK) Consortium	Harper VanSteenhouse, Yumi Kasai, David Gray, Carol Clugston, Anna Dominiczak and Alex Alderton, Roberto Amato, Sonia Goncalves, Ewan Harrison, David K. Jackson, Ian Johnston, Dominic Kwiatkowski, Cordelia Langford, John Sillitoe on behalf of the Wellcome Sanger Institute COVID-19 Surveillance Team
EPI_ISL_957305	Lighthouse Lab in Milton Keynes	Wellcome Sanger Institute for the COVID-19 Genomics UK (COG-UK) Consortium	The Lighthouse Lab in Milton Keynes and Alex Alderton, Roberto Amato, Sonia Goncalves, Ewan Harrison, David K. Jackson, Ian Johnston, Dominic Kwiatkowski, Cordelia Langford, John Sillitoe on behalf of the Wellcome Sanger Institute COVID-19 Surveillance Team
EPI_ISL_957306	Lighthouse Lab in Glasgow	Wellcome Sanger Institute for the COVID-19 Genomics UK (COG-UK) Consortium	Harper VanSteenhouse, Yumi Kasai, David Gray, Carol Clugston, Anna Dominiczak and Alex Alderton, Roberto Amato, Sonia Goncalves, Ewan Harrison, David K. Jackson, Ian Johnston, Dominic Kwiatkowski, Cordelia Langford, John Sillitoe on behalf of the Wellcome Sanger Institute COVID-19 Surveillance Team
EPI_ISL_957307	Lighthouse Lab in Milton Keynes	Wellcome Sanger Institute for the COVID-19 Genomics UK (COG-UK) Consortium	The Lighthouse Lab in Milton Keynes and Alex Alderton, Roberto Amato, Sonia Goncalves, Ewan Harrison, David K. Jackson, Ian Johnston, Dominic Kwiatkowski, Cordelia Langford, John Sillitoe on behalf of the Wellcome Sanger Institute COVID-19 Surveillance Team
EPI_ISL_957308	Lighthouse Lab in Glasgow	Wellcome Sanger Institute for the COVID-19 Genomics UK (COG-UK) Consortium	Harper VanSteenhouse, Yumi Kasai, David Gray, Carol Clugston, Anna Dominiczak and Alex Alderton, Roberto Amato, Sonia Goncalves, Ewan Harrison, David K. Jackson, Ian Johnston, Dominic Kwiatkowski, Cordelia Langford, John Sillitoe on behalf of the Wellcome Sanger Institute COVID-19 Surveillance Team
EPI_ISL_957309	Lighthouse Lab in Milton Keynes	Wellcome Sanger Institute for the COVID-19 Genomics UK (COG-UK) Consortium	The Lighthouse Lab in Milton Keynes and Alex Alderton, Roberto Amato, Sonia Goncalves, Ewan Harrison, David K. Jackson, Ian Johnston, Dominic Kwiatkowski, Cordelia Langford, John Sillitoe on behalf of the Wellcome Sanger Institute COVID-19 Surveillance Team
EPI_ISL_957310, EPI_ISL_957311, EPI_ISL_957312	Lighthouse Lab in Glasgow	Wellcome Sanger Institute for the COVID-19 Genomics UK (COG-UK) Consortium	Harper VanSteenhouse, Yumi Kasai, David Gray, Carol Clugston, Anna Dominiczak and Alex Alderton, Roberto Amato, Sonia Goncalves, Ewan Harrison, David K. Jackson, Ian Johnston, Dominic Kwiatkowski, Cordelia Langford, John Sillitoe on behalf of the Wellcome Sanger Institute COVID-19 Surveillance Team
EPI_ISL_957313	Lighthouse Lab in Milton Keynes	Wellcome Sanger Institute for the COVID-19 Genomics UK (COG-UK) Consortium	The Lighthouse Lab in Milton Keynes and Alex Alderton, Roberto Amato, Sonia Goncalves, Ewan Harrison, David K. Jackson, Ian Johnston, Dominic Kwiatkowski, Cordelia Langford, John Sillitoe on behalf of the Wellcome Sanger Institute COVID-19 Surveillance Team
EPI_ISL_957314, EPI_ISL_957315	Lighthouse Lab in Glasgow	Wellcome Sanger Institute for the COVID-19 Genomics UK (COG-UK) Consortium	Harper VanSteenhouse, Yumi Kasai, David Gray, Carol Clugston, Anna Dominiczak and Alex Alderton, Roberto Amato, Sonia Goncalves, Ewan Harrison, David K. Jackson, Ian Johnston, Dominic Kwiatkowski, Cordelia Langford, John Sillitoe on behalf of the Wellcome Sanger Institute COVID-19 Surveillance Team
EPI_ISL_957316, EPI_ISL_957317, EPI_ISL_957318	Lighthouse Lab in Milton Keynes	Wellcome Sanger Institute for the COVID-19 Genomics UK (COG-UK) Consortium	The Lighthouse Lab in Milton Keynes and Alex Alderton, Roberto Amato, Sonia Goncalves, Ewan Harrison, David K. Jackson, Ian Johnston, Dominic Kwiatkowski, Cordelia Langford, John Sillitoe on behalf of the Wellcome Sanger Institute COVID-19 Surveillance Team
EPI_ISL_957319, EPI_ISL_957320	Lighthouse Lab in Glasgow	Wellcome Sanger Institute for the COVID-19 Genomics UK (COG-UK) Consortium	Harper VanSteenhouse, Yumi Kasai, David Gray, Carol Clugston, Anna Dominiczak and Alex Alderton, Roberto Amato, Sonia Goncalves, Ewan Harrison, David K. Jackson, Ian Johnston, Dominic Kwiatkowski, Cordelia Langford, John Sillitoe on behalf of the Wellcome Sanger Institute COVID-19 Surveillance Team
EPI_ISL_957321	Lighthouse Lab in Milton Keynes	Wellcome Sanger Institute for the COVID-19 Genomics UK (COG-UK) Consortium	The Lighthouse Lab in Milton Keynes and Alex Alderton, Roberto Amato, Sonia Goncalves, Ewan Harrison, David K. Jackson, Ian Johnston, Dominic Kwiatkowski, Cordelia Langford, John Sillitoe on behalf of the Wellcome Sanger Institute COVID-19 Surveillance Team
EPI_ISL_957322, EPI_ISL_957323	Lighthouse Lab in Glasgow	Wellcome Sanger Institute for the COVID-19 Genomics UK (COG-UK) Consortium	Harper VanSteenhouse, Yumi Kasai, David Gray, Carol Clugston, Anna Dominiczak and Alex Alderton, Roberto Amato, Sonia Goncalves, Ewan Harrison, David K. Jackson, Ian Johnston, Dominic Kwiatkowski, Cordelia Langford, John Sillitoe on behalf of the Wellcome Sanger Institute COVID-19 Surveillance Team
EPI_ISL_957324, EPI_ISL_957325, EPI_ISL_957326	Lighthouse Lab in Milton Keynes	Wellcome Sanger Institute for the COVID-19 Genomics UK (COG-UK) Consortium	The Lighthouse Lab in Milton Keynes and Alex Alderton, Roberto Amato, Sonia Goncalves, Ewan Harrison, David K. Jackson, Ian Johnston, Dominic Kwiatkowski, Cordelia Langford, John Sillitoe on behalf of the Wellcome Sanger Institute COVID-19 Surveillance Team
EPI_ISL_957327, EPI_ISL_957328	Lighthouse Lab in Glasgow	Wellcome Sanger Institute for the COVID-19 Genomics UK (COG-UK) Consortium	Harper VanSteenhouse, Yumi Kasai, David Gray, Carol Clugston, Anna Dominiczak and Alex Alderton, Roberto Amato, Sonia Goncalves, Ewan Harrison, David K. Jackson, Ian Johnston, Dominic Kwiatkowski, Cordelia Langford, John Sillitoe on behalf of the Wellcome Sanger Institute COVID-19 Surveillance Team
EPI_ISL_957329	Lighthouse Lab in Milton Keynes	Wellcome Sanger Institute for the COVID-19 Genomics UK (COG-UK) Consortium	The Lighthouse Lab in Milton Keynes and Alex Alderton, Roberto Amato, Sonia Goncalves, Ewan Harrison, David K. Jackson, Ian Johnston, Dominic Kwiatkowski, Cordelia Langford, John Sillitoe on behalf of the Wellcome Sanger Institute COVID-19 Surveillance Team
EPI_ISL_957330, EPI_ISL_957331	Lighthouse Lab in Glasgow	Wellcome Sanger Institute for the COVID-19 Genomics UK (COG-UK) Consortium	Harper VanSteenhouse, Yumi Kasai, David Gray, Carol Clugston, Anna Dominiczak and Alex Alderton, Roberto Amato, Sonia Goncalves, Ewan Harrison, David K. Jackson, Ian Johnston, Dominic Kwiatkowski, Cordelia Langford, John Sillitoe on behalf of the Wellcome Sanger Institute COVID-19 Surveillance Team
EPI_ISL_957332, EPI_ISL_957333	Lighthouse Lab in Milton Keynes	Wellcome Sanger Institute for the COVID-19 Genomics UK (COG-UK) Consortium	The Lighthouse Lab in Milton Keynes and Alex Alderton, Roberto Amato, Sonia Goncalves, Ewan Harrison, David K. Jackson, Ian Johnston, Dominic Kwiatkowski, Cordelia Langford, John Sillitoe on behalf of the Wellcome Sanger Institute COVID-19 Surveillance Team
EPI_ISL_957334	Lighthouse Lab in Glasgow	Wellcome Sanger Institute for the COVID-19 Genomics UK (COG-UK) Consortium	Harper VanSteenhouse, Yumi Kasai, David Gray, Carol Clugston, Anna Dominiczak and Alex Alderton, Roberto Amato, Sonia Goncalves, Ewan Harrison, David K. Jackson, Ian Johnston, Dominic Kwiatkowski, Cordelia Langford, John Sillitoe on behalf of the Wellcome Sanger Institute COVID-19 Surveillance Team
EPI_ISL_957335	Lighthouse Lab in Milton Keynes	Wellcome Sanger Institute for the COVID-19 Genomics UK (COG-UK) Consortium	The Lighthouse Lab in Milton Keynes and Alex Alderton, Roberto Amato, Sonia Goncalves, Ewan Harrison, David K. Jackson, Ian Johnston, Dominic Kwiatkowski, Cordelia Langford, John Sillitoe on behalf of the Wellcome Sanger Institute COVID-19 Surveillance Team
EPI_ISL_957336, EPI_ISL_957337	Lighthouse Lab in Glasgow	Wellcome Sanger Institute for the COVID-19 Genomics UK (COG-UK) Consortium	Harper VanSteenhouse, Yumi Kasai, David Gray, Carol Clugston, Anna Dominiczak and Alex Alderton, Roberto Amato, Sonia Goncalves, Ewan Harrison, David K. Jackson, Ian Johnston, Dominic Kwiatkowski, Cordelia Langford, John Sillitoe on behalf of the Wellcome Sanger Institute COVID-19 Surveillance Team
EPI_ISL_957338, EPI_ISL_957339, EPI_ISL_957340, EPI_ISL_957341, EPI_ISL_957342, EPI_ISL_957343, EPI_ISL_957344, EPI_ISL_957345, EPI_ISL_957346, EPI_ISL_957347, EPI_ISL_957348, EPI_ISL_957349, EPI_ISL_957350, EPI_ISL_957351, EPI_ISL_957352, EPI_ISL_957353, EPI_ISL_957354, EPI_ISL_957355, EPI_ISL_957356, EPI_ISL_957357, EPI_ISL_957358, EPI_ISL_957359, EPI_ISL_957360, EPI_ISL_957361, EPI_ISL_957362, EPI_ISL_957363, EPI_ISL_957364, EPI_ISL_957365, EPI_ISL_957366, EPI_ISL_957367, EPI_ISL_957368, EPI_ISL_957369, EPI_ISL_957370, EPI_ISL_957371, EPI_ISL_957372, EPI_ISL_957373,			

EPI_ISL_957374, EPI_ISL_957375, EPI_ISL_957376, EPI_ISL_957377, EPI_ISL_957378, EPI_ISL_957379, EPI_ISL_957380, EPI_ISL_957381, EPI_ISL_957382, EPI_ISL_957383, EPI_ISL_957384, EPI_ISL_957385, EPI_ISL_957386, EPI_ISL_957387, EPI_ISL_957388, EPI_ISL_957389, EPI_ISL_957390, EPI_ISL_957391, EPI_ISL_957392, EPI_ISL_957393, EPI_ISL_957394, EPI_ISL_957395, EPI_ISL_957396, EPI_ISL_957397, EPI_ISL_957398, EPI_ISL_957399, EPI_ISL_957400, EPI_ISL_957401, EPI_ISL_957402, EPI_ISL_957403, EPI_ISL_957404, EPI_ISL_957405, EPI_ISL_957406, EPI_ISL_957407, EPI_ISL_957408, EPI_ISL_957409, EPI_ISL_957410, EPI_ISL_957411, EPI_ISL_957412, EPI_ISL_957413, EPI_ISL_957414, EPI_ISL_957415, EPI_ISL_957416, EPI_ISL_957417, EPI_ISL_957418, EPI_ISL_957419, EPI_ISL_957420, EPI_ISL_957421, EPI_ISL_957422, EPI_ISL_957424, EPI_ISL_957425, EPI_ISL_957426, EPI_ISL_957427, EPI_ISL_957428, EPI_ISL_957429, EPI_ISL_957430, EPI_ISL_957431, EPI_ISL_957432, EPI_ISL_957433, EPI_ISL_957434, EPI_ISL_957435, EPI_ISL_957436, EPI_ISL_957437, EPI_ISL_957438, EPI_ISL_957439, EPI_ISL_957440, EPI_ISL_957441, EPI_ISL_957442, EPI_ISL_957443, EPI_ISL_957444, EPI_ISL_957445, EPI_ISL_957446, EPI_ISL_957447, EPI_ISL_957448, EPI_ISL_957449, EPI_ISL_957450, EPI_ISL_957451, EPI_ISL_957452, EPI_ISL_957453, EPI_ISL_957454, EPI_ISL_957455, EPI_ISL_957456, EPI_ISL_957457, EPI_ISL_957458, EPI_ISL_957459, EPI_ISL_957460, EPI_ISL_957461, EPI_ISL_957462, EPI_ISL_957463, EPI_ISL_957464, EPI_ISL_957465, EPI_ISL_957466, EPI_ISL_957467, EPI_ISL_957468, EPI_ISL_957469, EPI_ISL_957470, EPI_ISL_957471, EPI_ISL_957472, EPI_ISL_957473, EPI_ISL_957474, EPI_ISL_957475, EPI_ISL_957476, EPI_ISL_957477, EPI_ISL_957478, EPI_ISL_957479, EPI_ISL_957480, EPI_ISL_957481, EPI_ISL_957482, EPI_ISL_957483, EPI_ISL_957484, EPI_ISL_957485, EPI_ISL_957486, EPI_ISL_957487, EPI_ISL_957488, EPI_ISL_957489, EPI_ISL_957490, EPI_ISL_957491, EPI_ISL_957492, EPI_ISL_957493, EPI_ISL_957494, EPI_ISL_957495, EPI_ISL_957496, EPI_ISL_957497, EPI_ISL_957498, EPI_ISL_957499, EPI_ISL_957500, EPI_ISL_957501, EPI_ISL_957502, EPI_ISL_957503, EPI_ISL_957504, EPI_ISL_957505, EPI_ISL_957506, EPI_ISL_957507, EPI_ISL_957508, EPI_ISL_957509, EPI_ISL_957510, EPI_ISL_957511, EPI_ISL_957512, EPI_ISL_957513, EPI_ISL_957514, EPI_ISL_957515, EPI_ISL_957516, EPI_ISL_957517, EPI_ISL_957518, EPI_ISL_957519, EPI_ISL_957520, EPI_ISL_957521, EPI_ISL_957522, EPI_ISL_957523, EPI_ISL_957524, EPI_ISL_957525, EPI_ISL_957526, EPI_ISL_957527, EPI_ISL_957528, EPI_ISL_957529, EPI_ISL_957530, EPI_ISL_957531, EPI_ISL_957532, EPI_ISL_957533, EPI_ISL_957534, EPI_ISL_957535, EPI_ISL_957536, EPI_ISL_957537, EPI_ISL_957538, EPI_ISL_957539, EPI_ISL_957540, EPI_ISL_957541, EPI_ISL_957542, EPI_ISL_957543, EPI_ISL_957544, EPI_ISL_957545, EPI_ISL_957546, EPI_ISL_957547, EPI_ISL_957548, EPI_ISL_957549, EPI_ISL_957550, EPI_ISL_957551, EPI_ISL_957552, EPI_ISL_957553, EPI_ISL_957554, EPI_ISL_957555, EPI_ISL_957556, EPI_ISL_957557, EPI_ISL_957558, EPI_ISL_957559, EPI_ISL_957560, EPI_ISL_957561, EPI_ISL_957562, EPI_ISL_957563, EPI_ISL_957564, EPI_ISL_957565, EPI_ISL_957566, EPI_ISL_957567, EPI_ISL_957568, EPI_ISL_957569, EPI_ISL_957570, EPI_ISL_957571, EPI_ISL_957572, EPI_ISL_957573, EPI_ISL_957574, EPI_ISL_957575, EPI_ISL_957576, EPI_ISL_957577, EPI_ISL_957578, EPI_ISL_957579, EPI_ISL_957580, EPI_ISL_957581, EPI_ISL_957582, EPI_ISL_957583, EPI_ISL_957584, EPI_ISL_957585, EPI_ISL_957586, EPI_ISL_957587, EPI_ISL_957588, EPI_ISL_957589, EPI_ISL_957590, EPI_ISL_957591, EPI_ISL_957592, EPI_ISL_957593, EPI_ISL_957594, EPI_ISL_957595, EPI_ISL_957596, EPI_ISL_957597, EPI_ISL_957598, EPI_ISL_957599, EPI_ISL_957600, EPI_ISL_957601, EPI_ISL_957602, EPI_ISL_957603, EPI_ISL_957604, EPI_ISL_957605, EPI_ISL_957606, EPI_ISL_957607, EPI_ISL_957608, EPI_ISL_957609, EPI_ISL_957610, EPI_ISL_957611			
see above	Lighthouse Lab in Milton Keynes	Wellcome Sanger Institute for the COVID-19 Genomics UK (COG-UK) Consortium	The Lighthouse Lab in Milton Keynes and Alex Alderton, Roberto Amato, Sonia Goncalves, Ewan Harrison, David K. Jackson, Ian Johnston, Dominic Kwiatkowski, Cordelia Langford, John Sillitoe on behalf of the Wellcome Sanger Institute COVID-19 Surveillance Team
EPI_ISL_957612	Lighthouse Lab in Cambridge	Wellcome Sanger Institute for the COVID-19 Genomics UK (COG-UK) Consortium	Rob Howes, The Lighthouse Lab in Cambridge and Alex Alderton, Roberto Amato, Sonia Goncalves, Ewan Harrison, David K. Jackson, Ian Johnston, Dominic Kwiatkowski, Cordelia Langford, John Sillitoe on behalf of the Wellcome Sanger Institute COVID-19 Surveillance Team
EPI_ISL_957614	Lighthouse Lab in Milton Keynes	Wellcome Sanger Institute for the COVID-19 Genomics UK (COG-UK) Consortium	The Lighthouse Lab in Milton Keynes and Alex Alderton, Roberto Amato, Sonia Goncalves, Ewan Harrison, David K. Jackson, Ian Johnston, Dominic Kwiatkowski, Cordelia Langford, John Sillitoe on behalf of the Wellcome Sanger Institute COVID-19 Surveillance Team
EPI_ISL_957615, EPI_ISL_957617, EPI_ISL_957618	Lighthouse Lab in Cambridge	Wellcome Sanger Institute for the COVID-19 Genomics UK (COG-UK) Consortium	Rob Howes, The Lighthouse Lab in Cambridge and Alex Alderton, Roberto Amato, Sonia Goncalves, Ewan Harrison, David K. Jackson, Ian Johnston, Dominic Kwiatkowski, Cordelia Langford, John Sillitoe on behalf of the Wellcome Sanger Institute COVID-19 Surveillance Team
EPI_ISL_957619	Lighthouse Lab in Milton Keynes	Wellcome Sanger Institute for the COVID-19 Genomics UK (COG-UK) Consortium	The Lighthouse Lab in Milton Keynes and Alex Alderton, Roberto Amato, Sonia Goncalves, Ewan Harrison, David K. Jackson, Ian Johnston, Dominic Kwiatkowski, Cordelia Langford, John Sillitoe on behalf of the Wellcome Sanger Institute COVID-19 Surveillance Team
EPI_ISL_957622	Lighthouse Lab in Cambridge	Wellcome Sanger Institute for the COVID-19 Genomics UK (COG-UK) Consortium	Rob Howes, The Lighthouse Lab in Cambridge and Alex Alderton, Roberto Amato, Sonia Goncalves, Ewan Harrison, David K. Jackson, Ian Johnston, Dominic Kwiatkowski, Cordelia Langford, John Sillitoe on behalf of the Wellcome Sanger Institute COVID-19 Surveillance Team
EPI_ISL_957623	Lighthouse Lab in Milton Keynes	Wellcome Sanger Institute for the COVID-19 Genomics UK (COG-UK) Consortium	The Lighthouse Lab in Milton Keynes and Alex Alderton, Roberto Amato, Sonia Goncalves, Ewan Harrison, David K. Jackson, Ian Johnston, Dominic Kwiatkowski, Cordelia Langford, John Sillitoe on behalf of the Wellcome Sanger Institute COVID-19 Surveillance Team
EPI_ISL_957625, EPI_ISL_957626	Lighthouse Lab in Cambridge	Wellcome Sanger Institute for the COVID-19 Genomics UK (COG-UK) Consortium	Rob Howes, The Lighthouse Lab in Cambridge and Alex Alderton, Roberto Amato, Sonia Goncalves, Ewan Harrison, David K. Jackson, Ian Johnston, Dominic Kwiatkowski, Cordelia Langford, John Sillitoe on behalf of the Wellcome Sanger Institute COVID-19 Surveillance Team
EPI_ISL_957628, EPI_ISL_957629, EPI_ISL_957631, EPI_ISL_957632	Lighthouse Lab in Milton Keynes	Wellcome Sanger Institute for the COVID-19 Genomics UK (COG-UK) Consortium	The Lighthouse Lab in Milton Keynes and Alex Alderton, Roberto Amato, Sonia Goncalves, Ewan Harrison, David K. Jackson, Ian Johnston, Dominic Kwiatkowski, Cordelia Langford, John Sillitoe on behalf of the Wellcome Sanger Institute COVID-19 Surveillance Team
EPI_ISL_957633	Lighthouse Lab in Glasgow	Wellcome Sanger Institute for the COVID-19 Genomics UK (COG-UK) Consortium	Harper VanSteenhouse, Yumi Kasai, David Gray, Carol Clugston, Anna Dominiczak and Alex Alderton, Roberto Amato, Sonia Goncalves, Ewan Harrison, David K. Jackson, Ian Johnston, Dominic Kwiatkowski, Cordelia Langford, John Sillitoe on behalf of the Wellcome Sanger Institute COVID-19 Surveillance Team
EPI_ISL_957634, EPI_ISL_957636	Lighthouse Lab in Cambridge	Wellcome Sanger Institute for the COVID-19 Genomics UK (COG-UK) Consortium	Rob Howes, The Lighthouse Lab in Cambridge and Alex Alderton, Roberto Amato, Sonia Goncalves, Ewan Harrison, David K. Jackson, Ian Johnston, Dominic Kwiatkowski, Cordelia Langford, John Sillitoe on behalf of the Wellcome Sanger Institute COVID-19 Surveillance Team
EPI_ISL_957637	Lighthouse Lab in Glasgow	Wellcome Sanger Institute for the COVID-19 Genomics UK (COG-UK) Consortium	Harper VanSteenhouse, Yumi Kasai, David Gray, Carol Clugston, Anna Dominiczak and Alex Alderton, Roberto Amato, Sonia Goncalves, Ewan Harrison, David K. Jackson, Ian Johnston, Dominic Kwiatkowski, Cordelia Langford, John Sillitoe on behalf of the Wellcome Sanger Institute COVID-19 Surveillance Team
EPI_ISL_957638, EPI_ISL_957639, EPI_ISL_957640, EPI_ISL_957642, EPI_ISL_957644, EPI_ISL_957646, EPI_ISL_957647, EPI_ISL_957648	Lighthouse Lab in Cambridge	Wellcome Sanger Institute for the COVID-19 Genomics UK (COG-UK) Consortium	Rob Howes, The Lighthouse Lab in Cambridge and Alex Alderton, Roberto Amato, Sonia Goncalves, Ewan Harrison, David K. Jackson, Ian Johnston, Dominic Kwiatkowski, Cordelia Langford, John Sillitoe on behalf of the Wellcome Sanger Institute COVID-19 Surveillance Team
EPI_ISL_957649, EPI_ISL_957651, EPI_ISL_957653	Lighthouse Lab in Glasgow	Wellcome Sanger Institute for the COVID-19 Genomics UK (COG-UK) Consortium	Harper VanSteenhouse, Yumi Kasai, David Gray, Carol Clugston, Anna Dominiczak and Alex Alderton, Roberto Amato, Sonia Goncalves, Ewan Harrison, David K. Jackson, Ian Johnston, Dominic Kwiatkowski, Cordelia Langford, John Sillitoe on behalf of the Wellcome Sanger Institute COVID-19 Surveillance Team
EPI_ISL_957657, EPI_ISL_957658	Lighthouse Lab in Cambridge	Wellcome Sanger Institute for the COVID-19 Genomics UK (COG-UK) Consortium	Rob Howes, The Lighthouse Lab in Cambridge and Alex Alderton, Roberto Amato, Sonia Goncalves, Ewan Harrison, David K. Jackson, Ian Johnston, Dominic Kwiatkowski, Cordelia Langford, John Sillitoe on behalf of the Wellcome Sanger Institute COVID-19 Surveillance Team
EPI_ISL_957659	Lighthouse Lab in Milton Keynes	Wellcome Sanger Institute for the COVID-19 Genomics UK (COG-UK) Consortium	The Lighthouse Lab in Milton Keynes and Alex Alderton, Roberto Amato, Sonia Goncalves, Ewan Harrison, David K. Jackson, Ian Johnston, Dominic Kwiatkowski, Cordelia Langford, John Sillitoe on behalf of the Wellcome Sanger Institute COVID-19 Surveillance Team
EPI_ISL_957661	Lighthouse Lab in Cambridge	Wellcome Sanger Institute for the COVID-19 Genomics UK (COG-UK) Consortium	Rob Howes, The Lighthouse Lab in Cambridge and Alex Alderton, Roberto Amato, Sonia Goncalves, Ewan Harrison, David K. Jackson, Ian Johnston, Dominic Kwiatkowski, Cordelia Langford, John Sillitoe on behalf of the Wellcome Sanger Institute COVID-19 Surveillance Team
EPI_ISL_957662	Lighthouse Lab in Milton Keynes	Wellcome Sanger Institute for the COVID-19 Genomics UK (COG-UK) Consortium	The Lighthouse Lab in Milton Keynes and Alex Alderton, Roberto Amato, Sonia Goncalves, Ewan Harrison, David K. Jackson, Ian Johnston, Dominic Kwiatkowski, Cordelia Langford, John Sillitoe on behalf of the Wellcome Sanger Institute COVID-19 Surveillance Team
EPI_ISL_957663, EPI_ISL_957664	Lighthouse Lab in Glasgow	Wellcome Sanger Institute for the COVID-19 Genomics UK (COG-UK) Consortium	Harper VanSteenhouse, Yumi Kasai, David Gray, Carol Clugston, Anna Dominiczak and Alex Alderton, Roberto Amato, Sonia Goncalves, Ewan Harrison, David K. Jackson, Ian Johnston, Dominic Kwiatkowski, Cordelia Langford, John Sillitoe on behalf of the Wellcome Sanger Institute COVID-19 Surveillance Team
EPI_ISL_957665	Lighthouse Lab in Cambridge	Wellcome Sanger Institute for the COVID-19 Genomics UK (COG-UK) Consortium	Rob Howes, The Lighthouse Lab in Cambridge and Alex Alderton, Roberto Amato, Sonia Goncalves, Ewan Harrison, David K. Jackson, Ian Johnston, Dominic Kwiatkowski, Cordelia Langford, John Sillitoe on behalf of the Wellcome Sanger Institute COVID-19 Surveillance Team
EPI_ISL_957666	Lighthouse Lab in Glasgow	Wellcome Sanger Institute for the COVID-19 Genomics UK (COG-UK) Consortium	Harper VanSteenhouse, Yumi Kasai, David Gray, Carol Clugston, Anna Dominiczak and Alex Alderton, Roberto Amato, Sonia Goncalves, Ewan Harrison, David K. Jackson, Ian Johnston, Dominic Kwiatkowski, Cordelia Langford, John Sillitoe on behalf of the Wellcome Sanger Institute COVID-19 Surveillance Team
EPI_ISL_957667, EPI_ISL_957668	Lighthouse Lab in Milton Keynes	Wellcome Sanger Institute for the COVID-19 Genomics UK (COG-UK) Consortium	The Lighthouse Lab in Milton Keynes and Alex Alderton, Roberto Amato, Sonia Goncalves, Ewan Harrison, David K. Jackson, Ian Johnston, Dominic Kwiatkowski, Cordelia Langford, John Sillitoe on behalf of the Wellcome Sanger Institute COVID-19 Surveillance Team
EPI_ISL_957669	Lighthouse Lab in Glasgow	Wellcome Sanger Institute for the COVID-19 Genomics UK (COG-UK) Consortium	Harper VanSteenhouse, Yumi Kasai, David Gray, Carol Clugston, Anna Dominiczak and Alex Alderton, Roberto Amato, Sonia Goncalves, Ewan Harrison, David K. Jackson, Ian Johnston, Dominic Kwiatkowski, Cordelia Langford, John Sillitoe on behalf of the Wellcome Sanger Institute COVID-19 Surveillance Team
EPI_ISL_957671	Lighthouse Lab in Milton Keynes	Wellcome Sanger Institute for the COVID-19 Genomics UK (COG-UK) Consortium	The Lighthouse Lab in Milton Keynes and Alex Alderton, Roberto Amato, Sonia Goncalves, Ewan Harrison, David K. Jackson, Ian Johnston, Dominic Kwiatkowski, Cordelia Langford, John Sillitoe on behalf of the Wellcome Sanger Institute COVID-19 Surveillance Team
EPI_ISL_957673, EPI_ISL_957674	Lighthouse Lab in Glasgow	Wellcome Sanger Institute for the COVID-19 Genomics UK (COG-UK) Consortium	Harper VanSteenhouse, Yumi Kasai, David Gray, Carol Clugston, Anna Dominiczak and Alex Alderton, Roberto Amato, Sonia Goncalves, Ewan Harrison, David K. Jackson, Ian Johnston, Dominic Kwiatkowski, Cordelia Langford, John Sillitoe on behalf of the Wellcome Sanger Institute COVID-19 Surveillance Team
EPI_ISL_957676	Lighthouse Lab in Cambridge	Wellcome Sanger Institute for the COVID-19 Genomics UK	Rob Howes, The Lighthouse Lab in Cambridge and Alex Alderton, Roberto Amato, Sonia Goncalves, Ewan Harrison, David K. Jackson, Ian Johnston,

		(COG-UK) Consortium	Dominic Kwiatkowski, Cordelia Langford, John Sillitoe on behalf of the Wellcome Sanger Institute COVID-19 Surveillance Team
EPI_ISL_957678, EPI_ISL_957679, EPI_ISL_957681, EPI_ISL_957682	Lighthouse Lab in Glasgow	Wellcome Sanger Institute for the COVID-19 Genomics UK (COG-UK) Consortium	Harper VanSteenhouse, Yumi Kasai, David Gray, Carol Clugston, Anna Dominiczak and Alex Alderton, Roberto Amato, Sonia Goncalves, Ewan Harrison, David K. Jackson, Ian Johnston, Dominic Kwiatkowski, Cordelia Langford, John Sillitoe on behalf of the Wellcome Sanger Institute COVID-19 Surveillance Team
EPI_ISL_957684, EPI_ISL_957685	Lighthouse Lab in Milton Keynes	Wellcome Sanger Institute for the COVID-19 Genomics UK (COG-UK) Consortium	The Lighthouse Lab in Milton Keynes and Alex Alderton, Roberto Amato, Sonia Goncalves, Ewan Harrison, David K. Jackson, Ian Johnston, Dominic Kwiatkowski, Cordelia Langford, John Sillitoe on behalf of the Wellcome Sanger Institute COVID-19 Surveillance Team
EPI_ISL_957688, EPI_ISL_957689	Lighthouse Lab in Cambridge	Wellcome Sanger Institute for the COVID-19 Genomics UK (COG-UK) Consortium	Rob Howes, The Lighthouse Lab in Cambridge and Alex Alderton, Roberto Amato, Sonia Goncalves, Ewan Harrison, David K. Jackson, Ian Johnston, Dominic Kwiatkowski, Cordelia Langford, John Sillitoe on behalf of the Wellcome Sanger Institute COVID-19 Surveillance Team
EPI_ISL_957690, EPI_ISL_957692	Lighthouse Lab in Milton Keynes	Wellcome Sanger Institute for the COVID-19 Genomics UK (COG-UK) Consortium	The Lighthouse Lab in Milton Keynes and Alex Alderton, Roberto Amato, Sonia Goncalves, Ewan Harrison, David K. Jackson, Ian Johnston, Dominic Kwiatkowski, Cordelia Langford, John Sillitoe on behalf of the Wellcome Sanger Institute COVID-19 Surveillance Team
EPI_ISL_957693, EPI_ISL_957694	Lighthouse Lab in Glasgow	Wellcome Sanger Institute for the COVID-19 Genomics UK (COG-UK) Consortium	Harper VanSteenhouse, Yumi Kasai, David Gray, Carol Clugston, Anna Dominiczak and Alex Alderton, Roberto Amato, Sonia Goncalves, Ewan Harrison, David K. Jackson, Ian Johnston, Dominic Kwiatkowski, Cordelia Langford, John Sillitoe on behalf of the Wellcome Sanger Institute COVID-19 Surveillance Team
EPI_ISL_957695	Lighthouse Lab in Milton Keynes	Wellcome Sanger Institute for the COVID-19 Genomics UK (COG-UK) Consortium	The Lighthouse Lab in Milton Keynes and Alex Alderton, Roberto Amato, Sonia Goncalves, Ewan Harrison, David K. Jackson, Ian Johnston, Dominic Kwiatkowski, Cordelia Langford, John Sillitoe on behalf of the Wellcome Sanger Institute COVID-19 Surveillance Team
EPI_ISL_957696	Lighthouse Lab in Cambridge	Wellcome Sanger Institute for the COVID-19 Genomics UK (COG-UK) Consortium	Rob Howes, The Lighthouse Lab in Cambridge and Alex Alderton, Roberto Amato, Sonia Goncalves, Ewan Harrison, David K. Jackson, Ian Johnston, Dominic Kwiatkowski, Cordelia Langford, John Sillitoe on behalf of the Wellcome Sanger Institute COVID-19 Surveillance Team
EPI_ISL_957697	Lighthouse Lab in Glasgow	Wellcome Sanger Institute for the COVID-19 Genomics UK (COG-UK) Consortium	Harper VanSteenhouse, Yumi Kasai, David Gray, Carol Clugston, Anna Dominiczak and Alex Alderton, Roberto Amato, Sonia Goncalves, Ewan Harrison, David K. Jackson, Ian Johnston, Dominic Kwiatkowski, Cordelia Langford, John Sillitoe on behalf of the Wellcome Sanger Institute COVID-19 Surveillance Team
EPI_ISL_957699	Lighthouse Lab in Milton Keynes	Wellcome Sanger Institute for the COVID-19 Genomics UK (COG-UK) Consortium	The Lighthouse Lab in Milton Keynes and Alex Alderton, Roberto Amato, Sonia Goncalves, Ewan Harrison, David K. Jackson, Ian Johnston, Dominic Kwiatkowski, Cordelia Langford, John Sillitoe on behalf of the Wellcome Sanger Institute COVID-19 Surveillance Team
EPI_ISL_957702, EPI_ISL_957703, EPI_ISL_957704	Lighthouse Lab in Cambridge	Wellcome Sanger Institute for the COVID-19 Genomics UK (COG-UK) Consortium	Rob Howes, The Lighthouse Lab in Cambridge and Alex Alderton, Roberto Amato, Sonia Goncalves, Ewan Harrison, David K. Jackson, Ian Johnston, Dominic Kwiatkowski, Cordelia Langford, John Sillitoe on behalf of the Wellcome Sanger Institute COVID-19 Surveillance Team
EPI_ISL_957705	Lighthouse Lab in Milton Keynes	Wellcome Sanger Institute for the COVID-19 Genomics UK (COG-UK) Consortium	The Lighthouse Lab in Milton Keynes and Alex Alderton, Roberto Amato, Sonia Goncalves, Ewan Harrison, David K. Jackson, Ian Johnston, Dominic Kwiatkowski, Cordelia Langford, John Sillitoe on behalf of the Wellcome Sanger Institute COVID-19 Surveillance Team
EPI_ISL_957706	Lighthouse Lab in Cambridge	Wellcome Sanger Institute for the COVID-19 Genomics UK (COG-UK) Consortium	Rob Howes, The Lighthouse Lab in Cambridge and Alex Alderton, Roberto Amato, Sonia Goncalves, Ewan Harrison, David K. Jackson, Ian Johnston, Dominic Kwiatkowski, Cordelia Langford, John Sillitoe on behalf of the Wellcome Sanger Institute COVID-19 Surveillance Team
EPI_ISL_957708	Lighthouse Lab in Glasgow	Wellcome Sanger Institute for the COVID-19 Genomics UK (COG-UK) Consortium	Harper VanSteenhouse, Yumi Kasai, David Gray, Carol Clugston, Anna Dominiczak and Alex Alderton, Roberto Amato, Sonia Goncalves, Ewan Harrison, David K. Jackson, Ian Johnston, Dominic Kwiatkowski, Cordelia Langford, John Sillitoe on behalf of the Wellcome Sanger Institute COVID-19 Surveillance Team
EPI_ISL_957709	Lighthouse Lab in Cambridge	Wellcome Sanger Institute for the COVID-19 Genomics UK (COG-UK) Consortium	Rob Howes, The Lighthouse Lab in Cambridge and Alex Alderton, Roberto Amato, Sonia Goncalves, Ewan Harrison, David K. Jackson, Ian Johnston, Dominic Kwiatkowski, Cordelia Langford, John Sillitoe on behalf of the Wellcome Sanger Institute COVID-19 Surveillance Team
EPI_ISL_957710, EPI_ISL_957711, EPI_ISL_957712	Lighthouse Lab in Milton Keynes	Wellcome Sanger Institute for the COVID-19 Genomics UK (COG-UK) Consortium	The Lighthouse Lab in Milton Keynes and Alex Alderton, Roberto Amato, Sonia Goncalves, Ewan Harrison, David K. Jackson, Ian Johnston, Dominic Kwiatkowski, Cordelia Langford, John Sillitoe on behalf of the Wellcome Sanger Institute COVID-19 Surveillance Team
EPI_ISL_957713	Lighthouse Lab in Glasgow	Wellcome Sanger Institute for the COVID-19 Genomics UK (COG-UK) Consortium	Harper VanSteenhouse, Yumi Kasai, David Gray, Carol Clugston, Anna Dominiczak and Alex Alderton, Roberto Amato, Sonia Goncalves, Ewan Harrison, David K. Jackson, Ian Johnston, Dominic Kwiatkowski, Cordelia Langford, John Sillitoe on behalf of the Wellcome Sanger Institute COVID-19 Surveillance Team
EPI_ISL_957714	Lighthouse Lab in Cambridge	Wellcome Sanger Institute for the COVID-19 Genomics UK (COG-UK) Consortium	Rob Howes, The Lighthouse Lab in Cambridge and Alex Alderton, Roberto Amato, Sonia Goncalves, Ewan Harrison, David K. Jackson, Ian Johnston, Dominic Kwiatkowski, Cordelia Langford, John Sillitoe on behalf of the Wellcome Sanger Institute COVID-19 Surveillance Team
EPI_ISL_957715	Lighthouse Lab in Glasgow	Wellcome Sanger Institute for the COVID-19 Genomics UK (COG-UK) Consortium	Harper VanSteenhouse, Yumi Kasai, David Gray, Carol Clugston, Anna Dominiczak and Alex Alderton, Roberto Amato, Sonia Goncalves, Ewan Harrison, David K. Jackson, Ian Johnston, Dominic Kwiatkowski, Cordelia Langford, John Sillitoe on behalf of the Wellcome Sanger Institute COVID-19 Surveillance Team
EPI_ISL_957716	Lighthouse Lab in Cambridge	Wellcome Sanger Institute for the COVID-19 Genomics UK (COG-UK) Consortium	Rob Howes, The Lighthouse Lab in Cambridge and Alex Alderton, Roberto Amato, Sonia Goncalves, Ewan Harrison, David K. Jackson, Ian Johnston, Dominic Kwiatkowski, Cordelia Langford, John Sillitoe on behalf of the Wellcome Sanger Institute COVID-19 Surveillance Team
EPI_ISL_957717, EPI_ISL_957718	Lighthouse Lab in Glasgow	Wellcome Sanger Institute for the COVID-19 Genomics UK (COG-UK) Consortium	Harper VanSteenhouse, Yumi Kasai, David Gray, Carol Clugston, Anna Dominiczak and Alex Alderton, Roberto Amato, Sonia Goncalves, Ewan Harrison, David K. Jackson, Ian Johnston, Dominic Kwiatkowski, Cordelia Langford, John Sillitoe on behalf of the Wellcome Sanger Institute COVID-19 Surveillance Team
EPI_ISL_957719	Lighthouse Lab in Cambridge	Wellcome Sanger Institute for the COVID-19 Genomics UK (COG-UK) Consortium	Rob Howes, The Lighthouse Lab in Cambridge and Alex Alderton, Roberto Amato, Sonia Goncalves, Ewan Harrison, David K. Jackson, Ian Johnston, Dominic Kwiatkowski, Cordelia Langford, John Sillitoe on behalf of the Wellcome Sanger Institute COVID-19 Surveillance Team
EPI_ISL_957720	Lighthouse Lab in Glasgow	Wellcome Sanger Institute for the COVID-19 Genomics UK (COG-UK) Consortium	Harper VanSteenhouse, Yumi Kasai, David Gray, Carol Clugston, Anna Dominiczak and Alex Alderton, Roberto Amato, Sonia Goncalves, Ewan Harrison, David K. Jackson, Ian Johnston, Dominic Kwiatkowski, Cordelia Langford, John Sillitoe on behalf of the Wellcome Sanger Institute COVID-19 Surveillance Team
EPI_ISL_957724	Lighthouse Lab in Milton Keynes	Wellcome Sanger Institute for the COVID-19 Genomics UK (COG-UK) Consortium	The Lighthouse Lab in Milton Keynes and Alex Alderton, Roberto Amato, Sonia Goncalves, Ewan Harrison, David K. Jackson, Ian Johnston, Dominic Kwiatkowski, Cordelia Langford, John Sillitoe on behalf of the Wellcome Sanger Institute COVID-19 Surveillance Team
EPI_ISL_957725	Lighthouse Lab in Glasgow	Wellcome Sanger Institute for the COVID-19 Genomics UK (COG-UK) Consortium	Harper VanSteenhouse, Yumi Kasai, David Gray, Carol Clugston, Anna Dominiczak and Alex Alderton, Roberto Amato, Sonia Goncalves, Ewan Harrison, David K. Jackson, Ian Johnston, Dominic Kwiatkowski, Cordelia Langford, John Sillitoe on behalf of the Wellcome Sanger Institute COVID-19 Surveillance Team
EPI_ISL_957727	Lighthouse Lab in Milton Keynes	Wellcome Sanger Institute for the COVID-19 Genomics UK (COG-UK) Consortium	The Lighthouse Lab in Milton Keynes and Alex Alderton, Roberto Amato, Sonia Goncalves, Ewan Harrison, David K. Jackson, Ian Johnston, Dominic Kwiatkowski, Cordelia Langford, John Sillitoe on behalf of the Wellcome Sanger Institute COVID-19 Surveillance Team
EPI_ISL_957728	Lighthouse Lab in Cambridge	Wellcome Sanger Institute for the COVID-19 Genomics UK (COG-UK) Consortium	Rob Howes, The Lighthouse Lab in Cambridge and Alex Alderton, Roberto Amato, Sonia Goncalves, Ewan Harrison, David K. Jackson, Ian Johnston, Dominic Kwiatkowski, Cordelia Langford, John Sillitoe on behalf of the Wellcome Sanger Institute COVID-19 Surveillance Team
EPI_ISL_957729, EPI_ISL_957730	Lighthouse Lab in Glasgow	Wellcome Sanger Institute for the COVID-19 Genomics UK (COG-UK) Consortium	Harper VanSteenhouse, Yumi Kasai, David Gray, Carol Clugston, Anna Dominiczak and Alex Alderton, Roberto Amato, Sonia Goncalves, Ewan Harrison, David K. Jackson, Ian Johnston, Dominic Kwiatkowski, Cordelia Langford, John Sillitoe on behalf of the Wellcome Sanger Institute COVID-19 Surveillance Team
EPI_ISL_957731	Lighthouse Lab in Cambridge	Wellcome Sanger Institute for the COVID-19 Genomics UK (COG-UK) Consortium	Rob Howes, The Lighthouse Lab in Cambridge and Alex Alderton, Roberto Amato, Sonia Goncalves, Ewan Harrison, David K. Jackson, Ian Johnston, Dominic Kwiatkowski, Cordelia Langford, John Sillitoe on behalf of the Wellcome Sanger Institute COVID-19 Surveillance Team
EPI_ISL_957732	Lighthouse Lab in Glasgow	Wellcome Sanger Institute for the COVID-19 Genomics UK (COG-UK) Consortium	Harper VanSteenhouse, Yumi Kasai, David Gray, Carol Clugston, Anna Dominiczak and Alex Alderton, Roberto Amato, Sonia Goncalves, Ewan Harrison, David K. Jackson, Ian Johnston, Dominic Kwiatkowski, Cordelia Langford, John Sillitoe on behalf of the Wellcome Sanger Institute COVID-19 Surveillance Team
EPI_ISL_957733, EPI_ISL_957734	Lighthouse Lab in Cambridge	Wellcome Sanger Institute for the COVID-19 Genomics UK (COG-UK) Consortium	Rob Howes, The Lighthouse Lab in Cambridge and Alex Alderton, Roberto Amato, Sonia Goncalves, Ewan Harrison, David K. Jackson, Ian Johnston, Dominic Kwiatkowski, Cordelia Langford, John Sillitoe on behalf of the Wellcome Sanger Institute COVID-19 Surveillance Team

EPI_ISL_957736	Lighthouse Lab in Glasgow	Wellcome Sanger Institute for the COVID-19 Genomics UK (COG-UK) Consortium	Harper VanSteenhouse, Yumi Kasai, David Gray, Carol Clugston, Anna Dominiczak and Alex Alderton, Roberto Amato, Sonia Goncalves, Ewan Harrison, David K. Jackson, Ian Johnston, Dominic Kwiatkowski, Cordelia Langford, John Sillitoe on behalf of the Wellcome Sanger Institute COVID-19 Surveillance Team
EPI_ISL_957737, EPI_ISL_957738, EPI_ISL_957739, EPI_ISL_957742	Lighthouse Lab in Cambridge	Wellcome Sanger Institute for the COVID-19 Genomics UK (COG-UK) Consortium	Rob Howes, The Lighthouse Lab in Cambridge and Alex Alderton, Roberto Amato, Sonia Goncalves, Ewan Harrison, David K. Jackson, Ian Johnston, Dominic Kwiatkowski, Cordelia Langford, John Sillitoe on behalf of the Wellcome Sanger Institute COVID-19 Surveillance Team
EPI_ISL_957743	Lighthouse Lab in Glasgow	Wellcome Sanger Institute for the COVID-19 Genomics UK (COG-UK) Consortium	Harper VanSteenhouse, Yumi Kasai, David Gray, Carol Clugston, Anna Dominiczak and Alex Alderton, Roberto Amato, Sonia Goncalves, Ewan Harrison, David K. Jackson, Ian Johnston, Dominic Kwiatkowski, Cordelia Langford, John Sillitoe on behalf of the Wellcome Sanger Institute COVID-19 Surveillance Team
EPI_ISL_957744	Lighthouse Lab in Milton Keynes	Wellcome Sanger Institute for the COVID-19 Genomics UK (COG-UK) Consortium	The Lighthouse Lab in Milton Keynes and Alex Alderton, Roberto Amato, Sonia Goncalves, Ewan Harrison, David K. Jackson, Ian Johnston, Dominic Kwiatkowski, Cordelia Langford, John Sillitoe on behalf of the Wellcome Sanger Institute COVID-19 Surveillance Team
EPI_ISL_957746	Lighthouse Lab in Cambridge	Wellcome Sanger Institute for the COVID-19 Genomics UK (COG-UK) Consortium	Rob Howes, The Lighthouse Lab in Cambridge and Alex Alderton, Roberto Amato, Sonia Goncalves, Ewan Harrison, David K. Jackson, Ian Johnston, Dominic Kwiatkowski, Cordelia Langford, John Sillitoe on behalf of the Wellcome Sanger Institute COVID-19 Surveillance Team
EPI_ISL_957749, EPI_ISL_957750	Lighthouse Lab in Glasgow	Wellcome Sanger Institute for the COVID-19 Genomics UK (COG-UK) Consortium	Harper VanSteenhouse, Yumi Kasai, David Gray, Carol Clugston, Anna Dominiczak and Alex Alderton, Roberto Amato, Sonia Goncalves, Ewan Harrison, David K. Jackson, Ian Johnston, Dominic Kwiatkowski, Cordelia Langford, John Sillitoe on behalf of the Wellcome Sanger Institute COVID-19 Surveillance Team
EPI_ISL_957751, EPI_ISL_957752, EPI_ISL_957754	Lighthouse Lab in Cambridge	Wellcome Sanger Institute for the COVID-19 Genomics UK (COG-UK) Consortium	Rob Howes, The Lighthouse Lab in Cambridge and Alex Alderton, Roberto Amato, Sonia Goncalves, Ewan Harrison, David K. Jackson, Ian Johnston, Dominic Kwiatkowski, Cordelia Langford, John Sillitoe on behalf of the Wellcome Sanger Institute COVID-19 Surveillance Team
EPI_ISL_957756, EPI_ISL_957758	Lighthouse Lab in Glasgow	Wellcome Sanger Institute for the COVID-19 Genomics UK (COG-UK) Consortium	Harper VanSteenhouse, Yumi Kasai, David Gray, Carol Clugston, Anna Dominiczak and Alex Alderton, Roberto Amato, Sonia Goncalves, Ewan Harrison, David K. Jackson, Ian Johnston, Dominic Kwiatkowski, Cordelia Langford, John Sillitoe on behalf of the Wellcome Sanger Institute COVID-19 Surveillance Team
EPI_ISL_957760, EPI_ISL_957761, EPI_ISL_957762	Lighthouse Lab in Milton Keynes	Wellcome Sanger Institute for the COVID-19 Genomics UK (COG-UK) Consortium	The Lighthouse Lab in Milton Keynes and Alex Alderton, Roberto Amato, Sonia Goncalves, Ewan Harrison, David K. Jackson, Ian Johnston, Dominic Kwiatkowski, Cordelia Langford, John Sillitoe on behalf of the Wellcome Sanger Institute COVID-19 Surveillance Team
EPI_ISL_957763, EPI_ISL_957764	Lighthouse Lab in Cambridge	Wellcome Sanger Institute for the COVID-19 Genomics UK (COG-UK) Consortium	Rob Howes, The Lighthouse Lab in Cambridge and Alex Alderton, Roberto Amato, Sonia Goncalves, Ewan Harrison, David K. Jackson, Ian Johnston, Dominic Kwiatkowski, Cordelia Langford, John Sillitoe on behalf of the Wellcome Sanger Institute COVID-19 Surveillance Team
EPI_ISL_957765	Lighthouse Lab in Glasgow	Wellcome Sanger Institute for the COVID-19 Genomics UK (COG-UK) Consortium	Harper VanSteenhouse, Yumi Kasai, David Gray, Carol Clugston, Anna Dominiczak and Alex Alderton, Roberto Amato, Sonia Goncalves, Ewan Harrison, David K. Jackson, Ian Johnston, Dominic Kwiatkowski, Cordelia Langford, John Sillitoe on behalf of the Wellcome Sanger Institute COVID-19 Surveillance Team
EPI_ISL_957767	Lighthouse Lab in Milton Keynes	Wellcome Sanger Institute for the COVID-19 Genomics UK (COG-UK) Consortium	The Lighthouse Lab in Milton Keynes and Alex Alderton, Roberto Amato, Sonia Goncalves, Ewan Harrison, David K. Jackson, Ian Johnston, Dominic Kwiatkowski, Cordelia Langford, John Sillitoe on behalf of the Wellcome Sanger Institute COVID-19 Surveillance Team
EPI_ISL_957768, EPI_ISL_957770, EPI_ISL_957773, EPI_ISL_957774	Lighthouse Lab in Glasgow	Wellcome Sanger Institute for the COVID-19 Genomics UK (COG-UK) Consortium	Harper VanSteenhouse, Yumi Kasai, David Gray, Carol Clugston, Anna Dominiczak and Alex Alderton, Roberto Amato, Sonia Goncalves, Ewan Harrison, David K. Jackson, Ian Johnston, Dominic Kwiatkowski, Cordelia Langford, John Sillitoe on behalf of the Wellcome Sanger Institute COVID-19 Surveillance Team
EPI_ISL_957775	Lighthouse Lab in Milton Keynes	Wellcome Sanger Institute for the COVID-19 Genomics UK (COG-UK) Consortium	The Lighthouse Lab in Milton Keynes and Alex Alderton, Roberto Amato, Sonia Goncalves, Ewan Harrison, David K. Jackson, Ian Johnston, Dominic Kwiatkowski, Cordelia Langford, John Sillitoe on behalf of the Wellcome Sanger Institute COVID-19 Surveillance Team
EPI_ISL_957776	Lighthouse Lab in Cambridge	Wellcome Sanger Institute for the COVID-19 Genomics UK (COG-UK) Consortium	Rob Howes, The Lighthouse Lab in Cambridge and Alex Alderton, Roberto Amato, Sonia Goncalves, Ewan Harrison, David K. Jackson, Ian Johnston, Dominic Kwiatkowski, Cordelia Langford, John Sillitoe on behalf of the Wellcome Sanger Institute COVID-19 Surveillance Team
EPI_ISL_957777	Lighthouse Lab in Milton Keynes	Wellcome Sanger Institute for the COVID-19 Genomics UK (COG-UK) Consortium	The Lighthouse Lab in Milton Keynes and Alex Alderton, Roberto Amato, Sonia Goncalves, Ewan Harrison, David K. Jackson, Ian Johnston, Dominic Kwiatkowski, Cordelia Langford, John Sillitoe on behalf of the Wellcome Sanger Institute COVID-19 Surveillance Team
EPI_ISL_957778	Lighthouse Lab in Glasgow	Wellcome Sanger Institute for the COVID-19 Genomics UK (COG-UK) Consortium	Harper VanSteenhouse, Yumi Kasai, David Gray, Carol Clugston, Anna Dominiczak and Alex Alderton, Roberto Amato, Sonia Goncalves, Ewan Harrison, David K. Jackson, Ian Johnston, Dominic Kwiatkowski, Cordelia Langford, John Sillitoe on behalf of the Wellcome Sanger Institute COVID-19 Surveillance Team
EPI_ISL_957779, EPI_ISL_957781, EPI_ISL_957782, EPI_ISL_957784	Lighthouse Lab in Cambridge	Wellcome Sanger Institute for the COVID-19 Genomics UK (COG-UK) Consortium	Rob Howes, The Lighthouse Lab in Cambridge and Alex Alderton, Roberto Amato, Sonia Goncalves, Ewan Harrison, David K. Jackson, Ian Johnston, Dominic Kwiatkowski, Cordelia Langford, John Sillitoe on behalf of the Wellcome Sanger Institute COVID-19 Surveillance Team
EPI_ISL_957785	Lighthouse Lab in Glasgow	Wellcome Sanger Institute for the COVID-19 Genomics UK (COG-UK) Consortium	Harper VanSteenhouse, Yumi Kasai, David Gray, Carol Clugston, Anna Dominiczak and Alex Alderton, Roberto Amato, Sonia Goncalves, Ewan Harrison, David K. Jackson, Ian Johnston, Dominic Kwiatkowski, Cordelia Langford, John Sillitoe on behalf of the Wellcome Sanger Institute COVID-19 Surveillance Team
EPI_ISL_957786	Lighthouse Lab in Milton Keynes	Wellcome Sanger Institute for the COVID-19 Genomics UK (COG-UK) Consortium	The Lighthouse Lab in Milton Keynes and Alex Alderton, Roberto Amato, Sonia Goncalves, Ewan Harrison, David K. Jackson, Ian Johnston, Dominic Kwiatkowski, Cordelia Langford, John Sillitoe on behalf of the Wellcome Sanger Institute COVID-19 Surveillance Team
EPI_ISL_957788, EPI_ISL_957789	Lighthouse Lab in Cambridge	Wellcome Sanger Institute for the COVID-19 Genomics UK (COG-UK) Consortium	Rob Howes, The Lighthouse Lab in Cambridge and Alex Alderton, Roberto Amato, Sonia Goncalves, Ewan Harrison, David K. Jackson, Ian Johnston, Dominic Kwiatkowski, Cordelia Langford, John Sillitoe on behalf of the Wellcome Sanger Institute COVID-19 Surveillance Team
EPI_ISL_957790, EPI_ISL_957791	Lighthouse Lab in Milton Keynes	Wellcome Sanger Institute for the COVID-19 Genomics UK (COG-UK) Consortium	The Lighthouse Lab in Milton Keynes and Alex Alderton, Roberto Amato, Sonia Goncalves, Ewan Harrison, David K. Jackson, Ian Johnston, Dominic Kwiatkowski, Cordelia Langford, John Sillitoe on behalf of the Wellcome Sanger Institute COVID-19 Surveillance Team
EPI_ISL_957792, EPI_ISL_957793, EPI_ISL_957794	Lighthouse Lab in Glasgow	Wellcome Sanger Institute for the COVID-19 Genomics UK (COG-UK) Consortium	Harper VanSteenhouse, Yumi Kasai, David Gray, Carol Clugston, Anna Dominiczak and Alex Alderton, Roberto Amato, Sonia Goncalves, Ewan Harrison, David K. Jackson, Ian Johnston, Dominic Kwiatkowski, Cordelia Langford, John Sillitoe on behalf of the Wellcome Sanger Institute COVID-19 Surveillance Team
EPI_ISL_957795	Lighthouse Lab in Milton Keynes	Wellcome Sanger Institute for the COVID-19 Genomics UK (COG-UK) Consortium	The Lighthouse Lab in Milton Keynes and Alex Alderton, Roberto Amato, Sonia Goncalves, Ewan Harrison, David K. Jackson, Ian Johnston, Dominic Kwiatkowski, Cordelia Langford, John Sillitoe on behalf of the Wellcome Sanger Institute COVID-19 Surveillance Team
EPI_ISL_957796	Lighthouse Lab in Glasgow	Wellcome Sanger Institute for the COVID-19 Genomics UK (COG-UK) Consortium	Harper VanSteenhouse, Yumi Kasai, David Gray, Carol Clugston, Anna Dominiczak and Alex Alderton, Roberto Amato, Sonia Goncalves, Ewan Harrison, David K. Jackson, Ian Johnston, Dominic Kwiatkowski, Cordelia Langford, John Sillitoe on behalf of the Wellcome Sanger Institute COVID-19 Surveillance Team
EPI_ISL_957798	Lighthouse Lab in Cambridge	Wellcome Sanger Institute for the COVID-19 Genomics UK (COG-UK) Consortium	Rob Howes, The Lighthouse Lab in Cambridge and Alex Alderton, Roberto Amato, Sonia Goncalves, Ewan Harrison, David K. Jackson, Ian Johnston, Dominic Kwiatkowski, Cordelia Langford, John Sillitoe on behalf of the Wellcome Sanger Institute COVID-19 Surveillance Team
EPI_ISL_957800, EPI_ISL_957801	Lighthouse Lab in Milton Keynes	Wellcome Sanger Institute for the COVID-19 Genomics UK (COG-UK) Consortium	The Lighthouse Lab in Milton Keynes and Alex Alderton, Roberto Amato, Sonia Goncalves, Ewan Harrison, David K. Jackson, Ian Johnston, Dominic Kwiatkowski, Cordelia Langford, John Sillitoe on behalf of the Wellcome Sanger Institute COVID-19 Surveillance Team
EPI_ISL_957802	Lighthouse Lab in Glasgow	Wellcome Sanger Institute for the COVID-19 Genomics UK (COG-UK) Consortium	Harper VanSteenhouse, Yumi Kasai, David Gray, Carol Clugston, Anna Dominiczak and Alex Alderton, Roberto Amato, Sonia Goncalves, Ewan Harrison, David K. Jackson, Ian Johnston, Dominic Kwiatkowski, Cordelia Langford, John Sillitoe on behalf of the Wellcome Sanger Institute COVID-19 Surveillance Team
EPI_ISL_957803, EPI_ISL_957804	Lighthouse Lab in Milton Keynes	Wellcome Sanger Institute for the COVID-19 Genomics UK (COG-UK) Consortium	The Lighthouse Lab in Milton Keynes and Alex Alderton, Roberto Amato, Sonia Goncalves, Ewan Harrison, David K. Jackson, Ian Johnston, Dominic Kwiatkowski, Cordelia Langford, John Sillitoe on behalf of the Wellcome Sanger Institute COVID-19 Surveillance Team
EPI_ISL_957805	Lighthouse Lab in Cambridge	Wellcome Sanger Institute for the COVID-19 Genomics UK (COG-UK) Consortium	Rob Howes, The Lighthouse Lab in Cambridge and Alex Alderton, Roberto Amato, Sonia Goncalves, Ewan Harrison, David K. Jackson, Ian Johnston, Dominic Kwiatkowski, Cordelia Langford, John Sillitoe on behalf of the Wellcome Sanger Institute COVID-19 Surveillance Team
EPI_ISL_957806	Lighthouse Lab in Glasgow	Wellcome Sanger Institute for the COVID-19 Genomics UK (COG-UK) Consortium	Harper VanSteenhouse, Yumi Kasai, David Gray, Carol Clugston, Anna Dominiczak and Alex Alderton, Roberto Amato, Sonia Goncalves, Ewan Harrison, David K. Jackson, Ian Johnston, Dominic Kwiatkowski, Cordelia Langford, John Sillitoe on behalf of the Wellcome Sanger Institute COVID-19 Surveillance

			Team
EPI_ISL_957807, EPI_ISL_957808, EPI_ISL_957809	Lighthouse Lab in Cambridge	Wellcome Sanger Institute for the COVID-19 Genomics UK (COG-UK) Consortium	Rob Howes, The Lighthouse Lab in Cambridge and Alex Alderton, Roberto Amato, Sonia Goncalves, Ewan Harrison, David K. Jackson, Ian Johnston, Dominic Kwiatkowski, Cordelia Langford, John Sillitoe on behalf of the Wellcome Sanger Institute COVID-19 Surveillance Team
EPI_ISL_957810	Lighthouse Lab in Milton Keynes	Wellcome Sanger Institute for the COVID-19 Genomics UK (COG-UK) Consortium	The Lighthouse Lab in Milton Keynes and Alex Alderton, Roberto Amato, Sonia Goncalves, Ewan Harrison, David K. Jackson, Ian Johnston, Dominic Kwiatkowski, Cordelia Langford, John Sillitoe on behalf of the Wellcome Sanger Institute COVID-19 Surveillance Team
EPI_ISL_957811, EPI_ISL_957812	Lighthouse Lab in Cambridge	Wellcome Sanger Institute for the COVID-19 Genomics UK (COG-UK) Consortium	Rob Howes, The Lighthouse Lab in Cambridge and Alex Alderton, Roberto Amato, Sonia Goncalves, Ewan Harrison, David K. Jackson, Ian Johnston, Dominic Kwiatkowski, Cordelia Langford, John Sillitoe on behalf of the Wellcome Sanger Institute COVID-19 Surveillance Team
EPI_ISL_957814	Lighthouse Lab in Glasgow	Wellcome Sanger Institute for the COVID-19 Genomics UK (COG-UK) Consortium	Harper VanSteenhouse, Yumi Kasai, David Gray, Carol Clugston, Anna Dominiczak and Alex Alderton, Roberto Amato, Sonia Goncalves, Ewan Harrison, David K. Jackson, Ian Johnston, Dominic Kwiatkowski, Cordelia Langford, John Sillitoe on behalf of the Wellcome Sanger Institute COVID-19 Surveillance Team
EPI_ISL_957815	Lighthouse Lab in Cambridge	Wellcome Sanger Institute for the COVID-19 Genomics UK (COG-UK) Consortium	Rob Howes, The Lighthouse Lab in Cambridge and Alex Alderton, Roberto Amato, Sonia Goncalves, Ewan Harrison, David K. Jackson, Ian Johnston, Dominic Kwiatkowski, Cordelia Langford, John Sillitoe on behalf of the Wellcome Sanger Institute COVID-19 Surveillance Team
EPI_ISL_957816	Lighthouse Lab in Milton Keynes	Wellcome Sanger Institute for the COVID-19 Genomics UK (COG-UK) Consortium	The Lighthouse Lab in Milton Keynes and Alex Alderton, Roberto Amato, Sonia Goncalves, Ewan Harrison, David K. Jackson, Ian Johnston, Dominic Kwiatkowski, Cordelia Langford, John Sillitoe on behalf of the Wellcome Sanger Institute COVID-19 Surveillance Team
EPI_ISL_957818	Lighthouse Lab in Cambridge	Wellcome Sanger Institute for the COVID-19 Genomics UK (COG-UK) Consortium	Rob Howes, The Lighthouse Lab in Cambridge and Alex Alderton, Roberto Amato, Sonia Goncalves, Ewan Harrison, David K. Jackson, Ian Johnston, Dominic Kwiatkowski, Cordelia Langford, John Sillitoe on behalf of the Wellcome Sanger Institute COVID-19 Surveillance Team
EPI_ISL_957819	Lighthouse Lab in Glasgow	Wellcome Sanger Institute for the COVID-19 Genomics UK (COG-UK) Consortium	Harper VanSteenhouse, Yumi Kasai, David Gray, Carol Clugston, Anna Dominiczak and Alex Alderton, Roberto Amato, Sonia Goncalves, Ewan Harrison, David K. Jackson, Ian Johnston, Dominic Kwiatkowski, Cordelia Langford, John Sillitoe on behalf of the Wellcome Sanger Institute COVID-19 Surveillance Team
EPI_ISL_957820, EPI_ISL_957821	Lighthouse Lab in Cambridge	Wellcome Sanger Institute for the COVID-19 Genomics UK (COG-UK) Consortium	Rob Howes, The Lighthouse Lab in Cambridge and Alex Alderton, Roberto Amato, Sonia Goncalves, Ewan Harrison, David K. Jackson, Ian Johnston, Dominic Kwiatkowski, Cordelia Langford, John Sillitoe on behalf of the Wellcome Sanger Institute COVID-19 Surveillance Team
EPI_ISL_957823	Lighthouse Lab in Milton Keynes	Wellcome Sanger Institute for the COVID-19 Genomics UK (COG-UK) Consortium	The Lighthouse Lab in Milton Keynes and Alex Alderton, Roberto Amato, Sonia Goncalves, Ewan Harrison, David K. Jackson, Ian Johnston, Dominic Kwiatkowski, Cordelia Langford, John Sillitoe on behalf of the Wellcome Sanger Institute COVID-19 Surveillance Team
EPI_ISL_957824	Lighthouse Lab in Glasgow	Wellcome Sanger Institute for the COVID-19 Genomics UK (COG-UK) Consortium	Harper VanSteenhouse, Yumi Kasai, David Gray, Carol Clugston, Anna Dominiczak and Alex Alderton, Roberto Amato, Sonia Goncalves, Ewan Harrison, David K. Jackson, Ian Johnston, Dominic Kwiatkowski, Cordelia Langford, John Sillitoe on behalf of the Wellcome Sanger Institute COVID-19 Surveillance Team
EPI_ISL_957825	Lighthouse Lab in Cambridge	Wellcome Sanger Institute for the COVID-19 Genomics UK (COG-UK) Consortium	Rob Howes, The Lighthouse Lab in Cambridge and Alex Alderton, Roberto Amato, Sonia Goncalves, Ewan Harrison, David K. Jackson, Ian Johnston, Dominic Kwiatkowski, Cordelia Langford, John Sillitoe on behalf of the Wellcome Sanger Institute COVID-19 Surveillance Team
EPI_ISL_957827	Lighthouse Lab in Milton Keynes	Wellcome Sanger Institute for the COVID-19 Genomics UK (COG-UK) Consortium	The Lighthouse Lab in Milton Keynes and Alex Alderton, Roberto Amato, Sonia Goncalves, Ewan Harrison, David K. Jackson, Ian Johnston, Dominic Kwiatkowski, Cordelia Langford, John Sillitoe on behalf of the Wellcome Sanger Institute COVID-19 Surveillance Team
EPI_ISL_957831, EPI_ISL_957833, EPI_ISL_957834	Lighthouse Lab in Cambridge	Wellcome Sanger Institute for the COVID-19 Genomics UK (COG-UK) Consortium	Rob Howes, The Lighthouse Lab in Cambridge and Alex Alderton, Roberto Amato, Sonia Goncalves, Ewan Harrison, David K. Jackson, Ian Johnston, Dominic Kwiatkowski, Cordelia Langford, John Sillitoe on behalf of the Wellcome Sanger Institute COVID-19 Surveillance Team
EPI_ISL_957835	Lighthouse Lab in Milton Keynes	Wellcome Sanger Institute for the COVID-19 Genomics UK (COG-UK) Consortium	The Lighthouse Lab in Milton Keynes and Alex Alderton, Roberto Amato, Sonia Goncalves, Ewan Harrison, David K. Jackson, Ian Johnston, Dominic Kwiatkowski, Cordelia Langford, John Sillitoe on behalf of the Wellcome Sanger Institute COVID-19 Surveillance Team
EPI_ISL_957836, EPI_ISL_957837, EPI_ISL_957839	Lighthouse Lab in Glasgow	Wellcome Sanger Institute for the COVID-19 Genomics UK (COG-UK) Consortium	Harper VanSteenhouse, Yumi Kasai, David Gray, Carol Clugston, Anna Dominiczak and Alex Alderton, Roberto Amato, Sonia Goncalves, Ewan Harrison, David K. Jackson, Ian Johnston, Dominic Kwiatkowski, Cordelia Langford, John Sillitoe on behalf of the Wellcome Sanger Institute COVID-19 Surveillance Team
EPI_ISL_957840	Lighthouse Lab in Cambridge	Wellcome Sanger Institute for the COVID-19 Genomics UK (COG-UK) Consortium	Rob Howes, The Lighthouse Lab in Cambridge and Alex Alderton, Roberto Amato, Sonia Goncalves, Ewan Harrison, David K. Jackson, Ian Johnston, Dominic Kwiatkowski, Cordelia Langford, John Sillitoe on behalf of the Wellcome Sanger Institute COVID-19 Surveillance Team
EPI_ISL_957842, EPI_ISL_957843	Lighthouse Lab in Milton Keynes	Wellcome Sanger Institute for the COVID-19 Genomics UK (COG-UK) Consortium	The Lighthouse Lab in Milton Keynes and Alex Alderton, Roberto Amato, Sonia Goncalves, Ewan Harrison, David K. Jackson, Ian Johnston, Dominic Kwiatkowski, Cordelia Langford, John Sillitoe on behalf of the Wellcome Sanger Institute COVID-19 Surveillance Team
EPI_ISL_957845	Lighthouse Lab in Glasgow	Wellcome Sanger Institute for the COVID-19 Genomics UK (COG-UK) Consortium	Harper VanSteenhouse, Yumi Kasai, David Gray, Carol Clugston, Anna Dominiczak and Alex Alderton, Roberto Amato, Sonia Goncalves, Ewan Harrison, David K. Jackson, Ian Johnston, Dominic Kwiatkowski, Cordelia Langford, John Sillitoe on behalf of the Wellcome Sanger Institute COVID-19 Surveillance Team
EPI_ISL_957846	Lighthouse Lab in Cambridge	Wellcome Sanger Institute for the COVID-19 Genomics UK (COG-UK) Consortium	Rob Howes, The Lighthouse Lab in Cambridge and Alex Alderton, Roberto Amato, Sonia Goncalves, Ewan Harrison, David K. Jackson, Ian Johnston, Dominic Kwiatkowski, Cordelia Langford, John Sillitoe on behalf of the Wellcome Sanger Institute COVID-19 Surveillance Team
EPI_ISL_957847, EPI_ISL_957848	Lighthouse Lab in Glasgow	Wellcome Sanger Institute for the COVID-19 Genomics UK (COG-UK) Consortium	Harper VanSteenhouse, Yumi Kasai, David Gray, Carol Clugston, Anna Dominiczak and Alex Alderton, Roberto Amato, Sonia Goncalves, Ewan Harrison, David K. Jackson, Ian Johnston, Dominic Kwiatkowski, Cordelia Langford, John Sillitoe on behalf of the Wellcome Sanger Institute COVID-19 Surveillance Team
EPI_ISL_957849, EPI_ISL_957850	Lighthouse Lab in Milton Keynes	Wellcome Sanger Institute for the COVID-19 Genomics UK (COG-UK) Consortium	The Lighthouse Lab in Milton Keynes and Alex Alderton, Roberto Amato, Sonia Goncalves, Ewan Harrison, David K. Jackson, Ian Johnston, Dominic Kwiatkowski, Cordelia Langford, John Sillitoe on behalf of the Wellcome Sanger Institute COVID-19 Surveillance Team
EPI_ISL_957851	Lighthouse Lab in Glasgow	Wellcome Sanger Institute for the COVID-19 Genomics UK (COG-UK) Consortium	Harper VanSteenhouse, Yumi Kasai, David Gray, Carol Clugston, Anna Dominiczak and Alex Alderton, Roberto Amato, Sonia Goncalves, Ewan Harrison, David K. Jackson, Ian Johnston, Dominic Kwiatkowski, Cordelia Langford, John Sillitoe on behalf of the Wellcome Sanger Institute COVID-19 Surveillance Team
EPI_ISL_957852	Lighthouse Lab in Cambridge	Wellcome Sanger Institute for the COVID-19 Genomics UK (COG-UK) Consortium	Rob Howes, The Lighthouse Lab in Cambridge and Alex Alderton, Roberto Amato, Sonia Goncalves, Ewan Harrison, David K. Jackson, Ian Johnston, Dominic Kwiatkowski, Cordelia Langford, John Sillitoe on behalf of the Wellcome Sanger Institute COVID-19 Surveillance Team
EPI_ISL_957853, EPI_ISL_957854	Lighthouse Lab in Glasgow	Wellcome Sanger Institute for the COVID-19 Genomics UK (COG-UK) Consortium	Harper VanSteenhouse, Yumi Kasai, David Gray, Carol Clugston, Anna Dominiczak and Alex Alderton, Roberto Amato, Sonia Goncalves, Ewan Harrison, David K. Jackson, Ian Johnston, Dominic Kwiatkowski, Cordelia Langford, John Sillitoe on behalf of the Wellcome Sanger Institute COVID-19 Surveillance Team
EPI_ISL_957855, EPI_ISL_957857	Lighthouse Lab in Cambridge	Wellcome Sanger Institute for the COVID-19 Genomics UK (COG-UK) Consortium	Rob Howes, The Lighthouse Lab in Cambridge and Alex Alderton, Roberto Amato, Sonia Goncalves, Ewan Harrison, David K. Jackson, Ian Johnston, Dominic Kwiatkowski, Cordelia Langford, John Sillitoe on behalf of the Wellcome Sanger Institute COVID-19 Surveillance Team
EPI_ISL_957858	Lighthouse Lab in Milton Keynes	Wellcome Sanger Institute for the COVID-19 Genomics UK (COG-UK) Consortium	The Lighthouse Lab in Milton Keynes and Alex Alderton, Roberto Amato, Sonia Goncalves, Ewan Harrison, David K. Jackson, Ian Johnston, Dominic Kwiatkowski, Cordelia Langford, John Sillitoe on behalf of the Wellcome Sanger Institute COVID-19 Surveillance Team
EPI_ISL_957860	Lighthouse Lab in Glasgow	Wellcome Sanger Institute for the COVID-19 Genomics UK (COG-UK) Consortium	Harper VanSteenhouse, Yumi Kasai, David Gray, Carol Clugston, Anna Dominiczak and Alex Alderton, Roberto Amato, Sonia Goncalves, Ewan Harrison, David K. Jackson, Ian Johnston, Dominic Kwiatkowski, Cordelia Langford, John Sillitoe on behalf of the Wellcome Sanger Institute COVID-19 Surveillance Team
EPI_ISL_957861	Lighthouse Lab in Cambridge	Wellcome Sanger Institute for the COVID-19 Genomics UK (COG-UK) Consortium	Rob Howes, The Lighthouse Lab in Cambridge and Alex Alderton, Roberto Amato, Sonia Goncalves, Ewan Harrison, David K. Jackson, Ian Johnston, Dominic Kwiatkowski, Cordelia Langford, John Sillitoe on behalf of the Wellcome Sanger Institute COVID-19 Surveillance Team
EPI_ISL_957864, EPI_ISL_957865	Lighthouse Lab in Glasgow	Wellcome Sanger Institute for the COVID-19 Genomics UK (COG-UK) Consortium	Harper VanSteenhouse, Yumi Kasai, David Gray, Carol Clugston, Anna Dominiczak and Alex Alderton, Roberto Amato, Sonia Goncalves, Ewan Harrison, David K. Jackson, Ian Johnston, Dominic Kwiatkowski, Cordelia Langford, John Sillitoe on behalf of the Wellcome Sanger Institute COVID-19 Surveillance Team
EPI_ISL_957869, EPI_ISL_957870	Lighthouse Lab in Cambridge	Wellcome Sanger Institute for the COVID-19 Genomics UK	Rob Howes, The Lighthouse Lab in Cambridge and Alex Alderton, Roberto Amato, Sonia Goncalves, Ewan Harrison, David K. Jackson, Ian Johnston,

		(COG-UK) Consortium	Dominic Kwiatkowski, Cordelia Langford, John Sillitoe on behalf of the Wellcome Sanger Institute COVID-19 Surveillance Team
EPI_ISL_957871	Lighthouse Lab in Glasgow	Wellcome Sanger Institute for the COVID-19 Genomics UK (COG-UK) Consortium	Harper VanSteenhouse, Yumi Kasai, David Gray, Carol Clugston, Anna Dominiczak and Alex Alderton, Roberto Amato, Sonia Goncalves, Ewan Harrison, David K. Jackson, Ian Johnston, Dominic Kwiatkowski, Cordelia Langford, John Sillitoe on behalf of the Wellcome Sanger Institute COVID-19 Surveillance Team
EPI_ISL_957873, EPI_ISL_957874	Lighthouse Lab in Cambridge	Wellcome Sanger Institute for the COVID-19 Genomics UK (COG-UK) Consortium	Rob Howes, The Lighthouse Lab in Cambridge and Alex Alderton, Roberto Amato, Sonia Goncalves, Ewan Harrison, David K. Jackson, Ian Johnston, Dominic Kwiatkowski, Cordelia Langford, John Sillitoe on behalf of the Wellcome Sanger Institute COVID-19 Surveillance Team
EPI_ISL_957875	Lighthouse Lab in Glasgow	Wellcome Sanger Institute for the COVID-19 Genomics UK (COG-UK) Consortium	Harper VanSteenhouse, Yumi Kasai, David Gray, Carol Clugston, Anna Dominiczak and Alex Alderton, Roberto Amato, Sonia Goncalves, Ewan Harrison, David K. Jackson, Ian Johnston, Dominic Kwiatkowski, Cordelia Langford, John Sillitoe on behalf of the Wellcome Sanger Institute COVID-19 Surveillance Team
EPI_ISL_957876	Lighthouse Lab in Cambridge	Wellcome Sanger Institute for the COVID-19 Genomics UK (COG-UK) Consortium	Rob Howes, The Lighthouse Lab in Cambridge and Alex Alderton, Roberto Amato, Sonia Goncalves, Ewan Harrison, David K. Jackson, Ian Johnston, Dominic Kwiatkowski, Cordelia Langford, John Sillitoe on behalf of the Wellcome Sanger Institute COVID-19 Surveillance Team
EPI_ISL_957879	Lighthouse Lab in Glasgow	Wellcome Sanger Institute for the COVID-19 Genomics UK (COG-UK) Consortium	Harper VanSteenhouse, Yumi Kasai, David Gray, Carol Clugston, Anna Dominiczak and Alex Alderton, Roberto Amato, Sonia Goncalves, Ewan Harrison, David K. Jackson, Ian Johnston, Dominic Kwiatkowski, Cordelia Langford, John Sillitoe on behalf of the Wellcome Sanger Institute COVID-19 Surveillance Team
EPI_ISL_957880, EPI_ISL_957881, EPI_ISL_957882, EPI_ISL_957883	Lighthouse Lab in Cambridge	Wellcome Sanger Institute for the COVID-19 Genomics UK (COG-UK) Consortium	Rob Howes, The Lighthouse Lab in Cambridge and Alex Alderton, Roberto Amato, Sonia Goncalves, Ewan Harrison, David K. Jackson, Ian Johnston, Dominic Kwiatkowski, Cordelia Langford, John Sillitoe on behalf of the Wellcome Sanger Institute COVID-19 Surveillance Team
EPI_ISL_957884	Lighthouse Lab in Glasgow	Wellcome Sanger Institute for the COVID-19 Genomics UK (COG-UK) Consortium	Harper VanSteenhouse, Yumi Kasai, David Gray, Carol Clugston, Anna Dominiczak and Alex Alderton, Roberto Amato, Sonia Goncalves, Ewan Harrison, David K. Jackson, Ian Johnston, Dominic Kwiatkowski, Cordelia Langford, John Sillitoe on behalf of the Wellcome Sanger Institute COVID-19 Surveillance Team
EPI_ISL_957885	Lighthouse Lab in Milton Keynes	Wellcome Sanger Institute for the COVID-19 Genomics UK (COG-UK) Consortium	The Lighthouse Lab in Milton Keynes and Alex Alderton, Roberto Amato, Sonia Goncalves, Ewan Harrison, David K. Jackson, Ian Johnston, Dominic Kwiatkowski, Cordelia Langford, John Sillitoe on behalf of the Wellcome Sanger Institute COVID-19 Surveillance Team
EPI_ISL_957887	Lighthouse Lab in Cambridge	Wellcome Sanger Institute for the COVID-19 Genomics UK (COG-UK) Consortium	Rob Howes, The Lighthouse Lab in Cambridge and Alex Alderton, Roberto Amato, Sonia Goncalves, Ewan Harrison, David K. Jackson, Ian Johnston, Dominic Kwiatkowski, Cordelia Langford, John Sillitoe on behalf of the Wellcome Sanger Institute COVID-19 Surveillance Team
EPI_ISL_957888	Lighthouse Lab in Milton Keynes	Wellcome Sanger Institute for the COVID-19 Genomics UK (COG-UK) Consortium	The Lighthouse Lab in Milton Keynes and Alex Alderton, Roberto Amato, Sonia Goncalves, Ewan Harrison, David K. Jackson, Ian Johnston, Dominic Kwiatkowski, Cordelia Langford, John Sillitoe on behalf of the Wellcome Sanger Institute COVID-19 Surveillance Team
EPI_ISL_957889	Lighthouse Lab in Glasgow	Wellcome Sanger Institute for the COVID-19 Genomics UK (COG-UK) Consortium	Harper VanSteenhouse, Yumi Kasai, David Gray, Carol Clugston, Anna Dominiczak and Alex Alderton, Roberto Amato, Sonia Goncalves, Ewan Harrison, David K. Jackson, Ian Johnston, Dominic Kwiatkowski, Cordelia Langford, John Sillitoe on behalf of the Wellcome Sanger Institute COVID-19 Surveillance Team
EPI_ISL_957890, EPI_ISL_957891	Lighthouse Lab in Cambridge	Wellcome Sanger Institute for the COVID-19 Genomics UK (COG-UK) Consortium	Rob Howes, The Lighthouse Lab in Cambridge and Alex Alderton, Roberto Amato, Sonia Goncalves, Ewan Harrison, David K. Jackson, Ian Johnston, Dominic Kwiatkowski, Cordelia Langford, John Sillitoe on behalf of the Wellcome Sanger Institute COVID-19 Surveillance Team
EPI_ISL_957893	Lighthouse Lab in Milton Keynes	Wellcome Sanger Institute for the COVID-19 Genomics UK (COG-UK) Consortium	The Lighthouse Lab in Milton Keynes and Alex Alderton, Roberto Amato, Sonia Goncalves, Ewan Harrison, David K. Jackson, Ian Johnston, Dominic Kwiatkowski, Cordelia Langford, John Sillitoe on behalf of the Wellcome Sanger Institute COVID-19 Surveillance Team
EPI_ISL_957894	Lighthouse Lab in Glasgow	Wellcome Sanger Institute for the COVID-19 Genomics UK (COG-UK) Consortium	Harper VanSteenhouse, Yumi Kasai, David Gray, Carol Clugston, Anna Dominiczak and Alex Alderton, Roberto Amato, Sonia Goncalves, Ewan Harrison, David K. Jackson, Ian Johnston, Dominic Kwiatkowski, Cordelia Langford, John Sillitoe on behalf of the Wellcome Sanger Institute COVID-19 Surveillance Team
EPI_ISL_957896, EPI_ISL_957898	Lighthouse Lab in Cambridge	Wellcome Sanger Institute for the COVID-19 Genomics UK (COG-UK) Consortium	Rob Howes, The Lighthouse Lab in Cambridge and Alex Alderton, Roberto Amato, Sonia Goncalves, Ewan Harrison, David K. Jackson, Ian Johnston, Dominic Kwiatkowski, Cordelia Langford, John Sillitoe on behalf of the Wellcome Sanger Institute COVID-19 Surveillance Team
EPI_ISL_957899, EPI_ISL_957900, EPI_ISL_957901	Lighthouse Lab in Milton Keynes	Wellcome Sanger Institute for the COVID-19 Genomics UK (COG-UK) Consortium	The Lighthouse Lab in Milton Keynes and Alex Alderton, Roberto Amato, Sonia Goncalves, Ewan Harrison, David K. Jackson, Ian Johnston, Dominic Kwiatkowski, Cordelia Langford, John Sillitoe on behalf of the Wellcome Sanger Institute COVID-19 Surveillance Team
EPI_ISL_957902, EPI_ISL_957903, EPI_ISL_957905, EPI_ISL_957906, EPI_ISL_957908	Lighthouse Lab in Cambridge	Wellcome Sanger Institute for the COVID-19 Genomics UK (COG-UK) Consortium	Rob Howes, The Lighthouse Lab in Cambridge and Alex Alderton, Roberto Amato, Sonia Goncalves, Ewan Harrison, David K. Jackson, Ian Johnston, Dominic Kwiatkowski, Cordelia Langford, John Sillitoe on behalf of the Wellcome Sanger Institute COVID-19 Surveillance Team
EPI_ISL_957909, EPI_ISL_957910, EPI_ISL_957911, EPI_ISL_957912, EPI_ISL_957913, EPI_ISL_957914, EPI_ISL_957915, EPI_ISL_957916, EPI_ISL_957917, EPI_ISL_957918, EPI_ISL_957919, EPI_ISL_957920, EPI_ISL_957922, EPI_ISL_957923, EPI_ISL_957924, EPI_ISL_957925, EPI_ISL_957926, EPI_ISL_957927, EPI_ISL_957928, EPI_ISL_957929, EPI_ISL_957930, EPI_ISL_957931, EPI_ISL_957932, EPI_ISL_957933, EPI_ISL_957934, EPI_ISL_957935, EPI_ISL_957936, EPI_ISL_957937, EPI_ISL_957938, EPI_ISL_957939, EPI_ISL_957940, EPI_ISL_957941, EPI_ISL_957942, EPI_ISL_957943, EPI_ISL_957944, EPI_ISL_957945, EPI_ISL_957946, EPI_ISL_957947, EPI_ISL_957948, EPI_ISL_957949, EPI_ISL_957950, EPI_ISL_957951, EPI_ISL_957952, EPI_ISL_957953, EPI_ISL_957954, EPI_ISL_957955, EPI_ISL_957956, EPI_ISL_957957, EPI_ISL_957958, EPI_ISL_957959, EPI_ISL_957960, EPI_ISL_957963, EPI_ISL_957964, EPI_ISL_957965, EPI_ISL_957967, EPI_ISL_957968, EPI_ISL_957969, EPI_ISL_957970, EPI_ISL_957972, EPI_ISL_957973, EPI_ISL_957974, EPI_ISL_957975, EPI_ISL_957976, EPI_ISL_957977, EPI_ISL_957978, EPI_ISL_957979, EPI_ISL_957980, EPI_ISL_957981, EPI_ISL_957982, EPI_ISL_957983, EPI_ISL_957984, EPI_ISL_957985, EPI_ISL_957986, EPI_ISL_957987, EPI_ISL_957988, EPI_ISL_957989, EPI_ISL_957990, EPI_ISL_957991, EPI_ISL_957992, EPI_ISL_957993, EPI_ISL_957994, EPI_ISL_957995, EPI_ISL_957996, EPI_ISL_957997, EPI_ISL_957998, EPI_ISL_957999, EPI_ISL_958000, EPI_ISL_958001, EPI_ISL_958002, EPI_ISL_958003, EPI_ISL_958004, EPI_ISL_958005, EPI_ISL_958006, EPI_ISL_958007, EPI_ISL_958008, EPI_ISL_958009, EPI_ISL_958010, EPI_ISL_958011, EPI_ISL_958012, EPI_ISL_958013, EPI_ISL_958014, EPI_ISL_958015, EPI_ISL_958016, EPI_ISL_958017, EPI_ISL_958018, EPI_ISL_958019, EPI_ISL_958020, EPI_ISL_958021, EPI_ISL_958022, EPI_ISL_958023, EPI_ISL_958024, EPI_ISL_958025, EPI_ISL_958026, EPI_ISL_958027, EPI_ISL_958028, EPI_ISL_958029, EPI_ISL_958030, EPI_ISL_958031, EPI_ISL_958032, EPI_ISL_958033, EPI_ISL_958034, EPI_ISL_958035, EPI_ISL_958036, EPI_ISL_958037, EPI_ISL_958038, EPI_ISL_958039, EPI_ISL_958040, EPI_ISL_958041, EPI_ISL_958042, EPI_ISL_958043, EPI_ISL_958045, EPI_ISL_958046, EPI_ISL_958047, EPI_ISL_958048, EPI_ISL_958049, EPI_ISL_958050, EPI_ISL_958051, EPI_ISL_958052, EPI_ISL_958053, EPI_ISL_958054, EPI_ISL_958055, EPI_ISL_958056, EPI_ISL_958057, EPI_ISL_958058, EPI_ISL_958059, EPI_ISL_958060, EPI_ISL_958061, EPI_ISL_958062, EPI_ISL_958063, EPI_ISL_958064, EPI_ISL_958065, EPI_ISL_958066, EPI_ISL_958067, EPI_ISL_958068, EPI_ISL_958069, EPI_ISL_958070, EPI_ISL_958071, EPI_ISL_958072, EPI_ISL_958073, EPI_ISL_958074, EPI_ISL_958075, EPI_ISL_958076, EPI_ISL_958077, EPI_ISL_958078, EPI_ISL_958079, EPI_ISL_958080, EPI_ISL_958081, EPI_ISL_958082, EPI_ISL_958083, EPI_ISL_958084, EPI_ISL_958085, EPI_ISL_958086, EPI_ISL_958087, EPI_ISL_958088, EPI_ISL_958089, EPI_ISL_958090, EPI_ISL_958091, EPI_ISL_958092, EPI_ISL_958093, EPI_ISL_958094, EPI_ISL_958095, EPI_ISL_958096, EPI_ISL_958097, EPI_ISL_958098, EPI_ISL_958099, EPI_ISL_958100, EPI_ISL_958101, EPI_ISL_958102, EPI_ISL_958103, EPI_ISL_958104, EPI_ISL_958105, EPI_ISL_958106, EPI_ISL_958107, EPI_ISL_958108, EPI_ISL_958109, EPI_ISL_958110, EPI_ISL_958111, EPI_ISL_958112, EPI_ISL_958113, EPI_ISL_958114, EPI_ISL_958115, EPI_ISL_958116, EPI_ISL_958117, EPI_ISL_958118, EPI_ISL_958119, EPI_ISL_958120, EPI_ISL_958121, EPI_ISL_958122, EPI_ISL_958123, EPI_ISL_958124, EPI_ISL_958125, EPI_ISL_958126, EPI_ISL_958127, EPI_ISL_958128, EPI_ISL_958129, EPI_ISL_958130, EPI_ISL_958131, EPI_ISL_958132, EPI_ISL_958133, EPI_ISL_958134, EPI_ISL_958135, EPI_ISL_958136, EPI_ISL_958137, EPI_ISL_958138, EPI_ISL_958139, EPI_ISL_958140, EPI_ISL_958141, EPI_ISL_958142, EPI_ISL_958143, EPI_ISL_958144, EPI_ISL_958145, EPI_ISL_958146, EPI_ISL_958147, EPI_ISL_958148, EPI_ISL_958149, EPI_ISL_958150, EPI_ISL_958151, EPI_ISL_958152, EPI_ISL_958153, EPI_ISL_958154, EPI_ISL_958155, EPI_ISL_958156, EPI_ISL_958157, EPI_ISL_958158, EPI_ISL_958159, EPI_ISL_958160, EPI_ISL_958161, EPI_ISL_958162, EPI_ISL_958163, EPI_ISL_958164, EPI_ISL_958165, EPI_ISL_958166, EPI_ISL_958167, EPI_ISL_958168, EPI_ISL_958169, EPI_ISL_958170, EPI_ISL_958171, EPI_ISL_958172, EPI_ISL_958173, EPI_ISL_958174, EPI_ISL_958175, EPI_ISL_958176, EPI_ISL_958177, EPI_ISL_958178, EPI_ISL_958179, EPI_ISL_958180, EPI_ISL_958181, EPI_ISL_958182, EPI_ISL_958183, EPI_ISL_958184, EPI_ISL_958185, EPI_ISL_958186, EPI_ISL_958187, EPI_ISL_958188, EPI_ISL_958189, EPI_ISL_958190, EPI_ISL_958191, EPI_ISL_958192, EPI_ISL_958193, EPI_ISL_958195, EPI_ISL_958196, EPI_ISL_958197, EPI_ISL_958198, EPI_ISL_958199, EPI_ISL_958200, EPI_ISL_958201, EPI_ISL_958202, EPI_ISL_958203, EPI_ISL_958204, EPI_ISL_958205, EPI_ISL_958207, EPI_ISL_958208, EPI_ISL_958209, EPI_ISL_958210, EPI_ISL_958211, EPI_ISL_958212, EPI_ISL_958213, EPI_ISL_958214, EPI_ISL_958567, EPI_ISL_958568, EPI_ISL_958570, EPI_ISL_958571, EPI_ISL_958573, EPI_ISL_958574, EPI_ISL_958575, EPI_ISL_958576, EPI_ISL_958577, EPI_ISL_958580, EPI_ISL_958581, EPI_ISL_958582, EPI_ISL_958583, EPI_ISL_958584, EPI_ISL_958585, EPI_ISL_958588, EPI_ISL_958589, EPI_ISL_958592, EPI_ISL_958593, EPI_ISL_958594, EPI_ISL_958596, EPI_ISL_958597, EPI_ISL_958598, EPI_ISL_958599, EPI_ISL_958601, EPI_ISL_958603, EPI_ISL_958604, EPI_ISL_958605, EPI_ISL_958606, EPI_ISL_958607, EPI_ISL_958608, EPI_ISL_958609, EPI_ISL_958611, EPI_ISL_958612, EPI_ISL_958613, EPI_ISL_958614, EPI_ISL_958615, EPI_ISL_958616, EPI_ISL_958617, EPI_ISL_958618, EPI_ISL_958619, EPI_ISL_958620, EPI_ISL_958621, EPI_ISL_958622, EPI_ISL_958623, EPI_ISL_958625, EPI_ISL_958626, EPI_ISL_958627, EPI_ISL_958628, EPI_ISL_958629, EPI_ISL_958630, EPI_ISL_958631, EPI_ISL_958635, EPI_ISL_958637, EPI_ISL_958640, EPI_ISL_958642, EPI_ISL_958643, EPI_ISL_958644, EPI_ISL_958645, EPI_ISL_958647, EPI_ISL_958648, EPI_ISL_958649, EPI_ISL_958651, EPI_ISL_958652, EPI_ISL_958653, EPI_ISL_958654, EPI_ISL_958655, EPI_ISL_958656, EPI_ISL_958657, EPI_ISL_958658, EPI_ISL_958660, EPI_ISL_958661, EPI_ISL_958664, EPI_ISL_958665, EPI_ISL_958666, EPI_ISL_958667, EPI_ISL_958668, EPI_ISL_958669, EPI_ISL_958672, EPI_ISL_958674, EPI_ISL_958675, EPI_ISL_958676, EPI_ISL_958677, EPI_ISL_958678, EPI_ISL_958679, EPI_ISL_958681, EPI_ISL_958682, EPI_ISL_958683, EPI_ISL_958685, EPI_ISL_958686, EPI_ISL_958687, EPI_ISL_958688, EPI_ISL_958689, EPI_ISL_958692, EPI_ISL_958693, EPI_ISL_958694, EPI_ISL_958695, EPI_ISL_958697, EPI_ISL_958698, EPI_ISL_958699, EPI_ISL_958700, EPI_ISL_958701, EPI_ISL_958702, EPI_ISL_958703, EPI_ISL_958704, EPI_ISL_958707, EPI_ISL_958708, EPI_ISL_958709, EPI_ISL_958710, EPI_ISL_958711, EPI_ISL_958712, EPI_ISL_958714, EPI_ISL_958717, EPI_ISL_958718, EPI_ISL_958719, EPI_ISL_958720, EPI_ISL_958721, EPI_ISL_958722, EPI_ISL_958724, EPI_ISL_958727, EPI_ISL_958728, EPI_ISL_958729, EPI_ISL_958730, EPI_ISL_958732, EPI_ISL_958734, EPI_ISL_958736, EPI_ISL_958737, EPI_ISL_958738, EPI_ISL_958739, EPI_ISL_958740, EPI_ISL_958741, EPI_ISL_958742, EPI_ISL_958743, EPI_ISL_958744, EPI_ISL_958746, EPI_ISL_958749, EPI_ISL_958750, EPI_ISL_958752, EPI_ISL_958753, EPI_ISL_958755, EPI_ISL_958757, EPI_ISL_958758, EPI_ISL_958759, EPI_ISL_958761, EPI_ISL_958762, EPI_ISL_958764, EPI_ISL_958765, EPI_ISL_958766, EPI_ISL_958768, EPI_ISL_958770, EPI_ISL_958771, EPI_ISL_958772, EPI_ISL_958773, EPI_ISL_958774, EPI_ISL_958776, EPI_ISL_958777, EPI_ISL_958778, EPI_ISL_958780, EPI_ISL_958781, EPI_ISL_958782, EPI_ISL_958783, EPI_ISL_958785, EPI_ISL_958786, EPI_ISL_958788, EPI_ISL_958790, EPI_ISL_958794, EPI_ISL_958795, EPI_ISL_958796, EPI_ISL_958797, EPI_ISL_958798, EPI_ISL_958799, EPI_ISL_958800, EPI_ISL_958801, EPI_ISL_958802, EPI_ISL_958804, EPI_ISL_958805, EPI_ISL_958806, EPI_ISL_958809, EPI_ISL_958810, EPI_ISL_958812, EPI_ISL_958813, EPI_ISL_958814, EPI_ISL_958816, EPI_ISL_958817, EPI_ISL_958818, EPI_ISL_958819, EPI_ISL_958820, EPI_ISL_958821, EPI_ISL_958822, EPI_ISL_958824, EPI_ISL_958825, EPI_ISL_958827, EPI_ISL_958829, EPI_ISL_958830, EPI_ISL_958831, EPI_ISL_958832, EPI_ISL_958834, EPI_ISL_958835, EPI_ISL_958837, EPI_ISL_958838, EPI_ISL_958839, EPI_ISL_958841, EPI_ISL_958843, EPI_ISL_958844, EPI_ISL_958845, EPI_ISL_958846, EPI_ISL_958847, EPI_ISL_958848, EPI_ISL_958850, EPI_ISL_958852, EPI_ISL_958853, EPI_ISL_958854, EPI_ISL_958855, EPI_ISL_958856, EPI_ISL_958857, EPI_ISL_958858, EPI_ISL_958859, EPI_ISL_958860, EPI_ISL_958861, EPI_ISL_958862, EPI_ISL_958864, EPI_ISL_958865, EPI_ISL_958867, EPI_ISL_958868, EPI_ISL_958869, EPI_ISL_958870, EPI_ISL_958871, EPI_ISL_958872, EPI_ISL_958874, EPI_ISL_958875, EPI_ISL_958876, EPI_ISL_958877, EPI_ISL_958879, EPI_ISL_958880, EPI_ISL_958881, EPI_ISL_958882, EPI_ISL_958883, EPI_ISL_958886, EPI_ISL_958888, EPI_ISL_958890, EPI_ISL_958892, EPI_ISL_958894, EPI_ISL_958896, EPI_ISL_958897, EPI_ISL_958899, EPI_ISL_958900, EPI_ISL_958901, EPI_ISL_958905, EPI_ISL_958906, EPI_ISL_958907, EPI_ISL_958909, EPI_ISL_958910, EPI_ISL_958913, EPI_ISL_958914			
see above	Lighthouse Lab in Milton Keynes	Wellcome Sanger Institute for the COVID-19 Genomics UK (COG-UK) Consortium	The Lighthouse Lab in Milton Keynes and Alex Alderton, Roberto Amato, Sonia Goncalves, Ewan Harrison, David K. Jackson, Ian Johnston, Dominic Kwiatkowski, Cordelia Langford, John Sillitoe on behalf of the Wellcome Sanger Institute COVID-19 Surveillance Team
EPI_ISL_959419, EPI_ISL_959432, EPI_ISL_959433, EPI_ISL_959434,	Servicio de Microbiología, Hospital Universitario Son Espases	SeqCOVID-SPAIN consortium/IBV(CSIC)	Carla López-Causapé, Jordi Reina, Antonio Oliver and SeqCOVID-SPAIN consortium

EPI_ISL_959442, EPI_ISL_959447, EPI_ISL_959448, EPI_ISL_959449			
EPI_ISL_959604, EPI_ISL_959605, EPI_ISL_959606	Institute of Virology, Biomedical Research Center of the Slovak Academy of Sciences, Bratislava	Faculty of Natural Sciences, Comenius University, Bratislava	Broa Brejová, Viktória abanová, Kristína Boršová, Viktória Hodorová, Sabina Fumaová Havlíková, Juraj Kopáek, Martina Liková, ubomíra Lukáiková, Martina Neboháová, Monika Sláviková, Tomáš Vina, Jozef Nosek, Boris Klempa
EPI_ISL_959607	Institute of Virology, Biomedical Research Center of the Slovak Academy of Sciences, Bratislava	Faculty of Natural Sciences, Comenius University, Bratislava	Viktória abanová, Kristína Boršová, Broa Brejová, Viktória Hodorová, Sabina Fumaová Havlíková, Juraj Kopáek, Martina Liková, ubomíra Lukáiková, Martina Neboháová, Monika Sláviková, Tomáš Vina, Jozef Nosek, Boris Klempa
EPI_ISL_959608	Institute of Virology, Biomedical Research Center of the Slovak Academy of Sciences, Bratislava	Faculty of Natural Sciences, Comenius University, Bratislava	Kristína Boršová, Viktória abanová, Broa Brejová, Viktória Hodorová, Sabina Fumaová Havlíková, Juraj Kopáek, Martina Liková, ubomíra Lukáiková, Martina Neboháová, Monika Sláviková, Tomáš Vina, Boris Klempa, Jozef Nosek
EPI_ISL_959609	Institute of Virology, Biomedical Research Center of the Slovak Academy of Sciences, Bratislava	Faculty of Natural Sciences, Comenius University, Bratislava	Viktória abanová, Kristína Boršová, Broa Brejová, Viktória Hodorová, Sabina Fumaová Havlíková, Juraj Kopáek, Martina Liková, ubomíra Lukáiková, Martina Neboháová, Monika Sláviková, Tomáš Vina, Jozef Nosek, Boris Klempa
EPI_ISL_959610	Institute of Virology, Biomedical Research Center of the Slovak Academy of Sciences, Bratislava	Faculty of Natural Sciences, Comenius University, Bratislava	Kristína Boršová, Viktória abanová, Broa Brejová, Viktória Hodorová, Sabina Fumaová Havlíková, Juraj Kopáek, Martina Liková, ubomíra Lukáiková, Martina Neboháová, Monika Sláviková, Tomáš Vina, Boris Klempa, Jozef Nosek
EPI_ISL_959611	Institute of Virology, Biomedical Research Center of the Slovak Academy of Sciences, Bratislava	Faculty of Natural Sciences, Comenius University, Bratislava	Viktória abanová, Kristína Boršová, Broa Brejová, Viktória Hodorová, Sabina Fumaová Havlíková, Juraj Kopáek, Martina Liková, ubomíra Lukáiková, Martina Neboháová, Monika Sláviková, Tomáš Vina, Jozef Nosek, Boris Klempa
EPI_ISL_959612	Institute of Virology, Biomedical Research Center of the Slovak Academy of Sciences, Bratislava	Faculty of Natural Sciences, Comenius University, Bratislava	Kristína Boršová, Viktória abanová, Broa Brejová, Viktória Hodorová, Sabina Fumaová Havlíková, Juraj Kopáek, Martina Liková, ubomíra Lukáiková, Martina Neboháová, Monika Sláviková, Tomáš Vina, Boris Klempa, Jozef Nosek
EPI_ISL_959613	Institute of Virology, Biomedical Research Center of the Slovak Academy of Sciences, Bratislava	Faculty of Natural Sciences, Comenius University, Bratislava	Viktória abanová, Kristína Boršová, Broa Brejová, Viktória Hodorová, Sabina Fumaová Havlíková, Juraj Kopáek, Martina Liková, ubomíra Lukáiková, Martina Neboháová, Monika Sláviková, Tomáš Vina, Jozef Nosek, Boris Klempa
EPI_ISL_959614	Institute of Virology, Biomedical Research Center of the Slovak Academy of Sciences, Bratislava	Faculty of Natural Sciences, Comenius University, Bratislava	Kristína Boršová, Viktória abanová, Broa Brejová, Viktória Hodorová, Sabina Fumaová Havlíková, Juraj Kopáek, Martina Liková, ubomíra Lukáiková, Martina Neboháová, Monika Sláviková, Tomáš Vina, Boris Klempa, Jozef Nosek
EPI_ISL_959615, EPI_ISL_959616	Institute of Virology, Biomedical Research Center of the Slovak Academy of Sciences, Bratislava	Faculty of Natural Sciences, Comenius University, Bratislava	Broa Brejová, Viktória abanová, Kristína Boršová, Viktória Hodorová, Sabina Fumaová Havlíková, Juraj Kopáek, Martina Liková, ubomíra Lukáiková, Martina Neboháová, Monika Sláviková, Tomáš Vina, Jozef Nosek, Boris Klempa
EPI_ISL_959617	Institute of Virology, Biomedical Research Center of the Slovak Academy of Sciences, Bratislava	Faculty of Natural Sciences, Comenius University, Bratislava	Viktória abanová, Kristína Boršová, Broa Brejová, Viktória Hodorová, Sabina Fumaová Havlíková, Juraj Kopáek, Martina Liková, ubomíra Lukáiková, Martina Neboháová, Monika Sláviková, Tomáš Vina, Jozef Nosek, Boris Klempa
EPI_ISL_959618	Institute of Virology, Biomedical Research Center of the Slovak Academy of Sciences, Bratislava	Faculty of Natural Sciences, Comenius University, Bratislava	Kristína Boršová, Viktória abanová, Broa Brejová, Viktória Hodorová, Sabina Fumaová Havlíková, Juraj Kopáek, Martina Liková, ubomíra Lukáiková, Martina Neboháová, Monika Sláviková, Tomáš Vina, Boris Klempa, Jozef Nosek
EPI_ISL_959619	Institute of Virology, Biomedical Research Center of the Slovak Academy of Sciences, Bratislava	Faculty of Natural Sciences, Comenius University, Bratislava	Broa Brejová, Viktória abanová, Kristína Boršová, Viktória Hodorová, Sabina Fumaová Havlíková, Juraj Kopáek, Martina Liková, ubomíra Lukáiková, Martina Neboháová, Monika Sláviková, Tomáš Vina, Jozef Nosek, Boris Klempa
EPI_ISL_959620	Institute of Virology, Biomedical Research Center of the Slovak Academy of Sciences, Bratislava	Faculty of Natural Sciences, Comenius University, Bratislava	Viktória abanová, Kristína Boršová, Broa Brejová, Viktória Hodorová, Sabina Fumaová Havlíková, Juraj Kopáek, Martina Liková, ubomíra Lukáiková, Martina Neboháová, Monika Sláviková, Tomáš Vina, Jozef Nosek, Boris Klempa
EPI_ISL_959626	Institute of Virology, Biomedical Research Center of the Slovak Academy of Sciences, Bratislava	Faculty of Natural Sciences, Comenius University, Bratislava	Kristína Boršová, Viktória abanová, Broa Brejová, Viktória Hodorová, Sabina Fumaová Havlíková, Juraj Kopáek, Martina Liková, ubomíra Lukáiková, Martina Neboháová, Monika Sláviková, Tomáš Vina, Boris Klempa, Jozef Nosek
EPI_ISL_959627, EPI_ISL_959628	Institute of Virology, Biomedical Research Center of the Slovak Academy of Sciences, Bratislava	Faculty of Natural Sciences, Comenius University, Bratislava	Viktória abanová, Kristína Boršová, Broa Brejová, Viktória Hodorová, Sabina Fumaová Havlíková, Juraj Kopáek, Martina Liková, ubomíra Lukáiková, Martina Neboháová, Monika Sláviková, Tomáš Vina, Jozef Nosek, Boris Klempa
EPI_ISL_959629	Institute of Virology, Biomedical Research Center of the Slovak Academy of Sciences, Bratislava	Faculty of Natural Sciences, Comenius University, Bratislava	Broa Brejová, Viktória abanová, Kristína Boršová, Viktória Hodorová, Sabina Fumaová Havlíková, Juraj Kopáek, Martina Liková, ubomíra Lukáiková, Martina Neboháová, Monika Sláviková, Tomáš Vina, Jozef Nosek, Boris Klempa
EPI_ISL_959630	Institute of Virology, Biomedical Research Center of the Slovak Academy of Sciences, Bratislava	Faculty of Natural Sciences, Comenius University, Bratislava	Kristína Boršová, Viktória abanová, Broa Brejová, Viktória Hodorová, Sabina Fumaová Havlíková, Juraj Kopáek, Martina Liková, ubomíra Lukáiková, Martina Neboháová, Monika Sláviková, Tomáš Vina, Boris Klempa, Jozef Nosek
EPI_ISL_959631	Institute of Virology, Biomedical Research Center of the Slovak Academy of Sciences, Bratislava	Faculty of Natural Sciences, Comenius University, Bratislava	Broa Brejová, Viktória abanová, Kristína Boršová, Viktória Hodorová, Sabina Fumaová Havlíková, Juraj Kopáek, Martina Liková, ubomíra Lukáiková, Martina Neboháová, Monika Sláviková, Tomáš Vina, Jozef Nosek, Boris Klempa
EPI_ISL_959701, EPI_ISL_959702, EPI_ISL_959703, EPI_ISL_959704, EPI_ISL_959705, EPI_ISL_959706, EPI_ISL_959707, EPI_ISL_959708, EPI_ISL_959709, EPI_ISL_959710, EPI_ISL_959711, EPI_ISL_959712, EPI_ISL_959852, EPI_ISL_959853, EPI_ISL_959860, EPI_ISL_959861			
see above	National Virus Reference Laboratory	National Virus Reference Laboratory	Michael Carr, Gabriel Gonzalez, Jonathan Dean, Cillian F De Gascun
EPI_ISL_960333, EPI_ISL_960385, EPI_ISL_960403	University of Wisconsin-Madison AIDS Vaccine Research Laboratories	University of Wisconsin-Madison AIDS Vaccine Research Laboratories	Gage Moreno, Katarina Braun, et al. AIDS Vaccine Research Laboratories
EPI_ISL_960893	Institute of Medical Microbiology and Hospital Hygiene	Institute of Medical Microbiology and Hospital Hygiene	Prof. Dr. Achim Kaasch, Aljoscha Tersteegen
EPI_ISL_961747	Dipartimento Prevenzione Avezzano-Servizio Igiene epidemiologia Sanità Pubblica	Istituto Zooprofilattico Sperimentale dell'Abruzzo e Molise "G. Caporale"	Lorusso A, Marcacci M, Di Domenico M, Ancora M, Curini V, Mangone I, Rinaldi A, Scialabba S, Di Pasquale A, Cammà C, Puglia I, Calistri P, Savini G
EPI_ISL_962445	Seattle Flu Study	Seattle Flu Study	Deborah A. Nickerson, Chris D. Frazar, Jover Lee, Benjamin Pelle, Erica Ryke, Matthew Richardson, Amanda Adler, Elisabeth Brandstetter, Peter D. Han, Kairsten Fay, Misja Ilcisin, Kirsten Lacombe, Thomas R. Sibley, Melissa Truong, Caitlin R. Wolf, Michael Boeckh, Janet A. Englund, Michael Famulare, Barry R. Lutz, Mark J. Rieder, Lea M. Starita, Matthew Thompson, Jay Shendure, Trevor Bedford, Helen Y. Chu
EPI_ISL_962449, EPI_ISL_962450, EPI_ISL_962451	Seattle Flu Study	Seattle Flu Study	Deborah A. Nickerson, Chris D. Frazar, Jover Lee, Benjamin Pelle, Erica Ryke, Matthew Richardson, Amanda Adler, Elisabeth Brandstetter, Peter D. Han, Kairsten Fay, Misja Ilcisin, Kirsten Lacombe, Thomas R. Sibley, Melissa Truong, Caitlin R. Wolf, Karen Cowgill, Stephanie Schrag, Jeff Duchin, Michael Boeckh, Janet A. Englund, Michael Famulare, Barry R. Lutz, Mark J. Rieder, Lea M. Starita, Matthew Thompson, Helen Y. Chu, Trevor Bedford, Jay Shendure
EPI_ISL_962458	Seattle Flu Study	Seattle Flu Study	Deborah A. Nickerson, Chris D. Frazar, Jover Lee, Benjamin Pelle, Erica Ryke, Matthew Richardson, Amanda Adler, Elisabeth Brandstetter, Peter D. Han, Kairsten Fay, Misja Ilcisin, Kirsten Lacombe, Thomas R. Sibley, Melissa Truong, Caitlin R. Wolf, Michael Boeckh, Janet A. Englund, Michael Famulare, Barry R. Lutz, Mark J. Rieder, Lea M. Starita, Matthew Thompson, Jay Shendure, Trevor Bedford, Helen Y. Chu
EPI_ISL_962474, EPI_ISL_962475, EPI_ISL_962476	Seattle Flu Study	Seattle Flu Study	Deborah A. Nickerson, Chris D. Frazar, Jover Lee, Benjamin Pelle, Erica Ryke, Matthew Richardson, Amanda Adler, Elisabeth Brandstetter, Peter D. Han, Kairsten Fay, Misja Ilcisin, Kirsten Lacombe, Thomas R. Sibley, Melissa Truong, Caitlin R. Wolf, Karen Cowgill, Stephanie Schrag, Jeff Duchin, Michael Boeckh, Janet A. Englund, Michael Famulare, Barry R. Lutz, Mark J. Rieder, Lea M. Starita, Matthew Thompson, Helen Y. Chu, Trevor Bedford, Jay Shendure
EPI_ISL_962477	Seattle Flu Study	Seattle Flu Study	Deborah A. Nickerson, Chris D. Frazar, Jover Lee, Benjamin Pelle, Erica Ryke, Matthew Richardson, Amanda Adler, Elisabeth Brandstetter, Peter D. Han, Kairsten Fay, Misja Ilcisin, Kirsten Lacombe, Thomas R. Sibley, Melissa Truong, Caitlin R. Wolf, Michael Boeckh, Janet A. Englund, Michael Famulare, Barry R. Lutz, Mark J. Rieder, Lea M. Starita, Matthew Thompson, Jay Shendure, Trevor Bedford, Helen Y. Chu
EPI_ISL_962478, EPI_ISL_962479, EPI_ISL_962480	Seattle Flu Study	Seattle Flu Study	Deborah A. Nickerson, Chris D. Frazar, Jover Lee, Benjamin Pelle, Erica Ryke, Matthew Richardson, Amanda Adler, Elisabeth Brandstetter, Peter D. Han, Kairsten Fay, Misja Ilcisin, Kirsten Lacombe, Thomas R. Sibley, Melissa Truong, Caitlin R. Wolf, Karen Cowgill, Stephanie Schrag, Jeff Duchin, Michael Boeckh, Janet A. Englund, Michael Famulare, Barry R. Lutz, Mark J. Rieder, Lea M. Starita, Matthew Thompson, Helen Y. Chu, Trevor Bedford, Jay Shendure
EPI_ISL_962848, EPI_ISL_962849, EPI_ISL_962850, EPI_ISL_962851	Los Angeles County PHL	Los Angeles County PHL	P. Hemarajata et al.
EPI_ISL_962975, EPI_ISL_962976, EPI_ISL_962977, EPI_ISL_962978, EPI_ISL_962979, EPI_ISL_962980, EPI_ISL_962981, EPI_ISL_962982, EPI_ISL_962983, EPI_ISL_962984, EPI_ISL_962985, EPI_ISL_962986, EPI_ISL_962987, EPI_ISL_962988, EPI_ISL_962989, EPI_ISL_962990, EPI_ISL_962991, EPI_ISL_962992, EPI_ISL_962993, EPI_ISL_962994, EPI_ISL_962995, EPI_ISL_962996, EPI_ISL_962997, EPI_ISL_962998, EPI_ISL_962999, EPI_ISL_963000, EPI_ISL_963001, EPI_ISL_963002, EPI_ISL_963003, EPI_ISL_963004, EPI_ISL_963005, EPI_ISL_963006, EPI_ISL_963007, EPI_ISL_963008, EPI_ISL_963009, EPI_ISL_963010,			

EPI_ISL_963011, EPI_ISL_963012, EPI_ISL_963013, EPI_ISL_963014, EPI_ISL_963015, EPI_ISL_963016, EPI_ISL_963017, EPI_ISL_963018, EPI_ISL_963019, EPI_ISL_963020, EPI_ISL_963021, EPI_ISL_963022, EPI_ISL_963023, EPI_ISL_963024, EPI_ISL_963025, EPI_ISL_963026, EPI_ISL_963027, EPI_ISL_963028, EPI_ISL_963029, EPI_ISL_963030, EPI_ISL_963031, EPI_ISL_963032, EPI_ISL_963033, EPI_ISL_963034, EPI_ISL_963035, EPI_ISL_963036, EPI_ISL_963037, EPI_ISL_963038, EPI_ISL_963039, EPI_ISL_963040, EPI_ISL_963041, EPI_ISL_963042, EPI_ISL_963043, EPI_ISL_963044, EPI_ISL_963045, EPI_ISL_963046, EPI_ISL_963047, EPI_ISL_963048, EPI_ISL_963049, EPI_ISL_963050, EPI_ISL_963051, EPI_ISL_963052, EPI_ISL_963053, EPI_ISL_963054, EPI_ISL_963055, EPI_ISL_963056, EPI_ISL_963057, EPI_ISL_963058, EPI_ISL_963059, EPI_ISL_963060, EPI_ISL_963062, EPI_ISL_963063, EPI_ISL_963064, EPI_ISL_963065, EPI_ISL_963066, EPI_ISL_963067, EPI_ISL_963068, EPI_ISL_963069, EPI_ISL_963070, EPI_ISL_963071, EPI_ISL_963072, EPI_ISL_963073, EPI_ISL_963074, EPI_ISL_963075, EPI_ISL_963076, EPI_ISL_963077, EPI_ISL_963078, EPI_ISL_963079, EPI_ISL_963080, EPI_ISL_963081, EPI_ISL_963082, EPI_ISL_963083, EPI_ISL_963084, EPI_ISL_963085, EPI_ISL_963086, EPI_ISL_963087, EPI_ISL_963088, EPI_ISL_963089, EPI_ISL_963090, EPI_ISL_963091, EPI_ISL_963092, EPI_ISL_963093, EPI_ISL_963094, EPI_ISL_963095, EPI_ISL_963096, EPI_ISL_963097, EPI_ISL_963098, EPI_ISL_963099, EPI_ISL_963100, EPI_ISL_963101, EPI_ISL_963102, EPI_ISL_963103, EPI_ISL_963104, EPI_ISL_963105, EPI_ISL_963107, EPI_ISL_963108, EPI_ISL_963109, EPI_ISL_963110, EPI_ISL_963111, EPI_ISL_963112, EPI_ISL_963113, EPI_ISL_963114, EPI_ISL_963115, EPI_ISL_963116, EPI_ISL_963117, EPI_ISL_963118, EPI_ISL_963119, EPI_ISL_963120, EPI_ISL_963121, EPI_ISL_963122, EPI_ISL_963123, EPI_ISL_963124, EPI_ISL_963125, EPI_ISL_963126, EPI_ISL_963127, EPI_ISL_963128, EPI_ISL_963129, EPI_ISL_963130, EPI_ISL_963131, EPI_ISL_963132, EPI_ISL_963133, EPI_ISL_963134, EPI_ISL_963135, EPI_ISL_963136, EPI_ISL_963137, EPI_ISL_963138, EPI_ISL_963139, EPI_ISL_963140, EPI_ISL_963141, EPI_ISL_963142, EPI_ISL_963143, EPI_ISL_963144, EPI_ISL_963145, EPI_ISL_963146, EPI_ISL_963147, EPI_ISL_963148, EPI_ISL_963149, EPI_ISL_963150, EPI_ISL_963151, EPI_ISL_963152, EPI_ISL_963153, EPI_ISL_963154, EPI_ISL_963155, EPI_ISL_963156, EPI_ISL_963157, EPI_ISL_963158, EPI_ISL_963159, EPI_ISL_963160, EPI_ISL_963161, EPI_ISL_963162, EPI_ISL_963163, EPI_ISL_963164, EPI_ISL_963165, EPI_ISL_963166, EPI_ISL_963167, EPI_ISL_963168, EPI_ISL_963169, EPI_ISL_963170, EPI_ISL_963171, EPI_ISL_963172, EPI_ISL_963173, EPI_ISL_963174, EPI_ISL_963175, EPI_ISL_963176, EPI_ISL_963177, EPI_ISL_963178, EPI_ISL_963179, EPI_ISL_963180, EPI_ISL_963181, EPI_ISL_963182, EPI_ISL_963183, EPI_ISL_963184, EPI_ISL_963185, EPI_ISL_963186, EPI_ISL_963187, EPI_ISL_963188, EPI_ISL_963189, EPI_ISL_963190, EPI_ISL_963191, EPI_ISL_963192, EPI_ISL_963193, EPI_ISL_963194, EPI_ISL_963195, EPI_ISL_963196, EPI_ISL_963197, EPI_ISL_963198, EPI_ISL_963199, EPI_ISL_963200, EPI_ISL_963201, EPI_ISL_963202, EPI_ISL_963203, EPI_ISL_963204, EPI_ISL_963205, EPI_ISL_963206, EPI_ISL_963207, EPI_ISL_963208, EPI_ISL_963209, EPI_ISL_963210, EPI_ISL_963211, EPI_ISL_963212, EPI_ISL_963213, EPI_ISL_963214, EPI_ISL_963215, EPI_ISL_963216, EPI_ISL_963217, EPI_ISL_963218, EPI_ISL_963219, EPI_ISL_963220, EPI_ISL_963221, EPI_ISL_963222, EPI_ISL_963223, EPI_ISL_963224, EPI_ISL_963225, EPI_ISL_963226, EPI_ISL_963227, EPI_ISL_963228, EPI_ISL_963229, EPI_ISL_963230, EPI_ISL_963231, EPI_ISL_963232, EPI_ISL_963233, EPI_ISL_963234, EPI_ISL_963235, EPI_ISL_963236, EPI_ISL_963237, EPI_ISL_963238, EPI_ISL_963239, EPI_ISL_963240, EPI_ISL_963241, EPI_ISL_963242, EPI_ISL_963243, EPI_ISL_963244, EPI_ISL_963245, EPI_ISL_963246, EPI_ISL_963247, EPI_ISL_963248, EPI_ISL_963249, EPI_ISL_963250, EPI_ISL_963251, EPI_ISL_963252, EPI_ISL_963253, EPI_ISL_963254, EPI_ISL_963255, EPI_ISL_963256, EPI_ISL_963257, EPI_ISL_963258, EPI_ISL_963259, EPI_ISL_963260, EPI_ISL_963261, EPI_ISL_963262, EPI_ISL_963263, EPI_ISL_963264, EPI_ISL_963265, EPI_ISL_963266, EPI_ISL_963267, EPI_ISL_963268, EPI_ISL_963269, EPI_ISL_963270, EPI_ISL_963271, EPI_ISL_963272, EPI_ISL_963273, EPI_ISL_963274, EPI_ISL_963275, EPI_ISL_963276, EPI_ISL_963277, EPI_ISL_963278, EPI_ISL_963279, EPI_ISL_963280, EPI_ISL_963281, EPI_ISL_963282, EPI_ISL_963283, EPI_ISL_963284, EPI_ISL_963285, EPI_ISL_963286, EPI_ISL_963287, EPI_ISL_963288, EPI_ISL_963289, EPI_ISL_963290, EPI_ISL_963291, EPI_ISL_963292, EPI_ISL_963293, EPI_ISL_963294, EPI_ISL_963295, EPI_ISL_963297, EPI_ISL_963298, EPI_ISL_963299, EPI_ISL_963300, EPI_ISL_963301, EPI_ISL_963302, EPI_ISL_963303, EPI_ISL_963304, EPI_ISL_963305, EPI_ISL_963306, EPI_ISL_963307, EPI_ISL_963308, EPI_ISL_963309, EPI_ISL_963310, EPI_ISL_963311, EPI_ISL_963312, EPI_ISL_963313, EPI_ISL_963314, EPI_ISL_963315, EPI_ISL_963316, EPI_ISL_963317, EPI_ISL_963318, EPI_ISL_963319, EPI_ISL_963320, EPI_ISL_963322, EPI_ISL_963323

see above	Lighthouse Lab in Alderley Park	Wellcome Sanger Institute for the COVID-19 Genomics UK (COG-UK) Consortium	Jacquelyn Wynn, Mairead Hyland, The Lighthouse Lab in Alderley Park and Alex Alderton, Roberto Amato, Sonia Goncalves, Ewan Harrison, David K. Jackson, Ian Johnston, Dominic Kwiatkowski, Cordelia Langford, John Sillitoe on behalf of the Wellcome Sanger Institute COVID-19 Surveillance Team
EPI_ISL_963324	Lighthouse Lab in Milton Keynes	Wellcome Sanger Institute for the COVID-19 Genomics UK (COG-UK) Consortium	The Lighthouse Lab in Milton Keynes and Alex Alderton, Roberto Amato, Sonia Goncalves, Ewan Harrison, David K. Jackson, Ian Johnston, Dominic Kwiatkowski, Cordelia Langford, John Sillitoe on behalf of the Wellcome Sanger Institute COVID-19 Surveillance Team
EPI_ISL_963326	Lighthouse Lab in Glasgow	Wellcome Sanger Institute for the COVID-19 Genomics UK (COG-UK) Consortium	Harper VanSteenhouse, Yumi Kasai, David Gray, Carol Clugston, Anna Dominiczak and Alex Alderton, Roberto Amato, Sonia Goncalves, Ewan Harrison, David K. Jackson, Ian Johnston, Dominic Kwiatkowski, Cordelia Langford, John Sillitoe on behalf of the Wellcome Sanger Institute COVID-19 Surveillance Team
EPI_ISL_963327, EPI_ISL_963328, EPI_ISL_963329	Lighthouse Lab in Milton Keynes	Wellcome Sanger Institute for the COVID-19 Genomics UK (COG-UK) Consortium	The Lighthouse Lab in Milton Keynes and Alex Alderton, Roberto Amato, Sonia Goncalves, Ewan Harrison, David K. Jackson, Ian Johnston, Dominic Kwiatkowski, Cordelia Langford, John Sillitoe on behalf of the Wellcome Sanger Institute COVID-19 Surveillance Team
EPI_ISL_963330	Lighthouse Lab in Glasgow	Wellcome Sanger Institute for the COVID-19 Genomics UK (COG-UK) Consortium	Harper VanSteenhouse, Yumi Kasai, David Gray, Carol Clugston, Anna Dominiczak and Alex Alderton, Roberto Amato, Sonia Goncalves, Ewan Harrison, David K. Jackson, Ian Johnston, Dominic Kwiatkowski, Cordelia Langford, John Sillitoe on behalf of the Wellcome Sanger Institute COVID-19 Surveillance Team
EPI_ISL_963331, EPI_ISL_963332, EPI_ISL_963333, EPI_ISL_963334, EPI_ISL_963335, EPI_ISL_963336, EPI_ISL_963337, EPI_ISL_963338, EPI_ISL_963339, EPI_ISL_963340	Lighthouse Lab in Milton Keynes	Wellcome Sanger Institute for the COVID-19 Genomics UK (COG-UK) Consortium	The Lighthouse Lab in Milton Keynes and Alex Alderton, Roberto Amato, Sonia Goncalves, Ewan Harrison, David K. Jackson, Ian Johnston, Dominic Kwiatkowski, Cordelia Langford, John Sillitoe on behalf of the Wellcome Sanger Institute COVID-19 Surveillance Team
EPI_ISL_963341	Lighthouse Lab in Glasgow	Wellcome Sanger Institute for the COVID-19 Genomics UK (COG-UK) Consortium	Harper VanSteenhouse, Yumi Kasai, David Gray, Carol Clugston, Anna Dominiczak and Alex Alderton, Roberto Amato, Sonia Goncalves, Ewan Harrison, David K. Jackson, Ian Johnston, Dominic Kwiatkowski, Cordelia Langford, John Sillitoe on behalf of the Wellcome Sanger Institute COVID-19 Surveillance Team
EPI_ISL_963342, EPI_ISL_963343	Lighthouse Lab in Milton Keynes	Wellcome Sanger Institute for the COVID-19 Genomics UK (COG-UK) Consortium	The Lighthouse Lab in Milton Keynes and Alex Alderton, Roberto Amato, Sonia Goncalves, Ewan Harrison, David K. Jackson, Ian Johnston, Dominic Kwiatkowski, Cordelia Langford, John Sillitoe on behalf of the Wellcome Sanger Institute COVID-19 Surveillance Team
EPI_ISL_963344, EPI_ISL_963345, EPI_ISL_963346	Lighthouse Lab in Glasgow	Wellcome Sanger Institute for the COVID-19 Genomics UK (COG-UK) Consortium	Harper VanSteenhouse, Yumi Kasai, David Gray, Carol Clugston, Anna Dominiczak and Alex Alderton, Roberto Amato, Sonia Goncalves, Ewan Harrison, David K. Jackson, Ian Johnston, Dominic Kwiatkowski, Cordelia Langford, John Sillitoe on behalf of the Wellcome Sanger Institute COVID-19 Surveillance Team
EPI_ISL_963347	Lighthouse Lab in Milton Keynes	Wellcome Sanger Institute for the COVID-19 Genomics UK (COG-UK) Consortium	The Lighthouse Lab in Milton Keynes and Alex Alderton, Roberto Amato, Sonia Goncalves, Ewan Harrison, David K. Jackson, Ian Johnston, Dominic Kwiatkowski, Cordelia Langford, John Sillitoe on behalf of the Wellcome Sanger Institute COVID-19 Surveillance Team
EPI_ISL_963348	Lighthouse Lab in Glasgow	Wellcome Sanger Institute for the COVID-19 Genomics UK (COG-UK) Consortium	Harper VanSteenhouse, Yumi Kasai, David Gray, Carol Clugston, Anna Dominiczak and Alex Alderton, Roberto Amato, Sonia Goncalves, Ewan Harrison, David K. Jackson, Ian Johnston, Dominic Kwiatkowski, Cordelia Langford, John Sillitoe on behalf of the Wellcome Sanger Institute COVID-19 Surveillance Team
EPI_ISL_963349, EPI_ISL_963350, EPI_ISL_963351, EPI_ISL_963352, EPI_ISL_963353, EPI_ISL_963354	Lighthouse Lab in Milton Keynes	Wellcome Sanger Institute for the COVID-19 Genomics UK (COG-UK) Consortium	The Lighthouse Lab in Milton Keynes and Alex Alderton, Roberto Amato, Sonia Goncalves, Ewan Harrison, David K. Jackson, Ian Johnston, Dominic Kwiatkowski, Cordelia Langford, John Sillitoe on behalf of the Wellcome Sanger Institute COVID-19 Surveillance Team
EPI_ISL_963355, EPI_ISL_963356, EPI_ISL_963357, EPI_ISL_963358	Lighthouse Lab in Glasgow	Wellcome Sanger Institute for the COVID-19 Genomics UK (COG-UK) Consortium	Harper VanSteenhouse, Yumi Kasai, David Gray, Carol Clugston, Anna Dominiczak and Alex Alderton, Roberto Amato, Sonia Goncalves, Ewan Harrison, David K. Jackson, Ian Johnston, Dominic Kwiatkowski, Cordelia Langford, John Sillitoe on behalf of the Wellcome Sanger Institute COVID-19 Surveillance Team

EPI_ISL_963359, EPI_ISL_963360, EPI_ISL_963361, EPI_ISL_963362, EPI_ISL_963363, EPI_ISL_963364, EPI_ISL_963365, EPI_ISL_963366, EPI_ISL_963367, EPI_ISL_963368, EPI_ISL_963369

see above	Lighthouse Lab in Milton Keynes	Wellcome Sanger Institute for the COVID-19 Genomics UK (COG-UK) Consortium	The Lighthouse Lab in Milton Keynes and Alex Alderton, Roberto Amato, Sonia Goncalves, Ewan Harrison, David K. Jackson, Ian Johnston, Dominic Kwiatkowski, Cordelia Langford, John Sillitoe on behalf of the Wellcome Sanger Institute COVID-19 Surveillance Team
EPI_ISL_963370, EPI_ISL_963371	Lighthouse Lab in Glasgow	Wellcome Sanger Institute for the COVID-19 Genomics UK (COG-UK) Consortium	Harper VanSteenhouse, Yumi Kasai, David Gray, Carol Clugston, Anna Dominiczak and Alex Alderton, Roberto Amato, Sonia Goncalves, Ewan Harrison, David K. Jackson, Ian Johnston, Dominic Kwiatkowski, Cordelia Langford, John Sillitoe on behalf of the Wellcome Sanger Institute COVID-19 Surveillance Team
EPI_ISL_963372, EPI_ISL_963373, EPI_ISL_963374, EPI_ISL_963375	Lighthouse Lab in Milton Keynes	Wellcome Sanger Institute for the COVID-19 Genomics UK (COG-UK) Consortium	The Lighthouse Lab in Milton Keynes and Alex Alderton, Roberto Amato, Sonia Goncalves, Ewan Harrison, David K. Jackson, Ian Johnston, Dominic Kwiatkowski, Cordelia Langford, John Sillitoe on behalf of the Wellcome Sanger Institute COVID-19 Surveillance Team
EPI_ISL_963376	Lighthouse Lab in Glasgow	Wellcome Sanger Institute for the COVID-19 Genomics UK (COG-UK) Consortium	Harper VanSteenhouse, Yumi Kasai, David Gray, Carol Clugston, Anna Dominiczak and Alex Alderton, Roberto Amato, Sonia Goncalves, Ewan Harrison, David K. Jackson, Ian Johnston, Dominic Kwiatkowski, Cordelia Langford, John Sillitoe on behalf of the Wellcome Sanger Institute COVID-19 Surveillance Team
EPI_ISL_963377, EPI_ISL_963378, EPI_ISL_963379	Lighthouse Lab in Milton Keynes	Wellcome Sanger Institute for the COVID-19 Genomics UK (COG-UK) Consortium	The Lighthouse Lab in Milton Keynes and Alex Alderton, Roberto Amato, Sonia Goncalves, Ewan Harrison, David K. Jackson, Ian Johnston, Dominic Kwiatkowski, Cordelia Langford, John Sillitoe on behalf of the Wellcome Sanger Institute COVID-19 Surveillance Team
EPI_ISL_963380	Lighthouse Lab in Glasgow	Wellcome Sanger Institute for the COVID-19 Genomics UK (COG-UK) Consortium	Harper VanSteenhouse, Yumi Kasai, David Gray, Carol Clugston, Anna Dominiczak and Alex Alderton, Roberto Amato, Sonia Goncalves, Ewan Harrison, David K. Jackson, Ian Johnston, Dominic Kwiatkowski, Cordelia Langford, John Sillitoe on behalf of the Wellcome Sanger Institute COVID-19 Surveillance Team
EPI_ISL_963381, EPI_ISL_963382, EPI_ISL_963383, EPI_ISL_963384, EPI_ISL_963385	Lighthouse Lab in Milton Keynes	Wellcome Sanger Institute for the COVID-19 Genomics UK (COG-UK) Consortium	The Lighthouse Lab in Milton Keynes and Alex Alderton, Roberto Amato, Sonia Goncalves, Ewan Harrison, David K. Jackson, Ian Johnston, Dominic Kwiatkowski, Cordelia Langford, John Sillitoe on behalf of the Wellcome Sanger Institute COVID-19 Surveillance Team
EPI_ISL_963386	Lighthouse Lab in Glasgow	Wellcome Sanger Institute for the COVID-19 Genomics UK (COG-UK) Consortium	Harper VanSteenhouse, Yumi Kasai, David Gray, Carol Clugston, Anna Dominiczak and Alex Alderton, Roberto Amato, Sonia Goncalves, Ewan Harrison, David K. Jackson, Ian Johnston, Dominic Kwiatkowski, Cordelia Langford, John Sillitoe on behalf of the Wellcome Sanger Institute COVID-19 Surveillance Team

EPI_ISL_963387, EPI_ISL_963388, EPI_ISL_963389, EPI_ISL_963390, EPI_ISL_963391, EPI_ISL_963392, EPI_ISL_963393, EPI_ISL_963394, EPI_ISL_963395, EPI_ISL_963396	Lighthouse Lab in Milton Keynes	Wellcome Sanger Institute for the COVID-19 Genomics UK (COG-UK) Consortium	The Lighthouse Lab in Milton Keynes and Alex Alderton, Roberto Amato, Sonia Goncalves, Ewan Harrison, David K. Jackson, Ian Johnston, Dominic Kwiatkowski, Cordelia Langford, John Sillitoe on behalf of the Wellcome Sanger Institute COVID-19 Surveillance Team
EPI_ISL_963397	Lighthouse Lab in Glasgow	Wellcome Sanger Institute for the COVID-19 Genomics UK (COG-UK) Consortium	Harper VanSteenhouse, Yumi Kasai, David Gray, Carol Clugston, Anna Dominiczak and Alex Alderton, Roberto Amato, Sonia Goncalves, Ewan Harrison, David K. Jackson, Ian Johnston, Dominic Kwiatkowski, Cordelia Langford, John Sillitoe on behalf of the Wellcome Sanger Institute COVID-19 Surveillance Team
EPI_ISL_963398, EPI_ISL_963399, EPI_ISL_963400, EPI_ISL_963401, EPI_ISL_963402, EPI_ISL_963403, EPI_ISL_963404, EPI_ISL_963405, EPI_ISL_963406, EPI_ISL_963407, EPI_ISL_963409, EPI_ISL_963410, EPI_ISL_963411, EPI_ISL_963412, EPI_ISL_963413, EPI_ISL_963414, EPI_ISL_963415, EPI_ISL_963416, EPI_ISL_963417, EPI_ISL_963418			
see above	Lighthouse Lab in Milton Keynes	Wellcome Sanger Institute for the COVID-19 Genomics UK (COG-UK) Consortium	The Lighthouse Lab in Milton Keynes and Alex Alderton, Roberto Amato, Sonia Goncalves, Ewan Harrison, David K. Jackson, Ian Johnston, Dominic Kwiatkowski, Cordelia Langford, John Sillitoe on behalf of the Wellcome Sanger Institute COVID-19 Surveillance Team
EPI_ISL_963419	Lighthouse Lab in Glasgow	Wellcome Sanger Institute for the COVID-19 Genomics UK (COG-UK) Consortium	Harper VanSteenhouse, Yumi Kasai, David Gray, Carol Clugston, Anna Dominiczak and Alex Alderton, Roberto Amato, Sonia Goncalves, Ewan Harrison, David K. Jackson, Ian Johnston, Dominic Kwiatkowski, Cordelia Langford, John Sillitoe on behalf of the Wellcome Sanger Institute COVID-19 Surveillance Team
EPI_ISL_963420	Lighthouse Lab in Milton Keynes	Wellcome Sanger Institute for the COVID-19 Genomics UK (COG-UK) Consortium	The Lighthouse Lab in Milton Keynes and Alex Alderton, Roberto Amato, Sonia Goncalves, Ewan Harrison, David K. Jackson, Ian Johnston, Dominic Kwiatkowski, Cordelia Langford, John Sillitoe on behalf of the Wellcome Sanger Institute COVID-19 Surveillance Team
EPI_ISL_963421	Lighthouse Lab in Glasgow	Wellcome Sanger Institute for the COVID-19 Genomics UK (COG-UK) Consortium	Harper VanSteenhouse, Yumi Kasai, David Gray, Carol Clugston, Anna Dominiczak and Alex Alderton, Roberto Amato, Sonia Goncalves, Ewan Harrison, David K. Jackson, Ian Johnston, Dominic Kwiatkowski, Cordelia Langford, John Sillitoe on behalf of the Wellcome Sanger Institute COVID-19 Surveillance Team
EPI_ISL_963422, EPI_ISL_963423, EPI_ISL_963424	Lighthouse Lab in Milton Keynes	Wellcome Sanger Institute for the COVID-19 Genomics UK (COG-UK) Consortium	The Lighthouse Lab in Milton Keynes and Alex Alderton, Roberto Amato, Sonia Goncalves, Ewan Harrison, David K. Jackson, Ian Johnston, Dominic Kwiatkowski, Cordelia Langford, John Sillitoe on behalf of the Wellcome Sanger Institute COVID-19 Surveillance Team
EPI_ISL_963425, EPI_ISL_963426	Lighthouse Lab in Glasgow	Wellcome Sanger Institute for the COVID-19 Genomics UK (COG-UK) Consortium	Harper VanSteenhouse, Yumi Kasai, David Gray, Carol Clugston, Anna Dominiczak and Alex Alderton, Roberto Amato, Sonia Goncalves, Ewan Harrison, David K. Jackson, Ian Johnston, Dominic Kwiatkowski, Cordelia Langford, John Sillitoe on behalf of the Wellcome Sanger Institute COVID-19 Surveillance Team
EPI_ISL_963427, EPI_ISL_963429, EPI_ISL_963430	Lighthouse Lab in Milton Keynes	Wellcome Sanger Institute for the COVID-19 Genomics UK (COG-UK) Consortium	The Lighthouse Lab in Milton Keynes and Alex Alderton, Roberto Amato, Sonia Goncalves, Ewan Harrison, David K. Jackson, Ian Johnston, Dominic Kwiatkowski, Cordelia Langford, John Sillitoe on behalf of the Wellcome Sanger Institute COVID-19 Surveillance Team
EPI_ISL_963431	Lighthouse Lab in Glasgow	Wellcome Sanger Institute for the COVID-19 Genomics UK (COG-UK) Consortium	Harper VanSteenhouse, Yumi Kasai, David Gray, Carol Clugston, Anna Dominiczak and Alex Alderton, Roberto Amato, Sonia Goncalves, Ewan Harrison, David K. Jackson, Ian Johnston, Dominic Kwiatkowski, Cordelia Langford, John Sillitoe on behalf of the Wellcome Sanger Institute COVID-19 Surveillance Team
EPI_ISL_963432	Lighthouse Lab in Milton Keynes	Wellcome Sanger Institute for the COVID-19 Genomics UK (COG-UK) Consortium	The Lighthouse Lab in Milton Keynes and Alex Alderton, Roberto Amato, Sonia Goncalves, Ewan Harrison, David K. Jackson, Ian Johnston, Dominic Kwiatkowski, Cordelia Langford, John Sillitoe on behalf of the Wellcome Sanger Institute COVID-19 Surveillance Team
EPI_ISL_963433, EPI_ISL_963434	Lighthouse Lab in Glasgow	Wellcome Sanger Institute for the COVID-19 Genomics UK (COG-UK) Consortium	Harper VanSteenhouse, Yumi Kasai, David Gray, Carol Clugston, Anna Dominiczak and Alex Alderton, Roberto Amato, Sonia Goncalves, Ewan Harrison, David K. Jackson, Ian Johnston, Dominic Kwiatkowski, Cordelia Langford, John Sillitoe on behalf of the Wellcome Sanger Institute COVID-19 Surveillance Team
EPI_ISL_963435, EPI_ISL_963436, EPI_ISL_963437, EPI_ISL_963438, EPI_ISL_963439, EPI_ISL_963440, EPI_ISL_963441, EPI_ISL_963442, EPI_ISL_963443, EPI_ISL_963444, EPI_ISL_963445, EPI_ISL_963446, EPI_ISL_963447, EPI_ISL_963448			
see above	Lighthouse Lab in Milton Keynes	Wellcome Sanger Institute for the COVID-19 Genomics UK (COG-UK) Consortium	The Lighthouse Lab in Milton Keynes and Alex Alderton, Roberto Amato, Sonia Goncalves, Ewan Harrison, David K. Jackson, Ian Johnston, Dominic Kwiatkowski, Cordelia Langford, John Sillitoe on behalf of the Wellcome Sanger Institute COVID-19 Surveillance Team
EPI_ISL_963449	Lighthouse Lab in Glasgow	Wellcome Sanger Institute for the COVID-19 Genomics UK (COG-UK) Consortium	Harper VanSteenhouse, Yumi Kasai, David Gray, Carol Clugston, Anna Dominiczak and Alex Alderton, Roberto Amato, Sonia Goncalves, Ewan Harrison, David K. Jackson, Ian Johnston, Dominic Kwiatkowski, Cordelia Langford, John Sillitoe on behalf of the Wellcome Sanger Institute COVID-19 Surveillance Team
EPI_ISL_963450, EPI_ISL_963451	Lighthouse Lab in Milton Keynes	Wellcome Sanger Institute for the COVID-19 Genomics UK (COG-UK) Consortium	The Lighthouse Lab in Milton Keynes and Alex Alderton, Roberto Amato, Sonia Goncalves, Ewan Harrison, David K. Jackson, Ian Johnston, Dominic Kwiatkowski, Cordelia Langford, John Sillitoe on behalf of the Wellcome Sanger Institute COVID-19 Surveillance Team
EPI_ISL_963452	Lighthouse Lab in Glasgow	Wellcome Sanger Institute for the COVID-19 Genomics UK (COG-UK) Consortium	Harper VanSteenhouse, Yumi Kasai, David Gray, Carol Clugston, Anna Dominiczak and Alex Alderton, Roberto Amato, Sonia Goncalves, Ewan Harrison, David K. Jackson, Ian Johnston, Dominic Kwiatkowski, Cordelia Langford, John Sillitoe on behalf of the Wellcome Sanger Institute COVID-19 Surveillance Team
EPI_ISL_963453, EPI_ISL_963454, EPI_ISL_963455, EPI_ISL_963456, EPI_ISL_963457	Lighthouse Lab in Milton Keynes	Wellcome Sanger Institute for the COVID-19 Genomics UK (COG-UK) Consortium	The Lighthouse Lab in Milton Keynes and Alex Alderton, Roberto Amato, Sonia Goncalves, Ewan Harrison, David K. Jackson, Ian Johnston, Dominic Kwiatkowski, Cordelia Langford, John Sillitoe on behalf of the Wellcome Sanger Institute COVID-19 Surveillance Team
EPI_ISL_963458	Lighthouse Lab in Glasgow	Wellcome Sanger Institute for the COVID-19 Genomics UK (COG-UK) Consortium	Harper VanSteenhouse, Yumi Kasai, David Gray, Carol Clugston, Anna Dominiczak and Alex Alderton, Roberto Amato, Sonia Goncalves, Ewan Harrison, David K. Jackson, Ian Johnston, Dominic Kwiatkowski, Cordelia Langford, John Sillitoe on behalf of the Wellcome Sanger Institute COVID-19 Surveillance Team
EPI_ISL_963459, EPI_ISL_963460, EPI_ISL_963461, EPI_ISL_963462, EPI_ISL_963463, EPI_ISL_963464, EPI_ISL_963465, EPI_ISL_963466, EPI_ISL_963467, EPI_ISL_963468, EPI_ISL_963469, EPI_ISL_963470, EPI_ISL_963472, EPI_ISL_963473, EPI_ISL_963474			
see above	Lighthouse Lab in Milton Keynes	Wellcome Sanger Institute for the COVID-19 Genomics UK (COG-UK) Consortium	The Lighthouse Lab in Milton Keynes and Alex Alderton, Roberto Amato, Sonia Goncalves, Ewan Harrison, David K. Jackson, Ian Johnston, Dominic Kwiatkowski, Cordelia Langford, John Sillitoe on behalf of the Wellcome Sanger Institute COVID-19 Surveillance Team
EPI_ISL_963475	Lighthouse Lab in Glasgow	Wellcome Sanger Institute for the COVID-19 Genomics UK (COG-UK) Consortium	Harper VanSteenhouse, Yumi Kasai, David Gray, Carol Clugston, Anna Dominiczak and Alex Alderton, Roberto Amato, Sonia Goncalves, Ewan Harrison, David K. Jackson, Ian Johnston, Dominic Kwiatkowski, Cordelia Langford, John Sillitoe on behalf of the Wellcome Sanger Institute COVID-19 Surveillance Team
EPI_ISL_963476, EPI_ISL_963477, EPI_ISL_963478, EPI_ISL_963479, EPI_ISL_963480	Lighthouse Lab in Milton Keynes	Wellcome Sanger Institute for the COVID-19 Genomics UK (COG-UK) Consortium	The Lighthouse Lab in Milton Keynes and Alex Alderton, Roberto Amato, Sonia Goncalves, Ewan Harrison, David K. Jackson, Ian Johnston, Dominic Kwiatkowski, Cordelia Langford, John Sillitoe on behalf of the Wellcome Sanger Institute COVID-19 Surveillance Team
EPI_ISL_963481	Lighthouse Lab in Glasgow	Wellcome Sanger Institute for the COVID-19 Genomics UK (COG-UK) Consortium	Harper VanSteenhouse, Yumi Kasai, David Gray, Carol Clugston, Anna Dominiczak and Alex Alderton, Roberto Amato, Sonia Goncalves, Ewan Harrison, David K. Jackson, Ian Johnston, Dominic Kwiatkowski, Cordelia Langford, John Sillitoe on behalf of the Wellcome Sanger Institute COVID-19 Surveillance Team
EPI_ISL_963482, EPI_ISL_963483, EPI_ISL_963484, EPI_ISL_963485, EPI_ISL_963486, EPI_ISL_963487, EPI_ISL_963488	Lighthouse Lab in Milton Keynes	Wellcome Sanger Institute for the COVID-19 Genomics UK (COG-UK) Consortium	The Lighthouse Lab in Milton Keynes and Alex Alderton, Roberto Amato, Sonia Goncalves, Ewan Harrison, David K. Jackson, Ian Johnston, Dominic Kwiatkowski, Cordelia Langford, John Sillitoe on behalf of the Wellcome Sanger Institute COVID-19 Surveillance Team
EPI_ISL_963489, EPI_ISL_963490	Lighthouse Lab in Glasgow	Wellcome Sanger Institute for the COVID-19 Genomics UK (COG-UK) Consortium	Harper VanSteenhouse, Yumi Kasai, David Gray, Carol Clugston, Anna Dominiczak and Alex Alderton, Roberto Amato, Sonia Goncalves, Ewan Harrison, David K. Jackson, Ian Johnston, Dominic Kwiatkowski, Cordelia Langford, John Sillitoe on behalf of the Wellcome Sanger Institute COVID-19 Surveillance Team
EPI_ISL_963491, EPI_ISL_963492, EPI_ISL_963493, EPI_ISL_963494	Lighthouse Lab in Milton Keynes	Wellcome Sanger Institute for the COVID-19 Genomics UK (COG-UK) Consortium	The Lighthouse Lab in Milton Keynes and Alex Alderton, Roberto Amato, Sonia Goncalves, Ewan Harrison, David K. Jackson, Ian Johnston, Dominic Kwiatkowski, Cordelia Langford, John Sillitoe on behalf of the Wellcome Sanger Institute COVID-19 Surveillance Team

EPI_ISL_963495	Lighthouse Lab in Glasgow	Wellcome Sanger Institute for the COVID-19 Genomics UK (COG-UK) Consortium	Harper VanSteenhouse, Yumi Kasai, David Gray, Carol Clugston, Anna Dominiczak and Alex Alderton, Roberto Amato, Sonia Goncalves, Ewan Harrison, David K. Jackson, Ian Johnston, Dominic Kwiatkowski, Cordelia Langford, John Sillitoe on behalf of the Wellcome Sanger Institute COVID-19 Surveillance Team
EPI_ISL_963496, EPI_ISL_963497, EPI_ISL_963498, EPI_ISL_963499, EPI_ISL_963500, EPI_ISL_963501, EPI_ISL_963502, EPI_ISL_963503, EPI_ISL_963504, EPI_ISL_963505, EPI_ISL_963506, EPI_ISL_963507, EPI_ISL_963508			
see above	Lighthouse Lab in Milton Keynes	Wellcome Sanger Institute for the COVID-19 Genomics UK (COG-UK) Consortium	The Lighthouse Lab in Milton Keynes and Alex Alderton, Roberto Amato, Sonia Goncalves, Ewan Harrison, David K. Jackson, Ian Johnston, Dominic Kwiatkowski, Cordelia Langford, John Sillitoe on behalf of the Wellcome Sanger Institute COVID-19 Surveillance Team
EPI_ISL_963509	Lighthouse Lab in Glasgow	Wellcome Sanger Institute for the COVID-19 Genomics UK (COG-UK) Consortium	Harper VanSteenhouse, Yumi Kasai, David Gray, Carol Clugston, Anna Dominiczak and Alex Alderton, Roberto Amato, Sonia Goncalves, Ewan Harrison, David K. Jackson, Ian Johnston, Dominic Kwiatkowski, Cordelia Langford, John Sillitoe on behalf of the Wellcome Sanger Institute COVID-19 Surveillance Team
EPI_ISL_963510	Lighthouse Lab in Milton Keynes	Wellcome Sanger Institute for the COVID-19 Genomics UK (COG-UK) Consortium	The Lighthouse Lab in Milton Keynes and Alex Alderton, Roberto Amato, Sonia Goncalves, Ewan Harrison, David K. Jackson, Ian Johnston, Dominic Kwiatkowski, Cordelia Langford, John Sillitoe on behalf of the Wellcome Sanger Institute COVID-19 Surveillance Team
EPI_ISL_963511	Lighthouse Lab in Glasgow	Wellcome Sanger Institute for the COVID-19 Genomics UK (COG-UK) Consortium	Harper VanSteenhouse, Yumi Kasai, David Gray, Carol Clugston, Anna Dominiczak and Alex Alderton, Roberto Amato, Sonia Goncalves, Ewan Harrison, David K. Jackson, Ian Johnston, Dominic Kwiatkowski, Cordelia Langford, John Sillitoe on behalf of the Wellcome Sanger Institute COVID-19 Surveillance Team
EPI_ISL_963512, EPI_ISL_963513, EPI_ISL_963514, EPI_ISL_963515, EPI_ISL_963516, EPI_ISL_963517, EPI_ISL_963518, EPI_ISL_963519	Lighthouse Lab in Milton Keynes	Wellcome Sanger Institute for the COVID-19 Genomics UK (COG-UK) Consortium	The Lighthouse Lab in Milton Keynes and Alex Alderton, Roberto Amato, Sonia Goncalves, Ewan Harrison, David K. Jackson, Ian Johnston, Dominic Kwiatkowski, Cordelia Langford, John Sillitoe on behalf of the Wellcome Sanger Institute COVID-19 Surveillance Team
EPI_ISL_963520, EPI_ISL_963521	Lighthouse Lab in Glasgow	Wellcome Sanger Institute for the COVID-19 Genomics UK (COG-UK) Consortium	Harper VanSteenhouse, Yumi Kasai, David Gray, Carol Clugston, Anna Dominiczak and Alex Alderton, Roberto Amato, Sonia Goncalves, Ewan Harrison, David K. Jackson, Ian Johnston, Dominic Kwiatkowski, Cordelia Langford, John Sillitoe on behalf of the Wellcome Sanger Institute COVID-19 Surveillance Team
EPI_ISL_963522, EPI_ISL_963523, EPI_ISL_963525, EPI_ISL_963526, EPI_ISL_963527, EPI_ISL_963528	Lighthouse Lab in Milton Keynes	Wellcome Sanger Institute for the COVID-19 Genomics UK (COG-UK) Consortium	The Lighthouse Lab in Milton Keynes and Alex Alderton, Roberto Amato, Sonia Goncalves, Ewan Harrison, David K. Jackson, Ian Johnston, Dominic Kwiatkowski, Cordelia Langford, John Sillitoe on behalf of the Wellcome Sanger Institute COVID-19 Surveillance Team
EPI_ISL_963529, EPI_ISL_963530	Lighthouse Lab in Glasgow	Wellcome Sanger Institute for the COVID-19 Genomics UK (COG-UK) Consortium	Harper VanSteenhouse, Yumi Kasai, David Gray, Carol Clugston, Anna Dominiczak and Alex Alderton, Roberto Amato, Sonia Goncalves, Ewan Harrison, David K. Jackson, Ian Johnston, Dominic Kwiatkowski, Cordelia Langford, John Sillitoe on behalf of the Wellcome Sanger Institute COVID-19 Surveillance Team
EPI_ISL_963531, EPI_ISL_963532, EPI_ISL_963533, EPI_ISL_963534, EPI_ISL_963535, EPI_ISL_963536, EPI_ISL_963537	Lighthouse Lab in Milton Keynes	Wellcome Sanger Institute for the COVID-19 Genomics UK (COG-UK) Consortium	The Lighthouse Lab in Milton Keynes and Alex Alderton, Roberto Amato, Sonia Goncalves, Ewan Harrison, David K. Jackson, Ian Johnston, Dominic Kwiatkowski, Cordelia Langford, John Sillitoe on behalf of the Wellcome Sanger Institute COVID-19 Surveillance Team
EPI_ISL_963538	Lighthouse Lab in Glasgow	Wellcome Sanger Institute for the COVID-19 Genomics UK (COG-UK) Consortium	Harper VanSteenhouse, Yumi Kasai, David Gray, Carol Clugston, Anna Dominiczak and Alex Alderton, Roberto Amato, Sonia Goncalves, Ewan Harrison, David K. Jackson, Ian Johnston, Dominic Kwiatkowski, Cordelia Langford, John Sillitoe on behalf of the Wellcome Sanger Institute COVID-19 Surveillance Team
EPI_ISL_963539, EPI_ISL_963540, EPI_ISL_963541, EPI_ISL_963542, EPI_ISL_963543, EPI_ISL_963544, EPI_ISL_963545, EPI_ISL_963546, EPI_ISL_963547, EPI_ISL_963548, EPI_ISL_963549, EPI_ISL_963550, EPI_ISL_963551, EPI_ISL_963552, EPI_ISL_963553			
see above	Lighthouse Lab in Milton Keynes	Wellcome Sanger Institute for the COVID-19 Genomics UK (COG-UK) Consortium	The Lighthouse Lab in Milton Keynes and Alex Alderton, Roberto Amato, Sonia Goncalves, Ewan Harrison, David K. Jackson, Ian Johnston, Dominic Kwiatkowski, Cordelia Langford, John Sillitoe on behalf of the Wellcome Sanger Institute COVID-19 Surveillance Team
EPI_ISL_963554	Lighthouse Lab in Glasgow	Wellcome Sanger Institute for the COVID-19 Genomics UK (COG-UK) Consortium	Harper VanSteenhouse, Yumi Kasai, David Gray, Carol Clugston, Anna Dominiczak and Alex Alderton, Roberto Amato, Sonia Goncalves, Ewan Harrison, David K. Jackson, Ian Johnston, Dominic Kwiatkowski, Cordelia Langford, John Sillitoe on behalf of the Wellcome Sanger Institute COVID-19 Surveillance Team
EPI_ISL_963556, EPI_ISL_963557, EPI_ISL_963558	Lighthouse Lab in Milton Keynes	Wellcome Sanger Institute for the COVID-19 Genomics UK (COG-UK) Consortium	The Lighthouse Lab in Milton Keynes and Alex Alderton, Roberto Amato, Sonia Goncalves, Ewan Harrison, David K. Jackson, Ian Johnston, Dominic Kwiatkowski, Cordelia Langford, John Sillitoe on behalf of the Wellcome Sanger Institute COVID-19 Surveillance Team
EPI_ISL_963559, EPI_ISL_963560	Lighthouse Lab in Glasgow	Wellcome Sanger Institute for the COVID-19 Genomics UK (COG-UK) Consortium	Harper VanSteenhouse, Yumi Kasai, David Gray, Carol Clugston, Anna Dominiczak and Alex Alderton, Roberto Amato, Sonia Goncalves, Ewan Harrison, David K. Jackson, Ian Johnston, Dominic Kwiatkowski, Cordelia Langford, John Sillitoe on behalf of the Wellcome Sanger Institute COVID-19 Surveillance Team
EPI_ISL_963561	Lighthouse Lab in Milton Keynes	Wellcome Sanger Institute for the COVID-19 Genomics UK (COG-UK) Consortium	The Lighthouse Lab in Milton Keynes and Alex Alderton, Roberto Amato, Sonia Goncalves, Ewan Harrison, David K. Jackson, Ian Johnston, Dominic Kwiatkowski, Cordelia Langford, John Sillitoe on behalf of the Wellcome Sanger Institute COVID-19 Surveillance Team
EPI_ISL_963562	Lighthouse Lab in Glasgow	Wellcome Sanger Institute for the COVID-19 Genomics UK (COG-UK) Consortium	Harper VanSteenhouse, Yumi Kasai, David Gray, Carol Clugston, Anna Dominiczak and Alex Alderton, Roberto Amato, Sonia Goncalves, Ewan Harrison, David K. Jackson, Ian Johnston, Dominic Kwiatkowski, Cordelia Langford, John Sillitoe on behalf of the Wellcome Sanger Institute COVID-19 Surveillance Team
EPI_ISL_963563, EPI_ISL_963564, EPI_ISL_963565, EPI_ISL_963566, EPI_ISL_963567, EPI_ISL_963568, EPI_ISL_963569	Lighthouse Lab in Milton Keynes	Wellcome Sanger Institute for the COVID-19 Genomics UK (COG-UK) Consortium	The Lighthouse Lab in Milton Keynes and Alex Alderton, Roberto Amato, Sonia Goncalves, Ewan Harrison, David K. Jackson, Ian Johnston, Dominic Kwiatkowski, Cordelia Langford, John Sillitoe on behalf of the Wellcome Sanger Institute COVID-19 Surveillance Team
EPI_ISL_963570	Lighthouse Lab in Glasgow	Wellcome Sanger Institute for the COVID-19 Genomics UK (COG-UK) Consortium	Harper VanSteenhouse, Yumi Kasai, David Gray, Carol Clugston, Anna Dominiczak and Alex Alderton, Roberto Amato, Sonia Goncalves, Ewan Harrison, David K. Jackson, Ian Johnston, Dominic Kwiatkowski, Cordelia Langford, John Sillitoe on behalf of the Wellcome Sanger Institute COVID-19 Surveillance Team
EPI_ISL_963571, EPI_ISL_963572	Lighthouse Lab in Milton Keynes	Wellcome Sanger Institute for the COVID-19 Genomics UK (COG-UK) Consortium	The Lighthouse Lab in Milton Keynes and Alex Alderton, Roberto Amato, Sonia Goncalves, Ewan Harrison, David K. Jackson, Ian Johnston, Dominic Kwiatkowski, Cordelia Langford, John Sillitoe on behalf of the Wellcome Sanger Institute COVID-19 Surveillance Team
EPI_ISL_963573	Lighthouse Lab in Glasgow	Wellcome Sanger Institute for the COVID-19 Genomics UK (COG-UK) Consortium	Harper VanSteenhouse, Yumi Kasai, David Gray, Carol Clugston, Anna Dominiczak and Alex Alderton, Roberto Amato, Sonia Goncalves, Ewan Harrison, David K. Jackson, Ian Johnston, Dominic Kwiatkowski, Cordelia Langford, John Sillitoe on behalf of the Wellcome Sanger Institute COVID-19 Surveillance Team
EPI_ISL_963574, EPI_ISL_963575	Lighthouse Lab in Milton Keynes	Wellcome Sanger Institute for the COVID-19 Genomics UK (COG-UK) Consortium	The Lighthouse Lab in Milton Keynes and Alex Alderton, Roberto Amato, Sonia Goncalves, Ewan Harrison, David K. Jackson, Ian Johnston, Dominic Kwiatkowski, Cordelia Langford, John Sillitoe on behalf of the Wellcome Sanger Institute COVID-19 Surveillance Team
EPI_ISL_963576	Lighthouse Lab in Glasgow	Wellcome Sanger Institute for the COVID-19 Genomics UK (COG-UK) Consortium	Harper VanSteenhouse, Yumi Kasai, David Gray, Carol Clugston, Anna Dominiczak and Alex Alderton, Roberto Amato, Sonia Goncalves, Ewan Harrison, David K. Jackson, Ian Johnston, Dominic Kwiatkowski, Cordelia Langford, John Sillitoe on behalf of the Wellcome Sanger Institute COVID-19 Surveillance Team
EPI_ISL_963577, EPI_ISL_963578, EPI_ISL_963579, EPI_ISL_963580	Lighthouse Lab in Milton Keynes	Wellcome Sanger Institute for the COVID-19 Genomics UK (COG-UK) Consortium	The Lighthouse Lab in Milton Keynes and Alex Alderton, Roberto Amato, Sonia Goncalves, Ewan Harrison, David K. Jackson, Ian Johnston, Dominic Kwiatkowski, Cordelia Langford, John Sillitoe on behalf of the Wellcome Sanger Institute COVID-19 Surveillance Team
EPI_ISL_963581	Lighthouse Lab in Glasgow	Wellcome Sanger Institute for the COVID-19 Genomics UK (COG-UK) Consortium	Harper VanSteenhouse, Yumi Kasai, David Gray, Carol Clugston, Anna Dominiczak and Alex Alderton, Roberto Amato, Sonia Goncalves, Ewan Harrison, David K. Jackson, Ian Johnston, Dominic Kwiatkowski, Cordelia Langford, John Sillitoe on behalf of the Wellcome Sanger Institute COVID-19 Surveillance Team
EPI_ISL_963582, EPI_ISL_963583	Lighthouse Lab in Milton Keynes	Wellcome Sanger Institute for the COVID-19 Genomics UK (COG-UK) Consortium	The Lighthouse Lab in Milton Keynes and Alex Alderton, Roberto Amato, Sonia Goncalves, Ewan Harrison, David K. Jackson, Ian Johnston, Dominic Kwiatkowski, Cordelia Langford, John Sillitoe on behalf of the Wellcome Sanger Institute COVID-19 Surveillance Team

EPI_ISL_963584	Lighthouse Lab in Glasgow	Wellcome Sanger Institute for the COVID-19 Genomics UK (COG-UK) Consortium	Harper VanSteenhouse, Yumi Kasai, David Gray, Carol Clugston, Anna Dominiczak and Alex Alderton, Roberto Amato, Sonia Goncalves, Ewan Harrison, David K. Jackson, Ian Johnston, Dominic Kwiatkowski, Cordelia Langford, John Sillitoe on behalf of the Wellcome Sanger Institute COVID-19 Surveillance Team
EPI_ISL_963585, EPI_ISL_963586, EPI_ISL_963587, EPI_ISL_963588, EPI_ISL_963589, EPI_ISL_963590	Lighthouse Lab in Milton Keynes	Wellcome Sanger Institute for the COVID-19 Genomics UK (COG-UK) Consortium	The Lighthouse Lab in Milton Keynes and Alex Alderton, Roberto Amato, Sonia Goncalves, Ewan Harrison, David K. Jackson, Ian Johnston, Dominic Kwiatkowski, Cordelia Langford, John Sillitoe on behalf of the Wellcome Sanger Institute COVID-19 Surveillance Team
EPI_ISL_963591	Lighthouse Lab in Glasgow	Wellcome Sanger Institute for the COVID-19 Genomics UK (COG-UK) Consortium	Harper VanSteenhouse, Yumi Kasai, David Gray, Carol Clugston, Anna Dominiczak and Alex Alderton, Roberto Amato, Sonia Goncalves, Ewan Harrison, David K. Jackson, Ian Johnston, Dominic Kwiatkowski, Cordelia Langford, John Sillitoe on behalf of the Wellcome Sanger Institute COVID-19 Surveillance Team
EPI_ISL_963592, EPI_ISL_963593	Lighthouse Lab in Milton Keynes	Wellcome Sanger Institute for the COVID-19 Genomics UK (COG-UK) Consortium	The Lighthouse Lab in Milton Keynes and Alex Alderton, Roberto Amato, Sonia Goncalves, Ewan Harrison, David K. Jackson, Ian Johnston, Dominic Kwiatkowski, Cordelia Langford, John Sillitoe on behalf of the Wellcome Sanger Institute COVID-19 Surveillance Team
EPI_ISL_963594	Lighthouse Lab in Glasgow	Wellcome Sanger Institute for the COVID-19 Genomics UK (COG-UK) Consortium	Harper VanSteenhouse, Yumi Kasai, David Gray, Carol Clugston, Anna Dominiczak and Alex Alderton, Roberto Amato, Sonia Goncalves, Ewan Harrison, David K. Jackson, Ian Johnston, Dominic Kwiatkowski, Cordelia Langford, John Sillitoe on behalf of the Wellcome Sanger Institute COVID-19 Surveillance Team
EPI_ISL_963595, EPI_ISL_963596, EPI_ISL_963597, EPI_ISL_963598, EPI_ISL_963599, EPI_ISL_963600	Lighthouse Lab in Milton Keynes	Wellcome Sanger Institute for the COVID-19 Genomics UK (COG-UK) Consortium	The Lighthouse Lab in Milton Keynes and Alex Alderton, Roberto Amato, Sonia Goncalves, Ewan Harrison, David K. Jackson, Ian Johnston, Dominic Kwiatkowski, Cordelia Langford, John Sillitoe on behalf of the Wellcome Sanger Institute COVID-19 Surveillance Team
EPI_ISL_963601	Lighthouse Lab in Glasgow	Wellcome Sanger Institute for the COVID-19 Genomics UK (COG-UK) Consortium	Harper VanSteenhouse, Yumi Kasai, David Gray, Carol Clugston, Anna Dominiczak and Alex Alderton, Roberto Amato, Sonia Goncalves, Ewan Harrison, David K. Jackson, Ian Johnston, Dominic Kwiatkowski, Cordelia Langford, John Sillitoe on behalf of the Wellcome Sanger Institute COVID-19 Surveillance Team
EPI_ISL_963602	Lighthouse Lab in Milton Keynes	Wellcome Sanger Institute for the COVID-19 Genomics UK (COG-UK) Consortium	The Lighthouse Lab in Milton Keynes and Alex Alderton, Roberto Amato, Sonia Goncalves, Ewan Harrison, David K. Jackson, Ian Johnston, Dominic Kwiatkowski, Cordelia Langford, John Sillitoe on behalf of the Wellcome Sanger Institute COVID-19 Surveillance Team
EPI_ISL_963603	Lighthouse Lab in Glasgow	Wellcome Sanger Institute for the COVID-19 Genomics UK (COG-UK) Consortium	Harper VanSteenhouse, Yumi Kasai, David Gray, Carol Clugston, Anna Dominiczak and Alex Alderton, Roberto Amato, Sonia Goncalves, Ewan Harrison, David K. Jackson, Ian Johnston, Dominic Kwiatkowski, Cordelia Langford, John Sillitoe on behalf of the Wellcome Sanger Institute COVID-19 Surveillance Team
EPI_ISL_963604, EPI_ISL_963605, EPI_ISL_963606	Lighthouse Lab in Milton Keynes	Wellcome Sanger Institute for the COVID-19 Genomics UK (COG-UK) Consortium	The Lighthouse Lab in Milton Keynes and Alex Alderton, Roberto Amato, Sonia Goncalves, Ewan Harrison, David K. Jackson, Ian Johnston, Dominic Kwiatkowski, Cordelia Langford, John Sillitoe on behalf of the Wellcome Sanger Institute COVID-19 Surveillance Team
EPI_ISL_963607	Lighthouse Lab in Glasgow	Wellcome Sanger Institute for the COVID-19 Genomics UK (COG-UK) Consortium	Harper VanSteenhouse, Yumi Kasai, David Gray, Carol Clugston, Anna Dominiczak and Alex Alderton, Roberto Amato, Sonia Goncalves, Ewan Harrison, David K. Jackson, Ian Johnston, Dominic Kwiatkowski, Cordelia Langford, John Sillitoe on behalf of the Wellcome Sanger Institute COVID-19 Surveillance Team
EPI_ISL_963608, EPI_ISL_963609, EPI_ISL_963610, EPI_ISL_963611, EPI_ISL_963612, EPI_ISL_963613, EPI_ISL_963614, EPI_ISL_963615, EPI_ISL_963616, EPI_ISL_963617	Lighthouse Lab in Milton Keynes	Wellcome Sanger Institute for the COVID-19 Genomics UK (COG-UK) Consortium	The Lighthouse Lab in Milton Keynes and Alex Alderton, Roberto Amato, Sonia Goncalves, Ewan Harrison, David K. Jackson, Ian Johnston, Dominic Kwiatkowski, Cordelia Langford, John Sillitoe on behalf of the Wellcome Sanger Institute COVID-19 Surveillance Team
EPI_ISL_963618	Lighthouse Lab in Glasgow	Wellcome Sanger Institute for the COVID-19 Genomics UK (COG-UK) Consortium	Harper VanSteenhouse, Yumi Kasai, David Gray, Carol Clugston, Anna Dominiczak and Alex Alderton, Roberto Amato, Sonia Goncalves, Ewan Harrison, David K. Jackson, Ian Johnston, Dominic Kwiatkowski, Cordelia Langford, John Sillitoe on behalf of the Wellcome Sanger Institute COVID-19 Surveillance Team
EPI_ISL_963619, EPI_ISL_963620, EPI_ISL_963621, EPI_ISL_963622, EPI_ISL_963623, EPI_ISL_963624, EPI_ISL_963625	Lighthouse Lab in Milton Keynes	Wellcome Sanger Institute for the COVID-19 Genomics UK (COG-UK) Consortium	The Lighthouse Lab in Milton Keynes and Alex Alderton, Roberto Amato, Sonia Goncalves, Ewan Harrison, David K. Jackson, Ian Johnston, Dominic Kwiatkowski, Cordelia Langford, John Sillitoe on behalf of the Wellcome Sanger Institute COVID-19 Surveillance Team
EPI_ISL_963626	Lighthouse Lab in Glasgow	Wellcome Sanger Institute for the COVID-19 Genomics UK (COG-UK) Consortium	Harper VanSteenhouse, Yumi Kasai, David Gray, Carol Clugston, Anna Dominiczak and Alex Alderton, Roberto Amato, Sonia Goncalves, Ewan Harrison, David K. Jackson, Ian Johnston, Dominic Kwiatkowski, Cordelia Langford, John Sillitoe on behalf of the Wellcome Sanger Institute COVID-19 Surveillance Team
EPI_ISL_963627, EPI_ISL_963628, EPI_ISL_963629, EPI_ISL_963630, EPI_ISL_963631, EPI_ISL_963632	Lighthouse Lab in Milton Keynes	Wellcome Sanger Institute for the COVID-19 Genomics UK (COG-UK) Consortium	The Lighthouse Lab in Milton Keynes and Alex Alderton, Roberto Amato, Sonia Goncalves, Ewan Harrison, David K. Jackson, Ian Johnston, Dominic Kwiatkowski, Cordelia Langford, John Sillitoe on behalf of the Wellcome Sanger Institute COVID-19 Surveillance Team
EPI_ISL_963633	Lighthouse Lab in Glasgow	Wellcome Sanger Institute for the COVID-19 Genomics UK (COG-UK) Consortium	Harper VanSteenhouse, Yumi Kasai, David Gray, Carol Clugston, Anna Dominiczak and Alex Alderton, Roberto Amato, Sonia Goncalves, Ewan Harrison, David K. Jackson, Ian Johnston, Dominic Kwiatkowski, Cordelia Langford, John Sillitoe on behalf of the Wellcome Sanger Institute COVID-19 Surveillance Team
EPI_ISL_963634, EPI_ISL_963635, EPI_ISL_963636, EPI_ISL_963637, EPI_ISL_963638	Lighthouse Lab in Milton Keynes	Wellcome Sanger Institute for the COVID-19 Genomics UK (COG-UK) Consortium	The Lighthouse Lab in Milton Keynes and Alex Alderton, Roberto Amato, Sonia Goncalves, Ewan Harrison, David K. Jackson, Ian Johnston, Dominic Kwiatkowski, Cordelia Langford, John Sillitoe on behalf of the Wellcome Sanger Institute COVID-19 Surveillance Team
EPI_ISL_963639	Lighthouse Lab in Glasgow	Wellcome Sanger Institute for the COVID-19 Genomics UK (COG-UK) Consortium	Harper VanSteenhouse, Yumi Kasai, David Gray, Carol Clugston, Anna Dominiczak and Alex Alderton, Roberto Amato, Sonia Goncalves, Ewan Harrison, David K. Jackson, Ian Johnston, Dominic Kwiatkowski, Cordelia Langford, John Sillitoe on behalf of the Wellcome Sanger Institute COVID-19 Surveillance Team
EPI_ISL_963640	Lighthouse Lab in Milton Keynes	Wellcome Sanger Institute for the COVID-19 Genomics UK (COG-UK) Consortium	The Lighthouse Lab in Milton Keynes and Alex Alderton, Roberto Amato, Sonia Goncalves, Ewan Harrison, David K. Jackson, Ian Johnston, Dominic Kwiatkowski, Cordelia Langford, John Sillitoe on behalf of the Wellcome Sanger Institute COVID-19 Surveillance Team
EPI_ISL_963641	Lighthouse Lab in Glasgow	Wellcome Sanger Institute for the COVID-19 Genomics UK (COG-UK) Consortium	Harper VanSteenhouse, Yumi Kasai, David Gray, Carol Clugston, Anna Dominiczak and Alex Alderton, Roberto Amato, Sonia Goncalves, Ewan Harrison, David K. Jackson, Ian Johnston, Dominic Kwiatkowski, Cordelia Langford, John Sillitoe on behalf of the Wellcome Sanger Institute COVID-19 Surveillance Team
EPI_ISL_963642, EPI_ISL_963643, EPI_ISL_963644, EPI_ISL_963645, EPI_ISL_963646, EPI_ISL_963647, EPI_ISL_963648	Lighthouse Lab in Milton Keynes	Wellcome Sanger Institute for the COVID-19 Genomics UK (COG-UK) Consortium	The Lighthouse Lab in Milton Keynes and Alex Alderton, Roberto Amato, Sonia Goncalves, Ewan Harrison, David K. Jackson, Ian Johnston, Dominic Kwiatkowski, Cordelia Langford, John Sillitoe on behalf of the Wellcome Sanger Institute COVID-19 Surveillance Team
EPI_ISL_963649	Lighthouse Lab in Glasgow	Wellcome Sanger Institute for the COVID-19 Genomics UK (COG-UK) Consortium	Harper VanSteenhouse, Yumi Kasai, David Gray, Carol Clugston, Anna Dominiczak and Alex Alderton, Roberto Amato, Sonia Goncalves, Ewan Harrison, David K. Jackson, Ian Johnston, Dominic Kwiatkowski, Cordelia Langford, John Sillitoe on behalf of the Wellcome Sanger Institute COVID-19 Surveillance Team
EPI_ISL_963650	Lighthouse Lab in Milton Keynes	Wellcome Sanger Institute for the COVID-19 Genomics UK (COG-UK) Consortium	The Lighthouse Lab in Milton Keynes and Alex Alderton, Roberto Amato, Sonia Goncalves, Ewan Harrison, David K. Jackson, Ian Johnston, Dominic Kwiatkowski, Cordelia Langford, John Sillitoe on behalf of the Wellcome Sanger Institute COVID-19 Surveillance Team

EPI_ISL_963652, EPI_ISL_963653, EPI_ISL_963655, EPI_ISL_963656, EPI_ISL_963657, EPI_ISL_963660, EPI_ISL_963661, EPI_ISL_963662, EPI_ISL_963663, EPI_ISL_963664, EPI_ISL_963666, EPI_ISL_963667, EPI_ISL_963668, EPI_ISL_963669, EPI_ISL_963674, EPI_ISL_963675, EPI_ISL_963678, EPI_ISL_963680, EPI_ISL_963682, EPI_ISL_963683, EPI_ISL_963684, EPI_ISL_963688, EPI_ISL_963691, EPI_ISL_963692, EPI_ISL_963693, EPI_ISL_963696, EPI_ISL_963697, EPI_ISL_963698, EPI_ISL_963699, EPI_ISL_963702, EPI_ISL_963705, EPI_ISL_963708, EPI_ISL_963710, EPI_ISL_963711, EPI_ISL_963712, EPI_ISL_963713, EPI_ISL_963714, EPI_ISL_963715, EPI_ISL_963716, EPI_ISL_963717, EPI_ISL_963719, EPI_ISL_963722, EPI_ISL_963723, EPI_ISL_963725, EPI_ISL_963728, EPI_ISL_963729, EPI_ISL_963730, EPI_ISL_963739, EPI_ISL_963742, EPI_ISL_963743, EPI_ISL_963744, EPI_ISL_963745, EPI_ISL_963746, EPI_ISL_963748, EPI_ISL_963749, EPI_ISL_963751, EPI_ISL_963752, EPI_ISL_963755, EPI_ISL_963756, EPI_ISL_963757, EPI_ISL_963760, EPI_ISL_963762, EPI_ISL_963763, EPI_ISL_963767, EPI_ISL_963770, EPI_ISL_963771, EPI_ISL_963776, EPI_ISL_963777, EPI_ISL_963778, EPI_ISL_963782, EPI_ISL_963784, EPI_ISL_963785,

EPI_ISL_963787, EPI_ISL_963788, EPI_ISL_963791, EPI_ISL_963792, EPI_ISL_963793, EPI_ISL_963795, EPI_ISL_963796, EPI_ISL_963797, EPI_ISL_963799, EPI_ISL_963801, EPI_ISL_963802, EPI_ISL_963804, EPI_ISL_963809, EPI_ISL_963811, EPI_ISL_963812, EPI_ISL_963817, EPI_ISL_963818, EPI_ISL_963820, EPI_ISL_963827, EPI_ISL_963828, EPI_ISL_963829, EPI_ISL_963830, EPI_ISL_963834, EPI_ISL_963836, EPI_ISL_963837, EPI_ISL_963840, EPI_ISL_963842, EPI_ISL_963843, EPI_ISL_963847, EPI_ISL_963848, EPI_ISL_963854, EPI_ISL_963856, EPI_ISL_963858, EPI_ISL_963860, EPI_ISL_963862, EPI_ISL_963871, EPI_ISL_963876, EPI_ISL_963885, EPI_ISL_963886, EPI_ISL_963887, EPI_ISL_963888, EPI_ISL_963892, EPI_ISL_963894, EPI_ISL_963897, EPI_ISL_963898, EPI_ISL_963899, EPI_ISL_963905, EPI_ISL_963907, EPI_ISL_963910, EPI_ISL_963912, EPI_ISL_963913, EPI_ISL_963917, EPI_ISL_963918, EPI_ISL_963919, EPI_ISL_963920, EPI_ISL_963924, EPI_ISL_963926, EPI_ISL_963928, EPI_ISL_963929, EPI_ISL_963930, EPI_ISL_963931, EPI_ISL_963933, EPI_ISL_963935, EPI_ISL_963937, EPI_ISL_963939, EPI_ISL_963940, EPI_ISL_963943, EPI_ISL_963944, EPI_ISL_963946, EPI_ISL_963948, EPI_ISL_963950, EPI_ISL_963951, EPI_ISL_963954, EPI_ISL_963956, EPI_ISL_963957, EPI_ISL_963959, EPI_ISL_963961, EPI_ISL_963963, EPI_ISL_963965, EPI_ISL_963966, EPI_ISL_963967, EPI_ISL_963969, EPI_ISL_963972, EPI_ISL_963974, EPI_ISL_963975, EPI_ISL_963982, EPI_ISL_963983, EPI_ISL_963985, EPI_ISL_963986, EPI_ISL_963987, EPI_ISL_963988, EPI_ISL_963989, EPI_ISL_963990, EPI_ISL_963991, EPI_ISL_963992, EPI_ISL_963993, EPI_ISL_963994, EPI_ISL_963995, EPI_ISL_963996, EPI_ISL_963997, EPI_ISL_963998, EPI_ISL_963999, EPI_ISL_964000, EPI_ISL_964001, EPI_ISL_964002, EPI_ISL_964003, EPI_ISL_964004, EPI_ISL_964005, EPI_ISL_964006, EPI_ISL_964007, EPI_ISL_964008, EPI_ISL_964009, EPI_ISL_964010, EPI_ISL_964011, EPI_ISL_964012, EPI_ISL_964013, EPI_ISL_964014, EPI_ISL_964015, EPI_ISL_964016, EPI_ISL_964017, EPI_ISL_964018, EPI_ISL_964019, EPI_ISL_964020, EPI_ISL_964021, EPI_ISL_964022, EPI_ISL_964023, EPI_ISL_964024, EPI_ISL_964025, EPI_ISL_964026, EPI_ISL_964027, EPI_ISL_964028, EPI_ISL_964029, EPI_ISL_964030, EPI_ISL_964031, EPI_ISL_964032, EPI_ISL_964033, EPI_ISL_964034, EPI_ISL_964035, EPI_ISL_964036, EPI_ISL_964037, EPI_ISL_964038, EPI_ISL_964039, EPI_ISL_964040, EPI_ISL_964041, EPI_ISL_964042, EPI_ISL_964043, EPI_ISL_964044, EPI_ISL_964045, EPI_ISL_964046, EPI_ISL_964047, EPI_ISL_964048, EPI_ISL_964049, EPI_ISL_964050, EPI_ISL_964051, EPI_ISL_964052, EPI_ISL_964053, EPI_ISL_964054, EPI_ISL_964055, EPI_ISL_964056, EPI_ISL_964057, EPI_ISL_964058, EPI_ISL_964059, EPI_ISL_964060, EPI_ISL_964061, EPI_ISL_964062, EPI_ISL_964063, EPI_ISL_964064, EPI_ISL_964065, EPI_ISL_964066, EPI_ISL_964068, EPI_ISL_964069, EPI_ISL_964070, EPI_ISL_964071, EPI_ISL_964072, EPI_ISL_964073, EPI_ISL_964074, EPI_ISL_964075, EPI_ISL_964076, EPI_ISL_964077, EPI_ISL_964078, EPI_ISL_964079, EPI_ISL_964080, EPI_ISL_964081, EPI_ISL_964082, EPI_ISL_964083, EPI_ISL_964084, EPI_ISL_964085, EPI_ISL_964086, EPI_ISL_964088, EPI_ISL_964089, EPI_ISL_964090, EPI_ISL_964091, EPI_ISL_964092, EPI_ISL_964093, EPI_ISL_964094, EPI_ISL_964095, EPI_ISL_964096, EPI_ISL_964097, EPI_ISL_964098, EPI_ISL_964099, EPI_ISL_964100, EPI_ISL_964101, EPI_ISL_964102, EPI_ISL_964103, EPI_ISL_964104, EPI_ISL_964105, EPI_ISL_964106, EPI_ISL_964108, EPI_ISL_964109, EPI_ISL_964110, EPI_ISL_964111, EPI_ISL_964112, EPI_ISL_964113, EPI_ISL_964114, EPI_ISL_964115, EPI_ISL_964116, EPI_ISL_964117, EPI_ISL_964118, EPI_ISL_964119, EPI_ISL_964120, EPI_ISL_964121, EPI_ISL_964122, EPI_ISL_964123, EPI_ISL_964124, EPI_ISL_964125, EPI_ISL_964126, EPI_ISL_964127, EPI_ISL_964128, EPI_ISL_964129, EPI_ISL_964130, EPI_ISL_964131, EPI_ISL_964132, EPI_ISL_964133, EPI_ISL_964134, EPI_ISL_964135, EPI_ISL_964136, EPI_ISL_964137, EPI_ISL_964138, EPI_ISL_964139, EPI_ISL_964140, EPI_ISL_964141, EPI_ISL_964142, EPI_ISL_964143, EPI_ISL_964144, EPI_ISL_964145, EPI_ISL_964146, EPI_ISL_964147, EPI_ISL_964148, EPI_ISL_964149, EPI_ISL_964150, EPI_ISL_964151, EPI_ISL_964152, EPI_ISL_964153, EPI_ISL_964154, EPI_ISL_964155, EPI_ISL_964156, EPI_ISL_964157, EPI_ISL_964158, EPI_ISL_964159, EPI_ISL_964160, EPI_ISL_964161, EPI_ISL_964162, EPI_ISL_964163, EPI_ISL_964164, EPI_ISL_964165, EPI_ISL_964166, EPI_ISL_964167, EPI_ISL_964168, EPI_ISL_964169, EPI_ISL_964170, EPI_ISL_964171, EPI_ISL_964172, EPI_ISL_964173, EPI_ISL_964174, EPI_ISL_964175, EPI_ISL_964176, EPI_ISL_964177, EPI_ISL_964178, EPI_ISL_964179, EPI_ISL_964180, EPI_ISL_964181, EPI_ISL_964182, EPI_ISL_964183, EPI_ISL_964184, EPI_ISL_964185, EPI_ISL_964186, EPI_ISL_964187, EPI_ISL_964188, EPI_ISL_964189, EPI_ISL_964190, EPI_ISL_964191, EPI_ISL_964192, EPI_ISL_964193, EPI_ISL_964194, EPI_ISL_964195, EPI_ISL_964196, EPI_ISL_964197, EPI_ISL_964198, EPI_ISL_964199, EPI_ISL_964200, EPI_ISL_964201, EPI_ISL_964202, EPI_ISL_964203, EPI_ISL_964204, EPI_ISL_964205, EPI_ISL_964206, EPI_ISL_964207, EPI_ISL_964208, EPI_ISL_964209, EPI_ISL_964210, EPI_ISL_964211, EPI_ISL_964212, EPI_ISL_964213, EPI_ISL_964214, EPI_ISL_964215, EPI_ISL_964216, EPI_ISL_964217, EPI_ISL_964218, EPI_ISL_964220, EPI_ISL_964221, EPI_ISL_964222, EPI_ISL_964223, EPI_ISL_964224, EPI_ISL_964225, EPI_ISL_964226, EPI_ISL_964227, EPI_ISL_964228, EPI_ISL_964229, EPI_ISL_964230, EPI_ISL_964231, EPI_ISL_964232, EPI_ISL_964233, EPI_ISL_964235, EPI_ISL_964237, EPI_ISL_964238, EPI_ISL_964239, EPI_ISL_964240, EPI_ISL_964241, EPI_ISL_964242, EPI_ISL_964243, EPI_ISL_964244, EPI_ISL_964245, EPI_ISL_964246, EPI_ISL_964247, EPI_ISL_964248, EPI_ISL_964249, EPI_ISL_964250, EPI_ISL_964251, EPI_ISL_964252, EPI_ISL_964253, EPI_ISL_964255, EPI_ISL_964256, EPI_ISL_964258, EPI_ISL_964260, EPI_ISL_964261, EPI_ISL_964263, EPI_ISL_964264, EPI_ISL_964266, EPI_ISL_964268, EPI_ISL_964269, EPI_ISL_964271, EPI_ISL_964273, EPI_ISL_964274, EPI_ISL_964276, EPI_ISL_964277, EPI_ISL_964279, EPI_ISL_964280, EPI_ISL_964282, EPI_ISL_964283, EPI_ISL_964285, EPI_ISL_964286, EPI_ISL_964288, EPI_ISL_964290, EPI_ISL_964291, EPI_ISL_964293, EPI_ISL_964294, EPI_ISL_964296, EPI_ISL_964298, EPI_ISL_964299, EPI_ISL_964301, EPI_ISL_964302, EPI_ISL_964304, EPI_ISL_964306, EPI_ISL_964307, EPI_ISL_964309, EPI_ISL_964310, EPI_ISL_964311, EPI_ISL_964312, EPI_ISL_964313, EPI_ISL_964314, EPI_ISL_964315, EPI_ISL_964316, EPI_ISL_964317, EPI_ISL_964318, EPI_ISL_964319, EPI_ISL_964320, EPI_ISL_964321, EPI_ISL_964322, EPI_ISL_964323, EPI_ISL_964324, EPI_ISL_964325, EPI_ISL_964326, EPI_ISL_964327, EPI_ISL_964328, EPI_ISL_964329, EPI_ISL_964330, EPI_ISL_964331, EPI_ISL_964332, EPI_ISL_964333, EPI_ISL_964334, EPI_ISL_964335, EPI_ISL_964336, EPI_ISL_964337, EPI_ISL_964338, EPI_ISL_964339, EPI_ISL_964340, EPI_ISL_964341, EPI_ISL_964342, EPI_ISL_964343, EPI_ISL_964344, EPI_ISL_964345

see above	Lighthouse Lab in Alderley Park	Wellcome Sanger Institute for the COVID-19 Genomics UK (COG-UK) Consortium	Jacquelyn Wynn, Mairead Hyland, The Lighthouse Lab in Alderley Park and Alex Alderton, Roberto Amato, Sonia Goncalves, Ewan Harrison, David K. Jackson, Ian Johnston, Dominic Kwiatkowski, Cordelia Langford, John Sillitoe on behalf of the Wellcome Sanger Institute COVID-19 Surveillance Team
EPI_ISL_964346, EPI_ISL_964347	Lighthouse Lab in Milton Keynes	Wellcome Sanger Institute for the COVID-19 Genomics UK (COG-UK) Consortium	The Lighthouse Lab in Milton Keynes and Alex Alderton, Roberto Amato, Sonia Goncalves, Ewan Harrison, David K. Jackson, Ian Johnston, Dominic Kwiatkowski, Cordelia Langford, John Sillitoe on behalf of the Wellcome Sanger Institute COVID-19 Surveillance Team
EPI_ISL_964348, EPI_ISL_964349	Lighthouse Lab in Glasgow	Wellcome Sanger Institute for the COVID-19 Genomics UK (COG-UK) Consortium	Harper VanSteenhouse, Yumi Kasai, David Gray, Carol Clugston, Anna Dominiczak and Alex Alderton, Roberto Amato, Sonia Goncalves, Ewan Harrison, David K. Jackson, Ian Johnston, Dominic Kwiatkowski, Cordelia Langford, John Sillitoe on behalf of the Wellcome Sanger Institute COVID-19 Surveillance Team
EPI_ISL_964350	Lighthouse Lab in Milton Keynes	Wellcome Sanger Institute for the COVID-19 Genomics UK (COG-UK) Consortium	The Lighthouse Lab in Milton Keynes and Alex Alderton, Roberto Amato, Sonia Goncalves, Ewan Harrison, David K. Jackson, Ian Johnston, Dominic Kwiatkowski, Cordelia Langford, John Sillitoe on behalf of the Wellcome Sanger Institute COVID-19 Surveillance Team
EPI_ISL_964351, EPI_ISL_964352	Lighthouse Lab in Glasgow	Wellcome Sanger Institute for the COVID-19 Genomics UK (COG-UK) Consortium	Harper VanSteenhouse, Yumi Kasai, David Gray, Carol Clugston, Anna Dominiczak and Alex Alderton, Roberto Amato, Sonia Goncalves, Ewan Harrison, David K. Jackson, Ian Johnston, Dominic Kwiatkowski, Cordelia Langford, John Sillitoe on behalf of the Wellcome Sanger Institute COVID-19 Surveillance Team
EPI_ISL_964353, EPI_ISL_964354	Lighthouse Lab in Milton Keynes	Wellcome Sanger Institute for the COVID-19 Genomics UK (COG-UK) Consortium	The Lighthouse Lab in Milton Keynes and Alex Alderton, Roberto Amato, Sonia Goncalves, Ewan Harrison, David K. Jackson, Ian Johnston, Dominic Kwiatkowski, Cordelia Langford, John Sillitoe on behalf of the Wellcome Sanger Institute COVID-19 Surveillance Team
EPI_ISL_964355	Lighthouse Lab in Glasgow	Wellcome Sanger Institute for the COVID-19 Genomics UK (COG-UK) Consortium	Harper VanSteenhouse, Yumi Kasai, David Gray, Carol Clugston, Anna Dominiczak and Alex Alderton, Roberto Amato, Sonia Goncalves, Ewan Harrison, David K. Jackson, Ian Johnston, Dominic Kwiatkowski, Cordelia Langford, John Sillitoe on behalf of the Wellcome Sanger Institute COVID-19 Surveillance Team
EPI_ISL_964356	Lighthouse Lab in Milton Keynes	Wellcome Sanger Institute for the COVID-19 Genomics UK (COG-UK) Consortium	The Lighthouse Lab in Milton Keynes and Alex Alderton, Roberto Amato, Sonia Goncalves, Ewan Harrison, David K. Jackson, Ian Johnston, Dominic Kwiatkowski, Cordelia Langford, John Sillitoe on behalf of the Wellcome Sanger Institute COVID-19 Surveillance Team
EPI_ISL_964357, EPI_ISL_964358, EPI_ISL_964359, EPI_ISL_964360	Lighthouse Lab in Glasgow	Wellcome Sanger Institute for the COVID-19 Genomics UK (COG-UK) Consortium	Harper VanSteenhouse, Yumi Kasai, David Gray, Carol Clugston, Anna Dominiczak and Alex Alderton, Roberto Amato, Sonia Goncalves, Ewan Harrison, David K. Jackson, Ian Johnston, Dominic Kwiatkowski, Cordelia Langford, John Sillitoe on behalf of the Wellcome Sanger Institute COVID-19 Surveillance Team
EPI_ISL_964361	Lighthouse Lab in Milton Keynes	Wellcome Sanger Institute for the COVID-19 Genomics UK (COG-UK) Consortium	The Lighthouse Lab in Milton Keynes and Alex Alderton, Roberto Amato, Sonia Goncalves, Ewan Harrison, David K. Jackson, Ian Johnston, Dominic Kwiatkowski, Cordelia Langford, John Sillitoe on behalf of the Wellcome Sanger Institute COVID-19 Surveillance Team
EPI_ISL_964362	Lighthouse Lab in Glasgow	Wellcome Sanger Institute for the COVID-19 Genomics UK (COG-UK) Consortium	Harper VanSteenhouse, Yumi Kasai, David Gray, Carol Clugston, Anna Dominiczak and Alex Alderton, Roberto Amato, Sonia Goncalves, Ewan Harrison, David K. Jackson, Ian Johnston, Dominic Kwiatkowski, Cordelia Langford, John Sillitoe on behalf of the Wellcome Sanger Institute COVID-19 Surveillance Team
EPI_ISL_964363	Lighthouse Lab in Milton Keynes	Wellcome Sanger Institute for the COVID-19 Genomics UK (COG-UK) Consortium	The Lighthouse Lab in Milton Keynes and Alex Alderton, Roberto Amato, Sonia Goncalves, Ewan Harrison, David K. Jackson, Ian Johnston, Dominic Kwiatkowski, Cordelia Langford, John Sillitoe on behalf of the Wellcome Sanger Institute COVID-19 Surveillance Team
EPI_ISL_964364	Lighthouse Lab in Glasgow	Wellcome Sanger Institute for the COVID-19 Genomics UK (COG-UK) Consortium	Harper VanSteenhouse, Yumi Kasai, David Gray, Carol Clugston, Anna Dominiczak and Alex Alderton, Roberto Amato, Sonia Goncalves, Ewan Harrison, David K. Jackson, Ian Johnston, Dominic Kwiatkowski, Cordelia Langford, John Sillitoe on behalf of the Wellcome Sanger Institute COVID-19 Surveillance Team
EPI_ISL_964365	Lighthouse Lab in Milton Keynes	Wellcome Sanger Institute for the COVID-19 Genomics UK (COG-UK) Consortium	The Lighthouse Lab in Milton Keynes and Alex Alderton, Roberto Amato, Sonia Goncalves, Ewan Harrison, David K. Jackson, Ian Johnston, Dominic Kwiatkowski, Cordelia Langford, John Sillitoe on behalf of the Wellcome Sanger Institute COVID-19 Surveillance Team
EPI_ISL_964366, EPI_ISL_964367, EPI_ISL_964368, EPI_ISL_964369	Lighthouse Lab in Glasgow	Wellcome Sanger Institute for the COVID-19 Genomics UK (COG-UK) Consortium	Harper VanSteenhouse, Yumi Kasai, David Gray, Carol Clugston, Anna Dominiczak and Alex Alderton, Roberto Amato, Sonia Goncalves, Ewan Harrison, David K. Jackson, Ian Johnston, Dominic Kwiatkowski, Cordelia Langford, John Sillitoe on behalf of the Wellcome Sanger Institute COVID-19 Surveillance Team
EPI_ISL_964370	Lighthouse Lab in Milton Keynes	Wellcome Sanger Institute for the COVID-19 Genomics UK (COG-UK) Consortium	The Lighthouse Lab in Milton Keynes and Alex Alderton, Roberto Amato, Sonia Goncalves, Ewan Harrison, David K. Jackson, Ian Johnston, Dominic Kwiatkowski, Cordelia Langford, John Sillitoe on behalf of the Wellcome Sanger Institute COVID-19 Surveillance Team
EPI_ISL_964371, EPI_ISL_964372	Lighthouse Lab in Glasgow	Wellcome Sanger Institute for the COVID-19 Genomics UK (COG-UK) Consortium	Harper VanSteenhouse, Yumi Kasai, David Gray, Carol Clugston, Anna Dominiczak and Alex Alderton, Roberto Amato, Sonia Goncalves, Ewan Harrison, David K. Jackson, Ian Johnston, Dominic Kwiatkowski, Cordelia Langford, John Sillitoe on behalf of the Wellcome Sanger Institute COVID-19 Surveillance Team
EPI_ISL_964373, EPI_ISL_964374	Lighthouse Lab in Milton Keynes	Wellcome Sanger Institute for the COVID-19 Genomics UK (COG-UK) Consortium	The Lighthouse Lab in Milton Keynes and Alex Alderton, Roberto Amato, Sonia Goncalves, Ewan Harrison, David K. Jackson, Ian Johnston, Dominic Kwiatkowski, Cordelia Langford, John Sillitoe on behalf of the Wellcome Sanger Institute COVID-19 Surveillance Team
EPI_ISL_964375	Lighthouse Lab in Glasgow	Wellcome Sanger Institute for the COVID-19 Genomics UK (COG-UK) Consortium	Harper VanSteenhouse, Yumi Kasai, David Gray, Carol Clugston, Anna Dominiczak and Alex Alderton, Roberto Amato, Sonia Goncalves, Ewan Harrison, David K. Jackson, Ian Johnston, Dominic Kwiatkowski, Cordelia Langford, John Sillitoe on behalf of the Wellcome Sanger Institute COVID-19 Surveillance Team
EPI_ISL_964376	Lighthouse Lab in Milton Keynes	Wellcome Sanger Institute for the COVID-19 Genomics UK (COG-UK) Consortium	The Lighthouse Lab in Milton Keynes and Alex Alderton, Roberto Amato, Sonia Goncalves, Ewan Harrison, David K. Jackson, Ian Johnston, Dominic Kwiatkowski, Cordelia Langford, John Sillitoe on behalf of the Wellcome Sanger Institute COVID-19 Surveillance Team
EPI_ISL_964377, EPI_ISL_964378	Lighthouse Lab in Glasgow	Wellcome Sanger Institute for the COVID-19 Genomics UK (COG-UK) Consortium	Harper VanSteenhouse, Yumi Kasai, David Gray, Carol Clugston, Anna Dominiczak and Alex Alderton, Roberto Amato, Sonia Goncalves, Ewan Harrison, David K. Jackson, Ian Johnston, Dominic Kwiatkowski, Cordelia Langford, John Sillitoe on behalf of the Wellcome Sanger Institute COVID-19 Surveillance Team

EPI_ISL_964379	Lighthouse Lab in Milton Keynes	Wellcome Sanger Institute for the COVID-19 Genomics UK (COG-UK) Consortium	The Lighthouse Lab in Milton Keynes and Alex Alderton, Roberto Amato, Sonia Goncalves, Ewan Harrison, David K. Jackson, Ian Johnston, Dominic Kwiatkowski, Cordelia Langford, John Sillitoe on behalf of the Wellcome Sanger Institute COVID-19 Surveillance Team
EPI_ISL_964380	Lighthouse Lab in Glasgow	Wellcome Sanger Institute for the COVID-19 Genomics UK (COG-UK) Consortium	Harper VanSteenhouse, Yumi Kasai, David Gray, Carol Clugston, Anna Dominiczak and Alex Alderton, Roberto Amato, Sonia Goncalves, Ewan Harrison, David K. Jackson, Ian Johnston, Dominic Kwiatkowski, Cordelia Langford, John Sillitoe on behalf of the Wellcome Sanger Institute COVID-19 Surveillance Team
EPI_ISL_964381	Lighthouse Lab in Milton Keynes	Wellcome Sanger Institute for the COVID-19 Genomics UK (COG-UK) Consortium	The Lighthouse Lab in Milton Keynes and Alex Alderton, Roberto Amato, Sonia Goncalves, Ewan Harrison, David K. Jackson, Ian Johnston, Dominic Kwiatkowski, Cordelia Langford, John Sillitoe on behalf of the Wellcome Sanger Institute COVID-19 Surveillance Team
EPI_ISL_964382	Lighthouse Lab in Glasgow	Wellcome Sanger Institute for the COVID-19 Genomics UK (COG-UK) Consortium	Harper VanSteenhouse, Yumi Kasai, David Gray, Carol Clugston, Anna Dominiczak and Alex Alderton, Roberto Amato, Sonia Goncalves, Ewan Harrison, David K. Jackson, Ian Johnston, Dominic Kwiatkowski, Cordelia Langford, John Sillitoe on behalf of the Wellcome Sanger Institute COVID-19 Surveillance Team
EPI_ISL_964383	Lighthouse Lab in Milton Keynes	Wellcome Sanger Institute for the COVID-19 Genomics UK (COG-UK) Consortium	The Lighthouse Lab in Milton Keynes and Alex Alderton, Roberto Amato, Sonia Goncalves, Ewan Harrison, David K. Jackson, Ian Johnston, Dominic Kwiatkowski, Cordelia Langford, John Sillitoe on behalf of the Wellcome Sanger Institute COVID-19 Surveillance Team
EPI_ISL_964384	Lighthouse Lab in Glasgow	Wellcome Sanger Institute for the COVID-19 Genomics UK (COG-UK) Consortium	Harper VanSteenhouse, Yumi Kasai, David Gray, Carol Clugston, Anna Dominiczak and Alex Alderton, Roberto Amato, Sonia Goncalves, Ewan Harrison, David K. Jackson, Ian Johnston, Dominic Kwiatkowski, Cordelia Langford, John Sillitoe on behalf of the Wellcome Sanger Institute COVID-19 Surveillance Team
EPI_ISL_964385, EPI_ISL_964386	Lighthouse Lab in Milton Keynes	Wellcome Sanger Institute for the COVID-19 Genomics UK (COG-UK) Consortium	The Lighthouse Lab in Milton Keynes and Alex Alderton, Roberto Amato, Sonia Goncalves, Ewan Harrison, David K. Jackson, Ian Johnston, Dominic Kwiatkowski, Cordelia Langford, John Sillitoe on behalf of the Wellcome Sanger Institute COVID-19 Surveillance Team
EPI_ISL_964387, EPI_ISL_964388	Lighthouse Lab in Glasgow	Wellcome Sanger Institute for the COVID-19 Genomics UK (COG-UK) Consortium	Harper VanSteenhouse, Yumi Kasai, David Gray, Carol Clugston, Anna Dominiczak and Alex Alderton, Roberto Amato, Sonia Goncalves, Ewan Harrison, David K. Jackson, Ian Johnston, Dominic Kwiatkowski, Cordelia Langford, John Sillitoe on behalf of the Wellcome Sanger Institute COVID-19 Surveillance Team
EPI_ISL_964389	Lighthouse Lab in Milton Keynes	Wellcome Sanger Institute for the COVID-19 Genomics UK (COG-UK) Consortium	The Lighthouse Lab in Milton Keynes and Alex Alderton, Roberto Amato, Sonia Goncalves, Ewan Harrison, David K. Jackson, Ian Johnston, Dominic Kwiatkowski, Cordelia Langford, John Sillitoe on behalf of the Wellcome Sanger Institute COVID-19 Surveillance Team
EPI_ISL_964390, EPI_ISL_964391	Lighthouse Lab in Glasgow	Wellcome Sanger Institute for the COVID-19 Genomics UK (COG-UK) Consortium	Harper VanSteenhouse, Yumi Kasai, David Gray, Carol Clugston, Anna Dominiczak and Alex Alderton, Roberto Amato, Sonia Goncalves, Ewan Harrison, David K. Jackson, Ian Johnston, Dominic Kwiatkowski, Cordelia Langford, John Sillitoe on behalf of the Wellcome Sanger Institute COVID-19 Surveillance Team
EPI_ISL_964392	Lighthouse Lab in Milton Keynes	Wellcome Sanger Institute for the COVID-19 Genomics UK (COG-UK) Consortium	The Lighthouse Lab in Milton Keynes and Alex Alderton, Roberto Amato, Sonia Goncalves, Ewan Harrison, David K. Jackson, Ian Johnston, Dominic Kwiatkowski, Cordelia Langford, John Sillitoe on behalf of the Wellcome Sanger Institute COVID-19 Surveillance Team
EPI_ISL_964393	Lighthouse Lab in Glasgow	Wellcome Sanger Institute for the COVID-19 Genomics UK (COG-UK) Consortium	Harper VanSteenhouse, Yumi Kasai, David Gray, Carol Clugston, Anna Dominiczak and Alex Alderton, Roberto Amato, Sonia Goncalves, Ewan Harrison, David K. Jackson, Ian Johnston, Dominic Kwiatkowski, Cordelia Langford, John Sillitoe on behalf of the Wellcome Sanger Institute COVID-19 Surveillance Team
EPI_ISL_964394, EPI_ISL_964395	Lighthouse Lab in Milton Keynes	Wellcome Sanger Institute for the COVID-19 Genomics UK (COG-UK) Consortium	The Lighthouse Lab in Milton Keynes and Alex Alderton, Roberto Amato, Sonia Goncalves, Ewan Harrison, David K. Jackson, Ian Johnston, Dominic Kwiatkowski, Cordelia Langford, John Sillitoe on behalf of the Wellcome Sanger Institute COVID-19 Surveillance Team
EPI_ISL_964396, EPI_ISL_964397	Lighthouse Lab in Glasgow	Wellcome Sanger Institute for the COVID-19 Genomics UK (COG-UK) Consortium	Harper VanSteenhouse, Yumi Kasai, David Gray, Carol Clugston, Anna Dominiczak and Alex Alderton, Roberto Amato, Sonia Goncalves, Ewan Harrison, David K. Jackson, Ian Johnston, Dominic Kwiatkowski, Cordelia Langford, John Sillitoe on behalf of the Wellcome Sanger Institute COVID-19 Surveillance Team
EPI_ISL_964398	Lighthouse Lab in Milton Keynes	Wellcome Sanger Institute for the COVID-19 Genomics UK (COG-UK) Consortium	The Lighthouse Lab in Milton Keynes and Alex Alderton, Roberto Amato, Sonia Goncalves, Ewan Harrison, David K. Jackson, Ian Johnston, Dominic Kwiatkowski, Cordelia Langford, John Sillitoe on behalf of the Wellcome Sanger Institute COVID-19 Surveillance Team
EPI_ISL_964399	Lighthouse Lab in Glasgow	Wellcome Sanger Institute for the COVID-19 Genomics UK (COG-UK) Consortium	Harper VanSteenhouse, Yumi Kasai, David Gray, Carol Clugston, Anna Dominiczak and Alex Alderton, Roberto Amato, Sonia Goncalves, Ewan Harrison, David K. Jackson, Ian Johnston, Dominic Kwiatkowski, Cordelia Langford, John Sillitoe on behalf of the Wellcome Sanger Institute COVID-19 Surveillance Team
EPI_ISL_964400	Lighthouse Lab in Milton Keynes	Wellcome Sanger Institute for the COVID-19 Genomics UK (COG-UK) Consortium	The Lighthouse Lab in Milton Keynes and Alex Alderton, Roberto Amato, Sonia Goncalves, Ewan Harrison, David K. Jackson, Ian Johnston, Dominic Kwiatkowski, Cordelia Langford, John Sillitoe on behalf of the Wellcome Sanger Institute COVID-19 Surveillance Team
EPI_ISL_964401	Lighthouse Lab in Glasgow	Wellcome Sanger Institute for the COVID-19 Genomics UK (COG-UK) Consortium	Harper VanSteenhouse, Yumi Kasai, David Gray, Carol Clugston, Anna Dominiczak and Alex Alderton, Roberto Amato, Sonia Goncalves, Ewan Harrison, David K. Jackson, Ian Johnston, Dominic Kwiatkowski, Cordelia Langford, John Sillitoe on behalf of the Wellcome Sanger Institute COVID-19 Surveillance Team
EPI_ISL_964402, EPI_ISL_964403	Lighthouse Lab in Milton Keynes	Wellcome Sanger Institute for the COVID-19 Genomics UK (COG-UK) Consortium	The Lighthouse Lab in Milton Keynes and Alex Alderton, Roberto Amato, Sonia Goncalves, Ewan Harrison, David K. Jackson, Ian Johnston, Dominic Kwiatkowski, Cordelia Langford, John Sillitoe on behalf of the Wellcome Sanger Institute COVID-19 Surveillance Team
EPI_ISL_964404	Lighthouse Lab in Glasgow	Wellcome Sanger Institute for the COVID-19 Genomics UK (COG-UK) Consortium	Harper VanSteenhouse, Yumi Kasai, David Gray, Carol Clugston, Anna Dominiczak and Alex Alderton, Roberto Amato, Sonia Goncalves, Ewan Harrison, David K. Jackson, Ian Johnston, Dominic Kwiatkowski, Cordelia Langford, John Sillitoe on behalf of the Wellcome Sanger Institute COVID-19 Surveillance Team
EPI_ISL_964405, EPI_ISL_964406	Lighthouse Lab in Milton Keynes	Wellcome Sanger Institute for the COVID-19 Genomics UK (COG-UK) Consortium	The Lighthouse Lab in Milton Keynes and Alex Alderton, Roberto Amato, Sonia Goncalves, Ewan Harrison, David K. Jackson, Ian Johnston, Dominic Kwiatkowski, Cordelia Langford, John Sillitoe on behalf of the Wellcome Sanger Institute COVID-19 Surveillance Team
EPI_ISL_964407, EPI_ISL_964408, EPI_ISL_964409, EPI_ISL_964410, EPI_ISL_964411	Lighthouse Lab in Glasgow	Wellcome Sanger Institute for the COVID-19 Genomics UK (COG-UK) Consortium	Harper VanSteenhouse, Yumi Kasai, David Gray, Carol Clugston, Anna Dominiczak and Alex Alderton, Roberto Amato, Sonia Goncalves, Ewan Harrison, David K. Jackson, Ian Johnston, Dominic Kwiatkowski, Cordelia Langford, John Sillitoe on behalf of the Wellcome Sanger Institute COVID-19 Surveillance Team
EPI_ISL_964412	Lighthouse Lab in Milton Keynes	Wellcome Sanger Institute for the COVID-19 Genomics UK (COG-UK) Consortium	The Lighthouse Lab in Milton Keynes and Alex Alderton, Roberto Amato, Sonia Goncalves, Ewan Harrison, David K. Jackson, Ian Johnston, Dominic Kwiatkowski, Cordelia Langford, John Sillitoe on behalf of the Wellcome Sanger Institute COVID-19 Surveillance Team
EPI_ISL_964413	Lighthouse Lab in Glasgow	Wellcome Sanger Institute for the COVID-19 Genomics UK (COG-UK) Consortium	Harper VanSteenhouse, Yumi Kasai, David Gray, Carol Clugston, Anna Dominiczak and Alex Alderton, Roberto Amato, Sonia Goncalves, Ewan Harrison, David K. Jackson, Ian Johnston, Dominic Kwiatkowski, Cordelia Langford, John Sillitoe on behalf of the Wellcome Sanger Institute COVID-19 Surveillance Team
EPI_ISL_964414	Lighthouse Lab in Milton Keynes	Wellcome Sanger Institute for the COVID-19 Genomics UK (COG-UK) Consortium	The Lighthouse Lab in Milton Keynes and Alex Alderton, Roberto Amato, Sonia Goncalves, Ewan Harrison, David K. Jackson, Ian Johnston, Dominic Kwiatkowski, Cordelia Langford, John Sillitoe on behalf of the Wellcome Sanger Institute COVID-19 Surveillance Team
EPI_ISL_964415, EPI_ISL_964416, EPI_ISL_964417	Lighthouse Lab in Glasgow	Wellcome Sanger Institute for the COVID-19 Genomics UK (COG-UK) Consortium	Harper VanSteenhouse, Yumi Kasai, David Gray, Carol Clugston, Anna Dominiczak and Alex Alderton, Roberto Amato, Sonia Goncalves, Ewan Harrison, David K. Jackson, Ian Johnston, Dominic Kwiatkowski, Cordelia Langford, John Sillitoe on behalf of the Wellcome Sanger Institute COVID-19 Surveillance Team
EPI_ISL_964418, EPI_ISL_964419, EPI_ISL_964420	Lighthouse Lab in Milton Keynes	Wellcome Sanger Institute for the COVID-19 Genomics UK (COG-UK) Consortium	The Lighthouse Lab in Milton Keynes and Alex Alderton, Roberto Amato, Sonia Goncalves, Ewan Harrison, David K. Jackson, Ian Johnston, Dominic Kwiatkowski, Cordelia Langford, John Sillitoe on behalf of the Wellcome Sanger Institute COVID-19 Surveillance Team
EPI_ISL_964421, EPI_ISL_964422	Lighthouse Lab in Glasgow	Wellcome Sanger Institute for the COVID-19 Genomics UK (COG-UK) Consortium	Harper VanSteenhouse, Yumi Kasai, David Gray, Carol Clugston, Anna Dominiczak and Alex Alderton, Roberto Amato, Sonia Goncalves, Ewan Harrison, David K. Jackson, Ian Johnston, Dominic Kwiatkowski, Cordelia Langford, John Sillitoe on behalf of the Wellcome Sanger Institute COVID-19 Surveillance Team
EPI_ISL_964423	Lighthouse Lab in Milton Keynes	Wellcome Sanger Institute for the COVID-19 Genomics UK (COG-UK) Consortium	The Lighthouse Lab in Milton Keynes and Alex Alderton, Roberto Amato, Sonia Goncalves, Ewan Harrison, David K. Jackson, Ian Johnston, Dominic Kwiatkowski, Cordelia Langford, John Sillitoe on behalf of the Wellcome Sanger Institute COVID-19 Surveillance Team
EPI_ISL_964424, EPI_ISL_964425	Lighthouse Lab in Glasgow	Wellcome Sanger Institute for the COVID-19 Genomics UK	Harper VanSteenhouse, Yumi Kasai, David Gray, Carol Clugston, Anna Dominiczak and Alex Alderton, Roberto Amato, Sonia Goncalves, Ewan Harrison,

		(COG-UK) Consortium	David K. Jackson, Ian Johnston, Dominic Kwiatkowski, Cordelia Langford, John Sillitoe on behalf of the Wellcome Sanger Institute COVID-19 Surveillance Team
EPI_ISL_964426	Lighthouse Lab in Milton Keynes	Wellcome Sanger Institute for the COVID-19 Genomics UK (COG-UK) Consortium	The Lighthouse Lab in Milton Keynes and Alex Alderton, Roberto Amato, Sonia Goncalves, Ewan Harrison, David K. Jackson, Ian Johnston, Dominic Kwiatkowski, Cordelia Langford, John Sillitoe on behalf of the Wellcome Sanger Institute COVID-19 Surveillance Team
EPI_ISL_964427	Lighthouse Lab in Glasgow	Wellcome Sanger Institute for the COVID-19 Genomics UK (COG-UK) Consortium	Harper VanSteenhouse, Yumi Kasai, David Gray, Carol Clugston, Anna Dominiczak and Alex Alderton, Roberto Amato, Sonia Goncalves, Ewan Harrison, David K. Jackson, Ian Johnston, Dominic Kwiatkowski, Cordelia Langford, John Sillitoe on behalf of the Wellcome Sanger Institute COVID-19 Surveillance Team
EPI_ISL_964428, EPI_ISL_964429	Lighthouse Lab in Milton Keynes	Wellcome Sanger Institute for the COVID-19 Genomics UK (COG-UK) Consortium	The Lighthouse Lab in Milton Keynes and Alex Alderton, Roberto Amato, Sonia Goncalves, Ewan Harrison, David K. Jackson, Ian Johnston, Dominic Kwiatkowski, Cordelia Langford, John Sillitoe on behalf of the Wellcome Sanger Institute COVID-19 Surveillance Team
EPI_ISL_964430	Lighthouse Lab in Glasgow	Wellcome Sanger Institute for the COVID-19 Genomics UK (COG-UK) Consortium	Harper VanSteenhouse, Yumi Kasai, David Gray, Carol Clugston, Anna Dominiczak and Alex Alderton, Roberto Amato, Sonia Goncalves, Ewan Harrison, David K. Jackson, Ian Johnston, Dominic Kwiatkowski, Cordelia Langford, John Sillitoe on behalf of the Wellcome Sanger Institute COVID-19 Surveillance Team
EPI_ISL_964431, EPI_ISL_964432	Lighthouse Lab in Milton Keynes	Wellcome Sanger Institute for the COVID-19 Genomics UK (COG-UK) Consortium	The Lighthouse Lab in Milton Keynes and Alex Alderton, Roberto Amato, Sonia Goncalves, Ewan Harrison, David K. Jackson, Ian Johnston, Dominic Kwiatkowski, Cordelia Langford, John Sillitoe on behalf of the Wellcome Sanger Institute COVID-19 Surveillance Team
EPI_ISL_964433, EPI_ISL_964434	Lighthouse Lab in Glasgow	Wellcome Sanger Institute for the COVID-19 Genomics UK (COG-UK) Consortium	Harper VanSteenhouse, Yumi Kasai, David Gray, Carol Clugston, Anna Dominiczak and Alex Alderton, Roberto Amato, Sonia Goncalves, Ewan Harrison, David K. Jackson, Ian Johnston, Dominic Kwiatkowski, Cordelia Langford, John Sillitoe on behalf of the Wellcome Sanger Institute COVID-19 Surveillance Team
EPI_ISL_964435	Lighthouse Lab in Milton Keynes	Wellcome Sanger Institute for the COVID-19 Genomics UK (COG-UK) Consortium	The Lighthouse Lab in Milton Keynes and Alex Alderton, Roberto Amato, Sonia Goncalves, Ewan Harrison, David K. Jackson, Ian Johnston, Dominic Kwiatkowski, Cordelia Langford, John Sillitoe on behalf of the Wellcome Sanger Institute COVID-19 Surveillance Team
EPI_ISL_964436, EPI_ISL_964437	Lighthouse Lab in Glasgow	Wellcome Sanger Institute for the COVID-19 Genomics UK (COG-UK) Consortium	Harper VanSteenhouse, Yumi Kasai, David Gray, Carol Clugston, Anna Dominiczak and Alex Alderton, Roberto Amato, Sonia Goncalves, Ewan Harrison, David K. Jackson, Ian Johnston, Dominic Kwiatkowski, Cordelia Langford, John Sillitoe on behalf of the Wellcome Sanger Institute COVID-19 Surveillance Team
EPI_ISL_964438	Lighthouse Lab in Milton Keynes	Wellcome Sanger Institute for the COVID-19 Genomics UK (COG-UK) Consortium	The Lighthouse Lab in Milton Keynes and Alex Alderton, Roberto Amato, Sonia Goncalves, Ewan Harrison, David K. Jackson, Ian Johnston, Dominic Kwiatkowski, Cordelia Langford, John Sillitoe on behalf of the Wellcome Sanger Institute COVID-19 Surveillance Team
EPI_ISL_964439, EPI_ISL_964440, EPI_ISL_964441, EPI_ISL_964442	Lighthouse Lab in Glasgow	Wellcome Sanger Institute for the COVID-19 Genomics UK (COG-UK) Consortium	Harper VanSteenhouse, Yumi Kasai, David Gray, Carol Clugston, Anna Dominiczak and Alex Alderton, Roberto Amato, Sonia Goncalves, Ewan Harrison, David K. Jackson, Ian Johnston, Dominic Kwiatkowski, Cordelia Langford, John Sillitoe on behalf of the Wellcome Sanger Institute COVID-19 Surveillance Team
EPI_ISL_964443, EPI_ISL_964444, EPI_ISL_964445	Lighthouse Lab in Milton Keynes	Wellcome Sanger Institute for the COVID-19 Genomics UK (COG-UK) Consortium	The Lighthouse Lab in Milton Keynes and Alex Alderton, Roberto Amato, Sonia Goncalves, Ewan Harrison, David K. Jackson, Ian Johnston, Dominic Kwiatkowski, Cordelia Langford, John Sillitoe on behalf of the Wellcome Sanger Institute COVID-19 Surveillance Team
EPI_ISL_964446	Lighthouse Lab in Glasgow	Wellcome Sanger Institute for the COVID-19 Genomics UK (COG-UK) Consortium	Harper VanSteenhouse, Yumi Kasai, David Gray, Carol Clugston, Anna Dominiczak and Alex Alderton, Roberto Amato, Sonia Goncalves, Ewan Harrison, David K. Jackson, Ian Johnston, Dominic Kwiatkowski, Cordelia Langford, John Sillitoe on behalf of the Wellcome Sanger Institute COVID-19 Surveillance Team
EPI_ISL_964447, EPI_ISL_964448	Lighthouse Lab in Milton Keynes	Wellcome Sanger Institute for the COVID-19 Genomics UK (COG-UK) Consortium	The Lighthouse Lab in Milton Keynes and Alex Alderton, Roberto Amato, Sonia Goncalves, Ewan Harrison, David K. Jackson, Ian Johnston, Dominic Kwiatkowski, Cordelia Langford, John Sillitoe on behalf of the Wellcome Sanger Institute COVID-19 Surveillance Team
EPI_ISL_964449	Lighthouse Lab in Glasgow	Wellcome Sanger Institute for the COVID-19 Genomics UK (COG-UK) Consortium	Harper VanSteenhouse, Yumi Kasai, David Gray, Carol Clugston, Anna Dominiczak and Alex Alderton, Roberto Amato, Sonia Goncalves, Ewan Harrison, David K. Jackson, Ian Johnston, Dominic Kwiatkowski, Cordelia Langford, John Sillitoe on behalf of the Wellcome Sanger Institute COVID-19 Surveillance Team
EPI_ISL_964450	Lighthouse Lab in Milton Keynes	Wellcome Sanger Institute for the COVID-19 Genomics UK (COG-UK) Consortium	The Lighthouse Lab in Milton Keynes and Alex Alderton, Roberto Amato, Sonia Goncalves, Ewan Harrison, David K. Jackson, Ian Johnston, Dominic Kwiatkowski, Cordelia Langford, John Sillitoe on behalf of the Wellcome Sanger Institute COVID-19 Surveillance Team
EPI_ISL_964451, EPI_ISL_964452	Lighthouse Lab in Glasgow	Wellcome Sanger Institute for the COVID-19 Genomics UK (COG-UK) Consortium	Harper VanSteenhouse, Yumi Kasai, David Gray, Carol Clugston, Anna Dominiczak and Alex Alderton, Roberto Amato, Sonia Goncalves, Ewan Harrison, David K. Jackson, Ian Johnston, Dominic Kwiatkowski, Cordelia Langford, John Sillitoe on behalf of the Wellcome Sanger Institute COVID-19 Surveillance Team
EPI_ISL_964453, EPI_ISL_964454, EPI_ISL_964455	Lighthouse Lab in Milton Keynes	Wellcome Sanger Institute for the COVID-19 Genomics UK (COG-UK) Consortium	The Lighthouse Lab in Milton Keynes and Alex Alderton, Roberto Amato, Sonia Goncalves, Ewan Harrison, David K. Jackson, Ian Johnston, Dominic Kwiatkowski, Cordelia Langford, John Sillitoe on behalf of the Wellcome Sanger Institute COVID-19 Surveillance Team
EPI_ISL_964456, EPI_ISL_964457, EPI_ISL_964458	Lighthouse Lab in Glasgow	Wellcome Sanger Institute for the COVID-19 Genomics UK (COG-UK) Consortium	Harper VanSteenhouse, Yumi Kasai, David Gray, Carol Clugston, Anna Dominiczak and Alex Alderton, Roberto Amato, Sonia Goncalves, Ewan Harrison, David K. Jackson, Ian Johnston, Dominic Kwiatkowski, Cordelia Langford, John Sillitoe on behalf of the Wellcome Sanger Institute COVID-19 Surveillance Team
EPI_ISL_964459	Lighthouse Lab in Milton Keynes	Wellcome Sanger Institute for the COVID-19 Genomics UK (COG-UK) Consortium	The Lighthouse Lab in Milton Keynes and Alex Alderton, Roberto Amato, Sonia Goncalves, Ewan Harrison, David K. Jackson, Ian Johnston, Dominic Kwiatkowski, Cordelia Langford, John Sillitoe on behalf of the Wellcome Sanger Institute COVID-19 Surveillance Team
EPI_ISL_964460	Lighthouse Lab in Glasgow	Wellcome Sanger Institute for the COVID-19 Genomics UK (COG-UK) Consortium	Harper VanSteenhouse, Yumi Kasai, David Gray, Carol Clugston, Anna Dominiczak and Alex Alderton, Roberto Amato, Sonia Goncalves, Ewan Harrison, David K. Jackson, Ian Johnston, Dominic Kwiatkowski, Cordelia Langford, John Sillitoe on behalf of the Wellcome Sanger Institute COVID-19 Surveillance Team
EPI_ISL_964461, EPI_ISL_964462, EPI_ISL_964463, EPI_ISL_964464	Lighthouse Lab in Milton Keynes	Wellcome Sanger Institute for the COVID-19 Genomics UK (COG-UK) Consortium	The Lighthouse Lab in Milton Keynes and Alex Alderton, Roberto Amato, Sonia Goncalves, Ewan Harrison, David K. Jackson, Ian Johnston, Dominic Kwiatkowski, Cordelia Langford, John Sillitoe on behalf of the Wellcome Sanger Institute COVID-19 Surveillance Team
EPI_ISL_964465	Lighthouse Lab in Glasgow	Wellcome Sanger Institute for the COVID-19 Genomics UK (COG-UK) Consortium	Harper VanSteenhouse, Yumi Kasai, David Gray, Carol Clugston, Anna Dominiczak and Alex Alderton, Roberto Amato, Sonia Goncalves, Ewan Harrison, David K. Jackson, Ian Johnston, Dominic Kwiatkowski, Cordelia Langford, John Sillitoe on behalf of the Wellcome Sanger Institute COVID-19 Surveillance Team
EPI_ISL_964466, EPI_ISL_964467	Lighthouse Lab in Milton Keynes	Wellcome Sanger Institute for the COVID-19 Genomics UK (COG-UK) Consortium	The Lighthouse Lab in Milton Keynes and Alex Alderton, Roberto Amato, Sonia Goncalves, Ewan Harrison, David K. Jackson, Ian Johnston, Dominic Kwiatkowski, Cordelia Langford, John Sillitoe on behalf of the Wellcome Sanger Institute COVID-19 Surveillance Team
EPI_ISL_964468, EPI_ISL_964469, EPI_ISL_964470, EPI_ISL_964471, EPI_ISL_964472, EPI_ISL_964473, EPI_ISL_964474, EPI_ISL_964475, EPI_ISL_964476, EPI_ISL_964477	Lighthouse Lab in Glasgow	Wellcome Sanger Institute for the COVID-19 Genomics UK (COG-UK) Consortium	Harper VanSteenhouse, Yumi Kasai, David Gray, Carol Clugston, Anna Dominiczak and Alex Alderton, Roberto Amato, Sonia Goncalves, Ewan Harrison, David K. Jackson, Ian Johnston, Dominic Kwiatkowski, Cordelia Langford, John Sillitoe on behalf of the Wellcome Sanger Institute COVID-19 Surveillance Team
EPI_ISL_964478, EPI_ISL_964479	Lighthouse Lab in Milton Keynes	Wellcome Sanger Institute for the COVID-19 Genomics UK (COG-UK) Consortium	The Lighthouse Lab in Milton Keynes and Alex Alderton, Roberto Amato, Sonia Goncalves, Ewan Harrison, David K. Jackson, Ian Johnston, Dominic Kwiatkowski, Cordelia Langford, John Sillitoe on behalf of the Wellcome Sanger Institute COVID-19 Surveillance Team
EPI_ISL_964480	Lighthouse Lab in Glasgow	Wellcome Sanger Institute for the COVID-19 Genomics UK (COG-UK) Consortium	Harper VanSteenhouse, Yumi Kasai, David Gray, Carol Clugston, Anna Dominiczak and Alex Alderton, Roberto Amato, Sonia Goncalves, Ewan Harrison, David K. Jackson, Ian Johnston, Dominic Kwiatkowski, Cordelia Langford, John Sillitoe on behalf of the Wellcome Sanger Institute COVID-19 Surveillance Team
EPI_ISL_964481	Lighthouse Lab in Milton Keynes	Wellcome Sanger Institute for the COVID-19 Genomics UK (COG-UK) Consortium	The Lighthouse Lab in Milton Keynes and Alex Alderton, Roberto Amato, Sonia Goncalves, Ewan Harrison, David K. Jackson, Ian Johnston, Dominic Kwiatkowski, Cordelia Langford, John Sillitoe on behalf of the Wellcome Sanger Institute COVID-19 Surveillance Team
EPI_ISL_964482, EPI_ISL_964483, EPI_ISL_964484	Lighthouse Lab in Glasgow	Wellcome Sanger Institute for the COVID-19 Genomics UK (COG-UK) Consortium	Harper VanSteenhouse, Yumi Kasai, David Gray, Carol Clugston, Anna Dominiczak and Alex Alderton, Roberto Amato, Sonia Goncalves, Ewan Harrison, David K. Jackson, Ian Johnston, Dominic Kwiatkowski, Cordelia Langford, John Sillitoe on behalf of the Wellcome Sanger Institute COVID-19 Surveillance

			Team
EPI_ISL_964485, EPI_ISL_964486	Lighthouse Lab in Milton Keynes	Wellcome Sanger Institute for the COVID-19 Genomics UK (COG-UK) Consortium	The Lighthouse Lab in Milton Keynes and Alex Alderton, Roberto Amato, Sonia Goncalves, Ewan Harrison, David K. Jackson, Ian Johnston, Dominic Kwiatkowski, Cordelia Langford, John Sillitoe on behalf of the Wellcome Sanger Institute COVID-19 Surveillance Team
EPI_ISL_964487, EPI_ISL_964488, EPI_ISL_964489, EPI_ISL_964490	Lighthouse Lab in Glasgow	Wellcome Sanger Institute for the COVID-19 Genomics UK (COG-UK) Consortium	Harper VanSteenhouse, Yumi Kasai, David Gray, Carol Clugston, Anna Dominiczak and Alex Alderton, Roberto Amato, Sonia Goncalves, Ewan Harrison, David K. Jackson, Ian Johnston, Dominic Kwiatkowski, Cordelia Langford, John Sillitoe on behalf of the Wellcome Sanger Institute COVID-19 Surveillance Team
EPI_ISL_964491, EPI_ISL_964492	Lighthouse Lab in Milton Keynes	Wellcome Sanger Institute for the COVID-19 Genomics UK (COG-UK) Consortium	The Lighthouse Lab in Milton Keynes and Alex Alderton, Roberto Amato, Sonia Goncalves, Ewan Harrison, David K. Jackson, Ian Johnston, Dominic Kwiatkowski, Cordelia Langford, John Sillitoe on behalf of the Wellcome Sanger Institute COVID-19 Surveillance Team
EPI_ISL_964493, EPI_ISL_964494	Lighthouse Lab in Glasgow	Wellcome Sanger Institute for the COVID-19 Genomics UK (COG-UK) Consortium	Harper VanSteenhouse, Yumi Kasai, David Gray, Carol Clugston, Anna Dominiczak and Alex Alderton, Roberto Amato, Sonia Goncalves, Ewan Harrison, David K. Jackson, Ian Johnston, Dominic Kwiatkowski, Cordelia Langford, John Sillitoe on behalf of the Wellcome Sanger Institute COVID-19 Surveillance Team
EPI_ISL_964495, EPI_ISL_964496, EPI_ISL_964497, EPI_ISL_964498, EPI_ISL_964499	Lighthouse Lab in Milton Keynes	Wellcome Sanger Institute for the COVID-19 Genomics UK (COG-UK) Consortium	The Lighthouse Lab in Milton Keynes and Alex Alderton, Roberto Amato, Sonia Goncalves, Ewan Harrison, David K. Jackson, Ian Johnston, Dominic Kwiatkowski, Cordelia Langford, John Sillitoe on behalf of the Wellcome Sanger Institute COVID-19 Surveillance Team
EPI_ISL_964500, EPI_ISL_964501	Lighthouse Lab in Glasgow	Wellcome Sanger Institute for the COVID-19 Genomics UK (COG-UK) Consortium	Harper VanSteenhouse, Yumi Kasai, David Gray, Carol Clugston, Anna Dominiczak and Alex Alderton, Roberto Amato, Sonia Goncalves, Ewan Harrison, David K. Jackson, Ian Johnston, Dominic Kwiatkowski, Cordelia Langford, John Sillitoe on behalf of the Wellcome Sanger Institute COVID-19 Surveillance Team
EPI_ISL_964502	Lighthouse Lab in Milton Keynes	Wellcome Sanger Institute for the COVID-19 Genomics UK (COG-UK) Consortium	The Lighthouse Lab in Milton Keynes and Alex Alderton, Roberto Amato, Sonia Goncalves, Ewan Harrison, David K. Jackson, Ian Johnston, Dominic Kwiatkowski, Cordelia Langford, John Sillitoe on behalf of the Wellcome Sanger Institute COVID-19 Surveillance Team
EPI_ISL_964503, EPI_ISL_964504	Lighthouse Lab in Glasgow	Wellcome Sanger Institute for the COVID-19 Genomics UK (COG-UK) Consortium	Harper VanSteenhouse, Yumi Kasai, David Gray, Carol Clugston, Anna Dominiczak and Alex Alderton, Roberto Amato, Sonia Goncalves, Ewan Harrison, David K. Jackson, Ian Johnston, Dominic Kwiatkowski, Cordelia Langford, John Sillitoe on behalf of the Wellcome Sanger Institute COVID-19 Surveillance Team
EPI_ISL_964505, EPI_ISL_964506, EPI_ISL_964507	Lighthouse Lab in Milton Keynes	Wellcome Sanger Institute for the COVID-19 Genomics UK (COG-UK) Consortium	The Lighthouse Lab in Milton Keynes and Alex Alderton, Roberto Amato, Sonia Goncalves, Ewan Harrison, David K. Jackson, Ian Johnston, Dominic Kwiatkowski, Cordelia Langford, John Sillitoe on behalf of the Wellcome Sanger Institute COVID-19 Surveillance Team
EPI_ISL_964508, EPI_ISL_964509	Lighthouse Lab in Glasgow	Wellcome Sanger Institute for the COVID-19 Genomics UK (COG-UK) Consortium	Harper VanSteenhouse, Yumi Kasai, David Gray, Carol Clugston, Anna Dominiczak and Alex Alderton, Roberto Amato, Sonia Goncalves, Ewan Harrison, David K. Jackson, Ian Johnston, Dominic Kwiatkowski, Cordelia Langford, John Sillitoe on behalf of the Wellcome Sanger Institute COVID-19 Surveillance Team
EPI_ISL_964510	Lighthouse Lab in Milton Keynes	Wellcome Sanger Institute for the COVID-19 Genomics UK (COG-UK) Consortium	The Lighthouse Lab in Milton Keynes and Alex Alderton, Roberto Amato, Sonia Goncalves, Ewan Harrison, David K. Jackson, Ian Johnston, Dominic Kwiatkowski, Cordelia Langford, John Sillitoe on behalf of the Wellcome Sanger Institute COVID-19 Surveillance Team
EPI_ISL_964511, EPI_ISL_964512	Lighthouse Lab in Glasgow	Wellcome Sanger Institute for the COVID-19 Genomics UK (COG-UK) Consortium	Harper VanSteenhouse, Yumi Kasai, David Gray, Carol Clugston, Anna Dominiczak and Alex Alderton, Roberto Amato, Sonia Goncalves, Ewan Harrison, David K. Jackson, Ian Johnston, Dominic Kwiatkowski, Cordelia Langford, John Sillitoe on behalf of the Wellcome Sanger Institute COVID-19 Surveillance Team
EPI_ISL_964513	Lighthouse Lab in Milton Keynes	Wellcome Sanger Institute for the COVID-19 Genomics UK (COG-UK) Consortium	The Lighthouse Lab in Milton Keynes and Alex Alderton, Roberto Amato, Sonia Goncalves, Ewan Harrison, David K. Jackson, Ian Johnston, Dominic Kwiatkowski, Cordelia Langford, John Sillitoe on behalf of the Wellcome Sanger Institute COVID-19 Surveillance Team
EPI_ISL_964514	Lighthouse Lab in Glasgow	Wellcome Sanger Institute for the COVID-19 Genomics UK (COG-UK) Consortium	Harper VanSteenhouse, Yumi Kasai, David Gray, Carol Clugston, Anna Dominiczak and Alex Alderton, Roberto Amato, Sonia Goncalves, Ewan Harrison, David K. Jackson, Ian Johnston, Dominic Kwiatkowski, Cordelia Langford, John Sillitoe on behalf of the Wellcome Sanger Institute COVID-19 Surveillance Team
EPI_ISL_964515	Lighthouse Lab in Milton Keynes	Wellcome Sanger Institute for the COVID-19 Genomics UK (COG-UK) Consortium	The Lighthouse Lab in Milton Keynes and Alex Alderton, Roberto Amato, Sonia Goncalves, Ewan Harrison, David K. Jackson, Ian Johnston, Dominic Kwiatkowski, Cordelia Langford, John Sillitoe on behalf of the Wellcome Sanger Institute COVID-19 Surveillance Team
EPI_ISL_964516	Lighthouse Lab in Glasgow	Wellcome Sanger Institute for the COVID-19 Genomics UK (COG-UK) Consortium	Harper VanSteenhouse, Yumi Kasai, David Gray, Carol Clugston, Anna Dominiczak and Alex Alderton, Roberto Amato, Sonia Goncalves, Ewan Harrison, David K. Jackson, Ian Johnston, Dominic Kwiatkowski, Cordelia Langford, John Sillitoe on behalf of the Wellcome Sanger Institute COVID-19 Surveillance Team
EPI_ISL_964517	Lighthouse Lab in Milton Keynes	Wellcome Sanger Institute for the COVID-19 Genomics UK (COG-UK) Consortium	The Lighthouse Lab in Milton Keynes and Alex Alderton, Roberto Amato, Sonia Goncalves, Ewan Harrison, David K. Jackson, Ian Johnston, Dominic Kwiatkowski, Cordelia Langford, John Sillitoe on behalf of the Wellcome Sanger Institute COVID-19 Surveillance Team
EPI_ISL_964518	Lighthouse Lab in Glasgow	Wellcome Sanger Institute for the COVID-19 Genomics UK (COG-UK) Consortium	Harper VanSteenhouse, Yumi Kasai, David Gray, Carol Clugston, Anna Dominiczak and Alex Alderton, Roberto Amato, Sonia Goncalves, Ewan Harrison, David K. Jackson, Ian Johnston, Dominic Kwiatkowski, Cordelia Langford, John Sillitoe on behalf of the Wellcome Sanger Institute COVID-19 Surveillance Team
EPI_ISL_964519	Lighthouse Lab in Milton Keynes	Wellcome Sanger Institute for the COVID-19 Genomics UK (COG-UK) Consortium	The Lighthouse Lab in Milton Keynes and Alex Alderton, Roberto Amato, Sonia Goncalves, Ewan Harrison, David K. Jackson, Ian Johnston, Dominic Kwiatkowski, Cordelia Langford, John Sillitoe on behalf of the Wellcome Sanger Institute COVID-19 Surveillance Team
EPI_ISL_964520, EPI_ISL_964521	Lighthouse Lab in Glasgow	Wellcome Sanger Institute for the COVID-19 Genomics UK (COG-UK) Consortium	Harper VanSteenhouse, Yumi Kasai, David Gray, Carol Clugston, Anna Dominiczak and Alex Alderton, Roberto Amato, Sonia Goncalves, Ewan Harrison, David K. Jackson, Ian Johnston, Dominic Kwiatkowski, Cordelia Langford, John Sillitoe on behalf of the Wellcome Sanger Institute COVID-19 Surveillance Team
EPI_ISL_964522	Lighthouse Lab in Milton Keynes	Wellcome Sanger Institute for the COVID-19 Genomics UK (COG-UK) Consortium	The Lighthouse Lab in Milton Keynes and Alex Alderton, Roberto Amato, Sonia Goncalves, Ewan Harrison, David K. Jackson, Ian Johnston, Dominic Kwiatkowski, Cordelia Langford, John Sillitoe on behalf of the Wellcome Sanger Institute COVID-19 Surveillance Team
EPI_ISL_964523	Lighthouse Lab in Glasgow	Wellcome Sanger Institute for the COVID-19 Genomics UK (COG-UK) Consortium	Harper VanSteenhouse, Yumi Kasai, David Gray, Carol Clugston, Anna Dominiczak and Alex Alderton, Roberto Amato, Sonia Goncalves, Ewan Harrison, David K. Jackson, Ian Johnston, Dominic Kwiatkowski, Cordelia Langford, John Sillitoe on behalf of the Wellcome Sanger Institute COVID-19 Surveillance Team
EPI_ISL_964524	Lighthouse Lab in Milton Keynes	Wellcome Sanger Institute for the COVID-19 Genomics UK (COG-UK) Consortium	The Lighthouse Lab in Milton Keynes and Alex Alderton, Roberto Amato, Sonia Goncalves, Ewan Harrison, David K. Jackson, Ian Johnston, Dominic Kwiatkowski, Cordelia Langford, John Sillitoe on behalf of the Wellcome Sanger Institute COVID-19 Surveillance Team
EPI_ISL_964525, EPI_ISL_964526, EPI_ISL_964527, EPI_ISL_964528	Lighthouse Lab in Glasgow	Wellcome Sanger Institute for the COVID-19 Genomics UK (COG-UK) Consortium	Harper VanSteenhouse, Yumi Kasai, David Gray, Carol Clugston, Anna Dominiczak and Alex Alderton, Roberto Amato, Sonia Goncalves, Ewan Harrison, David K. Jackson, Ian Johnston, Dominic Kwiatkowski, Cordelia Langford, John Sillitoe on behalf of the Wellcome Sanger Institute COVID-19 Surveillance Team
EPI_ISL_964529, EPI_ISL_964530, EPI_ISL_964531	Lighthouse Lab in Milton Keynes	Wellcome Sanger Institute for the COVID-19 Genomics UK (COG-UK) Consortium	The Lighthouse Lab in Milton Keynes and Alex Alderton, Roberto Amato, Sonia Goncalves, Ewan Harrison, David K. Jackson, Ian Johnston, Dominic Kwiatkowski, Cordelia Langford, John Sillitoe on behalf of the Wellcome Sanger Institute COVID-19 Surveillance Team
EPI_ISL_964532, EPI_ISL_964533	Lighthouse Lab in Glasgow	Wellcome Sanger Institute for the COVID-19 Genomics UK (COG-UK) Consortium	Harper VanSteenhouse, Yumi Kasai, David Gray, Carol Clugston, Anna Dominiczak and Alex Alderton, Roberto Amato, Sonia Goncalves, Ewan Harrison, David K. Jackson, Ian Johnston, Dominic Kwiatkowski, Cordelia Langford, John Sillitoe on behalf of the Wellcome Sanger Institute COVID-19 Surveillance Team
EPI_ISL_964534, EPI_ISL_964535, EPI_ISL_964536	Lighthouse Lab in Milton Keynes	Wellcome Sanger Institute for the COVID-19 Genomics UK (COG-UK) Consortium	The Lighthouse Lab in Milton Keynes and Alex Alderton, Roberto Amato, Sonia Goncalves, Ewan Harrison, David K. Jackson, Ian Johnston, Dominic Kwiatkowski, Cordelia Langford, John Sillitoe on behalf of the Wellcome Sanger Institute COVID-19 Surveillance Team
EPI_ISL_964537	Lighthouse Lab in Glasgow	Wellcome Sanger Institute for the COVID-19 Genomics UK (COG-UK) Consortium	Harper VanSteenhouse, Yumi Kasai, David Gray, Carol Clugston, Anna Dominiczak and Alex Alderton, Roberto Amato, Sonia Goncalves, Ewan Harrison, David K. Jackson, Ian Johnston, Dominic Kwiatkowski, Cordelia Langford, John Sillitoe on behalf of the Wellcome Sanger Institute COVID-19 Surveillance Team
EPI_ISL_964538	Lighthouse Lab in Milton Keynes	Wellcome Sanger Institute for the COVID-19 Genomics UK	The Lighthouse Lab in Milton Keynes and Alex Alderton, Roberto Amato, Sonia Goncalves, Ewan Harrison, David K. Jackson, Ian Johnston, Dominic

		(COG-UK) Consortium	Kwiatkowski, Cordelia Langford, John Sillitoe on behalf of the Wellcome Sanger Institute COVID-19 Surveillance Team
EPI_ISL_964539, EPI_ISL_964540, EPI_ISL_964541, EPI_ISL_964542	Lighthouse Lab in Glasgow	Wellcome Sanger Institute for the COVID-19 Genomics UK (COG-UK) Consortium	Harper VanSteenhouse, Yumi Kasai, David Gray, Carol Clugston, Anna Dominiczak and Alex Alderton, Roberto Amato, Sonia Goncalves, Ewan Harrison, David K. Jackson, Ian Johnston, Dominic Kwiatkowski, Cordelia Langford, John Sillitoe on behalf of the Wellcome Sanger Institute COVID-19 Surveillance Team
EPI_ISL_964543, EPI_ISL_964544, EPI_ISL_964545, EPI_ISL_964546, EPI_ISL_964547, EPI_ISL_964548, EPI_ISL_964549, EPI_ISL_964550, EPI_ISL_964551, EPI_ISL_964552, EPI_ISL_964553, EPI_ISL_964554, EPI_ISL_964555, EPI_ISL_964556, EPI_ISL_964557, EPI_ISL_964558, EPI_ISL_964559, EPI_ISL_964560, EPI_ISL_964561, EPI_ISL_964562, EPI_ISL_964563, EPI_ISL_964564, EPI_ISL_964565, EPI_ISL_964566, EPI_ISL_964567, EPI_ISL_964568, EPI_ISL_964569, EPI_ISL_964570, EPI_ISL_964571, EPI_ISL_964572, EPI_ISL_964573, EPI_ISL_964574, EPI_ISL_964575, EPI_ISL_964576, EPI_ISL_964577, EPI_ISL_964578, EPI_ISL_964579, EPI_ISL_964580, EPI_ISL_964581, EPI_ISL_964582, EPI_ISL_964583, EPI_ISL_964584, EPI_ISL_964585, EPI_ISL_964586, EPI_ISL_964587, EPI_ISL_964588, EPI_ISL_964589, EPI_ISL_964590, EPI_ISL_964591, EPI_ISL_964592, EPI_ISL_964593, EPI_ISL_964594, EPI_ISL_964595, EPI_ISL_964596, EPI_ISL_964597, EPI_ISL_964598, EPI_ISL_964599, EPI_ISL_964600, EPI_ISL_964601, EPI_ISL_964602, EPI_ISL_964603, EPI_ISL_964604, EPI_ISL_964605, EPI_ISL_964606, EPI_ISL_964607, EPI_ISL_964608, EPI_ISL_964609, EPI_ISL_964610, EPI_ISL_964611, EPI_ISL_964612, EPI_ISL_964613, EPI_ISL_964614, EPI_ISL_964615, EPI_ISL_964616, EPI_ISL_964617, EPI_ISL_964618, EPI_ISL_964619, EPI_ISL_964620, EPI_ISL_964621, EPI_ISL_964622, EPI_ISL_964623, EPI_ISL_964624, EPI_ISL_964625, EPI_ISL_964626, EPI_ISL_964627, EPI_ISL_964628, EPI_ISL_964629, EPI_ISL_964630, EPI_ISL_964631, EPI_ISL_964632, EPI_ISL_964633, EPI_ISL_964634, EPI_ISL_964635, EPI_ISL_964636, EPI_ISL_964637, EPI_ISL_964638, EPI_ISL_964639, EPI_ISL_964640, EPI_ISL_964641, EPI_ISL_964642, EPI_ISL_964643, EPI_ISL_964644, EPI_ISL_964645, EPI_ISL_964646, EPI_ISL_964647, EPI_ISL_964648, EPI_ISL_964649, EPI_ISL_964650, EPI_ISL_964651, EPI_ISL_964652, EPI_ISL_964653, EPI_ISL_964654, EPI_ISL_964655, EPI_ISL_964656, EPI_ISL_964657, EPI_ISL_964658, EPI_ISL_964659, EPI_ISL_964660, EPI_ISL_964661, EPI_ISL_964662, EPI_ISL_964663, EPI_ISL_964664, EPI_ISL_964665, EPI_ISL_964666, EPI_ISL_964667, EPI_ISL_964668, EPI_ISL_964669, EPI_ISL_964670, EPI_ISL_964671, EPI_ISL_964672, EPI_ISL_964673, EPI_ISL_964674, EPI_ISL_964675, EPI_ISL_964676, EPI_ISL_964677, EPI_ISL_964678, EPI_ISL_964679, EPI_ISL_964680, EPI_ISL_964681, EPI_ISL_964682, EPI_ISL_964683, EPI_ISL_964684, EPI_ISL_964685, EPI_ISL_964686, EPI_ISL_964687, EPI_ISL_964688, EPI_ISL_964689, EPI_ISL_964690, EPI_ISL_964691, EPI_ISL_964692, EPI_ISL_964693, EPI_ISL_964694, EPI_ISL_964695, EPI_ISL_964696, EPI_ISL_964697, EPI_ISL_964698, EPI_ISL_964699, EPI_ISL_964700, EPI_ISL_964701, EPI_ISL_964702, EPI_ISL_964703, EPI_ISL_964704, EPI_ISL_964705, EPI_ISL_964706, EPI_ISL_964707, EPI_ISL_964708, EPI_ISL_964709, EPI_ISL_964711, EPI_ISL_964712, EPI_ISL_964713, EPI_ISL_964714, EPI_ISL_964715, EPI_ISL_964716, EPI_ISL_964717, EPI_ISL_964718, EPI_ISL_964719, EPI_ISL_964720, EPI_ISL_964721, EPI_ISL_964722, EPI_ISL_964723, EPI_ISL_964724, EPI_ISL_964725, EPI_ISL_964727, EPI_ISL_964728, EPI_ISL_964729, EPI_ISL_964730, EPI_ISL_964731, EPI_ISL_964732, EPI_ISL_964733, EPI_ISL_964734, EPI_ISL_964735, EPI_ISL_964736, EPI_ISL_964737, EPI_ISL_964738, EPI_ISL_964739, EPI_ISL_964740, EPI_ISL_964741, EPI_ISL_964742, EPI_ISL_964743, EPI_ISL_964744, EPI_ISL_964745, EPI_ISL_964746, EPI_ISL_964747, EPI_ISL_964748, EPI_ISL_964750, EPI_ISL_964751, EPI_ISL_964752, EPI_ISL_964753, EPI_ISL_964754, EPI_ISL_964755, EPI_ISL_964756, EPI_ISL_964757, EPI_ISL_964758, EPI_ISL_964760, EPI_ISL_964761, EPI_ISL_964762, EPI_ISL_964763, EPI_ISL_964764, EPI_ISL_964765, EPI_ISL_964766, EPI_ISL_964767, EPI_ISL_964768, EPI_ISL_964769, EPI_ISL_964770, EPI_ISL_964771, EPI_ISL_964772, EPI_ISL_964773, EPI_ISL_964774, EPI_ISL_964775, EPI_ISL_964776, EPI_ISL_964777, EPI_ISL_964778, EPI_ISL_964779, EPI_ISL_964780, EPI_ISL_964781, EPI_ISL_964782, EPI_ISL_964783, EPI_ISL_964784, EPI_ISL_964785, EPI_ISL_964786, EPI_ISL_964787, EPI_ISL_964788, EPI_ISL_964789, EPI_ISL_964790, EPI_ISL_964791, EPI_ISL_964792, EPI_ISL_964793, EPI_ISL_964794, EPI_ISL_964795, EPI_ISL_964796, EPI_ISL_964797, EPI_ISL_964798, EPI_ISL_964799, EPI_ISL_964800, EPI_ISL_964801, EPI_ISL_964802, EPI_ISL_964803, EPI_ISL_964804, EPI_ISL_964805, EPI_ISL_964806, EPI_ISL_964807, EPI_ISL_964808, EPI_ISL_964809, EPI_ISL_964810, EPI_ISL_964811, EPI_ISL_964812, EPI_ISL_964813, EPI_ISL_964814, EPI_ISL_964815, EPI_ISL_964816, EPI_ISL_964818, EPI_ISL_964819, EPI_ISL_964820, EPI_ISL_964821, EPI_ISL_964822, EPI_ISL_964823, EPI_ISL_964824, EPI_ISL_964825, EPI_ISL_964826, EPI_ISL_964827, EPI_ISL_964828, EPI_ISL_964829, EPI_ISL_964830, EPI_ISL_964831, EPI_ISL_964832, EPI_ISL_964833, EPI_ISL_964834, EPI_ISL_964835, EPI_ISL_964836, EPI_ISL_964837, EPI_ISL_964838, EPI_ISL_964839, EPI_ISL_964840, EPI_ISL_964841, EPI_ISL_964842, EPI_ISL_964843, EPI_ISL_964844, EPI_ISL_964845, EPI_ISL_964846, EPI_ISL_964847, EPI_ISL_964848, EPI_ISL_964849, EPI_ISL_964850, EPI_ISL_964851, EPI_ISL_964852, EPI_ISL_964853, EPI_ISL_964854, EPI_ISL_964855, EPI_ISL_964856, EPI_ISL_964857, EPI_ISL_964858, EPI_ISL_964859, EPI_ISL_964860, EPI_ISL_964861, EPI_ISL_964862, EPI_ISL_964863, EPI_ISL_964864, EPI_ISL_964865, EPI_ISL_964866, EPI_ISL_964867, EPI_ISL_964868, EPI_ISL_964869, EPI_ISL_964870, EPI_ISL_964871, EPI_ISL_964872, EPI_ISL_964873, EPI_ISL_964874, EPI_ISL_964875			
see above	Lighthouse Lab in Alderley Park	Wellcome Sanger Institute for the COVID-19 Genomics UK (COG-UK) Consortium	Jacquelyn Wynn, Mairead Hyland, The Lighthouse Lab in Alderley Park and Alex Alderton, Roberto Amato, Sonia Goncalves, Ewan Harrison, David K. Jackson, Ian Johnston, Dominic Kwiatkowski, Cordelia Langford, John Sillitoe on behalf of the Wellcome Sanger Institute COVID-19 Surveillance Team
EPI_ISL_964876	Lighthouse Lab in Milton Keynes	Wellcome Sanger Institute for the COVID-19 Genomics UK (COG-UK) Consortium	The Lighthouse Lab in Milton Keynes and Alex Alderton, Roberto Amato, Sonia Goncalves, Ewan Harrison, David K. Jackson, Ian Johnston, Dominic Kwiatkowski, Cordelia Langford, John Sillitoe on behalf of the Wellcome Sanger Institute COVID-19 Surveillance Team
EPI_ISL_964988	Furst Medical Laboratory	Norwegian Institute of Public Health, Department of Virology	Kathrine Stene-Johansen, Kamilla Heddeland Instefjord, Hilde Elshaug, Ignacio Garcia Llorente, Serina B Engebretsen, Atiya R Ali,Marie Paulsen Madsen, Rasmus Riis Kopperud, Hilde Vollan, Karoline Bragstad, Olav Hungnes
EPI_ISL_964994	Akershus University Hospital, Department for Microbiology and Infectious Disease Control	Norwegian Institute of Public Health, Department of Virology	Kathrine Stene-Johansen, Kamilla Heddeland Instefjord, Hilde Elshaug, Ignacio Garcia Llorente, Serina B Engebretsen, Atiya R Ali,Marie Paulsen Madsen, Rasmus Riis Kopperud, Hilde Vollan, Karoline Bragstad, Olav Hungnes
EPI_ISL_965031	Laboratorio Biologia Molecolare Sars Cov2 - UOC Laboratorio Analisi - Servizio Medicina di Laboratorio, Ospedale "San Francesco" - ATS-ASSL Nuoro	Laboratorio specialistico UOC Ematologia - Ospedale "San Francesco" - ATS-ASSL Nuoro	Piras Giovanna, Asproni Rosanna, Malune Paolo, Fiamma Maura, Monne Maria Itria, Palmas Angelo Domenico, Lo Maglio Iana, Mameli Giuseppe
EPI_ISL_965044	Wyoming Public Health Laboratory	Wyoming Public Health Laboratory	Noah Hull, Taylor Fearing, Lynette Gumbleton, Channing Weber, Ashley Norberg, Bailey Bowcutt, and Wanda Manley
EPI_ISL_965122, EPI_ISL_965124	Viral Respiratory Lab, National Institute for Biomedical Research (INRB)	Pathogen Sequencing Lab, National Institute for Biomedical Research (INRB)	Placide Mbala-Kingebeni, Edith Nkwembe, Eddy Kinganda-Lusamaki, Amuri Aziza, Francisca Muyembe Mawete, Emmanuel Lokilo Lofiko, Jean-Claude Makangara Cigolo, Catherine Pratt, Matthias Pauthner, Josh Quick, Allison Black, James Hadfield, Trevor Bedford, Ian Goodfellow, Andrew Rambaut, Nick Loman, Kristian Andersen, Michael Wiley, Steve Ahuka-Mundeke, Jean-Jacques Muyembe Tamfum
EPI_ISL_965155, EPI_ISL_965156	Mary Eliza Mahoney HC	NJ_PHEL	Lindsey Bodnar, Shiv Verma, Dana Woell, Byeong Jeong
EPI_ISL_965224	OSPEDALE SS. ANNUNZIATA CHIETI	Istituto Zooprofilattico Sperimentale dell'Abruzzo e Molise "G. Caporale"	Lorusso A, Marcacci M, Di Domenico M, Ancora M, Curini V, Mangone I, Rinaldi A, Scialabba S, Di Pasquale A, Cammà C, Puglia I, Calistri P, Savini G
EPI_ISL_965245	OSPEDALE CIVILE TERAMO, DIV. PNEUMOLOGIA, PIA	Istituto Zooprofilattico Sperimentale dell'Abruzzo e Molise "G. Caporale"	Lorusso A, Marcacci M, Di Domenico M, Ancora M, Curini V, Mangone I, Rinaldi A, Scialabba S, Di Pasquale A, Cammà C, Puglia I, Calistri P, Savini G
EPI_ISL_965246	OSP CIV GIULIANOVA, MED INT	Istituto Zooprofilattico Sperimentale dell'Abruzzo e Molise "G. Caporale"	Lorusso A, Marcacci M, Di Domenico M, Ancora M, Curini V, Mangone I, Rinaldi A, Scialabba S, Di Pasquale A, Cammà C, Puglia I, Calistri P, Savini G
EPI_ISL_965247, EPI_ISL_965248, EPI_ISL_965249, EPI_ISL_965250, EPI_ISL_965251, EPI_ISL_965252, EPI_ISL_965266	SIESP CHIETI	Istituto Zooprofilattico Sperimentale dell'Abruzzo e Molise "G. Caporale"	Lorusso A, Marcacci M, Di Domenico M, Ancora M, Curini V, Mangone I, Rinaldi A, Scialabba S, Di Pasquale A, Cammà C, Puglia I, Calistri P, Savini G
EPI_ISL_965276	OSP CIV ATRI	Istituto Zooprofilattico Sperimentale dell'Abruzzo e Molise "G. Caporale"	Lorusso A, Marcacci M, Di Domenico M, Ancora M, Curini V, Mangone I, Rinaldi A, Scialabba S, Di Pasquale A, Cammà C, Puglia I, Calistri P, Savini G
EPI_ISL_965314	Synlab	GIGA Medical Genomics	Keith Durkin, Maria Artesi, Sébastien Bontems, Raphaël Boreux, Bouchra Boujemla, Cécile Meex, Pierrette Melin, Marie-Pierre Hayette, Vincent Bours
EPI_ISL_965397	Clinique N.D de Grâce Gosselies	GIGA Medical Genomics	Keith Durkin, Maria Artesi, Sébastien Bontems, Raphaël Boreux, Bouchra Boujemla, Cécile Meex, Pierrette Melin, Marie-Pierre Hayette, Vincent Bours
EPI_ISL_965403, EPI_ISL_965404, EPI_ISL_965433	Synlab	GIGA Medical Genomics	Keith Durkin, Maria Artesi, Sébastien Bontems, Raphaël Boreux, Bouchra Boujemla, Cécile Meex, Pierrette Melin, Marie-Pierre Hayette, Vincent Bours
EPI_ISL_965451	Clinique N.D de Grâce Gosselies	GIGA Medical Genomics	Keith Durkin, Maria Artesi, Sébastien Bontems, Raphaël Boreux, Bouchra Boujemla, Cécile Meex, Pierrette Melin, Marie-Pierre Hayette, Vincent Bours
EPI_ISL_965470, EPI_ISL_965499, EPI_ISL_965508, EPI_ISL_965521, EPI_ISL_965524	Synlab	GIGA Medical Genomics	Keith Durkin, Maria Artesi, Sébastien Bontems, Raphaël Boreux, Bouchra Boujemla, Cécile Meex, Pierrette Melin, Marie-Pierre Hayette, Vincent Bours
EPI_ISL_965855, EPI_ISL_965856, EPI_ISL_965857, EPI_ISL_965858, EPI_ISL_965859, EPI_ISL_965860, EPI_ISL_965861, EPI_ISL_965862, EPI_ISL_965864, EPI_ISL_965865, EPI_ISL_965866, EPI_ISL_965867, EPI_ISL_965868, EPI_ISL_965869, EPI_ISL_965870, EPI_ISL_965871, EPI_ISL_965872, EPI_ISL_965873, EPI_ISL_965874, EPI_ISL_965875, EPI_ISL_965876, EPI_ISL_965880, EPI_ISL_965881, EPI_ISL_965882, EPI_ISL_965883, EPI_ISL_965884, EPI_ISL_965886, EPI_ISL_965887, EPI_ISL_965888, EPI_ISL_965889, EPI_ISL_965904			
see above	Massachusetts State Public Health Laboratory	Massachusetts State Public Health Laboratory	Andrew Lang, Timelia Fink, Glen Gallagher, Sandra Smole
EPI_ISL_965957	Consejería de Sanidad y Asuntos Sociales de Castilla La Mancha	Instituto de Salud Carlos III	Iglesias-Caballero, M. Sandonís,V. Vázquez, S. Camarero, S. Pozo, F. Casas, I. Jiménez, P. Zaballos, A. Monzón, S. Varona, S. Cuesta, I. Gutiérrez, G.
EPI_ISL_966285	Baylor Scott & White - Irving	Baylor Scott & White - Temple	Ari Rao, Linden Morales, Kimberly Walker, Marcus Volz, Shelby Hendrickson
EPI_ISL_966310, EPI_ISL_966313	Baylor Scott & White - Temple	Baylor Scott & White - Temple	Ari Rao, Linden Morales, Kimberly Walker, Marcus Volz, Shelby Hendrickson
EPI_ISL_966334, EPI_ISL_966335, EPI_ISL_966336, EPI_ISL_966337, EPI_ISL_966339, EPI_ISL_966340, EPI_ISL_966341, EPI_ISL_966342, EPI_ISL_966343, EPI_ISL_966344, EPI_ISL_966345			
see above	Kansas Health and Environmental Lab	Kansas Health and Environmental Lab	Mike Grose, Paige Drury, Carissa Robertson, Ben Olsen, and Phil Adam
EPI_ISL_966382, EPI_ISL_966384	DOHMH Jamaica	New York City Public Health Laboratory	Jade Wang, et al.
EPI_ISL_966386	DOHMH Corona	New York City Public Health Laboratory	Jade Wang, et al.
EPI_ISL_966387, EPI_ISL_966388	DOHMH Jamaica	New York City Public Health Laboratory	Jade Wang, et al.
EPI_ISL_966391	OCME Office Of Chief Medical Examiner	New York City Public Health Laboratory	Jade Wang, et al.

EPI_ISL_966395	DOHMH Jamaica	New York City Public Health Laboratory	Jade Wang, et al.
EPI_ISL_966397, EPI_ISL_966400	OCME Office Of Chief Medical Examiner	New York City Public Health Laboratory	Jade Wang, et al.
EPI_ISL_966401	Department of Homeless Services	New York City Public Health Laboratory	Jade Wang, et al.
EPI_ISL_966402, EPI_ISL_966403	OCME Office Of Chief Medical Examiner	New York City Public Health Laboratory	Jade Wang, et al.
EPI_ISL_966404	DOHMH PHL	New York City Public Health Laboratory	Jade Wang, et al.
EPI_ISL_966405	DOHMH Jamaica	New York City Public Health Laboratory	Jade Wang, et al.
EPI_ISL_966411	OCME Office Of Chief Medical Examiner	New York City Public Health Laboratory	Jade Wang, et al.
EPI_ISL_966412	DOHMH Jamaica	New York City Public Health Laboratory	Jade Wang, et al.
EPI_ISL_966413	DOHMH Central Harlem	New York City Public Health Laboratory	Jade Wang, et al.
EPI_ISL_966414	DOHMH Morrisania	New York City Public Health Laboratory	Jade Wang, et al.
EPI_ISL_966415	DOHMH Corona	New York City Public Health Laboratory	Jade Wang, et al.
EPI_ISL_966416	DOHMH Central Harlem	New York City Public Health Laboratory	Jade Wang, et al.
EPI_ISL_966417, EPI_ISL_966418, EPI_ISL_966419, EPI_ISL_966429, EPI_ISL_966430, EPI_ISL_966431	OCME Office Of Chief Medical Examiner	New York City Public Health Laboratory	Jade Wang, et al.
EPI_ISL_966432, EPI_ISL_966433	DOHMH Jamaica	New York City Public Health Laboratory	Jade Wang, et al.
EPI_ISL_966434, EPI_ISL_966435, EPI_ISL_966436, EPI_ISL_966437	DOHMH Morrisania	New York City Public Health Laboratory	Jade Wang, et al.
EPI_ISL_966438, EPI_ISL_966439, EPI_ISL_966440, EPI_ISL_966441, EPI_ISL_966442	OCME Office Of Chief Medical Examiner	New York City Public Health Laboratory	Jade Wang, et al.
EPI_ISL_966443	DOHMH Fort Greene	New York City Public Health Laboratory	Jade Wang, et al.
EPI_ISL_966444	DOHMH PHL	New York City Public Health Laboratory	Jade Wang, et al.
EPI_ISL_966445, EPI_ISL_966446	DOHMH Corona	New York City Public Health Laboratory	Jade Wang, et al.
EPI_ISL_966447, EPI_ISL_966448, EPI_ISL_966449	DOHMH Jamaica	New York City Public Health Laboratory	Jade Wang, et al.
EPI_ISL_966450	DOHMH Central Harlem	New York City Public Health Laboratory	Jade Wang, et al.
EPI_ISL_966451	DOHMH Morrisania	New York City Public Health Laboratory	Jade Wang, et al.
EPI_ISL_966453, EPI_ISL_966454, EPI_ISL_966455, EPI_ISL_966456, EPI_ISL_966457, EPI_ISL_966458	OCME Office Of Chief Medical Examiner	New York City Public Health Laboratory	Jade Wang, et al.
EPI_ISL_966459	DOHMH Corona	New York City Public Health Laboratory	Jade Wang, et al.
EPI_ISL_966460	DOHMH Morrisania	New York City Public Health Laboratory	Jade Wang, et al.
EPI_ISL_966788, EPI_ISL_966789, EPI_ISL_966790, EPI_ISL_966791, EPI_ISL_966792, EPI_ISL_966793, EPI_ISL_966794, EPI_ISL_966795, EPI_ISL_966796, EPI_ISL_966797, EPI_ISL_966798, EPI_ISL_966799, EPI_ISL_966800, EPI_ISL_966801, EPI_ISL_966802, EPI_ISL_966803, EPI_ISL_966804, EPI_ISL_966805, EPI_ISL_966806			
see above	Maine HETL	Tewhey Lab, The Jackson Laboratory	Matluk,N., Dewey,H., Iosue,F., Barter,M., Lynch,R., Munger,H. and Tewhey,R.
EPI_ISL_966852, EPI_ISL_966853, EPI_ISL_966854, EPI_ISL_966855, EPI_ISL_966856, EPI_ISL_966857, EPI_ISL_966858, EPI_ISL_966859, EPI_ISL_966860, EPI_ISL_966861, EPI_ISL_966862, EPI_ISL_966863, EPI_ISL_966864, EPI_ISL_966865, EPI_ISL_966866, EPI_ISL_966867, EPI_ISL_966868, EPI_ISL_966869, EPI_ISL_966870, EPI_ISL_966871, EPI_ISL_966872, EPI_ISL_966873, EPI_ISL_966874, EPI_ISL_966875, EPI_ISL_966876, EPI_ISL_966877, EPI_ISL_966878, EPI_ISL_966879, EPI_ISL_966880, EPI_ISL_966881, EPI_ISL_966882, EPI_ISL_966883, EPI_ISL_966884, EPI_ISL_966885, EPI_ISL_966886, EPI_ISL_966887, EPI_ISL_966888, EPI_ISL_966889, EPI_ISL_966890, EPI_ISL_966891, EPI_ISL_966892, EPI_ISL_966893, EPI_ISL_966894, EPI_ISL_966895, EPI_ISL_966896, EPI_ISL_966897, EPI_ISL_966898, EPI_ISL_966899, EPI_ISL_966900, EPI_ISL_966901, EPI_ISL_966902, EPI_ISL_966903, EPI_ISL_966904, EPI_ISL_966905, EPI_ISL_966906, EPI_ISL_966907, EPI_ISL_966908, EPI_ISL_966909, EPI_ISL_966910, EPI_ISL_966911, EPI_ISL_966912, EPI_ISL_966913, EPI_ISL_966914, EPI_ISL_966915, EPI_ISL_966916, EPI_ISL_966917, EPI_ISL_966918, EPI_ISL_966919, EPI_ISL_966920, EPI_ISL_966921, EPI_ISL_966922, EPI_ISL_966923, EPI_ISL_966924, EPI_ISL_966925, EPI_ISL_966926, EPI_ISL_966927, EPI_ISL_966928			
see above	Helix/Illumina	Respiratory Viruses Branch, Division of Viral Diseases, Centers for Disease Control and Prevention	Peter W. Cook,Dakota Howard,Dhwani Batra,Ben L. Rambo-Martin,Eileen de Feo,Jan Antico,Christine Tran,Matthew Tolentino,Shannon Wickline,Kim Gietzen,Brad Sickler,Jingtao Liu,Eric Allen,Phil Febbo,Summer Galloway,Nicole L. Washington,Simon White,Geraint Levan,Kelly Schiabor Barrett,Elizabeth Cirulli,Alexandre Bolze,Ary Ascencio,Charlotte Rivera-Garcia,Ryan Cho,Jason Nguyen,Sherry Wang,Jimmy Ramirez,Tyler Cassens,Efren Sandoval,Magnus Isaksson,William Lee,David Becker,Marc Laurent,James Lu,Clinton R. Paden,Suxiang Tong,Duncan MacCannell,
EPI_ISL_967770	State Laboratories Division, Hawaii State Department of Health	State Laboratories Division, Hawaii State Department of Health	Pamela O'Brien, Drew Kuwazaki, Ayana Garnet, Razvan Sultana, Edward Desmond
EPI_ISL_968078	Monterey County Public Health Laboratory	Monterey County Public Health Laboratory	Monterey County Public Health Laboratory
EPI_ISL_968089	Institute for Medical Research, Infectious Disease Research Centre, National Institutes of Health, Ministry of Health Malaysia	Institute for Medical Research, Infectious Disease Research Centre, National Institutes of Health, Ministry of Health Malaysia	Suppiah J, Kamel K, Azizan MA, Thayan R
EPI_ISL_969018, EPI_ISL_969047, EPI_ISL_969048, EPI_ISL_969049, EPI_ISL_969068, EPI_ISL_969069, EPI_ISL_969072, EPI_ISL_969073, EPI_ISL_969074	KEMRI-Wellcome Trust Research Programme/KEMRI-CGMR-C Kilifi	KEMRI-Wellcome Trust Research Programme/KEMRI-CGMR-C Kilifi	Githinji et al
EPI_ISL_969455	Lighthouse Lab in Glasgow	Wellcome Sanger Institute for the COVID-19 Genomics UK (COG-UK) Consortium	Harper VanSteenhouse, Yumi Kasai, David Gray, Carol Clugston, Anna Dominiczak and Alex Alderton, Roberto Amato, Sonia Goncalves, Ewan Harrison, David K. Jackson, Ian Johnston, Dominic Kwiatkowski, Cordelia Langford, John Sillitoe on behalf of the Wellcome Sanger Institute COVID-19 Surveillance Team
EPI_ISL_969457, EPI_ISL_969458, EPI_ISL_969459, EPI_ISL_969460, EPI_ISL_969461, EPI_ISL_969462, EPI_ISL_969463, EPI_ISL_969464, EPI_ISL_969465, EPI_ISL_969466, EPI_ISL_969467, EPI_ISL_969468, EPI_ISL_969469, EPI_ISL_969471, EPI_ISL_969472, EPI_ISL_969473, EPI_ISL_969474, EPI_ISL_969475, EPI_ISL_969476, EPI_ISL_969477, EPI_ISL_969478, EPI_ISL_969479, EPI_ISL_969480, EPI_ISL_969481, EPI_ISL_969482, EPI_ISL_969483, EPI_ISL_969484, EPI_ISL_969485, EPI_ISL_969486, EPI_ISL_969487, EPI_ISL_969488, EPI_ISL_969489, EPI_ISL_969490, EPI_ISL_969491, EPI_ISL_969492, EPI_ISL_969493, EPI_ISL_969494, EPI_ISL_969495, EPI_ISL_969496, EPI_ISL_969497, EPI_ISL_969498, EPI_ISL_969500, EPI_ISL_969501, EPI_ISL_969502, EPI_ISL_969503, EPI_ISL_969504, EPI_ISL_969505, EPI_ISL_969506, EPI_ISL_969507, EPI_ISL_969508, EPI_ISL_969510, EPI_ISL_969511, EPI_ISL_969512, EPI_ISL_969513, EPI_ISL_969514, EPI_ISL_969515, EPI_ISL_969516, EPI_ISL_969517, EPI_ISL_969518, EPI_ISL_969519, EPI_ISL_969520, EPI_ISL_969521, EPI_ISL_969522, EPI_ISL_969523, EPI_ISL_969524, EPI_ISL_969525, EPI_ISL_969526, EPI_ISL_969527, EPI_ISL_969528, EPI_ISL_969529, EPI_ISL_969530, EPI_ISL_969531, EPI_ISL_969532, EPI_ISL_969533, EPI_ISL_969534, EPI_ISL_969535, EPI_ISL_969536, EPI_ISL_969537, EPI_ISL_969538, EPI_ISL_969539, EPI_ISL_969540, EPI_ISL_969541, EPI_ISL_969542, EPI_ISL_969543, EPI_ISL_969544, EPI_ISL_969545, EPI_ISL_969546, EPI_ISL_969548, EPI_ISL_969549, EPI_ISL_969550, EPI_ISL_969551, EPI_ISL_969552, EPI_ISL_969553, EPI_ISL_969554, EPI_ISL_969555, EPI_ISL_969556, EPI_ISL_969557, EPI_ISL_969558, EPI_ISL_969559, EPI_ISL_969560, EPI_ISL_969561, EPI_ISL_969562, EPI_ISL_969563, EPI_ISL_969564, EPI_ISL_969565, EPI_ISL_969566, EPI_ISL_969567, EPI_ISL_969568, EPI_ISL_969569, EPI_ISL_969570, EPI_ISL_969571, EPI_ISL_969572, EPI_ISL_969573, EPI_ISL_969574, EPI_ISL_969575, EPI_ISL_969576, EPI_ISL_969577, EPI_ISL_969578, EPI_ISL_969579, EPI_ISL_969580, EPI_ISL_969581, EPI_ISL_969582, EPI_ISL_969583, EPI_ISL_969584, EPI_ISL_969585, EPI_ISL_969586, EPI_ISL_969587, EPI_ISL_969588, EPI_ISL_969589, EPI_ISL_969591, EPI_ISL_969592, EPI_ISL_969593, EPI_ISL_969594, EPI_ISL_969595, EPI_ISL_969596, EPI_ISL_969597, EPI_ISL_969598, EPI_ISL_969599, EPI_ISL_969600, EPI_ISL_969602, EPI_ISL_969603, EPI_ISL_969604, EPI_ISL_969605, EPI_ISL_969606, EPI_ISL_969607, EPI_ISL_969608, EPI_ISL_969609, EPI_ISL_969610, EPI_ISL_969611, EPI_ISL_969612, EPI_ISL_969613, EPI_ISL_969614, EPI_ISL_969615, EPI_ISL_969616, EPI_ISL_969617, EPI_ISL_969618, EPI_ISL_969619, EPI_ISL_969620, EPI_ISL_969621, EPI_ISL_969622, EPI_ISL_969623, EPI_ISL_969624, EPI_ISL_969625, EPI_ISL_969626, EPI_ISL_969627, EPI_ISL_969628, EPI_ISL_969629, EPI_ISL_969630, EPI_ISL_969631, EPI_ISL_969632, EPI_ISL_969633, EPI_ISL_969634, EPI_ISL_969635, EPI_ISL_969636, EPI_ISL_969637, EPI_ISL_969638, EPI_ISL_969639, EPI_ISL_969640, EPI_ISL_969641, EPI_ISL_969642, EPI_ISL_969643, EPI_ISL_969644, EPI_ISL_969645, EPI_ISL_969646, EPI_ISL_969647, EPI_ISL_969649, EPI_ISL_969650, EPI_ISL_969651, EPI_ISL_969652, EPI_ISL_969653, EPI_ISL_969654, EPI_ISL_969655, EPI_ISL_969656, EPI_ISL_969657, EPI_ISL_969658, EPI_ISL_969659, EPI_ISL_969660, EPI_ISL_969661, EPI_ISL_969662, EPI_ISL_969663, EPI_ISL_969664, EPI_ISL_969665, EPI_ISL_969666, EPI_ISL_969667, EPI_ISL_969668, EPI_ISL_969669, EPI_ISL_969670, EPI_ISL_969671, EPI_ISL_969672, EPI_ISL_969673, EPI_ISL_969674, EPI_ISL_969675, EPI_ISL_969677, EPI_ISL_969679, EPI_ISL_969682, EPI_ISL_969683,			

EPI_ISL_969685, EPI_ISL_969688, EPI_ISL_969690, EPI_ISL_969691, EPI_ISL_969693, EPI_ISL_969695, EPI_ISL_969697, EPI_ISL_969699, EPI_ISL_969701, EPI_ISL_969702, EPI_ISL_969704, EPI_ISL_969706, EPI_ISL_969708, EPI_ISL_969710, EPI_ISL_969712, EPI_ISL_969714, EPI_ISL_969716, EPI_ISL_969718, EPI_ISL_969720, EPI_ISL_969722, EPI_ISL_969724, EPI_ISL_969726, EPI_ISL_969728, EPI_ISL_969730, EPI_ISL_969732, EPI_ISL_969734, EPI_ISL_969736, EPI_ISL_969739, EPI_ISL_969742, EPI_ISL_969744, EPI_ISL_969746, EPI_ISL_969748, EPI_ISL_969750, EPI_ISL_969752, EPI_ISL_969754, EPI_ISL_969756, EPI_ISL_969759, EPI_ISL_969760, EPI_ISL_969762, EPI_ISL_969764, EPI_ISL_969766, EPI_ISL_969768, EPI_ISL_969769, EPI_ISL_969771, EPI_ISL_969774, EPI_ISL_969776, EPI_ISL_969779, EPI_ISL_969780, EPI_ISL_969782, EPI_ISL_969785, EPI_ISL_969786, EPI_ISL_969789, EPI_ISL_969791, EPI_ISL_969793, EPI_ISL_969795, EPI_ISL_969797, EPI_ISL_969799, EPI_ISL_969801, EPI_ISL_969802, EPI_ISL_969804, EPI_ISL_969806, EPI_ISL_969808, EPI_ISL_969809, EPI_ISL_969812, EPI_ISL_969813, EPI_ISL_969815, EPI_ISL_969817, EPI_ISL_969819, EPI_ISL_969821, EPI_ISL_969823, EPI_ISL_969824, EPI_ISL_969826, EPI_ISL_969828, EPI_ISL_969831, EPI_ISL_969833, EPI_ISL_969834, EPI_ISL_969836, EPI_ISL_969838, EPI_ISL_969840, EPI_ISL_969841, EPI_ISL_969843, EPI_ISL_969846, EPI_ISL_969848, EPI_ISL_969849, EPI_ISL_969852, EPI_ISL_969853, EPI_ISL_969856, EPI_ISL_969858, EPI_ISL_969859, EPI_ISL_969861, EPI_ISL_969863, EPI_ISL_969865, EPI_ISL_969867, EPI_ISL_969869, EPI_ISL_969871, EPI_ISL_969873, EPI_ISL_969875, EPI_ISL_969877, EPI_ISL_969879, EPI_ISL_969881, EPI_ISL_969883, EPI_ISL_969886, EPI_ISL_969887, EPI_ISL_969889, EPI_ISL_969891, EPI_ISL_969893, EPI_ISL_969895, EPI_ISL_969897, EPI_ISL_969899, EPI_ISL_969901, EPI_ISL_969903, EPI_ISL_969906, EPI_ISL_969907, EPI_ISL_969909, EPI_ISL_969911, EPI_ISL_969914, EPI_ISL_969916, EPI_ISL_969917, EPI_ISL_969919, EPI_ISL_969921, EPI_ISL_969923, EPI_ISL_969925, EPI_ISL_969928, EPI_ISL_969930, EPI_ISL_969932, EPI_ISL_969934, EPI_ISL_969936, EPI_ISL_969937, EPI_ISL_969939, EPI_ISL_969940, EPI_ISL_969943, EPI_ISL_969945, EPI_ISL_969947, EPI_ISL_969948, EPI_ISL_969951, EPI_ISL_969952, EPI_ISL_969955, EPI_ISL_969956, EPI_ISL_969958, EPI_ISL_969961, EPI_ISL_969963, EPI_ISL_969965, EPI_ISL_969967, EPI_ISL_969969, EPI_ISL_969971, EPI_ISL_969974, EPI_ISL_969975, EPI_ISL_969977, EPI_ISL_969979, EPI_ISL_969982, EPI_ISL_969983, EPI_ISL_969986, EPI_ISL_969988, EPI_ISL_969990, EPI_ISL_969992, EPI_ISL_969994, EPI_ISL_969996, EPI_ISL_969998, EPI_ISL_970000, EPI_ISL_970002, EPI_ISL_970004, EPI_ISL_970005, EPI_ISL_970007, EPI_ISL_970009, EPI_ISL_970011, EPI_ISL_970013, EPI_ISL_970015, EPI_ISL_970017, EPI_ISL_970019, EPI_ISL_970022, EPI_ISL_970024, EPI_ISL_970026, EPI_ISL_970028, EPI_ISL_970030, EPI_ISL_970033, EPI_ISL_970035, EPI_ISL_970037, EPI_ISL_970039, EPI_ISL_970041, EPI_ISL_970043, EPI_ISL_970045, EPI_ISL_970047, EPI_ISL_970049, EPI_ISL_970050, EPI_ISL_970052, EPI_ISL_970057, EPI_ISL_970059, EPI_ISL_970061, EPI_ISL_970064, EPI_ISL_970066, EPI_ISL_970068, EPI_ISL_970070, EPI_ISL_970073, EPI_ISL_970075, EPI_ISL_970076, EPI_ISL_970079, EPI_ISL_970080, EPI_ISL_970082, EPI_ISL_970084, EPI_ISL_970086, EPI_ISL_970087, EPI_ISL_970089, EPI_ISL_970091, EPI_ISL_970094, EPI_ISL_970096, EPI_ISL_970098, EPI_ISL_970099, EPI_ISL_970101, EPI_ISL_970103, EPI_ISL_970105, EPI_ISL_970107, EPI_ISL_970109, EPI_ISL_970111, EPI_ISL_970113, EPI_ISL_970115, EPI_ISL_970117, EPI_ISL_970119, EPI_ISL_970120, EPI_ISL_970122, EPI_ISL_970124, EPI_ISL_970126, EPI_ISL_970128, EPI_ISL_970130, EPI_ISL_970132, EPI_ISL_970134, EPI_ISL_970136, EPI_ISL_970138, EPI_ISL_970140, EPI_ISL_970145, EPI_ISL_970147, EPI_ISL_970148, EPI_ISL_970150, EPI_ISL_970152, EPI_ISL_970153, EPI_ISL_970155, EPI_ISL_970157, EPI_ISL_970160, EPI_ISL_970161, EPI_ISL_970163, EPI_ISL_970165, EPI_ISL_970166, EPI_ISL_970168, EPI_ISL_970170, EPI_ISL_970172, EPI_ISL_970173, EPI_ISL_970175, EPI_ISL_970177, EPI_ISL_970181, EPI_ISL_970183, EPI_ISL_970184, EPI_ISL_970186, EPI_ISL_970188, EPI_ISL_970190, EPI_ISL_970192, EPI_ISL_970195, EPI_ISL_970197, EPI_ISL_970198, EPI_ISL_970200, EPI_ISL_970202, EPI_ISL_970205, EPI_ISL_970206, EPI_ISL_970209, EPI_ISL_970211, EPI_ISL_970213, EPI_ISL_970215, EPI_ISL_970217, EPI_ISL_970219, EPI_ISL_970221, EPI_ISL_970224, EPI_ISL_970225, EPI_ISL_970227, EPI_ISL_970229, EPI_ISL_970231, EPI_ISL_970233, EPI_ISL_970235, EPI_ISL_970237, EPI_ISL_970239, EPI_ISL_970242, EPI_ISL_970247, EPI_ISL_970249, EPI_ISL_970251, EPI_ISL_970252, EPI_ISL_970254, EPI_ISL_970256, EPI_ISL_970258, EPI_ISL_970260, EPI_ISL_970262, EPI_ISL_970264, EPI_ISL_970266, EPI_ISL_970268, EPI_ISL_970270, EPI_ISL_970272, EPI_ISL_970274, EPI_ISL_970275, EPI_ISL_970278, EPI_ISL_970279, EPI_ISL_970281, EPI_ISL_970283, EPI_ISL_970285, EPI_ISL_970287, EPI_ISL_970288, EPI_ISL_970291, EPI_ISL_970292, EPI_ISL_970295, EPI_ISL_970297, EPI_ISL_970299, EPI_ISL_970301, EPI_ISL_970303, EPI_ISL_970306, EPI_ISL_970307, EPI_ISL_970309, EPI_ISL_970312, EPI_ISL_970314, EPI_ISL_970316, EPI_ISL_970319, EPI_ISL_970321, EPI_ISL_970323, EPI_ISL_970324, EPI_ISL_970326, EPI_ISL_970328, EPI_ISL_970330, EPI_ISL_970332, EPI_ISL_970334, EPI_ISL_970337, EPI_ISL_970338, EPI_ISL_970340, EPI_ISL_970342, EPI_ISL_970344, EPI_ISL_970346, EPI_ISL_970348, EPI_ISL_970350, EPI_ISL_970352, EPI_ISL_970354, EPI_ISL_970356, EPI_ISL_970358, EPI_ISL_970360, EPI_ISL_970362, EPI_ISL_970364, EPI_ISL_970365, EPI_ISL_970368, EPI_ISL_970370, EPI_ISL_970371, EPI_ISL_970373, EPI_ISL_970376, EPI_ISL_970377, EPI_ISL_970380, EPI_ISL_970382, EPI_ISL_970383, EPI_ISL_970386, EPI_ISL_970387, EPI_ISL_970390, EPI_ISL_970392, EPI_ISL_970394, EPI_ISL_970396, EPI_ISL_970398, EPI_ISL_970400, EPI_ISL_970402, EPI_ISL_970404, EPI_ISL_970406, EPI_ISL_970409, EPI_ISL_970411, EPI_ISL_970412, EPI_ISL_970415, EPI_ISL_970416, EPI_ISL_970419, EPI_ISL_970421, EPI_ISL_970422, EPI_ISL_970424, EPI_ISL_970426, EPI_ISL_970428, EPI_ISL_970430, EPI_ISL_970432, EPI_ISL_970435, EPI_ISL_970436, EPI_ISL_970439, EPI_ISL_970441, EPI_ISL_970443, EPI_ISL_970444, EPI_ISL_970446, EPI_ISL_970448, EPI_ISL_970450, EPI_ISL_970452, EPI_ISL_970454, EPI_ISL_970457, EPI_ISL_970460, EPI_ISL_970462, EPI_ISL_970464, EPI_ISL_970466, EPI_ISL_970468, EPI_ISL_970470, EPI_ISL_970473, EPI_ISL_970475, EPI_ISL_970477, EPI_ISL_970478, EPI_ISL_970481, EPI_ISL_970483, EPI_ISL_970486, EPI_ISL_970488, EPI_ISL_970490, EPI_ISL_970492, EPI_ISL_970493, EPI_ISL_970495, EPI_ISL_970497, EPI_ISL_970499, EPI_ISL_970500, EPI_ISL_970502, EPI_ISL_970504, EPI_ISL_970506, EPI_ISL_970508, EPI_ISL_970510, EPI_ISL_970512, EPI_ISL_970513, EPI_ISL_970515, EPI_ISL_970517, EPI_ISL_970519, EPI_ISL_970521, EPI_ISL_970523, EPI_ISL_970525, EPI_ISL_970527, EPI_ISL_970528, EPI_ISL_970530, EPI_ISL_970533, EPI_ISL_970535, EPI_ISL_970537, EPI_ISL_970540, EPI_ISL_970541, EPI_ISL_970543, EPI_ISL_970546, EPI_ISL_970548, EPI_ISL_970551, EPI_ISL_970553, EPI_ISL_970555, EPI_ISL_970558, EPI_ISL_970560, EPI_ISL_970562, EPI_ISL_970564, EPI_ISL_970566, EPI_ISL_970568, EPI_ISL_970569, EPI_ISL_970571, EPI_ISL_970573, EPI_ISL_970575, EPI_ISL_970577, EPI_ISL_970579, EPI_ISL_970581, EPI_ISL_970583, EPI_ISL_970585			
see above	Lighthouse Lab in Alderley Park	Wellcome Sanger Institute for the COVID-19 Genomics UK (COG-UK) Consortium	Jacquelyn Wynn, Mairead Hyland, The Lighthouse Lab in Alderley Park and Alex Alderton, Roberto Amato, Sonia Goncalves, Ewan Harrison, David K. Jackson, Ian Johnston, Dominic Kwiatkowski, Cordelia Langford, John Sillitoe on behalf of the Wellcome Sanger Institute COVID-19 Surveillance Team
EPI_ISL_970587, EPI_ISL_970597, EPI_ISL_970616, EPI_ISL_970621, EPI_ISL_970628, EPI_ISL_970635, EPI_ISL_970639, EPI_ISL_970645, EPI_ISL_970664, EPI_ISL_970673, EPI_ISL_970680, EPI_ISL_970681, EPI_ISL_970687, EPI_ISL_970701, EPI_ISL_970708, EPI_ISL_970709, EPI_ISL_970715, EPI_ISL_970724, EPI_ISL_970732, EPI_ISL_970744, EPI_ISL_970747, EPI_ISL_970751, EPI_ISL_970753, EPI_ISL_970768, EPI_ISL_970775, EPI_ISL_970790			
see above	Lighthouse Lab in Milton Keynes	Wellcome Sanger Institute for the COVID-19 Genomics UK (COG-UK) Consortium	The Lighthouse Lab in Milton Keynes and Alex Alderton, Roberto Amato, Sonia Goncalves, Ewan Harrison, David K. Jackson, Ian Johnston, Dominic Kwiatkowski, Cordelia Langford, John Sillitoe on behalf of the Wellcome Sanger Institute COVID-19 Surveillance Team
EPI_ISL_970812, EPI_ISL_970819, EPI_ISL_970820, EPI_ISL_970824	Department of Virus and Microbiological Special Diagnostics, Statens Serum Institut, Copenhagen, Denmark	Aalborg University	Danish Covid-19 Genome Consortium
EPI_ISL_970826	Lighthouse Lab in Milton Keynes	Wellcome Sanger Institute for the COVID-19 Genomics UK (COG-UK) Consortium	The Lighthouse Lab in Milton Keynes and Alex Alderton, Roberto Amato, Sonia Goncalves, Ewan Harrison, David K. Jackson, Ian Johnston, Dominic Kwiatkowski, Cordelia Langford, John Sillitoe on behalf of the Wellcome Sanger Institute COVID-19 Surveillance Team
EPI_ISL_970830, EPI_ISL_970831, EPI_ISL_970832, EPI_ISL_970837, EPI_ISL_970844, EPI_ISL_970850, EPI_ISL_970851, EPI_ISL_970859	Department of Virus and Microbiological Special Diagnostics, Statens Serum Institut, Copenhagen, Denmark	Aalborg University	Danish Covid-19 Genome Consortium
EPI_ISL_970860	Lighthouse Lab in Milton Keynes	Wellcome Sanger Institute for the COVID-19 Genomics UK (COG-UK) Consortium	The Lighthouse Lab in Milton Keynes and Alex Alderton, Roberto Amato, Sonia Goncalves, Ewan Harrison, David K. Jackson, Ian Johnston, Dominic Kwiatkowski, Cordelia Langford, John Sillitoe on behalf of the Wellcome Sanger Institute COVID-19 Surveillance Team
EPI_ISL_970862, EPI_ISL_970863, EPI_ISL_970874	Department of Virus and Microbiological Special Diagnostics, Statens Serum Institut, Copenhagen, Denmark	Aalborg University	Danish Covid-19 Genome Consortium
EPI_ISL_970875	Lighthouse Lab in Milton Keynes	Wellcome Sanger Institute for the COVID-19 Genomics UK (COG-UK) Consortium	The Lighthouse Lab in Milton Keynes and Alex Alderton, Roberto Amato, Sonia Goncalves, Ewan Harrison, David K. Jackson, Ian Johnston, Dominic Kwiatkowski, Cordelia Langford, John Sillitoe on behalf of the Wellcome Sanger Institute COVID-19 Surveillance Team
EPI_ISL_970880, EPI_ISL_970882, EPI_ISL_970885, EPI_ISL_970886, EPI_ISL_970891, EPI_ISL_970892, EPI_ISL_970895, EPI_ISL_970905, EPI_ISL_970908, EPI_ISL_970910, EPI_ISL_970927, EPI_ISL_970928, EPI_ISL_970929, EPI_ISL_970930, EPI_ISL_970932, EPI_ISL_970938, EPI_ISL_970939, EPI_ISL_970943, EPI_ISL_970946, EPI_ISL_970949, EPI_ISL_970950, EPI_ISL_970952, EPI_ISL_970953, EPI_ISL_970959, EPI_ISL_970960, EPI_ISL_970969, EPI_ISL_970970, EPI_ISL_970973, EPI_ISL_970975, EPI_ISL_970976, EPI_ISL_970978, EPI_ISL_970980			
see above	Department of Virus and Microbiological Special Diagnostics, Statens Serum Institut, Copenhagen, Denmark	Aalborg University	Danish Covid-19 Genome Consortium
EPI_ISL_970986	Lighthouse Lab in Milton Keynes	Wellcome Sanger Institute for the COVID-19 Genomics UK (COG-UK) Consortium	The Lighthouse Lab in Milton Keynes and Alex Alderton, Roberto Amato, Sonia Goncalves, Ewan Harrison, David K. Jackson, Ian Johnston, Dominic Kwiatkowski, Cordelia Langford, John Sillitoe on behalf of the Wellcome Sanger Institute COVID-19 Surveillance Team
EPI_ISL_970987, EPI_ISL_970989, EPI_ISL_970990, EPI_ISL_970991, EPI_ISL_970994, EPI_ISL_970996, EPI_ISL_970999, EPI_ISL_971000, EPI_ISL_971004, EPI_ISL_971009, EPI_ISL_971014, EPI_ISL_971021, EPI_ISL_971022, EPI_ISL_971032, EPI_ISL_971033, EPI_ISL_971035			
see above	Department of Virus and Microbiological Special Diagnostics, Statens Serum Institut, Copenhagen, Denmark	Aalborg University	Danish Covid-19 Genome Consortium
EPI_ISL_971037, EPI_ISL_971042, EPI_ISL_971044	Lighthouse Lab in Milton Keynes	Wellcome Sanger Institute for the COVID-19 Genomics UK (COG-UK) Consortium	The Lighthouse Lab in Milton Keynes and Alex Alderton, Roberto Amato, Sonia Goncalves, Ewan Harrison, David K. Jackson, Ian Johnston, Dominic Kwiatkowski, Cordelia Langford, John Sillitoe on behalf of the Wellcome Sanger Institute COVID-19 Surveillance Team
EPI_ISL_971045	Department of Virus and Microbiological Special Diagnostics, Statens Serum Institut, Copenhagen, Denmark	Aalborg University	Danish Covid-19 Genome Consortium
EPI_ISL_971047, EPI_ISL_971053, EPI_ISL_971060, EPI_ISL_971071, EPI_ISL_971076	Lighthouse Lab in Milton Keynes	Wellcome Sanger Institute for the COVID-19 Genomics UK (COG-UK) Consortium	The Lighthouse Lab in Milton Keynes and Alex Alderton, Roberto Amato, Sonia Goncalves, Ewan Harrison, David K. Jackson, Ian Johnston, Dominic Kwiatkowski, Cordelia Langford, John Sillitoe on behalf of the Wellcome Sanger Institute COVID-19 Surveillance Team
EPI_ISL_971081, EPI_ISL_971092	Department of Virus and Microbiological Special Diagnostics, Statens Serum Institut, Copenhagen, Denmark	Aalborg University	Danish Covid-19 Genome Consortium
EPI_ISL_971093, EPI_ISL_971096	Lighthouse Lab in Milton Keynes	Wellcome Sanger Institute for the COVID-19 Genomics UK (COG-UK) Consortium	The Lighthouse Lab in Milton Keynes and Alex Alderton, Roberto Amato, Sonia Goncalves, Ewan Harrison, David K. Jackson, Ian Johnston, Dominic Kwiatkowski, Cordelia Langford, John Sillitoe on behalf of the Wellcome Sanger Institute COVID-19 Surveillance Team
EPI_ISL_971100	Department of Virus and Microbiological Special Diagnostics, Statens Serum Institut, Copenhagen, Denmark	Aalborg University	Danish Covid-19 Genome Consortium
EPI_ISL_971105	Lighthouse Lab in Milton Keynes	Wellcome Sanger Institute for the COVID-19 Genomics UK (COG-UK) Consortium	The Lighthouse Lab in Milton Keynes and Alex Alderton, Roberto Amato, Sonia Goncalves, Ewan Harrison, David K. Jackson, Ian Johnston, Dominic Kwiatkowski, Cordelia Langford, John Sillitoe on behalf of the Wellcome Sanger Institute COVID-19 Surveillance Team
EPI_ISL_971112, EPI_ISL_971115, EPI_ISL_971121, EPI_ISL_971124	Department of Virus and Microbiological Special Diagnostics, Statens Serum Institut, Copenhagen, Denmark	Aalborg University	Danish Covid-19 Genome Consortium
EPI_ISL_971125, EPI_ISL_971135, EPI_ISL_971142	Lighthouse Lab in Milton Keynes	Wellcome Sanger Institute for the COVID-19 Genomics UK (COG-UK) Consortium	The Lighthouse Lab in Milton Keynes and Alex Alderton, Roberto Amato, Sonia Goncalves, Ewan Harrison, David K. Jackson, Ian Johnston, Dominic Kwiatkowski, Cordelia Langford, John Sillitoe on behalf of the Wellcome Sanger Institute COVID-19 Surveillance Team
EPI_ISL_971148, EPI_ISL_971158, EPI_ISL_971163, EPI_ISL_971166	Department of Virus and Microbiological Special Diagnostics, Statens Serum Institut, Copenhagen, Denmark	Aalborg University	Danish Covid-19 Genome Consortium

EPI_ISL_971168, EPI_ISL_971171, EPI_ISL_971174, EPI_ISL_971176, EPI_ISL_971183	Lighthouse Lab in Milton Keynes	Wellcome Sanger Institute for the COVID-19 Genomics UK (COG-UK) Consortium	The Lighthouse Lab in Milton Keynes and Alex Alderton, Roberto Amato, Sonia Goncalves, Ewan Harrison, David K. Jackson, Ian Johnston, Dominic Kwiatkowski, Cordelia Langford, John Sillitoe on behalf of the Wellcome Sanger Institute COVID-19 Surveillance Team
EPI_ISL_971184, EPI_ISL_971188	Department of Virus and Microbiological Special Diagnostics, Statens Serum Institut, Copenhagen, Denmark	Aalborg University	Danish Covid-19 Genome Consortium
EPI_ISL_971191	Lighthouse Lab in Milton Keynes	Wellcome Sanger Institute for the COVID-19 Genomics UK (COG-UK) Consortium	The Lighthouse Lab in Milton Keynes and Alex Alderton, Roberto Amato, Sonia Goncalves, Ewan Harrison, David K. Jackson, Ian Johnston, Dominic Kwiatkowski, Cordelia Langford, John Sillitoe on behalf of the Wellcome Sanger Institute COVID-19 Surveillance Team
EPI_ISL_971193	Department of Virus and Microbiological Special Diagnostics, Statens Serum Institut, Copenhagen, Denmark	Aalborg University	Danish Covid-19 Genome Consortium
EPI_ISL_971194	Lighthouse Lab in Milton Keynes	Wellcome Sanger Institute for the COVID-19 Genomics UK (COG-UK) Consortium	The Lighthouse Lab in Milton Keynes and Alex Alderton, Roberto Amato, Sonia Goncalves, Ewan Harrison, David K. Jackson, Ian Johnston, Dominic Kwiatkowski, Cordelia Langford, John Sillitoe on behalf of the Wellcome Sanger Institute COVID-19 Surveillance Team
EPI_ISL_971198	Department of Virus and Microbiological Special Diagnostics, Statens Serum Institut, Copenhagen, Denmark	Aalborg University	Danish Covid-19 Genome Consortium
EPI_ISL_971206	Lighthouse Lab in Milton Keynes	Wellcome Sanger Institute for the COVID-19 Genomics UK (COG-UK) Consortium	The Lighthouse Lab in Milton Keynes and Alex Alderton, Roberto Amato, Sonia Goncalves, Ewan Harrison, David K. Jackson, Ian Johnston, Dominic Kwiatkowski, Cordelia Langford, John Sillitoe on behalf of the Wellcome Sanger Institute COVID-19 Surveillance Team
EPI_ISL_971214, EPI_ISL_971220, EPI_ISL_971229	Department of Virus and Microbiological Special Diagnostics, Statens Serum Institut, Copenhagen, Denmark	Aalborg University	Danish Covid-19 Genome Consortium
EPI_ISL_971231	Lighthouse Lab in Milton Keynes	Wellcome Sanger Institute for the COVID-19 Genomics UK (COG-UK) Consortium	The Lighthouse Lab in Milton Keynes and Alex Alderton, Roberto Amato, Sonia Goncalves, Ewan Harrison, David K. Jackson, Ian Johnston, Dominic Kwiatkowski, Cordelia Langford, John Sillitoe on behalf of the Wellcome Sanger Institute COVID-19 Surveillance Team
EPI_ISL_971232	Department of Virus and Microbiological Special Diagnostics, Statens Serum Institut, Copenhagen, Denmark	Aalborg University	Danish Covid-19 Genome Consortium
EPI_ISL_971239	Lighthouse Lab in Milton Keynes	Wellcome Sanger Institute for the COVID-19 Genomics UK (COG-UK) Consortium	The Lighthouse Lab in Milton Keynes and Alex Alderton, Roberto Amato, Sonia Goncalves, Ewan Harrison, David K. Jackson, Ian Johnston, Dominic Kwiatkowski, Cordelia Langford, John Sillitoe on behalf of the Wellcome Sanger Institute COVID-19 Surveillance Team
EPI_ISL_971241	Department of Virus and Microbiological Special Diagnostics, Statens Serum Institut, Copenhagen, Denmark	Aalborg University	Danish Covid-19 Genome Consortium
EPI_ISL_971242	Lighthouse Lab in Milton Keynes	Wellcome Sanger Institute for the COVID-19 Genomics UK (COG-UK) Consortium	The Lighthouse Lab in Milton Keynes and Alex Alderton, Roberto Amato, Sonia Goncalves, Ewan Harrison, David K. Jackson, Ian Johnston, Dominic Kwiatkowski, Cordelia Langford, John Sillitoe on behalf of the Wellcome Sanger Institute COVID-19 Surveillance Team
EPI_ISL_971250	Department of Virus and Microbiological Special Diagnostics, Statens Serum Institut, Copenhagen, Denmark	Aalborg University	Danish Covid-19 Genome Consortium
EPI_ISL_971255, EPI_ISL_971261, EPI_ISL_971269, EPI_ISL_971281	Lighthouse Lab in Milton Keynes	Wellcome Sanger Institute for the COVID-19 Genomics UK (COG-UK) Consortium	The Lighthouse Lab in Milton Keynes and Alex Alderton, Roberto Amato, Sonia Goncalves, Ewan Harrison, David K. Jackson, Ian Johnston, Dominic Kwiatkowski, Cordelia Langford, John Sillitoe on behalf of the Wellcome Sanger Institute COVID-19 Surveillance Team
EPI_ISL_971290	Department of Virus and Microbiological Special Diagnostics, Statens Serum Institut, Copenhagen, Denmark	Aalborg University	Danish Covid-19 Genome Consortium
EPI_ISL_971292	Lighthouse Lab in Milton Keynes	Wellcome Sanger Institute for the COVID-19 Genomics UK (COG-UK) Consortium	The Lighthouse Lab in Milton Keynes and Alex Alderton, Roberto Amato, Sonia Goncalves, Ewan Harrison, David K. Jackson, Ian Johnston, Dominic Kwiatkowski, Cordelia Langford, John Sillitoe on behalf of the Wellcome Sanger Institute COVID-19 Surveillance Team
EPI_ISL_971293	Department of Virus and Microbiological Special Diagnostics, Statens Serum Institut, Copenhagen, Denmark	Aalborg University	Danish Covid-19 Genome Consortium
EPI_ISL_971297	Lighthouse Lab in Milton Keynes	Wellcome Sanger Institute for the COVID-19 Genomics UK (COG-UK) Consortium	The Lighthouse Lab in Milton Keynes and Alex Alderton, Roberto Amato, Sonia Goncalves, Ewan Harrison, David K. Jackson, Ian Johnston, Dominic Kwiatkowski, Cordelia Langford, John Sillitoe on behalf of the Wellcome Sanger Institute COVID-19 Surveillance Team
EPI_ISL_971299, EPI_ISL_971306, EPI_ISL_971315, EPI_ISL_971326	Department of Virus and Microbiological Special Diagnostics, Statens Serum Institut, Copenhagen, Denmark	Aalborg University	Danish Covid-19 Genome Consortium
EPI_ISL_971327, EPI_ISL_971333	Lighthouse Lab in Milton Keynes	Wellcome Sanger Institute for the COVID-19 Genomics UK (COG-UK) Consortium	The Lighthouse Lab in Milton Keynes and Alex Alderton, Roberto Amato, Sonia Goncalves, Ewan Harrison, David K. Jackson, Ian Johnston, Dominic Kwiatkowski, Cordelia Langford, John Sillitoe on behalf of the Wellcome Sanger Institute COVID-19 Surveillance Team
EPI_ISL_971335	Department of Virus and Microbiological Special Diagnostics, Statens Serum Institut, Copenhagen, Denmark	Aalborg University	Danish Covid-19 Genome Consortium
EPI_ISL_971339, EPI_ISL_971347	Lighthouse Lab in Milton Keynes	Wellcome Sanger Institute for the COVID-19 Genomics UK (COG-UK) Consortium	The Lighthouse Lab in Milton Keynes and Alex Alderton, Roberto Amato, Sonia Goncalves, Ewan Harrison, David K. Jackson, Ian Johnston, Dominic Kwiatkowski, Cordelia Langford, John Sillitoe on behalf of the Wellcome Sanger Institute COVID-19 Surveillance Team
EPI_ISL_971353, EPI_ISL_971355	Department of Virus and Microbiological Special Diagnostics, Statens Serum Institut, Copenhagen, Denmark	Aalborg University	Danish Covid-19 Genome Consortium
EPI_ISL_971360	Lighthouse Lab in Milton Keynes	Wellcome Sanger Institute for the COVID-19 Genomics UK (COG-UK) Consortium	The Lighthouse Lab in Milton Keynes and Alex Alderton, Roberto Amato, Sonia Goncalves, Ewan Harrison, David K. Jackson, Ian Johnston, Dominic Kwiatkowski, Cordelia Langford, John Sillitoe on behalf of the Wellcome Sanger Institute COVID-19 Surveillance Team
EPI_ISL_971361	Department of Virus and Microbiological Special Diagnostics, Statens Serum Institut, Copenhagen, Denmark	Aalborg University	Danish Covid-19 Genome Consortium
EPI_ISL_971364, EPI_ISL_971365, EPI_ISL_971370	Lighthouse Lab in Milton Keynes	Wellcome Sanger Institute for the COVID-19 Genomics UK (COG-UK) Consortium	The Lighthouse Lab in Milton Keynes and Alex Alderton, Roberto Amato, Sonia Goncalves, Ewan Harrison, David K. Jackson, Ian Johnston, Dominic Kwiatkowski, Cordelia Langford, John Sillitoe on behalf of the Wellcome Sanger Institute COVID-19 Surveillance Team
EPI_ISL_971375, EPI_ISL_971381	Department of Virus and Microbiological Special Diagnostics, Statens Serum Institut, Copenhagen, Denmark	Aalborg University	Danish Covid-19 Genome Consortium
EPI_ISL_971385, EPI_ISL_971394	Lighthouse Lab in Milton Keynes	Wellcome Sanger Institute for the COVID-19 Genomics UK (COG-UK) Consortium	The Lighthouse Lab in Milton Keynes and Alex Alderton, Roberto Amato, Sonia Goncalves, Ewan Harrison, David K. Jackson, Ian Johnston, Dominic Kwiatkowski, Cordelia Langford, John Sillitoe on behalf of the Wellcome Sanger Institute COVID-19 Surveillance Team

EPI_ISL_971404, EPI_ISL_971418, EPI_ISL_971431, EPI_ISL_971437, EPI_ISL_971439, EPI_ISL_971447, EPI_ISL_971461, EPI_ISL_971467, EPI_ISL_971470, EPI_ISL_971480, EPI_ISL_971485, EPI_ISL_971494, EPI_ISL_971496, EPI_ISL_971497, EPI_ISL_971500, EPI_ISL_971502, EPI_ISL_971503, EPI_ISL_971504, EPI_ISL_971508, EPI_ISL_971509, EPI_ISL_971513, EPI_ISL_971515, EPI_ISL_971516, EPI_ISL_971529, EPI_ISL_971534, EPI_ISL_971539, EPI_ISL_971542, EPI_ISL_971547, EPI_ISL_971548, EPI_ISL_971549, EPI_ISL_971556, EPI_ISL_971557, EPI_ISL_971558, EPI_ISL_971565, EPI_ISL_971575, EPI_ISL_971578, EPI_ISL_971580, EPI_ISL_971581, EPI_ISL_971591, EPI_ISL_971615, EPI_ISL_971616, EPI_ISL_971623, EPI_ISL_971625, EPI_ISL_971628, EPI_ISL_971632, EPI_ISL_971633, EPI_ISL_971635, EPI_ISL_971638, EPI_ISL_971640, EPI_ISL_971648, EPI_ISL_971649, EPI_ISL_971650, EPI_ISL_971673, EPI_ISL_971674, EPI_ISL_971680, EPI_ISL_971684, EPI_ISL_971686, EPI_ISL_971697, EPI_ISL_971701, EPI_ISL_971705, EPI_ISL_971708, EPI_ISL_971710, EPI_ISL_971711, EPI_ISL_971714, EPI_ISL_971715, EPI_ISL_971716, EPI_ISL_971723, EPI_ISL_971724, EPI_ISL_971730, EPI_ISL_971733, EPI_ISL_971736, EPI_ISL_971737, EPI_ISL_971739, EPI_ISL_971742, EPI_ISL_971743, EPI_ISL_971745, EPI_ISL_971751, EPI_ISL_971755, EPI_ISL_971757, EPI_ISL_971761, EPI_ISL_971768, EPI_ISL_971769, EPI_ISL_971785, EPI_ISL_971808, EPI_ISL_971815, EPI_ISL_971822, EPI_ISL_971832, EPI_ISL_971849, EPI_ISL_971854, EPI_ISL_971856, EPI_ISL_971857, EPI_ISL_971859, EPI_ISL_971861, EPI_ISL_971865, EPI_ISL_971867, EPI_ISL_971876, EPI_ISL_971877, EPI_ISL_971880, EPI_ISL_971886, EPI_ISL_971899, EPI_ISL_971906, EPI_ISL_971908, EPI_ISL_971910, EPI_ISL_971915, EPI_ISL_971925, EPI_ISL_971931, EPI_ISL_971933, EPI_ISL_971934, EPI_ISL_971938, EPI_ISL_971942, EPI_ISL_971943, EPI_ISL_971946, EPI_ISL_971954, EPI_ISL_971960, EPI_ISL_971962, EPI_ISL_971963, EPI_ISL_971966, EPI_ISL_971968, EPI_ISL_971970, EPI_ISL_971971, EPI_ISL_971973, EPI_ISL_971975, EPI_ISL_971978, EPI_ISL_971981, EPI_ISL_971985, EPI_ISL_971986, EPI_ISL_971988, EPI_ISL_971990, EPI_ISL_971996, EPI_ISL_971999, EPI_ISL_972006, EPI_ISL_972008, EPI_ISL_972015, EPI_ISL_972016, EPI_ISL_972018, EPI_ISL_972023, EPI_ISL_972032, EPI_ISL_972034, EPI_ISL_972046, EPI_ISL_972049, EPI_ISL_972053, EPI_ISL_972054, EPI_ISL_972060, EPI_ISL_972068, EPI_ISL_972074, EPI_ISL_972080, EPI_ISL_972085, EPI_ISL_972088, EPI_ISL_972090, EPI_ISL_972092, EPI_ISL_972093, EPI_ISL_972101, EPI_ISL_972102, EPI_ISL_972105, EPI_ISL_972114, EPI_ISL_972119, EPI_ISL_972123, EPI_ISL_972130, EPI_ISL_972131, EPI_ISL_972132, EPI_ISL_972136, EPI_ISL_972138, EPI_ISL_972143, EPI_ISL_972150, EPI_ISL_972156, EPI_ISL_972164, EPI_ISL_972169, EPI_ISL_972170, EPI_ISL_972172, EPI_ISL_972184, EPI_ISL_972190, EPI_ISL_972193, EPI_ISL_972194, EPI_ISL_972195, EPI_ISL_972196, EPI_ISL_972197, EPI_ISL_972198, EPI_ISL_972201, EPI_ISL_972217, EPI_ISL_972222, EPI_ISL_972223, EPI_ISL_972241, EPI_ISL_972258, EPI_ISL_972261, EPI_ISL_972266, EPI_ISL_972267, EPI_ISL_972271, EPI_ISL_972274, EPI_ISL_972280, EPI_ISL_972288, EPI_ISL_972293, EPI_ISL_972309, EPI_ISL_972312, EPI_ISL_972313, EPI_ISL_972314, EPI_ISL_972324, EPI_ISL_972328, EPI_ISL_972332, EPI_ISL_972336, EPI_ISL_972338, EPI_ISL_972346, EPI_ISL_972348, EPI_ISL_972349, EPI_ISL_972351, EPI_ISL_972354, EPI_ISL_972358, EPI_ISL_972370, EPI_ISL_972375, EPI_ISL_972376, EPI_ISL_972377, EPI_ISL_972378, EPI_ISL_972381, EPI_ISL_972387, EPI_ISL_972391, EPI_ISL_972393, EPI_ISL_972394, EPI_ISL_972397, EPI_ISL_972406, EPI_ISL_972407, EPI_ISL_972423, EPI_ISL_972426, EPI_ISL_972431, EPI_ISL_972435, EPI_ISL_972436, EPI_ISL_972437, EPI_ISL_972438, EPI_ISL_972443, EPI_ISL_972448, EPI_ISL_972465, EPI_ISL_972466, EPI_ISL_972468, EPI_ISL_972471, EPI_ISL_972472, EPI_ISL_972474, EPI_ISL_972477, EPI_ISL_972478, EPI_ISL_972486, EPI_ISL_972488, EPI_ISL_972491, EPI_ISL_972493, EPI_ISL_972512, EPI_ISL_972513, EPI_ISL_972517, EPI_ISL_972528, EPI_ISL_972529, EPI_ISL_972530, EPI_ISL_972533, EPI_ISL_972536, EPI_ISL_972539, EPI_ISL_972545, EPI_ISL_972546, EPI_ISL_972547,

EPI_ISL_972549, EPI_ISL_972552, EPI_ISL_972555, EPI_ISL_972556, EPI_ISL_972560, EPI_ISL_972565, EPI_ISL_972569, EPI_ISL_972574, EPI_ISL_972576, EPI_ISL_972577, EPI_ISL_972579, EPI_ISL_972580, EPI_ISL_972586, EPI_ISL_972590, EPI_ISL_972591, EPI_ISL_972592, EPI_ISL_972597, EPI_ISL_972601, EPI_ISL_972609, EPI_ISL_972610, EPI_ISL_972612, EPI_ISL_972614, EPI_ISL_972615, EPI_ISL_972625, EPI_ISL_972627, EPI_ISL_972639, EPI_ISL_972644, EPI_ISL_972661, EPI_ISL_972663, EPI_ISL_972673, EPI_ISL_972674, EPI_ISL_972680, EPI_ISL_972686, EPI_ISL_972687, EPI_ISL_972689, EPI_ISL_972690, EPI_ISL_972692, EPI_ISL_972707, EPI_ISL_972712, EPI_ISL_972713, EPI_ISL_972715, EPI_ISL_972718, EPI_ISL_972727, EPI_ISL_972728, EPI_ISL_972730, EPI_ISL_972737, EPI_ISL_972742, EPI_ISL_972743, EPI_ISL_972744, EPI_ISL_972751, EPI_ISL_972755, EPI_ISL_972758, EPI_ISL_972759, EPI_ISL_972763, EPI_ISL_972768, EPI_ISL_972769, EPI_ISL_972771, EPI_ISL_972772, EPI_ISL_972773, EPI_ISL_972774, EPI_ISL_972776, EPI_ISL_972777, EPI_ISL_972785, EPI_ISL_972787, EPI_ISL_972790, EPI_ISL_972791, EPI_ISL_972807, EPI_ISL_972809, EPI_ISL_972812, EPI_ISL_972813, EPI_ISL_972819, EPI_ISL_972821, EPI_ISL_972822, EPI_ISL_972825, EPI_ISL_972833, EPI_ISL_972834, EPI_ISL_972843, EPI_ISL_972848, EPI_ISL_972851, EPI_ISL_972853, EPI_ISL_972855, EPI_ISL_972858, EPI_ISL_972860, EPI_ISL_972872, EPI_ISL_972876, EPI_ISL_972881, EPI_ISL_972887, EPI_ISL_972888, EPI_ISL_972892, EPI_ISL_972895, EPI_ISL_972897, EPI_ISL_972898, EPI_ISL_972900, EPI_ISL_972903, EPI_ISL_972907, EPI_ISL_972911, EPI_ISL_972918, EPI_ISL_972922, EPI_ISL_972936, EPI_ISL_972938, EPI_ISL_972940, EPI_ISL_972941, EPI_ISL_972945, EPI_ISL_972948, EPI_ISL_972949, EPI_ISL_972957, EPI_ISL_972960, EPI_ISL_972962, EPI_ISL_972965, EPI_ISL_972975, EPI_ISL_972995, EPI_ISL_972998, EPI_ISL_973001, EPI_ISL_973014, EPI_ISL_973020, EPI_ISL_973024, EPI_ISL_973026, EPI_ISL_973027, EPI_ISL_973030, EPI_ISL_973038, EPI_ISL_973040, EPI_ISL_973047, EPI_ISL_973048, EPI_ISL_973052, EPI_ISL_973053, EPI_ISL_973055, EPI_ISL_973060, EPI_ISL_973063, EPI_ISL_973064, EPI_ISL_973069, EPI_ISL_973071, EPI_ISL_973073, EPI_ISL_973076, EPI_ISL_973082, EPI_ISL_973085, EPI_ISL_973086, EPI_ISL_973094, EPI_ISL_973096, EPI_ISL_973097, EPI_ISL_973111, EPI_ISL_973115, EPI_ISL_973120, EPI_ISL_973122, EPI_ISL_973123, EPI_ISL_973127, EPI_ISL_973130, EPI_ISL_973132, EPI_ISL_973135, EPI_ISL_973137, EPI_ISL_973143, EPI_ISL_973160, EPI_ISL_973167, EPI_ISL_973179, EPI_ISL_973182, EPI_ISL_973186, EPI_ISL_973189, EPI_ISL_973191, EPI_ISL_973195, EPI_ISL_973196, EPI_ISL_973199, EPI_ISL_973206, EPI_ISL_973208, EPI_ISL_973216, EPI_ISL_973224, EPI_ISL_973225, EPI_ISL_973229, EPI_ISL_973230, EPI_ISL_973232, EPI_ISL_973236, EPI_ISL_973238, EPI_ISL_973249, EPI_ISL_973250, EPI_ISL_973251, EPI_ISL_973256, EPI_ISL_973260, EPI_ISL_973264, EPI_ISL_973266, EPI_ISL_973273, EPI_ISL_973281, EPI_ISL_973288, EPI_ISL_973289, EPI_ISL_973296, EPI_ISL_973302, EPI_ISL_973304, EPI_ISL_973312, EPI_ISL_973317, EPI_ISL_973324, EPI_ISL_973328, EPI_ISL_973333, EPI_ISL_973338, EPI_ISL_973340, EPI_ISL_973341, EPI_ISL_973345, EPI_ISL_973349, EPI_ISL_973354, EPI_ISL_973358, EPI_ISL_973359, EPI_ISL_973363, EPI_ISL_973365, EPI_ISL_973368, EPI_ISL_973373, EPI_ISL_973383, EPI_ISL_973384, EPI_ISL_973396, EPI_ISL_973398, EPI_ISL_973399, EPI_ISL_973404, EPI_ISL_973406, EPI_ISL_973410, EPI_ISL_973412, EPI_ISL_973422, EPI_ISL_973423, EPI_ISL_973430, EPI_ISL_973432, EPI_ISL_973445, EPI_ISL_973447, EPI_ISL_973453, EPI_ISL_973456, EPI_ISL_973459, EPI_ISL_973461, EPI_ISL_973465, EPI_ISL_973467, EPI_ISL_973468, EPI_ISL_973471, EPI_ISL_973474, EPI_ISL_973484, EPI_ISL_973486, EPI_ISL_973487, EPI_ISL_973495, EPI_ISL_973499, EPI_ISL_973505, EPI_ISL_973508, EPI_ISL_973516, EPI_ISL_973518, EPI_ISL_973540, EPI_ISL_973553, EPI_ISL_973570, EPI_ISL_973580, EPI_ISL_973584, EPI_ISL_973586, EPI_ISL_973595, EPI_ISL_973599, EPI_ISL_973633, EPI_ISL_973636, EPI_ISL_973638, EPI_ISL_973641, EPI_ISL_973654, EPI_ISL_973664, EPI_ISL_973674, EPI_ISL_973677, EPI_ISL_973693, EPI_ISL_973694, EPI_ISL_973699, EPI_ISL_973706, EPI_ISL_973711, EPI_ISL_973713, EPI_ISL_973732, EPI_ISL_973741, EPI_ISL_973748, EPI_ISL_973762, EPI_ISL_973766, EPI_ISL_973772, EPI_ISL_973784, EPI_ISL_973796, EPI_ISL_973817, EPI_ISL_973820, EPI_ISL_973824, EPI_ISL_973829, EPI_ISL_973831, EPI_ISL_973833			
see above	Department of Virus and Microbiological Special Diagnostics, Statens Serum Institut, Copenhagen, Denmark	Aalborg University	Danish Covid-19 Genome Consortium
EPI_ISL_974853	Laboratorio de Virología / Hospital Universitario Central de Asturias (HUCA)	Laboratorio de Virología / Hospital Universitario Central de Asturias (HUCA)	Castelló C, Gómez de Oña J, Boga JA, Rojo S, Alvarez-Arguelles ME, Abreu F, Costales I, Sandoval M, Perez-Martínez Z, Martín-Rodríguez G, Coto E, Melón S
EPI_ISL_977417	Hospital de Cabueñes	Laboratorio de Virología / Hospital Universitario Central de Asturias (HUCA)	Castelló C, Gómez de Oña J, Boga JA, Rojo S, Alvarez-Arguelles ME, Abreu F, Costales I, Sandoval M, Perez-Martínez Z, Martín-Rodríguez G, Coto E, Melón S
EPI_ISL_977489	UPA Dr. Akira Tada	Instituto Adolfo Lutz, Interdiciplinary Procedures Center, Strategic Laboratory	Claudio Tavares Sacchi, Claudia Regina Gonçalves, Erica Valessa Ramos Gomes, Karoline Rodrigues Campos
EPI_ISL_977492	Lab voor klinische biologie	Lab voor klinische biologie	Hannelore Hamerlinck, Marija Janevska, Bruno Verhasselt
EPI_ISL_977500	LATE - Laboratório de Técnicas Especiais - Hospital Israelita Albert Einstein	LATE - Laboratório de Técnicas Especiais - Hospital Israelita Albert Einstein	Deyvid Amgarten, Fernanda de Mello Malta, Raquel Riyuzo, Ana Paula Moreira Salles, Pedro Henrique Sebe Rodrigues, João Renato Rebello Pinho
EPI_ISL_977501	Hospital de Cabueñes	Laboratorio de Virología HUCA	Castelló C, Gómez de Oña J, Boga JA, Rojo S, Alvarez-Arguelles ME, Abreu F, Costales I, Sandoval M, Perez-Martínez Z, Martín-Rodríguez G, Coto E, Melón S
EPI_ISL_977502	Hospital Oriente de Asturias	Laboratorio de Virología HUCA	Sandoval M, Castelló C, Gómez de Oña J, Boga JA, Rojo S, Alvarez-Arguelles ME, Abreu F, Costales I, Perez-Martínez Z, Martín-Rodríguez G, Coto E, Melón S
EPI_ISL_977505, EPI_ISL_977506	Laboratorio de Virología HUCA	Laboratorio de Virología HUCA	Sandoval M, Castelló C, Gómez de Oña J, Boga JA, Rojo S, Alvarez-Arguelles ME, Abreu F, Costales I, Perez-Martínez Z, Martín-Rodríguez G, Coto E, Melón S
EPI_ISL_977507	Hospital Valle del Nalon	Laboratorio de Virología HUCA	Sandoval M, Castelló C, Gómez de Oña J, Boga JA, Rojo S, Alvarez-Arguelles ME, Abreu F, Costales I, Perez-Martínez Z, Martín-Rodríguez G, Coto E, Melón S
EPI_ISL_977509	Hospital Valle del Nalón	Laboratorio de Virología HUCA	Sandoval M, Castelló C, Gómez de Oña J, Boga JA, Rojo S, Alvarez-Arguelles ME, Abreu F, Costales I, Perez-Martínez Z, Martín-Rodríguez G, Coto E, Melón S
EPI_ISL_977510, EPI_ISL_977511, EPI_ISL_977512	Laboratorio de Virología HUCA	Laboratorio de Virología HUCA	Sandoval M, Castelló C, Gómez de Oña J, Boga JA, Rojo S, Alvarez-Arguelles ME, Abreu F, Costales I, Perez-Martínez Z, Martín-Rodríguez G, Coto E, Melón S
EPI_ISL_977537	Biolab Diagnostic Laboratories	Biolab Diagnostic Laboratories	Issa Abu-Dayyeh, Ahmad Tibi, Lama Hussein, Shayma Ali, Badia Saddedin, Amid Abdelnour
EPI_ISL_977566, EPI_ISL_977567, EPI_ISL_977575, EPI_ISL_977576, EPI_ISL_977577, EPI_ISL_977578, EPI_ISL_977579, EPI_ISL_977580	University of Michigan Clinical Microbiology Laboratory	Lauring Lab, University of Michigan, Department of Microbiology and Immunology	Valesano
EPI_ISL_977613, EPI_ISL_977614, EPI_ISL_977615, EPI_ISL_977620	SC (UCO) Igiene e Sanità Pubblica (funzione integrata con SC Microbiologia e Virologia) e Laboratory of Molecular Virology of the International Centre for Genetic Engineering and Biotechnology (ICGEB)	ARGO Laboratorio Genomica ed Epigenomica	Licastro D, Dal Monego S, Degasperi M, Marcello A, D'Agaro P, Pipan C
EPI_ISL_977660, EPI_ISL_977665	Auburn Sports Medicine	Synergy Laboratories	Megan Cornwell
EPI_ISL_978373, EPI_ISL_978374, EPI_ISL_978375, EPI_ISL_978376, EPI_ISL_978377, EPI_ISL_978481, EPI_ISL_978482, EPI_ISL_978483, EPI_ISL_978484, EPI_ISL_978485, EPI_ISL_978486, EPI_ISL_978487			
see above	Arizona State Public Health Laboratory	Arizona State Public Health Laboratory	Trung Huynh, Jessica Escobar, Katherine Fullerton, Nobuko Fukushima, Stacy White, Linda Getsinger, Victor Waddell
EPI_ISL_978555, EPI_ISL_978556, EPI_ISL_978557, EPI_ISL_978558, EPI_ISL_978559, EPI_ISL_978560, EPI_ISL_978561, EPI_ISL_978562, EPI_ISL_978563, EPI_ISL_978564, EPI_ISL_978565, EPI_ISL_978566, EPI_ISL_978567, EPI_ISL_978568, EPI_ISL_978569, EPI_ISL_978570, EPI_ISL_978571, EPI_ISL_978572, EPI_ISL_978573, EPI_ISL_978574, EPI_ISL_978575, EPI_ISL_978576, EPI_ISL_978577, EPI_ISL_978578, EPI_ISL_978579, EPI_ISL_978580, EPI_ISL_978581, EPI_ISL_978582, EPI_ISL_978583, EPI_ISL_978584, EPI_ISL_978585, EPI_ISL_978586, EPI_ISL_978587, EPI_ISL_978588, EPI_ISL_978589, EPI_ISL_978590, EPI_ISL_978591, EPI_ISL_978592, EPI_ISL_978593, EPI_ISL_978594, EPI_ISL_978595, EPI_ISL_978596, EPI_ISL_978597, EPI_ISL_978598, EPI_ISL_978599, EPI_ISL_978600, EPI_ISL_978601, EPI_ISL_978602, EPI_ISL_978603, EPI_ISL_978604, EPI_ISL_978605, EPI_ISL_978606, EPI_ISL_978607, EPI_ISL_978608, EPI_ISL_978609, EPI_ISL_978610, EPI_ISL_978611, EPI_ISL_978612, EPI_ISL_978613, EPI_ISL_978614, EPI_ISL_978615, EPI_ISL_978616, EPI_ISL_978617, EPI_ISL_978618, EPI_ISL_978619, EPI_ISL_978620			
see above	Helix/Illumina	Respiratory Viruses Branch, Division of Viral Diseases, Centers for Disease Control and Prevention	Peter W. Cook,Dakota Howard,Dhwani Batra,Ben L. Rambo-Martin,Eileen de Feo,Jan Antico,Christine Tran,Matthew Tolentino,Shannon Wickline,Kim Gietzen,Brad Sickler,Jingtao Liu,Eric Allen,Phil Febbo,Summer Galloway,Nicole L. Washington,Simon White,Geraint Levan,Kelly Schiabor Barrett,Elizabeth Cirulli,Alexandre Bolze,Ary Ascencio,Charlotte Rivera-Garcia,Ryan Cho,Jason Nguyen,Sherry Wang,Jimmy Ramirez,Tyler Cassens,Efren Sandoval,Magnus Isaksson,William Lee,David Becker,Marc Laurent,James Lu,Clinton R. Paden,Suxiang Tong,Duncan MacCannell,
EPI_ISL_978889, EPI_ISL_978918, EPI_ISL_978919, EPI_ISL_978954, EPI_ISL_978984, EPI_ISL_978988	Centre for Dengue Research and AICBU, Department of Immunology and Molecular Medicine	Centre for Dengue Research and AICBU, Department of Immunology and Molecular Medicine	Chandima Jeewandara, Deshni Jayathilaka, Dinuka Ariyaratne, Tibutius Thanesh Pramanayagam, Diyanath Ranasinghe, Laksiri Gomes, Gathsaurie Neelika Malavige
EPI_ISL_979317, EPI_ISL_979319, EPI_ISL_979320	Cadham Provincial laboratory	National Microbiology Laboratory (NML)	Anna Majer, Shari Tyson, Grace Seo, Philip Mabon, Elsie Grudeski, Rhiannon Huzarewich, Russell Mandes, Anneliese Landgraff, Jennifer Tanner, Natalie Knox, Morag Graham, Gary Van Domselaar, Paul Van Caeseele, Jared Bullard, David Alexander, Kerry Dust, Nathalie Bastien, Yan Li, Timothy Booth, Darian Hole, Madison Chapel, Kirsten Biggar, CanCOGeN's metadata curation team, Public Health Agency of Canada CanCOGeN team
EPI_ISL_979356	Victorian Infectious Diseases Reference Laboratory (VIDRL)	VIDRL and MDU-PHL	Caly L., Seemann T., Sait, M.L., Druce J., Sherry, N.L.
EPI_ISL_979357, EPI_ISL_979358, EPI_ISL_979359	Microbiological Diagnostic Unit - Public Health Laboratory (MDU-PHL)	MDU-PHL	Seemann T., Sait, M.L., Sherry, N.L.
EPI_ISL_979429, EPI_ISL_979430, EPI_ISL_979431, EPI_ISL_979432, EPI_ISL_979433, EPI_ISL_979434, EPI_ISL_979435, EPI_ISL_979436, EPI_ISL_979437, EPI_ISL_979438, EPI_ISL_979439, EPI_ISL_979440, EPI_ISL_979441, EPI_ISL_979442, EPI_ISL_979443, EPI_ISL_979444, EPI_ISL_979445, EPI_ISL_979446, EPI_ISL_979447, EPI_ISL_979448, EPI_ISL_979449, EPI_ISL_979450, EPI_ISL_979451, EPI_ISL_979452			
see above	New Mexico Department of Health Scientific Laboratory	New Mexico Department of Health Scientific Laboratory	Ellie Johnson, Anastacia Griego-Fisher, D'eldra Malone, Jennifer Benoit
EPI_ISL_979457	State Hygienic Laboratory at the University of Iowa	State Hygienic Laboratory at the University of Iowa	Valerie Reeb, Erik Twait, Wes Hottel, Alankar Kampoowale
EPI_ISL_979803, EPI_ISL_979804, EPI_ISL_979893, EPI_ISL_979959, EPI_ISL_979960, EPI_ISL_979961, EPI_ISL_979963, EPI_ISL_979964, EPI_ISL_979965, EPI_ISL_979966, EPI_ISL_979967			
see above	National Institute of Infectious Diseases-Prof. Dr. Matei Bals	National Institute of Infectious Diseases-Prof. Dr. Matei Bals	Leontina Banica, Marius Surleac, Corina Casangiu, Petre Milu, Andreea Tudor, Simona Paraschiv, Dan Otelea

	Molecular Diagnostics Laboratory	Molecular Diagnostics Laboratory	
EPI_ISL_979974, EPI_ISL_979975, EPI_ISL_979976, EPI_ISL_979977, EPI_ISL_979978, EPI_ISL_979979, EPI_ISL_979980, EPI_ISL_979981, EPI_ISL_979982, EPI_ISL_979983, EPI_ISL_979984, EPI_ISL_979985, EPI_ISL_979986, EPI_ISL_979987, EPI_ISL_979988, EPI_ISL_979989, EPI_ISL_979990, EPI_ISL_979991, EPI_ISL_979992, EPI_ISL_979993, EPI_ISL_979994, EPI_ISL_979995, EPI_ISL_979996, EPI_ISL_979997, EPI_ISL_979998, EPI_ISL_979999, EPI_ISL_980000, EPI_ISL_980001, EPI_ISL_980002, EPI_ISL_980003, EPI_ISL_980004, EPI_ISL_980005, EPI_ISL_980006, EPI_ISL_980007, EPI_ISL_980008, EPI_ISL_980009, EPI_ISL_980010, EPI_ISL_980011, EPI_ISL_980012, EPI_ISL_980013, EPI_ISL_980014, EPI_ISL_980015, EPI_ISL_980016, EPI_ISL_980017, EPI_ISL_980018, EPI_ISL_980019, EPI_ISL_980020, EPI_ISL_980021, EPI_ISL_980022, EPI_ISL_980023, EPI_ISL_980024, EPI_ISL_980025, EPI_ISL_980026, EPI_ISL_980027, EPI_ISL_980028, EPI_ISL_980029, EPI_ISL_980030, EPI_ISL_980031, EPI_ISL_980032, EPI_ISL_980033, EPI_ISL_980034, EPI_ISL_980035, EPI_ISL_980036, EPI_ISL_980037, EPI_ISL_980038, EPI_ISL_980039, EPI_ISL_980040, EPI_ISL_980041, EPI_ISL_980042, EPI_ISL_980043, EPI_ISL_980044, EPI_ISL_980045, EPI_ISL_980046, EPI_ISL_980047, EPI_ISL_980048, EPI_ISL_980049, EPI_ISL_980050, EPI_ISL_980051, EPI_ISL_980052, EPI_ISL_980053, EPI_ISL_980054, EPI_ISL_980055, EPI_ISL_980056, EPI_ISL_980057, EPI_ISL_980058, EPI_ISL_980059, EPI_ISL_980060, EPI_ISL_980061, EPI_ISL_980062, EPI_ISL_980063, EPI_ISL_980064, EPI_ISL_980065, EPI_ISL_980066, EPI_ISL_980067, EPI_ISL_980068, EPI_ISL_980069, EPI_ISL_980070, EPI_ISL_980071, EPI_ISL_980072, EPI_ISL_980073, EPI_ISL_980074, EPI_ISL_980075, EPI_ISL_980076, EPI_ISL_980077, EPI_ISL_980078, EPI_ISL_980079, EPI_ISL_980080, EPI_ISL_980081, EPI_ISL_980082, EPI_ISL_980083, EPI_ISL_980084, EPI_ISL_980085, EPI_ISL_980086, EPI_ISL_980087, EPI_ISL_980088, EPI_ISL_980089, EPI_ISL_980090, EPI_ISL_980091, EPI_ISL_980092, EPI_ISL_980093, EPI_ISL_980094, EPI_ISL_980095, EPI_ISL_980096, EPI_ISL_980097, EPI_ISL_980098, EPI_ISL_980099, EPI_ISL_980100, EPI_ISL_980101, EPI_ISL_980102, EPI_ISL_980103, EPI_ISL_980104, EPI_ISL_980105, EPI_ISL_980106, EPI_ISL_980107, EPI_ISL_980108, EPI_ISL_980109, EPI_ISL_980110, EPI_ISL_980111, EPI_ISL_980112, EPI_ISL_980113, EPI_ISL_980114, EPI_ISL_980115, EPI_ISL_980116, EPI_ISL_980117, EPI_ISL_980118, EPI_ISL_980119, EPI_ISL_980120, EPI_ISL_980121, EPI_ISL_980122, EPI_ISL_980123, EPI_ISL_980124, EPI_ISL_980125, EPI_ISL_980126, EPI_ISL_980127, EPI_ISL_980128, EPI_ISL_980129, EPI_ISL_980130, EPI_ISL_980131, EPI_ISL_980132, EPI_ISL_980133, EPI_ISL_980134, EPI_ISL_980135, EPI_ISL_980136, EPI_ISL_980137, EPI_ISL_980138, EPI_ISL_980139, EPI_ISL_980140, EPI_ISL_980141, EPI_ISL_980142, EPI_ISL_980143, EPI_ISL_980144, EPI_ISL_980145, EPI_ISL_980146, EPI_ISL_980147, EPI_ISL_980148, EPI_ISL_980149, EPI_ISL_980150, EPI_ISL_980151, EPI_ISL_980152, EPI_ISL_980153, EPI_ISL_980154, EPI_ISL_980155, EPI_ISL_980156, EPI_ISL_980157, EPI_ISL_980158, EPI_ISL_980159, EPI_ISL_980160, EPI_ISL_980161, EPI_ISL_980162, EPI_ISL_980163, EPI_ISL_980164, EPI_ISL_980165, EPI_ISL_980166, EPI_ISL_980167, EPI_ISL_980168, EPI_ISL_980169, EPI_ISL_980170, EPI_ISL_980171, EPI_ISL_980172, EPI_ISL_980173, EPI_ISL_980174, EPI_ISL_980175, EPI_ISL_980176, EPI_ISL_980177, EPI_ISL_980178, EPI_ISL_980179, EPI_ISL_980180, EPI_ISL_980181, EPI_ISL_980182, EPI_ISL_980183, EPI_ISL_980184, EPI_ISL_980185, EPI_ISL_980186, EPI_ISL_980187, EPI_ISL_980188, EPI_ISL_980189, EPI_ISL_980190, EPI_ISL_980191, EPI_ISL_980192, EPI_ISL_980193, EPI_ISL_980194, EPI_ISL_980195, EPI_ISL_980196, EPI_ISL_980197, EPI_ISL_980198, EPI_ISL_980199, EPI_ISL_980200, EPI_ISL_980201, EPI_ISL_980202, EPI_ISL_980203, EPI_ISL_980204, EPI_ISL_980205, EPI_ISL_980206, EPI_ISL_980207, EPI_ISL_980208, EPI_ISL_980209, EPI_ISL_980210, EPI_ISL_980211, EPI_ISL_980212, EPI_ISL_980213, EPI_ISL_980214, EPI_ISL_980215, EPI_ISL_980216, EPI_ISL_980217, EPI_ISL_980218, EPI_ISL_980219, EPI_ISL_980220, EPI_ISL_980221, EPI_ISL_980222, EPI_ISL_980223, EPI_ISL_980224, EPI_ISL_980225, EPI_ISL_980226, EPI_ISL_980227, EPI_ISL_980228, EPI_ISL_980229, EPI_ISL_980230, EPI_ISL_980231, EPI_ISL_980232, EPI_ISL_980233, EPI_ISL_980234			
see above	Lighthouse Lab in Milton Keynes	Wellcome Sanger Institute for the COVID-19 Genomics UK (COG-UK) Consortium	The Lighthouse Lab in Milton Keynes and Alex Alderton, Roberto Amato, Sonia Goncalves, Ewan Harrison, David K. Jackson, Ian Johnston, Dominic Kwiatkowski, Cordelia Langford, John Sillitoe on behalf of the Wellcome Sanger Institute COVID-19 Surveillance Team
EPI_ISL_980280, EPI_ISL_980356, EPI_ISL_980416, EPI_ISL_980419, EPI_ISL_980433, EPI_ISL_980449, EPI_ISL_980512	Lighthouse Lab in Cambridge	Wellcome Sanger Institute for the COVID-19 Genomics UK (COG-UK) Consortium	Rob Howes, The Lighthouse Lab in Cambridge and Alex Alderton, Roberto Amato, Sonia Goncalves, Ewan Harrison, David K. Jackson, Ian Johnston, Dominic Kwiatkowski, Cordelia Langford, John Sillitoe on behalf of the Wellcome Sanger Institute COVID-19 Surveillance Team
EPI_ISL_980551, EPI_ISL_980552, EPI_ISL_980553, EPI_ISL_980554, EPI_ISL_980555, EPI_ISL_980556, EPI_ISL_980557, EPI_ISL_980558, EPI_ISL_980559, EPI_ISL_980560, EPI_ISL_980561, EPI_ISL_980562, EPI_ISL_980563, EPI_ISL_980564, EPI_ISL_980565, EPI_ISL_980566, EPI_ISL_980567, EPI_ISL_980568, EPI_ISL_980569, EPI_ISL_980570, EPI_ISL_980571, EPI_ISL_980572, EPI_ISL_980573, EPI_ISL_980574, EPI_ISL_980575, EPI_ISL_980576, EPI_ISL_980577, EPI_ISL_980578, EPI_ISL_980579, EPI_ISL_980580, EPI_ISL_980581, EPI_ISL_980582, EPI_ISL_980583, EPI_ISL_980584, EPI_ISL_980585, EPI_ISL_980586, EPI_ISL_980587, EPI_ISL_980588, EPI_ISL_980589, EPI_ISL_980590, EPI_ISL_980591, EPI_ISL_980592, EPI_ISL_980593, EPI_ISL_980594, EPI_ISL_980595, EPI_ISL_980596, EPI_ISL_980597, EPI_ISL_980598, EPI_ISL_980599, EPI_ISL_980600, EPI_ISL_980601, EPI_ISL_980602, EPI_ISL_980603, EPI_ISL_980604, EPI_ISL_980605, EPI_ISL_980606, EPI_ISL_980607, EPI_ISL_980608, EPI_ISL_980609, EPI_ISL_980610, EPI_ISL_980611, EPI_ISL_980612, EPI_ISL_980613, EPI_ISL_980614, EPI_ISL_980615, EPI_ISL_980616, EPI_ISL_980617, EPI_ISL_980618, EPI_ISL_980619, EPI_ISL_980620, EPI_ISL_980621, EPI_ISL_980622, EPI_ISL_980623, EPI_ISL_980624, EPI_ISL_980625, EPI_ISL_980626, EPI_ISL_980627, EPI_ISL_980628, EPI_ISL_980629, EPI_ISL_980630, EPI_ISL_980631, EPI_ISL_980632, EPI_ISL_980633, EPI_ISL_980634, EPI_ISL_980635, EPI_ISL_980636, EPI_ISL_980637, EPI_ISL_980638, EPI_ISL_980639, EPI_ISL_980640, EPI_ISL_980641, EPI_ISL_980642, EPI_ISL_980643, EPI_ISL_980644, EPI_ISL_980645, EPI_ISL_980646, EPI_ISL_980647, EPI_ISL_980648, EPI_ISL_980649, EPI_ISL_980650, EPI_ISL_980651, EPI_ISL_980652, EPI_ISL_980653, EPI_ISL_980654, EPI_ISL_980655, EPI_ISL_980656, EPI_ISL_980657, EPI_ISL_980658, EPI_ISL_980659, EPI_ISL_980660, EPI_ISL_980661, EPI_ISL_980662, EPI_ISL_980663, EPI_ISL_980664, EPI_ISL_980665, EPI_ISL_980666, EPI_ISL_980667, EPI_ISL_980668, EPI_ISL_980669, EPI_ISL_980670, EPI_ISL_980671, EPI_ISL_980672, EPI_ISL_980673, EPI_ISL_980674, EPI_ISL_980675, EPI_ISL_980676, EPI_ISL_980677, EPI_ISL_980678, EPI_ISL_980679, EPI_ISL_980680, EPI_ISL_980681, EPI_ISL_980682, EPI_ISL_980683, EPI_ISL_980684, EPI_ISL_980685, EPI_ISL_980686, EPI_ISL_980687, EPI_ISL_980688, EPI_ISL_980689, EPI_ISL_980690, EPI_ISL_980691, EPI_ISL_980692, EPI_ISL_980693, EPI_ISL_980694, EPI_ISL_980695, EPI_ISL_980696, EPI_ISL_980697, EPI_ISL_980698, EPI_ISL_980699, EPI_ISL_980700, EPI_ISL_980701, EPI_ISL_980702, EPI_ISL_980703, EPI_ISL_980704, EPI_ISL_980705, EPI_ISL_980706, EPI_ISL_980707, EPI_ISL_980708, EPI_ISL_980709, EPI_ISL_980710, EPI_ISL_980711, EPI_ISL_980712, EPI_ISL_980713, EPI_ISL_980714, EPI_ISL_980715, EPI_ISL_980716, EPI_ISL_980717, EPI_ISL_980718, EPI_ISL_980719, EPI_ISL_980720, EPI_ISL_980721, EPI_ISL_980722, EPI_ISL_980723, EPI_ISL_980724, EPI_ISL_980725, EPI_ISL_980726, EPI_ISL_980727, EPI_ISL_980728, EPI_ISL_980729, EPI_ISL_980730, EPI_ISL_980731, EPI_ISL_980732, EPI_ISL_980733, EPI_ISL_980734, EPI_ISL_980735, EPI_ISL_980736, EPI_ISL_980737, EPI_ISL_980738, EPI_ISL_980739, EPI_ISL_980740, EPI_ISL_980741, EPI_ISL_980742, EPI_ISL_980743, EPI_ISL_980744, EPI_ISL_980745, EPI_ISL_980746, EPI_ISL_980747, EPI_ISL_980748, EPI_ISL_980749, EPI_ISL_980750, EPI_ISL_980751, EPI_ISL_980752, EPI_ISL_980753, EPI_ISL_980754, EPI_ISL_980755, EPI_ISL_980756, EPI_ISL_980757, EPI_ISL_980758, EPI_ISL_980759, EPI_ISL_980760, EPI_ISL_980761, EPI_ISL_980762, EPI_ISL_980763, EPI_ISL_980764, EPI_ISL_980765, EPI_ISL_980766, EPI_ISL_980767, EPI_ISL_980768, EPI_ISL_980769, EPI_ISL_980770, EPI_ISL_980771, EPI_ISL_980772, EPI_ISL_980773, EPI_ISL_980774, EPI_ISL_980775, EPI_ISL_980776, EPI_ISL_980777, EPI_ISL_980778, EPI_ISL_980779, EPI_ISL_980780, EPI_ISL_980781, EPI_ISL_980782, EPI_ISL_980783, EPI_ISL_980784, EPI_ISL_980785, EPI_ISL_980786, EPI_ISL_980787, EPI_ISL_980788, EPI_ISL_980789, EPI_ISL_980790, EPI_ISL_980791, EPI_ISL_980792, EPI_ISL_980793, EPI_ISL_980794, EPI_ISL_980795, EPI_ISL_980796, EPI_ISL_980797, EPI_ISL_980798, EPI_ISL_980799, EPI_ISL_980800, EPI_ISL_980801, EPI_ISL_980802, EPI_ISL_980803, EPI_ISL_980804, EPI_ISL_980805, EPI_ISL_980806			
see above	Lighthouse Lab in Milton Keynes	Wellcome Sanger Institute for the COVID-19 Genomics UK (COG-UK) Consortium	The Lighthouse Lab in Milton Keynes and Alex Alderton, Roberto Amato, Sonia Goncalves, Ewan Harrison, David K. Jackson, Ian Johnston, Dominic Kwiatkowski, Cordelia Langford, John Sillitoe on behalf of the Wellcome Sanger Institute COVID-19 Surveillance Team
EPI_ISL_980843, EPI_ISL_980857, EPI_ISL_980858, EPI_ISL_980860, EPI_ISL_980861, EPI_ISL_980864, EPI_ISL_980868, EPI_ISL_980870, EPI_ISL_980885, EPI_ISL_980887, EPI_ISL_980888, EPI_ISL_980893, EPI_ISL_980900, EPI_ISL_980901, EPI_ISL_980903, EPI_ISL_980911, EPI_ISL_980917, EPI_ISL_980923, EPI_ISL_980930, EPI_ISL_980965, EPI_ISL_980966, EPI_ISL_980967, EPI_ISL_980968, EPI_ISL_980969, EPI_ISL_980970, EPI_ISL_980971, EPI_ISL_980972, EPI_ISL_980973, EPI_ISL_980975, EPI_ISL_980976, EPI_ISL_980989, EPI_ISL_980990, EPI_ISL_980991, EPI_ISL_980992, EPI_ISL_980993, EPI_ISL_980994, EPI_ISL_980995, EPI_ISL_980996, EPI_ISL_980997, EPI_ISL_980998, EPI_ISL_980999, EPI_ISL_981000, EPI_ISL_981001, EPI_ISL_981002, EPI_ISL_981003			
see above	Innovative Genomics Institute, UC Berkeley	Innovative Genomics Institute, UC Berkeley	Stacia Wyman, Haridha Shivram, Phil Frankino, Liana Lareau
EPI_ISL_981060, EPI_ISL_981073, EPI_ISL_981074, EPI_ISL_981075, EPI_ISL_981078, EPI_ISL_981079, EPI_ISL_981080, EPI_ISL_981082, EPI_ISL_981085, EPI_ISL_981087, EPI_ISL_981089, EPI_ISL_981090, EPI_ISL_981091, EPI_ISL_981092, EPI_ISL_981096, EPI_ISL_981098, EPI_ISL_981099, EPI_ISL_981103, EPI_ISL_981104, EPI_ISL_981105, EPI_ISL_981106, EPI_ISL_981107, EPI_ISL_981110, EPI_ISL_981111, EPI_ISL_981112, EPI_ISL_981113, EPI_ISL_981119, EPI_ISL_981123, EPI_ISL_981124, EPI_ISL_981125, EPI_ISL_981126, EPI_ISL_981127, EPI_ISL_981147, EPI_ISL_981148, EPI_ISL_981149, EPI_ISL_981150, EPI_ISL_981151, EPI_ISL_981152, EPI_ISL_981153, EPI_ISL_981154, EPI_ISL_981155, EPI_ISL_981156, EPI_ISL_981157, EPI_ISL_981174, EPI_ISL_981179, EPI_ISL_981180, EPI_ISL_981181, EPI_ISL_981182, EPI_ISL_981183, EPI_ISL_981184, EPI_ISL_981185, EPI_ISL_981186, EPI_ISL_981187, EPI_ISL_981188, EPI_ISL_981189, EPI_ISL_981190			
see above	Johns Hopkins Hospital Department of Pathology	Johns Hopkins Hospital Department of Pathology	C. Paul Morris, Chun Huai Luo, Adannaya Amadi, Matthew Schwartz, Nicholas Gallagher, Heba H. Mostafa
EPI_ISL_981317, EPI_ISL_981318, EPI_ISL_981322, EPI_ISL_981323, EPI_ISL_981345, EPI_ISL_981346, EPI_ISL_981347, EPI_ISL_981348, EPI_ISL_981349, EPI_ISL_981351, EPI_ISL_981353			
see above	Hospital Universitari Vall d'Hebron - Vall d'Hebron Institut de Rercerca	Hospital Universitari Vall d'Hebron - Vall d'Hebron Institut de Rercerca	Cristina Andrés, Maria Piñana, Josep F Abril, Damir Garcia-Cehic, Ariadna Rando, Juliana Esperalba, Maria Gema Codina, Carla Castillo, Maria Carmen Martin, Tomàs Pumarola, Josep Quer, Andrés Antón
EPI_ISL_981375, EPI_ISL_981377	Laboratory for Respiratory Viruses, Cantacuzino National Military-Medical Institute for Research and Development	Cantacuzino Institute Virology	Luiza Ustea, Nicoleta Paraschiv, Catalina Pascu, Mihaela Lazar
EPI_ISL_981378, EPI_ISL_981379	AZ Klina	AZ Klina	Dr. C. Vael
EPI_ISL_981390, EPI_ISL_981391, EPI_ISL_981392, EPI_ISL_981393, EPI_ISL_981394, EPI_ISL_981396, EPI_ISL_981397, EPI_ISL_981398, EPI_ISL_981399, EPI_ISL_981400	CH.INTERCOMMUNAL DE CRETEIL	Department of Virology, Henri Mondor University Hospital, Assistance Publique Hôpitaux de Paris, Université Paris-Est Créteil, INSERM U955	Christophe Rodriguez, Slim Fourati, Vanessa Demontant, Guillaume Gricourt, Melissa N'Debi, Alexandre Soulier, Elisabeth Trawinski, Jean-Michel Pawlotsky
EPI_ISL_981401, EPI_ISL_981402, EPI_ISL_981403, EPI_ISL_981404, EPI_ISL_981405	Groupe LCD	Department of Virology, Henri Mondor University Hospital, Assistance Publique Hôpitaux de Paris, Université Paris-Est Créteil, INSERM U955	Christophe Rodriguez, Slim Fourati, Vanessa Demontant, Guillaume Gricourt, Melissa N'Debi, Alexandre Soulier, Elisabeth Trawinski, Jean-Michel Pawlotsky
EPI_ISL_981406, EPI_ISL_981407, EPI_ISL_981408	CH.INTERCOMMUNAL DE CRETEIL	Department of Virology, Henri Mondor University Hospital, Assistance Publique Hôpitaux de Paris, Université Paris-Est Créteil, INSERM U955	Christophe Rodriguez, Slim Fourati, Vanessa Demontant, Guillaume Gricourt, Melissa N'Debi, Alexandre Soulier, Elisabeth Trawinski, Jean-Michel Pawlotsky
EPI_ISL_981409, EPI_ISL_981410, EPI_ISL_981411, EPI_ISL_981412, EPI_ISL_981413, EPI_ISL_981414, EPI_ISL_981415, EPI_ISL_981416, EPI_ISL_981417, EPI_ISL_981418, EPI_ISL_981419, EPI_ISL_981420, EPI_ISL_981421, EPI_ISL_981422, EPI_ISL_981423, EPI_ISL_981424, EPI_ISL_981425, EPI_ISL_981426, EPI_ISL_981427, EPI_ISL_981428, EPI_ISL_981429, EPI_ISL_981430, EPI_ISL_981431, EPI_ISL_981432, EPI_ISL_981433, EPI_ISL_981434, EPI_ISL_981435, EPI_ISL_981436, EPI_ISL_981437, EPI_ISL_981438, EPI_ISL_981439, EPI_ISL_981440, EPI_ISL_981441, EPI_ISL_981442, EPI_ISL_981443, EPI_ISL_981444, EPI_ISL_981445, EPI_ISL_981446, EPI_ISL_981447, EPI_ISL_981448, EPI_ISL_981449, EPI_ISL_981450, EPI_ISL_981451			
see above	Groupe LCD	Department of Virology, Henri Mondor University Hospital, Assistance Publique Hôpitaux de Paris, Université Paris-Est Créteil, INSERM U955	Christophe Rodriguez, Slim Fourati, Vanessa Demontant, Guillaume Gricourt, Melissa N'Debi, Alexandre Soulier, Elisabeth Trawinski, Jean-Michel Pawlotsky
EPI_ISL_981452, EPI_ISL_981453	CH.INTERCOMMUNAL DE CRETEIL	Department of Virology, Henri Mondor University Hospital, Assistance Publique Hôpitaux de Paris, Université Paris-Est Créteil, INSERM U955	Christophe Rodriguez, Slim Fourati, Vanessa Demontant, Guillaume Gricourt, Melissa N'Debi, Alexandre Soulier, Elisabeth Trawinski, Jean-Michel Pawlotsky
EPI_ISL_981454, EPI_ISL_981455, EPI_ISL_981456, EPI_ISL_981457, EPI_ISL_981458, EPI_ISL_981459, EPI_ISL_981460, EPI_ISL_981461, EPI_ISL_981482, EPI_ISL_981483, EPI_ISL_981484			
see above	CHI VILLENEUVE ST GEORGES	Department of Virology, Henri Mondor University Hospital, Assistance Publique Hôpitaux de Paris, Université Paris-Est	Christophe Rodriguez, Slim Fourati, Vanessa Demontant, Guillaume Gricourt, Melissa N'Debi, Alexandre Soulier, Elisabeth Trawinski, Jean-Michel Pawlotsky

		Créteil, INSERM U955	
EPI_ISL_981485	INSTITUT GUSTAVE ROUSSY	Department of Virology, Henri Mondor University Hospital, Assistance Publique Hôpitaux de Paris, Université Paris-Est Créteil, INSERM U955	Christophe Rodriguez, Slim Fourati, Vanessa Demontant, Guillaume Gricourt, Melissa N'Debi, Alexandre Soulier, Elisabeth Trawinski, Jean-Michel Pawlotsky
EPI_ISL_981487	CH.INTERCOMMUNAL DE CRETEIL	Department of Virology, Henri Mondor University Hospital, Assistance Publique Hôpitaux de Paris, Université Paris-Est Créteil, INSERM U955	Christophe Rodriguez, Slim Fourati, Vanessa Demontant, Guillaume Gricourt, Melissa N'Debi, Alexandre Soulier, Elisabeth Trawinski, Jean-Michel Pawlotsky
EPI_ISL_981489, EPI_ISL_981490, EPI_ISL_981491	Groupe LCD	Department of Virology, Henri Mondor University Hospital, Assistance Publique Hôpitaux de Paris, Université Paris-Est Créteil, INSERM U955	Christophe Rodriguez, Slim Fourati, Vanessa Demontant, Guillaume Gricourt, Melissa N'Debi, Alexandre Soulier, Elisabeth Trawinski, Jean-Michel Pawlotsky
EPI_ISL_981492	LABM BIOLAM CLINIQUE ESTREE	Department of Virology, Henri Mondor University Hospital, Assistance Publique Hôpitaux de Paris, Université Paris-Est Créteil, INSERM U955	Christophe Rodriguez, Slim Fourati, Vanessa Demontant, Guillaume Gricourt, Melissa N'Debi, Alexandre Soulier, Elisabeth Trawinski, Jean-Michel Pawlotsky
EPI_ISL_981493, EPI_ISL_981494, EPI_ISL_981495, EPI_ISL_981496	Groupe LCD	Department of Virology, Henri Mondor University Hospital, Assistance Publique Hôpitaux de Paris, Université Paris-Est Créteil, INSERM U955	Christophe Rodriguez, Slim Fourati, Vanessa Demontant, Guillaume Gricourt, Melissa N'Debi, Alexandre Soulier, Elisabeth Trawinski, Jean-Michel Pawlotsky
EPI_ISL_981497	LABM BIOLAM CLINIQUE ESTREE	Department of Virology, Henri Mondor University Hospital, Assistance Publique Hôpitaux de Paris, Université Paris-Est Créteil, INSERM U955	Christophe Rodriguez, Slim Fourati, Vanessa Demontant, Guillaume Gricourt, Melissa N'Debi, Alexandre Soulier, Elisabeth Trawinski, Jean-Michel Pawlotsky
EPI_ISL_981498, EPI_ISL_981499, EPI_ISL_981500, EPI_ISL_981501, EPI_ISL_981502, EPI_ISL_981503, EPI_ISL_981504, EPI_ISL_981505, EPI_ISL_981506, EPI_ISL_981507, EPI_ISL_981508, EPI_ISL_981509, EPI_ISL_981510, EPI_ISL_981511, EPI_ISL_981512, EPI_ISL_981513, EPI_ISL_981514, EPI_ISL_981515, EPI_ISL_981516, EPI_ISL_981517, EPI_ISL_981518, EPI_ISL_981519, EPI_ISL_981520, EPI_ISL_981521, EPI_ISL_981522, EPI_ISL_981523, EPI_ISL_981524, EPI_ISL_981525, EPI_ISL_981526, EPI_ISL_981527, EPI_ISL_981528, EPI_ISL_981529, EPI_ISL_981530, EPI_ISL_981531			
see above	Groupe LCD	Department of Virology, Henri Mondor University Hospital, Assistance Publique Hôpitaux de Paris, Université Paris-Est Créteil, INSERM U955	Christophe Rodriguez, Slim Fourati, Vanessa Demontant, Guillaume Gricourt, Melissa N'Debi, Alexandre Soulier, Elisabeth Trawinski, Jean-Michel Pawlotsky
EPI_ISL_981532	Hôpital Pitié-Salpêtrière	Department of Virology, Henri Mondor University Hospital, Assistance Publique Hôpitaux de Paris, Université Paris-Est Créteil, INSERM U955	Christophe Rodriguez, Slim Fourati, Vanessa Demontant, Guillaume Gricourt, Melissa N'Debi, Alexandre Soulier, Elisabeth Trawinski, Jean-Michel Pawlotsky
EPI_ISL_981533, EPI_ISL_981534	Hôpital Avicenne	Department of Virology, Henri Mondor University Hospital, Assistance Publique Hôpitaux de Paris, Université Paris-Est Créteil, INSERM U955	Christophe Rodriguez, Slim Fourati, Vanessa Demontant, Guillaume Gricourt, Melissa N'Debi, Alexandre Soulier, Elisabeth Trawinski, Jean-Michel Pawlotsky
EPI_ISL_981535, EPI_ISL_981536, EPI_ISL_981537	Hôpital Pitié-Salpêtrière	Department of Virology, Henri Mondor University Hospital, Assistance Publique Hôpitaux de Paris, Université Paris-Est Créteil, INSERM U955	Christophe Rodriguez, Slim Fourati, Vanessa Demontant, Guillaume Gricourt, Melissa N'Debi, Alexandre Soulier, Elisabeth Trawinski, Jean-Michel Pawlotsky
EPI_ISL_981549, EPI_ISL_981550, EPI_ISL_981552, EPI_ISL_981553, EPI_ISL_981554, EPI_ISL_981555, EPI_ISL_981556, EPI_ISL_981558, EPI_ISL_981559, EPI_ISL_981560, EPI_ISL_981561, EPI_ISL_981562, EPI_ISL_981563, EPI_ISL_981564, EPI_ISL_981565, EPI_ISL_981566, EPI_ISL_981567, EPI_ISL_981568, EPI_ISL_981569, EPI_ISL_981570, EPI_ISL_981571, EPI_ISL_981572, EPI_ISL_981573, EPI_ISL_981574, EPI_ISL_981575, EPI_ISL_981576, EPI_ISL_981577, EPI_ISL_981578, EPI_ISL_981579, EPI_ISL_981580, EPI_ISL_981581, EPI_ISL_981582, EPI_ISL_981583, EPI_ISL_981584, EPI_ISL_981585, EPI_ISL_981586, EPI_ISL_981587, EPI_ISL_981588, EPI_ISL_981589, EPI_ISL_981590, EPI_ISL_981591, EPI_ISL_981592, EPI_ISL_981593, EPI_ISL_981594, EPI_ISL_981595, EPI_ISL_981596, EPI_ISL_981597, EPI_ISL_981598, EPI_ISL_981599, EPI_ISL_981600, EPI_ISL_981601, EPI_ISL_981602, EPI_ISL_981603, EPI_ISL_981604, EPI_ISL_981605, EPI_ISL_981606, EPI_ISL_981607, EPI_ISL_981608, EPI_ISL_981609, EPI_ISL_981610, EPI_ISL_981642, EPI_ISL_981643, EPI_ISL_981651, EPI_ISL_981652, EPI_ISL_981653, EPI_ISL_981655, EPI_ISL_981656, EPI_ISL_981657, EPI_ISL_981658, EPI_ISL_981660, EPI_ISL_981661, EPI_ISL_981662, EPI_ISL_981663, EPI_ISL_981664, EPI_ISL_981665, EPI_ISL_981666, EPI_ISL_981667, EPI_ISL_981668, EPI_ISL_981669, EPI_ISL_981670, EPI_ISL_981671, EPI_ISL_981672, EPI_ISL_981673, EPI_ISL_981674, EPI_ISL_981675, EPI_ISL_981676, EPI_ISL_981677, EPI_ISL_981678, EPI_ISL_981679, EPI_ISL_981680, EPI_ISL_981681, EPI_ISL_981682, EPI_ISL_981683, EPI_ISL_981684, EPI_ISL_981685, EPI_ISL_981686, EPI_ISL_981687, EPI_ISL_981688, EPI_ISL_981689, EPI_ISL_981696, EPI_ISL_981697, EPI_ISL_981698, EPI_ISL_981699, EPI_ISL_981700, EPI_ISL_981701, EPI_ISL_981702, EPI_ISL_981703, EPI_ISL_981704, EPI_ISL_981706, EPI_ISL_981707, EPI_ISL_981708, EPI_ISL_981709, EPI_ISL_981713, EPI_ISL_981714, EPI_ISL_981715, EPI_ISL_981716, EPI_ISL_981717, EPI_ISL_981719, EPI_ISL_981721, EPI_ISL_981722, EPI_ISL_981723, EPI_ISL_981724, EPI_ISL_981725, EPI_ISL_981726, EPI_ISL_981727, EPI_ISL_981728, EPI_ISL_981729, EPI_ISL_981730, EPI_ISL_981731, EPI_ISL_981732, EPI_ISL_981733, EPI_ISL_981734, EPI_ISL_981735, EPI_ISL_981736, EPI_ISL_981737, EPI_ISL_981738, EPI_ISL_981739, EPI_ISL_981740, EPI_ISL_981741, EPI_ISL_981742, EPI_ISL_981743, EPI_ISL_981744, EPI_ISL_981745, EPI_ISL_981747, EPI_ISL_981748, EPI_ISL_981749, EPI_ISL_981750, EPI_ISL_981751, EPI_ISL_981752, EPI_ISL_981753, EPI_ISL_981754, EPI_ISL_981755, EPI_ISL_981756, EPI_ISL_981757, EPI_ISL_981758, EPI_ISL_981759, EPI_ISL_981760, EPI_ISL_981761, EPI_ISL_981762, EPI_ISL_981763, EPI_ISL_981764, EPI_ISL_981765, EPI_ISL_981766, EPI_ISL_981767, EPI_ISL_981768, EPI_ISL_981769, EPI_ISL_981770, EPI_ISL_981771, EPI_ISL_981772, EPI_ISL_981773, EPI_ISL_981774, EPI_ISL_981775, EPI_ISL_981776, EPI_ISL_981777, EPI_ISL_981778, EPI_ISL_981779, EPI_ISL_981780, EPI_ISL_981781, EPI_ISL_981782, EPI_ISL_981783, EPI_ISL_981784, EPI_ISL_981785, EPI_ISL_981786, EPI_ISL_981787, EPI_ISL_981788, EPI_ISL_981789, EPI_ISL_981790, EPI_ISL_981791, EPI_ISL_981792, EPI_ISL_981793, EPI_ISL_981794, EPI_ISL_981795, EPI_ISL_981796, EPI_ISL_981797, EPI_ISL_981798, EPI_ISL_981799, EPI_ISL_981800, EPI_ISL_981801, EPI_ISL_981802, EPI_ISL_981803, EPI_ISL_981804, EPI_ISL_981805, EPI_ISL_981806, EPI_ISL_981807, EPI_ISL_981827, EPI_ISL_981828, EPI_ISL_981829, EPI_ISL_981830, EPI_ISL_981831, EPI_ISL_981832, EPI_ISL_981833, EPI_ISL_981834, EPI_ISL_981835, EPI_ISL_981837, EPI_ISL_981838, EPI_ISL_981839, EPI_ISL_981840, EPI_ISL_981841, EPI_ISL_981842, EPI_ISL_981843, EPI_ISL_981844, EPI_ISL_981845, EPI_ISL_981846, EPI_ISL_981847, EPI_ISL_981848, EPI_ISL_981849			
see above	University Hospitals of Geneva, Laboratory of Virology	HUG, Laboratory of Virology and the Health2030 Genome Center	Samuel Cordey, Ana Rita Goncalves, Laurent Kaiser, Lorenzo Cerutti, Henri Pegeot, Melyssa Elies, Deborah Penet, Keith Harshman, Ioannis Xenarios, Emmanouil Dermitzakis
EPI_ISL_982002, EPI_ISL_982019, EPI_ISL_982022, EPI_ISL_982029, EPI_ISL_982038, EPI_ISL_982056, EPI_ISL_982065, EPI_ISL_982106, EPI_ISL_982113	TGen North	TGen North	"Jolene Bowers, Megan Folkerts, Chris French, Hayley Yaglom, Ashlyn Pfeiffer, Darrin Lemmer, Dave Engelthaler, The Arizona COVID Genomics Union (ACGU)"
EPI_ISL_982118	CH.INTERCOMMUNAL DE CRETEIL	Department of Virology, Henri Mondor University Hospital, Assistance Publique Hôpitaux de Paris, Université Paris-Est Créteil, INSERM U955	Christophe Rodriguez, Slim Fourati, Vanessa Demontant, Guillaume Gricourt, Melissa N'Debi, Alexandre Soulier, Elisabeth Trawinski, Jean-Michel Pawlotsky
EPI_ISL_982120, EPI_ISL_982121, EPI_ISL_982125, EPI_ISL_982127	Hôpital Henri Mondor	Department of Virology, Henri Mondor University Hospital, Assistance Publique Hôpitaux de Paris, Université Paris-Est Créteil, INSERM U955	Christophe Rodriguez, Slim Fourati, Vanessa Demontant, Guillaume Gricourt, Melissa N'Debi, Alexandre Soulier, Elisabeth Trawinski, Jean-Michel Pawlotsky
EPI_ISL_982128	CHU SUD AMIENS	Department of Virology, Henri Mondor University Hospital, Assistance Publique Hôpitaux de Paris, Université Paris-Est Créteil, INSERM U955	Christophe Rodriguez, Slim Fourati, Vanessa Demontant, Guillaume Gricourt, Melissa N'Debi, Alexandre Soulier, Elisabeth Trawinski, Jean-Michel Pawlotsky
EPI_ISL_982130, EPI_ISL_982131, EPI_ISL_982132, EPI_ISL_982134, EPI_ISL_982135, EPI_ISL_982136, EPI_ISL_982137, EPI_ISL_982138, EPI_ISL_982139, EPI_ISL_982140, EPI_ISL_982141			
see above	Hôpital Henri Mondor	Department of Virology, Henri Mondor University Hospital, Assistance Publique Hôpitaux de Paris, Université Paris-Est Créteil, INSERM U955	Christophe Rodriguez, Slim Fourati, Vanessa Demontant, Guillaume Gricourt, Melissa N'Debi, Alexandre Soulier, Elisabeth Trawinski, Jean-Michel Pawlotsky
EPI_ISL_982142, EPI_ISL_982143, EPI_ISL_982144	CHU SUD AMIENS	Department of Virology, Henri Mondor University Hospital, Assistance Publique Hôpitaux de Paris, Université Paris-Est Créteil, INSERM U955	Christophe Rodriguez, Slim Fourati, Vanessa Demontant, Guillaume Gricourt, Melissa N'Debi, Alexandre Soulier, Elisabeth Trawinski, Jean-Michel Pawlotsky
EPI_ISL_982145	Groupe LCD	Department of Virology, Henri Mondor University Hospital, Assistance Publique Hôpitaux de Paris, Université Paris-Est Créteil, INSERM U955	Christophe Rodriguez, Slim Fourati, Vanessa Demontant, Guillaume Gricourt, Melissa N'Debi, Alexandre Soulier, Elisabeth Trawinski, Jean-Michel Pawlotsky
EPI_ISL_982146	CHU SUD AMIENS	Department of Virology, Henri Mondor University Hospital, Assistance Publique Hôpitaux de Paris, Université Paris-Est Créteil, INSERM U955	Christophe Rodriguez, Slim Fourati, Vanessa Demontant, Guillaume Gricourt, Melissa N'Debi, Alexandre Soulier, Elisabeth Trawinski, Jean-Michel Pawlotsky
EPI_ISL_982147	CH.INTERCOMMUNAL DE CRETEIL	Department of Virology, Henri Mondor University Hospital,	Christophe Rodriguez, Slim Fourati, Vanessa Demontant, Guillaume Gricourt, Melissa N'Debi, Alexandre Soulier, Elisabeth Trawinski, Jean-Michel

		Assistance Publique Hôpitaux de Paris, Université Paris-Est Créteil, INSERM U955	Pawlotsky
EPI_ISL_982148, EPI_ISL_982149	Hôpital Henri Mondor	Department of Virology, Henri Mondor University Hospital, Assistance Publique Hôpitaux de Paris, Université Paris-Est Créteil, INSERM U955	Christophe Rodriguez, Slim Fourati, Vanessa Demontant, Guillaume Gricourt, Melissa N'Debi, Alexandre Soulier, Elisabeth Trawinski, Jean-Michel Pawlotsky
EPI_ISL_982154	CHU SUD AMIENS	Department of Virology, Henri Mondor University Hospital, Assistance Publique Hôpitaux de Paris, Université Paris-Est Créteil, INSERM U955	Christophe Rodriguez, Slim Fourati, Vanessa Demontant, Guillaume Gricourt, Melissa N'Debi, Alexandre Soulier, Elisabeth Trawinski, Jean-Michel Pawlotsky
EPI_ISL_982155	Hôpital Henri Mondor	Department of Virology, Henri Mondor University Hospital, Assistance Publique Hôpitaux de Paris, Université Paris-Est Créteil, INSERM U955	Christophe Rodriguez, Slim Fourati, Vanessa Demontant, Guillaume Gricourt, Melissa N'Debi, Alexandre Soulier, Elisabeth Trawinski, Jean-Michel Pawlotsky
EPI_ISL_982158	CHI VILLENEUVE ST GEORGES	Department of Virology, Henri Mondor University Hospital, Assistance Publique Hôpitaux de Paris, Université Paris-Est Créteil, INSERM U955	Christophe Rodriguez, Slim Fourati, Vanessa Demontant, Guillaume Gricourt, Melissa N'Debi, Alexandre Soulier, Elisabeth Trawinski, Jean-Michel Pawlotsky
EPI_ISL_982159	Hôpital Necker-Enfants malades	Department of Virology, Henri Mondor University Hospital, Assistance Publique Hôpitaux de Paris, Université Paris-Est Créteil, INSERM U955	Christophe Rodriguez, Slim Fourati, Vanessa Demontant, Guillaume Gricourt, Melissa N'Debi, Alexandre Soulier, Elisabeth Trawinski, Jean-Michel Pawlotsky
EPI_ISL_982164	Hôpital Henri Mondor	Department of Virology, Henri Mondor University Hospital, Assistance Publique Hôpitaux de Paris, Université Paris-Est Créteil, INSERM U955	Christophe Rodriguez, Slim Fourati, Vanessa Demontant, Guillaume Gricourt, Melissa N'Debi, Alexandre Soulier, Elisabeth Trawinski, Jean-Michel Pawlotsky
EPI_ISL_982165	CHU SUD AMIENS	Department of Virology, Henri Mondor University Hospital, Assistance Publique Hôpitaux de Paris, Université Paris-Est Créteil, INSERM U955	Christophe Rodriguez, Slim Fourati, Vanessa Demontant, Guillaume Gricourt, Melissa N'Debi, Alexandre Soulier, Elisabeth Trawinski, Jean-Michel Pawlotsky
EPI_ISL_982166, EPI_ISL_982169, EPI_ISL_982170, EPI_ISL_982171	Hôpital Henri Mondor	Department of Virology, Henri Mondor University Hospital, Assistance Publique Hôpitaux de Paris, Université Paris-Est Créteil, INSERM U955	Christophe Rodriguez, Slim Fourati, Vanessa Demontant, Guillaume Gricourt, Melissa N'Debi, Alexandre Soulier, Elisabeth Trawinski, Jean-Michel Pawlotsky
EPI_ISL_982174, EPI_ISL_982175, EPI_ISL_982176	CHU SUD AMIENS	Department of Virology, Henri Mondor University Hospital, Assistance Publique Hôpitaux de Paris, Université Paris-Est Créteil, INSERM U955	Christophe Rodriguez, Slim Fourati, Vanessa Demontant, Guillaume Gricourt, Melissa N'Debi, Alexandre Soulier, Elisabeth Trawinski, Jean-Michel Pawlotsky
EPI_ISL_982177, EPI_ISL_982178	Hôpital Henri Mondor	Department of Virology, Henri Mondor University Hospital, Assistance Publique Hôpitaux de Paris, Université Paris-Est Créteil, INSERM U955	Christophe Rodriguez, Slim Fourati, Vanessa Demontant, Guillaume Gricourt, Melissa N'Debi, Alexandre Soulier, Elisabeth Trawinski, Jean-Michel Pawlotsky
EPI_ISL_982179	CHI VILLENEUVE ST GEORGES	Department of Virology, Henri Mondor University Hospital, Assistance Publique Hôpitaux de Paris, Université Paris-Est Créteil, INSERM U955	Christophe Rodriguez, Slim Fourati, Vanessa Demontant, Guillaume Gricourt, Melissa N'Debi, Alexandre Soulier, Elisabeth Trawinski, Jean-Michel Pawlotsky
EPI_ISL_982180	Hôpital Henri Mondor	Department of Virology, Henri Mondor University Hospital, Assistance Publique Hôpitaux de Paris, Université Paris-Est Créteil, INSERM U955	Christophe Rodriguez, Slim Fourati, Vanessa Demontant, Guillaume Gricourt, Melissa N'Debi, Alexandre Soulier, Elisabeth Trawinski, Jean-Michel Pawlotsky
EPI_ISL_982181	CH.INTERCOMMUNAL DE CRETEIL	Department of Virology, Henri Mondor University Hospital, Assistance Publique Hôpitaux de Paris, Université Paris-Est Créteil, INSERM U955	Christophe Rodriguez, Slim Fourati, Vanessa Demontant, Guillaume Gricourt, Melissa N'Debi, Alexandre Soulier, Elisabeth Trawinski, Jean-Michel Pawlotsky
EPI_ISL_982203, EPI_ISL_982204, EPI_ISL_982205, EPI_ISL_982206, EPI_ISL_982207, EPI_ISL_982208, EPI_ISL_982209, EPI_ISL_982210, EPI_ISL_982211, EPI_ISL_982212, EPI_ISL_982213, EPI_ISL_982214, EPI_ISL_982215, EPI_ISL_982216, EPI_ISL_982217, EPI_ISL_982218, EPI_ISL_982219, EPI_ISL_982220, EPI_ISL_982221, EPI_ISL_982222, EPI_ISL_982223, EPI_ISL_982224, EPI_ISL_982225			
see above	Hôpital Henri Mondor	Department of Virology, Henri Mondor University Hospital, Assistance Publique Hôpitaux de Paris, Université Paris-Est Créteil, INSERM U955	Christophe Rodriguez, Slim Fourati, Vanessa Demontant, Guillaume Gricourt, Melissa N'Debi, Alexandre Soulier, Elisabeth Trawinski, Jean-Michel Pawlotsky
EPI_ISL_982226, EPI_ISL_982227	CH.INTERCOMMUNAL DE CRETEIL	Department of Virology, Henri Mondor University Hospital, Assistance Publique Hôpitaux de Paris, Université Paris-Est Créteil, INSERM U955	Christophe Rodriguez, Slim Fourati, Vanessa Demontant, Guillaume Gricourt, Melissa N'Debi, Alexandre Soulier, Elisabeth Trawinski, Jean-Michel Pawlotsky
EPI_ISL_982274, EPI_ISL_982281, EPI_ISL_982282	Lab voor klinische biologie	Lab voor klinische biologie	Hannelore Hamerlinck, Marija Janevska, Bruno Verhasselt
EPI_ISL_982299	BCCDC Public Health Laboratory	BCCDC Public Health Laboratory	Prystajecky Natalie, John Tyson, Dan Fornika, Shannon Russell, Kim Macdonald, Kimia Kamelian, Ana Pacagnella, Corrinne Ng, Loretta Janz, Robert Azana, Mel Krajden
EPI_ISL_982862, EPI_ISL_982863, EPI_ISL_982864, EPI_ISL_982865, EPI_ISL_982870, EPI_ISL_982872, EPI_ISL_982873, EPI_ISL_982874, EPI_ISL_982875, EPI_ISL_982876, EPI_ISL_982887, EPI_ISL_982888, EPI_ISL_982895, EPI_ISL_982897, EPI_ISL_982900, EPI_ISL_982901, EPI_ISL_982904, EPI_ISL_982905, EPI_ISL_982906, EPI_ISL_982908, EPI_ISL_982911, EPI_ISL_982912, EPI_ISL_982913, EPI_ISL_982914, EPI_ISL_982915, EPI_ISL_982916, EPI_ISL_982917, EPI_ISL_982918, EPI_ISL_982919, EPI_ISL_982920, EPI_ISL_982921			
see above	MEPHI Aix Marseille University (AMU)	MEPHI Aix Marseille University (AMU)	Anthony LEVASSEUR
EPI_ISL_983096, EPI_ISL_983098	Microbiology and Virology Unit, Florence Careggi University Hospital	Microbiology and Virology Unit, Florence Careggi University Hospital	Vincenzo Di Pilato, Marco Coppi, Fabio Morecchiato, Noemi Aiezza, Ilaria Baccani, Alberto Antonelli, Emanuele Gori, Gian Maria Rossolini
EPI_ISL_983326	Laboratorio di Genetica Medica Ospedale Belcolle	INMI Lazzaro Spallanzani IRCCS	F Messina, C.E.M Gruber, B Bartolini, E Giombini, M Rueca, O Butera, F Natoni, G Pessina, A Di Caro, MR Capobianchi
EPI_ISL_983327	Dipartimento di Prevenzione ASL Roma 4	INMI Lazzaro Spallanzani IRCCS	O Butera, F Messina, CEM Gruber, B Bartolini, E Giombini, M Rueca, S Ursino, MR Capobianchi, A Di Caro
EPI_ISL_983374	Utah Public Health Laboratory	Utah Public Health Laboratory	Erin L. Young, Kelly F. Oakeson, Tara Gallagher
EPI_ISL_983439, EPI_ISL_983443, EPI_ISL_983447, EPI_ISL_983448, EPI_ISL_983449, EPI_ISL_983450, EPI_ISL_983451, EPI_ISL_983452, EPI_ISL_983453, EPI_ISL_983454, EPI_ISL_983455, EPI_ISL_983456, EPI_ISL_983457, EPI_ISL_983458, EPI_ISL_983459, EPI_ISL_983460			
see above	URMC LABS	Wadsworth Center, New York State Department of Health	Kirsten St. George, Daryl M. Lamson, Alexis Russel, Matthew Shudt, Melissa A Leisner, Jonathan Plitnick, Navjot Singh, John Kelly, Erasmus Schneider, Erica Lasek-Nesselquist
EPI_ISL_983588, EPI_ISL_983589, EPI_ISL_983590, EPI_ISL_983591, EPI_ISL_983592, EPI_ISL_983593, EPI_ISL_983594, EPI_ISL_983595, EPI_ISL_983596, EPI_ISL_983597, EPI_ISL_983598			
see above	Texas Department of State Health Services	Texas Department of State Health Services	Bonnie Oh, Anita Pokharel, James Daniel Bonser, Myong Koag, Chung Wang, Rachel Lee, Grace Kubin, Rashmi Tuladhar, Mayela Pedrueza, Maliha Rahman, Jenny Zhang
EPI_ISL_983640, EPI_ISL_983641, EPI_ISL_983642, EPI_ISL_983643, EPI_ISL_983644, EPI_ISL_983645, EPI_ISL_983646, EPI_ISL_983647	University of Michigan Clinical Microbiology Laboratory	Lauring Lab, University of Michigan, Department of Microbiology and Immunology	Valesano
EPI_ISL_983649, EPI_ISL_983650, EPI_ISL_983651, EPI_ISL_983653, EPI_ISL_983654, EPI_ISL_983655, EPI_ISL_983656, EPI_ISL_983657, EPI_ISL_983658, EPI_ISL_983659, EPI_ISL_983660, EPI_ISL_983661, EPI_ISL_983662, EPI_ISL_983663, EPI_ISL_983664, EPI_ISL_983665, EPI_ISL_983666, EPI_ISL_983667, EPI_ISL_983668, EPI_ISL_983669, EPI_ISL_983670, EPI_ISL_983671, EPI_ISL_983672, EPI_ISL_983673, EPI_ISL_983674, EPI_ISL_983675, EPI_ISL_983676, EPI_ISL_983677, EPI_ISL_983678, EPI_ISL_983679, EPI_ISL_983680, EPI_ISL_983681, EPI_ISL_983682, EPI_ISL_983683, EPI_ISL_983684, EPI_ISL_983685,			

EPI_ISL_983686, EPI_ISL_983687, EPI_ISL_983688, EPI_ISL_983689, EPI_ISL_983690, EPI_ISL_983691			
see above	Vault Health	Minnesota Department of Health, Public Health Laboratory	Alexandra Lorentz, Jacob Garfin, Matt Plumb, and Xiong Wang
EPI_ISL_983708, EPI_ISL_983709, EPI_ISL_983712, EPI_ISL_983714, EPI_ISL_983715, EPI_ISL_983716, EPI_ISL_983721, EPI_ISL_983722, EPI_ISL_983723, EPI_ISL_983724, EPI_ISL_983725, EPI_ISL_983732, EPI_ISL_983738, EPI_ISL_983739, EPI_ISL_983740, EPI_ISL_983741, EPI_ISL_983742, EPI_ISL_983743, EPI_ISL_983744, EPI_ISL_983745, EPI_ISL_983746			
see above	Colorado Department of Public Health and Environment	Colorado Department of Puplic Health and Environment	Laura Bankers, Molly C. Hetherington-Rauth, Diana Ir, Shannon Ely, Shannon R. Matzinger, Sarah Elizabeth Totten, Emily A. Travanty
EPI_ISL_983863, EPI_ISL_983864, EPI_ISL_983865, EPI_ISL_983866, EPI_ISL_983867, EPI_ISL_983868, EPI_ISL_983869	LACEN-RS/CEVS/SES-RS	State Center for Health Surveillance. Rio Grande do Sul State Secretary of Health (CEVS)	Aline Campos, Cynthia Molina, Lara Crescente, Leticia Garay, Ludmila Fiorenzano Baethgen, Richard Salvato, Tatiana Gregianini
