# Supplementary material for: Detection and characterization of the SARS-CoV-2 lineage B.1.526 in New York
Source: Nat Commun. 2021 Aug 9;12:4886. doi: 10.1038/s41467-021-25168-4 (PMC8352861; doi:10.1038/s41467-021-25168-4)
Supplement: Supplementary file 8 — Supplementary Data 4 [file 41467_2021_25168_MOESM8_ESM.zip › GISAID_acknowledements_tables/gisaid_hcov-19_acknowledgement_table_2021_02_12_23-7.pdf]

We gratefully acknowledge the following Authors from the Originating laboratories responsible for obtaining the specimens, as well as the Submitting laboratories where the genome data were generated and shared via GISAID, on which this research is based.

All Submitters of data may be contacted directly via www.gisaid.org

Authors are sorted alphabetically.

Accession ID	Originating Laboratory	Submitting Laboratory	Authors
EPI_ISL_534759, EPI_ISL_534760, EPI_ISL_534761, EPI_ISL_534762	Queens Medical Centre, Clinical Microbiology Department / DeepSeq Nottingham	COVID-19 Genomics UK (COG-UK) Consortium	Gemma Clark, Wendy Smith, Manjinder Khakh, Vicki M Fleming, Michelle M Lister, Hannah Howson-Wells, Jonathan Ball, Patrick McClure, Joseph Chappell, Theocharis Tsoleridis, Nadine Holmes, Matthew Carlisle, Christopher Moore, Fei Sang, Johnny Debebe, Victoria Wright, Matthew Loose
EPI_ISL_536656, EPI_ISL_536657	University of Wisconsin-Madison Campus AIDS Vaccine Research Laboratories	University of Wisconsin-Madison AIDS Vaccine Research Laboratories	Gage Moreno, Katarina Braun, et al. AIDS Vaccine Research Laboratories
EPI_ISL_539327	Area of Virology, Serology and Virology Division (SAViD), New South Wales Health Pathology Randwick	Area of Virology, Serology and Virology Division (SAViD), New South Wales Health Pathology Randwick	Rawlinson, W., Bull, R., Deveson, I., Van Hal, S.
EPI_ISL_540701, EPI_ISL_540702, EPI_ISL_540703, EPI_ISL_540704, EPI_ISL_540705, EPI_ISL_540706, EPI_ISL_540707, EPI_ISL_540708, EPI_ISL_540709, EPI_ISL_540710, EPI_ISL_540711, EPI_ISL_540712, EPI_ISL_540713, EPI_ISL_540714, EPI_ISL_540715, EPI_ISL_540716, EPI_ISL_540717, EPI_ISL_540718			
see above	Queens Medical Centre, Clinical Microbiology Department / DeepSeq Nottingham	COVID-19 Genomics UK (COG-UK) Consortium	Gemma Clark, Wendy Smith, Manjinder Khakh, Vicki M Fleming, Michelle M Lister, Hannah Howson-Wells, Jonathan Ball, Patrick McClure, Joseph Chappell, Theocharis Tsoleridis, Nadine Holmes, Matthew Carlisle, Christopher Moore, Fei Sang, Johnny Debebe, Victoria Wright, Matthew Loose
EPI_ISL_540731, EPI_ISL_540743, EPI_ISL_540750, EPI_ISL_540773	Virology Department, Sheffield Teaching Hospitals NHS Foundation Trust/Department of Infection, Immunity and Cardiovascular Disease, The Medical School, University of Sheffield	COVID-19 Genomics UK (COG-UK) Consortium	Thushan de Silva, Matthew Parker, Nikki Smith, Adri Angyal, Rebecca Brown, Luke Green, Rachel Tucker, Paul Parsons, Danielle Groves, Katie Johnson, Laura Carrilero, Alex Keeley, Dave Partridge, Matthew Wyles, Benjamin Lindsey, Mehmet Yavuz, Mohammad Raza, Cariad Evans
EPI_ISL_540886, EPI_ISL_540887, EPI_ISL_540888, EPI_ISL_540889, EPI_ISL_540891, EPI_ISL_540892, EPI_ISL_540893	Virology Department, Royal Infirmary of Edinburgh, NHS Lothian / School of Biological Sciences, University of Edinburgh / Institute of Genetics and Molecular Medicine, University of Edinburgh	COVID-19 Genomics UK (COG-UK) Consortium	McHugh M, Dewar R, Rooke S, Gallagher M, Balcaza C, O'Toole Á, Scher E, Hill V, McCrone JT, Colquhoun R, Yu X, Jackson B, Rambaut A, Williams TC, Templeton K
EPI_ISL_541540, EPI_ISL_541541, EPI_ISL_541542, EPI_ISL_541543, EPI_ISL_541544, EPI_ISL_541545, EPI_ISL_541546, EPI_ISL_541547, EPI_ISL_541548, EPI_ISL_541549, EPI_ISL_541550			
see above	University of Wisconsin-Madison AIDS Vaccine Research Laboratories	University of Wisconsin-Madison AIDS Vaccine Research Laboratories	Gage Moreno, Katarina Braun, et al. AIDS Vaccine Research Laboratories
EPI_ISL_541551, EPI_ISL_541552, EPI_ISL_541553, EPI_ISL_541554, EPI_ISL_541555, EPI_ISL_541556, EPI_ISL_541557, EPI_ISL_541558, EPI_ISL_541559, EPI_ISL_541560, EPI_ISL_541561, EPI_ISL_541562, EPI_ISL_541563, EPI_ISL_541564, EPI_ISL_541565			
see above	University of Wisconsin-Madison Campus AIDS Vaccine Research Laboratories	University of Wisconsin-Madison AIDS Vaccine Research Laboratories	Gage Moreno, Katarina Braun, et al. AIDS Vaccine Research Laboratories
EPI_ISL_541566, EPI_ISL_541567, EPI_ISL_541569, EPI_ISL_541570, EPI_ISL_541571, EPI_ISL_541572, EPI_ISL_541573, EPI_ISL_541574, EPI_ISL_541575, EPI_ISL_541576, EPI_ISL_541577, EPI_ISL_541578, EPI_ISL_541579, EPI_ISL_541580, EPI_ISL_541581, EPI_ISL_541582, EPI_ISL_541583, EPI_ISL_541584, EPI_ISL_541585, EPI_ISL_541586, EPI_ISL_541587, EPI_ISL_541588, EPI_ISL_541589, EPI_ISL_541590, EPI_ISL_541591, EPI_ISL_541592, EPI_ISL_541593, EPI_ISL_541594, EPI_ISL_541595, EPI_ISL_541596, EPI_ISL_541597, EPI_ISL_541598, EPI_ISL_541599, EPI_ISL_541600, EPI_ISL_541601, EPI_ISL_541602, EPI_ISL_541603, EPI_ISL_541604, EPI_ISL_541605, EPI_ISL_541606, EPI_ISL_541607, EPI_ISL_541608, EPI_ISL_541609, EPI_ISL_541610, EPI_ISL_541611, EPI_ISL_541612, EPI_ISL_541613, EPI_ISL_541614, EPI_ISL_541615, EPI_ISL_541616, EPI_ISL_541617, EPI_ISL_541618, EPI_ISL_541619, EPI_ISL_541620, EPI_ISL_541621, EPI_ISL_541622, EPI_ISL_541623, EPI_ISL_541624, EPI_ISL_541625, EPI_ISL_541626, EPI_ISL_541627, EPI_ISL_541628, EPI_ISL_541629, EPI_ISL_541630, EPI_ISL_541631, EPI_ISL_541632, EPI_ISL_541633, EPI_ISL_541634, EPI_ISL_541635, EPI_ISL_541636, EPI_ISL_541637, EPI_ISL_541638, EPI_ISL_541639, EPI_ISL_541640, EPI_ISL_541641, EPI_ISL_541642, EPI_ISL_541643, EPI_ISL_541644, EPI_ISL_541645, EPI_ISL_541646, EPI_ISL_541647, EPI_ISL_541648			
see above	University of Wisconsin-Madison AIDS Vaccine Research Laboratories	University of Wisconsin-Madison AIDS Vaccine Research Laboratories	Gage Moreno, Katarina Braun, et al. AIDS Vaccine Research Laboratories
EPI_ISL_545032, EPI_ISL_545033	Pathology West - NSW Health Pathology	NSW Health Pathology - Institute of Clinical Pathology and Medical Research; Westmead Hospital; University of Sydney	CIDM-PH et al.
EPI_ISL_545034, EPI_ISL_545035, EPI_ISL_545036	Histopath	NSW Health Pathology - Institute of Clinical Pathology and Medical Research; Westmead Hospital; University of Sydney	CIDM-PH et al.
EPI_ISL_545037	Sydney South West Pathology Service (SSWPS) - Concord Repatriation General Hospital - NSW Health Pathology	NSW Health Pathology - Institute of Clinical Pathology and Medical Research; Westmead Hospital; University of Sydney	CIDM-PH et al.

EPI_ISL_545038	Pathology West - NSW Health Pathology	NSW Health Pathology - Institute of Clinical Pathology and Medical Research; Westmead Hospital; University of Sydney	CIDM-PH et al.
EPI_ISL_545039	Sydney South West Pathology Service (SSWPS) - Royal Prince Alfred Hospital - NSW Health Pathology	NSW Health Pathology - Institute of Clinical Pathology and Medical Research; Westmead Hospital; University of Sydney	CIDM-PH et al.
EPI_ISL_545042	Sydney South West Pathology Service (SSWPS) - Liverpool Hospital - NSW Health Pathology	NSW Health Pathology - Institute of Clinical Pathology and Medical Research; Westmead Hospital; University of Sydney	CIDM-PH et al.
EPI_ISL_545046, EPI_ISL_545047	Pathology West - NSW Health Pathology	NSW Health Pathology - Institute of Clinical Pathology and Medical Research; Westmead Hospital; University of Sydney	CIDM-PH et al.
EPI_ISL_545048	South Eastern Area Laboratory Services (SEALS)	NSW Health Pathology - Institute of Clinical Pathology and Medical Research; Westmead Hospital; University of Sydney	CIDM-PH et al.
EPI_ISL_545049	Sydney South West Pathology Service (SSWPS) - Concord Repatriation General Hospital - NSW Health Pathology	NSW Health Pathology - Institute of Clinical Pathology and Medical Research; Westmead Hospital; University of Sydney	CIDM-PH et al.
EPI_ISL_547819, EPI_ISL_547820, EPI_ISL_547821, EPI_ISL_547822, EPI_ISL_547823, EPI_ISL_547824, EPI_ISL_547825, EPI_ISL_547826, EPI_ISL_547827, EPI_ISL_547828, EPI_ISL_547829, EPI_ISL_547830, EPI_ISL_547831, EPI_ISL_547832, EPI_ISL_547833, EPI_ISL_547834, EPI_ISL_547835, EPI_ISL_547836, EPI_ISL_547837, EPI_ISL_547838, EPI_ISL_547839, EPI_ISL_547840, EPI_ISL_547841, EPI_ISL_547842, EPI_ISL_547843, EPI_ISL_547844, EPI_ISL_547845, EPI_ISL_547846, EPI_ISL_547847, EPI_ISL_547848, EPI_ISL_547849, EPI_ISL_547850, EPI_ISL_547851, EPI_ISL_547852, EPI_ISL_547853, EPI_ISL_547854, EPI_ISL_547855, EPI_ISL_547856, EPI_ISL_547857, EPI_ISL_547858, EPI_ISL_547859, EPI_ISL_547860, EPI_ISL_547861, EPI_ISL_547862, EPI_ISL_547863, EPI_ISL_547864, EPI_ISL_547865, EPI_ISL_547866, EPI_ISL_547867, EPI_ISL_547868, EPI_ISL_547869			
see above	Gundersen Molecular Diagnostics Laboratory	Kabara Cancer Research Institute	Craig S. Richmond, Paraic A. Kenny
EPI_ISL_548082	Middlemore Hospital	Institute of Environmental Science and Research (ESR)	Xiaoyun Ren, Matt Storey, Nikki Freed, Muhammad Faisal, Jing Wang, Hermes Perez, Anja Werno, Antje van der Linden, Arlo Upton, Chris Mansell, David Hammer, Dragana Drinkovic, Gary McAuliffe, Hana Sofia Andersson, James Ussher, Jill Sherwood, Josh Freeman, Julia Howard, Juliet Elvy, Mary DeAlmeida, Matt Blakiston, Matthew Rogers, Max Bloomfield, Michael Addidle, Michelle Balm, Sally Roberts, Sarah Jefferies, Sharmini Muttaiyah, Susan Morpeth, Susan Taylor, Timothy Blackmore, Vani Sathyendran, Veronica Playle, Virginia Hope, Erasmus Smit, Lauren Jelly, Olin Silander, Joep de Ligt
EPI_ISL_548084	LabPLUS	Institute of Environmental Science and Research (ESR)	Xiaoyun Ren, Matt Storey, Nikki Freed, Muhammad Faisal, Jing Wang, Hermes Perez, Anja Werno, Antje van der Linden, Arlo Upton, Chris Mansell, David Hammer, Dragana Drinkovic, Gary McAuliffe, Hana Sofia Andersson, James Ussher, Jill Sherwood, Josh Freeman, Julia Howard, Juliet Elvy, Mary DeAlmeida, Matt Blakiston, Matthew Rogers, Max Bloomfield, Michael Addidle, Michelle Balm, Sally Roberts, Sarah Jefferies, Sharmini Muttaiyah, Susan Morpeth, Susan Taylor, Timothy Blackmore, Vani Sathyendran, Veronica Playle, Virginia Hope, Erasmus Smit, Lauren Jelly, Olin Silander, Joep de Ligt
EPI_ISL_548972, EPI_ISL_548974, EPI_ISL_548975, EPI_ISL_548976	National Public Health Laboratory, National Centre for Infectious Diseases	National Public Health Laboratory, National Centre for Infectious Diseases	Mak TM, Octavia S, Zhou Z, Cui L, Lin RTP
EPI_ISL_549334	Queens Medical Centre, Clinical Microbiology Department / DeepSeq Nottingham	COVID-19 Genomics UK (COG-UK) Consortium	Gemma Clark, Wendy Smith, Manjinder Khakh, Vicki M Fleming, Michelle M Lister, Hannah Howson-Wells, Jonathan Ball, Patrick McClure, Joseph Chappell, Theocharis Tsoleridis, Nadine Holmes, Matthew Carlisle, Christopher Moore, Fei Sang, Johnny Debebe, Victoria Wright, Matthew Loose
EPI_ISL_549341	Centre for Enzyme Innovation, University of Portsmouth / Translational Research Laboratory, Portsmouth Hospitals NHS Trust	COVID-19 Genomics UK (COG-UK) Consortium	Angela Beckett,Yann Bourgeois,Garry Scarlett,Sharon Glaysher,Scott Elliott,Kelly Bicknell,Robert Impey,Allyson Lloyd,Sarah Wyllie,Ethan Butcher,Anoop Chauhan,Samuel Robson
EPI_ISL_549354	Liverpool Clinical Laboratories	COVID-19 Genomics UK (COG-UK) Consortium	Sam Haldenby, Anita Lucaci, Steve Paterson, Julian Hiscox, Alistair Darby, M Almsaud, A Alrezaihi, Muhannad Alruwaili, Stuart D Armstrong, Jones Benjamin, Eleanor G Bentley, Anu Chawla, Jordan J Clark, Angela Cowell, Richard Eccles, Isabel García-Dorival, Matthew Gemmell, Alessandro Gerada, PKF Gilmore, Richard Gregory, Ximeng Han, Catherine Hartley, Margaret Hughes, Miren Iturriza-Gomara, James Johnson, L Luu, Jenifer Manson, Charlotte Nelson, Elaine O'Toole, Cassie Olateju, Rebekah Penrice-Randal , Lucille Rainbow, N.P Randle, Trevor Ian Robinson, Parul Sharma, Ghada T Shawli, James P Stewart, Neil Swainston, Ecaterina Vamos, Joanne Watts, Mark Whitehead
EPI_ISL_549355	Quadram Institute Bioscience	COVID-19 Genomics UK (COG-UK) Consortium	Dave J. Baker, Gemma L. Kay, Alp Aydin, Thanh Le-Viet, Steven Rudder, Ana P. Tedim, Anastasia Kolyva, Maria Diaz, Leonardo de Oliveira Martins, Nabil-Fareed Alikhan, Lizzie Meadows, Rachael Stanley, Ngozi Elumogo, Muhammed Yasir, Nicholas M. Thomson, Alexander J Trotter, Rachel Gilroy, Samuel Bloomfield, Claire Stuart, Andrew Bell, Reenesh Prakash, Samir Dervisevic, Alison E. Mather, John Wain, Mark Webber, Andrew J. Page, Justin O'Grady
EPI_ISL_549356	Lincolnshire Hospitals and DeepSeq Nottingham	COVID-19 Genomics UK (COG-UK) Consortium	Nichola Duckworth, Tim Sloan, Sarah Walsh, Jonathan Ball, Patrick McClure, Joeseph Chappell, Nadine Holmes, Matthew Carlisle, Christopher Moore, Fei Sang, Johnny Debebe, Victoria Wright, Matthew Loose
EPI_ISL_549360, EPI_ISL_549364, EPI_ISL_549366	Centre for Enzyme Innovation, University of Portsmouth / Translational Research Laboratory, Portsmouth Hospitals NHS Trust	COVID-19 Genomics UK (COG-UK) Consortium	Angela Beckett,Yann Bourgeois,Garry Scarlett,Sharon Glaysher,Scott Elliott,Kelly Bicknell,Robert Impey,Allyson Lloyd,Sarah Wyllie,Ethan Butcher,Anoop Chauhan,Samuel Robson
EPI_ISL_549371, EPI_ISL_549372	Lincolnshire Hospitals and DeepSeq Nottingham	COVID-19 Genomics UK (COG-UK) Consortium	Nichola Duckworth, Tim Sloan, Sarah Walsh, Jonathan Ball, Patrick McClure, Joeseph Chappell, Nadine Holmes, Matthew Carlisle, Christopher Moore, Fei Sang, Johnny Debebe, Victoria Wright, Matthew Loose
EPI_ISL_549380	Queens Medical Centre, Clinical Microbiology Department / DeepSeq Nottingham	COVID-19 Genomics UK (COG-UK) Consortium	Gemma Clark, Wendy Smith, Manjinder Khakh, Vicki M Fleming, Michelle M Lister, Hannah Howson-Wells, Jonathan Ball, Patrick McClure, Joseph Chappell, Theocharis Tsoleridis, Nadine Holmes, Matthew Carlisle, Christopher Moore, Fei Sang, Johnny Debebe, Victoria Wright, Matthew Loose
EPI_ISL_549387	Lincolnshire Hospitals and DeepSeq Nottingham	COVID-19 Genomics UK (COG-UK) Consortium	Nichola Duckworth, Tim Sloan, Sarah Walsh, Jonathan Ball, Patrick McClure, Joeseph Chappell, Nadine Holmes, Matthew Carlisle, Christopher Moore, Fei Sang, Johnny Debebe, Victoria Wright, Matthew Loose
EPI_ISL_549461,	Centre for Enzyme	COVID-19 Genomics UK	Angela Beckett,Yann Bourgeois,Garry Scarlett,Sharon Glaysher,Scott Elliott,Kelly Bicknell,Robert Impey,Allyson Lloyd,Sarah Wyllie,Ethan Butcher,Anoop Chauhan,Samuel Robson

EPI_ISL_549466, EPI_ISL_549469, EPI_ISL_549470, EPI_ISL_549471, EPI_ISL_549472, EPI_ISL_549473, EPI_ISL_549474, EPI_ISL_549475, EPI_ISL_549476	Innovation, University of Portsmouth / Translational Research Laboratory, Portsmouth Hospitals NHS Trust	(COG-UK) Consortium	
EPI_ISL_549479, EPI_ISL_549480, EPI_ISL_549481	Quadram Institute Bioscience	COVID-19 Genomics UK (COG-UK) Consortium	Dave J. Baker, Gemma L. Kay, Alp Aydin, Thanh Le-Viet, Steven Rudder, Ana P. Tedim, Anastasia Kolyva, Maria Diaz, Leonardo de Oliveira Martins, Nabil-Fareed Alikhan, Lizzie Meadows, Rachael Stanley, Ngozi Elumogo, Muhammed Yasir, Nicholas M. Thomson, Alexander J Trotter, Rachel Gilroy, Samuel Bloomfield, Claire Stuart, Andrew Bell, Reenesh Prakash, Samir Dervisevic, Alison E. Mather, John Wain, Mark Webber, Andrew J. Page, Justin O'Grady
EPI_ISL_549491, EPI_ISL_549492, EPI_ISL_549493, EPI_ISL_549494, EPI_ISL_549495, EPI_ISL_549496, EPI_ISL_549497, EPI_ISL_549498, EPI_ISL_549499, EPI_ISL_549500, EPI_ISL_549501, EPI_ISL_549502, EPI_ISL_549503, EPI_ISL_549504			
see above	Queens Medical Centre, Clinical Microbiology Department / DeepSeq Nottingham	COVID-19 Genomics UK (COG-UK) Consortium	Gemma Clark, Wendy Smith, Manjinder Khakh, Vicki M Fleming, Michelle M Lister, Hannah Howson-Wells, Jonathan Ball, Patrick McClure, Joseph Chappell, Theocharis Tsoleridis, Nadine Holmes, Matthew Carlisle, Christopher Moore, Fei Sang, Johnny Debebe, Victoria Wright, Matthew Loose
EPI_ISL_549519, EPI_ISL_549520, EPI_ISL_549521, EPI_ISL_549522	Lincolnshire Hospitals and DeepSeq Nottingham	COVID-19 Genomics UK (COG-UK) Consortium	Nichola Duckworth, Tim Sloan, Sarah Walsh, Jonathan Ball, Patrick McClure, Joeseph Chappell, Nadine Holmes, Matthew Carlisle, Christopher Moore, Fei Sang, Johnny Debebe, Victoria Wright, Matthew Loose
EPI_ISL_549555, EPI_ISL_549566, EPI_ISL_549576, EPI_ISL_549586, EPI_ISL_549595, EPI_ISL_549610, EPI_ISL_549617, EPI_ISL_549657, EPI_ISL_549708, EPI_ISL_549721, EPI_ISL_549724, EPI_ISL_549737, EPI_ISL_549738, EPI_ISL_549791			
see above	Lighthouse Lab in Glasgow	Wellcome Sanger Institute for the COVID-19 Genomics UK (COG-UK) consortium	Harper VanSteenhouse, Yumi Kasai, David Gray, Carol Clugston, Anna Dominiczak and Alex Alderton, Roberto Amato, Sonia Goncalves, Ewan Harrison, David K. Jackson, Ian Johnston, Dominic Kwiatkowski, Cordelia Langford, John Sillitoe on behalf of the Wellcome Sanger Institute COVID-19 Surveillance Team
EPI_ISL_559899, EPI_ISL_559903, EPI_ISL_559905, EPI_ISL_559906, EPI_ISL_559907, EPI_ISL_559908, EPI_ISL_559909, EPI_ISL_559913, EPI_ISL_559915, EPI_ISL_559916, EPI_ISL_559917, EPI_ISL_559918, EPI_ISL_559919, EPI_ISL_559924, EPI_ISL_559925, EPI_ISL_559926, EPI_ISL_559929, EPI_ISL_559933, EPI_ISL_559934, EPI_ISL_559936, EPI_ISL_559940, EPI_ISL_559941, EPI_ISL_559942, EPI_ISL_559943, EPI_ISL_559944, EPI_ISL_559945, EPI_ISL_559946			
see above	Virology Department, Sheffield Teaching Hospitals NHS Foundation Trust/Department of Infection, Immunity and Cardiovascular Disease, The Medical School, University of Sheffield	COVID-19 Genomics UK (COG-UK) Consortium	Thushan de Silva, Matthew Parker, Nikki Smith, Adri Angyal, Rebecca Brown, Luke Green, Rachel Tucker, Paul Parsons, Danielle Groves, Katie Johnson, Laura Carrilero, Alex Keeley, Dave Partridge, Matthew Wyles, Benjamin Lindsey, Mehmet Yavuz, Mohammad Raza, Cariad Evans
EPI_ISL_559979, EPI_ISL_559980, EPI_ISL_559981, EPI_ISL_559982	Virology Department, Royal Infirmary of Edinburgh, NHS Lothian / School of Biological Sciences, University of Edinburgh / Institute of Genetics and Molecular Medicine, University of Edinburgh	COVID-19 Genomics UK (COG-UK) Consortium	McHugh M, Dewar R, Rooke S, Gallagher M, Balcaza C, O'Toole Á, Scher E, Hill V, McCrone JT, Colquhoun R, Yu X, Jackson B, Rambaut A, Williams TC, Templeton K
EPI_ISL_559983	Centre for Enzyme Innovation, University of Portsmouth / Translational Research Laboratory, Portsmouth Hospitals NHS Trust	COVID-19 Genomics UK (COG-UK) Consortium	Angela Beckett,Yann Bourgeois,Garry Scarlett,Sharon Glaysher,Scott Elliott,Kelly Bicknell,Robert Impey,Allyson Lloyd,Sarah Wyllie,Ethan Butcher,Anoop Chauhan,Samuel Robson
EPI_ISL_560083, EPI_ISL_560086, EPI_ISL_560087, EPI_ISL_560089, EPI_ISL_560090, EPI_ISL_560095, EPI_ISL_560097, EPI_ISL_560098, EPI_ISL_560099, EPI_ISL_560100, EPI_ISL_560101, EPI_ISL_560102, EPI_ISL_560105, EPI_ISL_560106, EPI_ISL_560107, EPI_ISL_560108, EPI_ISL_560115, EPI_ISL_560116, EPI_ISL_560118, EPI_ISL_560119, EPI_ISL_560120, EPI_ISL_560121, EPI_ISL_560123, EPI_ISL_560130, EPI_ISL_560132, EPI_ISL_560133, EPI_ISL_560134, EPI_ISL_560135, EPI_ISL_560136, EPI_ISL_560138, EPI_ISL_560141, EPI_ISL_560142, EPI_ISL_560144, EPI_ISL_560145, EPI_ISL_560146, EPI_ISL_560148, EPI_ISL_560149, EPI_ISL_560151, EPI_ISL_560152, EPI_ISL_560153, EPI_ISL_560157, EPI_ISL_560158, EPI_ISL_560160, EPI_ISL_560161, EPI_ISL_560162, EPI_ISL_560165, EPI_ISL_560166, EPI_ISL_560167, EPI_ISL_560168, EPI_ISL_560169, EPI_ISL_560170, EPI_ISL_560171, EPI_ISL_560172, EPI_ISL_560173, EPI_ISL_560174, EPI_ISL_560175, EPI_ISL_560177, EPI_ISL_560178, EPI_ISL_560179, EPI_ISL_560180, EPI_ISL_560181, EPI_ISL_560182, EPI_ISL_560183, EPI_ISL_560184, EPI_ISL_560186, EPI_ISL_560187, EPI_ISL_560188, EPI_ISL_560189, EPI_ISL_560191, EPI_ISL_560192, EPI_ISL_560193, EPI_ISL_560194, EPI_ISL_560196, EPI_ISL_560198, EPI_ISL_560200, EPI_ISL_560203, EPI_ISL_560204, EPI_ISL_560208, EPI_ISL_560209, EPI_ISL_560210, EPI_ISL_560211, EPI_ISL_560212, EPI_ISL_560213, EPI_ISL_560214, EPI_ISL_560215, EPI_ISL_560216, EPI_ISL_560217, EPI_ISL_560218, EPI_ISL_560220, EPI_ISL_560221, EPI_ISL_560222, EPI_ISL_560223, EPI_ISL_560224, EPI_ISL_560225, EPI_ISL_560226, EPI_ISL_560227, EPI_ISL_560228, EPI_ISL_560229, EPI_ISL_560230, EPI_ISL_560231, EPI_ISL_560232, EPI_ISL_560233, EPI_ISL_560234, EPI_ISL_560235, EPI_ISL_560236, EPI_ISL_560237, EPI_ISL_560238, EPI_ISL_560239, EPI_ISL_560240, EPI_ISL_560241, EPI_ISL_560242, EPI_ISL_560243, EPI_ISL_560244, EPI_ISL_560245, EPI_ISL_560246, EPI_ISL_560247, EPI_ISL_560248, EPI_ISL_560249, EPI_ISL_560250, EPI_ISL_560251, EPI_ISL_560252, EPI_ISL_560253, EPI_ISL_560254, EPI_ISL_560255, EPI_ISL_560256, EPI_ISL_560257, EPI_ISL_560258, EPI_ISL_560259, EPI_ISL_560260, EPI_ISL_560261, EPI_ISL_560262, EPI_ISL_560263, EPI_ISL_560264, EPI_ISL_560265, EPI_ISL_560266, EPI_ISL_560267, EPI_ISL_560268, EPI_ISL_560269, EPI_ISL_560270, EPI_ISL_560271, EPI_ISL_560272, EPI_ISL_560273, EPI_ISL_560274, EPI_ISL_560275, EPI_ISL_560276, EPI_ISL_560277, EPI_ISL_560279, EPI_ISL_560280, EPI_ISL_560281, EPI_ISL_560282, EPI_ISL_560283, EPI_ISL_560284, EPI_ISL_560285, EPI_ISL_560286, EPI_ISL_560287, EPI_ISL_560288, EPI_ISL_560289, EPI_ISL_560290, EPI_ISL_560291, EPI_ISL_560292, EPI_ISL_560293, EPI_ISL_560294, EPI_ISL_560295, EPI_ISL_560296, EPI_ISL_560297, EPI_ISL_560298, EPI_ISL_560299, EPI_ISL_560300, EPI_ISL_560301, EPI_ISL_560302, EPI_ISL_560303, EPI_ISL_560304			
see above	Wales Specialist Virology Centre Sequencing lab: Pathogen Genomics Unit	COVID-19 Genomics UK (COG-UK) Consortium	Catherine Moore, Johnathan Evans, Laura Gifford, Malorie Perry, Simon Cottrell, Angela Marchbank, Alec Birchley, Alexander Adams, Amy Gaskin, Bree Gatica-Wilcox, Jason Coombes, Joel Southgate, Lauren Gilbert, Lee Graham, Nicole Pacchiarini, Sara Kumziene-Summerhayes, Sarah Taylor, Sophie Jones, Sara Rey, Matthew Bull, Joanne Watkins, Sally Corden, Tom Connor
EPI_ISL_560426, EPI_ISL_560428, EPI_ISL_560446, EPI_ISL_560447, EPI_ISL_560448, EPI_ISL_560449, EPI_ISL_560450, EPI_ISL_560451, EPI_ISL_560452, EPI_ISL_560453, EPI_ISL_560454, EPI_ISL_560464, EPI_ISL_560465, EPI_ISL_560466, EPI_ISL_560467, EPI_ISL_560469, EPI_ISL_560473, EPI_ISL_560474, EPI_ISL_560475, EPI_ISL_560480, EPI_ISL_560482, EPI_ISL_560523, EPI_ISL_560526, EPI_ISL_560528, EPI_ISL_560529, EPI_ISL_560530, EPI_ISL_560531, EPI_ISL_560532, EPI_ISL_560533, EPI_ISL_560536, EPI_ISL_560537, EPI_ISL_560538, EPI_ISL_560539, EPI_ISL_560540, EPI_ISL_560553			
see above	Viollier AG	Department of Biosystems Science and Engineering, ETH Zürich	Christian Beisel, Sarah Nadeau, Ivan Topolsky, Pedro Ferreira, Philipp Jablonski, Susana Posada-Céspedes, Tobias Schär, Ina Nissen, Natascha Santacroce, Elodie Burcklen, Christiane Beckmann, Maurice Redondo, Olivier Kobel, Christoph Noppen, Sophie Seidel, Noemie Santamaria de Souza, Niko Beerenwinkel, Tanja Stadler
EPI_ISL_560817, EPI_ISL_560818, EPI_ISL_560819, EPI_ISL_560820, EPI_ISL_560821, EPI_ISL_560822, EPI_ISL_560823, EPI_ISL_560824, EPI_ISL_560825, EPI_ISL_560826, EPI_ISL_560827, EPI_ISL_560828, EPI_ISL_560829			
see above	Maryland Public Health Laboratory	Maryland Public Health Laboratory	Maryland Department of Health Laboratories Administration
EPI_ISL_561398, EPI_ISL_561427, EPI_ISL_561439, EPI_ISL_561441, EPI_ISL_561447, EPI_ISL_561451, EPI_ISL_561452, EPI_ISL_561461, EPI_ISL_561463, EPI_ISL_561473, EPI_ISL_561484, EPI_ISL_561518, EPI_ISL_561537, EPI_ISL_561587, EPI_ISL_561598, EPI_ISL_561608, EPI_ISL_561659			
see above	Microbiological Diagnostic Unit - Public Health Laboratory (MDU-PHL)	MDU-PHL	Seemann, T., Schultz M. B., Sait, M., Sherry, N.
EPI_ISL_561682	Victorian Infectious Diseases Reference Laboratory (VIDRL)	VIDRL and MDU-PHL	Caly, L., Seemann, T., Sait, M., Schultz, M. B., Druce J., Sherry, N.

EPI_ISL_561691, EPI_ISL_561744, EPI_ISL_561795, EPI_ISL_561812, EPI_ISL_561834, EPI_ISL_561874, EPI_ISL_561892, EPI_ISL_561926, EPI_ISL_561929, EPI_ISL_561958, EPI_ISL_561984, EPI_ISL_561985, EPI_ISL_562064, EPI_ISL_562075			
see above	Microbiological Diagnostic Unit - Public Health Laboratory (MDU-PHL)	MDU-PHL	Seemann, T., Schultz M. B., Sait, M., Sherry, N.
EPI_ISL_562104	Victorian Infectious Diseases Reference Laboratory (VIDRL)	VIDRL and MDU-PHL	Caly, L., Seemann, T., Sait, M., Schultz, M. B., Druce J., Sherry, N.
EPI_ISL_562128, EPI_ISL_562151, EPI_ISL_562201, EPI_ISL_562218, EPI_ISL_562243, EPI_ISL_562252	Microbiological Diagnostic Unit - Public Health Laboratory (MDU-PHL)	MDU-PHL	Seemann, T., Schultz M. B., Sait, M., Sherry, N.
EPI_ISL_562261, EPI_ISL_562272, EPI_ISL_562280	Victorian Infectious Diseases Reference Laboratory (VIDRL)	VIDRL and MDU-PHL	Caly, L., Seemann, T., Sait, M., Schultz, M. B., Druce J., Sherry, N.
EPI_ISL_562295	Microbiological Diagnostic Unit - Public Health Laboratory (MDU-PHL)	MDU-PHL	Seemann, T., Schultz M. B., Sait, M., Sherry, N.
EPI_ISL_562335	Victorian Infectious Diseases Reference Laboratory (VIDRL)	VIDRL and MDU-PHL	Caly, L., Seemann, T., Sait, M., Schultz, M. B., Druce J., Sherry, N.
EPI_ISL_562418, EPI_ISL_562421, EPI_ISL_562423, EPI_ISL_562450, EPI_ISL_562481, EPI_ISL_562493, EPI_ISL_562494, EPI_ISL_562496, EPI_ISL_562508, EPI_ISL_562516, EPI_ISL_562519, EPI_ISL_562556, EPI_ISL_562575, EPI_ISL_562594, EPI_ISL_562615, EPI_ISL_562620, EPI_ISL_562622, EPI_ISL_562724, EPI_ISL_562725, EPI_ISL_562726, EPI_ISL_562756			
see above	Microbiological Diagnostic Unit - Public Health Laboratory (MDU-PHL)	MDU-PHL	Seemann, T., Schultz M. B., Sait, M., Sherry, N.
EPI_ISL_563499	Victorian Infectious Diseases Reference Laboratory (VIDRL)	VIDRL and MDU-PHL	Caly, L., Seemann, T., Sait, M., Schultz, M. B., Druce J., Sherry, N.
EPI_ISL_563505, EPI_ISL_563507, EPI_ISL_563509, EPI_ISL_563517, EPI_ISL_563518, EPI_ISL_563519, EPI_ISL_563521, EPI_ISL_563526, EPI_ISL_563534, EPI_ISL_563535, EPI_ISL_563538, EPI_ISL_563540, EPI_ISL_563543, EPI_ISL_563548, EPI_ISL_563552, EPI_ISL_563553, EPI_ISL_563554, EPI_ISL_563557, EPI_ISL_563558, EPI_ISL_563562, EPI_ISL_563563, EPI_ISL_563566, EPI_ISL_563571, EPI_ISL_563573, EPI_ISL_563576, EPI_ISL_563582, EPI_ISL_563584, EPI_ISL_563585, EPI_ISL_563587, EPI_ISL_563589, EPI_ISL_563590, EPI_ISL_563592, EPI_ISL_563593, EPI_ISL_563595, EPI_ISL_563596, EPI_ISL_563597, EPI_ISL_563599, EPI_ISL_563600, EPI_ISL_563601, EPI_ISL_563602, EPI_ISL_563603, EPI_ISL_563605, EPI_ISL_563607, EPI_ISL_563615			
see above	Microbiological Diagnostic Unit - Public Health Laboratory (MDU-PHL)	MDU-PHL	Seemann, T., Schultz M. B., Sait, M., Sherry, N.
EPI_ISL_563723	Victorian Infectious Diseases Reference Laboratory (VIDRL)	VIDRL and MDU-PHL	Caly, L., Seemann, T., Sait, M., Schultz, M. B., Druce J., Sherry, N.
EPI_ISL_563725, EPI_ISL_563727, EPI_ISL_563733, EPI_ISL_563735, EPI_ISL_563736, EPI_ISL_563737, EPI_ISL_563738, EPI_ISL_563747, EPI_ISL_563765, EPI_ISL_563769	Microbiological Diagnostic Unit - Public Health Laboratory (MDU-PHL)	MDU-PHL	Seemann, T., Schultz M. B., Sait, M., Sherry, N.
EPI_ISL_563792	Victorian Infectious Diseases Reference Laboratory (VIDRL)	VIDRL and MDU-PHL	Caly, L., Seemann, T., Sait, M., Schultz, M. B., Druce J., Sherry, N.
EPI_ISL_563809	Microbiological Diagnostic Unit - Public Health Laboratory (MDU-PHL)	MDU-PHL	Seemann, T., Schultz M. B., Sait, M., Sherry, N.
EPI_ISL_563811	Victorian Infectious Diseases Reference Laboratory (VIDRL)	VIDRL and MDU-PHL	Caly, L., Seemann, T., Sait, M., Schultz, M. B., Druce J., Sherry, N.
EPI_ISL_563821, EPI_ISL_563823, EPI_ISL_563825, EPI_ISL_563829, EPI_ISL_563834, EPI_ISL_563835	Microbiological Diagnostic Unit - Public Health Laboratory (MDU-PHL)	MDU-PHL	Seemann, T., Schultz M. B., Sait, M., Sherry, N.
EPI_ISL_563840	Victorian Infectious Diseases Reference Laboratory (VIDRL)	VIDRL and MDU-PHL	Caly, L., Seemann, T., Sait, M., Schultz, M. B., Druce J., Sherry, N.
EPI_ISL_563841, EPI_ISL_563842, EPI_ISL_563843, EPI_ISL_563844, EPI_ISL_563846, EPI_ISL_563849, EPI_ISL_563850, EPI_ISL_563851, EPI_ISL_563852, EPI_ISL_563856, EPI_ISL_563857, EPI_ISL_563861, EPI_ISL_563863, EPI_ISL_563864, EPI_ISL_563865, EPI_ISL_563867, EPI_ISL_563868, EPI_ISL_563869, EPI_ISL_563870, EPI_ISL_563871, EPI_ISL_563872, EPI_ISL_563873, EPI_ISL_563874, EPI_ISL_563875, EPI_ISL_563877, EPI_ISL_563878, EPI_ISL_563882, EPI_ISL_563884, EPI_ISL_563901, EPI_ISL_563927, EPI_ISL_563929			
see above	Microbiological Diagnostic Unit - Public Health Laboratory (MDU-PHL)	MDU-PHL	Seemann, T., Schultz M. B., Sait, M., Sherry, N.
EPI_ISL_563938	Victorian Infectious Diseases Reference Laboratory (VIDRL)	VIDRL and MDU-PHL	Caly, L., Seemann, T., Sait, M., Schultz, M. B., Druce J., Sherry, N.
EPI_ISL_563939, EPI_ISL_563941, EPI_ISL_563942, EPI_ISL_563945, EPI_ISL_563948, EPI_ISL_563949, EPI_ISL_563950, EPI_ISL_563951, EPI_ISL_563954, EPI_ISL_563955, EPI_ISL_563956, EPI_ISL_563957, EPI_ISL_563959, EPI_ISL_563960, EPI_ISL_563961, EPI_ISL_563964			

see above	Microbiological Diagnostic Unit - Public Health Laboratory (MDU-PHL)	MDU-PHL	Seemann, T., Schultz M. B., Sait, M., Sherry, N.
EPI_ISL_563973, EPI_ISL_563974, EPI_ISL_563975	Victorian Infectious Diseases Reference Laboratory (VIDRL)	VIDRL and MDU-PHL	Caly, L., Seemann, T., Sait, M., Schultz, M. B., Druce J., Sherry, N.
EPI_ISL_566110, EPI_ISL_566111, EPI_ISL_566112, EPI_ISL_566113, EPI_ISL_566114, EPI_ISL_566115, EPI_ISL_566116, EPI_ISL_566117, EPI_ISL_566118, EPI_ISL_566119, EPI_ISL_566120, EPI_ISL_566121, EPI_ISL_566122, EPI_ISL_566123, EPI_ISL_566124, EPI_ISL_566125, EPI_ISL_566126, EPI_ISL_566127, EPI_ISL_566128, EPI_ISL_566129, EPI_ISL_566130, EPI_ISL_566131, EPI_ISL_566132, EPI_ISL_566133, EPI_ISL_566134, EPI_ISL_566135, EPI_ISL_566136, EPI_ISL_566137, EPI_ISL_566138, EPI_ISL_566139, EPI_ISL_566140, EPI_ISL_566141, EPI_ISL_566142, EPI_ISL_566143, EPI_ISL_566144, EPI_ISL_566145, EPI_ISL_566146, EPI_ISL_566147, EPI_ISL_566148, EPI_ISL_566149, EPI_ISL_566150, EPI_ISL_566151, EPI_ISL_566152, EPI_ISL_566153, EPI_ISL_566154, EPI_ISL_566155, EPI_ISL_566156, EPI_ISL_566157, EPI_ISL_566158, EPI_ISL_566159, EPI_ISL_566160, EPI_ISL_566161, EPI_ISL_566162, EPI_ISL_566163, EPI_ISL_566164, EPI_ISL_566165, EPI_ISL_566166, EPI_ISL_566167, EPI_ISL_566168, EPI_ISL_566169, EPI_ISL_566170, EPI_ISL_566171, EPI_ISL_566172, EPI_ISL_566173, EPI_ISL_566174, EPI_ISL_566175, EPI_ISL_566176, EPI_ISL_566177, EPI_ISL_566178, EPI_ISL_566179, EPI_ISL_566180, EPI_ISL_566181, EPI_ISL_566182, EPI_ISL_566183, EPI_ISL_566184, EPI_ISL_566185, EPI_ISL_566186, EPI_ISL_566187, EPI_ISL_566188, EPI_ISL_566189, EPI_ISL_566190, EPI_ISL_566191, EPI_ISL_566192, EPI_ISL_566193, EPI_ISL_566194, EPI_ISL_566195, EPI_ISL_566196, EPI_ISL_566197, EPI_ISL_566198, EPI_ISL_566199, EPI_ISL_566200, EPI_ISL_566201, EPI_ISL_566202, EPI_ISL_566203, EPI_ISL_566204, EPI_ISL_566205, EPI_ISL_566206, EPI_ISL_566207, EPI_ISL_566208, EPI_ISL_566209, EPI_ISL_566210, EPI_ISL_566211, EPI_ISL_566212, EPI_ISL_566213, EPI_ISL_566214, EPI_ISL_566215, EPI_ISL_566216, EPI_ISL_566217, EPI_ISL_566218, EPI_ISL_566219, EPI_ISL_566220, EPI_ISL_566221, EPI_ISL_566222, EPI_ISL_566223, EPI_ISL_566224, EPI_ISL_566225, EPI_ISL_566226, EPI_ISL_566227, EPI_ISL_566228, EPI_ISL_566229, EPI_ISL_566230, EPI_ISL_566231, EPI_ISL_566232, EPI_ISL_566233, EPI_ISL_566234, EPI_ISL_566235, EPI_ISL_566236, EPI_ISL_566237, EPI_ISL_566238, EPI_ISL_566239, EPI_ISL_566240, EPI_ISL_566241, EPI_ISL_566242, EPI_ISL_566243, EPI_ISL_566244, EPI_ISL_566245, EPI_ISL_566246, EPI_ISL_566247, EPI_ISL_566248, EPI_ISL_566249, EPI_ISL_566250, EPI_ISL_566251, EPI_ISL_566252, EPI_ISL_566253, EPI_ISL_566254, EPI_ISL_566255, EPI_ISL_566256, EPI_ISL_566257, EPI_ISL_566258, EPI_ISL_566259, EPI_ISL_566260, EPI_ISL_566261, EPI_ISL_566262, EPI_ISL_566263, EPI_ISL_566264, EPI_ISL_566265, EPI_ISL_566266, EPI_ISL_566267, EPI_ISL_566268, EPI_ISL_566269, EPI_ISL_566270, EPI_ISL_566271, EPI_ISL_566272, EPI_ISL_566273, EPI_ISL_566274, EPI_ISL_566275, EPI_ISL_566276, EPI_ISL_566277, EPI_ISL_566278, EPI_ISL_566279, EPI_ISL_566280, EPI_ISL_566281, EPI_ISL_566282, EPI_ISL_566283, EPI_ISL_566284, EPI_ISL_566285, EPI_ISL_566286, EPI_ISL_566287, EPI_ISL_566288, EPI_ISL_566289, EPI_ISL_566290, EPI_ISL_566291, EPI_ISL_566292, EPI_ISL_566293, EPI_ISL_566294, EPI_ISL_566295, EPI_ISL_566296, EPI_ISL_566297, EPI_ISL_566298, EPI_ISL_566299, EPI_ISL_566300, EPI_ISL_566301, EPI_ISL_566302, EPI_ISL_566303, EPI_ISL_566304, EPI_ISL_566305, EPI_ISL_566306, EPI_ISL_566307, EPI_ISL_566308, EPI_ISL_566309, EPI_ISL_566310, EPI_ISL_566311, EPI_ISL_566312, EPI_ISL_566313, EPI_ISL_566314, EPI_ISL_566315, EPI_ISL_566316, EPI_ISL_566317, EPI_ISL_566318, EPI_ISL_566319, EPI_ISL_566320, EPI_ISL_566321, EPI_ISL_566322, EPI_ISL_566323, EPI_ISL_566324, EPI_ISL_566325, EPI_ISL_566326, EPI_ISL_566327, EPI_ISL_566328, EPI_ISL_566329, EPI_ISL_566330, EPI_ISL_566331, EPI_ISL_566332, EPI_ISL_566333, EPI_ISL_566334, EPI_ISL_566335, EPI_ISL_566336, EPI_ISL_566337, EPI_ISL_566338, EPI_ISL_566339, EPI_ISL_566340, EPI_ISL_566341, EPI_ISL_566342, EPI_ISL_566343, EPI_ISL_566344, EPI_ISL_566345, EPI_ISL_566346, EPI_ISL_566347, EPI_ISL_566348, EPI_ISL_566349, EPI_ISL_566350, EPI_ISL_566351, EPI_ISL_566352, EPI_ISL_566353, EPI_ISL_566354, EPI_ISL_566355, EPI_ISL_566356, EPI_ISL_566357, EPI_ISL_566358, EPI_ISL_566359, EPI_ISL_566360, EPI_ISL_566361, EPI_ISL_566362, EPI_ISL_566363, EPI_ISL_566364, EPI_ISL_566365, EPI_ISL_566366, EPI_ISL_566367, EPI_ISL_566368, EPI_ISL_566369, EPI_ISL_566370, EPI_ISL_566371, EPI_ISL_566372, EPI_ISL_566373, EPI_ISL_566374, EPI_ISL_566375, EPI_ISL_566376, EPI_ISL_566377, EPI_ISL_566378, EPI_ISL_566379, EPI_ISL_566380, EPI_ISL_566381, EPI_ISL_566382, EPI_ISL_566383, EPI_ISL_566384, EPI_ISL_566385, EPI_ISL_566386, EPI_ISL_566387, EPI_ISL_566388, EPI_ISL_566389, EPI_ISL_566390, EPI_ISL_566391, EPI_ISL_566392, EPI_ISL_566393, EPI_ISL_566394, EPI_ISL_566395, EPI_ISL_566396, EPI_ISL_566397, EPI_ISL_566398, EPI_ISL_566399, EPI_ISL_566400, EPI_ISL_566401, EPI_ISL_566402, EPI_ISL_566403, EPI_ISL_566404, EPI_ISL_566405, EPI_ISL_566406, EPI_ISL_566407, EPI_ISL_566408, EPI_ISL_566409, EPI_ISL_566410, EPI_ISL_566411, EPI_ISL_566412, EPI_ISL_566413, EPI_ISL_566414, EPI_ISL_566415, EPI_ISL_566416, EPI_ISL_566417, EPI_ISL_566418, EPI_ISL_566419, EPI_ISL_566420			
see above	Lighthouse Lab in Alderley Park	Wellcome Sanger Institute for the COVID-19 Genomics UK (COG-UK) consortium	Jacquelyn Wynn, Mairead Hyland, The Lighthouse Lab in Alderley Park and Alex Alderton, Roberto Amato, Sonia Goncalves, Ewan Harrison, David K. Jackson, Ian Johnston, Dominic Kwiatkowski, Cordelia Langford, John Sillitoe on behalf of the Wellcome Sanger Institute COVID-19 Surveillance Team
EPI_ISL_566421, EPI_ISL_566424, EPI_ISL_566425, EPI_ISL_566426, EPI_ISL_566430, EPI_ISL_566431, EPI_ISL_566432, EPI_ISL_566433, EPI_ISL_566434, EPI_ISL_566435, EPI_ISL_566436, EPI_ISL_566437, EPI_ISL_566438, EPI_ISL_566439, EPI_ISL_566440, EPI_ISL_566441, EPI_ISL_566442, EPI_ISL_566443, EPI_ISL_566446, EPI_ISL_566448, EPI_ISL_566449, EPI_ISL_566450, EPI_ISL_566452, EPI_ISL_566458, EPI_ISL_566459, EPI_ISL_566461, EPI_ISL_566462, EPI_ISL_566463, EPI_ISL_566464, EPI_ISL_566466, EPI_ISL_566468, EPI_ISL_566470, EPI_ISL_566471, EPI_ISL_566472, EPI_ISL_566475, EPI_ISL_566476, EPI_ISL_566477, EPI_ISL_566478, EPI_ISL_566480, EPI_ISL_566481, EPI_ISL_566482, EPI_ISL_566483, EPI_ISL_566484, EPI_ISL_566486, EPI_ISL_566487, EPI_ISL_566488, EPI_ISL_566489, EPI_ISL_566491, EPI_ISL_566496, EPI_ISL_566497, EPI_ISL_566498, EPI_ISL_566499, EPI_ISL_566501, EPI_ISL_566503, EPI_ISL_566505, EPI_ISL_566506, EPI_ISL_566507, EPI_ISL_566509, EPI_ISL_566511, EPI_ISL_566512, EPI_ISL_566513, EPI_ISL_566514, EPI_ISL_566516, EPI_ISL_566517, EPI_ISL_566520, EPI_ISL_566521, EPI_ISL_566522, EPI_ISL_566523, EPI_ISL_566525, EPI_ISL_566526, EPI_ISL_566527, EPI_ISL_566529, EPI_ISL_566532, EPI_ISL_566533, EPI_ISL_566535, EPI_ISL_566536, EPI_ISL_566538, EPI_ISL_566539, EPI_ISL_566540, EPI_ISL_566541, EPI_ISL_566542, EPI_ISL_566545, EPI_ISL_566546, EPI_ISL_566549, EPI_ISL_566550, EPI_ISL_566551, EPI_ISL_566552, EPI_ISL_566553, EPI_ISL_566554, EPI_ISL_566555, EPI_ISL_566556, EPI_ISL_566557, EPI_ISL_566558, EPI_ISL_566561, EPI_ISL_566562, EPI_ISL_566563, EPI_ISL_566564, EPI_ISL_566565, EPI_ISL_566566, EPI_ISL_566568, EPI_ISL_566569, EPI_ISL_566570, EPI_ISL_566571, EPI_ISL_566572, EPI_ISL_566574, EPI_ISL_566577, EPI_ISL_566578, EPI_ISL_566581, EPI_ISL_566584, EPI_ISL_566588, EPI_ISL_566590, EPI_ISL_566592, EPI_ISL_566593, EPI_ISL_566594, EPI_ISL_566595, EPI_ISL_566596, EPI_ISL_566597, EPI_ISL_566598, EPI_ISL_566599, EPI_ISL_566600, EPI_ISL_566601, EPI_ISL_566602, EPI_ISL_566604, EPI_ISL_566606, EPI_ISL_566607, EPI_ISL_566613, EPI_ISL_566614, EPI_ISL_566615, EPI_ISL_566616, EPI_ISL_566617, EPI_ISL_566618, EPI_ISL_566619, EPI_ISL_566620, EPI_ISL_566621, EPI_ISL_566623, EPI_ISL_566624, EPI_ISL_566627, EPI_ISL_566629, EPI_ISL_566630, EPI_ISL_566632, EPI_ISL_566633, EPI_ISL_566634, EPI_ISL_566635, EPI_ISL_566636, EPI_ISL_566639, EPI_ISL_566640, EPI_ISL_566641, EPI_ISL_566644, EPI_ISL_566646, EPI_ISL_566647, EPI_ISL_566648, EPI_ISL_566649, EPI_ISL_566650, EPI_ISL_566651, EPI_ISL_566652, EPI_ISL_566653, EPI_ISL_566654, EPI_ISL_566655, EPI_ISL_566656, EPI_ISL_566660, EPI_ISL_566663, EPI_ISL_566664, EPI_ISL_566668, EPI_ISL_566669, EPI_ISL_566670, EPI_ISL_566671, EPI_ISL_566678, EPI_ISL_566679, EPI_ISL_566684, EPI_ISL_566686, EPI_ISL_566689, EPI_ISL_566691, EPI_ISL_566695, EPI_ISL_566696, EPI_ISL_566702, EPI_ISL_566704, EPI_ISL_566706, EPI_ISL_566709, EPI_ISL_566712, EPI_ISL_566714, EPI_ISL_566715, EPI_ISL_566716, EPI_ISL_566717, EPI_ISL_566718, EPI_ISL_566719, EPI_ISL_566720, EPI_ISL_566722, EPI_ISL_566723, EPI_ISL_566724, EPI_ISL_566733, EPI_ISL_566736, EPI_ISL_566737, EPI_ISL_566738, EPI_ISL_566739, EPI_ISL_566743, EPI_ISL_566745, EPI_ISL_566747, EPI_ISL_566750, EPI_ISL_566753, EPI_ISL_566754, EPI_ISL_566756, EPI_ISL_566758, EPI_ISL_566760, EPI_ISL_566761, EPI_ISL_566762, EPI_ISL_566766, EPI_ISL_566767, EPI_ISL_566769, EPI_ISL_566770, EPI_ISL_566779, EPI_ISL_566783, EPI_ISL_566785, EPI_ISL_566786, EPI_ISL_566787, EPI_ISL_566789, EPI_ISL_566790, EPI_ISL_566792, EPI_ISL_566802, EPI_ISL_566804, EPI_ISL_566806, EPI_ISL_566808, EPI_ISL_566810, EPI_ISL_566812, EPI_ISL_566815, EPI_ISL_566816, EPI_ISL_566817, EPI_ISL_566819, EPI_ISL_566821, EPI_ISL_566822, EPI_ISL_566823, EPI_ISL_566824, EPI_ISL_566826, EPI_ISL_566832, EPI_ISL_566833, EPI_ISL_566834, EPI_ISL_566840, EPI_ISL_566841, EPI_ISL_566842, EPI_ISL_566844, EPI_ISL_566845, EPI_ISL_566848, EPI_ISL_566849, EPI_ISL_566851, EPI_ISL_566858, EPI_ISL_566859, EPI_ISL_566860, EPI_ISL_566861, EPI_ISL_566862, EPI_ISL_566863, EPI_ISL_566867, EPI_ISL_566868, EPI_ISL_566872, EPI_ISL_566873, EPI_ISL_566876, EPI_ISL_566881, EPI_ISL_566886, EPI_ISL_566888, EPI_ISL_566895, EPI_ISL_566897, EPI_ISL_566898, EPI_ISL_566899, EPI_ISL_566903, EPI_ISL_566904, EPI_ISL_566905, EPI_ISL_566910, EPI_ISL_566911, EPI_ISL_566914, EPI_ISL_566915, EPI_ISL_566916, EPI_ISL_566922, EPI_ISL_566925, EPI_ISL_566927, EPI_ISL_566932, EPI_ISL_566933, EPI_ISL_566937, EPI_ISL_566940, EPI_ISL_566945, EPI_ISL_566947, EPI_ISL_566950, EPI_ISL_566953, EPI_ISL_566957, EPI_ISL_566959, EPI_ISL_566964, EPI_ISL_566965, EPI_ISL_566969, EPI_ISL_566971, EPI_ISL_566972, EPI_ISL_566977, EPI_ISL_566992, EPI_ISL_566994, EPI_ISL_566996			
see above	Lighthouse Lab in Milton Keynes	Wellcome Sanger Institute for the COVID-19 Genomics UK (COG-UK) consortium	The Lighthouse Lab in Milton Keynes and Alex Alderton, Roberto Amato, Sonia Goncalves, Ewan Harrison, David K. Jackson, Ian Johnston, Dominic Kwiatkowski, Cordelia Langford, John Sillitoe on behalf of the Wellcome Sanger Institute COVID-19 Surveillance Team
EPI_ISL_566999, EPI_ISL_567000, EPI_ISL_567001, EPI_ISL_567002, EPI_ISL_567003, EPI_ISL_567004, EPI_ISL_567005, EPI_ISL_567006, EPI_ISL_567007, EPI_ISL_567008, EPI_ISL_567009, EPI_ISL_567010, EPI_ISL_567011, EPI_ISL_567012, EPI_ISL_567013, EPI_ISL_567014, EPI_ISL_567015, EPI_ISL_567016, EPI_ISL_567017, EPI_ISL_567018, EPI_ISL_567019, EPI_ISL_567020, EPI_ISL_567021, EPI_ISL_567022, EPI_ISL_567023			
see above	Lighthouse Lab in Glasgow	Wellcome Sanger Institute for the COVID-19 Genomics UK (COG-UK) consortium	Harper VanSteenhouse, Yumi Kasai, David Gray, Carol Clugston, Anna Dominiczak and Alex Alderton, Roberto Amato, Sonia Goncalves, Ewan Harrison, David K. Jackson, Ian Johnston, Dominic Kwiatkowski, Cordelia Langford, John Sillitoe on behalf of the Wellcome Sanger Institute COVID-19 Surveillance Team
EPI_ISL_567024	Lighthouse Lab in Glasgow	Wellcome Sanger Institute for the COVID-19 Genomics UK (COG-UK) Consortium	Harper VanSteenhouse, Yumi Kasai, David Gray, Carol Clugston, Anna Dominiczak and Alex Alderton, Roberto Amato, Sonia Goncalves, Ewan Harrison, David K. Jackson, Ian Johnston, Dominic Kwiatkowski, Cordelia Langford, John Sillitoe on behalf of the Wellcome Sanger Institute COVID-19 Surveillance Team
EPI_ISL_567025, EPI_ISL_567026, EPI_ISL_567027, EPI_ISL_567028, EPI_ISL_567029, EPI_ISL_567030, EPI_ISL_567031, EPI_ISL_567032, EPI_ISL_567033, EPI_ISL_567034, EPI_ISL_567035, EPI_ISL_567036, EPI_ISL_567037, EPI_ISL_567038, EPI_ISL_567039, EPI_ISL_567040, EPI_ISL_567041, EPI_ISL_567042, EPI_ISL_567043, EPI_ISL_567044, EPI_ISL_567045, EPI_ISL_567046, EPI_ISL_567047, EPI_ISL_567048, EPI_ISL_567049, EPI_ISL_567050, EPI_ISL_567051, EPI_ISL_567052, EPI_ISL_567053, EPI_ISL_567054, EPI_ISL_567055, EPI_ISL_567056, EPI_ISL_567057, EPI_ISL_567058			
see above	Lighthouse Lab in Glasgow	Wellcome Sanger Institute for the COVID-19 Genomics UK (COG-UK) consortium	Harper VanSteenhouse, Yumi Kasai, David Gray, Carol Clugston, Anna Dominiczak and Alex Alderton, Roberto Amato, Sonia Goncalves, Ewan Harrison, David K. Jackson, Ian Johnston, Dominic Kwiatkowski, Cordelia Langford, John Sillitoe on behalf of the Wellcome Sanger Institute COVID-19 Surveillance Team
EPI_ISL_567059	Lighthouse Lab in Glasgow	Wellcome Sanger Institute for the COVID-19 Genomics UK (COG-UK) Consortium	Harper VanSteenhouse, Yumi Kasai, David Gray, Carol Clugston, Anna Dominiczak and Alex Alderton, Roberto Amato, Sonia Goncalves, Ewan Harrison, David K. Jackson, Ian Johnston, Dominic Kwiatkowski, Cordelia Langford, John Sillitoe on behalf of the Wellcome Sanger Institute COVID-19 Surveillance Team
EPI_ISL_567060, EPI_ISL_567061, EPI_ISL_567062, EPI_ISL_567063, EPI_ISL_567064, EPI_ISL_567066, EPI_ISL_567067, EPI_ISL_567068, EPI_ISL_567069, EPI_ISL_567070, EPI_ISL_567071, EPI_ISL_567072, EPI_ISL_567073, EPI_ISL_567074, EPI_ISL_567075, EPI_ISL_567076, EPI_ISL_567077, EPI_ISL_567078, EPI_ISL_567079, EPI_ISL_567081, EPI_ISL_567082, EPI_ISL_567083, EPI_ISL_567084, EPI_ISL_567085, EPI_ISL_567086, EPI_ISL_567087, EPI_ISL_567088, EPI_ISL_567089, EPI_ISL_567090, EPI_ISL_567091, EPI_ISL_567092, EPI_ISL_567093, EPI_ISL_567094, EPI_ISL_567095, EPI_ISL_567096, EPI_ISL_567097, EPI_ISL_567098, EPI_ISL_567099, EPI_ISL_567100, EPI_ISL_567101, EPI_ISL_567102, EPI_ISL_567103, EPI_ISL_567104, EPI_ISL_567105, EPI_ISL_567106, EPI_ISL_567107, EPI_ISL_567108, EPI_ISL_567109, EPI_ISL_567110, EPI_ISL_567111, EPI_ISL_567112, EPI_ISL_567113, EPI_ISL_567114, EPI_ISL_567116, EPI_ISL_567117, EPI_ISL_567118, EPI_ISL_567119, EPI_ISL_567120, EPI_ISL_567121, EPI_ISL_567122, EPI_ISL_567123, EPI_ISL_567124, EPI_ISL_567125, EPI_ISL_567126, EPI_ISL_567127, EPI_ISL_567128, EPI_ISL_567129, EPI_ISL_567130, EPI_ISL_567131, EPI_ISL_567132, EPI_ISL_567133, EPI_ISL_567134, EPI_ISL_567135, EPI_ISL_567136, EPI_ISL_567137, EPI_ISL_567138, EPI_ISL_567139, EPI_ISL_567140, EPI_ISL_567141, EPI_ISL_567142, EPI_ISL_567143, EPI_ISL_567144, EPI_ISL_567145, EPI_ISL_567146, EPI_ISL_567147, EPI_ISL_567148, EPI_ISL_567149, EPI_ISL_567151, EPI_ISL_567152, EPI_ISL_567153, EPI_ISL_567154, EPI_ISL_567155, EPI_ISL_567156, EPI_ISL_567157, EPI_ISL_567158, EPI_ISL_567159, EPI_ISL_567160, EPI_ISL_567161, EPI_ISL_567162, EPI_ISL_567163, EPI_ISL_567164, EPI_ISL_567166, EPI_ISL_567167, EPI_ISL_567168, EPI_ISL_567169, EPI_ISL_567170, EPI_ISL_567171, EPI_ISL_567172, EPI_ISL_567173, EPI_ISL_567174, EPI_ISL_567175, EPI_ISL_567176, EPI_ISL_567177, EPI_ISL_567178, EPI_ISL_567179, EPI_ISL_567180, EPI_ISL_567181, EPI_ISL_567182, EPI_ISL_567183			
see above	Lighthouse Lab in Glasgow	Wellcome Sanger Institute for the COVID-19 Genomics UK (COG-UK) consortium	Harper VanSteenhouse, Yumi Kasai, David Gray, Carol Clugston, Anna Dominiczak and Alex Alderton, Roberto Amato, Sonia Goncalves, Ewan Harrison, David K. Jackson, Ian Johnston, Dominic Kwiatkowski, Cordelia Langford, John Sillitoe on behalf of the Wellcome Sanger Institute COVID-19 Surveillance Team
EPI_ISL_567184	Lighthouse Lab in Glasgow	Wellcome Sanger Institute for the COVID-19 Genomics UK (COG-UK) Consortium	Harper VanSteenhouse, Yumi Kasai, David Gray, Carol Clugston, Anna Dominiczak and Alex Alderton, Roberto Amato, Sonia Goncalves, Ewan Harrison, David K. Jackson, Ian Johnston, Dominic Kwiatkowski, Cordelia Langford, John Sillitoe on behalf of the Wellcome Sanger Institute COVID-19 Surveillance Team
EPI_ISL_567185, EPI_ISL_567186, EPI_ISL_567187, EPI_ISL_567188, EPI_ISL_567189, EPI_ISL_567190, EPI_ISL_567191, EPI_ISL_567192, EPI_ISL_567193, EPI_ISL_567194, EPI_ISL_567195, EPI_ISL_567196, EPI_ISL_567197, EPI_ISL_567198, EPI_ISL_567199, EPI_ISL_567200, EPI_ISL_567201			
see above	Lighthouse Lab in Glasgow	Wellcome Sanger Institute for the COVID-19 Genomics	Harper VanSteenhouse, Yumi Kasai, David Gray, Carol Clugston, Anna Dominiczak and Alex Alderton, Roberto Amato, Sonia Goncalves, Ewan Harrison, David K. Jackson, Ian Johnston, Dominic Kwiatkowski, Cordelia Langford, John Sillitoe on behalf of the Wellcome Sanger Institute COVID-19 Surveillance Team

		UK (COG-UK) consortium	
EPI_ISL_567202	Lighthouse Lab in Glasgow	Wellcome Sanger Institute for the COVID-19 Genomics UK (COG-UK) Consortium	Harper VanSteenhouse, Yumi Kasai, David Gray, Carol Clugston, Anna Dominiczak and Alex Alderton, Roberto Amato, Sonia Goncalves, Ewan Harrison, David K. Jackson, Ian Johnston, Dominic Kwiatkowski, Cordelia Langford, John Sillitoe on behalf of the Wellcome Sanger Institute COVID-19 Surveillance Team
EPI_ISL_567203, EPI_ISL_567204, EPI_ISL_567205, EPI_ISL_567206, EPI_ISL_567207, EPI_ISL_567208, EPI_ISL_567209, EPI_ISL_567210, EPI_ISL_567211, EPI_ISL_567212, EPI_ISL_567213, EPI_ISL_567214, EPI_ISL_567215, EPI_ISL_567216, EPI_ISL_567217, EPI_ISL_567218, EPI_ISL_567219, EPI_ISL_567220, EPI_ISL_567221, EPI_ISL_567222, EPI_ISL_567223, EPI_ISL_567224, EPI_ISL_567225, EPI_ISL_567226, EPI_ISL_567227, EPI_ISL_567228, EPI_ISL_567229, EPI_ISL_567230			
see above	Lighthouse Lab in Glasgow	Wellcome Sanger Institute for the COVID-19 Genomics UK (COG-UK) consortium	Harper VanSteenhouse, Yumi Kasai, David Gray, Carol Clugston, Anna Dominiczak and Alex Alderton, Roberto Amato, Sonia Goncalves, Ewan Harrison, David K. Jackson, Ian Johnston, Dominic Kwiatkowski, Cordelia Langford, John Sillitoe on behalf of the Wellcome Sanger Institute COVID-19 Surveillance Team
EPI_ISL_567231	Lighthouse Lab in Glasgow	Wellcome Sanger Institute for the COVID-19 Genomics UK (COG-UK) Consortium	Harper VanSteenhouse, Yumi Kasai, David Gray, Carol Clugston, Anna Dominiczak and Alex Alderton, Roberto Amato, Sonia Goncalves, Ewan Harrison, David K. Jackson, Ian Johnston, Dominic Kwiatkowski, Cordelia Langford, John Sillitoe on behalf of the Wellcome Sanger Institute COVID-19 Surveillance Team
EPI_ISL_567232, EPI_ISL_567233, EPI_ISL_567234, EPI_ISL_567235, EPI_ISL_567236, EPI_ISL_567237, EPI_ISL_567238, EPI_ISL_567239, EPI_ISL_567240, EPI_ISL_567241, EPI_ISL_567242, EPI_ISL_567244, EPI_ISL_567245, EPI_ISL_567246, EPI_ISL_567247, EPI_ISL_567249, EPI_ISL_567251, EPI_ISL_567252, EPI_ISL_567253, EPI_ISL_567255, EPI_ISL_567256, EPI_ISL_567257, EPI_ISL_567258, EPI_ISL_567259, EPI_ISL_567261, EPI_ISL_567262, EPI_ISL_567263, EPI_ISL_567264, EPI_ISL_567265, EPI_ISL_567266, EPI_ISL_567267, EPI_ISL_567268, EPI_ISL_567269, EPI_ISL_567270, EPI_ISL_567271, EPI_ISL_567272, EPI_ISL_567273, EPI_ISL_567275, EPI_ISL_567276, EPI_ISL_567277, EPI_ISL_567278, EPI_ISL_567279, EPI_ISL_567280, EPI_ISL_567281, EPI_ISL_567282, EPI_ISL_567283, EPI_ISL_567284, EPI_ISL_567285, EPI_ISL_567286, EPI_ISL_567287, EPI_ISL_567288, EPI_ISL_567290, EPI_ISL_567291, EPI_ISL_567292, EPI_ISL_567293, EPI_ISL_567295, EPI_ISL_567296, EPI_ISL_567297, EPI_ISL_567298, EPI_ISL_567299, EPI_ISL_567300, EPI_ISL_567301, EPI_ISL_567302, EPI_ISL_567303, EPI_ISL_567304, EPI_ISL_567305, EPI_ISL_567306, EPI_ISL_567307, EPI_ISL_567308, EPI_ISL_567309, EPI_ISL_567310, EPI_ISL_567311, EPI_ISL_567312, EPI_ISL_567313, EPI_ISL_567314, EPI_ISL_567315, EPI_ISL_567316, EPI_ISL_567317, EPI_ISL_567318, EPI_ISL_567319, EPI_ISL_567320, EPI_ISL_567321, EPI_ISL_567322, EPI_ISL_567323, EPI_ISL_567324, EPI_ISL_567325, EPI_ISL_567326, EPI_ISL_567327, EPI_ISL_567328, EPI_ISL_567329, EPI_ISL_567330, EPI_ISL_567331, EPI_ISL_567332, EPI_ISL_567333, EPI_ISL_567334, EPI_ISL_567335, EPI_ISL_567336, EPI_ISL_567337, EPI_ISL_567339, EPI_ISL_567340, EPI_ISL_567341, EPI_ISL_567342, EPI_ISL_567343, EPI_ISL_567344, EPI_ISL_567345, EPI_ISL_567346, EPI_ISL_567347, EPI_ISL_567348, EPI_ISL_567349, EPI_ISL_567350, EPI_ISL_567351, EPI_ISL_567352, EPI_ISL_567353, EPI_ISL_567354, EPI_ISL_567356, EPI_ISL_567357, EPI_ISL_567359, EPI_ISL_567360, EPI_ISL_567361, EPI_ISL_567362, EPI_ISL_567364, EPI_ISL_567365, EPI_ISL_567366, EPI_ISL_567367, EPI_ISL_567368, EPI_ISL_567369, EPI_ISL_567370, EPI_ISL_567371, EPI_ISL_567372, EPI_ISL_567373, EPI_ISL_567374, EPI_ISL_567376, EPI_ISL_567377, EPI_ISL_567378, EPI_ISL_567379, EPI_ISL_567380, EPI_ISL_567381, EPI_ISL_567383, EPI_ISL_567384, EPI_ISL_567385, EPI_ISL_567386, EPI_ISL_567387, EPI_ISL_567388, EPI_ISL_567389, EPI_ISL_567391, EPI_ISL_567392, EPI_ISL_567393, EPI_ISL_567394, EPI_ISL_567395, EPI_ISL_567397, EPI_ISL_567399, EPI_ISL_567400, EPI_ISL_567401, EPI_ISL_567402, EPI_ISL_567404, EPI_ISL_567405, EPI_ISL_567406, EPI_ISL_567407, EPI_ISL_567408, EPI_ISL_567409, EPI_ISL_567410, EPI_ISL_567411, EPI_ISL_567412, EPI_ISL_567413, EPI_ISL_567414, EPI_ISL_567415, EPI_ISL_567416, EPI_ISL_567417, EPI_ISL_567418, EPI_ISL_567419, EPI_ISL_567420, EPI_ISL_567421, EPI_ISL_567422, EPI_ISL_567423, EPI_ISL_567424, EPI_ISL_567425, EPI_ISL_567426, EPI_ISL_567428, EPI_ISL_567430, EPI_ISL_567431, EPI_ISL_567432, EPI_ISL_567433, EPI_ISL_567434, EPI_ISL_567436, EPI_ISL_567437, EPI_ISL_567438, EPI_ISL_567439, EPI_ISL_567440, EPI_ISL_567441, EPI_ISL_567442, EPI_ISL_567443, EPI_ISL_567444, EPI_ISL_567445, EPI_ISL_567447, EPI_ISL_567448, EPI_ISL_567449, EPI_ISL_567450, EPI_ISL_567451, EPI_ISL_567452, EPI_ISL_567454, EPI_ISL_567455, EPI_ISL_567456, EPI_ISL_567457, EPI_ISL_567458, EPI_ISL_567459, EPI_ISL_567460, EPI_ISL_567461, EPI_ISL_567462, EPI_ISL_567463, EPI_ISL_567465, EPI_ISL_567466, EPI_ISL_567467, EPI_ISL_567468, EPI_ISL_567469, EPI_ISL_567470, EPI_ISL_567471, EPI_ISL_567472, EPI_ISL_567474, EPI_ISL_567475, EPI_ISL_567476, EPI_ISL_567477, EPI_ISL_567478, EPI_ISL_567479, EPI_ISL_567480, EPI_ISL_567481, EPI_ISL_567483, EPI_ISL_567484, EPI_ISL_567485, EPI_ISL_567486, EPI_ISL_567487, EPI_ISL_567488, EPI_ISL_567490, EPI_ISL_567491, EPI_ISL_567492, EPI_ISL_567493, EPI_ISL_567494, EPI_ISL_567495, EPI_ISL_567496, EPI_ISL_567497, EPI_ISL_567498, EPI_ISL_567499, EPI_ISL_567500, EPI_ISL_567502, EPI_ISL_567503, EPI_ISL_567504, EPI_ISL_567505, EPI_ISL_567506, EPI_ISL_567507, EPI_ISL_567508, EPI_ISL_567509, EPI_ISL_567510, EPI_ISL_567511, EPI_ISL_567512, EPI_ISL_567513, EPI_ISL_567514, EPI_ISL_567515, EPI_ISL_567516, EPI_ISL_567517, EPI_ISL_567518, EPI_ISL_567519, EPI_ISL_567520, EPI_ISL_567521, EPI_ISL_567523, EPI_ISL_567525, EPI_ISL_567526, EPI_ISL_567527, EPI_ISL_567528, EPI_ISL_567529, EPI_ISL_567530, EPI_ISL_567531, EPI_ISL_567532			
see above	Lighthouse Lab in Glasgow	Wellcome Sanger Institute for the COVID-19 Genomics UK (COG-UK) consortium	Harper VanSteenhouse, Yumi Kasai, David Gray, Carol Clugston, Anna Dominiczak and Alex Alderton, Roberto Amato, Sonia Goncalves, Ewan Harrison, David K. Jackson, Ian Johnston, Dominic Kwiatkowski, Cordelia Langford, John Sillitoe on behalf of the Wellcome Sanger Institute COVID-19 Surveillance Team
EPI_ISL_567533, EPI_ISL_567534, EPI_ISL_567535, EPI_ISL_567536, EPI_ISL_567537, EPI_ISL_567538, EPI_ISL_567539, EPI_ISL_567540, EPI_ISL_567541, EPI_ISL_567542, EPI_ISL_567543, EPI_ISL_567544, EPI_ISL_567545, EPI_ISL_567546, EPI_ISL_567547, EPI_ISL_567548, EPI_ISL_567549, EPI_ISL_567550, EPI_ISL_567551, EPI_ISL_567552, EPI_ISL_567553, EPI_ISL_567554, EPI_ISL_567555, EPI_ISL_567556, EPI_ISL_567557, EPI_ISL_567558, EPI_ISL_567559, EPI_ISL_567560, EPI_ISL_567561, EPI_ISL_567562, EPI_ISL_567564, EPI_ISL_567565, EPI_ISL_567566, EPI_ISL_567567, EPI_ISL_567568, EPI_ISL_567569, EPI_ISL_567570, EPI_ISL_567571, EPI_ISL_567572, EPI_ISL_567573, EPI_ISL_567574, EPI_ISL_567575, EPI_ISL_567576, EPI_ISL_567577, EPI_ISL_567578, EPI_ISL_567579, EPI_ISL_567580, EPI_ISL_567581, EPI_ISL_567582, EPI_ISL_567583, EPI_ISL_567584, EPI_ISL_567585, EPI_ISL_567586, EPI_ISL_567587, EPI_ISL_567588, EPI_ISL_567589, EPI_ISL_567590, EPI_ISL_567591, EPI_ISL_567592, EPI_ISL_567593, EPI_ISL_567594, EPI_ISL_567595, EPI_ISL_567596, EPI_ISL_567597, EPI_ISL_567598, EPI_ISL_567599, EPI_ISL_567600, EPI_ISL_567601, EPI_ISL_567602, EPI_ISL_567603, EPI_ISL_567604, EPI_ISL_567605, EPI_ISL_567606, EPI_ISL_567607, EPI_ISL_567608, EPI_ISL_567609, EPI_ISL_567610, EPI_ISL_567611, EPI_ISL_567612, EPI_ISL_567613, EPI_ISL_567614, EPI_ISL_567615, EPI_ISL_567616, EPI_ISL_567617, EPI_ISL_567618, EPI_ISL_567619, EPI_ISL_567620, EPI_ISL_567621, EPI_ISL_567622, EPI_ISL_567623, EPI_ISL_567624, EPI_ISL_567625, EPI_ISL_567626, EPI_ISL_567627, EPI_ISL_567628, EPI_ISL_567629, EPI_ISL_567630, EPI_ISL_567631, EPI_ISL_567632, EPI_ISL_567633, EPI_ISL_567634, EPI_ISL_567635, EPI_ISL_567636, EPI_ISL_567637, EPI_ISL_567638, EPI_ISL_567639, EPI_ISL_567640, EPI_ISL_567641, EPI_ISL_567642, EPI_ISL_567643, EPI_ISL_567644, EPI_ISL_567645, EPI_ISL_567646, EPI_ISL_567647, EPI_ISL_567648, EPI_ISL_567649, EPI_ISL_567650, EPI_ISL_567651, EPI_ISL_567652, EPI_ISL_567653, EPI_ISL_567654, EPI_ISL_567655, EPI_ISL_567656, EPI_ISL_567657, EPI_ISL_567658, EPI_ISL_567659, EPI_ISL_567660, EPI_ISL_567661, EPI_ISL_567662, EPI_ISL_567663, EPI_ISL_567664, EPI_ISL_567665, EPI_ISL_567666, EPI_ISL_567667, EPI_ISL_567668, EPI_ISL_567669, EPI_ISL_567670, EPI_ISL_567671, EPI_ISL_567672, EPI_ISL_567673, EPI_ISL_567674, EPI_ISL_567675, EPI_ISL_567676, EPI_ISL_567677, EPI_ISL_567678, EPI_ISL_567679, EPI_ISL_567680, EPI_ISL_567681, EPI_ISL_567682, EPI_ISL_567683, EPI_ISL_567684, EPI_ISL_567685, EPI_ISL_567686, EPI_ISL_567687, EPI_ISL_567688, EPI_ISL_567689, EPI_ISL_567690, EPI_ISL_567691, EPI_ISL_567692, EPI_ISL_567693, EPI_ISL_567694, EPI_ISL_567695, EPI_ISL_567696, EPI_ISL_567697, EPI_ISL_567698, EPI_ISL_567699, EPI_ISL_567700, EPI_ISL_567701, EPI_ISL_567702, EPI_ISL_567703, EPI_ISL_567704, EPI_ISL_567705, EPI_ISL_567706, EPI_ISL_567707, EPI_ISL_567708, EPI_ISL_567709, EPI_ISL_567710, EPI_ISL_567711, EPI_ISL_567712, EPI_ISL_567713, EPI_ISL_567714, EPI_ISL_567715, EPI_ISL_567716, EPI_ISL_567717, EPI_ISL_567718, EPI_ISL_567719, EPI_ISL_567720, EPI_ISL_567721, EPI_ISL_567722, EPI_ISL_567723, EPI_ISL_567724, EPI_ISL_567725, EPI_ISL_567726, EPI_ISL_567727, EPI_ISL_567728, EPI_ISL_567729, EPI_ISL_567730, EPI_ISL_567731, EPI_ISL_567732, EPI_ISL_567733, EPI_ISL_567734, EPI_ISL_567735, EPI_ISL_567736, EPI_ISL_567737, EPI_ISL_567738, EPI_ISL_567739, EPI_ISL_567740, EPI_ISL_567741, EPI_ISL_567742, EPI_ISL_567743, EPI_ISL_567744, EPI_ISL_567745, EPI_ISL_567746, EPI_ISL_567747, EPI_ISL_567748, EPI_ISL_567749, EPI_ISL_567750, EPI_ISL_567751, EPI_ISL_567752, EPI_ISL_567753, EPI_ISL_567754, EPI_ISL_567755, EPI_ISL_567756, EPI_ISL_567757, EPI_ISL_567758, EPI_ISL_567759, EPI_ISL_567760, EPI_ISL_567761, EPI_ISL_567762, EPI_ISL_567763, EPI_ISL_567764, EPI_ISL_567765, EPI_ISL_567766, EPI_ISL_567767, EPI_ISL_567768, EPI_ISL_567769, EPI_ISL_567770, EPI_ISL_567771, EPI_ISL_567772, EPI_ISL_567773, EPI_ISL_567774, EPI_ISL_567775, EPI_ISL_567776, EPI_ISL_567777, EPI_ISL_567778, EPI_ISL_567779, EPI_ISL_567780, EPI_ISL_567781, EPI_ISL_567782, EPI_ISL_567783, EPI_ISL_567784, EPI_ISL_567785, EPI_ISL_567786, EPI_ISL_567787, EPI_ISL_567788, EPI_ISL_567789, EPI_ISL_567790, EPI_ISL_567791, EPI_ISL_567792, EPI_ISL_567793, EPI_ISL_567794, EPI_ISL_567795, EPI_ISL_567796, EPI_ISL_567797, EPI_ISL_567798, EPI_ISL_567799, EPI_ISL_567800, EPI_ISL_567801, EPI_ISL_567802			
see above	Lighthouse Lab in Alderley Park	Wellcome Sanger Institute for the COVID-19 Genomics UK (COG-UK) consortium	Jacquelyn Wynn, Mairead Hyland, The Lighthouse Lab in Alderley Park and Alex Alderton, Roberto Amato, Sonia Goncalves, Ewan Harrison, David K. Jackson, Ian Johnston, Dominic Kwiatkowski, Cordelia Langford, John Sillitoe on behalf of the Wellcome Sanger Institute COVID-19 Surveillance Team
EPI_ISL_567803	Lighthouse Lab in Alderley Park	Wellcome Sanger Institute for the COVID-19 Genomics UK (COG-UK) Consortium	Jacquelyn Wynn, Mairead Hyland, The Lighthouse Lab in Alderley Park and Alex Alderton, Roberto Amato, Sonia Goncalves, Ewan Harrison, David K. Jackson, Ian Johnston, Dominic Kwiatkowski, Cordelia Langford, John Sillitoe on behalf of the Wellcome Sanger Institute COVID-19 Surveillance Team
EPI_ISL_567804, EPI_ISL_567805, EPI_ISL_567806, EPI_ISL_567807, EPI_ISL_567808, EPI_ISL_567809, EPI_ISL_567810	Lighthouse Lab in Alderley Park	Wellcome Sanger Institute for the COVID-19 Genomics UK (COG-UK) consortium	Jacquelyn Wynn, Mairead Hyland, The Lighthouse Lab in Alderley Park and Alex Alderton, Roberto Amato, Sonia Goncalves, Ewan Harrison, David K. Jackson, Ian Johnston, Dominic Kwiatkowski, Cordelia Langford, John Sillitoe on behalf of the Wellcome Sanger Institute COVID-19 Surveillance Team
EPI_ISL_567811	Lighthouse Lab in Alderley Park	Wellcome Sanger Institute for the COVID-19 Genomics UK (COG-UK) Consortium	Jacquelyn Wynn, Mairead Hyland, The Lighthouse Lab in Alderley Park and Alex Alderton, Roberto Amato, Sonia Goncalves, Ewan Harrison, David K. Jackson, Ian Johnston, Dominic Kwiatkowski, Cordelia Langford, John Sillitoe on behalf of the Wellcome Sanger Institute COVID-19 Surveillance Team
EPI_ISL_567812, EPI_ISL_567813, EPI_ISL_567814, EPI_ISL_567815, EPI_ISL_567816, EPI_ISL_567817, EPI_ISL_567818, EPI_ISL_567819, EPI_ISL_567820, EPI_ISL_567821, EPI_ISL_567822, EPI_ISL_567823, EPI_ISL_567824, EPI_ISL_567825, EPI_ISL_567826, EPI_ISL_567827, EPI_ISL_567828, EPI_ISL_567829, EPI_ISL_567830, EPI_ISL_567831, EPI_ISL_567832, EPI_ISL_567833, EPI_ISL_567834, EPI_ISL_567835, EPI_ISL_567836, EPI_ISL_567837, EPI_ISL_567838, EPI_ISL_567839, EPI_ISL_567840, EPI_ISL_567841, EPI_ISL_567842, EPI_ISL_567843, EPI_ISL_567844, EPI_ISL_567845, EPI_ISL_567846, EPI_ISL_567847, EPI_ISL_567848, EPI_ISL_567849, EPI_ISL_567850, EPI_ISL_567851, EPI_ISL_567852, EPI_ISL_567853, EPI_ISL_567854, EPI_ISL_567855, EPI_ISL_567856, EPI_ISL_567857, EPI_ISL_567858, EPI_ISL_567859, EPI_ISL_567860, EPI_ISL_567861, EPI_ISL_567862, EPI_ISL_567863, EPI_ISL_567864, EPI_ISL_567865, EPI_ISL_567866, EPI_ISL_567867, EPI_ISL_567868, EPI_ISL_567869, EPI_ISL_567870, EPI_ISL_567871, EPI_ISL_567872, EPI_ISL_567873, EPI_ISL_567874, EPI_ISL_567875, EPI_ISL_567876, EPI_ISL_567877, EPI_ISL_567878, EPI_ISL_567879, EPI_ISL_567880, EPI_ISL_567881, EPI_ISL_567882, EPI_ISL_567883, EPI_ISL_567884, EPI_ISL_567885, EPI_ISL_567893, EPI_ISL_567898, EPI_ISL_567899, EPI_ISL_567900, EPI_ISL_567904, EPI_ISL_567905, EPI_ISL_567908, EPI_ISL_567912, EPI_ISL_567914, EPI_ISL_567916, EPI_ISL_567922, EPI_ISL_567923, EPI_ISL_567925, EPI_ISL_567926, EPI_ISL_567929, EPI_ISL_567930, EPI_ISL_567934, EPI_ISL_567935, EPI_ISL_567941, EPI_ISL_567947, EPI_ISL_567948, EPI_ISL_567952, EPI_ISL_567954, EPI_ISL_567955, EPI_ISL_567956, EPI_ISL_567958, EPI_ISL_567959, EPI_ISL_567962, EPI_ISL_567966, EPI_ISL_567971, EPI_ISL_567972, EPI_ISL_567973, EPI_ISL_567976, EPI_ISL_567978, EPI_ISL_567979, EPI_ISL_567980, EPI_ISL_567983, EPI_ISL_567984, EPI_ISL_567985, EPI_ISL_567992, EPI_ISL_567999, EPI_ISL_568000, EPI_ISL_568004, EPI_ISL_568011, EPI_ISL_568018, EPI_ISL_568020, EPI_ISL_568023, EPI_ISL_568024, EPI_ISL_568027, EPI_ISL_568029, EPI_ISL_568037, EPI_ISL_568040, EPI_ISL_568043, EPI_ISL_568048, EPI_ISL_568052, EPI_ISL_568054, EPI_ISL_568055, EPI_ISL_568057, EPI_ISL_568059, EPI_ISL_568060, EPI_ISL_568064, EPI_ISL_568072, EPI_ISL_568073, EPI_ISL_568074, EPI_ISL_568075, EPI_ISL_568077, EPI_ISL_568080, EPI_ISL_568083, EPI_ISL_568088, EPI_ISL_568089, EPI_ISL_568093, EPI_ISL_568094, EPI_ISL_568095, EPI_ISL_568097, EPI_ISL_568098, EPI_ISL_568102, EPI_ISL_568109, EPI_ISL_568124, EPI_ISL_568127, EPI_ISL_568131, EPI_ISL_568136, EPI_ISL_568139, EPI_ISL_568142, EPI_ISL_568143, EPI_ISL_568144, EPI_ISL_568145, EPI_ISL_568147, EPI_ISL_568148, EPI_ISL_568151, EPI_ISL_568153, EPI_ISL_568155, EPI_ISL_568159, EPI_ISL_568164, EPI_ISL_568176, EPI_ISL_568187, EPI_ISL_568191, EPI_ISL_568194, EPI_ISL_568195, EPI_ISL_568197, EPI_ISL_568202, EPI_ISL_568204, EPI_ISL_568210, EPI_ISL_568212, EPI_ISL_568213, EPI_ISL_568214, EPI_ISL_568215, EPI_ISL_568216, EPI_ISL_568217, EPI_ISL_568219, EPI_ISL_568220			
see above	Lighthouse Lab in Alderley Park	Wellcome Sanger Institute for the COVID-19 Genomics UK (COG-UK) consortium	Jacquelyn Wynn, Mairead Hyland, The Lighthouse Lab in Alderley Park and Alex Alderton, Roberto Amato, Sonia Goncalves, Ewan Harrison, David K. Jackson, Ian Johnston, Dominic Kwiatkowski, Cordelia Langford, John Sillitoe on behalf of the Wellcome Sanger Institute COVID-19 Surveillance Team
EPI_ISL_568224, EPI_ISL_568226, EPI_ISL_568227, EPI_ISL_568231, EPI_ISL_568235, EPI_ISL_568237, EPI_ISL_568238, EPI_ISL_568242, EPI_ISL_568246, EPI_ISL_568247, EPI_ISL_568249, EPI_ISL_568250, EPI_ISL_568255, EPI_ISL_568257, EPI_ISL_568262, EPI_ISL_568263, EPI_ISL_568265, EPI_ISL_568266, EPI_ISL_568268, EPI_ISL_568269, EPI_ISL_568270, EPI_ISL_568271, EPI_ISL_568273, EPI_ISL_568275, EPI_ISL_568277, EPI_ISL_568278, EPI_ISL_568279, EPI_ISL_568286, EPI_ISL_568287			
see above	Lighthouse Lab in Glasgow	Wellcome Sanger Institute for the COVID-19 Genomics	Harper VanSteenhouse, Yumi Kasai, David Gray, Carol Clugston, Anna Dominiczak and Alex Alderton, Roberto Amato, Sonia Goncalves, Ewan Harrison, David K. Jackson, Ian Johnston, Dominic Kwiatkowski, Cordelia Langford, John Sillitoe on behalf of the Wellcome Sanger Institute COVID-19 Surveillance Team

		UK (COG-UK) consortium	
EPI_ISL_568590, EPI_ISL_568591, EPI_ISL_568592, EPI_ISL_568593, EPI_ISL_568594, EPI_ISL_568595, EPI_ISL_568596, EPI_ISL_568658, EPI_ISL_568659, EPI_ISL_568660, EPI_ISL_568661, EPI_ISL_568662, EPI_ISL_568663, EPI_ISL_568664, EPI_ISL_568665, EPI_ISL_568666, EPI_ISL_568667, EPI_ISL_568668, EPI_ISL_568669, EPI_ISL_568670, EPI_ISL_568671, EPI_ISL_568672, EPI_ISL_568673, EPI_ISL_568674, EPI_ISL_568675, EPI_ISL_568676, EPI_ISL_568677, EPI_ISL_568678, EPI_ISL_568679			
see above	Florida Bureau of Public Health Laboratories	Florida Bureau of Public Health Laboratories	Sarah Schmedes, Jason Blanton
EPI_ISL_569220, EPI_ISL_569230, EPI_ISL_569231, EPI_ISL_569232, EPI_ISL_569233, EPI_ISL_569234, EPI_ISL_569235, EPI_ISL_569236, EPI_ISL_569237, EPI_ISL_569238, EPI_ISL_569239, EPI_ISL_569240, EPI_ISL_569241			
see above	MEPHI, Aix Marseille University	MEPHI, Aix Marseille University	Anthony LEVASSEUR
EPI_ISL_569858	Area of Virology, Serology and Virology Division (SAViD), New South Wales Health Pathology Randwick	Area of Virology, Serology and Virology Division (SAViD), New South Wales Health Pathology Randwick	Rawlinson, W., Deveson, I., Bull, R., Van Hal, S.
EPI_ISL_569859, EPI_ISL_569860, EPI_ISL_569861, EPI_ISL_569862	CSIR-Indian Institute of Chemical Biology, MEDICA Supercpecialty Hospital Kolkata	CSIR-Indian Institute of Chemical Biology, MEDICA Supercpecialty Hospital Kolkata	Sujay Krishna Maity, Priyanka Mallick, Debaleena Bhowmik, Abhishake Lahiri, Dr. Aviral Roy, Dr. Soumen Saha, Dr. Arpita Ghosh Mitra, Dr. Rajesh Pandey, Dr. Sandip Paul, Dr. Partha Chakrabarti, Dr. Saikat Chakrabarti
EPI_ISL_572320, EPI_ISL_572321, EPI_ISL_572322, EPI_ISL_572323	IZSM	IZSM	Maurizio Viscardi, Lorena Cardillo, Giovanna Fusco
EPI_ISL_572399, EPI_ISL_572401, EPI_ISL_572403, EPI_ISL_572406	Quadram Institute Bioscience	COVID-19 Genomics UK (COG-UK) Consortium	Dave J. Baker, Gemma L. Kay, Alp Aydin, Thanh Le-Viet, Steven Rudder, Ana P. Tedim, Anastasia Kolyva, Maria Diaz, Leonardo de Oliveira Martins, Nabil-Fareed Alikhan, Lizzie Meadows, Rachael Stanley, Ngozi Elumogo, Muhammed Yasir, Nicholas M. Thomson, Alexander J Trotter, Rachel Gilroy, Samuel Bloomfield, Claire Stuart, Andrew Bell, Reenesh Prakash, Samir Dervisevic, Alison E. Mather, John Wain, Mark Webber, Andrew J. Page, Justin O'Grady
EPI_ISL_572409, EPI_ISL_572410	Northumbria University / South Tees Hospitals NHS Foundation Trust / North Cumbria Integrated Care NHS Foundation Trust / North Tees and Hartlepool NHS Foundation Trust / Newcastle Hospitals NHS Foundation Trust	COVID-19 Genomics UK (COG-UK) Consortium	Darren L Smith,Andrew Nelson,Matthew Bashton,Greg R Young,Joshua Loh,John Allan,Mohammad A Tariq,Giles S Holt,Gary Black,Wen C Yew,Lynn Dover,Paul Baker,Steve Liggett,Sarah Essex,Jane Greenaway,Debra Padgett,Clive Graham,Garren Scott,Edward Barton,Emma Swindells,Brendan Payne,Jennifer Collins,Yusri Taha,Gary Eltringham
EPI_ISL_572415	West of Scotland Specialist Virology Centre, NHSGGC / MRC-University of Glasgow Centre for Virus Research	COVID-19 Genomics UK (COG-UK) Consortium	Ana da Silva Filipe, Natasha Johnson, Kathy Smollett, Daniel Mair, Stephen Carmichael, Lily Tong, Jenna Nichols, Elihu Aranday-Cortes, Kyriaki Nomikou; Sarah McDonald, Marc Niebel, Patawee Asamaphan; Richard Orton, Joseph Hughes, Sreenu Vattipally, David L Robertson; Alasdair MacLean, Rory Gunson; Kathy Li, Igor Starinskij, Natasha Jesudason, Rajiv Shah, James Shepherd, Antonia Ho, Emma Thomson
EPI_ISL_572423, EPI_ISL_572430	Quadram Institute Bioscience	COVID-19 Genomics UK (COG-UK) Consortium	Dave J. Baker, Gemma L. Kay, Alp Aydin, Thanh Le-Viet, Steven Rudder, Ana P. Tedim, Anastasia Kolyva, Maria Diaz, Leonardo de Oliveira Martins, Nabil-Fareed Alikhan, Lizzie Meadows, Rachael Stanley, Ngozi Elumogo, Muhammed Yasir, Nicholas M. Thomson, Alexander J Trotter, Rachel Gilroy, Samuel Bloomfield, Claire Stuart, Andrew Bell, Reenesh Prakash, Samir Dervisevic, Alison E. Mather, John Wain, Mark Webber, Andrew J. Page, Justin O'Grady
EPI_ISL_572432, EPI_ISL_572433	Virology Department, Sheffield Teaching Hospitals NHS Foundation Trust/Department of Infection, Immunity and Cardiovascular Disease, The Medical School, University of Sheffield	COVID-19 Genomics UK (COG-UK) Consortium	Thushan de Silva, Matthew Parker, Nikki Smith, Adri Angyal, Rebecca Brown, Luke Green, Rachel Tucker, Paul Parsons, Danielle Groves, Katie Johnson, Laura Carrilero, Alex Keeley, Dave Partridge, Matthew Wyles, Benjamin Lindsey, Mehmet Yavuz, Mohammad Raza, Cariad Evans
EPI_ISL_572436	Liverpool Clinical Laboratories	COVID-19 Genomics UK (COG-UK) Consortium	Sam Haldenby, Anita Lucaci, Steve Paterson, Julian Hiscox, Alistair Darby, M Almsaud, A Alrezaihi, Muhannad Alruwaili, Stuart D Armstrong, Jones Benjamin, Eleanor G Bentley, Anu Chawla, Jordan J Clark, Angela Cowell, Richard Eccles, Isabel García-Dorival, Matthew Gemmell, Alessandro Gerada, PKF Gilmore, Richard Gregory, Ximeng Han, Catherine Hartley, Margaret Hughes, Miren Iturriza-Gomara, James Johnson, L Luu, Jenifer Manson, Charlotte Nelson, Elaine O'Toole, Cassie Olateju, Rebekah Penrice-Randal , Lucille Rainbow, N.P Randle, Trevor Ian Robinson, Parul Sharma, Ghada T Shawli, James P Stewart, Neil Swainston, Ecaterina Vamos, Joanne Watts, Mark Whitehead
EPI_ISL_572437, EPI_ISL_572440, EPI_ISL_572441, EPI_ISL_572447, EPI_ISL_572448, EPI_ISL_572449, EPI_ISL_572453, EPI_ISL_572454	Quadram Institute Bioscience	COVID-19 Genomics UK (COG-UK) Consortium	Dave J. Baker, Gemma L. Kay, Alp Aydin, Thanh Le-Viet, Steven Rudder, Ana P. Tedim, Anastasia Kolyva, Maria Diaz, Leonardo de Oliveira Martins, Nabil-Fareed Alikhan, Lizzie Meadows, Rachael Stanley, Ngozi Elumogo, Muhammed Yasir, Nicholas M. Thomson, Alexander J Trotter, Rachel Gilroy, Samuel Bloomfield, Claire Stuart, Andrew Bell, Reenesh Prakash, Samir Dervisevic, Alison E. Mather, John Wain, Mark Webber, Andrew J. Page, Justin O'Grady
EPI_ISL_572455	Wales Specialist Virology Centre Sequencing lab: Pathogen Genomics Unit	COVID-19 Genomics UK (COG-UK) Consortium	Catherine Moore, Johnathan Evans, Laura Gifford, Malorie Perry, Simon Cottrell, Angela Marchbank, Alec Birchley, Alexander Adams, Amy Gaskin, Bree Gatica-Wilcox, Jason Coombes, Joel Southgate, Lauren Gilbert, Lee Graham, Nicole Pacchiarini, Sara Kumziene-Summerhayes, Sarah Taylor, Sophie Jones, Sara Rey, Matthew Bull, Joanne Watkins, Sally Corden, Tom Connor
EPI_ISL_572456, EPI_ISL_572459, EPI_ISL_572463	Quadram Institute Bioscience	COVID-19 Genomics UK (COG-UK) Consortium	Dave J. Baker, Gemma L. Kay, Alp Aydin, Thanh Le-Viet, Steven Rudder, Ana P. Tedim, Anastasia Kolyva, Maria Diaz, Leonardo de Oliveira Martins, Nabil-Fareed Alikhan, Lizzie Meadows, Rachael Stanley, Ngozi Elumogo, Muhammed Yasir, Nicholas M. Thomson, Alexander J Trotter, Rachel Gilroy, Samuel Bloomfield, Claire Stuart, Andrew Bell, Reenesh Prakash, Samir Dervisevic, Alison E. Mather, John Wain, Mark Webber, Andrew J. Page, Justin O'Grady
EPI_ISL_572464	Northumbria University / South Tees Hospitals NHS Foundation Trust / North Cumbria Integrated Care NHS Foundation Trust / North Tees and Hartlepool NHS Foundation Trust / Newcastle Hospitals NHS Foundation Trust	COVID-19 Genomics UK (COG-UK) Consortium	Darren L Smith,Andrew Nelson,Matthew Bashton,Greg R Young,Joshua Loh,John Allan,Mohammad A Tariq,Giles S Holt,Gary Black,Wen C Yew,Lynn Dover,Paul Baker,Steve Liggett,Sarah Essex,Jane Greenaway,Debra Padgett,Clive Graham,Garren Scott,Edward Barton,Emma Swindells,Brendan Payne,Jennifer Collins,Yusri Taha,Gary Eltringham
EPI_ISL_572466	Liverpool Clinical Laboratories	COVID-19 Genomics UK (COG-UK) Consortium	Sam Haldenby, Anita Lucaci, Steve Paterson, Julian Hiscox, Alistair Darby, M Almsaud, A Alrezaihi, Muhannad Alruwaili, Stuart D Armstrong, Jones Benjamin, Eleanor G Bentley, Anu Chawla, Jordan J Clark, Angela Cowell, Richard Eccles, Isabel García-Dorival, Matthew Gemmell, Alessandro Gerada, PKF Gilmore, Richard Gregory, Ximeng Han, Catherine Hartley, Margaret Hughes, Miren Iturriza-Gomara, James Johnson, L Luu, Jenifer Manson, Charlotte Nelson, Elaine O'Toole, Cassie Olateju, Rebekah Penrice-Randal , Lucille Rainbow, N.P Randle, Trevor Ian Robinson, Parul Sharma, Ghada T Shawli, James P Stewart, Neil Swainston, Ecaterina Vamos, Joanne Watts, Mark Whitehead
EPI_ISL_572468	Wales Specialist Virology Centre Sequencing lab:	COVID-19 Genomics UK (COG-UK) Consortium	Catherine Moore, Johnathan Evans, Laura Gifford, Malorie Perry, Simon Cottrell, Angela Marchbank, Alec Birchley, Alexander Adams, Amy Gaskin, Bree Gatica-Wilcox, Jason Coombes, Joel Southgate, Lauren Gilbert, Lee Graham, Nicole Pacchiarini, Sara Kumziene-Summerhayes, Sarah Taylor, Sophie Jones, Sara Rey, Matthew Bull, Joanne Watkins, Sally Corden, Tom Connor

	Pathogen Genomics Unit		
EPI_ISL_572470	West of Scotland Specialist Virology Centre, NHSGGC / MRC-University of Glasgow Centre for Virus Research	COVID-19 Genomics UK (COG-UK) Consortium	Ana da Silva Filipe, Natasha Johnson, Kathy Smollett, Daniel Mair, Stephen Carmichael, Lily Tong, Jenna Nichols, Elihu Aranday-Cortes, Kyriaki Nomikou; Sarah McDonald, Marc Niebel, Patawee Asamaphan; Richard Orton, Joseph Hughes, Sreenu Vattipally, David L Robertson; Alasdair MacLean, Rory Gunson; Kathy Li, Igor Starinskij, Natasha Jesudason, Rajiv Shah, James Shepherd, Antonia Ho, Emma Thomson
EPI_ISL_572471, EPI_ISL_572472	Department of Pathology, University of Cambridge	COVID-19 Genomics UK (COG-UK) Consortium	Aminu S. Jahun, Yasmin Chaudhry, Grant Hall, Iliana Georgana, Myra Hosmillo, Martin D. Curran, Malte Pinckert, Surendra Parmar, Ian Goodfellow
EPI_ISL_572477	Wales Specialist Virology Centre Sequencing lab: Pathogen Genomics Unit	COVID-19 Genomics UK (COG-UK) Consortium	Catherine Moore, Johnathan Evans, Laura Gifford, Malorie Perry, Simon Cottrell, Angela Marchbank, Alec Birchley, Alexander Adams, Amy Gaskin, Bree Gatica-Wilcox, Jason Coombes, Joel Southgate, Lauren Gilbert, Lee Graham, Nicole Pacchiarini, Sara Kumziene-Summerhayes, Sarah Taylor, Sophie Jones, Sara Rey, Matthew Bull, Joanne Watkins, Sally Corden, Tom Connor
EPI_ISL_572479	Liverpool Clinical Laboratories	COVID-19 Genomics UK (COG-UK) Consortium	Sam Haldenby, Anita Lucaci, Steve Paterson, Julian Hiscox, Alistair Darby, M Almsaud, A Alrezaihi, Muhannad Alruwaili, Stuart D Armstrong, Jones Benjamin, Eleanor G Bentley, Anu Chawla, Jordan J Clark, Angela Cowell, Richard Eccles, Isabel García-Dorival, Matthew Gemmell, Alessandro Gerada, PKF Gilmore, Richard Gregory, Ximeng Han, Catherine Hartley, Margaret Hughes, Miren Iturriza-Gomara, James Johnson, L Luu, Jenifer Manson, Charlotte Nelson, Elaine O'Toole, Cassie Olateju, Rebekah Penrice-Randal , Lucille Rainbow, N.P Randle, Trevor Ian Robinson, Parul Sharma, Ghada T Shawli, James P Stewart, Neil Swainston, Ecaterina Vamos, Joanne Watts, Mark Whitehead
EPI_ISL_572484	Department of Pathology, University of Cambridge	COVID-19 Genomics UK (COG-UK) Consortium	Aminu S. Jahun, Yasmin Chaudhry, Grant Hall, Iliana Georgana, Myra Hosmillo, Martin D. Curran, Malte Pinckert, Surendra Parmar, Ian Goodfellow
EPI_ISL_572485	Quadram Institute Bioscience	COVID-19 Genomics UK (COG-UK) Consortium	Dave J. Baker, Gemma L. Kay, Alp Aydin, Thanh Le-Viet, Steven Rudder, Ana P. Tedim, Anastasia Kolyva, Maria Diaz, Leonardo de Oliveira Martins, Nabil-Fareed Alikhan, Lizzie Meadows, Rachael Stanley, Ngozi Elumogo, Muhammed Yasir, Nicholas M. Thomson, Alexander J Trotter, Rachel Gilroy, Samuel Bloomfield, Claire Stuart, Andrew Bell, Reenesh Prakash, Samir Dervisevic, Alison E. Mather, John Wain, Mark Webber, Andrew J. Page, Justin O'Grady
EPI_ISL_572489	Northumbria University / South Tees Hospitals NHS Foundation Trust / North Cumbria Integrated Care NHS Foundation Trust / North Tees and Hartlepool NHS Foundation Trust / Newcastle Hospitals NHS Foundation Trust	COVID-19 Genomics UK (COG-UK) Consortium	Darren L Smith,Andrew Nelson,Matthew Bashton,Greg R Young,Joshua Loh,John Allan,Mohammad A Tariq,Giles S Holt,Gary Black,Wen C Yew,Lynn Dover,Paul Baker,Steve Liggett,Sarah Essex,Jane Greenaway,Debra Padgett,Clive Graham,Garren Scott,Edward Barton,Emma Swindells,Brendan Payne,Jennifer Collins,Yusri Taha,Gary Eltringham
EPI_ISL_572492, EPI_ISL_572493, EPI_ISL_572495, EPI_ISL_572497, EPI_ISL_572499, EPI_ISL_572503, EPI_ISL_572504, EPI_ISL_572505	Quadram Institute Bioscience	COVID-19 Genomics UK (COG-UK) Consortium	Dave J. Baker, Gemma L. Kay, Alp Aydin, Thanh Le-Viet, Steven Rudder, Ana P. Tedim, Anastasia Kolyva, Maria Diaz, Leonardo de Oliveira Martins, Nabil-Fareed Alikhan, Lizzie Meadows, Rachael Stanley, Ngozi Elumogo, Muhammed Yasir, Nicholas M. Thomson, Alexander J Trotter, Rachel Gilroy, Samuel Bloomfield, Claire Stuart, Andrew Bell, Reenesh Prakash, Samir Dervisevic, Alison E. Mather, John Wain, Mark Webber, Andrew J. Page, Justin O'Grady
EPI_ISL_572510	Lincolnshire Hospitals and DeepSeq Nottingham	COVID-19 Genomics UK (COG-UK) Consortium	Nichola Duckworth, Tim Sloan, Sarah Walsh, Jonathan Ball, Patrick McClure, Joeseph Chappell, Nadine Holmes, Matthew Carlisle, Christopher Moore, Fei Sang, Johnny Debebe, Victoria Wright, Matthew Loose
EPI_ISL_572516	Virology Department, Sheffield Teaching Hospitals NHS Foundation Trust/Department of Infection, Immunity and Cardiovascular Disease, The Medical School, University of Sheffield	COVID-19 Genomics UK (COG-UK) Consortium	Thushan de Silva, Matthew Parker, Nikki Smith, Adri Angyal, Rebecca Brown, Luke Green, Rachel Tucker, Paul Parsons, Danielle Groves, Katie Johnson, Laura Carrilero, Alex Keeley, Dave Partridge, Matthew Wyles, Benjamin Lindsey, Mehmet Yavuz, Mohammad Raza, Cariad Evans
EPI_ISL_572518	West of Scotland Specialist Virology Centre, NHSGGC / MRC-University of Glasgow Centre for Virus Research	COVID-19 Genomics UK (COG-UK) Consortium	Ana da Silva Filipe, Natasha Johnson, Kathy Smollett, Daniel Mair, Stephen Carmichael, Lily Tong, Jenna Nichols, Elihu Aranday-Cortes, Kyriaki Nomikou; Sarah McDonald, Marc Niebel, Patawee Asamaphan; Richard Orton, Joseph Hughes, Sreenu Vattipally, David L Robertson; Alasdair MacLean, Rory Gunson; Kathy Li, Igor Starinskij, Natasha Jesudason, Rajiv Shah, James Shepherd, Antonia Ho, Emma Thomson
EPI_ISL_572519	Department of Pathology, University of Cambridge	COVID-19 Genomics UK (COG-UK) Consortium	Aminu S. Jahun, Yasmin Chaudhry, Grant Hall, Iliana Georgana, Myra Hosmillo, Martin D. Curran, Malte Pinckert, Surendra Parmar, Ian Goodfellow
EPI_ISL_572520	Quadram Institute Bioscience	COVID-19 Genomics UK (COG-UK) Consortium	Dave J. Baker, Gemma L. Kay, Alp Aydin, Thanh Le-Viet, Steven Rudder, Ana P. Tedim, Anastasia Kolyva, Maria Diaz, Leonardo de Oliveira Martins, Nabil-Fareed Alikhan, Lizzie Meadows, Rachael Stanley, Ngozi Elumogo, Muhammed Yasir, Nicholas M. Thomson, Alexander J Trotter, Rachel Gilroy, Samuel Bloomfield, Claire Stuart, Andrew Bell, Reenesh Prakash, Samir Dervisevic, Alison E. Mather, John Wain, Mark Webber, Andrew J. Page, Justin O'Grady
EPI_ISL_572523	West of Scotland Specialist Virology Centre, NHSGGC / MRC-University of Glasgow Centre for Virus Research	COVID-19 Genomics UK (COG-UK) Consortium	Ana da Silva Filipe, Natasha Johnson, Kathy Smollett, Daniel Mair, Stephen Carmichael, Lily Tong, Jenna Nichols, Elihu Aranday-Cortes, Kyriaki Nomikou; Sarah McDonald, Marc Niebel, Patawee Asamaphan; Richard Orton, Joseph Hughes, Sreenu Vattipally, David L Robertson; Alasdair MacLean, Rory Gunson; Kathy Li, Igor Starinskij, Natasha Jesudason, Rajiv Shah, James Shepherd, Antonia Ho, Emma Thomson
EPI_ISL_572524	Department of Pathology, University of Cambridge	COVID-19 Genomics UK (COG-UK) Consortium	Aminu S. Jahun, Yasmin Chaudhry, Grant Hall, Iliana Georgana, Myra Hosmillo, Martin D. Curran, Malte Pinckert, Surendra Parmar, Ian Goodfellow
EPI_ISL_572526	Quadram Institute Bioscience	COVID-19 Genomics UK (COG-UK) Consortium	Dave J. Baker, Gemma L. Kay, Alp Aydin, Thanh Le-Viet, Steven Rudder, Ana P. Tedim, Anastasia Kolyva, Maria Diaz, Leonardo de Oliveira Martins, Nabil-Fareed Alikhan, Lizzie Meadows, Rachael Stanley, Ngozi Elumogo, Muhammed Yasir, Nicholas M. Thomson, Alexander J Trotter, Rachel Gilroy, Samuel Bloomfield, Claire Stuart, Andrew Bell, Reenesh Prakash, Samir Dervisevic, Alison E. Mather, John Wain, Mark Webber, Andrew J. Page, Justin O'Grady
EPI_ISL_572527	Lincolnshire Hospitals and DeepSeq Nottingham	COVID-19 Genomics UK (COG-UK) Consortium	Nichola Duckworth, Tim Sloan, Sarah Walsh, Jonathan Ball, Patrick McClure, Joeseph Chappell, Nadine Holmes, Matthew Carlisle, Christopher Moore, Fei Sang, Johnny Debebe, Victoria Wright, Matthew Loose
EPI_ISL_572528	West of Scotland Specialist Virology Centre, NHSGGC / MRC-University of Glasgow Centre for Virus Research	COVID-19 Genomics UK (COG-UK) Consortium	Ana da Silva Filipe, Natasha Johnson, Kathy Smollett, Daniel Mair, Stephen Carmichael, Lily Tong, Jenna Nichols, Elihu Aranday-Cortes, Kyriaki Nomikou; Sarah McDonald, Marc Niebel, Patawee Asamaphan; Richard Orton, Joseph Hughes, Sreenu Vattipally, David L Robertson; Alasdair MacLean, Rory Gunson; Kathy Li, Igor Starinskij, Natasha Jesudason, Rajiv Shah, James Shepherd, Antonia Ho, Emma Thomson
EPI_ISL_572533	Quadram Institute Bioscience	COVID-19 Genomics UK (COG-UK) Consortium	Dave J. Baker, Gemma L. Kay, Alp Aydin, Thanh Le-Viet, Steven Rudder, Ana P. Tedim, Anastasia Kolyva, Maria Diaz, Leonardo de Oliveira Martins, Nabil-Fareed Alikhan, Lizzie Meadows, Rachael Stanley, Ngozi Elumogo, Muhammed Yasir, Nicholas M. Thomson, Alexander J Trotter, Rachel Gilroy, Samuel Bloomfield, Claire Stuart, Andrew Bell, Reenesh Prakash, Samir Dervisevic, Alison E. Mather, John Wain, Mark Webber, Andrew J. Page, Justin O'Grady
EPI_ISL_572534	West of Scotland Specialist Virology Centre, NHSGGC / MRC-University of Glasgow Centre for Virus Research	COVID-19 Genomics UK (COG-UK) Consortium	Ana da Silva Filipe, Natasha Johnson, Kathy Smollett, Daniel Mair, Stephen Carmichael, Lily Tong, Jenna Nichols, Elihu Aranday-Cortes, Kyriaki Nomikou; Sarah McDonald, Marc Niebel, Patawee Asamaphan; Richard Orton, Joseph Hughes, Sreenu Vattipally, David L Robertson; Alasdair MacLean, Rory Gunson; Kathy Li, Igor Starinskij, Natasha Jesudason, Rajiv Shah, James Shepherd, Antonia Ho, Emma Thomson
EPI_ISL_572541	Department of Pathology, University of Cambridge	COVID-19 Genomics UK (COG-UK) Consortium	Aminu S. Jahun, Yasmin Chaudhry, Grant Hall, Iliana Georgana, Myra Hosmillo, Martin D. Curran, Malte Pinckert, Surendra Parmar, Ian Goodfellow
EPI_ISL_572546, EPI_ISL_572548,	Quadram Institute Bioscience	COVID-19 Genomics UK (COG-UK) Consortium	Dave J. Baker, Gemma L. Kay, Alp Aydin, Thanh Le-Viet, Steven Rudder, Ana P. Tedim, Anastasia Kolyva, Maria Diaz, Leonardo de Oliveira Martins, Nabil-Fareed Alikhan, Lizzie Meadows, Rachael Stanley, Ngozi Elumogo, Muhammed Yasir, Nicholas M. Thomson, Alexander J Trotter, Rachel Gilroy, Samuel Bloomfield, Claire Stuart, Andrew Bell, Reenesh Prakash, Samir Dervisevic, Alison E. Mather, John Wain, Mark Webber, Andrew J. Page, Justin O'Grady

EPI_ISL_572549, EPI_ISL_572550, EPI_ISL_572551, EPI_ISL_572553, EPI_ISL_572556			
EPI_ISL_572558, EPI_ISL_572564	Wales Specialist Virology Centre Sequencing lab: Pathogen Genomics Unit	COVID-19 Genomics UK (COG-UK) Consortium	Catherine Moore, Johnathan Evans, Laura Gifford, Malorie Perry, Simon Cottrell, Angela Marchbank, Alec Birchley, Alexander Adams, Amy Gaskin, Bree Gatica-Wilcox, Jason Coombes, Joel Southgate, Lauren Gilbert, Lee Graham, Nicole Pacchiarini, Sara Kumziene-Summerhayes, Sarah Taylor, Sophie Jones, Sara Rey, Matthew Bull, Joanne Watkins, Sally Corden, Tom Connor
EPI_ISL_572568	Northumbria University / South Tees Hospitals NHS Foundation Trust / North Cumbria Integrated Care NHS Foundation Trust / North Tees and Hartlepool NHS Foundation Trust / Newcastle Hospitals NHS Foundation Trust	COVID-19 Genomics UK (COG-UK) Consortium	Darren L Smith,Andrew Nelson,Matthew Bashton,Greg R Young,Joshua Loh,John Allan,Mohammad A Tariq,Giles S Holt,Gary Black,Wen C Yew,Lynn Dover,Paul Baker,Steve Liggett,Sarah Essex,Jane Greenaway,Debra Padgett,Clive Graham,Garren Scott,Edward Barton,Emma Swindells,Brendan Payne,Jennifer Collins,Yusri Taha,Gary Eltringham
EPI_ISL_572569	Quadram Institute Bioscience	COVID-19 Genomics UK (COG-UK) Consortium	Dave J. Baker, Gemma L. Kay, Alp Aydin, Thanh Le-Viet, Steven Rudder, Ana P. Tedim, Anastasia Kolyva, Maria Diaz, Leonardo de Oliveira Martins, Nabil-Fareed Alikhan, Lizzie Meadows, Rachael Stanley, Ngozi Elumogo, Muhammed Yasir, Nicholas M. Thomson, Alexander J Trotter, Rachel Gilroy, Samuel Bloomfield, Claire Stuart, Andrew Bell, Reenesh Prakash, Samir Dervisevic, Alison E. Mather, John Wain, Mark Webber, Andrew J. Page, Justin O'Grady
EPI_ISL_572570, EPI_ISL_572571	Northumbria University / South Tees Hospitals NHS Foundation Trust / North Cumbria Integrated Care NHS Foundation Trust / North Tees and Hartlepool NHS Foundation Trust / Newcastle Hospitals NHS Foundation Trust	COVID-19 Genomics UK (COG-UK) Consortium	Darren L Smith,Andrew Nelson,Matthew Bashton,Greg R Young,Joshua Loh,John Allan,Mohammad A Tariq,Giles S Holt,Gary Black,Wen C Yew,Lynn Dover,Paul Baker,Steve Liggett,Sarah Essex,Jane Greenaway,Debra Padgett,Clive Graham,Garren Scott,Edward Barton,Emma Swindells,Brendan Payne,Jennifer Collins,Yusri Taha,Gary Eltringham
EPI_ISL_572580	Wales Specialist Virology Centre Sequencing lab: Pathogen Genomics Unit	COVID-19 Genomics UK (COG-UK) Consortium	Catherine Moore, Johnathan Evans, Laura Gifford, Malorie Perry, Simon Cottrell, Angela Marchbank, Alec Birchley, Alexander Adams, Amy Gaskin, Bree Gatica-Wilcox, Jason Coombes, Joel Southgate, Lauren Gilbert, Lee Graham, Nicole Pacchiarini, Sara Kumziene-Summerhayes, Sarah Taylor, Sophie Jones, Sara Rey, Matthew Bull, Joanne Watkins, Sally Corden, Tom Connor
EPI_ISL_572581	Northumbria University / South Tees Hospitals NHS Foundation Trust / North Cumbria Integrated Care NHS Foundation Trust / North Tees and Hartlepool NHS Foundation Trust / Newcastle Hospitals NHS Foundation Trust	COVID-19 Genomics UK (COG-UK) Consortium	Darren L Smith,Andrew Nelson,Matthew Bashton,Greg R Young,Joshua Loh,John Allan,Mohammad A Tariq,Giles S Holt,Gary Black,Wen C Yew,Lynn Dover,Paul Baker,Steve Liggett,Sarah Essex,Jane Greenaway,Debra Padgett,Clive Graham,Garren Scott,Edward Barton,Emma Swindells,Brendan Payne,Jennifer Collins,Yusri Taha,Gary Eltringham
EPI_ISL_572583	Quadram Institute Bioscience	COVID-19 Genomics UK (COG-UK) Consortium	Dave J. Baker, Gemma L. Kay, Alp Aydin, Thanh Le-Viet, Steven Rudder, Ana P. Tedim, Anastasia Kolyva, Maria Diaz, Leonardo de Oliveira Martins, Nabil-Fareed Alikhan, Lizzie Meadows, Rachael Stanley, Ngozi Elumogo, Muhammed Yasir, Nicholas M. Thomson, Alexander J Trotter, Rachel Gilroy, Samuel Bloomfield, Claire Stuart, Andrew Bell, Reenesh Prakash, Samir Dervisevic, Alison E. Mather, John Wain, Mark Webber, Andrew J. Page, Justin O'Grady
EPI_ISL_572585	West of Scotland Specialist Virology Centre, NHSGGC / MRC-University of Glasgow Centre for Virus Research	COVID-19 Genomics UK (COG-UK) Consortium	Ana da Silva Filipe, Natasha Johnson, Kathy Smollett, Daniel Mair, Stephen Carmichael, Lily Tong, Jenna Nichols, Elihu Aranday-Cortes, Kyriaki Nomikou; Sarah McDonald, Marc Niebel, Patawee Asamaphan; Richard Orton, Joseph Hughes, Sreenu Vattipally, David L Robertson; Alasdair MacLean, Rory Gunson; Kathy Li, Igor Starinskij, Natasha Jesudason, Rajiv Shah, James Shepherd, Antonia Ho, Emma Thomson
EPI_ISL_572591	Quadram Institute Bioscience	COVID-19 Genomics UK (COG-UK) Consortium	Dave J. Baker, Gemma L. Kay, Alp Aydin, Thanh Le-Viet, Steven Rudder, Ana P. Tedim, Anastasia Kolyva, Maria Diaz, Leonardo de Oliveira Martins, Nabil-Fareed Alikhan, Lizzie Meadows, Rachael Stanley, Ngozi Elumogo, Muhammed Yasir, Nicholas M. Thomson, Alexander J Trotter, Rachel Gilroy, Samuel Bloomfield, Claire Stuart, Andrew Bell, Reenesh Prakash, Samir Dervisevic, Alison E. Mather, John Wain, Mark Webber, Andrew J. Page, Justin O'Grady
EPI_ISL_572592	Department of Pathology, University of Cambridge	COVID-19 Genomics UK (COG-UK) Consortium	Aminu S. Jahun, Yasmin Chaudhry, Grant Hall, Iliana Georgana, Myra Hosmillo, Martin D. Curran, Malte Pinckert, Surendra Parmar, Ian Goodfellow
EPI_ISL_572597	Northumbria University / South Tees Hospitals NHS Foundation Trust / North Cumbria Integrated Care NHS Foundation Trust / North Tees and Hartlepool NHS Foundation Trust / Newcastle Hospitals NHS Foundation Trust	COVID-19 Genomics UK (COG-UK) Consortium	Darren L Smith,Andrew Nelson,Matthew Bashton,Greg R Young,Joshua Loh,John Allan,Mohammad A Tariq,Giles S Holt,Gary Black,Wen C Yew,Lynn Dover,Paul Baker,Steve Liggett,Sarah Essex,Jane Greenaway,Debra Padgett,Clive Graham,Garren Scott,Edward Barton,Emma Swindells,Brendan Payne,Jennifer Collins,Yusri Taha,Gary Eltringham
EPI_ISL_572600, EPI_ISL_572602	Quadram Institute Bioscience	COVID-19 Genomics UK (COG-UK) Consortium	Dave J. Baker, Gemma L. Kay, Alp Aydin, Thanh Le-Viet, Steven Rudder, Ana P. Tedim, Anastasia Kolyva, Maria Diaz, Leonardo de Oliveira Martins, Nabil-Fareed Alikhan, Lizzie Meadows, Rachael Stanley, Ngozi Elumogo, Muhammed Yasir, Nicholas M. Thomson, Alexander J Trotter, Rachel Gilroy, Samuel Bloomfield, Claire Stuart, Andrew Bell, Reenesh Prakash, Samir Dervisevic, Alison E. Mather, John Wain, Mark Webber, Andrew J. Page, Justin O'Grady
EPI_ISL_572604	Liverpool Clinical Laboratories	COVID-19 Genomics UK (COG-UK) Consortium	Sam Haldenby, Anita Lucaci, Steve Paterson, Julian Hiscox, Alistair Darby, M Almsaud, A Alrezaihi, Muhannad Alruwaili, Stuart D Armstrong, Jones Benjamin, Eleanor G Bentley, Anu Chawla, Jordan J Clark, Angela Cowell, Richard Eccles, Isabel García-Dorival, Matthew Gemmell, Alessandro Gerada, PKF Gilmore, Richard Gregory, Ximeng Han, Catherine Hartley, Margaret Hughes, Miren Iturriza-Gomara, James Johnson, L Luu, Jenifer Manson, Charlotte Nelson, Elaine O'Toole, Cassie Olateju, Rebekah Penrice-Randal , Lucille Rainbow, N.P Randle, Trevor Ian Robinson, Parul Sharma, Ghada T Shawli, James P Stewart, Neil Swainston, Ecaterina Vamos, Joanne Watts, Mark Whitehead
EPI_ISL_572615	Quadram Institute Bioscience	COVID-19 Genomics UK (COG-UK) Consortium	Dave J. Baker, Gemma L. Kay, Alp Aydin, Thanh Le-Viet, Steven Rudder, Ana P. Tedim, Anastasia Kolyva, Maria Diaz, Leonardo de Oliveira Martins, Nabil-Fareed Alikhan, Lizzie Meadows, Rachael Stanley, Ngozi Elumogo, Muhammed Yasir, Nicholas M. Thomson, Alexander J Trotter, Rachel Gilroy, Samuel Bloomfield, Claire Stuart, Andrew Bell, Reenesh Prakash, Samir Dervisevic, Alison E. Mather, John Wain, Mark Webber, Andrew J. Page, Justin O'Grady
EPI_ISL_572616	Department of Pathology, University of Cambridge	COVID-19 Genomics UK (COG-UK) Consortium	Aminu S. Jahun, Yasmin Chaudhry, Grant Hall, Iliana Georgana, Myra Hosmillo, Martin D. Curran, Malte Pinckert, Surendra Parmar, Ian Goodfellow
EPI_ISL_572620, EPI_ISL_572623, EPI_ISL_572624, EPI_ISL_572625, EPI_ISL_572626, EPI_ISL_572633, EPI_ISL_572634, EPI_ISL_572635, EPI_ISL_572636, EPI_ISL_572637, EPI_ISL_572638, EPI_ISL_572639, EPI_ISL_572640, EPI_ISL_572641			
see above	West of Scotland Specialist Virology Centre, NHSGGC / MRC-University of Glasgow Centre for Virus Research	COVID-19 Genomics UK (COG-UK) Consortium	Ana da Silva Filipe, Natasha Johnson, Kathy Smollett, Daniel Mair, Stephen Carmichael, Lily Tong, Jenna Nichols, Elihu Aranday-Cortes, Kyriaki Nomikou; Sarah McDonald, Marc Niebel, Patawee Asamaphan; Richard Orton, Joseph Hughes, Sreenu Vattipally, David L Robertson; Alasdair MacLean, Rory Gunson; Kathy Li, Igor Starinskij, Natasha Jesudason, Rajiv Shah, James Shepherd, Antonia Ho, Emma Thomson
EPI_ISL_572642	Department of Pathology, University of Cambridge	COVID-19 Genomics UK (COG-UK) Consortium	Aminu S. Jahun, Yasmin Chaudhry, Grant Hall, Iliana Georgana, Myra Hosmillo, Martin D. Curran, Malte Pinckert, Surendra Parmar, Ian Goodfellow
EPI_ISL_572643	Wales Specialist Virology Centre Sequencing lab: Pathogen Genomics Unit	COVID-19 Genomics UK (COG-UK) Consortium	Catherine Moore, Johnathan Evans, Laura Gifford, Malorie Perry, Simon Cottrell, Angela Marchbank, Alec Birchley, Alexander Adams, Amy Gaskin, Bree Gatica-Wilcox, Jason Coombes, Joel Southgate, Lauren Gilbert, Lee Graham, Nicole Pacchiarini, Sara Kumziene-Summerhayes, Sarah Taylor, Sophie Jones, Sara Rey, Matthew Bull, Joanne Watkins, Sally Corden, Tom Connor

EPI_ISL_572646	Quadram Institute Bioscience	COVID-19 Genomics UK (COG-UK) Consortium	Dave J. Baker, Gemma L. Kay, Alp Aydin, Thanh Le-Viet, Steven Rudder, Ana P. Tedim, Anastasia Kolyva, Maria Diaz, Leonardo de Oliveira Martins, Nabil-Fareed Alikhan, Lizzie Meadows, Rachael Stanley, Ngozi Elumogo, Muhammed Yasir, Nicholas M. Thomson, Alexander J Trotter, Rachel Gilroy, Samuel Bloomfield, Claire Stuart, Andrew Bell, Reenesh Prakash, Samir Dervisevic, Alison E. Mather, John Wain, Mark Webber, Andrew J. Page, Justin O'Grady
EPI_ISL_572647	Wales Specialist Virology Centre Sequencing lab: Pathogen Genomics Unit	COVID-19 Genomics UK (COG-UK) Consortium	Catherine Moore, Johnathan Evans, Laura Gifford, Malorie Perry, Simon Cottrell, Angela Marchbank, Alec Birchley, Alexander Adams, Amy Gaskin, Bree Gatica-Wilcox, Jason Coombes, Joel Southgate, Lauren Gilbert, Lee Graham, Nicole Pacchiarini, Sara Kumziene-Summerhayes, Sarah Taylor, Sophie Jones, Sara Rey, Matthew Bull, Joanne Watkins, Sally Corden, Tom Connor
EPI_ISL_572649, EPI_ISL_572653, EPI_ISL_572655, EPI_ISL_572656, EPI_ISL_572658, EPI_ISL_572660, EPI_ISL_572665, EPI_ISL_572666, EPI_ISL_572670, EPI_ISL_572671, EPI_ISL_572673			
see above	Quadram Institute Bioscience	COVID-19 Genomics UK (COG-UK) Consortium	Dave J. Baker, Gemma L. Kay, Alp Aydin, Thanh Le-Viet, Steven Rudder, Ana P. Tedim, Anastasia Kolyva, Maria Diaz, Leonardo de Oliveira Martins, Nabil-Fareed Alikhan, Lizzie Meadows, Rachael Stanley, Ngozi Elumogo, Muhammed Yasir, Nicholas M. Thomson, Alexander J Trotter, Rachel Gilroy, Samuel Bloomfield, Claire Stuart, Andrew Bell, Reenesh Prakash, Samir Dervisevic, Alison E. Mather, John Wain, Mark Webber, Andrew J. Page, Justin O'Grady
EPI_ISL_572684, EPI_ISL_572685	University College London, Great Ormond Street Hospital for Children NHS Foundation Trust, Imperial College Healthcare NHS Trust	COVID-19 Genomics UK (COG-UK) Consortium	Sergi Castellano, Rachel Williams, Mark Kristiansen, Paola Resende Silva, Sunando Roy, Tony Brooks, Helena Tutill, Paola Niola, Patricia Dyal, Charlotte Williams, Leysa Forrest, Yasmin Panchbhaya, Jacqueline Findlay, Samuel Weeks, Julianne Brown, Kathryn Harris, Paul Randell, James Price, Alison Holmes, Judith Breuer
EPI_ISL_572686	West of Scotland Specialist Virology Centre, NHSGGC / MRC-University of Glasgow Centre for Virus Research	COVID-19 Genomics UK (COG-UK) Consortium	Ana da Silva Filipe, Natasha Johnson, Kathy Smollett, Daniel Mair, Stephen Carmichael, Lily Tong, Jenna Nichols, Elihu Aranday-Cortes, Kyriaki Nomikou; Sarah McDonald, Marc Niebel, Patawee Asamaphan; Richard Orton, Joseph Hughes, Sreenu Vattipally, David L Robertson; Alasdair MacLean, Rory Gunson; Kathy Li, Igor Starinskij, Natasha Jesudason, Rajiv Shah, James Shepherd, Antonia Ho, Emma Thomson
EPI_ISL_572703, EPI_ISL_572704	Northumbria University / South Tees Hospitals NHS Foundation Trust / North Cumbria Integrated Care NHS Foundation Trust / North Tees and Hartlepool NHS Foundation Trust / Newcastle Hospitals NHS Foundation Trust	COVID-19 Genomics UK (COG-UK) Consortium	Darren L Smith,Andrew Nelson,Matthew Bashton,Greg R Young,Joshua Loh,John Allan,Mohammad A Tariq,Giles S Holt,Gary Black,Wen C Yew,Lynn Dover,Paul Baker,Steve Liggett,Sarah Essex,Jane Greenaway,Debra Padgett,Clive Graham,Garren Scott,Edward Barton,Emma Swindells,Brendan Payne,Jennifer Collins,Yusri Taha,Gary Eltringham
EPI_ISL_572711	Quadram Institute Bioscience	COVID-19 Genomics UK (COG-UK) Consortium	Dave J. Baker, Gemma L. Kay, Alp Aydin, Thanh Le-Viet, Steven Rudder, Ana P. Tedim, Anastasia Kolyva, Maria Diaz, Leonardo de Oliveira Martins, Nabil-Fareed Alikhan, Lizzie Meadows, Rachael Stanley, Ngozi Elumogo, Muhammed Yasir, Nicholas M. Thomson, Alexander J Trotter, Rachel Gilroy, Samuel Bloomfield, Claire Stuart, Andrew Bell, Reenesh Prakash, Samir Dervisevic, Alison E. Mather, John Wain, Mark Webber, Andrew J. Page, Justin O'Grady
EPI_ISL_572713	Northumbria University / South Tees Hospitals NHS Foundation Trust / North Cumbria Integrated Care NHS Foundation Trust / North Tees and Hartlepool NHS Foundation Trust / Newcastle Hospitals NHS Foundation Trust	COVID-19 Genomics UK (COG-UK) Consortium	Darren L Smith,Andrew Nelson,Matthew Bashton,Greg R Young,Joshua Loh,John Allan,Mohammad A Tariq,Giles S Holt,Gary Black,Wen C Yew,Lynn Dover,Paul Baker,Steve Liggett,Sarah Essex,Jane Greenaway,Debra Padgett,Clive Graham,Garren Scott,Edward Barton,Emma Swindells,Brendan Payne,Jennifer Collins,Yusri Taha,Gary Eltringham
EPI_ISL_572714	Oxford Viromics, NDM, University of Oxford; Oxford University Hospitals; Basingstoke and North Hampshire Hospital	COVID-19 Genomics UK (COG-UK) Consortium	Tanya Golubchik, David Bonsall, George Macintyre, Amy Trebes, Mariateresa de Cesare, Catrin Moore, Alex Mobbs, Anita Justice, Robert Shaw, Monique Andersson, Timothy Peto, Emma Wise, Nathan Moore, Jessica Lynch, Nick Cortes, Matilde Mori, Stephen Kidd, David Buck, John Todd, Christophe Fraser
EPI_ISL_572716	Centre for Enzyme Innovation, University of Portsmouth / Translational Research Laboratory, Portsmouth Hospitals NHS Trust	COVID-19 Genomics UK (COG-UK) Consortium	Angela Beckett,Yann Bourgeois,Garry Scarlett,Sharon Glaysher,Scott Elliott,Kelly Bicknell,Robert Impey,Allyson Lloyd,Sarah Wyllie,Ethan Butcher,Anoop Chauhan,Samuel Robson
EPI_ISL_572717, EPI_ISL_572718	Quadram Institute Bioscience	COVID-19 Genomics UK (COG-UK) Consortium	Dave J. Baker, Gemma L. Kay, Alp Aydin, Thanh Le-Viet, Steven Rudder, Ana P. Tedim, Anastasia Kolyva, Maria Diaz, Leonardo de Oliveira Martins, Nabil-Fareed Alikhan, Lizzie Meadows, Rachael Stanley, Ngozi Elumogo, Muhammed Yasir, Nicholas M. Thomson, Alexander J Trotter, Rachel Gilroy, Samuel Bloomfield, Claire Stuart, Andrew Bell, Reenesh Prakash, Samir Dervisevic, Alison E. Mather, John Wain, Mark Webber, Andrew J. Page, Justin O'Grady
EPI_ISL_572721	Centre for Enzyme Innovation, University of Portsmouth / Translational Research Laboratory, Portsmouth Hospitals NHS Trust	COVID-19 Genomics UK (COG-UK) Consortium	Angela Beckett,Yann Bourgeois,Garry Scarlett,Sharon Glaysher,Scott Elliott,Kelly Bicknell,Robert Impey,Allyson Lloyd,Sarah Wyllie,Ethan Butcher,Anoop Chauhan,Samuel Robson
EPI_ISL_572725	University College London, Great Ormond Street Hospital for Children NHS Foundation Trust, Imperial College Healthcare NHS Trust	COVID-19 Genomics UK (COG-UK) Consortium	Sergi Castellano, Rachel Williams, Mark Kristiansen, Paola Resende Silva, Sunando Roy, Tony Brooks, Helena Tutill, Paola Niola, Patricia Dyal, Charlotte Williams, Leysa Forrest, Yasmin Panchbhaya, Jacqueline Findlay, Samuel Weeks, Julianne Brown, Kathryn Harris, Paul Randell, James Price, Alison Holmes, Judith Breuer
EPI_ISL_572730, EPI_ISL_572735	Quadram Institute Bioscience	COVID-19 Genomics UK (COG-UK) Consortium	Dave J. Baker, Gemma L. Kay, Alp Aydin, Thanh Le-Viet, Steven Rudder, Ana P. Tedim, Anastasia Kolyva, Maria Diaz, Leonardo de Oliveira Martins, Nabil-Fareed Alikhan, Lizzie Meadows, Rachael Stanley, Ngozi Elumogo, Muhammed Yasir, Nicholas M. Thomson, Alexander J Trotter, Rachel Gilroy, Samuel Bloomfield, Claire Stuart, Andrew Bell, Reenesh Prakash, Samir Dervisevic, Alison E. Mather, John Wain, Mark Webber, Andrew J. Page, Justin O'Grady
EPI_ISL_572737, EPI_ISL_572738	Liverpool Clinical Laboratories	COVID-19 Genomics UK (COG-UK) Consortium	Sam Haldenby, Anita Lucaci, Steve Paterson, Julian Hiscox, Alistair Darby, M Almsaud, A Alrezaihi, Muhannad Alruwaili, Stuart D Armstrong, Jones Benjamin, Eleanor G Bentley, Anu Chawla, Jordan J Clark, Angela Cowell, Richard Eccles, Isabel García-Dorival, Matthew Gemmell, Alessandro Gerada, PKF Gilmore, Richard Gregory, Ximeng Han, Catherine Hartley, Margaret Hughes, Miren Iturriza-Gomara, James Johnson, L Luu, Jenifer Manson, Charlotte Nelson, Elaine O'Toole, Cassie Olateju, Rebekah Penrice-Randal , Lucille Rainbow, N.P Randle, Trevor Ian Robinson, Parul Sharma, Ghada T Shawli, James P Stewart, Neil Swainston, Ecaterina Vamos, Joanne Watts, Mark Whitehead
EPI_ISL_572742, EPI_ISL_572745	Quadram Institute Bioscience	COVID-19 Genomics UK (COG-UK) Consortium	Dave J. Baker, Gemma L. Kay, Alp Aydin, Thanh Le-Viet, Steven Rudder, Ana P. Tedim, Anastasia Kolyva, Maria Diaz, Leonardo de Oliveira Martins, Nabil-Fareed Alikhan, Lizzie Meadows, Rachael Stanley, Ngozi Elumogo, Muhammed Yasir, Nicholas M. Thomson, Alexander J Trotter, Rachel Gilroy, Samuel Bloomfield, Claire Stuart, Andrew Bell, Reenesh Prakash, Samir Dervisevic, Alison E. Mather, John Wain, Mark Webber, Andrew J. Page, Justin O'Grady
EPI_ISL_572746	West of Scotland Specialist Virology Centre, NHSGGC / MRC-University of Glasgow Centre for Virus Research	COVID-19 Genomics UK (COG-UK) Consortium	Ana da Silva Filipe, Natasha Johnson, Kathy Smollett, Daniel Mair, Stephen Carmichael, Lily Tong, Jenna Nichols, Elihu Aranday-Cortes, Kyriaki Nomikou; Sarah McDonald, Marc Niebel, Patawee Asamaphan; Richard Orton, Joseph Hughes, Sreenu Vattipally, David L Robertson; Alasdair MacLean, Rory Gunson; Kathy Li, Igor Starinskij, Natasha Jesudason, Rajiv Shah, James Shepherd, Antonia Ho, Emma Thomson
EPI_ISL_572749, EPI_ISL_572755, EPI_ISL_572758, EPI_ISL_572759	Quadram Institute Bioscience	COVID-19 Genomics UK (COG-UK) Consortium	Dave J. Baker, Gemma L. Kay, Alp Aydin, Thanh Le-Viet, Steven Rudder, Ana P. Tedim, Anastasia Kolyva, Maria Diaz, Leonardo de Oliveira Martins, Nabil-Fareed Alikhan, Lizzie Meadows, Rachael Stanley, Ngozi Elumogo, Muhammed Yasir, Nicholas M. Thomson, Alexander J Trotter, Rachel Gilroy, Samuel Bloomfield, Claire Stuart, Andrew Bell, Reenesh Prakash, Samir Dervisevic, Alison E. Mather, John Wain, Mark Webber, Andrew J. Page, Justin O'Grady

EPI_ISL_572763, EPI_ISL_572765	West of Scotland Specialist Virology Centre, NHSGGC / MRC-University of Glasgow Centre for Virus Research	COVID-19 Genomics UK (COG-UK) Consortium	Ana da Silva Filipe, Natasha Johnson, Kathy Smollett, Daniel Mair, Stephen Carmichael, Lily Tong, Jenna Nichols, Elihu Aranday-Cortes, Kyriaki Nomikou; Sarah McDonald, Marc Niebel, Patawee Asamaphan; Richard Orton, Joseph Hughes, Sreenu Vattipally, David L Robertson; Alasdair MacLean, Rory Gunson; Kathy Li, Igor Starinskij, Natasha Jesudason, Rajiv Shah, James Shepherd, Antonia Ho, Emma Thomson
EPI_ISL_572767	Virology Department, Sheffield Teaching Hospitals NHS Foundation Trust/Department of Infection, Immunity and Cardiovascular Disease, The Medical School, University of Sheffield	COVID-19 Genomics UK (COG-UK) Consortium	Thushan de Silva, Matthew Parker, Nikki Smith, Adri Angyal, Rebecca Brown, Luke Green, Rachel Tucker, Paul Parsons, Danielle Groves, Katie Johnson, Laura Carrilero, Alex Keeley, Dave Partridge, Matthew Wyles, Benjamin Lindsey, Mehmet Yavuz, Mohammad Raza, Cariad Evans
EPI_ISL_572768, EPI_ISL_572775, EPI_ISL_572780	Quadram Institute Bioscience	COVID-19 Genomics UK (COG-UK) Consortium	Dave J. Baker, Gemma L. Kay, Alp Aydin, Thanh Le-Viet, Steven Rudder, Ana P. Tedim, Anastasia Kolyva, Maria Diaz, Leonardo de Oliveira Martins, Nabil-Fareed Alikhan, Lizzie Meadows, Rachael Stanley, Ngozi Elumogo, Muhammed Yasir, Nicholas M. Thomson, Alexander J Trotter, Rachel Gilroy, Samuel Bloomfield, Claire Stuart, Andrew Bell, Reenesh Prakash, Samir Dervisevic, Alison E. Mather, John Wain, Mark Webber, Andrew J. Page, Justin O'Grady
EPI_ISL_572781, EPI_ISL_572784	Wales Specialist Virology Centre Sequencing lab: Pathogen Genomics Unit	COVID-19 Genomics UK (COG-UK) Consortium	Catherine Moore, Johnathan Evans, Laura Gifford, Malorie Perry, Simon Cottrell, Angela Marchbank, Alec Birchley, Alexander Adams, Amy Gaskin, Bree Gatica-Wilcox, Jason Coombes, Joel Southgate, Lauren Gilbert, Lee Graham, Nicole Pacchiarini, Sara Kumziene-Summerhayes, Sarah Taylor, Sophie Jones, Sara Rey, Matthew Bull, Joanne Watkins, Sally Corden, Tom Connor
EPI_ISL_572786, EPI_ISL_572787	Quadram Institute Bioscience	COVID-19 Genomics UK (COG-UK) Consortium	Dave J. Baker, Gemma L. Kay, Alp Aydin, Thanh Le-Viet, Steven Rudder, Ana P. Tedim, Anastasia Kolyva, Maria Diaz, Leonardo de Oliveira Martins, Nabil-Fareed Alikhan, Lizzie Meadows, Rachael Stanley, Ngozi Elumogo, Muhammed Yasir, Nicholas M. Thomson, Alexander J Trotter, Rachel Gilroy, Samuel Bloomfield, Claire Stuart, Andrew Bell, Reenesh Prakash, Samir Dervisevic, Alison E. Mather, John Wain, Mark Webber, Andrew J. Page, Justin O'Grady
EPI_ISL_572788	West of Scotland Specialist Virology Centre, NHSGGC / MRC-University of Glasgow Centre for Virus Research	COVID-19 Genomics UK (COG-UK) Consortium	Ana da Silva Filipe, Natasha Johnson, Kathy Smollett, Daniel Mair, Stephen Carmichael, Lily Tong, Jenna Nichols, Elihu Aranday-Cortes, Kyriaki Nomikou; Sarah McDonald, Marc Niebel, Patawee Asamaphan; Richard Orton, Joseph Hughes, Sreenu Vattipally, David L Robertson; Alasdair MacLean, Rory Gunson; Kathy Li, Igor Starinskij, Natasha Jesudason, Rajiv Shah, James Shepherd, Antonia Ho, Emma Thomson
EPI_ISL_572789, EPI_ISL_572791	Quadram Institute Bioscience	COVID-19 Genomics UK (COG-UK) Consortium	Dave J. Baker, Gemma L. Kay, Alp Aydin, Thanh Le-Viet, Steven Rudder, Ana P. Tedim, Anastasia Kolyva, Maria Diaz, Leonardo de Oliveira Martins, Nabil-Fareed Alikhan, Lizzie Meadows, Rachael Stanley, Ngozi Elumogo, Muhammed Yasir, Nicholas M. Thomson, Alexander J Trotter, Rachel Gilroy, Samuel Bloomfield, Claire Stuart, Andrew Bell, Reenesh Prakash, Samir Dervisevic, Alison E. Mather, John Wain, Mark Webber, Andrew J. Page, Justin O'Grady
EPI_ISL_572792	Department of Pathology, University of Cambridge	COVID-19 Genomics UK (COG-UK) Consortium	Aminu S. Jahun, Yasmin Chaudhry, Grant Hall, Iliana Georgana, Myra Hosmillo, Martin D. Curran, Malte Pinckert, Surendra Parmar, Ian Goodfellow
EPI_ISL_572794	West of Scotland Specialist Virology Centre, NHSGGC / MRC-University of Glasgow Centre for Virus Research	COVID-19 Genomics UK (COG-UK) Consortium	Ana da Silva Filipe, Natasha Johnson, Kathy Smollett, Daniel Mair, Stephen Carmichael, Lily Tong, Jenna Nichols, Elihu Aranday-Cortes, Kyriaki Nomikou; Sarah McDonald, Marc Niebel, Patawee Asamaphan; Richard Orton, Joseph Hughes, Sreenu Vattipally, David L Robertson; Alasdair MacLean, Rory Gunson; Kathy Li, Igor Starinskij, Natasha Jesudason, Rajiv Shah, James Shepherd, Antonia Ho, Emma Thomson
EPI_ISL_572797	Quadram Institute Bioscience	COVID-19 Genomics UK (COG-UK) Consortium	Dave J. Baker, Gemma L. Kay, Alp Aydin, Thanh Le-Viet, Steven Rudder, Ana P. Tedim, Anastasia Kolyva, Maria Diaz, Leonardo de Oliveira Martins, Nabil-Fareed Alikhan, Lizzie Meadows, Rachael Stanley, Ngozi Elumogo, Muhammed Yasir, Nicholas M. Thomson, Alexander J Trotter, Rachel Gilroy, Samuel Bloomfield, Claire Stuart, Andrew Bell, Reenesh Prakash, Samir Dervisevic, Alison E. Mather, John Wain, Mark Webber, Andrew J. Page, Justin O'Grady
EPI_ISL_572798	Northumbria University / South Tees Hospitals NHS Foundation Trust / North Cumbria Integrated Care NHS Foundation Trust / North Tees and Hartlepool NHS Foundation Trust / Newcastle Hospitals NHS Foundation Trust	COVID-19 Genomics UK (COG-UK) Consortium	Darren L Smith,Andrew Nelson,Matthew Bashton,Greg R Young,Joshua Loh,John Allan,Mohammad A Tariq,Giles S Holt,Gary Black,Wen C Yew,Lynn Dover,Paul Baker,Steve Liggett,Sarah Essex,Jane Greenaway,Debra Padgett,Clive Graham,Garren Scott,Edward Barton,Emma Swindells,Brendan Payne,Jennifer Collins,Yusri Taha,Gary Eltringham
EPI_ISL_572800	University College London, Great Ormond Street Hospital for Children NHS Foundation Trust, Imperial College Healthcare NHS Trust	COVID-19 Genomics UK (COG-UK) Consortium	Sergi Castellano, Rachel Williams, Mark Kristiansen, Paola Resende Silva, Sunando Roy, Tony Brooks, Helena Tutill, Paola Niola, Patricia Dyal, Charlotte Williams, Leysa Forrest, Yasmin Panchbhaya, Jacqueline Findlay, Samuel Weeks, Julianne Brown, Kathryn Harris, Paul Randell, James Price, Alison Holmes, Judith Breuer
EPI_ISL_572804, EPI_ISL_572806	Quadram Institute Bioscience	COVID-19 Genomics UK (COG-UK) Consortium	Dave J. Baker, Gemma L. Kay, Alp Aydin, Thanh Le-Viet, Steven Rudder, Ana P. Tedim, Anastasia Kolyva, Maria Diaz, Leonardo de Oliveira Martins, Nabil-Fareed Alikhan, Lizzie Meadows, Rachael Stanley, Ngozi Elumogo, Muhammed Yasir, Nicholas M. Thomson, Alexander J Trotter, Rachel Gilroy, Samuel Bloomfield, Claire Stuart, Andrew Bell, Reenesh Prakash, Samir Dervisevic, Alison E. Mather, John Wain, Mark Webber, Andrew J. Page, Justin O'Grady
EPI_ISL_572808, EPI_ISL_572809	University College London, Great Ormond Street Hospital for Children NHS Foundation Trust, Imperial College Healthcare NHS Trust	COVID-19 Genomics UK (COG-UK) Consortium	Sergi Castellano, Rachel Williams, Mark Kristiansen, Paola Resende Silva, Sunando Roy, Tony Brooks, Helena Tutill, Paola Niola, Patricia Dyal, Charlotte Williams, Leysa Forrest, Yasmin Panchbhaya, Jacqueline Findlay, Samuel Weeks, Julianne Brown, Kathryn Harris, Paul Randell, James Price, Alison Holmes, Judith Breuer
EPI_ISL_572811, EPI_ISL_572812, EPI_ISL_572819	Quadram Institute Bioscience	COVID-19 Genomics UK (COG-UK) Consortium	Dave J. Baker, Gemma L. Kay, Alp Aydin, Thanh Le-Viet, Steven Rudder, Ana P. Tedim, Anastasia Kolyva, Maria Diaz, Leonardo de Oliveira Martins, Nabil-Fareed Alikhan, Lizzie Meadows, Rachael Stanley, Ngozi Elumogo, Muhammed Yasir, Nicholas M. Thomson, Alexander J Trotter, Rachel Gilroy, Samuel Bloomfield, Claire Stuart, Andrew Bell, Reenesh Prakash, Samir Dervisevic, Alison E. Mather, John Wain, Mark Webber, Andrew J. Page, Justin O'Grady
EPI_ISL_572822	Liverpool Clinical Laboratories	COVID-19 Genomics UK (COG-UK) Consortium	Sam Haldenby, Anita Lucaci, Steve Paterson, Julian Hiscox, Alistair Darby, M Almsaud, A Alrezaihi, Muhannad Alruwaili, Stuart D Armstrong, Jones Benjamin, Eleanor G Bentley, Anu Chawla, Jordan J Clark, Angela Cowell, Richard Eccles, Isabel García-Dorival, Matthew Gemmell, Alessandro Gerada, PKF Gilmore, Richard Gregory, Ximeng Han, Catherine Hartley, Margaret Hughes, Miren Iturriza-Gomara, James Johnson, L Luu, Jenifer Manson, Charlotte Nelson, Elaine O'Toole, Cassie Olateju, Rebekah Penrice-Randal , Lucille Rainbow, N.P Randle, Trevor Ian Robinson, Parul Sharma, Ghada T Shawli, James P Stewart, Neil Swainston, Ecaterina Vamos, Joanne Watts, Mark Whitehead
EPI_ISL_572823, EPI_ISL_572825, EPI_ISL_572826	Quadram Institute Bioscience	COVID-19 Genomics UK (COG-UK) Consortium	Dave J. Baker, Gemma L. Kay, Alp Aydin, Thanh Le-Viet, Steven Rudder, Ana P. Tedim, Anastasia Kolyva, Maria Diaz, Leonardo de Oliveira Martins, Nabil-Fareed Alikhan, Lizzie Meadows, Rachael Stanley, Ngozi Elumogo, Muhammed Yasir, Nicholas M. Thomson, Alexander J Trotter, Rachel Gilroy, Samuel Bloomfield, Claire Stuart, Andrew Bell, Reenesh Prakash, Samir Dervisevic, Alison E. Mather, John Wain, Mark Webber, Andrew J. Page, Justin O'Grady
EPI_ISL_572829	University College London, Great Ormond Street Hospital for Children NHS Foundation Trust, Imperial College Healthcare NHS Trust	COVID-19 Genomics UK (COG-UK) Consortium	Sergi Castellano, Rachel Williams, Mark Kristiansen, Paola Resende Silva, Sunando Roy, Tony Brooks, Helena Tutill, Paola Niola, Patricia Dyal, Charlotte Williams, Leysa Forrest, Yasmin Panchbhaya, Jacqueline Findlay, Samuel Weeks, Julianne Brown, Kathryn Harris, Paul Randell, James Price, Alison Holmes, Judith Breuer
EPI_ISL_572831	Quadram Institute Bioscience	COVID-19 Genomics UK (COG-UK) Consortium	Dave J. Baker, Gemma L. Kay, Alp Aydin, Thanh Le-Viet, Steven Rudder, Ana P. Tedim, Anastasia Kolyva, Maria Diaz, Leonardo de Oliveira Martins, Nabil-Fareed Alikhan, Lizzie Meadows, Rachael Stanley, Ngozi Elumogo, Muhammed Yasir, Nicholas M. Thomson, Alexander J Trotter, Rachel Gilroy, Samuel Bloomfield, Claire Stuart, Andrew Bell, Reenesh Prakash, Samir Dervisevic, Alison E. Mather, John Wain, Mark Webber, Andrew J. Page, Justin O'Grady
EPI_ISL_572832	West of Scotland Specialist Virology Centre, NHSGGC / MRC-University of Glasgow Centre for Virus Research	COVID-19 Genomics UK (COG-UK) Consortium	Ana da Silva Filipe, Natasha Johnson, Kathy Smollett, Daniel Mair, Stephen Carmichael, Lily Tong, Jenna Nichols, Elihu Aranday-Cortes, Kyriaki Nomikou; Sarah McDonald, Marc Niebel, Patawee Asamaphan; Richard Orton, Joseph Hughes, Sreenu Vattipally, David L Robertson; Alasdair MacLean, Rory Gunson; Kathy Li, Igor Starinskij, Natasha Jesudason, Rajiv Shah, James Shepherd, Antonia Ho, Emma Thomson

EPI_ISL_572835	Quadram Institute Bioscience	COVID-19 Genomics UK (COG-UK) Consortium	Dave J. Baker, Gemma L. Kay, Alp Aydin, Thanh Le-Viet, Steven Rudder, Ana P. Tedim, Anastasia Kolyva, Maria Diaz, Leonardo de Oliveira Martins, Nabil-Fareed Alikhan, Lizzie Meadows, Rachael Stanley, Ngozi Elumogo, Muhammed Yasir, Nicholas M. Thomson, Alexander J Trotter, Rachel Gilroy, Samuel Bloomfield, Claire Stuart, Andrew Bell, Reenesh Prakash, Samir Dervisevic, Alison E. Mather, John Wain, Mark Webber, Andrew J. Page, Justin O'Grady
EPI_ISL_572837	University College London, Great Ormond Street Hospital for Children NHS Foundation Trust, Imperial College Healthcare NHS Trust	COVID-19 Genomics UK (COG-UK) Consortium	Sergi Castellano, Rachel Williams, Mark Kristiansen, Paola Resende Silva, Sunando Roy, Tony Brooks, Helena Tutill, Paola Niola, Patricia Dyal, Charlotte Williams, Leysa Forrest, Yasmin Panchbhaya, Jacqueline Findlay, Samuel Weeks, Julianne Brown, Kathryn Harris, Paul Randell, James Price, Alison Holmes, Judith Breuer
EPI_ISL_572844, EPI_ISL_572847	Quadram Institute Bioscience	COVID-19 Genomics UK (COG-UK) Consortium	Dave J. Baker, Gemma L. Kay, Alp Aydin, Thanh Le-Viet, Steven Rudder, Ana P. Tedim, Anastasia Kolyva, Maria Diaz, Leonardo de Oliveira Martins, Nabil-Fareed Alikhan, Lizzie Meadows, Rachael Stanley, Ngozi Elumogo, Muhammed Yasir, Nicholas M. Thomson, Alexander J Trotter, Rachel Gilroy, Samuel Bloomfield, Claire Stuart, Andrew Bell, Reenesh Prakash, Samir Dervisevic, Alison E. Mather, John Wain, Mark Webber, Andrew J. Page, Justin O'Grady
EPI_ISL_572851, EPI_ISL_572852	Department of Pathology, University of Cambridge	COVID-19 Genomics UK (COG-UK) Consortium	Aminu S. Jahun, Yasmin Chaudhry, Grant Hall, Iliana Georgana, Myra Hosmillo, Martin D. Curran, Malte Pinckert, Surendra Parmar, Ian Goodfellow
EPI_ISL_572860, EPI_ISL_572861	Oxford Viromics, NDM, University of Oxford; Oxford University Hospitals; Basingstoke and North Hampshire Hospital	COVID-19 Genomics UK (COG-UK) Consortium	Tanya Golubchik, David Bonsall, George Macintyre, Amy Trebes, Mariateresa de Cesare, Catrin Moore, Alex Mobbs, Anita Justice, Robert Shaw, Monique Andersson, Timothy Peto, Emma Wise, Nathan Moore, Jessica Lynch, Nick Cortes, Matilde Mori, Stephen Kidd, David Buck, John Todd, Christophe Fraser
EPI_ISL_572862, EPI_ISL_572863, EPI_ISL_572864, EPI_ISL_572865, EPI_ISL_572866, EPI_ISL_572867, EPI_ISL_572868	Department of Pathology, University of Cambridge	COVID-19 Genomics UK (COG-UK) Consortium	Aminu S. Jahun, Yasmin Chaudhry, Grant Hall, Iliana Georgana, Myra Hosmillo, Martin D. Curran, Malte Pinckert, Surendra Parmar, Ian Goodfellow
EPI_ISL_572877, EPI_ISL_572878	Wales Specialist Virology Centre Sequencing lab: Pathogen Genomics Unit	COVID-19 Genomics UK (COG-UK) Consortium	Catherine Moore, Johnathan Evans, Laura Gifford, Malorie Perry, Simon Cottrell, Angela Marchbank, Alec Birchley, Alexander Adams, Amy Gaskin, Bree Gatica-Wilcox, Jason Coombes, Joel Southgate, Lauren Gilbert, Lee Graham, Nicole Pacchiarini, Sara Kumziene-Summerhayes, Sarah Taylor, Sophie Jones, Sara Rey, Matthew Bull, Joanne Watkins, Sally Corden, Tom Connor
EPI_ISL_572891	Oxford Viromics, NDM, University of Oxford; Oxford University Hospitals; Basingstoke and North Hampshire Hospital	COVID-19 Genomics UK (COG-UK) Consortium	Tanya Golubchik, David Bonsall, George Macintyre, Amy Trebes, Mariateresa de Cesare, Catrin Moore, Alex Mobbs, Anita Justice, Robert Shaw, Monique Andersson, Timothy Peto, Emma Wise, Nathan Moore, Jessica Lynch, Nick Cortes, Matilde Mori, Stephen Kidd, David Buck, John Todd, Christophe Fraser
EPI_ISL_572898, EPI_ISL_572899, EPI_ISL_572900, EPI_ISL_572901	Quadram Institute Bioscience	COVID-19 Genomics UK (COG-UK) Consortium	Dave J. Baker, Gemma L. Kay, Alp Aydin, Thanh Le-Viet, Steven Rudder, Ana P. Tedim, Anastasia Kolyva, Maria Diaz, Leonardo de Oliveira Martins, Nabil-Fareed Alikhan, Lizzie Meadows, Rachael Stanley, Ngozi Elumogo, Muhammed Yasir, Nicholas M. Thomson, Alexander J Trotter, Rachel Gilroy, Samuel Bloomfield, Claire Stuart, Andrew Bell, Reenesh Prakash, Samir Dervisevic, Alison E. Mather, John Wain, Mark Webber, Andrew J. Page, Justin O'Grady
EPI_ISL_572903	Oxford Viromics, NDM, University of Oxford; Oxford University Hospitals; Basingstoke and North Hampshire Hospital	COVID-19 Genomics UK (COG-UK) Consortium	Tanya Golubchik, David Bonsall, George Macintyre, Amy Trebes, Mariateresa de Cesare, Catrin Moore, Alex Mobbs, Anita Justice, Robert Shaw, Monique Andersson, Timothy Peto, Emma Wise, Nathan Moore, Jessica Lynch, Nick Cortes, Matilde Mori, Stephen Kidd, David Buck, John Todd, Christophe Fraser
EPI_ISL_572905, EPI_ISL_572906, EPI_ISL_572907, EPI_ISL_572908	Liverpool Clinical Laboratories	COVID-19 Genomics UK (COG-UK) Consortium	Sam Haldenby, Anita Lucaci, Steve Paterson, Julian Hiscox, Alistair Darby, M Almsaud, A Alrezaihi, Muhannad Alruwaili, Stuart D Armstrong, Jones Benjamin, Eleanor G Bentley, Anu Chawla, Jordan J Clark, Angela Cowell, Richard Eccles, Isabel García-Dorival, Matthew Gemmell, Alessandro Gerada, PKF Gilmore, Richard Gregory, Ximeng Han, Catherine Hartley, Margaret Hughes, Miren Iturriza-Gomara, James Johnson, L Luu, Jenifer Manson, Charlotte Nelson, Elaine O'Toole, Cassie Olateju, Rebekah Penrice-Randal , Lucille Rainbow, N.P Randle, Trevor Ian Robinson, Parul Sharma, Ghada T Shawli, James P Stewart, Neil Swainston, Ecaterina Vamos, Joanne Watts, Mark Whitehead
EPI_ISL_572928	Virology Department, Sheffield Teaching Hospitals NHS Foundation Trust/Department of Infection, Immunity and Cardiovascular Disease, The Medical School, University of Sheffield	COVID-19 Genomics UK (COG-UK) Consortium	Thushan de Silva, Matthew Parker, Nikki Smith, Adri Angyal, Rebecca Brown, Luke Green, Rachel Tucker, Paul Parsons, Danielle Groves, Katie Johnson, Laura Carrilero, Alex Keeley, Dave Partridge, Matthew Wyles, Benjamin Lindsey, Mehmet Yavuz, Mohammad Raza, Cariad Evans
EPI_ISL_572931	Wales Specialist Virology Centre Sequencing lab: Pathogen Genomics Unit	COVID-19 Genomics UK (COG-UK) Consortium	Catherine Moore, Johnathan Evans, Laura Gifford, Malorie Perry, Simon Cottrell, Angela Marchbank, Alec Birchley, Alexander Adams, Amy Gaskin, Bree Gatica-Wilcox, Jason Coombes, Joel Southgate, Lauren Gilbert, Lee Graham, Nicole Pacchiarini, Sara Kumziene-Summerhayes, Sarah Taylor, Sophie Jones, Sara Rey, Matthew Bull, Joanne Watkins, Sally Corden, Tom Connor
EPI_ISL_572937	Oxford Viromics, NDM, University of Oxford; Oxford University Hospitals; Basingstoke and North Hampshire Hospital	COVID-19 Genomics UK (COG-UK) Consortium	Tanya Golubchik, David Bonsall, George Macintyre, Amy Trebes, Mariateresa de Cesare, Catrin Moore, Alex Mobbs, Anita Justice, Robert Shaw, Monique Andersson, Timothy Peto, Emma Wise, Nathan Moore, Jessica Lynch, Nick Cortes, Matilde Mori, Stephen Kidd, David Buck, John Todd, Christophe Fraser
EPI_ISL_572939, EPI_ISL_572940, EPI_ISL_572941	Quadram Institute Bioscience	COVID-19 Genomics UK (COG-UK) Consortium	Dave J. Baker, Gemma L. Kay, Alp Aydin, Thanh Le-Viet, Steven Rudder, Ana P. Tedim, Anastasia Kolyva, Maria Diaz, Leonardo de Oliveira Martins, Nabil-Fareed Alikhan, Lizzie Meadows, Rachael Stanley, Ngozi Elumogo, Muhammed Yasir, Nicholas M. Thomson, Alexander J Trotter, Rachel Gilroy, Samuel Bloomfield, Claire Stuart, Andrew Bell, Reenesh Prakash, Samir Dervisevic, Alison E. Mather, John Wain, Mark Webber, Andrew J. Page, Justin O'Grady
EPI_ISL_572942	Northumbria University / South Tees Hospitals NHS Foundation Trust / North Cumbria Integrated Care NHS Foundation Trust / North Tees and Hartlepool NHS Foundation Trust / Newcastle Hospitals NHS Foundation Trust	COVID-19 Genomics UK (COG-UK) Consortium	Darren L Smith,Andrew Nelson,Matthew Bashton,Greg R Young,Joshua Loh,John Allan,Mohammad A Tariq,Giles S Holt,Gary Black,Wen C Yew,Lynn Dover,Paul Baker,Steve Liggett,Sarah Essex,Jane Greenaway,Debra Padgett,Clive Graham,Garren Scott,Edward Barton,Emma Swindells,Brendan Payne,Jennifer Collins,Yusri Taha,Gary Eltringham
EPI_ISL_572943, EPI_ISL_572944	Lincolnshire Hospitals and DeepSeq Nottingham	COVID-19 Genomics UK (COG-UK) Consortium	Nichola Duckworth, Tim Sloan, Sarah Walsh, Jonathan Ball, Patrick McClure, Joeseph Chappell, Nadine Holmes, Matthew Carlisle, Christopher Moore, Fei Sang, Johnny Debebe, Victoria Wright, Matthew Loose
EPI_ISL_572951	Wales Specialist Virology Centre Sequencing lab: Pathogen Genomics Unit	COVID-19 Genomics UK (COG-UK) Consortium	Catherine Moore, Johnathan Evans, Laura Gifford, Malorie Perry, Simon Cottrell, Angela Marchbank, Alec Birchley, Alexander Adams, Amy Gaskin, Bree Gatica-Wilcox, Jason Coombes, Joel Southgate, Lauren Gilbert, Lee Graham, Nicole Pacchiarini, Sara Kumziene-Summerhayes, Sarah Taylor, Sophie Jones, Sara Rey, Matthew Bull, Joanne Watkins, Sally Corden, Tom Connor

EPI_ISL_572954	Quadram Institute Bioscience	COVID-19 Genomics UK (COG-UK) Consortium	Dave J. Baker, Gemma L. Kay, Alp Aydin, Thanh Le-Viet, Steven Rudder, Ana P. Tedim, Anastasia Kolyva, Maria Diaz, Leonardo de Oliveira Martins, Nabil-Fareed Alikhan, Lizzie Meadows, Rachael Stanley, Ngozi Elumogo, Muhammed Yasir, Nicholas M. Thomson, Alexander J Trotter, Rachel Gilroy, Samuel Bloomfield, Claire Stuart, Andrew Bell, Reenesh Prakash, Samir Dervisevic, Alison E. Mather, John Wain, Mark Webber, Andrew J. Page, Justin O'Grady
EPI_ISL_572956	Virology Department, Sheffield Teaching Hospitals NHS Foundation Trust/Department of Infection, Immunity and Cardiovascular Disease, The Medical School, University of Sheffield	COVID-19 Genomics UK (COG-UK) Consortium	Thushan de Silva, Matthew Parker, Nikki Smith, Adri Angyal, Rebecca Brown, Luke Green, Rachel Tucker, Paul Parsons, Danielle Groves, Katie Johnson, Laura Carrilero, Alex Keeley, Dave Partridge, Matthew Wyles, Benjamin Lindsey, Mehmet Yavuz, Mohammad Raza, Cariad Evans
EPI_ISL_573081, EPI_ISL_573082, EPI_ISL_573083	Oxford Viromics, NDM, University of Oxford; Oxford University Hospitals; Basingstoke and North Hampshire Hospital	COVID-19 Genomics UK (COG-UK) Consortium	Tanya Golubchik, David Bonsall, George Macintyre, Amy Trebes, Mariateresa de Cesare, Catrin Moore, Alex Mobbs, Anita Justice, Robert Shaw, Monique Andersson, Timothy Peto, Emma Wise, Nathan Moore, Jessica Lynch, Nick Cortes, Matilde Mori, Stephen Kidd, David Buck, John Todd, Christophe Fraser
EPI_ISL_573085, EPI_ISL_573086, EPI_ISL_573087, EPI_ISL_573088, EPI_ISL_573089, EPI_ISL_573090, EPI_ISL_573091, EPI_ISL_573092, EPI_ISL_573093, EPI_ISL_573094, EPI_ISL_573095, EPI_ISL_573096, EPI_ISL_573097, EPI_ISL_573098, EPI_ISL_573099, EPI_ISL_573100, EPI_ISL_573101, EPI_ISL_573102, EPI_ISL_573103, EPI_ISL_573104, EPI_ISL_573105, EPI_ISL_573106, EPI_ISL_573107, EPI_ISL_573108, EPI_ISL_573109, EPI_ISL_573110, EPI_ISL_573111, EPI_ISL_573112, EPI_ISL_573113, EPI_ISL_573114, EPI_ISL_573115, EPI_ISL_573116, EPI_ISL_573117, EPI_ISL_573118, EPI_ISL_573119, EPI_ISL_573120, EPI_ISL_573121, EPI_ISL_573122, EPI_ISL_573123, EPI_ISL_573124, EPI_ISL_573125, EPI_ISL_573126, EPI_ISL_573127, EPI_ISL_573128, EPI_ISL_573129, EPI_ISL_573130, EPI_ISL_573131			
see above	Quadram Institute Bioscience	COVID-19 Genomics UK (COG-UK) Consortium	Dave J. Baker, Gemma L. Kay, Alp Aydin, Thanh Le-Viet, Steven Rudder, Ana P. Tedim, Anastasia Kolyva, Maria Diaz, Leonardo de Oliveira Martins, Nabil-Fareed Alikhan, Lizzie Meadows, Rachael Stanley, Ngozi Elumogo, Muhammed Yasir, Nicholas M. Thomson, Alexander J Trotter, Rachel Gilroy, Samuel Bloomfield, Claire Stuart, Andrew Bell, Reenesh Prakash, Samir Dervisevic, Alison E. Mather, John Wain, Mark Webber, Andrew J. Page, Justin O'Grady
EPI_ISL_573138, EPI_ISL_573139, EPI_ISL_573140, EPI_ISL_573141, EPI_ISL_573142, EPI_ISL_573143, EPI_ISL_573144, EPI_ISL_573146, EPI_ISL_573148, EPI_ISL_573149, EPI_ISL_573153			
see above	Department of Pathology, University of Cambridge	COVID-19 Genomics UK (COG-UK) Consortium	Aminu S. Jahun, Yasmin Chaudhry, Grant Hall, Iliana Georgana, Myra Hosmillo, Martin D. Curran, Malte Pinckert, Surendra Parmar, Ian Goodfellow
EPI_ISL_573195, EPI_ISL_573198, EPI_ISL_573199, EPI_ISL_573200, EPI_ISL_573201, EPI_ISL_573203, EPI_ISL_573206, EPI_ISL_573207, EPI_ISL_573208, EPI_ISL_573210, EPI_ISL_573211, EPI_ISL_573212, EPI_ISL_573213, EPI_ISL_573214, EPI_ISL_573215, EPI_ISL_573216, EPI_ISL_573217, EPI_ISL_573218, EPI_ISL_573219, EPI_ISL_573221, EPI_ISL_573222, EPI_ISL_573223, EPI_ISL_573224, EPI_ISL_573225, EPI_ISL_573227, EPI_ISL_573231, EPI_ISL_573232			
see above	Oxford Viromics, NDM, University of Oxford; Oxford University Hospitals; Basingstoke and North Hampshire Hospital	COVID-19 Genomics UK (COG-UK) Consortium	Tanya Golubchik, David Bonsall, George Macintyre, Amy Trebes, Mariateresa de Cesare, Catrin Moore, Alex Mobbs, Anita Justice, Robert Shaw, Monique Andersson, Timothy Peto, Emma Wise, Nathan Moore, Jessica Lynch, Nick Cortes, Matilde Mori, Stephen Kidd, David Buck, John Todd, Christophe Fraser
EPI_ISL_573255, EPI_ISL_573257, EPI_ISL_573261, EPI_ISL_573262, EPI_ISL_573263, EPI_ISL_573264	Centre for Enzyme Innovation, University of Portsmouth / Translational Research Laboratory, Portsmouth Hospitals NHS Trust	COVID-19 Genomics UK (COG-UK) Consortium	Angela Beckett,Yann Bourgeois,Garry Scarlett,Sharon Glaysher,Scott Elliott,Kelly Bicknell,Robert Impey,Allyson Lloyd,Sarah Wyllie,Ethan Butcher,Anoop Chauhan,Samuel Robson
EPI_ISL_573277, EPI_ISL_573278, EPI_ISL_573293, EPI_ISL_573298, EPI_ISL_573305, EPI_ISL_573382, EPI_ISL_573383, EPI_ISL_573384, EPI_ISL_573385, EPI_ISL_573386, EPI_ISL_573387, EPI_ISL_573388, EPI_ISL_573389, EPI_ISL_573390, EPI_ISL_573391, EPI_ISL_573392, EPI_ISL_573393, EPI_ISL_573394, EPI_ISL_573395, EPI_ISL_573396, EPI_ISL_573397, EPI_ISL_573398, EPI_ISL_573399, EPI_ISL_573400, EPI_ISL_573401, EPI_ISL_573402, EPI_ISL_573403, EPI_ISL_573404, EPI_ISL_573405, EPI_ISL_573406, EPI_ISL_573407			
see above	Northumbria University / South Tees Hospitals NHS Foundation Trust / North Cumbria Integrated Care NHS Foundation Trust / North Tees and Hartlepool NHS Foundation Trust / Newcastle Hospitals NHS Foundation Trust	COVID-19 Genomics UK (COG-UK) Consortium	Darren L Smith,Andrew Nelson,Matthew Bashton,Greg R Young,Joshua Loh,John Allan,Mohammad A Tariq,Giles S Holt,Gary Black,Wen C Yew,Lynn Dover,Paul Baker,Steve Liggett,Sarah Essex,Jane Greenaway,Debra Padgett,Clive Graham,Garren Scott,Edward Barton,Emma Swindells,Brendan Payne,Jennifer Collins,Yusri Taha,Gary Eltringham
EPI_ISL_573416	Quadram Institute Bioscience	COVID-19 Genomics UK (COG-UK) Consortium	Dave J. Baker, Gemma L. Kay, Alp Aydin, Thanh Le-Viet, Steven Rudder, Ana P. Tedim, Anastasia Kolyva, Maria Diaz, Leonardo de Oliveira Martins, Nabil-Fareed Alikhan, Lizzie Meadows, Rachael Stanley, Ngozi Elumogo, Muhammed Yasir, Nicholas M. Thomson, Alexander J Trotter, Rachel Gilroy, Samuel Bloomfield, Claire Stuart, Andrew Bell, Reenesh Prakash, Samir Dervisevic, Alison E. Mather, John Wain, Mark Webber, Andrew J. Page, Justin O'Grady
EPI_ISL_573437, EPI_ISL_573438, EPI_ISL_573439, EPI_ISL_573440, EPI_ISL_573441, EPI_ISL_573442, EPI_ISL_573443, EPI_ISL_573444, EPI_ISL_573445, EPI_ISL_573446	Lincolnshire Hospitals and DeepSeq Nottingham	COVID-19 Genomics UK (COG-UK) Consortium	Nichola Duckworth, Tim Sloan, Sarah Walsh, Jonathan Ball, Patrick McClure, Joeseph Chappell, Nadine Holmes, Matthew Carlisle, Christopher Moore, Fei Sang, Johnny Debebe, Victoria Wright, Matthew Loose
EPI_ISL_573652, EPI_ISL_573653, EPI_ISL_573654, EPI_ISL_573655, EPI_ISL_573656, EPI_ISL_573657, EPI_ISL_573658, EPI_ISL_573659, EPI_ISL_573660, EPI_ISL_573661, EPI_ISL_573662, EPI_ISL_573663, EPI_ISL_573664, EPI_ISL_573665, EPI_ISL_573666, EPI_ISL_573667, EPI_ISL_573668, EPI_ISL_573669, EPI_ISL_573670, EPI_ISL_573671, EPI_ISL_573672, EPI_ISL_573673, EPI_ISL_573674, EPI_ISL_573675, EPI_ISL_573676, EPI_ISL_573677, EPI_ISL_573678, EPI_ISL_573679, EPI_ISL_573680, EPI_ISL_573681, EPI_ISL_573682, EPI_ISL_573683, EPI_ISL_573684, EPI_ISL_573685			
see above	University College London, Great Ormond Street Hospital for Children NHS Foundation Trust, Imperial College Healthcare NHS Trust	COVID-19 Genomics UK (COG-UK) Consortium	Sergi Castellano, Rachel Williams, Mark Kristiansen, Paola Resende Silva, Sunando Roy, Tony Brooks, Helena Tutill, Paola Niola, Patricia Dyal, Charlotte Williams, Leysa Forrest, Yasmin Panchbhaya, Jacqueline Findlay, Samuel Weeks, Julianne Brown, Kathryn Harris, Paul Randell, James Price, Alison Holmes, Judith Breuer
EPI_ISL_573692, EPI_ISL_573694, EPI_ISL_573695, EPI_ISL_573699, EPI_ISL_573700, EPI_ISL_573709, EPI_ISL_573713, EPI_ISL_573719, EPI_ISL_573726, EPI_ISL_573727, EPI_ISL_573735, EPI_ISL_573741, EPI_ISL_573750, EPI_ISL_573753, EPI_ISL_573754			
see above	Virology Department, Sheffield Teaching Hospitals NHS Foundation Trust/Department of Infection, Immunity and Cardiovascular Disease, The Medical School, University of Sheffield	COVID-19 Genomics UK (COG-UK) Consortium	Thushan de Silva, Matthew Parker, Nikki Smith, Adri Angyal, Rebecca Brown, Luke Green, Rachel Tucker, Paul Parsons, Danielle Groves, Katie Johnson, Laura Carrilero, Alex Keeley, Dave Partridge, Matthew Wyles, Benjamin Lindsey, Mehmet Yavuz, Mohammad Raza, Cariad Evans
EPI_ISL_573764	University College London, Great Ormond Street	COVID-19 Genomics UK (COG-UK) Consortium	Sergi Castellano, Rachel Williams, Mark Kristiansen, Paola Resende Silva, Sunando Roy, Tony Brooks, Helena Tutill, Paola Niola, Patricia Dyal, Charlotte Williams, Leysa Forrest, Yasmin Panchbhaya, Jacqueline Findlay, Samuel Weeks, Julianne Brown, Kathryn Harris, Paul Randell, James Price, Alison Holmes, Judith Breuer

	Hospital for Children NHS Foundation Trust, Imperial College Healthcare NHS Trust		
EPI_ISL_573770, EPI_ISL_573771, EPI_ISL_573780, EPI_ISL_573781, EPI_ISL_573782, EPI_ISL_573783, EPI_ISL_573784, EPI_ISL_573785, EPI_ISL_573786, EPI_ISL_573787, EPI_ISL_573788, EPI_ISL_573789, EPI_ISL_573790, EPI_ISL_573791, EPI_ISL_573792, EPI_ISL_573793			
see above	West of Scotland Specialist Virology Centre, NHSGGC / MRC-University of Glasgow Centre for Virus Research	COVID-19 Genomics UK (COG-UK) Consortium	Ana da Silva Filipe, Natasha Johnson, Kathy Smollett, Daniel Mair, Stephen Carmichael, Lily Tong, Jenna Nichols, Elihu Aranday-Cortes, Kyriaki Nomikou; Sarah McDonald, Marc Niebel, Patawee Asamaphan; Richard Orton, Joseph Hughes, Sreenu Vattipally, David L Robertson; Alasdair MacLean, Rory Gunson; Kathy Li, Igor Starinskij, Natasha Jesudason, Rajiv Shah, James Shepherd, Antonia Ho, Emma Thomson
EPI_ISL_573794	Virology Department, Royal Infirmary of Edinburgh, NHS Lothian / School of Biological Sciences, University of Edinburgh / Institute of Genetics and Molecular Medicine, University of Edinburgh	COVID-19 Genomics UK (COG-UK) Consortium	McHugh M, Dewar R, Rooke S, Gallagher M, Balcaza C, O'Toole Á, Scher E, Hill V, McCrone JT, Colquhoun R, Yu X, Jackson B, Rambaut A, Williams TC, Templeton K
EPI_ISL_573856, EPI_ISL_573857, EPI_ISL_573858, EPI_ISL_573859, EPI_ISL_573860, EPI_ISL_573861, EPI_ISL_573862, EPI_ISL_573863, EPI_ISL_573864, EPI_ISL_573865	University College London, Great Ormond Street Hospital for Children NHS Foundation Trust, Imperial College Healthcare NHS Trust	COVID-19 Genomics UK (COG-UK) Consortium	Sergi Castellano, Rachel Williams, Mark Kristiansen, Paola Resende Silva, Sunando Roy, Tony Brooks, Helena Tutill, Paola Niola, Patricia Dyal, Charlotte Williams, Leysa Forrest, Yasmin Panchbhaya, Jacqueline Findlay, Samuel Weeks, Julianne Brown, Kathryn Harris, Paul Randell, James Price, Alison Holmes, Judith Breuer
EPI_ISL_573869	Oxford Viromics, NDM, University of Oxford; Oxford University Hospitals; Basingstoke and North Hampshire Hospital	COVID-19 Genomics UK (COG-UK) Consortium	Tanya Golubchik, David Bonsall, George Macintyre, Amy Trebes, Mariateresa de Cesare, Catrin Moore, Alex Mobbs, Anita Justice, Robert Shaw, Monique Andersson, Timothy Peto, Emma Wise, Nathan Moore, Jessica Lynch, Nick Cortes, Matilde Mori, Stephen Kidd, David Buck, John Todd, Christophe Fraser
EPI_ISL_573870	Centre for Enzyme Innovation, University of Portsmouth / Translational Research Laboratory, Portsmouth Hospitals NHS Trust	COVID-19 Genomics UK (COG-UK) Consortium	Angela Beckett,Yann Bourgeois,Garry Scarlett,Sharon Glaysher,Scott Elliott,Kelly Bicknell,Robert Impey,Allyson Lloyd,Sarah Wyllie,Ethan Butcher,Anoop Chauhan,Samuel Robson
EPI_ISL_573871	Northumbria University / South Tees Hospitals NHS Foundation Trust / North Cumbria Integrated Care NHS Foundation Trust / North Tees and Hartlepool NHS Foundation Trust / Newcastle Hospitals NHS Foundation Trust	COVID-19 Genomics UK (COG-UK) Consortium	Darren L Smith,Andrew Nelson,Matthew Bashton,Greg R Young,Joshua Loh,John Allan,Mohammad A Tariq,Giles S Holt,Gary Black,Wen C Yew,Lynn Dover,Paul Baker,Steve Liggett,Sarah Essex,Jane Greenaway,Debra Padgett,Clive Graham,Garren Scott,Edward Barton,Emma Swindells,Brendan Payne,Jennifer Collins,Yusri Taha,Gary Eltringham
EPI_ISL_573875, EPI_ISL_573877, EPI_ISL_573880, EPI_ISL_573883, EPI_ISL_573884, EPI_ISL_573885, EPI_ISL_573887, EPI_ISL_573888, EPI_ISL_573889, EPI_ISL_573891, EPI_ISL_573893, EPI_ISL_573894, EPI_ISL_573895, EPI_ISL_573896, EPI_ISL_573898, EPI_ISL_573900, EPI_ISL_573901, EPI_ISL_573903, EPI_ISL_573904, EPI_ISL_573905, EPI_ISL_573908, EPI_ISL_573909, EPI_ISL_573910, EPI_ISL_573911, EPI_ISL_573912, EPI_ISL_573914, EPI_ISL_573915, EPI_ISL_573918, EPI_ISL_573919, EPI_ISL_573920, EPI_ISL_573921, EPI_ISL_573923, EPI_ISL_573924, EPI_ISL_573925, EPI_ISL_573926, EPI_ISL_573927, EPI_ISL_573928, EPI_ISL_573929, EPI_ISL_573930, EPI_ISL_573931, EPI_ISL_573932, EPI_ISL_573934, EPI_ISL_573938, EPI_ISL_573939, EPI_ISL_573940, EPI_ISL_573941, EPI_ISL_573944, EPI_ISL_573948, EPI_ISL_573950, EPI_ISL_573951, EPI_ISL_573954, EPI_ISL_573956, EPI_ISL_573958, EPI_ISL_573960, EPI_ISL_573961, EPI_ISL_573962, EPI_ISL_573963, EPI_ISL_573964, EPI_ISL_573967, EPI_ISL_573969, EPI_ISL_573970, EPI_ISL_573971, EPI_ISL_573972, EPI_ISL_573973, EPI_ISL_573974, EPI_ISL_573975, EPI_ISL_573976, EPI_ISL_573978, EPI_ISL_573982, EPI_ISL_573983, EPI_ISL_573988, EPI_ISL_573989, EPI_ISL_573990, EPI_ISL_573991, EPI_ISL_573992, EPI_ISL_573994, EPI_ISL_573995, EPI_ISL_573996, EPI_ISL_573998, EPI_ISL_573999, EPI_ISL_574001, EPI_ISL_574006, EPI_ISL_574007, EPI_ISL_574008, EPI_ISL_574009, EPI_ISL_574011, EPI_ISL_574013, EPI_ISL_574016, EPI_ISL_574031, EPI_ISL_574037, EPI_ISL_574041, EPI_ISL_574058, EPI_ISL_574065, EPI_ISL_574066, EPI_ISL_574068, EPI_ISL_574072, EPI_ISL_574074, EPI_ISL_574080, EPI_ISL_574091, EPI_ISL_574092, EPI_ISL_574099, EPI_ISL_574100, EPI_ISL_574116, EPI_ISL_574138, EPI_ISL_574166, EPI_ISL_574184, EPI_ISL_574191, EPI_ISL_574193, EPI_ISL_574217, EPI_ISL_574224, EPI_ISL_574228, EPI_ISL_574230, EPI_ISL_574237, EPI_ISL_574244			
see above	Wales Specialist Virology Centre Sequencing lab: Pathogen Genomics Unit	COVID-19 Genomics UK (COG-UK) Consortium	Catherine Moore, Johnathan Evans, Laura Gifford, Malorie Perry, Simon Cottrell, Angela Marchbank, Alec Birchley, Alexander Adams, Amy Gaskin, Bree Gatica-Wilcox, Jason Coombes, Joel Southgate, Lauren Gilbert, Lee Graham, Nicole Pacchiarini, Sara Kumziene-Summerhayes, Sarah Taylor, Sophie Jones, Sara Rey, Matthew Bull, Joanne Watkins, Sally Corden, Tom Connor
EPI_ISL_574258	University College London, Great Ormond Street Hospital for Children NHS Foundation Trust, Imperial College Healthcare NHS Trust	COVID-19 Genomics UK (COG-UK) Consortium	Sergi Castellano, Rachel Williams, Mark Kristiansen, Paola Resende Silva, Sunando Roy, Tony Brooks, Helena Tutill, Paola Niola, Patricia Dyal, Charlotte Williams, Leysa Forrest, Yasmin Panchbhaya, Jacqueline Findlay, Samuel Weeks, Julianne Brown, Kathryn Harris, Paul Randell, James Price, Alison Holmes, Judith Breuer
EPI_ISL_574431	Hospital IESS Babahoyo	Institute of Microbiology, Universidad San Francisco de Quito	Belén Prado-Vivar, Sully Márquez, Juan José Guadalupe, Monica Becerra-Wong, Fernanda Zurita, Bernardo Gutiérrez, Francisco Cordova, Ninfa Henriquez, Killen Briones-Zamora, Killen Briones-Claudette, Verónica Barragán, Patricio Rojas-Silva, Gabriel Trueba, Michelle Grunauer, Paúl Cárdenas
EPI_ISL_574676, EPI_ISL_574677, EPI_ISL_574678	Seattle Flu Study	Seattle Flu Study	Deborah A. Nickerson, Chris D. Frazar, Jover Lee, Benjamin Pelle, Matthew Richardson, Amanda Adler, Elisabeth Brandstetter, Peter D. Han, Kairsten Fay, Misja Ilcisin, Kirsten Lacombe, Thomas R. Sibley, Melissa Truong, Caitlin R. Wolf, Karen Cowgill, Stephanie Schrag, Jeff Duchin, Michael Boeckh, Janet A. Englund, Michael Famulare, Barry R. Lutz, Mark J. Rieder, Lea M. Starita, Matthew Thompson, Helen Y. Chu, Trevor Bedford, Jay Shendure
EPI_ISL_574679	Seattle Flu Study	Seattle Flu Study	Deborah A. Nickerson, Chris D. Frazar, Jover Lee, Benjamin Pelle, Matthew Richardson, Amanda Adler, Elisabeth Brandstetter, Peter D. Han, Kairsten Fay, Misja Ilcisin, Kirsten Lacombe, Thomas R. Sibley, Melissa Truong, Caitlin R. Wolf, Michael Boeckh, Janet A. Englund, Michael Famulare, Barry R. Lutz, Mark J. Rieder, Lea M. Starita, Matthew Thompson, Jay Shendure, Trevor Bedford, Helen Y. Chu
EPI_ISL_574680, EPI_ISL_574681	Seattle Flu Study	Seattle Flu Study	Deborah A. Nickerson, Chris D. Frazar, Jover Lee, Benjamin Pelle, Matthew Richardson, Amanda Adler, Elisabeth Brandstetter, Peter D. Han, Kairsten Fay, Misja Ilcisin, Kirsten Lacombe, Thomas R. Sibley, Melissa Truong, Caitlin R. Wolf, Karen Cowgill, Stephanie Schrag, Jeff Duchin, Michael Boeckh, Janet A. Englund, Michael Famulare, Barry R. Lutz, Mark J. Rieder, Lea M. Starita, Matthew Thompson, Helen Y. Chu, Trevor Bedford, Jay Shendure
EPI_ISL_574682	Seattle Flu Study	Seattle Flu Study	Deborah A. Nickerson, Chris D. Frazar, Jover Lee, Benjamin Pelle, Matthew Richardson, Amanda Adler, Elisabeth Brandstetter, Peter D. Han, Kairsten Fay, Misja Ilcisin, Kirsten Lacombe, Thomas R. Sibley, Melissa Truong, Caitlin R. Wolf, Michael Boeckh, Janet A. Englund, Michael Famulare, Barry R. Lutz, Mark J. Rieder, Lea M. Starita, Matthew Thompson, Jay Shendure, Trevor Bedford, Helen Y. Chu
EPI_ISL_574683	Seattle Flu Study	Seattle Flu Study	Deborah A. Nickerson, Chris D. Frazar, Jover Lee, Benjamin Pelle, Matthew Richardson, Amanda Adler, Elisabeth Brandstetter, Peter D. Han, Kairsten Fay, Misja Ilcisin, Kirsten Lacombe, Thomas R. Sibley, Melissa Truong, Caitlin R. Wolf, Karen Cowgill, Stephanie Schrag, Jeff Duchin, Michael Boeckh, Janet A. Englund, Michael Famulare, Barry R. Lutz, Mark J. Rieder, Lea M. Starita, Matthew Thompson, Helen Y. Chu, Trevor Bedford, Jay Shendure
EPI_ISL_574684	Seattle Flu Study	Seattle Flu Study	Deborah A. Nickerson, Chris D. Frazar, Jover Lee, Benjamin Pelle, Matthew Richardson, Amanda Adler, Elisabeth Brandstetter, Peter D. Han, Kairsten Fay, Misja Ilcisin, Kirsten Lacombe, Thomas R. Sibley, Melissa Truong, Caitlin R. Wolf, Michael Boeckh, Janet A. Englund, Michael Famulare, Barry R. Lutz, Mark J. Rieder, Lea M. Starita, Matthew Thompson, Jay Shendure, Trevor Bedford, Helen Y. Chu

EPI_ISL_574685, EPI_ISL_574686, EPI_ISL_574687, EPI_ISL_574688	Seattle Flu Study	Seattle Flu Study	Deborah A. Nickerson, Chris D. Frazar, Jover Lee, Benjamin Pelle, Matthew Richardson, Amanda Adler, Elisabeth Brandstetter, Peter D. Han, Kairsten Fay, Misja Ilcisin, Kirsten Lacombe, Thomas R. Sibley, Melissa Truong, Caitlin R. Wolf, Karen Cowgill, Stephanie Schrag, Jeff Duchin, Michael Boeckh, Janet A. Englund, Michael Famulare, Barry R. Lutz, Mark J. Rieder, Lea M. Starita, Matthew Thompson, Helen Y. Chu, Trevor Bedford, Jay Shendure
EPI_ISL_574697	Respiratory Virus Unit, Microbiology Services Colindale, Public Health England	Respiratory Virus Unit, Microbiology Services Colindale, Public Health England	PHE Covid Sequencing Team
EPI_ISL_575399, EPI_ISL_575400, EPI_ISL_575401, EPI_ISL_575402, EPI_ISL_575403, EPI_ISL_575404, EPI_ISL_575405, EPI_ISL_575406, EPI_ISL_575407, EPI_ISL_575408, EPI_ISL_575409, EPI_ISL_575410, EPI_ISL_575411, EPI_ISL_575412, EPI_ISL_575413, EPI_ISL_575414, EPI_ISL_575415, EPI_ISL_575416, EPI_ISL_575417, EPI_ISL_575418, EPI_ISL_575419, EPI_ISL_575420, EPI_ISL_575421, EPI_ISL_575422, EPI_ISL_575423, EPI_ISL_575424, EPI_ISL_575425, EPI_ISL_575426, EPI_ISL_575427, EPI_ISL_575428, EPI_ISL_575429, EPI_ISL_575430, EPI_ISL_575431, EPI_ISL_575432, EPI_ISL_575433, EPI_ISL_575434, EPI_ISL_575435, EPI_ISL_575436, EPI_ISL_575437, EPI_ISL_575438, EPI_ISL_575439, EPI_ISL_575440, EPI_ISL_575441, EPI_ISL_575442, EPI_ISL_575443, EPI_ISL_575444, EPI_ISL_575445, EPI_ISL_575446, EPI_ISL_575447, EPI_ISL_575448, EPI_ISL_575449, EPI_ISL_575450, EPI_ISL_575451, EPI_ISL_575452, EPI_ISL_575453, EPI_ISL_575454, EPI_ISL_575455, EPI_ISL_575456, EPI_ISL_575457, EPI_ISL_575458, EPI_ISL_575459, EPI_ISL_575460, EPI_ISL_575461, EPI_ISL_575462, EPI_ISL_575463, EPI_ISL_575464, EPI_ISL_575465, EPI_ISL_575466, EPI_ISL_575467, EPI_ISL_575468, EPI_ISL_575469, EPI_ISL_575470, EPI_ISL_575471, EPI_ISL_575472, EPI_ISL_575473, EPI_ISL_575474, EPI_ISL_575475, EPI_ISL_575476, EPI_ISL_575477, EPI_ISL_575478, EPI_ISL_575479, EPI_ISL_575480, EPI_ISL_575481, EPI_ISL_575482, EPI_ISL_575483, EPI_ISL_575484, EPI_ISL_575485, EPI_ISL_575486, EPI_ISL_575487, EPI_ISL_575488, EPI_ISL_575489, EPI_ISL_575490, EPI_ISL_575491, EPI_ISL_575492, EPI_ISL_575493, EPI_ISL_575494, EPI_ISL_575495, EPI_ISL_575496, EPI_ISL_575497, EPI_ISL_575498, EPI_ISL_575499, EPI_ISL_575500, EPI_ISL_575501, EPI_ISL_575502, EPI_ISL_575503, EPI_ISL_575504, EPI_ISL_575505, EPI_ISL_575506, EPI_ISL_575507, EPI_ISL_575508, EPI_ISL_575509, EPI_ISL_575510, EPI_ISL_575511, EPI_ISL_575512, EPI_ISL_575513, EPI_ISL_575514, EPI_ISL_575515, EPI_ISL_575516, EPI_ISL_575517, EPI_ISL_575518, EPI_ISL_575519, EPI_ISL_575520, EPI_ISL_575521, EPI_ISL_575522, EPI_ISL_575523, EPI_ISL_575524, EPI_ISL_575525, EPI_ISL_575526, EPI_ISL_575527, EPI_ISL_575528, EPI_ISL_575529, EPI_ISL_575530, EPI_ISL_575531, EPI_ISL_575532, EPI_ISL_575533, EPI_ISL_575534, EPI_ISL_575535			
see above	Lighthouse Lab in Alderley Park	Wellcome Sanger Institute for the COVID-19 Genomics UK (COG-UK) consortium	Jacquelyn Wynn, Mairead Hyland, The Lighthouse Lab in Alderley Park and Alex Alderton, Roberto Amato, Sonia Goncalves, Ewan Harrison, David K. Jackson, Ian Johnston, Dominic Kwiatkowski, Cordelia Langford, John Sillitoe on behalf of the Wellcome Sanger Institute COVID-19 Surveillance Team
EPI_ISL_575536	Lighthouse Lab in Alderley Park	Wellcome Sanger Institute for the COVID-19 Genomics UK (COG-UK) Consortium	Jacquelyn Wynn, Mairead Hyland, The Lighthouse Lab in Alderley Park and Alex Alderton, Roberto Amato, Sonia Goncalves, Ewan Harrison, David K. Jackson, Ian Johnston, Dominic Kwiatkowski, Cordelia Langford, John Sillitoe on behalf of the Wellcome Sanger Institute COVID-19 Surveillance Team
EPI_ISL_575537, EPI_ISL_575538, EPI_ISL_575539, EPI_ISL_575540, EPI_ISL_575541, EPI_ISL_575542, EPI_ISL_575543, EPI_ISL_575544, EPI_ISL_575545, EPI_ISL_575546, EPI_ISL_575547, EPI_ISL_575548, EPI_ISL_575549, EPI_ISL_575550, EPI_ISL_575551, EPI_ISL_575552, EPI_ISL_575553, EPI_ISL_575554, EPI_ISL_575555, EPI_ISL_575556, EPI_ISL_575557, EPI_ISL_575558, EPI_ISL_575559, EPI_ISL_575560, EPI_ISL_575561, EPI_ISL_575562, EPI_ISL_575563, EPI_ISL_575564, EPI_ISL_575565, EPI_ISL_575566, EPI_ISL_575567, EPI_ISL_575568, EPI_ISL_575569, EPI_ISL_575570, EPI_ISL_575571, EPI_ISL_575572, EPI_ISL_575573, EPI_ISL_575574, EPI_ISL_575575, EPI_ISL_575576, EPI_ISL_575577, EPI_ISL_575578, EPI_ISL_575579, EPI_ISL_575580, EPI_ISL_575581, EPI_ISL_575582, EPI_ISL_575583, EPI_ISL_575584, EPI_ISL_575585, EPI_ISL_575586, EPI_ISL_575587, EPI_ISL_575588, EPI_ISL_575589, EPI_ISL_575590, EPI_ISL_575591, EPI_ISL_575592, EPI_ISL_575593, EPI_ISL_575594, EPI_ISL_575595, EPI_ISL_575596, EPI_ISL_575597, EPI_ISL_575598, EPI_ISL_575599, EPI_ISL_575600, EPI_ISL_575601, EPI_ISL_575602, EPI_ISL_575603, EPI_ISL_575604, EPI_ISL_575605, EPI_ISL_575606, EPI_ISL_575607, EPI_ISL_575608, EPI_ISL_575609, EPI_ISL_575610, EPI_ISL_575611, EPI_ISL_575612, EPI_ISL_575613, EPI_ISL_575614, EPI_ISL_575615, EPI_ISL_575616, EPI_ISL_575617, EPI_ISL_575618, EPI_ISL_575619, EPI_ISL_575620, EPI_ISL_575621, EPI_ISL_575622, EPI_ISL_575623, EPI_ISL_575624, EPI_ISL_575625, EPI_ISL_575626, EPI_ISL_575627, EPI_ISL_575628, EPI_ISL_575629, EPI_ISL_575630, EPI_ISL_575631, EPI_ISL_575632, EPI_ISL_575633, EPI_ISL_575634, EPI_ISL_575635, EPI_ISL_575636, EPI_ISL_575637, EPI_ISL_575638, EPI_ISL_575639, EPI_ISL_575640, EPI_ISL_575641, EPI_ISL_575642, EPI_ISL_575643, EPI_ISL_575644, EPI_ISL_575645, EPI_ISL_575646, EPI_ISL_575647, EPI_ISL_575648, EPI_ISL_575649, EPI_ISL_575650, EPI_ISL_575651, EPI_ISL_575652, EPI_ISL_575653, EPI_ISL_575654, EPI_ISL_575655, EPI_ISL_575656, EPI_ISL_575657, EPI_ISL_575658, EPI_ISL_575659, EPI_ISL_575660, EPI_ISL_575661, EPI_ISL_575662, EPI_ISL_575663, EPI_ISL_575664, EPI_ISL_575665, EPI_ISL_575666, EPI_ISL_575667, EPI_ISL_575668, EPI_ISL_575669, EPI_ISL_575670, EPI_ISL_575671, EPI_ISL_575672, EPI_ISL_575673, EPI_ISL_575674, EPI_ISL_575675, EPI_ISL_575676, EPI_ISL_575677, EPI_ISL_575678, EPI_ISL_575679, EPI_ISL_575680, EPI_ISL_575681, EPI_ISL_575682, EPI_ISL_575683, EPI_ISL_575684, EPI_ISL_575685, EPI_ISL_575686, EPI_ISL_575687, EPI_ISL_575688, EPI_ISL_575689, EPI_ISL_575690, EPI_ISL_575691, EPI_ISL_575692, EPI_ISL_575693, EPI_ISL_575694, EPI_ISL_575695, EPI_ISL_575696, EPI_ISL_575697, EPI_ISL_575698, EPI_ISL_575699, EPI_ISL_575700, EPI_ISL_575701, EPI_ISL_575702, EPI_ISL_575703, EPI_ISL_575704, EPI_ISL_575705, EPI_ISL_575706, EPI_ISL_575707, EPI_ISL_575708, EPI_ISL_575709, EPI_ISL_575710, EPI_ISL_575711, EPI_ISL_575712, EPI_ISL_575713, EPI_ISL_575714, EPI_ISL_575715, EPI_ISL_575716, EPI_ISL_575717, EPI_ISL_575718, EPI_ISL_575719, EPI_ISL_575720, EPI_ISL_575721, EPI_ISL_575722, EPI_ISL_575723, EPI_ISL_575724, EPI_ISL_575725, EPI_ISL_575726, EPI_ISL_575727, EPI_ISL_575728, EPI_ISL_575729, EPI_ISL_575730, EPI_ISL_575731, EPI_ISL_575732, EPI_ISL_575733, EPI_ISL_575734, EPI_ISL_575735, EPI_ISL_575736, EPI_ISL_575737, EPI_ISL_575738, EPI_ISL_575739, EPI_ISL_575740, EPI_ISL_575741, EPI_ISL_575742, EPI_ISL_575743, EPI_ISL_575744, EPI_ISL_575745, EPI_ISL_575746, EPI_ISL_575747, EPI_ISL_575748, EPI_ISL_575749, EPI_ISL_575750, EPI_ISL_575751, EPI_ISL_575752, EPI_ISL_575753, EPI_ISL_575754, EPI_ISL_575755, EPI_ISL_575756, EPI_ISL_575757, EPI_ISL_575758, EPI_ISL_575759, EPI_ISL_575760			
see above	Lighthouse Lab in Alderley Park	Wellcome Sanger Institute for the COVID-19 Genomics UK (COG-UK) consortium	Jacquelyn Wynn, Mairead Hyland, The Lighthouse Lab in Alderley Park and Alex Alderton, Roberto Amato, Sonia Goncalves, Ewan Harrison, David K. Jackson, Ian Johnston, Dominic Kwiatkowski, Cordelia Langford, John Sillitoe on behalf of the Wellcome Sanger Institute COVID-19 Surveillance Team
EPI_ISL_575982, EPI_ISL_575983	Lighthouse Lab in Alderley Park	Wellcome Sanger Institute for the COVID-19 Genomics UK (COG-UK) consortium	Jacquelyn Wynn, Mairead Hyland, The Lighthouse Lab in Alderley Park and Alex Alderton, Roberto Amato, Sonia Goncalves, Ewan Harrison, David K. Jackson, Ian Johnston, Dominic Kwiatkowski, Cordelia Langford, John Sillitoe on behalf of the Wellcome Sanger Institute COVID-19 Surveillance Team (http://www.sanger.ac.uk/covid-team)
EPI_ISL_575984, EPI_ISL_575985	Lighthouse Lab in Milton Keynes	Wellcome Sanger Institute for the COVID-19 Genomics UK (COG-UK) consortium	The Lighthouse Lab in Milton Keynes and Alex Alderton, Roberto Amato, Sonia Goncalves, Ewan Harrison, David K. Jackson, Ian Johnston, Dominic Kwiatkowski, Cordelia Langford, John Sillitoe on behalf of the Wellcome Sanger Institute COVID-19 Surveillance Team (http://www.sanger.ac.uk/covid-team)
EPI_ISL_575995, EPI_ISL_575996, EPI_ISL_575997, EPI_ISL_575998, EPI_ISL_575999, EPI_ISL_576000, EPI_ISL_576001, EPI_ISL_576002, EPI_ISL_576003, EPI_ISL_576004, EPI_ISL_576005, EPI_ISL_576006, EPI_ISL_576007, EPI_ISL_576008, EPI_ISL_576009, EPI_ISL_576010, EPI_ISL_576011, EPI_ISL_576012, EPI_ISL_576013, EPI_ISL_576014, EPI_ISL_576015, EPI_ISL_576016, EPI_ISL_576017, EPI_ISL_576018, EPI_ISL_576019, EPI_ISL_576020, EPI_ISL_576021, EPI_ISL_576022, EPI_ISL_576023, EPI_ISL_576024, EPI_ISL_576025, EPI_ISL_576026, EPI_ISL_576027, EPI_ISL_576028, EPI_ISL_576029, EPI_ISL_576030, EPI_ISL_576031, EPI_ISL_576032, EPI_ISL_576033, EPI_ISL_576034, EPI_ISL_576035, EPI_ISL_576036, EPI_ISL_576037, EPI_ISL_576038, EPI_ISL_576039, EPI_ISL_576040, EPI_ISL_576041, EPI_ISL_576042, EPI_ISL_576043, EPI_ISL_576044, EPI_ISL_576045, EPI_ISL_576046, EPI_ISL_576047, EPI_ISL_576048, EPI_ISL_576049			
see above	Lighthouse Lab in Glasgow	Wellcome Sanger Institute for the COVID-19 Genomics UK (COG-UK) consortium	Harper VanSteenhouse, Yumi Kasai, David Gray, Carol Clugston, Anna Dominiczak and Alex Alderton, Roberto Amato, Sonia Goncalves, Ewan Harrison, David K. Jackson, Ian Johnston, Dominic Kwiatkowski, Cordelia Langford, John Sillitoe on behalf of the Wellcome Sanger Institute COVID-19 Surveillance Team (http://www.sanger.ac.uk/covid-team)
EPI_ISL_576050	Lighthouse Lab in Glasgow	Wellcome Sanger Institute for the COVID-19 Genomics UK (COG-UK) Consortium	Harper VanSteenhouse, Yumi Kasai, David Gray, Carol Clugston, Anna Dominiczak and Alex Alderton, Roberto Amato, Sonia Goncalves, Ewan Harrison, David K. Jackson, Ian Johnston, Dominic Kwiatkowski, Cordelia Langford, John Sillitoe on behalf of the Wellcome Sanger Institute COVID-19 Surveillance Team
EPI_ISL_576051, EPI_ISL_576052, EPI_ISL_576054, EPI_ISL_576055, EPI_ISL_576056, EPI_ISL_576057, EPI_ISL_576058, EPI_ISL_576059, EPI_ISL_576060, EPI_ISL_576061, EPI_ISL_576062, EPI_ISL_576063			
see above	Lighthouse Lab in Glasgow	Wellcome Sanger Institute for the COVID-19 Genomics UK (COG-UK) consortium	Harper VanSteenhouse, Yumi Kasai, David Gray, Carol Clugston, Anna Dominiczak and Alex Alderton, Roberto Amato, Sonia Goncalves, Ewan Harrison, David K. Jackson, Ian Johnston, Dominic Kwiatkowski, Cordelia Langford, John Sillitoe on behalf of the Wellcome Sanger Institute COVID-19 Surveillance Team (http://www.sanger.ac.uk/covid-team)
EPI_ISL_576064	Lighthouse Lab in Glasgow	Wellcome Sanger Institute for the COVID-19 Genomics UK (COG-UK) Consortium	Harper VanSteenhouse, Yumi Kasai, David Gray, Carol Clugston, Anna Dominiczak and Alex Alderton, Roberto Amato, Sonia Goncalves, Ewan Harrison, David K. Jackson, Ian Johnston, Dominic Kwiatkowski, Cordelia Langford, John Sillitoe on behalf of the Wellcome Sanger Institute COVID-19 Surveillance Team
EPI_ISL_576065, EPI_ISL_576066, EPI_ISL_576067, EPI_ISL_576068, EPI_ISL_576069, EPI_ISL_576071, EPI_ISL_576072, EPI_ISL_576073, EPI_ISL_576074, EPI_ISL_576075, EPI_ISL_576077, EPI_ISL_576078, EPI_ISL_576079, EPI_ISL_576080, EPI_ISL_576081, EPI_ISL_576083, EPI_ISL_576084, EPI_ISL_576085, EPI_ISL_576086, EPI_ISL_576087			
see above	Lighthouse Lab in Glasgow	Wellcome Sanger Institute for the COVID-19 Genomics UK (COG-UK) consortium	Harper VanSteenhouse, Yumi Kasai, David Gray, Carol Clugston, Anna Dominiczak and Alex Alderton, Roberto Amato, Sonia Goncalves, Ewan Harrison, David K. Jackson, Ian Johnston, Dominic Kwiatkowski, Cordelia Langford, John Sillitoe on behalf of the Wellcome Sanger Institute COVID-19 Surveillance Team (http://www.sanger.ac.uk/covid-team)
EPI_ISL_576088	Lighthouse Lab in Glasgow	Wellcome Sanger Institute for the COVID-19 Genomics UK (COG-UK) Consortium	Harper VanSteenhouse, Yumi Kasai, David Gray, Carol Clugston, Anna Dominiczak and Alex Alderton, Roberto Amato, Sonia Goncalves, Ewan Harrison, David K. Jackson, Ian Johnston, Dominic Kwiatkowski, Cordelia Langford, John Sillitoe on behalf of the Wellcome Sanger Institute COVID-19 Surveillance Team
EPI_ISL_576089, EPI_ISL_576090, EPI_ISL_576091, EPI_ISL_576092, EPI_ISL_576093	Lighthouse Lab in Glasgow	Wellcome Sanger Institute for the COVID-19 Genomics UK (COG-UK) consortium	Harper VanSteenhouse, Yumi Kasai, David Gray, Carol Clugston, Anna Dominiczak and Alex Alderton, Roberto Amato, Sonia Goncalves, Ewan Harrison, David K. Jackson, Ian Johnston, Dominic Kwiatkowski, Cordelia Langford, John Sillitoe on behalf of the Wellcome Sanger Institute COVID-19 Surveillance Team (http://www.sanger.ac.uk/covid-team)
EPI_ISL_576142, EPI_ISL_576143, EPI_ISL_576144	unknown	Public Health Virology Laboratory, Forensic and Scientific Services (PHV-FSS)	Son Nguyen et al.

EPI_ISL_576189, EPI_ISL_576190, EPI_ISL_576191	GA Department of Public Health Laboratory	Pathogen Discovery, Respiratory Viruses Branch, Division of Viral Diseases, Centers for Disease Control and Prevention	Ying Tao, Jing Zhang, Brian Lynch, Yan Li, Krista Queen, Anna Uehara, Clinton R. Paden, Peter Cook, Haibin Wang, Suxiang Tong
EPI_ISL_576228, EPI_ISL_576229, EPI_ISL_576230, EPI_ISL_576231, EPI_ISL_576232, EPI_ISL_576233, EPI_ISL_576234, EPI_ISL_576235, EPI_ISL_576236, EPI_ISL_576238, EPI_ISL_576240, EPI_ISL_576241, EPI_ISL_576242, EPI_ISL_576244, EPI_ISL_576245, EPI_ISL_576246, EPI_ISL_576247, EPI_ISL_576248, EPI_ISL_576249, EPI_ISL_576250, EPI_ISL_576251, EPI_ISL_576256			
see above	Minnesota Department of Health, Public Health Laboratory	Minnesota Department of Health, Public Health Laboratory	Matt Plumb, Jacob Garfin, Alexandra Lorentz, and Xiong Wang
EPI_ISL_576358, EPI_ISL_576359, EPI_ISL_576360, EPI_ISL_576361, EPI_ISL_576362	Texas Department of State Health Services	Texas Department of State Health Services	Rashmi Tuladhar, Bonnie Oh, Mayela Pedrueza, Jenny Zhang, Maliha Rahman, Anita Pokharel, Myong Koag, Chun Wang, Rachel Lee, Grace Kubin
EPI_ISL_576609, EPI_ISL_576610, EPI_ISL_576612, EPI_ISL_576617, EPI_ISL_576618, EPI_ISL_576629, EPI_ISL_576630, EPI_ISL_576631, EPI_ISL_576632, EPI_ISL_576633, EPI_ISL_576634, EPI_ISL_576635, EPI_ISL_576646, EPI_ISL_576659, EPI_ISL_576831, EPI_ISL_576832, EPI_ISL_576833			
see above	Oxford Viromics, NDM, University of Oxford; Oxford University Hospitals; Basingstoke and North Hampshire Hospital	COVID-19 Genomics UK (COG-UK) Consortium	Tanya Golubchik, David Bonsall, George Macintyre, Amy Trebes, Mariateresa de Cesare, Catrin Moore, Alex Mobbs, Anita Justice, Robert Shaw, Monique Andersson, Timothy Peto, Emma Wise, Nathan Moore, Jessica Lynch, Nick Cortes, Matilde Mori, Stephen Kidd, David Buck, John Todd, Christophe Fraser
EPI_ISL_576965, EPI_ISL_576966, EPI_ISL_576967, EPI_ISL_576968, EPI_ISL_576969, EPI_ISL_576970, EPI_ISL_576971, EPI_ISL_576972, EPI_ISL_576973, EPI_ISL_576974, EPI_ISL_576975, EPI_ISL_576976, EPI_ISL_576977, EPI_ISL_576980			
see above	Liverpool Clinical Laboratories	COVID-19 Genomics UK (COG-UK) Consortium	Sam Haldenby, Anita Lucaci, Steve Paterson, Julian Hiscox, Alistair Darby, M Almsaud, A Alrezaihi, Muhannad Alruwaili, Stuart D Armstrong, Jones Benjamin, Eleanor G Bentley, Anu Chawla, Jordan J Clark, Angela Cowell, Richard Eccles, Isabel García-Dorival, Matthew Gemmell, Alessandro Gerada, PKF Gilmore, Richard Gregory, Ximeng Han, Catherine Hartley, Margaret Hughes, Miren Iturriza-Gomara, James Johnson, L Luu, Jenifer Manson, Charlotte Nelson, Elaine O'Toole, Cassie Olateju, Rebekah Penrice-Randal , Lucille Rainbow, N.P Randle, Trevor Ian Robinson, Parul Sharma, Ghada T Shawli, James P Stewart, Neil Swainston, Ecaterina Vamos, Joanne Watts, Mark Whitehead
EPI_ISL_577040, EPI_ISL_577044, EPI_ISL_577046, EPI_ISL_577049, EPI_ISL_577051, EPI_ISL_577066, EPI_ISL_577196, EPI_ISL_577197, EPI_ISL_577199, EPI_ISL_577200, EPI_ISL_577204, EPI_ISL_577207, EPI_ISL_577209, EPI_ISL_577216			
see above	Centre for Enzyme Innovation, University of Portsmouth / Translational Research Laboratory, Portsmouth Hospitals NHS Trust	COVID-19 Genomics UK (COG-UK) Consortium	Angela Beckett,Yann Bourgeois,Garry Scarlett,Sharon Glaysher,Scott Elliott,Kelly Bicknell,Robert Impey,Allyson Lloyd,Sarah Wyllie,Ethan Butcher,Anoop Chauhan,Samuel Robson
EPI_ISL_577273, EPI_ISL_577274, EPI_ISL_577275, EPI_ISL_577276, EPI_ISL_577277, EPI_ISL_577278, EPI_ISL_577279, EPI_ISL_577280, EPI_ISL_577281, EPI_ISL_577282, EPI_ISL_577283, EPI_ISL_577284, EPI_ISL_577285, EPI_ISL_577286, EPI_ISL_577287, EPI_ISL_577288, EPI_ISL_577289, EPI_ISL_577290, EPI_ISL_577291, EPI_ISL_577292, EPI_ISL_577293, EPI_ISL_577294, EPI_ISL_577295, EPI_ISL_577296, EPI_ISL_577297			
see above	University of Exeter	COVID-19 Genomics UK (COG-UK) Consortium	Ben Temperton,Aaron Jeffries,Michelle Michelsen,Joanna Warwick-Dugdale,Audrey Farbos,Robyn Manley,Stephen Michell,Jane Masoli
EPI_ISL_577303, EPI_ISL_577312, EPI_ISL_577322, EPI_ISL_577331	Centre for Enzyme Innovation, University of Portsmouth / Translational Research Laboratory, Portsmouth Hospitals NHS Trust	COVID-19 Genomics UK (COG-UK) Consortium	Angela Beckett,Yann Bourgeois,Garry Scarlett,Sharon Glaysher,Scott Elliott,Kelly Bicknell,Robert Impey,Allyson Lloyd,Sarah Wyllie,Ethan Butcher,Anoop Chauhan,Samuel Robson
EPI_ISL_577347, EPI_ISL_577348, EPI_ISL_577350, EPI_ISL_577351, EPI_ISL_577352, EPI_ISL_577353, EPI_ISL_577354	Virology Department, Royal Infirmary of Edinburgh, NHS Lothian / School of Biological Sciences, University of Edinburgh / Institute of Genetics and Molecular Medicine, University of Edinburgh	COVID-19 Genomics UK (COG-UK) Consortium	McHugh M, Dewar R, Rooke S, Gallagher M, Balcaza C, O'Toole Á, Scher E, Hill V, McCrone JT, Colquhoun R, Yu X, Jackson B, Rambaut A, Williams TC, Templeton K
EPI_ISL_577374, EPI_ISL_577375, EPI_ISL_577376, EPI_ISL_577377, EPI_ISL_577378, EPI_ISL_577379, EPI_ISL_577380	University of Exeter	COVID-19 Genomics UK (COG-UK) Consortium	Ben Temperton,Aaron Jeffries,Michelle Michelsen,Joanna Warwick-Dugdale,Audrey Farbos,Robyn Manley,Stephen Michell,Jane Masoli
EPI_ISL_577419, EPI_ISL_577460, EPI_ISL_577482, EPI_ISL_577485	Wales Specialist Virology Centre Sequencing lab: Pathogen Genomics Unit	COVID-19 Genomics UK (COG-UK) Consortium	Catherine Moore, Johnathan Evans, Laura Gifford, Malorie Perry, Simon Cottrell, Angela Marchbank, Alec Birchley, Alexander Adams, Amy Gaskin, Bree Gatica-Wilcox, Jason Coombes, Joel Southgate, Lauren Gilbert, Lee Graham, Nicole Pacchiarini, Sara Kumziene-Summerhayes, Sarah Taylor, Sophie Jones, Sara Rey, Matthew Bull, Joanne Watkins, Sally Corden, Tom Connor
EPI_ISL_577548, EPI_ISL_577558, EPI_ISL_577559, EPI_ISL_577563, EPI_ISL_577585, EPI_ISL_577586, EPI_ISL_577587, EPI_ISL_577591, EPI_ISL_577592, EPI_ISL_577593, EPI_ISL_577595, EPI_ISL_577596			
see above	Michigan Department of Health and Human Services, Bureau of Laboratories	Michigan Department of Health and Human Services, Bureau of Laboratories	Blankenship HM, Riner D, Soehnlen MK
EPI_ISL_577630	The National Institute of Public Health	State Veterinary Institute Prague	Nagy,A;Jirincova,H;Novakova,L;Trnka,D;Vecerova,J
EPI_ISL_577641, EPI_ISL_577642, EPI_ISL_577643, EPI_ISL_577644, EPI_ISL_577645, EPI_ISL_577646, EPI_ISL_577648, EPI_ISL_577661, EPI_ISL_577663, EPI_ISL_577664, EPI_ISL_577666, EPI_ISL_577667, EPI_ISL_577668, EPI_ISL_577669, EPI_ISL_577670, EPI_ISL_577674, EPI_ISL_577675, EPI_ISL_577676, EPI_ISL_577678, EPI_ISL_577703, EPI_ISL_577704, EPI_ISL_577705, EPI_ISL_577708, EPI_ISL_577716, EPI_ISL_577730, EPI_ISL_577731			
see above	NIV Influenza	NIV Influenza	Potdar V
EPI_ISL_577816, EPI_ISL_577817, EPI_ISL_577818, EPI_ISL_577819, EPI_ISL_577820, EPI_ISL_577821, EPI_ISL_577822, EPI_ISL_577823, EPI_ISL_577824, EPI_ISL_577825, EPI_ISL_577826, EPI_ISL_577827, EPI_ISL_577828			
see above	Dutch COVID-19 response team	Erasmus Medical Center	OH consortium
EPI_ISL_577863, EPI_ISL_577864, EPI_ISL_577865, EPI_ISL_577866, EPI_ISL_577964, EPI_ISL_577965, EPI_ISL_577966, EPI_ISL_577967, EPI_ISL_577968, EPI_ISL_577969, EPI_ISL_577970, EPI_ISL_577971, EPI_ISL_577972, EPI_ISL_577973, EPI_ISL_577974, EPI_ISL_577975, EPI_ISL_577976, EPI_ISL_577977, EPI_ISL_577978, EPI_ISL_577979, EPI_ISL_578035, EPI_ISL_578043, EPI_ISL_578044, EPI_ISL_578047, EPI_ISL_578048			

see above	Dutch COVID-19 response team	Erasmus Medical Center	Bas Oude Munnink, Reina Sikkema, David Nieuwenhuijse, Irina Chestakova, Anne van der Linden, Marjan Boter, Emmanuelle Munger, Corine GeurtsvanKessel, Annemiek van der Eijk, Richard Molenkamp, Marion Koopmans, on behalf of the Dutch national COVID-19 response team.
EPI_ISL_578080	CSIR-Indian Institute of Chemical Biology, MEDICA Supercpecialty Hospital Kolkata	CSIR-Indian Institute of Chemical Biology, MEDICA Supercpecialty Hospital Kolkata	Sujay Krishna Maity, Priyanka Mallick, Debaleena Bhowmik, Abhishake Lahiri, Dr. AviralRoy, Dr. Soumen Saha, Dr. Arpita Ghosh Mitra, Dr. Rajesh Pandey, Dr. Sandip Paul, Dr.Partha Chakrabarti, Dr. Saikat Chakrabarti
EPI_ISL_578081, EPI_ISL_578168, EPI_ISL_578175, EPI_ISL_578178, EPI_ISL_578179, EPI_ISL_578180, EPI_ISL_578181, EPI_ISL_578182, EPI_ISL_578183, EPI_ISL_578184	CSIR-Indian Institute of Chemical Biology, MEDICA Supercpecialty Hospital Kolkata	CSIR-Indian Institute of Chemical Biology, MEDICA Supercpecialty Hospital Kolkata	Sujay Krishna Maity, Priyanka Mallick, Debaleena Bhowmik, Abhishake Lahiri, Dr. Aviral Roy, Dr. Soumen Saha, Dr. Arpita Ghosh Mitra, Dr. Rajesh Pandey, Dr. Sandip Paul, Dr. Partha Chakrabarti, Dr. Saikat Chakrabarti
EPI_ISL_578202, EPI_ISL_578214, EPI_ISL_578215, EPI_ISL_578216, EPI_ISL_578218, EPI_ISL_578221, EPI_ISL_578222, EPI_ISL_578223, EPI_ISL_578224, EPI_ISL_578225, EPI_ISL_578231, EPI_ISL_578239, EPI_ISL_578244, EPI_ISL_578247, EPI_ISL_578259, EPI_ISL_578261, EPI_ISL_578263, EPI_ISL_578266, EPI_ISL_578267, EPI_ISL_578268, EPI_ISL_578269, EPI_ISL_578270, EPI_ISL_578271, EPI_ISL_578273, EPI_ISL_578274, EPI_ISL_578280, EPI_ISL_578281, EPI_ISL_578284, EPI_ISL_578285, EPI_ISL_578286, EPI_ISL_578287, EPI_ISL_578288, EPI_ISL_578289, EPI_ISL_578290, EPI_ISL_578291, EPI_ISL_578292, EPI_ISL_578293, EPI_ISL_578294, EPI_ISL_578296, EPI_ISL_578299, EPI_ISL_578300, EPI_ISL_578301, EPI_ISL_578311, EPI_ISL_578314, EPI_ISL_578319, EPI_ISL_578320			
see above	National Virus Reference Laboratory	National Virus Reference Laboratory	Michael Carr, Gabriel Gonzalez, Jonathan Dean, Suzie Coughlan, Cillian F De Gascun
EPI_ISL_579093, EPI_ISL_579094, EPI_ISL_579095	North Shore Hospital	Institute of Environmental Science and Research (ESR)	Xiaoyun Ren, Matt Storey, Nikki Freed, Muhammad Faisal, Jing Wang, Hermes Perez, Anja Werno, Antje van der Linden, Arlo Upton, Chris Mansell, David Hammer, Dragana Drinkovic, Gary McAuliffe, Hana Sofia Andersson, James Ussher, Jill Sherwood, Josh Freeman, Julia Howard, Juliet Elvy, Mary DeAlmeida, Matt Blakiston, Matthew Rogers, Max Bloomfield, Michael Addidle, Michelle Balm, Sally Roberts, Sarah Jefferies, Sharmini Muttaiyah, Susan Morpeth, Susan Taylor, Timothy Blackmore, Vani Sathyendran, Veronica Playle, Virginia Hope, Erasmus Smit, Lauren Jelly, Olin Silander, Joep de Ligt
EPI_ISL_579098	LabPLUS	Institute of Environmental Science and Research (ESR)	Xiaoyun Ren, Matt Storey, Nikki Freed, Muhammad Faisal, Jing Wang, Hermes Perez, Anja Werno, Antje van der Linden, Arlo Upton, Chris Mansell, David Hammer, Dragana Drinkovic, Gary McAuliffe, Hana Sofia Andersson, James Ussher, Jill Sherwood, Josh Freeman, Julia Howard, Juliet Elvy, Mary DeAlmeida, Matt Blakiston, Matthew Rogers, Max Bloomfield, Michael Addidle, Michelle Balm, Sally Roberts, Sarah Jefferies, Sharmini Muttaiyah, Susan Morpeth, Susan Taylor, Timothy Blackmore, Vani Sathyendran, Veronica Playle, Virginia Hope, Erasmus Smit, Lauren Jelly, Olin Silander, Joep de Ligt
EPI_ISL_579099	LabTests	Institute of Environmental Science and Research (ESR)	Xiaoyun Ren, Matt Storey, Nikki Freed, Muhammad Faisal, Jing Wang, Hermes Perez, Anja Werno, Antje van der Linden, Arlo Upton, Chris Mansell, David Hammer, Dragana Drinkovic, Gary McAuliffe, Hana Sofia Andersson, James Ussher, Jill Sherwood, Josh Freeman, Julia Howard, Juliet Elvy, Mary DeAlmeida, Matt Blakiston, Matthew Rogers, Max Bloomfield, Michael Addidle, Michelle Balm, Sally Roberts, Sarah Jefferies, Sharmini Muttaiyah, Susan Morpeth, Susan Taylor, Timothy Blackmore, Vani Sathyendran, Veronica Playle, Virginia Hope, Erasmus Smit, Lauren Jelly, Olin Silander, Joep de Ligt
EPI_ISL_579101, EPI_ISL_579102	Middlemore Hospital	Institute of Environmental Science and Research (ESR)	Xiaoyun Ren, Matt Storey, Nikki Freed, Muhammad Faisal, Jing Wang, Hermes Perez, Anja Werno, Antje van der Linden, Arlo Upton, Chris Mansell, David Hammer, Dragana Drinkovic, Gary McAuliffe, Hana Sofia Andersson, James Ussher, Jill Sherwood, Josh Freeman, Julia Howard, Juliet Elvy, Mary DeAlmeida, Matt Blakiston, Matthew Rogers, Max Bloomfield, Michael Addidle, Michelle Balm, Sally Roberts, Sarah Jefferies, Sharmini Muttaiyah, Susan Morpeth, Susan Taylor, Timothy Blackmore, Vani Sathyendran, Veronica Playle, Virginia Hope, Erasmus Smit, Lauren Jelly, Olin Silander, Joep de Ligt
EPI_ISL_581318	Lighthouse Lab in Milton Keynes	Wellcome Sanger Institute for the COVID-19 Genomics UK (COG-UK) consortium	The Lighthouse Lab in Milton Keynes and Alex Alderton, Roberto Amato, Sonia Goncalves, Ewan Harrison, David K. Jackson, Ian Johnston, Dominic Kwiatkowski, Cordelia Langford, John Sillitoe on behalf of the Wellcome Sanger Institute COVID-19 Surveillance Team
EPI_ISL_581367	Lighthouse Lab in Alderley Park	Wellcome Sanger Institute for the COVID-19 Genomics UK (COG-UK) consortium	Jacquelyn Wynn, Mairead Hyland, The Lighthouse Lab in Alderley Park and Alex Alderton, Roberto Amato, Sonia Goncalves, Ewan Harrison, David K. Jackson, Ian Johnston, Dominic Kwiatkowski, Cordelia Langford, John Sillitoe on behalf of the Wellcome Sanger Institute COVID-19 Surveillance Team
EPI_ISL_581371, EPI_ISL_581374, EPI_ISL_581375, EPI_ISL_581376	Lighthouse Lab in Milton Keynes	Wellcome Sanger Institute for the COVID-19 Genomics UK (COG-UK) consortium	The Lighthouse Lab in Milton Keynes and Alex Alderton, Roberto Amato, Sonia Goncalves, Ewan Harrison, David K. Jackson, Ian Johnston, Dominic Kwiatkowski, Cordelia Langford, John Sillitoe on behalf of the Wellcome Sanger Institute COVID-19 Surveillance Team
EPI_ISL_581377	Lighthouse Lab in Glasgow	Wellcome Sanger Institute for the COVID-19 Genomics UK (COG-UK) consortium	Harper VanSteenhouse, Yumi Kasai, David Gray, Carol Clugston, Anna Dominiczak and Alex Alderton, Roberto Amato, Sonia Goncalves, Ewan Harrison, David K. Jackson, Ian Johnston, Dominic Kwiatkowski, Cordelia Langford, John Sillitoe on behalf of the Wellcome Sanger Institute COVID-19 Surveillance Team
EPI_ISL_581378, EPI_ISL_581379, EPI_ISL_581380	Lighthouse Lab in Alderley Park	Wellcome Sanger Institute for the COVID-19 Genomics UK (COG-UK) consortium	Jacquelyn Wynn, Mairead Hyland, The Lighthouse Lab in Alderley Park and Alex Alderton, Roberto Amato, Sonia Goncalves, Ewan Harrison, David K. Jackson, Ian Johnston, Dominic Kwiatkowski, Cordelia Langford, John Sillitoe on behalf of the Wellcome Sanger Institute COVID-19 Surveillance Team
EPI_ISL_582034, EPI_ISL_582035, EPI_ISL_582038, EPI_ISL_582041, EPI_ISL_582042, EPI_ISL_582044, EPI_ISL_582045, EPI_ISL_582050, EPI_ISL_582051, EPI_ISL_582072, EPI_ISL_582073, EPI_ISL_582074, EPI_ISL_582075, EPI_ISL_582076, EPI_ISL_582077, EPI_ISL_582078, EPI_ISL_582079, EPI_ISL_582080, EPI_ISL_582081, EPI_ISL_582082, EPI_ISL_582083, EPI_ISL_582084, EPI_ISL_582085, EPI_ISL_582086, EPI_ISL_582087			
see above	Servicio de Microbiología. Hospital Universitario Donostia. OSI Donostialdea. Área de Enfermedades Infecciosas, Grupo de Infección Respiratoria y Resistencia Antimicrobiana. Instituto de Investigación Sanitaria Biodonostia	SeqCOVID-SPAIN consortium/IBV(CSIC)	Gustavo Cilla, Milagrosa Montes, Luis Piñeiro, Jose Maria Marimón and SeqCOVID-SPAIN consortium
EPI_ISL_582111, EPI_ISL_582113, EPI_ISL_582116, EPI_ISL_582122	CNR Virus des Infections Respiratoires - France SUD	CNR Virus des Infections Respiratoires - France SUD	Antonin Bal, Gregory Destras, Gwendolyne Burfin, Hadrien Règue, Alexandre Gaymard, Maude Bouscambert-Duchamp, Florence Morfin-Sherpa, Martine Valette, Bruno Lina, Laurence Josset
EPI_ISL_582128, EPI_ISL_582129, EPI_ISL_582130, EPI_ISL_582131, EPI_ISL_582132	Antwerp University Hospital	Institute of Tropical Medicine	Philippe Selhorst, Colin Anthony
EPI_ISL_582508	CNR Virus des Infections Respiratoires - France SUD	CNR Virus des Infections Respiratoires - France SUD	Antonin Bal, Gregory Destras, Gwendolyne Burfin, Hadrien Règue, Alexandre Gaymard, Maude Bouscambert-Duchamp, Florence Morfin-Sherpa, Martine Valette, Bruno Lina, Laurence Josset
EPI_ISL_582664, EPI_ISL_582665, EPI_ISL_582666, EPI_ISL_582667, EPI_ISL_582668, EPI_ISL_582669, EPI_ISL_582670, EPI_ISL_582671, EPI_ISL_582672, EPI_ISL_582673, EPI_ISL_582674, EPI_ISL_582675, EPI_ISL_582676			
see above	Sheikh Khalifa Medical City	Molecular/Surveillance lab Sheikh Khalifa Medical City	Amirtharaj Francis, Sajeed Abdul, Hala Imambaccus, Sahar Almarzooqi, Hiba Saud, Stefan Weber
EPI_ISL_582771, EPI_ISL_582772, EPI_ISL_582773,	Klinisk mikrobiologi Linkoping	The Public Health Agency of Sweden	Anna-Malin Linde, Maria Lind Karlberg, Mattias Haukland, Reza Advani, Olov Svartstrom, Oskar Karlsson Lindsjo, Sandra Broddesson, Petra Edquist, Mia Brytting, Anna Risberg, Karin Tegmark-Wisell

EPI_ISL_582774, EPI_ISL_582775			
EPI_ISL_582938, EPI_ISL_582943, EPI_ISL_582944, EPI_ISL_582945, EPI_ISL_582946, EPI_ISL_582947, EPI_ISL_582948, EPI_ISL_582951, EPI_ISL_582954	County of Santa Clara Public Health Department	Chan-Zuckerberg Biohub	CZB Cliahub Consortium
EPI_ISL_583027, EPI_ISL_583028, EPI_ISL_583029, EPI_ISL_583030, EPI_ISL_583031, EPI_ISL_583032, EPI_ISL_583033, EPI_ISL_583034, EPI_ISL_583035, EPI_ISL_583036, EPI_ISL_583037, EPI_ISL_583038, EPI_ISL_583039, EPI_ISL_583040, EPI_ISL_583041, EPI_ISL_583042, EPI_ISL_583043, EPI_ISL_583044			
see above	Orange County Public Health Lab	Chan-Zuckerberg Biohub	CZB Cliahub Consortium
EPI_ISL_583329, EPI_ISL_583331, EPI_ISL_583337, EPI_ISL_583344, EPI_ISL_583347, EPI_ISL_583348, EPI_ISL_583349, EPI_ISL_583350, EPI_ISL_583351, EPI_ISL_583352, EPI_ISL_583353, EPI_ISL_583354, EPI_ISL_583355, EPI_ISL_583356, EPI_ISL_583357, EPI_ISL_583358, EPI_ISL_583359, EPI_ISL_583360, EPI_ISL_583361, EPI_ISL_583363, EPI_ISL_583364, EPI_ISL_583365, EPI_ISL_583366, EPI_ISL_583367, EPI_ISL_583368, EPI_ISL_583369, EPI_ISL_583370, EPI_ISL_583371, EPI_ISL_583372, EPI_ISL_583373, EPI_ISL_583374, EPI_ISL_583378, EPI_ISL_583382, EPI_ISL_583384, EPI_ISL_583385, EPI_ISL_583386, EPI_ISL_583387, EPI_ISL_583388, EPI_ISL_583389, EPI_ISL_583390, EPI_ISL_583391, EPI_ISL_583392, EPI_ISL_583393, EPI_ISL_583394, EPI_ISL_583395, EPI_ISL_583396, EPI_ISL_583397, EPI_ISL_583398, EPI_ISL_583399, EPI_ISL_583400, EPI_ISL_583401, EPI_ISL_583402, EPI_ISL_583403			
see above	University of Michigan Clinical Microbiology Laboratory	Lauring Lab, University of Michigan, Department of Microbiology and Immunology	Valesano
EPI_ISL_583481	Institute of Virology, Biomedical Research Center of the Slovak Academy of Sciences, Bratislava	Faculty of Natural Sciences, Comenius University, Bratislava	Viktória Hodorová, Kristína Boršová, Broa Brejová, Viktória abanová, Dominika Friová, Sabina Fumaová Havlíková, Juraj Kopáek, Martina Liková, ubomíra Lukáiková, Martina Neboháová, Monika Sláviková, Edita Staroová, Elena Tichá, Tomáš Vina, Jozef Nosek, Boris Klempa
EPI_ISL_583482	Institute of Virology, Biomedical Research Center of the Slovak Academy of Sciences, Bratislava	Faculty of Natural Sciences, Comenius University, Bratislava	Kristína Boršová, Viktória Hodorová, Broa Brejová, Viktória abanová, Dominika Friová, Sabina Fumaová Havlíková, Juraj Kopáek, Martina Liková, ubomíra Lukáiková, Martina Neboháová, Monika Sláviková, Edita Staroová, Elena Tichá, Tomáš Vina, Boris Klempa, Jozef Nosek
EPI_ISL_583483	Institute of Virology, Biomedical Research Center of the Slovak Academy of Sciences, Bratislava	Faculty of Natural Sciences, Comenius University, Bratislava	Viktória Hodorová, Kristína Boršová, Broa Brejová, Viktória abanová, Dominika Friová, Sabina Fumaová Havlíková, Juraj Kopáek, Martina Liková, ubomíra Lukáiková, Martina Neboháová, Monika Sláviková, Edita Staroová, Elena Tichá, Tomáš Vina, Jozef Nosek, Boris Klempa
EPI_ISL_583484	Institute of Virology, Biomedical Research Center of the Slovak Academy of Sciences, Bratislava	Faculty of Natural Sciences, Comenius University, Bratislava	Kristína Boršová, Viktória Hodorová, Broa Brejová, Viktória abanová, Dominika Friová, Sabina Fumaová Havlíková, Juraj Kopáek, Martina Liková, ubomíra Lukáiková, Martina Neboháová, Monika Sláviková, Edita Staroová, Elena Tichá, Tomáš Vina, Boris Klempa, Jozef Nosek
EPI_ISL_583485	Institute of Virology, Biomedical Research Center of the Slovak Academy of Sciences, Bratislava	Faculty of Natural Sciences, Comenius University, Bratislava	Viktória Hodorová, Kristína Boršová, Broa Brejová, Viktória abanová, Dominika Friová, Sabina Fumaová Havlíková, Juraj Kopáek, Martina Liková, ubomíra Lukáiková, Martina Neboháová, Monika Sláviková, Edita Staroová, Elena Tichá, Tomáš Vina, Jozef Nosek, Boris Klempa
EPI_ISL_583486	Institute of Virology, Biomedical Research Center of the Slovak Academy of Sciences, Bratislava	Faculty of Natural Sciences, Comenius University, Bratislava	Kristína Boršová, Viktória Hodorová, Broa Brejová, Viktória abanová, Dominika Friová, Sabina Fumaová Havlíková, Juraj Kopáek, Martina Liková, ubomíra Lukáiková, Martina Neboháová, Monika Sláviková, Edita Staroová, Elena Tichá, Tomáš Vina, Boris Klempa, Jozef Nosek
EPI_ISL_583487, EPI_ISL_583488	Institute of Virology, Biomedical Research Center of the Slovak Academy of Sciences, Bratislava	Faculty of Natural Sciences, Comenius University, Bratislava	Broa Brejová, Viktória Hodorová, Kristína Boršová, Viktória abanová, Dominika Friová, Sabina Fumaová Havlíková, Juraj Kopáek, Martina Liková, ubomíra Lukáiková, Martina Neboháová, Monika Sláviková, Edita Staroová, Elena Tichá, Tomáš Vina, Jozef Nosek, Boris Klempa
EPI_ISL_583489	Institute of Virology, Biomedical Research Center of the Slovak Academy of Sciences, Bratislava	Faculty of Natural Sciences, Comenius University, Bratislava	Viktória Hodorová, Kristína Boršová, Broa Brejová, Viktória abanová, Dominika Friová, Sabina Fumaová Havlíková, Juraj Kopáek, Martina Liková, ubomíra Lukáiková, Martina Neboháová, Monika Sláviková, Edita Staroová, Elena Tichá, Tomáš Vina, Jozef Nosek, Boris Klempa
EPI_ISL_583524	Michigan Department of Health and Human Services, Bureau of Laboratories	Michigan Department of Health and Human Services, Bureau of Laboratories	Blankenship HM, Riner D, Soehnlen MK
EPI_ISL_583993	Respiratory Virus Unit, Microbiology Services Colindale, Public Health England	Respiratory Virus Unit, Microbiology Services Colindale, Public Health England	PHE Covid Sequencing Team
EPI_ISL_584072	IZSM	IZSM	Maurizio Viscardi, Lorena Cardillo, Giovanna Fusco
EPI_ISL_584097	University of Michigan Clinical Microbiology Laboratory	Lauring Lab, University of Michigan, Department of Microbiology and Immunology	Valesano
EPI_ISL_584165, EPI_ISL_584166	Quadram Institute Bioscience	COVID-19 Genomics UK (COG-UK) Consortium	Dave J. Baker, Gemma L. Kay, Alp Aydin, Thanh Le-Viet, Steven Rudder, Ana P. Tedim, Anastasia Kolyva, Maria Diaz, Leonardo de Oliveira Martins, Nabil-Fareed Alikhan, Lizzie Meadows, Rachael Stanley, Ngozi Elumogo, Muhammed Yasir, Nicholas M. Thomson, Alexander J Trotter, Rachel Gilroy, Samuel Bloomfield, Claire Stuart, Andrew Bell, Reenesh Prakash, Samir Dervisevic, Alison E. Mather, John Wain, Mark Webber, Andrew J. Page, Justin O'Grady
EPI_ISL_584370, EPI_ISL_584371, EPI_ISL_584372, EPI_ISL_584377	Virology Department, Sheffield Teaching Hospitals NHS Foundation Trust/Department of Infection, Immunity and Cardiovascular Disease, The Medical School, University of Sheffield	COVID-19 Genomics UK (COG-UK) Consortium	Thushan de Silva, Matthew Parker, Nikki Smith, Adri Angyal, Rebecca Brown, Luke Green, Rachel Tucker, Paul Parsons, Danielle Groves, Katie Johnson, Laura Carrilero, Alex Keeley, Dave Partridge, Matthew Wyles, Benjamin Lindsey, Mehmet Yavuz, Mohammad Raza, Cariad Evans
EPI_ISL_584675, EPI_ISL_584676,	University College London, Great Ormond Street	COVID-19 Genomics UK (COG-UK) Consortium	Sergi Castellano, Rachel Williams, Mark Kristiansen, Paola Resende Silva, Sunando Roy, Tony Brooks, Helena Tutill, Paola Niola, Patricia Dyal, Charlotte Williams, Leysa Forrest, Yasmin Panchbhaya, Jacqueline Findlay, Samuel Weeks, Julianne Brown, Kathryn Harris, Paul Randell, James Price, Alison Holmes, Judith Breuer

EPI_ISL_584677	Hospital for Children NHS Foundation Trust, Imperial College Healthcare NHS Trust		
EPI_ISL_585024, EPI_ISL_585026, EPI_ISL_585035, EPI_ISL_585036, EPI_ISL_585037, EPI_ISL_585038, EPI_ISL_585041, EPI_ISL_585071, EPI_ISL_585090	Virology Department, Sheffield Teaching Hospitals NHS Foundation Trust/Department of Infection, Immunity and Cardiovascular Disease, The Medical School, University of Sheffield	COVID-19 Genomics UK (COG-UK) Consortium	Thushan de Silva, Matthew Parker, Nikki Smith, Adri Angyal, Rebecca Brown, Luke Green, Rachel Tucker, Paul Parsons, Danielle Groves, Katie Johnson, Laura Carrilero, Alex Keeley, Dave Partridge, Matthew Wyles, Benjamin Lindsey, Mehmet Yavuz, Mohammad Raza, Cariad Evans
EPI_ISL_585187, EPI_ISL_585191, EPI_ISL_585192, EPI_ISL_585193, EPI_ISL_585194, EPI_ISL_585196, EPI_ISL_585197, EPI_ISL_585198, EPI_ISL_585199, EPI_ISL_585200, EPI_ISL_585204, EPI_ISL_585205, EPI_ISL_585206, EPI_ISL_585207, EPI_ISL_585208, EPI_ISL_585209, EPI_ISL_585211, EPI_ISL_585213, EPI_ISL_585214, EPI_ISL_585215, EPI_ISL_585216, EPI_ISL_585217, EPI_ISL_585218, EPI_ISL_585219, EPI_ISL_585220, EPI_ISL_585221, EPI_ISL_585222			
see above	Regional Virus Laboratory, Belfast Health and Social Care Trust	COVID-19 Genomics UK (COG-UK) Consortium	Conall McCaughey, James McKenna, Tanya Curran, Susan Feeney, Alison Watt, Ciara Cox, Mairead Connor, Zoltan Molnar, David Simpson, Derek Fairley
EPI_ISL_585261, EPI_ISL_585262, EPI_ISL_585263, EPI_ISL_585264, EPI_ISL_585265, EPI_ISL_585266, EPI_ISL_585272	West of Scotland Specialist Virology Centre, NHSGGC / MRC-University of Glasgow Centre for Virus Research	COVID-19 Genomics UK (COG-UK) Consortium	Ana da Silva Filipe, Natasha Johnson, Kathy Smollett, Daniel Mair, Stephen Carmichael, Lily Tong, Jenna Nichols, Elihu Aranday-Cortes, Kyriaki Nomikou; Sarah McDonald, Marc Niebel, Patawee Asamaphan; Richard Orton, Joseph Hughes, Sreenu Vattipally, David L Robertson; Alasdair MacLean, Rory Gunson; Kathy Li, Igor Starinskij, Natasha Jesudason, Rajiv Shah, James Shepherd, Antonia Ho, Emma Thomson
EPI_ISL_585297, EPI_ISL_585298, EPI_ISL_585299, EPI_ISL_585300, EPI_ISL_585301, EPI_ISL_585302, EPI_ISL_585303, EPI_ISL_585304, EPI_ISL_585421	Lighthouse Lab in Glasgow / MRC-University of Glasgow Centre for Virus Research	COVID-19 Genomics UK (COG-UK) Consortium	Ana da Silva Filipe, Natasha Johnson, Kathy Smollett, Daniel Mair, Stephen Carmichael, Lily Tong, Jenna Nichols, Elihu Aranday-Cortes, Kyriaki Nomikou; Sarah McDonald, Marc Niebel, Patawee Asamaphan; Harper VanSteenhouse, Yumi Kasai, David Gray, Carol Clugston, Anna Dominiczak; Alasdair MacLean, Rory Gunson; Richard Orton, Joseph Hughes, Sreenu Vattipally, David L Robertson; Sharif Shaaban, Matthew Holden; Kathy Li, Natasha Jesudason, Rajiv Shah, James Shepherd, Antonia Ho, Emma Thomson
EPI_ISL_585424	West of Scotland Specialist Virology Centre, NHSGGC / MRC-University of Glasgow Centre for Virus Research	COVID-19 Genomics UK (COG-UK) Consortium	Ana da Silva Filipe, Natasha Johnson, Kathy Smollett, Daniel Mair, Stephen Carmichael, Lily Tong, Jenna Nichols, Elihu Aranday-Cortes, Kyriaki Nomikou; Sarah McDonald, Marc Niebel, Patawee Asamaphan; Richard Orton, Joseph Hughes, Sreenu Vattipally, David L Robertson; Alasdair MacLean, Rory Gunson; Kathy Li, Igor Starinskij, Natasha Jesudason, Rajiv Shah, James Shepherd, Antonia Ho, Emma Thomson
EPI_ISL_585426	Virology Department, Royal Infirmary of Edinburgh, NHS Lothian / School of Biological Sciences, University of Edinburgh / Institute of Genetics and Molecular Medicine, University of Edinburgh	COVID-19 Genomics UK (COG-UK) Consortium	McHugh M, Dewar R, Rooke S, Gallagher M, Balcaza C, O'Toole Á, Scher E, Hill V, McCrone JT, Colquhoun R, Yu X, Jackson B, Rambaut A, Williams TC, Templeton K
EPI_ISL_585626	Virology Department, Sheffield Teaching Hospitals NHS Foundation Trust/Department of Infection, Immunity and Cardiovascular Disease, The Medical School, University of Sheffield	COVID-19 Genomics UK (COG-UK) Consortium	Thushan de Silva, Matthew Parker, Nikki Smith, Adri Angyal, Rebecca Brown, Luke Green, Rachel Tucker, Paul Parsons, Danielle Groves, Katie Johnson, Laura Carrilero, Alex Keeley, Dave Partridge, Matthew Wyles, Benjamin Lindsey, Mehmet Yavuz, Mohammad Raza, Cariad Evans
EPI_ISL_585797	Wales Specialist Virology Centre Sequencing lab: Pathogen Genomics Unit	COVID-19 Genomics UK (COG-UK) Consortium	Catherine Moore, Johnathan Evans, Laura Gifford, Malorie Perry, Simon Cottrell, Angela Marchbank, Alec Birchley, Alexander Adams, Amy Gaskin, Bree Gatica-Wilcox, Jason Coombes, Joel Southgate, Lauren Gilbert, Lee Graham, Nicole Pacchiarini, Sara Kumziene-Summerhayes, Sarah Taylor, Sophie Jones, Sara Rey, Matthew Bull, Joanne Watkins, Sally Corden, Tom Connor
EPI_ISL_589801	Lighthouse Lab in Milton Keynes	Wellcome Sanger Institute for the COVID-19 Genomics UK (COG-UK) consortium	The Lighthouse Lab in Milton Keynes and Alex Alderton, Roberto Amato, Sonia Goncalves, Ewan Harrison, David K. Jackson, Ian Johnston, Dominic Kwiatkowski, Cordelia Langford, John Sillitoe on behalf of the Wellcome Sanger Institute COVID-19 Surveillance Team (http://www.sanger.ac.uk/covid-team)
EPI_ISL_589802	Lighthouse Lab in Alderley Park	Wellcome Sanger Institute for the COVID-19 Genomics UK (COG-UK) consortium	Jacquelyn Wynn, Mairead Hyland, The Lighthouse Lab in Alderley Park and Alex Alderton, Roberto Amato, Sonia Goncalves, Ewan Harrison, David K. Jackson, Ian Johnston, Dominic Kwiatkowski, Cordelia Langford, John Sillitoe on behalf of the Wellcome Sanger Institute COVID-19 Surveillance Team (http://www.sanger.ac.uk/covid-team)
EPI_ISL_589803, EPI_ISL_589804, EPI_ISL_589805, EPI_ISL_589806, EPI_ISL_589807, EPI_ISL_589808, EPI_ISL_589809, EPI_ISL_589810, EPI_ISL_589811, EPI_ISL_589812, EPI_ISL_589813, EPI_ISL_589814, EPI_ISL_589815, EPI_ISL_589816, EPI_ISL_589817, EPI_ISL_589818, EPI_ISL_589819, EPI_ISL_589820, EPI_ISL_589821, EPI_ISL_589822, EPI_ISL_589823, EPI_ISL_589824, EPI_ISL_589825, EPI_ISL_589826, EPI_ISL_589827, EPI_ISL_589828, EPI_ISL_589829, EPI_ISL_589830, EPI_ISL_589831, EPI_ISL_589832, EPI_ISL_589833, EPI_ISL_589834, EPI_ISL_589835, EPI_ISL_589836, EPI_ISL_589837, EPI_ISL_589838, EPI_ISL_589839, EPI_ISL_589840, EPI_ISL_589841, EPI_ISL_589842, EPI_ISL_589843, EPI_ISL_589844, EPI_ISL_589845, EPI_ISL_589846, EPI_ISL_589847, EPI_ISL_589848, EPI_ISL_589849, EPI_ISL_589850, EPI_ISL_589851, EPI_ISL_589852, EPI_ISL_589853, EPI_ISL_589854, EPI_ISL_589855, EPI_ISL_589856, EPI_ISL_589857, EPI_ISL_589858, EPI_ISL_589859, EPI_ISL_589860, EPI_ISL_589861, EPI_ISL_589862, EPI_ISL_589863, EPI_ISL_589864, EPI_ISL_589865, EPI_ISL_589866, EPI_ISL_589867, EPI_ISL_589868, EPI_ISL_589869, EPI_ISL_589870, EPI_ISL_589871, EPI_ISL_589872, EPI_ISL_589873, EPI_ISL_589874, EPI_ISL_589875, EPI_ISL_589876, EPI_ISL_589877, EPI_ISL_589878, EPI_ISL_589879, EPI_ISL_589880, EPI_ISL_589881, EPI_ISL_589882, EPI_ISL_589883, EPI_ISL_589884, EPI_ISL_589885, EPI_ISL_589886, EPI_ISL_589887, EPI_ISL_589888, EPI_ISL_589889, EPI_ISL_589890, EPI_ISL_589891, EPI_ISL_589892, EPI_ISL_589893, EPI_ISL_589894, EPI_ISL_589895, EPI_ISL_589896, EPI_ISL_589897, EPI_ISL_589898, EPI_ISL_589899, EPI_ISL_589900, EPI_ISL_589901, EPI_ISL_589902, EPI_ISL_589903, EPI_ISL_589904, EPI_ISL_589905, EPI_ISL_589906, EPI_ISL_589907, EPI_ISL_589908, EPI_ISL_589909, EPI_ISL_589910, EPI_ISL_589911, EPI_ISL_589912, EPI_ISL_589913, EPI_ISL_589914, EPI_ISL_589915, EPI_ISL_589916, EPI_ISL_589917, EPI_ISL_589918, EPI_ISL_589919, EPI_ISL_589920, EPI_ISL_589921, EPI_ISL_589922, EPI_ISL_589923, EPI_ISL_589924, EPI_ISL_589925, EPI_ISL_589926, EPI_ISL_589927, EPI_ISL_589928, EPI_ISL_589929, EPI_ISL_589930, EPI_ISL_589931, EPI_ISL_589932, EPI_ISL_589933, EPI_ISL_589934, EPI_ISL_589935			
see above	Lighthouse Lab in Glasgow	Wellcome Sanger Institute for the COVID-19 Genomics UK (COG-UK) consortium	Harper VanSteenhouse, Yumi Kasai, David Gray, Carol Clugston, Anna Dominiczak and Alex Alderton, Roberto Amato, Sonia Goncalves, Ewan Harrison, David K. Jackson, Ian Johnston, Dominic Kwiatkowski, Cordelia Langford, John Sillitoe on behalf of the Wellcome Sanger Institute COVID-19 Surveillance Team (http://www.sanger.ac.uk/covid-team)
EPI_ISL_589936	Lighthouse Lab in Glasgow	Wellcome Sanger Institute for the COVID-19 Genomics UK (COG-UK) Consortium	Harper VanSteenhouse, Yumi Kasai, David Gray, Carol Clugston, Anna Dominiczak and Alex Alderton, Roberto Amato, Sonia Goncalves, Ewan Harrison, David K. Jackson, Ian Johnston, Dominic Kwiatkowski, Cordelia Langford, John Sillitoe on behalf of the Wellcome Sanger Institute COVID-19 Surveillance Team
EPI_ISL_589937, EPI_ISL_589938, EPI_ISL_589939, EPI_ISL_589940, EPI_ISL_589941, EPI_ISL_589942, EPI_ISL_589943, EPI_ISL_589944, EPI_ISL_589945, EPI_ISL_589946, EPI_ISL_589947, EPI_ISL_589948, EPI_ISL_589949, EPI_ISL_589950, EPI_ISL_589951, EPI_ISL_589952, EPI_ISL_589953, EPI_ISL_589954, EPI_ISL_589955, EPI_ISL_589956, EPI_ISL_589957, EPI_ISL_589958, EPI_ISL_589959, EPI_ISL_589960, EPI_ISL_589961, EPI_ISL_589962, EPI_ISL_589963, EPI_ISL_589964, EPI_ISL_589965, EPI_ISL_589966, EPI_ISL_589967, EPI_ISL_589968, EPI_ISL_589969, EPI_ISL_589970, EPI_ISL_589971, EPI_ISL_589972, EPI_ISL_589973, EPI_ISL_589974, EPI_ISL_589975, EPI_ISL_589976, EPI_ISL_589977, EPI_ISL_589978, EPI_ISL_589979, EPI_ISL_589980, EPI_ISL_589981, EPI_ISL_589982, EPI_ISL_589983, EPI_ISL_589984, EPI_ISL_589985, EPI_ISL_589986, EPI_ISL_589987, EPI_ISL_589988, EPI_ISL_589990, EPI_ISL_589991,			

EPI_ISL_589992, EPI_ISL_589993, EPI_ISL_589994, EPI_ISL_589995, EPI_ISL_589996, EPI_ISL_589997, EPI_ISL_589998, EPI_ISL_589999, EPI_ISL_590000, EPI_ISL_590001, EPI_ISL_590002, EPI_ISL_590003, EPI_ISL_590004, EPI_ISL_590005, EPI_ISL_590006, EPI_ISL_590007, EPI_ISL_590008, EPI_ISL_590009, EPI_ISL_590010, EPI_ISL_590011, EPI_ISL_590012, EPI_ISL_590013, EPI_ISL_590014, EPI_ISL_590015, EPI_ISL_590016, EPI_ISL_590017, EPI_ISL_590018, EPI_ISL_590019, EPI_ISL_590020, EPI_ISL_590021, EPI_ISL_590022, EPI_ISL_590023, EPI_ISL_590024, EPI_ISL_590025, EPI_ISL_590026, EPI_ISL_590027, EPI_ISL_590028, EPI_ISL_590029, EPI_ISL_590030, EPI_ISL_590031, EPI_ISL_590032, EPI_ISL_590033, EPI_ISL_590034, EPI_ISL_590035, EPI_ISL_590036, EPI_ISL_590037, EPI_ISL_590038, EPI_ISL_590039, EPI_ISL_590040, EPI_ISL_590041, EPI_ISL_590042, EPI_ISL_590043, EPI_ISL_590044, EPI_ISL_590045			
see above	Lighthouse Lab in Glasgow	Wellcome Sanger Institute for the COVID-19 Genomics UK (COG-UK) consortium	Harper VanSteenhouse, Yumi Kasai, David Gray, Carol Clugston, Anna Dominiczak and Alex Alderton, Roberto Amato, Sonia Goncalves, Ewan Harrison, David K. Jackson, Ian Johnston, Dominic Kwiatkowski, Cordelia Langford, John Sillitoe on behalf of the Wellcome Sanger Institute COVID-19 Surveillance Team (http://www.sanger.ac.uk/covid-team)
EPI_ISL_590046	Lighthouse Lab in Glasgow	Wellcome Sanger Institute for the COVID-19 Genomics UK (COG-UK) Consortium	Harper VanSteenhouse, Yumi Kasai, David Gray, Carol Clugston, Anna Dominiczak and Alex Alderton, Roberto Amato, Sonia Goncalves, Ewan Harrison, David K. Jackson, Ian Johnston, Dominic Kwiatkowski, Cordelia Langford, John Sillitoe on behalf of the Wellcome Sanger Institute COVID-19 Surveillance Team
EPI_ISL_590047, EPI_ISL_590048, EPI_ISL_590049, EPI_ISL_590050, EPI_ISL_590051, EPI_ISL_590052, EPI_ISL_590053, EPI_ISL_590054, EPI_ISL_590055, EPI_ISL_590056, EPI_ISL_590057, EPI_ISL_590058, EPI_ISL_590059, EPI_ISL_590060, EPI_ISL_590061, EPI_ISL_590062, EPI_ISL_590063, EPI_ISL_590064, EPI_ISL_590065, EPI_ISL_590066, EPI_ISL_590067, EPI_ISL_590068, EPI_ISL_590069, EPI_ISL_590070, EPI_ISL_590071, EPI_ISL_590072, EPI_ISL_590073, EPI_ISL_590074, EPI_ISL_590075, EPI_ISL_590076, EPI_ISL_590077, EPI_ISL_590078, EPI_ISL_590079, EPI_ISL_590080, EPI_ISL_590081, EPI_ISL_590082, EPI_ISL_590083, EPI_ISL_590084, EPI_ISL_590085, EPI_ISL_590086, EPI_ISL_590087, EPI_ISL_590088, EPI_ISL_590089, EPI_ISL_590090, EPI_ISL_590091, EPI_ISL_590092, EPI_ISL_590093, EPI_ISL_590094, EPI_ISL_590095, EPI_ISL_590096, EPI_ISL_590097, EPI_ISL_590098, EPI_ISL_590099, EPI_ISL_590100, EPI_ISL_590101, EPI_ISL_590102, EPI_ISL_590103, EPI_ISL_590104, EPI_ISL_590105, EPI_ISL_590106, EPI_ISL_590107, EPI_ISL_590108, EPI_ISL_590109, EPI_ISL_590110, EPI_ISL_590111, EPI_ISL_590112, EPI_ISL_590113, EPI_ISL_590114, EPI_ISL_590115, EPI_ISL_590116, EPI_ISL_590117, EPI_ISL_590118, EPI_ISL_590119, EPI_ISL_590120, EPI_ISL_590121, EPI_ISL_590122, EPI_ISL_590123, EPI_ISL_590124, EPI_ISL_590125, EPI_ISL_590126, EPI_ISL_590127, EPI_ISL_590128, EPI_ISL_590129, EPI_ISL_590130, EPI_ISL_590131, EPI_ISL_590132, EPI_ISL_590133, EPI_ISL_590135, EPI_ISL_590136, EPI_ISL_590137, EPI_ISL_590138, EPI_ISL_590139, EPI_ISL_590140, EPI_ISL_590141, EPI_ISL_590142, EPI_ISL_590143, EPI_ISL_590144, EPI_ISL_590145, EPI_ISL_590146, EPI_ISL_590147, EPI_ISL_590148, EPI_ISL_590149, EPI_ISL_590150, EPI_ISL_590151, EPI_ISL_590152, EPI_ISL_590153, EPI_ISL_590154, EPI_ISL_590155, EPI_ISL_590156, EPI_ISL_590157, EPI_ISL_590158, EPI_ISL_590159, EPI_ISL_590160, EPI_ISL_590161, EPI_ISL_590162, EPI_ISL_590163, EPI_ISL_590164, EPI_ISL_590165, EPI_ISL_590166, EPI_ISL_590167, EPI_ISL_590168, EPI_ISL_590169, EPI_ISL_590170, EPI_ISL_590171			
see above	Lighthouse Lab in Glasgow	Wellcome Sanger Institute for the COVID-19 Genomics UK (COG-UK) consortium	Harper VanSteenhouse, Yumi Kasai, David Gray, Carol Clugston, Anna Dominiczak and Alex Alderton, Roberto Amato, Sonia Goncalves, Ewan Harrison, David K. Jackson, Ian Johnston, Dominic Kwiatkowski, Cordelia Langford, John Sillitoe on behalf of the Wellcome Sanger Institute COVID-19 Surveillance Team (http://www.sanger.ac.uk/covid-team)
EPI_ISL_590172	Lighthouse Lab in Milton Keynes	Wellcome Sanger Institute for the COVID-19 Genomics UK (COG-UK) consortium	The Lighthouse Lab in Milton Keynes and Alex Alderton, Roberto Amato, Sonia Goncalves, Ewan Harrison, David K. Jackson, Ian Johnston, Dominic Kwiatkowski, Cordelia Langford, John Sillitoe on behalf of the Wellcome Sanger Institute COVID-19 Surveillance Team (http://www.sanger.ac.uk/covid-team)
EPI_ISL_590176, EPI_ISL_590177	Lighthouse Lab in Glasgow	Wellcome Sanger Institute for the COVID-19 Genomics UK (COG-UK) consortium	Harper VanSteenhouse, Yumi Kasai, David Gray, Carol Clugston, Anna Dominiczak and Alex Alderton, Roberto Amato, Sonia Goncalves, Ewan Harrison, David K. Jackson, Ian Johnston, Dominic Kwiatkowski, Cordelia Langford, John Sillitoe on behalf of the Wellcome Sanger Institute COVID-19 Surveillance Team (http://www.sanger.ac.uk/covid-team)
EPI_ISL_590887, EPI_ISL_590888	Vestfold Hospital, Toensberg Department of Microbiology	Norwegian Institute of Public Health, Department of Virology	Kathrine Stene-Johansen, Kamilla Heddeland Instefjord, Hilde Elshaug, Rasmus Riis Kopperud, Hilde Vollan, Karoline Bragstad, Olav Hungnes
EPI_ISL_590889, EPI_ISL_590890, EPI_ISL_590891, EPI_ISL_590892	Akershus University Hospital, Department for Microbiology and Infectious Disease Control	Norwegian Institute of Public Health, Department of Virology	Kathrine Stene-Johansen, Kamilla Heddeland Instefjord, Hilde Elshaug, Rasmus Riis Kopperud, Hilde Vollan, Karoline Bragstad, Olav Hungnes
EPI_ISL_590893	Medical Microbiology Unit, Department for Laboratory Medicine, Drammen Hospital, Vestre Viken Health Trust,	Norwegian Institute of Public Health, Department of Virology	Kathrine Stene-Johansen, Kamilla Heddeland Instefjord, Hilde Elshaug, Rasmus Riis Kopperud, Hilde Vollan, Karoline Bragstad, Olav Hungnes
EPI_ISL_590896, EPI_ISL_590897	Ostfold Hospital Trust - Kalnes, Centre for Laboratory Medicine, Section for gene technology and infection serology	Norwegian Institute of Public Health, Department of Virology	Kathrine Stene-Johansen, Kamilla Heddeland Instefjord, Hilde Elshaug, Rasmus Riis Kopperud, Hilde Vollan, Karoline Bragstad, Olav Hungnes
EPI_ISL_590927, EPI_ISL_590928, EPI_ISL_590930	Oslo University Hospital, Department of Medical Microbiology	Norwegian Institute of Public Health, Department of Virology	Kathrine Stene-Johansen, Kamilla Heddeland Instefjord, Hilde Elshaug, Rasmus Riis Kopperud, Hilde Vollan, Karoline Bragstad, Olav Hungnes
EPI_ISL_590932	Medical Microbiology Unit, Department for Laboratory Medicine, Drammen Hospital, Vestre Viken Health Trust,	Norwegian Institute of Public Health, Department of Virology	Kathrine Stene-Johansen, Kamilla Heddeland Instefjord, Hilde Elshaug, Rasmus Riis Kopperud, Hilde Vollan, Karoline Bragstad, Olav Hungnes
EPI_ISL_590935	Oslo University Hospital, Department of Medical Microbiology	Norwegian Institute of Public Health, Department of Virology	Kathrine Stene-Johansen, Kamilla Heddeland Instefjord, Hilde Elshaug, Rasmus Riis Kopperud, Hilde Vollan, Karoline Bragstad, Olav Hungnes
EPI_ISL_590936	Hospital of Southern Norway - Kristiansand, Department of Medical Microbiology	Norwegian Institute of Public Health, Department of Virology	Kathrine Stene-Johansen, Kamilla Heddeland Instefjord, Hilde Elshaug, Rasmus Riis Kopperud, Hilde Vollan, Karoline Bragstad, Olav Hungnes
EPI_ISL_590982	Vestfold Hospital, Toensberg Department of Microbiology	Norwegian Institute of Public Health, Department of Virology	Kathrine Stene-Johansen, Kamilla Heddeland Instefjord, Hilde Elshaug, Rasmus Riis Kopperud, Hilde Vollan, Karoline Bragstad, Olav Hungnes
EPI_ISL_591493	Pathology North - Royal North Shore Hospital - NSW Health Pathology	NSW Health Pathology - Institute of Clinical Pathology and Medical Research; Westmead Hospital; University of Sydney	CIDM-PH et al.
EPI_ISL_591496	Pathology West - NSW Health Pathology	NSW Health Pathology - Institute of Clinical Pathology and Medical Research; Westmead Hospital; University of Sydney	CIDM-PH et al.
EPI_ISL_591502	South Eastern Area Laboratory Services (SEALS)	NSW Health Pathology - Institute of Clinical Pathology and Medical Research; Westmead Hospital; University of Sydney	CIDM-PH et al.
EPI_ISL_591582, EPI_ISL_591589, EPI_ISL_591604, EPI_ISL_591629	Microbiological Diagnostic Unit - Public Health Laboratory (MDU-PHL)	MDU-PHL	Seemann T., Schultz, M. B., Sait, M., Sherry, N.

EPI_ISL_591645	Victorian Infectious Diseases Reference Laboratory (VIDRL)	VIDRL and MDU-PHL	Caly L., Seemann T., Sait, M., Schultz, M. B., Druce J., Sherry, N.
EPI_ISL_591646	Microbiological Diagnostic Unit - Public Health Laboratory (MDU-PHL)	MDU-PHL	Seemann T., Schultz, M. B., Sait, M., Sherry, N.
EPI_ISL_591653, EPI_ISL_591667	Victorian Infectious Diseases Reference Laboratory (VIDRL)	VIDRL and MDU-PHL	Caly L., Seemann T., Sait, M., Schultz, M. B., Druce J., Sherry, N.
EPI_ISL_591757	Microbiological Diagnostic Unit - Public Health Laboratory (MDU-PHL)	MDU-PHL	Seemann T., Schultz, M. B., Sait, M., Sherry, N.
EPI_ISL_591760	Victorian Infectious Diseases Reference Laboratory (VIDRL)	VIDRL and MDU-PHL	Caly L., Seemann T., Sait, M., Schultz, M. B., Druce J., Sherry, N.
EPI_ISL_591814, EPI_ISL_592310, EPI_ISL_592342, EPI_ISL_592344, EPI_ISL_592345, EPI_ISL_592347, EPI_ISL_592348, EPI_ISL_592349	Microbiological Diagnostic Unit - Public Health Laboratory (MDU-PHL)	MDU-PHL	Seemann T., Schultz, M. B., Sait, M., Sherry, N.
EPI_ISL_592509, EPI_ISL_592600	Victorian Infectious Diseases Reference Laboratory (VIDRL)	VIDRL and MDU-PHL	Caly L., Seemann T., Sait, M., Schultz, M. B., Druce J., Sherry, N.
EPI_ISL_592646, EPI_ISL_592681, EPI_ISL_592734, EPI_ISL_592762, EPI_ISL_592788, EPI_ISL_592935, EPI_ISL_592965	Microbiological Diagnostic Unit - Public Health Laboratory (MDU-PHL)	MDU-PHL	Seemann T., Schultz, M. B., Sait, M., Sherry, N.
EPI_ISL_592979	Victorian Infectious Diseases Reference Laboratory (VIDRL)	VIDRL and MDU-PHL	Caly L., Seemann T., Sait, M., Schultz, M. B., Druce J., Sherry, N.
EPI_ISL_592988, EPI_ISL_592989, EPI_ISL_593227, EPI_ISL_593230, EPI_ISL_593231, EPI_ISL_593241	Microbiological Diagnostic Unit - Public Health Laboratory (MDU-PHL)	MDU-PHL	Seemann T., Schultz, M. B., Sait, M., Sherry, N.
EPI_ISL_593617, EPI_ISL_593644	unknown	Public Health Virology Laboratory, Forensic and Scientific Services (PHV-FSS)	Son Nguyen et al.
EPI_ISL_593729, EPI_ISL_593730, EPI_ISL_593731, EPI_ISL_593732, EPI_ISL_593733, EPI_ISL_593734, EPI_ISL_593735, EPI_ISL_593736, EPI_ISL_593737, EPI_ISL_593739, EPI_ISL_593740, EPI_ISL_593750			
see above	South Eastern Area Laboratory Services (SEALS)	NSW Health Pathology - Institute of Clinical Pathology and Medical Research; Westmead Hospital; University of Sydney	CIDM-PH et al.
EPI_ISL_593770	Pathology West - NSW Health Pathology	NSW Health Pathology - Institute of Clinical Pathology and Medical Research; Westmead Hospital; University of Sydney	CIDM-PH et al.
EPI_ISL_593829, EPI_ISL_593831, EPI_ISL_593834, EPI_ISL_593843, EPI_ISL_593846, EPI_ISL_593848, EPI_ISL_593850	Respiratory Virus Unit, Microbiology Services Colindale, Public Health England	Respiratory Virus Unit, Microbiology Services Colindale, Public Health England	PHE Covid Sequencing Team
EPI_ISL_593902	Sentinelles, Limay	National Reference Center for Viruses of Respiratory Infections, Institut Pasteur, Paris	Sylvie Behillil, Fabiana Gambaro, Etienne Simon-Lorière, Vincent Enouf, Maud Vanpeene, Sylvie van der Werf
EPI_ISL_593904, EPI_ISL_593905, EPI_ISL_593906, EPI_ISL_593907, EPI_ISL_593909	Labo Analyses Med, Sarcelles	National Reference Center for Viruses of Respiratory Infections, Institut Pasteur, Paris	Sylvie Behillil, Fabiana Gambaro, Etienne Simon-Lorière, Vincent Enouf, Maud Vanpeene, Sylvie van der Werf
EPI_ISL_593910	Hospital, Boulogne-Billancourt	National Reference Center for Viruses of Respiratory Infections, Institut Pasteur, Paris	Sylvie Behillil, Fabiana Gambaro, Etienne Simon-Lorière, Vincent Enouf, Maud Vanpeene, Sylvie van der Werf
EPI_ISL_593924,	Labo Analyses Med,	National Reference Center	Sylvie Behillil, Fabiana Gambaro, Etienne Simon-Lorière, Vincent Enouf, Maud Vanpeene, Sylvie van der Werf

EPI_ISL_593926, EPI_ISL_593927, EPI_ISL_593928	Puteaux	for Viruses of Respiratory Infections, Institut Pasteur, Paris	
EPI_ISL_594149, EPI_ISL_594150, EPI_ISL_594151, EPI_ISL_594152, EPI_ISL_594153	Klinisk mikrobiologi Linkoping	The Public Health Agency of Sweden	Anna-Malin Linde, Maria Lind Karlberg, Mattias Haukland, Reza Advani, Olov Svartstrom, Oskar Karlsson Lindsjo, Sandra Broddesson, Petra Edquist, Mia Brytting, Anna Risberg, Karin Tegmark-Wisell
EPI_ISL_594312, EPI_ISL_594321, EPI_ISL_594322, EPI_ISL_594323, EPI_ISL_594324, EPI_ISL_594325, EPI_ISL_594326	Florida Bureau of Public Health Laboratories	Florida Bureau of Public Health Laboratories	Sarah Schmedes, Jason Blanton
EPI_ISL_594516, EPI_ISL_594517, EPI_ISL_594518, EPI_ISL_594519, EPI_ISL_594520, EPI_ISL_594521	University of Birmingham	COVID-19 Genomics UK (COG-UK) Consortium	Institute of Microbiology, University of Birmingham: Claire McMurray, Joanne Stockton, Samuel Nicholls, Radoslaw Poplawski, Will Rowe, Josh Quick, Nicholas Loman. University of Birmingham Testing Laboratory: Celina M Whalley, Andrew Bosworth, Charlotte Poxon, Kasun Wanigasooriya, Oliver Pickles, Mike Kidd, Alex Richter, Andrew D Beggs PHE Heartlands Lab: Husam Osman, Andrew Bosworth. Queen Elizabeth Hospital: Anna Casey
EPI_ISL_594849	Liverpool Clinical Laboratories	COVID-19 Genomics UK (COG-UK) Consortium	Sam Haldenby, Anita Lucaci, Steve Paterson, Julian Hiscox, Alistair Darby, M Almsaud, A Alrezaihi, Muhannad Alruwaili, Stuart D Armstrong, Jones Benjamin, Eleanor G Bentley, Anu Chawla, Jordan J Clark, Angela Cowell, Richard Eccles, Isabel García-Dorival, Matthew Gemmell, Alessandro Gerada, PKF Gilmore, Richard Gregory, Ximeng Han, Catherine Hartley, Margaret Hughes, Miren Iturriza-Gomara, James Johnson, L Luu, Jenifer Manson, Charlotte Nelson, Elaine O'Toole, Cassie Olateju, Rebekah Penrice-Randal , Lucille Rainbow, N.P Randle, Trevor Ian Robinson, Parul Sharma, Ghada T Shawli, James P Stewart, Neil Swainston, Ecaterina Vamos, Joanne Watts, Mark Whitehead
EPI_ISL_596232	WHO National Influenza Centre Russian Federation	WHO National Influenza Centre Russian Federation	Andrey Komissarov, Artem Fadeev, Anna Ivanova, Mariia Sergeeva, Kseniya Komissarova, Dmitry Bazhenov, Daria Danilenko
EPI_ISL_596235, EPI_ISL_596236	WHO National Influenza Centre Russian Federation	WHO National Influenza Centre Russian Federation	Andrey Komissarov, Artem Fadeev, Anna Ivanova, Kseniya Komissarova, Dmitry Bazhenov, Daria Danilenko
EPI_ISL_596268, EPI_ISL_596269, EPI_ISL_596272, EPI_ISL_596273, EPI_ISL_596274, EPI_ISL_596280, EPI_ISL_596281, EPI_ISL_596282, EPI_ISL_596284, EPI_ISL_596285, EPI_ISL_596286, EPI_ISL_596287, EPI_ISL_596288, EPI_ISL_596289, EPI_ISL_596290, EPI_ISL_596291, EPI_ISL_596292, EPI_ISL_596293, EPI_ISL_596294, EPI_ISL_596303, EPI_ISL_596304, EPI_ISL_596309			
see above	HELIX LCC	WHO National Influenza Centre Russian Federation	Andrey Komissarov, Artem Fadeev, Anna Ivanova, Kseniya Komissarova, Dmitry Bazhenov, Daria Danilenko
EPI_ISL_596387	Seattle Flu Study	Seattle Flu Study	Deborah A. Nickerson, Chris D. Frazar, Jover Lee, Benjamin Pelle, Matthew Richardson, Amanda Adler, Elisabeth Brandstetter, Peter D. Han, Kairsten Fay, Misja Ilcisin, Kirsten Lacombe, Thomas R. Sibley, Melissa Truong, Caitlin R. Wolf, Michael Boeckh, Janet A. Englund, Michael Famulare, Barry R. Lutz, Mark J. Rieder, Lea M. Starita, Matthew Thompson, Jay Shendure, Trevor Bedford, Helen Y. Chu
EPI_ISL_596388, EPI_ISL_596389, EPI_ISL_596390, EPI_ISL_596391	Seattle Flu Study	Seattle Flu Study	Deborah A. Nickerson, Chris D. Frazar, Jover Lee, Benjamin Pelle, Matthew Richardson, Amanda Adler, Elisabeth Brandstetter, Peter D. Han, Kairsten Fay, Misja Ilcisin, Kirsten Lacombe, Thomas R. Sibley, Melissa Truong, Caitlin R. Wolf, Karen Cowgill, Stephanie Schrag, Jeff Duchin, Michael Boeckh, Janet A. Englund, Michael Famulare, Barry R. Lutz, Mark J. Rieder, Lea M. Starita, Matthew Thompson, Helen Y. Chu, Trevor Bedford, Jay Shendure
EPI_ISL_596734	PathWest Laboratory Medicine WA	PathWest Laboratory Medicine WA Microbial Surveillance Unit	PathWest Laboratory Medicine WA Microbial Surveillance Unit
EPI_ISL_596890, EPI_ISL_596892, EPI_ISL_596893, EPI_ISL_596895, EPI_ISL_596897, EPI_ISL_596898, EPI_ISL_596899, EPI_ISL_596900, EPI_ISL_596901, EPI_ISL_596902, EPI_ISL_596904, EPI_ISL_596909, EPI_ISL_596910, EPI_ISL_596911, EPI_ISL_596912, EPI_ISL_596913, EPI_ISL_596914, EPI_ISL_596915, EPI_ISL_596916, EPI_ISL_596917, EPI_ISL_596918, EPI_ISL_596919, EPI_ISL_596920, EPI_ISL_596921, EPI_ISL_596922, EPI_ISL_596923, EPI_ISL_596924, EPI_ISL_596925, EPI_ISL_596926, EPI_ISL_596927			
see above	National Virus Reference Laboratory	National Virus Reference Laboratory	Michael Carr, Gabriel Gonzalez, Jonathan Dean, Daniel Hare, Cillian F De Gascun
EPI_ISL_601478, EPI_ISL_601490, EPI_ISL_601491, EPI_ISL_601507, EPI_ISL_601513, EPI_ISL_601520, EPI_ISL_601529, EPI_ISL_601536, EPI_ISL_601539, EPI_ISL_601546, EPI_ISL_601564, EPI_ISL_601597, EPI_ISL_601599, EPI_ISL_601612, EPI_ISL_601630, EPI_ISL_601635, EPI_ISL_601637, EPI_ISL_601640, EPI_ISL_601645, EPI_ISL_601647, EPI_ISL_601649			
see above	Lighthouse Lab in Milton Keynes	Wellcome Sanger Institute for the COVID-19 Genomics UK (COG-UK) consortium	The Lighthouse Lab in Milton Keynes and Alex Alderton, Roberto Amato, Sonia Goncalves, Ewan Harrison, David K. Jackson, Ian Johnston, Dominic Kwiatkowski, Cordelia Langford, John Sillitoe on behalf of the Wellcome Sanger Institute COVID-19 Surveillance Team (http://www.sanger.ac.uk/covid-team)
EPI_ISL_601667	Lighthouse Lab in Milton Keynes	Wellcome Sanger Institute for the COVID-19 Genomics UK (COG-UK) Consortium	The Lighthouse Lab in Milton Keynes and Alex Alderton, Roberto Amato, Sonia Goncalves, Ewan Harrison, David K. Jackson, Ian Johnston, Dominic Kwiatkowski, Cordelia Langford, John Sillitoe on behalf of the Wellcome Sanger Institute COVID-19 Surveillance Team
EPI_ISL_601669, EPI_ISL_601671, EPI_ISL_601686, EPI_ISL_601694, EPI_ISL_601706, EPI_ISL_601711, EPI_ISL_601737, EPI_ISL_601745, EPI_ISL_601746, EPI_ISL_601751, EPI_ISL_601752, EPI_ISL_601761, EPI_ISL_601774, EPI_ISL_601784			
see above	Lighthouse Lab in Milton Keynes	Wellcome Sanger Institute for the COVID-19 Genomics UK (COG-UK) consortium	The Lighthouse Lab in Milton Keynes and Alex Alderton, Roberto Amato, Sonia Goncalves, Ewan Harrison, David K. Jackson, Ian Johnston, Dominic Kwiatkowski, Cordelia Langford, John Sillitoe on behalf of the Wellcome Sanger Institute COVID-19 Surveillance Team (http://www.sanger.ac.uk/covid-team)
EPI_ISL_601785	Lighthouse Lab in Cambridge	Wellcome Sanger Institute for the COVID-19 Genomics UK (COG-UK) consortium	Rob Howes, The Lighthouse Lab in Cambridge and Alex Alderton, Roberto Amato, Sonia Goncalves, Ewan Harrison, David K. Jackson, Ian Johnston, Dominic Kwiatkowski, Cordelia Langford, John Sillitoe on behalf of the Wellcome Sanger Institute COVID-19 Surveillance Team (http://www.sanger.ac.uk/covid-team)
EPI_ISL_601787, EPI_ISL_601788, EPI_ISL_601789, EPI_ISL_601790, EPI_ISL_601791, EPI_ISL_601792, EPI_ISL_601793, EPI_ISL_601794, EPI_ISL_601795, EPI_ISL_601796	Lighthouse Lab in Glasgow	Wellcome Sanger Institute for the COVID-19 Genomics UK (COG-UK) consortium	Harper VanSteenhouse, Yumi Kasai, David Gray, Carol Clugston, Anna Dominiczak and Alex Alderton, Roberto Amato, Sonia Goncalves, Ewan Harrison, David K. Jackson, Ian Johnston, Dominic Kwiatkowski, Cordelia Langford, John Sillitoe on behalf of the Wellcome Sanger Institute COVID-19 Surveillance Team (http://www.sanger.ac.uk/covid-team)
EPI_ISL_601797	Lighthouse Lab in Glasgow	Wellcome Sanger Institute for the COVID-19 Genomics UK (COG-UK) Consortium	Harper VanSteenhouse, Yumi Kasai, David Gray, Carol Clugston, Anna Dominiczak and Alex Alderton, Roberto Amato, Sonia Goncalves, Ewan Harrison, David K. Jackson, Ian Johnston, Dominic Kwiatkowski, Cordelia Langford, John Sillitoe on behalf of the Wellcome Sanger Institute COVID-19 Surveillance Team
EPI_ISL_601798, EPI_ISL_601799, EPI_ISL_601800, EPI_ISL_601801, EPI_ISL_601802, EPI_ISL_601803, EPI_ISL_601804, EPI_ISL_601805, EPI_ISL_601806, EPI_ISL_601807, EPI_ISL_601808, EPI_ISL_601809, EPI_ISL_601810, EPI_ISL_601811, EPI_ISL_601812, EPI_ISL_601813, EPI_ISL_601814, EPI_ISL_601815, EPI_ISL_601816, EPI_ISL_601817, EPI_ISL_601818, EPI_ISL_601819, EPI_ISL_601820, EPI_ISL_601821, EPI_ISL_601822, EPI_ISL_601823, EPI_ISL_601824, EPI_ISL_601825, EPI_ISL_601826, EPI_ISL_601827, EPI_ISL_601828, EPI_ISL_601829, EPI_ISL_601830, EPI_ISL_601831, EPI_ISL_601832, EPI_ISL_601833			
see above	Lighthouse Lab in Glasgow	Wellcome Sanger Institute	Harper VanSteenhouse, Yumi Kasai, David Gray, Carol Clugston, Anna Dominiczak and Alex Alderton, Roberto Amato, Sonia Goncalves, Ewan Harrison, David K. Jackson, Ian Johnston, Dominic Kwiatkowski, Cordelia Langford, John Sillitoe on

		for the COVID-19 Genomics UK (COG-UK) consortium	behalf of the Wellcome Sanger Institute COVID-19 Surveillance Team (http://www.sanger.ac.uk/covid-team)
EPI_ISL_601834	Lighthouse Lab in Glasgow	Wellcome Sanger Institute for the COVID-19 Genomics UK (COG-UK) Consortium	Harper VanSteenhouse, Yumi Kasai, David Gray, Carol Clugston, Anna Dominiczak and Alex Alderton, Roberto Amato, Sonia Goncalves, Ewan Harrison, David K. Jackson, Ian Johnston, Dominic Kwiatkowski, Cordelia Langford, John Sillitoe on behalf of the Wellcome Sanger Institute COVID-19 Surveillance Team
EPI_ISL_601835, EPI_ISL_601836, EPI_ISL_601837, EPI_ISL_601838, EPI_ISL_601839, EPI_ISL_601840, EPI_ISL_601841, EPI_ISL_601842, EPI_ISL_601843, EPI_ISL_601844, EPI_ISL_601845, EPI_ISL_601846, EPI_ISL_601847, EPI_ISL_601848, EPI_ISL_601849, EPI_ISL_601850, EPI_ISL_601851, EPI_ISL_601852, EPI_ISL_601853, EPI_ISL_601854, EPI_ISL_601855, EPI_ISL_601856, EPI_ISL_601857, EPI_ISL_601858, EPI_ISL_601859, EPI_ISL_601860, EPI_ISL_601861, EPI_ISL_601862, EPI_ISL_601863, EPI_ISL_601864, EPI_ISL_601865, EPI_ISL_601866, EPI_ISL_601867, EPI_ISL_601868, EPI_ISL_601869, EPI_ISL_601870, EPI_ISL_601871, EPI_ISL_601872, EPI_ISL_601873, EPI_ISL_601874, EPI_ISL_601875, EPI_ISL_601876, EPI_ISL_601877, EPI_ISL_601878, EPI_ISL_601879, EPI_ISL_601880, EPI_ISL_601881, EPI_ISL_601882, EPI_ISL_601883, EPI_ISL_601884, EPI_ISL_601885, EPI_ISL_601886, EPI_ISL_601887, EPI_ISL_601888, EPI_ISL_601889, EPI_ISL_601890, EPI_ISL_601891, EPI_ISL_601892, EPI_ISL_601893, EPI_ISL_601894, EPI_ISL_601895, EPI_ISL_601896, EPI_ISL_601897, EPI_ISL_601898, EPI_ISL_601899, EPI_ISL_601900, EPI_ISL_601901, EPI_ISL_601902, EPI_ISL_601903, EPI_ISL_601904, EPI_ISL_601905, EPI_ISL_601906, EPI_ISL_601907, EPI_ISL_601908, EPI_ISL_601909, EPI_ISL_601910, EPI_ISL_601911, EPI_ISL_601912, EPI_ISL_601913, EPI_ISL_601914, EPI_ISL_601915, EPI_ISL_601916, EPI_ISL_601917, EPI_ISL_601918, EPI_ISL_601919, EPI_ISL_601920, EPI_ISL_601921, EPI_ISL_601923, EPI_ISL_601924, EPI_ISL_601925, EPI_ISL_601926, EPI_ISL_601927, EPI_ISL_601928, EPI_ISL_601929, EPI_ISL_601930, EPI_ISL_601931, EPI_ISL_601932, EPI_ISL_601933, EPI_ISL_601934, EPI_ISL_601935, EPI_ISL_601936, EPI_ISL_601937, EPI_ISL_601938, EPI_ISL_601939, EPI_ISL_601940, EPI_ISL_601941, EPI_ISL_601942, EPI_ISL_601943, EPI_ISL_601944, EPI_ISL_601945, EPI_ISL_601946, EPI_ISL_601947, EPI_ISL_601948, EPI_ISL_601949			
see above	Lighthouse Lab in Glasgow	Wellcome Sanger Institute for the COVID-19 Genomics UK (COG-UK) consortium	Harper VanSteenhouse, Yumi Kasai, David Gray, Carol Clugston, Anna Dominiczak and Alex Alderton, Roberto Amato, Sonia Goncalves, Ewan Harrison, David K. Jackson, Ian Johnston, Dominic Kwiatkowski, Cordelia Langford, John Sillitoe on behalf of the Wellcome Sanger Institute COVID-19 Surveillance Team (http://www.sanger.ac.uk/covid-team)
EPI_ISL_601950	Lighthouse Lab in Glasgow	Wellcome Sanger Institute for the COVID-19 Genomics UK (COG-UK) Consortium	Harper VanSteenhouse, Yumi Kasai, David Gray, Carol Clugston, Anna Dominiczak and Alex Alderton, Roberto Amato, Sonia Goncalves, Ewan Harrison, David K. Jackson, Ian Johnston, Dominic Kwiatkowski, Cordelia Langford, John Sillitoe on behalf of the Wellcome Sanger Institute COVID-19 Surveillance Team
EPI_ISL_601951, EPI_ISL_601952, EPI_ISL_601953, EPI_ISL_601954, EPI_ISL_601955, EPI_ISL_601956, EPI_ISL_601957, EPI_ISL_601958, EPI_ISL_601959, EPI_ISL_601960, EPI_ISL_601961, EPI_ISL_601962, EPI_ISL_601963, EPI_ISL_601964, EPI_ISL_601965, EPI_ISL_601966, EPI_ISL_601967, EPI_ISL_601968, EPI_ISL_601969, EPI_ISL_601970, EPI_ISL_601971, EPI_ISL_601972, EPI_ISL_601973, EPI_ISL_601974, EPI_ISL_601975, EPI_ISL_601976, EPI_ISL_601977, EPI_ISL_601978, EPI_ISL_601979, EPI_ISL_601980, EPI_ISL_601981, EPI_ISL_601982, EPI_ISL_601983, EPI_ISL_601984, EPI_ISL_601985, EPI_ISL_601986, EPI_ISL_601987, EPI_ISL_601988, EPI_ISL_601989, EPI_ISL_601990, EPI_ISL_601991, EPI_ISL_601992, EPI_ISL_601993, EPI_ISL_601994, EPI_ISL_601995, EPI_ISL_601996, EPI_ISL_601997, EPI_ISL_601998, EPI_ISL_601999, EPI_ISL_602000, EPI_ISL_602001, EPI_ISL_602002, EPI_ISL_602003, EPI_ISL_602004, EPI_ISL_602005			
see above	Lighthouse Lab in Glasgow	Wellcome Sanger Institute for the COVID-19 Genomics UK (COG-UK) consortium	Harper VanSteenhouse, Yumi Kasai, David Gray, Carol Clugston, Anna Dominiczak and Alex Alderton, Roberto Amato, Sonia Goncalves, Ewan Harrison, David K. Jackson, Ian Johnston, Dominic Kwiatkowski, Cordelia Langford, John Sillitoe on behalf of the Wellcome Sanger Institute COVID-19 Surveillance Team (http://www.sanger.ac.uk/covid-team)
EPI_ISL_602006	Lighthouse Lab in Glasgow	Wellcome Sanger Institute for the COVID-19 Genomics UK (COG-UK) Consortium	Harper VanSteenhouse, Yumi Kasai, David Gray, Carol Clugston, Anna Dominiczak and Alex Alderton, Roberto Amato, Sonia Goncalves, Ewan Harrison, David K. Jackson, Ian Johnston, Dominic Kwiatkowski, Cordelia Langford, John Sillitoe on behalf of the Wellcome Sanger Institute COVID-19 Surveillance Team
EPI_ISL_602007, EPI_ISL_602008, EPI_ISL_602009, EPI_ISL_602010, EPI_ISL_602011, EPI_ISL_602012, EPI_ISL_602013, EPI_ISL_602014	Lighthouse Lab in Glasgow	Wellcome Sanger Institute for the COVID-19 Genomics UK (COG-UK) consortium	Harper VanSteenhouse, Yumi Kasai, David Gray, Carol Clugston, Anna Dominiczak and Alex Alderton, Roberto Amato, Sonia Goncalves, Ewan Harrison, David K. Jackson, Ian Johnston, Dominic Kwiatkowski, Cordelia Langford, John Sillitoe on behalf of the Wellcome Sanger Institute COVID-19 Surveillance Team (http://www.sanger.ac.uk/covid-team)
EPI_ISL_602015	Lighthouse Lab in Glasgow	Wellcome Sanger Institute for the COVID-19 Genomics UK (COG-UK) Consortium	Harper VanSteenhouse, Yumi Kasai, David Gray, Carol Clugston, Anna Dominiczak and Alex Alderton, Roberto Amato, Sonia Goncalves, Ewan Harrison, David K. Jackson, Ian Johnston, Dominic Kwiatkowski, Cordelia Langford, John Sillitoe on behalf of the Wellcome Sanger Institute COVID-19 Surveillance Team
EPI_ISL_602016, EPI_ISL_602017, EPI_ISL_602018, EPI_ISL_602019, EPI_ISL_602020, EPI_ISL_602021, EPI_ISL_602022, EPI_ISL_602023, EPI_ISL_602024, EPI_ISL_602025, EPI_ISL_602026, EPI_ISL_602030, EPI_ISL_602031, EPI_ISL_602032, EPI_ISL_602033, EPI_ISL_602034, EPI_ISL_602035, EPI_ISL_602036, EPI_ISL_602037, EPI_ISL_602038, EPI_ISL_602039, EPI_ISL_602040, EPI_ISL_602041, EPI_ISL_602042			
see above	Lighthouse Lab in Glasgow	Wellcome Sanger Institute for the COVID-19 Genomics UK (COG-UK) consortium	Harper VanSteenhouse, Yumi Kasai, David Gray, Carol Clugston, Anna Dominiczak and Alex Alderton, Roberto Amato, Sonia Goncalves, Ewan Harrison, David K. Jackson, Ian Johnston, Dominic Kwiatkowski, Cordelia Langford, John Sillitoe on behalf of the Wellcome Sanger Institute COVID-19 Surveillance Team (http://www.sanger.ac.uk/covid-team)
EPI_ISL_602336, EPI_ISL_602337, EPI_ISL_602338, EPI_ISL_602339, EPI_ISL_602340, EPI_ISL_602342, EPI_ISL_602344, EPI_ISL_602345, EPI_ISL_602348, EPI_ISL_602349, EPI_ISL_602350, EPI_ISL_602351, EPI_ISL_602352, EPI_ISL_602353, EPI_ISL_602360, EPI_ISL_602361, EPI_ISL_602362, EPI_ISL_602363, EPI_ISL_602364, EPI_ISL_602365, EPI_ISL_602366, EPI_ISL_602367, EPI_ISL_602368, EPI_ISL_602369, EPI_ISL_602370, EPI_ISL_602371, EPI_ISL_602372, EPI_ISL_602374, EPI_ISL_602375, EPI_ISL_602376, EPI_ISL_602404			
see above	HELIX LLC	WHO National Influenza Centre Russian Federation	Andrey Komissarov, Artem Fadeev, Kseniya Komissarova, Anna Ivanova, Dmitry Bazhenov, Daria Danilenko
EPI_ISL_602413, EPI_ISL_602414, EPI_ISL_602415, EPI_ISL_602416	WHO National Influenza Centre Russian Federation	WHO National Influenza Centre Russian Federation	Andrey Komissarov, Artem Fadeev, Kseniya Komissarova, Anna Ivanova, Dmitry Bazhenov, Daria Danilenko
EPI_ISL_602417, EPI_ISL_602418, EPI_ISL_602424, EPI_ISL_602427, EPI_ISL_602428, EPI_ISL_602429, EPI_ISL_602442, EPI_ISL_602443, EPI_ISL_602453, EPI_ISL_602457, EPI_ISL_602460			
see above	HELIX LLC	WHO National Influenza Centre Russian Federation	Andrey Komissarov, Artem Fadeev, Kseniya Komissarova, Anna Ivanova, Dmitry Bazhenov, Daria Danilenko
EPI_ISL_602761, EPI_ISL_602762, EPI_ISL_602763, EPI_ISL_602764, EPI_ISL_602765, EPI_ISL_602767, EPI_ISL_602769, EPI_ISL_602770, EPI_ISL_602771, EPI_ISL_602772, EPI_ISL_602773, EPI_ISL_602774, EPI_ISL_602775, EPI_ISL_602776, EPI_ISL_602777, EPI_ISL_602778, EPI_ISL_602782, EPI_ISL_602783, EPI_ISL_602784, EPI_ISL_602785, EPI_ISL_602786, EPI_ISL_602787, EPI_ISL_602788, EPI_ISL_602789, EPI_ISL_602790, EPI_ISL_602791, EPI_ISL_602792, EPI_ISL_602793, EPI_ISL_602794, EPI_ISL_602795, EPI_ISL_602796, EPI_ISL_602797, EPI_ISL_602798, EPI_ISL_602799, EPI_ISL_602800, EPI_ISL_602801, EPI_ISL_602802, EPI_ISL_602803, EPI_ISL_602804, EPI_ISL_602805, EPI_ISL_602806, EPI_ISL_602807, EPI_ISL_602808, EPI_ISL_602809, EPI_ISL_602810, EPI_ISL_602811, EPI_ISL_602812, EPI_ISL_602813, EPI_ISL_602814, EPI_ISL_602815, EPI_ISL_602816, EPI_ISL_602817, EPI_ISL_602818, EPI_ISL_602819, EPI_ISL_602820, EPI_ISL_602821, EPI_ISL_602822, EPI_ISL_602823, EPI_ISL_602824, EPI_ISL_602825, EPI_ISL_602826, EPI_ISL_602827, EPI_ISL_602828, EPI_ISL_602829			
see above	NHLS-IALCH	KRISP, KZN Research Innovation and Sequencing Platform	Giandhari J, Pillay S, Lessells R, Mdlalose K, York D, Khan S, Tegally H, Wilkinson E, de Oliveira T
EPI_ISL_602958, EPI_ISL_602959, EPI_ISL_602960, EPI_ISL_602961, EPI_ISL_602966, EPI_ISL_602967, EPI_ISL_602968, EPI_ISL_602969, EPI_ISL_602970, EPI_ISL_602971	Minnesota Department of Health, Public Health Laboratory	Minnesota Department of Health, Public Health Laboratory	Matt Plumb, Jacob Garfin, Alexandra Lorentz, and Xiong Wang
EPI_ISL_603046	MDU-PHL, The Peter Doherty Institute for Infection and Immunity	MDU-PHL, The Peter Doherty Institute for Infection and Immunity	Seemann,T., Caly,L., Sait,M., Schultz,M.B., Druce,J., Sherry,N.
EPI_ISL_605408, EPI_ISL_605409, EPI_ISL_605410, EPI_ISL_605411, EPI_ISL_605412, EPI_ISL_605413, EPI_ISL_605414, EPI_ISL_605415, EPI_ISL_605416, EPI_ISL_605417, EPI_ISL_605418, EPI_ISL_605419, EPI_ISL_605420, EPI_ISL_605421, EPI_ISL_605422, EPI_ISL_605423, EPI_ISL_605424, EPI_ISL_605425, EPI_ISL_605426, EPI_ISL_605427, EPI_ISL_605428, EPI_ISL_605429, EPI_ISL_605430, EPI_ISL_605431, EPI_ISL_605432, EPI_ISL_605433, EPI_ISL_605434, EPI_ISL_605435, EPI_ISL_605436, EPI_ISL_605437, EPI_ISL_605438, EPI_ISL_605439, EPI_ISL_605440, EPI_ISL_605441, EPI_ISL_605442, EPI_ISL_605443,			

EPI_ISL_605444, EPI_ISL_605445, EPI_ISL_605446, EPI_ISL_605447, EPI_ISL_605448, EPI_ISL_605449, EPI_ISL_605450, EPI_ISL_605451, EPI_ISL_605452, EPI_ISL_605453, EPI_ISL_605454, EPI_ISL_605455, EPI_ISL_605456, EPI_ISL_605457, EPI_ISL_605458, EPI_ISL_605459, EPI_ISL_605460, EPI_ISL_605461, EPI_ISL_605462, EPI_ISL_605485, EPI_ISL_605486, EPI_ISL_605487, EPI_ISL_605488, EPI_ISL_605489, EPI_ISL_605490, EPI_ISL_605511, EPI_ISL_605512, EPI_ISL_605513, EPI_ISL_605514, EPI_ISL_605515, EPI_ISL_605516, EPI_ISL_605517, EPI_ISL_605518, EPI_ISL_605519, EPI_ISL_605524, EPI_ISL_605525, EPI_ISL_605526, EPI_ISL_605527, EPI_ISL_605529, EPI_ISL_605530, EPI_ISL_605531, EPI_ISL_605532, EPI_ISL_605533, EPI_ISL_605535, EPI_ISL_605536, EPI_ISL_605537, EPI_ISL_605538, EPI_ISL_605539, EPI_ISL_605540, EPI_ISL_605541, EPI_ISL_605542, EPI_ISL_605543, EPI_ISL_605544, EPI_ISL_605545, EPI_ISL_605546, EPI_ISL_605547, EPI_ISL_605548, EPI_ISL_605549, EPI_ISL_605550, EPI_ISL_605551, EPI_ISL_605552, EPI_ISL_605553, EPI_ISL_605554, EPI_ISL_605555, EPI_ISL_605556, EPI_ISL_605557, EPI_ISL_605558, EPI_ISL_605559, EPI_ISL_605560, EPI_ISL_605561, EPI_ISL_605562, EPI_ISL_605563, EPI_ISL_605564, EPI_ISL_605566, EPI_ISL_605568, EPI_ISL_605569, EPI_ISL_605571, EPI_ISL_605572, EPI_ISL_605575, EPI_ISL_605576, EPI_ISL_605577, EPI_ISL_605579, EPI_ISL_605580, EPI_ISL_605581, EPI_ISL_605582, EPI_ISL_605584, EPI_ISL_605586, EPI_ISL_605588, EPI_ISL_605589, EPI_ISL_605590, EPI_ISL_605591, EPI_ISL_605592, EPI_ISL_605593, EPI_ISL_605595, EPI_ISL_605597, EPI_ISL_605598, EPI_ISL_605599, EPI_ISL_605600, EPI_ISL_605601, EPI_ISL_605602, EPI_ISL_605606, EPI_ISL_605607, EPI_ISL_605608, EPI_ISL_605611, EPI_ISL_605612, EPI_ISL_605613, EPI_ISL_605617, EPI_ISL_605618, EPI_ISL_605619, EPI_ISL_605620, EPI_ISL_605621, EPI_ISL_605622, EPI_ISL_605623, EPI_ISL_605624, EPI_ISL_605625, EPI_ISL_605626, EPI_ISL_605627, EPI_ISL_605628, EPI_ISL_605629, EPI_ISL_605630, EPI_ISL_605631, EPI_ISL_605632, EPI_ISL_605633, EPI_ISL_605635, EPI_ISL_605636, EPI_ISL_605637, EPI_ISL_605638, EPI_ISL_605639, EPI_ISL_605640, EPI_ISL_605641, EPI_ISL_605642, EPI_ISL_605643, EPI_ISL_605644, EPI_ISL_605645, EPI_ISL_605646, EPI_ISL_605647, EPI_ISL_605648, EPI_ISL_605649, EPI_ISL_605650, EPI_ISL_605651, EPI_ISL_605652, EPI_ISL_605653, EPI_ISL_605654, EPI_ISL_605655, EPI_ISL_605656, EPI_ISL_605657, EPI_ISL_605658, EPI_ISL_605659, EPI_ISL_605660, EPI_ISL_605661, EPI_ISL_605662, EPI_ISL_605663, EPI_ISL_605664, EPI_ISL_605665, EPI_ISL_605666, EPI_ISL_605667, EPI_ISL_605668, EPI_ISL_605669, EPI_ISL_605670, EPI_ISL_605672, EPI_ISL_605673, EPI_ISL_605674, EPI_ISL_605675, EPI_ISL_605678, EPI_ISL_605679, EPI_ISL_605680, EPI_ISL_605681, EPI_ISL_605683, EPI_ISL_605692, EPI_ISL_605698, EPI_ISL_605700, EPI_ISL_605702, EPI_ISL_605703, EPI_ISL_605704, EPI_ISL_605707, EPI_ISL_605708, EPI_ISL_605709, EPI_ISL_605710, EPI_ISL_605711, EPI_ISL_605712, EPI_ISL_605713, EPI_ISL_605715, EPI_ISL_605716, EPI_ISL_605717, EPI_ISL_605719, EPI_ISL_605720, EPI_ISL_605722, EPI_ISL_605723, EPI_ISL_605724, EPI_ISL_605725, EPI_ISL_605726, EPI_ISL_605727, EPI_ISL_605728, EPI_ISL_605729, EPI_ISL_605730, EPI_ISL_605731, EPI_ISL_605732, EPI_ISL_605733, EPI_ISL_605734, EPI_ISL_605735, EPI_ISL_605736, EPI_ISL_605743, EPI_ISL_605744, EPI_ISL_605745, EPI_ISL_605746, EPI_ISL_605747, EPI_ISL_605748, EPI_ISL_605749, EPI_ISL_605750, EPI_ISL_605751, EPI_ISL_605752, EPI_ISL_605753, EPI_ISL_605754, EPI_ISL_605755, EPI_ISL_605756, EPI_ISL_605757, EPI_ISL_605764, EPI_ISL_605769, EPI_ISL_605771, EPI_ISL_605772, EPI_ISL_605773, EPI_ISL_605774, EPI_ISL_605775, EPI_ISL_605776, EPI_ISL_605777, EPI_ISL_605778, EPI_ISL_605779			
see above	University of Wisconsin-Madison AIDS Vaccine Research Laboratories	University of Wisconsin-Madison AIDS Vaccine Research Laboratories	Gage Moreno, Katarina Braun, et al. AIDS Vaccine Research Laboratories
EPI_ISL_609803, EPI_ISL_609804, EPI_ISL_609808, EPI_ISL_609810, EPI_ISL_609812, EPI_ISL_609814, EPI_ISL_609815, EPI_ISL_609819, EPI_ISL_609823	Unity Health Toronto	Ontario Institute for Cancer Research	Ramzi Fattouh, Larissa M. Matukas, Yan Chen,Mark Downing, Trina Otterman, Karel Boissinot, Wai Sum Siu, Zhi Cui, Le Luu, Samira Mubareka, TIBDN, Ilinca Lungu, Bernard Lam, Jeremy Johns, Paul Krzyzanowski, Richard de Borja, Felicia Vincelli, Philip Zuzarte, Jared T. Simpson
EPI_ISL_610218, EPI_ISL_610219	Department of Health Technology and Informatics, The Hong Kong Polytechnic University	Department of Health Technology and Informatics, The Hong Kong Polytechnic University	Siu,G.K.-H., Lee,L.-K., Leung,K.S.-S., Leung,J.S.-L., Ng,T.T.-L., Chan,C.T.-M., Tam,K.K.-G., Lao,H.-Y., Wu,A.K.-L., Yau,M.C.-Y., Lai,Y.W.-M., Fung,K.S.-C., Chau,S.K.-Y., Wong,B.K.-C., To,W.-K., Luk,K., Ho,A.Y.-M., Que,T.-L., Yip,K.-T., Yam,W.C., Shum,D.H.-K., Yip,S.P.
EPI_ISL_611539, EPI_ISL_611540, EPI_ISL_611551, EPI_ISL_611552, EPI_ISL_611607, EPI_ISL_611615, EPI_ISL_611616, EPI_ISL_611617, EPI_ISL_611618, EPI_ISL_611619, EPI_ISL_611620, EPI_ISL_611641, EPI_ISL_611701, EPI_ISL_611702, EPI_ISL_611703			
see above	University of Exeter	COVID-19 Genomics UK (COG-UK) Consortium	Ben Temperton,Aaron Jeffries,Michelle Michelsen,Joanna Warwick-Dugdale,Audrey Farbos,Robyn Manley,Stephen Michell,Jane Masoli
EPI_ISL_611766	Northumbria University / South Tees Hospitals NHS Foundation Trust / North Cumbria Integrated Care NHS Foundation Trust / North Tees and Hartlepool NHS Foundation Trust / Newcastle Hospitals NHS Foundation Trust	COVID-19 Genomics UK (COG-UK) Consortium	Darren L Smith,Andrew Nelson,Matthew Bashton,Greg R Young,Joshua Loh,John Allan,Mohammad A Tariq,Giles S Holt,Gary Black,Wen C Yew,Lynn Dover,Paul Baker,Steve Liggett,Sarah Essex,Jane Greenaway,Debra Padgett,Clive Graham,Garren Scott,Edward Barton,Emma Swindells,Brendan Payne,Jennifer Collins,Yusri Taha,Gary Eltringham
EPI_ISL_612056, EPI_ISL_612057, EPI_ISL_612095, EPI_ISL_612096, EPI_ISL_612395, EPI_ISL_612396, EPI_ISL_612397, EPI_ISL_612398, EPI_ISL_612399	University of Exeter	COVID-19 Genomics UK (COG-UK) Consortium	Ben Temperton,Aaron Jeffries,Michelle Michelsen,Joanna Warwick-Dugdale,Audrey Farbos,Robyn Manley,Stephen Michell,Jane Masoli
EPI_ISL_612548	Regional Virus Laboratory, Belfast Health and Social Care Trust	COVID-19 Genomics UK (COG-UK) Consortium	Conall McCaughey, James McKenna, Tanya Curran, Susan Feeney, Alison Watt, Ciara Cox, Mairead Connor, Zoltan Molnar, David Simpson, Derek Fairley
EPI_ISL_613477	Public Health Laboratory - Infectious Disease Lab, Minnesota Department of Health Infectious Disease Laboratory Submission Group	Minnesota Department of Health, Public Health Laboratory	Plumb,M., Garfin,J., Lorentz,A., Wang,X.
EPI_ISL_614048, EPI_ISL_614055, EPI_ISL_614056, EPI_ISL_614057, EPI_ISL_614058, EPI_ISL_614059, EPI_ISL_614060, EPI_ISL_614061, EPI_ISL_614062, EPI_ISL_614128, EPI_ISL_614130, EPI_ISL_614131, EPI_ISL_614132, EPI_ISL_614133, EPI_ISL_614134			
see above	Virginia DCLS	Virginia DCLS	Virginia DCLS
EPI_ISL_614301	Faroese National Reference Laboratory for Fish and Animal Diseases	Faroese National Reference Laboratory for Fish and Animal Diseases	Maria Marjunardóttir Dahl, Petra Elisabeth Petersen, Debes Hammershaimb Christiansen
EPI_ISL_615105, EPI_ISL_615106, EPI_ISL_615107	Halmstad klinisk mikrobiologi	The Public Health Agency of Sweden	Anna-Malin Linde, Maria Lind Karlberg, Mattias Haukland, Reza Advani, Olov Svartstrom, Oskar Karlsson Lindsjo, Sandra Broddesson, Petra Edquist, Mia Brytting, Anna Risberg, Karin Tegmark-Wisell
EPI_ISL_615166, EPI_ISL_615167, EPI_ISL_615168, EPI_ISL_615169, EPI_ISL_615170, EPI_ISL_615171, EPI_ISL_615172, EPI_ISL_615173, EPI_ISL_615174, EPI_ISL_615175, EPI_ISL_615176, EPI_ISL_615177, EPI_ISL_615179, EPI_ISL_615180, EPI_ISL_615182, EPI_ISL_615183, EPI_ISL_615184, EPI_ISL_615185, EPI_ISL_615186, EPI_ISL_615194, EPI_ISL_615195, EPI_ISL_615196, EPI_ISL_615197, EPI_ISL_615198, EPI_ISL_615199, EPI_ISL_615200, EPI_ISL_615201, EPI_ISL_615202, EPI_ISL_615203, EPI_ISL_615204, EPI_ISL_615205, EPI_ISL_615206, EPI_ISL_615207, EPI_ISL_615208, EPI_ISL_615209, EPI_ISL_615210, EPI_ISL_615211, EPI_ISL_615212, EPI_ISL_615214, EPI_ISL_615215, EPI_ISL_615216, EPI_ISL_615217, EPI_ISL_615218, EPI_ISL_615219, EPI_ISL_615220, EPI_ISL_615221, EPI_ISL_615222, EPI_ISL_615223, EPI_ISL_615224, EPI_ISL_615225, EPI_ISL_615226, EPI_ISL_615227, EPI_ISL_615228, EPI_ISL_615229, EPI_ISL_615230, EPI_ISL_615231, EPI_ISL_615232, EPI_ISL_615233, EPI_ISL_615234, EPI_ISL_615235, EPI_ISL_615236, EPI_ISL_615237, EPI_ISL_615238, EPI_ISL_615239, EPI_ISL_615240, EPI_ISL_615241, EPI_ISL_615242, EPI_ISL_615243, EPI_ISL_615244, EPI_ISL_615245, EPI_ISL_615246, EPI_ISL_615247, EPI_ISL_615248, EPI_ISL_615249, EPI_ISL_615250, EPI_ISL_615251, EPI_ISL_615252, EPI_ISL_615253, EPI_ISL_615254, EPI_ISL_615255, EPI_ISL_615256, EPI_ISL_615257, EPI_ISL_615258, EPI_ISL_615259, EPI_ISL_615260, EPI_ISL_615261, EPI_ISL_615262, EPI_ISL_615263, EPI_ISL_615264, EPI_ISL_615265, EPI_ISL_615266, EPI_ISL_615267, EPI_ISL_615268, EPI_ISL_615269, EPI_ISL_615270, EPI_ISL_615271, EPI_ISL_615272, EPI_ISL_615273, EPI_ISL_615274, EPI_ISL_615275, EPI_ISL_615276, EPI_ISL_615277, EPI_ISL_615278, EPI_ISL_615279, EPI_ISL_615280, EPI_ISL_615281, EPI_ISL_615282, EPI_ISL_615283, EPI_ISL_615284, EPI_ISL_615285, EPI_ISL_615286, EPI_ISL_615287, EPI_ISL_615288, EPI_ISL_615289, EPI_ISL_615290, EPI_ISL_615291, EPI_ISL_615292, EPI_ISL_615293, EPI_ISL_615294, EPI_ISL_615295, EPI_ISL_615296, EPI_ISL_615297, EPI_ISL_615298, EPI_ISL_615299, EPI_ISL_615300, EPI_ISL_615302, EPI_ISL_615303, EPI_ISL_615304, EPI_ISL_615305, EPI_ISL_615306, EPI_ISL_615307, EPI_ISL_615308, EPI_ISL_615309, EPI_ISL_615310, EPI_ISL_615311, EPI_ISL_615312, EPI_ISL_615313, EPI_ISL_615314, EPI_ISL_615315, EPI_ISL_615316, EPI_ISL_615317, EPI_ISL_615318, EPI_ISL_615319, EPI_ISL_615320, EPI_ISL_615321, EPI_ISL_615328, EPI_ISL_615329, EPI_ISL_615330, EPI_ISL_615331, EPI_ISL_615333, EPI_ISL_615334, EPI_ISL_615335, EPI_ISL_615336, EPI_ISL_615337, EPI_ISL_615338, EPI_ISL_615339, EPI_ISL_615340, EPI_ISL_615341, EPI_ISL_615342, EPI_ISL_615343, EPI_ISL_615344, EPI_ISL_615345, EPI_ISL_615346, EPI_ISL_615347, EPI_ISL_615348, EPI_ISL_615349, EPI_ISL_615350, EPI_ISL_615351, EPI_ISL_615352, EPI_ISL_615353, EPI_ISL_615354, EPI_ISL_615355, EPI_ISL_615356, EPI_ISL_615357, EPI_ISL_615358, EPI_ISL_615359, EPI_ISL_615360, EPI_ISL_615361, EPI_ISL_615362, EPI_ISL_615363, EPI_ISL_615364, EPI_ISL_615365, EPI_ISL_615366, EPI_ISL_615367, EPI_ISL_615368, EPI_ISL_615369, EPI_ISL_615370, EPI_ISL_615371, EPI_ISL_615372, EPI_ISL_615373, EPI_ISL_615374, EPI_ISL_615375, EPI_ISL_615376, EPI_ISL_615377, EPI_ISL_615378, EPI_ISL_615379, EPI_ISL_615380, EPI_ISL_615381, EPI_ISL_615382, EPI_ISL_615383, EPI_ISL_615384, EPI_ISL_615385, EPI_ISL_615386, EPI_ISL_615387, EPI_ISL_615388, EPI_ISL_615389, EPI_ISL_615390, EPI_ISL_615391, EPI_ISL_615392, EPI_ISL_615393, EPI_ISL_615394, EPI_ISL_615395, EPI_ISL_615396, EPI_ISL_615600, EPI_ISL_615601, EPI_ISL_615602,			

EPI_ISL_615603, EPI_ISL_615604, EPI_ISL_615605, EPI_ISL_615606, EPI_ISL_615608, EPI_ISL_615626, EPI_ISL_615628, EPI_ISL_615629, EPI_ISL_615630, EPI_ISL_615631, EPI_ISL_615632, EPI_ISL_615635, EPI_ISL_615652, EPI_ISL_615653, EPI_ISL_615654, EPI_ISL_615655, EPI_ISL_615656, EPI_ISL_615657, EPI_ISL_615658, EPI_ISL_615659, EPI_ISL_615660, EPI_ISL_615661, EPI_ISL_615662, EPI_ISL_615663, EPI_ISL_615664, EPI_ISL_615665, EPI_ISL_615666, EPI_ISL_615667, EPI_ISL_615668, EPI_ISL_615669, EPI_ISL_615674, EPI_ISL_615684, EPI_ISL_615685, EPI_ISL_615686, EPI_ISL_615753, EPI_ISL_615754, EPI_ISL_615755, EPI_ISL_615756, EPI_ISL_615757, EPI_ISL_615758, EPI_ISL_615759, EPI_ISL_615760, EPI_ISL_615761, EPI_ISL_615762, EPI_ISL_615763, EPI_ISL_615764, EPI_ISL_615765, EPI_ISL_615766, EPI_ISL_615767, EPI_ISL_615768, EPI_ISL_615769, EPI_ISL_615770, EPI_ISL_615771, EPI_ISL_615772, EPI_ISL_615773, EPI_ISL_615774, EPI_ISL_615775, EPI_ISL_615776, EPI_ISL_615777, EPI_ISL_615778, EPI_ISL_615779, EPI_ISL_615780, EPI_ISL_615781, EPI_ISL_615782, EPI_ISL_615783, EPI_ISL_615784, EPI_ISL_615785, EPI_ISL_615786, EPI_ISL_615787, EPI_ISL_615788, EPI_ISL_615789, EPI_ISL_615790, EPI_ISL_615791, EPI_ISL_615792, EPI_ISL_615793, EPI_ISL_615794, EPI_ISL_615795, EPI_ISL_615796, EPI_ISL_615797, EPI_ISL_615798, EPI_ISL_615799, EPI_ISL_615800, EPI_ISL_615801, EPI_ISL_615802, EPI_ISL_615803, EPI_ISL_615804, EPI_ISL_615805, EPI_ISL_615806, EPI_ISL_615807, EPI_ISL_615808, EPI_ISL_615809, EPI_ISL_615810, EPI_ISL_615811, EPI_ISL_615812, EPI_ISL_615813, EPI_ISL_615814, EPI_ISL_615815, EPI_ISL_615816, EPI_ISL_615817, EPI_ISL_615818, EPI_ISL_615819, EPI_ISL_615820, EPI_ISL_615821, EPI_ISL_615822, EPI_ISL_615823, EPI_ISL_615824, EPI_ISL_615825, EPI_ISL_615826, EPI_ISL_615827, EPI_ISL_615828, EPI_ISL_615829, EPI_ISL_615830, EPI_ISL_615831, EPI_ISL_615832, EPI_ISL_615833, EPI_ISL_615834, EPI_ISL_615835, EPI_ISL_615836, EPI_ISL_615837, EPI_ISL_615838, EPI_ISL_615839, EPI_ISL_615840, EPI_ISL_615841, EPI_ISL_615842, EPI_ISL_615843, EPI_ISL_615844, EPI_ISL_615845, EPI_ISL_615846, EPI_ISL_615847, EPI_ISL_615848, EPI_ISL_615849, EPI_ISL_615850, EPI_ISL_615851, EPI_ISL_615852, EPI_ISL_615853, EPI_ISL_615854, EPI_ISL_615855, EPI_ISL_615856, EPI_ISL_615857, EPI_ISL_615858, EPI_ISL_615859, EPI_ISL_615860, EPI_ISL_615861, EPI_ISL_615862, EPI_ISL_615863, EPI_ISL_615864, EPI_ISL_615865, EPI_ISL_615866, EPI_ISL_615867, EPI_ISL_615868, EPI_ISL_615869, EPI_ISL_615870, EPI_ISL_615871, EPI_ISL_615872, EPI_ISL_615873, EPI_ISL_615874, EPI_ISL_615875, EPI_ISL_615876, EPI_ISL_615877, EPI_ISL_615878, EPI_ISL_615879, EPI_ISL_615880, EPI_ISL_615881, EPI_ISL_615882, EPI_ISL_615883, EPI_ISL_615884, EPI_ISL_615885, EPI_ISL_615886, EPI_ISL_615887, EPI_ISL_615888, EPI_ISL_615889, EPI_ISL_615890, EPI_ISL_615891, EPI_ISL_615892, EPI_ISL_615893, EPI_ISL_615894, EPI_ISL_615895, EPI_ISL_615896, EPI_ISL_615897, EPI_ISL_615898, EPI_ISL_615899, EPI_ISL_615900, EPI_ISL_615901, EPI_ISL_615902, EPI_ISL_615903, EPI_ISL_615904, EPI_ISL_615905, EPI_ISL_620853, EPI_ISL_621177, EPI_ISL_621178, EPI_ISL_621179, EPI_ISL_621180, EPI_ISL_621181, EPI_ISL_621182, EPI_ISL_621184, EPI_ISL_621185, EPI_ISL_621536, EPI_ISL_621537, EPI_ISL_621541, EPI_ISL_621548, EPI_ISL_621568, EPI_ISL_621569, EPI_ISL_621570, EPI_ISL_621736, EPI_ISL_621737, EPI_ISL_621738, EPI_ISL_621745, EPI_ISL_621752, EPI_ISL_621753, EPI_ISL_621754, EPI_ISL_621755, EPI_ISL_621756, EPI_ISL_621757, EPI_ISL_621758, EPI_ISL_621761, EPI_ISL_621762, EPI_ISL_621763, EPI_ISL_621764, EPI_ISL_621765, EPI_ISL_621766, EPI_ISL_621767, EPI_ISL_621768, EPI_ISL_621769, EPI_ISL_621770, EPI_ISL_621771, EPI_ISL_621772, EPI_ISL_621773, EPI_ISL_621774, EPI_ISL_621775, EPI_ISL_621776, EPI_ISL_621777, EPI_ISL_621779, EPI_ISL_621780, EPI_ISL_621781, EPI_ISL_621782, EPI_ISL_621783, EPI_ISL_621784, EPI_ISL_621785, EPI_ISL_621786, EPI_ISL_621787, EPI_ISL_621788, EPI_ISL_621789, EPI_ISL_621790, EPI_ISL_621791, EPI_ISL_621792, EPI_ISL_621793, EPI_ISL_621794, EPI_ISL_621795, EPI_ISL_621798, EPI_ISL_621803, EPI_ISL_621814, EPI_ISL_621818, EPI_ISL_621819, EPI_ISL_621827, EPI_ISL_621833, EPI_ISL_621835, EPI_ISL_621836, EPI_ISL_621837, EPI_ISL_621838, EPI_ISL_621839, EPI_ISL_621846, EPI_ISL_621847, EPI_ISL_621848, EPI_ISL_621849, EPI_ISL_621850, EPI_ISL_621851, EPI_ISL_621866, EPI_ISL_621870, EPI_ISL_621871, EPI_ISL_621872, EPI_ISL_621873, EPI_ISL_621874, EPI_ISL_621875, EPI_ISL_621876, EPI_ISL_621877, EPI_ISL_621878, EPI_ISL_621879, EPI_ISL_621880, EPI_ISL_621881, EPI_ISL_621882, EPI_ISL_621883, EPI_ISL_621884, EPI_ISL_621886, EPI_ISL_621887, EPI_ISL_621889, EPI_ISL_621890, EPI_ISL_621891, EPI_ISL_621893, EPI_ISL_621894, EPI_ISL_621895, EPI_ISL_621896, EPI_ISL_621897, EPI_ISL_621898, EPI_ISL_621899, EPI_ISL_621900, EPI_ISL_621901, EPI_ISL_621902, EPI_ISL_621903, EPI_ISL_621904, EPI_ISL_621905, EPI_ISL_621906, EPI_ISL_621907, EPI_ISL_621908, EPI_ISL_621909, EPI_ISL_621910, EPI_ISL_621911, EPI_ISL_621912, EPI_ISL_621913, EPI_ISL_621914, EPI_ISL_621915, EPI_ISL_621917, EPI_ISL_621918, EPI_ISL_621919, EPI_ISL_621920, EPI_ISL_621921, EPI_ISL_621927, EPI_ISL_621930, EPI_ISL_621948, EPI_ISL_621986, EPI_ISL_621987, EPI_ISL_621988, EPI_ISL_621990, EPI_ISL_621991, EPI_ISL_621992, EPI_ISL_621993, EPI_ISL_621994, EPI_ISL_621995, EPI_ISL_621997, EPI_ISL_621998, EPI_ISL_621999, EPI_ISL_622000, EPI_ISL_622001, EPI_ISL_622002, EPI_ISL_622003, EPI_ISL_622004, EPI_ISL_622005, EPI_ISL_622006, EPI_ISL_622007, EPI_ISL_622008, EPI_ISL_622009, EPI_ISL_622010, EPI_ISL_622011, EPI_ISL_622012, EPI_ISL_622013, EPI_ISL_622014, EPI_ISL_622015, EPI_ISL_622016, EPI_ISL_622017, EPI_ISL_622018, EPI_ISL_622019, EPI_ISL_622020, EPI_ISL_622021, EPI_ISL_622022, EPI_ISL_622023, EPI_ISL_622024, EPI_ISL_622025, EPI_ISL_622026, EPI_ISL_622027, EPI_ISL_622028, EPI_ISL_622029, EPI_ISL_622030, EPI_ISL_622031, EPI_ISL_622032, EPI_ISL_622033, EPI_ISL_622034, EPI_ISL_622035, EPI_ISL_622036, EPI_ISL_622037, EPI_ISL_622038, EPI_ISL_622039, EPI_ISL_622040, EPI_ISL_622041, EPI_ISL_622042, EPI_ISL_622043, EPI_ISL_622044, EPI_ISL_622045, EPI_ISL_622046, EPI_ISL_622047, EPI_ISL_622048, EPI_ISL_622049, EPI_ISL_622050, EPI_ISL_622051, EPI_ISL_622052, EPI_ISL_622053, EPI_ISL_622054, EPI_ISL_622055, EPI_ISL_622056, EPI_ISL_622057, EPI_ISL_622058, EPI_ISL_622059, EPI_ISL_622060, EPI_ISL_622061, EPI_ISL_622062, EPI_ISL_622063, EPI_ISL_622064, EPI_ISL_622065, EPI_ISL_622066, EPI_ISL_622067, EPI_ISL_622068, EPI_ISL_622069, EPI_ISL_622070, EPI_ISL_622071, EPI_ISL_622072, EPI_ISL_622073, EPI_ISL_622074, EPI_ISL_622075, EPI_ISL_622076, EPI_ISL_622077, EPI_ISL_622090, EPI_ISL_622091, EPI_ISL_622092, EPI_ISL_622093, EPI_ISL_622094, EPI_ISL_622095, EPI_ISL_622096, EPI_ISL_622097, EPI_ISL_622098, EPI_ISL_622099, EPI_ISL_622100, EPI_ISL_622101, EPI_ISL_622102, EPI_ISL_622103, EPI_ISL_622104, EPI_ISL_622105, EPI_ISL_622106, EPI_ISL_622107, EPI_ISL_622108, EPI_ISL_622109, EPI_ISL_622110, EPI_ISL_622111, EPI_ISL_622112, EPI_ISL_622113, EPI_ISL_622114, EPI_ISL_622115, EPI_ISL_622116, EPI_ISL_622117, EPI_ISL_622118, EPI_ISL_622119, EPI_ISL_622120, EPI_ISL_622121, EPI_ISL_622122, EPI_ISL_622123, EPI_ISL_622124, EPI_ISL_622125, EPI_ISL_622126, EPI_ISL_622127, EPI_ISL_622128, EPI_ISL_622129, EPI_ISL_622130, EPI_ISL_622131, EPI_ISL_622132, EPI_ISL_622133, EPI_ISL_622134, EPI_ISL_622135, EPI_ISL_622136, EPI_ISL_622137, EPI_ISL_622138, EPI_ISL_622139, EPI_ISL_622140, EPI_ISL_622141, EPI_ISL_622142, EPI_ISL_622143, EPI_ISL_622144, EPI_ISL_622145, EPI_ISL_622146, EPI_ISL_622147, EPI_ISL_622148, EPI_ISL_622149, EPI_ISL_622165, EPI_ISL_622166, EPI_ISL_622167, EPI_ISL_622168, EPI_ISL_622186, EPI_ISL_622187, EPI_ISL_622188, EPI_ISL_622189, EPI_ISL_622190, EPI_ISL_622191, EPI_ISL_622192, EPI_ISL_622193, EPI_ISL_622194, EPI_ISL_622195, EPI_ISL_622196, EPI_ISL_622197, EPI_ISL_622198, EPI_ISL_622199, EPI_ISL_622200, EPI_ISL_622243, EPI_ISL_622244, EPI_ISL_622245, EPI_ISL_622246, EPI_ISL_622247, EPI_ISL_622248, EPI_ISL_622249, EPI_ISL_622250, EPI_ISL_622251, EPI_ISL_622252, EPI_ISL_622253, EPI_ISL_622254, EPI_ISL_622255, EPI_ISL_622256, EPI_ISL_622257, EPI_ISL_622258, EPI_ISL_622259, EPI_ISL_622748, EPI_ISL_622749, EPI_ISL_622750, EPI_ISL_622751, EPI_ISL_622752, EPI_ISL_622753, EPI_ISL_622754, EPI_ISL_622755, EPI_ISL_622756, EPI_ISL_622757, EPI_ISL_622758, EPI_ISL_622759, EPI_ISL_622760			
see above	Department of Virus and Microbiological Special Diagnostics, Statens Serum Institut, Denmark	Albertsen lab, Department of Chemistry and Bioscience, Aalborg University, Denmark	Danish Covid-19 Genome Consortia
EPI_ISL_622805, EPI_ISL_622806	PathLab Bay of Plenty	Institute of Environmental Science and Research (ESR)	Xiaoyun Ren, Matt Storey, Nikki Freed, Muhammad Faisal, Jing Wang, Hermes Perez, Anja Werno, Antje van der Linden, Arlo Upton, Chris Mansell, David Hammer, Dragana Drinkovic, Gary McAuliffe, Hana Sofia Andersson, James Ussher, Jill Sherwood, Josh Freeman, Julia Howard, Juliet Elvy, Mary DeAlmeida, Matt Blakiston, Matthew Rogers, Max Bloomfield, Michael Addidle, Michelle Balm, Sally Roberts, Sarah Jefferies, Sharmini Muttaiyah, Susan Morpeth, Susan Taylor, Timothy Blackmore, Vani Sathyendran, Veronica Playle, Virginia Hope, Erasmus Smit, Lauren Jelly, Olin Silander, Joep de Ligt
EPI_ISL_625522, EPI_ISL_625523, EPI_ISL_625524, EPI_ISL_625525, EPI_ISL_625526, EPI_ISL_625527, EPI_ISL_625528, EPI_ISL_625529, EPI_ISL_625530, EPI_ISL_625531, EPI_ISL_625532, EPI_ISL_625533, EPI_ISL_625534, EPI_ISL_625535, EPI_ISL_625536, EPI_ISL_625537, EPI_ISL_625538, EPI_ISL_625539, EPI_ISL_625540, EPI_ISL_625541, EPI_ISL_625542, EPI_ISL_625544, EPI_ISL_625545			
see above	Alameda County Public Health Lab	Chan-Zuckerberg Biohub	CZB Cliahub Consortium
EPI_ISL_625660	San Joaquin County Public Health Lab	Chan-Zuckerberg Biohub	CZB Cliahub Consortium
EPI_ISL_625669	UCSF Clinical Microbiology Laboratory	Chan-Zuckerberg Biohub	CZB Cliahub Consortium
EPI_ISL_625673	Laboratory of Molecular Medicine, University of Magallanes	Centro Asistencial Docente y de Investigacion, Universidad de Magallanes	Jorge Gonzalez, Jacqueline Aldridge, Diego Alvarez, Marcelo Navarrete
EPI_ISL_626574, EPI_ISL_626576, EPI_ISL_626577, EPI_ISL_626578, EPI_ISL_626579, EPI_ISL_626580, EPI_ISL_626581, EPI_ISL_626582, EPI_ISL_626583, EPI_ISL_626584	The National Institute of Public Health	State Veterinary Institute Prague	Nagy,A;Jirincova,H;Novakova,L;Trnka,D;Vecerova,J
EPI_ISL_626806, EPI_ISL_627064, EPI_ISL_627312	University of Exeter	COVID-19 Genomics UK (COG-UK) Consortium	Ben Temperton,Aaron Jeffries,Michelle Michelsen,Joanna Warwick-Dugdale,Audrey Farbos,Robyn Manley,Stephen Michell,Jane Masoli
EPI_ISL_627350, EPI_ISL_627355, EPI_ISL_627359, EPI_ISL_627360	West of Scotland Specialist Virology Centre, NHSGGC / MRC-University of Glasgow Centre for Virus Research	COVID-19 Genomics UK (COG-UK) Consortium	Ana da Silva Filipe, Natasha Johnson, Kathy Smollett, Daniel Mair, Stephen Carmichael, Lily Tong, Jenna Nichols, Elihu Aranday-Cortes, Kyriaki Nomikou; Sarah McDonald, Marc Niebel, Patawee Asamaphan; Richard Orton, Joseph Hughes, Sreenu Vattipally, David L Robertson; Alasdair MacLean, Rory Gunson; Kathy Li, Igor Starinskij, Natasha Jesudason, Rajiv Shah, James Shepherd, Antonia Ho, Emma Thomson
EPI_ISL_627441, EPI_ISL_627444	University of Exeter	COVID-19 Genomics UK (COG-UK) Consortium	Ben Temperton,Aaron Jeffries,Michelle Michelsen,Joanna Warwick-Dugdale,Audrey Farbos,Robyn Manley,Stephen Michell,Jane Masoli
EPI_ISL_629001	South Eastern Area Laboratory Services (SEALS)	NSW Health Pathology - Institute of Clinical Pathology and Medical Research; Westmead Hospital; University of Sydney	CIDM-PH et al.
EPI_ISL_631365, EPI_ISL_631366, EPI_ISL_631367, EPI_ISL_631368, EPI_ISL_631369, EPI_ISL_631370, EPI_ISL_631371, EPI_ISL_631372, EPI_ISL_631373, EPI_ISL_631374, EPI_ISL_631375, EPI_ISL_631376			
see above	ZOTZ KLIMAS MVZ Düsseldorf-Centrum GbR ÜBAG für Labormedizin, Genetik, Zytologie, Pathologie	Center of Medical Microbiology, Virology, and Hospital Hygiene, University of Duesseldorf	Maximilian Damagnez, Alexander Dilthey, Ashley-Jane Duplessis, Patrick Finzer, Katrin Hoffmann, Torsten Houwaart, Lisanna Hülse, Malte Kohns Vasconcelos, Marek Korencak, Nadine Lübke, Jessica Nicolai, Klaus Pfeffer, Daniel Strelow, Jörg Timm, Andreas Walker, Tobias Wienemann, Rainer Zotz
EPI_ISL_631378, EPI_ISL_631379, EPI_ISL_631383	University Hospital Cologne	Center of Medical Microbiology, Virology, and Hospital Hygiene, University	Maximilian Damagnez, Alexander Dilthey, Ashley-Jane Duplessis, Eva Heger, Torsten Houwaart, Rolf Kaiser, Florian Klein, Elena Knops, Malte Kohns Vasconcelos, Jessica Nicolai, Klaus Pfeffer, Gibran Rubio Quintanares, Saleta Sierra-Aragón, Daniel Strelow, Jörg Timm, Andreas Walker, Tobias Wienemann

		of Duesseldorf	
EPI_ISL_631387	ZOTZ KLIMAS MVZ Düsseldorf-Centrum GbR ÜBAG für Labormedizin, Genetik, Zytologie, Pathologie	Center of Medical Microbiology, Virology, and Hospital Hygiene, University of Duesseldorf	Maximilian Damagnez, Alexander Dilthey, Ashley-Jane Duplessis, Patrick Finzer, Katrin Hoffmann, Torsten Houwaart, Lisanna Hülse, Malte Kohns Vasconcelos, Marek Korencak, Nadine Lübke, Jessica Nicolai, Klaus Pfeffer, Daniel Strelow, Jörg Timm, Andreas Walker, Tobias Wienemann, Rainer Zotz
EPI_ISL_631441, EPI_ISL_631442, EPI_ISL_631443, EPI_ISL_631444, EPI_ISL_631445, EPI_ISL_631446, EPI_ISL_631447, EPI_ISL_631448, EPI_ISL_631449, EPI_ISL_631450, EPI_ISL_631451, EPI_ISL_631452, EPI_ISL_631453, EPI_ISL_631454, EPI_ISL_631494, EPI_ISL_631495, EPI_ISL_631496			
see above	Wisconsin State Laboratory of Hygiene Communicable Disease Division	Wisconsin State Laboratory of Hygiene Communicable Disease Division	Kelsey R. Florek, Abigail C. Shockey
EPI_ISL_632327, EPI_ISL_632346, EPI_ISL_632387, EPI_ISL_632388, EPI_ISL_632391, EPI_ISL_632405, EPI_ISL_632409, EPI_ISL_632417, EPI_ISL_632460, EPI_ISL_632495, EPI_ISL_632496, EPI_ISL_632497, EPI_ISL_632498, EPI_ISL_632499, EPI_ISL_632500, EPI_ISL_632501, EPI_ISL_632503, EPI_ISL_632504, EPI_ISL_632547, EPI_ISL_632548, EPI_ISL_632578, EPI_ISL_632579, EPI_ISL_632580, EPI_ISL_632581, EPI_ISL_632582, EPI_ISL_632583, EPI_ISL_632589, EPI_ISL_632762, EPI_ISL_632763			
see above	Dutch COVID-19 response team	Erasmus Medical Center	Bas Oude Munnink, David Nieuwenhuijse, Reina Sikkema, Claudia Schapendonk, Irina Chestakova, Anne van der Linden, Theo Bestebroer, Stefan van Nieuwkoop, Mark Pronk, Pascal Lexmond, Corien Swaan, Manon Haverkate, Madelief Mollers, Mart Stein, Sandra Kengne Kamga Mobou, Jeroen van Kampen, Jolanda Voermans, Aura Timen, Corine GeurtsvanKessel, Annemiek van der Eijk, Richard Molenkamp, Marion Koopmans, on behalf of the Dutch national COVID-19 response team.
EPI_ISL_632890, EPI_ISL_632891, EPI_ISL_632892, EPI_ISL_632893, EPI_ISL_632894, EPI_ISL_632895, EPI_ISL_632896, EPI_ISL_632897, EPI_ISL_632898	University of Wisconsin-Madison AIDS Vaccine Research Laboratories	University of Wisconsin-Madison AIDS Vaccine Research Laboratories	Gage Moreno, Katarina Braun, et al. AIDS Vaccine Research Laboratories
EPI_ISL_632934	Department of Acute Infectious Diseases Control and Prevention,Yunnan Provincial Center for Disease Control and Prevention	Department of Acute Infectious Diseases Control and Prevention,Yunnan Provincial Center for Disease Control and Prevention	Meiling Zhang,Jienan Zhou,Senquan Jia,Xiaonan Zhao,Xiaoqing Fu
EPI_ISL_632935	Department of Acute Infectious Diseases Control and Prevention, Yunnan Provincial Center for Disease Control and Prevention	Department of Acute Infectious Diseases Control and Prevention, Yunnan Provincial Center for Disease Control and Prevention	Meiling Zhang, Jienan Zhou, Senquan Jia, Xiaonan Zhao, Xiaoqing Fu
EPI_ISL_632976, EPI_ISL_632977, EPI_ISL_632978, EPI_ISL_632979	Empire City Laboratories	New York City Public Health Laboratory	Jade Wang, et al.
EPI_ISL_632981	DOHMH Corona	New York City Public Health Laboratory	Jade Wang, et al.
EPI_ISL_632994	DOHMH Crown Heights	New York City Public Health Laboratory	Jade Wang, et al.
EPI_ISL_633035	DOHMH PHL	New York City Public Health Laboratory	Jade Wang, et al.
EPI_ISL_633036	DOHMH Morrisania	New York City Public Health Laboratory	Jade Wang, et al.
EPI_ISL_633037	DOHMH Central Harlem	New York City Public Health Laboratory	Jade Wang, et al.
EPI_ISL_633038	DOHMH PHL	New York City Public Health Laboratory	Jade Wang, et al.
EPI_ISL_635946, EPI_ISL_635947, EPI_ISL_635948, EPI_ISL_635949, EPI_ISL_635950, EPI_ISL_635951, EPI_ISL_635952, EPI_ISL_635967, EPI_ISL_635968, EPI_ISL_635969, EPI_ISL_635970, EPI_ISL_635971, EPI_ISL_635972, EPI_ISL_635976, EPI_ISL_635977, EPI_ISL_635982, EPI_ISL_636031, EPI_ISL_636032, EPI_ISL_636033, EPI_ISL_636034, EPI_ISL_636036, EPI_ISL_636037, EPI_ISL_636038, EPI_ISL_636039, EPI_ISL_636041, EPI_ISL_636046, EPI_ISL_636047, EPI_ISL_636051, EPI_ISL_636052, EPI_ISL_636057, EPI_ISL_636063, EPI_ISL_636066, EPI_ISL_636067, EPI_ISL_636068, EPI_ISL_636125			
see above	San Diego County Public Health Laboratory	Andersen lab at Scripps Research	SEARCH Alliance San Diego with Tracy Basler, Jovan Shephard, Brett Austin
EPI_ISL_636589	Dutch COVID-19 response team	National Institute for Public Health and the Environment (RIVM)	Adam Meijer, Harry Vennema, Jeroen Cremer, Sharon van den Brink, Bas van der Veer, AnneMarie van den Brandt, Florian Zwagemaker, Dennis Schmitz, Chantal Reusken, on behalf of the national COVID-19 response team
EPI_ISL_636892, EPI_ISL_636893, EPI_ISL_636894, EPI_ISL_636895, EPI_ISL_636896, EPI_ISL_636897, EPI_ISL_636898, EPI_ISL_636899, EPI_ISL_636900, EPI_ISL_636901, EPI_ISL_636902, EPI_ISL_636903, EPI_ISL_636904, EPI_ISL_636905, EPI_ISL_636906			
see above	Lithuanian University of Health Sciences Hospital, Department of Laboratory Medicine	Lithuanian University of Health Sciences, Molecular cardiology lab.	Lukas Zemaitis, Ingrida Olendrait, Arnoldas Pautienius, Kamile Tamusauskaite, Dovydas Gecys, Laura Pareckaite, Vaiva Lesauskaite, Astra Vitkauskiene
EPI_ISL_637015	Department of Infectious Diseases and Immunology, National Hospital Organization Nagoya Medical Center	Clinical Research Center, National Hospital Organization Nagoya Medical Center	Yoshihiro Nakata, Hirotaka Ode, Mai Kubota, Masakazu Matsuda, Kazuhiro Matsuoka, Miho Nakasuji, Mikiko Mori, Mayumi Imahashi, Yoshiyuki Yokomaku, Yasumasa Iwatani
EPI_ISL_637495	Centre for Enzyme Innovation, University of Portsmouth / Translational Research Laboratory, Portsmouth Hospitals NHS Trust	COVID-19 Genomics UK (COG-UK) Consortium	Angela Beckett,Yann Bourgeois,Garry Scarlett,Sharon Glaysher,Scott Elliott,Kelly Bicknell,Robert Impey,Allyson Lloyd,Sarah Wyllie,Ethan Butcher,Anoop Chauhan,Samuel Robson
EPI_ISL_639645, EPI_ISL_639647	E. Gulbja Laboratorija	Latvian Biomedical Research and Study Centre	Ivars Silamielis, Kaspars Megnis, Monta Ustinova, ikita Zrelovs, Vita Rovte, Mikus Gavars, Dmitrijs Perminovs, Uga Dumpis, Jnis Kloviš

EPI_ISL_639651	Centrl Laboratorija	Latvian Biomedical Research and Study Centre	Ivars Silamielis, Kaspars Megnis, Monta Ustinova, ikita Zrelovs, Vita Rovte, Stella Lapia, Jana Oste, Marta Priedte, Uga Dumpis, Jnis Kloviš
EPI_ISL_639664, EPI_ISL_639665	E. Gulbja Laboratorija	Latvian Biomedical Research and Study Centre	Ivars Silamielis, Kaspars Megnis, Monta Ustinova, ikita Zrelovs, Vita Rovte, Mikus Gavars, Dmitrijs Perminovs, Uga Dumpis, Jnis Kloviš
EPI_ISL_639825, EPI_ISL_639828, EPI_ISL_639829, EPI_ISL_639830, EPI_ISL_639831, EPI_ISL_639834, EPI_ISL_639835, EPI_ISL_639837, EPI_ISL_639841, EPI_ISL_639843, EPI_ISL_639845, EPI_ISL_639846, EPI_ISL_639847, EPI_ISL_639849, EPI_ISL_639851, EPI_ISL_639852, EPI_ISL_639854, EPI_ISL_639860, EPI_ISL_639863, EPI_ISL_639864			
see above	National Virus Reference Laboratory	National Virus Reference Laboratory	Michael Carr, Gabriel Gonzalez, Jonathan Dean, Daniel Hare, Cillian F De Gascun
EPI_ISL_639948, EPI_ISL_639949	HELIX LLC	WHO National Influenza Centre Russian Federation	Andrey Komissarov, Artem Fadeev, Kseniya Komissarova, Anna Ivanova, Dmitry Bazhenov, Daria Danilenko
EPI_ISL_639978	Centre Hospitalier Saint Joseph Saint Luc	CNR Virus des Infections Respiratoires - France SUD	Antonin Bal, Gregory Destras, Gwendolyne Burfin, Hadrien Règue, Alexandre Gaymard, Maude Bouscambert-Duchamp, Florence Morfin-Sherpa, Martine Valette, Bruno Lina, Laurence Josset
EPI_ISL_640116	False Bay Hospital wc FBH	NHLS/UCT	Arash Iranzadeh, Deelan Doolabh, Lynn Tyers, Bruna Galvao, Innocent Mudau, Marvin Hsiao, Kruger Marais, Diana Hardie, Stephen Korsman, Carolyn Williamson
EPI_ISL_640117	2 Military Hospital wc MAA	NHLS/UCT	Arash Iranzadeh, Deelan Doolabh, Lynn Tyers, Bruna Galvao, Innocent Mudau, Marvin Hsiao, Kruger Marais, Diana Hardie, Stephen Korsman, Carolyn Williamson
EPI_ISL_640119, EPI_ISL_640120	Groote Schuur Hospital wc GSH	NHLS/UCT	Arash Iranzadeh, Deelan Doolabh, Lynn Tyers, Bruna Galvao, Innocent Mudau, Marvin Hsiao, Kruger Marais, Diana Hardie, Stephen Korsman, Carolyn Williamson
EPI_ISL_640121	False Bay Hospital wc FBH	NHLS/UCT	Arash Iranzadeh, Deelan Doolabh, Lynn Tyers, Bruna Galvao, Innocent Mudau, Marvin Hsiao, Kruger Marais, Diana Hardie, Stephen Korsman, Carolyn Williamson
EPI_ISL_640583	Victorian Infectious Diseases Reference Laboratory (VIDRL)	VIDRL and MDU-PHL	Caly L., Seemann T., Sait, M.L., Schultz M.B., Druce J., Sherry, N.L.
EPI_ISL_640969, EPI_ISL_641143, EPI_ISL_641166, EPI_ISL_641197	Microbiological Diagnostic Unit - Public Health Laboratory (MDU-PHL)	MDU-PHL	Seemann T., Schultz M.B., Sait, M.L., Sherry, N.L.
EPI_ISL_641447, EPI_ISL_641448, EPI_ISL_641476, EPI_ISL_641477, EPI_ISL_641478, EPI_ISL_641479, EPI_ISL_641480, EPI_ISL_641481, EPI_ISL_641482, EPI_ISL_641483, EPI_ISL_641484, EPI_ISL_641485, EPI_ISL_641486, EPI_ISL_641487, EPI_ISL_641488, EPI_ISL_641489, EPI_ISL_641490, EPI_ISL_641491, EPI_ISL_641492, EPI_ISL_641493, EPI_ISL_641494, EPI_ISL_641495			
see above	Department of Virus and Microbiological Special Diagnostics, Statens Serum Institut, Copenhagen, Denmark	Albertsen lab, Department of Chemistry and Bioscience, Aalborg University, Denmark	Thomas Bruun Rasmussen, Jannik Fonager, Morten Rasmussen
EPI_ISL_641557, EPI_ISL_641558, EPI_ISL_641559, EPI_ISL_641560, EPI_ISL_641561, EPI_ISL_641562, EPI_ISL_641563, EPI_ISL_641564, EPI_ISL_641565, EPI_ISL_641566, EPI_ISL_641567, EPI_ISL_641568, EPI_ISL_641569, EPI_ISL_641570, EPI_ISL_641571, EPI_ISL_641572			
see above	Department of Clinical Microbiology	GIGA Medical Genomics	Keith Durkin, Maria Artesi, Sébastien Bontems, Raphaël Boreux, Bouchra Boujemla, Cécile Meex, Pierrette Melin, Marie-Pierre Hayette, Vincent Bours
EPI_ISL_644382, EPI_ISL_644393, EPI_ISL_644394, EPI_ISL_644395, EPI_ISL_644400, EPI_ISL_644401, EPI_ISL_644413, EPI_ISL_644414, EPI_ISL_644415, EPI_ISL_644416, EPI_ISL_644417, EPI_ISL_644418, EPI_ISL_644419, EPI_ISL_644420, EPI_ISL_644421, EPI_ISL_644422, EPI_ISL_644423, EPI_ISL_644424, EPI_ISL_644426, EPI_ISL_644427, EPI_ISL_644428, EPI_ISL_644429, EPI_ISL_644430, EPI_ISL_644431, EPI_ISL_644432, EPI_ISL_644433, EPI_ISL_644434, EPI_ISL_644435, EPI_ISL_644436, EPI_ISL_644437, EPI_ISL_644438			
see above	MEPHI, Aix Marseille University	MEPHI, Aix Marseille University	Anthony LEVASSEUR
EPI_ISL_644610	National Virus Reference Laboratory	National Virus Reference Laboratory	Michael Carr, Gabriel Gonzalez, Jonathan Dean, Daniel Hare, Cillian F De Gascun
EPI_ISL_644842, EPI_ISL_644843, EPI_ISL_644844, EPI_ISL_644845, EPI_ISL_644846, EPI_ISL_644847, EPI_ISL_644848, EPI_ISL_644849, EPI_ISL_644850, EPI_ISL_644851, EPI_ISL_644852, EPI_ISL_644853, EPI_ISL_644854, EPI_ISL_644855, EPI_ISL_644858, EPI_ISL_644863, EPI_ISL_644864, EPI_ISL_644866, EPI_ISL_644867, EPI_ISL_644868, EPI_ISL_644869, EPI_ISL_644870, EPI_ISL_644871, EPI_ISL_644872, EPI_ISL_644873, EPI_ISL_644874, EPI_ISL_644875, EPI_ISL_644876, EPI_ISL_644877, EPI_ISL_644878, EPI_ISL_644879, EPI_ISL_644880, EPI_ISL_644881			
see above	Virginia DCLS	Virginia DCLS	Virginia DCLS
EPI_ISL_645116	National Public Health Laboratory, National Centre for Infectious Diseases	National Public Health Laboratory, National Centre for Infectious Diseases	Tze Minn Mak, Sophie Octavia, Zhenyang Zhou, Lin Cui, Raymond Tzer Pin Lin
EPI_ISL_648016, EPI_ISL_648017	MS Public Health Laboratory	Pathogen Discovery, Respiratory Viruses Branch, Division of Viral Diseases, Centers for Disease Control and Prevention	Yan Li, Jing Zhang, Ying Tao, Brian Lynch, Krista Queen, Anna Montmayeur, Anna Uehara, Clinton R. Paden, Rachel Marine, Haibin Wang, Suxiang Tong
EPI_ISL_648128	UHAS COVID-19 Lab	UHAS COVID-19 Lab	Kwabena O. Duedu, Jones Gyamfi, Reuben Ayivor-Djanie, John O. Gyapong and the UHAS COVID-19 Lab Team
EPI_ISL_648170	The Public Health Agency of Sweden	The Public Health Agency of Sweden	Anna-Malin Linde, Maria Lind Karlberg, Mattias Haukland, Reza Advani, Olov Svartstrom, Oskar Karlsson Lindsjo, Sandra Broddesson, Petra Edquist, Mia Brytting, Anna Risberg, Karin Tegmark-Wisell
EPI_ISL_648381, EPI_ISL_648382, EPI_ISL_648463, EPI_ISL_648465, EPI_ISL_648466	Santa Clara County Public Health Laboratory	Chan-Zuckerberg Biohub	CZB Cliahub Consortium
EPI_ISL_648501, EPI_ISL_648502, EPI_ISL_648503	Orange County Public Health Lab	Chan-Zuckerberg Biohub	CZB Cliahub Consortium
EPI_ISL_648545, EPI_ISL_648546, EPI_ISL_648547, EPI_ISL_648548, EPI_ISL_648549	Madera County Department of Public Health	Chan-Zuckerberg Biohub	CZB Cliahub Consortium
EPI_ISL_649048	San Diego County Public Health Laboratory	Andersen lab at Scripps Research	SEARCH Alliance San Diego with Tracy Basler, Jovan Shephard, Brett Austin
EPI_ISL_650850	West of Scotland Specialist Virology Centre, NHSGGC / MRC-University of Glasgow Centre for Virus Research	COVID-19 Genomics UK (COG-UK) Consortium	Ana da Silva Filipe, Natasha Johnson, Kathy Smollett, Daniel Mair, Stephen Carmichael, Alice Broos, Lily Tong, Jenna Nichols, Kyriaki Nomikou; Sarah McDonald; Richard Orton, Joseph Hughes, Sreenu Vattipally, David L Robertson; Alasdair MacLean, Rory Gunson; Sharif Shaaban, Matthew Holden; Rachel Blacow, Guy Mollett, Kathy Li, James Shepherd, Antonia Ho, Emma Thomson

EPI_ISL_653916	Diagnostic- and Research Institute of Pathology, Medical University of Graz	Diagnostic- and Research Institute of Pathology, Medical University of Graz	Karl Kashofer, Peter Regitnig, Martin Zacharias, Gregor Gorkiewicz
EPI_ISL_654021, EPI_ISL_654022, EPI_ISL_654023, EPI_ISL_654024, EPI_ISL_654025, EPI_ISL_654027, EPI_ISL_654028, EPI_ISL_654029, EPI_ISL_654030, EPI_ISL_654031, EPI_ISL_654033, EPI_ISL_654034, EPI_ISL_654036, EPI_ISL_654037, EPI_ISL_654038, EPI_ISL_654040, EPI_ISL_654041, EPI_ISL_654042, EPI_ISL_654044, EPI_ISL_654045, EPI_ISL_654046, EPI_ISL_654047, EPI_ISL_654050, EPI_ISL_654051, EPI_ISL_654052, EPI_ISL_654063, EPI_ISL_654064, EPI_ISL_654065, EPI_ISL_654066, EPI_ISL_654067, EPI_ISL_654068, EPI_ISL_654069, EPI_ISL_654070, EPI_ISL_654071, EPI_ISL_654072, EPI_ISL_654073, EPI_ISL_654074, EPI_ISL_654075, EPI_ISL_654076, EPI_ISL_654077, EPI_ISL_654078, EPI_ISL_654079, EPI_ISL_654080, EPI_ISL_654091, EPI_ISL_654092, EPI_ISL_654093, EPI_ISL_654094, EPI_ISL_654095, EPI_ISL_654096, EPI_ISL_654181, EPI_ISL_654196, EPI_ISL_654197, EPI_ISL_654198, EPI_ISL_654199, EPI_ISL_654200, EPI_ISL_654348			
see above	Hospital General Universitario Gregorio Marañón	SeqCOVID-SPAIN consortium/IBV(CSIC)	Darío García de Viedma, Laura Pérez-Lago, Marta Herranz, Jon Sicilia, Julia Suárez, Pilar Catalán, Patricia Muñoz and SeqCOVID-SPAIN consortium
EPI_ISL_654483, EPI_ISL_654484, EPI_ISL_654485, EPI_ISL_654486, EPI_ISL_654487, EPI_ISL_654488	Servicio de Microbiología. Hospital Clínico Universitario de Valencia	SeqCOVID-SPAIN consortium/IBV(CSIC)	David Navarro Ortega, Eliseo Albert Vicent, Ignacio Torres and SeqCOVID-SPAIN consortium
EPI_ISL_654513, EPI_ISL_654524	Servicio de Microbiología, Laboratori Clínic Metropolitana Nord. Hospital Universitari Germans Trias i Pujol. Institut d'Investigació en Ciències de la Salut Germans Trias i Pujol (IGTP)	SeqCOVID-SPAIN consortium/IBV(CSIC)	Elisa Martró, Antoni E. Bordoy, Anna Not, Adrián Antuori, Anabel Fernández, Nona Romaní and SeqCOVID-SPAIN consortium
EPI_ISL_654893, EPI_ISL_654894, EPI_ISL_654895, EPI_ISL_654896, EPI_ISL_654897, EPI_ISL_654898	Klinisk mikrobiologi	The Public Health Agency of Sweden	Anna-Malin Linde, Maria Lind Karlberg, Mattias Haukland, Reza Advani, Olov Svartstrom, Oskar Karlsson Lindsjo, Sandra Broddesson, Petra Edquist, Mia Brytting, Anna Risberg, Karin Tegmark-Wisell
EPI_ISL_660314	Servicio de Microbiología, Laboratori Clínic Metropolitana Nord. Hospital Universitari Germans Trias i Pujol. Institut d'Investigació en Ciències de la Salut Germans Trias i Pujol (IGTP)	SeqCOVID-SPAIN consortium/IBV(CSIC)	Elisa Martró, Antoni E. Bordoy, Anna Not, Adrián Antuori, Anabel Fernández, Nona Romaní and SeqCOVID-SPAIN consortium
EPI_ISL_660427, EPI_ISL_660428, EPI_ISL_660431	Klinisk Mikrobiologi	The Public Health Agency of Sweden	Anna-Malin Linde, Maria Lind Karlberg, Mattias Haukland, Reza Advani, Olov Svartstrom, Oskar Karlsson Lindsjo, Sandra Broddesson, Petra Edquist, Mia Brytting, Anna Risberg, Karin Tegmark-Wisell
EPI_ISL_660456, EPI_ISL_660458, EPI_ISL_660459, EPI_ISL_660460, EPI_ISL_660461, EPI_ISL_660462, EPI_ISL_660463, EPI_ISL_660481, EPI_ISL_660482, EPI_ISL_660483, EPI_ISL_660484, EPI_ISL_660485, EPI_ISL_660486, EPI_ISL_660487, EPI_ISL_660494, EPI_ISL_660496, EPI_ISL_660498, EPI_ISL_660501, EPI_ISL_660502, EPI_ISL_660509, EPI_ISL_660510, EPI_ISL_660517, EPI_ISL_660524, EPI_ISL_660525, EPI_ISL_660528			
see above	Laboratoire de Microbiologie CHU Souro Sanou	Centre Muraz	Abdoul-Salam Ouedraogo, Yacouba Sawadogo, Essia Belarbi, Grit Schubert, Fabian Leendertz, Arsène Zongo, Soumeya Ouangraoua, Zekiba Tarnagda, Lassana Sangaré, Halidou Tinto
EPI_ISL_660795, EPI_ISL_660796, EPI_ISL_660797, EPI_ISL_660798, EPI_ISL_660799, EPI_ISL_660800, EPI_ISL_660801	Gundersen Molecular Diagnostics Laboratory	Kabara Cancer Research Institute	Craig S. Richmond, Paraic A. Kenny
EPI_ISL_660802, EPI_ISL_660803, EPI_ISL_660804, EPI_ISL_660805	Gundersen Clinical Microbiology Laboratory	Kabara Cancer Research Institute	Craig S. Richmond, Paraic A. Kenny
EPI_ISL_660806, EPI_ISL_660807, EPI_ISL_660808, EPI_ISL_660809, EPI_ISL_660810, EPI_ISL_660811, EPI_ISL_660812, EPI_ISL_660813, EPI_ISL_660814, EPI_ISL_660815, EPI_ISL_660816, EPI_ISL_660817, EPI_ISL_660818, EPI_ISL_660819			
see above	Gundersen Molecular Diagnostics Laboratory	Kabara Cancer Research Institute	Craig S. Richmond, Paraic A. Kenny
EPI_ISL_661278	Klinisk mikrobiologi	The Public Health Agency of Sweden	Department of Microbiology, The Public Health Agency of Sweden
EPI_ISL_661286	Gavle klinisk mikrobiologi	The Public Health Agency of Sweden	Department of Microbiology, The Public Health Agency of Sweden
EPI_ISL_664233, EPI_ISL_664449, EPI_ISL_665153, EPI_ISL_665240	University College London Hospital	COVID-19 Genomics UK (COG-UK) Consortium	Judith Heaney, Matthew Byott, Catherine Houlihan, Dan Frampton, Stuart Kirk, Moira Spyer and Eleni Nastouli
EPI_ISL_666637, EPI_ISL_666638, EPI_ISL_666639, EPI_ISL_666640, EPI_ISL_666641, EPI_ISL_666642, EPI_ISL_666643, EPI_ISL_666644, EPI_ISL_666645, EPI_ISL_666646, EPI_ISL_666647, EPI_ISL_666648, EPI_ISL_666649, EPI_ISL_666650, EPI_ISL_666651, EPI_ISL_666652			
see above	ZOTZ KLIMAS MVZ Düsseldorf-Centrum GbR ÜBAG für Labormedizin, Genetik, Zytologie, Pathologie	Center of Medical Microbiology, Virology, and Hospital Hygiene, University of Duesseldorf	Maximilian Damagnez, Alexander Dilthey, Ashley-Jane Duplessis, Patrick Finzer, Katrin Hoffmann, Torsten Houwaart, Lisanna Hülse, Malte Kohns Vasconcelos, Marek Korencak, Nadine Lübke, Jessica Nicolai, Klaus Pfeffer, Daniel Strelow, Jörg Timm, Andreas Walker, Tobias Wienemann, Rainer Zotz
EPI_ISL_666856	Florida Bureau of Public Health Laboratories	Florida Bureau of Public Health Laboratories	Sarah Schmedes, Jason Blanton
EPI_ISL_666995, EPI_ISL_666996, EPI_ISL_666997, EPI_ISL_666998	San Diego County Public Health Laboratory	Andersen lab at Scripps Research	SEARCH Alliance San Diego with Tracy Basler, Jovan Shephard, Brett Austin

EPI_ISL_667449, EPI_ISL_667450, EPI_ISL_667451, EPI_ISL_667452, EPI_ISL_667453, EPI_ISL_667558	OHSU Lab Services Molecular Microbiology Lab	Oregon SARS-CoV-2 Genome Sequencing Center	Brendan L. O'Connell, Ruth V. Nichols, Sally Grindstaff, Alec J. Hirsch, Donna Hansel, Guang Fan, Daniel N. Streblow, William B. Messer, Andrew C. Adey, Benjamin N. Bimber, Brian J. O'Roak
EPI_ISL_667809	Laboratory Medicine	Department of Laboratory Medicine, Lin-Kou Chang Gung Memorial Hospital, Taoyuan, Taiwan	Kuo-Chien Tsao, Yu-Nong Gong, Shu-Li Yang, Yi-Chun Liu, Chung-Guei Huang, Mei-Jen Hsiao, Po-Wei Huang, Cheng-Ta Yang, Cheng-Hsun Chiu, Peng-Nien Huang, Kuo-Ming Lee, Guang-Wu Chen, Shin-Ru Shih
EPI_ISL_670651, EPI_ISL_670652, EPI_ISL_670653, EPI_ISL_670654, EPI_ISL_670655, EPI_ISL_670656, EPI_ISL_670657, EPI_ISL_670658, EPI_ISL_670672, EPI_ISL_670673, EPI_ISL_670674, EPI_ISL_670700, EPI_ISL_670767, EPI_ISL_671232, EPI_ISL_671233			
see above	Department of Virus and Microbiological Special Diagnostics, Statens Serum Institut, Copenhagen, Denmark	Albertsen Lab, Department of Chemistry and Bioscience, Aalborg University, Denmark	Danish Covid-19 Genome Consortium
EPI_ISL_671674	DOHMH Corona	New York City Public Health Laboratory	Jade Wang, et al.
EPI_ISL_671675	DOHMH Jamaica	New York City Public Health Laboratory	Jade Wang, et al.
EPI_ISL_671825, EPI_ISL_671826, EPI_ISL_671827, EPI_ISL_671828, EPI_ISL_671829, EPI_ISL_671830, EPI_ISL_671831, EPI_ISL_671832, EPI_ISL_671833	Hospital de la Santa Creu i Sant Pau. Servicio de Microbiología	SeqCOVID-SPAIN consortium/IBV(CSIC)	Ferran Navarro, Núria Rabella, Elisenda Miró and SeqCOVID-SPAIN consortium
EPI_ISL_671964	CHU Purpan - Laboratoire de Virologie - Institut Fédératif de Biologie	CHU Purpan - Laboratoire de Virologie - Institut Fédératif de Biologie	Latour J., Ranger N., Dubois M., Carcenac R., Harter A., Boyer P., Tremeaux P., Izopet J.
EPI_ISL_671974	Laboratorio de Virología y Microbiología Molecular, Depto. de Microbiología, Facultad de Medicina, Universidad de El Salvador/INS-laboratorio de Ref. Ministerio de Salud	Laboratorio de Virología y Microbiología Molecular, Depto. de Microbiología, Facultad de Medicina, Universidad de El Salvador/INS-laboratorio de Ref. Ministerio de Salud	Rivera NR, Ortega-Pérez C A, Xochitl Sandoval López, Carlos Hernández Ávila
EPI_ISL_672489	Contra Costa County Public Health Lab	Chan-Zuckerberg Biohub	CZB Cliahub Consortium
EPI_ISL_672637	PathWest Laboratory Medicine WA	PathWest Laboratory Medicine WA Microbial Surveillance Unit	PathWest Laboratory Medicine WA Microbial Surveillance Unit
EPI_ISL_676531, EPI_ISL_676532	Klinisk mikrobiologi	The Public Health Agency of Sweden	Department of Microbiology, The Public Health Agency of Sweden
EPI_ISL_676610	Texas Department of State Health Services	Texas Department of State Health Services	Rashmi Tuladhar, Bonnie Oh, Jenny Zhang, Maliha Rahman, Anita Pokharel, Myong Koag, Chung Wang, Rachel Lee, Grace Kubin, Mayela Pedrueza, James Daniel Bonser
EPI_ISL_677099, EPI_ISL_677100, EPI_ISL_677101, EPI_ISL_677102, EPI_ISL_677103	Wadsworth Center, New York State Department.of Health	Wadsworth Center, New York State Department.of Health	Kirsten St. George, Daryl M. Lamson, Alexis Russel, Jonathan Plitnick, Navjot Singh, John Kelly, Sara Griesemer, Erasmus Schneider, Erica Lasek-Nesselquist
EPI_ISL_677243, EPI_ISL_677262, EPI_ISL_677277, EPI_ISL_677279, EPI_ISL_677315, EPI_ISL_677316, EPI_ISL_677317	Colorado Department of Public Health and Environment	Colorado Department of Puplic Health and Environment	Laura Bankers, Molly Hetherington-Rauth, Shannon Ely, Shannon R. Matzinger, Sarah Elizabeth Totten, Emily A. Travanty
EPI_ISL_677343, EPI_ISL_677350, EPI_ISL_677413, EPI_ISL_677414, EPI_ISL_677415, EPI_ISL_677416, EPI_ISL_677417, EPI_ISL_677418, EPI_ISL_677419, EPI_ISL_677420, EPI_ISL_677421, EPI_ISL_677450			
see above	University of Wisconsin-Madison AIDS Vaccine Research Laboratories	University of Wisconsin-Madison AIDS Vaccine Research Laboratories	Gage Moreno, Katarina Braun, et al. AIDS Vaccine Research Laboratories
EPI_ISL_677634	Virology Unit, Institut Pasteur de Madagascar	Virology Unit, Institut Pasteur de Madagascar	Christian Ranaivoson, Cara E. Brook, Vida Ahyong, Soa Fy Andriamandimby, Vololoniaina Raharinosy, Tsiry Randriambolamanantsoa, Helisoa Razafimanjato, Norosoa Razanajatovo, Michelle Tan, Cristina M. Tato, Joseph L. DeRisi, Jean-Michel Heraud, Philippe Dussart
EPI_ISL_677648, EPI_ISL_677649, EPI_ISL_677650, EPI_ISL_677651, EPI_ISL_677652, EPI_ISL_677653, EPI_ISL_677654, EPI_ISL_677655, EPI_ISL_677656, EPI_ISL_677657	Colorado Department of Public Health and Environment	Colorado Department of Puplic Health and Environment	Laura Bankers, Molly C. Hetherington-Rauth, Shannon Ely, Shannon R. Matzinger, Sarah Elizabeth Totten, Emily A. Travanty
EPI_ISL_677659	University of	University of	Gage Moreno, Katarina Braun, et al. AIDS Vaccine Research Laboratories

	Wisconsin-Madison AIDS Vaccine Research Laboratories	Wisconsin-Madison AIDS Vaccine Research Laboratories	
EPI_ISL_677671	Wadsworth Center, New York State Department.of Health	Wadsworth Center, New York State Department.of Health	Kirsten St. George, Daryl M. Lamson, Alexis Russel, Jonathan Plitnick, Navjot Singh, John Kelly, Sara Griesemer, Erasmus Schneider, Erica Lasek-Nesselquist
EPI_ISL_677696, EPI_ISL_677699	Vanda Pharmaceuticals Clinical Site	Vanda Pharmaceuticals	Vanda Pharmaceuticals
EPI_ISL_678260	General Hospital - Struga	Research Center for Genetic Engineering and Biotechnology "Georgi D. Efremov" , Macedonian Academy of Sciences and Arts	RCGEB - MASA
EPI_ISL_678343, EPI_ISL_678344, EPI_ISL_678346, EPI_ISL_678358	Area of Virology, Serology and Virology Division (SAViD), New South Wales Health Pathology Randwick	Virology Research Laboratory; Area of Virology, Serology and Virology Division (SAViD), New South Wales Health Pathology Randwick	Foster, C.; Au, J.; Ruiz Silva, M.; Deveson, I.; Bull, R.; Van Hal, S.; Rawlinson, W.
EPI_ISL_678829	Respiratory Virus Unit, Microbiology Services Colindale, Public Health England	COVID-19 Genomics UK (COG-UK) Consortium	PHE Covid Sequencing Team
EPI_ISL_679409, EPI_ISL_679410, EPI_ISL_679411	University College London Hospital	COVID-19 Genomics UK (COG-UK) Consortium	Judith Heaney, Matthew Byott, Catherine Houlihan, Dan Frampton, Stuart Kirk, Moira Spyer and Eleni Nastouli
EPI_ISL_680258, EPI_ISL_680265, EPI_ISL_680301, EPI_ISL_680303	Regional Virus Laboratory, Belfast Health and Social Care Trust	COVID-19 Genomics UK (COG-UK) Consortium	Conall McCaughey, James McKenna, Tanya Curran, Susan Feeney, Alison Watt, Ciara Cox, Mairead Connor, Zoltan Molnar, David Simpson, Derek Fairley
EPI_ISL_680497	Virology Department, Royal Infirmary of Edinburgh, NHS Lothian / School of Biological Sciences, University of Edinburgh / Institute of Genetics and Molecular Medicine, University of Edinburgh	COVID-19 Genomics UK (COG-UK) Consortium	McHugh M, Dewar R, Rooke S, Gallagher M, Balcaza C, O'Toole Á, Scher E, Hill V, McCrone JT, Colquhoun R, Yu X, Jackson B, Rambaut A, Williams TC, Templeton K
EPI_ISL_681301, EPI_ISL_681309	Communicable Disease Laboratory, Public Health Directorate	Communicable Disease Laboratory, Public Health Directorate	Alwasti,H., Altaif,Z., AlHujairi,Z., AlAbbas,Z.
EPI_ISL_681936, EPI_ISL_682034, EPI_ISL_682036, EPI_ISL_682038, EPI_ISL_682044, EPI_ISL_682045, EPI_ISL_682046, EPI_ISL_682047, EPI_ISL_682048, EPI_ISL_682049, EPI_ISL_682055, EPI_ISL_682056			
see above	UPMC Clinical Microbiology Laboratory	Microbial Genomic Epidemiology Laboratory, University of Pittsburgh	Mustapha M. Mustapha, Jane W. Marsh, Dan Snyder, Marissa P. Griffith, Stephanie L. Mitchell, Vatsala R. Srinivasa, Kady D. Waggle, Chinelo Ezeonwuku, Vaughn S. Cooper, Lee H. Harrison
EPI_ISL_682300, EPI_ISL_682303	Communicable Disease Laboratory, Public Health Directorate	Communicable Disease Laboratory, Public Health Directorate	Alwasti,H., Altaif,Z., AlHujairi,Z., AlAbbas,Z.
EPI_ISL_683601, EPI_ISL_683602, EPI_ISL_683603	Servicio de Microbiología, Laboratori Clínic Metropolitana Nord. Hospital Universitari Germans Trias i Pujol. Institut d'Investigació en Ciències de la Salut Germans Trias i Pujol (IGTP)	SeqCOVID-SPAIN consortium/IBV(CSIC)	Elisa Martró, Antoni E. Bordoy, Anna Not, Adrián Antuori, Anabel Fernández, Nona Romaní, Verónica Saludes, Cristina Casañ and SeqCOVID-SPAIN consortium
EPI_ISL_684004	Utah Public Health Laboratory	Utah Public Health Laboratory	Erin Young, Kelly Oakeson
EPI_ISL_692746, EPI_ISL_692747, EPI_ISL_692748, EPI_ISL_692749, EPI_ISL_692750, EPI_ISL_692751, EPI_ISL_692752, EPI_ISL_692753, EPI_ISL_692754	CNR Virus des Infections Respiratoires - France SUD	CNR Virus des Infections Respiratoires - France SUD	Antonin Bal, Gregory Destras, Gwendolyne Burfin, Solenne Brun, Martine Valette, Bruno Lina, Laurence Josset
EPI_ISL_693299	Department of Microbiology, Yokohama City University School of Medicine	Department of Microbiology, Yokohama City University School of Medicine	Kei Miyakawa, Ryo Saji, Kazuya Sakai, Reo Matsumura, Mototsugu Nishii, Ichiro Takeuchi, Akihide Ryo
EPI_ISL_693479, EPI_ISL_693481	Central Public Health Laboratory	National Public Health Laboratory, National Centre for Infectious Diseases	Tze Minn Mak, Sophie Octavia, Zhenyang Zhou, Esorom Daoni, Theresa Palou, Lin Cui, Raymond Tzer Pin Lin
EPI_ISL_693488, EPI_ISL_693489, EPI_ISL_693491, EPI_ISL_693492, EPI_ISL_693493, EPI_ISL_693494, EPI_ISL_693495, EPI_ISL_693496, EPI_ISL_693497, EPI_ISL_693498, EPI_ISL_693499, EPI_ISL_693500			
see above	CNR Virus des Infections Respiratoires - France SUD	CNR Virus des Infections Respiratoires - France SUD	Antonin Bal, Gregory Destras, Gwendolyne Burfin, Quentin Semanas, Martine Valette, Bruno Lina, Laurence Josset
EPI_ISL_693718,	Delaware Public Health	Delaware Public Health	Gregory Hovan

EPI_ISL_693720, EPI_ISL_693745	Laboratory	Laboratory	
EPI_ISL_693763	Hospital	National Reference Center for Viruses of Respiratory Infections, Institut Pasteur, Paris	Marion Barbet, Sylvie Behillil, Méline Bizard, Angela Brisebarre, Camille Capel, Etienne Simon-Lorière, Vincent Enouf, Maud Vanpeene, Sylvie van der Werf, Gisèle Lagathu
EPI_ISL_699642	South Eastern Area Laboratory Services (SEALS)	NSW Health Pathology - Institute of Clinical Pathology and Medical Research; Westmead Hospital; University of Sydney	CIDM-PH et al.
EPI_ISL_700251, EPI_ISL_700252, EPI_ISL_700253, EPI_ISL_700254, EPI_ISL_700255, EPI_ISL_700256, EPI_ISL_700257, EPI_ISL_700258, EPI_ISL_700259, EPI_ISL_700260, EPI_ISL_700261, EPI_ISL_700262, EPI_ISL_700263, EPI_ISL_700264, EPI_ISL_700265, EPI_ISL_700266, EPI_ISL_700267, EPI_ISL_700268, EPI_ISL_700269, EPI_ISL_700270, EPI_ISL_700271, EPI_ISL_700272, EPI_ISL_700273, EPI_ISL_700274, EPI_ISL_700275, EPI_ISL_700276, EPI_ISL_700277, EPI_ISL_700278, EPI_ISL_700279, EPI_ISL_700280, EPI_ISL_700281, EPI_ISL_700282, EPI_ISL_700283, EPI_ISL_700284, EPI_ISL_700285, EPI_ISL_700286, EPI_ISL_700287, EPI_ISL_700288, EPI_ISL_700289, EPI_ISL_700290, EPI_ISL_700291, EPI_ISL_700292, EPI_ISL_700293, EPI_ISL_700294, EPI_ISL_700295			
see above	Hematopathology Laboratory, ACTREC, TMC	Hematopathology Laboratory, ACTREC, TMC	Hematopathology Laboratory, ACTREC
EPI_ISL_700328, EPI_ISL_700332, EPI_ISL_700344, EPI_ISL_700345, EPI_ISL_700346	Child Health Research Foundation	Child Health Research Foundation	Senjuti Saha, Afroza Akter Tanni, Syed Muktadir Al Sium, Roly Malaker, Sharmistha Goswami, Arif Mohammad Tanmoy, Md Hafizur Rahman, Samir K Saha
EPI_ISL_700532	Kwamandlenkosi Clinic wc KMN	NHLS/UCT	Arash Iranzadeh, Deelan Doolabh, Lynn Tyers, Bruna Galvao, Innocent Mudau, Marvin Hsiao, Kruger Marais, Diana Hardie, Stephen Korsman, Carolyn Williamson
EPI_ISL_700699, EPI_ISL_700701, EPI_ISL_700724, EPI_ISL_700732, EPI_ISL_700734	Texas Department of State Health Services	Texas Department of State Health Services	Rashmi Tuladhar, Bonnie Oh, Jenny Zhang, Maliha Rahman, Anita Pokharel, Myong Koag, Chung Wang, Rachel Lee, Grace Kubin, Mayela Pedrueza, James Daniel Bonser
EPI_ISL_706464	Wales Specialist Virology Centre Sequencing lab: Pathogen Genomics Unit	COVID-19 Genomics UK (COG-UK) Consortium	Catherine Moore, Johnathan Evans, Laura Gifford, Malorie Perry, Simon Cottrell, Angela Marchbank, Alec Birchley, Alexander Adams, Amy Gaskin, Bree Gatica-Wilcox, Jason Coombes, Joel Southgate, Lauren Gilbert, Lee Graham, Nicole Pacchiarini, Sara Kumziene-Summerhayes, Sarah Taylor, Sophie Jones, Sara Rey, Matthew Bull, Joanne Watkins, Sally Corden, Tom Connor
EPI_ISL_707887	Los Angeles County Public Health Laboratory	Los Angeles County Public Health Laboratory	P. Hemarajata et al.
EPI_ISL_708342	Michigan Department of Health and Human Services, Bureau of Laboratories	Michigan Department of Health and Human Services, Bureau of Laboratories	Blankenship HM, Riner D, Soehnlen MK
EPI_ISL_708396, EPI_ISL_708397, EPI_ISL_708433, EPI_ISL_708434, EPI_ISL_708435	Delaware Public Health Lab	Delaware Public Health Lab	Gregory Hovan
EPI_ISL_708812	World Medical Hospital	National Institute of Health, Department of Medical Sciences, Ministry of Public Health, Thailand	Pilailuk Okada; Siripaporn Phuygun; Thanutsapa Thanadachakul; Sittiporn Parnmen; Pakorn Piromtong; Warawan Wongboot; Sunthareeya Waicharoen; Malinee Chittaganpitch
EPI_ISL_710132, EPI_ISL_710142, EPI_ISL_710152, EPI_ISL_710154, EPI_ISL_710167, EPI_ISL_710169, EPI_ISL_710170, EPI_ISL_710224, EPI_ISL_710225, EPI_ISL_710226, EPI_ISL_710227, EPI_ISL_710228, EPI_ISL_710229, EPI_ISL_710230, EPI_ISL_710231, EPI_ISL_710232, EPI_ISL_710233, EPI_ISL_710234, EPI_ISL_710235, EPI_ISL_710236, EPI_ISL_710252, EPI_ISL_710254, EPI_ISL_710256, EPI_ISL_710257, EPI_ISL_710264, EPI_ISL_710272, EPI_ISL_710273, EPI_ISL_710275, EPI_ISL_710276, EPI_ISL_710280, EPI_ISL_710282, EPI_ISL_710294, EPI_ISL_710298, EPI_ISL_710333, EPI_ISL_710334, EPI_ISL_710335, EPI_ISL_710336, EPI_ISL_710337, EPI_ISL_710338, EPI_ISL_710339, EPI_ISL_710340, EPI_ISL_710341, EPI_ISL_710342, EPI_ISL_710343, EPI_ISL_710344, EPI_ISL_710345, EPI_ISL_710346, EPI_ISL_710347			
see above	Colorado Department of Public Health and Environment	Colorado Department of Puplic Health and Environment	Laura Bankers, Molly C. Hetherington-Rauth, Shannon Ely, Shannon R. Matzinger, Sarah Elizabeth Totten, Emily A. Travanty
EPI_ISL_714929	Department of Virus and Microbiological Special Diagnostics, Statens Serum Institut, Copenhagen, Denmark	Albertsen Lab, Department of Chemistry and Bioscience, Aalborg University, Denmark	Danish Covid-19 Genome Consortium
EPI_ISL_717700	Trinidad Public Health Laboratory	Carrington Lab, Department of PreClinical Sciences, Faculty of Medical Sciences, The University of the West Indies	Nikita S. D. Sahadeo, Arianne Brown-Jordan, Sarah Hill, Vernie Ramkissoon, Naresh Nandram, Avery Hinds, Jerome Foster, Stanley Giddings, Karla Georges, Marsha Ivey, Rahul Naidu, Risha Singh, SueMin Nathaniel, Rajini Haraksingh, Jaya Jayaraman, Chinna Chinnadurai, Adesh Ramsubhag, Nuno Faria, Oliver Pybus, Christopher Oura, Gabriel Escobar, Christine V. F. Carrington
EPI_ISL_717773, EPI_ISL_717774, EPI_ISL_717775	UW Virology Lab	UW Virology Lab	Pavitra Roychoudhury, Hong Xie, Lasata Shrestha, Michelle Lin, Meei-Li Huang, Keith R Jerome, Alexander Greninger
EPI_ISL_717975	Institute of Virology, Biomedical Research Center of the Slovak Academy of Sciences, Bratislava	Faculty of Natural Sciences, Comenius University, Bratislava	Broa Brejová, Viktória Hodorová, Kristína Boršová, Viktória abanová, Dominika Friová, Sabina Fumaová Havlíková, Juraj Kopáek, Martina Liková, ubomíra Lukáiková, Martina Neboháová, Monika Sláviková, Edita Staroová, Elena Tichá, Tomáš Vina, Jozef Nosek, Boris Klempa
EPI_ISL_718229, EPI_ISL_718233	Hospital	National Reference Center for Viruses of Respiratory Infections, Institut Pasteur, Paris	Marion Barbet, Sylvie Behillil, Méline Bizard, Angela Brisebarre, Camille Capel, Etienne Simon-Lorière, Vincent Enouf, Maud Vanpeene, Sylvie van der Werf, Gisèle Lagathu
EPI_ISL_718282	Institute for Medical Research, Infectious Disease Research Centre, National Institutes of Health, Ministry of Health Malaysia	Institute for Medical Research, Infectious Disease Research Centre, National Institutes of Health, Ministry of Health Malaysia	Suppiah J, Kamel K, Mohd-Zawawi Z, Thayan R

EPI_ISL_722375, EPI_ISL_722465, EPI_ISL_722816, EPI_ISL_722817, EPI_ISL_722818, EPI_ISL_722819, EPI_ISL_722820, EPI_ISL_722821, EPI_ISL_722822, EPI_ISL_722823	Dutch COVID-19 response team	Erasmus Medical Center	Bas Oude Munnink, Reina Sikkema, David Nieuwenhuijse, Irina Chestakova, Anne van der Linden, Marjan Boter, Emmanuelle Munger, Corine GeurtsvanKessel, Annemiek van der Eijk, Richard Molenkamp, Marion Koopmans, on behalf of the Dutch national COVID-19 response team.
EPI_ISL_723067, EPI_ISL_723068, EPI_ISL_723069, EPI_ISL_723070, EPI_ISL_723071, EPI_ISL_723072, EPI_ISL_723073, EPI_ISL_723074, EPI_ISL_723075	Hematopathology Laboratory, ACTREC, TMC	Hematopathology Laboratory, ACTREC, TMC	Hematopathology Laboratory, ACTREC
EPI_ISL_729343, EPI_ISL_729351, EPI_ISL_729352, EPI_ISL_729359, EPI_ISL_729375, EPI_ISL_729608	A. Krumbholz, Labor Dr. Krause und Kollegen MVZ GmbH, Kiel	Charité Universitätsmedizin Berlin, Institut für Virologie	Victor M Corman, Barbara Mühlemann, Jörn Beheim-Schwarzbach, Talitha Veith, Julia Schneider, Terry Jones, Christian Drosten
EPI_ISL_729735, EPI_ISL_729736, EPI_ISL_729741, EPI_ISL_729743, EPI_ISL_729744, EPI_ISL_729745, EPI_ISL_729748, EPI_ISL_729749, EPI_ISL_729750, EPI_ISL_729752, EPI_ISL_729754, EPI_ISL_729760, EPI_ISL_729773, EPI_ISL_729779, EPI_ISL_729780			
see above	Connecticut Department of Health	Grubaugh Lab - Yale School of Public Health	Joseph Fauver, Tara Alpert, Anderson Brito, Annie Watkins, Anne Wyllie, Chantal Vogels, Mary Petrone, Chaney Kalinich, Isabel Ott, Arnau Casanovas, Catherine Muenker, Adam Moore, Alice Lu, Maria Tokuyama, Patrick Wong, Peiwen Lu, Saad Omer, Richard Martinello, Allison Nelson, Shelli Farhadian, Akiko Iwasaki, Charlese Dela Cruz, Albert Ko, Nathan Grubaugh
EPI_ISL_729871, EPI_ISL_729906	Instituto de Medicina Tropical, Universidad Nacional Toribio Rodríguez de Mendoza de Amazonas	Laboratorio de Genómica Microbiana, Universidad Peruana Cayetano Heredia	Pablo Tsukayama, Alejandra Dávila-Barclay, Luis González, Pedro E. Romero, Brenda Ayzanoa, Janet Huancachoque, Pool Marcos, Stella Chenet, Rafael Tapia, Cecilia Pajuelo, Carla Montenegro
EPI_ISL_730115, EPI_ISL_730116	San Diego County Public Health Laboratory	Andersen lab at Scripps Research	SEARCH Alliance San Diego with Tracy Basler, Jovan Shephard, Brett Austin
EPI_ISL_730229	Biolab Diagnostic Laboratories	Andersen lab at Scripps Research	Issa Abu-Dayyeh, Ahmad Tibi, Lama Hussein, Lina Mohammad, Zein Naber, Amid Abdelnour with SEARCH Alliance San Diego
EPI_ISL_730341	San Diego County Public Health Laboratory	Andersen lab at Scripps Research	SEARCH Alliance San Diego with Tracy Basler, Jovan Shephard, Brett Austin
EPI_ISL_730569	Gazi University Faculty of Medicine, Medical Virology Laboratory	Gazi University Faculty of Medicine, Medical Virology Laboratory	Erdem ahin, Hager Muftah, Selin Yiit, Shaknoza Sarzhanova, Özlem Güzel Tunçcan, Murat Dizbay, Il Fidan, Kayhan Çalar, Gülendam Bozday
EPI_ISL_730591	Princess Margaret Hospital	Hong Kong Department of Health	Mak Gannon C.K., Lam Edman T.K., Chan Rickjason C.W., Tsang Dominic N.C.
EPI_ISL_730602	Tseung Kwan O Hospital	Hong Kong Department of Health	Mak Gannon C.K., Lam Edman T.K., Chan Rickjason C.W., Tsang Dominic N.C.
EPI_ISL_730604, EPI_ISL_730605, EPI_ISL_730609	Tuen Mun Hospital	Hong Kong Department of Health	Mak Gannon C.K., Lam Edman T.K., Chan Rickjason C.W., Tsang Dominic N.C.
EPI_ISL_730612, EPI_ISL_730613	United Christian Hospital	Hong Kong Department of Health	Mak Gannon C.K., Lam Edman T.K., Chan Rickjason C.W., Tsang Dominic N.C.
EPI_ISL_732750, EPI_ISL_732769, EPI_ISL_732770, EPI_ISL_732771, EPI_ISL_732783, EPI_ISL_732815	Centro de Investigación Biomédica de La Rioja - Hospital San Pedro Logroño	SeqCOVID-SPAIN consortium/IBV(CSIC)	María de Toro, José Manuel Azcona Gutiérrez, María Pilar Bea Escudero, Miriam Blasco Alberdi and SeqCOVID-SPAIN consortium
EPI_ISL_733046, EPI_ISL_733071, EPI_ISL_733075	HELIX LLC	WHO National Influenza Centre Russian Federation	Andrey Komissarov, Artem Fadeev, Anna Ivanova, Kseniya Komissarova, Dmitry Bazhenov, Daria Danilenko, Ksenia Safina, Elena Nabieva, Georgii Bazykin, Dmitry Lioznov
EPI_ISL_733186, EPI_ISL_733194, EPI_ISL_733203, EPI_ISL_733204, EPI_ISL_733214	Pathogenic Microorganisms Variability Laboratory	WHO National Influenza Centre Russian Federation	Andrey Komissarov, Artem Fadeev, Anna Ivanova, Kseniya Komissarova, Dmitry Bazhenov, Daria Danilenko, Ksenia Safina, Elena Nabieva, Georgii Bazykin, Nadezhda Kuznetsova, Elena Shidlovskaya, Sergey Alkhovsky, Tatyana Vishnevskaya, Elizaveta Divisenko, Alexey Shchetinin, Maria Nikiforova, Andrey Pochtovyy, Evgeny Usachev, Elena Vokalova, Maxim Rubalsky, Oleg Rubalsky, Artem Tkachuk, Vladimir Gushchin, Alexander Gintsburg, Dmitry Lioznov
EPI_ISL_733230	UMMC-Health	WHO National Influenza Centre Russian Federation	Andrey Komissarov, Artem Fadeev, Anna Ivanova, Kseniya Komissarova, Dmitry Bazhenov, Tatiana Platonova, Daria Danilenko, Ksenia Safina, Elena Nabieva, Georgii Bazykin, Dmitry Lioznov
EPI_ISL_733298, EPI_ISL_733299, EPI_ISL_733395	WHO National Influenza Centre Russian Federation	WHO National Influenza Centre Russian Federation	Andrey Komissarov, Artem Fadeev, Anna Ivanova, Kseniya Komissarova, Dmitry Bazhenov, Daria Danilenko, Ksenia Safina, Elena Nabieva, Georgii Bazykin, Dmitry Lioznov
EPI_ISL_734923, EPI_ISL_734924, EPI_ISL_734925, EPI_ISL_734926, EPI_ISL_734927, EPI_ISL_734928, EPI_ISL_734929, EPI_ISL_734930, EPI_ISL_734931, EPI_ISL_734932, EPI_ISL_734933, EPI_ISL_734934, EPI_ISL_734935, EPI_ISL_734936, EPI_ISL_734937, EPI_ISL_734938, EPI_ISL_734939, EPI_ISL_734940, EPI_ISL_734941, EPI_ISL_734942, EPI_ISL_734943, EPI_ISL_734944, EPI_ISL_734945, EPI_ISL_734946, EPI_ISL_734947, EPI_ISL_734948, EPI_ISL_734949, EPI_ISL_734950, EPI_ISL_734951, EPI_ISL_734952, EPI_ISL_734953, EPI_ISL_734954, EPI_ISL_734955, EPI_ISL_734956, EPI_ISL_734957, EPI_ISL_734958, EPI_ISL_734959, EPI_ISL_734960, EPI_ISL_734961, EPI_ISL_734962, EPI_ISL_734963, EPI_ISL_734964, EPI_ISL_734965, EPI_ISL_734966, EPI_ISL_734967, EPI_ISL_734968, EPI_ISL_734969, EPI_ISL_734970, EPI_ISL_734971, EPI_ISL_734972, EPI_ISL_734973, EPI_ISL_734974, EPI_ISL_734975, EPI_ISL_734976, EPI_ISL_734977, EPI_ISL_734978, EPI_ISL_734979, EPI_ISL_734980, EPI_ISL_734981, EPI_ISL_734982, EPI_ISL_734983, EPI_ISL_734984, EPI_ISL_734985, EPI_ISL_734986, EPI_ISL_734987, EPI_ISL_734988, EPI_ISL_734989, EPI_ISL_734990			
see above	UZ Leuven, National Reference Laboratory for Coronaviruses, Laboratory Medicine, Leuven, Belgium	KU Leuven, Rega Institute, Clinical and Epidemiological Virology	Tony Wawina-Bokalanga, Joan Marti-Carerras, Bert Vanmechelen, Piet Maes

EPI_ISL_737061, EPI_ISL_737062, EPI_ISL_737063, EPI_ISL_737064, EPI_ISL_737065, EPI_ISL_737066, EPI_ISL_737067, EPI_ISL_737068, EPI_ISL_737234, EPI_ISL_737235	Department of Virology and Immunology, University of Helsinki and Helsinki University Hospital, Huslab Finland	Department of Virology, Faculty of Medicine, University of Helsinki, Helsinki, Finland	Teemu Smura, Ravi Kant, Phuoc Truong, Hussein Alburkat, Hannimari Kallio-Kokko, Jenni Virtanen, Maija Suvanto, Sari Hannula, Harri Kangas, Pekka Ellonen, Olli Vapalahti
EPI_ISL_738223, EPI_ISL_738224, EPI_ISL_738225, EPI_ISL_738226	UZ Leuven, National Reference Laboratory for Coronaviruses, Laboratory Medicine, Leuven, Belgium	KU Leuven, Rega Institute, Clinical and Epidemiological Virology	Tony Wawina-Bokalanga, Joan Marti-Carerras, Bert Vanmechelen, Piet Maes
EPI_ISL_738541	Napa-Solano-Yolo- Marin County (NSYM) Public Health Laboratories	Chan-Zuckerberg Biohub	CZB Cliahub Consortium
EPI_ISL_738554, EPI_ISL_738797, EPI_ISL_738932	Alameda County Public Health Lab	Chan-Zuckerberg Biohub	CZB Cliahub Consortium
EPI_ISL_738952	Napa-Solano-Yolo- Marin County (NSYM) Public Health Laboratories	Chan-Zuckerberg Biohub	CZB Cliahub Consortium
EPI_ISL_738990	County of San Luis Obispo Public Health Laboratory	Chan-Zuckerberg Biohub	CZB Cliahub Consortium
EPI_ISL_739062, EPI_ISL_739110	Alameda County Public Health Lab	Chan-Zuckerberg Biohub	CZB Cliahub Consortium
EPI_ISL_739169	Napa-Solano-Yolo- Marin County (NSYM) Public Health Laboratories	Chan-Zuckerberg Biohub	CZB Cliahub Consortium
EPI_ISL_739173	Alameda County Public Health Lab	Chan-Zuckerberg Biohub	CZB Cliahub Consortium
EPI_ISL_739181, EPI_ISL_739223, EPI_ISL_739339	Napa-Solano-Yolo- Marin County (NSYM) Public Health Laboratories	Chan-Zuckerberg Biohub	CZB Cliahub Consortium
EPI_ISL_739444	Alameda County Public Health Lab	Chan-Zuckerberg Biohub	CZB Cliahub Consortium
EPI_ISL_739469	Napa-Solano-Yolo- Marin County (NSYM) Public Health Laboratories	Chan-Zuckerberg Biohub	CZB Cliahub Consortium
EPI_ISL_739492	County of San Luis Obispo Public Health Laboratory	Chan-Zuckerberg Biohub	CZB Cliahub Consortium
EPI_ISL_739507, EPI_ISL_739535	Alameda County Public Health Lab	Chan-Zuckerberg Biohub	CZB Cliahub Consortium
EPI_ISL_739546	Monterey County Public Health Lab	Chan-Zuckerberg Biohub	CZB Cliahub Consortium
EPI_ISL_739557	Napa-Solano-Yolo- Marin County (NSYM) Public Health Laboratories	Chan-Zuckerberg Biohub	CZB Cliahub Consortium
EPI_ISL_739583	Alameda County Public Health Lab	Chan-Zuckerberg Biohub	CZB Cliahub Consortium
EPI_ISL_739588	County of San Luis Obispo Public Health Laboratory	Chan-Zuckerberg Biohub	CZB Cliahub Consortium
EPI_ISL_739623, EPI_ISL_739634	Alameda County Public Health Lab	Chan-Zuckerberg Biohub	CZB Cliahub Consortium
EPI_ISL_739947, EPI_ISL_744291, EPI_ISL_744528, EPI_ISL_744531, EPI_ISL_744822, EPI_ISL_744936, EPI_ISL_744941	Laboratoire national de santé, Microbiology, Virology	Laboratoire national de santé, Microbiology, Microbial Genomics Platform	Anke Wienecke-Baldacchino, Catherine Ragimbeau,Jessica Tapp, Fatu Djabi, Lise Pignon, Raoul Salmon, Tamir Abdelrahman
EPI_ISL_745554, EPI_ISL_745664	Ginkgo Bioworks Clinical Laboratory	Utah Public Health Laboratory	Erin L. Young, Kelly Oakeson, Tara Gallagher, Michael T. Pyne, E. Susan Slechta, Melanie A. Mallory, Jeffrey B. Stevenson, Salika M. Shakir, David R. Hillyard, Malaika McKenzie-Bennett, James McGann, Jim Griffin, Keith Robison, Alex Plocik, Becky Schilling, Martha Pierson, Rebecca Littlefield, Michelle Spencer, Birgitte Simen
EPI_ISL_746481, EPI_ISL_746500, EPI_ISL_746509, EPI_ISL_746536, EPI_ISL_746537	Genetica Molecular and Subdepartamento de Virologia ISP Chile	Instituto de Salud Publica de Chile	Javier Tognarelli, Barbara Parra, Loredana Arata, Jaime Lagos, Gisselle Barra, Patricia Bustos, Rodrigo Fasce, Andres Castillo, Jorge Fernandez
EPI_ISL_747265, EPI_ISL_747266, EPI_ISL_747268, EPI_ISL_747274, EPI_ISL_747275, EPI_ISL_747276, EPI_ISL_747281, EPI_ISL_747285, EPI_ISL_747286, EPI_ISL_747288, EPI_ISL_747291, EPI_ISL_747292, EPI_ISL_747293, EPI_ISL_747294, EPI_ISL_747298, EPI_ISL_747299, EPI_ISL_747303, EPI_ISL_747304, EPI_ISL_747305, EPI_ISL_747306, EPI_ISL_747319, EPI_ISL_747336, EPI_ISL_747337, EPI_ISL_747338, EPI_ISL_747339, EPI_ISL_747374			
see above	Division of Emerging Infectious Diseases, Bureau of Infectious Diseases	Division of Emerging Infectious Diseases, Bureau of Infectious Diseases	Ae Kyung Park, Il-Hwan Kim, Heui Man Kim, Jeong-Min Kim, Namjoo Lee, Chaeyoung Lee, Sang Hee Woo, Eun-Jin Kim

	Diagnosis Control, Korea Disease Control and Prevention Agency	Diagnosis Control, Korea Disease Control and Prevention Agency	
EPI_ISL_748136, EPI_ISL_748137	Department of Virus and Microbiological Special Diagnostics, Statens Serum Institut, Copenhagen, Denmark	Albertsen Lab, Department of Chemistry and Bioscience, Aalborg University, Denmark	Danish Covid-19 Genome Consortium
EPI_ISL_752842, EPI_ISL_752843, EPI_ISL_752904, EPI_ISL_752905, EPI_ISL_752906, EPI_ISL_752907, EPI_ISL_752908, EPI_ISL_752909, EPI_ISL_752910, EPI_ISL_752927, EPI_ISL_752946, EPI_ISL_752998, EPI_ISL_752999, EPI_ISL_753000, EPI_ISL_753001, EPI_ISL_753022, EPI_ISL_753023			
see above	State Laboratories Division, Hawaii State Department of Health	State Laboratories Division, Hawaii State Department of Health	Pamela O'Brien, Sabrina Diemert, Drew Kuwazaki, Razvan Sultana, Edward Desmond
EPI_ISL_754180	Department for Virology, Molecular Biology and Genome Research, R. G. Lugar Center for Public Health Research, National Center for Disease Control and Public Health (NCDC) of Georgia.	Department for Virology, Molecular Biology and Genome Research, R. G. Lugar Center for Public Health Research, National Center for Disease Control and Public Health (NCDC) of Georgia.	Salome Javashvili, Nino Berishvili, Tata Imnadze, Giorgi Tomashvili, Ana Papkiauri, Meri Pantsulaia, Gvantsa Brachveli, Gvantsa Chanturia, Ann Machablishvili, Nato Kotaria, Marine Murtskhvaladze, Lela Sabadze, Mari Gavashelidze, Tamar Jashiashvili, Tea Tevdoradze, Ketevan Sidamonidze, Ekaterine Khmaladze, Ekaterine Zhghenti, Roena Sukhiashvili, Mariam Zakalashvili, Lela Urushadze, Magda Dgebuadze, Davit Tsaguria, Ekaterine Zangaladze, Adam Kotorashvili, Maia Alkhazashvili, Irma Burjanadze, Anna Kasradze, Khatuna Zakhashvili, Paata Imnadze, Amiran Gamkrelidze.
EPI_ISL_754856	CHU - Hôpital Cavale Blanche - Labo. de Virologie	National Reference Center for Viruses of Respiratory Infections, Institut Pasteur, Paris	Marion Barbet, Sylvie Behillil, Méline Bizard, Angela Brisebarre, Camille Capel, Etienne Simon-Lorière, Vincent Enouf, Maud Vanpeene, Sylvie van der Werf, Pilorge léa
EPI_ISL_754912	Laboratory Diagnostics and Clinical Immunology of Developmental Age, Medical University of Warsaw	genXone SA, Research & Development Laboratory; The Faculty of Mathematics, Informatics and Mechanics of the University of Warsaw	Maciej Sykulski, Grzegorz Nowicki, Monika Makowska-Woniak, Jakub Grabowski, Natalia Drwska-Matelska, ukasz Krych, Micha Kaszuba, Anna Gambin, Urszula Demkow
EPI_ISL_754925, EPI_ISL_754958, EPI_ISL_754987, EPI_ISL_755049, EPI_ISL_755050	California Department of Public Health	California Department of Public Health	CDPH IDLB COVIDNet
EPI_ISL_755118, EPI_ISL_755120, EPI_ISL_755122, EPI_ISL_755123, EPI_ISL_755125, EPI_ISL_755128, EPI_ISL_755142, EPI_ISL_755144, EPI_ISL_755214, EPI_ISL_755242, EPI_ISL_755244, EPI_ISL_755246, EPI_ISL_755247, EPI_ISL_755248, EPI_ISL_755251, EPI_ISL_755257, EPI_ISL_755258, EPI_ISL_755259, EPI_ISL_755260, EPI_ISL_755262, EPI_ISL_755266, EPI_ISL_755267, EPI_ISL_755268			
see above	Biolab Diagnostic Laboratories	Andersen lab at Scripps Research	Issa Abu-Dayyeh, Ahmad Tibi, Lama Hussein, Lina Mohammad, Zein Naber, Amid Abdelnour with SEARCH Alliance San Diego
EPI_ISL_755334, EPI_ISL_755336, EPI_ISL_755337, EPI_ISL_755338, EPI_ISL_755339, EPI_ISL_755340, EPI_ISL_755341, EPI_ISL_755342, EPI_ISL_755343, EPI_ISL_755344, EPI_ISL_755345, EPI_ISL_755346, EPI_ISL_755347, EPI_ISL_755348, EPI_ISL_755349, EPI_ISL_755350, EPI_ISL_755351, EPI_ISL_755352, EPI_ISL_755353, EPI_ISL_755354, EPI_ISL_755355, EPI_ISL_755356, EPI_ISL_755357, EPI_ISL_755358, EPI_ISL_755359, EPI_ISL_755360, EPI_ISL_755361, EPI_ISL_755362, EPI_ISL_755363, EPI_ISL_755364, EPI_ISL_755365, EPI_ISL_755366, EPI_ISL_755367, EPI_ISL_755368, EPI_ISL_755369			
see above	Maine Health and Environmental Testing Laboratory	Tewhey Lab, The Jackson Laboratory	Matluk,N., Dewey,H., Iosue,F., Barter,M., Lynch,R., Munger,H. and Tewhey,R.
EPI_ISL_755755, EPI_ISL_755756, EPI_ISL_755757, EPI_ISL_755758, EPI_ISL_755759, EPI_ISL_755809	Toronto Invasive Bacterial Diseases Network	McMaster University	Allison McGeer, Patryk Aftanas, Hooman Derakhshani, Angel Li, Kuganya Nirmalarajah, Emily Panousis, Ahmed Draia, Jalees Nasir, Michael Surette, Samira Mubareka, Andrew G. McArthur
EPI_ISL_755966, EPI_ISL_755967, EPI_ISL_755968, EPI_ISL_756181, EPI_ISL_756182, EPI_ISL_756183, EPI_ISL_756249, EPI_ISL_756250, EPI_ISL_756251, EPI_ISL_756252	Department of Virology and Immunology, University of Helsinki and Helsinki University Hospital, Huslab Finland	Department of Virology, Faculty of Medicine, University of Helsinki, Helsinki, Finland	Teemu Smura, Ravi Kant, Phuoc Truong, Hussein Alburkat, Hannimari Kallio-Kokko, Jenni Virtanen, Maija Suvanto, Sari Hannula, Harri Kangas, Pekka Ellonen, Olli Vapalahti
EPI_ISL_756374	CUMC - CHI Bergan Mercy	Creighton University School of Medicine, Departments of Medical Microbiology and Pharmacology and Neuroscience	Michael Belshan, Morgan A. Raine, Anne V. Cheng, Christopher J. Destache, Richard V. Goering, Jacob A. Siedlik, Holly A. Stessman
EPI_ISL_756376	National Institute of Health Research and Development	National Institute of Health Research and Development	Ramadhany,R; Rukminiati,Y; Agustiningsih; Adam,K; Wibowo,HA; Pawestri,HA; Subangkit;Puspa,KD; Nugraha,AA; Ikawati,HD; Pangesti,KNA; Soekarso,T; Susilarini,NK; Hariastuti,NI; Nikmah,UA;Mursinah; Febriyani,A; Herman,R; Susanti,N;Herna; Febriyanti,T; Nurhadi,M; Kurniawati,J; Kipuw,NL; Muna,F; Indalao,IL; Rizki,A; Puspandari,N; Setiawaty,Vivi
EPI_ISL_757287	National Institute of Health Research and Development	National Institute of Health Research and Development	Rukminiati,Y;Agustiningsih;Adam,K;Wibowo,HA;Ramadhany,R;Pawestri,HA;Subangkit;Puspa,KD;Nugraha,AA;Ikawati,HD;Pangesti,KNA;Soekarso,T;Susilarini,NK;Hariastuti,NI;Nikmah,UA;Mursinah;Febriyani,A;Herman,R;Susanti,N;Herna;Febriyanti,T;Nurhadi,M;Kurniawati,J;Kipuw,NL;Muna,F;Indalao,IL;Rizki,A;Puspandari,N;Setiawaty,V
EPI_ISL_759702	RS UNS Surakarta	Universitas Sebelas Maret (UNS), Rumah sakit Universitas Sebelas Maret (RS-UNS) Surakarta, National Institute of Health Research and Development, Indonesian Ministry of Health	Betty Suryawati, Yulia sari, hartono, maryani, Afif Avicena Gufron, Hana Apsari Pawestri, Kartika, Dewi Puspa, Hartanti Dian Ika, Arie Ardiansyah Nugraha, Vivi Setiawaty
EPI_ISL_760135, EPI_ISL_760157	Division of Emerging Infectious Diseases, Bureau of Infectious Diseases	Division of Emerging Infectious Diseases, Bureau of Infectious Diseases	Ae Kyung Park, Il-Hwan Kim, Heui Man Kim, Jeong-Min Kim, Namjoo Lee, Chaeyoung Lee, Sang Hee Woo, Eun-Jin Kim

	Diagnosis Control, Korea Disease Control and Prevention Agency	Diagnosis Control, Korea Disease Control and Prevention Agency	
EPI_ISL_763064	RS Kasih Ibu, Surakarta	Universitas Sebelas Maret (UNS); Rumah Sakit UNS (RS-UNS); National Institute of Health Research and Development, Indonesian Ministry of Health.	Betty Suryawati, Yulia Sari, Hartono, Maryani, Afif A Ghufron, Revi G H Novika, Hana A Pawestri, Kartika, Dewi Puspa, Hartanti Dian Ika, Arie A Nugraha, Vivi Setiawaty.
EPI_ISL_763306	Dutch COVID-19 response team	Erasmus Medical Center	Bas Oude Munnink, Reina Sikkema, David Nieuwenhuijse, Irina Chestakova, Anne van der Linden, Marjan Boter, Emmanuelle Munger, Corine GeurtsvanKessel, Annemiek van der Eijk, Richard Molenkamp, Marion Koopmans, on behalf of the Dutch national COVID-19 response team.
EPI_ISL_765482, EPI_ISL_765486, EPI_ISL_765502, EPI_ISL_765503, EPI_ISL_765504, EPI_ISL_765505, EPI_ISL_765506, EPI_ISL_765507, EPI_ISL_765508	Wadsworth Center, New York State Department.of Health	Wadsworth Center, New York State Department.of Health	Kirsten St. George, Daryl M. Lamson, Alexis Russel, Matthew Shudt, Melissa A Leisner, Jonathan Plitnick, Navjot Singh, John Kelly, Sara Griesemer, Erasmus Schneider, Erica Lasek-Nesselquist
EPI_ISL_765584, EPI_ISL_765585, EPI_ISL_765587, EPI_ISL_765588, EPI_ISL_765590	Brigham and Womens Hospital	Infectious Disease Program, Broad Institute of Harvard and MIT	Lemieux,J.E., Siddle,K.J., Shaw,B., Adams,G., Pierce,V., Turbett,S., Anahtar,M., Branda,J., Slater,D., Harris,J., Lin,A.E., Gladden-Young,A., Lagerborg,K., Rudy,M., DeRuff,K., Carter,A., Normandin,E., Bauer,M., Reilly,S., Tomkins-Tinch,C., Loreth,C., Chaluvadi,S., Neumann,A., Cusick,C., Chapman,S.B., Gnirke,A., Flowers,K., Cerrato,F., Birren,B.W., Gallagher,G., Smole,S., Park,D.J., MacInnis,B.L., Ryan,E., LaRocque,R., Rosenberg,E. and Sabeti,P.C.
EPI_ISL_765722, EPI_ISL_765726, EPI_ISL_765728	Massachusetts General Hospital	Infectious Disease Program, Broad Institute of Harvard and MIT	Lemieux,J.E., Siddle,K.J., Shaw,B., Adams,G., Pierce,V., Turbett,S., Anahtar,M., Branda,J., Slater,D., Harris,J., Lin,A.E., Gladden-Young,A., Lagerborg,K., Rudy,M., DeRuff,K., Carter,A., Normandin,E., Bauer,M., Reilly,S., Tomkins-Tinch,C., Loreth,C., Chaluvadi,S., Neumann,A., Cusick,C., Chapman,S.B., Gnirke,A., Flowers,K., Cerrato,F., Birren,B.W., Gallagher,G., Smole,S., Park,D.J., MacInnis,B.L., Ryan,E., LaRocque,R., Rosenberg,E. and Sabeti,P.C.
EPI_ISL_766592, EPI_ISL_766593, EPI_ISL_766625, EPI_ISL_766626, EPI_ISL_766627, EPI_ISL_766628, EPI_ISL_766629	Klinisk mikrobiologi	The Public Health Agency of Sweden	Department of Microbiology, The Public Health Agency of Sweden
EPI_ISL_768765, EPI_ISL_768767, EPI_ISL_768768	AIID	Irish Coronavirus Sequencing Consortium - National Virus Reference Laboratory	Michael Carr, Gabriel Gonzalez, Alejandro Abner Garcia Leon, Patrick Mallon
EPI_ISL_769991	Laboratorio Clinico Labin	Inciensa, Instituto Costarricense de Investigación y Enseñanza en Nutrición y Salud	Francisco Duarte, Hebleen Porras, Claudio Soto-Garita, Estela Cordero, Adriana Godínez, Melany Calderón & Mariel López
EPI_ISL_769994	Area De Salud Catedral Noreste	Inciensa, Instituto Costarricense de Investigación y Enseñanza en Nutrición y Salud	Francisco Duarte, Hebleen Porras, Claudio Soto-Garita, Estela Cordero, Adriana Godínez, Melany Calderón & Mariel López
EPI_ISL_770028	Hospital Dr. Raul Blanco Cervantes	Inciensa, Instituto Costarricense de Investigación y Enseñanza en Nutrición y Salud	Francisco Duarte, Hebleen Porras, Claudio Soto-Garita, Estela Cordero, Adriana Godínez, Melany Calderón & Mariel López
EPI_ISL_770035	E. Gulbja Laboratorija	Latvian Biomedical Research and Study Centre	Ivars Silamielis, Kaspars Megnis, Monta Ustinova, Jnis Pjalkovskis, ikita Zrelovs, Vita Rovte, Mikus Gavars, Dmitrijs Perminovs, Uga Dumpis, Jnis Kloviš
EPI_ISL_770039, EPI_ISL_770042	Centrl Laboratorija	Latvian Biomedical Research and Study Centre	Ivars Silamielis, Kaspars Megnis, Monta Ustinova, Jnis Pjalkovskis, ikita Zrelovs, Vita Rovte, Stella Lapia, Jana Oste, Marta Priedte, Uga Dumpis, Jnis Kloviš
EPI_ISL_770043, EPI_ISL_770044, EPI_ISL_770046	E. Gulbja Laboratorija	Latvian Biomedical Research and Study Centre	Ivars Silamielis, Kaspars Megnis, Monta Ustinova, Jnis Pjalkovskis, ikita Zrelovs, Vita Rovte, Mikus Gavars, Dmitrijs Perminovs, Uga Dumpis, Jnis Kloviš
EPI_ISL_770048, EPI_ISL_770049, EPI_ISL_770052	Centrl Laboratorija	Latvian Biomedical Research and Study Centre	Ivars Silamielis, Kaspars Megnis, Monta Ustinova, Jnis Pjalkovskis, ikita Zrelovs, Vita Rovte, Stella Lapia, Jana Oste, Marta Priedte, Uga Dumpis, Jnis Kloviš
EPI_ISL_770053	E. Gulbja Laboratorija	Latvian Biomedical Research and Study Centre	Ivars Silamielis, Kaspars Megnis, Monta Ustinova, Jnis Pjalkovskis, ikita Zrelovs, Vita Rovte, Mikus Gavars, Dmitrijs Perminovs, Uga Dumpis, Jnis Kloviš
EPI_ISL_770054	Centrl Laboratorija	Latvian Biomedical Research and Study Centre	Ivars Silamielis, Kaspars Megnis, Monta Ustinova, Jnis Pjalkovskis, ikita Zrelovs, Vita Rovte, Stella Lapia, Jana Oste, Marta Priedte, Uga Dumpis, Jnis Kloviš
EPI_ISL_770055	E. Gulbja Laboratorija	Latvian Biomedical Research and Study Centre	Ivars Silamielis, Kaspars Megnis, Monta Ustinova, Jnis Pjalkovskis, ikita Zrelovs, Vita Rovte, Mikus Gavars, Dmitrijs Perminovs, Uga Dumpis, Jnis Kloviš
EPI_ISL_770056	Centrl Laboratorija	Latvian Biomedical Research and Study Centre	Ivars Silamielis, Kaspars Megnis, Monta Ustinova, Jnis Pjalkovskis, ikita Zrelovs, Vita Rovte, Stella Lapia, Jana Oste, Marta Priedte, Uga Dumpis, Jnis Kloviš
EPI_ISL_770057	E. Gulbja Laboratorija	Latvian Biomedical Research and Study Centre	Ivars Silamielis, Kaspars Megnis, Monta Ustinova, Jnis Pjalkovskis, ikita Zrelovs, Vita Rovte, Mikus Gavars, Dmitrijs Perminovs, Uga Dumpis, Jnis Kloviš
EPI_ISL_770058	Centrl Laboratorija	Latvian Biomedical Research and Study Centre	Ivars Silamielis, Kaspars Megnis, Monta Ustinova, Jnis Pjalkovskis, ikita Zrelovs, Vita Rovte, Stella Lapia, Jana Oste, Marta Priedte, Uga Dumpis, Jnis Kloviš
EPI_ISL_770060	Latvijas Infektoloijas centrs	Latvian Biomedical Research and Study Centre	Ivars Silamielis, Kaspars Megnis, Monta Ustinova, Jnis Pjalkovskis, ikita Zrelovs, Vita Rovte, Jeena Storoženko, Tatjana Kolupajeva, Oksana Savicka, Uga Dumpis, Jnis Kloviš
EPI_ISL_770720,	ZOTZ KLIMAS MVZ	Center of Medical	Maximilian Damagnez, Alexander Dilthey, Ashley-Jane Duplessis, Patrick Finzer, Katrin Hoffmann, Torsten Houwaart, Lisanna Hülse, Malte Kohns Vasconcelos, Marek Korencak, Nadine Lübke, Jessica Nicolai, Klaus Pfeffer, Daniel Strelow, Jörg

EPI_ISL_770721, EPI_ISL_770722, EPI_ISL_770723, EPI_ISL_770724	Düsseldorf-Centrum GbR ÜBAG für Labormedizin, Genetik, Zytologie, Pathologie	Microbiology, Virology, and Hospital Hygiene, University of Duesseldorf	Timm, Andreas Walker, Tobias Wienemann, Rainer Zotz
EPI_ISL_776982, EPI_ISL_776983, EPI_ISL_776984, EPI_ISL_776985, EPI_ISL_776986, EPI_ISL_776987, EPI_ISL_776988, EPI_ISL_776989, EPI_ISL_776990, EPI_ISL_776991, EPI_ISL_776992, EPI_ISL_776993, EPI_ISL_776995, EPI_ISL_776996, EPI_ISL_778704, EPI_ISL_778705, EPI_ISL_778706, EPI_ISL_778707, EPI_ISL_778708, EPI_ISL_778709, EPI_ISL_778710, EPI_ISL_778711, EPI_ISL_778712, EPI_ISL_778713, EPI_ISL_778714, EPI_ISL_778715, EPI_ISL_778716, EPI_ISL_778717, EPI_ISL_778718, EPI_ISL_778719, EPI_ISL_778720, EPI_ISL_778721			
see above	Istituto Zooprofilattico Sperimentale del Mezzogiorno	TIGEM	Patrizia Annunziata, Andrea Ballabio, Valentina Bouche, Davide Cacchiarelli (CorrespAuthor), Pellegrino Cerino, Chiara Colantuono, Lucio Di Filippo, Antonio Grimaldi, Antonio Limone, Gabriella Loconte, Anna Manfredi, Francesco Panariello, Biancamaria Pierri, Marcello Salvi, Lucia Vassallo
EPI_ISL_779566	Microbiological Diagnostic Unit - Public Health Laboratory (MDU-PHL)	MDU-PHL	Seemann T., Sait, M.L., Sherry, N.L.
EPI_ISL_789781, EPI_ISL_789791, EPI_ISL_789830, EPI_ISL_789842, EPI_ISL_789847, EPI_ISL_789853, EPI_ISL_789856, EPI_ISL_789866, EPI_ISL_789872, EPI_ISL_789879, EPI_ISL_789884, EPI_ISL_789891, EPI_ISL_789892, EPI_ISL_789898, EPI_ISL_789910, EPI_ISL_789918, EPI_ISL_789932, EPI_ISL_789939, EPI_ISL_789940, EPI_ISL_789947, EPI_ISL_789948, EPI_ISL_789957, EPI_ISL_789958, EPI_ISL_789960, EPI_ISL_789967, EPI_ISL_789971, EPI_ISL_789973, EPI_ISL_789974, EPI_ISL_789979, EPI_ISL_789980, EPI_ISL_789984, EPI_ISL_789985, EPI_ISL_789986, EPI_ISL_789989, EPI_ISL_790029, EPI_ISL_790031, EPI_ISL_790047, EPI_ISL_790049, EPI_ISL_790050, EPI_ISL_790051, EPI_ISL_790052, EPI_ISL_790053, EPI_ISL_790054, EPI_ISL_790055, EPI_ISL_790056, EPI_ISL_790057, EPI_ISL_790058, EPI_ISL_790059, EPI_ISL_790060, EPI_ISL_790061, EPI_ISL_790062, EPI_ISL_790063, EPI_ISL_790064, EPI_ISL_790065, EPI_ISL_790066, EPI_ISL_790067, EPI_ISL_790068, EPI_ISL_790069, EPI_ISL_790070, EPI_ISL_790076, EPI_ISL_790079, EPI_ISL_790080, EPI_ISL_790081, EPI_ISL_790084, EPI_ISL_790085, EPI_ISL_790086, EPI_ISL_790092, EPI_ISL_790095, EPI_ISL_790097, EPI_ISL_790098, EPI_ISL_790101, EPI_ISL_790102, EPI_ISL_790103, EPI_ISL_790112, EPI_ISL_790113, EPI_ISL_790115, EPI_ISL_790116, EPI_ISL_790117, EPI_ISL_790118, EPI_ISL_790119, EPI_ISL_790120, EPI_ISL_790121, EPI_ISL_790122, EPI_ISL_790123, EPI_ISL_790124, EPI_ISL_790125, EPI_ISL_790126, EPI_ISL_790127, EPI_ISL_790128, EPI_ISL_790129, EPI_ISL_790130, EPI_ISL_790131, EPI_ISL_790132, EPI_ISL_790134, EPI_ISL_790137, EPI_ISL_790138, EPI_ISL_790139, EPI_ISL_790141, EPI_ISL_790142, EPI_ISL_790143, EPI_ISL_790144, EPI_ISL_790145, EPI_ISL_790146, EPI_ISL_790148, EPI_ISL_790150, EPI_ISL_790151, EPI_ISL_790154, EPI_ISL_790155, EPI_ISL_790156, EPI_ISL_790157, EPI_ISL_790159, EPI_ISL_790160, EPI_ISL_790161, EPI_ISL_790162, EPI_ISL_790163, EPI_ISL_790164, EPI_ISL_790165, EPI_ISL_790166, EPI_ISL_790168, EPI_ISL_790171, EPI_ISL_790187, EPI_ISL_790195, EPI_ISL_790200, EPI_ISL_790201, EPI_ISL_790247, EPI_ISL_790252, EPI_ISL_790263			
see above	Houston Methodist Hospital	Houston Methodist Hospital	S. Wesley Long, Randall J. Olsen, Paul A. Christensen, David W. Bernard, James J. Davis, Maulik Shukla, Marcus Nguyen, Matthew Ojeda Saavedra, Prasanti Yerramilli, Layne Pruitt, Sishir Subedi, Heather Hendrickson, and James M. Musser
EPI_ISL_792023	Plateforme COVID IDF	National Reference Center for Viruses of Respiratory Infections, Institut Pasteur, Paris	Marion Barbet, Sylvie Behillil, Méline Bizard, Angela Brisebarre, Camille Capel, Etienne Simon-Lorière, Vincent Enouf, Maud Vanpeene, Sylvie van der Werf,Jacques Fourgeaud
EPI_ISL_792612, EPI_ISL_792613	LACEN-PB	Laboratory of Respiratory Viruses and Measles, Oswaldo Cruz Institute, FIOCRUZ	Paola Resende, Luciana Appolinario, Fernando Motta, Anna Carolina Paixao, Ana Carolina Mendonca, João Felipe Bezerra, Romero Henrique Teixeira de Vasconcelos, Dalane Loudal Florentino Teixeira, Thiago Franco de Oliveira Carneiro, Marilda Siqueira
EPI_ISL_792687, EPI_ISL_792700	The National Institute of Public Health	State Veterinary Institute Prague	Nagy,A;Jirincova,H;Trnka,D;Vecerova,J
EPI_ISL_796028, EPI_ISL_796029, EPI_ISL_796033, EPI_ISL_796039, EPI_ISL_796040, EPI_ISL_796041, EPI_ISL_796042, EPI_ISL_796054	Institute of Virology, University of Cologne	Institute of Virology, University of Cologne	Saleta Sierra, Gibran Rubio, Zevanya Tessalonica, Dominik Aschenmeier, Eva Heger, Elena Knops, Rolf Kaiser, Maximilian Damagnez, Andreas Walker, Jörg Timm, Alexander Dilthey, Martin Däumer, Alex Thielen
EPI_ISL_801387, EPI_ISL_801388	Laboratorio de Ecologia de Doencas Transmissiveis na Amazonia, Instituto Leonidas e Maria Deane - Fiocruz Amazonia	Laboratorio de Ecologia de Doencas Transmissiveis na Amazonia, Instituto Leonidas e Maria Deane - Fiocruz Amazonia	Valdinete Nascimento, Victor Souza, André Corado, Fernanda Nascimento, George Silva, Ágatha Costa, Debora Duarte, Luciana Gonçalves, Maria Júlia Brandão, Michele Jesus, Felipe Naveca
EPI_ISL_801400, EPI_ISL_801401	Laboratório Central de Saúde Pública do Amazonas - LACEN-AM	Laboratorio de Ecologia de Doencas Transmissiveis na Amazonia, Instituto Leonidas e Maria Deane - Fiocruz Amazonia	Valdinete Nascimento, Victor Souza, André Corado, Fernanda Nascimento, George Silva, Ágatha Costa, Debora Duarte, Luciana Gonçalves, Maria Júlia Brandão, Michele Jesus, Felipe Naveca
EPI_ISL_801871, EPI_ISL_801873, EPI_ISL_801883, EPI_ISL_801899, EPI_ISL_801900, EPI_ISL_801934, EPI_ISL_802238, EPI_ISL_802239, EPI_ISL_802240, EPI_ISL_802241, EPI_ISL_802242, EPI_ISL_802243, EPI_ISL_802244, EPI_ISL_802245, EPI_ISL_802246, EPI_ISL_802247, EPI_ISL_802248, EPI_ISL_802249, EPI_ISL_802250, EPI_ISL_802251, EPI_ISL_802252, EPI_ISL_802253, EPI_ISL_802254, EPI_ISL_802255			
see above	MSHS Clinical Microbiology Laboratories	MSHS Pathogen Surveillance Program	Ana S. Gonzalez-Reiche, Hala Alshammary, Mitchell J. Sullivan, Brianne Ciferri, Ajay Obla, Angela Amoako, Mahmoud Awawda, Elena Hirsch, Ashley S. Salimbangon, Levy Sominsky, Katherine Beach, Kayla Russo, Charles Gleason, Shelcie Fabre, Giulio Kleiner, Zenab Khan, Bremy Alburquerque, Adriana van de Guchte, Komal Srivastava, Matthew M. Hernandez, Jayeeta Dutta, Denise Jurczyszak, Emily Ferreri, Rachel Chernet, Nancy Francoeur, Betsaida Salom Melo, Irina Oussenko, Gintaras Deikus, Juan Soto, Shwetha Hara Sridhar, Ying-Chih Wang, Kathryn Twyman, Andrew Kasarskis, Deena R. Altman, Robert Sebra, Adolfo García-Sastre, Marta Luksza, Gopi Patel, Sarah Schaefer, Melissa Gitman, Michael D. Nowak, Alberto Paniz-Mondolfi, Emilia Mia Sordillo, Viviana Simon, Harm van Bakel
EPI_ISL_803142, EPI_ISL_803143, EPI_ISL_803144, EPI_ISL_803145, EPI_ISL_803146, EPI_ISL_803147, EPI_ISL_803148, EPI_ISL_803149, EPI_ISL_803150, EPI_ISL_803151, EPI_ISL_803152, EPI_ISL_803153, EPI_ISL_803154, EPI_ISL_803155, EPI_ISL_803156, EPI_ISL_803157, EPI_ISL_803158, EPI_ISL_803159, EPI_ISL_803160, EPI_ISL_803161, EPI_ISL_803162, EPI_ISL_803163, EPI_ISL_803164, EPI_ISL_803165, EPI_ISL_803166, EPI_ISL_803167, EPI_ISL_803168, EPI_ISL_803169, EPI_ISL_803170, EPI_ISL_803171, EPI_ISL_803172, EPI_ISL_803173, EPI_ISL_803174, EPI_ISL_803175, EPI_ISL_803176, EPI_ISL_803177, EPI_ISL_803178, EPI_ISL_803179, EPI_ISL_803180, EPI_ISL_803181, EPI_ISL_803182, EPI_ISL_803183, EPI_ISL_803184, EPI_ISL_803185, EPI_ISL_803186, EPI_ISL_803187, EPI_ISL_803188, EPI_ISL_803189, EPI_ISL_803190, EPI_ISL_803191, EPI_ISL_803192, EPI_ISL_803193, EPI_ISL_803194, EPI_ISL_803195, EPI_ISL_803229, EPI_ISL_803230, EPI_ISL_803231, EPI_ISL_803238, EPI_ISL_803239, EPI_ISL_803240, EPI_ISL_803241, EPI_ISL_803242, EPI_ISL_803243, EPI_ISL_803244, EPI_ISL_803245, EPI_ISL_803246, EPI_ISL_803247, EPI_ISL_803341, EPI_ISL_803342, EPI_ISL_803343, EPI_ISL_803344, EPI_ISL_803345, EPI_ISL_803346, EPI_ISL_803347, EPI_ISL_803348, EPI_ISL_803349, EPI_ISL_803350, EPI_ISL_803351, EPI_ISL_803494, EPI_ISL_803495, EPI_ISL_803496, EPI_ISL_803497, EPI_ISL_803498, EPI_ISL_803499, EPI_ISL_803500, EPI_ISL_803501, EPI_ISL_803502, EPI_ISL_803503, EPI_ISL_803504, EPI_ISL_803505, EPI_ISL_803506, EPI_ISL_803509, EPI_ISL_803510, EPI_ISL_803511, EPI_ISL_803523, EPI_ISL_803524, EPI_ISL_803525, EPI_ISL_803526, EPI_ISL_803527, EPI_ISL_803528, EPI_ISL_803529, EPI_ISL_803538, EPI_ISL_803539, EPI_ISL_803540, EPI_ISL_803742, EPI_ISL_803743, EPI_ISL_803744, EPI_ISL_803745, EPI_ISL_803746, EPI_ISL_803747			
see above	Wisconsin State Laboratory of Hygiene Communicable Disease Division	Wisconsin State Laboratory of Hygiene Communicable Disease Division	Kelsey R. Florek, Abigail C. Shockey
EPI_ISL_804964, EPI_ISL_804968, EPI_ISL_804969, EPI_ISL_804971, EPI_ISL_804972, EPI_ISL_804973, EPI_ISL_804974, EPI_ISL_804975, EPI_ISL_804976, EPI_ISL_804978, EPI_ISL_804979			
see above	Hospital Comarcal de Melilla	Instituto de Salud Carlos III	Iglesias-Caballero, M. Molinero Calamita, M. González-Esguevillas, M. Camarero, S. Pozo, F. Casas, I. Jiménez, P. Jiménez, M. Zaballos, A. Monzón, S. Varona, S. Juliá, M. Cuesta, I, J. López
EPI_ISL_806899, EPI_ISL_806900, EPI_ISL_806901, EPI_ISL_806902, EPI_ISL_806903, EPI_ISL_806904, EPI_ISL_806905, EPI_ISL_806906, EPI_ISL_806907, EPI_ISL_806908, EPI_ISL_806909, EPI_ISL_806910, EPI_ISL_806911, EPI_ISL_806912, EPI_ISL_806913, EPI_ISL_806914, EPI_ISL_806915, EPI_ISL_806916			
see above	Washington State Department of Health	Seattle Flu Study	Deborah A. Nickerson, Chris D. Frazar, Jover Lee, Benjamin Pelle, Matthew Richardson, Amanda Adler, Elisabeth Brandstetter, Peter D. Han, Kairsten Fay, Misja Ilcisin, Kirsten Lacombe, Thomas R. Sibley, Melissa Truong, Caitlin R. Wolf, Romesh Gautom, Geoff Melly, Brian Hiatt, Philip Dykema, Scott Lindquist, Michael Boeckh, Janet A. Englund, Michael Famulare, Barry R. Lutz, Mark J. Rieder, Lea M. Starita, Matthew Thompson, Helen Y. Chu, Jay Shendure, Trevor Bedford
EPI_ISL_810980, EPI_ISL_811013	MRCG at LSHTM Genomics lab	MRCG at LSHTM Genomics lab	Abdul Karim sesay, Abdoulie Kanteh, Jarra Manneh, Mariama Kujabi, Bakary Sanyang
EPI_ISL_812688, EPI_ISL_812689, EPI_ISL_812690, EPI_ISL_812692, EPI_ISL_812694, EPI_ISL_812699, EPI_ISL_812700, EPI_ISL_812702, EPI_ISL_812704, EPI_ISL_812705, EPI_ISL_812706, EPI_ISL_812707, EPI_ISL_812708, EPI_ISL_812709, EPI_ISL_812710, EPI_ISL_812711			
see above	United States Air Force School of Aerospace Medicine	United States Air Force School of Aerospace Medicine	Anthony Fries, Jennifer Meyer, Amanda Javorina, Sarah Purves, William Gruner, Clarise Starr, Elizabeth Macias

EPI_ISL_813052	University of Birmingham	COVID-19 Genomics UK (COG-UK) Consortium	Institute of Microbiology, University of Birmingham: Claire McMurray, Joanne Stockton, Samuel Nicholls, Radoslaw Poplawski, Will Rowe, Josh Quick, Nicholas Loman. University of Birmingham Testing Laboratory: Celina M Whalley, Andrew Bosworth, Charlotte Poxon, Kasun Wanigasooriya, Oliver Pickles, Mike Kidd, Alex Richter, Andrew D Beggs PHE Heartlands Lab: Husam Osman, Andrew Bosworth. Queen Elizabeth Hospital: Anna Casey
EPI_ISL_813154, EPI_ISL_813155, EPI_ISL_813156, EPI_ISL_813157, EPI_ISL_813158, EPI_ISL_813159, EPI_ISL_813162, EPI_ISL_813163, EPI_ISL_813164, EPI_ISL_813165	Department of Pathology, University of Cambridge	COVID-19 Genomics UK (COG-UK) Consortium	Aminu S. Jahun, Yasmin Chaudhry, Grant Hall, Iliana Georgana, Myra Hosmillo, Martin D. Curran, Malte Pinckert, Surendra Parmar, Ian Goodfellow
EPI_ISL_815356	Centogene	Centogene	Peter Bauer, Krishna Kumar Kandaswamy, Vivi Hue-Trang Lieu
EPI_ISL_824487, EPI_ISL_824488, EPI_ISL_824489, EPI_ISL_824490, EPI_ISL_824491	Hospital Universitari Vall d'Hebron - Vall d'Hebron Institut de Rercerca	Hospital Universitari Vall d'Hebron	Cristina Andrés, Maria Piñana, Josep F Abril, Damir Garcia-Cehic, Ariadna Rando, Juliana Esperalba, Maria Gema Codina, Carla Castillo, Maria Carmen Martin, Tomàs Pumarola, Josep Quer, Andrés Antón
EPI_ISL_825028, EPI_ISL_825029, EPI_ISL_825030, EPI_ISL_825031, EPI_ISL_825032	Infectious Diseases, NC SLPH COVID-19 Response Team	Infectious Diseases, NC SLPH COVID-19 Response Team	Miller,M.C., Chase,K.
EPI_ISL_825553	Respiratory Virus Unit, National Infection Service, Public Health England	COVID-19 Genomics UK (COG-UK) Consortium	PHE Covid Sequencing Team
EPI_ISL_826834, EPI_ISL_826836	INSPI-CRN DE INFLUENZA Y OTROS VIRUS RESPIRATORIOS	Instituto de Salud Publica de Chile	Javier Tognarelli, Barbara Parra, Loredana Arata, Jaime Lagos, Gisselle Barra, Alfredo Bruno, Domenica de Mora, Solon Narvaez, Jimmy Garcez, Michelle Paez, Martiza Olmedo, Manuel Gonzalez, Patricia Bustos, Rodrigo Fasce, Andres Castillo, Jorge Fernandez
EPI_ISL_826898, EPI_ISL_826899, EPI_ISL_826900, EPI_ISL_826901, EPI_ISL_826902	deCODE genetics	deCODE genetics	Daniel F Gudbjartsson; Agnar Helgason; Hakon Jonsson; Olafur T Magnusson; Pall Melsted; Gudmundur L Norddahl; Jona Saemundsdottir; Asgeir Sigurdsson; Patrick Sulem; Arna B Agustsdottir; Hannes Eggertsson; Berglind Eiriksdottir; Run Fridriksdottir; Elisabet E Gardarsdottir; Gudmundur Georgsson; Olafia S Gretarsdottir; Kjartan R Gudmundsson; Thora R Gunnarsdottir; Arnaldur Gylfason; Hilma Holm; Brynjar O Jensson; Aslaug Jonasdottir; Kamilla S Josefsdottir; Thordur Kristjansson; Droplaug N Magnusdottir; Solvi Rognvaldsson; Louise le Roux; Gudrun Sigmundsdottir; Gardar Sveinbjornsson; Kristin E Sveinsdottir; Maney Sveinsdottir; Emil A Thorarensen; Bjarni Thorbjornsson; Gisli Masson; Ingileif Jonsdottir; Alma Moller; Thorolfur Gudnason; Karl G Kristinsson; Unnur Thorsteinsdottir; Kari Stefansson
EPI_ISL_826916	The National University Hospital of Iceland	deCODE genetics	Daniel F Gudbjartsson; Agnar Helgason; Hakon Jonsson; Olafur T Magnusson; Pall Melsted; Gudmundur L Norddahl; Jona Saemundsdottir; Asgeir Sigurdsson; Patrick Sulem; Arna B Agustsdottir; Hannes Eggertsson; Berglind Eiriksdottir; Run Fridriksdottir; Elisabet E Gardarsdottir; Gudmundur Georgsson; Olafia S Gretarsdottir; Kjartan R Gudmundsson; Thora R Gunnarsdottir; Arnaldur Gylfason; Hilma Holm; Brynjar O Jensson; Aslaug Jonasdottir; Kamilla S Josefsdottir; Thordur Kristjansson; Droplaug N Magnusdottir; Solvi Rognvaldsson; Louise le Roux; Gudrun Sigmundsdottir; Gardar Sveinbjornsson; Kristin E Sveinsdottir; Maney Sveinsdottir; Emil A Thorarensen; Bjarni Thorbjornsson; Gisli Masson; Ingileif Jonsdottir; Alma Moller; Thorolfur Gudnason; Karl G Kristinsson; Unnur Thorsteinsdottir; Kari Stefansson
EPI_ISL_826922, EPI_ISL_826923, EPI_ISL_826924, EPI_ISL_827060, EPI_ISL_827061, EPI_ISL_827062, EPI_ISL_827072, EPI_ISL_827073, EPI_ISL_827338, EPI_ISL_827341, EPI_ISL_827639, EPI_ISL_827643, EPI_ISL_827790, EPI_ISL_827791, EPI_ISL_828210, EPI_ISL_828254, EPI_ISL_828304, EPI_ISL_828308			
see above	deCODE genetics	deCODE genetics	Daniel F Gudbjartsson; Agnar Helgason; Hakon Jonsson; Olafur T Magnusson; Pall Melsted; Gudmundur L Norddahl; Jona Saemundsdottir; Asgeir Sigurdsson; Patrick Sulem; Arna B Agustsdottir; Hannes Eggertsson; Berglind Eiriksdottir; Run Fridriksdottir; Elisabet E Gardarsdottir; Gudmundur Georgsson; Olafia S Gretarsdottir; Kjartan R Gudmundsson; Thora R Gunnarsdottir; Arnaldur Gylfason; Hilma Holm; Brynjar O Jensson; Aslaug Jonasdottir; Kamilla S Josefsdottir; Thordur Kristjansson; Droplaug N Magnusdottir; Solvi Rognvaldsson; Louise le Roux; Gudrun Sigmundsdottir; Gardar Sveinbjornsson; Kristin E Sveinsdottir; Maney Sveinsdottir; Emil A Thorarensen; Bjarni Thorbjornsson; Gisli Masson; Ingileif Jonsdottir; Alma Moller; Thorolfur Gudnason; Karl G Kristinsson; Unnur Thorsteinsdottir; Kari Stefansson
EPI_ISL_828326, EPI_ISL_828327	The National University Hospital of Iceland	deCODE genetics	Daniel F Gudbjartsson; Agnar Helgason; Hakon Jonsson; Olafur T Magnusson; Pall Melsted; Gudmundur L Norddahl; Jona Saemundsdottir; Asgeir Sigurdsson; Patrick Sulem; Arna B Agustsdottir; Hannes Eggertsson; Berglind Eiriksdottir; Run Fridriksdottir; Elisabet E Gardarsdottir; Gudmundur Georgsson; Olafia S Gretarsdottir; Kjartan R Gudmundsson; Thora R Gunnarsdottir; Arnaldur Gylfason; Hilma Holm; Brynjar O Jensson; Aslaug Jonasdottir; Kamilla S Josefsdottir; Thordur Kristjansson; Droplaug N Magnusdottir; Solvi Rognvaldsson; Louise le Roux; Gudrun Sigmundsdottir; Gardar Sveinbjornsson; Kristin E Sveinsdottir; Maney Sveinsdottir; Emil A Thorarensen; Bjarni Thorbjornsson; Gisli Masson; Ingileif Jonsdottir; Alma Moller; Thorolfur Gudnason; Karl G Kristinsson; Unnur Thorsteinsdottir; Kari Stefansson
EPI_ISL_828534	deCODE genetics	deCODE genetics	Daniel F Gudbjartsson; Agnar Helgason; Hakon Jonsson; Olafur T Magnusson; Pall Melsted; Gudmundur L Norddahl; Jona Saemundsdottir; Asgeir Sigurdsson; Patrick Sulem; Arna B Agustsdottir; Hannes Eggertsson; Berglind Eiriksdottir; Run Fridriksdottir; Elisabet E Gardarsdottir; Gudmundur Georgsson; Olafia S Gretarsdottir; Kjartan R Gudmundsson; Thora R Gunnarsdottir; Arnaldur Gylfason; Hilma Holm; Brynjar O Jensson; Aslaug Jonasdottir; Kamilla S Josefsdottir; Thordur Kristjansson; Droplaug N Magnusdottir; Solvi Rognvaldsson; Louise le Roux; Gudrun Sigmundsdottir; Gardar Sveinbjornsson; Kristin E Sveinsdottir; Maney Sveinsdottir; Emil A Thorarensen; Bjarni Thorbjornsson; Gisli Masson; Ingileif Jonsdottir; Alma Moller; Thorolfur Gudnason; Karl G Kristinsson; Unnur Thorsteinsdottir; Kari Stefansson
EPI_ISL_828641, EPI_ISL_828775, EPI_ISL_828776	The National University Hospital of Iceland	deCODE genetics	Daniel F Gudbjartsson; Agnar Helgason; Hakon Jonsson; Olafur T Magnusson; Pall Melsted; Gudmundur L Norddahl; Jona Saemundsdottir; Asgeir Sigurdsson; Patrick Sulem; Arna B Agustsdottir; Hannes Eggertsson; Berglind Eiriksdottir; Run Fridriksdottir; Elisabet E Gardarsdottir; Gudmundur Georgsson; Olafia S Gretarsdottir; Kjartan R Gudmundsson; Thora R Gunnarsdottir; Arnaldur Gylfason; Hilma Holm; Brynjar O Jensson; Aslaug Jonasdottir; Kamilla S Josefsdottir; Thordur Kristjansson; Droplaug N Magnusdottir; Solvi Rognvaldsson; Louise le Roux; Gudrun Sigmundsdottir; Gardar Sveinbjornsson; Kristin E Sveinsdottir; Maney Sveinsdottir; Emil A Thorarensen; Bjarni Thorbjornsson; Gisli Masson; Ingileif Jonsdottir; Alma Moller; Thorolfur Gudnason; Karl G Kristinsson; Unnur Thorsteinsdottir; Kari Stefansson
EPI_ISL_828777, EPI_ISL_828778	deCODE genetics	deCODE genetics	Daniel F Gudbjartsson; Agnar Helgason; Hakon Jonsson; Olafur T Magnusson; Pall Melsted; Gudmundur L Norddahl; Jona Saemundsdottir; Asgeir Sigurdsson; Patrick Sulem; Arna B Agustsdottir; Hannes Eggertsson; Berglind Eiriksdottir; Run Fridriksdottir; Elisabet E Gardarsdottir; Gudmundur Georgsson; Olafia S Gretarsdottir; Kjartan R Gudmundsson; Thora R Gunnarsdottir; Arnaldur Gylfason; Hilma Holm; Brynjar O Jensson; Aslaug Jonasdottir; Kamilla S Josefsdottir; Thordur Kristjansson; Droplaug N Magnusdottir; Solvi Rognvaldsson; Louise le Roux; Gudrun Sigmundsdottir; Gardar Sveinbjornsson; Kristin E Sveinsdottir; Maney Sveinsdottir; Emil A Thorarensen; Bjarni Thorbjornsson; Gisli Masson; Ingileif Jonsdottir; Alma Moller; Thorolfur Gudnason; Karl G Kristinsson; Unnur Thorsteinsdottir; Kari Stefansson
EPI_ISL_828779	The National University Hospital of Iceland	deCODE genetics	Daniel F Gudbjartsson; Agnar Helgason; Hakon Jonsson; Olafur T Magnusson; Pall Melsted; Gudmundur L Norddahl; Jona Saemundsdottir; Asgeir Sigurdsson; Patrick Sulem; Arna B Agustsdottir; Hannes Eggertsson; Berglind Eiriksdottir; Run Fridriksdottir; Elisabet E Gardarsdottir; Gudmundur Georgsson; Olafia S Gretarsdottir; Kjartan R Gudmundsson; Thora R Gunnarsdottir; Arnaldur Gylfason; Hilma Holm; Brynjar O Jensson; Aslaug Jonasdottir; Kamilla S Josefsdottir; Thordur Kristjansson; Droplaug N Magnusdottir; Solvi Rognvaldsson; Louise le Roux; Gudrun Sigmundsdottir; Gardar Sveinbjornsson; Kristin E Sveinsdottir; Maney Sveinsdottir; Emil A Thorarensen; Bjarni Thorbjornsson; Gisli Masson; Ingileif Jonsdottir; Alma Moller; Thorolfur Gudnason; Karl G Kristinsson; Unnur Thorsteinsdottir; Kari Stefansson
EPI_ISL_828781, EPI_ISL_828782, EPI_ISL_828783, EPI_ISL_828784, EPI_ISL_829025, EPI_ISL_829026, EPI_ISL_829031, EPI_ISL_829032, EPI_ISL_829033, EPI_ISL_829034, EPI_ISL_829035, EPI_ISL_829430			
see above	deCODE genetics	deCODE genetics	Daniel F Gudbjartsson; Agnar Helgason; Hakon Jonsson; Olafur T Magnusson; Pall Melsted; Gudmundur L Norddahl; Jona Saemundsdottir; Asgeir Sigurdsson; Patrick Sulem; Arna B Agustsdottir; Hannes Eggertsson; Berglind Eiriksdottir; Run Fridriksdottir; Elisabet E Gardarsdottir; Gudmundur Georgsson; Olafia S Gretarsdottir; Kjartan R Gudmundsson; Thora R Gunnarsdottir; Arnaldur Gylfason; Hilma Holm; Brynjar O Jensson; Aslaug Jonasdottir; Kamilla S Josefsdottir; Thordur Kristjansson; Droplaug N Magnusdottir; Solvi Rognvaldsson; Louise le Roux; Gudrun Sigmundsdottir; Gardar Sveinbjornsson; Kristin E Sveinsdottir; Maney Sveinsdottir; Emil A Thorarensen; Bjarni Thorbjornsson; Gisli Masson; Ingileif Jonsdottir; Alma Moller; Thorolfur Gudnason; Karl G Kristinsson; Unnur Thorsteinsdottir; Kari Stefansson
EPI_ISL_829471, EPI_ISL_829707, EPI_ISL_829708, EPI_ISL_829709	The National University Hospital of Iceland	deCODE genetics	Daniel F Gudbjartsson; Agnar Helgason; Hakon Jonsson; Olafur T Magnusson; Pall Melsted; Gudmundur L Norddahl; Jona Saemundsdottir; Asgeir Sigurdsson; Patrick Sulem; Arna B Agustsdottir; Hannes Eggertsson; Berglind Eiriksdottir; Run Fridriksdottir; Elisabet E Gardarsdottir; Gudmundur Georgsson; Olafia S Gretarsdottir; Kjartan R Gudmundsson; Thora R Gunnarsdottir; Arnaldur Gylfason; Hilma Holm; Brynjar O Jensson; Aslaug Jonasdottir; Kamilla S Josefsdottir; Thordur Kristjansson; Droplaug N Magnusdottir; Solvi Rognvaldsson; Louise le Roux; Gudrun Sigmundsdottir; Gardar Sveinbjornsson; Kristin E Sveinsdottir; Maney Sveinsdottir; Emil A Thorarensen; Bjarni Thorbjornsson; Gisli Masson; Ingileif Jonsdottir; Alma Moller; Thorolfur Gudnason; Karl G Kristinsson; Unnur Thorsteinsdottir; Kari Stefansson
EPI_ISL_829919, EPI_ISL_829920, EPI_ISL_829921, EPI_ISL_829922, EPI_ISL_829924, EPI_ISL_829926, EPI_ISL_829927, EPI_ISL_829928, EPI_ISL_830189, EPI_ISL_830190, EPI_ISL_830281, EPI_ISL_830283, EPI_ISL_830287, EPI_ISL_830290, EPI_ISL_830292, EPI_ISL_830295, EPI_ISL_830297, EPI_ISL_830485, EPI_ISL_830491, EPI_ISL_830495			
see above	deCODE genetics	deCODE genetics	Daniel F Gudbjartsson; Agnar Helgason; Hakon Jonsson; Olafur T Magnusson; Pall Melsted; Gudmundur L Norddahl; Jona Saemundsdottir; Asgeir Sigurdsson; Patrick Sulem; Arna B Agustsdottir; Hannes Eggertsson; Berglind Eiriksdottir; Run Fridriksdottir; Elisabet E Gardarsdottir; Gudmundur Georgsson; Olafia S Gretarsdottir; Kjartan R Gudmundsson; Thora R Gunnarsdottir; Arnaldur Gylfason; Hilma Holm; Brynjar O Jensson; Aslaug Jonasdottir; Kamilla S Josefsdottir; Thordur Kristjansson; Droplaug N Magnusdottir; Solvi Rognvaldsson; Louise le Roux; Gudrun Sigmundsdottir; Gardar Sveinbjornsson; Kristin E Sveinsdottir; Maney Sveinsdottir; Emil A Thorarensen; Bjarni Thorbjornsson; Gisli Masson; Ingileif Jonsdottir; Alma

			Moller; Thorolfur Gudnason; Karl G Kristinsson; Unnur Thorsteinsdottir; Kari Stefansson
EPI_ISL_830737, EPI_ISL_830799, EPI_ISL_830800, EPI_ISL_830801, EPI_ISL_830802, EPI_ISL_830803, EPI_ISL_830804, EPI_ISL_830805, EPI_ISL_830806, EPI_ISL_830807, EPI_ISL_830808, EPI_ISL_830809, EPI_ISL_830810, EPI_ISL_830811, EPI_ISL_830987, EPI_ISL_830988, EPI_ISL_831005			
see above	University Hospital Basel, Clinical Virology	University Hospital Basel, Clinical Bacteriology	Tim Roloff, Madlen Stange, Helena MB Seth-Smith, Alfredo Mari, Karoline Leuzinger, Julia Bielicki, Manuel Battegay, Hans Hirsch, Adrian Egli
EPI_ISL_831033, EPI_ISL_831136, EPI_ISL_831137, EPI_ISL_831138, EPI_ISL_831139, EPI_ISL_831140, EPI_ISL_831141, EPI_ISL_831142, EPI_ISL_831143, EPI_ISL_831144, EPI_ISL_831145, EPI_ISL_831146, EPI_ISL_831147, EPI_ISL_831148, EPI_ISL_831149, EPI_ISL_831150, EPI_ISL_831151, EPI_ISL_831152, EPI_ISL_831153, EPI_ISL_831154, EPI_ISL_831155, EPI_ISL_831156, EPI_ISL_831157, EPI_ISL_831158, EPI_ISL_831159, EPI_ISL_831160, EPI_ISL_831161, EPI_ISL_831162, EPI_ISL_831163, EPI_ISL_831164, EPI_ISL_831165, EPI_ISL_831166, EPI_ISL_831167, EPI_ISL_831168, EPI_ISL_831169			
see above	Hospital Universitario La Paz (Madrid)	SeqCOVID-SPAIN consortium/IBV(CSIC)	María Rodríguez-Tejedor, Elias Dahdouh, Fernando Lázaro-Perona, Jesús Mingorance and SeqCOVID-SPAIN consortium
EPI_ISL_831940, EPI_ISL_832009	Laboratório de Microbiologia Molecular - Universidade FEEVALE	Universidade Federal de Ciências da Saúde de Porto Alegre	Vinícius Bonetti Franceschi, Amanda de Menezes Mayer, Gabriel Dickin Caldana, Carla Andretta Moreira Neves, Patrícia Aline Gröhs Ferrareze, Gabriela Bettella Cybis, Ricardo Ariel Zimerman, Lívia Kmetzsch, Fernando Rosado Spilki, Claudia Elizabeth Thompson
EPI_ISL_832103, EPI_ISL_832104	Hospital Universitari Germans Trias i Pujol(HUGTiP)/Fundació Lluita contra la SIDA (FLSida)	IrsiCaixa AIDS Research Lab	Marc Noguera-Julian, Mariona Parera, Maria Pilar Armengol, Marta Massanella, Ester Ballana, Lidia Ruiz, Nuria Izquierdo, Jorge Carrillo, Roger Paredes, Julia Blanco, Joaquim Segalés, Bonaventura Clotet
EPI_ISL_833040	RSUD Dr. Abdul Aziz Singkawang	National Institute of Health Research and Development	Subangkit;Pawestri,HA;Ikawati,HD;Nugraha,AA;Puspa,KD;Pangesti,KNA;Soekarso,T;Junus,HN;Puspandari,N;Setiawaty,V
EPI_ISL_833332	National Jail Management and Penology Training Institute	Research Institute for Tropical Medicine	Hannah Leah Morito, Othoniel Jan Onza, John Leonard Chan, Ma Angelica Tujan, Francisco Gerardo Polotan, Inez Andrea Medado, Kirstyn Brunker, Edelwisa Mercado, Daria Manalo, Catalino Demetria
EPI_ISL_837247, EPI_ISL_837248, EPI_ISL_837250, EPI_ISL_837252, EPI_ISL_837255, EPI_ISL_837285, EPI_ISL_837286, EPI_ISL_837287, EPI_ISL_837288, EPI_ISL_837289, EPI_ISL_837290, EPI_ISL_837291, EPI_ISL_837292, EPI_ISL_837293, EPI_ISL_837294, EPI_ISL_837295, EPI_ISL_837296, EPI_ISL_837297, EPI_ISL_837298, EPI_ISL_837299, EPI_ISL_837300, EPI_ISL_837301, EPI_ISL_837302, EPI_ISL_837303, EPI_ISL_837304, EPI_ISL_837305, EPI_ISL_837306, EPI_ISL_837307, EPI_ISL_837308, EPI_ISL_837309, EPI_ISL_837310, EPI_ISL_837311, EPI_ISL_837312, EPI_ISL_837313, EPI_ISL_837314, EPI_ISL_837315, EPI_ISL_837316, EPI_ISL_837317			
see above	Istituto Zooprofilattico Sperimentale del Mezzogiorno	TIGEM	Patrizia Annunziata, Andrea Ballabio, Valentina Bouche, Davide Cacchiarelli (CorrespAuthor), Pellegrino Cerino, Chiara Colantuono, Lucio Di Filippo, Antonio Grimaldi, Antonio Limone, Gabriella Loconte, Anna Manfredi, Francesco Panariello, Biancamaria Pierri, Marcello Salvi, Lucia Vassallo
EPI_ISL_842838, EPI_ISL_842839, EPI_ISL_842840, EPI_ISL_842841, EPI_ISL_842842, EPI_ISL_842843, EPI_ISL_842858, EPI_ISL_842859	Barts Health NHS Trust	COVID-19 Genomics UK (COG-UK) Consortium	CUTINO-MOGUEL, Maria-Teresa; HARRINGTON, David; OWOYEMI, Dola; SHYLINI, Raghavendran; BROAD, Claire; KELE, Beatrix
EPI_ISL_849033, EPI_ISL_849034, EPI_ISL_849035, EPI_ISL_849036, EPI_ISL_849037, EPI_ISL_849038, EPI_ISL_849039, EPI_ISL_849040, EPI_ISL_849041, EPI_ISL_849042, EPI_ISL_849043, EPI_ISL_849044, EPI_ISL_849045, EPI_ISL_849046, EPI_ISL_849047, EPI_ISL_849048, EPI_ISL_849049, EPI_ISL_849050, EPI_ISL_849051, EPI_ISL_849052, EPI_ISL_849053, EPI_ISL_849054, EPI_ISL_849055, EPI_ISL_849056, EPI_ISL_849057, EPI_ISL_849058, EPI_ISL_849059, EPI_ISL_849060, EPI_ISL_849061, EPI_ISL_849062, EPI_ISL_849063, EPI_ISL_849064, EPI_ISL_849065, EPI_ISL_849066, EPI_ISL_849067, EPI_ISL_849068, EPI_ISL_849069, EPI_ISL_849070, EPI_ISL_849071, EPI_ISL_849072, EPI_ISL_849073, EPI_ISL_849074, EPI_ISL_849075, EPI_ISL_849076, EPI_ISL_849077, EPI_ISL_849078, EPI_ISL_849079, EPI_ISL_849080, EPI_ISL_849081, EPI_ISL_849082, EPI_ISL_849083, EPI_ISL_849084			
see above	Florida Bureau of Public Health Laboratories	Florida Bureau of Public Health Laboratories	Sarah Schmedes, Jason Blanton
EPI_ISL_849693	unknown	PHV-FSS	Son Nguyen et al.
EPI_ISL_852584, EPI_ISL_852594	Max von Pettenkofer Institute, Virology, National Reference Center for Retroviruses, LMU München	Laboratory for Functional Genome Analysis, Dept. Genomics, Gene Center of the LMU Munich	Max Muenchhoff, Stefan Krebs, Alexander Graf, Oliver Keppler, Helmut Blum
EPI_ISL_853298	UPMC Clinical Microbiology Laboratory	Microbial Genome Sequencing Center; Microbial Genomic Epidemiology Laboratory	Mustapha M. Mustapha, Jane W. Marsh, Dan Snyder, Marissa P. Griffith, Stephanie L. Mitchell, Vatsala R. Srinivasa, Kady D. Waggle, Chinelo Ezeonwuku, Vaughn S. Cooper, Lee H. Harrison
EPI_ISL_855479, EPI_ISL_855480, EPI_ISL_855481, EPI_ISL_855482, EPI_ISL_855483, EPI_ISL_855484, EPI_ISL_855485, EPI_ISL_855487	Servicio de Microbiología, Laboratori Clínic Metropolitana Nord. Hospital Universitari Germans Trias i Pujol. Institut d'Investigació en Ciències de la Salut Germans Trias i Pujol (IGTP)	SeqCOVID-SPAIN consortium/IBV(CSIC)	Elisa Martró, Antoni E. Bordoy, Anna Not, Adrián Antuori, Anabel Fernández, Nona Romaní, Verónica Saludes, Cristina Casañ and SeqCOVID-SPAIN consortium
EPI_ISL_855557, EPI_ISL_855558, EPI_ISL_855559, EPI_ISL_855560, EPI_ISL_855561, EPI_ISL_855562, EPI_ISL_855563, EPI_ISL_855564, EPI_ISL_855565, EPI_ISL_855566, EPI_ISL_855567, EPI_ISL_855568, EPI_ISL_855569, EPI_ISL_855570, EPI_ISL_855571, EPI_ISL_855572			
see above	Department of Virology, Principal Military Hospital of Instruction of Tunis	Bundeswehr Institute of Microbiology	Susann Handrick, Malena Bestehorn-Willmann, Simone Eckstein, Mathias C. Walter, Markus H. Antwerpen, Habiba Naija, Kilian Stoecker, Roman Wölfel & Mohamed Ben Moussa
EPI_ISL_856682, EPI_ISL_856694, EPI_ISL_856700	Lab. Microbiologia e Virologia, Cotugno, A.O. dei Colli	Lab. Microbiologia e Virologia, Cotugno, A.O. dei Colli	Luigi Atripaldi, Claudia Tiberio, Anna Perfetti
EPI_ISL_859682, EPI_ISL_859683, EPI_ISL_859695, EPI_ISL_859696, EPI_ISL_859697, EPI_ISL_859755, EPI_ISL_859756, EPI_ISL_859757	BTC, Khalifa University	BTC, Khalifa University	Al Safar et al
EPI_ISL_860095	Lab. Microbiologia e Virologia, Cotugno, A.O. dei Colli	Lab. Microbiologia e Virologia, Cotugno, A.O. dei Colli	Luigi Atripaldi, Claudia Tiberio, Anna Perfetti
EPI_ISL_860140,	Keio University School of	Keio University School of	Kenjiro Kosaki, Yuka Iwasaki, Hirotsugu Ishizu, Haruhiko Siomi, Kodai Abe

EPI_ISL_860142	Medicine	Medicine	
EPI_ISL_860550	St.Olavs hospital/NTNU	IKOM, NTNU	Aleksandr Ianevski, Svein Arne Nordbo, Denis Kainov
EPI_ISL_860794	Lab. Microbiologia e Virologia, Cotugno, A.O. dei Colli	Lab. Microbiologia e Virologia, Cotugno, A.O. dei Colli	Luigi Atripaldi, Claudia Tiberio, Anna Perfetti
EPI_ISL_862589, EPI_ISL_862590, EPI_ISL_862591, EPI_ISL_862592, EPI_ISL_862593, EPI_ISL_862595, EPI_ISL_862596, EPI_ISL_862597, EPI_ISL_862598, EPI_ISL_862599, EPI_ISL_862600, EPI_ISL_862601, EPI_ISL_862602, EPI_ISL_862603, EPI_ISL_862604, EPI_ISL_862605, EPI_ISL_862606, EPI_ISL_862607, EPI_ISL_862608, EPI_ISL_862609, EPI_ISL_862610, EPI_ISL_862611, EPI_ISL_862612, EPI_ISL_862613, EPI_ISL_862614, EPI_ISL_862615, EPI_ISL_862616, EPI_ISL_862617, EPI_ISL_862618, EPI_ISL_862619, EPI_ISL_862620, EPI_ISL_862621, EPI_ISL_862622, EPI_ISL_862623, EPI_ISL_862624, EPI_ISL_862625, EPI_ISL_862626			
see above	Hospital Comarcal de Melilla	Instituto de Salud Carlos III	Iglesias-Caballero, M. Camarero, S. Molinero Calamita, M. González-Esguevillas, M. Pozo, F. Casas, I. Jiménez, P. Jiménez, M. Zaballos, A. Monzón, S. Varona, S. Juliá, M. Cuesta, I. López, J.
EPI_ISL_862815	Multidisciplinary Research Unit, DHR-ICMR, Institute of Medical Sciences, Banaras Hindu University, Varanasi	Multidisciplinary Research Unit, DHR-ICMR, Institute of Medical Sciences, Banaras Hindu University, Varanasi	Royana Singh, Priyoneel Basu, Ashish, Nitish Kumar Singh, Abhay Kumar Yadav, Manpreet Kaur, Deepa Devadas, Chetan Sahni, Gulshan Kumar, Tribhuwan Mohan Mohapatra, Richa Arya, Prashant Singh, Jay Prakash Maurya, Surendra Pratap Mishra
EPI_ISL_876820, EPI_ISL_876821, EPI_ISL_876886, EPI_ISL_876887, EPI_ISL_876888, EPI_ISL_876889, EPI_ISL_876890, EPI_ISL_876891, EPI_ISL_876892, EPI_ISL_876893, EPI_ISL_876894, EPI_ISL_876895, EPI_ISL_876896, EPI_ISL_876897, EPI_ISL_876898, EPI_ISL_876899, EPI_ISL_877120, EPI_ISL_877124, EPI_ISL_877125			
see above	Quest Diagnostics	Quest Diagnostics	Rosenthal,S.H., Gerasimova,A., Kagan,R.M., Anderson, B., Hua, M., Liu Y., Bernstein, L.E., Livingston, K.E., Perez, A., Shalhout, D.F., Shlyakhter, I.A., Owen, R., Tanpaiboon, P., Lacbawan, F.
EPI_ISL_882684, EPI_ISL_882686, EPI_ISL_882748, EPI_ISL_882754	1.AO Universitaria 'S. Giovanni di Dio e Ruggi D'Aragona, Scuola Medica Salernitana' Hospital / 2.UOC di Virologia e Microbiologia, Università della Campania 'L. Vanvitelli' / 3.AO Universitaria 'Federico II' Napoli Hospital / 4.AORN 'San Giuseppe Moscati' Avellino Hospital / 5.AO 'San Pio - presidio G. Rummo' Benevento Hospital / 6.AO 'Sant'Anna e San Sebastiano' Caserta Hospital / 7.PO 'Maria Santissima Addolorata' Eboli Hospital / 8.Biogem Istituto di Ricerche Genetiche	1. Genome Research Center for Health (CRGS) / 2. Laboratory of Molecular Medicine and Genomics(LMMGe) / 3. Center for Research in Pure and Applied Mathematics (CRMPA)	Giorgio Giurato, Francesca Rizzo, Alessandro Weisz, Gianluigi Franci, Giovanni Nassa, Pasquale Pagliano, Roberta Tarallo, Elena Alexandrova, Ylenia D'Agostino, Carlo Ferravante, Jessica Lamberti, Viola Melone, Domenico Memoli, Valeria Mirici Cappa, Domenico Palumbo, Giovanni Pecoraro, Assunta Sellitto, Oriana Strianese, Ilaria Terenzi, Giuseppe Fenza, Aniello Gentile, Antonello Saccomanno, Sonia Amabile, Teresa Rocco, Annamaria Salvati, Emilia Vaccaro, Massimiliano Galdiero, Michele Cennamo, Giuseppe Portella, Maria Grazia Foti, Mariarosaria Ingino, Maria Landi, Maurizio Fumi, Vincenzo Rocco, Rita Greco, Vittoria Letizia, Arnolfo Petruzziello, Maddalena Schioppa, Gregorio Goffredi, Francesca Marciano, Michele Caraglia, Alessia Cossu, Marianna Scrima
EPI_ISL_884308, EPI_ISL_884309, EPI_ISL_884310, EPI_ISL_884311, EPI_ISL_884408, EPI_ISL_884409, EPI_ISL_884410, EPI_ISL_884411, EPI_ISL_884412	Infectious Diseases, Quest Diagnostics	Infectious Diseases, Quest Diagnostics	Rosenthal,S.H., Gerasimova,A., Kagan,R.M., Anderson,B., Bernstein,L.E., Livingston,K.E., Hua,M., Liu,Y., Shalhout,D.F., Owen,R., Lacbawan,F.
EPI_ISL_885150	Hospital General Universitario Gregorio Marañón	Hospital General Universitario Gregorio Marañón	Sergio Buenestado Serrano, Pedro Sola Campoy, Laura Pérez-Lago, Pilar Catalán, Patricia Muñoz, Darío García de Viedma.
EPI_ISL_887109	Institute of Medical Microbiology and Virology, University Hospital Carl Gustav Carus, TU Dresden	DRESDEN-concept Genome Center, CMCB, TU Dresden	Beil J., Brown T., Büttner L., Gscheidel N., Hochauf-Stange K., Klemroth S., Lindemann D., Mehnert G., Petzold A., Reinhardt S., Rost F., Sameith K., Winkler S.
EPI_ISL_889372	Olomouc University Hospital	Institute of Applied Biotechnologies a.s.	Petr Klempt, Ondej Brzo, Martin Kašný, Kateina Kvapilová, Petr Kvapil
EPI_ISL_891267	Institute of Biocides and Medical Ecology, Belgarde, Serbia	Virology department Institute of microbiology and immunology Faculty of Medicine University of Belgrade	Banko Ana, Miljanovic Danijela, Milicevic Ognjen, Loncar Ana, Abazovic Dzihan, Despot Dragana
EPI_ISL_892228, EPI_ISL_892229	Lighthouse Lab in Glasgow	Wellcome Sanger Institute for the COVID-19 Genomics UK (COG-UK) Consortium	Harper VanSteenhouse, Yumi Kasai, David Gray, Carol Clugston, Anna Dominiczak and Alex Alderton, Roberto Amato, Sonia Goncalves, Ewan Harrison, David K. Jackson, Ian Johnston, Dominic Kwiatkowski, Cordelia Langford, John Sillitoe on behalf of the Wellcome Sanger Institute COVID-19 Surveillance Team
EPI_ISL_896182, EPI_ISL_900058, EPI_ISL_900069, EPI_ISL_900096, EPI_ISL_900100, EPI_ISL_900475, EPI_ISL_900489	MEPHI, Aix Marseille University	MEPHI, Aix Marseille University	Anthony LEVASSEUR
EPI_ISL_902742, EPI_ISL_902745	Hospital Universitari Germans Trias i Pujol (HUGTiP) / Fundació Lluita contra la SIDA (FLSida)	IrsiCaixa - Can Ruti CovidSeq	Fundació irsiCaixa. Hospital Universitari Germans Trias i Pujol(HUGTiP), 2a planta, maternal Ctra Canyet s/n, Badalona Marta Massanella, Ester Ballana, Lidia Ruiz, Nuria Izquierdo, Jorge Carrillo, Roger Paredes, Julia Blanco, Joaquim Segalés, Bonaventura Clotet
EPI_ISL_903336, EPI_ISL_903337, EPI_ISL_903338	University of Iowa Hospitals & Clinics, Microbiology Laboratory	University or Iowa, Lung Biology and Cystic Fibrosis Research Center, Pezzulo Lab	Miguel E Ortiz, Alejandro A Pezzulo
EPI_ISL_906063, EPI_ISL_906064	Tilia Laboratories s.r.o.	Tilia Laboratories s.r.o.	Sona Pekova, MD, PhD.

EPI_ISL_909772	National Institute of Health Research and Development	National Institute of Health Research and Development	Holy Arif Wibowo; Ririn Ramadhany; Yuni Rukminiati; Agustiningsih; Kindi Adam; Hana Apsari Pawestri; Subangkit; Kartika Dewi Puspa; Arie Ardiansyah Nugraha; Hartanti Dian Ikawati; Krisna Nur Andriana Pangesti; Triyani Soekarso; Ni Ketut Susilarini; Nur Ika Hariastuti; Uli Alfi Nikmah; Mursinah; Asri Febriyani; Reni Herman; Nike Susanti; Herna; Tati Febriyanti; Juwita Kurniawati; Natalie Laurencia Kipuw; Fauzul Muna; Irene Lorinda Indalao; Aulia Rizki; Nelly Puspandari; vivi Setiawaty
EPI_ISL_910161, EPI_ISL_910162, EPI_ISL_910163, EPI_ISL_910164, EPI_ISL_910165, EPI_ISL_910166, EPI_ISL_910167, EPI_ISL_910168, EPI_ISL_910169, EPI_ISL_910170, EPI_ISL_910171, EPI_ISL_910172, EPI_ISL_910173, EPI_ISL_910174, EPI_ISL_910175, EPI_ISL_910176, EPI_ISL_910177, EPI_ISL_910178, EPI_ISL_910179, EPI_ISL_910180, EPI_ISL_910181, EPI_ISL_910182, EPI_ISL_910183, EPI_ISL_910184, EPI_ISL_910185, EPI_ISL_910186, EPI_ISL_910187, EPI_ISL_910188, EPI_ISL_910189, EPI_ISL_910190, EPI_ISL_910191, EPI_ISL_910192, EPI_ISL_910193, EPI_ISL_910194, EPI_ISL_910195, EPI_ISL_910196, EPI_ISL_910197, EPI_ISL_910198, EPI_ISL_910199, EPI_ISL_910200, EPI_ISL_910201, EPI_ISL_910202, EPI_ISL_910203, EPI_ISL_910204, EPI_ISL_910205, EPI_ISL_910206, EPI_ISL_910207, EPI_ISL_910208, EPI_ISL_910209, EPI_ISL_910210			
see above	CSIR-Centre for Cellular and Molecular Biology	CSIR-Centre for Cellular and Molecular Biology	Payel Mukherjee,Pratheusa Maccha,Namami Gaur,Lamuk Zaveri,Tulasi Nagabandi,Purushotham Vodnala,Blessy B John,Viswagithe S L,B Himasri,Sofia Banu,Priya Singh,Archana Bharadwaj Siva,Karthik Bharadwaj Tallapaka,Rakesh K Mishra,Divya Tej Sowpati
EPI_ISL_912187, EPI_ISL_912188, EPI_ISL_912189	California Institute of Technology	Chan-Zuckerberg Biohub	CZB Cliahub Consortium
EPI_ISL_915395, EPI_ISL_915396, EPI_ISL_915397	Keio University School of Medicine	Keio University School of Medicine	Kenjiro Kosaki, Yuka Iwasaki, Hirotsugu Ishizu, Haruhiko Siomi, Kodai Abe
EPI_ISL_918126	Lighthouse Lab in Glasgow	Wellcome Sanger Institute for the COVID-19 Genomics UK (COG-UK) Consortium	Harper VanSteenhouse, Yumi Kasai, David Gray, Carol Clugston, Anna Dominiczak and Alex Alderton, Roberto Amato, Sonia Goncalves, Ewan Harrison, David K. Jackson, Ian Johnston, Dominic Kwiatkowski, Cordelia Langford, John Sillitoe on behalf of the Wellcome Sanger Institute COVID-19 Surveillance Team
EPI_ISL_924428	Virology Department, Sheffield Teaching Hospitals NHS Foundation Trust/Department of Infection, Immunity and Cardiovascular Disease, The Medical School, University of Sheffield	COVID-19 Genomics UK (COG-UK) Consortium	Thushan de Silva, Matthew Parker, Nikki Smith, Adri Angyal, Rebecca Brown, Luke Green, Rachel Tucker, Paul Parsons, Danielle Groves, Katie Johnson, Laura Carrilero, Alex Keeley, Dave Partridge, Matthew Wyles, Benjamin Lindsey, Mehmet Yavuz, Mohammad Raza, Cariad Evans
EPI_ISL_925429, EPI_ISL_925430, EPI_ISL_925431, EPI_ISL_925432, EPI_ISL_925433, EPI_ISL_925434	Department of Clinical Microbiology	GIGA Medical Genomics	Keith Durkin, Maria Artesi, Sébastien Bontems, Raphaël Boreux, Bouchra Boujemla, Cécile Meex, Pierrette Melin, Marie-Pierre Hayette, Vincent Bours
EPI_ISL_935367, EPI_ISL_935368, EPI_ISL_935369, EPI_ISL_935370, EPI_ISL_935371, EPI_ISL_935372, EPI_ISL_935373, EPI_ISL_935374, EPI_ISL_935375, EPI_ISL_935376, EPI_ISL_935377, EPI_ISL_935378, EPI_ISL_935379, EPI_ISL_935380, EPI_ISL_935381, EPI_ISL_935382, EPI_ISL_935385, EPI_ISL_935386, EPI_ISL_935387, EPI_ISL_935388, EPI_ISL_935399, EPI_ISL_935400, EPI_ISL_935402, EPI_ISL_935403, EPI_ISL_935404			
see above	Florida Bureau of Public Health Laboratories	Florida Bureau of Public Health Laboratories	Sarah Schmedes, Jason Blanton
EPI_ISL_935791, EPI_ISL_935817, EPI_ISL_935818, EPI_ISL_935819, EPI_ISL_935820, EPI_ISL_935821, EPI_ISL_935829, EPI_ISL_935830, EPI_ISL_935834, EPI_ISL_935837, EPI_ISL_935838, EPI_ISL_935839, EPI_ISL_935842, EPI_ISL_935843, EPI_ISL_935844, EPI_ISL_935845, EPI_ISL_935847, EPI_ISL_935848, EPI_ISL_935851			
see above	Cadham Provincial laboratory	National Microbiology Laboratory (NML)	Anna Majer, Shari Tyson, Grace Seo, Philip Mabon, Elsie Grudeski, Rhiannon Huzarewich, Russell Mandes, Anneliese Landgraff, Jennifer Tanner, Natalie Knox, Morag Graham, Gary Van Domselaar, Paul Van Caeseele, Jared Bullard, David Alexander, Kerry Dust, Nathalie Bastien, Yan Li, Timothy Booth, Darian Hole, Madison Chapel, Kirsten Biggar, CanCOGeN's metadata curation team, Public Health Agency of Canada CanCOGeN team
EPI_ISL_936837, EPI_ISL_936838, EPI_ISL_936839, EPI_ISL_936840, EPI_ISL_936841, EPI_ISL_936842, EPI_ISL_936843	Northwestern Memorial Hospital	Ozer Lab	Ramon Lorenzo-Redondo, Lacy M. Simons, Chad J. Achenbach, Lawrence J. Jennings, Michael G. Ison, Judd F. Hultquist, Egon A. Ozer
EPI_ISL_937030, EPI_ISL_937031, EPI_ISL_937036, EPI_ISL_937043, EPI_ISL_937044, EPI_ISL_937051, EPI_ISL_937052, EPI_ISL_937059, EPI_ISL_937064, EPI_ISL_937069, EPI_ISL_937080, EPI_ISL_937092, EPI_ISL_937094, EPI_ISL_937113			
see above	Quest Diagnostics	Quest Diagnostics	Rosenthal,S.H., Gerasimova,A., Kagan,R.M., Anderson, B., Livingston, K.E., Hua, M., Liu Y., Shalhout, D.F., Owen, R., Lacbawan, F.
EPI_ISL_940205, EPI_ISL_940206, EPI_ISL_940207, EPI_ISL_940394	Hôpital Bichat Claude Bernard, Laboratoire de Virologie	IAME UMR1137 Inserm, Université de Paris, Hôpital Bichat	Antoine Bridier-Nahmias, Amélie Recoing, Quentin Le Hingrat, Lena Daniel, Siham Hamri, Gilles Collin, Alexandre Storto, Mélanie Bertine, Charlotte Charpentier, Nadhira Houhou-Fidouh, Diane Descamps, Benoit Visseaux
EPI_ISL_940973, EPI_ISL_940974, EPI_ISL_940975	Centers for Disease Control and Prevention, Dengue Branch	Centers for Disease Control and Prevention, Dengue Branch	Gilberto A. Santiago, Glenda Gonzalez, Betzabel Flores, Keyla Charriez, Gabriela Paz-Bailey, Jorge L. Munoz-Jordan
EPI_ISL_941273	NCSLPH	NCSLPH	Chase K, Miller MC, Greene S, Glover W
EPI_ISL_941929, EPI_ISL_941940	Florida Bureau of Public Health Laboratories	Florida Bureau of Public Health Laboratories	Sarah Schmedes, Jason Blanton
EPI_ISL_942010	Centers for Disease Control and Prevention, Dengue Branch	Centers for Disease Control and Prevention, Dengue Branch	Gilberto A. Santiago, Glenda Gonzalez, Betzabel Flores, Keyla Charriez, Gabriela Paz-Bailey, Jorge L. Munoz-Jordan
EPI_ISL_949197, EPI_ISL_949218, EPI_ISL_949225, EPI_ISL_949242, EPI_ISL_949243	Departamento de Microbiología, CDB, Hospital Clínic, Barcelona	SeqCOVID-SPAIN consortium/IBV(CSIC)	Andrea Vergara, Mikel Martínez, Elisa Rubio, Jéssica Navero, Aida Peiró and SeqCOVID-SPAIN consortium
EPI_ISL_955148	University of Sarajevo, Veterinary Faculty, Laboratory for Molecular Diagnostic and Research Laboratory	University of Sarajevo, Veterinary Faculty, Laboratory for Molecular Diagnostic and Research Laboratory	Goleti T., Goleti Š., Softi A., Ali-Šeho A., Jaži A., Šabi E., Terzi I., Nicevi M., Hodži A.
EPI_ISL_956404	General Hospital - Strumica	Research Center for Genetic Engineering and Biotechnology "Georgi D. Efremov" , Macedonian Academy of Sciences and Arts	Aleksandar J. Dimovski, Dijana Plasheska-Karanfilska, Predrag Noveski, Gjorgji Bozinovski, Milena Jakimovska

EPI_ISL_959907, EPI_ISL_959910, EPI_ISL_959912, EPI_ISL_959998, EPI_ISL_959999, EPI_ISL_960000, EPI_ISL_960001, EPI_ISL_960002, EPI_ISL_960003, EPI_ISL_960004, EPI_ISL_960005, EPI_ISL_960006, EPI_ISL_960007, EPI_ISL_960008, EPI_ISL_960009, EPI_ISL_960010, EPI_ISL_960011, EPI_ISL_960012, EPI_ISL_960013, EPI_ISL_960014, EPI_ISL_960015, EPI_ISL_960016, EPI_ISL_960017, EPI_ISL_960018, EPI_ISL_960019, EPI_ISL_960020, EPI_ISL_960021			
see above	University Medical Center Hamburg Eppendorf	Heinrich Pette Institute, Leibniz Institute for Experimental Virology	Alexis Robitaille, Thomas Günther, Johannes Knobloch, Martin Aepfelbacher, Nicole Fischer, Adam Grundhoff
EPI_ISL_970355, EPI_ISL_970357, EPI_ISL_970359, EPI_ISL_970361, EPI_ISL_970363, EPI_ISL_970366, EPI_ISL_970367, EPI_ISL_970369, EPI_ISL_970372, EPI_ISL_970374, EPI_ISL_970375, EPI_ISL_970378, EPI_ISL_970379, EPI_ISL_970381, EPI_ISL_970384, EPI_ISL_970385, EPI_ISL_970388, EPI_ISL_970389, EPI_ISL_970391, EPI_ISL_970393, EPI_ISL_970395, EPI_ISL_970397, EPI_ISL_970399, EPI_ISL_970401, EPI_ISL_970403, EPI_ISL_970405, EPI_ISL_970407, EPI_ISL_970408, EPI_ISL_970410, EPI_ISL_970413, EPI_ISL_970414, EPI_ISL_970417, EPI_ISL_970418, EPI_ISL_970420, EPI_ISL_970423, EPI_ISL_970425, EPI_ISL_970427, EPI_ISL_970429, EPI_ISL_970431, EPI_ISL_970433, EPI_ISL_970434, EPI_ISL_970437, EPI_ISL_970438, EPI_ISL_970440, EPI_ISL_970442, EPI_ISL_970445, EPI_ISL_970447, EPI_ISL_970449, EPI_ISL_970451, EPI_ISL_970453, EPI_ISL_970455, EPI_ISL_970456, EPI_ISL_970458, EPI_ISL_970459, EPI_ISL_970461, EPI_ISL_970463, EPI_ISL_970465, EPI_ISL_970467, EPI_ISL_970471, EPI_ISL_970472, EPI_ISL_970474, EPI_ISL_970476, EPI_ISL_970479, EPI_ISL_970480, EPI_ISL_970482, EPI_ISL_970484, EPI_ISL_970485, EPI_ISL_970487, EPI_ISL_970489, EPI_ISL_970491, EPI_ISL_970494, EPI_ISL_970496, EPI_ISL_970498, EPI_ISL_970501, EPI_ISL_970503, EPI_ISL_970505, EPI_ISL_970507, EPI_ISL_970509, EPI_ISL_970511, EPI_ISL_970514, EPI_ISL_970516, EPI_ISL_970518, EPI_ISL_970520, EPI_ISL_970522, EPI_ISL_970524, EPI_ISL_970526, EPI_ISL_970529, EPI_ISL_970531, EPI_ISL_970532, EPI_ISL_970534, EPI_ISL_970536, EPI_ISL_970538, EPI_ISL_970539, EPI_ISL_970542, EPI_ISL_970544, EPI_ISL_970545, EPI_ISL_970547, EPI_ISL_970549, EPI_ISL_970550, EPI_ISL_970552, EPI_ISL_970554, EPI_ISL_970556, EPI_ISL_970557, EPI_ISL_970559, EPI_ISL_970561, EPI_ISL_970563, EPI_ISL_970565, EPI_ISL_970567, EPI_ISL_970570, EPI_ISL_970572, EPI_ISL_970574, EPI_ISL_970576, EPI_ISL_970578, EPI_ISL_970580, EPI_ISL_970582, EPI_ISL_970584, EPI_ISL_970586, EPI_ISL_970588, EPI_ISL_970590, EPI_ISL_970592, EPI_ISL_970594, EPI_ISL_970596, EPI_ISL_970598, EPI_ISL_970600, EPI_ISL_970603, EPI_ISL_970604, EPI_ISL_970606, EPI_ISL_970608, EPI_ISL_970610, EPI_ISL_970611, EPI_ISL_970613, EPI_ISL_970615, EPI_ISL_970617, EPI_ISL_970620, EPI_ISL_970622, EPI_ISL_970624, EPI_ISL_970626, EPI_ISL_970627, EPI_ISL_970629, EPI_ISL_970631, EPI_ISL_970634, EPI_ISL_970636, EPI_ISL_970638, EPI_ISL_970640, EPI_ISL_970642, EPI_ISL_970644, EPI_ISL_970646, EPI_ISL_970649, EPI_ISL_970651, EPI_ISL_970653, EPI_ISL_970655, EPI_ISL_970657, EPI_ISL_970659, EPI_ISL_970662, EPI_ISL_970663, EPI_ISL_970665, EPI_ISL_970667, EPI_ISL_970669, EPI_ISL_970671, EPI_ISL_970672, EPI_ISL_970674, EPI_ISL_970676, EPI_ISL_970678, EPI_ISL_970679, EPI_ISL_970682, EPI_ISL_970684, EPI_ISL_970686, EPI_ISL_970688, EPI_ISL_970691, EPI_ISL_970693, EPI_ISL_970695, EPI_ISL_970696, EPI_ISL_970698, EPI_ISL_970700, EPI_ISL_970702, EPI_ISL_970705, EPI_ISL_970707, EPI_ISL_970710, EPI_ISL_970712, EPI_ISL_970714, EPI_ISL_970716, EPI_ISL_970718, EPI_ISL_970719, EPI_ISL_970721, EPI_ISL_970723, EPI_ISL_970725, EPI_ISL_970728, EPI_ISL_970730, EPI_ISL_970731, EPI_ISL_970733, EPI_ISL_970735, EPI_ISL_970737, EPI_ISL_970740, EPI_ISL_970742, EPI_ISL_970743, EPI_ISL_970745, EPI_ISL_970748, EPI_ISL_970750, EPI_ISL_970752, EPI_ISL_970754, EPI_ISL_970756, EPI_ISL_970759, EPI_ISL_970761, EPI_ISL_970763, EPI_ISL_970765, EPI_ISL_970767, EPI_ISL_970769, EPI_ISL_970771, EPI_ISL_970773, EPI_ISL_970776, EPI_ISL_970778, EPI_ISL_970780, EPI_ISL_970783, EPI_ISL_970785, EPI_ISL_970787, EPI_ISL_970789, EPI_ISL_970791, EPI_ISL_970793, EPI_ISL_970795, EPI_ISL_970798, EPI_ISL_970799, EPI_ISL_970801, EPI_ISL_970804, EPI_ISL_970806, EPI_ISL_970809, EPI_ISL_970818, EPI_ISL_970840, EPI_ISL_970856, EPI_ISL_970878, EPI_ISL_970902, EPI_ISL_970931, EPI_ISL_970947, EPI_ISL_970965, EPI_ISL_970995, EPI_ISL_971012, EPI_ISL_971036, EPI_ISL_971040, EPI_ISL_971043, EPI_ISL_971046, EPI_ISL_971049, EPI_ISL_971052, EPI_ISL_971054, EPI_ISL_971057, EPI_ISL_971061, EPI_ISL_971064, EPI_ISL_971068, EPI_ISL_971072, EPI_ISL_971075, EPI_ISL_971077, EPI_ISL_971080, EPI_ISL_971084, EPI_ISL_971086, EPI_ISL_971089, EPI_ISL_971091, EPI_ISL_971094, EPI_ISL_971097, EPI_ISL_971101, EPI_ISL_971104, EPI_ISL_971107, EPI_ISL_971111, EPI_ISL_971114, EPI_ISL_971116, EPI_ISL_971120, EPI_ISL_971122, EPI_ISL_971126, EPI_ISL_971129, EPI_ISL_971132, EPI_ISL_971137, EPI_ISL_971139, EPI_ISL_971141, EPI_ISL_971144, EPI_ISL_971147, EPI_ISL_971150, EPI_ISL_971154, EPI_ISL_971157, EPI_ISL_971160, EPI_ISL_971162, EPI_ISL_971167, EPI_ISL_971170, EPI_ISL_971173, EPI_ISL_971177, EPI_ISL_971179, EPI_ISL_971182, EPI_ISL_971187, EPI_ISL_971189, EPI_ISL_971192, EPI_ISL_971196, EPI_ISL_971199, EPI_ISL_971202, EPI_ISL_971205, EPI_ISL_971208, EPI_ISL_971212, EPI_ISL_971216, EPI_ISL_971219, EPI_ISL_971222, EPI_ISL_971225, EPI_ISL_971227, EPI_ISL_971230, EPI_ISL_971233			
see above	BCCDC Public Health Laboratory	BCCDC Public Health Laboratory	Prystajecky Natalie, Linda Hoang, Dan Fornika, John Tyson, Shannon Russell, Kim Macdonald, Kimia Kamelian, Ana Pacagnella, Corrinne Ng, Loretta Janz, Robert Azana Terry Snutch, Mel Krajden
EPI_ISL_976912, EPI_ISL_976913, EPI_ISL_976914, EPI_ISL_976915, EPI_ISL_976917, EPI_ISL_976918, EPI_ISL_976921, EPI_ISL_976922, EPI_ISL_976924, EPI_ISL_976926, EPI_ISL_976928, EPI_ISL_976929, EPI_ISL_976930, EPI_ISL_976931			
see above	University of Massachusetts Medical School	Infectious Disease Program, Broad Institute of Harvard and MIT	Lemieux,J.E., Siddle,K.J., Ward,D., Ellison,R., Adams,G., Gladden-Young,A., Lagerborg,K., Rudy,M., DeRuff,K., Carter,A., Normandin,E., Bauer,M., Reilly,S., Tomkins-Tinch,C., Loreth,C., Chaluvadi,S., Birren,B.W., Gallagher,G., Smole,S., Park,D.J., MacInnis,B.L., and Sabeti,P.C.
EPI_ISL_979292, EPI_ISL_979293	Cadham Provincial laboratory	National Microbiology Laboratory (NML)	Anna Majer, Shari Tyson, Grace Seo, Philip Mabon, Elsie Grudeski, Rhiannon Huzarewich, Russell Mandes, Anneliese Landgraff, Jennifer Tanner, Natalie Knox, Morag Graham, Gary Van Domselaar, Paul Van Caeseele, Jared Bullard, David Alexander, Kerry Dust, Nathalie Bastien, Yan Li, Timothy Booth, Darian Hole, Madison Chapel, Kirsten Biggar, CanCOGeN's metadata curation team, Public Health Agency of Canada CanCOGeN team
