# Supplementary material for: Detection and characterization of the SARS-CoV-2 lineage B.1.526 in New York
Source: Nat Commun. 2021 Aug 9;12:4886. doi: 10.1038/s41467-021-25168-4 (PMC8352861; doi:10.1038/s41467-021-25168-4)
Supplement: Supplementary file 8 — Supplementary Data 4 [file 41467_2021_25168_MOESM8_ESM.zip › GISAID_acknowledements_tables/gisaid_hcov-19_acknowledgement_table_2021_02_12_16-10.pdf]

We gratefully acknowledge the following Authors from the Originating laboratories responsible for obtaining the specimens, as well as the Submitting laboratories where the genome data were generated and shared via GISAID, on which this research is based.

All Submitters of data may be contacted directly via www.gisaid.org

Authors are sorted alphabetically.

Accession ID	Originating Laboratory	Submitting Laboratory	Authors
EPI_ISL_427391	Genomic Laboratory (GLAB) (Conjoint lab of Health Directorate of Istanbul and Istanbul Technical University)	Genomic Laboratory (GLAB), Istanbul Technical University	Ilker Karacan, Tugba Kizilboga Akgun, Bugra Agaoglu, Gizem Alkurt, Jale Yildiz, Betsi Köse, Elifnaz Çelik, Mehtap Aydn, Levent Doganay, Gizem Dinler Doganay
EPI_ISL_427427, EPI_ISL_427437, EPI_ISL_427441, EPI_ISL_427450, EPI_ISL_427457	University of Wisconsin-Madison AIDS Vaccine Research Laboratories	University of Wisconsin-Madison AIDS Vaccine Research Laboratories	Gage Moreno, Katarina Braun, et al. AIDS Vaccine Research Laboratories
EPI_ISL_428346, EPI_ISL_428368	Genomic Laboratory (GLAB) (Conjoint lab of Health Directorate of Istanbul and Istanbul Technical University)	Genomic Laboratory (GLAB), Istanbul Technical University	Ilker Karacan, Tugba Kizilboga Akgun, Bugra Agaoglu, Gizem Alkurt, Jale Yildiz, Betsi Köse, Elifnaz Çelik, Arzu Irvem, Yasemin Kendir Demirkol, Ozlem Akgun Dogan, Mehtap Aydn, Levent Doganay, Gizem Dinler Doganay
EPI_ISL_428486, EPI_ISL_428487	District Surveillence Unit	Department of Neurovirology, National Institute of Mental Health and Neuroscience (NIMHANS)	Chitra Pattabiraman, Vijayalakshmi Reddy, Harsha PK, Risha Rasheed, Shafeeq S Hameed, Manjunatha Venkataswamy, Anita Desai, Ravi Vasanthapuram
EPI_ISL_428846, EPI_ISL_428847, EPI_ISL_428848, EPI_ISL_428849, EPI_ISL_428850	National Public Health Laboratory, National Centre for Infectious Diseases	National Public Health Laboratory, National Centre for Infectious Diseases	Mak TM, Octavia S, Chavatte JM, Cui L, Lin RTP
EPI_ISL_428935, EPI_ISL_428936	University of Wisconsin-Madison AIDS Vaccine Research Laboratories	University of Wisconsin-Madison AIDS Vaccine Research Laboratories	Gage Moreno, Katarina Braun, et al. AIDS Vaccine Research Laboratories
EPI_ISL_429845, EPI_ISL_429846, EPI_ISL_429847	Gundersen Molecular Diagnostics Laboratory	Kabara Cancer Research Institute	Craig S. Richmond, Paraic A. Kenny
EPI_ISL_429874	Microbiology, Virology and Biemergency Laboratory-ASST FBF Sacco	Microbiology, Virology and Biemergency Laboratory-ASST FBF Sacco	Rimoldi SG, Stefani F
EPI_ISL_430092, EPI_ISL_430093, EPI_ISL_430094, EPI_ISL_430095, EPI_ISL_430096, EPI_ISL_430097, EPI_ISL_430098, EPI_ISL_430099, EPI_ISL_430100, EPI_ISL_430101, EPI_ISL_430102, EPI_ISL_430103, EPI_ISL_430104, EPI_ISL_430105, EPI_ISL_430106, EPI_ISL_430107, EPI_ISL_430108, EPI_ISL_430109, EPI_ISL_430110, EPI_ISL_430111			
see above	WHO National Influenza Centre Russian Federation	WHO National Influenza Centre Russian Federation	Andrey Komissarov, Artem Fadeev, Mariia Sergeeva, Anna Ivanova, Daria Danilenko
EPI_ISL_430358, EPI_ISL_430416, EPI_ISL_430417, EPI_ISL_430418, EPI_ISL_430419, EPI_ISL_430420, EPI_ISL_430421, EPI_ISL_430422, EPI_ISL_430424, EPI_ISL_430425, EPI_ISL_430426, EPI_ISL_430427, EPI_ISL_430428, EPI_ISL_430429, EPI_ISL_430430, EPI_ISL_430431, EPI_ISL_430432, EPI_ISL_430433			
see above	NYU Langone Health	Departments of Pathology and Medicine, New York University School of Medicine	Maria Aguero-Rosenfeld, Brendan Belovarac, Margaret Black, Ludovic Boytard, John Cadley, Paolo Cotzia, John Chen, Dacia Dimartino, Xiaojun Feng, Tatyana Gindin, Emily Guzman, Adriana Heguy, Megan Hogan, Emily Huang, George Jour, Lawrence H. Lin, Raven Luther, Andrew Lytle, Christian Marier, Matthew T. Maurano, Mark J. Mulligan, Peter Meyn, Raquel Ordonez Ciriza, Iman Osman, Jared Pinnell, Vanessa Raabe, Sitharam Ramaswami, Amy Rapkiewicz, Andre M. Ribeiro-dos-Santos, Marie Samanovic-Golden, Antonio Serrano, Guomiao Shen, Matija Snuderl, Theodore Vougiouklakis, Nick Vulpescu, Gael Westby, Paul Zappile, Yutong Zhang
EPI_ISL_430682, EPI_ISL_430683, EPI_ISL_430684, EPI_ISL_430686	Microbiological Diagnostic Unit Public Health Laboratory	Microbiological Diagnostic Unit Public Health Laboratory	Seemann T., Schultz M., Sait, M., Sherry, N.
EPI_ISL_430692, EPI_ISL_430697, EPI_ISL_430698, EPI_ISL_430699, EPI_ISL_430706, EPI_ISL_430707, EPI_ISL_430708	Victorian Infectious Diseases Reference Laboratory (VIDRL)	Microbiological Diagnostic Unit Public Health Laboratory and Victorian Infectious Diseases Reference Laboratory, The Peter Doherty Institute for Infection and Immunity	Caly L., Seemann T., Sait, M., Schultz M., Druce J., Sherry, N.
EPI_ISL_430715	Microbiological Diagnostic Unit Public Health Laboratory	Microbiological Diagnostic Unit Public Health Laboratory	Seemann T., Schultz M., Sait, M., Sherry, N.
EPI_ISL_430814, EPI_ISL_430815, EPI_ISL_430816, EPI_ISL_430817, EPI_ISL_430818	Laboratorio de Virología del Hospital de Niños Dr. Ricardo Gutierrez	Área de Secuenciación del Laboratorio de Virología del Hospital de Niños Dr. Ricardo Gutierrez on behalf of 'Proyecto Argentino Interinstitucional de genomica de SARS-CoV-2' (PAIS Consortium)	Nabaes Jodar, MS; Goya, S; Natale, MI; Lusso, S; Gravis, E; Mistchenko, AS; Valinotto, LE; Viegas, M.
EPI_ISL_431087, EPI_ISL_431089, EPI_ISL_431090, EPI_ISL_431091, EPI_ISL_431092, EPI_ISL_431093, EPI_ISL_431094, EPI_ISL_431096	Yale COVID-19 Biorepository	Grubaugh Lab - Yale School of Public Health	Joseph Fauver, Tara Alpert, Anderson Brito, Anne Wyllie, Chantal Vogels, Mary Petrone, Cole Jensen, Chaney Kalinich, Isabel Ott, Arnau Casanovas, Catherine Muenker, Adam Moore, Alice Lu, Maria Tokuyama, Patrick Wong, Peiwen Lu, Saad Omer, Richard Martinello, Allison Nelson, Shelli Farhadian, Akiko Iwasaki, Charlese Dela Cruz, Albert Ko, Nathan Grubaugh
EPI_ISL_432585, EPI_ISL_432729, EPI_ISL_432762, EPI_ISL_432769, EPI_ISL_432780, EPI_ISL_432823, EPI_ISL_432866	Virology Department, Sheffield Teaching Hospitals NHS Foundation Trust / Virology Department, Sheffield Teaching Hospitals NHS Foundation Trust	COVID-19 Genomics UK (COG-UK) Consortium	Thushan de Silva, Matthew Parker,Adri Angyal, Rebecca Brown, Luke Green, Rachel Tucker, Paul Parsons, Danielle Groves, Alex Keeley, Dave Partridge, Matthew Wyles, Benjamin Lindsey, Mehmet Yavuz, Mohammad Raza, Cariad Evans
EPI_ISL_433069, EPI_ISL_433070, EPI_ISL_433071, EPI_ISL_433073, EPI_ISL_433080, EPI_ISL_433083, EPI_ISL_433085, EPI_ISL_433086, EPI_ISL_433087, EPI_ISL_433088, EPI_ISL_433089, EPI_ISL_433090, EPI_ISL_433091, EPI_ISL_433092, EPI_ISL_433093, EPI_ISL_433094, EPI_ISL_433095, EPI_ISL_433096, EPI_ISL_433097, EPI_ISL_433098, EPI_ISL_433099, EPI_ISL_433100, EPI_ISL_433101, EPI_ISL_433102, EPI_ISL_433103, EPI_ISL_433104, EPI_ISL_433105, EPI_ISL_433106, EPI_ISL_433107, EPI_ISL_433108, EPI_ISL_433109, EPI_ISL_433110, EPI_ISL_433111, EPI_ISL_433112, EPI_ISL_433113, EPI_ISL_433114, EPI_ISL_433115, EPI_ISL_433116, EPI_ISL_433117, EPI_ISL_433118, EPI_ISL_433119, EPI_ISL_433120, EPI_ISL_433121, EPI_ISL_433122, EPI_ISL_433123, EPI_ISL_433124, EPI_ISL_433125, EPI_ISL_433126, EPI_ISL_433127, EPI_ISL_433128, EPI_ISL_433129, EPI_ISL_433130, EPI_ISL_433131, EPI_ISL_433132, EPI_ISL_433133, EPI_ISL_433134, EPI_ISL_433135, EPI_ISL_433136, EPI_ISL_433137, EPI_ISL_433138, EPI_ISL_433139, EPI_ISL_433140, EPI_ISL_433141, EPI_ISL_433142, EPI_ISL_433143, EPI_ISL_433144, EPI_ISL_433145, EPI_ISL_433146, EPI_ISL_433147, EPI_ISL_433148, EPI_ISL_433149, EPI_ISL_433150, EPI_ISL_433151, EPI_ISL_433152, EPI_ISL_433153, EPI_ISL_433154, EPI_ISL_433155, EPI_ISL_433156, EPI_ISL_433157, EPI_ISL_433158, EPI_ISL_433159, EPI_ISL_433160, EPI_ISL_433161, EPI_ISL_433162, EPI_ISL_433163, EPI_ISL_433164, EPI_ISL_433165, EPI_ISL_433166, EPI_ISL_433167, EPI_ISL_433168, EPI_ISL_433169, EPI_ISL_433170, EPI_ISL_433171, EPI_ISL_433172, EPI_ISL_433173, EPI_ISL_433174, EPI_ISL_433175, EPI_ISL_433176, EPI_ISL_433177, EPI_ISL_433178, EPI_ISL_433179, EPI_ISL_433180, EPI_ISL_433181, EPI_ISL_433182, EPI_ISL_433183, EPI_ISL_433184, EPI_ISL_433185, EPI_ISL_433186, EPI_ISL_433187, EPI_ISL_433188, EPI_ISL_433189, EPI_ISL_433190, EPI_ISL_433191, EPI_ISL_433192, EPI_ISL_433193, EPI_ISL_433194, EPI_ISL_433195, EPI_ISL_433196, EPI_ISL_433197			
see above	Virology Department, Royal Infirmary of Edinburgh, NHS Lothian / School of Biological Sciences, University of Edinburgh / Institute of Genetics and Molecular Medicine, University of Edinburgh	COVID-19 Genomics UK (COG-UK) Consortium	McHugh M, Dewar R, Rooke S, Gallagher M, Balcaza C, O'Toole A, Hill V, McCrone JT, Colquhoun R, Yu X, Jackson B, Rambaut A, Williams TC, Templeton K
EPI_ISL_433273	West of Scotland Specialist Virology Centre, NHSGGC / MRC-University of Glasgow Centre for Virus Research	COVID-19 Genomics UK (COG-UK) Consortium	Ana da Silva Filipe, Natasha Johnson, Kathy Smollett, Daniel Mair, Stephen Carmichael, Lily Tong, Jenna Nichols, Elihu Aranday-Cortes, Kirstyn Brunker, Yasmin Parr, Kyriaki Nomikou; Sarah McDonald, Marc Niebel, Patawee Asamaphan; Richard Orton, Joseph Hughes, Sreenu Vattipally, David L Robertson; Alasdair MacLean, Rory Gunson; Kathy Li, Natasha Jesudason, Rajiv Shah, James Shepherd, Antonia Ho, Emma Thomson
EPI_ISL_433449, EPI_ISL_433450, EPI_ISL_433452, EPI_ISL_433453, EPI_ISL_433454, EPI_ISL_433462, EPI_ISL_433464	Virology Department, Royal Infirmary of Edinburgh, NHS Lothian / School of Biological Sciences, University of Edinburgh / Institute of Genetics and Molecular Medicine, University of Edinburgh	COVID-19 Genomics UK (COG-UK) Consortium	McHugh M, Dewar R, Rooke S, Gallagher M, Balcaza C, O'Toole A, Hill V, McCrone JT, Colquhoun R, Yu X, Jackson B, Rambaut A, Williams TC, Templeton K

EPI_ISL_433466, EPI_ISL_433467, EPI_ISL_433468, EPI_ISL_433469, EPI_ISL_433470, EPI_ISL_433471, EPI_ISL_433472, EPI_ISL_433473, EPI_ISL_433474, EPI_ISL_433475, EPI_ISL_433476, EPI_ISL_433477, EPI_ISL_433478, EPI_ISL_433479, EPI_ISL_433480, EPI_ISL_433481, EPI_ISL_433482, EPI_ISL_433483, EPI_ISL_433484, EPI_ISL_433485, EPI_ISL_433486, EPI_ISL_433487, EPI_ISL_433489, EPI_ISL_433490, EPI_ISL_433491, EPI_ISL_433492, EPI_ISL_433493, EPI_ISL_433494, EPI_ISL_433495, EPI_ISL_433496, EPI_ISL_433497, EPI_ISL_433953, EPI_ISL_433955, EPI_ISL_433956, EPI_ISL_433958, EPI_ISL_433970, EPI_ISL_433971, EPI_ISL_433974, EPI_ISL_433975, EPI_ISL_433976, EPI_ISL_433977, EPI_ISL_433978, EPI_ISL_433980, EPI_ISL_433981, EPI_ISL_433982, EPI_ISL_433983, EPI_ISL_433986, EPI_ISL_433988, EPI_ISL_433989, EPI_ISL_433990, EPI_ISL_433991, EPI_ISL_433992, EPI_ISL_433995, EPI_ISL_433996, EPI_ISL_433997, EPI_ISL_433999, EPI_ISL_434000, EPI_ISL_434001, EPI_ISL_434003, EPI_ISL_434004, EPI_ISL_434005, EPI_ISL_434006, EPI_ISL_434008, EPI_ISL_434009, EPI_ISL_434010, EPI_ISL_434011, EPI_ISL_434012, EPI_ISL_434013, EPI_ISL_434015, EPI_ISL_434016, EPI_ISL_434017, EPI_ISL_434018, EPI_ISL_434019, EPI_ISL_434020, EPI_ISL_434021, EPI_ISL_434022, EPI_ISL_434023, EPI_ISL_434024, EPI_ISL_434025, EPI_ISL_434026, EPI_ISL_434027, EPI_ISL_434028, EPI_ISL_434029, EPI_ISL_434030, EPI_ISL_434031, EPI_ISL_434032, EPI_ISL_434033, EPI_ISL_434034, EPI_ISL_434035, EPI_ISL_434036, EPI_ISL_434037, EPI_ISL_434038, EPI_ISL_434039, EPI_ISL_434040, EPI_ISL_434041, EPI_ISL_434042, EPI_ISL_434043, EPI_ISL_434044, EPI_ISL_434045, EPI_ISL_434046, EPI_ISL_434047, EPI_ISL_434048, EPI_ISL_434049, EPI_ISL_434050, EPI_ISL_434051, EPI_ISL_434052, EPI_ISL_434053, EPI_ISL_434054, EPI_ISL_434055, EPI_ISL_434056, EPI_ISL_434057, EPI_ISL_434058, EPI_ISL_434059, EPI_ISL_434060, EPI_ISL_434061, EPI_ISL_434062			
see above	Department of Pathology, University of Cambridge	COVID-19 Genomics UK (COG-UK) Consortium	Luke W Meredith, M. Estee Torok , Myra Hosmillo, William L. Hamilton, Martin D. Curran, Theresa Feltwell, Grant Hall, Anna Yakovleva, Fahad A Khokhar, Charlotte J. Houldcroft, Laura G Caller, Aminu S. Jahun, Sarah L. Caddy, Ian Goodfellow
EPI_ISL_434358	Lab voor klinische biologie	Onderzoeksgroep Virologie	Nick Vereecke, Laurens Lambrechts, Marthe Pauwels, Jozefien De Clercq, Bruno Verhasselt, Linos Vandekerckhove, Hans Nauwynck, Sebastiaan Theuns
EPI_ISL_434378, EPI_ISL_434381	Hospital AZ Rivierenland	Institute of Tropical Medicine	Philippe Selhorst, Colin Anthony,
EPI_ISL_434384	Hospital AZ Rivierenland	Institute of Tropical Medicine	Philippe Selhorst, Colin Anthony
EPI_ISL_434487, EPI_ISL_434489, EPI_ISL_434491, EPI_ISL_434498, EPI_ISL_434499, EPI_ISL_434500, EPI_ISL_434501, EPI_ISL_434502, EPI_ISL_434504, EPI_ISL_434506, EPI_ISL_434510, EPI_ISL_434512, EPI_ISL_434513, EPI_ISL_434514, EPI_ISL_434515			
see above	Laboratoire National de Sante, Microbiology, Virology	Laboratoire National de Sante, Microbiology, Epidemiology and Microbial Genomics	Anke Wienecke-Baldacchino, Ardashel Latsuzbaia, Jessica Tapp, Catherine Ragimbeau, Guillaume Fournier, Tamir Abdelrahman, Trung Nguyen Nguyen, Joel Mossong
EPI_ISL_434572	The National Institute of Public Health Center for Epidemiology and Microbiology	The National Institute of Public Health Center for Epidemiology and Microbiology	Alexander Nagy, Helena Jirincova, Ludmila Novakova, Dusan Trnka, Jaromira Vecerova
EPI_ISL_434597, EPI_ISL_434598, EPI_ISL_434599, EPI_ISL_434600	Virginia DCLS	Virginia DCLS	Virginia DCLS
EPI_ISL_434667	Ulltuna Vardcentral	The Public Health Agency of Sweden	Heidi Lindback, Oskar Karlsson Lindsjo, Maria Lind Karlberg, Anna-Malin Linde, Olov Svartstrom, Anna Risberg, Theresa Enkirch, Mia Brytting, Karin Tegmark-Wisell
EPI_ISL_434668	Kungsors VC	The Public Health Agency of Sweden	Jessica Karlsson, Oskar Karlsson Lindsjo, Maria Lind Karlberg, Anna-Malin Linde, Olov Svartstrom, Anna Risberg, Theresa Enkirch, Mia Brytting, Karin Tegmark-Wisell
EPI_ISL_434669	Lakargruppen	The Public Health Agency of Sweden	Boris Klanger, Oskar Karlsson Lindsjo, Maria Lind Karlberg, Anna-Malin Linde, Olov Svartstrom, Anna Risberg, Theresa Enkirch, Mia Brytting, Karin Tegmark-Wisell
EPI_ISL_434670	Narhalsan Molnlycke, Barn och ungdomsmedicin	The Public Health Agency of Sweden	Mats Reimer, Oskar Karlsson Lindsjo, Maria Lind Karlberg, Anna-Malin Linde, Olov Svartstrom, Anna Risberg, Theresa Enkirch, Mia Brytting, Karin Tegmark-Wisell
EPI_ISL_434671	Narhalsan Sjobo vardcentral	The Public Health Agency of Sweden	Lovisa Hjerten, Oskar Karlsson Lindsjo, Maria Lind Karlberg, Anna-Malin Linde, Olov Svartstrom, Anna Risberg, Theresa Enkirch, Mia Brytting, Karin Tegmark-Wisell
EPI_ISL_434672	Surbrunns VC	The Public Health Agency of Sweden	Erik Embring, Oskar Karlsson Lindsjo, Maria Lind Karlberg, Anna-Malin Linde, Olov Svartstrom, Anna Risberg, Theresa Enkirch, Mia Brytting, Karin Tegmark-Wisell
EPI_ISL_434673	Omtanken Grimmered	The Public Health Agency of Sweden	Bernd Sengpiel, Oskar Karlsson Lindsjo, Maria Lind Karlberg, Anna-Malin Linde, Olov Svartstrom, Anna Risberg, Theresa Enkirch, Mia Brytting, Karin Tegmark-Wisell
EPI_ISL_435037, EPI_ISL_435038, EPI_ISL_435039, EPI_ISL_435040, EPI_ISL_435041, EPI_ISL_435042, EPI_ISL_435043, EPI_ISL_435044	LSUHS Emerging Viral Threat Laboratory	Microbial Genome Sequencing Center	Jeremy P. Kamil, John A. Vanchiere, Rona S. Scott, Camille F. Abshire, Abida Siddiqa, Byeong-Jae Lee, Chan-ki Min, Md Maksudul Alam, Monica Gestal-Cartele, Edna Ondari, Adam Greer, Malgorzata Bienkowska-Haba, Katarzyna Zwolinska, Jason M. Bodily, Andrew D. Yurochko, Paul M. Weinberger, Christopher G. Kevil, Martin J. Sapp, Daniel J. Snyder, Vaughn S. Cooper
EPI_ISL_435049	B.J. Medical College and Civil hospital	Gujarat Biotechnology Research Centre	Pinal Trivedi, Maharshi Pandya, Amit Kanani, Akanksha Verma, Nitin Savaliya, Raghawendra Kumar, Dinesh Kumar, Zuber Saiyed, Dipa Kinariwala, Disha Patel, Binita Aring, Geeta Vaghela, Sonia Barve, Bhavesh Modi, Kairavi Joshi, Gaurishankar Shrimali, Nidhi Sood, Pranay Shah, R D Dixit, Snehal Bagatharia, Kamlesh J Upadhyay, Ramesh Pandit, Tejas Shah, Ankit Hinsu, Pritesh Sabara, Apurvasinh Puvar, Janvi Raval, Monika Gandhi, Neha Rajpara, Chaitanya Joshi, Madhvi Joshi
EPI_ISL_435050	B.J. Medical College and Civil hospital	Gujarat Biotechnology Research Centre	Ankit Hinsu, Pritesh Sabara, Apurvasinh Puvar, Janvi Raval, Monika Gandhi, Pinal Trivedi, Maharshi Pandya, Amit Kanani, Akanksha Verma, Nitin Savaliya, Raghawendra Kumar, Dinesh Kumar, Zuber Saiyed, Dipa Kinariwala, Disha Patel, Binita Aring, Geeta Vaghela, Sonia Barve, Bhavesh Modi, Kairavi Joshi, Gaurishankar Shrimali, Nidhi Sood, Pranay Shah, R D Dixit, Snehal Bagatharia, Kamlesh J Upadhyay, Ramesh Pandit, Tejas Shah, Dipeshwari Shewale, Chaitanya Joshi, Madhvi Joshi
EPI_ISL_435051	B.J. Medical College and Civil hospital	Gujarat Biotechnology Research Centre	Pritesh Sabara, Apurvasinh Puvar, Janvi Raval, Monika Gandhi, Pinal Trivedi, Maharshi Pandya, Amit Kanani, Akanksha Verma, Nitin Savaliya, Raghawendra Kumar, Dinesh Kumar, Zuber Saiyed, Dipa Kinariwala, Disha Patel, Binita Aring, Geeta Vaghela, Sonia Barve, Bhavesh Modi, Kairavi Joshi, Gaurishankar Shrimali, Nidhi Sood, Pranay Shah, R D Dixit, Snehal Bagatharia, Kamlesh J Upadhyay, Ramesh Pandit, Tejas Shah, Ankit Hinsu, Vasudha Sharma, Chaitanya Joshi, Madhvi Joshi
EPI_ISL_435052	B.J. Medical College and Civil hospital	Gujarat Biotechnology Research Centre	Apurvasinh Puvar, Janvi Raval, Monika Gandhi, Pinal Trivedi, Maharshi Pandya, Amit Kanani, Akanksha Verma, Nitin Savaliya, Raghawendra Kumar, Dinesh Kumar, Zuber Saiyed, Dipa Kinariwala, Disha Patel, Binita Aring, Geeta Vaghela, Sonia Barve, Bhavesh Modi, Kairavi Joshi, Gaurishankar Shrimali, Nidhi Sood, Pranay Shah, R D Dixit, Snehal Bagatharia, Kamlesh J Upadhyay, Ramesh Pandit, Tejas Shah, Ankit Hinsu, Pritesh Sabara, Pooja P Doshi, Chaitanya Joshi, Madhvi Joshi
EPI_ISL_435054	B.J. Medical College and Civil hospital	Gujarat Biotechnology Research Centre	Monika Gandhi, Pinal Trivedi, Maharshi Pandya, Amit Kanani, Akanksha Verma, Nitin Savaliya, Raghawendra Kumar, Dinesh Kumar, Zuber Saiyed, Dipa Kinariwala, Disha Patel, Binita Aring, Geeta Vaghela, Sonia Barve, Bhavesh Modi, Kairavi Joshi, Gaurishankar Shrimali, Nidhi Sood, Pranay Shah, R D Dixit, Snehal Bagatharia, Kamlesh J Upadhyay, Ramesh Pandit, Tejas Shah, Ankit Hinsu, Pritesh Sabara, Apurvasinh Puvar, Janvi Raval, Priti Pandita, Chaitanya Joshi, Madhvi Joshi
EPI_ISL_435165, EPI_ISL_435166, EPI_ISL_435167, EPI_ISL_435168	Viral Respiratory Lab, National Institute for Biomedical Research (INRB)	Pathogen Sequencing Lab, National Institute for Biomedical Research (INRB)	Placide Mbala-Kingebeni, Edith Nkwembe, Eddy Kinganda-Lusamaki, Amuri Aziza, Francisca Muyembe Mawete, Catherine Pratt, Matthias Pauthner, Josh Quick, Allison Black, James Hadfield, Trevor Bedford, Ian Goodfellow, Andrew Rambaut, Nick Loman, Kristian Andersen, Michael Wiley, Steve Ahuka-Mundeke, Jean-Jacques Muyembe Tamfum
EPI_ISL_435493, EPI_ISL_435494, EPI_ISL_435495, EPI_ISL_435496, EPI_ISL_435501, EPI_ISL_435504, EPI_ISL_435505, EPI_ISL_435506, EPI_ISL_435507, EPI_ISL_435508, EPI_ISL_435509, EPI_ISL_435510, EPI_ISL_435511, EPI_ISL_435512, EPI_ISL_435513, EPI_ISL_435514, EPI_ISL_435529, EPI_ISL_435530, EPI_ISL_435531, EPI_ISL_435532, EPI_ISL_435533, EPI_ISL_435535			
see above	NYU Langone Health	Departments of Pathology and Medicine, New York University School of Medicine	Maria Aguero-Rosenfeld, Brendan Belovarac, Margaret Black, Ludovic Boytard, John Cadley, Paolo Cotzia, John Chen, Dacia Dimartino, Xiaojun Feng, Tatyana Gindin, Emily Guzman, Adriana Heguy, Megan Hogan, Emily Huang, George Jour, Lawrence H. Lin, Raven Luther, Andrew Lytle, Christian Marier, Matthew T. Maurano, Mark J. Mulligan, Peter Meyn, Raquel Ordonez Ciriza, Iman Osman, Jared Pinnell, Vanessa Raabe, Sitharam Ramaswami, Amy Rapkiewicz, Andre M. Ribeiro-dos-Santos, Marie Samanovic-Golden, Antonio Serrano, Guomiao Shen, Matija Snuderl, Theodore Vougiouklakis, Nick Vulpescu, Gael Westby, Paul Zappile, Yutong Zhang
EPI_ISL_435686, EPI_ISL_435687, EPI_ISL_435688, EPI_ISL_435689, EPI_ISL_435690	National Public Health Laboratory, National Centre for Infectious Diseases	National Public Health Laboratory, National Centre for Infectious Diseases	Mak Tze Minn, Octavia Sophie, Chavatte Jean-Marc, Cui Lin, Lin Raymond Tzer Pin
EPI_ISL_435722	NYU Langone Health	Departments of Pathology and Medicine, New York University School of Medicine	Maria Aguero-Rosenfeld, Brendan Belovarac, Margaret Black, Ludovic Boytard, John Cadley, Paolo Cotzia, John Chen, Dacia Dimartino, Xiaojun Feng, Tatyana Gindin, Emily Guzman, Adriana Heguy, Megan Hogan, Emily Huang, George Jour, Lawrence H. Lin, Raven Luther, Andrew Lytle, Christian Marier,

			Matthew T. Maurano, Mark J. Mulligan, Peter Meyn, Raquel Ordonez Ciriza, Iman Osman, Jared Pinnell, Vanessa Raabe, Sitharam Ramaswami, Amy Rapkiewicz, Andre M. Ribeiro-dos-Santos, Marie Samanovic-Golden, Antonio Serrano, Guomiao Shen, Matija Snuderl, Theodore Vougiouklakis, Nick Vulpescu, Gael Westby, Paul Zappile, Yutong Zhang
EPI_ISL_436127, EPI_ISL_436128, EPI_ISL_436129, EPI_ISL_436130, EPI_ISL_436131, EPI_ISL_436132	Victorian Infectious Diseases Reference Laboratory (VIDRL)	Microbiological Diagnostic Unit Public Health Laboratory and Victorian Infectious Diseases Reference Laboratory, Doherty Institute	Caly L., Seemann T., Sait, M., Schultz M., Druce J., Sherry, N.
EPI_ISL_436156, EPI_ISL_436157	District Surveillance Unit	Department of Neurovirology, National Institute of Mental Health and Neuroscience (NIMHANS)	Chitra Pattabiraman, Vijayalakshmi Reddy, Harsha PK, Risha Rasheed, Shafeeq S Hameed, Manjunatha Venkataswamy, Anita Desai, Ravi Vasanthapuram
EPI_ISL_436194, EPI_ISL_436412	Viral Respiratory Lab, National Institute for Biomedical Research (INRB)	Pathogen Sequencing Lab, National Institute for Biomedical Research (INRB)	Placide Mbala-Kingebeni, Edith Nkwembe, Eddy Kinganda-Lusamaki, Amuri Aziza, Francisca Muyembe Mawete, Catherine Pratt, Matthias Pauthner, Josh Quick, Allison Black, James Hadfield, Trevor Bedford, Ian Goodfellow, Andrew Rambaut, Nick Loman, Kristian Andersen, Michael Wiley, Steve Ahuka-Mundeke, Jean-Jacques Muyembe Tamfum
EPI_ISL_436450, EPI_ISL_436451, EPI_ISL_436452, EPI_ISL_436453, EPI_ISL_436454, EPI_ISL_436455, EPI_ISL_436458, EPI_ISL_436462, EPI_ISL_436463	National Centre for Disease control (NCDC)	NCDC/CSIR-IGIB	Pramod Kumar#, Rajesh Pandey#, Pooja Sharma, Mahesh S Dhar, Vivekanand A, Bharathram Uppili, Himanshu Vashisht, Saruchi Wadhwa, Nishu Tyagi, Uma Sharma, Priyanka Singh, Hemlata Lall, Meena Datta, Poonam Gupta, Nidhi Saini, Aarti Tewari, Bibhash Nandi, Dhirendra Kumar, Satyabrata Bag, Varun Jaiswal, Hema Gogia, Preeti Madan, Simrita Singh, Prateek Singh, Debasis Dash, Mitali Mukerji, Manju Bala, Sandhya Kabra, Sujeet Singh, Mohammed Faruq, Anurag Agrawal*, Partha Rakshit*
EPI_ISL_436523, EPI_ISL_436524, EPI_ISL_436525, EPI_ISL_436526, EPI_ISL_436527, EPI_ISL_436528, EPI_ISL_436529, EPI_ISL_436530, EPI_ISL_436531, EPI_ISL_436532, EPI_ISL_436533, EPI_ISL_436534, EPI_ISL_436535, EPI_ISL_436536, EPI_ISL_436537, EPI_ISL_436538, EPI_ISL_436539, EPI_ISL_436540, EPI_ISL_436541, EPI_ISL_436542, EPI_ISL_436543, EPI_ISL_436544, EPI_ISL_436545, EPI_ISL_436546, EPI_ISL_436547, EPI_ISL_436548, EPI_ISL_436549, EPI_ISL_436550, EPI_ISL_436551, EPI_ISL_436552, EPI_ISL_436553, EPI_ISL_436554, EPI_ISL_436555, EPI_ISL_436556, EPI_ISL_436557, EPI_ISL_436558			
see above	Florida Bureau of Public Health Laboratories	Florida Bureau of Public Health Laboratories	Sarah Schmedes, Jason Blanton
EPI_ISL_436591, EPI_ISL_436592, EPI_ISL_436593, EPI_ISL_436594, EPI_ISL_436595, EPI_ISL_436596, EPI_ISL_436597, EPI_ISL_436598, EPI_ISL_436599, EPI_ISL_436600, EPI_ISL_436601, EPI_ISL_436602, EPI_ISL_436603, EPI_ISL_436604, EPI_ISL_436605, EPI_ISL_436607, EPI_ISL_436608, EPI_ISL_436609, EPI_ISL_436610, EPI_ISL_436611, EPI_ISL_436612			
see above	University of Wisconsin-Madison AIDS Vaccine Research Laboratories	University of Wisconsin-Madison AIDS Vaccine Research Laboratories	Gage Moreno, Katarina Braun, et al. AIDS Vaccine Research Laboratories
EPI_ISL_436668, EPI_ISL_436669, EPI_ISL_436670, EPI_ISL_436671	County of Santa Clara Public Health Department	Chan-Zuckerberg Biohub	CZB Cliahub Consortium
EPI_ISL_436715, EPI_ISL_436716, EPI_ISL_436717	Genomics and Computational Biology Lab, Scientific Research Institute of Physical-Chemical Medicine, FMBA of Russia	Genomics and Computational Biology Lab, Scientific Research Institute of Physical-Chemical Medicine, FMBA of Russia	A. Pavlenko, O. Guskova, K. Klimina, V. Veselovsky, A. Manolov, D. Fedorov, V. Govorun and E. Ilina
EPI_ISL_436918, EPI_ISL_436919, EPI_ISL_436920, EPI_ISL_436921, EPI_ISL_436922, EPI_ISL_436923, EPI_ISL_436924, EPI_ISL_436925	Utah Public Health Laboratory	Utah Public Health Laboratory	Erin Young, Kelly Oakeson
EPI_ISL_436926	x^2	Utah Public Health Laboratory	Erin Young, Kelly Oakeson
EPI_ISL_436927, EPI_ISL_436928, EPI_ISL_436929, EPI_ISL_436930, EPI_ISL_436931, EPI_ISL_436932, EPI_ISL_436933, EPI_ISL_436934, EPI_ISL_436935, EPI_ISL_436936, EPI_ISL_436937, EPI_ISL_436938			
see above	Utah Public Health Laboratory	Utah Public Health Laboratory	Erin Young, Kelly Oakeson
EPI_ISL_437065, EPI_ISL_437066, EPI_ISL_437067	County of Santa Clara Public Health	Chan-Zuckerberg Biohub	CZB Cliahub Consortium
EPI_ISL_437188	RSUD Dr. Soetomo	Institute of Tropical Disease, Universitas Airlangga	Krisnoadi Rahardjo, Aldise M Nastri, Jezzy R Dewantari, Rima R Prasetya, Joni Wahyuhadi, Gatot Soegiarto, Laksmi Wulandari, Retno A Setyoningrum, Resti Y Meliana, Yohko K Shimizu, Mitsuhiro Nishimura, Yasuko Mori, Soetjipto, Kazufumi Shimizu, Maria I Lusida
EPI_ISL_437193, EPI_ISL_437194, EPI_ISL_437195, EPI_ISL_437196	Viral Respiratory Lab, National Institute for Biomedical Research (INRB)	Pathogen Sequencing Lab, National Institute for Biomedical Research (INRB)	Placide Mbala-Kingebeni, Edith Nkwembe, Eddy Kinganda-Lusamaki, Amuri Aziza, Francisca Muyembe Mawete, Catherine Pratt, Matthias Pauthner, Josh Quick, Allison Black, James Hadfield, Trevor Bedford, Ian Goodfellow, Andrew Rambaut, Nick Loman, Kristian Andersen, Michael Wiley, Steve Ahuka-Mundeke, Jean-Jacques Muyembe Tamfum
EPI_ISL_437209, EPI_ISL_437227, EPI_ISL_437296, EPI_ISL_437297	Max von Pettenkofer Institute, Virology, National Reference Center for Retroviruses, LMU München	Laboratory for Functional Genome Analysis, Dept. Genomics, Gene Center of the LMU Munich	Max Muenchhoff, Stefan Krebs, Alexander Graf, Oliver Keppler, Helmut Blum
EPI_ISL_437300, EPI_ISL_437301, EPI_ISL_437302, EPI_ISL_437303	Diagnostic- and Research Institute of Pathology, Medical University of Graz	Diagnostic- and Research Institute of Pathology, Medical University of Graz	Karl Kashofer, Peter Regitnig, Martin Zacharias, Gregor Gorkiewicz
EPI_ISL_437337, EPI_ISL_437338, EPI_ISL_437339, EPI_ISL_437340, EPI_ISL_437341, EPI_ISL_437342, EPI_ISL_437343, EPI_ISL_437344, EPI_ISL_437345, EPI_ISL_437346, EPI_ISL_437347, EPI_ISL_437348			
see above	Viral Respiratory Lab, National Institute for Biomedical Research (INRB)	Pathogen Sequencing Lab, National Institute for Biomedical Research (INRB)	Placide Mbala-Kingebeni, Edith Nkwembe, Eddy Kinganda-Lusamaki, Amuri Aziza, Francisca Muyembe Mawete, Catherine Pratt, Matthias Pauthner, Josh Quick, Allison Black, James Hadfield, Trevor Bedford, Ian Goodfellow, Andrew Rambaut, Nick Loman, Kristian Andersen, Michael Wiley, Steve Ahuka-Mundeke, Jean-Jacques Muyembe Tamfum
EPI_ISL_437349	Ecole nationale vétérinaire d'Alfort-laboratoire de santé animale Anses UMR 1161 de virologie ENVA-Anses-INRAE	Institut Pasteur CIBU-ERI	Sophie Le Poder, Corinne Sailleau, Marine Dumarest, Bernard Klonjkowski, Stéphan Zientara
EPI_ISL_437350, EPI_ISL_437351, EPI_ISL_437352, EPI_ISL_437353, EPI_ISL_437354, EPI_ISL_437355, EPI_ISL_437356, EPI_ISL_437357, EPI_ISL_437358	Viral Respiratory Lab, National Institute for Biomedical Research (INRB)	Pathogen Sequencing Lab, National Institute for Biomedical Research (INRB)	Placide Mbala-Kingebeni, Edith Nkwembe, Eddy Kinganda-Lusamaki, Amuri Aziza, Francisca Muyembe Mawete, Catherine Pratt, Matthias Pauthner, Josh Quick, Allison Black, James Hadfield, Trevor Bedford, Ian Goodfellow, Andrew Rambaut, Nick Loman, Kristian Andersen, Michael Wiley, Steve Ahuka-Mundeke, Jean-Jacques Muyembe Tamfum
EPI_ISL_437378, EPI_ISL_437379, EPI_ISL_437380, EPI_ISL_437381, EPI_ISL_437382, EPI_ISL_437383, EPI_ISL_437384, EPI_ISL_437385, EPI_ISL_437386	Minnesota Department of Health, Public Health Laboratory	Minnesota Department of Health, Public Health Laboratory	Matt Plumb, Jacob Garfin, and Xiong Wang
EPI_ISL_437387, EPI_ISL_437388, EPI_ISL_437389, EPI_ISL_437390, EPI_ISL_437391, EPI_ISL_437392, EPI_ISL_437403, EPI_ISL_437404, EPI_ISL_437405, EPI_ISL_437406, EPI_ISL_437407, EPI_ISL_437408, EPI_ISL_437409, EPI_ISL_437410, EPI_ISL_437417, EPI_ISL_437418, EPI_ISL_437419, EPI_ISL_437420, EPI_ISL_437424, EPI_ISL_437425, EPI_ISL_437430			
see above	Virginia DCLS	Virginia DCLS	Virginia DCLS
EPI_ISL_437433	Bozeman Health Deaconess Hospital	Wiedenheft lab, Montana State University	Artem Nemudryi, Anna Nemudraia, Kevin Surya, Tanner Wiegand, Murat Buyukyoruk, Royce Wilkinson, Blake Wiedenheft
EPI_ISL_437524, EPI_ISL_437528, EPI_ISL_437530, EPI_ISL_437531, EPI_ISL_437532, EPI_ISL_437533, EPI_ISL_437534	OHSU Lab Services Molecular Microbiology Lab	Oregon SARS-CoV-2 Genome Sequencing Center	Brendan L. O'Connell, Ruth V. Nichols, Alec J. Hirsch, Guang Fan, Daniel N. Streblow, William B. Messer, Andrew C. Adey, Benjamin N. Bimber, Brian J. O'Roak
EPI_ISL_437578, EPI_ISL_437580	Scripps Medical Laboratory	Andersen lab at Scripps Research	SEARCH Alliance San Diego with Michael Quigley, Ellen Stefanski, Ian Mchardy
EPI_ISL_437765, EPI_ISL_437766, EPI_ISL_437769, EPI_ISL_437770, EPI_ISL_437771, EPI_ISL_437772, EPI_ISL_437773, EPI_ISL_437774, EPI_ISL_437775, EPI_ISL_437776, EPI_ISL_437777, EPI_ISL_437778, EPI_ISL_437779, EPI_ISL_437780, EPI_ISL_437781, EPI_ISL_437782, EPI_ISL_437783, EPI_ISL_437784, EPI_ISL_437785, EPI_ISL_437790, EPI_ISL_437791, EPI_ISL_437792, EPI_ISL_437793, EPI_ISL_437794, EPI_ISL_437795, EPI_ISL_437801, EPI_ISL_437802			

see above	Virginia DCLS	Virginia DCLS	Virginia DCLS
EPI_ISL_437912	Child Health Research Foundation	Child Health Research Lab	Senjuti Saha, Roly Malaker, Md Saiful Islam Sajib, Md Hasanuzzaman, Md Hafizur Rahman, Md Shahidul Islam, Zabed B Ahmed, Maksuda Islam, Samir K Saha
EPI_ISL_438149, EPI_ISL_438150, EPI_ISL_438151, EPI_ISL_438153, EPI_ISL_438156, EPI_ISL_438157, EPI_ISL_438158, EPI_ISL_438161	Seattle Flu Study	Seattle Flu Study	Chu et al
EPI_ISL_438176, EPI_ISL_438177, EPI_ISL_438178, EPI_ISL_438179, EPI_ISL_438180, EPI_ISL_438181, EPI_ISL_438182, EPI_ISL_438183, EPI_ISL_438184, EPI_ISL_438185, EPI_ISL_438186, EPI_ISL_438187, EPI_ISL_438188, EPI_ISL_438189, EPI_ISL_438190, EPI_ISL_438191, EPI_ISL_438192, EPI_ISL_438193, EPI_ISL_438194, EPI_ISL_438195, EPI_ISL_438196, EPI_ISL_438197, EPI_ISL_438198, EPI_ISL_438199, EPI_ISL_438200, EPI_ISL_438201, EPI_ISL_438202, EPI_ISL_438206, EPI_ISL_438208			
see above	Washington State Department of Health	Seattle Flu Study	Chu et al
EPI_ISL_438550, EPI_ISL_438551, EPI_ISL_438552, EPI_ISL_438560, EPI_ISL_438565, EPI_ISL_438567, EPI_ISL_438569, EPI_ISL_438571, EPI_ISL_438573, EPI_ISL_438579	Department of Pathology, University of Cambridge	COVID-19 Genomics UK (COG-UK) Consortium	Luke W Meredith, M. Est©e Trk , Myra Hosmillo, William L. Hamilton, Martin D. Curran, Theresa Feltwell, Grant Hall, Anna Yakovleva, Fahad A Khokhar, Charlotte J. Houldcroft, Laura G Caller, Aminu S. Jahun, Sarah L. Caddy, Ian Goodfellow
EPI_ISL_438887, EPI_ISL_438890, EPI_ISL_438891, EPI_ISL_438892, EPI_ISL_438893, EPI_ISL_438894, EPI_ISL_438895, EPI_ISL_438897, EPI_ISL_438899, EPI_ISL_438900, EPI_ISL_438901, EPI_ISL_438902, EPI_ISL_438903, EPI_ISL_438905, EPI_ISL_438907, EPI_ISL_438908, EPI_ISL_438910, EPI_ISL_438912, EPI_ISL_438915, EPI_ISL_438916, EPI_ISL_438917, EPI_ISL_438918, EPI_ISL_438920, EPI_ISL_438921, EPI_ISL_438923, EPI_ISL_438924, EPI_ISL_438925, EPI_ISL_438929, EPI_ISL_438931, EPI_ISL_438932, EPI_ISL_438934, EPI_ISL_438935, EPI_ISL_438937, EPI_ISL_438938, EPI_ISL_438939, EPI_ISL_438940, EPI_ISL_438941, EPI_ISL_438942, EPI_ISL_438943, EPI_ISL_438944, EPI_ISL_438945, EPI_ISL_438946			
see above	West of Scotland Specialist Virology Centre, NHSGGC / MRC-University of Glasgow Centre for Virus Research	COVID-19 Genomics UK (COG-UK) Consortium	Ana da Silva Filipe, Natasha Johnson, Kathy Smollett, Daniel Mair, Stephen Carmichael, Lily Tong, Jenna Nichols, Elihu Aranday-Cortes, Kirstyn Brunker, Yasmin Parr, Kyriaki Nomikou; Sarah McDonald, Marc Niebel, Patawee Asamaphan; Richard Orton, Joseph Hughes, Sreenu Vattipally, David L Robertson; Alasdair MacLean, Rory Gunson; Kathy Li, Natasha Jesudason, Rajiv Shah, James Shepherd, Antonia Ho, Emma Thomson
EPI_ISL_438962, EPI_ISL_438963, EPI_ISL_438964, EPI_ISL_438965, EPI_ISL_438966, EPI_ISL_438967	Keio University School of Medicine	Keio University School of Medicine	Kenjiro Kosaki
EPI_ISL_438973, EPI_ISL_438974, EPI_ISL_438975, EPI_ISL_438979	West of Scotland Specialist Virology Centre, NHSGGC / MRC-University of Glasgow Centre for Virus Research	COVID-19 Genomics UK (COG-UK) Consortium	Ana da Silva Filipe, Natasha Johnson, Kathy Smollett, Daniel Mair, Stephen Carmichael, Lily Tong, Jenna Nichols, Elihu Aranday-Cortes, Kirstyn Brunker, Yasmin Parr, Kyriaki Nomikou; Sarah McDonald, Marc Niebel, Patawee Asamaphan; Richard Orton, Joseph Hughes, Sreenu Vattipally, David L Robertson; Alasdair MacLean, Rory Gunson; Kathy Li, Natasha Jesudason, Rajiv Shah, James Shepherd, Antonia Ho, Emma Thomson
EPI_ISL_439149, EPI_ISL_439150, EPI_ISL_439151, EPI_ISL_439152, EPI_ISL_439160, EPI_ISL_439268, EPI_ISL_439269, EPI_ISL_439271, EPI_ISL_439272, EPI_ISL_439273, EPI_ISL_439274, EPI_ISL_439275, EPI_ISL_439276, EPI_ISL_439277, EPI_ISL_439278, EPI_ISL_439279, EPI_ISL_439280, EPI_ISL_439281, EPI_ISL_439282, EPI_ISL_439283, EPI_ISL_439284, EPI_ISL_439285, EPI_ISL_439286, EPI_ISL_439287			
see above	Virology Department, Royal Infirmary of Edinburgh, NHS Lothian / School of Biological Sciences, University of Edinburgh / Institute of Genetics and Molecular Medicine, University of Edinburgh	COVID-19 Genomics UK (COG-UK) Consortium	McHugh M, Dewar R, Rooke S, Gallagher M, Balcaza C, O'ÄôToole Å, Scher E, Hill V, McCrone JT, Colquhoun R, Yu X, Jackson B, Rambaut A, Williams TC, Templeton K
EPI_ISL_439786, EPI_ISL_439790, EPI_ISL_439791, EPI_ISL_439794, EPI_ISL_439795, EPI_ISL_439801, EPI_ISL_439803, EPI_ISL_439804, EPI_ISL_439806, EPI_ISL_439808, EPI_ISL_439810, EPI_ISL_439811, EPI_ISL_439813, EPI_ISL_439814, EPI_ISL_439815, EPI_ISL_439816, EPI_ISL_439817, EPI_ISL_439818, EPI_ISL_439819, EPI_ISL_439820, EPI_ISL_439822, EPI_ISL_439823, EPI_ISL_439824, EPI_ISL_439825, EPI_ISL_439826, EPI_ISL_439827, EPI_ISL_439828, EPI_ISL_439829, EPI_ISL_439830, EPI_ISL_439831, EPI_ISL_439832, EPI_ISL_439833, EPI_ISL_439834, EPI_ISL_439835, EPI_ISL_439836, EPI_ISL_439837, EPI_ISL_439838, EPI_ISL_439839, EPI_ISL_439840, EPI_ISL_439841, EPI_ISL_439842, EPI_ISL_439843, EPI_ISL_439844, EPI_ISL_439845, EPI_ISL_439846, EPI_ISL_439847, EPI_ISL_439848, EPI_ISL_439849, EPI_ISL_439850, EPI_ISL_439851, EPI_ISL_439852, EPI_ISL_439853, EPI_ISL_439854, EPI_ISL_439855, EPI_ISL_439856, EPI_ISL_439857, EPI_ISL_439858, EPI_ISL_439859, EPI_ISL_439860, EPI_ISL_439861, EPI_ISL_439862, EPI_ISL_440852, EPI_ISL_440853, EPI_ISL_440854, EPI_ISL_440855, EPI_ISL_440856, EPI_ISL_440857, EPI_ISL_440858, EPI_ISL_440859, EPI_ISL_440860, EPI_ISL_440861, EPI_ISL_440862, EPI_ISL_440863, EPI_ISL_440868, EPI_ISL_440884, EPI_ISL_440885, EPI_ISL_440886, EPI_ISL_440887, EPI_ISL_440888, EPI_ISL_440889, EPI_ISL_440890, EPI_ISL_440893, EPI_ISL_440894, EPI_ISL_440895, EPI_ISL_440896, EPI_ISL_440912, EPI_ISL_440914, EPI_ISL_440915, EPI_ISL_440918, EPI_ISL_440919, EPI_ISL_440920, EPI_ISL_440921			
see above	Liverpool Clinical Laboratories	COVID-19 Genomics UK (COG-UK) Consortium	Sam Haldenby, Anita Lucaci, Steve Paterson, Julian Hiscox, Alistair Darby, M Almsaud, A Alrezaihi, Muhannad Alruwaili, Stuart D Armstrong, Jones Benjamin , Eleanor G Bentley, Anu Chawla, Jordan J Clark, Angela Cowell, Richard Eccles, Isabel Garca-Dorival, Matthew Gemmell, Alessandro Gerada, PKF Gilmore, Richard Gregory, Ximeng Han, Catherine Hartley, Margaret Hughes, Miren Iturriza-Gomara, James Johnson, L Luu, Jenifer Manson , Charlotte Nelson, Elaine O'ÄôToole, Cassie Olateju, Rebekah Penrice-Randal¬†, Lucille Rainbow, N.P Randle, Trevor Ian Robinson, Parul Sharma, Ghada T Shawli, James P Stewart , Neil Swainston, Ecaterina Vamos, Joanne Watts, Mark Whitehead
EPI_ISL_441461, EPI_ISL_441462, EPI_ISL_441463, EPI_ISL_441464, EPI_ISL_441465, EPI_ISL_441504, EPI_ISL_441505, EPI_ISL_441506, EPI_ISL_441507, EPI_ISL_441508, EPI_ISL_441509, EPI_ISL_441510, EPI_ISL_441511, EPI_ISL_441512, EPI_ISL_441513, EPI_ISL_441514, EPI_ISL_441515, EPI_ISL_441521, EPI_ISL_441540, EPI_ISL_441541, EPI_ISL_441542, EPI_ISL_441543, EPI_ISL_441847, EPI_ISL_441849, EPI_ISL_441850			
see above	Queens Medical Centre, Clinical Microbiology Department / DeepSeq Nottingham	COVID-19 Genomics UK (COG-UK) Consortium	Gemma Clark, Wendy Smith, Manjinder Khakh, Hannah Howson-Wells, Jonathan Ball, Patrick McClure, Joseph Chappell, Theocharis Tsoleridis, Nadine Holmes, Matthew Carlisle, Christopher Moore, Fei Sang, Johnny Debebe, Victoria Wright, Matthew Loose
EPI_ISL_441931, EPI_ISL_441944, EPI_ISL_441948, EPI_ISL_441969, EPI_ISL_441972, EPI_ISL_441986, EPI_ISL_441989, EPI_ISL_441990, EPI_ISL_441994, EPI_ISL_442010, EPI_ISL_442020, EPI_ISL_442032, EPI_ISL_442040, EPI_ISL_442042, EPI_ISL_442361, EPI_ISL_442367, EPI_ISL_442406, EPI_ISL_442487, EPI_ISL_442500, EPI_ISL_442520			
see above	Virology Department, Sheffield Teaching Hospitals NHS Foundation Trust/Department of Infection, Immunity and Cardiovascular Disease, The Medical School, University of Sheffield	COVID-19 Genomics UK (COG-UK) Consortium	Thushan de Silva, Matthew Parker, Nikki Smith, Adri Angyal, Rebecca Brown, Luke Green, Rachel Tucker, Paul Parsons, Danielle Groves, Katie Johnson, Laura Carrilero, Alex Keeley, Dave Partridge, Matthew Wyles, Benjamin Lindsey, Mehmet Yavuz, Mohammad Raza, Cariad Evans
EPI_ISL_442526, EPI_ISL_442539, EPI_ISL_442567, EPI_ISL_442590, EPI_ISL_442602, EPI_ISL_442603, EPI_ISL_442619, EPI_ISL_443055, EPI_ISL_443075, EPI_ISL_443101, EPI_ISL_443106, EPI_ISL_443117			
see above	Department of Pathology, University of Cambridge	Wellcome Sanger Institute for the COVID-19 Genomics UK (COG-UK) consortium	Luke W Meredith, M. Estée Török , Myra Hosmillo, William L. Hamilton, Martin D. Curran, Theresa Feltwell, Grant Hall, Anna Yakovleva, Fahad A Khokhar, Charlotte J. Houldcroft, Laura G Caller, Aminu S. Jahun, Sarah L. Caddy, Ian Goodfellow, Alex Alderton, Roberto Amato, Sonia Goncalves, Ewan Harrison, David K. Jackson, Ian Johnston, Dominic Kwiatkowski, Cordelia Langford, John Sillitoe on behalf of the Wellcome Sanger Institute COVID-19 Surveillance Team (http://www.sanger.ac.uk/covid-team)
EPI_ISL_443191, EPI_ISL_443247	National Public Health Laboratory, National Centre for Infectious Diseases	National Public Health Laboratory, National Centre for Infectious Diseases	Mak Tze Minn, Octavia Sophie, Chavatte Jean-Marc, Cui Lin, Lin Raymond Tzer Pin
EPI_ISL_443305	LABM GH nord Essonne de Longjumeau - BP 125	National Reference Center for Viruses of Respiratory Infections, Institut Pasteur, Paris	Mélanie Albert, Marion Barbet, Sylvie Behillil, Méline Bizard, Angela Brisebarre, Flora Donati, Etienne Simon-Lorière, Vincent Enouf, Maud Vanpeene, Sylvie van der Werf
EPI_ISL_443308	Plaisance	National Reference Center for Viruses of Respiratory Infections, Institut Pasteur, Paris	Mélanie Albert, Marion Barbet, Sylvie Behillil, Méline Bizard, Angela Brisebarre, Flora Donati, Etienne Simon-Lorière, Vincent Enouf, Maud Vanpeene, Sylvie van der Werf
EPI_ISL_443314	LABM GH nord Essonne de Longjumeau - BP 125	National Reference Center for Viruses of Respiratory Infections, Institut Pasteur, Paris	Mélanie Albert, Marion Barbet, Sylvie Behillil, Méline Bizard, Angela Brisebarre, Flora Donati, Etienne Simon-Lorière, Vincent Enouf, Maud Vanpeene, Sylvie van der Werf
EPI_ISL_443318, EPI_ISL_443319, EPI_ISL_443321, EPI_ISL_443323, EPI_ISL_443324, EPI_ISL_443325, EPI_ISL_443326, EPI_ISL_443327, EPI_ISL_443328, EPI_ISL_443329, EPI_ISL_443330, EPI_ISL_443331, EPI_ISL_443332, EPI_ISL_443334, EPI_ISL_443335, EPI_ISL_443336, EPI_ISL_443337, EPI_ISL_443338, EPI_ISL_443339, EPI_ISL_443340, EPI_ISL_443341, EPI_ISL_443342, EPI_ISL_443343, EPI_ISL_443344, EPI_ISL_443345, EPI_ISL_443346, EPI_ISL_443347, EPI_ISL_443348, EPI_ISL_443349, EPI_ISL_443350, EPI_ISL_443351, EPI_ISL_443352, EPI_ISL_443354, EPI_ISL_443355, EPI_ISL_443357, EPI_ISL_443358, EPI_ISL_443359, EPI_ISL_443360, EPI_ISL_443361, EPI_ISL_443362, EPI_ISL_443363, EPI_ISL_443364, EPI_ISL_443365, EPI_ISL_443368, EPI_ISL_443369, EPI_ISL_443370, EPI_ISL_443371, EPI_ISL_443372, EPI_ISL_443374, EPI_ISL_443375, EPI_ISL_443376, EPI_ISL_443377, EPI_ISL_443378, EPI_ISL_443379, EPI_ISL_443380, EPI_ISL_443382, EPI_ISL_443383, EPI_ISL_443384, EPI_ISL_443385, EPI_ISL_443386, EPI_ISL_443387, EPI_ISL_443388, EPI_ISL_443389, EPI_ISL_443390, EPI_ISL_443391, EPI_ISL_443392, EPI_ISL_443393, EPI_ISL_443394, EPI_ISL_443395, EPI_ISL_443396, EPI_ISL_443397, EPI_ISL_443398, EPI_ISL_443399, EPI_ISL_443401, EPI_ISL_443402, EPI_ISL_443403, EPI_ISL_443404, EPI_ISL_443405, EPI_ISL_443406, EPI_ISL_443407, EPI_ISL_443408, EPI_ISL_443409, EPI_ISL_443410, EPI_ISL_443411, EPI_ISL_443412, EPI_ISL_443413, EPI_ISL_443414, EPI_ISL_443415, EPI_ISL_443416, EPI_ISL_443417, EPI_ISL_443418, EPI_ISL_443419, EPI_ISL_443420, EPI_ISL_443421, EPI_ISL_443422, EPI_ISL_443423, EPI_ISL_443424, EPI_ISL_443426, EPI_ISL_443429, EPI_ISL_443431, EPI_ISL_443432, EPI_ISL_443433, EPI_ISL_443434, EPI_ISL_443435, EPI_ISL_443436, EPI_ISL_443438, EPI_ISL_443439, EPI_ISL_443441, EPI_ISL_443442, EPI_ISL_443443, EPI_ISL_443444, EPI_ISL_443445, EPI_ISL_443446, EPI_ISL_443447, EPI_ISL_443448, EPI_ISL_443449, EPI_ISL_443450, EPI_ISL_443451, EPI_ISL_443452, EPI_ISL_443453, EPI_ISL_443454, EPI_ISL_443455, EPI_ISL_443456, EPI_ISL_443457, EPI_ISL_443458, EPI_ISL_443459, EPI_ISL_443460, EPI_ISL_443462, EPI_ISL_443463, EPI_ISL_443464, EPI_ISL_443466, EPI_ISL_443468, EPI_ISL_443469, EPI_ISL_443470, EPI_ISL_443471, EPI_ISL_443472, EPI_ISL_443473, EPI_ISL_443474, EPI_ISL_443475, EPI_ISL_443476, EPI_ISL_443477, EPI_ISL_443478, EPI_ISL_443479, EPI_ISL_443480,			

EPI_ISL_443481, EPI_ISL_443482, EPI_ISL_443483, EPI_ISL_443484, EPI_ISL_443485, EPI_ISL_443486, EPI_ISL_443488, EPI_ISL_443489, EPI_ISL_443490, EPI_ISL_443491, EPI_ISL_443492, EPI_ISL_443493, EPI_ISL_443494, EPI_ISL_443495, EPI_ISL_443496, EPI_ISL_443497, EPI_ISL_443498, EPI_ISL_443499, EPI_ISL_443500, EPI_ISL_443501, EPI_ISL_443502, EPI_ISL_443503, EPI_ISL_443505, EPI_ISL_443506, EPI_ISL_443507, EPI_ISL_443508, EPI_ISL_443509, EPI_ISL_443510, EPI_ISL_443511, EPI_ISL_443512, EPI_ISL_443513, EPI_ISL_443514, EPI_ISL_443515, EPI_ISL_443516, EPI_ISL_443517, EPI_ISL_443518, EPI_ISL_443519, EPI_ISL_443520, EPI_ISL_443522, EPI_ISL_443523, EPI_ISL_443524, EPI_ISL_443525, EPI_ISL_443526, EPI_ISL_443527, EPI_ISL_443528, EPI_ISL_443529, EPI_ISL_443530, EPI_ISL_443531, EPI_ISL_443532, EPI_ISL_443533, EPI_ISL_443534, EPI_ISL_443535, EPI_ISL_443536, EPI_ISL_443537, EPI_ISL_443538, EPI_ISL_443539, EPI_ISL_443541, EPI_ISL_443542, EPI_ISL_443543, EPI_ISL_443544, EPI_ISL_443546, EPI_ISL_443547, EPI_ISL_443548, EPI_ISL_443549, EPI_ISL_443550, EPI_ISL_443551, EPI_ISL_443553, EPI_ISL_443554, EPI_ISL_443555, EPI_ISL_443556, EPI_ISL_443557, EPI_ISL_443558, EPI_ISL_443559, EPI_ISL_443560, EPI_ISL_443561, EPI_ISL_443562, EPI_ISL_443564, EPI_ISL_443565, EPI_ISL_443566, EPI_ISL_443567, EPI_ISL_443568, EPI_ISL_443569, EPI_ISL_443570, EPI_ISL_443571, EPI_ISL_443572, EPI_ISL_443573, EPI_ISL_443574, EPI_ISL_443575, EPI_ISL_443576, EPI_ISL_443577, EPI_ISL_443578, EPI_ISL_443579, EPI_ISL_443580, EPI_ISL_443581, EPI_ISL_443582, EPI_ISL_443583, EPI_ISL_443584, EPI_ISL_443585, EPI_ISL_443586, EPI_ISL_443588, EPI_ISL_443589, EPI_ISL_443590, EPI_ISL_443591, EPI_ISL_443593, EPI_ISL_443594, EPI_ISL_443595, EPI_ISL_443596, EPI_ISL_443597, EPI_ISL_443598, EPI_ISL_443599, EPI_ISL_443600, EPI_ISL_443601, EPI_ISL_443602, EPI_ISL_443603, EPI_ISL_443604, EPI_ISL_443605, EPI_ISL_443606, EPI_ISL_443607, EPI_ISL_443608, EPI_ISL_443609, EPI_ISL_443610, EPI_ISL_443611, EPI_ISL_443612, EPI_ISL_443613, EPI_ISL_443614, EPI_ISL_443615, EPI_ISL_443616, EPI_ISL_443617, EPI_ISL_443618, EPI_ISL_443619, EPI_ISL_443620, EPI_ISL_443621, EPI_ISL_443622, EPI_ISL_443623, EPI_ISL_443624, EPI_ISL_443625, EPI_ISL_443629, EPI_ISL_443630, EPI_ISL_443631, EPI_ISL_443632, EPI_ISL_443633, EPI_ISL_443634, EPI_ISL_443635, EPI_ISL_443636, EPI_ISL_443637, EPI_ISL_443638, EPI_ISL_443639, EPI_ISL_443640, EPI_ISL_443641, EPI_ISL_443642, EPI_ISL_443643, EPI_ISL_443644, EPI_ISL_443645, EPI_ISL_443646, EPI_ISL_443647, EPI_ISL_443648, EPI_ISL_443649, EPI_ISL_443650, EPI_ISL_443651, EPI_ISL_443652, EPI_ISL_443653, EPI_ISL_443654, EPI_ISL_443655, EPI_ISL_443656, EPI_ISL_443657, EPI_ISL_443658, EPI_ISL_443659, EPI_ISL_443660, EPI_ISL_443661, EPI_ISL_443662, EPI_ISL_443663, EPI_ISL_443664, EPI_ISL_443665, EPI_ISL_443666, EPI_ISL_443667, EPI_ISL_443668, EPI_ISL_443669, EPI_ISL_443671, EPI_ISL_443672, EPI_ISL_443674, EPI_ISL_443675, EPI_ISL_443676, EPI_ISL_443677, EPI_ISL_443678, EPI_ISL_443679, EPI_ISL_443680, EPI_ISL_443681, EPI_ISL_443682, EPI_ISL_443683, EPI_ISL_443684, EPI_ISL_443685

see above	Department of Pathology, University of Cambridge	Wellcome Sanger Institute for the COVID-19 Genomics UK (COG-UK) consortium	Luke W Meredith, M. Estée Török , Myra Hosmillo, William L. Hamilton, Martin D. Curran, Theresa Feltwell, Grant Hall, Anna Yakovleva, Fahad A Khokhar, Charlotte J. Houldcroft, Laura G Caller, Aminu S. Jahun, Sarah L. Caddy, Ian Goodfellow; and Alex Alderton, Roberto Amato, Sonia Goncalves, Ewan Harrison, David K. Jackson, Ian Johnston, Dominic Kwiatkowski, Cordelia Langford, John Sillitoe on behalf of the Wellcome Sanger Institute COVID-19 Surveillance Team (http://www.sanger.ac.uk/covid-team)

EPI_ISL_444155, EPI_ISL_444156, EPI_ISL_444183, EPI_ISL_444184, EPI_ISL_444185, EPI_ISL_444186, EPI_ISL_444187, EPI_ISL_444188, EPI_ISL_444189, EPI_ISL_444190, EPI_ISL_444191, EPI_ISL_444192, EPI_ISL_444193, EPI_ISL_444194, EPI_ISL_444195, EPI_ISL_444196, EPI_ISL_444197, EPI_ISL_444198, EPI_ISL_444199, EPI_ISL_444200, EPI_ISL_444201, EPI_ISL_444202, EPI_ISL_444203, EPI_ISL_444204, EPI_ISL_444205, EPI_ISL_444206, EPI_ISL_444207, EPI_ISL_444208, EPI_ISL_444209, EPI_ISL_444210, EPI_ISL_444211, EPI_ISL_444212, EPI_ISL_444213, EPI_ISL_444214, EPI_ISL_444215, EPI_ISL_444216, EPI_ISL_444217, EPI_ISL_444218, EPI_ISL_444219, EPI_ISL_444220, EPI_ISL_444221, EPI_ISL_444222, EPI_ISL_444223, EPI_ISL_444224, EPI_ISL_444225, EPI_ISL_444226, EPI_ISL_444227, EPI_ISL_444228, EPI_ISL_444229, EPI_ISL_444230, EPI_ISL_444231, EPI_ISL_444232, EPI_ISL_444233, EPI_ISL_444234, EPI_ISL_444235, EPI_ISL_444236, EPI_ISL_444237, EPI_ISL_444238, EPI_ISL_444239, EPI_ISL_444240, EPI_ISL_444241, EPI_ISL_444242, EPI_ISL_444243, EPI_ISL_444244, EPI_ISL_444245, EPI_ISL_444246, EPI_ISL_444247, EPI_ISL_444248, EPI_ISL_444249, EPI_ISL_444250, EPI_ISL_444251, EPI_ISL_444252, EPI_ISL_444253, EPI_ISL_444254, EPI_ISL_444255, EPI_ISL_444256, EPI_ISL_444257, EPI_ISL_444258, EPI_ISL_444259, EPI_ISL_444260, EPI_ISL_444261, EPI_ISL_444262, EPI_ISL_444263, EPI_ISL_444264, EPI_ISL_444265, EPI_ISL_444266

see above	University College London, Great Ormond Street Hospital for Children NHS Foundation Trust, Imperial College Healthcare NHS Trust	COVID-19 Genomics UK (COG-UK) Consortium	Sergi Castellano, Rachel Williams, Mark Kristiansen, Paola Resende Silva, Sunando Roy, Tony Brooks, Helena Tutill, Paola Niola, Patricia Dyal, Charlotte Williams, Leysa Forrest, Yasmin Panchbhaya, Jacqueline Findlay, Sam Weeks, Julianne Brown, Kathryn Harris, Paul Randell, James Price, Alison Holmes, Judith Breuer

EPI_ISL_444279, EPI_ISL_444280, EPI_ISL_444281, EPI_ISL_444282, EPI_ISL_444283, EPI_ISL_444284, EPI_ISL_444285, EPI_ISL_444286, EPI_ISL_444287, EPI_ISL_444288, EPI_ISL_444289, EPI_ISL_444290, EPI_ISL_444291, EPI_ISL_444292, EPI_ISL_444293, EPI_ISL_444294

see above	University of Birmingham	COVID-19 Genomics UK (COG-UK) Consortium	Loman Lab: Claire McMurray, Joanne Stockton, Samuel Nicholls, Radoslaw Poplawski, Will Rowe, Josh Quick, Nicholas Loman // UHB Lab: Celina M Whalley, Andrew Bosworth, Charlotte Poxon, Kasun Wanigasooriya, Oliver Pickles, Mike Kidd, Alex Richter, Andrew D Beggs // PHE Heartlands Lab: Husam Osman, Andrew Bosworth

EPI_ISL_444318, EPI_ISL_444319, EPI_ISL_444321, EPI_ISL_444322, EPI_ISL_444397, EPI_ISL_444398, EPI_ISL_444399, EPI_ISL_444400, EPI_ISL_444401, EPI_ISL_444402, EPI_ISL_444403, EPI_ISL_444404, EPI_ISL_444405, EPI_ISL_444406, EPI_ISL_444411, EPI_ISL_444412, EPI_ISL_444418, EPI_ISL_444420, EPI_ISL_444421, EPI_ISL_444422, EPI_ISL_444423, EPI_ISL_444424, EPI_ISL_444425, EPI_ISL_444426, EPI_ISL_444427, EPI_ISL_444428, EPI_ISL_444429, EPI_ISL_444430, EPI_ISL_444431, EPI_ISL_444433, EPI_ISL_444435, EPI_ISL_444436, EPI_ISL_444437, EPI_ISL_444438, EPI_ISL_444439, EPI_ISL_444440, EPI_ISL_444441, EPI_ISL_444442, EPI_ISL_444443, EPI_ISL_444446, EPI_ISL_444447, EPI_ISL_444448, EPI_ISL_444449, EPI_ISL_444450, EPI_ISL_444451, EPI_ISL_444452, EPI_ISL_444453

see above	Department of Pathology, University of Cambridge	COVID-19 Genomics UK (COG-UK) Consortium	Luke W Meredith, M. Estée Török , Myra Hosmillo, William L. Hamilton, Martin D. Curran, Theresa Feltwell, Grant Hall, Anna Yakovleva, Fahad A Khokhar, Charlotte J. Houldcroft, Laura G Caller, Aminu S. Jahun, Sarah L. Caddy, Ian Goodfellow
EPI_ISL_444787	NYU Langone Health	Departments of Pathology and Medicine, New York University School of Medicine	Maria Aguero-Rosenfeld, Brendan Belovarac, Margaret Black, Ludovic Boytard, John Cadley, Paolo Cotzia, John Chen, Dacia Dimartino, Xiaojun Feng, Tatyana Gindin, Emily Guzman, Adriana Heguy, Megan Hogan, Emily Huang, George Jour, Alireza Khodadadi-Jamayran, Lawrence H. Lin, Raven Luther, Andrew Lytle, Christian Marier, Matthew T. Maurano, Mark J. Mulligan, Peter Meyn, Raquel Ordonez Ciriza, Iman Osman, Jared Pinnell, Vanessa Raabe, Sitharam Ramaswami, Amy Rapkiewicz, Andre M. Ribeiro-dos-Santos, Marie Samanovic-Golden, Antonio Serrano, Guomiao Shen, Matija Snuderl, Theodore Vougiouklakis, Nick Vulpescu, Gael Westby, Paul Zappile, Yutong Zhang
EPI_ISL_444969	Guangzhou Eighth People's Hospital (Jiahe Sector)	Institute of Human Virology, Zhongshan School of Medicine, Sun Yat-sen University	Junsong Zhang, Fei Yu, Jun Liu, Huimin Fan, Ruosu Ying, Feng Huang, Ting Pan, Bingfeng Liu,Yiwen Zhang, Xu Zhang, Mang Shi, Fengyu Hu, Fang Li, Kai Deng, Hui Zhang

EPI_ISL_445003, EPI_ISL_445004, EPI_ISL_445005, EPI_ISL_445006, EPI_ISL_445007, EPI_ISL_445008, EPI_ISL_445009, EPI_ISL_445010, EPI_ISL_445011, EPI_ISL_445012, EPI_ISL_445013, EPI_ISL_445014

see above	Florida Bureau of Public Health Laboratories	Florida Bureau of Public Health Laboratories	Sarah Schmedes, Jason Blanton
EPI_ISL_445065	Laboratoire National de Sante, Microbiology, Virology	Laboratoire National de Sante, Microbiology, Epidemiology and Microbial Genomics	Anke Wienecke-Baldacchino, Ardashel Latsuzbaia, Jessica Tapp, Catherine Ragimbeau, Guillaume Fournier, Tamir Abdelrahman, Trung Nguyen Nguyen, Joel Mossong
EPI_ISL_445086	Laboratory Diagnostic, Veterinary Specialized Institute Kraljevo	Laboratory Diagnostic, Veterinary Specialized Institute Kraljevo	Vidanovic,D., Tesovic,B., Sekler,M., Dmitric,M., Debeljak,Z., Matovic,K., Vaskovic,N., Petrovic,T., Volkening,J. and Alfonso,C.L.
EPI_ISL_445231	Uppsala Narakut Aleris	The Public Health Agency of Sweden	Annika Nilsson, Oskar Karlsson Lindsjo, Maria Lind Karlberg, Anna-Malin Linde, Olov Svartstrom, Anna Risberg, Theresa Enkirch, Mia Brytting, Karin Tegmark-Wisell
EPI_ISL_445232	Kungsors VC	The Public Health Agency of Sweden	Jessica Karlsson, Oskar Karlsson Lindsjo, Maria Lind Karlberg, Anna-Malin Linde, Olov Svartstrom, Anna Risberg, Theresa Enkirch, Mia Brytting, Karin Tegmark-Wisell
EPI_ISL_445233	Vardcentralen Brinken	The Public Health Agency of Sweden	Agnes Wigh, Oskar Karlsson Lindsjo, Maria Lind Karlberg, Anna-Malin Linde, Olov Svartstrom, Anna Risberg, Theresa Enkirch, Mia Brytting, Karin Tegmark-Wisell
EPI_ISL_445239	Uppsala Narakut Aleris	The Public Health Agency of Sweden	Annika Nilsson, Oskar Karlsson Lindsjo, Maria Lind Karlberg, Anna-Malin Linde, Olov Svartstrom, Anna Risberg, Theresa Enkirch, Mia Brytting, Karin Tegmark-Wisell

EPI_ISL_445734, EPI_ISL_445735, EPI_ISL_445736, EPI_ISL_445739, EPI_ISL_445742, EPI_ISL_445743, EPI_ISL_445749, EPI_ISL_445753, EPI_ISL_445754, EPI_ISL_445755, EPI_ISL_445759, EPI_ISL_445760, EPI_ISL_446023, EPI_ISL_446024, EPI_ISL_446025, EPI_ISL_446026, EPI_ISL_446027, EPI_ISL_446028, EPI_ISL_446029, EPI_ISL_446030, EPI_ISL_446031, EPI_ISL_446081, EPI_ISL_446082, EPI_ISL_446083, EPI_ISL_446084, EPI_ISL_446085, EPI_ISL_446086, EPI_ISL_446087, EPI_ISL_446088, EPI_ISL_446089, EPI_ISL_446090, EPI_ISL_446091, EPI_ISL_446255, EPI_ISL_446256, EPI_ISL_446257, EPI_ISL_446258, EPI_ISL_446259, EPI_ISL_446260, EPI_ISL_446261, EPI_ISL_446262, EPI_ISL_446263, EPI_ISL_446264, EPI_ISL_446265, EPI_ISL_446441, EPI_ISL_446444, EPI_ISL_446465, EPI_ISL_446467, EPI_ISL_446471, EPI_ISL_446473, EPI_ISL_446478, EPI_ISL_446480, EPI_ISL_446481, EPI_ISL_446482, EPI_ISL_446483, EPI_ISL_446487, EPI_ISL_446488, EPI_ISL_446489, EPI_ISL_446490, EPI_ISL_446491, EPI_ISL_446546, EPI_ISL_446547, EPI_ISL_446548, EPI_ISL_446549, EPI_ISL_446550, EPI_ISL_446551, EPI_ISL_446552, EPI_ISL_446553, EPI_ISL_446554, EPI_ISL_446555, EPI_ISL_446588, EPI_ISL_446589, EPI_ISL_446590, EPI_ISL_446591, EPI_ISL_446592, EPI_ISL_446593, EPI_ISL_446594, EPI_ISL_446595, EPI_ISL_446596, EPI_ISL_446597, EPI_ISL_446598, EPI_ISL_446599, EPI_ISL_446600, EPI_ISL_446601, EPI_ISL_446602, EPI_ISL_446603, EPI_ISL_446604, EPI_ISL_446605, EPI_ISL_446606, EPI_ISL_446607, EPI_ISL_446609, EPI_ISL_446610, EPI_ISL_446611, EPI_ISL_446612, EPI_ISL_446613, EPI_ISL_446614, EPI_ISL_446615, EPI_ISL_446616, EPI_ISL_446617, EPI_ISL_446618, EPI_ISL_446652, EPI_ISL_446653, EPI_ISL_446654, EPI_ISL_446655, EPI_ISL_446656, EPI_ISL_446657, EPI_ISL_446658, EPI_ISL_446659, EPI_ISL_446660, EPI_ISL_446661, EPI_ISL_446662, EPI_ISL_446663, EPI_ISL_446664, EPI_ISL_446665, EPI_ISL_446666, EPI_ISL_446667, EPI_ISL_446668, EPI_ISL_446669, EPI_ISL_446670, EPI_ISL_446671, EPI_ISL_446685, EPI_ISL_446686, EPI_ISL_446687, EPI_ISL_446688, EPI_ISL_446689, EPI_ISL_446690, EPI_ISL_446691, EPI_ISL_446692, EPI_ISL_446693, EPI_ISL_446694, EPI_ISL_446695, EPI_ISL_446696, EPI_ISL_446697, EPI_ISL_446698, EPI_ISL_446699, EPI_ISL_446700, EPI_ISL_446701, EPI_ISL_446702, EPI_ISL_446703, EPI_ISL_446704, EPI_ISL_446706, EPI_ISL_446707, EPI_ISL_446708, EPI_ISL_446709, EPI_ISL_446710, EPI_ISL_446711, EPI_ISL_446712, EPI_ISL_446713, EPI_ISL_446714, EPI_ISL_446715, EPI_ISL_446716, EPI_ISL_446717, EPI_ISL_446718, EPI_ISL_446719, EPI_ISL_446720, EPI_ISL_446721, EPI_ISL_446722, EPI_ISL_446723, EPI_ISL_446724, EPI_ISL_446725, EPI_ISL_446726, EPI_ISL_446727, EPI_ISL_446728, EPI_ISL_446729, EPI_ISL_446730, EPI_ISL_446731, EPI_ISL_446732, EPI_ISL_446733, EPI_ISL_446734, EPI_ISL_446735, EPI_ISL_446736, EPI_ISL_446737, EPI_ISL_446738, EPI_ISL_446739, EPI_ISL_446740, EPI_ISL_446741, EPI_ISL_446742, EPI_ISL_446743, EPI_ISL_446744, EPI_ISL_446745, EPI_ISL_446746, EPI_ISL_446747, EPI_ISL_446748, EPI_ISL_446749, EPI_ISL_446750, EPI_ISL_446751, EPI_ISL_446752, EPI_ISL_446753, EPI_ISL_446754, EPI_ISL_446755, EPI_ISL_446756, EPI_ISL_446757, EPI_ISL_446764, EPI_ISL_446765, EPI_ISL_446766, EPI_ISL_446767, EPI_ISL_446769, EPI_ISL_446777, EPI_ISL_446778, EPI_ISL_446779, EPI_ISL_446780, EPI_ISL_446781, EPI_ISL_446786, EPI_ISL_446787, EPI_ISL_446788, EPI_ISL_446799, EPI_ISL_446800, EPI_ISL_446801, EPI_ISL_446802, EPI_ISL_446803, EPI_ISL_446804, EPI_ISL_446805, EPI_ISL_446806, EPI_ISL_446807, EPI_ISL_446808, EPI_ISL_446809, EPI_ISL_446810, EPI_ISL_446811, EPI_ISL_446812, EPI_ISL_446813, EPI_ISL_446814, EPI_ISL_446815, EPI_ISL_446816, EPI_ISL_446817, EPI_ISL_446818, EPI_ISL_446819, EPI_ISL_446820, EPI_ISL_446821, EPI_ISL_446822, EPI_ISL_446823, EPI_ISL_446824, EPI_ISL_446825, EPI_ISL_446826, EPI_ISL_446827, EPI_ISL_446828, EPI_ISL_446829, EPI_ISL_446830, EPI_ISL_446831, EPI_ISL_446832, EPI_ISL_446833, EPI_ISL_446834, EPI_ISL_446835, EPI_ISL_446836, EPI_ISL_446837, EPI_ISL_446838, EPI_ISL_446839, EPI_ISL_446840, EPI_ISL_446841, EPI_ISL_446842, EPI_ISL_446843, EPI_ISL_446844, EPI_ISL_446845, EPI_ISL_446846, EPI_ISL_446847, EPI_ISL_446848, EPI_ISL_446849, EPI_ISL_446850, EPI_ISL_446852, EPI_ISL_446853, EPI_ISL_446854, EPI_ISL_446855, EPI_ISL_446856, EPI_ISL_446857, EPI_ISL_446858, EPI_ISL_446859, EPI_ISL_446860, EPI_ISL_446861, EPI_ISL_446862, EPI_ISL_446863, EPI_ISL_446864, EPI_ISL_446868, EPI_ISL_446869, EPI_ISL_446872, EPI_ISL_446873, EPI_ISL_446874, EPI_ISL_446875, EPI_ISL_446876, EPI_ISL_446877, EPI_ISL_446878, EPI_ISL_446879, EPI_ISL_446880, EPI_ISL_446881, EPI_ISL_446882, EPI_ISL_446883, EPI_ISL_446884, EPI_ISL_446885, EPI_ISL_446886, EPI_ISL_446887, EPI_ISL_446888, EPI_ISL_446889, EPI_ISL_446890, EPI_ISL_446891, EPI_ISL_446892, EPI_ISL_446893, EPI_ISL_446894, EPI_ISL_446895, EPI_ISL_446898, EPI_ISL_446899, EPI_ISL_446900, EPI_ISL_446901, EPI_ISL_446902, EPI_ISL_446903, EPI_ISL_446904, EPI_ISL_446905, EPI_ISL_446907, EPI_ISL_446908, EPI_ISL_446909, EPI_ISL_446910, EPI_ISL_446911, EPI_ISL_446912, EPI_ISL_446913, EPI_ISL_446914, EPI_ISL_446915, EPI_ISL_446916, EPI_ISL_446917, EPI_ISL_446918, EPI_ISL_446919, EPI_ISL_446920, EPI_ISL_446921, EPI_ISL_446922, EPI_ISL_446923, EPI_ISL_446924, EPI_ISL_446925, EPI_ISL_446926, EPI_ISL_446927, EPI_ISL_446928, EPI_ISL_446929, EPI_ISL_446930, EPI_ISL_446931, EPI_ISL_446932, EPI_ISL_446933, EPI_ISL_446934, EPI_ISL_446935, EPI_ISL_446936, EPI_ISL_446937, EPI_ISL_446938, EPI_ISL_446939, EPI_ISL_446940, EPI_ISL_446941, EPI_ISL_446942, EPI_ISL_446943, EPI_ISL_446944, EPI_ISL_446945, EPI_ISL_446963, EPI_ISL_446964, EPI_ISL_446965, EPI_ISL_446966, EPI_ISL_446975, EPI_ISL_446976, EPI_ISL_446977, EPI_ISL_446978, EPI_ISL_446979, EPI_ISL_446980, EPI_ISL_446981, EPI_ISL_446982, EPI_ISL_446983, EPI_ISL_446984, EPI_ISL_446985, EPI_ISL_446991, EPI_ISL_446992, EPI_ISL_446993, EPI_ISL_446994, EPI_ISL_446995

see above	Wales Specialist Virology Centre	Public Health Wales Microbiology Cardiff	Catherine Moore, Johnathan Evans, Laura Gifford, Malorie Perry, Simon Cottrell, Alec Birchley, Alexander Adams, Amy Gaskin, Bree Gatica-Wilcox, Jason Coombes, Lauren Gilbert, Lee Graham, Nicole Pacchiarini, Sara Kumziene-Summerhayes, Sarah Taylor, Sophie Jones, Sara Rey, Matthew Bull, Joanne Watkins, Sally Corden, Tom Connor

EPI_ISL_447075	Michigan Department of Health and Human Services, Bureau of Laboratories	Michigan Department of Health and Human Services, Bureau of Laboratories	Blankenship HM, Riner D, Soehnlen MK
EPI_ISL_447137, EPI_ISL_447138, EPI_ISL_447140, EPI_ISL_447142, EPI_ISL_447143, EPI_ISL_447144, EPI_ISL_447145, EPI_ISL_447146, EPI_ISL_447147, EPI_ISL_447148, EPI_ISL_447149, EPI_ISL_447150, EPI_ISL_447151, EPI_ISL_447152, EPI_ISL_447153, EPI_ISL_447154, EPI_ISL_447155, EPI_ISL_447156, EPI_ISL_447157, EPI_ISL_447158, EPI_ISL_447159, EPI_ISL_447160, EPI_ISL_447161, EPI_ISL_447162			
see above	Department of Clinical Microbiology	GIGA Medical Genomics	Keith Durkin, Maria Artesi, Sébastien Bontems, Raphaël Boreux, Cécile Meex, Pierrette Melin, Marie-Pierre Hayette, Vincent Bours.
EPI_ISL_447190	Michigan Department of Health and Human Services, Bureau of Laboratories	Michigan Department of Health and Human Services, Bureau of Laboratories	Blankenship HM, Riner D, Soehnlen MK
EPI_ISL_447334, EPI_ISL_447335, EPI_ISL_447336, EPI_ISL_447337, EPI_ISL_447338, EPI_ISL_447339, EPI_ISL_447340, EPI_ISL_447341, EPI_ISL_447342, EPI_ISL_447343, EPI_ISL_447344, EPI_ISL_447345, EPI_ISL_447346, EPI_ISL_447347, EPI_ISL_447348, EPI_ISL_447349, EPI_ISL_447350, EPI_ISL_447351, EPI_ISL_447352			
see above	Clinical Virology Unit, Hadassah Hebrew University Medical Center	Stern Lab	Stern Lab
EPI_ISL_447457, EPI_ISL_447458, EPI_ISL_447459, EPI_ISL_447460, EPI_ISL_447461, EPI_ISL_447462, EPI_ISL_447463, EPI_ISL_447464, EPI_ISL_447465, EPI_ISL_447466, EPI_ISL_447467, EPI_ISL_447468, EPI_ISL_447469			
see above	Clinical Microbiology Laboratory, Sheba Medical Center	Stern Lab	Stern Lab
EPI_ISL_447580	CSIR-Centre for Cellular and Molecular Biology	CSIR-Centre for Cellular and Molecular Biology	Tulasi Nagabandi, Namami Gaur, Sakshi Shambhavi, Lamuk Zaveri, Shagufta Khan, Purushotham Vodnala, Payel Mukherjee, Sofia Banu, Priya Singh, Dhiviya Vedagiri, Divya Gupta, Vishal Sah, Santosh Kumar Kuncha, Krishnan Harinivas Harshan, Archana Bharadwaj Siva, Karthik Bharadwaj Tallapaka, Rakesh K Mishra, Divya Tej Sowpati
EPI_ISL_447581	CSIR-Centre for Cellular and Molecular Biology	CSIR-Centre for Cellular and Molecular Biology	Sakshi Shambhavi, Lamuk Zaveri, Shagufta Khan, Namami Gaur, Tulasi Nagabandi, Purushotham Vodnala, Payel Mukherjee, Sofia Banu, Priya Singh, Dhiviya Vedagiri, Divya Gupta, Vishal Sah, Santosh Kumar Kuncha, Krishnan Harinivas Harshan, Archana Bharadwaj Siva, Karthik Bharadwaj Tallapaka, Rakesh K Mishra, Divya Tej Sowpati
EPI_ISL_447582, EPI_ISL_447583	CSIR-Centre for Cellular and Molecular Biology	CSIR-Centre for Cellular and Molecular Biology	Tulasi Nagabandi, Namami Gaur, Sakshi Shambhavi, Lamuk Zaveri, Shagufta Khan, Purushotham Vodnala, Payel Mukherjee, Sofia Banu, Priya Singh, Dhiviya Vedagiri, Divya Gupta, Vishal Sah, Santosh Kumar Kuncha, Krishnan Harinivas Harshan, Archana Bharadwaj Siva, Karthik Bharadwaj Tallapaka, Rakesh K Mishra, Divya Tej Sowpati
EPI_ISL_447584, EPI_ISL_447585, EPI_ISL_447586	Tamil Nadu Veterinary and Animal Sciences University	CSIR-Centre for Cellular and Molecular Biology	K Kaveri, S Sivasubramanian, S Vennila, P Padmapriya, R Kiruba, S Magesh, G Dhinakar Raj, G Ravi Kumar, Payel Mukherjee, Tulasi Nagabandi, Namami Gaur, Sakshi Shambhavi, Lamuk Zaveri, Shagufta Khan, Purushotham Vodnala, Sofia Banu, Priya Singh, Dhiviya Vedagiri, Divya Gupta, Vishal Sah, Santosh Kumar Kuncha, Krishnan Harinivas Harshan, Archana Bharadwaj Siva, Karthik Bharadwaj Tallapaka, Kumarasamy Thangaraj, Rakesh K Mishra, Divya Tej Sowpati
EPI_ISL_447621	Department of Laboratory Medicine, National Taiwan University Hospital	Microbial Genomics Core Lab, National Taiwan University Centers of Genomic and Precision Medicine	Shiou-Hwei Yeh, You-Yu Lin, Ya-Yun Lai, Chiao-Ling Li, Shan-Chwen Chang, Pei-Jer Chen, Sui-Yuan Chang
EPI_ISL_447859	CSIR-Centre for Cellular and Molecular Biology	CSIR-Centre for Cellular and Molecular Biology	Payel Mukherjee, Sofia Banu, Priya Singh, Dhiviya Vedagiri, Divya Gupta, Vishal Sah, Santosh Kumar Kuncha, Krishnan Harinivas Harshan, Archana Bharadwaj Siva, Karthik Bharadwaj Tallapaka, Shagufta Khan, Lamuk Zaveri, Namami Gaur, Sakshi Shambhavi, Tulasi Nagabandi, Purushotham Vodnala, Rakesh K Mishra, Divya Tej Sowpati
EPI_ISL_447860, EPI_ISL_447861	CSIR-Centre for Cellular and Molecular Biology	CSIR-Centre for Cellular and Molecular Biology	Tulasi Nagabandi, Namami Gaur, Sakshi Shambhavi, Lamuk Zaveri, Shagufta Khan, Purushotham Vodnala, Payel Mukherjee, Sofia Banu, Priya Singh, Dhiviya Vedagiri, Divya Gupta, Vishal Sah, Santosh Kumar Kuncha, Krishnan Harinivas Harshan, Archana Bharadwaj Siva, Karthik Bharadwaj Tallapaka, Rakesh K Mishra, Divya Tej Sowpati
EPI_ISL_447863, EPI_ISL_447864	CSIR-Centre for Cellular and Molecular Biology	CSIR-Centre for Cellular and Molecular Biology	Payel Mukherjee, Sofia Banu, Priya Singh, Dhiviya Vedagiri, Divya Gupta, Vishal Sah, Santosh Kumar Kuncha, Krishnan Harinivas Harshan, Archana Bharadwaj Siva, Karthik Bharadwaj Tallapaka, Shagufta Khan, Lamuk Zaveri, Namami Gaur, Sakshi Shambhavi, Tulasi Nagabandi, Purushotham Vodnala, Rakesh K Mishra, Divya Tej Sowpati
EPI_ISL_447865, EPI_ISL_447866	CSIR-Centre for Cellular and Molecular Biology	CSIR-Centre for Cellular and Molecular Biology	Sofia Banu, Payel Mukherjee, Priya Singh, Dhiviya Vedagiri, Divya Gupta, Vishal Sah, Santosh Kumar Kuncha, Krishnan Harinivas Harshan, Archana Bharadwaj Siva, Karthik Bharadwaj Tallapaka, Shagufta Khan, Lamuk Zaveri, Namami Gaur, Sakshi Shambhavi, Tulasi Nagabandi, Purushotham Vodnala, Rakesh K Mishra, Divya Tej Sowpati
EPI_ISL_447899	Microbiology	Microbiology	Saha,S., Malaker,R., Sajib,M.S.I., Hasanuzzaman,M., Rahman,H., Islam,M.S., Ahmed,Z.B., Islam,M. and Saha,S.K.
EPI_ISL_448174, EPI_ISL_448175, EPI_ISL_448178, EPI_ISL_448179, EPI_ISL_448180, EPI_ISL_448181, EPI_ISL_448182, EPI_ISL_448183, EPI_ISL_448184, EPI_ISL_448185, EPI_ISL_448186, EPI_ISL_448187, EPI_ISL_448188, EPI_ISL_448189, EPI_ISL_448190, EPI_ISL_448191			
see above	West of Scotland Specialist Virology Centre, NHSGGC / MRC-University of Glasgow Centre for Virus Research	COVID-19 Genomics UK (COG-UK) Consortium	Ana da Silva Filipe, Natasha Johnson, Kathy Smollett, Daniel Mair, Stephen Carmichael, Lily Tong, Jenna Nichols, Elihu Aranday-Cortes, Kirstyn Brunker, Yasmin Parr, Kyriaki Nomikou, Sarah McDonald, Marc Niebel, Patawee Asamaphan, Richard Orton, Joseph Hughes, Sreenu Vattipally, David L Robertson, Alasdair MacLean, Rory Gunson, Kathy Li, Natasha Jesudason, Rajiv Shah, James Shepherd, Antonia Ho, Emma Thomson
EPI_ISL_448223, EPI_ISL_448224, EPI_ISL_448225, EPI_ISL_448226, EPI_ISL_448227, EPI_ISL_448229, EPI_ISL_448230, EPI_ISL_448239, EPI_ISL_448245, EPI_ISL_448268, EPI_ISL_448269, EPI_ISL_448271, EPI_ISL_448272, EPI_ISL_448273, EPI_ISL_448274, EPI_ISL_448275, EPI_ISL_448276, EPI_ISL_448277, EPI_ISL_448278, EPI_ISL_448279, EPI_ISL_448280, EPI_ISL_448281, EPI_ISL_448282, EPI_ISL_448283, EPI_ISL_448286, EPI_ISL_448287, EPI_ISL_448288, EPI_ISL_448289, EPI_ISL_448290, EPI_ISL_448291, EPI_ISL_448292, EPI_ISL_448293, EPI_ISL_448295, EPI_ISL_448296, EPI_ISL_448297, EPI_ISL_448298, EPI_ISL_448299, EPI_ISL_448300, EPI_ISL_448301, EPI_ISL_448302, EPI_ISL_448303, EPI_ISL_448304, EPI_ISL_448305, EPI_ISL_448306			
see above	Quadram Institute Bioscience	COVID-19 Genomics UK (COG-UK) Consortium	Dave J. Baker, Gemma L. Kay, Alp Aydin, Thanh Le-Viet, Steven Rudder, Ana P. Tedim, Anastasia Kolyva, Maria Diaz, Leonardo de Oliveira Martins, Nabil-Fareed Alikhan, Lizzie Meadows, Rachael Stanley, Ngozi Elumogo, Muhammed Yasir, Nicholas M. Thomson, Alexander J Trotter, Rachel Gilroy, Samuel Bloomfield, Claire Stuart, Andrew Bell, Reenesh Prakash, Samir Dervisevic, Alison E. Mather, John Wain, Mark Webber, Andrew J. Page, Justin O'Grady
EPI_ISL_448434, EPI_ISL_448435, EPI_ISL_448436, EPI_ISL_448437	Queens Medical Centre, Clinical Microbiology Department / DeepSeq Nottingham	COVID-19 Genomics UK (COG-UK) Consortium	Gemma Clark, Wendy Smith, Manjinder Khakh, Hannah Howson-Wells, Jonathan Ball, Patrick McClure, Joseph Chappell, Theocharis Tsoleridis, Nadine Holmes, Matthew Carlisle, Christopher Moore, Fei Sang, Johnny Debebe, Victoria Wright, Matthew Loose
EPI_ISL_448826, EPI_ISL_448827, EPI_ISL_448828, EPI_ISL_448829, EPI_ISL_448830, EPI_ISL_448831, EPI_ISL_448832, EPI_ISL_448833, EPI_ISL_448834, EPI_ISL_448835, EPI_ISL_448836, EPI_ISL_448837, EPI_ISL_448838, EPI_ISL_448839, EPI_ISL_448840, EPI_ISL_448841, EPI_ISL_448842, EPI_ISL_448843, EPI_ISL_448845, EPI_ISL_448847, EPI_ISL_448848, EPI_ISL_448849, EPI_ISL_448851, EPI_ISL_448852, EPI_ISL_448853, EPI_ISL_448854, EPI_ISL_448855, EPI_ISL_448856, EPI_ISL_448857, EPI_ISL_448858, EPI_ISL_448859, EPI_ISL_448860, EPI_ISL_448861, EPI_ISL_448862, EPI_ISL_448863, EPI_ISL_448867, EPI_ISL_448868, EPI_ISL_448869, EPI_ISL_448870, EPI_ISL_448871, EPI_ISL_448872, EPI_ISL_448873, EPI_ISL_448874, EPI_ISL_448875, EPI_ISL_448876, EPI_ISL_448877, EPI_ISL_448878, EPI_ISL_448879, EPI_ISL_448880, EPI_ISL_448881, EPI_ISL_448882, EPI_ISL_448883, EPI_ISL_448884, EPI_ISL_448885, EPI_ISL_448886, EPI_ISL_448887, EPI_ISL_448888, EPI_ISL_448889, EPI_ISL_448890, EPI_ISL_448891, EPI_ISL_448892, EPI_ISL_448893, EPI_ISL_448895			
see above	Virology Department, Sheffield Teaching Hospitals NHS Foundation Trust/Department of Infection, Immunity and Cardiovascular Disease, The Medical School, University of Sheffield	COVID-19 Genomics UK (COG-UK) Consortium	Thushan de Silva, Matthew Parker, Nikki Smith, Adri Angyal, Rebecca Brown, Luke Green, Rachel Tucker, Paul Parsons, Danielle Groves, Katie Johnson, Laura Carrilero, Alex Keeley, Dave Partridge, Matthew Wyles, Benjamin Lindsey, Mehmet Yavuz, Mohammad Raza, Cariad Evans
EPI_ISL_448896	Virology Laboratory, Castle Hill Hospital, Hull University Teaching Hospitals NHS Trust/Department of Infection, Immunity and Cardiovascular Disease, The Medical School, University of Sheffield	COVID-19 Genomics UK (COG-UK) Consortium	Thushan de Silva, Matthew Parker, Nikki Smith, Adri Angyal, Rebecca Brown, Luke Green, Rachel Tucker, Paul Parsons, Danielle Groves, Katie Johnson, Laura Carrilero, Alex Keeley, Dave Partridge, Matthew Wyles, Benjamin Lindsey, Mehmet Yavuz, Mohammad Raza, Cariad Evans
EPI_ISL_448897, EPI_ISL_448898, EPI_ISL_448899, EPI_ISL_448900, EPI_ISL_448901	Virology Department, Sheffield Teaching Hospitals NHS Foundation Trust/Department of Infection, Immunity and Cardiovascular Disease, The Medical School, University of Sheffield	COVID-19 Genomics UK (COG-UK) Consortium	Thushan de Silva, Matthew Parker, Nikki Smith, Adri Angyal, Rebecca Brown, Luke Green, Rachel Tucker, Paul Parsons, Danielle Groves, Katie Johnson, Laura Carrilero, Alex Keeley, Dave Partridge, Matthew Wyles, Benjamin Lindsey, Mehmet Yavuz, Mohammad Raza, Cariad Evans
EPI_ISL_448902	Virology Laboratory, Castle Hill Hospital, Hull University Teaching Hospitals NHS Trust/Department of Infection, Immunity and Cardiovascular Disease, The Medical School, University of Sheffield	COVID-19 Genomics UK (COG-UK) Consortium	Thushan de Silva, Matthew Parker, Nikki Smith, Adri Angyal, Rebecca Brown, Luke Green, Rachel Tucker, Paul Parsons, Danielle Groves, Katie Johnson, Laura Carrilero, Alex Keeley, Dave Partridge, Matthew Wyles, Benjamin Lindsey, Mehmet Yavuz, Mohammad Raza, Cariad Evans

EPI_ISL_448903, EPI_ISL_448904, EPI_ISL_448905, EPI_ISL_448907, EPI_ISL_448908, EPI_ISL_448909, EPI_ISL_448910, EPI_ISL_448911, EPI_ISL_448912, EPI_ISL_448913, EPI_ISL_448914, EPI_ISL_448915, EPI_ISL_448916, EPI_ISL_448917			
see above	Virology Department, Sheffield Teaching Hospitals NHS Foundation Trust/Department of Infection, Immunity and Cardiovascular Disease, The Medical School, University of Sheffield	COVID-19 Genomics UK (COG-UK) Consortium	Thushan de Silva, Matthew Parker, Nikki Smith, Adri Angyal, Rebecca Brown, Luke Green, Rachel Tucker, Paul Parsons, Danielle Groves, Katie Johnson, Laura Carrilero, Alex Keeley, Dave Partridge, Matthew Wyles, Benjamin Lindsey, Mehmet Yavuz, Mohammad Raza, Cariad Evans
EPI_ISL_448979, EPI_ISL_448980, EPI_ISL_448984, EPI_ISL_448985, EPI_ISL_448986, EPI_ISL_448990, EPI_ISL_448992, EPI_ISL_448994, EPI_ISL_448995, EPI_ISL_448998, EPI_ISL_448999, EPI_ISL_449001, EPI_ISL_449002, EPI_ISL_449003, EPI_ISL_449004, EPI_ISL_449005, EPI_ISL_449007, EPI_ISL_449008, EPI_ISL_449010, EPI_ISL_449012, EPI_ISL_449015, EPI_ISL_449016, EPI_ISL_449020, EPI_ISL_449026, EPI_ISL_449028, EPI_ISL_449030, EPI_ISL_449101, EPI_ISL_449103, EPI_ISL_449108, EPI_ISL_449135, EPI_ISL_449148, EPI_ISL_449149, EPI_ISL_449150, EPI_ISL_449151, EPI_ISL_449152, EPI_ISL_449153, EPI_ISL_449154, EPI_ISL_449155, EPI_ISL_449156, EPI_ISL_449157, EPI_ISL_449158, EPI_ISL_449159, EPI_ISL_449160, EPI_ISL_449161, EPI_ISL_449162, EPI_ISL_449163, EPI_ISL_449164, EPI_ISL_449165, EPI_ISL_449166, EPI_ISL_449167, EPI_ISL_449168, EPI_ISL_449169, EPI_ISL_449170, EPI_ISL_449171, EPI_ISL_449172, EPI_ISL_449173, EPI_ISL_449174, EPI_ISL_449175			
see above	Quadram Institute Bioscience	COVID-19 Genomics UK (COG-UK) Consortium	Dave J. Baker, Gemma L. Kay, Alp Aydin, Thanh Le-Viet, Steven Rudder, Ana P. Tedim, Anastasia Kolyva, Maria Diaz, Leonardo de Oliveira Martins, Nabil-Fareed Alikhan, Lizzie Meadows, Rachael Stanley, Ngozi Elumogo, Muhammed Yasir, Nicholas M. Thomson, Alexander J Trotter, Rachel Gilroy, Samuel Bloomfield, Claire Stuart, Andrew Bell, Reenesh Prakash, Samir Dervisevic, Alison E. Mather, John Wain, Mark Webber, Andrew J. Page, Justin O'Grady
EPI_ISL_449188, EPI_ISL_449224, EPI_ISL_449225, EPI_ISL_449226, EPI_ISL_449227, EPI_ISL_449228, EPI_ISL_449229, EPI_ISL_449230, EPI_ISL_449231	West of Scotland Specialist Virology Centre, NHSGGC / MRC-University of Glasgow Centre for Virus Research	COVID-19 Genomics UK (COG-UK) Consortium	Ana da Silva Filipe, Natasha Johnson, Kathy Smollett, Daniel Mair, Stephen Carmichael, Lily Tong, Jenna Nichols, Elihu Aranday-Cortes, Kirstyn Brunker, Yasmin Parr, Kyriaki Nomikou, Sarah McDonald, Marc Niebel, Patawee Asamaphan, Richard Orton, Joseph Hughes, Sreenu Vattipally, David L Robertson, Alasdair MacLean, Rory Gunson, Kathy Li, Natasha Jesudason, Rajiv Shah, James Shepherd, Antonia Ho, Emma Thomson
EPI_ISL_449273, EPI_ISL_449274, EPI_ISL_449275, EPI_ISL_449276, EPI_ISL_449277	Virology Department, Royal Infirmary of Edinburgh, NHS Lothian / School of Biological Sciences, University of Edinburgh / Institute of Genetics and Molecular Medicine, University of Edinburgh	COVID-19 Genomics UK (COG-UK) Consortium	McHugh M, Dewar R, Rooke S, Gallagher M, Balcaza C, O'Toole Á, Scher E, Hill V, McCrone JT, Colquhoun R, Yu X, Jackson B, Rambaut A, Williams TC, Templeton K
EPI_ISL_449330, EPI_ISL_449331, EPI_ISL_449332, EPI_ISL_449625	Liverpool Clinical Laboratories	COVID-19 Genomics UK (COG-UK) Consortium	Sam Haldenby, Anita Lucaci, Steve Paterson, Julian Hiscox, Alistair Darby, M Almsaud, A Alrezaihi, Muhannad Alruwaili, Stuart D Armstrong, Jones Benjamin , Eleanor G Bentley, Anu Chawla, Jordan J Clark, Angela Cowell, Richard Eccles, Isabel García-Dorival, Matthew Gemmell, Alessandro Gerada, PKF Gilmore, Richard Gregory, Ximeng Han, Catherine Hartley, Margaret Hughes, Miren Iturriza-Gomara, James Johnson, L Luu, Jenifer Manson , Charlotte Nelson, Elaine O'Toole, Cassie Olateju, Rebekah Penrice-Randal , Lucille Rainbow, N.P Randle, Trevor Ian Robinson, Parul Sharma, Ghada T Shawli, James P Stewart , Neil Swainston, Ecaterina Vamos, Joanne Watts, Mark Whitehead
EPI_ISL_449661, EPI_ISL_449662, EPI_ISL_449663, EPI_ISL_449664, EPI_ISL_449665, EPI_ISL_449666, EPI_ISL_449667, EPI_ISL_449668, EPI_ISL_449669, EPI_ISL_449670, EPI_ISL_449671, EPI_ISL_449672, EPI_ISL_449673, EPI_ISL_449674, EPI_ISL_449675, EPI_ISL_449676, EPI_ISL_449677, EPI_ISL_449678, EPI_ISL_449679, EPI_ISL_449680, EPI_ISL_449681, EPI_ISL_449682, EPI_ISL_449683, EPI_ISL_449684, EPI_ISL_449685, EPI_ISL_449686, EPI_ISL_449687, EPI_ISL_449688, EPI_ISL_449689, EPI_ISL_449690, EPI_ISL_449691, EPI_ISL_449692, EPI_ISL_449693, EPI_ISL_449694, EPI_ISL_449695, EPI_ISL_449696, EPI_ISL_449697, EPI_ISL_449698, EPI_ISL_449699, EPI_ISL_449700, EPI_ISL_449701, EPI_ISL_449702, EPI_ISL_449703, EPI_ISL_449704, EPI_ISL_449705, EPI_ISL_449706, EPI_ISL_449707, EPI_ISL_449708, EPI_ISL_449709, EPI_ISL_449710, EPI_ISL_449711, EPI_ISL_449712, EPI_ISL_449713, EPI_ISL_449714, EPI_ISL_449715, EPI_ISL_449716, EPI_ISL_449717, EPI_ISL_449718, EPI_ISL_449719, EPI_ISL_449720, EPI_ISL_449721, EPI_ISL_449722, EPI_ISL_449723, EPI_ISL_449724, EPI_ISL_449725, EPI_ISL_449726, EPI_ISL_449727, EPI_ISL_449728, EPI_ISL_449729			
see above	University College London, Great Ormond Street Hospital for Children NHS Foundation Trust, Imperial College Healthcare NHS Trust	COVID-19 Genomics UK (COG-UK) Consortium	Sergi Castellano, Rachel Williams, Mark Kristiansen, Paola Resende Silva, Sunando Roy, Tony Brooks, Helena Tutill, Paola Niola, Patricia Dyal, Charlotte Williams, Leysa Forrest, Yasmin Panchbhaya, Jacqueline Findlay, Sam Weeks, Julianne Brown, Kathryn Harris, Paul Randell, James Price, Alison Holmes, Judith Breuer
EPI_ISL_449799	National Laboratory for Health, Environment and Food	National Laboratory for Health, Environment and Food	Mahnic A., Hedzet S., Janezic S., Duh D., Zavrsnik J., Blazun Vosner H., Rupnik M.
EPI_ISL_449902, EPI_ISL_449903, EPI_ISL_449904, EPI_ISL_449905, EPI_ISL_449906, EPI_ISL_449907, EPI_ISL_449908, EPI_ISL_449909, EPI_ISL_449910, EPI_ISL_449911, EPI_ISL_449912, EPI_ISL_449913, EPI_ISL_449915, EPI_ISL_449916, EPI_ISL_449917, EPI_ISL_449918, EPI_ISL_449920, EPI_ISL_449922, EPI_ISL_449923, EPI_ISL_449925, EPI_ISL_449926, EPI_ISL_449927, EPI_ISL_449928, EPI_ISL_449929, EPI_ISL_449930, EPI_ISL_449931, EPI_ISL_449932, EPI_ISL_449933, EPI_ISL_449934, EPI_ISL_449935, EPI_ISL_449936, EPI_ISL_449937, EPI_ISL_449938, EPI_ISL_449939, EPI_ISL_449940, EPI_ISL_449941, EPI_ISL_449942, EPI_ISL_449943, EPI_ISL_449944, EPI_ISL_449945, EPI_ISL_449946, EPI_ISL_449947, EPI_ISL_449948, EPI_ISL_449949, EPI_ISL_449950, EPI_ISL_449951, EPI_ISL_449952, EPI_ISL_449953, EPI_ISL_449954, EPI_ISL_449955, EPI_ISL_449956, EPI_ISL_449957, EPI_ISL_449958, EPI_ISL_449959, EPI_ISL_449960, EPI_ISL_449962, EPI_ISL_449963, EPI_ISL_449964, EPI_ISL_449965, EPI_ISL_449966, EPI_ISL_449967, EPI_ISL_449968, EPI_ISL_449969, EPI_ISL_449970, EPI_ISL_449971, EPI_ISL_449972, EPI_ISL_449973, EPI_ISL_449974, EPI_ISL_449975, EPI_ISL_449976, EPI_ISL_449977, EPI_ISL_449978, EPI_ISL_449979, EPI_ISL_449980, EPI_ISL_449981, EPI_ISL_449982, EPI_ISL_449983, EPI_ISL_449984, EPI_ISL_449985, EPI_ISL_449986, EPI_ISL_449987, EPI_ISL_449988, EPI_ISL_449989, EPI_ISL_449990, EPI_ISL_449991, EPI_ISL_449992, EPI_ISL_449994, EPI_ISL_449995, EPI_ISL_449996, EPI_ISL_449997, EPI_ISL_449998, EPI_ISL_449999, EPI_ISL_450000, EPI_ISL_450001, EPI_ISL_450002, EPI_ISL_450003			
see above	Washington State Department of Health	Seattle Flu Study	Chu et al
EPI_ISL_450241, EPI_ISL_450242, EPI_ISL_450243, EPI_ISL_450244, EPI_ISL_450245, EPI_ISL_450246, EPI_ISL_450247	WHO National Influenza Centre Russian Federation	WHO National Influenza Centre Russian Federation	Andrey Komissarov, Artem Fadeev, Mariia Sergeeva, Anna Ivanova, Tamila Musaeva, Ksenia Komissarova, Mariia Timofeeva, Veronica Eder, Mariia Pisareva, Daria Danilenko
EPI_ISL_450518, EPI_ISL_450519	E. Gulbja Laboratorija	Latvian Biomedical Research and Study Centre	Ivars Silamielis, Kaspars Megnis, Monta Ustinova, ikita Zrelovs, Vita Rovte, Mikus Gavars, Dmitrijs Perminovs, Uga Dumpis, Jnis Kloviš
EPI_ISL_450524	Centrl Laboratorija	Latvian Biomedical Research and Study Centre	Ivars Silamielis, Kaspars Megnis, Monta Ustinova, ikita Zrelovs, Vita Rovte, Stella Lapia, Jana Oste, Marta Priedte, Uga Dumpis, Jnis Kloviš
EPI_ISL_450562, EPI_ISL_450563, EPI_ISL_450564, EPI_ISL_450565, EPI_ISL_450566, EPI_ISL_450567, EPI_ISL_450568	Utah Public Health Laboratory	Utah Public Health Laboratory	Erin Young, Kelly Oakeson
EPI_ISL_450648, EPI_ISL_450653	Laboratoire de microbiologie, Hopital de Verdun	Smith Laboratory, Centre de Recherche CHU Sainte-Justine	Martin Smith, Marieke Rozendaal, Ivan Pavlov
EPI_ISL_450701, EPI_ISL_450702, EPI_ISL_450703, EPI_ISL_450704, EPI_ISL_450705, EPI_ISL_450706, EPI_ISL_450707, EPI_ISL_450709, EPI_ISL_450710, EPI_ISL_450711, EPI_ISL_450712, EPI_ISL_450713, EPI_ISL_450714, EPI_ISL_450715, EPI_ISL_450716, EPI_ISL_450717, EPI_ISL_450718, EPI_ISL_450719, EPI_ISL_450720, EPI_ISL_450722			
see above	University of Wisconsin-Madison AIDS Vaccine Research Laboratories	University of Wisconsin-Madison AIDS Vaccine Research Laboratories	Gage Moreno, Katarina Braun, et al. AIDS Vaccine Research Laboratories
EPI_ISL_450813	Bla Kustens halsocentral	The Public Health Agency of Sweden	Olof Norrby, Anna-Malin Linde, Maria Lind Karlberg, Oskar Karlsson Lindsjo, Olov Svartstrom, Anna Risberg, Theresa Enkirch, Mia Brytting, Karin Tegmark-Wisell
EPI_ISL_451180, EPI_ISL_451181, EPI_ISL_451182	Lab voor klinische biologie	Onderzoeksgroep Virologie	Nick Vereecke, Laurens Lambrechts, Marthe Pauwels, Jozefien De Clercq, Bruno Verhasselt, Linos Vandekerckhove, Hans Nauwynck, Sebastiaan Theuns
EPI_ISL_451614, EPI_ISL_451615, EPI_ISL_451616, EPI_ISL_451617, EPI_ISL_451618, EPI_ISL_451619, EPI_ISL_451620, EPI_ISL_451621, EPI_ISL_451622, EPI_ISL_451623, EPI_ISL_451624, EPI_ISL_451625, EPI_ISL_451626, EPI_ISL_451627, EPI_ISL_451628, EPI_ISL_451629, EPI_ISL_451637, EPI_ISL_451638, EPI_ISL_451639, EPI_ISL_451640			
see above	Pathology West - NSW Health Pathology	NSW Health Pathology - Institute of Clinical Pathology and Medical Research; Westmead Hospital; University of Sydney	CIDM-PH et al.
EPI_ISL_452035, EPI_ISL_452036, EPI_ISL_452037, EPI_ISL_452039, EPI_ISL_452040, EPI_ISL_452041, EPI_ISL_452042, EPI_ISL_452043, EPI_ISL_452045, EPI_ISL_452046, EPI_ISL_452047, EPI_ISL_452048, EPI_ISL_452049, EPI_ISL_452050, EPI_ISL_452051, EPI_ISL_452052, EPI_ISL_452053, EPI_ISL_452054, EPI_ISL_452055, EPI_ISL_452056, EPI_ISL_452057, EPI_ISL_452058, EPI_ISL_452080, EPI_ISL_452082, EPI_ISL_452092, EPI_ISL_452093, EPI_ISL_452094, EPI_ISL_452095, EPI_ISL_452096			
see above	Department of Clinical Microbiology, Copenhagen University Hospital, Hvidovre, Kettegaard Alle 30, 2650 Hvidovre.	Albertsen lab, Department of Chemistry and Bioscience, Aalborg University, Denmark	Rasmus Kirkegaard
EPI_ISL_452151	CUB Hopital Erasme Laboratoire d'Anatomie Pathologique	CUB Hopital Erasme Laboratoire d'Anatomie Pathologique	Prof. Isabelle Salmon, Dr.Nikcy D'Haene
EPI_ISL_452160, EPI_ISL_452161, EPI_ISL_452162, EPI_ISL_452163, EPI_ISL_452164, EPI_ISL_452165, EPI_ISL_452166, EPI_ISL_452167, EPI_ISL_452168, EPI_ISL_452169, EPI_ISL_452170, EPI_ISL_452171, EPI_ISL_452172, EPI_ISL_452173, EPI_ISL_452174, EPI_ISL_452175, EPI_ISL_452176, EPI_ISL_452177			
see above	Utah Public Health Laboratory	Utah Public Health Laboratory	Erin Young, Kelly Oakeson
EPI_ISL_452178, EPI_ISL_452179	Laboratory Medicine	Department of Laboratory Medicine, Lin-Kou Chang Gung	Kuo-Chien Tsao, Yu-Nong Gong, Shu-Li Yang, Yi-Chun Liu, Chung-Guei Huang, Mei-Jen Hsiao, Po-Wei Huang, Cheng-Ta Yang, Cheng-Hsun Chiu,

		Memorial Hospital, Taoyuan, Taiwan	Peng-Nien Huang, Kuo-Ming Lee, Guang-Wu Chen, Shin-Ru Shih
EPI_ISL_452192, EPI_ISL_452193, EPI_ISL_452194, EPI_ISL_452195, EPI_ISL_452196, EPI_ISL_452197, EPI_ISL_452211, EPI_ISL_452212	NIV Influenza	NIV Influenza	Potdar V
EPI_ISL_452235	Narhalsan Backa vardcentral	The Public Health Agency of Sweden	Mats Olsson, Anna-Malin Linde, Maria Lind Karlberg, Oskar Karlsson Lindsjo, Olov Svartstrom, Anna Risberg, Theresa Enkirch, Mia Brytting, Karin Tegmark-Wisell
EPI_ISL_452236	VC Sorgenfrimottagningen	The Public Health Agency of Sweden	Lisa Esbjornsson Klemendz, Anna-Malin Linde, Maria Lind Karlberg, Oskar Karlsson Lindsjo, Olov Svartstrom, Anna Risberg, Theresa Enkirch, Mia Brytting, Karin Tegmark-Wisell
EPI_ISL_452787, EPI_ISL_452788	ICAR-National Institute of High Security Animal Diseases	ICAR-National Institute of High Security Animal Diseases	Anamika Mishra, Ashutosh Aasdev, Sandeep Bhatia, Harshad Murugkar, Chakradhar Tosh, Niranjan Mishra, Shanmugasundaram Nagarajan, Katherukamem Rajukumar, Richa Sood, G Venkatesh, Atul Kumar Pateriya, Manoj Kumar, Shashi Bhushan Sudhakar, Fateh Singh, Sethil Kumar D, Senmannan Kalaiyarasu, Pradeep Gandhale, Naveen Kumar, Chandan Kumar Dubey, Sushil Tripathi, Sandeep Kumar Jhade, Meghna Tripathi, Suman Kumari Shah, Pushpendra Singh, Pushpendra Namdeo, Suman Mishra, Rupal Singh, Vishnupriya Patil, Dipesh Kumar Nayak, Vijendra Pal Singh, Ashwin Ashok Raut
EPI_ISL_452813, EPI_ISL_452816, EPI_ISL_452817, EPI_ISL_452818, EPI_ISL_452819, EPI_ISL_452820, EPI_ISL_452821, EPI_ISL_452822, EPI_ISL_452823, EPI_ISL_452833, EPI_ISL_452834, EPI_ISL_452835			
see above	Virginia DCLS	Virginia DCLS	Virginia DCLS
EPI_ISL_453041, EPI_ISL_453044, EPI_ISL_453046, EPI_ISL_453047, EPI_ISL_453048, EPI_ISL_453049, EPI_ISL_453050, EPI_ISL_453051, EPI_ISL_453052, EPI_ISL_453053, EPI_ISL_453054, EPI_ISL_453055, EPI_ISL_453056, EPI_ISL_453057, EPI_ISL_453058, EPI_ISL_453059, EPI_ISL_453060, EPI_ISL_453061, EPI_ISL_453062, EPI_ISL_453063, EPI_ISL_453064, EPI_ISL_453065, EPI_ISL_453066, EPI_ISL_453067, EPI_ISL_453068, EPI_ISL_453069, EPI_ISL_453070, EPI_ISL_453071, EPI_ISL_453072, EPI_ISL_453073, EPI_ISL_453074, EPI_ISL_453091, EPI_ISL_453093, EPI_ISL_453094			
see above	West of Scotland Specialist Virology Centre, NHSGGC / MRC-University of Glasgow Centre for Virus Research	COVID-19 Genomics UK (COG-UK) Consortium	Ana da Silva Filipe, Natasha Johnson, Kathy Smollett, Daniel Mair, Stephen Carmichael, Lily Tong, Jenna Nichols, Elihu Aranday-Cortes, Kirstyn Brunker, Yasmin Parr, Kyriaki Nomikou; Sarah McDonald, Marc Niebel, Patawee Asamaphan; Richard Orton, Joseph Hughes, Sreenu Vattipally, David L Robertson; Alasdair MacLean, Rory Gunson; Kathy Li, Natasha Jesudason, Rajiv Shah, James Shepherd, Antonia Ho, Emma Thomson
EPI_ISL_453102, EPI_ISL_453103, EPI_ISL_453104, EPI_ISL_453105, EPI_ISL_453106, EPI_ISL_453107, EPI_ISL_453108, EPI_ISL_453109	Virology Department, Royal Infirmary of Edinburgh, NHS Lothian / School of Biological Sciences, University of Edinburgh / Institute of Genetics and Molecular Medicine, University of Edinburgh	COVID-19 Genomics UK (COG-UK) Consortium	McHugh M, Dewar R, Rooke S, Gallagher M, Balcaza C, O'Toole Á, Scher E, Hill V, McCrone JT, Colquhoun R, Yu X, Jackson B, Rambaut A, Williams TC, Templeton K
EPI_ISL_453196, EPI_ISL_453197, EPI_ISL_453198, EPI_ISL_453199, EPI_ISL_453200, EPI_ISL_453201, EPI_ISL_453202, EPI_ISL_453203, EPI_ISL_453204, EPI_ISL_453205, EPI_ISL_453206, EPI_ISL_453207, EPI_ISL_453208, EPI_ISL_453210, EPI_ISL_453211, EPI_ISL_453212, EPI_ISL_453213, EPI_ISL_453214, EPI_ISL_453231, EPI_ISL_453232, EPI_ISL_453233, EPI_ISL_453234, EPI_ISL_453235, EPI_ISL_453236, EPI_ISL_453237, EPI_ISL_453238, EPI_ISL_453239, EPI_ISL_453417, EPI_ISL_453418, EPI_ISL_453419, EPI_ISL_453420, EPI_ISL_453421, EPI_ISL_453422, EPI_ISL_453423, EPI_ISL_453424, EPI_ISL_453425			
see above	Liverpool Clinical Laboratories	COVID-19 Genomics UK (COG-UK) Consortium	Sam Haldenby, Anita Lucaci, Steve Paterson, Julian Hiscox, Alistair Darby, M Almsaud, A Alrezaihi, Muhannad Alruwaili, Stuart D Armstrong, Jones Benjamin , Eleanor G Bentley, Anu Chawla, Jordan J Clark, Angela Cowell, Richard Eccles, Isabel García-Dorival, Matthew Gemmell, Alessandro Gerada, PKF Gilmore, Richard Gregory, Ximeng Han, Catherine Hartley, Margaret Hughes, Miren Iturriza-Gomara, James Johnson, L Luu, Jenifer Manson , Charlotte Nelson, Elaine O'Toole, Cassie Olateju, Rebekah Penrice-Randal , Lucille Rainbow, N.P Randle, Trevor Ian Robinson, Parul Sharma, Ghada T Shawli, James P Stewart , Neil Swainston, Ecaterina Vamos, Joanne Watts, Mark Whitehead
EPI_ISL_453460, EPI_ISL_453461, EPI_ISL_453462, EPI_ISL_453464	University College London, Great Ormond Street Hospital for Children NHS Foundation Trust, Imperial College Healthcare NHS Trust	COVID-19 Genomics UK (COG-UK) Consortium	Sergi Castellano, Rachel Williams, Mark Kristiansen, Paola Resende Silva, Sunando Roy, Tony Brooks, Helena Tutill, Paola Niola, Patricia Dyal, Charlotte Williams, Leysa Forrest, Yasmin Panchbhaya, Jacqueline Findlay, Sam Weeks, Julianne Brown, Kathryn Harris, Paul Randell, James Price, Alison Holmes, Judith Breuer
EPI_ISL_453719, EPI_ISL_453767, EPI_ISL_453771, EPI_ISL_453772	Virology Department, Sheffield Teaching Hospitals NHS Foundation Trust/Department of Infection, Immunity and Cardiovascular Disease, The Medical School, University of Sheffield	COVID-19 Genomics UK (COG-UK) Consortium	Thushan de Silva, Matthew Parker, Nikki Smith, Adri Angyal, Rebecca Brown, Luke Green, Rachel Tucker, Paul Parsons, Danielle Groves, Katie Johnson, Laura Carrilero, Alex Keeley, Dave Partridge, Matthew Wyles, Benjamin Lindsey, Mehmet Yavuz, Mohammad Raza, Cariad Evans
EPI_ISL_453997, EPI_ISL_454105, EPI_ISL_454106, EPI_ISL_454108, EPI_ISL_454109, EPI_ISL_454110, EPI_ISL_454111, EPI_ISL_454112, EPI_ISL_454113, EPI_ISL_454114, EPI_ISL_454115, EPI_ISL_454117, EPI_ISL_454119, EPI_ISL_454121, EPI_ISL_454122, EPI_ISL_454125, EPI_ISL_454178, EPI_ISL_454179, EPI_ISL_454180, EPI_ISL_454181, EPI_ISL_454182, EPI_ISL_454183, EPI_ISL_454184, EPI_ISL_454185, EPI_ISL_454186, EPI_ISL_454187, EPI_ISL_454188, EPI_ISL_454189, EPI_ISL_454190, EPI_ISL_454191, EPI_ISL_454192, EPI_ISL_454193, EPI_ISL_454194, EPI_ISL_454195, EPI_ISL_454196, EPI_ISL_454270, EPI_ISL_454290, EPI_ISL_454291, EPI_ISL_454292, EPI_ISL_454293, EPI_ISL_454299			
see above	unknown	Instituto Nacional de Saude (INSA)	Borges et al
EPI_ISL_454498, EPI_ISL_454499, EPI_ISL_454500, EPI_ISL_454508	RSE "National Center for Biotechnology"	RSE "National Center for Biotechnology"	Alexandr Shevtsov, Ilyas Akhmetollayev, Viktoriya Lutsay, Asylulan Amirgazin, Askar Abdaliyev, Akbota Rakhmetova, Zabira Aushakhmetova, Ruslan Kalendar, Yerlan Ramankulov
EPI_ISL_454521, EPI_ISL_454522, EPI_ISL_454523, EPI_ISL_454557, EPI_ISL_454558	NIV Influenza	NIV Influenza	Potdar V
EPI_ISL_454576, EPI_ISL_454577, EPI_ISL_454579, EPI_ISL_454580	Laboratory of virology, National Center of Expertise	Laboratory of molecular-genetic research, National Center of Expertise, Kazakhstan National Center for Biotechnology, Kazakhstan	Abdaliyev Askar, Shevtsov Alexandr, Akhmetollayev Ilyas, Kalendar Ruslan, Rakhmetova Akbota, , Lutsay Viktoriya, Amirgazin Asylulan, Aushakhmetova Zabira, Ramankulov Yerlan
EPI_ISL_454610, EPI_ISL_454611, EPI_ISL_454612, EPI_ISL_454613	Alameda County Public Health Lab	Chan-Zuckerberg Biohub	CZB Cliahub Consortium
EPI_ISL_454643	VI-US Virgin Islands Department of Health	Pathogen Discovery, Respiratory Viruses Branch, Division of Viral Diseases, Centers for Disease Control and Prevention	Jing Zhang, Ying Tao, Clinton R. Paden, Anna Uehara, Krista Queen, Yan Li, Haibin Wang, Zachary Weiner, Bettina Bankamp, Suxiang Tong
EPI_ISL_454660, EPI_ISL_454661, EPI_ISL_454662, EPI_ISL_454663, EPI_ISL_454664, EPI_ISL_454665, EPI_ISL_454666	County of Santa Clara Public Health Department	Chan-Zuckerberg Biohub	CZB Cliahub Consortium
EPI_ISL_454707, EPI_ISL_454708, EPI_ISL_454709, EPI_ISL_454710, EPI_ISL_454711, EPI_ISL_454712, EPI_ISL_454713, EPI_ISL_454714, EPI_ISL_454715, EPI_ISL_454716, EPI_ISL_454717, EPI_ISL_454718, EPI_ISL_454719, EPI_ISL_454720, EPI_ISL_454721, EPI_ISL_454722, EPI_ISL_454723, EPI_ISL_454724, EPI_ISL_454725, EPI_ISL_454726, EPI_ISL_454727, EPI_ISL_454728, EPI_ISL_454729, EPI_ISL_454730, EPI_ISL_454731			
see above	Utah Public Health Laboratory	Utah Public Health Laboratory	Erin Young, Kelly Oakeson
EPI_ISL_454863, EPI_ISL_454864, EPI_ISL_454865, EPI_ISL_454866, EPI_ISL_454867	Translational Health Science and Technology Institute -ESIC medical college and hospital, Faridabad	THSTI Bioassay laboratory	Saurabh Kumar, Jigme Wangchuk, Anil Kumar Pandey, Asim Das, Guruprasad R. Medigeshi
EPI_ISL_455038, EPI_ISL_455039, EPI_ISL_455040, EPI_ISL_455049, EPI_ISL_455056, EPI_ISL_455059, EPI_ISL_455095	Pathology West - NSW Health Pathology	NSW Health Pathology - Institute of Clinical Pathology and Medical Research; Westmead Hospital; University of Sydney	CIDM-PH et al.
EPI_ISL_455097	South Eastern Area Laboratory Services	NSW Health Pathology - Institute of Clinical Pathology and Medical Research; Westmead Hospital; University of Sydney	CIDM-PH et al.
EPI_ISL_455101	Jourcentralen	The Public Health Agency of Sweden	Salvatore Ascione, Anna-Malin Linde, Maria Lind Karlberg, Oskar Karlsson Lindsjo, Olov Svartstrom, Anna Risberg, Theresa Enkirch, Mia Brytting, Karin Tegmark-Wisell

EPI_ISL_455269, EPI_ISL_455270, EPI_ISL_455271, EPI_ISL_455272, EPI_ISL_455273, EPI_ISL_455274, EPI_ISL_455275, EPI_ISL_455276, EPI_ISL_455277, EPI_ISL_455278, EPI_ISL_455279			
see above	Dutch COVID-19 response team	Erasmus Medical Center	Bas Oude Munnink, David Nieuwenhuijse, Reina Sikkema, Claudia Schapendonk, Irina Chestakova, Anne van der Linden, Theo Bestebroer, Stefan van Nieuwkoop, Mark Pronk, Pascal Lexmond, Corien Swaan, Manon Haverkate, Madelief Mollers, Mart Stein, Sandra Kengne Kamga Mobou, Jeroen van Kampen, Jolanda Voermans, Aura Timen, Corine GeurtsvanKessel, Annemiek van der Eijk, Richard Molenkamp, Marion Koopmans, on behalf of the Dutch national COVID-19 response team.
EPI_ISL_455647	ICMR-National Institute of Cholera and Enteric Diseases	National Institute of Biomedical Genomics	Arindam Maitra, Mamta Chawla Sarkar, Sreedhar Chinnaswamy, Hasina Banu, Ananya Chatterjee, Shanta Dutta, Saumitra Das
EPI_ISL_455681	National Institute of Health, WHO Regional Reference Laboratory for Polio Eradication, Virology Department	National Institute of Health, WHO Regional Reference Laboratory for Polio Eradication, Virology Department	Sharif,S., Khurshid,A., Mahmood,N., Arshad,Y., Salman,M., Ikram,A., Badar,N., Umair,M., Tamim,S., Angez,M., Alam,M. and Ahad,A.
EPI_ISL_456094, EPI_ISL_456095, EPI_ISL_456096, EPI_ISL_456097, EPI_ISL_456098, EPI_ISL_456099, EPI_ISL_456100, EPI_ISL_456101, EPI_ISL_456102, EPI_ISL_456103, EPI_ISL_456104, EPI_ISL_456105, EPI_ISL_456106			
see above	Laboratory of Respiratory Viruses and Measles, Oswaldo Cruz Institute, FIOCRUZ	Laboratory of Respiratory Viruses and Measles, Oswaldo Cruz Institute, FIOCRUZ	Paola Resende, Luciana Appolinario, Fernando Motta, Aline Mattos, Milene Miranda, Cristiana Garcia, Braulia Caetano, Maria Ogrzewalska, Jonathan Lopes, Marilda Siqueira
EPI_ISL_456108	NYU Langone Health	Departments of Pathology and Medicine, New York University School of Medicine	Maria Aguero-Rosenfeld, Brendan Belovarac, Margaret Black, Ludovic Boytard, John Cadley, Paolo Cotzia, John Chen, Dacia Dimartino, Xiaojun Feng, Tatyana Gindin, Emily Guzman, Adriana Heguy, Megan Hogan, Emily Huang, George Jour, Alireza Khodadadi-Jamayran, Lawrence H. Lin, Raven Luther, Andrew Lytle, Christian Marier, Matthew T. Maurano, Mark J. Mulligan, Peter Meyn, Raquel Ordonez Ciriza, Iman Osman, Jared Pinnell, Vanessa Raabe, Sitharam Ramaswami, Amy Rapkiewicz, Andre M. Ribeiro-dos-Santos, Marie Samanovic-Golden, Antonio Serrano, Guomiao Shen, Matija Snuderl, Theodore Vougiouklakis, Nick Vulpescu, Gael Westby, Paul Zappile, Yutong Zhang
EPI_ISL_456372	Canterbury Health Laboratories	Institute of Environmental Science and Research (ESR)	Matt Storey, Xiaoyun Ren, Anja Werno, Antje van der Linden, Arlo Upton, Chris Mansell, David Hammer, Dragana Drinkovic, Erasmus Smit, Gary McAuliffe, Hana Sofia Andersson, James Ussher, Jill Sherwood, Josh Freeman, Julia Howard, Juliet Elvy, Mary DeAlmeida, Matt Blakiston, Matthew Rogers, Max Bloomfield, Michael Addidle, Michelle Balm, Sally Roberts, Sarah Jefferies, Sharmini Muttaiyah, Susan Morpeth, Susan Taylor, Timothy Blackmore, Vani Sathyendran, Veronica Playle, Virginia Hope, Erasmus Smit, Lauren Jelly, Joep de Ligt
EPI_ISL_456377, EPI_ISL_456378	MedLab Central Ltd	Institute of Environmental Science and Research (ESR)	Matt Storey, Xiaoyun Ren, Anja Werno, Antje van der Linden, Arlo Upton, Chris Mansell, David Hammer, Dragana Drinkovic, Erasmus Smit, Gary McAuliffe, Hana Sofia Andersson, James Ussher, Jill Sherwood, Josh Freeman, Julia Howard, Juliet Elvy, Mary DeAlmeida, Matt Blakiston, Matthew Rogers, Max Bloomfield, Michael Addidle, Michelle Balm, Sally Roberts, Sarah Jefferies, Sharmini Muttaiyah, Susan Morpeth, Susan Taylor, Timothy Blackmore, Vani Sathyendran, Veronica Playle, Virginia Hope, Erasmus Smit, Lauren Jelly, Joep de Ligt
EPI_ISL_456379, EPI_ISL_456380, EPI_ISL_456381, EPI_ISL_456382, EPI_ISL_456383, EPI_ISL_456384	LabPLUS	Institute of Environmental Science and Research (ESR)	Matt Storey, Xiaoyun Ren, Anja Werno, Antje van der Linden, Arlo Upton, Chris Mansell, David Hammer, Dragana Drinkovic, Erasmus Smit, Gary McAuliffe, Hana Sofia Andersson, James Ussher, Jill Sherwood, Josh Freeman, Julia Howard, Juliet Elvy, Mary DeAlmeida, Matt Blakiston, Matthew Rogers, Max Bloomfield, Michael Addidle, Michelle Balm, Sally Roberts, Sarah Jefferies, Sharmini Muttaiyah, Susan Morpeth, Susan Taylor, Timothy Blackmore, Vani Sathyendran, Veronica Playle, Virginia Hope, Erasmus Smit, Lauren Jelly, Joep de Ligt
EPI_ISL_456385, EPI_ISL_456386, EPI_ISL_456387	Middlemore Hospital	Institute of Environmental Science and Research (ESR)	Matt Storey, Xiaoyun Ren, Anja Werno, Antje van der Linden, Arlo Upton, Chris Mansell, David Hammer, Dragana Drinkovic, Erasmus Smit, Gary McAuliffe, Hana Sofia Andersson, James Ussher, Jill Sherwood, Josh Freeman, Julia Howard, Juliet Elvy, Mary DeAlmeida, Matt Blakiston, Matthew Rogers, Max Bloomfield, Michael Addidle, Michelle Balm, Sally Roberts, Sarah Jefferies, Sharmini Muttaiyah, Susan Morpeth, Susan Taylor, Timothy Blackmore, Vani Sathyendran, Veronica Playle, Virginia Hope, Erasmus Smit, Lauren Jelly, Joep de Ligt
EPI_ISL_456407	unknown	Research Center Of Tropical and Infectious Of Medical Sciences	Mollaei,H.R., Aghaei-Afshar,A., Kalantar-Neyestanaki,D.
EPI_ISL_456411, EPI_ISL_456412, EPI_ISL_456413, EPI_ISL_456414, EPI_ISL_456415, EPI_ISL_456416, EPI_ISL_456417, EPI_ISL_456418, EPI_ISL_456419, EPI_ISL_456420, EPI_ISL_456422, EPI_ISL_456423, EPI_ISL_456424, EPI_ISL_456425, EPI_ISL_456426, EPI_ISL_456428, EPI_ISL_456429, EPI_ISL_456430, EPI_ISL_456431, EPI_ISL_456432, EPI_ISL_456433, EPI_ISL_456434, EPI_ISL_456435, EPI_ISL_456436, EPI_ISL_456437, EPI_ISL_456438, EPI_ISL_456439, EPI_ISL_456440, EPI_ISL_456441, EPI_ISL_456442, EPI_ISL_456443			
see above	Victorian Infectious Diseases Reference Laboratory (VIDRL)	Microbiological Diagnostic Unit Public Health Laboratory and Victorian Infectious Diseases Reference Laboratory, Doherty Institute	Caly L., Seemann T., Sait, M., Schultz M., Druce J., Sherry, N.
EPI_ISL_456599, EPI_ISL_456607, EPI_ISL_456611, EPI_ISL_456612	National Health Laboratory, Timor-Leste	Microbiological Diagnostic Unit Public Health Laboratory, The Peter Doherty Institute for Infection and Immunity	Soares da Silva, E., Dolores de Jesus da Costa, M., Salles de Sousa, A., Jayanti Pereira Tilman, A., Antonia da Costa, E., Barreto, I., Marr, I., Wapling, J., Francis, J., Ximenes, J., Canisia, D., Freeman, K., Dakh, F., Douglas, N., Baird, R., Caly, L., Seemann, T., Sait, M., Schultz, M., Sherry, N.
EPI_ISL_456763, EPI_ISL_456764, EPI_ISL_456765, EPI_ISL_456766, EPI_ISL_456774, EPI_ISL_456880, EPI_ISL_456881	West of Scotland Specialist Virology Centre, NHSGGC / MRC-University of Glasgow Centre for Virus Research	COVID-19 Genomics UK (COG-UK) Consortium	Ana da Silva Filipe, Natasha Johnson, Kathy Smollett, Daniel Mair, Stephen Carmichael, Lily Tong, Jenna Nichols, Elihu Aranday-Cortes, Kirstyn Brunker, Yasmin Parr, Kyriaki Nomikou; Sarah McDonald, Marc Niebel, Patawee Asamaphan; Richard Orton, Joseph Hughes, Sreenu Vattipally, David L Robertson; Alasdair MacLean, Rory Gunson; Kathy Li, Natasha Jesudason, Rajiv Shah, James Shepherd, Antonia Ho, Emma Thomson
EPI_ISL_456916, EPI_ISL_456917, EPI_ISL_456918, EPI_ISL_456919, EPI_ISL_456920, EPI_ISL_456921, EPI_ISL_456922, EPI_ISL_456923, EPI_ISL_456924, EPI_ISL_456925, EPI_ISL_456926, EPI_ISL_456927, EPI_ISL_456928, EPI_ISL_456939			
see above	Virology Department, Royal Infirmary of Edinburgh, NHS Lothian / School of Biological Sciences, University of Edinburgh / Institute of Genetics and Molecular Medicine, University of Edinburgh	COVID-19 Genomics UK (COG-UK) Consortium	McHugh M, Dewar R, Rooke S, Gallagher M, Balcaza C, O'Toole Á, Scher E, Hill V, McCrone JT, Colquhoun R, Yu X, Jackson B, Rambaut A, Williams TC, Templeton K
EPI_ISL_457053, EPI_ISL_457055, EPI_ISL_457056, EPI_ISL_457057, EPI_ISL_457061, EPI_ISL_457062, EPI_ISL_457064, EPI_ISL_457070, EPI_ISL_457073, EPI_ISL_457075, EPI_ISL_457081, EPI_ISL_457083, EPI_ISL_457090, EPI_ISL_457094, EPI_ISL_457095, EPI_ISL_457100, EPI_ISL_457102, EPI_ISL_457104, EPI_ISL_457106, EPI_ISL_457107, EPI_ISL_457108, EPI_ISL_457109, EPI_ISL_457110, EPI_ISL_457111, EPI_ISL_457112, EPI_ISL_457113, EPI_ISL_457114, EPI_ISL_457115, EPI_ISL_457116, EPI_ISL_457117, EPI_ISL_457118, EPI_ISL_457178, EPI_ISL_457186, EPI_ISL_457187, EPI_ISL_457188, EPI_ISL_457189, EPI_ISL_457190, EPI_ISL_457191, EPI_ISL_457192, EPI_ISL_457193, EPI_ISL_457194, EPI_ISL_457195, EPI_ISL_457196, EPI_ISL_457197, EPI_ISL_457198, EPI_ISL_457199, EPI_ISL_457200, EPI_ISL_457201, EPI_ISL_457202, EPI_ISL_457203, EPI_ISL_457204, EPI_ISL_457205, EPI_ISL_457206, EPI_ISL_457207, EPI_ISL_457208, EPI_ISL_457209, EPI_ISL_457210, EPI_ISL_457211, EPI_ISL_457212, EPI_ISL_457213, EPI_ISL_457214, EPI_ISL_457218, EPI_ISL_457219, EPI_ISL_457220, EPI_ISL_457221			
see above	University of Exeter	COVID-19 Genomics UK (COG-UK) Consortium	Ben Temperton,Aaron Jeffries,Michelle Michelsen,Joanna Warwick-Dugdale,Audrey Farbos,Robyn Manley,Stephen Michell,Jane Masoli
EPI_ISL_457270, EPI_ISL_457271, EPI_ISL_457272, EPI_ISL_457273, EPI_ISL_457274, EPI_ISL_457275, EPI_ISL_457276, EPI_ISL_457277, EPI_ISL_457278, EPI_ISL_457279, EPI_ISL_457280, EPI_ISL_457281, EPI_ISL_457282, EPI_ISL_457283, EPI_ISL_457284, EPI_ISL_457285, EPI_ISL_457286, EPI_ISL_457287, EPI_ISL_457288, EPI_ISL_457289, EPI_ISL_457290, EPI_ISL_457291, EPI_ISL_457292, EPI_ISL_457293, EPI_ISL_457294, EPI_ISL_457295, EPI_ISL_457296, EPI_ISL_457297, EPI_ISL_457298, EPI_ISL_457299, EPI_ISL_457300, EPI_ISL_457301, EPI_ISL_457302			
see above	University College London, Great Ormond Street Hospital for Children NHS Foundation Trust, Imperial College Healthcare NHS Trust	COVID-19 Genomics UK (COG-UK) Consortium	Sergi Castellano, Rachel Williams, Mark Kristiansen, Paola Resende Silva, Sunando Roy, Tony Brooks, Helena Tutill, Paola Niola, Patricia Dyal, Charlotte Williams, Leysa Forrest, Yasmin Panchbhaya, Jacqueline Findlay, Sam Weeks, Julianne Brown, Kathryn Harris, Paul Randell, James Price, Alison Holmes, Judith Breuer
EPI_ISL_457308, EPI_ISL_457309, EPI_ISL_457310	Northumbria University / South Tees Hospitals NHS Foundation Trust / North Cumbria Integrated Care NHS Foundation Trust / North Tees and Hartlepool NHS Foundation Trust / Newcastle Hospitals NHS Foundation Trust	COVID-19 Genomics UK (COG-UK) Consortium	Darren L Smith,Andrew Nelson,Matthew Bashton,Greg R Young,Joshua Loh,John Allan,Mohammad A Tariq,Giles S Holt,Gary Black,Wen C Yew,Lynn Dover,Paul Baker,Steve Liggett,Sarah Essex,Jane Greenaway,Debra Padgett,Clive Graham,Garren Scott,Edward Barton,Emma Swindells,Brendan Payne,Jennifer Collins,Yusri Taha,Gary Eltringham
EPI_ISL_457584, EPI_ISL_457603, EPI_ISL_457604, EPI_ISL_457611, EPI_ISL_457612, EPI_ISL_457624, EPI_ISL_457626, EPI_ISL_457629, EPI_ISL_457639, EPI_ISL_457648, EPI_ISL_457654, EPI_ISL_457661, EPI_ISL_457663, EPI_ISL_457665, EPI_ISL_457676, EPI_ISL_457679, EPI_ISL_457683, EPI_ISL_457684, EPI_ISL_457685			
see above	Virology Department, Sheffield Teaching Hospitals NHS Foundation Trust/Department of Infection, Immunity and Cardiovascular Disease, The Medical School, University of Sheffield	COVID-19 Genomics UK (COG-UK) Consortium	Thushan de Silva, Matthew Parker, Nikki Smith, Adri Angyal, Rebecca Brown, Luke Green, Rachel Tucker, Paul Parsons, Danielle Groves, Katie Johnson, Laura Carrilero, Alex Keeley, Dave Partridge, Matthew Wyles, Benjamin Lindsey, Mehmet Yavuz, Mohammad Raza, Cariad Evans
EPI_ISL_457849, EPI_ISL_457850, EPI_ISL_457851, EPI_ISL_457852, EPI_ISL_457853, EPI_ISL_457854, EPI_ISL_457915, EPI_ISL_457916, EPI_ISL_457917, EPI_ISL_457918, EPI_ISL_457919, EPI_ISL_457920, EPI_ISL_457921, EPI_ISL_457922, EPI_ISL_457923, EPI_ISL_457924, EPI_ISL_457925, EPI_ISL_457926, EPI_ISL_457927, EPI_ISL_457928, EPI_ISL_457929, EPI_ISL_457930, EPI_ISL_457931			
see above	KEMRI-CGMR-C	KEMRI-Wellcome Trust Research Programme/KEMRI-CGMR-C Kilifi	Githinji G. et al 2020

EPI_ISL_457969, EPI_ISL_457970, EPI_ISL_457971	Laboratorio de Biología Molecular Asociación Española Primera en Salud	Departments of Pathology and Medicine, New York University School of Medicine	Maria Victoria Elizondo, Maria Noel Zubillaga, Gonzalo Manrique, Paul Zappile, Gael Westby, Matthew T Maurano, Christian Marier, Adriana Heguy
EPI_ISL_457982	Oman-NIC	Department of Microbiology and Immunology-SQUH	Fahad Zadjali, Samira Al-Maruqi, Amina Al Jardani, Khulood Al-Mammary, Hanan Al-kindi, Fatma BaAlawi, Hamida AL Barwani, Zeyana AL-Dahmani, Intisar Al-Shukri, Aisha Al-Busaidi, Aisha Al-Amri, Ahlam Al-Amri, Mohammed Al-Tobi, Samiha Al Kharusi, Abdulla Balkhair
EPI_ISL_458066, EPI_ISL_458067, EPI_ISL_458068	Osmania Medical College	CSIR-Centre for Cellular and Molecular Biology	Shashikala Reddy, Mahboob Khan,Payel Mukherjee, Sofia Banu, Priya Singh, Dhiviya Vedagiri, Divya Gupta, Vishal Sah, Santosh Kumar Kuncha, Krishnan Harinivas Harshan, Archana Bharadwaj Siva, Karthik Bharadwaj Tallapaka, Shagufta Khan, Lamuk Zaveri, Namami Gaur, Sakshi Shambhavi, Tulasi Nagabandi, Purushotham Vodnala, Rakesh K Mishra, Divya Tej Sowpati
EPI_ISL_458075, EPI_ISL_458077	CSIR-Centre for Cellular and Molecular Biology	CSIR-Centre for Cellular and Molecular Biology	Dhiviya Vedagiri, Divya Gupta, Vishal Sah, Payel Mukherjee, Sofia Banu, Priya Singh, Santosh Kumar Kuncha, Archana Bharadwaj Siva, Karthik Bharadwaj Tallapaka, Shagufta Khan, Lamuk Zaveri, Namami Gaur, Sakshi Shambhavi, Tulasi Nagabandi, Purushotham Vodnala, Rakesh K Mishra, Divya Tej Sowpati, Krishnan Harinivas Harshan
EPI_ISL_458120	Oman National Influenza Centre	Department of Microbiology and Immunology-SQUH	Fahad Zadjali, Samira Al-Maruqi, Amina Al Jardani, Khulood Al-Mammary, Hanan Al-kindi, Fatma BaAlawi, Hamida AL Barwani, Zeyana AL-Dahmani, Intisar Al-Shukri, Aisha Al-Busaidi, Aisha Al-Amri, Ahlam Al-Amri, Mohammed Al-Tobi, Samiha Al Kharusi, Abdulla Balkhair
EPI_ISL_458137	Oman National Influenza Centre	Department of Microbiology and Immunology	Fahad Zadjali, Samira Al-Maruqi, Amina Al Jardani, Khulood Al-Mammary, Hanan Al-kindi, Fatma BaAlawi, Hamida AL Barwani, Zeyana AL-Dahmani, Intisar Al-Shukri, Aisha Al-Busaidi, Aisha Al-Amri, Ahlam Al-Amri, Mohammed Al-Tobi, Samiha Al Kharusi, Abdulla Balkhair
EPI_ISL_458143, EPI_ISL_458144	Evandro Chagas Institute	Evandro Chagas Institute	Santos, M.C.; Silva, A.M.; Junior, W.D.C.; Barbagelata, L.S.; Ferreira, J.A.; Sousa, E.M.A.; da Silva, P.S.; Resque, H.R; Martins, L.C.; Sousa Junior, E.C.;Viana, G.M.R
EPI_ISL_458246, EPI_ISL_458247, EPI_ISL_458251, EPI_ISL_458258, EPI_ISL_458259, EPI_ISL_458268, EPI_ISL_458273, EPI_ISL_458284	Scripps Medical Laboratory	Andersen lab at Scripps Research	SEARCH Alliance San Diego with Michael Quigley, Ellen Stefanski, Ian Mchardy
EPI_ISL_458303, EPI_ISL_458305, EPI_ISL_458306, EPI_ISL_458309, EPI_ISL_458311, EPI_ISL_458315, EPI_ISL_458316, EPI_ISL_458317, EPI_ISL_458320, EPI_ISL_458326, EPI_ISL_458332, EPI_ISL_458336, EPI_ISL_458341, EPI_ISL_458343, EPI_ISL_458348, EPI_ISL_458349, EPI_ISL_458353, EPI_ISL_458359, EPI_ISL_458361, EPI_ISL_458368, EPI_ISL_458370, EPI_ISL_458371, EPI_ISL_458373, EPI_ISL_458374, EPI_ISL_458375, EPI_ISL_458377, EPI_ISL_458380, EPI_ISL_458381, EPI_ISL_458384, EPI_ISL_458386, EPI_ISL_458387, EPI_ISL_458391, EPI_ISL_458395, EPI_ISL_458396, EPI_ISL_458398, EPI_ISL_458401, EPI_ISL_458405, EPI_ISL_458409, EPI_ISL_458416, EPI_ISL_458419, EPI_ISL_458422, EPI_ISL_458439, EPI_ISL_458443, EPI_ISL_458444, EPI_ISL_458449, EPI_ISL_458450, EPI_ISL_458451, EPI_ISL_458456, EPI_ISL_458457, EPI_ISL_458459, EPI_ISL_458463, EPI_ISL_458467, EPI_ISL_458468, EPI_ISL_458473, EPI_ISL_458475, EPI_ISL_458477, EPI_ISL_458478, EPI_ISL_458480, EPI_ISL_458482, EPI_ISL_458498, EPI_ISL_458500, EPI_ISL_458501, EPI_ISL_458507, EPI_ISL_458509, EPI_ISL_458511, EPI_ISL_458512, EPI_ISL_458719, EPI_ISL_458720, EPI_ISL_458721, EPI_ISL_458723, EPI_ISL_458724, EPI_ISL_458725, EPI_ISL_458726, EPI_ISL_458727, EPI_ISL_458728, EPI_ISL_458729, EPI_ISL_458730, EPI_ISL_458731, EPI_ISL_458732, EPI_ISL_458733, EPI_ISL_458735, EPI_ISL_458736, EPI_ISL_458737, EPI_ISL_458739, EPI_ISL_458740, EPI_ISL_458741, EPI_ISL_458743, EPI_ISL_458744, EPI_ISL_458745, EPI_ISL_458746, EPI_ISL_458747, EPI_ISL_458748, EPI_ISL_458749, EPI_ISL_458750, EPI_ISL_458751, EPI_ISL_458752, EPI_ISL_458753, EPI_ISL_458755, EPI_ISL_458756, EPI_ISL_458757, EPI_ISL_458758, EPI_ISL_458759, EPI_ISL_458760, EPI_ISL_458761, EPI_ISL_458763, EPI_ISL_458767, EPI_ISL_458771, EPI_ISL_458772, EPI_ISL_458773, EPI_ISL_458774, EPI_ISL_458775, EPI_ISL_458776, EPI_ISL_458777, EPI_ISL_458779, EPI_ISL_458780, EPI_ISL_458781, EPI_ISL_458782, EPI_ISL_458783, EPI_ISL_458784, EPI_ISL_458785			
see above	PHE South West Regional Laboratory, National Infection Service	Wellcome Sanger Institute for the COVID-19 Genomics UK (COG-UK) consortium	Stephanie Hutchings, Hannah Pymont, Dr Peter Muir, Barry Vipond, Rich Hopes; and Alex Alderton, Roberto Amato, Sonia Goncalves, Ewan Harrison, David K. Jackson, Ian Johnston, Dominic Kwiatkowski, Cordelia Langford, John Sillitoe on behalf of the Wellcome Sanger Institute COVID-19 Surveillance Team (http://www.sanger.ac.uk/covid-team)
EPI_ISL_458786	PHE South West Regional Laboratory, National Infection Service	Wellcome Sanger Institute for the COVID-19 Genomics UK (COG-UK) Consortium	Stephanie Hutchings, Hannah Pymont, Dr Peter Muir, Barry Vipond, Rich Hopes; and Alex Alderton, Roberto Amato, Sonia Goncalves, Ewan Harrison, David K. Jackson, Ian Johnston, Dominic Kwiatkowski, Cordelia Langford, John Sillitoe on behalf of the Wellcome Sanger Institute COVID-19 Surveillance Team
EPI_ISL_458787, EPI_ISL_458789, EPI_ISL_458790, EPI_ISL_458791, EPI_ISL_458792, EPI_ISL_458793, EPI_ISL_458794, EPI_ISL_458795, EPI_ISL_458796, EPI_ISL_458797, EPI_ISL_458798, EPI_ISL_458799, EPI_ISL_458803, EPI_ISL_458804, EPI_ISL_458805, EPI_ISL_458806, EPI_ISL_458807, EPI_ISL_458811, EPI_ISL_458812, EPI_ISL_458813, EPI_ISL_458814, EPI_ISL_458815, EPI_ISL_458816, EPI_ISL_458818, EPI_ISL_458819, EPI_ISL_458822, EPI_ISL_458824, EPI_ISL_458826, EPI_ISL_458827, EPI_ISL_458829, EPI_ISL_458831, EPI_ISL_458832, EPI_ISL_458833, EPI_ISL_458835, EPI_ISL_458836, EPI_ISL_458838, EPI_ISL_458839, EPI_ISL_458840, EPI_ISL_458842, EPI_ISL_458843, EPI_ISL_458844, EPI_ISL_458845, EPI_ISL_458847, EPI_ISL_458848, EPI_ISL_458850, EPI_ISL_458852, EPI_ISL_458853, EPI_ISL_458854, EPI_ISL_458856, EPI_ISL_458857, EPI_ISL_458858, EPI_ISL_458859, EPI_ISL_458861, EPI_ISL_458863, EPI_ISL_458865, EPI_ISL_458866, EPI_ISL_458867, EPI_ISL_458868, EPI_ISL_458870, EPI_ISL_458871			
see above	PHE South West Regional Laboratory, National Infection Service	Wellcome Sanger Institute for the COVID-19 Genomics UK (COG-UK) consortium	Stephanie Hutchings, Hannah Pymont, Dr Peter Muir, Barry Vipond, Rich Hopes; and Alex Alderton, Roberto Amato, Sonia Goncalves, Ewan Harrison, David K. Jackson, Ian Johnston, Dominic Kwiatkowski, Cordelia Langford, John Sillitoe on behalf of the Wellcome Sanger Institute COVID-19 Surveillance Team (http://www.sanger.ac.uk/covid-team)
EPI_ISL_458872	PHE South West Regional Laboratory, National Infection Service	Wellcome Sanger Institute for the COVID-19 Genomics UK (COG-UK) Consortium	Stephanie Hutchings, Hannah Pymont, Dr Peter Muir, Barry Vipond, Rich Hopes; and Alex Alderton, Roberto Amato, Sonia Goncalves, Ewan Harrison, David K. Jackson, Ian Johnston, Dominic Kwiatkowski, Cordelia Langford, John Sillitoe on behalf of the Wellcome Sanger Institute COVID-19 Surveillance Team
EPI_ISL_458873, EPI_ISL_458874, EPI_ISL_458876, EPI_ISL_458877, EPI_ISL_458878, EPI_ISL_458879, EPI_ISL_458880, EPI_ISL_458881, EPI_ISL_458882, EPI_ISL_458883, EPI_ISL_458884, EPI_ISL_458885, EPI_ISL_458886, EPI_ISL_458887, EPI_ISL_458888, EPI_ISL_458889, EPI_ISL_458891, EPI_ISL_458892, EPI_ISL_458893, EPI_ISL_458896, EPI_ISL_458897, EPI_ISL_458898, EPI_ISL_458900, EPI_ISL_458901, EPI_ISL_458903, EPI_ISL_458905, EPI_ISL_458907, EPI_ISL_458909, EPI_ISL_458910, EPI_ISL_458911			
see above	PHE South West Regional Laboratory, National Infection Service	Wellcome Sanger Institute for the COVID-19 Genomics UK (COG-UK) consortium	Stephanie Hutchings, Hannah Pymont, Dr Peter Muir, Barry Vipond, Rich Hopes; and Alex Alderton, Roberto Amato, Sonia Goncalves, Ewan Harrison, David K. Jackson, Ian Johnston, Dominic Kwiatkowski, Cordelia Langford, John Sillitoe on behalf of the Wellcome Sanger Institute COVID-19 Surveillance Team (http://www.sanger.ac.uk/covid-team)
EPI_ISL_459324, EPI_ISL_459334, EPI_ISL_459341, EPI_ISL_459344, EPI_ISL_459346, EPI_ISL_459350, EPI_ISL_459351, EPI_ISL_459361, EPI_ISL_459362, EPI_ISL_459371, EPI_ISL_459373, EPI_ISL_459382, EPI_ISL_459384, EPI_ISL_459392, EPI_ISL_459395, EPI_ISL_459396, EPI_ISL_459400			
see above	Regional Virus Laboratory, Belfast Health and Social Care Trust	Wellcome Sanger Institute for the COVID-19 Genomics UK (COG-UK) consortium	Conall McCaughey, James McKenna, Tanya Curran, Susan Feeney, Alison Watt, Ciara Cox, Mairead Connor, Zoltan Molnar, David Simpson, Derek Fairley; and Alex Alderton, Roberto Amato, Sonia Goncalves, Ewan Harrison, David K. Jackson, Ian Johnston, Dominic Kwiatkowski, Cordelia Langford, John Sillitoe on behalf of the Wellcome Sanger Institute COVID-19 Surveillance Team (http://www.sanger.ac.uk/covid-team)
EPI_ISL_459897	Laboratoire National de Sante, Microbiology, Virology	Laboratoire National de Sante, Microbiology, Epidemiology and Microbial Genomics	Anke Wienecke-Baldacchino, Jessica Tapp, Guillaume Fournier, Tamir Abdelrahman, Trung Nguyen Nguyen, Catherine Ragimbeau
EPI_ISL_459910	Zoonotic and Exotic infection Diseases Division, Harbin Veterinary Research Institute, CAAS	Zoonotic and Exotic infection Diseases Division, Harbin Veterinary Resrarch Institute, CAAS	Jinliang Wang, Lei Shuai, Chong Wang, Renqiang Liu, Xijun He, Xianfeng Zhang, Ziruo Sun, Dan Shan, Jinying Ge, Xijun Wang, Gongxun Zhong, Zhiyuan Wen, Zhigao Bu
EPI_ISL_459979, EPI_ISL_459980, EPI_ISL_459981, EPI_ISL_459982	Institut Pasteur du Maroc	Institut Pasteur du Maroc	Marion Barbet, Sylvie Behillil, Méline Bizard, Angela Brisebarre, Camille Capel, Etienne Simon-Lorière, Vincent Enouf, Maud Vanpeene, Sylvie van der Werf, Latifa Anga, Abdellah Faouzi, Anass Abbad, Mjid Eloualid, Jalal Nourlil, Anderrahmane Maaroufi
EPI_ISL_460584	Michigan Department of Health and Human Services, Bureau of Laboratories	Michigan Department of Health and Human Services, Bureau of Laboratories	Blankenship HM, Riner D, Soehnlen MK
EPI_ISL_460617, EPI_ISL_460618, EPI_ISL_460619	unknown	Physiology	Pence,S., Caykara,B., Pence,H.H., Tekin,S., Yiyit,N., Cevher Keskin,B., Kara,A.
EPI_ISL_460639, EPI_ISL_460640, EPI_ISL_460657, EPI_ISL_460658, EPI_ISL_460659, EPI_ISL_460735, EPI_ISL_460736, EPI_ISL_460756, EPI_ISL_460757, EPI_ISL_460758, EPI_ISL_460759, EPI_ISL_460760, EPI_ISL_460761, EPI_ISL_460762, EPI_ISL_460790, EPI_ISL_460803, EPI_ISL_460804, EPI_ISL_460805, EPI_ISL_460806, EPI_ISL_460807, EPI_ISL_460808, EPI_ISL_460809, EPI_ISL_460810, EPI_ISL_460811, EPI_ISL_460812, EPI_ISL_460813, EPI_ISL_460814, EPI_ISL_460819, EPI_ISL_460823, EPI_ISL_460831, EPI_ISL_460832, EPI_ISL_460842, EPI_ISL_460844, EPI_ISL_460845, EPI_ISL_460846, EPI_ISL_460847, EPI_ISL_460848, EPI_ISL_460850, EPI_ISL_460851, EPI_ISL_460852, EPI_ISL_460853, EPI_ISL_460927, EPI_ISL_460928, EPI_ISL_460936, EPI_ISL_460940, EPI_ISL_461014, EPI_ISL_461015, EPI_ISL_461022, EPI_ISL_461027, EPI_ISL_461028, EPI_ISL_461040, EPI_ISL_461046, EPI_ISL_461048, EPI_ISL_461049, EPI_ISL_461050, EPI_ISL_461070, EPI_ISL_461073, EPI_ISL_461124, EPI_ISL_461133, EPI_ISL_461134, EPI_ISL_461135, EPI_ISL_461136, EPI_ISL_461137, EPI_ISL_461138, EPI_ISL_461172, EPI_ISL_461241, EPI_ISL_461243, EPI_ISL_461292, EPI_ISL_461314, EPI_ISL_461316, EPI_ISL_461317, EPI_ISL_461318, EPI_ISL_461319, EPI_ISL_461320, EPI_ISL_461321, EPI_ISL_461322, EPI_ISL_461323, EPI_ISL_461324, EPI_ISL_461338, EPI_ISL_461344, EPI_ISL_461345, EPI_ISL_461346, EPI_ISL_461347, EPI_ISL_461348, EPI_ISL_461349			
see above	Dutch COVID-19 response team	Erasmus Medical Center	Bas Oude Munnink, David Nieuwenhuijse, Reina Sikkema, Claudia Schapendonk, Irina Chestakova, Anne van der Linden, Theo Bestebroer, Stefan van Nieuwkoop, Mark Pronk, Pascal Lexmond, Corien Swaan, Manon Haverkate, Madelief Mollers, Mart Stein, Sandra Kengne Kamga Mobou, Jeroen van Kampen, Jolanda Voermans, Aura Timen, Corine GeurtsvanKessel, Annemiek van der Eijk, Richard Molenkamp, Marion Koopmans, on behalf of the Dutch national COVID-19 response team.
EPI_ISL_461795, EPI_ISL_461796, EPI_ISL_461797, EPI_ISL_461798, EPI_ISL_461799	Northumbria University / South Tees Hospitals NHS Foundation Trust / North Cumbria Integrated Care NHS Foundation Trust / North Tees and Hartlepool NHS Foundation Trust / Newcastle Hospitals NHS Foundation Trust	COVID-19 Genomics UK (COG-UK) Consortium	Darren L Smith,Andrew Nelson,Matthew Bashton,Greg R Young,Joshua Loh,John Allan,Mohammad A Tariq,Giles S Holt,Gary Black,Wen C Yew,Lynn Dover,Paul Baker,Steve Liggett,Sarah Essex,Jane Greenaway,Debra Padgett,Clive Graham,Garren Scott,Edward Barton,Emma Swindells,Brendan Payne,Jennifer Collins,Yusri Taha,Gary Eltringham

EPI_ISL_462000, EPI_ISL_462001, EPI_ISL_462004, EPI_ISL_462007, EPI_ISL_462008, EPI_ISL_462010, EPI_ISL_462011, EPI_ISL_462012, EPI_ISL_462013, EPI_ISL_462014, EPI_ISL_462016, EPI_ISL_462017, EPI_ISL_462018, EPI_ISL_462019, EPI_ISL_462022, EPI_ISL_462023, EPI_ISL_462026, EPI_ISL_462027, EPI_ISL_462029, EPI_ISL_462031, EPI_ISL_462032, EPI_ISL_462037, EPI_ISL_462040, EPI_ISL_462041, EPI_ISL_462042, EPI_ISL_462045, EPI_ISL_462046, EPI_ISL_462048, EPI_ISL_462049, EPI_ISL_462050, EPI_ISL_462053, EPI_ISL_462054, EPI_ISL_462060, EPI_ISL_462061, EPI_ISL_462062, EPI_ISL_462064, EPI_ISL_462066, EPI_ISL_462067, EPI_ISL_462068, EPI_ISL_462071, EPI_ISL_462072, EPI_ISL_462073, EPI_ISL_462074, EPI_ISL_462075, EPI_ISL_462077, EPI_ISL_462079, EPI_ISL_462080, EPI_ISL_462081, EPI_ISL_462082, EPI_ISL_462083			
see above	Virology Department, Sheffield Teaching Hospitals NHS Foundation Trust/Department of Infection, Immunity and Cardiovascular Disease, The Medical School, University of Sheffield	COVID-19 Genomics UK (COG-UK) Consortium	Thushan de Silva, Matthew Parker, Nikki Smith, Adri Angyal, Rebecca Brown, Luke Green, Rachel Tucker, Paul Parsons, Danielle Groves, Katie Johnson, Laura Carrilero, Alex Keeley, Dave Partridge, Matthew Wyles, Benjamin Lindsey, Mehmet Yavuz, Mohammad Raza, Cariad Evans
EPI_ISL_462276, EPI_ISL_462288, EPI_ISL_462289, EPI_ISL_462331, EPI_ISL_462344, EPI_ISL_462347, EPI_ISL_462380, EPI_ISL_462388, EPI_ISL_462394, EPI_ISL_462397, EPI_ISL_462399, EPI_ISL_462403, EPI_ISL_462408, EPI_ISL_462417, EPI_ISL_462423, EPI_ISL_462424, EPI_ISL_462425, EPI_ISL_462426, EPI_ISL_462430			
see above	National Public Health Laboratory, National Centre for Infectious Diseases	National Public Health Laboratory, National Centre for Infectious Diseases	Mak TM, Octavia S, Chavatte JM, Cui L, Lin RTP
EPI_ISL_462702, EPI_ISL_462703, EPI_ISL_462704, EPI_ISL_462705, EPI_ISL_462706, EPI_ISL_462707, EPI_ISL_462708, EPI_ISL_462709, EPI_ISL_462710, EPI_ISL_462711, EPI_ISL_462712, EPI_ISL_462713, EPI_ISL_462714, EPI_ISL_462715, EPI_ISL_462716, EPI_ISL_462717			
see above	Michigan Department of Health and Human Services, Bureau of Laboratories	Michigan Department of Health and Human Services, Bureau of Laboratories	Blankenship HM, Riner D, Soehnlen MK
EPI_ISL_463302	United Christian Hospital	Hong Kong Department of Health	Mak Gannon C.K., Cheng Peter K.C., Lam Edman T.K., Chan Rickjason C.W., Tsang Dominic N.C.
EPI_ISL_463330, EPI_ISL_463363, EPI_ISL_463364, EPI_ISL_463365, EPI_ISL_463366, EPI_ISL_463367, EPI_ISL_463368	Washington State Department of Health	Seattle Flu Study	Chu et al
EPI_ISL_463747	Department of Molecular Virology, Cyprus Institute of Neurology and Genetics	Department of Molecular Virology, Cyprus Institute of Neurology and Genetics	Jan Richter, George Krashias, Christina Tryfonos, Stavros Bashiardes, Dana Koptides, Christina Christodoulou
EPI_ISL_463914, EPI_ISL_463926, EPI_ISL_463953, EPI_ISL_463961, EPI_ISL_463963, EPI_ISL_463964, EPI_ISL_463965, EPI_ISL_463966, EPI_ISL_463969	Laboratoire de microbiologie, Hopital de Verdun	Smith Laboratory, Centre de Recherche CHU Sainte-Justine	Martin Smith, Marieke Rozendaal, Ivan Pavlov
EPI_ISL_463995, EPI_ISL_463998, EPI_ISL_464019, EPI_ISL_464057, EPI_ISL_464058	Unity Health Toronto	Ontario Institute for Cancer Research	Ramzi Fattouh,Larissa M. Matukas,Mark Downing,Annette Gower,Karel Boissinot,Samira Mubareka,TIBDN,Ilinca Lungu,Bernard Lam,Jeremy Johns,Paul Krzyzanowski,Richard de Borja,Philip Zuzarte,Jared Simpson
EPI_ISL_465165, EPI_ISL_465166, EPI_ISL_465167, EPI_ISL_465168, EPI_ISL_465169, EPI_ISL_465170, EPI_ISL_465171, EPI_ISL_465172, EPI_ISL_465173, EPI_ISL_465174, EPI_ISL_465175, EPI_ISL_465176, EPI_ISL_465177, EPI_ISL_465178, EPI_ISL_465179, EPI_ISL_465180, EPI_ISL_465181, EPI_ISL_465182, EPI_ISL_465183, EPI_ISL_465184, EPI_ISL_465185, EPI_ISL_465186, EPI_ISL_465187, EPI_ISL_465188, EPI_ISL_465189, EPI_ISL_465190, EPI_ISL_465191, EPI_ISL_465192, EPI_ISL_465193, EPI_ISL_465194, EPI_ISL_465195, EPI_ISL_465196, EPI_ISL_465197, EPI_ISL_465198, EPI_ISL_465199, EPI_ISL_465200, EPI_ISL_465201, EPI_ISL_465202, EPI_ISL_465203, EPI_ISL_465204, EPI_ISL_465205, EPI_ISL_465206, EPI_ISL_465207, EPI_ISL_465208, EPI_ISL_465209, EPI_ISL_465210, EPI_ISL_465211, EPI_ISL_465212, EPI_ISL_465213, EPI_ISL_465214, EPI_ISL_465215, EPI_ISL_465216, EPI_ISL_465217, EPI_ISL_465218, EPI_ISL_465219, EPI_ISL_465220, EPI_ISL_465221, EPI_ISL_465222, EPI_ISL_465223, EPI_ISL_465225, EPI_ISL_465226, EPI_ISL_465227, EPI_ISL_465228, EPI_ISL_465229, EPI_ISL_465230, EPI_ISL_465231, EPI_ISL_465232, EPI_ISL_465233, EPI_ISL_465234, EPI_ISL_465235, EPI_ISL_465236, EPI_ISL_465237, EPI_ISL_465238, EPI_ISL_465239, EPI_ISL_465240, EPI_ISL_465241, EPI_ISL_465242, EPI_ISL_465243, EPI_ISL_465244, EPI_ISL_465245, EPI_ISL_465246, EPI_ISL_465247, EPI_ISL_465248, EPI_ISL_465249, EPI_ISL_465250, EPI_ISL_465251, EPI_ISL_465252, EPI_ISL_465253, EPI_ISL_465254, EPI_ISL_465255, EPI_ISL_465256, EPI_ISL_465257, EPI_ISL_465258, EPI_ISL_465259, EPI_ISL_465260, EPI_ISL_465261, EPI_ISL_465264, EPI_ISL_465265, EPI_ISL_465266, EPI_ISL_465267, EPI_ISL_465268, EPI_ISL_465269, EPI_ISL_465270, EPI_ISL_465271, EPI_ISL_465272, EPI_ISL_465273, EPI_ISL_465274, EPI_ISL_465279, EPI_ISL_465280, EPI_ISL_465282, EPI_ISL_465284, EPI_ISL_465285, EPI_ISL_465286, EPI_ISL_465292, EPI_ISL_465293, EPI_ISL_465295, EPI_ISL_465296, EPI_ISL_465297, EPI_ISL_465308, EPI_ISL_465309, EPI_ISL_465310, EPI_ISL_465313, EPI_ISL_465314, EPI_ISL_465320, EPI_ISL_465321, EPI_ISL_465322, EPI_ISL_465323, EPI_ISL_465324, EPI_ISL_465336, EPI_ISL_465343, EPI_ISL_465345, EPI_ISL_465346, EPI_ISL_465347, EPI_ISL_465351, EPI_ISL_465352, EPI_ISL_465353, EPI_ISL_465354, EPI_ISL_465356, EPI_ISL_465396, EPI_ISL_465397, EPI_ISL_465398, EPI_ISL_465400, EPI_ISL_465406, EPI_ISL_465410, EPI_ISL_465431, EPI_ISL_465447, EPI_ISL_466376, EPI_ISL_466377, EPI_ISL_466378, EPI_ISL_466379, EPI_ISL_466380, EPI_ISL_466381, EPI_ISL_466382, EPI_ISL_466383, EPI_ISL_466384, EPI_ISL_466385, EPI_ISL_466386, EPI_ISL_466387, EPI_ISL_466388, EPI_ISL_466389, EPI_ISL_466390, EPI_ISL_466391, EPI_ISL_466392, EPI_ISL_466395, EPI_ISL_466396, EPI_ISL_466397, EPI_ISL_466398, EPI_ISL_466399, EPI_ISL_466402, EPI_ISL_466403, EPI_ISL_466404, EPI_ISL_466405, EPI_ISL_466406, EPI_ISL_466420, EPI_ISL_466426, EPI_ISL_466427, EPI_ISL_466432, EPI_ISL_466433, EPI_ISL_466434, EPI_ISL_466435, EPI_ISL_466436, EPI_ISL_466437, EPI_ISL_466438, EPI_ISL_466439, EPI_ISL_466440, EPI_ISL_466441, EPI_ISL_466442, EPI_ISL_466443, EPI_ISL_466444, EPI_ISL_466445, EPI_ISL_466446, EPI_ISL_466447, EPI_ISL_466448, EPI_ISL_466449, EPI_ISL_466450, EPI_ISL_466451, EPI_ISL_466452, EPI_ISL_466453, EPI_ISL_466454, EPI_ISL_466455, EPI_ISL_466456, EPI_ISL_466457, EPI_ISL_466458, EPI_ISL_466460, EPI_ISL_466461, EPI_ISL_466462, EPI_ISL_466463, EPI_ISL_466464, EPI_ISL_466465, EPI_ISL_466466, EPI_ISL_466478, EPI_ISL_466479, EPI_ISL_466480, EPI_ISL_466481, EPI_ISL_466482, EPI_ISL_466483, EPI_ISL_466484, EPI_ISL_466485, EPI_ISL_466486, EPI_ISL_466487, EPI_ISL_466488, EPI_ISL_466489, EPI_ISL_466490, EPI_ISL_466491, EPI_ISL_466492, EPI_ISL_466493, EPI_ISL_466494, EPI_ISL_466495, EPI_ISL_466496, EPI_ISL_466497, EPI_ISL_466498, EPI_ISL_466499, EPI_ISL_466500, EPI_ISL_466501, EPI_ISL_466502, EPI_ISL_466503, EPI_ISL_466504, EPI_ISL_466505, EPI_ISL_466506, EPI_ISL_466507, EPI_ISL_466508, EPI_ISL_466509, EPI_ISL_466510, EPI_ISL_466511, EPI_ISL_466512, EPI_ISL_466513, EPI_ISL_466514, EPI_ISL_466515, EPI_ISL_466516, EPI_ISL_466517, EPI_ISL_466518, EPI_ISL_466519, EPI_ISL_466520, EPI_ISL_466521, EPI_ISL_466522, EPI_ISL_466523, EPI_ISL_466524, EPI_ISL_466525, EPI_ISL_466526, EPI_ISL_466527, EPI_ISL_466528, EPI_ISL_466529, EPI_ISL_466530, EPI_ISL_466531, EPI_ISL_466532, EPI_ISL_466533, EPI_ISL_466534, EPI_ISL_466535, EPI_ISL_466536, EPI_ISL_466537, EPI_ISL_466538, EPI_ISL_466540, EPI_ISL_466541, EPI_ISL_466542, EPI_ISL_466543, EPI_ISL_466544, EPI_ISL_466545, EPI_ISL_466546, EPI_ISL_466547, EPI_ISL_466548, EPI_ISL_466549, EPI_ISL_466550, EPI_ISL_466551, EPI_ISL_466552, EPI_ISL_466553, EPI_ISL_466554, EPI_ISL_466555, EPI_ISL_466556, EPI_ISL_466557, EPI_ISL_466558, EPI_ISL_466560, EPI_ISL_466561, EPI_ISL_466562, EPI_ISL_466563, EPI_ISL_466565, EPI_ISL_466566, EPI_ISL_466567, EPI_ISL_466568, EPI_ISL_466569, EPI_ISL_466570, EPI_ISL_466571, EPI_ISL_466572, EPI_ISL_466573, EPI_ISL_466574, EPI_ISL_466575, EPI_ISL_466576, EPI_ISL_466577, EPI_ISL_466578, EPI_ISL_466579, EPI_ISL_466580, EPI_ISL_466581, EPI_ISL_466582, EPI_ISL_466583, EPI_ISL_466584, EPI_ISL_466585, EPI_ISL_466586, EPI_ISL_466587, EPI_ISL_466588, EPI_ISL_466589, EPI_ISL_466590, EPI_ISL_466591, EPI_ISL_466592, EPI_ISL_466593, EPI_ISL_466594, EPI_ISL_466595, EPI_ISL_466596, EPI_ISL_466597, EPI_ISL_466598, EPI_ISL_466599, EPI_ISL_466600, EPI_ISL_466601, EPI_ISL_466602, EPI_ISL_466603, EPI_ISL_466604, EPI_ISL_466605, EPI_ISL_466606, EPI_ISL_466607, EPI_ISL_466608, EPI_ISL_466609, EPI_ISL_466610, EPI_ISL_466611, EPI_ISL_466612, EPI_ISL_466614, EPI_ISL_466616, EPI_ISL_466617, EPI_ISL_466618, EPI_ISL_466619, EPI_ISL_466620, EPI_ISL_466621, EPI_ISL_466622, EPI_ISL_466623, EPI_ISL_466624, EPI_ISL_466625			
see above	Respiratory Virus Unit, Microbiology Services Colindale, Public Health England	Respiratory Virus Unit, Microbiology Services Colindale, Public Health England	PHE Covid Sequencing Team
EPI_ISL_466875, EPI_ISL_466876, EPI_ISL_466877, EPI_ISL_466878, EPI_ISL_466879, EPI_ISL_466880, EPI_ISL_466881, EPI_ISL_466882, EPI_ISL_466883, EPI_ISL_466884, EPI_ISL_466885, EPI_ISL_466886, EPI_ISL_466887, EPI_ISL_466889			
see above	Max von Pettenkofer Institute, Virology, National Reference Center for Retroviruses, LMU München	Laboratory for Functional Genome Analysis, Dept. Genomics, Gene Center of the LMU Munich	Max Muenchhoff, Stefan Krebs, Alexander Graf, Oliver Keppler, Helmut Blum
EPI_ISL_467262, EPI_ISL_467265, EPI_ISL_467266, EPI_ISL_467267, EPI_ISL_467268, EPI_ISL_467269, EPI_ISL_467272, EPI_ISL_467274, EPI_ISL_467276, EPI_ISL_467277, EPI_ISL_467280, EPI_ISL_467281, EPI_ISL_467282, EPI_ISL_467283, EPI_ISL_467284, EPI_ISL_467285, EPI_ISL_467288, EPI_ISL_467289, EPI_ISL_467290, EPI_ISL_467291, EPI_ISL_467292, EPI_ISL_467296			
see above	Hospital Clínico Universitario de Santiago de Compostela	SeqCOVID-SPAIN consortium/IBV(CSIC)	José Javier Costa Alcalde, Antonio Aguilera Guirao, Mª Luisa Pérez del Molino Bernal, Amparo Coira Nieto, Gema Barbeito Castiñeiras, Rocio Trastoy Pena and SeqCOVID-SPAIN consortium
EPI_ISL_467347, EPI_ISL_467348, EPI_ISL_467349, EPI_ISL_467350, EPI_ISL_467351	Laboratory of Respiratory Viruses and Measles, Oswaldo Cruz Institute, FIOCRUZ	Laboratory of Respiratory Viruses and Measles, Oswaldo Cruz Institute, FIOCRUZ	Paola Resende, Luciana Appolinario, Fernando Motta, Anna Carolina Paixão, Ana Carolina Mendonça, Aline Mattos, Milene Miranda, Cristiana Garcia, Braulia Caetano, Maria Ogrzewalska, Jonathan Lopes, Marilda Siqueira
EPI_ISL_467516	CAPRISA	KRISP, KZN Research Innovation and Sequencing Platform	Giandhari J, Pillay S, Lessells R, Chimukangara B, Mdlalose K, York D, Khan S, Tegally H, Wilkinson E, de Oliveira T
EPI_ISL_467782, EPI_ISL_467783, EPI_ISL_467784, EPI_ISL_467785, EPI_ISL_467786, EPI_ISL_467787, EPI_ISL_467788, EPI_ISL_467789, EPI_ISL_467790, EPI_ISL_467791, EPI_ISL_467792, EPI_ISL_467943, EPI_ISL_467944			
see above	Virginia DCLS	Virginia DCLS	Virginia DCLS
EPI_ISL_467963	San Diego County Public Health Laboratory	Andersen lab at Scripps Research	SEARCH Alliance San Diego with Michael Quigley, Ellen Stefanski, Ian Mchardy
EPI_ISL_467972	Scripps Medical Laboratory	Andersen lab at Scripps Research	SEARCH Alliance San Diego with Michael Quigley, Ellen Stefanski, Ian Mchardy
EPI_ISL_468066	Physiology, Istanbul Medeniyet University	Physiology, Istanbul Medeniyet University	Pence,S., Caykara,B., Pence,H.H., Tekin,S., Yiyit,N., Cevher Keskin,B. and Kara,A.
EPI_ISL_468071, EPI_ISL_468072	Child Health Research Foundation	Child Health Research Foundation	Senjuti Saha, Roly Malaker, Md Saiful Islam Sajib, Hafizur Rahman, Maksuda Islam, Samir K Saha
EPI_ISL_468080, EPI_ISL_468081, EPI_ISL_468082, EPI_ISL_468083, EPI_ISL_468084, EPI_ISL_468086	OHSU Lab Services Molecular Microbiology Lab	Oregon SARS-CoV-2 Genome Sequencing Center	Brendan L. O'Connell, Ruth V. Nichols, Alec J. Hirsch, Guang Fan, Daniel N. Streblow, William B. Messer, Andrew C. Adey, Benjamin N. Bimber, Brian J. O'Roak
EPI_ISL_468151, EPI_ISL_468152, EPI_ISL_468153, EPI_ISL_468154, EPI_ISL_468155	[Romania, Bucharest] National Institute for Infectious Diseases "Prof. Dr. Matei Bal"	[Romania, Bucharest] National Institute for Infectious Diseases "Prof. Dr. Matei Bal"	Leontina Banica, Marius Cotic, Corina Casangiu, Marius Surleac, Simona Paraschiv

EPI_ISL_468202, EPI_ISL_468203, EPI_ISL_468204, EPI_ISL_468205, EPI_ISL_468206, EPI_ISL_468207, EPI_ISL_468209, EPI_ISL_468210, EPI_ISL_468211, EPI_ISL_468212, EPI_ISL_468213, EPI_ISL_468214, EPI_ISL_468215, EPI_ISL_468216, EPI_ISL_468217, EPI_ISL_468218, EPI_ISL_468219, EPI_ISL_468220, EPI_ISL_468221, EPI_ISL_468222, EPI_ISL_468224, EPI_ISL_468225			
see above	Viollier AG	Department of Biosystems Science and Engineering, ETH Zürich	Christian Beisel, Sarah Nadeau, Ivan Topolsky, Pedro Ferreira, Philipp Jablonski, Susana Posada-Céspedes, Tobias Schär, Ina Nissen, Natascha Santacroce, Elodie Burcklen, Christiane Beckmann, Maurice Redondo, Olivier Kobel, Christoph Noppen, Sophie Seidel, Noemie Santamaria de Souza, Niko Beerenwinkel, Tanja Stadler
EPI_ISL_468316	UPA Vila Assis	Instituto Adolfo Lutz, Interdiciplinary Procedures Center, Strategic Laboratory	Claudio Tavares Sacchi, Claudia Regina Gonçalves, Erica Valessa Ramos Gomes
EPI_ISL_468318	Hospital Universitario da USP	Instituto Adolfo Lutz, Interdiciplinary Procedures Center, Strategic Laboratory	Claudio Tavares Sacchi, Claudia Regina Gonçalves, Erica Valessa Ramos Gomes
EPI_ISL_468319	Vigilancia Epidemiologica de São Bernardo do Campo	Instituto Adolfo Lutz, Interdiciplinary Procedures Center, Strategic Laboratory	Claudio Tavares Sacchi, Claudia Regina Gonçalves, Erica Valessa Ramos Gomes
EPI_ISL_468320	Secretaria Municipal de Saude de Hortolandia	Instituto Adolfo Lutz, Interdiciplinary Procedures Center, Strategic Laboratory	Claudio Tavares Sacchi, Claudia Regina Gonçalves, Erica Valessa Ramos Gomes
EPI_ISL_468373, EPI_ISL_468374, EPI_ISL_468375, EPI_ISL_468376, EPI_ISL_468377, EPI_ISL_468378, EPI_ISL_468379, EPI_ISL_468380, EPI_ISL_468381, EPI_ISL_468382	Alameda County Public Health Lab	Chan-Zuckerberg Biohub	CZB Cliahub Consortium
EPI_ISL_468419, EPI_ISL_468420	County of San Luis Obispo Public Health Laboratory	Chan-Zuckerberg Biohub	CZB Cliahub Consortium
EPI_ISL_468460, EPI_ISL_468461	Humboldt County Public Health Laboratory	Chan-Zuckerberg Biohub	CZB Cliahub Consortium
EPI_ISL_468543, EPI_ISL_468544, EPI_ISL_468545, EPI_ISL_468546, EPI_ISL_468547	San Joaquin County Public Health Lab	Chan-Zuckerberg Biohub	CZB Cliahub Consortium
EPI_ISL_468592, EPI_ISL_468593, EPI_ISL_468594, EPI_ISL_468595, EPI_ISL_468596, EPI_ISL_468597, EPI_ISL_468598, EPI_ISL_468599	Orange County Public Health Lab	Chan-Zuckerberg Biohub	CZB Cliahub Consortium
EPI_ISL_468721	University of Florida	University of Florida	Stephenson,C.J., Subramaniam,K., Waltzek,T.B., Lauzardo,M., Gibson,J.C., Morris,J.G., Lednicky,J.A.
EPI_ISL_468952, EPI_ISL_468957, EPI_ISL_468958, EPI_ISL_468966, EPI_ISL_468984, EPI_ISL_468995, EPI_ISL_469015	Servicio de Microbiología, Hospital Universitario Son Espases	SeqCOVID-SPAIN consortium/IBV(CSIC)	Carla López-Causapé, Jordi Reina, Antonio Oliver and SeqCOVID-SPAIN consortium
EPI_ISL_469017	LNR National Reference Laboratory, Mohammed VI University of Health Sciences	Medical Biotechnology Laboratory, Rabat Medical and Pharmacy School, Mohammed The Vth University in Rabat	Meriem LAAMARTI, Souad KARTTI, Rokaia LAAMRTI , M.W. CHEMAO-ELFIHRI, Loubna ALLAM, Mouna OUADGHIRI, Imane SMYEJ, Jalila RAHOUI, Houda BENRAHMA, Jalil El Atar, Idrissa Diawara, Rachid EL JAOUDI, Laila SBABOU, Chakib NEJJARI, Saaid AMZAZI, Rachid MENTAG, Lahcen BELYAMANI and Azeddine IBRAHIMI
EPI_ISL_469080, EPI_ISL_469081, EPI_ISL_469082, EPI_ISL_469083, EPI_ISL_469084, EPI_ISL_469085, EPI_ISL_469086, EPI_ISL_469087, EPI_ISL_469088, EPI_ISL_469089, EPI_ISL_469090, EPI_ISL_469091, EPI_ISL_469092, EPI_ISL_469093, EPI_ISL_469094, EPI_ISL_469095, EPI_ISL_469117, EPI_ISL_469128, EPI_ISL_469130, EPI_ISL_469131, EPI_ISL_469134, EPI_ISL_469135, EPI_ISL_469136, EPI_ISL_469137, EPI_ISL_469138, EPI_ISL_469140, EPI_ISL_469141, EPI_ISL_469142, EPI_ISL_469144, EPI_ISL_469145, EPI_ISL_469148, EPI_ISL_469149, EPI_ISL_469150, EPI_ISL_469151, EPI_ISL_469152, EPI_ISL_469153, EPI_ISL_469154, EPI_ISL_469155			
see above	National Public Health Laboratory, National Centre for Infectious Diseases	National Public Health Laboratory, National Centre for Infectious Diseases	Mak TM, Octavia S, Chavatte JM, Cui L, Lin RTP
EPI_ISL_469296	Keio University Hospital	Keio University Hospital	Kenjiro Kosaki
EPI_ISL_469302, EPI_ISL_469305, EPI_ISL_469306, EPI_ISL_469307, EPI_ISL_469309, EPI_ISL_469311, EPI_ISL_469314, EPI_ISL_469317, EPI_ISL_469320	NU-OMICS DNA Sequencing research facility, Northumbria University	Wellcome Sanger Institute for the COVID-19 Genomics UK (COG-UK) consortium	Chris Duncan, Sheia Waugh, Shirelle Burton-Fanning, Gary Eltringham, Jennifer Collins, Brendan Payne, Yusri Taha, Emma Swindells, Jane Greenaway, Edward Barton, Garren Scott, Debra Padgett, Clive Graham, Sarah Essex, Steve Ligget, Paul Baker, Lynn Dover, Wen Yew, Gary Black, John Allan, Joshua Loh, Greg Young, Matthew Bashton, Andrew Nelson, Darren Smith and Alex Alderton, Roberto Amato, Sonia Goncalves, Ewan Harrison, David K. Jackson, Ian Johnston, Dominic Kwiatkowski, Cordelia Langford, John Sillitoe on behalf of the Wellcome Sanger Institute COVID-19 Surveillance Team (http://www.sanger.ac.uk/covid-team)
EPI_ISL_469531, EPI_ISL_469534, EPI_ISL_469537, EPI_ISL_469538, EPI_ISL_469539, EPI_ISL_469542, EPI_ISL_469544, EPI_ISL_469545, EPI_ISL_469546, EPI_ISL_469550, EPI_ISL_469552, EPI_ISL_469555, EPI_ISL_469557, EPI_ISL_469559, EPI_ISL_469562, EPI_ISL_469564, EPI_ISL_469566, EPI_ISL_469569, EPI_ISL_469570, EPI_ISL_469571, EPI_ISL_469572, EPI_ISL_469575, EPI_ISL_469576, EPI_ISL_469578, EPI_ISL_469581, EPI_ISL_469582, EPI_ISL_469585, EPI_ISL_469587, EPI_ISL_469590, EPI_ISL_469591, EPI_ISL_469592, EPI_ISL_469598, EPI_ISL_469599, EPI_ISL_469600, EPI_ISL_469602, EPI_ISL_469604, EPI_ISL_469606, EPI_ISL_469607, EPI_ISL_469612, EPI_ISL_469613, EPI_ISL_469615, EPI_ISL_469616, EPI_ISL_469618, EPI_ISL_469621, EPI_ISL_469623, EPI_ISL_469625, EPI_ISL_469628, EPI_ISL_469629, EPI_ISL_469630, EPI_ISL_469635, EPI_ISL_469638, EPI_ISL_469639, EPI_ISL_469644, EPI_ISL_469646, EPI_ISL_469647, EPI_ISL_469652, EPI_ISL_469653, EPI_ISL_469654, EPI_ISL_469656, EPI_ISL_469659, EPI_ISL_469661, EPI_ISL_469664, EPI_ISL_469666, EPI_ISL_469668, EPI_ISL_469671, EPI_ISL_469674, EPI_ISL_469676, EPI_ISL_469677, EPI_ISL_469680, EPI_ISL_469682, EPI_ISL_469683, EPI_ISL_469687, EPI_ISL_469688, EPI_ISL_469689, EPI_ISL_469690, EPI_ISL_469691, EPI_ISL_469694, EPI_ISL_469695, EPI_ISL_469699, EPI_ISL_469700, EPI_ISL_469702, EPI_ISL_469703, EPI_ISL_469706, EPI_ISL_469709, EPI_ISL_469711, EPI_ISL_469712, EPI_ISL_469714, EPI_ISL_469715, EPI_ISL_469716, EPI_ISL_469717, EPI_ISL_469719, EPI_ISL_469720, EPI_ISL_469721, EPI_ISL_469722, EPI_ISL_469724, EPI_ISL_469727, EPI_ISL_469728, EPI_ISL_469729, EPI_ISL_469730, EPI_ISL_469731, EPI_ISL_469732, EPI_ISL_469734, EPI_ISL_469736, EPI_ISL_469737, EPI_ISL_469738, EPI_ISL_469740, EPI_ISL_469744, EPI_ISL_469746, EPI_ISL_469747, EPI_ISL_469748, EPI_ISL_469753, EPI_ISL_469754, EPI_ISL_469756, EPI_ISL_469757, EPI_ISL_469759, EPI_ISL_469760, EPI_ISL_469762, EPI_ISL_469763, EPI_ISL_469766, EPI_ISL_469768, EPI_ISL_469770, EPI_ISL_469772, EPI_ISL_469773, EPI_ISL_469774, EPI_ISL_469775, EPI_ISL_469776, EPI_ISL_469780, EPI_ISL_469781, EPI_ISL_469782, EPI_ISL_469783, EPI_ISL_469786, EPI_ISL_469787, EPI_ISL_469789, EPI_ISL_469790, EPI_ISL_469791, EPI_ISL_469794, EPI_ISL_469795, EPI_ISL_469796, EPI_ISL_469798, EPI_ISL_469799, EPI_ISL_469801, EPI_ISL_469802, EPI_ISL_469803			
see above	PHE South West Regional Laboratory, National Infection Service	Wellcome Sanger Institute for the COVID-19 Genomics UK (COG-UK) consortium	Stephanie Hutchings, Hannah Pymont, Dr Peter Muir, Barry Vipond, Rich Hopes; and Alex Alderton, Roberto Amato, Sonia Goncalves, Ewan Harrison, David K. Jackson, Ian Johnston, Dominic Kwiatkowski, Cordelia Langford, John Sillitoe on behalf of the Wellcome Sanger Institute COVID-19 Surveillance Team (http://www.sanger.ac.uk/covid-team)
EPI_ISL_469850, EPI_ISL_469857, EPI_ISL_469859, EPI_ISL_469862, EPI_ISL_469864, EPI_ISL_469865, EPI_ISL_469868, EPI_ISL_469872, EPI_ISL_469876, EPI_ISL_469879, EPI_ISL_469881, EPI_ISL_469885			
see above	Regional Virus Laboratory, Belfast Health and Social Care Trust	Wellcome Sanger Institute for the COVID-19 Genomics UK (COG-UK) consortium	Conall McCaughey, James McKenna, Tanya Curran, Susan Feeney, Alison Watt, Ciara Cox, Mairead Connor, Zoltan Molnar, David Simpson, Derek Fairley; and Alex Alderton, Roberto Amato, Sonia Goncalves, Ewan Harrison, David K. Jackson, Ian Johnston, Dominic Kwiatkowski, Cordelia Langford, John Sillitoe on behalf of the Wellcome Sanger Institute COVID-19 Surveillance Team (http://www.sanger.ac.uk/covid-team)
EPI_ISL_469916	PHE South West Regional Laboratory, National Infection Service	Wellcome Sanger Institute for the COVID-19 Genomics UK (COG-UK) consortium	Stephanie Hutchings, Hannah Pymont, Dr Peter Muir, Barry Vipond, Rich Hopes; and Alex Alderton, Roberto Amato, Sonia Goncalves, Ewan Harrison, David K. Jackson, Ian Johnston, Dominic Kwiatkowski, Cordelia Langford, John Sillitoe on behalf of the Wellcome Sanger Institute COVID-19 Surveillance Team (http://www.sanger.ac.uk/covid-team)
EPI_ISL_469918, EPI_ISL_469919, EPI_ISL_469922, EPI_ISL_469927	Virology Department, Sheffield Teaching Hospitals NHS Foundation Trust / Department of Infection, Immunity and Cardiovascular Disease, The Medical School, University of Sheffield	Wellcome Sanger Institute for the COVID-19 Genomics UK (COG-UK) consortium	Thushan de Silva, Matthew Parker,Adri Angyal, Rebecca Brown, Luke Green, Rachel Tucker, Paul Parsons, Danielle Groves, Alex Keeley, Dave Partridge, Matthew Wyles, Benjamin Lindsey, Mehmet Yavuz, Mohammad Raza, Cariad Evans and Alex Alderton, Roberto Amato, Sonia Goncalves, Ewan Harrison, David K. Jackson, Ian Johnston, Dominic Kwiatkowski, Cordelia Langford, John Sillitoe on behalf of the Wellcome Sanger Institute COVID-19 Surveillance Team (http://www.sanger.ac.uk/covid-team)
EPI_ISL_470017, EPI_ISL_470018, EPI_ISL_470020, EPI_ISL_470022, EPI_ISL_470023, EPI_ISL_470024, EPI_ISL_470030, EPI_ISL_470039, EPI_ISL_470040, EPI_ISL_470043, EPI_ISL_470053, EPI_ISL_470054, EPI_ISL_470057, EPI_ISL_470059, EPI_ISL_470064, EPI_ISL_470070, EPI_ISL_470074, EPI_ISL_470082, EPI_ISL_470083, EPI_ISL_470087			
see above	Regional Virus Laboratory, Belfast Health and Social Care Trust	Wellcome Sanger Institute for the COVID-19 Genomics UK (COG-UK) consortium	Conall McCaughey, James McKenna, Tanya Curran, Susan Feeney, Alison Watt, Ciara Cox, Mairead Connor, Zoltan Molnar, David Simpson, Derek Fairley; and Alex Alderton, Roberto Amato, Sonia Goncalves, Ewan Harrison, David K. Jackson, Ian Johnston, Dominic Kwiatkowski, Cordelia Langford, John Sillitoe on behalf of the Wellcome Sanger Institute COVID-19 Surveillance Team (http://www.sanger.ac.uk/covid-team)
EPI_ISL_470090, EPI_ISL_470091, EPI_ISL_470092, EPI_ISL_470093, EPI_ISL_470094, EPI_ISL_470095, EPI_ISL_470101, EPI_ISL_470102, EPI_ISL_470103, EPI_ISL_470104, EPI_ISL_470105, EPI_ISL_470106, EPI_ISL_470108, EPI_ISL_470109, EPI_ISL_470111, EPI_ISL_470112, EPI_ISL_470114, EPI_ISL_470115,			

EPI_ISL_470116, EPI_ISL_470119, EPI_ISL_470121, EPI_ISL_470124, EPI_ISL_470125, EPI_ISL_470126, EPI_ISL_470127, EPI_ISL_470128, EPI_ISL_470129, EPI_ISL_470130, EPI_ISL_470133, EPI_ISL_470134, EPI_ISL_470139, EPI_ISL_470141, EPI_ISL_470144, EPI_ISL_470147, EPI_ISL_470149, EPI_ISL_470153, EPI_ISL_470155, EPI_ISL_470156, EPI_ISL_470158, EPI_ISL_470159, EPI_ISL_470161, EPI_ISL_470162, EPI_ISL_470165, EPI_ISL_470166, EPI_ISL_470167, EPI_ISL_470168, EPI_ISL_470171, EPI_ISL_470176, EPI_ISL_470177, EPI_ISL_470179, EPI_ISL_470180, EPI_ISL_470182, EPI_ISL_470185, EPI_ISL_470187, EPI_ISL_470188, EPI_ISL_470190, EPI_ISL_470191, EPI_ISL_470199, EPI_ISL_470200, EPI_ISL_470202, EPI_ISL_470206, EPI_ISL_470209, EPI_ISL_470210, EPI_ISL_470212, EPI_ISL_470215, EPI_ISL_470217, EPI_ISL_470218, EPI_ISL_470221, EPI_ISL_470223, EPI_ISL_470224, EPI_ISL_470229, EPI_ISL_470232, EPI_ISL_470233, EPI_ISL_470234, EPI_ISL_470235, EPI_ISL_470236, EPI_ISL_470237, EPI_ISL_470238, EPI_ISL_470239, EPI_ISL_470241, EPI_ISL_470243, EPI_ISL_470244, EPI_ISL_470250, EPI_ISL_470251, EPI_ISL_470253, EPI_ISL_470254, EPI_ISL_470255, EPI_ISL_470256, EPI_ISL_470257, EPI_ISL_470260, EPI_ISL_470261, EPI_ISL_470263, EPI_ISL_470269, EPI_ISL_470272, EPI_ISL_470273, EPI_ISL_470277, EPI_ISL_470278, EPI_ISL_470279, EPI_ISL_470280, EPI_ISL_470282, EPI_ISL_470283, EPI_ISL_470284, EPI_ISL_470286, EPI_ISL_470287, EPI_ISL_470288, EPI_ISL_470289, EPI_ISL_470292, EPI_ISL_470293, EPI_ISL_470294, EPI_ISL_470298, EPI_ISL_470299, EPI_ISL_470303, EPI_ISL_470304, EPI_ISL_470305, EPI_ISL_470307, EPI_ISL_470308, EPI_ISL_470310, EPI_ISL_470311, EPI_ISL_470313, EPI_ISL_470314, EPI_ISL_470325, EPI_ISL_470326, EPI_ISL_470330, EPI_ISL_470331, EPI_ISL_470333, EPI_ISL_470334, EPI_ISL_470336, EPI_ISL_470338, EPI_ISL_470339, EPI_ISL_470340, EPI_ISL_470344, EPI_ISL_470345, EPI_ISL_470346, EPI_ISL_470350, EPI_ISL_470351, EPI_ISL_470353, EPI_ISL_470354, EPI_ISL_470355			
see above	Department of Pathology, University of Cambridge	Wellcome Sanger Institute for the COVID-19 Genomics UK (COG-UK) consortium	Luke W Meredith, M. Estée Török , Myra Hosmillo, William L. Hamilton, Martin D. Curran, Theresa Feltwell, Grant Hall, Anna Yakovleva, Fahad A Khokhar, Charlotte J. Houldcroft, Laura G Caller, Aminu S. Jahun, Sarah L. Caddy, Ian Goodfellow; and Alex Alderton, Roberto Amato, Sonia Goncalves, Ewan Harrison, David K. Jackson, Ian Johnston, Dominic Kwiatkowski, Cordelia Langford, John Sillitoe on behalf of the Wellcome Sanger Institute COVID-19 Surveillance Team (http://www.sanger.ac.uk/covid-team)
EPI_ISL_470541, EPI_ISL_470542, EPI_ISL_470543, EPI_ISL_470544, EPI_ISL_470554, EPI_ISL_470555, EPI_ISL_470556, EPI_ISL_470557, EPI_ISL_470558, EPI_ISL_470559, EPI_ISL_470560, EPI_ISL_470561, EPI_ISL_470562, EPI_ISL_470563, EPI_ISL_470564, EPI_ISL_470566			
see above	Utah Public Health Laboratory	Utah Public Health Laboratory	Erin Young, Kelly Oakeson
EPI_ISL_470589, EPI_ISL_470591, EPI_ISL_470592, EPI_ISL_470596	Simile	Bioinformatics Laboratory / LNCC	Alexandra Gerber, Ana Paula Guimarães, Luiz Gonzaga Paula de Almeida, Ronaldo da Silva Francisco Junior, Mariane Talon, Filipe Romero, Átila Duque Rossi, Terezinha Marta Pereira, working group UFRJ, Jaqueline Goes de Jesus, Ingra Morales Claro, Ester Cerdeira Sabino, Nuno Rodrigues Faria, CADDE-group, Laboratorio Hermes Pardini, Laboratorio Simile, working group UFMG, Amilcar Tanuri, Carolina Voloch, Renato Santana Aguiar e Ana Tereza Vasconcelos
EPI_ISL_470630, EPI_ISL_470631, EPI_ISL_470632, EPI_ISL_470633, EPI_ISL_470634, EPI_ISL_470635, EPI_ISL_470636, EPI_ISL_470637, EPI_ISL_470638	Laboratorio de Virologia Molecular / UFRJ	Bioinformatics Laboratory / LNCC	Alexandra Gerber, Ana Paula Guimarães, Luiz Gonzaga Paula de Almeida, Ronaldo da Silva Francisco Junior, Mariane Talon, Filipe Romero, Átila Duque Rossi, Terezinha Marta Pereira, working group UFRJ, Jaqueline Goes de Jesus, Ingra Morales Claro, Ester Cerdeira Sabino, Nuno Rodrigues Faria, CADDE-group, Laboratorio Hermes Pardini, Laboratorio Simile, working group UFMG, Amilcar Tanuri, Carolina Voloch, Renato Santana Aguiar e Ana Tereza Vasconcelos
EPI_ISL_470656, EPI_ISL_470657, EPI_ISL_470658, EPI_ISL_470659, EPI_ISL_470660, EPI_ISL_470661, EPI_ISL_470662, EPI_ISL_470663, EPI_ISL_470664, EPI_ISL_470665, EPI_ISL_470666, EPI_ISL_470667, EPI_ISL_470678, EPI_ISL_470679, EPI_ISL_470680, EPI_ISL_470681, EPI_ISL_470682, EPI_ISL_470684, EPI_ISL_470685, EPI_ISL_470686, EPI_ISL_470687, EPI_ISL_470688, EPI_ISL_470689, EPI_ISL_470690, EPI_ISL_470691, EPI_ISL_470692, EPI_ISL_470693, EPI_ISL_470694, EPI_ISL_470695, EPI_ISL_470696, EPI_ISL_470697, EPI_ISL_470698, EPI_ISL_470699, EPI_ISL_470700, EPI_ISL_470701, EPI_ISL_470702, EPI_ISL_470703, EPI_ISL_470704, EPI_ISL_470705, EPI_ISL_470706, EPI_ISL_470707, EPI_ISL_470708, EPI_ISL_470709, EPI_ISL_470710, EPI_ISL_470711			
see above	Utah Public Health Laboratory	Utah Public Health Laboratory	Erin Young, Kelly Oakeson
EPI_ISL_470719, EPI_ISL_470729, EPI_ISL_470730, EPI_ISL_470731, EPI_ISL_470732, EPI_ISL_470733, EPI_ISL_470734, EPI_ISL_470735, EPI_ISL_470736, EPI_ISL_470742, EPI_ISL_470743, EPI_ISL_470744			
see above	Utah Public Health Laboratory	Utah Public Health Laboratory	Heidi Butz, Erin Young, Kelly Oakeson
EPI_ISL_470837, EPI_ISL_470838, EPI_ISL_470855	PathWest Laboratory Medicine WA	PathWest Laboratory Medicine WA	Chisha Sikazwe, Jurissa Lang, Avram Levy, David Smith and David Speers
EPI_ISL_470877	Department for Virology, Molecular Biology and Genome Research, R. G. Lugar Center for Public Health Research, National Center for Disease Control and Public Health (NCDC) of Georgia.	Department for Virology, Molecular Biology and Genome Research, R. G. Lugar Center for Public Health Research, National Center for Disease Control and Public Health (NCDC) of Georgia.	Gvantsa Brachveli, Meri Pantsulaia, Giorgi Tomashvili, Gvantsa Chanturia, Ann Machablishvili, Nato Kotaria, Marine Murtskhvaladze, Lela Sabadze, Mari Gavashelidze, Ana Papkiauri, Tata Imnadze, Tamar Jashiashvili, Tea Tevdoradze, Ketevan Sidamonidze, Ekaterine Khmaladze, Ekaterine Zhghenti, Roena Sukhiashvili, Mariam Zakalashvili, Lela Urushadze, Magda Dgebuadze, Davit Tsaguria, Ekaterine Zangaladze, Nino Berishvili, Adam Kotorashvili, Maia Alkhazashvili, Irma Burjanadze, Anna Kasradze, Khatuna Zakhashvili, Paata Imnadze, Amiran Gamkrelidze.
EPI_ISL_471172	Unilabs Laboratory Medicine	Norwegian Institute of Public Health, Department of Virology	Kathrine Stene-Johansen, Kamilla Heddeland Instefjord, Hilde Elshaug, Rasmus Riis Kopperud, Karoline Bragstad, Olav Hungnes
EPI_ISL_471189, EPI_ISL_471190, EPI_ISL_471191, EPI_ISL_471192, EPI_ISL_471193, EPI_ISL_471209, EPI_ISL_471226, EPI_ISL_471231, EPI_ISL_471236, EPI_ISL_471239, EPI_ISL_471247			
see above	Wisconsin State Laboratory of Hygiene Communicable Disease Division	Wisconsin State Laboratory of Hygiene Communicable Disease Division	Kelsey R. Florek, Abigail C. Shockey
EPI_ISL_471267	Hospital IESS Babahoyo	Institute of Microbiology, Universidad San Francisco de Quito	Sully Márquez, Belén Prado-Vivar, Juan José Guadalupe, Bernardo Gutiérrez, Francisco Cordova, Ninfa Henriquez, Killen Briones-Zamora, Killen Briones-Claudette, Verónica Barragán, Patricio Rojas-Silva, Gabriel Trueba, Michelle Grunauer, Paúl Cárdenas
EPI_ISL_471268	Hospital IESS Babahoyo	Institute of Microbiology, Universidad San Francisco de Quito	Belén Prado-Vivar, Sully Márquez, Juan José Guadalupe, Bernardo Gutiérrez, Francisco Cordova, Ninfa Henriquez, Killen Briones-Zamora, Killen Briones-Claudette, Verónica Barragán, Patricio Rojas-Silva, Gabriel Trueba, Michelle Grunauer, Paúl Cárdenas
EPI_ISL_471457, EPI_ISL_471458, EPI_ISL_471459, EPI_ISL_471460	Centre de Virologie des Maladies Tropicales	Functional Genomic Platform/Service Analyses Biologique/UATRS/ Centre National Pour la Recherche Scientifique Et Technique (CNRST)	Hicham EL ANNAZ, Elmostafa EL FAHIME, Marouane MELLOUL, Youssef AKHOUAD, Mly Abdelaziz ELALAOUI, Ahmed REGGAD, Sanaa ALAOUI-Amine, Rachid ABI, Rida TAGAJDID, Zohour KASMY, Safae ELKOCHRI, Nadia TOUIL, Farida HILALI, Abdelkader LAATIRIS, Abdelillah LARAQUI, Tahar BAJJOU , Yassine SEKHSOKH , Idriss-Amine LAHLOU, Mostafa ELOUENNASS, Khalid ENNIBI
EPI_ISL_471549	Hospital Municipal Carmen Prudente	Instituto Adolfo Lutz, Interdiciplinary Procedures Center, Strategic Laboratory	Claudio Tavares Sacchi, Claudia Regina Gonçalves, Erica Valessa Ramos Gomes
EPI_ISL_471556	Pronto Socorro Jose Ibrahin	Instituto Adolfo Lutz, Interdiciplinary Procedures Center, Strategic Laboratory	Claudio Tavares Sacchi, Claudia Regina Gonçalves, Erica Valessa Ramos Gomes
EPI_ISL_471586	CSIR-Centre for Cellular and Molecular Biology	CSIR-Centre for Cellular and Molecular Biology	Lamuk Zaveri, Shagufta Khan, Namami Gaur, Sakshi Shambhavi, Tulasi Nagabandi, Purushotham Vodnala, Payel Mukherjee, Sofia Banu, Priya Singh, Dhiviya Vedagiri, Divya Gupta, Vishal Sah, Santosh Kumar Kuncha, Krishnan Harinivas Harshan, Archana Bharadwaj Siva, Karthik Bharadwaj Tallapaka,Zeba Rizvi, Zuberwasim Sayyad, Kakade Aishwarya Arun, Amrutha H C, Ananga Ghosh, Rakesh K Mishra, Divya Tej Sowpati
EPI_ISL_471587, EPI_ISL_471645, EPI_ISL_471646	CSIR-Centre for Cellular and Molecular Biology	CSIR-Centre for Cellular and Molecular Biology	Dhiviya Vedagiri, Divya Gupta, Vishal Sah, Payel Mukherjee, Sofia Banu, Priya Singh, Santosh Kumar Kuncha, Archana Bharadwaj Siva, Karthik Bharadwaj Tallapaka, Shagufta Khan, Lamuk Zaveri, Namami Gaur, Sakshi Shambhavi, Tulasi Nagabandi, Purushotham Vodnala, Rakesh K Mishra, Divya Tej Sowpati, Krishnan Harinivas Harshan
EPI_ISL_471739, EPI_ISL_471740, EPI_ISL_471741, EPI_ISL_471742, EPI_ISL_471748, EPI_ISL_471749, EPI_ISL_471750, EPI_ISL_471751, EPI_ISL_471752, EPI_ISL_471775, EPI_ISL_471788, EPI_ISL_471796, EPI_ISL_471799, EPI_ISL_471845, EPI_ISL_471848, EPI_ISL_471849, EPI_ISL_471851, EPI_ISL_471852, EPI_ISL_471853, EPI_ISL_471854, EPI_ISL_471855, EPI_ISL_471856, EPI_ISL_471858, EPI_ISL_471862, EPI_ISL_471885			
see above	Michigan Department of Health and Human Services, Bureau of Laboratories	Michigan Department of Health and Human Services, Bureau of Laboratories	Blankenship HM, Riner D, Soehnlen MK
EPI_ISL_471955	University of Exeter	COVID-19 Genomics UK (COG-UK) Consortium	Ben Temperton,Aaron Jeffries,Michelle Michelsen,Joanna Warwick-Dugdale,Audrey Farbos,Robyn Manley,Stephen Michell,Jane Masoli
EPI_ISL_471996, EPI_ISL_472000, EPI_ISL_472001	Liverpool Clinical Laboratories	COVID-19 Genomics UK (COG-UK) Consortium	Sam Haldenby, Anita Lucaci, Steve Paterson, Julian Hiscox, Alistair Darby, M Almsaud, A Alrezaihi, Muhannad Alruwaili, Stuart D Armstrong, Jones Benjamin, Eleanor G Bentley, Anu Chawla, Jordan J Clark, Angela Cowell, Richard Eccles, Isabel García-Dorival, Matthew Gemmell, Alessandro Gerada, PKF Gilmore, Richard Gregory, Ximeng Han, Catherine Hartley, Margaret Hughes, Miren Iturriza-Gomara, James Johnson, L Luu, Jenifer Manson, Charlotte Nelson, Elaine O'Toole, Cassie Olateju, Rebekah Penrice-Randal , Lucille Rainbow, N.P Randle, Trevor Ian Robinson, Parul Sharma, Ghada T Shawli, James P Stewart, Neil Swainston, Ecaterina Vamos, Joanne Watts, Mark Whitehead
EPI_ISL_472269, EPI_ISL_472270	Northumbria University / South Tees Hospitals NHS Foundation Trust / North Cumbria Integrated Care NHS Foundation Trust / North Tees and Hartlepool NHS Foundation Trust / Newcastle Hospitals NHS Foundation Trust	COVID-19 Genomics UK (COG-UK) Consortium	Darren L Smith,Andrew Nelson,Matthew Bashton,Greg R Young,Joshua Loh,John Allan,Mohammad A Tariq,Giles S Holt,Gary Black,Wen C Yew,Lynn Dover,Paul Baker,Steve Liggett,Sarah Essex,Jane Greenaway,Debra Padgett,Clive Graham,Garren Scott,Edward Barton,Emma Swindells,Brendan Payne,Jennifer Collins,Yusri Taha,Gary Eltringham
EPI_ISL_472409, EPI_ISL_472410	Queens Medical Centre, Clinical Microbiology Department / DeepSeq Nottingham	COVID-19 Genomics UK (COG-UK) Consortium	Gemma Clark, Wendy Smith, Manjinder Khakh, Vicki M Fleming, Michelle M Lister, Hannah Howson-Wells, Jonathan Ball, Patrick McClure, Joseph Chappell, Theocharis Tsoleridis, Nadine Holmes, Matthew Carlisle, Christopher Moore, Fei Sang, Johnny Debebe, Victoria Wright, Matthew Loose
EPI_ISL_472441, EPI_ISL_472451, EPI_ISL_472452, EPI_ISL_472462, EPI_ISL_472471, EPI_ISL_472481, EPI_ISL_472500, EPI_ISL_472506, EPI_ISL_472517, EPI_ISL_472529, EPI_ISL_472535, EPI_ISL_472541, EPI_ISL_472543, EPI_ISL_472553, EPI_ISL_472567, EPI_ISL_472585, EPI_ISL_472605, EPI_ISL_472647, EPI_ISL_472670, EPI_ISL_472676, EPI_ISL_472703, EPI_ISL_472717, EPI_ISL_472722, EPI_ISL_472731, EPI_ISL_472734, EPI_ISL_472884, EPI_ISL_472890, EPI_ISL_472907, EPI_ISL_472913, EPI_ISL_472942, EPI_ISL_472954, EPI_ISL_472955, EPI_ISL_472956, EPI_ISL_472957, EPI_ISL_472959, EPI_ISL_472960,			

EPI_ISL_472971, EPI_ISL_472972, EPI_ISL_472976, EPI_ISL_472977, EPI_ISL_472994, EPI_ISL_473009, EPI_ISL_473020, EPI_ISL_473034, EPI_ISL_473050, EPI_ISL_473144, EPI_ISL_473288			
see above	Wales Specialist Virology Centre Sequencing lab: Pathogen Genomics Unit	COVID-19 Genomics UK (COG-UK) Consortium	Catherine Moore, Johnathan Evans, Laura Gifford, Malorie Perry, Simon Cottrell, Angela Marchbank, Alec Birchley, Alexander Adams, Amy Gaskin, Bree Gatica-Wilcox, Jason Coombes, Joel Southgate, Lauren Gilbert, Lee Graham, Nicole Pacchiarini, Sara Kumziene-Summerhayes, Sarah Taylor, Sophie Jones, Sara Rey, Matthew Bull, Joanne Watkins, Sally Corden, Tom Connor
EPI_ISL_473513, EPI_ISL_473514, EPI_ISL_473515, EPI_ISL_473516, EPI_ISL_473517, EPI_ISL_473518, EPI_ISL_473519, EPI_ISL_473520, EPI_ISL_473521, EPI_ISL_473522, EPI_ISL_473523, EPI_ISL_473524, EPI_ISL_473525, EPI_ISL_473526, EPI_ISL_473527, EPI_ISL_473528, EPI_ISL_473529, EPI_ISL_473530, EPI_ISL_473531, EPI_ISL_473532, EPI_ISL_473533, EPI_ISL_473534, EPI_ISL_473535, EPI_ISL_473536, EPI_ISL_473537, EPI_ISL_473538, EPI_ISL_473539, EPI_ISL_473540, EPI_ISL_473541, EPI_ISL_473542, EPI_ISL_473543, EPI_ISL_473544, EPI_ISL_473545, EPI_ISL_473546, EPI_ISL_473547, EPI_ISL_473548, EPI_ISL_473549, EPI_ISL_473550, EPI_ISL_473551, EPI_ISL_473569			
see above	West of Scotland Specialist Virology Centre, NHSGGC / MRC-University of Glasgow Centre for Virus Research	COVID-19 Genomics UK (COG-UK) Consortium	Ana da Silva Filipe, Natasha Johnson, Kathy Smollett, Daniel Mair, Stephen Carmichael, Lily Tong, Jenna Nichols, Elihu Aranday-Cortes, Kirstyn Brunker, Yasmin Parr, Alice Broos, Kyriaki Nomikou; Sarah McDonald, Marc Niebel, Patawee Asamaphan; Richard Orton, Joseph Hughes, Sreenu Vattipally, David L Robertson; Alasdair MacLean, Rory Gunson; Kathy Li, Natasha Jesudason, Rajiv Shah, James Shepherd, Antonia Ho, Emma Thomson
EPI_ISL_473789, EPI_ISL_473791, EPI_ISL_473792, EPI_ISL_473793, EPI_ISL_473794, EPI_ISL_473824	Virology Department, Royal Infirmary of Edinburgh, NHS Lothian / School of Biological Sciences, University of Edinburgh / Institute of Genetics and Molecular Medicine, University of Edinburgh	COVID-19 Genomics UK (COG-UK) Consortium	McHugh M, Dewar R, Rooke S, Gallagher M, Balcaza C, O'Toole Á, Scher E, Hill V, McCrone JT, Colquhoun R, Yu X, Jackson B, Rambaut A, Williams TC, Templeton K
EPI_ISL_474320, EPI_ISL_474330, EPI_ISL_474331, EPI_ISL_474332, EPI_ISL_474333, EPI_ISL_474334, EPI_ISL_474335, EPI_ISL_474336, EPI_ISL_474337, EPI_ISL_474338, EPI_ISL_474339, EPI_ISL_474340, EPI_ISL_474341, EPI_ISL_474342, EPI_ISL_474343, EPI_ISL_474344, EPI_ISL_474345, EPI_ISL_474346, EPI_ISL_474347, EPI_ISL_474348, EPI_ISL_474349, EPI_ISL_474350, EPI_ISL_474351, EPI_ISL_474352, EPI_ISL_474353, EPI_ISL_474354, EPI_ISL_474355, EPI_ISL_474356, EPI_ISL_474357, EPI_ISL_474358, EPI_ISL_474359, EPI_ISL_474360, EPI_ISL_474361, EPI_ISL_474362, EPI_ISL_474363, EPI_ISL_474364, EPI_ISL_474365, EPI_ISL_474366, EPI_ISL_474367, EPI_ISL_474368, EPI_ISL_474369, EPI_ISL_474370, EPI_ISL_474371, EPI_ISL_474372, EPI_ISL_474373, EPI_ISL_474374, EPI_ISL_474375, EPI_ISL_474376, EPI_ISL_474377, EPI_ISL_474378, EPI_ISL_474379, EPI_ISL_474380, EPI_ISL_474381, EPI_ISL_474382, EPI_ISL_474383, EPI_ISL_474384, EPI_ISL_474385, EPI_ISL_474386, EPI_ISL_474387, EPI_ISL_474388, EPI_ISL_474389, EPI_ISL_474390, EPI_ISL_474391, EPI_ISL_474392, EPI_ISL_474393, EPI_ISL_474394, EPI_ISL_474395, EPI_ISL_474396, EPI_ISL_474397, EPI_ISL_474398, EPI_ISL_474399, EPI_ISL_474400, EPI_ISL_474401, EPI_ISL_474402, EPI_ISL_474405, EPI_ISL_474407, EPI_ISL_474408, EPI_ISL_474409, EPI_ISL_474410, EPI_ISL_474413, EPI_ISL_474414, EPI_ISL_474415, EPI_ISL_474416, EPI_ISL_474417, EPI_ISL_474418, EPI_ISL_474419			
see above	Wales Specialist Virology Centre Sequencing lab: Pathogen Genomics Unit	COVID-19 Genomics UK (COG-UK) Consortium	Catherine Moore, Johnathan Evans, Laura Gifford, Malorie Perry, Simon Cottrell, Angela Marchbank, Alec Birchley, Alexander Adams, Amy Gaskin, Bree Gatica-Wilcox, Jason Coombes, Joel Southgate, Lauren Gilbert, Lee Graham, Nicole Pacchiarini, Sara Kumziene-Summerhayes, Sarah Taylor, Sophie Jones, Sara Rey, Matthew Bull, Joanne Watkins, Sally Corden, Tom Connor
EPI_ISL_474970, EPI_ISL_474971, EPI_ISL_474982, EPI_ISL_474983, EPI_ISL_474990, EPI_ISL_474998, EPI_ISL_475006, EPI_ISL_475007, EPI_ISL_475008	Israel Central Virology laboratory	Israel Central Virology laboratory	Neta Zuckerman, Efrat Dahan Bucris, Oran Erster, Ella Mendelson, Michal Mandelboim
EPI_ISL_475125	Halmstad klinisk mikrobiologi	The Public Health Agency of Sweden	Oskar Karlsson Lindsjo, Maria Lind Karlberg, Mattias Haukland, Reza Advani, Olov Svartstrom, Anna-Malin Linde, Sandra Broddesson, Petra Edquist, Shamam Muradrasoli, Anna Risberg, Karin Tegmark-Wisell
EPI_ISL_475126	Umea klinisk mikrobiologi	The Public Health Agency of Sweden	Oskar Karlsson Lindsjo, Maria Lind Karlberg, Mattias Haukland, Reza Advani, Olov Svartstrom, Anna-Malin Linde, Sandra Broddesson, Petra Edquist, Shamam Muradrasoli, Anna Risberg, Karin Tegmark-Wisell
EPI_ISL_475157, EPI_ISL_475158, EPI_ISL_475159	Halmstad klinisk mikrobiologi	The Public Health Agency of Sweden	Oskar Karlsson Lindsjo, Maria Lind Karlberg, Mattias Haukland, Reza Advani, Olov Svartstrom, Anna-Malin Linde, Sandra Broddesson, Petra Edquist, Shamam Muradrasoli, Anna Risberg, Karin Tegmark-Wisell
EPI_ISL_475333	Centre for Enzyme Innovation, University of Portsmouth / Translational Research Laboratory, Portsmouth Hospitals NHS Trust	COVID-19 Genomics UK (COG-UK) Consortium	Angela Beckett,Yann Bourgeois,Garry Scarlett,Sharon Glaysher,Scott Elliott,Kelly Bicknell,Robert Impey,Allyson Lloyd,Sarah Wyllie,Ethan Butcher,Anoop Chauhan,Samuel Robson
EPI_ISL_475540	Bla Kustens halsocentral	The Public Health Agency of Sweden	Oskar Karlsson Lindsjo, Maria Lind Karlberg, Mattias Haukland, Reza Advani, Olov Svartstrom, Anna-Malin Linde, Sandra Broddesson, Mia Brytting, Anna Risberg, Karin Tegmark-Wisell
EPI_ISL_475625, EPI_ISL_475643, EPI_ISL_475662, EPI_ISL_475672	Cedars-Sinai Medical Center, Department of Pathology & Laboratory Medicine, Molecular Pathology Laboratory	Cedars-Sinai Medical Center, Molecular Pathology Laboratory of Department of Pathology & Laboratory Medicine and Genomic Core	Wenjuan Zhang, John Paul Govindavari, Brian Davis, Stephanie Chen, Jong Taek Kim, Jianbo Song, Jean Lopategui, Jasmine T Plummer, Eric Vail
EPI_ISL_475915	Klinikum Wels-Grieskirchen	Bergthaler laboratory, CeMM Research Center for Molecular Medicine of the Austrian Academy of Sciences	Alexandra Popa, Benedikt Agerer, Henrique Colaco, Lukas Endler, Jakob-Wendelin Genger, Alexander Lercher, Mark Smyth, Thomas Penz, Michael Schuster, Jan Laine, Martin Senekowitsch, Judith Aberle, Stephan Aberle, Peter Hufnagl, Daniela Schmid, Franz Allerberger, Elisabeth Puchhammer-Stoeckl, Manfred Nairz, Guenter Weiss, Gregor Hörmann, Kinga Rigler-Hohenwarter, Rainer Gattringer, Wegene Borena, Dorothee von Laer, Christoph Bock, Andreas Bergthaler
EPI_ISL_475973, EPI_ISL_475974, EPI_ISL_475975, EPI_ISL_475976, EPI_ISL_475977, EPI_ISL_475978, EPI_ISL_475979, EPI_ISL_475980	National Public Health Laboratory, National Centre for Infectious Diseases	National Public Health Laboratory, National Centre for Infectious Diseases	Mak TM, Octavia S, Chavatte JM, Cui L, Lin RTP
EPI_ISL_476077	University of Debrecen, Department of Medical Microbiology	National Laboratory of Virology, Szentágothai Research Centre	Endre Gábor Tóth, Balázs Somogyi, Brigitta Zana, Eszter Csoma, Ferenc Jakab, Gábor Kemenesi
EPI_ISL_476131, EPI_ISL_476132, EPI_ISL_476133, EPI_ISL_476134	Viollier AG	Department of Biosystems Science and Engineering, ETH Zürich	Christian Beisel, Sarah Nadeau, Ivan Topolsky, Pedro Ferreira, Philipp Jablonski, Susana Posada-Céspedes, Tobias Schär, Ina Nissen, Natascha Santacroce, Elodie Burcklen, Christiane Beckmann, Maurice Redondo, Olivier Kobel, Christoph Noppen, Sophie Seidel, Noemie Santamaria de Souza, Niko Beerenwinkel, Tanja Stadler
EPI_ISL_476163, EPI_ISL_476165, EPI_ISL_476166, EPI_ISL_476167, EPI_ISL_476337, EPI_ISL_476338, EPI_ISL_476339, EPI_ISL_476340, EPI_ISL_476341, EPI_ISL_476342, EPI_ISL_476343, EPI_ISL_476344, EPI_ISL_476345, EPI_ISL_476390, EPI_ISL_476392, EPI_ISL_476393, EPI_ISL_476394, EPI_ISL_476395, EPI_ISL_476396, EPI_ISL_476397, EPI_ISL_476398, EPI_ISL_476399			
see above	Laboratório de Patologia Clínica - UNICAMP	Laboratório de Estudos de Vírus Emergentes - UNICAMP	José Luiz Proença-Modena, Magnun Nueldo Nunes dos Santos, Angelica Schreiber, Julia Forato,Camila Simeoni, Marcilio Jorge Fumagalli, Mariene Ribeiro Amorim, Darlan da Silva Candido, Nuno Rodrigues Faria, Julien Theze, Luiz Gonzaga,Jaqueline Goes Jesus e William Marciel de Souza
EPI_ISL_476503, EPI_ISL_476509	Laboratoire de microbiologie, Hopital de Verdun	Smith Laboratory, Centre de Recherche CHU Sainte-Justine	Martin Smith, Marieke Rozendaal, Ivan Pavlov
EPI_ISL_476830, EPI_ISL_476831	Laboratoire des Fièvres Hémorragiques Virales du Benin	Charité-Universitätsmedizin Berlin	Yadouleton, Anges; Sander Anna-Lena; Moreira-Soto Andres; Drexler, Jan Felix
EPI_ISL_476837	National Influenza Centre for Northern Greece	National Influenza Centre for Northern Greece	Maria Christoforidi
EPI_ISL_476983, EPI_ISL_476984, EPI_ISL_476985, EPI_ISL_476986, EPI_ISL_476987, EPI_ISL_476988, EPI_ISL_476989, EPI_ISL_476990	KU Leuven, Rega Institute, Clinical and Epidemiological Virology	KU Leuven, Rega Institute, Clinical and Epidemiological Virology	Tony Wawina-Bokalanga, Joan Marti-Carerras, Bert Vanmechelen, Piet Maes
EPI_ISL_477160	Laboratory of Dr. John Lednicky	University of Florida	John A. Lednicky, Chang-Yu Wu, and John Glenn Morris, Jr.
EPI_ISL_477163	Laboratory of Dr. John Lednicky	University of Florida	John A. Lednicky, Maha A. Elbadry, Kuttichantran Subramaniam, Thomas B. Waltzek, John Glenn Morris, Jr.
EPI_ISL_477182, EPI_ISL_477184	Department of Laboratory Medicine Tan Tock Seng Hospital	Department of Laboratory Medicine Tan Tock Seng Hospital	Chen YYC, Zair X, Li C, Tang WY, Maurer-Stroh S, Barkham TMS, Nagarajan N, Sessions OM
EPI_ISL_477890, EPI_ISL_477891, EPI_ISL_477897, EPI_ISL_477898, EPI_ISL_477899, EPI_ISL_477901, EPI_ISL_477902, EPI_ISL_477903, EPI_ISL_477904, EPI_ISL_477905, EPI_ISL_477906, EPI_ISL_477908, EPI_ISL_477909, EPI_ISL_477910, EPI_ISL_477911, EPI_ISL_477912, EPI_ISL_477914, EPI_ISL_477915, EPI_ISL_477916, EPI_ISL_477917, EPI_ISL_477918, EPI_ISL_477919, EPI_ISL_477920, EPI_ISL_477921, EPI_ISL_477922, EPI_ISL_477923, EPI_ISL_477924, EPI_ISL_477925, EPI_ISL_477926, EPI_ISL_477927, EPI_ISL_477928, EPI_ISL_477929, EPI_ISL_477930, EPI_ISL_477931, EPI_ISL_477932, EPI_ISL_477933, EPI_ISL_477934, EPI_ISL_477935, EPI_ISL_477936, EPI_ISL_477937, EPI_ISL_477938, EPI_ISL_477939, EPI_ISL_477940, EPI_ISL_477941, EPI_ISL_477942, EPI_ISL_477943, EPI_ISL_477944, EPI_ISL_477945, EPI_ISL_477946, EPI_ISL_477947, EPI_ISL_477948, EPI_ISL_477949, EPI_ISL_477950, EPI_ISL_477951, EPI_ISL_477952, EPI_ISL_477953, EPI_ISL_477954, EPI_ISL_477955, EPI_ISL_477956, EPI_ISL_477957, EPI_ISL_477958, EPI_ISL_477959, EPI_ISL_477960, EPI_ISL_477961, EPI_ISL_477962, EPI_ISL_477963, EPI_ISL_477964, EPI_ISL_477965, EPI_ISL_477966, EPI_ISL_477967, EPI_ISL_477968, EPI_ISL_477969, EPI_ISL_477970, EPI_ISL_477971, EPI_ISL_477972, EPI_ISL_477973, EPI_ISL_477974, EPI_ISL_477975, EPI_ISL_477976, EPI_ISL_477977, EPI_ISL_477978, EPI_ISL_477979, EPI_ISL_477980, EPI_ISL_477981, EPI_ISL_477982, EPI_ISL_477983, EPI_ISL_477984, EPI_ISL_477985, EPI_ISL_477986, EPI_ISL_477987, EPI_ISL_477988, EPI_ISL_477989, EPI_ISL_477990, EPI_ISL_477991, EPI_ISL_477992, EPI_ISL_477993, EPI_ISL_477994, EPI_ISL_477995, EPI_ISL_477996, EPI_ISL_477997, EPI_ISL_477998, EPI_ISL_477999, EPI_ISL_478000, EPI_ISL_478001, EPI_ISL_478002, EPI_ISL_478003, EPI_ISL_478004, EPI_ISL_478005,			

EPI_ISL_478006, EPI_ISL_478007, EPI_ISL_478008, EPI_ISL_478009, EPI_ISL_478010, EPI_ISL_478011, EPI_ISL_478012, EPI_ISL_478013, EPI_ISL_478014, EPI_ISL_478015, EPI_ISL_478016, EPI_ISL_478017, EPI_ISL_478018, EPI_ISL_478019, EPI_ISL_478020, EPI_ISL_478021, EPI_ISL_478022, EPI_ISL_478023, EPI_ISL_478024, EPI_ISL_478025, EPI_ISL_478026, EPI_ISL_478027, EPI_ISL_478028			
see above	West of Scotland Specialist Virology Centre, NHSGGC / MRC-University of Glasgow Centre for Virus Research	COVID-19 Genomics UK (COG-UK) Consortium	Ana da Silva Filipe, Natasha Johnson, Kathy Smollett, Daniel Mair, Stephen Carmichael, Lily Tong, Jenna Nichols, Elihu Aranday-Cortes, Kirstyn Brunker, Yasmin Parr, Alice Broos, Kyriaki Nomikou; Sarah McDonald, Marc Niebel, Patawee Asamaphan; Richard Orton, Joseph Hughes, Sreenu Vattipally, David L Robertson; Alasdair MacLean, Rory Gunson; Kathy Li, Natasha Jesudason, Rajiv Shah, James Shepherd, Antonia Ho, Emma Thomson
EPI_ISL_478276, EPI_ISL_478278, EPI_ISL_478281, EPI_ISL_478282	University of Exeter	COVID-19 Genomics UK (COG-UK) Consortium	Ben Temperton,Aaron Jeffries,Michelle Michelsen,Joanna Warwick-Dugdale,Audrey Farbos,Robyn Manley,Stephen Michell,Jane Masoli
EPI_ISL_478405, EPI_ISL_478406, EPI_ISL_478407, EPI_ISL_478408, EPI_ISL_478409, EPI_ISL_478410, EPI_ISL_478471, EPI_ISL_478472	University College London, Great Ormond Street Hospital for Children NHS Foundation Trust, Imperial College Healthcare NHS Trust	COVID-19 Genomics UK (COG-UK) Consortium	Sergi Castellano, Rachel Williams, Mark Kristiansen, Paola Resende Silva, Sunando Roy, Tony Brooks, Helena Tutill, Paola Niola, Patricia Dyal, Charlotte Williams, Leysa Forrest, Yasmin Panchbhaya, Jacqueline Findlay, Samuel Weeks, Julianne Brown, Kathryn Harris, Paul Randell, James Price, Alison Holmes, Judith Breuer
EPI_ISL_478668	Northumbria University / South Tees Hospitals NHS Foundation Trust / North Cumbria Integrated Care NHS Foundation Trust / North Tees and Hartlepool NHS Foundation Trust / Newcastle Hospitals NHS Foundation Trust	COVID-19 Genomics UK (COG-UK) Consortium	Darren L Smith,Andrew Nelson,Matthew Bashton,Greg R Young,Joshua Loh,John Allan,Mohammad A Tariq,Giles S Holt,Gary Black,Wen C Yew,Lynn Dover,Paul Baker,Steve Liggett,Sarah Essex,Jane Greenaway,Debra Padgett,Clive Graham,Garren Scott,Edward Barton,Emma Swindells,Brendan Payne,Jennifer Collins,Yusri Taha,Gary Eltringham
EPI_ISL_478712, EPI_ISL_478713	Sydney South West Pathology Service (SSWPS) - Liverpool Hospital - NSW Health Pathology	NSW Health Pathology - Institute of Clinical Pathology and Medical Research; Westmead Hospital; University of Sydney	CIDM-PH et al.
EPI_ISL_478725, EPI_ISL_478726, EPI_ISL_478727	Queens Medical Centre, Clinical Microbiology Department / DeepSeq Nottingham	COVID-19 Genomics UK (COG-UK) Consortium	Gemma Clark, Wendy Smith, Manjinder Khakh, Vicki M Fleming, Michelle M Lister, Hannah Howson-Wells, Jonathan Ball, Patrick McClure, Joseph Chappell, Theocharis Tsoleridis, Nadine Holmes, Matthew Carlisle, Christopher Moore, Fei Sang, Johnny Debebe, Victoria Wright, Matthew Loose
EPI_ISL_478731, EPI_ISL_478732, EPI_ISL_478734, EPI_ISL_478736, EPI_ISL_478738, EPI_ISL_478740, EPI_ISL_478741, EPI_ISL_478744, EPI_ISL_478746, EPI_ISL_478747, EPI_ISL_478748, EPI_ISL_478751, EPI_ISL_478754, EPI_ISL_478757, EPI_ISL_478759, EPI_ISL_478761, EPI_ISL_478766, EPI_ISL_478768, EPI_ISL_478769, EPI_ISL_478771, EPI_ISL_478773, EPI_ISL_478774, EPI_ISL_478775, EPI_ISL_478776, EPI_ISL_478777, EPI_ISL_478779, EPI_ISL_478783, EPI_ISL_478785, EPI_ISL_478786, EPI_ISL_478787, EPI_ISL_478789, EPI_ISL_478790, EPI_ISL_478792, EPI_ISL_478795, EPI_ISL_478797, EPI_ISL_478799, EPI_ISL_478801, EPI_ISL_478802, EPI_ISL_478806, EPI_ISL_478808, EPI_ISL_478809, EPI_ISL_478810, EPI_ISL_478812, EPI_ISL_478813, EPI_ISL_478814, EPI_ISL_478816, EPI_ISL_478819, EPI_ISL_478820, EPI_ISL_478823, EPI_ISL_478824, EPI_ISL_478826, EPI_ISL_478827, EPI_ISL_478828, EPI_ISL_478830, EPI_ISL_478832, EPI_ISL_478834, EPI_ISL_478835, EPI_ISL_478836, EPI_ISL_478838, EPI_ISL_478839, EPI_ISL_478841, EPI_ISL_478843, EPI_ISL_478844, EPI_ISL_478845, EPI_ISL_478847, EPI_ISL_478848, EPI_ISL_478850, EPI_ISL_478852, EPI_ISL_478854, EPI_ISL_478855, EPI_ISL_478857, EPI_ISL_478858, EPI_ISL_478859, EPI_ISL_478860, EPI_ISL_478861, EPI_ISL_478863, EPI_ISL_478864, EPI_ISL_478865, EPI_ISL_478867, EPI_ISL_478868, EPI_ISL_478869, EPI_ISL_478872, EPI_ISL_478873, EPI_ISL_478874, EPI_ISL_478881, EPI_ISL_478884, EPI_ISL_478891, EPI_ISL_478918, EPI_ISL_478919, EPI_ISL_478926, EPI_ISL_478929, EPI_ISL_478930, EPI_ISL_478931, EPI_ISL_478932, EPI_ISL_478933, EPI_ISL_478935, EPI_ISL_478936, EPI_ISL_478938, EPI_ISL_478940, EPI_ISL_478942, EPI_ISL_478943, EPI_ISL_478945, EPI_ISL_478948, EPI_ISL_478949, EPI_ISL_478950, EPI_ISL_478951, EPI_ISL_478952, EPI_ISL_478954, EPI_ISL_478956, EPI_ISL_478959, EPI_ISL_478960, EPI_ISL_478962, EPI_ISL_478976, EPI_ISL_478982, EPI_ISL_478987, EPI_ISL_478989, EPI_ISL_478992, EPI_ISL_478994, EPI_ISL_478999, EPI_ISL_479029, EPI_ISL_479033, EPI_ISL_479035, EPI_ISL_479037, EPI_ISL_479038, EPI_ISL_479041, EPI_ISL_479043, EPI_ISL_479045, EPI_ISL_479049, EPI_ISL_479051, EPI_ISL_479052, EPI_ISL_479053, EPI_ISL_479054, EPI_ISL_479058, EPI_ISL_479059, EPI_ISL_479061, EPI_ISL_479062, EPI_ISL_479067, EPI_ISL_479068, EPI_ISL_479072, EPI_ISL_479073, EPI_ISL_479075, EPI_ISL_479078, EPI_ISL_479079, EPI_ISL_479083, EPI_ISL_479084, EPI_ISL_479086, EPI_ISL_479089, EPI_ISL_479090, EPI_ISL_479096, EPI_ISL_479101, EPI_ISL_479103, EPI_ISL_479109, EPI_ISL_479110, EPI_ISL_479112, EPI_ISL_479117, EPI_ISL_479119, EPI_ISL_479121, EPI_ISL_479123, EPI_ISL_479124, EPI_ISL_479127, EPI_ISL_479130, EPI_ISL_479131, EPI_ISL_479135, EPI_ISL_479136, EPI_ISL_479140, EPI_ISL_479143, EPI_ISL_479145, EPI_ISL_479147, EPI_ISL_479149, EPI_ISL_479151, EPI_ISL_479153, EPI_ISL_479157, EPI_ISL_479160, EPI_ISL_479163, EPI_ISL_479165, EPI_ISL_479166, EPI_ISL_479170, EPI_ISL_479172, EPI_ISL_479174			
see above	Oxford Viromics, NDM, University of Oxford; Oxford University Hospitals; Basingstoke and North Hampshire Hospital	COVID-19 Genomics UK (COG-UK) Consortium	Tanya Golubchik, David Bonsall, George Macintyre, Amy Trebes, Mariateresa de Cesare, Catrin Moore, Alex Mobbs, Anita Justice, Robert Shaw, Monique Andersson, Timothy Peto, Emma Wise, Nathan Moore, Jessica Lynch, Nick Cortes, Matilde Mori, Stephen Kidd, David Buck, John Todd, Christophe Fraser
EPI_ISL_479378, EPI_ISL_479428	Wales Specialist Virology Centre Sequencing lab: Pathogen Genomics Unit	COVID-19 Genomics UK (COG-UK) Consortium	Catherine Moore, Johnathan Evans, Laura Gifford, Malorie Perry, Simon Cottrell, Angela Marchbank, Alec Birchley, Alexander Adams, Amy Gaskin, Bree Gatica-Wilcox, Jason Coombes, Joel Southgate, Lauren Gilbert, Lee Graham, Nicole Pacchiarini, Sara Kumziene-Summerhayes, Sarah Taylor, Sophie Jones, Sara Rey, Matthew Bull, Joanne Watkins, Sally Corden, Tom Connor
EPI_ISL_479493, EPI_ISL_479494, EPI_ISL_479495	NIV Influenza	NIV Influenza	Potdar V
EPI_ISL_479575, EPI_ISL_479576, EPI_ISL_479577, EPI_ISL_479578, EPI_ISL_479596, EPI_ISL_479597, EPI_ISL_479598, EPI_ISL_479599, EPI_ISL_479600, EPI_ISL_479601, EPI_ISL_479602, EPI_ISL_479603			
see above	National Public Health Laboratory, National Centre for Infectious Diseases	National Public Health Laboratory, National Centre for Infectious Diseases	Mak TM, Octavia S, Zhou Z, Chavatte JM, Cui L, Lin RTP
EPI_ISL_479763, EPI_ISL_479768, EPI_ISL_479769, EPI_ISL_479770, EPI_ISL_479771, EPI_ISL_479772, EPI_ISL_479773, EPI_ISL_479774, EPI_ISL_479775	University of Miami Immunology and Histocompatibility Laboratory	University of Miami Immunology and Histocompatibility Laboratory	Emilio Margolles-Clark, PhD and Phillip Ruiz, MD, PhD
EPI_ISL_479790	Laboratory of Molecular Virology of the International Centre for Genetic Engineering and Biotechnology (ICGEB)	ARGO Open Lab Platform for Genome Sequencing	Licastro, D, Rajasekharan S, Dal Monego S, Segat L, D'Agaro P, Salton F, Confalonieri P, Confalonieri M, Marcello A
EPI_ISL_480228, EPI_ISL_480229, EPI_ISL_480230, EPI_ISL_480231, EPI_ISL_480232, EPI_ISL_480233, EPI_ISL_480234	Genomic Laboratory (GLAB) (Conjoint lab of Health Directorate of Istanbul and Istanbul Technical University)	Genomic Laboratory (GLAB), Istanbul Technical University	Ilker Karacan, Tugba Kizilboga Akgun, Bugra Agaoglu, Gizem Alkurt, Jale Yildiz, Betsi Köse, Elifnaz Çelik, Arzu Irvem, Yasemin Kendir Demirkol, Ozlem Akgun Dogan, Mehtap Aydn, Levent Doganay, Gizem Dinler Doganay
EPI_ISL_480300, EPI_ISL_480309	National Reference Laboratory "Influenza and acute respiratory diseases"	NRL-HIV	Ivan Ivanov, Ivailo Alexiev, Ivva Philipova
EPI_ISL_480345	Microbial Genomics Laboratory, Institut Pasteur de Montevideo	Microbial Genomics Laboratory, Institut Pasteur de Montevideo	Cecilia Salazar, Marianoel Pereira, Ignacio Ferrés, Gonzalo Moratorio, Pilar Moreno, Gregorio Iraola
EPI_ISL_480399, EPI_ISL_480400, EPI_ISL_480401	University of Wisconsin-Madison AIDS Vaccine Research Laboratories	University of Wisconsin-Madison AIDS Vaccine Research Laboratories	Gage Moreno, Katarina Braun, et al. AIDS Vaccine Research Laboratories
EPI_ISL_480587	Microbiological Diagnostic Unit - Public Health Laboratory (MDU-PHL)	MDU-PHL	Seemann T., Schultz M., Sait, M., Sherry, N.
EPI_ISL_480590, EPI_ISL_480591, EPI_ISL_480592, EPI_ISL_480593	Victorian Infectious Diseases Reference Laboratory (VIDRL)	VIDRL and MDU-PHL	Caly L., Seemann T., Sait, M., Schultz M., Druce J., Sherry, N.
EPI_ISL_480618	Microbiological Diagnostic Unit - Public Health Laboratory (MDU-PHL)	MDU-PHL	Seemann T., Schultz M., Sait, M., Sherry, N.
EPI_ISL_480857, EPI_ISL_480858, EPI_ISL_480859, EPI_ISL_480860, EPI_ISL_480861, EPI_ISL_480862, EPI_ISL_480863, EPI_ISL_480865, EPI_ISL_480871, EPI_ISL_480872, EPI_ISL_480873			
see above	Florida Bureau of Public Health Laboratories	Florida Bureau of Public Health Laboratories	Sarah Schmedes, Jason Blanton
EPI_ISL_481070, EPI_ISL_481076, EPI_ISL_481082, EPI_ISL_481101	Hospital General Universitario Gregorio Marañón	SeqCOVID-SPAIN consortium/IBV(CSIC)	Laura Pérez-Lago, Marta Herranz, Jon Sicilia, Julia Suárez, Pilar Catalán, Patricia Muñoz, Darío García de Viedma and SeqCOVID-SPAIN consortium
EPI_ISL_481216	Medical Microbiology Unit, Department for Laboratory Medicine, Drammen Hospital, Vestre Viken Health Trust,	Norwegian Institute of Public Health, Department of Virology	Kathrine Stene-Johansen, Kamilla Heddeland Instefjord, Hilde Elshaug, Rasmus Riis Kopperud, Karoline Bragstad, Olav Hungnes
EPI_ISL_481218	Oslo University Hospital, Department of Medical Microbiology	Norwegian Institute of Public Health, Department of Virology	Kathrine Stene-Johansen, Kamilla Heddeland Instefjord, Hilde Elshaug, Rasmus Riis Kopperud, Karoline Bragstad, Olav Hungnes
EPI_ISL_481225	Lab voor klinische biologie	Onderzoeksgroep Virologie	Laurens Lambrechts, Nick Vereecke, Marthe Pauwels, Bruno Verhasselt, Linos Vandekerckhove, Hans Nauwynck, Sebastiaan Theuns
EPI_ISL_481228, EPI_ISL_481229	Lab voor klinische biologie	Onderzoeksgroep Virologie	Nick Vereecke, Laurens Lambrechts, Marthe Pauwels, Bruno Verhasselt, Linos Vandekerckhove, Hans Nauwynck, Sebastiaan Theuns
EPI_ISL_481236, EPI_ISL_481237, EPI_ISL_481238, EPI_ISL_481239,	Institut Pasteur Dakar	Institut Pasteur de Dakar	Ndongo Dia, Moussa Moise Diagne, Mamadou Diop, Marie Henriette Dior Ndione, Mamadou Malado Jallow, Safietou Sanke, Ousmane Faye, Amadou Alpha Sall.

EPI_ISL_481240, EPI_ISL_481243			
EPI_ISL_481244, EPI_ISL_481245, EPI_ISL_481246, EPI_ISL_481247, EPI_ISL_481248	Hospital IESS Babahoyo	Institute of Microbiology, Universidad San Francisco de Quito	Belén Prado-Vivar, Sully Márquez, Juan José Guadalupe, Monica Becerra-Wong, Carla Torres, Bernardo Gutiérrez, Francisco Cordova, Ninfa Henriquez, Killen Briones-Zamora, Killen Briones-Claudette, Verónica Barragán, Patricio Rojas-Silva, Gabriel Trueba, Michelle Grunauer, Paúl Cárdenas
EPI_ISL_481513, EPI_ISL_481551, EPI_ISL_481552, EPI_ISL_481553, EPI_ISL_481554, EPI_ISL_481555, EPI_ISL_481556, EPI_ISL_481557, EPI_ISL_481558, EPI_ISL_481559, EPI_ISL_481560, EPI_ISL_481561, EPI_ISL_481562, EPI_ISL_481563, EPI_ISL_481564, EPI_ISL_481566, EPI_ISL_481567, EPI_ISL_481568, EPI_ISL_481572, EPI_ISL_481573, EPI_ISL_481574, EPI_ISL_481575, EPI_ISL_481576, EPI_ISL_481577, EPI_ISL_481578, EPI_ISL_481579, EPI_ISL_481580, EPI_ISL_481581, EPI_ISL_481582, EPI_ISL_481583, EPI_ISL_481584, EPI_ISL_481585, EPI_ISL_481586, EPI_ISL_481587, EPI_ISL_481588, EPI_ISL_481589, EPI_ISL_481590, EPI_ISL_481591, EPI_ISL_481592, EPI_ISL_481593, EPI_ISL_481594			
see above	Department of Virology and Immunology, University of Helsinki and Helsinki University Hospital, Huslab Finland	Department of Virology, Faculty of Medicine, University of Helsinki, Helsinki, Finland	Teemu Smura, Hannimari Kallio-Kokko, Jenni Virtanen, Maija Suvanto, Sari Hannula, Harri Kangas, Pekka Ellonen, Olli Vapalahti
EPI_ISL_481758	Dr. Georges-L.-Dumont University Hospital Centre	National Microbiology Laboratory	Anna Majer, Shari Tyson, Grace Seo, Kristyn Burak, Philip Mabon, Elsie Grudeski, Rhiannon Huzarewich, Russell Mandes, Jennifer Tanner, Natalie Knox, Morag Graham, Gary Van Domselaar, Richard Garceau, Guillaume Desnoyers, Nathalie Bastien, Yan Li, Timothy Booth
EPI_ISL_481767, EPI_ISL_481771, EPI_ISL_481784, EPI_ISL_481791, EPI_ISL_481794, EPI_ISL_481796, EPI_ISL_481797, EPI_ISL_481803, EPI_ISL_481817, EPI_ISL_481818, EPI_ISL_481820, EPI_ISL_481834, EPI_ISL_481837, EPI_ISL_481842, EPI_ISL_481847, EPI_ISL_481851, EPI_ISL_481854, EPI_ISL_481858, EPI_ISL_481869, EPI_ISL_481883, EPI_ISL_481884, EPI_ISL_481885, EPI_ISL_481887, EPI_ISL_481902, EPI_ISL_481906, EPI_ISL_481909, EPI_ISL_481924, EPI_ISL_481932, EPI_ISL_481935, EPI_ISL_481949, EPI_ISL_481953, EPI_ISL_481954, EPI_ISL_481955, EPI_ISL_481957, EPI_ISL_481958, EPI_ISL_481960, EPI_ISL_481963, EPI_ISL_481966, EPI_ISL_481977, EPI_ISL_481983, EPI_ISL_481988, EPI_ISL_481992, EPI_ISL_482003, EPI_ISL_482008, EPI_ISL_482009, EPI_ISL_482010, EPI_ISL_482019			
see above	PHE South West Regional Laboratory, National Infection Service	Wellcome Sanger Institute for the COVID-19 Genomics UK (COG-UK) consortium	Stephanie Hutchings, Hannah Pymont, Dr Peter Muir, Barry Vipond, Rich Hopes; and Alex Alderton, Roberto Amato, Sonia Goncalves, Ewan Harrison, David K. Jackson, Ian Johnston, Dominic Kwiatkowski, Cordelia Langford, John Sillitoe on behalf of the Wellcome Sanger Institute COVID-19 Surveillance Team (http://www.sanger.ac.uk/covid-team)
EPI_ISL_482034, EPI_ISL_482035, EPI_ISL_482037, EPI_ISL_482039, EPI_ISL_482040, EPI_ISL_482043, EPI_ISL_482045, EPI_ISL_482048, EPI_ISL_482051	Regional Virus Laboratory, Belfast Health and Social Care Trust	Wellcome Sanger Institute for the COVID-19 Genomics UK (COG-UK) consortium	Conall McCaughey, James McKenna, Tanya Curran, Susan Feeney, Alison Watt, Ciara Cox, Mairead Connor, Zoltan Molnar, David Simpson, Derek Fairley; and Alex Alderton, Roberto Amato, Sonia Goncalves, Ewan Harrison, David K. Jackson, Ian Johnston, Dominic Kwiatkowski, Cordelia Langford, John Sillitoe on behalf of the Wellcome Sanger Institute COVID-19 Surveillance Team (http://www.sanger.ac.uk/covid-team)
EPI_ISL_482057	The Department of Microbiology, Torbay and South Devon NHS Foundation Trust	Wellcome Sanger Institute for the COVID-19 Genomics UK (COG-UK) consortium	Amy Hurd, Sophie Lloyd, Anthony Mogridge, Jack Howe, Helen Brown, Gary Booth, Mel Brown, Cheryl Bailiss, Michelle Harrison and Alex Alderton, Roberto Amato, Sonia Goncalves, Ewan Harrison, David K. Jackson, Ian Johnston, Dominic Kwiatkowski, Cordelia Langford, John Sillitoe on behalf of the Wellcome Sanger Institute COVID-19 Surveillance Team (http://www.sanger.ac.uk/covid-team)
EPI_ISL_482070, EPI_ISL_482082, EPI_ISL_482091, EPI_ISL_482102	Regional Virus Laboratory, Belfast Health and Social Care Trust	Wellcome Sanger Institute for the COVID-19 Genomics UK (COG-UK) consortium	Conall McCaughey, James McKenna, Tanya Curran, Susan Feeney, Alison Watt, Ciara Cox, Mairead Connor, Zoltan Molnar, David Simpson, Derek Fairley; and Alex Alderton, Roberto Amato, Sonia Goncalves, Ewan Harrison, David K. Jackson, Ian Johnston, Dominic Kwiatkowski, Cordelia Langford, John Sillitoe on behalf of the Wellcome Sanger Institute COVID-19 Surveillance Team (http://www.sanger.ac.uk/covid-team)
EPI_ISL_482125, EPI_ISL_482126, EPI_ISL_482128	The Department of Microbiology, Torbay and South Devon NHS Foundation Trust	Wellcome Sanger Institute for the COVID-19 Genomics UK (COG-UK) consortium	Amy Hurd, Sophie Lloyd, Anthony Mogridge, Jack Howe, Helen Brown, Gary Booth, Mel Brown, Cheryl Bailiss, Michelle Harrison and Alex Alderton, Roberto Amato, Sonia Goncalves, Ewan Harrison, David K. Jackson, Ian Johnston, Dominic Kwiatkowski, Cordelia Langford, John Sillitoe on behalf of the Wellcome Sanger Institute COVID-19 Surveillance Team (http://www.sanger.ac.uk/covid-team)
EPI_ISL_482132, EPI_ISL_482133, EPI_ISL_482135, EPI_ISL_482156	Regional Virus Laboratory, Belfast Health and Social Care Trust	Wellcome Sanger Institute for the COVID-19 Genomics UK (COG-UK) consortium	Conall McCaughey, James McKenna, Tanya Curran, Susan Feeney, Alison Watt, Ciara Cox, Mairead Connor, Zoltan Molnar, David Simpson, Derek Fairley; and Alex Alderton, Roberto Amato, Sonia Goncalves, Ewan Harrison, David K. Jackson, Ian Johnston, Dominic Kwiatkowski, Cordelia Langford, John Sillitoe on behalf of the Wellcome Sanger Institute COVID-19 Surveillance Team (http://www.sanger.ac.uk/covid-team)
EPI_ISL_482339, EPI_ISL_482340, EPI_ISL_482341, EPI_ISL_482342, EPI_ISL_482346, EPI_ISL_482347, EPI_ISL_482348, EPI_ISL_482349, EPI_ISL_482350, EPI_ISL_482351, EPI_ISL_482352, EPI_ISL_482353, EPI_ISL_482354, EPI_ISL_482355, EPI_ISL_482356, EPI_ISL_482357, EPI_ISL_482446, EPI_ISL_482447			
see above	Providence St. Joseph Health Molecular Genomics Laboratory	Providence St. Joseph Health Molecular Genomics Laboratory	Alexa K Dowdell, Brian D Piening, Fred L Robinson, Carlo B Bifulco, Mary Campbell
EPI_ISL_482775	Medical Ain Shams Research Institute (MASRI), Ain Shams University	Medical Ain Shams Research Institute (MASRI), Ain Shams University	Hesham Elghazaly, Sara Hassan Agwa, Ahmad Moustafa, Hala Hafez, Sara Elnakeep, Shaimaa Moustafa, Aya Mohamed, Reham Mamdouh, Ghada Ismael, Ashraf Omar, Osama Mansour, Mahmoud Elmeitini
EPI_ISL_482967	Mayo Clinic & Mayo Clinic Laboratories	Minnesota Department of Health, Public Health Laboratory	Matt Plumb, Jacob Garfin, and Xiong Wang
EPI_ISL_483139, EPI_ISL_483140	Robert Koch Institute, ZBS1 Highly Pathogenic Viruses, Berlin, Germany	Robert Koch Institute, Bioinformatics MF1, Berlin, Germany	Janine Michel, Andrea Thuermer, Oliver Drechsel, Rene Kmiecinski, Stephan Fuchs, Max v. Kleist, Andreas Nitsche
EPI_ISL_483162, EPI_ISL_483163, EPI_ISL_483164	San Diego County Public Health Laboratory	Andersen lab at Scripps Research	SEARCH Alliance San Diego with Tracy Basler, Jovan Shephard, Brett Austin
EPI_ISL_483182, EPI_ISL_483185, EPI_ISL_483186, EPI_ISL_483238, EPI_ISL_483239, EPI_ISL_483240, EPI_ISL_483241, EPI_ISL_483242, EPI_ISL_483243, EPI_ISL_483244, EPI_ISL_483245, EPI_ISL_483246, EPI_ISL_483247, EPI_ISL_483248, EPI_ISL_483249, EPI_ISL_483250, EPI_ISL_483251, EPI_ISL_483254, EPI_ISL_483256, EPI_ISL_483282, EPI_ISL_483283, EPI_ISL_483286, EPI_ISL_483288, EPI_ISL_483338, EPI_ISL_483344, EPI_ISL_483345, EPI_ISL_483349, EPI_ISL_483353, EPI_ISL_483457, EPI_ISL_483458, EPI_ISL_483460, EPI_ISL_483461, EPI_ISL_483462, EPI_ISL_483465, EPI_ISL_483468			
see above	UC San Diego Center for Advanced Laboratory Medicine	Andersen lab at Scripps Research	SEARCH Alliance San Diego with David Pride, Ji H Shin
EPI_ISL_483594, EPI_ISL_483596, EPI_ISL_483597, EPI_ISL_483609	National Public Health Laboratory, National Centre for Infectious Diseases	National Public Health Laboratory, National Centre for Infectious Diseases	Mak TM, Octavia S, Zhou Z, Chavatte JM, Cui L, Lin RTP
EPI_ISL_483670, EPI_ISL_483672, EPI_ISL_483673, EPI_ISL_483675, EPI_ISL_483676, EPI_ISL_483679, EPI_ISL_483681, EPI_ISL_483684	University Hospital Zurich	Department of Biosystems Science and Engineering, ETH Zürich	Christian Beisel, Sarah Nadeau, Ivan Topolsky, Pedro Ferreira, Philipp Jablonski, Susana Posada-Céspedes, Tobias Schär, Ina Nissen, Natascha Santacroce, Elodie Burcklen, Julia Martinez-Gomez, Phil Cheng, Mitch Levesque, Philipp Bosshard, Niko Beerenwinkel, Tanja Stadler
EPI_ISL_483724	Israel Central Virology laboratory	Israel Central Virology laboratory	Neta Zuckerman, Efrat Dahan Bucris, Oran Erster, Ella Mendelson, Michal Mandelboim
EPI_ISL_484405, EPI_ISL_484406	Lincolnshire Hospitals and DeepSeq Nottingham	COVID-19 Genomics UK (COG-UK) Consortium	Nichola Duckworth, Tim Sloan, Sarah Walsh, Jonathan Ball, Patrick McClure, Joeseph Chappell, Nadine Holmes, Matthew Carlisle, Christopher Moore, Fei Sang, Johnny Debebe, Victoria Wright, Matthew Loose
EPI_ISL_484418, EPI_ISL_484423	Centre for Enzyme Innovation, University of Portsmouth / Translational Research Laboratory, Portsmouth Hospitals NHS Trust	COVID-19 Genomics UK (COG-UK) Consortium	Angela Beckett,Yann Bourgeois,Garry Scarlett,Sharon Glaysher,Scott Elliott,Kelly Bicknell,Robert Impey,Allyson Lloyd,Sarah Wyllie,Ethan Butcher,Anoop Chauhan,Samuel Robson
EPI_ISL_484575, EPI_ISL_484576, EPI_ISL_484577, EPI_ISL_484578, EPI_ISL_484579, EPI_ISL_484580, EPI_ISL_484581, EPI_ISL_484582, EPI_ISL_484583, EPI_ISL_484584, EPI_ISL_484585, EPI_ISL_484586, EPI_ISL_484587, EPI_ISL_484588, EPI_ISL_484589, EPI_ISL_484590, EPI_ISL_484591, EPI_ISL_484592, EPI_ISL_484593, EPI_ISL_484594, EPI_ISL_484595, EPI_ISL_484596, EPI_ISL_484597, EPI_ISL_484598, EPI_ISL_484599, EPI_ISL_484600, EPI_ISL_484601, EPI_ISL_484602, EPI_ISL_484603, EPI_ISL_484604, EPI_ISL_484605, EPI_ISL_484606, EPI_ISL_484607, EPI_ISL_484608, EPI_ISL_484609, EPI_ISL_484610, EPI_ISL_484611, EPI_ISL_484612, EPI_ISL_484613, EPI_ISL_484614, EPI_ISL_484615, EPI_ISL_484616, EPI_ISL_484617, EPI_ISL_484618, EPI_ISL_484619, EPI_ISL_484620, EPI_ISL_484621, EPI_ISL_484622, EPI_ISL_484623, EPI_ISL_484624, EPI_ISL_484625, EPI_ISL_484626, EPI_ISL_484627, EPI_ISL_484628, EPI_ISL_484629, EPI_ISL_484630, EPI_ISL_484631, EPI_ISL_484632, EPI_ISL_484633, EPI_ISL_484634, EPI_ISL_484635, EPI_ISL_484636, EPI_ISL_484637, EPI_ISL_484638, EPI_ISL_484639, EPI_ISL_484640, EPI_ISL_484641, EPI_ISL_484642, EPI_ISL_484643, EPI_ISL_484644, EPI_ISL_484645, EPI_ISL_484646, EPI_ISL_484647, EPI_ISL_484648, EPI_ISL_484649, EPI_ISL_484650, EPI_ISL_484651			
see above	West of Scotland Specialist Virology Centre, NHSGGC / MRC-University of Glasgow Centre for Virus Research	COVID-19 Genomics UK (COG-UK) Consortium	Ana da Silva Filipe, Natasha Johnson, Kathy Smollett, Daniel Mair, Stephen Carmichael, Lily Tong, Jenna Nichols, Elihu Aranday-Cortes, Kirstyn Brunker, Yasmin Parr, Alice Broos, Kyriaki Nomikou; Sarah McDonald, Marc Niebel, Patawee Asamaphan; Richard Orton, Joseph Hughes, Sreenu Vattipally, David L Robertson; Alasdair MacLean, Rory Gunson; Kathy Li, Natasha Jesudason, Rajiv Shah, James Shepherd, Antonia Ho, Emma Thomson
EPI_ISL_485117, EPI_ISL_485118, EPI_ISL_485119, EPI_ISL_485120, EPI_ISL_485121, EPI_ISL_485122, EPI_ISL_485123, EPI_ISL_485124, EPI_ISL_485125, EPI_ISL_485126, EPI_ISL_485127, EPI_ISL_485128, EPI_ISL_485129, EPI_ISL_485130, EPI_ISL_485131, EPI_ISL_485132, EPI_ISL_485133, EPI_ISL_485134, EPI_ISL_485135, EPI_ISL_485136, EPI_ISL_485137, EPI_ISL_485138, EPI_ISL_485139, EPI_ISL_485140, EPI_ISL_485141, EPI_ISL_485142, EPI_ISL_485143, EPI_ISL_485144, EPI_ISL_485145, EPI_ISL_485146, EPI_ISL_485147, EPI_ISL_485148, EPI_ISL_485149, EPI_ISL_485150, EPI_ISL_485151, EPI_ISL_485152, EPI_ISL_485153, EPI_ISL_485154, EPI_ISL_485155, EPI_ISL_485156, EPI_ISL_485157, EPI_ISL_485158, EPI_ISL_485159, EPI_ISL_485160, EPI_ISL_485161, EPI_ISL_485162, EPI_ISL_485163, EPI_ISL_485164, EPI_ISL_485165, EPI_ISL_485166, EPI_ISL_485167, EPI_ISL_485168, EPI_ISL_485169, EPI_ISL_485170, EPI_ISL_485171, EPI_ISL_485172, EPI_ISL_485173, EPI_ISL_485174, EPI_ISL_485175, EPI_ISL_485176, EPI_ISL_485177, EPI_ISL_485178, EPI_ISL_485179, EPI_ISL_485180, EPI_ISL_485181, EPI_ISL_485182, EPI_ISL_485183, EPI_ISL_485184, EPI_ISL_485185, EPI_ISL_485186, EPI_ISL_485187, EPI_ISL_485188, EPI_ISL_485189, EPI_ISL_485190, EPI_ISL_485191, EPI_ISL_485192, EPI_ISL_485193, EPI_ISL_485194, EPI_ISL_485195, EPI_ISL_485196, EPI_ISL_485197, EPI_ISL_485198, EPI_ISL_485199, EPI_ISL_485200, EPI_ISL_485201, EPI_ISL_485202, EPI_ISL_485203, EPI_ISL_485204, EPI_ISL_485205, EPI_ISL_485206, EPI_ISL_485207, EPI_ISL_485208, EPI_ISL_485209, EPI_ISL_485210, EPI_ISL_485212, EPI_ISL_485248, EPI_ISL_485260, EPI_ISL_485261, EPI_ISL_485262			
see above	River Road Testing Lab	Ginkgo Bioworks Clinical Laboratory	Rebecca C. Christofferson, Stephania A. Cormier, Luan V. Dinh, E. Handly Mayton, Hollis R. O'Neil, Thaya Stoufflet, Malaika Mckenzie-Bennett, James

			McGann, Jim Griffin, Keith Robison, Alex Plocik, Becky Schilling, Rebecca Littlefield, Michelle Spencer, Birgitte Simen
EPI_ISL_485391	University of Ulsan College of Medicine and Asan Medical Center	University of Ulsan College of Medicine and Asan Medical Center	Kuenyoul Park, Jaewoong Lee, Kihyun Lee, Jiwon Jung, Sung-Han Kim, Jina Lee, Mauricio Chalita, Seok-Hwan Yoon, Jongsik Chun, Kyu-Hwa Hur, Heungsup Sung, Mi-Na Kim, and Hae Kyung Lee
EPI_ISL_485579, EPI_ISL_485580, EPI_ISL_485581, EPI_ISL_485585, EPI_ISL_485586, EPI_ISL_485587, EPI_ISL_485588, EPI_ISL_485589, EPI_ISL_485590, EPI_ISL_485591, EPI_ISL_485592, EPI_ISL_485593, EPI_ISL_485594, EPI_ISL_485595, EPI_ISL_485597			
see above	Instituto de diagnóstico y Referencia Epidemiologicos (INDRE)	Instituto de diagnóstico y Referencia Epidemiologicos (INDRE)	Barrera-Badillo,G., Ramirez-Gonzalez,E.
EPI_ISL_485603	Division of Infectious Disease	Steininger Lab	Jakob Thannesberger, Ingeborg Klymiuk, Nicolas Rascovan, Lorenz Schubert, Oliver Robak, Christoph Steininger
EPI_ISL_485886, EPI_ISL_485887, EPI_ISL_485888, EPI_ISL_485889, EPI_ISL_485890, EPI_ISL_485891, EPI_ISL_485899, EPI_ISL_485900	River Road Testing Lab	Ginkgo Bioworks Clinical Laboratory	Rebecca C. Christofferson, Stephania A. Cormier, Luan V. Dinh, E. Handly Mayton, Hollis R. O'Neil, Thaya Stoufflet, Malaika Mckenzie-Bennett, James McGann, Jim Griffin, Keith Robison, Alex Plocik, Becky Schilling, Rebecca Littlefield, Michelle Spencer, Birgitte Simen
EPI_ISL_485949, EPI_ISL_485950, EPI_ISL_485951, EPI_ISL_485952, EPI_ISL_485953, EPI_ISL_485954, EPI_ISL_485955, EPI_ISL_485956, EPI_ISL_485957, EPI_ISL_485958, EPI_ISL_485959, EPI_ISL_485960, EPI_ISL_485961, EPI_ISL_485963, EPI_ISL_485964, EPI_ISL_485967, EPI_ISL_485968, EPI_ISL_485969, EPI_ISL_485972, EPI_ISL_485973, EPI_ISL_485983, EPI_ISL_485984, EPI_ISL_485985, EPI_ISL_485990, EPI_ISL_485991, EPI_ISL_485992, EPI_ISL_485993, EPI_ISL_486025, EPI_ISL_486026, EPI_ISL_486027, EPI_ISL_486029, EPI_ISL_486032, EPI_ISL_486036, EPI_ISL_486108, EPI_ISL_486109, EPI_ISL_486110			
see above	UW Virology Lab	UW Virology Lab	Pavitra Roychoudhury, Hong Xie, Lasata Shrestha, Amin Addetia, Truong Nguyen, Victoria M Rachleff, Meei-Li Huang, Keith R Jerome, Alexander Greninger
EPI_ISL_486120, EPI_ISL_486121, EPI_ISL_486122, EPI_ISL_486123, EPI_ISL_486124, EPI_ISL_486125, EPI_ISL_486126, EPI_ISL_486127, EPI_ISL_486128, EPI_ISL_486129, EPI_ISL_486130, EPI_ISL_486131, EPI_ISL_486132, EPI_ISL_486184, EPI_ISL_486185, EPI_ISL_486186, EPI_ISL_486187, EPI_ISL_486188, EPI_ISL_486189, EPI_ISL_486190, EPI_ISL_486218, EPI_ISL_486219, EPI_ISL_486220, EPI_ISL_486221			
see above	Orange County Public Health Laboratory	Chan-Zuckerberg Biohub	CZB Cliahub Consortium
EPI_ISL_486292	San Joaquin County Public Health Lab	Chan-Zuckerberg Biohub	CZB Cliahub Consortium
EPI_ISL_486382	District Surveillence Unit	Department of Neurovirology, National Institute of Mental Health and Neuroscience (NIMHANS)	Chitra Pattabiraman, Vijayalakshmi Reddy, Harsha PK, Risha Rasheed, Shafeeq S Hameed, Manjunatha Venkataswamy, Anita Desai, Ravi Vasanthapuram
EPI_ISL_486392	Victoria Hospital	Department of Neurovirology, National Institute of Mental Health and Neuroscience (NIMHANS)	Chitra Pattabiraman, Vijayalakshmi Reddy, Harsha PK, Risha Rasheed, Shafeeq S Hameed, Manjunatha Venkataswamy, Anita Desai, Ravi Vasanthapuram
EPI_ISL_486393	SJMCH	Department of Neurovirology, National Institute of Mental Health and Neuroscience (NIMHANS)	Chitra Pattabiraman, Vijayalakshmi Reddy, Harsha PK, Risha Rasheed, Shafeeq S Hameed, Manjunatha Venkataswamy, Anita Desai, Ravi Vasanthapuram
EPI_ISL_487098	Nigeria Centre for Disease Control (NCDC)	African Centre of Excellence for Genomics of Infectious Diseases (ACEGID), Redeemer's University, Ede, Osun State, Nigeria	Oluniyi P.E., Ajogbasile F.V., Kayode A., Oguzie J., Olawoye I., Uwanibe J., Olumade T., Folarin O.A., Ihekweazu C., Happi C.T.
EPI_ISL_487462, EPI_ISL_487463, EPI_ISL_487464, EPI_ISL_487465, EPI_ISL_487466	CICM-Mali	Bundeswehr Institut of Microbiology	Kouriba, Dürr, Sangaré, Rehn, Traoré, Bestehorn-Willmann, Walter, Quedraogo, Zimmermann, Maiga, Heitzer, Sogodogo, Antwerpen, Wölfel
EPI_ISL_487636, EPI_ISL_487637, EPI_ISL_487638, EPI_ISL_487639, EPI_ISL_487640, EPI_ISL_487641, EPI_ISL_487642, EPI_ISL_487643, EPI_ISL_487645, EPI_ISL_487646	University College London, Great Ormond Street Hospital for Children NHS Foundation Trust, Imperial College Healthcare NHS Trust	Wellcome Sanger Institute for the COVID-19 Genomics UK (COG-UK) consortium	Sergi Castellano, Rachel Williams, Mark Kristiansen, Paola Resende Silva, Sunando Roy, Tony Brooks, Helena Tutill, Paola Niola, Patricia Dyal, Charlotte Williams, Leysa Forrest, Yasmin Panchbhaya, Jacqueline Findlay, Sam Weeks, Julianne Brown, Kathryn Harris, Paul Randell, James Price, Alison Holmes, Judith Breuer and Alex Alderton, Roberto Amato, Sonia Goncalves, Ewan Harrison, David K. Jackson, Ian Johnston, Dominic Kwiatkowski, Cordelia Langford, John Sillitoe on behalf of the Wellcome Sanger Institute COVID-19 Surveillance Team (http://www.sanger.ac.uk/covid-team)
EPI_ISL_487647, EPI_ISL_487650, EPI_ISL_487656, EPI_ISL_487660, EPI_ISL_487662, EPI_ISL_487672, EPI_ISL_487675, EPI_ISL_487676, EPI_ISL_487679, EPI_ISL_487681, EPI_ISL_487687, EPI_ISL_487692, EPI_ISL_487694, EPI_ISL_487696, EPI_ISL_487704, EPI_ISL_487711, EPI_ISL_487713, EPI_ISL_487715, EPI_ISL_487722, EPI_ISL_487729, EPI_ISL_487738, EPI_ISL_487747, EPI_ISL_487748, EPI_ISL_487750, EPI_ISL_487753, EPI_ISL_487765, EPI_ISL_487769, EPI_ISL_487773, EPI_ISL_487784, EPI_ISL_487785, EPI_ISL_487792, EPI_ISL_487796, EPI_ISL_487803, EPI_ISL_487814, EPI_ISL_487833, EPI_ISL_487836, EPI_ISL_487840, EPI_ISL_487842, EPI_ISL_487847, EPI_ISL_487848, EPI_ISL_487853, EPI_ISL_487855, EPI_ISL_487863, EPI_ISL_487864, EPI_ISL_487870, EPI_ISL_487880, EPI_ISL_487887, EPI_ISL_487894, EPI_ISL_487901, EPI_ISL_487902, EPI_ISL_487909, EPI_ISL_487929, EPI_ISL_487937, EPI_ISL_487942, EPI_ISL_487943, EPI_ISL_487950, EPI_ISL_487961, EPI_ISL_487972, EPI_ISL_487974, EPI_ISL_487980, EPI_ISL_487998, EPI_ISL_488002			
see above	Virology Department, Royal Infirmary of Edinburgh, NHS Lothian / School of Biological Sciences, University of Edinburgh	Wellcome Sanger Institute for the COVID-19 Genomics UK (COG-UK) consortium	McHugh M, Dewar R, Rooke S, O'Toole Á, Scher E, Hill V, McCrone JT, Colquhoun R, Yu X, Jackson B, Rambaut A, Templeton K and Alex Alderton, Roberto Amato, Sonia Goncalves, Ewan Harrison, David K. Jackson, Ian Johnston, Dominic Kwiatkowski, Cordelia Langford, John Sillitoe on behalf of the Wellcome Sanger Institute COVID-19 Surveillance Team (http://www.sanger.ac.uk/covid-team)
EPI_ISL_488035, EPI_ISL_488059	NU-OMICS DNA Sequencing research facility, Northumbria University	Wellcome Sanger Institute for the COVID-19 Genomics UK (COG-UK) consortium	Chris Duncan, Sheia Waugh, Shirelle Burton-Fanning, Gary Eltringham, Jennifer Collins, Brendan Payne, Yusri Taha, Emma Swindells, Jane Greenaway, Edward Barton, Garren Scott, Debra Padgett, Clive Graham, Sarah Essex, Steve Ligget, Paul Baker, Lynn Dover, Wen Yew, Gary Black, John Allan, Joshua Loh, Greg Young, Matthew Bashton, Andrew Nelson, Darren Smith and Alex Alderton, Roberto Amato, Sonia Goncalves, Ewan Harrison, David K. Jackson, Ian Johnston, Dominic Kwiatkowski, Cordelia Langford, John Sillitoe on behalf of the Wellcome Sanger Institute COVID-19 Surveillance Team (http://www.sanger.ac.uk/covid-team)
EPI_ISL_488194, EPI_ISL_488200, EPI_ISL_488204, EPI_ISL_488218, EPI_ISL_488224, EPI_ISL_488226, EPI_ISL_488230, EPI_ISL_488237, EPI_ISL_488238, EPI_ISL_488239, EPI_ISL_488265, EPI_ISL_488266, EPI_ISL_488269, EPI_ISL_488278, EPI_ISL_488291, EPI_ISL_488295, EPI_ISL_488296, EPI_ISL_488298, EPI_ISL_488300, EPI_ISL_488309, EPI_ISL_488311, EPI_ISL_488326, EPI_ISL_488334, EPI_ISL_488340, EPI_ISL_488345, EPI_ISL_488350, EPI_ISL_488359, EPI_ISL_488361, EPI_ISL_488365, EPI_ISL_488372, EPI_ISL_488373, EPI_ISL_488374, EPI_ISL_488385, EPI_ISL_488386, EPI_ISL_488400, EPI_ISL_488408, EPI_ISL_488416, EPI_ISL_488422, EPI_ISL_488424, EPI_ISL_488445, EPI_ISL_488446, EPI_ISL_488451, EPI_ISL_488453			
see above	PHE South West Regional Laboratory, National Infection Service	Wellcome Sanger Institute for the COVID-19 Genomics UK (COG-UK) consortium	Stephanie Hutchings, Hannah Pymont, Dr Peter Muir, Barry Vipond, Rich Hopes; and Alex Alderton, Roberto Amato, Sonia Goncalves, Ewan Harrison, David K. Jackson, Ian Johnston, Dominic Kwiatkowski, Cordelia Langford, John Sillitoe on behalf of the Wellcome Sanger Institute COVID-19 Surveillance Team (http://www.sanger.ac.uk/covid-team)
EPI_ISL_488845, EPI_ISL_488853, EPI_ISL_488864	Department of Medical Microbiology, Western Sussex Hospitals NHS Foundation Trust, St Richard's Hospital	Wellcome Sanger Institute for the COVID-19 Genomics UK (COG-UK) consortium	Manasa Mutingwende, Sarah Lowdon, Olga Podplomyk, Michelle Erkiert, Jonathan Lewis, Paul Randell and Alex Alderton, Roberto Amato, Sonia Goncalves, Ewan Harrison, David K. Jackson, Ian Johnston, Dominic Kwiatkowski, Cordelia Langford, John Sillitoe on behalf of the Wellcome Sanger Institute COVID-19 Surveillance Team (http://www.sanger.ac.uk/covid-team)
EPI_ISL_489161, EPI_ISL_489163, EPI_ISL_489164, EPI_ISL_489169, EPI_ISL_489170, EPI_ISL_489171, EPI_ISL_489177, EPI_ISL_489178, EPI_ISL_489179, EPI_ISL_489185, EPI_ISL_489186, EPI_ISL_489188, EPI_ISL_489189, EPI_ISL_489190, EPI_ISL_489191, EPI_ISL_489192, EPI_ISL_489195, EPI_ISL_489196, EPI_ISL_489197, EPI_ISL_489198, EPI_ISL_489201, EPI_ISL_489202, EPI_ISL_489204, EPI_ISL_489205, EPI_ISL_489206, EPI_ISL_489207, EPI_ISL_489210, EPI_ISL_489211, EPI_ISL_489214, EPI_ISL_489215, EPI_ISL_489216, EPI_ISL_489217, EPI_ISL_489219, EPI_ISL_489221, EPI_ISL_489223, EPI_ISL_489224, EPI_ISL_489228, EPI_ISL_489232, EPI_ISL_489234, EPI_ISL_489237, EPI_ISL_489241, EPI_ISL_489243, EPI_ISL_489248, EPI_ISL_489249, EPI_ISL_489252, EPI_ISL_489254, EPI_ISL_489255, EPI_ISL_489256, EPI_ISL_489262, EPI_ISL_489266, EPI_ISL_489269, EPI_ISL_489275, EPI_ISL_489300, EPI_ISL_489306, EPI_ISL_489309, EPI_ISL_489312, EPI_ISL_489314, EPI_ISL_489315, EPI_ISL_489317, EPI_ISL_489318, EPI_ISL_489328, EPI_ISL_489333, EPI_ISL_489341, EPI_ISL_489342, EPI_ISL_489344, EPI_ISL_489345, EPI_ISL_489348, EPI_ISL_489349, EPI_ISL_489354, EPI_ISL_489357, EPI_ISL_489360, EPI_ISL_489366, EPI_ISL_489369, EPI_ISL_489375, EPI_ISL_489377			
see above	Regional Virus Laboratory, Belfast Health and Social Care Trust	Wellcome Sanger Institute for the COVID-19 Genomics UK (COG-UK) consortium	Conall McCaughey, James McKenna, Tanya Curran, Susan Feeney, Alison Watt, Ciara Cox, Mairead Connor, Zoltan Molnar, David Simpson, Derek Fairley; and Alex Alderton, Roberto Amato, Sonia Goncalves, Ewan Harrison, David K. Jackson, Ian Johnston, Dominic Kwiatkowski, Cordelia Langford, John Sillitoe on behalf of the Wellcome Sanger Institute COVID-19 Surveillance Team (http://www.sanger.ac.uk/covid-team)
EPI_ISL_489710, EPI_ISL_489727, EPI_ISL_489728, EPI_ISL_489729, EPI_ISL_489730, EPI_ISL_489732, EPI_ISL_489734, EPI_ISL_489735, EPI_ISL_489736, EPI_ISL_489737, EPI_ISL_489738, EPI_ISL_489739, EPI_ISL_489740, EPI_ISL_489741, EPI_ISL_489742, EPI_ISL_489743, EPI_ISL_489745, EPI_ISL_489746, EPI_ISL_489747, EPI_ISL_489748, EPI_ISL_489749, EPI_ISL_489750, EPI_ISL_489756, EPI_ISL_489757, EPI_ISL_489758, EPI_ISL_489759, EPI_ISL_489760, EPI_ISL_489761, EPI_ISL_489762, EPI_ISL_489765, EPI_ISL_489766, EPI_ISL_489767, EPI_ISL_489768, EPI_ISL_489769, EPI_ISL_489770, EPI_ISL_489771, EPI_ISL_489772, EPI_ISL_489773, EPI_ISL_489774, EPI_ISL_489776			
see above	Florida Bureau of Public Health Laboratories	Florida Bureau of Public Health Laboratories	Sarah Schmedes, Jason Blanton
EPI_ISL_490019	South Eastern Area Laboratory Services (SEALS)	NSW Health Pathology - Institute of Clinical Pathology and Medical Research; Westmead Hospital; University of Sydney	CIDM-PH et al.
EPI_ISL_490205, EPI_ISL_490207	München Klinik Schwabing	MGZ Medical Genetics Center	Dieter A. Wolf, Elke Holinski-Feder
EPI_ISL_490563, EPI_ISL_490564, EPI_ISL_490565, EPI_ISL_490566, EPI_ISL_490567, EPI_ISL_490568, EPI_ISL_490569, EPI_ISL_490570, EPI_ISL_490571, EPI_ISL_490572, EPI_ISL_490573, EPI_ISL_490574, EPI_ISL_490575, EPI_ISL_490576, EPI_ISL_490577, EPI_ISL_490578, EPI_ISL_490579, EPI_ISL_490580			
see above	Lincolnshire Hospitals and DeepSeq Nottingham	COVID-19 Genomics UK (COG-UK) Consortium	Nichola Duckworth, Tim Sloan, Sarah Walsh, Jonathan Ball, Patrick McClure, Joeseph Chappell, Nadine Holmes, Matthew Carlisle, Christopher Moore, Fei

			Sang, Johnny Debebe, Victoria Wright, Matthew Loose
EPI_ISL_490613, EPI_ISL_490621, EPI_ISL_490635	Virology Department, Sheffield Teaching Hospitals NHS Foundation Trust/Department of Infection, Immunity and Cardiovascular Disease, The Medical School, University of Sheffield	COVID-19 Genomics UK (COG-UK) Consortium	Thushan de Silva, Matthew Parker, Nikki Smith, Adri Angyal, Rebecca Brown, Luke Green, Rachel Tucker, Paul Parsons, Danielle Groves, Katie Johnson, Laura Carrilero, Alex Keeley, Dave Partridge, Matthew Wyles, Benjamin Lindsey, Mehmet Yavuz, Mohammad Raza, Cariad Evans
EPI_ISL_490683, EPI_ISL_490684, EPI_ISL_490685, EPI_ISL_490686, EPI_ISL_490687, EPI_ISL_490688, EPI_ISL_490689, EPI_ISL_490690, EPI_ISL_490691, EPI_ISL_490692	West of Scotland Specialist Virology Centre, NHSGGC / MRC-University of Glasgow Centre for Virus Research	COVID-19 Genomics UK (COG-UK) Consortium	Ana da Silva Filipe, Natasha Johnson, Kathy Smollett, Daniel Mair, Stephen Carmichael, Lily Tong, Jenna Nichols, Elihu Aranday-Cortes, Kirstyn Brunker, Yasmin Parr, Alice Broos, Kyriaki Nomikou; Sarah McDonald, Marc Niebel, Patawee Asamaphan; Richard Orton, Joseph Hughes, Sreenu Vattipally, David L Robertson; Alasdair MacLean, Rory Gunson; Kathy Li, Natasha Jesudason, Rajiv Shah, James Shepherd, Antonia Ho, Emma Thomson
EPI_ISL_490765, EPI_ISL_490780	Wales Specialist Virology Centre Sequencing lab: Pathogen Genomics Unit	COVID-19 Genomics UK (COG-UK) Consortium	Catherine Moore, Johnathan Evans, Laura Gifford, Malorie Perry, Simon Cottrell, Angela Marchbank, Alec Birchley, Alexander Adams, Amy Gaskin, Bree Gatica-Wilcox, Jason Coombes, Joel Southgate, Lauren Gilbert, Lee Graham, Nicole Pacchiarini, Sara Kumziene-Summerhayes, Sarah Taylor, Sophie Jones, Sara Rey, Matthew Bull, Joanne Watkins, Sally Corden, Tom Connor
EPI_ISL_491002	UW Virology Lab	UW Virology Lab	Pavitra Roychoudhury, Hong Xie, Lasata Shrestha, Amin Addetia, Truong Nguyen, Victoria M Rachleff, Meei-Li Huang, Keith R Jerome, Alexander Greninger
EPI_ISL_491058, EPI_ISL_491059	Suceava County Emergency Hospital	"Stefan cel Mare" University Metagenomics Lab	Lobiuc Andrei, Antoniadis Panagiotis et al.
EPI_ISL_491060, EPI_ISL_491061	Suceava County Emergency Hospital	"Stefan cel Mare" University Metagenomics Lab	Lobiuc Andrei et al.
EPI_ISL_491062, EPI_ISL_491063, EPI_ISL_491064, EPI_ISL_491065	Suceava County Emergency Hospital	"Stefan cel Mare" University Metagenomics Lab	Lobiuc Andrei, Antoniadis Panagiotis et al.
EPI_ISL_491066, EPI_ISL_491067	Suceava County Emergency Hospital	"Stefan cel Mare" University Metagenomics Lab	Lobiuc Andrei et al.
EPI_ISL_491105, EPI_ISL_491106	SC Department of Health and Environmental Control	SC Department of Health and Environmental Control	Flores,H.
EPI_ISL_491123, EPI_ISL_491129	Oman-National Influenza Center	Biotechnology & OMICs Laboratory	Samira Al-Mahruqi, Abdul Latif Khan, Samiha Al-Kharusi, Adil Khan , Ahmed Al-Rawahi, Sajjad Asaf, Amina Al-Jardani, Hanan Al-Kindi, Intisar Al-Shukri, Ahlam Al-Amri, Aisha Al-Amri, Aisha Al-Busaidi, Adil Al-Wahaibi, Seif Al-Abri, Ahmed Al-Harrasi
EPI_ISL_491137, EPI_ISL_491139, EPI_ISL_491144	Oman-National Influenza Center	Biotechnology & OMICs Laboratory	Samiha Al-Kharusi, Sajjad Asaf, Abdul Latif Khan, Samira Al-Mahruqi, Adil Khan, Ahmed Al-Rawahi, Amina Al-Jardani, Hanan Al-Kindi, Intisar Al-Shukri, Ahlam Al-Amri, Aisha Al-Amri, Aisha Al-Busaidi, Adil Al-Wahaibi, Seif Al-Abri, Ahmed Al-Harrasi
EPI_ISL_491148, EPI_ISL_491151, EPI_ISL_491158	Oman-National Influenza Center	Biotechnology & OMICs Laboratory	Abdul Latif Khan, Samira Al-Mahruqi, Ahmed Al-Harrasi, Samiha Al-Kharusi, Adil Khan, Ahmed Al-Rawahi, Sajjad Asaf, Amina Al-Jardani, Hanan Al-Kindi, Intisar Al-Shukri, Ahlam Al-Amri, Aisha Al-Amri, Aisha Al-Busaidi, Adil Al-Wahaibi, Seif Al-Abri.
EPI_ISL_491167	Oman-National Influenza Center	Biotechnology & OMICs Laboratory	Sajjad Asaf, Samiha Al-Kharusi, Ahmed Al-Harrasi, Samira Al-Mahruqi, Adil Khan, Ahmed Al-Rawahi, Abdul Latif Khan, Amina Al-Jardani, Hanan Al-Kindi, Intisar Al-Shukri, Ahlam Al-Amri, Aisha Al-Amri, Aisha Al-Busaidi, Adil Al-Wahaibi, Seif Al-Abri.
EPI_ISL_491458	Laboratorio de Referencia Nacional de Virus Respiratorio. Instituto Nacional de Salud Perú	Laboratorio de Referencia Nacional de Biotecnología y Biología Molecular. Instituto Nacional de Salud Perú	Carlos Padilla Rojas, Karolyn Vega Chozo, Priscila Lope Pari, Omar Caceres Rey, Marco Galarza Perez, Maribel Huaringa Nuñez, Johanna Balbuena Torrez, Henri Bailon Calderon, Nancy Rojas Serrano
EPI_ISL_491499, EPI_ISL_491502, EPI_ISL_491516, EPI_ISL_491518, EPI_ISL_491519, EPI_ISL_491524, EPI_ISL_491525, EPI_ISL_491536, EPI_ISL_491548, EPI_ISL_491549, EPI_ISL_491551, EPI_ISL_491553, EPI_ISL_491555, EPI_ISL_491557, EPI_ISL_491560, EPI_ISL_491563, EPI_ISL_491565, EPI_ISL_491566			
see above	Virology Department, Royal Infirmary of Edinburgh, NHS Lothian / School of Biological Sciences, University of Edinburgh	Wellcome Sanger Institute for the COVID-19 Genomics UK (COG-UK) consortium	McHugh M, Dewar R, Rooke S, O'Toole Á, Scher E, Hill V, McCrone JT, Colquhoun R, Yu X, Jackson B, Rambaut A, Templeton K and Alex Alderton, Roberto Amato, Sonia Goncalves, Ewan Harrison, David K. Jackson, Ian Johnston, Dominic Kwiatkowski, Cordelia Langford, John Sillitoe on behalf of the Wellcome Sanger Institute COVID-19 Surveillance Team (http://www.sanger.ac.uk/covid-team)
EPI_ISL_491572	Virology Department, Royal Infirmary of Edinburgh, NHS Lothian / School of Biological Sciences, University of Edinburgh	Wellcome Sanger Institute for the COVID-19 Genomics UK (COG-UK) Consortium	McHugh M, Dewar R, Rooke S, O'Toole Á, Scher E, Hill V, McCrone JT, Colquhoun R, Yu X, Jackson B, Rambaut A, Templeton K and Alex Alderton, Roberto Amato, Sonia Goncalves, Ewan Harrison, David K. Jackson, Ian Johnston, Dominic Kwiatkowski, Cordelia Langford, John Sillitoe on behalf of the Wellcome Sanger Institute COVID-19 Surveillance Team
EPI_ISL_491581, EPI_ISL_491587, EPI_ISL_491588, EPI_ISL_491591, EPI_ISL_491599	Virology Department, Royal Infirmary of Edinburgh, NHS Lothian / School of Biological Sciences, University of Edinburgh	Wellcome Sanger Institute for the COVID-19 Genomics UK (COG-UK) consortium	McHugh M, Dewar R, Rooke S, O'Toole Á, Scher E, Hill V, McCrone JT, Colquhoun R, Yu X, Jackson B, Rambaut A, Templeton K and Alex Alderton, Roberto Amato, Sonia Goncalves, Ewan Harrison, David K. Jackson, Ian Johnston, Dominic Kwiatkowski, Cordelia Langford, John Sillitoe on behalf of the Wellcome Sanger Institute COVID-19 Surveillance Team (http://www.sanger.ac.uk/covid-team)
EPI_ISL_491608	Virology Department, Royal Infirmary of Edinburgh, NHS Lothian / School of Biological Sciences, University of Edinburgh	Wellcome Sanger Institute for the COVID-19 Genomics UK (COG-UK) Consortium	McHugh M, Dewar R, Rooke S, O'Toole Á, Scher E, Hill V, McCrone JT, Colquhoun R, Yu X, Jackson B, Rambaut A, Templeton K and Alex Alderton, Roberto Amato, Sonia Goncalves, Ewan Harrison, David K. Jackson, Ian Johnston, Dominic Kwiatkowski, Cordelia Langford, John Sillitoe on behalf of the Wellcome Sanger Institute COVID-19 Surveillance Team
EPI_ISL_491610	Virology Department, Royal Infirmary of Edinburgh, NHS Lothian / School of Biological Sciences, University of Edinburgh	Wellcome Sanger Institute for the COVID-19 Genomics UK (COG-UK) consortium	McHugh M, Dewar R, Rooke S, O'Toole Á, Scher E, Hill V, McCrone JT, Colquhoun R, Yu X, Jackson B, Rambaut A, Templeton K and Alex Alderton, Roberto Amato, Sonia Goncalves, Ewan Harrison, David K. Jackson, Ian Johnston, Dominic Kwiatkowski, Cordelia Langford, John Sillitoe on behalf of the Wellcome Sanger Institute COVID-19 Surveillance Team (http://www.sanger.ac.uk/covid-team)
EPI_ISL_491611	Virology Department, Royal Infirmary of Edinburgh, NHS Lothian / School of Biological Sciences, University of Edinburgh	Wellcome Sanger Institute for the COVID-19 Genomics UK (COG-UK) Consortium	McHugh M, Dewar R, Rooke S, O'Toole Á, Scher E, Hill V, McCrone JT, Colquhoun R, Yu X, Jackson B, Rambaut A, Templeton K and Alex Alderton, Roberto Amato, Sonia Goncalves, Ewan Harrison, David K. Jackson, Ian Johnston, Dominic Kwiatkowski, Cordelia Langford, John Sillitoe on behalf of the Wellcome Sanger Institute COVID-19 Surveillance Team
EPI_ISL_491612	Virology Department, Royal Infirmary of Edinburgh, NHS Lothian / School of Biological Sciences, University of Edinburgh	Wellcome Sanger Institute for the COVID-19 Genomics UK (COG-UK) consortium	McHugh M, Dewar R, Rooke S, O'Toole Á, Scher E, Hill V, McCrone JT, Colquhoun R, Yu X, Jackson B, Rambaut A, Templeton K and Alex Alderton, Roberto Amato, Sonia Goncalves, Ewan Harrison, David K. Jackson, Ian Johnston, Dominic Kwiatkowski, Cordelia Langford, John Sillitoe on behalf of the Wellcome Sanger Institute COVID-19 Surveillance Team (http://www.sanger.ac.uk/covid-team)
EPI_ISL_491616	Virology Department, Royal Infirmary of Edinburgh, NHS Lothian / School of Biological Sciences, University of Edinburgh	Wellcome Sanger Institute for the COVID-19 Genomics UK (COG-UK) Consortium	McHugh M, Dewar R, Rooke S, O'Toole Á, Scher E, Hill V, McCrone JT, Colquhoun R, Yu X, Jackson B, Rambaut A, Templeton K and Alex Alderton, Roberto Amato, Sonia Goncalves, Ewan Harrison, David K. Jackson, Ian Johnston, Dominic Kwiatkowski, Cordelia Langford, John Sillitoe on behalf of the Wellcome Sanger Institute COVID-19 Surveillance Team
EPI_ISL_491618, EPI_ISL_491620, EPI_ISL_491623, EPI_ISL_491625, EPI_ISL_491628, EPI_ISL_491630, EPI_ISL_491635, EPI_ISL_491638, EPI_ISL_491639, EPI_ISL_491642, EPI_ISL_491646, EPI_ISL_491649, EPI_ISL_491654, EPI_ISL_491657, EPI_ISL_491658, EPI_ISL_491661, EPI_ISL_491664, EPI_ISL_491669, EPI_ISL_491670, EPI_ISL_491681, EPI_ISL_491686, EPI_ISL_491687, EPI_ISL_491691, EPI_ISL_491692, EPI_ISL_491696			
see above	Virology Department, Royal Infirmary of Edinburgh, NHS Lothian / School of Biological Sciences, University of Edinburgh	Wellcome Sanger Institute for the COVID-19 Genomics UK (COG-UK) consortium	McHugh M, Dewar R, Rooke S, O'Toole Á, Scher E, Hill V, McCrone JT, Colquhoun R, Yu X, Jackson B, Rambaut A, Templeton K and Alex Alderton, Roberto Amato, Sonia Goncalves, Ewan Harrison, David K. Jackson, Ian Johnston, Dominic Kwiatkowski, Cordelia Langford, John Sillitoe on behalf of the Wellcome Sanger Institute COVID-19 Surveillance Team (http://www.sanger.ac.uk/covid-team)
EPI_ISL_491909	Naval Infectious Diseases Diagnostic Laboratory	Naval Medical Research Center Biological Defense Research Directorate	Logan Voegtly, Regina Cer, Lindsay Glang, Victor Sugiharto, Francisco Malgon Bautista, Hua Wei Chen, Dessiree Pena-Gomez, Megan Schilling, Adrian Paskey, Kyle Long, Mark Simons, Kimberly Bishop-Lilly
EPI_ISL_491935	Centro de Investigaciones, Universidad de Especialidades Espíritu Santo	Institute of Microbiology, Universidad San Francisco de Quito	Derly Andrade, Juan Carlos Fernandez, Belén Prado-Vivar, Sully Márquez, Juan José Guadalupe, Monica Becerra-Wong, Bernardo Gutiérrez, Gabriel Morey, Ruben Armas, Jose Pedro Barberan, Fernando Espinoza, Edith Lopez, Verónica Barragán, Patricio Rojas-Silva, Gabriel Trueba, Michelle Grunauer, Paúl Cárdenas
EPI_ISL_491943	Naval Infectious Diseases Diagnostic Laboratory	Naval Medical Research Center Biological Defense Research Directorate	Logan Voegtly, Regina Cer, Lindsay Glang, Victor Sugiharto, Francisco Malgon Bautista, Hua Wei Chen, Dessiree Pena-Gomez, Megan Schilling, Adrian Paskey, Kyle Long, Mark Simons, Kimberly Bishop-Lilly
EPI_ISL_492032	Instituto de Biologia do Exército	Laboratório Metabolismo Macromolecular FirminoTorres de Castro, Instituto de Biofísica Carlos Chagas Filho, Universidade Federal do Rio de Janeiro	Bianca Catarina Azevedo Cabral, Aline Rosa Vianna de Souza , Marcos Dornelas-Ribeiro, Tatiana LS Nogueira, Nádia Vaez Gonçalves da Cruz, Caleb GM Santos, Elizabeth Valentin, Marcio da Costa Cipitelli, Virginia Sara Grancieri do Amaral, Rodrigo Soares de Moura Neto, Clarissa Damaso, Rosane Silva
EPI_ISL_492034, EPI_ISL_492042	Instituto de Biologia do Exército	Laboratório Metabolismo Macromolecular FirminoTorres de Castro, Instituto de Biofísica Carlos Chagas Filho,	Bianca Catarina Azevedo Cabral, Aline Rosa Vianna de Souza, Nádia Vaez Gonçalves da Cruz, Caleb GM Santos, Marcos Dornelas-Ribeiro, Tatiana LS Nogueira, Elizabeth Valentin, Marcio da Costa Cipitelli, Virginia Sara Grancieri do Amaral, Rodrigo Soares de Moura Neto, Clarissa Damaso, Rosane Silva

		Universidade Federal do Rio de Janeiro	
EPI_ISL_492062, EPI_ISL_492063	Alaska State Virology Laboratory	Alaska State Virology Laboratory	Chen J et al with Pathogenomics group Dagdag R, Redlinger M, Milton E, George W, Kovalenko A, Drown DM, Bortz E
EPI_ISL_492070, EPI_ISL_492071, EPI_ISL_492072, EPI_ISL_492073	1. ViroGenetics - BSL3 Laboratory of Virology, Maopolska Centre of Biotechnology, Jagiellonian University; 2. II Department of Internal Medicine, Faculty of Medicine, Jagiellonian University Medical College; 3. Narodowy Instytut Zdrowia Publicznego - Pastwowy Zakad Higieny (NIZP-PZH)	1. ViroGenetics - BSL3 Laboratory of Virology, Maopolska Centre of Biotechnology, Jagiellonian University; 2. II Department of Internal Medicine, Faculty of Medicine, Jagiellonian University Medical College; 3. Narodowy Instytut Zdrowia Publicznego - Pastwowy Zakad Higieny (NIZP-PZH).	Katarzyna Pancer, Marek Sanak, Aleksandra A. Zasada, Magdalena Rzeczkowska, Tomasz Wokowicz, Katarzyna Zacharczuk, Agnieszka Koakowska-Kulesza, Katarzyna Owczarek, Aleksandra Milewska, Natalia Wolaniuk, Ewelina Hallman-Szeliska, Pawe P abaj, Wojciech Branicki, Krzysztof Pyr
EPI_ISL_492113	SA Pathology	SA Pathology	Lex Leong, Chuan Kok Lim, Mark Turra, Ivan Bastian, Geoff Higgins
EPI_ISL_492211, EPI_ISL_492217, EPI_ISL_492218, EPI_ISL_492219, EPI_ISL_492220, EPI_ISL_492222, EPI_ISL_492236, EPI_ISL_492240, EPI_ISL_492243, EPI_ISL_492256, EPI_ISL_492262, EPI_ISL_492266, EPI_ISL_492267, EPI_ISL_492268, EPI_ISL_492269, EPI_ISL_492279, EPI_ISL_492288, EPI_ISL_492307, EPI_ISL_492308, EPI_ISL_492318, EPI_ISL_492337, EPI_ISL_492346, EPI_ISL_492353, EPI_ISL_492359, EPI_ISL_492361, EPI_ISL_492366, EPI_ISL_492377, EPI_ISL_492388, EPI_ISL_492389, EPI_ISL_492393, EPI_ISL_492394, EPI_ISL_492398, EPI_ISL_492409, EPI_ISL_492412, EPI_ISL_492424, EPI_ISL_492430, EPI_ISL_492432, EPI_ISL_492438, EPI_ISL_492439, EPI_ISL_492441, EPI_ISL_492443			
see above	PHE South West Regional Laboratory, National Infection Service	Wellcome Sanger Institute for the COVID-19 Genomics UK (COG-UK) consortium	Stephanie Hutchings, Hannah Pymont, Dr Peter Muir, Barry Vipond, Rich Hopes; and Alex Alderton, Roberto Amato, Sonia Goncalves, Ewan Harrison, David K. Jackson, Ian Johnston, Dominic Kwiatkowski, Cordelia Langford, John Sillitoe on behalf of the Wellcome Sanger Institute COVID-19 Surveillance Team (http://www.sanger.ac.uk/covid-team)
EPI_ISL_492495	University College London, Great Ormond Street Hospital for Children NHS Foundation Trust, Imperial College Healthcare NHS Trust	Wellcome Sanger Institute for the COVID-19 Genomics UK (COG-UK) Consortium	Sergi Castellano, Rachel Williams, Mark Kristiansen, Paola Resende Silva, Sunando Roy, Tony Brooks, Helena Tutill, Paola Niola, Patricia Dyal, Charlotte Williams, Leysa Forrest, Yasmin Panchbhaya, Jacqueline Findlay, Sam Weeks, Julianne Brown, Kathryn Harris, Paul Randell, James Price, Alison Holmes, Judith Breuer and Alex Alderton, Roberto Amato, Sonia Goncalves, Ewan Harrison, David K. Jackson, Ian Johnston, Dominic Kwiatkowski, Cordelia Langford, John Sillitoe on behalf of the Wellcome Sanger Institute COVID-19 Surveillance Team
EPI_ISL_492496, EPI_ISL_492498, EPI_ISL_492499, EPI_ISL_492500, EPI_ISL_492501, EPI_ISL_492502, EPI_ISL_492503, EPI_ISL_492504	University College London, Great Ormond Street Hospital for Children NHS Foundation Trust, Imperial College Healthcare NHS Trust	Wellcome Sanger Institute for the COVID-19 Genomics UK (COG-UK) consortium	Sergi Castellano, Rachel Williams, Mark Kristiansen, Paola Resende Silva, Sunando Roy, Tony Brooks, Helena Tutill, Paola Niola, Patricia Dyal, Charlotte Williams, Leysa Forrest, Yasmin Panchbhaya, Jacqueline Findlay, Sam Weeks, Julianne Brown, Kathryn Harris, Paul Randell, James Price, Alison Holmes, Judith Breuer and Alex Alderton, Roberto Amato, Sonia Goncalves, Ewan Harrison, David K. Jackson, Ian Johnston, Dominic Kwiatkowski, Cordelia Langford, John Sillitoe on behalf of the Wellcome Sanger Institute COVID-19 Surveillance Team (http://www.sanger.ac.uk/covid-team)
EPI_ISL_492514	PHE South West Regional Laboratory, National Infection Service	Wellcome Sanger Institute for the COVID-19 Genomics UK (COG-UK) Consortium	Stephanie Hutchings, Hannah Pymont, Dr Peter Muir, Barry Vipond, Rich Hopes; and Alex Alderton, Roberto Amato, Sonia Goncalves, Ewan Harrison, David K. Jackson, Ian Johnston, Dominic Kwiatkowski, Cordelia Langford, John Sillitoe on behalf of the Wellcome Sanger Institute COVID-19 Surveillance Team
EPI_ISL_492539, EPI_ISL_492561, EPI_ISL_492567, EPI_ISL_492569, EPI_ISL_492577, EPI_ISL_492604, EPI_ISL_492623, EPI_ISL_492629, EPI_ISL_492655, EPI_ISL_492662, EPI_ISL_492710, EPI_ISL_492736			
see above	PHE South West Regional Laboratory, National Infection Service	Wellcome Sanger Institute for the COVID-19 Genomics UK (COG-UK) consortium	Stephanie Hutchings, Hannah Pymont, Dr Peter Muir, Barry Vipond, Rich Hopes; and Alex Alderton, Roberto Amato, Sonia Goncalves, Ewan Harrison, David K. Jackson, Ian Johnston, Dominic Kwiatkowski, Cordelia Langford, John Sillitoe on behalf of the Wellcome Sanger Institute COVID-19 Surveillance Team (http://www.sanger.ac.uk/covid-team)
EPI_ISL_492746, EPI_ISL_492748	University College London, Great Ormond Street Hospital for Children NHS Foundation Trust, Imperial College Healthcare NHS Trust	Wellcome Sanger Institute for the COVID-19 Genomics UK (COG-UK) Consortium	Sergi Castellano, Rachel Williams, Mark Kristiansen, Paola Resende Silva, Sunando Roy, Tony Brooks, Helena Tutill, Paola Niola, Patricia Dyal, Charlotte Williams, Leysa Forrest, Yasmin Panchbhaya, Jacqueline Findlay, Sam Weeks, Julianne Brown, Kathryn Harris, Paul Randell, James Price, Alison Holmes, Judith Breuer and Alex Alderton, Roberto Amato, Sonia Goncalves, Ewan Harrison, David K. Jackson, Ian Johnston, Dominic Kwiatkowski, Cordelia Langford, John Sillitoe on behalf of the Wellcome Sanger Institute COVID-19 Surveillance Team
EPI_ISL_492749	University College London, Great Ormond Street Hospital for Children NHS Foundation Trust, Imperial College Healthcare NHS Trust	Wellcome Sanger Institute for the COVID-19 Genomics UK (COG-UK) consortium	Sergi Castellano, Rachel Williams, Mark Kristiansen, Paola Resende Silva, Sunando Roy, Tony Brooks, Helena Tutill, Paola Niola, Patricia Dyal, Charlotte Williams, Leysa Forrest, Yasmin Panchbhaya, Jacqueline Findlay, Sam Weeks, Julianne Brown, Kathryn Harris, Paul Randell, James Price, Alison Holmes, Judith Breuer and Alex Alderton, Roberto Amato, Sonia Goncalves, Ewan Harrison, David K. Jackson, Ian Johnston, Dominic Kwiatkowski, Cordelia Langford, John Sillitoe on behalf of the Wellcome Sanger Institute COVID-19 Surveillance Team (http://www.sanger.ac.uk/covid-team)
EPI_ISL_492750	University College London, Great Ormond Street Hospital for Children NHS Foundation Trust, Imperial College Healthcare NHS Trust	Wellcome Sanger Institute for the COVID-19 Genomics UK (COG-UK) Consortium	Sergi Castellano, Rachel Williams, Mark Kristiansen, Paola Resende Silva, Sunando Roy, Tony Brooks, Helena Tutill, Paola Niola, Patricia Dyal, Charlotte Williams, Leysa Forrest, Yasmin Panchbhaya, Jacqueline Findlay, Sam Weeks, Julianne Brown, Kathryn Harris, Paul Randell, James Price, Alison Holmes, Judith Breuer and Alex Alderton, Roberto Amato, Sonia Goncalves, Ewan Harrison, David K. Jackson, Ian Johnston, Dominic Kwiatkowski, Cordelia Langford, John Sillitoe on behalf of the Wellcome Sanger Institute COVID-19 Surveillance Team
EPI_ISL_492751, EPI_ISL_492752, EPI_ISL_492753, EPI_ISL_492754, EPI_ISL_492755, EPI_ISL_492757	University College London, Great Ormond Street Hospital for Children NHS Foundation Trust, Imperial College Healthcare NHS Trust	Wellcome Sanger Institute for the COVID-19 Genomics UK (COG-UK) consortium	Sergi Castellano, Rachel Williams, Mark Kristiansen, Paola Resende Silva, Sunando Roy, Tony Brooks, Helena Tutill, Paola Niola, Patricia Dyal, Charlotte Williams, Leysa Forrest, Yasmin Panchbhaya, Jacqueline Findlay, Sam Weeks, Julianne Brown, Kathryn Harris, Paul Randell, James Price, Alison Holmes, Judith Breuer and Alex Alderton, Roberto Amato, Sonia Goncalves, Ewan Harrison, David K. Jackson, Ian Johnston, Dominic Kwiatkowski, Cordelia Langford, John Sillitoe on behalf of the Wellcome Sanger Institute COVID-19 Surveillance Team (http://www.sanger.ac.uk/covid-team)
EPI_ISL_492758	University College London, Great Ormond Street Hospital for Children NHS Foundation Trust, Imperial College Healthcare NHS Trust	Wellcome Sanger Institute for the COVID-19 Genomics UK (COG-UK) Consortium	Sergi Castellano, Rachel Williams, Mark Kristiansen, Paola Resende Silva, Sunando Roy, Tony Brooks, Helena Tutill, Paola Niola, Patricia Dyal, Charlotte Williams, Leysa Forrest, Yasmin Panchbhaya, Jacqueline Findlay, Sam Weeks, Julianne Brown, Kathryn Harris, Paul Randell, James Price, Alison Holmes, Judith Breuer and Alex Alderton, Roberto Amato, Sonia Goncalves, Ewan Harrison, David K. Jackson, Ian Johnston, Dominic Kwiatkowski, Cordelia Langford, John Sillitoe on behalf of the Wellcome Sanger Institute COVID-19 Surveillance Team
EPI_ISL_492759, EPI_ISL_492760, EPI_ISL_492761, EPI_ISL_492762, EPI_ISL_492763	University College London, Great Ormond Street Hospital for Children NHS Foundation Trust, Imperial College Healthcare NHS Trust	Wellcome Sanger Institute for the COVID-19 Genomics UK (COG-UK) consortium	Sergi Castellano, Rachel Williams, Mark Kristiansen, Paola Resende Silva, Sunando Roy, Tony Brooks, Helena Tutill, Paola Niola, Patricia Dyal, Charlotte Williams, Leysa Forrest, Yasmin Panchbhaya, Jacqueline Findlay, Sam Weeks, Julianne Brown, Kathryn Harris, Paul Randell, James Price, Alison Holmes, Judith Breuer and Alex Alderton, Roberto Amato, Sonia Goncalves, Ewan Harrison, David K. Jackson, Ian Johnston, Dominic Kwiatkowski, Cordelia Langford, John Sillitoe on behalf of the Wellcome Sanger Institute COVID-19 Surveillance Team (http://www.sanger.ac.uk/covid-team)
EPI_ISL_492841, EPI_ISL_492849	Department of Medical Microbiology, Western Sussex Hospitals NHS Foundation Trust, St Richard's Hospital	Wellcome Sanger Institute for the COVID-19 Genomics UK (COG-UK) consortium	Manasa Mutingwende, Sarah Lowdon, Olga Podplomyk, Michelle Erkiert, Jonathan Lewis, Paul Randell and Alex Alderton, Roberto Amato, Sonia Goncalves, Ewan Harrison, David K. Jackson, Ian Johnston, Dominic Kwiatkowski, Cordelia Langford, John Sillitoe on behalf of the Wellcome Sanger Institute COVID-19 Surveillance Team (http://www.sanger.ac.uk/covid-team)
EPI_ISL_492856	Royal Free Hospital / Health Services Laboratories	Wellcome Sanger Institute for the COVID-19 Genomics UK (COG-UK) consortium	Tanzina Haque, Tabitha Mahungu, Dianne Irish, Cate Goodlad, Jenny Cross, Judith Heaney and Alex Alderton, Roberto Amato, Sonia Goncalves, Ewan Harrison, David K. Jackson, Ian Johnston, Dominic Kwiatkowski, Cordelia Langford, John Sillitoe on behalf of the Wellcome Sanger Institute COVID-19 Surveillance Team (http://www.sanger.ac.uk/covid-team)
EPI_ISL_492875, EPI_ISL_492886, EPI_ISL_492897, EPI_ISL_492903	Department of Medical Microbiology, Western Sussex Hospitals NHS Foundation Trust, St Richard's Hospital	Wellcome Sanger Institute for the COVID-19 Genomics UK (COG-UK) consortium	Manasa Mutingwende, Sarah Lowdon, Olga Podplomyk, Michelle Erkiert, Jonathan Lewis, Paul Randell and Alex Alderton, Roberto Amato, Sonia Goncalves, Ewan Harrison, David K. Jackson, Ian Johnston, Dominic Kwiatkowski, Cordelia Langford, John Sillitoe on behalf of the Wellcome Sanger Institute COVID-19 Surveillance Team (http://www.sanger.ac.uk/covid-team)
EPI_ISL_492905, EPI_ISL_492911	Royal Free Hospital / Health Services Laboratories	Wellcome Sanger Institute for the COVID-19 Genomics UK (COG-UK) consortium	Tanzina Haque, Tabitha Mahungu, Dianne Irish, Cate Goodlad, Jenny Cross, Judith Heaney and Alex Alderton, Roberto Amato, Sonia Goncalves, Ewan Harrison, David K. Jackson, Ian Johnston, Dominic Kwiatkowski, Cordelia Langford, John Sillitoe on behalf of the Wellcome Sanger Institute COVID-19 Surveillance Team (http://www.sanger.ac.uk/covid-team)
EPI_ISL_492915	Department of Medical Microbiology, Western Sussex Hospitals NHS Foundation Trust, St Richard's Hospital	Wellcome Sanger Institute for the COVID-19 Genomics UK (COG-UK) consortium	Manasa Mutingwende, Sarah Lowdon, Olga Podplomyk, Michelle Erkiert, Jonathan Lewis, Paul Randell and Alex Alderton, Roberto Amato, Sonia Goncalves, Ewan Harrison, David K. Jackson, Ian Johnston, Dominic Kwiatkowski, Cordelia Langford, John Sillitoe on behalf of the Wellcome Sanger Institute COVID-19 Surveillance Team (http://www.sanger.ac.uk/covid-team)
EPI_ISL_492916, EPI_ISL_492917, EPI_ISL_492918, EPI_ISL_492919, EPI_ISL_492920, EPI_ISL_492921, EPI_ISL_492922, EPI_ISL_492923, EPI_ISL_492925, EPI_ISL_492927, EPI_ISL_492928, EPI_ISL_492932, EPI_ISL_492934, EPI_ISL_492935, EPI_ISL_492936, EPI_ISL_492937, EPI_ISL_492938, EPI_ISL_492939, EPI_ISL_492940, EPI_ISL_492942, EPI_ISL_492943, EPI_ISL_492945, EPI_ISL_492946, EPI_ISL_492947, EPI_ISL_492948, EPI_ISL_492950, EPI_ISL_492953, EPI_ISL_492955, EPI_ISL_492956, EPI_ISL_492959, EPI_ISL_492961, EPI_ISL_492962, EPI_ISL_492963, EPI_ISL_492964, EPI_ISL_492965, EPI_ISL_492966, EPI_ISL_492968, EPI_ISL_492969, EPI_ISL_492970, EPI_ISL_492971, EPI_ISL_492972, EPI_ISL_492973, EPI_ISL_492974, EPI_ISL_492976, EPI_ISL_492977			
see above	NU-OMICS DNA Sequencing research facility, Northumbria University	Wellcome Sanger Institute for the COVID-19 Genomics UK (COG-UK) consortium	Chris Duncan, Sheia Waugh, Shirelle Burton-Fanning, Gary Eltringham, Jennifer Collins, Brendan Payne, Yusri Taha, Emma Swindells, Jane Greenaway, Edward Barton, Garren Scott, Debra Padgett, Clive Graham, Sarah Essex, Steve Ligget, Paul Baker, Lynn Dover, Wen Yew, Gary Black, John Allan, Joshua Loh, Greg Young, Matthew Bashton, Andrew Nelson, Darren Smith and Alex Alderton, Roberto Amato, Sonia Goncalves, Ewan Harrison, David K. Jackson, Ian Johnston, Dominic Kwiatkowski, Cordelia Langford, John Sillitoe on behalf of the Wellcome Sanger Institute COVID-19 Surveillance Team (http://www.sanger.ac.uk/covid-team)

EPI_ISL_492983	IRCCS Sacro Cuore Don Calabria Hospital, Department of Infectious, Tropical Diseases & Microbiology	University of Verona, Department of Biotechnology	Antonio Mori, Michela Deiana, Elena Pomari, Chiara Piubelli; Giulia Lopatriello, Luca Marcolungo, Cristina Beltrami, Chiara Degli Esposti, Emanuela Cosentino, Massimo Delledonne
EPI_ISL_493336	Instituto de Diagnostico y Referencia Epidemiologicos (INDRE)	Instituto de Diagnostico y Referencia Epidemiologicos (INDRE)	Gisela Barrera-Badillo , Abril Rodriguez-Maldonado, Claudia Wong-Arambula , Natividad Cruz-Ortiz, Tatiana Nunez-Garcia, Dayanira Arellano-Suarez, Fabiola Garces-Ayala, Edgar Mendieta-Condado, Lucia Hernandez-Rivas, Irma Lopez-Martinez, Ernesto Ramirez-Gonzalez.
EPI_ISL_493391, EPI_ISL_493392, EPI_ISL_493393, EPI_ISL_493410, EPI_ISL_493413, EPI_ISL_493416, EPI_ISL_493418, EPI_ISL_493422, EPI_ISL_493423, EPI_ISL_493424	National Public Health Laboratory, National Centre for Infectious Diseases	National Public Health Laboratory, National Centre for Infectious Diseases	Mak TM, Octavia S, Zhou Z, Chavatte JM, Cui L, Lin RTP
EPI_ISL_493468, EPI_ISL_493469, EPI_ISL_493470, EPI_ISL_493471	Northumbria University / South Tees Hospitals NHS Foundation Trust / North Cumbria Integrated Care NHS Foundation Trust / North Tees and Hartlepool NHS Foundation Trust / Newcastle Hospitals NHS Foundation Trust	COVID-19 Genomics UK (COG-UK) Consortium	Darren L Smith,Andrew Nelson,Matthew Bashton,Greg R Young,Joshua Loh,John Allan,Mohammad A Tariq,Giles S Holt,Gary Black,Wen C Yew,Lynn Dover,Paul Baker,Steve Liggett,Sarah Essex,Jane Greenaway,Debra Padgett,Clive Graham,Garren Scott,Edward Barton,Emma Swindells,Brendan Payne,Jennifer Collins,Yusri Taha,Gary Eltringham
EPI_ISL_493549, EPI_ISL_493550, EPI_ISL_493551, EPI_ISL_493552, EPI_ISL_493553, EPI_ISL_493554, EPI_ISL_493555, EPI_ISL_493556, EPI_ISL_493557, EPI_ISL_493558, EPI_ISL_493559, EPI_ISL_493560, EPI_ISL_493561, EPI_ISL_493562, EPI_ISL_493603, EPI_ISL_493604, EPI_ISL_493605			
see above	Lincolnshire Hospitals and DeepSeq Nottingham	COVID-19 Genomics UK (COG-UK) Consortium	Nichola Duckworth, Tim Sloan, Sarah Walsh, Jonathan Ball, Patrick McClure, Joeseph Chappell, Nadine Holmes, Matthew Carlisle, Christopher Moore, Fei Sang, Johnny Debebe, Victoria Wright, Matthew Loose
EPI_ISL_493637	Centre for Enzyme Innovation, University of Portsmouth / Translational Research Laboratory, Portsmouth Hospitals NHS Trust	COVID-19 Genomics UK (COG-UK) Consortium	Angela Beckett,Yann Bourgeois,Garry Scarlett,Sharon Glaysher,Scott Elliott,Kelly Bicknell,Robert Impey,Allyson Lloyd,Sarah Wyllie,Ethan Butcher,Anoop Chauhan,Samuel Robson
EPI_ISL_493787, EPI_ISL_493788, EPI_ISL_493789, EPI_ISL_493791, EPI_ISL_493792, EPI_ISL_493793, EPI_ISL_493794, EPI_ISL_493795, EPI_ISL_493796, EPI_ISL_493797, EPI_ISL_493798, EPI_ISL_493799, EPI_ISL_493800, EPI_ISL_493801, EPI_ISL_493802, EPI_ISL_493803, EPI_ISL_493804, EPI_ISL_493805, EPI_ISL_493806, EPI_ISL_493807, EPI_ISL_493808, EPI_ISL_493809, EPI_ISL_493810, EPI_ISL_493811, EPI_ISL_493812, EPI_ISL_493813, EPI_ISL_493814, EPI_ISL_493815, EPI_ISL_493816, EPI_ISL_493817, EPI_ISL_493818, EPI_ISL_493819, EPI_ISL_493820, EPI_ISL_493821, EPI_ISL_493822, EPI_ISL_493823, EPI_ISL_493824			
see above	West of Scotland Specialist Virology Centre, NHSGGC / MRC-University of Glasgow Centre for Virus Research	COVID-19 Genomics UK (COG-UK) Consortium	Ana da Silva Filipe, Natasha Johnson, Kathy Smollett, Daniel Mair, Stephen Carmichael, Lily Tong, Jenna Nichols, Elihu Aranday-Cortes, Kirstyn Brunker, Yasmin Parr, Alice Broos, Kyriaki Nomikou; Sarah McDonald, Marc Niebel, Patawee Asamaphan; Richard Orton, Joseph Hughes, Sreenu Vattipally, David L Robertson; Alasdair MacLean, Rory Gunson; Kathy Li, Natasha Jesudason, Rajiv Shah, James Shepherd, Antonia Ho, Emma Thomson
EPI_ISL_493975, EPI_ISL_493983, EPI_ISL_494149, EPI_ISL_494151, EPI_ISL_494154, EPI_ISL_494157, EPI_ISL_494158, EPI_ISL_494159, EPI_ISL_494160, EPI_ISL_494164, EPI_ISL_494172, EPI_ISL_494178, EPI_ISL_494179, EPI_ISL_494181, EPI_ISL_494193, EPI_ISL_494196, EPI_ISL_494203, EPI_ISL_494205, EPI_ISL_494216, EPI_ISL_494217, EPI_ISL_494222, EPI_ISL_494227, EPI_ISL_494229, EPI_ISL_494233, EPI_ISL_494235, EPI_ISL_494236, EPI_ISL_494245, EPI_ISL_494251, EPI_ISL_494252, EPI_ISL_494257, EPI_ISL_494258, EPI_ISL_494260, EPI_ISL_494261, EPI_ISL_494280, EPI_ISL_494291, EPI_ISL_494298, EPI_ISL_494299, EPI_ISL_494302, EPI_ISL_494305, EPI_ISL_494319, EPI_ISL_494321, EPI_ISL_494326, EPI_ISL_494327, EPI_ISL_494328, EPI_ISL_494332, EPI_ISL_494336, EPI_ISL_494341, EPI_ISL_494350, EPI_ISL_494351, EPI_ISL_494353, EPI_ISL_494355, EPI_ISL_494356, EPI_ISL_494361, EPI_ISL_494367, EPI_ISL_494370			
see above	Wales Specialist Virology Centre Sequencing lab: Pathogen Genomics Unit	COVID-19 Genomics UK (COG-UK) Consortium	Catherine Moore, Johnathan Evans, Laura Gifford, Malorie Perry, Simon Cottrell, Angela Marchbank, Alec Birchley, Alexander Adams, Amy Gaskin, Bree Gatica-Wilcox, Jason Coombes, Joel Southgate, Lauren Gilbert, Lee Graham, Nicole Pacchiarini, Sara Kumziene-Summerhayes, Sarah Taylor, Sophie Jones, Sara Rey, Matthew Bull, Joanne Watkins, Sally Corden, Tom Connor
EPI_ISL_494383, EPI_ISL_494386, EPI_ISL_494444, EPI_ISL_494446, EPI_ISL_494448, EPI_ISL_494628	San Diego County Public Health Laboratory	Andersen lab at Scripps Research	SEARCH Alliance San Diego with Tracy Basler, Jovan Shephard, Brett Austin
EPI_ISL_495086, EPI_ISL_495090, EPI_ISL_495091	Department of Medical Microbiology, Western Sussex Hospitals NHS Foundation Trust, St Richard's Hospital	Wellcome Sanger Institute for the COVID-19 Genomics UK (COG-UK) consortium	Manasa Mutingwende, Sarah Lowdon, Olga Podplomyk, Michelle Erkiert, Jonathan Lewis, Paul Randell and Alex Alderton, Roberto Amato, Sonia Goncalves, Ewan Harrison, David K. Jackson, Ian Johnston, Dominic Kwiatkowski, Cordelia Langford, John Sillitoe on behalf of the Wellcome Sanger Institute COVID-19 Surveillance Team (http://www.sanger.ac.uk/covid-team)
EPI_ISL_495108	PHE South West Regional Laboratory, National Infection Service	Wellcome Sanger Institute for the COVID-19 Genomics UK (COG-UK) consortium	Stephanie Hutchings, Hannah Pymont, Dr Peter Muir, Barry Vipond, Rich Hopes; and Alex Alderton, Roberto Amato, Sonia Goncalves, Ewan Harrison, David K. Jackson, Ian Johnston, Dominic Kwiatkowski, Cordelia Langford, John Sillitoe on behalf of the Wellcome Sanger Institute COVID-19 Surveillance Team (http://www.sanger.ac.uk/covid-team)
EPI_ISL_495563, EPI_ISL_495572, EPI_ISL_495574, EPI_ISL_495576, EPI_ISL_495577, EPI_ISL_495578, EPI_ISL_495579, EPI_ISL_495580, EPI_ISL_495581, EPI_ISL_495582, EPI_ISL_495583, EPI_ISL_495588			
see above	University of Michigan Clinical Microbiology Laboratory	Lauring Lab, University of Michigan, Department of Microbiology and Immunology	Valesano et al.
EPI_ISL_495600, EPI_ISL_495605, EPI_ISL_495609	Mayo Clinic & Mayo Clinic Laboratories	Minnesota Department of Health, Public Health Laboratory	Matt Plumb, Jacob Garfin, and Xiong Wang
EPI_ISL_495656	Seattle Flu Study	Seattle Flu Study	Deborah A. Nickerson, Chris D. Frazar, Jover Lee, Benjamin Pelle, Matthew Richardson, Amanda Adler, Elisabeth Brandstetter, Peter D. Han, Kairsten Fay, Misja Ilcisin, Kirsten Lacombe, Thomas R. Sibley, Melissa Truong, Caitlin R. Wolf, Karen Cowgill, Stephanie Schrag, Jeff Duchin, Michael Boeckh, Janet A. Englund, Michael Famulare, Barry R. Lutz, Mark J. Rieder, Lea M. Starita, Matthew Thompson, Helen Y. Chu, Trevor Bedford, Jay Shendure
EPI_ISL_496521, EPI_ISL_496530, EPI_ISL_496546, EPI_ISL_496547, EPI_ISL_496548	B.J. Govt. Medical College	National Centre For Cell Science	Dhiraj Paul, Kunal Jani, Radha Chauhan, Janesh Kumar, Vasudevan Seshadri, Girdhari Lal, Rajesh Karyakarte, Suvarna Joshi, Murlidhar Tambe, Sourav Sen, Santosh Karade, Kavita Bala Anand, Shelinder Pal Singh Shergill, Rajiv Mohan Gupta, Manoj Kumar Bhat, Arvind Sahu, Maharashtra COVID-19 Study Group, DBT's PAN-INDIA 1000 SARS-CoV2 RNA genome sequencing consortium, Yogesh S Shouche
EPI_ISL_496901, EPI_ISL_496902, EPI_ISL_496903, EPI_ISL_496904, EPI_ISL_496905, EPI_ISL_496906, EPI_ISL_496907, EPI_ISL_496908, EPI_ISL_496909, EPI_ISL_496910, EPI_ISL_496911, EPI_ISL_496912, EPI_ISL_496913, EPI_ISL_496914, EPI_ISL_496915			
see above	Gorgas Memorial Laboratory of Health Studies	Gorgas Memorial Laboratory of Health Studies	Danilo Franco, Claudia Gonzalez Sandra Lopez-Verges, Alexander A Martinez
EPI_ISL_497865	Department of Microbiology, The University of Hong Kong	Department of Microbiology, The University of Hong Kong	Kelvin K.W. To, Kwok-Yung Yuen
EPI_ISL_497889, EPI_ISL_497890, EPI_ISL_497891	B.J. Govt. Medical College	National Centre For Cell Science	Dhiraj Paul, Kunal Jani, Radha Chauhan, Janesh Kumar, Vasudevan Seshadri, Girdhari Lal, Rajesh Karyakarte, Suvarna Joshi, Murlidhar Tambe, Sourav Sen, Santosh Karade, Kavita Bala Anand, Shelinder Pal Singh Shergill, Rajiv Mohan Gupta, Manoj Kumar Bhat, Arvind Sahu, Maharashtra COVID-19 Study Group, DBT's PAN-INDIA 1000 SARS-CoV2 RNA genome sequencing consortium, Yogesh S Shouche
EPI_ISL_498630, EPI_ISL_498631, EPI_ISL_498632, EPI_ISL_498633, EPI_ISL_498634, EPI_ISL_498637, EPI_ISL_498638	Utah Public Health Laboratory	Utah Public Health Laboratory	Heidi Butz, Erin Young, Kelly Oakeson
EPI_ISL_498705, EPI_ISL_498706, EPI_ISL_498707, EPI_ISL_498708, EPI_ISL_498709, EPI_ISL_498710, EPI_ISL_498711, EPI_ISL_498712	Quest Diagnostics	Quest Diagnostics	Rosenthal,S.H., Gerasimova,A., Kagan,R.M. and Owen, R.
EPI_ISL_499083	Instituto de Virologia "Dr. J. M. Vanella", Facultad de Ciencias Medicas, Universidad Nacional de Cordoba. Laboratorio Central de la Provincia de Cordoba, Argentina. Ministerio de Salud de la provincia de Cordoba, Argentina.	Laboratorio de Virología, Hospital de Niños Ricardo Gutiérrez, CABA, Argentina.	Sandra Gallego, Brenda Konigheim, Sebastian Blanco, Lorena Spinsanti, Javier Aguilar, Adrian Diaz, Gonzalo Castro, Gabriela Barbas, Mercedes Nabaes, Stephanie Goya, Monica Natale, Silvina Lusso, Mariana Viegas.
EPI_ISL_499309, EPI_ISL_499310, EPI_ISL_499311, EPI_ISL_499312, EPI_ISL_499313, EPI_ISL_499314	Centre for Enzyme Innovation, University of Portsmouth / Translational Research Laboratory, Portsmouth Hospitals NHS Trust	COVID-19 Genomics UK (COG-UK) Consortium	Angela Beckett,Yann Bourgeois,Garry Scarlett,Sharon Glaysher,Scott Elliott,Kelly Bicknell,Robert Impey,Allyson Lloyd,Sarah Wyllie,Ethan Butcher,Anoop Chauhan,Samuel Robson
EPI_ISL_500171, EPI_ISL_500187,	Hospital Universitario Virgen de las Nieves de Granada-SAS	SeqCOVID-SPAIN consortium/IBV(CSIC)	Mercedes Pérez Ruiz, Sara Sanbonmatsu Gámez, Irene Pedrosa Corral, José M. Navarro-Marí and SeqCOVID-SPAIN consortium

EPI_ISL_500188, EPI_ISL_500189, EPI_ISL_500190, EPI_ISL_500191, EPI_ISL_500204, EPI_ISL_500220, EPI_ISL_500226, EPI_ISL_500227			
EPI_ISL_500478, EPI_ISL_500479, EPI_ISL_500480	LACEN/PE	WallauLab, Aggeu Magalhaes Institute	Marcelo Henrique Santos Paiva, Duschinka Ribeiro Duarte Guedes, Cássia Docena, Matheus Filgueira Bezerra, Filipe Zimmer Dezordi, Laís Ceschini Machado, Larissa Krokovsky, Elisama Helvecio, Alexandre Freitas da Silva, Luydson Richardson Silva Vasconcelos, Antonio Mauro Rezende, Severino Jefferson Ribeiro da Silva, Kamila Gaudêncio da Silva Sales, Bruna Santos Lima Figueiredo de Sá, Derciliano Lopes da Cruz, Claudio Eduardo Cavalcanti, Armando de Menezes Neto, Caroline Targino Alves da Silva, Renata Pessôa Germano Mendes, Maria Almerice Lopes da Silva, Tiago Gräf, Paola Cristina Resende, Gonzalo Bello, Michelle da Silva Barros, Wheverton Ricardo Correia do Nascimento, Rodrigo Moraes Loyo Arcoverde, Luciane Caroline Albuquerque Bezerra, Sinval Pinto Brandão Filho, Constância Flávia Junqueira Ayres, Gabriel Luz Wallau
EPI_ISL_500522	Mayo Clinic Laboratories	University of Washington Virology Lab	Pavitra Roychoudhury, Hong Xie, Lasata Shrestha, Amin Addetia, Truong Nguyen, Victoria M Rachleff, Meei-Li Huang, Keith R Jerome, Alexander Greninger
EPI_ISL_500541	Singapore General Hospital	Department of Microbiology	Nurdyana Abdul Rahman, Kun Lee Lim, Chenhao Li, Kian Sing Chan, Lynette Oon, Kern Rei Chng, Niranjan Nagarajan, Karrie Ko
EPI_ISL_500801, EPI_ISL_500802, EPI_ISL_500803, EPI_ISL_500804, EPI_ISL_500805, EPI_ISL_500806, EPI_ISL_500807, EPI_ISL_500808, EPI_ISL_500809	National Institute for Biological Standards and Control	National Institute for Biological Standards and Control	Javier Martin, Dimitra Klapsa, Thomas Wilton
EPI_ISL_500833, EPI_ISL_500834, EPI_ISL_500835, EPI_ISL_500836, EPI_ISL_500837, EPI_ISL_500838, EPI_ISL_500839, EPI_ISL_500840, EPI_ISL_500841, EPI_ISL_500842, EPI_ISL_500843, EPI_ISL_500844, EPI_ISL_500845, EPI_ISL_500846, EPI_ISL_500847, EPI_ISL_500848, EPI_ISL_500849, EPI_ISL_500850, EPI_ISL_500851, EPI_ISL_500852, EPI_ISL_500853, EPI_ISL_500854, EPI_ISL_500855, EPI_ISL_500856, EPI_ISL_500857, EPI_ISL_500858			
see above	Virginia DCLS	Virginia DCLS	Virginia DCLS
EPI_ISL_500873, EPI_ISL_500874, EPI_ISL_500875	LACEN/PE	WallauLab, Aggeu Magalhaes Institute	Marcelo Henrique Santos Paiva, Duschinka Ribeiro Duarte Guedes, Cássia Docena, Matheus Filgueira Bezerra, Filipe Zimmer Dezordi, Laís Ceschini Machado, Larissa Krokovsky, Elisama Helvecio, Alexandre Freitas da Silva, Luydson Richardson Silva Vasconcelos, Antonio Mauro Rezende, Severino Jefferson Ribeiro da Silva, Kamila Gaudêncio da Silva Sales, Bruna Santos Lima Figueiredo de Sá, Derciliano Lopes da Cruz, Claudio Eduardo Cavalcanti, Armando de Menezes Neto, Caroline Targino Alves da Silva, Renata Pessôa Germano Mendes, Maria Almerice Lopes da Silva, Tiago Gräf, Paola Cristina Resende, Gonzalo Bello, Michelle da Silva Barros, Wheverton Ricardo Correia do Nascimento, Rodrigo Moraes Loyo Arcoverde, Luciane Caroline Albuquerque Bezerra, Sinval Pinto Brandão Filho, Constância Flávia Junqueira Ayres, Gabriel Luz Wallau
EPI_ISL_501151	University of Washington Virology Lab	University of Washington Virology Lab	Pavitra Roychoudhury, Hong Xie, Lasata Shrestha, Amin Addetia, Truong Nguyen, Victoria M Rachleff, Meei-Li Huang, Keith R Jerome, Alexander Greninger
EPI_ISL_501172, EPI_ISL_501173	Baylor College of Medicine	Baylor College of Medicine: HGSC	Vasanthi Avadhanula, Erin Nicholson, David Henke, Pedro Piedra, Harsha Doddapaneni, Donna Muzny, Qingchang Meng, Hsu Chao, Zeineen Momin, Hua Shen, George Weissenberger, Kavya Kottapalli, Yimiti Meiheerguli, Sejal Salvi, Ginger Metcalf, Vipin Menon, Sara J.J. Cregeen, Matthew C. Ross, Tulin Ayvaz, Richard Sucgang, Kristi L. Hoffman, Matthew Wong, Joseph F. Petrosino
EPI_ISL_501616, EPI_ISL_501618, EPI_ISL_501619, EPI_ISL_501623	Lab Microbiology, Pathology Department, William Harvey Hospital	Wellcome Sanger Institute for the COVID-19 Genomics UK (COG-UK) consortium	Samuel Moses, Hannah Lowe, Felicity Ryan and Alex Alderton, Roberto Amato, Sonia Goncalves, Ewan Harrison, David K. Jackson, Ian Johnston, Dominic Kwiatkowski, Cordelia Langford, John Sillitoe on behalf of the Wellcome Sanger Institute COVID-19 Surveillance Team (http://www.sanger.ac.uk/covid-team)
EPI_ISL_507040, EPI_ISL_507045, EPI_ISL_507094, EPI_ISL_507095, EPI_ISL_507096	University College London Hospital	COVID-19 Genomics UK (COG-UK) Consortium	Judith Heaney, Matthew Byott, Catherine Houlihan, Dan Frampton, Stuart Kirk, Moira Spyer and Eleni Nastouli
EPI_ISL_507201	Virology Department, Royal Infirmary of Edinburgh, NHS Lothian / School of Biological Sciences, University of Edinburgh / Institute of Genetics and Molecular Medicine, University of Edinburgh	COVID-19 Genomics UK (COG-UK) Consortium	McHugh M, Dewar R, Rooke S, Gallagher M, Balcaza C, O'Toole Á, Scher E, Hill V, McCrone JT, Colquhoun R, Yu X, Jackson B, Rambaut A, Williams TC, Templeton K
EPI_ISL_507934, EPI_ISL_507935, EPI_ISL_507936	Minnesota Department of Health, Public Health Laboratory	Minnesota Department of Health, Public Health Laboratory	Matt Plumb, Jacob Garfin, and Xiong Wang
EPI_ISL_508017, EPI_ISL_508036, EPI_ISL_508060, EPI_ISL_508072, EPI_ISL_508074, EPI_ISL_508076	New Mexico Department of Health Scientific Laboratory Division	Center for Global Health, University of New Mexico Health Sciences Center	Daryl Domman, Kurt Schwalm, Twila Kunde, Joseph Hicks, Michael Edwards, Darrell Dinwiddie
EPI_ISL_508339, EPI_ISL_508340, EPI_ISL_508407	Institute of Post Graduate Medical Education & Research	National Institute of Biomedical Genomics	Arindam Maitra, Aritra Biswas, Jayeeta Haldar, Raja Ray, Monimoy Banerjee, Saumitra Das
EPI_ISL_509085, EPI_ISL_509088, EPI_ISL_509089, EPI_ISL_509090, EPI_ISL_509091	OHSU Lab Services Molecular Microbiology Lab	Oregon SARS-CoV-2 Genome Sequencing Center	Brendan L. O'Connell, Ruth V. Nichols, Sally B. Grindstaff, Alec J. Hirsch, Guang Fan, Daniel N. Streblow, William B. Messer, Andrew C. Adey, Benjamin N. Bimber, Brian J. O'Roak
EPI_ISL_509412, EPI_ISL_509417	Acibadem Labcell Cellular Therapy Laboratory	Acibadem Mehmet Ali Aydinlar University School of Medicine, Medical Genetics Department	Ozden Hatirnaz Ng, Sezer Akyoney, Ilayda Sahin, Gunseli Bayram Akcapinar, Ozkan Ozdemir, Derya Dilek Kancagi, Gozde Sir Karakus, Bulut Yurtsever, Cihan Tastan, Ercument Ovali, Ugur Ozbek
EPI_ISL_509426	Microbiology and Immunology, University of South Alabama	Microbiology and Immunology, University of South Alabama	Wood,R.R., Roberts,R.A., Houserova,D., Borchert,G.M., Fouty,B., Rayner,J.O.
EPI_ISL_509498	Area of Virology, Serology and Virology Division (SAViD), New South Wales Health Pathology Randwick	Area of Virology, Serology and Virology Division (SAViD), New South Wales Health Pathology Randwick	Rawlinson, W.
EPI_ISL_509575, EPI_ISL_509577	Utah Public Health Laboratory	Utah Public Health Laboratory	Heidi Butz, Erin Young, Kelly Oakeson
EPI_ISL_509689	Utah Public Health Laboratory	Pathogen Discovery, Respiratory Viruses Branch, Division of Viral Diseases, Centers for Disease Control and Prevention	Ying Tao, Jing Zhang, Krista Queen, Anna Uehara, Yan Li, Clinton Paden, Haibin Wang, Suxiang Tong
EPI_ISL_509704	Wisconsin Department of Health Services	Pathogen Discovery, Respiratory Viruses Branch, Division of Viral Diseases, Centers for Disease Control and Prevention	Ying Tao, Jing Zhang, Krista Queen, Anna Uehara, Yan Li, Clinton Paden, Haibin Wang, Suxiang Tong
EPI_ISL_509707	Utah Public Health Laboratory	Pathogen Discovery, Respiratory Viruses Branch, Division of Viral Diseases, Centers for Disease Control and Prevention	Jing Zhang, Ying Tao, Krista Queen, Anna Uehara, Yan Li, Clinton Paden, Haibin Wang, Suxiang Tong
EPI_ISL_510044, EPI_ISL_510045, EPI_ISL_510046, EPI_ISL_510047, EPI_ISL_510048, EPI_ISL_510049, EPI_ISL_510050, EPI_ISL_510051, EPI_ISL_510052, EPI_ISL_510053, EPI_ISL_510054, EPI_ISL_510055			
see above	Servicio de Microbiología. HRU de Málaga. Servicio Andaluz de Salud	SeqCOVID-SPAIN consortium/IBV(CSIC)	Inmaculada de Toro Peinado. MªConcepción Mediavilla Gradolph. Begoña Palop Borrás and SeqCOVID-SPAIN consortium
EPI_ISL_510139, EPI_ISL_510147	Hospital General Universitario Gregorio Marañón	SeqCOVID-SPAIN consortium/IBV(CSIC)	Laura Pérez-Lago, Marta Herranz, Jon Sicilia, Julia Suárez, Pilar Catalán, Patricia Muñoz, Darío García de Viedma and SeqCOVID-SPAIN consortium
EPI_ISL_510333, EPI_ISL_510334, EPI_ISL_510335, EPI_ISL_510336, EPI_ISL_510341, EPI_ISL_510342	Servicio de Microbiología, Hospital Miguel Servet, Zaragoza	SeqCOVID-SPAIN consortium/IBV(CSIC)	Antonio Rezusta López, Alexander Tristancho Baró, Ana Milagro, Yolanda Gracia Grataloup, Nieves Martínez Cameo and SeqCOVID-SPAIN consortium
EPI_ISL_510441	Hospital Universitario Virgen de las Nieves de Granada-SAS	SeqCOVID-SPAIN consortium/IBV(CSIC)	Mercedes Pérez Ruiz, Sara Sanbonmatsu Gámez, Irene Pedrosa Corral, José M. Navarro-Marí and SeqCOVID-SPAIN consortium
EPI_ISL_510548	Division of Viral Diseases, Center for Laboratory Control of	Division of Viral Diseases, Center for Laboratory Control of	Jeong-Min Kim, Yoon-Seok Chung, Namjoo Lee, Sang Hee Woo, Hye-Jun Jo, Heui Man Kim, Jun-Sub Kim, Myung Guk Han

	Infectious Diseases, Korea Centers for Diseases Control and Prevention	Infectious Diseases, Korea Centers for Diseases Control and Prevention	
EPI_ISL_510931, EPI_ISL_510933, EPI_ISL_510947, EPI_ISL_510948, EPI_ISL_510949, EPI_ISL_510950	Instituto Nacional de Saude (INSA)	Instituto Nacional de Saude (INSA)	Borges et al
EPI_ISL_511501, EPI_ISL_511502, EPI_ISL_511503, EPI_ISL_511504, EPI_ISL_511506, EPI_ISL_511507	Instituto Nacional de Saude (INSA)	Instituto Nacional de Saude (INSA) and Instituto Gulbenkian de Ciencia (IGC)	Borges et al
EPI_ISL_511876	Johns Hopkins Hospital Department of Pathology	Johns Hopkins Hospital Department of Pathology	Peter M. Thielen, Thomas Mehoke, Shirlee Wohl, Srividya Ramakrishnan, Melanie Kirsche, Amanda Ernlund, Craig Howser, Kristina Zudock, Oluwaseun Falade-Nwulia, Norah Sadowski, Paul Morris, Mark Hopkins, Yunfan Fan, Nidia Trovao, Victoria Gniazdowski, Michael C. Schatz, Stuart C. Ray, Winston Timp, Heba H. Mostafa
EPI_ISL_511898	National Hospital of Tropical Diseases	Oxford University Clinical Research Unit, Hanoi, Vietnam	Nguyen Thi Tam, Van Dinh Trang, Nguyen Thi Hong Thuong, Vu Thi Ngoc Bich, Nguyen Thu Trang, Nguyen Thi Ngoc Diep, Le Nguyen Minh Hoa, Pham Ngoc Thach, H. Rogier van Doorn, on behalf of the OUCRU COVID-19 research group
EPI_ISL_512227	San Diego County Public Health Laboratory	Andersen lab at Scripps Research	SEARCH Alliance San Diego with Tracy Basler, Jovan Shephard, Brett Austin
EPI_ISL_512413, EPI_ISL_512426	Centre for Enzyme Innovation, University of Portsmouth / Translational Research Laboratory, Portsmouth Hospitals NHS Trust	COVID-19 Genomics UK (COG-UK) Consortium	Angela Beckett,Yann Bourgeois,Garry Scarlett,Sharon Glaysher,Scott Elliott,Kelly Bicknell,Robert Impey,Allyson Lloyd,Sarah Wyllie,Ethan Butcher,Anoop Chauhan,Samuel Robson
EPI_ISL_512505	Wales Specialist Virology Centre Sequencing lab: Pathogen Genomics Unit	COVID-19 Genomics UK (COG-UK) Consortium	Catherine Moore, Johnathan Evans, Laura Gifford, Malorie Perry, Simon Cottrell, Angela Marchbank, Alec Birchley, Alexander Adams, Amy Gaskin, Bree Gatica-Wilcox, Jason Coombes, Joel Southgate, Lauren Gilbert, Lee Graham, Nicole Pacchiarini, Sara Kumziene-Summerhayes, Sarah Taylor, Sophie Jones, Sara Rey, Matthew Bull, Joanne Watkins, Sally Corden, Tom Connor
EPI_ISL_512758	PathWest Laboratory Medicine WA	PathWest Laboratory Medicine WA Microbial Surveillance Unit	PathWest Laboratory Medicine WA Microbial Surveillance Unit
EPI_ISL_512779, EPI_ISL_512781	Utah Public Health Laboratory, Utah Public Health Laboratory Infectious Disease submission group	Utah Public Health Laboratory, Utah Public Health Laboratory Infectious Disease submission group	Butz,H.A., Young,E.L., Oakeson,K.
EPI_ISL_512820	Kenema Government Hospital, Ministry of Health and Sanitation	Kenema Government Hospital, Ministry of Health and Sanitation	Goba,A., Momoh,M., Sandi,J., Tomkins-Tinch,C., Siddle,K., Mehta,S., Oluniyi,P., Jalloh,S., Park,D., Andersen,K., Garry,R., Happi,C., Grant,D., Olawoye,I.
EPI_ISL_512908, EPI_ISL_512909, EPI_ISL_512910, EPI_ISL_512911, EPI_ISL_512912, EPI_ISL_512913, EPI_ISL_512914, EPI_ISL_512916, EPI_ISL_512917	Pathogen Genomics Lab King Abdullah University of Science and Technology(KAUST)	Pathogen Genomics Lab King Abdullah University of Science and Technology(KAUST)	Sharif Hala, Fadwa Alofi, Sara Mfarrej, Amit Kumar Subudhi, Rahul P Salunke, Fathia Ben Rached, Amanda Ooi, Luke Esau, Afrah Alsomali, Asim Khogeer, Jumana Taha, Abdulaziz Alahmadi, Kahled Algithami, Raeece Naeem, Anwar Hashem, Naif Almontashiri, Arnab Pain
EPI_ISL_512918, EPI_ISL_512919, EPI_ISL_512941, EPI_ISL_512942, EPI_ISL_512943	Pathogen Genomics Lab King Abdullah University of Science and Technology(KAUST)	Pathogen Genomics Lab King Abdullah University of Science and Technology(KAUST)	Fadwa Alofi, Sharif Hala, Rahul P Salunke, Sara Mfarrej, Amit Kumar Subudhi, Fathia Ben Rached, Amanda, Luke, Afrah Alsomali, Asim Khogeer, Jumana Taha, Abdulaziz Alahmadi, Kahled Algithami, Raeece Naeem, Anwar Hashem, Naif Almontashiri, Arnab Pain
EPI_ISL_513227, EPI_ISL_513228, EPI_ISL_513229, EPI_ISL_513230, EPI_ISL_513231, EPI_ISL_513234, EPI_ISL_513235, EPI_ISL_513236, EPI_ISL_513237, EPI_ISL_513238, EPI_ISL_513240, EPI_ISL_513241, EPI_ISL_513242, EPI_ISL_513243, EPI_ISL_513244, EPI_ISL_513245, EPI_ISL_513246, EPI_ISL_513247			
see above	Pathogen Genomics Lab King Abdullah University of Science and Technology(KAUST)	Pathogen Genomics Lab King Abdullah University of Science and Technology(KAUST)	Amit Kumar Subudhi, Rahul P Salunke, Sara Mfarrej, Sharif Hala, Fadwa Alofi, Fathia Ben Rached, Afrah Alsomali, Asim Khogeer, Nashwa Al-khotani, Raeece Naeem, Anwar Hashem, Naif Almontashiri, Arnab Pain
EPI_ISL_513294	Baylor College of Medicine	Baylor College of Medicine: HGSC	Vasanthi Avadhanula, Erin Nicholson, David Henke, Pedro Piedra, Harsha Doddapaneni, Donna Muzny, Qingchang Meng, Hsu Chao, Zeineen Momin, Hua Shen, George Weissenberger, Kavya Kottapalli, Yimiti Meiheerguli, Sejal Salvi, Ginger Metcalf, Vipin Menon, Sara J.J. Cregeen, Matthew C. Ross, Tulin Ayvaz, Richard Sucgang, Kristi L. Hoffman, Matthew Wong, Joseph F. Petrosino
EPI_ISL_513515, EPI_ISL_513516, EPI_ISL_513517, EPI_ISL_513518, EPI_ISL_513530, EPI_ISL_513531, EPI_ISL_513532, EPI_ISL_513533, EPI_ISL_513534, EPI_ISL_513535, EPI_ISL_513536, EPI_ISL_513537, EPI_ISL_513538, EPI_ISL_513539, EPI_ISL_513540, EPI_ISL_513541, EPI_ISL_513542, EPI_ISL_513543, EPI_ISL_513544, EPI_ISL_513545, EPI_ISL_513546, EPI_ISL_513547, EPI_ISL_513548, EPI_ISL_513549, EPI_ISL_513550, EPI_ISL_513551			
see above	Programa de Oncovirologia, Instituto Nacional de Câncer	Programa de Oncovirologia, Instituto Nacional de Câncer	Juliana D. Siqueira, Livia R. Goes, Brunna M. Alves, Claudia Cicala,James Arthos, João P.B. Viola, Andreia C. de Melo, Marcelo A. Soares
EPI_ISL_513878, EPI_ISL_513879, EPI_ISL_513880, EPI_ISL_513881, EPI_ISL_513882	San Francisco Public Health Laboratory	Chan-Zuckerberg Biohub	CZB Cliahub Consortium
EPI_ISL_514432	Prof. Massimo Zollo CEINGE TASK-FORCE COVID19 - Regione Campania	Prof. Massimo Zollo CEINGE TASK-FORCE COVID19 - Regione Campania	Veronica Ferrucci, Dae young Kong, Fatemeh asadzadeh, Laura Marrone, Roberto Siciliano, Rino Cerino, Giovanna Fusco, Marika Comegna, Angelo Boccia, Maurizio Viscardi, Giorgia Borriello, Sergio Brandi, Claudia Tiberio, Luigi Atripaldi, Giovanni Paolella, Giuseppe Castaldo, Stefano Pascarella, Martina Bianchi, Lorenzo Chiariotti, Jae Myun Lee, Jae Ho Jung, Kyong Seop Yun, Hong Yeoul Kim and Massimo Zollo
EPI_ISL_514481, EPI_ISL_514483, EPI_ISL_514484	Centre for Enzyme Innovation, University of Portsmouth / Translational Research Laboratory, Portsmouth Hospitals NHS Trust	COVID-19 Genomics UK (COG-UK) Consortium	Angela Beckett,Yann Bourgeois,Garry Scarlett,Sharon Glaysher,Scott Elliott,Kelly Bicknell,Robert Impey,Allyson Lloyd,Sarah Wyllie,Ethan Butcher,Anoop Chauhan,Samuel Robson
EPI_ISL_514673	Nevada State Public Health Laboratory	Nevada State Public Health Laboratory	Richard Tillet, Joel R. Sevinsky, Paul Hartley, Heather Kerwin, David Jackson, Subhash C. Verma, Cyprian Rosetto, Andrew Gorzalski, Chris Laverdure, Natalie Crawford, Stephanie Van Hooser, and Mark Pandori
EPI_ISL_514750	Pirogov Russian National Research Medical University	Pirogov Russian National Research Medical University, Research and Development	Blagodatskikh, K.A.
EPI_ISL_514751	CoronaNet Lab- TaskForce Regione Campania, CEINGE Biotecnologie Avanzate, Via G. Salvatore	CoronaNet Lab- TaskForce Regione Campania, CEINGE Biotecnologie Avanzate, Via G. Salvatore	Zollo,M., Ferrucci,V., Kong,Dy., Asadzadeh,F., Marrone,L.,Siciliano,R., Cerino,R., Fusco,G., Comegna,M., Boccia,A.,Viscardi,M., Borriello,G., Brandi,S., Tiberio,C., Atripaldi,L.,Paolella,G., Castaldo,G., Pascarella,S., Bianchi,M., Chiariotti,L.,Lee,J.M., Jung,J.H., Yun,K.S. and Kim,H.Y.
EPI_ISL_515204, EPI_ISL_515205, EPI_ISL_515207, EPI_ISL_515209, EPI_ISL_515210, EPI_ISL_515212, EPI_ISL_515213, EPI_ISL_515219, EPI_ISL_515221, EPI_ISL_515223, EPI_ISL_515224, EPI_ISL_515232, EPI_ISL_515233, EPI_ISL_515237, EPI_ISL_515238, EPI_ISL_515239, EPI_ISL_515240, EPI_ISL_515241, EPI_ISL_515242, EPI_ISL_515244			
see above	Laboratoire de microbiologie, Hopital de Verdun	Smith Laboratory, Centre de Recherche CHU Sainte-Justine	Martin Smith, Marieke Rozendaal, Ivan Pavlov
EPI_ISL_515355, EPI_ISL_515356, EPI_ISL_515357, EPI_ISL_515358, EPI_ISL_515359, EPI_ISL_515360, EPI_ISL_515361, EPI_ISL_515362, EPI_ISL_515363, EPI_ISL_515364, EPI_ISL_515365, EPI_ISL_515366, EPI_ISL_515367, EPI_ISL_515368, EPI_ISL_515369, EPI_ISL_515370, EPI_ISL_515371, EPI_ISL_515372, EPI_ISL_515373, EPI_ISL_515374, EPI_ISL_515375, EPI_ISL_515376, EPI_ISL_515377, EPI_ISL_515378, EPI_ISL_515379, EPI_ISL_515380, EPI_ISL_515381, EPI_ISL_515382, EPI_ISL_515383, EPI_ISL_515384, EPI_ISL_515385			
see above	Nevada State Public Health Laboratory	Nevada State Public Health Laboratory	Richard Tillett, Joel R. Sevinsky, Paul Hartley, Heather Kerwin, David Jackson, Subhash C. Verma, Cyprian Rossetto, Andrew Gorzalski, Chris Laverdure, Natalie Crawford, Stephanie Van Hooser, and Mark Pandori
EPI_ISL_515552	Hospital Municipal do Tatuape Carmino Caricchio	Instituto Adolfo Lutz, Interdiciplinary Procedures Center, Strategic Laboratory	Claudio Tavares Sacchi, Claudia Regina Gonçalves, Erica Valessa Ramos Gomes
EPI_ISL_515553	Hospital Municipal Dr. Ignacio Proença de Gouvea	Instituto Adolfo Lutz, Interdiciplinary Procedures Center, Strategic Laboratory	Claudio Tavares Sacchi, Claudia Regina Gonçalves, Erica Valessa Ramos Gomes
EPI_ISL_515557	Hospital Municipal Dr. Moysés Deutsch	Instituto Adolfo Lutz, Interdiciplinary Procedures Center, Strategic Laboratory	Claudio Tavares Sacchi, Claudia Regina Gonçalves, Erica Valessa Ramos Gomes
EPI_ISL_515559, EPI_ISL_515560	Hospital Sao Paulo de Ensino da Unifesp	Instituto Adolfo Lutz, Interdiciplinary Procedures Center, Strategic Laboratory	Claudio Tavares Sacchi, Claudia Regina Gonçalves, Erica Valessa Ramos Gomes

EPI_ISL_515561	Hospital Montemagno	Instituto Adolfo Lutz, Interdiciplinary Procedures Center, Strategic Laboratory	Claudio Tavares Sacchi, Claudia Regina Gonçalves, Erica Valessa Ramos Gomes
EPI_ISL_515562	Hospital Municipal Doutor Alexandre Zaio	Instituto Adolfo Lutz, Interdiciplinary Procedures Center, Strategic Laboratory	Claudio Tavares Sacchi, Claudia Regina Gonçalves, Erica Valessa Ramos Gomes
EPI_ISL_515563	Hospital Municipal Dr. Jose Soares Hungria	Instituto Adolfo Lutz, Interdiciplinary Procedures Center, Strategic Laboratory	Claudio Tavares Sacchi, Claudia Regina Gonçalves, Erica Valessa Ramos Gomes
EPI_ISL_515933	BIMS	Department of Neurovirology, National Institute of Mental Health and Neuroscience (NIMHANS)	Chitra Pattabiraman,Vijayalakshmi Reddy, Harsha PK, Risha Rasheed, Pramada Prasad, Shafeeq S Hameed, Manjunatha Venkataswamy, Anita Desai, Ravi Vasanthapuram
EPI_ISL_515934, EPI_ISL_515935, EPI_ISL_515936	DH	Department of Neurovirology, National Institute of Mental Health and Neuroscience (NIMHANS)	Chitra Pattabiraman,Vijayalakshmi Reddy, Harsha PK, Risha Rasheed, Pramada Prasad, Shafeeq S Hameed, Manjunatha Venkataswamy, Anita Desai, Ravi Vasanthapuram
EPI_ISL_515937	BIMS	Department of Neurovirology, National Institute of Mental Health and Neuroscience (NIMHANS)	Chitra Pattabiraman,Vijayalakshmi Reddy, Harsha PK, Risha Rasheed, Pramada Prasad, Shafeeq S Hameed, Manjunatha Venkataswamy, Anita Desai, Ravi Vasanthapuram
EPI_ISL_516076, EPI_ISL_516077, EPI_ISL_516078	VICTORIA HOSPITAL	Department of Neurovirology, National Institute of Mental Health and Neuroscience (NIMHANS)	Chitra Pattabiraman,Vijayalakshmi Reddy, Harsha PK, Risha Rasheed, Pramada Prasad, Shafeeq S Hameed, Manjunatha Venkataswamy, Anita Desai, Ravi Vasanthapuram
EPI_ISL_516960, EPI_ISL_516961, EPI_ISL_516962, EPI_ISL_516970, EPI_ISL_516971, EPI_ISL_516972, EPI_ISL_516973	King Georges Medical University	CSIR-National Botanical Research Institute	Priti Prasad, Shantanu Prakash, Kishan Sahu, Babita Singh, Suruchi Shukla, Hricha Mishra, Danish Nasar Khan , Om Prakash, MLB Bhatt, SK Barik, Mehar H.Asif,Samir V. Sawant,Amita Jain, Sumit Kr. Bag
EPI_ISL_517528, EPI_ISL_517529, EPI_ISL_517530, EPI_ISL_517532, EPI_ISL_517533, EPI_ISL_517534, EPI_ISL_517535, EPI_ISL_517536, EPI_ISL_517537, EPI_ISL_517538, EPI_ISL_517539, EPI_ISL_517540, EPI_ISL_517541			
see above	Centre for Enzyme Innovation, University of Portsmouth / Translational Research Laboratory, Portsmouth Hospitals NHS Trust	COVID-19 Genomics UK (COG-UK) Consortium	Angela Beckett,Yann Bourgeois,Garry Scarlett,Sharon Glaysher,Scott Elliott,Kelly Bicknell,Robert Impey,Allyson Lloyd,Sarah Wyllie,Ethan Butcher,Anoop Chauhan,Samuel Robson
EPI_ISL_517779, EPI_ISL_517780, EPI_ISL_517781, EPI_ISL_517787	Florida Bureau of Public Health Laboratories	Florida Bureau of Public Health Laboratories	Sarah Schmedes, Jason Blanton
EPI_ISL_518821	Oman-National Influenza Center	Biotechnology & OMICs Laboratory, Natural & Medical Sciences Research Center, University of Nizwa	Samira Al-Mahruqi, Abdul Latif Khan, Samiha Al-Kharusi, Adil Khan , Ahmed Al-Rawahi, Sajjad Asaf, Amina Al-Jardani, Hanan Al-Kindi, Intisar Al-Shukri, Adil Al-Wahaibi, Seif Al-Abri, Ahmed Al-Harrasi
EPI_ISL_519947	Microbiological Diagnostic Unit - Public Health Laboratory (MDU-PHL)	MDU-PHL	Seemann T., Schultz M., Sait, M., Sherry, N.
EPI_ISL_520664, EPI_ISL_520665, EPI_ISL_520668, EPI_ISL_520669, EPI_ISL_520670, EPI_ISL_520671, EPI_ISL_520717, EPI_ISL_520734, EPI_ISL_520735, EPI_ISL_520736, EPI_ISL_520737			
see above	Mohammed Bin Rashid University of Medicine and Health Sciences	Al Jalila Genomics Center	Ahmad Abou Tayoun, Tom Loney, Hamda Khansaheb, Sathishkumar Ramaswamy, Divinlal Harilal, Zulfa Omar Deesi, Rupa Murthy Varghese, Hanan Al Suwaidi, Abdulmajeed Alkhaja, Mohammed Uddin, Rifat Hamoudi, Rabih Halwani, Abiola Catherine Senok, Qutayba Hamid, Norbert Nowotny, Alawi Alsheikh-Ali
EPI_ISL_521870, EPI_ISL_521871	Victorian Infectious Diseases Reference Laboratory (VIDRL)	VIDRL and MDU-PHL	Caly L., Seemann T., Sait, M., Schultz M., Druce J., Sherry, N.
EPI_ISL_522562, EPI_ISL_522563, EPI_ISL_522564, EPI_ISL_522565, EPI_ISL_522573, EPI_ISL_522575, EPI_ISL_522585, EPI_ISL_522592, EPI_ISL_522595, EPI_ISL_522598, EPI_ISL_522599, EPI_ISL_522600, EPI_ISL_522601, EPI_ISL_522602, EPI_ISL_522603, EPI_ISL_522605, EPI_ISL_522607, EPI_ISL_522609, EPI_ISL_522612, EPI_ISL_522613, EPI_ISL_522614, EPI_ISL_522617, EPI_ISL_522622, EPI_ISL_522630, EPI_ISL_522633, EPI_ISL_522634, EPI_ISL_522638, EPI_ISL_522643, EPI_ISL_522646, EPI_ISL_522658, EPI_ISL_522666, EPI_ISL_522667, EPI_ISL_522668, EPI_ISL_522669, EPI_ISL_522670, EPI_ISL_522671, EPI_ISL_522672, EPI_ISL_522673, EPI_ISL_522698, EPI_ISL_522699, EPI_ISL_522700, EPI_ISL_522701, EPI_ISL_522702, EPI_ISL_522703, EPI_ISL_522704, EPI_ISL_522707, EPI_ISL_522713, EPI_ISL_522714, EPI_ISL_522715, EPI_ISL_522718, EPI_ISL_522719			
see above	Royal Hobart Hospital Microbiology Department	MDU-PHL	Cooley L., van Haeften R., Seemann T., Sait M., Schultz, M.B., Sherry N.
EPI_ISL_522868	ULSS9 Distretto di Bussolengo	Istituto Zooprofilattico Sperimentale delle Venezie	Adelaide Milani, Alessia Schivo, Annalisa Salviato, Erika Giorgia Quaranta, Ambra Pastori, Bianca Zecchin, Alice Fusaro, Isabella Monne, Calogero Terregino, Antonia Ricci
EPI_ISL_523122, EPI_ISL_523136, EPI_ISL_523157, EPI_ISL_523158, EPI_ISL_523159, EPI_ISL_523160, EPI_ISL_523173, EPI_ISL_523208, EPI_ISL_523235, EPI_ISL_523236, EPI_ISL_523420, EPI_ISL_523421, EPI_ISL_523422, EPI_ISL_523423, EPI_ISL_523428, EPI_ISL_523479, EPI_ISL_523480, EPI_ISL_523481, EPI_ISL_523482, EPI_ISL_523506, EPI_ISL_523574, EPI_ISL_523575, EPI_ISL_523607, EPI_ISL_523693			
see above	Dutch COVID-19 response team	Erasmus Medical Center	Bas Oude Munnink, David Nieuwenhuijse, Reina Sikkema, Claudia Schapendonk, Irina Chestakova, Anne van der Linden, Theo Bestebroer, Stefan van Nieuwkoop, Mark Pronk, Pascal Lexmond, Corien Swaan, Manon Haverkate, Madelief Mollers, Mart Stein, Sandra Kengne Kamga Mobou, Jeroen van Kampen, Jolanda Voermans, Aura Timen, Corine GeurtsvanKessel, Annemiek van der Eijk, Richard Molenkamp, Marion Koopmans, on behalf of the Dutch national COVID-19 response team.
EPI_ISL_523953	Mohammed Bin Rashid University of Medicine and Health Sciences	Al Jalila Genomics Center	Ahmad Abou Tayoun, Tom Loney, Hamda Khansaheb, Sathishkumar Ramaswamy, Divinlal Harilal, Zulfa Omar Deesi, Rupa Murthy Varghese, Hanan Al Suwaidi, Abdulmajeed Alkhaja, Mohammed Uddin, Rifat Hamoudi, Rabih Halwani, Abiola Catherine Senok, Qutayba Hamid, Norbert Nowotny, Alawi Alsheikh-Ali
EPI_ISL_523958	Pronto Socorro Municipal de Perus	Instituto Adolfo Lutz, Interdiciplinary Procedures Center, Strategic Laboratory	Claudio Tavares Sacchi, Claudia Regina Gonçalves, Erica Valessa Ramos Gomes
EPI_ISL_523961	Pronto Socorro Municipal 21 de Junho	Instituto Adolfo Lutz, Interdiciplinary Procedures Center, Strategic Laboratory	Claudio Tavares Sacchi, Claudia Regina Gonçalves, Erica Valessa Ramos Gomes
EPI_ISL_523967	Hospital Sancta Maggiore	Instituto Adolfo Lutz, Interdiciplinary Procedures Center, Strategic Laboratory	Claudio Tavares Sacchi, Claudia Regina Gonçalves, Erica Valessa Ramos Gomes
EPI_ISL_523983	UPA Campo Limpo	Instituto Adolfo Lutz, Interdiciplinary Procedures Center, Strategic Laboratory	Claudio Tavares Sacchi, Claudia Regina Gonçalves, Erica Valessa Ramos Gomes
EPI_ISL_523984	Ama Dr Jose Soares Hungria	Instituto Adolfo Lutz, Interdiciplinary Procedures Center, Strategic Laboratory	Claudio Tavares Sacchi, Claudia Regina Gonçalves, Erica Valessa Ramos Gomes
EPI_ISL_523985	Hospital Municipal Dr. Benedicto Montenegro	Instituto Adolfo Lutz, Interdiciplinary Procedures Center, Strategic Laboratory	Claudio Tavares Sacchi, Claudia Regina Gonçalves, Erica Valessa Ramos Gomes
EPI_ISL_523990	AMA Jardim Peri	Instituto Adolfo Lutz, Interdiciplinary Procedures Center, Strategic Laboratory	Claudio Tavares Sacchi, Claudia Regina Gonçalves, Erica Valessa Ramos Gomes
EPI_ISL_523993	UPA Campo Limpo	Instituto Adolfo Lutz, Interdiciplinary Procedures Center, Strategic Laboratory	Claudio Tavares Sacchi, Claudia Regina Gonçalves, Erica Valessa Ramos Gomes
EPI_ISL_524048	WHO National Influenza Centre Russian Federation	WHO National Influenza Centre Russian Federation	Andrey Komissarov, Artem Fadeev, Mariia Sergeeva, Anna Ivanova, Daria Danilenko
EPI_ISL_524069	Texas Department of State Health Services	Texas Department of State Health Services	Rashmi Tuladhar, Bonnie Oh, Cara Akrout, Jenny Zhang, Maliha Rahman, Anita Pokharel, Myong Koag, Chun Wang, Rachel Lee, Grace Kubin
EPI_ISL_524465	PS Municipal Dr. Caetano Virgílio Neto	Instituto Adolfo Lutz, Interdiciplinary Procedures Center, Strategic Laboratory	Claudio Tavares Sacchi, Claudia Regina Gonçalves, Erica Valessa Ramos Gomes
EPI_ISL_524787	Evandro Chagas Institute	Evandro Chagas Institute	Santos, M.C.; Silva, A.M.; Junior, W.D.C.; Barbagelata, L.S.; Ferreira, J.A.; Sousa, E.M.A.; da Silva, P.S.; Resque, H.R; Martins, L.C.; Sousa Junior, E.C.;Viana, G.M.R
EPI_ISL_524877, EPI_ISL_524878	MD PHL	MD PHL	Maryland Department of Health Laboratories Administration

EPI_ISL_525474	Centre for Dengue Research	Centre for Dengue Research, USJ, SL	Chandima Jeewandara, Deshni Jayathilaka, Dinuka Ariyaratne, Laksiri Gomes, Diyanath Ranasinghe, Dinuka Guruge, Ruwan Wijayamuni, Gathsaurie Neelika Malavige
EPI_ISL_525674, EPI_ISL_525675, EPI_ISL_525676, EPI_ISL_525679, EPI_ISL_525681, EPI_ISL_525682, EPI_ISL_525683, EPI_ISL_525684, EPI_ISL_525685	Wadsworth Center, New York State Department of Health	Wadsworth Center, New York State Department of Health	Kirsten St. George, Daryl M. Lamson, Sara Griesemer, Jonathan Plitnick, Navjot Singh, Matthew D. Shudt, Erica Lasek-Nesselquist
EPI_ISL_526144, EPI_ISL_526145	South Eastern Area Laboratory Services (SEALS)	NSW Health Pathology - Institute of Clinical Pathology and Medical Research; Westmead Hospital; University of Sydney	CIDM-PH et al.
EPI_ISL_526216, EPI_ISL_526217, EPI_ISL_526229, EPI_ISL_526230, EPI_ISL_526232, EPI_ISL_526233, EPI_ISL_526234	Hungarian Defence Forces Military Medical Centre	National Laboratory of Virology, Szentágothai Research Centre	Endre Gábor Tóth, Balázs Somogyi, Bálint Eszenyi, Ferenc Jakab, Gábor Kemenesi
EPI_ISL_526433	Centre for Enzyme Innovation, University of Portsmouth / Translational Research Laboratory, Portsmouth Hospitals NHS Trust	COVID-19 Genomics UK (COG-UK) Consortium	Angela Beckett,Yann Bourgeois,Garry Scarlett,Sharon Glaysher,Scott Elliott,Kelly Bicknell,Robert Impey,Allyson Lloyd,Sarah Wyllie,Ethan Butcher,Anoop Chauhan,Samuel Robson
EPI_ISL_526463, EPI_ISL_526464, EPI_ISL_526465, EPI_ISL_526466, EPI_ISL_526467, EPI_ISL_526468, EPI_ISL_526469, EPI_ISL_526475, EPI_ISL_526476, EPI_ISL_526477, EPI_ISL_526478, EPI_ISL_526536			
see above	Virology Department, Royal Infirmary of Edinburgh, NHS Lothian / School of Biological Sciences, University of Edinburgh / Institute of Genetics and Molecular Medicine, University of Edinburgh	COVID-19 Genomics UK (COG-UK) Consortium	McHugh M, Dewar R, Rooke S, Gallagher M, Balcaza C, O'Toole Á, Scher E, Hill V, McCrone JT, Colquhoun R, Yu X, Jackson B, Rambaut A, Williams TC, Templeton K
EPI_ISL_526537, EPI_ISL_526538, EPI_ISL_526539, EPI_ISL_526540, EPI_ISL_526541	Respiratory Virus Unit, Microbiology Services Colindale, Public Health England	Respiratory Virus Unit, Microbiology Services Colindale, Public Health England	PHE Covid Sequencing Team
EPI_ISL_526687	Faith Laboratory, Immunology Institute, Icahn School of Medicine at Mount Sinai	van Bakel Laboratory, Genetics and Genomics Sciences, Icahn School of Medicine at Mount Sinai	Graham J. Britton, Alice Chen-Liaw, Francesca Cossarini, Alexandra Livanos, Matthew P. Spindler, Tamar Plitt, Joseph Eggers, Ilaria Mogno, Ana S. Gonzalez-Reiche, Sophia Sui, Michael Tankelevich, Lauren Tal Grinspan, Rebekah E. Dixon, Divya Jha, Gustavo Martinez-Delgado, Fatima Amanat, Daisy Hoagland, Benjamin R. tenOever, Marla C. Dubinsky, Miriam Merad, Harm Van Bakel, Florian Krammer, Gerold Bongers, Saurabh Mehandru and Jeremiah J. Faith
EPI_ISL_526754	Respiratory Virus Unit, Microbiology Services Colindale, Public Health England	Respiratory Virus Unit, Microbiology Services Colindale, Public Health England	PHE Covid Sequencing Team
EPI_ISL_526841, EPI_ISL_526842, EPI_ISL_526843	Virginia DCLS	Virginia DCLS	Virginia DCLS
EPI_ISL_526962, EPI_ISL_526963, EPI_ISL_526964, EPI_ISL_526965	Instituto Nacional de Salud, Bogotá, Colombia	Instituto Nacional de Salud, Bogotá, Colombia	Katherine Laiton-Donato, Diego A. Álvarez-Díaz, Carlos Franco-Muñoz, Jonathan Reales, Diego Andrés Prada, Jeadran Malagón-Rojas, Felix Betzler, Wendy K. Jo, Edmilson F. de Oliveira-Filho, Carolina Ferro, Diana Marcela Walteros-Acero, Franklin Prieto, Carlos Andrés Durán, Martha Lucia Ospina Martinez, Marcela Mercado-Reyes
EPI_ISL_527059	Area of Virology, Serology and Virology Division (SAViD), New South Wales Health Pathology Randwick	Area of Virology, Serology and Virology Division (SAViD), New South Wales Health Pathology Randwick	Rawlinson, W.
EPI_ISL_527329, EPI_ISL_527330, EPI_ISL_527331, EPI_ISL_527332, EPI_ISL_527333, EPI_ISL_527334, EPI_ISL_527335, EPI_ISL_527336, EPI_ISL_527337, EPI_ISL_527338, EPI_ISL_527339			
see above	Respiratory Virus Unit, Microbiology Services Colindale, Public Health England	Respiratory Virus Unit, Microbiology Services Colindale, Public Health England	PHE Covid Sequencing Team
EPI_ISL_527403, EPI_ISL_527406, EPI_ISL_527407, EPI_ISL_527412, EPI_ISL_527413, EPI_ISL_527418, EPI_ISL_527419, EPI_ISL_527422, EPI_ISL_527429, EPI_ISL_527432, EPI_ISL_527435, EPI_ISL_527438, EPI_ISL_527440, EPI_ISL_527444, EPI_ISL_527462, EPI_ISL_527463, EPI_ISL_527470, EPI_ISL_527476, EPI_ISL_527484			
see above	Colorado State University - Ebel Lab	Colorado State University - Ebel Lab	Greg Ebel et al.
EPI_ISL_527716	MN PHL Division, Minnesota Department of Health	Pathogen Discovery, Respiratory Viruses Branch, Division of Viral Diseases, Centers for Disease Control and Prevention	Krista Queen, Brian Lynch, Yan Li, Anna Montmayeur, Jing Zhang, Ying Tao, Anna Uehara, Rachel Marine, Clinton R. Paden, Haibin Wang, Suxiang Tong
EPI_ISL_527718, EPI_ISL_527719, EPI_ISL_527721, EPI_ISL_527722	MN PHL Division, Minnesota Department of Health	Pathogen Discovery, Respiratory Viruses Branch, Division of Viral Diseases, Centers for Disease Control and Prevention	Yan Li, Anna Montmayeur, Jing Zhang, Krista Queen, Ying Tao, Anna Uehara, Rachel Marine, Clinton R. Paden, Haibin Wang, Suxiang Tong
EPI_ISL_527724	MN PHL Division, Minnesota Department of Health	Pathogen Discovery, Respiratory Viruses Branch, Division of Viral Diseases, Centers for Disease Control and Prevention	Krista Queen, Brian Lynch, Yan Li, Anna Montmayeur, Jing Zhang, Ying Tao, Anna Uehara, Rachel Marine, Clinton R. Paden, Haibin Wang, Suxiang Tong
EPI_ISL_527725, EPI_ISL_527726, EPI_ISL_527727, EPI_ISL_527728, EPI_ISL_527729, EPI_ISL_527730, EPI_ISL_527732	MN PHL Division, Minnesota Department of Health	Pathogen Discovery, Respiratory Viruses Branch, Division of Viral Diseases, Centers for Disease Control and Prevention	Yan Li, Anna Montmayeur, Jing Zhang, Krista Queen, Ying Tao, Anna Uehara, Rachel Marine, Clinton R. Paden, Haibin Wang, Suxiang Tong
EPI_ISL_527787, EPI_ISL_527789	Laboratorio de Referencia Nacional de Virus Respiratorio. Centro Nacional de Salud Publica. Instituto Nacional de Salud Peru.	Laboratorio de Referencia Nacional de Biotecnología y Biología Molecular. Centro Nacional de Salud Publica. Instituto Nacional de Salud Peru.	Carlos Padilla Rojas, Karolyn Vega Chozo, Priscila Lope Pari, Omar Caceres Rey, Marco Galarza Perez, Maribel Huaringa Nuñez, Johanna Balbuena Torres, Henri Bailon Calderon, Nancy Rojas Serrano.
EPI_ISL_527856	Hospital Municipal Prof. Waldomiro de Paula	Instituto Adolfo Lutz, Interdiciplinary Procedures Center, Strategic Laboratory	Claudio Tavares Sacchi, Claudia Regina Gonçalves, Erica Valessa Ramos Gomes
EPI_ISL_527857	Hospital Regional Vale do Ribeira	Instituto Adolfo Lutz, Interdiciplinary Procedures Center, Strategic Laboratory	Claudio Tavares Sacchi, Claudia Regina Gonçalves, Erica Valessa Ramos Gomes
EPI_ISL_527860	Hospital Municipal de Parelheiros Josanias Castanha Braga	Instituto Adolfo Lutz, Interdiciplinary Procedures Center, Strategic Laboratory	Claudio Tavares Sacchi, Claudia Regina Gonçalves, Erica Valessa Ramos Gomes
EPI_ISL_527868	Hospital e Maternidade do Braz	Instituto Adolfo Lutz, Interdiciplinary Procedures Center, Strategic Laboratory	Claudio Tavares Sacchi, Claudia Regina Gonçalves, Erica Valessa Ramos Gomes
EPI_ISL_528388, EPI_ISL_528389, EPI_ISL_528396, EPI_ISL_528397, EPI_ISL_528398, EPI_ISL_528399, EPI_ISL_528400, EPI_ISL_528401, EPI_ISL_528402, EPI_ISL_528403, EPI_ISL_528404, EPI_ISL_528406, EPI_ISL_528430			
see above	Respiratory Virus Unit, Microbiology Services Colindale, Public Health England	Respiratory Virus Unit, Microbiology Services Colindale, Public Health England	PHE Covid Sequencing Team
EPI_ISL_528607, EPI_ISL_528608	National Genomics Core-Center for DNA Fingerprinting and Diagnostics	National Genomics Core- Center for DNA Fingerprinting and Diagnostics (NGC-CDFD)- DBT's PAN-INDIA-1000 Genome consortium	Ashwin Dalal, Bala Pratyusha, Heena Shah, G Shashikanth, Vinay Donipadi, K.Manohar, Madhumohan Rao,Neeraj Kumar, Niteen Pathak, Pradipta Hore, Rahul Baroi, Sayantan Goswami, Shaffiqu T S, Shalini Aricthota, Sobhan Babu, R Harinarayanan, Rashna Bhandari, Murali Dharan Bashyam, Debashish Mitra, Divya Vashisht
EPI_ISL_528609, EPI_ISL_528610	National Genomics Core-Center for DNA Fingerprinting and Diagnostics	National Genomics Core- Center for DNA Fingerprinting and Diagnostics (NGC-CDFD)- DBT's PAN-INDIA-1000 Genome consortium	Divya Vashisht, Bala Pratyusha, Heena Shah, G Shashikanth, Vinay Donipadi, K.Manohar, Madhumohan Rao, SPR Prasad, Yogesh Patidar, Arjita Jaiswal, Arpita Singh, Devanshi Gupta, Romila Moirangthem, Sanjana Sarkar, Shivani Yadav, R Harinarayanan, Rashna Bhandari, Murali Dharan Bashyam, Debashish Mitra, Ashwin Dalal

EPI_ISL_528950	Respiratory Virus Unit, Microbiology Services Colindale, Public Health England	Respiratory Virus Unit, Microbiology Services Colindale, Public Health England	PHE Covid Sequencing Team
EPI_ISL_529018	Ospedale "Giuseppe Mazzini"-Teramo	Istituto Zooprofilattico Sperimentale dell'Abruzzo e Molise "G.Caporale"	Lorusso A, Marcacci M, Di Domenico M, Curini V, Ancora M, Cammà C, Rinaldi A, Mangone I, Di Pasquale A, Puglia I, Savini G.
EPI_ISL_529035	Wadsworth Center, New York State Department of Health	Wadsworth Center, New York State Department of Health	Kirsten St. George, Daryl M. Lamson, Sara Griesemer, Jonathan Plitnick, Navjot Singh, Matthew D. Shudt, Erica Lasek-Nesselquist
EPI_ISL_529152	Department of Immunology, The Scripps Research Institute	Andersen lab at Scripps Research	Quigley, M., Stefanski, E., Mchardy, I. with SEARCH Alliance San Diego
EPI_ISL_529177	South Carolina Department of Health and Environmental Control	South Carolina Department of Health and Environmental Control	Haley V. Flores
EPI_ISL_529206, EPI_ISL_529207	Utah Public Health Laboratory	Utah Public Health Laboratory	Erin Young, Kelly Oakeson
EPI_ISL_529272, EPI_ISL_529344, EPI_ISL_529345, EPI_ISL_529346, EPI_ISL_529347, EPI_ISL_529348, EPI_ISL_529349, EPI_ISL_529350, EPI_ISL_529351, EPI_ISL_529352, EPI_ISL_529373, EPI_ISL_529374, EPI_ISL_529375, EPI_ISL_529431, EPI_ISL_529432, EPI_ISL_529465, EPI_ISL_529481, EPI_ISL_529511, EPI_ISL_529525, EPI_ISL_529672, EPI_ISL_529673			
see above	Centre for Enzyme Innovation, University of Portsmouth / Translational Research Laboratory, Portsmouth Hospitals NHS Trust	COVID-19 Genomics UK (COG-UK) Consortium	Angela Beckett,Yann Bourgeois,Garry Scarlett,Sharon Glaysher,Scott Elliott,Kelly Bicknell,Robert Impey,Allyson Lloyd,Sarah Wyllie,Ethan Butcher,Anoop Chauhan,Samuel Robson
EPI_ISL_529822	Michigan Department of Health and Human Services, Bureau of Laboratories	Michigan Department of Health and Human Services, Bureau of Laboratories	Blankenship HM, Riner D, Soehnlen MK
EPI_ISL_529986, EPI_ISL_529987, EPI_ISL_529988	Hospital Universitario 12 de Octubre	Hospital Universitario 12 de Octubre	Raúl Recio, Sara González, Esther Viedma, Elias Dahdouh, Fernando Lázaro, Natalia Stella, Julio García, Juan Carlos Galán, Rafael Cantón, Mª Dolores Folgueira, Rafael Delgado, Jesús Mingorance
EPI_ISL_529996, EPI_ISL_529997, EPI_ISL_530003, EPI_ISL_530004	Hospital Universitario 12 de Octubre	Hospital Universitario 12 de Octubre	Sara González, Esther Viedma, Raúl Recio, Elias Dahdouh, Fernando Lázaro, Natalia Stella, Julio García, Juan Carlos Galán, Rafael Cantón, Mª Dolores Folgueira, Rafael Delgado, Jesús Mingorance
EPI_ISL_530007, EPI_ISL_530008, EPI_ISL_530009, EPI_ISL_530010, EPI_ISL_530011, EPI_ISL_530012, EPI_ISL_530013, EPI_ISL_530014, EPI_ISL_530015, EPI_ISL_530016, EPI_ISL_530017, EPI_ISL_530018, EPI_ISL_530019, EPI_ISL_530020, EPI_ISL_530021, EPI_ISL_530022, EPI_ISL_530023			
see above	Hospital Universitario 12 de Octubre	Hospital Universitario 12 de Octubre	Esther Viedma, Raúl Recio, Sara González, Elias Dahdouh, Fernando Lázaro, Natalia Stella, Julio García, Juan Carlos Galán, Rafael Cantón, Mª Dolores Folgueira, Rafael Delgado, Jesús Mingorance
EPI_ISL_530066, EPI_ISL_530067, EPI_ISL_530068, EPI_ISL_530069	Hospital Universitario La Paz	Hospital Universitario La Paz	Elias Dahdouh, Sara González, Raúl Recio, Fernando Lázaro, Esther Viedma, Natalia Stella, Julio García, Juan Carlos Galán, Rafael Cantón, Mª Dolores Folgueira, Rafael Delgado, Jesús Mingorance
EPI_ISL_530105, EPI_ISL_530106, EPI_ISL_530107, EPI_ISL_530111	Hospital Universitario Ramón y Cajal	Hospital Universitario La Paz	Raúl Recio, Sara González, Elias Dahdouh, Fernando Lázaro, Esther Viedma, Natalia Stella, Julio García, Juan Carlos Galán, Rafael Cantón, Mª Dolores Folgueira, Rafael Delgado, Jesús Mingorance
EPI_ISL_530238, EPI_ISL_530277	Queensland Health Forensic and Scientific Services, Public Health Virology	Public Health Virology Laboratory, Forensic and Scientific Services, Queensland Health	Son Nguyen et al
EPI_ISL_534329	Hospital Universitario 12 de Octubre	Hospital Universitario 12 de Octubre	Esther Viedma, Raúl Recio, Sara González, Elias Dahdouh, Fernando Lázaro, Natalia Stella, Julio García, Juan Carlos Galán, Rafael Cantón, Ma Dolores Folgueira, Rafael Delgado, Jesús Mingorance
EPI_ISL_534763, EPI_ISL_534764, EPI_ISL_534765, EPI_ISL_534766, EPI_ISL_534767, EPI_ISL_534768, EPI_ISL_534771, EPI_ISL_534772, EPI_ISL_534777, EPI_ISL_534778, EPI_ISL_534779, EPI_ISL_534781, EPI_ISL_534785, EPI_ISL_534786, EPI_ISL_534792, EPI_ISL_534793, EPI_ISL_534802, EPI_ISL_534803, EPI_ISL_534806, EPI_ISL_534808, EPI_ISL_534809, EPI_ISL_534810, EPI_ISL_534814, EPI_ISL_534828, EPI_ISL_534834, EPI_ISL_534895, EPI_ISL_535022, EPI_ISL_535023			
see above	Oxford Viromics, NDM, University of Oxford; Oxford University Hospitals; Basingstoke and North Hampshire Hospital	COVID-19 Genomics UK (COG-UK) Consortium	Tanya Golubchik, David Bonsall, George Macintyre, Amy Trebes, Mariateresa de Cesare, Catrin Moore, Alex Mobbs, Anita Justice, Robert Shaw, Monique Andersson, Timothy Peto, Emma Wise, Nathan Moore, Jessica Lynch, Nick Cortes, Matilde Mori, Stephen Kidd, David Buck, John Todd, Christophe Fraser
EPI_ISL_535027, EPI_ISL_535028, EPI_ISL_535029, EPI_ISL_535030, EPI_ISL_535031, EPI_ISL_535032, EPI_ISL_535033, EPI_ISL_535034, EPI_ISL_535035, EPI_ISL_535036, EPI_ISL_535037, EPI_ISL_535038			
see above	Centre for Enzyme Innovation, University of Portsmouth / Translational Research Laboratory, Portsmouth Hospitals NHS Trust	COVID-19 Genomics UK (COG-UK) Consortium	Angela Beckett,Yann Bourgeois,Garry Scarlett,Sharon Glaysher,Scott Elliott,Kelly Bicknell,Robert Impey,Allyson Lloyd,Sarah Wyllie,Ethan Butcher,Anoop Chauhan,Samuel Robson
EPI_ISL_536398	Lithuanian University of Health Sciences Hospital, Department of Laboratory Medicine	Lithuanian University of Health Sciences, Molecular cardiology lab.	Lukas Zemaitis, Arnoldas Pautienius, Kamile Tamusauskaite, Dovydas Gecys, Vaiva Lesauskaite, Astra Vitkauskiene
EPI_ISL_536415, EPI_ISL_536416, EPI_ISL_536422	National Public Health Laboratory, National Centre for Infectious Diseases	National Public Health Laboratory, National Centre for Infectious Diseases	Mak TM, Octavia S, Zhou Z, Cui L, Lin RTP
EPI_ISL_537327, EPI_ISL_537328, EPI_ISL_537329, EPI_ISL_537330, EPI_ISL_537331, EPI_ISL_537332, EPI_ISL_537333, EPI_ISL_537334, EPI_ISL_537335, EPI_ISL_537336, EPI_ISL_537337, EPI_ISL_537338, EPI_ISL_537339, EPI_ISL_537340, EPI_ISL_537341, EPI_ISL_537342, EPI_ISL_537343, EPI_ISL_537344, EPI_ISL_537345, EPI_ISL_537346, EPI_ISL_537347, EPI_ISL_537348, EPI_ISL_537349, EPI_ISL_537350, EPI_ISL_537351, EPI_ISL_537352, EPI_ISL_537353, EPI_ISL_537354, EPI_ISL_537355, EPI_ISL_537356, EPI_ISL_537357, EPI_ISL_537358, EPI_ISL_537359, EPI_ISL_537360, EPI_ISL_537361, EPI_ISL_537362, EPI_ISL_537363, EPI_ISL_537364, EPI_ISL_537365, EPI_ISL_537366, EPI_ISL_537367, EPI_ISL_537368, EPI_ISL_537369, EPI_ISL_537370, EPI_ISL_537371, EPI_ISL_537372, EPI_ISL_537373, EPI_ISL_537374, EPI_ISL_537375, EPI_ISL_537376, EPI_ISL_537377, EPI_ISL_537378, EPI_ISL_537379			
see above	Universidad de León	SeqCOVID-SPAIN consortium/IBV(CSIC)	Ana Carvajal, Vicente Martín, Héctor Argüello, Juan M. Fregeneda, Tania Fernández-Villa, Antonio J. Molina and SeqCOVID-SPAIN consortium
EPI_ISL_537468, EPI_ISL_537470, EPI_ISL_537514, EPI_ISL_537515, EPI_ISL_537533, EPI_ISL_537562, EPI_ISL_537570, EPI_ISL_537579, EPI_ISL_537585, EPI_ISL_537594, EPI_ISL_537600, EPI_ISL_537605			
see above	UCLA Pathology Clinical Microbiology Lab	Kruglyak Lab	Guo et al.
EPI_ISL_537609, EPI_ISL_537610, EPI_ISL_537611, EPI_ISL_537612, EPI_ISL_537613, EPI_ISL_537614, EPI_ISL_537615, EPI_ISL_537616, EPI_ISL_537617	Universidad de León	SeqCOVID-SPAIN consortium/IBV(CSIC)	Ana Carvajal, Vicente Martín, Héctor Argüello, Juan M. Fregeneda, Tania Fernández-Villa, Antonio J. Molina and SeqCOVID-SPAIN consortium
EPI_ISL_538129, EPI_ISL_538130, EPI_ISL_538131, EPI_ISL_538133, EPI_ISL_538601	Servicio de Microbiología y Parasitología clínica. UCEIMP. Hospital Universitario Virgen del Rocío/IBIS/CSIC/US	SeqCOVID-SPAIN consortium/IBV(CSIC)	Guillermo Martín Gutiérrez, Ángel Rodríguez Villodres, Lidia Gálvez Benitez, Verónica González Galán, Javier Aznar Martín and SeqCOVID-SPAIN consortium
EPI_ISL_538751, EPI_ISL_538754, EPI_ISL_538794, EPI_ISL_538798, EPI_ISL_538812, EPI_ISL_538821, EPI_ISL_538831, EPI_ISL_538837, EPI_ISL_538848, EPI_ISL_538871, EPI_ISL_538879, EPI_ISL_538891, EPI_ISL_538907, EPI_ISL_538930, EPI_ISL_538936, EPI_ISL_538946, EPI_ISL_538953, EPI_ISL_538960, EPI_ISL_539122			
see above	Leeds Teaching Hospitals NHS Trust and Public Health England, National Infection Service (Leeds laboratory)	Wellcome Sanger Institute for the COVID-19 Genomics UK (COG-UK) consortium	Louissa Macfarlane-Smith, Holli Carden, Katherine L. Harper, Antony Hale and Alex Alderton, Roberto Amato, Sonia Goncalves, Ewan Harrison, David K. Jackson, Ian Johnston, Dominic Kwiatkowski, Cordelia Langford, John Sillitoe on behalf of the Wellcome Sanger Institute COVID-19 Surveillance Team
EPI_ISL_539795, EPI_ISL_539796	Wyoming Public Health Laboratory	Wyoming Public Health Laboratory	Noah Hull, Rob Christensen, Jim Mildenberger, Joel Sevinsky, Cari Sloma, and Wanda Manley
EPI_ISL_539873	Center for Microbiology and Cell Biology, Instituto Venezolano de Investigaciones Científicas (CMBC, IVIC)	Center for Microbiology and Cell Biology, Instituto Venezolano de Investigaciones Científicas (CMBC, IVIC)	Loureiro,C.L., Jaspe,R.C., D'Angelo,P., Garzaro,D., Rodriguez,L., Alarcon,V., Delgado,M., Aguilar,M., Rangel,H.R., Pujol,F.H.
EPI_ISL_540437	WI State Laboratory of Hygiene	Pathogen Discovery, Respiratory Viruses Branch, Division of Viral Diseases, Centers for Disease Control and Prevention	Yan Li, Jing Zhang, Anna Montmayeur, Krista Queen,Ying Tao, Anna Uehara, Clinton R. Paden, Rachel Marine, Haibin Wang, Suxiang Tong
EPI_ISL_540439	WVDHHR - Office of Laboratory Services	Pathogen Discovery, Respiratory Viruses Branch, Division of Viral Diseases, Centers for Disease Control and Prevention	Ying Tao, Yan Li, Jing Zhang, Krista Queen, Anna Uehara, Clinton R. Paden, Haibin Wang, Suxiang Tong
EPI_ISL_540441	NC State Laboratory of Public Health	Pathogen Discovery, Respiratory Viruses Branch, Division of Viral Diseases, Centers for Disease Control and Prevention	Yan Li, Jing Zhang, Anna Montmayeur, Krista Queen,Ying Tao, Anna Uehara, Clinton R. Paden, Rachel Marine, Haibin Wang, Suxiang Tong

EPI_ISL_541055	Hospital Clínico Universitario de Santiago de Compostela	SeqCOVID-SPAIN consortium/Institute of Biomedicine of Valencia, IBV-CSIC	José Javier Costa Alcalde, Antonio Aguilera Guirao, Mª Luisa Pérez del Molino Bernal, Amparo Coira Nieto, Gema Barbeito Castiñeiras, Rocio Trastoy Pena and SeqCOVID-SPAIN consortium
EPI_ISL_541146, EPI_ISL_541147, EPI_ISL_541148, EPI_ISL_541149, EPI_ISL_541150, EPI_ISL_541151, EPI_ISL_541153, EPI_ISL_541154, EPI_ISL_541241	Florida Bureau of Public Health Laboratories, Florida Department of Health	Florida Bureau of Public Health Laboratories, Florida Department of Health	Schmedes,S., Blanton,J.
EPI_ISL_541352, EPI_ISL_541353, EPI_ISL_541355, EPI_ISL_541356	Laboratory of Respiratory Viruses and Measles, Oswaldo Cruz Institute, FIOCRUZ	Laboratory of Respiratory Viruses and Measles, Oswaldo Cruz Institute, FIOCRUZ	Paola Resende, Luciana Appolinario, Fernando Motta, Anna Carolina Paixão, Ana Carolina Mendonça, Jonathan Lopes, Marilda Siqueira
EPI_ISL_541682, EPI_ISL_541683	National Institute of Virology, NIV Influenza	National Institute of Virology, NIV Influenza	Potdar V
EPI_ISL_541754, EPI_ISL_541757, EPI_ISL_541759, EPI_ISL_541776, EPI_ISL_541778, EPI_ISL_541780	Barts Health NHS Trust	Wellcome Sanger Institute for the COVID-19 Genomics UK (COG-UK) consortium	Teresa Cutino-Moguel, Mark Hopkins, Beatrix Kele, David Harrington and Alex Alderton, Roberto Amato, Sonia Goncalves, Ewan Harrison, David K. Jackson, Ian Johnston, Dominic Kwiatkowski, Cordelia Langford, John Sillitoe on behalf of the Wellcome Sanger Institute COVID-19 Surveillance Team
EPI_ISL_541847, EPI_ISL_541871, EPI_ISL_541872, EPI_ISL_541873, EPI_ISL_541874, EPI_ISL_541875, EPI_ISL_541876, EPI_ISL_541877	Lithuanian University of Health Sciences Hospital, Department of Laboratory Medicine	Lithuanian University of Health Sciences, Laboratory of Molecular Cardiology	Lukas Zemaitis, Arnoldas Pautienius, Kamile Tamusauskaite, Dovydas Gecys, Laura Pareckaite, Vaiva Lesauskaite, Astra Vitkauskiene
EPI_ISL_541880, EPI_ISL_541881	Hospital General Universitario Gregorio Marañón	SeqCOVID-SPAIN consortium/IBV(CSIC)	Laura Pérez-Lago, Marta Herranz, Jon Sicilia, Julia Suárez, Pilar Catalán, Patricia Muñoz, Darío García de Viedma and SeqCOVID-SPAIN consortium
EPI_ISL_542491, EPI_ISL_542493, EPI_ISL_542496, EPI_ISL_542516, EPI_ISL_542525, EPI_ISL_542526, EPI_ISL_542532, EPI_ISL_542537, EPI_ISL_542541, EPI_ISL_542544, EPI_ISL_542550, EPI_ISL_542551, EPI_ISL_542555, EPI_ISL_542558, EPI_ISL_542559, EPI_ISL_542560, EPI_ISL_542561, EPI_ISL_542564, EPI_ISL_542565, EPI_ISL_542566, EPI_ISL_542574, EPI_ISL_542579, EPI_ISL_542580, EPI_ISL_542584, EPI_ISL_542586, EPI_ISL_542591, EPI_ISL_542595, EPI_ISL_542602, EPI_ISL_542607, EPI_ISL_542614, EPI_ISL_542619, EPI_ISL_542620, EPI_ISL_542622, EPI_ISL_542623, EPI_ISL_542624, EPI_ISL_542625, EPI_ISL_542626, EPI_ISL_542627, EPI_ISL_542628, EPI_ISL_542629, EPI_ISL_542630, EPI_ISL_542633, EPI_ISL_542634, EPI_ISL_542637, EPI_ISL_542638, EPI_ISL_542642, EPI_ISL_542643, EPI_ISL_542644, EPI_ISL_542645, EPI_ISL_542647, EPI_ISL_542648, EPI_ISL_542649, EPI_ISL_542651, EPI_ISL_542653, EPI_ISL_542654, EPI_ISL_542655, EPI_ISL_542656, EPI_ISL_542659, EPI_ISL_542663, EPI_ISL_542665, EPI_ISL_542667, EPI_ISL_542669, EPI_ISL_542672, EPI_ISL_542696, EPI_ISL_542697, EPI_ISL_542698, EPI_ISL_542701, EPI_ISL_542702, EPI_ISL_542705, EPI_ISL_542706, EPI_ISL_542709, EPI_ISL_542724, EPI_ISL_542727, EPI_ISL_542728, EPI_ISL_542732, EPI_ISL_542742, EPI_ISL_542758, EPI_ISL_542779, EPI_ISL_542780, EPI_ISL_542781, EPI_ISL_542782, EPI_ISL_542783, EPI_ISL_542784, EPI_ISL_542785, EPI_ISL_542786, EPI_ISL_542787, EPI_ISL_542788, EPI_ISL_542789, EPI_ISL_542790, EPI_ISL_542794, EPI_ISL_542802, EPI_ISL_542808, EPI_ISL_542878, EPI_ISL_542880, EPI_ISL_542900, EPI_ISL_542901, EPI_ISL_542902, EPI_ISL_542903, EPI_ISL_542904, EPI_ISL_542906, EPI_ISL_542918			
see above	Houston Methodist Hospital	Houston Methodist Hospital	S. Wesley Long, Randall J. Olsen, Paul A. Christensen, David W. Bernard, James J. Davis, Maulik Shukla, Marcus Nguyen, Matthew Ojeda Saavedra, Concepcion C. Cantu, Prasanti Yerramilli, Layne Pruitt, Sishir Subedi, Hung-Che Kuo, Heather Hendrickson, Ghazaleh Eskandari, Hoang A. T. Nguyen, J. Hunter Long, Muthiah Kumaraswami, Jule Goike, Daniel Boutz, Jimmy Gollihar, Jason S. McLellan, Chia-Wei Chou, Kamyab Javanmardi, Ilya J. Finkelstein, and James M. Musser
EPI_ISL_542950	TriCore Reference Laboratories	Center for Global Health, University of New Mexico Health Sciences Center	Daryl Domman, Kurt Schwalm, Twila Kunde, Joseph Hicks, Michael Edwards, Darrell Dinwiddie
EPI_ISL_543863, EPI_ISL_543910, EPI_ISL_543956, EPI_ISL_543957, EPI_ISL_543958, EPI_ISL_543959, EPI_ISL_543960, EPI_ISL_543961, EPI_ISL_543962	Houston Methodist Hospital	Houston Methodist Hospital	S. Wesley Long, Randall J. Olsen, Paul A. Christensen, David W. Bernard, James J. Davis, Maulik Shukla, Marcus Nguyen, Matthew Ojeda Saavedra, Concepcion C. Cantu, Prasanti Yerramilli, Layne Pruitt, Sishir Subedi, Hung-Che Kuo, Heather Hendrickson, Ghazaleh Eskandari, Hoang A. T. Nguyen, J. Hunter Long, Muthiah Kumaraswami, Jule Goike, Daniel Boutz, Jimmy Gollihar, Jason S. McLellan, Chia-Wei Chou, Kamyab Javanmardi, Ilya J. Finkelstein, and James M. Musser
EPI_ISL_545015	South Eastern Area Laboratory Services (SEALS)	NSW Health Pathology - Institute of Clinical Pathology and Medical Research; Westmead Hospital; University of Sydney	CIDM-PH et al.
EPI_ISL_545770, EPI_ISL_545881, EPI_ISL_545893, EPI_ISL_545897, EPI_ISL_545898, EPI_ISL_545899, EPI_ISL_545900, EPI_ISL_545901, EPI_ISL_545902, EPI_ISL_545903, EPI_ISL_545904, EPI_ISL_545905, EPI_ISL_545906, EPI_ISL_545910, EPI_ISL_545913, EPI_ISL_545914, EPI_ISL_545915, EPI_ISL_545916, EPI_ISL_545917, EPI_ISL_545918, EPI_ISL_545919, EPI_ISL_545920, EPI_ISL_545921, EPI_ISL_545922, EPI_ISL_545923, EPI_ISL_545924, EPI_ISL_545925, EPI_ISL_545926, EPI_ISL_545927, EPI_ISL_545928, EPI_ISL_545929, EPI_ISL_545930, EPI_ISL_545931, EPI_ISL_545932, EPI_ISL_546075, EPI_ISL_546280, EPI_ISL_546288, EPI_ISL_546295, EPI_ISL_546305, EPI_ISL_546315, EPI_ISL_546324, EPI_ISL_546334, EPI_ISL_546342, EPI_ISL_546352, EPI_ISL_546368, EPI_ISL_546386, EPI_ISL_546413, EPI_ISL_546439, EPI_ISL_546457, EPI_ISL_546462, EPI_ISL_546471, EPI_ISL_546495, EPI_ISL_546511			
see above	Houston Methodist Hospital	Houston Methodist Hospital	S. Wesley Long, Randall J. Olsen, Paul A. Christensen, David W. Bernard, James J. Davis, Maulik Shukla, Marcus Nguyen, Matthew Ojeda Saavedra, Concepcion C. Cantu, Prasanti Yerramilli, Layne Pruitt, Sishir Subedi, Hung-Che Kuo, Heather Hendrickson, Ghazaleh Eskandari, Hoang A. T. Nguyen, J. Hunter Long, Muthiah Kumaraswami, Jule Goike, Daniel Boutz, Jimmy Gollihar, Jason S. McLellan, Chia-Wei Chou, Kamyab Javanmardi, Ilya J. Finkelstein, and James M. Musser
EPI_ISL_547520, EPI_ISL_547521	Dutch COVID-19 response team	National Institute for Public Health and the Environment (RIVM)	Adam Meijer, Harry Vennema, Jeroen Cremer, Sharon van den Brink, Bas van der Veer, AnneMarie van den Brandt, Florian Zwagemaker, Dennis Schmitz, Chantal Reusken, on behalf of the national COVID-19 response team
EPI_ISL_548244	Faith Laboratory, Immunology Institute, Icahn School of Medicine at Mount Sinai	van Bakel Laboratory, Genetics and Genomics Sciences, Icahn School of Medicine at Mount Sinai	Graham J. Britton, Alice Chen-Liaw, Francesca Cossarini, Alexandra Livanos, Matthew P. Spindler, Tamar Plitt, Joseph Eggers, Ilaria Mogno, Ana S. Gonzalez-Reiche, Sophia Sui, Michael Tankelevich, Lauren Tal Grinspan, Rebekah E. Dixon, Divya Jha, Gustavo Martinez-Delgado, Fatima Amanat, Daisy Hoagland, Benjamin R. tenOever, Marla C. Dubinsky, Miriam Merad, Harm Van Bakel, Florian Krammer, Gerold Bongers, Saurabh Mehandru and Jeremiah J. Faith
EPI_ISL_548249	Halmstad klinisk mikrobiologi	The Public Health Agency of Sweden	Anna-Malin Linde, Maria Lind Karlberg, Mattias Haukland, Reza Advani, Olov Svartstrom, Oskar Karlsson Lindsjo, Sandra Broddesson, Petra Edquist, Mia Brytting, Anna Risberg, Karin Tegmark-Wisell
EPI_ISL_548390, EPI_ISL_548473, EPI_ISL_548480	Ventura County Public Health Lab	Chan-Zuckerberg Biohub	CZB Cliahub Consortium
EPI_ISL_548950, EPI_ISL_548953, EPI_ISL_548954	Max von Pettenkofer Institute, Virology, National Reference Center for Retroviruses, LMU München	Laboratory for Functional Genome Analysis, Dept. Genomics, Gene Center of the LMU Munich	Max Muenchhoff, Stefan Krebs, Alexander Graf, Oliver Keppler, Helmut Blum
EPI_ISL_549019	KWR Watercycle Research Institute	Erasmus Medical Center	Ray Izquierdo-Lara, Goffe Elsinga, Leo Heijnen, Bas B. Oude Munnink, Claudia M. E. Schapendonk, David Nieuwenhuijse, Matthijs Kon, Lu Lu, Frank M. Aarestrup, Samantha Lycett, Gertjan Medema, Marion P.G. Koopmans, Miranda de Graaf
EPI_ISL_549178, EPI_ISL_549179, EPI_ISL_549180	Florida Bureau of Public Health Laboratories	Florida Bureau of Public Health Laboratories	Sarah Schmedes, Jason Blanton
EPI_ISL_559973	Virology Department, Royal Infirmary of Edinburgh, NHS Lothian / School of Biological Sciences, University of Edinburgh / Institute of Genetics and Molecular Medicine, University of Edinburgh	COVID-19 Genomics UK (COG-UK) Consortium	McHugh M, Dewar R, Rooke S, Gallagher M, Balcaza C, O'Toole Á, Scher E, Hill V, McCrone JT, Colquhoun R, Yu X, Jackson B, Rambaut A, Williams TC, Templeton K
EPI_ISL_560335, EPI_ISL_560357	TriCore Reference Laboratories	Center for Global Health, University of New Mexico Health Sciences Center	Daryl Domman, Kurt Schwalm, Twila Kunde, Joseph Hicks, Michael Edwards, Darrell Dinwiddie
EPI_ISL_560388, EPI_ISL_560389	National Health Laboratory	Botswana Institute for Technology Research and Innovation	Kefentse Arnold Tumedi, Madisa Mine, Dineo Emang Tshiamo. Gape Nyepetsi, Thongbotho Mphoyakgosi, Maitshwarelo Ignatius Matsheka
EPI_ISL_560585	Hopital	National Reference Center for Viruses of Respiratory Infections, Institut Pasteur, Paris	Sylvie Behillil, Fabiana Gambaro, Etienne Simon-Lorière, Vincent Enouf, Maud Vanpeene, Sylvie van der Werf
EPI_ISL_560630, EPI_ISL_560631, EPI_ISL_560632	Hospital	National Reference Center for Viruses of Respiratory Infections, Institut Pasteur, Paris	Sylvie Behillil, Fabiana Gambaro, Etienne Simon-Lorière, Vincent Enouf, Maud Vanpeene, Sylvie van der Werf
EPI_ISL_560991	RSUD Dr. Soetomo	Institute of Tropical Disease, Universitas Airlangga	Rima R Prasetya, Krisnoadi Rahardjo, Aldise M Nastri, Jezzy R Dewantari, Joni Wahyuhadi, Gatot Soegiarto, Laksmi Wulandari, Retno A Setyoningrum, Resti Yudhawati, Yohko K Shimizu, Mitsuhiro Nishimura, Yasuko Mori, Soetjipto, Kazufumi Shimizu, Maria I Lusida
EPI_ISL_561428, EPI_ISL_561462,	Victorian Infectious Diseases Reference Laboratory (VIDRL)	VIDRL and MDU-PHL	Caly, L., Seemann, T., Sait, M., Schultz, M. B., Druce J., Sherry, N.

EPI_ISL_561470, EPI_ISL_561893, EPI_ISL_561935, EPI_ISL_561936, EPI_ISL_561946, EPI_ISL_562080, EPI_ISL_562106, EPI_ISL_562216			
EPI_ISL_562579	Microbiological Diagnostic Unit - Public Health Laboratory (MDU-PHL)	MDU-PHL	Seemann, T., Schultz M. B., Sait, M., Sherry, N.
EPI_ISL_562696, EPI_ISL_562697, EPI_ISL_563808, EPI_ISL_563866, EPI_ISL_563925, EPI_ISL_563977	Victorian Infectious Diseases Reference Laboratory (VIDRL)	VIDRL and MDU-PHL	Caly, L., Seemann, T., Sait, M., Schultz, M. B., Druce J., Sherry, N.
EPI_ISL_565835, EPI_ISL_565836, EPI_ISL_565837, EPI_ISL_565838, EPI_ISL_565840, EPI_ISL_565841, EPI_ISL_565878, EPI_ISL_565879, EPI_ISL_565880, EPI_ISL_565881, EPI_ISL_565882, EPI_ISL_565883, EPI_ISL_565884, EPI_ISL_565885, EPI_ISL_565886, EPI_ISL_565887, EPI_ISL_565888, EPI_ISL_565889, EPI_ISL_565926, EPI_ISL_565927, EPI_ISL_565933, EPI_ISL_565935, EPI_ISL_565936, EPI_ISL_565940, EPI_ISL_565941, EPI_ISL_565942, EPI_ISL_565943, EPI_ISL_565944, EPI_ISL_565945, EPI_ISL_565946, EPI_ISL_565947, EPI_ISL_565949, EPI_ISL_565950, EPI_ISL_565964, EPI_ISL_565970, EPI_ISL_565977, EPI_ISL_566010			
see above	Michigan Department of Health and Human Services, Bureau of Laboratories	Michigan Department of Health and Human Services, Bureau of Laboratories	Blankenship HM, Riner D, Soehnlen MK
EPI_ISL_568561, EPI_ISL_568562	Department of Infectious Diseases and Immunology, National Hospital Organization Nagoya Medical Center	Clinical Research Center, National Hospital Organization Nagoya Medical Center	Yoshihiro Nakata, Hirotaka Ode, Mai Kubota, Masakazu Matsuda, Kazuhiro Matsuoka, Nakasuji Miho, Mikiko Mori, Mayumi Imahashi, Yoshiyuki Yokomaku, Yasumasa Iwatani
EPI_ISL_568701, EPI_ISL_568703, EPI_ISL_568704, EPI_ISL_568705, EPI_ISL_568706, EPI_ISL_568707, EPI_ISL_568708	KEMRI-Wellcome Trust Research Programme/KEMRI-CGMR-C Kilifi	KEMRI-Wellcome Trust Research Programme/KEMRI-CGMR-C Kilifi	Githinji et al 2020
EPI_ISL_568898, EPI_ISL_568899, EPI_ISL_568900, EPI_ISL_568901, EPI_ISL_568902, EPI_ISL_568903, EPI_ISL_568904, EPI_ISL_568905, EPI_ISL_568906, EPI_ISL_568907, EPI_ISL_568908, EPI_ISL_568923, EPI_ISL_568924, EPI_ISL_568925, EPI_ISL_568926, EPI_ISL_568927, EPI_ISL_568928, EPI_ISL_568929, EPI_ISL_568930, EPI_ISL_568931, EPI_ISL_568932, EPI_ISL_568933, EPI_ISL_568934, EPI_ISL_568935, EPI_ISL_568936, EPI_ISL_568937, EPI_ISL_568938, EPI_ISL_568939, EPI_ISL_568940, EPI_ISL_568941, EPI_ISL_568942, EPI_ISL_568943, EPI_ISL_568944, EPI_ISL_568945, EPI_ISL_568946			
see above	MEPHI, Aix Marseille University	MEPHI, Aix Marseille University	Anthony LEVASSEUR
EPI_ISL_569664, EPI_ISL_569665, EPI_ISL_569666	Lee Lab	Lee Lab	Sung Yong Park, Gina Faraci, Pamela M. Ward, Jane F. Emerson, and Ha Youn Lee
EPI_ISL_569841	Omsk Research Institute of Natural Focal Infections	WHO National Influenza Centre Russian Federation	Artem Fadeev, Ekaterina Gradoboeva, Ekaterina Savkina, Daria Nashatyreva, Elena Poleshchuk, Aleksei Vasilenko, Valery Yakimenko, Andrey Komissarov
EPI_ISL_570027, EPI_ISL_570174, EPI_ISL_570176, EPI_ISL_570189, EPI_ISL_570190, EPI_ISL_570191, EPI_ISL_570193, EPI_ISL_570194, EPI_ISL_570970	UW Virology Lab	UW Virology Lab	Pavitra Roychoudhury, Hong Xie, Lasata Shrestha, Amin Addetia, Victoria M Rachleff, Meei-Li Huang, Keith R Jerome, Alexander Greninger
EPI_ISL_572345	LACEN/PE	WallauLab, Aggeu Magalhaes Institute	Marcelo Henrique Santos Paiva, Duschinka Ribeiro Duarte Guedes, Cássia Docena, Matheus Filgueira Bezerra, Filipe Zimmer Dezordi, Laís Ceschini Machado, Larissa Krokovsky, Elisama Helvecio, Alexandre Freitas da Silva, Luydson Richardson Silva Vasconcelos, Antonio Mauro Rezende, Severino Jefferson Ribeiro da Silva, Kamila Gaudêncio da Silva Sales, Bruna Santos Lima Figueiredo de Sá, Derciliano Lopes da Cruz, Claudio Eduardo Cavalcanti, Armando de Menezes Neto, Caroline Targino Alves da Silva, Renata Pessôa Germano Mendes, Maria Almerice Lopes da Silva, Tiago Gräf, Paola Cristina Resende, Gonzalo Bello0, Michelle da Silva Barros, Wheverton Ricardo Correia do Nascimento,, Rodrigo Moraes Loyo Arcoverde, Luciane Caroline Albuquerque Bezerra, Sinval Pinto Brandão Filho, Constância Flávia Junqueira Ayres, Gabriel Luz Wallau
EPI_ISL_574577	Hospital Municipal Dr. Ignacio Proença de Gouvea	Instituto Adolfo Lutz, Interdiciplinary Procedures Center, Strategic Laboratory	Claudio Tavares Sacchi, Claudia Regina Gonçalves, Erica Valessa Ramos Gomes, Karoline Rodrigues Campos
EPI_ISL_574609	RSJPD Harapan Kita	Eijkman Institute for Molecular Biology, Ministry of Research and Technology/National Agency for Research and Innovation	Frilasita A Yudhaputri, Edison Johar, Hidayat Trimarsanto, Iskandar A Adnan, Willy Agustine, David H Muljono, Safarina G Malik, Herawati Sudoyo, Khin Saw Myint, Amin Soebandrio
EPI_ISL_574859, EPI_ISL_574860, EPI_ISL_574861	Institute for Infectious Diseases, University of Bern	Institute for Infectious Diseases, University of Bern	Michel C Koch, Christian Baumann, Miguel A Terrazos Miani, Cora Sägesser, Stephen L Leib, Peter Keller, Franziska Suter-Riniker, Alban Ramette
EPI_ISL_575073, EPI_ISL_575097, EPI_ISL_575115, EPI_ISL_575240, EPI_ISL_575241, EPI_ISL_575242, EPI_ISL_575243, EPI_ISL_575244, EPI_ISL_575245	Utah Public Health Laboratory	Utah Public Health Laboratory	Erin Young, Kelly Oakeson
EPI_ISL_575330	National Institute for Viral Disease Control and Prevention, China CDC	National Institute for Viral Disease Control and Prevention, China CDC	Rongbao Gao, Kang Xiao, Qinqin Song, ZhiQiang Xia, Dong Xia, Juan Song, Haijun Du, Yuan He, Shuai Pang, Xuancheng Lu, Guizhen Wu, Geogia Fu Gao, Jun Han
EPI_ISL_576363, EPI_ISL_576364, EPI_ISL_576365	Utah Public Health Laboratory, Utah Public Health Laboratory Infectious Disease submission group	Utah Public Health Laboratory, Utah Public Health Laboratory Infectious Disease submission group	Young,E.L., Oakeson,K., Sangster,A., Hirschi,B., Butz,H.
EPI_ISL_576498, EPI_ISL_576499, EPI_ISL_576500, EPI_ISL_576501, EPI_ISL_576502, EPI_ISL_576503, EPI_ISL_576504, EPI_ISL_576505, EPI_ISL_576506, EPI_ISL_576507, EPI_ISL_576508, EPI_ISL_576509, EPI_ISL_576510, EPI_ISL_576511, EPI_ISL_576512, EPI_ISL_576513			
see above	UW Virology Lab	UW Virology Lab	Pavitra Roychoudhury, Hong Xie, Lasata Shrestha, Amin Addetia, Victoria M Rachleff, Meei-Li Huang, Keith R Jerome, Alexander Greninger
EPI_ISL_577566	Michigan Department of Health and Human Services, Bureau of Laboratories	Michigan Department of Health and Human Services, Bureau of Laboratories	Blankenship HM, Riner D, Soehnlen MK
EPI_ISL_577598	Utah Public Health Laboratory, Utah Public Health Laboratory Infectious Disease submission group	Utah Public Health Laboratory	Young,E.L., Oakeson,K., Sangster,A., Hirschi,B., Butz,H.
EPI_ISL_577599	Utah Public Health Laboratory, Utah Public Health Laboratory Infectious Disease submission group	Utah Public Health Laboratory, Utah Public Health Laboratory Infectious Disease submission group	Young,E.L., Oakeson,K., Sangster,A., Hirschi,B., Butz,H.
EPI_ISL_577600	Utah Public Health Laboratory, Utah Public Health Laboratory Infectious Disease submission group	Utah Public Health Laboratory	Young,E.L., Oakeson,K., Sangster,A., Hirschi,B., Butz,H.
EPI_ISL_577601	Utah Public Health Laboratory, Utah Public Health Laboratory Infectious Disease submission group	Utah Public Health Laboratory, Utah Public Health Laboratory Infectious Disease submission group	Young,E.L., Oakeson,K., Sangster,A., Hirschi,B., Butz,H.
EPI_ISL_577737	Institute of Virology, Biomedical Research Center of the Slovak Academy of Sciences, Bratislava	Faculty of Natural Sciences, Comenius University, Bratislava	Kristína Boršová, Viktória Hodorová, Broa Brejová, Viktória abanová, Dominika Friová, Sabina Fumaová Havlíková, Juraj Kopáek, Martina Liková, ubomíra Lukáiková, Martina Neboháová, Monika Sláviková, Edita Staroová, Elena Tichá, Tomáš Vina, Boris Klempa, Jozef Nosek
EPI_ISL_578156, EPI_ISL_578157, EPI_ISL_578158, EPI_ISL_578159	University of Michigan Clinical Microbiology Laboratory	Lauring Lab, University of Michigan, Department of Microbiology and Immunology	Valesano
EPI_ISL_578350, EPI_ISL_578351, EPI_ISL_578352, EPI_ISL_578353, EPI_ISL_578354, EPI_ISL_578355, EPI_ISL_578445, EPI_ISL_578446, EPI_ISL_578447, EPI_ISL_578448, EPI_ISL_578449, EPI_ISL_578450, EPI_ISL_578452, EPI_ISL_578453, EPI_ISL_578454, EPI_ISL_578455, EPI_ISL_578456, EPI_ISL_578457, EPI_ISL_578458, EPI_ISL_578459, EPI_ISL_578460, EPI_ISL_578461, EPI_ISL_578462, EPI_ISL_578463, EPI_ISL_578464, EPI_ISL_578465, EPI_ISL_578466, EPI_ISL_578467, EPI_ISL_578468, EPI_ISL_578474, EPI_ISL_578475, EPI_ISL_578476, EPI_ISL_578477, EPI_ISL_578478, EPI_ISL_578479, EPI_ISL_578480, EPI_ISL_578481, EPI_ISL_578482, EPI_ISL_578483, EPI_ISL_578484, EPI_ISL_578486, EPI_ISL_578487, EPI_ISL_578492, EPI_ISL_578493, EPI_ISL_578499, EPI_ISL_578500, EPI_ISL_578504, EPI_ISL_578505, EPI_ISL_578507, EPI_ISL_578546			
see above	Wisconsin State Laboratory of Hygiene Communicable	Wisconsin State Laboratory of Hygiene Communicable	Kelsey R. Florek, Abigail C. Shockey

	Disease Division	Disease Division	
EPI_ISL_579420	Wellington SCL (WN)	Institute of Environmental Science and Research (ESR)	Xiaoyun Ren, Matt Storey, Nikki Freed, Muhammad Faisal, Jing Wang, Hermes Perez, Anja Werno, Antje van der Linden, Arlo Upton, Chris Mansell, David Hammer, Dragana Drinkovic, Gary McAuliffe, Hana Sofia Andersson, James Ussher, Jill Sherwood, Josh Freeman, Julia Howard, Juliet Elvy, Mary DeAlmeida, Matt Blakiston, Matthew Rogers, Max Bloomfield, Michael Addidle, Michelle Balm, Sally Roberts, Sarah Jefferies, Sharmini Muttaiyah, Susan Morpeth, Susan Taylor, Timothy Blackmore, Vani Sathyendran, Veronica Playle, Virginia Hope, Erasmus Smit, Lauren Jelly, Olin Silander, Joep de Ligt
EPI_ISL_579502, EPI_ISL_579503, EPI_ISL_579504	Canterbury Health Laboratories	Institute of Environmental Science and Research (ESR)	Xiaoyun Ren, Matt Storey, Nikki Freed, Muhammad Faisal, Jing Wang, Hermes Perez, Anja Werno, Antje van der Linden, Arlo Upton, Chris Mansell, David Hammer, Dragana Drinkovic, Gary McAuliffe, Hana Sofia Andersson, James Ussher, Jill Sherwood, Josh Freeman, Julia Howard, Juliet Elvy, Mary DeAlmeida, Matt Blakiston, Matthew Rogers, Max Bloomfield, Michael Addidle, Michelle Balm, Sally Roberts, Sarah Jefferies, Sharmini Muttaiyah, Susan Morpeth, Susan Taylor, Timothy Blackmore, Vani Sathyendran, Veronica Playle, Virginia Hope, Erasmus Smit, Lauren Jelly, Olin Silander, Joep de Ligt
EPI_ISL_579518, EPI_ISL_579519, EPI_ISL_579520, EPI_ISL_579521, EPI_ISL_579522, EPI_ISL_579523, EPI_ISL_579524, EPI_ISL_579525, EPI_ISL_579526, EPI_ISL_579527, EPI_ISL_579529, EPI_ISL_579530, EPI_ISL_579531, EPI_ISL_579655, EPI_ISL_579656, EPI_ISL_579662, EPI_ISL_579663, EPI_ISL_579665, EPI_ISL_579721, EPI_ISL_579722, EPI_ISL_579723, EPI_ISL_579724, EPI_ISL_579725, EPI_ISL_579726, EPI_ISL_579727, EPI_ISL_579728, EPI_ISL_579729, EPI_ISL_579730, EPI_ISL_579731, EPI_ISL_579732, EPI_ISL_579733, EPI_ISL_579734, EPI_ISL_579735, EPI_ISL_579736, EPI_ISL_579737, EPI_ISL_579738, EPI_ISL_579739, EPI_ISL_579740, EPI_ISL_579741, EPI_ISL_579742, EPI_ISL_579743, EPI_ISL_579744, EPI_ISL_579745, EPI_ISL_579746, EPI_ISL_579747, EPI_ISL_579748, EPI_ISL_579749, EPI_ISL_579750, EPI_ISL_579751, EPI_ISL_579752, EPI_ISL_579753, EPI_ISL_579754, EPI_ISL_579755, EPI_ISL_579756, EPI_ISL_579757, EPI_ISL_579758			
see above	QEII Health Sciences Centre	National Microbiology Laboratory (NML)	Anna Majer, Shari Tyson, Grace Seo, Philip Mabon, Darian Hole, Elsie Grudeski, Rhiannon Huzarewich, Russell Mandes, Anneliese Landgraff, Jennifer Tanner, Natalie Knox, Morag Graham, Gary Van Domselaar, Todd Hatchette, Jason LeBlanc, Nathalie Bastien, Yan Li, Timothy Booth, CanCOGeN's metadata curation team, Public Health Agency of Canada's CanCOGeN team
EPI_ISL_581465	Medizinische Klinik Innere Medizin I, Universitätsklinikum Tübingen	NGS Competence Center Tübingen, Institut für Medizinische Mikrobiologie und Hygiene, Universitätsklinikum Tübingen	Angel Angelov
EPI_ISL_581699, EPI_ISL_581700, EPI_ISL_581701, EPI_ISL_581702, EPI_ISL_581703, EPI_ISL_581704, EPI_ISL_581705, EPI_ISL_581706, EPI_ISL_581707, EPI_ISL_581708, EPI_ISL_581709, EPI_ISL_581710, EPI_ISL_581711, EPI_ISL_581712, EPI_ISL_581713, EPI_ISL_581714			
see above	University Hospital Basel, Clinical Virology	University Hospital Basel, Clinical Bacteriology	Madlen Stange, Alfredo Mari, Tim Roloff, Helena MB Seth-Smith, Michael Schweitzer, Myrta Brunner, Karoline Leuzinger, Kirstine K. Soegaard, Alexander Gensch, Sarah Tschudin-Sutter, Simon Fuchs, Julia Bielicki, Hans Pargger, Martin Siegemund, Christian Nickel, Roland Bingisser, Michael Osthoff, Stefano Bassetti, Rita Schneider-Sliwa, Manuel Battegay, Hans Hirsch, Adrian Egli
EPI_ISL_582206, EPI_ISL_582209	TriCore Reference Laboratories	Center for Global Health, University of New Mexico Health Sciences Center	Daryl Domman, Kurt Schwalm, Twila Kunde, Joseph Hicks, Michael Edwards, Darrell Dinwiddie
EPI_ISL_582318, EPI_ISL_582355, EPI_ISL_582362, EPI_ISL_582388, EPI_ISL_582407	Cadham Provincial Laboratory	National Microbiology Laboratory (NML)	Anna Majer, Shari Tyson, Grace Seo, Philip Mabon, Elsie Grudeski, Rhiannon Huzarewich, Russell Mandes, Anneliese Landgraff, Jennifer Tanner, Natalie Knox, Morag Graham, Gary Van Domselaar, Paul Van Caeseele, Jared Bullard, David Alexander, Kerry Dust, Nathalie Bastien, Yan Li, Timothy Booth, Darian Hole, Madison Chapel, CanCOGeN's metadata curation team, Public Health Agency of Canada CanCOGeN team
EPI_ISL_582515	unknown	Instituto Nacional de Saude (INSA) and Instituto Gulbenkian de Ciencia (IGC)	Borges et al
EPI_ISL_582528, EPI_ISL_582529	Veterinary Specialized Institute "Kraljevo", Serbia	Veterinary Specialized Institute "Kraljevo", Serbia	Vidanovic,D., Tesovic,B., Knezevic,A., Jovanovic,T., Jankovic,M., Sekler,M., Banovic Djeri,B., Petrovic,T., Volkening,J., Afonso,C.
EPI_ISL_582629, EPI_ISL_582630, EPI_ISL_582631, EPI_ISL_582632, EPI_ISL_582633, EPI_ISL_582634	Sheikh Khalifa Medical City	Molecular/Surveillance lab Sheikh Khalifa Medical City	Amirtharaj Francis, Sajeed Abdul, Hala Imambaccus, Sahar Almarzooqi, Hiba Saud, Stefan Weber
EPI_ISL_583426	Garcia-Sastre Laboratory, Department of Microbiology, Icahn School of Medicine at Mount Sinai	van Bakel Laboratory, Genetics and Genomics Sciences, Icahn School of Medicine at Mount Sinai	Teresa Aydillo, Ana S. Gonzalez-Reiche, Sadaf Aslam, Adriana van de Guchte, Zenab Khan, Ajay Obla, Jayeeta Dutta, Harm van Bakel, Judith Aberg, Adolfo García-Sastre, Gunjan Shah, Tobias Hohl, Genovefa Papanicolaou, Miguel-Angel Perales, Kent Sepkowitz, Ngolela Esther Babady, and Mini Kamboj
EPI_ISL_583427, EPI_ISL_583433	Memorial Sloan Kettering Cancer Center	van Bakel Laboratory, Genetics and Genomics Sciences, Icahn School of Medicine at Mount Sinai	Teresa Aydillo, Ana S. Gonzalez-Reiche, Sadaf Aslam, Adriana van de Guchte, Zenab Khan, Ajay Obla, Jayeeta Dutta, Harm van Bakel, Judith Aberg, Adolfo García-Sastre, Gunjan Shah, Tobias Hohl, Genovefa Papanicolaou, Miguel-Angel Perales, Kent Sepkowitz, Ngolela Esther Babady, and Mini Kamboj
EPI_ISL_583436	Garcia-Sastre Laboratory, Department of Microbiology, Icahn School of Medicine at Mount Sinai	van Bakel Laboratory, Genetics and Genomics Sciences, Icahn School of Medicine at Mount Sinai	Teresa Aydillo, Ana S. Gonzalez-Reiche, Sadaf Aslam, Adriana van de Guchte, Zenab Khan, Ajay Obla, Jayeeta Dutta, Harm van Bakel, Judith Aberg, Adolfo García-Sastre, Gunjan Shah, Tobias Hohl, Genovefa Papanicolaou, Miguel-Angel Perales, Kent Sepkowitz, Ngolela Esther Babady, and Mini Kamboj
EPI_ISL_583439	Memorial Sloan Kettering Cancer Center	van Bakel Laboratory, Genetics and Genomics Sciences, Icahn School of Medicine at Mount Sinai	Teresa Aydillo, Ana S. Gonzalez-Reiche, Sadaf Aslam, Adriana van de Guchte, Zenab Khan, Ajay Obla, Jayeeta Dutta, Harm van Bakel, Judith Aberg, Adolfo García-Sastre, Gunjan Shah, Tobias Hohl, Genovefa Papanicolaou, Miguel-Angel Perales, Kent Sepkowitz, Ngolela Esther Babady, and Mini Kamboj
EPI_ISL_583441	Garcia-Sastre Laboratory, Department of Microbiology, Icahn School of Medicine at Mount Sinai	van Bakel Laboratory, Genetics and Genomics Sciences, Icahn School of Medicine at Mount Sinai	Teresa Aydillo, Ana S. Gonzalez-Reiche, Sadaf Aslam, Adriana van de Guchte, Zenab Khan, Ajay Obla, Jayeeta Dutta, Harm van Bakel, Judith Aberg, Adolfo García-Sastre, Gunjan Shah, Tobias Hohl, Genovefa Papanicolaou, Miguel-Angel Perales, Kent Sepkowitz, Ngolela Esther Babady, and Mini Kamboj
EPI_ISL_583468, EPI_ISL_583474	Memorial Sloan Kettering Cancer Center	van Bakel Laboratory, Genetics and Genomics Sciences, Icahn School of Medicine at Mount Sinai	Teresa Aydillo, Ana S. Gonzalez-Reiche, Sadaf Aslam, Adriana van de Guchte, Zenab Khan, Ajay Obla, Jayeeta Dutta, Harm van Bakel, Judith Aberg, Adolfo García-Sastre, Gunjan Shah, Tobias Hohl, Genovefa Papanicolaou, Miguel-Angel Perales, Kent Sepkowitz, Ngolela Esther Babady, and Mini Kamboj
EPI_ISL_583490	Hospital Estadual Sumare	Instituto Adolfo Lutz, Interdiciplinary Procedures Center, Strategic Laboratory	Claudio Tavares Sacchi, Claudia Regina Gonçalves, Erica Valessa Ramos Gomes, Karoline Rodrigues Campos
EPI_ISL_583491	Centro de Saude Esf IV Zona Rual Domingos de SJ Rio Pardo	Instituto Adolfo Lutz, Interdiciplinary Procedures Center, Strategic Laboratory	Claudio Tavares Sacchi, Claudia Regina Gonçalves, Erica Valessa Ramos Gomes, Karoline Rodrigues Campos
EPI_ISL_583492	Santa Casa Anna Cintra	Instituto Adolfo Lutz, Interdiciplinary Procedures Center, Strategic Laboratory	Claudio Tavares Sacchi, Claudia Regina Gonçalves, Erica Valessa Ramos Gomes, Karoline Rodrigues Campos
EPI_ISL_583634, EPI_ISL_583641, EPI_ISL_583642, EPI_ISL_583643, EPI_ISL_583650, EPI_ISL_583651, EPI_ISL_583659, EPI_ISL_583660, EPI_ISL_583661, EPI_ISL_583667, EPI_ISL_583668, EPI_ISL_583672, EPI_ISL_583673, EPI_ISL_583679, EPI_ISL_583680, EPI_ISL_583687, EPI_ISL_583688			
see above	Austrian Agency for Health and Food Safety (AGES)	Bergthaler laboratory, CeMM Research Center for Molecular Medicine of the Austrian Academy of Sciences	Alexandra Popa, Benedikt Agerer, Henrique Colaco, Lukas Endler, Jakob-Wendelin Genger, Alexander Lercher, Mark Smyth, Thomas Penz, Michael Schuster, Jan Laine, Martin Senekowitsch, Judith Aberle, Stephan Aberle, Peter Hufnagl, Daniela Schmid, Franz Allerberger, Elisabeth Puchhammer-Stoeckl, Manfred Nairz, Guenter Weiss, Gregor Hörmann, Kinga Rigler-Hohenwarter, Rainer Gattringer, Wegene Borena, Dorothee von Laer, Gernot Walder, Peter Obrist, Christian Paar, Sabine Sussitz-Rack, Gunther Vogl, Adi Steinrigl, Christoph Bock, Andreas Bergthaler
EPI_ISL_583698, EPI_ISL_583699, EPI_ISL_583700, EPI_ISL_583709, EPI_ISL_583715, EPI_ISL_583724	Center for Virology, Medical University of Vienna	Bergthaler laboratory, CeMM Research Center for Molecular Medicine of the Austrian Academy of Sciences	Alexandra Popa, Benedikt Agerer, Henrique Colaco, Lukas Endler, Jakob-Wendelin Genger, Alexander Lercher, Mark Smyth, Thomas Penz, Michael Schuster, Jan Laine, Martin Senekowitsch, Judith Aberle, Stephan Aberle, Peter Hufnagl, Daniela Schmid, Franz Allerberger, Elisabeth Puchhammer-Stoeckl, Manfred Nairz, Guenter Weiss, Gregor Hörmann, Kinga Rigler-Hohenwarter, Rainer Gattringer, Wegene Borena, Dorothee von Laer, Gernot Walder, Peter Obrist, Christian Paar, Sabine Sussitz-Rack, Gunther Vogl, Adi Steinrigl, Christoph Bock, Andreas Bergthaler
EPI_ISL_583840, EPI_ISL_583841, EPI_ISL_583842, EPI_ISL_583843, EPI_ISL_583844	Dr. Gernot Walder GmbH	Bergthaler laboratory, CeMM Research Center for Molecular Medicine of the Austrian Academy of Sciences	Alexandra Popa, Benedikt Agerer, Henrique Colaco, Lukas Endler, Jakob-Wendelin Genger, Alexander Lercher, Mark Smyth, Thomas Penz, Michael Schuster, Jan Laine, Martin Senekowitsch, Judith Aberle, Stephan Aberle, Peter Hufnagl, Daniela Schmid, Franz Allerberger, Elisabeth Puchhammer-Stoeckl, Manfred Nairz, Guenter Weiss, Gregor Hörmann, Kinga Rigler-Hohenwarter, Rainer Gattringer, Wegene Borena, Dorothee von Laer, Gernot Walder, Peter Obrist, Christian Paar, Sabine Sussitz-Rack, Gunther Vogl, Adi Steinrigl, Christoph Bock, Andreas Bergthaler
EPI_ISL_583855, EPI_ISL_583863, EPI_ISL_583864, EPI_ISL_583865, EPI_ISL_583866, EPI_ISL_583868	Institute for Laboratory Diagnostics and Microbiology, Klinikum Klagenfurt am Worthersee	Bergthaler laboratory, CeMM Research Center for Molecular Medicine of the Austrian Academy of Sciences	Alexandra Popa, Benedikt Agerer, Henrique Colaco, Lukas Endler, Jakob-Wendelin Genger, Alexander Lercher, Mark Smyth, Thomas Penz, Michael Schuster, Jan Laine, Martin Senekowitsch, Judith Aberle, Stephan Aberle, Peter Hufnagl, Daniela Schmid, Franz Allerberger, Elisabeth Puchhammer-Stoeckl, Manfred Nairz, Guenter Weiss, Gregor Hörmann, Kinga Rigler-Hohenwarter, Rainer Gattringer, Wegene Borena, Dorothee von Laer, Gernot Walder, Peter Obrist, Christian Paar, Sabine Sussitz-Rack, Gunther Vogl, Adi Steinrigl, Christoph Bock, Andreas Bergthaler

EPI_ISL_583915	Victoria Hospital	Department of Neurovirology, National Institute of Mental Health and Neuroscience (NIMHANS)	Chitra Pattabiraman,Vijayalakshmi Reddy, Harsha PK, Risha Rasheed, Pramada Prasad, Shafeeq S Hameed, Manjunatha Venkataswamy, Anita Desai, Ravi Vasanthapuram
EPI_ISL_583917	Servicio de Microbiología. Hospital General Universitario de Castellón	SeqCOVID-SPAIN consortium/IBV (CSIC)	Rosario Moreno, María Dolores Tirado and SeqCOVID-SPAIN consortium
EPI_ISL_583918, EPI_ISL_583919, EPI_ISL_583921, EPI_ISL_583922, EPI_ISL_583923, EPI_ISL_583924	Servicio de Microbiología. Hospital General Universitario de Castellón	SeqCOVID-SPAIN consortium/IBV(CSIC)	Rosario Moreno, María Dolores Tirado and SeqCOVID-SPAIN consortium
EPI_ISL_584443, EPI_ISL_584444, EPI_ISL_584445, EPI_ISL_584446, EPI_ISL_584588, EPI_ISL_584589, EPI_ISL_584590	UHCW / University of Warwick	COVID-19 Genomics UK (COG-UK) Consortium	Richard Stark, Chrystala Constantinidou, Meera Unnikrishnan, Laura Baxter, Jeff Cheng, Grace Taylor-Joyce, Hannah Elizabeth Bridgewater, Lucy Frost, Sarojini Pandey, Paul Brown, Tauqeer Alam, Sascha Ott, Dimitris Grammatopoulos
EPI_ISL_584606, EPI_ISL_584607, EPI_ISL_584608, EPI_ISL_584614	Liverpool Clinical Laboratories	COVID-19 Genomics UK (COG-UK) Consortium	Sam Haldenby, Anita Lucaci, Steve Paterson, Julian Hiscox, Alistair Darby, M Almsaud, A Alrezaihi, Muhannad Alruwaili, Stuart D Armstrong, Jones Benjamin, Eleanor G Bentley, Anu Chawla, Jordan J Clark, Angela Cowell, Richard Eccles, Isabel García-Dorival, Matthew Gemmell, Alessandro Gerada, PKF Gilmore, Richard Gregory, Ximeng Han, Catherine Hartley, Margaret Hughes, Miren Iturriza-Gomara, James Johnson, L Luu, Jenifer Manson, Charlotte Nelson, Elaine O'Toole, Cassie Olateju, Rebekah Penrice-Randal , Lucille Rainbow, N.P Randle, Trevor Ian Robinson, Parul Sharma, Ghada T Shawli, James P Stewart, Neil Swainston, Ecaterina Vamos, Joanne Watts, Mark Whitehead
EPI_ISL_586212	Wales Specialist Virology Centre Sequencing lab: Pathogen Genomics Unit	COVID-19 Genomics UK (COG-UK) Consortium	Catherine Moore, Johnathan Evans, Laura Gifford, Malorie Perry, Simon Cottrell, Angela Marchbank, Alec Birchley, Alexander Adams, Amy Gaskin, Bree Gatica-Wilcox, Jason Coombes, Joel Southgate, Lauren Gilbert, Lee Graham, Nicole Pacchiarini, Sara Kumziene-Summerhayes, Sarah Taylor, Sophie Jones, Sara Rey, Matthew Bull, Joanne Watkins, Sally Corden, Tom Connor
EPI_ISL_586272, EPI_ISL_586273, EPI_ISL_586280, EPI_ISL_586281, EPI_ISL_586308, EPI_ISL_586311, EPI_ISL_586328, EPI_ISL_586329, EPI_ISL_586334, EPI_ISL_586480, EPI_ISL_586482, EPI_ISL_586483, EPI_ISL_586484, EPI_ISL_586485, EPI_ISL_586486, EPI_ISL_586487, EPI_ISL_586488, EPI_ISL_586490, EPI_ISL_586491, EPI_ISL_586492, EPI_ISL_586493, EPI_ISL_586494, EPI_ISL_586495, EPI_ISL_586496, EPI_ISL_586498, EPI_ISL_586499, EPI_ISL_586500, EPI_ISL_586501, EPI_ISL_586502, EPI_ISL_586504			
see above	Toronto Invasive Bacterial Diseases Network	McMaster University	Allison McGeer, Patryk Aftanas, Hooman Derakhshani, Angel Li, Kuganya Nirmalarajah, Emily Panousis, Ahmed Draia, Jalees Nasir, Michael Surette, Samira Mubareka, Andrew G. McArthur
EPI_ISL_590749	University of Michigan Clinical Microbiology Laboratory	Lauring Lab, University of Michigan, Department of Microbiology and Immunology	Valesano
EPI_ISL_590777, EPI_ISL_590778, EPI_ISL_590779, EPI_ISL_590780, EPI_ISL_590781, EPI_ISL_590782, EPI_ISL_590783, EPI_ISL_590784, EPI_ISL_590785, EPI_ISL_590786, EPI_ISL_590787, EPI_ISL_590788, EPI_ISL_590789, EPI_ISL_590790, EPI_ISL_590791, EPI_ISL_590792, EPI_ISL_590793, EPI_ISL_590794, EPI_ISL_590795, EPI_ISL_590796, EPI_ISL_590797, EPI_ISL_590798, EPI_ISL_590799, EPI_ISL_590800, EPI_ISL_590801, EPI_ISL_590802, EPI_ISL_590803, EPI_ISL_590804, EPI_ISL_590805, EPI_ISL_590806, EPI_ISL_590807, EPI_ISL_590808, EPI_ISL_590809, EPI_ISL_590810, EPI_ISL_590811, EPI_ISL_590812, EPI_ISL_590813, EPI_ISL_590814, EPI_ISL_590815, EPI_ISL_590816, EPI_ISL_590817, EPI_ISL_590818, EPI_ISL_590819, EPI_ISL_590820, EPI_ISL_590821, EPI_ISL_590822			
see above	Minnesota Department of Health, Public Health Laboratory	Minnesota Department of Health, Public Health Laboratory	Matt Plumb, Jacob Garfin, Alexandra Lorentz, and Xiong Wang
EPI_ISL_590832	Institute of Medical Virology, University of Zurich	Institute of Medical Virology, University of Zurich	Marie O. Pohl, Idoia Busnadiego, Verena Kufner, Stefan Schmutz, Maryam Zaheri, Irene Abela, Alexandra Trkola, Michael Huber, Silke Stertz, Benjamin G. Hale
EPI_ISL_590837	Minnesota Department of Health, Public Health Laboratory	Minnesota Department of Health, Public Health Laboratory	Matt Plumb, Jacob Garfin, Alexandra Lorentz, and Xiong Wang
EPI_ISL_591076, EPI_ISL_591077, EPI_ISL_591078, EPI_ISL_591079, EPI_ISL_591091	Department of Pathology, University of Cambridge	Wellcome Sanger Institute for the COVID-19 Genomics UK (COG-UK) consortium	Luke W Meredith, M. Estée Török , Myra Hosmillo, William L. Hamilton, Martin D. Curran, Theresa Feltwell, Grant Hall, Anna Yakovleva, Fahad A Khokhar, Charlotte J. Houldcroft, Laura G Caller, Aminu S. Jahun, Sarah L. Caddy, Ian Goodfellow; and Alex Alderton, Roberto Amato, Sonia Goncalves, Ewan Harrison, David K. Jackson, Ian Johnston, Dominic Kwiatkowski, Cordelia Langford, John Sillitoe on behalf of the Wellcome Sanger Institute COVID-19 Surveillance Team
EPI_ISL_591179, EPI_ISL_591181, EPI_ISL_591258, EPI_ISL_591259, EPI_ISL_591260, EPI_ISL_591261, EPI_ISL_591262, EPI_ISL_591263, EPI_ISL_591264, EPI_ISL_591265, EPI_ISL_591266, EPI_ISL_591267, EPI_ISL_591268			
see above	Toronto Invasive Bacterial Diseases Network	McMaster University	Allison McGeer, Patryk Aftanas, Hooman Derakhshani, Angel Li, Kuganya Nirmalarajah, Emily Panousis, Ahmed Draia, Jalees Nasir, Michael Surette, Samira Mubareka, Andrew G. McArthur
EPI_ISL_591520	Programa de Virología, Facultad de Medicina UC	Center for Mathematical Modeling and Center for Genome Regulation. Santiago, Chile	Gaggero A, Valiente F, Gaete A, Travisany D, Palma R, Urra C, Varas M, Allende ML, Maass A, González M, Ferres M.
EPI_ISL_591540	St.Johns Hospital	Department of Neurovirology, National Institute of Mental Health and Neuroscience (NIMHANS)	Chitra Pattabiraman,Vijayalakshmi Reddy, Harsha PK, Risha Rasheed, Pramada Prasad, Shafeeq S Hameed, Manjunatha Venkataswamy, Anita Desai, Ravi Vasanthapuram
EPI_ISL_593548	Eastern Ontario Regional Laboratory Association	McMaster University	Leanne Mortimer, Hooman Derakhshani, Emily Panousis, Ahmed Draia, Jalees Nasir, Robert Slinger, Andrew G. McArthur
EPI_ISL_594102, EPI_ISL_594103, EPI_ISL_594104, EPI_ISL_594105, EPI_ISL_594106, EPI_ISL_594107, EPI_ISL_594108, EPI_ISL_594109, EPI_ISL_594110, EPI_ISL_594111, EPI_ISL_594112, EPI_ISL_594113, EPI_ISL_594114, EPI_ISL_594115, EPI_ISL_594116, EPI_ISL_594117			
see above	Utah Public Health Laboratory	Utah Public Health Laboratory	Erin Young, Kelly Oakeson
EPI_ISL_594185	Department of Pathology, School of Medicine, Imam Khomeini Hospital, Tehran University of Medical Sciences	Genetics Research Center, University of Social Welfare and Rehabilitation Sciences	Zohreh Fattahi, Marzieh Mohseni, Khadijeh Jalalvand, Azam Ghaziasadi, Seyedeh elham Mortazavi, Ali Jafarpour, Azar Hadadi, Alireza Abdollahi, Ali Jafarpour, Azam Ghaziasad, Seyedeh elham Mortazavi, Saber Soltani, Reza Najafipour, Kimia Kahrizi, Seyed Mohammad Jazayeri, Hossein Najmabadi
EPI_ISL_594207	Michigan Department of Health and Human Services, Bureau of Laboratories	Michigan Department of Health and Human Services, Bureau of Laboratories	Blankenship HM, Riner D, Soehnlen MK
EPI_ISL_596662	St.Vincent's University Hospital	St.Vincent's University Hospital	Mary Lucey, Guerrino Macori, Niamh Mullane, Una Sutton-Fitzpatrick, Gabriel Gonzalez, Suzie Coughlan, Aisling Purcell, Lynda Fenelon, Séamus Fanning, Kirsten Schaffer
EPI_ISL_596681, EPI_ISL_596704, EPI_ISL_596758, EPI_ISL_596763, EPI_ISL_596765, EPI_ISL_596771, EPI_ISL_596787, EPI_ISL_596873, EPI_ISL_596875, EPI_ISL_596881	PathWest Laboratory Medicine WA	PathWest Laboratory Medicine WA Microbial Surveillance Unit	PathWest Laboratory Medicine WA Microbial Surveillance Unit
EPI_ISL_600443, EPI_ISL_600444, EPI_ISL_600454, EPI_ISL_600484	Institute of Epidemiology Disease Control And Research	Institute for Developing Science and Health Initiatives	Lauren Cowley, Mokibul Hassan Afrad, Sadia Isfat Ara Rahman, Md. Mahfuz-Al-mamun, Firadausi Qadri, Tahmina Shirin
EPI_ISL_602294, EPI_ISL_602295, EPI_ISL_602296, EPI_ISL_602298	Evangelisches Klinikum Bethel, Institut für Laboratoriumsmedizin, Mikrobiologie und Hygiene	Bielefeld University	David Brandt, Tobias Busche, Markus Haak, Jörn Kalinowski, Levin-Joe Klages, Christiane Scherer, Alexander Sczyrba, Marina Simunovic, Svenja Vinke
EPI_ISL_602485	Institute for Virology, University Hospital Essen	Center of Medical Microbiology, Virology, and Hospital Hygiene, University of Duesseldorf	Olympia E. Anastasiou, Ulf Dittmer, Maximilian Damagnez, Alexander Dilthey, Torsten Houwaart, Lisanna Hülse, Malte Kohns Vasconcelos, Nadine Lübke, Jessica Nicolai, Klaus Pfeffer, Daniel Strelow, Jörg Timm, Andreas Walker, Tobias Wienemann
EPI_ISL_602540, EPI_ISL_602546, EPI_ISL_602548, EPI_ISL_602549	Center of Medical Microbiology, Virology, and Hospital Hygiene, University of Duesseldorf	Center of Medical Microbiology, Virology, and Hospital Hygiene, University of Duesseldorf	Maximilian Damagnez, Alexander Dilthey, Torsten Houwaart, Malte Kohns Vasconcelos, Marek Korencak, Nadine Lübke, Jessica Nicolai, Klaus Pfeffer, Hendrik Streeck, Daniel Strelow, Jörg Timm, Andreas Walker, Tobias Wienemann
EPI_ISL_603058	Utah Public Health Laboratory, Utah Public Health Laboratory Infectious Disease submission group	Utah Public Health Laboratory, Utah Public Health Laboratory Infectious Disease submission group	Young,E.L., Oakeson,K.
EPI_ISL_603097, EPI_ISL_603098, EPI_ISL_603099, EPI_ISL_603100, EPI_ISL_603101, EPI_ISL_603102, EPI_ISL_603103, EPI_ISL_603104, EPI_ISL_603105, EPI_ISL_603106, EPI_ISL_603107, EPI_ISL_603108, EPI_ISL_603109, EPI_ISL_603110, EPI_ISL_603111, EPI_ISL_603122			
see above	Lithuanian University of Health Sciences Hospital, Department of Laboratory Medicine	Lithuanian University of Health Sciences, Molecular cardiology lab.	Lukas Zemaitis, Ingrida Olendrait, Arnoldas Pautienius, Kamile Tamusauskaite, Dovydas Gecys, Laura Pareckaite, Vaiva Lesauskaite, Astra Vitkauskiene
EPI_ISL_605862, EPI_ISL_605872	PathWest Laboratory Medicine WA	PathWest Laboratory Medicine WA Microbial Surveillance Unit	PathWest Laboratory Medicine WA Microbial Surveillance Unit

EPI_ISL_613706	Laboratorio Biologia Molecolare Sars Cov2 - UOC Laboratorio Analisi - Servizio Medicina di Laboratorio, Ospedale "San Francesco" - ATS-ASSL Nuoro	Laboratorio specialistico UOC Ematologia - Ospedale "San Francesco" - ATS-ASSL Nuoro	Piras Giovanna, Fancello Tatiana, Asproni Rosanna, Fiamma Maura, Monne Maria Itria, Toja Alessandro, Sanna Filomena, Floris Anna Rita, Sulis Vincenzo, Palmas Angelo Domenico, Casu Gavino, Lo Maglio Iana, Mameli Giuseppe
EPI_ISL_614281	CHRU Pontchaillou - Laboratoire de Virologie 2, rue Henri Le Guilloux	National Reference Center for Viruses of Respiratory Infections, Institut Pasteur, Paris	Marion Barbet, Sylvie Behillil, Méline Bizard, Angela Brisebarre, Camille Capel, Etienne Simon-Lorière, Vincent Enouf, Maud Vanpeene, Sylvie van der Werf, Gisèle Lagathu
EPI_ISL_614406, EPI_ISL_614412, EPI_ISL_614450, EPI_ISL_614711, EPI_ISL_614712, EPI_ISL_614713, EPI_ISL_614714, EPI_ISL_614715, EPI_ISL_614716, EPI_ISL_614717, EPI_ISL_614718, EPI_ISL_614719, EPI_ISL_614720, EPI_ISL_614721, EPI_ISL_614722, EPI_ISL_614723, EPI_ISL_614725, EPI_ISL_618496, EPI_ISL_618511, EPI_ISL_618517, EPI_ISL_618525, EPI_ISL_618595, EPI_ISL_618609, EPI_ISL_618626, EPI_ISL_618627, EPI_ISL_618628, EPI_ISL_618629, EPI_ISL_618630, EPI_ISL_618631, EPI_ISL_618632, EPI_ISL_618633, EPI_ISL_618634, EPI_ISL_618635, EPI_ISL_618636, EPI_ISL_618637, EPI_ISL_618638, EPI_ISL_618639, EPI_ISL_618640, EPI_ISL_618641, EPI_ISL_618642, EPI_ISL_618647, EPI_ISL_618648, EPI_ISL_618649, EPI_ISL_618650, EPI_ISL_618651, EPI_ISL_618652, EPI_ISL_618653, EPI_ISL_618654, EPI_ISL_618655, EPI_ISL_618656, EPI_ISL_618657, EPI_ISL_618658, EPI_ISL_618659, EPI_ISL_618660, EPI_ISL_618661, EPI_ISL_619017, EPI_ISL_619018, EPI_ISL_619272, EPI_ISL_619273, EPI_ISL_619321, EPI_ISL_619328, EPI_ISL_619332, EPI_ISL_619337, EPI_ISL_619344, EPI_ISL_619378, EPI_ISL_619384, EPI_ISL_619389, EPI_ISL_619398, EPI_ISL_619405, EPI_ISL_619840, EPI_ISL_622644			
see above	Department of Virus and Microbiological Special Diagnostics, Statens Serum Institut, Denmark	Albertsen lab, Department of Chemistry and Bioscience, Aalborg University, Denmark	Danish Covid-19 Genome Consortia
EPI_ISL_623105	Simile Medicina Diagnóstica	Bioinformatics Laboratory / LNCC	Carolina M Voloch, Ronaldo S Francisco Jr, Luiz G P de Almeida, Otavio J. Brustolini, Cynthia C Cardoso, Alexandra L Gerber, Ana Paula de C Guimarães, Diana Mariani, Covid19-UFRJ Workgroup, Luís Cristóvão Pôrto, Renato S Aguiar, Terezinha M P P Castiñeiras, Orlando C. Ferreira, Amilcar Tanuri, Ana Tereza R de Vasconcelos
EPI_ISL_623108, EPI_ISL_623140, EPI_ISL_623152, EPI_ISL_623156	Laboratorio de Virologia Molecular / UFRJ	Bioinformatics Laboratory / LNCC	Carolina M Voloch, Ronaldo S Francisco Jr, Luiz G P de Almeida, Otavio J. Brustolini, Cynthia C Cardoso, Alexandra L Gerber, Ana Paula de C Guimarães, Diana Mariani, Covid19-UFRJ Workgroup, Luís Cristóvão Pôrto, Renato S Aguiar, Terezinha M P P Castiñeiras, Orlando C. Ferreira, Amilcar Tanuri, Ana Tereza R de Vasconcelos
EPI_ISL_626211, EPI_ISL_626212, EPI_ISL_626213, EPI_ISL_626214, EPI_ISL_626215	Institute for Virology, University Hospital Essen	Center of Medical Microbiology, Virology, and Hospital Hygiene, University of Duesseldorf	Olympia E. Anastasiou, Ulf Dittmer, Maximilian Damagnez, Alexander Dilthey, Torsten Houwaart, Lisanna Hülse, Malte Kohns Vasconcelos, Nadine Lübke, Jessica Nicolai, Klaus Pfeffer, Daniel Strelow, Jörg Timm, Andreas Walker, Tobias Wienemann
EPI_ISL_626385, EPI_ISL_626386, EPI_ISL_626387, EPI_ISL_626388, EPI_ISL_626389, EPI_ISL_626390, EPI_ISL_626391, EPI_ISL_626392, EPI_ISL_626393, EPI_ISL_626394, EPI_ISL_626395, EPI_ISL_626396, EPI_ISL_626397, EPI_ISL_626398, EPI_ISL_626399, EPI_ISL_626400, EPI_ISL_626401, EPI_ISL_626402, EPI_ISL_626409, EPI_ISL_626410, EPI_ISL_626411			
see above	Northwestern Memorial Hospital	Ozer Lab	Ramon Lorenzo-Redondo, Hannah H. Nam, Scott C. Roberts, Lacy M. Simons, Chad J. Achenbach, Lawrence J. Jennings, Chao Qi, Alan R. Hauser, Michael G. Ison, Judd F. Hultquist, Egon A. Ozer
EPI_ISL_626627	National Centre for Communicable Disease (NCCD) National Influenza Center	National Centre for Communicable Disease (NCCD) National Influenza Center	Naranzul Ts,Darmaa B,Bayasgalan N,Ankhbayar S, Nyamkhuu D, Erden-Ochir Ts,Nymadawa P Chang-Seon Song
EPI_ISL_629102, EPI_ISL_629103, EPI_ISL_629104, EPI_ISL_629105	Laboratoire du Centre Hospitalier Annecy Genevois	CNR Virus des Infections Respiratoires - France SUD	Antonin Bal, Gregory Destras, Gwendolyne Burfin, Hadrien Règue, Quentin Semanas, Martine Valette, Bruno Lina, Hélène Petitprez, Bruno Chanzy, Laurence Josset
EPI_ISL_631393, EPI_ISL_631394, EPI_ISL_631395	Wisconsin State Laboratory of Hygiene Communicable Disease Division	Wisconsin State Laboratory of Hygiene Communicable Disease Division	Kelsey R. Florek, Abigail C. Shockey
EPI_ISL_631712, EPI_ISL_631713	Jamaica Hospital Medical Center	New York City Public Health Laboratory	Jade Wang, et al.
EPI_ISL_631714, EPI_ISL_631715	Flushing Hospital Medical Center	New York City Public Health Laboratory	Jade Wang, et al.
EPI_ISL_631716, EPI_ISL_631717, EPI_ISL_631718, EPI_ISL_631723	Jamaica Hospital Medical Center	New York City Public Health Laboratory	Jade Wang, et al.
EPI_ISL_631724, EPI_ISL_631725	Flushing Hospital Medical Center	New York City Public Health Laboratory	Jade Wang, et al.
EPI_ISL_631726	Jamaica Hospital Medical Center	New York City Public Health Laboratory	Jade Wang, et al.
EPI_ISL_631727	Flushing Hospital Medical Center	New York City Public Health Laboratory	Jade Wang, et al.
EPI_ISL_631728, EPI_ISL_631729, EPI_ISL_631730, EPI_ISL_631731	Jamaica Hospital Medical Center	New York City Public Health Laboratory	Jade Wang, et al.
EPI_ISL_631732	Flushing Hospital Medical Center	New York City Public Health Laboratory	Jade Wang, et al.
EPI_ISL_631733, EPI_ISL_631734, EPI_ISL_631735, EPI_ISL_631736, EPI_ISL_631737, EPI_ISL_631738, EPI_ISL_631739, EPI_ISL_631740, EPI_ISL_631741, EPI_ISL_631742, EPI_ISL_631772			
see above	Jamaica Hospital Medical Center	New York City Public Health Laboratory	Jade Wang, et al.
EPI_ISL_631773, EPI_ISL_631774	Flushing Hospital Medical Center	New York City Public Health Laboratory	Jade Wang, et al.
EPI_ISL_631775, EPI_ISL_631776	Jamaica Hospital Medical Center	New York City Public Health Laboratory	Jade Wang, et al.
EPI_ISL_631907, EPI_ISL_631908	Flushing Hospital Medical Center	New York City Public Health Laboratory	Jade Wang, et al.
EPI_ISL_631909, EPI_ISL_631910	Jamaica Hospital Medical Center	New York City Public Health Laboratory	Jade Wang, et al.
EPI_ISL_632938	Jena University Hospital; Institute for Infectious Diseases and Infection Control	Institute of infectious medicine & hospital hygiene, CaSe-Group	Spott, Riccardo; Marquet, Mike; Pletz, Matthias W.; Brandt, Christian
EPI_ISL_635267, EPI_ISL_635268, EPI_ISL_635299, EPI_ISL_636460	Institute of Microbiology and Immunology, Faculty of Medicine, University of Ljubljana	Institute of Microbiology and Immunology, Faculty of Medicine, University of Ljubljana	Tomaž Mark Zorec, Samo Zakotnik, Miša Korva, Tatjana Avši - Županc, Mario Poljak
EPI_ISL_636462, EPI_ISL_636463	ULSS6 Euganea	Istituto Zooprofilattico Sperimentale delle Venezie	Adelaide Milani, Alessia Schivo, Annalisa Salviato, Erika Giorgia Quaranta, Ambra Pastori, Bianca Zecchin, Alice Fusaro, Isabella Monne, Calogero Terregino, Antonia Ricci
EPI_ISL_636544, EPI_ISL_636547, EPI_ISL_636554	Dutch COVID-19 response team	National Institute for Public Health and the Environment (RIVM)	Adam Meijer, Harry Vennema, Jeroen Cremer, Sharon van den Brink, Bas van der Veer, AnneMarie van den Brandt, Florian Zwagemaker, Dennis Schmitz, Chantal Reusken, on behalf of the national COVID-19 response team
EPI_ISL_636961	Pathogen Genomics Lab King Abdullah University of Science and Technology(KAUST)	Pathogen Genomics Lab King Abdullah University of Science and Technology(KAUST)	Fadwa Alofi, Sharif Hala, Rahul P Salunke, Sara Mfarrej, Amit Kumar Subudhi, Fathia Ben Rached, Amanda, Luke, Afrah Alsomali, Asim Khogeer, Jumana Taha, Abdulaziz Alahmadi, Kahled Algithami, Raeece Naeem, Anwar Hashem, Naif Almontashiri, Arnab Pain
EPI_ISL_636994	Department of Infectious Diseases and Immunology, National Hospital Organization Nagoya Medical Center	Clinical Research Center, National Hospital Organization Nagoya Medical Center	Yoshihiro Nakata, Hirotaka Ode, Mai Kubota, Masakazu Matsuda, Kazuhiro Matsuoka, Miho Nakasuji, Mikiko Mori, Mayumi Imahashi, Yoshiyuki Yokomaku, Yasumasa Iwatani
EPI_ISL_640096, EPI_ISL_640097, EPI_ISL_640098, EPI_ISL_640099	Vanguard CHC wc VGC	NHLS/UCT	Arash Iranzadeh, Deelan Doolabh, Lynn Tyers, Bruna Galvao, Innocent Mudau, Marvin Hsiao, Kruger Marais, Diana Hardie, Stephen Korsman, Carolyn Williamson
EPI_ISL_640222, EPI_ISL_640223, EPI_ISL_640226, EPI_ISL_640228, EPI_ISL_640229, EPI_ISL_640230, EPI_ISL_640232, EPI_ISL_640235, EPI_ISL_640237, EPI_ISL_640238, EPI_ISL_640239, EPI_ISL_640240, EPI_ISL_640241, EPI_ISL_640247, EPI_ISL_640248, EPI_ISL_640250, EPI_ISL_640253, EPI_ISL_640256, EPI_ISL_640259, EPI_ISL_640264, EPI_ISL_640268, EPI_ISL_640269			
see above	MVZ Laborärzte Singen	MVZ Laborärzte Singen	Jonas Schmidt, Frithjof Blessing, Sandro Berghaus, Folker Wenzel
EPI_ISL_641537, EPI_ISL_641541	CHU de Saint-Étienne Hôpital Nord	CNR Virus des Infections Respiratoires - France SUD	Antonin Bal, Gregory Destras, Gwendolyne Burfin, Hadrien Règue, Quentin Semanas, Martine Valette, Bruno Lina, Issam Bechri, Manon Vogrig, Marine Delorme, Bruno Pozzetto, Thomas Bourlet, Sylvie Gonzalo, Sylvie Pillet, Laurence Josset
EPI_ISL_641547	CHU Clermont-Ferrand	CNR Virus des Infections Respiratoires - France SUD	Antonin Bal, Gregory Destras, Gwendolyne Burfin, Hadrien Règue, Quentin Semanas, Martine Valette, Bruno Lina, Christine Archimbaud, Amélie Brebion, Hélène Chabrolles, Martine Chambon, Audrey Mirand, Christel Regagnon, Maxime Bisseux, Patricia Combes, Cécile Henquell, Laurence Josset
EPI_ISL_641554	CHU Toulouse	CNR Virus des Infections Respiratoires - France SUD	Antonin Bal, Gregory Destras, Gwendolyne Burfin, Hadrien Règue, Quentin Semanas, Martine Valette, Bruno Lina, Jean Michel Mansuy, Laurence Josset
EPI_ISL_644210, EPI_ISL_644212, EPI_ISL_644213, EPI_ISL_644219, EPI_ISL_644220, EPI_ISL_644226, EPI_ISL_644232, EPI_ISL_644235, EPI_ISL_644248, EPI_ISL_644261, EPI_ISL_644262, EPI_ISL_644279, EPI_ISL_644286, EPI_ISL_644288, EPI_ISL_644289, EPI_ISL_644290, EPI_ISL_644291, EPI_ISL_644293,			

EPI_ISL_644296, EPI_ISL_644305, EPI_ISL_644313, EPI_ISL_644314, EPI_ISL_644318, EPI_ISL_644322, EPI_ISL_644324, EPI_ISL_644327, EPI_ISL_644328, EPI_ISL_644331, EPI_ISL_644333, EPI_ISL_644334			
see above	CEPHR / Vincent's Hospital	Irish Coronavirus Sequencing Consortium - National Virus Reference Laboratory	Michael Carr, Gabriel Gonzalez, Alejandro Abner Garcia Leon, Patrick Mallon
EPI_ISL_644725, EPI_ISL_644726, EPI_ISL_644727, EPI_ISL_644728, EPI_ISL_644729	Osmania Medical College	CSIR-Centre for Cellular and Molecular Biology	Dr.V.Sudha Rani,Dr.S.Pavani,Dr.Satyaprasad,Dr.P.Shashikala Reddy,Pratheusa Maccha,Shagufta Khan,Lamuk Zaveri,Namami Gaur,Sakshi Shambhavi,Tulasi Nagabandi,Nikhil Hajirnis,M Soujanya Reddy,Purushotham Vodnala,Blessy B John,Viswagithe S L,B Himasri,Payel Mukherjee,Sofia Banu,Priya Singh,Archana Bharadwaj Siva,Karthik Bharadwaj Tallapaka,Rakesh K Mishra,Divya Tej Sowpati
EPI_ISL_644731	Osmania Medical College	CSIR-Centre for Cellular and Molecular Biology	Dr.V.Sudha Rani,Dr.S.Pavani,Dr.Satyaprasad,Dr.P.Shashikala Reddy,Namami Gaur,Sakshi Shambhavi,Lamuk Zaveri,Shagufta Khan,Nikhil Hajirnis,M Soujanya Reddy,Pratheusa Maccha,Tulasi Nagabandi,Purushotham Vodnala,Blessy B John,Viswagithe S L,B Himasri,Payel Mukherjee,Sofia Banu,Priya Singh,Archana Bharadwaj Siva,Karthik Bharadwaj Tallapaka,Rakesh K Mishra,Divya Tej Sowpati
EPI_ISL_645029, EPI_ISL_645030, EPI_ISL_645066, EPI_ISL_645067, EPI_ISL_645072, EPI_ISL_645073, EPI_ISL_645074	Human Genome Variation Research Group, Malopolska Centre of Biotechnology	Human Genome Variation Research Group, Malopolska Centre of Biotechnology	Kowalski,M., Pospiech,E., Klajmon,A., Gromowski,T., Pisarek,A., Marszalek,K., Kopera,K., Foremny,J., Swadzba,J., Sanak,M., Owczarek,K., Dabrowska,A., Szczepanski,A., Botwina,P., Labaj,P.P., Pyrc,K., Branicki,W.
EPI_ISL_645218	CHU Nantes	CNR Virus des Infections Respiratoires - France SUD	Antonin Bal, Louise Castain, Gregory Destras, Gwendolyne Burfin, Hadrien Règue, Quentin Semanas, Martine Valette, Bruno Lina, Celine Bressollette, Laurence Josset
EPI_ISL_648046, EPI_ISL_648047, EPI_ISL_648048, EPI_ISL_648049, EPI_ISL_648050, EPI_ISL_648051, EPI_ISL_648052, EPI_ISL_648053, EPI_ISL_648054, EPI_ISL_648055, EPI_ISL_648056, EPI_ISL_648057, EPI_ISL_648058, EPI_ISL_648059, EPI_ISL_648060, EPI_ISL_648061, EPI_ISL_648112, EPI_ISL_648113, EPI_ISL_648114, EPI_ISL_648115, EPI_ISL_648116, EPI_ISL_648117, EPI_ISL_648118, EPI_ISL_648119, EPI_ISL_648120, EPI_ISL_648121, EPI_ISL_648610, EPI_ISL_648611, EPI_ISL_648612, EPI_ISL_648613			
see above	Department of Laboratory Medicine, Tan Tock Seng Hospital	Department of Laboratory Medicine, Tan Tock Seng Hospital	Chen YYC, Zair X, Lim JX, Li C, Tang WY, Maurer-Stroh S, Barkham TMS, Nagarajan N, Sessions OM
EPI_ISL_649184, EPI_ISL_649185	CHU Nantes	CNR Virus des Infections Respiratoires - France SUD	Antonin Bal, Louise Castain, Gregory Destras, Gwendolyne Burfin, Hadrien Règue, Quentin Semanas, Martine Valette, Bruno Lina, Celine Bressollette, Laurence Josset
EPI_ISL_649949, EPI_ISL_649950	CHU de Saint-Étienne Hôpital Nord	CNR Virus des Infections Respiratoires - France SUD	Antonin Bal, Gregory Destras, Gwendolyne Burfin, Hadrien Règue, Quentin Semanas, Martine Valette, Bruno Lina, Issam Bechri, Manon Vogrig, Marine Delorme, Bruno Pozzetto, Thomas Bourlet, Sylvie Gonzalo, Sylvie Pillet, Laurence Josset
EPI_ISL_653110, EPI_ISL_653111, EPI_ISL_653112, EPI_ISL_653114, EPI_ISL_653115, EPI_ISL_653116, EPI_ISL_653118, EPI_ISL_653119, EPI_ISL_653120, EPI_ISL_653121, EPI_ISL_653122, EPI_ISL_653123, EPI_ISL_653124, EPI_ISL_653125, EPI_ISL_653126, EPI_ISL_653127, EPI_ISL_653128, EPI_ISL_653129, EPI_ISL_653130, EPI_ISL_653131, EPI_ISL_653132, EPI_ISL_653133, EPI_ISL_653134, EPI_ISL_653135, EPI_ISL_653136, EPI_ISL_653137, EPI_ISL_653138, EPI_ISL_653139, EPI_ISL_653140, EPI_ISL_653141, EPI_ISL_653142, EPI_ISL_653144, EPI_ISL_653145, EPI_ISL_653146, EPI_ISL_653147, EPI_ISL_653148			
see above	Florida Bureau of Public Health Laboratories	Florida Bureau of Public Health Laboratories	Sarah Schmedes, Jason Blanton
EPI_ISL_653749, EPI_ISL_653825	Instituto Nacional de Salud, Bogotá, Colombia	Instituto Nacional de Salud, Bogotá, Colombia	Katherine Laiton-Donato, Diego A. Álvarez-Díaz, Carlos Franco-Muñoz, Mauricio Pacheco-Montealegre, Jonathan Reales, Diego Andrés Prada, Jose A. Usme-Ciro, Zulma M. Cucunubá, Christian Julian VillabonaArenas, Liz Villabona-Arenas, Sussy Echeverria, Astrid C. Flórez, Carolina Ferro, Diana Marcela Walteros-Acero, Franklin Prieto, Carlos Andrés Durán, Martha Lucia Ospina Martinez, Marcela Mercado-Reyes
EPI_ISL_654633, EPI_ISL_654634, EPI_ISL_654674	Servicio de Microbiología, Hospital Universitario Central de Asturias	SeqCOVID-SPAIN consortium/IBV(CSIC)	Cristián Castelló Abietar, Jose A. Boga, Susana Rojo-Alba, Marta Elena Álvarez-Argüelles, Santiago Melón and SeqCOVID-SPAIN consortium
EPI_ISL_654930, EPI_ISL_654931, EPI_ISL_654932, EPI_ISL_654933, EPI_ISL_654934, EPI_ISL_654935, EPI_ISL_654936	Texas Department of State Health Services	Texas Department of State Health Services	Rashmi Tuladhar, Bonnie Oh, Jenny Zhang, Maliha Rahman, Anita Pokharel, Myong Koag, Chung Wang, Rachel Lee, Grace Kubin, Mayela Pedrueza, James Daniel Bonser
EPI_ISL_660111	Infectious Diseases and Tropical Medicine Research Center, Infectious Diseases and Tropical Medicine Research Center	Infectious Diseases and Tropical Medicine Research Center, Infectious Diseases and Tropical Medicine Research Center	Haghjooy Javanmard,S., Ahangarzadeh,S., Shariati,L., Ataei,B., Aboutalebian,S.
EPI_ISL_660112	Infectious Diseases and Tropical Medicine Research Center, Infectious Diseases and Tropical Medicine Research Center	Infectious Diseases and Tropical Medicine Research Center, Infectious Diseases and Tropical Medicine Research Center	Ahangarzadeh,S., Haghjooy Javanmard,S., Aboutalebian,S., Ataei,B., Shariati,L.
EPI_ISL_660113	Infectious Diseases and Tropical Medicine Research Center, Infectious Diseases and Tropical Medicine Research Center	Infectious Diseases and Tropical Medicine Research Center, Infectious Diseases and Tropical Medicine Research Center	Ahangarzadeh,S., Ataei,B., Shariati,L., Haghjooy javanmard,S.
EPI_ISL_660114, EPI_ISL_660115	Infectious Diseases and Tropical Medicine Research Center, Infectious Diseases and Tropical Medicine Research Center	Infectious Diseases and Tropical Medicine Research Center, Infectious Diseases and Tropical Medicine Research Center	Ahangarzadeh,S., Ataei,B., Shariati,L., Haghjooy Javanmard,S.
EPI_ISL_660116	Infectious Diseases and Tropical Medicine Research Center, Infectious Diseases and Tropical Medicine Research Center	Infectious Diseases and Tropical Medicine Research Center, Infectious Diseases and Tropical Medicine Research Center	Ahangarzadeh,S., Haghjooy Javanmard,S., Aboutalebian,S., Ataei,B., Shariati,L.
EPI_ISL_660117, EPI_ISL_660118	Infectious Diseases and Tropical Medicine Research Center, Infectious Diseases and Tropical Medicine Research Center	Infectious Diseases and Tropical Medicine Research Center, Infectious Diseases and Tropical Medicine Research Center	Haghjooy Javanmard,S., Ahangarzadeh,S., Shariati,L., Ataei,B., Aboutalebian,S.
EPI_ISL_660343	CHU de Saint-Étienne Hôpital Nord	CNR Virus des Infections Respiratoires - France SUD	Antonin Bal, Gregory Destras, Gwendolyne Burfin, Hadrien Règue, Quentin Semanas, Martine Valette, Bruno Lina, Issam Bechri, Manon Vogrig, Marine Delorme, Bruno Pozzetto, Thomas Bourlet, Sylvie Gonzalo, Sylvie Pillet, Laurence Josset
EPI_ISL_660363, EPI_ISL_660364	CHU Clermont-Ferrand	CNR Virus des Infections Respiratoires - France SUD	Antonin Bal, Gregory Destras, Gwendolyne Burfin, Hadrien Règue, Quentin Semanas, Martine Valette, Bruno Lina, Christine Archimbaud, Amélie Brebion, Hélène Chabrolles, Martine Chambon, Audrey Mirand, Christel Regagnon, Maxime Bisseux, Patricia Combes, Cécile Henquell, Laurence Josset
EPI_ISL_660665, EPI_ISL_660666, EPI_ISL_660667, EPI_ISL_660668	CHU Toulouse	CNR Virus des Infections Respiratoires - France SUD	Antonin Bal, Gregory Destras, Gwendolyne Burfin, Hadrien Règue, Quentin Semanas, Martine Valette, Bruno Lina, Jean Michel Mansuy, Laurence Josset
EPI_ISL_661268, EPI_ISL_661270	Russian Academy of Sciences, Federal Research Center for Virology and Microbiology	Russian Academy of Sciences, Federal Research Center for Virology and Microbiology	Titov,I., Nefedeva,M., Egorova,I., Malogolovkin,A.
EPI_ISL_663284	CHU Nantes	CNR Virus des Infections Respiratoires - France SUD	Antonin Bal, Louise Castain, Gregory Destras, Gwendolyne Burfin, Hadrien Règue, Quentin Semanas, Martine Valette, Bruno Lina, Virginie Ferré, Celine Bressollette, Laurence Josset
EPI_ISL_664105, EPI_ISL_664106	Dept. of Microbiology and Infection Control, Akershus University Hospital HF	Dept. of Microbiology and Infection Control, Akershus University Hospital HF	Hege Vangstein Aamot, Alexander Hesselberg Løvestad, Silje Bakken Jørgensen, Nina Handal, Ole Herman Ambur
EPI_ISL_664187, EPI_ISL_664225, EPI_ISL_665146, EPI_ISL_665147, EPI_ISL_665148	University College London Hospital	COVID-19 Genomics UK (COG-UK) Consortium	Judith Heaney, Matthew Byott, Catherine Houlihan, Dan Frampton, Stuart Kirk, Moira Spyer and Eleni Nastouli
EPI_ISL_665254, EPI_ISL_666568, EPI_ISL_666593, EPI_ISL_666594	Dept. of Microbiology and Infection Control, Akershus University Hospital HF	Dept. of Microbiology and Infection Control, Akershus University Hospital HF	Hege Vangstein Aamot, Alexander Hesselberg Løvestad, Silje Bakken Jørgensen, Nina Handal, Ole Herman Ambur
EPI_ISL_666673, EPI_ISL_666674, EPI_ISL_666675, EPI_ISL_666676, EPI_ISL_666677	Laboratoire du Centre Hospitalier Annecy Genevois	CNR Virus des Infections Respiratoires - France SUD	Antonin Bal, Gregory Destras, Gwendolyne Burfin, Hadrien Règue, Quentin Semanas, Martine Valette, Bruno Lina, Hélène Petitprez, Bruno Chanzy, Laurence Josset
EPI_ISL_667081, EPI_ISL_667082	OHSU Lab Services Molecular Microbiology Lab	Oregon SARS-CoV-2 Genome Sequencing Center	Brendan L. O'Connell, Ruth V. Nichols, Sally Grindstaff, Alec J. Hirsch, Donna Hansel, Guang Fan, Daniel N. Streblow, William B. Messer, Andrew C. Adey, Benjamin N. Bimber, Brian J. O'Roak
EPI_ISL_671359, EPI_ISL_671360, EPI_ISL_671361, EPI_ISL_671362, EPI_ISL_671363, EPI_ISL_671364, EPI_ISL_671365, EPI_ISL_671366, EPI_ISL_671367, EPI_ISL_671368, EPI_ISL_671369, EPI_ISL_671370, EPI_ISL_671371, EPI_ISL_671372, EPI_ISL_671381, EPI_ISL_671382, EPI_ISL_671383, EPI_ISL_671384, EPI_ISL_671385, EPI_ISL_671386, EPI_ISL_671387, EPI_ISL_671388, EPI_ISL_671389, EPI_ISL_671390, EPI_ISL_671391, EPI_ISL_671392, EPI_ISL_671393, EPI_ISL_671394, EPI_ISL_671395, EPI_ISL_671396, EPI_ISL_671397, EPI_ISL_671398, EPI_ISL_671399, EPI_ISL_671400, EPI_ISL_671401, EPI_ISL_671402, EPI_ISL_671403			
see above	National Virus Reference Laboratory	Irish Coronavirus Sequencing Consortium - Teagasc	Calum Walsh, Fiona Crispie, John Kenny, Paul Cotter

		Moorepark	
EPI_ISL_671932, EPI_ISL_671933	National Virus Reference Laboratory	National Virus Reference Laboratory	Michael Carr, Gabriel Gonzalez, Jonathan Dean, Daniel Hare, Cillian F De Gascun
EPI_ISL_672027, EPI_ISL_672049, EPI_ISL_672074, EPI_ISL_672127, EPI_ISL_672128, EPI_ISL_672129, EPI_ISL_672130, EPI_ISL_672131, EPI_ISL_672132, EPI_ISL_672133, EPI_ISL_672140, EPI_ISL_672141			
see above	The Ashley Laboratory, Stanford University	Chan-Zuckerberg Biohub	CZB Cliahub Consortium
EPI_ISL_672564, EPI_ISL_672565, EPI_ISL_672566, EPI_ISL_672567	Utah Public Health Laboratory	Utah Public Health Laboratory	Erin Young, Kelly Oakeson
EPI_ISL_672582	Infectious Diseases and Tropical Medicine Research Center, Infectious Diseases and Tropical Medicine Research Center	Infectious Diseases and Tropical Medicine Research Center, Infectious Diseases and Tropical Medicine Research Center	Ahangarzadeh,S., Haghjooy Javanmard,S., Shoaei,P., Ataei,B., Shariati,L.
EPI_ISL_672675, EPI_ISL_672676, EPI_ISL_672677	DB Diagnosticos do Brasil	Laboratório de Parasitologia Médica - Instituto de Medicina Tropical - Universidade de São Paulo	Brazil-UK Centre for Arbovirus Discovery Diagnosis Genomics and Epidemiology (CADDE) Genomic Network - Instituto de Medicina Tropical
EPI_ISL_672688, EPI_ISL_672689, EPI_ISL_672690, EPI_ISL_672691, EPI_ISL_672692, EPI_ISL_672693, EPI_ISL_672694, EPI_ISL_672695, EPI_ISL_672696, EPI_ISL_672697, EPI_ISL_672698, EPI_ISL_672699, EPI_ISL_672700			
see above	Hospital das Clínicas da Faculdade de Medicina da Universidade de São Paulo (HC-FMUSP)	Laboratório de Parasitologia Médica - Instituto de Medicina Tropical - Universidade de São Paulo	Brazil-UK Centre for Arbovirus Discovery Diagnosis Genomics and Epidemiology (CADDE) Genomic Network - Instituto de Medicina Tropical
EPI_ISL_672713, EPI_ISL_672714, EPI_ISL_672715, EPI_ISL_672716, EPI_ISL_672717, EPI_ISL_672718, EPI_ISL_672719, EPI_ISL_672720, EPI_ISL_672721	Institute of Tropical Medicine at the University of São Paulo (IMT-USP)	Laboratório de Parasitologia Médica - Instituto de Medicina Tropical - Universidade de São Paulo	Brazil-UK Centre for Arbovirus Discovery Diagnosis Genomics and Epidemiology (CADDE) Genomic Network - Instituto de Medicina Tropical
EPI_ISL_672722, EPI_ISL_672723, EPI_ISL_672724, EPI_ISL_672725, EPI_ISL_672726, EPI_ISL_672727, EPI_ISL_672728, EPI_ISL_672729, EPI_ISL_672730, EPI_ISL_672731, EPI_ISL_672732			
see above	Hospital das Clínicas da Faculdade de Medicina da Universidade de São Paulo (HC-FMUSP)	Laboratório de Parasitologia Médica - Instituto de Medicina Tropical - Universidade de São Paulo	Brazil-UK Centre for Arbovirus Discovery Diagnosis Genomics and Epidemiology (CADDE) Genomic Network - Instituto de Medicina Tropical
EPI_ISL_672746, EPI_ISL_672747, EPI_ISL_672748, EPI_ISL_672749, EPI_ISL_672750, EPI_ISL_672751	Institute of Tropical Medicine at the University of São Paulo (IMT-USP)	Laboratório de Parasitologia Médica - Instituto de Medicina Tropical - Universidade de São Paulo	Brazil-UK Centre for Arbovirus Discovery Diagnosis Genomics and Epidemiology (CADDE) Genomic Network - Instituto de Medicina Tropical
EPI_ISL_676524	Uppsala klinisk mikrobiologi	The Public Health Agency of Sweden	Department of Microbiology, The Public Health Agency of Sweden
EPI_ISL_676543, EPI_ISL_676544, EPI_ISL_676545, EPI_ISL_676546, EPI_ISL_676547, EPI_ISL_676548, EPI_ISL_676549, EPI_ISL_676550, EPI_ISL_676551, EPI_ISL_676552, EPI_ISL_676553, EPI_ISL_676554, EPI_ISL_676555, EPI_ISL_676556, EPI_ISL_676557, EPI_ISL_676558, EPI_ISL_676559, EPI_ISL_676560, EPI_ISL_676561, EPI_ISL_676562, EPI_ISL_676563, EPI_ISL_676564, EPI_ISL_676565, EPI_ISL_676566, EPI_ISL_676567, EPI_ISL_676568, EPI_ISL_676569, EPI_ISL_676570, EPI_ISL_676571, EPI_ISL_676572, EPI_ISL_676573			
see above	CNR Virus des Infections Respiratoires - France SUD	CNR Virus des Infections Respiratoires - France SUD	Antonin Bal, Gregory Destras, Gwendolyne Burfin, Solenne Brun, Alexandre Gaymard, Maude Bouscambert-Duchamp, Florence Morfin-Sherpa, Martine Valette, Bruno Lina, Laurence Josset
EPI_ISL_676593, EPI_ISL_676594, EPI_ISL_676595, EPI_ISL_676596	Scientific Veterinary Institute Novi Sad	Veterinary Specialized Institute "Kraljevo", Serbia	Vidanovic,D., Tesovic,B., Knezevic,A., Jovanovic,T., Jankovic,M., Sekler,M., Banovic Djeri,B., Petrovic,T., Volkening,J., Afonso,C.
EPI_ISL_676649	Wadsworth Center, New York State Department of Health	Wadsworth Center, New York State Department of Health	Kirsten St. George, Daryl M. Lamson, Sara Griesemer, Jonathan Plitnick, Navjot Singh, Matthew D. Shudt, Erica Lasek-Nesselquist
EPI_ISL_676658, EPI_ISL_676691, EPI_ISL_676695, EPI_ISL_676840, EPI_ISL_676841, EPI_ISL_676842, EPI_ISL_676843, EPI_ISL_676845, EPI_ISL_676846, EPI_ISL_676847, EPI_ISL_676848, EPI_ISL_676849, EPI_ISL_676850, EPI_ISL_676851, EPI_ISL_676852, EPI_ISL_676853, EPI_ISL_676854, EPI_ISL_676855, EPI_ISL_676856, EPI_ISL_676857, EPI_ISL_676858, EPI_ISL_676859, EPI_ISL_676860, EPI_ISL_676861, EPI_ISL_676862, EPI_ISL_676863, EPI_ISL_676864, EPI_ISL_676865, EPI_ISL_676967, EPI_ISL_676969, EPI_ISL_676970, EPI_ISL_676978, EPI_ISL_677665			
see above	Wadsworth Center, New York State Department.of Health	Wadsworth Center, New York State Department.of Health	Kirsten St. George, Daryl M. Lamson, Alexis Russel, Jonathan Plitnick, Navjot Singh, John Kelly, Sara Griesemer, Erasmus Schneider, Erica Lasek-Nesselquist
EPI_ISL_677678, EPI_ISL_677680, EPI_ISL_677682, EPI_ISL_677689	Vanda Pharmaceuticals Clinical Site	Vanda Pharmaceuticals	Vanda Pharmaceuticals
EPI_ISL_677937	Pathogen Genomics Lab King Abdullah University of Science and Technology(KAUST)	Pathogen Genomics Lab King Abdullah University of Science and Technology(KAUST)	Sara Mfarrej, Raeece Naeem, Raushan Nugmanova, Olga Douvropoulou, Amanda Ooi, Luke Esau, Sharif Hala, Afrah Alsomali, Asim Khogeer, Fadwa Alofi,Jumana Taha, Abdulaziz Alahmadi, Kahled Algithami, Anwar Hashem, Naif Almontashiri, Arnab Pain
EPI_ISL_678045, EPI_ISL_678051, EPI_ISL_678053, EPI_ISL_678097, EPI_ISL_678098, EPI_ISL_678099, EPI_ISL_678104, EPI_ISL_678105, EPI_ISL_678106, EPI_ISL_678107, EPI_ISL_678108, EPI_ISL_678109, EPI_ISL_678110, EPI_ISL_678111, EPI_ISL_678112, EPI_ISL_678113			
see above	Pathogen Genomics Lab King Abdullah University of Science and Technology(KAUST)	Pathogen Genomics Lab King Abdullah University of Science and Technology(KAUST)	Amanda Ooi, Luke Esau, Sara Mfarrej, Raeece Naeem, Sharif Hala, Asim Khogeer, Fadwa Alofi, Afrah Alsomali, Jumana Taha, Abdulaziz Alahmadi, Kahled Algithami, Anwar Hashem, Naif Almontashiri, Arnab Pain
EPI_ISL_678114, EPI_ISL_678115, EPI_ISL_678118, EPI_ISL_678121, EPI_ISL_678122, EPI_ISL_678123	Pathogen Genomics Lab King Abdullah University of Science and Technology(KAUST)	Pathogen Genomics Lab King Abdullah University of Science and Technology(KAUST)	Luke Esau, Amanda Ooi, Sharif Hala, Raeece Naeem, Sara Mfarrej, Asim Khogeer, Fadwa Alofi, Afrah Alsomali, Jumana Taha, Abdulaziz Alahmadi, Kahled Algithami, Anwar Hashem, Naif Almontashiri, Arnab Pain
EPI_ISL_678143	Pathogen Genomics Lab King Abdullah University of Science and Technology(KAUST)	Pathogen Genomics Lab King Abdullah University of Science and Technology(KAUST)	Sara Mfarrej, Olga Douvropoulou, Raushan Nugmanova, Sharif Hala, Raeece Naeem, Asim Khogeer,Fadwa Alofi, Afrah Alsomali, Jumana Taha, Abdulaziz Alahmadi, Kahled Algithami, Anwar Hashem, Naif Almontashiri, Arnab Pain
EPI_ISL_678273	Mikrobiologie, RARI	Mikrobiologie, RARI	Krasnov,Y.M., Naryshkina,E.A., Guseva,N.P., Sosedova,E.A., Fedorov,A.V., Badanin,D.V., Sharapova,N.A., Portenko,S.A., Shcherbakova,S.A., Kutyrev,V.V.
EPI_ISL_678298	Area of Virology, Serology and Virology Division (SAViD), New South Wales Health Pathology Randwick	Virology Research Laboratory; Area of Virology, Serology and Virology Division (SAViD), New South Wales Health Pathology Randwick	Foster, C.; Au, J.; Ruiz Silva, M.; Deveson, I.; Bull, R.; Van Hal, S.; Rawlinson, W.
EPI_ISL_681677, EPI_ISL_681678	Molecular Medicine Laboratory, University of Magallanes	Centro Asistencial Docente y de Investigacion, Universidad de Magallanes	Jorge González, Jacqueline Aldridge, Diego Alvarez, Marco Montes de Oca, Hermy Alvarez, Roberto Uribe-Paredes, Marcelo Navarrete
EPI_ISL_681753, EPI_ISL_681755, EPI_ISL_681756	University Hospital Limerick	Irish Coronavirus Sequencing Consortium - Teagasc Moorepark	Paul Cotter, Fiona Crispie, Amy Fitzpatrick, John Kenny, Carolyn Meaney, Patrick Stapleton, Calum Walsh
EPI_ISL_681890, EPI_ISL_681891, EPI_ISL_681894, EPI_ISL_681895, EPI_ISL_681896, EPI_ISL_681897, EPI_ISL_681898, EPI_ISL_681899, EPI_ISL_681900, EPI_ISL_681901, EPI_ISL_681902, EPI_ISL_681903, EPI_ISL_681904, EPI_ISL_681905			
see above	National Virus Reference Laboratory	Irish Coronavirus Sequencing Consortium - Teagasc Moorepark	Paul Cotter, Fiona Crispie, John Kenny, Matthew McCabe, Calum Walsh
EPI_ISL_681939, EPI_ISL_681949, EPI_ISL_681958, EPI_ISL_681959, EPI_ISL_681961, EPI_ISL_681962, EPI_ISL_681963, EPI_ISL_681964, EPI_ISL_681967, EPI_ISL_681969, EPI_ISL_681970, EPI_ISL_681971, EPI_ISL_681972, EPI_ISL_681973, EPI_ISL_681974, EPI_ISL_681975, EPI_ISL_681976, EPI_ISL_681977, EPI_ISL_681978, EPI_ISL_681979, EPI_ISL_681980, EPI_ISL_681981, EPI_ISL_681982, EPI_ISL_681983, EPI_ISL_681984, EPI_ISL_681985, EPI_ISL_681986, EPI_ISL_681991, EPI_ISL_681992, EPI_ISL_681995			
see above	UPMC Clinical Microbiology Laboratory	Microbial Genomic Epidemiology Laboratory, University of Pittsburgh	Mustapha M. Mustapha, Jane W. Marsh, Dan Snyder, Marissa P. Griffith, Stephanie L. Mitchell, Vatsala R. Srinivasa, Kady D. Waggle, Chinelo Ezeonwuku, Vaughn S. Cooper, Lee H. Harrison
EPI_ISL_683446, EPI_ISL_683447, EPI_ISL_683448	Texas Department of State Health Services	Texas Department of State Health Services	Rashmi Tuladhar, Bonnie Oh, Jenny Zhang, Maliha Rahman, Anita Pokharel, Myong Koag, Chung Wang, Rachel Lee, Grace Kubin, Mayela Pedrueza, James Daniel Bonser
EPI_ISL_692959, EPI_ISL_692960, EPI_ISL_692961, EPI_ISL_692962	Massachusetts State Public Health Laboratory	Massachusetts State Public Health Laboratory	Andrew Lang, Timelia Fink, Glen Gallagher, Sandra Smole
EPI_ISL_693548, EPI_ISL_693549	Instituto Nacional de Saude (INSA)	Instituto Nacional de Saude (INSA)	Borges et al

EPI_ISL_694105, EPI_ISL_694106, EPI_ISL_694107, EPI_ISL_694108, EPI_ISL_694109, EPI_ISL_694110, EPI_ISL_694111, EPI_ISL_694112, EPI_ISL_694113, EPI_ISL_694114, EPI_ISL_694115, EPI_ISL_694116, EPI_ISL_694117, EPI_ISL_694118, EPI_ISL_694119, EPI_ISL_694120, EPI_ISL_694121, EPI_ISL_694122, EPI_ISL_694123, EPI_ISL_694124, EPI_ISL_694125, EPI_ISL_694126, EPI_ISL_694127, EPI_ISL_694128, EPI_ISL_694129, EPI_ISL_694130, EPI_ISL_694131, EPI_ISL_694132, EPI_ISL_694133, EPI_ISL_694134, EPI_ISL_694135, EPI_ISL_694136, EPI_ISL_694137, EPI_ISL_694138, EPI_ISL_694148, EPI_ISL_694149, EPI_ISL_694150, EPI_ISL_694151, EPI_ISL_694152, EPI_ISL_694153, EPI_ISL_694154, EPI_ISL_694155			
see above	AZ SPHL, Arizona Department of Health Services	TGen North	Jolene Bowers, Megan Folkerts, Chris French, Hayley Yaglom, Ashlyn Pfeiffer, Darrin Lemmer, Dave Engelthaler, The Arizona COVID Genomics Union (ACGU)
EPI_ISL_695806, EPI_ISL_695807, EPI_ISL_695809, EPI_ISL_695810, EPI_ISL_695812, EPI_ISL_695819, EPI_ISL_695821, EPI_ISL_695876, EPI_ISL_695895, EPI_ISL_695897, EPI_ISL_695900, EPI_ISL_695901, EPI_ISL_695903, EPI_ISL_695904, EPI_ISL_695905, EPI_ISL_695906, EPI_ISL_695908, EPI_ISL_695909, EPI_ISL_695914, EPI_ISL_695916, EPI_ISL_695920, EPI_ISL_695924, EPI_ISL_695925, EPI_ISL_695929, EPI_ISL_695931, EPI_ISL_695932, EPI_ISL_695934, EPI_ISL_695935, EPI_ISL_695938, EPI_ISL_695942, EPI_ISL_695943, EPI_ISL_695945, EPI_ISL_695946, EPI_ISL_695947, EPI_ISL_695950, EPI_ISL_695952, EPI_ISL_695953, EPI_ISL_695954, EPI_ISL_695957, EPI_ISL_695958, EPI_ISL_695959, EPI_ISL_695960, EPI_ISL_695961, EPI_ISL_695963, EPI_ISL_695964, EPI_ISL_695966, EPI_ISL_695968, EPI_ISL_695970, EPI_ISL_695976, EPI_ISL_695977, EPI_ISL_695978, EPI_ISL_695980, EPI_ISL_695981, EPI_ISL_695982, EPI_ISL_695984, EPI_ISL_695987, EPI_ISL_695989, EPI_ISL_695990, EPI_ISL_695992, EPI_ISL_695993, EPI_ISL_695998, EPI_ISL_696002, EPI_ISL_696003, EPI_ISL_696004, EPI_ISL_696005, EPI_ISL_696006, EPI_ISL_696009, EPI_ISL_696010, EPI_ISL_696011, EPI_ISL_696012, EPI_ISL_696013, EPI_ISL_696016, EPI_ISL_696108, EPI_ISL_696123, EPI_ISL_696124, EPI_ISL_696125, EPI_ISL_696126, EPI_ISL_696128, EPI_ISL_696129, EPI_ISL_696130, EPI_ISL_696135, EPI_ISL_696138, EPI_ISL_696144, EPI_ISL_696158, EPI_ISL_696167, EPI_ISL_696168, EPI_ISL_696173, EPI_ISL_696274, EPI_ISL_696292, EPI_ISL_696293, EPI_ISL_696294, EPI_ISL_696306, EPI_ISL_696309			
see above	Sonora Quest Laboratories, Laboratory Sciences of Arizona	TGen North	Jolene Bowers, Megan Folkerts, Chris French, Hayley Yaglom, Ashlyn Pfeiffer, Darrin Lemmer, Dave Engelthaler, The Arizona COVID Genomics Union (ACGU)
EPI_ISL_700351	Laboratoire de virologie, CHU de Grenoble - CS 10217 - 38043 Grenoble cedex 12	CNR Virus des Infections Respiratoires - France SUD	Antonin Bal, Gregory Destras, Gwendolyne Burfin, Hadrien Règue, Quentin Semanas, Martine Valette, Bruno Lina, Sylvie Larrat, Laurence Josset
EPI_ISL_700379, EPI_ISL_700382, EPI_ISL_700383, EPI_ISL_700384, EPI_ISL_700385, EPI_ISL_700389, EPI_ISL_700390, EPI_ISL_700391	Centre hospitalier Métropole Savoie	CNR Virus des Infections Respiratoires - France SUD	Antonin Bal, Carine Dumollard, Gregory Destras, Gwendolyne Burfin, Hadrien Règue, Quentin Semanas, Martine Valette, Bruno Lina, Jérôme Grosjean, Laurence Josset
EPI_ISL_700413, EPI_ISL_700527	Vanguard CHC wc VGC	NHLS/UCT	Arash Iranzadeh, Deelan Doolabh, Lynn Tyers, Bruna Galvao, Innocent Mudau, Marvin Hsiao, Kruger Marais, Diana Hardie, Stephen Korsman, Carolyn Williamson
EPI_ISL_700552	Dr Abdurahman CDC wc DAC	NHLS/UCT	Arash Iranzadeh, Deelan Doolabh, Lynn Tyers, Bruna Galvao, Innocent Mudau, Marvin Hsiao, Kruger Marais, Diana Hardie, Stephen Korsman, Carolyn Williamson
EPI_ISL_707693	Los Angeles County Public Health Laboratory	Los Angeles County Public Health Laboratory	P. Hemarajata et al.
EPI_ISL_708019	Virology, Universitätsklinikum des Saarlandes	Epigenetics, Saarland University	Kathrin Kattler, Markus Vogelgesang, Stefan Lohse, Sascha Tierling, Sigrun Smola, Jörn Walter
EPI_ISL_708506, EPI_ISL_708726	Los Angeles County Public Health Laboratory	Los Angeles County Public Health Laboratory	P. Hemarajata et al.
EPI_ISL_710492	Virology (microbiology), Hospital Universitario Central de Asturias	Virology (microbiology), Hospital Universitario Central de Asturias	Castello,C., Gomez-deOna,J., Martin-Rodriguez,G., Abreu,F., Costales,I., Boga,J.A., Coto,E., Rojo,S., Alvarez-Argueelles,M.E., Perez-Martinez,Z., Sandoval,M., Melon,S.
EPI_ISL_717726, EPI_ISL_717727, EPI_ISL_717728, EPI_ISL_717729, EPI_ISL_717735, EPI_ISL_717740, EPI_ISL_717742, EPI_ISL_717745, EPI_ISL_717747, EPI_ISL_717749, EPI_ISL_717750, EPI_ISL_717751, EPI_ISL_717754, EPI_ISL_717756, EPI_ISL_717757, EPI_ISL_717759, EPI_ISL_717760, EPI_ISL_717762, EPI_ISL_717764			
see above	Kingston Health Sciences Centre and Queen's University	Ontario Institute for Cancer Research	Prameet M. Sheth,Calvin Sjaarda,Robert Colautti,Katya Douchant,Ilinca Lungu,Bernard Lam,Paul Krzyzanowski,Michael Laszloffy,Lawrence E. Heisler,Richard de Borja,Jared T. Simpson
EPI_ISL_717810, EPI_ISL_717811, EPI_ISL_717840, EPI_ISL_717842	Laboratorio de Virologia Molecular / UFRJ	Bioinformatics Laboratory / LNCC	Carolina M Voloch, Ronaldo da Silva F Jr, Luiz G P de Almeida, Cynthia C Cardoso, Otavio Bustrolini, Alexandra L Gerber, Ana Paula de C Guimarães, Diana Mariani, Andréa Cony Cavalcanti, Claudia dos Santos Rodrigues, Terezinha M P P Castiñeira, Amilcar Tanuri, Ana Tereza R de Vasconcelos
EPI_ISL_718141, EPI_ISL_718145, EPI_ISL_718151, EPI_ISL_718153, EPI_ISL_718155, EPI_ISL_718160, EPI_ISL_718221	Ministry of Health Hospitals	Institute of Health and Community Medicine	David Perera, Ooi Mong How, Chua Hock Hin, Tonnii Sia Loong Loong, Wong Jyn Shan, Wong Kiing Aik, Chan Chia Jui
EPI_ISL_721992, EPI_ISL_722014, EPI_ISL_722032, EPI_ISL_722033, EPI_ISL_722034, EPI_ISL_722035, EPI_ISL_722045, EPI_ISL_722054, EPI_ISL_722083, EPI_ISL_722085, EPI_ISL_722101, EPI_ISL_722114, EPI_ISL_722127			
see above	Hospital das Clínicas Universidade de São Paulo Medical School	Laboratório de Parasitologia Médica - Instituto de Medicina Tropical - Universidade de São Paulo	Brazil-UK Centre for Arbovirus Discovery Diagnosis Genomics and Epidemiology (CADDE) Genomic Network - Instituto de Medicina Tropical
EPI_ISL_722130, EPI_ISL_722131, EPI_ISL_722132, EPI_ISL_722133	Instituto de Medicina Tropical Universidade de São Paulo	Laboratório de Parasitologia Médica - Instituto de Medicina Tropical - Universidade de São Paulo	Brazil-UK Centre for Arbovirus Discovery Diagnosis Genomics and Epidemiology (CADDE) Genomic Network - Instituto de Medicina Tropical
EPI_ISL_722135, EPI_ISL_722138, EPI_ISL_722141, EPI_ISL_722144, EPI_ISL_722145, EPI_ISL_722149, EPI_ISL_722150, EPI_ISL_722151, EPI_ISL_722152, EPI_ISL_722153, EPI_ISL_722154, EPI_ISL_722157, EPI_ISL_722158, EPI_ISL_722160, EPI_ISL_722162, EPI_ISL_722163, EPI_ISL_722165, EPI_ISL_722166, EPI_ISL_722167, EPI_ISL_722168			
see above	DB Diagnosticos do Brasil	Laboratório de Parasitologia Médica - Instituto de Medicina Tropical - Universidade de São Paulo	Brazil-UK Centre for Arbovirus Discovery Diagnosis Genomics and Epidemiology (CADDE) Genomic Network - Instituto de Medicina Tropical
EPI_ISL_722236	Servicio de Microbiología. Hospital General Universitario de Castellón	SeqCOVID-SPAIN consortium/IBV(CSIC)	Rosario Moreno Muñoz, María Dolores Tirado Balaguer and SeqCOVID-SPAIN consortium
EPI_ISL_723077, EPI_ISL_723081, EPI_ISL_723116, EPI_ISL_723117	Institute of Medical Genetics and Applied Genomics	Institute of Medical Genetics and Applied Genomics	Caspar Gross, Tina Ganzenmüller, Siri Göpel, Michaela Pogoda, Daniela Bezdan, Michael Sonnabend, Angel Angelov, Nicolas Casadei, Stephan Ossowski, Thomas Iftner, Michael Bitzer
EPI_ISL_728346, EPI_ISL_728348, EPI_ISL_728350, EPI_ISL_728351, EPI_ISL_728354, EPI_ISL_728356, EPI_ISL_728357	Institute of medical Microbiology and hospital Hygiene	Institute of medical Microbiology and hospital Hygiene	Prof. Dr. Achim Kaasch, Aljoscha Tersteegen
EPI_ISL_729995	Nigeria Centre for Disease Control (NCDC)	African Centre of Excellence for Genomics of Infectious Diseases (ACEGID), Redeemer's University, Ede, Osun State, Nigeria	Oluniyi P.E. et al
EPI_ISL_731942, EPI_ISL_731943, EPI_ISL_731944	Instituto Nacional de Saude (INSA)	Instituto Nacional de Saude (INSA)	Borges et al
EPI_ISL_732554, EPI_ISL_732555	Bundeswehr Institute of Microbiology	Bundeswehr Institute of Microbiology	Markus Antwerpen, Alexandra Rehn, Mathias Walter, Malena Bestehorn-Willmann, Sabine Zange, Enrico Georgi, Roman Wölfel
EPI_ISL_732748, EPI_ISL_732766	Centro de Investigación Biomédica de La Rioja - Hospital San Pedro Logroño	SeqCOVID-SPAIN consortium/IBV(CSIC)	María de Toro, José Manuel Azcona Gutiérrez, María Pilar Bea Escudero, Miriam Blasco Alberdi and SeqCOVID-SPAIN consortium
EPI_ISL_733482	Omsk Research Institute of Natural Focal Infections	WHO National Influenza Centre Russian Federation	Andrey Komissarov, Artem Fadeev, Anna Ivanova, Kseniya Komissarova, Dmitry Bazhenov, Daria Danilenko, Ekaterina Gradoboeva, Elena Poleshchuk, Aleksei Vasilenko, Valery Yakimenko, Ksenia Safina, Elena Nabieva, Georgii Bazykin, Dmitry Lioznov
EPI_ISL_734568, EPI_ISL_734569, EPI_ISL_734570, EPI_ISL_734571, EPI_ISL_734572, EPI_ISL_734573, EPI_ISL_734574, EPI_ISL_734575, EPI_ISL_734576, EPI_ISL_734577, EPI_ISL_734578, EPI_ISL_734579, EPI_ISL_734580, EPI_ISL_734581, EPI_ISL_734582, EPI_ISL_734583, EPI_ISL_734584, EPI_ISL_734585, EPI_ISL_734586, EPI_ISL_734587, EPI_ISL_734588, EPI_ISL_734589, EPI_ISL_734590, EPI_ISL_734591, EPI_ISL_734592, EPI_ISL_734593, EPI_ISL_734594, EPI_ISL_734595, EPI_ISL_734596, EPI_ISL_734597, EPI_ISL_734598, EPI_ISL_734599, EPI_ISL_734600, EPI_ISL_734601, EPI_ISL_734602, EPI_ISL_734603, EPI_ISL_734604, EPI_ISL_734605, EPI_ISL_734606, EPI_ISL_734607, EPI_ISL_734608, EPI_ISL_734609, EPI_ISL_734610, EPI_ISL_734611, EPI_ISL_734612, EPI_ISL_734613, EPI_ISL_734614, EPI_ISL_734615, EPI_ISL_734616, EPI_ISL_734617, EPI_ISL_734618, EPI_ISL_734619, EPI_ISL_734620, EPI_ISL_734621, EPI_ISL_734622, EPI_ISL_734623, EPI_ISL_734624, EPI_ISL_734625, EPI_ISL_734626, EPI_ISL_734627, EPI_ISL_734628, EPI_ISL_734629, EPI_ISL_734630, EPI_ISL_734631, EPI_ISL_734736, EPI_ISL_735241, EPI_ISL_735242, EPI_ISL_735243			
see above	UZ Leuven, National Reference Laboratory for Coronaviruses, Laboratory Medicine, Leuven, Belgium	KU Leuven, Rega Institute, Clinical and Epidemiological Virology	Tony Wawina-Bokalanga, Joan Marti-Carerras, Bert Vanmechelen, Piet Maes
EPI_ISL_735259, EPI_ISL_735260,	Genomic Laboratory (GLAB) (Conjoint lab of Health	Genomic Laboratory (GLAB), Istanbul Technical University	Ilker Karacan, Tugba Kizilboga Akgun, Nihat Bugra Agaoglu, Payam Zolfagharian, Mehtap Aydin, Gizem Alkurt, Jale Yildiz, Betsi Kose, Nisan Denizce Can,

EPI_ISL_735261, EPI_ISL_735262	Directorate of Istanbul and Istanbul Technical University)		Ayse Serra Ozel, Nilsun Altunal, Arzu Irvem, Yasemin Kendir Demirkol, Ozlem Akgun Dogan, Levent Doganay, Gizem Dinler Doganay
EPI_ISL_737206	National Reference Laboratory, Nigeria Centre for Disease Control.	National Reference Laboratory, Nigeria Centre for Disease Control, Gaduwa,Abuja, Nigeria	Dr Ndodo Nnaemeka, Olusola Akanbi, Chimaobi Chukwu, Dr Adesuyi Omoare, Grace Esebanmen, Nwando Mba, Shirlee Wohl, Anthony Ahumibe, Celestina Obiekea, Catherine Okoi, Dr Chinwe Ochu, Dr Chikwe Ihekweazu.
EPI_ISL_738211, EPI_ISL_738212, EPI_ISL_738213, EPI_ISL_738241, EPI_ISL_738242	UZ Leuven, National Reference Laboratory for Coronaviruses, Laboratory Medicine, Leuven, Belgium	KU Leuven, Rega Institute, Clinical and Epidemiological Virology	Tony Wawina-Bokalanga, Joan Marti-Carerras, Bert Vanmechelen, Piet Maes
EPI_ISL_738244, EPI_ISL_738245, EPI_ISL_738246, EPI_ISL_738248, EPI_ISL_738250, EPI_ISL_738251, EPI_ISL_738252, EPI_ISL_738253, EPI_ISL_738254, EPI_ISL_738282	Connecticut Veterans' Affairs Hospital	Grubaugh Lab - Yale School of Public Health	Joseph Fauver, Tara Alpert, Chantal Vogels, Mary Petrone, Isabel Ott, Ellen Foxman, Shaili Gupta, Danielle Plank, Nathan Grubaugh
EPI_ISL_751205, EPI_ISL_751207	Pathogen Genomics Lab King Abdullah University of Science and Technology(KAUST)	Pathogen Genomics Lab King Abdullah University of Science and Technology(KAUST)	Fathia Ben Rached, Sharif Hala, Raushan Nugmanova, Olga Douvropoulou, Sara Mfarrej, Raeece Naeem, Rahul P Salunke, Asim Khogeer, Fadwa Alofi, Afrah Alsomali, Jumana Taha, Abdulaziz Alahmadi, Kahled Algithami, Anwar Hashem, Naif Almontashiri, Arnab Pain
EPI_ISL_751211	Pathogen Genomics Lab King Abdullah University of Science and Technology(KAUST)	Pathogen Genomics Lab King Abdullah University of Science and Technology(KAUST)	Sara Mfarrej, Luke Esau, Amanda Ooi, Sharif Hala, Raeece Naeem, Asim Khogeer, Fadwa Alofi, Afrah Alsomali, Jumana Taha, Abdulaziz Alahmadi, Kahled Algithami, Anwar Hashem, Naif Almontashiri, Arnab Pain
EPI_ISL_751221	Pathogen Genomics Lab King Abdullah University of Science and Technology(KAUST)	Pathogen Genomics Lab King Abdullah University of Science and Technology(KAUST)	Sharif Hala, Fathia Ben Rached, Sara Mfarrej, Olga Douvropoulou, Raushan Nugmanova, Raeece Naeem, Rahul P Salunke, Afrah Alsomali, Fadwa Alofi, Asim Khogeer, Jumana Taha, Abdulaziz Alahmadi, Kahled Algithami, Anwar Hashem, Naif Almontashiri, Arnab Pain
EPI_ISL_751223, EPI_ISL_751238	Pathogen Genomics Lab King Abdullah University of Science and Technology(KAUST)	Pathogen Genomics Lab King Abdullah University of Science and Technology(KAUST)	Sara Mfarrej, Amanda Ooi, Luke Esau, Sharif Hala, Raeece Naeem, Asim Khogeer, Fadwa Alofi, Afrah Alsomali, Jumana Taha, Abdulaziz Alahmadi, Kahled Algithami, Anwar Hashem, Naif Almontashiri, Arnab Pain
EPI_ISL_751322, EPI_ISL_751334, EPI_ISL_751446, EPI_ISL_751447	IRCCS Sacro Cuore Don Calabria Hospital, Department of Infectious, Tropical Diseases & Microbiology	University of Verona, Department of Biotechnology	Antonio Mori, Michela Deiana, Elena Pomari, Chiara Piubelli; Giulia Lopatriello, Luca Marcolungo, Cristina Beltrami, Chiara Degli Esposti, Emanuela Cosentino, Massimo Delledonne
EPI_ISL_751502	Pathogen Genomics Lab King Abdullah University of Science and Technology(KAUST)	Pathogen Genomics Lab King Abdullah University of Science and Technology(KAUST)	Amit Kumar Subudhi, Sharif Hala, Sara Mfarrej, Rahul P Salunke, Raushan Nugmanova, Olga Douvropoulou, Raeece Naeem, Afrah Alsomali, Fadwa Alofi, Asim Khogeer, Jumana Taha, Abdulaziz Alahmadi, Kahled Algithami, Anwar Hashem, Naif Almontashiri, Arnab Pain
EPI_ISL_751503	Pathogen Genomics Lab King Abdullah University of Science and Technology(KAUST)	Pathogen Genomics Lab King Abdullah University of Science and Technology(KAUST)	Sara Mfarrej, Luke Esau, Amanda Ooi, Sharif Hala, Raeece Naeem, Asim Khogeer, Fadwa Alofi, Afrah Alsomali, Jumana Taha, Abdulaziz Alahmadi, Kahled Algithami, Anwar Hashem, Naif Almontashiri, Arnab Pain
EPI_ISL_752610, EPI_ISL_752611, EPI_ISL_752621, EPI_ISL_752622, EPI_ISL_752623, EPI_ISL_752624, EPI_ISL_752625, EPI_ISL_752631, EPI_ISL_752632, EPI_ISL_752633, EPI_ISL_752636, EPI_ISL_752637, EPI_ISL_752638, EPI_ISL_752640, EPI_ISL_752641, EPI_ISL_752643, EPI_ISL_752668			
see above	State Laboratories Division, Hawaii State Department of Health	State Laboratories Division, Hawaii State Department of Health	Pamela O'Brien, Sabrina Diemert, Drew Kuwazaki, Razvan Sultana, Edward Desmond
EPI_ISL_753752, EPI_ISL_753753, EPI_ISL_753759, EPI_ISL_753797, EPI_ISL_753798, EPI_ISL_753836, EPI_ISL_753946, EPI_ISL_753947, EPI_ISL_753948, EPI_ISL_754036, EPI_ISL_754052, EPI_ISL_754053			
see above	Charité Universitätsmedizin Berlin, Institut für Virologie/Labor Berlin	Charité Universitätsmedizin Berlin, Institut für Virologie	Victor M Corman, Jörn Beheim-Schwarzbach, Barbara Mühlemann, Julia Schneider, Talitha Veith, Terry Jones, Christian Drosten
EPI_ISL_754676	University of Wisconsin-Madison AIDS Vaccine Research Laboratories	University of Wisconsin-Madison AIDS Vaccine Research Laboratories	Gage Moreno, Katarina Braun, et al. AIDS Vaccine Research Laboratories
EPI_ISL_754746	Emory Molecular Diagnostics Laboratory, Emory Healthcare	Piantadosi Lab, Emory Department of Pathology	Ahmed Babiker, Anne Piantadosi
EPI_ISL_755038, EPI_ISL_755039, EPI_ISL_755040, EPI_ISL_755041	California Department of Public Health	California Department of Public Health	CDPH IDLB COVIDNet
EPI_ISL_759955	Primasatya Husada Citra Hospital	Institute of Tropical Disease, Universitas Airlangga	Aldise M Nastri, Jezzy R Dewantari, Rima R Prasetya, Krisnoadi Rahardjo, Pudji Djanuartono, Gatot Soegiarto, Laksmi Wulandari, Resti Yudhawati, Soetjipto, Yasuko Mori, Maria I Lusida, Kazufumi Shimizu
EPI_ISL_759962	RSUD Dr. Soetomo	Institute of Tropical Disease, Universitas Airlangga	Krisnoadi Rahardjo, Aldise M Nastri, Jezzy R Dewantari, Rima R Prasetya, Joni Wahyuhadi, Gatot Soegiarto, Laksmi Wulandari, Resti Yudhawati, Yasuko Mori, Soetjipto, Kazufumi Shimizu ,Maria I Lusida
EPI_ISL_763352	National Virus Reference Laboratory	Irish Coronavirus Sequencing Consortium - Teagasc Moorepark	Calum Walsh, Fiona Crispie, John Kenny, Paul Cotter
EPI_ISL_766037	Puskesmas Palmerah	Eijkman Institute for Molecular Biology, Ministry of Research and Technology/National Agency for Research and Innovation	Frilasita A Yudhaputri, Edison Johar, Hidayat Trimarsanto, David H Muljono, Safarina G Malik, Khin Saw Myint, Amin Soebandrio
EPI_ISL_766038	RSJPD Harapan Kita	Eijkman Institute for Molecular Biology, Ministry of Research and Technology/National Agency for Research and Innovation	Frilasita A Yudhaputri, Edison Johar, Hidayat Trimarsanto, David H Muljono, Safarina G Malik, Khin Saw Myint, Amin Soebandrio
EPI_ISL_766039	RS Bhayangkara Tk. III	Eijkman Institute for Molecular Biology, Ministry of Research and Technology/National Agency for Research and Innovation	Frilasita A Yudhaputri, Edison Johar, Hidayat Trimarsanto, David H Muljono, Safarina G Malik, Khin Saw Myint, Amin Soebandrio
EPI_ISL_766040	RS Tingkat II Moh. Ridwan Meuraksa	Eijkman Institute for Molecular Biology, Ministry of Research and Technology/National Agency for Research and Innovation	Frilasita A Yudhaputri, Edison Johar, Hidayat Trimarsanto, David H Muljono, Safarina G Malik, Khin Saw Myint, Amin Soebandrio
EPI_ISL_766041	RS Polri	Eijkman Institute for Molecular Biology, Ministry of Research and Technology/National Agency for Research and Innovation	Frilasita A Yudhaputri, Edison Johar, Hidayat Trimarsanto, David H Muljono, Safarina G Malik, Khin Saw Myint, Amin Soebandrio
EPI_ISL_766042	RS Sari Asih Ciledug	Eijkman Institute for Molecular Biology, Ministry of Research and Technology/National Agency for Research and Innovation	Frilasita A Yudhaputri, Edison Johar, Hidayat Trimarsanto, David H Muljono, Safarina G Malik, Khin Saw Myint, Amin Soebandrio
EPI_ISL_766048	RS Bhayangkara Tk. III	Eijkman Institute for Molecular Biology, Ministry of Research and Technology/National Agency for Research and Innovation	Frilasita A Yudhaputri, Edison Johar, Hidayat Trimarsanto, David H Muljono, Safarina G Malik, Khin Saw Myint, Amin Soebandrio
EPI_ISL_767716, EPI_ISL_767719, EPI_ISL_767720, EPI_ISL_767722, EPI_ISL_767723, EPI_ISL_767729, EPI_ISL_767730, EPI_ISL_767731, EPI_ISL_767732, EPI_ISL_767733, EPI_ISL_767734, EPI_ISL_767735, EPI_ISL_767770, EPI_ISL_767771, EPI_ISL_767772, EPI_ISL_767773, EPI_ISL_767774, EPI_ISL_767775, EPI_ISL_767776, EPI_ISL_767779, EPI_ISL_767780, EPI_ISL_767781, EPI_ISL_767786, EPI_ISL_767799, EPI_ISL_767800, EPI_ISL_767801, EPI_ISL_767802, EPI_ISL_767806, EPI_ISL_767807, EPI_ISL_767808, EPI_ISL_767809, EPI_ISL_767822, EPI_ISL_767823, EPI_ISL_767824, EPI_ISL_767832, EPI_ISL_767833, EPI_ISL_768745, EPI_ISL_768746, EPI_ISL_768747, EPI_ISL_768748			
see above	National Virus Reference Laboratory	Irish Coronavirus Sequencing Consortium - Teagasc Moorepark	Calum Walsh, Genuity Ireland
EPI_ISL_768759	AIID	Irish Coronavirus Sequencing Consortium - National Virus Reference Laboratory	Michael Carr, Gabriel Gonzalez, Alejandro Abner Garcia Leon, Patrick Mallon
EPI_ISL_770129	Instituto Nacional de Investigación y Tecnología Agraria y Alimentaria (INIA)/ Departamento de Mejora Genética Animal	Instituto Nacional de Investigación y Tecnología Agraria y Alimentaria (INIA)/ Departamento de Mejora Genética Animal	Oscar González-Recio, Mónica Gutiérrez-Rivas, Ramón Peiró-Pastor, Miguel Ángel Jiménez-Clavero, Jovita Fernández-Pinero and SeqCOVID-SPAIN consortium
EPI_ISL_775687, EPI_ISL_776504, EPI_ISL_776508, EPI_ISL_776511, EPI_ISL_776512	University Medical Center Hamburg Eppendorf	Heinrich Pette Institute, Leibniz Institute for Experimental Virology	Alexis Robitaille, Thomas Günther, Johannes Knobloch, Martin Aepfelbacher, Nicole Fischer, Adam Grundhoff
EPI_ISL_776693	UW Virology Lab	UW Virology Lab	Pavitra Roychoudhury, Hong Xie, Lasata Shrestha, Meei-Li Huang, Keith R Jerome, Alexander Greninger
EPI_ISL_776711	Institute of medical Microbiology and hospital Hygiene	Institute of medical Microbiology and hospital Hygiene	Prof. Dr. Achim Kaasch, Aljoscha Tersteegen

EPI_ISL_776750	Instituto Adolfo Lutz - Central	Instituto Adolfo Lutz, Interdiciplinary Procedures Center, Strategic Laboratory	Claudio Tavares Sacchi, Claudia Regina Gonçalves, Erica Valessa Ramos Gomes, Karoline Rodrigues Campos
EPI_ISL_776771	Institute of medical Microbiology and hospital Hygiene	Institute of medical Microbiology and hospital Hygiene	Prof. Dr. Achim Kaasch, Aljoscha Tersteegen
EPI_ISL_778645	Hospital General Universitario Gregorio Marañón	Hospital General Universitario Gregorio Marañón	Sergio Buenestado Serrano, Pedro Sola Campoy, Laura Perez-Lago, Pilar Catalán, Patricia Muñoz, Darío García de Viedma
EPI_ISL_778659, EPI_ISL_778660, EPI_ISL_778661, EPI_ISL_778662, EPI_ISL_778663, EPI_ISL_778664, EPI_ISL_778665, EPI_ISL_778666, EPI_ISL_778667, EPI_ISL_778668, EPI_ISL_778669, EPI_ISL_778670, EPI_ISL_778671, EPI_ISL_778672, EPI_ISL_778729, EPI_ISL_778730, EPI_ISL_778731, EPI_ISL_778732, EPI_ISL_778733, EPI_ISL_778734			
see above	Istituto Zooprofilattico Sperimentale del Mezzogiorno	TIGEM	Patrizia Annunziata, Andrea Ballabio, Valentina Bouche, Davide Cacchiarelli (CorrespAuthor), Pellegrino Cerino, Chiara Colantuono, Lucio Di Filippo, Antonio Grimaldi, Antonio Limone, Gabriella Loconte, Anna Manfredi, Francesco Panariello, Biancamaria Pierri, Marcello Salvi, Lucia Vassallo
EPI_ISL_788944, EPI_ISL_788962, EPI_ISL_788963, EPI_ISL_788964	Ospedale "Di Venere"	Beaconlab (Bioinformatics, Evolution and Comparative Genomics lab), Dept of Biosciences, University on Milan	Iacobellis M, d'Avenia M, Piluscio R, Parisi A, Chiara M, Manzari C, Pesole G
EPI_ISL_789102, EPI_ISL_789103	Ohio Department of Health Laboratory	Ohio Department of Health Laboratory	Holmes, Jennifer; Eric Brandt, Keoni Omura, Glen McGillivary, Caitlin McDonnell, Kirtana Ramadugu, Erica Leasure, Kelsey Florek, Heather Blankenship, Quanta Brown, and Tammy Bannerman
EPI_ISL_791664, EPI_ISL_791665, EPI_ISL_791666, EPI_ISL_791667, EPI_ISL_791668, EPI_ISL_791669, EPI_ISL_791670, EPI_ISL_791671, EPI_ISL_791672, EPI_ISL_791673, EPI_ISL_791674, EPI_ISL_791675, EPI_ISL_791676, EPI_ISL_791677, EPI_ISL_791678, EPI_ISL_791679, EPI_ISL_791680, EPI_ISL_791681, EPI_ISL_791682, EPI_ISL_791683, EPI_ISL_791684, EPI_ISL_791685, EPI_ISL_791686, EPI_ISL_791687, EPI_ISL_791688, EPI_ISL_791689, EPI_ISL_791690, EPI_ISL_791691, EPI_ISL_791692, EPI_ISL_791693, EPI_ISL_791694, EPI_ISL_791695, EPI_ISL_791696, EPI_ISL_791697, EPI_ISL_791698, EPI_ISL_791699, EPI_ISL_791700, EPI_ISL_791701, EPI_ISL_791702, EPI_ISL_791703, EPI_ISL_791704, EPI_ISL_791705, EPI_ISL_791706, EPI_ISL_791707, EPI_ISL_791708, EPI_ISL_791709, EPI_ISL_791710, EPI_ISL_791711, EPI_ISL_791712, EPI_ISL_791713, EPI_ISL_791714, EPI_ISL_791715, EPI_ISL_791716, EPI_ISL_791717, EPI_ISL_791718, EPI_ISL_791719, EPI_ISL_791720, EPI_ISL_791721, EPI_ISL_791722, EPI_ISL_791723, EPI_ISL_791724, EPI_ISL_791725, EPI_ISL_791726, EPI_ISL_791727, EPI_ISL_791728			
see above	Massachusetts State Public Health Laboratory	Infectious Disease Program, Broad Institute of Harvard and MIT	Lemieux,J.E., Siddle,K.J., Shaw,B., Adams,G., Pierce,V., Turbett,S., Anahtar,M., Branda,J., Slater,D., Harris,J., Lin,A.E., Gladden-Young,A., Lagerborg,K., Rudy,M., DeRuff,K., Carter,A., Normandin,E., Bauer,M., Reilly,S., Tomkins-Tinch,C., Loreth,C., Chaluvadi,S., Neumann,A., Cusick,C., Chapman,S.B., Gnirke,A., Flowers,K., Cerrato,F., Birren,B.W., Gallagher,G., Smole,S., Park,D.J., MacInnis,B.L., Ryan,E., LaRocque,R., Rosenberg,E. and Sabeti,P.C.
EPI_ISL_791764, EPI_ISL_791765, EPI_ISL_791766, EPI_ISL_791767, EPI_ISL_791768, EPI_ISL_791769, EPI_ISL_791770, EPI_ISL_791771, EPI_ISL_791772, EPI_ISL_791773, EPI_ISL_791774, EPI_ISL_791775, EPI_ISL_791776, EPI_ISL_791777, EPI_ISL_791778, EPI_ISL_791779, EPI_ISL_791780, EPI_ISL_791781, EPI_ISL_791782, EPI_ISL_791783, EPI_ISL_791784, EPI_ISL_791785, EPI_ISL_791786, EPI_ISL_791787, EPI_ISL_791788, EPI_ISL_791789, EPI_ISL_791790, EPI_ISL_791791, EPI_ISL_791792, EPI_ISL_791793, EPI_ISL_791794, EPI_ISL_791795, EPI_ISL_791796, EPI_ISL_791797, EPI_ISL_791798, EPI_ISL_791799, EPI_ISL_791800, EPI_ISL_791801, EPI_ISL_791802, EPI_ISL_791803, EPI_ISL_791804, EPI_ISL_791805, EPI_ISL_791806, EPI_ISL_791807, EPI_ISL_791808, EPI_ISL_791809, EPI_ISL_791810, EPI_ISL_791811, EPI_ISL_791817			
see above	Massachusetts General Hospital	Infectious Disease Program, Broad Institute of Harvard and MIT	Lemieux,J.E., Siddle,K.J., Shaw,B., Adams,G., Pierce,V., Turbett,S., Anahtar,M., Branda,J., Slater,D., Harris,J., Lin,A.E., Gladden-Young,A., Lagerborg,K., Rudy,M., DeRuff,K., Carter,A., Normandin,E., Bauer,M., Reilly,S., Tomkins-Tinch,C., Loreth,C., Chaluvadi,S., Neumann,A., Cusick,C., Chapman,S.B., Gnirke,A., Flowers,K., Cerrato,F., Birren,B.W., Gallagher,G., Smole,S., Park,D.J., MacInnis,B.L., Ryan,E., LaRocque,R., Rosenberg,E. and Sabeti,P.C.
EPI_ISL_792108	Instituto Adolfo Lutz - Central	Instituto Adolfo Lutz, Interdiciplinary Procedures Center, Strategic Laboratory	Claudio Tavares Sacchi, Claudia Regina Gonçalves, Erica Valessa Ramos Gomes, Karoline Rodrigues Campos
EPI_ISL_792320, EPI_ISL_792326, EPI_ISL_792327, EPI_ISL_792328, EPI_ISL_792329	Instituto de Investigaciones Biomédicas en Retrovirus y SIDA	Área de Secuenciación del Laboratorio de Virología del Hospital de Niños Dr. Ricardo Gutierrez on behalf of 'Proyecto Argentino Interinstitucional de genomica de SARS-CoV-2' (PAIS Consortium)	Nabaes Jodar, MS; Goya, S; Natale, MI; Lusso, S; Seery, V; Remes Lenicov, F; Salomón, H; Valinotto, LE; Viegas, M.
EPI_ISL_792333, EPI_ISL_792343, EPI_ISL_792345, EPI_ISL_792346, EPI_ISL_792348	Laboratorio del Hospital Interzonal General de Agudos Evita	Área de Secuenciación del Laboratorio de Virología del Hospital de Niños Dr. Ricardo Gutierrez on behalf of 'Proyecto Argentino Interinstitucional de genomica de SARS-CoV-2' (PAIS Consortium)	Nabaes Jodar, MS; Goya, S; Natale, MI; Lusso, S; Desimone, I; Luczac, E; Serrano, L; Grossi, O; Musto, A; Valinotto, LE; Viegas, M.
EPI_ISL_792461, EPI_ISL_792465, EPI_ISL_792472	Laboratorio del Hospital Regional Ushuaia Gdor. Ernesto Campos	Hospital Regional Ushuaia - Centro Austral De Investigaciones Científicas - Universidad Nacional De Tierra Del Fuego on behalf of 'Proyecto Argentino Interinstitucional de genomica de SARS-CoV-2' (PAIS Consortium)	Ceballos, SG; Nardi, CF; Gramundi, ID; Gallego, F; De Roccis, CA; Castro, G; Cáceres, SB; Yulan, CB; Boutureira, MF.
EPI_ISL_792495, EPI_ISL_792497	Laboratorio Central Mg. Luis Alfredo Pianciola	Hospital Regional Ushuaia - Centro Austral De Investigaciones Científicas - Universidad Nacional De Tierra Del Fuego on behalf of 'Proyecto Argentino Interinstitucional de genomica de SARS-CoV-2' (PAIS Consortium)	Ceballos, SG; Nardi, CF; Gramundi, ID; Gallego, F; Pinto, C; Ziehm, MC; Mazzeo, M; Pianciola, L.
EPI_ISL_792504, EPI_ISL_792505, EPI_ISL_792506, EPI_ISL_792511	Laboratorio de Inmunología del Hospital Perrando e Instituto de Medicina Regional de la UNNE	Grupo de Genómica y Bioinformática del Instituto de Investigación de la Cadena Láctea CONICET-INTA on behalf of 'Proyecto Argentino Interinstitucional de genomica de SARS-CoV-2' (PAIS Consortium)	Eberhardt, MF; Irazoqui, JM; Foussal, MD; Deluca, G; Ayala, NA; Gómez, MV; Giusiano, G; Lucero, H; Marin, M; Lescano, L; Cayré, A; Amadio, AF
EPI_ISL_792576, EPI_ISL_792577	LACEN-PB	Laboratory of Respiratory Viruses and Measles, Oswaldo Cruz Institute, FIOCRUZ	Paola Resende, Luciana Appolinario, Fernando Motta, Anna Carolina Paixao, Ana Carolina Mendonca, João Felipe Bezerra, Romero Henrique Teixeira de Vasconcelos, Dalane Loudal Florentino Teixeira, Thiago Franco de Oliveira Carneiro, Marilda Siqueira
EPI_ISL_796090, EPI_ISL_796091, EPI_ISL_796092	Servicio de Microbiología. Hospital General Universitario de Castellón	SeqCOVID-SPAIN consortium/IBV(CSIC)	Rosario Moreno Muñoz, María Dolores Tirado Balaguer and SeqCOVID-SPAIN consortium
EPI_ISL_796127	Laboratorio de Microbiología. Hospital General Universitario de Elda, Alicante	SeqCOVID-SPAIN consortium/IBV(CSIC)	Mª Isabel Gascón Ros, Cristina Torregrosa Hetland, Eva Pastor Boix, Paloma Cascales Ramos and SeqCOVID-SPAIN consortium
EPI_ISL_796154, EPI_ISL_796155, EPI_ISL_796156, EPI_ISL_796157	Servicio de Microbiología. Hospital General Universitario de Castellón	SeqCOVID-SPAIN consortium/IBV(CSIC)	Rosario Moreno Muñoz, María Dolores Tirado Balaguer and SeqCOVID-SPAIN consortium
EPI_ISL_801541	Laboratory of Molecular Virology, Pontificia Universidad Católica de Chile	MSHS Pathogen Surveillance Program	Leonardo I. Almonacid, Ana S. Gonzalez-Reiche, Matthew M. Hernandez, Jorge Levican, Ana Maria Contreras, Carlos Palma, Tamara García-Salum, Zenab Khan, Adriana van De Guchte, Ajay Obla, Jayeeta Dutta, Bremy Alburquerque, Eileen Serrano, Constanza Maldonado, M. Belen Leyton, Erick Salinas, Hala Alshammary, Juan Soto, Shwetha Hara Sridhar, Ying-Chih Wang, Melissa Smith, Robert Sebra, Marcela Ferres, Adolfo Garcia-Sastre, Edward C. Holmes, Viviana Simon, Harm van Bakel, Rafael A. Medina.
EPI_ISL_801584	Laboratory of Molecular Virology, Pontificia Universidad Católica de Chile	MSHS Pathogen Surveillance Program	Leonardo I. Almonacid, Ana S. Gonzalez-Reiche, Matthew M. Hernandez, Jorge Levican, Tamara García-Salum, Zenab Khan, Adriana van De Guchte, Ajay Obla, Jayeeta Dutta, Bremy Alburquerque, Eileen Serrano, Erick Salinas, Hala Alshammary, Juan Soto, Shwetha Hara Sridhar, Ying-Chih Wang, Melissa Smith, Robert Sebra, Adolfo Garcia-Sastre, Edward C. Holmes, Viviana Simon, Harm van Bakel, Rafael A. Medina
EPI_ISL_801706, EPI_ISL_801707, EPI_ISL_801708, EPI_ISL_801709, EPI_ISL_801710, EPI_ISL_801711, EPI_ISL_801712	Laboratory of Molecular Virology, Pontificia Universidad Católica de Chile	MSHS Pathogen Surveillance Program	Leonardo I. Almonacid, Ana S. Gonzalez-Reiche, Matthew M. Hernandez, Jorge Levican, Ana Maria Contreras, Carlos Palma, Tamara García-Salum, Zenab Khan, Adriana van De Guchte, Ajay Obla, Jayeeta Dutta, Bremy Alburquerque, Eileen Serrano, Constanza Maldonado, M. Belen Leyton, Erick Salinas, Hala Alshammary, Juan Soto, Shwetha Hara Sridhar, Ying-Chih Wang, Melissa Smith, Robert Sebra, Marcela Ferres, Adolfo Garcia-Sastre, Edward C. Holmes, Viviana Simon, Harm van Bakel, Rafael A. Medina.
EPI_ISL_801894, EPI_ISL_801912, EPI_ISL_801928, EPI_ISL_801938, EPI_ISL_802016, EPI_ISL_802017, EPI_ISL_802018, EPI_ISL_802019, EPI_ISL_802020, EPI_ISL_802021, EPI_ISL_802022, EPI_ISL_802023, EPI_ISL_802024, EPI_ISL_802025, EPI_ISL_802026, EPI_ISL_802027, EPI_ISL_802028, EPI_ISL_802029, EPI_ISL_802030			
see above	MSHS Clinical Microbiology Laboratories	MSHS Pathogen Surveillance Program	Ana S. Gonzalez-Reiche, Hala Alshammary, Mitchell J. Sullivan, Brianne Ciferri, Ajay Obla, Angela Amoako, Mahmoud Awawda, Elena Hirsch, Ashley S. Salimbangon, Levy Sominsky, Katherine Beach, Kayla Russo, Charles Gleason, Shelcie Fabre, Giulio Kleiner, Zenab Khan, Bremy Alburquerque, Adriana van de Guchte, Komal Srivastava, Matthew M. Hernandez, Jayeeta Dutta, Denise Jurczyszak, Emily Ferreri, Rachel Chernet, Nancy Francoeur, Betsaida Salom Melo, Irina Oussenko, Gintaras Deikus, Juan Soto, Shwetha Hara Sridhar, Ying-Chih Wang, Kathryn Twyman, Andrew Kasarskis, Deena R. Altman, Robert Sebra, Adolfo García-Sastre, Marta Luksza, Gopi Patel, Sarah Schaefer, Melissa Gitman, Michael D. Nowak, Alberto Paniz-Mondolfi, Emilia Mia Sordillo, Viviana Simon, Harm van Bakel

EPI_ISL_804379, EPI_ISL_804388, EPI_ISL_804391, EPI_ISL_804404, EPI_ISL_804407, EPI_ISL_804408, EPI_ISL_804423, EPI_ISL_804425, EPI_ISL_804430, EPI_ISL_804437, EPI_ISL_804440, EPI_ISL_804441, EPI_ISL_804453, EPI_ISL_804454, EPI_ISL_804461			
see above	Dutch COVID-19 response team	National Institute for Public Health and the Environment (RIVM)	Adam Meijer, Harry Vennema, Dirk Eggink, Matthijs Welkers, Jeroen Cremer, Sharon van den Brink, Bas van der Veer, AnneMarie van den Brandt, Florian Zwagemaker, Dennis Schmitz, Chantal Reusken, on behalf of the national COVID-19 response team
EPI_ISL_804986, EPI_ISL_804994, EPI_ISL_804996, EPI_ISL_804997, EPI_ISL_805000, EPI_ISL_805005, EPI_ISL_805011, EPI_ISL_805012, EPI_ISL_805075, EPI_ISL_805078, EPI_ISL_805127, EPI_ISL_805133, EPI_ISL_805140, EPI_ISL_805149, EPI_ISL_805166, EPI_ISL_805181, EPI_ISL_805185, EPI_ISL_805186, EPI_ISL_805188, EPI_ISL_805191, EPI_ISL_805193, EPI_ISL_805202, EPI_ISL_805208, EPI_ISL_805214, EPI_ISL_805215, EPI_ISL_805220, EPI_ISL_805225, EPI_ISL_805226, EPI_ISL_805228, EPI_ISL_805229, EPI_ISL_805231, EPI_ISL_805234, EPI_ISL_805235, EPI_ISL_805238, EPI_ISL_805240, EPI_ISL_805241, EPI_ISL_805242, EPI_ISL_805244, EPI_ISL_805247, EPI_ISL_805249, EPI_ISL_805253, EPI_ISL_805257, EPI_ISL_805260, EPI_ISL_805261, EPI_ISL_805262, EPI_ISL_805263, EPI_ISL_805264, EPI_ISL_805265, EPI_ISL_805267, EPI_ISL_805268, EPI_ISL_805269, EPI_ISL_805270, EPI_ISL_805271, EPI_ISL_805272, EPI_ISL_805275, EPI_ISL_805276, EPI_ISL_805277, EPI_ISL_805279, EPI_ISL_805280, EPI_ISL_805282, EPI_ISL_805284, EPI_ISL_805285, EPI_ISL_805286, EPI_ISL_805287, EPI_ISL_805288, EPI_ISL_805289, EPI_ISL_805290, EPI_ISL_805291, EPI_ISL_805293, EPI_ISL_805294, EPI_ISL_805295, EPI_ISL_805297, EPI_ISL_805298, EPI_ISL_805299, EPI_ISL_805300, EPI_ISL_805302, EPI_ISL_805304, EPI_ISL_805305, EPI_ISL_805307, EPI_ISL_805308, EPI_ISL_805309, EPI_ISL_805313, EPI_ISL_805314, EPI_ISL_805317, EPI_ISL_805318, EPI_ISL_805319, EPI_ISL_805320, EPI_ISL_805321, EPI_ISL_805322, EPI_ISL_805323, EPI_ISL_805326, EPI_ISL_805327, EPI_ISL_805328, EPI_ISL_805332, EPI_ISL_805333, EPI_ISL_805334, EPI_ISL_805335, EPI_ISL_805336, EPI_ISL_805338, EPI_ISL_805340, EPI_ISL_805342, EPI_ISL_805343, EPI_ISL_805345, EPI_ISL_805347, EPI_ISL_805348, EPI_ISL_805349, EPI_ISL_805350, EPI_ISL_805352, EPI_ISL_805353, EPI_ISL_805354, EPI_ISL_805355, EPI_ISL_805356, EPI_ISL_805358, EPI_ISL_805360, EPI_ISL_805364, EPI_ISL_805365, EPI_ISL_805366, EPI_ISL_805367, EPI_ISL_805369, EPI_ISL_805370, EPI_ISL_805371, EPI_ISL_805373, EPI_ISL_805374, EPI_ISL_805375, EPI_ISL_805380, EPI_ISL_805382, EPI_ISL_805383, EPI_ISL_805388, EPI_ISL_805389, EPI_ISL_805391, EPI_ISL_805392, EPI_ISL_805393, EPI_ISL_805397, EPI_ISL_805398, EPI_ISL_805399, EPI_ISL_805401, EPI_ISL_805402, EPI_ISL_805403, EPI_ISL_805404, EPI_ISL_805406, EPI_ISL_805407, EPI_ISL_805408, EPI_ISL_805412, EPI_ISL_805413, EPI_ISL_805414, EPI_ISL_805415, EPI_ISL_805416, EPI_ISL_805419, EPI_ISL_805421, EPI_ISL_805422, EPI_ISL_805425, EPI_ISL_805428, EPI_ISL_805432, EPI_ISL_805433, EPI_ISL_805439, EPI_ISL_805440, EPI_ISL_805441, EPI_ISL_805442, EPI_ISL_805443, EPI_ISL_805445, EPI_ISL_805447, EPI_ISL_805448, EPI_ISL_805451, EPI_ISL_805461, EPI_ISL_805463, EPI_ISL_805467, EPI_ISL_805470, EPI_ISL_805471, EPI_ISL_805473, EPI_ISL_805474, EPI_ISL_805483, EPI_ISL_805493, EPI_ISL_805495, EPI_ISL_805502, EPI_ISL_805509, EPI_ISL_805513, EPI_ISL_805517, EPI_ISL_805520, EPI_ISL_805521, EPI_ISL_805522, EPI_ISL_805523, EPI_ISL_805525, EPI_ISL_805530, EPI_ISL_805536, EPI_ISL_805541, EPI_ISL_805545, EPI_ISL_805546, EPI_ISL_805551, EPI_ISL_805556, EPI_ISL_805558, EPI_ISL_805560, EPI_ISL_805563, EPI_ISL_805573, EPI_ISL_805574, EPI_ISL_805587, EPI_ISL_805588, EPI_ISL_805599, EPI_ISL_805602, EPI_ISL_805611, EPI_ISL_805619, EPI_ISL_805623, EPI_ISL_805624, EPI_ISL_805628, EPI_ISL_805632, EPI_ISL_805635, EPI_ISL_805637, EPI_ISL_805648, EPI_ISL_805651, EPI_ISL_805654, EPI_ISL_805656, EPI_ISL_805658, EPI_ISL_805661, EPI_ISL_805663, EPI_ISL_805666, EPI_ISL_805670, EPI_ISL_805671, EPI_ISL_805674, EPI_ISL_805685, EPI_ISL_805692, EPI_ISL_805698, EPI_ISL_805702, EPI_ISL_805703, EPI_ISL_805705, EPI_ISL_805710, EPI_ISL_805711, EPI_ISL_805712, EPI_ISL_805716, EPI_ISL_805727, EPI_ISL_805728, EPI_ISL_805752, EPI_ISL_805758, EPI_ISL_805761, EPI_ISL_805766, EPI_ISL_805770, EPI_ISL_805779, EPI_ISL_805784, EPI_ISL_805795, EPI_ISL_805805, EPI_ISL_805822, EPI_ISL_805826, EPI_ISL_805843, EPI_ISL_805844, EPI_ISL_805846, EPI_ISL_805850, EPI_ISL_805852, EPI_ISL_805854, EPI_ISL_805855, EPI_ISL_805857, EPI_ISL_805860, EPI_ISL_805871, EPI_ISL_805880, EPI_ISL_805881, EPI_ISL_805886, EPI_ISL_805891, EPI_ISL_805893, EPI_ISL_805898, EPI_ISL_805899, EPI_ISL_805903, EPI_ISL_805904, EPI_ISL_805908, EPI_ISL_805911, EPI_ISL_805913, EPI_ISL_805917, EPI_ISL_805918, EPI_ISL_805921, EPI_ISL_805929, EPI_ISL_805930, EPI_ISL_805932, EPI_ISL_805943, EPI_ISL_805946, EPI_ISL_805948, EPI_ISL_805949, EPI_ISL_805954, EPI_ISL_805963, EPI_ISL_805974, EPI_ISL_805975, EPI_ISL_805976, EPI_ISL_805981, EPI_ISL_805991, EPI_ISL_805994, EPI_ISL_805995, EPI_ISL_806005, EPI_ISL_806006, EPI_ISL_806009, EPI_ISL_806012, EPI_ISL_806013, EPI_ISL_806015, EPI_ISL_806017, EPI_ISL_806018, EPI_ISL_806019, EPI_ISL_806027, EPI_ISL_806029, EPI_ISL_806030, EPI_ISL_806037, EPI_ISL_806043, EPI_ISL_806048, EPI_ISL_806050, EPI_ISL_806054, EPI_ISL_806057, EPI_ISL_806058, EPI_ISL_806061, EPI_ISL_806070, EPI_ISL_806073, EPI_ISL_806079, EPI_ISL_806080, EPI_ISL_806086, EPI_ISL_806088, EPI_ISL_806089, EPI_ISL_806093, EPI_ISL_806100, EPI_ISL_806101, EPI_ISL_806103, EPI_ISL_806104, EPI_ISL_806107, EPI_ISL_806111, EPI_ISL_806113, EPI_ISL_806114, EPI_ISL_806115, EPI_ISL_806116, EPI_ISL_806117, EPI_ISL_806119, EPI_ISL_806122, EPI_ISL_806127, EPI_ISL_806129, EPI_ISL_806131, EPI_ISL_806132, EPI_ISL_806133, EPI_ISL_806134, EPI_ISL_806136, EPI_ISL_806137, EPI_ISL_806139, EPI_ISL_806140, EPI_ISL_806149, EPI_ISL_806152, EPI_ISL_806154, EPI_ISL_806156, EPI_ISL_806158, EPI_ISL_806161, EPI_ISL_806162, EPI_ISL_806163, EPI_ISL_806166, EPI_ISL_806169, EPI_ISL_806171, EPI_ISL_806174, EPI_ISL_806176, EPI_ISL_806184, EPI_ISL_806186, EPI_ISL_806187, EPI_ISL_806192, EPI_ISL_806199, EPI_ISL_806200, EPI_ISL_806206, EPI_ISL_806209, EPI_ISL_806212, EPI_ISL_806221, EPI_ISL_806222, EPI_ISL_806231, EPI_ISL_806234, EPI_ISL_806239, EPI_ISL_806240, EPI_ISL_806243, EPI_ISL_806244, EPI_ISL_806246, EPI_ISL_806247, EPI_ISL_806251, EPI_ISL_806253, EPI_ISL_806255, EPI_ISL_806256, EPI_ISL_806257, EPI_ISL_806258, EPI_ISL_806260, EPI_ISL_806261, EPI_ISL_806266, EPI_ISL_806267, EPI_ISL_806268, EPI_ISL_806270, EPI_ISL_806272, EPI_ISL_806280, EPI_ISL_806282, EPI_ISL_806283, EPI_ISL_806296, EPI_ISL_806298, EPI_ISL_806301, EPI_ISL_806310, EPI_ISL_806311, EPI_ISL_806320, EPI_ISL_806327, EPI_ISL_806328, EPI_ISL_806331, EPI_ISL_806332, EPI_ISL_806341, EPI_ISL_806346, EPI_ISL_806348, EPI_ISL_806349, EPI_ISL_806352, EPI_ISL_806359, EPI_ISL_806363, EPI_ISL_806368, EPI_ISL_806370, EPI_ISL_806374, EPI_ISL_806375, EPI_ISL_806377, EPI_ISL_806378, EPI_ISL_806382, EPI_ISL_806385, EPI_ISL_806386, EPI_ISL_806387, EPI_ISL_806390, EPI_ISL_806391, EPI_ISL_806392, EPI_ISL_806396, EPI_ISL_806397, EPI_ISL_806400, EPI_ISL_806402, EPI_ISL_806403, EPI_ISL_806407, EPI_ISL_806411, EPI_ISL_806419, EPI_ISL_806421, EPI_ISL_806422, EPI_ISL_806424, EPI_ISL_806425, EPI_ISL_806427, EPI_ISL_806430, EPI_ISL_806436, EPI_ISL_806440, EPI_ISL_806445, EPI_ISL_806449, EPI_ISL_806451, EPI_ISL_806458, EPI_ISL_806459, EPI_ISL_806460, EPI_ISL_806471, EPI_ISL_806476, EPI_ISL_806477, EPI_ISL_806480, EPI_ISL_806482, EPI_ISL_806483, EPI_ISL_806488, EPI_ISL_806495, EPI_ISL_806498, EPI_ISL_806499, EPI_ISL_806506, EPI_ISL_806508, EPI_ISL_806509, EPI_ISL_806516, EPI_ISL_806520, EPI_ISL_806524			
see above	Alberta Precision Labs (APL)	Alberta Precision Labs (APL)	Gordon P, Lam LG, Pabbaraju K, Wong A, Ma R, Li V, Melin A, Tipples G, Berenger B, Zelyas N, Kellner J, Bernier F, Chui L, Croxen M
EPI_ISL_806534, EPI_ISL_806535, EPI_ISL_806536, EPI_ISL_806537, EPI_ISL_806542	Charité Universitätsmedizin Berlin, Institut für Virologie/Labor Berlin	Charité Universitätsmedizin Berlin, Institut für Virologie	Victor M Corman, Jörn Beheim-Schwarzbach, Barbara Mühlemann, Julia Schneider, Talitha Veith, Cornelia Schlee, Tomasz Zemojtel, Terry Jones, Christian Drosten
EPI_ISL_812325, EPI_ISL_812340	United States Air Force School of Aerospace Medicine	United States Air Force School of Aerospace Medicine	Anthony Fries, Jennifer Meyer, Amanda Javorina, Sarah Purves, William Gruner, Clarise Starr, Elizabeth Macias
EPI_ISL_814074	Ministry of Health Turkey	Ministry of Health Turkey	Fatma Bayrakdar, Yasemin Cogun, Süleyman Yalcin, Aye Baak Alta, Gülay Korukluolu
EPI_ISL_816693, EPI_ISL_816757, EPI_ISL_816758, EPI_ISL_816759, EPI_ISL_816760, EPI_ISL_816761, EPI_ISL_816762, EPI_ISL_816763, EPI_ISL_816764, EPI_ISL_816765, EPI_ISL_816766, EPI_ISL_816767, EPI_ISL_816768, EPI_ISL_816769, EPI_ISL_816770, EPI_ISL_816771, EPI_ISL_816772, EPI_ISL_816773, EPI_ISL_816774, EPI_ISL_816789			
see above	Bioinformatics and Biostatistics Lab, Advanced Sequencing Facility	COVID-19 Genomics UK (COG-UK) Consortium	Aengus Stewart,Jerome Nicod,Chelsea Sawyer,Laura Cubitt,Harshil Patel,Margaret Crawford
EPI_ISL_819325, EPI_ISL_819326, EPI_ISL_819327, EPI_ISL_819328, EPI_ISL_819329, EPI_ISL_819330	Hospital Universitari Vall d'Hebron - Vall d'Hebron Institut de Rercerca	Hospital Universitari Vall d'Hebron	Cristina Andrés, Maria Piñana, Josep F Abril, Damir Garcia-Cehic, Ariadna Rando, Juliana Esperalba, Maria Gema Codina, Carla Castillo, Maria Carmen Martin, Tomàs Pumarola, Josep Quer, Andrés Antón
EPI_ISL_825834, EPI_ISL_825837, EPI_ISL_825863, EPI_ISL_825864, EPI_ISL_825865, EPI_ISL_825868, EPI_ISL_825869, EPI_ISL_825870, EPI_ISL_825871, EPI_ISL_825872, EPI_ISL_825873, EPI_ISL_825874, EPI_ISL_825879, EPI_ISL_826262			
see above	Laboratoire de santé publique du Québec	Laboratoire de santé publique du Québec	Sandrine Moreira, Ioannis Ragoussis, Guillaume Bourque, Jesse Shapiro, Mark Lathrop and Michel Roger on behalf of the CoVSeQ research group (http://covseq.ca/researchgroup)
EPI_ISL_826578, EPI_ISL_826579, EPI_ISL_826580, EPI_ISL_826582, EPI_ISL_826583, EPI_ISL_826584, EPI_ISL_826585, EPI_ISL_826586, EPI_ISL_826587, EPI_ISL_826594, EPI_ISL_826595, EPI_ISL_826596, EPI_ISL_826597, EPI_ISL_826598, EPI_ISL_826599, EPI_ISL_826600, EPI_ISL_826601, EPI_ISL_826602, EPI_ISL_826610, EPI_ISL_826611, EPI_ISL_826612, EPI_ISL_826613, EPI_ISL_826614, EPI_ISL_826615, EPI_ISL_826616, EPI_ISL_826617, EPI_ISL_826618, EPI_ISL_826619, EPI_ISL_826620, EPI_ISL_826629, EPI_ISL_826630, EPI_ISL_826631, EPI_ISL_826632, EPI_ISL_826633, EPI_ISL_826634			
see above	Montefiore Medical Center	Albert Einstein College of Medicine, Dept. of Microbiology & Immunology, Chandran lab	J. Maximilian Fels, Saad Khan, Ryan Forster, Karin A. Skalina, Surksha Sirichand, Amy S. Fox, Aviv Bergman, William B. Mitchell, Lucia R. Wolgast, Wendy Szymczak, Robert H. Bortz III, M. Eugenia Dieterle, Catalina Florez, Denise Haslwanter, Rohit K. Jangra, Ethan Laudermilch, Ariel S. Wirchnianski, Jason Barnhill, David L. Goldman, Hnin Khine, D. Yitzchak Goldstein, Johanna P. Daily, Kartik Chandran, Libusha Kelly
EPI_ISL_831043, EPI_ISL_831044, EPI_ISL_831045, EPI_ISL_831046, EPI_ISL_831047, EPI_ISL_831048, EPI_ISL_831054, EPI_ISL_831055, EPI_ISL_831056, EPI_ISL_831057, EPI_ISL_831058, EPI_ISL_831059, EPI_ISL_831060, EPI_ISL_831061, EPI_ISL_831062, EPI_ISL_831063, EPI_ISL_831064			
see above	Hospital Universitario La Paz (Madrid)	SeqCOVID-SPAIN consortium/IBV(CSIC)	María Rodríguez-Tejedor, Elias Dahdouh, Fernando Lázaro-Perona, Jesús Mingorance and SeqCOVID-SPAIN consortium
EPI_ISL_837600, EPI_ISL_837604, EPI_ISL_837647, EPI_ISL_837648, EPI_ISL_837649, EPI_ISL_837650, EPI_ISL_837651, EPI_ISL_837652, EPI_ISL_837653, EPI_ISL_837654, EPI_ISL_837655, EPI_ISL_837656, EPI_ISL_837657, EPI_ISL_837658, EPI_ISL_837659, EPI_ISL_837660, EPI_ISL_837661, EPI_ISL_837662, EPI_ISL_837663, EPI_ISL_837664, EPI_ISL_837665, EPI_ISL_837666, EPI_ISL_837667			
see above	Instituto Nacional de Enfermedades Respiratorias (INER)	Instituto Nacional de Enfermedades Respiratorias (INER)	Celia Boukadida, Margarita Matías-Florentino, Alma Rincón-Rubio, Hector Esteban Paz-Juárez, Olivia Briceño, Edgar Sevilla-Reyes, Fidencio Mejía-Nepomuceno, Mario Mújica-Sánchez, Eduardo Becerril-Vargas, José Arturo Martínez-Orozco, Alejandra Hernández-Terán, Jorge Salas-Hernández, Santiago Ávila-Ríos, Joel Armando Vázquez-Pérez
EPI_ISL_842805, EPI_ISL_842806, EPI_ISL_842807, EPI_ISL_842808, EPI_ISL_842811, EPI_ISL_842834	Barts Health NHS Trust	COVID-19 Genomics UK (COG-UK) Consortium	CUTINO-MOGUEL, Maria-Teresa; HARRINGTON, David; OWOYEMI, Dola; SHYLINI, Raghavendran; BROAD, Claire; KELE, Beatrix
EPI_ISL_848590, EPI_ISL_848591, EPI_ISL_848592, EPI_ISL_848593, EPI_ISL_848594, EPI_ISL_848621, EPI_ISL_848622, EPI_ISL_848623	Evandro Chagas Institute	Evandro Chagas Institute	Santos, M.C.; Silva, A.M.; Junior, W.D.C.; Barbagelata, L.S.; Ferreira, J.A.; Sousa, E.M.A.; da Silva, P.S.; Pinheiro, K.C.; L.C.; Sousa Junior, E.C.
EPI_ISL_849185	Servicio Virosis Respiratorias-Departamento Virología-INEI	Instituto Nacional Enfermedades Infecciosas C.G.Malbran	Baumeister E., Avaro M., Benedetti E., Russo M., Dattero ME, Pontoriero A., Cisterna D., Molina V., Perandones C., Tuduri E., Lorenzo F., Poklepovich T., Campos J.
EPI_ISL_849690	unknown	PHV-FSS	Son Nguyen et al.
EPI_ISL_850181, EPI_ISL_850182	Division of Emerging Infectious Diseases, Bureau of Infectious Diseases Diagnosis Control, Korea Disease Control and Prevention Agency	Division of Emerging Infectious Diseases, Bureau of Infectious Diseases Diagnosis Control, Korea Disease Control and Prevention Agency	Ae Kyung Park, Il-Hwan Kim, Heui Man Kim, Jeong-Min Kim, Namjoo Lee, Chaeyoung Lee, Sang Hee Woo, Eun-Jin Kim
EPI_ISL_850946, EPI_ISL_850947	National Institute for Viral Disease Control and Prevention, China CDC	National Institute for Viral Disease Control and Prevention, China CDC	Xiang Zhao, Yenan Feng, Zhixiao Chen, Yao Meng, Yuchao Wu, Yang Song, Ji Wang, Kai Nie, Yong Zhang, Yanhai Wang, Weimin Zhou, Wenjie Tan, Jun Han, Shiwen Wang, Wenbo Xu, Cao Chen, Dayan Wang
EPI_ISL_852661	Institute of Virology, Medical Center, University of Freiburg, Freiburg, Germany	Institute of Virology, Clinial Virus Genomics, Medical Center, University of Freiburg, Freiburg, Germany	Jonas Fuchs, Lisa Kern, Sandra Reuter, Hajo Grundmann, Marcus Panning
EPI_ISL_853275, EPI_ISL_853276, EPI_ISL_853279, EPI_ISL_853280,	UPMC Clinical Microbiology Laboratory	Microbial Genome Sequencing Center; Microbial Genomic Epidemiology Laboratory	Mustapha M. Mustapha, Jane W. Marsh, Dan Snyder, Marissa P. Griffith, Stephanie L. Mitchell, Vatsala R. Srinivasa, Kady D. Waggle, Chinelo Ezeonwuku, Vaughn S. Cooper, Lee H. Harrison

EPI_ISL_853281, EPI_ISL_853282, EPI_ISL_853283			
EPI_ISL_853406	Laboratoire de santé publique du Québec	Laboratoire de santé publique du Québec	Sandrine Moreira, Ioannis Ragoussis, Guillaume Bourque, Jesse Shapiro, Mark Lathrop and Michel Roger on behalf of the CoVSeQ research group (http://covseq.ca/researchgroup)
EPI_ISL_853546, EPI_ISL_853547, EPI_ISL_853548, EPI_ISL_853549, EPI_ISL_853550	Santa Clara County Public Health Laboratory	Chan-Zuckerberg Biohub	CZB Cliahub Consortium
EPI_ISL_853835, EPI_ISL_853883	Center for Virology, Medical University of Vienna	Bergthaler laboratory, CeMM Research Center for Molecular Medicine of the Austrian Academy of Sciences	Lukas Endler, Alexandra Popa, Benedikt Agerer, Jakob-Wendelin Genger, Alexander Lercher, Anna Schedl, Thomas Penz, Michael Schuster, Jan Laine, Martin Senekowitsch, Christoph Bock, Andreas Bergthaler
EPI_ISL_854477, EPI_ISL_854478, EPI_ISL_854479, EPI_ISL_854480, EPI_ISL_854481, EPI_ISL_854482, EPI_ISL_854483, EPI_ISL_854484, EPI_ISL_854485, EPI_ISL_854486, EPI_ISL_854487, EPI_ISL_854488, EPI_ISL_854489, EPI_ISL_854490, EPI_ISL_854491, EPI_ISL_854492, EPI_ISL_854493, EPI_ISL_854494, EPI_ISL_854495, EPI_ISL_854496, EPI_ISL_854497, EPI_ISL_854498, EPI_ISL_854499, EPI_ISL_854500, EPI_ISL_854501, EPI_ISL_854502, EPI_ISL_854503, EPI_ISL_854504, EPI_ISL_854505, EPI_ISL_854506, EPI_ISL_854507, EPI_ISL_854508, EPI_ISL_854509, EPI_ISL_854510, EPI_ISL_854511, EPI_ISL_854512, EPI_ISL_854513, EPI_ISL_854514			
see above	Alberta Precision Labs (APL)	Alberta Precision Labs (APL)	Gordon P, Lam LG, Pabbaraju K, Wong A, Ma R, Li V, Melin A, Tipples G, Berenger B, Zelyas N, Kellner J, Bernier F, Chui L, Croxen M
EPI_ISL_860093, EPI_ISL_860094	MEPHI, Aix Marseille University	MEPHI, Aix Marseille University	Anthony Levasseur
EPI_ISL_861420	Instituto de Diagnostico y Referencia Epidemiologicos (INDRE)	Instituto de Diagnostico y Referencia Epidemiologicos (INDRE)	Abril Rodriguez-Maldonado, Gisela Barrera-Badillo ,Claudia Wong-Arambula , Natividad Cruz-Ortiz, Tatiana Nunez-Garcia, Dayanira Arellano-Suarez, Fabiola Garces-Ayala, Adnan-Araiza Rodríguez, Edgar Mendieta-Condado, Lucia Hernandez-Rivas, Irma Lopez-Martinez, Ernesto Ramirez-Gonzalez.
EPI_ISL_861629, EPI_ISL_861630, EPI_ISL_861631, EPI_ISL_861632	Instituto Adolfo Lutz - Central	Instituto Adolfo Lutz, Interdiciplinary Procedures Center, Strategic Laboratory	Claudio Tavares Sacchi, Claudia Regina Gonçalves, Erica Valessa Ramos Gomes, Karoline Rodrigues Campos
EPI_ISL_861889	Genomika Einstein	LATE - Laboratório de Técnicas Especiais - Hospital Israelita Albert Einstein	Deyvid Amgarten, Fernanda de Mello Malta, Raquel Riyuzo, Ana Paula Moreira Salles, Pedro Henrique Sebe Rodrigues, João Bosco Oliveira Filho, João Renato Rebello Pinho
EPI_ISL_861896	LATE - Laboratório de Técnicas Especiais - Hospital Israelita Albert Einstein	LATE - Laboratório de Técnicas Especiais - Hospital Israelita Albert Einstein	Deyvid Amgarten, Fernanda de Mello Malta, Raquel Riyuzo, Ana Paula Moreira Salles, Pedro Henrique Sebe Rodrigues, João Renato Rebello Pinho
EPI_ISL_861911, EPI_ISL_861914	Genomika Einstein	LATE - Laboratório de Técnicas Especiais - Hospital Israelita Albert Einstein	Deyvid Amgarten, Fernanda de Mello Malta, Raquel Riyuzo, Ana Paula Moreira Salles, Pedro Henrique Sebe Rodrigues, João Bosco Oliveira Filho, João Renato Rebello Pinho
EPI_ISL_862858	Isehara Kyodo Hospital	Fujita health University, School of Medicine, Department of Microbiology	Masahiro suzuki, Yohei Doi
EPI_ISL_862866	Nara Medical University Hospital	Fujita health University, School of Medicine, Department of Microbiology	Masahiro suzuki, Yohei Doi
EPI_ISL_862869, EPI_ISL_862870	Saiseikai Niigata Hospital	Fujita health University, School of Medicine, Department of Microbiology	Masahiro suzuki, Yohei Doi
EPI_ISL_862872	Japanese Red Cross Narita Hospital	Fujita health University, School of Medicine, Department of Microbiology	Masahiro suzuki, Yohei Doi
EPI_ISL_865899	University College London, Great Ormond Street Hospital for Children NHS Foundation Trust, Imperial College Healthcare NHS Trust	COVID-19 Genomics UK (COG-UK) Consortium	Sergi Castellano, Rachel Williams, Mark Kristiansen, Paola Resende Silva, Sunando Roy, Tony Brooks, Helena Tutill, Paola Niola, Patricia Dyal, Charlotte Williams, Leysa Forrest, Yasmin Panchbhaya, Jacqueline Findlay, Samuel Weeks, Julianne Brown, Kathryn Harris, Paul Randell, James Price, Alison Holmes, Judith Breuer
EPI_ISL_869148, EPI_ISL_869149, EPI_ISL_869150, EPI_ISL_869151, EPI_ISL_869152, EPI_ISL_869153, EPI_ISL_869154, EPI_ISL_869155, EPI_ISL_869156, EPI_ISL_869157, EPI_ISL_869158, EPI_ISL_869159, EPI_ISL_869160, EPI_ISL_869161, EPI_ISL_869162, EPI_ISL_869163, EPI_ISL_869164, EPI_ISL_869165			
see above	National Virus Reference Laboratory	Irish Coronavirus Sequencing Consortium - Teagasc Moorepark	Calum Walsh, Genuity Ireland
EPI_ISL_872028, EPI_ISL_872029, EPI_ISL_872030, EPI_ISL_872031, EPI_ISL_872032	Ospedale "Di Venere"	Beaconlab (Bioinformatics, Evolution and Comparative Genomics lab), Dept of Biosciences, University on Milan	Iacobellis M, d'Avenia M, Piluscio R, Parisi A, Chiara M, Manzari C, Pesole G
EPI_ISL_872092	Instituto de Diagnostico y Referencia Epidemiologicos (INDRE)	Instituto de Diagnostico y Referencia Epidemiologicos (INDRE)	Ernesto Ramirez-Gonzalez, Adnan-Araiza Rodríguez,Claudia Wong-Arambula, Gisela Barrera-Badillo , Abril Rodriguez-Maldonado, Natividad Cruz-Ortiz, Tatiana Nunez-Garcia, Dayanira Arellano-Suarez, Fabiola Garces-Ayala, Edgar Mendieta-Condado, Lucia Hernandez-Rivas, Irma Lopez-Martinez.
EPI_ISL_876034, EPI_ISL_876039, EPI_ISL_876040	Montefiore Medical Center	Albert Einstein College of Medicine, Dept. of Microbiology & Immunology, Chandran lab	J. Maximilian Fels, Saad Khan, Ryan Forster, Karin A. Skalina, Surksha Sirichand, Amy S. Fox, Aviv Bergman, William B. Mitchell, Lucia R. Wolgast, Wendy Szymczak, Robert H. Bortz III, M. Eugenia Dieterle, Catalina Florez, Denise Haslwanter, Rohit K. Jangra, Ethan Laudermilch, Ariel S. Wirchnianski, Jason Barnhill, David L. Goldman, Hnin Khine, D. Yitzchak Goldstein, Johanna P. Daily, Kartik Chandran, Libusha Kelly
EPI_ISL_876971, EPI_ISL_877024, EPI_ISL_877025, EPI_ISL_877026, EPI_ISL_877027, EPI_ISL_877028, EPI_ISL_877029, EPI_ISL_877030, EPI_ISL_877031	1.AO Universitaria 'S. Giovanni di Dio e Ruggi D'Aragona, Scuola Medica Salernitana' Hospital / 2.UOC di Virologia e Microbiologia, Università della Campania 'L. Vanvitelli' / 3.AO Universitaria 'Federico II' Napoli Hospital / 4.AORN 'San Giuseppe Moscati' Avellino Hospital / 5.AO 'San Pio - presidio G. Rummo' Benevento Hospital / 6.AO 'Sant'Anna e San Sebastiano' Caserta Hospital / 7.PO 'Maria Santissima Addolorata' Eboli Hospital / 8.Biogem Istituto di Ricerche Genetiche	1. Genome Research Center for Health (CRGS) / 2. Laboratory of Molecular Medicine and Genomics(LMMGe) / 3. Center for Research in Pure and Applied Mathematics (CRMPA)	Giorgio Giurato (Corresponding Author), Francesca Rizzo (Corresponding Author), Alessandro Weisz (Corresponding Author), Gianluigi Franci, Giovanni Nassa, Pasquale Pagliano, Roberta Tarallo, Elena Alexandrova, Ylenia D'Agostino, Carlo Ferravante, Jessica Lamberti, Viola Melone, Domenico Memoli, Valeria Mirici Cappa, Domenico Palumbo, Giovanni Pecoraro, Assunta Sellitto, Oriana Strianese, Ilaria Terenzi, Giuseppe Fenza, Aniello Gentile, Antonello Saccomanno, Sonia Amabile, Teresa Rocco, Annamaria Salvati, Emilia Vaccaro, Massimiliano Galdiero, Michele Cennamo, Giuseppe Portella, Maria Grazia Foti, Mariarosaria Ingino, Maria Landi, Maurizio Fumi, Vincenzo Rocco, Rita Greco, Vittoria Letizia, Arnolfo Petruzziello, Maddalena Schioppa, Gregorio Goffredi, Francesca Marciano, Michele Caraglia, Alessia Cossu, Marianna Scrima
EPI_ISL_877597, EPI_ISL_877598, EPI_ISL_877599	Clinical Molecular Microbiology Laboratory, UNC Hospital	Dirk Dittmer	Razia Moorad , Justin T. Landis , Brent A. Eason, Melissa B. Miller, Linda Pluta, Dirk Dittmer, Angelica Juarez, Cecilia Thompson , Cameroon Grant, Evelyn Hoffman, Patricio Cano, Jason Wong, Carolina Caro-Vegas, Blossom Damania.
EPI_ISL_883169, EPI_ISL_883170	Hannover Medical School, Institute of Virology	Hannover Medical School, Institute of Virology	Lars Steinbrück, Jasper Götting
EPI_ISL_884564, EPI_ISL_884565, EPI_ISL_884566, EPI_ISL_884567	Molecular Microbiology & Immunology, University of Missouri	Molecular Microbiology & Immunology, University of Missouri	Tang,C.Y., Li,T., Hang,J., Lidl,G.M., Wan,X.-F.
EPI_ISL_887184, EPI_ISL_887192, EPI_ISL_887194, EPI_ISL_887197, EPI_ISL_887208, EPI_ISL_887210, EPI_ISL_887213, EPI_ISL_887220, EPI_ISL_887223, EPI_ISL_887224, EPI_ISL_887227, EPI_ISL_887233, EPI_ISL_887234, EPI_ISL_887236, EPI_ISL_887249, EPI_ISL_887251, EPI_ISL_887262, EPI_ISL_887268, EPI_ISL_887300, EPI_ISL_887304, EPI_ISL_887316, EPI_ISL_887355, EPI_ISL_887365, EPI_ISL_887369			
see above	Protzer Lab	Protzer Lab, Gagneur Lab, Robert Koch Institut	Ulrike Protzer, Dieter Hoffmann, Eva Schulte, Andrea Theumer, Oliver Drechsel, Max von Kleist,Aleksandar Radonic,Stephan Fuchs, Alexander Karollus, Julien Gagneur
EPI_ISL_887507, EPI_ISL_888680	1.AO Universitaria 'S. Giovanni di Dio e Ruggi D'Aragona, Scuola Medica Salernitana' Hospital / 2.UOC di Virologia e Microbiologia, Università della Campania 'L. Vanvitelli' / 3.AO Universitaria 'Federico II' Napoli Hospital / 4.AORN 'San Giuseppe Moscati' Avellino Hospital / 5.AO 'San Pio - presidio G. Rummo' Benevento Hospital / 6.AO 'Sant'Anna e San Sebastiano' Caserta Hospital / 7.PO 'Maria Santissima Addolorata' Eboli Hospital / 8.Biogem Istituto di Ricerche Genetiche	1. Genome Research Center for Health (CRGS) / 2. Laboratory of Molecular Medicine and Genomics(LMMGe) / 3. Center for Research in Pure and Applied Mathematics (CRMPA)	Giorgio Giurato, Francesca Rizzo, Alessandro Weisz, Gianluigi Franci, Giovanni Nassa, Pasquale Pagliano, Roberta Tarallo, Elena Alexandrova, Ylenia D'Agostino, Carlo Ferravante, Jessica Lamberti, Viola Melone, Domenico Memoli, Valeria Mirici Cappa, Domenico Palumbo, Giovanni Pecoraro, Assunta Sellitto, Oriana Strianese, Ilaria Terenzi, Giuseppe Fenza, Aniello Gentile, Antonello Saccomanno, Sonia Amabile, Teresa Rocco, Annamaria Salvati, Emilia Vaccaro, Massimiliano Galdiero, Michele Cennamo, Giuseppe Portella, Maria Grazia Foti, Mariarosaria Ingino, Maria Landi, Maurizio Fumi, Vincenzo Rocco, Rita Greco, Vittoria Letizia, Arnolfo Petruzziello, Maddalena Schioppa, Gregorio Goffredi, Francesca Marciano, Michele Caraglia, Alessia Cossu, Marianna Scrima

EPI_ISL_895739, EPI_ISL_895745, EPI_ISL_895748, EPI_ISL_895753, EPI_ISL_895755, EPI_ISL_895758, EPI_ISL_895772, EPI_ISL_895777, EPI_ISL_895787, EPI_ISL_895822, EPI_ISL_895831			
see above	Molecular biology division, Institute of Clinical Biochemistry and Diagnostics, Charles University, Faculty of Medicine in Hradec Králové and University Hospital Hradec Králové	Molecular biology division, Institute of Clinical Biochemistry and Diagnostics, Charles University, Faculty of Medicine in Hradec Králové and University Hospital Hradec Králové	Helena Kovaíková, Petr Brož, Ivana Baranová, Kateina Hrochová, Tereza Baková, Jitka Novotná, Kateina Pehlíková, Vladimír Palika. Cooperation project with BioVendor-R&D and bioinformatics company BIOXSYS s r.o.
EPI_ISL_900138, EPI_ISL_900216, EPI_ISL_900306, EPI_ISL_900363	MEPHI, Aix Marseille University	MEPHI, Aix Marseille University	Anthony LEVASSEUR
EPI_ISL_903070	Washington State Department of Health	Seattle Flu Study	Deborah A. Nickerson, Chris D. Frazar, Jover Lee, Benjamin Pelle, Erica Ryke, Matthew Richardson, Amanda Adler, Elisabeth Brandstetter, Peter D. Han, Kairsten Fay, Misja Ilcisin, Kirsten Lacombe, Thomas R. Sibley, Melissa Truong, Caitlin R. Wolf, Romesh Gautom, Geoff Melly, Brian Hiatt, Philip Dykema, Scott Lindquist, Michael Boeckh, Janet A. Englund, Michael Famulare, Barry R. Lutz, Mark J. Rieder, Lea M. Starita, Matthew Thompson, Helen Y. Chu, Jay Shendure, Trevor Bedford
EPI_ISL_904025, EPI_ISL_904026, EPI_ISL_904034	DB Diagnosticos do Brasil	Laboratório de Parasitologia Médica - Instituto de Medicina Tropical - Universidade de São Paulo	Brazil-UK Centre for Arbovirus Discovery Diagnosis Genomics and Epidemiology (CADDE) Genomic Network - Instituto de Medicina Tropical
EPI_ISL_906065	Day Hospital de Ermelino Matarazzo	Instituto Adolfo Lutz, Interdiciplinary Procedures Center, Strategic Laboratory	Claudio Tavares Sacchi, Claudia Regina Gonçalves, Erica Valessa Ramos Gomes, Karoline Rodrigues Campos
EPI_ISL_914040, EPI_ISL_914041, EPI_ISL_914042, EPI_ISL_914044, EPI_ISL_914045, EPI_ISL_914046	TGen North	TGen North	"Jolene Bowers, Megan Folkerts, Chris French, Hayley Yaglom, Ashlyn Pfeiffer, Darrin Lemmer, Dave Engelthaler, The Arizona COVID Genomics Union (ACGU)"
EPI_ISL_914971, EPI_ISL_914972, EPI_ISL_914990, EPI_ISL_914993, EPI_ISL_915009, EPI_ISL_915010, EPI_ISL_915011, EPI_ISL_915012, EPI_ISL_915013, EPI_ISL_915014, EPI_ISL_915015, EPI_ISL_915016, EPI_ISL_915017, EPI_ISL_915018, EPI_ISL_915019, EPI_ISL_915020, EPI_ISL_915021, EPI_ISL_915022, EPI_ISL_915023, EPI_ISL_915024, EPI_ISL_915025, EPI_ISL_915026, EPI_ISL_915027, EPI_ISL_915028, EPI_ISL_915029, EPI_ISL_915030, EPI_ISL_915031, EPI_ISL_915032, EPI_ISL_915033, EPI_ISL_915034, EPI_ISL_915035, EPI_ISL_915036, EPI_ISL_915037, EPI_ISL_915038, EPI_ISL_915039, EPI_ISL_915040, EPI_ISL_915041, EPI_ISL_915042, EPI_ISL_915043, EPI_ISL_915044, EPI_ISL_915045, EPI_ISL_915046, EPI_ISL_915047, EPI_ISL_915048, EPI_ISL_915049, EPI_ISL_915050, EPI_ISL_915051, EPI_ISL_915052, EPI_ISL_915053, EPI_ISL_915054, EPI_ISL_915055, EPI_ISL_915056, EPI_ISL_915057, EPI_ISL_915058, EPI_ISL_915059, EPI_ISL_915060, EPI_ISL_915061, EPI_ISL_915062, EPI_ISL_915063, EPI_ISL_915064, EPI_ISL_915065, EPI_ISL_915066, EPI_ISL_915067, EPI_ISL_915068, EPI_ISL_915069, EPI_ISL_915070, EPI_ISL_915071, EPI_ISL_915072, EPI_ISL_915073, EPI_ISL_915074, EPI_ISL_915075, EPI_ISL_915076, EPI_ISL_915077, EPI_ISL_915078, EPI_ISL_915079			
see above	QEII Health Sciences Centre	National Microbiology Laboratory (NML)	Anna Majer, Shari Tyson, Grace Seo, Philip Mabon, Elsie Grudeski, Rhiannon Huzarewich, Russell Mandes, Anneliese Landgraff, Jennifer Tanner, Natalie Knox, Morag Graham, Gary Van Domselaar, Todd Hatchette, Jason LeBlanc, Janice Pettipas, Dan Gaston, Nathalie Bastien, Yan Li, Timothy Booth, Darian Hole, Madison Chapel, CanCOGeN's metadata curation team, Public Health Agency of Canada CanCOGeN team
EPI_ISL_915387	Keio University School of Medicine	Keio University School of Medicine	Kenjiro Kosaki, Yuka Iwasaki, Hirotsugu Ishizu, Haruhiko Siomi, Kodai Abe
EPI_ISL_918471, EPI_ISL_918481, EPI_ISL_918482	1.AO Universitaria 'S. Giovanni di Dio e Ruggi D'Aragona, Scuola Medica Salernitana' Hospital / 2.UOC di Virologia e Microbiologia, Università della Campania 'L. Vanvitelli' / 3.AO Universitaria 'Federico II' Napoli Hospital / 4.AORN 'San Giuseppe Moscati' Avellino Hospital / 5.AO 'San Pio - presidio G. Rummo' Benevento Hospital / 6.AO 'Sant'Anna e San Sebastiano' Caserta Hospital / 7.PO 'Maria Santissima Addolorata' Eboli Hospital / 8.Biogem Istituto di Ricerche Genetiche	1. Genome Research Center for Health (CRGS) / 2. Laboratory of Molecular Medicine and Genomics(LMMGe) / 3. Center for Research in Pure and Applied Mathematics (CRMPA)	Giorgio Giurato (Corresponding Author), Francesca Rizzo (Corresponding Author), Alessandro Weisz (Corresponding Author), Gianluigi Franci, Giovanni Nassa, Pasquale Pagliano, Roberta Tarallo, Elena Alexandrova, Ylenia D'Agostino, Carlo Ferravante, Jessica Lamberti, Viola Melone, Domenico Memoli, Valeria Mirici Cappa, Domenico Palumbo, Giovanni Pecoraro, Assunta Sellitto, Oriana Strianese, Ilaria Terenzi, Giuseppe Fenza, Aniello Gentile, Antonello Saccomanno, Sonia Amabile, Teresa Rocco, Annamaria Salvati, Emilia Vaccaro, Massimiliano Galdiero, Michele Cennamo, Giuseppe Portella, Maria Grazia Foti, Mariarosaria Ingino, Maria Landi, Maurizio Fumi, Vincenzo Rocco, Rita Greco, Vittoria Letizia, Arnolfo Petruzziello, Maddalena Schioppa, Gregorio Goffredi, Francesca Marciano, Michele Caraglia, Alessia Cossu, Marianna Scrima
EPI_ISL_918536	LACEN - Laboratório Central de Saúde Pública do Ceara	Evandro Chagas Institute	Santos, M.C.; Silva, A.M.; Junior, W.D.C.; Barbagelata, L.S.; Ferreira, J.A.; Sousa, E.M.A.; da Silva, P.S.; Pinheiro, K.C.; L.C.; Sousa Junior, E.C.
EPI_ISL_933630	Toronto Invasive Bacterial Diseases Network	McMaster University	Allison McGeer, Patryk Aftanas, Hooman Derakhshani, Angel Li, Kuganya Nirmalarajah, Emily Panousis, Ahmed Draia, Jalees Nasir, Michael Surette, Samira Mubareka, Andrew G. McArthur
EPI_ISL_937533, EPI_ISL_937537, EPI_ISL_937538	Thai Red Cross Emerging Infectious Diseases Health Science Centre, Chulalongkorn Hospital, Faculty of Medicine, Chulalongkorn University	Thai Red Cross Emerging Infectious Diseases Center and Faculty of Medicine, Chulalongkorn University	Opass Putcharoen, Watsamon Jantarabenjakul, Pattama Torvorapanit, Gompol Suwanpimolkul, Leilani Paitoonpong, Sopon Iamsirithaworn, Sininat Petcharat, Yutthana Joyjinda, Weenassarin Ampoot, Apaporn Rodpan, Thiravat Hemachudha, Supaporn Wacharapluesadee
EPI_ISL_939670	Division of Infectious Diseases, University Hospital Zürich	Institute of Medical Virology, University of Zurich	Stefan Schmutz, Maryam Zaheri, Verena Kufner, Annette Audigé, Maria Grünberg, Kevin Steiner, Jon Huder, Cyril Shah, Riccarda Capaul, Guido Bloemberg, Jürg Böni, Michael Huber, Alexandra Trkola
EPI_ISL_940382, EPI_ISL_940423, EPI_ISL_940424, EPI_ISL_940428	Hôpital Bichat Claude Bernard, Laboratoire de Virologie	IAME UMR1137 Inserm, Université de Paris, Hôpital Bichat	Antoine Bridier-Nahmias, Amélie Recoing, Quentin Le Hingrat, Lena Daniel, Siham Hamri, Gilles Collin, Alexandre Storto, Mélanie Bertine, Charlotte Charpentier, Nadhira Houhou-Fidouh, Diane Descamps, Benoit Visseaux
EPI_ISL_941941, EPI_ISL_941946, EPI_ISL_941952, EPI_ISL_941954, EPI_ISL_941959, EPI_ISL_941966, EPI_ISL_941967, EPI_ISL_941999, EPI_ISL_942005	Centro de Investigaciones en Microbiología y Biotecnología-UR (CIMBIUR), Facultad de Ciencias Naturales, Universidad del Rosario, Bogotá, Colombia	Centro de Investigaciones en Microbiología y Biotecnología-UR (CIMBIUR), Facultad de Ciencias Naturales, Universidad del Rosario, Bogotá, Colombia Instituto Nacional de Salud, Bogotá, Colombia Icahn School of Medicine at Mount Sinai, New York, USA	Luz Helena Patiño, Marina Muñoz, Nathalia Ballesteros, Carolina Hernández, Carolina Flórez, Sergio Gomez, Adriana van de Guchte, Zenab Khan, Jayeeta Dutta, Hala Alejel Alshammary, Ana S. Gonzalez-Reiche, Matthew M. Hernandez, Emilia Mia Sordillo, Viviana Simon, Harm van Bakel, Alberto Paniz-Mondolfi, Juan David Ramírez
EPI_ISL_943592	Lacen_RS	State Center for Health Surveillance. Rio Grande do Sul State Secretary of Health	Aline Campos, Amanda da Silva, Anelise Schaurich, Claudia Dornelles, Cynthia Molina, Fernanda Godinho, Lara Crescente, Leticia Garay, Regina Barcellos, Richard Salvato, Tatiana Gregianini, Vagner Fonseca
EPI_ISL_943614, EPI_ISL_943615, EPI_ISL_943616, EPI_ISL_943621, EPI_ISL_943622, EPI_ISL_943623, EPI_ISL_943625, EPI_ISL_943629, EPI_ISL_943630, EPI_ISL_943633, EPI_ISL_943635			
see above	Utah Public Health Laboratory	Utah Public Health Laboratory	Erin L. Young, Kelly F. Oakeson, Tara Gallagher
EPI_ISL_944635, EPI_ISL_944637	Centro de Investigaciones en Microbiología y Biotecnología-UR (CIMBIUR), Facultad de Ciencias Naturales, Universidad del Rosario, Bogotá, Colombia	Centro de Investigaciones en Microbiología y Biotecnología-UR (CIMBIUR), Facultad de Ciencias Naturales, Universidad del Rosario, Bogotá, Colombia Instituto Nacional de Salud, Bogotá, Colombia Icahn School of Medicine at Mount Sinai, New York, USA	Luz Helena Patiño, Marina Muñoz, Nathalia Ballesteros, Carolina Hernández, Carolina Flórez, Sergio Gomez, Adriana van de Guchte, Zenab Khan, Jayeeta Dutta, Hala Alejel Alshammary, Ana S. Gonzalez-Reiche, Matthew M. Hernandez, Emilia Mia Sordillo, Viviana Simon, Harm van Bakel, Alberto Paniz-Mondolfi, Juan David Ramírez
EPI_ISL_949125	Virology, National Health Laboratory Service, Charlotte Maxeke Johannesburg Academic Institute	Virology, National Health Laboratory Service, Charlotte Maxeke Johannesburg Academic Institute	Kathleen Subramoney, Mushal Ali, Ashard Ismail, Thabo Mohale, Florette K. Treurnicht
EPI_ISL_953286, EPI_ISL_953287, EPI_ISL_953288, EPI_ISL_953289, EPI_ISL_953300, EPI_ISL_953303, EPI_ISL_953304, EPI_ISL_953305, EPI_ISL_953306, EPI_ISL_953328, EPI_ISL_953329, EPI_ISL_953332, EPI_ISL_953339, EPI_ISL_953340, EPI_ISL_953342, EPI_ISL_953343, EPI_ISL_953345, EPI_ISL_953346, EPI_ISL_953359, EPI_ISL_953361, EPI_ISL_953362, EPI_ISL_953363, EPI_ISL_953364, EPI_ISL_953368, EPI_ISL_953369, EPI_ISL_953370, EPI_ISL_953372			
see above	Bioinformatics and Biostatistics Lab, Advanced Sequencing Facility	COVID-19 Genomics UK (COG-UK) Consortium	Aengus Stewart,Jerome Nicod,Chelsea Sawyer,Laura Cubitt,Harshil Patel,Margaret Crawford
EPI_ISL_954303, EPI_ISL_954304, EPI_ISL_954305, EPI_ISL_954306, EPI_ISL_954307, EPI_ISL_954308, EPI_ISL_954309, EPI_ISL_954310, EPI_ISL_954311, EPI_ISL_954312, EPI_ISL_954313, EPI_ISL_954314, EPI_ISL_954315, EPI_ISL_954316, EPI_ISL_954317, EPI_ISL_954318, EPI_ISL_954319, EPI_ISL_954320, EPI_ISL_954321, EPI_ISL_954322, EPI_ISL_954323, EPI_ISL_954324, EPI_ISL_954325, EPI_ISL_954326, EPI_ISL_954327, EPI_ISL_954328, EPI_ISL_954329, EPI_ISL_954330, EPI_ISL_954331, EPI_ISL_954332, EPI_ISL_954333, EPI_ISL_954334, EPI_ISL_954335, EPI_ISL_954336, EPI_ISL_954337, EPI_ISL_954338, EPI_ISL_954339, EPI_ISL_954340, EPI_ISL_954341, EPI_ISL_954342, EPI_ISL_954343, EPI_ISL_954344, EPI_ISL_954345, EPI_ISL_954346, EPI_ISL_954347, EPI_ISL_954348, EPI_ISL_954349, EPI_ISL_954350, EPI_ISL_954351, EPI_ISL_954352, EPI_ISL_954353, EPI_ISL_954354, EPI_ISL_954355, EPI_ISL_954356, EPI_ISL_954357, EPI_ISL_954358, EPI_ISL_954359, EPI_ISL_954360, EPI_ISL_954361, EPI_ISL_954362, EPI_ISL_954363, EPI_ISL_954364			
see above	Laboratoire de santé publique du Québec	Laboratoire de santé publique du Québec	Sandrine Moreira, Ioannis Ragoussis, Guillaume Bourque, Jesse Shapiro, Mark Lathrop and Michel Roger on behalf of the CoVSeQ research group (http://covseq.ca/researchgroup)
EPI_ISL_955234	HE CMN LA RAZA	BIOBANCO / COCTI	Borja-Aburto VH, Grajales-Muñiz C, Santacruz Tinoco CE, Rojas-Mendoza T, Ochoa Carrera LA, Sánchez A, Grande R, Isa P, Taboada B, López S, Arias C, Muñoz-Medina JE
EPI_ISL_955235	UMF 23 UNIDAD MORELOS	BIOBANCO / COCTI	Borja-Aburto VH, Grajales-Muñiz C, Santacruz Tinoco CE, Rojas-Mendoza T, Ochoa Carrera LA, Sánchez A, Grande R, Isa P, Taboada B, López S, Arias

			C, Muñoz-Medina JE
EPI_ISL_955236	HGR 1 VICENTE GUERRERO	BIOBANCO / COCTI	Borja-Aburto VH, Grajales-Muñiz C, Santacruz Tinoco CE, Rojas-Mendoza T, Ochoa Carrera LA, Sánchez A, Grande R, Isa P, Taboada B, López S, Arias C, Muñoz-Medina JE
EPI_ISL_955237	UMF 75 NETZAHUALCOYOTL	BIOBANCO / COCTI	Borja-Aburto VH, Grajales-Muñiz C, Santacruz Tinoco CE, Rojas-Mendoza T, Ochoa Carrera LA, Sánchez A, Grande R, Isa P, Taboada B, López S, Arias C, Muñoz-Medina JE
EPI_ISL_955238	HI CMN LA RAZA	BIOBANCO / COCTI	Borja-Aburto VH, Grajales-Muñiz C, Santacruz Tinoco CE, Rojas-Mendoza T, Ochoa Carrera LA, Sánchez A, Grande R, Isa P, Taboada B, López S, Arias C, Muñoz-Medina JE
EPI_ISL_955239	HE CMN LA RAZA	BIOBANCO / COCTI	Borja-Aburto VH, Grajales-Muñiz C, Santacruz Tinoco CE, Rojas-Mendoza T, Ochoa Carrera LA, Sánchez A, Grande R, Isa P, Taboada B, López S, Arias C, Muñoz-Medina JE
EPI_ISL_960307	NLZOH, Laboratory for Virology	NLZOH, Laboratory for Virology	Katarina Prosenc (Laboratory for Virology), Cesare Camma (IZSAM), Erik Alm (ECDC)
EPI_ISL_961798, EPI_ISL_961799	Laboratorio de Infectología, Servicio de Infectología, Hospital Universitario Dr. José Eleuterio González - Universidad Autónoma de Nuevo León	Laboratorio de Infectología Molecular, Departamento de Bioquímica y Medicina Molecular,Facultad de Medicina - Universidad Autónoma de Nuevo León	Kame A. Galán-Huerta, María F. Herrera-Saldivar, Natalia Martínez-Acuña, Sonia A. Lozano-Sepúlveda, Daniel Arellanos-Soto, Ana M. Rivas-Estilla, Paola Bocanegra-Ibarias, Samantha M. Flores-Treviño, Elvira Garza-González, Eduardo Perez-Alba, Laura Nuzzolo-Shihadeh, Adrian Camacho-Ortiz
EPI_ISL_965564, EPI_ISL_965566, EPI_ISL_965612	Dutch COVID-19 response team	Medical Microbiology, Maastricht University Medical Centre	Jozef Dingemans*, Brian van der Veer*, Erik Beuken, Carmen Reumkens, Lieke van Alphen, Christian Hoebe, Paul Savelkoul
EPI_ISL_966365, EPI_ISL_966366, EPI_ISL_966367	Kentucky State Public Health Lab	Kentucky State Public Health Lab	Stephanie Lunn, Karim George, Joshua Tobias, William Grooms, Vaneet Arora, Matthew Johnson, Rachel Zinner, Rhonda Lucas
EPI_ISL_968141, EPI_ISL_968145, EPI_ISL_968148, EPI_ISL_968150, EPI_ISL_968176	Clinical Molecular Microbiology Laboratory, UNC Hospital	Dirk Dittmer	Justin T. Landis , Razia Moorad , Brent A. Eason, Melissa B. Miller, Linda Pluta, Dirk Dittmer, Angelica Juarez, Cecilia Thompson, Shawn Hawken, Cameroon Grant, Evelyn Hoffman, Patricio Cano, Jason Wong, Carolina Caro-Vegas, Ryan McNamara, Blossom Damania.
EPI_ISL_976907, EPI_ISL_976908, EPI_ISL_976909	Thai Red Cross Emerging Infectious Diseases Health Science Centre, Chulalongkorn Hospital, Faculty of Medicine, Chulalongkorn University	Thai Red Cross Emerging Infectious Diseases Center and Faculty of Medicine, Chulalongkorn University	Opass Putcharoen, Watsamon Jantarabenjakul, Pattama Torvorapanit, Gompol Suwanpimolkul, Leilani Paitoonpong, Sopon Iamsirithaworn, Sininat Petcharat, Yutthana Joyjinda, Weenassarin Ampoot, Apaporn Rodpan, Thiravat Hemachudha, Supaporn Wacharapluesadee
EPI_ISL_977094, EPI_ISL_977095, EPI_ISL_977099, EPI_ISL_977100	Massachusetts General Hospital	Infectious Disease Program, Broad Institute of Harvard and MIT	Lemieux,J.E., Siddle,K.J., Shaw,B., Adams,G., Pierce,V., Turbett,S., Anahtar,M., Branda,J., Slater,D., Harris,J., Lin,A.E., Gladden-Young,A., Lagerborg,K., Rudy,M., DeRuff,K., Carter,A., Normandin,E., Bauer,M., Reilly,S., Tomkins-Tinch,C., Loreth,C., Chaluvadi,S., Neumann,A., Cusick,C., Chapman,S.B., Gnirke,A., Flowers,K., Cerrato,F., Birren,B.W., Gallagher,G., Smole,S., Park,D.J., MacInnis,B.L., Ryan,E., LaRocque,R., Rosenberg,E. and Sabeti,P.C.
EPI_ISL_978489, EPI_ISL_978505, EPI_ISL_978525, EPI_ISL_978526	Central Public Health Laboratory - LACEN -Bahia, Salvador, Brazil	Central Public Health Laboratory - LACEN -Bahia, Salvador, Brazil	Stephane Tosta, Luciana Oliveira, Vanessa Nardy,Patrícia Cajado,Marcela Gómez, Breno Dominguez, Jaqueline Gomes, Vagner Fonseca,Marta Giovanetti,Luiz Alcantara, Felicidade Pereira, Arabela Leal
EPI_ISL_981041	Hospital Bariloche	Laboratorio Central Mg. Luis Alfredo Pianciola on behalf of 'Proyecto Argentino Interinstitucional de genomica de SARS-CoV-2' (PAIS Consortium)	L Pianciola, M Mazzeo, C Ziehm, C Pintos, M Fernandez, J Ousset, M Nabaes, M Viegas.
EPI_ISL_981204, EPI_ISL_981205, EPI_ISL_981206, EPI_ISL_981207, EPI_ISL_981208, EPI_ISL_981209, EPI_ISL_981210, EPI_ISL_981211, EPI_ISL_981212, EPI_ISL_981213, EPI_ISL_981214, EPI_ISL_981215			
see above	National Virus Reference Laboratory	Irish Coronavirus Sequencing Consortium - Helixworks	Sachin Chalapati, Conor Crosbie, Nimesh Pinnamaneni
