# Supplementary material for: Detection and characterization of the SARS-CoV-2 lineage B.1.526 in New York
Source: Nat Commun. 2021 Aug 9;12:4886. doi: 10.1038/s41467-021-25168-4 (PMC8352861; doi:10.1038/s41467-021-25168-4)
Supplement: Supplementary file 8 — Supplementary Data 4 [file 41467_2021_25168_MOESM8_ESM.zip › GISAID_acknowledements_tables/gisaid_hcov-19_acknowledgement_table_2021_02_13_010-7.pdf]

We gratefully acknowledge the following Authors from the Originating laboratories responsible for obtaining the specimens, as well as the Submitting laboratories where the genome data were generated and shared via GISAID, on which this research is based.

All Submitters of data may be contacted directly via www.gisaid.org

Authors are sorted alphabetically.

Accession ID	Originating Laboratory	Submitting Laboratory	Authors
EPI_ISL_771370	SA Pathology	SA Pathology	Lex Leong, Julien Soubrier, Chuan Kok Lim, Song Gao, Mark Turra, Karin Kassahn, Ivan Bastian, Geoff Higgins
EPI_ISL_775242, EPI_ISL_775245	Area of Virology, Serology and Virology Division (SAViD), New South Wales Health Pathology Randwick	Area of Virology, Serology and Virology Division (SAViD), New South Wales Health Pathology Randwick	Rawlinson, W., Bull, R., Deveson, I.
EPI_ISL_779154	Yale Pathology Lab	Grubaugh Lab - Yale School of Public Health	Tara Alpert, Joseph Fauver, Chen Liu, Pei Hui, Jianhui Wang, Susan Bell and Han Zhou, Anderson Brito, Mallery Breban, Anne Wyllie, Chantal Vogels, Mary Petrone, Chaney Kalinich, Isabel Ott, Arnau Casanovas, Catherine Muenker, Adam Moore, Alice Lu, Maria Tokuyama, Patrick Wong, Peiwen Lu, Saad Omer, Richard Martinello, Allison Nelson, Shelli Farhadian, Akiko Iwasaki, Charlese Dela Cruz, Albert Ko, Nathan Grubaugh
EPI_ISL_779398	Douglass Hanly Moir Pathology	NSW Health Pathology - Institute of Clinical Pathology and Medical Research; Westmead Hospital; University of Sydney	CIDM-PH et al.
EPI_ISL_788909, EPI_ISL_788910, EPI_ISL_788911, EPI_ISL_788912, EPI_ISL_788913, EPI_ISL_788914, EPI_ISL_788915, EPI_ISL_788916, EPI_ISL_788917, EPI_ISL_788918, EPI_ISL_788942, EPI_ISL_791097, EPI_ISL_791098, EPI_ISL_791099, EPI_ISL_791100, EPI_ISL_791101, EPI_ISL_791102, EPI_ISL_791103, EPI_ISL_791104, EPI_ISL_791105, EPI_ISL_791106			
see above	University of Wisconsin-Madison AIDS Vaccine Research Laboratories	University of Wisconsin-Madison AIDS Vaccine Research Laboratories	Gage Moreno, Katarina Braun, et al. AIDS Vaccine Research Laboratories
EPI_ISL_791194, EPI_ISL_791197, EPI_ISL_791198, EPI_ISL_791223, EPI_ISL_791224, EPI_ISL_791225, EPI_ISL_791226, EPI_ISL_791227, EPI_ISL_791228, EPI_ISL_791229, EPI_ISL_791230, EPI_ISL_791231, EPI_ISL_791232, EPI_ISL_791233, EPI_ISL_791234, EPI_ISL_791235, EPI_ISL_791236, EPI_ISL_791237, EPI_ISL_791238, EPI_ISL_791239, EPI_ISL_791240, EPI_ISL_791241, EPI_ISL_791242, EPI_ISL_791243, EPI_ISL_791244, EPI_ISL_791245, EPI_ISL_791246, EPI_ISL_791247, EPI_ISL_791248, EPI_ISL_791254, EPI_ISL_791255, EPI_ISL_791256, EPI_ISL_791257, EPI_ISL_791258, EPI_ISL_791259, EPI_ISL_791260, EPI_ISL_791261, EPI_ISL_791262, EPI_ISL_791263, EPI_ISL_791264, EPI_ISL_791265, EPI_ISL_791266, EPI_ISL_791267, EPI_ISL_791268			
see above	Respiratory Virus Unit, National Infection Service, Public Health England	COVID-19 Genomics UK (COG-UK) Consortium	PHE Covid Sequencing Team
EPI_ISL_791430	Johns Hopkins Hospital Department of Pathology	Johns Hopkins Hospital Department of Pathology	C. Paul Morris, Chun Huai Luo, Adannaya Amadi, Nicholas Gallagher, Heba H. Mostafa
EPI_ISL_792089	Toronto Invasive Bacterial Diseases Network	McMaster University	Allison McGeer, Patryk Aftanas, Hooman Derakhshani, Angel Li, Kuganya Nirmalarajah, Emily Panousis, Ahmed Draia, Jalees Nasir, Michael Surette, Samira Mubareka, Andrew G. McArthur
EPI_ISL_792551	Centre for Dengue Research and AICBU, Department of Immunology and Molecular Medicine	Centre for Dengue Research and AICBU, Department of Immunology and Molecular Medicine	Chandima Jeewandara, Deshni Jayathilaka, Dinuka Ariyaratne, Diyanath Ranasinghe, Laksiri Gomes, Gathsaurie Neelika Malavige
EPI_ISL_794308	Wadsworth Center, New York State Department.of Health	Wadsworth Center, New York State Department.of Health	Kirsten St. George, Daryl M. Lamson, Alexis Russel, Matthew Shudt, Melissa A Leisner, Jonathan Plitnick, Navjot Singh, John Kelly, Sara Griesemer, Erasmus Schneider, Erica Lasek-Nesselquist
EPI_ISL_794626	Middlemore Hospital	Institute of Environmental Science and Research (ESR)	Xiaoyun Ren, Matt Storey, Nikki Freed, Muhammad Faisal, Jing Wang, Hermes Perez, Anja Werno, Antje van der Linden, Arlo Upton, Chris Mansell, David Hammer, Dragana Drinkovic, Gary McAuliffe, Hana Sofia Andersson, James Ussher, Jill Sherwood, Josh Freeman, Julia Howard, Juliet Elvy, Mary DeAlmeida, Matt Blakiston, Matthew Rogers, Max Bloomfield, Michael Addidle, Michelle Balm, Sally Roberts, Sarah Jefferies, Sharmini Muttaiyah, Susan Morpeth, Susan Taylor, Timothy Blackmore, Vani Sathyendran, Veronica Playle, Virginia Hope, Erasmus Smit, Lauren Jelly, Olin Silander, Joep de Ligt
EPI_ISL_794639, EPI_ISL_794669	USC Clinical Lab	Los Angeles County PHL	P. Hemarajata et al.
EPI_ISL_794744	National Institute of Infectious Diseases-Prof. Dr. Matei Bals Molecular Diagnostics Laboratory	National Institute of Infectious Diseases-Prof. Dr. Matei Bals Molecular Diagnostics Laboratory	Leontina Banica, Marius Surleac, Corina Casangiu, Petre Milu, Andreea Tudor, Simona Paraschiv, Dan Otelea
EPI_ISL_796023, EPI_ISL_796024, EPI_ISL_796025, EPI_ISL_796026	Hebei Provincial Center for Disease Control and Prevention, Shijiazhuang, Hebei Province; National Institute for Viral Disease Control and Prevention, China CDC	Hebei Provincial Center for Disease Control and Prevention, Shijiazhuang, Hebei Province; National Institute for Viral Disease Control and Prevention, China CDC	Shunxiang Qi, Xiang Zhao, Nankun Liu, George F. Gao, Yang Song, Wenbo Xu, Qi Li
EPI_ISL_796736, EPI_ISL_796765	Instituto Nacional de Saude (INSA)	Instituto Nacional de Saude (INSA)	Borges et al
EPI_ISL_797970	Lighthouse Lab in Glasgow	Wellcome Sanger Institute for the COVID-19 Genomics UK (COG-UK) Consortium	Harper VanSteenhouse, Yumi Kasai, David Gray, Carol Clugston, Anna Dominiczak and Alex Alderton, Roberto Amato, Sonia Goncalves, Ewan Harrison, David K. Jackson, Ian Johnston, Dominic Kwiatkowski, Cordelia Langford, John Sillitoe on behalf of the Wellcome Sanger Institute COVID-19 Surveillance Team
EPI_ISL_802498, EPI_ISL_802499, EPI_ISL_802500	Wadsworth Center, New York State Department.of Health	Wadsworth Center, New York State Department.of Health	Kirsten St. George, Daryl M. Lamson, Alexis Russel, Matthew Shudt, Melissa A Leisner, Jonathan Plitnick, Navjot Singh, John Kelly, Sara Griesemer, Erasmus Schneider, Erica Lasek-Nesselquist
EPI_ISL_803994, EPI_ISL_803995, EPI_ISL_803996, EPI_ISL_803997, EPI_ISL_804000, EPI_ISL_804001, EPI_ISL_804002, EPI_ISL_804003, EPI_ISL_804004, EPI_ISL_804005	National Public Health Laboratory, National Centre for Infectious Diseases	National Public Health Laboratory, National Centre for Infectious Diseases	Tze Minn Mak, Sophie Octavia, Zhenyang Zhou, Lin Cui, Raymond Tzer Pin Lin
EPI_ISL_804217, EPI_ISL_804225, EPI_ISL_804276	Respiratory Virus Unit, National Infection Service, Public Health England	COVID-19 Genomics UK (COG-UK) Consortium	PHE Covid Sequencing Team
EPI_ISL_804945, EPI_ISL_804948	Wadsworth Center, New York State Department.of Health	Wadsworth Center, New York State Department.of Health	Kirsten St. George, Daryl M. Lamson, Alexis Russel, Matthew Shudt, Melissa A Leisner, Jonathan Plitnick, Navjot Singh, John Kelly, Sara Griesemer, Erasmus Schneider, Erica Lasek-Nesselquist
EPI_ISL_810965, EPI_ISL_810966	PathWest Laboratory Medicine WA	PathWest Laboratory Medicine WA Microbial Surveillance Unit	PathWest Laboratory Medicine WA Microbial Surveillance Unit
EPI_ISL_811121	Respiratory Virus Unit, National Infection Service, Public Health England	COVID-19 Genomics UK (COG-UK) Consortium	PHE Covid Sequencing Team
EPI_ISL_811140, EPI_ISL_811141, EPI_ISL_811142	Ministry of Health Turkey	Ministry of Health Turkey	Fatma Bayrakdar, Yasemin Cogun, Süleyman Yalcin, Aye Baak Alta, Gülay Korukluolu
EPI_ISL_811200, EPI_ISL_811213, EPI_ISL_811216, EPI_ISL_811221, EPI_ISL_811222, EPI_ISL_811226, EPI_ISL_811235, EPI_ISL_811254, EPI_ISL_811256, EPI_ISL_811257, EPI_ISL_811258, EPI_ISL_811260, EPI_ISL_811261, EPI_ISL_811263, EPI_ISL_811268, EPI_ISL_811273, EPI_ISL_811289, EPI_ISL_811295, EPI_ISL_811297, EPI_ISL_811316, EPI_ISL_811317, EPI_ISL_811338, EPI_ISL_811347, EPI_ISL_811348, EPI_ISL_811355, EPI_ISL_811356, EPI_ISL_811364, EPI_ISL_811365, EPI_ISL_811374, EPI_ISL_811376, EPI_ISL_811384, EPI_ISL_811387, EPI_ISL_811388, EPI_ISL_811391, EPI_ISL_811405, EPI_ISL_811415, EPI_ISL_811417, EPI_ISL_811423, EPI_ISL_811424, EPI_ISL_811426, EPI_ISL_811427, EPI_ISL_811432, EPI_ISL_811453			
see above	Lighthouse Lab in Glasgow	Wellcome Sanger Institute for the COVID-19 Genomics UK (COG-UK) Consortium	Harper VanSteenhouse, Yumi Kasai, David Gray, Carol Clugston, Anna Dominiczak and Alex Alderton, Roberto Amato, Sonia Goncalves, Ewan Harrison, David K. Jackson, Ian Johnston, Dominic Kwiatkowski, Cordelia Langford, John Sillitoe on behalf of the Wellcome Sanger Institute COVID-19 Surveillance Team
EPI_ISL_812257	Analytica Medizinische Laboratorien AG	Institute of Medical Virology, University of Zurich	Stefan Schmutz, Maryam Zaheri, Verena Kufner, Annette Audigé, Maria Grünberg, Kevin Steiner, Jon Huder, Cyril Shah, Riccarda Capaul, Guido Bloemberg, Jürg Böni, Michael Huber, Alexandra Trkola

EPI_ISL_812423	Royal Darwin Hospital Pathology	MDU-PHL	Meumann, E., Caly L., Seemann T., Sait, M.L., Druce J., Sherry, N.L.
EPI_ISL_812772, EPI_ISL_812773	Ministry of Health Turkey	Ministry of Health Turkey	Fatma Bayrakdar, Yasemin Cogun, Süleyman Yalcin, Aye Baak Alta, Gülay Korukluolu
EPI_ISL_813826, EPI_ISL_813829, EPI_ISL_813830, EPI_ISL_813831	Liverpool Clinical Laboratories	COVID-19 Genomics UK (COG-UK) Consortium	Sam Haldenby, Anita Lucaci, Steve Paterson, Julian Hiscox, Alistair Darby, M Almsaud, A Alrezaihi, Muhannad Alruwaili, Stuart D Armstrong, Jones Benjamin, Eleanor G Bentley, Anu Chawla, Jordan J Clark, Angela Cowell, Richard Eccles, Isabel García-Dorival, Matthew Gemmell, Alessandro Gerada, PKF Gilmore, Richard Gregory, Ximeng Han, Catherine Hartley, Margaret Hughes, Miren Iturriza-Gomara, James Johnson, L Luu, Jenifer Manson, Charlotte Nelson, Elaine O'Toole, Cassie Olateju, Rebekah Penrice-Randal , Lucille Rainbow, N.P Randle, Trevor Ian Robinson, Parul Sharma, Ghada T Shawli, James P Stewart, Neil Swainston, Ecaterina Vamos, Joanne Watts, Mark Whitehead
EPI_ISL_813995, EPI_ISL_814010, EPI_ISL_814014, EPI_ISL_814021, EPI_ISL_814034, EPI_ISL_814035	Hospital General Universitario Gregorio Marañón	SeqCOVID-SPAIN consortium/IBV(CSIC)	Darío García de Viedma, Laura Pérez-Lago, Marta Herranz, Jon Sicilia, Julia Suárez, Pilar Catalán, Patricia Muñoz and SeqCOVID-SPAIN consortium
EPI_ISL_816244, EPI_ISL_816253, EPI_ISL_816284, EPI_ISL_816299, EPI_ISL_816307, EPI_ISL_816358, EPI_ISL_816369, EPI_ISL_816376, EPI_ISL_816378, EPI_ISL_816387, EPI_ISL_816389, EPI_ISL_816391, EPI_ISL_816433, EPI_ISL_816455, EPI_ISL_816493, EPI_ISL_816518, EPI_ISL_816524, EPI_ISL_816559, EPI_ISL_816623, EPI_ISL_816639, EPI_ISL_816648, EPI_ISL_816652			
see above	Virology Department, Sheffield Teaching Hospitals NHS Foundation Trust/Department of Infection, Immunity and Cardiovascular Disease, The Medical School, University of Sheffield	COVID-19 Genomics UK (COG-UK) Consortium	Thushan de Silva, Matthew Parker, Nikki Smith, Adri Angyal, Rebecca Brown, Luke Green, Rachel Tucker, Paul Parsons, Danielle Groves, Katie Johnson, Laura Carrilero, Alex Keeley, Dave Partridge, Matthew Wyles, Benjamin Lindsey, Mehmet Yavuz, Mohammad Raza, Cariad Evans
EPI_ISL_817135, EPI_ISL_817137, EPI_ISL_817138, EPI_ISL_817146, EPI_ISL_817147, EPI_ISL_817149, EPI_ISL_817159, EPI_ISL_817160, EPI_ISL_817161, EPI_ISL_817162, EPI_ISL_817163, EPI_ISL_817164, EPI_ISL_817165, EPI_ISL_817166, EPI_ISL_817167, EPI_ISL_817168, EPI_ISL_817169, EPI_ISL_817170, EPI_ISL_817171, EPI_ISL_817172, EPI_ISL_817173, EPI_ISL_817174, EPI_ISL_817175, EPI_ISL_817176, EPI_ISL_817264, EPI_ISL_817269, EPI_ISL_817272, EPI_ISL_817273, EPI_ISL_817278, EPI_ISL_817279, EPI_ISL_817281, EPI_ISL_817282, EPI_ISL_817288, EPI_ISL_817290, EPI_ISL_817291, EPI_ISL_817292, EPI_ISL_817296, EPI_ISL_817297, EPI_ISL_817299, EPI_ISL_817301, EPI_ISL_817303, EPI_ISL_817306, EPI_ISL_817308, EPI_ISL_817312, EPI_ISL_817315, EPI_ISL_817316, EPI_ISL_817317, EPI_ISL_817333, EPI_ISL_817334, EPI_ISL_817335, EPI_ISL_817375, EPI_ISL_817376, EPI_ISL_817383, EPI_ISL_817384, EPI_ISL_817385, EPI_ISL_817386, EPI_ISL_817392, EPI_ISL_817398, EPI_ISL_817399, EPI_ISL_817401, EPI_ISL_817404, EPI_ISL_817405, EPI_ISL_817413, EPI_ISL_817414, EPI_ISL_817415, EPI_ISL_817416, EPI_ISL_817417, EPI_ISL_817418, EPI_ISL_817419, EPI_ISL_817487, EPI_ISL_817496, EPI_ISL_817497, EPI_ISL_817499, EPI_ISL_817501, EPI_ISL_817508, EPI_ISL_817509, EPI_ISL_817510, EPI_ISL_817511, EPI_ISL_817530, EPI_ISL_817532, EPI_ISL_817537, EPI_ISL_817538, EPI_ISL_817555, EPI_ISL_817556, EPI_ISL_817557, EPI_ISL_817558, EPI_ISL_817559, EPI_ISL_817560, EPI_ISL_817561, EPI_ISL_817562, EPI_ISL_817563, EPI_ISL_817564, EPI_ISL_817565, EPI_ISL_817566, EPI_ISL_817567, EPI_ISL_817568, EPI_ISL_817569, EPI_ISL_817570, EPI_ISL_817571, EPI_ISL_817572, EPI_ISL_817573, EPI_ISL_817574, EPI_ISL_817575, EPI_ISL_817576, EPI_ISL_817577, EPI_ISL_817578, EPI_ISL_817579, EPI_ISL_817580, EPI_ISL_817581, EPI_ISL_817582, EPI_ISL_817583, EPI_ISL_817584, EPI_ISL_817585, EPI_ISL_817586, EPI_ISL_817587, EPI_ISL_817588, EPI_ISL_817589, EPI_ISL_817590, EPI_ISL_817591, EPI_ISL_817592, EPI_ISL_817593, EPI_ISL_817594, EPI_ISL_817595, EPI_ISL_817596, EPI_ISL_817597, EPI_ISL_817598, EPI_ISL_817599, EPI_ISL_817600, EPI_ISL_817601, EPI_ISL_817602, EPI_ISL_817603, EPI_ISL_817604, EPI_ISL_817605, EPI_ISL_817606, EPI_ISL_817607, EPI_ISL_817608, EPI_ISL_817609, EPI_ISL_817610, EPI_ISL_817611, EPI_ISL_817612, EPI_ISL_817613, EPI_ISL_817614, EPI_ISL_817615, EPI_ISL_817616, EPI_ISL_817617, EPI_ISL_817618, EPI_ISL_817619, EPI_ISL_817620, EPI_ISL_817621, EPI_ISL_817622, EPI_ISL_817623, EPI_ISL_817624, EPI_ISL_817625, EPI_ISL_817626, EPI_ISL_817627, EPI_ISL_817628, EPI_ISL_817629, EPI_ISL_817630, EPI_ISL_817631, EPI_ISL_817632, EPI_ISL_817633, EPI_ISL_817634, EPI_ISL_817635, EPI_ISL_817636, EPI_ISL_817637, EPI_ISL_817638, EPI_ISL_817639, EPI_ISL_817640, EPI_ISL_817641, EPI_ISL_817642, EPI_ISL_817643, EPI_ISL_817644, EPI_ISL_817645, EPI_ISL_817646, EPI_ISL_817647, EPI_ISL_817648, EPI_ISL_817649, EPI_ISL_817650, EPI_ISL_817651, EPI_ISL_817652, EPI_ISL_818123, EPI_ISL_818124, EPI_ISL_818125, EPI_ISL_818126, EPI_ISL_818127, EPI_ISL_818128, EPI_ISL_818129, EPI_ISL_818130, EPI_ISL_818131, EPI_ISL_818132, EPI_ISL_818133, EPI_ISL_818134, EPI_ISL_818135, EPI_ISL_818136, EPI_ISL_818137, EPI_ISL_818138, EPI_ISL_818139, EPI_ISL_818140, EPI_ISL_818141, EPI_ISL_818142, EPI_ISL_818143, EPI_ISL_818144, EPI_ISL_818145, EPI_ISL_818146, EPI_ISL_818147, EPI_ISL_818148, EPI_ISL_818149, EPI_ISL_818150, EPI_ISL_818151, EPI_ISL_818152, EPI_ISL_818153, EPI_ISL_818154, EPI_ISL_818155, EPI_ISL_818156, EPI_ISL_818157, EPI_ISL_818158, EPI_ISL_818159, EPI_ISL_818160, EPI_ISL_818161, EPI_ISL_818162, EPI_ISL_818163, EPI_ISL_818164, EPI_ISL_818165, EPI_ISL_818166, EPI_ISL_818167, EPI_ISL_818168, EPI_ISL_818169, EPI_ISL_818170, EPI_ISL_818171, EPI_ISL_818172, EPI_ISL_818173, EPI_ISL_818174, EPI_ISL_818175, EPI_ISL_818176, EPI_ISL_818177, EPI_ISL_818178, EPI_ISL_818179, EPI_ISL_818180, EPI_ISL_818181, EPI_ISL_818182, EPI_ISL_818183, EPI_ISL_818184, EPI_ISL_818185, EPI_ISL_818186, EPI_ISL_818187, EPI_ISL_818188, EPI_ISL_818189, EPI_ISL_818190, EPI_ISL_818191, EPI_ISL_818192, EPI_ISL_818193, EPI_ISL_818194, EPI_ISL_818195, EPI_ISL_818196, EPI_ISL_818197, EPI_ISL_818198, EPI_ISL_818199, EPI_ISL_818200, EPI_ISL_818201, EPI_ISL_818202, EPI_ISL_818203, EPI_ISL_818204, EPI_ISL_818205, EPI_ISL_818206, EPI_ISL_818207, EPI_ISL_818208, EPI_ISL_818209, EPI_ISL_818210, EPI_ISL_818211, EPI_ISL_818212, EPI_ISL_818213, EPI_ISL_818214, EPI_ISL_818215, EPI_ISL_818216, EPI_ISL_818217, EPI_ISL_818218, EPI_ISL_818219, EPI_ISL_818220, EPI_ISL_818221, EPI_ISL_818222, EPI_ISL_818223, EPI_ISL_818224, EPI_ISL_818225, EPI_ISL_818226, EPI_ISL_818227, EPI_ISL_818228, EPI_ISL_818229, EPI_ISL_818230, EPI_ISL_818231, EPI_ISL_818232, EPI_ISL_818233, EPI_ISL_818234, EPI_ISL_818235, EPI_ISL_818236, EPI_ISL_818237, EPI_ISL_818238, EPI_ISL_818239, EPI_ISL_818240, EPI_ISL_818241, EPI_ISL_818242, EPI_ISL_818243, EPI_ISL_818244, EPI_ISL_818245, EPI_ISL_818246, EPI_ISL_818247, EPI_ISL_818248, EPI_ISL_818249, EPI_ISL_818250, EPI_ISL_818251, EPI_ISL_818252, EPI_ISL_818253, EPI_ISL_818254, EPI_ISL_818255, EPI_ISL_818256, EPI_ISL_818257, EPI_ISL_818258, EPI_ISL_818259, EPI_ISL_818260, EPI_ISL_818261, EPI_ISL_818262, EPI_ISL_818263, EPI_ISL_818264, EPI_ISL_818265, EPI_ISL_818266, EPI_ISL_818267, EPI_ISL_818268, EPI_ISL_818269, EPI_ISL_818270, EPI_ISL_818271, EPI_ISL_818272, EPI_ISL_818273, EPI_ISL_818274, EPI_ISL_818275, EPI_ISL_818276, EPI_ISL_818277, EPI_ISL_818278, EPI_ISL_818279, EPI_ISL_818280, EPI_ISL_818281, EPI_ISL_818282, EPI_ISL_818283, EPI_ISL_818284, EPI_ISL_818285, EPI_ISL_818286, EPI_ISL_818287, EPI_ISL_818288, EPI_ISL_818289, EPI_ISL_818290, EPI_ISL_818291, EPI_ISL_818292, EPI_ISL_818293, EPI_ISL_818294, EPI_ISL_818295, EPI_ISL_818296, EPI_ISL_818297, EPI_ISL_818298, EPI_ISL_818299, EPI_ISL_818300, EPI_ISL_818301, EPI_ISL_818302, EPI_ISL_818303, EPI_ISL_818304, EPI_ISL_818305, EPI_ISL_818306, EPI_ISL_818307, EPI_ISL_818308, EPI_ISL_818309, EPI_ISL_818310, EPI_ISL_818311, EPI_ISL_818312, EPI_ISL_818313, EPI_ISL_818314, EPI_ISL_818315, EPI_ISL_818316, EPI_ISL_818317, EPI_ISL_818318, EPI_ISL_818319, EPI_ISL_818320, EPI_ISL_818321, EPI_ISL_818322, EPI_ISL_818323, EPI_ISL_818324, EPI_ISL_818325, EPI_ISL_818326, EPI_ISL_818327, EPI_ISL_818328, EPI_ISL_818329, EPI_ISL_818330, EPI_ISL_818331, EPI_ISL_818332, EPI_ISL_818333, EPI_ISL_818334, EPI_ISL_818335, EPI_ISL_818336, EPI_ISL_818337, EPI_ISL_818338, EPI_ISL_818339, EPI_ISL_818340, EPI_ISL_818341, EPI_ISL_818343, EPI_ISL_818344, EPI_ISL_818345, EPI_ISL_818346, EPI_ISL_818347, EPI_ISL_818348, EPI_ISL_818349, EPI_ISL_818350, EPI_ISL_818351, EPI_ISL_818352, EPI_ISL_818353, EPI_ISL_818354, EPI_ISL_818355, EPI_ISL_818356, EPI_ISL_818357, EPI_ISL_818358, EPI_ISL_818359, EPI_ISL_818360, EPI_ISL_818361, EPI_ISL_818362, EPI_ISL_818363, EPI_ISL_818364, EPI_ISL_818365, EPI_ISL_818366, EPI_ISL_818367, EPI_ISL_818368, EPI_ISL_818369, EPI_ISL_818370, EPI_ISL_818371, EPI_ISL_818372, EPI_ISL_818373, EPI_ISL_818374, EPI_ISL_818375, EPI_ISL_818376, EPI_ISL_818377, EPI_ISL_818378, EPI_ISL_818379, EPI_ISL_818380, EPI_ISL_818381, EPI_ISL_818382, EPI_ISL_818383, EPI_ISL_818384, EPI_ISL_818385, EPI_ISL_818386, EPI_ISL_818387, EPI_ISL_818388, EPI_ISL_818389, EPI_ISL_818390, EPI_ISL_818391, EPI_ISL_818392, EPI_ISL_818393, EPI_ISL_818394, EPI_ISL_818395, EPI_ISL_818396, EPI_ISL_818397, EPI_ISL_818398, EPI_ISL_818399, EPI_ISL_818400, EPI_ISL_818401, EPI_ISL_818402, EPI_ISL_818403, EPI_ISL_818404, EPI_ISL_818405, EPI_ISL_818406, EPI_ISL_818407, EPI_ISL_818408, EPI_ISL_818409, EPI_ISL_818410, EPI_ISL_818411, EPI_ISL_818412, EPI_ISL_818413, EPI_ISL_818414, EPI_ISL_818415, EPI_ISL_818416, EPI_ISL_818417, EPI_ISL_818418, EPI_ISL_818419, EPI_ISL_818420, EPI_ISL_818421, EPI_ISL_818422, EPI_ISL_818423, EPI_ISL_818424, EPI_ISL_818425, EPI_ISL_818426, EPI_ISL_818427, EPI_ISL_818428, EPI_ISL_818429, EPI_ISL_818430, EPI_ISL_818431, EPI_ISL_818432, EPI_ISL_818433, EPI_ISL_818434, EPI_ISL_818435, EPI_ISL_818436, EPI_ISL_818437, EPI_ISL_818438, EPI_ISL_818439, EPI_ISL_818440, EPI_ISL_818441, EPI_ISL_818442, EPI_ISL_818443, EPI_ISL_818444, EPI_ISL_818445, EPI_ISL_818446, EPI_ISL_818447, EPI_ISL_818448, EPI_ISL_818449, EPI_ISL_818450, EPI_ISL_818451, EPI_ISL_818452, EPI_ISL_818453, EPI_ISL_818454, EPI_ISL_818455, EPI_ISL_818456, EPI_ISL_818457, EPI_ISL_818458, EPI_ISL_818459, EPI_ISL_818460, EPI_ISL_818461, EPI_ISL_818462, EPI_ISL_818463, EPI_ISL_818464, EPI_ISL_818465, EPI_ISL_818466, EPI_ISL_818467, EPI_ISL_818468, EPI_ISL_818469, EPI_ISL_818470, EPI_ISL_818471, EPI_ISL_818472, EPI_ISL_818473, EPI_ISL_818474, EPI_ISL_818475, EPI_ISL_818476, EPI_ISL_818477, EPI_ISL_818478, EPI_ISL_818479, EPI_ISL_818480, EPI_ISL_818481, EPI_ISL_818482, EPI_ISL_818483, EPI_ISL_818484, EPI_ISL_818485, EPI_ISL_818486, EPI_ISL_818487, EPI_ISL_818488, EPI_ISL_818489, EPI_ISL_818490, EPI_ISL_818491, EPI_ISL_818492, EPI_ISL_818493, EPI_ISL_818494, EPI_ISL_818495, EPI_ISL_818496, EPI_ISL_818497, EPI_ISL_818498, EPI_ISL_818499, EPI_ISL_818500, EPI_ISL_818501, EPI_ISL_818502, EPI_ISL_818503, EPI_ISL_818504, EPI_ISL_818505, EPI_ISL_818506, EPI_ISL_818507, EPI_ISL_818508, EPI_ISL_818509, EPI_ISL_818510, EPI_ISL_818511, EPI_ISL_818512, EPI_ISL_818513, EPI_ISL_818514, EPI_ISL_818515, EPI_ISL_818516, EPI_ISL_818517, EPI_ISL_818518, EPI_ISL_818519, EPI_ISL_818520, EPI_ISL_818521, EPI_ISL_818522, EPI_ISL_818523, EPI_ISL_818524, EPI_ISL_818525, EPI_ISL_818526, EPI_ISL_818527, EPI_ISL_818528, EPI_ISL_818529, EPI_ISL_818530, EPI_ISL_818531, EPI_ISL_818532, EPI_ISL_818533, EPI_ISL_818534, EPI_ISL_818535, EPI_ISL_818536, EPI_ISL_818537, EPI_ISL_818538, EPI_ISL_818539, EPI_ISL_818540, EPI_ISL_818541, EPI_ISL_818542, EPI_ISL_818543, EPI_ISL_818544, EPI_ISL_818545, EPI_ISL_818546, EPI_ISL_818547, EPI_ISL_818548, EPI_ISL_818549, EPI_ISL_818550, EPI_ISL_818551, EPI_ISL_818552, EPI_ISL_818553, EPI_ISL_818554, EPI_ISL_818555, EPI_ISL_818556, EPI_ISL_818557, EPI_ISL_818558, EPI_ISL_818559, EPI_ISL_818560, EPI_ISL_818561, EPI_ISL_818562, EPI_ISL_818563, EPI_ISL_818564, EPI_ISL_818565, EPI_ISL_818566, EPI_ISL_818567, EPI_ISL_818568, EPI_ISL_818569, EPI_ISL_818570, EPI_ISL_818571, EPI_ISL_818572, EPI_ISL_818573, EPI_ISL_818574, EPI_ISL_818575, EPI_ISL_818576, EPI_ISL_818577, EPI_ISL_818578, EPI_ISL_818579, EPI_ISL_818580, EPI_ISL_818581, EPI_ISL_818582, EPI_ISL_818583, EPI_ISL_818584, EPI_ISL_818585, EPI_ISL_818586, EPI_ISL_818587, EPI_ISL_818588, EPI_ISL_818589, EPI_ISL_818590, EPI_ISL_818591, EPI_ISL_818592, EPI_ISL_818593, EPI_ISL_818594, EPI_ISL_818595, EPI_ISL_818596, EPI_ISL_818597, EPI_ISL_818598, EPI_ISL_818599, EPI_ISL_818600, EPI_ISL_818601, EPI_ISL_818602, EPI_ISL_818603, EPI_ISL_818604, EPI_ISL_818605, EPI_ISL_818606, EPI_ISL_818607, EPI_ISL_818608, EPI_ISL_818609, EPI_ISL_818610, EPI_ISL_818611, EPI_ISL_818612, EPI_ISL_818613, EPI_ISL_818614, EPI_ISL_818615, EPI_ISL_818616, EPI_ISL_818617, EPI_ISL_818618, EPI_ISL_818619, EPI_ISL_818620, EPI_ISL_818621, EPI_ISL_818622, EPI_ISL_818623, EPI_ISL_818624, EPI_ISL_818625, EPI_ISL_818626, EPI_ISL_818627, EPI_ISL_818628, EPI_ISL_818629, EPI_ISL_818630, EPI_ISL_818631, EPI_ISL_818632, EPI_ISL_818633, EPI_ISL_818634, EPI_ISL_818635, EPI_ISL_818636, EPI_ISL_818637, EPI_ISL_818638, EPI_ISL_818639, EPI_ISL_818640, EPI_ISL_818641, EPI_ISL_818642, EPI_ISL_818643, EPI_ISL_818644, EPI_ISL_818645, EPI_ISL_818646, EPI_ISL_818647, EPI_ISL_818648, EPI_ISL_818649, EPI_ISL_818650, EPI_ISL_818651, EPI_ISL_818652, EPI_ISL_818653, EPI_ISL_818654, EPI_ISL_818655, EPI_ISL_818656, EPI_ISL_818657, EPI_ISL_818658, EPI_ISL_818659, EPI_ISL_818660, EPI_ISL_818661, EPI_ISL_818662, EPI_ISL_818663, EPI_ISL_818664, EPI_ISL_818665, EPI_ISL_818666, EPI_ISL_818667, EPI_ISL_818668, EPI_ISL_818669, EPI_ISL_818670, EPI_ISL_818671, EPI_ISL_818672, EPI_ISL_818673, EPI_ISL_818674, EPI_ISL_818675, EPI_ISL_818676, EPI_ISL_818677, EPI_ISL_818678, EPI_ISL_818679, EPI_ISL_818680, EPI_ISL_818681, EPI_ISL_818682, EPI_ISL_818683, EPI_ISL_818684, EPI_ISL_818685, EPI_ISL_818686, EPI_ISL_818687, EPI_ISL_818688, EPI_ISL_818689, EPI_ISL_818690, EPI_ISL_818691, EPI_ISL_818692, EPI_ISL_818693, EPI_ISL_818694, EPI_ISL_818695, EPI_ISL_818696, EPI_ISL_818697, EPI_ISL_818698, EPI_ISL_818699, EPI_ISL_818700, EPI_ISL_818701, EPI_ISL_818702, EPI_ISL_818703, EPI_ISL_818704, EPI_ISL_818705, EPI_ISL_818706, EPI_ISL_818707, EPI_ISL_818708, EPI_ISL_818709, EPI_ISL_818710, EPI_ISL_818711, EPI_ISL_818712, EPI_ISL_818713, EPI_ISL_818714, EPI_ISL_818715, EPI_ISL_818716, EPI_ISL_818717, EPI_ISL_818718, EPI_ISL_818719, EPI_ISL_818720, EPI_ISL_818721, EPI_ISL_818722, EPI_ISL_818723, EPI_ISL_818724, EPI_ISL_818725, EPI_ISL_818726, EPI_ISL_818727, EPI_ISL_818728, EPI_ISL_818729, EPI_ISL_818730, EPI_ISL_818731, EPI_ISL_818732, EPI_ISL_818733, EPI_ISL_818734, EPI_ISL_818735, EPI_ISL_818736, EPI_ISL_818737, EPI_ISL_818738, EPI_ISL_818739, EPI_ISL_818740, EPI_ISL_818741, EPI_ISL_818742, EPI_ISL_818743, EPI_ISL_818744, EPI_ISL_818745, EPI_ISL_818746, EPI_ISL_818747, EPI_ISL_818748, EPI_ISL_818749, EPI_ISL_818750, EPI_ISL_818751, EPI_ISL_818752, EPI_ISL_818753, EPI_ISL_818754, EPI_ISL_818755, EPI_ISL_818756, EPI_ISL_818757, EPI_ISL_818758, EPI_ISL_818759, EPI_ISL_818760, EPI_ISL_818761, EPI_ISL_818762, EPI_ISL_818763, EPI_ISL_818764, EPI_ISL_818765, EPI_ISL_818766, EPI_ISL_818767, EPI_ISL_818768, EPI_ISL_818769, EPI_ISL_818770, EPI_ISL_818771, EPI_ISL_818772, EPI_ISL_818773, EPI_ISL_818774, EPI_ISL_818775, EPI_ISL_818776, EPI_ISL_818777, EPI_ISL_818778, EPI_ISL_818779, EPI_ISL_818780, EPI_ISL_818781, EPI_ISL_818782, EPI_ISL_818783, EPI_ISL_818784, EPI_ISL_818785, EPI_ISL_818786, EPI_ISL_818787, EPI_ISL_818788, EPI_ISL_818789, EPI_ISL_818790, EPI_ISL_818791, EPI_ISL_818792, EPI_ISL_818793, EPI_ISL_818794, EPI_ISL_818795, EPI_ISL_818796, EPI_ISL_818797, EPI_ISL_818798, EPI_ISL_818799, EPI_ISL_818800, EPI_ISL_818801, EPI_ISL_818802, EPI_ISL_818803, EPI_ISL_818804, EPI_ISL_818805, EPI_ISL_818806, EPI_ISL_818807, EPI_ISL_818808, EPI_ISL_818809, EPI_ISL_818810, EPI_ISL_818811, EPI_ISL_818812, EPI_ISL_818813, EPI_ISL_818814, EPI_ISL_818815, EPI_ISL_818816, EPI_ISL_818817, EPI_ISL_818818, EPI_ISL_818819, EPI_ISL_818820, EPI_ISL_818821, EPI_ISL_818822, EPI_ISL_818823, EPI_ISL_818824, EPI_ISL_818825, EPI_ISL_818826, EPI_ISL_818827, EPI_ISL_818828, EPI_ISL_818829, EPI_ISL_818830, EPI_ISL_818831, EPI_ISL_818832, EPI_ISL_818833, EPI_ISL_818834, EPI_ISL_818835, EPI_ISL_818836, EPI_ISL_818837, EPI_ISL_818838, EPI_ISL_818839, EPI_ISL_818840, EPI_ISL_818841, EPI_ISL_818842, EPI_ISL_818843, EPI_ISL_818844, EPI_ISL_818845, EPI_ISL_818846, EPI_ISL_818847, EPI_ISL_818848, EPI_ISL_818849, EPI_ISL_818850, EPI_ISL_818851, EPI_ISL_818852, EPI_ISL_818853, EPI_ISL_818854, EPI_ISL_818855, EPI_ISL_818856, EPI_ISL_818857, EPI_ISL_818858, EPI_ISL_818859, EPI_ISL_818860, EPI_ISL_818861, EPI_ISL_818862, EPI_ISL_818863, EPI_ISL_818864, EPI_ISL_818865, EPI_ISL_818866, EPI_ISL_818867, EPI_ISL_818868, EPI_ISL_818869, EPI_ISL_818870, EPI_ISL_818871, EPI_ISL_818872, EPI_ISL_818873, EPI_ISL_818874, EPI_ISL_818875, EPI_ISL_818876, EPI_ISL_818877, EPI_ISL_818878, EPI_ISL_818879, EPI_ISL_818880, EPI_ISL_818881, EPI_ISL_818882, EPI_ISL_818883, EPI_ISL_818884, EPI_ISL_818885, EPI_ISL_818886, EPI_ISL_818887, EPI_ISL_818888, EPI_ISL_818889, EPI_ISL_818890, EPI_ISL_818891, EPI_ISL_818892, EPI_ISL_818893, EPI_ISL_818894, EPI_ISL_818895, EPI_ISL_818896, EPI_ISL_818897, EPI_ISL_818898, EPI_ISL_818899, EPI_ISL_818900, EPI_ISL_818901, EPI_ISL_818902, EPI_ISL_818903, EPI_ISL_818904, EPI_ISL_818905, EPI_ISL_818906, EPI_ISL_818907, EPI_ISL_818908, EPI_ISL_818909, EPI_ISL_818910, EPI_ISL_818911, EPI_ISL_818912, EPI_ISL_818913, EPI_ISL_818914, EPI_ISL_818915, EPI_ISL_818916, EPI_ISL_818917, EPI_ISL_818918, EPI_ISL_818919, EPI_ISL_818920, EPI_ISL_818921, EPI_ISL_818922, EPI_ISL_818923, EPI_ISL_818924, EPI_ISL_818925, EPI_ISL_818926, EPI_ISL_818927, EPI_ISL_818928, EPI_ISL_818929, EPI_ISL_818930, EPI_ISL_818931, EPI_ISL_818932, EPI_ISL_818933, EPI_ISL_818934, EPI_ISL_818935, EPI_ISL_818936, EPI_ISL_818937, EPI_ISL_818938, EPI_ISL_818939, EPI_ISL_818940, EPI_ISL_818941, EPI_ISL_818942, EPI_ISL_818943, EPI_ISL_818944, EPI_ISL_818945, EPI_ISL_818946, EPI_ISL_818947, EPI_ISL_818948, EPI_ISL_818949, EPI_ISL_818950, EPI_ISL_818951, EPI_ISL_818952, EPI_ISL_818953, EPI_ISL_818954, EPI_ISL_818955, EPI_ISL_818956, EPI_ISL_818957, EPI_ISL_818958, EPI_ISL_818959, EPI_ISL_818960, EPI_ISL_818961, EPI_ISL_818962, EPI_ISL_818963, EPI_ISL_818964, EPI_ISL_818965, EPI_ISL_818966, EPI_ISL_818967, EPI_ISL_818968, EPI_ISL_818969, EPI_ISL_818970, EPI_ISL_818971, EPI_ISL_818972, EPI_ISL_818973, EPI_ISL_818974, EPI_ISL_818975, EPI_ISL_818976, EPI_ISL_818977, EPI_ISL_818978, EPI_ISL_818979, EPI_ISL_818980, EPI_ISL_818981, EPI_ISL_818982, EPI_ISL_818983, EPI_ISL_818984, EPI_ISL_818985, EPI_ISL_818986, EPI_ISL_818987, EPI_ISL_818988, EPI_ISL_818989, EPI_ISL_818990, EPI_ISL_818991, EPI_ISL_818992, EPI_ISL_818993, EPI_ISL_818994, EPI_ISL_818995, EPI_ISL_818996, EPI_ISL_818997, EPI_ISL_818998, EPI_ISL_818999, EPI_ISL_819000, EPI_ISL_819001, EPI_ISL_819002, EPI_ISL_819003, EPI_ISL_819004, EPI_ISL_819005, EPI_ISL_819006, EPI_ISL_819007, EPI_ISL_819008, EPI_ISL_819009, EPI_ISL_819010, EPI_ISL_819011, EPI_ISL_819012, EPI_ISL_819013, EPI_ISL_819014, EPI_ISL_819015, EPI_ISL_819016, EPI_ISL_819017, EPI_ISL_819018, EPI_ISL_819019, EPI_ISL_819020, EPI_ISL_819021, EPI_ISL_819022, EPI_ISL_819023, EPI_ISL_819024, EPI_ISL_819025, EPI_ISL_819026, EPI_ISL_819027, EPI_ISL_819028, EPI_ISL_819029, EPI_ISL_819030, EPI_ISL_819031, EPI_ISL_819032, EPI_ISL_819033, EPI_ISL_819034, EPI_ISL_819035, EPI_ISL_819036, EPI_ISL_819037, EPI_ISL_819038, EPI_ISL_819039, EPI_ISL_819040, EPI_ISL_819041, EPI_ISL_819042, EPI_ISL_819043, EPI_ISL_819044, EPI_ISL_819045, EPI_ISL_819046, EPI_ISL_819047, EPI_ISL_819048, EPI_ISL_819049, EPI_ISL_819050, EPI_ISL_819051, EPI_ISL_819052, EPI_ISL_819053, EPI_ISL_819054, EPI_ISL_819055, EPI_ISL_819056, EPI_ISL_819057, EPI_ISL_819058, EPI_ISL_819059, EPI_ISL_819060, EPI_ISL_819061, EPI_ISL_819062, EPI_ISL_819063, EPI_ISL_819064, EPI_ISL_819065, EPI_ISL_819066, EPI_ISL_819067, EPI_ISL_819068, EPI_ISL_819069, EPI_ISL_819070, EPI_ISL_819071, EPI_ISL_819072, EPI_ISL_819073, EPI_ISL_819074, EPI_ISL_819075, EPI_ISL_819076, EPI_ISL_819077, EPI_ISL_819078, EPI_ISL_819079, EPI_ISL_819080, EPI_ISL_819081, EPI_ISL_819082, EPI_ISL_819083, EPI_ISL_819084, EPI_ISL_819085, EPI_ISL_819086, EPI_ISL_819087, EPI_ISL_819088, EPI_ISL_819089, EPI_ISL_819090, EPI_ISL_819091, EPI_ISL_819092, EPI_ISL_819093, EPI_ISL_819094, EPI_ISL_819095, EPI_ISL_819096, EPI_ISL_819097, EPI_ISL_819098, EPI_ISL_819099, EPI_ISL_819100, EPI_ISL_819101, EPI_ISL_819102, EPI_ISL_819103, EPI_ISL_819104, EPI_ISL_819105, EPI_ISL_819106, EPI_ISL_819107, EPI_ISL_819108, EPI_ISL_819109, EPI_ISL_819110, EPI_ISL_819111, EPI_ISL_819112, EPI_ISL_819113, EPI_ISL_819114, EPI_ISL_819115, EPI_ISL_819116, EPI_ISL_819117, EPI_ISL_819118, EPI_ISL_819119, EPI_ISL_819120, EPI_ISL_819121, EPI_ISL_819122, EPI_ISL_819123			
see above	Department of Virus and Microbiological Special Diagnostics, Statens Serum Institut, Copenhagen, Denmark	Albertsen Lab, Department of Chemistry and Bioscience, Aalborg University, Denmark	Danish Covid-19 Genome Consortium

EPI_ISL_819166	Servicio de Microbiología, Hospital Universitario Son Espases	SeqCOVID-SPAIN consortium/IBV(CSIC)	Carla López-Causapé, Jordi Reina, Antonio Oliver and SeqCOVID-SPAIN consortium
EPI_ISL_819205, EPI_ISL_819207, EPI_ISL_819209, EPI_ISL_819210, EPI_ISL_819213, EPI_ISL_819215, EPI_ISL_819216, EPI_ISL_819217, EPI_ISL_819218, EPI_ISL_819219, EPI_ISL_819220, EPI_ISL_819221, EPI_ISL_819222, EPI_ISL_819223, EPI_ISL_819236, EPI_ISL_819237, EPI_ISL_819238, EPI_ISL_819239, EPI_ISL_819241, EPI_ISL_819242, EPI_ISL_819243, EPI_ISL_819244, EPI_ISL_819245, EPI_ISL_819268, EPI_ISL_819272, EPI_ISL_819273, EPI_ISL_819274, EPI_ISL_819275, EPI_ISL_819276, EPI_ISL_819277, EPI_ISL_819278, EPI_ISL_819279, EPI_ISL_819280, EPI_ISL_819281, EPI_ISL_819282, EPI_ISL_819283, EPI_ISL_819284, EPI_ISL_819285, EPI_ISL_819286, EPI_ISL_819287, EPI_ISL_819288, EPI_ISL_819289, EPI_ISL_819290, EPI_ISL_819291, EPI_ISL_819292			
see above	Wyoming Public Health Laboratory	Wyoming Public Health Laboratory	Noah Hull, Taylor Fearing, Lynette Gumbleton, Channing Weber, Ashley Norberg, Bailey Bowcutt, and Wanda Manley
EPI_ISL_819384, EPI_ISL_819414, EPI_ISL_819417, EPI_ISL_819420, EPI_ISL_819429, EPI_ISL_819445, EPI_ISL_819446	Quadram Institute Bioscience	COVID-19 Genomics UK (COG-UK) Consortium	Dave J. Baker, Gemma L. Kay, Alp Aydin, Thanh Le-Viet, Steven Rudder, Ana P. Tedim, Anastasia Kolyva, Maria Diaz, Leonardo de Oliveira Martins, Nabil-Fareed Alikhan, Lizzie Meadows, Rachael Stanley, Ngozi Elumogo, Muhammed Yasir, Nicholas M. Thomson, Alexander J Trotter, Rachel Gilroy, Samuel Bloomfield, Claire Stuart, Andrew Bell, Reenesh Prakash, Samir Dervisevic, Alison E. Mather, John Wain, Mark Webber, Andrew J. Page, Justin O'Grady
EPI_ISL_820588, EPI_ISL_820591, EPI_ISL_820593	Queens Medical Centre, Clinical Microbiology Department / DeepSeq Nottingham	COVID-19 Genomics UK (COG-UK) Consortium	Gemma Clark, Wendy Smith, Manjinder Khakh, Vicki M Fleming, Michelle M Lister, Hannah Howson-Wells, Jonathan Ball, Patrick McClure, Joseph Chappell, Theocharis Tsoleridis, Nadine Holmes, Matthew Carlisle, Christopher Moore, Fei Sang, Johnny Debebe, Victoria Wright, Matthew Loose
EPI_ISL_821032, EPI_ISL_821037, EPI_ISL_821056, EPI_ISL_821061, EPI_ISL_821062, EPI_ISL_821069, EPI_ISL_821077, EPI_ISL_821098, EPI_ISL_821105, EPI_ISL_821142, EPI_ISL_821150, EPI_ISL_821160, EPI_ISL_821195, EPI_ISL_821198, EPI_ISL_821207, EPI_ISL_821225, EPI_ISL_821227, EPI_ISL_821241, EPI_ISL_821245, EPI_ISL_821249, EPI_ISL_821257, EPI_ISL_821258, EPI_ISL_821266, EPI_ISL_821269, EPI_ISL_821621			
see above	Lighthouse Lab in Glasgow	Wellcome Sanger Institute for the COVID-19 Genomics UK (COG-UK) Consortium	Harper VanSteenhouse, Yumi Kasai, David Gray, Carol Clugston, Anna Dominiczak and Alex Alderton, Roberto Amato, Sonia Goncalves, Ewan Harrison, David K. Jackson, Ian Johnston, Dominic Kwiatkowski, Cordelia Langford, John Sillitoe on behalf of the Wellcome Sanger Institute COVID-19 Surveillance Team
EPI_ISL_824096, EPI_ISL_824097, EPI_ISL_824098, EPI_ISL_824099, EPI_ISL_824100, EPI_ISL_824141, EPI_ISL_824145, EPI_ISL_824146, EPI_ISL_824147, EPI_ISL_824148, EPI_ISL_824149, EPI_ISL_824150, EPI_ISL_824153, EPI_ISL_824154, EPI_ISL_824253, EPI_ISL_824254			
see above	Dutch COVID-19 response team	National Institute for Public Health and the Environment (RIVM)	Adam Meijer, Harry Vennema, Jeroen Cremer, Sharon van den Brink, Bas van der Veer, AnneMarie van den Brandt, Florian Zwagemaker, Dennis Schmitz, Chantal Reusken, on behalf of the national COVID-19 response team
EPI_ISL_824842, EPI_ISL_824843, EPI_ISL_824844, EPI_ISL_824845, EPI_ISL_824846, EPI_ISL_824847, EPI_ISL_824848, EPI_ISL_824849, EPI_ISL_824850, EPI_ISL_824851	Department of Clinical Microbiology	GIGA Medical Genomics	Keith Durkin, Maria Artesi, Sébastien Bontems, Raphaël Boreux, Bouchra Boujemla, Cécile Meex, Pierrette Melin, Marie-Pierre Hayette, Vincent Bours
EPI_ISL_824939, EPI_ISL_824940	Arizona State Public Health Laboratory	Arizona State Public Health Laboratory	Trung Huynh, Jessica Escobar, Katherine Fullerton, Nobuko Fukushima, Stacy White, Linda Getsinger, Victor Waddell
EPI_ISL_824953, EPI_ISL_824954, EPI_ISL_824955, EPI_ISL_824956, EPI_ISL_824957, EPI_ISL_824958, EPI_ISL_824959, EPI_ISL_824960, EPI_ISL_824961, EPI_ISL_824962, EPI_ISL_824963, EPI_ISL_824964, EPI_ISL_824965, EPI_ISL_824974, EPI_ISL_824975, EPI_ISL_824976, EPI_ISL_824977			
see above	Maryland Public Health Laboratory	Maryland Public Health Laboratory	Maryland Department of Health Laboratories Administration
EPI_ISL_827396	The National University Hospital of Iceland	deCODE genetics	Daniel F Gudbjartsson; Agnar Helgason; Hakon Jonsson; Olafur T Magnusson; Pall Melsted; Gudmundur L Norddahl; Jona Saemundsdottir; Asgeir Sigurdsson; Patrick Sulem; Arna B Agustsdottir; Hannes Eggertsson; Berglind Eiriksdottir; Run Fridriksdottir; Elisabet E Gardarsdottir; Gudmundur Georgsson; Olafia S Gretarsdottir; Kjartan R Gudmundsson; Thora R Gunnarsdottir; Arnaldur Gylfason; Hilma Holm; Brynjar O Jensson; Aslaug Jonasdottir; Kamilla S Josefsdottir; Thordur Kristjansson; Droplaug N Magnusdottir; Solvi Rognvaldsson; Louise le Roux; Gudrun Sigmundsdottir; Gardar Sveinbjornsson; Kristin E Sveinsdottir; Maney Sveinsdottir; Emil A Thorarensen; Bjarni Thorbjornsson; Gisli Masson; Ingileif Jonsdottir; Alma Moller; Thorolfur Gudnason; Karl G Kristinsson; Unnur Thorsteinsdottir; Kari Stefansson
EPI_ISL_827545, EPI_ISL_827546, EPI_ISL_827554, EPI_ISL_827862, EPI_ISL_827921, EPI_ISL_827922, EPI_ISL_827923, EPI_ISL_827925, EPI_ISL_828255, EPI_ISL_828256, EPI_ISL_829703, EPI_ISL_829972, EPI_ISL_830194			
see above	deCODE genetics	deCODE genetics	Daniel F Gudbjartsson; Agnar Helgason; Hakon Jonsson; Olafur T Magnusson; Pall Melsted; Gudmundur L Norddahl; Jona Saemundsdottir; Asgeir Sigurdsson; Patrick Sulem; Arna B Agustsdottir; Hannes Eggertsson; Berglind Eiriksdottir; Run Fridriksdottir; Elisabet E Gardarsdottir; Gudmundur Georgsson; Olafia S Gretarsdottir; Kjartan R Gudmundsson; Thora R Gunnarsdottir; Arnaldur Gylfason; Hilma Holm; Brynjar O Jensson; Aslaug Jonasdottir; Kamilla S Josefsdottir; Thordur Kristjansson; Droplaug N Magnusdottir; Solvi Rognvaldsson; Louise le Roux; Gudrun Sigmundsdottir; Gardar Sveinbjornsson; Kristin E Sveinsdottir; Maney Sveinsdottir; Emil A Thorarensen; Bjarni Thorbjornsson; Gisli Masson; Ingileif Jonsdottir; Alma Moller; Thorolfur Gudnason; Karl G Kristinsson; Unnur Thorsteinsdottir; Kari Stefansson
EPI_ISL_830244, EPI_ISL_830246, EPI_ISL_830248, EPI_ISL_830250, EPI_ISL_830252, EPI_ISL_830253, EPI_ISL_830255, EPI_ISL_830257, EPI_ISL_830259, EPI_ISL_830261	Wadsworth Center, New York State Department of Health	Wadsworth Center, New York State Department of Health	Kirsten St. George, Daryl M. Lamson, Alexis Russel, Matthew Shudt, Melissa A Leisner, Jonathan Plitnick, Navjot Singh, John Kelly, Erasmus Schneider, Erica Lasek-Nesselquist
EPI_ISL_830567	deCODE genetics	deCODE genetics	Daniel F Gudbjartsson; Agnar Helgason; Hakon Jonsson; Olafur T Magnusson; Pall Melsted; Gudmundur L Norddahl; Jona Saemundsdottir; Asgeir Sigurdsson; Patrick Sulem; Arna B Agustsdottir; Hannes Eggertsson; Berglind Eiriksdottir; Run Fridriksdottir; Elisabet E Gardarsdottir; Gudmundur Georgsson; Olafia S Gretarsdottir; Kjartan R Gudmundsson; Thora R Gunnarsdottir; Arnaldur Gylfason; Hilma Holm; Brynjar O Jensson; Aslaug Jonasdottir; Kamilla S Josefsdottir; Thordur Kristjansson; Droplaug N Magnusdottir; Solvi Rognvaldsson; Louise le Roux; Gudrun Sigmundsdottir; Gardar Sveinbjornsson; Kristin E Sveinsdottir; Maney Sveinsdottir; Emil A Thorarensen; Bjarni Thorbjornsson; Gisli Masson; Ingileif Jonsdottir; Alma Moller; Thorolfur Gudnason; Karl G Kristinsson; Unnur Thorsteinsdottir; Kari Stefansson
EPI_ISL_830572, EPI_ISL_830574, EPI_ISL_830576, EPI_ISL_830577, EPI_ISL_830578, EPI_ISL_830629, EPI_ISL_830630, EPI_ISL_830631, EPI_ISL_830632, EPI_ISL_830633	NORTHWELL HEALTH LABORATORIES	Wadsworth Center, New York State Department of Health	Kirsten St. George, Daryl M. Lamson, Alexis Russel, Matthew Shudt, Melissa A Leisner, Jonathan Plitnick, Navjot Singh, John Kelly, Erasmus Schneider, Erica Lasek-Nesselquist
EPI_ISL_830634	MARY IMOGENE BASSETT HOSPITAL	Wadsworth Center, New York State Department of Health	Kirsten St. George, Daryl M. Lamson, Alexis Russel, Matthew Shudt, Melissa A Leisner, Jonathan Plitnick, Navjot Singh, John Kelly, Erasmus Schneider, Erica Lasek-Nesselquist
EPI_ISL_830635, EPI_ISL_830636, EPI_ISL_830637, EPI_ISL_830638, EPI_ISL_830639, EPI_ISL_830640, EPI_ISL_830641, EPI_ISL_830642	NORTHWELL HEALTH LABORATORIES	Wadsworth Center, New York State Department of Health	Kirsten St. George, Daryl M. Lamson, Alexis Russel, Matthew Shudt, Melissa A Leisner, Jonathan Plitnick, Navjot Singh, John Kelly, Erasmus Schneider, Erica Lasek-Nesselquist
EPI_ISL_830664	SUNY UPSTATE MEDICAL UNIVERSITY	Wadsworth Center, New York State Department of Health	Kirsten St. George, Daryl M. Lamson, Alexis Russel, Matthew Shudt, Melissa A Leisner, Jonathan Plitnick, Navjot Singh, John Kelly, Erasmus Schneider, Erica Lasek-Nesselquist
EPI_ISL_830699, EPI_ISL_830700, EPI_ISL_830701, EPI_ISL_830702, EPI_ISL_830703, EPI_ISL_830707, EPI_ISL_830708, EPI_ISL_830717, EPI_ISL_830718, EPI_ISL_830719	ALBANY MEDICAL CENTER HOSPITAL CLINICAL LABORATORIES	Wadsworth Center, New York State Department of Health	Kirsten St. George, Daryl M. Lamson, Alexis Russel, Matthew Shudt, Melissa A Leisner, Jonathan Plitnick, Navjot Singh, John Kelly, Erasmus Schneider, Erica Lasek-Nesselquist
EPI_ISL_830724, EPI_ISL_830725	ACUTIS DIAGNOSTICS	Wadsworth Center, New York State Department of Health	Kirsten St. George, Daryl M. Lamson, Alexis Russel, Matthew Shudt, Melissa A Leisner, Jonathan Plitnick, Navjot Singh, John Kelly, Erasmus Schneider, Erica Lasek-Nesselquist
EPI_ISL_831289, EPI_ISL_831295, EPI_ISL_831296, EPI_ISL_831310, EPI_ISL_831324, EPI_ISL_831327, EPI_ISL_831333	Texas Department of State Health Services	Texas Department of State Health Services	Anita Pokharel, Bonnie Oh, James Daniel Bonser, Rashmi Tuladhar, Mayela Pedrueza, Jenny Zhang, Maliha Rahman, Myong Koag, Chung Wang, Rachel Lee, Grace Kubin

EPI_ISL_831475, EPI_ISL_831477, EPI_ISL_831482, EPI_ISL_831485, EPI_ISL_831488, EPI_ISL_831491, EPI_ISL_831492, EPI_ISL_831493, EPI_ISL_831494, EPI_ISL_831497, EPI_ISL_831551, EPI_ISL_831555, EPI_ISL_831556, EPI_ISL_831557, EPI_ISL_831558, EPI_ISL_831559, EPI_ISL_831560, EPI_ISL_831561, EPI_ISL_831562, EPI_ISL_831563, EPI_ISL_831564, EPI_ISL_831565, EPI_ISL_831566, EPI_ISL_831567, EPI_ISL_831568, EPI_ISL_831569, EPI_ISL_831570, EPI_ISL_831571, EPI_ISL_831574, EPI_ISL_831576, EPI_ISL_831587, EPI_ISL_831588, EPI_ISL_831589, EPI_ISL_831590, EPI_ISL_831591, EPI_ISL_831592, EPI_ISL_831593, EPI_ISL_831594, EPI_ISL_831595, EPI_ISL_831596, EPI_ISL_831597, EPI_ISL_831598, EPI_ISL_831599, EPI_ISL_831600, EPI_ISL_831601, EPI_ISL_831602, EPI_ISL_831603, EPI_ISL_831604, EPI_ISL_831605, EPI_ISL_831606, EPI_ISL_831607, EPI_ISL_831608, EPI_ISL_831609, EPI_ISL_831610, EPI_ISL_831611, EPI_ISL_831612, EPI_ISL_831613, EPI_ISL_831614, EPI_ISL_831615, EPI_ISL_831616, EPI_ISL_831617, EPI_ISL_831618, EPI_ISL_831619, EPI_ISL_831620, EPI_ISL_831622			
see above	University of Wisconsin-Madison AIDS Vaccine Research Laboratories	University of Wisconsin-Madison AIDS Vaccine Research Laboratories	Gage Moreno, Katarina Braun, et al. AIDS Vaccine Research Laboratories
EPI_ISL_831909	New Mexico Department of Health Scientific Laboratory	New Mexico Department of Health Scientific Laboratory	Ellie Johnson, Anastacia Griego-Fisher, D'eldra Malone
EPI_ISL_831943	Unilabs, Mikrobiologiska laboratoriet	The Public Health Agency of Sweden	Department of Microbiology, The Public Health Agency of Sweden
EPI_ISL_832003, EPI_ISL_832004	Mikrobiologen	The Public Health Agency of Sweden	Department of Microbiology, The Public Health Agency of Sweden
EPI_ISL_832005	Klinisk Mikrobiologi	The Public Health Agency of Sweden	Department of Microbiology, The Public Health Agency of Sweden
EPI_ISL_832018	University of Wisconsin-Madison AIDS Vaccine Research Laboratories	University of Wisconsin-Madison AIDS Vaccine Research Laboratories	Gage Moreno, Katarina Braun, et al. AIDS Vaccine Research Laboratories
EPI_ISL_832121, EPI_ISL_832124	Clinical Molecular Microbiology Laboratory, UNC Hospitals	Jeremy Wang	Jeremy Wang, Alexander Rubinsteyn, Colleen Rice, Jason Smedberg, Melissa Miller, Corbin Jones, Robert Hagan
EPI_ISL_832208, EPI_ISL_832209	Department of Clinical Microbiology	GIGA Medical Genomics	Keith Durkin, Maria Artesi, Sébastien Bontems, Raphaël Boreux, Bouchra Boujemla, Cécile Meex, Pierrette Melin, Marie-Pierre Hayette, Vincent Bours
EPI_ISL_832978, EPI_ISL_832979, EPI_ISL_832980, EPI_ISL_832981, EPI_ISL_832982, EPI_ISL_832983, EPI_ISL_832984, EPI_ISL_832985	Maine HETL	Tewhey Lab, The Jackson Laboratory	Matluk,N., Dewey,H., Iosue,F., Barter,M., Lynch,R., Munger,H. and Tewhey,R.
EPI_ISL_833041	Defence Services Medical Research Center, Biological Research Laboratory	Defence Services Medical Research Center, Biological Research Laboratory	Khine Zaw Oo, Nay Myo Aung, Ko Ko Win, Phyo Kyaw Aung, Zaw Win Htun, Sat Paing Htoo, Kyaw Wanna, Thein Zaw, Kyee Myint, Ko Ko Lwin
EPI_ISL_833188	Department of Clinical Microbiology	GIGA Medical Genomics	Keith Durkin, Maria Artesi, Sébastien Bontems, Raphaël Boreux, Bouchra Boujemla, Cécile Meex, Pierrette Melin, Marie-Pierre Hayette, Vincent Bours
EPI_ISL_833206, EPI_ISL_833213	Department of Virology and Immunology, University of Helsinki and Helsinki University Hospital, Huslab Finland	Department of Virology, Faculty of Medicine, University of Helsinki, Helsinki, Finland	Teemu Smura, Ravi Kant, Phuoc Truong, Hussein Alburkat, Hannimari Kallio-Kokko, Jenni Virtanen, Maija Suvanto, Fathiah Zakham, Essi Korhonen, Sari Hannula, Harri Kangas, Pekka Ellonen, Olli Vapalahti
EPI_ISL_833415, EPI_ISL_833416	USC Clinical Lab	Los Angeles County PHL	P. Hemarajata et al.
EPI_ISL_833944, EPI_ISL_833956, EPI_ISL_833966, EPI_ISL_833968, EPI_ISL_833995, EPI_ISL_833997, EPI_ISL_834009, EPI_ISL_834022, EPI_ISL_834029, EPI_ISL_834042, EPI_ISL_834069, EPI_ISL_834082, EPI_ISL_834104, EPI_ISL_834134, EPI_ISL_834144, EPI_ISL_834197, EPI_ISL_834210, EPI_ISL_834214, EPI_ISL_834216, EPI_ISL_834219, EPI_ISL_834221, EPI_ISL_834225, EPI_ISL_834229, EPI_ISL_834232, EPI_ISL_834255, EPI_ISL_834260, EPI_ISL_834273, EPI_ISL_834275, EPI_ISL_834276, EPI_ISL_834282, EPI_ISL_834304, EPI_ISL_834308, EPI_ISL_834309, EPI_ISL_834314, EPI_ISL_834317, EPI_ISL_834321, EPI_ISL_834327, EPI_ISL_834330, EPI_ISL_834333, EPI_ISL_834335, EPI_ISL_834336, EPI_ISL_834338, EPI_ISL_834345, EPI_ISL_834348, EPI_ISL_834349, EPI_ISL_834355, EPI_ISL_834359, EPI_ISL_834375, EPI_ISL_834387, EPI_ISL_834392, EPI_ISL_834395, EPI_ISL_834398, EPI_ISL_834399, EPI_ISL_834403, EPI_ISL_834411, EPI_ISL_834414, EPI_ISL_834415, EPI_ISL_834418, EPI_ISL_834421, EPI_ISL_834426, EPI_ISL_834434, EPI_ISL_834438, EPI_ISL_834450, EPI_ISL_834461, EPI_ISL_834463, EPI_ISL_834471, EPI_ISL_834472, EPI_ISL_834476, EPI_ISL_834480, EPI_ISL_834481, EPI_ISL_834482, EPI_ISL_834489, EPI_ISL_834493, EPI_ISL_834498, EPI_ISL_834505, EPI_ISL_834506, EPI_ISL_834509, EPI_ISL_834510, EPI_ISL_834527, EPI_ISL_834536, EPI_ISL_834538, EPI_ISL_834542, EPI_ISL_834544, EPI_ISL_834550, EPI_ISL_834552			
see above	Lighthouse Lab in Alderley Park	Wellcome Sanger Institute for the COVID-19 Genomics UK (COG-UK) Consortium	Jacquelyn Wynn, Mairead Hyland, The Lighthouse Lab in Alderley Park and Alex Alderton, Roberto Amato, Sonia Goncalves, Ewan Harrison, David K. Jackson, Ian Johnston, Dominic Kwiatkowski, Cordelia Langford, John Sillitoe on behalf of the Wellcome Sanger Institute COVID-19 Surveillance Team
EPI_ISL_834558, EPI_ISL_834561, EPI_ISL_834568, EPI_ISL_834579, EPI_ISL_834581, EPI_ISL_834588, EPI_ISL_834592, EPI_ISL_834593, EPI_ISL_834599, EPI_ISL_834600, EPI_ISL_834603, EPI_ISL_834604, EPI_ISL_834608, EPI_ISL_834612, EPI_ISL_834619, EPI_ISL_834623, EPI_ISL_834629, EPI_ISL_834632, EPI_ISL_834639, EPI_ISL_834642, EPI_ISL_834645, EPI_ISL_834648, EPI_ISL_834658, EPI_ISL_834670, EPI_ISL_834672, EPI_ISL_834673, EPI_ISL_834683, EPI_ISL_834686, EPI_ISL_834687, EPI_ISL_834689, EPI_ISL_834690, EPI_ISL_834691, EPI_ISL_834692, EPI_ISL_834695, EPI_ISL_834699, EPI_ISL_834703, EPI_ISL_834708, EPI_ISL_834710, EPI_ISL_834714, EPI_ISL_834717, EPI_ISL_834718, EPI_ISL_834726, EPI_ISL_834733, EPI_ISL_834734, EPI_ISL_834738, EPI_ISL_834741, EPI_ISL_834751, EPI_ISL_834754, EPI_ISL_834756, EPI_ISL_834757, EPI_ISL_834760, EPI_ISL_834764, EPI_ISL_834765, EPI_ISL_834767, EPI_ISL_834782, EPI_ISL_834793			
see above	Lighthouse Lab in Glasgow	Wellcome Sanger Institute for the COVID-19 Genomics UK (COG-UK) Consortium	Harper VanSteenhouse, Yumi Kasai, David Gray, Carol Clugston, Anna Dominiczak and Alex Alderton, Roberto Amato, Sonia Goncalves, Ewan Harrison, David K. Jackson, Ian Johnston, Dominic Kwiatkowski, Cordelia Langford, John Sillitoe on behalf of the Wellcome Sanger Institute COVID-19 Surveillance Team
EPI_ISL_834799, EPI_ISL_834811, EPI_ISL_834813, EPI_ISL_834816, EPI_ISL_834827, EPI_ISL_834828, EPI_ISL_834830, EPI_ISL_834836, EPI_ISL_834838, EPI_ISL_834844, EPI_ISL_834856, EPI_ISL_834858, EPI_ISL_834863, EPI_ISL_834864, EPI_ISL_834871, EPI_ISL_834882, EPI_ISL_834884, EPI_ISL_834887, EPI_ISL_834890, EPI_ISL_834895, EPI_ISL_834897, EPI_ISL_834898, EPI_ISL_834899, EPI_ISL_834902, EPI_ISL_834905, EPI_ISL_834911, EPI_ISL_834912, EPI_ISL_834915, EPI_ISL_834916, EPI_ISL_834918, EPI_ISL_834928, EPI_ISL_834942, EPI_ISL_834943, EPI_ISL_834946, EPI_ISL_834951, EPI_ISL_834952, EPI_ISL_834953, EPI_ISL_834965, EPI_ISL_834974, EPI_ISL_834978, EPI_ISL_834984, EPI_ISL_834988, EPI_ISL_834994, EPI_ISL_834996, EPI_ISL_835003, EPI_ISL_835005, EPI_ISL_835009, EPI_ISL_835013, EPI_ISL_835018, EPI_ISL_835024, EPI_ISL_835026, EPI_ISL_835028, EPI_ISL_835033, EPI_ISL_835036, EPI_ISL_835053, EPI_ISL_835066, EPI_ISL_835069, EPI_ISL_835077, EPI_ISL_835080, EPI_ISL_835083, EPI_ISL_835085, EPI_ISL_835088, EPI_ISL_835102, EPI_ISL_835103, EPI_ISL_835107, EPI_ISL_835109, EPI_ISL_835117, EPI_ISL_835120, EPI_ISL_835122, EPI_ISL_835133, EPI_ISL_835138			
see above	Lighthouse Lab in Alderley Park	Wellcome Sanger Institute for the COVID-19 Genomics UK (COG-UK) Consortium	Jacquelyn Wynn, Mairead Hyland, The Lighthouse Lab in Alderley Park and Alex Alderton, Roberto Amato, Sonia Goncalves, Ewan Harrison, David K. Jackson, Ian Johnston, Dominic Kwiatkowski, Cordelia Langford, John Sillitoe on behalf of the Wellcome Sanger Institute COVID-19 Surveillance Team
EPI_ISL_835149, EPI_ISL_835153, EPI_ISL_835154, EPI_ISL_835174, EPI_ISL_835179, EPI_ISL_835181, EPI_ISL_835189, EPI_ISL_835192, EPI_ISL_835198, EPI_ISL_835200, EPI_ISL_835202, EPI_ISL_835205, EPI_ISL_835214, EPI_ISL_835219, EPI_ISL_835222, EPI_ISL_835223, EPI_ISL_835231, EPI_ISL_835243, EPI_ISL_835246, EPI_ISL_835253, EPI_ISL_835257, EPI_ISL_835259, EPI_ISL_835260, EPI_ISL_835268, EPI_ISL_835289, EPI_ISL_835298, EPI_ISL_835300, EPI_ISL_835303, EPI_ISL_835310, EPI_ISL_835313, EPI_ISL_835316, EPI_ISL_835320, EPI_ISL_835321, EPI_ISL_835334, EPI_ISL_835338, EPI_ISL_835339, EPI_ISL_835354, EPI_ISL_835357, EPI_ISL_835359, EPI_ISL_835361, EPI_ISL_835362, EPI_ISL_835365, EPI_ISL_835366, EPI_ISL_835369, EPI_ISL_835375, EPI_ISL_835384, EPI_ISL_835385, EPI_ISL_835389, EPI_ISL_835400, EPI_ISL_835403, EPI_ISL_835409, EPI_ISL_835410, EPI_ISL_835415, EPI_ISL_835416, EPI_ISL_835420, EPI_ISL_835421, EPI_ISL_835433, EPI_ISL_835439, EPI_ISL_835447, EPI_ISL_835448, EPI_ISL_835454, EPI_ISL_835457, EPI_ISL_835464, EPI_ISL_835465, EPI_ISL_835470, EPI_ISL_835475, EPI_ISL_835481, EPI_ISL_835486, EPI_ISL_835487, EPI_ISL_835488, EPI_ISL_835493, EPI_ISL_835497, EPI_ISL_835499, EPI_ISL_835500, EPI_ISL_835502, EPI_ISL_835503, EPI_ISL_835505, EPI_ISL_835506, EPI_ISL_835507, EPI_ISL_835515, EPI_ISL_835518, EPI_ISL_835527, EPI_ISL_835530, EPI_ISL_835531, EPI_ISL_835533, EPI_ISL_835535, EPI_ISL_835539, EPI_ISL_835540, EPI_ISL_835541, EPI_ISL_835547, EPI_ISL_835548, EPI_ISL_835553, EPI_ISL_835555, EPI_ISL_835564, EPI_ISL_835570, EPI_ISL_835573, EPI_ISL_835574, EPI_ISL_835576, EPI_ISL_835577, EPI_ISL_835579, EPI_ISL_835582, EPI_ISL_835589, EPI_ISL_835590, EPI_ISL_835592, EPI_ISL_835597, EPI_ISL_835600, EPI_ISL_835603, EPI_ISL_835610, EPI_ISL_835614, EPI_ISL_835616, EPI_ISL_835618, EPI_ISL_835622, EPI_ISL_835623, EPI_ISL_835631, EPI_ISL_835643, EPI_ISL_835646, EPI_ISL_835647, EPI_ISL_835651, EPI_ISL_835659, EPI_ISL_835664, EPI_ISL_835671, EPI_ISL_835673, EPI_ISL_835677, EPI_ISL_835678, EPI_ISL_835682, EPI_ISL_835683, EPI_ISL_835686, EPI_ISL_835687, EPI_ISL_835688, EPI_ISL_835692, EPI_ISL_835695, EPI_ISL_835701, EPI_ISL_835703, EPI_ISL_835707			
see above	Lighthouse Lab in Glasgow	Wellcome Sanger Institute for the COVID-19 Genomics UK (COG-UK) Consortium	Harper VanSteenhouse, Yumi Kasai, David Gray, Carol Clugston, Anna Dominiczak and Alex Alderton, Roberto Amato, Sonia Goncalves, Ewan Harrison, David K. Jackson, Ian Johnston, Dominic Kwiatkowski, Cordelia Langford, John Sillitoe on behalf of the Wellcome Sanger Institute COVID-19 Surveillance Team
EPI_ISL_836075, EPI_ISL_836077, EPI_ISL_836078, EPI_ISL_836079, EPI_ISL_836083, EPI_ISL_836084, EPI_ISL_836088, EPI_ISL_836089, EPI_ISL_836095, EPI_ISL_836101, EPI_ISL_836105, EPI_ISL_836106, EPI_ISL_836107, EPI_ISL_836108, EPI_ISL_836111, EPI_ISL_836112, EPI_ISL_836115, EPI_ISL_836120, EPI_ISL_836128, EPI_ISL_836129, EPI_ISL_836131, EPI_ISL_836132, EPI_ISL_836137, EPI_ISL_836138, EPI_ISL_836142, EPI_ISL_836144, EPI_ISL_836147, EPI_ISL_836149, EPI_ISL_836154, EPI_ISL_836155, EPI_ISL_836156, EPI_ISL_836160, EPI_ISL_836162, EPI_ISL_836165, EPI_ISL_836168, EPI_ISL_836169, EPI_ISL_836174, EPI_ISL_836178, EPI_ISL_836180, EPI_ISL_836184, EPI_ISL_836190, EPI_ISL_836192, EPI_ISL_836197, EPI_ISL_836198, EPI_ISL_836201, EPI_ISL_836203, EPI_ISL_836211, EPI_ISL_836212, EPI_ISL_836218, EPI_ISL_836223, EPI_ISL_836225, EPI_ISL_836228, EPI_ISL_836230, EPI_ISL_836231, EPI_ISL_836233, EPI_ISL_836234, EPI_ISL_836238, EPI_ISL_836241, EPI_ISL_836247, EPI_ISL_836248, EPI_ISL_836250, EPI_ISL_836251, EPI_ISL_836252, EPI_ISL_836261, EPI_ISL_836262, EPI_ISL_836266, EPI_ISL_836268, EPI_ISL_836274, EPI_ISL_836275, EPI_ISL_836282, EPI_ISL_836284, EPI_ISL_836285, EPI_ISL_836291, EPI_ISL_836292, EPI_ISL_836293, EPI_ISL_836298, EPI_ISL_836305, EPI_ISL_836306, EPI_ISL_836307, EPI_ISL_836308, EPI_ISL_836310, EPI_ISL_836311, EPI_ISL_836313, EPI_ISL_836314, EPI_ISL_836315, EPI_ISL_836318, EPI_ISL_836319, EPI_ISL_836320, EPI_ISL_836321, EPI_ISL_836323, EPI_ISL_836328, EPI_ISL_836329, EPI_ISL_836333, EPI_ISL_836336, EPI_ISL_836338, EPI_ISL_836342, EPI_ISL_836343, EPI_ISL_836351, EPI_ISL_836352, EPI_ISL_836356, EPI_ISL_836357, EPI_ISL_836358, EPI_ISL_836362, EPI_ISL_836363, EPI_ISL_836366, EPI_ISL_836367, EPI_ISL_836371, EPI_ISL_836373, EPI_ISL_836377, EPI_ISL_836378, EPI_ISL_836387, EPI_ISL_836390, EPI_ISL_836395, EPI_ISL_836396, EPI_ISL_836403, EPI_ISL_836406, EPI_ISL_836407, EPI_ISL_836409, EPI_ISL_836410, EPI_ISL_836415, EPI_ISL_836416, EPI_ISL_836418			
see above	Lighthouse Lab in Milton Keynes	Wellcome Sanger Institute for the COVID-19 Genomics UK (COG-UK) Consortium	The Lighthouse Lab in Milton Keynes and Alex Alderton, Roberto Amato, Sonia Goncalves, Ewan Harrison, David K. Jackson, Ian Johnston, Dominic Kwiatkowski, Cordelia Langford, John Sillitoe on behalf of the Wellcome Sanger Institute COVID-19 Surveillance Team
EPI_ISL_836421, EPI_ISL_836422, EPI_ISL_836425, EPI_ISL_836426, EPI_ISL_836427, EPI_ISL_836428, EPI_ISL_836429, EPI_ISL_836430, EPI_ISL_836433, EPI_ISL_836434, EPI_ISL_836435, EPI_ISL_836436, EPI_ISL_836437, EPI_ISL_836438, EPI_ISL_836440, EPI_ISL_836441, EPI_ISL_836442, EPI_ISL_836443, EPI_ISL_836446, EPI_ISL_836447, EPI_ISL_836448, EPI_ISL_836449, EPI_ISL_836450, EPI_ISL_836452, EPI_ISL_836453, EPI_ISL_836454, EPI_ISL_836455, EPI_ISL_836456, EPI_ISL_836457, EPI_ISL_836458, EPI_ISL_836459, EPI_ISL_836460, EPI_ISL_836461, EPI_ISL_836462, EPI_ISL_836463, EPI_ISL_836464, EPI_ISL_836465, EPI_ISL_836467, EPI_ISL_836468, EPI_ISL_836469, EPI_ISL_836470, EPI_ISL_836472, EPI_ISL_836473, EPI_ISL_836474, EPI_ISL_836475, EPI_ISL_836476, EPI_ISL_836477, EPI_ISL_836478, EPI_ISL_836479, EPI_ISL_836480, EPI_ISL_836481, EPI_ISL_836484, EPI_ISL_836485, EPI_ISL_836486, EPI_ISL_836487, EPI_ISL_836488, EPI_ISL_836489, EPI_ISL_836490, EPI_ISL_836492, EPI_ISL_836493, EPI_ISL_836495, EPI_ISL_836497, EPI_ISL_836498, EPI_ISL_836499, EPI_ISL_836502, EPI_ISL_836503, EPI_ISL_836505, EPI_ISL_836507, EPI_ISL_836508, EPI_ISL_836509, EPI_ISL_836510, EPI_ISL_836511, EPI_ISL_836512, EPI_ISL_836513, EPI_ISL_836514, EPI_ISL_836515, EPI_ISL_836516, EPI_ISL_836518, EPI_ISL_836519, EPI_ISL_836520, EPI_ISL_836521, EPI_ISL_836522, EPI_ISL_836523, EPI_ISL_836524, EPI_ISL_836525, EPI_ISL_836526, EPI_ISL_836529, EPI_ISL_836531, EPI_ISL_836532, EPI_ISL_836533, EPI_ISL_836534, EPI_ISL_836535, EPI_ISL_836537, EPI_ISL_836538, EPI_ISL_836539, EPI_ISL_836540, EPI_ISL_836541, EPI_ISL_836542, EPI_ISL_836543, EPI_ISL_836544, EPI_ISL_836545, EPI_ISL_836546, EPI_ISL_836547, EPI_ISL_836549, EPI_ISL_836550, EPI_ISL_836551, EPI_ISL_836552, EPI_ISL_836553, EPI_ISL_836554, EPI_ISL_836555, EPI_ISL_836557, EPI_ISL_836558, EPI_ISL_836559, EPI_ISL_836560, EPI_ISL_836561, EPI_ISL_836563, EPI_ISL_836564, EPI_ISL_836565, EPI_ISL_836566, EPI_ISL_836568, EPI_ISL_836569, EPI_ISL_836570, EPI_ISL_836571, EPI_ISL_836573, EPI_ISL_836574, EPI_ISL_836575, EPI_ISL_836577, EPI_ISL_836578, EPI_ISL_836579, EPI_ISL_836580, EPI_ISL_836581, EPI_ISL_836582, EPI_ISL_836583, EPI_ISL_836584, EPI_ISL_836586, EPI_ISL_836587, EPI_ISL_836588, EPI_ISL_836589, EPI_ISL_836590, EPI_ISL_836591, EPI_ISL_836593, EPI_ISL_836594, EPI_ISL_836598, EPI_ISL_836599, EPI_ISL_836600, EPI_ISL_836601, EPI_ISL_836602, EPI_ISL_836604, EPI_ISL_836605, EPI_ISL_836606, EPI_ISL_836607, EPI_ISL_836608, EPI_ISL_836609, EPI_ISL_836610, EPI_ISL_836612, EPI_ISL_836613, EPI_ISL_836614, EPI_ISL_836615, EPI_ISL_836616, EPI_ISL_836618, EPI_ISL_836619, EPI_ISL_836621, EPI_ISL_836622, EPI_ISL_836624, EPI_ISL_836625, EPI_ISL_836628, EPI_ISL_836629, EPI_ISL_836630, EPI_ISL_836631, EPI_ISL_836632, EPI_ISL_836633, EPI_ISL_836634, EPI_ISL_836635, EPI_ISL_836637, EPI_ISL_836638, EPI_ISL_836639, EPI_ISL_836640, EPI_ISL_836641, EPI_ISL_836642, EPI_ISL_836643, EPI_ISL_836646, EPI_ISL_836647, EPI_ISL_836648, EPI_ISL_836649, EPI_ISL_836650, EPI_ISL_836653, EPI_ISL_836654, EPI_ISL_836658, EPI_ISL_836659, EPI_ISL_836660, EPI_ISL_836661, EPI_ISL_836662, EPI_ISL_836663, EPI_ISL_836664, EPI_ISL_836666, EPI_ISL_836667, EPI_ISL_836668, EPI_ISL_836669,			

EPI_ISL_836670, EPI_ISL_836671, EPI_ISL_836672, EPI_ISL_836673, EPI_ISL_836674, EPI_ISL_836675, EPI_ISL_836676, EPI_ISL_836677, EPI_ISL_836678, EPI_ISL_836680, EPI_ISL_836684, EPI_ISL_836685, EPI_ISL_836687, EPI_ISL_836690, EPI_ISL_836691, EPI_ISL_836692, EPI_ISL_836693, EPI_ISL_836694, EPI_ISL_836696, EPI_ISL_836698, EPI_ISL_836699, EPI_ISL_836700, EPI_ISL_836701, EPI_ISL_836702, EPI_ISL_836703, EPI_ISL_836704, EPI_ISL_836705, EPI_ISL_836706, EPI_ISL_836707, EPI_ISL_836708, EPI_ISL_836709, EPI_ISL_836710, EPI_ISL_836711, EPI_ISL_836714, EPI_ISL_836715, EPI_ISL_836716, EPI_ISL_836717, EPI_ISL_836718, EPI_ISL_836719, EPI_ISL_836720, EPI_ISL_836721, EPI_ISL_836723, EPI_ISL_836724, EPI_ISL_836726, EPI_ISL_836727, EPI_ISL_836728, EPI_ISL_836729, EPI_ISL_836730, EPI_ISL_836731, EPI_ISL_836732, EPI_ISL_836733, EPI_ISL_836734, EPI_ISL_836735, EPI_ISL_836736, EPI_ISL_836737, EPI_ISL_836738, EPI_ISL_836739, EPI_ISL_836740, EPI_ISL_836741, EPI_ISL_836742, EPI_ISL_836743, EPI_ISL_836744, EPI_ISL_836745, EPI_ISL_836746, EPI_ISL_836747			
see above	Lighthouse Lab in Cambridge	Wellcome Sanger Institute for the COVID-19 Genomics UK (COG-UK) Consortium	Rob Howes, The Lighthouse Lab in Cambridge and Alex Alderton, Roberto Amato, Sonia Goncalves, Ewan Harrison, David K. Jackson, Ian Johnston, Dominic Kwiatkowski, Cordelia Langford, John Sillitoe on behalf of the Wellcome Sanger Institute COVID-19 Surveillance Team
EPI_ISL_836748, EPI_ISL_836749, EPI_ISL_836750, EPI_ISL_836752, EPI_ISL_836753, EPI_ISL_836755, EPI_ISL_836756, EPI_ISL_836757, EPI_ISL_836758, EPI_ISL_836760, EPI_ISL_836761, EPI_ISL_836762, EPI_ISL_836763, EPI_ISL_836764			
see above	Lighthouse Lab in Glasgow	Wellcome Sanger Institute for the COVID-19 Genomics UK (COG-UK) Consortium	Harper VanSteenhouse, Yumi Kasai, David Gray, Carol Clugston, Anna Dominiczak and Alex Alderton, Roberto Amato, Sonia Goncalves, Ewan Harrison, David K. Jackson, Ian Johnston, Dominic Kwiatkowski, Cordelia Langford, John Sillitoe on behalf of the Wellcome Sanger Institute COVID-19 Surveillance Team
EPI_ISL_836766	Lighthouse Lab in Alderley Park	Wellcome Sanger Institute for the COVID-19 Genomics UK (COG-UK) Consortium	Jacquelyn Wynn, Mairead Hyland, The Lighthouse Lab in Alderley Park and Alex Alderton, Roberto Amato, Sonia Goncalves, Ewan Harrison, David K. Jackson, Ian Johnston, Dominic Kwiatkowski, Cordelia Langford, John Sillitoe on behalf of the Wellcome Sanger Institute COVID-19 Surveillance Team
EPI_ISL_836767, EPI_ISL_836768	Lighthouse Lab in Glasgow	Wellcome Sanger Institute for the COVID-19 Genomics UK (COG-UK) Consortium	Harper VanSteenhouse, Yumi Kasai, David Gray, Carol Clugston, Anna Dominiczak and Alex Alderton, Roberto Amato, Sonia Goncalves, Ewan Harrison, David K. Jackson, Ian Johnston, Dominic Kwiatkowski, Cordelia Langford, John Sillitoe on behalf of the Wellcome Sanger Institute COVID-19 Surveillance Team
EPI_ISL_836769	Lighthouse Lab in Alderley Park	Wellcome Sanger Institute for the COVID-19 Genomics UK (COG-UK) Consortium	Jacquelyn Wynn, Mairead Hyland, The Lighthouse Lab in Alderley Park and Alex Alderton, Roberto Amato, Sonia Goncalves, Ewan Harrison, David K. Jackson, Ian Johnston, Dominic Kwiatkowski, Cordelia Langford, John Sillitoe on behalf of the Wellcome Sanger Institute COVID-19 Surveillance Team
EPI_ISL_836771, EPI_ISL_836773, EPI_ISL_836774, EPI_ISL_836775, EPI_ISL_836776, EPI_ISL_836779, EPI_ISL_836780, EPI_ISL_836782, EPI_ISL_836783, EPI_ISL_836785, EPI_ISL_836786, EPI_ISL_836787, EPI_ISL_836789, EPI_ISL_836790, EPI_ISL_836791, EPI_ISL_836793, EPI_ISL_836794, EPI_ISL_836796, EPI_ISL_836798, EPI_ISL_836799, EPI_ISL_836800, EPI_ISL_836801			
see above	Lighthouse Lab in Glasgow	Wellcome Sanger Institute for the COVID-19 Genomics UK (COG-UK) Consortium	Harper VanSteenhouse, Yumi Kasai, David Gray, Carol Clugston, Anna Dominiczak and Alex Alderton, Roberto Amato, Sonia Goncalves, Ewan Harrison, David K. Jackson, Ian Johnston, Dominic Kwiatkowski, Cordelia Langford, John Sillitoe on behalf of the Wellcome Sanger Institute COVID-19 Surveillance Team
EPI_ISL_836802	Lighthouse Lab in Alderley Park	Wellcome Sanger Institute for the COVID-19 Genomics UK (COG-UK) Consortium	Jacquelyn Wynn, Mairead Hyland, The Lighthouse Lab in Alderley Park and Alex Alderton, Roberto Amato, Sonia Goncalves, Ewan Harrison, David K. Jackson, Ian Johnston, Dominic Kwiatkowski, Cordelia Langford, John Sillitoe on behalf of the Wellcome Sanger Institute COVID-19 Surveillance Team
EPI_ISL_836804, EPI_ISL_836805, EPI_ISL_836808, EPI_ISL_836809, EPI_ISL_836810, EPI_ISL_836812, EPI_ISL_836815, EPI_ISL_836816	Lighthouse Lab in Glasgow	Wellcome Sanger Institute for the COVID-19 Genomics UK (COG-UK) Consortium	Harper VanSteenhouse, Yumi Kasai, David Gray, Carol Clugston, Anna Dominiczak and Alex Alderton, Roberto Amato, Sonia Goncalves, Ewan Harrison, David K. Jackson, Ian Johnston, Dominic Kwiatkowski, Cordelia Langford, John Sillitoe on behalf of the Wellcome Sanger Institute COVID-19 Surveillance Team
EPI_ISL_836817	Lighthouse Lab in Alderley Park	Wellcome Sanger Institute for the COVID-19 Genomics UK (COG-UK) Consortium	Jacquelyn Wynn, Mairead Hyland, The Lighthouse Lab in Alderley Park and Alex Alderton, Roberto Amato, Sonia Goncalves, Ewan Harrison, David K. Jackson, Ian Johnston, Dominic Kwiatkowski, Cordelia Langford, John Sillitoe on behalf of the Wellcome Sanger Institute COVID-19 Surveillance Team
EPI_ISL_836818	Lighthouse Lab in Glasgow	Wellcome Sanger Institute for the COVID-19 Genomics UK (COG-UK) Consortium	Harper VanSteenhouse, Yumi Kasai, David Gray, Carol Clugston, Anna Dominiczak and Alex Alderton, Roberto Amato, Sonia Goncalves, Ewan Harrison, David K. Jackson, Ian Johnston, Dominic Kwiatkowski, Cordelia Langford, John Sillitoe on behalf of the Wellcome Sanger Institute COVID-19 Surveillance Team
EPI_ISL_836819	Lighthouse Lab in Alderley Park	Wellcome Sanger Institute for the COVID-19 Genomics UK (COG-UK) Consortium	Jacquelyn Wynn, Mairead Hyland, The Lighthouse Lab in Alderley Park and Alex Alderton, Roberto Amato, Sonia Goncalves, Ewan Harrison, David K. Jackson, Ian Johnston, Dominic Kwiatkowski, Cordelia Langford, John Sillitoe on behalf of the Wellcome Sanger Institute COVID-19 Surveillance Team
EPI_ISL_836821, EPI_ISL_836823, EPI_ISL_836824, EPI_ISL_836826, EPI_ISL_836828, EPI_ISL_836830, EPI_ISL_836832, EPI_ISL_836833, EPI_ISL_836835, EPI_ISL_836838, EPI_ISL_836839			
see above	Lighthouse Lab in Glasgow	Wellcome Sanger Institute for the COVID-19 Genomics UK (COG-UK) Consortium	Harper VanSteenhouse, Yumi Kasai, David Gray, Carol Clugston, Anna Dominiczak and Alex Alderton, Roberto Amato, Sonia Goncalves, Ewan Harrison, David K. Jackson, Ian Johnston, Dominic Kwiatkowski, Cordelia Langford, John Sillitoe on behalf of the Wellcome Sanger Institute COVID-19 Surveillance Team
EPI_ISL_836840	Lighthouse Lab in Alderley Park	Wellcome Sanger Institute for the COVID-19 Genomics UK (COG-UK) Consortium	Jacquelyn Wynn, Mairead Hyland, The Lighthouse Lab in Alderley Park and Alex Alderton, Roberto Amato, Sonia Goncalves, Ewan Harrison, David K. Jackson, Ian Johnston, Dominic Kwiatkowski, Cordelia Langford, John Sillitoe on behalf of the Wellcome Sanger Institute COVID-19 Surveillance Team
EPI_ISL_836841, EPI_ISL_836844, EPI_ISL_836845, EPI_ISL_836846, EPI_ISL_836852, EPI_ISL_836853, EPI_ISL_836854, EPI_ISL_836855, EPI_ISL_836856, EPI_ISL_836860, EPI_ISL_836861, EPI_ISL_836865, EPI_ISL_836868, EPI_ISL_836869, EPI_ISL_836871, EPI_ISL_836872, EPI_ISL_836873, EPI_ISL_836874, EPI_ISL_836875			
see above	Lighthouse Lab in Glasgow	Wellcome Sanger Institute for the COVID-19 Genomics UK (COG-UK) Consortium	Harper VanSteenhouse, Yumi Kasai, David Gray, Carol Clugston, Anna Dominiczak and Alex Alderton, Roberto Amato, Sonia Goncalves, Ewan Harrison, David K. Jackson, Ian Johnston, Dominic Kwiatkowski, Cordelia Langford, John Sillitoe on behalf of the Wellcome Sanger Institute COVID-19 Surveillance Team
EPI_ISL_836876, EPI_ISL_836878	Lighthouse Lab in Alderley Park	Wellcome Sanger Institute for the COVID-19 Genomics UK (COG-UK) Consortium	Jacquelyn Wynn, Mairead Hyland, The Lighthouse Lab in Alderley Park and Alex Alderton, Roberto Amato, Sonia Goncalves, Ewan Harrison, David K. Jackson, Ian Johnston, Dominic Kwiatkowski, Cordelia Langford, John Sillitoe on behalf of the Wellcome Sanger Institute COVID-19 Surveillance Team
EPI_ISL_836879, EPI_ISL_836880, EPI_ISL_836881, EPI_ISL_836882, EPI_ISL_836884, EPI_ISL_836885, EPI_ISL_836886, EPI_ISL_836888, EPI_ISL_836889, EPI_ISL_836892, EPI_ISL_836894, EPI_ISL_836896, EPI_ISL_836899, EPI_ISL_836901, EPI_ISL_836904, EPI_ISL_836905			
see above	Lighthouse Lab in Glasgow	Wellcome Sanger Institute for the COVID-19 Genomics UK (COG-UK) Consortium	Harper VanSteenhouse, Yumi Kasai, David Gray, Carol Clugston, Anna Dominiczak and Alex Alderton, Roberto Amato, Sonia Goncalves, Ewan Harrison, David K. Jackson, Ian Johnston, Dominic Kwiatkowski, Cordelia Langford, John Sillitoe on behalf of the Wellcome Sanger Institute COVID-19 Surveillance Team
EPI_ISL_836906	Lighthouse Lab in Alderley Park	Wellcome Sanger Institute for the COVID-19 Genomics UK (COG-UK) Consortium	Jacquelyn Wynn, Mairead Hyland, The Lighthouse Lab in Alderley Park and Alex Alderton, Roberto Amato, Sonia Goncalves, Ewan Harrison, David K. Jackson, Ian Johnston, Dominic Kwiatkowski, Cordelia Langford, John Sillitoe on behalf of the Wellcome Sanger Institute COVID-19 Surveillance Team
EPI_ISL_836911, EPI_ISL_836912	Lighthouse Lab in Glasgow	Wellcome Sanger Institute for the COVID-19 Genomics UK (COG-UK) Consortium	Harper VanSteenhouse, Yumi Kasai, David Gray, Carol Clugston, Anna Dominiczak and Alex Alderton, Roberto Amato, Sonia Goncalves, Ewan Harrison, David K. Jackson, Ian Johnston, Dominic Kwiatkowski, Cordelia Langford, John Sillitoe on behalf of the Wellcome Sanger Institute COVID-19 Surveillance Team
EPI_ISL_836914	Lighthouse Lab in Alderley Park	Wellcome Sanger Institute for the COVID-19 Genomics UK (COG-UK) Consortium	Jacquelyn Wynn, Mairead Hyland, The Lighthouse Lab in Alderley Park and Alex Alderton, Roberto Amato, Sonia Goncalves, Ewan Harrison, David K. Jackson, Ian Johnston, Dominic Kwiatkowski, Cordelia Langford, John Sillitoe on behalf of the Wellcome Sanger Institute COVID-19 Surveillance Team
EPI_ISL_836915, EPI_ISL_836916, EPI_ISL_836917, EPI_ISL_836918, EPI_ISL_836919, EPI_ISL_836923, EPI_ISL_836925, EPI_ISL_836927, EPI_ISL_836932, EPI_ISL_836933, EPI_ISL_836934			
see above	Lighthouse Lab in Glasgow	Wellcome Sanger Institute for the COVID-19 Genomics UK (COG-UK) Consortium	Harper VanSteenhouse, Yumi Kasai, David Gray, Carol Clugston, Anna Dominiczak and Alex Alderton, Roberto Amato, Sonia Goncalves, Ewan Harrison, David K. Jackson, Ian Johnston, Dominic Kwiatkowski, Cordelia Langford, John Sillitoe on behalf of the Wellcome Sanger Institute COVID-19 Surveillance Team
EPI_ISL_836935	Lighthouse Lab in Alderley Park	Wellcome Sanger Institute for the COVID-19 Genomics UK (COG-UK) Consortium	Jacquelyn Wynn, Mairead Hyland, The Lighthouse Lab in Alderley Park and Alex Alderton, Roberto Amato, Sonia Goncalves, Ewan Harrison, David K. Jackson, Ian Johnston, Dominic Kwiatkowski, Cordelia Langford, John Sillitoe on behalf of the Wellcome Sanger Institute COVID-19 Surveillance Team
EPI_ISL_836937, EPI_ISL_836938, EPI_ISL_836939, EPI_ISL_836941, EPI_ISL_836943, EPI_ISL_836945, EPI_ISL_836950, EPI_ISL_836951, EPI_ISL_836952, EPI_ISL_836953, EPI_ISL_836958, EPI_ISL_836959, EPI_ISL_836960, EPI_ISL_836961			
see above	Lighthouse Lab in Glasgow	Wellcome Sanger Institute for the COVID-19 Genomics UK (COG-UK) Consortium	Harper VanSteenhouse, Yumi Kasai, David Gray, Carol Clugston, Anna Dominiczak and Alex Alderton, Roberto Amato, Sonia Goncalves, Ewan Harrison, David K. Jackson, Ian Johnston, Dominic Kwiatkowski, Cordelia Langford, John Sillitoe on behalf of the Wellcome Sanger Institute COVID-19 Surveillance Team
EPI_ISL_836962	Lighthouse Lab in Alderley Park	Wellcome Sanger Institute for the COVID-19 Genomics UK (COG-UK) Consortium	Jacquelyn Wynn, Mairead Hyland, The Lighthouse Lab in Alderley Park and Alex Alderton, Roberto Amato, Sonia Goncalves, Ewan Harrison, David K. Jackson, Ian Johnston, Dominic Kwiatkowski, Cordelia Langford, John Sillitoe on behalf of the Wellcome Sanger Institute COVID-19 Surveillance Team
EPI_ISL_836963, EPI_ISL_836964,	Lighthouse Lab in Glasgow	Wellcome Sanger Institute for the COVID-19 Genomics UK	Harper VanSteenhouse, Yumi Kasai, David Gray, Carol Clugston, Anna Dominiczak and Alex Alderton, Roberto Amato, Sonia Goncalves, Ewan Harrison,

EPI_ISL_836965, EPI_ISL_836966, EPI_ISL_836967, EPI_ISL_836968, EPI_ISL_836970, EPI_ISL_836972		(COG-UK) Consortium	David K. Jackson, Ian Johnston, Dominic Kwiatkowski, Cordelia Langford, John Sillitoe on behalf of the Wellcome Sanger Institute COVID-19 Surveillance Team
EPI_ISL_837241, EPI_ISL_837242	Respiratory Virus Unit, National Infection Service, Public Health England	COVID-19 Genomics UK (COG-UK) Consortium	PHE Covid Sequencing Team
EPI_ISL_837844, EPI_ISL_837845, EPI_ISL_837850, EPI_ISL_837903, EPI_ISL_837906, EPI_ISL_837907, EPI_ISL_837910, EPI_ISL_837912, EPI_ISL_837920, EPI_ISL_837921, EPI_ISL_837922, EPI_ISL_837929, EPI_ISL_837931, EPI_ISL_837934, EPI_ISL_837935, EPI_ISL_837936, EPI_ISL_837940, EPI_ISL_837942, EPI_ISL_837943, EPI_ISL_837955, EPI_ISL_837971			
see above	Department of Pathology, University of Cambridge	COVID-19 Genomics UK (COG-UK) Consortium	Aminu S. Jahun, Yasmin Chaudhry, Grant Hall, Iliana Georgana, Myra Hosmillo, Martin D. Curran, Malte Pinckert, Surendra Parmar, Ian Goodfellow
EPI_ISL_838340	University of Exeter	COVID-19 Genomics UK (COG-UK) Consortium	Ben Temperton,Aaron Jeffries,Michelle Michelsen,Joanna Warwick-Dugdale,Audrey Farbos,Robyn Manley,Stephen Michell,Jane Masoli
EPI_ISL_838864, EPI_ISL_838865, EPI_ISL_838866, EPI_ISL_838867, EPI_ISL_838868, EPI_ISL_838869, EPI_ISL_838870, EPI_ISL_838871, EPI_ISL_838872, EPI_ISL_838873, EPI_ISL_838874, EPI_ISL_838875, EPI_ISL_838876, EPI_ISL_838877, EPI_ISL_838878, EPI_ISL_838879, EPI_ISL_838880, EPI_ISL_838881, EPI_ISL_838882, EPI_ISL_838883, EPI_ISL_838884, EPI_ISL_838885, EPI_ISL_838886, EPI_ISL_838887, EPI_ISL_838888, EPI_ISL_838889, EPI_ISL_838890, EPI_ISL_838891, EPI_ISL_838892, EPI_ISL_838893, EPI_ISL_838894, EPI_ISL_838906, EPI_ISL_838907, EPI_ISL_838908, EPI_ISL_838924, EPI_ISL_838925, EPI_ISL_839044, EPI_ISL_839070, EPI_ISL_839071, EPI_ISL_839072, EPI_ISL_839073, EPI_ISL_839117, EPI_ISL_839156, EPI_ISL_839172, EPI_ISL_839176, EPI_ISL_839177, EPI_ISL_839178, EPI_ISL_839179, EPI_ISL_839180, EPI_ISL_839181, EPI_ISL_839182, EPI_ISL_839183, EPI_ISL_839186, EPI_ISL_839187, EPI_ISL_839189, EPI_ISL_839192, EPI_ISL_839195, EPI_ISL_839210, EPI_ISL_839214, EPI_ISL_839231			
see above	University College London, Great Ormond Street Hospital for Children NHS Foundation Trust, Imperial College Healthcare NHS Trust	COVID-19 Genomics UK (COG-UK) Consortium	Sergi Castellano, Rachel Williams, Mark Kristiansen, Paola Resende Silva, Sunando Roy, Tony Brooks, Helena Tutill, Paola Niola, Patricia Dyal, Charlotte Williams, Leysa Forrest, Yasmin Panchbhaya, Jacqueline Findlay, Samuel Weeks, Julianne Brown, Kathryn Harris, Paul Randell, James Price, Alison Holmes, Judith Breuer
EPI_ISL_839823, EPI_ISL_839824, EPI_ISL_839840, EPI_ISL_839841, EPI_ISL_839842, EPI_ISL_839843, EPI_ISL_839844, EPI_ISL_839845, EPI_ISL_839846, EPI_ISL_839847, EPI_ISL_839848, EPI_ISL_839849, EPI_ISL_839850, EPI_ISL_839851, EPI_ISL_839852, EPI_ISL_839853, EPI_ISL_839854, EPI_ISL_839866, EPI_ISL_839899, EPI_ISL_839907, EPI_ISL_839908, EPI_ISL_839913, EPI_ISL_839914, EPI_ISL_839916, EPI_ISL_839918, EPI_ISL_839921, EPI_ISL_839932, EPI_ISL_839933, EPI_ISL_839934, EPI_ISL_839935, EPI_ISL_839936, EPI_ISL_839937, EPI_ISL_839938, EPI_ISL_839974, EPI_ISL_839975, EPI_ISL_839976, EPI_ISL_839977, EPI_ISL_839978, EPI_ISL_839979, EPI_ISL_839980			
see above	Quadram Institute Bioscience	COVID-19 Genomics UK (COG-UK) Consortium	Dave J. Baker, Gemma L. Kay, Alp Aydin, Thanh Le-Viet, Steven Rudder, Ana P. Tedim, Anastasia Kolyva, Maria Diaz, Leonardo de Oliveira Martins, Nabil-Fareed Alikhan, Lizzie Meadows, Rachael Stanley, Ngozi Elumogo, Muhammed Yasir, Nicholas M. Thomson, Alexander J Trotter, Rachel Gilroy, Samuel Bloomfield, Claire Stuart, Andrew Bell, Reenesh Prakash, Samir Dervisevic, Alison E. Mather, John Wain, Mark Webber, Andrew J. Page, Justin O'Grady
EPI_ISL_839997	Queens Medical Centre, Clinical Microbiology Department / DeepSeq Nottingham	COVID-19 Genomics UK (COG-UK) Consortium	Gemma Clark, Wendy Smith, Manjinder Khakh, Vicki M Fleming, Michelle M Lister, Hannah Howson-Wells, Jonathan Ball, Patrick McClure, Joseph Chappell, Theocharis Tsoleridis, Nadine Holmes, Matthew Carlisle, Christopher Moore, Fei Sang, Johnny Debebe, Victoria Wright, Matthew Loose
EPI_ISL_840135, EPI_ISL_840136, EPI_ISL_840137, EPI_ISL_840138, EPI_ISL_840140, EPI_ISL_840141, EPI_ISL_840142, EPI_ISL_840143, EPI_ISL_840144	Lincolnshire Hospitals and DeepSeq Nottingham	COVID-19 Genomics UK (COG-UK) Consortium	Nichola Duckworth, Tim Sloan, Sarah Walsh, Jonathan Ball, Patrick McClure, Joeseph Chappell, Nadine Holmes, Matthew Carlisle, Christopher Moore, Fei Sang, Johnny Debebe, Victoria Wright, Matthew Loose
EPI_ISL_840694	Originating lab: Wales Specialist Virology Centre Sequencing lab: Pathogen Genomics Unit	Public Health Wales Microbiology Cardiff Wales Specialist Virology Centre	Catherine Moore, Johnathan Evans, Laura Gifford, Malorie Perry, Simon Cottrell, Angela Marchbank, Alec Birchley, Alexander Adams, Amy Gaskin, Bree Gatica-Wilcox, Jason Coombes, Joel Southgate, Lauren Gilbert, Lee Graham, Nicole Pacchiarini, Sara Kumziene-Summerhayes, Sarah Taylor, Sophie Jones, Sara Rey, Matthew Bull, Joanne Watkins, Sally Corden, Tom Connor
EPI_ISL_840843, EPI_ISL_840849, EPI_ISL_840852, EPI_ISL_840855, EPI_ISL_840856, EPI_ISL_840857, EPI_ISL_840858, EPI_ISL_840859, EPI_ISL_840881, EPI_ISL_840907, EPI_ISL_840908, EPI_ISL_840951, EPI_ISL_840961, EPI_ISL_840963, EPI_ISL_840964, EPI_ISL_840965, EPI_ISL_840968, EPI_ISL_840969, EPI_ISL_840970, EPI_ISL_840971, EPI_ISL_840974, EPI_ISL_840975, EPI_ISL_840976, EPI_ISL_840977, EPI_ISL_840978, EPI_ISL_840979, EPI_ISL_840981, EPI_ISL_840982, EPI_ISL_840983, EPI_ISL_840984, EPI_ISL_840985, EPI_ISL_840989, EPI_ISL_840990, EPI_ISL_840991, EPI_ISL_840992, EPI_ISL_840993, EPI_ISL_840994, EPI_ISL_840995, EPI_ISL_840996, EPI_ISL_840997, EPI_ISL_840998, EPI_ISL_841000, EPI_ISL_841002, EPI_ISL_841004, EPI_ISL_841032, EPI_ISL_841040, EPI_ISL_841043, EPI_ISL_841044, EPI_ISL_841051, EPI_ISL_841052, EPI_ISL_841060, EPI_ISL_841086, EPI_ISL_841098, EPI_ISL_841166, EPI_ISL_841168, EPI_ISL_841169, EPI_ISL_841170, EPI_ISL_841172, EPI_ISL_841174, EPI_ISL_841175, EPI_ISL_841176, EPI_ISL_841178, EPI_ISL_841179, EPI_ISL_841183, EPI_ISL_841184, EPI_ISL_841185, EPI_ISL_841186, EPI_ISL_841193, EPI_ISL_841195, EPI_ISL_841204, EPI_ISL_841208, EPI_ISL_841214, EPI_ISL_841307, EPI_ISL_841308, EPI_ISL_841309, EPI_ISL_841310, EPI_ISL_841311, EPI_ISL_841312, EPI_ISL_841313, EPI_ISL_841316			
see above	Wales Specialist Virology Centre Sequencing lab: Pathogen Genomics Unit	Public Health Wales Microbiology Cardiff Wales Specialist Virology Centre	Catherine Moore, Johnathan Evans, Laura Gifford, Malorie Perry, Simon Cottrell, Angela Marchbank, Alec Birchley, Alexander Adams, Amy Gaskin, Bree Gatica-Wilcox, Jason Coombes, Joel Southgate, Lauren Gilbert, Lee Graham, Nicole Pacchiarini, Sara Kumziene-Summerhayes, Sarah Taylor, Sophie Jones, Sara Rey, Matthew Bull, Joanne Watkins, Sally Corden, Tom Connor
EPI_ISL_841332, EPI_ISL_841334, EPI_ISL_841375, EPI_ISL_841376, EPI_ISL_841377, EPI_ISL_841387, EPI_ISL_841388, EPI_ISL_841389, EPI_ISL_841390, EPI_ISL_841498, EPI_ISL_841506, EPI_ISL_841512, EPI_ISL_841586, EPI_ISL_841588, EPI_ISL_841590, EPI_ISL_841596, EPI_ISL_841611			
see above	Originating lab: Wales Specialist Virology Centre Sequencing lab: Pathogen Genomics Unit	Public Health Wales Microbiology Cardiff Wales Specialist Virology Centre	Catherine Moore, Johnathan Evans, Laura Gifford, Malorie Perry, Simon Cottrell, Angela Marchbank, Alec Birchley, Alexander Adams, Amy Gaskin, Bree Gatica-Wilcox, Jason Coombes, Joel Southgate, Lauren Gilbert, Lee Graham, Nicole Pacchiarini, Sara Kumziene-Summerhayes, Sarah Taylor, Sophie Jones, Sara Rey, Matthew Bull, Joanne Watkins, Sally Corden, Tom Connor
EPI_ISL_842208, EPI_ISL_842215, EPI_ISL_842216, EPI_ISL_842221, EPI_ISL_842224, EPI_ISL_842226, EPI_ISL_842239, EPI_ISL_842241, EPI_ISL_842246, EPI_ISL_842251, EPI_ISL_842258, EPI_ISL_842261, EPI_ISL_842280, EPI_ISL_842287, EPI_ISL_842288, EPI_ISL_842289, EPI_ISL_842294, EPI_ISL_842300, EPI_ISL_842301, EPI_ISL_842303, EPI_ISL_842305, EPI_ISL_842324, EPI_ISL_842329, EPI_ISL_842330, EPI_ISL_842334, EPI_ISL_842340			
see above	Virology Department, Sheffield Teaching Hospitals NHS Foundation Trust/Department of Infection, Immunity and Cardiovascular Disease, The Medical School, University of Sheffield	COVID-19 Genomics UK (COG-UK) Consortium	Thushan de Silva, Matthew Parker, Nikki Smith, Adri Angyal, Rebecca Brown, Luke Green, Rachel Tucker, Paul Parsons, Danielle Groves, Katie Johnson, Laura Carrilero, Alex Keeley, Dave Partridge, Matthew Wyles, Benjamin Lindsey, Mehmet Yavuz, Mohammad Raza, Cariad Evans
EPI_ISL_842638, EPI_ISL_842640	University Hospital of Northern Norway, Department for Microbiology and Infectious Disease Control	Norwegian Institute of Public Health, Department of Virology	Kathrine Stene-Johansen, Kamilla Heddeland Instefjord, Hilde Elshaug, Atiya R Ali,Marie Paulsen Madsen, Rasmus Riis Kopperud, Hilde Vollan, Karoline Bragstad, Olav Hungnes
EPI_ISL_842641	Unilabs Laboratory Medicine	Norwegian Institute of Public Health, Department of Virology	Kathrine Stene-Johansen, Kamilla Heddeland Instefjord, Hilde Elshaug, Atiya R Ali,Marie Paulsen Madsen, Rasmus Riis Kopperud, Hilde Vollan, Karoline Bragstad, Olav Hungnes
EPI_ISL_842645	Hospital of Southern Norway - Kristiansand, Department of Medical Microbiology	Norwegian Institute of Public Health, Department of Virology	Kathrine Stene-Johansen, Kamilla Heddeland Instefjord, Hilde Elshaug, Atiya R Ali,Marie Paulsen Madsen, Rasmus Riis Kopperud, Hilde Vollan, Karoline Bragstad, Olav Hungnes
EPI_ISL_843201, EPI_ISL_843202, EPI_ISL_843203, EPI_ISL_843204, EPI_ISL_843205, EPI_ISL_843206, EPI_ISL_843207, EPI_ISL_843208, EPI_ISL_843209, EPI_ISL_843210, EPI_ISL_843211, EPI_ISL_843212, EPI_ISL_843214, EPI_ISL_843215, EPI_ISL_843217, EPI_ISL_843218, EPI_ISL_843219, EPI_ISL_843220, EPI_ISL_843221, EPI_ISL_843222, EPI_ISL_843223, EPI_ISL_843224, EPI_ISL_843225, EPI_ISL_843226, EPI_ISL_843227, EPI_ISL_843228, EPI_ISL_843229, EPI_ISL_843230, EPI_ISL_843231, EPI_ISL_843232, EPI_ISL_843233, EPI_ISL_843234, EPI_ISL_843235, EPI_ISL_843236, EPI_ISL_843237, EPI_ISL_843238, EPI_ISL_843239, EPI_ISL_843240, EPI_ISL_843241, EPI_ISL_843242, EPI_ISL_843243, EPI_ISL_843244, EPI_ISL_843245, EPI_ISL_843246, EPI_ISL_843247, EPI_ISL_843248, EPI_ISL_843249, EPI_ISL_843250, EPI_ISL_843251, EPI_ISL_843252, EPI_ISL_843253, EPI_ISL_843254, EPI_ISL_843255, EPI_ISL_843256, EPI_ISL_843257, EPI_ISL_843258, EPI_ISL_843259, EPI_ISL_843260, EPI_ISL_843261, EPI_ISL_843262, EPI_ISL_843263, EPI_ISL_843264, EPI_ISL_843265, EPI_ISL_843266, EPI_ISL_843267, EPI_ISL_843268, EPI_ISL_843269, EPI_ISL_843271, EPI_ISL_843272, EPI_ISL_843273, EPI_ISL_843274, EPI_ISL_843275, EPI_ISL_843276, EPI_ISL_843277, EPI_ISL_843278, EPI_ISL_843279, EPI_ISL_843280, EPI_ISL_843281, EPI_ISL_843282, EPI_ISL_843283, EPI_ISL_843284, EPI_ISL_843285, EPI_ISL_843286, EPI_ISL_843287, EPI_ISL_843288, EPI_ISL_843289, EPI_ISL_843290, EPI_ISL_843291, EPI_ISL_843292, EPI_ISL_843293, EPI_ISL_843294, EPI_ISL_843295, EPI_ISL_843296, EPI_ISL_843297, EPI_ISL_843298, EPI_ISL_843299, EPI_ISL_843300, EPI_ISL_843301, EPI_ISL_843303, EPI_ISL_843304, EPI_ISL_843305, EPI_ISL_843306, EPI_ISL_843307, EPI_ISL_843308, EPI_ISL_843309, EPI_ISL_843310, EPI_ISL_843311, EPI_ISL_843312, EPI_ISL_843313, EPI_ISL_843314, EPI_ISL_843315, EPI_ISL_843317, EPI_ISL_843318, EPI_ISL_843319, EPI_ISL_843320, EPI_ISL_843321, EPI_ISL_843322, EPI_ISL_843323, EPI_ISL_843324, EPI_ISL_843325, EPI_ISL_843326, EPI_ISL_843327, EPI_ISL_843328, EPI_ISL_843329, EPI_ISL_843330, EPI_ISL_843331, EPI_ISL_843332, EPI_ISL_843333, EPI_ISL_843334, EPI_ISL_843335, EPI_ISL_843336, EPI_ISL_843337, EPI_ISL_843338, EPI_ISL_843339, EPI_ISL_843340, EPI_ISL_843342, EPI_ISL_843343, EPI_ISL_843344, EPI_ISL_843345, EPI_ISL_843346, EPI_ISL_843347, EPI_ISL_843348, EPI_ISL_843349, EPI_ISL_843350, EPI_ISL_843351, EPI_ISL_843352, EPI_ISL_843353, EPI_ISL_843354, EPI_ISL_843355, EPI_ISL_843356, EPI_ISL_843357, EPI_ISL_843358, EPI_ISL_843359, EPI_ISL_843361, EPI_ISL_843362, EPI_ISL_843363, EPI_ISL_843364, EPI_ISL_843365, EPI_ISL_843366, EPI_ISL_843367, EPI_ISL_843368, EPI_ISL_843369, EPI_ISL_843371, EPI_ISL_843372, EPI_ISL_843373, EPI_ISL_843374, EPI_ISL_843375, EPI_ISL_843376, EPI_ISL_843377, EPI_ISL_843378, EPI_ISL_843379, EPI_ISL_843380, EPI_ISL_843381, EPI_ISL_843382, EPI_ISL_843383, EPI_ISL_843385, EPI_ISL_843386, EPI_ISL_843387, EPI_ISL_843388, EPI_ISL_843389, EPI_ISL_843390, EPI_ISL_843391, EPI_ISL_843392, EPI_ISL_843393, EPI_ISL_843394, EPI_ISL_843395, EPI_ISL_843396, EPI_ISL_843397, EPI_ISL_843398, EPI_ISL_843399, EPI_ISL_843400, EPI_ISL_843401, EPI_ISL_843402, EPI_ISL_843403, EPI_ISL_843404, EPI_ISL_843405, EPI_ISL_843406, EPI_ISL_843407, EPI_ISL_843408, EPI_ISL_843409, EPI_ISL_843410, EPI_ISL_843411, EPI_ISL_843412, EPI_ISL_843413, EPI_ISL_843414, EPI_ISL_843415, EPI_ISL_843416, EPI_ISL_843417, EPI_ISL_843418, EPI_ISL_843419, EPI_ISL_843420, EPI_ISL_843421, EPI_ISL_843422, EPI_ISL_843423, EPI_ISL_843424, EPI_ISL_843425, EPI_ISL_843426, EPI_ISL_843427, EPI_ISL_843428, EPI_ISL_843429, EPI_ISL_843430, EPI_ISL_843431, EPI_ISL_843432, EPI_ISL_843433, EPI_ISL_843434, EPI_ISL_843435, EPI_ISL_843436, EPI_ISL_843437, EPI_ISL_843438, EPI_ISL_843439, EPI_ISL_843440, EPI_ISL_843441, EPI_ISL_843442, EPI_ISL_843443, EPI_ISL_843444, EPI_ISL_843445, EPI_ISL_843446, EPI_ISL_843447, EPI_ISL_843448, EPI_ISL_843449, EPI_ISL_843450, EPI_ISL_843451, EPI_ISL_843452, EPI_ISL_843453, EPI_ISL_843454, EPI_ISL_843455, EPI_ISL_843456, EPI_ISL_843457, EPI_ISL_843458, EPI_ISL_843459, EPI_ISL_843460, EPI_ISL_843461, EPI_ISL_843462, EPI_ISL_843463, EPI_ISL_843464, EPI_ISL_843465, EPI_ISL_843466, EPI_ISL_843467, EPI_ISL_843468, EPI_ISL_843469, EPI_ISL_843470, EPI_ISL_843471, EPI_ISL_843472, EPI_ISL_843473, EPI_ISL_843474, EPI_ISL_843475, EPI_ISL_843476, EPI_ISL_843477, EPI_ISL_843478, EPI_ISL_843479, EPI_ISL_843480, EPI_ISL_843481, EPI_ISL_843482, EPI_ISL_843483, EPI_ISL_843484, EPI_ISL_843485, EPI_ISL_843486, EPI_ISL_843487, EPI_ISL_843488, EPI_ISL_843489, EPI_ISL_843490, EPI_ISL_843491, EPI_ISL_843492, EPI_ISL_843493, EPI_ISL_843494, EPI_ISL_843495, EPI_ISL_843496, EPI_ISL_843497, EPI_ISL_843498, EPI_ISL_843499, EPI_ISL_843500, EPI_ISL_843501, EPI_ISL_843502, EPI_ISL_843503, EPI_ISL_843504, EPI_ISL_843505, EPI_ISL_843506, EPI_ISL_843507, EPI_ISL_843508, EPI_ISL_843509, EPI_ISL_843510, EPI_ISL_843511, EPI_ISL_843512, EPI_ISL_843513, EPI_ISL_843514, EPI_ISL_843515, EPI_ISL_843516, EPI_ISL_843517, EPI_ISL_843518, EPI_ISL_843519, EPI_ISL_843520, EPI_ISL_843521, EPI_ISL_843522, EPI_ISL_843523, EPI_ISL_843524, EPI_ISL_843525, EPI_ISL_843526, EPI_ISL_843527, EPI_ISL_843528, EPI_ISL_843529, EPI_ISL_843530, EPI_ISL_843531, EPI_ISL_843532, EPI_ISL_843533, EPI_ISL_843534, EPI_ISL_843535, EPI_ISL_843536, EPI_ISL_843537, EPI_ISL_843538, EPI_ISL_843539, EPI_ISL_843540, EPI_ISL_843541, EPI_ISL_843542, EPI_ISL_843543, EPI_ISL_843544, EPI_ISL_843545, EPI_ISL_843546, EPI_ISL_843548, EPI_ISL_843549, EPI_ISL_843550, EPI_ISL_843551, EPI_ISL_843552, EPI_ISL_843553, EPI_ISL_843554, EPI_ISL_843555, EPI_ISL_843556, EPI_ISL_843557, EPI_ISL_843558, EPI_ISL_843559, EPI_ISL_843560, EPI_ISL_843561, EPI_ISL_843562, EPI_ISL_843563, EPI_ISL_843564, EPI_ISL_843565, EPI_ISL_843566, EPI_ISL_843567, EPI_ISL_843568, EPI_ISL_843569, EPI_ISL_843570,			

EPI_ISL_843571, EPI_ISL_843572, EPI_ISL_843573, EPI_ISL_843575, EPI_ISL_843576, EPI_ISL_843577, EPI_ISL_843578, EPI_ISL_843579, EPI_ISL_843580, EPI_ISL_843581, EPI_ISL_843582, EPI_ISL_843583, EPI_ISL_843584, EPI_ISL_843585, EPI_ISL_843586, EPI_ISL_843587, EPI_ISL_843588, EPI_ISL_843589, EPI_ISL_843590, EPI_ISL_843592, EPI_ISL_843593, EPI_ISL_843594, EPI_ISL_843596, EPI_ISL_843597, EPI_ISL_843599, EPI_ISL_843600, EPI_ISL_843601, EPI_ISL_843602, EPI_ISL_843603, EPI_ISL_843604, EPI_ISL_843605, EPI_ISL_843606, EPI_ISL_843607, EPI_ISL_843609, EPI_ISL_843610, EPI_ISL_843611, EPI_ISL_843612, EPI_ISL_843613, EPI_ISL_843614, EPI_ISL_843672, EPI_ISL_843700, EPI_ISL_843789, EPI_ISL_843803, EPI_ISL_843912, EPI_ISL_843925, EPI_ISL_843973, EPI_ISL_844000, EPI_ISL_844008, EPI_ISL_844026, EPI_ISL_844067, EPI_ISL_844108, EPI_ISL_844113, EPI_ISL_844120, EPI_ISL_844129, EPI_ISL_844544, EPI_ISL_844546, EPI_ISL_844547, EPI_ISL_844548, EPI_ISL_844549, EPI_ISL_844550, EPI_ISL_844551, EPI_ISL_844554, EPI_ISL_844555, EPI_ISL_844578, EPI_ISL_844579, EPI_ISL_844580, EPI_ISL_844581, EPI_ISL_844582, EPI_ISL_844583, EPI_ISL_844584, EPI_ISL_844585, EPI_ISL_844586, EPI_ISL_844587, EPI_ISL_844588, EPI_ISL_844589, EPI_ISL_844590, EPI_ISL_844591, EPI_ISL_844592, EPI_ISL_844593, EPI_ISL_844594, EPI_ISL_844595, EPI_ISL_844596, EPI_ISL_844597, EPI_ISL_844598, EPI_ISL_844599, EPI_ISL_844600, EPI_ISL_844601, EPI_ISL_844602, EPI_ISL_844603, EPI_ISL_844604, EPI_ISL_844605, EPI_ISL_844606, EPI_ISL_844607, EPI_ISL_844608, EPI_ISL_844609, EPI_ISL_844610, EPI_ISL_844611, EPI_ISL_844612, EPI_ISL_844613, EPI_ISL_844614, EPI_ISL_844615, EPI_ISL_844616, EPI_ISL_844617, EPI_ISL_844618, EPI_ISL_844619, EPI_ISL_844620, EPI_ISL_844621, EPI_ISL_844622, EPI_ISL_844623, EPI_ISL_844624, EPI_ISL_844625, EPI_ISL_844626, EPI_ISL_844627, EPI_ISL_844628, EPI_ISL_844629, EPI_ISL_844630, EPI_ISL_844631, EPI_ISL_844632, EPI_ISL_844633, EPI_ISL_844634, EPI_ISL_844640, EPI_ISL_844641, EPI_ISL_844642, EPI_ISL_844643, EPI_ISL_844644, EPI_ISL_844645, EPI_ISL_844646, EPI_ISL_844647, EPI_ISL_844648, EPI_ISL_844649, EPI_ISL_844650, EPI_ISL_844651, EPI_ISL_844652, EPI_ISL_844656, EPI_ISL_844657, EPI_ISL_844658, EPI_ISL_844659, EPI_ISL_844660, EPI_ISL_844661, EPI_ISL_844662, EPI_ISL_844663, EPI_ISL_844664, EPI_ISL_844665, EPI_ISL_844668, EPI_ISL_844671, EPI_ISL_844672, EPI_ISL_844673, EPI_ISL_844674, EPI_ISL_844675, EPI_ISL_844677, EPI_ISL_844678, EPI_ISL_844679, EPI_ISL_844682, EPI_ISL_844683, EPI_ISL_844684, EPI_ISL_844685, EPI_ISL_844686, EPI_ISL_844687, EPI_ISL_844688, EPI_ISL_844689, EPI_ISL_844690, EPI_ISL_844691, EPI_ISL_844692, EPI_ISL_844695, EPI_ISL_844696, EPI_ISL_844697, EPI_ISL_844698, EPI_ISL_844699, EPI_ISL_844700, EPI_ISL_844701, EPI_ISL_844703, EPI_ISL_844704, EPI_ISL_844705, EPI_ISL_844709, EPI_ISL_844710, EPI_ISL_844711, EPI_ISL_844712, EPI_ISL_844713, EPI_ISL_844714, EPI_ISL_844715, EPI_ISL_844719, EPI_ISL_844720, EPI_ISL_844721, EPI_ISL_844722, EPI_ISL_844723, EPI_ISL_844724, EPI_ISL_844725, EPI_ISL_844726, EPI_ISL_844727, EPI_ISL_844728, EPI_ISL_844729, EPI_ISL_844739, EPI_ISL_844740, EPI_ISL_844741, EPI_ISL_844742, EPI_ISL_844743, EPI_ISL_844744, EPI_ISL_844745, EPI_ISL_844746, EPI_ISL_844748, EPI_ISL_844749, EPI_ISL_844750, EPI_ISL_844751, EPI_ISL_844752, EPI_ISL_844753, EPI_ISL_844754, EPI_ISL_844755, EPI_ISL_844756, EPI_ISL_844757, EPI_ISL_844760, EPI_ISL_844761, EPI_ISL_844762, EPI_ISL_844764, EPI_ISL_844765, EPI_ISL_844769, EPI_ISL_844770, EPI_ISL_844771, EPI_ISL_844772, EPI_ISL_844773, EPI_ISL_844774, EPI_ISL_844775, EPI_ISL_844776, EPI_ISL_844777, EPI_ISL_844778, EPI_ISL_844779, EPI_ISL_844780, EPI_ISL_844781, EPI_ISL_844782, EPI_ISL_844783, EPI_ISL_844784, EPI_ISL_844785, EPI_ISL_844786, EPI_ISL_844787, EPI_ISL_844789, EPI_ISL_844790, EPI_ISL_844791, EPI_ISL_844792, EPI_ISL_844793, EPI_ISL_844794, EPI_ISL_844795, EPI_ISL_844796, EPI_ISL_844797, EPI_ISL_844798, EPI_ISL_844799, EPI_ISL_844800, EPI_ISL_844801, EPI_ISL_844802, EPI_ISL_844803, EPI_ISL_844804, EPI_ISL_844806, EPI_ISL_844809, EPI_ISL_844810, EPI_ISL_844811, EPI_ISL_844812, EPI_ISL_844813, EPI_ISL_844814, EPI_ISL_844815, EPI_ISL_844816, EPI_ISL_844817, EPI_ISL_844819, EPI_ISL_844820, EPI_ISL_844822, EPI_ISL_844823, EPI_ISL_844825, EPI_ISL_844826, EPI_ISL_844827, EPI_ISL_844828, EPI_ISL_844829, EPI_ISL_844830, EPI_ISL_844831, EPI_ISL_844832, EPI_ISL_844833, EPI_ISL_844835, EPI_ISL_844838, EPI_ISL_844839, EPI_ISL_844840, EPI_ISL_844841, EPI_ISL_844850, EPI_ISL_844851, EPI_ISL_844852, EPI_ISL_844853, EPI_ISL_844854, EPI_ISL_844855, EPI_ISL_844856, EPI_ISL_844857, EPI_ISL_844858, EPI_ISL_844859, EPI_ISL_844866, EPI_ISL_844867, EPI_ISL_844868, EPI_ISL_844869, EPI_ISL_844870, EPI_ISL_844871, EPI_ISL_844872, EPI_ISL_844873, EPI_ISL_844874, EPI_ISL_844875, EPI_ISL_844876, EPI_ISL_844877, EPI_ISL_844878, EPI_ISL_844879, EPI_ISL_844880, EPI_ISL_844881, EPI_ISL_844882, EPI_ISL_844883, EPI_ISL_844884, EPI_ISL_844885, EPI_ISL_844886, EPI_ISL_844887, EPI_ISL_844888, EPI_ISL_844889, EPI_ISL_844890, EPI_ISL_844891, EPI_ISL_844892, EPI_ISL_844893, EPI_ISL_844894, EPI_ISL_844895, EPI_ISL_844896, EPI_ISL_844897, EPI_ISL_844898, EPI_ISL_844899, EPI_ISL_844900, EPI_ISL_844901, EPI_ISL_844902, EPI_ISL_844903, EPI_ISL_844904, EPI_ISL_844905, EPI_ISL_844906, EPI_ISL_844907, EPI_ISL_844908, EPI_ISL_844909, EPI_ISL_844910, EPI_ISL_844913, EPI_ISL_844914, EPI_ISL_844915, EPI_ISL_844916, EPI_ISL_844917, EPI_ISL_844918, EPI_ISL_844919, EPI_ISL_844920, EPI_ISL_844921, EPI_ISL_844922, EPI_ISL_844923, EPI_ISL_844924, EPI_ISL_844925, EPI_ISL_844926, EPI_ISL_844927, EPI_ISL_844928, EPI_ISL_844929, EPI_ISL_844930, EPI_ISL_844933, EPI_ISL_844934, EPI_ISL_844935, EPI_ISL_844936, EPI_ISL_844937, EPI_ISL_844938, EPI_ISL_844939, EPI_ISL_844940, EPI_ISL_844941, EPI_ISL_844942, EPI_ISL_844943, EPI_ISL_844944, EPI_ISL_844945, EPI_ISL_844946, EPI_ISL_844947, EPI_ISL_844949, EPI_ISL_844950, EPI_ISL_844952, EPI_ISL_844959, EPI_ISL_844960, EPI_ISL_844961, EPI_ISL_844962, EPI_ISL_844963, EPI_ISL_844964, EPI_ISL_844965, EPI_ISL_844966, EPI_ISL_844967, EPI_ISL_844968, EPI_ISL_844969, EPI_ISL_844970, EPI_ISL_844971, EPI_ISL_844972, EPI_ISL_844973, EPI_ISL_844974, EPI_ISL_844975, EPI_ISL_844976, EPI_ISL_844977, EPI_ISL_844979, EPI_ISL_844980, EPI_ISL_844982, EPI_ISL_844983, EPI_ISL_844984, EPI_ISL_844985, EPI_ISL_844986, EPI_ISL_844987, EPI_ISL_844989, EPI_ISL_844990, EPI_ISL_844994, EPI_ISL_844995, EPI_ISL_844996, EPI_ISL_844997, EPI_ISL_845081, EPI_ISL_845082, EPI_ISL_845083, EPI_ISL_845084, EPI_ISL_845085, EPI_ISL_845086, EPI_ISL_845087, EPI_ISL_845088, EPI_ISL_845089, EPI_ISL_845090, EPI_ISL_845091, EPI_ISL_845092, EPI_ISL_845093, EPI_ISL_845094, EPI_ISL_845095, EPI_ISL_845096, EPI_ISL_845097, EPI_ISL_845098, EPI_ISL_845099, EPI_ISL_845100, EPI_ISL_845101, EPI_ISL_845102, EPI_ISL_845103, EPI_ISL_845104, EPI_ISL_845105, EPI_ISL_845106, EPI_ISL_845107, EPI_ISL_845108, EPI_ISL_845109, EPI_ISL_845110, EPI_ISL_845111, EPI_ISL_845112, EPI_ISL_845113, EPI_ISL_845114, EPI_ISL_845115, EPI_ISL_845116, EPI_ISL_845117, EPI_ISL_845118, EPI_ISL_845119, EPI_ISL_845120, EPI_ISL_845121, EPI_ISL_845122, EPI_ISL_845123, EPI_ISL_845124, EPI_ISL_845125, EPI_ISL_845126, EPI_ISL_845127, EPI_ISL_845128, EPI_ISL_845129, EPI_ISL_845130, EPI_ISL_845131, EPI_ISL_845132, EPI_ISL_845133, EPI_ISL_845134, EPI_ISL_845135, EPI_ISL_845136, EPI_ISL_845137, EPI_ISL_845138, EPI_ISL_845139, EPI_ISL_845140, EPI_ISL_845141, EPI_ISL_845142, EPI_ISL_845143, EPI_ISL_845144, EPI_ISL_845145, EPI_ISL_845146, EPI_ISL_845147, EPI_ISL_845148, EPI_ISL_845149, EPI_ISL_845150, EPI_ISL_845151, EPI_ISL_845152, EPI_ISL_845153, EPI_ISL_845154, EPI_ISL_845155, EPI_ISL_845156, EPI_ISL_845157, EPI_ISL_845158, EPI_ISL_845159, EPI_ISL_845160, EPI_ISL_845161, EPI_ISL_845162, EPI_ISL_845163, EPI_ISL_845164, EPI_ISL_845165, EPI_ISL_845166, EPI_ISL_845167, EPI_ISL_845168, EPI_ISL_845169, EPI_ISL_845170, EPI_ISL_845171, EPI_ISL_845172, EPI_ISL_845173, EPI_ISL_845174, EPI_ISL_845175, EPI_ISL_845176, EPI_ISL_845177, EPI_ISL_845178, EPI_ISL_845179, EPI_ISL_845180, EPI_ISL_845181, EPI_ISL_845182, EPI_ISL_845183, EPI_ISL_845184, EPI_ISL_845185, EPI_ISL_845186, EPI_ISL_845187, EPI_ISL_845188, EPI_ISL_845189, EPI_ISL_845190, EPI_ISL_845191, EPI_ISL_845192, EPI_ISL_845193, EPI_ISL_845194, EPI_ISL_845195, EPI_ISL_845196, EPI_ISL_845197, EPI_ISL_845198, EPI_ISL_845199, EPI_ISL_845200, EPI_ISL_845201, EPI_ISL_845202, EPI_ISL_845203, EPI_ISL_845204, EPI_ISL_845205, EPI_ISL_845206, EPI_ISL_845207, EPI_ISL_845208, EPI_ISL_845209, EPI_ISL_845210, EPI_ISL_845211, EPI_ISL_845212, EPI_ISL_845213, EPI_ISL_845214, EPI_ISL_845215, EPI_ISL_845216, EPI_ISL_845217, EPI_ISL_845218, EPI_ISL_845219, EPI_ISL_845220, EPI_ISL_845221, EPI_ISL_845222, EPI_ISL_845223, EPI_ISL_845224, EPI_ISL_845225, EPI_ISL_845226, EPI_ISL_845227, EPI_ISL_845228, EPI_ISL_845229, EPI_ISL_845230, EPI_ISL_845231, EPI_ISL_845232, EPI_ISL_845233, EPI_ISL_845234, EPI_ISL_845235, EPI_ISL_845236, EPI_ISL_845237, EPI_ISL_845238, EPI_ISL_845239, EPI_ISL_845240, EPI_ISL_845241, EPI_ISL_845242, EPI_ISL_845243, EPI_ISL_845244, EPI_ISL_845245, EPI_ISL_845246, EPI_ISL_845247, EPI_ISL_845248, EPI_ISL_845249, EPI_ISL_845250, EPI_ISL_845251, EPI_ISL_845252, EPI_ISL_845253, EPI_ISL_845254, EPI_ISL_845255, EPI_ISL_845256, EPI_ISL_845257, EPI_ISL_845258, EPI_ISL_845259, EPI_ISL_845260, EPI_ISL_845261, EPI_ISL_845262, EPI_ISL_845263, EPI_ISL_845264, EPI_ISL_845265, EPI_ISL_845266, EPI_ISL_845267, EPI_ISL_845268, EPI_ISL_845269, EPI_ISL_845270, EPI_ISL_845271, EPI_ISL_845272, EPI_ISL_845273, EPI_ISL_845274, EPI_ISL_845281, EPI_ISL_845282, EPI_ISL_845283, EPI_ISL_845284, EPI_ISL_845285, EPI_ISL_845286, EPI_ISL_845287, EPI_ISL_845288, EPI_ISL_845289, EPI_ISL_845290, EPI_ISL_845291, EPI_ISL_845292, EPI_ISL_845293, EPI_ISL_845294, EPI_ISL_845295, EPI_ISL_845296, EPI_ISL_845297, EPI_ISL_845298, EPI_ISL_845299, EPI_ISL_845300, EPI_ISL_845301, EPI_ISL_845302, EPI_ISL_845303, EPI_ISL_845304, EPI_ISL_845305, EPI_ISL_845306, EPI_ISL_845307, EPI_ISL_845308, EPI_ISL_845309, EPI_ISL_845310, EPI_ISL_845311, EPI_ISL_845312, EPI_ISL_845313, EPI_ISL_845314, EPI_ISL_845315, EPI_ISL_845316, EPI_ISL_845317, EPI_ISL_845318, EPI_ISL_845319, EPI_ISL_845320, EPI_ISL_845321, EPI_ISL_845322, EPI_ISL_845323, EPI_ISL_845324, EPI_ISL_845325, EPI_ISL_845326, EPI_ISL_845327, EPI_ISL_845328, EPI_ISL_845329, EPI_ISL_845330, EPI_ISL_845331, EPI_ISL_845332, EPI_ISL_845333, EPI_ISL_845334, EPI_ISL_845335, EPI_ISL_845336, EPI_ISL_845337, EPI_ISL_845338, EPI_ISL_845339, EPI_ISL_845340, EPI_ISL_845341, EPI_ISL_845342, EPI_ISL_845343, EPI_ISL_845344, EPI_ISL_845345, EPI_ISL_845346, EPI_ISL_845347, EPI_ISL_845348, EPI_ISL_845349, EPI_ISL_845350, EPI_ISL_845351, EPI_ISL_845352, EPI_ISL_845353, EPI_ISL_845354, EPI_ISL_845355, EPI_ISL_845356, EPI_ISL_845357, EPI_ISL_845358, EPI_ISL_845359, EPI_ISL_845360, EPI_ISL_845361, EPI_ISL_845362, EPI_ISL_845363, EPI_ISL_845364, EPI_ISL_845365, EPI_ISL_845366, EPI_ISL_845367, EPI_ISL_845368, EPI_ISL_845369, EPI_ISL_845370, EPI_ISL_845371, EPI_ISL_845372, EPI_ISL_845373, EPI_ISL_845374, EPI_ISL_845375, EPI_ISL_845376, EPI_ISL_845400, EPI_ISL_845401, EPI_ISL_845402, EPI_ISL_845403, EPI_ISL_845404, EPI_ISL_845405, EPI_ISL_845406, EPI_ISL_845407, EPI_ISL_845408, EPI_ISL_845409, EPI_ISL_845410, EPI_ISL_845411, EPI_ISL_845412, EPI_ISL_845413, EPI_ISL_845414, EPI_ISL_845415, EPI_ISL_845416, EPI_ISL_845417, EPI_ISL_845418, EPI_ISL_845419, EPI_ISL_845420, EPI_ISL_845421, EPI_ISL_845422, EPI_ISL_845423, EPI_ISL_845424, EPI_ISL_845425, EPI_ISL_845426, EPI_ISL_845427, EPI_ISL_845428, EPI_ISL_845429, EPI_ISL_845430, EPI_ISL_845431, EPI_ISL_845432, EPI_ISL_845433, EPI_ISL_845434, EPI_ISL_845435, EPI_ISL_845436, EPI_ISL_845437, EPI_ISL_845438, EPI_ISL_845439, EPI_ISL_845440, EPI_ISL_845441, EPI_ISL_845442, EPI_ISL_845443, EPI_ISL_845444, EPI_ISL_845445, EPI_ISL_845446, EPI_ISL_845447, EPI_ISL_845448, EPI_ISL_845449, EPI_ISL_845450, EPI_ISL_845451, EPI_ISL_845452, EPI_ISL_845453, EPI_ISL_845454, EPI_ISL_845455, EPI_ISL_845456, EPI_ISL_845457, EPI_ISL_845458, EPI_ISL_845459, EPI_ISL_845460, EPI_ISL_845461, EPI_ISL_845462, EPI_ISL_845463, EPI_ISL_845464, EPI_ISL_845465, EPI_ISL_845466, EPI_ISL_845467, EPI_ISL_845468, EPI_ISL_845469, EPI_ISL_845470, EPI_ISL_845471, EPI_ISL_845472, EPI_ISL_845473, EPI_ISL_845474, EPI_ISL_845475, EPI_ISL_845476, EPI_ISL_845477, EPI_ISL_845478, EPI_ISL_845479, EPI_ISL_845480, EPI_ISL_845481, EPI_ISL_845482, EPI_ISL_845483, EPI_ISL_845484, EPI_ISL_845485, EPI_ISL_845486, EPI_ISL_845487, EPI_ISL_845488, EPI_ISL_845489, EPI_ISL_845490, EPI_ISL_845491, EPI_ISL_845492, EPI_ISL_845493, EPI_ISL_845494, EPI_ISL_845495, EPI_ISL_845496, EPI_ISL_845497, EPI_ISL_845501, EPI_ISL_845502, EPI_ISL_845503, EPI_ISL_845504, EPI_ISL_845505, EPI_ISL_845506, EPI_ISL_845507, EPI_ISL_845508, EPI_ISL_845509, EPI_ISL_845510, EPI_ISL_845511, EPI_ISL_845512, EPI_ISL_845513, EPI_ISL_845514, EPI_ISL_845515, EPI_ISL_845516, EPI_ISL_845517, EPI_ISL_845518, EPI_ISL_845519, EPI_ISL_845520, EPI_ISL_845521, EPI_ISL_845522, EPI_ISL_845523, EPI_ISL_845524, EPI_ISL_845525, EPI_ISL_845526, EPI_ISL_845527, EPI_ISL_845528, EPI_ISL_845529, EPI_ISL_845530, EPI_ISL_845531, EPI_ISL_845532, EPI_ISL_845533, EPI_ISL_845534, EPI_ISL_845535, EPI_ISL_845536, EPI_ISL_845537, EPI_ISL_845538, EPI_ISL_845539, EPI_ISL_845540, EPI_ISL_845541, EPI_ISL_845542

see above	Department of Virus and Microbiological Special Diagnostics, Statens Serum Institut, Copenhagen, Denmark	Albertsen Lab, Department of Chemistry and Bioscience, Aalborg University, Denmark	Danish Covid-19 Genome Consortium

EPI_ISL_845573, EPI_ISL_845576, EPI_ISL_845579, EPI_ISL_845582, EPI_ISL_845595, EPI_ISL_845597, EPI_ISL_845599, EPI_ISL_845601, EPI_ISL_845603, EPI_ISL_845607, EPI_ISL_845609

see above	KU Leuven, Rega Institute, Clinical and Epidemiological Virology	KU Leuven, Rega Institute, Clinical and Epidemiological Virology	Tony Wawina-Bokalanga, Bert Vanmechelen, Joan Marti-Carerras, Piet Maes
EPI_ISL_845652, EPI_ISL_845653	Laboratorio de Salud Pública - Secretaría Distrital de Salud	Instituto Nacional de Salud - Dirección de Investigación en Salud Pública	Katherine Laiton-Donato, Diego A. Álvarez-Díaz, Carlos Franco-Muñoz, Mauricio Pacheco-Montealegre, Maria T. Herrera-Sepúlveda, Jonathan Reales, Sheryll Corchuelo, Julian Naizaque, Gerardo Santamaría, Paola Muñoz-Laiton, Diego Andrés Prada, Magdalena Wiesner, Martha Lucia Ospina Martinez, Marcela Mercado-Reyes
EPI_ISL_845760	Emory Molecular Diagnostics Laboratory, Emory Healthcare	Piantadosi Lab, Emory Department of Pathology	Ahmed Babiker, Anne Piantadosi

EPI_ISL_845861, EPI_ISL_845862, EPI_ISL_845863, EPI_ISL_845864, EPI_ISL_845865, EPI_ISL_845866, EPI_ISL_845867, EPI_ISL_845868, EPI_ISL_845869, EPI_ISL_845870, EPI_ISL_845871

see above	TGen North	TGen North	Jolene Bowers, Megan Folkerts, Chris French, Hayley Yaglom, Ashlyn Pfeiffer, Darrin Lemmer, Dave Engelthaler, The Arizona COVID Genomics Union (ACGU)
EPI_ISL_846528, EPI_ISL_846529, EPI_ISL_846530, EPI_ISL_846531, EPI_ISL_846532, EPI_ISL_846533, EPI_ISL_846535	Lighthouse Lab in Glasgow	Wellcome Sanger Institute for the COVID-19 Genomics UK (COG-UK) Consortium	Harper VanSteenhouse, Yumi Kasai, David Gray, Carol Clugston, Anna Dominiczak and Alex Alderton, Roberto Amato, Sonia Goncalves, Ewan Harrison, David K. Jackson, Ian Johnston, Dominic Kwiatkowski, Cordelia Langford, John Sillitoe on behalf of the Wellcome Sanger Institute COVID-19 Surveillance Team
EPI_ISL_846537	Lighthouse Lab in Alderley Park	Wellcome Sanger Institute for the COVID-19 Genomics UK (COG-UK) Consortium	Jacquelyn Wynn, Mairead Hyland, The Lighthouse Lab in Alderley Park and Alex Alderton, Roberto Amato, Sonia Goncalves, Ewan Harrison, David K. Jackson, Ian Johnston, Dominic Kwiatkowski, Cordelia Langford, John Sillitoe on behalf of the Wellcome Sanger Institute COVID-19 Surveillance Team
EPI_ISL_846541, EPI_ISL_846552	Lab voor klinische biologie	Lab voor klinische biologie	Hannelore Hamerlinck, Bruno Verhasselt
EPI_ISL_846596	Respiratory Virus Unit, National Infection Service, Public Health England	COVID-19 Genomics UK (COG-UK) Consortium	PHE Covid Sequencing Team
EPI_ISL_846636	Hopital	National Reference Center for Viruses of Respiratory Infections, Institut Pasteur, Paris	Marion Barbet, Sylvie Behillil, Méline Bizard, Angela Brisebarre, Camille Capel, Etienne Simon-Lorière, Vincent Enouf, Maud Vanpeene, Sylvie van der Werf,Farfour Eric
EPI_ISL_846642, EPI_ISL_846643, EPI_ISL_846645	Labo Analyses Med	National Reference Center for Viruses of Respiratory Infections, Institut Pasteur, Paris	Marion Barbet, Sylvie Behillil, Méline Bizard, Angela Brisebarre, Camille Capel, Etienne Simon-Lorière, Vincent Enouf, Maud Vanpeene, Sylvie van der Werf
EPI_ISL_846646, EPI_ISL_846647	Labo Analyses Med	National Reference Center for Viruses of Respiratory Infections, Institut Pasteur, Paris	Marion Barbet, Sylvie Behillil, Méline Bizard, Angela Brisebarre, Camille Capel, Etienne Simon-Lorière, Vincent Enouf, Maud Vanpeene, Sylvie van der Werf,Coignard Catherine
EPI_ISL_846650	Hopital	National Reference Center for Viruses of Respiratory Infections, Institut Pasteur, Paris	Marion Barbet, Sylvie Behillil, Méline Bizard, Angela Brisebarre, Camille Capel, Etienne Simon-Lorière, Vincent Enouf, Maud Vanpeene, Sylvie van der Werf,Farfour Eric

EPI_ISL_846779, EPI_ISL_846781, EPI_ISL_846812, EPI_ISL_846813, EPI_ISL_846814, EPI_ISL_846815, EPI_ISL_846816, EPI_ISL_846817, EPI_ISL_846860, EPI_ISL_846877, EPI_ISL_846884, EPI_ISL_846886, EPI_ISL_846888, EPI_ISL_846889, EPI_ISL_846893, EPI_ISL_846898, EPI_ISL_846904, EPI_ISL_846910, EPI_ISL_847125, EPI_ISL_847126, EPI_ISL_847127, EPI_ISL_847128, EPI_ISL_847129, EPI_ISL_847130, EPI_ISL_847131, EPI_ISL_847132, EPI_ISL_847133, EPI_ISL_847134, EPI_ISL_847135, EPI_ISL_847136, EPI_ISL_847137, EPI_ISL_847138, EPI_ISL_847139, EPI_ISL_847140, EPI_ISL_847141, EPI_ISL_847142, EPI_ISL_847143, EPI_ISL_847144, EPI_ISL_847145, EPI_ISL_847146, EPI_ISL_847147, EPI_ISL_847148, EPI_ISL_847149, EPI_ISL_847150, EPI_ISL_847151, EPI_ISL_847152, EPI_ISL_847153, EPI_ISL_847154, EPI_ISL_847155, EPI_ISL_847156, EPI_ISL_847157, EPI_ISL_847158, EPI_ISL_847159, EPI_ISL_847160,

EPI_ISL_847161, EPI_ISL_847162, EPI_ISL_847163, EPI_ISL_847164, EPI_ISL_847165, EPI_ISL_847166, EPI_ISL_847167, EPI_ISL_847199, EPI_ISL_847200, EPI_ISL_847201, EPI_ISL_847202, EPI_ISL_847203, EPI_ISL_847204, EPI_ISL_847233, EPI_ISL_847234, EPI_ISL_847235, EPI_ISL_847236, EPI_ISL_847237, EPI_ISL_847244, EPI_ISL_847245, EPI_ISL_847246, EPI_ISL_847247, EPI_ISL_847248, EPI_ISL_847249, EPI_ISL_847252, EPI_ISL_847253, EPI_ISL_847254, EPI_ISL_847255, EPI_ISL_847262, EPI_ISL_847263, EPI_ISL_847264, EPI_ISL_847266, EPI_ISL_847270, EPI_ISL_847280, EPI_ISL_847332, EPI_ISL_847335, EPI_ISL_847337, EPI_ISL_847351, EPI_ISL_847352, EPI_ISL_847353, EPI_ISL_847358, EPI_ISL_847366, EPI_ISL_847377, EPI_ISL_847486, EPI_ISL_847487, EPI_ISL_847488, EPI_ISL_847489, EPI_ISL_847490, EPI_ISL_847491, EPI_ISL_847492, EPI_ISL_847493, EPI_ISL_847494, EPI_ISL_847495, EPI_ISL_847496, EPI_ISL_847497, EPI_ISL_847498, EPI_ISL_847499, EPI_ISL_847500, EPI_ISL_847501, EPI_ISL_847502, EPI_ISL_847503, EPI_ISL_847504, EPI_ISL_847505, EPI_ISL_847506, EPI_ISL_847507, EPI_ISL_847508, EPI_ISL_847509			
see above	Department of Virus and Microbiological Special Diagnostics, Statens Serum Institut, Copenhagen, Denmark	Albertsen Lab, Department of Chemistry and Bioscience, Aalborg University, Denmark	Danish Covid-19 Genome Consortium
EPI_ISL_848003	Michigan Department of Health and Human Services, Bureau of Laboratories	Michigan Department of Health and Human Services, Bureau of Laboratories	Blankenship HM, Riner D, Soehnlen MK
EPI_ISL_849336, EPI_ISL_849337, EPI_ISL_849338, EPI_ISL_849339, EPI_ISL_849340, EPI_ISL_849341, EPI_ISL_849342, EPI_ISL_849343, EPI_ISL_849344, EPI_ISL_849345, EPI_ISL_849346, EPI_ISL_849347, EPI_ISL_849348, EPI_ISL_849349			
see above	Delaware Public Health Lab	Delaware Public Health Lab	Gregory Hovan
EPI_ISL_849778, EPI_ISL_849779, EPI_ISL_849781, EPI_ISL_849782, EPI_ISL_849784, EPI_ISL_849785, EPI_ISL_849786, EPI_ISL_849789, EPI_ISL_849791	Utah Public Health Laboratory	Utah Public Health Laboratory	Erin L. Young, Kelly F. Oakeson, Tara Gallagher
EPI_ISL_849922	Orange County Public Health Lab	Chan-Zuckerberg Biohub	CZB Cliahub Consortium
EPI_ISL_850517, EPI_ISL_850518	Helix/Illumina	Genomics and Discovery, Respiratory Viruses Branch, Division of Viral Diseases, Centers for Disease Control and Prevention	Peter W. Cook, Dhwani Batra, Ben L. Rambo-Martin Eileen de Feo, Jan Antico, Christine Tran, Matthew Tolentino, Shannon Wickline, Kim Gietzen, Brad Sickler, Jingtao Liu, Eric Allen, Phil Febbo, Summer Galloway, Nicole L. Washington, Simon White, Geraint Levan, Kelly Schiabor Barrett, Elizabeth Cirulli, Alexandre Bolze, Ary Ascencio, Charlotte Rivera-Garcia, Ryan Cho, Jason Nguyen, Sherry Wang, Jimmy Ramirez, Tyler Cassens, Efren Sandoval, Magnus Isaksson, William Lee, David Becker, Marc Laurent, James Lu, Clinton R. Paden, Suxiang Tong, Duncan MacCannell
EPI_ISL_850673	The National Institute of Public Health	State Veterinary Institute Prague	Nagy,A;Jirincova,H;Trnka,D;Vecerova,J;Trinklova,M
EPI_ISL_850678, EPI_ISL_850680, EPI_ISL_850681, EPI_ISL_850682, EPI_ISL_850683	The National Institute of Public Health	State Veterinary Institute Prague	Nagy,A;Jirincova,H;Trnka,D;Vecerova,J
EPI_ISL_850817, EPI_ISL_850818, EPI_ISL_850819, EPI_ISL_850820, EPI_ISL_850821, EPI_ISL_850822, EPI_ISL_850823, EPI_ISL_850824, EPI_ISL_850825, EPI_ISL_850826, EPI_ISL_850827, EPI_ISL_850828, EPI_ISL_850829, EPI_ISL_850830, EPI_ISL_850831, EPI_ISL_850832, EPI_ISL_850833, EPI_ISL_850834, EPI_ISL_850835, EPI_ISL_850836, EPI_ISL_850837, EPI_ISL_850838, EPI_ISL_850839, EPI_ISL_850840, EPI_ISL_850841, EPI_ISL_850842, EPI_ISL_850843, EPI_ISL_850844, EPI_ISL_850845, EPI_ISL_850846, EPI_ISL_850847, EPI_ISL_850848, EPI_ISL_850849, EPI_ISL_850850, EPI_ISL_850851, EPI_ISL_850852, EPI_ISL_850853, EPI_ISL_850854, EPI_ISL_850855, EPI_ISL_850856, EPI_ISL_850857, EPI_ISL_850858, EPI_ISL_850859, EPI_ISL_850860, EPI_ISL_850861, EPI_ISL_850862, EPI_ISL_850863, EPI_ISL_850864, EPI_ISL_850865, EPI_ISL_850866, EPI_ISL_850867, EPI_ISL_850868, EPI_ISL_850869, EPI_ISL_850870, EPI_ISL_850871, EPI_ISL_850872, EPI_ISL_850873, EPI_ISL_850874, EPI_ISL_850875, EPI_ISL_850876, EPI_ISL_850877, EPI_ISL_850878, EPI_ISL_850879, EPI_ISL_850880, EPI_ISL_850881, EPI_ISL_850882, EPI_ISL_850883, EPI_ISL_850884, EPI_ISL_850885, EPI_ISL_850886, EPI_ISL_850887, EPI_ISL_850888, EPI_ISL_850889, EPI_ISL_850890, EPI_ISL_850891, EPI_ISL_850892, EPI_ISL_850893			
see above	Helix / Illumina	Genomics and Discovery, Respiratory Viruses Branch, Division of Viral Diseases, Centers for Disease Control and Prevention	Peter W. Cook, Dhwani Batra, Ben L. Rambo-Martin Eileen de Feo, Jan Antico, Christine Tran, Matthew Tolentino, Shannon Wickline, Kim Gietzen, Brad Sickler, Jingtao Liu, Eric Allen, Phil Febbo, Summer Galloway, Nicole L. Washington, Simon White, Geraint Levan, Kelly Schiabor Barrett, Elizabeth Cirulli, Alexandre Bolze, Ary Ascencio, Charlotte Rivera-Garcia, Ryan Cho, Jason Nguyen, Sherry Wang, Jimmy Ramirez, Tyler Cassens, Efren Sandoval, Magnus Isaksson, William Lee, David Becker, Marc Laurent, James Lu, Clinton R. Paden, Suxiang Tong, Duncan MacCannell
EPI_ISL_850952, EPI_ISL_850953, EPI_ISL_850954, EPI_ISL_850955, EPI_ISL_850956, EPI_ISL_850957, EPI_ISL_850958, EPI_ISL_850959, EPI_ISL_850960	Helix/Illumina	Genomics and Discovery, Respiratory Viruses Branch, Division of Viral Diseases, Centers for Disease Control and Prevention	Peter W. Cook, Dhwani Batra, Ben L. Rambo-Martin Eileen de Feo, Jan Antico, Christine Tran, Matthew Tolentino, Shannon Wickline, Kim Gietzen, Brad Sickler, Jingtao Liu, Eric Allen, Phil Febbo, Summer Galloway, Nicole L. Washington, Simon White, Geraint Levan, Kelly Schiabor Barrett, Elizabeth Cirulli, Alexandre Bolze, Ary Ascencio, Charlotte Rivera-Garcia, Ryan Cho, Jason Nguyen, Sherry Wang, Jimmy Ramirez, Tyler Cassens, Efren Sandoval, Magnus Isaksson, William Lee, David Becker, Marc Laurent, James Lu, Clinton R. Paden, Suxiang Tong, Duncan MacCannell
EPI_ISL_852383	Lighthouse Lab in Glasgow	Wellcome Sanger Institute for the COVID-19 Genomics UK (COG-UK) Consortium	Harper VanSteenhouse, Yumi Kasai, David Gray, Carol Clugston, Anna Dominiczak and Alex Alderton, Roberto Amato, Sonia Goncalves, Ewan Harrison, David K. Jackson, Ian Johnston, Dominic Kwiatkowski, Cordelia Langford, John Sillitoe on behalf of the Wellcome Sanger Institute COVID-19 Surveillance Team
EPI_ISL_852858, EPI_ISL_852859, EPI_ISL_852860	Florida Bureau of Public Health Laboratories	Florida Bureau of Public Health Laboratories	Sarah Schmedes, Jason Blanton
EPI_ISL_853004, EPI_ISL_853005	Hospital General Universitario Gregorio Marañón	SeqCOVID-SPAIN consortium/IBV(CSIC)	Darío García de Viedma, Laura Pérez-Lago, Pedro J Sola-Campoy, Sergio Buenestado-Serrano, Marta Herranz, Victor Manuel de la Cueva, Julia Suárez, Pilar Catalán, Patricia Muñoz and SeqCOVID-SPAIN consortium
EPI_ISL_853648, EPI_ISL_853668, EPI_ISL_853669	THE MARY IMOGENE BASSETT HOSPITAL	Wadsworth Center, New York State Department of Health	Kirsten St. George, Daryl M. Lamson, Alexis Russel, Matthew Shudt, Melissa A Leisner, Jonathan Plitnick, Navjot Singh, John Kelly, Erasmus Schneider, Erica Lasek-Nesselquist
EPI_ISL_853670, EPI_ISL_853671, EPI_ISL_853672, EPI_ISL_853673, EPI_ISL_853674, EPI_ISL_853675	NORTHWELL HEALTH LABORATORIES	Wadsworth Center, New York State Department of Health	Kirsten St. George, Daryl M. Lamson, Alexis Russel, Matthew Shudt, Melissa A Leisner, Jonathan Plitnick, Navjot Singh, John Kelly, Erasmus Schneider, Erica Lasek-Nesselquist
EPI_ISL_853676, EPI_ISL_853677, EPI_ISL_853678, EPI_ISL_853679, EPI_ISL_853680, EPI_ISL_853681, EPI_ISL_853682	ACUTIS DIAGNOSTICS	Wadsworth Center, New York State Department of Health	Kirsten St. George, Daryl M. Lamson, Alexis Russel, Matthew Shudt, Melissa A Leisner, Jonathan Plitnick, Navjot Singh, John Kelly, Erasmus Schneider, Erica Lasek-Nesselquist
EPI_ISL_853712	NORTHWELL HEALTH LABORATORIES	Wadsworth Center, New York State Department of Health	Kirsten St. George, Daryl M. Lamson, Alexis Russel, Matthew Shudt, Melissa A Leisner, Jonathan Plitnick, Navjot Singh, John Kelly, Erasmus Schneider, Erica Lasek-Nesselquist
EPI_ISL_854306, EPI_ISL_854321, EPI_ISL_854322, EPI_ISL_854323, EPI_ISL_854324, EPI_ISL_854325, EPI_ISL_854326, EPI_ISL_854327, EPI_ISL_854328, EPI_ISL_854329, EPI_ISL_854330, EPI_ISL_854331, EPI_ISL_854332, EPI_ISL_854333, EPI_ISL_854334, EPI_ISL_854335, EPI_ISL_854336, EPI_ISL_854337, EPI_ISL_854338, EPI_ISL_854339, EPI_ISL_854340, EPI_ISL_854341, EPI_ISL_854342, EPI_ISL_854343, EPI_ISL_854344, EPI_ISL_854345, EPI_ISL_854346, EPI_ISL_854347, EPI_ISL_854348, EPI_ISL_854349, EPI_ISL_854370, EPI_ISL_854371, EPI_ISL_854404			
see above	URMC LABS	Wadsworth Center, New York State Department of Health	Kirsten St. George, Daryl M. Lamson, Alexis Russel, Matthew Shudt, Melissa A Leisner, Jonathan Plitnick, Navjot Singh, John Kelly, Erasmus Schneider, Erica Lasek-Nesselquist
EPI_ISL_854843, EPI_ISL_854850, EPI_ISL_854862, EPI_ISL_854867, EPI_ISL_854885, EPI_ISL_854890, EPI_ISL_854892, EPI_ISL_854898, EPI_ISL_854943, EPI_ISL_854963, EPI_ISL_854964, EPI_ISL_854965, EPI_ISL_854967, EPI_ISL_855115, EPI_ISL_855129, EPI_ISL_855164			
see above	Quest Diagnostics	Quest Diagnostics	Rosenthal,S.H., Gerasimova,A., Kagan,R.M., Anderson, B., Hua, M., Liu Y., Bernstein, L.E., Livingston, K.E., Perez, A., Shalhout, D.F., Shlyakhter, I.A., Owen, R., Tanpaiboon, P., Lacbawan, F.
EPI_ISL_855386	Hospital	National Reference Center for Viruses of Respiratory Infections, Institut Pasteur, Paris	Marion Barbet, Sylvie Behillil, Méline Bizard, Angela Brisebarre, Camille Capel, Etienne Simon-Lorière, Vincent Enouf, Maud Vanpeene, Sylvie van der Werf
EPI_ISL_855588	Respiratory Virus Unit, National Infection Service, Public Health England	COVID-19 Genomics UK (COG-UK) Consortium	PHE Covid Sequencing Team
EPI_ISL_855949, EPI_ISL_855951, EPI_ISL_855953, EPI_ISL_855955, EPI_ISL_855957, EPI_ISL_855959, EPI_ISL_855960, EPI_ISL_855962, EPI_ISL_855964, EPI_ISL_855966, EPI_ISL_855968			
see above	Lab voor klinische biologie	Onderzoeksgroep Virologie	Nick Vereecke, Laurens Lambrechts, Marthe Pauwels, Bruno Verhasselt, Linos Vandekerckhove, Hans Nauwynck, Sebastiaan Theuns
EPI_ISL_856292, EPI_ISL_856293, EPI_ISL_856294, EPI_ISL_856295, EPI_ISL_856296, EPI_ISL_856297, EPI_ISL_856298, EPI_ISL_856299, EPI_ISL_856300, EPI_ISL_856301, EPI_ISL_856302, EPI_ISL_856303, EPI_ISL_856304, EPI_ISL_856305, EPI_ISL_856306, EPI_ISL_856307, EPI_ISL_856308, EPI_ISL_856309, EPI_ISL_856310, EPI_ISL_856311, EPI_ISL_856312, EPI_ISL_856313, EPI_ISL_856314, EPI_ISL_856315, EPI_ISL_856316, EPI_ISL_856317, EPI_ISL_856318, EPI_ISL_856319, EPI_ISL_856320, EPI_ISL_856321, EPI_ISL_856322, EPI_ISL_856323, EPI_ISL_856324, EPI_ISL_856325, EPI_ISL_856326, EPI_ISL_856327, EPI_ISL_856328, EPI_ISL_856329, EPI_ISL_856330, EPI_ISL_856331, EPI_ISL_856332, EPI_ISL_856333, EPI_ISL_856334, EPI_ISL_856335, EPI_ISL_856336, EPI_ISL_856337, EPI_ISL_856338, EPI_ISL_856339, EPI_ISL_856340, EPI_ISL_856341, EPI_ISL_856342, EPI_ISL_856343, EPI_ISL_856344, EPI_ISL_856345, EPI_ISL_856346, EPI_ISL_856347, EPI_ISL_856348, EPI_ISL_856349, EPI_ISL_856350, EPI_ISL_856351, EPI_ISL_856352, EPI_ISL_856353, EPI_ISL_856354, EPI_ISL_856355, EPI_ISL_856356, EPI_ISL_856357, EPI_ISL_856358, EPI_ISL_856359, EPI_ISL_856360, EPI_ISL_856361, EPI_ISL_856362, EPI_ISL_856363,			

EPI_ISL_856364, EPI_ISL_856365, EPI_ISL_856366, EPI_ISL_856367, EPI_ISL_856368, EPI_ISL_856369, EPI_ISL_856370, EPI_ISL_856371, EPI_ISL_856372, EPI_ISL_856373, EPI_ISL_856374, EPI_ISL_856375, EPI_ISL_856376, EPI_ISL_856377, EPI_ISL_856378, EPI_ISL_856379, EPI_ISL_856380, EPI_ISL_856381, EPI_ISL_856382, EPI_ISL_856383, EPI_ISL_856384, EPI_ISL_856385, EPI_ISL_856386, EPI_ISL_856387, EPI_ISL_856388, EPI_ISL_856389, EPI_ISL_856390, EPI_ISL_856391, EPI_ISL_856392, EPI_ISL_856393, EPI_ISL_856394, EPI_ISL_856395, EPI_ISL_856396, EPI_ISL_856397, EPI_ISL_856398, EPI_ISL_856399, EPI_ISL_856400, EPI_ISL_856401, EPI_ISL_856402, EPI_ISL_856403, EPI_ISL_856404, EPI_ISL_856405, EPI_ISL_856406, EPI_ISL_856407, EPI_ISL_856408, EPI_ISL_856409, EPI_ISL_856410, EPI_ISL_856411, EPI_ISL_856412, EPI_ISL_856413, EPI_ISL_856414, EPI_ISL_856415, EPI_ISL_856416, EPI_ISL_856417, EPI_ISL_856418, EPI_ISL_856419, EPI_ISL_856420, EPI_ISL_856421, EPI_ISL_856422, EPI_ISL_856423, EPI_ISL_856424, EPI_ISL_856425, EPI_ISL_856426, EPI_ISL_856427, EPI_ISL_856428, EPI_ISL_856429, EPI_ISL_856430, EPI_ISL_856431, EPI_ISL_856432, EPI_ISL_856433, EPI_ISL_856434, EPI_ISL_856435, EPI_ISL_856436, EPI_ISL_856437, EPI_ISL_856438, EPI_ISL_856439, EPI_ISL_856440, EPI_ISL_856441, EPI_ISL_856442, EPI_ISL_856443, EPI_ISL_856444, EPI_ISL_856445, EPI_ISL_856446, EPI_ISL_856447, EPI_ISL_856448, EPI_ISL_856449, EPI_ISL_856450, EPI_ISL_856451, EPI_ISL_856452, EPI_ISL_856453, EPI_ISL_856454, EPI_ISL_856455, EPI_ISL_856456, EPI_ISL_856457, EPI_ISL_856458, EPI_ISL_856459, EPI_ISL_856460, EPI_ISL_856461, EPI_ISL_856462, EPI_ISL_856463, EPI_ISL_856464, EPI_ISL_856465, EPI_ISL_856466, EPI_ISL_856467, EPI_ISL_856468, EPI_ISL_856469, EPI_ISL_856470, EPI_ISL_856471, EPI_ISL_856472, EPI_ISL_856473, EPI_ISL_856474, EPI_ISL_856475, EPI_ISL_856476, EPI_ISL_856477, EPI_ISL_856478, EPI_ISL_856479, EPI_ISL_856480, EPI_ISL_856481, EPI_ISL_856482, EPI_ISL_856483, EPI_ISL_856484, EPI_ISL_856485, EPI_ISL_856486, EPI_ISL_856487, EPI_ISL_856488, EPI_ISL_856489, EPI_ISL_856490, EPI_ISL_856491, EPI_ISL_856492, EPI_ISL_856493, EPI_ISL_856494, EPI_ISL_856495, EPI_ISL_856496, EPI_ISL_856497, EPI_ISL_856498, EPI_ISL_856499, EPI_ISL_856500, EPI_ISL_856501, EPI_ISL_856502, EPI_ISL_856503, EPI_ISL_856504, EPI_ISL_856505, EPI_ISL_856506, EPI_ISL_856507, EPI_ISL_856508, EPI_ISL_856509, EPI_ISL_856510, EPI_ISL_856511, EPI_ISL_856512, EPI_ISL_856513, EPI_ISL_856514, EPI_ISL_856515, EPI_ISL_856516, EPI_ISL_856517, EPI_ISL_856518, EPI_ISL_856519, EPI_ISL_856520, EPI_ISL_856521, EPI_ISL_856522, EPI_ISL_856523, EPI_ISL_856524, EPI_ISL_856525, EPI_ISL_856526, EPI_ISL_856527, EPI_ISL_856528, EPI_ISL_856529, EPI_ISL_856530, EPI_ISL_856531, EPI_ISL_856532, EPI_ISL_856533, EPI_ISL_856534, EPI_ISL_856535, EPI_ISL_856536, EPI_ISL_856537, EPI_ISL_856538, EPI_ISL_856539, EPI_ISL_856540, EPI_ISL_856541, EPI_ISL_856542, EPI_ISL_856543, EPI_ISL_856544, EPI_ISL_856545, EPI_ISL_856546, EPI_ISL_856547, EPI_ISL_856548, EPI_ISL_856549, EPI_ISL_856550, EPI_ISL_856551, EPI_ISL_856552, EPI_ISL_856553, EPI_ISL_856554, EPI_ISL_856555, EPI_ISL_856556, EPI_ISL_856557, EPI_ISL_856558, EPI_ISL_856559, EPI_ISL_856560, EPI_ISL_856561, EPI_ISL_856562, EPI_ISL_856563, EPI_ISL_856564, EPI_ISL_856565, EPI_ISL_856566, EPI_ISL_856567, EPI_ISL_856568, EPI_ISL_856569, EPI_ISL_856570, EPI_ISL_856571, EPI_ISL_856572, EPI_ISL_856573, EPI_ISL_856574, EPI_ISL_856575, EPI_ISL_856576, EPI_ISL_856577, EPI_ISL_856578, EPI_ISL_856579, EPI_ISL_856580, EPI_ISL_856581, EPI_ISL_856582, EPI_ISL_856583, EPI_ISL_856584, EPI_ISL_856585, EPI_ISL_856586, EPI_ISL_856587, EPI_ISL_856588, EPI_ISL_856589, EPI_ISL_856590, EPI_ISL_856591, EPI_ISL_856592, EPI_ISL_856593, EPI_ISL_856594, EPI_ISL_856595, EPI_ISL_856596, EPI_ISL_856597, EPI_ISL_856598, EPI_ISL_856599, EPI_ISL_856600, EPI_ISL_856601, EPI_ISL_856602, EPI_ISL_856603, EPI_ISL_856604, EPI_ISL_856605, EPI_ISL_856606, EPI_ISL_856607, EPI_ISL_856608, EPI_ISL_856609, EPI_ISL_856610, EPI_ISL_856611			
see above	Department of Virus and Microbiological Special Diagnostics, Statens Serum Institut, Copenhagen, Denmark	Aalborg University	Danish Covid-19 Genome Consortium
EPI_ISL_856934, EPI_ISL_856938, EPI_ISL_856939, EPI_ISL_856940, EPI_ISL_856943, EPI_ISL_856944, EPI_ISL_856961, EPI_ISL_856962, EPI_ISL_856963, EPI_ISL_856964, EPI_ISL_856965, EPI_ISL_856966, EPI_ISL_856967			
see above	Wyoming Public Health Laboratory	Wyoming Public Health Laboratory	Noah Hull, Taylor Fearing, Lynette Gumbleton, Channing Weber, Ashley Norberg, Bailey Bowcutt, and Wanda Manley
EPI_ISL_857046	Colorado Department of Public Health and Environment	Colorado Department of Puplic Health and Environment	Laura Bankers, Molly C. Hetherington-Rauth, Diana Ir, Shannon Ely, Shannon R. Matzinger, Sarah Elizabeth Totten, Emily A. Travanty
EPI_ISL_857062	OCME Office Of Chief Medical Examiner	New York City Public Health Laboratory	Jade Wang, et al.
EPI_ISL_857063, EPI_ISL_857064, EPI_ISL_857065	DOHMH PHL	New York City Public Health Laboratory	Jade Wang, et al.
EPI_ISL_857066	OCME Office Of Chief Medical Examiner	New York City Public Health Laboratory	Jade Wang, et al.
EPI_ISL_857194, EPI_ISL_857200	DOHMH Central Harlem	New York City Public Health Laboratory	Jade Wang, et al.
EPI_ISL_857201	DOHMH Jamaica	New York City Public Health Laboratory	Jade Wang, et al.
EPI_ISL_857203	DOHMH Riverside	New York City Public Health Laboratory	Jade Wang, et al.
EPI_ISL_857206	DOHMH PHL	New York City Public Health Laboratory	Jade Wang, et al.
EPI_ISL_857207, EPI_ISL_857208	DOHMH Morrisania	New York City Public Health Laboratory	Jade Wang, et al.
EPI_ISL_857209	DOHMH Central Harlem	New York City Public Health Laboratory	Jade Wang, et al.
EPI_ISL_857210, EPI_ISL_857211, EPI_ISL_857212, EPI_ISL_857213, EPI_ISL_857214, EPI_ISL_857215, EPI_ISL_857216, EPI_ISL_857217	DOHMH Jamaica	New York City Public Health Laboratory	Jade Wang, et al.
EPI_ISL_857218, EPI_ISL_857219, EPI_ISL_857220	DOHMH Corona	New York City Public Health Laboratory	Jade Wang, et al.
EPI_ISL_857221	DOHMH Fort Greene	New York City Public Health Laboratory	Jade Wang, et al.
EPI_ISL_857222, EPI_ISL_857223, EPI_ISL_857224	DOHMH Jamaica	New York City Public Health Laboratory	Jade Wang, et al.
EPI_ISL_857225	DOHMH Corona	New York City Public Health Laboratory	Jade Wang, et al.
EPI_ISL_857226	DOHMH Jamaica	New York City Public Health Laboratory	Jade Wang, et al.
EPI_ISL_858086, EPI_ISL_858122, EPI_ISL_858136, EPI_ISL_858143, EPI_ISL_858144, EPI_ISL_858146, EPI_ISL_858147, EPI_ISL_858149, EPI_ISL_858150, EPI_ISL_858152, EPI_ISL_858153, EPI_ISL_858154, EPI_ISL_858155, EPI_ISL_858156, EPI_ISL_858157			
see above	Lighthouse Lab in Alderley Park	Wellcome Sanger Institute for the COVID-19 Genomics UK (COG-UK) Consortium	Jacquelyn Wynn, Mairead Hyland, The Lighthouse Lab in Alderley Park and Alex Alderton, Roberto Amato, Sonia Goncalves, Ewan Harrison, David K. Jackson, Ian Johnston, Dominic Kwiatkowski, Cordelia Langford, John Sillitoe on behalf of the Wellcome Sanger Institute COVID-19 Surveillance Team
EPI_ISL_858160	Lighthouse Lab in Glasgow	Wellcome Sanger Institute for the COVID-19 Genomics UK (COG-UK) Consortium	Harper VanSteenhouse, Yumi Kasai, David Gray, Carol Clugston, Anna Dominiczak and Alex Alderton, Roberto Amato, Sonia Goncalves, Ewan Harrison, David K. Jackson, Ian Johnston, Dominic Kwiatkowski, Cordelia Langford, John Sillitoe on behalf of the Wellcome Sanger Institute COVID-19 Surveillance Team
EPI_ISL_858216	Lighthouse Lab in Alderley Park	Wellcome Sanger Institute for the COVID-19 Genomics UK (COG-UK) Consortium	Jacquelyn Wynn, Mairead Hyland, The Lighthouse Lab in Alderley Park and Alex Alderton, Roberto Amato, Sonia Goncalves, Ewan Harrison, David K. Jackson, Ian Johnston, Dominic Kwiatkowski, Cordelia Langford, John Sillitoe on behalf of the Wellcome Sanger Institute COVID-19 Surveillance Team
EPI_ISL_858241, EPI_ISL_858246, EPI_ISL_858263, EPI_ISL_858272, EPI_ISL_858278, EPI_ISL_858285	Lighthouse Lab in Glasgow	Wellcome Sanger Institute for the COVID-19 Genomics UK (COG-UK) Consortium	Harper VanSteenhouse, Yumi Kasai, David Gray, Carol Clugston, Anna Dominiczak and Alex Alderton, Roberto Amato, Sonia Goncalves, Ewan Harrison, David K. Jackson, Ian Johnston, Dominic Kwiatkowski, Cordelia Langford, John Sillitoe on behalf of the Wellcome Sanger Institute COVID-19 Surveillance Team
EPI_ISL_858287	Lighthouse Lab in Alderley Park	Wellcome Sanger Institute for the COVID-19 Genomics UK (COG-UK) Consortium	Jacquelyn Wynn, Mairead Hyland, The Lighthouse Lab in Alderley Park and Alex Alderton, Roberto Amato, Sonia Goncalves, Ewan Harrison, David K. Jackson, Ian Johnston, Dominic Kwiatkowski, Cordelia Langford, John Sillitoe on behalf of the Wellcome Sanger Institute COVID-19 Surveillance Team
EPI_ISL_858290, EPI_ISL_858303	Lighthouse Lab in Glasgow	Wellcome Sanger Institute for the COVID-19 Genomics UK (COG-UK) Consortium	Harper VanSteenhouse, Yumi Kasai, David Gray, Carol Clugston, Anna Dominiczak and Alex Alderton, Roberto Amato, Sonia Goncalves, Ewan Harrison, David K. Jackson, Ian Johnston, Dominic Kwiatkowski, Cordelia Langford, John Sillitoe on behalf of the Wellcome Sanger Institute COVID-19 Surveillance Team
EPI_ISL_858307, EPI_ISL_858308, EPI_ISL_858309	Lighthouse Lab in Alderley Park	Wellcome Sanger Institute for the COVID-19 Genomics UK (COG-UK) Consortium	Jacquelyn Wynn, Mairead Hyland, The Lighthouse Lab in Alderley Park and Alex Alderton, Roberto Amato, Sonia Goncalves, Ewan Harrison, David K. Jackson, Ian Johnston, Dominic Kwiatkowski, Cordelia Langford, John Sillitoe on behalf of the Wellcome Sanger Institute COVID-19 Surveillance Team
EPI_ISL_858310, EPI_ISL_858312, EPI_ISL_858318	Lighthouse Lab in Glasgow	Wellcome Sanger Institute for the COVID-19 Genomics UK (COG-UK) Consortium	Harper VanSteenhouse, Yumi Kasai, David Gray, Carol Clugston, Anna Dominiczak and Alex Alderton, Roberto Amato, Sonia Goncalves, Ewan Harrison, David K. Jackson, Ian Johnston, Dominic Kwiatkowski, Cordelia Langford, John Sillitoe on behalf of the Wellcome Sanger Institute COVID-19 Surveillance Team
EPI_ISL_858324, EPI_ISL_858325, EPI_ISL_858329	Lighthouse Lab in Alderley Park	Wellcome Sanger Institute for the COVID-19 Genomics UK (COG-UK) Consortium	Jacquelyn Wynn, Mairead Hyland, The Lighthouse Lab in Alderley Park and Alex Alderton, Roberto Amato, Sonia Goncalves, Ewan Harrison, David K. Jackson, Ian Johnston, Dominic Kwiatkowski, Cordelia Langford, John Sillitoe on behalf of the Wellcome Sanger Institute COVID-19 Surveillance Team
EPI_ISL_858330	Lighthouse Lab in Glasgow	Wellcome Sanger Institute for the COVID-19 Genomics UK (COG-UK) Consortium	Harper VanSteenhouse, Yumi Kasai, David Gray, Carol Clugston, Anna Dominiczak and Alex Alderton, Roberto Amato, Sonia Goncalves, Ewan Harrison, David K. Jackson, Ian Johnston, Dominic Kwiatkowski, Cordelia Langford, John Sillitoe on behalf of the Wellcome Sanger Institute COVID-19 Surveillance Team
EPI_ISL_858332, EPI_ISL_858334	Lighthouse Lab in Alderley Park	Wellcome Sanger Institute for the COVID-19 Genomics UK (COG-UK) Consortium	Jacquelyn Wynn, Mairead Hyland, The Lighthouse Lab in Alderley Park and Alex Alderton, Roberto Amato, Sonia Goncalves, Ewan Harrison, David K. Jackson, Ian Johnston, Dominic Kwiatkowski, Cordelia Langford, John Sillitoe on behalf of the Wellcome Sanger Institute COVID-19 Surveillance Team
EPI_ISL_858339, EPI_ISL_858341	Lighthouse Lab in Glasgow	Wellcome Sanger Institute for the COVID-19 Genomics UK (COG-UK) Consortium	Harper VanSteenhouse, Yumi Kasai, David Gray, Carol Clugston, Anna Dominiczak and Alex Alderton, Roberto Amato, Sonia Goncalves, Ewan Harrison, David K. Jackson, Ian Johnston, Dominic Kwiatkowski, Cordelia Langford, John Sillitoe on behalf of the Wellcome Sanger Institute COVID-19 Surveillance Team

EPI_ISL_858343	Lighthouse Lab in Alderley Park	Wellcome Sanger Institute for the COVID-19 Genomics UK (COG-UK) Consortium	Jacquelyn Wynn, Mairead Hyland, The Lighthouse Lab in Alderley Park and Alex Alderton, Roberto Amato, Sonia Goncalves, Ewan Harrison, David K. Jackson, Ian Johnston, Dominic Kwiatkowski, Cordelia Langford, John Sillitoe on behalf of the Wellcome Sanger Institute COVID-19 Surveillance Team
EPI_ISL_858350, EPI_ISL_858351	Lighthouse Lab in Glasgow	Wellcome Sanger Institute for the COVID-19 Genomics UK (COG-UK) Consortium	Harper VanSteenhouse, Yumi Kasai, David Gray, Carol Clugston, Anna Dominiczak and Alex Alderton, Roberto Amato, Sonia Goncalves, Ewan Harrison, David K. Jackson, Ian Johnston, Dominic Kwiatkowski, Cordelia Langford, John Sillitoe on behalf of the Wellcome Sanger Institute COVID-19 Surveillance Team
EPI_ISL_858354	Lighthouse Lab in Alderley Park	Wellcome Sanger Institute for the COVID-19 Genomics UK (COG-UK) Consortium	Jacquelyn Wynn, Mairead Hyland, The Lighthouse Lab in Alderley Park and Alex Alderton, Roberto Amato, Sonia Goncalves, Ewan Harrison, David K. Jackson, Ian Johnston, Dominic Kwiatkowski, Cordelia Langford, John Sillitoe on behalf of the Wellcome Sanger Institute COVID-19 Surveillance Team
EPI_ISL_858363	Lighthouse Lab in Glasgow	Wellcome Sanger Institute for the COVID-19 Genomics UK (COG-UK) Consortium	Harper VanSteenhouse, Yumi Kasai, David Gray, Carol Clugston, Anna Dominiczak and Alex Alderton, Roberto Amato, Sonia Goncalves, Ewan Harrison, David K. Jackson, Ian Johnston, Dominic Kwiatkowski, Cordelia Langford, John Sillitoe on behalf of the Wellcome Sanger Institute COVID-19 Surveillance Team
EPI_ISL_858368, EPI_ISL_858370	Lighthouse Lab in Alderley Park	Wellcome Sanger Institute for the COVID-19 Genomics UK (COG-UK) Consortium	Jacquelyn Wynn, Mairead Hyland, The Lighthouse Lab in Alderley Park and Alex Alderton, Roberto Amato, Sonia Goncalves, Ewan Harrison, David K. Jackson, Ian Johnston, Dominic Kwiatkowski, Cordelia Langford, John Sillitoe on behalf of the Wellcome Sanger Institute COVID-19 Surveillance Team
EPI_ISL_858371	Lighthouse Lab in Glasgow	Wellcome Sanger Institute for the COVID-19 Genomics UK (COG-UK) Consortium	Harper VanSteenhouse, Yumi Kasai, David Gray, Carol Clugston, Anna Dominiczak and Alex Alderton, Roberto Amato, Sonia Goncalves, Ewan Harrison, David K. Jackson, Ian Johnston, Dominic Kwiatkowski, Cordelia Langford, John Sillitoe on behalf of the Wellcome Sanger Institute COVID-19 Surveillance Team
EPI_ISL_858375	Lighthouse Lab in Alderley Park	Wellcome Sanger Institute for the COVID-19 Genomics UK (COG-UK) Consortium	Jacquelyn Wynn, Mairead Hyland, The Lighthouse Lab in Alderley Park and Alex Alderton, Roberto Amato, Sonia Goncalves, Ewan Harrison, David K. Jackson, Ian Johnston, Dominic Kwiatkowski, Cordelia Langford, John Sillitoe on behalf of the Wellcome Sanger Institute COVID-19 Surveillance Team
EPI_ISL_858378, EPI_ISL_858385, EPI_ISL_858386, EPI_ISL_858391, EPI_ISL_858392, EPI_ISL_858395, EPI_ISL_858398, EPI_ISL_858399	Lighthouse Lab in Glasgow	Wellcome Sanger Institute for the COVID-19 Genomics UK (COG-UK) Consortium	Harper VanSteenhouse, Yumi Kasai, David Gray, Carol Clugston, Anna Dominiczak and Alex Alderton, Roberto Amato, Sonia Goncalves, Ewan Harrison, David K. Jackson, Ian Johnston, Dominic Kwiatkowski, Cordelia Langford, John Sillitoe on behalf of the Wellcome Sanger Institute COVID-19 Surveillance Team
EPI_ISL_858400, EPI_ISL_858403	Lighthouse Lab in Alderley Park	Wellcome Sanger Institute for the COVID-19 Genomics UK (COG-UK) Consortium	Jacquelyn Wynn, Mairead Hyland, The Lighthouse Lab in Alderley Park and Alex Alderton, Roberto Amato, Sonia Goncalves, Ewan Harrison, David K. Jackson, Ian Johnston, Dominic Kwiatkowski, Cordelia Langford, John Sillitoe on behalf of the Wellcome Sanger Institute COVID-19 Surveillance Team
EPI_ISL_858425, EPI_ISL_858427, EPI_ISL_858429, EPI_ISL_858430, EPI_ISL_858431, EPI_ISL_858432, EPI_ISL_858433, EPI_ISL_858434, EPI_ISL_858435, EPI_ISL_858436, EPI_ISL_858437, EPI_ISL_858440, EPI_ISL_858441, EPI_ISL_858444, EPI_ISL_858446, EPI_ISL_858447, EPI_ISL_858448, EPI_ISL_858451, EPI_ISL_858452, EPI_ISL_858453, EPI_ISL_858454, EPI_ISL_858455, EPI_ISL_858456, EPI_ISL_858457, EPI_ISL_858459, EPI_ISL_858460, EPI_ISL_858462, EPI_ISL_858463, EPI_ISL_858465, EPI_ISL_858466, EPI_ISL_858467, EPI_ISL_858468, EPI_ISL_858470, EPI_ISL_858472, EPI_ISL_858474, EPI_ISL_858475, EPI_ISL_858476, EPI_ISL_858480, EPI_ISL_858482, EPI_ISL_858483, EPI_ISL_858484, EPI_ISL_858487, EPI_ISL_858488, EPI_ISL_858489, EPI_ISL_858490, EPI_ISL_858492, EPI_ISL_858493, EPI_ISL_858494, EPI_ISL_858495, EPI_ISL_858496, EPI_ISL_858497, EPI_ISL_858498, EPI_ISL_858499, EPI_ISL_858500, EPI_ISL_858502, EPI_ISL_858503, EPI_ISL_858504, EPI_ISL_858505, EPI_ISL_858506, EPI_ISL_858507, EPI_ISL_858508, EPI_ISL_858509, EPI_ISL_858510, EPI_ISL_858511, EPI_ISL_858512, EPI_ISL_858513, EPI_ISL_858514, EPI_ISL_858515, EPI_ISL_858517, EPI_ISL_858518, EPI_ISL_858519, EPI_ISL_858520, EPI_ISL_858522, EPI_ISL_858524, EPI_ISL_858525, EPI_ISL_858526, EPI_ISL_858527, EPI_ISL_858528, EPI_ISL_858529, EPI_ISL_858530, EPI_ISL_858532, EPI_ISL_858533, EPI_ISL_858534, EPI_ISL_858535, EPI_ISL_858536, EPI_ISL_858537, EPI_ISL_858538, EPI_ISL_858539, EPI_ISL_858540, EPI_ISL_858541, EPI_ISL_858542, EPI_ISL_858543, EPI_ISL_858544, EPI_ISL_858545, EPI_ISL_858546, EPI_ISL_858547, EPI_ISL_858548, EPI_ISL_858549, EPI_ISL_858550, EPI_ISL_858551, EPI_ISL_858552, EPI_ISL_858553, EPI_ISL_858554, EPI_ISL_858555, EPI_ISL_858556, EPI_ISL_858558, EPI_ISL_858559, EPI_ISL_858560, EPI_ISL_858561, EPI_ISL_858562, EPI_ISL_858564, EPI_ISL_858565, EPI_ISL_858566, EPI_ISL_858567, EPI_ISL_858568, EPI_ISL_858569, EPI_ISL_858570, EPI_ISL_858571			
see above	Lighthouse Lab in Glasgow	Wellcome Sanger Institute for the COVID-19 Genomics UK (COG-UK) Consortium	Harper VanSteenhouse, Yumi Kasai, David Gray, Carol Clugston, Anna Dominiczak and Alex Alderton, Roberto Amato, Sonia Goncalves, Ewan Harrison, David K. Jackson, Ian Johnston, Dominic Kwiatkowski, Cordelia Langford, John Sillitoe on behalf of the Wellcome Sanger Institute COVID-19 Surveillance Team
EPI_ISL_860186	Bangalore Medical College and Research Institute	Department of Neurovirology, National Institute of Mental Health and Neurosciences (NIMHANS)	Chitra Pattabiraman, Pramada Prasad, Anson Kunjumon George, Risha Rasheed, Darshan Sreenivas, Nakka Vijay Kiran Reddy, Anita S Desai, V Ravi
EPI_ISL_860196	National Institute of Mental Health and Neurosciences (NIMHANS)	Department of Neurovirology, National Institute of Mental Health and Neurosciences (NIMHANS)	Chitra Pattabiraman, Pramada Prasad, Anson Kunjumon George, Risha Rasheed, Darshan Sreenivas, Nakka Vijay Kiran Reddy, Anita S Desai, V Ravi
EPI_ISL_860197	BBMP Urban PHC	Department of Neurovirology, National Institute of Mental Health and Neurosciences (NIMHANS)	Chitra Pattabiraman, Pramada Prasad, Anson Kunjumon George, Risha Rasheed, Darshan Sreenivas, Nakka Vijay Kiran Reddy, Anita S Desai, V Ravi
EPI_ISL_860215	Norwegian Institute of Public Health, Department of Virology	Norwegian Institute of Public Health, Department of Virology	Kathrine Stene-Johansen, Kamilla Heddeland Instefjord, Hilde Elshaug, Atiya R Ali,Marie Paulsen Madsen, Rasmus Riis Kopperud, Hilde Vollan, Karoline Bragstad, Olav Hungnes
EPI_ISL_860219, EPI_ISL_860232	Akershus University Hospital, Department for Microbiology and Infectious Disease Control	Norwegian Institute of Public Health, Department of Virology	Kathrine Stene-Johansen, Kamilla Heddeland Instefjord, Hilde Elshaug, Atiya R Ali,Marie Paulsen Madsen, Rasmus Riis Kopperud, Hilde Vollan, Karoline Bragstad, Olav Hungnes
EPI_ISL_860243, EPI_ISL_860249, EPI_ISL_860250, EPI_ISL_860252	University Hospitals of Geneva, Laboratory of Virology	HUG, Laboratory of Virology and Universitätsspital Basel	Samuel Cordey, Ana Rita Goncalves, Laurent Kaiser, Tim Roloff, Madlen Stange, Helena MB Seth-Smith, Alfredo Mari, Karoline Leuzinger, Julia Bielicki, Manuel Battegay, Hans Hirsch, Adrian Egli
EPI_ISL_860261	Nordland Hospital - Bodo, Laboratory Department, Molecular Biology Unit	Norwegian Institute of Public Health, Department of Virology	Kathrine Stene-Johansen, Kamilla Heddeland Instefjord, Hilde Elshaug, Atiya R Ali,Marie Paulsen Madsen, Rasmus Riis Kopperud, Hilde Vollan, Karoline Bragstad, Olav Hungnes
EPI_ISL_860264, EPI_ISL_860265, EPI_ISL_860266, EPI_ISL_860267	Vestfold Hospital, Toensberg Department of Microbiology	Norwegian Institute of Public Health, Department of Virology	Kathrine Stene-Johansen, Kamilla Heddeland Instefjord, Hilde Elshaug, Atiya R Ali,Marie Paulsen Madsen, Rasmus Riis Kopperud, Hilde Vollan, Karoline Bragstad, Olav Hungnes
EPI_ISL_860272	Ostfold Hospital Trust - Kalnes, Centre for Laboratory Medicine, Section for gene technology and infection serology	Norwegian Institute of Public Health, Department of Virology	Kathrine Stene-Johansen, Kamilla Heddeland Instefjord, Hilde Elshaug, Atiya R Ali,Marie Paulsen Madsen, Rasmus Riis Kopperud, Hilde Vollan, Karoline Bragstad, Olav Hungnes
EPI_ISL_860275	Foerde Hospital, Department of Microbiology	Norwegian Institute of Public Health, Department of Virology	Kathrine Stene-Johansen, Kamilla Heddeland Instefjord, Hilde Elshaug, Atiya R Ali,Marie Paulsen Madsen, Rasmus Riis Kopperud, Hilde Vollan, Karoline Bragstad, Olav Hungnes
EPI_ISL_860284, EPI_ISL_860286, EPI_ISL_860287	Department of Medical Microbiology, St. Olavs hospital	Norwegian Institute of Public Health, Department of Virology	Kathrine Stene-Johansen, Kamilla Heddeland Instefjord, Hilde Elshaug, Atiya R Ali,Marie Paulsen Madsen, Rasmus Riis Kopperud, Hilde Vollan, Karoline Bragstad, Olav Hungnes
EPI_ISL_860295	Hospital of Southern Norway - Kristiansand, Department of Medical Microbiology	Norwegian Institute of Public Health, Department of Virology	Kathrine Stene-Johansen, Kamilla Heddeland Instefjord, Hilde Elshaug, Atiya R Ali,Marie Paulsen Madsen, Rasmus Riis Kopperud, Hilde Vollan, Karoline Bragstad, Olav Hungnes
EPI_ISL_860304, EPI_ISL_860305, EPI_ISL_860306, EPI_ISL_860307, EPI_ISL_860309, EPI_ISL_860310, EPI_ISL_860311, EPI_ISL_860312	Unit 17: Influenza & Other Respiratory Viruses, German National Influenza Center	Project group Epidemiology of Highly Pathogenic Microorganisms, Robert Koch-Institute	Andreas Sachse, Grit Schubert, Essia Belarbi, Sébastien Calvignac-Spencer, Thorsten Wolff, Ralf Dürrwald, Djin-Ye Oh, Marianne Wedde
EPI_ISL_860668, EPI_ISL_860669, EPI_ISL_860670, EPI_ISL_860672, EPI_ISL_860673, EPI_ISL_860678, EPI_ISL_860679	Respiratory Virus Unit, National Infection Service, Public Health England	COVID-19 Genomics UK (COG-UK) Consortium	PHE Covid Sequencing Team
EPI_ISL_860879, EPI_ISL_860880, EPI_ISL_860885	Labo Analyses Med	National Reference Center for Viruses of Respiratory Infections, Institut Pasteur, Paris	Marion Barbet, Sylvie Behillil, Méline Bizard, Angela Brisebarre, Camille Capel, Etienne Simon-Lorière, Vincent Enouf, Maud Vanpeene, Sylvie van der Werf,Lefaure Brieuc
EPI_ISL_860937, EPI_ISL_860947, EPI_ISL_860949, EPI_ISL_860964, EPI_ISL_860987, EPI_ISL_860996, EPI_ISL_860998, EPI_ISL_861028, EPI_ISL_861031, EPI_ISL_861037, EPI_ISL_861068, EPI_ISL_861087, EPI_ISL_861098, EPI_ISL_861099			
see above	Johns Hopkins Hospital Department of Pathology	Johns Hopkins Hospital Department of Pathology	C. Paul Morris, Chun Huai Luo, Adannaya Amadi, Nicholas Gallagher, Heba H. Mostafa
EPI_ISL_861115, EPI_ISL_861116, EPI_ISL_861117	Wadsworth Center, New York State Department of Health	Wadsworth Center, New York State Department of Health	Kirsten St. George, Daryl M. Lamson, Alexis Russel, Matthew Shudt, Melissa A Leisner, Jonathan Plitnick, Navjot Singh, John Kelly, Erasmus Schneider, Erica Lasek-Nesselquist
EPI_ISL_861180	BIO-REFERENCE LABORATORIES	Wadsworth Center, New York State Department of Health	Kirsten St. George, Daryl M. Lamson, Alexis Russel, Matthew Shudt, Melissa A Leisner, Jonathan Plitnick, Navjot Singh, John Kelly, Erasmus Schneider, Erica Lasek-Nesselquist

EPI_ISL_861360, EPI_ISL_861361, EPI_ISL_861362, EPI_ISL_861363, EPI_ISL_861364, EPI_ISL_861365, EPI_ISL_861366, EPI_ISL_861367, EPI_ISL_861368, EPI_ISL_861370, EPI_ISL_861371, EPI_ISL_861372, EPI_ISL_861381, EPI_ISL_861388, EPI_ISL_861390, EPI_ISL_861391, EPI_ISL_861398			
see above	WESTCHESTER MEDICAL CENTER	Wadsworth Center, New York State Department of Health	Kirsten St. George, Daryl M. Lamson, Alexis Russel, Matthew Shudt, Melissa A Leisner, Jonathan Plitnick, Navjot Singh, John Kelly, Erasmus Schneider, Erica Lasek-Nesselquist
EPI_ISL_861427, EPI_ISL_861429, EPI_ISL_861431	Wyoming Public Health Laboratory	Wyoming Public Health Laboratory	Noah Hull, Taylor Fearing, Lynette Gumbleton, Channing Weber, Ashley Norberg, Bailey Bowcutt, and Wanda Manley
EPI_ISL_861472, EPI_ISL_861473	Gundersen Molecular Diagnostics Laboratory	Kabara Cancer Research Institute	Craig S. Richmond, Paraic A. Kenny
EPI_ISL_861550, EPI_ISL_861572	Instituto Nacional de Saude (INSA)	Instituto Nacional de Saude (INSA)	Borges et al
EPI_ISL_861683	Complexo Hospitalar Padre Bento de Guarulhos	Instituto Adolfo Lutz, Interdiciplinary Procedures Center, Strategic Laboratory	Claudio Tavares Sacchi, Claudia Regina Gonçalves, Erica Valessa Ramos Gomes, Karoline Rodrigues Campos
EPI_ISL_861686, EPI_ISL_861690, EPI_ISL_861693	Los Angeles County PHL	Los Angeles County PHL	P. Hemarajata et al.
EPI_ISL_861742, EPI_ISL_861744	Tempus	Grubaugh Lab - Yale School of Public Health	Tara Alpert, Joseph Fauver, Anderson Brito, Mallery Breban, Anne Wyllie, Chantal Vogels, Mary Petrone, Annie Watkins, Chaney Kalinich, Isabel Ott, Nathan Grubaugh
EPI_ISL_862037	Johns Hopkins Hospital Department of Pathology	Johns Hopkins Hospital Department of Pathology	C. Paul Morris, Chun Huai Luo, Adannaya Amadi, Matthew Schwartz, Nicholas Gallagher, Heba H. Mostafa
EPI_ISL_862051	Multidisciplinary Research Unit, DHR-ICMR, Institute of Medical Sciences, Banaras Hindu University	Multidisciplinary Research Unit, DHR-ICMR, Institute of Medical Sciences, Banaras Hindu University	Priyoneel Basu, Ashish, Nitish Kumar Singh, Abhay Kumar Yadav, Manpreet Kaur, Arup Acharjee, Deepa Devadas, Chetan Sahni, Sanjay Kumar, Tribhuwan Mohan Mahapatra, Richa Arya, Prashant Singh, Jay Prakash Maurya, Surendra Pratap Mishra, Royana Singh
EPI_ISL_862153, EPI_ISL_862155, EPI_ISL_862156	Charité Universitätsmedizin Berlin, Institut fur Virologie/Labor Berlin	Charité Universitätsmedizin Berlin, Institut für Virologie	Victor M Corman, Barbara Mühlemann, Jörn Beheim-Schwarzbach, Tobias Bleicker, Julia Tesch, Talitha Veith, Julia Schneider, Terry Jones, Christian Drosten
EPI_ISL_862746, EPI_ISL_862747, EPI_ISL_862750, EPI_ISL_862753, EPI_ISL_862755, EPI_ISL_862762, EPI_ISL_862764, EPI_ISL_862767, EPI_ISL_862768, EPI_ISL_862770, EPI_ISL_862778			
see above	Utah Public Health Laboratory, Utah Public Health Laboratory Infectious Disease submission group	Utah Public Health Laboratory, Utah Public Health Laboratory Infectious Disease submission group	Young,E.L., Oakeson,K.F., Gallagher,T.
EPI_ISL_862831	National Institute of Infectious Diseases-Prof. Dr. Matei Bals Molecular Diagnostics Laboratory	National Institute of Infectious Diseases-Prof. Dr. Matei Bals Molecular Diagnostics Laboratory	Leontina Banica, Marius Surleac, Corina Casangiu, Petre Milu, Andreea Tudor, Simona Paraschiv, Dan Otelea
EPI_ISL_864368	Lighthouse Lab in Milton Keynes	Wellcome Sanger Institute for the COVID-19 Genomics UK (COG-UK) Consortium	The Lighthouse Lab in Milton Keynes and Alex Alderton, Roberto Amato, Sonia Goncalves, Ewan Harrison, David K. Jackson, Ian Johnston, Dominic Kwiatkowski, Cordelia Langford, John Sillitoe on behalf of the Wellcome Sanger Institute COVID-19 Surveillance Team
EPI_ISL_864755, EPI_ISL_864756, EPI_ISL_864758, EPI_ISL_864762, EPI_ISL_864764, EPI_ISL_864774, EPI_ISL_864776, EPI_ISL_864778, EPI_ISL_864780, EPI_ISL_864805, EPI_ISL_864819, EPI_ISL_864821, EPI_ISL_864828, EPI_ISL_864832, EPI_ISL_864834, EPI_ISL_864838, EPI_ISL_864850, EPI_ISL_864856			
see above	Department of Pathology, University of Cambridge	COVID-19 Genomics UK (COG-UK) Consortium	Aminu S. Jahun, Yasmin Chaudhry, Grant Hall, Iliana Georgana, Myra Hosmillo, Martin D. Curran, Malte Pinckert, Surendra Parmar, Ian Goodfellow
EPI_ISL_864996, EPI_ISL_864997, EPI_ISL_864998, EPI_ISL_864999	West of Scotland Specialist Virology Centre, NHSGGC / MRC-University of Glasgow Centre for Virus Research	COVID-19 Genomics UK (COG-UK) Consortium	Ana da Silva Filipe, Natasha Johnson, Kathy Smollett, Daniel Mair, Stephen Carmichael, Alice Broos, Lily Tong, Jenna Nichols, Kyriaki Nomikou; Sarah McDonald; Richard Orton, Joseph Hughes, Sreenu Vattipally, David L Robertson; Alasdair MacLean, Rory Gunson; Sharif Shaaban, Matthew Holden; Rachel Blacow, Guy Mollett, Kathy Li, James Shepherd, Antonia Ho, Emma Thomson
EPI_ISL_865007, EPI_ISL_865010, EPI_ISL_865011	Lighthouse Lab in Glasgow / MRC-University of Glasgow Centre for Virus Research	COVID-19 Genomics UK (COG-UK) Consortium	Ana da Silva Filipe, Natasha Johnson, Kathy Smollett, Daniel Mair, Stephen Carmichael, Alice Broos, Lily Tong, Jenna Nichols, Kyriaki Nomikou; Sarah McDonald; Harper VanSteenhouse, Yumi Kasai, David Gray, Carol Clugston, Anna Dominiczak; Alasdair MacLean, Rory Gunson; Richard Orton, Joseph Hughes, Sreenu Vattipally, David L Robertson; Sharif Shaaban, Matthew Holden; Kathy Li, James Shepherd, Antonia Ho, Emma Thomson
EPI_ISL_865095, EPI_ISL_865102	Virology Department, Royal Infirmary of Edinburgh, NHS Lothian / School of Biological Sciences, University of Edinburgh / Institute of Genetics and Molecular Medicine, University of Edinburgh	COVID-19 Genomics UK (COG-UK) Consortium	McHugh M, Dewar R, Rooke S, Gallagher M, Balcaza C, O'Toole Á, Scher E, Hill V, McCrone JT, Colquhoun R, Yu X, Jackson B, Rambaut A, Williams TC, Templeton K
EPI_ISL_865181, EPI_ISL_865182, EPI_ISL_865185, EPI_ISL_865186, EPI_ISL_865187, EPI_ISL_865188, EPI_ISL_865189, EPI_ISL_865192, EPI_ISL_865193, EPI_ISL_865194, EPI_ISL_865196, EPI_ISL_865197, EPI_ISL_865199, EPI_ISL_865200, EPI_ISL_865201, EPI_ISL_865202, EPI_ISL_865334, EPI_ISL_865335, EPI_ISL_865337, EPI_ISL_865450, EPI_ISL_865478, EPI_ISL_865482, EPI_ISL_865483			
see above	Liverpool Clinical Laboratories	COVID-19 Genomics UK (COG-UK) Consortium	Sam Haldenby, Anita Lucaci, Steve Paterson, Julian Hiscox, Alistair Darby, M Almsaud, A Alrezaihi, Muhannad Alruwaili, Stuart D Armstrong, Jones Benjamin, Eleanor G Bentley, Anu Chawla, Jordan J Clark, Angela Cowell, Richard Eccles, Isabel García-Dorival, Matthew Gemmell, Alessandro Gerada, PKF Gilmore, Richard Gregory, Ximeng Han, Catherine Hartley, Margaret Hughes, Miren Iturriza-Gomara, James Johnson, L Luu, Jenifer Manson, Charlotte Nelson, Elaine O'Toole, Cassie Olateju, Rebekah Penrice-Randal , Lucille Rainbow, N.P Randle, Trevor Ian Robinson, Parul Sharma, Ghada T Shawli, James P Stewart, Neil Swainston, Ecaterina Vamos, Joanne Watts, Mark Whitehead
EPI_ISL_865500	Barts Health NHS Trust	COVID-19 Genomics UK (COG-UK) Consortium	CUTINO-MOGUEL, Maria-Teresa; HARRINGTON, David; OWOYEMI, Dola; KULASEGARAN-SHYLINI, Raghavendran; BROAD, Claire; KELE, Beatrix
EPI_ISL_866076	University College London Hospital	COVID-19 Genomics UK (COG-UK) Consortium	Judith Heaney, Matthew Byott, Catherine Houlihan, Dan Frampton, Stuart Kirk, Moira Spyer and Eleni Nastouli
EPI_ISL_866187	University College London, Great Ormond Street Hospital for Children NHS Foundation Trust, Imperial College Healthcare NHS Trust	COVID-19 Genomics UK (COG-UK) Consortium	Sergi Castellano, Rachel Williams, Mark Kristiansen, Paola Resende Silva, Sunando Roy, Tony Brooks, Helena Tutill, Paola Niola, Patricia Dyal, Charlotte Williams, Leysa Forrest, Yasmin Panchbhaya, Jacqueline Findlay, Samuel Weeks, Julianne Brown, Kathryn Harris, Paul Randell, James Price, Alison Holmes, Judith Breuer
EPI_ISL_866353, EPI_ISL_866360, EPI_ISL_866361, EPI_ISL_866370, EPI_ISL_866389, EPI_ISL_866390, EPI_ISL_866391, EPI_ISL_866392	Regional Virus Laboratory, Belfast Health and Social Care Trust	COVID-19 Genomics UK (COG-UK) Consortium	Conall McCaughey, James McKenna, Tanya Curran, Susan Feeney, Alison Watt, Ciara Cox, Mairead Connor, Zoltan Molnar, David Simpson, Derek Fairley
EPI_ISL_867030, EPI_ISL_867032, EPI_ISL_867033	Lincolnshire Hospitals and DeepSeq Nottingham	COVID-19 Genomics UK (COG-UK) Consortium	Nichola Duckworth, Tim Sloan, Sarah Walsh, Jonathan Ball, Patrick McClure, Joeseph Chappell, Nadine Holmes, Matthew Carlisle, Christopher Moore, Fei Sang, Johnny Debebe, Victoria Wright, Matthew Loose
EPI_ISL_867100, EPI_ISL_867101, EPI_ISL_867133, EPI_ISL_867134, EPI_ISL_867137, EPI_ISL_867138, EPI_ISL_867140, EPI_ISL_867141, EPI_ISL_867142, EPI_ISL_867144, EPI_ISL_867145, EPI_ISL_867147, EPI_ISL_867148, EPI_ISL_867150, EPI_ISL_867166			
see above	Oxford Viromics, NDM, University of Oxford; Oxford University Hospitals; Basingstoke and North Hampshire Hospital	COVID-19 Genomics UK (COG-UK) Consortium	Tanya Golubchik, David Bonsall, George Macintyre, Amy Trebes, Mariateresa de Cesare, Catrin Moore, Alex Mobbs, Anita Justice, Robert Shaw, Monique Andersson, Timothy Peto, Emma Wise, Nathan Moore, Jessica Lynch, Nick Cortes, Matilde Mori, Stephen Kidd, David Buck, John Todd, Christophe Fraser
EPI_ISL_867186, EPI_ISL_867206, EPI_ISL_867207	Originating lab: Wales Specialist Virology Centre Sequencing lab: Pathogen Genomics Unit	Public Health Wales Microbiology Cardiff Wales Specialist Virology Centre	Catherine Moore, Johnathan Evans, Laura Gifford, Malorie Perry, Simon Cottrell, Angela Marchbank, Alec Birchley, Alexander Adams, Amy Gaskin, Bree Gatica-Wilcox, Jason Coombes, Joel Southgate, Lauren Gilbert, Lee Graham, Nicole Pacchiarini, Sara Kumziene-Summerhayes, Sarah Taylor, Sophie Jones, Sara Rey, Matthew Bull, Joanne Watkins, Sally Corden, Tom Connor
EPI_ISL_867990, EPI_ISL_868312, EPI_ISL_868313, EPI_ISL_868315, EPI_ISL_868316, EPI_ISL_868326, EPI_ISL_868327, EPI_ISL_868349, EPI_ISL_868350	Centre for Enzyme Innovation, University of Portsmouth / Translational Research Laboratory, Portsmouth Hospitals NHS Trust	COVID-19 Genomics UK (COG-UK) Consortium	Angela Beckett,Yann Bourgeois,Garry Scarlett,Sharon Glaysher,Scott Elliott,Kelly Bicknell,Robert Impey,Allyson Lloyd,Sarah Wyllie,Ethan Butcher,Anoop Chauhan,Samuel Robson
EPI_ISL_868357, EPI_ISL_868607, EPI_ISL_868704	Virology Department, Sheffield Teaching Hospitals NHS Foundation Trust/Department of Infection, Immunity and Cardiovascular Disease, The Medical School, University of Sheffield	COVID-19 Genomics UK (COG-UK) Consortium	Thushan de Silva, Matthew Parker, Nikki Smith, Adri Angyal, Rebecca Brown, Luke Green, Rachel Tucker, Paul Parsons, Danielle Groves, Katie Johnson, Laura Carrilero, Alex Keeley, Dave Partridge, Matthew Wyles, Benjamin Lindsey, Mehmet Yavuz, Mohammad Raza, Cariad Evans
EPI_ISL_869083, EPI_ISL_869124, EPI_ISL_869125, EPI_ISL_869126,	Charité Universitätsmedizin Berlin, Institut für Virologie/Labor Berlin	Charité Universitätsmedizin Berlin, Institut für Virologie	Victor M Corman, Barbara Mühlemann, Jörn Beheim-Schwarzbach, Tobias Bleicker, Julia Tesch, Talitha Veith, Julia Schneider, Terry Jones, Christian Drosten

EPI_ISL_869127, EPI_ISL_869128, EPI_ISL_869129, EPI_ISL_869130			
EPI_ISL_869182, EPI_ISL_869183	New Mexico Department of Health Scientific Laboratory	Center for Global Health, University of New Mexico Health Sciences Center	Daryl Domman, Kurt Schwalm, Twila Kunde, Joseph Hicks, Anastacia Griego, Michael Edwards, Darrell Dinwiddie
EPI_ISL_871133, EPI_ISL_871134, EPI_ISL_871135, EPI_ISL_871136, EPI_ISL_871137, EPI_ISL_871138, EPI_ISL_871139, EPI_ISL_871140, EPI_ISL_871141, EPI_ISL_871142, EPI_ISL_871143, EPI_ISL_871144, EPI_ISL_871145, EPI_ISL_871146, EPI_ISL_871147, EPI_ISL_871148, EPI_ISL_871149, EPI_ISL_871150, EPI_ISL_871151, EPI_ISL_871152, EPI_ISL_871153, EPI_ISL_871154, EPI_ISL_871155, EPI_ISL_871156, EPI_ISL_871157, EPI_ISL_871158, EPI_ISL_871159, EPI_ISL_871160, EPI_ISL_871161, EPI_ISL_871162, EPI_ISL_871163, EPI_ISL_871164, EPI_ISL_871165, EPI_ISL_871166, EPI_ISL_871167, EPI_ISL_871168, EPI_ISL_871169, EPI_ISL_871170, EPI_ISL_871171, EPI_ISL_871172, EPI_ISL_871173, EPI_ISL_871174, EPI_ISL_871175, EPI_ISL_871176, EPI_ISL_871177, EPI_ISL_871178, EPI_ISL_871179, EPI_ISL_871180, EPI_ISL_871181, EPI_ISL_871182, EPI_ISL_871183, EPI_ISL_871184, EPI_ISL_871185, EPI_ISL_871186, EPI_ISL_871187, EPI_ISL_871188, EPI_ISL_871189, EPI_ISL_871190, EPI_ISL_871191, EPI_ISL_871192, EPI_ISL_871193, EPI_ISL_871194, EPI_ISL_871195, EPI_ISL_871196, EPI_ISL_871197, EPI_ISL_871198, EPI_ISL_871199, EPI_ISL_871200, EPI_ISL_871201, EPI_ISL_871202, EPI_ISL_871203, EPI_ISL_871204, EPI_ISL_871205, EPI_ISL_871206, EPI_ISL_871207, EPI_ISL_871208, EPI_ISL_871209, EPI_ISL_871210, EPI_ISL_871211, EPI_ISL_871212, EPI_ISL_871213, EPI_ISL_871214, EPI_ISL_871215, EPI_ISL_871216, EPI_ISL_871217, EPI_ISL_871218, EPI_ISL_871219, EPI_ISL_871220, EPI_ISL_871221, EPI_ISL_871222, EPI_ISL_871223, EPI_ISL_871224, EPI_ISL_871225, EPI_ISL_871226, EPI_ISL_871227, EPI_ISL_871228, EPI_ISL_871229, EPI_ISL_871230, EPI_ISL_871231, EPI_ISL_871232, EPI_ISL_871233, EPI_ISL_871234, EPI_ISL_871235, EPI_ISL_871236, EPI_ISL_871237, EPI_ISL_871238, EPI_ISL_871239, EPI_ISL_871240, EPI_ISL_871241, EPI_ISL_871242, EPI_ISL_871243, EPI_ISL_871244, EPI_ISL_871245, EPI_ISL_871246, EPI_ISL_871247, EPI_ISL_871248, EPI_ISL_871249, EPI_ISL_871250, EPI_ISL_871251, EPI_ISL_871252, EPI_ISL_871253, EPI_ISL_871254, EPI_ISL_871255, EPI_ISL_871256, EPI_ISL_871257, EPI_ISL_871258, EPI_ISL_871259, EPI_ISL_871260, EPI_ISL_871261, EPI_ISL_871262, EPI_ISL_871263, EPI_ISL_871264, EPI_ISL_871265, EPI_ISL_871266, EPI_ISL_871267, EPI_ISL_871268, EPI_ISL_871269, EPI_ISL_871270, EPI_ISL_871271, EPI_ISL_871272, EPI_ISL_871273, EPI_ISL_871274, EPI_ISL_871275, EPI_ISL_871276, EPI_ISL_871277, EPI_ISL_871278, EPI_ISL_871279, EPI_ISL_871280, EPI_ISL_871281, EPI_ISL_871282, EPI_ISL_871283, EPI_ISL_871284, EPI_ISL_871285, EPI_ISL_871286, EPI_ISL_871287, EPI_ISL_871288, EPI_ISL_871289, EPI_ISL_871290, EPI_ISL_871291, EPI_ISL_871292, EPI_ISL_871293, EPI_ISL_871294, EPI_ISL_871295, EPI_ISL_871296, EPI_ISL_871297, EPI_ISL_871298, EPI_ISL_871299, EPI_ISL_871300, EPI_ISL_871301, EPI_ISL_871302, EPI_ISL_871303, EPI_ISL_871304, EPI_ISL_871305, EPI_ISL_871306, EPI_ISL_871307, EPI_ISL_871308, EPI_ISL_871309, EPI_ISL_871310, EPI_ISL_871311, EPI_ISL_871312, EPI_ISL_871313, EPI_ISL_871314, EPI_ISL_871315, EPI_ISL_871316, EPI_ISL_871317, EPI_ISL_871318, EPI_ISL_871319, EPI_ISL_871320, EPI_ISL_871321, EPI_ISL_871322, EPI_ISL_871323, EPI_ISL_871324, EPI_ISL_871326, EPI_ISL_871327, EPI_ISL_871328, EPI_ISL_871329, EPI_ISL_871330, EPI_ISL_871331, EPI_ISL_871332, EPI_ISL_871333, EPI_ISL_871334, EPI_ISL_871335, EPI_ISL_871336, EPI_ISL_871337, EPI_ISL_871338, EPI_ISL_871339, EPI_ISL_871340, EPI_ISL_871341, EPI_ISL_871342, EPI_ISL_871343, EPI_ISL_871344, EPI_ISL_871345, EPI_ISL_871346, EPI_ISL_871347, EPI_ISL_871348, EPI_ISL_871349, EPI_ISL_871350, EPI_ISL_871351, EPI_ISL_871352, EPI_ISL_871353, EPI_ISL_871354, EPI_ISL_871355, EPI_ISL_871356, EPI_ISL_871357, EPI_ISL_871358, EPI_ISL_871359, EPI_ISL_871360, EPI_ISL_871361, EPI_ISL_871362, EPI_ISL_871363, EPI_ISL_871364, EPI_ISL_871365, EPI_ISL_871366, EPI_ISL_871367, EPI_ISL_871368, EPI_ISL_871369, EPI_ISL_871370, EPI_ISL_871371, EPI_ISL_871372, EPI_ISL_871373, EPI_ISL_871374, EPI_ISL_871375, EPI_ISL_871376, EPI_ISL_871377, EPI_ISL_871378, EPI_ISL_871379, EPI_ISL_871380, EPI_ISL_871381, EPI_ISL_871382, EPI_ISL_871383, EPI_ISL_871384, EPI_ISL_871385, EPI_ISL_871386, EPI_ISL_871387, EPI_ISL_871388, EPI_ISL_871389, EPI_ISL_871390, EPI_ISL_871391, EPI_ISL_871392, EPI_ISL_871393, EPI_ISL_871394, EPI_ISL_871395, EPI_ISL_871396, EPI_ISL_871397, EPI_ISL_871398, EPI_ISL_871399, EPI_ISL_871400, EPI_ISL_871401, EPI_ISL_871402, EPI_ISL_871403, EPI_ISL_871404, EPI_ISL_871405, EPI_ISL_871406, EPI_ISL_871407, EPI_ISL_871408, EPI_ISL_871409, EPI_ISL_871410, EPI_ISL_871411, EPI_ISL_871412, EPI_ISL_871413, EPI_ISL_871414, EPI_ISL_871415, EPI_ISL_871416, EPI_ISL_871417, EPI_ISL_871418, EPI_ISL_871419, EPI_ISL_871420, EPI_ISL_871421, EPI_ISL_871422, EPI_ISL_871423, EPI_ISL_871424, EPI_ISL_871425, EPI_ISL_871426, EPI_ISL_871427, EPI_ISL_871428, EPI_ISL_871429, EPI_ISL_871430, EPI_ISL_871431, EPI_ISL_871432, EPI_ISL_871433, EPI_ISL_871434, EPI_ISL_871435, EPI_ISL_871436, EPI_ISL_871437, EPI_ISL_871438, EPI_ISL_871439, EPI_ISL_871440, EPI_ISL_871441, EPI_ISL_871442, EPI_ISL_871443, EPI_ISL_871444, EPI_ISL_871445, EPI_ISL_871446, EPI_ISL_871447, EPI_ISL_871448, EPI_ISL_871449, EPI_ISL_871450, EPI_ISL_871451, EPI_ISL_871452, EPI_ISL_871453, EPI_ISL_871454, EPI_ISL_871455, EPI_ISL_871456, EPI_ISL_871457			
see above	Department of Virus and Microbiological Special Diagnostics, Statens Serum Institut, Copenhagen, Denmark	Aalborg University	Danish Covid-19 Genome Consortium
EPI_ISL_871841, EPI_ISL_871871	Wyoming Public Health Laboratory	Wyoming Public Health Laboratory	Noah Hull, Taylor Fearing, Lynette Gumbleton, Channing Weber, Ashley Norberg, Bailey Bowcutt, and Wanda Manley
EPI_ISL_871890	Hospital Universitario Marqués de Valdecilla - IDIVAL (Santander, Cantabria)	SeqCOVID-SPAIN consortium/IBV(CSIC)	Mónica Gozalo Margüello, María Eliecer Cano García, Jose Manuel Méndez Legaza, Daniel Pablo Marcos, Jesús Rodríguez Rodríguez, María Siller Ruiz and SeqCOVID-SPAIN consortium
EPI_ISL_871959	Servicio de Microbiología Clínica (Complejo Hospitalario de Navarra, Pamplona), Instituto de Investigación Sanitaria de Navarra (IdiSNA)	SeqCOVID-SPAIN consortium/IBV(CSIC)	Carmen Ezpeleta Baquedano, Ana Navascués, Ana Miqueleiz and SeqCOVID-SPAIN consortium
EPI_ISL_872095, EPI_ISL_872096	Wyoming Public Health Laboratory	Wyoming Public Health Laboratory	Noah Hull, Taylor Fearing, Lynette Gumbleton, Channing Weber, Ashley Norberg, Bailey Bowcutt, and Wanda Manley
EPI_ISL_872239	Labo Analyses Med	National Reference Center for Viruses of Respiratory Infections, Institut Pasteur, Paris	Marion Barbet, Sylvie Behillil, Méline Bizard, Angela Brisebarre, Camille Capel, Etienne Simon-Lorière, Vincent Enouf, Maud Vanpeene, Sylvie van der Werf,Rouah Raquel
EPI_ISL_872298	Hopital	National Reference Center for Viruses of Respiratory Infections, Institut Pasteur, Paris	Marion Barbet, Sylvie Behillil, Méline Bizard, Angela Brisebarre, Camille Capel, Etienne Simon-Lorière, Vincent Enouf, Maud Vanpeene, Sylvie van der Werf,Florin CéCile
EPI_ISL_872573	Laverty Pathology	NSW Health Pathology - Institute of Clinical Pathology and Medical Research; Westmead Hospital; University of Sydney	CIDM-PH et al.
EPI_ISL_872577	South Eastern Area Laboratory Services (SEALS)	NSW Health Pathology - Institute of Clinical Pathology and Medical Research; Westmead Hospital; University of Sydney	CIDM-PH et al.
EPI_ISL_873049, EPI_ISL_873050, EPI_ISL_873051, EPI_ISL_873052, EPI_ISL_873053, EPI_ISL_873054, EPI_ISL_873056, EPI_ISL_873058, EPI_ISL_873059, EPI_ISL_873060, EPI_ISL_873061, EPI_ISL_873064			
see above	University of Michigan Clinical Microbiology Laboratory	Lauring Lab, University of Michigan, Department of Microbiology and Immunology	Valesano
EPI_ISL_873164	Medical Laboratory Sciences, Arab American University	Medical Laboratory Sciences, Arab American University	Dumaidi,k., Al-Jawabreh,A., Ereqat,S., Al-Jawabreh,H., Nasereddin,A.
EPI_ISL_873185	Microbiology Division, South Carolina Department of Health and Environmental Control (SC DHEC)	Microbiology Division, South Carolina Department of Health and Environmental Control (SC DHEC)	Flores,H., Freeman,J.
EPI_ISL_873197, EPI_ISL_873201, EPI_ISL_873207, EPI_ISL_873208	North Dakota Department of Health, Public Health Laboratory	North Dakota Department of Health, Public Health Laboratory	Lisa Wingerter
EPI_ISL_875511	National Virus Reference Laboratory	National Virus Reference Laboratory	Michael Carr, Gabriel Gonzalez, Jonathan Dean, Cillian F De Gascun
EPI_ISL_875517	Institute of Virology, Biomedical Research Center of the Slovak Academy of Sciences, Bratislava	Faculty of Natural Sciences, Comenius University, Bratislava	Broa Brejová, Viktória abanová, Kristína Boršová, Viktória Hodorová, Sabina Fumaová Havlíková, Juraj Kopáek, Martina Liková, ubomíra Lukáiková, Martina Neboháová, Monika Sláviková, Tomáš Vina, Jozef Nosek, Boris Klempa
EPI_ISL_875533	Institute of Virology, Biomedical Research Center of the Slovak Academy of Sciences, Bratislava	Faculty of Natural Sciences, Comenius University, Bratislava	Viktória abanová, Kristína Boršová, Broa Brejová, Viktória Hodorová, Sabina Fumaová Havlíková, Juraj Kopáek, Martina Liková, ubomíra Lukáiková, Martina Neboháová, Monika Sláviková, Tomáš Vina, Jozef Nosek, Boris Klempa
EPI_ISL_875534	Institute of Virology, Biomedical Research Center of the Slovak Academy of Sciences, Bratislava	Faculty of Natural Sciences, Comenius University, Bratislava	Kristína Boršová, Viktória abanová, Broa Brejová, Viktória Hodorová, Sabina Fumaová Havlíková, Juraj Kopáek, Martina Liková, ubomíra Lukáiková, Martina Neboháová, Monika Sláviková, Tomáš Vina, Boris Klempa, Jozef Nosek
EPI_ISL_875561, EPI_ISL_875562, EPI_ISL_875563, EPI_ISL_875565	Ohio Department of Health Laboratory	Ohio Department of Health Laboratory	Holmes, Jennifer; Eric Brandt, Keoni Omura, Glen McGillivary, Caitlin McDonnell, Kirtana Ramadugu, Erica Leasure, Kelsey Florek, Heather Blankenship, Quanta Brown, and Tammy Bannerman
EPI_ISL_875688	National Influenza Center - Instituto Adolfo Lutz	Instituto Adolfo Lutz, Interdiciplinary Procedures Center, Strategic Laboratory	Claudio Tavares Sacchi, Claudia Regina Gonçalves, Erica Valessa Ramos Gomes, Karoline Rodrigues Campos, Katia Correa de Oliveira Santos, Ana Lucia de Carvalho Avelino, Clovis Roberto Abe Constantino
EPI_ISL_875871, EPI_ISL_875872	Ohio Department of Health Laboratory	Ohio Department of Health Laboratory	Holmes, Jennifer; Eric Brandt, Keoni Omura, Glen McGillivary, Caitlin McDonnell, Kirtana Ramadugu, Erica Leasure, Kelsey Florek, Heather Blankenship, Quanta Brown, and Tammy Bannerman
EPI_ISL_876046, EPI_ISL_876114, EPI_ISL_876176, EPI_ISL_876193, EPI_ISL_876197, EPI_ISL_876199	Massachusetts State Public Health Laboratory	Massachusetts State Public Health Laboratory	Andrew Lang, Timelia Fink, Glen Gallagher, Sandra Smole
EPI_ISL_876519	DOHMH Jamaica	New York City Public Health Laboratory	Jade Wang, et al.
EPI_ISL_876627, EPI_ISL_876642, EPI_ISL_876659, EPI_ISL_876669, EPI_ISL_876690, EPI_ISL_876694	Helix/Illumina	Genomics and Discovery, Respiratory Viruses Branch, Division of Viral Diseases, Centers for Disease Control and Prevention	Peter W. Cook,Dhwani Batra,Ben L. Rambo-Martin,Eileen de Feo,Jan Antico,Christine Tran,Matthew Tolentino,Shannon Wickline,Kim Gietzen,Brad Sickler,Jingtao Liu,Eric Allen,Phil Febbo,Summer Galloway,Nicole L. Washington,Simon White,Geraint Levan,Kelly Schiabor Barrett,Elizabeth Cirulli,Alexandre Bolze,Ary Ascencio,Charlotte Rivera-Garcia,Ryan Cho,Jason Nguyen,Sherry Wang,Jimmy Ramirez,Tyler Cassens,Efren Sandoval,Magnus Isaksson,William Lee,David Becker,Marc Laurent,James Lu,Clinton R. Paden,Suxiang Tong,Duncan MacCannell,
EPI_ISL_878440, EPI_ISL_878443, EPI_ISL_878445, EPI_ISL_878448, EPI_ISL_878450, EPI_ISL_878452, EPI_ISL_878455, EPI_ISL_878457, EPI_ISL_878460, EPI_ISL_878463, EPI_ISL_878465, EPI_ISL_878468, EPI_ISL_878470, EPI_ISL_878472, EPI_ISL_878474			
see above	Biolab Diagnostic Laboratories	Andersen lab at Scripps Research	Issa Abu-Dayyeh, Ahmad Tibi, Lama Hussein, Lina Mohammad, Zein Naber, Amid Abdelnour with SEARCH Alliance San Diego
EPI_ISL_878784, EPI_ISL_878794	Rady's Childrens Hospital	Andersen lab at Scripps Research	SEARCH Alliance San Diego with Nanda Radamchar, David Dimmock, Linda Luo, Christina Clarke, Kathryn Bouic, Teresa Mueller, Denise Malicki
EPI_ISL_882315, EPI_ISL_882324, EPI_ISL_882329, EPI_ISL_882338, EPI_ISL_882340, EPI_ISL_882347, EPI_ISL_882357, EPI_ISL_882370, EPI_ISL_882378, EPI_ISL_882405, EPI_ISL_882430, EPI_ISL_882487, EPI_ISL_882530, EPI_ISL_882540, EPI_ISL_882556, EPI_ISL_882567, EPI_ISL_882575, EPI_ISL_882591, EPI_ISL_882597			

see above	Lighthouse Lab in Alderley Park	Wellcome Sanger Institute for the COVID-19 Genomics UK (COG-UK) Consortium	Jacquelyn Wynn, Mairead Hyland, The Lighthouse Lab in Alderley Park and Alex Alderton, Roberto Amato, Sonia Goncalves, Ewan Harrison, David K. Jackson, Ian Johnston, Dominic Kwiatkowski, Cordelia Langford, John Sillitoe on behalf of the Wellcome Sanger Institute COVID-19 Surveillance Team
EPI_ISL_882640	Azerbaijan National Hematology Center Division of Medical Genetics	Azerbaijan National Hematology Center Division of Medical Genetics	Aghayev Agha Rza
EPI_ISL_882641	Azerbaijan National Hematology Center Division of Medical Genetics	Azerbaijan National Hematology Center Division of Medical Genetics	Aghayev Agha Rza, Bayraml Ramin
EPI_ISL_882645	Medical Research Center, Faculty of Medicine, Syarif Hidayatullah State Islamic University Jakarta	Medical Research Center, Faculty of Medicine, Syarif Hidayatullah State Islamic University Jakarta	Chris Adhiyanto, Erike A Suwarsono, Laifa Hendarmin, Zeti Harriyati, Endah Wulandari, Flori Ratna Sari, Fika Ekayanti, Hari Hendarto
EPI_ISL_882772	Office of Diseases Prevention and Control Region 4 Saraburi	COVID-19 Network Investigations (CONI) Alliance	Kamolthip Atsawawaranunt, Elizabeth Batty, Wasun Chantratita, Thanat Chookajorn, Stefan Fernandez, Angkana Huang, Anthony R. Jones, Khajohn Joonsalak, Chonticha Klungtong, Theerarat Kochakarn, Prayuth Kaewmalang, Amornmas Kongklieng, Namfon Kotanan, Krittikorn Kumpornsin, Duangkamon Loesbanluechai, Wudtichai Manasatienkij, Anek Mungaomklang, Bhakbhoom Panthan, Pukkaporn Parnwijitkul, Ekawat Pasomsub, Vichan Pawun, Kingkan Rakmanee, Insee Sensorn, Janjira Thaipadungpanit, Arporn Wangwiwatsin, Treewat Watthanachockchai
EPI_ISL_882792, EPI_ISL_882793, EPI_ISL_882794	Institute for Urban Disease Control and Prevention	COVID-19 Network Investigations (CONI) Alliance	Kamolthip Atsawawaranunt, Elizabeth Batty, Wasun Chantratita, Thanat Chookajorn, Stefan Fernandez, Angkana Huang, Anthony R. Jones, Khajohn Joonsalak, Chonticha Klungtong, Theerarat Kochakarn, Prayuth Kaewmalang, Amornmas Kongklieng, Namfon Kotanan, Krittikorn Kumpornsin, Duangkamon Loesbanluechai, Wudtichai Manasatienkij, Anek Mungaomklang, Bhakbhoom Panthan, Pukkaporn Parnwijitkul, Ekawat Pasomsub, Vichan Pawun, Kingkan Rakmanee, Insee Sensorn, Janjira Thaipadungpanit, Arporn Wangwiwatsin, Treewat Watthanachockchai
EPI_ISL_882795, EPI_ISL_882796, EPI_ISL_882797, EPI_ISL_882798, EPI_ISL_882799	Office of Diseases Prevention and Control Region 4 Saraburi	COVID-19 Network Investigations (CONI) Alliance	Kamolthip Atsawawaranunt, Elizabeth Batty, Wasun Chantratita, Thanat Chookajorn, Stefan Fernandez, Angkana Huang, Anthony R. Jones, Khajohn Joonsalak, Chonticha Klungtong, Theerarat Kochakarn, Prayuth Kaewmalang, Amornmas Kongklieng, Namfon Kotanan, Krittikorn Kumpornsin, Duangkamon Loesbanluechai, Wudtichai Manasatienkij, Anek Mungaomklang, Bhakbhoom Panthan, Pukkaporn Parnwijitkul, Ekawat Pasomsub, Vichan Pawun, Kingkan Rakmanee, Insee Sensorn, Janjira Thaipadungpanit, Arporn Wangwiwatsin, Treewat Watthanachockchai
EPI_ISL_882927	INMI Lazzaro Spallanzani IRCCS	INMI Lazzaro Spallanzani IRCCS	M Rueca, E Giombini, C.E.M Gruber, B Bartolini, O Butera, F Messina, A Di Caro, MR Capobianchi
EPI_ISL_882932	INMI Lazzaro Spallanzani IRCCS	INMI Lazzaro Spallanzani IRCCS	F Messina, O Butera, E Giombini, M Rueca, B Bartolini, C.E.M Gruber, MR Capobianchi, A Di Caro
EPI_ISL_882943	The National Institute of Public Health	State Veterinary Institute Prague	Nagy,A;Jirincova,H;Vecerova,JTrnka,D
EPI_ISL_883003	Maryland Public Health Laboratory	Maryland Public Health Laboratory	Maryland Department of Health Laboratories Administration
EPI_ISL_883290	SIESP CHIETI-DRIVE IN CHIETI	Istituto Zooprofilattico Sperimentale dell'Abruzzo e Molise "G. Caporale"	Lorusso A, Marcacci M, Di Domenico M, Ancora M, Curini V, Mangone I, Rinaldi A, Scialabba S, Di Pasquale A, Cammà C, Puglia I, Calistri P, Savini G
EPI_ISL_883291	RP Guardiagrele-Ospedale di Comunità	Istituto Zooprofilattico Sperimentale dell'Abruzzo e Molise "G. Caporale"	Lorusso A, Marcacci M, Di Domenico M, Ancora M, Curini V, Mangone I, Rinaldi A, Scialabba S, Di Pasquale A, Cammà C, Puglia I, Calistri P, Savini G
EPI_ISL_883292	SIESP CHIETI-DRIVE IN ORTONA	Istituto Zooprofilattico Sperimentale dell'Abruzzo e Molise "G. Caporale"	Lorusso A, Marcacci M, Di Domenico M, Ancora M, Curini V, Mangone I, Rinaldi A, Scialabba S, Di Pasquale A, Cammà C, Puglia I, Calistri P, Savini G
EPI_ISL_883297	RP Guardiagrele-Ospedale di Comunità	Istituto Zooprofilattico Sperimentale dell'Abruzzo e Molise "G. Caporale"	Lorusso A, Marcacci M, Di Domenico M, Ancora M, Curini V, Mangone I, Rinaldi A, Scialabba S, Di Pasquale A, Cammà C, Puglia I, Calistri P, Savini G
EPI_ISL_883298, EPI_ISL_883299	SIESP DIPARTIMENTO DI PREVENZIONE CHIE	Istituto Zooprofilattico Sperimentale dell'Abruzzo e Molise "G. Caporale"	Lorusso A, Marcacci M, Di Domenico M, Ancora M, Curini V, Mangone I, Rinaldi A, Scialabba S, Di Pasquale A, Cammà C, Puglia I, Calistri P, Savini G
EPI_ISL_883300	SIESP CHIETI-DRIVE IN CHIETI	Istituto Zooprofilattico Sperimentale dell'Abruzzo e Molise "G. Caporale"	Lorusso A, Marcacci M, Di Domenico M, Ancora M, Curini V, Mangone I, Rinaldi A, Scialabba S, Di Pasquale A, Cammà C, Puglia I, Calistri P, Savini G
EPI_ISL_883448, EPI_ISL_883449, EPI_ISL_883450, EPI_ISL_883451, EPI_ISL_883454, EPI_ISL_883455, EPI_ISL_883457	NORTHWELL HEALTH LABORATORIES	Wadsworth Center, New York State Department of Health	Kirsten St. George, Daryl M. Lamson, Alexis Russel, Matthew Shudt, Melissa A Leisner, Jonathan Plitnick, Navjot Singh, John Kelly, Erasmus Schneider, Erica Lasek-Nesselquist
EPI_ISL_884000	Labo Analyses Med	National Reference Center for Viruses of Respiratory Infections, Institut Pasteur, Paris	Marion Barbet, Sylvie Behillil, Méline Bizard, Angela Brisebarre, Camille Capel, Etienne Simon-Lorière, Vincent Enouf, Maud Vanpeene, Sylvie van der Werf,Rousset Dominique
EPI_ISL_884254	Maryland Public Health Laboratory	Maryland Public Health Laboratory	Maryland Department of Health Laboratories Administration
EPI_ISL_884860	Bacteriology, Georgia Public Health Laboratory (GPHL)	Bacteriology, Georgia Public Health Laboratory (GPHL)	Reeves,S., Edwards,J., Dixey,C., Parrott,T.
EPI_ISL_885049	Santa Clara County Public Health Laboratory	Chan-Zuckerberg Biohub	CZB Cliahub Consortium
EPI_ISL_886161, EPI_ISL_886168, EPI_ISL_886187, EPI_ISL_886193, EPI_ISL_886206, EPI_ISL_886208, EPI_ISL_886209, EPI_ISL_886211, EPI_ISL_886217, EPI_ISL_886229, EPI_ISL_886231, EPI_ISL_886250, EPI_ISL_886251, EPI_ISL_886257, EPI_ISL_886258, EPI_ISL_886268, EPI_ISL_886272, EPI_ISL_886277, EPI_ISL_886290, EPI_ISL_886291, EPI_ISL_886299, EPI_ISL_886300, EPI_ISL_886301, EPI_ISL_886309, EPI_ISL_886311, EPI_ISL_886317, EPI_ISL_886320, EPI_ISL_886326, EPI_ISL_886332, EPI_ISL_886340, EPI_ISL_886346, EPI_ISL_886349, EPI_ISL_886373, EPI_ISL_886377, EPI_ISL_886379, EPI_ISL_886381, EPI_ISL_886383, EPI_ISL_886390, EPI_ISL_886395, EPI_ISL_886397, EPI_ISL_886398, EPI_ISL_886399, EPI_ISL_886400, EPI_ISL_886413, EPI_ISL_886417, EPI_ISL_886421, EPI_ISL_886422, EPI_ISL_886429, EPI_ISL_886444, EPI_ISL_886446, EPI_ISL_886450, EPI_ISL_886454, EPI_ISL_886477, EPI_ISL_886485, EPI_ISL_886491, EPI_ISL_886492, EPI_ISL_886497, EPI_ISL_886503, EPI_ISL_886508, EPI_ISL_886513, EPI_ISL_886526, EPI_ISL_886527, EPI_ISL_886543, EPI_ISL_886544, EPI_ISL_886550, EPI_ISL_886551, EPI_ISL_886559, EPI_ISL_886560, EPI_ISL_886561, EPI_ISL_886563, EPI_ISL_886581, EPI_ISL_886586, EPI_ISL_886589, EPI_ISL_886593, EPI_ISL_886596, EPI_ISL_886612, EPI_ISL_886619, EPI_ISL_886627, EPI_ISL_886631, EPI_ISL_886651, EPI_ISL_886654, EPI_ISL_886658, EPI_ISL_886665, EPI_ISL_886675, EPI_ISL_886691, EPI_ISL_886692, EPI_ISL_886693, EPI_ISL_886694, EPI_ISL_886712, EPI_ISL_886714, EPI_ISL_886715, EPI_ISL_886717, EPI_ISL_886722, EPI_ISL_886727, EPI_ISL_886731, EPI_ISL_886732, EPI_ISL_886742, EPI_ISL_886744, EPI_ISL_886752, EPI_ISL_886754, EPI_ISL_886756, EPI_ISL_886763, EPI_ISL_886766, EPI_ISL_886772, EPI_ISL_886782, EPI_ISL_886786, EPI_ISL_886797, EPI_ISL_886798, EPI_ISL_886801, EPI_ISL_886803, EPI_ISL_886804, EPI_ISL_886805, EPI_ISL_886806, EPI_ISL_886810, EPI_ISL_886815, EPI_ISL_886826, EPI_ISL_886843, EPI_ISL_886844, EPI_ISL_886846, EPI_ISL_886862, EPI_ISL_886872, EPI_ISL_886878, EPI_ISL_886889, EPI_ISL_886891, EPI_ISL_886893, EPI_ISL_886899, EPI_ISL_886909, EPI_ISL_886916, EPI_ISL_886919, EPI_ISL_886920, EPI_ISL_886921, EPI_ISL_886924, EPI_ISL_886925, EPI_ISL_886928, EPI_ISL_886931, EPI_ISL_886937, EPI_ISL_886938, EPI_ISL_886942, EPI_ISL_886945, EPI_ISL_886951, EPI_ISL_886960, EPI_ISL_886977, EPI_ISL_886979, EPI_ISL_886984, EPI_ISL_886987, EPI_ISL_886998, EPI_ISL_886999, EPI_ISL_887000, EPI_ISL_887002, EPI_ISL_887003, EPI_ISL_887004, EPI_ISL_887005, EPI_ISL_887024, EPI_ISL_887027, EPI_ISL_887031, EPI_ISL_887035, EPI_ISL_887036, EPI_ISL_887039, EPI_ISL_887044, EPI_ISL_887047, EPI_ISL_887075, EPI_ISL_887078, EPI_ISL_887079, EPI_ISL_887081, EPI_ISL_887102			
see above	Labcorp	Genomics and Discovery, Respiratory Viruses Branch, Division of Viral Diseases, Centers for Disease Control and Prevention	Peter W. Cook,Dhwani Batra,Ben L. Rambo-Martin,Summer Galloway,Brian Krueger,Minoo Agarwal,Eyad Almasri,Debbie Boles,Ayla Burns,Nuthawin Charoensri,Oren Cohen,Susan Countryman,Mary Ann Cristobal,Bobbi Croy,Suzanne Dale,Hrushikesh Deshmukh,Amanda Douglas,Vincent Drouillon,Marcia Eisenberg,Howard Engler,Rama Ghatti,Prashant Gupta,Susan Hicks,Jake Humphrey,Lax Iyer,Manoj Jain,Mohan Kolli,Tim Kuphal,Stanley Letovsky,Michael Levandoski,Craig Lukasik,Jonathan Meltzer,Brian Norvell,Mindy Nye,Scott Parker,Christos Petropoulos,John Pruitt,Steven Ragan,Scott Ryan,Mike Sapeta,Jana Schroth,Suresh Babu Selvaraju,Goran Stevovic,Amanda Suchanek,Andrea Throop,Lyndon Tilson,Thomas Urban,Joe Voshell,Kimberly Wagner,Jonathan Williams,Mary Williamson,Qian Zeng,Tricia Zwiefelhofer,Clinton R. Paden,Suxiang Tong,Duncan MacCannell,
EPI_ISL_887534	Johns Hopkins Hospital Department of Pathology	Johns Hopkins Hospital Department of Pathology	C. Paul Morris, Chun Huai Luo, Adannaya Amadi, Matthew Schwartz, Nicholas Gallagher, Heba H. Mostafa
EPI_ISL_887591, EPI_ISL_887618, EPI_ISL_887620, EPI_ISL_887724, EPI_ISL_887746, EPI_ISL_887773, EPI_ISL_887792, EPI_ISL_887824, EPI_ISL_887909, EPI_ISL_887913, EPI_ISL_887936, EPI_ISL_887939, EPI_ISL_887970, EPI_ISL_887978, EPI_ISL_887980, EPI_ISL_888004, EPI_ISL_888023, EPI_ISL_888053, EPI_ISL_888091, EPI_ISL_888097, EPI_ISL_888160, EPI_ISL_888176, EPI_ISL_888188, EPI_ISL_888207, EPI_ISL_888254, EPI_ISL_888263, EPI_ISL_888286, EPI_ISL_888509, EPI_ISL_888548, EPI_ISL_888557, EPI_ISL_888564, EPI_ISL_888581, EPI_ISL_888588			
see above	Labcorp	Genomics and Discovery, Respiratory Viruses Branch, Division of Viral Diseases, Centers for Disease Control and Prevention	Peter W. Cook,Dhwani Batra,Ben L. Rambo-Martin,Summer Galloway,Brian Krueger,Minoo Agarwal,Eyad Almasri,Debbie Boles,Ayla Burns,Nuthawin Charoensri,Oren Cohen,Susan Countryman,Mary Ann Cristobal,Bobbi Croy,Suzanne Dale,Hrushikesh Deshmukh,Amanda Douglas,Vincent Drouillon,Marcia Eisenberg,Howard Engler,Rama Ghatti,Prashant Gupta,Susan Hicks,Jake Humphrey,Lax Iyer,Manoj Jain,Mohan Kolli,Tim Kuphal,Stanley Letovsky,Michael Levandoski,Craig Lukasik,Jonathan Meltzer,Brian Norvell,Mindy Nye,Scott Parker,Christos Petropoulos,John Pruitt,Steven Ragan,Scott Ryan,Mike Sapeta,Jana Schroth,Suresh Babu Selvaraju,Goran Stevovic,Amanda Suchanek,Andrea Throop,Lyndon Tilson,Thomas Urban,Joe Voshell,Kimberly Wagner,Jonathan Williams,Mary Williamson,Qian Zeng,Tricia Zwiefelhofer,Clinton R. Paden,Suxiang Tong,Duncan MacCannell,
EPI_ISL_888853	Michigan Department of Health and Human Services, Bureau of Laboratories	Michigan Department of Health and Human Services, Bureau of Laboratories	Blankenship HM, Riner D, Soehnlen MK
EPI_ISL_888997	RSU Asysyifaa	Eijkman Institute for Molecular Biology, Ministry of Research	Sukma Oktavianthi, Willy Agustine, Edison Johar, Hidayat Trimarsanto, Iskandar Adnan, Lydia V. Panggalo, Frilasita A Yudhaputri, Safarina G Malik, Khin

		and Technology/National Agency for Research and Innovation	Saw Myint, Amin Soebandrio
EPI_ISL_888998	RSUD Cileungsi	Eijkman Institute for Molecular Biology, Ministry of Research and Technology/National Agency for Research and Innovation	Sukma Oktavianthi, Willy Agustine, Edison Johar, Hidayat Trimarsanto, Iskandar Adnan, Lydia V. Panggalo, Frilasita A Yudhaputri, Safarina G Malik, Khin Saw Myint, Amin Soebandrio
EPI_ISL_888999	RS Mitra Keluarga Cibubur	Eijkman Institute for Molecular Biology, Ministry of Research and Technology/National Agency for Research and Innovation	Sukma Oktavianthi, Willy Agustine, Edison Johar, Hidayat Trimarsanto, Iskandar Adnan, Lydia V. Panggalo, Frilasita A Yudhaputri, Safarina G Malik, Khin Saw Myint, Amin Soebandrio
EPI_ISL_889018	RSU Medika Dramaga	Eijkman Institute for Molecular Biology, Ministry of Research and Technology/National Agency for Research and Innovation	Lydia V. Panggalo, Sukma Oktavianthi, Willy Agustine, Edison Johar, Hidayat Trimarsanto, Iskandar Adnan, Frilasita A Yudhaputri, Safarina G Malik, Khin Saw Myint, Amin Soebandrio
EPI_ISL_889019	RSIA Sayyidah	Eijkman Institute for Molecular Biology, Ministry of Research and Technology/National Agency for Research and Innovation	Lydia V. Panggalo, Sukma Oktavianthi, Willy Agustine, Edison Johar, Hidayat Trimarsanto, Iskandar Adnan, Frilasita A Yudhaputri, Safarina G Malik, Khin Saw Myint, Amin Soebandrio
EPI_ISL_889568, EPI_ISL_889569, EPI_ISL_889570, EPI_ISL_889571, EPI_ISL_889572, EPI_ISL_889573, EPI_ISL_889574, EPI_ISL_889575, EPI_ISL_889576, EPI_ISL_889577, EPI_ISL_889578, EPI_ISL_889579, EPI_ISL_889580, EPI_ISL_889581, EPI_ISL_889582, EPI_ISL_889583, EPI_ISL_889584, EPI_ISL_889585, EPI_ISL_889586, EPI_ISL_889587, EPI_ISL_889588, EPI_ISL_889589, EPI_ISL_889590, EPI_ISL_889591, EPI_ISL_889592, EPI_ISL_889593, EPI_ISL_889594, EPI_ISL_889595, EPI_ISL_889596, EPI_ISL_889597, EPI_ISL_889598, EPI_ISL_889599, EPI_ISL_889600, EPI_ISL_889601, EPI_ISL_889602, EPI_ISL_889603, EPI_ISL_889604, EPI_ISL_889605, EPI_ISL_889606			
see above	LSUHS Emerging Viral Threat Laboratory	Microbial Genome Sequencing Center	Jeremy P. Kamil, Jennifer L. Carroll, Camille F. Abshire, Maarten Van Diest, Mohammed N.A. Siddiquey, Andrew D. Yurochko, Martin J. Sapp, Rona S. Scott, Christopher G. Kevil, Daniel J. Snyder, Vaughn S. Cooper, John A. Vanchiere
EPI_ISL_890307, EPI_ISL_890310, EPI_ISL_890337, EPI_ISL_890341	KU Leuven, Rega Institute, Clinical and Epidemiological Virology	KU Leuven, Rega Institute, Clinical and Epidemiological Virology	Tony Wawina-Bokalanga, Bert Vanmechelen, Joan Marti-Carerras, Piet Maes
EPI_ISL_891138	The Jackson Laboratory	The Jackson Laboratory	Lloyd M, Sanderson B, Srivastava A, Maurya R, Renzette N, Omerza G, Kelly K, Li L, Wei C L, Adams M
EPI_ISL_891254	IHU Méditerranée Infection	IHU Méditerranée Infection	Philippe Colson et al.
EPI_ISL_891255	IHU Méditerranée Infection	19-21, boulevard Jean Moulin, 13005 Marseille	Philippe Colson et al.
EPI_ISL_892060	Lighthouse Lab in Alderley Park	Wellcome Sanger Institute for the COVID-19 Genomics UK (COG-UK) Consortium	Jacquelyn Wynn, Mairead Hyland, The Lighthouse Lab in Alderley Park and Alex Alderton, Roberto Amato, Sonia Goncalves, Ewan Harrison, David K. Jackson, Ian Johnston, Dominic Kwiatkowski, Cordelia Langford, John Sillitoe on behalf of the Wellcome Sanger Institute COVID-19 Surveillance Team
EPI_ISL_892211	Lighthouse Lab in Glasgow	Wellcome Sanger Institute for the COVID-19 Genomics UK (COG-UK) Consortium	Harper VanSteenhouse, Yumi Kasai, David Gray, Carol Clugston, Anna Dominiczak and Alex Alderton, Roberto Amato, Sonia Goncalves, Ewan Harrison, David K. Jackson, Ian Johnston, Dominic Kwiatkowski, Cordelia Langford, John Sillitoe on behalf of the Wellcome Sanger Institute COVID-19 Surveillance Team
EPI_ISL_893748, EPI_ISL_893777	Institute of Virology, Medical Center, University of Freiburg, Freiburg, Germany	Institute of Virology, Clinial Virus Genomics, Medical Center, University of Freiburg, Freiburg, Germany	Jonas Fuchs, Lisa Kern, Sandra Reuter, Hajo Grundmann, Marcus Panning
EPI_ISL_894172, EPI_ISL_894173, EPI_ISL_894174, EPI_ISL_894183, EPI_ISL_894187	KU Leuven, Rega Institute, Clinical and Epidemiological Virology	KU Leuven, Rega Institute, Clinical and Epidemiological Virology	Tony Wawina-Bokalanga, Bert Vanmechelen, Joan Marti-Carerras, Piet Maes
EPI_ISL_896077	Labormedizinisches Zentrum Dr Risch	University Hospital Basel, Clinical Bacteriology	Tim Roloff, Madlen Stange, Helena MB Seth-Smith, Alfredo Mari, Karoline Leuzinger, Julia Bielicki, Nadia Wohlwend,Martin Risch, Lorenz Risch, Manuel Battegay, Hans Hirsch, Adrian Egli
EPI_ISL_896078	Viollier AG	University Hospital Basel, Clinical Bacteriology	Tim Roloff, Madlen Stange, Helena MB Seth-Smith, Alfredo Mari, Karoline Leuzinger, Julia Bielicki, Christiane Beckmann, Manuel Battegay, Hans Hirsch, Adrian Egli
EPI_ISL_896079, EPI_ISL_896080, EPI_ISL_896081, EPI_ISL_896082, EPI_ISL_896083, EPI_ISL_896092, EPI_ISL_896097	Labormedizinisches Zentrum Dr Risch	University Hospital Basel, Clinical Bacteriology	Tim Roloff, Madlen Stange, Helena MB Seth-Smith, Alfredo Mari, Karoline Leuzinger, Julia Bielicki, Nadia Wohlwend,Martin Risch, Lorenz Risch, Manuel Battegay, Hans Hirsch, Adrian Egli
EPI_ISL_896098	Rothen Medizinische Laboratorien AG	University Hospital Basel, Clinical Bacteriology	Tim Roloff, Madlen Stange, Helena MB Seth-Smith, Alfredo Mari, Karoline Leuzinger, Julia Bielicki, Ingrid Steffen, Manuel Battegay, Hans Hirsch, Adrian Egli
EPI_ISL_896106, EPI_ISL_896115	Labormedizinisches Zentrum Dr Risch	University Hospital Basel, Clinical Bacteriology	Tim Roloff, Madlen Stange, Helena MB Seth-Smith, Alfredo Mari, Karoline Leuzinger, Julia Bielicki, Nadia Wohlwend,Martin Risch, Lorenz Risch, Manuel Battegay, Hans Hirsch, Adrian Egli
EPI_ISL_896155, EPI_ISL_896168, EPI_ISL_896205	MEPHI, Aix Marseille University	MEPHI, Aix Marseille University	Anthony LEVASSEUR
EPI_ISL_896294, EPI_ISL_896298	New York Presbyterian Hospital	Wadsworth Center, New York State Department of Health	Kirsten St. George, Daryl M. Lamson, Alexis Russel, Matthew Shudt, Melissa A Leisner, Jonathan Plitnick, Navjot Singh, John Kelly, Erasmus Schneider, Erica Lasek-Nesselquist
EPI_ISL_896301	STONY BROOK UNIVERSITY HOSPITAL	Wadsworth Center, New York State Department of Health	Kirsten St. George, Daryl M. Lamson, Alexis Russel, Matthew Shudt, Melissa A Leisner, Jonathan Plitnick, Navjot Singh, John Kelly, Erasmus Schneider, Erica Lasek-Nesselquist
EPI_ISL_896304, EPI_ISL_896338, EPI_ISL_896339, EPI_ISL_896340, EPI_ISL_896341, EPI_ISL_896342, EPI_ISL_896347, EPI_ISL_896348, EPI_ISL_896350, EPI_ISL_896351, EPI_ISL_896352, EPI_ISL_896353, EPI_ISL_896354, EPI_ISL_896355, EPI_ISL_896356, EPI_ISL_896357, EPI_ISL_896358, EPI_ISL_896359, EPI_ISL_896360, EPI_ISL_896361, EPI_ISL_896362, EPI_ISL_896363, EPI_ISL_896364, EPI_ISL_896365			
see above	New York Presbyterian Hospital	Wadsworth Center, New York State Department of Health	Kirsten St. George, Daryl M. Lamson, Alexis Russel, Matthew Shudt, Melissa A Leisner, Jonathan Plitnick, Navjot Singh, John Kelly, Erasmus Schneider, Erica Lasek-Nesselquist
EPI_ISL_896382, EPI_ISL_896383, EPI_ISL_896384, EPI_ISL_896386, EPI_ISL_896389, EPI_ISL_896391, EPI_ISL_896392, EPI_ISL_896393, EPI_ISL_896394, EPI_ISL_896395, EPI_ISL_896430, EPI_ISL_896431, EPI_ISL_896432, EPI_ISL_896433, EPI_ISL_896434, EPI_ISL_896435, EPI_ISL_896436, EPI_ISL_896437, EPI_ISL_896438, EPI_ISL_896439, EPI_ISL_896440, EPI_ISL_896441, EPI_ISL_896442, EPI_ISL_896443, EPI_ISL_896444, EPI_ISL_896445, EPI_ISL_896446			
see above	Columbia University Irving Medical Center	Wadsworth Center, New York State Department of Health	Kirsten St. George, Daryl M. Lamson, Alexis Russel, Matthew Shudt, Melissa A Leisner, Jonathan Plitnick, Navjot Singh, John Kelly, Erasmus Schneider, Erica Lasek-Nesselquist
EPI_ISL_899027, EPI_ISL_899047, EPI_ISL_899048, EPI_ISL_899062, EPI_ISL_899095, EPI_ISL_899115, EPI_ISL_899129, EPI_ISL_899218, EPI_ISL_899219, EPI_ISL_899220, EPI_ISL_899221, EPI_ISL_899222, EPI_ISL_899223, EPI_ISL_899224, EPI_ISL_899225, EPI_ISL_899226, EPI_ISL_899227, EPI_ISL_899228, EPI_ISL_899229, EPI_ISL_899230, EPI_ISL_899231, EPI_ISL_899232, EPI_ISL_899233, EPI_ISL_899234, EPI_ISL_899235, EPI_ISL_899237, EPI_ISL_899318, EPI_ISL_899319, EPI_ISL_899320, EPI_ISL_899321, EPI_ISL_899322, EPI_ISL_899323, EPI_ISL_899324, EPI_ISL_899439, EPI_ISL_899440, EPI_ISL_899441, EPI_ISL_899564, EPI_ISL_899565, EPI_ISL_899566, EPI_ISL_899567, EPI_ISL_899568, EPI_ISL_899686, EPI_ISL_899687, EPI_ISL_899688, EPI_ISL_899689, EPI_ISL_899690, EPI_ISL_899691, EPI_ISL_899692, EPI_ISL_899693, EPI_ISL_899694, EPI_ISL_899695, EPI_ISL_899696, EPI_ISL_899697, EPI_ISL_899698, EPI_ISL_899699, EPI_ISL_899783, EPI_ISL_899784, EPI_ISL_899785, EPI_ISL_899786, EPI_ISL_899974, EPI_ISL_899985, EPI_ISL_899986, EPI_ISL_899987			
see above	Viollier AG	Department of Biosystems Science and Engineering, ETH Zürich	Christian Beisel, Sarah Nadeau, Chaoran Chen, Ivan Topolsky, Philipp Jablonski, Lara Fuhrmann, David Dreifuss, Katharina Jahn, Tobias Schär, Ina Nissen, Natascha Santacroce, Elodie Burcklen, Christiane Beckmann, Maurice Redondo, Olivier Kobel, Christoph Noppen, Sophie Seidel, Noemie Santamaria de Souza, Niko Beerenwinkel, Tanja Stadler
EPI_ISL_900110, EPI_ISL_900317, EPI_ISL_900468	MEPHI, Aix Marseille University	MEPHI, Aix Marseille University	Anthony LEVASSEUR
EPI_ISL_900522	Novabio	CNR Virus des Infections Respiratoires - France SUD	Antonin Bal, Gregory Destras, Gwendolyne Burfin, Hadrien Règue, Quentin Semanas, Martine Valette, Bruno Lina, Sylvie Larrat, Laurence Josset
EPI_ISL_903064, EPI_ISL_903065	Seattle Flu Study	Seattle Flu Study	Deborah A. Nickerson, Chris D. Frazar, Jover Lee, Benjamin Pelle, Erica Ryke, Matthew Richardson, Amanda Adler, Elisabeth Brandstetter, Peter D. Han, Kairsten Fay, Misja Ilcisin, Kirsten Lacombe, Thomas R. Sibley, Melissa Truong, Caitlin R. Wolf, Michael Boeckh, Janet A. Englund, Michael Famulare, Barry R. Lutz, Mark J. Rieder, Lea M. Starita, Matthew Thompson, Jay Shendure, Trevor Bedford, Helen Y. Chu
EPI_ISL_903136, EPI_ISL_903137, EPI_ISL_903138, EPI_ISL_903139, EPI_ISL_903140, EPI_ISL_903141, EPI_ISL_903142, EPI_ISL_903143, EPI_ISL_903144, EPI_ISL_903145, EPI_ISL_903146, EPI_ISL_903147, EPI_ISL_903148, EPI_ISL_903150, EPI_ISL_903152, EPI_ISL_903163, EPI_ISL_903165, EPI_ISL_903166, EPI_ISL_903167, EPI_ISL_903168, EPI_ISL_903169			
see above	Washington State Department of Health	Seattle Flu Study	Deborah A. Nickerson, Chris D. Frazar, Jover Lee, Benjamin Pelle, Erica Ryke, Matthew Richardson, Amanda Adler, Elisabeth Brandstetter, Peter D. Han,

			Kairsten Fay, Misja Ilcisin, Kirsten Lacombe, Thomas R. Sibley, Melissa Truong, Caitlin R. Wolf, Romesh Gautom, Geoff Melly, Brian Hiatt, Philip Dykema, Scott Lindquist, Michael Boeckh, Janet A. Englund, Michael Famulare, Barry R. Lutz, Mark J. Rieder, Lea M. Starita, Matthew Thompson, Helen Y. Chu, Jay Shendure, Trevor Bedford
EPI_ISL_903180, EPI_ISL_903181, EPI_ISL_903182, EPI_ISL_903183, EPI_ISL_903184	Seattle Flu Study	Seattle Flu Study	Deborah A. Nickerson, Chris D. Frazar, Jover Lee, Benjamin Pelle, Erica Ryke, Matthew Richardson, Amanda Adler, Elisabeth Brandstetter, Peter D. Han, Kairsten Fay, Misja Ilcisin, Kirsten Lacombe, Thomas R. Sibley, Melissa Truong, Caitlin R. Wolf, Karen Cowgill, Stephanie Schrag, Jeff Duchin, Michael Boeckh, Janet A. Englund, Michael Famulare, Barry R. Lutz, Mark J. Rieder, Lea M. Starita, Matthew Thompson, Helen Y. Chu, Trevor Bedford, Jay Shendure
EPI_ISL_903237, EPI_ISL_903291, EPI_ISL_903293, EPI_ISL_903295, EPI_ISL_903296, EPI_ISL_903297, EPI_ISL_903298, EPI_ISL_903300, EPI_ISL_903301, EPI_ISL_903302, EPI_ISL_903307, EPI_ISL_903308, EPI_ISL_903310			
see above	M Health Fairview	Minnesota Department of Health, Public Health Laboratory	Alexandra Lorentz, Jacob Garfin, Matt Plumb, and Xiong Wang
EPI_ISL_903670, EPI_ISL_903685	GA Department of Public Health Laboratory	Genomics and Discovery, Respiratory Viruses Branch, Division of Viral Diseases, Centers for Disease Control and Prevention	Krista Queen, Yan Li, Ying Tao, Jing Zhang, Anna Uehara, Anna Montmayeur, Clinton R. Paden, Peter W. Cook, Rachel Marine, Mili Sheth, Jasmine Padilla, Sarah Nobles, Mark Burroughs, Lori Rowe, Haibin Wang, Ben L. Rambo-Martin, Dhwani Batra, Justin Lee, Suxiang Tong
EPI_ISL_903822, EPI_ISL_903865	WI State Laboratory of Hygiene	Genomics and Discovery, Respiratory Viruses Branch, Division of Viral Diseases, Centers for Disease Control and Prevention	Krista Queen, Yan Li, Ying Tao, Jing Zhang, Anna Uehara, Anna Montmayeur, Clinton R. Paden, Peter W. Cook, Rachel Marine, Mili Sheth, Jasmine Padilla, Sarah Nobles, Mark Burroughs, Lori Rowe, Haibin Wang, Ben L. Rambo-Martin, Dhwani Batra, Justin Lee, Suxiang Tong
EPI_ISL_904050, EPI_ISL_904051, EPI_ISL_904052, EPI_ISL_904053, EPI_ISL_904054, EPI_ISL_904055, EPI_ISL_904056, EPI_ISL_904057	New Mexico Department of Health Scientific Laboratory	New Mexico Department of Health Scientific Laboratory	Ellie Johnson, Anastacia Griego-Fisher, D'eldra Malone
EPI_ISL_904270, EPI_ISL_904373, EPI_ISL_904381, EPI_ISL_904540, EPI_ISL_904541, EPI_ISL_904542, EPI_ISL_904543, EPI_ISL_904544, EPI_ISL_904545, EPI_ISL_904546, EPI_ISL_904547			
see above	Dutch COVID-19 response team	Erasmus Medical Center	Bas Oude Munnink, Reina Sikkema, David Nieuwenhuijse, Irina Chestakova, Anne van der Linden, Marjan Boter, Emmanuelle Munger, Corine GeurtsvanKessel, Annemiek van der Eijk, Richard Molenkamp, Marion Koopmans, on behalf of the Dutch national COVID-19 response team.
EPI_ISL_904629, EPI_ISL_904632, EPI_ISL_904633, EPI_ISL_904648, EPI_ISL_904655	Servicio de Microbiología, Laboratori Clínic Metropolitana Nord. Hospital Universitari Germans Trias i Pujol. Institut d'Investigació en Ciències de la Salut Germans Trias i Pujol (IGTP)	IrsiCaixa - Can Ruti CovidSeq	Marc Noguera-Julian, Mariona Parera, Maria Casadellà, Pilar Armengol, Francesc Catala-Moll, Roger Paredes, Bonaventura Clotet Elisa Martró, Verónica Saludes, Anna Not, Ana Pérez, Montserrat Giménez, Ignacio Blanco, Cristina Casañ, Antoni E. Bordoy, Adrián Antuori
EPI_ISL_904982, EPI_ISL_904984, EPI_ISL_904985, EPI_ISL_904991, EPI_ISL_904993, EPI_ISL_904996, EPI_ISL_905010, EPI_ISL_905012, EPI_ISL_905013, EPI_ISL_905014, EPI_ISL_905015, EPI_ISL_905016, EPI_ISL_905018, EPI_ISL_905019, EPI_ISL_905020, EPI_ISL_905021, EPI_ISL_905022, EPI_ISL_905023, EPI_ISL_905024, EPI_ISL_905025, EPI_ISL_905026, EPI_ISL_905027, EPI_ISL_905028, EPI_ISL_905030, EPI_ISL_905268, EPI_ISL_905275, EPI_ISL_905276, EPI_ISL_905282, EPI_ISL_905284, EPI_ISL_905285, EPI_ISL_905286, EPI_ISL_905287, EPI_ISL_905288, EPI_ISL_905361, EPI_ISL_905362, EPI_ISL_905384, EPI_ISL_905385, EPI_ISL_905386, EPI_ISL_905388, EPI_ISL_905391, EPI_ISL_905393, EPI_ISL_905424, EPI_ISL_905439, EPI_ISL_905440, EPI_ISL_905441, EPI_ISL_905442, EPI_ISL_905443, EPI_ISL_905444, EPI_ISL_905445, EPI_ISL_905446, EPI_ISL_905447, EPI_ISL_905448, EPI_ISL_905449, EPI_ISL_905459, EPI_ISL_905460, EPI_ISL_905461, EPI_ISL_905481, EPI_ISL_905501, EPI_ISL_905513, EPI_ISL_905516, EPI_ISL_905519, EPI_ISL_905520, EPI_ISL_905521, EPI_ISL_905522, EPI_ISL_905533, EPI_ISL_905568, EPI_ISL_905592, EPI_ISL_905593, EPI_ISL_905594, EPI_ISL_905595, EPI_ISL_905596, EPI_ISL_905610, EPI_ISL_905641, EPI_ISL_905642, EPI_ISL_905714, EPI_ISL_905715, EPI_ISL_905718, EPI_ISL_905728			
see above	Dutch COVID-19 response team	National Institute for Public Health and the Environment (RIVM)	Adam Meijer, Harry Vennema, Dirk Eggink, Jeroen Cremer, Sharon van den Brink, Bas van der Veer, AnneMarie van den Brandt, Florian Zwagemaker, Dennis Schmitz, Chantal Reusken, on behalf of the national COVID-19 response team
EPI_ISL_905740	NZOZ LM Diagnostyka Czstochowa	National Institute of Public Health - National Institute of Hygiene	Wokowicz Tomasz, Zacharczuk Katarzyna
EPI_ISL_905914, EPI_ISL_905915, EPI_ISL_905916, EPI_ISL_905917, EPI_ISL_905918, EPI_ISL_905919, EPI_ISL_905920, EPI_ISL_905921, EPI_ISL_905922, EPI_ISL_905923, EPI_ISL_905924, EPI_ISL_905925, EPI_ISL_905926			
see above	OHSU Lab Services Molecular Microbiology Lab	Oregon SARS-CoV-2 Genome Sequencing Center	Brendan L. O'Connell, Sally Grindstaff, Kayla Carter, Ruth V. Nichols, Alec J. Hirsch, Donna Hansel, Guang Fan, Xuan, Qin, Daniel N. Streblow, William B. Messer, Andrew C. Adey, Benjamin N. Bimber, Brian J. O'Roak
EPI_ISL_906098	Shimantik Pathology and Diagnostic Center	Child Health Research Foundation	Senjuti Saha, Arif Mohammad Tanmoy, Syed Muktadir Al Sium, Afroza Akter Tanni, Sharmistha Goswami, Roly Malaker, Md Hafizur Rahman, Md. Parvej Alam, Md. Mobarok Karim, Samir K Saha
EPI_ISL_906547	HOSPITAL DEPARTAMENTAL DE VILLAVICENCIO E.S.E.	Instituto Nacional de Salud- Dirección de Investigación en Salud Pública, Universidad de los Andes- Applied genomics research group, Vicerrectoria de Investigación y Creación, Universidad de los Andes- Systems and Computing Engineering Department	Katherine Laiton-Donato, Diego A. Álvarez-Díaz, Carlos Franco-Muñoz, Mauricio Pacheco-Montealegre, Héctor Alejandro Ruiz-Moreno, Maria T. Herrera-Sepúlveda, Diego Andrés Prada, Jhonnatan Reales-González, Sheryll Corchuelo, Julian Naizaque, Gerardo Santamaría Jorge Duitama, Laura Natalia Gonzalez, Jorge Ivan Diaz, Silvia Restrepo-Restrepo, Magdalena Wiesner, Martha Lucia Ospina Martinez, Marcela Mercado-Reyes
EPI_ISL_906573, EPI_ISL_906574	Maine Health and Environmental Testing Laboratory (Maine HETL)	Tewhey Lab, The Jackson Laboratory	Matluk,N., Dewey,H., Iosue,F., Barter,M., Lynch,R., Munger,H. and Tewhey,R.
EPI_ISL_906728	Hematology Laboratory, Section of Molecular Diagnostics, University Clinical Centre, Medical University of Gdansk	Laboratory of Recombinant Vaccines	Lukasz Rabalski, Maciej Kosinski, Maciej Grzybek, Adam Sodol, Aneta Szulc, Krzysztof Lewandowski, Ewa Milosz, Marlena Robakowska, Boguslaw Szewczyk, Krystyna Bienkowska-Szewczyk
EPI_ISL_906780, EPI_ISL_906781, EPI_ISL_906790, EPI_ISL_906792	Center for Genome Research and Biocomputing	Center for Genome Research and Biocomputing	Oregon State University TRACE Project
EPI_ISL_906800, EPI_ISL_906802	Nordland Hospital - Bodo, Laboratory Department, Molecular Biology Unit	Norwegian Institute of Public Health, Department of Virology	Kathrine Stene-Johansen, Kamilla Heddeland Instefjord, Hilde Elshaug, Atiya R Ali,Marie Paulsen Madsen, Rasmus Riis Kopperud, Hilde Vollan, Karoline Bragstad, Olav Hungnes
EPI_ISL_906824	Department of Medical Microbiology - section Molde, Molde Hospital	Norwegian Institute of Public Health, Department of Virology	Kathrine Stene-Johansen, Kamilla Heddeland Instefjord, Hilde Elshaug, Atiya R Ali,Marie Paulsen Madsen, Rasmus Riis Kopperud, Hilde Vollan, Karoline Bragstad, Olav Hungnes
EPI_ISL_906830	Furst Medical Laboratory	Norwegian Institute of Public Health, Department of Virology	Kathrine Stene-Johansen, Kamilla Heddeland Instefjord, Hilde Elshaug, Atiya R Ali,Marie Paulsen Madsen, Rasmus Riis Kopperud, Hilde Vollan, Karoline Bragstad, Olav Hungnes
EPI_ISL_906831	Hospital of Southern Norway - Kristiansand, Department of Medical Microbiology	Norwegian Institute of Public Health, Department of Virology	Kathrine Stene-Johansen, Kamilla Heddeland Instefjord, Hilde Elshaug, Atiya R Ali,Marie Paulsen Madsen, Rasmus Riis Kopperud, Hilde Vollan, Karoline Bragstad, Olav Hungnes
EPI_ISL_906832	Oslo University Hospital, Department of Medical Microbiology	Norwegian Institute of Public Health, Department of Virology	Kathrine Stene-Johansen, Kamilla Heddeland Instefjord, Hilde Elshaug, Atiya R Ali,Marie Paulsen Madsen, Rasmus Riis Kopperud, Hilde Vollan, Karoline Bragstad, Olav Hungnes
EPI_ISL_910016, EPI_ISL_910338, EPI_ISL_910339	Laboratory for Respiratory Viruses, Cantacuzino National Military-Medical Institute for Research and Development	Cantacuzino Institute Virology	Luiza Ustea, Nicoleta Paraschiv, Mihaela Lazar
EPI_ISL_910888, EPI_ISL_910889, EPI_ISL_910890, EPI_ISL_910891, EPI_ISL_910892, EPI_ISL_910893, EPI_ISL_910894, EPI_ISL_910895, EPI_ISL_910896, EPI_ISL_910897, EPI_ISL_910898, EPI_ISL_910899, EPI_ISL_910900, EPI_ISL_910901			
see above	Laboratoire national de sante, Microbiology, Virology	Laboratoire national de sante, Microbiology, Microbial Genomics Platform	Anke Wienecke-Baldacchino, Catherine Ragimbeau,Jessica Tapp, Fatu Djabi, Lise Pignon, Raoul Salmon, Tamir Abdelrahman
EPI_ISL_911650, EPI_ISL_911651, EPI_ISL_911652, EPI_ISL_911653, EPI_ISL_911654, EPI_ISL_911655, EPI_ISL_911656, EPI_ISL_911657, EPI_ISL_911665, EPI_ISL_911666, EPI_ISL_911667, EPI_ISL_911668, EPI_ISL_911669			
see above	Texas Department of State Health Services (TXDSHS)	Texas Department of State Health Services (TXDSHS)	Bonnie Oh, Anita Pokharel, James Daniel Bonser, Myong Koag, Chung Wang, Rachel Lee, Grace Kubin, Rashmi Tuladhar, Mayela Pedrueza, Maliha Rahman, Jenny Zhang
EPI_ISL_911923	IL Department of Public Health Chicago Laboratory/Tempus Labs	Pathogen Discovery, Respiratory Viruses Branch, Division of Viral Diseases, Centers for Disease Control and Prevention	Ying Tao, Yan Li, Jing Zhang, Krista Queen, Anna Uehara, Peter Cook, Clinton R. Paden, Haibin Wang, Suxiang Tong
EPI_ISL_912163, EPI_ISL_912164	Connecticut Department of Health	Grubaugh Lab - Yale School of Public Health	Tara Alpert, Joseph Fauver, Anderson Brito, Mallery Breban, Anne Wyllie, Chantal Vogels, Mary Petrone, Annie Watkins, Chaney Kalinich, Isabel Ott,

			Nathan Grubaugh
EPI_ISL_912272, EPI_ISL_912293, EPI_ISL_912318, EPI_ISL_912325, EPI_ISL_912330, EPI_ISL_912339, EPI_ISL_912349, EPI_ISL_912351	Hospital General Universitario Gregorio Marañón	SeqCOVID-SPAIN consortium / IBV (CSIC)	Darío García de Viedma, Laura Pérez-Lago, Pedro J Sola-Campoy, Sergio Buenestado-Serrano, Marta Herranz, Victor Manuel de la Cueva, Julia Suárez, Pilar Catalán, Patricia Muñoz and SeqCOVID-SPAIN consortium
EPI_ISL_912376, EPI_ISL_912377, EPI_ISL_912378	Fondation Congolaise pour la recherche medicale (FCRM), Francine Ntoumi	NGS Competence Center Tuebingen, Institut für Medizinische Mikrobiologie und Hygiene, Universitaetsklinikum Tübingen	Angel Angelov
EPI_ISL_912406, EPI_ISL_912410, EPI_ISL_912418, EPI_ISL_912421, EPI_ISL_912422, EPI_ISL_912427, EPI_ISL_912429, EPI_ISL_912453	KU Leuven, Rega Institute, Clinical and Epidemiological Virology	KU Leuven, Rega Institute, Clinical and Epidemiological Virology	Tony Wawina-Bokalanga, Bert Vanmechelen, Joan Marti-Carerras, Piet Maes
EPI_ISL_912645, EPI_ISL_912650, EPI_ISL_912658, EPI_ISL_912669, EPI_ISL_912706, EPI_ISL_912718, EPI_ISL_912722, EPI_ISL_912723, EPI_ISL_912724, EPI_ISL_912727, EPI_ISL_912728, EPI_ISL_912729, EPI_ISL_912730, EPI_ISL_912731, EPI_ISL_912732, EPI_ISL_912733			
see above	Hôpital Henri Mondor	Department of Virology, Henri Mondor University Hospital, Assistance Publique Hôpitaux de Paris, Université Paris-Est Créteil, INSERM U955	Christophe Rodriguez, Slim Fourati, Vanessa Demontant, Guillaume Gricourt, Melissa N'Debi, Alexandre Soulier, Elisabeth Trawinski, Jean-Michel Pawlotsky
EPI_ISL_913059	Consejería de Sanidad y Asuntos Sociales	Instituto de Salud Carlos III	Iglesias-Caballero, M. Camarero, S. Sandonís,V. Vázquez, S. Pozo, F. Casas, I. Jiménez, P. Zaballos, A. Monzón, S. Varona, S. Cuesta, I. Gutiérrez, G.
EPI_ISL_913298, EPI_ISL_913299	Klinisk Mikrobiologi	The Public Health Agency of Sweden	Anna-Malin Linde, Maria Lind Karlberg, Carlo Berg, Oskar Karlsson Lindsjo, Sofia Stamouli, Reza Advani, Mattias Haukland, Petra Holmstrom, Noura Walai, Petra Edquist, Mia Brytting, Anna Risberg, Karin Tegmark-Wisell
EPI_ISL_913312, EPI_ISL_913330	Mikrobiologen	The Public Health Agency of Sweden	Anna-Malin Linde, Maria Lind Karlberg, Carlo Berg, Oskar Karlsson Lindsjo, Sofia Stamouli, Reza Advani, Mattias Haukland, Petra Holmstrom, Noura Walai, Petra Edquist, Mia Brytting, Anna Risberg, Karin Tegmark-Wisell
EPI_ISL_913334, EPI_ISL_913335, EPI_ISL_913336	Klinisk Mikrobiologi	The Public Health Agency of Sweden	Anna-Malin Linde, Maria Lind Karlberg, Carlo Berg, Oskar Karlsson Lindsjo, Sofia Stamouli, Reza Advani, Mattias Haukland, Petra Holmstrom, Noura Walai, Petra Edquist, Mia Brytting, Anna Risberg, Karin Tegmark-Wisell
EPI_ISL_913410	Massachusetts State Public Health Laboratory	Massachusetts State Public Health Laboratory	Andrew Lang, Timelia Fink, Glen Gallagher, Sandra Smole
EPI_ISL_913568	M Health Fairview	Minnesota Department of Health, Public Health Laboratory	Alexandra Lorentz, Jacob Garfin, Matt Plumb, and Xiong Wang
EPI_ISL_913647, EPI_ISL_913649, EPI_ISL_913650, EPI_ISL_913655, EPI_ISL_913656, EPI_ISL_913657, EPI_ISL_913658, EPI_ISL_913659, EPI_ISL_913660, EPI_ISL_913661, EPI_ISL_913662, EPI_ISL_913663, EPI_ISL_913664, EPI_ISL_913665			
see above	Michigan Department of Health and Human Services, Bureau of Laboratories	Michigan Department of Health and Human Services, Bureau of Laboratories	Blankenship HM, Riner D, Soehnlen MK
EPI_ISL_913713, EPI_ISL_913714, EPI_ISL_913715, EPI_ISL_913716, EPI_ISL_913717, EPI_ISL_913718, EPI_ISL_913719	Minnesota Department of Health, Public Health Laboratory	Minnesota Department of Health, Public Health Laboratory	Alexandra Lorentz, Jacob Garfin, Matt Plumb, and Xiong Wang
EPI_ISL_913732, EPI_ISL_913733, EPI_ISL_913734, EPI_ISL_913735, EPI_ISL_913736	M Health Fairview	Minnesota Department of Health, Public Health Laboratory	Alexandra Lorentz, Jacob Garfin, Matt Plumb, and Xiong Wang
EPI_ISL_913778, EPI_ISL_913818, EPI_ISL_913842, EPI_ISL_913859, EPI_ISL_913866, EPI_ISL_913870, EPI_ISL_913882, EPI_ISL_913888, EPI_ISL_913889, EPI_ISL_913905, EPI_ISL_913910			
see above	TGen North	TGen North	"Jolene Bowers, Megan Folkerts, Chris French, Hayley Yaglom, Ashlyn Pfeiffer, Darrin Lemmer, Dave Engelthaler, The Arizona COVID Genomics Union (ACGU)"
EPI_ISL_913990	IL Department of Public Health Chicago Laboratory	Pathogen Discovery, Respiratory Viruses Branch, Division of Viral Diseases, Centers for Disease Control and Prevention	Ying Tao, Yan Li, Jing Zhang, Krista Queen, Anna Uehara, Peter Cook, Clinton R. Paden, Haibin Wang, Suxiang Tong
EPI_ISL_914036, EPI_ISL_914037	CT-Dr. Katherine A. Kelley State Public Health Lab	Pathogen Discovery, Respiratory Viruses Branch, Division of Viral Diseases, Centers for Disease Control and Prevention	Ying Tao, Yan Li, Jing Zhang, Krista Queen, Anna Uehara, Peter Cook, Clinton R. Paden, Haibin Wang, Suxiang Tong
EPI_ISL_914820	AREA DE SALUD SAN JUAN-SAN DIEGO-CONCEPCION 2	Inciensa, Instituto Costarricense de Investigación y Enseñanza en Nutrición y Salud	Francisco Duarte, Hebleen Porras, Claudio Soto-Garita, Estela Cordero, Adriana Godínez, Melany Calderón & Mariel López
EPI_ISL_914824	TAMIZAJE COMUNITARIO - PASO CANOAS	Inciensa, Instituto Costarricense de Investigación y Enseñanza en Nutrición y Salud	Francisco Duarte, Hebleen Porras, Claudio Soto-Garita, Estela Cordero, Adriana Godínez, Melany Calderón & Mariel López
EPI_ISL_918174	Department of Infectious Diseases and Immunology, National Hospital Organization Nagoya Medical Center	Clinical Research Center, National Hospital Organization Nagoya Medical Center	Yoshihiro Nakata, Hirotaka Ode, Mai Kubota, Masakazu Matsuda, Kazuhiro Matsuoka, Miho Nakasuji, Mikiko Mori, Mayumi Imahashi, Yoshiyuki Yokomaku, Yasumasa Iwatani
EPI_ISL_918273, EPI_ISL_918274, EPI_ISL_918275, EPI_ISL_918276	Hospital Universitari Vall d'Hebron - Vall d'Hebron Institut de Rercerca	Hospital Universitari Vall d'Hebron	Cristina Andrés, Maria Piñana, Josep F Abril, Damir Garcia-Cehic, Ariadna Rando, Juliana Esperalba, Maria Gema Codina, Carla Castillo, Maria Carmen Martin, Tomàs Pumarola, Josep Quer, Andrés Antón
EPI_ISL_918348	Institute of Virology, Medical Center, University of Freiburg, Freiburg, Germany	Institute of Virology, Clinial Virus Genomics, Medical Center, University of Freiburg, Freiburg, Germany	Jonas Fuchs, Lisa Kern, Sandra Reuter, Hajo Grundmann, Marcus Panning
EPI_ISL_918434, EPI_ISL_918436, EPI_ISL_918437, EPI_ISL_918441, EPI_ISL_918442	AIID	Irish Coronavirus Sequencing Consortium-Teagasc Grange	Matthew McCabe, Aljandro Abner Garcia Leon, Fiona Crispie, Calum Walsh, Michael Carr, John Kenny, Paul Cotter, Patrick Mallon, Gabriel Gonzalez
EPI_ISL_918541, EPI_ISL_918542	LACEN - Laboratório Central de Saúde Pública do Ceara	Evandro Chagas Institute	Santos, M.C.; Silva, A.M.; Junior, W.D.C.; Barbagelata, L.S.; Ferreira, J.A.; Sousa, E.M.A.; da Silva, P.S.; Pinheiro, K.C.; L.C.; Sousa Junior, E.C.
EPI_ISL_918584, EPI_ISL_918738, EPI_ISL_918739, EPI_ISL_918741, EPI_ISL_918742, EPI_ISL_918743, EPI_ISL_918744, EPI_ISL_918746, EPI_ISL_918747, EPI_ISL_918907, EPI_ISL_918908			
see above	University of Birmingham	COVID-19 Genomics UK (COG-UK) Consortium	Institute of Microbiology, University of Birmingham: Claire McMurray, Joanne Stockton, Sam Nicholls, Radoslaw Poplawski, Will Rowe, Josh Quick, Nicholas Loman. University of Birmingham Testing Laboratory: Celina M Whalley, Andrew Bosworth, Charlotte Poxon, Kasun Wanigasooriya, Oliver Pickles, Mike Kidd, Alex Richter, Andrew D Beggs PHE Heartlands Lab: Husam Osman, Andrew Bosworth. Queen Elizabeth Hospital: Anna Casey
EPI_ISL_919455, EPI_ISL_919459, EPI_ISL_919461	Liverpool Clinical Laboratories	COVID-19 Genomics UK (COG-UK) Consortium	Sam Haldenby, Anita Lucaci, Steve Paterson, Julian Hiscox, Alistair Darby, M Almsaud, A Alrezaihi, Muhannad Alruwaili, Stuart D Armstrong, Jones Benjamin, Eleanor G Bentley, Anu Chawla, Jordan J Clark, Angela Cowell, Richard Eccles, Isabel García-Dorival, Matthew Gemmell, Alessandro Gerada, PKF Gilmore, Richard Gregory, Ximeng Han, Catherine Hartley, Margaret Hughes, Miren Iturriza-Gomara, James Johnson, L Luu, Jenifer Manson, Charlotte Nelson, Elaine O'Toole, Cassie Olateju, Rebekah Penrice-Randal , Lucille Rainbow, N.P Randle, Trevor Ian Robinson, Parul Sharma, Ghada T Shawli, James P Stewart, Neil Swainston, Ecaterina Vamos, Joanne Watts, Mark Whitehead
EPI_ISL_919798, EPI_ISL_919799, EPI_ISL_919800, EPI_ISL_919801, EPI_ISL_919802, EPI_ISL_919805, EPI_ISL_919814, EPI_ISL_919815, EPI_ISL_919816, EPI_ISL_919817, EPI_ISL_919818, EPI_ISL_919819, EPI_ISL_919821, EPI_ISL_919822, EPI_ISL_919823, EPI_ISL_919824, EPI_ISL_919825, EPI_ISL_919843, EPI_ISL_919844			
see above	Barts Health NHS Trust	COVID-19 Genomics UK (COG-UK) Consortium	CUTINO-MOGUEL, Maria-Teresa; HARRINGTON, David; OWOYEMI, Dola; KULASEGARAN-SHYLINI, Raghavendran; BROAD, Claire; KELE, Beatrix
EPI_ISL_919970, EPI_ISL_919973, EPI_ISL_919978, EPI_ISL_919979, EPI_ISL_919980, EPI_ISL_919981, EPI_ISL_919985, EPI_ISL_919987, EPI_ISL_920040, EPI_ISL_920047	University College London, Great Ormond Street Hospital for Children NHS Foundation Trust, Imperial College Healthcare NHS Trust	COVID-19 Genomics UK (COG-UK) Consortium	Sergi Castellano, Rachel Williams, Mark Kristiansen, Paola Resende Silva, Sunando Roy, Tony Brooks, Helena Tutill, Paola Niola, Patricia Dyal, Charlotte Williams, Leysa Forrest, Yasmin Panchbhaya, Jacqueline Findlay, Samuel Weeks, Julianne Brown, Kathryn Harris, Paul Randell, James Price, Alison Holmes, Judith Breuer

EPI_ISL_920711	University College London Hospital	COVID-19 Genomics UK (COG-UK) Consortium	Judith Heaney, Matthew Byott, Catherine Houlihan, Dan Frampton, Stuart Kirk, Moira Spyer and Eleni Nastouli
EPI_ISL_920849	University College London, Great Ormond Street Hospital for Children NHS Foundation Trust, Imperial College Healthcare NHS Trust	COVID-19 Genomics UK (COG-UK) Consortium	Sergi Castellano, Rachel Williams, Mark Kristiansen, Paola Resende Silva, Sunando Roy, Tony Brooks, Helena Tutill, Paola Niola, Patricia Dyal, Charlotte Williams, Leysa Forrest, Yasmin Panchbhaya, Jacqueline Findlay, Samuel Weeks, Julianne Brown, Kathryn Harris, Paul Randell, James Price, Alison Holmes, Judith Breuer
EPI_ISL_921020, EPI_ISL_921021, EPI_ISL_921023, EPI_ISL_921080, EPI_ISL_921081, EPI_ISL_921082, EPI_ISL_921083, EPI_ISL_921084, EPI_ISL_921099, EPI_ISL_921103, EPI_ISL_921104, EPI_ISL_921108, EPI_ISL_921112, EPI_ISL_921123, EPI_ISL_921124			
see above	Regional Virus Laboratory, Belfast Health and Social Care Trust	COVID-19 Genomics UK (COG-UK) Consortium	Conall McCaughey, James McKenna, Tanya Curran, Susan Feeney, Alison Watt, Ciara Cox, Mairead Connor, Zoltan Molnar, David Simpson, Derek Fairley
EPI_ISL_921277, EPI_ISL_921287, EPI_ISL_921289, EPI_ISL_921290, EPI_ISL_921291, EPI_ISL_921292, EPI_ISL_921294, EPI_ISL_921296, EPI_ISL_921299, EPI_ISL_921407, EPI_ISL_921408, EPI_ISL_921409, EPI_ISL_921410, EPI_ISL_921411, EPI_ISL_921412, EPI_ISL_921413, EPI_ISL_921438, EPI_ISL_921440, EPI_ISL_921441, EPI_ISL_921443, EPI_ISL_921444, EPI_ISL_921445, EPI_ISL_921447, EPI_ISL_921448, EPI_ISL_921449			
see above	Northumbria University / South Tees Hospitals NHS Foundation Trust / North Cumbria Integrated Care NHS Foundation Trust / North Tees and Hartlepool NHS Foundation Trust / Newcastle Hospitals NHS Foundation Trust	COVID-19 Genomics UK (COG-UK) Consortium	Darren L Smith,Andrew Nelson,Matthew Bashton,Greg R Young,Joshua Loh,John Allan,Mohammad A Tariq,Giles S Holt,Gary Black,Wen C Yew,Lynn Dover,Paul Baker,Steve Liggett,Sarah Essex,Jane Greenaway,Debra Padgett,Clive Graham,Garren Scott,Edward Barton,Emma Swindells,Brendan Payne,Jennifer Collins,Yusri Taha,Gary Eltringham
EPI_ISL_922175, EPI_ISL_922178, EPI_ISL_922183, EPI_ISL_922188, EPI_ISL_922192, EPI_ISL_922249, EPI_ISL_922283, EPI_ISL_922285, EPI_ISL_922304, EPI_ISL_922309, EPI_ISL_922310, EPI_ISL_922317, EPI_ISL_922321, EPI_ISL_922339, EPI_ISL_922340, EPI_ISL_922341			
see above	Oxford Viromics, NDM, University of Oxford; Oxford University Hospitals; Basingstoke and North Hampshire Hospital	COVID-19 Genomics UK (COG-UK) Consortium	Tanya Golubchik, David Bonsall, George Macintyre, Amy Trebes, Mariateresa de Cesare, Catrin Moore, Alex Mobbs, Anita Justice, Robert Shaw, Monique Andersson, Timothy Peto, Emma Wise, Nathan Moore, Jessica Lynch, Nick Cortes, Matilde Mori, Stephen Kidd, David Buck, John Todd, Christophe Fraser
EPI_ISL_923577, EPI_ISL_923580	Centre for Enzyme Innovation, University of Portsmouth / Translational Research Laboratory, Portsmouth Hospitals NHS Trust	COVID-19 Genomics UK (COG-UK) Consortium	Angela Beckett,Salman Goudarzi,Christopher Fearn,Kate Cook,Katie Loveson,Sharon Glaysher,Scott Elliott,Samuel Robson
EPI_ISL_924079, EPI_ISL_924107, EPI_ISL_924132, EPI_ISL_924292, EPI_ISL_924342, EPI_ISL_924400	Virology Department, Sheffield Teaching Hospitals NHS Foundation Trust/Department of Infection, Immunity and Cardiovascular Disease, The Medical School, University of Sheffield	COVID-19 Genomics UK (COG-UK) Consortium	Thushan de Silva, Matthew Parker, Nikki Smith, Adri Angyal, Rebecca Brown, Luke Green, Rachel Tucker, Paul Parsons, Danielle Groves, Katie Johnson, Laura Carrilero, Alex Keeley, Dave Partridge, Matthew Wyles, Benjamin Lindsey, Mehmet Yavuz, Mohammad Raza, Cariad Evans
EPI_ISL_924622, EPI_ISL_924630, EPI_ISL_924634, EPI_ISL_924893, EPI_ISL_924894, EPI_ISL_924896, EPI_ISL_924900, EPI_ISL_924906	Bioinformatics and Biostatistics Lab, Advanced Sequencing Facility	COVID-19 Genomics UK (COG-UK) Consortium	Aengus Stewart,Jerome Nicod,Chelsea Sawyer,Laura Cubitt,Harshil Patel,Margaret Crawford
EPI_ISL_925064	TXDSHS	TXDSHS	Bonnie Oh, Anita Pokharel, James Daniel Bonser, Myong Koag, Chung Wang, Rachel Lee, Grace Kubin, Rashmi Tuladhar, Mayela Pedrueza, Maliha Rahman, Jenny Zhang
EPI_ISL_925398	Department of Clinical Microbiology	GIGA Medical Genomics	Keith Durkin, Maria Artesi, Sébastien Bontems, Raphaël Boreux, Bouchra Boujemla, Cécile Meex, Pierrette Melin, Marie-Pierre Hayette, Vincent Bours
EPI_ISL_925513, EPI_ISL_925515, EPI_ISL_925516, EPI_ISL_925517, EPI_ISL_925518, EPI_ISL_925519, EPI_ISL_925527, EPI_ISL_925529, EPI_ISL_925530, EPI_ISL_925531, EPI_ISL_925532, EPI_ISL_925533, EPI_ISL_925832			
see above	Arizona State Public Health Laboratory	Arizona State Public Health Laboratory	Trung Huynh, Jessica Escobar, Katherine Fullerton, Nobuko Fukushima, Stacy White, Linda Getsinger, Victor Waddell
EPI_ISL_925924, EPI_ISL_925933, EPI_ISL_925949, EPI_ISL_926010, EPI_ISL_926025, EPI_ISL_926041, EPI_ISL_926082, EPI_ISL_926104, EPI_ISL_926151, EPI_ISL_926237, EPI_ISL_926238, EPI_ISL_926265, EPI_ISL_926321, EPI_ISL_926410, EPI_ISL_926442, EPI_ISL_926505, EPI_ISL_926546, EPI_ISL_926593, EPI_ISL_926596, EPI_ISL_926609, EPI_ISL_926620, EPI_ISL_926632, EPI_ISL_926667, EPI_ISL_926716, EPI_ISL_926791, EPI_ISL_926795, EPI_ISL_926818, EPI_ISL_926844, EPI_ISL_926885, EPI_ISL_926936, EPI_ISL_927056, EPI_ISL_927075, EPI_ISL_927103, EPI_ISL_927125, EPI_ISL_927338, EPI_ISL_927356, EPI_ISL_927366, EPI_ISL_927432, EPI_ISL_927480, EPI_ISL_927543, EPI_ISL_927546, EPI_ISL_927601, EPI_ISL_927614, EPI_ISL_927639, EPI_ISL_927668, EPI_ISL_927698, EPI_ISL_927709, EPI_ISL_927740, EPI_ISL_927774, EPI_ISL_927792, EPI_ISL_927830, EPI_ISL_927832, EPI_ISL_927858, EPI_ISL_927878, EPI_ISL_927933, EPI_ISL_927978, EPI_ISL_927979, EPI_ISL_927987, EPI_ISL_928003, EPI_ISL_928026, EPI_ISL_928038, EPI_ISL_928062, EPI_ISL_928064, EPI_ISL_928085, EPI_ISL_928100, EPI_ISL_928137, EPI_ISL_928213, EPI_ISL_928218, EPI_ISL_928237, EPI_ISL_928298, EPI_ISL_928365, EPI_ISL_928434, EPI_ISL_928480, EPI_ISL_928481, EPI_ISL_928483, EPI_ISL_928529, EPI_ISL_928537, EPI_ISL_928720, EPI_ISL_928727, EPI_ISL_928768, EPI_ISL_928834, EPI_ISL_928867, EPI_ISL_928894, EPI_ISL_928932, EPI_ISL_928983, EPI_ISL_929020, EPI_ISL_929064, EPI_ISL_929066, EPI_ISL_929119, EPI_ISL_929202, EPI_ISL_929262, EPI_ISL_929279, EPI_ISL_929309, EPI_ISL_929370, EPI_ISL_929371, EPI_ISL_929390, EPI_ISL_929449, EPI_ISL_929469, EPI_ISL_929533, EPI_ISL_929677, EPI_ISL_929722, EPI_ISL_929726, EPI_ISL_929734, EPI_ISL_929744, EPI_ISL_929748, EPI_ISL_929827, EPI_ISL_929861, EPI_ISL_929881, EPI_ISL_929887, EPI_ISL_929917, EPI_ISL_929923, EPI_ISL_929940, EPI_ISL_929966, EPI_ISL_930004, EPI_ISL_930021, EPI_ISL_930040, EPI_ISL_930090, EPI_ISL_930096, EPI_ISL_930173, EPI_ISL_930259, EPI_ISL_930270, EPI_ISL_930280, EPI_ISL_930303, EPI_ISL_930356, EPI_ISL_930390, EPI_ISL_930455, EPI_ISL_930461, EPI_ISL_930494, EPI_ISL_930509, EPI_ISL_930510			
see above	Department of Virus and Microbiological Special Diagnostics, Statens Serum Institut, Copenhagen, Denmark	Aalborg University	Danish Covid-19 Genome Consortium
EPI_ISL_931574, EPI_ISL_931575, EPI_ISL_931576, EPI_ISL_931577, EPI_ISL_931578, EPI_ISL_931579, EPI_ISL_931580, EPI_ISL_931581, EPI_ISL_931582, EPI_ISL_931583	Utah Public Health Laboratory	Utah Public Health Laboratory	Erin L. Young, Kelly F. Oakeson, Tara Gallagher
EPI_ISL_932832, EPI_ISL_933425	Lighthouse Lab in Glasgow	Wellcome Sanger Institute for the COVID-19 Genomics UK (COG-UK) Consortium	Harper VanSteenhouse, Yumi Kasai, David Gray, Carol Clugston, Anna Dominiczak and Alex Alderton, Roberto Amato, Sonia Goncalves, Ewan Harrison, David K. Jackson, Ian Johnston, Dominic Kwiatkowski, Cordelia Langford, John Sillitoe on behalf of the Wellcome Sanger Institute COVID-19 Surveillance Team
EPI_ISL_934180, EPI_ISL_934181, EPI_ISL_934188, EPI_ISL_934189, EPI_ISL_934190, EPI_ISL_934191, EPI_ISL_934192, EPI_ISL_934193, EPI_ISL_934194, EPI_ISL_934202, EPI_ISL_934204, EPI_ISL_934205, EPI_ISL_934206, EPI_ISL_934207, EPI_ISL_934208, EPI_ISL_934209, EPI_ISL_934210, EPI_ISL_934211, EPI_ISL_934212, EPI_ISL_934213, EPI_ISL_934214, EPI_ISL_934215, EPI_ISL_934216, EPI_ISL_934217, EPI_ISL_934218, EPI_ISL_934219, EPI_ISL_934220, EPI_ISL_934221, EPI_ISL_934223, EPI_ISL_934224, EPI_ISL_934225, EPI_ISL_934226, EPI_ISL_934227, EPI_ISL_934228			
see above	Vilnius university hospital Santaros Klinikos, Center of Laboratory Medicine	Vilnius university hospital Santaros Klinikos, Center of Laboratory Medicine	Ingrida Olendraite, Daniel Naumovas, Rimvydas Norvilas, Dovile Ezerskyte, Justinas Slikas, Gytis Dudas
EPI_ISL_934364, EPI_ISL_934365	Synlab Medilab, Mikrobiologi	The Public Health Agency of Sweden	Anna-Malin Linde, Maria Lind Karlberg, Carlo Berg, Oskar Karlsson Lindsjo, Sofia Stamouli, Reza Advani, Mattias Haukland, Petra Holmstrom, Noura Walai, Petra Edquist, Mia Brytting, Anna Risberg, Karin Tegmark-Wisell
EPI_ISL_935211	KU Leuven, Rega Institute, Clinical and Epidemiological Virology	KU Leuven, Rega Institute, Clinical and Epidemiological Virology	Tony Wawina-Bokalanga, Bert Vanmechelen, Joan Marti-Carerras, Piet Maes
EPI_ISL_935359, EPI_ISL_935360	Florida Bureau of Public Health Laboratories	Florida Bureau of Public Health Laboratories	Sarah Schmedes, Jason Blanton
EPI_ISL_935988, EPI_ISL_935989, EPI_ISL_935990	GLENS FALLS HOSPITAL LABORATORY	Wadsworth Center, New York State Department of Health	Kirsten St. George, Daryl M. Lamson, Alexis Russel, Matthew Shudt, Melissa A Leisner, Jonathan Plitnick, Navjot Singh, John Kelly, Erasmus Schneider, Erica Lasek-Nesselquist
EPI_ISL_936048	ADIRONDACK MEDICAL CENTER	Wadsworth Center, New York State Department of Health	Kirsten St. George, Daryl M. Lamson, Alexis Russel, Matthew Shudt, Melissa A Leisner, Jonathan Plitnick, Navjot Singh, John Kelly, Erasmus Schneider, Erica Lasek-Nesselquist
EPI_ISL_936078, EPI_ISL_936079, EPI_ISL_936080, EPI_ISL_936081, EPI_ISL_936083, EPI_ISL_936095, EPI_ISL_936096, EPI_ISL_936097, EPI_ISL_936101	KALEIDA CENTER FOR LABORATORY MEDICINE	Wadsworth Center, New York State Department of Health	Kirsten St. George, Daryl M. Lamson, Alexis Russel, Matthew Shudt, Melissa A Leisner, Jonathan Plitnick, Navjot Singh, John Kelly, Erasmus Schneider, Erica Lasek-Nesselquist
EPI_ISL_936142	SUNY UPSTATE MEDICAL UNIVERSITY	Wadsworth Center, New York State Department of Health	Kirsten St. George, Daryl M. Lamson, Alexis Russel, Matthew Shudt, Melissa A Leisner, Jonathan Plitnick, Navjot Singh, John Kelly, Erasmus Schneider, Erica Lasek-Nesselquist
EPI_ISL_936144	KALEIDA CENTER FOR LABORATORY MEDICINE	Wadsworth Center, New York State Department of Health	Kirsten St. George, Daryl M. Lamson, Alexis Russel, Matthew Shudt, Melissa A Leisner, Jonathan Plitnick, Navjot Singh, John Kelly, Erasmus Schneider, Erica Lasek-Nesselquist
EPI_ISL_936391, EPI_ISL_936395, EPI_ISL_936426, EPI_ISL_936431, EPI_ISL_936433, EPI_ISL_936435, EPI_ISL_936436, EPI_ISL_936442, EPI_ISL_936445, EPI_ISL_936447, EPI_ISL_936449			

see above	TGen North	TGen North	Jolene Bowers, Megan Folkerts, Chris French, Hayley Yaglom, Ashlyn Pfeiffer, Darrin Lemmer, Dave Engelthaler, The Arizona COVID Genomics Union (ACGU)
EPI_ISL_936493	Santa Clara County Public Health Laboratory	Santa Clara County Public Health Laboratory	Santa Clara County Public Health Department
EPI_ISL_936867, EPI_ISL_936875, EPI_ISL_936876, EPI_ISL_936877, EPI_ISL_936878, EPI_ISL_936879, EPI_ISL_936880, EPI_ISL_936881, EPI_ISL_936885, EPI_ISL_936886, EPI_ISL_936887, EPI_ISL_936889, EPI_ISL_936893, EPI_ISL_936895, EPI_ISL_936897, EPI_ISL_936898, EPI_ISL_936899, EPI_ISL_936900, EPI_ISL_936901, EPI_ISL_936902, EPI_ISL_936903, EPI_ISL_936904			
see above	Northwestern Memorial Hospital	Ozer Lab	Ramon Lorenzo-Redondo, Lacy M. Simons, Chad J. Achenbach, Lawrence J. Jennings, Michael G. Ison, Judd F. Hultquist, Egon A. Ozer
EPI_ISL_940716, EPI_ISL_940717, EPI_ISL_940718, EPI_ISL_940719, EPI_ISL_940720, EPI_ISL_940721, EPI_ISL_940722, EPI_ISL_940733	City of Milwaukee Health Department Laboratory	City of Milwaukee Health Department Laboratory	Sanjib Bhattacharyya
EPI_ISL_940777	NSPI-CRN de Influenza y otros virus respiratorios	INSPI-Centro de Investigación Multidisciplinaria de la DTIDI	Leandro Patiño, Doménica de Mora, Maritza Olmedo, Andrés Carrazco-Montalvo, Orson Mestanza, Mary Regato-Arrata, Melissa Zambrano, Manuel González, Alfredo Bruno, Alberto Orlando.
EPI_ISL_940781	INSPI-CRN de Influenza y otros virus respiratorios	INSPI-Centro de Investigación Multidisciplinaria de la DTIDI	Leandro Patiño, Doménica de Mora, Maritza Olmedo, Andrés Carrazco-Montalvo, Orson Mestanza, Mary Regato-Arrata, Melissa Zambrano, Manuel González, Alfredo Bruno, Alberto Orlando
EPI_ISL_940821	INSPI-CRN de Influenza y otros virus respiratorios	INSPI-Centro de Investigación Multidisciplinaria de la DTIDI	Leandro Patiño, Doménica de Mora, Maritza Olmedo, Andrés Carrazco-Montalvo, Orson Mestanza, Mary Regato-Arrata, Melissa Zambrano, Manuel González, Alfredo Bruno, Alberto Orlando.
EPI_ISL_941226, EPI_ISL_941227	Laboratorio de Microbiología. Hospital General Universitario de Elda, Alicante	SeqCOVID-SPAIN consortium/IBV(CSIC)	Mª Isabel Gascón Ros, Cristina Torregrosa Hetland, Eva Pastor Boix, Paloma Cascales Ramos and SeqCOVID-SPAIN consortium
EPI_ISL_941288, EPI_ISL_941292	Nigeria Centre for Disease Control (NCDC)	African Centre of Excellence for Genomics of Infectious Diseases (ACEGID), Redeemer's University	Oluniyi P.E. et al
EPI_ISL_941373, EPI_ISL_941374, EPI_ISL_941446, EPI_ISL_941447, EPI_ISL_941448, EPI_ISL_941449, EPI_ISL_941450, EPI_ISL_941628	Instituto Nacional de Saude (INSA)	Instituto Nacional de Saude (INSA)	Borges et al
EPI_ISL_941926	Nigeria Centre for Disease Control (NCDC)	African Centre of Excellence for Genomics of Infectious Diseases (ACEGID), Redeemer's University	Oluniyi P.E. et al
EPI_ISL_942013	Wyoming Public Health Laboratory	Wyoming Public Health Laboratory	Noah Hull, Taylor Fearing, Lynette Gumbleton, Channing Weber, Ashley Norberg, Bailey Bowcutt, and Wanda Manley
EPI_ISL_942788, EPI_ISL_942789, EPI_ISL_942790, EPI_ISL_942791, EPI_ISL_942792, EPI_ISL_942793, EPI_ISL_942794	Gundersen Molecular Diagnostics Laboratory	Kabara Cancer Research Institute	Craig S. Richmond, Paraic A. Kenny
EPI_ISL_942960, EPI_ISL_942971	National Institute of Health Research and Development	National Institute of Health Research and Development	Subangkit, Hana Apsari Pawestri, Kartika Dewi Puspa, Arie Ardiansyah Nugraha, Hartanti Dian Ikawati, Krisna Nur Andriana Pangesti, Yuni Rukminiati, Ririn Ramadhany, Agustiningsih, Kindi Adam, Holy Arif Wibowo, Triyani Soekarso, Ni Ketut Susilarini, Nurika Hariastuti, Ully Alfi Nikmah, Reni Herman, Nike Susanti, Herna, Tati Febriyanti, Natalie Laurencia Kipuw, Fauzul Muna, Irene Lorinda Indalao, Nelly Puspandari, Vivi Setiawaty.
EPI_ISL_942999, EPI_ISL_943183, EPI_ISL_943184, EPI_ISL_943242, EPI_ISL_943395, EPI_ISL_943396, EPI_ISL_943397, EPI_ISL_943478	Dutch COVID-19 response team	National Institute for Public Health and the Environment (RIVM)	Adam Meijer, Harry Vennema, Dirk Eggink, Jeroen Cremer, Sharon van den Brink, Bas van der Veer, AnneMarie van den Brandt, Florian Zwagemaker, Dennis Schmitz, Chantal Reusken, on behalf of the national COVID-19 response team
EPI_ISL_943823	Utah Public Health Laboratory	Utah Public Health Laboratory	Erin L. Young, Kelly F. Oakeson, Tara Gallagher
EPI_ISL_944124, EPI_ISL_944127, EPI_ISL_944128, EPI_ISL_944131, EPI_ISL_944135, EPI_ISL_944137, EPI_ISL_944145, EPI_ISL_944148, EPI_ISL_944152, EPI_ISL_944153, EPI_ISL_944154, EPI_ISL_944155, EPI_ISL_944157, EPI_ISL_944158, EPI_ISL_944159, EPI_ISL_944162, EPI_ISL_944163, EPI_ISL_944166, EPI_ISL_944167, EPI_ISL_944168, EPI_ISL_944169, EPI_ISL_944170, EPI_ISL_944171, EPI_ISL_944173, EPI_ISL_944175, EPI_ISL_944176			
see above	National Health Laboratory Service, South Africa	KRISP, KZN Research Innovation and Sequencing Platform	Laguda-Akingba O, Giandhari J, Pillay S, Lessells R, Mdlalose K, York D, Khan S, Emmanuel SJ, Tegally H, Wilkinson E, de Oliveira T
EPI_ISL_944208	Israel Central Virology laboratory	Israel National Consortium for SARS-CoV-2 sequencing	Neta Zuckerman, Efrat Dahan Bucris, Michal Mandelboim, Dana Bar-Ilan, Oran Erster, Tzvia Mann, Omer Murik, David A. Zeevi, Assaf Rokney, Joseph Jaffe, Eva Nachum, Maya Davidovich Cohen, Ephraim Fass, Gal Zizelski Valenci, Mor Rubinstein, Efrat Rorman, Israel Nissan, Efrat Glick-Saar, Omri Nayshool, Gideon Rechavi, Ella Mendelson, Orna Mor
EPI_ISL_944614	Instituto Nacional de Medicina Genomica	Instituto Nacional de Medicina Genomica	Hidalgo-Miranda A, Mendoza-Vargas A, Reyes-Grajeda JP, Cisneros-Villanueva M, Cedro-Tanda A,Peñaloza-Figueroa F, Herrera-Montalvo LA
EPI_ISL_944691, EPI_ISL_944713	Department of Biochemistry, Cell and Molecular Biology, West African Centre for Cell Biology of Infectious Pathogens (WACCBIP), University of Ghana	Department of Biochemistry, Cell and Molecular Biology, West African Centre for Cell Biology of Infectious Pathogens (WACCBIP), University of Ghana	Morang'a,C.M., Ngoi,J.M., Quansah,E.B., Saiid,S., Amuzu,D.S., Asante,I., Bonney,J.H., Bonney,E., Odoom,J.K., Ndam,N.T., Tei-Maya,F., Adusei-Poku,M., Ofori-Boadu,L., Ampofo,W.K., Amenga-Etego,L.N., Quashie,P., Bediako,Y., Awandare,G.A.
EPI_ISL_945057, EPI_ISL_945071, EPI_ISL_945074, EPI_ISL_945075, EPI_ISL_945077, EPI_ISL_945078, EPI_ISL_945079, EPI_ISL_945081, EPI_ISL_945086, EPI_ISL_945097, EPI_ISL_945104			
see above	Lighthouse Lab in Milton Keynes	Wellcome Sanger Institute for the COVID-19 Genomics UK (COG-UK) Consortium	The Lighthouse Lab in Milton Keynes and Alex Alderton, Roberto Amato, Sonia Goncalves, Ewan Harrison, David K. Jackson, Ian Johnston, Dominic Kwiatkowski, Cordelia Langford, John Sillitoe on behalf of the Wellcome Sanger Institute COVID-19 Surveillance Team
EPI_ISL_945107	Lighthouse Lab in Cambridge	Wellcome Sanger Institute for the COVID-19 Genomics UK (COG-UK) Consortium	Rob Howes, The Lighthouse Lab in Cambridge and Alex Alderton, Roberto Amato, Sonia Goncalves, Ewan Harrison, David K. Jackson, Ian Johnston, Dominic Kwiatkowski, Cordelia Langford, John Sillitoe on behalf of the Wellcome Sanger Institute COVID-19 Surveillance Team
EPI_ISL_945110, EPI_ISL_945127, EPI_ISL_945132, EPI_ISL_945135, EPI_ISL_945137	Lighthouse Lab in Milton Keynes	Wellcome Sanger Institute for the COVID-19 Genomics UK (COG-UK) Consortium	The Lighthouse Lab in Milton Keynes and Alex Alderton, Roberto Amato, Sonia Goncalves, Ewan Harrison, David K. Jackson, Ian Johnston, Dominic Kwiatkowski, Cordelia Langford, John Sillitoe on behalf of the Wellcome Sanger Institute COVID-19 Surveillance Team
EPI_ISL_945144	Lighthouse Lab in Glasgow	Wellcome Sanger Institute for the COVID-19 Genomics UK (COG-UK) Consortium	Harper VanSteenhouse, Yumi Kasai, David Gray, Carol Clugston, Anna Dominiczak and Alex Alderton, Roberto Amato, Sonia Goncalves, Ewan Harrison, David K. Jackson, Ian Johnston, Dominic Kwiatkowski, Cordelia Langford, John Sillitoe on behalf of the Wellcome Sanger Institute COVID-19 Surveillance Team
EPI_ISL_945151, EPI_ISL_945154, EPI_ISL_945158	Lighthouse Lab in Milton Keynes	Wellcome Sanger Institute for the COVID-19 Genomics UK (COG-UK) Consortium	The Lighthouse Lab in Milton Keynes and Alex Alderton, Roberto Amato, Sonia Goncalves, Ewan Harrison, David K. Jackson, Ian Johnston, Dominic Kwiatkowski, Cordelia Langford, John Sillitoe on behalf of the Wellcome Sanger Institute COVID-19 Surveillance Team
EPI_ISL_945162	Lighthouse Lab in Glasgow	Wellcome Sanger Institute for the COVID-19 Genomics UK (COG-UK) Consortium	Harper VanSteenhouse, Yumi Kasai, David Gray, Carol Clugston, Anna Dominiczak and Alex Alderton, Roberto Amato, Sonia Goncalves, Ewan Harrison, David K. Jackson, Ian Johnston, Dominic Kwiatkowski, Cordelia Langford, John Sillitoe on behalf of the Wellcome Sanger Institute COVID-19 Surveillance Team
EPI_ISL_945165, EPI_ISL_945167, EPI_ISL_945168, EPI_ISL_945173, EPI_ISL_945182, EPI_ISL_945187, EPI_ISL_945194, EPI_ISL_945196, EPI_ISL_945200, EPI_ISL_945206, EPI_ISL_945211, EPI_ISL_945213, EPI_ISL_945214, EPI_ISL_945215, EPI_ISL_945216, EPI_ISL_945219, EPI_ISL_945225, EPI_ISL_945227, EPI_ISL_945229, EPI_ISL_945233, EPI_ISL_945242, EPI_ISL_945245, EPI_ISL_945259, EPI_ISL_945260, EPI_ISL_945262, EPI_ISL_945263, EPI_ISL_945264, EPI_ISL_945265, EPI_ISL_945267, EPI_ISL_945272, EPI_ISL_945274, EPI_ISL_945280, EPI_ISL_945281, EPI_ISL_945283, EPI_ISL_945284, EPI_ISL_945286, EPI_ISL_945290, EPI_ISL_945291, EPI_ISL_945294, EPI_ISL_945296, EPI_ISL_945298, EPI_ISL_945300, EPI_ISL_945302, EPI_ISL_945303, EPI_ISL_945308, EPI_ISL_945312, EPI_ISL_945316, EPI_ISL_945317, EPI_ISL_945318, EPI_ISL_945326, EPI_ISL_945327, EPI_ISL_945328, EPI_ISL_945330, EPI_ISL_945348, EPI_ISL_945349, EPI_ISL_945353, EPI_ISL_945354, EPI_ISL_945355, EPI_ISL_945359, EPI_ISL_945369, EPI_ISL_945370, EPI_ISL_945371			
see above	Lighthouse Lab in Milton Keynes	Wellcome Sanger Institute for the COVID-19 Genomics UK (COG-UK) Consortium	The Lighthouse Lab in Milton Keynes and Alex Alderton, Roberto Amato, Sonia Goncalves, Ewan Harrison, David K. Jackson, Ian Johnston, Dominic Kwiatkowski, Cordelia Langford, John Sillitoe on behalf of the Wellcome Sanger Institute COVID-19 Surveillance Team
EPI_ISL_947323	RS Siloam Kebon Jeruk	Eijkman Institute for Molecular Biology, Ministry of Research	Sukma Oktavianthi, Willy Agustine, Edison Johar, Hidayat Trimarsanto, Iskandar Adnan, Lydia V. Panggalo, Frilasita A Yudhaputri, Safarina G Malik, Khin

		and Technology/National Agency for Research and Innovation	Saw Myint, Amin Soebandrio
EPI_ISL_949406	University of Birmingham	COVID-19 Genomics UK (COG-UK) Consortium	Institute of Microbiology, University of Birmingham: Claire McMurray, Joanne Stockton, Samuel Nicholls, Radoslaw Poplawski, Will Rowe, Josh Quick, Nicholas Loman. University of Birmingham Testing Laboratory: Celina M Whalley, Andrew Bosworth, Charlotte Poxon, Kasun Wanigasooriya, Oliver Pickles, Mike Kidd, Alex Richter, Andrew D Beggs PHE Heartlands Lab: Husam Osman, Andrew Bosworth. Queen Elizabeth Hospital: Anna Casey
EPI_ISL_949755, EPI_ISL_949767	Barts Health NHS Trust	COVID-19 Genomics UK (COG-UK) Consortium	CUTINO-MOGUEL, Maria-Teresa; HARRINGTON, David; OWOYEMI, Dola; KULASEGARAN-SHYLINI, Raghavendran; BROAD, Claire; KELE, Beatrix
EPI_ISL_950094, EPI_ISL_950111, EPI_ISL_950146, EPI_ISL_950151	University College London, Great Ormond Street Hospital for Children NHS Foundation Trust, Imperial College Healthcare NHS Trust	COVID-19 Genomics UK (COG-UK) Consortium	Sergi Castellano, Rachel Williams, Mark Kristiansen, Paola Resende Silva, Sunando Roy, Tony Brooks, Helena Tutill, Paola Niola, Patricia Dyal, Charlotte Williams, Leysa Forrest, Yasmin Panchbhaya, Jacqueline Findlay, Samuel Weeks, Julianne Brown, Kathryn Harris, Paul Randell, James Price, Alison Holmes, Judith Breuer
EPI_ISL_950288, EPI_ISL_950482, EPI_ISL_950483, EPI_ISL_950484, EPI_ISL_950485, EPI_ISL_950486, EPI_ISL_950487, EPI_ISL_950489, EPI_ISL_950490, EPI_ISL_950491, EPI_ISL_950492, EPI_ISL_950493			
see above	Northumbria University / South Tees Hospitals NHS Foundation Trust / North Cumbria Integrated Care NHS Foundation Trust / North Tees and Hartlepool NHS Foundation Trust / Newcastle Hospitals NHS Foundation Trust	COVID-19 Genomics UK (COG-UK) Consortium	Darren L Smith,Andrew Nelson,Matthew Bashton,Greg R Young,Joshua Loh,John Allan,Mohammad A Tariq,Giles S Holt,Gary Black,Wen C Yew,Lynn Dover,Paul Baker,Steve Liggett,Sarah Essex,Jane Greenaway,Debra Padgett,Clive Graham,Garren Scott,Edward Barton,Emma Swindells,Brendan Payne,Jennifer Collins,Yusri Taha,Gary Eltringham
EPI_ISL_951084, EPI_ISL_951085, EPI_ISL_951087, EPI_ISL_951088, EPI_ISL_951089, EPI_ISL_951090, EPI_ISL_951093, EPI_ISL_951126, EPI_ISL_951131, EPI_ISL_951132, EPI_ISL_951134, EPI_ISL_951136, EPI_ISL_951138, EPI_ISL_951139, EPI_ISL_951144, EPI_ISL_951145, EPI_ISL_951147, EPI_ISL_951148, EPI_ISL_951149, EPI_ISL_951153, EPI_ISL_951155, EPI_ISL_951159, EPI_ISL_951173, EPI_ISL_951178, EPI_ISL_951183, EPI_ISL_951185, EPI_ISL_951187, EPI_ISL_951191, EPI_ISL_951192, EPI_ISL_951198, EPI_ISL_951200, EPI_ISL_951214, EPI_ISL_951229, EPI_ISL_951230, EPI_ISL_951319, EPI_ISL_951384, EPI_ISL_951386			
see above	Oxford Viromics, NDM, University of Oxford; Oxford University Hospitals; Basingstoke and North Hampshire Hospital	COVID-19 Genomics UK (COG-UK) Consortium	Tanya Golubchik, David Bonsall, George Macintyre, Amy Trebes, Mariateresa de Cesare, Catrin Moore, Alex Mobbs, Anita Justice, Robert Shaw, Monique Andersson, Timothy Peto, Emma Wise, Nathan Moore, Jessica Lynch, Nick Cortes, Matilde Mori, Stephen Kidd, David Buck, John Todd, Christophe Fraser
EPI_ISL_952420, EPI_ISL_952423	Centre for Enzyme Innovation, University of Portsmouth / Translational Research Laboratory, Portsmouth Hospitals NHS Trust	COVID-19 Genomics UK (COG-UK) Consortium	Angela Beckett,Salman Goudarzi,Christopher Fearn,Kate Cook,Katie Loveson,Sharon Glaysher,Scott Elliott,Samuel Robson
EPI_ISL_953176, EPI_ISL_953184, EPI_ISL_953188, EPI_ISL_953189, EPI_ISL_953190, EPI_ISL_953192, EPI_ISL_953200, EPI_ISL_953201, EPI_ISL_953202, EPI_ISL_953212, EPI_ISL_953223, EPI_ISL_953225, EPI_ISL_953229			
see above	Bioinformatics and Biostatistics Lab, Advanced Sequencing Facility	COVID-19 Genomics UK (COG-UK) Consortium	Aengus Stewart,Jerome Nicod,Chelsea Sawyer,Laura Cubitt,Harshil Patel,Margaret Crawford
EPI_ISL_954805	Hospital General Universitario de Ciudad Real	Instituto de Salud Carlos III	Iglesias-Caballero, M. Camarero, S. Sandonís,V. Vázquez, S. Pozo, F. Casas, I. Jiménez, P. Zaballos, A. Monzón, S. Varona, S. Cuesta, I. Illescas, S.
EPI_ISL_954820, EPI_ISL_954877, EPI_ISL_954885	Colorado Department of Public Health and Environment	Colorado Department of Puplic Health and Environment	Laura Bankers, Molly C. Hetherington-Rauth, Diana Ir, Shannon Ely, Shannon R. Matzinger, Sarah Elizabeth Totten, Emily A. Travanty
EPI_ISL_955271	American Esoteric Laboratory	Pathogen Discovery, Respiratory Viruses Branch, Division of Viral Diseases, Centers for Disease Control and Prevention	Ying Tao, Jing Zhang, Yan Li, Krista Queen, Anna Uehara, Peter Cook, Clinton R. Paden, Haibin Wang, Suxiang Tong
EPI_ISL_955393, EPI_ISL_955394, EPI_ISL_955395, EPI_ISL_955396, EPI_ISL_955397, EPI_ISL_955398, EPI_ISL_955399, EPI_ISL_955400, EPI_ISL_955401, EPI_ISL_955402, EPI_ISL_955403, EPI_ISL_955404, EPI_ISL_955405, EPI_ISL_955406, EPI_ISL_955407, EPI_ISL_955408, EPI_ISL_955409, EPI_ISL_955410, EPI_ISL_955412, EPI_ISL_955413, EPI_ISL_955414, EPI_ISL_955415, EPI_ISL_955416			
see above	Alameda County Public Health Lab	Chan-Zuckerberg Biohub	CZB Cliahub Consortium
EPI_ISL_955434, EPI_ISL_955435, EPI_ISL_955436, EPI_ISL_955437, EPI_ISL_955438, EPI_ISL_955439, EPI_ISL_955440, EPI_ISL_955441, EPI_ISL_955442, EPI_ISL_955443, EPI_ISL_955444, EPI_ISL_955445, EPI_ISL_955446, EPI_ISL_955448, EPI_ISL_955449, EPI_ISL_955450, EPI_ISL_955451, EPI_ISL_955454, EPI_ISL_955455, EPI_ISL_955456, EPI_ISL_955457, EPI_ISL_955458, EPI_ISL_955459, EPI_ISL_955460, EPI_ISL_955461, EPI_ISL_955462, EPI_ISL_955463, EPI_ISL_955468, EPI_ISL_955469			
see above	Orange County Public Health Lab	Chan-Zuckerberg Biohub	CZB Cliahub Consortium
EPI_ISL_955764, EPI_ISL_955767, EPI_ISL_955769, EPI_ISL_955770, EPI_ISL_955771, EPI_ISL_955772, EPI_ISL_955773, EPI_ISL_955774, EPI_ISL_955775, EPI_ISL_955776	Humboldt County Public Health Laboratory	Chan-Zuckerberg Biohub	CZB Cliahub Consortium
EPI_ISL_956280	Institute of Tropical Disease	Institute of Tropical Disease, Universitas Airlangga	Rima R Prasetya, Krisnoadi Rahardjo, Aldise M Nastri, Jezzy R Dewantari, Gatot Soegiarto, Laksmi Wulandari, Resti Yudhawati, Soetjipto, Yasuko Mori, Maria I Lusida, Kazufumi Shimizu
EPI_ISL_956281	Siti Khodijah Hospital	Institute of Tropical Disease, Universitas Airlangga	Maria I Lusida, Krisnoadi Rahardjo, Aldise M Nastri, Jezzy R Dewantari, Rima R Prasetya, Muhammad Hamdan, Gatot Soegiarto, Laksmi Wulandari, Resti Yudhawati, Soetjipto, Yasuko Mori, Kazufumi Shimizu
EPI_ISL_956297	Instituto Nacional de Salud- Dirección de Redes de Laboratorios de Salud Pública	Instituto Nacional de Salud- Dirección de Investigación en Salud Pública	Katherine Laiton-Donato, Diego A. Álvarez-Díaz, Carlos Franco-Muñoz, Mauricio Pacheco-Montealegre, Hector Alejandro Ruiz-Moreno, Maria T. Herrera-Sepúlveda, Diego Andrés Prada, Jhonnatan Reales-González, Sheryll Corchuelo, Julian Naizaque, Gerardo Santamaría, Magdalena Wiesner, Martha Lucia Ospina Martinez, Marcela Mercado-Reyes
EPI_ISL_956315	Siti Khodijah Hospital	Institute of Tropical Disease, Universitas Airlangga	Kazufumi Shimizu, Krisnoadi Rahardjo, Aldise M Nastri, Jezzy R Dewantari, Rima R Prasetya, Muhammad Hamdan, Gatot Soegiarto, Laksmi Wulandari, Resti Yudhawati, Yasuko Mori, Soetjipto, Maria I Lusida
EPI_ISL_959369	Viollier AG	University Hospital Basel, Clinical Bacteriology	Tim Roloff, Madlen Stange, Helena MB Seth-Smith, Alfredo Mari, Karoline Leuzinger, Julia Bielicki, Christiane Beckmann, Manuel Battegay, Hans Hirsch, Adrian Egli
EPI_ISL_960408, EPI_ISL_960410, EPI_ISL_960412, EPI_ISL_960437, EPI_ISL_960439	The National Institute of Public Health	State Veterinary Institute Prague	Nagy,A;Vecerova,J;Cernikova,L;Stara,M;Jirincova,H;Trnka,D
EPI_ISL_960459, EPI_ISL_960462	Istituto Zooprofilattico Sperimentale del Mezzogiorno	TIGEM	Patrizia Annunziata, Andrea Ballabio, Valentina Bouche, Davide Cacchiarelli, Pellegrino Cerino, Chiara Colantuono, Maria Concetta Cuomo, Denise Di Concilio, Lucio Di Filippo, Antonio Grimaldi, Antonio Limone, Anna Manfredi, Francesco Panariello, Biancamaria Pierri, Marcello Salvi
EPI_ISL_961207, EPI_ISL_961253	Hospital General Universitario de Alicante - Instituto de Investigación Sanitaria y Biomédica de Alicante	SeqCOVID-SPAIN consortium/IBV(CSIC)	Maripaz Ventero Martín, Carmen Molina Pardines and SeqCOVID-SPAIN consortium
EPI_ISL_961482, EPI_ISL_961492, EPI_ISL_961552, EPI_ISL_961553	Michigan Department of Health and Human Services, Bureau of Laboratories	Michigan Department of Health and Human Services, Bureau of Laboratories	Blankenship HM, Riner D, Soehnlen MK
EPI_ISL_961583, EPI_ISL_961652	Hôpital Georges L. Dumont	National Microbiology Laboratory (NML)	Anna Majer, Shari Tyson, Grace Seo, Philip Mabon, Elsie Grudeski, Rhiannon Huzarewich, Russell Mandes, Anneliese Landgraff, Jennifer Tanner, Natalie Knox, Morag Graham, Gary Van Domselaar, Richard Garceau, Guillaume Desnoyers, Nathalie Bastien, Yan Li, Timothy Booth, Darian Hole, Madison Chapel, Kirsten Biggar, CanCOGeN's metadata curation team, Public Health Agency of Canada CanCOGeN team
EPI_ISL_961898, EPI_ISL_961927, EPI_ISL_961937, EPI_ISL_961967, EPI_ISL_962130, EPI_ISL_962131, EPI_ISL_962132, EPI_ISL_962133, EPI_ISL_962134, EPI_ISL_962135, EPI_ISL_962136, EPI_ISL_962137, EPI_ISL_962140			
see above	Illinois Department of Public Health	Gagnon Lab, Southern Illinois University	Keith Gagnon
EPI_ISL_962918	Servicio de Microbiología, Laboratori Clínic Metropolitana Nord. Hospital Universitari Germans Trias i Pujol. Institut d'Investigació en Ciències de la Salut Germans Trias i Pujol (IGTP)	SeqCOVID-SPAIN consortium/IBV(CSIC)	Elisa Martró, Antoni E. Bordoy, Anna Not, Adrián Antuori, Anabel Fernández, Nona Romaní, Verónica Saludes, Cristina Casañ and SeqCOVID-SPAIN consortium
EPI_ISL_965029	Laboratorio de Virologia HUCA	Laboratorio de Virologia HUCA	Castelló C, Gómez de Oña J, Boga JA, Rojo S, Alvarez-Arguelles ME, Abreu F, Costales I, Sandoval M, Perez-Martínez Z, Martín-Rodríguez G, Coto E, Melón S

EPI_ISL_965033	Laboratorio de Virología HUCA	Laboratorio de Virología HUCA	Castelló C, Gómez de Oña J, Boga JA, Rojo S, Alvarez-Arguelles ME, Abreu F, Costales I, Sandoval M, Perez-Martínez Z, Martín-Rodríguez G, Coto E, Melón S
EPI_ISL_965186, EPI_ISL_965188, EPI_ISL_965198, EPI_ISL_965199, EPI_ISL_965200, EPI_ISL_965201, EPI_ISL_965202, EPI_ISL_965203, EPI_ISL_965204, EPI_ISL_965205, EPI_ISL_965206, EPI_ISL_965207, EPI_ISL_965208, EPI_ISL_965209, EPI_ISL_965210			
see above	Virginia Division of Consolidated Laboratory Services	Virginia Division of Consolidated Laboratory Services	Virginia DCLS
EPI_ISL_965572, EPI_ISL_965573, EPI_ISL_965605, EPI_ISL_965618, EPI_ISL_965641, EPI_ISL_965652, EPI_ISL_965659, EPI_ISL_965671, EPI_ISL_965692, EPI_ISL_965699, EPI_ISL_965721, EPI_ISL_965798, EPI_ISL_965809			
see above	Dutch COVID-19 response team	Medical Microbiology, Maastricht University Medical Centre	Jozef Dingemans*, Brian van der Veer*, Erik Beuken, Carmen Reumkens, Lieke van Alphen, Christian Hoebe, Paul Savelkoul
EPI_ISL_966769, EPI_ISL_966770, EPI_ISL_966771, EPI_ISL_966772, EPI_ISL_966773, EPI_ISL_966774, EPI_ISL_966775, EPI_ISL_966776	Maine HETL	Tewhey Lab, The Jackson Laboratory	Matluk,N., Dewey,H., Iosue,F., Barter,M., Lynch,R., Munger,H. and Tewhey,R.
EPI_ISL_967588, EPI_ISL_967593, EPI_ISL_967649, EPI_ISL_967680, EPI_ISL_967681, EPI_ISL_967701, EPI_ISL_967731, EPI_ISL_967732, EPI_ISL_967733	State Laboratories Division, Hawaii State Department of Health	State Laboratories Division, Hawaii State Department of Health	Pamela O'Brien, Drew Kuwazaki, Ayana Garnet, Razvan Sultana, Edward Desmond
EPI_ISL_967929, EPI_ISL_967946, EPI_ISL_967954, EPI_ISL_967968, EPI_ISL_967996, EPI_ISL_968019	TGen North	Sonora Quest Laboratories	"Jolene Bowers, Megan Folkerts, Chris French, Hayley Yaglom, Ashlyn Pfeiffer, Darrin Lemmer, Dave Engelthaler, The Arizona COVID Genomics Union (ACGU)"
EPI_ISL_968853, EPI_ISL_968856, EPI_ISL_968858	KEMRI-Wellcome Trust Research Programme/KEMRI-CGMR-C Kilifi	KEMRI-Wellcome Trust Research Programme/KEMRI-CGMR-C Kilifi	Githinji et al
EPI_ISL_970868, EPI_ISL_970901, EPI_ISL_970984, EPI_ISL_971051, EPI_ISL_971210, EPI_ISL_971217, EPI_ISL_971226, EPI_ISL_971280, EPI_ISL_971341, EPI_ISL_971366, EPI_ISL_971387, EPI_ISL_971477, EPI_ISL_971518, EPI_ISL_971527, EPI_ISL_971553, EPI_ISL_971579, EPI_ISL_971614, EPI_ISL_971642, EPI_ISL_971655, EPI_ISL_971706, EPI_ISL_971738, EPI_ISL_971773, EPI_ISL_971784, EPI_ISL_971790, EPI_ISL_971805, EPI_ISL_971819, EPI_ISL_971834, EPI_ISL_971885, EPI_ISL_971914, EPI_ISL_971920, EPI_ISL_971939, EPI_ISL_971951, EPI_ISL_971987, EPI_ISL_971991, EPI_ISL_971995, EPI_ISL_971997, EPI_ISL_972027, EPI_ISL_972058, EPI_ISL_972116, EPI_ISL_972117, EPI_ISL_972118, EPI_ISL_972153, EPI_ISL_972207, EPI_ISL_972212, EPI_ISL_972213, EPI_ISL_972236, EPI_ISL_972302, EPI_ISL_972307, EPI_ISL_972341, EPI_ISL_972452, EPI_ISL_972459, EPI_ISL_972501, EPI_ISL_972525, EPI_ISL_972544, EPI_ISL_972559, EPI_ISL_972587, EPI_ISL_972602, EPI_ISL_972662, EPI_ISL_972668, EPI_ISL_972697, EPI_ISL_972702, EPI_ISL_972766, EPI_ISL_972779, EPI_ISL_972810, EPI_ISL_972846, EPI_ISL_972861, EPI_ISL_972891, EPI_ISL_972905, EPI_ISL_972924, EPI_ISL_972954, EPI_ISL_973009, EPI_ISL_973019, EPI_ISL_973080, EPI_ISL_973105, EPI_ISL_973124, EPI_ISL_973145, EPI_ISL_973165, EPI_ISL_973252, EPI_ISL_973277, EPI_ISL_973278, EPI_ISL_973332, EPI_ISL_973346, EPI_ISL_973443, EPI_ISL_973544, EPI_ISL_973578, EPI_ISL_973601, EPI_ISL_973607, EPI_ISL_973635, EPI_ISL_973656, EPI_ISL_973704, EPI_ISL_973780, EPI_ISL_973799, EPI_ISL_973827			
see above	Department of Virus and Microbiological Special Diagnostics, Statens Serum Institut, Copenhagen, Denmark	Aalborg University	Danish Covid-19 Genome Consortium
EPI_ISL_977653	Michigan Department of Health and Human Services, Bureau of Laboratories	Michigan Department of Health and Human Services, Bureau of Laboratories	Blankenship HM, Riner D, Soehnlen MK
EPI_ISL_978152, EPI_ISL_978153, EPI_ISL_978154, EPI_ISL_978155, EPI_ISL_978156, EPI_ISL_978157	Chiu Laboratory, University of California, San Francisco	Chiu Laboratory, University of California, San Francisco	Charles Chiu, Xianding (Wayne) Deng, Candace Wang, Venice Servellita, Jill Hacker, Debra Wadford
EPI_ISL_978409, EPI_ISL_978410	Arizona State Public Health Laboratory	Arizona State Public Health Laboratory	Trung Huynh, Jessica Escobar, Katherine Fullerton, Nobuko Fukushima, Stacy White, Linda Getsinger, Victor Waddell
EPI_ISL_979695, EPI_ISL_979698, EPI_ISL_979699, EPI_ISL_979700, EPI_ISL_979701, EPI_ISL_979702, EPI_ISL_979703, EPI_ISL_979704, EPI_ISL_979707	Orange County Public Health Lab	Chan-Zuckerberg Biohub	CZB Cliahub Consortium
EPI_ISL_981953	Microbiology Service, Hospital Universitario Clinico San Cecilio, Granada	Microbiology Service, Hospital Universitario Clinico San Cecilio, Granada	Adolfo de Salazar, Natalia Chueca, Laura Viñuela, Ana Fuentes, Federico García
EPI_ISL_982005, EPI_ISL_982072, EPI_ISL_982093, EPI_ISL_982102, EPI_ISL_982107	TGen North	Sonora Quest Laboratories	"Jolene Bowers, Megan Folkerts, Chris French, Hayley Yaglom, Ashlyn Pfeiffer, Darrin Lemmer, Dave Engelthaler, The Arizona COVID Genomics Union (ACGU)"
EPI_ISL_982369, EPI_ISL_982371, EPI_ISL_982372, EPI_ISL_982373, EPI_ISL_982375, EPI_ISL_982376, EPI_ISL_982377, EPI_ISL_982378, EPI_ISL_982379, EPI_ISL_982380, EPI_ISL_982381, EPI_ISL_982382, EPI_ISL_982383			
see above	M Health Fairview	Minnesota Department of Health, Public Health Laboratory	Alexandra Lorentz, Jacob Garfin, Matt Plumb, and Xiong Wang
EPI_ISL_982427, EPI_ISL_982428, EPI_ISL_982429, EPI_ISL_982430, EPI_ISL_982431, EPI_ISL_982432, EPI_ISL_982433, EPI_ISL_982434, EPI_ISL_982435, EPI_ISL_982440, EPI_ISL_982445, EPI_ISL_982446, EPI_ISL_982448, EPI_ISL_982456, EPI_ISL_982459, EPI_ISL_982462, EPI_ISL_982465, EPI_ISL_982467, EPI_ISL_982468, EPI_ISL_982470, EPI_ISL_982472, EPI_ISL_982478, EPI_ISL_982480, EPI_ISL_982481, EPI_ISL_982486, EPI_ISL_982487, EPI_ISL_982489, EPI_ISL_982492, EPI_ISL_982495			
see above	MONTEFIORE MEDICAL CENTER LABORATORIES	Wadsworth Center, New York State Department of Health	Kirsten St. George, Daryl M. Lamson, Alexis Russel, Matthew Shudt, Melissa A Leisner, Jonathan Plitnick, Navjot Singh, John Kelly, Erasmus Schneider, Erica Lasek-Nesselquist
EPI_ISL_982534, EPI_ISL_982542, EPI_ISL_982566, EPI_ISL_982571, EPI_ISL_982574, EPI_ISL_982575, EPI_ISL_982586, EPI_ISL_982592, EPI_ISL_982593, EPI_ISL_982594, EPI_ISL_982601, EPI_ISL_982602, EPI_ISL_982605, EPI_ISL_982606, EPI_ISL_982616, EPI_ISL_982650, EPI_ISL_982669, EPI_ISL_982678, EPI_ISL_982696, EPI_ISL_982697, EPI_ISL_982698, EPI_ISL_982699, EPI_ISL_982700, EPI_ISL_982701, EPI_ISL_982728, EPI_ISL_982736, EPI_ISL_982739, EPI_ISL_982741, EPI_ISL_982748, EPI_ISL_982749, EPI_ISL_982750, EPI_ISL_982759			
see above	US Air Force School of Aerospace Medicine	US Air Force School of Aerospace Medicine	Anthony Fries, Jennifer Meyer, William Gruner, William Buggele, Amanda Javorina, Sarah Purves, Clarise Starr, Elizabeth Macias
EPI_ISL_982854, EPI_ISL_982855, EPI_ISL_982856	Kentucky State Public Health Lab	Kentucky State Public Health Lab	Stephanie Lunn, Karim George, Joshua Tobias, William Grooms, Vaneet Arora, Matthew Johnson, Rachel Zinner, Rhonda Lucas
EPI_ISL_982857, EPI_ISL_982858, EPI_ISL_982859, EPI_ISL_983081	Gravity Diagnostics	Kentucky State Public Health Lab	Stephanie Lunn, Karim George, Joshua Tobias, William Grooms, Vaneet Arora, Matthew Johnson, Rachel Zinner, Rhonda Lucas
EPI_ISL_983099, EPI_ISL_983102, EPI_ISL_983111, EPI_ISL_983112, EPI_ISL_983132, EPI_ISL_983133, EPI_ISL_983135, EPI_ISL_983137, EPI_ISL_983140, EPI_ISL_983141, EPI_ISL_983147, EPI_ISL_983149, EPI_ISL_983156, EPI_ISL_983162, EPI_ISL_983163, EPI_ISL_983164, EPI_ISL_983167, EPI_ISL_983170			
see above	MONTEFIORE MEDICAL CENTER LABORATORIES	Wadsworth Center, New York State Department of Health	Kirsten St. George, Daryl M. Lamson, Alexis Russel, Matthew Shudt, Melissa A Leisner, Jonathan Plitnick, Navjot Singh, John Kelly, Erasmus Schneider, Erica Lasek-Nesselquist
EPI_ISL_983245	SUNY UPSTATE MEDICAL UNIVERSITY	Wadsworth Center, New York State Department of Health	Kirsten St. George, Daryl M. Lamson, Alexis Russel, Matthew Shudt, Melissa A Leisner, Jonathan Plitnick, Navjot Singh, John Kelly, Erasmus Schneider, Erica Lasek-Nesselquist
EPI_ISL_983247	KALEIDA CENTER FOR LABORATORY MEDICINE	Wadsworth Center, New York State Department of Health	Kirsten St. George, Daryl M. Lamson, Alexis Russel, Matthew Shudt, Melissa A Leisner, Jonathan Plitnick, Navjot Singh, John Kelly, Erasmus Schneider, Erica Lasek-Nesselquist
EPI_ISL_983248	SUNY UPSTATE MEDICAL UNIVERSITY	Wadsworth Center, New York State Department of Health	Kirsten St. George, Daryl M. Lamson, Alexis Russel, Matthew Shudt, Melissa A Leisner, Jonathan Plitnick, Navjot Singh, John Kelly, Erasmus Schneider, Erica Lasek-Nesselquist
EPI_ISL_983296, EPI_ISL_983297, EPI_ISL_983298, EPI_ISL_983299	KALEIDA CENTER FOR LABORATORY MEDICINE	Wadsworth Center, New York State Department of Health	Kirsten St. George, Daryl M. Lamson, Alexis Russel, Matthew Shudt, Melissa A Leisner, Jonathan Plitnick, Navjot Singh, John Kelly, Erasmus Schneider, Erica Lasek-Nesselquist
EPI_ISL_983300, EPI_ISL_983301, EPI_ISL_983302, EPI_ISL_983303, EPI_ISL_983304, EPI_ISL_983305, EPI_ISL_983306, EPI_ISL_983307, EPI_ISL_983308, EPI_ISL_983309, EPI_ISL_983310, EPI_ISL_983311, EPI_ISL_983312, EPI_ISL_983313, EPI_ISL_983314, EPI_ISL_983315, EPI_ISL_983316, EPI_ISL_983317, EPI_ISL_983318, EPI_ISL_983319			
see above	SUNY UPSTATE MEDICAL UNIVERSITY	Wadsworth Center, New York State Department of Health	Kirsten St. George, Daryl M. Lamson, Alexis Russel, Matthew Shudt, Melissa A Leisner, Jonathan Plitnick, Navjot Singh, John Kelly, Erasmus Schneider, Erica Lasek-Nesselquist

EPI_ISL_983375, EPI_ISL_983376, EPI_ISL_983377, EPI_ISL_983378, EPI_ISL_983379, EPI_ISL_983380, EPI_ISL_983381	Utah Public Health Laboratory	Utah Public Health Laboratory	Erin L. Young, Kelly F. Oakeson, Tara Gallagher
EPI_ISL_983826, EPI_ISL_983827, EPI_ISL_983828, EPI_ISL_983829, EPI_ISL_983830, EPI_ISL_983831, EPI_ISL_983832, EPI_ISL_983833, EPI_ISL_983834, EPI_ISL_983835	Colorado Department of Public Health and Environment	Colorado Department of Puplic Health and Environment	Laura Bankers, Molly C. Hetherington-Rauth, Diana Ir, Shannon Ely, Shannon R. Matzinger, Sarah Elizabeth Totten, Emily A. Travanty
