# Supplementary material for: Detection and characterization of the SARS-CoV-2 lineage B.1.526 in New York
Source: Nat Commun. 2021 Aug 9;12:4886. doi: 10.1038/s41467-021-25168-4 (PMC8352861; doi:10.1038/s41467-021-25168-4)
Supplement: Supplementary file 8 — Supplementary Data 4 [file 41467_2021_25168_MOESM8_ESM.zip › GISAID_acknowledements_tables/gisaid_hcov-19_acknowledgement_table_2021_02_12_16-3.pdf]

We gratefully acknowledge the following Authors from the Originating laboratories responsible for obtaining the specimens, as well as the Submitting laboratories where the genome data were generated and shared via GISAID, on which this research is based.

All Submitters of data may be contacted directly via www.gisaid.org

Authors are sorted alphabetically.

Accession ID	Originating Laboratory	Submitting Laboratory	Authors
EPI_ISL_415710	WHO National Influenza Centre Russian Federation	WHO National Influenza Centre Russian Federation	Andrey Komissarov, Artem Fadeev, Anna Ivanova, Daria Danilenko
EPI_ISL_416426	Virological Research Group, Szentágothai Research Centre, University of Pécs	Bioinformatics Research Group, Szentágothai Research Centre, University of Pécs	Péter Urbán, Endre Gábor Tóth, Gábor Kemenesi, Róbert Herczeg, Attila Gyenesei, Ferenc Jakab
EPI_ISL_416457	Andersen Lab, The Scripps Research Institute	Andersen Lab, The Scripps Research Institute	Mark Zeller, Catie Anderson, Emily Spender, Sarah Topol, Raphaelle Klitting, Refugio Robles-Sikisaka, Karthik Gangavarapu, Laura Nicholson, Kristian Andersen
EPI_ISL_416481	R. G. Lugar Center for Public Health Research, National Center for Disease Control and Public Health (NCDC) of Georgia.	R. G. Lugar Center for Public Health Research, National Center for Disease Control and Public Health (NCDC) of Georgia.	Gvantsa Chanturia, Marine Murtskhvaladze, Nato Kotaria, Ann Machablishvili, Lela Sabadze, Mari Gavashelidze, Ana Papkiauri, Meri Pantsulaia, Gvantsa Brachveli, Tata Imnadze, Tamar Jashiashvili, Tea Tevdoradze, Ketevan Sidamonidze, Ekaterine Khmaladze, Ekaterine Zhghenti, Roena Sukhiashvili, Mariam Zakalashvili, Lela Urushadze, Magda Dgebuadze, Giorgi Tomashvili, Davit Tsaguria, Ekaterine Zangaladze, Nino Berishvili, Adam Kotorashvili, Maia Alkhazashvili, Irma Burjanadze, Anna Kasradze, Khatuna Zakhashvili, Paata Imnadze, Amiran Gamkrelidze.
EPI_ISL_416489, EPI_ISL_416491, EPI_ISL_416492	University of Wisconsin-Madison AIDS Vaccine Research Laboratories	University of Wisconsin-Madison AIDS Vaccine Research Laboratories	Gage Moreno, Katarina Braun, et al. AIDS Vaccine Research Laboratories
EPI_ISL_416514, EPI_ISL_416515, EPI_ISL_416516, EPI_ISL_416517, EPI_ISL_416518	Victorian Infectious Diseases Reference Laboratory (VIDRL)	Victorian Infectious Diseases Reference Laboratory and Microbiological Diagnostic Unit Public Health Laboratory, Doherty Institute	Caly L., Seemann T., Schultz M., Taiaroa, G., Druce J.
EPI_ISL_416538	Wellington Hospital	Institute of Environmental Science and Research (ESR)	Wellington SCL, Wellington Hospital, Riddiford Street, Newtown, Wellington 6021, New Zealand
EPI_ISL_416539	Wellington Hospital	Institute of Environmental Science and Research (ESR)	Matt Storey, Xiaoyun Ren, Craig Thornley, Maxim Bloomfield, Erasmus Smit, Lauren Jelly, Joep de Ligt
EPI_ISL_416683, EPI_ISL_416685, EPI_ISL_416704, EPI_ISL_416711, EPI_ISL_416713, EPI_ISL_416715, EPI_ISL_416717	UW Virology Lab	UW Virology Lab	Pavitra Roychoudhury, Hong Xie, Keith Jerome, Alexander Greninger
EPI_ISL_416830, EPI_ISL_416831, EPI_ISL_416832	NYU Langone Health	Department of Pathology and Medicine, New York University School of Medicine	John Chen, Dacia Dimartino, Xiaojun Feng, Adriana Heguy, Megan Hogan, Emily Huang, George Jour, Christian Marier, Matt Maurano, Mark Mulligan, Peter Meyn, Marie Samanovic-Golden, Amy Rapkiewicz, Guomiao Shen, Matija Snuderl, Gael Westby, Paul Zappile
EPI_ISL_417020, EPI_ISL_417021, EPI_ISL_417022, EPI_ISL_417023, EPI_ISL_417025	Department of Clinical Microbiology	GIGA Medical Genomics	Durkin Keith, Artesi Maria, Bontems Sébastien, Boreux Raphaël, Meex Cécile, Melin Pierrette, Hayette Marie-Pierre, Bours Vincent.
EPI_ISL_417034	Laboratorio de Ecologia de Doencas Transmissiveis na Amazonia, Instituto Leonidas e Maria Deane - Fiocruz Amazonia	Laboratorio de Ecologia de Doencas Transmissiveis na Amazonia, Instituto Leonidas e Maria Deane - Fiocruz Amazonia	Valdinete Nascimento, André Corado, Fernanda Nascimento, Ágatha Costa, Debora Duarte, Luciana Gonçalves, Michele Jesus, Sérgio Luz, Felipe Naveca
EPI_ISL_417374, EPI_ISL_417375, EPI_ISL_417376, EPI_ISL_417377, EPI_ISL_417379, EPI_ISL_417382	UW Virology Lab	UW Virology Lab	Pavitra Roychoudhury, Hong Xie, Keith Jerome, Alexander Greninger
EPI_ISL_417408	Centre for Infectious Diseases and Microbiology Public Health	NSW Health Pathology - Institute of Clinical Pathology and Medical Research; Westmead Hospital; University of Sydney	Carter I, Rahman H, Holmes EC, O'Sullivan MV, Sintchenko V, Chen SC, Maddocks S, Kok J, Dwyer DE, Rockett R, Eden J-S, Lam C, Gray K, Timms V, Gall M, Arnott A and Sadsad R for the 2019-nCoV Study Group
EPI_ISL_417409	Centre for Infectious Diseases and Microbiology Public Health	NSW Health Pathology - Institute of Clinical Pathology and Medical Research; Westmead Hospital; University of Sydney	Rahman H, Holmes EC, O'Sullivan MV, Sintchenko V, Chen SC, Maddocks S, Kok J, Dwyer DE, Rockett R, Eden J-S, Lam C, Gray K, Timms V, Gall M, Arnott A, Sadsad R and Carter I for the 2019-nCoV Study Group
EPI_ISL_417410	Centre for Infectious Diseases and Microbiology Public Health	NSW Health Pathology - Institute of Clinical Pathology and Medical Research; Westmead Hospital; University of Sydney	Holmes EC, O'Sullivan MV, Sintchenko V, Chen SC, Maddocks S, Kok J, Dwyer DE, Rockett R, Eden J-S, Lam C, Gray K, Timms V, Gall M, Arnott A, Sadsad R, Carter I and Rahman H for the 2019-nCoV Study Group
EPI_ISL_417411	Centre for Infectious Diseases and Microbiology Public Health	NSW Health Pathology - Institute of Clinical Pathology and Medical Research; Westmead Hospital; University of Sydney	O'Sullivan MV, Sintchenko V, Chen SC, Maddocks S, Kok J, Dwyer DE, Rockett R, Eden J-S, Lam C, Gray K, Timms V, Gall M, Arnott A, Sadsad R, Carter I, Rahman H and Holmes EC for the 2019-nCoV Study Group
EPI_ISL_417412	Centre for Infectious Diseases and Microbiology Public Health	NSW Health Pathology - Institute of Clinical Pathology and Medical Research; Westmead Hospital; University of Sydney	Sintchenko V, Chen SC, Maddocks S, Kok J, Dwyer DE, Rockett R, Eden J-S, Lam C, Gray K, Timms V, Gall M, Arnott A, Sadsad R, Carter I, Rahman H, Holmes EC and O'Sullivan MV for the 2019-nCoV Study Group
EPI_ISL_417413	Ministry of Health Turkey	Ministry of Health Turkey	Fatma Bayrakdar,Aye Baak Alta,Yasemin Cogun,Gülay Korukluolu,Selçuk Klç
EPI_ISL_417435, EPI_ISL_417436, EPI_ISL_417437, EPI_ISL_417438, EPI_ISL_417439, EPI_ISL_417440, EPI_ISL_417441, EPI_ISL_417442	Viral Respiratory Lab, National Institute for Biomedical Research (INRB)	Pathogen Sequencing Lab, National Institute for Biomedical Research (INRB)	Placide Mbala-Kingebeni, Edith Nkwembe, Eddy Kinganda-Lusamaki, Amuri Aziza, Catherine Pratt, Matthias Pauthner, Josh Quick, Allison Black, James Hadfield, Trevor Bedford, Ian Goodfellow, Nick Loman, Kristian Andersen, Michael Wiley, Steve Ahuka-Mundeke, Jean-Jacques Muyembe Tamfum
EPI_ISL_417467, EPI_ISL_417468	Center of Medical Microbiology, Virology, and Hospital Hygiene, University of Duesseldorf	Center of Medical Microbiology, Virology, and Hospital Hygiene, University of Duesseldorf	Ortwin Adams, Marcel Andree, Alexander Dilthey, Torsten Feldt, Sandra Hauka, Torsten Houwaart, Björn-Erik Jensen, Detlef Kindgen-Milles, Malte Kohns Vasconcelos, Klaus Pfeffer, Tina Senff, Daniel Strelow, Jörg Timm, Andreas Walker, Tobias Wienemann
EPI_ISL_417504, EPI_ISL_417505, EPI_ISL_417506, EPI_ISL_417507, EPI_ISL_417508, EPI_ISL_417510, EPI_ISL_417512, EPI_ISL_417513, EPI_ISL_417515, EPI_ISL_417516	University of Wisconsin-Madison AIDS Vaccine Research Laboratories	University of Wisconsin-Madison AIDS Vaccine Research Laboratories	Gage Moreno, Katarina Braun, et al. AIDS Vaccine Research Laboratories
EPI_ISL_417526, EPI_ISL_417527, EPI_ISL_417528, EPI_ISL_417529, EPI_ISL_417530, EPI_ISL_417531, EPI_ISL_417532, EPI_ISL_417533, EPI_ISL_417534	Laboratoire Nationale de Santé, Microbiology, Virology	Laboratoire Nationale de Santé, Microbiology, Epidemiology and Microbial Genomics	Anke Wienecke-Baldacchino, Ardashel Latsuzbaia, Jessica Tapp, Catherine Ragimbeau, Guillaume Fournier, Tamir Abdelrahman, Trung Nguyen Nguyen, Joel Mossong
EPI_ISL_417536, EPI_ISL_417537, EPI_ISL_417538, EPI_ISL_417539, EPI_ISL_417540, EPI_ISL_417541, EPI_ISL_417542, EPI_ISL_417543, EPI_ISL_417544, EPI_ISL_417545, EPI_ISL_417546, EPI_ISL_417547, EPI_ISL_417548			
see above	deCODE genetics	deCODE genetics	Daniel F Gudbjartsson; Agnar Helgason; Hakon Jonsson; Olafur T Magnusson; Pall Melsted; Gudmundur L Norddahl; Jona Saemundsdottir; Asgeir Sigurdsson; Patrick Sulem; Arna B Agustsdottir; Berglind Eiriksdottir; Run Fridriksdottir; Elisabet E Gardarsdottir; Gudmundur Georgsson; Olafia S Gretarsdottir; Kjartan R Gudmundsson; Thora R Gunnarsdottir; Arnaldur Gylfason; Hilma Holm; Brynjar O Jensson; Aslaug Jonasdottir; Kamilla S Josefsdottir; Thordur Kristjansson; Droplaug N Magnusdottir; Louise le Roux; Gudrun Sigmundsdottir; Gardar Sveinbjornsson; Kristin E Sveinsdottir; Maney

			Sveinsdottir; Emil A Thorarensen; Bjarni Thorbjornsson; Gisli Masson; Ingileif Jonsdottir; Alma Moller; Thorolfur Gudnason; Karl G Kristinsson; Unnur Thorsteinsdottir; Kari Stefansson
EPI_ISL_417550, EPI_ISL_417551	The National University Hospital of Iceland	deCODE genetics	Daniel F Gudbjartsson; Agnar Helgason; Hakon Jonsson; Olafur T Magnusson; Pall Melsted; Gudmundur L Norddahl; Jona Saemundsdottir; Asgeir Sigurdsson; Patrick Sulem; Arna B Agustsdottir; Berglind Eiriksdottir; Run Fridriksdottir; Elisabet E Gardarsdottir; Gudmundur Georgsson; Olafia S Gretarsdottir; Kjartan R Gudmundsson; Thora R Gunnarsdottir; Arnaldur Gylfason; Hilma Holm; Brynjar O Jensson; Aslaug Jonasdottir; Kamilla S Josefsdottir; Thordur Kristjansson; Droplaug N Magnusdottir; Louise le Roux; Gudrun Sigmundsdottir; Gardar Sveinbjornsson; Kristin E Sveinsdottir; Maney Sveinsdottir; Emil A Thorarensen; Bjarni Thorbjornsson; Gisli Masson; Ingileif Jonsdottir; Alma Moller; Thorolfur Gudnason; Karl G Kristinsson; Unnur Thorsteinsdottir; Kari Stefansson
EPI_ISL_417552	deCODE genetics	deCODE genetics	Daniel F Gudbjartsson; Agnar Helgason; Hakon Jonsson; Olafur T Magnusson; Pall Melsted; Gudmundur L Norddahl; Jona Saemundsdottir; Asgeir Sigurdsson; Patrick Sulem; Arna B Agustsdottir; Berglind Eiriksdottir; Run Fridriksdottir; Elisabet E Gardarsdottir; Gudmundur Georgsson; Olafia S Gretarsdottir; Kjartan R Gudmundsson; Thora R Gunnarsdottir; Arnaldur Gylfason; Hilma Holm; Brynjar O Jensson; Aslaug Jonasdottir; Kamilla S Josefsdottir; Thordur Kristjansson; Droplaug N Magnusdottir; Louise le Roux; Gudrun Sigmundsdottir; Gardar Sveinbjornsson; Kristin E Sveinsdottir; Maney Sveinsdottir; Emil A Thorarensen; Bjarni Thorbjornsson; Gisli Masson; Ingileif Jonsdottir; Alma Moller; Thorolfur Gudnason; Karl G Kristinsson; Unnur Thorsteinsdottir; Kari Stefansson
EPI_ISL_417553, EPI_ISL_417554, EPI_ISL_417555, EPI_ISL_417556, EPI_ISL_417557, EPI_ISL_417558, EPI_ISL_417559, EPI_ISL_417560, EPI_ISL_417561, EPI_ISL_417562, EPI_ISL_417563, EPI_ISL_417564, EPI_ISL_417565, EPI_ISL_417566, EPI_ISL_417567, EPI_ISL_417568, EPI_ISL_417569, EPI_ISL_417570, EPI_ISL_417571, EPI_ISL_417572, EPI_ISL_417573, EPI_ISL_417574, EPI_ISL_417575, EPI_ISL_417576, EPI_ISL_417577, EPI_ISL_417578, EPI_ISL_417579, EPI_ISL_417580, EPI_ISL_417581, EPI_ISL_417582, EPI_ISL_417583, EPI_ISL_417584, EPI_ISL_417585, EPI_ISL_417586, EPI_ISL_417587, EPI_ISL_417588, EPI_ISL_417589			
see above	The National University Hospital of Iceland	deCODE genetics	Daniel F Gudbjartsson; Agnar Helgason; Hakon Jonsson; Olafur T Magnusson; Pall Melsted; Gudmundur L Norddahl; Jona Saemundsdottir; Asgeir Sigurdsson; Patrick Sulem; Arna B Agustsdottir; Berglind Eiriksdottir; Run Fridriksdottir; Elisabet E Gardarsdottir; Gudmundur Georgsson; Olafia S Gretarsdottir; Kjartan R Gudmundsson; Thora R Gunnarsdottir; Arnaldur Gylfason; Hilma Holm; Brynjar O Jensson; Aslaug Jonasdottir; Kamilla S Josefsdottir; Thordur Kristjansson; Droplaug N Magnusdottir; Louise le Roux; Gudrun Sigmundsdottir; Gardar Sveinbjornsson; Kristin E Sveinsdottir; Maney Sveinsdottir; Emil A Thorarensen; Bjarni Thorbjornsson; Gisli Masson; Ingileif Jonsdottir; Alma Moller; Thorolfur Gudnason; Karl G Kristinsson; Unnur Thorsteinsdottir; Kari Stefansson
EPI_ISL_417590	deCODE genetics	deCODE genetics	Daniel F Gudbjartsson; Agnar Helgason; Hakon Jonsson; Olafur T Magnusson; Pall Melsted; Gudmundur L Norddahl; Jona Saemundsdottir; Asgeir Sigurdsson; Patrick Sulem; Arna B Agustsdottir; Berglind Eiriksdottir; Run Fridriksdottir; Elisabet E Gardarsdottir; Gudmundur Georgsson; Olafia S Gretarsdottir; Kjartan R Gudmundsson; Thora R Gunnarsdottir; Arnaldur Gylfason; Hilma Holm; Brynjar O Jensson; Aslaug Jonasdottir; Kamilla S Josefsdottir; Thordur Kristjansson; Droplaug N Magnusdottir; Louise le Roux; Gudrun Sigmundsdottir; Gardar Sveinbjornsson; Kristin E Sveinsdottir; Maney Sveinsdottir; Emil A Thorarensen; Bjarni Thorbjornsson; Gisli Masson; Ingileif Jonsdottir; Alma Moller; Thorolfur Gudnason; Karl G Kristinsson; Unnur Thorsteinsdottir; Kari Stefansson
EPI_ISL_417591, EPI_ISL_417592, EPI_ISL_417593, EPI_ISL_417594, EPI_ISL_417595, EPI_ISL_417596, EPI_ISL_417597, EPI_ISL_417598, EPI_ISL_417599, EPI_ISL_417600, EPI_ISL_417601, EPI_ISL_417602, EPI_ISL_417603, EPI_ISL_417604, EPI_ISL_417605, EPI_ISL_417606, EPI_ISL_417607, EPI_ISL_417608, EPI_ISL_417609, EPI_ISL_417610, EPI_ISL_417611, EPI_ISL_417612, EPI_ISL_417613, EPI_ISL_417614, EPI_ISL_417615, EPI_ISL_417616, EPI_ISL_417617			
see above	The National University Hospital of Iceland	deCODE genetics	Daniel F Gudbjartsson; Agnar Helgason; Hakon Jonsson; Olafur T Magnusson; Pall Melsted; Gudmundur L Norddahl; Jona Saemundsdottir; Asgeir Sigurdsson; Patrick Sulem; Arna B Agustsdottir; Berglind Eiriksdottir; Run Fridriksdottir; Elisabet E Gardarsdottir; Gudmundur Georgsson; Olafia S Gretarsdottir; Kjartan R Gudmundsson; Thora R Gunnarsdottir; Arnaldur Gylfason; Hilma Holm; Brynjar O Jensson; Aslaug Jonasdottir; Kamilla S Josefsdottir; Thordur Kristjansson; Droplaug N Magnusdottir; Louise le Roux; Gudrun Sigmundsdottir; Gardar Sveinbjornsson; Kristin E Sveinsdottir; Maney Sveinsdottir; Emil A Thorarensen; Bjarni Thorbjornsson; Gisli Masson; Ingileif Jonsdottir; Alma Moller; Thorolfur Gudnason; Karl G Kristinsson; Unnur Thorsteinsdottir; Kari Stefansson
EPI_ISL_417618	deCODE genetics	deCODE genetics	Daniel F Gudbjartsson; Agnar Helgason; Hakon Jonsson; Olafur T Magnusson; Pall Melsted; Gudmundur L Norddahl; Jona Saemundsdottir; Asgeir Sigurdsson; Patrick Sulem; Arna B Agustsdottir; Berglind Eiriksdottir; Run Fridriksdottir; Elisabet E Gardarsdottir; Gudmundur Georgsson; Olafia S Gretarsdottir; Kjartan R Gudmundsson; Thora R Gunnarsdottir; Arnaldur Gylfason; Hilma Holm; Brynjar O Jensson; Aslaug Jonasdottir; Kamilla S Josefsdottir; Thordur Kristjansson; Droplaug N Magnusdottir; Louise le Roux; Gudrun Sigmundsdottir; Gardar Sveinbjornsson; Kristin E Sveinsdottir; Maney Sveinsdottir; Emil A Thorarensen; Bjarni Thorbjornsson; Gisli Masson; Ingileif Jonsdottir; Alma Moller; Thorolfur Gudnason; Karl G Kristinsson; Unnur Thorsteinsdottir; Kari Stefansson
EPI_ISL_417619, EPI_ISL_417620, EPI_ISL_417621, EPI_ISL_417622, EPI_ISL_417623, EPI_ISL_417624, EPI_ISL_417625, EPI_ISL_417626, EPI_ISL_417627, EPI_ISL_417628, EPI_ISL_417629, EPI_ISL_417630, EPI_ISL_417631, EPI_ISL_417632, EPI_ISL_417633, EPI_ISL_417634, EPI_ISL_417635, EPI_ISL_417636, EPI_ISL_417637, EPI_ISL_417638, EPI_ISL_417639, EPI_ISL_417640, EPI_ISL_417641, EPI_ISL_417642, EPI_ISL_417643, EPI_ISL_417644, EPI_ISL_417645, EPI_ISL_417646, EPI_ISL_417647, EPI_ISL_417648, EPI_ISL_417649, EPI_ISL_417650, EPI_ISL_417651, EPI_ISL_417652, EPI_ISL_417653, EPI_ISL_417654			
see above	The National University Hospital of Iceland	deCODE genetics	Daniel F Gudbjartsson; Agnar Helgason; Hakon Jonsson; Olafur T Magnusson; Pall Melsted; Gudmundur L Norddahl; Jona Saemundsdottir; Asgeir Sigurdsson; Patrick Sulem; Arna B Agustsdottir; Berglind Eiriksdottir; Run Fridriksdottir; Elisabet E Gardarsdottir; Gudmundur Georgsson; Olafia S Gretarsdottir; Kjartan R Gudmundsson; Thora R Gunnarsdottir; Arnaldur Gylfason; Hilma Holm; Brynjar O Jensson; Aslaug Jonasdottir; Kamilla S Josefsdottir; Thordur Kristjansson; Droplaug N Magnusdottir; Louise le Roux; Gudrun Sigmundsdottir; Gardar Sveinbjornsson; Kristin E Sveinsdottir; Maney Sveinsdottir; Emil A Thorarensen; Bjarni Thorbjornsson; Gisli Masson; Ingileif Jonsdottir; Alma Moller; Thorolfur Gudnason; Karl G Kristinsson; Unnur Thorsteinsdottir; Kari Stefansson
EPI_ISL_417666, EPI_ISL_417667, EPI_ISL_417668, EPI_ISL_417669, EPI_ISL_417670, EPI_ISL_417671, EPI_ISL_417672, EPI_ISL_417676	deCODE genetics	deCODE genetics	Daniel F Gudbjartsson; Agnar Helgason; Hakon Jonsson; Olafur T Magnusson; Pall Melsted; Gudmundur L Norddahl; Jona Saemundsdottir; Asgeir Sigurdsson; Patrick Sulem; Arna B Agustsdottir; Berglind Eiriksdottir; Run Fridriksdottir; Elisabet E Gardarsdottir; Gudmundur Georgsson; Olafia S Gretarsdottir; Kjartan R Gudmundsson; Thora R Gunnarsdottir; Arnaldur Gylfason; Hilma Holm; Brynjar O Jensson; Aslaug Jonasdottir; Kamilla S Josefsdottir; Thordur Kristjansson; Droplaug N Magnusdottir; Louise le Roux; Gudrun Sigmundsdottir; Gardar Sveinbjornsson; Kristin E Sveinsdottir; Maney Sveinsdottir; Emil A Thorarensen; Bjarni Thorbjornsson; Gisli Masson; Ingileif Jonsdottir; Alma Moller; Thorolfur Gudnason; Karl G Kristinsson; Unnur Thorsteinsdottir; Kari Stefansson
EPI_ISL_417678, EPI_ISL_417680, EPI_ISL_417685, EPI_ISL_417699, EPI_ISL_417700, EPI_ISL_417703, EPI_ISL_417706, EPI_ISL_417709, EPI_ISL_417712, EPI_ISL_417716, EPI_ISL_417717, EPI_ISL_417724, EPI_ISL_417733, EPI_ISL_417737, EPI_ISL_417740, EPI_ISL_417742, EPI_ISL_417743, EPI_ISL_417746, EPI_ISL_417750, EPI_ISL_417752, EPI_ISL_417753, EPI_ISL_417754, EPI_ISL_417762, EPI_ISL_417763, EPI_ISL_417764, EPI_ISL_417766, EPI_ISL_417774, EPI_ISL_417808, EPI_ISL_417809, EPI_ISL_417813, EPI_ISL_417814, EPI_ISL_417815, EPI_ISL_417816, EPI_ISL_417818, EPI_ISL_417819, EPI_ISL_417820, EPI_ISL_417821, EPI_ISL_417822, EPI_ISL_417823, EPI_ISL_417824, EPI_ISL_417825, EPI_ISL_417826, EPI_ISL_417827, EPI_ISL_417829, EPI_ISL_417830, EPI_ISL_417831, EPI_ISL_417832, EPI_ISL_417833, EPI_ISL_417834, EPI_ISL_417835, EPI_ISL_417836, EPI_ISL_417837, EPI_ISL_417838, EPI_ISL_417839, EPI_ISL_417864			
see above	The National University Hospital of Iceland	deCODE genetics	Daniel F Gudbjartsson; Agnar Helgason; Hakon Jonsson; Olafur T Magnusson; Pall Melsted; Gudmundur L Norddahl; Jona Saemundsdottir; Asgeir Sigurdsson; Patrick Sulem; Arna B Agustsdottir; Berglind Eiriksdottir; Run Fridriksdottir; Elisabet E Gardarsdottir; Gudmundur Georgsson; Olafia S Gretarsdottir; Kjartan R Gudmundsson; Thora R Gunnarsdottir; Arnaldur Gylfason; Hilma Holm; Brynjar O Jensson; Aslaug Jonasdottir; Kamilla S Josefsdottir; Thordur Kristjansson; Droplaug N Magnusdottir; Louise le Roux; Gudrun Sigmundsdottir; Gardar Sveinbjornsson; Kristin E Sveinsdottir; Maney Sveinsdottir; Emil A Thorarensen; Bjarni Thorbjornsson; Gisli Masson; Ingileif Jonsdottir; Alma Moller; Thorolfur Gudnason; Karl G Kristinsson; Unnur Thorsteinsdottir; Kari Stefansson
EPI_ISL_417918	Department of Medical Microbiology, University Malaya Medical Centre	Department of Medical Microbiology, Faculty of Medicine, University of Malaya	Yoong Min CHONG, Sasheela PONNAMPALAVANAR, Sharifah Faridah SYED OMAR, Adeeba KAMARULZAMAN,Vijayan MUNUSAMY, Chee Kuan WONG, Cindy Shuan Ju TEH, I-Ching SAM, Yoke Fun Chan, University Malaya Medical Centre COVID Team
EPI_ISL_417925	Laboratório Simili	Bioinformatics Laboratory / LNCC	Filipe Romero, Ana Paula Guimarães, Mariane Talon, Luiz Gonzaga Paula de Almeida, Ronaldo da Silva, Francisco Junior, Diana Mariani, Lídia Boullosa, Alexandra Gerber, Jaqueline Goes de Jesus, Ingra Morales Claro, Ester Cerdeira Sabino, Nuno Rodrigues Faria, Terezinha Marta Pereira, Pinto Castiñeiras, Isabela de Carvalho Leitão, Rafael de Mello Galliez, Cássia Cristina Alves Gonçalves, Érica Ramos dos Santos Nascimento, Richard Araújo Maia, Mauro Teixeira,Cristiano Xavier Lima, Orlando Ferreira Jr., Rodrigo Brindeiro, Luciana Jesus Costa e André Felipe Santos, Laboratorio Hermes Pardini, Laboratorio Simile, Amilcar Tanuri, Renato Santana Aguiar e Ana Tereza Vasconcelos
EPI_ISL_417926	Laboratório Simili	Bioinformatics Laboratory / LNCC	Filipe Romero, Ana Paula Guimarães, Mariane Talon, Luiz Gonzaga Paula de Almeida, Ronaldo da Silva Francisco Junior, Diana Mariani, Lídia Boullosa, Alexandra Gerber, Jaqueline Goes de Jesus, Ingra Morales Claro, Ester Cerdeira Sabino, Nuno Rodrigues Faria, Terezinha Marta Pereira, Pinto Castiñeiras, Isabela de Carvalho Leitão, Rafael de Mello Galliez, Cássia Cristina Alves Gonçalves, Érica Ramos dos Santos Nascimento, Richard Araújo Maia, Mauro Teixeira,Cristiano Xavier Lima, Orlando Ferreira Jr., Rodrigo Brindeiro, Luciana Jesus Costa e André Felipe Santos, Laboratorio Hermes Pardini, Laboratorio Simile, Amilcar Tanuri, Renato Santana Aguiar e Ana Tereza Vasconcelos

EPI_ISL_417931, EPI_ISL_417932, EPI_ISL_417933, EPI_ISL_417935	UCSF Clinical Microbiology Laboratory	Chan-Zuckerberg Biohub	Shaun Arevalo, Josh Batson, Olga Botvinnik, Gloria Castaneda, Angela Detweiler, David Dynerman, Samantha Hao, Jack Kamm, Amy Kistler, G. Renuka Kumar, Chaz Langelier, Lucy Li, Steve Miller, Lusajo Mwakibete, Norma Neff, Angela Pisco, Maira Phelps, Michelle Tan, Chunyu Zhao
EPI_ISL_417936	Laboratório Hermes Pardini	Bioinformatics Laboratory - LNCC	Filipe Romero, Ana Paula Guimarães, Mariane Talon, Luiz Gonzaga Paula de Almeida, Ronaldo da Silva Francisco Junior, Diana Mariani, Lídia Boullosa, Alexandra Gerber, Jaqueline Goes de Jesus, Ingra Morales Claro, Ester Cerdeira Sabino, Nuno Rodrigues Faria, Terezinha Marta Pereira, Pinto Castiñeiras, Isabela de Carvalho Leitão, Rafael de Mello Galliez, Cássia Cristina Alves Gonçalves, Érica Ramos dos Santos Nascimento, Richard Araújo Maia, Mauro Teixeira,Cristiano Xavier Lima, Orlando Ferreira Jr., Rodrigo Brindeiro, Luciana Jesus Costa e André Felipe Santos, Laboratorio Hermes Pardini, Laboratorio Simile, Amilcar Tanuri, Renato Santana Aguiar e Ana Tereza Vasconcelos
EPI_ISL_417937, EPI_ISL_417938, EPI_ISL_417939	UCSF Clinical Microbiology Laboratory	Chan-Zuckerberg Biohub	Shaun Arevalo, Josh Batson, Olga Botvinnik, Gloria Castaneda, Angela Detweiler, David Dynerman, Samantha Hao, Jack Kamm, Amy Kistler, G. Renuka Kumar, Chaz Langelier, Lucy Li, Steve Miller, Lusajo Mwakibete, Norma Neff, Angela Pisco, Maira Phelps, Michelle Tan, Chunyu Zhao
EPI_ISL_417940	Laboratório Hermes Pardini	Bioinformatics Laboratory - LNCC	Filipe Romero, Ana Paula Guimarães, Mariane Talon, Luiz Gonzaga Paula de Almeida, Ronaldo da Silva Francisco Junior, Diana Mariani, Lídia Boullosa,Alexandra Gerber, Jaqueline Goes de Jesus, Ingra Morales Claro, Ester Cerdeira Sabino, Nuno Rodrigues Faria, Terezinha Marta Pereira, Pinto Castiñeiras, Isabela de Carvalho Leitão, Rafael de Mello Galliez, Cássia Alves Gonçalves, Érica Ramos dos Santos Nascimento, Richard Araújo Maia, Mauro Teixeira,Cristiano Xavier Lima, Orlando Ferreira Jr., Rodrigo Brindeiro, Luciana Jesus Costa e André Felipe Santos, Laboratorio Hermes Pardini, Laboratorio Simile, Amilcar Tanuri, Renato Santana Aguiar e Ana Tereza Vasconcelos
EPI_ISL_417941, EPI_ISL_417942	Viral Respiratory Lab, National Institute for Biomedical Research (INRB)	Pathogen Sequencing Lab, National Institute for Biomedical Research (INRB)	Placide Mbala-Kingebeni, Edith Nkwembe, Eddy Kinganda-Lusamaki, Amuri Aziza, Catherine Pratt, Matthias Pauthner, Josh Quick, Allison Black, James Hadfield, Trevor Bedford, Ian Goodfellow, Nick Loman, Kristian Andersen, Michael Wiley, Steve Ahuka-Mundeke, Jean-Jacques Muyembe Tamfum
EPI_ISL_417943	Laboratório Hermes Pardini	Bioinformatics Laboratory	Filipe Romero, Ana Paula Guimarães, Mariane Talon, Luiz Gonzaga Paula de Almeida, Ronaldo da Silva Francisco Junior, Diana Mariani, Lídia Boullosa,Alexandra Gerber, Jaqueline Goes de Jesus, Ingra Morales Claro, Ester Cerdeira Sabino, Nuno Rodrigues Faria, Terezinha Marta Pereira, Pinto Castiñeiras, Isabela de Carvalho Leitão, Rafael de Mello Galliez, Cássia Alves Gonçalves, Érica Ramos dos Santos Nascimento, Richard Araújo Maia, Mauro Teixeira,Cristiano Xavier Lima, Orlando Ferreira Jr., Rodrigo Brindeiro, Luciana Jesus Costa e André Felipe Santos, Laboratorio Hermes Pardini, Laboratorio Simile, Amilcar Tanuri, Renato Santana Aguiar e Ana Tereza Vasconcelos
EPI_ISL_417944	Viral Respiratory Lab, National Institute for Biomedical Research (INRB)	Pathogen Sequencing Lab, National Institute for Biomedical Research (INRB)	Placide Mbala-Kingebeni, Edith Nkwembe, Eddy Kinganda-Lusamaki, Amuri Aziza, Catherine Pratt, Matthias Pauthner, Josh Quick, Allison Black, James Hadfield, Trevor Bedford, Ian Goodfellow, Nick Loman, Kristian Andersen, Michael Wiley, Steve Ahuka-Mundeke, Jean-Jacques Muyembe Tamfum
EPI_ISL_417945	Laboratório Hermes Pardini	Bioinformatics Laboratory - LNCC	Filipe Romero, Ana Paula Guimarães, Mariane Talon, Luiz Gonzaga Paula de Almeida, Ronaldo da Silva Francisco Junior, Diana Mariani, Lídia Boullosa,Alexandra Gerber, Jaqueline Goes de Jesus, Ingra Morales Claro, Ester Cerdeira Sabino, Nuno Rodrigues Faria, Terezinha Marta Pereira, Pinto Castiñeiras, Isabela de Carvalho Leitão, Rafael de Mello Galliez, Cássia Alves Gonçalves, Érica Ramos dos Santos Nascimento, Richard Araújo Maia, Mauro Teixeira,Cristiano Xavier Lima, Orlando Ferreira Jr., Rodrigo Brindeiro, Luciana Jesus Costa e André Felipe Santos, Laboratorio Hermes Pardini, Laboratorio Simile, Amilcar Tanuri, Renato Santana Aguiar e Ana Tereza Vasconcelos
EPI_ISL_417946	Viral Respiratory Lab, National Institute for Biomedical Research (INRB)	Pathogen Sequencing Lab, National Institute for Biomedical Research (INRB)	Placide Mbala-Kingebeni, Edith Nkwembe, Eddy Kinganda-Lusamaki, Amuri Aziza, Catherine Pratt, Matthias Pauthner, Josh Quick, Allison Black, James Hadfield, Trevor Bedford, Ian Goodfellow, Nick Loman, Kristian Andersen, Michael Wiley, Steve Ahuka-Mundeke, Jean-Jacques Muyembe Tamfum
EPI_ISL_417949	Laboratório Hermes Pardini	Bioinformatics Laboratory - LNCC	Filipe Romero, Ana Paula Guimarães, Mariane Talon, Luiz Gonzaga Paula de Almeida, Ronaldo da Silva Francisco Junior, Diana Mariani, Lídia Boullosa,Alexandra Gerber, Jaqueline Goes de Jesus, Ingra Morales Claro, Ester Cerdeira Sabino, Nuno Rodrigues Faria, Terezinha Marta Pereira, Pinto Castiñeiras, Isabela de Carvalho Leitão, Rafael de Mello Galliez, Cássia Alves Gonçalves, Érica Ramos dos Santos Nascimento, Richard Araújo Maia, Mauro Teixeira,Cristiano Xavier Lima, Orlando Ferreira Jr., Rodrigo Brindeiro, Luciana Jesus Costa e André Felipe Santos, Laboratorio Hermes Pardini, Laboratorio Simile, Amilcar Tanuri, Renato Santana Aguiar e Ana Tereza Vasconcelos
EPI_ISL_417951, EPI_ISL_417953	Universidade Federal do Rio de Janeiro	Bioinformatics Laboratory - LNCC	Filipe Romero, Ana Paula Guimarães, Mariane Talon, Luiz Gonzaga Paula de Almeida, Ronaldo da Silva Francisco Junior, Diana Mariani, Lídia Boullosa,Alexandra Gerber, Jaqueline Goes de Jesus, Ingra Morales Claro, Ester Cerdeira Sabino, Nuno Rodrigues Faria, Terezinha Marta Pereira, Pinto Castiñeiras, Isabela de Carvalho Leitão, Rafael de Mello Galliez, Cássia Alves Gonçalves, Érica Ramos dos Santos Nascimento, Richard Araújo Maia, Mauro Teixeira,Cristiano Xavier Lima, Orlando Ferreira Jr., Rodrigo Brindeiro, Luciana Jesus Costa e André Felipe Santos, Laboratorio Hermes Pardini, Laboratorio Simile, Amilcar Tanuri, Renato Santana Aguiar e Ana Tereza Vasconcelos
EPI_ISL_417970	Utah Public Health Laboratory	Utah Public Health Laboratory	Erin Young, Kelly Oakeson
EPI_ISL_418009	HSE Ilha Terceira - Angra do Heroismo	Instituto Nacional de Saude (INSA)	Guiomar et al
EPI_ISL_418017	CHMT	Instituto Nacional de Saude (INSA)	Guiomar et al
EPI_ISL_418018	H Garcia de Orta	Instituto Nacional de Saude (INSA)	Guiomar et al
EPI_ISL_418019, EPI_ISL_418020, EPI_ISL_418021, EPI_ISL_418022	H Braga	Instituto Nacional de Saude (INSA)	Guiomar et al
EPI_ISL_418023	H Evora	Instituto Nacional de Saude (INSA)	Guiomar et al
EPI_ISL_418024	CHUA - Faro	Instituto Nacional de Saude (INSA)	Guiomar et al
EPI_ISL_418025	H Santarem	Instituto Nacional de Saude (INSA)	Guiomar et al
EPI_ISL_418026	H Dr. Nelio Mendonca - Funchal	Instituto Nacional de Saude (INSA)	Guiomar et al
EPI_ISL_418027	CHTMAD	Instituto Nacional de Saude (INSA)	Guiomar et al
EPI_ISL_418029, EPI_ISL_418030, EPI_ISL_418031, EPI_ISL_418032, EPI_ISL_418033, EPI_ISL_418034, EPI_ISL_418037, EPI_ISL_418038, EPI_ISL_418040, EPI_ISL_418046, EPI_ISL_418047, EPI_ISL_418048, EPI_ISL_418050, EPI_ISL_418052, EPI_ISL_418053, EPI_ISL_418054, EPI_ISL_418063, EPI_ISL_418064, EPI_ISL_418067, EPI_ISL_418071, EPI_ISL_418072, EPI_ISL_418073, EPI_ISL_418074, EPI_ISL_418075, EPI_ISL_418076, EPI_ISL_418077, EPI_ISL_418078, EPI_ISL_418079, EPI_ISL_418080, EPI_ISL_418081, EPI_ISL_418082			
see above	UW Virology Lab	UW Virology Lab	Pavitra Roychoudhury, Hong Xie, Keith Jerome, Alexander Greninger
EPI_ISL_418101, EPI_ISL_418102, EPI_ISL_418103, EPI_ISL_418104, EPI_ISL_418105, EPI_ISL_418126, EPI_ISL_418127, EPI_ISL_418128, EPI_ISL_418129, EPI_ISL_418130, EPI_ISL_418131, EPI_ISL_418132, EPI_ISL_418133, EPI_ISL_418134, EPI_ISL_418135, EPI_ISL_418136, EPI_ISL_418137, EPI_ISL_418138, EPI_ISL_418139, EPI_ISL_418140, EPI_ISL_418148, EPI_ISL_418149, EPI_ISL_418150, EPI_ISL_418151, EPI_ISL_418152, EPI_ISL_418153, EPI_ISL_418154, EPI_ISL_418155, EPI_ISL_418156, EPI_ISL_418157, EPI_ISL_418158, EPI_ISL_418159, EPI_ISL_418160, EPI_ISL_418161, EPI_ISL_418162, EPI_ISL_418163, EPI_ISL_418164, EPI_ISL_418165			
see above	Wales Specialist Virology Centre	Public Health Wales Microbiology Cardiff	Catherine Moore, Joanne Watkins, Sally Corden, Sara Rey, Matt Bull, Tom Connor
EPI_ISL_418183	Virological Research Group, Szentágothai Research Centre	Bioinformatics Research Group, Szentágothai Research Centre	Péter Urbán, Endre Gábor Tóth, Gábor Kemenesi, Róbert Herczeg, Attila Gyenesei, Ferenc Jakab
EPI_ISL_418184	Gundersen Molecular Diagnostics Laboratory	Kabara Cancer Research Institute	Craig S. Richmond & Paraic A. Kenny
EPI_ISL_418185	Gundersen Molecular Diagnostic Laboratory	Kabara Cancer Research Institute	Craig S. Richmond & Paraic A. Kenny
EPI_ISL_418190, EPI_ISL_418191, EPI_ISL_418192, EPI_ISL_418193, EPI_ISL_418194, EPI_ISL_418195, EPI_ISL_418197, EPI_ISL_418198, EPI_ISL_418199, EPI_ISL_418200, EPI_ISL_418201, EPI_ISL_418202, EPI_ISL_418203, EPI_ISL_418204			
see above	NYU Langone Health	Department of Pathology and Medicine, New York University School of Medicine	Margaret Black, John Cadley, Paolo Cotzia, John Chen, Dacia Dimartino, Xiaojun Feng, Adriana Heguy, Megan Hogan, Emily Huang, George Jour, Christian Marier, Matthew T. Maurano, Mark J. Mulligan, Peter Meyn, Jared Pinnell, Amy Rapkiewicz, Marie Samanovic-Golden, Antonio Serrano, Guomiao Shen, Matija Snuderl, Nick Vulpescu, Gael Westby, Paul Zappile
EPI_ISL_418231	Centre Hospitalier Compiègne Laboratoire de Biologie	National Reference Center for Viruses of Respiratory Infections, Institut Pasteur, Paris	Mélanie Albert, Marion Barbet, Sylvie Behillil, Méline Bizard, Angela Brisebarre, Flora Donati, Etienne Simon-Lorière, Vincent Enouf, Maud Vanpeene, Sylvie van der Werf, Raulin Olivia
EPI_ISL_418232, EPI_ISL_418233	Service des Urgences	National Reference Center for Viruses of Respiratory Infections, Institut Pasteur, Paris	Mélanie Albert, Marion Barbet, Sylvie Behillil, Méline Bizard, Angela Brisebarre, Flora Donati, Etienne Simon-Lorière, Vincent Enouf, Maud Vanpeene, Sylvie van der Werf, Boubkeur
EPI_ISL_418235	Cabinet médical	National Reference Center for Viruses of Respiratory Infections, Institut Pasteur, Paris	Mélanie Albert, Marion Barbet, Sylvie Behillil, Méline Bizard, Angela Brisebarre, Flora Donati, Etienne Simon-Lorière, Vincent Enouf, Maud Vanpeene, Sylvie van der Werf

EPI_ISL_418236, EPI_ISL_418237, EPI_ISL_418238, EPI_ISL_418239	Centre Hospitalier Compiègne Laboratoire de Biologie	National Reference Center for Viruses of Respiratory Infections, Institut Pasteur, Paris	Mélanie Albert, Marion Barbet, Sylvie Behillil, Méline Bizard, Angela Brisebarre, Flora Donati, Etienne Simon-Lorière, Vincent Enouf, Maud Vanpeene, Sylvie van der Werf, Raulin Olivia
EPI_ISL_418240	LABM GH nord Essonne	National Reference Center for Viruses of Respiratory Infections, Institut Pasteur, Paris	Mélanie Albert, Marion Barbet, Sylvie Behillil, Méline Bizard, Angela Brisebarre, Flora Donati, Etienne Simon-Lorière, Vincent Enouf, Maud Vanpeene, Sylvie van der Werf, Christine Lambert
EPI_ISL_418254	NYU Langone Health	Department of Pathology and Medicine, New York University School of Medicine	Margaret Black, John Cadley, Paolo Cotzia, John Chen, Dacia Dimartino, Xiaojun Feng, Adriana Heguy, Megan Hogan, Emily Huang, George Jour, Christian Marier, Matthew T. Maurano, Mark J. Mulligan, Peter Meyn, Jared Pinnell, Amy Rapkiewicz, Marie Samanovic-Golden, Antonio Serrano, Guomiao Shen, Matija Snuderl, Nick Vulpescu, Gael Westby, Paul Zappile
EPI_ISL_418257	Ospedale Civile Giuseppe Mazzini, Teramo	Istituto Zooprofilattico Sperimentale dell'Abruzzo e Molise "G.Caporale"	Lorusso A, Marcacci M, Di Domenico M, Puglia I, Curini V, Ancora M, Di Pasquale A, Rinaldi A, Mangone I, Cammà C, Savini G.
EPI_ISL_418260, EPI_ISL_418261	Ospedale Civile Giuseppe Mazzini	Istituto Zooprofilattico Sperimentale dell'Abruzzo e Molise "G. Caporale"	Lorusso A, Marcacci M, Di Domenico M, Puglia I, Curini V, Ancora M, Di Pasquale A, Rinaldi A, Mangone I, Cammà C, Savini G.
EPI_ISL_418263, EPI_ISL_418264, EPI_ISL_418265	Laboratory of Microbiology, Department of Medicine, National and Kapodistrian University of Athens, Greece	Laboratory of Biology, Department of Medicine, Democritus University of Thrace, Greece	Maria Bampali, Elisavet Gatzidou, Nikolaos Dovrolis, Stavroula Veletza, Nikolaos Spanakis, Ioannis Karakasiliotis
EPI_ISL_418286, EPI_ISL_418287, EPI_ISL_418288, EPI_ISL_418289, EPI_ISL_418290, EPI_ISL_418291, EPI_ISL_418292, EPI_ISL_418293, EPI_ISL_418294, EPI_ISL_418295, EPI_ISL_418296, EPI_ISL_418297, EPI_ISL_418298, EPI_ISL_418299, EPI_ISL_418300			
see above	Virology Department, Sheffield Teaching Hospitals NHS Foundation Trust	Department of Infection, Immunity and Cardiovascular Disease, The Florey Institute, The Medical School, University of Sheffield	Thushan de Silva, Matthew Parker, Adri Angyal, Rebecca Brown, Rachel Tucker, Paul Parsons, Danielle Groves, Alex Keeley, Dave Partridge, Matthew Wyles, Benjamin Lindsey, Mehmet Yavuz, Mohammad Raza, Cariad Evans
EPI_ISL_418412	Centre Hospitalier des Vals d'Ardeche	CNR Virus des Infections Respiratoires - France SUD	Antonin Bal, Gregory Destras, Gwendolyne Burfin, Solenne Brun, Carine Moustaud, Raphaelle Lamy, Alexandre Gaymard, Maude Bouscambert-Duchamp, Florence Morfin-Sherpa, Martine Valette, Bruno Lina, Laurence Josset
EPI_ISL_418413	Centre Hospitalier de Macon	CNR Virus des Infections Respiratoires - France SUD	Antonin Bal, Gregory Destras, Gwendolyne Burfin, Solenne Brun, Carine Moustaud, Raphaelle Lamy, Alexandre Gaymard, Maude Bouscambert-Duchamp, Florence Morfin-Sherpa, Martine Valette, Bruno Lina, Laurence Josset
EPI_ISL_418414, EPI_ISL_418415	Centre Hospitalier de Valence	CNR Virus des Infections Respiratoires - France SUD	Antonin Bal, Gregory Destras, Gwendolyne Burfin, Solenne Brun, Carine Moustaud, Raphaelle Lamy, Alexandre Gaymard, Maude Bouscambert-Duchamp, Florence Morfin-Sherpa, Martine Valette, Bruno Lina, Laurence Josset
EPI_ISL_418416	GH Les Portes du Sud	CNR Virus des Infections Respiratoires - France SUD	Antonin Bal, Gregory Destras, Gwendolyne Burfin, Solenne Brun, Carine Moustaud, Raphaelle Lamy, Alexandre Gaymard, Maude Bouscambert-Duchamp, Florence Morfin-Sherpa, Martine Valette, Bruno Lina, Laurence Josset
EPI_ISL_418417	Centre Hospitalier de Valence	CNR Virus des Infections Respiratoires - France SUD	Antonin Bal, Gregory Destras, Gwendolyne Burfin, Solenne Brun, Carine Moustaud, Raphaelle Lamy, Alexandre Gaymard, Maude Bouscambert-Duchamp, Florence Morfin-Sherpa, Martine Valette, Bruno Lina, Laurence Josset
EPI_ISL_418418, EPI_ISL_418419	Centre Hospitalier Saint Joseph Saint Luc	CNR Virus des Infections Respiratoires - France SUD	Antonin Bal, Gregory Destras, Gwendolyne Burfin, Solenne Brun, Carine Moustaud, Raphaelle Lamy, Alexandre Gaymard, Maude Bouscambert-Duchamp, Florence Morfin-Sherpa, Martine Valette, Bruno Lina, Laurence Josset
EPI_ISL_418420, EPI_ISL_418421, EPI_ISL_418422, EPI_ISL_418423, EPI_ISL_418424, EPI_ISL_418425	Institut des Agents Infectieux (IAI), Hospices Civils de Lyon	CNR Virus des Infections Respiratoires - France SUD	Antonin Bal, Gregory Destras, Gwendolyne Burfin, Solenne Brun, Carine Moustaud, Raphaelle Lamy, Alexandre Gaymard, Maude Bouscambert-Duchamp, Florence Morfin-Sherpa, Martine Valette, Bruno Lina, Laurence Josset
EPI_ISL_418426	Centre Hospitalier de Bourg en Bresse	CNR Virus des Infections Respiratoires - France SUD	Antonin Bal, Gregory Destras, Gwendolyne Burfin, Solenne Brun, Carine Moustaud, Raphaelle Lamy, Alexandre Gaymard, Maude Bouscambert-Duchamp, Florence Morfin-Sherpa, Martine Valette, Bruno Lina, Laurence Josset
EPI_ISL_418427	Hopital Privé de l'Est Lyonnais	CNR Virus des Infections Respiratoires - France SUD	Antonin Bal, Gregory Destras, Gwendolyne Burfin, Solenne Brun, Carine Moustaud, Raphaelle Lamy, Alexandre Gaymard, Maude Bouscambert-Duchamp, Florence Morfin-Sherpa, Martine Valette, Bruno Lina, Laurence Josset
EPI_ISL_418428	Centre Hospitalier Lucien Hussel	CNR Virus des Infections Respiratoires - France SUD	Antonin Bal, Gregory Destras, Gwendolyne Burfin, Solenne Brun, Carine Moustaud, Raphaelle Lamy, Alexandre Gaymard, Maude Bouscambert-Duchamp, Florence Morfin-Sherpa, Martine Valette, Bruno Lina, Laurence Josset
EPI_ISL_418429, EPI_ISL_418430, EPI_ISL_418431, EPI_ISL_418432	Institut des Agents Infectieux (IAI), Hospices Civils de Lyon	CNR Virus des Infections Respiratoires - France SUD	Antonin Bal, Gregory Destras, Gwendolyne Burfin, Solenne Brun, Carine Moustaud, Raphaelle Lamy, Alexandre Gaymard, Maude Bouscambert-Duchamp, Florence Morfin-Sherpa, Martine Valette, Bruno Lina, Laurence Josset
EPI_ISL_418624, EPI_ISL_418626, EPI_ISL_418628, EPI_ISL_418629, EPI_ISL_418630, EPI_ISL_418632, EPI_ISL_418633	Department of Clinical Microbiology	GIGA Medical Genomics	Keith Durkin, Maria Artesi, Sébastien Bontems, Raphaël Boreux, Cécile Meex, Pierrette Melin, Marie-Pierre Hayette, Vincent Bours.
EPI_ISL_418672, EPI_ISL_418673, EPI_ISL_418674, EPI_ISL_418675, EPI_ISL_418677, EPI_ISL_418678, EPI_ISL_418679, EPI_ISL_418680, EPI_ISL_418681, EPI_ISL_418682, EPI_ISL_418683, EPI_ISL_418684, EPI_ISL_418685, EPI_ISL_418686, EPI_ISL_418687, EPI_ISL_418688, EPI_ISL_418689, EPI_ISL_418690, EPI_ISL_418691, EPI_ISL_418692, EPI_ISL_418693, EPI_ISL_418694, EPI_ISL_418695, EPI_ISL_418696, EPI_ISL_418697, EPI_ISL_418698, EPI_ISL_418699, EPI_ISL_418700, EPI_ISL_418701, EPI_ISL_418702, EPI_ISL_418703, EPI_ISL_418704, EPI_ISL_418705, EPI_ISL_418706, EPI_ISL_418707, EPI_ISL_418708, EPI_ISL_418709, EPI_ISL_418710, EPI_ISL_418711, EPI_ISL_418712, EPI_ISL_418713, EPI_ISL_418714, EPI_ISL_418715, EPI_ISL_418716, EPI_ISL_418717, EPI_ISL_418718, EPI_ISL_418719, EPI_ISL_418720, EPI_ISL_418721, EPI_ISL_418722, EPI_ISL_418723, EPI_ISL_418724, EPI_ISL_418725, EPI_ISL_418728, EPI_ISL_418729, EPI_ISL_418730, EPI_ISL_418733, EPI_ISL_418735, EPI_ISL_418736, EPI_ISL_418737, EPI_ISL_418738, EPI_ISL_418739, EPI_ISL_418740, EPI_ISL_418741, EPI_ISL_418743, EPI_ISL_418745, EPI_ISL_418746, EPI_ISL_418748, EPI_ISL_418749, EPI_ISL_418750, EPI_ISL_418751, EPI_ISL_418752, EPI_ISL_418754, EPI_ISL_418755, EPI_ISL_418756, EPI_ISL_418757, EPI_ISL_418758, EPI_ISL_418759, EPI_ISL_418761, EPI_ISL_418763, EPI_ISL_418764, EPI_ISL_418765, EPI_ISL_418766, EPI_ISL_418767, EPI_ISL_418768, EPI_ISL_418769, EPI_ISL_418770			
see above	Respiratory Virus Unit, Microbiology Services Colindale, Public Health England	Respiratory Virus Unit, Microbiology Services Colindale, Public Health England	Monica Galiano, Shahjahan Miah, Angie Lackenby, Omolola Akinbami, Tiina Talts, Leena Bhaw, Richard Myers, Steven Platt, Kirstin Edwards, Jonathan Hubb, Joanna Ellis, Maria Zambon
EPI_ISL_418802, EPI_ISL_418803, EPI_ISL_418804	Pathology Queensland	Public Health Virology Laboratory	Bixing Huang, Alyssa Pyke, Amanda De Jong, Andrew Van Den Hurk, Carmel Taylor, David Warrilow, Doris Genge, Elisabeth Gamez, Glen Hewitson, Ian Maxwell Mackay, Inga Sultana, Jamie McMahon, Jean Barcelon, Judy Northill, Mitchell Finger, Natalie Simpson, Neelima Nair, Peter Burtonclay, Peter Moore, Sarah Wheatley, Sean Moody, Sonja Hall-Mendelin, Timothy Gardam, and Frederick Moore
EPI_ISL_418860	Hospital Universitari Vall d'Hebron (HUVH) - Vall d'Hebron Research Institute (VHIR)	Hospital Universitari Vall d'Hebron (HUVH) - Vall d'Hebron Research Institute (VHIR)	Cristina Andrés, Dàmir Garcia-Cehic, Maria Piñana, Mercedes Guerrero-Murillo, Ariadna Rando, Tomàs Pumarola, Maria Gema Codina, Andrés Antón, Josep Quer
EPI_ISL_418864	Virginia DCLS	Virginia DCLS	Virginia DCLS
EPI_ISL_418869, EPI_ISL_418870, EPI_ISL_418872, EPI_ISL_418873, EPI_ISL_418874, EPI_ISL_418875, EPI_ISL_418876, EPI_ISL_418884, EPI_ISL_418886, EPI_ISL_418888, EPI_ISL_418889, EPI_ISL_418890, EPI_ISL_418892, EPI_ISL_418897, EPI_ISL_418899, EPI_ISL_418900, EPI_ISL_418903, EPI_ISL_418904, EPI_ISL_418905, EPI_ISL_418906, EPI_ISL_418907, EPI_ISL_418908, EPI_ISL_418909, EPI_ISL_418910, EPI_ISL_418911, EPI_ISL_418912, EPI_ISL_418913, EPI_ISL_418914, EPI_ISL_418921, EPI_ISL_418922, EPI_ISL_418923, EPI_ISL_418927, EPI_ISL_418928, EPI_ISL_418929, EPI_ISL_418930, EPI_ISL_418931, EPI_ISL_418932, EPI_ISL_418933, EPI_ISL_418934, EPI_ISL_418935, EPI_ISL_418936, EPI_ISL_418937, EPI_ISL_418938, EPI_ISL_418939, EPI_ISL_418940, EPI_ISL_418942, EPI_ISL_418943, EPI_ISL_418944, EPI_ISL_418945, EPI_ISL_418947, EPI_ISL_418948, EPI_ISL_418949, EPI_ISL_418950, EPI_ISL_418951, EPI_ISL_418952, EPI_ISL_418953, EPI_ISL_418954, EPI_ISL_418955			
see above	UW Virology Lab	UW Virology Lab	Pavitra Roychoudhury, Hong Xie, Keith Jerome, Alexander Greninger
EPI_ISL_418956, EPI_ISL_418957, EPI_ISL_418958	Virginia DCLS	Virginia DCLS	Virginia DCLS
EPI_ISL_418959	Universidade Federal do Rio de Janeiro - UFRJ	Bioinformatics Laboratory - LNCC	Filipe Romero, Ana Paula Guimarães, Mariane Talon, Luiz Gonzaga Paula de Almeida, Ronaldo da Silva Francisco Junior, Diana Mariani, Lídia Boullosa, Alexandra Gerber, Jaqueline Goes de Jesus, Ingra Morales Claro, Ester Cerdeira Sabino, Nuno Rodrigues Faria, Terezinha Marta Pereira, Pinto Castiñeiras, Isabela de Carvalho Leitão, Rafael de Mello Galliez, Cássia Cristina Alves Gonçalves, Érica Ramos dos Santos Nascimento, Richard Araújo Maia, Mauro Teixeira,Cristiano Xavier Lima, Orlando Ferreira Jr., Rodrigo Brindeiro, Luciana Jesus Costa e André Felipe Santos, Laboratorio Hermes Pardini, Laboratorio Simile, Amilcar Tanuri, Renato Santana Aguiar, e Ana Tereza Vasconcelos
EPI_ISL_418968, EPI_ISL_418970, EPI_ISL_418971, EPI_ISL_418972, EPI_ISL_418973, EPI_ISL_418974, EPI_ISL_418975, EPI_ISL_418976, EPI_ISL_418977, EPI_ISL_418978, EPI_ISL_418979, EPI_ISL_418980			
see above	NYU Langone Health	Department of Pathology and Medicine, New York University School of Medicine	Maria Aguero-Rosenfeld, Margaret Black, John Cadley, Paolo Cotzia, John Chen, Dacia Dimartino, Xiaojun Feng, Adriana Heguy, Megan Hogan, Emily Huang, George Jour, Christian Marier, Matthew T. Maurano, Mark J. Mulligan, Peter Meyn, Jared Pinnell, Sitharam Ramaswami, Amy Rapkiewicz, Marie

			Samanovic-Golden, Antonio Serrano, Guomiao Shen, Matija Snuderl, Nick Vulpescu, Gael Westby, Paul Zappile, Yutong Zhang
EPI_ISL_419168	Centre Hospitalier de Valence	CNR Virus des Infections Respiratoires - France SUD	Antonin Bal, Gregory Destras, Gwendolyne Burfin, Solenne Brun, Carine Moustaud, Raphaelle Lamy, Alexandre Gaymard, Maude Bouscambert-Duchamp, Florence Morfin-Sherpa, Martine Valette, Bruno Lina, Laurence Josset
EPI_ISL_419256	Virginia DCLS	Virginia DCLS	Virginia DCLS
EPI_ISL_419304, EPI_ISL_419305	Saitama Prefectural Institute of Public Health	Pathogen Genomics Center, National Institute of Infectious Diseases	Tsuyoshi Sekizuka, Michiyo Shinohara, Tsuyoshi Kishimoto, Kentaro Itokawa, Rina Tanaka, Masanori Hashino, Hajime Kamiya, Motoi Suzuki, Makoto Kuroda
EPI_ISL_419386, EPI_ISL_419387	Hospital Prof. Doutor Fernando Fonseca, EPE	Instituto Gulbenkian de Ciência	João Costa, Cathy Paulino, Joao Sobral, Susana Ladeiro, Ricardo Leite
EPI_ISL_419398, EPI_ISL_419399, EPI_ISL_419400, EPI_ISL_419401, EPI_ISL_419402, EPI_ISL_419403, EPI_ISL_419404, EPI_ISL_419405, EPI_ISL_419406, EPI_ISL_419407, EPI_ISL_419408, EPI_ISL_419409, EPI_ISL_419410, EPI_ISL_419411, EPI_ISL_419412, EPI_ISL_419413, EPI_ISL_419414, EPI_ISL_419415, EPI_ISL_419416, EPI_ISL_419417, EPI_ISL_419418, EPI_ISL_419419, EPI_ISL_419420, EPI_ISL_419421, EPI_ISL_419422, EPI_ISL_419423, EPI_ISL_419424, EPI_ISL_419425, EPI_ISL_419426, EPI_ISL_419427, EPI_ISL_419428, EPI_ISL_419429, EPI_ISL_419430, EPI_ISL_419431, EPI_ISL_419432, EPI_ISL_419433, EPI_ISL_419434, EPI_ISL_419435, EPI_ISL_419436, EPI_ISL_419438, EPI_ISL_419439, EPI_ISL_419440, EPI_ISL_419441, EPI_ISL_419442, EPI_ISL_419443, EPI_ISL_419444, EPI_ISL_419448, EPI_ISL_419449, EPI_ISL_419450, EPI_ISL_419451			
see above	Wales Specialist Virology Centre	Public Health Wales Microbiology Cardiff	Catherine Moore, Joanne Watkins, Sally Corden, Sara Rey, Matt Bull, Tom Connor
EPI_ISL_419521, EPI_ISL_419522, EPI_ISL_419523, EPI_ISL_419524, EPI_ISL_419525, EPI_ISL_419526, EPI_ISL_419527	Yale Clinical Virology Laboratory	Grubaugh Lab - Yale School of Public Health	Joseph Fauver, Anderson Brito, Tara Alpert, Chantal Vogels, Ellen Foxman, Albert Ko, Marie Landry, Nathan Grubaugh
EPI_ISL_419542, EPI_ISL_419543, EPI_ISL_419544, EPI_ISL_419545, EPI_ISL_419546, EPI_ISL_419547, EPI_ISL_419548, EPI_ISL_419549, EPI_ISL_419550, EPI_ISL_419551, EPI_ISL_419552			
see above	Center of Medical Microbiology, Virology, and Hospital Hygiene, University of Duesseldorf	Center of Medical Microbiology, Virology, and Hospital Hygiene, University of Duesseldorf	Ortwin Adams, Marcel Andree, Alexander Dilthey, Torsten Feldt, Sandra Hauka, Torsten Houwaart, Björn-Erik Jensen, Detlef Kindgen-Milles, Malte Kohns Vasconcelos, Klaus Pfeffer, Tina Senff, Daniel Strelow, Jörg Timm, Andreas Walker, Tobias Wienemann
EPI_ISL_419564, EPI_ISL_419567, EPI_ISL_419574, EPI_ISL_419576, EPI_ISL_419583, EPI_ISL_419588, EPI_ISL_419589, EPI_ISL_419592, EPI_ISL_419593, EPI_ISL_419594, EPI_ISL_419597, EPI_ISL_419599, EPI_ISL_419601, EPI_ISL_419603, EPI_ISL_419604			
see above	Laboratoire National de Santé, Microbiology, Virology	Laboratoire National de Santé, Microbiology, Epidemiology and Microbial Genomics	Anke Wienecke-Baldacchino, Ardashel Latsuzbaia, Jessica Tapp, Catherine Ragimbeau, Guillaume Fournier, Tamir Abdelrahman, Trung Nguyen Nguyen, Joel Mossong
EPI_ISL_419663, EPI_ISL_419669, EPI_ISL_419670	Center for Virology, Medical University of Vienna	Bergthaler laboratory, CeMM Research Center for Molecular Medicine of the Austrian Academy of Sciences	Alexandra Popa, Benedikt Agerer, Henrique Colaco, Lukas Endler, Jakob-Wendelin Genger, Alexander Lercher, Mark Smyth, Thomas Penz, Michael Schuster, Judith Aberle, Stephan Aberle, Elisabeth Puchhammer-Stöckl, Christoph Bock, Andreas Bergthaler
EPI_ISL_419696, EPI_ISL_419697, EPI_ISL_419698, EPI_ISL_419699, EPI_ISL_419700, EPI_ISL_419701, EPI_ISL_419702, EPI_ISL_419703, EPI_ISL_419704, EPI_ISL_419705	NYU Langone Health	Departments of Pathology and Medicine, New York University School of Medicine	Maria Aguero-Rosenfeld, Margaret Black, John Cadley, Paolo Cotzia, John Chen, Dacia Dimartino, Xiaojun Feng, Adriana Heguy, Megan Hogan, Emily Huang, George Jour, Christian Marier, Matthew T. Maurano, Mark J. Mulligan, Peter Meyn, Jared Pinnell, Sitharam Ramaswami, Amy Rapkiewicz, Marie Samanovic-Golden, Antonio Serrano, Guomiao Shen, Matija Snuderl, Nick Vulpescu, Gael Westby, Paul Zappile, Yutong Zhang
EPI_ISL_419714, EPI_ISL_419715, EPI_ISL_419716, EPI_ISL_419717, EPI_ISL_419718, EPI_ISL_419719, EPI_ISL_419724	Microbiological Diagnostic Unit Public Health Laboratory	Microbiological Diagnostic Unit Public Health Laboratory	Seemann T., Schultz M., Sait, M., Sherry, N.
EPI_ISL_419795, EPI_ISL_419796, EPI_ISL_419797, EPI_ISL_419798, EPI_ISL_419800, EPI_ISL_419801, EPI_ISL_419804, EPI_ISL_419805, EPI_ISL_419806, EPI_ISL_419807, EPI_ISL_419808, EPI_ISL_419809, EPI_ISL_419810, EPI_ISL_419811, EPI_ISL_419812, EPI_ISL_419813, EPI_ISL_419814, EPI_ISL_419815, EPI_ISL_419817, EPI_ISL_419818, EPI_ISL_419819, EPI_ISL_419820, EPI_ISL_419821, EPI_ISL_419822, EPI_ISL_419836, EPI_ISL_419837, EPI_ISL_419838, EPI_ISL_419839, EPI_ISL_419840, EPI_ISL_419841, EPI_ISL_419842, EPI_ISL_419843, EPI_ISL_419844, EPI_ISL_419845, EPI_ISL_419846, EPI_ISL_419847, EPI_ISL_419848, EPI_ISL_419849, EPI_ISL_419850, EPI_ISL_419851, EPI_ISL_419852, EPI_ISL_419853, EPI_ISL_419854, EPI_ISL_419855, EPI_ISL_419856, EPI_ISL_419857, EPI_ISL_419858, EPI_ISL_419859, EPI_ISL_419860, EPI_ISL_419861, EPI_ISL_419862, EPI_ISL_419863, EPI_ISL_419864, EPI_ISL_419865, EPI_ISL_419866, EPI_ISL_419867, EPI_ISL_419868, EPI_ISL_419869, EPI_ISL_419870, EPI_ISL_419871, EPI_ISL_419872, EPI_ISL_419873, EPI_ISL_419874, EPI_ISL_419875			
see above	Victorian Infectious Diseases Reference Laboratory (VIDRL)	Victorian Infectious Diseases Reference Laboratory and Microbiological Diagnostic Unit Public Health Laboratory, Doherty Institute	Caly L., Seemann T., Sait, M., Schultz M., Druce J., Sherry, N.
EPI_ISL_420018	Virginia DCLS	Virginia DCLS	Virginia DCLS
EPI_ISL_420038	Sentinelles network	National Reference Center for Viruses of Respiratory Infections, Institut Pasteur, Paris	Mélanie Albert, Marion Barbet, Sylvie Behillil, Méline Bizard, Angela Brisebarre, Flora Donati, Etienne Simon-Lorière, Vincent Enouf, Maud Vanpeene, Sylvie van der Werf
EPI_ISL_420041	CH Compiègne Laboratoire de Biologie	National Reference Center for Viruses of Respiratory Infections, Institut Pasteur, Paris	Mélanie Albert, Marion Barbet, Sylvie Behillil, Méline Bizard, Angela Brisebarre, Flora Donati, Etienne Simon-Lorière, Vincent Enouf, Maud Vanpeene, Sylvie van der Werf, Raulin Olivia
EPI_ISL_420042	Service de Biologie clinique	National Reference Center for Viruses of Respiratory Infections, Institut Pasteur, Paris	Mélanie Albert, Marion Barbet, Sylvie Behillil, Méline Bizard, Angela Brisebarre, Flora Donati, Etienne Simon-Lorière, Vincent Enouf, Maud Vanpeene, Sylvie van der Werf
EPI_ISL_420043	CMIP	National Reference Center for Viruses of Respiratory Infections, Institut Pasteur, Paris	Mélanie Albert, Marion Barbet, Sylvie Behillil, Méline Bizard, Angela Brisebarre, Flora Donati, Etienne Simon-Lorière, Vincent Enouf, Maud Vanpeene, Sylvie van der Werf
EPI_ISL_420044	CH Jean de Navarre Laboratoire de Biologie	National Reference Center for Viruses of Respiratory Infections, Institut Pasteur, Paris	Mélanie Albert, Marion Barbet, Sylvie Behillil, Méline Bizard, Angela Brisebarre, Flora Donati, Etienne Simon-Lorière, Vincent Enouf, Maud Vanpeene, Sylvie van der Werf
EPI_ISL_420045	Sentinelles network	National Reference Center for Viruses of Respiratory Infections, Institut Pasteur, Paris	Mélanie Albert, Marion Barbet, Sylvie Behillil, Méline Bizard, Angela Brisebarre, Flora Donati, Etienne Simon-Lorière, Vincent Enouf, Maud Vanpeene, Sylvie van der Werf
EPI_ISL_420046, EPI_ISL_420047	Résidence Villa Caroline	National Reference Center for Viruses of Respiratory Infections, Institut Pasteur, Paris	Mélanie Albert, Marion Barbet, Sylvie Behillil, Méline Bizard, Angela Brisebarre, Flora Donati, Etienne Simon-Lorière, Vincent Enouf, Maud Vanpeene, Sylvie van der Werf
EPI_ISL_420048	Service de Biologie Médicale - BP 125	National Reference Center for Viruses of Respiratory Infections, Institut Pasteur, Paris	Mélanie Albert, Marion Barbet, Sylvie Behillil, Méline Bizard, Angela Brisebarre, Flora Donati, Etienne Simon-Lorière, Vincent Enouf, Maud Vanpeene, Sylvie van der Werf, Christine Lambert
EPI_ISL_420055	Sentinelles network	National Reference Center for Viruses of Respiratory Infections, Institut Pasteur, Paris	Mélanie Albert, Marion Barbet, Sylvie Behillil, Méline Bizard, Angela Brisebarre, Flora Donati, Etienne Simon-Lorière, Vincent Enouf, Maud Vanpeene, Sylvie van der Werf
EPI_ISL_420069, EPI_ISL_420070, EPI_ISL_420071	Institut Pasteur Dakar	Institut Pasteur de Dakar	Ndongo Dia, Moussa Moise Diagne, Mamadou Diop, Ousmane Faye, Amadou Alpha Sall
EPI_ISL_420072	Institut Pasteur Dakar	Institut Pasteur de Dakar	Ndongo Dia, Moussa Moise Diagne, Mamadou Diop, Ousmane Faye , Amadou Alpha Sall
EPI_ISL_420080	WHO National Influenza Centre Russian Federation	WHO National Influenza Centre Russian Federation	Andrey Komissarov, Artem Fadeev, Anna Ivanova, Daria Danilenko
EPI_ISL_420081	WHO National Influenza Centre Russian Federation	WHO National Influenza Centre Russian Federation	Andrey Komissarov, Artem Fadeev, Mariia Sergeeva, Anna Ivanova, Daria Danilenko
EPI_ISL_420083	Centers for Disease Control, R.O.C. (Taiwan)	Centers for Disease Control, R.O.C. (Taiwan)	Ji-Rong Yang, Yu-Chi Lin, Jung-Jung Mu, Ming-Tsan Liu
EPI_ISL_420090, EPI_ISL_420091, EPI_ISL_420092, EPI_ISL_420096, EPI_ISL_420097	Yale Clinical Virology Laboratory	Grubaugh Lab - Yale School of Public Health	Joseph Fauver, Anderson Brito, Tara Alpert, Chantal Vogels, Ellen Foxman, Albert Ko, Marie Landry, Nathan Grubaugh
EPI_ISL_420130	Servicio de Microbiologia. Hospital Clinico Universitario de Valencia	Sequencing and Bioinformatics Service and Molecular Epidemiology Research Group. FISABIO-Public Health	David Navarro, Loreto Ferrús Abad, Maria Alma Bracho, Griselda De Marco, Beatriz Beamud, Lidia Ruiz Roldan, Marta Pla Diaz, Neris Garcia-Gonzalez, Inma Galán Vendrell, Paula Ruiz-Hueso, Mariana Reyes-Prieto, Vicente Soriano Chirona, Sandra Carbo, Ivan Ansari, Llúcia Martínez-Priego, Giuseppe D'Auria, Fernando Gonzalez-Candelas
EPI_ISL_420131	Servicio de Microbiologia. Hospital Clinico Universitario de	Sequencing and Bioinformatics Service and Molecular	Paula Ruiz-Hueso,Loreto Ferrús Abad, Maria Alma Bracho, Griselda De Marco, Beatriz Beamud, Sandra Carbo, Lidia Ruiz Roldan, Marta Pla Diaz, Neris

	Valencia	Epidemiology Research Group. FISABIO-Public Health	Garcia-Gonzalez, Inma Galán Vendrell, Mariana Reyes-Prieto, Vicente Soriano Chirona, Ivan Ansari, David Navarro, Llúcia Martínez-Priego, Giuseppe D'Auria, Fernando Gonzalez-Candelas
EPI_ISL_420132	Servicio de Microbiologia. Hospital Clinico Universitario de Valencia	Sequencing and Bioinformatics Service and Molecular Epidemiology Research Group. FISABIO-Public Health	Giuseppe D'Auria,Sandra Carbo, Loreto Ferrús Abad, Maria Alma Bracho, Griselda De Marco, Beatriz Beamud, Lidia Ruiz Roldan, Marta Pla Diaz, Neris Garcia-Gonzalez, Inma Galán Vendrell, Paula Ruiz-Hueso, Mariana Reyes-Prieto, Vicente Soriano Chirona, Ivan Ansari, David Navarro, Llúcia Martínez-Priego, Fernando Gonzalez-Candelas
EPI_ISL_420151	Nordland Hospital - Bodo, Laboratory Department, Molecular Biology Unit	Norwegian Institute of Public Health, Department of Virology	Kathrine Stene-Johansen, Kamilla Heddeland Instefjord, Hilde Elshaug, Karoline Bragstad, Olav Hungnes
EPI_ISL_420152, EPI_ISL_420153	University Hospital of Northern Norway, Department for Microbiology and Infectious Disease Control	Norwegian Institute of Public Health, Department of Virology	Kathrine Stene-Johansen, Kamilla Heddeland Instefjord, Hilde Elshaug, Karoline Bragstad, Olav Hungnes
EPI_ISL_420177, EPI_ISL_420288, EPI_ISL_420289, EPI_ISL_420290, EPI_ISL_420291	Virology Department, Sheffield Teaching Hospitals NHS Foundation Trust	Department of Infection, Immunity and Cardiovascular Disease, The Florey Institute, The Medical School, University of Sheffield	Thushan de Silva, Matthew Parker, Adri Angyal, Rebecca Brown, Rachel Tucker, Paul Parsons, Luke Green, Danielle Groves, Alex Keeley, Dave Partridge, Matthew Wyles, Benjamin Lindsey, Mehmet Yavuz, Mohammad Raza, Cariad Evans
EPI_ISL_420296, EPI_ISL_420297, EPI_ISL_420299, EPI_ISL_420300, EPI_ISL_420301, EPI_ISL_420302	NYU Langone Health	Departments of Pathology and Medicine, New York University School of Medicine	Maria Aguero-Rosenfeld, Margaret Black, John Cadley, Paolo Cotzia, John Chen, Dacia Dimartino, Xiaojun Feng, Adriana Heguy, Megan Hogan, Emily Huang, George Jour, Christian Marier, Matthew T. Maurano, Mark J. Mulligan, Peter Meyn, Jared Pinnell, Sitharam Ramaswami, Amy Rapkiewicz, Marie Samanovic-Golden, Antonio Serrano, Guomiao Shen, Matija Snuderl, Nick Vulpescu, Gael Westby, Paul Zappile, Yutong Zhang
EPI_ISL_420303	Alaska State Virology Laboratory	Alaska State Virology Laboratory	Chen, J
EPI_ISL_420304, EPI_ISL_420305, EPI_ISL_420306	Alaska State Virology Laboratory	Alaska State Virology Laboratory	Chen, J.
EPI_ISL_420310	University Hospital of Northern Norway, Department for Microbiology and Infectious Disease Control	Norwegian Institute of Public Health, Department of Virology	Kathrine Stene-Johansen, Kamilla Heddeland Instefjord, Hilde Elshaug, Karoline Bragstad, Olav Hungnes
EPI_ISL_420311, EPI_ISL_420312	Akershus University Hospital, Department for Microbiology and Infectious Disease Control	Norwegian Institute of Public Health, Department of Virology	Kathrine Stene-Johansen, Kamilla Heddeland Instefjord, Hilde Elshaug, Karoline Bragstad, Olav Hungnes
EPI_ISL_420358, EPI_ISL_420359, EPI_ISL_420360, EPI_ISL_420361, EPI_ISL_420362	KU Leuven, Clinical and Epidemiological Virology	KU Leuven, Clinical and Epidemiological Virology	Joan Marti-Carreras, Bert Vanmechelen, Tony Wawina, Piet Maes
EPI_ISL_420465, EPI_ISL_420466, EPI_ISL_420467, EPI_ISL_420468, EPI_ISL_420469, EPI_ISL_420470, EPI_ISL_420471, EPI_ISL_420472, EPI_ISL_420473, EPI_ISL_420474, EPI_ISL_420476, EPI_ISL_420477, EPI_ISL_420480, EPI_ISL_420481, EPI_ISL_420482, EPI_ISL_420483, EPI_ISL_420485			
see above	Respiratory Virus Unit, Microbiology Services Colindale, Public Health England	Respiratory Virus Unit, Microbiology Services Colindale, Public Health England	Monica Galiano, Shahjahan Miah, Angie Lackenby, Omolola Akinbami, Tiina Talts, Leena Bhaw, Richard Myers, Steven Platt, Kirstin Edwards, Jonathan Hubb, Joanna Ellis, Maria Zambon
EPI_ISL_420536	Department of Microbiology, PathWest QEII Medical Centre	Department of Microbiology, PathWest QEII Medical Centre	Chisha Sikazwe, Jurissa Lang, Avram Levy, David Speers and David Smith
EPI_ISL_420563	Ospedale Civile Giuseppe Mazzini	Istituto Zooprofilattico Sperimentale dell'Abruzzo e Molise "G. Caporale"	Lorusso A, Marcacci M, Di Domenico M, Ancora M, Curini V, Mangone I, Rinaldi A, Di Pasquale A, Cammà C, Puglia I, Savini G
EPI_ISL_420570, EPI_ISL_420571, EPI_ISL_420572, EPI_ISL_420573, EPI_ISL_420574, EPI_ISL_420575, EPI_ISL_420576, EPI_ISL_420577, EPI_ISL_420578, EPI_ISL_420579, EPI_ISL_420580, EPI_ISL_420581, EPI_ISL_420582, EPI_ISL_420584, EPI_ISL_420585, EPI_ISL_420587, EPI_ISL_420588, EPI_ISL_420589, EPI_ISL_420590, EPI_ISL_420591			
see above	NYU Langone Health	Departments of Pathology and Medicine, New York University School of Medicine	Maria Aguero-Rosenfeld, Brendan Belovarac, Margaret Black, Ludovic Boytard, John Cadley, Paolo Cotzia, John Chen, Dacia Dimartino, Xiaojun Feng, Tatyana Gindin, Adriana Heguy, Megan Hogan, Emily Huang, George Jour, Andrew Lytle, Christian Marier, Matthew T. Maurano, Mark J. Mulligan, Peter Meyn, Iman Osman, Jared Pinnell, Sitharam Ramaswami, Amy Rapkiewicz, Marie Samanovic-Golden, Antonio Serrano, Guomiao Shen, Matija Snuderl, Theodore Vougiouklakis, Nick Vulpescu, Gael Westby, Paul Zappile, Yutong Zhang
EPI_ISL_420599	Servicio Virosis Respiratorias-Departamento Virología-INEI	Instituto Nacional Enfermedades Infecciosas C.G.Malbran	Baumeister E., Avaro M., Benedetti E., Russo M., Dattero ME, Pontoriero A., Cisterna D., Molina V., Perandones C., Tuduri E., Lorenzo F., Poklepovich T., Campos J.
EPI_ISL_420904, EPI_ISL_420905, EPI_ISL_420906, EPI_ISL_420907	Max von Pettenkofer Institute, Virology, National Reference Center for Retroviruses, LMU Munich	Laboratory for Functional Genome Analysis, Dept. Genomics, Gene Center of the LMU Munich	Max Muenchhoff, Stefan Krebs, Alexander Graf, Ashok Varadharajan, Oliver Keppler, Helmut Blum
EPI_ISL_420917	Wales Specialist Virology Centre	Public Health Wales Microbiology Cardiff	Catherine Moore, Joanne Watkins, Sally Corden, Malorie Perry, Simon Cottrell Sara Rey, Matt Bull, Tom Connor
EPI_ISL_421221	Hangzhou Center for Diseases Control and Prevention	Hangzhou Center for Diseases Control and Prevention	Jun Li, Haoqiu Wang, Lingfeng Mao, Hua Yu, Xinfen Yu, Zhou Sun, Xin Qian, Shuchang Chen, Junfang Chen, Xuchu Wang
EPI_ISL_421275	Russian State Collection of Viruses	Pathogenic Microorganisms Variability Laboratory	Alexey Shchetinin, Maria Nikiforova, Nadezhda Kuznetsova, Ekaterina Aksenova, Marina Kunda, Natalia Ryzhova, Olga Voronina, Inna Dolzhikova, Daria Grousova, Andrey Botikov, Denis Logunov, Alexander Gintsburg, Vladimir Gushchin
EPI_ISL_421300, EPI_ISL_421302, EPI_ISL_421310, EPI_ISL_421311, EPI_ISL_421314	University of Wisconsin-Madison AIDS Vaccine Research Laboratories	University of Wisconsin-Madison AIDS Vaccine Research Laboratories	Gage Moreno, Katarina Braun, et al. AIDS Vaccine Research Laboratories
EPI_ISL_421347	Wyoming Public Health Laboratory	Center for Global Health, University of New Mexico Health Sciences Center	Daryl Domman, Kurt Schwalm, Rob Christensen, Wanda Manley, Cari Sloma, Noah Hull, Darrell Dinwiddie
EPI_ISL_421348, EPI_ISL_421349, EPI_ISL_421350, EPI_ISL_421357, EPI_ISL_421358, EPI_ISL_421359, EPI_ISL_421361, EPI_ISL_421362, EPI_ISL_421363, EPI_ISL_421364, EPI_ISL_421366, EPI_ISL_421367, EPI_ISL_421368, EPI_ISL_421369, EPI_ISL_421370, EPI_ISL_421371, EPI_ISL_421372, EPI_ISL_421373, EPI_ISL_421374, EPI_ISL_421375, EPI_ISL_421376, EPI_ISL_421377, EPI_ISL_421378, EPI_ISL_421379, EPI_ISL_421380, EPI_ISL_421381, EPI_ISL_421383, EPI_ISL_421384, EPI_ISL_421388, EPI_ISL_421389, EPI_ISL_421390, EPI_ISL_421391, EPI_ISL_421392, EPI_ISL_421393, EPI_ISL_421394, EPI_ISL_421395, EPI_ISL_421396, EPI_ISL_421397, EPI_ISL_421398, EPI_ISL_421399, EPI_ISL_421400, EPI_ISL_421401, EPI_ISL_421402, EPI_ISL_421403, EPI_ISL_421404, EPI_ISL_421405, EPI_ISL_421406, EPI_ISL_421407, EPI_ISL_421408, EPI_ISL_421409, EPI_ISL_421410, EPI_ISL_421411, EPI_ISL_421412, EPI_ISL_421413, EPI_ISL_421414, EPI_ISL_421416, EPI_ISL_421417, EPI_ISL_421418, EPI_ISL_421419, EPI_ISL_421420, EPI_ISL_421421, EPI_ISL_421422, EPI_ISL_421423, EPI_ISL_421424, EPI_ISL_421425, EPI_ISL_421426, EPI_ISL_421427, EPI_ISL_421428, EPI_ISL_421429, EPI_ISL_421430, EPI_ISL_421431, EPI_ISL_421432, EPI_ISL_421433, EPI_ISL_421434, EPI_ISL_421435			
see above	MSHS Clinical Microbiology Laboratories	MSHS Pathogen Surveillance Program	Ana S. Gonzalez-Reiche, Mitchell Sullivan, Ajay Obla, Gopi Patel, Emilia Sordillo, Melissa Gitman, Alberto Paniz-mondolfi, Matthew Hernandez, Shelcie Fabre, Jose Polanco, Zenab Khan, Bremy Albuquerque, Jayeeta Dutta, Juan Soto, Shwetha Sridhar Hara, Ying-Chih Wang, Melissa Smith, Robert Sebra, Lisa Miorin, Wen-chun Liu, Randy Albrecht, Judith Aberg, Florian Krammer, Adolfo Garcia-Sarstre, Viviana Simon, Harm van Bakel
EPI_ISL_421446, EPI_ISL_421447, EPI_ISL_421448	H Guimaraes	Instituto Nacional de Saude (INSA)	Guiomar et al
EPI_ISL_421449	H Dr. Nelio Mendonca - Funchal	Instituto Nacional de Saude (INSA)	Guiomar et al
EPI_ISL_421450, EPI_ISL_421451, EPI_ISL_421452	Instituto Nacional de Saude (INSA)	Instituto Nacional de Saude (INSA)	Guiomar et al
EPI_ISL_421453	CHTMAD	Instituto Nacional de Saude (INSA)	Guiomar et al
EPI_ISL_421454	H Beatriz Angelo	Instituto Nacional de Saude (INSA)	Guiomar et al
EPI_ISL_421455	CH Barreiro Montijo	Instituto Nacional de Saude (INSA)	Guiomar et al
EPI_ISL_421457	H Dr Nelio Mendonca - Funchal	Instituto Nacional de Saude (INSA)	Guiomar et al
EPI_ISL_421464	CHTMAD	Instituto Nacional de Saude (INSA)	Guiomar et al
EPI_ISL_421482, EPI_ISL_421483	CH VN Gaia - Espinho	Instituto Nacional de Saude (INSA)	Guiomar et al
EPI_ISL_421505	Service de Biologie Médicale - BP 125	National Reference Center for Viruses of Respiratory	Mélanie Albert, Marion Barbet, Sylvie Behillil, Méline Bizard, Angela Brisebarre, Flora Donati, Etienne Simon-Lorière, Vincent Enouf, Maud Vanpeene,

		Infections, Institut Pasteur, Paris	Sylvie van der Werf, Christine Lambert
EPI_ISL_421516	Servicio de Microbiologia. Hospital Clinico Universitario de Valencia	Sequencing and Bioinformatics Service and Molecular Epidemiology Research Group. FISABIO-Public Health	Loreto Ferrús Abad, Maria Alma Bracho, Griselda De Marco, Beatriz Beamud, Lidia Ruiz Roldan, Marta Pla Diaz, Neris Garcia-Gonzalez, Inma Galán Vendrell, Paula Ruiz-Hueso, Mariana Reyes-Prieto, Vicente Soriano Chirona, David Navarro, Llúcia Martínez-Priego, Giuseppe D'Auria, Fernando Gonzalez-Candelas
EPI_ISL_421517	Servicio de Microbiologia. Hospital Clinico Universitario de Valencia	Sequencing and Bioinformatics Service and Molecular Epidemiology Research Group. FISABIO-Public Health	Sandra Carbo, Loreto Ferrús Abad, Maria Alma Bracho, Griselda De Marco, Beatriz Beamud, Lidia Ruiz Roldan, Marta Pla Diaz, Neris Garcia-Gonzalez, Inma Galán Vendrell, Paula Ruiz-Hueso, Mariana Reyes-Prieto, Vicente Soriano Chirona, Ivan Ansari, David Navarro, Llúcia Martínez-Priego, Giuseppe D'Auria, Fernando Gonzalez-Candelas
EPI_ISL_421518	Servicio de Microbiologia. Hospital Clinico Universitario de Valencia	Sequencing and Bioinformatics Service and Molecular Epidemiology Research Group. FISABIO-Public Health	Inma Galán Vendrell, Paula Ruiz-Hueso, Sandra Carbo, Loreto Ferrús Abad, Maria Alma Bracho, Griselda De Marco, Beatriz Beamud, Lidia Ruiz Roldan, Marta Pla Diaz, Neris Garcia-Gonzalez, Mariana Reyes-Prieto, Vicente Soriano Chirona, Ivan Ansari, David Navarro, Llúcia Martínez-Priego, Giuseppe D'Auria, Fernando Gonzalez-Candelas
EPI_ISL_421531	State Key Laboratory of Agricultural Microbiology	State Key Laboratory of Agricultural Microbiology	Meilin Jin
EPI_ISL_421543, EPI_ISL_421544, EPI_ISL_421545, EPI_ISL_421546, EPI_ISL_421547, EPI_ISL_421550, EPI_ISL_421551	Wyoming Public Health Laboratory	Center for Global Health, University of New Mexico Health Sciences Center	Daryl Domman, Kurt Schwalm, Rob Christensen, Wanda Manley, Cari Sloma, Noah Hull, Darrell Dinwiddie
EPI_ISL_421560, EPI_ISL_421561	Utah Public Health Laboratory	Utah Public Health Laboratory	Erin Young, Kelly Oakeson
EPI_ISL_421577, EPI_ISL_421578, EPI_ISL_421579, EPI_ISL_421580, EPI_ISL_421581, EPI_ISL_421582, EPI_ISL_421583, EPI_ISL_421584, EPI_ISL_421585, EPI_ISL_421586, EPI_ISL_421587, EPI_ISL_421588, EPI_ISL_421589, EPI_ISL_421590, EPI_ISL_421591, EPI_ISL_421592			
see above	NYU Langone Health	Departments of Pathology and Medicine, New York University School of Medicine	Maria Aguero-Rosenfeld, Brendan Belovarac, Margaret Black, Ludovic Boytard, John Cadley, Paolo Cotzia, John Chen, Dacia Dimartino, Xiaojun Feng, Tatyana Gindin, Adriana Heguy, Megan Hogan, Emily Huang, George Jour, Andrew Lytle, Christian Marier, Matthew T. Maurano, Mark J. Mulligan, Peter Meyn, Iman Osman, Jared Pinnell, Sitharam Ramaswami, Amy Rapkiewicz, Marie Samanovic-Golden, Antonio Serrano, Guomiao Shen, Matija Snuderl, Theodore Vougiouklakis, Nick Vulpescu, Gael Westby, Paul Zappile, Yutong Zhang
EPI_ISL_421627, EPI_ISL_421630, EPI_ISL_421631, EPI_ISL_421633	MSHS Clinical Microbiology Laboratories	MSHS Pathogen Surveillance Program	Ana S. Gonzalez-Reiche, Mitchell Sullivan, Ajay Obla, Gopi Patel, Emilia Sordillo, Melissa Gitman, Alberto Paniz-mondolfi, Matthew Hernandez, Shelcie Fabre, Jose Polanco, Zenab Khan, Bremy Albuquerque, Jayeeta Dutta, Juan Soto, Shwetha Sridhar Hara, Ying-Chih Wang, Melissa Smith, Robert Sebra, Lisa Miorin, Wen-chun Liu, Randy Albrecht, Judith Aberg, Florian Krammer, Adolfo Garcia-Sarstre, Viviana Simon, Harm van Bakel
EPI_ISL_421652	Dasman Diabetes Institute	Dasman Diabetes Institute	Fahd Al-Mulla, Rasheeba Iqbal, Sumi John, Ebaa Al-Ozairi, Qais Al-Duwairi
EPI_ISL_421683, EPI_ISL_421684, EPI_ISL_421685, EPI_ISL_421686, EPI_ISL_421687, EPI_ISL_421688, EPI_ISL_421689, EPI_ISL_421690, EPI_ISL_421691, EPI_ISL_421692	Minnesota Department of Health, Public Health Laboratory	Minnesota Department of Health, Public Health Laboratory	Matt Plumb, Jacob Garfin, Xiong Wang
EPI_ISL_421704, EPI_ISL_421705, EPI_ISL_421706, EPI_ISL_421707, EPI_ISL_421708, EPI_ISL_421709, EPI_ISL_421710, EPI_ISL_421711, EPI_ISL_421712, EPI_ISL_421713, EPI_ISL_421714, EPI_ISL_421715, EPI_ISL_421716, EPI_ISL_421717, EPI_ISL_421718, EPI_ISL_421719, EPI_ISL_421720, EPI_ISL_421721, EPI_ISL_421722, EPI_ISL_421723, EPI_ISL_421724, EPI_ISL_421725, EPI_ISL_421726, EPI_ISL_421727, EPI_ISL_421728, EPI_ISL_421729, EPI_ISL_421730, EPI_ISL_421731, EPI_ISL_421732, EPI_ISL_421733			
see above	NYU Langone Health	Departments of Pathology and Medicine, New York University School of Medicine	Maria Aguero-Rosenfeld, Brendan Belovarac, Margaret Black, Ludovic Boytard, John Cadley, Paolo Cotzia, John Chen, Dacia Dimartino, Xiaojun Feng, Tatyana Gindin, Adriana Heguy, Megan Hogan, Emily Huang, George Jour, Andrew Lytle, Christian Marier, Matthew T. Maurano, Mark J. Mulligan, Peter Meyn, Iman Osman, Jared Pinnell, Sitharam Ramaswami, Amy Rapkiewicz, Marie Samanovic-Golden, Antonio Serrano, Guomiao Shen, Matija Snuderl, Theodore Vougiouklakis, Nick Vulpescu, Gael Westby, Paul Zappile, Yutong Zhang
EPI_ISL_421734, EPI_ISL_421735, EPI_ISL_421736, EPI_ISL_421737, EPI_ISL_421738, EPI_ISL_421739, EPI_ISL_421740, EPI_ISL_421741, EPI_ISL_421742, EPI_ISL_421743, EPI_ISL_421744, EPI_ISL_421745, EPI_ISL_421746, EPI_ISL_421747, EPI_ISL_421748, EPI_ISL_421749, EPI_ISL_421750, EPI_ISL_421751, EPI_ISL_421752, EPI_ISL_421753, EPI_ISL_421754, EPI_ISL_421755, EPI_ISL_421756, EPI_ISL_421757, EPI_ISL_421758, EPI_ISL_421759, EPI_ISL_421760, EPI_ISL_421761, EPI_ISL_421762, EPI_ISL_421763			
see above	Laboratoire National de Sante, Microbiology, Virology	Laboratoire National de Sante, Microbiology, Epidemiology and Microbial Genomics	Anke Wienecke-Baldacchino, Ardashel Latsuzbaia, Jessica Tapp, Catherine Ragimbeau, Guillaume Fournier, Tamir Abdelrahman, Trung Nguyen Nguyen, Joel Mossong
EPI_ISL_422413, EPI_ISL_422414, EPI_ISL_422415, EPI_ISL_422416, EPI_ISL_422417	Department of Laboratory Medicine, National Taiwan University Hospital	Microbial Genomics Core Lab, National Taiwan University Centers of Genomic and Precision Medicine	Shiou-Hwei Yeh, You-Yu Lin, Ya-Yun Lai, Chiao-Ling Li, Shan-Chwen Chang, Pei-Jer Chen, Sui-Yuan Chang
EPI_ISL_422424	Jaber Al Ahmad Al Sabah Hospital	Dasman diabetes Institute	Fahd Al-Mulla, Rasheeba Iqbal, Sumi John, Ebaa Al-Ozairi, Qais Al-Duwairi
EPI_ISL_422426, EPI_ISL_422427	JABER AL AHMAD AL SABAH HOSPITAL - KUWAIT CITY	Dasman Diabetes Institute	Fahd Al-Mulla, Rasheeba Iqbal, Sumi John, Ebaa Al-Ozairi, Qais Al-Duwairi
EPI_ISL_422645, EPI_ISL_422649, EPI_ISL_422650, EPI_ISL_422651, EPI_ISL_422652, EPI_ISL_422653, EPI_ISL_422654, EPI_ISL_422655, EPI_ISL_422656, EPI_ISL_422657, EPI_ISL_422658, EPI_ISL_422659, EPI_ISL_422664, EPI_ISL_422665, EPI_ISL_422666, EPI_ISL_422667, EPI_ISL_422668, EPI_ISL_422669, EPI_ISL_422670, EPI_ISL_422671, EPI_ISL_422672, EPI_ISL_422673, EPI_ISL_422674, EPI_ISL_422703, EPI_ISL_422704, EPI_ISL_422705, EPI_ISL_422814, EPI_ISL_422815, EPI_ISL_422816, EPI_ISL_422817, EPI_ISL_422846, EPI_ISL_422847, EPI_ISL_422848, EPI_ISL_422849, EPI_ISL_422850, EPI_ISL_422851, EPI_ISL_422852, EPI_ISL_422853, EPI_ISL_422854, EPI_ISL_422855, EPI_ISL_422856, EPI_ISL_422857, EPI_ISL_422858, EPI_ISL_422888, EPI_ISL_422889, EPI_ISL_422890, EPI_ISL_422901, EPI_ISL_422906, EPI_ISL_422907, EPI_ISL_422908, EPI_ISL_422909, EPI_ISL_422910, EPI_ISL_422911, EPI_ISL_422912, EPI_ISL_422913			
see above	Dutch COVID-19 response team	Erasmus Medical Center	Bas Oude Munnink, David Nieuwenhuijse, Reina Sikkema, Claudia Schapendonk, Irina Chestakova, Anne van der Linden, Theo Bestebroer, Stefan van Nieuwkoop, Mark Pronk, Pascal Lexmond, Corien Swaan, Manon Haverkate, Madelief Mollers, Mart Stein, Sandra Kengne Kamga Mobou, Jeroen van Kampen, Jolanda Voermans, Aura Timen, Corine GeurtsvanKessel, Annemiek van der Eijk, Richard Molenkamp, Marion Koopmans, on behalf of the Dutch national COVID-19 response team.
EPI_ISL_422962, EPI_ISL_422963, EPI_ISL_422964, EPI_ISL_422965, EPI_ISL_422966, EPI_ISL_422967, EPI_ISL_422969, EPI_ISL_422970, EPI_ISL_422971, EPI_ISL_422972, EPI_ISL_422973, EPI_ISL_422974, EPI_ISL_422975, EPI_ISL_422976, EPI_ISL_422977, EPI_ISL_422978, EPI_ISL_422979, EPI_ISL_422980, EPI_ISL_422981, EPI_ISL_422982, EPI_ISL_422983, EPI_ISL_422984, EPI_ISL_422985, EPI_ISL_422986, EPI_ISL_422987, EPI_ISL_422988, EPI_ISL_422989, EPI_ISL_422990, EPI_ISL_422991, EPI_ISL_422992, EPI_ISL_422993, EPI_ISL_422994, EPI_ISL_422995, EPI_ISL_422996, EPI_ISL_422997, EPI_ISL_422998, EPI_ISL_422999, EPI_ISL_423000, EPI_ISL_423008, EPI_ISL_423009, EPI_ISL_423010, EPI_ISL_423024, EPI_ISL_423025, EPI_ISL_423026, EPI_ISL_423027, EPI_ISL_423028, EPI_ISL_423029, EPI_ISL_423030, EPI_ISL_423031, EPI_ISL_423032, EPI_ISL_423033			
see above	UW Virology Lab	UW Virology Lab	Pavitra Roychoudhury, Hong Xie, Keith Jerome, Alexander Greninger
EPI_ISL_423043	Ramathibodi Hospital	COVID-19 Network Investigations (CONI) Alliance	Elizabeth Batty, Wasun Chantratita, Thanat Chookajorn, Stefan Fernandez, Angkana Huang, Anthony R. Jones, Khajohn Joonsalak, Chonticha Klungtong, Theerarat Kochakarn, Namfon Kotanan, Krittikorn Kumpornsin, Wudtichai Manasatienkij, Bhakbhoom Panthan, Ekawat Pasomsub, Insee Sensorn, Arporn Wangwiwatsin
EPI_ISL_423053, EPI_ISL_423054, EPI_ISL_423637, EPI_ISL_423854, EPI_ISL_423855, EPI_ISL_423856, EPI_ISL_423857, EPI_ISL_423858, EPI_ISL_423859, EPI_ISL_423860, EPI_ISL_423861, EPI_ISL_423862, EPI_ISL_423863, EPI_ISL_423864, EPI_ISL_423867, EPI_ISL_423868, EPI_ISL_423869, EPI_ISL_423870, EPI_ISL_423871, EPI_ISL_423872, EPI_ISL_423873, EPI_ISL_423874, EPI_ISL_423875, EPI_ISL_423876, EPI_ISL_423877, EPI_ISL_423878, EPI_ISL_423881, EPI_ISL_423883, EPI_ISL_423884, EPI_ISL_423887, EPI_ISL_423888, EPI_ISL_423890, EPI_ISL_423891, EPI_ISL_423892, EPI_ISL_423893, EPI_ISL_423894, EPI_ISL_423895, EPI_ISL_423896, EPI_ISL_423897, EPI_ISL_423898, EPI_ISL_423900, EPI_ISL_423901, EPI_ISL_423902, EPI_ISL_423903, EPI_ISL_423904, EPI_ISL_423905, EPI_ISL_423907, EPI_ISL_423909, EPI_ISL_423911, EPI_ISL_423912, EPI_ISL_423913, EPI_ISL_423914, EPI_ISL_423915, EPI_ISL_423916, EPI_ISL_423917, EPI_ISL_423924, EPI_ISL_423925, EPI_ISL_423926, EPI_ISL_423927, EPI_ISL_423935, EPI_ISL_423936, EPI_ISL_423940, EPI_ISL_423941, EPI_ISL_423948, EPI_ISL_423953, EPI_ISL_423955, EPI_ISL_423956, EPI_ISL_423964			
see above	Respiratory Virus Unit, Microbiology Services Colindale, Public Health England	Respiratory Virus Unit, Microbiology Services Colindale, Public Health England	Monica Galiano, Shahjahan Miah, Angie Lackenby, Omolola Akinbami, Tiina Talts, Leena Bhaw, Richard Myers, Steven Platt, Kirstin Edwards, Jonathan Hubb, Joanna Ellis, Maria Zambon
EPI_ISL_424175, EPI_ISL_424176, EPI_ISL_424177, EPI_ISL_424178, EPI_ISL_424179, EPI_ISL_424180, EPI_ISL_424181, EPI_ISL_424182, EPI_ISL_424183, EPI_ISL_424184, EPI_ISL_424185, EPI_ISL_424255			
see above	UW Virology Lab	UW Virology Lab	Pavitra Roychoudhury, Hong Xie, Keith Jerome, Alexander Greninger
EPI_ISL_424364, EPI_ISL_424365	National Influenza Center, Indian Council of Medical Research - National Institute of Virology	Indian Council of Medical Research-National Institute of Virology, Microbial Containment Complex	Pragya D. Yadav, Varsha Potdar, Savita Patil, Dimpal A. Nyayanit, Triparna Majumdar, Manohar. L. Chaudhary, Gururaj Deshpande, Padinjaremattathil Thankappan Ullas, Anita Shete-Aich, Hitesh Dighe, Sreelekshmy Mohandas, Gajanan Sapkal, Atanu Basu, Amita Jain, Bharti Malhotra, Deepika Chaudhary, Sarah Cherian, Priya Abraham
EPI_ISL_424366	Vaccine Research, Development and Application Center, Erciyes University	Gen Era Diagnostics Inc.	Shaikh Terkis Islam Pavel, Hazel Yetiskin, Gunsu Aydin, Can Holyavkin, Muhammet Ali Uygut, Zehra B Dursun, lhami Celik, Alper Iseri, Aykut Ozdarendeli

EPI_ISL_424377	deCODE genetics	deCODE genetics	Daniel F Gudbjartsson; Agnar Helgason; Hakon Jonsson; Olafur T Magnusson; Pall Melsted; Gudmundur L Norddahl; Jona Saemundsdottir; Asgeir Sigurdsson; Patrick Sulem; Arna B Agustsdottir; Berglind Eiriksdottir; Run Fridriksdottir; Elisabet E Gardarsdottir; Gudmundur Georgsson; Olafia S Gretarsdottir; Kjartan R Gudmundsson; Thora R Gunnarsdottir; Arnaldur Gylfason; Hilma Holm; Brynjar O Jensson; Aslaug Jonasdottir; Kamilla S Josefsdottir; Thordur Kristjansson; Droplaug N Magnusdottir; Louise le Roux; Gudrun Sigmundsdottir; Gardar Sveinbjornsson; Kristin E Sveinsdottir; Maney Sveinsdottir; Emil A Thorarensen; Bjarni Thorbjornsson; Gisli Masson; Ingileif Jonsdottir; Alma Moller; Thorolfur Gudnason; Karl G Kristinsson; Unnur Thorsteinsdottir; Kari Stefansson
EPI_ISL_424403	The National University Hospital of Iceland	deCODE genetics	Daniel F Gudbjartsson; Agnar Helgason; Hakon Jonsson; Olafur T Magnusson; Pall Melsted; Gudmundur L Norddahl; Jona Saemundsdottir; Asgeir Sigurdsson; Patrick Sulem; Arna B Agustsdottir; Berglind Eiriksdottir; Run Fridriksdottir; Elisabet E Gardarsdottir; Gudmundur Georgsson; Olafia S Gretarsdottir; Kjartan R Gudmundsson; Thora R Gunnarsdottir; Arnaldur Gylfason; Hilma Holm; Brynjar O Jensson; Aslaug Jonasdottir; Kamilla S Josefsdottir; Thordur Kristjansson; Droplaug N Magnusdottir; Louise le Roux; Gudrun Sigmundsdottir; Gardar Sveinbjornsson; Kristin E Sveinsdottir; Maney Sveinsdottir; Emil A Thorarensen; Bjarni Thorbjornsson; Gisli Masson; Ingileif Jonsdottir; Alma Moller; Thorolfur Gudnason; Karl G Kristinsson; Unnur Thorsteinsdottir; Kari Stefansson
EPI_ISL_424626, EPI_ISL_424627	Instituto Nacional de Enfermedades Respiratorias	Instituto Nacional de Enfermedades Respiratorias	Joel Armando Vázquez Pérez, Celia Boukadida, Santiago Avila Ríos, Mario Mújica Sánchez, José Arturo Martínez Orozco, Eduardo Becerril Vargas, Jorge Salas Hernández, Irma López Martínez, Lucia Hernández Rivas, Gisela Barrera Badillo, Edgar Mendieta Condado, Fabiola Garcés Ayala, Adnan Araiza Rodríguez, José Ernesto Ramírez González, Victor Hugo Borja Aburto, Concepción Grajales Muñiz, Cesar Raúl González Bonilla, Carolina González Torres, Francisco Javier Gaytán Cervantes, José Esteban Muñoz Medina, Guillermo M. Ruiz-Palacios, Pilar Ramos Cervantes, Violeta Ibarra Gonzalez, Fernando Ledesma Barrientos, Luis Alberto García Andrade, Alfredo Ponce de León Garduño, Blanca Taboada, Alejandro Sánchez, Pavel Isa, Ricardo Grande, Gloria Vázquez, Francisco Pulido, Carlos F. Arias.
EPI_ISL_424628	Department of Clinical Microbiology	GIGA Medical Genomics	Keith Durkin, Maria Artesi, Sébastien Bontems, Raphaël Boreux, Cécile Meex, Pierrette Melin, Marie-Pierre Hayette, Vincent Bours.
EPI_ISL_424668, EPI_ISL_424669, EPI_ISL_424671	Arizona State University Health Services	Arizona State University	Rabia Maqsood, LaRinda A. Holland, Emily A. Kaelin, Bereket Estifanos, Nicholas J. Mellor, Jason Steel, Lily I. Wu, Arvind Varsani, Rolf U. Halden, Brenda G. Hogue, Matthew Scotch, Efrem S. Lim
EPI_ISL_424703	Klinisk mikrobiologi, Region Västerbotten	Unit for Biological Agents, Department for CBRN Defence and Security, Swedish Defence Research Agency	FOI Bioinformatics team
EPI_ISL_424969, EPI_ISL_424970, EPI_ISL_424971, EPI_ISL_424972, EPI_ISL_424973, EPI_ISL_424974, EPI_ISL_424975, EPI_ISL_424978	Laboratory Medicine	Department of Laboratory Medicine, Lin-Kou Chang Gung Memorial Hospital, Taoyuan, Taiwan	Kuo-Chien Tsao, Yu-Nong Gong, Shu-Li Yang, Yi-Chun Liu, Chung-Guei Huang, Mei-Jen Hsiao, Po-Wei Huang, Cheng-Ta Yang, Cheng-Hsun Chiu, Peng-Nien Huang, Kuo-Ming Lee, Guang-Wu Chen, Shin-Ru Shih
EPI_ISL_425120, EPI_ISL_425121, EPI_ISL_425122, EPI_ISL_425123, EPI_ISL_425124, EPI_ISL_425125, EPI_ISL_425126	Center of Medical Microbiology, Virology, and Hospital Hygiene, University of Duesseldorf	Center of Medical Microbiology, Virology, and Hospital Hygiene, University of Duesseldorf	Ortwin Adams, Marcel Andree, Alexander Dilthey, Torsten Feldt, Sandra Hauka, Torsten Houwaart, Björn-Erik Jensen, Detlef Kindgen-Milles, Malte Kohns Vasconcelos, Klaus Pfeffer, Tina Senff, Daniel Strelow, Jörg Timm, Andreas Walker, Tobias Wienemann
EPI_ISL_425187	Servicio de Microbiología. Consorcio Hospital General Universitario de Valencia	Sequencing and Bioinformatics Service and Molecular Epidemiology Research Group. FISABIO-Public Health	Loreto Ferrús Abad, Paula Ruiz-Hueso, Mariana Reyes-Prieto, Vicente Soriano Chirona, Ivan Ansari, David Navarro, Maria Alma Bracho, Griselda De Marco, Beatriz Beamud, Lidia Ruiz Roldan, Marta Pla Diaz, Neris Garcia-Gonzalez, Inma Galán Vendrell, Sandra Carbo, Llúcia Martínez-Priego, Giuseppe D'Auria, Fernando Gonzalez-Candelas
EPI_ISL_425188	Servicio de Microbiología. Consorcio Hospital General Universitario de Valencia	Sequencing and Bioinformatics Service and Molecular Epidemiology Research Group. FISABIO-Public Health	Paula Ruiz-Hueso, Mariana Reyes-Prieto, Vicente Soriano Chirona, Ivan Ansari, David Navarro, Maria Alma Bracho, Griselda De Marco, Beatriz Beamud, Lidia Ruiz Roldan, Marta Pla Diaz, Neris Garcia-Gonzalez, Inma Galán Vendrell, Sandra Carbo, Loreto Ferrús Abad, Llúcia Martínez-Priego, Giuseppe D'Auria, Fernando Gonzalez-Candelas
EPI_ISL_425189	Servicio de Microbiología. Consorcio Hospital General Universitario de Valencia	Sequencing and Bioinformatics Service and Molecular Epidemiology Research Group. FISABIO-Public Health	Mariana Reyes-Prieto, Vicente Soriano Chirona, Ivan Ansari, David Navarro, Maria Alma Bracho, Griselda De Marco, Beatriz Beamud, Lidia Ruiz Roldan, Marta Pla Diaz, Neris Garcia-Gonzalez, Inma Galán Vendrell, Sandra Carbo, Loreto Ferrús Abad, Paula Ruiz-Hueso, Llúcia Martínez-Priego, Giuseppe D'Auria, Fernando Gonzalez-Candelas
EPI_ISL_425190	Servicio de Microbiología. Consorcio Hospital General Universitario de Valencia	Sequencing and Bioinformatics Service and Molecular Epidemiology Research Group. FISABIO-Public Health	Vicente Soriano Chirona, Ivan Ansari, David Navarro, Maria Alma Bracho, Griselda De Marco, Beatriz Beamud, Lidia Ruiz Roldan, Marta Pla Diaz, Neris Garcia-Gonzalez, Inma Galán Vendrell, Sandra Carbo, Loreto Ferrús Abad, Paula Ruiz-Hueso, Mariana Reyes-Prieto, Llúcia Martínez-Priego, Giuseppe D'Auria, Fernando Gonzalez-Candelas
EPI_ISL_425191	Servicio de Microbiología. Consorcio Hospital General Universitario de Valencia	Sequencing and Bioinformatics Service and Molecular Epidemiology Research Group. FISABIO-Public Health	Ivan Ansari, David Navarro, Maria Alma Bracho, Griselda De Marco, Beatriz Beamud, Lidia Ruiz Roldan, Marta Pla Diaz, Neris Garcia-Gonzalez, Inma Galán Vendrell, Sandra Carbo, Loreto Ferrús Abad, Paula Ruiz-Hueso, Mariana Reyes-Prieto, Vicente Soriano Chirona, Llúcia Martínez-Priego, Giuseppe D'Auria, Fernando Gonzalez-Candelas
EPI_ISL_425192	Servicio de Microbiología. Consorcio Hospital General Universitario de Valencia	Sequencing and Bioinformatics Service and Molecular Epidemiology Research Group. FISABIO-Public Health	David Navarro, Maria Alma Bracho, Griselda De Marco, Beatriz Beamud, Lidia Ruiz Roldan, Marta Pla Diaz, Neris Garcia-Gonzalez, Inma Galán Vendrell, Sandra Carbo, Loreto Ferrús Abad, Paula Ruiz-Hueso, Mariana Reyes-Prieto, Vicente Soriano Chirona, Ivan Ansari, Llúcia Martínez-Priego, Giuseppe D'Auria, Fernando Gonzalez-Candelas
EPI_ISL_425193	Servicio de Microbiología. Consorcio Hospital General Universitario de Valencia	Sequencing and Bioinformatics Service and Molecular Epidemiology Research Group. FISABIO-Public Health	Maria Alma Bracho, Griselda De Marco, Beatriz Beamud, Lidia Ruiz Roldan, Marta Pla Diaz, Neris Garcia-Gonzalez, Inma Galán Vendrell, Sandra Carbo, Loreto Ferrús Abad, Paula Ruiz-Hueso, Mariana Reyes-Prieto, Vicente Soriano Chirona, Ivan Ansari, David Navarro, Llúcia Martínez-Priego, Giuseppe D'Auria, Fernando Gonzalez-Candelas
EPI_ISL_425194	Servicio de Microbiología. Consorcio Hospital General Universitario de Valencia	Sequencing and Bioinformatics Service and Molecular Epidemiology Research Group. FISABIO-Public Health	Griselda De Marco, Beatriz Beamud, Lidia Ruiz Roldan, Marta Pla Diaz, Neris Garcia-Gonzalez, Inma Galán Vendrell, Sandra Carbo, Loreto Ferrús Abad, Paula Ruiz-Hueso, Mariana Reyes-Prieto, Vicente Soriano Chirona, Ivan Ansari, David Navarro, Maria Alma Bracho, Llúcia Martínez-Priego, Giuseppe D'Auria, Fernando Gonzalez-Candelas
EPI_ISL_425195	Servicio de Microbiología. Consorcio Hospital General Universitario de Valencia	Sequencing and Bioinformatics Service and Molecular Epidemiology Research Group. FISABIO-Public Health	Beatriz Beamud, Lidia Ruiz Roldan, Marta Pla Diaz, Neris Garcia-Gonzalez, Inma Galán Vendrell, Sandra Carbo, Loreto Ferrús Abad, Paula Ruiz-Hueso, Mariana Reyes-Prieto, Vicente Soriano Chirona, Ivan Ansari, David Navarro, Maria Alma Bracho, Griselda De Marco, Llúcia Martínez-Priego, Giuseppe D'Auria, Fernando Gonzalez-Candelas
EPI_ISL_425196	Servicio de Microbiología. Consorcio Hospital General Universitario de Valencia	Sequencing and Bioinformatics Service and Molecular Epidemiology Research Group. FISABIO-Public Health	Lidia Ruiz Roldan, Marta Pla Diaz, Neris Garcia-Gonzalez, Inma Galán Vendrell, Sandra Carbo, Loreto Ferrús Abad, Paula Ruiz-Hueso, Mariana Reyes-Prieto, Vicente Soriano Chirona, Ivan Ansari, David Navarro, Maria Alma Bracho, Griselda De Marco, Beatriz Beamud, Llúcia Martínez-Priego, Giuseppe D'Auria, Fernando Gonzalez-Candelas
EPI_ISL_425197	Servicio de Microbiología. Consorcio Hospital General Universitario de Valencia	Sequencing and Bioinformatics Service and Molecular Epidemiology Research Group. FISABIO-Public Health	Marta Pla Diaz, Neris Garcia-Gonzalez, Inma Galán Vendrell, Sandra Carbo, Loreto Ferrús Abad, Paula Ruiz-Hueso, Mariana Reyes-Prieto, Vicente Soriano Chirona, Ivan Ansari, David Navarro, Maria Alma Bracho, Griselda De Marco, Beatriz Beamud, Lidia Ruiz Roldan, Llúcia Martínez-Priego, Giuseppe D'Auria, Fernando Gonzalez-Candelas
EPI_ISL_425198	Servicio de Microbiología. Consorcio Hospital General Universitario de Valencia	Sequencing and Bioinformatics Service and Molecular Epidemiology Research Group. FISABIO-Public Health	Neris Garcia-Gonzalez, Inma Galán Vendrell, Sandra Carbo, Loreto Ferrús Abad, Paula Ruiz-Hueso, Mariana Reyes-Prieto, Vicente Soriano Chirona, Ivan Ansari, David Navarro, Maria Alma Bracho, Griselda De Marco, Beatriz Beamud, Lidia Ruiz Roldan, Marta Pla Diaz, Llúcia Martínez-Priego, Giuseppe D'Auria, Fernando Gonzalez-Candelas
EPI_ISL_425200	Servicio de Microbiologia. Hospital Clinico Universitario de Valencia	Sequencing and Bioinformatics Service and Molecular Epidemiology Research Group. FISABIO-Public Health	Paula Ruiz-Hueso, Mariana Reyes-Prieto, Vicente Soriano Chirona, Maria Alma Bracho, Griselda De Marco, Beatriz Beamud, Lidia Ruiz Roldan, Marta Pla Diaz,Neris Garcia-Gonzalez, Loreto Ferrús Abad, Maria Dolores Ocete, Llúcia Martínez-Priego, Inma Galán Vendrell, Concepcion Gimeno, Giuseppe D'Auria, Fernando Gonzalez-Candelas
EPI_ISL_425215	Servicio de Microbiología. Consorcio Hospital General Universitario de Valencia	Sequencing and Bioinformatics Service and Molecular Epidemiology Research Group. FISABIO-Public Health	Beatriz Beamud, Lidia Ruiz Roldan, Marta Pla Diaz, Neris Garcia-Gonzalez, Inma Galán Vendrell, Sandra Carbo, Loreto Ferrús Abad, Paula Ruiz-Hueso, Mariana Reyes-Prieto, Vicente Soriano Chirona, Ivan Ansari, David Navarro, Maria Alma Bracho, Griselda De Marco, Llúcia Martínez-Priego, Giuseppe D'Auria, Fernando Gonzalez-Candelas
EPI_ISL_425216	Servicio de Microbiología. Consorcio Hospital General Universitario de Valencia	Sequencing and Bioinformatics Service and Molecular Epidemiology Research Group. FISABIO-Public Health	Lidia Ruiz Roldan, Marta Pla Diaz, Neris Garcia-Gonzalez, Inma Galán Vendrell, Sandra Carbo, Loreto Ferrús Abad, Paula Ruiz-Hueso, Mariana Reyes-Prieto, Vicente Soriano Chirona, Ivan Ansari, David Navarro, Maria Alma Bracho, Griselda De Marco, Beatriz Beamud, Llúcia Martínez-Priego, Giuseppe D'Auria, Fernando Gonzalez-Candelas

EPI_ISL_425217	Servicio de Microbiología. Consorcio Hospital General Universitario de Valencia	Sequencing and Bioinformatics Service and Molecular Epidemiology Research Group. FISABIO-Public Health	Marta Pla Diaz, Neris Garcia-Gonzalez, Inma Galán Vendrell, Sandra Carbo, Loreto Ferrús Abad, Paula Ruiz-Hueso, Mariana Reyes-Prieto, Vicente Soriano Chirona, Ivan Ansari, David Navarro, Maria Alma Bracho, Griselda De Marco, Beatriz Beamud, Lidia Ruiz Roldan, Llúcia Martínez-Priego, Giuseppe D'Auria, Fernando Gonzalez-Candelas
EPI_ISL_425218, EPI_ISL_425219	Servicio de Microbiología. Consorcio Hospital General Universitario de Valencia	Sequencing and Bioinformatics Service and Molecular Epidemiology Research Group. FISABIO-Public Health	Neris Garcia-Gonzalez, Inma Galán Vendrell, Sandra Carbo, Loreto Ferrús Abad, Paula Ruiz-Hueso, Mariana Reyes-Prieto, Vicente Soriano Chirona, Ivan Ansari, David Navarro, Maria Alma Bracho, Griselda De Marco, Beatriz Beamud, Lidia Ruiz Roldan, Marta Pla Diaz, Llúcia Martínez-Priego, Giuseppe D'Auria, Fernando Gonzalez-Candelas
EPI_ISL_425220	Servicio de Microbiología. Consorcio Hospital General Universitario de Valencia	Sequencing and Bioinformatics Service and Molecular Epidemiology Research Group. FISABIO-Public Health	Inma Galán Vendrell, Sandra Carbo, Loreto Ferrús Abad, Paula Ruiz-Hueso, Mariana Reyes-Prieto, Vicente Soriano Chirona, Ivan Ansari, David Navarro, Maria Alma Bracho, Griselda De Marco, Beatriz Beamud, Lidia Ruiz Roldan, Marta Pla Diaz, Neris Garcia-Gonzalez, Llúcia Martínez-Priego, Giuseppe D'Auria, Fernando Gonzalez-Candelas
EPI_ISL_425221	Servicio de Microbiología. Consorcio Hospital General Universitario de Valencia	Sequencing and Bioinformatics Service and Molecular Epidemiology Research Group. FISABIO-Public Health	Sandra Carbo, Loreto Ferrús Abad, Paula Ruiz-Hueso, Mariana Reyes-Prieto, Vicente Soriano Chirona, Ivan Ansari, David Navarro, Maria Alma Bracho, Griselda De Marco, Beatriz Beamud, Lidia Ruiz Roldan, Marta Pla Diaz, Neris Garcia-Gonzalez, Inma Galán Vendrell, Llúcia Martínez-Priego, Giuseppe D'Auria, Fernando Gonzalez-Candelas
EPI_ISL_425222	Servicio de Microbiología. Consorcio Hospital General Universitario de Valencia	Sequencing and Bioinformatics Service and Molecular Epidemiology Research Group. FISABIO-Public Health	Loreto Ferrús Abad, Paula Ruiz-Hueso, Mariana Reyes-Prieto, Vicente Soriano Chirona, Ivan Ansari, David Navarro, Maria Alma Bracho, Griselda De Marco, Beatriz Beamud, Lidia Ruiz Roldan, Marta Pla Diaz, Neris Garcia-Gonzalez, Inma Galán Vendrell, Sandra Carbo, Llúcia Martínez-Priego, Giuseppe D'Auria, Fernando Gonzalez-Candelas
EPI_ISL_425223	Servicio de Microbiología. Consorcio Hospital General Universitario de Valencia	Sequencing and Bioinformatics Service and Molecular Epidemiology Research Group. FISABIO-Public Health	Paula Ruiz-Hueso, Mariana Reyes-Prieto, Vicente Soriano Chirona, Ivan Ansari, David Navarro, Maria Alma Bracho, Griselda De Marco, Beatriz Beamud, Lidia Ruiz Roldan, Marta Pla Diaz, Neris Garcia-Gonzalez, Inma Galán Vendrell, Sandra Carbo, Loreto Ferrús Abad, Llúcia Martínez-Priego, Giuseppe D'Auria, Fernando Gonzalez-Candelas
EPI_ISL_425284, EPI_ISL_425341, EPI_ISL_425342, EPI_ISL_425343, EPI_ISL_425344, EPI_ISL_425345, EPI_ISL_425347, EPI_ISL_425348, EPI_ISL_425349, EPI_ISL_425350, EPI_ISL_425351, EPI_ISL_425352, EPI_ISL_425353, EPI_ISL_425354, EPI_ISL_425355, EPI_ISL_425356, EPI_ISL_425357, EPI_ISL_425358, EPI_ISL_425359, EPI_ISL_425362, EPI_ISL_425363, EPI_ISL_425364, EPI_ISL_425365, EPI_ISL_425366, EPI_ISL_425367, EPI_ISL_425368, EPI_ISL_425375, EPI_ISL_425376, EPI_ISL_425377, EPI_ISL_425378, EPI_ISL_425379, EPI_ISL_425380, EPI_ISL_425381, EPI_ISL_425382, EPI_ISL_425383, EPI_ISL_425384, EPI_ISL_425385, EPI_ISL_425389, EPI_ISL_425393, EPI_ISL_425395, EPI_ISL_425396, EPI_ISL_425397, EPI_ISL_425403, EPI_ISL_425406, EPI_ISL_425408, EPI_ISL_425409, EPI_ISL_425411, EPI_ISL_425412, EPI_ISL_425413, EPI_ISL_425416, EPI_ISL_425417, EPI_ISL_425418, EPI_ISL_425420			
see above	Department of Pathology, University of Cambridge	COVID-19 Genomics UK (COG-UK) Consortium	Luke W Meredith, M. Estee Torok , Myra Hosmillo, William L. Hamilton, Martin D. Curran, Theresa Feltwell, Anna Yakovleva, Charlotte J. Houldcroft, Aminu S. Jahun, Sarah L. Caddy, Ian Goodfellow
EPI_ISL_425478, EPI_ISL_425479, EPI_ISL_425480, EPI_ISL_425481, EPI_ISL_425482, EPI_ISL_425483, EPI_ISL_425484, EPI_ISL_425485, EPI_ISL_425486, EPI_ISL_425487, EPI_ISL_425488, EPI_ISL_425489, EPI_ISL_425490, EPI_ISL_425491, EPI_ISL_425492, EPI_ISL_425493, EPI_ISL_425494, EPI_ISL_425495, EPI_ISL_425501, EPI_ISL_425502, EPI_ISL_425503, EPI_ISL_425504, EPI_ISL_425505, EPI_ISL_425644, EPI_ISL_425645			
see above	Queens Medical Centre, Clinical Microbiology Department / DeepSeq Nottingham	COVID-19 Genomics UK (COG-UK) Consortium	Gemma Clark, Wendy Smith, Manjinder Khakh, Hannah Howson-Wells, Jonathan Ball, Patrick McClure, Joseph Chappell, Theocharis Tsoleridis, Nadine Holmes, Matthew Carlisle, Christopher Moore, Fei Sang, Johnny Debebe, Victoria Wright, Matthew Loose
EPI_ISL_425657, EPI_ISL_425658, EPI_ISL_425665, EPI_ISL_425667, EPI_ISL_425669, EPI_ISL_425673, EPI_ISL_425675, EPI_ISL_425676, EPI_ISL_425677, EPI_ISL_425678, EPI_ISL_425679, EPI_ISL_425680, EPI_ISL_425681, EPI_ISL_425682, EPI_ISL_425684, EPI_ISL_425685, EPI_ISL_425686, EPI_ISL_425687, EPI_ISL_425688, EPI_ISL_425691, EPI_ISL_425693, EPI_ISL_425694, EPI_ISL_425695, EPI_ISL_425696, EPI_ISL_425697, EPI_ISL_425698, EPI_ISL_425700, EPI_ISL_425701, EPI_ISL_425702, EPI_ISL_425703, EPI_ISL_425704, EPI_ISL_425705, EPI_ISL_425706, EPI_ISL_425707, EPI_ISL_425708, EPI_ISL_425710, EPI_ISL_425711, EPI_ISL_425712, EPI_ISL_425713, EPI_ISL_425714, EPI_ISL_425715, EPI_ISL_425716, EPI_ISL_425717, EPI_ISL_425718, EPI_ISL_425719, EPI_ISL_425721, EPI_ISL_425722, EPI_ISL_425723, EPI_ISL_425725, EPI_ISL_425726, EPI_ISL_425727, EPI_ISL_425729, EPI_ISL_425731, EPI_ISL_425732, EPI_ISL_425734, EPI_ISL_425736, EPI_ISL_425737, EPI_ISL_425740, EPI_ISL_425741, EPI_ISL_425743, EPI_ISL_425747			
see above	West of Scotland Specialist Virology Centre, NHSGGC / MRC-University of Glasgow Centre for Virus Research	COVID-19 Genomics UK (COG-UK) Consortium	Ana da Silva Filipe, Kathy Smollett, Stephen Carmichael, Natasha Johnson, Daniel Mair, Lily Tong, Jenna Nichols; Sarah McDonald; Richard Orton, Joseph Hughes, Sreenu Vattipally, David L Robertson; Kathy Li, Natasha Jesudason, Rajiv Shah, James Shepherd, Antonia Ho, Emma Thomson; Alasdair MacLean, Rory Gunson.
EPI_ISL_425831, EPI_ISL_425850, EPI_ISL_425851, EPI_ISL_425852, EPI_ISL_425853, EPI_ISL_425854, EPI_ISL_425855, EPI_ISL_425856, EPI_ISL_425857, EPI_ISL_425858, EPI_ISL_425860, EPI_ISL_425861, EPI_ISL_425862, EPI_ISL_425865, EPI_ISL_425906			
see above	Virology Department, Royal Infirmary of Edinburgh, NHS Lothian / School of Biological Sciences, University of Edinburgh / Institute of Genetics and Molecular Medicine, University of Edinburgh	COVID-19 Genomics UK (COG-UK) Consortium	McHugh M, Dewar R, Rooke S, Gallagher M, Balcaza C, O'Toole A, Hill V, McCrone JT, Colquhoun R, Yu X, Jackson B, Scher E, Rambaut A, Williams TC, Templeton K
EPI_ISL_426297, EPI_ISL_426306, EPI_ISL_426316, EPI_ISL_426317, EPI_ISL_426319, EPI_ISL_426323	Wadsworth Center, New York State Department.of Health	Wadsworth Center, New York State Department.of Health	Kirsten St. George, Daryl M. Lamson, Sara Griesemer, Jonathan Plitnick, Navjot Singh, Matthew D. Shudt, Erica Lasek-Nesselquist
EPI_ISL_426357, EPI_ISL_426359, EPI_ISL_426360	Laboratory of Molecular Genetics, 2nd Faculty of Medicine, Charles University in Prague, Prague, Czech Republic	Laboratory of Molecular Genetics, 2nd Faculty of Medicine, Charles University in Prague, Prague, Czech Republic	Lenka Kramna, Katerina Polackova, Ondrej Cinek
EPI_ISL_426405	Queen Mary Hospital	Hong Kong Department of Health	Mak Gannon C.K., Cheng Peter K.C., Lam Edman T.K., Chan Rickjason C.W., Tsang Dominic N.C.
EPI_ISL_426406	Queen Elizabeth Hospital	Hong Kong Department of Health	Mak Gannon C.K., Cheng Peter K.C., Lam Edman T.K., Chan Rickjason C.W., Tsang Dominic N.C.
EPI_ISL_426407	Caritas Medical Centre	Hong Kong Department of Health	Mak Gannon C.K., Cheng Peter K.C., Lam Edman T.K., Chan Rickjason C.W., Tsang Dominic N.C.
EPI_ISL_426408	Ruttonjee Hospital	Hong Kong Department of Health	Mak Gannon C.K., Cheng Peter K.C., Lam Edman T.K., Chan Rickjason C.W., Tsang Dominic N.C.
EPI_ISL_426476, EPI_ISL_426477, EPI_ISL_426478	Microbial Genomics Laboratory, Institut Pasteur Montevideo	Microbial Genomics Laboratory, Institut Pasteur Montevideo, Uruguay	Cecilia Salazar, Florencia Díaz-Viraqué, Marianoel Pereira, Pilar Moreno, Gonzalo Moratorio, Gregorio Iraola
EPI_ISL_426479, EPI_ISL_426480	Microbial Genomics Laboratory, Institut Pasteur Montevideo	Microbial Genomics Laboratory, Institut Pasteur Montevideo	Cecilia Salazar, Florencia Díaz-Viraqué, Marianoel Pereira, Pilar Moreno, Gonzalo Moratorio, Gregorio Iraola
EPI_ISL_426481, EPI_ISL_426482	Microbial Genomics Laboratory, Institut Pasteur Montevideo, Uruguay	Microbial Genomics Laboratory, Institut Pasteur Montevideo	Cecilia Salazar, Florencia Díaz-Viraqué, Marianoel Pereira, Pilar Moreno, Gonzalo Moratorio, Gregorio Iraola
EPI_ISL_426499, EPI_ISL_426500, EPI_ISL_426501, EPI_ISL_426502, EPI_ISL_426503, EPI_ISL_426504, EPI_ISL_426505, EPI_ISL_426506, EPI_ISL_426507, EPI_ISL_426509, EPI_ISL_426510			
see above	TGen North	TGen North	Jolene Bowers, Megan Folkerts, Darrin Lemmer, Dave Engelthaler
EPI_ISL_426527, EPI_ISL_426529, EPI_ISL_426530	AZ SPHL, Arizona Department of Health Services	TGen North	Jolene Bowers, Megan Folkerts, Darrin Lemmer, Dave Engelthaler
EPI_ISL_426535	TGen North	TGen North	Jolene Bowers, Megan Folkerts, Darrin Lemmer, Dave Engelthaler
EPI_ISL_426537	AZ SPHL, Arizona Department of Health Services	TGen North	Jolene Bowers, Megan Folkerts, Darrin Lemmer, Dave Engelthaler
EPI_ISL_426583	Microbial Genomics Laboratory, Institut Pasteur Monteivdeo	Microbial Genomics Laboratory, Institut Pasteur Montevideo, Uruguay	Cecilia Salazar, Florencia Díaz-Viraqué, Marianoel Pereira, Pilar Moreno, Gonzalo Moratorio, Gregorio Iraola
EPI_ISL_426630	TSGH-CP molecular lab	TSGH-CP molecular lab	Cherng-Lih Perng, Ming-Jr Jian, Chih-Kai Chang, Jung-Chung Lin, Kuo-Ming Yeh, Chien-Wen Chen, Sheng-Kang Chiu, Hsing-Yi Chung, Shih-Hung Tsai, Kuo-Sheng Hung, Feng-Yee Chang, Hung-Sheng Shang
EPI_ISL_426631, EPI_ISL_426632	TSGH-CP molecular lab	TSGH-CP molecular lab	Cherng-Lih Perng, Ming-Jr Jian, Chih-Kai Chang, Jung-Chung Lin, Kuo-Ming Yeh, Chien-Wen Chen, Sheng-Kang Chiu, Hsing-Yi Chung, Shih-Hung Tsai, Kuo-Sheng Hung, Tien-Yao Chang, Feng-Yee Chang, Hung-Sheng Shang
EPI_ISL_426637, EPI_ISL_426638, EPI_ISL_426639, EPI_ISL_426640, EPI_ISL_426641, EPI_ISL_426642, EPI_ISL_427042	Victorian Infectious Diseases Reference Laboratory (VIDRL)	Microbiological Diagnostic Unit Public Health Laboratory and Victorian Infectious Diseases Reference Laboratory, Doherty Institute	Caly L., Seemann T., Sait, M., Schultz M., Druce J., Sherry, N.
EPI_ISL_427043	Laboratory of Microbiology, Medical School, National and Kapodistrian University of Athens	Laboratory of Biology, Department of Medicine, Democritus University of Thrace	Bampali,M., Dovrolis,N., Gatzidou,E., Froukala,E., Stavropoulou,A., Veletza,S., Tsakris,A., Spanakis,N. and Karakasiliotis,I.

EPI_ISL_427047, EPI_ISL_427050, EPI_ISL_427079	Victorian Infectious Diseases Reference Laboratory (VIDRL)	Microbiological Diagnostic Unit Public Health Laboratory and Victorian Infectious Diseases Reference Laboratory, Doherty Institute	Caly L., Seemann T., Sait, M., Schultz M., Druce J., Sherry, N.
EPI_ISL_427281	Minnesota Department of Health, Public Health Laboratory	Minnesota Department of Health, Public Health Laboratory	Matt Plumb, Jacob Garfin and Xiong Wang
EPI_ISL_427292	LACEN-AL - Laboratorio Central de Alagoas	Instituto Oswaldo Cruz FIOCRUZ - Laboratory of Respiratory Viruses and Measles (LVRS)	Paola Resende, Fernando Motta, Luciana Appolinario, Sunando Roy, Aline Mattos, Milene Miranda, Cristiana Garcia, Braulia Caetano, Maria Ogrzewalska, Priscila Born, Jonathan Lopes, Marilda Siqueira
EPI_ISL_427393	TSGH-CP molecular lab	TSGH-CP molecular lab	Cherng-Lih Perng, Ming-Jr Jian, Chih-Kai Chang, Jung-Chung Lin, Kuo-Ming Yeh, Chien-Wen Chen, Sheng-Kang Chiu, Hsing-Yi Chung, Shih-Hung Tsai, Kuo-Sheng Hung, Tien-Yao Chang, Feng-Yee Chang, Hung-Sheng Shang
EPI_ISL_427515, EPI_ISL_427516, EPI_ISL_427517, EPI_ISL_427518, EPI_ISL_427519, EPI_ISL_427520, EPI_ISL_427521	NYU Langone Health	Departments of Pathology and Medicine, New York University School of Medicine	Maria Aguero-Rosenfeld, Brendan Belovarac, Margaret Black, Ludovic Boytard, John Cadley, Paolo Cotzia, John Chen, Dacia Dimartino, Xiaojun Feng, Tatyana Gindin, Emily Guzman, Adriana Heguy, Megan Hogan, Emily Huang, George Jour, Andrew Lytle, Christian Marier, Matthew T. Maurano, Mark J. Mulligan, Peter Meyn, Iman Osman, Jared Pinnell, Vanessa Raabe, Sitharam Ramaswami, Amy Rapkiewicz, Marie Samanovic-Golden, Antonio Serrano, Guomiao Shen, Matija Snuderl, Theodore Vougiouklakis, Nick Vulpescu, Gael Westby, Paul Zappile, Yutong Zhang
EPI_ISL_427553, EPI_ISL_427554, EPI_ISL_427555, EPI_ISL_427556, EPI_ISL_427557, EPI_ISL_427558, EPI_ISL_427559, EPI_ISL_427560, EPI_ISL_427561, EPI_ISL_427562, EPI_ISL_427563, EPI_ISL_427564, EPI_ISL_427565, EPI_ISL_427566, EPI_ISL_427567, EPI_ISL_427568, EPI_ISL_427569, EPI_ISL_427570, EPI_ISL_427571, EPI_ISL_427572, EPI_ISL_427573, EPI_ISL_427574, EPI_ISL_427575, EPI_ISL_427576, EPI_ISL_427577, EPI_ISL_427578, EPI_ISL_427579, EPI_ISL_427580, EPI_ISL_427581, EPI_ISL_427582, EPI_ISL_427583, EPI_ISL_427584, EPI_ISL_427585, EPI_ISL_427586, EPI_ISL_427587, EPI_ISL_427588, EPI_ISL_427589, EPI_ISL_427590, EPI_ISL_427591, EPI_ISL_427592, EPI_ISL_427593, EPI_ISL_427594, EPI_ISL_427595, EPI_ISL_427596, EPI_ISL_427597, EPI_ISL_427598, EPI_ISL_427599, EPI_ISL_427600, EPI_ISL_427601, EPI_ISL_427602, EPI_ISL_427603, EPI_ISL_427604, EPI_ISL_427605, EPI_ISL_427606, EPI_ISL_427607, EPI_ISL_427608, EPI_ISL_427609, EPI_ISL_427610, EPI_ISL_427611, EPI_ISL_427612, EPI_ISL_427613, EPI_ISL_427614, EPI_ISL_427615, EPI_ISL_427616, EPI_ISL_427617, EPI_ISL_427618			
see above	NewYork-Presbyterian & Mason Lab	Mason Lab	Daniel J. Butler, Christopher Mozsary, Cem Meydan, David Danko, Jonathan Foox, Joel Rosiene, Alon Shaiber, Matthew MacKay, Ebrahim Afshinnekoo, Fritz J. Sedlazeck, Nikolay A. Ivanov, Maria Sierra, Craig D. Westover, Krista Ryon, Benjamin Young, Chandrima Bhattacharya, Phyllis Ruggiero, Justyna Gawrys, Iman Hajirasouliha, Dmitry Meleshko, Mirella Salvatore, Dong Xu, Jenny Xiang, John Sipley, Lin Cong, Arryn Craney, Priya Velu, Lars F. Westblade, Massimo Loda, Shawn Levy, Melissa Cushing, Marcin Imielinski, Hanna Rennert, Christopher E. Mason
EPI_ISL_427661	Centre for Infectious Diseases and Microbiology Public Health	NSW Health Pathology - Institute of Clinical Pathology and Medical Research; Westmead Hospital; University of Sydney	Sim E, Bachmann N, Rockett R, Lam C, Gray K, Timms V, Gall M, Arnott A, Sadsad R, Draper J, Carter I, Holmes EC, O'Sullivan MV, Byun R, Sintchenko V, Chen SC, Eden JS, Maddocks S, Kok J, Propenko M, Sorrell T, Chang S, Basile K, Dwyer DE for the 2019-nCoV Study Group
EPI_ISL_427662	Centre for Infectious Diseases and Microbiology Public Health	NSW Health Pathology - Institute of Clinical Pathology and Medical Research; Westmead Hospital; University of Sydney	Lam C, Gray K, Timms, V, Gall M, Arnott A, Sadsad R, Draper J, Sim E, Bachmann N, Rockett R, Carter I, Holmes EC, O'Sullivan MV, Byun R, Sintchenko V, Chen SC, Eden JS, Maddocks S, Kok J, Propenko M, Sorrell T, Chang S, Basile K, Dwyer DE for the 2019-nCoV Study Group
EPI_ISL_427663	Centre for Infectious Diseases and Microbiology Public Health	NSW Health Pathology - Institute of Clinical Pathology and Medical Research; Westmead Hospital; University of Sydney	Timms V, Gall M, Arnott A, Sadsad R, Draper J, Sim E, Bachmann N, Rockett R, Lam C, Gray K, Carter I, Holmes EC, O'Sullivan MV, Byun R, Sintchenko V, Chen SC, Eden JS, Maddocks S, Kok J, Propenko M, Sorrell T, Chang S, Basile K, Dwyer DE for the 2019-nCoV Study Group
EPI_ISL_427666, EPI_ISL_427675	Centre for Infectious Diseases and Microbiology Public Health	NSW Health Pathology - Institute of Clinical Pathology and Medical Research; Westmead Hospital; University of Sydney	Bachmann N, Rockett R, Lam C, Gray K, Timms V, Gall M, Arnott A, Sadsad R, Draper J, Sim E, Carter I, Holmes EC, O'Sullivan MV, Byun R, Sintchenko V, Chen SC, Eden JS, Maddocks S, Kok J, Propenko M, Sorrell T, Chang S, Basile K, Dwyer DE for the 2019-nCoV Study Group
EPI_ISL_427677	Centre for Infectious Diseases and Microbiology Public Health	NSW Health Pathology - Institute of Clinical Pathology and Medical Research; Westmead Hospital; University of Sydney	Lam C, Gray K, Timms, V, Gall M, Arnott A, Sadsad R, Draper J, Sim E, Bachmann N, Rockett R, Carter I, Holmes EC, O'Sullivan MV, Byun R, Sintchenko V, Chen SC, Eden JS, Maddocks S, Kok J, Propenko M, Sorrell T, Chang S, Basile K, Dwyer DE for the 2019-nCoV Study Group
EPI_ISL_427687	Centre for Infectious Diseases and Microbiology Public Health	NSW Health Pathology - Institute of Clinical Pathology and Medical Research; Westmead Hospital; University of Sydney	Gray K, Timms V, Gall M, Arnott A, Sadsad R, Draper J, Sim E, Bachmann N, Rockett R, Lam C, Carter I, Holmes EC, O'Sullivan MV, Byun R, Sintchenko V, Chen SC, Eden JS, Maddocks S, Kok J, Propenko M, Sorrell T, Chang S, Basile K, Dwyer DE for the 2019-nCoV Study Group
EPI_ISL_427702	Centre for Infectious Diseases and Microbiology Public Health	NSW Health Pathology - Institute of Clinical Pathology and Medical Research; Westmead Hospital; University of Sydney	Arnott A, Sadsad R, Draper J, Sim E, Bachmann N, Rockett R, Lam C, Gray K, Timms V, Gall M, Carter I, Holmes EC, O'Sullivan MV, Byun R, Sintchenko V, Chen SC, Eden JS, Maddocks S, Kok J, Propenko M, Sorrell T, Chang S, Basile K, Dwyer DE for the 2019-nCoV Study Group
EPI_ISL_427703	Centre for Infectious Diseases and Microbiology Public Health	NSW Health Pathology - Institute of Clinical Pathology and Medical Research; Westmead Hospital; University of Sydney	Gray K, Timms V, Gall M, Arnott A, Sadsad R, Draper J, Sim E, Bachmann N, Rockett R, Lam C, Carter I, Holmes EC, O'Sullivan MV, Byun R, Sintchenko V, Chen SC, Eden JS, Maddocks S, Kok J, Propenko M, Sorrell T, Chang S, Basile K, Dwyer DE for the 2019-nCoV Study Group
EPI_ISL_427716	Centre for Infectious Diseases and Microbiology Public Health	NSW Health Pathology - Institute of Clinical Pathology and Medical Research; Westmead Hospital; University of Sydney	Lam C, Gray K, Timms, V, Gall M, Arnott A, Sadsad R, Draper J, Sim E, Bachmann N, Rockett R, Carter I, Holmes EC, O'Sullivan MV, Byun R, Sintchenko V, Chen SC, Eden JS, Maddocks S, Kok J, Propenko M, Sorrell T, Chang S, Basile K, Dwyer DE for the 2019-nCoV Study Group
EPI_ISL_427717	ACT Pathology, The Canberra Hospital	NSW Health Pathology - Institute of Clinical Pathology and Medical Research; Westmead Hospital; University of Sydney	Sim E, Bachmann N, Rockett R, Lam C, Gray K, Timms V, Gall M, Arnott A, Sadsad R, Draper J, Carter I, Holmes EC, O'Sullivan MV, Byun R, Sintchenko V, Chen SC, Eden JS, Maddocks S, Kok J, Propenko M, Sorrell T, Chang S, Basile K, Dwyer DE for the 2019-nCoV Study Group
EPI_ISL_427719	ACT Pathology, The Canberra Hospital	NSW Health Pathology - Institute of Clinical Pathology and Medical Research; Westmead Hospital; University of Sydney	Timms V, Gall M, Arnott A, Sadsad R, Draper J, Sim E, Bachmann N, Rockett R, Lam C, Gray K, Carter I, Holmes EC, O'Sullivan MV, Byun R, Sintchenko V, Chen SC, Eden JS, Maddocks S, Kok J, Propenko M, Sorrell T, Chang S, Basile K, Dwyer DE for the 2019-nCoV Study Group
EPI_ISL_427721	ACT Pathology, The Canberra Hospital	NSW Health Pathology - Institute of Clinical Pathology and Medical Research; Westmead Hospital; University of Sydney	Sim E, Bachmann N, Rockett R, Lam C, Gray K, Timms V, Gall M, Arnott A, Sadsad R, Draper J, Carter I, Holmes EC, O'Sullivan MV, Byun R, Sintchenko V, Chen SC, Eden JS, Maddocks S, Kok J, Propenko M, Sorrell T, Chang S, Basile K, Dwyer DE for the 2019-nCoV Study Group
EPI_ISL_427726	Centre for Infectious Diseases and Microbiology Public Health	NSW Health Pathology - Institute of Clinical Pathology and Medical Research; Westmead Hospital; University of Sydney	Rockett R, Lam C, Gray K, Timms V, Gall M, Arnott A, Sadsad R, Draper J, Sim E, Bachmann N, Carter I, Holmes EC, O'Sullivan MV, Byun R, Sintchenko V, Chen SC, Eden JS, Maddocks S, Kok J, Propenko M, Sorrell T, Chang S, Basile K, Dwyer DE for the 2019-nCoV Study Group
EPI_ISL_427727	Centre for Infectious Diseases and Microbiology Public Health	NSW Health Pathology - Institute of Clinical Pathology and Medical Research; Westmead Hospital; University of Sydney	Lam C, Gray K, Timms, V, Gall M, Arnott A, Sadsad R, Draper J, Sim E, Bachmann N, Rockett R, Carter I, Holmes EC, O'Sullivan MV, Byun R, Sintchenko V, Chen SC, Eden JS, Maddocks S, Kok J, Propenko M, Sorrell T, Chang S, Basile K, Dwyer DE for the 2019-nCoV Study Group
EPI_ISL_427728	Centre for Infectious Diseases and Microbiology Public Health	NSW Health Pathology - Institute of Clinical Pathology and Medical Research; Westmead Hospital; University of Sydney	Rockett R, Lam C, Gray K, Timms V, Gall M, Arnott A, Sadsad R, Draper J, Sim E, Bachmann N, Carter I, Holmes EC, O'Sullivan MV, Byun R, Sintchenko V, Chen SC, Eden JS, Maddocks S, Kok J, Propenko M, Sorrell T, Chang S, Basile K, Dwyer DE for the 2019-nCoV Study Group
EPI_ISL_427729	Centre for Infectious Diseases and Microbiology Public Health	NSW Health Pathology - Institute of Clinical Pathology and Medical Research; Westmead Hospital; University of Sydney	Gall M, Arnott A, Sadsad R, Draper J, Sim E, Bachmann N, Rockett R, Lam C, Gray K, Timms V, Carter I, Holmes EC, O'Sullivan MV, Byun R, Sintchenko V, Chen SC, Eden JS, Maddocks S, Kok J, Propenko M, Sorrell T, Chang S, Basile K, Dwyer DE for the 2019-nCoV Study Group
EPI_ISL_427730, EPI_ISL_427731, EPI_ISL_427732	Centre for Infectious Diseases and Microbiology Public Health	NSW Health Pathology - Institute of Clinical Pathology and Medical Research; Westmead Hospital; University of Sydney	Gray K, Timms V, Gall M, Arnott A, Sadsad R, Draper J, Sim E, Bachmann N, Rockett R, Lam C, Carter I, Holmes EC, O'Sullivan MV, Byun R, Sintchenko V, Chen SC, Eden JS, Maddocks S, Kok J, Propenko M, Sorrell T, Chang S, Basile K, Dwyer DE for the 2019-nCoV Study Group
EPI_ISL_427733	Centre for Infectious Diseases and Microbiology Public Health	NSW Health Pathology - Institute of Clinical Pathology and Medical Research; Westmead Hospital; University of Sydney	Gall M, Arnott A, Sadsad R, Draper J, Sim E, Bachmann N, Rockett R, Lam C, Gray K, Timms V, Carter I, Holmes EC, O'Sullivan MV, Byun R, Sintchenko V, Chen SC, Eden JS, Maddocks S, Kok J, Propenko M, Sorrell T, Chang S, Basile K, Dwyer DE for the 2019-nCoV Study Group
EPI_ISL_427734	Centre for Infectious Diseases and Microbiology Public Health	NSW Health Pathology - Institute of Clinical Pathology and Medical Research; Westmead Hospital; University of Sydney	Timms V, Gall M, Arnott A, Sadsad R, Draper J, Sim E, Bachmann N, Rockett R, Lam C, Gray K, Carter I, Holmes EC, O'Sullivan MV, Byun R, Sintchenko V, Chen SC, Eden JS, Maddocks S, Kok J, Propenko M, Sorrell T, Chang S, Basile K, Dwyer DE for the 2019-nCoV Study Group
EPI_ISL_427735	Centre for Infectious Diseases and Microbiology Public Health	NSW Health Pathology - Institute of Clinical Pathology and Medical Research; Westmead Hospital; University of Sydney	Arnott A, Sadsad R, Draper J, Sim E, Bachmann N, Rockett R, Lam C, Gray K, Timms V, Gall M, Carter I, Holmes EC, O'Sullivan MV, Byun R, Sintchenko V, Chen SC, Eden JS, Maddocks S, Kok J, Propenko M, Sorrell T, Chang S, Basile K, Dwyer DE for the 2019-nCoV Study Group
EPI_ISL_428148	Klinisk mikrobiologi, Region Västerbotten	Unit for Biological Agents, Department for CBRN Defence and Security, Swedish Defence Research Agency	FOI bioinformatics team
EPI_ISL_428229, EPI_ISL_428230	TSGH-CP molecular lab	TSGH-CP molecular lab	Cherng-Lih Perng, Ming-Jr Jian, Chih-Kai Chang, Jung-Chung Lin, Kuo-Ming Yeh, Chien-Wen Chen, Sheng-Kang Chiu, Hsing-Yi Chung, Shih-Hung Tsai, Kuo-Sheng Hung, Tien-Yao Chang, Feng-Yee Chang, Hung-Sheng Shang
EPI_ISL_428232, EPI_ISL_428233	Hematology Laboratory, Section of Molecular Diagnostics, University Clinical Centre, Medical University of Gdansk	Department of Virology, Faculty of Medicine, University of Helsinki, Helsinki, Finland	Marlena Robakowska, Aneta Szulc, Maciej Grzybek, Olli Vapalahti, Teemu Smura
EPI_ISL_428253, EPI_ISL_428254, EPI_ISL_428255, EPI_ISL_428256, EPI_ISL_428259, EPI_ISL_428260, EPI_ISL_428261, EPI_ISL_428262, EPI_ISL_428263, EPI_ISL_428345	University of Wisconsin-Madison AIDS Vaccine Research Laboratories	University of Wisconsin-Madison AIDS Vaccine Research Laboratories	Gage Moreno, Katarina Braun, et al. AIDS Vaccine Research Laboratories
EPI_ISL_428370, EPI_ISL_428371,	Yale Clinical Virology Laboratory	Grubaugh Lab - Yale School of Public Health	Joseph Fauver, Anderson Brito, Tara Alpert, Chantal Vogels, Ellen Foxman, Albert Ko, Marie Landry, Nathan Grubaugh

EPI_ISL_428372, EPI_ISL_428381			
EPI_ISL_428490, EPI_ISL_428491	Centers for Disease Control, R.O.C. (Taiwan)	Centers for Disease Control, R.O.C. (Taiwan)	Ji-Rong Yang, Yu-Chi Lin, Jung-Jung Mu, Ming-Tsan Liu
EPI_ISL_428670	Centre for Dengue Research	Centre for Dengue Research	Chandima Jeewandara, Dinuka Ariyaratne, Laksiri Gomes, Deshni Jayathilaka, Ananda Wijewickrama, Eranga Narangoda, Damayanthi Idampitiya, Neelika Malaige
EPI_ISL_428712, EPI_ISL_428713, EPI_ISL_428714, EPI_ISL_428715, EPI_ISL_428716	Ministry of Health Turkey	Ministry of Health Turkey	Fatma Bayrakdar,Aye Baak Alta,Yasemin Cogun,Gülay Korukluolu,Selçuk Klç
EPI_ISL_428824, EPI_ISL_428825, EPI_ISL_428826, EPI_ISL_428827	National Public Health Laboratory, National Centre for Infectious Diseases	National Public Health Laboratory, National Centre for Infectious Diseases	Mak TM, Octavia S, Chavatte JM, Cui L, Lin RTP
EPI_ISL_428855	MRCG at LSHTM Geomics lab	MRCG at LSHTM Genomics lab	Sesay et al
EPI_ISL_428875, EPI_ISL_428881	State Research Center of Virology and Biotechnology VECTOR, Department of Collection of Microorganisms	State Research Center of Virology and Biotechnology VECTOR, Department of Collection of Microorganisms	Sergey A. Bodnev, Oleg V. Pyankov, Tatyana V. Tregubchak, Alexander N. Shvalov, Elena V. Gavrilova, Rinat A. Maksyutov
EPI_ISL_428882	State Research Center of Virology and Biotechnology VECTOR, Department of Collection of Microorganisms	State Research Center of Virology and Biotechnology VECTOR, Department of Collection of Microorganisms	Oleg V. Pyankov, Sergey A. Bodnev, Tatyana V. Tregubchak, Alexander N. Shvalov, Elena V. Gavrilova, Rinat A. Maksyutov
EPI_ISL_429007	UCSF Clinical Microbiology Laboratory	Chan-Zuckerberg Biohub	CZB Cliahub Consortium
EPI_ISL_429116	Klinisk mikrobiologi och vardhygien Halmstad	The Public Health Agency of Sweden	Arne Kotz, Olov Svartstrom, Maria Lind Karlberg, Anna-Malin Linde, Oskar Karlsson Lindsjo, Anna Risberg, Shaman Muradrasoli, Karin Tegmark-Wisell
EPI_ISL_429162, EPI_ISL_429163	The Public Health Agency of Sweden	The Public Health Agency of Sweden	Olov Svartstrom, Maria Lind Karlberg, Anna-Malin Linde, Oskar Karlsson Lindsjo, Anna Risberg, Shaman Muradrasoli, Karin Tegmark-Wisell
EPI_ISL_429168, EPI_ISL_429169, EPI_ISL_429170, EPI_ISL_429171	Ramathibodi Hospital	COVID-19 Network Investigations (CONI) Alliance	Elizabeth Batty, Wasun Chantratita, Thanat Chookajorn, Stefan Fernandez, Angkana Huang, Poramate Jiaranai, Anthony R. Jones, Khajohn Joonsalak, Chonticha Klungtong, Theerarat Kochakarn, Namfon Kotanan, Krittikorn Kumpornsin, Wudtichai Manasatienkij, Bhakbhoom Panthan, Ekawat Pasomsub, Kingkan Rakmanee, Insee Sensorn, Janjira Thaipadungpanit, Arporn Wangwiwatsin,Treewat Watthanachockchai
EPI_ISL_429196, EPI_ISL_429208, EPI_ISL_429209, EPI_ISL_429210, EPI_ISL_429213	University Hospitals of Geneva Laboratory of Virology	University Hospitals of Geneva Laboratory of Virology	Laubscher F.
EPI_ISL_429226, EPI_ISL_429227	Presidio Ospedaliero Santo Spirito	Istituto Zooprofilattico Sperimentale dell'Abruzzo e Molise "G. Caporale"	Lorusso A, Marcacci M, Di Domenico M, Ancora M, Curini V, Mangone I, Rinaldi A, Di Pasquale A, Camma C, Puglia I, Savini G
EPI_ISL_429298, EPI_ISL_429301, EPI_ISL_429302, EPI_ISL_429303, EPI_ISL_429304, EPI_ISL_429305, EPI_ISL_429306, EPI_ISL_429307, EPI_ISL_429308, EPI_ISL_429309, EPI_ISL_429310, EPI_ISL_429311, EPI_ISL_429312, EPI_ISL_429313, EPI_ISL_429314, EPI_ISL_429315, EPI_ISL_429316, EPI_ISL_429317, EPI_ISL_429318, EPI_ISL_429320, EPI_ISL_429321, EPI_ISL_429322, EPI_ISL_429323, EPI_ISL_429324, EPI_ISL_429325, EPI_ISL_429326, EPI_ISL_429327, EPI_ISL_429328, EPI_ISL_429329, EPI_ISL_429330, EPI_ISL_429331, EPI_ISL_429332			
see above	Department of Clinical Microbiology, Copenhagen University Hospital, Hvidovre, Kettegaard Alle 30, 2650 Hvidovre.	Albertsen lab, Department of Chemistry and Bioscience, Aalborg University, Denmark	Rasmus Kirkegaard
EPI_ISL_429582	Department of Virus and Microbiological Special Diagnostics, Statens Serum Institut, Copenhagen, Denmark, Artillerivej 5, 2300 Copenahgen S	Albertsen lab, Department of Chemistry and Bioscience, Aalborg University, Denmark	Rasmus Kirkegaard
EPI_ISL_429673, EPI_ISL_429675, EPI_ISL_429676, EPI_ISL_429678, EPI_ISL_429679, EPI_ISL_429680, EPI_ISL_429681, EPI_ISL_429682, EPI_ISL_429683, EPI_ISL_429684, EPI_ISL_429685, EPI_ISL_429686, EPI_ISL_429687, EPI_ISL_429688, EPI_ISL_429689, EPI_ISL_429690, EPI_ISL_429691, EPI_ISL_429692, EPI_ISL_429694, EPI_ISL_429696, EPI_ISL_429697			
see above	Central Public Health Laboratory/Octávio Magalhães Institute (IOM) from the Ezequiel Dias Foundation (FUNED)	Instituto Octávio Magalhães / Fundação Ezequiel Dias (IOM/Funed)	Talita Adelino, Joilson Xavier, Marta Giovanetti, Vagner Fonseca, Marcos Vinícius Silva, Luiz Carlos Junior Alcantara, Marluce Aparecida Assunção Oliveira
EPI_ISL_429707, EPI_ISL_429738, EPI_ISL_429748, EPI_ISL_429750	Laboratoire National de Sante, Microbiology, Virology	Laboratoire National de Sante, Microbiology, Epidemiology and Microbial Genomics	Anke Wienecke-Baldacchino, Ardashel Latsuzbaia, Jessica Tapp, Catherine Ragimbeau, Guillaume Fournier, Tamir Abdelrahman, Trung Nguyen Nguyen, Joel Mossong
EPI_ISL_429813, EPI_ISL_429814	Queen Elizabeth II Health Science Centre	National Microbiology Laboratory	Anna Majer, Shari Tyson, Grace Seo, Kristyn Burak, Philip Mabon, Elsie Grudeski, Rhiannon Huzarewich, Russell Mandes, Jennifer Tanner, Natalie Knox, Morag Graham, Gary Van Domselaar, Todd Hatchette, Jason LeBlanc, Nathalie Bastien, Yan Li, Timothy Booth, Matthew Gilmour
EPI_ISL_429815	Public Health Laboratory	National Microbiology Laboratory	Anna Majer, Shari Tyson, Grace Seo, Kristyn Burak, Philip Mabon, Elsie Grudeski, Rhiannon Huzarewich, Russell Mandes, Jennifer Tanner, Natalie Knox, Morag Graham, Gary Van Domselaar, Robert Needle, Yang Yu, Adel Malek, Laura Gilbert, George Zahariadis, Nathalie Bastien, Yan Li, Timothy Booth, Matthew Gilmour
EPI_ISL_429818, EPI_ISL_429819	Cadham Provincial Laboratory	National Microbiology Laboratory	Anna Majer, Shari Tyson, Grace Seo, Kristyn Burak, Philip Mabon, Elsie Grudeski, Rhiannon Huzarewich, Russell Mandes, Jennifer Tanner, Natalie Knox, Morag Graham, Gary Van Domselaar, Paul Van Caeseele, Jared Bullard, David Alexander, Kerry Dust, Nathalie Bastien, Yan Li, Timothy Booth, Matthew Gilmour
EPI_ISL_429865, EPI_ISL_429866, EPI_ISL_429867, EPI_ISL_429868, EPI_ISL_429869	Ministry of Health Turkey	Ministry of Health Turkey	Fatma Bayrakdar,Aye Baak Alta,Yasemin Cogun,Gülay Korukluolu,Selçuk Klç
EPI_ISL_429993, EPI_ISL_429994, EPI_ISL_429997, EPI_ISL_429999, EPI_ISL_430001, EPI_ISL_430002, EPI_ISL_430003, EPI_ISL_430004, EPI_ISL_430014	Biolab Diagnostic Laboratories	Andersen lab at Scripps Research	Issa Abu-Dayyeh, Ahmad Tibi, Lama Hussein, Lina Mohammad, Zein Naber, Amid Abdelnour with SEARCH Alliance San Diego
EPI_ISL_430019, EPI_ISL_430020, EPI_ISL_430021, EPI_ISL_430022, EPI_ISL_430023, EPI_ISL_430024, EPI_ISL_430025, EPI_ISL_430026, EPI_ISL_430027, EPI_ISL_430028, EPI_ISL_430029, EPI_ISL_430030, EPI_ISL_430031, EPI_ISL_430032, EPI_ISL_430033, EPI_ISL_430034, EPI_ISL_430035, EPI_ISL_430038			
see above	Utah Public Health Laboratory	Utah Public Health Laboratory	Erin Young, Kelly Oakeson
EPI_ISL_430116, EPI_ISL_430132, EPI_ISL_430140	Seattle Flu Study	Seattle Flu Study	Chu et al
EPI_ISL_430180, EPI_ISL_430181, EPI_ISL_430182, EPI_ISL_430188, EPI_ISL_430189, EPI_ISL_430190, EPI_ISL_430191, EPI_ISL_430192, EPI_ISL_430193, EPI_ISL_430194, EPI_ISL_430196, EPI_ISL_430197, EPI_ISL_430198, EPI_ISL_430199, EPI_ISL_430203, EPI_ISL_430204, EPI_ISL_430205, EPI_ISL_430206, EPI_ISL_430207, EPI_ISL_430208			
see above	Washington State Department of Health	Seattle Flu Study	Chu et al
EPI_ISL_430819	Center of Scientific Excellence for Influenza Viruses,National Research Centre (NRC), Egypt.	Center of Scientific Excellence for Influenza Viruses,National Research Centre (NRC), Egypt.	Mohamed Ahmed Ali, Ahmed Kandeil, Ahmed Mostafa, Rabeh El-Shesheny, Mahmoud Shehata, Wael Roshdy, Shymaa Showky Ahmed , Amal Naguib, Nancy M. El Guindy, Mokhtar Gomaa, Ahmed El-Taweel, Ahmed E Kayed, Yassmin Moatasim, Omnia Kutkat, Sara Mahmoud, Mina Kamel, Abo Shama, M Noura, Mohamed El Sayes
EPI_ISL_430820	Center of Scientific Excellence for Influenza Viruses, National Research Centre (NRC), Egypt.	Center of Scientific Excellence for Influenza Viruses, National Research Centre (NRC), Egypt.	Mohamed Ahmed Ali, Ahmed Kandeil, Ahmed Mostafa, Rabeh El-Shesheny, Mahmoud Shehata, Wael Roshdy, Shymaa Showky Ahmed , Amal Naguib, Mokhtar Gomaa, Ahmed El-Taweel, Ahmed E Kayed, Yassmin Moatasim, Omnia Kutkat, Sara Mahmoud, Mina Kamel, Abo Shama, M Noura, Mohamed El Sayes, Nancy M. El Guindy
EPI_ISL_430841	Praram 9 Hospital	National Institute of Health. Department of medical Sciences, Ministry of Public Health, Thailand	Pilailuk,Okada; Siripaporn,Phuygun; Thanutsapa,Thanadachakul; Sittiporn,Parnmen;Warawan,Wongboot; Sunthareeya,Waicharoen; Malinee,Chittaganpitch
EPI_ISL_430842	Central chest Institute of Thailand	National Institute of Health. Department of medical Sciences, Ministry of Public Health, Thailand	Pilailuk,Okada; Siripaporn,Phuygun; Thanutsapa,Thanadachakul; Sittiporn,Parnmen;Warawan,Wongboot; Sunthareeya,Waicharoen; Malinee,Chittaganpitch

EPI_ISL_430864	The Public Health Agency of Sweden	The Public Health Agency of Sweden	Oskar Karlsson Lindsjo, Maria Lind Karlberg, Anna-Malin Linde, Olov Svartstrom, Anna Risberg, Shaman Muradrasoli, Karin Tegmark-Wisell
EPI_ISL_431013	Alaska State Virology Laboratory	Alaska State Virology Laboratory	Jack Chen
EPI_ISL_431014, EPI_ISL_431016	Alaska State Virology Laboratory	Alaska State Virology Laboratory	Jack Chen, Ph.D.
EPI_ISL_431103	Department of Microbiology, Gandhi Medical College and Hospital, Secendrabad, Hyderabad, India	Department of Microbiology, Gandhi Medical College and Hospital, Secendrabad, Hyderabad, India	Nagamani K, Muttineni Radhakrishna, Thrilok Chander B, Raja Rao M, Kalyani Putty, Ravikumar P, Sunitha P, Pankaj Singh D, Anand Kumar K, Amit A. Upadhyay, Steven E. Bosinger, Rama Amara
EPI_ISL_431180, EPI_ISL_431240, EPI_ISL_431292, EPI_ISL_431780, EPI_ISL_431784	Fujian Center for Disease Control and Prevention	Fujian Center for Disease Control and Prevention	Lin Qi, Huang Zhimiao, Zhang Yanhua, Weng Yuwei
EPI_ISL_432640, EPI_ISL_432653, EPI_ISL_432710, EPI_ISL_432794, EPI_ISL_432810	Virology Department, Sheffield Teaching Hospitals NHS Foundation Trust / Virology Department, Sheffield Teaching Hospitals NHS Foundation Trust	COVID-19 Genomics UK (COG-UK) Consortium	Thushan de Silva, Matthew Parker,Adri Angyal, Rebecca Brown, Luke Green, Rachel Tucker, Paul Parsons, Danielle Groves, Alex Keeley, Dave Partridge, Matthew Wyles, Benjamin Lindsey, Mehmet Yavuz, Mohammad Raza, Cariad Evans
EPI_ISL_433274	West of Scotland Specialist Virology Centre, NHSGGC / MRC-University of Glasgow Centre for Virus Research	COVID-19 Genomics UK (COG-UK) Consortium	Ana da Silva Filipe, Natasha Johnson, Kathy Smollett, Daniel Mair, Stephen Carmichael, Lily Tong, Jenna Nichols, Elihu Aranday-Cortes, Kirstyn Brunker, Yasmin Parr, Kyriaki Nomikou; Sarah McDonald, Marc Niebel, Patawee Asamaphan; Richard Orton, Joseph Hughes, Sreenu Vattipally, David L Robertson; Alasdair MacLean, Rory Gunson; Kathy Li, Natasha Jesudason, Rajiv Shah, James Shepherd, Antonia Ho, Emma Thomson
EPI_ISL_434464, EPI_ISL_434477, EPI_ISL_434482, EPI_ISL_434483	Laboratory of Microbiology, Medical School, National and Kapodistrian University of Athens	Laboratory of Biology, Department of Medicine, Democritus University of Thrace	Kassela K., Bampali,M., Dovrolis,N., Gatzidou,E., Froukala,E., Stavropoulou,A., Veletza,S., Tsakris,A., Spanakis,N. and Karakasiliotis,I.
EPI_ISL_434516	Biolab Diagnostic Laboratories	Andersen lab at Scripps Research	Issa Abu-Dayyeh, Ahmad Tibi, Lama Hussein, Lina Mohammad, Zein Naber, Amid Abdelnour with SEARCH Alliance San Diego
EPI_ISL_434533, EPI_ISL_434535	Area de Salud Alajuela Sur	Inciensa, Instituto Costarricense de Investigación y Enseñanza en Nutrición y Salud	Francisco Duarte, Hebleen Porras, Claudio Soto-Garita, Estela Cordero, Adriana Godinez & Melany Calderon
EPI_ISL_434536	Hospital San Vicente de Paul	Inciensa, Instituto Costarricense de Investigación y Enseñanza en Nutrición y Salud	Francisco Duarte, Hebleen Porras, Claudio Soto-Garita, Estela Cordero, Adriana Godinez & Melany Calderon
EPI_ISL_434540	EBAIS Concepción Norte	Inciensa, Instituto Costarricense de Investigación y Enseñanza en Nutrición y Salud	Francisco Duarte, Hebleen Porras, Claudio Soto-Garita, Estela Cordero, Adriana Godinez & Melany Calderon
EPI_ISL_434647, EPI_ISL_434648	Victoria Vard och Halsa	The Public Health Agency of Sweden	Sarah Henriksson, Oskar Karlsson Lindsjo, Maria Lind Karlberg, Anna-Malin Linde, Olov Svartstrom, Anna Risberg, Theresa Enkirch, Mia Brytting, Karin Tegmark-Wisell
EPI_ISL_434649	Svardsjo VC	The Public Health Agency of Sweden	Tommy Janers, Oskar Karlsson Lindsjo, Maria Lind Karlberg, Anna-Malin Linde, Olov Svartstrom, Anna Risberg, Theresa Enkirch, Mia Brytting, Karin Tegmark-Wisell
EPI_ISL_434689, EPI_ISL_434690	Johns Hopkins Hospital Department of Pathology	Johns Hopkins Hospital Department of Pathology	Peter M. Thielen, Thomas Mehoke, Shirlee Wohl, Srividya Ramakrishnan, Melanie Kirsche, Amanda Ernlund, Oluwaseun Falade-Nwulia, Timothy Gilpatrick, Paul Morris, Norah Sadowski, Nídiá Trovao, Victoria Gniazdowski, Michael Schatz, Stuart C. Ray, Winston Timp, Heba Mostafa
EPI_ISL_434704, EPI_ISL_434705, EPI_ISL_434706	Praram 9 Hospital	National Institute of Health. Department of medical Sciences, Ministry of Public Health, Thailand	Pilailuk,Okada; Siripaporn,Phuygun; Thanutsapa,Thanadachakul; Sittiporn,Parnmen;Warawan,Wongboot; Sunthareeya,Waicharoen; Malinee,Chittaganpitch
EPI_ISL_434707	Thammasat University Hospital	National Institute of Health. Department of medical Sciences, Ministry of Public Health, Thailand	Pilailuk,Okada; Siripaporn,Phuygun; Thanutsapa,Thanadachakul; Sittiporn,Parnmen;Warawan,Wongboot; Sunthareeya,Waicharoen; Malinee,Chittaganpitch
EPI_ISL_434708	unknown	National Institute of Health. Department of medical Sciences, Ministry of Public Health, Thailand	Pilailuk,Okada; Siripaporn,Phuygun; Thanutsapa,Thanadachakul; Sittiporn,Parnmen;Warawan,Wongboot; Sunthareeya,Waicharoen; Malinee,Chittaganpitch
EPI_ISL_434712, EPI_ISL_434713, EPI_ISL_434714, EPI_ISL_434715, EPI_ISL_434716, EPI_ISL_434717, EPI_ISL_434718, EPI_ISL_434719, EPI_ISL_434720, EPI_ISL_434721, EPI_ISL_434723, EPI_ISL_434725, EPI_ISL_434728, EPI_ISL_434729, EPI_ISL_434731, EPI_ISL_434732, EPI_ISL_434754, EPI_ISL_434756, EPI_ISL_434757, EPI_ISL_434758, EPI_ISL_434759, EPI_ISL_434760, EPI_ISL_434761, EPI_ISL_434762, EPI_ISL_434763, EPI_ISL_434764, EPI_ISL_434765, EPI_ISL_434766, EPI_ISL_434767, EPI_ISL_434768, EPI_ISL_434769, EPI_ISL_434770, EPI_ISL_434772, EPI_ISL_434773, EPI_ISL_434774, EPI_ISL_434775, EPI_ISL_434776, EPI_ISL_434777, EPI_ISL_434778, EPI_ISL_434779, EPI_ISL_434780, EPI_ISL_434781, EPI_ISL_434782, EPI_ISL_434783, EPI_ISL_434786			
see above	Houston Methodist Hospital	Houston Methodist Hospital	S. Wesley Long, Randall J. Olsen, Paul A. Christensen, David W. Bernard, James J. Davis, Maulik Shukla, Marcus Nguyen, Matthew Ojeda Saavedra, Concepcion C. Cantu, Prasanti Yerramilli, Layne Pruitt, Sishir Subedi, Heather Hendrickson, Ghazaleh Eskandari, Muthiah Kumaraswami, Jason S. McLellan, Hakon Jonsson, Kari Stefansson, and James M. Musser
EPI_ISL_435061, EPI_ISL_435062, EPI_ISL_435064, EPI_ISL_435065, EPI_ISL_435066, EPI_ISL_435067, EPI_ISL_435068, EPI_ISL_435069, EPI_ISL_435070, EPI_ISL_435101, EPI_ISL_435102, EPI_ISL_435103, EPI_ISL_435104, EPI_ISL_435105, EPI_ISL_435106, EPI_ISL_435108, EPI_ISL_435109, EPI_ISL_435110			
see above	National Centre for Disease control (NCDC), CSIR-Institute of Genomics and Integrative Biology (CSIR-IGIB)	NCDC/CSIR-IGIB	Pramod Kumar, Rajesh Pandey, Pooja Sharma, Mahesh Dhar, Vivekanand A, Bharathram Uppili, Himanshu Vashisht, Saruchi Wadhwa, Nishu Tyagi, Uma Sharma, Priyanka Singh, Hemlata Lall, Meena Datta, Poonam Gupta, Nidhi Saini, Aarti Tewari, Bibhash Nandi, Dhirendra Kumar, Satyabrata Bag, Varun Jaiswal, Hema Gogia, Preeti Madan, Simrita Singh, Prateek Singh, Debasis Dash, Mitali Mukerji, Manju Bala, Sandhya Kabra, Sujeet Singh, Mohammed Faruq, Anurag Agrawal, Partha Rakshit
EPI_ISL_435124, EPI_ISL_435125, EPI_ISL_435127, EPI_ISL_435128, EPI_ISL_435132, EPI_ISL_435143	Mohammed Bin Rashid University of Medicine and Health Sciences	Al Jalila Genomics Center	Ahmad Abou Tayoun, Tom Loney, Hamda Khansaheb, Sathishkumar Ramaswamy, Divinlal Harilal, Zulfa Omar Deesi, Rupa Murthy Varghese, Hanan Al Suwaidi, Abdulmajeed Alkhaja, Mohammed Uddin, Rifat Hamoudi, Rabih Halwani, Abiola Catherine Senok, Qutayba Hamid, Norbert Nowotny, Alawi Alsheikh-Ali
EPI_ISL_435281	Medistra Hospital Jakarta	Eijkman Institute for Molecular Biology, Ministry of Research and Technology/National Agency for Research and Innovation	Edison Johar, Frilasita A Yudhaputri, Hidayat Trimarsanto, David H Muljono, Safarina G Malik, Khin Saw Myint, Amin Soebandrio
EPI_ISL_435315, EPI_ISL_435316, EPI_ISL_435317	National Hospital of Tropical Diseases	Oxford University Clinical Research Unit, Hanoi, Vietnam	Nguyen Thi Tam, Van Dinh Trang, Nguyen Thu Trang, Nguyen Thi Ngoc Diep, Le Nguyen Minh Hoa, Pham Ngoc Thach, H. Rogier van Doorn, on behalf of the OUCRU COVID-19 research group
EPI_ISL_435658, EPI_ISL_435659, EPI_ISL_435660, EPI_ISL_435661, EPI_ISL_435662, EPI_ISL_435663, EPI_ISL_435664, EPI_ISL_435665	Santa Clara County Public Health Department	Chiu Laboratory, University of California, San Francisco	Xianding Deng, Scot Federman, Wei Gu, Elsa Villarino, Brandon Bonin, Debra A. Wadford, and Charles Y. Chiu
EPI_ISL_435697, EPI_ISL_435698, EPI_ISL_435699, EPI_ISL_435700	National Public Health Laboratory, National Centre for Infectious Diseases	National Public Health Laboratory, National Centre for Infectious Diseases	Mak Tze Minn, Octavia Sophie, Chavatte Jean-Marc, Cui Lin, Lin Raymond Tzer Pin
EPI_ISL_436057, EPI_ISL_436058, EPI_ISL_436059, EPI_ISL_436064, EPI_ISL_436065, EPI_ISL_436071, EPI_ISL_436073, EPI_ISL_436080, EPI_ISL_436081	NYC Department of Health and Mental Hygiene	Pathogen Discovery, Respiratory Viruses Branch, Division of Viral Diseases, Centers for Disease Control and Prevention	Ying Tao, Krista Queen, Christy Harrison, Jennifer Rakeman, Clinton R. Paden, Jing Zhang, Anna Uehara, Yan Li, Haibin Wang, Jasmine Padilla, Justin Lee, Bettina Bankamp, Zachary Weiner, Suxiang Tong
EPI_ISL_436196	Servicio de Microbiología. Consorcio Hospital General Universitario de Valencia	Sequencing and Bioinformatics Service and Molecular Epidemiology Research Group. FISABIO-Public Health	Griselda De Marco, Beatriz Beamud, Lidia Ruiz Roldan, Marta Pla Diaz,Neris Garcia-Gonzalez, Loreto Ferrús Abad, Maria Dolores Ocete, Inma Galán Vendrell, Paula Ruiz-Hueso, Mariana Reyes-Prieto, Vicente Soriano Chirona, Maria Alma Bracho, Llúcia Martínez-Priego, Concepcion Gimeno, Giuseppe D'Auria, Fernando Gonzalez-Candelas
EPI_ISL_436197	Servicio de Microbiología. Consorcio Hospital General Universitario de Valencia	Sequencing and Bioinformatics Service and Molecular Epidemiology Research Group. FISABIO-Public Health	Beatriz Beamud, Lidia Ruiz Roldan, Marta Pla Diaz,Neris Garcia-Gonzalez, Loreto Ferrús Abad, Maria Dolores Ocete, Inma Galán Vendrell, Paula Ruiz-Hueso, Mariana Reyes-Prieto, Vicente Soriano Chirona, Maria Alma Bracho, Griselda De Marco, Llúcia Martínez-Priego, Concepcion Gimeno, Giuseppe D'Auria, Fernando Gonzalez-Candelas
EPI_ISL_436198	Servicio de Microbiología. Consorcio Hospital General Universitario de Valencia	Sequencing and Bioinformatics Service and Molecular Epidemiology Research Group. FISABIO-Public Health	Lidia Ruiz Roldan, Marta Pla Diaz,Neris Garcia-Gonzalez, Loreto Ferrús Abad, Maria Dolores Ocete, Inma Galán Vendrell, Paula Ruiz-Hueso, Mariana Reyes-Prieto, Vicente Soriano Chirona, Maria Alma Bracho, Griselda De Marco, Beatriz Beamud, Llúcia Martínez-Priego, Concepcion Gimeno, Giuseppe D'Auria, Fernando Gonzalez-Candelas

EPI_ISL_436199	Servicio de Microbiología. Consorcio Hospital General Universitario de Valencia	Sequencing and Bioinformatics Service and Molecular Epidemiology Research Group. FISABIO-Public Health	Marta Pla Diaz,Neris Garcia-Gonzalez, Loreto Ferrús Abad, Maria Dolores Ocete, Inma Galán Vendrell, Paula Ruiz-Hueso, Mariana Reyes-Prieto, Vicente Soriano Chirona, Maria Alma Bracho, Griselda De Marco, Beatriz Beamud, Lidia Ruiz Roldan, Llúcia Martínez-Priego, Concepcion Gimeno, Giuseppe D'Auria, Fernando Gonzalez-Candelas
EPI_ISL_436200	Servicio de Microbiología. Consorcio Hospital General Universitario de Valencia	Sequencing and Bioinformatics Service and Molecular Epidemiology Research Group. FISABIO-Public Health	Neris Garcia-Gonzalez, Loreto Ferrús Abad, Maria Dolores Ocete, Inma Galán Vendrell, Paula Ruiz-Hueso, Mariana Reyes-Prieto, Vicente Soriano Chirona, Maria Alma Bracho, Griselda De Marco, Beatriz Beamud, Lidia Ruiz Roldan, Marta Pla Diaz, Llúcia Martínez-Priego, Concepcion Gimeno, Giuseppe D'Auria, Fernando Gonzalez-Candelas
EPI_ISL_436201	Servicio de Microbiología. Consorcio Hospital General Universitario de Valencia	Sequencing and Bioinformatics Service and Molecular Epidemiology Research Group. FISABIO-Public Health	Loreto Ferrús Abad, Maria Dolores Ocete, Inma Galán Vendrell, Paula Ruiz-Hueso, Mariana Reyes-Prieto, Vicente Soriano Chirona, Maria Alma Bracho, Griselda De Marco, Beatriz Beamud, Lidia Ruiz Roldan, Marta Pla Diaz,Neris Garcia-Gonzalez, Llúcia Martínez-Priego, Concepcion Gimeno, Giuseppe D'Auria, Fernando Gonzalez-Candelas
EPI_ISL_436202	Servicio de Microbiología. Consorcio Hospital General Universitario de Valencia	Sequencing and Bioinformatics Service and Molecular Epidemiology Research Group. FISABIO-Public Health	Loreto Ferrús Abad, Maria Dolores Ocete,Inma Galán Vendrell, Paula Ruiz-Hueso, Mariana Reyes-Prieto, Vicente Soriano Chirona, Maria Alma Bracho, Griselda De Marco, Beatriz Beamud, Lidia Ruiz Roldan, Marta Pla Diaz,Neris Garcia-Gonzalez, Llúcia Martínez-Priego, Concepcion Gimeno, Giuseppe D'Auria, Fernando Gonzalez-Candelas
EPI_ISL_436203	Servicio de Microbiología. Consorcio Hospital General Universitario de Valencia	Sequencing and Bioinformatics Service and Molecular Epidemiology Research Group. FISABIO-Public Health	Maria Dolores Ocete, Inma Galán Vendrell, Paula Ruiz-Hueso, Mariana Reyes-Prieto, Vicente Soriano Chirona, Maria Alma Bracho, Griselda De Marco, Beatriz Beamud, Lidia Ruiz Roldan, Marta Pla Diaz,Neris Garcia-Gonzalez, Loreto Ferrús Abad, Llúcia Martínez-Priego, Concepcion Gimeno, Giuseppe D'Auria, Fernando Gonzalez-Candelas
EPI_ISL_436224	Servicio de Microbiología. Consorcio Hospital General Universitario de Valencia	Sequencing and Bioinformatics Service and Molecular Epidemiology Research Group. FISABIO-Public Health	Neris Garcia-Gonzalez, Loreto Ferrús Abad, Maria Dolores Ocete, Inma Galán Vendrell, Paula Ruiz-Hueso, Mariana Reyes-Prieto, Vicente Soriano Chirona, Maria Alma Bracho, Griselda De Marco, Beatriz Beamud, Lidia Ruiz Roldan, Marta Pla Diaz, Llúcia Martínez-Priego, Concepcion Gimeno, Giuseppe D'Auria, Fernando Gonzalez-Candelas
EPI_ISL_436225	Servicio de Microbiología. Consorcio Hospital General Universitario de Valencia	Sequencing and Bioinformatics Service and Molecular Epidemiology Research Group. FISABIO-Public Health	Loreto Ferrús Abad, Maria Dolores Ocete, Inma Galán Vendrell, Paula Ruiz-Hueso, Mariana Reyes-Prieto, Vicente Soriano Chirona, Maria Alma Bracho, Griselda De Marco, Beatriz Beamud, Lidia Ruiz Roldan, Marta Pla Diaz,Neris Garcia-Gonzalez, Llúcia Martínez-Priego, Concepcion Gimeno, Giuseppe D'Auria, Fernando Gonzalez-Candelas
EPI_ISL_436226	Servicio de Microbiología. Consorcio Hospital General Universitario de Valencia	Sequencing and Bioinformatics Service and Molecular Epidemiology Research Group. FISABIO-Public Health	Maria Dolores Ocete, Inma Galán Vendrell, Paula Ruiz-Hueso, Mariana Reyes-Prieto, Vicente Soriano Chirona, Maria Alma Bracho, Griselda De Marco, Beatriz Beamud, Lidia Ruiz Roldan, Marta Pla Diaz,Neris Garcia-Gonzalez, Loreto Ferrús Abad, Llúcia Martínez-Priego, Concepcion Gimeno, Giuseppe D'Auria, Fernando Gonzalez-Candelas
EPI_ISL_436227	Servicio de Microbiología. Consorcio Hospital General Universitario de Valencia	Sequencing and Bioinformatics Service and Molecular Epidemiology Research Group. FISABIO-Public Health	Griselda De Marco, Beatriz Beamud, Lidia Ruiz Roldan, Marta Pla Diaz,Neris Garcia-Gonzalez, Loreto Ferrús Abad, Maria Dolores Ocete, Inma Galán Vendrell, Paula Ruiz-Hueso, Mariana Reyes-Prieto, Vicente Soriano Chirona, Maria Alma Bracho, Llúcia Martínez-Priego, Concepcion Gimeno, Giuseppe D'Auria, Fernando Gonzalez-Candelas
EPI_ISL_436228	Servicio de Microbiología. Consorcio Hospital General Universitario de Valencia	Sequencing and Bioinformatics Service and Molecular Epidemiology Research Group. FISABIO-Public Health	Beatriz Beamud, Lidia Ruiz Roldan, Marta Pla Diaz,Neris Garcia-Gonzalez, Loreto Ferrús Abad, Maria Dolores Ocete, Inma Galán Vendrell, Paula Ruiz-Hueso, Mariana Reyes-Prieto, Vicente Soriano Chirona, Maria Alma Bracho, Griselda De Marco, Llúcia Martínez-Priego, Concepcion Gimeno, Giuseppe D'Auria, Fernando Gonzalez-Candelas
EPI_ISL_436229	Servicio de Microbiología. Consorcio Hospital General Universitario de Valencia	Sequencing and Bioinformatics Service and Molecular Epidemiology Research Group. FISABIO-Public Health	Lidia Ruiz Roldan, Marta Pla Diaz,Neris Garcia-Gonzalez, Loreto Ferrús Abad, Maria Dolores Ocete, Inma Galán Vendrell, Paula Ruiz-Hueso, Mariana Reyes-Prieto, Vicente Soriano Chirona, Maria Alma Bracho, Griselda De Marco, Beatriz Beamud, Llúcia Martínez-Priego, Concepcion Gimeno, Giuseppe D'Auria, Fernando Gonzalez-Candelas
EPI_ISL_436230	Servicio de Microbiología. Consorcio Hospital General Universitario de Valencia	Sequencing and Bioinformatics Service and Molecular Epidemiology Research Group. FISABIO-Public Health	Marta Pla Diaz,Neris Garcia-Gonzalez, Loreto Ferrús Abad, Maria Dolores Ocete, Inma Galán Vendrell, Paula Ruiz-Hueso, Mariana Reyes-Prieto, Vicente Soriano Chirona, Maria Alma Bracho, Griselda De Marco, Beatriz Beamud, Lidia Ruiz Roldan, Llúcia Martínez-Priego, Concepcion Gimeno, Giuseppe D'Auria, Fernando Gonzalez-Candelas
EPI_ISL_436231	Servicio de Microbiología. Consorcio Hospital General Universitario de Valencia	Sequencing and Bioinformatics Service and Molecular Epidemiology Research Group. FISABIO-Public Health	Neris Garcia-Gonzalez, Loreto Ferrús Abad, Maria Dolores Ocete, Inma Galán Vendrell, Paula Ruiz-Hueso, Mariana Reyes-Prieto, Vicente Soriano Chirona, Maria Alma Bracho, Griselda De Marco, Beatriz Beamud, Lidia Ruiz Roldan, Marta Pla Diaz, Llúcia Martínez-Priego, Concepcion Gimeno, Giuseppe D'Auria, Fernando Gonzalez-Candelas
EPI_ISL_436232	Servicio de Microbiología. Consorcio Hospital General Universitario de Valencia	Sequencing and Bioinformatics Service and Molecular Epidemiology Research Group. FISABIO-Public Health	Loreto Ferrús Abad, Maria Dolores Ocete, Inma Galán Vendrell, Paula Ruiz-Hueso, Mariana Reyes-Prieto, Vicente Soriano Chirona, Maria Alma Bracho, Griselda De Marco, Beatriz Beamud, Lidia Ruiz Roldan, Marta Pla Diaz,Neris Garcia-Gonzalez, Llúcia Martínez-Priego, Concepcion Gimeno, Giuseppe D'Auria, Fernando Gonzalez-Candelas
EPI_ISL_436233	Servicio de Microbiología. Consorcio Hospital General Universitario de Valencia	Sequencing and Bioinformatics Service and Molecular Epidemiology Research Group. FISABIO-Public Health	Loreto Ferrús Abad, Maria Dolores Ocete,Inma Galán Vendrell, Paula Ruiz-Hueso, Mariana Reyes-Prieto, Vicente Soriano Chirona, Maria Alma Bracho, Griselda De Marco, Beatriz Beamud, Lidia Ruiz Roldan, Marta Pla Diaz,Neris Garcia-Gonzalez, Llúcia Martínez-Priego, Concepcion Gimeno, Giuseppe D'Auria, Fernando Gonzalez-Candelas
EPI_ISL_436234	Servicio de Microbiología. Consorcio Hospital General Universitario de Valencia	Sequencing and Bioinformatics Service and Molecular Epidemiology Research Group. FISABIO-Public Health	Maria Dolores Ocete, Inma Galán Vendrell, Paula Ruiz-Hueso, Mariana Reyes-Prieto, Vicente Soriano Chirona, Maria Alma Bracho, Griselda De Marco, Beatriz Beamud, Lidia Ruiz Roldan, Marta Pla Diaz,Neris Garcia-Gonzalez, Loreto Ferrús Abad, Llúcia Martínez-Priego, Concepcion Gimeno, Giuseppe D'Auria, Fernando Gonzalez-Candelas
EPI_ISL_436235, EPI_ISL_436236, EPI_ISL_436237, EPI_ISL_436238, EPI_ISL_436239, EPI_ISL_436240, EPI_ISL_436241, EPI_ISL_436242, EPI_ISL_436243	Servicio de Microbiologia. Hospital Universitario Doctor Peset	Sequencing and Bioinformatics Service and Molecular Epidemiology Research Group. FISABIO-Public Health	Juan Alberola Enguídanos, Juan Jose Camarena Miñana, Rosa González Pellicer, Neris Garcia-Gonzalez, Inma Galán Vendrell, Sandra Carbo, Loreto Ferrús Abad, Paula Ruiz-Hueso, Mariana Reyes-Prieto, Vicente Soriano Chirona, Ivan Ansari, Maria Alma Bracho, Griselda De Marco, Beatriz Beamud, Lidia Ruiz Roldan, Marta Pla Diaz, Llúcia Martínez-Priego, Giuseppe D'Auria, Jose Miguel Nogueira Coito, Fernando Gonzalez-Candelas
EPI_ISL_436277	Servicio de Microbiología. Consorcio Hospital General Universitario de Valencia	Sequencing and Bioinformatics Service and Molecular Epidemiology Research Group. FISABIO-Public Health	Neris Garcia-Gonzalez, Loreto Ferrús Abad, Maria Dolores Ocete, Inma Galán Vendrell, Paula Ruiz-Hueso, Mariana Reyes-Prieto, Vicente Soriano Chirona, Maria Alma Bracho, Griselda De Marco, Beatriz Beamud, Lidia Ruiz Roldan, Marta Pla Diaz, Llúcia Martínez-Priego, Concepcion Gimeno, Giuseppe D'Auria, Fernando Gonzalez-Candelas
EPI_ISL_436279	Servicio de Microbiologia. Hospital Clinico Universitario de Valencia	Sequencing and Bioinformatics Service and Molecular Epidemiology Research Group. FISABIO-Public Health	Griselda De Marco, Beatriz Beamud, Lidia Ruiz Roldan, Marta Pla Diaz, Neris Garcia-Gonzalez, Inma Galán Vendrell, Sandra Carbo, Loreto Ferrús Abad, Paula Ruiz-Hueso, Mariana Reyes-Prieto, Vicente Soriano Chirona, Ivan Ansari, David Navarro, Maria Alma Bracho, Llúcia Martínez-Priego, Giuseppe D'Auria, Fernando Gonzalez-Candelas
EPI_ISL_436280	Servicio de Microbiologia. Hospital Clinico Universitario de Valencia	Sequencing and Bioinformatics Service and Molecular Epidemiology Research Group. FISABIO-Public Health	Beatriz Beamud, Lidia Ruiz Roldan, Marta Pla Diaz, Neris Garcia-Gonzalez, Inma Galán Vendrell, Sandra Carbo, Loreto Ferrús Abad, Paula Ruiz-Hueso, Mariana Reyes-Prieto, Vicente Soriano Chirona, Ivan Ansari, David Navarro, Maria Alma Bracho, Griselda De Marco, Llúcia Martínez-Priego, Giuseppe D'Auria, Fernando Gonzalez-Candelas
EPI_ISL_436283	Servicio de Microbiologia. Hospital Clinico Universitario de Valencia	Sequencing and Bioinformatics Service and Molecular Epidemiology Research Group. FISABIO-Public Health	Neris Garcia-Gonzalez, Inma Galán Vendrell, Sandra Carbo, Loreto Ferrús Abad, Paula Ruiz-Hueso, Mariana Reyes-Prieto, Vicente Soriano Chirona, Ivan Ansari, David Navarro, Maria Alma Bracho, Griselda De Marco, Beatriz Beamud, Lidia Ruiz Roldan, Marta Pla Diaz, Llúcia Martínez-Priego, Giuseppe D'Auria, Fernando Gonzalez-Candelas
EPI_ISL_436291	Servicio de Microbiologia. Hospital Clinico Universitario de Valencia	Sequencing and Bioinformatics Service and Molecular Epidemiology Research Group. FISABIO-Public Health	David Navarro, Maria Alma Bracho, Griselda De Marco, Beatriz Beamud, Lidia Ruiz Roldan, Marta Pla Diaz, Neris Garcia-Gonzalez, Inma Galán Vendrell, Sandra Carbo, Loreto Ferrús Abad, Paula Ruiz-Hueso, Mariana Reyes-Prieto, Vicente Soriano Chirona, Ivan Ansari, Llúcia Martínez-Priego, Giuseppe D'Auria, Fernando Gonzalez-Candelas
EPI_ISL_436301	Servicio de Microbiologia. Hospital Clinico Universitario de Valencia	Sequencing and Bioinformatics Service and Molecular Epidemiology Research Group. FISABIO-Public Health	Lidia Ruiz Roldan, Marta Pla Diaz, Neris Garcia-Gonzalez, Inma Galán Vendrell, Sandra Carbo, Loreto Ferrús Abad, Paula Ruiz-Hueso, Mariana Reyes-Prieto, Vicente Soriano Chirona, Ivan Ansari, David Navarro, Maria Alma Bracho, Griselda De Marco, Beatriz Beamud, Llúcia Martínez-Priego, Giuseppe D'Auria, Fernando Gonzalez-Candelas
EPI_ISL_436362	Servicio de Microbiologia. Hospital Clinico Universitario de Valencia	Sequencing and Bioinformatics Service and Molecular Epidemiology Research Group. FISABIO-Public Health	David Navarro, Maria Alma Bracho, Griselda De Marco, Beatriz Beamud, Lidia Ruiz Roldan, Marta Pla Diaz, Neris Garcia-Gonzalez, Inma Galán Vendrell, Sandra Carbo, Loreto Ferrús Abad, Paula Ruiz-Hueso, Mariana Reyes-Prieto, Vicente Soriano Chirona, Ivan Ansari, David Navarro, Llúcia Martínez-Priego, Giuseppe D'Auria, Fernando Gonzalez-Candelas
EPI_ISL_436363	Servicio de Microbiologia. Hospital Clinico Universitario de	Sequencing and Bioinformatics Service and Molecular	Griselda De Marco, Beatriz Beamud, Lidia Ruiz Roldan, Marta Pla Diaz, Neris Garcia-Gonzalez, Inma Galán Vendrell, Sandra Carbo, Loreto Ferrús Abad,

	Valencia	Epidemiology Research Group. FISABIO-Public Health	Paula Ruiz-Hueso, Mariana Reyes-Prieto, Vicente Soriano Chirona, Ivan Ansari, David Navarro, Maria Alma Bracho, Llúcia Martínez-Priego, Giuseppe D'Auria, Fernando Gonzalez-Candelas
EPI_ISL_436372, EPI_ISL_436373, EPI_ISL_436374, EPI_ISL_436375, EPI_ISL_436376, EPI_ISL_436377, EPI_ISL_436378, EPI_ISL_436379, EPI_ISL_436380, EPI_ISL_436381	Servicio de Microbiologia. Hospital Universitario Doctor Peset	Sequencing and Bioinformatics Service and Molecular Epidemiology Research Group. FISABIO-Public Health	Juan Alberola Enguídanos, Juan Jose Camarena Miñana, Rosa González Pellicer, Neris Garcia-Gonzalez, Inma Galán Vendrell, Sandra Carbo, Loreto Ferrús Abad, Paula Ruiz-Hueso, Mariana Reyes-Prieto, Vicente Soriano Chirona, Ivan Ansari, Maria Alma Bracho, Griselda De Marco, Beatriz Beamud, Lidia Ruiz Roldan, Marta Pla Diaz, Llúcia Martínez-Priego, Giuseppe D'Auria, Jose Miguel Nogueira Coito, Fernando Gonzalez-Candelas
EPI_ISL_436382	Servicio de Microbiología. Consorcio Hospital General Universitario de Valencia	Sequencing and Bioinformatics Service and Molecular Epidemiology Research Group. FISABIO-Public Health	Loreto Ferrús Abad, Maria Dolores Ocete,Inma Galán Vendrell, Paula Ruiz-Hueso, Mariana Reyes-Prieto, Vicente Soriano Chirona, Maria Alma Bracho, Griselda De Marco, Beatriz Beamud, Lidia Ruiz Roldan, Marta Pla Diaz,Neris Garcia-Gonzalez, Llúcia Martínez-Priego, Concepcion Gimeno, Giuseppe D'Auria, Fernando Gonzalez-Candelas
EPI_ISL_436383	Servicio de Microbiología. Consorcio Hospital General Universitario de Valencia	Sequencing and Bioinformatics Service and Molecular Epidemiology Research Group. FISABIO-Public Health	Maria Dolores Ocete, Inma Galán Vendrell, Paula Ruiz-Hueso, Mariana Reyes-Prieto, Vicente Soriano Chirona, Maria Alma Bracho, Griselda De Marco, Beatriz Beamud, Lidia Ruiz Roldan, Marta Pla Diaz,Neris Garcia-Gonzalez, Loreto Ferrús Abad, Llúcia Martínez-Priego, Concepcion Gimeno, Giuseppe D'Auria, Fernando Gonzalez-Candelas
EPI_ISL_436384	Servicio de Microbiología. Consorcio Hospital General Universitario de Valencia	Sequencing and Bioinformatics Service and Molecular Epidemiology Research Group. FISABIO-Public Health	Griselda De Marco, Beatriz Beamud, Lidia Ruiz Roldan, Marta Pla Diaz,Neris Garcia-Gonzalez, Loreto Ferrús Abad, Maria Dolores Ocete, Inma Galán Vendrell, Paula Ruiz-Hueso, Mariana Reyes-Prieto, Vicente Soriano Chirona, Maria Alma Bracho, Llúcia Martínez-Priego, Concepcion Gimeno, Giuseppe D'Auria, Fernando Gonzalez-Candelas
EPI_ISL_436385	Servicio de Microbiología. Consorcio Hospital General Universitario de Valencia	Sequencing and Bioinformatics Service and Molecular Epidemiology Research Group. FISABIO-Public Health	Beatriz Beamud, Lidia Ruiz Roldan, Marta Pla Diaz,Neris Garcia-Gonzalez, Loreto Ferrús Abad, Maria Dolores Ocete, Inma Galán Vendrell, Paula Ruiz-Hueso, Mariana Reyes-Prieto, Vicente Soriano Chirona, Maria Alma Bracho, Griselda De Marco, Llúcia Martínez-Priego, Concepcion Gimeno, Giuseppe D'Auria, Fernando Gonzalez-Candelas
EPI_ISL_436386	Servicio de Microbiología. Consorcio Hospital General Universitario de Valencia	Sequencing and Bioinformatics Service and Molecular Epidemiology Research Group. FISABIO-Public Health	Lidia Ruiz Roldan, Marta Pla Diaz,Neris Garcia-Gonzalez, Loreto Ferrús Abad, Maria Dolores Ocete, Inma Galán Vendrell, Paula Ruiz-Hueso, Mariana Reyes-Prieto, Vicente Soriano Chirona, Maria Alma Bracho, Griselda De Marco, Beatriz Beamud, Llúcia Martínez-Priego, Concepcion Gimeno, Giuseppe D'Auria, Fernando Gonzalez-Candelas
EPI_ISL_436387	Servicio de Microbiología. Consorcio Hospital General Universitario de Valencia	Sequencing and Bioinformatics Service and Molecular Epidemiology Research Group. FISABIO-Public Health	Marta Pla Diaz,Neris Garcia-Gonzalez, Loreto Ferrús Abad, Maria Dolores Ocete, Inma Galán Vendrell, Paula Ruiz-Hueso, Mariana Reyes-Prieto, Vicente Soriano Chirona, Maria Alma Bracho, Griselda De Marco, Beatriz Beamud, Lidia Ruiz Roldan, Llúcia Martínez-Priego, Concepcion Gimeno, Giuseppe D'Auria, Fernando Gonzalez-Candelas
EPI_ISL_436388	Servicio de Microbiología. Consorcio Hospital General Universitario de Valencia	Sequencing and Bioinformatics Service and Molecular Epidemiology Research Group. FISABIO-Public Health	Neris Garcia-Gonzalez, Loreto Ferrús Abad, Maria Dolores Ocete, Inma Galán Vendrell, Paula Ruiz-Hueso, Mariana Reyes-Prieto, Vicente Soriano Chirona, Maria Alma Bracho, Griselda De Marco, Beatriz Beamud, Lidia Ruiz Roldan, Marta Pla Diaz, Llúcia Martínez-Priego, Concepcion Gimeno, Giuseppe D'Auria, Fernando Gonzalez-Candelas
EPI_ISL_436389	Servicio de Microbiología. Consorcio Hospital General Universitario de Valencia	Sequencing and Bioinformatics Service and Molecular Epidemiology Research Group. FISABIO-Public Health	Loreto Ferrús Abad, Maria Dolores Ocete, Inma Galán Vendrell, Paula Ruiz-Hueso, Mariana Reyes-Prieto, Vicente Soriano Chirona, Maria Alma Bracho, Griselda De Marco, Beatriz Beamud, Lidia Ruiz Roldan, Marta Pla Diaz,Neris Garcia-Gonzalez, Llúcia Martínez-Priego, Concepcion Gimeno, Giuseppe D'Auria, Fernando Gonzalez-Candelas
EPI_ISL_436390	Servicio de Microbiología. Consorcio Hospital General Universitario de Valencia	Sequencing and Bioinformatics Service and Molecular Epidemiology Research Group. FISABIO-Public Health	Maria Dolores Ocete, Inma Galán Vendrell, Paula Ruiz-Hueso, Mariana Reyes-Prieto, Vicente Soriano Chirona, Maria Alma Bracho, Griselda De Marco, Beatriz Beamud, Lidia Ruiz Roldan, Marta Pla Diaz,Neris Garcia-Gonzalez, Loreto Ferrús Abad, Llúcia Martínez-Priego, Concepcion Gimeno, Giuseppe D'Auria, Fernando Gonzalez-Candelas
EPI_ISL_436391	Servicio de Microbiología. Consorcio Hospital General Universitario de Valencia	Sequencing and Bioinformatics Service and Molecular Epidemiology Research Group. FISABIO-Public Health	Griselda De Marco, Beatriz Beamud, Lidia Ruiz Roldan, Marta Pla Diaz,Neris Garcia-Gonzalez, Loreto Ferrús Abad, Maria Dolores Ocete, Inma Galán Vendrell, Paula Ruiz-Hueso, Mariana Reyes-Prieto, Vicente Soriano Chirona, Maria Alma Bracho, Llúcia Martínez-Priego, Concepcion Gimeno, Giuseppe D'Auria, Fernando Gonzalez-Candelas
EPI_ISL_436392	Servicio de Microbiología. Consorcio Hospital General Universitario de Valencia	Sequencing and Bioinformatics Service and Molecular Epidemiology Research Group. FISABIO-Public Health	Beatriz Beamud, Lidia Ruiz Roldan, Marta Pla Diaz,Neris Garcia-Gonzalez, Loreto Ferrús Abad, Maria Dolores Ocete, Inma Galán Vendrell, Paula Ruiz-Hueso, Mariana Reyes-Prieto, Vicente Soriano Chirona, Maria Alma Bracho, Griselda De Marco, Llúcia Martínez-Priego, Concepcion Gimeno, Giuseppe D'Auria, Fernando Gonzalez-Candelas
EPI_ISL_436393	Servicio de Microbiología. Consorcio Hospital General Universitario de Valencia	Sequencing and Bioinformatics Service and Molecular Epidemiology Research Group. FISABIO-Public Health	Lidia Ruiz Roldan, Marta Pla Diaz,Neris Garcia-Gonzalez, Loreto Ferrús Abad, Maria Dolores Ocete, Inma Galán Vendrell, Paula Ruiz-Hueso, Mariana Reyes-Prieto, Vicente Soriano Chirona, Maria Alma Bracho, Griselda De Marco, Beatriz Beamud, Llúcia Martínez-Priego, Concepcion Gimeno, Giuseppe D'Auria, Fernando Gonzalez-Candelas
EPI_ISL_436394	Servicio de Microbiología. Consorcio Hospital General Universitario de Valencia	Sequencing and Bioinformatics Service and Molecular Epidemiology Research Group. FISABIO-Public Health	Marta Pla Diaz,Neris Garcia-Gonzalez, Loreto Ferrús Abad, Maria Dolores Ocete, Inma Galán Vendrell, Paula Ruiz-Hueso, Mariana Reyes-Prieto, Vicente Soriano Chirona, Maria Alma Bracho, Griselda De Marco, Beatriz Beamud, Lidia Ruiz Roldan, Llúcia Martínez-Priego, Concepcion Gimeno, Giuseppe D'Auria, Fernando Gonzalez-Candelas
EPI_ISL_436395	Servicio de Microbiología. Consorcio Hospital General Universitario de Valencia	Sequencing and Bioinformatics Service and Molecular Epidemiology Research Group. FISABIO-Public Health	Neris Garcia-Gonzalez, Loreto Ferrús Abad, Maria Dolores Ocete, Inma Galán Vendrell, Paula Ruiz-Hueso, Mariana Reyes-Prieto, Vicente Soriano Chirona, Maria Alma Bracho, Griselda De Marco, Beatriz Beamud, Lidia Ruiz Roldan, Marta Pla Diaz, Llúcia Martínez-Priego, Concepcion Gimeno, Giuseppe D'Auria, Fernando Gonzalez-Candelas
EPI_ISL_436733, EPI_ISL_436734, EPI_ISL_436735, EPI_ISL_436736, EPI_ISL_436737, EPI_ISL_436738, EPI_ISL_436739, EPI_ISL_436740, EPI_ISL_436741, EPI_ISL_436742, EPI_ISL_436743			
see above	NYU Langone Health	Departments of Pathology and Medicine, New York University School of Medicine	Maria Aguero-Rosenfeld, Brendan Belovarac, Margaret Black, Ludovic Boytard, John Cadley, Paolo Cotzia, John Chen, Dacia Dimartino, Xiaojun Feng, Tatyana Gindin, Emily Guzman, Adriana Heguy, Megan Hogan, Emily Huang, George Jour, Alireza Khodadadi-Jamayran, Lawrence H. Lin, Raven Luther, Andrew Lytle, Christian Marier, Matthew T. Maurano, Mark J. Mulligan, Peter Meyn, Raquel Ordonez Ciriza, Iman Osman, Jared Pinnell, Vanessa Raabe, Sitharam Ramaswami, Amy Rapkiewicz, Andre M. Ribeiro-dos-Santos, Marie Samanovic-Golden, Antonio Serrano, Guomiao Shen, Matija Snuderl, Theodore Vougiouklakis, Nick Vulpescu, Gael Westby, Paul Zappile, Yutong Zhang
EPI_ISL_436817, EPI_ISL_436818, EPI_ISL_436819, EPI_ISL_436820, EPI_ISL_436821, EPI_ISL_436822, EPI_ISL_436823, EPI_ISL_436824, EPI_ISL_436889	Michigan Department of Health and Human Services, Bureau of Laboratories	Michigan Department of Health and Human Services, Bureau of Laboratories	Blankenship HM, Riner D, Soehnlen MK
EPI_ISL_437034	Department of Virus and Microbiological Special Diagnostics, Statens Serum Institut, Copenhagen, Denmark, Artillerivej 5, 2300 Copenahgen S	Albertsen lab, Department of Chemistry and Bioscience, Aalborg University, Denmark	Rasmus Kirkegaard
EPI_ISL_437108, EPI_ISL_437109, EPI_ISL_437110, EPI_ISL_437111, EPI_ISL_437113, EPI_ISL_437114, EPI_ISL_437115, EPI_ISL_437116, EPI_ISL_437117, EPI_ISL_437118, EPI_ISL_437119, EPI_ISL_437120, EPI_ISL_437122, EPI_ISL_437123, EPI_ISL_437131, EPI_ISL_437138			
see above	Michigan Department of Health and Human Services, Bureau of Laboratories	Michigan Department of Health and Human Services, Bureau of Laboratories	Blankenship HM, Riner D, Soehnlen MK
EPI_ISL_437205, EPI_ISL_437206, EPI_ISL_437234, EPI_ISL_437252, EPI_ISL_437253, EPI_ISL_437254	Max von Pettenkofer Institute, Virology, National Reference Center for Retroviruses, LMU München	Laboratory for Functional Genome Analysis, Dept. Genomics, Gene Center of the LMU Munich	Max Muenchhoff, Stefan Krebs, Alexander Graf, Oliver Keppler, Helmut Blum
EPI_ISL_437332	Ministry of Health Turkey	Ministry of Health Turkey	Fatma Bayrakdar,Aye Baak Alta,Yasemin Cogun,Süleyman Yalçn, Gülay Korukluolu,Selçuk Klç
EPI_ISL_437481	Pathogen Genomics Lab King Abdullah University of Science and Technology(KAUST)	Pathogen Genomics Lab King Abdullah University of Science and Technology(KAUST)	Sara Mfarrej,Raeece Naeem,Sharif Hala,Amit Subudhi,Fathia Rached,Arnab Pain
EPI_ISL_437519	The National Institute of Public Health Center for Epidemiology and Microbiology	The National Institute of Public Health Center for Epidemiology and Microbiology	Alexander Nagy, Helena Jirincova, Ludmila Novakova, Dusan Trnka, Jaromira Vecerova

EPI_ISL_437561, EPI_ISL_437584	Scripps Medical Laboratory	Andersen lab at Scripps Research	SEARCH Alliance San Diego with Michael Quigley, Ellen Stefanski, Ian Mchardy
EPI_ISL_437605, EPI_ISL_437606, EPI_ISL_437607	unknown	Faculty of Medicine	Rodpan,A., Joyjinda,Y., Wacharapluesadee,S., Buathong,R., Ghai,S., Petcharat,S., Bunprakob,S., Sirichan,N., Prasithsirikul,W., Mungaomklang,A., Plipat,T. and Hemachudha,T.
EPI_ISL_437625	Laboratory of Genomics & Bioinformatics, Institute of Immunology and Experimental Therapy, Polish Academy of Sciences Oddzial Mikrobiologii Wojewodzkiej Stacji Sanitarno-Epidemiologicznej.	Laboratory of Genomics & Bioinformatics, Institute of Immunology and Experimental Therapy, Polish Academy of Sciences	Dorota Kujawa, Aleksandra Herud, Dariusz Martynowski, Krzysztof Jakub Pawlik, Joanna Sikorska, Paulina Zebrowska, Grazyna Zalewska, Oskar Karpinski and Lukasz Laczmanski
EPI_ISL_437635, EPI_ISL_437680	Department of Virus and Microbiological Special Diagnostics, Statens Serum Institut, Copenhagen, Denmark, Artillerivej 5, 2300 Copenahgen S	Albertsen lab, Department of Chemistry and Bioscience, Aalborg University, Denmark	Rasmus Kirkegaard
EPI_ISL_437883, EPI_ISL_437884, EPI_ISL_437885, EPI_ISL_437886, EPI_ISL_437887, EPI_ISL_437889, EPI_ISL_437890, EPI_ISL_437891	Laboratory of Microbiology, Medical School, National and Kapodistrian University of Athens	Laboratory of Biology, Department of Medicine, Democritus University of Thrace	Kassela K., Dovrolis,N., Bampali,M., Gatzidou,E., Froukala,E., Stavropoulou,A., Veletza,S., Tsakris,A., Spanakis,N. and Karakasiliotis,I.
EPI_ISL_437943, EPI_ISL_437944, EPI_ISL_437945, EPI_ISL_437966, EPI_ISL_437970, EPI_ISL_437971, EPI_ISL_437972, EPI_ISL_437973	Universitaetsklinik für Innere Medizin II Innsbruck	Bergthaler laboratory, CeMM Research Center for Molecular Medicine of the Austrian Academy of Sciences	Alexandra Popa, Benedikt Agerer, Henrique Colaco, Lukas Endler, Jakob-Wendelin Genger, Alexander Lercher, Mark Smyth, Thomas Penz, Michael Schuster, Jan Laine, Martin Senekowitsch, Judith Aberle, Stephan Aberle, Elisabeth Puchhammer-Stoeckl, Manfred Nairz, Guenter Weiss, Wegene Borena, Dorothee von Laer, Christoph Bock, Andreas Bergthaler
EPI_ISL_437977	Institut für Virologie am Department für Hygiene, Mikrobiologie und Public Health	Bergthaler laboratory, CeMM Research Center for Molecular Medicine of the Austrian Academy of Sciences	Alexandra Popa, Benedikt Agerer, Henrique Colaco, Lukas Endler, Jakob-Wendelin Genger, Alexander Lercher, Mark Smyth, Thomas Penz, Michael Schuster, Jan Laine, Martin Senekowitsch, Judith Aberle, Stephan Aberle, Elisabeth Puchhammer-Stoeckl, Manfred Nairz, Guenter Weiss, Wegene Borena, Dorothee von Laer, Christoph Bock, Andreas Bergthaler
EPI_ISL_438040, EPI_ISL_438041, EPI_ISL_438042, EPI_ISL_438043, EPI_ISL_438044, EPI_ISL_438045, EPI_ISL_438046, EPI_ISL_438047, EPI_ISL_438048, EPI_ISL_438049, EPI_ISL_438050, EPI_ISL_438051, EPI_ISL_438052, EPI_ISL_438053, EPI_ISL_438054, EPI_ISL_438055, EPI_ISL_438056, EPI_ISL_438057, EPI_ISL_438058, EPI_ISL_438059, EPI_ISL_438060, EPI_ISL_438061, EPI_ISL_438062, EPI_ISL_438063, EPI_ISL_438064, EPI_ISL_438065			
see above	Center for Virology, Medical University of Vienna	Bergthaler laboratory, CeMM Research Center for Molecular Medicine of the Austrian Academy of Sciences	Alexandra Popa, Benedikt Agerer, Henrique Colaco, Lukas Endler, Jakob-Wendelin Genger, Alexander Lercher, Mark Smyth, Thomas Penz, Michael Schuster, Jan Laine, Martin Senekowitsch, Judith Aberle, Stephan Aberle, Elisabeth Puchhammer-Stoeckl, Manfred Nairz, Guenter Weiss, Wegene Borena, Dorothee von Laer, Christoph Bock, Andreas Bergthaler
EPI_ISL_438222, EPI_ISL_438226, EPI_ISL_438229, EPI_ISL_438231	Johns Hopkins Hospital Department of Pathology	Johns Hopkins Hospital Department of Pathology	Peter M. Thielen, Thomas Mehoke, Shirlee Wohl, Srividya Ramakrishnan, Melanie Kirsche,_Amanda Ernlund,_Oluwaseun Falade-Nwulia, Timothy Gilpatrick, Paul Morris, Norah Sadowski, N_di_ Trovao, Victoria Gniazdowski, Michael Schatz, Stuart C. Ray, Winston Timp, Heba Mostafa
EPI_ISL_438236, EPI_ISL_438246, EPI_ISL_438247	Johns Hopkins Hospital Department of Pathology	Johns Hopkins Hospital Department of Pathology	Peter M. Thielen, Thomas Mehoke, Shirlee Wohl, Srividya Ramakrishnan, Melanie Kirsche, Amanda Ernlund, Oluwaseun Falade-Nwulia, Timothy Gilpatrick, Paul Morris, Norah Sadowski, Nidia Trovao, Victoria Gniazdowski, Michael Schatz, Stuart C. Ray, Winston Timp, Heba Mostafa
EPI_ISL_438883, EPI_ISL_438885, EPI_ISL_438906	West of Scotland Specialist Virology Centre, NHSGGC / MRC-University of Glasgow Centre for Virus Research	COVID-19 Genomics UK (COG-UK) Consortium	Ana da Silva Filipe, Natasha Johnson, Kathy Smollett, Daniel Mair, Stephen Carmichael, Lily Tong, Jenna Nichols, Elihu Aranday-Cortes, Kirstyn Brunker, Yasmin Parr, Kyriaki Nomikou; Sarah McDonald, Marc Niebel, Patawee Asamaphan; Richard Orton, Joseph Hughes, Sreenu Vattipally, David L Robertson; Alasdair MacLean, Rory Gunson; Kathy Li, Natasha Jesudason, Rajiv Shah, James Shepherd, Antonia Ho, Emma Thomson
EPI_ISL_440006, EPI_ISL_440037, EPI_ISL_440098, EPI_ISL_440105, EPI_ISL_440119, EPI_ISL_440131, EPI_ISL_440134, EPI_ISL_440136, EPI_ISL_440139, EPI_ISL_440140, EPI_ISL_440148, EPI_ISL_440149, EPI_ISL_440151, EPI_ISL_440165, EPI_ISL_440168, EPI_ISL_440174, EPI_ISL_440175, EPI_ISL_440194, EPI_ISL_440196, EPI_ISL_440198, EPI_ISL_440200, EPI_ISL_440201, EPI_ISL_440204, EPI_ISL_440206, EPI_ISL_440215, EPI_ISL_440218, EPI_ISL_440220, EPI_ISL_440232, EPI_ISL_440233, EPI_ISL_440241, EPI_ISL_440242, EPI_ISL_440243, EPI_ISL_440248, EPI_ISL_440251, EPI_ISL_440253, EPI_ISL_440255, EPI_ISL_440266, EPI_ISL_440273, EPI_ISL_440276, EPI_ISL_440277			
see above	PHE South West Regional Laboratory, National Infection Service	Wellcome Sanger Institute for the COVID-19 Genomics UK (COG-UK) consortium	Stephanie Hutchings, Hannah Pymont, Dr Peter Muir, Barry Vipond, Rich Hopes, Alex Alderton, Roberto Amato, Sonia Goncalves, Ewan Harrison, David K. Jackson, Ian Johnston, Dominic Kwiatkowski, Cordelia Langford, John Sillitoe on behalf of the Wellcome Sanger Institute COVID-19 Surveillance Team (http://www.sanger.ac.uk/covid-team)
EPI_ISL_440288	Department of Pathology, University of Cambridge	Wellcome Sanger Institute for the COVID-19 Genomics UK (COG-UK) consortium	Luke W Meredith, M. Estée Török , Myra Hosmillo, William L. Hamilton, Martin D. Curran, Theresa Feltwell, Grant Hall, Anna Yakovleva, Fahad A Khokhar, Charlotte J. Houldcroft, Laura G Caller, Aminu S. Jahun, Sarah L. Caddy, Ian Goodfellow, Alex Alderton, Roberto Amato, Sonia Goncalves, Ewan Harrison, David K. Jackson, Ian Johnston, Dominic Kwiatkowski, Cordelia Langford, John Sillitoe on behalf of the Wellcome Sanger Institute COVID-19 Surveillance Team (http://www.sanger.ac.uk/covid-team)
EPI_ISL_440291, EPI_ISL_440299	PHE South West Regional Laboratory, National Infection Service	Wellcome Sanger Institute for the COVID-19 Genomics UK (COG-UK) consortium	Stephanie Hutchings, Hannah Pymont, Dr Peter Muir, Barry Vipond, Rich Hopes, Alex Alderton, Roberto Amato, Sonia Goncalves, Ewan Harrison, David K. Jackson, Ian Johnston, Dominic Kwiatkowski, Cordelia Langford, John Sillitoe on behalf of the Wellcome Sanger Institute COVID-19 Surveillance Team (http://www.sanger.ac.uk/covid-team)
EPI_ISL_440303, EPI_ISL_440308, EPI_ISL_440315, EPI_ISL_440317, EPI_ISL_440320, EPI_ISL_440326, EPI_ISL_440328, EPI_ISL_440329, EPI_ISL_440330, EPI_ISL_440334, EPI_ISL_440337, EPI_ISL_440340, EPI_ISL_440341, EPI_ISL_440349, EPI_ISL_440353, EPI_ISL_440356, EPI_ISL_440358, EPI_ISL_440368, EPI_ISL_440370, EPI_ISL_440376, EPI_ISL_440377, EPI_ISL_440379, EPI_ISL_440382, EPI_ISL_440384, EPI_ISL_440385, EPI_ISL_440386, EPI_ISL_440388, EPI_ISL_440390, EPI_ISL_440391, EPI_ISL_440392, EPI_ISL_440393, EPI_ISL_440394, EPI_ISL_440395, EPI_ISL_440398, EPI_ISL_440400, EPI_ISL_440401, EPI_ISL_440402, EPI_ISL_440405, EPI_ISL_440406, EPI_ISL_440410, EPI_ISL_440412, EPI_ISL_440414, EPI_ISL_440416, EPI_ISL_440417, EPI_ISL_440418, EPI_ISL_440420, EPI_ISL_440422, EPI_ISL_440424, EPI_ISL_440425, EPI_ISL_440428, EPI_ISL_440429, EPI_ISL_440430, EPI_ISL_440431, EPI_ISL_440432, EPI_ISL_440433, EPI_ISL_440434, EPI_ISL_440436, EPI_ISL_440437, EPI_ISL_440438, EPI_ISL_440441, EPI_ISL_440454			
see above	Department of Pathology, University of Cambridge	Wellcome Sanger Institute for the COVID-19 Genomics UK (COG-UK) consortium	Luke W Meredith, M. Estée Török , Myra Hosmillo, William L. Hamilton, Martin D. Curran, Theresa Feltwell, Grant Hall, Anna Yakovleva, Fahad A Khokhar, Charlotte J. Houldcroft, Laura G Caller, Aminu S. Jahun, Sarah L. Caddy, Ian Goodfellow, Alex Alderton, Roberto Amato, Sonia Goncalves, Ewan Harrison, David K. Jackson, Ian Johnston, Dominic Kwiatkowski, Cordelia Langford, John Sillitoe on behalf of the Wellcome Sanger Institute COVID-19 Surveillance Team (http://www.sanger.ac.uk/covid-team)
EPI_ISL_441409, EPI_ISL_441413, EPI_ISL_441414, EPI_ISL_441419, EPI_ISL_441420, EPI_ISL_441421, EPI_ISL_441422, EPI_ISL_441423, EPI_ISL_441425, EPI_ISL_441426, EPI_ISL_441428, EPI_ISL_441429, EPI_ISL_441430, EPI_ISL_441431, EPI_ISL_441432, EPI_ISL_441433, EPI_ISL_441434, EPI_ISL_441435, EPI_ISL_441436			
see above	Regional Virus Laboratory, Belfast Health and Social Care Trust	COVID-19 Genomics UK (COG-UK) Consortium	Conall McCaughey, James McKenna, Tanya Curran, Susan Feeney, Alison Watt, Ciara Cox, Mairead Connor, Zoltan Molnar, David Simpson, Derek Fairley
EPI_ISL_443188, EPI_ISL_443189, EPI_ISL_443193, EPI_ISL_443194, EPI_ISL_443197, EPI_ISL_443199, EPI_ISL_443200, EPI_ISL_443202, EPI_ISL_443203, EPI_ISL_443204, EPI_ISL_443205, EPI_ISL_443206, EPI_ISL_443207, EPI_ISL_443208, EPI_ISL_443209, EPI_ISL_443210, EPI_ISL_443211, EPI_ISL_443214, EPI_ISL_443215, EPI_ISL_443226, EPI_ISL_443227, EPI_ISL_443228, EPI_ISL_443229, EPI_ISL_443230, EPI_ISL_443231, EPI_ISL_443232, EPI_ISL_443233, EPI_ISL_443234, EPI_ISL_443235, EPI_ISL_443236, EPI_ISL_443237, EPI_ISL_443238, EPI_ISL_443239, EPI_ISL_443240, EPI_ISL_443241, EPI_ISL_443242, EPI_ISL_443243			
see above	National Public Health Laboratory, National Centre for Infectious Diseases	National Public Health Laboratory, National Centre for Infectious Diseases	Mak Tze Minn, Octavia Sophie, Chavatte Jean-Marc, Cui Lin, Lin Raymond Tzer Pin
EPI_ISL_443266, EPI_ISL_443267, EPI_ISL_443268	CHU - Hôpital Cavale Blanche - Labo. de Virologie	National Reference Center for Viruses of Respiratory Infections, Institut Pasteur, Paris	Mélanie Albert, Marion Barbet, Sylvie Behillil, Méline Bizard, Angela Brisebarre, Flora Donati, Etienne Simon-Lorière, Vincent Enouf, Maud Vanpeene, Sylvie van der Werf, Léa Pilorge
EPI_ISL_443300	Cabinet Médical	National Reference Center for Viruses of Respiratory Infections, Institut Pasteur, Paris	Mélanie Albert, Marion Barbet, Sylvie Behillil, Méline Bizard, Angela Brisebarre, Flora Donati, Etienne Simon-Lorière, Vincent Enouf, Maud Vanpeene, Sylvie van der Werf
EPI_ISL_444022	Baylor College of Medicine	Baylor College of Medicine: HGSC	Vasanthi Avadhanula, Erin Nicholson, David Henke, Pedro Piedra, Harsha Doddapaneni, Donna Muzny, Qingchang Meng, Hsu Chao, Zeineen Momin, Hua Shen, George Weissenberger, Kavya Kottapalli, Yimiti Meiheerguli, Sejal Salvi, Ginger Metcalf, Vipin Menon, Sara J.J. Cregeen, Matthew C. Ross, Tulin Ayvaz, Richard Sucgang, Kristi L. Hoffman, Matthew Wong, Joseph F. Petrosino
EPI_ISL_444275	Laboratory Medicine	Department of Laboratory Medicine, Lin-Kou Chang Gung Memorial Hospital, Taoyuan, Taiwan	Kuo-Chien Tsao, Yu-Nong Gong, Shu-Li Yang, Yi-Chun Liu, Chung-Guei Huang, Mei-Jen Hsiao, Po-Wei Huang, Cheng-Ta Yang, Cheng-Hsun Chiu, Peng-Nien Huang, Kuo-Ming Lee, Guang-Wu Chen, Shin-Ru Shih
EPI_ISL_444520, EPI_ISL_444521, EPI_ISL_444522, EPI_ISL_444523, EPI_ISL_444524, EPI_ISL_444525, EPI_ISL_444526, EPI_ISL_444527, EPI_ISL_444528, EPI_ISL_444529, EPI_ISL_444530, EPI_ISL_444531, EPI_ISL_444532, EPI_ISL_444535, EPI_ISL_444536, EPI_ISL_444537, EPI_ISL_444538, EPI_ISL_444539, EPI_ISL_444540, EPI_ISL_444541, EPI_ISL_444542, EPI_ISL_444549, EPI_ISL_444550, EPI_ISL_444552, EPI_ISL_444553, EPI_ISL_444554, EPI_ISL_444555, EPI_ISL_444556, EPI_ISL_444557, EPI_ISL_444558, EPI_ISL_444559, EPI_ISL_444560, EPI_ISL_444561, EPI_ISL_444562, EPI_ISL_444563, EPI_ISL_444564,			

EPI_ISL_444565, EPI_ISL_444566, EPI_ISL_444567, EPI_ISL_444568, EPI_ISL_444569, EPI_ISL_444570, EPI_ISL_444584, EPI_ISL_444585			
see above	Northwestern Memorial Hospital	Ozer Lab	Ramon Lorenzo-Redondo, Hannah H. Nam, Scott C. Roberts, Lacy M. Simons, Chad J. Achenbach, Lawrence J. Jennings, Chao Qi, Alan R. Hauser, Michael G. Ison, Judd F. Hultquist, Egon A. Ozer
EPI_ISL_444773, EPI_ISL_444774, EPI_ISL_444775, EPI_ISL_444776, EPI_ISL_444777, EPI_ISL_444778, EPI_ISL_444779, EPI_ISL_444780, EPI_ISL_444781, EPI_ISL_444782, EPI_ISL_444783, EPI_ISL_444784, EPI_ISL_444785, EPI_ISL_444786, EPI_ISL_444788, EPI_ISL_444789, EPI_ISL_444790, EPI_ISL_444791, EPI_ISL_444792			
see above	NYU Langone Health	Departments of Pathology and Medicine, New York University School of Medicine	Maria Aguero-Rosenfeld, Brendan Belovarac, Margaret Black, Ludovic Boytard, John Cadley, Paolo Cotzia, John Chen, Dacia Dimartino, Xiaojun Feng, Tatyana Gindin, Emily Guzman, Adriana Heguy, Megan Hogan, Emily Huang, George Jour, Alireza Khodadadi-Jamayran, Lawrence H. Lin, Raven Luther, Andrew Lytle, Christian Marier, Matthew T. Maurano, Mark J. Mulligan, Peter Meyn, Raquel Ordonez Ciriza, Iman Osman, Jared Pinnell, Vanessa Raabe, Sitharam Ramaswami, Amy Rapkiewicz, Andre M. Ribeiro-dos-Santos, Marie Samanovic-Golden, Antonio Serrano, Guomiao Shen, Matija Snuderl, Theodore Vougiouklakis, Nick Vulpescu, Gael Westby, Paul Zappile, Yutong Zhang
EPI_ISL_444897, EPI_ISL_444898, EPI_ISL_444899	Department of Virus and Microbiological Special Diagnostics, Statens Serum Institut, Copenhagen, Denmark, Artillerivej 5, 2300 Copenahgen S	Albertsen lab, Department of Chemistry and Bioscience, Aalborg University, Denmark	Rasmus Kirkegaard
EPI_ISL_444977	Hospital Universitari Vall d'Hebron - Vall d'Hebron Institut de Recerca	Hospital Universitari Vall d'Hebron	Cristina Andrés, Maria Piñana, Damir Garcia-Cehic, Mercedes Guerrero-Murillo, Ariadna Rando, Juliana Esperalba, Maria Gema Codina, Tomàs Pumarola, Josep Quer, Andrés Antón
EPI_ISL_445094, EPI_ISL_445100, EPI_ISL_445101	UC San Diego Center for Advanced Laboratory Medicine	Andersen lab at Scripps Research	SEARCH Alliance San Diego with David Pride, Ji H Shin
EPI_ISL_445165	Scripps Medical Laboratory	Andersen lab at Scripps Research	SEARCH Alliance San Diego with Michael Quigley, Ellen Stefanski, Ian Mchardy
EPI_ISL_445222	Saroledens Familjelakare	The Public Health Agency of Sweden	Katarina Jarbur, Oskar Karlsson Lindsjo, Maria Lind Karlberg, Anna-Malin Linde, Olov Svartstrom, Anna Risberg, Theresa Enkirch, Mia Brytting, Karin Tegmark-Wisell
EPI_ISL_445269	HOSPITAL REG.LAUTARO NAVARRO AVARIA	Instituto de Salud Publica de Chile	Andrés E Castillo, Bárbara Parra,Paz Tapia, Jaime Lagos, Loredana Arata, Alejandra Acevedo, Winston Andrade, Gabriel Leal, Carolina Tambley, Patricia Bustos, Rodrigo Fasce, Jorge Fernandez
EPI_ISL_445272	CLINICA CIUDAD DEL MAR	Instituto de Salud Publica de Chile	Andrés E Castillo, Bárbara Parra,Paz Tapia, Jaime Lagos, Loredana Arata, Alejandra Acevedo, Winston Andrade, Gabriel Leal, Carolina Tambley, Patricia Bustos, Rodrigo Fasce, Jorge Fernandez
EPI_ISL_445273, EPI_ISL_445274	LABORATORIO TORRE MEDICA LTDA.	Instituto de Salud Publica de Chile	Andrés E Castillo, Bárbara Parra,Paz Tapia, Jaime Lagos, Loredana Arata, Alejandra Acevedo, Winston Andrade, Gabriel Leal, Carolina Tambley, Patricia Bustos, Rodrigo Fasce, Jorge Fernandez
EPI_ISL_445275, EPI_ISL_445276	HOSPITAL CLINICO FUSAT	Instituto de Salud Publica de Chile	Andrés E Castillo, Bárbara Parra,Paz Tapia, Jaime Lagos, Loredana Arata, Alejandra Acevedo, Winston Andrade, Gabriel Leal, Carolina Tambley, Patricia Bustos, Rodrigo Fasce, Jorge Fernandez
EPI_ISL_445277	FUNDACION DE SALUD EL TENIENTE	Instituto de Salud Publica de Chile	Andrés E Castillo, Bárbara Parra,Paz Tapia, Jaime Lagos, Loredana Arata, Alejandra Acevedo, Winston Andrade, Gabriel Leal, Carolina Tambley, Patricia Bustos, Rodrigo Fasce, Jorge Fernandez
EPI_ISL_445278	LABORATORIO TORRE MEDICA LTDA.	Instituto de Salud Publica de Chile	Andrés E Castillo, Bárbara Parra,Paz Tapia, Jaime Lagos, Loredana Arata, Alejandra Acevedo, Winston Andrade, Gabriel Leal, Carolina Tambley, Patricia Bustos, Rodrigo Fasce, Jorge Fernandez
EPI_ISL_445279	LABORATORIO INMUNOLAB SPA	Instituto de Salud Publica de Chile	Andrés E Castillo, Bárbara Parra,Paz Tapia, Jaime Lagos, Loredana Arata, Alejandra Acevedo, Winston Andrade, Gabriel Leal, Carolina Tambley, Patricia Bustos, Rodrigo Fasce, Jorge Fernandez
EPI_ISL_445280	HOSPITAL REG.LAUTARO NAVARRO AVARIA	Instituto de Salud Publica de Chile	Andrés E Castillo, Bárbara Parra,Paz Tapia, Jaime Lagos, Loredana Arata, Alejandra Acevedo, Winston Andrade, Gabriel Leal, Carolina Tambley, Patricia Bustos, Rodrigo Fasce, Jorge Fernandez
EPI_ISL_445281	HOSPITAL CLINICO DEL SUR	Instituto de Salud Publica de Chile	Andrés E Castillo, Bárbara Parra,Paz Tapia, Jaime Lagos, Loredana Arata, Alejandra Acevedo, Winston Andrade, Gabriel Leal, Carolina Tambley, Patricia Bustos, Rodrigo Fasce, Jorge Fernandez
EPI_ISL_445304	HOSPITAL SAN JUAN DE DIOS	Instituto de Salud Publica de Chile	Andrés E Castillo, Bárbara Parra,Paz Tapia, Jaime Lagos, Loredana Arata, Alejandra Acevedo, Winston Andrade, Gabriel Leal, Carolina Tambley, Patricia Bustos, Rodrigo Fasce, Jorge Fernandez
EPI_ISL_445305	HOSPITAL DE CARABINEROS	Instituto de Salud Publica de Chile	Andrés E Castillo, Bárbara Parra,Paz Tapia, Jaime Lagos, Loredana Arata, Alejandra Acevedo, Winston Andrade, Gabriel Leal, Carolina Tambley, Patricia Bustos, Rodrigo Fasce, Jorge Fernandez
EPI_ISL_445306	CLINICA UC SAN CARLOS DE APOQUINDO	Instituto de Salud Publica de Chile	Andrés E Castillo, Bárbara Parra,Paz Tapia, Jaime Lagos, Loredana Arata, Alejandra Acevedo, Winston Andrade, Gabriel Leal, Carolina Tambley, Patricia Bustos, Rodrigo Fasce, Jorge Fernandez
EPI_ISL_445307	HOSPITAL SAN JOSE DE MAIPO	Instituto de Salud Publica de Chile	Andrés E Castillo, Bárbara Parra,Paz Tapia, Jaime Lagos, Loredana Arata, Alejandra Acevedo, Winston Andrade, Gabriel Leal, Carolina Tambley, Patricia Bustos, Rodrigo Fasce, Jorge Fernandez
EPI_ISL_445308	HOSPITAL EL CARMEN DR.LUIS VALENTIN F.	Instituto de Salud Publica de Chile	Andrés E Castillo, Bárbara Parra,Paz Tapia, Jaime Lagos, Loredana Arata, Alejandra Acevedo, Winston Andrade, Gabriel Leal, Carolina Tambley, Patricia Bustos, Rodrigo Fasce, Jorge Fernandez
EPI_ISL_445309	MUTUAL DE SEGURIDAD C.CH.C.	Instituto de Salud Publica de Chile	Andrés E Castillo, Bárbara Parra,Paz Tapia, Jaime Lagos, Loredana Arata, Alejandra Acevedo, Winston Andrade, Gabriel Leal, Carolina Tambley, Patricia Bustos, Rodrigo Fasce, Jorge Fernandez
EPI_ISL_445310	HOSPITAL DR.SOTERO DEL RIO	Instituto de Salud Publica de Chile	Andrés E Castillo, Bárbara Parra,Paz Tapia, Jaime Lagos, Loredana Arata, Alejandra Acevedo, Winston Andrade, Gabriel Leal, Carolina Tambley, Patricia Bustos, Rodrigo Fasce, Jorge Fernandez
EPI_ISL_445311	MEGASALUD S.A.	Instituto de Salud Publica de Chile	Andrés E Castillo, Bárbara Parra,Paz Tapia, Jaime Lagos, Loredana Arata, Alejandra Acevedo, Winston Andrade, Gabriel Leal, Carolina Tambley, Patricia Bustos, Rodrigo Fasce, Jorge Fernandez
EPI_ISL_445312	CLINICA UC SAN CARLOS DE APOQUINDO	Instituto de Salud Publica de Chile	Andrés E Castillo, Bárbara Parra,Paz Tapia, Jaime Lagos, Loredana Arata, Alejandra Acevedo, Winston Andrade, Gabriel Leal, Carolina Tambley, Patricia Bustos, Rodrigo Fasce, Jorge Fernandez
EPI_ISL_445313, EPI_ISL_445314	HOSPITAL EL CARMEN DR.LUIS VALENTIN F.	Instituto de Salud Publica de Chile	Andrés E Castillo, Bárbara Parra,Paz Tapia, Jaime Lagos, Loredana Arata, Alejandra Acevedo, Winston Andrade, Gabriel Leal, Carolina Tambley, Patricia Bustos, Rodrigo Fasce, Jorge Fernandez
EPI_ISL_445316	CESFAM BALMACEDA DE RENCA	Instituto de Salud Publica de Chile	Andrés E Castillo, Bárbara Parra,Paz Tapia, Jaime Lagos, Loredana Arata, Alejandra Acevedo, Winston Andrade, Gabriel Leal, Carolina Tambley, Patricia Bustos, Rodrigo Fasce, Jorge Fernandez
EPI_ISL_445317	UNIV.DE CHILE HOSP.CLINICO	Instituto de Salud Publica de Chile	Andrés E Castillo, Bárbara Parra,Paz Tapia, Jaime Lagos, Loredana Arata, Alejandra Acevedo, Winston Andrade, Gabriel Leal, Carolina Tambley, Patricia Bustos, Rodrigo Fasce, Jorge Fernandez
EPI_ISL_445319	HOSPITAL FELIX BULNES	Instituto de Salud Publica de Chile	Andrés E Castillo, Bárbara Parra,Paz Tapia, Jaime Lagos, Loredana Arata, Alejandra Acevedo, Winston Andrade, Gabriel Leal, Carolina Tambley, Patricia Bustos, Rodrigo Fasce, Jorge Fernandez
EPI_ISL_445320	C.C.SALUD FAMILIAR PADRE FELIX DONOSO G.	Instituto de Salud Publica de Chile	Andrés E Castillo, Bárbara Parra,Paz Tapia, Jaime Lagos, Loredana Arata, Alejandra Acevedo, Winston Andrade, Gabriel Leal, Carolina Tambley, Patricia Bustos, Rodrigo Fasce, Jorge Fernandez
EPI_ISL_445321	HOSPITAL FELIX BULNES	Instituto de Salud Publica de Chile	Andrés E Castillo, Bárbara Parra,Paz Tapia, Jaime Lagos, Loredana Arata, Alejandra Acevedo, Winston Andrade, Gabriel Leal, Carolina Tambley, Patricia Bustos, Rodrigo Fasce, Jorge Fernandez
EPI_ISL_445322	UNIV.DE CHILE HOSP.CLINICO	Instituto de Salud Publica de Chile	Andrés E Castillo, Bárbara Parra,Paz Tapia, Jaime Lagos, Loredana Arata, Alejandra Acevedo, Winston Andrade, Gabriel Leal, Carolina Tambley, Patricia Bustos, Rodrigo Fasce, Jorge Fernandez
EPI_ISL_445323	HOSP.ENFERMEDADES INFECCIOSAS	Instituto de Salud Publica de Chile	Andrés E Castillo, Bárbara Parra,Paz Tapia, Jaime Lagos, Loredana Arata, Alejandra Acevedo, Winston Andrade, Gabriel Leal, Carolina Tambley, Patricia

			Bustos, Rodrigo Fasce, Jorge Fernandez
EPI_ISL_445359	HOSP. SANTIAGO ORIENTE DR. LUIS TISNE B.	Instituto de Salud Publica de Chile	Andrés E Castillo, Bárbara Parra,Paz Tapia, Jaime Lagos, Loredana Arata, Alejandra Acevedo, Winston Andrade, Gabriel Leal, Carolina Tambley, Patricia Bustos, Rodrigo Fasce, Jorge Fernandez
EPI_ISL_445360	HOSPITAL DEL PROFESOR	Instituto de Salud Publica de Chile	Andrés E Castillo, Bárbara Parra,Paz Tapia, Jaime Lagos, Loredana Arata, Alejandra Acevedo, Winston Andrade, Gabriel Leal, Carolina Tambley, Patricia Bustos, Rodrigo Fasce, Jorge Fernandez
EPI_ISL_445361	CLINICA UC SAN CARLOS DE APOQUINDO	Instituto de Salud Publica de Chile	Andrés E Castillo, Bárbara Parra,Paz Tapia, Jaime Lagos, Loredana Arata, Alejandra Acevedo, Winston Andrade, Gabriel Leal, Carolina Tambley, Patricia Bustos, Rodrigo Fasce, Jorge Fernandez
EPI_ISL_445362	BUPA SERVICIOS CLINICOS S.A	Instituto de Salud Publica de Chile	Andrés E Castillo, Bárbara Parra,Paz Tapia, Jaime Lagos, Loredana Arata, Alejandra Acevedo, Winston Andrade, Gabriel Leal, Carolina Tambley, Patricia Bustos, Rodrigo Fasce, Jorge Fernandez
EPI_ISL_445364	HOSPITAL EL CARMEN DR.LUIS VALENTIN F.	Instituto de Salud Publica de Chile	Andrés E Castillo, Bárbara Parra,Paz Tapia, Jaime Lagos, Loredana Arata, Alejandra Acevedo, Winston Andrade, Gabriel Leal, Carolina Tambley, Patricia Bustos, Rodrigo Fasce, Jorge Fernandez
EPI_ISL_447059, EPI_ISL_447060, EPI_ISL_447092, EPI_ISL_447101, EPI_ISL_447114	Michigan Department of Health and Human Services, Bureau of Laboratories	Michigan Department of Health and Human Services, Bureau of Laboratories	Blankenship HM, Riner D, Soehnlen MK
EPI_ISL_447136	Department of Clinical Microbiology	GIGA Medical Genomics	Keith Durkin, Maria Artesi, Sébastien Bontems, Raphaël Boreux, Cécile Meex, Pierrette Melin, Marie-Pierre Hayette, Vincent Bours.
EPI_ISL_447169, EPI_ISL_447175, EPI_ISL_447177, EPI_ISL_447180, EPI_ISL_447183, EPI_ISL_447186, EPI_ISL_447191, EPI_ISL_447192, EPI_ISL_447194, EPI_ISL_447196, EPI_ISL_447200, EPI_ISL_447206, EPI_ISL_447214, EPI_ISL_447218, EPI_ISL_447228			
see above	Michigan Department of Health and Human Services, Bureau of Laboratories	Michigan Department of Health and Human Services, Bureau of Laboratories	Blankenship HM, Riner D, Soehnlen MK
EPI_ISL_447250	Central Virology Laboratory	Central Virology Laboratory	Neta Zuckerman, Efrat Bucris, Oran Erster, Danit Sofer, Orna Mor, Ella Mendelson, Michal Mandelboim
EPI_ISL_447254, EPI_ISL_447255	TSGH-CP molecular lab	TSGH-CP molecular lab	Cherng-Lih Perng, Ming-Jr JIAN, Chih-Kai Chang, Jung-Chung Lin, Kuo-Ming Yeh, Chien-Wen Chen, Sheng-Kang Chiu, Hsing-Yi Chung, Shih-Hung Tsai, Kuo-Sheng Hung, Tien-Yao Chang, Feng-Yee Chang, Hung-Sheng Shang
EPI_ISL_447324, EPI_ISL_447325, EPI_ISL_447326, EPI_ISL_447327, EPI_ISL_447328, EPI_ISL_447329	Clinical Virology Laboratory, Soroka Medical Center and the Faculty of Health Sciences, Ben-Gurion University of the Negev	Stern Lab	Stern Lab
EPI_ISL_447407, EPI_ISL_447408, EPI_ISL_447409, EPI_ISL_447410, EPI_ISL_447411, EPI_ISL_447412, EPI_ISL_447413, EPI_ISL_447414, EPI_ISL_447415, EPI_ISL_447416	Clinical Virology Unit, Hadassah Hebrew University Medical Center	Stern Lab	Stern Lab
EPI_ISL_447532	Hospital Universitari Vall d'Hebron - Vall d'Hebron Institut de Recerca	Hospital Universitari Vall d'Hebron	Cristina Andrés, Maria Piñana, Damir Garcia-Cehic, Mercedes Guerrero-Murillo, Ariadna Rando, Juliana Esperalba, Maria Gema Codina, Tomàs Pumarola, Josep Quer, Andrés Antón
EPI_ISL_447614	Department of Laboratory Medicine, National Taiwan University Hospital	Microbial Genomics Core Lab, National Taiwan University Centers of Genomic and Precision Medicine	Shiou-Hwei Yeh, You-Yu Lin, Ya-Yun Lai, Chiao-Ling Li, Shan-Chwen Chang, Pei-Jer Chen, Sui-Yuan Chang
EPI_ISL_447640, EPI_ISL_447641, EPI_ISL_447836	unknown	Department of Medicine	Kassela,K., Dovrolis,N., Bampali,M., Gatzidou,E., Froukala,E., Stavropoulou,A., Veletza,S., Tsakris,A., Spanakis,N. and Karakasiliotis,I.
EPI_ISL_448222	Pasteur Insitute Ho Chi Minh City	National Key Laboratory of Gene Technology, Institute of Biotechnology, Vietnam Academy of Science and Technology	Le Tung Lam, Nguyen Trung Hieu, Nguyen Hong Trang, Ho Thi Thuong, Nguyen Thi Ngoc Thao, Huynh Thi Kim Loan, Luu Thuy Tien, Tran Huyen Linh, Pham Duy Quang, Luong Chan Quang, Cao Minh Thang, Nguyen Vu Thuong, Hoang Ha, Chu Hoang Ha, Phan Trong Lan, Truong Nam Hai
EPI_ISL_448815, EPI_ISL_448816, EPI_ISL_448818, EPI_ISL_448824, EPI_ISL_448825	Oxford Viromics, NDM, University of Oxford; Oxford University Hospitals; Basingstoke and North Hampshire Hospital	COVID-19 Genomics UK (COG-UK) Consortium	Tanya Golubchik, David Bonsall, George Macintyre, Amy Trebes, Mariateresa de Cesare, Catrin Moore, Alex Mobbs, Anita Justice, Robert Shaw, Monique Andersson, Emma Wise, Nathan Moore, Jessica Lynch, Nick Cortes, Stephen Kidd, David Buck, John Todd, Christophe Fraser
EPI_ISL_450011, EPI_ISL_450012, EPI_ISL_450013, EPI_ISL_450014, EPI_ISL_450015, EPI_ISL_450016, EPI_ISL_450017, EPI_ISL_450018, EPI_ISL_450019, EPI_ISL_450020, EPI_ISL_450021, EPI_ISL_450022, EPI_ISL_450023, EPI_ISL_450024, EPI_ISL_450025, EPI_ISL_450043, EPI_ISL_450044, EPI_ISL_450045, EPI_ISL_450046, EPI_ISL_450047, EPI_ISL_450048, EPI_ISL_450049, EPI_ISL_450050, EPI_ISL_450051, EPI_ISL_450052, EPI_ISL_450053, EPI_ISL_450054			
see above	MSHS Clinical Microbiology Laboratories	MSHS Pathogen Surveillance Program	Ana S. Gonzalez-Reiche, Mitchell Sullivan, Ajay Obla, Gopi Patel, Emilia Sordillo, Melissa Gitman, Alberto Paniz-mondolfi, Matthew Hernandez, Shelcie Fabre, Jose Polanco, Zenab Khan, Bremy Alburquerque, Jayeeta Dutta, Juan Soto, Shwetha Sridhar Hara, Ying-Chih Wang, Melissa Smith, Robert Sebra, Lisa Miorin, Wen-chun Liu, Randy Albrecht, Judith Aberg, Florian Krammer, Adolfo Garcia-Sastre, Viviana Simon, Harm van Bakel
EPI_ISL_450193	SA Pathology	SA Pathology	Lex Leong
EPI_ISL_450214	unknown	Microbiological Diagnostic Unit Public Health Laboratory (MDU-PHL) and Victorian Infectious Disease Reference Laboratory (VIDRL)	Seemann,T., Lane,C.R., Sherry,N.L., Duchene,S., Goncalves da Silva,A., Caly,L., Sait,M., Ballard,S.A., Horan,K., Schultz,M.B., Hoang,T., Easton,M., Dougal,S., Stinear,T.P., Druce,J., Catton,M., Sutton,B., van Diemen,A., Alpren,C., Williamson,D.A., Howden,B.P.
EPI_ISL_450232, EPI_ISL_450238	UCSF Clinical Microbiology Laboratory	Chiu Laboratory, University of California, San Francisco	Xianding Deng, Scot Federman, Wei Gu, and Charles Y. Chiu
EPI_ISL_450296	National Institute for Communicable Diseases of the National Health Laboratory Service	National Institute for Communicable Diseases of the National Health Laboratory Service	Allam M, Ismail A, Khumalo Z, Kwenda S, van Heusden P, Mtshali P, Mnyameni F, Mohale T, Subramoney K, Bhiman JN
EPI_ISL_450325	NIV Pune	CSIR-Centre for Cellular and Molecular Biology	Dr V A Potdar, Dr ML Choudhary,Dr Priya Abraham,V. Vipat, S. Jadhav, U. Saha, H. Kengle, A. Awhale, A. Jagtap, A. Gondhalikar, V Malik, N Srivastava, S. Digraskar, P. Malsane, S. Hundekar, K. Patel, Yogesh Balakartik, M. Kakade, S. Jadhav, R. Gunjikar, V. Awtade, S. Bhorekar, P Shinde, S. Salve, B. Minhas S. Bharadwaj, H Kaushal Y. Gurav, S. Tomar,Payel Mukherjee, Sofia Banu, Priya Singh, Dhiviya Vedagiri, Divya Gupta, Vishal Sah, Santosh Kumar Kuncha, Krishnan Harinivas Harshan, Archana Bharadwaj Siva, Karthik Bharadwaj Tallapaka, Shagufta Khan, Lamuk Zaveri, Namami Gaur, Sakshi Shambhavi, Tulasi Nagabandi, Purushotham Vodnala,G. Aditya Kumar, Koushick Sivakumar, Pooja Ramesh Gupta, Rajan Kumar Jha, Shraddha Vijay Lahoti, Deepak Kumar, Devi Prasad Vijayashankara, Disha Nanda, Divya Das, Jotin Gogoi, Manish
EPI_ISL_450403	School of Public Health, The University of Hong Kong	School of Public Health, The University of Hong Kong	Sit,T.H.S., Brackman,C.J., Sims,L.D., Tsang,D.N.C., Chu,D.K.W., Perera,R.A.P.M., Poon,L.L.M. and Peiris,M.
EPI_ISL_450404	unknown	School of Public Health, The University of Hong Kong	Sit,T.H.S., Brackman,C.J., Sims,L.D., Tsang,D.N.C., Chu,D.K.W., Perera,R.A.P.M., Poon,L.L.M. and Peiris,M.
EPI_ISL_450468, EPI_ISL_450469, EPI_ISL_450474, EPI_ISL_450477, EPI_ISL_450478, EPI_ISL_450479, EPI_ISL_450480, EPI_ISL_450481	Stanford clinical virology lab	Chan-Zuckerberg Biohub	Benjamin Pinksy, Katharine Walter, Victoria N. Parikh, John Gorzynski, Hannah N. DeJong, Matthew T. Wheeler, Jason Andrews, Manuel Rivas, Carlos Bustamante, Euan Ashley, with CZB Cliahub Consortium
EPI_ISL_450506	Clinical Laboratory, Hospital Israelita Albert Einstein	Clinical Laboratory, Hospital Israelita Albert Einstein	Malta,F., Amgarten,D., de Oliveira,D.B.L., Araujo,D.B., Machado,R.R.G., Santana,R.A.F., Mangueira,C.L.P., Durigon,E.L. and Pinho,J.R.R.
EPI_ISL_450509, EPI_ISL_450516	Rafik Hariri University Hospital	Rafik Hariri University Hospital	Rita Feghali
EPI_ISL_450539	Utah Public Health Laboratory	Utah Public Health Laboratory	Erin Young, Kelly Oakeson
EPI_ISL_450578, EPI_ISL_450580, EPI_ISL_450581, EPI_ISL_450582, EPI_ISL_450583, EPI_ISL_450587	Michigan Department of Health and Human Services, Bureau of Laboratories	Michigan Department of Health and Human Services, Bureau of Laboratories	Blankenship HM; Riner D; Soehnlen MK
EPI_ISL_450602, EPI_ISL_450604,	Michigan Department of Health and Human Services, Bureau	Michigan Department of Health and Human Services, Bureau	Blankenship HM, Riner D, Soehnlen MK

EPI_ISL_450606, EPI_ISL_450607, EPI_ISL_450608, EPI_ISL_450610	of Laboratories	of Laboratories	
EPI_ISL_450700	University of Wisconsin-Madison AIDS Vaccine Research Laboratories	University of Wisconsin-Madison AIDS Vaccine Research Laboratories	Gage Moreno, Katarina Braun, et al. AIDS Vaccine Research Laboratories
EPI_ISL_450739	OUCRU/HTD	OUCRU/HTD	Nguyen Van Vinh Chau, Nguyen Thi Thu Hong, Nguyen Thi Han Ny, Le Nguyen Truc Nhu, Nghiem My Ngoc, Vo Thanh Lam, Nguyen Thanh Dung, Lam Minh Yen, Ngo Ngoc Quang Minh, Le Manh Hung, Nguyen Tri Dung, Dinh Nguyen Huy Man, Lam Anh Nguyet, Tran Chanh Xuan, Tran Tinh Hien, Nguyen Thanh Phong, Tran Nguyen Hoang Tu, Tran Tan Thanh, Nguyen Thanh Truong, Nguyen Tan Binh, Tang Chi Thuong, Guy Thwaites, and Le Van Tan, for OUCRU COVID-19 research group*
EPI_ISL_450796, EPI_ISL_450797	Jamaica Ministry of Health and Wellness	Pathogen Discovery, Respiratory Viruses Branch, Division of Viral Diseases, Centers for Disease Control and Prevention	Yan Li, Anna Montmayeur, Ying Tao, Krista Queen, Jing Zhang, Anna Uehara, Clinton R. Paden, Rachel Marine, Haibin Wang, Zachary Weiner, Bettina Bankamp, Suxiang Tong
EPI_ISL_450798	Jamaica Ministry of Health and Wellness	Pathogen Discovery, Respiratory Viruses Branch, Division of Viral Diseases, Centers for Disease Control and Prevention	Krista Queen, Yan Li, Anna Montmayeur, Ying Tao, Jing Zhang, Anna Uehara, Clinton R. Paden, Rachel Marine, Haibin Wang, Jasmine Padilla, Justin Lee, Zachary Weiner, Bettina Bankamp, Suxiang Tong
EPI_ISL_450799	Jamaica Ministry of Health and Wellness	Pathogen Discovery, Respiratory Viruses Branch, Division of Viral Diseases, Centers for Disease Control and Prevention	Yan Li, Anna Montmayeur, Ying Tao, Krista Queen, Jing Zhang, Anna Uehara, Clinton R. Paden, Rachel Marine, Haibin Wang, Zachary Weiner, Bettina Bankamp, Suxiang Tong
EPI_ISL_450803	PA Department of Health, Bureau of Laboratories	Pathogen Discovery, Respiratory Viruses Branch, Division of Viral Diseases, Centers for Disease Control and Prevention	Yan Li, Anna Montmayeur, Ying Tao, Krista Queen, Jing Zhang, Anna Uehara, Clinton R. Paden, Rachel Marine, Haibin Wang, Zachary Weiner, Bettina Bankamp, Suxiang Tong
EPI_ISL_450873	Evandro Chagas Institute	Evandro Chagas Institute	Santos, M.C.; Silva, A.M.; Junior, W.D.C.; Barbagelata, L.S.; Ferreira, J.A.; Sousa, E.M.A.; da Silva, P.S.; Martins, L.C.; Sousa Junior, E.C.; Viana, G.M.R
EPI_ISL_450878, EPI_ISL_450879, EPI_ISL_450880, EPI_ISL_450881, EPI_ISL_450886, EPI_ISL_450887, EPI_ISL_450888, EPI_ISL_450889, EPI_ISL_450894, EPI_ISL_450895, EPI_ISL_450907, EPI_ISL_450918, EPI_ISL_450919, EPI_ISL_450920, EPI_ISL_450921, EPI_ISL_450926, EPI_ISL_450927, EPI_ISL_450928, EPI_ISL_450929, EPI_ISL_450934, EPI_ISL_450935, EPI_ISL_450947, EPI_ISL_450958, EPI_ISL_450959, EPI_ISL_450960, EPI_ISL_450961, EPI_ISL_450966, EPI_ISL_450967, EPI_ISL_450968, EPI_ISL_450969, EPI_ISL_450974, EPI_ISL_450975, EPI_ISL_450987, EPI_ISL_450998, EPI_ISL_450999, EPI_ISL_451000, EPI_ISL_451001, EPI_ISL_451006, EPI_ISL_451007, EPI_ISL_451008, EPI_ISL_451009, EPI_ISL_451014, EPI_ISL_451015, EPI_ISL_451027, EPI_ISL_451038, EPI_ISL_451039, EPI_ISL_451040, EPI_ISL_451041, EPI_ISL_451046, EPI_ISL_451047, EPI_ISL_451048, EPI_ISL_451049, EPI_ISL_451054, EPI_ISL_451055, EPI_ISL_451067			
see above	Center of Excellence in Clinical Virology	Center of Excellence in Clinical Virology	Puenpa,J., Chansaenroj,J., Nilyanimit,P., Auphimai,C., Yorsaeng,R., Suwannakarn,K., Poovorawan,Y.
EPI_ISL_451077, EPI_ISL_451130, EPI_ISL_451131, EPI_ISL_451132, EPI_ISL_451147	SA Pathology	SA Pathology	Lex Leong, Chuan Kok Lim, Mark Turra, Ivan Bastian, Geoff Higgins
EPI_ISL_451694, EPI_ISL_451695, EPI_ISL_451696, EPI_ISL_451697, EPI_ISL_451698, EPI_ISL_451699, EPI_ISL_451700, EPI_ISL_451701, EPI_ISL_451702, EPI_ISL_451703, EPI_ISL_451704, EPI_ISL_451705, EPI_ISL_451706, EPI_ISL_451707, EPI_ISL_451708, EPI_ISL_451709, EPI_ISL_451710, EPI_ISL_451711, EPI_ISL_451712, EPI_ISL_451713, EPI_ISL_451714, EPI_ISL_451715, EPI_ISL_451716, EPI_ISL_451717, EPI_ISL_451718, EPI_ISL_451719, EPI_ISL_451720, EPI_ISL_451721, EPI_ISL_451722, EPI_ISL_451723, EPI_ISL_451724, EPI_ISL_451725, EPI_ISL_451726, EPI_ISL_451727, EPI_ISL_451728, EPI_ISL_451729, EPI_ISL_451730, EPI_ISL_451731, EPI_ISL_451732, EPI_ISL_451733, EPI_ISL_451734, EPI_ISL_451735, EPI_ISL_451736, EPI_ISL_451737, EPI_ISL_451738, EPI_ISL_451739			
see above	Viollier AG	Department of Biosystems Science and Engineering, ETH Zürich	Christian Beisel, Sarah Nadeau, Ivan Topolsky, Pedro Ferreira, Philipp Jablonski, Susana Posada-Céspedes, Tobias Schär, Ina Nissen, Natascha Santacroce, Elodie Burcklen, Christiane Beckmann, Maurice Redondo, Olivier Kobel, Christoph Noppen, Sophie Seidel, Noemie Santamaria de Souza, Niko Beerenwinkel, Tanja Stadler
EPI_ISL_451935	CUB Hopital Erasme Laboratoire d'Anatomie Pathologique	CUB Hopital Erasme Laboratoire d'Anatomie Pathologique	Prof. Isabelle Salmon, Dr. Nicky D'Haene
EPI_ISL_451937	Max von Pettenkofer Institute, Virology, National Reference Center for Retroviruses, LMU München	Laboratory for Functional Genome Analysis, Dept. Genomics, Gene Center of the LMU Munich	Max Muenchhoff, Stefan Krebs, Alexander Graf, Oliver Keppler, Helmut Blum
EPI_ISL_451958	Jamil-ur-Rahman Center for Genome Research, Dr. Panjwani Center for Molecular Medicine and Drug Research, International Center for Chemical and Biological Sciences, University of Karachi	Jamil-ur-Rahman Center for Genome Research, Dr. Panjwani Center for Molecular Medicine and Drug Research, International Center for Chemical and Biological Sciences, University of Karachi	Shakeel,M., Raza,S.A., Khan,S., Khan,B.A., Zahid,M., Qureshi,M.A.and Khan,I.A
EPI_ISL_451968	Federal Budget Institution of Science, State Research Center for Applied Microbiology & Biotechnology	Federal Budget Institution of Science, State Research Center for Applied Microbiology & Biotechnology	Dyatlov I, Shemyakin I, Khramov M, Bogun A, Kislichkina A, Frolov V, Shishkina L, Sizova A, Chekan L, Blagodatskikh S, Podkopaev Y, Kosilova I, Koroleva-Ushakova A, Tyurin E, Galkina E, Slukina N, Shaikhutdinova R, Kalmantayev T, Kalmantayeva O, Fursova N, Silkina M, Gorbatov A, Titareva G, Firstova V, Makarova M, Gapelchenkova T, Solovieva A, Slukin P, Dentovskaya S, Detushev K, Vagayskaya A, Kartsev N, Detusheva E, Zeninskaya N, Ivanov S, Kartseva A, Platonov M, Hlyntseva A, Khomyakov A, Chernysh S, Krasilnikova E, Ryabko A, Solomentsev V, Teymurazov M, Bakhteeva I, Borzilov A, Skryabin Y, Kanashenko M, Abaimova A, Kolchanova A, Novikova T, Goncharova J, Timofeev V, Kuzina E, Fursov M, Zhumakaev R, Marin M, Denisenko E, Trunyakova A, Kuzin V
EPI_ISL_452034	Department of Clinical Microbiology, Copenhagen University Hospital, Hvidovre, Kettegaard Alle 30, 2650 Hvidovre.	Albertsen lab, Department of Chemistry and Bioscience, Aalborg University, Denmark	Rasmus Kirkegaard
EPI_ISL_452119	VI-US Virgin Islands Department of Health	Pathogen Discovery, Respiratory Viruses Branch, Division of Viral Diseases, Centers for Disease Control and Prevention	Yan Li, Anna Montmayeur, Ying Tao, Krista Queen, Jing Zhang, Anna Uehara, Clinton R. Paden, Rachel Marine, Mary S. Keckler, Alison S. Laufer Halpin, Haibin Wang, Christopher A. Elkins, Zachary Weiner, Suxiang Tong
EPI_ISL_452202	NIV Influenza	NIV Influenza	Potdar V
EPI_ISL_452229	Narhalsan Backa vardcentral	The Public Health Agency of Sweden	Mats Olsson, Anna-Malin Linde, Maria Lind Karlberg, Oskar Karlsson Lindsjo, Olov Svartstrom, Anna Risberg, Theresa Enkirch, Mia Brytting, Karin Tegmark-Wisell
EPI_ISL_452285, EPI_ISL_452286, EPI_ISL_452288, EPI_ISL_452289, EPI_ISL_452290, EPI_ISL_452291, EPI_ISL_452292, EPI_ISL_452294, EPI_ISL_452295, EPI_ISL_452296, EPI_ISL_452297, EPI_ISL_452298, EPI_ISL_452299, EPI_ISL_452300, EPI_ISL_452301, EPI_ISL_452303, EPI_ISL_452315, EPI_ISL_452317, EPI_ISL_452318, EPI_ISL_452319, EPI_ISL_452321, EPI_ISL_452322, EPI_ISL_452323, EPI_ISL_452324			
see above	Michigan Department of Health and Human Services, Bureau of Laboratories	Michigan Department of Health and Human Services, Bureau of Laboratories	Blankenship HM, Riner D, Soehnlen MK
EPI_ISL_452328, EPI_ISL_452335, EPI_ISL_452343, EPI_ISL_452347, EPI_ISL_452348	Laboratory of Infectious Diseases Center of Beijing Ditan Hospital	Laboratory of Infectious Diseases Center of Beijing Ditan Hospital	Siyuan Yang, Chengjie Jie, Fengting Yu, Yunxia Tang, Liting Yan, Linghang Wang
EPI_ISL_452374, EPI_ISL_452375, EPI_ISL_452376, EPI_ISL_452377, EPI_ISL_452379, EPI_ISL_452380, EPI_ISL_452381, EPI_ISL_452382, EPI_ISL_452383, EPI_ISL_452384, EPI_ISL_452385, EPI_ISL_452386, EPI_ISL_452387, EPI_ISL_452388, EPI_ISL_452389, EPI_ISL_452390, EPI_ISL_452391, EPI_ISL_452392, EPI_ISL_452393, EPI_ISL_452394, EPI_ISL_452397, EPI_ISL_452398, EPI_ISL_452399, EPI_ISL_452400, EPI_ISL_452401, EPI_ISL_452402, EPI_ISL_452403, EPI_ISL_452404, EPI_ISL_452405, EPI_ISL_452406, EPI_ISL_452407, EPI_ISL_452408			
see above	Servicio de Microbiología. HRU de Málaga. Servicio Andaluz de Salud	SeqCOVID-SPAIN consortium/IBV(CSIC)	Inmaculada de Toro Peinado, Maria Concepción Mediavilla Gradolph, Begoña Palop Borrás and SeqCOVID-SPAIN consortium
EPI_ISL_452453	Hospital Universitario Puerta del Mar de Cádiz - INIBICA	SeqCOVID-SPAIN consortium/IBV(CSIC)	Salud Rodríguez-Pallares, Fátima-Galán-Sánchez, Manuel Rodrí-guez-Iglesias and SeqCOVID-SPAIN consortium
EPI_ISL_452500, EPI_ISL_452501, EPI_ISL_452502, EPI_ISL_452503, EPI_ISL_452504, EPI_ISL_452505, EPI_ISL_452506, EPI_ISL_452507, EPI_ISL_452508, EPI_ISL_452509, EPI_ISL_452510, EPI_ISL_452511, EPI_ISL_452512, EPI_ISL_452513, EPI_ISL_452514, EPI_ISL_452515, EPI_ISL_452516, EPI_ISL_452517, EPI_ISL_452518, EPI_ISL_452519			
see above	Clínica Universidad de Navarra. Servicio de Enfermedades Infecciosas y Microbiología clínica	SeqCOVID-SPAIN consortium/IBV(CSIC)	Mirian Fernández-Alonso, Jose Luis del Pozo and SeqCOVID-SPAIN consortium
EPI_ISL_453825, EPI_ISL_454002, EPI_ISL_454018, EPI_ISL_454024, EPI_ISL_454029, EPI_ISL_454030, EPI_ISL_454033, EPI_ISL_454034, EPI_ISL_454035, EPI_ISL_454036, EPI_ISL_454037, EPI_ISL_454038, EPI_ISL_454039, EPI_ISL_454040, EPI_ISL_454041, EPI_ISL_454042, EPI_ISL_454043, EPI_ISL_454044, EPI_ISL_454045, EPI_ISL_454046, EPI_ISL_454047, EPI_ISL_454048, EPI_ISL_454049, EPI_ISL_454050, EPI_ISL_454051, EPI_ISL_454052, EPI_ISL_454053, EPI_ISL_454054, EPI_ISL_454055, EPI_ISL_454056, EPI_ISL_454057, EPI_ISL_454059, EPI_ISL_454060, EPI_ISL_454061, EPI_ISL_454062, EPI_ISL_454063, EPI_ISL_454064, EPI_ISL_454065, EPI_ISL_454067, EPI_ISL_454068, EPI_ISL_454069, EPI_ISL_454070, EPI_ISL_454071, EPI_ISL_454127, EPI_ISL_454128, EPI_ISL_454225, EPI_ISL_454226, EPI_ISL_454227, EPI_ISL_454228, EPI_ISL_454229, EPI_ISL_454334, EPI_ISL_454335, EPI_ISL_454336, EPI_ISL_454337, EPI_ISL_454338, EPI_ISL_454339, EPI_ISL_454340, EPI_ISL_454341, EPI_ISL_454342			
see above	unknown	Instituto Nacional de Saude (INSA)	Borges et al

EPI_ISL_454434, EPI_ISL_454435	Maryland Department of Health Laboratories Administration	Maryland Department of Health Laboratories Administration	MDH Laboratories Administration
EPI_ISL_454528	NIV Influenza	NIV Influenza	Potdar V
EPI_ISL_454581, EPI_ISL_454583	University Hospital for Infectious Diseases "Dr. Fran Mihaljevi", Research Unit	University of Zagreb, Centre for research and knowledge transfer in biotechnology	Ivan-Christian Kurolt, Jelena Ivancic Jelecki, Anamarija Slovic
EPI_ISL_454635	County Of San Luis Obispo Public Health Laboratory	Chan-Zuckerberg Biohub	CZB Cliahub Consortium
EPI_ISL_454762, EPI_ISL_454763, EPI_ISL_454764, EPI_ISL_454765, EPI_ISL_454788	Dutch COVID-19 response team	National Institute for Public Health and the Environment (RIVM)	Adam Meijer, Harry Vennema, Jeroen Cremer, Sharon van den Brink, Pieter Overduin, Florian Zwagemaker, Dennis Schmitz, Chantal Reusken, on behalf of the national COVID-19 response team
EPI_ISL_454945, EPI_ISL_454946, EPI_ISL_454953, EPI_ISL_454954, EPI_ISL_454960, EPI_ISL_454969, EPI_ISL_454970, EPI_ISL_454971, EPI_ISL_454972, EPI_ISL_454977, EPI_ISL_454979, EPI_ISL_454984			
see above	Wuhan Chain Medical Labs (CMLabs)	State Key Laboratory of Biotherapy of Sichuan University	Baowen Du, Minjin Wang, Chao Tang, Chuan Chen, Yongzhao Zhou, Mingxia Yu, Hancheng Wei, Weimin Li, Jing-wen Lin, Jia Geng, Binwu Ying, Lu Chen
EPI_ISL_455207, EPI_ISL_455208, EPI_ISL_455209, EPI_ISL_455283	Dutch COVID-19 response team	Erasmus Medical Center	Bas Oude Munnink, David Nieuwenhuijse, Reina Sikkema, Claudia Schapendonk, Irina Chestakova, Anne van der Linden, Theo Bestebroer, Stefan van Nieuwkoop, Mark Pronk, Pascal Lexmond, Corien Swaan, Manon Haverkate, Madelief Mollers, Mart Stein, Sandra Kengne Kamga Mobou, Jeroen van Kampen, Jolanda Voermans, Aura Timen, Corine GeurtsvanKessel, Annemiek van der Eijk, Richard Molenkamp, Marion Koopmans, on behalf of the Dutch national COVID-19 response team.
EPI_ISL_455381, EPI_ISL_455384, EPI_ISL_455387, EPI_ISL_455388, EPI_ISL_455392, EPI_ISL_455393, EPI_ISL_455394, EPI_ISL_455395, EPI_ISL_455397, EPI_ISL_455398	Wuhan Chain Medical Labs (CMLabs)	State Key Laboratory of Biotherapy of Sichuan University	Baowen Du, Minjin Wang, Chao Tang, Chuan Chen, Yongzhao Zhou, Mingxia Yu, Hancheng Wei, Weimin Li, Jing-wen Lin, Jia Geng, Binwu Ying, Lu Chen
EPI_ISL_455442, EPI_ISL_455449, EPI_ISL_455451	1. ViroGenetics - BSL3 Laboratory of Virology, Maopolska Centre of Biotechnology, Jagiellonian University; 2. II Department of Internal Medicine, Faculty of Medicine, Jagiellonian University Medical College; 3. Narodowy Instytut Zdrowia Publicznego - Pastwowy Zakad Higieny (NIZP-PZH)	1. ViroGenetics - BSL3 Laboratory of Virology, Maopolska Centre of Biotechnology, Jagiellonian University; 2. II Department of Internal Medicine, Faculty of Medicine, Jagiellonian University Medical College; 3. Narodowy Instytut Zdrowia Publicznego - Pastwowy Zakad Higieny (NIZP-PZH).	Katarzyna Pancer, Marek Sanak, Aleksandra A. Zasada, Magdalena Rzeczkowska, Tomasz Wokowicz, Katarzyna Zacharczuk, Agnieszka Koakowska-Kulesza, Katarzyna Owczarek, Aleksandra Milewska, Natalia Wolaniuk, Ewelina Hallman-Szeliska, Pawe P abaj, Wojciech Branicki, Krzysztof Pyr
EPI_ISL_455454	Instituto de Diagnostico y Referencia Epidemiologicos (INDRE)	Instituto de Diagnostico y Referencia Epidemiologicos (INDRE)	Mendieta-Condado Edgar, Araiza-Rodriguez Adnan, Garces-Ayala Fabiola, , Rodriguez-Maldonado Abril, Wong-Arambula Claudia, Barrera-Badillo Gisela, Taboada Ramírez Blanca, Ramirez-Gonzalez Ernesto, Hernandez-Rivas Lucia, Lopez-Martinez Irma.
EPI_ISL_455583	Central Chest Institute of Thailand	National Institute of Health. Department of medical Sciences, Ministry of Public Health, Thailand	Pilailuk,Okada; Siripaporn,Phuygun; Thanutsapa,Thanadachakul; Sittiporn,Parnmen;Warawan,Wongboot; Sunthareeya,Waicharoen; Malinee,Chittaganpitch
EPI_ISL_455584	National Institute of Health. Department of medical Sciences, Ministry of Public Health, Thailand	National Institute of Health. Department of medical Sciences, Ministry of Public Health, Thailand	Pilailuk,Okada; Siripaporn,Phuygun; Thanutsapa,Thanadachakul; Sittiporn,Parnmen;Warawan,Wongboot; Sunthareeya,Waicharoen; Malinee,Chittaganpitch
EPI_ISL_455586	Siriraj hospital	National Institute of Health. Department of medical Sciences, Ministry of Public Health, Thailand	Pilailuk,Okada; Siripaporn,Phuygun; Thanutsapa,Thanadachakul; Sittiporn,Parnmen;Warawan,Wongboot; Sunthareeya,Waicharoen; Malinee,Chittaganpitch
EPI_ISL_455594	Central Chest Institute of Thailand	National Institute of Health. Department of medical Sciences, Ministry of Public Health, Thailand	Pilailuk,Okada; Siripaporn,Phuygun; Thanutsapa,Thanadachakul; Sittiporn,Parnmen;Warawan,Wongboot; Sunthareeya,Waicharoen; Malinee,Chittaganpitch
EPI_ISL_455694, EPI_ISL_455695, EPI_ISL_455696	National Hospital of Tropical Diseases	Oxford University Clinical Research Unit, Hanoi, Vietnam	Nguyen Thi Tam, Van Dinh Trang, Nguyen Thu Trang, Nguyen Thi Ngoc Diep, Le Nguyen Minh Hoa, Pham Ngoc Thach, H. Rogier van Doorn, on behalf of the OUCRU COVID-19 research group
EPI_ISL_455716, EPI_ISL_455717	National Hospital of Tropical Diseases	Oxford University Clinical Research Unit, Hanoi, Vietnam	Nguyen Thi Tam, Van Dinh Trang, Nguyen Thi Hong Thuong, Vu Thi Ngoc Bich, Nguyen Thu Trang, Nguyen Thi Ngoc Diep, Le Nguyen Minh Hoa, Pham Ngoc Thach, H. Rogier van Doorn, on behalf of the OUCRU COVID-19 research group
EPI_ISL_455937, EPI_ISL_455938, EPI_ISL_455939, EPI_ISL_455940, EPI_ISL_455941, EPI_ISL_455942, EPI_ISL_455943, EPI_ISL_455944, EPI_ISL_455945, EPI_ISL_455946, EPI_ISL_455947, EPI_ISL_455948			
see above	Ramathibodi Hospital	COVID-19 Network Investigations (CONI) Alliance	Elizabeth Batty, Wasun Chantratita, Thanat Chookajorn, Stefan Fernandez, Angkana Huang, Anthony R. Jones, Khajohn Joonsalak, Chonticha Klungtong, Theerarat Kochakarn, Namfon Kotanan, Krittikorn Kumpornsin, Wudtichai Manasatienkij, Bhakbhoom Panthan, Ekawat Pasomsub, Kingkan Rakmanee, Insee Sensorn, Janjira Thaipadungpanit, Arporn Wangwiwatsin,Treewat Watthanachockchai
EPI_ISL_456118, EPI_ISL_456119, EPI_ISL_456122, EPI_ISL_456124, EPI_ISL_456126, EPI_ISL_456127, EPI_ISL_456128, EPI_ISL_456132, EPI_ISL_456138, EPI_ISL_456139, EPI_ISL_456141, EPI_ISL_456142, EPI_ISL_456148, EPI_ISL_456149, EPI_ISL_456150, EPI_ISL_456155			
see above	Instituto Nacional de Salud - Unidad de Secuenciación y Análisis Genómico	Instituto Nacional de Salud, Universidad Cooperativa de Colombia, Instituto Alexander von Humboldt, Imperial College-London, London School of Hygiene & Tropical Medicine	Katherine Laiton-Donato, Diego A. Álvarez-Díaz, Carlos Franco-Muñoz, Jose A. Usme-Ciro, Gloria Puerto, Nicolas D. Franco-Sierra, Mailyn A.Gonzalez, Zulma M. Cucunubá, Christian Julian VillabonaArenas, Liz Villabona-Arenas, Sussy Echeverria, Astrid C. Flórez, Sergio Gomez-Rangel, Luz Dary Rodriguez, Juliana Barbosa, Erika Ospitia, Diana Marcela Walteros-Acero, Martha Lucia Ospina Martinez, Marcela Mercado-Reyes.
EPI_ISL_456159	Southern Community Labs Dunedin	Institute of Environmental Science and Research (ESR)	Matt Storey, Xiaoyun Ren, Anja Werno, Antje van der Linden, Arlo Upton, Chris Mansell, David Hammer, Dragana Drinkovic, Erasmus Smit, Gary McAuliffe, Hana Sofia Andersson, James Ussher, Jill Sherwood, Josh Freeman, Julia Howard, Juliet Elvy, Mary DeAlmeida, Matt Blakiston, Matthew Rogers, Max Bloomfield, Michael Addidle, Michelle Balm, Sally Roberts, Sarah Jefferies, Sharmini Muttaiyah, Susan Morpeth, Susan Taylor, Timothy Blackmore, Vani Sathyendran, Veronica Playle, Virginia Hope, Erasmus Smit, Lauren Jelly, Joep de Ligt
EPI_ISL_456160	Waikato Hospital	Institute of Environmental Science and Research (ESR)	Matt Storey, Xiaoyun Ren, Anja Werno, Antje van der Linden, Arlo Upton, Chris Mansell, David Hammer, Dragana Drinkovic, Erasmus Smit, Gary McAuliffe, Hana Sofia Andersson, James Ussher, Jill Sherwood, Josh Freeman, Julia Howard, Juliet Elvy, Mary DeAlmeida, Matt Blakiston, Matthew Rogers, Max Bloomfield, Michael Addidle, Michelle Balm, Sally Roberts, Sarah Jefferies, Sharmini Muttaiyah, Susan Morpeth, Susan Taylor, Timothy Blackmore, Vani Sathyendran, Veronica Playle, Virginia Hope, Erasmus Smit, Lauren Jelly, Joep de Ligt
EPI_ISL_456161	Wellington SCL	Institute of Environmental Science and Research (ESR)	Matt Storey, Xiaoyun Ren, Anja Werno, Antje van der Linden, Arlo Upton, Chris Mansell, David Hammer, Dragana Drinkovic, Erasmus Smit, Gary McAuliffe, Hana Sofia Andersson, James Ussher, Jill Sherwood, Josh Freeman, Julia Howard, Juliet Elvy, Mary DeAlmeida, Matt Blakiston, Matthew Rogers, Max Bloomfield, Michael Addidle, Michelle Balm, Sally Roberts, Sarah Jefferies, Sharmini Muttaiyah, Susan Morpeth, Susan Taylor, Timothy Blackmore, Vani Sathyendran, Veronica Playle, Virginia Hope, Erasmus Smit, Lauren Jelly, Joep de Ligt
EPI_ISL_456162	Southern Community Labs Dunedin	Institute of Environmental Science and Research (ESR)	Matt Storey, Xiaoyun Ren, Anja Werno, Antje van der Linden, Arlo Upton, Chris Mansell, David Hammer, Dragana Drinkovic, Erasmus Smit, Gary McAuliffe, Hana Sofia Andersson, James Ussher, Jill Sherwood, Josh Freeman, Julia Howard, Juliet Elvy, Mary DeAlmeida, Matt Blakiston, Matthew Rogers, Max Bloomfield, Michael Addidle, Michelle Balm, Sally Roberts, Sarah Jefferies, Sharmini Muttaiyah, Susan Morpeth, Susan Taylor, Timothy Blackmore, Vani Sathyendran, Veronica Playle, Virginia Hope, Erasmus Smit, Lauren Jelly, Joep de Ligt
EPI_ISL_456164, EPI_ISL_456165, EPI_ISL_456166	Wellington SCL	Institute of Environmental Science and Research (ESR)	Matt Storey, Xiaoyun Ren, Anja Werno, Antje van der Linden, Arlo Upton, Chris Mansell, David Hammer, Dragana Drinkovic, Erasmus Smit, Gary McAuliffe, Hana Sofia Andersson, James Ussher, Jill Sherwood, Josh Freeman, Julia Howard, Juliet Elvy, Mary DeAlmeida, Matt Blakiston, Matthew Rogers, Max Bloomfield, Michael Addidle, Michelle Balm, Sally Roberts, Sarah Jefferies, Sharmini Muttaiyah, Susan Morpeth, Susan Taylor, Timothy Blackmore, Vani Sathyendran, Veronica Playle, Virginia Hope, Erasmus Smit, Lauren Jelly, Joep de Ligt
EPI_ISL_456167	Southern Community Labs Dunedin	Institute of Environmental Science and Research (ESR)	Matt Storey, Xiaoyun Ren, Anja Werno, Antje van der Linden, Arlo Upton, Chris Mansell, David Hammer, Dragana Drinkovic, Erasmus Smit, Gary McAuliffe, Hana Sofia Andersson, James Ussher, Jill Sherwood, Josh Freeman, Julia Howard, Juliet Elvy, Mary DeAlmeida, Matt Blakiston, Matthew Rogers, Max Bloomfield, Michael Addidle, Michelle Balm, Sally Roberts, Sarah Jefferies, Sharmini Muttaiyah, Susan Morpeth, Susan Taylor, Timothy Blackmore, Vani Sathyendran, Veronica Playle, Virginia Hope, Erasmus Smit, Lauren Jelly, Joep de Ligt
EPI_ISL_456168	Waikato Hospital	Institute of Environmental Science and Research (ESR)	Matt Storey, Xiaoyun Ren, Anja Werno, Antje van der Linden, Arlo Upton, Chris Mansell, David Hammer, Dragana Drinkovic, Erasmus Smit, Gary McAuliffe, Hana Sofia Andersson, James Ussher, Jill Sherwood, Josh Freeman, Julia Howard, Juliet Elvy, Mary DeAlmeida, Matt Blakiston, Matthew Rogers, Max Bloomfield, Michael Addidle, Michelle Balm, Sally Roberts, Sarah Jefferies, Sharmini Muttaiyah, Susan Morpeth, Susan Taylor, Timothy

			Blackmore, Vani Sathyendran, Veronica Playle, Virginia Hope, Erasmus Smit, Lauren Jelly, Joep de Ligt
EPI_ISL_456172, EPI_ISL_456173, EPI_ISL_456174, EPI_ISL_456175, EPI_ISL_456176, EPI_ISL_456177, EPI_ISL_456178, EPI_ISL_456179, EPI_ISL_456180, EPI_ISL_456209	LabPLUS	Institute of Environmental Science and Research (ESR)	Matt Storey, Xiaoyun Ren, Anja Werno, Antje van der Linden, Arlo Upton, Chris Mansell, David Hammer, Dragana Drinkovic, Erasmus Smit, Gary McAuliffe, Hana Sofia Andersson, James Ussher, Jill Sherwood, Josh Freeman, Julia Howard, Juliet Elvy, Mary DeAlmeida, Matt Blakiston, Matthew Rogers, Max Bloomfield, Michael Addidle, Michelle Balm, Sally Roberts, Sarah Jefferies, Sharmini Muttaiyah, Susan Morpeth, Susan Taylor, Timothy Blackmore, Vani Sathyendran, Veronica Playle, Virginia Hope, Erasmus Smit, Lauren Jelly, Joep de Ligt
EPI_ISL_457124, EPI_ISL_457140	University of Exeter	COVID-19 Genomics UK (COG-UK) Consortium	Ben Temperton,Aaron Jeffries,Michelle Michelsen,Joanna Warwick-Dugdale,Audrey Farbos,Robyn Manley,Stephen Michell,Jane Masoli
EPI_ISL_457724	Department of Infectious Diseases, Istituto Superiore di Sanità, Roma , Italy	Army Medical and Veterinary Research Center	Paola Stefanelli, Alessandra Lo Presti, Stefano Fiore, Antonella Marchi, Eleonora Benedetti, Concetta Fabiani Silvia Fillo, Giovanni Faggioni, Riccardo De Sanctis, Antonella Fortunato, Anna Anselmo, Francesco Giordani, Vanessa Vera Fain, Nino D'Amore, Florigio Lista
EPI_ISL_457752, EPI_ISL_457753, EPI_ISL_457754, EPI_ISL_457755, EPI_ISL_457763, EPI_ISL_457770, EPI_ISL_457774, EPI_ISL_457785, EPI_ISL_457786, EPI_ISL_457789, EPI_ISL_457791, EPI_ISL_457792, EPI_ISL_457803, EPI_ISL_457806, EPI_ISL_457807, EPI_ISL_457812, EPI_ISL_457814, EPI_ISL_457819, EPI_ISL_457820			
see above	Johns Hopkins Hospital Department of Pathology	Johns Hopkins Hospital Department of Pathology	Peter M. Thielen, Thomas Mehoke, Shirlee Wohl, Srividya Ramakrishnan, Melanie Kirsche, Amanda Ernlund, Craig Howser, Kristina Zudock, Oluwaseun Falade-Nwulia, Norah Sadowski, Paul Morris, Mark Hopkins, Yunfan Fan, Nidia Trovao, Victoria Gniazdowski, Michael C. Schatz, Stuart C. Ray, Winston Timp, Heba H. Mostafa
EPI_ISL_457845	KEMRI-CGMR-C	KEMRI-Wellcome Trust Research Programme/KEMRI-CGMR-C Kilifi	Githinji G. et al 2020
EPI_ISL_457937, EPI_ISL_457938, EPI_ISL_457939	Oman-NIC	Oman-NIC	Samira Al-Maruqi, Fahad Zadjali, Amina Al Jardani, Khulood Al-Mammary, Hanan Al-kindi, Fatma BaAlawi, Hamida AL Barwani, Zeyana AL-Dahmani, Intisar Al-Shukri, Aisha Al-Busaidi, Aisha Al-Amri, Ahlam Al-Amri, Mohammed Al-Tobi, Samiha Al Kharusi, Abdulla Balkhair
EPI_ISL_457940, EPI_ISL_457941, EPI_ISL_457942, EPI_ISL_457943, EPI_ISL_457944, EPI_ISL_457945, EPI_ISL_457946, EPI_ISL_457947, EPI_ISL_457948, EPI_ISL_457949	Laboratorio de Biología Molecular Asociación Española Primera en Salud	Departments of Pathology and Medicine, New York University School of Medicine	Maria Victoria Elizondo, Maria Noel Zubillaga, Gonzalo Manrique, Paul Zappile, Gael Westby, Matthew T Maurano, Christian Marier, Adriana Heguy
EPI_ISL_457975, EPI_ISL_457976, EPI_ISL_457977, EPI_ISL_457978, EPI_ISL_457979, EPI_ISL_457980, EPI_ISL_457985	Oman-NIC	Oman-NIC	Samira Al-Maruqi, Fahad Zadjali, Amina Al Jardani, Khulood Al-Mammary, Hanan Al-kindi, Fatma BaAlawi, Hamida AL Barwani, Zeyana AL-Dahmani, Intisar Al-Shukri, Aisha Al-Busaidi, Aisha Al-Amri, Ahlam Al-Amri, Mohammed Al-Tobi, Samiha Al Kharusi, Abdulla Balkhair
EPI_ISL_458000	Centre For Biotechnology Research and Development	Centre For Biotechnology Research and Development	Matoke-Muhia,D.K., Symeker,S.L., Muuo,S.N., Ochwoto,M., Zablon,J.O., Kimotho,J., Waruhiu,C.N. and Michuki,G.N.
EPI_ISL_458017, EPI_ISL_458018, EPI_ISL_458019, EPI_ISL_458020, EPI_ISL_458021	NYU Langone Health	Departments of Pathology and Medicine, New York University School of Medicine	Maria Aguero-Rosenfeld, Brendan Belovarac, Margaret Black, Ludovic Boytard, John Cadley, Paolo Cotzia, John Chen, Dacia Dimartino, Xiaojun Feng, Tatyana Gindin, Emily Guzman, Adriana Heguy, Megan Hogan, Emily Huang, George Jour, Alireza Khodadadi-Jamayran, Lawrence H. Lin, Raven Luther, Andrew Lytle, Christian Marier, Matthew T. Maurano, Mark J. Mulligan, Peter Meyn, Raquel Ordonez Ciriza, Iman Osman, Jared Pinnell, Vanessa Raabe, Sitharam Ramaswami, Amy Rapkiewicz, Andre M. Ribeiro-dos-Santos, Marie Samanovic-Golden, Antonio Serrano, Guomiao Shen, Matija Snuderl, Theodore Vougiouklakis, Nick Vulpescu, Gael Westby, Paul Zappile, Yutong Zhang
EPI_ISL_458139	Evandro Chagas Institute	Evandro Chagas Institute	Santos, M.C.; Silva, A.M.; Junior, W.D.C.; Barbagelata, L.S.; Ferreira, J.A.; Sousa, E.M.A.; da Silva, P.S.; Resque, H.R; Martins, L.C.; Sousa Junior, E.C.;Viana, G.M.R
EPI_ISL_458230	KU Leuven, Rega Institute, Clinical and Epidemiological Virology	KU Leuven, Rega Institute, Clinical and Epidemiological Virology	Tony Wawina-Bokalanga, Bert Vanmechelen, Joan Marti-Carerras, Piet Maes
EPI_ISL_459856, EPI_ISL_459857, EPI_ISL_459858, EPI_ISL_459862	Center for Genome Regulation (CRG)	Center for Mathematical Modeling and Center for Genome Regulation. Santiago, Chile	Gaete A, Travisany D, Palma R, Urra C, Varas M, Allende ML, Maass A, González M.
EPI_ISL_459866	Kingston Health Sciences Center	Queen's Genomics Lab at Ongwanada (Q-GLO)	Sjaarda CP, Rustom N, Huang D, Perez-Patrigeon S, Hudson ML, Wong H,Guan H, Ayub M, Soares CN, Colautti R, Evans GA, Sheth P
EPI_ISL_459953	Institute for Medical Research, Infectious Disease Research Centre, National Institutes of Health, Ministry of Health Malaysia	Institute for Medical Research Infectious Disease Research Centre, National Institutes of Health, Ministry of Health Malaysia	Suppiah J, Mohd-Zawawi Z, Kamel KA, Ellan K, Kalyanasundram J, Mohd-Zain R, Thayan R
EPI_ISL_459954, EPI_ISL_459955	Institute for Medical Research, Infectious Disease Research Centre, National Institutes of Health, Ministry of Health Malaysia	Institute for Medical Research, Infectious Disease Research Centre, National Institutes of Health, Ministry of Health Malaysia	Suppiah J, Mohd-Zawawi Z, Kamel KA, Ellan K, Kalyanasundram J, Mohd-Zain R, Thayan R
EPI_ISL_459966, EPI_ISL_459967, EPI_ISL_459968, EPI_ISL_459970, EPI_ISL_459971, EPI_ISL_459976	Institut Pasteur du Maroc	Institut Pasteur du Maroc	Marion Barbet, Sylvie Behillil, Méline Bizard, Angela Brisebarre, Camille Capel, Etienne Simon-Lorière, Vincent Enouf, Maud Vanpeene, Sylvie van der Werf, Latifa Anga, Abdellah Faouzi, Anass Abbad, Mjid Eloualid, Jalal Nourlil, Anderrahmane Maaroufi
EPI_ISL_460045, EPI_ISL_460046, EPI_ISL_460047, EPI_ISL_460048, EPI_ISL_460049, EPI_ISL_460050, EPI_ISL_460051, EPI_ISL_460062, EPI_ISL_460063, EPI_ISL_460064, EPI_ISL_460065, EPI_ISL_460066, EPI_ISL_460068, EPI_ISL_460069, EPI_ISL_460070, EPI_ISL_460071, EPI_ISL_460072, EPI_ISL_460073, EPI_ISL_460075, EPI_ISL_460076, EPI_ISL_460077, EPI_ISL_460078			
see above	Minnesota Department of Health, Public Health Laboratory	Minnesota Department of Health, Public Health Laboratory	Matt Plumb, Jacob Garfin, and Xiong Wang
EPI_ISL_460178, EPI_ISL_460211, EPI_ISL_460224, EPI_ISL_460262, EPI_ISL_460344, EPI_ISL_460381, EPI_ISL_460413, EPI_ISL_460458	Massachusetts General Hospital	Infectious Disease Program, Broad Institute of Harvard and MIT	Lemieux,J.E., Siddle,K.J., Shaw,B., Adams,G., Pierce,V., Turbett,S., Anahtar,M., Branda,J., Slater,D., Harris,J., Lin,A.E., Gladden-Young,A., Lagerborg,K., Rudy,M., DeRuff,K., Carter,A., Normandin,E., Bauer,M., Reilly,S., Tomkins-Tinch,C., Loreth,C., Chaluvadi,S., Neumann,A., Cusick,C., Chapman,S.B., Gnirke,A., Flowers,K., Cerrato,F., Birren,B.W., Gallagher,G., Smole,S., Park,D.J., MacInnis,B.L., Ryan,E., LaRocque,R., Rosenberg,E., Sabeti,P.C.
EPI_ISL_460655, EPI_ISL_460666, EPI_ISL_460667, EPI_ISL_460698, EPI_ISL_460714, EPI_ISL_460720, EPI_ISL_460778, EPI_ISL_460779, EPI_ISL_460815, EPI_ISL_460816, EPI_ISL_460817, EPI_ISL_460818, EPI_ISL_461116, EPI_ISL_461259, EPI_ISL_461287			
see above	Dutch COVID-19 response team	Erasmus Medical Center	Bas Oude Munnink, David Nieuwenhuijse, Reina Sikkema, Claudia Schapendonk, Irina Chestakova, Anne van der Linden, Theo Bestebroer, Stefan van Nieuwkoop, Mark Pronk, Pascal Lexmond, Corien Swaan, Manon Haverkate, Madelief Mollers, Mart Stein, Sandra Kengne Kamga Mobou, Jeroen van Kampen, Jolanda Voermans, Aura Timen, Corine GeurtsvanKessel, Annemiek van der Eijk, Richard Molenkamp, Marion Koopmans, on behalf of the Dutch national COVID-19 response team.
EPI_ISL_462178, EPI_ISL_462179, EPI_ISL_462180, EPI_ISL_462181	KU Leuven, Rega Institute, Clinical and Epidemiological Virology	KU Leuven, Rega Institute, Clinical and Epidemiological Virology	Tony Wawina-Bokalanga, Bert Vanmechelen, Joan Marti-Carerras, Piet Maes
EPI_ISL_462284, EPI_ISL_462292, EPI_ISL_462387, EPI_ISL_462401, EPI_ISL_462405, EPI_ISL_462409, EPI_ISL_462416, EPI_ISL_462429, EPI_ISL_462432	National Public Health Laboratory, National Centre for Infectious Diseases	National Public Health Laboratory, National Centre for Infectious Diseases	Mak TM, Octavia S, Chavatte JM, Cui L, Lin RTP
EPI_ISL_462450	Clinical Center, University of Sarajevo	Charite Universitatsmedizin Berlin, Institute of Virology	Victor M Corman, Jorn Beheim-Schwarzbach, Barbara Muehlemann, Talitha Veith, Julia Schneider, Terry Jones, Amela Dedeic-Ljubovic, Irma Salimovic-Besic, Suzana Arapcic, Almedina Hadzihasanovic-Moro, Selma Mutevelic, Christian Drosten
EPI_ISL_463001	unknown	Clinical virology	Fares,W., Triki,H.
EPI_ISL_463311, EPI_ISL_463312	Queen Elizabeth Hospital	Hong Kong Department of Health	Mak Gannon C.K., Cheng Peter K.C., Lam Edman T.K., Chan Rickjason C.W., Tsang Dominic N.C.
EPI_ISL_463313	Central Health Medical Practice	Hong Kong Department of Health	Mak Gannon C.K., Cheng Peter K.C., Lam Edman T.K., Chan Rickjason C.W., Tsang Dominic N.C.

EPI_ISL_463315	Princess Margaret Hospital	Hong Kong Department of Health	Mak Gannon C.K., Cheng Peter K.C., Lam Edman T.K., Chan Rickjason C.W., Tsang Dominic N.C.
EPI_ISL_463975, EPI_ISL_463977, EPI_ISL_463984, EPI_ISL_463987, EPI_ISL_463989, EPI_ISL_463990	Toronto Invasive Bacterial Diseases Network	McMaster University	Allison McGeer, Patryk Aftanas, Angel Li, Kuganya Nirmalarajah, Samira Mubareka, Andrew G. McArthur
EPI_ISL_464009, EPI_ISL_464051	Unity Health Toronto	Ontario Institute for Cancer Research	Ramzi Fattouh,Larissa M. Matukas,Mark Downing,Annette Gower,Karel Boissinot,Samira Mubareka,TIBDN,Ilinca Lungu,Bernard Lam,Jeremy Johns,Paul Krzyzanowski,Richard de Borja,Philip Zuzarte,Jared Simpson
EPI_ISL_464092	Laboratory Medicine	Department of Laboratory Medicine, Lin-Kou Chang Gung Memorial Hospital, Taoyuan, Taiwan	Kuo-Chien Tsao, Yu-Nong Gong, Shu-Li Yang, Yi-Chun Liu, Chung-Guei Huang, Mei-Jen Hsiao, Po-Wei Huang, Cheng-Ta Yang, Cheng-Hsun Chiu, Peng-Nien Huang, Kuo-Ming Lee, Guang-Wu Chen, Shin-Ru Shih
EPI_ISL_464112, EPI_ISL_464116	National Health Laboratory Service (NHLS), Tygerberg	Division of Medical Virology, Stellenbosch University and National Health Laboratory Service (NHLS)	Susan Engelbrecht, Kayla Delaney, Bronwyn Kleinhans, Houriiyah Tegally, Eduan Wilkindon, Gert van Zyl, Wolfgang Preiser, Tulio de Oliveira
EPI_ISL_464877, EPI_ISL_465096, EPI_ISL_465099, EPI_ISL_465102, EPI_ISL_465114, EPI_ISL_465115, EPI_ISL_465116, EPI_ISL_465117, EPI_ISL_465118, EPI_ISL_465121, EPI_ISL_465133, EPI_ISL_465135, EPI_ISL_465136, EPI_ISL_465137, EPI_ISL_465138, EPI_ISL_465139, EPI_ISL_465142, EPI_ISL_465145, EPI_ISL_465146, EPI_ISL_465149, EPI_ISL_465154, EPI_ISL_465155, EPI_ISL_465156, EPI_ISL_465157, EPI_ISL_465158, EPI_ISL_465159, EPI_ISL_465160, EPI_ISL_465161, EPI_ISL_465721, EPI_ISL_465722, EPI_ISL_465723, EPI_ISL_465724, EPI_ISL_465725, EPI_ISL_465726, EPI_ISL_465727, EPI_ISL_465728, EPI_ISL_465729, EPI_ISL_465730, EPI_ISL_465731, EPI_ISL_465732, EPI_ISL_465733, EPI_ISL_465734, EPI_ISL_465735, EPI_ISL_465736, EPI_ISL_465737, EPI_ISL_465738, EPI_ISL_465739, EPI_ISL_465740, EPI_ISL_465741, EPI_ISL_465742, EPI_ISL_465743, EPI_ISL_465744, EPI_ISL_465745, EPI_ISL_465746, EPI_ISL_465747, EPI_ISL_465748, EPI_ISL_465749, EPI_ISL_465750, EPI_ISL_465751, EPI_ISL_465752, EPI_ISL_465753, EPI_ISL_465754, EPI_ISL_465755, EPI_ISL_465756, EPI_ISL_465757, EPI_ISL_465758, EPI_ISL_465759, EPI_ISL_465760, EPI_ISL_465761, EPI_ISL_465762, EPI_ISL_465763, EPI_ISL_465764, EPI_ISL_465765, EPI_ISL_465766, EPI_ISL_465767, EPI_ISL_465768, EPI_ISL_465770, EPI_ISL_465771, EPI_ISL_465772, EPI_ISL_465773, EPI_ISL_465774, EPI_ISL_465775, EPI_ISL_465776, EPI_ISL_465777, EPI_ISL_465778, EPI_ISL_465779, EPI_ISL_465780, EPI_ISL_465781, EPI_ISL_465782, EPI_ISL_465783, EPI_ISL_465784, EPI_ISL_465785, EPI_ISL_465786, EPI_ISL_465787, EPI_ISL_465788, EPI_ISL_465789, EPI_ISL_465790, EPI_ISL_465792, EPI_ISL_465793, EPI_ISL_465795, EPI_ISL_465797, EPI_ISL_465798			
see above	Respiratory Virus Unit, Microbiology Services Colindale, Public Health England	Respiratory Virus Unit, Microbiology Services Colindale, Public Health England	PHE Covid Sequencing Team
EPI_ISL_467055	Servicio de Microbiología, Hospital Universitario Son Espases	SeqCOVID-SPAIN consortium/IBV(CSIC)	Carla López-Causapé, Jordi Reina y Antonio Oliver and SeqCOVID-SPAIN consortium
EPI_ISL_467062	Hospital Universitario Virgen de las Nieves de Granada-SAS	SeqCOVID-SPAIN consortium/IBV(CSIC)	Mercedes Pérez Ruiz, Sara Sanbonmatsu Gámez, Irene Pedrosa Corral, José M. Navarro-Marí and SeqCOVID-SPAIN consortium
EPI_ISL_467064, EPI_ISL_467066, EPI_ISL_467067, EPI_ISL_467068, EPI_ISL_467069, EPI_ISL_467070, EPI_ISL_467071, EPI_ISL_467072, EPI_ISL_467073, EPI_ISL_467074, EPI_ISL_467075, EPI_ISL_467076, EPI_ISL_467077, EPI_ISL_467078, EPI_ISL_467079, EPI_ISL_467080, EPI_ISL_467081, EPI_ISL_467083, EPI_ISL_467084, EPI_ISL_467085			
see above	Hospital Universitario Puerta del Mar de Cádiz - INIBICA	SeqCOVID-SPAIN consortium/IBV(CSIC)	Salud Rodríguez-Pallares, Fátima-Galán-Sánchez, Manuel Rodríguez-Iglesias and SeqCOVID-SPAIN consortium
EPI_ISL_467086, EPI_ISL_467087, EPI_ISL_467088, EPI_ISL_467089	Hospital Universitario de Gran Canaria Dr. Negrín	SeqCOVID-SPAIN consortium/IBV(CSIC)	M. Carmen Pérez González, Francisco J. Chamizo López, Ana Bordes Benítez and SeqCOVID-SPAIN consortium
EPI_ISL_467092, EPI_ISL_467094, EPI_ISL_467095, EPI_ISL_467098, EPI_ISL_467099, EPI_ISL_467101, EPI_ISL_467102, EPI_ISL_467106, EPI_ISL_467108, EPI_ISL_467109, EPI_ISL_467110, EPI_ISL_467111, EPI_ISL_467115, EPI_ISL_467117, EPI_ISL_467118, EPI_ISL_467119, EPI_ISL_467120, EPI_ISL_467122, EPI_ISL_467123, EPI_ISL_467125, EPI_ISL_467127, EPI_ISL_467128, EPI_ISL_467130, EPI_ISL_467131, EPI_ISL_467133, EPI_ISL_467137, EPI_ISL_467138, EPI_ISL_467140, EPI_ISL_467143, EPI_ISL_467144, EPI_ISL_467145, EPI_ISL_467148, EPI_ISL_467149, EPI_ISL_467151, EPI_ISL_467152, EPI_ISL_467154, EPI_ISL_467155, EPI_ISL_467163, EPI_ISL_467166, EPI_ISL_467167, EPI_ISL_467168, EPI_ISL_467169, EPI_ISL_467170, EPI_ISL_467173, EPI_ISL_467174, EPI_ISL_467176, EPI_ISL_467178, EPI_ISL_467179, EPI_ISL_467181, EPI_ISL_467182, EPI_ISL_467183			
see above	Hospital Universitario Araba. Vitoria-Gasteiz	SeqCOVID-SPAIN consortium/IBV(CSIC)	Silvia Hernáez Crespo, Carmen Gómez González, Amaia Aguirre Quiñonero, Marina Fernández Torres, Mª Rosario Almela Ferrer, Mª Concepción Lecaroz Agara, Andrés Canut Blasco. and SeqCOVID-SPAIN consortium
EPI_ISL_467372	Arizona State University Health Services	Arizona State University	Peter T. Skidmore, Rabia Maqsood, LaRinda A. Holland, Emily A. Kaelin, Lily I. Wu, Arvind Varsani, Rolf U. Halden, Brenda G. Hogue, Matthew Scotch, Efrem S. Lim
EPI_ISL_467374	Dinkes Samarinda	Eijkman Institute for Molecular Biology, Ministry of Research and Technology/National Agency for Research and Innovation	Edison Johar, Frilasita A Yudhaputri, Hidayat Trimarsanto, David H Muljono, Safarina G Malik, Khin Saw Myint, Amin Soebandrio
EPI_ISL_467411, EPI_ISL_467412, EPI_ISL_467413, EPI_ISL_467414, EPI_ISL_467415, EPI_ISL_467416, EPI_ISL_467417, EPI_ISL_467418, EPI_ISL_467419, EPI_ISL_467420, EPI_ISL_467421, EPI_ISL_467422			
see above	NYU Langone Health	Departments of Pathology and Medicine, New York University School of Medicine	Maria Aguero-Rosenfeld, Brendan Belovarac, Margaret Black, Ludovic Boytard, John Cadley, Paolo Cotzia, John Chen, Dacia Dimartino, Xiaojun Feng, Tatyana Gindin, Emily Guzman, Adriana Heguy, Megan Hogan, Emily Huang, George Jour, Alireza Khodadadi-Jamayran, Lawrence H. Lin, Raven Luther, Andrew Lytle, Christian Marier, Matthew T. Maurano, Mark J. Mulligan, Peter Meyn, Raquel Ordonez Ciriza, Iman Osman, Jared Pinnell, Vanessa Raabe, Sitharam Ramaswami, Amy Rapkiewicz, Andre M. Ribeiro-dos-Santos, Marie Samanovic-Golden, Antonio Serrano, Guomiao Shen, Matija Snuderl, Theodore Vougiouklakis, Nick Vulpescu, Gael Westby, Paul Zappile, Yutong Zhang
EPI_ISL_467526, EPI_ISL_467527, EPI_ISL_467532, EPI_ISL_467594, EPI_ISL_467595, EPI_ISL_467601, EPI_ISL_467602, EPI_ISL_467603, EPI_ISL_467604, EPI_ISL_467605, EPI_ISL_467606, EPI_ISL_467607, EPI_ISL_467608, EPI_ISL_467609, EPI_ISL_467610, EPI_ISL_467611, EPI_ISL_467612, EPI_ISL_467613, EPI_ISL_467614, EPI_ISL_467615, EPI_ISL_467616, EPI_ISL_467619, EPI_ISL_467620, EPI_ISL_467621, EPI_ISL_467622, EPI_ISL_467623, EPI_ISL_467624			
see above	New Mexico Department of Health Scientific Laboratory Division	Center for Global Health, University of New Mexico Health Sciences Center	Daryl Domman, Kurt Schwalm, Twila Kunde, Joseph Hicks, Michael Edwards, Darrell Dinwiddie
EPI_ISL_467811, EPI_ISL_467812, EPI_ISL_467813, EPI_ISL_467814, EPI_ISL_467815, EPI_ISL_467816, EPI_ISL_467817, EPI_ISL_467818, EPI_ISL_467819, EPI_ISL_467820, EPI_ISL_467821, EPI_ISL_467822, EPI_ISL_467823, EPI_ISL_467824, EPI_ISL_467825, EPI_ISL_467826, EPI_ISL_467827, EPI_ISL_467828, EPI_ISL_467829, EPI_ISL_467830, EPI_ISL_467831, EPI_ISL_467833, EPI_ISL_467834, EPI_ISL_467835, EPI_ISL_467836, EPI_ISL_467837, EPI_ISL_467838, EPI_ISL_467840, EPI_ISL_467841, EPI_ISL_467842, EPI_ISL_467843, EPI_ISL_467858, EPI_ISL_467859, EPI_ISL_467860, EPI_ISL_467861, EPI_ISL_467863, EPI_ISL_467865, EPI_ISL_467869, EPI_ISL_467870, EPI_ISL_467871, EPI_ISL_467872, EPI_ISL_467875, EPI_ISL_467877, EPI_ISL_467879, EPI_ISL_467884, EPI_ISL_467885, EPI_ISL_467886, EPI_ISL_467887, EPI_ISL_467888, EPI_ISL_467889, EPI_ISL_467890, EPI_ISL_467891, EPI_ISL_467892, EPI_ISL_467893, EPI_ISL_467894, EPI_ISL_467895, EPI_ISL_467896, EPI_ISL_467897, EPI_ISL_467898, EPI_ISL_467899, EPI_ISL_467900, EPI_ISL_467901, EPI_ISL_467903, EPI_ISL_467906, EPI_ISL_467907			
see above	Quest Diagnostics	Quest Diagnostics	Anderson,B.P., Rosenthal,S.H., Gerasimova,A., Kagan,R.M. and Owen, R.
EPI_ISL_467952, EPI_ISL_467955, EPI_ISL_467969, EPI_ISL_467978	San Diego County Public Health Laboratory	Andersen lab at Scripps Research	SEARCH Alliance San Diego with Tracy Basler, Jovan Shephard, Brett Austin
EPI_ISL_467995, EPI_ISL_468000, EPI_ISL_468016	SA Pathology	SA Pathology	Lex Leong, Chuan Kok Lim, Mark Turra, Ivan Bastian, Geoff Higgins
EPI_ISL_468063, EPI_ISL_468064, EPI_ISL_468065	unknown	Computer Science and Engineering	Rouchka,E.C., Chariker,J.H., Chung,D., Ramirez,J., Palmer,K.E., Lasnik,A.B., Carrico,R., Arnold,F.W., Adcock,R.S., Zhang,M., Alejandro,B., Wolf,L.A., Hwang,J.Y., Park,J.W., Waigel,S., Zacharias,W.
EPI_ISL_468389, EPI_ISL_468390, EPI_ISL_468392, EPI_ISL_468393	County of San Luis Obispo Public Health Laboratory	Chan-Zuckerberg Biohub	CZB Cliahub Consortium
EPI_ISL_468495	Ventura County Public Health Lab	Chan-Zuckerberg Biohub	CZB Cliahub Consortium
EPI_ISL_468509, EPI_ISL_468510	San Joaquin County Public Health Lab	Chan-Zuckerberg Biohub	CZB Cliahub Consortium
EPI_ISL_468737, EPI_ISL_468743, EPI_ISL_468744, EPI_ISL_468745, EPI_ISL_468746	Lab voor klinische biologie	Onderzoeksgroep Virologie	Nick Vereecke, Laurens Lambrechts, Marthe Pauwels, Bruno Verhasselt, Linos Vandekerckhove, Hans Nauwynck, Sebastiaan Theuns
EPI_ISL_468760	Center for Genome Regulation (CRG)	Center for Mathematical Modeling and Center for Genome Regulation. Santiago, Chile	Gaete A, Travisany D, Palma R, Urra C, Varas M, Allende ML, Maass A, González M.
EPI_ISL_468765, EPI_ISL_468769, EPI_ISL_468778, EPI_ISL_468797, EPI_ISL_468801, EPI_ISL_468803, EPI_ISL_468818, EPI_ISL_468830, EPI_ISL_468834, EPI_ISL_468836, EPI_ISL_468840, EPI_ISL_468856			
see above	Servicio de Microbiología, Hospital Miguel Servet, Zaragoza	SeqCOVID-SPAIN consortium/IBV(CSIC)	Antonio Rezusta López, Alexander Tristancho Baró, Ana Milagro, Yolanda Gracia Grataloup, Nieves Martínez Cameo and SeqCOVID-SPAIN consortium
EPI_ISL_468914	Istituto Zooprofilattico Sperimentale Puglia e Basilicata; Dipartimento di Bioscienze, Biotecnologie e Biofarmaceutica dell'Università degli Studi di Bari "A.Moro"; Istituto di Biomembrane. Bioenergetica e Biotecnologie Molecolari del Consiglio Nazionale delle Ricerche di Bari	Beaconlab (Bioinformatics, Evolution and Comparative Genomics lab), Dept of Biosciences, University on Milan	Parisi A.,Pesole G., Manzari C., Chiara M.

EPI_ISL_468961, EPI_ISL_468970, EPI_ISL_468982, EPI_ISL_468985, EPI_ISL_468989, EPI_ISL_468990, EPI_ISL_469005	Servicio de Microbiología, Hospital Universitario Son Espases	SeqCOVID-SPAIN consortium/IBV(CSIC)	Carla López-Causapé, Jordi Reina, Antonio Oliver and SeqCOVID-SPAIN consortium
EPI_ISL_469016	Istituto Zooprofilattico Sperimentale Puglia e Basilicata; Dipartimento di Bioscienze, Biotecnologie e Biofarmaceutica dell'Università degli Studi di Bari "A.Moro"; Istituto di Biomembrane. Bioenergetica e Biotecnologie Molecolari del Consiglio Nazionale delle Ricerche di Bari	Beaconlab (Bioinformatics, Evolution and Comparative Genomics lab), Dept of Biosciences, University on Milan	Parisi A.,Pesole G., Manzari C., Chiara M.
EPI_ISL_469241, EPI_ISL_469242, EPI_ISL_469243, EPI_ISL_469244, EPI_ISL_469245, EPI_ISL_469246, EPI_ISL_469247, EPI_ISL_469248, EPI_ISL_469249, EPI_ISL_469250, EPI_ISL_469251, EPI_ISL_469252			
see above	Special Infectious Agents Unit	Special Infectious Agents Unit	Azhar,E.I., Hassan,A.M., Tolah,A.M., Uthman,N.A., Al-Sobahy,T.L., Farraj,S.A., El-Kafrawy,S.A.
EPI_ISL_469283, EPI_ISL_469284	Service de Virologie Hôpital Saint-Louis	Laboratory Cell Biology of Viral Infection-INSERM unit 944	Laurent Meertens, Lucie Bonnet-Madin, Séverine Mercier-Delarue, Maud SALMONA, Constance Delaugerre, Ali Amara
EPI_ISL_470540	Wisconsin State Laboratory of Hygiene Communicable Disease Division	Wisconsin State Laboratory of Hygiene Communicable Disease Division	Kelsey R Florek
EPI_ISL_470575, EPI_ISL_470580, EPI_ISL_470598, EPI_ISL_470652, EPI_ISL_470653, EPI_ISL_470654	Hermes Pardini	Bioinformatics Laboratory / LNCC	Alexandra Gerber, Ana Paula Guimarães, Luiz Gonzaga Paula de Almeida, Ronaldo da Silva Francisco Junior, Mariane Talon, Filipe Romero, Átila Duque Rossi, Terezinha Marta Pereira, working group UFRJ, Jaqueline Goes de Jesus, Ingra Morales Claro, Ester Cerdeira Sabino, Nuno Rodrigues Faria, CADDE-group, Laboratorio Hermes Pardini, Laboratorio Simile, working group UFMG, Amilcar Tanuri, Carolina Voloch, Renato Santana Aguiar e Ana Tereza Vasconcelos
EPI_ISL_471205, EPI_ISL_471207, EPI_ISL_471235, EPI_ISL_471249, EPI_ISL_471250, EPI_ISL_471251, EPI_ISL_471252	Wisconsin State Laboratory of Hygiene Communicable Disease Division	Wisconsin State Laboratory of Hygiene Communicable Disease Division	Kelsey R. Florek, Abigail C. Shockey
EPI_ISL_471472	Hospital Universitari Germans Trias i Pujol(HUGTiP)/Fundació Lluita contra la SIDA (FLSida)/IRTA-CReSA	IrsiCaixa AIDS Research Lab	Marc Noguera-Julian, Pilar Armengol, Jordi Rodón, Julia Vergara, Lidia Ruiz, Nuria Izquierdo, Jorge Carrillo, Roger Paredes, Albert Bensaid, Julia Blanco, Joaquim Segalés, Bonaventura Clotet
EPI_ISL_471553, EPI_ISL_471555	The National Institute of Public Health	State Veterinary Institute Prague and The National Institute of Public Health	Nagy,A;Jirincova,H;Novakova,L;Trnka,D;Vecerova,J
EPI_ISL_471677	Michigan Department of Health and Human Services, Bureau of Laboratories	Michigan Department of Health and Human Services, Bureau of Laboratories	Blankenship HM, Riner D, Soehnlen MK
EPI_ISL_471970	University of Exeter	COVID-19 Genomics UK (COG-UK) Consortium	Ben Temperton,Aaron Jeffries,Michelle Michelsen,Joanna Warwick-Dugdale,Audrey Farbos,Robyn Manley,Stephen Michell,Jane Masoli
EPI_ISL_472165, EPI_ISL_472166	Northumbria University / South Tees Hospitals NHS Foundation Trust / North Cumbria Integrated Care NHS Foundation Trust / North Tees and Hartlepool NHS Foundation Trust / Newcastle Hospitals NHS Foundation Trust	COVID-19 Genomics UK (COG-UK) Consortium	Darren L Smith,Andrew Nelson,Matthew Bashton,Greg R Young,Joshua Loh,John Allan,Mohammad A Tariq,Giles S Holt,Gary Black,Wen C Yew,Lynn Dover,Paul Baker,Steve Liggett,Sarah Essex,Jane Greenaway,Debra Padgett,Clive Graham,Garren Scott,Edward Barton,Emma Swindells,Brendan Payne,Jennifer Collins,Yusri Taha,Gary Eltringham
EPI_ISL_474224, EPI_ISL_474225, EPI_ISL_474226, EPI_ISL_474227, EPI_ISL_474228, EPI_ISL_474229, EPI_ISL_474230	Wales Specialist Virology Centre Sequencing lab: Pathogen Genomics Unit	COVID-19 Genomics UK (COG-UK) Consortium	Catherine Moore, Johnathan Evans, Laura Gifford, Malorie Perry, Simon Cottrell, Angela Marchbank, Alec Birchley, Alexander Adams, Amy Gaskin, Bree Gatica-Wilcox, Jason Coombes, Joel Southgate, Lauren Gilbert, Lee Graham, Nicole Pacchiarini, Sara Kumziene-Summerhayes, Sarah Taylor, Sophie Jones, Sara Rey, Matthew Bull, Joanne Watkins, Sally Corden, Tom Connor
EPI_ISL_474833, EPI_ISL_474834, EPI_ISL_474844, EPI_ISL_474852, EPI_ISL_474860, EPI_ISL_474861, EPI_ISL_474862, EPI_ISL_474863, EPI_ISL_474864, EPI_ISL_474865, EPI_ISL_474866, EPI_ISL_474867, EPI_ISL_474868, EPI_ISL_474869, EPI_ISL_474897, EPI_ISL_474909			
see above	Hospital Universitario Virgen de las Nieves de Granada-SAS	SeqCOVID-SPAIN consortium/IBV(CSIC)	Mercedes Pérez Ruiz, Sara Sanbonmatsu Gámez, Irene Pedrosa Corral, José M. Navarro-Marí and SeqCOVID-SPAIN consortium
EPI_ISL_474912, EPI_ISL_474913, EPI_ISL_474914, EPI_ISL_474915, EPI_ISL_474916, EPI_ISL_474917, EPI_ISL_474918	Hospital Universitario de Gran Canaria Dr. Negrín	SeqCOVID-SPAIN consortium/IBV(CSIC)	M. Carmen Pérez González, Francisco J. Chamizo López, Ana Bordes Benítez and SeqCOVID-SPAIN consortium
EPI_ISL_474920, EPI_ISL_474934, EPI_ISL_474936, EPI_ISL_474937, EPI_ISL_474938, EPI_ISL_474942, EPI_ISL_474948	Hospital Universitario Virgen de las Nieves de Granada-SAS	SeqCOVID-SPAIN consortium/IBV(CSIC)	Mercedes Pérez Ruiz, Sara Sanbonmatsu Gámez, Irene Pedrosa Corral, José M. Navarro-Marí and SeqCOVID-SPAIN consortium
EPI_ISL_474960, EPI_ISL_474965, EPI_ISL_474969, EPI_ISL_474973, EPI_ISL_474974, EPI_ISL_474975, EPI_ISL_474979, EPI_ISL_474980, EPI_ISL_474981, EPI_ISL_474986, EPI_ISL_474987, EPI_ISL_474988, EPI_ISL_474992, EPI_ISL_474993, EPI_ISL_474994, EPI_ISL_474999, EPI_ISL_475000, EPI_ISL_475001, EPI_ISL_475002, EPI_ISL_475003, EPI_ISL_475010, EPI_ISL_475013, EPI_ISL_475014, EPI_ISL_475015, EPI_ISL_475018, EPI_ISL_475019, EPI_ISL_475021, EPI_ISL_475022			
see above	Israel Central Virology laboratory	Israel Central Virology laboratory	Neta Zuckerman, Efrat Dahan Bucris, Oran Erster, Ella Mendelson, Michal Mandelboim
EPI_ISL_475060, EPI_ISL_475061, EPI_ISL_475062, EPI_ISL_475063, EPI_ISL_475064, EPI_ISL_475065, EPI_ISL_475066, EPI_ISL_475067, EPI_ISL_475068, EPI_ISL_475069, EPI_ISL_475070			
see above	Lab voor klinische biologie	Onderzoeksgroep Virologie	Laurens Lambrechts, Nick Vereecke, Marthe Pauwels, Bruno Verhasselt, Linos Vandekerckhove, Hans Nauwynck, Sebastiaan Theuns
EPI_ISL_475071, EPI_ISL_475072	Lab voor klinische biologie	Onderzoeksgroep Virologie	Nick Vereecke, Laurens Lambrechts, Marthe Pauwels, Bruno Verhasselt, Linos Vandekerckhove, Hans Nauwynck, Sebastiaan Theuns
EPI_ISL_475092	Skovde/Unilabs	The Public Health Agency of Sweden	Oskar Karlsson Lindsjo, Maria Lind Karlberg, Mattias Haukland, Reza Advani, Olov Svartstrom, Anna-Malin Linde, Sandra Broddesson, Petra Edquist, Shamam Muradrasoli, Anna Risberg, Karin Tegmark-Wisell
EPI_ISL_475093, EPI_ISL_475094	Halmstad klinisk mikrobiologi	The Public Health Agency of Sweden	Oskar Karlsson Lindsjo, Maria Lind Karlberg, Mattias Haukland, Reza Advani, Olov Svartstrom, Anna-Malin Linde, Sandra Broddesson, Petra Edquist, Shamam Muradrasoli, Anna Risberg, Karin Tegmark-Wisell
EPI_ISL_475095	Karolinska Universitetslaboratoriet	The Public Health Agency of Sweden	Oskar Karlsson Lindsjo, Maria Lind Karlberg, Mattias Haukland, Reza Advani, Olov Svartstrom, Anna-Malin Linde, Sandra Broddesson, Petra Edquist, Shamam Muradrasoli, Anna Risberg, Karin Tegmark-Wisell
EPI_ISL_475137, EPI_ISL_475138	Kalmar klinisk mikrobiologi	The Public Health Agency of Sweden	Oskar Karlsson Lindsjo, Maria Lind Karlberg, Mattias Haukland, Reza Advani, Olov Svartstrom, Anna-Malin Linde, Sandra Broddesson, Petra Edquist, Shamam Muradrasoli, Anna Risberg, Karin Tegmark-Wisell
EPI_ISL_475139	Orebro klinisk mikrobiologi	The Public Health Agency of Sweden	Oskar Karlsson Lindsjo, Maria Lind Karlberg, Mattias Haukland, Reza Advani, Olov Svartstrom, Anna-Malin Linde, Sandra Broddesson, Petra Edquist, Shamam Muradrasoli, Anna Risberg, Karin Tegmark-Wisell
EPI_ISL_475562, EPI_ISL_475563	Din Klinik	The Public Health Agency of Sweden	Oskar Karlsson Lindsjo, Maria Lind Karlberg, Mattias Haukland, Reza Advani, Olov Svartstrom, Anna-Malin Linde, Sandra Broddesson, Mia Brytting, Anna Risberg, Karin Tegmark-Wisell
EPI_ISL_475572	Imperial College London	Imperial College London	Jie Zhou, Wendy Barclay
EPI_ISL_475768	Universitaetsklinik für Innere Medizin II Innsbruck	Bergthaler laboratory, CeMM Research Center for Molecular Medicine of the Austrian Academy of Sciences	Alexandra Popa, Benedikt Agerer, Henrique Colaco, Lukas Endler, Jakob-Wendelin Genger, Alexander Lercher, Mark Smyth, Thomas Penz, Michael Schuster, Jan Laine, Martin Senekowitsch, Judith Aberle, Stephan Aberle, Peter Hufnagl, Daniela Schmid, Franz Allerberger, Elisabeth Puchhammer-Stoeckl, Manfred Nairz, Guenter Weiss, Gregor Hörmann, Kinga Rigler-Hohenwarter, Rainer Gattringer, Wegene Borena, Dorothee von Laer, Christoph Bock, Andreas Bergthaler
EPI_ISL_475771	Center for Virology, Medical University of Vienna	Bergthaler laboratory, CeMM Research Center for Molecular Medicine of the Austrian Academy of Sciences	Alexandra Popa, Benedikt Agerer, Henrique Colaco, Lukas Endler, Jakob-Wendelin Genger, Alexander Lercher, Mark Smyth, Thomas Penz, Michael Schuster, Jan Laine, Martin Senekowitsch, Judith Aberle, Stephan Aberle, Peter Hufnagl, Daniela Schmid, Franz Allerberger, Elisabeth

			Puchhammer-Stoeckl, Manfred Nairz, Guenter Weiss, Gregor Hörmann, Kinga Rigler-Hohenwarter, Rainer Gattringer, Wegene Borena, Dorothee von Laer, Christoph Bock, Andreas Bergthaler
EPI_ISL_475813, EPI_ISL_475814	Institut für Virologie am Department für Hygiene, Mikrobiologie und Public Health	Bergthaler laboratory, CeMM Research Center for Molecular Medicine of the Austrian Academy of Sciences	Alexandra Popa, Benedikt Agerer, Henrique Colaco, Lukas Endler, Jakob-Wendelin Genger, Alexander Lercher, Mark Smyth, Thomas Penz, Michael Schuster, Jan Laine, Martin Senekowitsch, Judith Aberle, Stephan Aberle, Peter Hufnagl, Daniela Schmid, Franz Allerberger, Elisabeth Puchhammer-Stoeckl, Manfred Nairz, Guenter Weiss, Gregor Hörmann, Kinga Rigler-Hohenwarter, Rainer Gattringer, Wegene Borena, Dorothee von Laer, Christoph Bock, Andreas Bergthaler
EPI_ISL_475863, EPI_ISL_475867, EPI_ISL_475874, EPI_ISL_475880	Austrian Agency for Health and Food Safety (AGES)	Bergthaler laboratory, CeMM Research Center for Molecular Medicine of the Austrian Academy of Sciences	Alexandra Popa, Benedikt Agerer, Henrique Colaco, Lukas Endler, Jakob-Wendelin Genger, Alexander Lercher, Mark Smyth, Thomas Penz, Michael Schuster, Jan Laine, Martin Senekowitsch, Judith Aberle, Stephan Aberle, Peter Hufnagl, Daniela Schmid, Franz Allerberger, Elisabeth Puchhammer-Stoeckl, Manfred Nairz, Guenter Weiss, Gregor Hörmann, Kinga Rigler-Hohenwarter, Rainer Gattringer, Wegene Borena, Dorothee von Laer, Christoph Bock, Andreas Bergthaler
EPI_ISL_475905, EPI_ISL_475906, EPI_ISL_475907, EPI_ISL_475908, EPI_ISL_475909	Zentralinstitut für medizinische und chemische Labordiagnostik, Universitätskliniken Insbruck	Bergthaler laboratory, CeMM Research Center for Molecular Medicine of the Austrian Academy of Sciences	Alexandra Popa, Benedikt Agerer, Henrique Colaco, Lukas Endler, Jakob-Wendelin Genger, Alexander Lercher, Mark Smyth, Thomas Penz, Michael Schuster, Jan Laine, Martin Senekowitsch, Judith Aberle, Stephan Aberle, Peter Hufnagl, Daniela Schmid, Franz Allerberger, Elisabeth Puchhammer-Stoeckl, Manfred Nairz, Guenter Weiss, Gregor Hörmann, Kinga Rigler-Hohenwarter, Rainer Gattringer, Wegene Borena, Dorothee von Laer, Christoph Bock, Andreas Bergthaler
EPI_ISL_475924	Institut für Virologie am Department für Hygiene, Mikrobiologie und Public Health	Bergthaler laboratory, CeMM Research Center for Molecular Medicine of the Austrian Academy of Sciences	Alexandra Popa, Benedikt Agerer, Henrique Colaco, Lukas Endler, Jakob-Wendelin Genger, Alexander Lercher, Mark Smyth, Thomas Penz, Michael Schuster, Jan Laine, Martin Senekowitsch, Judith Aberle, Stephan Aberle, Peter Hufnagl, Daniela Schmid, Franz Allerberger, Elisabeth Puchhammer-Stoeckl, Manfred Nairz, Guenter Weiss, Gregor Hörmann, Kinga Rigler-Hohenwarter, Rainer Gattringer, Wegene Borena, Dorothee von Laer, Christoph Bock, Andreas Bergthaler
EPI_ISL_475948, EPI_ISL_475949, EPI_ISL_475950, EPI_ISL_475961, EPI_ISL_475962, EPI_ISL_475963, EPI_ISL_475964	National Public Health Laboratory, National Centre for Infectious Diseases	National Public Health Laboratory, National Centre for Infectious Diseases	Mak TM, Octavia S, Chavatte JM, Cui L, Lin RTP
EPI_ISL_476175, EPI_ISL_476178, EPI_ISL_476179, EPI_ISL_476180, EPI_ISL_476181, EPI_ISL_476182, EPI_ISL_476183, EPI_ISL_476185, EPI_ISL_476186, EPI_ISL_476187, EPI_ISL_476189, EPI_ISL_476190, EPI_ISL_476191, EPI_ISL_476192, EPI_ISL_476193, EPI_ISL_476194, EPI_ISL_476195, EPI_ISL_476196, EPI_ISL_476197, EPI_ISL_476202, EPI_ISL_476211, EPI_ISL_476212, EPI_ISL_476213, EPI_ISL_476214, EPI_ISL_476224, EPI_ISL_476225, EPI_ISL_476226, EPI_ISL_476227, EPI_ISL_476228, EPI_ISL_476229			
see above	DB Diagnósticos do Brasil	Instituto de Medicina Tropical da Univesidade de São Paulo	Samples: Nelson Gaburo Jr; Sequencing: Ingra Morales Claro, Jaqueline Goes de Jesus, Erika Regina Manuli, Flavia Cristina da Silva Sales, Thais de Moura Coletti, Camila Alves Maia da Silva, Mariana Severo Ramundo, Giula Magalhaes Ferreira, Darlan da Silva Candido, Julien Theze, Nuno Faria, Ester Sabino
EPI_ISL_476428, EPI_ISL_476429, EPI_ISL_476433, EPI_ISL_476458, EPI_ISL_476471, EPI_ISL_476488	Hospital da Clínicas da Faculdade de Medicina da Universidade de São Paulo	Instituto de Medicina Tropical da Univesidade de São Paulo	Samples: Ingra Morales Claro, Erika Regina Manuli, Cecilia Salete Alencar, Carolina S. Lazar, Silvia F. Costa; Sequencing: Ingra Morales Claro, Jaqueline Goes de Jesus, Erika Regina Manuli, Flavia Cristina da Silva Sales, Thais de Moura Coletti, Camila Alves Maia da Silva, Mariana Severo Ramundo, Giula Magalhaes Ferreira, Darlan da Silva Candido, Julien Theze, Nuno Faria, Ester Sabino
EPI_ISL_476777, EPI_ISL_476779, EPI_ISL_476780, EPI_ISL_476793	Stanford clinical virology lab	Chan-Zuckerberg Biohub	Benjamin Pinksy, Katharine Walter, Victoria N. Parikh, John Gorzynski, Hannah N. DeJong, Matthew T. Wheeler, Jason Andrews, Manuel Rivas, Carlos Bustamante, Euan Ashley, with CZB Cliahub Consortium
EPI_ISL_476801, EPI_ISL_476803	Hong Kong Department of Health	School of Public Health, The University of Hong Kong	Dominic N.C. Tsang, Daniel K.W. Chu, Leo L.M. Poon, Malik Peiris
EPI_ISL_476806	Department of Laboratory Medicine Tan Tock Seng Hospital	Department of Laboratory Medicine Tan Tock Seng Hospital	Chen YYC, Zair X, Li C, Tang WY, Maurer-Stroh S, Barkham TMS, Nagarajan N, Sessions OM
EPI_ISL_476816, EPI_ISL_476817	Department of Laboratory Medicine, Tan Tock Seng Hospital	Department of Laboratory Medicine, Tan Tock Seng Hospital	Chen YYC, Zair X, Li C, Tang WY, Maurer-Stroh S, Barkham TMS, Nagarajan N, Sessions OM
EPI_ISL_476822, EPI_ISL_476823, EPI_ISL_476824, EPI_ISL_476833, EPI_ISL_476834	Laboratoire des Fièvres Hémorragiques Virales du Benin	Charité-Universitätsmedizin Berlin	Yadouleton, Anges; Sander Anna-Lena; Moreira-Soto Andres; Drexler, Jan Felix
EPI_ISL_476902, EPI_ISL_476905	UW Virology Lab	UW Virology Lab	Pavitra Roychoudhury, Hong Xie, Lasata Shrestha, Amin Addetia, Truong Nguyen, Victoria M Rachleff, Meei-Li Huang, Keith R Jerome, Alexander Greninger
EPI_ISL_477171	Department of Laboratory, Medicine Tan Tock Seng Hospital	Department of Laboratory, Medicine Tan Tock Seng Hospital	Chen YYC, Zair X, Li C, Tang WY, Maurer-Stroh S, Barkham TMS, Nagarajan N, Sessions OM
EPI_ISL_477172	Department of Laboratory Medicine Tan Tock Seng Hospital	Department of Laboratory Medicine Tan Tock Seng Hospital	Chen YYC, Zair X, Li C, Tang WY, Maurer-Stroh S, Barkham TMS, Nagarajan N, Sessions OM
EPI_ISL_477193, EPI_ISL_477194	Istituto Zooprofilattico Sperimentale Puglia e Basilicata;	Beaconlab (Bioinformatics, Evolution and Comparative Genomics lab), Dept of Biosciences, University on Mila	Parisi A.,Pesole G., Manzari C., Chiara M.
EPI_ISL_477291	Mayo Clinic & Mayo Clinic Laboratories	Minnesota Department of Health, Public Health Laboratory	Matt Plumb, Jacob Garfin, Kelly Pung, and Xiong Wang
EPI_ISL_477685, EPI_ISL_477686, EPI_ISL_477687, EPI_ISL_477690	UW Virology Lab	UW Virology Lab	Pavitra Roychoudhury, Hong Xie, Lasata Shrestha, Amin Addetia, Truong Nguyen, Victoria M Rachleff, Meei-Li Huang, Keith R Jerome, Alexander Greninger
EPI_ISL_477824	West of Scotland Specialist Virology Centre, NHSGGC / MRC-University of Glasgow Centre for Virus Research	COVID-19 Genomics UK (COG-UK) Consortium	Ana da Silva Filipe, Natasha Johnson, Kathy Smollett, Daniel Mair, Stephen Carmichael, Lily Tong, Jenna Nichols, Elihu Aranday-Cortes, Kirstyn Brunker, Yasmin Parr, Alice Broos, Kyriaki Nomikou; Sarah McDonald, Marc Niebel, Patawee Asamaphan; Richard Orton, Joseph Hughes, Sreenu Vattipally, David L Robertson; Alasdair MacLean, Rory Gunson; Kathy Li, Natasha Jesudason, Rajiv Shah, James Shepherd, Antonia Ho, Emma Thomson
EPI_ISL_478536, EPI_ISL_478537, EPI_ISL_478538, EPI_ISL_478539, EPI_ISL_478540, EPI_ISL_478541	Northumbria University / South Tees Hospitals NHS Foundation Trust / North Cumbria Integrated Care NHS Foundation Trust / North Tees and Hartlepool NHS Foundation Trust / Newcastle Hospitals NHS Foundation Trust	COVID-19 Genomics UK (COG-UK) Consortium	Darren L Smith,Andrew Nelson,Matthew Bashton,Greg R Young,Joshua Loh,John Allan,Mohammad A Tariq,Giles S Holt,Gary Black,Wen C Yew,Lynn Dover,Paul Baker,Steve Liggett,Sarah Essex,Jane Greenaway,Debra Padgett,Clive Graham,Garren Scott,Edward Barton,Emma Swindells,Brendan Payne,Jennifer Collins,Yusri Taha,Gary Eltringham
EPI_ISL_478696, EPI_ISL_478697, EPI_ISL_478698	South Eastern Area Laboratory Services (SEALS)	NSW Health Pathology - Institute of Clinical Pathology and Medical Research; Westmead Hospital; University of Sydney	CIDM-PH et al.
EPI_ISL_479214, EPI_ISL_479218	Virology Department, Sheffield Teaching Hospitals NHS Foundation Trust/Department of Infection, Immunity and Cardiovascular Disease, The Medical School, University of Sheffield	COVID-19 Genomics UK (COG-UK) Consortium	Thushan de Silva, Matthew Parker, Nikki Smith, Adri Angyal, Rebecca Brown, Luke Green, Rachel Tucker, Paul Parsons, Danielle Groves, Katie Johnson, Laura Carrilero, Alex Keeley, Dave Partridge, Matthew Wyles, Benjamin Lindsey, Mehmet Yavuz, Mohammad Raza, Cariad Evans
EPI_ISL_479664, EPI_ISL_479665, EPI_ISL_479666	Center for Genomics and System Biology, New York University	Center for Genomics and System Biology, New York University	Roder,A., Banakis,S., Johnson,K., Khalfan,M., Borenstein,E.S., Samanovic,M., Cornelius,A., Herati,R., Ulrich,R., Fleming,A., Kottkamp,A., Raabe,V., Mulligan,M.J., Gresham,D., Ghedin,E.
EPI_ISL_479861, EPI_ISL_479868	Department of Infectious Diseases, Kobe Institute of Health	Pathogen Genomics Center, National Institute of Infectious Diseases	Tsuyoshi Sekizuka, Ryohei Nomoto, Kentaro Itokawa, Rina Tanaka, Masanori Hashino, Hajime Kamiya, Motoi Suzuki, Makoto Kuroda
EPI_ISL_479881, EPI_ISL_479882, EPI_ISL_479883, EPI_ISL_479884	Sapporo City Institute of Public Health	Pathogen Genomics Center, National Institute of Infectious Diseases	Tsuyoshi Sekizuka, Asami Ohnishi, Kentaro Itokawa, Rina Tanaka, Masanori Hashino, Hajime Kamiya, Motoi Suzuki, Makoto Kuroda
EPI_ISL_479891, EPI_ISL_479892, EPI_ISL_479893, EPI_ISL_479894, EPI_ISL_479895	Tokyo Metropolitan Institute of Public Health	Pathogen Genomics Center, National Institute of Infectious Diseases	Tsuyoshi Sekizuka, Kenji Sadamasu, Takashi Chiba, Mami Nagashima, Kentaro Itokawa, Rina Tanaka, Masanori Hashino, Hajime Kamiya, Motoi Suzuki, Makoto Kuroda
EPI_ISL_479898, EPI_ISL_479899, EPI_ISL_479900	Gunma Prefectural Institute of Public Health and Environmental Sciences	Pathogen Genomics Center, National Institute of Infectious Diseases	Tsuyoshi Sekizuka, Hiroyuki Tsukagoshi, Kentaro Itokawa, Rina Tanaka, Masanori Hashino, Hajime Kamiya, Motoi Suzuki, Makoto Kuroda
EPI_ISL_479908	Himeji City Institute of Environment and Health	Pathogen Genomics Center, National Institute of Infectious Diseases	Tsuyoshi Sekizuka, Kentaro Itokawa, Rina Tanaka, Masanori Hashino, Hajime Kamiya, Motoi Suzuki, Makoto Kuroda

EPI_ISL_479924	Niigata City Public Health Research Institute	Pathogen Genomics Center, National Institute of Infectious Diseases	Tsuyoshi Sekizuka, Yurie Takahashi, Kentaro Itokawa, Rina Tanaka, Masanori Hashino, Hajime Kamiya, Motoi Suzuki, Makoto Kuroda
EPI_ISL_479933, EPI_ISL_479934, EPI_ISL_479935	Saitama Prefectural Institute of Public Health	Pathogen Genomics Center, National Institute of Infectious Diseases	Tsuyoshi Sekizuka, Hayato Ehara, Kentaro Itokawa, Rina Tanaka, Masanori Hashino, Hajime Kamiya, Motoi Suzuki, Makoto Kuroda
EPI_ISL_479937, EPI_ISL_479938, EPI_ISL_479939, EPI_ISL_479940	Ibaraki Prefectural Institute of Public Health	Pathogen Genomics Center, National Institute of Infectious Diseases	Tsuyoshi Sekizuka, Keiko Goto, Kentaro Itokawa, Rina Tanaka, Masanori Hashino, Hajime Kamiya, Motoi Suzuki, Makoto Kuroda
EPI_ISL_479967	Fukui Prefectural Institute of Public Health and Environmental Science	Pathogen Genomics Center, National Institute of Infectious Diseases	Tsuyoshi Sekizuka, Miho Toho, Kentaro Itokawa, Rina Tanaka, Masanori Hashino, Hajime Kamiya, Motoi Suzuki, Makoto Kuroda
EPI_ISL_480227	Tokyo Metropolitan Institute of Public Health	Pathogen Genomics Center, National Institute of Infectious Diseases	Tsuyoshi Sekizuka, Kenji Sadamasu, Takashi Chiba, Mami Nagashima, Kentaro Itokawa, Rina Tanaka, Masanori Hashino, Hajime Kamiya, Motoi Suzuki, Makoto Kuroda
EPI_ISL_480312, EPI_ISL_480313, EPI_ISL_480314	Hospital Mexico	Charité Virology-University of Costa Rica	Andres Moreira-Soto, Eugenia Corrales-Aguilar, Ignacio Postigo-Hidalgo, Teresita Somogyi, Jan Felix Drexler
EPI_ISL_480322, EPI_ISL_480323	Hospital Nacional de Niños	Charité Virology-University of Costa Rica	Andres Moreira-Soto, Eugenia Corrales-Aguilar, Ignacio Postigo-Hidalgo, Cristian Pérez Corrales, Andrei Montero Bonilla, Jan Felix Drexler
EPI_ISL_480377, EPI_ISL_480379, EPI_ISL_480380, EPI_ISL_480381, EPI_ISL_480382, EPI_ISL_480383, EPI_ISL_480384, EPI_ISL_480385, EPI_ISL_480387	University of Wisconsin-Madison AIDS Vaccine Research Laboratories	University of Wisconsin-Madison AIDS Vaccine Research Laboratories	Gage Moreno, Katarina Braun, et al. AIDS Vaccine Research Laboratories
EPI_ISL_480438	Laboratorio de Biología Molecular Asociación Española Primera en Salud	Departments of Pathology and Medicine, New York University School of Medicine	Maria Victoria Elizondo, Maria Noel Zubillaga, Gonzalo Manrique, Paul Zappile, Gael Westby, Matthew T Maurano, Christian Marier, Adriana Heguy
EPI_ISL_480953, EPI_ISL_480955, EPI_ISL_480956, EPI_ISL_480957, EPI_ISL_480958, EPI_ISL_480965, EPI_ISL_480966, EPI_ISL_480967, EPI_ISL_480973, EPI_ISL_480976, EPI_ISL_480977, EPI_ISL_480978, EPI_ISL_480983, EPI_ISL_480984, EPI_ISL_480987, EPI_ISL_480988, EPI_ISL_480991, EPI_ISL_480993, EPI_ISL_480994, EPI_ISL_480996, EPI_ISL_480997, EPI_ISL_480998, EPI_ISL_481002, EPI_ISL_481004, EPI_ISL_481006, EPI_ISL_481008, EPI_ISL_481011, EPI_ISL_481012, EPI_ISL_481014, EPI_ISL_481019, EPI_ISL_481021, EPI_ISL_481022, EPI_ISL_481023, EPI_ISL_481028, EPI_ISL_481032, EPI_ISL_481037, EPI_ISL_481039, EPI_ISL_481040			
see above	Servicio de Microbiología. Hospital Universitario Donostia. OSI Donostialdea. Área de Enfermedades Infecciosas, Grupo de Infección Respiratoria y Resistencia Antimicrobiana. Instituto de Investigación Sanitaria Biodonostia	SeqCOVID-SPAIN consortium/IBV(CSIC)	Gustavo Cilla, Milagrosa Montes, Luis Piñeiro, Jose Maria Marimón and SeqCOVID-SPAIN consortium
EPI_ISL_481043, EPI_ISL_481054	Hospital General Universitario Gregorio Marañón	SeqCOVID-SPAIN consortium/IBV(CSIC)	Laura Pérez-Lago, Marta Herranz, Jon Sicilia, Julia Suárez, Pilar Catalán, Patricia Muñoz, Darío García de Viedma and SeqCOVID-SPAIN consortium
EPI_ISL_481569, EPI_ISL_481570	Department of Virology and Immunology, University of Helsinki and Helsinki University Hospital, Huslab Finland	Department of Virology, Faculty of Medicine, University of Helsinki, Helsinki, Finland	Teemu Smura, Hannimari Kallio-Kokko, Jenni Virtanen, Maija Suvanto, Sari Hannula, Harri Kangas, Pekka Ellonen, Olli Vapalahti
EPI_ISL_482323, EPI_ISL_482463, EPI_ISL_482464	Providence St. Joseph Health Molecular Genomics Laboratory	Providence St. Joseph Health Molecular Genomics Laboratory	Alexa K Dowdell, Brian D Piening, Fred L Robinson, Carlo B Bifulco, Mary Campbell
EPI_ISL_482470	Queen Elizabeth II Health Science Centre	National Microbiology Laboratory	Anna Majer, Shari Tyson, Grace Seo, Kristyn Burak, Philip Mabon, Elsie Grudeski, Rhiannon Huzarewich, Russell Mandes, Jennifer Tanner, Natalie Knox, Morag Graham, Gary Van Domselaar, Todd Hatchette, Jason LeBlanc, Nathalie Bastien, Yan Li, Timothy Booth
EPI_ISL_482471, EPI_ISL_482472, EPI_ISL_482473	Dr. Georges-L.-Dumont University Hospital Centre	National Microbiology Laboratory	Anna Majer, Shari Tyson, Grace Seo, Kristyn Burak, Philip Mabon, Elsie Grudeski, Rhiannon Huzarewich, Russell Mandes, Jennifer Tanner, Natalie Knox, Morag Graham, Gary Van Domselaar, Richard Garceau, Guillaume Desnoyers, Nathalie Bastien, Yan Li, Timothy Booth
EPI_ISL_482475	Cadham Provincial Laboratory	National Microbiology Laboratory	Anna Majer, Shari Tyson, Grace Seo, Kristyn Burak, Philip Mabon, Elsie Grudeski, Rhiannon Huzarewich, Russell Mandes, Jennifer Tanner, Natalie Knox, Morag Graham, Gary Van Domselaar, Paul Van Caeseele, Jared Bullard, David Alexander, Kerry Dust, Nathalie Bastien, Yan Li, Timothy Booth,
EPI_ISL_482476, EPI_ISL_482477	Queen Elizabeth II Health Science Centre	National Microbiology Laboratory	Anna Majer, Shari Tyson, Grace Seo, Kristyn Burak, Philip Mabon, Elsie Grudeski, Rhiannon Huzarewich, Russell Mandes, Jennifer Tanner, Natalie Knox, Morag Graham, Gary Van Domselaar, Todd Hatchette, Jason LeBlanc, Nathalie Bastien, Yan Li, Timothy Booth
EPI_ISL_482478, EPI_ISL_482481, EPI_ISL_482482	Cadham Provincial Laboratory	National Microbiology Laboratory	Anna Majer, Shari Tyson, Grace Seo, Kristyn Burak, Philip Mabon, Elsie Grudeski, Rhiannon Huzarewich, Russell Mandes, Jennifer Tanner, Natalie Knox, Morag Graham, Gary Van Domselaar, Paul Van Caeseele, Jared Bullard, David Alexander, Kerry Dust, Nathalie Bastien, Yan Li, Timothy Booth,
EPI_ISL_482579, EPI_ISL_482580	Hangzhou Center for Diseases Control and Prevention	Hangzhou Center for Diseases Control and Prevention	Jun Li, Haoqiu Wang, Lingfeng Mao, Hua Yu, Xinfen Yu, Zhou Sun, Xin Qian, Shuchang Chen, Junfang Chen, Xuchu Wang
EPI_ISL_482677	Singapore General Hospital	Department of Microbiology	Nurdyana Abdul Rahman, Kun Lee Lim, Chenhao Li, Kian Sing Chan, Lynette Oon, Kern Rei Chng, Niranjan Nagarajan, Karrie Ko
EPI_ISL_482999, EPI_ISL_483000, EPI_ISL_483001, EPI_ISL_483003	Minnesota Department of Health, Public Health Laboratory	Minnesota Department of Health, Public Health Laboratory	Matt Plumb, Jacob Garfin, and Xiong Wang
EPI_ISL_483059	Hospital Universitari Germans Trias i Pujol	IrsiCaixa AIDS Research Lab	J. Segalés, M. Puig, J. Rodon, C. Avila-Nieto, J. Carrillo, G. Cantero, M.T. Terrón, S. Cruz, M. Parera ,M. Noguera-Julián, N. Izquierdo-Useros, V. Guallar, E. Vidal, A. Valencia, I. Blanco, J. Blanco, B. Clotet, J. Vergara-Alert
EPI_ISL_483077	SA Pathology	SA Pathology	Lex Leong, Chuan Kok Lim, Mark Turra, Ivan Bastian, Geoff Higgins
EPI_ISL_483544, EPI_ISL_483546, EPI_ISL_483549, EPI_ISL_483550	Kingdom of Bahrein Ministry of Health	Erasmus Medical Center	Bas Oude Munnink, David Nieuwenhuijse, Reina Sikkema, Fatema, Ebrahim Shehad, Amjad Ghanem Mohamed, Hashmeya Al Wasti, Claudia Schapendonk, Irina Chestakova, Anne van der Linden, Theo Bestebroer, Stefan van Nieuwkoop, Mark Pronk, Pascal Lexmond, Richard Molenkamp, Marion Koopmans, on behalf of the Dutch national COVID-19 response team.
EPI_ISL_483637	National Laboratory of Virology, Szentágothai Research Centre	National Laboratory of Virology, Szentágothai Research Centre	Endre Gábor Tóth, Balázs Somogyi, Ferenc Jakab, Gábor Kemenesi
EPI_ISL_483704, EPI_ISL_483708, EPI_ISL_483715, EPI_ISL_483717	Israel Central Virology laboratory	Israel Central Virology laboratory	Neta Zuckerman, Efrat Dahan Bucris, Oran Erster, Ella Mendelson, Michal Mandelboim
EPI_ISL_483922, EPI_ISL_483923, EPI_ISL_483924, EPI_ISL_483925, EPI_ISL_483926, EPI_ISL_483927, EPI_ISL_483928, EPI_ISL_483929, EPI_ISL_483930, EPI_ISL_483931, EPI_ISL_483932, EPI_ISL_483933, EPI_ISL_483934, EPI_ISL_483935, EPI_ISL_483936, EPI_ISL_483937, EPI_ISL_483938, EPI_ISL_483939, EPI_ISL_483940, EPI_ISL_483941, EPI_ISL_483942, EPI_ISL_483943, EPI_ISL_483944, EPI_ISL_483945, EPI_ISL_483946, EPI_ISL_483947, EPI_ISL_483948, EPI_ISL_483949, EPI_ISL_483950, EPI_ISL_483951, EPI_ISL_483952, EPI_ISL_483953, EPI_ISL_483954, EPI_ISL_483955, EPI_ISL_483957, EPI_ISL_483958, EPI_ISL_483969, EPI_ISL_484111, EPI_ISL_484197			
see above	Centre for Clinical Infection and Diagnostics Research and Genomics Innovation Unit, Guy's and St. Thomas' NHS Trust	COVID-19 Genomics UK (COG-UK) Consortium	Chloe Fisher, Luke Snell, Penny Cliff, Rahul Batra, Jonathan Edgeworth, Ali Raza Awan
EPI_ISL_484339	Queens Medical Centre, Clinical Microbiology Department / DeepSeq Nottingham	COVID-19 Genomics UK (COG-UK) Consortium	Gemma Clark, Wendy Smith, Manjinder Khakh, Vicki M Fleming, Michelle M Lister, Hannah Howson-Wells, Jonathan Ball, Patrick McClure, Joseph Chappell, Theocharis Tsoleridis, Nadine Holmes, Matthew Carlisle, Christopher Moore, Fei Sang, Johnny Debebe, Victoria Wright, Matthew Loose
EPI_ISL_484701, EPI_ISL_484705	Department of Clinical Microbiology	GIGA Medical Genomics	Keith Durkin, Maria Artesi, Sébastien Bontems, Raphaël Boreux, Cécile Meex, Axelle Chaslain, Céline Fombellida-Lopez, Pierrette Melin, Marie-Pierre Hayette, Vincent Bours.
EPI_ISL_485848, EPI_ISL_485856, EPI_ISL_485857, EPI_ISL_485858, EPI_ISL_485859, EPI_ISL_485860, EPI_ISL_485861, EPI_ISL_485862, EPI_ISL_485863, EPI_ISL_485864, EPI_ISL_485865, EPI_ISL_485866, EPI_ISL_485867, EPI_ISL_485868, EPI_ISL_485869, EPI_ISL_485870			
see above	Virginia DCLS	Virginia DCLS	Virginia DCLS
EPI_ISL_485971, EPI_ISL_486113	UW Virology Lab	UW Virology Lab	Pavitra Roychoudhury, Hong Xie, Lasata Shrestha, Amin Addetia, Truong Nguyen, Victoria M Rachleff, Meei-Li Huang, Keith R Jerome, Alexander Greninger
EPI_ISL_486422, EPI_ISL_486423, EPI_ISL_486431, EPI_ISL_486432,	Latvijas Infektoloijas centrs	Latvian Biomedical Research and Study Centre	Ivars Silamielis, Kaspars Megnis, Monta Ustinova, ikita Zrelovs, Vita Rovte, Jeena Storoženko, Tatjana Kolupajeva, Oksana Savicka, Uga Dumpis, Jnis Kloviš

EPI_ISL_486433, EPI_ISL_486434			
EPI_ISL_486650	Microbiology, Virology and Biemergency Laboratory-ASST FBF Sacco	Microbiology, Virology and Biemergency Laboratory-ASST FBF Sacco	Romeri F, Comandatore F, Mancon A, Micheli V, Rimoldi SG
EPI_ISL_488335	PHE South West Regional Laboratory, National Infection Service	Wellcome Sanger Institute for the COVID-19 Genomics UK (COG-UK) consortium	Stephanie Hutchings, Hannah Pymont, Dr Peter Muir, Barry Vipond, Rich Hopes; and Alex Alderton, Roberto Amato, Sonia Goncalves, Ewan Harrison, David K. Jackson, Ian Johnston, Dominic Kwiatkowski, Cordelia Langford, John Sillitoe on behalf of the Wellcome Sanger Institute COVID-19 Surveillance Team (http://www.sanger.ac.uk/covid-team)
EPI_ISL_488485, EPI_ISL_488508, EPI_ISL_488541, EPI_ISL_488588, EPI_ISL_488628, EPI_ISL_488707, EPI_ISL_488744, EPI_ISL_488749, EPI_ISL_488750, EPI_ISL_488757, EPI_ISL_488762, EPI_ISL_488766, EPI_ISL_488767, EPI_ISL_488772, EPI_ISL_488774, EPI_ISL_488775, EPI_ISL_488779, EPI_ISL_488785, EPI_ISL_488786, EPI_ISL_488792, EPI_ISL_488794, EPI_ISL_488797, EPI_ISL_488799, EPI_ISL_488801, EPI_ISL_488805, EPI_ISL_488809, EPI_ISL_488818, EPI_ISL_488823, EPI_ISL_488828			
see above	NU-OMICS DNA Sequencing research facility, Northumbria University	Wellcome Sanger Institute for the COVID-19 Genomics UK (COG-UK) consortium	Chris Duncan, Sheia Waugh, Shirelle Burton-Fanning, Gary Eltringham, Jennifer Collins, Brendan Payne, Yusri Taha, Emma Swindells, Jane Greenaway, Edward Barton, Garren Scott, Debra Padgett, Clive Graham, Sarah Essex, Steve Ligget, Paul Baker, Lynn Dover, Wen Yew, Gary Black, John Allan, Joshua Loh, Greg Young, Matthew Bashton, Andrew Nelson, Darren Smith and Alex Alderton, Roberto Amato, Sonia Goncalves, Ewan Harrison, David K. Jackson, Ian Johnston, Dominic Kwiatkowski, Cordelia Langford, John Sillitoe on behalf of the Wellcome Sanger Institute COVID-19 Surveillance Team (http://www.sanger.ac.uk/covid-team)
EPI_ISL_489626, EPI_ISL_489630, EPI_ISL_489662, EPI_ISL_489688, EPI_ISL_489692, EPI_ISL_489700	NHSGGC West of Scotland Specialist Virology Centre / MRC-University of Glasgow Centre for Virus Research	Wellcome Sanger Institute for the COVID-19 Genomics UK (COG-UK) consortium	Ana da Silva Filipe, Natasha Johnson, Kathy Smollett, Daniel Mair, Stephen Carmichael, Lily Tong, Jenna Nichols, Elihu Aranday-Cortes, Kirstyn Brunker, Yasmin Parr, Kyriaki Nomikou; Sarah McDonald, Marc Niebel, Patawee Asamaphan; Richard Orton, Joseph Hughes, Sreenu Vattipally, David L Robertson; Alasdair MacLean, Rory Gunson; Kathy Li, Natasha Jesudason, Rajiv Shah, James Shepherd, Antonia Ho, Alice Broos, Emma Thomson and Alex Alderton, Roberto Amato, Sonia Goncalves, Ewan Harrison, David K. Jackson, Ian Johnston, Dominic Kwiatkowski, Cordelia Langford, John Sillitoe on behalf of the Wellcome Sanger Institute COVID-19 Surveillance Team (http://www.sanger.ac.uk/covid-team)
EPI_ISL_489800, EPI_ISL_489801, EPI_ISL_489802, EPI_ISL_489803, EPI_ISL_489805, EPI_ISL_489808	Florida Bureau of Public Health Laboratories	Florida Bureau of Public Health Laboratories	Sarah Schmedes, Jason Blanton
EPI_ISL_490018	South Eastern Area Laboratory Services (SEALS)	NSW Health Pathology - Institute of Clinical Pathology and Medical Research; Westmead Hospital; University of Sydney	CIDM-PH et al.
EPI_ISL_490112	The National Institute of Public Health	The National Institute of Public Health and State Veterinary Institute Prague	Nagy,A;Jirincova,H;Novakova,L;Trnka,D;Vecerova,J
EPI_ISL_490988, EPI_ISL_490997, EPI_ISL_490998, EPI_ISL_491000	Mayo Clinic Laboratories	UW Virology Lab	Pavitra Roychoudhury, Hong Xie, Lasata Shrestha, Amin Addetia, Truong Nguyen, Victoria M Rachleff, Meei-Li Huang, Keith R Jerome, Alexander Greninger
EPI_ISL_491093	The National Institute of Public Health	The National Institute of Public Health and State Veterinary Institute Prague	Nagy,A; Jirincova,H; Novakova,L; Trnka,D; Vecerova,J
EPI_ISL_491100	SC Department of Health and Environmental Control	SC Department of Health and Environmental Control	Flores,H.
EPI_ISL_491117	The National Institute of Public Health	The National Institute of Public Health and State Veterinary Institute Prague	Nagy,A;Jirincova,H;Novakova,L;Trnka,D;Vecerova,J
EPI_ISL_491444	Area de Salud Escazu (Coopesana)	Inciensa, Instituto Costarricense de Investigación y Enseñanza en Nutrición y Salud	Francisco Duarte, Hebleen Brenes, Claudio Soto-Garita, Estela Cordero, Adriana Godinez & Melany Calderon
EPI_ISL_491445	Area de Salud Mata Redonda	Inciensa, Instituto Costarricense de Investigación y Enseñanza en Nutrición y Salud	Francisco Duarte, Hebleen Brenes, Claudio Soto-Garita, Estela Cordero, Adriana Godinez & Melany Calderon
EPI_ISL_491447	Hospital Fernando Escalante Pradilla	Inciensa, Instituto Costarricense de Investigación y Enseñanza en Nutrición y Salud	Francisco Duarte, Hebleen Brenes, Claudio Soto-Garita, Estela Cordero, Adriana Godinez & Melany Calderon
EPI_ISL_491448	Hospital San Rafael de Alajuela	Inciensa, Instituto Costarricense de Investigación y Enseñanza en Nutrición y Salud	Francisco Duarte, Hebleen Brenes, Claudio Soto-Garita, Estela Cordero, Adriana Godinez & Melany Calderon
EPI_ISL_491449	Area de Salud Alajuela Sur	Inciensa, Instituto Costarricense de Investigación y Enseñanza en Nutrición y Salud	Francisco Duarte, Hebleen Brenes, Claudio Soto-Garita, Estela Cordero, Adriana Godinez & Melany Calderon
EPI_ISL_491450, EPI_ISL_491454	Hospital San Juan de Dios	Inciensa, Instituto Costarricense de Investigación y Enseñanza en Nutrición y Salud	Francisco Duarte, Hebleen Brenes, Claudio Soto-Garita, Estela Cordero, Adriana Godinez & Melany Calderon
EPI_ISL_491476	BSL3 Lab, Pendik Veterinary Control Enstitue	Genomic Laboratory (GLAB), Istanbul Technical University	Mustafa HASOKSUZ, Fahriye SARAC, Osman ERGANIS, Serdar UZAR, Hakan ENUL, Cumhur ADIAY, Ahmet SAIT, Orbay SAYI, Kadir YESILBAG, Oguz KARABEY
EPI_ISL_492114, EPI_ISL_492115, EPI_ISL_492116, EPI_ISL_492121, EPI_ISL_492122, EPI_ISL_492172, EPI_ISL_492175, EPI_ISL_492176	SA Pathology	SA Pathology	Lex Leong, Chuan Kok Lim, Mark Turra, Ivan Bastian, Geoff Higgins
EPI_ISL_492536, EPI_ISL_492544, EPI_ISL_492606, EPI_ISL_492659	PHE South West Regional Laboratory, National Infection Service	Wellcome Sanger Institute for the COVID-19 Genomics UK (COG-UK) consortium	Stephanie Hutchings, Hannah Pymont, Dr Peter Muir, Barry Vipond, Rich Hopes; and Alex Alderton, Roberto Amato, Sonia Goncalves, Ewan Harrison, David K. Jackson, Ian Johnston, Dominic Kwiatkowski, Cordelia Langford, John Sillitoe on behalf of the Wellcome Sanger Institute COVID-19 Surveillance Team (http://www.sanger.ac.uk/covid-team)
EPI_ISL_492862	Royal Free Hospital / Health Services Laboratories	Wellcome Sanger Institute for the COVID-19 Genomics UK (COG-UK) Consortium	Tanzina Haque, Tabitha Mahungu, Dianne Irish, Cate Goodlad, Jenny Cross, Judith Heaney and Alex Alderton, Roberto Amato, Sonia Goncalves, Ewan Harrison, David K. Jackson, Ian Johnston, Dominic Kwiatkowski, Cordelia Langford, John Sillitoe on behalf of the Wellcome Sanger Institute COVID-19 Surveillance Team
EPI_ISL_492978	Department of Laboratory Medicine Tan Tock Seng Hospital	Department of Laboratory Medicine Tan Tock Seng Hospital	Chen YYC, Zair X, Li C, Tang WY, Maurer-Stroh S, Barkham TMS, Nagarajan N, Sessions OM
EPI_ISL_492980	IRCCS Sacro Cuore Don Calabria Hospital, Department of Infectious, Tropical Diseases & Microbiology	University of Verona, Department of Biotechnology	Antonio Mori, Michela Deiana, Elena Pomari, Chiara Piubelli; Giulia Lopatriello, Luca Marcolungo, Cristina Beltrami, Chiara Degli Esposti, Emanuela Cosentino, Massimo Delledonne
EPI_ISL_493198, EPI_ISL_493199, EPI_ISL_493200	Virology Lab,Department of Pathology, National Cheng Kung University Hospital	Virology Lab,Department of Pathology, National Cheng Kung University Hospital	Huey-Pin Tsai, et al
EPI_ISL_493328	INMI Lazzaro Spallanzani IRCCS	INMI Lazzaro Spallanzani IRCCS	Martina Rueca, Cesare E.M. Gruber, Barbara Bartolini, Francesco Messina, Maria R. Capobianchi, Antonino Di Caro
EPI_ISL_493741	West of Scotland Specialist Virology Centre, NHSGGC / MRC-University of Glasgow Centre for Virus Research	COVID-19 Genomics UK (COG-UK) Consortium	Ana da Silva Filipe, Natasha Johnson, Kathy Smollett, Daniel Mair, Stephen Carmichael, Lily Tong, Jenna Nichols, Elihu Aranday-Cortes, Kirstyn Brunker, Yasmin Parr, Alice Broos, Kyriaki Nomikou; Sarah McDonald, Marc Niebel, Patawee Asamaphan; Richard Orton, Joseph Hughes, Sreenu Vattipally, David L Robertson; Alasdair MacLean, Rory Gunson; Kathy Li, Natasha Jesudason, Rajiv Shah, James Shepherd, Antonia Ho, Emma Thomson
EPI_ISL_494575, EPI_ISL_494592	San Diego County Public Health Laboratory	Andersen lab at Scripps Research	SEARCH Alliance San Diego with Tracy Basler, Jovan Shephard, Brett Austin
EPI_ISL_495389	Florida Bureau of Public Health Laboratories	Florida Bureau of Public Health Laboratories	Sarah Schmedes, Jason Blanton
EPI_ISL_495610	Minnesota Department of Health, Public Health Laboratory	Minnesota Department of Health, Public Health Laboratory	Matt Plumb, Jacob Garfin, and Xiong Wang
EPI_ISL_496482	Dept. Infectious, Tropical Diseases & Microbiology, IRCCS Sacro Cuore Don Calabria Hospital	1) Dept. Infectious, Tropical Diseases & Microbiology, IRCCS Sacro Cuore Don Calabria Hospital; 2) Centro Piattaforme Tecnologiche, University of Verona; 3) Dept. Neurosciences, Biomedicine and Movement Sciences, University of Verona.	1) Antonio Mori, Michela Deiana, Elena Pomari, Chiara Piubelli; 2) Monica Castellucci and Francesca Griggio; 3) Giovanni Malerba

EPI_ISL_496644, EPI_ISL_496645, EPI_ISL_496657, EPI_ISL_496659, EPI_ISL_496668, EPI_ISL_496669, EPI_ISL_496674, EPI_ISL_496678, EPI_ISL_496680, EPI_ISL_496684, EPI_ISL_496689, EPI_ISL_496690, EPI_ISL_496694, EPI_ISL_496696, EPI_ISL_496704, EPI_ISL_496714, EPI_ISL_496718, EPI_ISL_496739, EPI_ISL_496747, EPI_ISL_496748			
see above	Gorgas Memorial Laboratory of Health Studies	Gorgas Memorial Laboratory of Health Studies	Danilo Franco, Claudia Gonzalez Sandra Lopez-Verges, Alexander A Martinez
EPI_ISL_496919	Minnesota Department of Health, Public Health Laboratory	Minnesota Department of Health, Public Health Laboratory	Matt Plumb, Jacob Garfin, and Xiong Wang
EPI_ISL_497736	Instituto Nacional de Salud, Bogotá, Colombia	Instituto Nacional de Salud, Bogotá, Colombia	Katherine Laiton-Donato, Diego A. Álvarez-Díaz, Carlos Franco-Muñoz, Jonathan Reales, Diego Andrés Prada, Jose A. Usme-Ciro, Nicolas D. Franco-Sierra, Zulma M. Cucunubá, Christian Julian VillabonaArenas, Liz Villabona-Arenas, Sussy Echeverria, Astrid C. Flórez, Carolina Ferro, Diana Marcela Walteros-Acero, Franklin Prieto, Carlos Andrés Durán, Martha Lucia Ospina Martinez, Marcela Mercado-Reyes
EPI_ISL_497773, EPI_ISL_497796, EPI_ISL_497799, EPI_ISL_497802, EPI_ISL_497805, EPI_ISL_497810, EPI_ISL_497820, EPI_ISL_497840, EPI_ISL_497845, EPI_ISL_497850, EPI_ISL_497864, EPI_ISL_497870			
see above	Department of Microbiology, The University of Hong Kong	Department of Microbiology, The University of Hong Kong	Kelvin K.W. To, Kwok-Yung Yuen
EPI_ISL_498018, EPI_ISL_498019, EPI_ISL_498020, EPI_ISL_498021, EPI_ISL_498022, EPI_ISL_498023, EPI_ISL_498024, EPI_ISL_498025, EPI_ISL_498026, EPI_ISL_498027, EPI_ISL_498028, EPI_ISL_498029, EPI_ISL_498030, EPI_ISL_498031, EPI_ISL_498032, EPI_ISL_498033			
see above	Division of Viral Diseases, Center for Laboratory Control of Infectious Diseases, Korea Centers for Diseases Control and Prevention	Division of Viral Diseases, Center for Laboratory Control of Infectious Diseases, Korea Centers for Diseases Control and Prevention	Jeong-Min Kim, Yoon-Seok Chung, Namjoo Lee, Sang Hee Woo, Hye-Jun Jo, Heui Man Kim, Jun-Sub Kim, Dong Hyun Song, Daesang Lee, Seong Tae Jeong, Myung Guk Han
EPI_ISL_498036	Division of Viral Diseases, Center for Laboratory Control of Infectious Diseases, Korea Centers for Diseases Control and Prevention	Division of Viral Diseases, Center for Laboratory Control of Infectious Diseases, Korea Centers for Diseases Control and Prevention	Jeong-Min Kim, Yoon-Seok Chung, Namjoo Lee, Sang Hee Woo, Hye-Jun Jo, Heui Man Kim, Jun-Sub Kim, Myung Guk Han
EPI_ISL_498037	Division of Viral Diseases, Center for Laboratory Control of Infectious Diseases, Korea Centers for Diseases Control and Prevention	Division of Viral Diseases, Center for Laboratory Control of Infectious Diseases, Korea Centers for Diseases Control and Prevention	Jeong-Min Kim, Yoon-Seok Chung, Namjoo Lee, Sang Hee Woo, Hye-Jun Jo, Heui Man Kim, Jun-Sub Kim, Dong Hyun Song, Daesang Lee, Seong Tae Jeong, Myung Guk Han
EPI_ISL_498176, EPI_ISL_498181, EPI_ISL_498186	OUCRU	OUCRU	Nguyen Van Vinh Chau, Nguyen Thi Thu Hong, Nguyen Thi Han Ny, Le Nguyen Truc Nhu, Nghiem My Ngoc, Vo Thanh Lam, Nguyen Thanh Dung, Lam Minh Yen, Ngo Ngoc Quang Minh, Le Manh Hung, Nguyen Tri Dung, Dinh Nguyen Huy Man, Lam Anh Nguyet, Tran Chanh Xuan, Tran Tinh Hien, Nguyen Thanh Phong, Tran Nguyen Hoang Tu, Tran Tan Thanh, Nguyen Thanh Truong, Nguyen Tan Binh, Tang Chi Thuong, Guy Thwaites, and Le Van Tan, for OUCRU COVID-19 research group*
EPI_ISL_498266	Ramathibodi Hospital	COVID-19 Network Investigations (CONI) Alliance	Elizabeth Batty, Wasun Chantratita, Thanat Chookajorn, Stefan Fernandez, Angkana Huang, Anthony R. Jones, Khajohn Joonsalak, Chonticha Klungtong, Theerarat Kochakarn, Namfon Kotanan, Krittikorn Kumpornsin, Wudtichai Manasatienkij, Bhakbhoom Panthan, Ekawat Pasomsub, Kingkan Rakmanee, Insee Sensorn, Janjira Thaipadungpanit, Arporn Wangwiwatsin,Treewat Watthanachockchai
EPI_ISL_498481, EPI_ISL_498492, EPI_ISL_498514, EPI_ISL_498529, EPI_ISL_498538, EPI_ISL_498542	ACT Pathology	Schwessinger Lab	Ashley Jones, Benjamin Schwessinger, Robert Lanfear, Robyn N Hall, Megan McDonald, Ming-Dao Chia, Kevin Murray, Craig Kennedy, Karina Kennedy
EPI_ISL_498552	Lebanese American University	Lebanese American University	Abi Habib,W., Abdallah,J., El Shesheny,R., Mokhbat,J., Webby,R.J., Goldstein,J. and Kayali,G.
EPI_ISL_499301, EPI_ISL_499302	Centre for Enzyme Innovation, University of Portsmouth / Translational Research Laboratory, Portsmouth Hospitals NHS Trust	COVID-19 Genomics UK (COG-UK) Consortium	Angela Beckett,Yann Bourgeois,Garry Scarlett,Sharon Glaysher,Scott Elliott,Kelly Bicknell,Robert Impey,Allyson Lloyd,Sarah Wyllie,Ethan Butcher,Anoop Chauhan,Samuel Robson
EPI_ISL_499460, EPI_ISL_499462, EPI_ISL_499463, EPI_ISL_499464, EPI_ISL_499467, EPI_ISL_499469, EPI_ISL_499473, EPI_ISL_499475, EPI_ISL_499476, EPI_ISL_499477, EPI_ISL_499481, EPI_ISL_499483, EPI_ISL_499486, EPI_ISL_499487, EPI_ISL_499488, EPI_ISL_499489, EPI_ISL_499496, EPI_ISL_499498, EPI_ISL_499499, EPI_ISL_499500, EPI_ISL_499501, EPI_ISL_499509, EPI_ISL_499511, EPI_ISL_499513, EPI_ISL_499514, EPI_ISL_499516, EPI_ISL_499518, EPI_ISL_499521, EPI_ISL_499522, EPI_ISL_499526, EPI_ISL_499527, EPI_ISL_499528, EPI_ISL_499529, EPI_ISL_499531, EPI_ISL_499532, EPI_ISL_499533, EPI_ISL_499540, EPI_ISL_499542, EPI_ISL_499544, EPI_ISL_499566, EPI_ISL_499567, EPI_ISL_499569, EPI_ISL_499570, EPI_ISL_499571, EPI_ISL_499573, EPI_ISL_499574, EPI_ISL_499575, EPI_ISL_499576, EPI_ISL_499577, EPI_ISL_499580, EPI_ISL_499581, EPI_ISL_499582, EPI_ISL_499583, EPI_ISL_499584, EPI_ISL_499590, EPI_ISL_499594, EPI_ISL_499600, EPI_ISL_499605, EPI_ISL_499607, EPI_ISL_499608, EPI_ISL_499611, EPI_ISL_499613, EPI_ISL_499616, EPI_ISL_499617, EPI_ISL_499621, EPI_ISL_499624, EPI_ISL_499627, EPI_ISL_499628, EPI_ISL_499632, EPI_ISL_499633, EPI_ISL_499634, EPI_ISL_499635, EPI_ISL_499636, EPI_ISL_499637, EPI_ISL_499639, EPI_ISL_499644, EPI_ISL_499646, EPI_ISL_499647, EPI_ISL_499648, EPI_ISL_499649, EPI_ISL_499650, EPI_ISL_499651, EPI_ISL_499652, EPI_ISL_499653, EPI_ISL_499654, EPI_ISL_499655, EPI_ISL_499656, EPI_ISL_499657, EPI_ISL_499658, EPI_ISL_499660, EPI_ISL_499661, EPI_ISL_499662, EPI_ISL_499700, EPI_ISL_499702, EPI_ISL_499703, EPI_ISL_499705, EPI_ISL_499707, EPI_ISL_499708, EPI_ISL_499709, EPI_ISL_499710, EPI_ISL_499711, EPI_ISL_499712, EPI_ISL_499713, EPI_ISL_499714, EPI_ISL_499715, EPI_ISL_499716, EPI_ISL_499717, EPI_ISL_499718, EPI_ISL_499719, EPI_ISL_499720, EPI_ISL_499721, EPI_ISL_499722, EPI_ISL_499723, EPI_ISL_499728, EPI_ISL_499729, EPI_ISL_499734, EPI_ISL_499735, EPI_ISL_499736, EPI_ISL_499737, EPI_ISL_499740, EPI_ISL_499746, EPI_ISL_499747, EPI_ISL_499751, EPI_ISL_499752, EPI_ISL_499753, EPI_ISL_499754, EPI_ISL_499757, EPI_ISL_499758, EPI_ISL_499760, EPI_ISL_499761, EPI_ISL_499762, EPI_ISL_499763, EPI_ISL_499764, EPI_ISL_499765, EPI_ISL_499767, EPI_ISL_499768, EPI_ISL_499769, EPI_ISL_499771, EPI_ISL_499772, EPI_ISL_499773, EPI_ISL_499774, EPI_ISL_499775			
see above	Liverpool Clinical Laboratories	COVID-19 Genomics UK (COG-UK) Consortium	Sam Haldenby, Anita Lucaci, Steve Paterson, Julian Hiscox, Alistair Darby, M Almsaud, A Alrezaihi, Muhannad Alruwaili, Stuart D Armstrong, Jones Benjamin, Eleanor G Bentley, Anu Chawla, Jordan J Clark, Angela Cowell, Richard Eccles, Isabel García-Dorival, Matthew Gemmell, Alessandro Gerada, PKF Gilmore, Richard Gregory, Ximeng Han, Catherine Hartley, Margaret Hughes, Miren Iturriza-Gomara, James Johnson, L Luu, Jenifer Manson, Charlotte Nelson, Elaine O'Toole, Cassie Olateju, Rebekah Penrice-Randal , Lucille Rainbow, N.P Randle, Trevor Ian Robinson, Parul Sharma, Ghada T Shawli, James P Stewart, Neil Swainston, Ecaterina Vamos, Joanne Watts, Mark Whitehead
EPI_ISL_500219	Hospital Clínico Universitario de Santiago de Compostela	SeqCOVID-SPAIN consortium/IBV(CSIC)	José Javier Costa Alcalde, Antonio Aguilera Guirao, Mª Luisa Pérez del Molino Bernal, Amparo Coira Nieto, Gema Barbeito Castiñeiras, Rocio Trastoy Pena and SeqCOVID-SPAIN consortium
EPI_ISL_500503, EPI_ISL_500504, EPI_ISL_500508, EPI_ISL_500509, EPI_ISL_500511, EPI_ISL_500513, EPI_ISL_500514, EPI_ISL_500516, EPI_ISL_500519, EPI_ISL_500520, EPI_ISL_500521, EPI_ISL_500526, EPI_ISL_500528, EPI_ISL_500537, EPI_ISL_500538			
see above	Mayo Clinic Laboratories	University of Washington Virology Lab	Pavitra Roychoudhury, Hong Xie, Lasata Shrestha, Amin Addetia, Truong Nguyen, Victoria M Rachleff, Meei-Li Huang, Keith R Jerome, Alexander Greninger
EPI_ISL_500596, EPI_ISL_500629, EPI_ISL_500630, EPI_ISL_500631, EPI_ISL_500643, EPI_ISL_500644, EPI_ISL_500646, EPI_ISL_500676	Area of Virology, Serology and Virology Division (SAViD), New South Wales Health Pathology Randwick	Area of Virology, Serology and Virology Division (SAViD), New South Wales Health Pathology Randwick	Rawlinson, W.
EPI_ISL_500716	BSL3 Lab Pendik Veterinary Control Institute	Department of Medicinal Genetics, Bursa Uluda University, Faculty of medicine By Sehime Gülsün Temel, Adem Alemdar, Kadir Yeilba	Mustafa HASOKSUZ, Fahriye SARAC, Osman ERGANIS, Serdar UZAR, Hakan ENUL, Cumhur ADIAY, Ahmet SAIT, Orbay SAYI, Kadir YESILBAG, Oguz KARABEY
EPI_ISL_500831	Virginia DCLS	Virginia DCLS	Virginia DCLS
EPI_ISL_501073, EPI_ISL_501078	Mayo Clinic Laboratories	University of Washington Virology Lab	Pavitra Roychoudhury, Hong Xie, Lasata Shrestha, Amin Addetia, Truong Nguyen, Victoria M Rachleff, Meei-Li Huang, Keith R Jerome, Alexander Greninger
EPI_ISL_501221, EPI_ISL_501223, EPI_ISL_501224	Department of Medical Microbiology, University Malaya Medical Centre	Department of Medical Microbiology, Faculty of Medicine, University of Malaya	Yoong Min CHONG, Jennifer Chong, I-Ching SAM, Yoke Fun CHAN, University Malaya Medical Centre COVID Team
EPI_ISL_507016, EPI_ISL_507017, EPI_ISL_507018, EPI_ISL_507019	unknown	Infectious Diseases Research, King Abdullah International Medical Research Center (KAIMRC)	Alghoribi,M.F.
EPI_ISL_507052, EPI_ISL_507053, EPI_ISL_507054, EPI_ISL_507055, EPI_ISL_507056, EPI_ISL_507085	University College London Hospital	COVID-19 Genomics UK (COG-UK) Consortium	Judith Heaney, Matthew Byott, Catherine Houlihan, Dan Frampton, Stuart Kirk, Moira Spyer and Eleni Nastouli
EPI_ISL_507291	WHO National Influenza Centre Russian Federation	WHO National Influenza Centre Russian Federation	Andrey Komissarov, Artem Fadeev, Mariia Sergeeva, Anna Ivanova, Daria Danilenko
EPI_ISL_507707, EPI_ISL_507721, EPI_ISL_507743	Michigan Department of Health and Human Services, Bureau of Laboratories	Michigan Department of Health and Human Services, Bureau of Laboratories	Blankenship HM, Riner D, Soehnlen MK
EPI_ISL_508124, EPI_ISL_508130, EPI_ISL_508143, EPI_ISL_508144	SA Pathology	SA Pathology	Lex Leong, Chuan Kok Lim, Mark Turra, Ivan Bastian, Geoff Higgins

EPI_ISL_508615, EPI_ISL_508616, EPI_ISL_508617, EPI_ISL_508618, EPI_ISL_508619, EPI_ISL_508620, EPI_ISL_508621, EPI_ISL_508622, EPI_ISL_508623, EPI_ISL_508624, EPI_ISL_508634, EPI_ISL_508636, EPI_ISL_508637, EPI_ISL_508638, EPI_ISL_508639, EPI_ISL_508641, EPI_ISL_508642, EPI_ISL_508643, EPI_ISL_508644, EPI_ISL_508645, EPI_ISL_508675, EPI_ISL_508678, EPI_ISL_508681, EPI_ISL_508682, EPI_ISL_508684			
see above	Departamento de Microbiología, CDB, Hospital Clínic, Barcelona	SeqCOVID-SPAIN consortium/IBV(CSIC)	Andrea Vergara, Mikel Martínez, Elisa Rubio, Jéssica Navero, Aida Peiró and SeqCOVID-SPAIN consortium
EPI_ISL_508770, EPI_ISL_508771, EPI_ISL_508773, EPI_ISL_508805, EPI_ISL_508807	Florida Bureau of Public Health Laboratories	Florida Bureau of Public Health Laboratories	Sarah Schmedes, Jason Blanton
EPI_ISL_508864	Division of Infectious Diseases and Hospital Epidemiology, University Hospital Zürich	Institute of Medical Virology, University of Zurich	Verena Kufner, Maryam Zaheri, Dana Weissberg, Jürg Böni, Silvana K. Rampini, Peter W. Schreiber, Irene A. Abela, Hugo Sax, Aline Wolfensberger, Michael Huber
EPI_ISL_508865, EPI_ISL_508867	Division of Infectious Diseases and Hospital Epidemiology, University Hospital Zürich	Institute of Medical Virology, University of Zurich	Maryam Zaheri, Verena Kufner, Dana Weissberg, Jürg Böni, Silvana K. Rampini, Peter W. Schreiber, Irene A. Abela, Hugo Sax, Aline Wolfensberger, Michael Huber
EPI_ISL_508868	Division of Infectious Diseases and Hospital Epidemiology, University Hospital Zürich	Institute of Medical Virology, University of Zurich	Verena Kufner, Maryam Zaheri, Dana Weissberg, Jürg Böni, Silvana K. Rampini, Peter W. Schreiber, Irene A. Abela, Hugo Sax, Aline Wolfensberger, Michael Huber
EPI_ISL_508869	Division of Infectious Diseases and Hospital Epidemiology, University Hospital Zürich	Institute of Medical Virology, University of Zurich	Maryam Zaheri, Verena Kufner, Dana Weissberg, Jürg Böni, Silvana K. Rampini, Peter W. Schreiber, Irene A. Abela, Hugo Sax, Aline Wolfensberger, Michael Huber
EPI_ISL_508871, EPI_ISL_508877	Institut des Agents Infectieux (IAI), Hospices Civils de Lyon	CNR Virus des Infections Respiratoires - France SUD	Antonin Bal, Gregory Destras, Gwendolyne Burfin, Solenne Brun, Carine Moustaud, Raphaelle Lamy, Alexandre Gaymard, Maude Bouscambert-Duchamp, Florence Morfin-Sherpa, Martine Valette, Bruno Lina, Laurence Josset
EPI_ISL_508878	GH Les Portes du Sud	CNR Virus des Infections Respiratoires - France SUD	Antonin Bal, Gregory Destras, Gwendolyne Burfin, Solenne Brun, Carine Moustaud, Raphaelle Lamy, Alexandre Gaymard, Maude Bouscambert-Duchamp, Florence Morfin-Sherpa, Martine Valette, Bruno Lina, Laurence Josset
EPI_ISL_508879, EPI_ISL_508880	Centre Hospitalier Saint Joseph Saint Luc	CNR Virus des Infections Respiratoires - France SUD	Antonin Bal, Gregory Destras, Gwendolyne Burfin, Solenne Brun, Carine Moustaud, Raphaelle Lamy, Alexandre Gaymard, Maude Bouscambert-Duchamp, Florence Morfin-Sherpa, Martine Valette, Bruno Lina, Laurence Josset
EPI_ISL_508881	Centre Hospitalier de Valence	CNR Virus des Infections Respiratoires - France SUD	Antonin Bal, Gregory Destras, Gwendolyne Burfin, Solenne Brun, Carine Moustaud, Raphaelle Lamy, Alexandre Gaymard, Maude Bouscambert-Duchamp, Florence Morfin-Sherpa, Martine Valette, Bruno Lina, Laurence Josset
EPI_ISL_508882, EPI_ISL_508883, EPI_ISL_508909	Institut des Agents Infectieux (IAI), Hospices Civils de Lyon	CNR Virus des Infections Respiratoires - France SUD	Antonin Bal, Gregory Destras, Gwendolyne Burfin, Solenne Brun, Carine Moustaud, Raphaelle Lamy, Alexandre Gaymard, Maude Bouscambert-Duchamp, Florence Morfin-Sherpa, Martine Valette, Bruno Lina, Laurence Josset
EPI_ISL_508998	Centre Hospitalier Pierre Oudot	CNR Virus des Infections Respiratoires - France SUD	Antonin Bal, Gregory Destras, Gwendolyne Burfin, Solenne Brun, Carine Moustaud, Raphaelle Lamy, Alexandre Gaymard, Maude Bouscambert-Duchamp, Florence Morfin-Sherpa, Martine Valette, Bruno Lina, Laurence Josset
EPI_ISL_508999, EPI_ISL_509000, EPI_ISL_509001	CNR Virus des Infections Respiratoires - France SUD	CNR Virus des Infections Respiratoires - France SUD	Antonin Bal, Gregory Destras, Gwendolyne Burfin, Solenne Brun, Carine Moustaud, Raphaelle Lamy, Alexandre Gaymard, Maude Bouscambert-Duchamp, Florence Morfin-Sherpa, Martine Valette, Bruno Lina, Laurence Josset
EPI_ISL_509003, EPI_ISL_509004	GH Les Portes du Sud	CNR Virus des Infections Respiratoires - France SUD	Antonin Bal, Gregory Destras, Gwendolyne Burfin, Solenne Brun, Carine Moustaud, Raphaelle Lamy, Alexandre Gaymard, Maude Bouscambert-Duchamp, Florence Morfin-Sherpa, Martine Valette, Bruno Lina, Laurence Josset
EPI_ISL_509501, EPI_ISL_509502, EPI_ISL_509504	Area of Virology, Serology and Virology Division (SAViD), New South Wales Health Pathology Randwick	Area of Virology, Serology and Virology Division (SAViD), New South Wales Health Pathology Randwick	Rawlinson, W.
EPI_ISL_509603, EPI_ISL_509604, EPI_ISL_509605, EPI_ISL_509606, EPI_ISL_509608, EPI_ISL_509609, EPI_ISL_509611	Servicio de Microbiología. HRU de Málaga. Servicio Andaluz de Salud	SeqCOVID-SPAIN consortium/IBV(CSIC)	Inmaculada de Toro Peinado. MªConcepción Mediavilla Gradolph. Begoña Palop Borrás and SeqCOVID-SPAIN consortium
EPI_ISL_509616, EPI_ISL_509617, EPI_ISL_509618	Hospital Universitario Araba. Vitoria-Gasteiz	SeqCOVID-SPAIN consortium/IBV(CSIC)	Silvia Hernáez Crespo, Carmen Gómez González, Amaia Aguirre Quiñonero, Marina Fernández Torres, Mª Rosario Almela Ferrer, Mª Concepción Lecaroz Agara, Andrés Canut Blasco and SeqCOVID-SPAIN consortium
EPI_ISL_509619, EPI_ISL_509620, EPI_ISL_509621, EPI_ISL_509622, EPI_ISL_509623, EPI_ISL_509624, EPI_ISL_509625, EPI_ISL_509626, EPI_ISL_509627, EPI_ISL_509628, EPI_ISL_509629, EPI_ISL_509630, EPI_ISL_509631, EPI_ISL_509632			
see above	Servicio de Microbiología. HRU de Málaga. Servicio Andaluz de Salud	SeqCOVID-SPAIN consortium/IBV(CSIC)	Inmaculada de Toro Peinado. MªConcepción Mediavilla Gradolph. Begoña Palop Borrás and SeqCOVID-SPAIN consortium
EPI_ISL_509696, EPI_ISL_509697, EPI_ISL_509698, EPI_ISL_509699, EPI_ISL_509700	Guatemala Ministry of Public Health	Pathogen Discovery, Respiratory Viruses Branch, Division of Viral Diseases, Centers for Disease Control and Prevention	Ying Tao, Jing Zhang, Krista Queen, Anna Uehara, Yan Li, Clinton Paden, Haibin Wang, Suxiang Tong
EPI_ISL_509710	Guatemala Ministry of Public Health	Pathogen Discovery, Respiratory Viruses Branch, Division of Viral Diseases, Centers for Disease Control and Prevention	Jing Zhang, Ying Tao, Krista Queen, Anna Uehara, Yan Li, Clinton Paden, Haibin Wang, Suxiang Tong
EPI_ISL_509723, EPI_ISL_509733, EPI_ISL_509736	Florida Bureau of Public Health Laboratories	Florida Bureau of Public Health Laboratories	Sarah Schmedes, Jason Blanton
EPI_ISL_510149, EPI_ISL_510171, EPI_ISL_510172, EPI_ISL_510173, EPI_ISL_510174, EPI_ISL_510175, EPI_ISL_510176, EPI_ISL_510177, EPI_ISL_510178, EPI_ISL_510179, EPI_ISL_510180, EPI_ISL_510181, EPI_ISL_510182, EPI_ISL_510183, EPI_ISL_510184, EPI_ISL_510185, EPI_ISL_510189, EPI_ISL_510190, EPI_ISL_510191, EPI_ISL_510192, EPI_ISL_510193, EPI_ISL_510194, EPI_ISL_510195, EPI_ISL_510196, EPI_ISL_510197, EPI_ISL_510198, EPI_ISL_510199, EPI_ISL_510200, EPI_ISL_510201, EPI_ISL_510202, EPI_ISL_510203, EPI_ISL_510204, EPI_ISL_510205, EPI_ISL_510206, EPI_ISL_510207, EPI_ISL_510208, EPI_ISL_510209, EPI_ISL_510210, EPI_ISL_510211, EPI_ISL_510212, EPI_ISL_510213, EPI_ISL_510214, EPI_ISL_510215, EPI_ISL_510216, EPI_ISL_510217, EPI_ISL_510218, EPI_ISL_510219			
see above	Hospital General Universitario Gregorio Marañón	SeqCOVID-SPAIN consortium/IBV(CSIC)	Laura Pérez-Lago, Marta Herranz, Jon Sicilia, Julia Suárez, Pilar Catalán, Patricia Muñoz, Darío García de Viedma and SeqCOVID-SPAIN consortium
EPI_ISL_510247, EPI_ISL_510248, EPI_ISL_510249, EPI_ISL_510250, EPI_ISL_510251, EPI_ISL_510252, EPI_ISL_510253, EPI_ISL_510254, EPI_ISL_510255, EPI_ISL_510256, EPI_ISL_510257, EPI_ISL_510258, EPI_ISL_510259, EPI_ISL_510260, EPI_ISL_510261, EPI_ISL_510262, EPI_ISL_510263, EPI_ISL_510264			
see above	Hospital de la Santa Creu i Sant Pau. Servicio de Microbiología	SeqCOVID-SPAIN consortium/IBV(CSIC)	Ferran Navarro, Núria Rabella, Elisenda Miró and SeqCOVID-SPAIN consortium
EPI_ISL_510277, EPI_ISL_510278, EPI_ISL_510279, EPI_ISL_510280, EPI_ISL_510281, EPI_ISL_510282, EPI_ISL_510283, EPI_ISL_510284, EPI_ISL_510285, EPI_ISL_510286, EPI_ISL_510287, EPI_ISL_510288, EPI_ISL_510289, EPI_ISL_510290, EPI_ISL_510291, EPI_ISL_510292, EPI_ISL_510293, EPI_ISL_510294, EPI_ISL_510295, EPI_ISL_510296, EPI_ISL_510297, EPI_ISL_510298, EPI_ISL_510299, EPI_ISL_510300, EPI_ISL_510301, EPI_ISL_510302, EPI_ISL_510303, EPI_ISL_510304			
see above	Hospital Clínico Universitario de Santiago de Compostela	SeqCOVID-SPAIN consortium/IBV(CSIC)	José Javier Costa Alcalde, Antonio Aguilera Guirao, Mª Luisa Pérez del Molino Bernal, Amparo Coira Nieto, Gema Barbeito Castiñeiras, Rocio Trastoy Pena and SeqCOVID-SPAIN consortium
EPI_ISL_510444	Hospital Universitario Virgen de las Nieves de Granada-SAS	SeqCOVID-SPAIN consortium/IBV(CSIC)	Mercedes Pérez Ruiz, Sara Sanbonmatsu Gámez, Irene Pedrosa Corral, José M. Navarro-Marí and SeqCOVID-SPAIN consortium
EPI_ISL_510529	School of Veterinary Medicine, Disease Control	School of Veterinary Medicine, Disease Control	Simulundu,E., Kapata,N., Mupeta,F., Kapata,P.C., Saasa,N., Changula,K., Muleya,W., Chitanga,S., Chambaro,H., Mubemba,B., Masahiro,K., Chanda,D., Mulenga,L., Fwoloshi,S., Shibemba,A.L., Kapaya,F., Zulu,P., Musonda,K., Monze,M., Sinyange,N., Liwewe,M.M., Kapin'a,M., Chipimo,P.J., Ngosa,W., Morales,A.N., Kayeyi,N., Malama,K., Tembo,J., Bates,M., Sawa,H., Takada,A., Nalubamba,K.S., Mukonka,V., Chilufya,C. and Zumla,A.
EPI_ISL_510959, EPI_ISL_510960, EPI_ISL_510961, EPI_ISL_510962, EPI_ISL_510988, EPI_ISL_511037, EPI_ISL_511040, EPI_ISL_511041, EPI_ISL_511042, EPI_ISL_511043, EPI_ISL_511044, EPI_ISL_511045, EPI_ISL_511046, EPI_ISL_511047, EPI_ISL_511048, EPI_ISL_511049, EPI_ISL_511050, EPI_ISL_511051, EPI_ISL_511052, EPI_ISL_511189			
see above	Instituto Nacional de Saude (INSA)	Instituto Nacional de Saude (INSA)	Borges et al
EPI_ISL_511210, EPI_ISL_511211, EPI_ISL_511212, EPI_ISL_511213, EPI_ISL_511214, EPI_ISL_511215, EPI_ISL_511216, EPI_ISL_511217, EPI_ISL_511218, EPI_ISL_511219, EPI_ISL_511220, EPI_ISL_511221, EPI_ISL_511257, EPI_ISL_511258, EPI_ISL_511259, EPI_ISL_511260, EPI_ISL_511261, EPI_ISL_511262, EPI_ISL_511263, EPI_ISL_511264, EPI_ISL_511265, EPI_ISL_511266, EPI_ISL_511267, EPI_ISL_511268, EPI_ISL_511269, EPI_ISL_511270, EPI_ISL_511271, EPI_ISL_511272, EPI_ISL_511273, EPI_ISL_511274, EPI_ISL_511275, EPI_ISL_511276, EPI_ISL_511277, EPI_ISL_511278, EPI_ISL_511279, EPI_ISL_511280, EPI_ISL_511281, EPI_ISL_511282, EPI_ISL_511283, EPI_ISL_511284, EPI_ISL_511285, EPI_ISL_511286, EPI_ISL_511287, EPI_ISL_511288, EPI_ISL_511289, EPI_ISL_511290, EPI_ISL_511293, EPI_ISL_511294, EPI_ISL_511295			
see above	Instituto Nacional de Saude (INSA) and Instituto Gulbenkian	Instituto Nacional de Saude (INSA) and Instituto Gulbenkian	Borges et al

	de Ciencia (IGC)	de Ciencia (IGC)	
EPI_ISL_511372, EPI_ISL_511374, EPI_ISL_511385, EPI_ISL_511389, EPI_ISL_511412, EPI_ISL_511417, EPI_ISL_511426, EPI_ISL_511427, EPI_ISL_511428, EPI_ISL_511429, EPI_ISL_511434, EPI_ISL_511441, EPI_ISL_511444, EPI_ISL_511447, EPI_ISL_511450, EPI_ISL_511452, EPI_ISL_511455, EPI_ISL_511459, EPI_ISL_511460, EPI_ISL_511462, EPI_ISL_511463, EPI_ISL_511465, EPI_ISL_511467, EPI_ISL_511471, EPI_ISL_511472, EPI_ISL_511473, EPI_ISL_511474, EPI_ISL_511477, EPI_ISL_511479, EPI_ISL_511642, EPI_ISL_511643, EPI_ISL_511645, EPI_ISL_511646			
see above	Instituto Nacional de Saude (INSA)	Instituto Nacional de Saude (INSA)	Borges et al
EPI_ISL_511893, EPI_ISL_511894, EPI_ISL_511895, EPI_ISL_511896	National Hospital of Tropical Diseases	Oxford University Clinical Research Unit, Hanoi, Vietnam	Nguyen Thi Tam, Van Dinh Trang, Nguyen Thi Hong Thuong, Vu Thi Ngoc Bich, Nguyen Thu Trang, Nguyen Thi Ngoc Diep, Le Nguyen Minh Hoa, Pham Ngoc Thach, H. Rogier van Doorn, on behalf of the OUCRU COVID-19 research group
EPI_ISL_512384, EPI_ISL_512385, EPI_ISL_512386, EPI_ISL_512387, EPI_ISL_512388	Centre for Enzyme Innovation, University of Portsmouth / Translational Research Laboratory, Portsmouth Hospitals NHS Trust	COVID-19 Genomics UK (COG-UK) Consortium	Angela Beckett,Yann Bourgeois,Garry Scarlett,Sharon Glaysher,Scott Elliott,Kelly Bicknell,Robert Impey,Allyson Lloyd,Sarah Wyllie,Ethan Butcher,Anoop Chauhan,Samuel Robson
EPI_ISL_512654	Hospital Dr. Rafael A. Calderon Guardia [San Jose/San Jose]	Inciensa, Instituto Costarricense de Investigación y Enseñanza en Nutrición y Salud	Francisco Duarte, Hebleen Porras, Claudio Soto-Garita, Estela Cordero, Adriana Godinez & Melany Calderon
EPI_ISL_512759, EPI_ISL_512760, EPI_ISL_512761	PathWest Laboratory Medicine WA	PathWest Laboratory Medicine WA Microbial Surveillance Unit	PathWest Laboratory Medicine WA Microbial Surveillance Unit
EPI_ISL_512848, EPI_ISL_512851, EPI_ISL_512852, EPI_ISL_512853, EPI_ISL_512854, EPI_ISL_512855, EPI_ISL_512856, EPI_ISL_512857, EPI_ISL_512858, EPI_ISL_512859, EPI_ISL_512860, EPI_ISL_512861, EPI_ISL_512862, EPI_ISL_512863, EPI_ISL_512864			
see above	Ramathibodi Hospital	COVID-19 Network Investigations (CONI) Alliance	Elizabeth Batty, Wasun Chantratita, Thanat Chookajorn, Stefan Fernandez, Angkana Huang, Anthony R. Jones, Khajohn Joonsalak, Chonticha Klungtong, Theerarat Kochakarn, Namfon Kotanan, Krittikorn Kumpornsin, Wudtichai Manasatienkij, Bhakbhoom Panthan, Ekawat Pasomsub, Kingkan Rakmanee, Insee Sensorn, Janjira Thaipadungpanit, Arporn Wangwiwatsin,Treewat Watthanachockchai
EPI_ISL_513298	Department of Infection Prevention and Infectious Diseases, University Hospital Regensburg	University Hospital Regensburg	Fritsch,J., Holzmann,T., Schneider-Brachert,W.
EPI_ISL_513310	Public Health, United States Air Force School of Aerospace Medicine	Public Health, United States Air Force School of Aerospace Medicine	Fries,A.C., Purves,S.M., Meyer,J.R., Javorina,A.K., Connors,B.C., Macias,E.A., Lambert,A.W., Chapleau,R.R. and Starr,C.R.
EPI_ISL_513428, EPI_ISL_513431, EPI_ISL_513433, EPI_ISL_513436, EPI_ISL_513437, EPI_ISL_513438, EPI_ISL_513439, EPI_ISL_513440, EPI_ISL_513441, EPI_ISL_513442, EPI_ISL_513443, EPI_ISL_513444, EPI_ISL_513445, EPI_ISL_513446, EPI_ISL_513447, EPI_ISL_513448, EPI_ISL_513449, EPI_ISL_513450, EPI_ISL_513451, EPI_ISL_513452, EPI_ISL_513453, EPI_ISL_513454, EPI_ISL_513455, EPI_ISL_513456, EPI_ISL_513457, EPI_ISL_513458, EPI_ISL_513459, EPI_ISL_513463, EPI_ISL_513466, EPI_ISL_513467, EPI_ISL_513468, EPI_ISL_513469, EPI_ISL_513470, EPI_ISL_513474, EPI_ISL_513475, EPI_ISL_513487, EPI_ISL_513488, EPI_ISL_513492			
see above	Maine HETL	Tewhey Lab, The Jackson Laboratory	Matluk,N., Dewey,H., Barter,M., Lynch,R., Munger,H. and Tewhey,R.
EPI_ISL_513511, EPI_ISL_513512	The National Institute of Public Health	The National Institute of Public Health and State Veterinary Institute Prague	Nagy,A;Jirincova,H;Novakova,L;Trnka,D;Vecerova,J
EPI_ISL_513773	County of Santa Clara Public Health Department	Chan-Zuckerberg Biohub	CZB Cliahub Consortium
EPI_ISL_514140	Florida Bureau of Public Health Laboratories	Florida Bureau of Public Health Laboratories	Sarah Schmedes, Jason Blanton
EPI_ISL_514308, EPI_ISL_514309, EPI_ISL_514310, EPI_ISL_514311	Israel Central Virology laboratory	Israel Central Virology laboratory	Neta Zuckerman, Efrat Dahan Bucris, Oran Erster, Ella Mendelson, Michal Mandelboim
EPI_ISL_514456, EPI_ISL_514457, EPI_ISL_514459, EPI_ISL_514460, EPI_ISL_514462, EPI_ISL_514463	Centre for Enzyme Innovation, University of Portsmouth / Translational Research Laboratory, Portsmouth Hospitals NHS Trust	COVID-19 Genomics UK (COG-UK) Consortium	Angela Beckett,Yann Bourgeois,Garry Scarlett,Sharon Glaysher,Scott Elliott,Kelly Bicknell,Robert Impey,Allyson Lloyd,Sarah Wyllie,Ethan Butcher,Anoop Chauhan,Samuel Robson
EPI_ISL_514637	M Health Fairview St. Joseph's Hospital	Minnesota Department of Health, Public Health Laboratory	Matt Plumb, Jacob Garfin, and Xiong Wang
EPI_ISL_514638	Mayo Clinic & Mayo Clinic Laboratories	Minnesota Department of Health, Public Health Laboratory	Matt Plumb, Jacob Garfin, and Xiong Wang
EPI_ISL_515300, EPI_ISL_515301, EPI_ISL_515302, EPI_ISL_515303, EPI_ISL_515304, EPI_ISL_515305, EPI_ISL_515306, EPI_ISL_515307, EPI_ISL_515308, EPI_ISL_515309, EPI_ISL_515310, EPI_ISL_515311, EPI_ISL_515312			
see above	Nevada State Public Health Laboratory	Nevada State Public Health Laboratory	Richard Tillett, Joel R. Sevinsky, Paul Hartley, Heather Kerwin, David Jackson, Subhash C. Verma, Cyprian Rossetto, Andrew Gorzalski, Chris Laverdure, Natalie Crawford, Stephanie Van Hooser, and Mark Pandori
EPI_ISL_515527	Hospital Santa Clara	Instituto Adolfo Lutz, Interdiciplinary Procedures Center, Strategic Laboratory	Claudio Tavares Sacchi, Claudia Regina Gonçalves, Erica Valessa Ramos Gomes
EPI_ISL_515894, EPI_ISL_515895	California Department of Public Health	California Department of Public Health	CDPH IDLB COVIDNet
EPI_ISL_516082	Biomedical Sciences and Public Health, Polytechnic University of Marche	Biomedical Sciences and Public Health, Polytechnic University of Marche	Bagnarelli,P., Caucci,S., Di Sante,L., Menzo,S., Alessandrini,F., Onofri,V., Turchi,C., Melchionda,F., Tagliabracci,A.
EPI_ISL_516269, EPI_ISL_516281, EPI_ISL_516282	Michigan Department of Health and Human Services, Bureau of Laboratories	Michigan Department of Health and Human Services, Bureau of Laboratories	Blankenship HM, Riner D, Soehnlen MK
EPI_ISL_516833, EPI_ISL_516835, EPI_ISL_516837, EPI_ISL_516838, EPI_ISL_516840, EPI_ISL_516841, EPI_ISL_516845, EPI_ISL_516850, EPI_ISL_516851, EPI_ISL_516852, EPI_ISL_516855, EPI_ISL_516856, EPI_ISL_516857, EPI_ISL_516859, EPI_ISL_516862, EPI_ISL_516863, EPI_ISL_516868			
see above	North West London Pathology, Imperial College Healthcare NHS Trust	Wellcome Sanger Institute for the COVID-19 Genomics UK (COG-UK) consortium	Ling Li, Paul Randell, David Muir, Frankie Bolt, Alison Holmes, James Price, Aileen Rowan, Graham Taylor, Anjna Badhan, Carolina Herrera and Alex Alderton, Roberto Amato, Sonia Goncalves, Ewan Harrison, David K. Jackson, Ian Johnston, Dominic Kwiatkowski, Cordelia Langford, John Sillitoe on behalf of the Wellcome Sanger Institute COVID-19 Surveillance Team (http://www.sanger.ac.uk/covid-team)
EPI_ISL_516922, EPI_ISL_516923	Department for Molecular Diagnostics, Centre for Medical Microbiology, Institute of Public Health of Montenegro	Charite Universitatsmedizin Berlin, Institut fur Virologie	Victor M Corman, Terry Jones, Jörn Beheim-Schwarzbach, Barbara Muehlemann, Talitha Veith, Julia Schneider, Marija Govedarica and Danijela Vujoševi, Christian Drosten
EPI_ISL_516948	King Georges Medical University	CSIR-National Botanical Research Institute	Priti Prasad, Shantanu Prakash, Kishan Sahu, Babita Singh, Suruchi Shukla, Hricha Mishra, Danish Nasar Khan , Om Prakash, MLB Bhatt, SK Barik, Mehar H.Asif,Samir V. Sawant,Amita Jain, Sumit Kr. Bag
EPI_ISL_516987	Laboratorio de Referencia Nacional de Virus Respiratorio. Centro Nacional de Salud Publica. Instituto Nacional de Salud Peru.	Laboratorio de Referencia Nacional de Biotecnología y Biología Molecular. Centro Nacional de Salud Publica. Instituto Nacional de Salud Peru.	Carlos Padilla Rojas, Karolyn Vega Chozo, Priscila Lope Pari, Omar Caceres Rey, Marco Galarza Perez, Maribel Huaringa Nuñez, Johanna Balbuena Torres, Henri Bailon Calderon, Nancy Rojas Serrano.
EPI_ISL_518868, EPI_ISL_518874, EPI_ISL_518875, EPI_ISL_518877, EPI_ISL_518879, EPI_ISL_518880, EPI_ISL_518881, EPI_ISL_518892, EPI_ISL_521153	Mayo Clinic & Mayo Clinic Laboratories	Minnesota Department of Health, Public Health Laboratory	Matt Plumb, Jacob Garfin, and Xiong Wang
EPI_ISL_521885	Victorian Infectious Diseases Reference Laboratory (VIDRL)	VIDRL and MDU-PHL	Caly L., Seemann T., Sait, M., Schultz M., Druce J., Sherry, N.
EPI_ISL_522566, EPI_ISL_522737, EPI_ISL_522742, EPI_ISL_522743	Royal Hobart Hospital Microbiology Department	MDU-PHL	Cooley L., van Haeften R., Seemann T., Sait M., Schultz, M.B., Sherry N.
EPI_ISL_523332, EPI_ISL_523470, EPI_ISL_523525	Dutch COVID-19 response team	Erasmus Medical Center	Bas Oude Munnink, David Nieuwenhuijse, Reina Sikkema, Claudia Schapendonk, Irina Chestakova, Anne van der Linden, Theo Bestebroer, Stefan van Nieuwkoop, Mark Pronk, Pascal Lexmond, Corien Swaan, Manon Haverkate, Madelief Mollers, Mart Stein, Sandra Kengne Kamga Mobou, Jeroen van Kampen, Jolanda Voermans, Aura Timen, Corine GeurtsvanKessel, Annemiek van der Eijk, Richard Molenkamp, Marion Koopmans, on behalf of the Dutch national COVID-19 response team.
EPI_ISL_523933, EPI_ISL_523939	Center of Medical Microbiology, Virology, and Hospital	Center of Medical Microbiology, Virology, and Hospital	Maximilian Damagnez, Alexander Dilthey, Torsten Houwaart, Malte Kohns Vasconcelos, Marek Korencak, Jessica Nicolai, Klaus Pfeffer, Hendrik Streeck,

	Hygiene, University of Duesseldorf	Hygiene, University of Duesseldorf	Daniel Strelow, Jörg Timm, Andreas Walker, Tobias Wienemann
EPI_ISL_523954, EPI_ISL_523979	Laboratorio de Referencia Nacional de Virus Respiratorio. Centro Nacional de Salud Publica. Instituto Nacional de Salud Peru.	Laboratorio de Referencia Nacional de Biotecnología y Biología Molecular. Centro Nacional de Salud Publica. Instituto Nacional de Salud Peru.	Carlos Padilla Rojas, Karolyn Vega Chozo, Priscila Lope Pari, Omar Caceres Rey, Marco Galarza Perez, Maribel Huaringa Nuñez, Johanna Balbuena Torres, Henri Bailon Calderon, Nancy Rojas Serrano.
EPI_ISL_524060, EPI_ISL_524061, EPI_ISL_524062, EPI_ISL_524063, EPI_ISL_524064, EPI_ISL_524065, EPI_ISL_524066	Texas Department of State Health Services	Texas Department of State Health Services	Rashmi Tuladhar, Bonnie Oh, Cara Akrout, Jenny Zhang, Maliha Rahman, Anita Pokharel, Myong Koag, Chun Wang, Rachel Lee, Grace Kubin
EPI_ISL_524462	Hospital Metropolitano	Instituto Adolfo Lutz, Interdiciplinary Procedures Center, Strategic Laboratory	Claudio Tavares Sacchi, Claudia Regina Gonçalves, Erica Valessa Ramos Gomes
EPI_ISL_524471	Laboratorio de Referencia Nacional de Virus Respiratorio. Centro Nacional de Salud Publica. Instituto Nacional de Salud Peru.	Laboratorio de Referencia Nacional de Biotecnología y Biología Molecular. Centro Nacional de Salud Publica. Instituto Nacional de Salud Peru.	Carlos Padilla Rojas, Karolyn Vega Chozo, Priscila Lope Pari, Omar Caceres Rey, Marco Galarza Perez, Maribel Huaringa Nuñez, Johanna Balbuena Torres, Henri Bailon Calderon, Nancy Rojas Serrano.
EPI_ISL_524627, EPI_ISL_524638, EPI_ISL_524645, EPI_ISL_524651, EPI_ISL_524652, EPI_ISL_524653, EPI_ISL_524654, EPI_ISL_524658, EPI_ISL_524660, EPI_ISL_524661, EPI_ISL_524662, EPI_ISL_524663, EPI_ISL_524668, EPI_ISL_524669, EPI_ISL_524670, EPI_ISL_524671, EPI_ISL_524673, EPI_ISL_524676			
see above	North West London Pathology, Imperial College Healthcare NHS Trust	Wellcome Sanger Institute for the COVID-19 Genomics UK (COG-UK) consortium	Ling Li, Paul Randell, David Muir, Frankie Bolt, Alison Holmes, James Price, Aileen Rowan, Graham Taylor, Anjna Badhan, Carolina Herrera and Alex Alderton, Roberto Amato, Sonia Goncalves, Ewan Harrison, David K. Jackson, Ian Johnston, Dominic Kwiatkowski, Cordelia Langford, John Sillitoe on behalf of the Wellcome Sanger Institute COVID-19 Surveillance Team (http://www.sanger.ac.uk/covid-team)
EPI_ISL_525537, EPI_ISL_525538	CNR Virus des Infections Respiratoires - France SUD	CNR Virus des Infections Respiratoires - France SUD	Antonin Bal, Gregory Destras, Gwendolyne Burfin, Solenne Brun, Alexandre Gaymard, Maude Bouscambert-Duchamp, Florence Morfin-Sherpa, Martine Valette, Bruno Lina, Laurence Josset
EPI_ISL_525539	Centre Hospitalier de Bourg en Bresse	CNR Virus des Infections Respiratoires - France SUD	Antonin Bal, Gregory Destras, Gwendolyne Burfin, Solenne Brun, Alexandre Gaymard, Maude Bouscambert-Duchamp, Florence Morfin-Sherpa, Martine Valette, Bruno Lina, Laurence Josset
EPI_ISL_525540, EPI_ISL_525541, EPI_ISL_525542	CNR Virus des Infections Respiratoires - France SUD	CNR Virus des Infections Respiratoires - France SUD	Antonin Bal, Gregory Destras, Gwendolyne Burfin, Solenne Brun, Alexandre Gaymard, Maude Bouscambert-Duchamp, Florence Morfin-Sherpa, Martine Valette, Bruno Lina, Laurence Josset
EPI_ISL_525553, EPI_ISL_525556, EPI_ISL_525557, EPI_ISL_525570	Istituto Zooprofilattico Sperimentale Puglia e Basilicata; Dipartimento di Bioscienze, Biotecnologie e Biofarmaceutica dell'Università degli Studi di Bari "A.Moro"; Istituto di Biomembrane. Bioenergetica e Biotecnologie Molecolari del Consiglio Nazionale delle Ricerche di Bari	Beaconlab (Bioinformatics, Evolution and Comparative Genomics lab), Dept of Biosciences, University on Milan	Parisi A.,Pesole G., Manzari C., Chiara M
EPI_ISL_525614, EPI_ISL_525615, EPI_ISL_525616, EPI_ISL_525617, EPI_ISL_525618, EPI_ISL_525619, EPI_ISL_525620, EPI_ISL_525621, EPI_ISL_525622	Wadsworth Center, New York State Department of Health	Wadsworth Center, New York State Department of Health	Kirsten St. George, Daryl M. Lamson, Sara Griesemer, Jonathan Plitnick, Navjot Singh, Matthew D. Shudt, Erica Lasek-Nesselquist
EPI_ISL_525764, EPI_ISL_525765, EPI_ISL_525766	Texas Department of State Health Services	Texas Department of State Health Services	Jenny Zhang, Rashmi Tuladhar, Bonnie Oh, Maliha Rahman, Anita Pokharel, Myong Koag, Chun Wang, Rachel Lee, Grace Kubin
EPI_ISL_526558, EPI_ISL_526581	Florida Bureau of Public Health Laboratories	Florida Bureau of Public Health Laboratories	Sarah Schmedes, Jason Blanton
EPI_ISL_526797, EPI_ISL_526798, EPI_ISL_526799, EPI_ISL_526801, EPI_ISL_526802, EPI_ISL_526803, EPI_ISL_526804, EPI_ISL_526805, EPI_ISL_526806, EPI_ISL_526807	Virginia DCLS	Virginia DCLS	Virginia DCLS
EPI_ISL_526942, EPI_ISL_526943, EPI_ISL_526944, EPI_ISL_526945, EPI_ISL_526946, EPI_ISL_526947, EPI_ISL_526948	Faroese National Reference Laboratory for Fish and Animal Diseases	Faroese National Reference Laboratory for Fish and Animal Diseases	Maria Marjunardóttir Dahl, Petra Elisabeth Petersen, Debes Hammershaimb Christiansen
EPI_ISL_527008	Area of Virology, Serology and Virology Division (SAViD), New South Wales Health Pathology Randwick	Area of Virology, Serology and Virology Division (SAViD), New South Wales Health Pathology Randwick	Rawlinson, W.
EPI_ISL_527759	Clinica Biblica	Inciensa, Instituto Costarricense de Investigación y Enseñanza en Nutrición y Salud	Francisco Duarte, Hebleen Porras, Claudio Soto-Garita, Estela Cordero, Adriana Godinez & Melany Calderon
EPI_ISL_528095, EPI_ISL_528096, EPI_ISL_528097, EPI_ISL_528098, EPI_ISL_528099, EPI_ISL_528100, EPI_ISL_528101, EPI_ISL_528102, EPI_ISL_528103, EPI_ISL_528104, EPI_ISL_528105, EPI_ISL_528106, EPI_ISL_528109, EPI_ISL_528110, EPI_ISL_528111, EPI_ISL_528112, EPI_ISL_528114, EPI_ISL_528115, EPI_ISL_528116, EPI_ISL_528117, EPI_ISL_528118, EPI_ISL_528119, EPI_ISL_528120, EPI_ISL_528121, EPI_ISL_528122, EPI_ISL_528123, EPI_ISL_528124, EPI_ISL_528125, EPI_ISL_528126, EPI_ISL_528127, EPI_ISL_528128, EPI_ISL_528129, EPI_ISL_528130, EPI_ISL_528131, EPI_ISL_528132, EPI_ISL_528133, EPI_ISL_528134, EPI_ISL_528135, EPI_ISL_528136, EPI_ISL_528137, EPI_ISL_528138, EPI_ISL_528139, EPI_ISL_528140, EPI_ISL_528141, EPI_ISL_528142, EPI_ISL_528143, EPI_ISL_528144, EPI_ISL_528145, EPI_ISL_528146, EPI_ISL_528147, EPI_ISL_528148, EPI_ISL_528149, EPI_ISL_528150, EPI_ISL_528151, EPI_ISL_528152, EPI_ISL_528153, EPI_ISL_528154, EPI_ISL_528155, EPI_ISL_528156, EPI_ISL_528157, EPI_ISL_528158, EPI_ISL_528159, EPI_ISL_528160, EPI_ISL_528161, EPI_ISL_528162, EPI_ISL_528163, EPI_ISL_528164, EPI_ISL_528165, EPI_ISL_528166, EPI_ISL_528167, EPI_ISL_528168, EPI_ISL_528169, EPI_ISL_528170, EPI_ISL_528171, EPI_ISL_528172, EPI_ISL_528173, EPI_ISL_528174, EPI_ISL_528175, EPI_ISL_528176, EPI_ISL_528177, EPI_ISL_528178, EPI_ISL_528179, EPI_ISL_528180, EPI_ISL_528181, EPI_ISL_528182, EPI_ISL_528183, EPI_ISL_528184, EPI_ISL_528185, EPI_ISL_528186, EPI_ISL_528187, EPI_ISL_528188, EPI_ISL_528189, EPI_ISL_528190, EPI_ISL_528191, EPI_ISL_528192, EPI_ISL_528193, EPI_ISL_528194, EPI_ISL_528195, EPI_ISL_528196, EPI_ISL_528197, EPI_ISL_528198, EPI_ISL_528199, EPI_ISL_528200, EPI_ISL_528201, EPI_ISL_528202, EPI_ISL_528203, EPI_ISL_528204, EPI_ISL_528205, EPI_ISL_528206, EPI_ISL_528207, EPI_ISL_528208, EPI_ISL_528209, EPI_ISL_528210, EPI_ISL_528211, EPI_ISL_528212, EPI_ISL_528213, EPI_ISL_528214, EPI_ISL_528215, EPI_ISL_528216, EPI_ISL_528217, EPI_ISL_528218, EPI_ISL_528219, EPI_ISL_528377, EPI_ISL_528378			
see above	University Hospital Basel, Clinical Virology	University Hospital Basel, Clinical Bacteriology	Madlen Stange, Alfredo Mari, Tim Roloff, Helena MB Seth-Smith, Michael Schweitzer, Myrta Brunner, Karoline Leuzinger, Kirstine K. Soegaard, Alexander Gensch, Sarah Tschudin-Sutter, Simon Fuchs, Julia Bielicki, Hans Pargger, Martin Siegemund, Christian Nickel, Roland Bingisser, Michael Osthoff, Stefano Bassetti, Rita Schneider-Sliwa, Manuel Battegay, Hans Hirsch, Adrian Egli
EPI_ISL_528637, EPI_ISL_528638	LVM/UFRJ	Bioinformatics Laboratory / LNCC	Gustavo D. P. Silva; M. Romário M. de Souza; Bruno B. Bezerra; Lucio A. Caldas; Fabio Limonte; Elena Cobos; Sharton V. A. Coelho; Luiz Almeida; Luiza Higga; Isadora A. Correa; Diana Marianni; Luciana B. Arruda; Marcelo Bozza; Orlando Ferreira; Wanderley de Souza; Ana Teresa R. Vasconcelos; Terezinha M. Castineiras; Amilcar Tanuri; Luciana J. Costa
EPI_ISL_528919	Ospedale Civile S. Liberatore-Atri	Istituto Zooprofilattico Sperimentale dell'Abruzzo e Molise "G.Caporale"	Lorusso A, Marcacci M, Di Domenico M, Curini V, Ancora M, Cammà C, Rinaldi A, Mangone I, Di Pasquale A, Puglia I, Savini G.
EPI_ISL_528920, EPI_ISL_528921	Presidio Ospedaliero "Santo Spirito"-Pescara	Istituto Zooprofilattico Sperimentale dell'Abruzzo e Molise "G.Caporale"	Lorusso A, Marcacci M, Di Domenico M, Curini V, Ancora M, Cammà C, Rinaldi A, Mangone I, Di Pasquale A, Puglia I, Savini G.
EPI_ISL_528922	Ospedale "Giuseppe Mazzini"-Teramo	Istituto Zooprofilattico Sperimentale dell'Abruzzo e Molise "G.Caporale"	Lorusso A, Marcacci M, Di Domenico M, Curini V, Ancora M, Cammà C, Rinaldi A, Mangone I, Di Pasquale A, Puglia I, Savini G.
EPI_ISL_528923	Ospedale Civile S. Liberatore-Atri	Istituto Zooprofilattico Sperimentale dell'Abruzzo e Molise "G.Caporale"	Lorusso A, Marcacci M, Di Domenico M, Curini V, Ancora M, Cammà C, Rinaldi A, Mangone I, Di Pasquale A, Puglia I, Savini G.
EPI_ISL_528924	Ospedale "Giuseppe Mazzini"-Teramo	Istituto Zooprofilattico Sperimentale dell'Abruzzo e Molise "G.Caporale"	Lorusso A, Marcacci M, Di Domenico M, Curini V, Ancora M, Cammà C, Rinaldi A, Mangone I, Di Pasquale A, Puglia I, Savini G.
EPI_ISL_528925	Ospedale Regionale San Salvatore-L'Aquila	Istituto Zooprofilattico Sperimentale dell'Abruzzo e Molise "G.Caporale"	Lorusso A, Marcacci M, Di Domenico M, Curini V, Ancora M, Cammà C, Rinaldi A, Mangone I, Di Pasquale A, Puglia I, Savini G.

EPI_ISL_529148	Democritus University of Thrace, Department of Medicine	Democritus University of Thrace, Department of Medicine	Kassela,K., Dovrolis,N., Bampali,M., Gatzidou,E., Froukala,E., Stavropoulou,A., Veletza,S., Tsakris,A., Spanakis,N., Karakasiliotis,I.
EPI_ISL_529172	South Carolina Department of Health and Environmental Control	South Carolina Department of Health and Environmental Control	Haley V. Flores
EPI_ISL_529202	Utah Public Health Laboratory	Utah Public Health Laboratory	Erin Young, Kelly Oakeson
EPI_ISL_529962	Universitas Airlangga Hospital	Institute of Tropical Disease, Universitas Airlangga	Jezzy R Dewantari, Rima R Prasetya, Krisnoadi Rahardjo, Aldise M Nastri, Nasronudin, Gatot Soegiarto, Laksmi Wulandari, Retno A Setyoningrum, Resti Yudhawati, Yohko K Shimizu, Mitsuhiro Nishimura, Yasuko Mori, Soetjipto, Kazufumi Shimizu, Maria I Lusida
EPI_ISL_530109, EPI_ISL_530110, EPI_ISL_530112, EPI_ISL_530113	Hospital Universitario Ramón y Cajal	Hospital Universitario La Paz	Raúl Recio, Sara González, Elias Dahdouh, Fernando Lázaro, Esther Viedma, Natalia Stella, Julio García, Juan Carlos Galán, Rafael Cantón, Mª Dolores Folgueira, Rafael Delgado, Jesús Mingorance
EPI_ISL_530347, EPI_ISL_530350	The National Institute of Public Health	State Veterinary Institute Prague	Nagy,A;Jirincova,H;Novakova,L;Trnka,D;Vecerova,J
EPI_ISL_534722	Respiratory Virus Unit, Microbiology Services Colindale, Public Health England	Respiratory Virus Unit, Microbiology Services Colindale, Public Health England	PHE Covid Sequencing Team
EPI_ISL_534891	Oxford Viromics, NDM, University of Oxford; Oxford University Hospitals; Basingstoke and North Hampshire Hospital	COVID-19 Genomics UK (COG-UK) Consortium	Tanya Golubchik, David Bonsall, George Macintyre, Amy Trebes, Mariateresa de Cesare, Catrin Moore, Alex Mobbs, Anita Justice, Robert Shaw, Monique Andersson, Timothy Peto, Emma Wise, Nathan Moore, Jessica Lynch, Nick Cortes, Matilde Mori, Stephen Kidd, David Buck, John Todd, Christophe Fraser
EPI_ISL_535793	Hôpital de Saint-Eustache	Laboratoire de santé publique du Québec	Sandrine Moreira, Ioannis Ragoussis, Guillaume Bourque, Jesse Shapiro, Mark Lathrop and Michel Roger
EPI_ISL_535795	Hôpital Notre-Dame	Laboratoire de santé publique du Québec	Sandrine Moreira, Ioannis Ragoussis, Guillaume Bourque, Jesse Shapiro, Mark Lathrop and Michel Roger
EPI_ISL_535796	Hôpital Charles-LeMoyne	Laboratoire de santé publique du Québec	Sandrine Moreira, Ioannis Ragoussis, Guillaume Bourque, Jesse Shapiro, Mark Lathrop and Michel Roger
EPI_ISL_535797	Hôpital Honoré-Mercier	Laboratoire de santé publique du Québec	Sandrine Moreira, Ioannis Ragoussis, Guillaume Bourque, Jesse Shapiro, Mark Lathrop and Michel Roger
EPI_ISL_535798	Hôpital régional de Saint-Jérôme	Laboratoire de santé publique du Québec	Sandrine Moreira, Ioannis Ragoussis, Guillaume Bourque, Jesse Shapiro, Mark Lathrop and Michel Roger
EPI_ISL_535799, EPI_ISL_535800	CSSS Haut-Richelieu/Rouville (Hôpital)	Laboratoire de santé publique du Québec	Sandrine Moreira, Ioannis Ragoussis, Guillaume Bourque, Jesse Shapiro, Mark Lathrop and Michel Roger
EPI_ISL_535801	CHU Sainte-Justine	Laboratoire de santé publique du Québec	Sandrine Moreira, Ioannis Ragoussis, Guillaume Bourque, Jesse Shapiro, Mark Lathrop and Michel Roger
EPI_ISL_535802, EPI_ISL_535806	CHUM - Microbiologie - Hôpital Saint-Luc	Laboratoire de santé publique du Québec	Sandrine Moreira, Ioannis Ragoussis, Guillaume Bourque, Jesse Shapiro, Mark Lathrop and Michel Roger
EPI_ISL_535807	Centre hospitalier régional du Grand Portage	Laboratoire de santé publique du Québec	Sandrine Moreira, Ioannis Ragoussis, Guillaume Bourque, Jesse Shapiro, Mark Lathrop and Michel Roger
EPI_ISL_535809	Hôpital de Hull	Laboratoire de santé publique du Québec	Sandrine Moreira, Ioannis Ragoussis, Guillaume Bourque, Jesse Shapiro, Mark Lathrop and Michel Roger
EPI_ISL_535810	Hôpital Sainte-Croix	Laboratoire de santé publique du Québec	Sandrine Moreira, Ioannis Ragoussis, Guillaume Bourque, Jesse Shapiro, Mark Lathrop and Michel Roger
EPI_ISL_535811	CUSM-Site Glen-LAB Microbiologie	Laboratoire de santé publique du Québec	Sandrine Moreira, Ioannis Ragoussis, Guillaume Bourque, Jesse Shapiro, Mark Lathrop and Michel Roger
EPI_ISL_535812	Hôpital du Sacré-Coeur de Montréal	Laboratoire de santé publique du Québec	Sandrine Moreira, Ioannis Ragoussis, Guillaume Bourque, Jesse Shapiro, Mark Lathrop and Michel Roger
EPI_ISL_535813	CSSS Haut-Richelieu/Rouville (Hôpital)	Laboratoire de santé publique du Québec	Sandrine Moreira, Ioannis Ragoussis, Guillaume Bourque, Jesse Shapiro, Mark Lathrop and Michel Roger
EPI_ISL_535814	CHUM - Microbiologie - Hôpital Saint-Luc	Laboratoire de santé publique du Québec	Sandrine Moreira, Ioannis Ragoussis, Guillaume Bourque, Jesse Shapiro, Mark Lathrop and Michel Roger
EPI_ISL_535815	CSSS Haut-Richelieu/Rouville (Hôpital)	Laboratoire de santé publique du Québec	Sandrine Moreira, Ioannis Ragoussis, Guillaume Bourque, Jesse Shapiro, Mark Lathrop and Michel Roger
EPI_ISL_535817	Hôpital Maisonneuve-Rosemont	Laboratoire de santé publique du Québec	Sandrine Moreira, Ioannis Ragoussis, Guillaume Bourque, Jesse Shapiro, Mark Lathrop and Michel Roger
EPI_ISL_535818	Hôpital Pierre-Boucher	Laboratoire de santé publique du Québec	Sandrine Moreira, Ioannis Ragoussis, Guillaume Bourque, Jesse Shapiro, Mark Lathrop and Michel Roger
EPI_ISL_535819	Hôpital Charles-LeMoyne	Laboratoire de santé publique du Québec	Sandrine Moreira, Ioannis Ragoussis, Guillaume Bourque, Jesse Shapiro, Mark Lathrop and Michel Roger
EPI_ISL_535820, EPI_ISL_535821	Hôtel-Dieu de Lévis	Laboratoire de santé publique du Québec	Sandrine Moreira, Ioannis Ragoussis, Guillaume Bourque, Jesse Shapiro, Mark Lathrop and Michel Roger
EPI_ISL_535822, EPI_ISL_535824, EPI_ISL_535825	CHUL-LABO MULTI / MICRO	Laboratoire de santé publique du Québec	Sandrine Moreira, Ioannis Ragoussis, Guillaume Bourque, Jesse Shapiro, Mark Lathrop and Michel Roger
EPI_ISL_535826, EPI_ISL_535827	CHUM - Microbiologie - Hôpital Saint-Luc	Laboratoire de santé publique du Québec	Sandrine Moreira, Ioannis Ragoussis, Guillaume Bourque, Jesse Shapiro, Mark Lathrop and Michel Roger
EPI_ISL_535828	CHUL-LABO MULTI / MICRO	Laboratoire de santé publique du Québec	Sandrine Moreira, Ioannis Ragoussis, Guillaume Bourque, Jesse Shapiro, Mark Lathrop and Michel Roger
EPI_ISL_535829	Hôpital de Chicoutimi	Laboratoire de santé publique du Québec	Sandrine Moreira, Ioannis Ragoussis, Guillaume Bourque, Jesse Shapiro, Mark Lathrop and Michel Roger
EPI_ISL_535830	CHUM - Microbiologie - Hôpital Saint-Luc	Laboratoire de santé publique du Québec	Sandrine Moreira, Ioannis Ragoussis, Guillaume Bourque, Jesse Shapiro, Mark Lathrop and Michel Roger
EPI_ISL_535831	Hôpital Maisonneuve-Rosemont	Laboratoire de santé publique du Québec	Sandrine Moreira, Ioannis Ragoussis, Guillaume Bourque, Jesse Shapiro, Mark Lathrop and Michel Roger
EPI_ISL_535832	CHUM - Microbiologie - Hôpital Saint-Luc	Laboratoire de santé publique du Québec	Sandrine Moreira, Ioannis Ragoussis, Guillaume Bourque, Jesse Shapiro, Mark Lathrop and Michel Roger
EPI_ISL_535833, EPI_ISL_535834, EPI_ISL_535835, EPI_ISL_535836	Hôtel-Dieu de Lévis	Laboratoire de santé publique du Québec	Sandrine Moreira, Ioannis Ragoussis, Guillaume Bourque, Jesse Shapiro, Mark Lathrop and Michel Roger
EPI_ISL_535837, EPI_ISL_535838	Hôpital régional de Saint-Jérôme	Laboratoire de santé publique du Québec	Sandrine Moreira, Ioannis Ragoussis, Guillaume Bourque, Jesse Shapiro, Mark Lathrop and Michel Roger
EPI_ISL_535839	Hôpital Cité de la Santé	Laboratoire de santé publique du Québec	Sandrine Moreira, Ioannis Ragoussis, Guillaume Bourque, Jesse Shapiro, Mark Lathrop and Michel Roger
EPI_ISL_535840	Hôpital Pierre-Boucher	Laboratoire de santé publique du Québec	Sandrine Moreira, Ioannis Ragoussis, Guillaume Bourque, Jesse Shapiro, Mark Lathrop and Michel Roger
EPI_ISL_535841	CSSS Haut-Richelieu/Rouville (Hôpital)	Laboratoire de santé publique du Québec	Sandrine Moreira, Ioannis Ragoussis, Guillaume Bourque, Jesse Shapiro, Mark Lathrop and Michel Roger
EPI_ISL_535842, EPI_ISL_535843, EPI_ISL_535844	Centre Hospitalier Régional de Lanaudière	Laboratoire de santé publique du Québec	Sandrine Moreira, Ioannis Ragoussis, Guillaume Bourque, Jesse Shapiro, Mark Lathrop and Michel Roger
EPI_ISL_535845	Centre de SSS D'Arthabaska-et-de-l'érable - Hôtel-Dieu	Laboratoire de santé publique du Québec	Sandrine Moreira, Ioannis Ragoussis, Guillaume Bourque, Jesse Shapiro, Mark Lathrop and Michel Roger
EPI_ISL_535846	CSSS Haut-Richelieu/Rouville (Hôpital)	Laboratoire de santé publique du Québec	Sandrine Moreira, Ioannis Ragoussis, Guillaume Bourque, Jesse Shapiro, Mark Lathrop and Michel Roger
EPI_ISL_535847	Hôpital du Suroît	Laboratoire de santé publique du Québec	Sandrine Moreira, Ioannis Ragoussis, Guillaume Bourque, Jesse Shapiro, Mark Lathrop and Michel Roger
EPI_ISL_535848	Hôpital Charles-LeMoyne	Laboratoire de santé publique du Québec	Sandrine Moreira, Ioannis Ragoussis, Guillaume Bourque, Jesse Shapiro, Mark Lathrop and Michel Roger
EPI_ISL_535849	Centre de SSS de Trois-Rivières	Laboratoire de santé publique du Québec	Sandrine Moreira, Ioannis Ragoussis, Guillaume Bourque, Jesse Shapiro, Mark Lathrop and Michel Roger
EPI_ISL_535850	Hôpital Pierre-Le Gardeur	Laboratoire de santé publique du Québec	Sandrine Moreira, Ioannis Ragoussis, Guillaume Bourque, Jesse Shapiro, Mark Lathrop and Michel Roger
EPI_ISL_535851	Hôpital Charles-LeMoyne	Laboratoire de santé publique du Québec	Sandrine Moreira, Ioannis Ragoussis, Guillaume Bourque, Jesse Shapiro, Mark Lathrop and Michel Roger
EPI_ISL_535852	Hôpital général du Lakeshore	Laboratoire de santé publique du Québec	Sandrine Moreira, Ioannis Ragoussis, Guillaume Bourque, Jesse Shapiro, Mark Lathrop and Michel Roger
EPI_ISL_535853	Hôpital Charles-LeMoyne	Laboratoire de santé publique du Québec	Sandrine Moreira, Ioannis Ragoussis, Guillaume Bourque, Jesse Shapiro, Mark Lathrop and Michel Roger
EPI_ISL_535854, EPI_ISL_535855	Centre de SSS de la Haute-Yamaska	Laboratoire de santé publique du Québec	Sandrine Moreira, Ioannis Ragoussis, Guillaume Bourque, Jesse Shapiro, Mark Lathrop and Michel Roger
EPI_ISL_535856	Hôpital de Verdun	Laboratoire de santé publique du Québec	Sandrine Moreira, Ioannis Ragoussis, Guillaume Bourque, Jesse Shapiro, Mark Lathrop and Michel Roger
EPI_ISL_535857	Hôpital Pierre-Boucher	Laboratoire de santé publique du Québec	Sandrine Moreira, Ioannis Ragoussis, Guillaume Bourque, Jesse Shapiro, Mark Lathrop and Michel Roger

EPI_ISL_535858	Hôpital de Gatineau	Laboratoire de santé publique du Québec	Sandrine Moreira, Ioannis Ragoussis, Guillaume Bourque, Jesse Shapiro, Mark Lathrop and Michel Roger
EPI_ISL_535859, EPI_ISL_535860	Hôpital Sainte-Croix	Laboratoire de santé publique du Québec	Sandrine Moreira, Ioannis Ragoussis, Guillaume Bourque, Jesse Shapiro, Mark Lathrop and Michel Roger
EPI_ISL_535861, EPI_ISL_535862	Hôpital Pierre-Le Gardeur	Laboratoire de santé publique du Québec	Sandrine Moreira, Ioannis Ragoussis, Guillaume Bourque, Jesse Shapiro, Mark Lathrop and Michel Roger
EPI_ISL_535870	Centre Hospitalier Régional de Lanaudière	Laboratoire de santé publique du Québec	Sandrine Moreira, Ioannis Ragoussis, Guillaume Bourque, Jesse Shapiro, Mark Lathrop and Michel Roger
EPI_ISL_535872	Centre hospitalier régional du Grand Portage	Laboratoire de santé publique du Québec	Sandrine Moreira, Ioannis Ragoussis, Guillaume Bourque, Jesse Shapiro, Mark Lathrop and Michel Roger
EPI_ISL_536527, EPI_ISL_536531, EPI_ISL_536533, EPI_ISL_536534, EPI_ISL_536535, EPI_ISL_536536, EPI_ISL_536537, EPI_ISL_536538, EPI_ISL_536539, EPI_ISL_536540, EPI_ISL_536541, EPI_ISL_536543, EPI_ISL_536544, EPI_ISL_536546, EPI_ISL_536551, EPI_ISL_536554, EPI_ISL_536560, EPI_ISL_536565			
see above	Instituto Nacional de Salud	Laboratorio de Infecciones Respiratorias Agudas	Eduardo Juscamayta Lopez, David Tarazona, Faviola Valdivia Guerrero, Nancy Rojas Serrano, Dennis Carhuaricra, Lenin Maturrano Hernandez, Ronnie Gavilan Chavez
EPI_ISL_537507, EPI_ISL_537568, EPI_ISL_537569, EPI_ISL_537571, EPI_ISL_537572, EPI_ISL_537581, EPI_ISL_537582, EPI_ISL_537586, EPI_ISL_537587, EPI_ISL_537595, EPI_ISL_537596, EPI_ISL_537598, EPI_ISL_537599, EPI_ISL_537603			
see above	UCLA Pathology Clinical Microbiology Lab	Kruglyak Lab	Guo et al.
EPI_ISL_537608	Servicio de Microbiología. Consorcio Hospital General Universitario de Valencia	SeqCOVID-SPAIN consortium/IBV(CSIC)	Paula Ruiz-Hueso, Mariana Reyes-Prieto, Vicente Soriano Chirona, Ivan Ansari, David Navarro, Maria Alma Bracho, Griselda De Marco, Beatriz Beamud, Lidia Ruiz Roldan, Marta Pla Diaz, Neris Garcia-Gonzalez, Inma Galán Vendrell, Sandra Carbo, Loreto Ferrús Abad, Llúcia Martínez-Priego, Giuseppe D'Auria, Fernando Gonzalez-Candelas and SeqCOVID-SPAIN consortium
EPI_ISL_537788, EPI_ISL_537789	Hospital Universitario de Gran Canaria Dr. Negrín	SeqCOVID-SPAIN consortium/IBV(CSIC)	M. Carmen Pérez González, Francisco J. Chamizo López, Ana Bordes Benítez and SeqCOVID-SPAIN consortium
EPI_ISL_537874, EPI_ISL_537875, EPI_ISL_537876, EPI_ISL_537877, EPI_ISL_537878, EPI_ISL_537880, EPI_ISL_537881, EPI_ISL_537882, EPI_ISL_537883, EPI_ISL_537884, EPI_ISL_537885, EPI_ISL_537886, EPI_ISL_537887, EPI_ISL_537888, EPI_ISL_537889, EPI_ISL_537890, EPI_ISL_537891, EPI_ISL_537892, EPI_ISL_537893, EPI_ISL_537894, EPI_ISL_537895, EPI_ISL_537896, EPI_ISL_537897, EPI_ISL_537898, EPI_ISL_537899, EPI_ISL_537900, EPI_ISL_537901, EPI_ISL_537902, EPI_ISL_537903, EPI_ISL_537905, EPI_ISL_537906, EPI_ISL_537907, EPI_ISL_537908, EPI_ISL_537909, EPI_ISL_537910, EPI_ISL_537911, EPI_ISL_537912, EPI_ISL_537913, EPI_ISL_537914, EPI_ISL_537915, EPI_ISL_537916, EPI_ISL_537917, EPI_ISL_537920, EPI_ISL_537928, EPI_ISL_537933, EPI_ISL_537937, EPI_ISL_537938, EPI_ISL_537939, EPI_ISL_537940, EPI_ISL_537941, EPI_ISL_537942, EPI_ISL_537943, EPI_ISL_537944, EPI_ISL_537945			
see above	Hospital Universitario Araba. Vitoria-Gasteiz	SeqCOVID-SPAIN consortium/IBV(CSIC)	Silvia Hernáez Crespo, Carmen Gómez González, Amaia Aguirre Quiñonero, Marina Fernández Torres, Ma Rosario Almela Ferrer, Ma Concepción Lecaroz Agara, Andrés Canut Blasco and SeqCOVID-SPAIN consortium
EPI_ISL_537985	Servicio de Microbiología. Hospital General Universitario de Castellón	SeqCOVID-SPAIN consortium/IBV(CSIC)	Rosario Moreno, María Dolores Tirado and SeqCOVID-SPAIN consortium
EPI_ISL_538032, EPI_ISL_538033, EPI_ISL_538034, EPI_ISL_538035, EPI_ISL_538036, EPI_ISL_538037, EPI_ISL_538038, EPI_ISL_538039, EPI_ISL_538040, EPI_ISL_538041, EPI_ISL_538042, EPI_ISL_538043, EPI_ISL_538044, EPI_ISL_538045, EPI_ISL_538046, EPI_ISL_538047, EPI_ISL_538048, EPI_ISL_538049, EPI_ISL_538050, EPI_ISL_538051, EPI_ISL_538052, EPI_ISL_538053, EPI_ISL_538054			
see above	Hospital Universitari i Politècnic La Fe de València	SeqCOVID-SPAIN consortium/IBV(CSIC)	María Dolores Gómez Ruiz, Eva González Barbera, Ana Gil Brusola, Salvador Giner Almaraz, José Luis López Hontangas and SeqCOVID-SPAIN consortium
EPI_ISL_538118	Hospital Universitario Araba. Vitoria-Gasteiz	SeqCOVID-SPAIN consortium/IBV(CSIC)	Silvia Hernáez Crespo, Carmen Gómez González, Amaia Aguirre Quiñonero, Marina Fernández Torres, Mª Rosario Almela Ferrer, Mª Concepción Lecaroz Agara, Andrés Canut Blasco and SeqCOVID-SPAIN consortium
EPI_ISL_538437, EPI_ISL_538438, EPI_ISL_538439, EPI_ISL_538440, EPI_ISL_538447, EPI_ISL_538448	Department of Laboratory Medicine, Tan Tock Seng Hospital	Department of Laboratory Medicine, Tan Tock Seng Hospital	Chen YYC, Zair X, Lim JX, Li C, Tang WY, Maurer-Stroh S, Barkham TMS, Nagarajan N, Sessions OM
EPI_ISL_538540, EPI_ISL_538548	Department of Microbiology, The University of Hong Kong	Department of Microbiology, The University of Hong Kong	Kelvin K.W. To, Kwok-Yung Yuen
EPI_ISL_538617, EPI_ISL_538618, EPI_ISL_538619, EPI_ISL_538620, EPI_ISL_538622, EPI_ISL_538623, EPI_ISL_538624, EPI_ISL_538625, EPI_ISL_538626, EPI_ISL_538627, EPI_ISL_538628, EPI_ISL_538629, EPI_ISL_538630, EPI_ISL_538631, EPI_ISL_538632, EPI_ISL_538633, EPI_ISL_538634, EPI_ISL_538635, EPI_ISL_538636, EPI_ISL_538637			
see above	Servicio de Microbiología. Hospital General Universitario de Castellón	SeqCOVID-SPAIN consortium/IBV(CSIC)	Rosario Moreno, María Dolores Tirado and SeqCOVID-SPAIN consortium
EPI_ISL_538673, EPI_ISL_538674, EPI_ISL_538675, EPI_ISL_538676, EPI_ISL_538677, EPI_ISL_538678, EPI_ISL_538679, EPI_ISL_538680, EPI_ISL_538681, EPI_ISL_538686	Hospital Universitario Virgen de las Nieves de Granada-SAS	SeqCOVID-SPAIN consortium/IBV(CSIC)	Mercedes Pérez Ruiz, Sara Sanbonmatsu Gámez, Irene Pedrosa Corral, José M. Navarro-Marí and SeqCOVID-SPAIN consortium
EPI_ISL_538881, EPI_ISL_539049, EPI_ISL_539174	Leeds Teaching Hospitals NHS Trust and Public Health England, National Infection Service (Leeds laboratory)	Wellcome Sanger Institute for the COVID-19 Genomics UK (COG-UK) consortium	Louissa Macfarlane-Smith, Holli Carden, Katherine L. Harper, Antony Hale and Alex Alderton, Roberto Amato, Sonia Goncalves, Ewan Harrison, David K. Jackson, Ian Johnston, Dominic Kwiatkowski, Cordelia Langford, John Sillitoe on behalf of the Wellcome Sanger Institute COVID-19 Surveillance Team
EPI_ISL_539230, EPI_ISL_539243	Servicio de Microbiologia. Hospital Clinico Universitario de Valencia	SeqCOVID-SPAIN consortium/IBV(CSIC)	Maria Dolores Ocete, Inma Galán Vendrell, Paula Ruiz-Hueso, Mariana Reyes-Prieto, Vicente Soriano Chirona, Maria Alma Bracho, Griselda De Marco, Beatriz Beamud, Lidia Ruiz Roldan, Marta Pla Diaz,Neris Garcia-Gonzalez, Loreto Ferrús Abad, Llúcia Martínez-Priego, Concepcion Gimeno, Giuseppe D'Auria, Fernando Gonzalez-Candelas and SeqCOVID-SPAIN consortium
EPI_ISL_539245	Hospital Universitari i Politècnic La Fe de València	SeqCOVID-SPAIN consortium/IBV(CSIC)	María Dolores Gómez Ruiz, Eva González Barbera, Ana Gil Brusola, Salvador Giner Almaraz, José Luis López Hontangas and SeqCOVID-SPAIN consortium
EPI_ISL_539291, EPI_ISL_539292, EPI_ISL_539293, EPI_ISL_539294, EPI_ISL_539295, EPI_ISL_539296, EPI_ISL_539297, EPI_ISL_539298	Servicio de Microbiología. Consorcio Hospital General Universitario de Valencia	SeqCOVID-SPAIN consortium/IBV(CSIC)	Paula Ruiz-Hueso, Mariana Reyes-Prieto, Vicente Soriano Chirona, Ivan Ansari, David Navarro, Maria Alma Bracho, Griselda De Marco, Beatriz Beamud, Lidia Ruiz Roldan, Marta Pla Diaz, Neris Garcia-Gonzalez, Inma Galán Vendrell, Sandra Carbo, Loreto Ferrús Abad, Llúcia Martínez-Priego, Giuseppe D'Auria, Fernando Gonzalez-Candelas and SeqCOVID-SPAIN consortium
EPI_ISL_539521	Hospital Universitario de Ceuta	Instituto de Salud Carlos III	Iglesias-Caballero, M. Molinero Calamita, M. González-Esguevillas, M. Camarero, S. Pozo, F. Casas, I. Jiménez, P. Jiménez, M. Zaballos, A. Monzón, S. Varona, S. Juliá, M. Cuesta, I, J. López
EPI_ISL_539522	Hospital Universitario de Ceuta	Instituto de Salud Carlos III	Iglesias-Caballero, M. Molinero Calamita, M. González-Esguevillas, M. Camarero, S. Pozo, F. Casas, I. Jiménez, P. Jiménez, M. Zaballos, A. Monzón, S. Varona, S. Juliá, M. Cuesta, I, G. Sánchez
EPI_ISL_539524	Gerencia de Asistencia Sanitaria de Soria	Instituto de Salud Carlos III	Iglesias-Caballero, M. Molinero Calamita, M. González-Esguevillas, M. Camarero, S. Pozo, F. Casas, I. Jiménez, P. Jiménez, M. Zaballos, A. Monzón, S. Varona, S. Juliá, M. Cuesta, I, C. Aldea
EPI_ISL_539781	National Institute of Public Health (Czech Republic)	State Veterinary Institute Prague	Nagy, A; Jirincova, H; Novakova, L; Trnka, D; Vecerova, J.
EPI_ISL_541072, EPI_ISL_541073	Hospital de la Santa Creu i Sant Pau. Servicio de Microbiología	SeqCOVID-SPAIN consortium/Institute of Biomedicine of Valencia, IBV-CSIC	Ferran Navarro, Núria Rabella, Elisenda Miró and SeqCOVID-SPAIN consortium
EPI_ISL_541242	Florida Bureau of Public Health Laboratories, Florida Department of Health	Florida Bureau of Public Health Laboratories, Florida Department of Health	Schmedes,S., Blanton,J.
EPI_ISL_541340, EPI_ISL_541341, EPI_ISL_541342	LACEN/PR	Laboratory of Respiratory Viruses and Measles, Oswaldo Cruz Institute, FIOCRUZ	Paola Resende, Luciana Appolinario, Fernando Motta, Anna Carolina Paixão, Ana Carolina Mendonça, Jonathan Lopes, Irina Riediger, Maria do Carmo Debur, Marilda Siqueira
EPI_ISL_541942	Health and Environmental Research Institute of Gwangju	Health & Environment Institute of Gwangju	Min Ji Kim, Ji-eun Lee
EPI_ISL_541958, EPI_ISL_541960, EPI_ISL_541963, EPI_ISL_541966	Servicio de Microbiología, Hospital Universitario Son Espases	SeqCOVID-SPAIN consortium/IBV(CSIC)	Carla López-Causapé, Jordi Reina, Antonio Oliver and SeqCOVID-SPAIN consortium
EPI_ISL_541970	Influenza Centre, University of Bergen	Norwegian Institute of Public Health, Department of Virology	Fan Zhou, Rebecca J Cox, Karl A Brokstad, Bjørn Blomberg, Kathrine Stene-Johansen, Kamilla Heddeland Instefjord, Hilde Elshaug, Rasmus Riis Kopperud, Hilde Synnøve Vollan, Karoline Bragstad, Olav Hungnes
EPI_ISL_542114, EPI_ISL_542117, EPI_ISL_542122, EPI_ISL_542123, EPI_ISL_542129, EPI_ISL_542130, EPI_ISL_542134, EPI_ISL_542135, EPI_ISL_542147, EPI_ISL_542148, EPI_ISL_542150, EPI_ISL_542159, EPI_ISL_542161, EPI_ISL_542169, EPI_ISL_542171, EPI_ISL_542178, EPI_ISL_542179, EPI_ISL_542204,			

EPI_ISL_542206			
see above	ASST GOM Niguarda	Dep. Of Oncology and Hemato-Oncology University of Milan	Claudia Alteri, Valeria Cento, Antonio Piralla, Valentino Costabile, Monica Tallarita, Luna Colagrossi, Silvia Renica, Federica Giardina, Federica Novazzi, Stefano Gaiarsa, Elisa Matarazzo, Maria Antonello, Chiara Vismara, Roberto Fumagalli, Oscar Massimiliano Epis, Massimo Puoti, Carlo Federico Perno, Fausto Baldanti
EPI_ISL_542325, EPI_ISL_542327, EPI_ISL_542333, EPI_ISL_542334, EPI_ISL_542335, EPI_ISL_542336, EPI_ISL_542337, EPI_ISL_542339, EPI_ISL_542340, EPI_ISL_542341, EPI_ISL_542343, EPI_ISL_542346, EPI_ISL_542348, EPI_ISL_542366, EPI_ISL_542378, EPI_ISL_542385, EPI_ISL_542393, EPI_ISL_542394			
see above	San Matteo Hospital Pavia	Dep. Of Oncology and Hemato-Oncology University of Milan	Claudia Alteri, Valeria Cento, Antonio Piralla, Valentino Costabile, Monica Tallarita, Luna Colagrossi, Silvia Renica, Federica Giardina, Federica Novazzi, Stefano Gaiarsa, Elisa Matarazzo, Maria Antonello, Chiara Vismara, Roberto Fumagalli, Oscar Massimiliano Epis, Massimo Puoti, Carlo Federico Perno, Fausto Baldanti
EPI_ISL_545707, EPI_ISL_545714	Houston Methodist Hospital	Houston Methodist Hospital	S. Wesley Long, Randall J. Olsen, Paul A. Christensen, David W. Bernard, James J. Davis, Maulik Shukla, Marcus Nguyen, Matthew Ojeda Saavedra, Concepcion C. Cantu, Prasanti Yerramilli, Layne Pruitt, Sishir Subedi, Hung-Che Kuo, Heather Hendrickson, Ghazaleh Eskandari, Hoang A. T. Nguyen, J. Hunter Long, Muthiah Kumaraswami, Jule Goike, Daniel Boutz, Jimmy Gollihar, Jason S. McLellan, Chia-Wei Chou, Kamyab Javanmardi, Ilya J. Finkelstein, and James M. Musser
EPI_ISL_547446, EPI_ISL_547447, EPI_ISL_547463, EPI_ISL_547464, EPI_ISL_547482, EPI_ISL_547492, EPI_ISL_547503, EPI_ISL_547504, EPI_ISL_547529	Dutch COVID-19 response team	National Institute for Public Health and the Environment (RIVM)	Adam Meijer, Harry Vennema, Jeroen Cremer, Sharon van den Brink, Bas van der Veer, AnneMarie van den Brandt, Florian Zwagemaker, Dennis Schmitz, Chantal Reusken, on behalf of the national COVID-19 response team
EPI_ISL_548349, EPI_ISL_548356	Ventura County Public Health Lab	Chan-Zuckerberg Biohub	CZB Cliahub Consortium
EPI_ISL_548961	Max von Pettenkofer Institute, Virology, National Reference Center for Retroviruses, LMU München	Laboratory for Functional Genome Analysis, Dept. Genomics, Gene Center of the LMU Munich	Max Muenchhoff, Stefan Krebs, Alexander Graf, Oliver Keppler, Helmut Blum
EPI_ISL_548984, EPI_ISL_548989, EPI_ISL_548998, EPI_ISL_549005	National Public Health Laboratory, National Centre for Infectious Diseases	National Public Health Laboratory, National Centre for Infectious Diseases	Mak TM, Octavia S, Zhou Z, Cui L, Lin RTP
EPI_ISL_549026	Klinisk mikrobiologi, Region Västerbotten	Unit for Biological Agents, Department for CBRN Defence and Security, Swedish Defence Research Agency	FOI Bioinformatics team
EPI_ISL_549359	Quadram Institute Bioscience	COVID-19 Genomics UK (COG-UK) Consortium	Dave J. Baker, Gemma L. Kay, Alp Aydin, Thanh Le-Viet, Steven Rudder, Ana P. Tedim, Anastasia Kolyva, Maria Diaz, Leonardo de Oliveira Martins, Nabil-Fareed Alikhan, Lizzie Meadows, Rachael Stanley, Ngozi Elumogo, Muhammed Yasir, Nicholas M. Thomson, Alexander J Trotter, Rachel Gilroy, Samuel Bloomfield, Claire Stuart, Andrew Bell, Reenesh Prakash, Samir Dervisevic, Alison E. Mather, John Wain, Mark Webber, Andrew J. Page, Justin O'Grady
EPI_ISL_560623	Hospital	National Reference Center for Viruses of Respiratory Infections, Institut Pasteur, Paris	Sylvie Behillil, Fabiana Gambaro, Etienne Simon-Lorière, Vincent Enouf, Maud Vanpeene, Sylvie van der Werf
EPI_ISL_560651, EPI_ISL_560652, EPI_ISL_560653, EPI_ISL_560654, EPI_ISL_560655, EPI_ISL_560656, EPI_ISL_560657, EPI_ISL_560658, EPI_ISL_560659, EPI_ISL_560660, EPI_ISL_560662, EPI_ISL_560664			
see above	Centre for Clinical Infection and Diagnostics Research and Genomics Innovation Unit, Guy's and St. Thomas' NHS Trust	Centre for Clinical Infection and Diagnostics Research and Genomics Innovation Unit, Guy's and St. Thomas' NHS Trust	Chloe Fisher, Luke Snell, Rahul Batra, Jonathan Edgeworth, Ali Raza Awan
EPI_ISL_561356, EPI_ISL_561357, EPI_ISL_561358, EPI_ISL_561359, EPI_ISL_561360	Delaware Public Health Lab	Delaware Public Health Lab	Gregory Hovan
EPI_ISL_561363	Centre for Clinical Infection and Diagnostics Research and Genomics Innovation Unit, Guy's and St. Thomas' NHS Trust	Centre for Clinical Infection and Diagnostics Research and Genomics Innovation Unit, Guy's and St. Thomas' NHS Trust	Chloe Fisher, Luke Snell, Rahul Batra, Jonathan Edgeworth, Ali Raza Awan
EPI_ISL_565863, EPI_ISL_565864, EPI_ISL_565865, EPI_ISL_565867, EPI_ISL_565868, EPI_ISL_565869	Michigan Department of Health and Human Services, Bureau of Laboratories	Michigan Department of Health and Human Services, Bureau of Laboratories	Blankenship HM, Riner D, Soehnlen MK
EPI_ISL_565912, EPI_ISL_565913, EPI_ISL_565914, EPI_ISL_565917, EPI_ISL_565918, EPI_ISL_565919	Servicio de Microbiología. HRU de Málaga. Servicio Andaluz de Salud	SeqCOVID-SPAIN consortium/IBV(CSIC)	Inmaculada de Toro Peinado, Ma Concepción Mediavilla Gradolph, Begoña Palop Borrás and SeqCOVID-SPAIN consortium
EPI_ISL_565961, EPI_ISL_566024, EPI_ISL_566028	Michigan Department of Health and Human Services, Bureau of Laboratories	Michigan Department of Health and Human Services, Bureau of Laboratories	Blankenship HM, Riner D, Soehnlen MK
EPI_ISL_568580	Ramathibodi Hospital	COVID-19 Network Investigations (CONI) Alliance	Elizabeth Batty, Wasun Chantratita, Thanat Chookajorn, Stefan Fernandez, Angkana Huang, Anthony R. Jones, Khajohn Joonsalak, Chonticha Klungtong, Theerarat Kochakarn, Namfon Kotanan, Krittikorn Kumpornsin, Wudtichai Manasatienkij, Bhakbhoom Panthan, Ekawat Pasomsub, Kingkan Rakmanee, Insee Sensorn, Janjira Thaipadungpanit, Arporn Wangwiwatsin,Treewat Watthanachockchai
EPI_ISL_568914, EPI_ISL_568919, EPI_ISL_568920, EPI_ISL_568921, EPI_ISL_568922, EPI_ISL_568956, EPI_ISL_568989, EPI_ISL_569254, EPI_ISL_569255, EPI_ISL_569256, EPI_ISL_569257, EPI_ISL_569258, EPI_ISL_569259, EPI_ISL_569260, EPI_ISL_569261, EPI_ISL_569262, EPI_ISL_569263, EPI_ISL_569264, EPI_ISL_569265, EPI_ISL_569266, EPI_ISL_569267, EPI_ISL_569268, EPI_ISL_569269, EPI_ISL_569270, EPI_ISL_569271, EPI_ISL_569331, EPI_ISL_569332, EPI_ISL_569338			
see above	MEPHI, Aix Marseille University	MEPHI, Aix Marseille University	Anthony LEVASSEUR
EPI_ISL_570201, EPI_ISL_570536, EPI_ISL_570537, EPI_ISL_570547, EPI_ISL_570806, EPI_ISL_570816, EPI_ISL_570895, EPI_ISL_570901, EPI_ISL_570912	UW Virology Lab	UW Virology Lab	Pavitra Roychoudhury, Hong Xie, Lasata Shrestha, Amin Addetia, Victoria M Rachleff, Meei-Li Huang, Keith R Jerome, Alexander Greninger
EPI_ISL_571027, EPI_ISL_571028, EPI_ISL_571032, EPI_ISL_571033, EPI_ISL_571036, EPI_ISL_571037, EPI_ISL_571078, EPI_ISL_571149, EPI_ISL_571150, EPI_ISL_571152, EPI_ISL_571156, EPI_ISL_571157, EPI_ISL_571178, EPI_ISL_571179, EPI_ISL_571180, EPI_ISL_571181, EPI_ISL_571182, EPI_ISL_571183, EPI_ISL_571184, EPI_ISL_571185, EPI_ISL_571186, EPI_ISL_571187, EPI_ISL_571188, EPI_ISL_571248, EPI_ISL_571249, EPI_ISL_571250, EPI_ISL_571251, EPI_ISL_571253, EPI_ISL_571255, EPI_ISL_571256, EPI_ISL_571259, EPI_ISL_571261, EPI_ISL_571262, EPI_ISL_571263, EPI_ISL_571264, EPI_ISL_571265, EPI_ISL_571266, EPI_ISL_571267, EPI_ISL_571268, EPI_ISL_571270, EPI_ISL_571286, EPI_ISL_571287, EPI_ISL_571288, EPI_ISL_571289, EPI_ISL_571290, EPI_ISL_571291, EPI_ISL_571292, EPI_ISL_571293, EPI_ISL_571294, EPI_ISL_571295, EPI_ISL_571296, EPI_ISL_571297, EPI_ISL_571299, EPI_ISL_571300, EPI_ISL_571301, EPI_ISL_571302, EPI_ISL_571303, EPI_ISL_571304, EPI_ISL_571305, EPI_ISL_571306, EPI_ISL_571307, EPI_ISL_571309, EPI_ISL_571310, EPI_ISL_571311, EPI_ISL_571312, EPI_ISL_571313, EPI_ISL_571314, EPI_ISL_571315, EPI_ISL_571316, EPI_ISL_571317, EPI_ISL_571318, EPI_ISL_571319, EPI_ISL_571321, EPI_ISL_571322, EPI_ISL_571324, EPI_ISL_571325, EPI_ISL_571327, EPI_ISL_571328, EPI_ISL_571329, EPI_ISL_571330, EPI_ISL_571332, EPI_ISL_571333, EPI_ISL_571334, EPI_ISL_571340, EPI_ISL_571341, EPI_ISL_571342, EPI_ISL_571343, EPI_ISL_571344, EPI_ISL_571345, EPI_ISL_571346, EPI_ISL_571347, EPI_ISL_571348, EPI_ISL_571349, EPI_ISL_571350, EPI_ISL_571351, EPI_ISL_571353, EPI_ISL_571354, EPI_ISL_571356, EPI_ISL_571357, EPI_ISL_571358, EPI_ISL_571359, EPI_ISL_571360, EPI_ISL_571361, EPI_ISL_571362, EPI_ISL_571363, EPI_ISL_571364, EPI_ISL_571365, EPI_ISL_571366, EPI_ISL_571367, EPI_ISL_571368, EPI_ISL_571369, EPI_ISL_571370, EPI_ISL_571371, EPI_ISL_571376, EPI_ISL_571377, EPI_ISL_571381, EPI_ISL_571382, EPI_ISL_571383, EPI_ISL_571384, EPI_ISL_571385, EPI_ISL_571386, EPI_ISL_571387, EPI_ISL_571388, EPI_ISL_571389, EPI_ISL_571390, EPI_ISL_571391, EPI_ISL_571392, EPI_ISL_571393, EPI_ISL_571394, EPI_ISL_571395, EPI_ISL_571396, EPI_ISL_571398, EPI_ISL_571399, EPI_ISL_571400, EPI_ISL_571401, EPI_ISL_571402, EPI_ISL_571403, EPI_ISL_571404, EPI_ISL_571405, EPI_ISL_571406, EPI_ISL_571407, EPI_ISL_571408, EPI_ISL_571409, EPI_ISL_571424, EPI_ISL_571425, EPI_ISL_571426, EPI_ISL_571427, EPI_ISL_571428, EPI_ISL_571429, EPI_ISL_571430, EPI_ISL_571431, EPI_ISL_571432, EPI_ISL_571434, EPI_ISL_571435, EPI_ISL_571436, EPI_ISL_571437, EPI_ISL_571438, EPI_ISL_571440, EPI_ISL_571473, EPI_ISL_571474, EPI_ISL_571475, EPI_ISL_571476, EPI_ISL_571477, EPI_ISL_571478, EPI_ISL_571479, EPI_ISL_571480, EPI_ISL_571481, EPI_ISL_571482, EPI_ISL_571483, EPI_ISL_571509, EPI_ISL_571510, EPI_ISL_571511, EPI_ISL_571512, EPI_ISL_571513, EPI_ISL_571514, EPI_ISL_571515, EPI_ISL_571516, EPI_ISL_571517, EPI_ISL_571518, EPI_ISL_571528, EPI_ISL_571529, EPI_ISL_571530, EPI_ISL_571531, EPI_ISL_571532, EPI_ISL_571545, EPI_ISL_571546, EPI_ISL_571548, EPI_ISL_571551, EPI_ISL_571552, EPI_ISL_571553, EPI_ISL_571554, EPI_ISL_571555, EPI_ISL_571556, EPI_ISL_571563, EPI_ISL_571565, EPI_ISL_571567, EPI_ISL_571568, EPI_ISL_571573, EPI_ISL_571592, EPI_ISL_571602, EPI_ISL_571603, EPI_ISL_571604, EPI_ISL_571606, EPI_ISL_571607, EPI_ISL_571608, EPI_ISL_571609, EPI_ISL_571610, EPI_ISL_571611, EPI_ISL_571614, EPI_ISL_571641, EPI_ISL_571642, EPI_ISL_571643, EPI_ISL_571644, EPI_ISL_571645, EPI_ISL_571646, EPI_ISL_571647, EPI_ISL_571648, EPI_ISL_571649, EPI_ISL_571650, EPI_ISL_571651, EPI_ISL_571652, EPI_ISL_571653, EPI_ISL_571654, EPI_ISL_571655, EPI_ISL_571656, EPI_ISL_571657, EPI_ISL_571658, EPI_ISL_571659, EPI_ISL_571660, EPI_ISL_571661, EPI_ISL_571662, EPI_ISL_571663, EPI_ISL_571664, EPI_ISL_571665, EPI_ISL_571666, EPI_ISL_571667, EPI_ISL_571668, EPI_ISL_571669, EPI_ISL_571670, EPI_ISL_571671, EPI_ISL_571672, EPI_ISL_571673, EPI_ISL_571674, EPI_ISL_571675, EPI_ISL_571676, EPI_ISL_571677, EPI_ISL_571678, EPI_ISL_571679, EPI_ISL_571680, EPI_ISL_571681, EPI_ISL_571682, EPI_ISL_571683, EPI_ISL_571684, EPI_ISL_571687, EPI_ISL_571688, EPI_ISL_571689, EPI_ISL_571690, EPI_ISL_571692, EPI_ISL_571693, EPI_ISL_571694, EPI_ISL_571695, EPI_ISL_571696, EPI_ISL_571697, EPI_ISL_571698, EPI_ISL_571699, EPI_ISL_571700, EPI_ISL_571701, EPI_ISL_571702, EPI_ISL_571703, EPI_ISL_571704, EPI_ISL_571705, EPI_ISL_571706, EPI_ISL_571707, EPI_ISL_571708, EPI_ISL_571709, EPI_ISL_571710, EPI_ISL_571711, EPI_ISL_571712, EPI_ISL_571713, EPI_ISL_571714, EPI_ISL_571715, EPI_ISL_571716, EPI_ISL_571717, EPI_ISL_571718, EPI_ISL_571719, EPI_ISL_571720, EPI_ISL_571721, EPI_ISL_571722, EPI_ISL_571723, EPI_ISL_571724, EPI_ISL_571725, EPI_ISL_571726, EPI_ISL_571728, EPI_ISL_571731, EPI_ISL_571732, EPI_ISL_571733, EPI_ISL_571734, EPI_ISL_571735, EPI_ISL_571738, EPI_ISL_571739, EPI_ISL_571740, EPI_ISL_571743, EPI_ISL_571746, EPI_ISL_571747, EPI_ISL_571748, EPI_ISL_571749, EPI_ISL_571750, EPI_ISL_571751, EPI_ISL_571753, EPI_ISL_571754, EPI_ISL_571755, EPI_ISL_571756, EPI_ISL_571757, EPI_ISL_571758, EPI_ISL_571759, EPI_ISL_571762, EPI_ISL_571764, EPI_ISL_571765, EPI_ISL_571768, EPI_ISL_571769, EPI_ISL_571773, EPI_ISL_571774, EPI_ISL_571777, EPI_ISL_571779, EPI_ISL_571780, EPI_ISL_571781, EPI_ISL_571782, EPI_ISL_571783, EPI_ISL_571784, EPI_ISL_571789, EPI_ISL_571797, EPI_ISL_571798, EPI_ISL_571799, EPI_ISL_571800, EPI_ISL_571801, EPI_ISL_571802, EPI_ISL_571821, EPI_ISL_571823, EPI_ISL_571843, EPI_ISL_571844, EPI_ISL_571901, EPI_ISL_571911,			

EPI_ISL_571912, EPI_ISL_571913, EPI_ISL_571916, EPI_ISL_571917, EPI_ISL_571918, EPI_ISL_571919, EPI_ISL_571920, EPI_ISL_571922, EPI_ISL_571923, EPI_ISL_571924, EPI_ISL_571927, EPI_ISL_571928, EPI_ISL_571929, EPI_ISL_571930, EPI_ISL_571931, EPI_ISL_571932, EPI_ISL_571933, EPI_ISL_571934, EPI_ISL_571935, EPI_ISL_571936, EPI_ISL_571937, EPI_ISL_571938, EPI_ISL_571939, EPI_ISL_571940, EPI_ISL_571941, EPI_ISL_571942, EPI_ISL_571943, EPI_ISL_571944, EPI_ISL_571945, EPI_ISL_571946, EPI_ISL_571947, EPI_ISL_571948, EPI_ISL_571949, EPI_ISL_571950, EPI_ISL_571951, EPI_ISL_571952, EPI_ISL_571953, EPI_ISL_571954, EPI_ISL_571955, EPI_ISL_571956, EPI_ISL_571957, EPI_ISL_571958, EPI_ISL_571959, EPI_ISL_571960, EPI_ISL_571961, EPI_ISL_571962, EPI_ISL_571963, EPI_ISL_571964, EPI_ISL_571965, EPI_ISL_571966, EPI_ISL_571968, EPI_ISL_571970, EPI_ISL_571971, EPI_ISL_571972, EPI_ISL_571973, EPI_ISL_571974, EPI_ISL_571975, EPI_ISL_571976, EPI_ISL_571977, EPI_ISL_571978, EPI_ISL_571979, EPI_ISL_571980, EPI_ISL_571981, EPI_ISL_571982, EPI_ISL_571983, EPI_ISL_571990, EPI_ISL_571994, EPI_ISL_571995, EPI_ISL_571996, EPI_ISL_571997, EPI_ISL_571998, EPI_ISL_571999, EPI_ISL_572000, EPI_ISL_572001, EPI_ISL_572002, EPI_ISL_572003, EPI_ISL_572004, EPI_ISL_572005, EPI_ISL_572006, EPI_ISL_572007, EPI_ISL_572008, EPI_ISL_572009, EPI_ISL_572010, EPI_ISL_572011, EPI_ISL_572012, EPI_ISL_572013, EPI_ISL_572014, EPI_ISL_572015, EPI_ISL_572016, EPI_ISL_572017, EPI_ISL_572018, EPI_ISL_572019, EPI_ISL_572020, EPI_ISL_572021, EPI_ISL_572022, EPI_ISL_572023, EPI_ISL_572024, EPI_ISL_572025, EPI_ISL_572026, EPI_ISL_572027, EPI_ISL_572028, EPI_ISL_572029, EPI_ISL_572030, EPI_ISL_572031, EPI_ISL_572032, EPI_ISL_572033, EPI_ISL_572034, EPI_ISL_572035, EPI_ISL_572036, EPI_ISL_572037, EPI_ISL_572038, EPI_ISL_572039, EPI_ISL_572040, EPI_ISL_572042, EPI_ISL_572044, EPI_ISL_572045, EPI_ISL_572046, EPI_ISL_572047, EPI_ISL_572049, EPI_ISL_572050, EPI_ISL_572051, EPI_ISL_572052, EPI_ISL_572053, EPI_ISL_572054, EPI_ISL_572055, EPI_ISL_572056, EPI_ISL_572057, EPI_ISL_572058, EPI_ISL_572059, EPI_ISL_572060, EPI_ISL_572061, EPI_ISL_572062, EPI_ISL_572063, EPI_ISL_572064, EPI_ISL_572065, EPI_ISL_572066, EPI_ISL_572067, EPI_ISL_572068, EPI_ISL_572069, EPI_ISL_572070, EPI_ISL_572071, EPI_ISL_572072, EPI_ISL_572073, EPI_ISL_572074, EPI_ISL_572075, EPI_ISL_572076, EPI_ISL_572077, EPI_ISL_572078, EPI_ISL_572079, EPI_ISL_572080, EPI_ISL_572081, EPI_ISL_572082, EPI_ISL_572083, EPI_ISL_572084, EPI_ISL_572085, EPI_ISL_572086, EPI_ISL_572087, EPI_ISL_572088, EPI_ISL_572089, EPI_ISL_572090, EPI_ISL_572091, EPI_ISL_572092, EPI_ISL_572093, EPI_ISL_572094, EPI_ISL_572095, EPI_ISL_572096, EPI_ISL_572097, EPI_ISL_572098, EPI_ISL_572099, EPI_ISL_572100, EPI_ISL_572101, EPI_ISL_572102, EPI_ISL_572103, EPI_ISL_572104, EPI_ISL_572107, EPI_ISL_572109, EPI_ISL_572111, EPI_ISL_572112, EPI_ISL_572113, EPI_ISL_572114, EPI_ISL_572115, EPI_ISL_572116, EPI_ISL_572117, EPI_ISL_572118, EPI_ISL_572119, EPI_ISL_572120, EPI_ISL_572121, EPI_ISL_572122, EPI_ISL_572123, EPI_ISL_572127, EPI_ISL_572128, EPI_ISL_572129, EPI_ISL_572130, EPI_ISL_572131, EPI_ISL_572132, EPI_ISL_572133, EPI_ISL_572134, EPI_ISL_572135, EPI_ISL_572137, EPI_ISL_572142, EPI_ISL_572147, EPI_ISL_572151, EPI_ISL_572153, EPI_ISL_572156, EPI_ISL_572157, EPI_ISL_572159, EPI_ISL_572160, EPI_ISL_572161, EPI_ISL_572162, EPI_ISL_572163, EPI_ISL_572164, EPI_ISL_572165, EPI_ISL_572166, EPI_ISL_572167, EPI_ISL_572170, EPI_ISL_572171, EPI_ISL_572173, EPI_ISL_572174, EPI_ISL_572175, EPI_ISL_572176, EPI_ISL_572178, EPI_ISL_572180, EPI_ISL_572181, EPI_ISL_572182, EPI_ISL_572183			
see above	Quest Diagnostics	Quest Diagnostics	Rosenthal,S.H., Gerasimova,A., Kagan,R.M., Anderson, B., Grover, D., Livingston, K.E., Hua, M., Liu Y., Shalhout, D.F., Owen, R., Lacbawan, F.
EPI_ISL_574292	Instituto Nacional de Investigación en Salud Pública	Instituto Nacional de Investigación en Salud Pública	Leandro Patino Patino, Domenica de Mora Coloma, Denisse Portugal, Alfredo Bruno Caicedo, Andrés Carrasco, Orson Mestanza, Manuel Gonzalez, Alberto Orlando Narvaez.
EPI_ISL_574581	Delaware Public Health Lab	Delaware Public Health Lab	Gregory Hovan
EPI_ISL_574800, EPI_ISL_574801, EPI_ISL_574802, EPI_ISL_574803, EPI_ISL_574804, EPI_ISL_574805, EPI_ISL_574806, EPI_ISL_574807, EPI_ISL_574808, EPI_ISL_574809, EPI_ISL_574810, EPI_ISL_574811, EPI_ISL_574812			
see above	Institute for Infectious Diseases, University of Bern	Institute for Infectious Diseases, University of Bern	Michel C Koch, Christian Baumann, Miguel A Terrazos Miani, Cora Sägesser, Stephen L Leib, Peter Keller, Franziska Suter-Riniker, Alban Ramette
EPI_ISL_574876, EPI_ISL_574877, EPI_ISL_574878, EPI_ISL_574883, EPI_ISL_574884, EPI_ISL_574885, EPI_ISL_574886, EPI_ISL_574887, EPI_ISL_574888, EPI_ISL_574905, EPI_ISL_574906, EPI_ISL_574907, EPI_ISL_574908, EPI_ISL_574909, EPI_ISL_574910, EPI_ISL_574913, EPI_ISL_574914, EPI_ISL_574915, EPI_ISL_574916, EPI_ISL_574917, EPI_ISL_574918, EPI_ISL_574919, EPI_ISL_574920, EPI_ISL_574931, EPI_ISL_574932, EPI_ISL_574933, EPI_ISL_574934, EPI_ISL_574936, EPI_ISL_574937, EPI_ISL_574938, EPI_ISL_574944, EPI_ISL_574945, EPI_ISL_574946, EPI_ISL_574947, EPI_ISL_574948, EPI_ISL_574949, EPI_ISL_574950, EPI_ISL_574951, EPI_ISL_574954, EPI_ISL_574955, EPI_ISL_574956, EPI_ISL_574957, EPI_ISL_574958, EPI_ISL_574959, EPI_ISL_574960, EPI_ISL_574961, EPI_ISL_574962, EPI_ISL_574963, EPI_ISL_574964, EPI_ISL_574965, EPI_ISL_574966, EPI_ISL_574967, EPI_ISL_574968, EPI_ISL_574969, EPI_ISL_574970, EPI_ISL_574971, EPI_ISL_574972, EPI_ISL_574977, EPI_ISL_574978, EPI_ISL_574979, EPI_ISL_574980, EPI_ISL_574981, EPI_ISL_574982, EPI_ISL_574983, EPI_ISL_574984, EPI_ISL_574985, EPI_ISL_574986, EPI_ISL_574987, EPI_ISL_574988, EPI_ISL_574989, EPI_ISL_574990, EPI_ISL_574991, EPI_ISL_574992, EPI_ISL_574993, EPI_ISL_574994, EPI_ISL_574995, EPI_ISL_574996, EPI_ISL_574997, EPI_ISL_574998, EPI_ISL_574999, EPI_ISL_575000, EPI_ISL_575003, EPI_ISL_575004, EPI_ISL_575005, EPI_ISL_575006, EPI_ISL_575007, EPI_ISL_575008, EPI_ISL_575009, EPI_ISL_575010, EPI_ISL_575011, EPI_ISL_575013, EPI_ISL_575014, EPI_ISL_575015, EPI_ISL_575016, EPI_ISL_575017, EPI_ISL_575018			
see above	Viollier AG	Department of Biosystems Science and Engineering, ETH Zürich	Christian Beisel, Sarah Nadeau, Ivan Topolsky, Pedro Ferreira, Philipp Jablonski, Susana Posada-Céspedes, Tobias Schär, Ina Nissen, Natascha Santacroce, Elodie Burcklen, Christiane Beckmann, Maurice Redondo, Olivier Kobel, Christoph Noppen, Sophie Seidel, Noemie Santamaria de Souza, Niko Beerenwinkel, Tanja Stadler
EPI_ISL_576171	PA Department of Health, Bureau of Laboratories	Pathogen Discovery, Respiratory Viruses Branch, Division of Viral Diseases, Centers for Disease Control and Prevention	Yan Li, Anna Montmayeur, Brian Lynch, Jing Zhang, Krista Queen, Ying Tao, Anna Uehara, Rachel Marine, Clinton R. Paden, Peter Cook, Haibin Wang, Suxiang Tong
EPI_ISL_578420, EPI_ISL_578422, EPI_ISL_578423, EPI_ISL_578424, EPI_ISL_578425, EPI_ISL_578470, EPI_ISL_578471	Wisconsin State Laboratory of Hygiene Communicable Disease Division	Wisconsin State Laboratory of Hygiene Communicable Disease Division	Kelsey R. Florek, Abigail C. Shockey
EPI_ISL_579060, EPI_ISL_579061	Canterbury Health Laboratories	Institute of Environmental Science and Research (ESR)	Xiaoyun Ren, Matt Storey, Nikki Freed, Muhammad Faisal, Jing Wang, Hermes Perez, Anja Werno, Antje van der Linden, Arlo Upton, Chris Mansell, David Hammer, Dragana Drinkovic, Gary McAuliffe, Hana Sofia Andersson, James Ussher, Jill Sherwood, Josh Freeman, Julia Howard, Juliet Elvy, Mary DeAlmeida, Matt Blakiston, Matthew Rogers, Max Bloomfield, Michael Addidle, Michelle Balm, Sally Roberts, Sarah Jefferies, Sharmini Muttaiyah, Susan Morpeth, Susan Taylor, Timothy Blackmore, Vani Sathyendran, Veronica Playle, Virginia Hope, Erasmus Smit, Lauren Jelly, Olin Silander, Joep de Ligt
EPI_ISL_579118	Waikato Hospital	Institute of Environmental Science and Research (ESR)	Xiaoyun Ren, Matt Storey, Nikki Freed, Muhammad Faisal, Jing Wang, Hermes Perez, Anja Werno, Antje van der Linden, Arlo Upton, Chris Mansell, David Hammer, Dragana Drinkovic, Gary McAuliffe, Hana Sofia Andersson, James Ussher, Jill Sherwood, Josh Freeman, Julia Howard, Juliet Elvy, Mary DeAlmeida, Matt Blakiston, Matthew Rogers, Max Bloomfield, Michael Addidle, Michelle Balm, Sally Roberts, Sarah Jefferies, Sharmini Muttaiyah, Susan Morpeth, Susan Taylor, Timothy Blackmore, Vani Sathyendran, Veronica Playle, Virginia Hope, Erasmus Smit, Lauren Jelly, Olin Silander, Joep de Ligt
EPI_ISL_579119, EPI_ISL_579120	LabPLUS	Institute of Environmental Science and Research (ESR)	Xiaoyun Ren, Matt Storey, Nikki Freed, Muhammad Faisal, Jing Wang, Hermes Perez, Anja Werno, Antje van der Linden, Arlo Upton, Chris Mansell, David Hammer, Dragana Drinkovic, Gary McAuliffe, Hana Sofia Andersson, James Ussher, Jill Sherwood, Josh Freeman, Julia Howard, Juliet Elvy, Mary DeAlmeida, Matt Blakiston, Matthew Rogers, Max Bloomfield, Michael Addidle, Michelle Balm, Sally Roberts, Sarah Jefferies, Sharmini Muttaiyah, Susan Morpeth, Susan Taylor, Timothy Blackmore, Vani Sathyendran, Veronica Playle, Virginia Hope, Erasmus Smit, Lauren Jelly, Olin Silander, Joep de Ligt
EPI_ISL_579517, EPI_ISL_579537	QEII Health Sciences Centre	National Microbiology Laboratory (NML)	Anna Majer, Shari Tyson, Grace Seo, Philip Mabon, Darian Hole, Elsie Grudeski, Rhiannon Huzarewich, Russell Mandes, Anneliese Landgraff, Jennifer Tanner, Natalie Knox, Morag Graham, Gary Van Domselaar, Todd Hatchette, Jason LeBlanc, Nathalie Bastien, Yan Li, Timothy Booth, CanCOGeN's metadata curation team, Public Health Agency of Canada's CanCOGeN team
EPI_ISL_581452	Medizinische Klinik Innere Medizin I, Universitätsklinikum Tübingen	NGS Competence Center Tübingen, Institut für Medizinische Mikrobiologie und Hygiene, Universitätsklinikum Tübingen	Angel Angelov
EPI_ISL_581719	University Hospital Basel, Clinical Virology	University Hospital Basel, Clinical Bacteriology	Madlen Stange, Alfredo Mari, Tim Roloff, Helena MB Seth-Smith, Michael Schweitzer, Myrta Brunner, Karoline Leuzinger, Kirstine K. Soegaard, Alexander Gensch, Sarah Tschudin-Sutter, Simon Fuchs, Julia Bielicki, Hans Pargger, Martin Siegemund, Christian Nickel, Roland Bingisser, Michael Osthoff, Stefano Bassetti, Rita Schneider-Sliwa, Manuel Battegay, Hans Hirsch, Adrian Egli
EPI_ISL_582127	Antwerp University Hospital	Institute of Tropical Medicine	Philippe Selhorst, Colin Anthony
EPI_ISL_582303, EPI_ISL_582304, EPI_ISL_582305	Cadham Provincial Laboratory	National Microbiology Laboratory (NML)	Anna Majer, Shari Tyson, Grace Seo, Philip Mabon, Elsie Grudeski, Rhiannon Huzarewich, Russell Mandes, Anneliese Landgraff, Jennifer Tanner, Natalie Knox, Morag Graham, Gary Van Domselaar, Paul Van Caeseele, Jared Bullard, David Alexander, Kerry Dust, Nathalie Bastien, Yan Li, Timothy Booth, Darian Hole, Madison Chapel, CanCOGeN's metadata curation team, Public Health Agency of Canada CanCOGeN team
EPI_ISL_582703, EPI_ISL_582707	Servicio de Microbiología, Hospital Universitario Cruces, Bilbao	SeqCOVID-SPAIN consortium/IBV(CSIC)	Maitane Aranzamendi Zaldumbide and SeqCOVID-SPAIN consortium
EPI_ISL_583461	Memorial Sloan Kettering Cancer Center	van Bakel Laboratory, Genetics and Genomics Sciences, Icahn School of Medicine at Mount Sinai	Teresa Aydillo, Ana S. Gonzalez-Reiche, Sadaf Aslam, Adriana van de Guchte, Zenab Khan, Ajay Obla, Jayeeta Dutta, Harm van Bakel, Judith Aberg, Adolfo García-Sastre, Gunjan Shah, Tobias Hohl, Genovefa Papanicolaou, Miguel-Angel Perales, Kent Sepkowitz, Ngolela Esther Babady, and Mini Kamboj
EPI_ISL_583463	Garcia-Sastre Laboratory, Department of Microbiology, Icahn School of Medicine at Mount Sinai	van Bakel Laboratory, Genetics and Genomics Sciences, Icahn School of Medicine at Mount Sinai	Teresa Aydillo, Ana S. Gonzalez-Reiche, Sadaf Aslam, Adriana van de Guchte, Zenab Khan, Ajay Obla, Jayeeta Dutta, Harm van Bakel, Judith Aberg, Adolfo García-Sastre, Gunjan Shah, Tobias Hohl, Genovefa Papanicolaou, Miguel-Angel Perales, Kent Sepkowitz, Ngolela Esther Babady, and Mini Kamboj
EPI_ISL_583469	Memorial Sloan Kettering Cancer Center	van Bakel Laboratory, Genetics and Genomics Sciences, Icahn School of Medicine at Mount Sinai	Teresa Aydillo, Ana S. Gonzalez-Reiche, Sadaf Aslam, Adriana van de Guchte, Zenab Khan, Ajay Obla, Jayeeta Dutta, Harm van Bakel, Judith Aberg, Adolfo García-Sastre, Gunjan Shah, Tobias Hohl, Genovefa Papanicolaou, Miguel-Angel Perales, Kent Sepkowitz, Ngolela Esther Babady, and Mini Kamboj
EPI_ISL_583558	Universitaetsklinik für Innere Medizin II Innsbruck	Bergthaler laboratory, CeMM Research Center for Molecular Medicine of the Austrian Academy of Sciences	Alexandra Popa, Benedikt Agerer, Henrique Colaco, Lukas Endler, Jakob-Wendelin Genger, Alexander Lercher, Mark Smyth, Thomas Penz, Michael Schuster, Jan Laine, Martin Senekowitsch, Judith Aberle, Stephan Aberle, Peter Hufnagl, Daniela Schmid, Franz Allerberger, Elisabeth Puchhammer-Stoeckl, Manfred Nairz, Guenter Weiss, Gregor Hörmann, Kinga Rigler-Hohenwarter, Rainer Gattringer, Wegene Borena, Dorothee von Laer, Gernot Walder, Peter Obrist, Christian Paar, Sabine Sussitz-Rack, Gunther Vogl, Adi Steinrigl, Christoph Bock, Andreas Bergthaler
EPI_ISL_583566	Center for Virology, Medical University of Vienna	Bergthaler laboratory, CeMM Research Center for Molecular Medicine of the Austrian Academy of Sciences	Alexandra Popa, Benedikt Agerer, Henrique Colaco, Lukas Endler, Jakob-Wendelin Genger, Alexander Lercher, Mark Smyth, Thomas Penz, Michael Schuster, Jan Laine, Martin Senekowitsch, Judith Aberle, Stephan Aberle, Peter Hufnagl, Daniela Schmid, Franz Allerberger, Elisabeth Puchhammer-Stoeckl, Manfred Nairz, Guenter Weiss, Gregor Hörmann, Kinga Rigler-Hohenwarter, Rainer Gattringer, Wegene Borena, Dorothee von Laer,

			Gernot Walder, Peter Obrist, Christian Paar, Sabine Sussitz-Rack, Gunther Vogl, Adi Steinrigl, Christoph Bock, Andreas Bergthaler
EPI_ISL_583577	Austrian Agency for Health and Food Safety (AGES)	Bergthaler laboratory, CeMM Research Center for Molecular Medicine of the Austrian Academy of Sciences	Alexandra Popa, Benedikt Agerer, Henrique Colaco, Lukas Endler, Jakob-Wendelin Genger, Alexander Lercher, Mark Smyth, Thomas Penz, Michael Schuster, Jan Laine, Martin Senekowitsch, Judith Aberle, Stephan Aberle, Peter Hufnagl, Daniela Schmid, Franz Allerberger, Elisabeth Puchhammer-Stoeckl, Manfred Nairz, Guenter Weiss, Gregor Hörmann, Kinga Rigler-Hohenwarter, Rainer Gattringer, Wegene Borena, Dorothee von Laer, Gernot Walder, Peter Obrist, Christian Paar, Sabine Sussitz-Rack, Gunther Vogl, Adi Steinrigl, Christoph Bock, Andreas Bergthaler
EPI_ISL_583583, EPI_ISL_583585, EPI_ISL_583596, EPI_ISL_583598	Institute for Medical and Chemical Laboratory Diagnostics, Kepler Universitätsklinikum	Bergthaler laboratory, CeMM Research Center for Molecular Medicine of the Austrian Academy of Sciences	Alexandra Popa, Benedikt Agerer, Henrique Colaco, Lukas Endler, Jakob-Wendelin Genger, Alexander Lercher, Mark Smyth, Thomas Penz, Michael Schuster, Jan Laine, Martin Senekowitsch, Judith Aberle, Stephan Aberle, Peter Hufnagl, Daniela Schmid, Franz Allerberger, Elisabeth Puchhammer-Stoeckl, Manfred Nairz, Guenter Weiss, Gregor Hörmann, Kinga Rigler-Hohenwarter, Rainer Gattringer, Wegene Borena, Dorothee von Laer, Gernot Walder, Peter Obrist, Christian Paar, Sabine Sussitz-Rack, Gunther Vogl, Adi Steinrigl, Christoph Bock, Andreas Bergthaler
EPI_ISL_583605, EPI_ISL_583609, EPI_ISL_583610, EPI_ISL_583611, EPI_ISL_583612	Institut für Virologie am Department für Hygiene, Mikrobiologie und Public Health	Bergthaler laboratory, CeMM Research Center for Molecular Medicine of the Austrian Academy of Sciences	Alexandra Popa, Benedikt Agerer, Henrique Colaco, Lukas Endler, Jakob-Wendelin Genger, Alexander Lercher, Mark Smyth, Thomas Penz, Michael Schuster, Jan Laine, Martin Senekowitsch, Judith Aberle, Stephan Aberle, Peter Hufnagl, Daniela Schmid, Franz Allerberger, Elisabeth Puchhammer-Stoeckl, Manfred Nairz, Guenter Weiss, Gregor Hörmann, Kinga Rigler-Hohenwarter, Rainer Gattringer, Wegene Borena, Dorothee von Laer, Gernot Walder, Peter Obrist, Christian Paar, Sabine Sussitz-Rack, Gunther Vogl, Adi Steinrigl, Christoph Bock, Andreas Bergthaler
EPI_ISL_583744, EPI_ISL_583745, EPI_ISL_583746, EPI_ISL_583747, EPI_ISL_583766, EPI_ISL_583767, EPI_ISL_583768, EPI_ISL_583769, EPI_ISL_583770, EPI_ISL_583771, EPI_ISL_583772, EPI_ISL_583773, EPI_ISL_583774, EPI_ISL_583775, EPI_ISL_583776, EPI_ISL_583777, EPI_ISL_583778, EPI_ISL_583779			
see above	Dr. Gernot Walder GmbH	Bergthaler laboratory, CeMM Research Center for Molecular Medicine of the Austrian Academy of Sciences	Alexandra Popa, Benedikt Agerer, Henrique Colaco, Lukas Endler, Jakob-Wendelin Genger, Alexander Lercher, Mark Smyth, Thomas Penz, Michael Schuster, Jan Laine, Martin Senekowitsch, Judith Aberle, Stephan Aberle, Peter Hufnagl, Daniela Schmid, Franz Allerberger, Elisabeth Puchhammer-Stoeckl, Manfred Nairz, Guenter Weiss, Gregor Hörmann, Kinga Rigler-Hohenwarter, Rainer Gattringer, Wegene Borena, Dorothee von Laer, Gernot Walder, Peter Obrist, Christian Paar, Sabine Sussitz-Rack, Gunther Vogl, Adi Steinrigl, Christoph Bock, Andreas Bergthaler
EPI_ISL_583879, EPI_ISL_583880, EPI_ISL_583881	Center for Virology, Medical University of Vienna	Bergthaler laboratory, CeMM Research Center for Molecular Medicine of the Austrian Academy of Sciences	Alexandra Popa, Benedikt Agerer, Henrique Colaco, Lukas Endler, Jakob-Wendelin Genger, Alexander Lercher, Mark Smyth, Thomas Penz, Michael Schuster, Jan Laine, Martin Senekowitsch, Judith Aberle, Stephan Aberle, Peter Hufnagl, Daniela Schmid, Franz Allerberger, Elisabeth Puchhammer-Stoeckl, Manfred Nairz, Guenter Weiss, Gregor Hörmann, Kinga Rigler-Hohenwarter, Rainer Gattringer, Wegene Borena, Dorothee von Laer, Gernot Walder, Peter Obrist, Christian Paar, Sabine Sussitz-Rack, Gunther Vogl, Adi Steinrigl, Christoph Bock, Andreas Bergthaler
EPI_ISL_586297, EPI_ISL_586298, EPI_ISL_586299, EPI_ISL_586356, EPI_ISL_586361, EPI_ISL_586369, EPI_ISL_586370, EPI_ISL_586373, EPI_ISL_586381, EPI_ISL_586393, EPI_ISL_586402, EPI_ISL_586406, EPI_ISL_586407, EPI_ISL_586409, EPI_ISL_586410, EPI_ISL_586411, EPI_ISL_586412, EPI_ISL_586413, EPI_ISL_586416, EPI_ISL_586418, EPI_ISL_586419, EPI_ISL_586422			
see above	Toronto Invasive Bacterial Diseases Network	McMaster University	Allison McGeer, Patryk Aftanas, Hooman Derakhshani, Angel Li, Kuganya Nirmalarajah, Emily Panousis, Ahmed Draia, Jalees Nasir, Michael Surette, Samira Mubareka, Andrew G. McArthur
EPI_ISL_590917	Unilabs Laboratory Medicine	Norwegian Institute of Public Health, Department of Virology	Kathrine Stene-Johansen, Kamilla Heddeland Instefjord, Hilde Elshaug, Rasmus Riis Kopperud, Hilde Vollan, Karoline Bragstad, Olav Hungnes
EPI_ISL_591124, EPI_ISL_591127, EPI_ISL_591128, EPI_ISL_591130, EPI_ISL_591138	Toronto Invasive Bacterial Diseases Network	McMaster University	Allison McGeer, Patryk Aftanas, Hooman Derakhshani, Angel Li, Kuganya Nirmalarajah, Emily Panousis, Ahmed Draia, Jalees Nasir, Michael Surette, Samira Mubareka, Andrew G. McArthur
EPI_ISL_593756, EPI_ISL_593757, EPI_ISL_593758, EPI_ISL_593759	Sydney South West Pathology Service (SSWPS) - Liverpool Hospital - NSW Health Pathology	NSW Health Pathology - Institute of Clinical Pathology and Medical Research; Westmead Hospital; University of Sydney	CIDM-PH et al.
EPI_ISL_594190, EPI_ISL_594191, EPI_ISL_594192	Michigan Department of Health and Human Services, Bureau of Laboratories	Michigan Department of Health and Human Services, Bureau of Laboratories	Blankenship HM, Riner D, Soehnlen MK
EPI_ISL_596505, EPI_ISL_596506	Palestinian Ministry of Health	Molecular Genetics Lab	Nouar Qutob, Zaidoun Salah, Damien Richard, Hisham Darwish, Husam Sallam, Issa Shtayeh, Osama Najjar, Mahmoud Ruzayqat, Dana Najjar, Francois Balloux, Lucy van Dorp
EPI_ISL_596824, EPI_ISL_596827, EPI_ISL_596829, EPI_ISL_596846, EPI_ISL_596847, EPI_ISL_596854, EPI_ISL_596865, EPI_ISL_596874, EPI_ISL_596877, EPI_ISL_596880	PathWest Laboratory Medicine WA	PathWest Laboratory Medicine WA Microbial Surveillance Unit	PathWest Laboratory Medicine WA Microbial Surveillance Unit
EPI_ISL_600436, EPI_ISL_600440	Institute of Epidemiology Disease Control And Research	Institute for Developing Science and Health Initiatives	Lauren Cowley, Mokibul Hassan Afrad, Sadia Isfat Ara Rahman, Md. Mahfuz-Al-mamun, Firadausi Qadri, Tahmina Shirin
EPI_ISL_602486, EPI_ISL_602487, EPI_ISL_602488, EPI_ISL_602489	Institute for Virology, University Hospital Essen	Center of Medical Microbiology, Virology, and Hospital Hygiene, University of Duesseldorf	Olympia E. Anastasiou, Ulf Dittmer, Maximilian Damagnez, Alexander Dilthey, Torsten Houwaart, Lisanna Hülse, Malte Kohns Vasconcelos, Nadine Lübke, Jessica Nicolai, Klaus Pfeffer, Daniel Strelow, Jörg Timm, Andreas Walker, Tobias Wienemann
EPI_ISL_602516	Center of Medical Microbiology, Virology, and Hospital Hygiene, University of Duesseldorf	Center of Medical Microbiology, Virology, and Hospital Hygiene, University of Duesseldorf	Maximilian Damagnez, Alexander Dilthey, Torsten Houwaart, Lisanna Hülse, Malte Kohns Vasconcelos, Marek Korencak, Nadine Lübke, Jessica Nicolai, Klaus Pfeffer, Hendrik Streeck, Daniel Strelow, Jörg Timm, Andreas Walker, Tobias Wienemann
EPI_ISL_602532	Institute for Virology, University Hospital Essen	Center of Medical Microbiology, Virology, and Hospital Hygiene, University of Duesseldorf	Olympia E. Anastasiou, Ulf Dittmer, Maximilian Damagnez, Alexander Dilthey, Torsten Houwaart, Lisanna Hülse, Malte Kohns Vasconcelos, Nadine Lübke, Jessica Nicolai, Klaus Pfeffer, Daniel Strelow, Jörg Timm, Andreas Walker, Tobias Wienemann
EPI_ISL_602534	Center of Medical Microbiology, Virology, and Hospital Hygiene, University of Duesseldorf	Center of Medical Microbiology, Virology, and Hospital Hygiene, University of Duesseldorf	Maximilian Damagnez, Alexander Dilthey, Torsten Houwaart, Malte Kohns Vasconcelos, Marek Korencak, Nadine Lübke, Jessica Nicolai, Klaus Pfeffer, Hendrik Streeck, Daniel Strelow, Jörg Timm, Andreas Walker, Tobias Wienemann
EPI_ISL_603759, EPI_ISL_603760, EPI_ISL_603761, EPI_ISL_603762, EPI_ISL_603763, EPI_ISL_603764, EPI_ISL_603765, EPI_ISL_603766, EPI_ISL_603767, EPI_ISL_603768, EPI_ISL_603769, EPI_ISL_603770, EPI_ISL_603771, EPI_ISL_603772, EPI_ISL_603773, EPI_ISL_603774, EPI_ISL_603775, EPI_ISL_603776, EPI_ISL_603777, EPI_ISL_603778, EPI_ISL_603779, EPI_ISL_603780, EPI_ISL_603781, EPI_ISL_603782, EPI_ISL_603783, EPI_ISL_603784, EPI_ISL_603785, EPI_ISL_603786, EPI_ISL_603787, EPI_ISL_603788, EPI_ISL_603789, EPI_ISL_603790, EPI_ISL_603791, EPI_ISL_603792, EPI_ISL_603793, EPI_ISL_603794, EPI_ISL_603795, EPI_ISL_603796, EPI_ISL_603797, EPI_ISL_603798, EPI_ISL_603799, EPI_ISL_603800, EPI_ISL_603801, EPI_ISL_603802, EPI_ISL_603803, EPI_ISL_603804, EPI_ISL_603805, EPI_ISL_603806, EPI_ISL_603807, EPI_ISL_603808, EPI_ISL_603809, EPI_ISL_603810, EPI_ISL_603811, EPI_ISL_603812, EPI_ISL_603813, EPI_ISL_603814, EPI_ISL_603815, EPI_ISL_603816, EPI_ISL_603817, EPI_ISL_603818, EPI_ISL_603819, EPI_ISL_603820, EPI_ISL_603821, EPI_ISL_603822, EPI_ISL_603823, EPI_ISL_603826, EPI_ISL_603827, EPI_ISL_603828, EPI_ISL_603829, EPI_ISL_603830, EPI_ISL_603831, EPI_ISL_603832, EPI_ISL_603833, EPI_ISL_603834, EPI_ISL_603835, EPI_ISL_603836, EPI_ISL_603837, EPI_ISL_603838, EPI_ISL_603839, EPI_ISL_603840, EPI_ISL_603841, EPI_ISL_603842, EPI_ISL_603843, EPI_ISL_603844, EPI_ISL_603845, EPI_ISL_603847, EPI_ISL_603849, EPI_ISL_603850, EPI_ISL_603851, EPI_ISL_603852, EPI_ISL_603853, EPI_ISL_603854, EPI_ISL_603855, EPI_ISL_603856, EPI_ISL_603857, EPI_ISL_603859, EPI_ISL_603862, EPI_ISL_603866, EPI_ISL_603872, EPI_ISL_603873, EPI_ISL_603874, EPI_ISL_603877, EPI_ISL_603878, EPI_ISL_603879, EPI_ISL_603880, EPI_ISL_603881, EPI_ISL_603882, EPI_ISL_603883, EPI_ISL_603884, EPI_ISL_603886, EPI_ISL_603888, EPI_ISL_603889, EPI_ISL_603890, EPI_ISL_603891, EPI_ISL_603893, EPI_ISL_603894, EPI_ISL_603895, EPI_ISL_603898, EPI_ISL_603899, EPI_ISL_603901, EPI_ISL_603903, EPI_ISL_603906, EPI_ISL_603908, EPI_ISL_603909, EPI_ISL_603910, EPI_ISL_603911, EPI_ISL_603914, EPI_ISL_603919, EPI_ISL_603920, EPI_ISL_603924, EPI_ISL_603927, EPI_ISL_603928, EPI_ISL_603934, EPI_ISL_603936, EPI_ISL_603937, EPI_ISL_603938, EPI_ISL_603939, EPI_ISL_603940, EPI_ISL_603941, EPI_ISL_603942, EPI_ISL_603943, EPI_ISL_603944, EPI_ISL_603945, EPI_ISL_603946, EPI_ISL_603947, EPI_ISL_603948, EPI_ISL_603949, EPI_ISL_603950, EPI_ISL_603951, EPI_ISL_603952, EPI_ISL_603953, EPI_ISL_603954, EPI_ISL_603955, EPI_ISL_603956, EPI_ISL_603957, EPI_ISL_603958, EPI_ISL_603959, EPI_ISL_603960, EPI_ISL_603961, EPI_ISL_603962, EPI_ISL_603963, EPI_ISL_603964, EPI_ISL_603965, EPI_ISL_603966, EPI_ISL_603967, EPI_ISL_603968, EPI_ISL_603969, EPI_ISL_603970, EPI_ISL_603971, EPI_ISL_603972, EPI_ISL_603973, EPI_ISL_603974, EPI_ISL_603975, EPI_ISL_603976, EPI_ISL_603977, EPI_ISL_603978, EPI_ISL_603979, EPI_ISL_603980, EPI_ISL_603981, EPI_ISL_603982, EPI_ISL_603983, EPI_ISL_603984, EPI_ISL_603985, EPI_ISL_603986, EPI_ISL_603987, EPI_ISL_603988, EPI_ISL_603989, EPI_ISL_603990, EPI_ISL_603991, EPI_ISL_603992, EPI_ISL_603993, EPI_ISL_603994, EPI_ISL_603995, EPI_ISL_603996, EPI_ISL_603997, EPI_ISL_603998, EPI_ISL_603999, EPI_ISL_604000, EPI_ISL_604001, EPI_ISL_604002, EPI_ISL_604003, EPI_ISL_604004, EPI_ISL_604005, EPI_ISL_604006, EPI_ISL_604007, EPI_ISL_604008, EPI_ISL_604009, EPI_ISL_604010, EPI_ISL_604011, EPI_ISL_604012, EPI_ISL_604013, EPI_ISL_604014, EPI_ISL_604015, EPI_ISL_604016, EPI_ISL_604017, EPI_ISL_604018, EPI_ISL_604020, EPI_ISL_604021, EPI_ISL_604022, EPI_ISL_604023, EPI_ISL_604024, EPI_ISL_604025, EPI_ISL_604026, EPI_ISL_604027, EPI_ISL_604028, EPI_ISL_604029, EPI_ISL_604030, EPI_ISL_604031, EPI_ISL_604032, EPI_ISL_604033, EPI_ISL_604034, EPI_ISL_604035, EPI_ISL_604036, EPI_ISL_604037, EPI_ISL_604038, EPI_ISL_604039, EPI_ISL_604040, EPI_ISL_604041, EPI_ISL_604042, EPI_ISL_604043, EPI_ISL_604044, EPI_ISL_604045, EPI_ISL_604046, EPI_ISL_604047, EPI_ISL_604048, EPI_ISL_604049, EPI_ISL_604050, EPI_ISL_604051, EPI_ISL_604052, EPI_ISL_604053, EPI_ISL_604054, EPI_ISL_604055, EPI_ISL_604056, EPI_ISL_604057, EPI_ISL_604058, EPI_ISL_604059, EPI_ISL_604060, EPI_ISL_604061, EPI_ISL_604063, EPI_ISL_604064, EPI_ISL_604065, EPI_ISL_604066, EPI_ISL_604067, EPI_ISL_604068, EPI_ISL_604069, EPI_ISL_604070, EPI_ISL_604071, EPI_ISL_604072, EPI_ISL_604073, EPI_ISL_604074, EPI_ISL_604075, EPI_ISL_604076, EPI_ISL_604077, EPI_ISL_604078, EPI_ISL_604079, EPI_ISL_604080, EPI_ISL_604081, EPI_ISL_604082, EPI_ISL_604083, EPI_ISL_604084, EPI_ISL_604085, EPI_ISL_604086, EPI_ISL_604087, EPI_ISL_604088, EPI_ISL_604089, EPI_ISL_604091, EPI_ISL_604092, EPI_ISL_604094, EPI_ISL_604095, EPI_ISL_604096, EPI_ISL_604097, EPI_ISL_604098, EPI_ISL_604099, EPI_ISL_604100, EPI_ISL_604102, EPI_ISL_604103, EPI_ISL_604104, EPI_ISL_604105, EPI_ISL_604106, EPI_ISL_604107, EPI_ISL_604108, EPI_ISL_604109, EPI_ISL_604110, EPI_ISL_604111, EPI_ISL_604112, EPI_ISL_604113, EPI_ISL_604114, EPI_ISL_604115, EPI_ISL_604116, EPI_ISL_604118, EPI_ISL_604120, EPI_ISL_604121, EPI_ISL_604124, EPI_ISL_604125, EPI_ISL_604126, EPI_ISL_604127, EPI_ISL_604128, EPI_ISL_604130, EPI_ISL_604133, EPI_ISL_604135, EPI_ISL_604137, EPI_ISL_604138, EPI_ISL_604140, EPI_ISL_604141, EPI_ISL_604142, EPI_ISL_604143, EPI_ISL_604146, EPI_ISL_604147, EPI_ISL_604148, EPI_ISL_604155, EPI_ISL_604158, EPI_ISL_604159, EPI_ISL_604160, EPI_ISL_604162, EPI_ISL_604164, EPI_ISL_604165, EPI_ISL_604166, EPI_ISL_604167, EPI_ISL_604168, EPI_ISL_604169, EPI_ISL_604171, EPI_ISL_604172, EPI_ISL_604175, EPI_ISL_604176, EPI_ISL_604177, EPI_ISL_604179, EPI_ISL_604180, EPI_ISL_604182, EPI_ISL_604183, EPI_ISL_604185, EPI_ISL_604186, EPI_ISL_604188, EPI_ISL_604192, EPI_ISL_604194, EPI_ISL_604195, EPI_ISL_604196, EPI_ISL_604197, EPI_ISL_604198, EPI_ISL_604200, EPI_ISL_604201, EPI_ISL_604202, EPI_ISL_604203, EPI_ISL_604204, EPI_ISL_604205, EPI_ISL_604206, EPI_ISL_604207, EPI_ISL_604209, EPI_ISL_604211, EPI_ISL_604212, EPI_ISL_604213, EPI_ISL_604214, EPI_ISL_604215, EPI_ISL_604216, EPI_ISL_604217, EPI_ISL_604218, EPI_ISL_604220, EPI_ISL_604221, EPI_ISL_604224, EPI_ISL_604226, EPI_ISL_604227, EPI_ISL_604230, EPI_ISL_604232, EPI_ISL_604234, EPI_ISL_604235, EPI_ISL_604236, EPI_ISL_604237, EPI_ISL_604238, EPI_ISL_604239, EPI_ISL_604240, EPI_ISL_604241, EPI_ISL_604243, EPI_ISL_604244, EPI_ISL_604245, EPI_ISL_604246, EPI_ISL_604247, EPI_ISL_604248, EPI_ISL_604249, EPI_ISL_604250, EPI_ISL_604251, EPI_ISL_604252, EPI_ISL_604253, EPI_ISL_604254, EPI_ISL_604255, EPI_ISL_604256, EPI_ISL_604257, EPI_ISL_604259, EPI_ISL_604260, EPI_ISL_604261, EPI_ISL_604262, EPI_ISL_604263, EPI_ISL_604265, EPI_ISL_604266, EPI_ISL_604267, EPI_ISL_604268, EPI_ISL_604269, EPI_ISL_604270, EPI_ISL_604271, EPI_ISL_604272, EPI_ISL_604273, EPI_ISL_604274, EPI_ISL_604275, EPI_ISL_604276, EPI_ISL_604277, EPI_ISL_604278, EPI_ISL_604293, EPI_ISL_604294, EPI_ISL_604295, EPI_ISL_604296, EPI_ISL_604297, EPI_ISL_604298, EPI_ISL_604299, EPI_ISL_604300, EPI_ISL_604301, EPI_ISL_604302, EPI_ISL_604303, EPI_ISL_604304, EPI_ISL_604306, EPI_ISL_604307, EPI_ISL_604309, EPI_ISL_604312, EPI_ISL_604313, EPI_ISL_604314, EPI_ISL_604315, EPI_ISL_604316, EPI_ISL_604317, EPI_ISL_604318, EPI_ISL_604319, EPI_ISL_604322, EPI_ISL_604323, EPI_ISL_604324, EPI_ISL_604325, EPI_ISL_604326, EPI_ISL_604327, EPI_ISL_604328, EPI_ISL_604329, EPI_ISL_604330, EPI_ISL_604331, EPI_ISL_604332, EPI_ISL_604333, EPI_ISL_604334, EPI_ISL_604335, EPI_ISL_604336, EPI_ISL_604337, EPI_ISL_604338, EPI_ISL_604339, EPI_ISL_604340, EPI_ISL_604341, EPI_ISL_604342, EPI_ISL_604343, EPI_ISL_604344, EPI_ISL_604345, EPI_ISL_604346, EPI_ISL_604347, EPI_ISL_604349, EPI_ISL_604350, EPI_ISL_604351, EPI_ISL_604353, EPI_ISL_604354, EPI_ISL_604355, EPI_ISL_604357, EPI_ISL_604358, EPI_ISL_604359, EPI_ISL_604360, EPI_ISL_604361, EPI_ISL_604362, EPI_ISL_604363,			

EPI_ISL_604364, EPI_ISL_604365, EPI_ISL_604366, EPI_ISL_604367, EPI_ISL_604368, EPI_ISL_604369, EPI_ISL_604370, EPI_ISL_604371, EPI_ISL_604372, EPI_ISL_604373, EPI_ISL_604374, EPI_ISL_604375, EPI_ISL_604376, EPI_ISL_604377, EPI_ISL_604378, EPI_ISL_604379, EPI_ISL_604381, EPI_ISL_604382, EPI_ISL_604384, EPI_ISL_604385, EPI_ISL_604386, EPI_ISL_604387, EPI_ISL_604388, EPI_ISL_604389, EPI_ISL_604392, EPI_ISL_604400, EPI_ISL_604403, EPI_ISL_604406, EPI_ISL_604407, EPI_ISL_604408, EPI_ISL_604410, EPI_ISL_604412, EPI_ISL_604413, EPI_ISL_604414, EPI_ISL_604415, EPI_ISL_604418, EPI_ISL_604420, EPI_ISL_604421, EPI_ISL_604423, EPI_ISL_604427, EPI_ISL_604428, EPI_ISL_604429, EPI_ISL_604431, EPI_ISL_604432, EPI_ISL_604435, EPI_ISL_604437, EPI_ISL_604438, EPI_ISL_604441, EPI_ISL_604443, EPI_ISL_604444, EPI_ISL_604446, EPI_ISL_604455, EPI_ISL_604457, EPI_ISL_604458, EPI_ISL_604459, EPI_ISL_604460, EPI_ISL_604461, EPI_ISL_604462, EPI_ISL_604463, EPI_ISL_604464, EPI_ISL_604465, EPI_ISL_604466, EPI_ISL_604467, EPI_ISL_604468, EPI_ISL_604469, EPI_ISL_604470, EPI_ISL_604471, EPI_ISL_604472, EPI_ISL_604473, EPI_ISL_604474, EPI_ISL_604475, EPI_ISL_604476, EPI_ISL_604477, EPI_ISL_604478, EPI_ISL_604479, EPI_ISL_604480, EPI_ISL_604481, EPI_ISL_604482, EPI_ISL_604483, EPI_ISL_604488, EPI_ISL_604496, EPI_ISL_604497, EPI_ISL_604501, EPI_ISL_604503, EPI_ISL_604504, EPI_ISL_604505, EPI_ISL_604506, EPI_ISL_604507, EPI_ISL_604509, EPI_ISL_604510, EPI_ISL_604511, EPI_ISL_604514, EPI_ISL_604515, EPI_ISL_604516, EPI_ISL_604517, EPI_ISL_604518, EPI_ISL_604519, EPI_ISL_604520, EPI_ISL_604521, EPI_ISL_604522, EPI_ISL_604523, EPI_ISL_604528, EPI_ISL_604529, EPI_ISL_604530, EPI_ISL_604534, EPI_ISL_604539, EPI_ISL_604541, EPI_ISL_604542, EPI_ISL_604544, EPI_ISL_604547, EPI_ISL_604549, EPI_ISL_604553, EPI_ISL_604554, EPI_ISL_604561, EPI_ISL_604565, EPI_ISL_604566, EPI_ISL_604567, EPI_ISL_604568, EPI_ISL_604569, EPI_ISL_604570, EPI_ISL_604571, EPI_ISL_604573, EPI_ISL_604574, EPI_ISL_604575, EPI_ISL_604576, EPI_ISL_604577, EPI_ISL_604621, EPI_ISL_604622, EPI_ISL_604626, EPI_ISL_604627, EPI_ISL_604628, EPI_ISL_604629, EPI_ISL_604630, EPI_ISL_604631, EPI_ISL_604632, EPI_ISL_604633, EPI_ISL_604634, EPI_ISL_604635, EPI_ISL_604636, EPI_ISL_604637, EPI_ISL_604638, EPI_ISL_604639, EPI_ISL_604640, EPI_ISL_604642, EPI_ISL_604643, EPI_ISL_604644, EPI_ISL_604645, EPI_ISL_604647, EPI_ISL_604648, EPI_ISL_604649, EPI_ISL_604650, EPI_ISL_604651, EPI_ISL_604652, EPI_ISL_604653, EPI_ISL_604654, EPI_ISL_604655, EPI_ISL_604656, EPI_ISL_604657, EPI_ISL_604658, EPI_ISL_604659, EPI_ISL_604660, EPI_ISL_604662, EPI_ISL_604663, EPI_ISL_604664, EPI_ISL_604665, EPI_ISL_604666, EPI_ISL_604667, EPI_ISL_604668, EPI_ISL_604669, EPI_ISL_604670, EPI_ISL_604671, EPI_ISL_604672, EPI_ISL_604673, EPI_ISL_604674, EPI_ISL_604675, EPI_ISL_604676, EPI_ISL_604677, EPI_ISL_604678, EPI_ISL_604679, EPI_ISL_604680, EPI_ISL_604681, EPI_ISL_604682, EPI_ISL_604683, EPI_ISL_604684, EPI_ISL_604685, EPI_ISL_604686, EPI_ISL_604687, EPI_ISL_604688, EPI_ISL_604689, EPI_ISL_604690, EPI_ISL_604691, EPI_ISL_604692, EPI_ISL_604693, EPI_ISL_604694, EPI_ISL_604695, EPI_ISL_604696, EPI_ISL_604697, EPI_ISL_604698, EPI_ISL_604699, EPI_ISL_604700, EPI_ISL_604701, EPI_ISL_604702, EPI_ISL_604703, EPI_ISL_604704, EPI_ISL_604705, EPI_ISL_604706, EPI_ISL_604707, EPI_ISL_604709, EPI_ISL_604710, EPI_ISL_604711, EPI_ISL_604712, EPI_ISL_604713, EPI_ISL_604714, EPI_ISL_604715, EPI_ISL_604716, EPI_ISL_604717, EPI_ISL_604718, EPI_ISL_604719, EPI_ISL_604720, EPI_ISL_604721, EPI_ISL_604722, EPI_ISL_604723, EPI_ISL_604724, EPI_ISL_604727, EPI_ISL_604729, EPI_ISL_604731, EPI_ISL_604732, EPI_ISL_604733, EPI_ISL_604734, EPI_ISL_604735, EPI_ISL_604736, EPI_ISL_604737, EPI_ISL_604738, EPI_ISL_604739, EPI_ISL_604740, EPI_ISL_604741, EPI_ISL_604742, EPI_ISL_604743, EPI_ISL_604744, EPI_ISL_604745, EPI_ISL_604746, EPI_ISL_604747, EPI_ISL_604748, EPI_ISL_604749, EPI_ISL_604750, EPI_ISL_604752, EPI_ISL_604753, EPI_ISL_604754, EPI_ISL_604755, EPI_ISL_604756, EPI_ISL_604757, EPI_ISL_604759, EPI_ISL_604760, EPI_ISL_604761, EPI_ISL_604763, EPI_ISL_604764, EPI_ISL_604765, EPI_ISL_604766, EPI_ISL_604767, EPI_ISL_604768, EPI_ISL_604769, EPI_ISL_604770, EPI_ISL_604772, EPI_ISL_604773, EPI_ISL_604774, EPI_ISL_604775, EPI_ISL_604776, EPI_ISL_604786, EPI_ISL_604792, EPI_ISL_604794, EPI_ISL_604796, EPI_ISL_604797, EPI_ISL_604798, EPI_ISL_604799, EPI_ISL_604802, EPI_ISL_604803, EPI_ISL_604804, EPI_ISL_604806, EPI_ISL_604807, EPI_ISL_604808, EPI_ISL_604810, EPI_ISL_604812, EPI_ISL_604813, EPI_ISL_604815, EPI_ISL_604816, EPI_ISL_604817, EPI_ISL_604818, EPI_ISL_604820, EPI_ISL_604821, EPI_ISL_604822, EPI_ISL_604824, EPI_ISL_604825, EPI_ISL_604826, EPI_ISL_604827, EPI_ISL_604828, EPI_ISL_604830, EPI_ISL_604833, EPI_ISL_604834, EPI_ISL_604840, EPI_ISL_604868, EPI_ISL_604869, EPI_ISL_604870, EPI_ISL_604871, EPI_ISL_604872, EPI_ISL_604873, EPI_ISL_604874, EPI_ISL_604875, EPI_ISL_604876, EPI_ISL_604877, EPI_ISL_604878, EPI_ISL_604879, EPI_ISL_604880, EPI_ISL_604881, EPI_ISL_604882, EPI_ISL_604883, EPI_ISL_604884, EPI_ISL_604885, EPI_ISL_604886, EPI_ISL_604887, EPI_ISL_604888, EPI_ISL_604889, EPI_ISL_604890, EPI_ISL_604891, EPI_ISL_604892, EPI_ISL_604893, EPI_ISL_604894, EPI_ISL_604895, EPI_ISL_604896, EPI_ISL_604897, EPI_ISL_604898, EPI_ISL_604899, EPI_ISL_604900, EPI_ISL_604901, EPI_ISL_604902, EPI_ISL_604903, EPI_ISL_604904, EPI_ISL_604905, EPI_ISL_604906, EPI_ISL_604907, EPI_ISL_604908, EPI_ISL_604909, EPI_ISL_604910, EPI_ISL_604911, EPI_ISL_604912, EPI_ISL_604913, EPI_ISL_604914, EPI_ISL_604915, EPI_ISL_604916, EPI_ISL_604917, EPI_ISL_604918, EPI_ISL_604921, EPI_ISL_604922, EPI_ISL_604923, EPI_ISL_604925, EPI_ISL_604926, EPI_ISL_604927, EPI_ISL_604928, EPI_ISL_604929, EPI_ISL_604930, EPI_ISL_604934, EPI_ISL_604935, EPI_ISL_604936, EPI_ISL_604938, EPI_ISL_604939, EPI_ISL_604940, EPI_ISL_604941, EPI_ISL_604942, EPI_ISL_604943, EPI_ISL_604945, EPI_ISL_604947, EPI_ISL_604948, EPI_ISL_604950, EPI_ISL_604951, EPI_ISL_604952, EPI_ISL_604953, EPI_ISL_604954, EPI_ISL_604955, EPI_ISL_604956, EPI_ISL_604957, EPI_ISL_604958, EPI_ISL_604959, EPI_ISL_604960, EPI_ISL_604992, EPI_ISL_604993, EPI_ISL_604994, EPI_ISL_604995, EPI_ISL_604996, EPI_ISL_604997, EPI_ISL_604998, EPI_ISL_604999, EPI_ISL_605000, EPI_ISL_605001, EPI_ISL_605002, EPI_ISL_605003, EPI_ISL_605004, EPI_ISL_605005, EPI_ISL_605006, EPI_ISL_605007, EPI_ISL_605008, EPI_ISL_605009, EPI_ISL_605010, EPI_ISL_605011, EPI_ISL_605012, EPI_ISL_605013, EPI_ISL_605014, EPI_ISL_605015, EPI_ISL_605016, EPI_ISL_605017, EPI_ISL_605018, EPI_ISL_605019, EPI_ISL_605020, EPI_ISL_605021, EPI_ISL_605022, EPI_ISL_605023, EPI_ISL_605024, EPI_ISL_605025, EPI_ISL_605037, EPI_ISL_605038, EPI_ISL_605039, EPI_ISL_605040, EPI_ISL_605042, EPI_ISL_605043, EPI_ISL_605046, EPI_ISL_605047, EPI_ISL_605048, EPI_ISL_605050, EPI_ISL_605053, EPI_ISL_605054, EPI_ISL_605055, EPI_ISL_605056, EPI_ISL_605057			
see above	Quest Diagnostics	Quest Diagnostics	Rosenthal,S.H., Gerasimova,A., Kagan,R.M., Anderson, B., Grover, D., Livingston, K.E., Hua, M., Liu Y., Shalhout, D.F., Owen, R., Lacbawan, F.
EPI_ISL_605059, EPI_ISL_605064, EPI_ISL_605068, EPI_ISL_605073, EPI_ISL_605079	National Virus Reference Laboratory	Irish Coronavirus Sequencing Consortium - Helixworks	Sachin Chalapati, Conor Crosbie, Nimesh Pinnamaneni
EPI_ISL_605926	Emergence des Maladies Virales, Centre Interdisciplinaire de Recherches Medicales de Franceville	Emergence des Maladies Virales, Centre Interdisciplinaire de Recherches Medicales de Franceville	Lekana-Douki,S.E., N'dilimabaka,N., Mbongo kama,E., Kandet Yattara,M., Mintsa Ndong,A., Ngonga Dikongo,A.M., Andeko,J.C., Zong Minko,O., Lekana-Douki,J.-B.
EPI_ISL_610164, EPI_ISL_610165, EPI_ISL_610166	Department of Health Technology and Informatics, The Hong Kong Polytechnic University	Department of Health Technology and Informatics, The Hong Kong Polytechnic University	Siu,G.K.-H., Lee,L.-K., Leung,K.S.-S., Leung,J.S.-L., Ng,T.T.-L., Chan,C.T.-M., Tam,K.K.-G., Lao,H.-Y., Wu,A.K.-L., Yau,M.C.-Y., Lai,Y.W.-M., Fung,K.S.-C., Chau,S.K.-Y., Wong,B.K.-C., To,W.-K., Luk,K., Ho,A.Y.-M., Que,T.-L., Yip,K.-T., Yam,W.C., Shum,D.H.-K., Yip,S.P.
EPI_ISL_613419, EPI_ISL_613422, EPI_ISL_613423, EPI_ISL_613425, EPI_ISL_613426, EPI_ISL_613427, EPI_ISL_613428, EPI_ISL_613430, EPI_ISL_613431	Institut Pasteur de la Guadeloupe	Institut Pasteur de la Guadeloupe	Marion Barbet, Sylvie Behillil, Méline Bizard, Angela Brisebarre, Camille Capel, Etienne Simon-Lorière, Vincent Enouf, Maud Vanpeene, Sylvie van der Werf, Stéphanie Guyomard, Sébastien Breurec, Antoine Talarmin
EPI_ISL_614403, EPI_ISL_614415, EPI_ISL_614416, EPI_ISL_614420, EPI_ISL_614421, EPI_ISL_614422, EPI_ISL_614439, EPI_ISL_614473, EPI_ISL_614600, EPI_ISL_614601, EPI_ISL_614602, EPI_ISL_614603, EPI_ISL_614604, EPI_ISL_614605, EPI_ISL_614606, EPI_ISL_614607, EPI_ISL_614608, EPI_ISL_614609, EPI_ISL_614610, EPI_ISL_614611, EPI_ISL_614612, EPI_ISL_614613, EPI_ISL_614614, EPI_ISL_614615, EPI_ISL_614616, EPI_ISL_614617, EPI_ISL_614618, EPI_ISL_614619, EPI_ISL_614620, EPI_ISL_614621, EPI_ISL_614622, EPI_ISL_614623, EPI_ISL_614624, EPI_ISL_614625, EPI_ISL_614626, EPI_ISL_614627, EPI_ISL_614628, EPI_ISL_614630, EPI_ISL_614633, EPI_ISL_614634, EPI_ISL_614635, EPI_ISL_614637, EPI_ISL_614638, EPI_ISL_614639, EPI_ISL_614640, EPI_ISL_614641, EPI_ISL_614642, EPI_ISL_614643, EPI_ISL_614644, EPI_ISL_614645, EPI_ISL_614646, EPI_ISL_614647, EPI_ISL_614648, EPI_ISL_614649, EPI_ISL_614650, EPI_ISL_614651, EPI_ISL_614652, EPI_ISL_614653, EPI_ISL_614654, EPI_ISL_614655, EPI_ISL_614658, EPI_ISL_614659, EPI_ISL_614660, EPI_ISL_614661, EPI_ISL_614662, EPI_ISL_614664, EPI_ISL_614665, EPI_ISL_614666, EPI_ISL_614667, EPI_ISL_614668, EPI_ISL_614675, EPI_ISL_614677, EPI_ISL_614678, EPI_ISL_614679, EPI_ISL_614681, EPI_ISL_614682, EPI_ISL_614683, EPI_ISL_614684, EPI_ISL_614685, EPI_ISL_614686, EPI_ISL_614687, EPI_ISL_614689, EPI_ISL_614690, EPI_ISL_614691, EPI_ISL_614692, EPI_ISL_614755, EPI_ISL_614757, EPI_ISL_615467, EPI_ISL_619455, EPI_ISL_619456, EPI_ISL_619492, EPI_ISL_619534, EPI_ISL_619556, EPI_ISL_619657, EPI_ISL_620145, EPI_ISL_620146, EPI_ISL_620147, EPI_ISL_620286, EPI_ISL_620302, EPI_ISL_622500, EPI_ISL_622511, EPI_ISL_622524, EPI_ISL_622679, EPI_ISL_622724			
see above	Department of Virus and Microbiological Special Diagnostics, Statens Serum Institut, Denmark	Albertsen lab, Department of Chemistry and Bioscience, Aalborg University, Denmark	Danish Covid-19 Genome Consortia
EPI_ISL_623104	Simile Medicina Diagnóstica	Bioinformatics Laboratory / LNCC	Carolina M Voloch, Ronaldo S Francisco Jr, Luiz G P de Almeida, Otavio J. Brustolini, Cynthia C Cardoso, Alexandra L Gerber, Ana Paula de C Guimarães, Diana Mariani, Covid19-UFRJ Workgroup, Luís Cristóvão Pôrto, Renato S Aguiar, Terezinha M P P Castiñeiras, Orlando C. Ferreira, Amilcar Tanuri, Ana Tereza R de Vasconcelos
EPI_ISL_626225, EPI_ISL_626226	Institute for Virology, University Hospital Essen	Center of Medical Microbiology, Virology, and Hospital Hygiene, University of Duesseldorf	Olympia E. Anastasiou, Ulf Dittmer, Maximilian Damagnez, Alexander Dilthey, Torsten Houwaart, Lisanna Hülse, Malte Kohns Vasconcelos, Nadine Lübke, Jessica Nicolai, Klaus Pfeffer, Daniel Strelow, Jörg Timm, Andreas Walker, Tobias Wienemann
EPI_ISL_626357, EPI_ISL_626358, EPI_ISL_626359, EPI_ISL_626360, EPI_ISL_626361, EPI_ISL_626362	Northwestern Memorial Hospital	Ozer Lab	Ramon Lorenzo-Redondo, Hannah H. Nam, Scott C. Roberts, Lacy M. Simons, Chad J. Achenbach, Lawrence J. Jennings, Chao Qi, Alan R. Hauser, Michael G. Ison, Judd F. Hultquist, Egon A. Ozer
EPI_ISL_626566	National Centre for Communication Disease (NCCD) National Influenza Center	National Centre for Communication Disease (NCCD) National Influenza Center	Naranzul Ts,Darmaa B,Bayasgalan N,Ankhbayar S,Tsogtbaatar B,Erden-Ochir Ts,Nymadawa P,Chang-Seon Song
EPI_ISL_629024	Centro de Biotecnología Vegetal, Universidad Andrés Bello, Center for Genome Regulation	Center for Mathematical Modeling and Center for Genome Regulation. Santiago, Chile	Bastias M, Sanhueza D, Travisany D, Allende ML, Maass A, González M, Bustos F, Arriagada G, Montecino, M, Orellana A, Castro E, Meneses C.
EPI_ISL_629088	Laboratoire du Centre Hospitalier Annecy Genevois	CNR Virus des Infections Respiratoires - France SUD	Antonin Bal, Gregory Destras, Gwendolyne Burfin, Hadrien Règue, Quentin Semanas, Martine Valette, Bruno Lina, Hélène Petitprez, Bruno Chanzy, Laurence Josset
EPI_ISL_631550, EPI_ISL_631551	Jamaica Hospital Medical Center	New York City Public Health Laboratory	Jade Wang, et al.
EPI_ISL_631552, EPI_ISL_631553	Brookdale University Hospital Medical Center	New York City Public Health Laboratory	Jade Wang, et al.
EPI_ISL_631554	Jamaica Hospital Medical Center	New York City Public Health Laboratory	Jade Wang, et al.
EPI_ISL_631555	Richmond University Medical Center	New York City Public Health Laboratory	Jade Wang, et al.
EPI_ISL_631556	NYC HH Jacobi Medical Center	New York City Public Health Laboratory	Jade Wang, et al.
EPI_ISL_631573	Brookdale University Hospital Medical Center	New York City Public Health Laboratory	Jade Wang, et al.
EPI_ISL_631574, EPI_ISL_631575	Jamaica Hospital Medical Center	New York City Public Health Laboratory	Jade Wang, et al.
EPI_ISL_631576	Richmond University Medical Center	New York City Public Health Laboratory	Jade Wang, et al.
EPI_ISL_631654, EPI_ISL_631655, EPI_ISL_631656, EPI_ISL_631657, EPI_ISL_631658, EPI_ISL_631659, EPI_ISL_631660, EPI_ISL_631661, EPI_ISL_631662, EPI_ISL_631663, EPI_ISL_631664, EPI_ISL_631665, EPI_ISL_631666, EPI_ISL_631667, EPI_ISL_631668, EPI_ISL_631669, EPI_ISL_631670, EPI_ISL_631671, EPI_ISL_631672			
see above	Texas Department of State Health Services	Texas Department of State Health Services	Rashmi Tuladhar, Bonnie Oh, Jenny Zhang, Maliha Rahman, Anita Pokharel, Myong Koag, Chung Wang, Rachel Lee, Grace Kubin, Mayela Pedrueza
EPI_ISL_631752	NYC HH Lincoln Medical And Mental Health Center	New York City Public Health Laboratory	Jade Wang, et al.
EPI_ISL_631764	Mount Sinai Hospital	New York City Public Health Laboratory	Jade Wang, et al.
EPI_ISL_631765	Bellevue Hospital Center	New York City Public Health Laboratory	Jade Wang, et al.
EPI_ISL_631785, EPI_ISL_631786	Mount Sinai Hospital	New York City Public Health Laboratory	Jade Wang, et al.

EPI_ISL_631787	Flushing Hospital Medical Center	New York City Public Health Laboratory	Jade Wang, et al.
EPI_ISL_631788, EPI_ISL_631789, EPI_ISL_631790, EPI_ISL_631791	Jamaica Hospital Medical Center	New York City Public Health Laboratory	Jade Wang, et al.
EPI_ISL_631792	Richmond University Medical Center	New York City Public Health Laboratory	Jade Wang, et al.
EPI_ISL_631793, EPI_ISL_631794	SUNY Downstate Medical Center	New York City Public Health Laboratory	Jade Wang, et al.
EPI_ISL_631795	Jamaica Hospital Medical Center	New York City Public Health Laboratory	Jade Wang, et al.
EPI_ISL_631796	Maimonides Medical Center	New York City Public Health Laboratory	Jade Wang, et al.
EPI_ISL_631797	OCME Office Of Chief Medical Examiner	New York City Public Health Laboratory	Jade Wang, et al.
EPI_ISL_631798	Jamaica Hospital Medical Center	New York City Public Health Laboratory	Jade Wang, et al.
EPI_ISL_631799	NYC HH Lincoln Medical And Mental Health Center	New York City Public Health Laboratory	Jade Wang, et al.
EPI_ISL_631800	Brookdale University Hospital Medical Center	New York City Public Health Laboratory	Jade Wang, et al.
EPI_ISL_631801	Richmond University Medical Center	New York City Public Health Laboratory	Jade Wang, et al.
EPI_ISL_631832	Metro Urgicare	New York City Public Health Laboratory	Jade Wang, et al.
EPI_ISL_631833	Jamaica Hospital Medical Center	New York City Public Health Laboratory	Jade Wang, et al.
EPI_ISL_631834	Mount Sinai Hospital	New York City Public Health Laboratory	Jade Wang, et al.
EPI_ISL_631835, EPI_ISL_631836	Jamaica Hospital Medical Center	New York City Public Health Laboratory	Jade Wang, et al.
EPI_ISL_631837	Richmond University Medical Center	New York City Public Health Laboratory	Jade Wang, et al.
EPI_ISL_631838	Bellevue Hospital Center	New York City Public Health Laboratory	Jade Wang, et al.
EPI_ISL_631839	Mount Sinai Hospital	New York City Public Health Laboratory	Jade Wang, et al.
EPI_ISL_631868, EPI_ISL_631869	Richmond University Medical Center	New York City Public Health Laboratory	Jade Wang, et al.
EPI_ISL_631915	Maimonides Medical Center	New York City Public Health Laboratory	Jade Wang, et al.
EPI_ISL_631978	Brookdale University Hospital Medical Center	New York City Public Health Laboratory	Jade Wang, et al.
EPI_ISL_631979	Jamaica Hospital Medical Center	New York City Public Health Laboratory	Jade Wang, et al.
EPI_ISL_631980	St Barnabas Hospital	New York City Public Health Laboratory	Jade Wang, et al.
EPI_ISL_631981	Brookdale University Hospital Medical Center	New York City Public Health Laboratory	Jade Wang, et al.
EPI_ISL_631994	Jamaica Hospital Medical Center	New York City Public Health Laboratory	Jade Wang, et al.
EPI_ISL_631995	Wyckoff Heights Medical Center	New York City Public Health Laboratory	Jade Wang, et al.
EPI_ISL_631996, EPI_ISL_631997	Jamaica Hospital Medical Center	New York City Public Health Laboratory	Jade Wang, et al.
EPI_ISL_631998, EPI_ISL_632085	Richmond University Medical Center	New York City Public Health Laboratory	Jade Wang, et al.
EPI_ISL_632086	Jamaica Hospital Medical Center	New York City Public Health Laboratory	Jade Wang, et al.
EPI_ISL_632087	Flushing Hospital Medical Center	New York City Public Health Laboratory	Jade Wang, et al.
EPI_ISL_632088	Jamaica Hospital Medical Center	New York City Public Health Laboratory	Jade Wang, et al.
EPI_ISL_632089	Brookdale University Hospital Medical Center	New York City Public Health Laboratory	Jade Wang, et al.
EPI_ISL_632090, EPI_ISL_632108, EPI_ISL_632109, EPI_ISL_632110	Jamaica Hospital Medical Center	New York City Public Health Laboratory	Jade Wang, et al.
EPI_ISL_632112, EPI_ISL_632113, EPI_ISL_632115	Richmond University Medical Center	New York City Public Health Laboratory	Jade Wang, et al.
EPI_ISL_632116	NYC HH Lincoln Medical And Mental Health Center	New York City Public Health Laboratory	Jade Wang, et al.
EPI_ISL_632119	Flushing Hospital Medical Center	New York City Public Health Laboratory	Jade Wang, et al.
EPI_ISL_632121	Bellevue Hospital Center	New York City Public Health Laboratory	Jade Wang, et al.
EPI_ISL_632123	Brookdale University Hospital Medical Center	New York City Public Health Laboratory	Jade Wang, et al.
EPI_ISL_632129	Wyckoff Heights Medical Center	New York City Public Health Laboratory	Jade Wang, et al.
EPI_ISL_632133, EPI_ISL_632140, EPI_ISL_632144	Flushing Hospital Medical Center	New York City Public Health Laboratory	Jade Wang, et al.
EPI_ISL_632154, EPI_ISL_632163	Richmond University Medical Center	New York City Public Health Laboratory	Jade Wang, et al.
EPI_ISL_632172	Jamaica Hospital Medical Center	New York City Public Health Laboratory	Jade Wang, et al.
EPI_ISL_632174	Flushing Hospital Medical Center	New York City Public Health Laboratory	Jade Wang, et al.
EPI_ISL_632847, EPI_ISL_632848, EPI_ISL_632850, EPI_ISL_632852, EPI_ISL_632853, EPI_ISL_632864, EPI_ISL_632865, EPI_ISL_632871, EPI_ISL_632873, EPI_ISL_632876, EPI_ISL_632881, EPI_ISL_632882			
see above	Idaho Bureau of Laboratories	Center for Global Health, University of New Mexico Health Sciences Center	Daryl Domman, Kurt Schwalm, Matthew Burns, Robert Voermans, Christopher Ball, Darrell Dinwiddie
EPI_ISL_632926, EPI_ISL_632927, EPI_ISL_632928	Cadham Provincial laboratory	Cadham Provincial laboratory	Anna Majer, Shari Tyson, Grace Seo, Philip Mabon, Elsie Grudeski, Rhiannon Huzarewich, Russell Mandes, Anneliese Landgraff, Jennifer Tanner, Natalie Knox, Morag Graham, Gary Van Domselaar, Paul Van Caeseele, Jared Bullard, David Alexander, Kerry Dust, Nathalie Bastien, Yan Li, Timothy Booth, Darian Hole, Madison Chapel, CanCOGeN's metadata curation team, Public Health Agency of Canada CanCOGeN team
EPI_ISL_632966, EPI_ISL_632967	Flushing Hospital Medical Center	New York City Public Health Laboratory	Jade Wang, et al.
EPI_ISL_635205, EPI_ISL_635220, EPI_ISL_635244, EPI_ISL_635245, EPI_ISL_635246, EPI_ISL_635247, EPI_ISL_635248, EPI_ISL_635249	Institute of Microbiology and Immunology, Faculty of Medicine, University of Ljubljana	Institute of Microbiology and Immunology, Faculty of Medicine, University of Ljubljana	Samo Zakotnik, Tomaž Mark Zorec, Miša Korva, Mario Poljak, Tatjana Avši - Županc
EPI_ISL_635250, EPI_ISL_635251	Institute of Microbiology and Immunology, Faculty of Medicine, University of Ljubljana	Institute of Microbiology and Immunology, Faculty of Medicine, University of Ljubljana	Tomaž Mark Zorec, Samo Zakotnik, Miša Korva, Tatjana Avši - Županc, Mario Poljak
EPI_ISL_636531	Dutch COVID-19 response team	National Institute for Public Health and the Environment (RIVM)	Adam Meijer, Harry Vennema, Jeroen Cremer, Sharon van den Brink, Bas van der Veer, AnneMarie van den Brandt, Florian Zwagemaker, Dennis Schmitz, Chantal Reusken, on behalf of the national COVID-19 response team
EPI_ISL_639794, EPI_ISL_639795,	unknown	Public Health Virology Laboratory, Forensic and Scientific	Son Nguyen et al.

EPI_ISL_639796		Services (PHV-FSS)	
EPI_ISL_640038	Knysna Hospital wc KNY	NHLS/UCT	Arash Iranzadeh, Deelan Doolabh, Lynn Tyers, Bruna Galvao, Innocent Mudau, Marvin Hsiao, Kruger Marais, Diana Hardie, Stephen Korsman, Carolyn Williamson
EPI_ISL_640060	Groote Schuur Hospital wc GSH	NHLS/UCT	Arash Iranzadeh, Deelan Doolabh, Lynn Tyers, Bruna Galvao, Innocent Mudau, Marvin Hsiao, Kruger Marais, Diana Hardie, Stephen Korsman, Carolyn Williamson
EPI_ISL_641519, EPI_ISL_641543	CHU de Saint-Étienne Hôpital Nord	CNR Virus des Infections Respiratoires - France SUD	Antonin Bal, Gregory Destras, Gwendolyne Burfin, Hadrien Règue, Quentin Semanas, Martine Valette, Bruno Lina, Issam Bechri, Manon Vogrig, Marine Delorme, Bruno Pozzetto, Thomas Bourlet, Sylvie Gonzalo, Sylvie Pillet, Laurence Josset
EPI_ISL_644403, EPI_ISL_644405, EPI_ISL_644409, EPI_ISL_644410	MEPHI, Aix Marseille University	MEPHI, Aix Marseille University	Anthony LEVASSEUR
EPI_ISL_644687	CHU Montpellier	CNR Virus des Infections Respiratoires - France SUD	Antonin Bal, Gregory Destras, Gwendolyne Burfin, Hadrien Règue, Quentin Semanas, Martine Valette, Bruno Lina, Michel Segondy, Vincent Foulongne, Laurence Josset
EPI_ISL_644702	CHU Nîmes	CNR Virus des Infections Respiratoires - France SUD	Antonin Bal, Gregory Destras, Gwendolyne Burfin, Hadrien Règue, Quentin Semanas, Martine Valette, Bruno Lina, Jean-Philippe Lavigne, Stephan Robin, Maxence Lotellier, Marie-Josée Carles, Laurence Josset
EPI_ISL_644710, EPI_ISL_644712	Unité des Virus Émergents	CNR Virus des Infections Respiratoires - France SUD	Antonin Bal, Gregory Destras, Gwendolyne Burfin, Hadrien Règue, Quentin Semanas, Martine Valette, Bruno Lina, Laetitia Ninove, Léa Luciani, Antoine Nougairède, Laurence Josset
EPI_ISL_645174	CHU de Limoges	CNR Virus des Infections Respiratoires - France SUD	Antonin Bal, Gregory Destras, Gwendolyne Burfin, Hadrien Règue, Quentin Semanas, Martine Valette, Bruno Lina, Sylvie Rogez, Laurence Josset
EPI_ISL_645190, EPI_ISL_645191	CHU Clermont-Ferrand	CNR Virus des Infections Respiratoires - France SUD	Antonin Bal, Gregory Destras, Gwendolyne Burfin, Hadrien Règue, Quentin Semanas, Martine Valette, Bruno Lina, Christine Archimbaud, Amélie Brebion, Hélène Chabrolles, Martine Chambon, Audrey Mirand, Christel Regagnon, Maxime Bisseux, Patricia Combes, Cécile Henquell, Laurence Josset
EPI_ISL_645197, EPI_ISL_645198	CHU Nîmes	CNR Virus des Infections Respiratoires - France SUD	Antonin Bal, Gregory Destras, Gwendolyne Burfin, Hadrien Règue, Quentin Semanas, Martine Valette, Bruno Lina, Jean-Philippe Lavigne, Stephan Robin, Maxence Lotellier, Marie-Josée Carles, Laurence Josset
EPI_ISL_648024, EPI_ISL_648025, EPI_ISL_648026	MS Public Health Laboratory	Pathogen Discovery, Respiratory Viruses Branch, Division of Viral Diseases, Centers for Disease Control and Prevention	Yan Li, Jing Zhang, Ying Tao, Brian Lynch, Krista Queen, Anna Montmayeur, Anna Uehara, Clinton R. Paden, Rachel Marine, Haibin Wang, Suxiang Tong
EPI_ISL_648075, EPI_ISL_648076, EPI_ISL_648077, EPI_ISL_648078, EPI_ISL_648079, EPI_ISL_648080	Department of Laboratory Medicine, Tan Tock Seng Hospital	Department of Laboratory Medicine, Tan Tock Seng Hospital	Chen YYC, Zair X, Lim JX, Li C, Tang WY, Maurer-Stroh S, Barkham TMS, Nagarajan N, Sessions OM
EPI_ISL_648380, EPI_ISL_648595	Laboratorio de Infectología Servicio de Infectología Hospital Universitario Dr. José Eleuterio González - Universidad Autónoma de Nuevo León	Laboratorio de Infectología Molecular Departamento de Bioquímica y Medicina Molecular Facultad de Medicina - Universidad Autónoma de Nuevo León	Kame A. Galán-Huerta, María F. Herrera-Saldivar, Natalia Martínez-Acuña, Sonia A. Lozano-Sepúlveda, Daniel Arellanos-Soto, Ana M. Rivas-Estilla, Paola Bocanegra-Ibarias, Samantha M. Flores-Treviño, Elvira Garza-González, Eduardo Perez-Alba, Laura Nuzzolo-Shihadeh, Adrian Camacho-Ortiz, Roberto Montes-de-Oca, Consuelo Treviño-Garza, Manuel E. de-la-O-Cavazos
EPI_ISL_653179, EPI_ISL_653277, EPI_ISL_653278	Florida Bureau of Public Health Laboratories	Florida Bureau of Public Health Laboratories	Sarah Schmedes, Jason Blanton
EPI_ISL_654016	Laboratory of Microbiology, National Reference Lab, Charles Nicolle Hospital; 2-University of Tunis ElManar, Faculty of Medicine of Tunis, LR99ES09, Tunis, Tunisia	1-Clinical and Experimental Pharmacology Lab, LR16SP02, National Center of Pharmacovigilance, University of Tunis El Manar, Tunis, Tunisia. 2-Neurodegenerative diseases and psychiatric troubles, LR18SP03, Razi Hospital, University of Tunis El Manar, Tunis, Tunisia. 3- Ministry of Health, National Observatory of New and Emerging Diseases, 1006, Tunis, Tunisia	Ilhem Boutiba-Ben Boubaker, Sameh Trabelsi, Nissaf Ben Alaya, Maher Kharrat, Alia Ben Kahla, Jalila Ben Khelil, Salma Abid, Sana Ferjani, Mouna Ben Sassi, Mouna Safer, Imen Mkada, Imen Kacem, Gaies Emna, Soumaya Rammeh, Riadh Daghfous, Riadh Gouider.
EPI_ISL_654187	Hospital General Universitario Gregorio Marañón	SeqCOVID-SPAIN consortium/IBV(CSIC)	Darío García de Viedma, Laura Pérez-Lago, Marta Herranz, Jon Sicilia, Julia Suárez, Pilar Catalán, Patricia Muñoz and SeqCOVID-SPAIN consortium
EPI_ISL_654673	Servicio de Microbiología, Hospital Universitario Central de Asturias	SeqCOVID-SPAIN consortium/IBV(CSIC)	Cristián Castelló Abietar, Jose A. Boga, Susana Rojo-Alba, Marta Elena Álvarez-Argüelles, Santiago Melón and SeqCOVID-SPAIN consortium
EPI_ISL_654754	Texas Department of State Health Services	Texas Department of State Health Services	Rashmi Tuladhar, Bonnie Oh, Jenny Zhang, Maliha Rahman, Anita Pokharel, Myong Koag, Chung Wang, Rachel Lee, Grace Kubin, Mayela Pedrueza, James Daniel Bonser
EPI_ISL_654867	Pasteur Institute in Ho Chi Minh city	Department of Microbiology and Immunology - Pasteur Institute in Ho Chi Minh city	Hoàng Minh, Cao Minh Thng, Phm Th Thu Hng, V Phm Hng Nhung, ào Huy Mnh, Hunh Th Kim Loan, Nguyn Hoàng Quân, Hunh Phng Tho, Hoàng Nh ào, Nguyn Trung Hiu, Nguyn Hoàng Anh, Nguyn Thu Ngc, Phm Th Nhung, La Hoàng Huy, ng Thanh Giang, Nguyn Th Thanh Thng, Trn Th Hng Kim, Nguyn Thanh Long, Phm Duy Quang, Lng Chn Quang, Hoàng Quc Cng, Phan Trng Lân, Nguyn V Thng
EPI_ISL_654868	Pasteur Institute in Ho Chi Minh city	Department of Microbiology and Immunology - Pasteur Institute in Ho Chi Minh city	ào Huy Mnh, Phm Th Thu Hng, V Phm Hng Nhung, Cao Minh Thng, Hunh Th Kim Loan, Nguyn Hoàng Quân, Hunh Phng Tho, Hoàng Nh ào, Nguyn Trung Hiu, Nguyn Hoàng Anh, Nguyn Thu Ngc, Phm Th Nhung, Nguyn Th Ngc Tho, Hà Bích Ngc, Nguyn Th Thanh Thng, Hoàng Minh, Trn Th Hng Kim, Nguyn Thanh Long, Phm Duy Quang, Lng Chn Quang, Hoàng Quc Cng, Nguyn V Thng, Phan Trng Lân
EPI_ISL_654869	Pasteur Institute in Ho Chi Minh city	Department of Microbiology and Immunology - Pasteur Institute in Ho Chi Minh city	Phm Th Nhung, ào Huy Mnh, Phm Th Thu Hng, V Phm Hng Nhung, Cao Minh Thng, Hunh Th Kim Loan, Nguyn Hoàng Quân, Hunh Phng Tho, Hoàng Nh ào, Nguyn Trung Hiu, Nguyn Hoàng Anh, Nguyn Thu Ngc, Nguyn Th Ngc Tho, ng Thanh Giang, Võ Th Trang ài, Hoàng Minh, Trn Th Hng Kim, Nguyn Thanh Long, Phm Duy Quang, Lng Chn Quang, Hoàng Quc Cng, Phan Trng Lân, Nguyn V Thng
EPI_ISL_654870	Pasteur Institute in Ho Chi Minh city	Department of Microbiology and Immunology - Pasteur Institute in Ho Chi Minh city	Trn Th Hng Kim, Phm Th Thu Hng, ào Huy Mnh, V Phm Hng Nhung, Cao Minh Thng, Hunh Th Kim Loan, Nguyn Hoàng Quân, Hunh Phng Tho, Hoàng Nh ào, Nguyn Trung Hiu, Nguyn Hoàng Anh, Nguyn Thu Ngc, Phm Th Nhung, Nguyn Th Ngc Tho, ng Thanh Giang, Nguyn Th Thanh Thng, Hoàng Kim Loan, Nguyn Thanh Long, Phm Duy Quang, Lng Chn Quang, Hoàng Quc Cng, Nguyn V Thng, Phan Trng Lân
EPI_ISL_654878	Pasteur Institute in Ho Chi Minh city	Department of Microbiology and Immunology - Pasteur Institute in Ho Chi Minh city	Phm Duy Quang, ào Huy Mnh, Phm Th Thu Hng, V Phm Hng Nhung, Cao Minh Thng, Hunh Th Kim Loan, Nguyn Hoàng Quân, Hunh Phng Tho, Hoàng Nh ào, Nguyn Trung Hiu, Nguyn Hoàng Anh, Nguyn Thu Ngc, Phm Th Nhung, Nguyn Th Ngc Tho, Viên ng Khánh Linh, Nguyn Th Thanh Thng, Hoàng Minh, Trn Th Hng Kim, Nguyn Thanh Long, Lng Chn Quang, Hoàng Quc Cng, Phan Trng Lân, Nguyn V Thng
EPI_ISL_654885	Pasteur Institute in Ho Chi Minh city	Department of Microbiology and Immunology - Pasteur Institute in Ho Chi Minh city	Nguyn V Thng, ào Huy Mnh, Phm Th Thu Hng, V Phm Hng Nhung, Cao Minh Thng, Hunh Th Kim Loan, Nguyn Hoàng Quân, Hunh Phng Tho, Hoàng Nh ào, Nguyn Trung Hiu, Nguyn Hoàng Anh, Nguyn Thu Ngc, Phm Th Nhung, Nguyn Th Ngc Tho, ng Thanh Giang, Hoàng Thùy Linh, Hoàng Minh, Trn Th Hng Kim, Nguyn Thanh Long, Phm Duy Quang, Lng Chn Quang, Hoàng Quc Cng, Phan Trng Lân
EPI_ISL_654888	Pasteur Institute in Ho Chi Minh city	Department of Microbiology and Immunology - Pasteur Institute in Ho Chi Minh city	Nguyn Hoàng Anh, Hoàng Quc Cng, ào Huy Mnh, Phm Th Thu Hng, V Phm Hng Nhung, Cao Minh Thng, Hunh Th Kim Loan, Nguyn Hoàng Quân, Hunh Phng Tho, Hoàng Nh ào, Nguyn Trung Hiu, Nguyn Thu Ngc, Phm Th Nhung, Nguyn Th Ngc Tho, ng Thanh Giang, Nguyn Th Thanh Thng, Hoàng Minh, Khu Vn Ngha, Nguyn Thanh Long, Phm Duy Quang, Lng Chn Quang, Phan Trng Lân, Nguyn V Thng
EPI_ISL_657460, EPI_ISL_657463	Servicio de Microbiología. Hospital General Universitario de Castellón	SeqCOVID-SPAIN consortium/IBV(CSIC)	Rosario Moreno Muñoz, María Dolores Tirado Balaguer and SeqCOVID-SPAIN consortium
EPI_ISL_660338, EPI_ISL_660339, EPI_ISL_660340, EPI_ISL_660341	CHU de Saint-Étienne Hôpital Nord	CNR Virus des Infections Respiratoires - France SUD	Antonin Bal, Gregory Destras, Gwendolyne Burfin, Hadrien Règue, Quentin Semanas, Martine Valette, Bruno Lina, Issam Bechri, Manon Vogrig, Marine Delorme, Bruno Pozzetto, Thomas Bourlet, Sylvie Gonzalo, Sylvie Pillet, Laurence Josset
EPI_ISL_660356, EPI_ISL_660357, EPI_ISL_660358	CHU Clermont-Ferrand	CNR Virus des Infections Respiratoires - France SUD	Antonin Bal, Gregory Destras, Gwendolyne Burfin, Hadrien Règue, Quentin Semanas, Martine Valette, Bruno Lina, Christine Archimbaud, Amélie Brebion, Hélène Chabrolles, Martine Chambon, Audrey Mirand, Christel Regagnon, Maxime Bisseux, Patricia Combes, Cécile Henquell, Laurence Josset
EPI_ISL_660385	Klinisk mikrobiologi Linkoping	The Public Health Agency of Sweden	Anna-Malin Linde, Maria Lind Karlberg, Mattias Haukland, Reza Advani, Olov Svartstrom, Oskar Karlsson Lindsjo, Sandra Broddesson, Petra Edquist, Mia Brytting, Anna Risberg, Karin Tegmark-Wisell
EPI_ISL_660675, EPI_ISL_660676, EPI_ISL_660677, EPI_ISL_660678,	CHU Montpellier	CNR Virus des Infections Respiratoires - France SUD	Antonin Bal, Gregory Destras, Gwendolyne Burfin, Hadrien Règue, Quentin Semanas, Martine Valette, Bruno Lina, Michel Segondy, Vincent Foulongne, Laurence Josset

EPI_ISL_660679, EPI_ISL_660680			
EPI_ISL_660713	CHU Nîmes	CNR Virus des Infections Respiratoires - France SUD	Antonin Bal, Gregory Destras, Gwendolyne Burfin, Hadrien Règue, Quentin Semanas, Martine Valette, Bruno Lina, Jean-Philippe Lavigne, Stephan Robin, Maxence Lotellier, Marie-Josée Carles, Laurence Josset
EPI_ISL_660734, EPI_ISL_660736	Unité des Virus Émergents	CNR Virus des Infections Respiratoires - France SUD	Antonin Bal, Gregory Destras, Gwendolyne Burfin, Hadrien Règue, Quentin Semanas, Martine Valette, Bruno Lina, Laetitia Ninove, Léa Luciani, Antoine Nougairède, Laurence Josset
EPI_ISL_661253, EPI_ISL_661254	Quest Diagnostics	Quest Diagnostics	Rosenthal,S.H., Gerasimova,A., Kagan,R.M., Anderson, B., Owen, R., Lacbawan, F.
EPI_ISL_663206	CHU de Limoges	CNR Virus des Infections Respiratoires - France SUD	Antonin Bal, Gregory Destras, Gwendolyne Burfin, Hadrien Règue, Quentin Semanas, Martine Valette, Bruno Lina, Sylvie Rogez, Laurence Josset
EPI_ISL_663251, EPI_ISL_663252, EPI_ISL_663253	CHU Nantes	CNR Virus des Infections Respiratoires - France SUD	Antonin Bal, Louise Castain, Gregory Destras, Gwendolyne Burfin, Hadrien Règue, Quentin Semanas, Martine Valette, Bruno Lina, Virginie Ferré, Celine Bressollette, Laurence Josset
EPI_ISL_664707	University College London Hospital	COVID-19 Genomics UK (COG-UK) Consortium	Judith Heaney, Matthew Byott, Catherine Houlihan, Dan Frampton, Stuart Kirk, Moira Spyer and Eleni Nastouli
EPI_ISL_666654	Laboratoire du Centre Hospitalier Annecy Genevois	CNR Virus des Infections Respiratoires - France SUD	Antonin Bal, Gregory Destras, Gwendolyne Burfin, Hadrien Règue, Quentin Semanas, Martine Valette, Bruno Lina, Hélène Petitprez, Bruno Chanzy, Laurence Josset
EPI_ISL_666683, EPI_ISL_666684, EPI_ISL_666685	Centre hospitalier Métropole Savoie	CNR Virus des Infections Respiratoires - France SUD	Antonin Bal, Carine Dumollard, Gregory Destras, Gwendolyne Burfin, Hadrien Règue, Quentin Semanas, Martine Valette, Bruno Lina, Jérôme Grosjean, Laurence Josset
EPI_ISL_666964	Quest Diagnostics	Quest Diagnostics	Rosenthal,S.H., Gerasimova,A., Kagan,R.M., Anderson, B., Owen, R., Lacbawan, F.
EPI_ISL_671374	National Virus Reference Laboratory	Irish Coronavirus Sequencing Consortium - Teagasc Moorepark	Calum Walsh, Fiona Crispie, John Kenny, Paul Cotter
EPI_ISL_671765	Santa Clara County Public Health Department	Chiu Laboratory, University of California, San Francisco	Xianding Deng, Scot Federman, Wei Gu, Elsa Villarino, Brandon Bonin, Debra A. Wadford, and Charles Y. Chiu
EPI_ISL_672158, EPI_ISL_672159	The Ashley Laboratory, Stanford University	Chan-Zuckerberg Biohub	CZB Cliahub Consortium
EPI_ISL_676703, EPI_ISL_676704, EPI_ISL_676737, EPI_ISL_676738, EPI_ISL_676739, EPI_ISL_676740, EPI_ISL_676742, EPI_ISL_676743, EPI_ISL_676744, EPI_ISL_676745, EPI_ISL_676746, EPI_ISL_676747			
see above	Wadsworth Center, New York State Department.of Health	Wadsworth Center, New York State Department.of Health	Kirsten St. George, Daryl M. Lamson, Alexis Russel, Jonathan Plitnick, Navjot Singh, John Kelly, Sara Griesemer, Erasmus Schneider, Erica Lasek-Nesselquist
EPI_ISL_677260	Colorado Department of Public Health and Environment	Colorado Department of Puplic Health and Environment	Laura Bankers, Molly Hetherington-Rauth, Shannon Ely, Shannon R. Matzinger, Sarah Elizabeth Totten, Emily A. Travanty
EPI_ISL_677664	Wadsworth Center, New York State Department.of Health	Wadsworth Center, New York State Department.of Health	Kirsten St. George, Daryl M. Lamson, Alexis Russel, Jonathan Plitnick, Navjot Singh, John Kelly, Sara Griesemer, Erasmus Schneider, Erica Lasek-Nesselquist
EPI_ISL_678275, EPI_ISL_678279, EPI_ISL_678287	Mikrobiologie, RARI	Mikrobiologie, RARI	Krasnov,Y.M., Naryshkina,E.A., Guseva,N.P., Sosedova,E.A., Fedorov,A.V., Badanin,D.V., Sharapova,N.A., Portenko,S.A., Shcherbakova,S.A., Kutyrev,V.V.
EPI_ISL_678300	Area of Virology, Serology and Virology Division (SAViD), New South Wales Health Pathology Randwick	Virology Research Laboratory; Area of Virology, Serology and Virology Division (SAViD), New South Wales Health Pathology Randwick	Foster, C.; Au, J.; Ruiz Silva, M.; Deveson, I.; Bull, R.; Van Hal, S.; Rawlinson, W.
EPI_ISL_678494, EPI_ISL_678495	CNR Virus des Infections Respiratoires - France SUD	CNR Virus des Infections Respiratoires - France SUD	Antonin Bal, Gregory Destras, Gwendolyne Burfin, Solenne Brun, Martine Valette, Bruno Lina, Laurence Josset
EPI_ISL_681999	UPMC Clinical Microbiology Laboratory	Microbial Genomic Epidemiology Laboratory, University of Pittsburgh	Mustapha M. Mustapha, Jane W. Marsh, Dan Snyder, Marissa P. Griffith, Stephanie L. Mitchell, Vatsala R. Srinivasa, Kady D. Waggle, Chinelo Ezeonwuku, Vaughn S. Cooper, Lee H. Harrison
EPI_ISL_683329	1-Laboratory of Microbiology, National Reference Lab, Charles Nicolle Hospital; 2-University of Tunis ElManar, Faculty of Medicine of Tunis, LR99ES09, Tunis, Tunisia	1-Clinical and Experimental Pharmacology Lab, LR16SP02, National Center of Pharmacovigilance, University of Tunis El Manar, Tunis, Tunisia. 2-Neurodegenerative diseases and psychiatric troubles, LR18SP03, Razi Hospital, University of Tunis El Manar, Tunis, Tunisia. 3- Ministry of Health, National Observatory of New and Emerging Diseases, 1006, Tunis, Tunisia	Ilhem Boutiba-Ben Boubaker, Sameh Trabelsi, Nissaf Ben Alaya, Maher Kharrat, Alia Ben Kahla, Jalila Ben Khelil, Salma Abid, Sana Ferjani, Asma Ferjani, Mouna Ben Sassi, Mouna Safer, Guedi Berrabeh,Salwa Mrabet, Hanen ElJebari, Gaies Emna, Riadh Daghfous, Riadh Gouider.
EPI_ISL_683665, EPI_ISL_683666, EPI_ISL_683674, EPI_ISL_683675	IdISSC/Hospital Clínico San Carlos de Madrid	SeqCOVID-SPAIN consortium/IBV(CSIC)	Alberto Delgado-Iribarren, Esther Culebras Lopez, Vicente Estrada Pérez, Jorge Matias-Guiu, Luis Ortega Medina, Silvia Sánchez Ramón, Ulises Gómez-Pinedo and SeqCOVID-SPAIN consortium
EPI_ISL_693359, EPI_ISL_693360, EPI_ISL_693361, EPI_ISL_693362, EPI_ISL_693363, EPI_ISL_693364, EPI_ISL_693365, EPI_ISL_693366, EPI_ISL_693367, EPI_ISL_693368, EPI_ISL_693369, EPI_ISL_693370, EPI_ISL_693371, EPI_ISL_693372, EPI_ISL_693373, EPI_ISL_693374, EPI_ISL_693375, EPI_ISL_693376, EPI_ISL_693377, EPI_ISL_693378, EPI_ISL_693379, EPI_ISL_693380, EPI_ISL_693381, EPI_ISL_693382, EPI_ISL_693383, EPI_ISL_693384, EPI_ISL_693385			
see above	Ramathibodi Hospital	COVID-19 Network Investigations (CONI) Alliance	Elizabeth Batty, Wasun Chantratita, Thanat Chookajorn, Stefan Fernandez, Angkana Huang, Anthony R. Jones, Khajohn Joonsalak, Chonticha Klungtong, Theerarat Kochakarn, Namfon Kotanan, Krittikorn Kumpornsin, Wudtichai Manasatienkij, Bhakbhoom Panthan, Ekawat Pasomsub, Kingkan Rakmanee, Insee Sensorn, Janjira Thaipadungpanit, Arporn Wangwiwatsin,Treewat Watthanachockchai
EPI_ISL_694048	TGen North	TGen North	Jolene Bowers, Megan Folkerts, Chris French, Hayley Yaglom, Ashlyn Pfeiffer, Darrin Lemmer, Dave Engelthaler, The Arizona COVID Genomics Union (ACGU)
EPI_ISL_694049	AZ SPHL, Arizona Department of Health Services	TGen North	Jolene Bowers, Megan Folkerts, Chris French, Hayley Yaglom, Ashlyn Pfeiffer, Darrin Lemmer, Dave Engelthaler, The Arizona COVID Genomics Union (ACGU)
EPI_ISL_694052	TGen North	TGen North	Jolene Bowers, Megan Folkerts, Chris French, Hayley Yaglom, Ashlyn Pfeiffer, Darrin Lemmer, Dave Engelthaler, The Arizona COVID Genomics Union (ACGU)
EPI_ISL_699464	Regional Medical Sciences Center 11/1 phuket	National Institute of Health, Department of Medical Sciences, Ministry of Public Health, Thailand	Pilailuk Okada; Siripaporn Phuygun; Thanutsapa Thanadachakul; Sittiporn Parnmen; Warawan Wongboot; Sunthareeya Waicharoen; Malinee Chittaganpitch
EPI_ISL_699657	1-Laboratory of Microbiology, National Reference Lab, Charles Nicolle Hospital; 2-University of Tunis ElManar, Faculty of Medicine of Tunis, LR99ES09, Tunis, Tunisia	1-Clinical and Experimental Pharmacology Lab, LR16SP02, National Center of Pharmacovigilance, University of Tunis El Manar, Tunis, Tunisia. 2-Neurodegenerative diseases and psychiatric troubles, LR18SP03, Razi Hospital, University of Tunis El Manar, Tunis, Tunisia. 3- Ministry of Health, National Observatory of New and Emerging Diseases, 1006, Tunis, Tunisia	Ilhem Boutiba-Ben Boubaker, Sameh Trabelsi, Nissaf Ben Alaya, Maher Kharrat, Alia Ben Kahla, Jalila Ben Khelil, Salma Abid, Sana Ferjani, Asma Ferjani, Mouna Ben Sassi, Mouna Safer, Guedi Berrabeh,Salwa Mrabet, Hanen ElJebari, Gaies Emna, Riadh Daghfous, Riadh Gouider.
EPI_ISL_700326	CHU Bordeaux	CNR Virus des Infections Respiratoires - France SUD	Antonin Bal, Gregory Destras, Gwendolyne Burfin, Hadrien Règue, Quentin Semanas, Martine Valette, Bruno Lina, Pantxika Bellecave, Camille Ciccone, Isabelle Garrigue, Marie-Edith Lafon, Pascale Trimoulet, Laurence Josset
EPI_ISL_700371	CHU Nantes	CNR Virus des Infections Respiratoires - France SUD	Antonin Bal, Louise Castain, Gregory Destras, Gwendolyne Burfin, Hadrien Règue, Quentin Semanas, Martine Valette, Bruno Lina, Virginie Ferré, Celine Bressollette, Laurence Josset
EPI_ISL_707782	CHU Poitiers	CNR Virus des Infections Respiratoires - France SUD	Antonin Bal, Gregory Destras, Gwendolyne Burfin, Hadrien Règue, Quentin Semanas, Martine Valette, Bruno Lina, Agnès Beby-Defaux, Magali Garcia, Clément Jousselin, Nicolas Lévêque, Laurence Josset
EPI_ISL_707949, EPI_ISL_707950, EPI_ISL_707951, EPI_ISL_707952, EPI_ISL_707953, EPI_ISL_707954	Virology, Universitätsklinikum des Saarlandes	Epigenetics, Saarland University	Kathrin Kattler, Markus Vogelgesang, Stefan Lohse, Sascha Tierling, Sigrun Smola, Jörn Walter

EPI_ISL_723079, EPI_ISL_723085, EPI_ISL_723086	Institute of Medical Genetics and Applied Genomics	Institute of Medical Genetics and Applied Genomics	Caspar Gross, Tina Ganzenmüller, Siri Göpel, Michaela Pogoda, Daniela Bezdan, Michael Sonnabend, Angel Angelov, Nicolas Casadei, Stephan Ossowski, Thomas Iftner, Michael Bitzer
EPI_ISL_729340, EPI_ISL_729466	A. Krumbholz, Labor Dr. Krause und Kollegen MVZ GmbH, Kiel	Charité Universitätsmedizin Berlin, Institut für Virologie	Victor M Corman, Barbara Mühlemann, Jörn Beheim-Schwarzbach, Talitha Veith, Julia Schneider, Terry Jones, Christian Drosten
EPI_ISL_729482, EPI_ISL_729483	Charité Universitätsmedizin Berlin, Institut für Virologie/Labor Berlin	Charité Universitätsmedizin Berlin, Institut für Virologie	Victor M Corman, Barbara Mühlemann, Jörn Beheim-Schwarzbach, Talitha Veith, Julia Schneider, Terry Jones, Christian Drosten
EPI_ISL_729507, EPI_ISL_729508, EPI_ISL_729509, EPI_ISL_729510, EPI_ISL_729511, EPI_ISL_729512, EPI_ISL_729513, EPI_ISL_729515, EPI_ISL_729516, EPI_ISL_729517, EPI_ISL_729518, EPI_ISL_729519, EPI_ISL_729520, EPI_ISL_729521, EPI_ISL_729549			
see above	A. Krumbholz, Labor Dr. Krause und Kollegen MVZ GmbH, Kiel	Charité Universitätsmedizin Berlin, Institut für Virologie	Victor M Corman, Barbara Mühlemann, Jörn Beheim-Schwarzbach, Talitha Veith, Julia Schneider, Terry Jones, Christian Drosten
EPI_ISL_729805, EPI_ISL_729806, EPI_ISL_729807	Laboratorio Central de Saude Publica do Estado do Rio Grande do Sul (LACEN-RS)	Laboratory of Respiratory Viruses and Measles, Oswaldo Cruz Institute, FIOCRUZ	Paola Resende, Luciana Appolinario, Fernando Motta, Anna Carolina Paixão, Ana Carolina Mendonça, Tatiana Schaffer Gregianini, Marilda Tereza Mar da Rosa, Marilda Siqueira
EPI_ISL_732545, EPI_ISL_732546	Bundeswehr Institute of Microbiology	Bundeswehr Institute of Microbiology	Markus Antwerpen, Alexandra Rehn, Mathias Walter, Malena Bestehorn-Willmann, Sabine Zange, Enrico Georgi, Roman Wölfel
EPI_ISL_734505, EPI_ISL_734506	UZ Leuven, National Reference Laboratory for Coronaviruses, Laboratory Medicine, Leuven, Belgium	KU Leuven, Rega Institute, Clinical and Epidemiological Virology	Tony Wawina-Bokalanga, Joan Marti-Carerras, Bert Vanmechelen, Piet Maes
EPI_ISL_735449, EPI_ISL_736775	UW Virology Lab	UW Virology Lab	Pavitra Roychoudhury, Hong Xie, Lasata Shrestha, Meei-Li Huang, Keith R Jerome, Alexander Greninger
EPI_ISL_738085	University Hospitals of Geneva, Laboratory of Virology	University Hospitals of Geneva, Laboratory of Virology	Cordey Samuel and Laubscher Florian
EPI_ISL_740250, EPI_ISL_740440, EPI_ISL_744478	Laboratoire national de santé, Microbiology, Virology	Laboratoire national de santé, Microbiology, Microbial Genomics Platform	Anke Wienecke-Baldacchino, Catherine Ragimbeau, Tamir Abdelrahman, Jessica Tapp, Fatu Djabi
EPI_ISL_751380, EPI_ISL_751381	IRCCS Sacro Cuore Don Calabria Hospital, Department of Infectious, Tropical Diseases & Microbiology	University of Verona, Department of Biotechnology	Antonio Mori, Michela Deiana, Elena Pomari, Chiara Piubelli; Giulia Lopatriello, Luca Marcolungo, Cristina Beltrami, Chiara Degli Esposti, Emanuela Cosentino, Massimo Delledonne
EPI_ISL_752608, EPI_ISL_752614, EPI_ISL_752615	State Laboratories Division, Hawaii State Department of Health	State Laboratories Division, Hawaii State Department of Health	Pamela O'Brien, Sabrina Diemert, Drew Kuwazaki, Razvan Sultana, Edward Desmond
EPI_ISL_753763, EPI_ISL_753799, EPI_ISL_753921, EPI_ISL_753922, EPI_ISL_754056, EPI_ISL_754058	Charité Universitätsmedizin Berlin, Institut für Virologie/Labor Berlin	Charité Universitätsmedizin Berlin, Institut für Virologie	Victor M Corman, Jörn Beheim-Schwarzbach, Barbara Mühlemann, Julia Schneider, Talitha Veith, Terry Jones, Christian Drosten
EPI_ISL_754749, EPI_ISL_754751, EPI_ISL_754752, EPI_ISL_754753, EPI_ISL_754754, EPI_ISL_754756, EPI_ISL_754757, EPI_ISL_754758, EPI_ISL_754759, EPI_ISL_754760, EPI_ISL_754761, EPI_ISL_754762, EPI_ISL_754763, EPI_ISL_754764, EPI_ISL_754765			
see above	Emory Molecular Diagnostics Laboratory, Emory Healthcare	Piantadosi Lab, Emory Department of Pathology	Ahmed Babiker, Anne Piantadosi
EPI_ISL_755794, EPI_ISL_755881	Toronto Invasive Bacterial Diseases Network	McMaster University	Allison McGeer, Patryk Aftanas, Hooman Derakhshani, Angel Li, Kuganya Nirmalarajah, Emily Panousis, Ahmed Draia, Jalees Nasir, Michael Surette, Samira Mubareka, Andrew G. McArthur
EPI_ISL_766032	RSUP Dr. Sardjito	Eijkman Institute for Molecular Biology, Ministry of Research and Technology/National Agency for Research and Innovation	Frilasita A Yudhaputri, Edison Johar, Hidayat Trimarsanto, David H Muljono, Safarina G Malik, Khin Saw Myint, Amin Soebandrio
EPI_ISL_775610, EPI_ISL_775615, EPI_ISL_775616, EPI_ISL_775618, EPI_ISL_775620, EPI_ISL_775622, EPI_ISL_775625, EPI_ISL_775629, EPI_ISL_775630, EPI_ISL_775634, EPI_ISL_775635, EPI_ISL_775637, EPI_ISL_775638, EPI_ISL_775639, EPI_ISL_775642, EPI_ISL_775645, EPI_ISL_775646, EPI_ISL_775647, EPI_ISL_775649, EPI_ISL_775651, EPI_ISL_775656, EPI_ISL_775659, EPI_ISL_775664, EPI_ISL_775665, EPI_ISL_775671, EPI_ISL_775672, EPI_ISL_775673, EPI_ISL_775674, EPI_ISL_775676, EPI_ISL_775677, EPI_ISL_775679, EPI_ISL_775680, EPI_ISL_775681, EPI_ISL_775682, EPI_ISL_775688, EPI_ISL_775694, EPI_ISL_775697, EPI_ISL_775699, EPI_ISL_775707, EPI_ISL_775709, EPI_ISL_775714, EPI_ISL_775715, EPI_ISL_775716, EPI_ISL_775718, EPI_ISL_775719, EPI_ISL_775720, EPI_ISL_775725, EPI_ISL_775727, EPI_ISL_775730, EPI_ISL_775736, EPI_ISL_775740, EPI_ISL_775742, EPI_ISL_775743, EPI_ISL_775744, EPI_ISL_775747, EPI_ISL_775748, EPI_ISL_775759, EPI_ISL_775763, EPI_ISL_775764, EPI_ISL_775767, EPI_ISL_775770, EPI_ISL_775775, EPI_ISL_775776, EPI_ISL_775777, EPI_ISL_775780, EPI_ISL_775781, EPI_ISL_775784, EPI_ISL_775792, EPI_ISL_775794, EPI_ISL_775796, EPI_ISL_775799, EPI_ISL_775801, EPI_ISL_775803, EPI_ISL_775804, EPI_ISL_775807, EPI_ISL_775808, EPI_ISL_775809, EPI_ISL_775811, EPI_ISL_775812, EPI_ISL_775814, EPI_ISL_775815, EPI_ISL_775818, EPI_ISL_775819, EPI_ISL_775822, EPI_ISL_775823, EPI_ISL_775824, EPI_ISL_775833, EPI_ISL_775835, EPI_ISL_775838, EPI_ISL_775839, EPI_ISL_775842, EPI_ISL_775846, EPI_ISL_775847, EPI_ISL_775854, EPI_ISL_775858, EPI_ISL_775859, EPI_ISL_775860, EPI_ISL_775861, EPI_ISL_775862, EPI_ISL_775867, EPI_ISL_775871, EPI_ISL_775872, EPI_ISL_775874, EPI_ISL_775875, EPI_ISL_775876, EPI_ISL_775878, EPI_ISL_775879, EPI_ISL_775889, EPI_ISL_775891, EPI_ISL_775895, EPI_ISL_775901, EPI_ISL_775902, EPI_ISL_775905, EPI_ISL_775906, EPI_ISL_775925, EPI_ISL_775926, EPI_ISL_775927, EPI_ISL_775928, EPI_ISL_775929, EPI_ISL_775930, EPI_ISL_775937, EPI_ISL_775938, EPI_ISL_776013, EPI_ISL_776014, EPI_ISL_776015, EPI_ISL_776016, EPI_ISL_776017, EPI_ISL_776018, EPI_ISL_776019, EPI_ISL_776020, EPI_ISL_776021, EPI_ISL_776022, EPI_ISL_776023, EPI_ISL_776025, EPI_ISL_776026, EPI_ISL_776027, EPI_ISL_776028, EPI_ISL_776029, EPI_ISL_776030, EPI_ISL_776031, EPI_ISL_776032, EPI_ISL_776033, EPI_ISL_776034, EPI_ISL_776035, EPI_ISL_776036, EPI_ISL_776037, EPI_ISL_776038, EPI_ISL_776039, EPI_ISL_776040, EPI_ISL_776041, EPI_ISL_776042, EPI_ISL_776043, EPI_ISL_776044, EPI_ISL_776045, EPI_ISL_776046, EPI_ISL_776047, EPI_ISL_776048, EPI_ISL_776049, EPI_ISL_776050, EPI_ISL_776051, EPI_ISL_776052, EPI_ISL_776053, EPI_ISL_776054, EPI_ISL_776055, EPI_ISL_776056, EPI_ISL_776057, EPI_ISL_776058, EPI_ISL_776059, EPI_ISL_776060, EPI_ISL_776061, EPI_ISL_776062, EPI_ISL_776063, EPI_ISL_776064, EPI_ISL_776065, EPI_ISL_776066, EPI_ISL_776067, EPI_ISL_776068, EPI_ISL_776069, EPI_ISL_776070, EPI_ISL_776071, EPI_ISL_776072, EPI_ISL_776073, EPI_ISL_776074, EPI_ISL_776075, EPI_ISL_776076, EPI_ISL_776077, EPI_ISL_776078, EPI_ISL_776079, EPI_ISL_776080, EPI_ISL_776081, EPI_ISL_776082, EPI_ISL_776083, EPI_ISL_776084, EPI_ISL_776085, EPI_ISL_776086, EPI_ISL_776087, EPI_ISL_776088, EPI_ISL_776089, EPI_ISL_776090, EPI_ISL_776091, EPI_ISL_776092, EPI_ISL_776093, EPI_ISL_776094, EPI_ISL_776095, EPI_ISL_776096, EPI_ISL_776097, EPI_ISL_776098, EPI_ISL_776099, EPI_ISL_776100, EPI_ISL_776101, EPI_ISL_776102, EPI_ISL_776103, EPI_ISL_776104, EPI_ISL_776105, EPI_ISL_776106, EPI_ISL_776107, EPI_ISL_776108, EPI_ISL_776109, EPI_ISL_776110, EPI_ISL_776111, EPI_ISL_776115, EPI_ISL_776116, EPI_ISL_776336, EPI_ISL_776418, EPI_ISL_776443, EPI_ISL_776549, EPI_ISL_776550, EPI_ISL_776551, EPI_ISL_776555, EPI_ISL_776559, EPI_ISL_776561			
see above	University Medical Center Hamburg Eppendorf	Heinrich Pette Institute, Leibniz Institute for Experimental Virology	Alexis Robitaille, Thomas Günther, Johannes Knobloch, Martin Aepfelbacher, Nicole Fischer, Adam Grundhoff
EPI_ISL_779169	Laboratorio de Infectología, Servicio de Infectología, Hospital Universitario Dr. José Eleuterio González - Universidad Autónoma de Nuevo León	Laboratorio de Infectología Molecular, Departamento de Bioquímica y Medicina Molecular,Facultad de Medicina - Universidad Autónoma de Nuevo León	Kame A. Galán-Huerta, María F. Herrera-Saldivar, Natalia Martínez-Acuña, Sonia A. Lozano-Sepúlveda, Daniel Arellanos-Soto, Ana M. Rivas-Estilla, Paola Bocanegra-Ibarias, Samantha M. Flores-Treviño, Elvira Garza-González, Eduardo Perez-Alba, Laura Nuzzolo-Shihadeh, Adrian Camacho-Ortiz
EPI_ISL_779739, EPI_ISL_779741, EPI_ISL_779742, EPI_ISL_779744, EPI_ISL_779746, EPI_ISL_779758, EPI_ISL_779759, EPI_ISL_779760, EPI_ISL_779761, EPI_ISL_779764	IdISSC/Hospital Clínico San Carlos de Madrid	SeqCOVID-SPAIN consortium/IBV(CSIC)	Alberto Delgado-Iribarren, Esther Culebras López, Vicente Estrada Pérez, Jorge Matias-Guiu, Luis Ortega Medina, Silvia Sánchez Ramón, Ulises Gómez-Pinedo and SeqCOVID-SPAIN consortium
EPI_ISL_780376, EPI_ISL_780378, EPI_ISL_780381, EPI_ISL_780411	Laboratory of Infectious Diseases, Department of Biomedical and Clinical Sciences L. Sacco, University of Milan	Laboratory of Infectious Diseases, Department of Biomedical and Clinical Sciences L. Sacco, University of Milan	Alessia Lai, Annalisa Bergna, Carla Della Ventura, Claudia Balotta, Massimo Galli, Gianguglielmo Zehender on behalf of SARS-CoV-2 ITALIAN RESEARCH ENTERPRISE-(SCIRE) Collaborative Group
EPI_ISL_792458	Laboratorio del Hospital Regional Ushuaia Gdor. Ernesto Campos	Hospital Regional Ushuaia - Centro Austral De Investigaciones Científicas - Universidad Nacional De Tierra Del Fuego on behalf of 'Proyecto Argentino Interinstitucional de genomica de SARS-CoV-2' (PAIS Consortium)	Ceballos, SG; Nardi, CF; Gramundi, ID; Gallego, F; De Roccis, CA; Castro, G; Cáceres, SB; Yulan, CB; Boutureira, MF.
EPI_ISL_794606	Central Public Health Laboratories, Egyptian Ministry of Health and Population	Central Public Health Laboratories, Egyptian Ministry of Health and Population	El-Shesheny,R., Roshdy,W.H., Kayed,A.E., Mostafa,A., Khalifa,M.K., Shehata,M., Shawky,S., Saleh,M., Gomaa,M., El Taweel,A., Mahmoud,S.H., Moatasim,Y., Kutkat,O., Kamel,M.N., Mahrous,N., El Sayes,M.A., El Guindy,N.M., Naguib,A., Kandeil,A., Kayali,G., Ali,M.A.
EPI_ISL_794678, EPI_ISL_794679, EPI_ISL_794680, EPI_ISL_794692, EPI_ISL_794694, EPI_ISL_794702, EPI_ISL_794704, EPI_ISL_794712, EPI_ISL_794714, EPI_ISL_794720	PathWest Laboratory Medicine WA	PathWest Laboratory Medicine WA Microbial Surveillance Unit	PathWest Laboratory Medicine WA Microbial Surveillance Unit
EPI_ISL_796067, EPI_ISL_796101, EPI_ISL_796102, EPI_ISL_796135, EPI_ISL_796136	Servicio de Microbiología. Hospital General Universitario de Castellón	SeqCOVID-SPAIN consortium/IBV(CSIC)	Rosario Moreno Muñoz, María Dolores Tirado Balaguer and SeqCOVID-SPAIN consortium
EPI_ISL_801549	Laboratory of Molecular Virology, Pontificia Universidad Católica de Chile	MSHS Pathogen Surveillance Program	Leonardo I. Almonacid, Ana S. Gonzalez-Reiche, Matthew M. Hernandez, Jorge Levican, Ana Maria Contreras, Carlos Palma, Tamara García-Salum, Zenab Khan, Adriana van De Guchte, Ajay Obla, Jayeeta Dutta, Bremy Alburquerque, Eileen Serrano, Constanza Maldonado, M. Belen Leyton, Erick Salinas, Hala Alshammary, Juan Soto, Shwetha Hara Sridhar, Ying-Chih Wang, Melissa Smith, Robert Sebra, Marcela Ferres, Adolfo Garcia-Sastre, Edward C. Holmes, Viviana Simon, Harm van Bakel, Rafael A. Medina.

EPI_ISL_801587, EPI_ISL_801588, EPI_ISL_801589, EPI_ISL_801590	Laboratory of Molecular Virology, Pontificia Universidad Católica de Chile	MSHS Pathogen Surveillance Program	Leonardo I. Almonacid, Ana S. Gonzalez-Reiche, Matthew M. Hernandez, Jorge Levican, Tamara García-Salum, Zenab Khan, Adriana van De Guchte, Ajay Obla, Jayeeta Dutta, Bremy Alburquerque, Eileen Serrano, Erick Salinas, Hala Alshammary, Juan Soto, Shwetha Hara Sridhar, Ying-Chih Wang, Melissa Smith, Robert Sebra, Adolfo Garcia-Sastre, Edward C. Holmes, Viviana Simon, Harm van Bakel, Rafael A. Medina
EPI_ISL_801600, EPI_ISL_801601, EPI_ISL_801602, EPI_ISL_801603, EPI_ISL_801605, EPI_ISL_801611, EPI_ISL_801612, EPI_ISL_801613	Laboratory of Molecular Virology, Pontificia Universidad Católica de Chile	MSHS Pathogen Surveillance Program	Leonardo I. Almonacid, Ana S. Gonzalez-Reiche, Matthew M. Hernandez, Jorge Levican, Ana Maria Contreras, Carlos Palma, Tamara García-Salum, Zenab Khan, Adriana van De Guchte, Ajay Obla, Jayeeta Dutta, Bremy Alburquerque, Eileen Serrano, Constanza Maldonado, M. Belen Leyton, Erick Salinas, Hala Alshammary, Juan Soto, Shwetha Hara Sridhar, Ying-Chih Wang, Melissa Smith, Robert Sebra, Marcela Ferres, Adolfo Garcia-Sastre, Edward C. Holmes, Viviana Simon, Harm van Bakel, Rafael A. Medina.
EPI_ISL_803306, EPI_ISL_803638	Wisconsin State Laboratory of Hygiene Communicable Disease Division	Wisconsin State Laboratory of Hygiene Communicable Disease Division	Kelsey R. Florek, Abigail C. Shockey
EPI_ISL_803886	Department of Medical Biotechnologies, University of Siena	Laboratory of Infectious Diseases, Department of Biomedical and Clinical Sciences L. Sacco, University of Milan	Ilaria Vicenti, Filippo Dragoni, Maurizio Zazzi, Maria Grazia Cusi, Alessia Lai, Annalisa Bergna, Carla Della Ventura, Claudia Balotta, Massimo Galli, Gianguglielmo Zehender on behalf of SARS-CoV-2 ITALIAN RESEARCH ENTERPRISE-(SCIRE) Collaborative Group
EPI_ISL_804406, EPI_ISL_804459	Dutch COVID-19 response team	National Institute for Public Health and the Environment (RIVM)	Adam Meijer, Harry Vennema, Dirk Eggink, Matthijs Welkers, Jeroen Cremer, Sharon van den Brink, Bas van der Veer, AnneMarie van den Brandt, Florian Zwagemaker, Dennis Schmitz, Chantal Reusken, on behalf of the national COVID-19 response team
EPI_ISL_804851, EPI_ISL_804862, EPI_ISL_804885, EPI_ISL_804888, EPI_ISL_804891, EPI_ISL_804893, EPI_ISL_804898, EPI_ISL_804899	DC Public Health Lab/ Dept. of Forensic Sciences	DC Public Health Lab/ Dept. of Forensic Sciences	Scott Nguyen, Elizabeth Zelaya, Connie Maza, Monica Mann, Brittany Hamilton, David Payne, Jocelyn Hauser
EPI_ISL_805020, EPI_ISL_805581, EPI_ISL_805679, EPI_ISL_805695, EPI_ISL_805801, EPI_ISL_805859, EPI_ISL_805897, EPI_ISL_805922, EPI_ISL_805953, EPI_ISL_806032, EPI_ISL_806197, EPI_ISL_806201, EPI_ISL_806215, EPI_ISL_806230, EPI_ISL_806245, EPI_ISL_806263, EPI_ISL_806284, EPI_ISL_806340, EPI_ISL_806383, EPI_ISL_806389, EPI_ISL_806409, EPI_ISL_806462			
see above	Alberta Precision Labs (APL)	Alberta Precision Labs (APL)	Gordon P, Lam LG, Pabbaraju K, Wong A, Ma R, Li V, Melin A, Tipples G, Berenger B, Zelyas N, Kellner J, Bernier F, Chui L, Croxen M
EPI_ISL_812237, EPI_ISL_812238, EPI_ISL_812239, EPI_ISL_812240, EPI_ISL_812241, EPI_ISL_812242, EPI_ISL_812243, EPI_ISL_812244	North Dakota Department of Health, Public Health Laboratory	North Dakota Department of Health, Public Health Laboratory	Lisa Wingerter
EPI_ISL_812321	United States Air Force School of Aerospace Medicine	United States Air Force School of Aerospace Medicine	Anthony Fries, Jennifer Meyer, Amanda Javorina, Sarah Purves, William Gruner, Clarise Starr, Elizabeth Macias
EPI_ISL_812957, EPI_ISL_812958, EPI_ISL_812959, EPI_ISL_812960, EPI_ISL_812961	Division of Pathogen Resource Management, Korea National Institute of Health, Korea Disease Control and Prevention Agency	Division of Pathogen Resource Management, Korea National Institute of Health, Korea Disease Control and Prevention Agency	Kim,S.T., Kim,S.Y., Choi,Y.S., Kim,E.-J., Kim,J.-M., Yun,M.-r., Choi,C.
EPI_ISL_812966	University Clinical Research Center, University of Sciences	University Clinical Research Center, University of Sciences	Diarra,B., Kone,A., Guindo,I., Bane,S., Diakite,M., Dao,S., Iknane,A.A., Doumbia,S.
EPI_ISL_812968	Department of Molecular Medicine, University of Padova	Department of Molecular Medicine, University of Padova	Lavezzo,E., Franchin,E., Barzon,L., Del Vecchio,C., Rossi,L.,Manganelli,R., Loregian,A., Abate,D., Sciro,M., De Canale,E.,Vanuzzo,M.C., Besutti,V., Saluzzo,F., Onelia,F., Pacenti,M.,Manuto,L., Parisi,S.G., Masi,G., Trevisan,M., Toppo,S. and Crisanti,A.
EPI_ISL_819301, EPI_ISL_819302	Hospital Universitari Vall d'Hebron - Vall d'Hebron Institut de Rercerca	Hospital Universitari Vall d'Hebron	Cristina Andrés, Maria Piñana, Josep F Abril, Damir Garcia-Cehic, Ariadna Rando, Juliana Esperalba, Maria Gema Codina, Carla Castillo, Maria Carmen Martin, Tomàs Pumarola, Josep Quer, Andrés Antón
EPI_ISL_831975	Uppsala klinisk mikrobiologi	The Public Health Agency of Sweden	Department of Microbiology, The Public Health Agency of Sweden
EPI_ISL_837608	Instituto Nacional de Enfermedades Respiratorias (INER)	Instituto Nacional de Enfermedades Respiratorias (INER)	Celia Boukadida, Margarita Matías-Florentino, Alma Rincón-Rubio, Hector Esteban Paz-Juárez, Olivia Briceño, Edgar Sevilla-Reyes, Fidencio Mejía-Nepomuceno, Mario Mújica-Sánchez, Eduardo Becerril-Vargas, José Arturo Martínez-Orozco, Alejandra Hernández-Terán, Jorge Salas-Hernández, Santiago Ávila-Ríos, Joel Armando Vázquez-Pérez
EPI_ISL_848085, EPI_ISL_848086, EPI_ISL_848096, EPI_ISL_848100, EPI_ISL_848106, EPI_ISL_848108, EPI_ISL_848109, EPI_ISL_848110, EPI_ISL_848111, EPI_ISL_848112, EPI_ISL_848113, EPI_ISL_848114, EPI_ISL_848115, EPI_ISL_848116, EPI_ISL_848117, EPI_ISL_848118, EPI_ISL_848119, EPI_ISL_848120, EPI_ISL_848121, EPI_ISL_848122, EPI_ISL_848123, EPI_ISL_848124, EPI_ISL_848125, EPI_ISL_848126, EPI_ISL_848146, EPI_ISL_848148, EPI_ISL_848149, EPI_ISL_848151, EPI_ISL_848152, EPI_ISL_848153, EPI_ISL_848154, EPI_ISL_848155, EPI_ISL_848156, EPI_ISL_848157, EPI_ISL_848158, EPI_ISL_848159, EPI_ISL_848160, EPI_ISL_848161, EPI_ISL_848162, EPI_ISL_848163			
see above	National Virus Reference Laboratory	Irish Coronavirus Sequencing Consortium - National University of Ireland Galway	Kate Reddington, Grainne Mc Andrew, Simone Coughlan
EPI_ISL_848205	Illinois Department of Public Health	Gagnon Lab, Southern Illinois University	Keith Gagnon
EPI_ISL_848599, EPI_ISL_848600, EPI_ISL_848601, EPI_ISL_848602	Evandro Chagas Institute	Evandro Chagas Institute	Santos, M.C.; Silva, A.M.; Junior, W.D.C.; Barbagelata, L.S.; Ferreira, J.A.; Sousa, E.M.A.; da Silva, P.S.; Pinheiro, K.C.; L.C.; Sousa Junior, E.C.
EPI_ISL_848634, EPI_ISL_848635, EPI_ISL_848636	North Dakota Department of Health, Public Health Laboratory	North Dakota Department of Health, Public Health Laboratory	Lisa Wingerter
EPI_ISL_849696, EPI_ISL_849698, EPI_ISL_849699, EPI_ISL_849700, EPI_ISL_849701, EPI_ISL_849702, EPI_ISL_849707, EPI_ISL_849708, EPI_ISL_849709, EPI_ISL_849724	unknown	PHV-FSS	Son Nguyen et al.
EPI_ISL_852724, EPI_ISL_852725, EPI_ISL_852726, EPI_ISL_852727, EPI_ISL_852728, EPI_ISL_852729, EPI_ISL_852730, EPI_ISL_852731, EPI_ISL_852732, EPI_ISL_852733, EPI_ISL_852775, EPI_ISL_852776, EPI_ISL_852777, EPI_ISL_852778, EPI_ISL_852779, EPI_ISL_852780, EPI_ISL_852781, EPI_ISL_852782, EPI_ISL_852783, EPI_ISL_852784			
see above	Institute of Virology, Medical Center, University of Freiburg, Freiburg, Germany	Institute of Virology, Clinial Virus Genomics, Medical Center, University of Freiburg, Freiburg, Germany	Jonas Fuchs, Lisa Kern, Sandra Reuter, Hajo Grundmann, Marcus Panning
EPI_ISL_853759, EPI_ISL_853882	Center for Virology, Medical University of Vienna	Bergthaler laboratory, CeMM Research Center for Molecular Medicine of the Austrian Academy of Sciences	Lukas Endler, Alexandra Popa, Benedikt Agerer, Jakob-Wendelin Genger, Alexander Lercher, Anna Schedl, Thomas Penz, Michael Schuster, Jan Laine, Martin Senekowitsch, Christoph Bock, Andreas Bergthaler
EPI_ISL_854461	Alberta Precision Labs (APL)	Alberta Precision Labs (APL)	Gordon P, Lam LG, Pabbaraju K, Wong A, Ma R, Li V, Melin A, Tipples G, Berenger B, Zelyas N, Kellner J, Bernier F, Chui L, Croxen M
EPI_ISL_855186, EPI_ISL_855187, EPI_ISL_855188, EPI_ISL_855189, EPI_ISL_855190, EPI_ISL_855191, EPI_ISL_855192, EPI_ISL_855193, EPI_ISL_855194, EPI_ISL_855195, EPI_ISL_855196, EPI_ISL_855197, EPI_ISL_855198, EPI_ISL_855199, EPI_ISL_855200, EPI_ISL_855201, EPI_ISL_855202, EPI_ISL_855203, EPI_ISL_855204, EPI_ISL_855205, EPI_ISL_855206, EPI_ISL_855207, EPI_ISL_855208, EPI_ISL_855209, EPI_ISL_855210, EPI_ISL_855211, EPI_ISL_855212, EPI_ISL_855213, EPI_ISL_855214, EPI_ISL_855215, EPI_ISL_855216, EPI_ISL_855217, EPI_ISL_855218, EPI_ISL_855219, EPI_ISL_855220, EPI_ISL_855221, EPI_ISL_855222, EPI_ISL_855223, EPI_ISL_855224, EPI_ISL_855225, EPI_ISL_855226, EPI_ISL_855227, EPI_ISL_855228, EPI_ISL_855229, EPI_ISL_855230, EPI_ISL_855231, EPI_ISL_855232, EPI_ISL_855233, EPI_ISL_855234, EPI_ISL_855235, EPI_ISL_855236, EPI_ISL_855237, EPI_ISL_855238, EPI_ISL_855239, EPI_ISL_855240, EPI_ISL_855241, EPI_ISL_855242, EPI_ISL_855243, EPI_ISL_855244, EPI_ISL_855245, EPI_ISL_855246, EPI_ISL_855247, EPI_ISL_855248, EPI_ISL_855249, EPI_ISL_855250, EPI_ISL_855251, EPI_ISL_855252, EPI_ISL_855253, EPI_ISL_855254, EPI_ISL_855255, EPI_ISL_855256, EPI_ISL_855257, EPI_ISL_855258, EPI_ISL_855259, EPI_ISL_855260, EPI_ISL_855261, EPI_ISL_855262, EPI_ISL_855263, EPI_ISL_855264, EPI_ISL_855265, EPI_ISL_855266, EPI_ISL_855267, EPI_ISL_855268, EPI_ISL_855269, EPI_ISL_855270, EPI_ISL_855271, EPI_ISL_855272, EPI_ISL_855273, EPI_ISL_855274, EPI_ISL_855275, EPI_ISL_855276, EPI_ISL_855277, EPI_ISL_855278, EPI_ISL_855279, EPI_ISL_855280, EPI_ISL_855281, EPI_ISL_855282, EPI_ISL_855283, EPI_ISL_855284, EPI_ISL_855285, EPI_ISL_855286, EPI_ISL_855287, EPI_ISL_855288, EPI_ISL_855289, EPI_ISL_855290, EPI_ISL_855291, EPI_ISL_855292, EPI_ISL_855293, EPI_ISL_855294, EPI_ISL_855295, EPI_ISL_855296, EPI_ISL_855297, EPI_ISL_855298, EPI_ISL_855299, EPI_ISL_855300, EPI_ISL_855301, EPI_ISL_855302, EPI_ISL_855303, EPI_ISL_855304, EPI_ISL_855305, EPI_ISL_855306, EPI_ISL_855307, EPI_ISL_855308, EPI_ISL_855309, EPI_ISL_855310, EPI_ISL_855311, EPI_ISL_855312, EPI_ISL_855313, EPI_ISL_855314, EPI_ISL_855315, EPI_ISL_855316, EPI_ISL_855317, EPI_ISL_855318, EPI_ISL_855319, EPI_ISL_855320, EPI_ISL_855321, EPI_ISL_855322, EPI_ISL_855323, EPI_ISL_855324, EPI_ISL_855325, EPI_ISL_855326, EPI_ISL_855327, EPI_ISL_855328, EPI_ISL_855329, EPI_ISL_855330, EPI_ISL_855331, EPI_ISL_855332, EPI_ISL_855333, EPI_ISL_855334, EPI_ISL_855335, EPI_ISL_855336, EPI_ISL_855337, EPI_ISL_855338, EPI_ISL_855339			
see above	Institute for Computational Biomedicine, Weill Cornell Medicine	Institute for Computational Biomedicine, Weill Cornell Medicine	Butler,D.J., Mozsary,C., Meydan,C., Danko,D., Foox,J., Rosiene,J., Shaiber,A., Afshinnekoo,E., MacKay,M., Sedlazeck,F.J., Ivanov,N.A., Sierra,M., Pohle,D., Zietz,M., Gisladottir,U., Ramlall,V., Westover,C.D., Ryon,K., Young,B., Bhattacharya,C., Ruggiero,P., Langhorst,B.W., Tanner,N., Gawrys,J., Meleshko,D., Xu,D., Steel,P.A.D., Shemesh,A.J., Xiang,J., Thierry-Mieg,J., Thierry-Mieg,D., Schwartz,R.E., Iftner,A., Bezdan,D., Sipley,J., Cong,L., Craney,A., Velu,P., Melnick,A.M., Hajirasouliha,I., Horner,S.M., Iftner,T., Salvatore,M., Loda,M., Westblade,L.F., Cushing,M., Levy,S., Wu,S., Tatonetti,N., Imielinski,M., Rennert,H., Mason,C.E.

EPI_ISL_856695	University of Zambia, School of Veterinary Medicine, Disease Control	University of Zambia, School of Veterinary Medicine, Disease Control	Simulundu,E., Kapata,N., Mupeta,F., Kapata,P.C., Saasa,N., Changula,K., Muleya,W., Chitanga,S., Chambaro,H., Mubemba,B., Masahiro,K., Chanda,D., Mulenga,L., Fwoloshi,S., Shibemba,A.L., Kapaya,F., Zulu,P., Musonda,K., Monze,M., Sinyange,N., Liwewe,M.M., Kapin'a,M., Chipimo,P.J., Ngosa,W., Morales,A.N., Kayeyi,N., Malama,K., Tembo,J., Bates,M., Sawa,H., Takada,A., Nalubamba,K.S., Mukonka,V., Chilufya,C. and Zumla,A.
EPI_ISL_856719	Infectious Diseases Unit, Department of Internal Medicine, Azienda Ospedaliera-Universitaria di Padova	Laboratory of Infectious Diseases, Department of Biomedical and Clinical Sciences L. Sacco, University of Milan	Anna Maria Cattelan, Lolita Sasset, Davide Leoni, Alessia Lai, Annalisa Bergna, Carla Della Ventura, Claudia Balotta, Massimo Galli, Gianguglielmo Zehender on behalf of SARS-CoV-2 ITALIAN RESEARCH ENTERPRISE-(SCIRE) Collaborative Group
EPI_ISL_856897	Department of Infectious Diseases, Istituto Superiore di Sanità, Roma, Italy; PO Cardarelli, Laboratorio Analisi Microbiologia e Virologia, Campobasso, Italy	Virology Laboratory, Scientific Department, Army Medical Center	Paola Stefanelli, Angela Di Martino, Alessandra Lo Presti, Stefano Fiore, Massimiliano Scutellà, Silvia Fillo, Giovanni Faggioni, Riccardo De Sanctis, Antonella Fortunato, Anna Anselmo, Francesco Giordani, Vanessa Vera Fain, Nino D'Amore, Florigio Lista
EPI_ISL_856907	Department of Infectious Diseases, Istituto Superiore di Sanità, Roma, Italy; AUSL Valle d'Aosta, Aosta, Italy	Virology Laboratory, Scientific Department, Army Medical Center	Paola Stefanelli, Angela Di Martino, Alessandra Lo Presti, Stefano Fiore, Massimo Di Benedetto, Silvia Fillo, Giovanni Faggioni, Riccardo De Sanctis, Antonella Fortunato, Anna Anselmo, Francesco Giordani, Vanessa Vera Fain, Nino D'Amore, Florigio Lista
EPI_ISL_857039	The Ashley Laboratory, Stanford University	Chan-Zuckerberg Biohub	CZB Cliahub Consortium
EPI_ISL_860711, EPI_ISL_860714	Ohio Department of Health Laboratory	Ohio Department of Health Laboratory	Holmes, Jennifer; Eric Brandt, Keoni Omura, Glen McGillivary, Caitlin McDonnell, Kirtana Ramadugu, Erica Leasure, Kelsey Florek, Heather Blankenship, Quanta Brown, and Tammy Bannerman
EPI_ISL_861532	Instituto Nacional de Saude (INSA)	Instituto Nacional de Saude (INSA)	Borges et al
EPI_ISL_861893	LATE - Laboratório de Técnicas Especiais - Hospital Israelita Albert Einstein	LATE - Laboratório de Técnicas Especiais - Hospital Israelita Albert Einstein	Deyvid Amgarten, Fernanda de Mello Malta, Raquel Riyuzo, Ana Paula Moreira Salles, Pedro Henrique Sebe Rodrigues, João Renato Rebello Pinho
EPI_ISL_862658	Hospital Universitario de Ceuta	Instituto de Salud Carlos III	Iglesias-Caballero, M. Camarero, S. Molinero Calamita, M. González-Esguevillas, M. Pozo, F. Casas, I. Jiménez, P. Jiménez, M. Zaballos, A. Monzón, S. Varona, S. Juliá, M. Cuesta, I. Hijano, S.
EPI_ISL_875676, EPI_ISL_875678, EPI_ISL_875679, EPI_ISL_875680, EPI_ISL_875681	Kansas Health and Environmental Lab	Kansas Health and Environmental Lab	Mike Grose, Carissa Robertson, Ben Olsen, and Phil Adam
EPI_ISL_876975, EPI_ISL_876976, EPI_ISL_876978, EPI_ISL_876983, EPI_ISL_876984, EPI_ISL_876985, EPI_ISL_876986, EPI_ISL_876987, EPI_ISL_876988, EPI_ISL_876989, EPI_ISL_876990, EPI_ISL_876991, EPI_ISL_876992, EPI_ISL_876993, EPI_ISL_876994, EPI_ISL_876995, EPI_ISL_876996, EPI_ISL_877036			
see above	1.AO Universitaria 'S. Giovanni di Dio e Ruggi D'Aragona, Scuola Medica Salernitana' Hospital / 2.UOC di Virologia e Microbiologia, Università della Campania 'L. Vanvitelli' / 3.AO Universitaria 'Federico II' Napoli Hospital / 4.AORN 'San Giuseppe Moscati' Avellino Hospital / 5.AO 'San Pio - presidio G. Rummo' Benevento Hospital / 6.AO 'Sant'Anna e San Sebastiano' Caserta Hospital / 7.PO 'Maria Santissima Addolorata' Eboli Hospital / 8.Biogem Istituto di Ricerche Genetiche	1. Genome Research Center for Health (CRGS) / 2. Laboratory of Molecular Medicine and Genomics(LMMGe) / 3. Center for Research in Pure and Applied Mathematics (CRMPA)	Giorgio Giurato (Corresponding Author), Francesca Rizzo (Corresponding Author), Alessandro Weisz (Corresponding Author), Gianluigi Franci, Giovanni Nassa, Pasquale Pagliano, Roberta Tarallo, Elena Alexandrova, Ylenia D'Agostino, Carlo Ferravante, Jessica Lamberti, Viola Melone, Domenico Memoli, Valeria Mirici Cappa, Domenico Palumbo, Giovanni Pecoraro, Assunta Sellitto, Oriana Strianese, Ilaria Terenzi, Giuseppe Fenza, Aniello Gentile, Antonello Saccomanno, Sonia Amabile, Teresa Rocco, Annamaria Salvati, Emilia Vaccaro, Massimiliano Galdiero, Michele Cennamo, Giuseppe Portella, Maria Grazia Foti, Mariarosaria Ingino, Maria Landi, Maurizio Fumi, Vincenzo Rocco, Rita Greco, Vittoria Letizia, Arnolfo Petruzziello, Maddalena Schioppa, Gregorio Goffredi, Francesca Marciano, Michele Caraglia, Alessia Cossu, Marianna Scrima
EPI_ISL_884531, EPI_ISL_884532	Molecular Microbiology & Immunology, University of Missouri	Molecular Microbiology & Immunology, University of Missouri	Tang,C.Y., Li,T., Hang,J., Lidl,G.M., Wan,X.-F.
EPI_ISL_887173, EPI_ISL_887176, EPI_ISL_887193, EPI_ISL_887198, EPI_ISL_887212, EPI_ISL_887216, EPI_ISL_887217, EPI_ISL_887219, EPI_ISL_887242, EPI_ISL_887252, EPI_ISL_887269, EPI_ISL_887297, EPI_ISL_887319, EPI_ISL_887324			
see above	Protzer Lab	Protzer Lab, Gagneur Lab, Robert Koch Institut	Ulrike Protzer, Dieter Hoffmann, Eva Schulte, Andrea Theumer, Oliver Drechsel, Max von Kleist,Aleksandar Radonic,Stephan Fuchs, Alexander Karollus, Julien Gagneur
EPI_ISL_888681	1.AO Universitaria 'S. Giovanni di Dio e Ruggi D'Aragona, Scuola Medica Salernitana' Hospital / 2.UOC di Virologia e Microbiologia, Università della Campania 'L. Vanvitelli' / 3.AO Universitaria 'Federico II' Napoli Hospital / 4.AORN 'San Giuseppe Moscati' Avellino Hospital / 5.AO 'San Pio - presidio G. Rummo' Benevento Hospital / 6.AO 'Sant'Anna e San Sebastiano' Caserta Hospital / 7.PO 'Maria Santissima Addolorata' Eboli Hospital / 8.Biogem Istituto di Ricerche Genetiche	1. Genome Research Center for Health (CRGS) / 2. Laboratory of Molecular Medicine and Genomics(LMMGe) / 3. Center for Research in Pure and Applied Mathematics (CRMPA)	Giorgio Giurato, Francesca Rizzo, Alessandro Weisz, Gianluigi Franci, Giovanni Nassa, Pasquale Pagliano, Roberta Tarallo, Elena Alexandrova, Ylenia D'Agostino, Carlo Ferravante, Jessica Lamberti, Viola Melone, Domenico Memoli, Valeria Mirici Cappa, Domenico Palumbo, Giovanni Pecoraro, Assunta Sellitto, Oriana Strianese, Ilaria Terenzi, Giuseppe Fenza, Aniello Gentile, Antonello Saccomanno, Sonia Amabile, Teresa Rocco, Annamaria Salvati, Emilia Vaccaro, Massimiliano Galdiero, Michele Cennamo, Giuseppe Portella, Maria Grazia Foti, Mariarosaria Ingino, Maria Landi, Maurizio Fumi, Vincenzo Rocco, Rita Greco, Vittoria Letizia, Arnolfo Petruzziello, Maddalena Schioppa, Gregorio Goffredi, Francesca Marciano, Michele Caraglia, Alessia Cossu, Marianna Scrima
EPI_ISL_888982	RS Pondok Indah Hospital - Pondok Indah	Eijkman Institute for Molecular Biology, Ministry of Research and Technology/National Agency for Research and Innovation	Edison Johar, Frilasita A Yudhaputri, Hidayat Trimarsanto, Iskandar Adnan, Lydia V. Panggalo, Sukma Oktavianthi, Willy Agustine, Safarina G Malik, Khin Saw Myint, Amin Soebandrio
EPI_ISL_889309, EPI_ISL_889310, EPI_ISL_889311, EPI_ISL_889312	The University Hospital Brno	Institute of Applied Biotechnologies a.s.	Petr Klempt, Ondej Brzo, Martin Kašný, Kateina Kvapilová, Martina Lengerová, Petr Kvapil
EPI_ISL_890376, EPI_ISL_890426, EPI_ISL_890430, EPI_ISL_890431, EPI_ISL_890432, EPI_ISL_890433, EPI_ISL_890434, EPI_ISL_890435, EPI_ISL_890436	Laboratoire de santé publique du Québec	Laboratoire de santé publique du Québec	Sandrine Moreira, Ioannis Ragoussis, Guillaume Bourque, Jesse Shapiro, Mark Lathrop and Michel Roger on behalf of the CoVSeQ research group (http://covseq.ca/researchgroup)
EPI_ISL_894218	1.AO Universitaria 'S. Giovanni di Dio e Ruggi D'Aragona, Scuola Medica Salernitana' Hospital / 2.UOC di Virologia e Microbiologia, Università della Campania 'L. Vanvitelli' / 3.AO Universitaria 'Federico II' Napoli Hospital / 4.AORN 'San Giuseppe Moscati' Avellino Hospital / 5.AO 'San Pio - presidio G. Rummo' Benevento Hospital / 6.AO 'Sant'Anna e San Sebastiano' Caserta Hospital / 7.PO 'Maria Santissima Addolorata' Eboli Hospital / 8.Biogem Istituto di Ricerche Genetiche	1. Genome Research Center for Health (CRGS) / 2. Laboratory of Molecular Medicine and Genomics(LMMGe) / 3. Center for Research in Pure and Applied Mathematics (CRMPA)	Giorgio Giurato, Francesca Rizzo, Alessandro Weisz, Gianluigi Franci, Giovanni Nassa, Pasquale Pagliano, Roberta Tarallo, Elena Alexandrova, Ylenia D'Agostino, Carlo Ferravante, Jessica Lamberti, Viola Melone, Domenico Memoli, Valeria Mirici Cappa, Domenico Palumbo, Giovanni Pecoraro, Assunta Sellitto, Oriana Strianese, Ilaria Terenzi, Giuseppe Fenza, Aniello Gentile, Antonello Saccomanno, Sonia Amabile, Teresa Rocco, Annamaria Salvati, Emilia Vaccaro, Massimiliano Galdiero, Michele Cennamo, Giuseppe Portella, Maria Grazia Foti, Mariarosaria Ingino, Maria Landi, Maurizio Fumi, Vincenzo Rocco, Rita Greco, Vittoria Letizia, Arnolfo Petruzziello, Maddalena Schioppa, Gregorio Goffredi, Francesca Marciano, Michele Caraglia, Alessia Cossu, Marianna Scrima
EPI_ISL_895735, EPI_ISL_895776, EPI_ISL_895779, EPI_ISL_895835, EPI_ISL_895837, EPI_ISL_895842, EPI_ISL_895847	Molecular biology division, Institute of Clinical Biochemistry and Diagnostics, Charles University, Faculty of Medicine in Hradec Králové and University Hospital Hradec Králové	Molecular biology division, Institute of Clinical Biochemistry and Diagnostics, Charles University, Faculty of Medicine in Hradec Králové and University Hospital Hradec Králové	Helena Kovaíková, Petr Brož, Ivana Baranová, Kateina Hrochová, Tereza Baková, Jitka Novotná, Kateina Pehlíková, Vladimír Palika. Cooperation project with BioVendor-R&D and bioinformatics company BIOXSYS s r.o.
EPI_ISL_900076, EPI_ISL_900111, EPI_ISL_900115, EPI_ISL_900144, EPI_ISL_900204, EPI_ISL_900226, EPI_ISL_900314, EPI_ISL_900359, EPI_ISL_900392, EPI_ISL_900420, EPI_ISL_900446			
see above	MEPHI, Aix Marseille University	MEPHI, Aix Marseille University	Anthony LEVASSEUR
EPI_ISL_904628	Servicio de Microbiología, Laboratori Clínic Metropolitana Nord. Hospital Universitari Germans Trias i Pujol. Institut d'Investigació en Ciències de la Salut Germans Trias i Pujol (IGTP)	IrsiCaixa - Can Ruti CovidSeq	Marc Noguera-Julian, Mariona Parera, Maria Casadellà, Pilar Armengol, Francesc Catala-Moll, Roger Paredes, Bonaventura Clotet Elisa Martró, Verónica Saludes, Anna Not, Ana Pérez, Montserrat Giménez, Ignacio Blanco, Cristina Casañ, Antoni E. Bordoy, Adrián Antuori
EPI_ISL_906760	Dr. Leonard A. Miller Centre for Health Services	National Microbiology Laboratory (NML)	Anna Majer, Shari Tyson, Grace Seo, Philip Mabon, Elsie Grudeski, Rhiannon Huzarewich, Russell Mandes, Anneliese Landgraff, Jennifer Tanner, Natalie Knox, Morag Graham, Gary Van Domselaar, Robert Needle, Yang Yu, Adel Malek, Laura Gilbert, George Zahariadis, Nathalie Bastien, Yan Li, Timothy Booth, Darian Hole, Madison Chapel, CanCOGeN's metadata curation team, Public Health Agency of Canada CanCOGeN team

EPI_ISL_913323	Klinisk mikrobiologi, Viruslab	The Public Health Agency of Sweden	Anna-Malin Linde, Maria Lind Karlberg, Carlo Berg, Oskar Karlsson Lindsjo, Sofia Stamouli, Reza Advani, Mattias Haukland, Petra Holmstrom, Noura Walai, Petra Edquist, Mia Brytting, Anna Risberg, Karin Tegmark-Wisell
EPI_ISL_918457, EPI_ISL_918458, EPI_ISL_918459, EPI_ISL_918460, EPI_ISL_918461, EPI_ISL_918462, EPI_ISL_918463, EPI_ISL_918470, EPI_ISL_918476	1.AO Universitaria 'S. Giovanni di Dio e Ruggi D'Aragona, Scuola Medica Salernitana' Hospital / 2.UOC di Virologia e Microbiologia, Università della Campania 'L. Vanvitelli' / 3.AO Universitaria 'Federico II' Napoli Hospital / 4.AORN 'San Giuseppe Moscati' Avellino Hospital / 5.AO 'San Pio - presidio G. Rummo' Benevento Hospital / 6.AO 'Sant'Anna e San Sebastiano' Caserta Hospital / 7.PO 'Maria Santissima Addolorata' Eboli Hospital / 8.Biogem Istituto di Ricerche Genetiche	1. Genome Research Center for Health (CRGS) / 2. Laboratory of Molecular Medicine and Genomics(LMMGe) / 3. Center for Research in Pure and Applied Mathematics (CRMPA)	Giorgio Giurato (Corresponding Author), Francesca Rizzo (Corresponding Author), Alessandro Weisz (Corresponding Author), Gianluigi Franci, Giovanni Nassa, Pasquale Pagliano, Roberta Tarallo, Elena Alexandrova, Ylenia D'Agostino, Carlo Ferravante, Jessica Lamberti, Viola Melone, Domenico Memoli, Valeria Mirici Cappa, Domenico Palumbo, Giovanni Pecoraro, Assunta Sellitto, Oriana Strianese, Ilaria Terenzi, Giuseppe Fenza, Aniello Gentile, Antonello Saccomanno, Sonia Amabile, Teresa Rocco, Annamaria Salvati, Emilia Vaccaro, Massimiliano Galdiero, Michele Cennamo, Giuseppe Portella, Maria Grazia Foti, Mariarosaria Ingino, Maria Landi, Maurizio Fumi, Vincenzo Rocco, Rita Greco, Vittoria Letizia, Arnolfo Petruzziello, Maddalena Schioppa, Gregorio Goffredi, Francesca Marciano, Michele Caraglia, Alessia Cossu, Marianna Scrima
EPI_ISL_933604	Toronto Invasive Bacterial Diseases Network	McMaster University	Allison McGeer, Patryk Aftanas, Hooman Derakhshani, Angel Li, Kuganya Nirmalarajah, Emily Panousis, Ahmed Draia, Jalees Nasir, Michael Surette, Samira Mubareka, Andrew G. McArthur
EPI_ISL_934322	Klinisk mikrobiologi, Viruslab	The Public Health Agency of Sweden	Anna-Malin Linde, Maria Lind Karlberg, Carlo Berg, Oskar Karlsson Lindsjo, Sofia Stamouli, Reza Advani, Mattias Haukland, Petra Holmstrom, Noura Walai, Petra Edquist, Mia Brytting, Anna Risberg, Karin Tegmark-Wisell
EPI_ISL_937539, EPI_ISL_937540, EPI_ISL_937541, EPI_ISL_937542, EPI_ISL_937543	Thai Red Cross Emerging Infectious Diseases Health Science Centre, Chulalongkorn Hospital, Faculty of Medicine, Chulalongkorn University	Thai Red Cross Emerging Infectious Diseases Center and Faculty of Medicine, Chulalongkorn University	Opass Putcharoen, Watsamon Jantarabenjakul, Pattama Torvorapanit, Gompol Suwanpimolkul, Leilani Paitoonpong, Sopon Iamsirithaworn, Sininat Petcharat, Yutthana Joyjinda, Weenassarin Ampoot, Apaporn Rodpan, Thiravat Hemachudha, Supaporn Wacharapluesadee
EPI_ISL_940430, EPI_ISL_940431, EPI_ISL_940432, EPI_ISL_940433, EPI_ISL_940434, EPI_ISL_940435, EPI_ISL_940436, EPI_ISL_940437, EPI_ISL_940438, EPI_ISL_940439, EPI_ISL_940440, EPI_ISL_940441, EPI_ISL_940442, EPI_ISL_940443, EPI_ISL_940444, EPI_ISL_940445, EPI_ISL_940446, EPI_ISL_940447, EPI_ISL_940448, EPI_ISL_940449, EPI_ISL_940450, EPI_ISL_940454			
see above	Hôpital Bichat Claude Bernard, Laboratoire de Virologie	IAME UMR1137 Inserm, Université de Paris, Hôpital Bichat	Antoine Bridier-Nahmias, Amélie Recoing, Quentin Le Hingrat, Lena Daniel, Siham Hamri, Gilles Collin, Alexandre Storto, Mélanie Bertine, Charlotte Charpentier, Nadhira Houhou-Fidouh, Diane Descamps, Benoit Visseaux
EPI_ISL_949249	Cliniques universitaires Saint-Luc	UCLouvain/IREC/MBLG	Jean Ruelle, Lysa Pinsmaye, Benoit Kabamba Mukadi
EPI_ISL_956390, EPI_ISL_956391, EPI_ISL_956392, EPI_ISL_956393, EPI_ISL_956394	Isolation - Virology Unit, Institut Pasteur du Cambodge; Sequencing - US National Institute of Allergy and Infectious Diseases Cambodia, US Naval Medical Research Unit -2, Cambodia National Institute for Public Health	Virology Unit, Institut Pasteur du Cambodge	Vireak Heang, Jennifer Bohl, Sophana Chea, Sreyngim Lay, Ly Sovann, Kraing Sidonn, Yi Sengdoeurn, Chin Savuth, Chau Darapheak, Veasna Duong, Jose A Garcia-Rivera, Jessica Manning, Erik A Karlsson
EPI_ISL_961010	Viral Respiratory Lab, National Institute for Biomedical Research (INRB)	Pathogen Sequencing Lab, National Institute for Biomedical Research (INRB)	Placide Mbala-Kingebeni, Edith Nkwembe, Eddy Kinganda-Lusamaki, Amuri Aziza, Francisca Muyembe Mawete, Emmanuel Lokilo Lofiko, Jean-Claude Makangara Cigolo, Catherine Pratt, Matthias Pauthner, Josh Quick, Allison Black, James Hadfield, Trevor Bedford, Ian Goodfellow, Andrew Rambaut, Nick Loman, Kristian Andersen, Michael Wiley, Steve Ahuka-Mundeke, Jean-Jacques Muyembe Tamfum
EPI_ISL_976894, EPI_ISL_976895	Thai Red Cross Emerging Infectious Diseases Health Science Centre, Chulalongkorn Hospital, Faculty of Medicine, Chulalongkorn University	Thai Red Cross Emerging Infectious Diseases Center and Faculty of Medicine, Chulalongkorn University	Opass Putcharoen, Watsamon Jantarabenjakul, Pattama Torvorapanit, Gompol Suwanpimolkul, Leilani Paitoonpong, Sopon Iamsirithaworn, Sininat Petcharat, Yutthana Joyjinda, Weenassarin Ampoot, Apaporn Rodpan, Thiravat Hemachudha, Supaporn Wacharapluesadee
EPI_ISL_978503, EPI_ISL_978511, EPI_ISL_978519, EPI_ISL_978520, EPI_ISL_978524, EPI_ISL_978533	Central Public Health Laboratory - LACEN -Bahia, Salvador, Brazil	Central Public Health Laboratory - LACEN -Bahia, Salvador, Brazil	Stephane Tosta, Luciana Oliveira, Vanessa Nardy,Patrícia Cajado,Marcela Gómez, Breno Dominguez, Jaqueline Gomes, Vagner Fonseca,Marta Giovanetti,Luiz Alcantara, Felicidade Pereira, Arabela Leal
EPI_ISL_979273, EPI_ISL_979278, EPI_ISL_979280, EPI_ISL_979283, EPI_ISL_979284, EPI_ISL_979287	Cadham Provincial laboratory	National Microbiology Laboratory (NML)	Anna Majer, Shari Tyson, Grace Seo, Philip Mabon, Elsie Grudeski, Rhiannon Huzarewich, Russell Mandes, Anneliese Landgraff, Jennifer Tanner, Natalie Knox, Morag Graham, Gary Van Domselaar, Paul Van Caeseele, Jared Bullard, David Alexander, Kerry Dust, Nathalie Bastien, Yan Li, Timothy Booth, Darian Hole, Madison Chapel, Kirsten Biggar, CanCOGeN's metadata curation team, Public Health Agency of Canada CanCOGeN team
