# Supplementary material for: Detection and characterization of the SARS-CoV-2 lineage B.1.526 in New York
Source: Nat Commun. 2021 Aug 9;12:4886. doi: 10.1038/s41467-021-25168-4 (PMC8352861; doi:10.1038/s41467-021-25168-4)
Supplement: Supplementary file 8 — Supplementary Data 4 [file 41467_2021_25168_MOESM8_ESM.zip › GISAID_acknowledements_tables/gisaid_hcov-19_acknowledgement_table_2021_02_13_010-6.pdf]

We gratefully acknowledge the following Authors from the Originating laboratories responsible for obtaining the specimens, as well as the Submitting laboratories where the genome data were generated and shared via GISAID, on which this research is based.

All Submitters of data may be contacted directly via www.gisaid.org

Authors are sorted alphabetically.

Accession ID	Originating Laboratory	Submitting Laboratory	Authors
EPI_ISL_767937	Pathology West - NSW Health Pathology	NSW Health Pathology - Institute of Clinical Pathology and Medical Research; Westmead Hospital; University of Sydney	CIDM-PH et al.
EPI_ISL_767938	Laverty Pathology	NSW Health Pathology - Institute of Clinical Pathology and Medical Research; Westmead Hospital; University of Sydney	CIDM-PH et al.
EPI_ISL_767939, EPI_ISL_767940	Sydney South West Pathology Service (SSWPS) - Liverpool Hospital - NSW Health Pathology	NSW Health Pathology - Institute of Clinical Pathology and Medical Research; Westmead Hospital; University of Sydney	CIDM-PH et al.
EPI_ISL_768815, EPI_ISL_768816	Respiratory Virus Unit, National Infection Service, Public Health England	COVID-19 Genomics UK (COG-UK) Consortium	PHE Covid Sequencing Team
EPI_ISL_770477, EPI_ISL_770496, EPI_ISL_770497	Wyoming Public Health Laboratory	Wyoming Public Health Laboratory	Noah Hull, Taylor Fearing, Lynette Gumbleton, Channing Weber, Ashley Norberg, Bailey Bowcutt, and Wanda Manley
EPI_ISL_771368, EPI_ISL_771369	SA Pathology	SA Pathology	Lex Leong, Julien Soubrier, Chuan Kok Lim, Song Gao, Mark Turra, Karin Kassahn, Ivan Bastian, Geoff Higgins
EPI_ISL_779151	Yale Clinical Virology Laboratory	Grubaugh Lab - Yale School of Public Health	Tara Alpert, Joseph Fauver, Anderson Brito, Mallery Breban, Anne Wyllie, Chantal Vogels, Mary Petrone, Chaney Kalinich, Isabel Ott, Arnau Casanovas, Catherine Muenker, Adam Moore, Alice Lu, Maria Tokuyama, Patrick Wong, Peiwen Lu, Saad Omer, Richard Martinello, Allison Nelson, Shelli Farhadian, Akiko Iwasaki, Charlese Dela Cruz, Albert Ko, Nathan Grubaugh
EPI_ISL_779153	Yale Pathology Lab	Grubaugh Lab - Yale School of Public Health	Tara Alpert, Joseph Fauver, Chen Liu, Pei Hui, Jianhui Wang, Susan Bell and Han Zhou, Anderson Brito, Mallery Breban, Anne Wyllie, Chantal Vogels, Mary Petrone, Chaney Kalinich, Isabel Ott, Arnau Casanovas, Catherine Muenker, Adam Moore, Alice Lu, Maria Tokuyama, Patrick Wong, Peiwen Lu, Saad Omer, Richard Martinello, Allison Nelson, Shelli Farhadian, Akiko Iwasaki, Charlese Dela Cruz, Albert Ko, Nathan Grubaugh
EPI_ISL_779401, EPI_ISL_779402	South Eastern Area Laboratory Services (SEALS)	NSW Health Pathology - Institute of Clinical Pathology and Medical Research; Westmead Hospital; University of Sydney	CIDM-PH et al.
EPI_ISL_779409, EPI_ISL_779410, EPI_ISL_779411	Royal Darwin Hospital Pathology	MDU-PHL	Meumann, E., Caly L., Seemann T., Sait, M.L., Druce J., Sherry, N.L.
EPI_ISL_779647	Microbiological Diagnostic Unit - Public Health Laboratory (MDU-PHL)	MDU-PHL	Seemann T., Sait, M.L., Sherry, N.L.
EPI_ISL_779648, EPI_ISL_779649	Victorian Infectious Diseases Reference Laboratory (VIDRL)	VIDRL and MDU-PHL	Caly L., Seemann T., Sait, M.L., Druce J., Sherry, N.L.
EPI_ISL_789061, EPI_ISL_789074, EPI_ISL_789075, EPI_ISL_789077, EPI_ISL_789078, EPI_ISL_789080, EPI_ISL_789081, EPI_ISL_789082, EPI_ISL_789094, EPI_ISL_789096, EPI_ISL_789097, EPI_ISL_789099, EPI_ISL_789100, EPI_ISL_789101			
see above	National Virus Reference Laboratory	National Virus Reference Laboratory	Michael Carr, Gabriel Gonzalez, Dana Alalwan, Jonathan Dean, Daniel Hare, Cillian F De Gascun
EPI_ISL_791107, EPI_ISL_791108, EPI_ISL_791109, EPI_ISL_791110, EPI_ISL_791111, EPI_ISL_791112, EPI_ISL_791113, EPI_ISL_791114, EPI_ISL_791115, EPI_ISL_791116, EPI_ISL_791117, EPI_ISL_791118, EPI_ISL_791119, EPI_ISL_791120, EPI_ISL_791121, EPI_ISL_791122, EPI_ISL_791123, EPI_ISL_791124, EPI_ISL_791125, EPI_ISL_791126, EPI_ISL_791127, EPI_ISL_791128, EPI_ISL_791129, EPI_ISL_791130, EPI_ISL_791131, EPI_ISL_791132, EPI_ISL_791133, EPI_ISL_791134, EPI_ISL_791135, EPI_ISL_791136, EPI_ISL_791137, EPI_ISL_791138, EPI_ISL_791140, EPI_ISL_791141, EPI_ISL_791142, EPI_ISL_791143, EPI_ISL_791145, EPI_ISL_791146, EPI_ISL_791147, EPI_ISL_791148, EPI_ISL_791149, EPI_ISL_791151, EPI_ISL_791156, EPI_ISL_791162			
see above	University of Wisconsin-Madison AIDS Vaccine Research Laboratories	University of Wisconsin-Madison AIDS Vaccine Research Laboratories	Gage Moreno, Katarina Braun, et al. AIDS Vaccine Research Laboratories
EPI_ISL_791279	South Eastern Area Laboratory Services (SEALS)	NSW Health Pathology - Institute of Clinical Pathology and Medical Research; Westmead Hospital; University of Sydney	CIDM-PH et al.
EPI_ISL_791327, EPI_ISL_791328, EPI_ISL_791329	University of Wisconsin-Madison AIDS Vaccine Research Laboratories	University of Wisconsin-Madison AIDS Vaccine Research Laboratories	Gage Moreno, Katarina Braun, et al. AIDS Vaccine Research Laboratories
EPI_ISL_791427	Johns Hopkins Hospital Department of Pathology	Johns Hopkins Hospital Department of Pathology	C. Paul Morris, Chun Huai Luo, Adannaya Amadi, Nicholas Gallagher, Heba H. Mostafa
EPI_ISL_792037	South Eastern Area Laboratory Services (SEALS)	NSW Health Pathology - Institute of Clinical Pathology and Medical Research; Westmead Hospital; University of Sydney	CIDM-PH et al.
EPI_ISL_792555	Centre for Dengue Research and AICBU, Department of Immunology and Molecular Medicine	Centre for Dengue Research and AICBU, Department of Immunology and Molecular Medicine	Chandima Jeewandara, Deshni Jayathilaka, Dinuka Ariyaratne, Diyanath Ranasinghe, Laksiri Gomes, Gathsaurie Neelika Malavige
EPI_ISL_792680, EPI_ISL_792681, EPI_ISL_792682, EPI_ISL_792683	Pathogen Genomics Center, National Institute of Infectious Diseases	Pathogen Genomics Center, National Institute of Infectious Diseases	Tsuyoshi Sekizuka, Kentaro Itokawa, Rina Tanaka, Masanori Hashino, Makoto Kuroda
EPI_ISL_794206, EPI_ISL_794207	Wadsworth Center, New York State Department.of Health	Wadsworth Center, New York State Department.of Health	Kirsten St. George, Daryl M. Lamson, Alexis Russel, Matthew Shudt, Melissa A Leisner, Jonathan Plitnick, Navjot Singh, John Kelly, Sara Griesemer, Erasmus Schneider, Erica Lasek-Nesselquist
EPI_ISL_794261, EPI_ISL_794262, EPI_ISL_794263, EPI_ISL_794264, EPI_ISL_794265, EPI_ISL_794266, EPI_ISL_794267, EPI_ISL_794268, EPI_ISL_794269, EPI_ISL_794270, EPI_ISL_794271, EPI_ISL_794272, EPI_ISL_794273, EPI_ISL_794274, EPI_ISL_794275, EPI_ISL_794276, EPI_ISL_794277, EPI_ISL_794278, EPI_ISL_794279, EPI_ISL_794280			
see above	WESTCHESTER MEDICAL CENTER	Wadsworth Center, New York State Department.of Health	Kirsten St. George, Daryl M. Lamson, Alexis Russel, Matthew Shudt, Melissa A Leisner, Jonathan Plitnick, Navjot Singh, John Kelly, Sara Griesemer, Erasmus Schneider, Erica Lasek-Nesselquist
EPI_ISL_794611, EPI_ISL_794612, EPI_ISL_794613, EPI_ISL_794614, EPI_ISL_794615, EPI_ISL_794616, EPI_ISL_794617, EPI_ISL_794618, EPI_ISL_794619, EPI_ISL_794623	LabPLUS	Institute of Environmental Science and Research (ESR)	Xiaoyun Ren, Matt Storey, Nikki Freed, Muhammad Faisal, Jing Wang, Hermes Perez, Anja Werno, Antje van der Linden, Arlo Upton, Chris Mansell, David Hammer, Dragana Drinkovic, Gary McAuliffe, Hana Sofia Andersson, James Ussher, Jill Sherwood, Josh Freeman, Julia Howard, Juliet Elvy, Mary DeAlmeida, Matt Blakiston, Matthew Rogers, Max Bloomfield, Michael Addidle, Michelle Balm, Sally Roberts, Sarah Jefferies, Sharmini Muttaiyah, Susan Morpeth, Susan Taylor, Timothy Blackmore, Vani Sathyendran, Veronica Playle, Virginia Hope, Erasmus Smit, Lauren Jelly, Olin Silander, Joep de Ligt
EPI_ISL_794625	Middlemore Hospital	Institute of Environmental Science and Research (ESR)	Xiaoyun Ren, Matt Storey, Nikki Freed, Muhammad Faisal, Jing Wang, Hermes Perez, Anja Werno, Antje van der Linden, Arlo Upton, Chris Mansell, David Hammer, Dragana Drinkovic, Gary McAuliffe, Hana Sofia Andersson, James Ussher, Jill Sherwood, Josh Freeman, Julia Howard, Juliet Elvy, Mary DeAlmeida, Matt Blakiston, Matthew Rogers, Max Bloomfield, Michael Addidle, Michelle Balm, Sally Roberts, Sarah Jefferies, Sharmini Muttaiyah, Susan Morpeth, Susan Taylor, Timothy Blackmore, Vani Sathyendran, Veronica Playle, Virginia Hope, Erasmus Smit, Lauren Jelly, Olin Silander, Joep de Ligt
EPI_ISL_794635, EPI_ISL_794636, EPI_ISL_794637, EPI_ISL_794638	USC Clinical Lab	Los Angeles County PHL	P. Hemarajata et al.
EPI_ISL_794675, EPI_ISL_794676	PathWest Laboratory Medicine WA	PathWest Laboratory Medicine WA Microbial Surveillance Unit	PathWest Laboratory Medicine WA Microbial Surveillance Unit
EPI_ISL_796012, EPI_ISL_796013, EPI_ISL_796014, EPI_ISL_796015, EPI_ISL_796016, EPI_ISL_796017, EPI_ISL_796018, EPI_ISL_796019, EPI_ISL_796020, EPI_ISL_796021, EPI_ISL_796022			

see above	Hebei Provincial Center for Disease Control and Prevention, Shijiazhuang, Hebei Province; National Institute for Viral Disease Control and Prevention, China CDC	Hebei Provincial Center for Disease Control and Prevention, Shijiazhuang, Hebei Province; National Institute for Viral Disease Control and Prevention, China CDC	Shunxiang Qi, Xiang Zhao, Nankun Liu, George F. Gao, Yang Song, Wenbo Xu, Qi Li
EPI_ISL_796755, EPI_ISL_796756, EPI_ISL_796757, EPI_ISL_796758, EPI_ISL_796762, EPI_ISL_796767	Instituto Nacional de Saude (INSA)	Instituto Nacional de Saude (INSA)	Borges et al
EPI_ISL_797405, EPI_ISL_797408	Lighthouse Lab in Glasgow	Wellcome Sanger Institute for the COVID-19 Genomics UK (COG-UK) Consortium	Harper VanSteenhouse, Yumi Kasai, David Gray, Carol Clugston, Anna Dominiczak and Alex Alderton, Roberto Amato, Sonia Goncalves, Ewan Harrison, David K. Jackson, Ian Johnston, Dominic Kwiatkowski, Cordelia Langford, John Sillitoe on behalf of the Wellcome Sanger Institute COVID-19 Surveillance Team
EPI_ISL_797411	Lighthouse Lab in Alderley Park	Wellcome Sanger Institute for the COVID-19 Genomics UK (COG-UK) Consortium	Jacquelyn Wynn, Mairead Hyland, The Lighthouse Lab in Alderley Park and Alex Alderton, Roberto Amato, Sonia Goncalves, Ewan Harrison, David K. Jackson, Ian Johnston, Dominic Kwiatkowski, Cordelia Langford, John Sillitoe on behalf of the Wellcome Sanger Institute COVID-19 Surveillance Team
EPI_ISL_797432	Lighthouse Lab in Glasgow	Wellcome Sanger Institute for the COVID-19 Genomics UK (COG-UK) Consortium	Harper VanSteenhouse, Yumi Kasai, David Gray, Carol Clugston, Anna Dominiczak and Alex Alderton, Roberto Amato, Sonia Goncalves, Ewan Harrison, David K. Jackson, Ian Johnston, Dominic Kwiatkowski, Cordelia Langford, John Sillitoe on behalf of the Wellcome Sanger Institute COVID-19 Surveillance Team
EPI_ISL_797438, EPI_ISL_797441	Lighthouse Lab in Alderley Park	Wellcome Sanger Institute for the COVID-19 Genomics UK (COG-UK) Consortium	Jacquelyn Wynn, Mairead Hyland, The Lighthouse Lab in Alderley Park and Alex Alderton, Roberto Amato, Sonia Goncalves, Ewan Harrison, David K. Jackson, Ian Johnston, Dominic Kwiatkowski, Cordelia Langford, John Sillitoe on behalf of the Wellcome Sanger Institute COVID-19 Surveillance Team
EPI_ISL_797445, EPI_ISL_797451, EPI_ISL_797459	Lighthouse Lab in Glasgow	Wellcome Sanger Institute for the COVID-19 Genomics UK (COG-UK) Consortium	Harper VanSteenhouse, Yumi Kasai, David Gray, Carol Clugston, Anna Dominiczak and Alex Alderton, Roberto Amato, Sonia Goncalves, Ewan Harrison, David K. Jackson, Ian Johnston, Dominic Kwiatkowski, Cordelia Langford, John Sillitoe on behalf of the Wellcome Sanger Institute COVID-19 Surveillance Team
EPI_ISL_797461, EPI_ISL_797462, EPI_ISL_797475, EPI_ISL_797484	Lighthouse Lab in Alderley Park	Wellcome Sanger Institute for the COVID-19 Genomics UK (COG-UK) Consortium	Jacquelyn Wynn, Mairead Hyland, The Lighthouse Lab in Alderley Park and Alex Alderton, Roberto Amato, Sonia Goncalves, Ewan Harrison, David K. Jackson, Ian Johnston, Dominic Kwiatkowski, Cordelia Langford, John Sillitoe on behalf of the Wellcome Sanger Institute COVID-19 Surveillance Team
EPI_ISL_797500	Lighthouse Lab in Glasgow	Wellcome Sanger Institute for the COVID-19 Genomics UK (COG-UK) Consortium	Harper VanSteenhouse, Yumi Kasai, David Gray, Carol Clugston, Anna Dominiczak and Alex Alderton, Roberto Amato, Sonia Goncalves, Ewan Harrison, David K. Jackson, Ian Johnston, Dominic Kwiatkowski, Cordelia Langford, John Sillitoe on behalf of the Wellcome Sanger Institute COVID-19 Surveillance Team
EPI_ISL_797504, EPI_ISL_797509	Lighthouse Lab in Alderley Park	Wellcome Sanger Institute for the COVID-19 Genomics UK (COG-UK) Consortium	Jacquelyn Wynn, Mairead Hyland, The Lighthouse Lab in Alderley Park and Alex Alderton, Roberto Amato, Sonia Goncalves, Ewan Harrison, David K. Jackson, Ian Johnston, Dominic Kwiatkowski, Cordelia Langford, John Sillitoe on behalf of the Wellcome Sanger Institute COVID-19 Surveillance Team
EPI_ISL_797514, EPI_ISL_797531	Lighthouse Lab in Glasgow	Wellcome Sanger Institute for the COVID-19 Genomics UK (COG-UK) Consortium	Harper VanSteenhouse, Yumi Kasai, David Gray, Carol Clugston, Anna Dominiczak and Alex Alderton, Roberto Amato, Sonia Goncalves, Ewan Harrison, David K. Jackson, Ian Johnston, Dominic Kwiatkowski, Cordelia Langford, John Sillitoe on behalf of the Wellcome Sanger Institute COVID-19 Surveillance Team
EPI_ISL_797553	Lighthouse Lab in Alderley Park	Wellcome Sanger Institute for the COVID-19 Genomics UK (COG-UK) Consortium	Jacquelyn Wynn, Mairead Hyland, The Lighthouse Lab in Alderley Park and Alex Alderton, Roberto Amato, Sonia Goncalves, Ewan Harrison, David K. Jackson, Ian Johnston, Dominic Kwiatkowski, Cordelia Langford, John Sillitoe on behalf of the Wellcome Sanger Institute COVID-19 Surveillance Team
EPI_ISL_797554	Lighthouse Lab in Glasgow	Wellcome Sanger Institute for the COVID-19 Genomics UK (COG-UK) Consortium	Harper VanSteenhouse, Yumi Kasai, David Gray, Carol Clugston, Anna Dominiczak and Alex Alderton, Roberto Amato, Sonia Goncalves, Ewan Harrison, David K. Jackson, Ian Johnston, Dominic Kwiatkowski, Cordelia Langford, John Sillitoe on behalf of the Wellcome Sanger Institute COVID-19 Surveillance Team
EPI_ISL_797559, EPI_ISL_797560	Lighthouse Lab in Alderley Park	Wellcome Sanger Institute for the COVID-19 Genomics UK (COG-UK) Consortium	Jacquelyn Wynn, Mairead Hyland, The Lighthouse Lab in Alderley Park and Alex Alderton, Roberto Amato, Sonia Goncalves, Ewan Harrison, David K. Jackson, Ian Johnston, Dominic Kwiatkowski, Cordelia Langford, John Sillitoe on behalf of the Wellcome Sanger Institute COVID-19 Surveillance Team
EPI_ISL_797563, EPI_ISL_797564	Lighthouse Lab in Glasgow	Wellcome Sanger Institute for the COVID-19 Genomics UK (COG-UK) Consortium	Harper VanSteenhouse, Yumi Kasai, David Gray, Carol Clugston, Anna Dominiczak and Alex Alderton, Roberto Amato, Sonia Goncalves, Ewan Harrison, David K. Jackson, Ian Johnston, Dominic Kwiatkowski, Cordelia Langford, John Sillitoe on behalf of the Wellcome Sanger Institute COVID-19 Surveillance Team
EPI_ISL_797575, EPI_ISL_797576, EPI_ISL_797586, EPI_ISL_797605	Lighthouse Lab in Alderley Park	Wellcome Sanger Institute for the COVID-19 Genomics UK (COG-UK) Consortium	Jacquelyn Wynn, Mairead Hyland, The Lighthouse Lab in Alderley Park and Alex Alderton, Roberto Amato, Sonia Goncalves, Ewan Harrison, David K. Jackson, Ian Johnston, Dominic Kwiatkowski, Cordelia Langford, John Sillitoe on behalf of the Wellcome Sanger Institute COVID-19 Surveillance Team
EPI_ISL_797615, EPI_ISL_797620, EPI_ISL_797627, EPI_ISL_797632	Lighthouse Lab in Glasgow	Wellcome Sanger Institute for the COVID-19 Genomics UK (COG-UK) Consortium	Harper VanSteenhouse, Yumi Kasai, David Gray, Carol Clugston, Anna Dominiczak and Alex Alderton, Roberto Amato, Sonia Goncalves, Ewan Harrison, David K. Jackson, Ian Johnston, Dominic Kwiatkowski, Cordelia Langford, John Sillitoe on behalf of the Wellcome Sanger Institute COVID-19 Surveillance Team
EPI_ISL_797635, EPI_ISL_797673	Lighthouse Lab in Alderley Park	Wellcome Sanger Institute for the COVID-19 Genomics UK (COG-UK) Consortium	Jacquelyn Wynn, Mairead Hyland, The Lighthouse Lab in Alderley Park and Alex Alderton, Roberto Amato, Sonia Goncalves, Ewan Harrison, David K. Jackson, Ian Johnston, Dominic Kwiatkowski, Cordelia Langford, John Sillitoe on behalf of the Wellcome Sanger Institute COVID-19 Surveillance Team
EPI_ISL_797677, EPI_ISL_797678, EPI_ISL_797685	Lighthouse Lab in Glasgow	Wellcome Sanger Institute for the COVID-19 Genomics UK (COG-UK) Consortium	Harper VanSteenhouse, Yumi Kasai, David Gray, Carol Clugston, Anna Dominiczak and Alex Alderton, Roberto Amato, Sonia Goncalves, Ewan Harrison, David K. Jackson, Ian Johnston, Dominic Kwiatkowski, Cordelia Langford, John Sillitoe on behalf of the Wellcome Sanger Institute COVID-19 Surveillance Team
EPI_ISL_797686	Lighthouse Lab in Alderley Park	Wellcome Sanger Institute for the COVID-19 Genomics UK (COG-UK) Consortium	Jacquelyn Wynn, Mairead Hyland, The Lighthouse Lab in Alderley Park and Alex Alderton, Roberto Amato, Sonia Goncalves, Ewan Harrison, David K. Jackson, Ian Johnston, Dominic Kwiatkowski, Cordelia Langford, John Sillitoe on behalf of the Wellcome Sanger Institute COVID-19 Surveillance Team
EPI_ISL_797704, EPI_ISL_797713, EPI_ISL_797716, EPI_ISL_797847, EPI_ISL_797867, EPI_ISL_797870, EPI_ISL_797914, EPI_ISL_797929, EPI_ISL_797945, EPI_ISL_797961, EPI_ISL_797980, EPI_ISL_797996			
see above	Lighthouse Lab in Glasgow	Wellcome Sanger Institute for the COVID-19 Genomics UK (COG-UK) Consortium	Harper VanSteenhouse, Yumi Kasai, David Gray, Carol Clugston, Anna Dominiczak and Alex Alderton, Roberto Amato, Sonia Goncalves, Ewan Harrison, David K. Jackson, Ian Johnston, Dominic Kwiatkowski, Cordelia Langford, John Sillitoe on behalf of the Wellcome Sanger Institute COVID-19 Surveillance Team
EPI_ISL_801519, EPI_ISL_801520	Instituto Nacional de Saude (INSA)	Instituto Nacional de Saude (INSA)	Borges et al
EPI_ISL_802428, EPI_ISL_802432, EPI_ISL_802437, EPI_ISL_802491, EPI_ISL_802492, EPI_ISL_802493, EPI_ISL_802494, EPI_ISL_802495, EPI_ISL_802496, EPI_ISL_802497	SARATOGA HOSPITAL LABORATORY	Wadsworth Center, New York State Department.of Health	Kirsten St. George, Daryl M. Lamson, Alexis Russel, Matthew Shudt, Melissa A Leisner, Jonathan Plitnick, Navjot Singh, John Kelly, Sara Griesemer, Erasmus Schneider, Erica Lasek-Nesselquist
EPI_ISL_803958, EPI_ISL_803967, EPI_ISL_803968, EPI_ISL_803989, EPI_ISL_803990, EPI_ISL_803991, EPI_ISL_803992, EPI_ISL_803993, EPI_ISL_803998	National Public Health Laboratory, National Centre for Infectious Diseases	National Public Health Laboratory, National Centre for Infectious Diseases	Tze Minn Mak, Sophie Octavia, Zhenyang Zhou, Lin Cui, Raymond Tzer Pin Lin
EPI_ISL_804249	Respiratory Virus Unit, National Infection Service, Public Health England	COVID-19 Genomics UK (COG-UK) Consortium	PHE Covid Sequencing Team
EPI_ISL_804981	BIO-REFERENCE LABORATORIES	Wadsworth Center, New York State Department of Health	Kirsten St. George, Daryl M. Lamson, Alexis Russel, Matthew Shudt, Melissa A Leisner, Jonathan Plitnick, Navjot Singh, John Kelly, Erasmus Schneider, Erica Lasek-Nesselquist
EPI_ISL_806725	Medlab Pathology	NSW Health Pathology - Institute of Clinical Pathology and Medical Research; Westmead Hospital; University of Sydney	CIDM-PH et al.
EPI_ISL_806726	4Cyte Pathology	NSW Health Pathology - Institute of Clinical Pathology and	CIDM-PH et al.

		Medical Research; Westmead Hospital; University of Sydney	
EPI_ISL_806728	Sydney South West Pathology Service (SSWPS) - Liverpool Hospital - NSW Health Pathology	NSW Health Pathology - Institute of Clinical Pathology and Medical Research; Westmead Hospital; University of Sydney	CIDM-PH et al.
EPI_ISL_806807	Gundersen Molecular Diagnostics Laboratory	Kabara Cancer Research Institute	Craig S. Richmond, Paraic A. Kenny
EPI_ISL_810964	PathWest Laboratory Medicine WA	PathWest Laboratory Medicine WA Microbial Surveillance Unit	PathWest Laboratory Medicine WA Microbial Surveillance Unit
EPI_ISL_811072, EPI_ISL_811106, EPI_ISL_811107, EPI_ISL_811108, EPI_ISL_811109, EPI_ISL_811110, EPI_ISL_811111, EPI_ISL_811112, EPI_ISL_811113, EPI_ISL_811114, EPI_ISL_811116			
see above	Respiratory Virus Unit, National Infection Service, Public Health England	COVID-19 Genomics UK (COG-UK) Consortium	PHE Covid Sequencing Team
EPI_ISL_811194, EPI_ISL_811195, EPI_ISL_811196	Lighthouse Lab in Glasgow	Wellcome Sanger Institute for the COVID-19 Genomics UK (COG-UK) Consortium	Harper VanSteenhouse, Yumi Kasai, David Gray, Carol Clugston, Anna Dominiczak and Alex Alderton, Roberto Amato, Sonia Goncalves, Ewan Harrison, David K. Jackson, Ian Johnston, Dominic Kwiatkowski, Cordelia Langford, John Sillitoe on behalf of the Wellcome Sanger Institute COVID-19 Surveillance Team
EPI_ISL_811197	Lighthouse Lab in Milton Keynes	Wellcome Sanger Institute for the COVID-19 Genomics UK (COG-UK) Consortium	The Lighthouse Lab in Milton Keynes and Alex Alderton, Roberto Amato, Sonia Goncalves, Ewan Harrison, David K. Jackson, Ian Johnston, Dominic Kwiatkowski, Cordelia Langford, John Sillitoe on behalf of the Wellcome Sanger Institute COVID-19 Surveillance Team
EPI_ISL_811198, EPI_ISL_811199, EPI_ISL_811201, EPI_ISL_811202, EPI_ISL_811203, EPI_ISL_811204	Lighthouse Lab in Glasgow	Wellcome Sanger Institute for the COVID-19 Genomics UK (COG-UK) Consortium	Harper VanSteenhouse, Yumi Kasai, David Gray, Carol Clugston, Anna Dominiczak and Alex Alderton, Roberto Amato, Sonia Goncalves, Ewan Harrison, David K. Jackson, Ian Johnston, Dominic Kwiatkowski, Cordelia Langford, John Sillitoe on behalf of the Wellcome Sanger Institute COVID-19 Surveillance Team
EPI_ISL_811205	Lighthouse Lab in Milton Keynes	Wellcome Sanger Institute for the COVID-19 Genomics UK (COG-UK) Consortium	The Lighthouse Lab in Milton Keynes and Alex Alderton, Roberto Amato, Sonia Goncalves, Ewan Harrison, David K. Jackson, Ian Johnston, Dominic Kwiatkowski, Cordelia Langford, John Sillitoe on behalf of the Wellcome Sanger Institute COVID-19 Surveillance Team
EPI_ISL_811206	Lighthouse Lab in Glasgow	Wellcome Sanger Institute for the COVID-19 Genomics UK (COG-UK) Consortium	Harper VanSteenhouse, Yumi Kasai, David Gray, Carol Clugston, Anna Dominiczak and Alex Alderton, Roberto Amato, Sonia Goncalves, Ewan Harrison, David K. Jackson, Ian Johnston, Dominic Kwiatkowski, Cordelia Langford, John Sillitoe on behalf of the Wellcome Sanger Institute COVID-19 Surveillance Team
EPI_ISL_811207	Lighthouse Lab in Milton Keynes	Wellcome Sanger Institute for the COVID-19 Genomics UK (COG-UK) Consortium	The Lighthouse Lab in Milton Keynes and Alex Alderton, Roberto Amato, Sonia Goncalves, Ewan Harrison, David K. Jackson, Ian Johnston, Dominic Kwiatkowski, Cordelia Langford, John Sillitoe on behalf of the Wellcome Sanger Institute COVID-19 Surveillance Team
EPI_ISL_811208, EPI_ISL_811209, EPI_ISL_811210	Lighthouse Lab in Glasgow	Wellcome Sanger Institute for the COVID-19 Genomics UK (COG-UK) Consortium	Harper VanSteenhouse, Yumi Kasai, David Gray, Carol Clugston, Anna Dominiczak and Alex Alderton, Roberto Amato, Sonia Goncalves, Ewan Harrison, David K. Jackson, Ian Johnston, Dominic Kwiatkowski, Cordelia Langford, John Sillitoe on behalf of the Wellcome Sanger Institute COVID-19 Surveillance Team
EPI_ISL_811211, EPI_ISL_811212, EPI_ISL_811215, EPI_ISL_811217	Lighthouse Lab in Milton Keynes	Wellcome Sanger Institute for the COVID-19 Genomics UK (COG-UK) Consortium	The Lighthouse Lab in Milton Keynes and Alex Alderton, Roberto Amato, Sonia Goncalves, Ewan Harrison, David K. Jackson, Ian Johnston, Dominic Kwiatkowski, Cordelia Langford, John Sillitoe on behalf of the Wellcome Sanger Institute COVID-19 Surveillance Team
EPI_ISL_811218, EPI_ISL_811219, EPI_ISL_811220, EPI_ISL_811223, EPI_ISL_811224, EPI_ISL_811225, EPI_ISL_811227, EPI_ISL_811228	Lighthouse Lab in Glasgow	Wellcome Sanger Institute for the COVID-19 Genomics UK (COG-UK) Consortium	Harper VanSteenhouse, Yumi Kasai, David Gray, Carol Clugston, Anna Dominiczak and Alex Alderton, Roberto Amato, Sonia Goncalves, Ewan Harrison, David K. Jackson, Ian Johnston, Dominic Kwiatkowski, Cordelia Langford, John Sillitoe on behalf of the Wellcome Sanger Institute COVID-19 Surveillance Team
EPI_ISL_811229	Lighthouse Lab in Milton Keynes	Wellcome Sanger Institute for the COVID-19 Genomics UK (COG-UK) Consortium	The Lighthouse Lab in Milton Keynes and Alex Alderton, Roberto Amato, Sonia Goncalves, Ewan Harrison, David K. Jackson, Ian Johnston, Dominic Kwiatkowski, Cordelia Langford, John Sillitoe on behalf of the Wellcome Sanger Institute COVID-19 Surveillance Team
EPI_ISL_811230, EPI_ISL_811231, EPI_ISL_811232, EPI_ISL_811233, EPI_ISL_811237, EPI_ISL_811238, EPI_ISL_811239, EPI_ISL_811240, EPI_ISL_811242, EPI_ISL_811243, EPI_ISL_811244			
see above	Lighthouse Lab in Glasgow	Wellcome Sanger Institute for the COVID-19 Genomics UK (COG-UK) Consortium	Harper VanSteenhouse, Yumi Kasai, David Gray, Carol Clugston, Anna Dominiczak and Alex Alderton, Roberto Amato, Sonia Goncalves, Ewan Harrison, David K. Jackson, Ian Johnston, Dominic Kwiatkowski, Cordelia Langford, John Sillitoe on behalf of the Wellcome Sanger Institute COVID-19 Surveillance Team
EPI_ISL_811245	Lighthouse Lab in Milton Keynes	Wellcome Sanger Institute for the COVID-19 Genomics UK (COG-UK) Consortium	The Lighthouse Lab in Milton Keynes and Alex Alderton, Roberto Amato, Sonia Goncalves, Ewan Harrison, David K. Jackson, Ian Johnston, Dominic Kwiatkowski, Cordelia Langford, John Sillitoe on behalf of the Wellcome Sanger Institute COVID-19 Surveillance Team
EPI_ISL_811246	Lighthouse Lab in Glasgow	Wellcome Sanger Institute for the COVID-19 Genomics UK (COG-UK) Consortium	Harper VanSteenhouse, Yumi Kasai, David Gray, Carol Clugston, Anna Dominiczak and Alex Alderton, Roberto Amato, Sonia Goncalves, Ewan Harrison, David K. Jackson, Ian Johnston, Dominic Kwiatkowski, Cordelia Langford, John Sillitoe on behalf of the Wellcome Sanger Institute COVID-19 Surveillance Team
EPI_ISL_811248	Lighthouse Lab in Milton Keynes	Wellcome Sanger Institute for the COVID-19 Genomics UK (COG-UK) Consortium	The Lighthouse Lab in Milton Keynes and Alex Alderton, Roberto Amato, Sonia Goncalves, Ewan Harrison, David K. Jackson, Ian Johnston, Dominic Kwiatkowski, Cordelia Langford, John Sillitoe on behalf of the Wellcome Sanger Institute COVID-19 Surveillance Team
EPI_ISL_811249, EPI_ISL_811250	Lighthouse Lab in Glasgow	Wellcome Sanger Institute for the COVID-19 Genomics UK (COG-UK) Consortium	Harper VanSteenhouse, Yumi Kasai, David Gray, Carol Clugston, Anna Dominiczak and Alex Alderton, Roberto Amato, Sonia Goncalves, Ewan Harrison, David K. Jackson, Ian Johnston, Dominic Kwiatkowski, Cordelia Langford, John Sillitoe on behalf of the Wellcome Sanger Institute COVID-19 Surveillance Team
EPI_ISL_811251	Lighthouse Lab in Milton Keynes	Wellcome Sanger Institute for the COVID-19 Genomics UK (COG-UK) Consortium	The Lighthouse Lab in Milton Keynes and Alex Alderton, Roberto Amato, Sonia Goncalves, Ewan Harrison, David K. Jackson, Ian Johnston, Dominic Kwiatkowski, Cordelia Langford, John Sillitoe on behalf of the Wellcome Sanger Institute COVID-19 Surveillance Team
EPI_ISL_811252, EPI_ISL_811253	Lighthouse Lab in Glasgow	Wellcome Sanger Institute for the COVID-19 Genomics UK (COG-UK) Consortium	Harper VanSteenhouse, Yumi Kasai, David Gray, Carol Clugston, Anna Dominiczak and Alex Alderton, Roberto Amato, Sonia Goncalves, Ewan Harrison, David K. Jackson, Ian Johnston, Dominic Kwiatkowski, Cordelia Langford, John Sillitoe on behalf of the Wellcome Sanger Institute COVID-19 Surveillance Team
EPI_ISL_811255	Lighthouse Lab in Milton Keynes	Wellcome Sanger Institute for the COVID-19 Genomics UK (COG-UK) Consortium	The Lighthouse Lab in Milton Keynes and Alex Alderton, Roberto Amato, Sonia Goncalves, Ewan Harrison, David K. Jackson, Ian Johnston, Dominic Kwiatkowski, Cordelia Langford, John Sillitoe on behalf of the Wellcome Sanger Institute COVID-19 Surveillance Team
EPI_ISL_811259	Lighthouse Lab in Glasgow	Wellcome Sanger Institute for the COVID-19 Genomics UK (COG-UK) Consortium	Harper VanSteenhouse, Yumi Kasai, David Gray, Carol Clugston, Anna Dominiczak and Alex Alderton, Roberto Amato, Sonia Goncalves, Ewan Harrison, David K. Jackson, Ian Johnston, Dominic Kwiatkowski, Cordelia Langford, John Sillitoe on behalf of the Wellcome Sanger Institute COVID-19 Surveillance Team
EPI_ISL_811262	Lighthouse Lab in Milton Keynes	Wellcome Sanger Institute for the COVID-19 Genomics UK (COG-UK) Consortium	The Lighthouse Lab in Milton Keynes and Alex Alderton, Roberto Amato, Sonia Goncalves, Ewan Harrison, David K. Jackson, Ian Johnston, Dominic Kwiatkowski, Cordelia Langford, John Sillitoe on behalf of the Wellcome Sanger Institute COVID-19 Surveillance Team
EPI_ISL_811264, EPI_ISL_811265, EPI_ISL_811266, EPI_ISL_811267	Lighthouse Lab in Glasgow	Wellcome Sanger Institute for the COVID-19 Genomics UK (COG-UK) Consortium	Harper VanSteenhouse, Yumi Kasai, David Gray, Carol Clugston, Anna Dominiczak and Alex Alderton, Roberto Amato, Sonia Goncalves, Ewan Harrison, David K. Jackson, Ian Johnston, Dominic Kwiatkowski, Cordelia Langford, John Sillitoe on behalf of the Wellcome Sanger Institute COVID-19 Surveillance Team
EPI_ISL_811269	Lighthouse Lab in Milton Keynes	Wellcome Sanger Institute for the COVID-19 Genomics UK (COG-UK) Consortium	The Lighthouse Lab in Milton Keynes and Alex Alderton, Roberto Amato, Sonia Goncalves, Ewan Harrison, David K. Jackson, Ian Johnston, Dominic Kwiatkowski, Cordelia Langford, John Sillitoe on behalf of the Wellcome Sanger Institute COVID-19 Surveillance Team
EPI_ISL_811270, EPI_ISL_811271, EPI_ISL_811272, EPI_ISL_811274, EPI_ISL_811276, EPI_ISL_811277, EPI_ISL_811278, EPI_ISL_811279	Lighthouse Lab in Glasgow	Wellcome Sanger Institute for the COVID-19 Genomics UK (COG-UK) Consortium	Harper VanSteenhouse, Yumi Kasai, David Gray, Carol Clugston, Anna Dominiczak and Alex Alderton, Roberto Amato, Sonia Goncalves, Ewan Harrison, David K. Jackson, Ian Johnston, Dominic Kwiatkowski, Cordelia Langford, John Sillitoe on behalf of the Wellcome Sanger Institute COVID-19 Surveillance Team
EPI_ISL_811280	Lighthouse Lab in Milton Keynes	Wellcome Sanger Institute for the COVID-19 Genomics UK (COG-UK) Consortium	The Lighthouse Lab in Milton Keynes and Alex Alderton, Roberto Amato, Sonia Goncalves, Ewan Harrison, David K. Jackson, Ian Johnston, Dominic Kwiatkowski, Cordelia Langford, John Sillitoe on behalf of the Wellcome Sanger Institute COVID-19 Surveillance Team
EPI_ISL_811281	Lighthouse Lab in Glasgow	Wellcome Sanger Institute for the COVID-19 Genomics UK	Harper VanSteenhouse, Yumi Kasai, David Gray, Carol Clugston, Anna Dominiczak and Alex Alderton, Roberto Amato, Sonia Goncalves, Ewan Harrison,

		(COG-UK) Consortium	David K. Jackson, Ian Johnston, Dominic Kwiatkowski, Cordelia Langford, John Sillitoe on behalf of the Wellcome Sanger Institute COVID-19 Surveillance Team
EPI_ISL_811282	Lighthouse Lab in Milton Keynes	Wellcome Sanger Institute for the COVID-19 Genomics UK (COG-UK) Consortium	The Lighthouse Lab in Milton Keynes and Alex Alderton, Roberto Amato, Sonia Goncalves, Ewan Harrison, David K. Jackson, Ian Johnston, Dominic Kwiatkowski, Cordelia Langford, John Sillitoe on behalf of the Wellcome Sanger Institute COVID-19 Surveillance Team
EPI_ISL_811283	Lighthouse Lab in Glasgow	Wellcome Sanger Institute for the COVID-19 Genomics UK (COG-UK) Consortium	Harper VanSteenhouse, Yumi Kasai, David Gray, Carol Clugston, Anna Dominiczak and Alex Alderton, Roberto Amato, Sonia Goncalves, Ewan Harrison, David K. Jackson, Ian Johnston, Dominic Kwiatkowski, Cordelia Langford, John Sillitoe on behalf of the Wellcome Sanger Institute COVID-19 Surveillance Team
EPI_ISL_811284, EPI_ISL_811285	Lighthouse Lab in Milton Keynes	Wellcome Sanger Institute for the COVID-19 Genomics UK (COG-UK) Consortium	The Lighthouse Lab in Milton Keynes and Alex Alderton, Roberto Amato, Sonia Goncalves, Ewan Harrison, David K. Jackson, Ian Johnston, Dominic Kwiatkowski, Cordelia Langford, John Sillitoe on behalf of the Wellcome Sanger Institute COVID-19 Surveillance Team
EPI_ISL_811286, EPI_ISL_811287, EPI_ISL_811288, EPI_ISL_811290, EPI_ISL_811291, EPI_ISL_811292, EPI_ISL_811294, EPI_ISL_811296, EPI_ISL_811298, EPI_ISL_811299	Lighthouse Lab in Glasgow	Wellcome Sanger Institute for the COVID-19 Genomics UK (COG-UK) Consortium	Harper VanSteenhouse, Yumi Kasai, David Gray, Carol Clugston, Anna Dominiczak and Alex Alderton, Roberto Amato, Sonia Goncalves, Ewan Harrison, David K. Jackson, Ian Johnston, Dominic Kwiatkowski, Cordelia Langford, John Sillitoe on behalf of the Wellcome Sanger Institute COVID-19 Surveillance Team
EPI_ISL_811300	Lighthouse Lab in Milton Keynes	Wellcome Sanger Institute for the COVID-19 Genomics UK (COG-UK) Consortium	The Lighthouse Lab in Milton Keynes and Alex Alderton, Roberto Amato, Sonia Goncalves, Ewan Harrison, David K. Jackson, Ian Johnston, Dominic Kwiatkowski, Cordelia Langford, John Sillitoe on behalf of the Wellcome Sanger Institute COVID-19 Surveillance Team
EPI_ISL_811301	Lighthouse Lab in Glasgow	Wellcome Sanger Institute for the COVID-19 Genomics UK (COG-UK) Consortium	Harper VanSteenhouse, Yumi Kasai, David Gray, Carol Clugston, Anna Dominiczak and Alex Alderton, Roberto Amato, Sonia Goncalves, Ewan Harrison, David K. Jackson, Ian Johnston, Dominic Kwiatkowski, Cordelia Langford, John Sillitoe on behalf of the Wellcome Sanger Institute COVID-19 Surveillance Team
EPI_ISL_811302	Lighthouse Lab in Milton Keynes	Wellcome Sanger Institute for the COVID-19 Genomics UK (COG-UK) Consortium	The Lighthouse Lab in Milton Keynes and Alex Alderton, Roberto Amato, Sonia Goncalves, Ewan Harrison, David K. Jackson, Ian Johnston, Dominic Kwiatkowski, Cordelia Langford, John Sillitoe on behalf of the Wellcome Sanger Institute COVID-19 Surveillance Team
EPI_ISL_811303	Lighthouse Lab in Glasgow	Wellcome Sanger Institute for the COVID-19 Genomics UK (COG-UK) Consortium	Harper VanSteenhouse, Yumi Kasai, David Gray, Carol Clugston, Anna Dominiczak and Alex Alderton, Roberto Amato, Sonia Goncalves, Ewan Harrison, David K. Jackson, Ian Johnston, Dominic Kwiatkowski, Cordelia Langford, John Sillitoe on behalf of the Wellcome Sanger Institute COVID-19 Surveillance Team
EPI_ISL_811304	Lighthouse Lab in Milton Keynes	Wellcome Sanger Institute for the COVID-19 Genomics UK (COG-UK) Consortium	The Lighthouse Lab in Milton Keynes and Alex Alderton, Roberto Amato, Sonia Goncalves, Ewan Harrison, David K. Jackson, Ian Johnston, Dominic Kwiatkowski, Cordelia Langford, John Sillitoe on behalf of the Wellcome Sanger Institute COVID-19 Surveillance Team
EPI_ISL_811305	Lighthouse Lab in Glasgow	Wellcome Sanger Institute for the COVID-19 Genomics UK (COG-UK) Consortium	Harper VanSteenhouse, Yumi Kasai, David Gray, Carol Clugston, Anna Dominiczak and Alex Alderton, Roberto Amato, Sonia Goncalves, Ewan Harrison, David K. Jackson, Ian Johnston, Dominic Kwiatkowski, Cordelia Langford, John Sillitoe on behalf of the Wellcome Sanger Institute COVID-19 Surveillance Team
EPI_ISL_811306, EPI_ISL_811307	Lighthouse Lab in Milton Keynes	Wellcome Sanger Institute for the COVID-19 Genomics UK (COG-UK) Consortium	The Lighthouse Lab in Milton Keynes and Alex Alderton, Roberto Amato, Sonia Goncalves, Ewan Harrison, David K. Jackson, Ian Johnston, Dominic Kwiatkowski, Cordelia Langford, John Sillitoe on behalf of the Wellcome Sanger Institute COVID-19 Surveillance Team
EPI_ISL_811308	Lighthouse Lab in Glasgow	Wellcome Sanger Institute for the COVID-19 Genomics UK (COG-UK) Consortium	Harper VanSteenhouse, Yumi Kasai, David Gray, Carol Clugston, Anna Dominiczak and Alex Alderton, Roberto Amato, Sonia Goncalves, Ewan Harrison, David K. Jackson, Ian Johnston, Dominic Kwiatkowski, Cordelia Langford, John Sillitoe on behalf of the Wellcome Sanger Institute COVID-19 Surveillance Team
EPI_ISL_811309, EPI_ISL_811311	Lighthouse Lab in Milton Keynes	Wellcome Sanger Institute for the COVID-19 Genomics UK (COG-UK) Consortium	The Lighthouse Lab in Milton Keynes and Alex Alderton, Roberto Amato, Sonia Goncalves, Ewan Harrison, David K. Jackson, Ian Johnston, Dominic Kwiatkowski, Cordelia Langford, John Sillitoe on behalf of the Wellcome Sanger Institute COVID-19 Surveillance Team
EPI_ISL_811313, EPI_ISL_811314	Lighthouse Lab in Glasgow	Wellcome Sanger Institute for the COVID-19 Genomics UK (COG-UK) Consortium	Harper VanSteenhouse, Yumi Kasai, David Gray, Carol Clugston, Anna Dominiczak and Alex Alderton, Roberto Amato, Sonia Goncalves, Ewan Harrison, David K. Jackson, Ian Johnston, Dominic Kwiatkowski, Cordelia Langford, John Sillitoe on behalf of the Wellcome Sanger Institute COVID-19 Surveillance Team
EPI_ISL_811315	Lighthouse Lab in Milton Keynes	Wellcome Sanger Institute for the COVID-19 Genomics UK (COG-UK) Consortium	The Lighthouse Lab in Milton Keynes and Alex Alderton, Roberto Amato, Sonia Goncalves, Ewan Harrison, David K. Jackson, Ian Johnston, Dominic Kwiatkowski, Cordelia Langford, John Sillitoe on behalf of the Wellcome Sanger Institute COVID-19 Surveillance Team
EPI_ISL_811318, EPI_ISL_811319, EPI_ISL_811320	Lighthouse Lab in Glasgow	Wellcome Sanger Institute for the COVID-19 Genomics UK (COG-UK) Consortium	Harper VanSteenhouse, Yumi Kasai, David Gray, Carol Clugston, Anna Dominiczak and Alex Alderton, Roberto Amato, Sonia Goncalves, Ewan Harrison, David K. Jackson, Ian Johnston, Dominic Kwiatkowski, Cordelia Langford, John Sillitoe on behalf of the Wellcome Sanger Institute COVID-19 Surveillance Team
EPI_ISL_811322	Lighthouse Lab in Milton Keynes	Wellcome Sanger Institute for the COVID-19 Genomics UK (COG-UK) Consortium	The Lighthouse Lab in Milton Keynes and Alex Alderton, Roberto Amato, Sonia Goncalves, Ewan Harrison, David K. Jackson, Ian Johnston, Dominic Kwiatkowski, Cordelia Langford, John Sillitoe on behalf of the Wellcome Sanger Institute COVID-19 Surveillance Team
EPI_ISL_811323, EPI_ISL_811324	Lighthouse Lab in Glasgow	Wellcome Sanger Institute for the COVID-19 Genomics UK (COG-UK) Consortium	Harper VanSteenhouse, Yumi Kasai, David Gray, Carol Clugston, Anna Dominiczak and Alex Alderton, Roberto Amato, Sonia Goncalves, Ewan Harrison, David K. Jackson, Ian Johnston, Dominic Kwiatkowski, Cordelia Langford, John Sillitoe on behalf of the Wellcome Sanger Institute COVID-19 Surveillance Team
EPI_ISL_811325	Lighthouse Lab in Milton Keynes	Wellcome Sanger Institute for the COVID-19 Genomics UK (COG-UK) Consortium	The Lighthouse Lab in Milton Keynes and Alex Alderton, Roberto Amato, Sonia Goncalves, Ewan Harrison, David K. Jackson, Ian Johnston, Dominic Kwiatkowski, Cordelia Langford, John Sillitoe on behalf of the Wellcome Sanger Institute COVID-19 Surveillance Team
EPI_ISL_811326, EPI_ISL_811327, EPI_ISL_811328	Lighthouse Lab in Glasgow	Wellcome Sanger Institute for the COVID-19 Genomics UK (COG-UK) Consortium	Harper VanSteenhouse, Yumi Kasai, David Gray, Carol Clugston, Anna Dominiczak and Alex Alderton, Roberto Amato, Sonia Goncalves, Ewan Harrison, David K. Jackson, Ian Johnston, Dominic Kwiatkowski, Cordelia Langford, John Sillitoe on behalf of the Wellcome Sanger Institute COVID-19 Surveillance Team
EPI_ISL_811329	Lighthouse Lab in Milton Keynes	Wellcome Sanger Institute for the COVID-19 Genomics UK (COG-UK) Consortium	The Lighthouse Lab in Milton Keynes and Alex Alderton, Roberto Amato, Sonia Goncalves, Ewan Harrison, David K. Jackson, Ian Johnston, Dominic Kwiatkowski, Cordelia Langford, John Sillitoe on behalf of the Wellcome Sanger Institute COVID-19 Surveillance Team
EPI_ISL_811330, EPI_ISL_811331	Lighthouse Lab in Glasgow	Wellcome Sanger Institute for the COVID-19 Genomics UK (COG-UK) Consortium	Harper VanSteenhouse, Yumi Kasai, David Gray, Carol Clugston, Anna Dominiczak and Alex Alderton, Roberto Amato, Sonia Goncalves, Ewan Harrison, David K. Jackson, Ian Johnston, Dominic Kwiatkowski, Cordelia Langford, John Sillitoe on behalf of the Wellcome Sanger Institute COVID-19 Surveillance Team
EPI_ISL_811332	Lighthouse Lab in Milton Keynes	Wellcome Sanger Institute for the COVID-19 Genomics UK (COG-UK) Consortium	The Lighthouse Lab in Milton Keynes and Alex Alderton, Roberto Amato, Sonia Goncalves, Ewan Harrison, David K. Jackson, Ian Johnston, Dominic Kwiatkowski, Cordelia Langford, John Sillitoe on behalf of the Wellcome Sanger Institute COVID-19 Surveillance Team
EPI_ISL_811333	Lighthouse Lab in Glasgow	Wellcome Sanger Institute for the COVID-19 Genomics UK (COG-UK) Consortium	Harper VanSteenhouse, Yumi Kasai, David Gray, Carol Clugston, Anna Dominiczak and Alex Alderton, Roberto Amato, Sonia Goncalves, Ewan Harrison, David K. Jackson, Ian Johnston, Dominic Kwiatkowski, Cordelia Langford, John Sillitoe on behalf of the Wellcome Sanger Institute COVID-19 Surveillance Team
EPI_ISL_811334, EPI_ISL_811335, EPI_ISL_811336	Lighthouse Lab in Milton Keynes	Wellcome Sanger Institute for the COVID-19 Genomics UK (COG-UK) Consortium	The Lighthouse Lab in Milton Keynes and Alex Alderton, Roberto Amato, Sonia Goncalves, Ewan Harrison, David K. Jackson, Ian Johnston, Dominic Kwiatkowski, Cordelia Langford, John Sillitoe on behalf of the Wellcome Sanger Institute COVID-19 Surveillance Team
EPI_ISL_811337	Lighthouse Lab in Glasgow	Wellcome Sanger Institute for the COVID-19 Genomics UK (COG-UK) Consortium	Harper VanSteenhouse, Yumi Kasai, David Gray, Carol Clugston, Anna Dominiczak and Alex Alderton, Roberto Amato, Sonia Goncalves, Ewan Harrison, David K. Jackson, Ian Johnston, Dominic Kwiatkowski, Cordelia Langford, John Sillitoe on behalf of the Wellcome Sanger Institute COVID-19 Surveillance Team
EPI_ISL_811339	Lighthouse Lab in Milton Keynes	Wellcome Sanger Institute for the COVID-19 Genomics UK (COG-UK) Consortium	The Lighthouse Lab in Milton Keynes and Alex Alderton, Roberto Amato, Sonia Goncalves, Ewan Harrison, David K. Jackson, Ian Johnston, Dominic Kwiatkowski, Cordelia Langford, John Sillitoe on behalf of the Wellcome Sanger Institute COVID-19 Surveillance Team
EPI_ISL_811340, EPI_ISL_811341	Lighthouse Lab in Glasgow	Wellcome Sanger Institute for the COVID-19 Genomics UK (COG-UK) Consortium	Harper VanSteenhouse, Yumi Kasai, David Gray, Carol Clugston, Anna Dominiczak and Alex Alderton, Roberto Amato, Sonia Goncalves, Ewan Harrison, David K. Jackson, Ian Johnston, Dominic Kwiatkowski, Cordelia Langford, John Sillitoe on behalf of the Wellcome Sanger Institute COVID-19 Surveillance

			Team
EPI_ISL_811342	Lighthouse Lab in Milton Keynes	Wellcome Sanger Institute for the COVID-19 Genomics UK (COG-UK) Consortium	The Lighthouse Lab in Milton Keynes and Alex Alderton, Roberto Amato, Sonia Goncalves, Ewan Harrison, David K. Jackson, Ian Johnston, Dominic Kwiatkowski, Cordelia Langford, John Sillitoe on behalf of the Wellcome Sanger Institute COVID-19 Surveillance Team
EPI_ISL_811343	Lighthouse Lab in Glasgow	Wellcome Sanger Institute for the COVID-19 Genomics UK (COG-UK) Consortium	Harper VanSteenhouse, Yumi Kasai, David Gray, Carol Clugston, Anna Dominiczak and Alex Alderton, Roberto Amato, Sonia Goncalves, Ewan Harrison, David K. Jackson, Ian Johnston, Dominic Kwiatkowski, Cordelia Langford, John Sillitoe on behalf of the Wellcome Sanger Institute COVID-19 Surveillance Team
EPI_ISL_811344	Lighthouse Lab in Milton Keynes	Wellcome Sanger Institute for the COVID-19 Genomics UK (COG-UK) Consortium	The Lighthouse Lab in Milton Keynes and Alex Alderton, Roberto Amato, Sonia Goncalves, Ewan Harrison, David K. Jackson, Ian Johnston, Dominic Kwiatkowski, Cordelia Langford, John Sillitoe on behalf of the Wellcome Sanger Institute COVID-19 Surveillance Team
EPI_ISL_811346, EPI_ISL_811349, EPI_ISL_811351, EPI_ISL_811352, EPI_ISL_811354, EPI_ISL_811357	Lighthouse Lab in Glasgow	Wellcome Sanger Institute for the COVID-19 Genomics UK (COG-UK) Consortium	Harper VanSteenhouse, Yumi Kasai, David Gray, Carol Clugston, Anna Dominiczak and Alex Alderton, Roberto Amato, Sonia Goncalves, Ewan Harrison, David K. Jackson, Ian Johnston, Dominic Kwiatkowski, Cordelia Langford, John Sillitoe on behalf of the Wellcome Sanger Institute COVID-19 Surveillance Team
EPI_ISL_811358, EPI_ISL_811359	Lighthouse Lab in Milton Keynes	Wellcome Sanger Institute for the COVID-19 Genomics UK (COG-UK) Consortium	The Lighthouse Lab in Milton Keynes and Alex Alderton, Roberto Amato, Sonia Goncalves, Ewan Harrison, David K. Jackson, Ian Johnston, Dominic Kwiatkowski, Cordelia Langford, John Sillitoe on behalf of the Wellcome Sanger Institute COVID-19 Surveillance Team
EPI_ISL_811360, EPI_ISL_811361	Lighthouse Lab in Glasgow	Wellcome Sanger Institute for the COVID-19 Genomics UK (COG-UK) Consortium	Harper VanSteenhouse, Yumi Kasai, David Gray, Carol Clugston, Anna Dominiczak and Alex Alderton, Roberto Amato, Sonia Goncalves, Ewan Harrison, David K. Jackson, Ian Johnston, Dominic Kwiatkowski, Cordelia Langford, John Sillitoe on behalf of the Wellcome Sanger Institute COVID-19 Surveillance Team
EPI_ISL_811362	Lighthouse Lab in Milton Keynes	Wellcome Sanger Institute for the COVID-19 Genomics UK (COG-UK) Consortium	The Lighthouse Lab in Milton Keynes and Alex Alderton, Roberto Amato, Sonia Goncalves, Ewan Harrison, David K. Jackson, Ian Johnston, Dominic Kwiatkowski, Cordelia Langford, John Sillitoe on behalf of the Wellcome Sanger Institute COVID-19 Surveillance Team
EPI_ISL_811363	Lighthouse Lab in Glasgow	Wellcome Sanger Institute for the COVID-19 Genomics UK (COG-UK) Consortium	Harper VanSteenhouse, Yumi Kasai, David Gray, Carol Clugston, Anna Dominiczak and Alex Alderton, Roberto Amato, Sonia Goncalves, Ewan Harrison, David K. Jackson, Ian Johnston, Dominic Kwiatkowski, Cordelia Langford, John Sillitoe on behalf of the Wellcome Sanger Institute COVID-19 Surveillance Team
EPI_ISL_811367, EPI_ISL_811368, EPI_ISL_811369	Lighthouse Lab in Milton Keynes	Wellcome Sanger Institute for the COVID-19 Genomics UK (COG-UK) Consortium	The Lighthouse Lab in Milton Keynes and Alex Alderton, Roberto Amato, Sonia Goncalves, Ewan Harrison, David K. Jackson, Ian Johnston, Dominic Kwiatkowski, Cordelia Langford, John Sillitoe on behalf of the Wellcome Sanger Institute COVID-19 Surveillance Team
EPI_ISL_811370, EPI_ISL_811371, EPI_ISL_811372	Lighthouse Lab in Glasgow	Wellcome Sanger Institute for the COVID-19 Genomics UK (COG-UK) Consortium	Harper VanSteenhouse, Yumi Kasai, David Gray, Carol Clugston, Anna Dominiczak and Alex Alderton, Roberto Amato, Sonia Goncalves, Ewan Harrison, David K. Jackson, Ian Johnston, Dominic Kwiatkowski, Cordelia Langford, John Sillitoe on behalf of the Wellcome Sanger Institute COVID-19 Surveillance Team
EPI_ISL_811373	Lighthouse Lab in Milton Keynes	Wellcome Sanger Institute for the COVID-19 Genomics UK (COG-UK) Consortium	The Lighthouse Lab in Milton Keynes and Alex Alderton, Roberto Amato, Sonia Goncalves, Ewan Harrison, David K. Jackson, Ian Johnston, Dominic Kwiatkowski, Cordelia Langford, John Sillitoe on behalf of the Wellcome Sanger Institute COVID-19 Surveillance Team
EPI_ISL_811375, EPI_ISL_811378, EPI_ISL_811379	Lighthouse Lab in Glasgow	Wellcome Sanger Institute for the COVID-19 Genomics UK (COG-UK) Consortium	Harper VanSteenhouse, Yumi Kasai, David Gray, Carol Clugston, Anna Dominiczak and Alex Alderton, Roberto Amato, Sonia Goncalves, Ewan Harrison, David K. Jackson, Ian Johnston, Dominic Kwiatkowski, Cordelia Langford, John Sillitoe on behalf of the Wellcome Sanger Institute COVID-19 Surveillance Team
EPI_ISL_811380, EPI_ISL_811381, EPI_ISL_811382, EPI_ISL_811383, EPI_ISL_811385	Lighthouse Lab in Milton Keynes	Wellcome Sanger Institute for the COVID-19 Genomics UK (COG-UK) Consortium	The Lighthouse Lab in Milton Keynes and Alex Alderton, Roberto Amato, Sonia Goncalves, Ewan Harrison, David K. Jackson, Ian Johnston, Dominic Kwiatkowski, Cordelia Langford, John Sillitoe on behalf of the Wellcome Sanger Institute COVID-19 Surveillance Team
EPI_ISL_811386	Lighthouse Lab in Glasgow	Wellcome Sanger Institute for the COVID-19 Genomics UK (COG-UK) Consortium	Harper VanSteenhouse, Yumi Kasai, David Gray, Carol Clugston, Anna Dominiczak and Alex Alderton, Roberto Amato, Sonia Goncalves, Ewan Harrison, David K. Jackson, Ian Johnston, Dominic Kwiatkowski, Cordelia Langford, John Sillitoe on behalf of the Wellcome Sanger Institute COVID-19 Surveillance Team
EPI_ISL_811389	Lighthouse Lab in Milton Keynes	Wellcome Sanger Institute for the COVID-19 Genomics UK (COG-UK) Consortium	The Lighthouse Lab in Milton Keynes and Alex Alderton, Roberto Amato, Sonia Goncalves, Ewan Harrison, David K. Jackson, Ian Johnston, Dominic Kwiatkowski, Cordelia Langford, John Sillitoe on behalf of the Wellcome Sanger Institute COVID-19 Surveillance Team
EPI_ISL_811390	Lighthouse Lab in Glasgow	Wellcome Sanger Institute for the COVID-19 Genomics UK (COG-UK) Consortium	Harper VanSteenhouse, Yumi Kasai, David Gray, Carol Clugston, Anna Dominiczak and Alex Alderton, Roberto Amato, Sonia Goncalves, Ewan Harrison, David K. Jackson, Ian Johnston, Dominic Kwiatkowski, Cordelia Langford, John Sillitoe on behalf of the Wellcome Sanger Institute COVID-19 Surveillance Team
EPI_ISL_811392	Lighthouse Lab in Milton Keynes	Wellcome Sanger Institute for the COVID-19 Genomics UK (COG-UK) Consortium	The Lighthouse Lab in Milton Keynes and Alex Alderton, Roberto Amato, Sonia Goncalves, Ewan Harrison, David K. Jackson, Ian Johnston, Dominic Kwiatkowski, Cordelia Langford, John Sillitoe on behalf of the Wellcome Sanger Institute COVID-19 Surveillance Team
EPI_ISL_811393	Lighthouse Lab in Glasgow	Wellcome Sanger Institute for the COVID-19 Genomics UK (COG-UK) Consortium	Harper VanSteenhouse, Yumi Kasai, David Gray, Carol Clugston, Anna Dominiczak and Alex Alderton, Roberto Amato, Sonia Goncalves, Ewan Harrison, David K. Jackson, Ian Johnston, Dominic Kwiatkowski, Cordelia Langford, John Sillitoe on behalf of the Wellcome Sanger Institute COVID-19 Surveillance Team
EPI_ISL_811394, EPI_ISL_811395	Lighthouse Lab in Milton Keynes	Wellcome Sanger Institute for the COVID-19 Genomics UK (COG-UK) Consortium	The Lighthouse Lab in Milton Keynes and Alex Alderton, Roberto Amato, Sonia Goncalves, Ewan Harrison, David K. Jackson, Ian Johnston, Dominic Kwiatkowski, Cordelia Langford, John Sillitoe on behalf of the Wellcome Sanger Institute COVID-19 Surveillance Team
EPI_ISL_811396	Lighthouse Lab in Glasgow	Wellcome Sanger Institute for the COVID-19 Genomics UK (COG-UK) Consortium	Harper VanSteenhouse, Yumi Kasai, David Gray, Carol Clugston, Anna Dominiczak and Alex Alderton, Roberto Amato, Sonia Goncalves, Ewan Harrison, David K. Jackson, Ian Johnston, Dominic Kwiatkowski, Cordelia Langford, John Sillitoe on behalf of the Wellcome Sanger Institute COVID-19 Surveillance Team
EPI_ISL_811397	Lighthouse Lab in Milton Keynes	Wellcome Sanger Institute for the COVID-19 Genomics UK (COG-UK) Consortium	The Lighthouse Lab in Milton Keynes and Alex Alderton, Roberto Amato, Sonia Goncalves, Ewan Harrison, David K. Jackson, Ian Johnston, Dominic Kwiatkowski, Cordelia Langford, John Sillitoe on behalf of the Wellcome Sanger Institute COVID-19 Surveillance Team
EPI_ISL_811398, EPI_ISL_811399	Lighthouse Lab in Glasgow	Wellcome Sanger Institute for the COVID-19 Genomics UK (COG-UK) Consortium	Harper VanSteenhouse, Yumi Kasai, David Gray, Carol Clugston, Anna Dominiczak and Alex Alderton, Roberto Amato, Sonia Goncalves, Ewan Harrison, David K. Jackson, Ian Johnston, Dominic Kwiatkowski, Cordelia Langford, John Sillitoe on behalf of the Wellcome Sanger Institute COVID-19 Surveillance Team
EPI_ISL_811400	Lighthouse Lab in Milton Keynes	Wellcome Sanger Institute for the COVID-19 Genomics UK (COG-UK) Consortium	The Lighthouse Lab in Milton Keynes and Alex Alderton, Roberto Amato, Sonia Goncalves, Ewan Harrison, David K. Jackson, Ian Johnston, Dominic Kwiatkowski, Cordelia Langford, John Sillitoe on behalf of the Wellcome Sanger Institute COVID-19 Surveillance Team
EPI_ISL_811401, EPI_ISL_811402, EPI_ISL_811403	Lighthouse Lab in Glasgow	Wellcome Sanger Institute for the COVID-19 Genomics UK (COG-UK) Consortium	Harper VanSteenhouse, Yumi Kasai, David Gray, Carol Clugston, Anna Dominiczak and Alex Alderton, Roberto Amato, Sonia Goncalves, Ewan Harrison, David K. Jackson, Ian Johnston, Dominic Kwiatkowski, Cordelia Langford, John Sillitoe on behalf of the Wellcome Sanger Institute COVID-19 Surveillance Team
EPI_ISL_811404, EPI_ISL_811406	Lighthouse Lab in Milton Keynes	Wellcome Sanger Institute for the COVID-19 Genomics UK (COG-UK) Consortium	The Lighthouse Lab in Milton Keynes and Alex Alderton, Roberto Amato, Sonia Goncalves, Ewan Harrison, David K. Jackson, Ian Johnston, Dominic Kwiatkowski, Cordelia Langford, John Sillitoe on behalf of the Wellcome Sanger Institute COVID-19 Surveillance Team
EPI_ISL_811407, EPI_ISL_811408	Lighthouse Lab in Glasgow	Wellcome Sanger Institute for the COVID-19 Genomics UK (COG-UK) Consortium	Harper VanSteenhouse, Yumi Kasai, David Gray, Carol Clugston, Anna Dominiczak and Alex Alderton, Roberto Amato, Sonia Goncalves, Ewan Harrison, David K. Jackson, Ian Johnston, Dominic Kwiatkowski, Cordelia Langford, John Sillitoe on behalf of the Wellcome Sanger Institute COVID-19 Surveillance Team
EPI_ISL_811409, EPI_ISL_811410	Lighthouse Lab in Milton Keynes	Wellcome Sanger Institute for the COVID-19 Genomics UK (COG-UK) Consortium	The Lighthouse Lab in Milton Keynes and Alex Alderton, Roberto Amato, Sonia Goncalves, Ewan Harrison, David K. Jackson, Ian Johnston, Dominic Kwiatkowski, Cordelia Langford, John Sillitoe on behalf of the Wellcome Sanger Institute COVID-19 Surveillance Team
EPI_ISL_811411, EPI_ISL_811412, EPI_ISL_811413, EPI_ISL_811416, EPI_ISL_811418, EPI_ISL_811419	Lighthouse Lab in Glasgow	Wellcome Sanger Institute for the COVID-19 Genomics UK (COG-UK) Consortium	Harper VanSteenhouse, Yumi Kasai, David Gray, Carol Clugston, Anna Dominiczak and Alex Alderton, Roberto Amato, Sonia Goncalves, Ewan Harrison, David K. Jackson, Ian Johnston, Dominic Kwiatkowski, Cordelia Langford, John Sillitoe on behalf of the Wellcome Sanger Institute COVID-19 Surveillance Team
EPI_ISL_811420	Lighthouse Lab in Milton Keynes	Wellcome Sanger Institute for the COVID-19 Genomics UK	The Lighthouse Lab in Milton Keynes and Alex Alderton, Roberto Amato, Sonia Goncalves, Ewan Harrison, David K. Jackson, Ian Johnston, Dominic

		(COG-UK) Consortium	Kwiatkowski, Cordelia Langford, John Sillitoe on behalf of the Wellcome Sanger Institute COVID-19 Surveillance Team
EPI_ISL_811421, EPI_ISL_811425, EPI_ISL_811428	Lighthouse Lab in Glasgow	Wellcome Sanger Institute for the COVID-19 Genomics UK (COG-UK) Consortium	Harper VanSteenhouse, Yumi Kasai, David Gray, Carol Clugston, Anna Dominiczak and Alex Alderton, Roberto Amato, Sonia Goncalves, Ewan Harrison, David K. Jackson, Ian Johnston, Dominic Kwiatkowski, Cordelia Langford, John Sillitoe on behalf of the Wellcome Sanger Institute COVID-19 Surveillance Team
EPI_ISL_811429, EPI_ISL_811430, EPI_ISL_811431, EPI_ISL_811433	Lighthouse Lab in Milton Keynes	Wellcome Sanger Institute for the COVID-19 Genomics UK (COG-UK) Consortium	The Lighthouse Lab in Milton Keynes and Alex Alderton, Roberto Amato, Sonia Goncalves, Ewan Harrison, David K. Jackson, Ian Johnston, Dominic Kwiatkowski, Cordelia Langford, John Sillitoe on behalf of the Wellcome Sanger Institute COVID-19 Surveillance Team
EPI_ISL_811434, EPI_ISL_811435	Lighthouse Lab in Glasgow	Wellcome Sanger Institute for the COVID-19 Genomics UK (COG-UK) Consortium	Harper VanSteenhouse, Yumi Kasai, David Gray, Carol Clugston, Anna Dominiczak and Alex Alderton, Roberto Amato, Sonia Goncalves, Ewan Harrison, David K. Jackson, Ian Johnston, Dominic Kwiatkowski, Cordelia Langford, John Sillitoe on behalf of the Wellcome Sanger Institute COVID-19 Surveillance Team
EPI_ISL_811436	Lighthouse Lab in Milton Keynes	Wellcome Sanger Institute for the COVID-19 Genomics UK (COG-UK) Consortium	The Lighthouse Lab in Milton Keynes and Alex Alderton, Roberto Amato, Sonia Goncalves, Ewan Harrison, David K. Jackson, Ian Johnston, Dominic Kwiatkowski, Cordelia Langford, John Sillitoe on behalf of the Wellcome Sanger Institute COVID-19 Surveillance Team
EPI_ISL_811437, EPI_ISL_811438, EPI_ISL_811439, EPI_ISL_811440, EPI_ISL_811441, EPI_ISL_811442, EPI_ISL_811443	Lighthouse Lab in Glasgow	Wellcome Sanger Institute for the COVID-19 Genomics UK (COG-UK) Consortium	Harper VanSteenhouse, Yumi Kasai, David Gray, Carol Clugston, Anna Dominiczak and Alex Alderton, Roberto Amato, Sonia Goncalves, Ewan Harrison, David K. Jackson, Ian Johnston, Dominic Kwiatkowski, Cordelia Langford, John Sillitoe on behalf of the Wellcome Sanger Institute COVID-19 Surveillance Team
EPI_ISL_811444, EPI_ISL_811445	Lighthouse Lab in Milton Keynes	Wellcome Sanger Institute for the COVID-19 Genomics UK (COG-UK) Consortium	The Lighthouse Lab in Milton Keynes and Alex Alderton, Roberto Amato, Sonia Goncalves, Ewan Harrison, David K. Jackson, Ian Johnston, Dominic Kwiatkowski, Cordelia Langford, John Sillitoe on behalf of the Wellcome Sanger Institute COVID-19 Surveillance Team
EPI_ISL_811446, EPI_ISL_811447, EPI_ISL_811448, EPI_ISL_811449, EPI_ISL_811450, EPI_ISL_811451	Lighthouse Lab in Glasgow	Wellcome Sanger Institute for the COVID-19 Genomics UK (COG-UK) Consortium	Harper VanSteenhouse, Yumi Kasai, David Gray, Carol Clugston, Anna Dominiczak and Alex Alderton, Roberto Amato, Sonia Goncalves, Ewan Harrison, David K. Jackson, Ian Johnston, Dominic Kwiatkowski, Cordelia Langford, John Sillitoe on behalf of the Wellcome Sanger Institute COVID-19 Surveillance Team
EPI_ISL_811452, EPI_ISL_811454, EPI_ISL_811455, EPI_ISL_811456	Lighthouse Lab in Milton Keynes	Wellcome Sanger Institute for the COVID-19 Genomics UK (COG-UK) Consortium	The Lighthouse Lab in Milton Keynes and Alex Alderton, Roberto Amato, Sonia Goncalves, Ewan Harrison, David K. Jackson, Ian Johnston, Dominic Kwiatkowski, Cordelia Langford, John Sillitoe on behalf of the Wellcome Sanger Institute COVID-19 Surveillance Team
EPI_ISL_811457, EPI_ISL_811458, EPI_ISL_811459, EPI_ISL_811460, EPI_ISL_811461, EPI_ISL_811462, EPI_ISL_811463, EPI_ISL_811464, EPI_ISL_811465, EPI_ISL_811466, EPI_ISL_811467, EPI_ISL_811468			
see above	Lighthouse Lab in Cambridge	Wellcome Sanger Institute for the COVID-19 Genomics UK (COG-UK) Consortium	Rob Howes, The Lighthouse Lab in Cambridge and Alex Alderton, Roberto Amato, Sonia Goncalves, Ewan Harrison, David K. Jackson, Ian Johnston, Dominic Kwiatkowski, Cordelia Langford, John Sillitoe on behalf of the Wellcome Sanger Institute COVID-19 Surveillance Team
EPI_ISL_811469	Lighthouse Lab in Milton Keynes	Wellcome Sanger Institute for the COVID-19 Genomics UK (COG-UK) Consortium	The Lighthouse Lab in Milton Keynes and Alex Alderton, Roberto Amato, Sonia Goncalves, Ewan Harrison, David K. Jackson, Ian Johnston, Dominic Kwiatkowski, Cordelia Langford, John Sillitoe on behalf of the Wellcome Sanger Institute COVID-19 Surveillance Team
EPI_ISL_811470, EPI_ISL_811471, EPI_ISL_811473, EPI_ISL_811474, EPI_ISL_811475, EPI_ISL_811476, EPI_ISL_811477	Lighthouse Lab in Cambridge	Wellcome Sanger Institute for the COVID-19 Genomics UK (COG-UK) Consortium	Rob Howes, The Lighthouse Lab in Cambridge and Alex Alderton, Roberto Amato, Sonia Goncalves, Ewan Harrison, David K. Jackson, Ian Johnston, Dominic Kwiatkowski, Cordelia Langford, John Sillitoe on behalf of the Wellcome Sanger Institute COVID-19 Surveillance Team
EPI_ISL_811478	Lighthouse Lab in Milton Keynes	Wellcome Sanger Institute for the COVID-19 Genomics UK (COG-UK) Consortium	The Lighthouse Lab in Milton Keynes and Alex Alderton, Roberto Amato, Sonia Goncalves, Ewan Harrison, David K. Jackson, Ian Johnston, Dominic Kwiatkowski, Cordelia Langford, John Sillitoe on behalf of the Wellcome Sanger Institute COVID-19 Surveillance Team
EPI_ISL_811479, EPI_ISL_811480, EPI_ISL_811481	Lighthouse Lab in Cambridge	Wellcome Sanger Institute for the COVID-19 Genomics UK (COG-UK) Consortium	Rob Howes, The Lighthouse Lab in Cambridge and Alex Alderton, Roberto Amato, Sonia Goncalves, Ewan Harrison, David K. Jackson, Ian Johnston, Dominic Kwiatkowski, Cordelia Langford, John Sillitoe on behalf of the Wellcome Sanger Institute COVID-19 Surveillance Team
EPI_ISL_811482	Lighthouse Lab in Milton Keynes	Wellcome Sanger Institute for the COVID-19 Genomics UK (COG-UK) Consortium	The Lighthouse Lab in Milton Keynes and Alex Alderton, Roberto Amato, Sonia Goncalves, Ewan Harrison, David K. Jackson, Ian Johnston, Dominic Kwiatkowski, Cordelia Langford, John Sillitoe on behalf of the Wellcome Sanger Institute COVID-19 Surveillance Team
EPI_ISL_811483	Lighthouse Lab in Cambridge	Wellcome Sanger Institute for the COVID-19 Genomics UK (COG-UK) Consortium	Rob Howes, The Lighthouse Lab in Cambridge and Alex Alderton, Roberto Amato, Sonia Goncalves, Ewan Harrison, David K. Jackson, Ian Johnston, Dominic Kwiatkowski, Cordelia Langford, John Sillitoe on behalf of the Wellcome Sanger Institute COVID-19 Surveillance Team
EPI_ISL_811484	Lighthouse Lab in Milton Keynes	Wellcome Sanger Institute for the COVID-19 Genomics UK (COG-UK) Consortium	The Lighthouse Lab in Milton Keynes and Alex Alderton, Roberto Amato, Sonia Goncalves, Ewan Harrison, David K. Jackson, Ian Johnston, Dominic Kwiatkowski, Cordelia Langford, John Sillitoe on behalf of the Wellcome Sanger Institute COVID-19 Surveillance Team
EPI_ISL_811485, EPI_ISL_811486	Lighthouse Lab in Cambridge	Wellcome Sanger Institute for the COVID-19 Genomics UK (COG-UK) Consortium	Rob Howes, The Lighthouse Lab in Cambridge and Alex Alderton, Roberto Amato, Sonia Goncalves, Ewan Harrison, David K. Jackson, Ian Johnston, Dominic Kwiatkowski, Cordelia Langford, John Sillitoe on behalf of the Wellcome Sanger Institute COVID-19 Surveillance Team
EPI_ISL_811487	Lighthouse Lab in Milton Keynes	Wellcome Sanger Institute for the COVID-19 Genomics UK (COG-UK) Consortium	The Lighthouse Lab in Milton Keynes and Alex Alderton, Roberto Amato, Sonia Goncalves, Ewan Harrison, David K. Jackson, Ian Johnston, Dominic Kwiatkowski, Cordelia Langford, John Sillitoe on behalf of the Wellcome Sanger Institute COVID-19 Surveillance Team
EPI_ISL_811488, EPI_ISL_811489, EPI_ISL_811491, EPI_ISL_811492, EPI_ISL_811493, EPI_ISL_811495, EPI_ISL_811496, EPI_ISL_811497	Lighthouse Lab in Cambridge	Wellcome Sanger Institute for the COVID-19 Genomics UK (COG-UK) Consortium	Rob Howes, The Lighthouse Lab in Cambridge and Alex Alderton, Roberto Amato, Sonia Goncalves, Ewan Harrison, David K. Jackson, Ian Johnston, Dominic Kwiatkowski, Cordelia Langford, John Sillitoe on behalf of the Wellcome Sanger Institute COVID-19 Surveillance Team
EPI_ISL_811498	Lighthouse Lab in Milton Keynes	Wellcome Sanger Institute for the COVID-19 Genomics UK (COG-UK) Consortium	The Lighthouse Lab in Milton Keynes and Alex Alderton, Roberto Amato, Sonia Goncalves, Ewan Harrison, David K. Jackson, Ian Johnston, Dominic Kwiatkowski, Cordelia Langford, John Sillitoe on behalf of the Wellcome Sanger Institute COVID-19 Surveillance Team
EPI_ISL_811499, EPI_ISL_811501	Lighthouse Lab in Cambridge	Wellcome Sanger Institute for the COVID-19 Genomics UK (COG-UK) Consortium	Rob Howes, The Lighthouse Lab in Cambridge and Alex Alderton, Roberto Amato, Sonia Goncalves, Ewan Harrison, David K. Jackson, Ian Johnston, Dominic Kwiatkowski, Cordelia Langford, John Sillitoe on behalf of the Wellcome Sanger Institute COVID-19 Surveillance Team
EPI_ISL_811502	Lighthouse Lab in Milton Keynes	Wellcome Sanger Institute for the COVID-19 Genomics UK (COG-UK) Consortium	The Lighthouse Lab in Milton Keynes and Alex Alderton, Roberto Amato, Sonia Goncalves, Ewan Harrison, David K. Jackson, Ian Johnston, Dominic Kwiatkowski, Cordelia Langford, John Sillitoe on behalf of the Wellcome Sanger Institute COVID-19 Surveillance Team
EPI_ISL_811503, EPI_ISL_811504, EPI_ISL_811505, EPI_ISL_811506, EPI_ISL_811507, EPI_ISL_811508, EPI_ISL_811509, EPI_ISL_811510	Lighthouse Lab in Cambridge	Wellcome Sanger Institute for the COVID-19 Genomics UK (COG-UK) Consortium	Rob Howes, The Lighthouse Lab in Cambridge and Alex Alderton, Roberto Amato, Sonia Goncalves, Ewan Harrison, David K. Jackson, Ian Johnston, Dominic Kwiatkowski, Cordelia Langford, John Sillitoe on behalf of the Wellcome Sanger Institute COVID-19 Surveillance Team
EPI_ISL_811511	Lighthouse Lab in Milton Keynes	Wellcome Sanger Institute for the COVID-19 Genomics UK (COG-UK) Consortium	The Lighthouse Lab in Milton Keynes and Alex Alderton, Roberto Amato, Sonia Goncalves, Ewan Harrison, David K. Jackson, Ian Johnston, Dominic Kwiatkowski, Cordelia Langford, John Sillitoe on behalf of the Wellcome Sanger Institute COVID-19 Surveillance Team
EPI_ISL_811512, EPI_ISL_811513, EPI_ISL_811514, EPI_ISL_811515, EPI_ISL_811517, EPI_ISL_811518, EPI_ISL_811520, EPI_ISL_811521, EPI_ISL_811522, EPI_ISL_811523	Lighthouse Lab in Cambridge	Wellcome Sanger Institute for the COVID-19 Genomics UK (COG-UK) Consortium	Rob Howes, The Lighthouse Lab in Cambridge and Alex Alderton, Roberto Amato, Sonia Goncalves, Ewan Harrison, David K. Jackson, Ian Johnston, Dominic Kwiatkowski, Cordelia Langford, John Sillitoe on behalf of the Wellcome Sanger Institute COVID-19 Surveillance Team
EPI_ISL_811525	Lighthouse Lab in Milton Keynes	Wellcome Sanger Institute for the COVID-19 Genomics UK (COG-UK) Consortium	The Lighthouse Lab in Milton Keynes and Alex Alderton, Roberto Amato, Sonia Goncalves, Ewan Harrison, David K. Jackson, Ian Johnston, Dominic Kwiatkowski, Cordelia Langford, John Sillitoe on behalf of the Wellcome Sanger Institute COVID-19 Surveillance Team
EPI_ISL_811526, EPI_ISL_811527, EPI_ISL_811528, EPI_ISL_811529, EPI_ISL_811531, EPI_ISL_811532, EPI_ISL_811533, EPI_ISL_811534, EPI_ISL_811535, EPI_ISL_811536, EPI_ISL_811537			
see above	Lighthouse Lab in Cambridge	Wellcome Sanger Institute for the COVID-19 Genomics UK (COG-UK) Consortium	Rob Howes, The Lighthouse Lab in Cambridge and Alex Alderton, Roberto Amato, Sonia Goncalves, Ewan Harrison, David K. Jackson, Ian Johnston, Dominic Kwiatkowski, Cordelia Langford, John Sillitoe on behalf of the Wellcome Sanger Institute COVID-19 Surveillance Team
EPI_ISL_811538	Lighthouse Lab in Milton Keynes	Wellcome Sanger Institute for the COVID-19 Genomics UK (COG-UK) Consortium	The Lighthouse Lab in Milton Keynes and Alex Alderton, Roberto Amato, Sonia Goncalves, Ewan Harrison, David K. Jackson, Ian Johnston, Dominic Kwiatkowski, Cordelia Langford, John Sillitoe on behalf of the Wellcome Sanger Institute COVID-19 Surveillance Team

EPI_ISL_811539, EPI_ISL_811540	Lighthouse Lab in Cambridge	Wellcome Sanger Institute for the COVID-19 Genomics UK (COG-UK) Consortium	Rob Howes, The Lighthouse Lab in Cambridge and Alex Alderton, Roberto Amato, Sonia Goncalves, Ewan Harrison, David K. Jackson, Ian Johnston, Dominic Kwiatkowski, Cordelia Langford, John Sillitoe on behalf of the Wellcome Sanger Institute COVID-19 Surveillance Team
EPI_ISL_811541	Lighthouse Lab in Milton Keynes	Wellcome Sanger Institute for the COVID-19 Genomics UK (COG-UK) Consortium	The Lighthouse Lab in Milton Keynes and Alex Alderton, Roberto Amato, Sonia Goncalves, Ewan Harrison, David K. Jackson, Ian Johnston, Dominic Kwiatkowski, Cordelia Langford, John Sillitoe on behalf of the Wellcome Sanger Institute COVID-19 Surveillance Team
EPI_ISL_811542, EPI_ISL_811543, EPI_ISL_811544	Lighthouse Lab in Cambridge	Wellcome Sanger Institute for the COVID-19 Genomics UK (COG-UK) Consortium	Rob Howes, The Lighthouse Lab in Cambridge and Alex Alderton, Roberto Amato, Sonia Goncalves, Ewan Harrison, David K. Jackson, Ian Johnston, Dominic Kwiatkowski, Cordelia Langford, John Sillitoe on behalf of the Wellcome Sanger Institute COVID-19 Surveillance Team
EPI_ISL_811545	Lighthouse Lab in Milton Keynes	Wellcome Sanger Institute for the COVID-19 Genomics UK (COG-UK) Consortium	The Lighthouse Lab in Milton Keynes and Alex Alderton, Roberto Amato, Sonia Goncalves, Ewan Harrison, David K. Jackson, Ian Johnston, Dominic Kwiatkowski, Cordelia Langford, John Sillitoe on behalf of the Wellcome Sanger Institute COVID-19 Surveillance Team
EPI_ISL_811546, EPI_ISL_811547	Lighthouse Lab in Cambridge	Wellcome Sanger Institute for the COVID-19 Genomics UK (COG-UK) Consortium	Rob Howes, The Lighthouse Lab in Cambridge and Alex Alderton, Roberto Amato, Sonia Goncalves, Ewan Harrison, David K. Jackson, Ian Johnston, Dominic Kwiatkowski, Cordelia Langford, John Sillitoe on behalf of the Wellcome Sanger Institute COVID-19 Surveillance Team
EPI_ISL_811549	Lighthouse Lab in Milton Keynes	Wellcome Sanger Institute for the COVID-19 Genomics UK (COG-UK) Consortium	The Lighthouse Lab in Milton Keynes and Alex Alderton, Roberto Amato, Sonia Goncalves, Ewan Harrison, David K. Jackson, Ian Johnston, Dominic Kwiatkowski, Cordelia Langford, John Sillitoe on behalf of the Wellcome Sanger Institute COVID-19 Surveillance Team
EPI_ISL_811550, EPI_ISL_811551, EPI_ISL_811552, EPI_ISL_811553, EPI_ISL_811554, EPI_ISL_811555	Lighthouse Lab in Cambridge	Wellcome Sanger Institute for the COVID-19 Genomics UK (COG-UK) Consortium	Rob Howes, The Lighthouse Lab in Cambridge and Alex Alderton, Roberto Amato, Sonia Goncalves, Ewan Harrison, David K. Jackson, Ian Johnston, Dominic Kwiatkowski, Cordelia Langford, John Sillitoe on behalf of the Wellcome Sanger Institute COVID-19 Surveillance Team
EPI_ISL_811556	Lighthouse Lab in Milton Keynes	Wellcome Sanger Institute for the COVID-19 Genomics UK (COG-UK) Consortium	The Lighthouse Lab in Milton Keynes and Alex Alderton, Roberto Amato, Sonia Goncalves, Ewan Harrison, David K. Jackson, Ian Johnston, Dominic Kwiatkowski, Cordelia Langford, John Sillitoe on behalf of the Wellcome Sanger Institute COVID-19 Surveillance Team
EPI_ISL_811557, EPI_ISL_811558	Lighthouse Lab in Cambridge	Wellcome Sanger Institute for the COVID-19 Genomics UK (COG-UK) Consortium	Rob Howes, The Lighthouse Lab in Cambridge and Alex Alderton, Roberto Amato, Sonia Goncalves, Ewan Harrison, David K. Jackson, Ian Johnston, Dominic Kwiatkowski, Cordelia Langford, John Sillitoe on behalf of the Wellcome Sanger Institute COVID-19 Surveillance Team
EPI_ISL_811559	Lighthouse Lab in Milton Keynes	Wellcome Sanger Institute for the COVID-19 Genomics UK (COG-UK) Consortium	The Lighthouse Lab in Milton Keynes and Alex Alderton, Roberto Amato, Sonia Goncalves, Ewan Harrison, David K. Jackson, Ian Johnston, Dominic Kwiatkowski, Cordelia Langford, John Sillitoe on behalf of the Wellcome Sanger Institute COVID-19 Surveillance Team
EPI_ISL_811560, EPI_ISL_811561, EPI_ISL_811562, EPI_ISL_811564	Lighthouse Lab in Cambridge	Wellcome Sanger Institute for the COVID-19 Genomics UK (COG-UK) Consortium	Rob Howes, The Lighthouse Lab in Cambridge and Alex Alderton, Roberto Amato, Sonia Goncalves, Ewan Harrison, David K. Jackson, Ian Johnston, Dominic Kwiatkowski, Cordelia Langford, John Sillitoe on behalf of the Wellcome Sanger Institute COVID-19 Surveillance Team
EPI_ISL_811565	Lighthouse Lab in Milton Keynes	Wellcome Sanger Institute for the COVID-19 Genomics UK (COG-UK) Consortium	The Lighthouse Lab in Milton Keynes and Alex Alderton, Roberto Amato, Sonia Goncalves, Ewan Harrison, David K. Jackson, Ian Johnston, Dominic Kwiatkowski, Cordelia Langford, John Sillitoe on behalf of the Wellcome Sanger Institute COVID-19 Surveillance Team
EPI_ISL_811566, EPI_ISL_811567, EPI_ISL_811568	Lighthouse Lab in Cambridge	Wellcome Sanger Institute for the COVID-19 Genomics UK (COG-UK) Consortium	Rob Howes, The Lighthouse Lab in Cambridge and Alex Alderton, Roberto Amato, Sonia Goncalves, Ewan Harrison, David K. Jackson, Ian Johnston, Dominic Kwiatkowski, Cordelia Langford, John Sillitoe on behalf of the Wellcome Sanger Institute COVID-19 Surveillance Team
EPI_ISL_811569	Lighthouse Lab in Milton Keynes	Wellcome Sanger Institute for the COVID-19 Genomics UK (COG-UK) Consortium	The Lighthouse Lab in Milton Keynes and Alex Alderton, Roberto Amato, Sonia Goncalves, Ewan Harrison, David K. Jackson, Ian Johnston, Dominic Kwiatkowski, Cordelia Langford, John Sillitoe on behalf of the Wellcome Sanger Institute COVID-19 Surveillance Team
EPI_ISL_811570, EPI_ISL_811571, EPI_ISL_811572, EPI_ISL_811574, EPI_ISL_811575, EPI_ISL_811576, EPI_ISL_811577, EPI_ISL_811578, EPI_ISL_811579, EPI_ISL_811581, EPI_ISL_811582, EPI_ISL_811583, EPI_ISL_811585, EPI_ISL_811586, EPI_ISL_811587, EPI_ISL_811588, EPI_ISL_811589, EPI_ISL_811590			
see above	Lighthouse Lab in Cambridge	Wellcome Sanger Institute for the COVID-19 Genomics UK (COG-UK) Consortium	Rob Howes, The Lighthouse Lab in Cambridge and Alex Alderton, Roberto Amato, Sonia Goncalves, Ewan Harrison, David K. Jackson, Ian Johnston, Dominic Kwiatkowski, Cordelia Langford, John Sillitoe on behalf of the Wellcome Sanger Institute COVID-19 Surveillance Team
EPI_ISL_811591	Lighthouse Lab in Milton Keynes	Wellcome Sanger Institute for the COVID-19 Genomics UK (COG-UK) Consortium	The Lighthouse Lab in Milton Keynes and Alex Alderton, Roberto Amato, Sonia Goncalves, Ewan Harrison, David K. Jackson, Ian Johnston, Dominic Kwiatkowski, Cordelia Langford, John Sillitoe on behalf of the Wellcome Sanger Institute COVID-19 Surveillance Team
EPI_ISL_811592, EPI_ISL_811593, EPI_ISL_811595, EPI_ISL_811596, EPI_ISL_811597, EPI_ISL_811598, EPI_ISL_811599, EPI_ISL_811600, EPI_ISL_811601, EPI_ISL_811602	Lighthouse Lab in Cambridge	Wellcome Sanger Institute for the COVID-19 Genomics UK (COG-UK) Consortium	Rob Howes, The Lighthouse Lab in Cambridge and Alex Alderton, Roberto Amato, Sonia Goncalves, Ewan Harrison, David K. Jackson, Ian Johnston, Dominic Kwiatkowski, Cordelia Langford, John Sillitoe on behalf of the Wellcome Sanger Institute COVID-19 Surveillance Team
EPI_ISL_811603	Lighthouse Lab in Milton Keynes	Wellcome Sanger Institute for the COVID-19 Genomics UK (COG-UK) Consortium	The Lighthouse Lab in Milton Keynes and Alex Alderton, Roberto Amato, Sonia Goncalves, Ewan Harrison, David K. Jackson, Ian Johnston, Dominic Kwiatkowski, Cordelia Langford, John Sillitoe on behalf of the Wellcome Sanger Institute COVID-19 Surveillance Team
EPI_ISL_811604, EPI_ISL_811605	Lighthouse Lab in Cambridge	Wellcome Sanger Institute for the COVID-19 Genomics UK (COG-UK) Consortium	Rob Howes, The Lighthouse Lab in Cambridge and Alex Alderton, Roberto Amato, Sonia Goncalves, Ewan Harrison, David K. Jackson, Ian Johnston, Dominic Kwiatkowski, Cordelia Langford, John Sillitoe on behalf of the Wellcome Sanger Institute COVID-19 Surveillance Team
EPI_ISL_811606	Lighthouse Lab in Milton Keynes	Wellcome Sanger Institute for the COVID-19 Genomics UK (COG-UK) Consortium	The Lighthouse Lab in Milton Keynes and Alex Alderton, Roberto Amato, Sonia Goncalves, Ewan Harrison, David K. Jackson, Ian Johnston, Dominic Kwiatkowski, Cordelia Langford, John Sillitoe on behalf of the Wellcome Sanger Institute COVID-19 Surveillance Team
EPI_ISL_811607, EPI_ISL_811608, EPI_ISL_811609	Lighthouse Lab in Cambridge	Wellcome Sanger Institute for the COVID-19 Genomics UK (COG-UK) Consortium	Rob Howes, The Lighthouse Lab in Cambridge and Alex Alderton, Roberto Amato, Sonia Goncalves, Ewan Harrison, David K. Jackson, Ian Johnston, Dominic Kwiatkowski, Cordelia Langford, John Sillitoe on behalf of the Wellcome Sanger Institute COVID-19 Surveillance Team
EPI_ISL_811610, EPI_ISL_811611	Lighthouse Lab in Milton Keynes	Wellcome Sanger Institute for the COVID-19 Genomics UK (COG-UK) Consortium	The Lighthouse Lab in Milton Keynes and Alex Alderton, Roberto Amato, Sonia Goncalves, Ewan Harrison, David K. Jackson, Ian Johnston, Dominic Kwiatkowski, Cordelia Langford, John Sillitoe on behalf of the Wellcome Sanger Institute COVID-19 Surveillance Team
EPI_ISL_811612, EPI_ISL_811613, EPI_ISL_811614, EPI_ISL_811615, EPI_ISL_811616, EPI_ISL_811619	Lighthouse Lab in Cambridge	Wellcome Sanger Institute for the COVID-19 Genomics UK (COG-UK) Consortium	Rob Howes, The Lighthouse Lab in Cambridge and Alex Alderton, Roberto Amato, Sonia Goncalves, Ewan Harrison, David K. Jackson, Ian Johnston, Dominic Kwiatkowski, Cordelia Langford, John Sillitoe on behalf of the Wellcome Sanger Institute COVID-19 Surveillance Team
EPI_ISL_811622	Lighthouse Lab in Milton Keynes	Wellcome Sanger Institute for the COVID-19 Genomics UK (COG-UK) Consortium	The Lighthouse Lab in Milton Keynes and Alex Alderton, Roberto Amato, Sonia Goncalves, Ewan Harrison, David K. Jackson, Ian Johnston, Dominic Kwiatkowski, Cordelia Langford, John Sillitoe on behalf of the Wellcome Sanger Institute COVID-19 Surveillance Team
EPI_ISL_811623, EPI_ISL_811624	Lighthouse Lab in Cambridge	Wellcome Sanger Institute for the COVID-19 Genomics UK (COG-UK) Consortium	Rob Howes, The Lighthouse Lab in Cambridge and Alex Alderton, Roberto Amato, Sonia Goncalves, Ewan Harrison, David K. Jackson, Ian Johnston, Dominic Kwiatkowski, Cordelia Langford, John Sillitoe on behalf of the Wellcome Sanger Institute COVID-19 Surveillance Team
EPI_ISL_811625, EPI_ISL_811626	Lighthouse Lab in Milton Keynes	Wellcome Sanger Institute for the COVID-19 Genomics UK (COG-UK) Consortium	The Lighthouse Lab in Milton Keynes and Alex Alderton, Roberto Amato, Sonia Goncalves, Ewan Harrison, David K. Jackson, Ian Johnston, Dominic Kwiatkowski, Cordelia Langford, John Sillitoe on behalf of the Wellcome Sanger Institute COVID-19 Surveillance Team
EPI_ISL_811628, EPI_ISL_811629, EPI_ISL_811630, EPI_ISL_811631, EPI_ISL_811632, EPI_ISL_811633	Lighthouse Lab in Cambridge	Wellcome Sanger Institute for the COVID-19 Genomics UK (COG-UK) Consortium	Rob Howes, The Lighthouse Lab in Cambridge and Alex Alderton, Roberto Amato, Sonia Goncalves, Ewan Harrison, David K. Jackson, Ian Johnston, Dominic Kwiatkowski, Cordelia Langford, John Sillitoe on behalf of the Wellcome Sanger Institute COVID-19 Surveillance Team
EPI_ISL_811634	Lighthouse Lab in Milton Keynes	Wellcome Sanger Institute for the COVID-19 Genomics UK (COG-UK) Consortium	The Lighthouse Lab in Milton Keynes and Alex Alderton, Roberto Amato, Sonia Goncalves, Ewan Harrison, David K. Jackson, Ian Johnston, Dominic Kwiatkowski, Cordelia Langford, John Sillitoe on behalf of the Wellcome Sanger Institute COVID-19 Surveillance Team
EPI_ISL_811635, EPI_ISL_811636, EPI_ISL_811637, EPI_ISL_811638, EPI_ISL_811639, EPI_ISL_811640, EPI_ISL_811641, EPI_ISL_811642, EPI_ISL_811643	Lighthouse Lab in Cambridge	Wellcome Sanger Institute for the COVID-19 Genomics UK (COG-UK) Consortium	Rob Howes, The Lighthouse Lab in Cambridge and Alex Alderton, Roberto Amato, Sonia Goncalves, Ewan Harrison, David K. Jackson, Ian Johnston, Dominic Kwiatkowski, Cordelia Langford, John Sillitoe on behalf of the Wellcome Sanger Institute COVID-19 Surveillance Team
EPI_ISL_811644	Lighthouse Lab in Milton Keynes	Wellcome Sanger Institute for the COVID-19 Genomics UK (COG-UK) Consortium	The Lighthouse Lab in Milton Keynes and Alex Alderton, Roberto Amato, Sonia Goncalves, Ewan Harrison, David K. Jackson, Ian Johnston, Dominic Kwiatkowski, Cordelia Langford, John Sillitoe on behalf of the Wellcome Sanger Institute COVID-19 Surveillance Team
EPI_ISL_811646, EPI_ISL_811647, EPI_ISL_811648, EPI_ISL_811649,	Lighthouse Lab in Cambridge	Wellcome Sanger Institute for the COVID-19 Genomics UK (COG-UK) Consortium	Rob Howes, The Lighthouse Lab in Cambridge and Alex Alderton, Roberto Amato, Sonia Goncalves, Ewan Harrison, David K. Jackson, Ian Johnston, Dominic Kwiatkowski, Cordelia Langford, John Sillitoe on behalf of the Wellcome Sanger Institute COVID-19 Surveillance Team

EPI_ISL_811652, EPI_ISL_811653, EPI_ISL_811654, EPI_ISL_811655, EPI_ISL_811656			
EPI_ISL_811657	Lighthouse Lab in Milton Keynes	Wellcome Sanger Institute for the COVID-19 Genomics UK (COG-UK) Consortium	The Lighthouse Lab in Milton Keynes and Alex Alderton, Roberto Amato, Sonia Goncalves, Ewan Harrison, David K. Jackson, Ian Johnston, Dominic Kwiatkowski, Cordelia Langford, John Sillitoe on behalf of the Wellcome Sanger Institute COVID-19 Surveillance Team
EPI_ISL_811658, EPI_ISL_811659	Lighthouse Lab in Cambridge	Wellcome Sanger Institute for the COVID-19 Genomics UK (COG-UK) Consortium	Rob Howes, The Lighthouse Lab in Cambridge and Alex Alderton, Roberto Amato, Sonia Goncalves, Ewan Harrison, David K. Jackson, Ian Johnston, Dominic Kwiatkowski, Cordelia Langford, John Sillitoe on behalf of the Wellcome Sanger Institute COVID-19 Surveillance Team
EPI_ISL_811660	Lighthouse Lab in Milton Keynes	Wellcome Sanger Institute for the COVID-19 Genomics UK (COG-UK) Consortium	The Lighthouse Lab in Milton Keynes and Alex Alderton, Roberto Amato, Sonia Goncalves, Ewan Harrison, David K. Jackson, Ian Johnston, Dominic Kwiatkowski, Cordelia Langford, John Sillitoe on behalf of the Wellcome Sanger Institute COVID-19 Surveillance Team
EPI_ISL_811662, EPI_ISL_811663	Lighthouse Lab in Cambridge	Wellcome Sanger Institute for the COVID-19 Genomics UK (COG-UK) Consortium	Rob Howes, The Lighthouse Lab in Cambridge and Alex Alderton, Roberto Amato, Sonia Goncalves, Ewan Harrison, David K. Jackson, Ian Johnston, Dominic Kwiatkowski, Cordelia Langford, John Sillitoe on behalf of the Wellcome Sanger Institute COVID-19 Surveillance Team
EPI_ISL_811664	Lighthouse Lab in Milton Keynes	Wellcome Sanger Institute for the COVID-19 Genomics UK (COG-UK) Consortium	The Lighthouse Lab in Milton Keynes and Alex Alderton, Roberto Amato, Sonia Goncalves, Ewan Harrison, David K. Jackson, Ian Johnston, Dominic Kwiatkowski, Cordelia Langford, John Sillitoe on behalf of the Wellcome Sanger Institute COVID-19 Surveillance Team
EPI_ISL_811665	Lighthouse Lab in Cambridge	Wellcome Sanger Institute for the COVID-19 Genomics UK (COG-UK) Consortium	Rob Howes, The Lighthouse Lab in Cambridge and Alex Alderton, Roberto Amato, Sonia Goncalves, Ewan Harrison, David K. Jackson, Ian Johnston, Dominic Kwiatkowski, Cordelia Langford, John Sillitoe on behalf of the Wellcome Sanger Institute COVID-19 Surveillance Team
EPI_ISL_811666	Lighthouse Lab in Milton Keynes	Wellcome Sanger Institute for the COVID-19 Genomics UK (COG-UK) Consortium	The Lighthouse Lab in Milton Keynes and Alex Alderton, Roberto Amato, Sonia Goncalves, Ewan Harrison, David K. Jackson, Ian Johnston, Dominic Kwiatkowski, Cordelia Langford, John Sillitoe on behalf of the Wellcome Sanger Institute COVID-19 Surveillance Team
EPI_ISL_811667, EPI_ISL_811668	Lighthouse Lab in Cambridge	Wellcome Sanger Institute for the COVID-19 Genomics UK (COG-UK) Consortium	Rob Howes, The Lighthouse Lab in Cambridge and Alex Alderton, Roberto Amato, Sonia Goncalves, Ewan Harrison, David K. Jackson, Ian Johnston, Dominic Kwiatkowski, Cordelia Langford, John Sillitoe on behalf of the Wellcome Sanger Institute COVID-19 Surveillance Team
EPI_ISL_811672, EPI_ISL_811673	Lighthouse Lab in Milton Keynes	Wellcome Sanger Institute for the COVID-19 Genomics UK (COG-UK) Consortium	The Lighthouse Lab in Milton Keynes and Alex Alderton, Roberto Amato, Sonia Goncalves, Ewan Harrison, David K. Jackson, Ian Johnston, Dominic Kwiatkowski, Cordelia Langford, John Sillitoe on behalf of the Wellcome Sanger Institute COVID-19 Surveillance Team
EPI_ISL_811674, EPI_ISL_811675, EPI_ISL_811676, EPI_ISL_811679, EPI_ISL_811680, EPI_ISL_811681, EPI_ISL_811683, EPI_ISL_811684, EPI_ISL_811685, EPI_ISL_811686, EPI_ISL_811687, EPI_ISL_811689, EPI_ISL_811690, EPI_ISL_811691, EPI_ISL_811692, EPI_ISL_811693, EPI_ISL_811694, EPI_ISL_811695			
see above	Lighthouse Lab in Cambridge	Wellcome Sanger Institute for the COVID-19 Genomics UK (COG-UK) Consortium	Rob Howes, The Lighthouse Lab in Cambridge and Alex Alderton, Roberto Amato, Sonia Goncalves, Ewan Harrison, David K. Jackson, Ian Johnston, Dominic Kwiatkowski, Cordelia Langford, John Sillitoe on behalf of the Wellcome Sanger Institute COVID-19 Surveillance Team
EPI_ISL_811696	Lighthouse Lab in Milton Keynes	Wellcome Sanger Institute for the COVID-19 Genomics UK (COG-UK) Consortium	The Lighthouse Lab in Milton Keynes and Alex Alderton, Roberto Amato, Sonia Goncalves, Ewan Harrison, David K. Jackson, Ian Johnston, Dominic Kwiatkowski, Cordelia Langford, John Sillitoe on behalf of the Wellcome Sanger Institute COVID-19 Surveillance Team
EPI_ISL_811697	Lighthouse Lab in Cambridge	Wellcome Sanger Institute for the COVID-19 Genomics UK (COG-UK) Consortium	Rob Howes, The Lighthouse Lab in Cambridge and Alex Alderton, Roberto Amato, Sonia Goncalves, Ewan Harrison, David K. Jackson, Ian Johnston, Dominic Kwiatkowski, Cordelia Langford, John Sillitoe on behalf of the Wellcome Sanger Institute COVID-19 Surveillance Team
EPI_ISL_811698	Lighthouse Lab in Milton Keynes	Wellcome Sanger Institute for the COVID-19 Genomics UK (COG-UK) Consortium	The Lighthouse Lab in Milton Keynes and Alex Alderton, Roberto Amato, Sonia Goncalves, Ewan Harrison, David K. Jackson, Ian Johnston, Dominic Kwiatkowski, Cordelia Langford, John Sillitoe on behalf of the Wellcome Sanger Institute COVID-19 Surveillance Team
EPI_ISL_811699, EPI_ISL_811700	Lighthouse Lab in Cambridge	Wellcome Sanger Institute for the COVID-19 Genomics UK (COG-UK) Consortium	Rob Howes, The Lighthouse Lab in Cambridge and Alex Alderton, Roberto Amato, Sonia Goncalves, Ewan Harrison, David K. Jackson, Ian Johnston, Dominic Kwiatkowski, Cordelia Langford, John Sillitoe on behalf of the Wellcome Sanger Institute COVID-19 Surveillance Team
EPI_ISL_811701, EPI_ISL_811702, EPI_ISL_811703	Lighthouse Lab in Milton Keynes	Wellcome Sanger Institute for the COVID-19 Genomics UK (COG-UK) Consortium	The Lighthouse Lab in Milton Keynes and Alex Alderton, Roberto Amato, Sonia Goncalves, Ewan Harrison, David K. Jackson, Ian Johnston, Dominic Kwiatkowski, Cordelia Langford, John Sillitoe on behalf of the Wellcome Sanger Institute COVID-19 Surveillance Team
EPI_ISL_811704, EPI_ISL_811705, EPI_ISL_811706, EPI_ISL_811707	Lighthouse Lab in Cambridge	Wellcome Sanger Institute for the COVID-19 Genomics UK (COG-UK) Consortium	Rob Howes, The Lighthouse Lab in Cambridge and Alex Alderton, Roberto Amato, Sonia Goncalves, Ewan Harrison, David K. Jackson, Ian Johnston, Dominic Kwiatkowski, Cordelia Langford, John Sillitoe on behalf of the Wellcome Sanger Institute COVID-19 Surveillance Team
EPI_ISL_811708	Lighthouse Lab in Milton Keynes	Wellcome Sanger Institute for the COVID-19 Genomics UK (COG-UK) Consortium	The Lighthouse Lab in Milton Keynes and Alex Alderton, Roberto Amato, Sonia Goncalves, Ewan Harrison, David K. Jackson, Ian Johnston, Dominic Kwiatkowski, Cordelia Langford, John Sillitoe on behalf of the Wellcome Sanger Institute COVID-19 Surveillance Team
EPI_ISL_811709, EPI_ISL_811710, EPI_ISL_811711, EPI_ISL_811712, EPI_ISL_811714, EPI_ISL_811715, EPI_ISL_811716, EPI_ISL_811717, EPI_ISL_811718, EPI_ISL_811719, EPI_ISL_811721, EPI_ISL_811722, EPI_ISL_811723, EPI_ISL_811724, EPI_ISL_811725			
see above	Lighthouse Lab in Cambridge	Wellcome Sanger Institute for the COVID-19 Genomics UK (COG-UK) Consortium	Rob Howes, The Lighthouse Lab in Cambridge and Alex Alderton, Roberto Amato, Sonia Goncalves, Ewan Harrison, David K. Jackson, Ian Johnston, Dominic Kwiatkowski, Cordelia Langford, John Sillitoe on behalf of the Wellcome Sanger Institute COVID-19 Surveillance Team
EPI_ISL_811726	Lighthouse Lab in Milton Keynes	Wellcome Sanger Institute for the COVID-19 Genomics UK (COG-UK) Consortium	The Lighthouse Lab in Milton Keynes and Alex Alderton, Roberto Amato, Sonia Goncalves, Ewan Harrison, David K. Jackson, Ian Johnston, Dominic Kwiatkowski, Cordelia Langford, John Sillitoe on behalf of the Wellcome Sanger Institute COVID-19 Surveillance Team
EPI_ISL_811728, EPI_ISL_811729, EPI_ISL_811730, EPI_ISL_811731, EPI_ISL_811732, EPI_ISL_811733, EPI_ISL_811734, EPI_ISL_811735	Lighthouse Lab in Cambridge	Wellcome Sanger Institute for the COVID-19 Genomics UK (COG-UK) Consortium	Rob Howes, The Lighthouse Lab in Cambridge and Alex Alderton, Roberto Amato, Sonia Goncalves, Ewan Harrison, David K. Jackson, Ian Johnston, Dominic Kwiatkowski, Cordelia Langford, John Sillitoe on behalf of the Wellcome Sanger Institute COVID-19 Surveillance Team
EPI_ISL_811736	Lighthouse Lab in Milton Keynes	Wellcome Sanger Institute for the COVID-19 Genomics UK (COG-UK) Consortium	The Lighthouse Lab in Milton Keynes and Alex Alderton, Roberto Amato, Sonia Goncalves, Ewan Harrison, David K. Jackson, Ian Johnston, Dominic Kwiatkowski, Cordelia Langford, John Sillitoe on behalf of the Wellcome Sanger Institute COVID-19 Surveillance Team
EPI_ISL_811737, EPI_ISL_811738, EPI_ISL_811739	Lighthouse Lab in Cambridge	Wellcome Sanger Institute for the COVID-19 Genomics UK (COG-UK) Consortium	Rob Howes, The Lighthouse Lab in Cambridge and Alex Alderton, Roberto Amato, Sonia Goncalves, Ewan Harrison, David K. Jackson, Ian Johnston, Dominic Kwiatkowski, Cordelia Langford, John Sillitoe on behalf of the Wellcome Sanger Institute COVID-19 Surveillance Team
EPI_ISL_811740, EPI_ISL_811741	Lighthouse Lab in Milton Keynes	Wellcome Sanger Institute for the COVID-19 Genomics UK (COG-UK) Consortium	The Lighthouse Lab in Milton Keynes and Alex Alderton, Roberto Amato, Sonia Goncalves, Ewan Harrison, David K. Jackson, Ian Johnston, Dominic Kwiatkowski, Cordelia Langford, John Sillitoe on behalf of the Wellcome Sanger Institute COVID-19 Surveillance Team
EPI_ISL_811742, EPI_ISL_811743, EPI_ISL_811744, EPI_ISL_811745, EPI_ISL_811746, EPI_ISL_811747, EPI_ISL_811748, EPI_ISL_811749, EPI_ISL_811751, EPI_ISL_811752	Lighthouse Lab in Cambridge	Wellcome Sanger Institute for the COVID-19 Genomics UK (COG-UK) Consortium	Rob Howes, The Lighthouse Lab in Cambridge and Alex Alderton, Roberto Amato, Sonia Goncalves, Ewan Harrison, David K. Jackson, Ian Johnston, Dominic Kwiatkowski, Cordelia Langford, John Sillitoe on behalf of the Wellcome Sanger Institute COVID-19 Surveillance Team
EPI_ISL_811754	Lighthouse Lab in Milton Keynes	Wellcome Sanger Institute for the COVID-19 Genomics UK (COG-UK) Consortium	The Lighthouse Lab in Milton Keynes and Alex Alderton, Roberto Amato, Sonia Goncalves, Ewan Harrison, David K. Jackson, Ian Johnston, Dominic Kwiatkowski, Cordelia Langford, John Sillitoe on behalf of the Wellcome Sanger Institute COVID-19 Surveillance Team
EPI_ISL_811755, EPI_ISL_811757, EPI_ISL_811758, EPI_ISL_811760, EPI_ISL_811761	Lighthouse Lab in Cambridge	Wellcome Sanger Institute for the COVID-19 Genomics UK (COG-UK) Consortium	Rob Howes, The Lighthouse Lab in Cambridge and Alex Alderton, Roberto Amato, Sonia Goncalves, Ewan Harrison, David K. Jackson, Ian Johnston, Dominic Kwiatkowski, Cordelia Langford, John Sillitoe on behalf of the Wellcome Sanger Institute COVID-19 Surveillance Team
EPI_ISL_811762	Lighthouse Lab in Milton Keynes	Wellcome Sanger Institute for the COVID-19 Genomics UK (COG-UK) Consortium	The Lighthouse Lab in Milton Keynes and Alex Alderton, Roberto Amato, Sonia Goncalves, Ewan Harrison, David K. Jackson, Ian Johnston, Dominic Kwiatkowski, Cordelia Langford, John Sillitoe on behalf of the Wellcome Sanger Institute COVID-19 Surveillance Team
EPI_ISL_811763, EPI_ISL_811764, EPI_ISL_811765, EPI_ISL_811766, EPI_ISL_811768, EPI_ISL_811769, EPI_ISL_811770, EPI_ISL_811771, EPI_ISL_811772, EPI_ISL_811773, EPI_ISL_811774, EPI_ISL_811775			
see above	Lighthouse Lab in Cambridge	Wellcome Sanger Institute for the COVID-19 Genomics UK (COG-UK) Consortium	Rob Howes, The Lighthouse Lab in Cambridge and Alex Alderton, Roberto Amato, Sonia Goncalves, Ewan Harrison, David K. Jackson, Ian Johnston, Dominic Kwiatkowski, Cordelia Langford, John Sillitoe on behalf of the Wellcome Sanger Institute COVID-19 Surveillance Team
EPI_ISL_811776	Lighthouse Lab in Milton Keynes	Wellcome Sanger Institute for the COVID-19 Genomics UK (COG-UK) Consortium	The Lighthouse Lab in Milton Keynes and Alex Alderton, Roberto Amato, Sonia Goncalves, Ewan Harrison, David K. Jackson, Ian Johnston, Dominic Kwiatkowski, Cordelia Langford, John Sillitoe on behalf of the Wellcome Sanger Institute COVID-19 Surveillance Team
EPI_ISL_811777, EPI_ISL_811778,	Lighthouse Lab in Cambridge	Wellcome Sanger Institute for the COVID-19 Genomics UK	Rob Howes, The Lighthouse Lab in Cambridge and Alex Alderton, Roberto Amato, Sonia Goncalves, Ewan Harrison, David K. Jackson, Ian Johnston,

EPI_ISL_811779, EPI_ISL_811780, EPI_ISL_811781, EPI_ISL_811782, EPI_ISL_811783, EPI_ISL_811784, EPI_ISL_811786		(COG-UK) Consortium	Dominic Kwiatkowski, Cordelia Langford, John Sillitoe on behalf of the Wellcome Sanger Institute COVID-19 Surveillance Team
EPI_ISL_811787	Lighthouse Lab in Milton Keynes	Wellcome Sanger Institute for the COVID-19 Genomics UK (COG-UK) Consortium	The Lighthouse Lab in Milton Keynes and Alex Alderton, Roberto Amato, Sonia Goncalves, Ewan Harrison, David K. Jackson, Ian Johnston, Dominic Kwiatkowski, Cordelia Langford, John Sillitoe on behalf of the Wellcome Sanger Institute COVID-19 Surveillance Team
EPI_ISL_811788, EPI_ISL_811789, EPI_ISL_811790, EPI_ISL_811791, EPI_ISL_811792, EPI_ISL_811793, EPI_ISL_811794, EPI_ISL_811795, EPI_ISL_811796, EPI_ISL_811797, EPI_ISL_811798, EPI_ISL_811799, EPI_ISL_811800, EPI_ISL_811801			
see above	Lighthouse Lab in Cambridge	Wellcome Sanger Institute for the COVID-19 Genomics UK (COG-UK) Consortium	Rob Howes, The Lighthouse Lab in Cambridge and Alex Alderton, Roberto Amato, Sonia Goncalves, Ewan Harrison, David K. Jackson, Ian Johnston, Dominic Kwiatkowski, Cordelia Langford, John Sillitoe on behalf of the Wellcome Sanger Institute COVID-19 Surveillance Team
EPI_ISL_811802	Lighthouse Lab in Milton Keynes	Wellcome Sanger Institute for the COVID-19 Genomics UK (COG-UK) Consortium	The Lighthouse Lab in Milton Keynes and Alex Alderton, Roberto Amato, Sonia Goncalves, Ewan Harrison, David K. Jackson, Ian Johnston, Dominic Kwiatkowski, Cordelia Langford, John Sillitoe on behalf of the Wellcome Sanger Institute COVID-19 Surveillance Team
EPI_ISL_811803, EPI_ISL_811804, EPI_ISL_811805, EPI_ISL_811806	Lighthouse Lab in Cambridge	Wellcome Sanger Institute for the COVID-19 Genomics UK (COG-UK) Consortium	Rob Howes, The Lighthouse Lab in Cambridge and Alex Alderton, Roberto Amato, Sonia Goncalves, Ewan Harrison, David K. Jackson, Ian Johnston, Dominic Kwiatkowski, Cordelia Langford, John Sillitoe on behalf of the Wellcome Sanger Institute COVID-19 Surveillance Team
EPI_ISL_811807	Lighthouse Lab in Milton Keynes	Wellcome Sanger Institute for the COVID-19 Genomics UK (COG-UK) Consortium	The Lighthouse Lab in Milton Keynes and Alex Alderton, Roberto Amato, Sonia Goncalves, Ewan Harrison, David K. Jackson, Ian Johnston, Dominic Kwiatkowski, Cordelia Langford, John Sillitoe on behalf of the Wellcome Sanger Institute COVID-19 Surveillance Team
EPI_ISL_812249	Cantonal Hospital Baden	Institute of Medical Virology, University of Zurich	Stefan Schmutz, Maryam Zaheri, Verena Kufner, Annette Audigé, Maria Grünberg, Kevin Steiner, Jon Huder, Cyril Shah, Riccarda Capaul, Guido Bloemberg, Jürg Böni, Michael Huber, Alexandra Trkola
EPI_ISL_812251, EPI_ISL_812252, EPI_ISL_812253, EPI_ISL_812254	Kantonsarztamt Bern	Institute of Medical Virology, University of Zurich	Stefan Schmutz, Maryam Zaheri, Verena Kufner, Annette Audigé, Maria Grünberg, Kevin Steiner, Jon Huder, Cyril Shah, Riccarda Capaul, Guido Bloemberg, Jürg Böni, Michael Huber, Alexandra Trkola
EPI_ISL_812255	Kantonsärztlicher Dienst Aargau	Institute of Medical Virology, University of Zurich	Stefan Schmutz, Maryam Zaheri, Verena Kufner, Annette Audigé, Maria Grünberg, Kevin Steiner, Jon Huder, Cyril Shah, Riccarda Capaul, Guido Bloemberg, Jürg Böni, Michael Huber, Alexandra Trkola
EPI_ISL_812434	Microbiological Diagnostic Unit - Public Health Laboratory (MDU-PHL)	MDU-PHL	Seemann T., Sait, M.L., Sherry, N.L.
EPI_ISL_812435, EPI_ISL_812436, EPI_ISL_812438, EPI_ISL_812439	Victorian Infectious Diseases Reference Laboratory (VIDRL)	VIDRL and MDU-PHL	Caly L., Seemann T., Sait, M.L., Druce J., Sherry, N.L.
EPI_ISL_813199	Department of Pathology, University of Cambridge	COVID-19 Genomics UK (COG-UK) Consortium	Aminu S. Jahun, Yasmin Chaudhry, Grant Hall, Iliana Georgana, Myra Hosmillo, Martin D. Curran, Malte Pinckert, Surendra Parmar, Ian Goodfellow
EPI_ISL_813816, EPI_ISL_813817, EPI_ISL_813818, EPI_ISL_813819, EPI_ISL_813820, EPI_ISL_813821, EPI_ISL_813822, EPI_ISL_813823, EPI_ISL_813824, EPI_ISL_813825, EPI_ISL_813827, EPI_ISL_813828			
see above	Liverpool Clinical Laboratories	COVID-19 Genomics UK (COG-UK) Consortium	Sam Haldenby, Anita Lucaci, Steve Paterson, Julian Hiscox, Alistair Darby, M Almsaud, A Alrezaihi, Muhannad Alruwaili, Stuart D Armstrong, Jones Benjamin, Eleanor G Bentley, Anu Chawla, Jordan J Clark, Angela Cowell, Richard Eccles, Isabel García-Dorival, Matthew Gemmell, Alessandro Gerada, PKF Gilmore, Richard Gregory, Ximeng Han, Catherine Hartley, Margaret Hughes, Miren Iturriza-Gomara, James Johnson, L Luu, Jenifer Manson, Charlotte Nelson, Elaine O'Toole, Cassie Olateju, Rebekah Penrice-Randal , Lucille Rainbow, N.P Randle, Trevor Ian Robinson, Parul Sharma, Ghada T Shawli, James P Stewart, Neil Swainston, Ecaterina Vamos, Joanne Watts, Mark Whitehead
EPI_ISL_813987, EPI_ISL_813988, EPI_ISL_813996, EPI_ISL_814000, EPI_ISL_814007, EPI_ISL_814011, EPI_ISL_814012, EPI_ISL_814013, EPI_ISL_814017, EPI_ISL_814023, EPI_ISL_814024, EPI_ISL_814025, EPI_ISL_814036, EPI_ISL_814041, EPI_ISL_814042, EPI_ISL_814043, EPI_ISL_814044, EPI_ISL_814045, EPI_ISL_814046, EPI_ISL_814047, EPI_ISL_814048, EPI_ISL_814049, EPI_ISL_814050			
see above	Hospital General Universitario Gregorio Marañón	SeqCOVID-SPAIN consortium/IBV(CSIC)	Darío García de Viedma, Laura Pérez-Lago, Marta Herranz, Jon Sicilia, Julia Suárez, Pilar Catalán, Patricia Muñoz and SeqCOVID-SPAIN consortium
EPI_ISL_816210, EPI_ISL_816211, EPI_ISL_816212, EPI_ISL_816216, EPI_ISL_816217, EPI_ISL_816218, EPI_ISL_816219	Centre for Enzyme Innovation, University of Portsmouth / Translational Research Laboratory, Portsmouth Hospitals NHS Trust	COVID-19 Genomics UK (COG-UK) Consortium	Angela Beckett,Yann Bourgeois,Garry Scarlett,Sharon Glaysher,Scott Elliott,Kelly Bicknell,Robert Impey,Allyson Lloyd,Sarah Wyllie,Ethan Butcher,Anoop Chauhan,Samuel Robson
EPI_ISL_816237, EPI_ISL_816243, EPI_ISL_816251, EPI_ISL_816260, EPI_ISL_816264, EPI_ISL_816267, EPI_ISL_816281, EPI_ISL_816286, EPI_ISL_816287, EPI_ISL_816293, EPI_ISL_816303, EPI_ISL_816309, EPI_ISL_816312, EPI_ISL_816315, EPI_ISL_816322, EPI_ISL_816326, EPI_ISL_816333, EPI_ISL_816334, EPI_ISL_816336, EPI_ISL_816338, EPI_ISL_816341, EPI_ISL_816355, EPI_ISL_816367, EPI_ISL_816394, EPI_ISL_816395, EPI_ISL_816399, EPI_ISL_816402, EPI_ISL_816408, EPI_ISL_816416, EPI_ISL_816436, EPI_ISL_816439, EPI_ISL_816450, EPI_ISL_816452, EPI_ISL_816458, EPI_ISL_816463, EPI_ISL_816470, EPI_ISL_816471, EPI_ISL_816498, EPI_ISL_816502, EPI_ISL_816509, EPI_ISL_816517, EPI_ISL_816520, EPI_ISL_816523, EPI_ISL_816526, EPI_ISL_816527, EPI_ISL_816543, EPI_ISL_816546, EPI_ISL_816552, EPI_ISL_816564, EPI_ISL_816565, EPI_ISL_816572, EPI_ISL_816573, EPI_ISL_816583, EPI_ISL_816588, EPI_ISL_816590, EPI_ISL_816591, EPI_ISL_816596, EPI_ISL_816603, EPI_ISL_816618, EPI_ISL_816622, EPI_ISL_816624, EPI_ISL_816632, EPI_ISL_816636, EPI_ISL_816649, EPI_ISL_816655			
see above	Virology Department, Sheffield Teaching Hospitals NHS Foundation Trust/Department of Infection, Immunity and Cardiovascular Disease, The Medical School, University of Sheffield	COVID-19 Genomics UK (COG-UK) Consortium	Thushan de Silva, Matthew Parker, Nikki Smith, Adri Angyal, Rebecca Brown, Luke Green, Rachel Tucker, Paul Parsons, Danielle Groves, Katie Johnson, Laura Carrilero, Alex Keeley, Dave Partridge, Matthew Wyles, Benjamin Lindsey, Mehmet Yavuz, Mohammad Raza, Cariad Evans
EPI_ISL_819267, EPI_ISL_819269, EPI_ISL_819270, EPI_ISL_819271	Wyoming Public Health Laboratory	Wyoming Public Health Laboratory	Noah Hull, Taylor Fearing, Lynette Gumbleton, Channing Weber, Ashley Norberg, Bailey Bowcutt, and Wanda Manley
EPI_ISL_819366, EPI_ISL_819375, EPI_ISL_819377, EPI_ISL_819391, EPI_ISL_819392, EPI_ISL_819393, EPI_ISL_819394, EPI_ISL_819395, EPI_ISL_819405, EPI_ISL_819406, EPI_ISL_819407, EPI_ISL_819408, EPI_ISL_819409, EPI_ISL_819410, EPI_ISL_819411, EPI_ISL_819412, EPI_ISL_819413, EPI_ISL_819415, EPI_ISL_819416, EPI_ISL_819418, EPI_ISL_819419, EPI_ISL_819421, EPI_ISL_819424, EPI_ISL_819425, EPI_ISL_819427, EPI_ISL_819440, EPI_ISL_819441, EPI_ISL_819442, EPI_ISL_819443, EPI_ISL_819444			
see above	Quadram Institute Bioscience	COVID-19 Genomics UK (COG-UK) Consortium	Dave J. Baker, Gemma L. Kay, Alp Aydin, Thanh Le-Viet, Steven Rudder, Ana P. Tedim, Anastasia Kolyva, Maria Diaz, Leonardo de Oliveira Martins, Nabil-Fareed Alikhan, Lizzie Meadows, Rachael Stanley, Ngozi Elumogo, Muhammed Yasir, Nicholas M. Thomson, Alexander J Trotter, Rachel Gilroy, Samuel Bloomfield, Claire Stuart, Andrew Bell, Reenesh Prakash, Samir Dervisevic, Alison E. Mather, John Wain, Mark Webber, Andrew J. Page, Justin O'Grady
EPI_ISL_820560	Queens Medical Centre, Clinical Microbiology Department / DeepSeq Nottingham	COVID-19 Genomics UK (COG-UK) Consortium	Gemma Clark, Wendy Smith, Manjinder Khakh, Vicki M Fleming, Michelle M Lister, Hannah Howson-Wells, Jonathan Ball, Patrick McClure, Joseph Chappell, Theocharis Tsoleridis, Nadine Holmes, Matthew Carlisle, Christopher Moore, Fei Sang, Johnny Debebe, Victoria Wright, Matthew Loose
EPI_ISL_820563, EPI_ISL_820565, EPI_ISL_820568, EPI_ISL_820570	Quadram Institute Bioscience	COVID-19 Genomics UK (COG-UK) Consortium	Dave J. Baker, Gemma L. Kay, Alp Aydin, Thanh Le-Viet, Steven Rudder, Ana P. Tedim, Anastasia Kolyva, Maria Diaz, Leonardo de Oliveira Martins, Nabil-Fareed Alikhan, Lizzie Meadows, Rachael Stanley, Ngozi Elumogo, Muhammed Yasir, Nicholas M. Thomson, Alexander J Trotter, Rachel Gilroy, Samuel Bloomfield, Claire Stuart, Andrew Bell, Reenesh Prakash, Samir Dervisevic, Alison E. Mather, John Wain, Mark Webber, Andrew J. Page, Justin O'Grady
EPI_ISL_820573, EPI_ISL_820575, EPI_ISL_820578, EPI_ISL_820580, EPI_ISL_820583, EPI_ISL_820586	Queens Medical Centre, Clinical Microbiology Department / DeepSeq Nottingham	COVID-19 Genomics UK (COG-UK) Consortium	Gemma Clark, Wendy Smith, Manjinder Khakh, Vicki M Fleming, Michelle M Lister, Hannah Howson-Wells, Jonathan Ball, Patrick McClure, Joseph Chappell, Theocharis Tsoleridis, Nadine Holmes, Matthew Carlisle, Christopher Moore, Fei Sang, Johnny Debebe, Victoria Wright, Matthew Loose
EPI_ISL_820648, EPI_ISL_820649, EPI_ISL_820650, EPI_ISL_820651, EPI_ISL_820652, EPI_ISL_820653, EPI_ISL_820654, EPI_ISL_820655, EPI_ISL_820656, EPI_ISL_820657, EPI_ISL_820658, EPI_ISL_820659, EPI_ISL_820660, EPI_ISL_820661, EPI_ISL_820662, EPI_ISL_820663, EPI_ISL_820664, EPI_ISL_820665, EPI_ISL_820666, EPI_ISL_820667, EPI_ISL_820668, EPI_ISL_820669, EPI_ISL_820670, EPI_ISL_820671, EPI_ISL_820672, EPI_ISL_820673, EPI_ISL_820674, EPI_ISL_820675, EPI_ISL_820676, EPI_ISL_820677, EPI_ISL_820678, EPI_ISL_820679, EPI_ISL_820680, EPI_ISL_820681, EPI_ISL_820682, EPI_ISL_820683, EPI_ISL_820684, EPI_ISL_820685, EPI_ISL_820686, EPI_ISL_820687, EPI_ISL_820688, EPI_ISL_820689, EPI_ISL_820690, EPI_ISL_820691, EPI_ISL_820692, EPI_ISL_820693, EPI_ISL_820694, EPI_ISL_820695, EPI_ISL_820696, EPI_ISL_820697, EPI_ISL_820698, EPI_ISL_820699, EPI_ISL_820700, EPI_ISL_820701, EPI_ISL_820702, EPI_ISL_820703, EPI_ISL_820704, EPI_ISL_820705, EPI_ISL_820706, EPI_ISL_820707, EPI_ISL_820708, EPI_ISL_820709, EPI_ISL_820710, EPI_ISL_820711, EPI_ISL_820712, EPI_ISL_820713, EPI_ISL_820714, EPI_ISL_820715, EPI_ISL_820716, EPI_ISL_820717, EPI_ISL_820718, EPI_ISL_820719, EPI_ISL_820720, EPI_ISL_820721, EPI_ISL_820722, EPI_ISL_820723, EPI_ISL_820724, EPI_ISL_820725, EPI_ISL_820726, EPI_ISL_820727, EPI_ISL_820728, EPI_ISL_820729, EPI_ISL_820730, EPI_ISL_820731, EPI_ISL_820732, EPI_ISL_820733, EPI_ISL_820734, EPI_ISL_820735, EPI_ISL_820736, EPI_ISL_820737, EPI_ISL_820738, EPI_ISL_820739, EPI_ISL_820740, EPI_ISL_820741, EPI_ISL_820742, EPI_ISL_820743, EPI_ISL_820744, EPI_ISL_820745, EPI_ISL_820746, EPI_ISL_820747, EPI_ISL_820748, EPI_ISL_820749, EPI_ISL_820750, EPI_ISL_820751, EPI_ISL_820752, EPI_ISL_820753, EPI_ISL_820754, EPI_ISL_820755, EPI_ISL_820756, EPI_ISL_820757, EPI_ISL_820758, EPI_ISL_820759, EPI_ISL_820760, EPI_ISL_820761, EPI_ISL_820762, EPI_ISL_820763, EPI_ISL_820764, EPI_ISL_820765, EPI_ISL_820766, EPI_ISL_820767, EPI_ISL_820768, EPI_ISL_820769, EPI_ISL_820770, EPI_ISL_820771, EPI_ISL_820772, EPI_ISL_820773, EPI_ISL_820774, EPI_ISL_820775, EPI_ISL_820776, EPI_ISL_820777, EPI_ISL_820778, EPI_ISL_820779, EPI_ISL_820780, EPI_ISL_820781, EPI_ISL_820782, EPI_ISL_820783, EPI_ISL_820784, EPI_ISL_820785, EPI_ISL_820786, EPI_ISL_820787, EPI_ISL_820788, EPI_ISL_820789, EPI_ISL_820790, EPI_ISL_820791, EPI_ISL_820792, EPI_ISL_820793, EPI_ISL_820794, EPI_ISL_820795, EPI_ISL_820796, EPI_ISL_820797, EPI_ISL_820798, EPI_ISL_820799, EPI_ISL_820800, EPI_ISL_820801, EPI_ISL_820802, EPI_ISL_820803, EPI_ISL_820804, EPI_ISL_820805, EPI_ISL_820806, EPI_ISL_820807, EPI_ISL_820808, EPI_ISL_820809, EPI_ISL_820810, EPI_ISL_820811, EPI_ISL_820812, EPI_ISL_820813, EPI_ISL_820814, EPI_ISL_820815, EPI_ISL_820816, EPI_ISL_820817, EPI_ISL_820818, EPI_ISL_820819, EPI_ISL_820820, EPI_ISL_820821, EPI_ISL_820822, EPI_ISL_820823, EPI_ISL_820824, EPI_ISL_820825, EPI_ISL_820826, EPI_ISL_820827,			

EPI_ISL_820828, EPI_ISL_820829, EPI_ISL_820830, EPI_ISL_820831, EPI_ISL_820832, EPI_ISL_820833, EPI_ISL_820834, EPI_ISL_820835, EPI_ISL_820836, EPI_ISL_820837, EPI_ISL_820838, EPI_ISL_820839, EPI_ISL_820840, EPI_ISL_820841, EPI_ISL_820842, EPI_ISL_820843, EPI_ISL_820844, EPI_ISL_820845, EPI_ISL_820846, EPI_ISL_820847, EPI_ISL_820848, EPI_ISL_820849, EPI_ISL_820850, EPI_ISL_820851, EPI_ISL_820852, EPI_ISL_820853, EPI_ISL_820854, EPI_ISL_820855, EPI_ISL_820856, EPI_ISL_820857, EPI_ISL_820858, EPI_ISL_820859, EPI_ISL_820860, EPI_ISL_820861, EPI_ISL_820862, EPI_ISL_820863, EPI_ISL_820864, EPI_ISL_820865, EPI_ISL_820866, EPI_ISL_820867, EPI_ISL_820868, EPI_ISL_820869, EPI_ISL_820870, EPI_ISL_820871, EPI_ISL_820872, EPI_ISL_820873, EPI_ISL_820874, EPI_ISL_820875, EPI_ISL_820876, EPI_ISL_820877, EPI_ISL_820878, EPI_ISL_820879, EPI_ISL_820880, EPI_ISL_820881, EPI_ISL_820882, EPI_ISL_820883, EPI_ISL_820884, EPI_ISL_820885, EPI_ISL_820886, EPI_ISL_820887, EPI_ISL_820888, EPI_ISL_820889, EPI_ISL_820890, EPI_ISL_820891, EPI_ISL_820892, EPI_ISL_820893, EPI_ISL_820894, EPI_ISL_820895, EPI_ISL_820896, EPI_ISL_820897, EPI_ISL_820898, EPI_ISL_820899, EPI_ISL_820900, EPI_ISL_820901, EPI_ISL_820902, EPI_ISL_820903, EPI_ISL_820904, EPI_ISL_820905, EPI_ISL_820906, EPI_ISL_820907, EPI_ISL_820908, EPI_ISL_820909, EPI_ISL_820910, EPI_ISL_820911, EPI_ISL_820912, EPI_ISL_820913, EPI_ISL_820914, EPI_ISL_820915, EPI_ISL_820916, EPI_ISL_820917, EPI_ISL_820918, EPI_ISL_820919, EPI_ISL_820920, EPI_ISL_820921, EPI_ISL_820922, EPI_ISL_820923, EPI_ISL_820924, EPI_ISL_820925, EPI_ISL_820926, EPI_ISL_820927, EPI_ISL_820928, EPI_ISL_820929, EPI_ISL_820930, EPI_ISL_820931, EPI_ISL_820932, EPI_ISL_820933, EPI_ISL_820934, EPI_ISL_820935, EPI_ISL_820936, EPI_ISL_820937, EPI_ISL_820938, EPI_ISL_820939, EPI_ISL_820940, EPI_ISL_820941, EPI_ISL_820942, EPI_ISL_820943, EPI_ISL_820944, EPI_ISL_820945, EPI_ISL_820946, EPI_ISL_820947, EPI_ISL_820948, EPI_ISL_820949, EPI_ISL_820950, EPI_ISL_820951, EPI_ISL_820952, EPI_ISL_820953, EPI_ISL_820954, EPI_ISL_820955, EPI_ISL_820956, EPI_ISL_820957, EPI_ISL_820958, EPI_ISL_820959, EPI_ISL_820960, EPI_ISL_820961, EPI_ISL_820962, EPI_ISL_820963, EPI_ISL_820964, EPI_ISL_820965, EPI_ISL_820966, EPI_ISL_820967, EPI_ISL_820968, EPI_ISL_820969, EPI_ISL_820970, EPI_ISL_820971, EPI_ISL_820972, EPI_ISL_820973, EPI_ISL_820974, EPI_ISL_820975, EPI_ISL_820976, EPI_ISL_820977, EPI_ISL_820978, EPI_ISL_820979, EPI_ISL_820980, EPI_ISL_820981, EPI_ISL_820982, EPI_ISL_820983, EPI_ISL_820984, EPI_ISL_820985, EPI_ISL_820986, EPI_ISL_820987, EPI_ISL_820988, EPI_ISL_820989, EPI_ISL_820990, EPI_ISL_820991, EPI_ISL_820992, EPI_ISL_820993, EPI_ISL_820994, EPI_ISL_820995, EPI_ISL_820996, EPI_ISL_820997, EPI_ISL_820998, EPI_ISL_820999, EPI_ISL_821000, EPI_ISL_821001			
see above	Lighthouse Lab in Milton Keynes	Wellcome Sanger Institute for the COVID-19 Genomics UK (COG-UK) Consortium	The Lighthouse Lab in Milton Keynes and Alex Alderton, Roberto Amato, Sonia Goncalves, Ewan Harrison, David K. Jackson, Ian Johnston, Dominic Kwiatkowski, Cordelia Langford, John Sillitoe on behalf of the Wellcome Sanger Institute COVID-19 Surveillance Team
EPI_ISL_821002, EPI_ISL_821003, EPI_ISL_821004, EPI_ISL_821005, EPI_ISL_821006, EPI_ISL_821007, EPI_ISL_821008, EPI_ISL_821009, EPI_ISL_821010, EPI_ISL_821011, EPI_ISL_821012, EPI_ISL_821013, EPI_ISL_821014, EPI_ISL_821015, EPI_ISL_821016, EPI_ISL_821017, EPI_ISL_821018, EPI_ISL_821019, EPI_ISL_821020, EPI_ISL_821021, EPI_ISL_821022, EPI_ISL_821023, EPI_ISL_821024, EPI_ISL_821025, EPI_ISL_821026, EPI_ISL_821027, EPI_ISL_821028, EPI_ISL_821029, EPI_ISL_821030, EPI_ISL_821031, EPI_ISL_821033, EPI_ISL_821034, EPI_ISL_821035, EPI_ISL_821036, EPI_ISL_821038, EPI_ISL_821040, EPI_ISL_821041, EPI_ISL_821042, EPI_ISL_821043, EPI_ISL_821044, EPI_ISL_821045, EPI_ISL_821046, EPI_ISL_821047, EPI_ISL_821048, EPI_ISL_821049, EPI_ISL_821050, EPI_ISL_821051, EPI_ISL_821052, EPI_ISL_821053, EPI_ISL_821054, EPI_ISL_821055, EPI_ISL_821057, EPI_ISL_821058, EPI_ISL_821059, EPI_ISL_821060, EPI_ISL_821063, EPI_ISL_821064, EPI_ISL_821065, EPI_ISL_821066, EPI_ISL_821067, EPI_ISL_821068, EPI_ISL_821070, EPI_ISL_821071, EPI_ISL_821072, EPI_ISL_821073, EPI_ISL_821074, EPI_ISL_821075, EPI_ISL_821076, EPI_ISL_821078, EPI_ISL_821079, EPI_ISL_821080, EPI_ISL_821081, EPI_ISL_821082, EPI_ISL_821083, EPI_ISL_821084, EPI_ISL_821085, EPI_ISL_821086, EPI_ISL_821087, EPI_ISL_821089, EPI_ISL_821090, EPI_ISL_821091, EPI_ISL_821092, EPI_ISL_821093, EPI_ISL_821094, EPI_ISL_821095, EPI_ISL_821096, EPI_ISL_821097, EPI_ISL_821099, EPI_ISL_821100, EPI_ISL_821101, EPI_ISL_821102, EPI_ISL_821103, EPI_ISL_821104, EPI_ISL_821106, EPI_ISL_821107, EPI_ISL_821108, EPI_ISL_821109, EPI_ISL_821110, EPI_ISL_821111, EPI_ISL_821112, EPI_ISL_821113, EPI_ISL_821114, EPI_ISL_821115, EPI_ISL_821116, EPI_ISL_821117, EPI_ISL_821118, EPI_ISL_821119, EPI_ISL_821120, EPI_ISL_821121, EPI_ISL_821122, EPI_ISL_821123, EPI_ISL_821124, EPI_ISL_821125, EPI_ISL_821126, EPI_ISL_821127, EPI_ISL_821129, EPI_ISL_821130, EPI_ISL_821131, EPI_ISL_821132, EPI_ISL_821133, EPI_ISL_821134, EPI_ISL_821135, EPI_ISL_821136, EPI_ISL_821137, EPI_ISL_821138, EPI_ISL_821141, EPI_ISL_821143, EPI_ISL_821144, EPI_ISL_821145, EPI_ISL_821146, EPI_ISL_821147, EPI_ISL_821148, EPI_ISL_821149, EPI_ISL_821151, EPI_ISL_821152, EPI_ISL_821153, EPI_ISL_821154, EPI_ISL_821155, EPI_ISL_821156, EPI_ISL_821157, EPI_ISL_821158, EPI_ISL_821159, EPI_ISL_821161, EPI_ISL_821162, EPI_ISL_821164, EPI_ISL_821166, EPI_ISL_821167, EPI_ISL_821168, EPI_ISL_821169, EPI_ISL_821170, EPI_ISL_821171, EPI_ISL_821173, EPI_ISL_821174, EPI_ISL_821175, EPI_ISL_821176, EPI_ISL_821177, EPI_ISL_821178, EPI_ISL_821179, EPI_ISL_821180, EPI_ISL_821181, EPI_ISL_821182, EPI_ISL_821183, EPI_ISL_821184, EPI_ISL_821185, EPI_ISL_821186, EPI_ISL_821187, EPI_ISL_821188, EPI_ISL_821189, EPI_ISL_821190, EPI_ISL_821191, EPI_ISL_821192, EPI_ISL_821193, EPI_ISL_821194, EPI_ISL_821196, EPI_ISL_821199, EPI_ISL_821200, EPI_ISL_821201, EPI_ISL_821202, EPI_ISL_821203, EPI_ISL_821204, EPI_ISL_821205, EPI_ISL_821206, EPI_ISL_821208, EPI_ISL_821209, EPI_ISL_821210, EPI_ISL_821211, EPI_ISL_821212, EPI_ISL_821213, EPI_ISL_821214, EPI_ISL_821215, EPI_ISL_821216, EPI_ISL_821217, EPI_ISL_821218, EPI_ISL_821219, EPI_ISL_821221, EPI_ISL_821222, EPI_ISL_821223, EPI_ISL_821224, EPI_ISL_821226, EPI_ISL_821228, EPI_ISL_821229, EPI_ISL_821230, EPI_ISL_821231, EPI_ISL_821232, EPI_ISL_821233, EPI_ISL_821234, EPI_ISL_821236, EPI_ISL_821237, EPI_ISL_821238, EPI_ISL_821239, EPI_ISL_821240, EPI_ISL_821242, EPI_ISL_821243, EPI_ISL_821244, EPI_ISL_821246, EPI_ISL_821247, EPI_ISL_821248, EPI_ISL_821250, EPI_ISL_821251, EPI_ISL_821252, EPI_ISL_821253, EPI_ISL_821254, EPI_ISL_821255, EPI_ISL_821256, EPI_ISL_821259, EPI_ISL_821260, EPI_ISL_821261, EPI_ISL_821262, EPI_ISL_821264, EPI_ISL_821265, EPI_ISL_821267, EPI_ISL_821268, EPI_ISL_821272, EPI_ISL_821273			
see above	Lighthouse Lab in Alderley Park	Wellcome Sanger Institute for the COVID-19 Genomics UK (COG-UK) Consortium	Jacquelyn Wynn, Mairead Hyland, The Lighthouse Lab in Alderley Park and Alex Alderton, Roberto Amato, Sonia Goncalves, Ewan Harrison, David K. Jackson, Ian Johnston, Dominic Kwiatkowski, Cordelia Langford, John Sillitoe on behalf of the Wellcome Sanger Institute COVID-19 Surveillance Team
EPI_ISL_821274	Lighthouse Lab in Milton Keynes	Wellcome Sanger Institute for the COVID-19 Genomics UK (COG-UK) Consortium	The Lighthouse Lab in Milton Keynes and Alex Alderton, Roberto Amato, Sonia Goncalves, Ewan Harrison, David K. Jackson, Ian Johnston, Dominic Kwiatkowski, Cordelia Langford, John Sillitoe on behalf of the Wellcome Sanger Institute COVID-19 Surveillance Team
EPI_ISL_821276, EPI_ISL_821279	Lighthouse Lab in Cambridge	Wellcome Sanger Institute for the COVID-19 Genomics UK (COG-UK) Consortium	Rob Howes, The Lighthouse Lab in Cambridge and Alex Alderton, Roberto Amato, Sonia Goncalves, Ewan Harrison, David K. Jackson, Ian Johnston, Dominic Kwiatkowski, Cordelia Langford, John Sillitoe on behalf of the Wellcome Sanger Institute COVID-19 Surveillance Team
EPI_ISL_821280, EPI_ISL_821281, EPI_ISL_821282	Lighthouse Lab in Milton Keynes	Wellcome Sanger Institute for the COVID-19 Genomics UK (COG-UK) Consortium	The Lighthouse Lab in Milton Keynes and Alex Alderton, Roberto Amato, Sonia Goncalves, Ewan Harrison, David K. Jackson, Ian Johnston, Dominic Kwiatkowski, Cordelia Langford, John Sillitoe on behalf of the Wellcome Sanger Institute COVID-19 Surveillance Team
EPI_ISL_821283	Lighthouse Lab in Alderley Park	Wellcome Sanger Institute for the COVID-19 Genomics UK (COG-UK) Consortium	Jacquelyn Wynn, Mairead Hyland, The Lighthouse Lab in Alderley Park and Alex Alderton, Roberto Amato, Sonia Goncalves, Ewan Harrison, David K. Jackson, Ian Johnston, Dominic Kwiatkowski, Cordelia Langford, John Sillitoe on behalf of the Wellcome Sanger Institute COVID-19 Surveillance Team
EPI_ISL_821284	Lighthouse Lab in Cambridge	Wellcome Sanger Institute for the COVID-19 Genomics UK (COG-UK) Consortium	Rob Howes, The Lighthouse Lab in Cambridge and Alex Alderton, Roberto Amato, Sonia Goncalves, Ewan Harrison, David K. Jackson, Ian Johnston, Dominic Kwiatkowski, Cordelia Langford, John Sillitoe on behalf of the Wellcome Sanger Institute COVID-19 Surveillance Team
EPI_ISL_821285	Lighthouse Lab in Alderley Park	Wellcome Sanger Institute for the COVID-19 Genomics UK (COG-UK) Consortium	Jacquelyn Wynn, Mairead Hyland, The Lighthouse Lab in Alderley Park and Alex Alderton, Roberto Amato, Sonia Goncalves, Ewan Harrison, David K. Jackson, Ian Johnston, Dominic Kwiatkowski, Cordelia Langford, John Sillitoe on behalf of the Wellcome Sanger Institute COVID-19 Surveillance Team
EPI_ISL_821286	Lighthouse Lab in Cambridge	Wellcome Sanger Institute for the COVID-19 Genomics UK (COG-UK) Consortium	Rob Howes, The Lighthouse Lab in Cambridge and Alex Alderton, Roberto Amato, Sonia Goncalves, Ewan Harrison, David K. Jackson, Ian Johnston, Dominic Kwiatkowski, Cordelia Langford, John Sillitoe on behalf of the Wellcome Sanger Institute COVID-19 Surveillance Team
EPI_ISL_821287	Lighthouse Lab in Milton Keynes	Wellcome Sanger Institute for the COVID-19 Genomics UK (COG-UK) Consortium	The Lighthouse Lab in Milton Keynes and Alex Alderton, Roberto Amato, Sonia Goncalves, Ewan Harrison, David K. Jackson, Ian Johnston, Dominic Kwiatkowski, Cordelia Langford, John Sillitoe on behalf of the Wellcome Sanger Institute COVID-19 Surveillance Team
EPI_ISL_821288	Lighthouse Lab in Alderley Park	Wellcome Sanger Institute for the COVID-19 Genomics UK (COG-UK) Consortium	Jacquelyn Wynn, Mairead Hyland, The Lighthouse Lab in Alderley Park and Alex Alderton, Roberto Amato, Sonia Goncalves, Ewan Harrison, David K. Jackson, Ian Johnston, Dominic Kwiatkowski, Cordelia Langford, John Sillitoe on behalf of the Wellcome Sanger Institute COVID-19 Surveillance Team
EPI_ISL_821289, EPI_ISL_821290	Lighthouse Lab in Cambridge	Wellcome Sanger Institute for the COVID-19 Genomics UK (COG-UK) Consortium	Rob Howes, The Lighthouse Lab in Cambridge and Alex Alderton, Roberto Amato, Sonia Goncalves, Ewan Harrison, David K. Jackson, Ian Johnston, Dominic Kwiatkowski, Cordelia Langford, John Sillitoe on behalf of the Wellcome Sanger Institute COVID-19 Surveillance Team
EPI_ISL_821292	Lighthouse Lab in Alderley Park	Wellcome Sanger Institute for the COVID-19 Genomics UK (COG-UK) Consortium	Jacquelyn Wynn, Mairead Hyland, The Lighthouse Lab in Alderley Park and Alex Alderton, Roberto Amato, Sonia Goncalves, Ewan Harrison, David K. Jackson, Ian Johnston, Dominic Kwiatkowski, Cordelia Langford, John Sillitoe on behalf of the Wellcome Sanger Institute COVID-19 Surveillance Team
EPI_ISL_821293	Lighthouse Lab in Milton Keynes	Wellcome Sanger Institute for the COVID-19 Genomics UK (COG-UK) Consortium	The Lighthouse Lab in Milton Keynes and Alex Alderton, Roberto Amato, Sonia Goncalves, Ewan Harrison, David K. Jackson, Ian Johnston, Dominic Kwiatkowski, Cordelia Langford, John Sillitoe on behalf of the Wellcome Sanger Institute COVID-19 Surveillance Team
EPI_ISL_821294	Lighthouse Lab in Alderley Park	Wellcome Sanger Institute for the COVID-19 Genomics UK (COG-UK) Consortium	Jacquelyn Wynn, Mairead Hyland, The Lighthouse Lab in Alderley Park and Alex Alderton, Roberto Amato, Sonia Goncalves, Ewan Harrison, David K. Jackson, Ian Johnston, Dominic Kwiatkowski, Cordelia Langford, John Sillitoe on behalf of the Wellcome Sanger Institute COVID-19 Surveillance Team
EPI_ISL_821295	Lighthouse Lab in Cambridge	Wellcome Sanger Institute for the COVID-19 Genomics UK (COG-UK) Consortium	Rob Howes, The Lighthouse Lab in Cambridge and Alex Alderton, Roberto Amato, Sonia Goncalves, Ewan Harrison, David K. Jackson, Ian Johnston, Dominic Kwiatkowski, Cordelia Langford, John Sillitoe on behalf of the Wellcome Sanger Institute COVID-19 Surveillance Team
EPI_ISL_821296, EPI_ISL_821297	Lighthouse Lab in Milton Keynes	Wellcome Sanger Institute for the COVID-19 Genomics UK (COG-UK) Consortium	The Lighthouse Lab in Milton Keynes and Alex Alderton, Roberto Amato, Sonia Goncalves, Ewan Harrison, David K. Jackson, Ian Johnston, Dominic Kwiatkowski, Cordelia Langford, John Sillitoe on behalf of the Wellcome Sanger Institute COVID-19 Surveillance Team
EPI_ISL_821298	Lighthouse Lab in Alderley Park	Wellcome Sanger Institute for the COVID-19 Genomics UK (COG-UK) Consortium	Jacquelyn Wynn, Mairead Hyland, The Lighthouse Lab in Alderley Park and Alex Alderton, Roberto Amato, Sonia Goncalves, Ewan Harrison, David K. Jackson, Ian Johnston, Dominic Kwiatkowski, Cordelia Langford, John Sillitoe on behalf of the Wellcome Sanger Institute COVID-19 Surveillance Team
EPI_ISL_821300, EPI_ISL_821301	Lighthouse Lab in Milton Keynes	Wellcome Sanger Institute for the COVID-19 Genomics UK (COG-UK) Consortium	The Lighthouse Lab in Milton Keynes and Alex Alderton, Roberto Amato, Sonia Goncalves, Ewan Harrison, David K. Jackson, Ian Johnston, Dominic Kwiatkowski, Cordelia Langford, John Sillitoe on behalf of the Wellcome Sanger Institute COVID-19 Surveillance Team
EPI_ISL_821303	Lighthouse Lab in Alderley Park	Wellcome Sanger Institute for the COVID-19 Genomics UK (COG-UK) Consortium	Jacquelyn Wynn, Mairead Hyland, The Lighthouse Lab in Alderley Park and Alex Alderton, Roberto Amato, Sonia Goncalves, Ewan Harrison, David K. Jackson, Ian Johnston, Dominic Kwiatkowski, Cordelia Langford, John Sillitoe on behalf of the Wellcome Sanger Institute COVID-19 Surveillance Team
EPI_ISL_821304, EPI_ISL_821306, EPI_ISL_821307	Lighthouse Lab in Milton Keynes	Wellcome Sanger Institute for the COVID-19 Genomics UK (COG-UK) Consortium	The Lighthouse Lab in Milton Keynes and Alex Alderton, Roberto Amato, Sonia Goncalves, Ewan Harrison, David K. Jackson, Ian Johnston, Dominic Kwiatkowski, Cordelia Langford, John Sillitoe on behalf of the Wellcome Sanger Institute COVID-19 Surveillance Team
EPI_ISL_821308	Lighthouse Lab in Alderley Park	Wellcome Sanger Institute for the COVID-19 Genomics UK (COG-UK) Consortium	Jacquelyn Wynn, Mairead Hyland, The Lighthouse Lab in Alderley Park and Alex Alderton, Roberto Amato, Sonia Goncalves, Ewan Harrison, David K. Jackson, Ian Johnston, Dominic Kwiatkowski, Cordelia Langford, John Sillitoe on behalf of the Wellcome Sanger Institute COVID-19 Surveillance Team
EPI_ISL_821309, EPI_ISL_821310	Lighthouse Lab in Cambridge	Wellcome Sanger Institute for the COVID-19 Genomics UK (COG-UK) Consortium	Rob Howes, The Lighthouse Lab in Cambridge and Alex Alderton, Roberto Amato, Sonia Goncalves, Ewan Harrison, David K. Jackson, Ian Johnston, Dominic Kwiatkowski, Cordelia Langford, John Sillitoe on behalf of the Wellcome Sanger Institute COVID-19 Surveillance Team
EPI_ISL_821314	Lighthouse Lab in Alderley Park	Wellcome Sanger Institute for the COVID-19 Genomics UK (COG-UK) Consortium	Jacquelyn Wynn, Mairead Hyland, The Lighthouse Lab in Alderley Park and Alex Alderton, Roberto Amato, Sonia Goncalves, Ewan Harrison, David K. Jackson, Ian Johnston, Dominic Kwiatkowski, Cordelia Langford, John Sillitoe on behalf of the Wellcome Sanger Institute COVID-19 Surveillance Team
EPI_ISL_821317, EPI_ISL_821319, EPI_ISL_821323, EPI_ISL_821324	Lighthouse Lab in Milton Keynes	Wellcome Sanger Institute for the COVID-19 Genomics UK (COG-UK) Consortium	The Lighthouse Lab in Milton Keynes and Alex Alderton, Roberto Amato, Sonia Goncalves, Ewan Harrison, David K. Jackson, Ian Johnston, Dominic Kwiatkowski, Cordelia Langford, John Sillitoe on behalf of the Wellcome Sanger Institute COVID-19 Surveillance Team
EPI_ISL_821327	Lighthouse Lab in Alderley Park	Wellcome Sanger Institute for the COVID-19 Genomics UK	Jacquelyn Wynn, Mairead Hyland, The Lighthouse Lab in Alderley Park and Alex Alderton, Roberto Amato, Sonia Goncalves, Ewan Harrison, David K.

		(COG-UK) Consortium	Jackson, Ian Johnston, Dominic Kwiatkowski, Cordelia Langford, John Sillitoe on behalf of the Wellcome Sanger Institute COVID-19 Surveillance Team
EPI_ISL_821328	Lighthouse Lab in Milton Keynes	Wellcome Sanger Institute for the COVID-19 Genomics UK (COG-UK) Consortium	The Lighthouse Lab in Milton Keynes and Alex Alderton, Roberto Amato, Sonia Goncalves, Ewan Harrison, David K. Jackson, Ian Johnston, Dominic Kwiatkowski, Cordelia Langford, John Sillitoe on behalf of the Wellcome Sanger Institute COVID-19 Surveillance Team
EPI_ISL_821329	Lighthouse Lab in Alderley Park	Wellcome Sanger Institute for the COVID-19 Genomics UK (COG-UK) Consortium	Jacquelyn Wynn, Mairead Hyland, The Lighthouse Lab in Alderley Park and Alex Alderton, Roberto Amato, Sonia Goncalves, Ewan Harrison, David K. Jackson, Ian Johnston, Dominic Kwiatkowski, Cordelia Langford, John Sillitoe on behalf of the Wellcome Sanger Institute COVID-19 Surveillance Team
EPI_ISL_821331	Lighthouse Lab in Milton Keynes	Wellcome Sanger Institute for the COVID-19 Genomics UK (COG-UK) Consortium	The Lighthouse Lab in Milton Keynes and Alex Alderton, Roberto Amato, Sonia Goncalves, Ewan Harrison, David K. Jackson, Ian Johnston, Dominic Kwiatkowski, Cordelia Langford, John Sillitoe on behalf of the Wellcome Sanger Institute COVID-19 Surveillance Team
EPI_ISL_821333	Lighthouse Lab in Cambridge	Wellcome Sanger Institute for the COVID-19 Genomics UK (COG-UK) Consortium	Rob Howes, The Lighthouse Lab in Cambridge and Alex Alderton, Roberto Amato, Sonia Goncalves, Ewan Harrison, David K. Jackson, Ian Johnston, Dominic Kwiatkowski, Cordelia Langford, John Sillitoe on behalf of the Wellcome Sanger Institute COVID-19 Surveillance Team
EPI_ISL_821334	Lighthouse Lab in Milton Keynes	Wellcome Sanger Institute for the COVID-19 Genomics UK (COG-UK) Consortium	The Lighthouse Lab in Milton Keynes and Alex Alderton, Roberto Amato, Sonia Goncalves, Ewan Harrison, David K. Jackson, Ian Johnston, Dominic Kwiatkowski, Cordelia Langford, John Sillitoe on behalf of the Wellcome Sanger Institute COVID-19 Surveillance Team
EPI_ISL_821335	Lighthouse Lab in Alderley Park	Wellcome Sanger Institute for the COVID-19 Genomics UK (COG-UK) Consortium	Jacquelyn Wynn, Mairead Hyland, The Lighthouse Lab in Alderley Park and Alex Alderton, Roberto Amato, Sonia Goncalves, Ewan Harrison, David K. Jackson, Ian Johnston, Dominic Kwiatkowski, Cordelia Langford, John Sillitoe on behalf of the Wellcome Sanger Institute COVID-19 Surveillance Team
EPI_ISL_821336	Lighthouse Lab in Milton Keynes	Wellcome Sanger Institute for the COVID-19 Genomics UK (COG-UK) Consortium	The Lighthouse Lab in Milton Keynes and Alex Alderton, Roberto Amato, Sonia Goncalves, Ewan Harrison, David K. Jackson, Ian Johnston, Dominic Kwiatkowski, Cordelia Langford, John Sillitoe on behalf of the Wellcome Sanger Institute COVID-19 Surveillance Team
EPI_ISL_821338, EPI_ISL_821339	Lighthouse Lab in Cambridge	Wellcome Sanger Institute for the COVID-19 Genomics UK (COG-UK) Consortium	Rob Howes, The Lighthouse Lab in Cambridge and Alex Alderton, Roberto Amato, Sonia Goncalves, Ewan Harrison, David K. Jackson, Ian Johnston, Dominic Kwiatkowski, Cordelia Langford, John Sillitoe on behalf of the Wellcome Sanger Institute COVID-19 Surveillance Team
EPI_ISL_821340, EPI_ISL_821341	Lighthouse Lab in Milton Keynes	Wellcome Sanger Institute for the COVID-19 Genomics UK (COG-UK) Consortium	The Lighthouse Lab in Milton Keynes and Alex Alderton, Roberto Amato, Sonia Goncalves, Ewan Harrison, David K. Jackson, Ian Johnston, Dominic Kwiatkowski, Cordelia Langford, John Sillitoe on behalf of the Wellcome Sanger Institute COVID-19 Surveillance Team
EPI_ISL_821342, EPI_ISL_821343	Lighthouse Lab in Cambridge	Wellcome Sanger Institute for the COVID-19 Genomics UK (COG-UK) Consortium	Rob Howes, The Lighthouse Lab in Cambridge and Alex Alderton, Roberto Amato, Sonia Goncalves, Ewan Harrison, David K. Jackson, Ian Johnston, Dominic Kwiatkowski, Cordelia Langford, John Sillitoe on behalf of the Wellcome Sanger Institute COVID-19 Surveillance Team
EPI_ISL_821344, EPI_ISL_821346	Lighthouse Lab in Milton Keynes	Wellcome Sanger Institute for the COVID-19 Genomics UK (COG-UK) Consortium	The Lighthouse Lab in Milton Keynes and Alex Alderton, Roberto Amato, Sonia Goncalves, Ewan Harrison, David K. Jackson, Ian Johnston, Dominic Kwiatkowski, Cordelia Langford, John Sillitoe on behalf of the Wellcome Sanger Institute COVID-19 Surveillance Team
EPI_ISL_821347	Lighthouse Lab in Alderley Park	Wellcome Sanger Institute for the COVID-19 Genomics UK (COG-UK) Consortium	Jacquelyn Wynn, Mairead Hyland, The Lighthouse Lab in Alderley Park and Alex Alderton, Roberto Amato, Sonia Goncalves, Ewan Harrison, David K. Jackson, Ian Johnston, Dominic Kwiatkowski, Cordelia Langford, John Sillitoe on behalf of the Wellcome Sanger Institute COVID-19 Surveillance Team
EPI_ISL_821348	Lighthouse Lab in Cambridge	Wellcome Sanger Institute for the COVID-19 Genomics UK (COG-UK) Consortium	Rob Howes, The Lighthouse Lab in Cambridge and Alex Alderton, Roberto Amato, Sonia Goncalves, Ewan Harrison, David K. Jackson, Ian Johnston, Dominic Kwiatkowski, Cordelia Langford, John Sillitoe on behalf of the Wellcome Sanger Institute COVID-19 Surveillance Team
EPI_ISL_821350	Lighthouse Lab in Milton Keynes	Wellcome Sanger Institute for the COVID-19 Genomics UK (COG-UK) Consortium	The Lighthouse Lab in Milton Keynes and Alex Alderton, Roberto Amato, Sonia Goncalves, Ewan Harrison, David K. Jackson, Ian Johnston, Dominic Kwiatkowski, Cordelia Langford, John Sillitoe on behalf of the Wellcome Sanger Institute COVID-19 Surveillance Team
EPI_ISL_821351	Lighthouse Lab in Alderley Park	Wellcome Sanger Institute for the COVID-19 Genomics UK (COG-UK) Consortium	Jacquelyn Wynn, Mairead Hyland, The Lighthouse Lab in Alderley Park and Alex Alderton, Roberto Amato, Sonia Goncalves, Ewan Harrison, David K. Jackson, Ian Johnston, Dominic Kwiatkowski, Cordelia Langford, John Sillitoe on behalf of the Wellcome Sanger Institute COVID-19 Surveillance Team
EPI_ISL_821352, EPI_ISL_821353	Lighthouse Lab in Milton Keynes	Wellcome Sanger Institute for the COVID-19 Genomics UK (COG-UK) Consortium	The Lighthouse Lab in Milton Keynes and Alex Alderton, Roberto Amato, Sonia Goncalves, Ewan Harrison, David K. Jackson, Ian Johnston, Dominic Kwiatkowski, Cordelia Langford, John Sillitoe on behalf of the Wellcome Sanger Institute COVID-19 Surveillance Team
EPI_ISL_821354	Lighthouse Lab in Cambridge	Wellcome Sanger Institute for the COVID-19 Genomics UK (COG-UK) Consortium	Rob Howes, The Lighthouse Lab in Cambridge and Alex Alderton, Roberto Amato, Sonia Goncalves, Ewan Harrison, David K. Jackson, Ian Johnston, Dominic Kwiatkowski, Cordelia Langford, John Sillitoe on behalf of the Wellcome Sanger Institute COVID-19 Surveillance Team
EPI_ISL_821356, EPI_ISL_821358, EPI_ISL_821359	Lighthouse Lab in Milton Keynes	Wellcome Sanger Institute for the COVID-19 Genomics UK (COG-UK) Consortium	The Lighthouse Lab in Milton Keynes and Alex Alderton, Roberto Amato, Sonia Goncalves, Ewan Harrison, David K. Jackson, Ian Johnston, Dominic Kwiatkowski, Cordelia Langford, John Sillitoe on behalf of the Wellcome Sanger Institute COVID-19 Surveillance Team
EPI_ISL_821360	Lighthouse Lab in Cambridge	Wellcome Sanger Institute for the COVID-19 Genomics UK (COG-UK) Consortium	Rob Howes, The Lighthouse Lab in Cambridge and Alex Alderton, Roberto Amato, Sonia Goncalves, Ewan Harrison, David K. Jackson, Ian Johnston, Dominic Kwiatkowski, Cordelia Langford, John Sillitoe on behalf of the Wellcome Sanger Institute COVID-19 Surveillance Team
EPI_ISL_821362	Lighthouse Lab in Milton Keynes	Wellcome Sanger Institute for the COVID-19 Genomics UK (COG-UK) Consortium	The Lighthouse Lab in Milton Keynes and Alex Alderton, Roberto Amato, Sonia Goncalves, Ewan Harrison, David K. Jackson, Ian Johnston, Dominic Kwiatkowski, Cordelia Langford, John Sillitoe on behalf of the Wellcome Sanger Institute COVID-19 Surveillance Team
EPI_ISL_821363, EPI_ISL_821365	Lighthouse Lab in Cambridge	Wellcome Sanger Institute for the COVID-19 Genomics UK (COG-UK) Consortium	Rob Howes, The Lighthouse Lab in Cambridge and Alex Alderton, Roberto Amato, Sonia Goncalves, Ewan Harrison, David K. Jackson, Ian Johnston, Dominic Kwiatkowski, Cordelia Langford, John Sillitoe on behalf of the Wellcome Sanger Institute COVID-19 Surveillance Team
EPI_ISL_821366	Lighthouse Lab in Alderley Park	Wellcome Sanger Institute for the COVID-19 Genomics UK (COG-UK) Consortium	Jacquelyn Wynn, Mairead Hyland, The Lighthouse Lab in Alderley Park and Alex Alderton, Roberto Amato, Sonia Goncalves, Ewan Harrison, David K. Jackson, Ian Johnston, Dominic Kwiatkowski, Cordelia Langford, John Sillitoe on behalf of the Wellcome Sanger Institute COVID-19 Surveillance Team
EPI_ISL_821367	Lighthouse Lab in Cambridge	Wellcome Sanger Institute for the COVID-19 Genomics UK (COG-UK) Consortium	Rob Howes, The Lighthouse Lab in Cambridge and Alex Alderton, Roberto Amato, Sonia Goncalves, Ewan Harrison, David K. Jackson, Ian Johnston, Dominic Kwiatkowski, Cordelia Langford, John Sillitoe on behalf of the Wellcome Sanger Institute COVID-19 Surveillance Team
EPI_ISL_821370, EPI_ISL_821371, EPI_ISL_821372	Lighthouse Lab in Alderley Park	Wellcome Sanger Institute for the COVID-19 Genomics UK (COG-UK) Consortium	Jacquelyn Wynn, Mairead Hyland, The Lighthouse Lab in Alderley Park and Alex Alderton, Roberto Amato, Sonia Goncalves, Ewan Harrison, David K. Jackson, Ian Johnston, Dominic Kwiatkowski, Cordelia Langford, John Sillitoe on behalf of the Wellcome Sanger Institute COVID-19 Surveillance Team
EPI_ISL_821373	Lighthouse Lab in Milton Keynes	Wellcome Sanger Institute for the COVID-19 Genomics UK (COG-UK) Consortium	The Lighthouse Lab in Milton Keynes and Alex Alderton, Roberto Amato, Sonia Goncalves, Ewan Harrison, David K. Jackson, Ian Johnston, Dominic Kwiatkowski, Cordelia Langford, John Sillitoe on behalf of the Wellcome Sanger Institute COVID-19 Surveillance Team
EPI_ISL_821374, EPI_ISL_821375	Lighthouse Lab in Cambridge	Wellcome Sanger Institute for the COVID-19 Genomics UK (COG-UK) Consortium	Rob Howes, The Lighthouse Lab in Cambridge and Alex Alderton, Roberto Amato, Sonia Goncalves, Ewan Harrison, David K. Jackson, Ian Johnston, Dominic Kwiatkowski, Cordelia Langford, John Sillitoe on behalf of the Wellcome Sanger Institute COVID-19 Surveillance Team
EPI_ISL_821377, EPI_ISL_821378, EPI_ISL_821379, EPI_ISL_821380, EPI_ISL_821381	Lighthouse Lab in Milton Keynes	Wellcome Sanger Institute for the COVID-19 Genomics UK (COG-UK) Consortium	The Lighthouse Lab in Milton Keynes and Alex Alderton, Roberto Amato, Sonia Goncalves, Ewan Harrison, David K. Jackson, Ian Johnston, Dominic Kwiatkowski, Cordelia Langford, John Sillitoe on behalf of the Wellcome Sanger Institute COVID-19 Surveillance Team
EPI_ISL_821382	Lighthouse Lab in Alderley Park	Wellcome Sanger Institute for the COVID-19 Genomics UK (COG-UK) Consortium	Jacquelyn Wynn, Mairead Hyland, The Lighthouse Lab in Alderley Park and Alex Alderton, Roberto Amato, Sonia Goncalves, Ewan Harrison, David K. Jackson, Ian Johnston, Dominic Kwiatkowski, Cordelia Langford, John Sillitoe on behalf of the Wellcome Sanger Institute COVID-19 Surveillance Team
EPI_ISL_821383	Lighthouse Lab in Milton Keynes	Wellcome Sanger Institute for the COVID-19 Genomics UK (COG-UK) Consortium	The Lighthouse Lab in Milton Keynes and Alex Alderton, Roberto Amato, Sonia Goncalves, Ewan Harrison, David K. Jackson, Ian Johnston, Dominic Kwiatkowski, Cordelia Langford, John Sillitoe on behalf of the Wellcome Sanger Institute COVID-19 Surveillance Team
EPI_ISL_821384	Lighthouse Lab in Alderley Park	Wellcome Sanger Institute for the COVID-19 Genomics UK (COG-UK) Consortium	Jacquelyn Wynn, Mairead Hyland, The Lighthouse Lab in Alderley Park and Alex Alderton, Roberto Amato, Sonia Goncalves, Ewan Harrison, David K. Jackson, Ian Johnston, Dominic Kwiatkowski, Cordelia Langford, John Sillitoe on behalf of the Wellcome Sanger Institute COVID-19 Surveillance Team
EPI_ISL_821385	Lighthouse Lab in Milton Keynes	Wellcome Sanger Institute for the COVID-19 Genomics UK (COG-UK) Consortium	The Lighthouse Lab in Milton Keynes and Alex Alderton, Roberto Amato, Sonia Goncalves, Ewan Harrison, David K. Jackson, Ian Johnston, Dominic Kwiatkowski, Cordelia Langford, John Sillitoe on behalf of the Wellcome Sanger Institute COVID-19 Surveillance Team
EPI_ISL_821386	Lighthouse Lab in Cambridge	Wellcome Sanger Institute for the COVID-19 Genomics UK (COG-UK) Consortium	Rob Howes, The Lighthouse Lab in Cambridge and Alex Alderton, Roberto Amato, Sonia Goncalves, Ewan Harrison, David K. Jackson, Ian Johnston, Dominic Kwiatkowski, Cordelia Langford, John Sillitoe on behalf of the Wellcome Sanger Institute COVID-19 Surveillance Team
EPI_ISL_821387, EPI_ISL_821388	Lighthouse Lab in Milton Keynes	Wellcome Sanger Institute for the COVID-19 Genomics UK (COG-UK) Consortium	The Lighthouse Lab in Milton Keynes and Alex Alderton, Roberto Amato, Sonia Goncalves, Ewan Harrison, David K. Jackson, Ian Johnston, Dominic Kwiatkowski, Cordelia Langford, John Sillitoe on behalf of the Wellcome Sanger Institute COVID-19 Surveillance Team
EPI_ISL_821389, EPI_ISL_821390	Lighthouse Lab in Alderley Park	Wellcome Sanger Institute for the COVID-19 Genomics UK (COG-UK) Consortium	Jacquelyn Wynn, Mairead Hyland, The Lighthouse Lab in Alderley Park and Alex Alderton, Roberto Amato, Sonia Goncalves, Ewan Harrison, David K. Jackson, Ian Johnston, Dominic Kwiatkowski, Cordelia Langford, John Sillitoe on behalf of the Wellcome Sanger Institute COVID-19 Surveillance Team

EPI_ISL_821391	Lighthouse Lab in Milton Keynes	Wellcome Sanger Institute for the COVID-19 Genomics UK (COG-UK) Consortium	The Lighthouse Lab in Milton Keynes and Alex Alderton, Roberto Amato, Sonia Goncalves, Ewan Harrison, David K. Jackson, Ian Johnston, Dominic Kwiatkowski, Cordelia Langford, John Sillitoe on behalf of the Wellcome Sanger Institute COVID-19 Surveillance Team
EPI_ISL_821392, EPI_ISL_821394	Lighthouse Lab in Cambridge	Wellcome Sanger Institute for the COVID-19 Genomics UK (COG-UK) Consortium	Rob Howes, The Lighthouse Lab in Cambridge and Alex Alderton, Roberto Amato, Sonia Goncalves, Ewan Harrison, David K. Jackson, Ian Johnston, Dominic Kwiatkowski, Cordelia Langford, John Sillitoe on behalf of the Wellcome Sanger Institute COVID-19 Surveillance Team
EPI_ISL_821395, EPI_ISL_821397	Lighthouse Lab in Milton Keynes	Wellcome Sanger Institute for the COVID-19 Genomics UK (COG-UK) Consortium	The Lighthouse Lab in Milton Keynes and Alex Alderton, Roberto Amato, Sonia Goncalves, Ewan Harrison, David K. Jackson, Ian Johnston, Dominic Kwiatkowski, Cordelia Langford, John Sillitoe on behalf of the Wellcome Sanger Institute COVID-19 Surveillance Team
EPI_ISL_821398	Lighthouse Lab in Alderley Park	Wellcome Sanger Institute for the COVID-19 Genomics UK (COG-UK) Consortium	Jacquelyn Wynn, Mairead Hyland, The Lighthouse Lab in Alderley Park and Alex Alderton, Roberto Amato, Sonia Goncalves, Ewan Harrison, David K. Jackson, Ian Johnston, Dominic Kwiatkowski, Cordelia Langford, John Sillitoe on behalf of the Wellcome Sanger Institute COVID-19 Surveillance Team
EPI_ISL_821400	Lighthouse Lab in Milton Keynes	Wellcome Sanger Institute for the COVID-19 Genomics UK (COG-UK) Consortium	The Lighthouse Lab in Milton Keynes and Alex Alderton, Roberto Amato, Sonia Goncalves, Ewan Harrison, David K. Jackson, Ian Johnston, Dominic Kwiatkowski, Cordelia Langford, John Sillitoe on behalf of the Wellcome Sanger Institute COVID-19 Surveillance Team
EPI_ISL_821401	Lighthouse Lab in Alderley Park	Wellcome Sanger Institute for the COVID-19 Genomics UK (COG-UK) Consortium	Jacquelyn Wynn, Mairead Hyland, The Lighthouse Lab in Alderley Park and Alex Alderton, Roberto Amato, Sonia Goncalves, Ewan Harrison, David K. Jackson, Ian Johnston, Dominic Kwiatkowski, Cordelia Langford, John Sillitoe on behalf of the Wellcome Sanger Institute COVID-19 Surveillance Team
EPI_ISL_821402	Lighthouse Lab in Milton Keynes	Wellcome Sanger Institute for the COVID-19 Genomics UK (COG-UK) Consortium	The Lighthouse Lab in Milton Keynes and Alex Alderton, Roberto Amato, Sonia Goncalves, Ewan Harrison, David K. Jackson, Ian Johnston, Dominic Kwiatkowski, Cordelia Langford, John Sillitoe on behalf of the Wellcome Sanger Institute COVID-19 Surveillance Team
EPI_ISL_821403	Lighthouse Lab in Alderley Park	Wellcome Sanger Institute for the COVID-19 Genomics UK (COG-UK) Consortium	Jacquelyn Wynn, Mairead Hyland, The Lighthouse Lab in Alderley Park and Alex Alderton, Roberto Amato, Sonia Goncalves, Ewan Harrison, David K. Jackson, Ian Johnston, Dominic Kwiatkowski, Cordelia Langford, John Sillitoe on behalf of the Wellcome Sanger Institute COVID-19 Surveillance Team
EPI_ISL_821404	Lighthouse Lab in Cambridge	Wellcome Sanger Institute for the COVID-19 Genomics UK (COG-UK) Consortium	Rob Howes, The Lighthouse Lab in Cambridge and Alex Alderton, Roberto Amato, Sonia Goncalves, Ewan Harrison, David K. Jackson, Ian Johnston, Dominic Kwiatkowski, Cordelia Langford, John Sillitoe on behalf of the Wellcome Sanger Institute COVID-19 Surveillance Team
EPI_ISL_821405, EPI_ISL_821406, EPI_ISL_821407	Lighthouse Lab in Milton Keynes	Wellcome Sanger Institute for the COVID-19 Genomics UK (COG-UK) Consortium	The Lighthouse Lab in Milton Keynes and Alex Alderton, Roberto Amato, Sonia Goncalves, Ewan Harrison, David K. Jackson, Ian Johnston, Dominic Kwiatkowski, Cordelia Langford, John Sillitoe on behalf of the Wellcome Sanger Institute COVID-19 Surveillance Team
EPI_ISL_821408	Lighthouse Lab in Cambridge	Wellcome Sanger Institute for the COVID-19 Genomics UK (COG-UK) Consortium	Rob Howes, The Lighthouse Lab in Cambridge and Alex Alderton, Roberto Amato, Sonia Goncalves, Ewan Harrison, David K. Jackson, Ian Johnston, Dominic Kwiatkowski, Cordelia Langford, John Sillitoe on behalf of the Wellcome Sanger Institute COVID-19 Surveillance Team
EPI_ISL_821409, EPI_ISL_821410	Lighthouse Lab in Milton Keynes	Wellcome Sanger Institute for the COVID-19 Genomics UK (COG-UK) Consortium	The Lighthouse Lab in Milton Keynes and Alex Alderton, Roberto Amato, Sonia Goncalves, Ewan Harrison, David K. Jackson, Ian Johnston, Dominic Kwiatkowski, Cordelia Langford, John Sillitoe on behalf of the Wellcome Sanger Institute COVID-19 Surveillance Team
EPI_ISL_821411	Lighthouse Lab in Cambridge	Wellcome Sanger Institute for the COVID-19 Genomics UK (COG-UK) Consortium	Rob Howes, The Lighthouse Lab in Cambridge and Alex Alderton, Roberto Amato, Sonia Goncalves, Ewan Harrison, David K. Jackson, Ian Johnston, Dominic Kwiatkowski, Cordelia Langford, John Sillitoe on behalf of the Wellcome Sanger Institute COVID-19 Surveillance Team
EPI_ISL_821412, EPI_ISL_821414, EPI_ISL_821415	Lighthouse Lab in Alderley Park	Wellcome Sanger Institute for the COVID-19 Genomics UK (COG-UK) Consortium	Jacquelyn Wynn, Mairead Hyland, The Lighthouse Lab in Alderley Park and Alex Alderton, Roberto Amato, Sonia Goncalves, Ewan Harrison, David K. Jackson, Ian Johnston, Dominic Kwiatkowski, Cordelia Langford, John Sillitoe on behalf of the Wellcome Sanger Institute COVID-19 Surveillance Team
EPI_ISL_821416	Lighthouse Lab in Cambridge	Wellcome Sanger Institute for the COVID-19 Genomics UK (COG-UK) Consortium	Rob Howes, The Lighthouse Lab in Cambridge and Alex Alderton, Roberto Amato, Sonia Goncalves, Ewan Harrison, David K. Jackson, Ian Johnston, Dominic Kwiatkowski, Cordelia Langford, John Sillitoe on behalf of the Wellcome Sanger Institute COVID-19 Surveillance Team
EPI_ISL_821417	Lighthouse Lab in Milton Keynes	Wellcome Sanger Institute for the COVID-19 Genomics UK (COG-UK) Consortium	The Lighthouse Lab in Milton Keynes and Alex Alderton, Roberto Amato, Sonia Goncalves, Ewan Harrison, David K. Jackson, Ian Johnston, Dominic Kwiatkowski, Cordelia Langford, John Sillitoe on behalf of the Wellcome Sanger Institute COVID-19 Surveillance Team
EPI_ISL_821418, EPI_ISL_821419	Lighthouse Lab in Alderley Park	Wellcome Sanger Institute for the COVID-19 Genomics UK (COG-UK) Consortium	Jacquelyn Wynn, Mairead Hyland, The Lighthouse Lab in Alderley Park and Alex Alderton, Roberto Amato, Sonia Goncalves, Ewan Harrison, David K. Jackson, Ian Johnston, Dominic Kwiatkowski, Cordelia Langford, John Sillitoe on behalf of the Wellcome Sanger Institute COVID-19 Surveillance Team
EPI_ISL_821422, EPI_ISL_821423, EPI_ISL_821424	Lighthouse Lab in Milton Keynes	Wellcome Sanger Institute for the COVID-19 Genomics UK (COG-UK) Consortium	The Lighthouse Lab in Milton Keynes and Alex Alderton, Roberto Amato, Sonia Goncalves, Ewan Harrison, David K. Jackson, Ian Johnston, Dominic Kwiatkowski, Cordelia Langford, John Sillitoe on behalf of the Wellcome Sanger Institute COVID-19 Surveillance Team
EPI_ISL_821425, EPI_ISL_821426	Lighthouse Lab in Alderley Park	Wellcome Sanger Institute for the COVID-19 Genomics UK (COG-UK) Consortium	Jacquelyn Wynn, Mairead Hyland, The Lighthouse Lab in Alderley Park and Alex Alderton, Roberto Amato, Sonia Goncalves, Ewan Harrison, David K. Jackson, Ian Johnston, Dominic Kwiatkowski, Cordelia Langford, John Sillitoe on behalf of the Wellcome Sanger Institute COVID-19 Surveillance Team
EPI_ISL_821428, EPI_ISL_821431, EPI_ISL_821432	Lighthouse Lab in Milton Keynes	Wellcome Sanger Institute for the COVID-19 Genomics UK (COG-UK) Consortium	The Lighthouse Lab in Milton Keynes and Alex Alderton, Roberto Amato, Sonia Goncalves, Ewan Harrison, David K. Jackson, Ian Johnston, Dominic Kwiatkowski, Cordelia Langford, John Sillitoe on behalf of the Wellcome Sanger Institute COVID-19 Surveillance Team
EPI_ISL_821433	Lighthouse Lab in Alderley Park	Wellcome Sanger Institute for the COVID-19 Genomics UK (COG-UK) Consortium	Jacquelyn Wynn, Mairead Hyland, The Lighthouse Lab in Alderley Park and Alex Alderton, Roberto Amato, Sonia Goncalves, Ewan Harrison, David K. Jackson, Ian Johnston, Dominic Kwiatkowski, Cordelia Langford, John Sillitoe on behalf of the Wellcome Sanger Institute COVID-19 Surveillance Team
EPI_ISL_821434	Lighthouse Lab in Milton Keynes	Wellcome Sanger Institute for the COVID-19 Genomics UK (COG-UK) Consortium	The Lighthouse Lab in Milton Keynes and Alex Alderton, Roberto Amato, Sonia Goncalves, Ewan Harrison, David K. Jackson, Ian Johnston, Dominic Kwiatkowski, Cordelia Langford, John Sillitoe on behalf of the Wellcome Sanger Institute COVID-19 Surveillance Team
EPI_ISL_821435	Lighthouse Lab in Cambridge	Wellcome Sanger Institute for the COVID-19 Genomics UK (COG-UK) Consortium	Rob Howes, The Lighthouse Lab in Cambridge and Alex Alderton, Roberto Amato, Sonia Goncalves, Ewan Harrison, David K. Jackson, Ian Johnston, Dominic Kwiatkowski, Cordelia Langford, John Sillitoe on behalf of the Wellcome Sanger Institute COVID-19 Surveillance Team
EPI_ISL_821436, EPI_ISL_821437	Lighthouse Lab in Alderley Park	Wellcome Sanger Institute for the COVID-19 Genomics UK (COG-UK) Consortium	Jacquelyn Wynn, Mairead Hyland, The Lighthouse Lab in Alderley Park and Alex Alderton, Roberto Amato, Sonia Goncalves, Ewan Harrison, David K. Jackson, Ian Johnston, Dominic Kwiatkowski, Cordelia Langford, John Sillitoe on behalf of the Wellcome Sanger Institute COVID-19 Surveillance Team
EPI_ISL_821438	Lighthouse Lab in Milton Keynes	Wellcome Sanger Institute for the COVID-19 Genomics UK (COG-UK) Consortium	The Lighthouse Lab in Milton Keynes and Alex Alderton, Roberto Amato, Sonia Goncalves, Ewan Harrison, David K. Jackson, Ian Johnston, Dominic Kwiatkowski, Cordelia Langford, John Sillitoe on behalf of the Wellcome Sanger Institute COVID-19 Surveillance Team
EPI_ISL_821439	Lighthouse Lab in Cambridge	Wellcome Sanger Institute for the COVID-19 Genomics UK (COG-UK) Consortium	Rob Howes, The Lighthouse Lab in Cambridge and Alex Alderton, Roberto Amato, Sonia Goncalves, Ewan Harrison, David K. Jackson, Ian Johnston, Dominic Kwiatkowski, Cordelia Langford, John Sillitoe on behalf of the Wellcome Sanger Institute COVID-19 Surveillance Team
EPI_ISL_821440	Lighthouse Lab in Alderley Park	Wellcome Sanger Institute for the COVID-19 Genomics UK (COG-UK) Consortium	Jacquelyn Wynn, Mairead Hyland, The Lighthouse Lab in Alderley Park and Alex Alderton, Roberto Amato, Sonia Goncalves, Ewan Harrison, David K. Jackson, Ian Johnston, Dominic Kwiatkowski, Cordelia Langford, John Sillitoe on behalf of the Wellcome Sanger Institute COVID-19 Surveillance Team
EPI_ISL_821441	Lighthouse Lab in Cambridge	Wellcome Sanger Institute for the COVID-19 Genomics UK (COG-UK) Consortium	Rob Howes, The Lighthouse Lab in Cambridge and Alex Alderton, Roberto Amato, Sonia Goncalves, Ewan Harrison, David K. Jackson, Ian Johnston, Dominic Kwiatkowski, Cordelia Langford, John Sillitoe on behalf of the Wellcome Sanger Institute COVID-19 Surveillance Team
EPI_ISL_821443, EPI_ISL_821445	Lighthouse Lab in Milton Keynes	Wellcome Sanger Institute for the COVID-19 Genomics UK (COG-UK) Consortium	The Lighthouse Lab in Milton Keynes and Alex Alderton, Roberto Amato, Sonia Goncalves, Ewan Harrison, David K. Jackson, Ian Johnston, Dominic Kwiatkowski, Cordelia Langford, John Sillitoe on behalf of the Wellcome Sanger Institute COVID-19 Surveillance Team
EPI_ISL_821446	Lighthouse Lab in Alderley Park	Wellcome Sanger Institute for the COVID-19 Genomics UK (COG-UK) Consortium	Jacquelyn Wynn, Mairead Hyland, The Lighthouse Lab in Alderley Park and Alex Alderton, Roberto Amato, Sonia Goncalves, Ewan Harrison, David K. Jackson, Ian Johnston, Dominic Kwiatkowski, Cordelia Langford, John Sillitoe on behalf of the Wellcome Sanger Institute COVID-19 Surveillance Team
EPI_ISL_821447	Lighthouse Lab in Cambridge	Wellcome Sanger Institute for the COVID-19 Genomics UK (COG-UK) Consortium	Rob Howes, The Lighthouse Lab in Cambridge and Alex Alderton, Roberto Amato, Sonia Goncalves, Ewan Harrison, David K. Jackson, Ian Johnston, Dominic Kwiatkowski, Cordelia Langford, John Sillitoe on behalf of the Wellcome Sanger Institute COVID-19 Surveillance Team
EPI_ISL_821448, EPI_ISL_821449	Lighthouse Lab in Milton Keynes	Wellcome Sanger Institute for the COVID-19 Genomics UK (COG-UK) Consortium	The Lighthouse Lab in Milton Keynes and Alex Alderton, Roberto Amato, Sonia Goncalves, Ewan Harrison, David K. Jackson, Ian Johnston, Dominic Kwiatkowski, Cordelia Langford, John Sillitoe on behalf of the Wellcome Sanger Institute COVID-19 Surveillance Team
EPI_ISL_821451	Lighthouse Lab in Alderley Park	Wellcome Sanger Institute for the COVID-19 Genomics UK (COG-UK) Consortium	Jacquelyn Wynn, Mairead Hyland, The Lighthouse Lab in Alderley Park and Alex Alderton, Roberto Amato, Sonia Goncalves, Ewan Harrison, David K. Jackson, Ian Johnston, Dominic Kwiatkowski, Cordelia Langford, John Sillitoe on behalf of the Wellcome Sanger Institute COVID-19 Surveillance Team
EPI_ISL_821452	Lighthouse Lab in Milton Keynes	Wellcome Sanger Institute for the COVID-19 Genomics UK (COG-UK) Consortium	The Lighthouse Lab in Milton Keynes and Alex Alderton, Roberto Amato, Sonia Goncalves, Ewan Harrison, David K. Jackson, Ian Johnston, Dominic Kwiatkowski, Cordelia Langford, John Sillitoe on behalf of the Wellcome Sanger Institute COVID-19 Surveillance Team
EPI_ISL_821453	Lighthouse Lab in Alderley Park	Wellcome Sanger Institute for the COVID-19 Genomics UK (COG-UK) Consortium	Jacquelyn Wynn, Mairead Hyland, The Lighthouse Lab in Alderley Park and Alex Alderton, Roberto Amato, Sonia Goncalves, Ewan Harrison, David K. Jackson, Ian Johnston, Dominic Kwiatkowski, Cordelia Langford, John Sillitoe on behalf of the Wellcome Sanger Institute COVID-19 Surveillance Team

EPI_ISL_821457, EPI_ISL_821458	Lighthouse Lab in Milton Keynes	Wellcome Sanger Institute for the COVID-19 Genomics UK (COG-UK) Consortium	The Lighthouse Lab in Milton Keynes and Alex Alderton, Roberto Amato, Sonia Goncalves, Ewan Harrison, David K. Jackson, Ian Johnston, Dominic Kwiatkowski, Cordelia Langford, John Sillitoe on behalf of the Wellcome Sanger Institute COVID-19 Surveillance Team
EPI_ISL_821459	Lighthouse Lab in Alderley Park	Wellcome Sanger Institute for the COVID-19 Genomics UK (COG-UK) Consortium	Jacquelyn Wynn, Mairead Hyland, The Lighthouse Lab in Alderley Park and Alex Alderton, Roberto Amato, Sonia Goncalves, Ewan Harrison, David K. Jackson, Ian Johnston, Dominic Kwiatkowski, Cordelia Langford, John Sillitoe on behalf of the Wellcome Sanger Institute COVID-19 Surveillance Team
EPI_ISL_821460, EPI_ISL_821461, EPI_ISL_821462	Lighthouse Lab in Milton Keynes	Wellcome Sanger Institute for the COVID-19 Genomics UK (COG-UK) Consortium	The Lighthouse Lab in Milton Keynes and Alex Alderton, Roberto Amato, Sonia Goncalves, Ewan Harrison, David K. Jackson, Ian Johnston, Dominic Kwiatkowski, Cordelia Langford, John Sillitoe on behalf of the Wellcome Sanger Institute COVID-19 Surveillance Team
EPI_ISL_821465, EPI_ISL_821466	Lighthouse Lab in Alderley Park	Wellcome Sanger Institute for the COVID-19 Genomics UK (COG-UK) Consortium	Jacquelyn Wynn, Mairead Hyland, The Lighthouse Lab in Alderley Park and Alex Alderton, Roberto Amato, Sonia Goncalves, Ewan Harrison, David K. Jackson, Ian Johnston, Dominic Kwiatkowski, Cordelia Langford, John Sillitoe on behalf of the Wellcome Sanger Institute COVID-19 Surveillance Team
EPI_ISL_821467, EPI_ISL_821468	Lighthouse Lab in Milton Keynes	Wellcome Sanger Institute for the COVID-19 Genomics UK (COG-UK) Consortium	The Lighthouse Lab in Milton Keynes and Alex Alderton, Roberto Amato, Sonia Goncalves, Ewan Harrison, David K. Jackson, Ian Johnston, Dominic Kwiatkowski, Cordelia Langford, John Sillitoe on behalf of the Wellcome Sanger Institute COVID-19 Surveillance Team
EPI_ISL_821469, EPI_ISL_821470	Lighthouse Lab in Alderley Park	Wellcome Sanger Institute for the COVID-19 Genomics UK (COG-UK) Consortium	Jacquelyn Wynn, Mairead Hyland, The Lighthouse Lab in Alderley Park and Alex Alderton, Roberto Amato, Sonia Goncalves, Ewan Harrison, David K. Jackson, Ian Johnston, Dominic Kwiatkowski, Cordelia Langford, John Sillitoe on behalf of the Wellcome Sanger Institute COVID-19 Surveillance Team
EPI_ISL_821472, EPI_ISL_821474, EPI_ISL_821475	Lighthouse Lab in Milton Keynes	Wellcome Sanger Institute for the COVID-19 Genomics UK (COG-UK) Consortium	The Lighthouse Lab in Milton Keynes and Alex Alderton, Roberto Amato, Sonia Goncalves, Ewan Harrison, David K. Jackson, Ian Johnston, Dominic Kwiatkowski, Cordelia Langford, John Sillitoe on behalf of the Wellcome Sanger Institute COVID-19 Surveillance Team
EPI_ISL_821476	Lighthouse Lab in Alderley Park	Wellcome Sanger Institute for the COVID-19 Genomics UK (COG-UK) Consortium	Jacquelyn Wynn, Mairead Hyland, The Lighthouse Lab in Alderley Park and Alex Alderton, Roberto Amato, Sonia Goncalves, Ewan Harrison, David K. Jackson, Ian Johnston, Dominic Kwiatkowski, Cordelia Langford, John Sillitoe on behalf of the Wellcome Sanger Institute COVID-19 Surveillance Team
EPI_ISL_821477, EPI_ISL_821478	Lighthouse Lab in Milton Keynes	Wellcome Sanger Institute for the COVID-19 Genomics UK (COG-UK) Consortium	The Lighthouse Lab in Milton Keynes and Alex Alderton, Roberto Amato, Sonia Goncalves, Ewan Harrison, David K. Jackson, Ian Johnston, Dominic Kwiatkowski, Cordelia Langford, John Sillitoe on behalf of the Wellcome Sanger Institute COVID-19 Surveillance Team
EPI_ISL_821479	Lighthouse Lab in Cambridge	Wellcome Sanger Institute for the COVID-19 Genomics UK (COG-UK) Consortium	Rob Howes, The Lighthouse Lab in Cambridge and Alex Alderton, Roberto Amato, Sonia Goncalves, Ewan Harrison, David K. Jackson, Ian Johnston, Dominic Kwiatkowski, Cordelia Langford, John Sillitoe on behalf of the Wellcome Sanger Institute COVID-19 Surveillance Team
EPI_ISL_821480	Lighthouse Lab in Milton Keynes	Wellcome Sanger Institute for the COVID-19 Genomics UK (COG-UK) Consortium	The Lighthouse Lab in Milton Keynes and Alex Alderton, Roberto Amato, Sonia Goncalves, Ewan Harrison, David K. Jackson, Ian Johnston, Dominic Kwiatkowski, Cordelia Langford, John Sillitoe on behalf of the Wellcome Sanger Institute COVID-19 Surveillance Team
EPI_ISL_821482, EPI_ISL_821483	Lighthouse Lab in Alderley Park	Wellcome Sanger Institute for the COVID-19 Genomics UK (COG-UK) Consortium	Jacquelyn Wynn, Mairead Hyland, The Lighthouse Lab in Alderley Park and Alex Alderton, Roberto Amato, Sonia Goncalves, Ewan Harrison, David K. Jackson, Ian Johnston, Dominic Kwiatkowski, Cordelia Langford, John Sillitoe on behalf of the Wellcome Sanger Institute COVID-19 Surveillance Team
EPI_ISL_821484	Lighthouse Lab in Cambridge	Wellcome Sanger Institute for the COVID-19 Genomics UK (COG-UK) Consortium	Rob Howes, The Lighthouse Lab in Cambridge and Alex Alderton, Roberto Amato, Sonia Goncalves, Ewan Harrison, David K. Jackson, Ian Johnston, Dominic Kwiatkowski, Cordelia Langford, John Sillitoe on behalf of the Wellcome Sanger Institute COVID-19 Surveillance Team
EPI_ISL_821486, EPI_ISL_821487	Lighthouse Lab in Milton Keynes	Wellcome Sanger Institute for the COVID-19 Genomics UK (COG-UK) Consortium	The Lighthouse Lab in Milton Keynes and Alex Alderton, Roberto Amato, Sonia Goncalves, Ewan Harrison, David K. Jackson, Ian Johnston, Dominic Kwiatkowski, Cordelia Langford, John Sillitoe on behalf of the Wellcome Sanger Institute COVID-19 Surveillance Team
EPI_ISL_821488	Lighthouse Lab in Cambridge	Wellcome Sanger Institute for the COVID-19 Genomics UK (COG-UK) Consortium	Rob Howes, The Lighthouse Lab in Cambridge and Alex Alderton, Roberto Amato, Sonia Goncalves, Ewan Harrison, David K. Jackson, Ian Johnston, Dominic Kwiatkowski, Cordelia Langford, John Sillitoe on behalf of the Wellcome Sanger Institute COVID-19 Surveillance Team
EPI_ISL_821490	Lighthouse Lab in Alderley Park	Wellcome Sanger Institute for the COVID-19 Genomics UK (COG-UK) Consortium	Jacquelyn Wynn, Mairead Hyland, The Lighthouse Lab in Alderley Park and Alex Alderton, Roberto Amato, Sonia Goncalves, Ewan Harrison, David K. Jackson, Ian Johnston, Dominic Kwiatkowski, Cordelia Langford, John Sillitoe on behalf of the Wellcome Sanger Institute COVID-19 Surveillance Team
EPI_ISL_821491, EPI_ISL_821492	Lighthouse Lab in Cambridge	Wellcome Sanger Institute for the COVID-19 Genomics UK (COG-UK) Consortium	Rob Howes, The Lighthouse Lab in Cambridge and Alex Alderton, Roberto Amato, Sonia Goncalves, Ewan Harrison, David K. Jackson, Ian Johnston, Dominic Kwiatkowski, Cordelia Langford, John Sillitoe on behalf of the Wellcome Sanger Institute COVID-19 Surveillance Team
EPI_ISL_821493	Lighthouse Lab in Alderley Park	Wellcome Sanger Institute for the COVID-19 Genomics UK (COG-UK) Consortium	Jacquelyn Wynn, Mairead Hyland, The Lighthouse Lab in Alderley Park and Alex Alderton, Roberto Amato, Sonia Goncalves, Ewan Harrison, David K. Jackson, Ian Johnston, Dominic Kwiatkowski, Cordelia Langford, John Sillitoe on behalf of the Wellcome Sanger Institute COVID-19 Surveillance Team
EPI_ISL_821494	Lighthouse Lab in Milton Keynes	Wellcome Sanger Institute for the COVID-19 Genomics UK (COG-UK) Consortium	The Lighthouse Lab in Milton Keynes and Alex Alderton, Roberto Amato, Sonia Goncalves, Ewan Harrison, David K. Jackson, Ian Johnston, Dominic Kwiatkowski, Cordelia Langford, John Sillitoe on behalf of the Wellcome Sanger Institute COVID-19 Surveillance Team
EPI_ISL_821496, EPI_ISL_821497	Lighthouse Lab in Alderley Park	Wellcome Sanger Institute for the COVID-19 Genomics UK (COG-UK) Consortium	Jacquelyn Wynn, Mairead Hyland, The Lighthouse Lab in Alderley Park and Alex Alderton, Roberto Amato, Sonia Goncalves, Ewan Harrison, David K. Jackson, Ian Johnston, Dominic Kwiatkowski, Cordelia Langford, John Sillitoe on behalf of the Wellcome Sanger Institute COVID-19 Surveillance Team
EPI_ISL_821498	Lighthouse Lab in Milton Keynes	Wellcome Sanger Institute for the COVID-19 Genomics UK (COG-UK) Consortium	The Lighthouse Lab in Milton Keynes and Alex Alderton, Roberto Amato, Sonia Goncalves, Ewan Harrison, David K. Jackson, Ian Johnston, Dominic Kwiatkowski, Cordelia Langford, John Sillitoe on behalf of the Wellcome Sanger Institute COVID-19 Surveillance Team
EPI_ISL_821500	Lighthouse Lab in Alderley Park	Wellcome Sanger Institute for the COVID-19 Genomics UK (COG-UK) Consortium	Jacquelyn Wynn, Mairead Hyland, The Lighthouse Lab in Alderley Park and Alex Alderton, Roberto Amato, Sonia Goncalves, Ewan Harrison, David K. Jackson, Ian Johnston, Dominic Kwiatkowski, Cordelia Langford, John Sillitoe on behalf of the Wellcome Sanger Institute COVID-19 Surveillance Team
EPI_ISL_821501	Lighthouse Lab in Milton Keynes	Wellcome Sanger Institute for the COVID-19 Genomics UK (COG-UK) Consortium	The Lighthouse Lab in Milton Keynes and Alex Alderton, Roberto Amato, Sonia Goncalves, Ewan Harrison, David K. Jackson, Ian Johnston, Dominic Kwiatkowski, Cordelia Langford, John Sillitoe on behalf of the Wellcome Sanger Institute COVID-19 Surveillance Team
EPI_ISL_821502, EPI_ISL_821503	Lighthouse Lab in Alderley Park	Wellcome Sanger Institute for the COVID-19 Genomics UK (COG-UK) Consortium	Jacquelyn Wynn, Mairead Hyland, The Lighthouse Lab in Alderley Park and Alex Alderton, Roberto Amato, Sonia Goncalves, Ewan Harrison, David K. Jackson, Ian Johnston, Dominic Kwiatkowski, Cordelia Langford, John Sillitoe on behalf of the Wellcome Sanger Institute COVID-19 Surveillance Team
EPI_ISL_821504	Lighthouse Lab in Milton Keynes	Wellcome Sanger Institute for the COVID-19 Genomics UK (COG-UK) Consortium	The Lighthouse Lab in Milton Keynes and Alex Alderton, Roberto Amato, Sonia Goncalves, Ewan Harrison, David K. Jackson, Ian Johnston, Dominic Kwiatkowski, Cordelia Langford, John Sillitoe on behalf of the Wellcome Sanger Institute COVID-19 Surveillance Team
EPI_ISL_821506	Lighthouse Lab in Cambridge	Wellcome Sanger Institute for the COVID-19 Genomics UK (COG-UK) Consortium	Rob Howes, The Lighthouse Lab in Cambridge and Alex Alderton, Roberto Amato, Sonia Goncalves, Ewan Harrison, David K. Jackson, Ian Johnston, Dominic Kwiatkowski, Cordelia Langford, John Sillitoe on behalf of the Wellcome Sanger Institute COVID-19 Surveillance Team
EPI_ISL_821507, EPI_ISL_821509	Lighthouse Lab in Milton Keynes	Wellcome Sanger Institute for the COVID-19 Genomics UK (COG-UK) Consortium	The Lighthouse Lab in Milton Keynes and Alex Alderton, Roberto Amato, Sonia Goncalves, Ewan Harrison, David K. Jackson, Ian Johnston, Dominic Kwiatkowski, Cordelia Langford, John Sillitoe on behalf of the Wellcome Sanger Institute COVID-19 Surveillance Team
EPI_ISL_821510	Lighthouse Lab in Cambridge	Wellcome Sanger Institute for the COVID-19 Genomics UK (COG-UK) Consortium	Rob Howes, The Lighthouse Lab in Cambridge and Alex Alderton, Roberto Amato, Sonia Goncalves, Ewan Harrison, David K. Jackson, Ian Johnston, Dominic Kwiatkowski, Cordelia Langford, John Sillitoe on behalf of the Wellcome Sanger Institute COVID-19 Surveillance Team
EPI_ISL_821511	Lighthouse Lab in Alderley Park	Wellcome Sanger Institute for the COVID-19 Genomics UK (COG-UK) Consortium	Jacquelyn Wynn, Mairead Hyland, The Lighthouse Lab in Alderley Park and Alex Alderton, Roberto Amato, Sonia Goncalves, Ewan Harrison, David K. Jackson, Ian Johnston, Dominic Kwiatkowski, Cordelia Langford, John Sillitoe on behalf of the Wellcome Sanger Institute COVID-19 Surveillance Team
EPI_ISL_821514, EPI_ISL_821515	Lighthouse Lab in Milton Keynes	Wellcome Sanger Institute for the COVID-19 Genomics UK (COG-UK) Consortium	The Lighthouse Lab in Milton Keynes and Alex Alderton, Roberto Amato, Sonia Goncalves, Ewan Harrison, David K. Jackson, Ian Johnston, Dominic Kwiatkowski, Cordelia Langford, John Sillitoe on behalf of the Wellcome Sanger Institute COVID-19 Surveillance Team
EPI_ISL_821516	Lighthouse Lab in Cambridge	Wellcome Sanger Institute for the COVID-19 Genomics UK (COG-UK) Consortium	Rob Howes, The Lighthouse Lab in Cambridge and Alex Alderton, Roberto Amato, Sonia Goncalves, Ewan Harrison, David K. Jackson, Ian Johnston, Dominic Kwiatkowski, Cordelia Langford, John Sillitoe on behalf of the Wellcome Sanger Institute COVID-19 Surveillance Team
EPI_ISL_821517	Lighthouse Lab in Milton Keynes	Wellcome Sanger Institute for the COVID-19 Genomics UK (COG-UK) Consortium	The Lighthouse Lab in Milton Keynes and Alex Alderton, Roberto Amato, Sonia Goncalves, Ewan Harrison, David K. Jackson, Ian Johnston, Dominic Kwiatkowski, Cordelia Langford, John Sillitoe on behalf of the Wellcome Sanger Institute COVID-19 Surveillance Team
EPI_ISL_821518	Lighthouse Lab in Alderley Park	Wellcome Sanger Institute for the COVID-19 Genomics UK (COG-UK) Consortium	Jacquelyn Wynn, Mairead Hyland, The Lighthouse Lab in Alderley Park and Alex Alderton, Roberto Amato, Sonia Goncalves, Ewan Harrison, David K. Jackson, Ian Johnston, Dominic Kwiatkowski, Cordelia Langford, John Sillitoe on behalf of the Wellcome Sanger Institute COVID-19 Surveillance Team
EPI_ISL_821519	Lighthouse Lab in Cambridge	Wellcome Sanger Institute for the COVID-19 Genomics UK (COG-UK) Consortium	Rob Howes, The Lighthouse Lab in Cambridge and Alex Alderton, Roberto Amato, Sonia Goncalves, Ewan Harrison, David K. Jackson, Ian Johnston, Dominic Kwiatkowski, Cordelia Langford, John Sillitoe on behalf of the Wellcome Sanger Institute COVID-19 Surveillance Team
EPI_ISL_821520, EPI_ISL_821521, EPI_ISL_821522	Lighthouse Lab in Milton Keynes	Wellcome Sanger Institute for the COVID-19 Genomics UK (COG-UK) Consortium	The Lighthouse Lab in Milton Keynes and Alex Alderton, Roberto Amato, Sonia Goncalves, Ewan Harrison, David K. Jackson, Ian Johnston, Dominic Kwiatkowski, Cordelia Langford, John Sillitoe on behalf of the Wellcome Sanger Institute COVID-19 Surveillance Team

EPI_ISL_821523	Lighthouse Lab in Cambridge	Wellcome Sanger Institute for the COVID-19 Genomics UK (COG-UK) Consortium	Rob Howes, The Lighthouse Lab in Cambridge and Alex Alderton, Roberto Amato, Sonia Goncalves, Ewan Harrison, David K. Jackson, Ian Johnston, Dominic Kwiatkowski, Cordelia Langford, John Sillitoe on behalf of the Wellcome Sanger Institute COVID-19 Surveillance Team
EPI_ISL_821524	Lighthouse Lab in Milton Keynes	Wellcome Sanger Institute for the COVID-19 Genomics UK (COG-UK) Consortium	The Lighthouse Lab in Milton Keynes and Alex Alderton, Roberto Amato, Sonia Goncalves, Ewan Harrison, David K. Jackson, Ian Johnston, Dominic Kwiatkowski, Cordelia Langford, John Sillitoe on behalf of the Wellcome Sanger Institute COVID-19 Surveillance Team
EPI_ISL_821526	Lighthouse Lab in Alderley Park	Wellcome Sanger Institute for the COVID-19 Genomics UK (COG-UK) Consortium	Jacquelyn Wynn, Mairead Hyland, The Lighthouse Lab in Alderley Park and Alex Alderton, Roberto Amato, Sonia Goncalves, Ewan Harrison, David K. Jackson, Ian Johnston, Dominic Kwiatkowski, Cordelia Langford, John Sillitoe on behalf of the Wellcome Sanger Institute COVID-19 Surveillance Team
EPI_ISL_821527	Lighthouse Lab in Milton Keynes	Wellcome Sanger Institute for the COVID-19 Genomics UK (COG-UK) Consortium	The Lighthouse Lab in Milton Keynes and Alex Alderton, Roberto Amato, Sonia Goncalves, Ewan Harrison, David K. Jackson, Ian Johnston, Dominic Kwiatkowski, Cordelia Langford, John Sillitoe on behalf of the Wellcome Sanger Institute COVID-19 Surveillance Team
EPI_ISL_821528	Lighthouse Lab in Alderley Park	Wellcome Sanger Institute for the COVID-19 Genomics UK (COG-UK) Consortium	Jacquelyn Wynn, Mairead Hyland, The Lighthouse Lab in Alderley Park and Alex Alderton, Roberto Amato, Sonia Goncalves, Ewan Harrison, David K. Jackson, Ian Johnston, Dominic Kwiatkowski, Cordelia Langford, John Sillitoe on behalf of the Wellcome Sanger Institute COVID-19 Surveillance Team
EPI_ISL_821529	Lighthouse Lab in Cambridge	Wellcome Sanger Institute for the COVID-19 Genomics UK (COG-UK) Consortium	Rob Howes, The Lighthouse Lab in Cambridge and Alex Alderton, Roberto Amato, Sonia Goncalves, Ewan Harrison, David K. Jackson, Ian Johnston, Dominic Kwiatkowski, Cordelia Langford, John Sillitoe on behalf of the Wellcome Sanger Institute COVID-19 Surveillance Team
EPI_ISL_821530, EPI_ISL_821532	Lighthouse Lab in Milton Keynes	Wellcome Sanger Institute for the COVID-19 Genomics UK (COG-UK) Consortium	The Lighthouse Lab in Milton Keynes and Alex Alderton, Roberto Amato, Sonia Goncalves, Ewan Harrison, David K. Jackson, Ian Johnston, Dominic Kwiatkowski, Cordelia Langford, John Sillitoe on behalf of the Wellcome Sanger Institute COVID-19 Surveillance Team
EPI_ISL_821533, EPI_ISL_821534	Lighthouse Lab in Alderley Park	Wellcome Sanger Institute for the COVID-19 Genomics UK (COG-UK) Consortium	Jacquelyn Wynn, Mairead Hyland, The Lighthouse Lab in Alderley Park and Alex Alderton, Roberto Amato, Sonia Goncalves, Ewan Harrison, David K. Jackson, Ian Johnston, Dominic Kwiatkowski, Cordelia Langford, John Sillitoe on behalf of the Wellcome Sanger Institute COVID-19 Surveillance Team
EPI_ISL_821535, EPI_ISL_821536, EPI_ISL_821537	Lighthouse Lab in Milton Keynes	Wellcome Sanger Institute for the COVID-19 Genomics UK (COG-UK) Consortium	The Lighthouse Lab in Milton Keynes and Alex Alderton, Roberto Amato, Sonia Goncalves, Ewan Harrison, David K. Jackson, Ian Johnston, Dominic Kwiatkowski, Cordelia Langford, John Sillitoe on behalf of the Wellcome Sanger Institute COVID-19 Surveillance Team
EPI_ISL_821538	Lighthouse Lab in Alderley Park	Wellcome Sanger Institute for the COVID-19 Genomics UK (COG-UK) Consortium	Jacquelyn Wynn, Mairead Hyland, The Lighthouse Lab in Alderley Park and Alex Alderton, Roberto Amato, Sonia Goncalves, Ewan Harrison, David K. Jackson, Ian Johnston, Dominic Kwiatkowski, Cordelia Langford, John Sillitoe on behalf of the Wellcome Sanger Institute COVID-19 Surveillance Team
EPI_ISL_821539, EPI_ISL_821540, EPI_ISL_821541, EPI_ISL_821542, EPI_ISL_821543	Lighthouse Lab in Milton Keynes	Wellcome Sanger Institute for the COVID-19 Genomics UK (COG-UK) Consortium	The Lighthouse Lab in Milton Keynes and Alex Alderton, Roberto Amato, Sonia Goncalves, Ewan Harrison, David K. Jackson, Ian Johnston, Dominic Kwiatkowski, Cordelia Langford, John Sillitoe on behalf of the Wellcome Sanger Institute COVID-19 Surveillance Team
EPI_ISL_821544	Lighthouse Lab in Cambridge	Wellcome Sanger Institute for the COVID-19 Genomics UK (COG-UK) Consortium	Rob Howes, The Lighthouse Lab in Cambridge and Alex Alderton, Roberto Amato, Sonia Goncalves, Ewan Harrison, David K. Jackson, Ian Johnston, Dominic Kwiatkowski, Cordelia Langford, John Sillitoe on behalf of the Wellcome Sanger Institute COVID-19 Surveillance Team
EPI_ISL_821545	Lighthouse Lab in Milton Keynes	Wellcome Sanger Institute for the COVID-19 Genomics UK (COG-UK) Consortium	The Lighthouse Lab in Milton Keynes and Alex Alderton, Roberto Amato, Sonia Goncalves, Ewan Harrison, David K. Jackson, Ian Johnston, Dominic Kwiatkowski, Cordelia Langford, John Sillitoe on behalf of the Wellcome Sanger Institute COVID-19 Surveillance Team
EPI_ISL_821547	Lighthouse Lab in Cambridge	Wellcome Sanger Institute for the COVID-19 Genomics UK (COG-UK) Consortium	Rob Howes, The Lighthouse Lab in Cambridge and Alex Alderton, Roberto Amato, Sonia Goncalves, Ewan Harrison, David K. Jackson, Ian Johnston, Dominic Kwiatkowski, Cordelia Langford, John Sillitoe on behalf of the Wellcome Sanger Institute COVID-19 Surveillance Team
EPI_ISL_821548	Lighthouse Lab in Alderley Park	Wellcome Sanger Institute for the COVID-19 Genomics UK (COG-UK) Consortium	Jacquelyn Wynn, Mairead Hyland, The Lighthouse Lab in Alderley Park and Alex Alderton, Roberto Amato, Sonia Goncalves, Ewan Harrison, David K. Jackson, Ian Johnston, Dominic Kwiatkowski, Cordelia Langford, John Sillitoe on behalf of the Wellcome Sanger Institute COVID-19 Surveillance Team
EPI_ISL_821549	Lighthouse Lab in Cambridge	Wellcome Sanger Institute for the COVID-19 Genomics UK (COG-UK) Consortium	Rob Howes, The Lighthouse Lab in Cambridge and Alex Alderton, Roberto Amato, Sonia Goncalves, Ewan Harrison, David K. Jackson, Ian Johnston, Dominic Kwiatkowski, Cordelia Langford, John Sillitoe on behalf of the Wellcome Sanger Institute COVID-19 Surveillance Team
EPI_ISL_821550, EPI_ISL_821551, EPI_ISL_821552, EPI_ISL_821553	Lighthouse Lab in Alderley Park	Wellcome Sanger Institute for the COVID-19 Genomics UK (COG-UK) Consortium	Jacquelyn Wynn, Mairead Hyland, The Lighthouse Lab in Alderley Park and Alex Alderton, Roberto Amato, Sonia Goncalves, Ewan Harrison, David K. Jackson, Ian Johnston, Dominic Kwiatkowski, Cordelia Langford, John Sillitoe on behalf of the Wellcome Sanger Institute COVID-19 Surveillance Team
EPI_ISL_821554	Lighthouse Lab in Milton Keynes	Wellcome Sanger Institute for the COVID-19 Genomics UK (COG-UK) Consortium	The Lighthouse Lab in Milton Keynes and Alex Alderton, Roberto Amato, Sonia Goncalves, Ewan Harrison, David K. Jackson, Ian Johnston, Dominic Kwiatkowski, Cordelia Langford, John Sillitoe on behalf of the Wellcome Sanger Institute COVID-19 Surveillance Team
EPI_ISL_821555	Lighthouse Lab in Cambridge	Wellcome Sanger Institute for the COVID-19 Genomics UK (COG-UK) Consortium	Rob Howes, The Lighthouse Lab in Cambridge and Alex Alderton, Roberto Amato, Sonia Goncalves, Ewan Harrison, David K. Jackson, Ian Johnston, Dominic Kwiatkowski, Cordelia Langford, John Sillitoe on behalf of the Wellcome Sanger Institute COVID-19 Surveillance Team
EPI_ISL_821556, EPI_ISL_821558	Lighthouse Lab in Alderley Park	Wellcome Sanger Institute for the COVID-19 Genomics UK (COG-UK) Consortium	Jacquelyn Wynn, Mairead Hyland, The Lighthouse Lab in Alderley Park and Alex Alderton, Roberto Amato, Sonia Goncalves, Ewan Harrison, David K. Jackson, Ian Johnston, Dominic Kwiatkowski, Cordelia Langford, John Sillitoe on behalf of the Wellcome Sanger Institute COVID-19 Surveillance Team
EPI_ISL_821559, EPI_ISL_821560, EPI_ISL_821561, EPI_ISL_821562	Lighthouse Lab in Milton Keynes	Wellcome Sanger Institute for the COVID-19 Genomics UK (COG-UK) Consortium	The Lighthouse Lab in Milton Keynes and Alex Alderton, Roberto Amato, Sonia Goncalves, Ewan Harrison, David K. Jackson, Ian Johnston, Dominic Kwiatkowski, Cordelia Langford, John Sillitoe on behalf of the Wellcome Sanger Institute COVID-19 Surveillance Team
EPI_ISL_821563, EPI_ISL_821564, EPI_ISL_821565	Lighthouse Lab in Cambridge	Wellcome Sanger Institute for the COVID-19 Genomics UK (COG-UK) Consortium	Rob Howes, The Lighthouse Lab in Cambridge and Alex Alderton, Roberto Amato, Sonia Goncalves, Ewan Harrison, David K. Jackson, Ian Johnston, Dominic Kwiatkowski, Cordelia Langford, John Sillitoe on behalf of the Wellcome Sanger Institute COVID-19 Surveillance Team
EPI_ISL_821566	Lighthouse Lab in Alderley Park	Wellcome Sanger Institute for the COVID-19 Genomics UK (COG-UK) Consortium	Jacquelyn Wynn, Mairead Hyland, The Lighthouse Lab in Alderley Park and Alex Alderton, Roberto Amato, Sonia Goncalves, Ewan Harrison, David K. Jackson, Ian Johnston, Dominic Kwiatkowski, Cordelia Langford, John Sillitoe on behalf of the Wellcome Sanger Institute COVID-19 Surveillance Team
EPI_ISL_821567	Lighthouse Lab in Milton Keynes	Wellcome Sanger Institute for the COVID-19 Genomics UK (COG-UK) Consortium	The Lighthouse Lab in Milton Keynes and Alex Alderton, Roberto Amato, Sonia Goncalves, Ewan Harrison, David K. Jackson, Ian Johnston, Dominic Kwiatkowski, Cordelia Langford, John Sillitoe on behalf of the Wellcome Sanger Institute COVID-19 Surveillance Team
EPI_ISL_821568	Lighthouse Lab in Cambridge	Wellcome Sanger Institute for the COVID-19 Genomics UK (COG-UK) Consortium	Rob Howes, The Lighthouse Lab in Cambridge and Alex Alderton, Roberto Amato, Sonia Goncalves, Ewan Harrison, David K. Jackson, Ian Johnston, Dominic Kwiatkowski, Cordelia Langford, John Sillitoe on behalf of the Wellcome Sanger Institute COVID-19 Surveillance Team
EPI_ISL_821569	Lighthouse Lab in Milton Keynes	Wellcome Sanger Institute for the COVID-19 Genomics UK (COG-UK) Consortium	The Lighthouse Lab in Milton Keynes and Alex Alderton, Roberto Amato, Sonia Goncalves, Ewan Harrison, David K. Jackson, Ian Johnston, Dominic Kwiatkowski, Cordelia Langford, John Sillitoe on behalf of the Wellcome Sanger Institute COVID-19 Surveillance Team
EPI_ISL_821570	Lighthouse Lab in Cambridge	Wellcome Sanger Institute for the COVID-19 Genomics UK (COG-UK) Consortium	Rob Howes, The Lighthouse Lab in Cambridge and Alex Alderton, Roberto Amato, Sonia Goncalves, Ewan Harrison, David K. Jackson, Ian Johnston, Dominic Kwiatkowski, Cordelia Langford, John Sillitoe on behalf of the Wellcome Sanger Institute COVID-19 Surveillance Team
EPI_ISL_821571, EPI_ISL_821572, EPI_ISL_821573, EPI_ISL_821574, EPI_ISL_821575	Lighthouse Lab in Milton Keynes	Wellcome Sanger Institute for the COVID-19 Genomics UK (COG-UK) Consortium	The Lighthouse Lab in Milton Keynes and Alex Alderton, Roberto Amato, Sonia Goncalves, Ewan Harrison, David K. Jackson, Ian Johnston, Dominic Kwiatkowski, Cordelia Langford, John Sillitoe on behalf of the Wellcome Sanger Institute COVID-19 Surveillance Team
EPI_ISL_821576, EPI_ISL_821577, EPI_ISL_821579	Lighthouse Lab in Cambridge	Wellcome Sanger Institute for the COVID-19 Genomics UK (COG-UK) Consortium	Rob Howes, The Lighthouse Lab in Cambridge and Alex Alderton, Roberto Amato, Sonia Goncalves, Ewan Harrison, David K. Jackson, Ian Johnston, Dominic Kwiatkowski, Cordelia Langford, John Sillitoe on behalf of the Wellcome Sanger Institute COVID-19 Surveillance Team
EPI_ISL_821580, EPI_ISL_821582	Lighthouse Lab in Milton Keynes	Wellcome Sanger Institute for the COVID-19 Genomics UK (COG-UK) Consortium	The Lighthouse Lab in Milton Keynes and Alex Alderton, Roberto Amato, Sonia Goncalves, Ewan Harrison, David K. Jackson, Ian Johnston, Dominic Kwiatkowski, Cordelia Langford, John Sillitoe on behalf of the Wellcome Sanger Institute COVID-19 Surveillance Team
EPI_ISL_821583	Lighthouse Lab in Cambridge	Wellcome Sanger Institute for the COVID-19 Genomics UK (COG-UK) Consortium	Rob Howes, The Lighthouse Lab in Cambridge and Alex Alderton, Roberto Amato, Sonia Goncalves, Ewan Harrison, David K. Jackson, Ian Johnston, Dominic Kwiatkowski, Cordelia Langford, John Sillitoe on behalf of the Wellcome Sanger Institute COVID-19 Surveillance Team
EPI_ISL_821584, EPI_ISL_821585, EPI_ISL_821586	Lighthouse Lab in Alderley Park	Wellcome Sanger Institute for the COVID-19 Genomics UK (COG-UK) Consortium	Jacquelyn Wynn, Mairead Hyland, The Lighthouse Lab in Alderley Park and Alex Alderton, Roberto Amato, Sonia Goncalves, Ewan Harrison, David K. Jackson, Ian Johnston, Dominic Kwiatkowski, Cordelia Langford, John Sillitoe on behalf of the Wellcome Sanger Institute COVID-19 Surveillance Team
EPI_ISL_821587	Lighthouse Lab in Milton Keynes	Wellcome Sanger Institute for the COVID-19 Genomics UK (COG-UK) Consortium	The Lighthouse Lab in Milton Keynes and Alex Alderton, Roberto Amato, Sonia Goncalves, Ewan Harrison, David K. Jackson, Ian Johnston, Dominic Kwiatkowski, Cordelia Langford, John Sillitoe on behalf of the Wellcome Sanger Institute COVID-19 Surveillance Team
EPI_ISL_821588	Lighthouse Lab in Cambridge	Wellcome Sanger Institute for the COVID-19 Genomics UK (COG-UK) Consortium	Rob Howes, The Lighthouse Lab in Cambridge and Alex Alderton, Roberto Amato, Sonia Goncalves, Ewan Harrison, David K. Jackson, Ian Johnston, Dominic Kwiatkowski, Cordelia Langford, John Sillitoe on behalf of the Wellcome Sanger Institute COVID-19 Surveillance Team
EPI_ISL_821589	Lighthouse Lab in Milton Keynes	Wellcome Sanger Institute for the COVID-19 Genomics UK	The Lighthouse Lab in Milton Keynes and Alex Alderton, Roberto Amato, Sonia Goncalves, Ewan Harrison, David K. Jackson, Ian Johnston, Dominic

		(COG-UK) Consortium	Kwiatkowski, Cordelia Langford, John Sillitoe on behalf of the Wellcome Sanger Institute COVID-19 Surveillance Team
EPI_ISL_821590, EPI_ISL_821592, EPI_ISL_821593	Lighthouse Lab in Cambridge	Wellcome Sanger Institute for the COVID-19 Genomics UK (COG-UK) Consortium	Rob Howes, The Lighthouse Lab in Cambridge and Alex Alderton, Roberto Amato, Sonia Goncalves, Ewan Harrison, David K. Jackson, Ian Johnston, Dominic Kwiatkowski, Cordelia Langford, John Sillitoe on behalf of the Wellcome Sanger Institute COVID-19 Surveillance Team
EPI_ISL_821594, EPI_ISL_821595	Lighthouse Lab in Milton Keynes	Wellcome Sanger Institute for the COVID-19 Genomics UK (COG-UK) Consortium	The Lighthouse Lab in Milton Keynes and Alex Alderton, Roberto Amato, Sonia Goncalves, Ewan Harrison, David K. Jackson, Ian Johnston, Dominic Kwiatkowski, Cordelia Langford, John Sillitoe on behalf of the Wellcome Sanger Institute COVID-19 Surveillance Team
EPI_ISL_821596	Lighthouse Lab in Cambridge	Wellcome Sanger Institute for the COVID-19 Genomics UK (COG-UK) Consortium	Rob Howes, The Lighthouse Lab in Cambridge and Alex Alderton, Roberto Amato, Sonia Goncalves, Ewan Harrison, David K. Jackson, Ian Johnston, Dominic Kwiatkowski, Cordelia Langford, John Sillitoe on behalf of the Wellcome Sanger Institute COVID-19 Surveillance Team
EPI_ISL_821597, EPI_ISL_821598	Lighthouse Lab in Milton Keynes	Wellcome Sanger Institute for the COVID-19 Genomics UK (COG-UK) Consortium	The Lighthouse Lab in Milton Keynes and Alex Alderton, Roberto Amato, Sonia Goncalves, Ewan Harrison, David K. Jackson, Ian Johnston, Dominic Kwiatkowski, Cordelia Langford, John Sillitoe on behalf of the Wellcome Sanger Institute COVID-19 Surveillance Team
EPI_ISL_821599, EPI_ISL_821600	Lighthouse Lab in Alderley Park	Wellcome Sanger Institute for the COVID-19 Genomics UK (COG-UK) Consortium	Jacquelyn Wynn, Mairead Hyland, The Lighthouse Lab in Alderley Park and Alex Alderton, Roberto Amato, Sonia Goncalves, Ewan Harrison, David K. Jackson, Ian Johnston, Dominic Kwiatkowski, Cordelia Langford, John Sillitoe on behalf of the Wellcome Sanger Institute COVID-19 Surveillance Team
EPI_ISL_821601, EPI_ISL_821603	Lighthouse Lab in Milton Keynes	Wellcome Sanger Institute for the COVID-19 Genomics UK (COG-UK) Consortium	The Lighthouse Lab in Milton Keynes and Alex Alderton, Roberto Amato, Sonia Goncalves, Ewan Harrison, David K. Jackson, Ian Johnston, Dominic Kwiatkowski, Cordelia Langford, John Sillitoe on behalf of the Wellcome Sanger Institute COVID-19 Surveillance Team
EPI_ISL_821604, EPI_ISL_821605	Lighthouse Lab in Cambridge	Wellcome Sanger Institute for the COVID-19 Genomics UK (COG-UK) Consortium	Rob Howes, The Lighthouse Lab in Cambridge and Alex Alderton, Roberto Amato, Sonia Goncalves, Ewan Harrison, David K. Jackson, Ian Johnston, Dominic Kwiatkowski, Cordelia Langford, John Sillitoe on behalf of the Wellcome Sanger Institute COVID-19 Surveillance Team
EPI_ISL_821606, EPI_ISL_821607	Lighthouse Lab in Alderley Park	Wellcome Sanger Institute for the COVID-19 Genomics UK (COG-UK) Consortium	Jacquelyn Wynn, Mairead Hyland, The Lighthouse Lab in Alderley Park and Alex Alderton, Roberto Amato, Sonia Goncalves, Ewan Harrison, David K. Jackson, Ian Johnston, Dominic Kwiatkowski, Cordelia Langford, John Sillitoe on behalf of the Wellcome Sanger Institute COVID-19 Surveillance Team
EPI_ISL_821608	Lighthouse Lab in Cambridge	Wellcome Sanger Institute for the COVID-19 Genomics UK (COG-UK) Consortium	Rob Howes, The Lighthouse Lab in Cambridge and Alex Alderton, Roberto Amato, Sonia Goncalves, Ewan Harrison, David K. Jackson, Ian Johnston, Dominic Kwiatkowski, Cordelia Langford, John Sillitoe on behalf of the Wellcome Sanger Institute COVID-19 Surveillance Team
EPI_ISL_821609, EPI_ISL_821610, EPI_ISL_821612	Lighthouse Lab in Milton Keynes	Wellcome Sanger Institute for the COVID-19 Genomics UK (COG-UK) Consortium	The Lighthouse Lab in Milton Keynes and Alex Alderton, Roberto Amato, Sonia Goncalves, Ewan Harrison, David K. Jackson, Ian Johnston, Dominic Kwiatkowski, Cordelia Langford, John Sillitoe on behalf of the Wellcome Sanger Institute COVID-19 Surveillance Team
EPI_ISL_821614	Lighthouse Lab in Alderley Park	Wellcome Sanger Institute for the COVID-19 Genomics UK (COG-UK) Consortium	Jacquelyn Wynn, Mairead Hyland, The Lighthouse Lab in Alderley Park and Alex Alderton, Roberto Amato, Sonia Goncalves, Ewan Harrison, David K. Jackson, Ian Johnston, Dominic Kwiatkowski, Cordelia Langford, John Sillitoe on behalf of the Wellcome Sanger Institute COVID-19 Surveillance Team
EPI_ISL_821615	Lighthouse Lab in Milton Keynes	Wellcome Sanger Institute for the COVID-19 Genomics UK (COG-UK) Consortium	The Lighthouse Lab in Milton Keynes and Alex Alderton, Roberto Amato, Sonia Goncalves, Ewan Harrison, David K. Jackson, Ian Johnston, Dominic Kwiatkowski, Cordelia Langford, John Sillitoe on behalf of the Wellcome Sanger Institute COVID-19 Surveillance Team
EPI_ISL_821616, EPI_ISL_821618, EPI_ISL_821620, EPI_ISL_821622, EPI_ISL_821623, EPI_ISL_821624	Lighthouse Lab in Cambridge	Wellcome Sanger Institute for the COVID-19 Genomics UK (COG-UK) Consortium	Rob Howes, The Lighthouse Lab in Cambridge and Alex Alderton, Roberto Amato, Sonia Goncalves, Ewan Harrison, David K. Jackson, Ian Johnston, Dominic Kwiatkowski, Cordelia Langford, John Sillitoe on behalf of the Wellcome Sanger Institute COVID-19 Surveillance Team
EPI_ISL_821625	Lighthouse Lab in Milton Keynes	Wellcome Sanger Institute for the COVID-19 Genomics UK (COG-UK) Consortium	The Lighthouse Lab in Milton Keynes and Alex Alderton, Roberto Amato, Sonia Goncalves, Ewan Harrison, David K. Jackson, Ian Johnston, Dominic Kwiatkowski, Cordelia Langford, John Sillitoe on behalf of the Wellcome Sanger Institute COVID-19 Surveillance Team
EPI_ISL_821669, EPI_ISL_821834, EPI_ISL_821842, EPI_ISL_821858, EPI_ISL_821861, EPI_ISL_821895, EPI_ISL_821903, EPI_ISL_821950, EPI_ISL_821967	Lighthouse Lab in Cambridge	Wellcome Sanger Institute for the COVID-19 Genomics UK (COG-UK) Consortium	Rob Howes, The Lighthouse Lab in Cambridge and Alex Alderton, Roberto Amato, Sonia Goncalves, Ewan Harrison, David K. Jackson, Ian Johnston, Dominic Kwiatkowski, Cordelia Langford, John Sillitoe on behalf of the Wellcome Sanger Institute COVID-19 Surveillance Team
EPI_ISL_821972, EPI_ISL_821973	Lighthouse Lab in Alderley Park	Wellcome Sanger Institute for the COVID-19 Genomics UK (COG-UK) Consortium	Jacquelyn Wynn, Mairead Hyland, The Lighthouse Lab in Alderley Park and Alex Alderton, Roberto Amato, Sonia Goncalves, Ewan Harrison, David K. Jackson, Ian Johnston, Dominic Kwiatkowski, Cordelia Langford, John Sillitoe on behalf of the Wellcome Sanger Institute COVID-19 Surveillance Team
EPI_ISL_821975	Lighthouse Lab in Glasgow	Wellcome Sanger Institute for the COVID-19 Genomics UK (COG-UK) Consortium	Harper VanSteenhouse, Yumi Kasai, David Gray, Carol Clugston, Anna Dominiczak and Alex Alderton, Roberto Amato, Sonia Goncalves, Ewan Harrison, David K. Jackson, Ian Johnston, Dominic Kwiatkowski, Cordelia Langford, John Sillitoe on behalf of the Wellcome Sanger Institute COVID-19 Surveillance Team
EPI_ISL_821987, EPI_ISL_821988, EPI_ISL_821991, EPI_ISL_821992	Lighthouse Lab in Alderley Park	Wellcome Sanger Institute for the COVID-19 Genomics UK (COG-UK) Consortium	Jacquelyn Wynn, Mairead Hyland, The Lighthouse Lab in Alderley Park and Alex Alderton, Roberto Amato, Sonia Goncalves, Ewan Harrison, David K. Jackson, Ian Johnston, Dominic Kwiatkowski, Cordelia Langford, John Sillitoe on behalf of the Wellcome Sanger Institute COVID-19 Surveillance Team
EPI_ISL_821993, EPI_ISL_821995	Lighthouse Lab in Glasgow	Wellcome Sanger Institute for the COVID-19 Genomics UK (COG-UK) Consortium	Harper VanSteenhouse, Yumi Kasai, David Gray, Carol Clugston, Anna Dominiczak and Alex Alderton, Roberto Amato, Sonia Goncalves, Ewan Harrison, David K. Jackson, Ian Johnston, Dominic Kwiatkowski, Cordelia Langford, John Sillitoe on behalf of the Wellcome Sanger Institute COVID-19 Surveillance Team
EPI_ISL_821996, EPI_ISL_821997	Lighthouse Lab in Alderley Park	Wellcome Sanger Institute for the COVID-19 Genomics UK (COG-UK) Consortium	Jacquelyn Wynn, Mairead Hyland, The Lighthouse Lab in Alderley Park and Alex Alderton, Roberto Amato, Sonia Goncalves, Ewan Harrison, David K. Jackson, Ian Johnston, Dominic Kwiatkowski, Cordelia Langford, John Sillitoe on behalf of the Wellcome Sanger Institute COVID-19 Surveillance Team
EPI_ISL_822001, EPI_ISL_822004, EPI_ISL_822005, EPI_ISL_822006, EPI_ISL_822011	Lighthouse Lab in Glasgow	Wellcome Sanger Institute for the COVID-19 Genomics UK (COG-UK) Consortium	Harper VanSteenhouse, Yumi Kasai, David Gray, Carol Clugston, Anna Dominiczak and Alex Alderton, Roberto Amato, Sonia Goncalves, Ewan Harrison, David K. Jackson, Ian Johnston, Dominic Kwiatkowski, Cordelia Langford, John Sillitoe on behalf of the Wellcome Sanger Institute COVID-19 Surveillance Team
EPI_ISL_822013, EPI_ISL_822019	Lighthouse Lab in Alderley Park	Wellcome Sanger Institute for the COVID-19 Genomics UK (COG-UK) Consortium	Jacquelyn Wynn, Mairead Hyland, The Lighthouse Lab in Alderley Park and Alex Alderton, Roberto Amato, Sonia Goncalves, Ewan Harrison, David K. Jackson, Ian Johnston, Dominic Kwiatkowski, Cordelia Langford, John Sillitoe on behalf of the Wellcome Sanger Institute COVID-19 Surveillance Team
EPI_ISL_822022, EPI_ISL_822023, EPI_ISL_822029	Lighthouse Lab in Glasgow	Wellcome Sanger Institute for the COVID-19 Genomics UK (COG-UK) Consortium	Harper VanSteenhouse, Yumi Kasai, David Gray, Carol Clugston, Anna Dominiczak and Alex Alderton, Roberto Amato, Sonia Goncalves, Ewan Harrison, David K. Jackson, Ian Johnston, Dominic Kwiatkowski, Cordelia Langford, John Sillitoe on behalf of the Wellcome Sanger Institute COVID-19 Surveillance Team
EPI_ISL_822030	Lighthouse Lab in Alderley Park	Wellcome Sanger Institute for the COVID-19 Genomics UK (COG-UK) Consortium	Jacquelyn Wynn, Mairead Hyland, The Lighthouse Lab in Alderley Park and Alex Alderton, Roberto Amato, Sonia Goncalves, Ewan Harrison, David K. Jackson, Ian Johnston, Dominic Kwiatkowski, Cordelia Langford, John Sillitoe on behalf of the Wellcome Sanger Institute COVID-19 Surveillance Team
EPI_ISL_822033, EPI_ISL_822035, EPI_ISL_822040, EPI_ISL_822041	Lighthouse Lab in Glasgow	Wellcome Sanger Institute for the COVID-19 Genomics UK (COG-UK) Consortium	Harper VanSteenhouse, Yumi Kasai, David Gray, Carol Clugston, Anna Dominiczak and Alex Alderton, Roberto Amato, Sonia Goncalves, Ewan Harrison, David K. Jackson, Ian Johnston, Dominic Kwiatkowski, Cordelia Langford, John Sillitoe on behalf of the Wellcome Sanger Institute COVID-19 Surveillance Team
EPI_ISL_822043	Lighthouse Lab in Alderley Park	Wellcome Sanger Institute for the COVID-19 Genomics UK (COG-UK) Consortium	Jacquelyn Wynn, Mairead Hyland, The Lighthouse Lab in Alderley Park and Alex Alderton, Roberto Amato, Sonia Goncalves, Ewan Harrison, David K. Jackson, Ian Johnston, Dominic Kwiatkowski, Cordelia Langford, John Sillitoe on behalf of the Wellcome Sanger Institute COVID-19 Surveillance Team
EPI_ISL_822044	Lighthouse Lab in Glasgow	Wellcome Sanger Institute for the COVID-19 Genomics UK (COG-UK) Consortium	Harper VanSteenhouse, Yumi Kasai, David Gray, Carol Clugston, Anna Dominiczak and Alex Alderton, Roberto Amato, Sonia Goncalves, Ewan Harrison, David K. Jackson, Ian Johnston, Dominic Kwiatkowski, Cordelia Langford, John Sillitoe on behalf of the Wellcome Sanger Institute COVID-19 Surveillance Team
EPI_ISL_822047	Lighthouse Lab in Alderley Park	Wellcome Sanger Institute for the COVID-19 Genomics UK (COG-UK) Consortium	Jacquelyn Wynn, Mairead Hyland, The Lighthouse Lab in Alderley Park and Alex Alderton, Roberto Amato, Sonia Goncalves, Ewan Harrison, David K. Jackson, Ian Johnston, Dominic Kwiatkowski, Cordelia Langford, John Sillitoe on behalf of the Wellcome Sanger Institute COVID-19 Surveillance Team
EPI_ISL_822048, EPI_ISL_822051, EPI_ISL_822053, EPI_ISL_822059	Lighthouse Lab in Glasgow	Wellcome Sanger Institute for the COVID-19 Genomics UK (COG-UK) Consortium	Harper VanSteenhouse, Yumi Kasai, David Gray, Carol Clugston, Anna Dominiczak and Alex Alderton, Roberto Amato, Sonia Goncalves, Ewan Harrison, David K. Jackson, Ian Johnston, Dominic Kwiatkowski, Cordelia Langford, John Sillitoe on behalf of the Wellcome Sanger Institute COVID-19 Surveillance Team
EPI_ISL_822060	Lighthouse Lab in Alderley Park	Wellcome Sanger Institute for the COVID-19 Genomics UK (COG-UK) Consortium	Jacquelyn Wynn, Mairead Hyland, The Lighthouse Lab in Alderley Park and Alex Alderton, Roberto Amato, Sonia Goncalves, Ewan Harrison, David K. Jackson, Ian Johnston, Dominic Kwiatkowski, Cordelia Langford, John Sillitoe on behalf of the Wellcome Sanger Institute COVID-19 Surveillance Team

EPI_ISL_822063	Lighthouse Lab in Glasgow	Wellcome Sanger Institute for the COVID-19 Genomics UK (COG-UK) Consortium	Harper VanSteenhouse, Yumi Kasai, David Gray, Carol Clugston, Anna Dominiczak and Alex Alderton, Roberto Amato, Sonia Goncalves, Ewan Harrison, David K. Jackson, Ian Johnston, Dominic Kwiatkowski, Cordelia Langford, John Sillitoe on behalf of the Wellcome Sanger Institute COVID-19 Surveillance Team
EPI_ISL_822065	Lighthouse Lab in Alderley Park	Wellcome Sanger Institute for the COVID-19 Genomics UK (COG-UK) Consortium	Jacquelyn Wynn, Mairead Hyland, The Lighthouse Lab in Alderley Park and Alex Alderton, Roberto Amato, Sonia Goncalves, Ewan Harrison, David K. Jackson, Ian Johnston, Dominic Kwiatkowski, Cordelia Langford, John Sillitoe on behalf of the Wellcome Sanger Institute COVID-19 Surveillance Team
EPI_ISL_822067, EPI_ISL_822069, EPI_ISL_822072, EPI_ISL_822075, EPI_ISL_822078	Lighthouse Lab in Glasgow	Wellcome Sanger Institute for the COVID-19 Genomics UK (COG-UK) Consortium	Harper VanSteenhouse, Yumi Kasai, David Gray, Carol Clugston, Anna Dominiczak and Alex Alderton, Roberto Amato, Sonia Goncalves, Ewan Harrison, David K. Jackson, Ian Johnston, Dominic Kwiatkowski, Cordelia Langford, John Sillitoe on behalf of the Wellcome Sanger Institute COVID-19 Surveillance Team
EPI_ISL_822080, EPI_ISL_822081	Lighthouse Lab in Alderley Park	Wellcome Sanger Institute for the COVID-19 Genomics UK (COG-UK) Consortium	Jacquelyn Wynn, Mairead Hyland, The Lighthouse Lab in Alderley Park and Alex Alderton, Roberto Amato, Sonia Goncalves, Ewan Harrison, David K. Jackson, Ian Johnston, Dominic Kwiatkowski, Cordelia Langford, John Sillitoe on behalf of the Wellcome Sanger Institute COVID-19 Surveillance Team
EPI_ISL_822083	Lighthouse Lab in Glasgow	Wellcome Sanger Institute for the COVID-19 Genomics UK (COG-UK) Consortium	Harper VanSteenhouse, Yumi Kasai, David Gray, Carol Clugston, Anna Dominiczak and Alex Alderton, Roberto Amato, Sonia Goncalves, Ewan Harrison, David K. Jackson, Ian Johnston, Dominic Kwiatkowski, Cordelia Langford, John Sillitoe on behalf of the Wellcome Sanger Institute COVID-19 Surveillance Team
EPI_ISL_822086, EPI_ISL_822094, EPI_ISL_822097	Lighthouse Lab in Alderley Park	Wellcome Sanger Institute for the COVID-19 Genomics UK (COG-UK) Consortium	Jacquelyn Wynn, Mairead Hyland, The Lighthouse Lab in Alderley Park and Alex Alderton, Roberto Amato, Sonia Goncalves, Ewan Harrison, David K. Jackson, Ian Johnston, Dominic Kwiatkowski, Cordelia Langford, John Sillitoe on behalf of the Wellcome Sanger Institute COVID-19 Surveillance Team
EPI_ISL_822098, EPI_ISL_822099	Lighthouse Lab in Glasgow	Wellcome Sanger Institute for the COVID-19 Genomics UK (COG-UK) Consortium	Harper VanSteenhouse, Yumi Kasai, David Gray, Carol Clugston, Anna Dominiczak and Alex Alderton, Roberto Amato, Sonia Goncalves, Ewan Harrison, David K. Jackson, Ian Johnston, Dominic Kwiatkowski, Cordelia Langford, John Sillitoe on behalf of the Wellcome Sanger Institute COVID-19 Surveillance Team
EPI_ISL_822104, EPI_ISL_822105	Lighthouse Lab in Alderley Park	Wellcome Sanger Institute for the COVID-19 Genomics UK (COG-UK) Consortium	Jacquelyn Wynn, Mairead Hyland, The Lighthouse Lab in Alderley Park and Alex Alderton, Roberto Amato, Sonia Goncalves, Ewan Harrison, David K. Jackson, Ian Johnston, Dominic Kwiatkowski, Cordelia Langford, John Sillitoe on behalf of the Wellcome Sanger Institute COVID-19 Surveillance Team
EPI_ISL_822113, EPI_ISL_822117, EPI_ISL_822119, EPI_ISL_822122, EPI_ISL_822127, EPI_ISL_822130	Lighthouse Lab in Glasgow	Wellcome Sanger Institute for the COVID-19 Genomics UK (COG-UK) Consortium	Harper VanSteenhouse, Yumi Kasai, David Gray, Carol Clugston, Anna Dominiczak and Alex Alderton, Roberto Amato, Sonia Goncalves, Ewan Harrison, David K. Jackson, Ian Johnston, Dominic Kwiatkowski, Cordelia Langford, John Sillitoe on behalf of the Wellcome Sanger Institute COVID-19 Surveillance Team
EPI_ISL_822135, EPI_ISL_822139	Lighthouse Lab in Alderley Park	Wellcome Sanger Institute for the COVID-19 Genomics UK (COG-UK) Consortium	Jacquelyn Wynn, Mairead Hyland, The Lighthouse Lab in Alderley Park and Alex Alderton, Roberto Amato, Sonia Goncalves, Ewan Harrison, David K. Jackson, Ian Johnston, Dominic Kwiatkowski, Cordelia Langford, John Sillitoe on behalf of the Wellcome Sanger Institute COVID-19 Surveillance Team
EPI_ISL_822140	Lighthouse Lab in Glasgow	Wellcome Sanger Institute for the COVID-19 Genomics UK (COG-UK) Consortium	Harper VanSteenhouse, Yumi Kasai, David Gray, Carol Clugston, Anna Dominiczak and Alex Alderton, Roberto Amato, Sonia Goncalves, Ewan Harrison, David K. Jackson, Ian Johnston, Dominic Kwiatkowski, Cordelia Langford, John Sillitoe on behalf of the Wellcome Sanger Institute COVID-19 Surveillance Team
EPI_ISL_822141, EPI_ISL_822142	Lighthouse Lab in Alderley Park	Wellcome Sanger Institute for the COVID-19 Genomics UK (COG-UK) Consortium	Jacquelyn Wynn, Mairead Hyland, The Lighthouse Lab in Alderley Park and Alex Alderton, Roberto Amato, Sonia Goncalves, Ewan Harrison, David K. Jackson, Ian Johnston, Dominic Kwiatkowski, Cordelia Langford, John Sillitoe on behalf of the Wellcome Sanger Institute COVID-19 Surveillance Team
EPI_ISL_822148	Lighthouse Lab in Glasgow	Wellcome Sanger Institute for the COVID-19 Genomics UK (COG-UK) Consortium	Harper VanSteenhouse, Yumi Kasai, David Gray, Carol Clugston, Anna Dominiczak and Alex Alderton, Roberto Amato, Sonia Goncalves, Ewan Harrison, David K. Jackson, Ian Johnston, Dominic Kwiatkowski, Cordelia Langford, John Sillitoe on behalf of the Wellcome Sanger Institute COVID-19 Surveillance Team
EPI_ISL_822150	Lighthouse Lab in Alderley Park	Wellcome Sanger Institute for the COVID-19 Genomics UK (COG-UK) Consortium	Jacquelyn Wynn, Mairead Hyland, The Lighthouse Lab in Alderley Park and Alex Alderton, Roberto Amato, Sonia Goncalves, Ewan Harrison, David K. Jackson, Ian Johnston, Dominic Kwiatkowski, Cordelia Langford, John Sillitoe on behalf of the Wellcome Sanger Institute COVID-19 Surveillance Team
EPI_ISL_822153, EPI_ISL_822156, EPI_ISL_822158, EPI_ISL_822159, EPI_ISL_822165, EPI_ISL_822169, EPI_ISL_822170	Lighthouse Lab in Glasgow	Wellcome Sanger Institute for the COVID-19 Genomics UK (COG-UK) Consortium	Harper VanSteenhouse, Yumi Kasai, David Gray, Carol Clugston, Anna Dominiczak and Alex Alderton, Roberto Amato, Sonia Goncalves, Ewan Harrison, David K. Jackson, Ian Johnston, Dominic Kwiatkowski, Cordelia Langford, John Sillitoe on behalf of the Wellcome Sanger Institute COVID-19 Surveillance Team
EPI_ISL_822173	Lighthouse Lab in Alderley Park	Wellcome Sanger Institute for the COVID-19 Genomics UK (COG-UK) Consortium	Jacquelyn Wynn, Mairead Hyland, The Lighthouse Lab in Alderley Park and Alex Alderton, Roberto Amato, Sonia Goncalves, Ewan Harrison, David K. Jackson, Ian Johnston, Dominic Kwiatkowski, Cordelia Langford, John Sillitoe on behalf of the Wellcome Sanger Institute COVID-19 Surveillance Team
EPI_ISL_822178, EPI_ISL_822182	Lighthouse Lab in Glasgow	Wellcome Sanger Institute for the COVID-19 Genomics UK (COG-UK) Consortium	Harper VanSteenhouse, Yumi Kasai, David Gray, Carol Clugston, Anna Dominiczak and Alex Alderton, Roberto Amato, Sonia Goncalves, Ewan Harrison, David K. Jackson, Ian Johnston, Dominic Kwiatkowski, Cordelia Langford, John Sillitoe on behalf of the Wellcome Sanger Institute COVID-19 Surveillance Team
EPI_ISL_822183, EPI_ISL_822184, EPI_ISL_822191, EPI_ISL_822193, EPI_ISL_822197, EPI_ISL_822199	Lighthouse Lab in Alderley Park	Wellcome Sanger Institute for the COVID-19 Genomics UK (COG-UK) Consortium	Jacquelyn Wynn, Mairead Hyland, The Lighthouse Lab in Alderley Park and Alex Alderton, Roberto Amato, Sonia Goncalves, Ewan Harrison, David K. Jackson, Ian Johnston, Dominic Kwiatkowski, Cordelia Langford, John Sillitoe on behalf of the Wellcome Sanger Institute COVID-19 Surveillance Team
EPI_ISL_822201	Lighthouse Lab in Glasgow	Wellcome Sanger Institute for the COVID-19 Genomics UK (COG-UK) Consortium	Harper VanSteenhouse, Yumi Kasai, David Gray, Carol Clugston, Anna Dominiczak and Alex Alderton, Roberto Amato, Sonia Goncalves, Ewan Harrison, David K. Jackson, Ian Johnston, Dominic Kwiatkowski, Cordelia Langford, John Sillitoe on behalf of the Wellcome Sanger Institute COVID-19 Surveillance Team
EPI_ISL_822202, EPI_ISL_822209	Lighthouse Lab in Alderley Park	Wellcome Sanger Institute for the COVID-19 Genomics UK (COG-UK) Consortium	Jacquelyn Wynn, Mairead Hyland, The Lighthouse Lab in Alderley Park and Alex Alderton, Roberto Amato, Sonia Goncalves, Ewan Harrison, David K. Jackson, Ian Johnston, Dominic Kwiatkowski, Cordelia Langford, John Sillitoe on behalf of the Wellcome Sanger Institute COVID-19 Surveillance Team
EPI_ISL_822214, EPI_ISL_822223	Lighthouse Lab in Glasgow	Wellcome Sanger Institute for the COVID-19 Genomics UK (COG-UK) Consortium	Harper VanSteenhouse, Yumi Kasai, David Gray, Carol Clugston, Anna Dominiczak and Alex Alderton, Roberto Amato, Sonia Goncalves, Ewan Harrison, David K. Jackson, Ian Johnston, Dominic Kwiatkowski, Cordelia Langford, John Sillitoe on behalf of the Wellcome Sanger Institute COVID-19 Surveillance Team
EPI_ISL_822224	Lighthouse Lab in Alderley Park	Wellcome Sanger Institute for the COVID-19 Genomics UK (COG-UK) Consortium	Jacquelyn Wynn, Mairead Hyland, The Lighthouse Lab in Alderley Park and Alex Alderton, Roberto Amato, Sonia Goncalves, Ewan Harrison, David K. Jackson, Ian Johnston, Dominic Kwiatkowski, Cordelia Langford, John Sillitoe on behalf of the Wellcome Sanger Institute COVID-19 Surveillance Team
EPI_ISL_822230, EPI_ISL_822237, EPI_ISL_822240, EPI_ISL_822242, EPI_ISL_822243, EPI_ISL_822244, EPI_ISL_822245, EPI_ISL_822247	Lighthouse Lab in Glasgow	Wellcome Sanger Institute for the COVID-19 Genomics UK (COG-UK) Consortium	Harper VanSteenhouse, Yumi Kasai, David Gray, Carol Clugston, Anna Dominiczak and Alex Alderton, Roberto Amato, Sonia Goncalves, Ewan Harrison, David K. Jackson, Ian Johnston, Dominic Kwiatkowski, Cordelia Langford, John Sillitoe on behalf of the Wellcome Sanger Institute COVID-19 Surveillance Team
EPI_ISL_822248	Lighthouse Lab in Alderley Park	Wellcome Sanger Institute for the COVID-19 Genomics UK (COG-UK) Consortium	Jacquelyn Wynn, Mairead Hyland, The Lighthouse Lab in Alderley Park and Alex Alderton, Roberto Amato, Sonia Goncalves, Ewan Harrison, David K. Jackson, Ian Johnston, Dominic Kwiatkowski, Cordelia Langford, John Sillitoe on behalf of the Wellcome Sanger Institute COVID-19 Surveillance Team
EPI_ISL_822249	Lighthouse Lab in Glasgow	Wellcome Sanger Institute for the COVID-19 Genomics UK (COG-UK) Consortium	Harper VanSteenhouse, Yumi Kasai, David Gray, Carol Clugston, Anna Dominiczak and Alex Alderton, Roberto Amato, Sonia Goncalves, Ewan Harrison, David K. Jackson, Ian Johnston, Dominic Kwiatkowski, Cordelia Langford, John Sillitoe on behalf of the Wellcome Sanger Institute COVID-19 Surveillance Team
EPI_ISL_822250	Lighthouse Lab in Alderley Park	Wellcome Sanger Institute for the COVID-19 Genomics UK (COG-UK) Consortium	Jacquelyn Wynn, Mairead Hyland, The Lighthouse Lab in Alderley Park and Alex Alderton, Roberto Amato, Sonia Goncalves, Ewan Harrison, David K. Jackson, Ian Johnston, Dominic Kwiatkowski, Cordelia Langford, John Sillitoe on behalf of the Wellcome Sanger Institute COVID-19 Surveillance Team
EPI_ISL_822253, EPI_ISL_822256, EPI_ISL_822260, EPI_ISL_822264, EPI_ISL_822265, EPI_ISL_822267, EPI_ISL_822268, EPI_ISL_822272, EPI_ISL_822275, EPI_ISL_822281, EPI_ISL_822282			
see above	Lighthouse Lab in Glasgow	Wellcome Sanger Institute for the COVID-19 Genomics UK (COG-UK) Consortium	Harper VanSteenhouse, Yumi Kasai, David Gray, Carol Clugston, Anna Dominiczak and Alex Alderton, Roberto Amato, Sonia Goncalves, Ewan Harrison, David K. Jackson, Ian Johnston, Dominic Kwiatkowski, Cordelia Langford, John Sillitoe on behalf of the Wellcome Sanger Institute COVID-19 Surveillance Team
EPI_ISL_822283	Lighthouse Lab in Alderley Park	Wellcome Sanger Institute for the COVID-19 Genomics UK	Jacquelyn Wynn, Mairead Hyland, The Lighthouse Lab in Alderley Park and Alex Alderton, Roberto Amato, Sonia Goncalves, Ewan Harrison, David K.

		(COG-UK) Consortium	Jackson, Ian Johnston, Dominic Kwiatkowski, Cordelia Langford, John Sillitoe on behalf of the Wellcome Sanger Institute COVID-19 Surveillance Team
EPI_ISL_822288	Lighthouse Lab in Glasgow	Wellcome Sanger Institute for the COVID-19 Genomics UK (COG-UK) Consortium	Harper VanSteenhouse, Yumi Kasai, David Gray, Carol Clugston, Anna Dominiczak and Alex Alderton, Roberto Amato, Sonia Goncalves, Ewan Harrison, David K. Jackson, Ian Johnston, Dominic Kwiatkowski, Cordelia Langford, John Sillitoe on behalf of the Wellcome Sanger Institute COVID-19 Surveillance Team
EPI_ISL_822806, EPI_ISL_822807, EPI_ISL_822808, EPI_ISL_822809, EPI_ISL_822810, EPI_ISL_822811, EPI_ISL_822812, EPI_ISL_822813, EPI_ISL_822814, EPI_ISL_822815, EPI_ISL_822816, EPI_ISL_822817, EPI_ISL_822818, EPI_ISL_822819, EPI_ISL_822820, EPI_ISL_822821, EPI_ISL_822822, EPI_ISL_822823, EPI_ISL_822824, EPI_ISL_822825, EPI_ISL_822826, EPI_ISL_822827, EPI_ISL_822828, EPI_ISL_822829, EPI_ISL_822830, EPI_ISL_822831, EPI_ISL_822832, EPI_ISL_822833, EPI_ISL_822834, EPI_ISL_822835, EPI_ISL_822836			
see above	Wales Specialist Virology Centre Sequencing lab: Pathogen Genomics Unit	COVID-19 Genomics UK (COG-UK) Consortium	Catherine Moore, Johnathan Evans, Laura Gifford, Malorie Perry, Simon Cottrell, Angela Marchbank, Alec Birchley, Alexander Adams, Amy Gaskin, Bree Gatica-Wilcox, Jason Coombes, Joel Southgate, Lauren Gilbert, Lee Graham, Nicole Pacchiarini, Sara Kumziene-Summerhayes, Sarah Taylor, Sophie Jones, Sara Rey, Matthew Bull, Joanne Watkins, Sally Corden, Tom Connor
EPI_ISL_824169, EPI_ISL_824209, EPI_ISL_824264, EPI_ISL_824267, EPI_ISL_824268, EPI_ISL_824270	Dutch COVID-19 response team	National Institute for Public Health and the Environment (RIVM)	Adam Meijer, Harry Vennema, Jeroen Cremer, Sharon van den Brink, Bas van der Veer, AnneMarie van den Brandt, Florian Zwagemaker, Dennis Schmitz, Chantal Reusken, on behalf of the national COVID-19 response team
EPI_ISL_824554	New Mexico Department of Health Scientific Laboratory	New Mexico Department of Health Scientific Laboratory	Ellie Johnson, Anastacia Griego-Fisher, D'eldra Malone
EPI_ISL_824832, EPI_ISL_824835, EPI_ISL_824836, EPI_ISL_824837, EPI_ISL_824838, EPI_ISL_824839, EPI_ISL_824840, EPI_ISL_824841	Department of Clinical Microbiology	GIGA Medical Genomics	Keith Durkin, Maria Artesi, Sébastien Bontems, Raphaël Boreux, Bouchra Boujemla, Cécile Meex, Pierrette Melin, Marie-Pierre Hayette, Vincent Bours
EPI_ISL_824943	Charité Universitätsmedizin Berlin, Institute of Virology, Charitéplatz 1, 10117 Berlin, Germany	Charité Universitätsmedizin Berlin, Institute of Virology, Charitéplatz 1, 10117 Berlin, Germany	Victor M Corman, Jörn Beheim-Schwarzbach, Tobias Bleicker, Julia Tesch, Barbara Mühlemann, Talitha Veith, Julia Schneider, Terry Jones, Christian Drosten
EPI_ISL_826466	Lighthouse Lab in Milton Keynes	Wellcome Sanger Institute for the COVID-19 Genomics UK (COG-UK) Consortium	The Lighthouse Lab in Milton Keynes and Alex Alderton, Roberto Amato, Sonia Goncalves, Ewan Harrison, David K. Jackson, Ian Johnston, Dominic Kwiatkowski, Cordelia Langford, John Sillitoe on behalf of the Wellcome Sanger Institute COVID-19 Surveillance Team
EPI_ISL_826467, EPI_ISL_826468, EPI_ISL_826469, EPI_ISL_826470, EPI_ISL_826471	Lighthouse Lab in Alderley Park	Wellcome Sanger Institute for the COVID-19 Genomics UK (COG-UK) Consortium	Jacquelyn Wynn, Mairead Hyland, The Lighthouse Lab in Alderley Park and Alex Alderton, Roberto Amato, Sonia Goncalves, Ewan Harrison, David K. Jackson, Ian Johnston, Dominic Kwiatkowski, Cordelia Langford, John Sillitoe on behalf of the Wellcome Sanger Institute COVID-19 Surveillance Team
EPI_ISL_826473	Lighthouse Lab in Cambridge	Wellcome Sanger Institute for the COVID-19 Genomics UK (COG-UK) Consortium	Rob Howes, The Lighthouse Lab in Cambridge and Alex Alderton, Roberto Amato, Sonia Goncalves, Ewan Harrison, David K. Jackson, Ian Johnston, Dominic Kwiatkowski, Cordelia Langford, John Sillitoe on behalf of the Wellcome Sanger Institute COVID-19 Surveillance Team
EPI_ISL_826474	Lighthouse Lab in Alderley Park	Wellcome Sanger Institute for the COVID-19 Genomics UK (COG-UK) Consortium	Jacquelyn Wynn, Mairead Hyland, The Lighthouse Lab in Alderley Park and Alex Alderton, Roberto Amato, Sonia Goncalves, Ewan Harrison, David K. Jackson, Ian Johnston, Dominic Kwiatkowski, Cordelia Langford, John Sillitoe on behalf of the Wellcome Sanger Institute COVID-19 Surveillance Team
EPI_ISL_826477	Lighthouse Lab in Glasgow	Wellcome Sanger Institute for the COVID-19 Genomics UK (COG-UK) Consortium	Harper VanSteenhouse, Yumi Kasai, David Gray, Carol Clugston, Anna Dominiczak and Alex Alderton, Roberto Amato, Sonia Goncalves, Ewan Harrison, David K. Jackson, Ian Johnston, Dominic Kwiatkowski, Cordelia Langford, John Sillitoe on behalf of the Wellcome Sanger Institute COVID-19 Surveillance Team
EPI_ISL_826478	Lighthouse Lab in Alderley Park	Wellcome Sanger Institute for the COVID-19 Genomics UK (COG-UK) Consortium	Jacquelyn Wynn, Mairead Hyland, The Lighthouse Lab in Alderley Park and Alex Alderton, Roberto Amato, Sonia Goncalves, Ewan Harrison, David K. Jackson, Ian Johnston, Dominic Kwiatkowski, Cordelia Langford, John Sillitoe on behalf of the Wellcome Sanger Institute COVID-19 Surveillance Team
EPI_ISL_826479	Lighthouse Lab in Glasgow	Wellcome Sanger Institute for the COVID-19 Genomics UK (COG-UK) Consortium	Harper VanSteenhouse, Yumi Kasai, David Gray, Carol Clugston, Anna Dominiczak and Alex Alderton, Roberto Amato, Sonia Goncalves, Ewan Harrison, David K. Jackson, Ian Johnston, Dominic Kwiatkowski, Cordelia Langford, John Sillitoe on behalf of the Wellcome Sanger Institute COVID-19 Surveillance Team
EPI_ISL_826726, EPI_ISL_826727, EPI_ISL_826728, EPI_ISL_826729, EPI_ISL_826730, EPI_ISL_826731, EPI_ISL_826740, EPI_ISL_827038, EPI_ISL_827041, EPI_ISL_827044, EPI_ISL_827896, EPI_ISL_827897, EPI_ISL_827898			
see above	deCODE genetics	deCODE genetics	Daniel F Gudbjartsson; Agnar Helgason; Hakon Jonsson; Olafur T Magnusson; Pall Melsted; Gudmundur L Norddahl; Jona Saemundsdottir; Asgeir Sigurdsson; Patrick Sulem; Arna B Agustsdottir; Hannes Eggertsson; Berglind Eiriksdottir; Run Fridriksdottir; Elisabet E Gardarsdottir; Gudmundur Georgsson; Olafia S Gretarsdottir; Kjartan R Gudmundsson; Thora R Gunnarsdottir; Arnaldur Gylfason; Hilma Holm; Brynjar O Jensson; Aslaug Jonasdottir; Kamilla S Josefsdottir; Thordur Kristjansson; Droplaug N Magnusdottir; Solvi Rognvaldsson; Louise le Roux; Gudrun Sigmundsdottir; Gardar Sveinbjornsson; Kristin E Sveinsdottir; Maney Sveinsdottir; Emil A Thorarensen; Bjarni Thorbjornsson; Gisli Masson; Ingileif Jonsdottir; Alma Moller; Thorolfur Gudnason; Karl G Kristinsson; Unnur Thorsteinsdottir; Kari Stefansson
EPI_ISL_827899	The National University Hospital of Iceland	deCODE genetics	Daniel F Gudbjartsson; Agnar Helgason; Hakon Jonsson; Olafur T Magnusson; Pall Melsted; Gudmundur L Norddahl; Jona Saemundsdottir; Asgeir Sigurdsson; Patrick Sulem; Arna B Agustsdottir; Hannes Eggertsson; Berglind Eiriksdottir; Run Fridriksdottir; Elisabet E Gardarsdottir; Gudmundur Georgsson; Olafia S Gretarsdottir; Kjartan R Gudmundsson; Thora R Gunnarsdottir; Arnaldur Gylfason; Hilma Holm; Brynjar O Jensson; Aslaug Jonasdottir; Kamilla S Josefsdottir; Thordur Kristjansson; Droplaug N Magnusdottir; Solvi Rognvaldsson; Louise le Roux; Gudrun Sigmundsdottir; Gardar Sveinbjornsson; Kristin E Sveinsdottir; Maney Sveinsdottir; Emil A Thorarensen; Bjarni Thorbjornsson; Gisli Masson; Ingileif Jonsdottir; Alma Moller; Thorolfur Gudnason; Karl G Kristinsson; Unnur Thorsteinsdottir; Kari Stefansson
EPI_ISL_827930, EPI_ISL_827931, EPI_ISL_827933, EPI_ISL_828523, EPI_ISL_828538, EPI_ISL_828785, EPI_ISL_828873, EPI_ISL_829062, EPI_ISL_829080, EPI_ISL_829316, EPI_ISL_829323, EPI_ISL_829325, EPI_ISL_829326, EPI_ISL_829743, EPI_ISL_830186, EPI_ISL_830318, EPI_ISL_830396			
see above	deCODE genetics	deCODE genetics	Daniel F Gudbjartsson; Agnar Helgason; Hakon Jonsson; Olafur T Magnusson; Pall Melsted; Gudmundur L Norddahl; Jona Saemundsdottir; Asgeir Sigurdsson; Patrick Sulem; Arna B Agustsdottir; Hannes Eggertsson; Berglind Eiriksdottir; Run Fridriksdottir; Elisabet E Gardarsdottir; Gudmundur Georgsson; Olafia S Gretarsdottir; Kjartan R Gudmundsson; Thora R Gunnarsdottir; Arnaldur Gylfason; Hilma Holm; Brynjar O Jensson; Aslaug Jonasdottir; Kamilla S Josefsdottir; Thordur Kristjansson; Droplaug N Magnusdottir; Solvi Rognvaldsson; Louise le Roux; Gudrun Sigmundsdottir; Gardar Sveinbjornsson; Kristin E Sveinsdottir; Maney Sveinsdottir; Emil A Thorarensen; Bjarni Thorbjornsson; Gisli Masson; Ingileif Jonsdottir; Alma Moller; Thorolfur Gudnason; Karl G Kristinsson; Unnur Thorsteinsdottir; Kari Stefansson
EPI_ISL_830628	NORTHWELL HEALTH LABORATORIES	Wadsworth Center, New York State Department of Health	Kirsten St. George, Daryl M. Lamson, Alexis Russel, Matthew Shudt, Melissa A Leisner, Jonathan Plitnick, Navjot Singh, John Kelly, Erasmus Schneider, Erica Lasek-Nesselquist
EPI_ISL_830648	Institute for Infectious Diseases, University of Bern, Switzerland	Institute for Infectious Diseases, University of Bern, Switzerland	Michel C Koch, Christian Baumann, Miguel A Terrazos Miani, Cora Sägesser, Pascal Bittel, Stephen L Leib, Peter Keller, Franziska Suter-Riniker, Alban Ramette
EPI_ISL_830669	SUNY UPSTATE MEDICAL UNIVERSITY	Wadsworth Center, New York State Department of Health	Kirsten St. George, Daryl M. Lamson, Alexis Russel, Matthew Shudt, Melissa A Leisner, Jonathan Plitnick, Navjot Singh, John Kelly, Erasmus Schneider, Erica Lasek-Nesselquist
EPI_ISL_830683, EPI_ISL_830684, EPI_ISL_830685, EPI_ISL_830686, EPI_ISL_830687, EPI_ISL_830688, EPI_ISL_830689, EPI_ISL_830690, EPI_ISL_830691, EPI_ISL_830692, EPI_ISL_830693, EPI_ISL_830694, EPI_ISL_830695, EPI_ISL_830696, EPI_ISL_830697, EPI_ISL_830698, EPI_ISL_830709, EPI_ISL_830710, EPI_ISL_830711, EPI_ISL_830712, EPI_ISL_830713, EPI_ISL_830714, EPI_ISL_830715, EPI_ISL_830716			
see above	ALBANY MEDICAL CENTER HOSPITAL CLINICAL LABORATORIES	Wadsworth Center, New York State Department of Health	Kirsten St. George, Daryl M. Lamson, Alexis Russel, Matthew Shudt, Melissa A Leisner, Jonathan Plitnick, Navjot Singh, John Kelly, Erasmus Schneider, Erica Lasek-Nesselquist
EPI_ISL_831552, EPI_ISL_831553, EPI_ISL_831554	University of Wisconsin-Madison AIDS Vaccine Research Laboratories	University of Wisconsin-Madison AIDS Vaccine Research Laboratories	Gage Moreno, Katarina Braun, et al. AIDS Vaccine Research Laboratories
EPI_ISL_832006, EPI_ISL_832007	Unilabs AB Skovde	The Public Health Agency of Sweden	Department of Microbiology, The Public Health Agency of Sweden
EPI_ISL_832090, EPI_ISL_832095	Santa Clara County Public Health Laboratory	Santa Clara County Public Health Laboratory	Santa Clara County Public Health Department
EPI_ISL_832851, EPI_ISL_832871, EPI_ISL_832872, EPI_ISL_832876, EPI_ISL_832877, EPI_ISL_832878, EPI_ISL_832879, EPI_ISL_832880, EPI_ISL_832881, EPI_ISL_832882, EPI_ISL_832883, EPI_ISL_832884, EPI_ISL_832913, EPI_ISL_832945, EPI_ISL_832946			
see above	Maine HETL	Tewhey Lab, The Jackson Laboratory	Matluk,N., Dewey,H., Iosue,F., Barter,M., Lynch,R., Munger,H. and Tewhey,R.
EPI_ISL_833208, EPI_ISL_833209,	Department of Virology and Immunology, University of Helsinki	Department of Virology, Faculty of Medicine, University of	Teemu Smura, Ravi Kant, Phuoc Truong, Hussein Alburkat, Hannimari Kallio-Kokko, Jenni Virtanen, Maija Suvanto, Fathiah Zakham, Essi Korhonen, Sari

EPI_ISL_833220, EPI_ISL_833221, EPI_ISL_833222, EPI_ISL_833223, EPI_ISL_833224, EPI_ISL_833225, EPI_ISL_833226 | and Helsinki University Hospital, Huslab Finland | Helsinki, Helsinki, Finland | Hannula, Harri Kangas, Pekka Ellonen, Olli Vapalahti

EPI_ISL_833344, EPI_ISL_833345, EPI_ISL_833346, EPI_ISL_833347, EPI_ISL_833348, EPI_ISL_833349, EPI_ISL_833350, EPI_ISL_833351, EPI_ISL_833352, EPI_ISL_833353, EPI_ISL_833354, EPI_ISL_833355

see above | Microbiology Division, South Carolina Department of Health and Environmental Control (SC DHEC) | Microbiology Division, South Carolina Department of Health and Environmental Control (SC DHEC) | Flores,H., Freeman,J.

EPI_ISL_833394, EPI_ISL_833395, EPI_ISL_833396, EPI_ISL_833397, EPI_ISL_833398, EPI_ISL_833399, EPI_ISL_833400 | St. Francis Medical Center | Los Angeles County PHL | P. Hemarajata et al.

EPI_ISL_833414 | USC Clinical Lab | Los Angeles County PHL | P. Hemarajata et al.

EPI_ISL_834213, EPI_ISL_834215, EPI_ISL_834217, EPI_ISL_834218, EPI_ISL_834220, EPI_ISL_834222, EPI_ISL_834224, EPI_ISL_834227, EPI_ISL_834230, EPI_ISL_834234, EPI_ISL_834235, EPI_ISL_834238, EPI_ISL_834239, EPI_ISL_834240, EPI_ISL_834241, EPI_ISL_834242, EPI_ISL_834243, EPI_ISL_834244, EPI_ISL_834245, EPI_ISL_834246, EPI_ISL_834247, EPI_ISL_834249, EPI_ISL_834251, EPI_ISL_834252, EPI_ISL_834253, EPI_ISL_834254, EPI_ISL_834257, EPI_ISL_834258, EPI_ISL_834259, EPI_ISL_834261, EPI_ISL_834262, EPI_ISL_834263, EPI_ISL_834265, EPI_ISL_834266, EPI_ISL_834267, EPI_ISL_834268, EPI_ISL_834269, EPI_ISL_834270, EPI_ISL_834272, EPI_ISL_834274, EPI_ISL_834277, EPI_ISL_834278, EPI_ISL_834279, EPI_ISL_834281, EPI_ISL_834284, EPI_ISL_834285, EPI_ISL_834286, EPI_ISL_834287, EPI_ISL_834289, EPI_ISL_834291, EPI_ISL_834292, EPI_ISL_834294, EPI_ISL_834295, EPI_ISL_834296, EPI_ISL_834297, EPI_ISL_834300, EPI_ISL_834301, EPI_ISL_834302, EPI_ISL_834303, EPI_ISL_834305, EPI_ISL_834306, EPI_ISL_834307, EPI_ISL_834310, EPI_ISL_834315, EPI_ISL_834316, EPI_ISL_834318, EPI_ISL_834319, EPI_ISL_834320, EPI_ISL_834322, EPI_ISL_834323, EPI_ISL_834324, EPI_ISL_834325, EPI_ISL_834326, EPI_ISL_834328, EPI_ISL_834329, EPI_ISL_834331, EPI_ISL_834332, EPI_ISL_834334, EPI_ISL_834337, EPI_ISL_834339, EPI_ISL_834340, EPI_ISL_834341, EPI_ISL_834342, EPI_ISL_834346, EPI_ISL_834347, EPI_ISL_834350, EPI_ISL_834351, EPI_ISL_834354, EPI_ISL_834356, EPI_ISL_834357, EPI_ISL_834358, EPI_ISL_834361, EPI_ISL_834362, EPI_ISL_834363, EPI_ISL_834364, EPI_ISL_834365, EPI_ISL_834366, EPI_ISL_834367, EPI_ISL_834368, EPI_ISL_834369, EPI_ISL_834370, EPI_ISL_834371, EPI_ISL_834372, EPI_ISL_834374, EPI_ISL_834379, EPI_ISL_834383, EPI_ISL_834384, EPI_ISL_834385, EPI_ISL_834386, EPI_ISL_834389, EPI_ISL_834390, EPI_ISL_834391, EPI_ISL_834393, EPI_ISL_834394, EPI_ISL_834396, EPI_ISL_834397, EPI_ISL_834400, EPI_ISL_834401, EPI_ISL_834402, EPI_ISL_834405, EPI_ISL_834406, EPI_ISL_834408, EPI_ISL_834410, EPI_ISL_834412, EPI_ISL_834413, EPI_ISL_834416, EPI_ISL_834419, EPI_ISL_834420, EPI_ISL_834422, EPI_ISL_834424, EPI_ISL_834425, EPI_ISL_834428, EPI_ISL_834429, EPI_ISL_834430, EPI_ISL_834431, EPI_ISL_834432, EPI_ISL_834433, EPI_ISL_834435, EPI_ISL_834436, EPI_ISL_834437, EPI_ISL_834439, EPI_ISL_834441, EPI_ISL_834442, EPI_ISL_834444, EPI_ISL_834446, EPI_ISL_834447, EPI_ISL_834451, EPI_ISL_834452, EPI_ISL_834453, EPI_ISL_834454, EPI_ISL_834455, EPI_ISL_834456, EPI_ISL_834457, EPI_ISL_834458, EPI_ISL_834460, EPI_ISL_834462, EPI_ISL_834464, EPI_ISL_834465, EPI_ISL_834469, EPI_ISL_834470, EPI_ISL_834473, EPI_ISL_834474, EPI_ISL_834475, EPI_ISL_834477, EPI_ISL_834478, EPI_ISL_834479, EPI_ISL_834483, EPI_ISL_834484, EPI_ISL_834487, EPI_ISL_834490, EPI_ISL_834491, EPI_ISL_834492, EPI_ISL_834495, EPI_ISL_834496, EPI_ISL_834501, EPI_ISL_834502, EPI_ISL_834504, EPI_ISL_834507, EPI_ISL_834508, EPI_ISL_834512, EPI_ISL_834513, EPI_ISL_834514, EPI_ISL_834515, EPI_ISL_834516, EPI_ISL_834518, EPI_ISL_834519, EPI_ISL_834520, EPI_ISL_834521, EPI_ISL_834522, EPI_ISL_834523, EPI_ISL_834524, EPI_ISL_834525, EPI_ISL_834526, EPI_ISL_834528, EPI_ISL_834530, EPI_ISL_834531, EPI_ISL_834532, EPI_ISL_834533, EPI_ISL_834534, EPI_ISL_834535, EPI_ISL_834537, EPI_ISL_834539, EPI_ISL_834540, EPI_ISL_834543, EPI_ISL_834546, EPI_ISL_834547, EPI_ISL_834548, EPI_ISL_834549, EPI_ISL_834551

see above | Lighthouse Lab in Alderley Park | Wellcome Sanger Institute for the COVID-19 Genomics UK (COG-UK) Consortium | Jacquelyn Wynn, Mairead Hyland, The Lighthouse Lab in Alderley Park and Alex Alderton, Roberto Amato, Sonia Goncalves, Ewan Harrison, David K. Jackson, Ian Johnston, Dominic Kwiatkowski, Cordelia Langford, John Sillitoe on behalf of the Wellcome Sanger Institute COVID-19 Surveillance Team

EPI_ISL_834562, EPI_ISL_834572, EPI_ISL_834583, EPI_ISL_834584, EPI_ISL_834640, EPI_ISL_834644, EPI_ISL_834646, EPI_ISL_834650, EPI_ISL_834729, EPI_ISL_834746, EPI_ISL_834761, EPI_ISL_834783, EPI_ISL_834791

see above | Lighthouse Lab in Glasgow | Wellcome Sanger Institute for the COVID-19 Genomics UK (COG-UK) Consortium | Harper VanSteenhouse, Yumi Kasai, David Gray, Carol Clugston, Anna Dominiczak and Alex Alderton, Roberto Amato, Sonia Goncalves, Ewan Harrison, David K. Jackson, Ian Johnston, Dominic Kwiatkowski, Cordelia Langford, John Sillitoe on behalf of the Wellcome Sanger Institute COVID-19 Surveillance Team

EPI_ISL_834798, EPI_ISL_834800, EPI_ISL_834802, EPI_ISL_834803, EPI_ISL_834805, EPI_ISL_834806, EPI_ISL_834807, EPI_ISL_834808, EPI_ISL_834809, EPI_ISL_834810, EPI_ISL_834812, EPI_ISL_834815, EPI_ISL_834817, EPI_ISL_834818, EPI_ISL_834819, EPI_ISL_834820, EPI_ISL_834821, EPI_ISL_834822, EPI_ISL_834824, EPI_ISL_834829, EPI_ISL_834831, EPI_ISL_834832, EPI_ISL_834835, EPI_ISL_834839, EPI_ISL_834840, EPI_ISL_834841, EPI_ISL_834842, EPI_ISL_834843, EPI_ISL_834845, EPI_ISL_834846, EPI_ISL_834847, EPI_ISL_834849, EPI_ISL_834850, EPI_ISL_834851, EPI_ISL_834852, EPI_ISL_834853, EPI_ISL_834854, EPI_ISL_834855, EPI_ISL_834857, EPI_ISL_834859, EPI_ISL_834860, EPI_ISL_834861, EPI_ISL_834862, EPI_ISL_834866, EPI_ISL_834867, EPI_ISL_834869, EPI_ISL_834872, EPI_ISL_834873, EPI_ISL_834875, EPI_ISL_834876, EPI_ISL_834877, EPI_ISL_834878, EPI_ISL_834879, EPI_ISL_834880, EPI_ISL_834881, EPI_ISL_834883, EPI_ISL_834885, EPI_ISL_834886, EPI_ISL_834888, EPI_ISL_834889, EPI_ISL_834891, EPI_ISL_834892, EPI_ISL_834893, EPI_ISL_834894, EPI_ISL_834896, EPI_ISL_834900, EPI_ISL_834901, EPI_ISL_834904, EPI_ISL_834906, EPI_ISL_834907, EPI_ISL_834908, EPI_ISL_834913, EPI_ISL_834914, EPI_ISL_834917, EPI_ISL_834919, EPI_ISL_834921, EPI_ISL_834922, EPI_ISL_834923, EPI_ISL_834924, EPI_ISL_834926, EPI_ISL_834927, EPI_ISL_834929, EPI_ISL_834930, EPI_ISL_834932, EPI_ISL_834934, EPI_ISL_834935, EPI_ISL_834936, EPI_ISL_834937, EPI_ISL_834938, EPI_ISL_834939, EPI_ISL_834940, EPI_ISL_834941, EPI_ISL_834944, EPI_ISL_834945, EPI_ISL_834947, EPI_ISL_834948, EPI_ISL_834950, EPI_ISL_834954, EPI_ISL_834955, EPI_ISL_834956, EPI_ISL_834957, EPI_ISL_834958, EPI_ISL_834960, EPI_ISL_834961, EPI_ISL_834962, EPI_ISL_834963, EPI_ISL_834964, EPI_ISL_834966, EPI_ISL_834967, EPI_ISL_834969, EPI_ISL_834970, EPI_ISL_834971, EPI_ISL_834973, EPI_ISL_834975, EPI_ISL_834977, EPI_ISL_834980, EPI_ISL_834982, EPI_ISL_834986, EPI_ISL_834987, EPI_ISL_834990, EPI_ISL_834992, EPI_ISL_834993, EPI_ISL_834995, EPI_ISL_834997, EPI_ISL_834998, EPI_ISL_834999, EPI_ISL_835000, EPI_ISL_835002, EPI_ISL_835006, EPI_ISL_835007, EPI_ISL_835011, EPI_ISL_835012, EPI_ISL_835014, EPI_ISL_835015, EPI_ISL_835019, EPI_ISL_835020, EPI_ISL_835021, EPI_ISL_835022, EPI_ISL_835023, EPI_ISL_835025, EPI_ISL_835027, EPI_ISL_835029, EPI_ISL_835030, EPI_ISL_835031, EPI_ISL_835032, EPI_ISL_835034, EPI_ISL_835035, EPI_ISL_835037, EPI_ISL_835039, EPI_ISL_835040, EPI_ISL_835042, EPI_ISL_835043, EPI_ISL_835044, EPI_ISL_835045, EPI_ISL_835046, EPI_ISL_835048, EPI_ISL_835049, EPI_ISL_835050, EPI_ISL_835051, EPI_ISL_835052, EPI_ISL_835054, EPI_ISL_835056, EPI_ISL_835057, EPI_ISL_835059, EPI_ISL_835061, EPI_ISL_835062, EPI_ISL_835063, EPI_ISL_835064, EPI_ISL_835070, EPI_ISL_835071, EPI_ISL_835072, EPI_ISL_835073, EPI_ISL_835074, EPI_ISL_835075, EPI_ISL_835076, EPI_ISL_835078, EPI_ISL_835079, EPI_ISL_835081, EPI_ISL_835082, EPI_ISL_835084, EPI_ISL_835086, EPI_ISL_835087, EPI_ISL_835089, EPI_ISL_835090, EPI_ISL_835091, EPI_ISL_835092, EPI_ISL_835093, EPI_ISL_835094, EPI_ISL_835095, EPI_ISL_835096, EPI_ISL_835097, EPI_ISL_835098, EPI_ISL_835099, EPI_ISL_835100, EPI_ISL_835101, EPI_ISL_835104, EPI_ISL_835105, EPI_ISL_835106, EPI_ISL_835111, EPI_ISL_835113, EPI_ISL_835116, EPI_ISL_835118, EPI_ISL_835119, EPI_ISL_835123, EPI_ISL_835124, EPI_ISL_835125, EPI_ISL_835126, EPI_ISL_835128, EPI_ISL_835129, EPI_ISL_835131, EPI_ISL_835134, EPI_ISL_835135, EPI_ISL_835136, EPI_ISL_835137, EPI_ISL_835139, EPI_ISL_835141, EPI_ISL_835142

see above | Lighthouse Lab in Alderley Park | Wellcome Sanger Institute for the COVID-19 Genomics UK (COG-UK) Consortium | Jacquelyn Wynn, Mairead Hyland, The Lighthouse Lab in Alderley Park and Alex Alderton, Roberto Amato, Sonia Goncalves, Ewan Harrison, David K. Jackson, Ian Johnston, Dominic Kwiatkowski, Cordelia Langford, John Sillitoe on behalf of the Wellcome Sanger Institute COVID-19 Surveillance Team

EPI_ISL_835145, EPI_ISL_835155, EPI_ISL_835177, EPI_ISL_835212, EPI_ISL_835235, EPI_ISL_835241, EPI_ISL_835247, EPI_ISL_835261, EPI_ISL_835282, EPI_ISL_835307, EPI_ISL_835319, EPI_ISL_835330, EPI_ISL_835333, EPI_ISL_835368, EPI_ISL_835378, EPI_ISL_835390

see above | Lighthouse Lab in Glasgow | Wellcome Sanger Institute for the COVID-19 Genomics UK (COG-UK) Consortium | Harper VanSteenhouse, Yumi Kasai, David Gray, Carol Clugston, Anna Dominiczak and Alex Alderton, Roberto Amato, Sonia Goncalves, Ewan Harrison, David K. Jackson, Ian Johnston, Dominic Kwiatkowski, Cordelia Langford, John Sillitoe on behalf of the Wellcome Sanger Institute COVID-19 Surveillance Team

EPI_ISL_835426, EPI_ISL_835427, EPI_ISL_835428, EPI_ISL_835429, EPI_ISL_835430, EPI_ISL_835431, EPI_ISL_835432, EPI_ISL_835434, EPI_ISL_835435, EPI_ISL_835436, EPI_ISL_835437, EPI_ISL_835438, EPI_ISL_835440, EPI_ISL_835441, EPI_ISL_835442, EPI_ISL_835443, EPI_ISL_835444, EPI_ISL_835445, EPI_ISL_835446, EPI_ISL_835449, EPI_ISL_835450, EPI_ISL_835451, EPI_ISL_835452, EPI_ISL_835453, EPI_ISL_835455, EPI_ISL_835456, EPI_ISL_835458, EPI_ISL_835459, EPI_ISL_835460, EPI_ISL_835461, EPI_ISL_835462, EPI_ISL_835463, EPI_ISL_835466, EPI_ISL_835467, EPI_ISL_835468, EPI_ISL_835469, EPI_ISL_835471, EPI_ISL_835472, EPI_ISL_835473, EPI_ISL_835476, EPI_ISL_835477, EPI_ISL_835478, EPI_ISL_835479, EPI_ISL_835480, EPI_ISL_835482, EPI_ISL_835484, EPI_ISL_835485, EPI_ISL_835489, EPI_ISL_835490, EPI_ISL_835491, EPI_ISL_835494, EPI_ISL_835496, EPI_ISL_835498, EPI_ISL_835501, EPI_ISL_835504, EPI_ISL_835508, EPI_ISL_835509, EPI_ISL_835510, EPI_ISL_835511, EPI_ISL_835512, EPI_ISL_835513, EPI_ISL_835514, EPI_ISL_835516, EPI_ISL_835517, EPI_ISL_835519, EPI_ISL_835520, EPI_ISL_835521, EPI_ISL_835522, EPI_ISL_835523, EPI_ISL_835524, EPI_ISL_835525, EPI_ISL_835526, EPI_ISL_835528, EPI_ISL_835529, EPI_ISL_835534, EPI_ISL_835536, EPI_ISL_835537, EPI_ISL_835538, EPI_ISL_835542, EPI_ISL_835543, EPI_ISL_835544, EPI_ISL_835545, EPI_ISL_835549, EPI_ISL_835551, EPI_ISL_835552, EPI_ISL_835554, EPI_ISL_835556, EPI_ISL_835557, EPI_ISL_835558, EPI_ISL_835559, EPI_ISL_835560, EPI_ISL_835561, EPI_ISL_835563, EPI_ISL_835565, EPI_ISL_835566, EPI_ISL_835567, EPI_ISL_835568, EPI_ISL_835569, EPI_ISL_835571, EPI_ISL_835572, EPI_ISL_835575, EPI_ISL_835578, EPI_ISL_835580, EPI_ISL_835581, EPI_ISL_835583, EPI_ISL_835584, EPI_ISL_835585, EPI_ISL_835586, EPI_ISL_835587, EPI_ISL_835588, EPI_ISL_835591, EPI_ISL_835593, EPI_ISL_835594, EPI_ISL_835595, EPI_ISL_835596, EPI_ISL_835598, EPI_ISL_835599, EPI_ISL_835601, EPI_ISL_835602, EPI_ISL_835604, EPI_ISL_835605, EPI_ISL_835606, EPI_ISL_835607, EPI_ISL_835608, EPI_ISL_835609, EPI_ISL_835611, EPI_ISL_835612, EPI_ISL_835613, EPI_ISL_835617, EPI_ISL_835619, EPI_ISL_835620, EPI_ISL_835621, EPI_ISL_835624, EPI_ISL_835625, EPI_ISL_835626, EPI_ISL_835627, EPI_ISL_835628, EPI_ISL_835629, EPI_ISL_835630, EPI_ISL_835632, EPI_ISL_835633, EPI_ISL_835634, EPI_ISL_835635, EPI_ISL_835636, EPI_ISL_835637, EPI_ISL_835638, EPI_ISL_835640, EPI_ISL_835641, EPI_ISL_835642, EPI_ISL_835644, EPI_ISL_835645, EPI_ISL_835648, EPI_ISL_835649, EPI_ISL_835650, EPI_ISL_835652, EPI_ISL_835653, EPI_ISL_835654, EPI_ISL_835656, EPI_ISL_835657, EPI_ISL_835658, EPI_ISL_835660, EPI_ISL_835661, EPI_ISL_835662, EPI_ISL_835663, EPI_ISL_835665, EPI_ISL_835666, EPI_ISL_835667, EPI_ISL_835668, EPI_ISL_835669, EPI_ISL_835670, EPI_ISL_835672, EPI_ISL_835674, EPI_ISL_835675, EPI_ISL_835676, EPI_ISL_835679, EPI_ISL_835681, EPI_ISL_835684, EPI_ISL_835685, EPI_ISL_835689, EPI_ISL_835690, EPI_ISL_835691, EPI_ISL_835693, EPI_ISL_835694, EPI_ISL_835696, EPI_ISL_835697, EPI_ISL_835699, EPI_ISL_835700, EPI_ISL_835702, EPI_ISL_835704, EPI_ISL_835705, EPI_ISL_835706, EPI_ISL_835708, EPI_ISL_835709

see above | Lighthouse Lab in Alderley Park | Wellcome Sanger Institute for the COVID-19 Genomics UK (COG-UK) Consortium | Jacquelyn Wynn, Mairead Hyland, The Lighthouse Lab in Alderley Park and Alex Alderton, Roberto Amato, Sonia Goncalves, Ewan Harrison, David K. Jackson, Ian Johnston, Dominic Kwiatkowski, Cordelia Langford, John Sillitoe on behalf of the Wellcome Sanger Institute COVID-19 Surveillance Team

EPI_ISL_835710, EPI_ISL_835711, EPI_ISL_835712, EPI_ISL_835713, EPI_ISL_835714, EPI_ISL_835715, EPI_ISL_835716, EPI_ISL_835717, EPI_ISL_835718, EPI_ISL_835719, EPI_ISL_835720, EPI_ISL_835721, EPI_ISL_835722, EPI_ISL_835723, EPI_ISL_835724, EPI_ISL_835725, EPI_ISL_835726, EPI_ISL_835727, EPI_ISL_835728, EPI_ISL_835729, EPI_ISL_835730, EPI_ISL_835731, EPI_ISL_835732, EPI_ISL_835733, EPI_ISL_835734, EPI_ISL_835735, EPI_ISL_835736, EPI_ISL_835737, EPI_ISL_835738, EPI_ISL_835739, EPI_ISL_835740, EPI_ISL_835741, EPI_ISL_835742, EPI_ISL_835743, EPI_ISL_835744, EPI_ISL_835745, EPI_ISL_835746, EPI_ISL_835747, EPI_ISL_835748, EPI_ISL_835749, EPI_ISL_835750, EPI_ISL_835751, EPI_ISL_835752, EPI_ISL_835753, EPI_ISL_835754, EPI_ISL_835755, EPI_ISL_835756, EPI_ISL_835757, EPI_ISL_835758, EPI_ISL_835759, EPI_ISL_835760, EPI_ISL_835761, EPI_ISL_835762, EPI_ISL_835763, EPI_ISL_835764, EPI_ISL_835765, EPI_ISL_835766, EPI_ISL_835767, EPI_ISL_835768, EPI_ISL_835769, EPI_ISL_835770, EPI_ISL_835771, EPI_ISL_835772, EPI_ISL_835773, EPI_ISL_835774, EPI_ISL_835775, EPI_ISL_835776, EPI_ISL_835777, EPI_ISL_835778, EPI_ISL_835779, EPI_ISL_835780, EPI_ISL_835781, EPI_ISL_835782, EPI_ISL_835783, EPI_ISL_835784, EPI_ISL_835785, EPI_ISL_835786, EPI_ISL_835787, EPI_ISL_835788, EPI_ISL_835789, EPI_ISL_835790, EPI_ISL_835791, EPI_ISL_835792, EPI_ISL_835793, EPI_ISL_835794, EPI_ISL_835795, EPI_ISL_835796, EPI_ISL_835797, EPI_ISL_835798, EPI_ISL_835799, EPI_ISL_835800, EPI_ISL_835801, EPI_ISL_835802, EPI_ISL_835803, EPI_ISL_835804, EPI_ISL_835805, EPI_ISL_835806, EPI_ISL_835807, EPI_ISL_835808, EPI_ISL_835809, EPI_ISL_835810, EPI_ISL_835811, EPI_ISL_835812, EPI_ISL_835813, EPI_ISL_835814, EPI_ISL_835815, EPI_ISL_835816, EPI_ISL_835817, EPI_ISL_835818, EPI_ISL_835819, EPI_ISL_835820, EPI_ISL_835821, EPI_ISL_835822, EPI_ISL_835823, EPI_ISL_835824, EPI_ISL_835825, EPI_ISL_835826, EPI_ISL_835827, EPI_ISL_835828, EPI_ISL_835829, EPI_ISL_835830, EPI_ISL_835831, EPI_ISL_835832, EPI_ISL_835833, EPI_ISL_835834, EPI_ISL_835835, EPI_ISL_835836, EPI_ISL_835837, EPI_ISL_835838, EPI_ISL_835839, EPI_ISL_835840, EPI_ISL_835841, EPI_ISL_835842, EPI_ISL_835843, EPI_ISL_835844, EPI_ISL_835845, EPI_ISL_835846, EPI_ISL_835847, EPI_ISL_835848, EPI_ISL_835849, EPI_ISL_835850, EPI_ISL_835851, EPI_ISL_835852, EPI_ISL_835853, EPI_ISL_835854, EPI_ISL_835855, EPI_ISL_835856, EPI_ISL_835857, EPI_ISL_835858, EPI_ISL_835859, EPI_ISL_835860, EPI_ISL_835861, EPI_ISL_835862, EPI_ISL_835863, EPI_ISL_835864, EPI_ISL_835865, EPI_ISL_835866, EPI_ISL_835867, EPI_ISL_835868, EPI_ISL_835869, EPI_ISL_835870, EPI_ISL_835871, EPI_ISL_835872, EPI_ISL_835873, EPI_ISL_835874, EPI_ISL_835875, EPI_ISL_835876, EPI_ISL_835877, EPI_ISL_835878, EPI_ISL_835879, EPI_ISL_835880, EPI_ISL_835881, EPI_ISL_835882, EPI_ISL_835883, EPI_ISL_835884, EPI_ISL_835885, EPI_ISL_835886, EPI_ISL_835887, EPI_ISL_835888, EPI_ISL_835889, EPI_ISL_835890, EPI_ISL_835891, EPI_ISL_835892, EPI_ISL_835893, EPI_ISL_835894, EPI_ISL_835895, EPI_ISL_835896, EPI_ISL_835897, EPI_ISL_835898, EPI_ISL_835899, EPI_ISL_835900, EPI_ISL_835901, EPI_ISL_835902, EPI_ISL_835903, EPI_ISL_835904, EPI_ISL_835905, EPI_ISL_835906, EPI_ISL_835907, EPI_ISL_835908, EPI_ISL_835909, EPI_ISL_835910, EPI_ISL_835911, EPI_ISL_835912, EPI_ISL_835913, EPI_ISL_835914, EPI_ISL_835915, EPI_ISL_835916, EPI_ISL_835917, EPI_ISL_835918, EPI_ISL_835919, EPI_ISL_835920, EPI_ISL_835921, EPI_ISL_835922, EPI_ISL_835923, EPI_ISL_835924, EPI_ISL_835925, EPI_ISL_835926, EPI_ISL_835927, EPI_ISL_835928, EPI_ISL_835929, EPI_ISL_835930, EPI_ISL_835931, EPI_ISL_835932, EPI_ISL_835933, EPI_ISL_835934, EPI_ISL_835935, EPI_ISL_835936, EPI_ISL_835937, EPI_ISL_835938, EPI_ISL_835939, EPI_ISL_835940, EPI_ISL_835941, EPI_ISL_835942, EPI_ISL_835943, EPI_ISL_835944, EPI_ISL_835945, EPI_ISL_835946, EPI_ISL_835947, EPI_ISL_835948, EPI_ISL_835949, EPI_ISL_835950, EPI_ISL_835951, EPI_ISL_835952, EPI_ISL_835953, EPI_ISL_835954, EPI_ISL_835955, EPI_ISL_835956, EPI_ISL_835957, EPI_ISL_835958, EPI_ISL_835959, EPI_ISL_835960, EPI_ISL_835961, EPI_ISL_835962, EPI_ISL_835963, EPI_ISL_835964, EPI_ISL_835965, EPI_ISL_835966, EPI_ISL_835967, EPI_ISL_835968, EPI_ISL_835969, EPI_ISL_835970, EPI_ISL_835971, EPI_ISL_835972, EPI_ISL_835973, EPI_ISL_835974, EPI_ISL_835975, EPI_ISL_835976, EPI_ISL_835977, EPI_ISL_835978, EPI_ISL_835979, EPI_ISL_835980, EPI_ISL_835981, EPI_ISL_835982, EPI_ISL_835983, EPI_ISL_835984, EPI_ISL_835985, EPI_ISL_835986, EPI_ISL_835987, EPI_ISL_835988, EPI_ISL_835989, EPI_ISL_835990, EPI_ISL_835991, EPI_ISL_835992, EPI_ISL_835993, EPI_ISL_835994, EPI_ISL_835995, EPI_ISL_835996, EPI_ISL_835997, EPI_ISL_835998, EPI_ISL_835999, EPI_ISL_836000, EPI_ISL_836001, EPI_ISL_836002, EPI_ISL_836003, EPI_ISL_836004, EPI_ISL_836005, EPI_ISL_836006, EPI_ISL_836007, EPI_ISL_836008, EPI_ISL_836009, EPI_ISL_836010, EPI_ISL_836011, EPI_ISL_836012, EPI_ISL_836013, EPI_ISL_836014, EPI_ISL_836015, EPI_ISL_836016, EPI_ISL_836017, EPI_ISL_836018, EPI_ISL_836019, EPI_ISL_836020, EPI_ISL_836021, EPI_ISL_836022, EPI_ISL_836023, EPI_ISL_836024, EPI_ISL_836025, EPI_ISL_836026, EPI_ISL_836027, EPI_ISL_836028, EPI_ISL_836029, EPI_ISL_836030, EPI_ISL_836031, EPI_ISL_836032, EPI_ISL_836033,

EPI_ISL_836034, EPI_ISL_836035, EPI_ISL_836036, EPI_ISL_836037, EPI_ISL_836038, EPI_ISL_836039, EPI_ISL_836040, EPI_ISL_836041, EPI_ISL_836042, EPI_ISL_836043, EPI_ISL_836044, EPI_ISL_836045, EPI_ISL_836046, EPI_ISL_836047, EPI_ISL_836048, EPI_ISL_836049, EPI_ISL_836050, EPI_ISL_836051, EPI_ISL_836052, EPI_ISL_836053, EPI_ISL_836054, EPI_ISL_836055, EPI_ISL_836056, EPI_ISL_836057, EPI_ISL_836058, EPI_ISL_836059, EPI_ISL_836060, EPI_ISL_836061, EPI_ISL_836062, EPI_ISL_836063, EPI_ISL_836064, EPI_ISL_836065, EPI_ISL_836066, EPI_ISL_836067, EPI_ISL_836068, EPI_ISL_836069, EPI_ISL_836070, EPI_ISL_836071, EPI_ISL_836072, EPI_ISL_836073, EPI_ISL_836074, EPI_ISL_836076, EPI_ISL_836080, EPI_ISL_836081, EPI_ISL_836082, EPI_ISL_836085, EPI_ISL_836086, EPI_ISL_836087, EPI_ISL_836090, EPI_ISL_836091, EPI_ISL_836092, EPI_ISL_836093, EPI_ISL_836094, EPI_ISL_836096, EPI_ISL_836097, EPI_ISL_836098, EPI_ISL_836099, EPI_ISL_836100, EPI_ISL_836102, EPI_ISL_836103, EPI_ISL_836104, EPI_ISL_836109, EPI_ISL_836110, EPI_ISL_836113, EPI_ISL_836114, EPI_ISL_836116, EPI_ISL_836117, EPI_ISL_836118, EPI_ISL_836119, EPI_ISL_836121, EPI_ISL_836122, EPI_ISL_836123, EPI_ISL_836124, EPI_ISL_836125, EPI_ISL_836126, EPI_ISL_836127, EPI_ISL_836130, EPI_ISL_836133, EPI_ISL_836134, EPI_ISL_836135, EPI_ISL_836136, EPI_ISL_836139, EPI_ISL_836140, EPI_ISL_836141, EPI_ISL_836145, EPI_ISL_836146, EPI_ISL_836148, EPI_ISL_836150, EPI_ISL_836151, EPI_ISL_836152, EPI_ISL_836153, EPI_ISL_836157, EPI_ISL_836158, EPI_ISL_836159, EPI_ISL_836161, EPI_ISL_836163, EPI_ISL_836164, EPI_ISL_836166, EPI_ISL_836167, EPI_ISL_836170, EPI_ISL_836171, EPI_ISL_836172, EPI_ISL_836173, EPI_ISL_836175, EPI_ISL_836176, EPI_ISL_836177, EPI_ISL_836179, EPI_ISL_836181, EPI_ISL_836182, EPI_ISL_836183, EPI_ISL_836185, EPI_ISL_836186, EPI_ISL_836187, EPI_ISL_836188, EPI_ISL_836189, EPI_ISL_836191, EPI_ISL_836193, EPI_ISL_836194, EPI_ISL_836195, EPI_ISL_836196, EPI_ISL_836199, EPI_ISL_836200, EPI_ISL_836202, EPI_ISL_836204, EPI_ISL_836205, EPI_ISL_836206, EPI_ISL_836207, EPI_ISL_836208, EPI_ISL_836209, EPI_ISL_836210, EPI_ISL_836213, EPI_ISL_836214, EPI_ISL_836215, EPI_ISL_836216, EPI_ISL_836217, EPI_ISL_836219, EPI_ISL_836220, EPI_ISL_836221, EPI_ISL_836222, EPI_ISL_836224, EPI_ISL_836226, EPI_ISL_836227, EPI_ISL_836229, EPI_ISL_836232, EPI_ISL_836235, EPI_ISL_836236, EPI_ISL_836237, EPI_ISL_836239, EPI_ISL_836240, EPI_ISL_836242, EPI_ISL_836243, EPI_ISL_836244, EPI_ISL_836245, EPI_ISL_836246, EPI_ISL_836249, EPI_ISL_836253, EPI_ISL_836254, EPI_ISL_836255, EPI_ISL_836256, EPI_ISL_836257, EPI_ISL_836258, EPI_ISL_836259, EPI_ISL_836260, EPI_ISL_836263, EPI_ISL_836264, EPI_ISL_836265, EPI_ISL_836267, EPI_ISL_836269, EPI_ISL_836270, EPI_ISL_836271, EPI_ISL_836272, EPI_ISL_836273, EPI_ISL_836276, EPI_ISL_836277, EPI_ISL_836278, EPI_ISL_836279, EPI_ISL_836280, EPI_ISL_836281, EPI_ISL_836283, EPI_ISL_836286, EPI_ISL_836287, EPI_ISL_836288, EPI_ISL_836289, EPI_ISL_836290, EPI_ISL_836294, EPI_ISL_836295, EPI_ISL_836296, EPI_ISL_836297, EPI_ISL_836299, EPI_ISL_836300, EPI_ISL_836301, EPI_ISL_836302, EPI_ISL_836303, EPI_ISL_836304, EPI_ISL_836309, EPI_ISL_836312, EPI_ISL_836316, EPI_ISL_836317, EPI_ISL_836322, EPI_ISL_836324, EPI_ISL_836325, EPI_ISL_836326, EPI_ISL_836327, EPI_ISL_836330, EPI_ISL_836331, EPI_ISL_836332, EPI_ISL_836334, EPI_ISL_836335, EPI_ISL_836337, EPI_ISL_836339, EPI_ISL_836340, EPI_ISL_836341, EPI_ISL_836344, EPI_ISL_836345, EPI_ISL_836346, EPI_ISL_836347, EPI_ISL_836348, EPI_ISL_836349, EPI_ISL_836350, EPI_ISL_836353, EPI_ISL_836354, EPI_ISL_836355, EPI_ISL_836359, EPI_ISL_836360, EPI_ISL_836361, EPI_ISL_836364, EPI_ISL_836365, EPI_ISL_836368, EPI_ISL_836369, EPI_ISL_836370, EPI_ISL_836372, EPI_ISL_836374, EPI_ISL_836375, EPI_ISL_836376, EPI_ISL_836379, EPI_ISL_836380, EPI_ISL_836381, EPI_ISL_836382, EPI_ISL_836383, EPI_ISL_836384, EPI_ISL_836385, EPI_ISL_836386, EPI_ISL_836388, EPI_ISL_836389, EPI_ISL_836391, EPI_ISL_836392, EPI_ISL_836393, EPI_ISL_836394, EPI_ISL_836397, EPI_ISL_836398, EPI_ISL_836399, EPI_ISL_836400, EPI_ISL_836401, EPI_ISL_836402, EPI_ISL_836404, EPI_ISL_836405, EPI_ISL_836408, EPI_ISL_836411, EPI_ISL_836412, EPI_ISL_836413, EPI_ISL_836414, EPI_ISL_836417, EPI_ISL_836419, EPI_ISL_836420			
see above	Lighthouse Lab in Milton Keynes	Wellcome Sanger Institute for the COVID-19 Genomics UK (COG-UK) Consortium	The Lighthouse Lab in Milton Keynes and Alex Alderton, Roberto Amato, Sonia Goncalves, Ewan Harrison, David K. Jackson, Ian Johnston, Dominic Kwiatkowski, Cordelia Langford, John Sillitoe on behalf of the Wellcome Sanger Institute COVID-19 Surveillance Team
EPI_ISL_836754, EPI_ISL_836765, EPI_ISL_836772, EPI_ISL_836777, EPI_ISL_836781, EPI_ISL_836788, EPI_ISL_836792, EPI_ISL_836795, EPI_ISL_836797, EPI_ISL_836803, EPI_ISL_836806, EPI_ISL_836807, EPI_ISL_836811, EPI_ISL_836813, EPI_ISL_836814, EPI_ISL_836822, EPI_ISL_836836, EPI_ISL_836837, EPI_ISL_836842, EPI_ISL_836843, EPI_ISL_836848, EPI_ISL_836851, EPI_ISL_836858, EPI_ISL_836859, EPI_ISL_836862, EPI_ISL_836863, EPI_ISL_836866, EPI_ISL_836867, EPI_ISL_836877, EPI_ISL_836883, EPI_ISL_836887, EPI_ISL_836890, EPI_ISL_836891, EPI_ISL_836897, EPI_ISL_836903			
see above	Lighthouse Lab in Alderley Park	Wellcome Sanger Institute for the COVID-19 Genomics UK (COG-UK) Consortium	Jacquelyn Wynn, Mairead Hyland, The Lighthouse Lab in Alderley Park and Alex Alderton, Roberto Amato, Sonia Goncalves, Ewan Harrison, David K. Jackson, Ian Johnston, Dominic Kwiatkowski, Cordelia Langford, John Sillitoe on behalf of the Wellcome Sanger Institute COVID-19 Surveillance Team
EPI_ISL_836907	Lighthouse Lab in Glasgow	Wellcome Sanger Institute for the COVID-19 Genomics UK (COG-UK) Consortium	Harper VanSteenhouse, Yumi Kasai, David Gray, Carol Clugston, Anna Dominiczak and Alex Alderton, Roberto Amato, Sonia Goncalves, Ewan Harrison, David K. Jackson, Ian Johnston, Dominic Kwiatkowski, Cordelia Langford, John Sillitoe on behalf of the Wellcome Sanger Institute COVID-19 Surveillance Team
EPI_ISL_836909, EPI_ISL_836910, EPI_ISL_836913, EPI_ISL_836920, EPI_ISL_836921, EPI_ISL_836922, EPI_ISL_836926, EPI_ISL_836928, EPI_ISL_836929, EPI_ISL_836936, EPI_ISL_836940, EPI_ISL_836946, EPI_ISL_836947, EPI_ISL_836948, EPI_ISL_836955, EPI_ISL_836975, EPI_ISL_836976			
see above	Lighthouse Lab in Alderley Park	Wellcome Sanger Institute for the COVID-19 Genomics UK (COG-UK) Consortium	Jacquelyn Wynn, Mairead Hyland, The Lighthouse Lab in Alderley Park and Alex Alderton, Roberto Amato, Sonia Goncalves, Ewan Harrison, David K. Jackson, Ian Johnston, Dominic Kwiatkowski, Cordelia Langford, John Sillitoe on behalf of the Wellcome Sanger Institute COVID-19 Surveillance Team
EPI_ISL_837052, EPI_ISL_837067, EPI_ISL_837077, EPI_ISL_837173, EPI_ISL_837174, EPI_ISL_837175, EPI_ISL_837239, EPI_ISL_837240	Respiratory Virus Unit, National Infection Service, Public Health England	COVID-19 Genomics UK (COG-UK) Consortium	PHE Covid Sequencing Team
EPI_ISL_837510, EPI_ISL_837526	UW Virology Lab	UW Virology Lab	Pavitra Roychoudhury, Hong Xie, Lasata Shrestha, Meei-Li Huang, Keith R Jerome, Alexander Greninger
EPI_ISL_837843, EPI_ISL_837846, EPI_ISL_837849, EPI_ISL_837852, EPI_ISL_837854, EPI_ISL_837855, EPI_ISL_837856, EPI_ISL_837857, EPI_ISL_837859, EPI_ISL_837860, EPI_ISL_837861, EPI_ISL_837862, EPI_ISL_837863, EPI_ISL_837864, EPI_ISL_837865, EPI_ISL_837869, EPI_ISL_837870, EPI_ISL_837871, EPI_ISL_837905, EPI_ISL_837908, EPI_ISL_837909, EPI_ISL_837911, EPI_ISL_837913, EPI_ISL_837914, EPI_ISL_837915, EPI_ISL_837916, EPI_ISL_837917, EPI_ISL_837918, EPI_ISL_837919, EPI_ISL_837923, EPI_ISL_837924, EPI_ISL_837925, EPI_ISL_837926, EPI_ISL_837927, EPI_ISL_837928, EPI_ISL_837930, EPI_ISL_837932, EPI_ISL_837933			
see above	Department of Pathology, University of Cambridge	COVID-19 Genomics UK (COG-UK) Consortium	Aminu S. Jahun, Yasmin Chaudhry, Grant Hall, Iliana Georgana, Myra Hosmillo, Martin D. Curran, Malte Pinckert, Surendra Parmar, Ian Goodfellow
EPI_ISL_838331, EPI_ISL_838334, EPI_ISL_838335, EPI_ISL_838336, EPI_ISL_838337, EPI_ISL_838338, EPI_ISL_838339	University of Exeter	COVID-19 Genomics UK (COG-UK) Consortium	Ben Temperton,Aaron Jeffries,Michelle Michelsen,Joanna Warwick-Dugdale,Audrey Farbos,Robyn Manley,Stephen Michell,Jane Masoli
EPI_ISL_838586, EPI_ISL_838587, EPI_ISL_838592, EPI_ISL_838593, EPI_ISL_838597, EPI_ISL_838598, EPI_ISL_838600, EPI_ISL_838602, EPI_ISL_838603, EPI_ISL_838605, EPI_ISL_838610, EPI_ISL_838612, EPI_ISL_838613, EPI_ISL_838618, EPI_ISL_838620, EPI_ISL_838622, EPI_ISL_838628, EPI_ISL_838633, EPI_ISL_838640, EPI_ISL_838641, EPI_ISL_838652, EPI_ISL_838653, EPI_ISL_838655, EPI_ISL_838667, EPI_ISL_838668, EPI_ISL_838669, EPI_ISL_838670, EPI_ISL_838671			
see above	Liverpool Clinical Laboratories	COVID-19 Genomics UK (COG-UK) Consortium	Sam Haldenby, Anita Lucaci, Steve Paterson, Julian Hiscox, Alistair Darby, M Almsaud, A Alrezaihi, Muhannad Alruwaili, Stuart D Armstrong, Jones Benjamin, Eleanor G Bentley, Anu Chawla, Jordan J Clark, Angela Cowell, Richard Eccles, Isabel García-Dorival, Matthew Gemmell, Alessandro Gerada, PKF Gilmore, Richard Gregory, Ximeng Han, Catherine Hartley, Margaret Hughes, Miren Iturriza-Gomara, James Johnson, L Luu, Jenifer Manson, Charlotte Nelson, Elaine O'Toole, Cassie Olateju, Rebekah Penrice-Randal , Lucille Rainbow, N.P Randle, Trevor Ian Robinson, Parul Sharma, Ghada T Shawli, James P Stewart, Neil Swainston, Ecaterina Vamos, Joanne Watts, Mark Whitehead
EPI_ISL_838807, EPI_ISL_838828, EPI_ISL_838829, EPI_ISL_838830, EPI_ISL_838831, EPI_ISL_838832, EPI_ISL_838833, EPI_ISL_838834, EPI_ISL_838835, EPI_ISL_838836, EPI_ISL_838837, EPI_ISL_838838, EPI_ISL_838839, EPI_ISL_838840, EPI_ISL_838841, EPI_ISL_838842, EPI_ISL_838843, EPI_ISL_838844, EPI_ISL_838846, EPI_ISL_838847, EPI_ISL_838848, EPI_ISL_838849, EPI_ISL_838850, EPI_ISL_838851, EPI_ISL_838852, EPI_ISL_838853, EPI_ISL_838855, EPI_ISL_838856, EPI_ISL_838857, EPI_ISL_838858, EPI_ISL_838859, EPI_ISL_838860, EPI_ISL_838861, EPI_ISL_838862, EPI_ISL_838863, EPI_ISL_838922, EPI_ISL_838923, EPI_ISL_839040, EPI_ISL_839045, EPI_ISL_839047, EPI_ISL_839048, EPI_ISL_839049, EPI_ISL_839050, EPI_ISL_839051, EPI_ISL_839052, EPI_ISL_839053, EPI_ISL_839054, EPI_ISL_839055, EPI_ISL_839056, EPI_ISL_839057, EPI_ISL_839058, EPI_ISL_839059, EPI_ISL_839060, EPI_ISL_839061, EPI_ISL_839062, EPI_ISL_839074, EPI_ISL_839075, EPI_ISL_839076, EPI_ISL_839077, EPI_ISL_839078, EPI_ISL_839079, EPI_ISL_839085, EPI_ISL_839086, EPI_ISL_839087, EPI_ISL_839088, EPI_ISL_839089, EPI_ISL_839090, EPI_ISL_839091, EPI_ISL_839092, EPI_ISL_839093, EPI_ISL_839094, EPI_ISL_839095, EPI_ISL_839096, EPI_ISL_839097, EPI_ISL_839098, EPI_ISL_839099, EPI_ISL_839100, EPI_ISL_839115, EPI_ISL_839118, EPI_ISL_839119, EPI_ISL_839120, EPI_ISL_839121, EPI_ISL_839122, EPI_ISL_839123, EPI_ISL_839124, EPI_ISL_839125, EPI_ISL_839184, EPI_ISL_839185, EPI_ISL_839198, EPI_ISL_839199, EPI_ISL_839201, EPI_ISL_839203, EPI_ISL_839204, EPI_ISL_839205, EPI_ISL_839206, EPI_ISL_839207, EPI_ISL_839208, EPI_ISL_839209, EPI_ISL_839211, EPI_ISL_839213, EPI_ISL_839216, EPI_ISL_839217, EPI_ISL_839221, EPI_ISL_839224, EPI_ISL_839225, EPI_ISL_839228, EPI_ISL_839229, EPI_ISL_839232			
see above	University College London, Great Ormond Street Hospital for Children NHS Foundation Trust, Imperial College Healthcare NHS Trust	COVID-19 Genomics UK (COG-UK) Consortium	Sergi Castellano, Rachel Williams, Mark Kristiansen, Paola Resende Silva, Sunando Roy, Tony Brooks, Helena Tutill, Paola Niola, Patricia Dyal, Charlotte Williams, Leysa Forrest, Yasmin Panchbhaya, Jacqueline Findlay, Samuel Weeks, Julianne Brown, Kathryn Harris, Paul Randell, James Price, Alison Holmes, Judith Breuer
EPI_ISL_839733, EPI_ISL_839734	Northumbria University / South Tees Hospitals NHS Foundation Trust / North Cumbria Integrated Care NHS Foundation Trust / North Tees and Hartlepool NHS Foundation Trust / Newcastle Hospitals NHS Foundation Trust	COVID-19 Genomics UK (COG-UK) Consortium	Darren L Smith,Andrew Nelson,Matthew Bashton,Greg R Young,Joshua Loh,John Allan,Mohammad A Tariq,Giles S Holt,Gary Black,Wen C Yew,Lynn Dover,Paul Baker,Steve Liggett,Sarah Essex,Jane Greenaway,Debra Padgett,Clive Graham,Garren Scott,Edward Barton,Emma Swindells,Brendan Payne,Jennifer Collins,Yusri Taha,Gary Eltringham
EPI_ISL_839825, EPI_ISL_839826, EPI_ISL_839827, EPI_ISL_839828, EPI_ISL_839829, EPI_ISL_839830, EPI_ISL_839831, EPI_ISL_839832, EPI_ISL_839833, EPI_ISL_839834, EPI_ISL_839835, EPI_ISL_839836, EPI_ISL_839837, EPI_ISL_839838, EPI_ISL_839839, EPI_ISL_839901, EPI_ISL_839902, EPI_ISL_839903, EPI_ISL_839904, EPI_ISL_839905, EPI_ISL_839906, EPI_ISL_839909, EPI_ISL_839910, EPI_ISL_839912, EPI_ISL_839915, EPI_ISL_839917, EPI_ISL_839919, EPI_ISL_839920, EPI_ISL_839922, EPI_ISL_839923, EPI_ISL_839924, EPI_ISL_839925, EPI_ISL_839926, EPI_ISL_839927, EPI_ISL_839928, EPI_ISL_839929, EPI_ISL_839930, EPI_ISL_839931			
see above	Quadram Institute Bioscience	COVID-19 Genomics UK (COG-UK) Consortium	Dave J. Baker, Gemma L. Kay, Alp Aydin, Thanh Le-Viet, Steven Rudder, Ana P. Tedim, Anastasia Kolyva, Maria Diaz, Leonardo de Oliveira Martins, Nabil-Fareed Alikhan, Lizzie Meadows, Rachael Stanley, Ngozi Elumogo, Muhammed Yasir, Nicholas M. Thomson, Alexander J Trotter, Rachel Gilroy, Samuel Bloomfield, Claire Stuart, Andrew Bell, Reenesh Prakash, Samir Dervisevic, Alison E. Mather, John Wain, Mark Webber, Andrew J. Page, Justin O'Grady
EPI_ISL_839995, EPI_ISL_839996	Queens Medical Centre, Clinical Microbiology Department / DeepSeq Nottingham	COVID-19 Genomics UK (COG-UK) Consortium	Gemma Clark, Wendy Smith, Manjinder Khakh, Vicki M Fleming, Michelle M Lister, Hannah Howson-Wells, Jonathan Ball, Patrick McClure, Joseph Chappell, Theocharis Tsoleridis, Nadine Holmes, Matthew Carlisle, Christopher Moore, Fei Sang, Johnny Debebe, Victoria Wright, Matthew Loose
EPI_ISL_840121, EPI_ISL_840122, EPI_ISL_840123, EPI_ISL_840124, EPI_ISL_840125, EPI_ISL_840126, EPI_ISL_840127, EPI_ISL_840128, EPI_ISL_840129, EPI_ISL_840130, EPI_ISL_840131, EPI_ISL_840132, EPI_ISL_840133, EPI_ISL_840134			
see above	Lincolnshire Hospitals and DeepSeq Nottingham	COVID-19 Genomics UK (COG-UK) Consortium	Nichola Duckworth, Tim Sloan, Sarah Walsh, Jonathan Ball, Patrick McClure, Joeseph Chappell, Nadine Holmes, Matthew Carlisle, Christopher Moore, Fei Sang, Johnny Debebe, Victoria Wright, Matthew Loose
EPI_ISL_840650, EPI_ISL_840652, EPI_ISL_840660, EPI_ISL_840661, EPI_ISL_840663, EPI_ISL_840664, EPI_ISL_840666, EPI_ISL_840673, EPI_ISL_840674, EPI_ISL_840675, EPI_ISL_840676, EPI_ISL_840677, EPI_ISL_840678, EPI_ISL_840679, EPI_ISL_840680, EPI_ISL_840681, EPI_ISL_840682, EPI_ISL_840683, EPI_ISL_840684, EPI_ISL_840685, EPI_ISL_840686, EPI_ISL_840687, EPI_ISL_840688, EPI_ISL_840689, EPI_ISL_840690, EPI_ISL_840691, EPI_ISL_840692, EPI_ISL_840693, EPI_ISL_840811, EPI_ISL_840812, EPI_ISL_840813, EPI_ISL_840814, EPI_ISL_840815			
see above	Originating lab: Wales Specialist Virology Centre Sequencing lab: Pathogen Genomics Unit	Public Health Wales Microbiology Cardiff Wales Specialist Virology Centre	Catherine Moore, Johnathan Evans, Laura Gifford, Malorie Perry, Simon Cottrell, Angela Marchbank, Alec Birchley, Alexander Adams, Amy Gaskin, Bree Gatica-Wilcox, Jason Coombes, Joel Southgate, Lauren Gilbert, Lee Graham, Nicole Pacchiarini, Sara Kumziene-Summerhayes, Sarah Taylor, Sophie

			Jones, Sara Rey, Matthew Bull, Joanne Watkins, Sally Corden, Tom Connor
EPI_ISL_840816, EPI_ISL_840819, EPI_ISL_840820, EPI_ISL_840821, EPI_ISL_840822, EPI_ISL_840824, EPI_ISL_840826, EPI_ISL_840827, EPI_ISL_840839, EPI_ISL_840840, EPI_ISL_840841, EPI_ISL_840842, EPI_ISL_840845, EPI_ISL_840846, EPI_ISL_840847, EPI_ISL_840848, EPI_ISL_840853, EPI_ISL_840916, EPI_ISL_840917, EPI_ISL_840918, EPI_ISL_840919, EPI_ISL_840922, EPI_ISL_840925, EPI_ISL_840926, EPI_ISL_840928, EPI_ISL_840929, EPI_ISL_840930, EPI_ISL_840932, EPI_ISL_840933, EPI_ISL_840934, EPI_ISL_840935, EPI_ISL_840936, EPI_ISL_840942, EPI_ISL_840944, EPI_ISL_840945, EPI_ISL_840947, EPI_ISL_840952, EPI_ISL_840954, EPI_ISL_840955, EPI_ISL_840956, EPI_ISL_840957, EPI_ISL_840958, EPI_ISL_840959, EPI_ISL_840960, EPI_ISL_840962, EPI_ISL_840966, EPI_ISL_840967, EPI_ISL_841041, EPI_ISL_841042, EPI_ISL_841059, EPI_ISL_841062, EPI_ISL_841063, EPI_ISL_841064, EPI_ISL_841081, EPI_ISL_841114, EPI_ISL_841115, EPI_ISL_841116, EPI_ISL_841117, EPI_ISL_841118, EPI_ISL_841119, EPI_ISL_841120, EPI_ISL_841121, EPI_ISL_841122, EPI_ISL_841123, EPI_ISL_841124, EPI_ISL_841125, EPI_ISL_841126, EPI_ISL_841127, EPI_ISL_841128, EPI_ISL_841129, EPI_ISL_841130, EPI_ISL_841131, EPI_ISL_841133, EPI_ISL_841134, EPI_ISL_841137, EPI_ISL_841138, EPI_ISL_841139, EPI_ISL_841140, EPI_ISL_841141, EPI_ISL_841142, EPI_ISL_841143, EPI_ISL_841144, EPI_ISL_841145, EPI_ISL_841146, EPI_ISL_841156, EPI_ISL_841157, EPI_ISL_841158, EPI_ISL_841159, EPI_ISL_841161, EPI_ISL_841162, EPI_ISL_841163, EPI_ISL_841164, EPI_ISL_841165, EPI_ISL_841167, EPI_ISL_841306			
see above	Wales Specialist Virology Centre Sequencing lab: Pathogen Genomics Unit	Public Health Wales Microbiology Cardiff Wales Specialist Virology Centre	Catherine Moore, Johnathan Evans, Laura Gifford, Malorie Perry, Simon Cottrell, Angela Marchbank, Alec Birchley, Alexander Adams, Amy Gaskin, Bree Gatica-Wilcox, Jason Coombes, Joel Southgate, Lauren Gilbert, Lee Graham, Nicole Pacchiarini, Sara Kumziene-Summerhayes, Sarah Taylor, Sophie Jones, Sara Rey, Matthew Bull, Joanne Watkins, Sally Corden, Tom Connor
EPI_ISL_841333, EPI_ISL_841362, EPI_ISL_841363, EPI_ISL_841364, EPI_ISL_841365, EPI_ISL_841366, EPI_ISL_841367, EPI_ISL_841368, EPI_ISL_841369, EPI_ISL_841370, EPI_ISL_841371, EPI_ISL_841372, EPI_ISL_841374, EPI_ISL_841508, EPI_ISL_841509, EPI_ISL_841510, EPI_ISL_841511, EPI_ISL_841591, EPI_ISL_841608, EPI_ISL_841610			
see above	Originating lab: Wales Specialist Virology Centre Sequencing lab: Pathogen Genomics Unit	Public Health Wales Microbiology Cardiff Wales Specialist Virology Centre	Catherine Moore, Johnathan Evans, Laura Gifford, Malorie Perry, Simon Cottrell, Angela Marchbank, Alec Birchley, Alexander Adams, Amy Gaskin, Bree Gatica-Wilcox, Jason Coombes, Joel Southgate, Lauren Gilbert, Lee Graham, Nicole Pacchiarini, Sara Kumziene-Summerhayes, Sarah Taylor, Sophie Jones, Sara Rey, Matthew Bull, Joanne Watkins, Sally Corden, Tom Connor
EPI_ISL_841922, EPI_ISL_841927, EPI_ISL_841945, EPI_ISL_842008, EPI_ISL_842015	Centre for Enzyme Innovation, University of Portsmouth / Translational Research Laboratory, Portsmouth Hospitals NHS Trust	COVID-19 Genomics UK (COG-UK) Consortium	Angela Beckett,Yann Bourgeois,Garry Scarlett,Sharon Glaysher,Scott Elliott,Kelly Bicknell,Robert Impey,Allyson Lloyd,Sarah Wyllie,Ethan Butcher,Anoop Chauhan,Samuel Robson
EPI_ISL_842285, EPI_ISL_842315, EPI_ISL_842322	Virology Department, Sheffield Teaching Hospitals NHS Foundation Trust/Department of Infection, Immunity and Cardiovascular Disease, The Medical School, University of Sheffield	COVID-19 Genomics UK (COG-UK) Consortium	Thushan de Silva, Matthew Parker, Nikki Smith, Adri Angyal, Rebecca Brown, Luke Green, Rachel Tucker, Paul Parsons, Danielle Groves, Katie Johnson, Laura Carrilero, Alex Keeley, Dave Partridge, Matthew Wyles, Benjamin Lindsey, Mehmet Yavuz, Mohammad Raza, Cariad Evans
EPI_ISL_843193	LabPLUS	Institute of Environmental Science and Research (ESR)	Xiaoyun Ren, Matt Storey, Nikki Freed, Muhammad Faisal, Jing Wang, Hermes Perez, Anja Werno, Antje van der Linden, Arlo Upton, Chris Mansell, David Hammer, Dragana Drinkovic, Gary McAuliffe, Hana Sofia Andersson, James Ussher, Jill Sherwood, Josh Freeman, Julia Howard, Juliet Elvy, Mary DeAlmeida, Matt Blakiston, Matthew Rogers, Max Bloomfield, Michael Addidle, Michelle Balm, Sally Roberts, Sarah Jefferies, Sharmini Muttaiyah, Susan Morpeth, Susan Taylor, Timothy Blackmore, Vani Sathyendran, Veronica Playle, Virginia Hope, Erasmus Smit, Lauren Jelly, Olin Silander, Joep de Ligt
EPI_ISL_845566, EPI_ISL_845567, EPI_ISL_845571, EPI_ISL_845574, EPI_ISL_845577, EPI_ISL_845581, EPI_ISL_845584, EPI_ISL_845591, EPI_ISL_845598, EPI_ISL_845600, EPI_ISL_845605			
see above	KU Leuven, Rega Institute, Clinical and Epidemiological Virology	KU Leuven, Rega Institute, Clinical and Epidemiological Virology	Tony Wawina-Bokalanga, Bert Vanmechelen, Joan Marti-Carerras, Piet Maes
EPI_ISL_845641, EPI_ISL_845642, EPI_ISL_845643, EPI_ISL_845644, EPI_ISL_845645, EPI_ISL_845648, EPI_ISL_845649, EPI_ISL_845651	Laboratorio de Salud Pública - Secretaría Distrital de Salud	Instituto Nacional de Salud - Dirección de Investigación en Salud Pública	Katherine Laiton-Donato, Diego A. Álvarez-Díaz, Carlos Franco-Muñoz, Mauricio Pacheco-Montealegre, Maria T. Herrera-Sepúlveda, Jonathan Reales, Sheryll Corchuelo, Julian Naizaque, Gerardo Santamaría, Paola Muñoz-Laiton, Diego Andrés Prada, Magdalena Wiesner, Martha Lucia Ospina Martinez, Marcela Mercado-Reyes
EPI_ISL_845751	Toronto Invasive Bacterial Diseases Network	McMaster University	Allison McGeer, Patryk Aftanas, Hooman Derakhshani, Angel Li, Kuganya Nirmalarajah, Emily Panousis, Ahmed Draia, Jalees Nasir, Michael Surette, Samira Mubareka, Andrew G. McArthur
EPI_ISL_845771	Emory Molecular Diagnostics Laboratory, Emory Healthcare	Piantadosi Lab, Emory Department of Pathology	Ahmed Babiker, Anne Piantadosi
EPI_ISL_846523, EPI_ISL_846524, EPI_ISL_846525	Lighthouse Lab in Alderley Park	Wellcome Sanger Institute for the COVID-19 Genomics UK (COG-UK) Consortium	Jacquelyn Wynn, Mairead Hyland, The Lighthouse Lab in Alderley Park and Alex Alderton, Roberto Amato, Sonia Goncalves, Ewan Harrison, David K. Jackson, Ian Johnston, Dominic Kwiatkowski, Cordelia Langford, John Sillitoe on behalf of the Wellcome Sanger Institute COVID-19 Surveillance Team
EPI_ISL_846527	Lighthouse Lab in Glasgow	Wellcome Sanger Institute for the COVID-19 Genomics UK (COG-UK) Consortium	Harper VanSteenhouse, Yumi Kasai, David Gray, Carol Clugston, Anna Dominiczak and Alex Alderton, Roberto Amato, Sonia Goncalves, Ewan Harrison, David K. Jackson, Ian Johnston, Dominic Kwiatkowski, Cordelia Langford, John Sillitoe on behalf of the Wellcome Sanger Institute COVID-19 Surveillance Team
EPI_ISL_846543	Lab voor klinische biologie	Lab voor klinische biologie	Hannelore Hamerlinck, Bruno Verhasselt
EPI_ISL_846595	Respiratory Virus Unit, National Infection Service, Public Health England	COVID-19 Genomics UK (COG-UK) Consortium	PHE Covid Sequencing Team
EPI_ISL_846631, EPI_ISL_846632, EPI_ISL_846633, EPI_ISL_846634, EPI_ISL_846635, EPI_ISL_846649	Hopital	National Reference Center for Viruses of Respiratory Infections, Institut Pasteur, Paris	Marion Barbet, Sylvie Behillil, Méline Bizard, Angela Brisebarre, Camille Capel, Etienne Simon-Lorière, Vincent Enouf, Maud Vanpeene, Sylvie van der Werf,Farfour Eric
EPI_ISL_847568, EPI_ISL_847590, EPI_ISL_847591, EPI_ISL_847592, EPI_ISL_847593, EPI_ISL_847758, EPI_ISL_847759	California Department of Public Health	Chiu Laboratory, University of California, San Francisco	Charles Chiu, Xianding (Wayne) Deng, Candace Wang, Brian Bushnell, Scot Federman, Jill Hacker, Debra Wadford
EPI_ISL_847825	Lab. Microbiologia e Virologia, Cotugno, A.O. dei Colli	Lab. Microbiologia e Virologia, Cotugno, A.O. dei Colli	Luigi Atripaldi, Claudia Tiberio, Anna Perfetti
EPI_ISL_847829	Tempus	Grubaugh Lab - Yale School of Public Health	Tara Alpert, Joseph Fauver, Anderson Brito, Mallery Breban, Anne Wyllie, Chantal Vogels, Mary Petrone, Chaney Kalinich, Isabel Ott, Nathan Grubaugh
EPI_ISL_847832, EPI_ISL_847833, EPI_ISL_847837	Yale New Haven Hospital	Grubaugh Lab - Yale School of Public Health	Tara Alpert, Joseph Fauver, Anderson Brito, Mallery Breban, Anne Wyllie, Chantal Vogels, Mary Petrone, Chaney Kalinich, Isabel Ott, Nathan Grubaugh
EPI_ISL_847844, EPI_ISL_847847, EPI_ISL_847852, EPI_ISL_847853, EPI_ISL_847855, EPI_ISL_847856, EPI_ISL_847857, EPI_ISL_847858, EPI_ISL_847859, EPI_ISL_847860, EPI_ISL_847861, EPI_ISL_847862, EPI_ISL_847863, EPI_ISL_847864, EPI_ISL_847865, EPI_ISL_847866, EPI_ISL_847867, EPI_ISL_847868, EPI_ISL_847869, EPI_ISL_847870, EPI_ISL_847871, EPI_ISL_847872, EPI_ISL_847920, EPI_ISL_847929, EPI_ISL_847930, EPI_ISL_847931, EPI_ISL_847932, EPI_ISL_847933, EPI_ISL_847934, EPI_ISL_847935, EPI_ISL_847936, EPI_ISL_847940, EPI_ISL_847941, EPI_ISL_847942, EPI_ISL_847943, EPI_ISL_847944, EPI_ISL_847945, EPI_ISL_847946, EPI_ISL_847947, EPI_ISL_847948, EPI_ISL_847949, EPI_ISL_847950, EPI_ISL_847951, EPI_ISL_847952			
see above	University Hospitals of Geneva, Laboratory of Virology	HUG, Laboratory of Virology and the Health2030 Genome Center	Samuel Cordey, Ana Rita Goncalves, Laurent Kaiser, Lorenzo Cerutti, Henri Pegeot, Melyssa Elies, Keith Harshman, Ioannis Xenarios, Emmanouil Dermitzakis
EPI_ISL_847998, EPI_ISL_848056	Michigan Department of Health and Human Services, Bureau of Laboratories	Michigan Department of Health and Human Services, Bureau of Laboratories	Blankenship HM, Riner D, Soehnlen MK
EPI_ISL_849326, EPI_ISL_849327, EPI_ISL_849328, EPI_ISL_849329, EPI_ISL_849330, EPI_ISL_849331, EPI_ISL_849332, EPI_ISL_849333, EPI_ISL_849334, EPI_ISL_849335	Delaware Public Health Lab	Delaware Public Health Lab	Gregory Hovan
EPI_ISL_849750, EPI_ISL_849751, EPI_ISL_849752	unknown	PHV-FSS	Son Nguyen et al.
EPI_ISL_849780	Utah Public Health Laboratory	Utah Public Health Laboratory	Erin L. Young, Kelly F. Oakeson, Tara Gallagher
EPI_ISL_849924	UCSF Clinical Microbiology Laboratory	Chan-Zuckerberg Biohub	CZB Cliahub Consortium
EPI_ISL_850519, EPI_ISL_850520, EPI_ISL_850521, EPI_ISL_850522, EPI_ISL_850523, EPI_ISL_850524, EPI_ISL_850525, EPI_ISL_850526, EPI_ISL_850527, EPI_ISL_850528, EPI_ISL_850529, EPI_ISL_850530, EPI_ISL_850531, EPI_ISL_850532, EPI_ISL_850533, EPI_ISL_850534, EPI_ISL_850535, EPI_ISL_850536, EPI_ISL_850537, EPI_ISL_850538, EPI_ISL_850539, EPI_ISL_850540, EPI_ISL_850541, EPI_ISL_850542, EPI_ISL_850543, EPI_ISL_850544, EPI_ISL_850545, EPI_ISL_850546, EPI_ISL_850547, EPI_ISL_850548, EPI_ISL_850549, EPI_ISL_850550, EPI_ISL_850551, EPI_ISL_850552, EPI_ISL_850553, EPI_ISL_850554,			

EPI_ISL_850555, EPI_ISL_850556, EPI_ISL_850557, EPI_ISL_850558, EPI_ISL_850559, EPI_ISL_850560, EPI_ISL_850561, EPI_ISL_850562, EPI_ISL_850563, EPI_ISL_850564, EPI_ISL_850565, EPI_ISL_850566, EPI_ISL_850567, EPI_ISL_850568, EPI_ISL_850569, EPI_ISL_850570, EPI_ISL_850571, EPI_ISL_850572, EPI_ISL_850573, EPI_ISL_850574, EPI_ISL_850575, EPI_ISL_850576, EPI_ISL_850577, EPI_ISL_850578, EPI_ISL_850579, EPI_ISL_850580, EPI_ISL_850581, EPI_ISL_850582, EPI_ISL_850583, EPI_ISL_850584, EPI_ISL_850585, EPI_ISL_850586, EPI_ISL_850587, EPI_ISL_850588, EPI_ISL_850589, EPI_ISL_850590, EPI_ISL_850591, EPI_ISL_850592, EPI_ISL_850593, EPI_ISL_850594, EPI_ISL_850595, EPI_ISL_850596, EPI_ISL_850597, EPI_ISL_850598, EPI_ISL_850599, EPI_ISL_850600			
see above	Helix/Illumina	Genomics and Discovery, Respiratory Viruses Branch, Division of Viral Diseases, Centers for Disease Control and Prevention	Peter W. Cook, Dhwani Batra, Ben L. Rambo-Martin Eileen de Feo, Jan Antico, Christine Tran, Matthew Tolentino, Shannon Wickline, Kim Gietzen, Brad Sickler, Jingtao Liu, Eric Allen, Phil Febbo, Summer Galloway, Nicole L. Washington, Simon White, Geraint Levan, Kelly Schiabor Barrett, Elizabeth Cirulli, Alexandre Bolze, Ary Ascencio, Charlotte Rivera-Garcia, Ryan Cho, Jason Nguyen, Sherry Wang, Jimmy Ramirez, Tyler Cassens, Efren Sandoval, Magnus Isaksson, William Lee, David Becker, Marc Laurent, James Lu, Clinton R. Paden, Suxiang Tong, Duncan MacCannell
EPI_ISL_850671, EPI_ISL_850672	The National Institute of Public Health	State Veterinary Institute Prague	Nagy,A;Jirincova,H;Trnka,D;Vecerova,J;Trinklova,M
EPI_ISL_850894, EPI_ISL_850895, EPI_ISL_850896, EPI_ISL_850897, EPI_ISL_850898, EPI_ISL_850899, EPI_ISL_850900, EPI_ISL_850901, EPI_ISL_850902, EPI_ISL_850903, EPI_ISL_850904, EPI_ISL_850905, EPI_ISL_850906, EPI_ISL_850907, EPI_ISL_850908, EPI_ISL_850909, EPI_ISL_850910, EPI_ISL_850911, EPI_ISL_850912, EPI_ISL_850913, EPI_ISL_850914, EPI_ISL_850915, EPI_ISL_850916, EPI_ISL_850917, EPI_ISL_850918, EPI_ISL_850919, EPI_ISL_850920, EPI_ISL_850921, EPI_ISL_850922, EPI_ISL_850923, EPI_ISL_850924, EPI_ISL_850925, EPI_ISL_850926, EPI_ISL_850927, EPI_ISL_850928, EPI_ISL_850929, EPI_ISL_850930, EPI_ISL_850931, EPI_ISL_850932, EPI_ISL_850933, EPI_ISL_850934, EPI_ISL_850935, EPI_ISL_850936, EPI_ISL_850937, EPI_ISL_850938, EPI_ISL_850939, EPI_ISL_850940, EPI_ISL_850941			
see above	Helix / Illumina	Genomics and Discovery, Respiratory Viruses Branch, Division of Viral Diseases, Centers for Disease Control and Prevention	Peter W. Cook, Dhwani Batra, Ben L. Rambo-Martin Eileen de Feo, Jan Antico, Christine Tran, Matthew Tolentino, Shannon Wickline, Kim Gietzen, Brad Sickler, Jingtao Liu, Eric Allen, Phil Febbo, Summer Galloway, Nicole L. Washington, Simon White, Geraint Levan, Kelly Schiabor Barrett, Elizabeth Cirulli, Alexandre Bolze, Ary Ascencio, Charlotte Rivera-Garcia, Ryan Cho, Jason Nguyen, Sherry Wang, Jimmy Ramirez, Tyler Cassens, Efren Sandoval, Magnus Isaksson, William Lee, David Becker, Marc Laurent, James Lu, Clinton R. Paden, Suxiang Tong, Duncan MacCannell
EPI_ISL_850961, EPI_ISL_850962, EPI_ISL_850963, EPI_ISL_850964, EPI_ISL_850965, EPI_ISL_850966, EPI_ISL_850967, EPI_ISL_850968, EPI_ISL_850969, EPI_ISL_850970, EPI_ISL_850971, EPI_ISL_850972, EPI_ISL_850973, EPI_ISL_850974, EPI_ISL_850975, EPI_ISL_850976, EPI_ISL_850977, EPI_ISL_850978, EPI_ISL_850979, EPI_ISL_850980, EPI_ISL_850981, EPI_ISL_850982, EPI_ISL_850983, EPI_ISL_850984, EPI_ISL_850985, EPI_ISL_850986, EPI_ISL_850987, EPI_ISL_850988, EPI_ISL_850989, EPI_ISL_850990, EPI_ISL_850991, EPI_ISL_850992, EPI_ISL_850993, EPI_ISL_850994, EPI_ISL_850995, EPI_ISL_850996, EPI_ISL_850997, EPI_ISL_850998, EPI_ISL_850999, EPI_ISL_851000, EPI_ISL_851001, EPI_ISL_851002, EPI_ISL_851003, EPI_ISL_851004, EPI_ISL_851005, EPI_ISL_851006, EPI_ISL_851021, EPI_ISL_851022, EPI_ISL_851023, EPI_ISL_851024, EPI_ISL_851025, EPI_ISL_851029, EPI_ISL_851030, EPI_ISL_851031, EPI_ISL_851032			
see above	Helix/Illumina	Genomics and Discovery, Respiratory Viruses Branch, Division of Viral Diseases, Centers for Disease Control and Prevention	Peter W. Cook, Dhwani Batra, Ben L. Rambo-Martin Eileen de Feo, Jan Antico, Christine Tran, Matthew Tolentino, Shannon Wickline, Kim Gietzen, Brad Sickler, Jingtao Liu, Eric Allen, Phil Febbo, Summer Galloway, Nicole L. Washington, Simon White, Geraint Levan, Kelly Schiabor Barrett, Elizabeth Cirulli, Alexandre Bolze, Ary Ascencio, Charlotte Rivera-Garcia, Ryan Cho, Jason Nguyen, Sherry Wang, Jimmy Ramirez, Tyler Cassens, Efren Sandoval, Magnus Isaksson, William Lee, David Becker, Marc Laurent, James Lu, Clinton R. Paden, Suxiang Tong, Duncan MacCannell
EPI_ISL_852047, EPI_ISL_852225	Lighthouse Lab in Alderley Park	Wellcome Sanger Institute for the COVID-19 Genomics UK (COG-UK) Consortium	Jacquelyn Wynn, Mairead Hyland, The Lighthouse Lab in Alderley Park and Alex Alderton, Roberto Amato, Sonia Goncalves, Ewan Harrison, David K. Jackson, Ian Johnston, Dominic Kwiatkowski, Cordelia Langford, John Sillitoe on behalf of the Wellcome Sanger Institute COVID-19 Surveillance Team
EPI_ISL_852567, EPI_ISL_852570	Max von Pettenkofer Institute, Virology, National Reference Center for Retroviruses, LMU München	Laboratory for Functional Genome Analysis, Dept. Genomics, Gene Center of the LMU Munich	Max Muenchhoff, Stefan Krebs, Alexander Graf, Oliver Keppler, Helmut Blum
EPI_ISL_852629, EPI_ISL_852630, EPI_ISL_852631, EPI_ISL_852632, EPI_ISL_852813, EPI_ISL_852819, EPI_ISL_852821, EPI_ISL_852823, EPI_ISL_852825, EPI_ISL_852828	Lab. Microbiologia e Virologia, Cotugno, A.O. dei Colli	Lab. Microbiologia e Virologia, Cotugno, A.O. dei Colli	Luigi Atripaldi, Claudia Tiberio, Anna Perfetti
EPI_ISL_853400	Charité Universitätsmedizin Berlin, Institut für Virologie/Labor Berlin	Charité Universitätsmedizin Berlin, Institut für Virologie	Victor M Corman, Julia Schneider, Barbara Mühlemann, Jörn Beheim-Schwarzbach, Talitha Veith, Julia Tesch, Tobias Bleicker, Terry Jones, Christian Drosten
EPI_ISL_853683	NORTHWELL HEALTH LABORATORIES	Wadsworth Center, New York State Department of Health	Kirsten St. George, Daryl M. Lamson, Alexis Russel, Matthew Shudt, Melissa A Leisner, Jonathan Plitnick, Navjot Singh, John Kelly, Erasmus Schneider, Erica Lasek-Nesselquist
EPI_ISL_853684, EPI_ISL_853685, EPI_ISL_853686, EPI_ISL_853687	ALBANY MEDICAL CENTER HOSPITAL CLINICAL LABORATORIES	Wadsworth Center, New York State Department of Health	Kirsten St. George, Daryl M. Lamson, Alexis Russel, Matthew Shudt, Melissa A Leisner, Jonathan Plitnick, Navjot Singh, John Kelly, Erasmus Schneider, Erica Lasek-Nesselquist
EPI_ISL_853727, EPI_ISL_853748, EPI_ISL_853760	Department of Microbiology, University Innsbruck	Bergthaler laboratory, CeMM Research Center for Molecular Medicine of the Austrian Academy of Sciences	Lukas Endler, Alexandra Popa, Benedikt Agerer, Jakob-Wendelin Genger, Alexander Lercher, Anna Schedl, Thomas Penz, Michael Schuster, Jan Laine, Martin Senekowitsch, Christoph Bock, Andreas Bergthaler
EPI_ISL_855353	Hospital	National Reference Center for Viruses of Respiratory Infections, Institut Pasteur, Paris	Marion Barbet, Sylvie Behillil, Méline Bizard, Angela Brisebarre, Camille Capel, Etienne Simon-Lorière, Vincent Enouf, Maud Vanpeene, Sylvie van der Werf,Pilorge Léa
EPI_ISL_855383, EPI_ISL_855384	Hospital	National Reference Center for Viruses of Respiratory Infections, Institut Pasteur, Paris	Marion Barbet, Sylvie Behillil, Méline Bizard, Angela Brisebarre, Camille Capel, Etienne Simon-Lorière, Vincent Enouf, Maud Vanpeene, Sylvie van der Werf
EPI_ISL_855387	Labo Analyses Med	National Reference Center for Viruses of Respiratory Infections, Institut Pasteur, Paris	Marion Barbet, Sylvie Behillil, Méline Bizard, Angela Brisebarre, Camille Capel, Etienne Simon-Lorière, Vincent Enouf, Maud Vanpeene, Sylvie van der Werf
EPI_ISL_856930, EPI_ISL_856931, EPI_ISL_856941, EPI_ISL_856942, EPI_ISL_856968, EPI_ISL_856969, EPI_ISL_856970	Wyoming Public Health Laboratory	Wyoming Public Health Laboratory	Noah Hull, Taylor Fearing, Lynette Gumbleton, Channing Weber, Ashley Norberg, Bailey Bowcutt, and Wanda Manley
EPI_ISL_857059, EPI_ISL_857060, EPI_ISL_857061	OCME Office Of Chief Medical Examiner	New York City Public Health Laboratory	Jade Wang, et al.
EPI_ISL_857314	Centers for Disease Control, R.O.C. (Taiwan)	Centers for Disease Control, R.O.C. (Taiwan)	Ji-Rong Yang, Yu-Chi Lin, Jung-Jung-Mu, Ming-Tsan Liu
EPI_ISL_858077, EPI_ISL_858085, EPI_ISL_858101, EPI_ISL_858142, EPI_ISL_858145, EPI_ISL_858148, EPI_ISL_858151, EPI_ISL_858207, EPI_ISL_858208, EPI_ISL_858209, EPI_ISL_858210, EPI_ISL_858211, EPI_ISL_858212, EPI_ISL_858213, EPI_ISL_858214, EPI_ISL_858215, EPI_ISL_858217, EPI_ISL_858218, EPI_ISL_858219, EPI_ISL_858220, EPI_ISL_858221, EPI_ISL_858222			
see above	Lighthouse Lab in Alderley Park	Wellcome Sanger Institute for the COVID-19 Genomics UK (COG-UK) Consortium	Jacquelyn Wynn, Mairead Hyland, The Lighthouse Lab in Alderley Park and Alex Alderton, Roberto Amato, Sonia Goncalves, Ewan Harrison, David K. Jackson, Ian Johnston, Dominic Kwiatkowski, Cordelia Langford, John Sillitoe on behalf of the Wellcome Sanger Institute COVID-19 Surveillance Team
EPI_ISL_858267	Lighthouse Lab in Glasgow	Wellcome Sanger Institute for the COVID-19 Genomics UK (COG-UK) Consortium	Harper VanSteenhouse, Yumi Kasai, David Gray, Carol Clugston, Anna Dominiczak and Alex Alderton, Roberto Amato, Sonia Goncalves, Ewan Harrison, David K. Jackson, Ian Johnston, Dominic Kwiatkowski, Cordelia Langford, John Sillitoe on behalf of the Wellcome Sanger Institute COVID-19 Surveillance Team
EPI_ISL_858268	Lighthouse Lab in Milton Keynes	Wellcome Sanger Institute for the COVID-19 Genomics UK (COG-UK) Consortium	The Lighthouse Lab in Milton Keynes and Alex Alderton, Roberto Amato, Sonia Goncalves, Ewan Harrison, David K. Jackson, Ian Johnston, Dominic Kwiatkowski, Cordelia Langford, John Sillitoe on behalf of the Wellcome Sanger Institute COVID-19 Surveillance Team
EPI_ISL_858269, EPI_ISL_858270, EPI_ISL_858296, EPI_ISL_858306, EPI_ISL_858311, EPI_ISL_858326, EPI_ISL_858328, EPI_ISL_858357, EPI_ISL_858381, EPI_ISL_858382	Lighthouse Lab in Alderley Park	Wellcome Sanger Institute for the COVID-19 Genomics UK (COG-UK) Consortium	Jacquelyn Wynn, Mairead Hyland, The Lighthouse Lab in Alderley Park and Alex Alderton, Roberto Amato, Sonia Goncalves, Ewan Harrison, David K. Jackson, Ian Johnston, Dominic Kwiatkowski, Cordelia Langford, John Sillitoe on behalf of the Wellcome Sanger Institute COVID-19 Surveillance Team
EPI_ISL_858383	Lighthouse Lab in Glasgow	Wellcome Sanger Institute for the COVID-19 Genomics UK (COG-UK) Consortium	Harper VanSteenhouse, Yumi Kasai, David Gray, Carol Clugston, Anna Dominiczak and Alex Alderton, Roberto Amato, Sonia Goncalves, Ewan Harrison, David K. Jackson, Ian Johnston, Dominic Kwiatkowski, Cordelia Langford, John Sillitoe on behalf of the Wellcome Sanger Institute COVID-19 Surveillance Team
EPI_ISL_858387, EPI_ISL_858388, EPI_ISL_858389, EPI_ISL_858393	Lighthouse Lab in Alderley Park	Wellcome Sanger Institute for the COVID-19 Genomics UK (COG-UK) Consortium	Jacquelyn Wynn, Mairead Hyland, The Lighthouse Lab in Alderley Park and Alex Alderton, Roberto Amato, Sonia Goncalves, Ewan Harrison, David K. Jackson, Ian Johnston, Dominic Kwiatkowski, Cordelia Langford, John Sillitoe on behalf of the Wellcome Sanger Institute COVID-19 Surveillance Team
EPI_ISL_858394	Lighthouse Lab in Glasgow	Wellcome Sanger Institute for the COVID-19 Genomics UK (COG-UK) Consortium	Harper VanSteenhouse, Yumi Kasai, David Gray, Carol Clugston, Anna Dominiczak and Alex Alderton, Roberto Amato, Sonia Goncalves, Ewan Harrison, David K. Jackson, Ian Johnston, Dominic Kwiatkowski, Cordelia Langford, John Sillitoe on behalf of the Wellcome Sanger Institute COVID-19 Surveillance

			Team
EPI_ISL_858397	Lighthouse Lab in Alderley Park	Wellcome Sanger Institute for the COVID-19 Genomics UK (COG-UK) Consortium	Jacquelyn Wynn, Mairead Hyland, The Lighthouse Lab in Alderley Park and Alex Alderton, Roberto Amato, Sonia Goncalves, Ewan Harrison, David K. Jackson, Ian Johnston, Dominic Kwiatkowski, Cordelia Langford, John Sillitoe on behalf of the Wellcome Sanger Institute COVID-19 Surveillance Team
EPI_ISL_858426, EPI_ISL_858428, EPI_ISL_858438, EPI_ISL_858439, EPI_ISL_858442, EPI_ISL_858443, EPI_ISL_858445, EPI_ISL_858449, EPI_ISL_858450, EPI_ISL_858458, EPI_ISL_858461, EPI_ISL_858464, EPI_ISL_858469, EPI_ISL_858471, EPI_ISL_858473, EPI_ISL_858477, EPI_ISL_858478, EPI_ISL_858479, EPI_ISL_858481, EPI_ISL_858485, EPI_ISL_858486, EPI_ISL_858491, EPI_ISL_858501			
see above	Lighthouse Lab in Glasgow	Wellcome Sanger Institute for the COVID-19 Genomics UK (COG-UK) Consortium	Harper VanSteenhouse, Yumi Kasai, David Gray, Carol Clugston, Anna Dominiczak and Alex Alderton, Roberto Amato, Sonia Goncalves, Ewan Harrison, David K. Jackson, Ian Johnston, Dominic Kwiatkowski, Cordelia Langford, John Sillitoe on behalf of the Wellcome Sanger Institute COVID-19 Surveillance Team
EPI_ISL_858516, EPI_ISL_858521, EPI_ISL_858523, EPI_ISL_858531, EPI_ISL_858557, EPI_ISL_858563, EPI_ISL_858572, EPI_ISL_858574, EPI_ISL_858576, EPI_ISL_858580, EPI_ISL_858581			
see above	Lighthouse Lab in Alderley Park	Wellcome Sanger Institute for the COVID-19 Genomics UK (COG-UK) Consortium	Jacquelyn Wynn, Mairead Hyland, The Lighthouse Lab in Alderley Park and Alex Alderton, Roberto Amato, Sonia Goncalves, Ewan Harrison, David K. Jackson, Ian Johnston, Dominic Kwiatkowski, Cordelia Langford, John Sillitoe on behalf of the Wellcome Sanger Institute COVID-19 Surveillance Team
EPI_ISL_858583, EPI_ISL_858584, EPI_ISL_858585, EPI_ISL_858586, EPI_ISL_858587, EPI_ISL_858588, EPI_ISL_858592, EPI_ISL_858594, EPI_ISL_858598, EPI_ISL_858600, EPI_ISL_858601, EPI_ISL_858604, EPI_ISL_858607, EPI_ISL_858608, EPI_ISL_858609, EPI_ISL_858610, EPI_ISL_858612, EPI_ISL_858613, EPI_ISL_858614, EPI_ISL_858615, EPI_ISL_858616, EPI_ISL_858618, EPI_ISL_858621, EPI_ISL_858623, EPI_ISL_858624, EPI_ISL_858626, EPI_ISL_858627, EPI_ISL_858628, EPI_ISL_858630, EPI_ISL_858633, EPI_ISL_858634, EPI_ISL_858635, EPI_ISL_858636, EPI_ISL_858637, EPI_ISL_858638, EPI_ISL_858640, EPI_ISL_858641, EPI_ISL_858642, EPI_ISL_858643			
see above	Lighthouse Lab in Glasgow	Wellcome Sanger Institute for the COVID-19 Genomics UK (COG-UK) Consortium	Harper VanSteenhouse, Yumi Kasai, David Gray, Carol Clugston, Anna Dominiczak and Alex Alderton, Roberto Amato, Sonia Goncalves, Ewan Harrison, David K. Jackson, Ian Johnston, Dominic Kwiatkowski, Cordelia Langford, John Sillitoe on behalf of the Wellcome Sanger Institute COVID-19 Surveillance Team
EPI_ISL_858645	Lighthouse Lab in Alderley Park	Wellcome Sanger Institute for the COVID-19 Genomics UK (COG-UK) Consortium	Jacquelyn Wynn, Mairead Hyland, The Lighthouse Lab in Alderley Park and Alex Alderton, Roberto Amato, Sonia Goncalves, Ewan Harrison, David K. Jackson, Ian Johnston, Dominic Kwiatkowski, Cordelia Langford, John Sillitoe on behalf of the Wellcome Sanger Institute COVID-19 Surveillance Team
EPI_ISL_858646, EPI_ISL_858650, EPI_ISL_858651, EPI_ISL_858652, EPI_ISL_858656, EPI_ISL_858657, EPI_ISL_858658, EPI_ISL_858659, EPI_ISL_858660, EPI_ISL_858661, EPI_ISL_858664, EPI_ISL_858665, EPI_ISL_858668			
see above	Lighthouse Lab in Glasgow	Wellcome Sanger Institute for the COVID-19 Genomics UK (COG-UK) Consortium	Harper VanSteenhouse, Yumi Kasai, David Gray, Carol Clugston, Anna Dominiczak and Alex Alderton, Roberto Amato, Sonia Goncalves, Ewan Harrison, David K. Jackson, Ian Johnston, Dominic Kwiatkowski, Cordelia Langford, John Sillitoe on behalf of the Wellcome Sanger Institute COVID-19 Surveillance Team
EPI_ISL_860184, EPI_ISL_860185	Bangalore Medical College and Research Institute	Department of Neurovirology, National Institute of Mental Health and Neurosciences (NIMHANS)	Chitra Pattabiraman, Pramada Prasad, Anson Kunjumon George, Risha Rasheed, Darshan Sreenivas, Nakka Vijay Kiran Reddy, Anita S Desai, V Ravi
EPI_ISL_860187, EPI_ISL_860188	Shimoga Institute of Medical Sciences	Department of Neurovirology, National Institute of Mental Health and Neurosciences (NIMHANS)	Chitra Pattabiraman, Pramada Prasad, Anson Kunjumon George, Risha Rasheed, Darshan Sreenivas, Nakka Vijay Kiran Reddy, Anita S Desai, V Ravi
EPI_ISL_860189	BBMP Urban PHC	Department of Neurovirology, National Institute of Mental Health and Neurosciences (NIMHANS)	Chitra Pattabiraman, Pramada Prasad, Anson Kunjumon George, Risha Rasheed, Darshan Sreenivas, Nakka Vijay Kiran Reddy, Anita S Desai, V Ravi
EPI_ISL_860190	Railway Hospital	Department of Neurovirology, National Institute of Mental Health and Neurosciences (NIMHANS)	Chitra Pattabiraman, Pramada Prasad, Anson Kunjumon George, Risha Rasheed, Darshan Sreenivas, Nakka Vijay Kiran Reddy, Anita S Desai, V Ravi
EPI_ISL_860191	National Institute of Mental Health and Neurosciences (NIMHANS)	Department of Neurovirology, National Institute of Mental Health and Neurosciences (NIMHANS)	Chitra Pattabiraman, Pramada Prasad, Anson Kunjumon George, Risha Rasheed, Darshan Sreenivas, Nakka Vijay Kiran Reddy, Anita S Desai, V Ravi
EPI_ISL_860192	Railway Hospital	Department of Neurovirology, National Institute of Mental Health and Neurosciences (NIMHANS)	Chitra Pattabiraman, Pramada Prasad, Anson Kunjumon George, Risha Rasheed, Darshan Sreenivas, Nakka Vijay Kiran Reddy, Anita S Desai, V Ravi
EPI_ISL_860193	Jayanagar General Hospital	Department of Neurovirology, National Institute of Mental Health and Neurosciences (NIMHANS)	Chitra Pattabiraman, Pramada Prasad, Anson Kunjumon George, Risha Rasheed, Darshan Sreenivas, Nakka Vijay Kiran Reddy, Anita S Desai, V Ravi
EPI_ISL_860194, EPI_ISL_860195	BBMP Urban PHC	Department of Neurovirology, National Institute of Mental Health and Neurosciences (NIMHANS)	Chitra Pattabiraman, Pramada Prasad, Anson Kunjumon George, Risha Rasheed, Darshan Sreenivas, Nakka Vijay Kiran Reddy, Anita S Desai, V Ravi
EPI_ISL_860210, EPI_ISL_860212	Akershus University Hospital, Department for Microbiology and Infectious Disease Control	Norwegian Institute of Public Health, Department of Virology	Kathrine Stene-Johansen, Kamilla Heddeland Instefjord, Hilde Elshaug, Atiya R Ali,Marie Paulsen Madsen, Rasmus Riis Kopperud, Hilde Vollan, Karoline Bragstad, Olav Hungnes
EPI_ISL_860241, EPI_ISL_860242, EPI_ISL_860248, EPI_ISL_860254	University Hospitals of Geneva, Laboratory of Virology	HUG, Laboratory of Virology and Universitätsspital Basel	Samuel Cordey, Ana Rita Goncalves, Laurent Kaiser, Tim Roloff, Madlen Stange, Helena MB Seth-Smith, Alfredo Mari, Karoline Leuzinger, Julia Bielicki, Manuel Battegay, Hans Hirsch, Adrian Egli
EPI_ISL_860259	Akershus University Hospital, Department for Microbiology and Infectious Disease Control	Norwegian Institute of Public Health, Department of Virology	Kathrine Stene-Johansen, Kamilla Heddeland Instefjord, Hilde Elshaug, Atiya R Ali,Marie Paulsen Madsen, Rasmus Riis Kopperud, Hilde Vollan, Karoline Bragstad, Olav Hungnes
EPI_ISL_860276	Foerde Hospital, Department of Microbiology	Norwegian Institute of Public Health, Department of Virology	Kathrine Stene-Johansen, Kamilla Heddeland Instefjord, Hilde Elshaug, Atiya R Ali,Marie Paulsen Madsen, Rasmus Riis Kopperud, Hilde Vollan, Karoline Bragstad, Olav Hungnes
EPI_ISL_860277	Akershus University Hospital, Department for Microbiology and Infectious Disease Control	Norwegian Institute of Public Health, Department of Virology	Kathrine Stene-Johansen, Kamilla Heddeland Instefjord, Hilde Elshaug, Atiya R Ali,Marie Paulsen Madsen, Rasmus Riis Kopperud, Hilde Vollan, Karoline Bragstad, Olav Hungnes
EPI_ISL_860285, EPI_ISL_860288	Department of Medical Microbiology, St. Olavs hospital	Norwegian Institute of Public Health, Department of Virology	Kathrine Stene-Johansen, Kamilla Heddeland Instefjord, Hilde Elshaug, Atiya R Ali,Marie Paulsen Madsen, Rasmus Riis Kopperud, Hilde Vollan, Karoline Bragstad, Olav Hungnes
EPI_ISL_860560, EPI_ISL_860561, EPI_ISL_860562, EPI_ISL_860563, EPI_ISL_860564, EPI_ISL_860565, EPI_ISL_860567, EPI_ISL_860568, EPI_ISL_860569, EPI_ISL_860570, EPI_ISL_860571, EPI_ISL_860572, EPI_ISL_860573, EPI_ISL_860574, EPI_ISL_860575, EPI_ISL_860577, EPI_ISL_860578, EPI_ISL_860582, EPI_ISL_860585, EPI_ISL_860587, EPI_ISL_860588, EPI_ISL_860589, EPI_ISL_860590, EPI_ISL_860591, EPI_ISL_860592, EPI_ISL_860593, EPI_ISL_860594			
see above	NHLS-IALCH	KRISP, KZn Research Innovation and Sequencing Platform	Giandhari J, Pillay S, Lessells R, Mdlalose K, York D, Khan S, Tegally H, Wilkinson E, de Oliveira T
EPI_ISL_860666, EPI_ISL_860667	Respiratory Virus Unit, National Infection Service, Public Health England	COVID-19 Genomics UK (COG-UK) Consortium	PHE Covid Sequencing Team
EPI_ISL_860881, EPI_ISL_860888	Labo Analyses Med	National Reference Center for Viruses of Respiratory Infections, Institut Pasteur, Paris	Marion Barbet, Sylvie Behillil, Méline Bizard, Angela Brisebarre, Camille Capel, Etienne Simon-Lorière, Vincent Enouf, Maud Vanpeene, Sylvie van der Werf,Lefaure Brieuc
EPI_ISL_860940, EPI_ISL_860943, EPI_ISL_860948, EPI_ISL_860951, EPI_ISL_860955, EPI_ISL_860962, EPI_ISL_860971, EPI_ISL_860976, EPI_ISL_860979, EPI_ISL_860981, EPI_ISL_860989, EPI_ISL_860992, EPI_ISL_860995, EPI_ISL_861018, EPI_ISL_861021, EPI_ISL_861023, EPI_ISL_861025, EPI_ISL_861056, EPI_ISL_861059, EPI_ISL_861060, EPI_ISL_861061, EPI_ISL_861062, EPI_ISL_861077, EPI_ISL_861079			
see above	Johns Hopkins Hospital Department of Pathology	Johns Hopkins Hospital Department of Pathology	C. Paul Morris, Chun Huai Luo, Adannaya Amadi, Nicholas Gallagher, Heba H. Mostafa
EPI_ISL_861110, EPI_ISL_861111, EPI_ISL_861112, EPI_ISL_861113, EPI_ISL_861114, EPI_ISL_861118, EPI_ISL_861120, EPI_ISL_861121, EPI_ISL_861122, EPI_ISL_861123, EPI_ISL_861124, EPI_ISL_861125, EPI_ISL_861126, EPI_ISL_861127, EPI_ISL_861128, EPI_ISL_861129, EPI_ISL_861130, EPI_ISL_861131, EPI_ISL_861132, EPI_ISL_861133			
see above	New York Presbyterian Hospital	Wadsworth Center, New York State Department of Health	Kirsten St. George, Daryl M. Lamson, Alexis Russel, Matthew Shudt, Melissa A Leisner, Jonathan Plitnick, Navjot Singh, John Kelly, Erasmus Schneider, Erica Lasek-Nesselquist
EPI_ISL_861177, EPI_ISL_861178, EPI_ISL_861181, EPI_ISL_861182	BIO-REFERENCE LABORATORIES	Wadsworth Center, New York State Department of Health	Kirsten St. George, Daryl M. Lamson, Alexis Russel, Matthew Shudt, Melissa A Leisner, Jonathan Plitnick, Navjot Singh, John Kelly, Erasmus Schneider, Erica Lasek-Nesselquist
EPI_ISL_861186, EPI_ISL_861188, EPI_ISL_861189, EPI_ISL_861190, EPI_ISL_861192, EPI_ISL_861193, EPI_ISL_861194, EPI_ISL_861195, EPI_ISL_861196, EPI_ISL_861197, EPI_ISL_861198			
see above	NORTH SHORE UNIVERSITY HOSPITAL	Wadsworth Center, New York State Department of Health	Kirsten St. George, Daryl M. Lamson, Alexis Russel, Matthew Shudt, Melissa A Leisner, Jonathan Plitnick, Navjot Singh, John Kelly, Erasmus Schneider, Erica Lasek-Nesselquist

EPI_ISL_861369	WESTCHESTER MEDICAL CENTER	Wadsworth Center, New York State Department of Health	Kirsten St. George, Daryl M. Lamson, Alexis Russel, Matthew Shudt, Melissa A Leisner, Jonathan Plitnick, Navjot Singh, John Kelly, Erasmus Schneider, Erica Lasek-Nesselquist
EPI_ISL_861414, EPI_ISL_861415, EPI_ISL_861416, EPI_ISL_861418	NORTH SHORE UNIVERSITY HOSPITAL	Wadsworth Center, New York State Department of Health	Kirsten St. George, Daryl M. Lamson, Alexis Russel, Matthew Shudt, Melissa A Leisner, Jonathan Plitnick, Navjot Singh, John Kelly, Erasmus Schneider, Erica Lasek-Nesselquist
EPI_ISL_861536, EPI_ISL_861539, EPI_ISL_861540, EPI_ISL_861557, EPI_ISL_861577	Instituto Nacional de Saude (INSA)	Instituto Nacional de Saude (INSA)	Borges et al
EPI_ISL_861681	Hospital Municipal Dr. Waldemar Tebaldi	Instituto Adolfo Lutz, Interdiciplinary Procedures Center, Strategic Laboratory	Claudio Tavares Sacchi, Claudia Regina Gonçalves, Erica Valessa Ramos Gomes, Karoline Rodrigues Campos
EPI_ISL_861684, EPI_ISL_861685	Hospital Pronto Socorro Itaquera	Instituto Adolfo Lutz, Interdiciplinary Procedures Center, Strategic Laboratory	Claudio Tavares Sacchi, Claudia Regina Gonçalves, Erica Valessa Ramos Gomes, Karoline Rodrigues Campos
EPI_ISL_861689, EPI_ISL_861692, EPI_ISL_861694, EPI_ISL_861695, EPI_ISL_861696, EPI_ISL_861697	Los Angeles County PHL	Los Angeles County PHL	P. Hemarajata et al.
EPI_ISL_861750, EPI_ISL_861771	Yale Clinical Virology Lab	Grubaugh Lab - Yale School of Public Health	Tara Alpert, Joseph Fauver, Anderson Brito, Mallery Breban, Anne Wyllie, Chantal Vogels, Mary Petrone, Chaney Kalinich, Isabel Ott, Nathan Grubaugh
EPI_ISL_862660	Charité Universitätsmedizin Berlin, Institut fur Virologie/Labor Berlin	Charité Universitätsmedizin Berlin, Institut für Virologie	Victor M Corman, Barbara Mühlemann, Jörn Beheim-Schwarzbach, Tobias Bleicker, Julia Tesch, Talitha Veith, Julia Schneider, Terry Jones, Christian Drosten
EPI_ISL_862665, EPI_ISL_862666, EPI_ISL_862667, EPI_ISL_862668, EPI_ISL_862714, EPI_ISL_862715	University of Michigan Clinical Microbiology Laboratory	Lauring Lab, University of Michigan, Department of Microbiology and Immunology	Valesano
EPI_ISL_862742, EPI_ISL_862743, EPI_ISL_862744, EPI_ISL_862751, EPI_ISL_862760, EPI_ISL_862763, EPI_ISL_862771, EPI_ISL_862773, EPI_ISL_862777	Utah Public Health Laboratory, Utah Public Health Laboratory Infectious Disease submission group	Utah Public Health Laboratory, Utah Public Health Laboratory Infectious Disease submission group	Young,E.L., Oakeson,K.F., Gallagher,T.
EPI_ISL_862786, EPI_ISL_862787, EPI_ISL_862788, EPI_ISL_862789, EPI_ISL_862790, EPI_ISL_862791, EPI_ISL_862792, EPI_ISL_862793, EPI_ISL_862794, EPI_ISL_862795, EPI_ISL_862796, EPI_ISL_862797, EPI_ISL_862798, EPI_ISL_862799, EPI_ISL_862800, EPI_ISL_862801, EPI_ISL_862802, EPI_ISL_862803, EPI_ISL_862804, EPI_ISL_862805, EPI_ISL_862806, EPI_ISL_862807, EPI_ISL_862808, EPI_ISL_862809, EPI_ISL_862810, EPI_ISL_862811, EPI_ISL_862812, EPI_ISL_862813			
see above	Cancer Biology Department, National Cancer Institute	Cancer Biology Department, National Cancer Institute	Zekri,A.N., Sedawy,M.G., Ahmed,O.S., Hafez,M.M., Soliman,H.K., Bahnassy,A.A., Elhosieny,F.W., Gad,A.E., Hamdy,M.S., Soliman,M.S., Soliman,L., Abouelhoda,M.
EPI_ISL_863534, EPI_ISL_863535, EPI_ISL_863536, EPI_ISL_863538, EPI_ISL_863541, EPI_ISL_863545, EPI_ISL_863548, EPI_ISL_863550, EPI_ISL_863554, EPI_ISL_863559, EPI_ISL_863563, EPI_ISL_863564, EPI_ISL_863568, EPI_ISL_863569, EPI_ISL_863571, EPI_ISL_863573, EPI_ISL_863575, EPI_ISL_863585, EPI_ISL_863596, EPI_ISL_863597, EPI_ISL_863598, EPI_ISL_863602, EPI_ISL_863607, EPI_ISL_863611, EPI_ISL_863613, EPI_ISL_863615, EPI_ISL_863617, EPI_ISL_863620, EPI_ISL_863624, EPI_ISL_863632, EPI_ISL_863634, EPI_ISL_863637, EPI_ISL_863638, EPI_ISL_863642, EPI_ISL_863646, EPI_ISL_863653, EPI_ISL_863657, EPI_ISL_863660, EPI_ISL_863669, EPI_ISL_863677, EPI_ISL_863678, EPI_ISL_863679, EPI_ISL_863681, EPI_ISL_863696, EPI_ISL_863699, EPI_ISL_863700, EPI_ISL_863705, EPI_ISL_863709, EPI_ISL_863711, EPI_ISL_863723, EPI_ISL_863725, EPI_ISL_863726, EPI_ISL_863733, EPI_ISL_863734, EPI_ISL_863741, EPI_ISL_863742, EPI_ISL_863747, EPI_ISL_863759, EPI_ISL_863761, EPI_ISL_863766, EPI_ISL_863773, EPI_ISL_863776, EPI_ISL_863781, EPI_ISL_863784, EPI_ISL_863785, EPI_ISL_863788, EPI_ISL_863789, EPI_ISL_863792, EPI_ISL_863793, EPI_ISL_863795, EPI_ISL_863796, EPI_ISL_863797, EPI_ISL_863802, EPI_ISL_863804, EPI_ISL_863806, EPI_ISL_863810, EPI_ISL_863812, EPI_ISL_863838, EPI_ISL_863842, EPI_ISL_863845, EPI_ISL_863847, EPI_ISL_863848, EPI_ISL_863850, EPI_ISL_863851, EPI_ISL_863856, EPI_ISL_863858, EPI_ISL_863860, EPI_ISL_863863, EPI_ISL_863865			
see above	Lighthouse Lab in Milton Keynes	Wellcome Sanger Institute for the COVID-19 Genomics UK (COG-UK) Consortium	The Lighthouse Lab in Milton Keynes and Alex Alderton, Roberto Amato, Sonia Goncalves, Ewan Harrison, David K. Jackson, Ian Johnston, Dominic Kwiatkowski, Cordelia Langford, John Sillitoe on behalf of the Wellcome Sanger Institute COVID-19 Surveillance Team
EPI_ISL_864543	Lighthouse Lab in Glasgow	Wellcome Sanger Institute for the COVID-19 Genomics UK (COG-UK) Consortium	Harper VanSteenhouse, Yumi Kasai, David Gray, Carol Clugston, Anna Dominiczak and Alex Alderton, Roberto Amato, Sonia Goncalves, Ewan Harrison, David K. Jackson, Ian Johnston, Dominic Kwiatkowski, Cordelia Langford, John Sillitoe on behalf of the Wellcome Sanger Institute COVID-19 Surveillance Team
EPI_ISL_864746, EPI_ISL_864747, EPI_ISL_864748, EPI_ISL_864749, EPI_ISL_864750, EPI_ISL_864751, EPI_ISL_864766, EPI_ISL_864768, EPI_ISL_864771, EPI_ISL_864773, EPI_ISL_864775, EPI_ISL_864777, EPI_ISL_864779			
see above	Department of Pathology, University of Cambridge	COVID-19 Genomics UK (COG-UK) Consortium	Aminu S. Jahun, Yasmin Chaudhry, Grant Hall, Iliana Georgana, Myra Hosmillo, Martin D. Curran, Malte Pinckert, Surendra Parmar, Ian Goodfellow
EPI_ISL_864981, EPI_ISL_864984, EPI_ISL_864985, EPI_ISL_864986, EPI_ISL_864987, EPI_ISL_864988, EPI_ISL_864989, EPI_ISL_864990, EPI_ISL_864991, EPI_ISL_864994, EPI_ISL_864995			
see above	West of Scotland Specialist Virology Centre, NHSGGC / MRC-University of Glasgow Centre for Virus Research	COVID-19 Genomics UK (COG-UK) Consortium	Ana da Silva Filipe, Natasha Johnson, Kathy Smollett, Daniel Mair, Stephen Carmichael, Alice Broos, Lily Tong, Jenna Nichols, Kyriaki Nomikou; Sarah McDonald; Richard Orton, Joseph Hughes, Sreenu Vattipally, David L Robertson; Alasdair MacLean, Rory Gunson; Sharif Shaaban, Matthew Holden; Rachel Blacow, Guy Mollett, Kathy Li, James Shepherd, Antonia Ho, Emma Thomson
EPI_ISL_865004, EPI_ISL_865005, EPI_ISL_865006, EPI_ISL_865009	Lighthouse Lab in Glasgow / MRC-University of Glasgow Centre for Virus Research	COVID-19 Genomics UK (COG-UK) Consortium	Ana da Silva Filipe, Natasha Johnson, Kathy Smollett, Daniel Mair, Stephen Carmichael, Alice Broos, Lily Tong, Jenna Nichols, Kyriaki Nomikou; Sarah McDonald; Harper VanSteenhouse, Yumi Kasai, David Gray, Carol Clugston, Anna Dominiczak; Alasdair MacLean, Rory Gunson; Richard Orton, Joseph Hughes, Sreenu Vattipally, David L Robertson; Sharif Shaaban, Matthew Holden; Kathy Li, James Shepherd, Antonia Ho, Emma Thomson
EPI_ISL_865013	West of Scotland Specialist Virology Centre, NHSGGC / MRC-University of Glasgow Centre for Virus Research	COVID-19 Genomics UK (COG-UK) Consortium	Ana da Silva Filipe, Natasha Johnson, Kathy Smollett, Daniel Mair, Stephen Carmichael, Alice Broos, Lily Tong, Jenna Nichols, Kyriaki Nomikou; Sarah McDonald; Richard Orton, Joseph Hughes, Sreenu Vattipally, David L Robertson; Alasdair MacLean, Rory Gunson; Sharif Shaaban, Matthew Holden; Rachel Blacow, Guy Mollett, Kathy Li, James Shepherd, Antonia Ho, Emma Thomson
EPI_ISL_865073, EPI_ISL_865094, EPI_ISL_865096, EPI_ISL_865097, EPI_ISL_865098, EPI_ISL_865099, EPI_ISL_865100, EPI_ISL_865101, EPI_ISL_865103, EPI_ISL_865104	Virology Department, Royal Infirmary of Edinburgh, NHS Lothian / School of Biological Sciences, University of Edinburgh / Institute of Genetics and Molecular Medicine, University of Edinburgh	COVID-19 Genomics UK (COG-UK) Consortium	McHugh M, Dewar R, Rooke S, Gallagher M, Balcaza C, O'Toole Á, Scher E, Hill V, McCrone JT, Colquhoun R, Yu X, Jackson B, Rambaut A, Williams TC, Templeton K
EPI_ISL_865178, EPI_ISL_865179, EPI_ISL_865183, EPI_ISL_865184, EPI_ISL_865190, EPI_ISL_865191, EPI_ISL_865198, EPI_ISL_865364, EPI_ISL_865365, EPI_ISL_865366, EPI_ISL_865367, EPI_ISL_865368, EPI_ISL_865369, EPI_ISL_865370, EPI_ISL_865383, EPI_ISL_865384, EPI_ISL_865385, EPI_ISL_865387, EPI_ISL_865388, EPI_ISL_865389, EPI_ISL_865390, EPI_ISL_865481			
see above	Liverpool Clinical Laboratories	COVID-19 Genomics UK (COG-UK) Consortium	Sam Haldenby, Anita Lucaci, Steve Paterson, Julian Hiscox, Alistair Darby, M Almsaud, A Alrezaihi, Muhannad Alruwaili, Stuart D Armstrong, Jones Benjamin, Eleanor G Bentley, Anu Chawla, Jordan J Clark, Angela Cowell, Richard Eccles, Isabel García-Dorival, Matthew Gemmell, Alessandro Gerada, PKF Gilmore, Richard Gregory, Ximeng Han, Catherine Hartley, Margaret Hughes, Miren Iturriza-Gomara, James Johnson, L Luu, Jenifer Manson, Charlotte Nelson, Elaine O'Toole, Cassie Olateju, Rebekah Penrice-Randal , Lucille Rainbow, N.P Randle, Trevor Ian Robinson, Parul Sharma, Ghada T Shawli, James P Stewart, Neil Swainston, Ecaterina Vamos, Joanne Watts, Mark Whitehead
EPI_ISL_865499, EPI_ISL_865571, EPI_ISL_865572, EPI_ISL_865574, EPI_ISL_865575	Barts Health NHS Trust	COVID-19 Genomics UK (COG-UK) Consortium	CUTINO-MOGUEL, Maria-Teresa; HARRINGTON, David; OWOYEMI, Dola; KULASEGARAN-SHYLINI, Raghavendran; BROAD, Claire; KELE, Beatrix
EPI_ISL_866147	University College London Hospital	COVID-19 Genomics UK (COG-UK) Consortium	Judith Heaney, Matthew Byott, Catherine Houlihan, Dan Frampton, Stuart Kirk, Moira Spyer and Eleni Nastouli
EPI_ISL_866188, EPI_ISL_866189, EPI_ISL_866190	University College London, Great Ormond Street Hospital for Children NHS Foundation Trust, Imperial College Healthcare NHS Trust	COVID-19 Genomics UK (COG-UK) Consortium	Sergi Castellano, Rachel Williams, Mark Kristiansen, Paola Resende Silva, Sunando Roy, Tony Brooks, Helena Tutill, Paola Niola, Patricia Dyal, Charlotte Williams, Leysa Forrest, Yasmin Panchbhaya, Jacqueline Findlay, Samuel Weeks, Julianne Brown, Kathryn Harris, Paul Randell, James Price, Alison Holmes, Judith Breuer
EPI_ISL_866346, EPI_ISL_866347, EPI_ISL_866348	Regional Virus Laboratory, Belfast Health and Social Care Trust	COVID-19 Genomics UK (COG-UK) Consortium	Conall McCaughey, James McKenna, Tanya Curran, Susan Feeney, Alison Watt, Ciara Cox, Mairead Connor, Zoltan Molnar, David Simpson, Derek Fairley
EPI_ISL_866520, EPI_ISL_866523, EPI_ISL_866528, EPI_ISL_866529, EPI_ISL_866531, EPI_ISL_866533, EPI_ISL_866534, EPI_ISL_866535, EPI_ISL_866536, EPI_ISL_866537, EPI_ISL_866538, EPI_ISL_866539, EPI_ISL_866540			

see above	Northumbria University / South Tees Hospitals NHS Foundation Trust / North Cumbria Integrated Care NHS Foundation Trust / North Tees and Hartlepool NHS Foundation Trust / Newcastle Hospitals NHS Foundation Trust	COVID-19 Genomics UK (COG-UK) Consortium	Darren L Smith,Andrew Nelson,Matthew Bashton,Greg R Young,Joshua Loh,John Allan,Mohammad A Tariq,Giles S Holt,Gary Black,Wen C Yew,Lynn Dover,Paul Baker,Steve Liggett,Sarah Essex,Jane Greenaway,Debra Padgett,Clive Graham,Garren Scott,Edward Barton,Emma Swindells,Brendan Payne,Jennifer Collins,Yusri Taha,Gary Eltringham
EPI_ISL_866588, EPI_ISL_866589, EPI_ISL_866590, EPI_ISL_866591, EPI_ISL_866592, EPI_ISL_866593, EPI_ISL_866594, EPI_ISL_866595, EPI_ISL_866596, EPI_ISL_866597, EPI_ISL_866598, EPI_ISL_866599, EPI_ISL_866600, EPI_ISL_866601, EPI_ISL_866602			
see above	Quadram Institute Bioscience	COVID-19 Genomics UK (COG-UK) Consortium	Dave J. Baker, Gemma L. Kay, Alp Aydin, Thanh Le-Viet, Steven Rudder, Ana P. Tedim, Anastasia Kolyva, Maria Diaz, Leonardo de Oliveira Martins, Nabil-Fareed Alikhan, Lizzie Meadows, Rachael Stanley, Ngozi Elumogo, Muhammed Yasir, Nicholas M. Thomson, Alexander J Trotter, Rachel Gilroy, Samuel Bloomfield, Claire Stuart, Andrew Bell, Reenesh Prakash, Samir Dervisevic, Alison E. Mather, John Wain, Mark Webber, Andrew J. Page, Justin O'Grady
EPI_ISL_866906	Queens Medical Centre, Clinical Microbiology Department / DeepSeq Nottingham	COVID-19 Genomics UK (COG-UK) Consortium	Gemma Clark, Wendy Smith, Manjinder Khakh, Vicki M Fleming, Michelle M Lister, Hannah Howson-Wells, Jonathan Ball, Patrick McClure, Joseph Chappell, Theocharis Tsoleridis, Nadine Holmes, Matthew Carlisle, Christopher Moore, Fei Sang, Johnny Debebe, Victoria Wright, Matthew Loose
EPI_ISL_867008, EPI_ISL_867009, EPI_ISL_867011, EPI_ISL_867012, EPI_ISL_867013, EPI_ISL_867014, EPI_ISL_867015, EPI_ISL_867016, EPI_ISL_867017, EPI_ISL_867018, EPI_ISL_867019, EPI_ISL_867020, EPI_ISL_867021, EPI_ISL_867022, EPI_ISL_867023, EPI_ISL_867024, EPI_ISL_867025, EPI_ISL_867026, EPI_ISL_867027, EPI_ISL_867028, EPI_ISL_867029, EPI_ISL_867031			
see above	Lincolnshire Hospitals and DeepSeq Nottingham	COVID-19 Genomics UK (COG-UK) Consortium	Nichola Duckworth, Tim Sloan, Sarah Walsh, Jonathan Ball, Patrick McClure, Joeseph Chappell, Nadine Holmes, Matthew Carlisle, Christopher Moore, Fei Sang, Johnny Debebe, Victoria Wright, Matthew Loose
EPI_ISL_867091, EPI_ISL_867092, EPI_ISL_867099, EPI_ISL_867103, EPI_ISL_867105, EPI_ISL_867106, EPI_ISL_867107, EPI_ISL_867108, EPI_ISL_867110, EPI_ISL_867111, EPI_ISL_867112, EPI_ISL_867113, EPI_ISL_867114, EPI_ISL_867118, EPI_ISL_867119, EPI_ISL_867120, EPI_ISL_867121, EPI_ISL_867123, EPI_ISL_867125, EPI_ISL_867127, EPI_ISL_867128, EPI_ISL_867129, EPI_ISL_867131, EPI_ISL_867132, EPI_ISL_867139, EPI_ISL_867151, EPI_ISL_867152, EPI_ISL_867163			
see above	Oxford Viromics, NDM, University of Oxford; Oxford University Hospitals; Basingstoke and North Hampshire Hospital	COVID-19 Genomics UK (COG-UK) Consortium	Tanya Golubchik, David Bonsall, George Macintyre, Amy Trebes, Mariateresa de Cesare, Catrin Moore, Alex Mobbs, Anita Justice, Robert Shaw, Monique Andersson, Timothy Peto, Emma Wise, Nathan Moore, Jessica Lynch, Nick Cortes, Matilde Mori, Stephen Kidd, David Buck, John Todd, Christophe Fraser
EPI_ISL_867203, EPI_ISL_867204, EPI_ISL_867205, EPI_ISL_867431, EPI_ISL_867432, EPI_ISL_867433, EPI_ISL_867434	Originating lab: Wales Specialist Virology Centre Sequencing lab: Pathogen Genomics Unit	Public Health Wales Microbiology Cardiff Wales Specialist Virology Centre	Catherine Moore, Johnathan Evans, Laura Gifford, Malorie Perry, Simon Cottrell, Angela Marchbank, Alec Birchley, Alexander Adams, Amy Gaskin, Bree Gatica-Wilcox, Jason Coombes, Joel Southgate, Lauren Gilbert, Lee Graham, Nicole Pacchiarini, Sara Kumziene-Summerhayes, Sarah Taylor, Sophie Jones, Sara Rey, Matthew Bull, Joanne Watkins, Sally Corden, Tom Connor
EPI_ISL_868311, EPI_ISL_868314, EPI_ISL_868317, EPI_ISL_868318, EPI_ISL_868319, EPI_ISL_868320, EPI_ISL_868321, EPI_ISL_868328, EPI_ISL_868351, EPI_ISL_868352	Centre for Enzyme Innovation, University of Portsmouth / Translational Research Laboratory, Portsmouth Hospitals NHS Trust	COVID-19 Genomics UK (COG-UK) Consortium	Angela Beckett,Yann Bourgeois,Garry Scarlett,Sharon Glaysher,Scott Elliott,Kelly Bicknell,Robert Impey,Allyson Lloyd,Sarah Wyllie,Ethan Butcher,Anoop Chauhan,Samuel Robson
EPI_ISL_868479	Virology Department, Sheffield Teaching Hospitals NHS Foundation Trust/Department of Infection, Immunity and Cardiovascular Disease, The Medical School, University of Sheffield	COVID-19 Genomics UK (COG-UK) Consortium	Thushan de Silva, Matthew Parker, Nikki Smith, Adri Angyal, Rebecca Brown, Luke Green, Rachel Tucker, Paul Parsons, Danielle Groves, Katie Johnson, Laura Carrilero, Alex Keeley, Dave Partridge, Matthew Wyles, Benjamin Lindsey, Mehmet Yavuz, Mohammad Raza, Cariad Evans
EPI_ISL_869045, EPI_ISL_869046, EPI_ISL_869048, EPI_ISL_869049, EPI_ISL_869050, EPI_ISL_869055, EPI_ISL_869057, EPI_ISL_869059, EPI_ISL_869060, EPI_ISL_869063, EPI_ISL_869064, EPI_ISL_869065, EPI_ISL_869066, EPI_ISL_869068, EPI_ISL_869070, EPI_ISL_869076			
see above	Bioinformatics and Biostatistics Lab, Advanced Sequencing Facility	COVID-19 Genomics UK (COG-UK) Consortium	Aengus Stewart,Jerome Nicod,Chelsea Sawyer,Laura Cubitt,Harshil Patel,Margaret Crawford
EPI_ISL_869133	Charité Universitätsmedizin Berlin, Institut für Virologie/Labor Berlin	Charité Universitätsmedizin Berlin, Institut für Virologie	Victor M Corman, Barbara Mühlemann, Jörn Beheim-Schwarzbach, Tobias Bleicker, Julia Tesch, Talitha Veith, Julia Schneider, Terry Jones, Christian Drosten
EPI_ISL_871832, EPI_ISL_871833, EPI_ISL_871834, EPI_ISL_871835, EPI_ISL_871836, EPI_ISL_871837, EPI_ISL_871838, EPI_ISL_871839	Jessa	Jessa	Brigitte Maes, Bert Cruys
EPI_ISL_872204, EPI_ISL_872282	Labo Analyses Med	National Reference Center for Viruses of Respiratory Infections, Institut Pasteur, Paris	Marion Barbet, Sylvie Behillil, Méline Bizard, Angela Brisebarre, Camille Capel, Etienne Simon-Lorière, Vincent Enouf, Maud Vanpeene, Sylvie van der Werf,Girard Sophie
EPI_ISL_872575	South Eastern Area Laboratory Services (SEALS)	NSW Health Pathology - Institute of Clinical Pathology and Medical Research; Westmead Hospital; University of Sydney	CIDM-PH et al.
EPI_ISL_872744	Rhode Island Department of Health	Infectious Disease Program, Broad Institute of Harvard and MIT	Lemieux,J.E., Siddle,K.J., Huard,R., King,E., Azevedo,K., Miller,A., Adams,G., Gladden-Young,A., Lagerborg,K., Rudy,M., DeRuff,K., Carter,A., Normandin,E., Bauer,M., Reilly,S., Tomkins-Tinch,C., Loreth,C., Chaluvadi,S., Birren,B.W., Gallagher,G., Smole,S., Park,D.J., MacInnis,B.L., and Sabeti,P.C.
EPI_ISL_872972, EPI_ISL_872973, EPI_ISL_872974, EPI_ISL_872975, EPI_ISL_872976, EPI_ISL_872977, EPI_ISL_872978, EPI_ISL_872979, EPI_ISL_872980, EPI_ISL_872981, EPI_ISL_872982, EPI_ISL_872983, EPI_ISL_872984, EPI_ISL_873027, EPI_ISL_873028, EPI_ISL_873029, EPI_ISL_873030, EPI_ISL_873031, EPI_ISL_873032, EPI_ISL_873033, EPI_ISL_873035, EPI_ISL_873036, EPI_ISL_873037, EPI_ISL_873038, EPI_ISL_873039			
see above	HELIX LLC	WHO National Influenza Centre Russian Federation	Andrey Komissarov, Artem Fadeev, Anna Ivanova, Kseniya Komissarova, Dmitry Bazhenov, Mikhail Bakaev, Daria Danilenko, Ksenia Safina, Elena Nabieva, Georgii Bazykin, Dmitry Lioznov
EPI_ISL_873041, EPI_ISL_873042, EPI_ISL_873043, EPI_ISL_873044, EPI_ISL_873045, EPI_ISL_873046, EPI_ISL_873047, EPI_ISL_873048	University of Michigan Clinical Microbiology Laboratory	Lauring Lab, University of Michigan, Department of Microbiology and Immunology	Valesano
EPI_ISL_873184, EPI_ISL_873186, EPI_ISL_873187, EPI_ISL_873188, EPI_ISL_873189, EPI_ISL_873190, EPI_ISL_873191, EPI_ISL_873192, EPI_ISL_873193	Microbiology Division, South Carolina Department of Health and Environmental Control (SC DHEC)	Microbiology Division, South Carolina Department of Health and Environmental Control (SC DHEC)	Flores,H., Freeman,J.
EPI_ISL_873204, EPI_ISL_873205	North Dakota Department of Health, Public Health Laboratory	North Dakota Department of Health, Public Health Laboratory	Lisa Wingerter
EPI_ISL_875512	National Virus Reference Laboratory	National Virus Reference Laboratory	Michael Carr, Gabriel Gonzalez, Jonathan Dean, Cillian F De Gascun
EPI_ISL_876101	Massachusetts State Public Health Laboratory	Massachusetts State Public Health Laboratory	Andrew Lang, Timelia Fink, Glen Gallagher, Sandra Smole
EPI_ISL_876631, EPI_ISL_876667, EPI_ISL_876679, EPI_ISL_876697, EPI_ISL_876707, EPI_ISL_876710, EPI_ISL_876717	Helix/Illumina	Genomics and Discovery, Respiratory Viruses Branch, Division of Viral Diseases, Centers for Disease Control and Prevention	Peter W. Cook,Dhwani Batra,Ben L. Rambo-Martin,Eileen de Feo,Jan Antico,Christine Tran,Matthew Tolentino,Shannon Wickline,Kim Gietzen,Brad Sickler,Jingtao Liu,Eric Allen,Phil Febbo,Summer Galloway,Nicole L. Washington,Simon White,Geraint Levan,Kelly Schiabor Barrett,Elizabeth Cirulli,Alexandre Bolze,Ary Ascencio,Charlotte Rivera-Garcia,Ryan Cho,Jason Nguyen,Sherry Wang,Jimmy Ramirez,Tyler Cassens,Efren Sandoval,Magnus Isaksson,William Lee,David Becker,Marc Laurent,James Lu,Clinton R. Paden,Suxiang Tong,Duncan MacCannell,
EPI_ISL_878495, EPI_ISL_878498, EPI_ISL_878500, EPI_ISL_878576, EPI_ISL_878579, EPI_ISL_878581	Biolab Diagnostic Laboratories	Andersen lab at Scripps Research	Issa Abu-Dayyeh, Ahmad Tibi, Lama Hussein, Lina Mohammad, Zein Naber, Amid Abdelnour with SEARCH Alliance San Diego
EPI_ISL_878622, EPI_ISL_878627	San Diego County Public Health Laboratory	Andersen lab at Scripps Research	SEARCH Alliance San Diego with Tracy Basler, Jovan Shephard, Brett Austin
EPI_ISL_878695, EPI_ISL_878744,	Rady's Childrens Hospital	Andersen lab at Scripps Research	SEARCH Alliance San Diego with Nanda Radamchar, David Dimmock, Linda Luo, Christina Clarke, Kathryn Bouic, Teresa Mueller, Denise Malicki

EPI_ISL_880176, EPI_ISL_880177, EPI_ISL_880178			
EPI_ISL_882301, EPI_ISL_882302, EPI_ISL_882304, EPI_ISL_882306, EPI_ISL_882310, EPI_ISL_882311, EPI_ISL_882312, EPI_ISL_882313, EPI_ISL_882317, EPI_ISL_882318, EPI_ISL_882319, EPI_ISL_882321, EPI_ISL_882322, EPI_ISL_882325, EPI_ISL_882326, EPI_ISL_882328, EPI_ISL_882330, EPI_ISL_882331, EPI_ISL_882334, EPI_ISL_882335, EPI_ISL_882336, EPI_ISL_882337, EPI_ISL_882339, EPI_ISL_882341, EPI_ISL_882342, EPI_ISL_882344, EPI_ISL_882345, EPI_ISL_882346, EPI_ISL_882348, EPI_ISL_882349, EPI_ISL_882350, EPI_ISL_882351, EPI_ISL_882353, EPI_ISL_882354, EPI_ISL_882355, EPI_ISL_882356, EPI_ISL_882358, EPI_ISL_882359, EPI_ISL_882360, EPI_ISL_882362, EPI_ISL_882365, EPI_ISL_882366, EPI_ISL_882367, EPI_ISL_882368, EPI_ISL_882369, EPI_ISL_882373, EPI_ISL_882374, EPI_ISL_882379, EPI_ISL_882380, EPI_ISL_882381, EPI_ISL_882382, EPI_ISL_882383, EPI_ISL_882384, EPI_ISL_882386, EPI_ISL_882387, EPI_ISL_882389, EPI_ISL_882391, EPI_ISL_882392, EPI_ISL_882394, EPI_ISL_882395, EPI_ISL_882396, EPI_ISL_882399, EPI_ISL_882400, EPI_ISL_882401, EPI_ISL_882403, EPI_ISL_882406, EPI_ISL_882407, EPI_ISL_882408, EPI_ISL_882410, EPI_ISL_882411, EPI_ISL_882412, EPI_ISL_882414, EPI_ISL_882416, EPI_ISL_882417, EPI_ISL_882419, EPI_ISL_882422, EPI_ISL_882423, EPI_ISL_882424, EPI_ISL_882425, EPI_ISL_882426, EPI_ISL_882428, EPI_ISL_882429, EPI_ISL_882431, EPI_ISL_882432, EPI_ISL_882433, EPI_ISL_882434, EPI_ISL_882435, EPI_ISL_882436, EPI_ISL_882439, EPI_ISL_882443, EPI_ISL_882444, EPI_ISL_882445, EPI_ISL_882446, EPI_ISL_882447, EPI_ISL_882448, EPI_ISL_882449, EPI_ISL_882450, EPI_ISL_882451, EPI_ISL_882453, EPI_ISL_882454, EPI_ISL_882455, EPI_ISL_882456, EPI_ISL_882457, EPI_ISL_882458, EPI_ISL_882459, EPI_ISL_882460, EPI_ISL_882461, EPI_ISL_882462, EPI_ISL_882463, EPI_ISL_882464, EPI_ISL_882465, EPI_ISL_882466, EPI_ISL_882467, EPI_ISL_882468, EPI_ISL_882469, EPI_ISL_882470, EPI_ISL_882472, EPI_ISL_882474, EPI_ISL_882477, EPI_ISL_882478, EPI_ISL_882479, EPI_ISL_882482, EPI_ISL_882483, EPI_ISL_882485, EPI_ISL_882486, EPI_ISL_882488, EPI_ISL_882489, EPI_ISL_882490, EPI_ISL_882492, EPI_ISL_882493, EPI_ISL_882494, EPI_ISL_882495, EPI_ISL_882496, EPI_ISL_882497, EPI_ISL_882498, EPI_ISL_882499, EPI_ISL_882500, EPI_ISL_882501, EPI_ISL_882502, EPI_ISL_882503, EPI_ISL_882504, EPI_ISL_882505, EPI_ISL_882507, EPI_ISL_882508, EPI_ISL_882509, EPI_ISL_882510, EPI_ISL_882511, EPI_ISL_882513, EPI_ISL_882514, EPI_ISL_882515, EPI_ISL_882516, EPI_ISL_882517, EPI_ISL_882519, EPI_ISL_882521, EPI_ISL_882522, EPI_ISL_882523, EPI_ISL_882524, EPI_ISL_882526, EPI_ISL_882527, EPI_ISL_882528, EPI_ISL_882529, EPI_ISL_882531, EPI_ISL_882532, EPI_ISL_882533, EPI_ISL_882535, EPI_ISL_882536, EPI_ISL_882537, EPI_ISL_882541, EPI_ISL_882543, EPI_ISL_882544, EPI_ISL_882545, EPI_ISL_882546, EPI_ISL_882547, EPI_ISL_882549, EPI_ISL_882551, EPI_ISL_882552, EPI_ISL_882553, EPI_ISL_882554, EPI_ISL_882555, EPI_ISL_882557, EPI_ISL_882558, EPI_ISL_882560, EPI_ISL_882561, EPI_ISL_882564, EPI_ISL_882565, EPI_ISL_882566, EPI_ISL_882568, EPI_ISL_882569, EPI_ISL_882570, EPI_ISL_882572, EPI_ISL_882573, EPI_ISL_882574, EPI_ISL_882576, EPI_ISL_882577, EPI_ISL_882578, EPI_ISL_882581, EPI_ISL_882582, EPI_ISL_882583, EPI_ISL_882584, EPI_ISL_882587, EPI_ISL_882588, EPI_ISL_882592, EPI_ISL_882593, EPI_ISL_882594, EPI_ISL_882596, EPI_ISL_882598, EPI_ISL_882599, EPI_ISL_882600, EPI_ISL_882601, EPI_ISL_882602, EPI_ISL_882603, EPI_ISL_882604			
see above	Lighthouse Lab in Alderley Park	Wellcome Sanger Institute for the COVID-19 Genomics UK (COG-UK) Consortium	Jacquelyn Wynn, Mairead Hyland, The Lighthouse Lab in Alderley Park and Alex Alderton, Roberto Amato, Sonia Goncalves, Ewan Harrison, David K. Jackson, Ian Johnston, Dominic Kwiatkowski, Cordelia Langford, John Sillitoe on behalf of the Wellcome Sanger Institute COVID-19 Surveillance Team
EPI_ISL_882612, EPI_ISL_882613, EPI_ISL_882615	Hospital Ramón y Cajal	Hospital Ramón y Cajal	José M Gonzalez-Alba, Concepción Rodríguez, Melanie Abreu, Laura Martínez, Val F Lanza, Luz Leticia Olavarrieta, Rafael Cantón, JC Galán
EPI_ISL_882637, EPI_ISL_882638, EPI_ISL_882639	Azerbaijan National Hematology Center Division of Medical Genetics	Azerbaijan National Hematology Center Division of Medical Genetics	Aghayev Agha Rza, Bayraml Ramin
EPI_ISL_882918	INMI Lazzaro Spallanzani IRCCS	INMI Lazzaro Spallanzani IRCCS	E Giombini, M. Rueca, B Bartolini, O Butera, C.E.M Gruber, F Messina, MR Capobianchi, A Di Caro
EPI_ISL_882920	INMI Lazzaro Spallanzani IRCCS	INMI Lazzaro Spallanzani IRCCS	M Rueca, E Giombini, C.E.M Gruber, B Bartolini, O Butera, F Messina, A Di Caro, MR Capobianchi
EPI_ISL_882926	INMI Lazzaro Spallanzani IRCCS	INMI Lazzaro Spallanzani IRCCS	B Bartolini, O Butera, C.E.M Gruber, M Rueca, F Messina, E Giombini, MR Capobianchi, A Di Caro
EPI_ISL_882928	INMI Lazzaro Spallanzani IRCCS	INMI Lazzaro Spallanzani IRCCS	F Messina, O Butera, E Giombini, M Rueca, B Bartolini, C.E.M Gruber, MR Capobianchi, A Di Caro
EPI_ISL_882929	INMI Lazzaro Spallanzani IRCCS	INMI Lazzaro Spallanzani IRCCS	C.E.M Gruber, B Bartolini, E Giombini, M Rueca, O Butera, F Messina, A Di Caro, MR Capobianchi
EPI_ISL_882930	INMI Lazzaro Spallanzani IRCCS	INMI Lazzaro Spallanzani IRCCS	E Giombini, M. Rueca, B Bartolini, O Butera, C.E.M Gruber, F Messina, MR Capobianchi, A Di Caro
EPI_ISL_882931	INMI Lazzaro Spallanzani IRCCS	INMI Lazzaro Spallanzani IRCCS	B Bartolini, O Butera, C.E.M Gruber, M Rueca, F Messina, E Giombini, MR Capobianchi, A Di Caro
EPI_ISL_882945, EPI_ISL_882946, EPI_ISL_882947, EPI_ISL_882948, EPI_ISL_882949	The National Institute of Public Health	State Veterinary Institute Prague	Nagy,A;Jirincova,H;Vecerova,JTrnka,D
EPI_ISL_883286, EPI_ISL_883287	SIESP DIPARTIMENTO DI PREVENZIONE CHIE	Istituto Zooprofilattico Sperimentale dell'Abruzzo e Molise "G. Caporale"	Lorusso A, Marcacci M, Di Domenico M, Ancora M, Curini V, Mangone I, Rinaldi A, Scialabba S, Di Pasquale A, Cammà C, Puglia I, Calistri P, Savini G
EPI_ISL_883288, EPI_ISL_883289, EPI_ISL_883296	SIESP CHIETI-DRIVE IN ORTONA	Istituto Zooprofilattico Sperimentale dell'Abruzzo e Molise "G. Caporale"	Lorusso A, Marcacci M, Di Domenico M, Ancora M, Curini V, Mangone I, Rinaldi A, Scialabba S, Di Pasquale A, Cammà C, Puglia I, Calistri P, Savini G
EPI_ISL_883332	Department of Homeless Services	New York City Public Health Laboratory	Jade Wang, et al.
EPI_ISL_883351, EPI_ISL_883352, EPI_ISL_883353	OCME Office Of Chief Medical Examiner	New York City Public Health Laboratory	Jade Wang, et al.
EPI_ISL_885022, EPI_ISL_885026, EPI_ISL_885027, EPI_ISL_885028, EPI_ISL_885029, EPI_ISL_885030, EPI_ISL_885031, EPI_ISL_885032, EPI_ISL_885035, EPI_ISL_885037, EPI_ISL_885038, EPI_ISL_885041, EPI_ISL_885042, EPI_ISL_885044, EPI_ISL_885045, EPI_ISL_885046, EPI_ISL_885047, EPI_ISL_885048, EPI_ISL_885050			
see above	Santa Clara County Public Health Laboratory	Chan-Zuckerberg Biohub	CZB Cliahub Consortium
EPI_ISL_886162, EPI_ISL_886163, EPI_ISL_886164, EPI_ISL_886166, EPI_ISL_886167, EPI_ISL_886173, EPI_ISL_886175, EPI_ISL_886183, EPI_ISL_886190, EPI_ISL_886196, EPI_ISL_886197, EPI_ISL_886198, EPI_ISL_886203, EPI_ISL_886222, EPI_ISL_886230, EPI_ISL_886234, EPI_ISL_886236, EPI_ISL_886239, EPI_ISL_886256, EPI_ISL_886278, EPI_ISL_886282, EPI_ISL_886296, EPI_ISL_886304, EPI_ISL_886307, EPI_ISL_886313, EPI_ISL_886315, EPI_ISL_886316, EPI_ISL_886323, EPI_ISL_886325, EPI_ISL_886328, EPI_ISL_886329, EPI_ISL_886334, EPI_ISL_886335, EPI_ISL_886368, EPI_ISL_886382, EPI_ISL_886389, EPI_ISL_886394, EPI_ISL_886408, EPI_ISL_886410, EPI_ISL_886415, EPI_ISL_886419, EPI_ISL_886425, EPI_ISL_886428, EPI_ISL_886441, EPI_ISL_886449, EPI_ISL_886453, EPI_ISL_886456, EPI_ISL_886458, EPI_ISL_886459, EPI_ISL_886462, EPI_ISL_886468, EPI_ISL_886480, EPI_ISL_886481, EPI_ISL_886484, EPI_ISL_886486, EPI_ISL_886490, EPI_ISL_886493, EPI_ISL_886500, EPI_ISL_886501, EPI_ISL_886507, EPI_ISL_886512, EPI_ISL_886517, EPI_ISL_886524, EPI_ISL_886536, EPI_ISL_886548, EPI_ISL_886549, EPI_ISL_886556, EPI_ISL_886573, EPI_ISL_886578, EPI_ISL_886585, EPI_ISL_886598, EPI_ISL_886601, EPI_ISL_886604, EPI_ISL_886605, EPI_ISL_886607, EPI_ISL_886611, EPI_ISL_886615, EPI_ISL_886621, EPI_ISL_886623, EPI_ISL_886629, EPI_ISL_886639, EPI_ISL_886641, EPI_ISL_886642, EPI_ISL_886643, EPI_ISL_886644, EPI_ISL_886648, EPI_ISL_886660, EPI_ISL_886664, EPI_ISL_886667, EPI_ISL_886670, EPI_ISL_886673, EPI_ISL_886679, EPI_ISL_886680, EPI_ISL_886681, EPI_ISL_886686, EPI_ISL_886698, EPI_ISL_886704, EPI_ISL_886708, EPI_ISL_886724, EPI_ISL_886733, EPI_ISL_886738, EPI_ISL_886739, EPI_ISL_886755, EPI_ISL_886760, EPI_ISL_886762, EPI_ISL_886769, EPI_ISL_886770, EPI_ISL_886780, EPI_ISL_886785, EPI_ISL_886787, EPI_ISL_886791, EPI_ISL_886792, EPI_ISL_886793, EPI_ISL_886795, EPI_ISL_886809, EPI_ISL_886811, EPI_ISL_886812, EPI_ISL_886831, EPI_ISL_886832, EPI_ISL_886845, EPI_ISL_886868, EPI_ISL_886877, EPI_ISL_886913, EPI_ISL_886922, EPI_ISL_886929, EPI_ISL_886930, EPI_ISL_886939, EPI_ISL_886943, EPI_ISL_886947, EPI_ISL_886962, EPI_ISL_886982, EPI_ISL_886985, EPI_ISL_886989, EPI_ISL_886995, EPI_ISL_887009, EPI_ISL_887014, EPI_ISL_887015, EPI_ISL_887021, EPI_ISL_887030, EPI_ISL_887040, EPI_ISL_887051, EPI_ISL_887056, EPI_ISL_887062, EPI_ISL_887069, EPI_ISL_887076, EPI_ISL_887090, EPI_ISL_887096, EPI_ISL_887100, EPI_ISL_887101, EPI_ISL_887104			
see above	Labcorp	Genomics and Discovery, Respiratory Viruses Branch, Division of Viral Diseases, Centers for Disease Control and Prevention	Peter W. Cook,Dhwani Batra,Ben L. Rambo-Martin,Summer Galloway,Brian Krueger,Minoo Agarwal,Eyad Almasri,Debbie Boles,Ayla Burns,Nuthawin Charoensri,Oren Cohen,Susan Countryman,Mary Ann Cristobal,Bobbi Croy,Suzanne Dale,Hrushikesh Deshmukh,Amanda Douglas,Vincent Drouillon,Marcia Eisenberg,Howard Engler,Rama Ghatti,Prashant Gupta,Susan Hicks,Jake Humphrey,Lax Iyer,Manoj Jain,Mohan Kolli,Tim Kuphal,Stanley Letovsky,Michael Levandoski,Craig Lukasik,Jonathan Meltzer,Brian Norvell,Mindy Nye,Scott Parker,Christos Petropoulos,John Pruitt,Steven Ragan,Scott Ryan,Mike Sapeta,Jana Schroth,Suresh Babu Selvaraju,Goran Stevovic,Amanda Suchanek,Andrea Throop,Lyndon Tilson,Thomas Urban,Joe Voshell,Kimberly Wagner,Jonathan Williams,Mary Williamson,Qian Zeng,Tricia Zwiefelhofer,Clinton R. Paden,Suxiang Tong,Duncan MacCannell,
EPI_ISL_887115, EPI_ISL_887118, EPI_ISL_887126, EPI_ISL_887127, EPI_ISL_887135, EPI_ISL_887142, EPI_ISL_887143	Institute of Medical Microbiology and Hospital Hygiene	Institute of Medical Microbiology and Hospital Hygiene	Prof. Dr. Achim Kaasch, Aljoscha Tersteegen
EPI_ISL_887531, EPI_ISL_887535, EPI_ISL_887536	Johns Hopkins Hospital Department of Pathology	Johns Hopkins Hospital Department of Pathology	C. Paul Morris, Chun Huai Luo, Adannaya Amadi, Matthew Schwartz, Nicholas Gallagher, Heba H. Mostafa
EPI_ISL_887595, EPI_ISL_887617, EPI_ISL_887619, EPI_ISL_887628, EPI_ISL_887641, EPI_ISL_887647, EPI_ISL_887651, EPI_ISL_887671, EPI_ISL_887674, EPI_ISL_887677, EPI_ISL_887679, EPI_ISL_887702, EPI_ISL_887716, EPI_ISL_887725, EPI_ISL_887729, EPI_ISL_887733, EPI_ISL_887739, EPI_ISL_887748, EPI_ISL_887753, EPI_ISL_887754, EPI_ISL_887756, EPI_ISL_887757, EPI_ISL_887758, EPI_ISL_887765, EPI_ISL_887784, EPI_ISL_887798, EPI_ISL_887807, EPI_ISL_887810, EPI_ISL_887817, EPI_ISL_887829, EPI_ISL_887841, EPI_ISL_887845, EPI_ISL_887852, EPI_ISL_887857, EPI_ISL_887866, EPI_ISL_887910, EPI_ISL_887917, EPI_ISL_887921, EPI_ISL_887923, EPI_ISL_887928, EPI_ISL_887945, EPI_ISL_887947, EPI_ISL_887949, EPI_ISL_887950, EPI_ISL_887966, EPI_ISL_887974, EPI_ISL_887975, EPI_ISL_887981, EPI_ISL_887984, EPI_ISL_887985, EPI_ISL_887993, EPI_ISL_887995, EPI_ISL_888007, EPI_ISL_888009, EPI_ISL_888012, EPI_ISL_888028, EPI_ISL_888032, EPI_ISL_888041, EPI_ISL_888054, EPI_ISL_888059, EPI_ISL_888060, EPI_ISL_888065, EPI_ISL_888071, EPI_ISL_888094, EPI_ISL_888100, EPI_ISL_888104, EPI_ISL_888112, EPI_ISL_888123, EPI_ISL_888125, EPI_ISL_888127, EPI_ISL_888157, EPI_ISL_888159, EPI_ISL_888173, EPI_ISL_888186, EPI_ISL_888196, EPI_ISL_888211, EPI_ISL_888219, EPI_ISL_888223, EPI_ISL_888234, EPI_ISL_888238, EPI_ISL_888304, EPI_ISL_888318, EPI_ISL_888324, EPI_ISL_888401, EPI_ISL_888450, EPI_ISL_888511, EPI_ISL_888515, EPI_ISL_888516, EPI_ISL_888522, EPI_ISL_888523, EPI_ISL_888524, EPI_ISL_888525, EPI_ISL_888526, EPI_ISL_888543, EPI_ISL_888545, EPI_ISL_888546, EPI_ISL_888553, EPI_ISL_888556, EPI_ISL_888559, EPI_ISL_888560, EPI_ISL_888563, EPI_ISL_888572, EPI_ISL_888587			
see above	Labcorp	Genomics and Discovery, Respiratory Viruses Branch, Division of Viral Diseases, Centers for Disease Control and Prevention	Peter W. Cook,Dhwani Batra,Ben L. Rambo-Martin,Summer Galloway,Brian Krueger,Minoo Agarwal,Eyad Almasri,Debbie Boles,Ayla Burns,Nuthawin Charoensri,Oren Cohen,Susan Countryman,Mary Ann Cristobal,Bobbi Croy,Suzanne Dale,Hrushikesh Deshmukh,Amanda Douglas,Vincent Drouillon,Marcia Eisenberg,Howard Engler,Rama Ghatti,Prashant Gupta,Susan Hicks,Jake Humphrey,Lax Iyer,Manoj Jain,Mohan Kolli,Tim Kuphal,Stanley Letovsky,Michael Levandoski,Craig Lukasik,Jonathan Meltzer,Brian Norvell,Mindy Nye,Scott Parker,Christos Petropoulos,John Pruitt,Steven Ragan,Scott Ryan,Mike Sapeta,Jana Schroth,Suresh Babu Selvaraju,Goran Stevovic,Amanda Suchanek,Andrea Throop,Lyndon Tilson,Thomas Urban,Joe Voshell,Kimberly Wagner,Jonathan Williams,Mary Williamson,Qian Zeng,Tricia Zwiefelhofer,Clinton R. Paden,Suxiang Tong,Duncan MacCannell,
EPI_ISL_889004	RS Mitra Keluarga Gading Serpong	Eijkman Institute for Molecular Biology, Ministry of Research and Technology/National Agency for Research and Innovation	Willy Agustine, Edison Johar, Hidayat Trimarsanto, Iskandar Adnan, Lydia V. Panggalo, Sukma Oktavianthi, Frilasita A Yudhaputri, Safarina G Malik, Khin Saw Myint, Amin Soebandrio

EPI_ISL_889552, EPI_ISL_889553, EPI_ISL_889554, EPI_ISL_889555, EPI_ISL_889556, EPI_ISL_889557, EPI_ISL_889558, EPI_ISL_889559, EPI_ISL_889560, EPI_ISL_889561, EPI_ISL_889562, EPI_ISL_889563, EPI_ISL_889564, EPI_ISL_889565, EPI_ISL_889566, EPI_ISL_889567			
see above	LSUHS Emerging Viral Threat Laboratory	Microbial Genome Sequencing Center	Jeremy P. Kamil, Jennifer L. Carroll, Camille F. Abshire, Maarten Van Diest, Mohammed N.A. Siddiquey, Andrew D. Yurochko, Martin J. Sapp, Rona S. Scott, Christopher G. Kevil, Daniel J. Snyder, Vaughn S. Cooper, John A. Vanchiere
EPI_ISL_890280, EPI_ISL_890283, EPI_ISL_890284, EPI_ISL_890287, EPI_ISL_890288, EPI_ISL_890291, EPI_ISL_890294, EPI_ISL_890298, EPI_ISL_890299, EPI_ISL_890304, EPI_ISL_890305, EPI_ISL_890306, EPI_ISL_890320, EPI_ISL_890322, EPI_ISL_890329, EPI_ISL_890333			
see above	KU Leuven, Rega Institute, Clinical and Epidemiological Virology	KU Leuven, Rega Institute, Clinical and Epidemiological Virology	Tony Wawina-Bokalanga, Bert Vanmechelen, Joan Marti-Carerras, Piet Maes
EPI_ISL_890352	Labo Analyses Med	National Reference Center for Viruses of Respiratory Infections, Institut Pasteur, Paris	Marion Barbet, Sylvie Behillil, Méline Bizard, Angela Brisebarre, Camille Capel, Etienne Simon-Lorière, Vincent Enouf, Maud Vanpeene, Sylvie van der Werf,Rousset Dominique
EPI_ISL_891137	The Jackson Laboratory	The Jackson Laboratory	Lloyd M, Sanderson B, Srivastava A, Maurya R, Renzette N, Omerza G, Kelly K, Li L, Wei C L, Adams M
EPI_ISL_892059, EPI_ISL_892061, EPI_ISL_892062, EPI_ISL_892063, EPI_ISL_892064, EPI_ISL_892065, EPI_ISL_892066	Lighthouse Lab in Alderley Park	Wellcome Sanger Institute for the COVID-19 Genomics UK (COG-UK) Consortium	Jacquelyn Wynn, Mairead Hyland, The Lighthouse Lab in Alderley Park and Alex Alderton, Roberto Amato, Sonia Goncalves, Ewan Harrison, David K. Jackson, Ian Johnston, Dominic Kwiatkowski, Cordelia Langford, John Sillitoe on behalf of the Wellcome Sanger Institute COVID-19 Surveillance Team
EPI_ISL_893746, EPI_ISL_893775	Institute of Virology, Medical Center, University of Freiburg, Freiburg, Germany	Institute of Virology, Clinial Virus Genomics, Medical Center, University of Freiburg, Freiburg, Germany	Jonas Fuchs, Lisa Kern, Sandra Reuter, Hajo Grundmann, Marcus Panning
EPI_ISL_894171	KU Leuven, Rega Institute, Clinical and Epidemiological Virology	KU Leuven, Rega Institute, Clinical and Epidemiological Virology	Tony Wawina-Bokalanga, Bert Vanmechelen, Joan Marti-Carerras, Piet Maes
EPI_ISL_896073	Labormedizinisches Zentrum Dr Risch	University Hospital Basel, Clinical Bacteriology	Tim Roloff, Madlen Stange, Helena MB Seth-Smith, Alfredo Mari, Karoline Leuzinger, Julia Bielicki, Nadia Wohlwend,Martin Risch, Lorenz Risch, Manuel Battegay, Hans Hirsch, Adrian Egli
EPI_ISL_896074, EPI_ISL_896075, EPI_ISL_896076	Viollier AG	University Hospital Basel, Clinical Bacteriology	Tim Roloff, Madlen Stange, Helena MB Seth-Smith, Alfredo Mari, Karoline Leuzinger, Julia Bielicki, Christiane Beckmann, Manuel Battegay, Hans Hirsch, Adrian Egli
EPI_ISL_896096, EPI_ISL_896108	Labormedizinisches Zentrum Dr Risch	University Hospital Basel, Clinical Bacteriology	Tim Roloff, Madlen Stange, Helena MB Seth-Smith, Alfredo Mari, Karoline Leuzinger, Julia Bielicki, Nadia Wohlwend,Martin Risch, Lorenz Risch, Manuel Battegay, Hans Hirsch, Adrian Egli
EPI_ISL_896110	Viollier AG	University Hospital Basel, Clinical Bacteriology	Tim Roloff, Madlen Stange, Helena MB Seth-Smith, Alfredo Mari, Karoline Leuzinger, Julia Bielicki, Christiane Beckmann, Manuel Battegay, Hans Hirsch, Adrian Egli
EPI_ISL_896167, EPI_ISL_896209	MEPHI, Aix Marseille University	MEPHI, Aix Marseille University	Anthony LEVASSEUR
EPI_ISL_896422, EPI_ISL_896423, EPI_ISL_896424, EPI_ISL_896425, EPI_ISL_896426, EPI_ISL_896427, EPI_ISL_896428, EPI_ISL_896429	Columbia University Irving Medical Center	Wadsworth Center, New York State Department of Health	Kirsten St. George, Daryl M. Lamson, Alexis Russel, Matthew Shudt, Melissa A Leisner, Jonathan Plitnick, Navjot Singh, John Kelly, Erasmus Schneider, Erica Lasek-Nesselquist
EPI_ISL_899006, EPI_ISL_899017, EPI_ISL_899050, EPI_ISL_899051, EPI_ISL_899085, EPI_ISL_899092, EPI_ISL_899101, EPI_ISL_899107, EPI_ISL_899108, EPI_ISL_899112, EPI_ISL_899113, EPI_ISL_899114, EPI_ISL_899127, EPI_ISL_899128, EPI_ISL_899131, EPI_ISL_899145, EPI_ISL_899146, EPI_ISL_899156, EPI_ISL_899162, EPI_ISL_899172, EPI_ISL_899197, EPI_ISL_899198, EPI_ISL_899199, EPI_ISL_899200, EPI_ISL_899201, EPI_ISL_899202, EPI_ISL_899203, EPI_ISL_899204, EPI_ISL_899205, EPI_ISL_899206, EPI_ISL_899207, EPI_ISL_899208, EPI_ISL_899209, EPI_ISL_899210, EPI_ISL_899211, EPI_ISL_899212, EPI_ISL_899213, EPI_ISL_899214, EPI_ISL_899215, EPI_ISL_899216, EPI_ISL_899217, EPI_ISL_899302, EPI_ISL_899303, EPI_ISL_899304, EPI_ISL_899305, EPI_ISL_899306, EPI_ISL_899307, EPI_ISL_899308, EPI_ISL_899309, EPI_ISL_899310, EPI_ISL_899311, EPI_ISL_899312, EPI_ISL_899313, EPI_ISL_899314, EPI_ISL_899315, EPI_ISL_899316, EPI_ISL_899317, EPI_ISL_899429, EPI_ISL_899430, EPI_ISL_899431, EPI_ISL_899432, EPI_ISL_899433, EPI_ISL_899434, EPI_ISL_899435, EPI_ISL_899436, EPI_ISL_899437, EPI_ISL_899438, EPI_ISL_899524, EPI_ISL_899525, EPI_ISL_899526, EPI_ISL_899527, EPI_ISL_899528, EPI_ISL_899549, EPI_ISL_899550, EPI_ISL_899551, EPI_ISL_899563, EPI_ISL_899631, EPI_ISL_899632, EPI_ISL_899633, EPI_ISL_899634, EPI_ISL_899635, EPI_ISL_899636, EPI_ISL_899637, EPI_ISL_899638, EPI_ISL_899639, EPI_ISL_899668, EPI_ISL_899669, EPI_ISL_899670, EPI_ISL_899671, EPI_ISL_899672, EPI_ISL_899673, EPI_ISL_899674, EPI_ISL_899675, EPI_ISL_899676, EPI_ISL_899677, EPI_ISL_899678, EPI_ISL_899679, EPI_ISL_899680, EPI_ISL_899681, EPI_ISL_899682, EPI_ISL_899683, EPI_ISL_899684, EPI_ISL_899685, EPI_ISL_899781, EPI_ISL_899782, EPI_ISL_899838, EPI_ISL_899839, EPI_ISL_899882, EPI_ISL_899883, EPI_ISL_899975, EPI_ISL_899976, EPI_ISL_899977, EPI_ISL_899978, EPI_ISL_899979, EPI_ISL_899980, EPI_ISL_899981, EPI_ISL_899982, EPI_ISL_899983, EPI_ISL_899984			
see above	Viollier AG	Department of Biosystems Science and Engineering, ETH Zürich	Christian Beisel, Sarah Nadeau, Chaoran Chen, Ivan Topolsky, Philipp Jablonski, Lara Fuhrmann, David Dreifuss, Katharina Jahn, Tobias Schär, Ina Nissen, Natascha Santacroce, Elodie Burcklen, Christiane Beckmann, Maurice Redondo, Olivier Kobel, Christoph Noppen, Sophie Seidel, Noemie Santamaria de Souza, Niko Beerenwinkel, Tanja Stadler
EPI_ISL_900511	Oriade	CNR Virus des Infections Respiratoires - France SUD	Antonin Bal, Gregory Destras, Gwendolyne Burfin, Hadrien Règue, Quentin Semanas, Martine Valette, Bruno Lina, Sylvie Larrat, Laurence Josset
EPI_ISL_900518	Bioesterel	CNR Virus des Infections Respiratoires - France SUD	Antonin Bal, Gregory Destras, Gwendolyne Burfin, Hadrien Règue, Quentin Semanas, Martine Valette, Bruno Lina, Sylvie Larrat, Laurence Josset
EPI_ISL_900519	Bioesterel	CNR Virus des Infections Respiratoires - France SUD	Antonin Bal, Géraldine Gonfrier, Gregory Destras, Gwendolyne Burfin, Hadrien Règue, Quentin Semanas, Martine Valette, Bruno Lina, Valérie Giordanengo, Laurence Josset
EPI_ISL_900524	Labosud	CNR Virus des Infections Respiratoires - France SUD	Antonin Bal, Gregory Destras, Gwendolyne Burfin, Hadrien Règue, Quentin Semanas, Martine Valette, Bruno Lina, Laurence Josset
EPI_ISL_900565	CNR Virus des Infections Respiratoires - France SUD	CNR Virus des Infections Respiratoires - France SUD	Antonin Bal, Gregory Destras, Gwendolyne Burfin, Hadrien Règue, Quentin Semanas, Martine Valette, Bruno Lina, Laurence Josset
EPI_ISL_902759, EPI_ISL_902760, EPI_ISL_902761, EPI_ISL_902762, EPI_ISL_902763, EPI_ISL_902764, EPI_ISL_902765, EPI_ISL_902766, EPI_ISL_902767	Department of Virology and Immunology, University of Helsinki and Helsinki University Hospital, Huslab Finland	Department of Virology, Faculty of Medicine, University of Helsinki, Helsinki, Finland	Teemu Smura, Ravi Kant, Phuoc Truong, Hussein Alburkat, Hannimari Kallio-Kokko, Jenni Virtanen, Maija Suvanto, Essi Korhonen, Sari Hannula, Harri Kangas, Hanna Liimatainen, Satu Kurkela, Hanna Jarva, Maija Lappalainen, Pekka Ellonen, Olli Vapalahti
EPI_ISL_903061, EPI_ISL_903062, EPI_ISL_903063, EPI_ISL_903092, EPI_ISL_903093, EPI_ISL_903094, EPI_ISL_903095, EPI_ISL_903096, EPI_ISL_903097, EPI_ISL_903098, EPI_ISL_903099, EPI_ISL_903100, EPI_ISL_903101, EPI_ISL_903102, EPI_ISL_903103			
see above	Seattle Flu Study	Seattle Flu Study	Deborah A. Nickerson, Chris D. Frazar, Jover Lee, Benjamin Pelle, Erica Ryke, Matthew Richardson, Amanda Adler, Elisabeth Brandstetter, Peter D. Han, Kairsten Fay, Misja Ilcisin, Kirsten Lacombe, Thomas R. Sibley, Melissa Truong, Caitlin R. Wolf, Karen Cowgill, Stephanie Schrag, Jeff Duchin, Michael Boeckh, Janet A. Englund, Michael Famulare, Barry R. Lutz, Mark J. Rieder, Lea M. Starita, Matthew Thompson, Helen Y. Chu, Trevor Bedford, Jay Shendure
EPI_ISL_903108, EPI_ISL_903110, EPI_ISL_903111, EPI_ISL_903113, EPI_ISL_903120, EPI_ISL_903121, EPI_ISL_903122, EPI_ISL_903123, EPI_ISL_903129, EPI_ISL_903130, EPI_ISL_903131, EPI_ISL_903132, EPI_ISL_903133, EPI_ISL_903134, EPI_ISL_903135, EPI_ISL_903149			
see above	Washington State Department of Health	Seattle Flu Study	Deborah A. Nickerson, Chris D. Frazar, Jover Lee, Benjamin Pelle, Erica Ryke, Matthew Richardson, Amanda Adler, Elisabeth Brandstetter, Peter D. Han, Kairsten Fay, Misja Ilcisin, Kirsten Lacombe, Thomas R. Sibley, Melissa Truong, Caitlin R. Wolf, Romesh Gautom, Geoff Melly, Brian Hiatt, Philip Dykema, Scott Lindquist, Michael Boeckh, Janet A. Englund, Michael Famulare, Barry R. Lutz, Mark J. Rieder, Lea M. Starita, Matthew Thompson, Helen Y. Chu, Jay Shendure, Trevor Bedford
EPI_ISL_903231, EPI_ISL_903238, EPI_ISL_903239, EPI_ISL_903281, EPI_ISL_903284, EPI_ISL_903285, EPI_ISL_903287, EPI_ISL_903289, EPI_ISL_903290, EPI_ISL_903292, EPI_ISL_903294, EPI_ISL_903299, EPI_ISL_903303, EPI_ISL_903304, EPI_ISL_903305, EPI_ISL_903306, EPI_ISL_903309, EPI_ISL_903311			
see above	M Health Fairview	Minnesota Department of Health, Public Health Laboratory	Alexandra Lorentz, Jacob Garfin, Matt Plumb, and Xiong Wang
EPI_ISL_903567, EPI_ISL_903614, EPI_ISL_903626	NM Dept. Health, Scientific Laboratory Division	Genomics and Discovery, Respiratory Viruses Branch, Division of Viral Diseases, Centers for Disease Control and Prevention	Krista Queen, Yan Li, Ying Tao, Jing Zhang, Anna Uehara, Anna Montmayeur, Clinton R. Paden, Peter W. Cook, Rachel Marine, Mili Sheth, Jasmine Padilla, Sarah Nobles, Mark Burroughs, Lori Rowe, Haibin Wang, Ben L. Rambo-Martin, Dhwani Batra, Justin Lee, Suxiang Tong
EPI_ISL_903668, EPI_ISL_903671, EPI_ISL_903672, EPI_ISL_903688, EPI_ISL_903717	SC Dept of Health and Env. Control-Bureau of Laboratories	Genomics and Discovery, Respiratory Viruses Branch, Division of Viral Diseases, Centers for Disease Control and Prevention	Krista Queen, Yan Li, Ying Tao, Jing Zhang, Anna Uehara, Anna Montmayeur, Clinton R. Paden, Peter W. Cook, Rachel Marine, Mili Sheth, Jasmine Padilla, Sarah Nobles, Mark Burroughs, Lori Rowe, Haibin Wang, Ben L. Rambo-Martin, Dhwani Batra, Justin Lee, Suxiang Tong
EPI_ISL_903752	LA Office of Public Health Laboratories	Genomics and Discovery, Respiratory Viruses Branch, Division of Viral Diseases, Centers for Disease Control and Prevention	Krista Queen, Yan Li, Ying Tao, Jing Zhang, Anna Uehara, Anna Montmayeur, Clinton R. Paden, Peter W. Cook, Rachel Marine, Mili Sheth, Jasmine Padilla, Sarah Nobles, Mark Burroughs, Lori Rowe, Haibin Wang, Ben L. Rambo-Martin, Dhwani Batra, Justin Lee, Suxiang Tong

EPI_ISL_903769, EPI_ISL_903804	NM Dept. Health, Scientific Laboratory Division	Genomics and Discovery, Respiratory Viruses Branch, Division of Viral Diseases, Centers for Disease Control and Prevention	Krista Queen, Yan Li, Ying Tao, Jing Zhang, Anna Uehara, Anna Montmayeur, Clinton R. Paden, Peter W. Cook, Rachel Marine, Mili Sheth, Jasmine Padilla, Sarah Nobles, Mark Burroughs, Lori Rowe, Haibin Wang, Ben L. Rambo-Martin, Dhwani Batra, Justin Lee, Suxiang Tong
EPI_ISL_903819	SC Dept of Health and Env. Control-Bureau of Laboratories	Genomics and Discovery, Respiratory Viruses Branch, Division of Viral Diseases, Centers for Disease Control and Prevention	Krista Queen, Yan Li, Ying Tao, Jing Zhang, Anna Uehara, Anna Montmayeur, Clinton R. Paden, Peter W. Cook, Rachel Marine, Mili Sheth, Jasmine Padilla, Sarah Nobles, Mark Burroughs, Lori Rowe, Haibin Wang, Ben L. Rambo-Martin, Dhwani Batra, Justin Lee, Suxiang Tong
EPI_ISL_903833	CDPH, Viral and Rickettsial Disease Laboratory	Genomics and Discovery, Respiratory Viruses Branch, Division of Viral Diseases, Centers for Disease Control and Prevention	Krista Queen, Yan Li, Ying Tao, Jing Zhang, Anna Uehara, Anna Montmayeur, Clinton R. Paden, Peter W. Cook, Rachel Marine, Mili Sheth, Jasmine Padilla, Sarah Nobles, Mark Burroughs, Lori Rowe, Haibin Wang, Ben L. Rambo-Martin, Dhwani Batra, Justin Lee, Suxiang Tong
EPI_ISL_903839	NM Dept. Health, Scientific Laboratory Division	Genomics and Discovery, Respiratory Viruses Branch, Division of Viral Diseases, Centers for Disease Control and Prevention	Krista Queen, Yan Li, Ying Tao, Jing Zhang, Anna Uehara, Anna Montmayeur, Clinton R. Paden, Peter W. Cook, Rachel Marine, Mili Sheth, Jasmine Padilla, Sarah Nobles, Mark Burroughs, Lori Rowe, Haibin Wang, Ben L. Rambo-Martin, Dhwani Batra, Justin Lee, Suxiang Tong
EPI_ISL_903844, EPI_ISL_903856	CDPH, Viral and Rickettsial Disease Laboratory	Genomics and Discovery, Respiratory Viruses Branch, Division of Viral Diseases, Centers for Disease Control and Prevention	Krista Queen, Yan Li, Ying Tao, Jing Zhang, Anna Uehara, Anna Montmayeur, Clinton R. Paden, Peter W. Cook, Rachel Marine, Mili Sheth, Jasmine Padilla, Sarah Nobles, Mark Burroughs, Lori Rowe, Haibin Wang, Ben L. Rambo-Martin, Dhwani Batra, Justin Lee, Suxiang Tong
EPI_ISL_903872	SC Dept of Health and Env. Control-Bureau of Laboratories	Genomics and Discovery, Respiratory Viruses Branch, Division of Viral Diseases, Centers for Disease Control and Prevention	Krista Queen, Yan Li, Ying Tao, Jing Zhang, Anna Uehara, Anna Montmayeur, Clinton R. Paden, Peter W. Cook, Rachel Marine, Mili Sheth, Jasmine Padilla, Sarah Nobles, Mark Burroughs, Lori Rowe, Haibin Wang, Ben L. Rambo-Martin, Dhwani Batra, Justin Lee, Suxiang Tong
EPI_ISL_903907	NM Dept. Health, Scientific Laboratory Division	Genomics and Discovery, Respiratory Viruses Branch, Division of Viral Diseases, Centers for Disease Control and Prevention	Krista Queen, Yan Li, Ying Tao, Jing Zhang, Anna Uehara, Anna Montmayeur, Clinton R. Paden, Peter W. Cook, Rachel Marine, Mili Sheth, Jasmine Padilla, Sarah Nobles, Mark Burroughs, Lori Rowe, Haibin Wang, Ben L. Rambo-Martin, Dhwani Batra, Justin Lee, Suxiang Tong
EPI_ISL_903913, EPI_ISL_903941	CDPH, Viral and Rickettsial Disease Laboratory	Genomics and Discovery, Respiratory Viruses Branch, Division of Viral Diseases, Centers for Disease Control and Prevention	Krista Queen, Yan Li, Ying Tao, Jing Zhang, Anna Uehara, Anna Montmayeur, Clinton R. Paden, Peter W. Cook, Rachel Marine, Mili Sheth, Jasmine Padilla, Sarah Nobles, Mark Burroughs, Lori Rowe, Haibin Wang, Ben L. Rambo-Martin, Dhwani Batra, Justin Lee, Suxiang Tong
EPI_ISL_904043, EPI_ISL_904044, EPI_ISL_904045, EPI_ISL_904046, EPI_ISL_904047, EPI_ISL_904048, EPI_ISL_904049	New Mexico Department of Health Scientific Laboratory	New Mexico Department of Health Scientific Laboratory	Ellie Johnson, Anastacia Griego-Fisher, D'eldra Malone
EPI_ISL_904143, EPI_ISL_904145, EPI_ISL_904149, EPI_ISL_904160, EPI_ISL_904165, EPI_ISL_904180, EPI_ISL_904191, EPI_ISL_904192, EPI_ISL_904200, EPI_ISL_904223, EPI_ISL_904224, EPI_ISL_904268, EPI_ISL_904269, EPI_ISL_904280, EPI_ISL_904376, EPI_ISL_904529, EPI_ISL_904530, EPI_ISL_904531, EPI_ISL_904532, EPI_ISL_904533, EPI_ISL_904534, EPI_ISL_904535, EPI_ISL_904536, EPI_ISL_904537, EPI_ISL_904538, EPI_ISL_904539			
see above	Dutch COVID-19 response team	Erasmus Medical Center	Bas Oude Munnink, Reina Sikkema, David Nieuwenhuijse, Irina Chestakova, Anne van der Linden, Marjan Boter, Emmanuelle Munger, Corine GeurtsvanKessel, Annemiek van der Eijk, Richard Molenkamp, Marion Koopmans, on behalf of the Dutch national COVID-19 response team.
EPI_ISL_904626, EPI_ISL_904630, EPI_ISL_904644, EPI_ISL_904649, EPI_ISL_904653	Servicio de Microbiología, Laboratori Clínic Metropolitana Nord. Hospital Universitari Germans Trias i Pujol. Institut d'Investigació en Ciències de la Salut Germans Trias i Pujol (IGTP)	IrsiCaixa - Can Ruti CovidSeq	Marc Noguera-Julian, Mariona Parera, Maria Casadellà, Pilar Armengol, Francesc Catala-Moll, Roger Paredes, Bonaventura Clotet Elisa Martró, Verónica Saludes, Anna Not, Ana Pérez, Montserrat Giménez, Ignacio Blanco, Cristina Casañ, Antoni E. Bordoy, Adrián Antuori
EPI_ISL_904743, EPI_ISL_904776, EPI_ISL_904973, EPI_ISL_905005, EPI_ISL_905006, EPI_ISL_905008, EPI_ISL_905009, EPI_ISL_905034, EPI_ISL_905049, EPI_ISL_905070, EPI_ISL_905105, EPI_ISL_905106, EPI_ISL_905235, EPI_ISL_905238, EPI_ISL_905339, EPI_ISL_905340, EPI_ISL_905351, EPI_ISL_905360, EPI_ISL_905376, EPI_ISL_905419, EPI_ISL_905420, EPI_ISL_905421, EPI_ISL_905422, EPI_ISL_905423, EPI_ISL_905432, EPI_ISL_905436, EPI_ISL_905437, EPI_ISL_905438, EPI_ISL_905488, EPI_ISL_905494, EPI_ISL_905524, EPI_ISL_905534, EPI_ISL_905541, EPI_ISL_905542, EPI_ISL_905543, EPI_ISL_905544, EPI_ISL_905566, EPI_ISL_905567, EPI_ISL_905661			
see above	Dutch COVID-19 response team	National Institute for Public Health and the Environment (RIVM)	Adam Meijer, Harry Vennema, Dirk Eggink, Jeroen Cremer, Sharon van den Brink, Bas van der Veer, AnneMarie van den Brandt, Florian Zwagemaker, Dennis Schmitz, Chantal Reusken, on behalf of the national COVID-19 response team
EPI_ISL_905866, EPI_ISL_905867, EPI_ISL_905868, EPI_ISL_905869, EPI_ISL_905870, EPI_ISL_905871, EPI_ISL_905872, EPI_ISL_905873, EPI_ISL_905874, EPI_ISL_905875, EPI_ISL_905876, EPI_ISL_905877, EPI_ISL_905878, EPI_ISL_905879, EPI_ISL_905880, EPI_ISL_905881, EPI_ISL_905882, EPI_ISL_905883, EPI_ISL_905884, EPI_ISL_905885, EPI_ISL_905886, EPI_ISL_905887, EPI_ISL_905888, EPI_ISL_905889, EPI_ISL_905890, EPI_ISL_905891, EPI_ISL_905892, EPI_ISL_905893, EPI_ISL_905894, EPI_ISL_905895, EPI_ISL_905896, EPI_ISL_905897, EPI_ISL_905898, EPI_ISL_905899, EPI_ISL_905907, EPI_ISL_905908, EPI_ISL_905909, EPI_ISL_905910, EPI_ISL_905911, EPI_ISL_905912, EPI_ISL_905913			
see above	OHSU Lab Services Molecular Microbiology Lab	Oregon SARS-CoV-2 Genome Sequencing Center	Brendan L. O'Connell, Sally Grindstaff, Kayla Carter, Ruth V. Nichols, Alec J. Hirsch, Donna Hansel, Guang Fan, Xuan, Qin, Daniel N. Streblow, William B. Messer, Andrew C. Adey, Benjamin N. Bimber, Brian J. O'Roak
EPI_ISL_906539	HOSPITAL DEPARTAMENTAL DE VILLAVICENCIO	Instituto Nacional de Salud- Dirección de Investigación en Salud Pública, Universidad de los Andes- Applied genomics research group, Vicerrectoria de Investigación y Creación, Universidad de los Andes- Systems and Computing Engineering Department	Katherine Laiton-Donato, Diego A. Álvarez-Díaz, Carlos Franco-Muñoz, Mauricio Pacheco-Montealegre, Héctor Alejandro Ruiz-Moreno, Maria T. Herrera-Sepúlveda, Diego Andrés Prada, Jhonnatan Reales-González, Sheryll Corchuelo, Julian Naizaque, Gerardo Santamaría Jorge Duitama, Laura Natalia Gonzalez, Jorge Ivan Diaz, Silvia Restrepo-Restrepo, Magdalena Wiesner, Martha Lucia Ospina Martinez, Marcela Mercado-Reyes
EPI_ISL_906712, EPI_ISL_906714, EPI_ISL_906725, EPI_ISL_906744, EPI_ISL_906746	Hematology Laboratory, Section of Molecular Diagnostics, University Clinical Centre, Medical University of Gdansk	Laboratory of Recombinant Vaccines	Lukasz Rabalski, Maciej Kosinski, Maciej Grzybek, Adam Sodol, Aneta Szulc, Krzysztof Lewandowski, Ewa Milosz, Marlena Robakowska, Boguslaw Szewczyk, Krystyna Bienkowska-Szewczyk
EPI_ISL_907093, EPI_ISL_907101, EPI_ISL_907102, EPI_ISL_907103, EPI_ISL_907104, EPI_ISL_907105, EPI_ISL_907106, EPI_ISL_907107, EPI_ISL_907108, EPI_ISL_907109, EPI_ISL_907110, EPI_ISL_907111, EPI_ISL_907112, EPI_ISL_907113, EPI_ISL_907114, EPI_ISL_907115, EPI_ISL_907116, EPI_ISL_907117, EPI_ISL_907118			
see above	Cancer Biology Department, National Cancer Institute	Cancer Biology Department, National Cancer Institute	Zekri,A.N., Sedawy,M.G., Ahmed,O.S., Hafez,M.M., Soliman,H.K., Bahnassy,A.A., Elhosieny,F.W., Gad,A.E., Hamdy,M.S., Soliman,M.S., Soliman,L., Abouelhoda,M.
EPI_ISL_910337	Laboratory for Respiratory Viruses, Cantacuzino National Military-Medical Institute for Research and Development	Cantacuzino Institute Virology	Luiza Ustea, Nicoleta Paraschiv, Mihaela Lazar
EPI_ISL_910585, EPI_ISL_910586, EPI_ISL_910587, EPI_ISL_910588, EPI_ISL_910589, EPI_ISL_910590, EPI_ISL_910591, EPI_ISL_910592, EPI_ISL_910593, EPI_ISL_910594, EPI_ISL_910595, EPI_ISL_910596, EPI_ISL_910597, EPI_ISL_910598, EPI_ISL_910599, EPI_ISL_910600, EPI_ISL_910601, EPI_ISL_910602, EPI_ISL_910603, EPI_ISL_910604, EPI_ISL_910605, EPI_ISL_910606, EPI_ISL_910607, EPI_ISL_910608, EPI_ISL_910609, EPI_ISL_910611, EPI_ISL_910612, EPI_ISL_910613, EPI_ISL_910614, EPI_ISL_910615, EPI_ISL_910616, EPI_ISL_910617, EPI_ISL_910618, EPI_ISL_910619, EPI_ISL_910620, EPI_ISL_910621, EPI_ISL_910622, EPI_ISL_910623, EPI_ISL_910624, EPI_ISL_910625, EPI_ISL_910626, EPI_ISL_910627, EPI_ISL_910628, EPI_ISL_910629, EPI_ISL_910630, EPI_ISL_910631, EPI_ISL_910632, EPI_ISL_910881, EPI_ISL_910882, EPI_ISL_910883, EPI_ISL_910884, EPI_ISL_910885, EPI_ISL_910886, EPI_ISL_910887			
see above	Laboratoire national de sante, Microbiology, Virology	Laboratoire national de sante, Microbiology, Microbial Genomics Platform	Anke Wienecke-Baldacchino, Catherine Ragimbeau,Jessica Tapp, Fatu Djabi, Lise Pignon, Raoul Salmon, Tamir Abdelrahman
EPI_ISL_911530, EPI_ISL_911531	M Health Fairview	Minnesota Department of Health, Public Health Laboratory	Alexandra Lorentz, Jacob Garfin, Matt Plumb, and Xiong Wang
EPI_ISL_911658, EPI_ISL_911659, EPI_ISL_911660, EPI_ISL_911661	Texas Department of State Health Services (TXDSHS)	Texas Department of State Health Services (TXDSHS)	Bonnie Oh, Anita Pokharel, James Daniel Bonser, Myong Koag, Chung Wang, Rachel Lee, Grace Kubin, Rashmi Tuladhar, Mayela Pedrueza, Maliha Rahman, Jenny Zhang
EPI_ISL_911826, EPI_ISL_911866, EPI_ISL_911867	Johns Hopkins Hospital Department of Pathology	Johns Hopkins Hospital Department of Pathology	C. Paul Morris, Chun Huai Luo, Adannaya Amadi, Matthew Schwartz, Nicholas Gallagher, Heba H. Mostafa
EPI_ISL_912170	Tempus	Grubaugh Lab - Yale School of Public Health	Tara Alpert, Joseph Fauver, Anderson Brito, Mallery Breban, Anne Wyllie, Chantal Vogels, Mary Petrone, Annie Watkins, Chaney Kalinich, Isabel Ott, Nathan Grubaugh
EPI_ISL_912375	Fondation Congolaise pour la recherche medicale (FCRM), Francine Ntoumi	NGS Competence Center Tuebingen, Institut für Medizinische Mikrobiologie und Hygiene, Universitaetsklinikum Tübingen	Angel Angelov

EPI_ISL_912454	KU Leuven, Rega Institute, Clinical and Epidemiological Virology	KU Leuven, Rega Institute, Clinical and Epidemiological Virology	Tony Wawina-Bokalanga, Bert Vanmechelen, Joan Marti-Carerras, Piet Maes
EPI_ISL_912484, EPI_ISL_912526	NHLS Universitas Academic	UFS Virology	PA Bester, MM Nyaga, P Nthiga, MT Mogotsi, D Goedhals, T de Oliveira
EPI_ISL_912691, EPI_ISL_912721, EPI_ISL_912901	Hôpital Henri Mondor	Department of Virology, Henri Mondor University Hospital, Assistance Publique Hôpitaux de Paris, Université Paris-Est Créteil, INSERM U955	Christophe Rodriguez, Slim Fourati, Vanessa Demontant, Guillaume Gricourt, Melissa N'Debi, Alexandre Soulier, Elisabeth Trawinski, Jean-Michel Pawlotsky
EPI_ISL_912924	Hôpital Pitié-Salpêtrière	Department of Virology, Henri Mondor University Hospital, Assistance Publique Hôpitaux de Paris, Université Paris-Est Créteil, INSERM U955	Christophe Rodriguez, Slim Fourati, Vanessa Demontant, Guillaume Gricourt, Melissa N'Debi, Alexandre Soulier, Elisabeth Trawinski, Jean-Michel Pawlotsky
EPI_ISL_912943, EPI_ISL_912978, EPI_ISL_912979, EPI_ISL_912980, EPI_ISL_912981	Hôpital Henri Mondor	Department of Virology, Henri Mondor University Hospital, Assistance Publique Hôpitaux de Paris, Université Paris-Est Créteil, INSERM U955	Christophe Rodriguez, Slim Fourati, Vanessa Demontant, Guillaume Gricourt, Melissa N'Debi, Alexandre Soulier, Elisabeth Trawinski, Jean-Michel Pawlotsky
EPI_ISL_913034, EPI_ISL_913035	Hospital San Pedro de Alcántara	Instituto de Salud Carlos III	Iglesias-Caballero, M. Camarero, S. Sandonís,V. Vázquez, S. Pozo, F. Casas, I. Jiménez, P. Zaballos, A. Monzón, S. Varona, S. Cuesta, I. Rodríguez, G.
EPI_ISL_913044, EPI_ISL_913045, EPI_ISL_913047	Hospital Comarcal Sierrallana	Instituto de Salud Carlos III	Iglesias-Caballero, M. Camarero, S. Sandonís,V. Vázquez, S. Pozo, F. Casas, I. Jiménez, P. Zaballos, A. Monzón, S. Varona, S. Cuesta, I. De Benito, I.
EPI_ISL_913065	Center for Virology	Center for Virology	Jeremy V. Camp, Irene Goerzer, Monika Redlberger-Fritz, Stephan W. Aberle
EPI_ISL_913256, EPI_ISL_913257	Synlab Medilab, Mikrobiologi	The Public Health Agency of Sweden	Anna-Malin Linde, Maria Lind Karlberg, Carlo Berg, Oskar Karlsson Lindsjo, Sofia Stamouli, Reza Advani, Mattias Haukland, Petra Holmstrom, Noura Walai, Petra Edquist, Mia Brytting, Anna Risberg, Karin Tegmark-Wisell
EPI_ISL_913275, EPI_ISL_913277, EPI_ISL_913281	Dynamic Code AB	The Public Health Agency of Sweden	Anna-Malin Linde, Maria Lind Karlberg, Carlo Berg, Oskar Karlsson Lindsjo, Sofia Stamouli, Reza Advani, Mattias Haukland, Petra Holmstrom, Noura Walai, Petra Edquist, Mia Brytting, Anna Risberg, Karin Tegmark-Wisell
EPI_ISL_913297	Klinisk mikrobiologi	The Public Health Agency of Sweden	Anna-Malin Linde, Maria Lind Karlberg, Carlo Berg, Oskar Karlsson Lindsjo, Sofia Stamouli, Reza Advani, Mattias Haukland, Petra Holmstrom, Noura Walai, Petra Edquist, Mia Brytting, Anna Risberg, Karin Tegmark-Wisell
EPI_ISL_913301, EPI_ISL_913303, EPI_ISL_913304	Synlab Medilab, Mikrobiologi	The Public Health Agency of Sweden	Anna-Malin Linde, Maria Lind Karlberg, Carlo Berg, Oskar Karlsson Lindsjo, Sofia Stamouli, Reza Advani, Mattias Haukland, Petra Holmstrom, Noura Walai, Petra Edquist, Mia Brytting, Anna Risberg, Karin Tegmark-Wisell
EPI_ISL_913309	Dynamic Code AB	The Public Health Agency of Sweden	Anna-Malin Linde, Maria Lind Karlberg, Carlo Berg, Oskar Karlsson Lindsjo, Sofia Stamouli, Reza Advani, Mattias Haukland, Petra Holmstrom, Noura Walai, Petra Edquist, Mia Brytting, Anna Risberg, Karin Tegmark-Wisell
EPI_ISL_913328	Klinisk mikrobiologi	The Public Health Agency of Sweden	Anna-Malin Linde, Maria Lind Karlberg, Carlo Berg, Oskar Karlsson Lindsjo, Sofia Stamouli, Reza Advani, Mattias Haukland, Petra Holmstrom, Noura Walai, Petra Edquist, Mia Brytting, Anna Risberg, Karin Tegmark-Wisell
EPI_ISL_913331, EPI_ISL_913332, EPI_ISL_913333	Mikrobiologen	The Public Health Agency of Sweden	Anna-Malin Linde, Maria Lind Karlberg, Carlo Berg, Oskar Karlsson Lindsjo, Sofia Stamouli, Reza Advani, Mattias Haukland, Petra Holmstrom, Noura Walai, Petra Edquist, Mia Brytting, Anna Risberg, Karin Tegmark-Wisell
EPI_ISL_913337, EPI_ISL_913338	Unilabs AB Skovde	The Public Health Agency of Sweden	Anna-Malin Linde, Maria Lind Karlberg, Carlo Berg, Oskar Karlsson Lindsjo, Sofia Stamouli, Reza Advani, Mattias Haukland, Petra Holmstrom, Noura Walai, Petra Edquist, Mia Brytting, Anna Risberg, Karin Tegmark-Wisell
EPI_ISL_913385, EPI_ISL_913386, EPI_ISL_913387, EPI_ISL_913388, EPI_ISL_913389, EPI_ISL_913390, EPI_ISL_913391, EPI_ISL_913392, EPI_ISL_913459	Dynamic Code AB	The Public Health Agency of Sweden	Anna-Malin Linde, Maria Lind Karlberg, Carlo Berg, Oskar Karlsson Lindsjo, Sofia Stamouli, Reza Advani, Mattias Haukland, Petra Holmstrom, Noura Walai, Petra Edquist, Mia Brytting, Anna Risberg, Karin Tegmark-Wisell
EPI_ISL_913465, EPI_ISL_913490, EPI_ISL_913491, EPI_ISL_913492	Klinisk mikrobiologi	The Public Health Agency of Sweden	Anna-Malin Linde, Maria Lind Karlberg, Carlo Berg, Oskar Karlsson Lindsjo, Sofia Stamouli, Reza Advani, Mattias Haukland, Petra Holmstrom, Noura Walai, Petra Edquist, Mia Brytting, Anna Risberg, Karin Tegmark-Wisell
EPI_ISL_913563, EPI_ISL_913564, EPI_ISL_913565, EPI_ISL_913566	M Health Fairview	Minnesota Department of Health, Public Health Laboratory	Alexandra Lorentz, Jacob Garfin, Matt Plumb, and Xiong Wang
EPI_ISL_913648	Michigan Department of Health and Human Services, Bureau of Laboratories	Michigan Department of Health and Human Services, Bureau of Laboratories	Blankenship HM, Riner D, Soehnlen MK
EPI_ISL_913711, EPI_ISL_913712	Minnesota Department of Health, Public Health Laboratory	Minnesota Department of Health, Public Health Laboratory	Alexandra Lorentz, Jacob Garfin, Matt Plumb, and Xiong Wang
EPI_ISL_914815, EPI_ISL_914816, EPI_ISL_914818	HOSPITAL DE NIÑOS DR. CARLOS SAENZ HERRERA	Inciensa, Instituto Costarricense de Investigación y Enseñanza en Nutrición y Salud	Francisco Duarte, Hebleen Porras, Claudio Soto-Garita, Estela Cordero, Adriana Godínez, Melany Calderón & Cristian Pérez-Corrales
EPI_ISL_914823	HOSPITAL DR. ENRIQUE BALTODANO BRICEÑO	Inciensa, Instituto Costarricense de Investigación y Enseñanza en Nutrición y Salud	Francisco Duarte, Hebleen Porras, Claudio Soto-Garita, Estela Cordero, Adriana Godínez, Melany Calderón & Mariel López
EPI_ISL_917936, EPI_ISL_917938	Los Angeles County PHL	Los Angeles County PHL	P. Hemarajata et al.
EPI_ISL_918435, EPI_ISL_918438, EPI_ISL_918439, EPI_ISL_918440	AIID	Irish Coronavirus Sequencing Consortium-Teagasc Grange	Matthew McCabe, Aljandro Abner Garcia Leon, Fiona Crispie, Calum Walsh, Michael Carr, John Kenny, Paul Cotter, Patrick Mallon, Gabriel Gonzalez
EPI_ISL_918557, EPI_ISL_918558, EPI_ISL_918559	LACEN - Laboratório Central de Saúde Pública do Amapa	Evandro Chagas Institute	Santos, M.C.; Silva, A.M.; Junior, W.D.C.; Barbagelata, L.S.; Ferreira, J.A.; Sousa, E.M.A.; da Silva, P.S.; Pinheiro, K.C.; L.C.; Sousa Junior, E.C.
EPI_ISL_918721, EPI_ISL_918745, EPI_ISL_918901, EPI_ISL_918902, EPI_ISL_918903, EPI_ISL_918906, EPI_ISL_918909, EPI_ISL_918910, EPI_ISL_918911, EPI_ISL_918958, EPI_ISL_918960, EPI_ISL_918964, EPI_ISL_918966, EPI_ISL_918967, EPI_ISL_918968			
see above	University of Birmingham	COVID-19 Genomics UK (COG-UK) Consortium	Institute of Microbiology, University of Birmingham: Claire McMurray, Joanne Stockton, Samuel Nicholls, Radoslaw Poplawski, Will Rowe, Josh Quick, Nicholas Loman. University of Birmingham Testing Laboratory: Celina M Whalley, Andrew Bosworth, Charlotte Poxon, Kasun Wanigasooriya, Oliver Pickles, Mike Kidd, Alex Richter, Andrew D Beggs PHE Heartlands Lab: Husam Osman, Andrew Bosworth. Queen Elizabeth Hospital: Anna Casey
EPI_ISL_919336	Virology Department, Royal Infirmary of Edinburgh, NHS Lothian / School of Biological Sciences, University of Edinburgh / Institute of Genetics and Molecular Medicine, University of Edinburgh	COVID-19 Genomics UK (COG-UK) Consortium	McHugh M, Dewar R, Rooke S, Gallagher M, Balcaza C, O'Toole Á, Scher E, Hill V, McCrone JT, Colquhoun R, Yu X, Jackson B, Rambaut A, Williams TC, Templeton K
EPI_ISL_919449, EPI_ISL_919450, EPI_ISL_919451, EPI_ISL_919454	Liverpool Clinical Laboratories	COVID-19 Genomics UK (COG-UK) Consortium	Sam Haldenby, Anita Lucaci, Steve Paterson, Julian Hiscox, Alistair Darby, M Almsaud, A Alrezaihi, Muhannad Alruwaili, Stuart D Armstrong, Jones Benjamin, Eleanor G Bentley, Anu Chawla, Jordan J Clark, Angela Cowell, Richard Eccles, Isabel García-Dorival, Matthew Gemmell, Alessandro Gerada, PKF Gilmore, Richard Gregory, Ximeng Han, Catherine Hartley, Margaret Hughes, Miren Iturriza-Gomara, James Johnson, L Luu, Jenifer Manson, Charlotte Nelson, Elaine O'Toole, Cassie Olateju, Rebekah Penrice-Randal , Lucille Rainbow, N.P Randle, Trevor Ian Robinson, Parul Sharma, Ghada T Shawli, James P Stewart, Neil Swainston, Ecaterina Vamos, Joanne Watts, Mark Whitehead
EPI_ISL_919806, EPI_ISL_919807, EPI_ISL_919808, EPI_ISL_919809, EPI_ISL_919810, EPI_ISL_919811, EPI_ISL_919812, EPI_ISL_919813, EPI_ISL_919842	Barts Health NHS Trust	COVID-19 Genomics UK (COG-UK) Consortium	CUTINO-MOGUEL, Maria-Teresa; HARRINGTON, David; OWOYEMI, Dola; KULASEGARAN-SHYLINI, Raghavendran; BROAD, Claire; KELE, Beatrix
EPI_ISL_919961, EPI_ISL_919962, EPI_ISL_919963, EPI_ISL_919964, EPI_ISL_919965, EPI_ISL_919966, EPI_ISL_919967, EPI_ISL_919968, EPI_ISL_919969, EPI_ISL_919971, EPI_ISL_919972, EPI_ISL_919974, EPI_ISL_919975, EPI_ISL_919976, EPI_ISL_919977			

see above	University College London, Great Ormond Street Hospital for Children NHS Foundation Trust, Imperial College Healthcare NHS Trust	COVID-19 Genomics UK (COG-UK) Consortium	Sergi Castellano, Rachel Williams, Mark Kristiansen, Paola Resende Silva, Sunando Roy, Tony Brooks, Helena Tutill, Paola Niola, Patricia Dyal, Charlotte Williams, Leysa Forrest, Yasmin Panchbhaya, Jacqueline Findlay, Samuel Weeks, Julianne Brown, Kathryn Harris, Paul Randell, James Price, Alison Holmes, Judith Breuer
EPI_ISL_920344, EPI_ISL_920457	University College London Hospital	COVID-19 Genomics UK (COG-UK) Consortium	Judith Heaney, Matthew Byott, Catherine Houlihan, Dan Frampton, Stuart Kirk, Moira Spyer and Eleni Nastouli
EPI_ISL_920840, EPI_ISL_920848	University College London, Great Ormond Street Hospital for Children NHS Foundation Trust, Imperial College Healthcare NHS Trust	COVID-19 Genomics UK (COG-UK) Consortium	Sergi Castellano, Rachel Williams, Mark Kristiansen, Paola Resende Silva, Sunando Roy, Tony Brooks, Helena Tutill, Paola Niola, Patricia Dyal, Charlotte Williams, Leysa Forrest, Yasmin Panchbhaya, Jacqueline Findlay, Samuel Weeks, Julianne Brown, Kathryn Harris, Paul Randell, James Price, Alison Holmes, Judith Breuer
EPI_ISL_921024, EPI_ISL_921033, EPI_ISL_921038, EPI_ISL_921039, EPI_ISL_921040, EPI_ISL_921041, EPI_ISL_921046, EPI_ISL_921047, EPI_ISL_921048, EPI_ISL_921049, EPI_ISL_921050, EPI_ISL_921051, EPI_ISL_921052, EPI_ISL_921053, EPI_ISL_921054, EPI_ISL_921055, EPI_ISL_921056, EPI_ISL_921057, EPI_ISL_921058, EPI_ISL_921059, EPI_ISL_921060, EPI_ISL_921061, EPI_ISL_921062, EPI_ISL_921063, EPI_ISL_921064, EPI_ISL_921065, EPI_ISL_921066, EPI_ISL_921067, EPI_ISL_921068, EPI_ISL_921069, EPI_ISL_921070, EPI_ISL_921071, EPI_ISL_921072, EPI_ISL_921073, EPI_ISL_921074, EPI_ISL_921075, EPI_ISL_921076, EPI_ISL_921077, EPI_ISL_921091, EPI_ISL_921092, EPI_ISL_921093, EPI_ISL_921094, EPI_ISL_921095, EPI_ISL_921098, EPI_ISL_921100, EPI_ISL_921102, EPI_ISL_921106, EPI_ISL_921107, EPI_ISL_921109, EPI_ISL_921110, EPI_ISL_921111, EPI_ISL_921113, EPI_ISL_921114, EPI_ISL_921115, EPI_ISL_921116, EPI_ISL_921117, EPI_ISL_921125, EPI_ISL_921131, EPI_ISL_921134			
see above	Regional Virus Laboratory, Belfast Health and Social Care Trust	COVID-19 Genomics UK (COG-UK) Consortium	Conall McCaughey, James McKenna, Tanya Curran, Susan Feeney, Alison Watt, Ciara Cox, Mairead Connor, Zoltan Molnar, David Simpson, Derek Fairley
EPI_ISL_921246, EPI_ISL_921250, EPI_ISL_921251, EPI_ISL_921283, EPI_ISL_921284, EPI_ISL_921285, EPI_ISL_921286, EPI_ISL_921419, EPI_ISL_921424, EPI_ISL_921425, EPI_ISL_921426, EPI_ISL_921427, EPI_ISL_921429, EPI_ISL_921430, EPI_ISL_921431, EPI_ISL_921432, EPI_ISL_921434			
see above	Northumbria University / South Tees Hospitals NHS Foundation Trust / North Cumbria Integrated Care NHS Foundation Trust / North Tees and Hartlepool NHS Foundation Trust / Newcastle Hospitals NHS Foundation Trust	COVID-19 Genomics UK (COG-UK) Consortium	Darren L Smith,Andrew Nelson,Matthew Bashton,Greg R Young,Joshua Loh,John Allan,Mohammad A Tariq,Giles S Holt,Gary Black,Wen C Yew,Lynn Dover,Paul Baker,Steve Liggett,Sarah Essex,Jane Greenaway,Debra Padgett,Clive Graham,Garren Scott,Edward Barton,Emma Swindells,Brendan Payne,Jennifer Collins,Yusri Taha,Gary Eltringham
EPI_ISL_922180, EPI_ISL_922242, EPI_ISL_922255, EPI_ISL_922286, EPI_ISL_922289, EPI_ISL_922308, EPI_ISL_922311, EPI_ISL_922327, EPI_ISL_922336, EPI_ISL_922337	Oxford Viromics, NDM, University of Oxford; Oxford University Hospitals; Basingstoke and North Hampshire Hospital	COVID-19 Genomics UK (COG-UK) Consortium	Tanya Golubchik, David Bonsall, George Macintyre, Amy Trebes, Mariateresa de Cesare, Catrin Moore, Alex Mobbs, Anita Justice, Robert Shaw, Monique Andersson, Timothy Peto, Emma Wise, Nathan Moore, Jessica Lynch, Nick Cortes, Matilde Mori, Stephen Kidd, David Buck, John Todd, Christophe Fraser
EPI_ISL_923242	Centre for Enzyme Innovation, University of Portsmouth / Translational Research Laboratory, Portsmouth Hospitals NHS Trust	COVID-19 Genomics UK (COG-UK) Consortium	Angela Beckett,Salman Goudarzi,Christopher Fearn,Kate Cook,Katie Loveson,Sharon Glaysher,Scott Elliott,Samuel Robson
EPI_ISL_924157, EPI_ISL_924333, EPI_ISL_924387, EPI_ISL_924402, EPI_ISL_924412	Virology Department, Sheffield Teaching Hospitals NHS Foundation Trust/Department of Infection, Immunity and Cardiovascular Disease, The Medical School, University of Sheffield	COVID-19 Genomics UK (COG-UK) Consortium	Thushan de Silva, Matthew Parker, Nikki Smith, Adri Angyal, Rebecca Brown, Luke Green, Rachel Tucker, Paul Parsons, Danielle Groves, Katie Johnson, Laura Carrilero, Alex Keeley, Dave Partridge, Matthew Wyles, Benjamin Lindsey, Mehmet Yavuz, Mohammad Raza, Cariad Evans
EPI_ISL_924461, EPI_ISL_924462, EPI_ISL_924464, EPI_ISL_924465, EPI_ISL_924466, EPI_ISL_924471, EPI_ISL_924473, EPI_ISL_924475, EPI_ISL_924480, EPI_ISL_924481, EPI_ISL_924482, EPI_ISL_924484, EPI_ISL_924486, EPI_ISL_924492, EPI_ISL_924495, EPI_ISL_924517, EPI_ISL_924518, EPI_ISL_924521, EPI_ISL_924528, EPI_ISL_924529, EPI_ISL_924530, EPI_ISL_924531, EPI_ISL_924533, EPI_ISL_924539, EPI_ISL_924540, EPI_ISL_924542, EPI_ISL_924544, EPI_ISL_924545, EPI_ISL_924547, EPI_ISL_924548, EPI_ISL_924550, EPI_ISL_924552, EPI_ISL_924553, EPI_ISL_924555, EPI_ISL_924556, EPI_ISL_924557, EPI_ISL_924558, EPI_ISL_924559, EPI_ISL_924560, EPI_ISL_924562, EPI_ISL_924563, EPI_ISL_924564, EPI_ISL_924565, EPI_ISL_924566, EPI_ISL_924567, EPI_ISL_924568, EPI_ISL_924570, EPI_ISL_924571, EPI_ISL_924572, EPI_ISL_924573, EPI_ISL_924574, EPI_ISL_924575, EPI_ISL_924576, EPI_ISL_924577, EPI_ISL_924578, EPI_ISL_924579, EPI_ISL_924580, EPI_ISL_924581, EPI_ISL_924582, EPI_ISL_924583, EPI_ISL_924584, EPI_ISL_924585, EPI_ISL_924586, EPI_ISL_924587, EPI_ISL_924588, EPI_ISL_924589, EPI_ISL_924590, EPI_ISL_924591, EPI_ISL_924592, EPI_ISL_924593, EPI_ISL_924594, EPI_ISL_924595, EPI_ISL_924596, EPI_ISL_924597, EPI_ISL_924598, EPI_ISL_924599, EPI_ISL_924600, EPI_ISL_924601, EPI_ISL_924602, EPI_ISL_924603, EPI_ISL_924604, EPI_ISL_924738			
see above	Bioinformatics and Biostatistics Lab, Advanced Sequencing Facility	COVID-19 Genomics UK (COG-UK) Consortium	Aengus Stewart,Jerome Nicod,Chelsea Sawyer,Laura Cubitt,Harshil Patel,Margaret Crawford
EPI_ISL_925397	Department of Clinical Microbiology	GIGA Medical Genomics	Keith Durkin, Maria Artesi, Sébastien Bontems, Raphaël Boreux, Bouchra Boujemla, Cécile Meex, Pierrette Melin, Marie-Pierre Hayette, Vincent Bours
EPI_ISL_925514, EPI_ISL_925520, EPI_ISL_925521	Arizona State Public Health Laboratory	Arizona State Public Health Laboratory	Trung Huynh, Jessica Escobar, Katherine Fullerton, Nobuko Fukushima, Stacy White, Linda Getsinger, Victor Waddell
EPI_ISL_925915	Nucleic Acid Testing, National Reference Laboratory	GIGA Medical Genomics	Yvan Butera, Keith Durkin, Maria Artesi, Bouchra Boujemla, Robert Rutayisire, Patrick Tuyisenge, Esperence Umumararungu, Sébastien Bontems, Marie-Pierre Hayette, Nathalie Renotte, Swaibu Gatare, Jacob Souopgui, Sabin Nsanzimana, Vincent Bours, Léon Mutesa
EPI_ISL_931570	Utah Public Health Laboratory	Utah Public Health Laboratory	Erin L. Young, Kelly F. Oakeson, Tara Gallagher
EPI_ISL_934174, EPI_ISL_934175, EPI_ISL_934176, EPI_ISL_934177, EPI_ISL_934178, EPI_ISL_934179, EPI_ISL_934187, EPI_ISL_934203, EPI_ISL_934222	Vilnius university hospital Santaros Klinikos, Center of Laboratory Medicine	Vilnius university hospital Santaros Klinikos, Center of Laboratory Medicine	Ingrida Olendraite, Daniel Naumovas, Rimvydas Norvilas, Dovile Ezerskyte, Justinas Slikas, Gytis Dudas
EPI_ISL_934345, EPI_ISL_934346, EPI_ISL_934347	Klinisk mikrobiologi	The Public Health Agency of Sweden	Anna-Malin Linde, Maria Lind Karlberg, Carlo Berg, Oskar Karlsson Lindsjo, Sofia Stamouli, Reza Advani, Mattias Haukland, Petra Holmstrom, Noura Walai, Petra Edquist, Mia Brytting, Anna Risberg, Karin Tegmark-Wisell
EPI_ISL_934377	Dynamic Code AB	The Public Health Agency of Sweden	Anna-Malin Linde, Maria Lind Karlberg, Carlo Berg, Oskar Karlsson Lindsjo, Sofia Stamouli, Reza Advani, Mattias Haukland, Petra Holmstrom, Noura Walai, Petra Edquist, Mia Brytting, Anna Risberg, Karin Tegmark-Wisell
EPI_ISL_934384	Klinisk mikrobiologi	The Public Health Agency of Sweden	Anna-Malin Linde, Maria Lind Karlberg, Carlo Berg, Oskar Karlsson Lindsjo, Sofia Stamouli, Reza Advani, Mattias Haukland, Petra Holmstrom, Noura Walai, Petra Edquist, Mia Brytting, Anna Risberg, Karin Tegmark-Wisell
EPI_ISL_934593, EPI_ISL_934594, EPI_ISL_934595, EPI_ISL_934596, EPI_ISL_934597, EPI_ISL_934598, EPI_ISL_934599, EPI_ISL_934600, EPI_ISL_934601, EPI_ISL_934602, EPI_ISL_934603, EPI_ISL_934604, EPI_ISL_934605, EPI_ISL_934606, EPI_ISL_934607, EPI_ISL_934608, EPI_ISL_934609, EPI_ISL_934610, EPI_ISL_934611, EPI_ISL_934612, EPI_ISL_934613, EPI_ISL_934614, EPI_ISL_934615, EPI_ISL_934616, EPI_ISL_934617, EPI_ISL_934618, EPI_ISL_934619, EPI_ISL_934620, EPI_ISL_934621, EPI_ISL_934622, EPI_ISL_934623, EPI_ISL_934624, EPI_ISL_934625, EPI_ISL_934626, EPI_ISL_934627, EPI_ISL_934628, EPI_ISL_934629, EPI_ISL_934630, EPI_ISL_934631, EPI_ISL_934632			
see above	Austrian Agency for Health and Food Safety (AGES)	Bergthaler laboratory, CeMM Research Center for Molecular Medicine of the Austrian Academy of Sciences	Lukas Endler, Anna Schedl, Thomas Penz, Benedikt Agerer, Maelle Le Moing, Michael Schuster, Bekir Erguner, Jan Laine, Martin Senekowitsch, Christoph Bock, Andreas Bergthaler
EPI_ISL_934637, EPI_ISL_934643	Department of Microbiology, University Innsbruck	Bergthaler laboratory, CeMM Research Center for Molecular Medicine of the Austrian Academy of Sciences	Lukas Endler, Anna Schedl, Thomas Penz, Benedikt Agerer, Maelle Le Moing, Michael Schuster, Bekir Erguner, Jan Laine, Martin Senekowitsch, Christoph Bock, Andreas Bergthaler
EPI_ISL_934650	Institute for Water Quality and Resource Management, Technical University Vienna	Bergthaler laboratory, CeMM Research Center for Molecular Medicine of the Austrian Academy of Sciences	Lukas Endler, Anna Schedl, Thomas Penz, Benedikt Agerer, Maelle Le Moing, Michael Schuster, Bekir Erguner, Jan Laine, Martin Senekowitsch, Christoph Bock, Andreas Bergthaler
EPI_ISL_935220, EPI_ISL_935223, EPI_ISL_935224	KU Leuven, Rega Institute, Clinical and Epidemiological Virology	KU Leuven, Rega Institute, Clinical and Epidemiological Virology	Tony Wawina-Bokalanga, Bert Vanmechelen, Joan Marti-Carerras, Piet Maes
EPI_ISL_935361, EPI_ISL_935363, EPI_ISL_935364	Florida Bureau of Public Health Laboratories	Florida Bureau of Public Health Laboratories	Sarah Schmedes, Jason Blanton
EPI_ISL_935711, EPI_ISL_935712, EPI_ISL_935713, EPI_ISL_935714, EPI_ISL_935715, EPI_ISL_935716, EPI_ISL_935717, EPI_ISL_935718, EPI_ISL_935719, EPI_ISL_935720, EPI_ISL_935721, EPI_ISL_935722			
see above	Houston Health Dept.	Houston Health Dept.	Ryker Penn, Pamela Brown, Adolpho Lara
EPI_ISL_935986, EPI_ISL_935987, EPI_ISL_935992	GLENS FALLS HOSPITAL LABORATORY	Wadsworth Center, New York State Department of Health	Kirsten St. George, Daryl M. Lamson, Alexis Russel, Matthew Shudt, Melissa A Leisner, Jonathan Plitnick, Navjot Singh, John Kelly, Erasmus Schneider, Erica Lasek-Nesselquist
EPI_ISL_936061, EPI_ISL_936067, EPI_ISL_936068, EPI_ISL_936070, EPI_ISL_936071, EPI_ISL_936072, EPI_ISL_936073, EPI_ISL_936074, EPI_ISL_936075, EPI_ISL_936076, EPI_ISL_936077, EPI_ISL_936084, EPI_ISL_936085, EPI_ISL_936087, EPI_ISL_936088, EPI_ISL_936090, EPI_ISL_936091, EPI_ISL_936094			
see above	KALEIDA CENTER FOR LABORATORY MEDICINE	Wadsworth Center, New York State Department of Health	Kirsten St. George, Daryl M. Lamson, Alexis Russel, Matthew Shudt, Melissa A Leisner, Jonathan Plitnick, Navjot Singh, John Kelly, Erasmus Schneider,

			Erica Lasek-Nesselquist
EPI_ISL_936861, EPI_ISL_936862, EPI_ISL_936863, EPI_ISL_936864, EPI_ISL_936865, EPI_ISL_936866, EPI_ISL_936868, EPI_ISL_936869, EPI_ISL_936870, EPI_ISL_936871, EPI_ISL_936872, EPI_ISL_936873, EPI_ISL_936874, EPI_ISL_936882, EPI_ISL_936883, EPI_ISL_936884, EPI_ISL_936896, EPI_ISL_936919, EPI_ISL_936920, EPI_ISL_936921, EPI_ISL_936922			
see above	Northwestern Memorial Hospital	Ozer Lab	Ramon Lorenzo-Redondo, Lacy M. Simons, Chad J. Achenbach, Lawrence J. Jennings, Michael G. Ison, Judd F. Hultquist, Egon A. Ozer
EPI_ISL_940715	City of Milwaukee Health Department Laboratory	City of Milwaukee Health Department Laboratory	Sanjib Bhattacharyya
EPI_ISL_940773	INSPI-CRN de Influenza y otros virus respiratorios	INSPI-Centro de Investigación Multidisciplinaria de la DTIDI	Leandro Patiño, Doménica de Mora, Maritza Olmedo, Andrés Carrazco-Montalvo, Orson Mestanza, Mary Regato-Arrata, Melissa Zambrano, Manuel González, Alfredo Bruno, Alberto Orlando.
EPI_ISL_941371, EPI_ISL_941372, EPI_ISL_941442, EPI_ISL_941443, EPI_ISL_941444, EPI_ISL_941445, EPI_ISL_941625, EPI_ISL_941626, EPI_ISL_941627	Instituto Nacional de Saude (INSA)	Instituto Nacional de Saude (INSA)	Borges et al
EPI_ISL_942770, EPI_ISL_942772, EPI_ISL_942773, EPI_ISL_942774, EPI_ISL_942775, EPI_ISL_942776, EPI_ISL_942777, EPI_ISL_942778, EPI_ISL_942779, EPI_ISL_942780, EPI_ISL_942781, EPI_ISL_942782, EPI_ISL_942783, EPI_ISL_942784, EPI_ISL_942785, EPI_ISL_942786, EPI_ISL_942787			
see above	Gundersen Molecular Diagnostics Laboratory	Kabara Cancer Research Institute	Craig S. Richmond, Paraic A. Kenny
EPI_ISL_942900, EPI_ISL_942901, EPI_ISL_942902, EPI_ISL_942903, EPI_ISL_942904, EPI_ISL_942905, EPI_ISL_942906, EPI_ISL_942907, EPI_ISL_942908, EPI_ISL_942909, EPI_ISL_942910, EPI_ISL_942911, EPI_ISL_942912, EPI_ISL_942913, EPI_ISL_942914, EPI_ISL_942915			
see above	Houston Health Dept.	Houston Health Dept.	Ryker Penn, Pamela Brown, Adolpho Lara
EPI_ISL_943153, EPI_ISL_943154, EPI_ISL_943200, EPI_ISL_943201, EPI_ISL_943202, EPI_ISL_943203, EPI_ISL_943204, EPI_ISL_943205, EPI_ISL_943245, EPI_ISL_943328, EPI_ISL_943408, EPI_ISL_943451, EPI_ISL_943452, EPI_ISL_943465, EPI_ISL_943489, EPI_ISL_943502, EPI_ISL_943514, EPI_ISL_943515			
see above	Dutch COVID-19 response team	National Institute for Public Health and the Environment (RIVM)	Adam Meijer, Harry Vennema, Dirk Eggink, Jeroen Cremer, Sharon van den Brink, Bas van der Veer, AnneMarie van den Brandt, Florian Zwagemaker, Dennis Schmitz, Chantal Reusken, on behalf of the national COVID-19 response team
EPI_ISL_943573	Servizo de Microbioloxía. Complexo Hospitalario Universitario de Santiago de Compostela	Servizo de Microbioloxía. Complexo Hospitalario Universitario de Santiago de Compostela	Antonio Aguilera, Gema Barbeito, Amparo Coira, José Costa, Rocio Trastoy, María Luisa Pérez del Molino.
EPI_ISL_943822	Utah Public Health Laboratory	Utah Public Health Laboratory	Erin L. Young, Kelly F. Oakeson, Tara Gallagher
EPI_ISL_944125, EPI_ISL_944133, EPI_ISL_944151, EPI_ISL_944156, EPI_ISL_944164, EPI_ISL_944174	National Health Laboratory Service, South Africa	KRISP, KZN Research Innovation and Sequencing Platform	Laguda-Akingba O, Giandhari J, Pillay S, Lessells R, Mdlalose K, York D, Khan S, Emmanuel SJ, Tegally H, Wilkinson E, de Oliveira T
EPI_ISL_944197, EPI_ISL_944198, EPI_ISL_944205, EPI_ISL_944213, EPI_ISL_944222, EPI_ISL_944241, EPI_ISL_944276, EPI_ISL_944277, EPI_ISL_944278, EPI_ISL_944378, EPI_ISL_944379, EPI_ISL_944382, EPI_ISL_944421, EPI_ISL_944422, EPI_ISL_944459, EPI_ISL_944460, EPI_ISL_944461, EPI_ISL_944462, EPI_ISL_944463			
see above	Israel Central Virology laboratory	Israel National Consortium for SARS-CoV-2 sequencing	Neta Zuckerman, Efrat Dahan Bucris, Michal Mandelboim, Dana Bar-Ilan, Oran Erster, Tzvia Mann, Omer Murik, David A. Zeevi, Assaf Rokney, Joseph Jaffe, Eva Nachum, Maya Davidovich Cohen, Ephraim Fass, Gal Zizelski Valenci, Mor Rubinstein, Efrat Rorman, Israel Nissan, Efrat Glick-Saar, Omri Nayshool, Gideon Rechavi, Ella Mendelson, Orna Mor
EPI_ISL_944652, EPI_ISL_944673, EPI_ISL_944683, EPI_ISL_944719, EPI_ISL_944726, EPI_ISL_944727	Department of Biochemistry, Cell and Molecular Biology, West African Centre for Cell Biology of Infectious Pathogens (WACCBIP), University of Ghana	Department of Biochemistry, Cell and Molecular Biology, West African Centre for Cell Biology of Infectious Pathogens (WACCBIP), University of Ghana	Morang'a,C.M., Ngoi,J.M., Quansah,E.B., Saiid,S., Amuzu,D.S., Asante,I., Bonney,J.H., Bonney,E., Odoom,J.K., Ndam,N.T., Tei-Maya,F., Adusei-Poku,M., Ofori-Boadu,L., Ampofo,W.K., Amenga-Etego,L.N., Quashie,P., Bediako,Y., Awandare,G.A.
EPI_ISL_945064	Lighthouse Lab in Milton Keynes	Wellcome Sanger Institute for the COVID-19 Genomics UK (COG-UK) Consortium	The Lighthouse Lab in Milton Keynes and Alex Alderton, Roberto Amato, Sonia Goncalves, Ewan Harrison, David K. Jackson, Ian Johnston, Dominic Kwiatkowski, Cordelia Langford, John Sillitoe on behalf of the Wellcome Sanger Institute COVID-19 Surveillance Team
EPI_ISL_945068, EPI_ISL_945070	Lighthouse Lab in Cambridge	Wellcome Sanger Institute for the COVID-19 Genomics UK (COG-UK) Consortium	Rob Howes, The Lighthouse Lab in Cambridge and Alex Alderton, Roberto Amato, Sonia Goncalves, Ewan Harrison, David K. Jackson, Ian Johnston, Dominic Kwiatkowski, Cordelia Langford, John Sillitoe on behalf of the Wellcome Sanger Institute COVID-19 Surveillance Team
EPI_ISL_945105, EPI_ISL_945159, EPI_ISL_945174	Lighthouse Lab in Milton Keynes	Wellcome Sanger Institute for the COVID-19 Genomics UK (COG-UK) Consortium	The Lighthouse Lab in Milton Keynes and Alex Alderton, Roberto Amato, Sonia Goncalves, Ewan Harrison, David K. Jackson, Ian Johnston, Dominic Kwiatkowski, Cordelia Langford, John Sillitoe on behalf of the Wellcome Sanger Institute COVID-19 Surveillance Team
EPI_ISL_945180	Lighthouse Lab in Cambridge	Wellcome Sanger Institute for the COVID-19 Genomics UK (COG-UK) Consortium	Rob Howes, The Lighthouse Lab in Cambridge and Alex Alderton, Roberto Amato, Sonia Goncalves, Ewan Harrison, David K. Jackson, Ian Johnston, Dominic Kwiatkowski, Cordelia Langford, John Sillitoe on behalf of the Wellcome Sanger Institute COVID-19 Surveillance Team
EPI_ISL_945295	Lighthouse Lab in Milton Keynes	Wellcome Sanger Institute for the COVID-19 Genomics UK (COG-UK) Consortium	The Lighthouse Lab in Milton Keynes and Alex Alderton, Roberto Amato, Sonia Goncalves, Ewan Harrison, David K. Jackson, Ian Johnston, Dominic Kwiatkowski, Cordelia Langford, John Sillitoe on behalf of the Wellcome Sanger Institute COVID-19 Surveillance Team
EPI_ISL_947326	RS Mitra Keluarga Gading Serpong	Eijkman Institute for Molecular Biology, Ministry of Research and Technology/National Agency for Research and Innovation	Willy Agustine, Edison Johar, Hidayat Trimarsanto, Iskandar Adnan, Lydia V. Panggalo, Sukma Oktavianthi, Frilasita A Yudhaputri, Safarina G Malik, Khin Saw Myint, Amin Soebandrio
EPI_ISL_947649	Lighthouse Lab in Alderley Park	Wellcome Sanger Institute for the COVID-19 Genomics UK (COG-UK) Consortium	Jacquelyn Wynn, Mairead Hyland, The Lighthouse Lab in Alderley Park and Alex Alderton, Roberto Amato, Sonia Goncalves, Ewan Harrison, David K. Jackson, Ian Johnston, Dominic Kwiatkowski, Cordelia Langford, John Sillitoe on behalf of the Wellcome Sanger Institute COVID-19 Surveillance Team
EPI_ISL_949748, EPI_ISL_949784	Barts Health NHS Trust	COVID-19 Genomics UK (COG-UK) Consortium	CUTINO-MOGUEL, Maria-Teresa; HARRINGTON, David; OWOYEMI, Dola; KULASEGARAN-SHYLINI, Raghavendran; BROAD, Claire; KELE, Beatrix
EPI_ISL_950048, EPI_ISL_950078, EPI_ISL_950082, EPI_ISL_950086, EPI_ISL_950121, EPI_ISL_950127, EPI_ISL_950134, EPI_ISL_950135, EPI_ISL_950153, EPI_ISL_950165	University College London, Great Ormond Street Hospital for Children NHS Foundation Trust, Imperial College Healthcare NHS Trust	COVID-19 Genomics UK (COG-UK) Consortium	Sergi Castellano, Rachel Williams, Mark Kristiansen, Paola Resende Silva, Sunando Roy, Tony Brooks, Helena Tutill, Paola Niola, Patricia Dyal, Charlotte Williams, Leysa Forrest, Yasmin Panchbhaya, Jacqueline Findlay, Samuel Weeks, Julianne Brown, Kathryn Harris, Paul Randell, James Price, Alison Holmes, Judith Breuer
EPI_ISL_950241, EPI_ISL_950243, EPI_ISL_950244, EPI_ISL_950245, EPI_ISL_950247, EPI_ISL_950248, EPI_ISL_950250, EPI_ISL_950253, EPI_ISL_950255, EPI_ISL_950256, EPI_ISL_950258, EPI_ISL_950259, EPI_ISL_950260, EPI_ISL_950262, EPI_ISL_950263, EPI_ISL_950265, EPI_ISL_950267, EPI_ISL_950460, EPI_ISL_950461, EPI_ISL_950462, EPI_ISL_950463, EPI_ISL_950465, EPI_ISL_950466, EPI_ISL_950467, EPI_ISL_950468, EPI_ISL_950469, EPI_ISL_950470, EPI_ISL_950471, EPI_ISL_950472, EPI_ISL_950473, EPI_ISL_950474, EPI_ISL_950475, EPI_ISL_950476, EPI_ISL_950477, EPI_ISL_950478, EPI_ISL_950480, EPI_ISL_950481			
see above	Northumbria University / South Tees Hospitals NHS Foundation Trust / North Cumbria Integrated Care NHS Foundation Trust / North Tees and Hartlepool NHS Foundation Trust / Newcastle Hospitals NHS Foundation Trust	COVID-19 Genomics UK (COG-UK) Consortium	Darren L Smith,Andrew Nelson,Matthew Bashton,Greg R Young,Joshua Loh,John Allan,Mohammad A Tariq,Giles S Holt,Gary Black,Wen C Yew,Lynn Dover,Paul Baker,Steve Liggett,Sarah Essex,Jane Greenaway,Debra Padgett,Clive Graham,Garren Scott,Edward Barton,Emma Swindells,Brendan Payne,Jennifer Collins,Yusri Taha,Gary Eltringham
EPI_ISL_950551, EPI_ISL_950582	Quadram Institute Bioscience	COVID-19 Genomics UK (COG-UK) Consortium	Dave J. Baker, Gemma L. Kay, Alp Aydin, Thanh Le-Viet, Steven Rudder, Ana P. Tedim, Anastasia Kolyva, Maria Diaz, Leonardo de Oliveira Martins, Nabil-Fareed Alikhan, Lizzie Meadows, Rachael Stanley, Ngozi Elumogo, Muhammed Yasir, Nicholas M. Thomson, Alexander J Trotter, Rachel Gilroy, Samuel Bloomfield, Claire Stuart, Andrew Bell, Reenesh Prakash, Samir Dervisevic, Alison E. Mather, John Wain, Mark Webber, Andrew J. Page, Justin O'Grady
EPI_ISL_950711	Lincolnshire Hospitals and DeepSeq Nottingham	COVID-19 Genomics UK (COG-UK) Consortium	Nichola Duckworth, Tim Sloan, Sarah Walsh, Jonathan Ball, Patrick McClure, Joeseph Chappell, Nadine Holmes, Matthew Carlisle, Christopher Moore, Fei Sang, Johnny Debebe, Victoria Wright, Matthew Loose
EPI_ISL_951068, EPI_ISL_951069, EPI_ISL_951070, EPI_ISL_951071, EPI_ISL_951072, EPI_ISL_951073, EPI_ISL_951074, EPI_ISL_951075, EPI_ISL_951077, EPI_ISL_951078, EPI_ISL_951082, EPI_ISL_951091, EPI_ISL_951101, EPI_ISL_951102, EPI_ISL_951103, EPI_ISL_951104, EPI_ISL_951105, EPI_ISL_951106, EPI_ISL_951107, EPI_ISL_951108, EPI_ISL_951109, EPI_ISL_951110, EPI_ISL_951111, EPI_ISL_951112, EPI_ISL_951114, EPI_ISL_951115, EPI_ISL_951116, EPI_ISL_951117, EPI_ISL_951118, EPI_ISL_951119, EPI_ISL_951120, EPI_ISL_951121, EPI_ISL_951122, EPI_ISL_951135, EPI_ISL_951137, EPI_ISL_951143, EPI_ISL_951161, EPI_ISL_951162, EPI_ISL_951188, EPI_ISL_951338, EPI_ISL_951381			
see above	Oxford Viromics, NDM, University of Oxford; Oxford University Hospitals; Basingstoke and North Hampshire Hospital	COVID-19 Genomics UK (COG-UK) Consortium	Tanya Golubchik, David Bonsall, George Macintyre, Amy Trebes, Mariateresa de Cesare, Catrin Moore, Alex Mobbs, Anita Justice, Robert Shaw, Monique Andersson, Timothy Peto, Emma Wise, Nathan Moore, Jessica Lynch, Nick Cortes, Matilde Mori, Stephen Kidd, David Buck, John Todd, Christophe Fraser

EPI_ISL_952430, EPI_ISL_952435	Centre for Enzyme Innovation, University of Portsmouth / Translational Research Laboratory, Portsmouth Hospitals NHS Trust	COVID-19 Genomics UK (COG-UK) Consortium	Angela Beckett,Salman Goudarzi,Christopher Fearn,Kate Cook,Katie Loveson,Sharon Glaysher,Scott Elliott,Samuel Robson
EPI_ISL_953174, EPI_ISL_953175, EPI_ISL_953179, EPI_ISL_953183, EPI_ISL_953186, EPI_ISL_953191, EPI_ISL_953194, EPI_ISL_953195, EPI_ISL_953196, EPI_ISL_953197, EPI_ISL_953198, EPI_ISL_953203, EPI_ISL_953204, EPI_ISL_953206, EPI_ISL_953208, EPI_ISL_953209, EPI_ISL_953211, EPI_ISL_953213, EPI_ISL_953215, EPI_ISL_953218, EPI_ISL_953220, EPI_ISL_953221, EPI_ISL_953224, EPI_ISL_953227, EPI_ISL_953228, EPI_ISL_953234, EPI_ISL_953235, EPI_ISL_953237			
see above	Bioinformatics and Biostatistics Lab, Advanced Sequencing Facility	COVID-19 Genomics UK (COG-UK) Consortium	Aengus Stewart,Jerome Nicod,Chelsea Sawyer,Laura Cubitt,Harshil Patel,Margaret Crawford
EPI_ISL_953427	National Institute of Health Research and Development	National Institute of Health Research and Development	Subangkit, Hana Apsari Pawestri, Kartika Dewi Puspa, Arie Ardiansyah Nugraha, Hartanti Dian Ikawati, Krisna Nur Andriana Pangesti, Yuni Rukminiati, Ririn Ramadhany, Agustiningsih, Kindi Adam, Holy Arif Wibowo, Triyani Soekarso, Ni Ketut Susilarini, Nurika Hariastuti, Ully Alfi Nikmah, Reni Herman, Nike Susanti, Herna, Tati Febriyanti, Natalie Laurencia Kipuw, Fauzul Muna, Irene Lorinda Indalao, Nelly Puspandari, Vivi Setiawaty
EPI_ISL_954218, EPI_ISL_954219	1.AO Universitaria 'S. Giovanni di Dio e Ruggi D'Aragona, Scuola Medica Salernitana' Hospital / 2.UOC di Virologia e Microbiologia, Università della Campania 'L. Vanvitelli' / 3.AO Universitaria 'Federico II' Napoli Hospital / 4.AORN 'San Giuseppe Moscati' Avellino Hospital / 5.AO 'San Pio - presidio G. Rummo' Benevento Hospital / 6.AO 'Sant'Anna e San Sebastiano' Caserta Hospital / 7.PO 'Maria Santissima Addolorata' Eboli Hospital / 8.Biogem Istituto di Ricerche Genetiche	1. Genome Research Center for Health (CRGS) / 2. Laboratory of Molecular Medicine and Genomics(LMMGe) / 3. Center for Research in Pure and Applied Mathematics (CRMPA)	Giorgio Giurato, Francesca Rizzo, Alessandro Weisz, Gianluigi Franci, Giovanni Nassa, Pasquale Pagliano, Roberta Tarallo, Elena Alexandrova, Ylenia D'Agostino, Carlo Ferravante, Jessica Lamberti, Viola Melone, Domenico Memoli, Valeria Mirici Cappa, Domenico Palumbo, Giovanni Pecoraro, Assunta Sellitto, Oriana Strianese, Ilaria Terenzi, Giuseppe Fenza, Aniello Gentile, Antonello Saccomanno, Sonia Amabile, Teresa Rocco, Annamaria Salvati, Emilia Vaccaro, Massimiliano Galdiero, Michele Cennamo, Giuseppe Portella, Maria Grazia Foti, Mariarosaria Ingino, Maria Landi, Maurizio Fumi, Vincenzo Rocco, Rita Greco, Vittoria Letizia, Arnolfo Petruzziello, Maddalena Schioppa, Gregorio Goffredi, Francesca Marciano, Michele Caraglia, Alessia Cossu, Marianna Scrima, Edmondo Adorisio, Morena D'Avenia, Michela Iacobellis, Rosanna Piluscio, Giorgio Dirani, Vittorio Sambri, Simona Semprini, Silvia Zanoli, Francesco Curcio, Stefania Marzinotto, Andreina Baj, Fausto Sessa.
EPI_ISL_954226, EPI_ISL_954297, EPI_ISL_954298, EPI_ISL_954299, EPI_ISL_954300	MRC/UVRI & LSHTM Uganda Research Unit	Where sequence data have been generated and submitted to GISAID	Matthew Cotten, Dan Lule Bugembe, My V.T. Phan, Isaac Sseeewanyana, Patrick Semanda, Susan Nabadda, Pontiano Kaleebu
EPI_ISL_954825, EPI_ISL_954829, EPI_ISL_954832, EPI_ISL_954835, EPI_ISL_954865, EPI_ISL_954867, EPI_ISL_954878, EPI_ISL_954881, EPI_ISL_954884, EPI_ISL_954895, EPI_ISL_954896, EPI_ISL_954897, EPI_ISL_954898, EPI_ISL_954899, EPI_ISL_954900, EPI_ISL_954901			
see above	Colorado Department of Public Health and Environment	Colorado Department of Puplic Health and Environment	Laura Bankers, Molly C. Hetherington-Rauth, Diana Ir, Shannon Ely, Shannon R. Matzinger, Sarah Elizabeth Totten, Emily A. Travanty
EPI_ISL_955231, EPI_ISL_955232, EPI_ISL_955258, EPI_ISL_955259, EPI_ISL_955260	BioReference Lab	NJ Public Health and Environmental Laboratories	Lindsey Bodnar, Shiv Verma, Dana Woell, Byeong Jeong
EPI_ISL_955392	Alameda County Public Health Lab	Chan-Zuckerberg Biohub	CZB Cliahub Consortium
EPI_ISL_955427, EPI_ISL_955428, EPI_ISL_955429, EPI_ISL_955430, EPI_ISL_955431, EPI_ISL_955432, EPI_ISL_955433	Orange County Public Health Lab	Chan-Zuckerberg Biohub	CZB Cliahub Consortium
EPI_ISL_955768	Humboldt County Public Health Laboratory	Chan-Zuckerberg Biohub	CZB Cliahub Consortium
EPI_ISL_955905	Pamela Youde Nethersole Eastern Hospital	Hong Kong Department of Health	Alan K.L. Tsang, Peter C.W. Yip, Edman T.K. Lam, Rickjason C.W. Chan, Dominic N.C. Tsang
EPI_ISL_956312	Institute of Tropical Disease	Institute of Tropical Disease, Universitas Airlangga	Krisnoadi Rahardjo, Aldise M Nastri, Jezzy R Dewantari, Rima R Prasetya, Gatot Soegiarto, Laksmi Wulandari, Resti Yudhawati, Yasuko Mori, Soetjipto, Kazufumi Shimizu, Maria I Lusida
EPI_ISL_956327, EPI_ISL_956328	Laboratory Medicine	Department of Laboratory Medicine, Lin-Kou Chang Gung Memorial Hospital, Taoyuan, Taiwan	Kuo-Chien Tsao, Yu-Nong Gong, Shu-Li Yang, Yi-Chun Liu, Chung-Guei Huang, Mei-Jen Hsiao, Po-Wei Huang, Cheng-Ta Yang, Cheng-Hsun Chiu, Peng-Nien Huang, Kuo-Ming Lee, Guang-Wu Chen, Shin-Ru Shih
EPI_ISL_956355, EPI_ISL_956356, EPI_ISL_956357, EPI_ISL_956358, EPI_ISL_956359, EPI_ISL_956360, EPI_ISL_956361, EPI_ISL_956362, EPI_ISL_956363, EPI_ISL_956364, EPI_ISL_956365, EPI_ISL_956366			
see above	Houston Health Department, Disease Prevention and Control	Houston Health Department, Disease Prevention and Control	Penn,R., Brown,P., Lara,A.
EPI_ISL_956405	General Hospital - Veles	Research Center for Genetic Engineering and Biotechnology "Georgi D. Efremov" , Macedonian Academy of Sciences and Arts	Aleksandar J. Dimovski, Dijana Plasheska-Karanfilska, Predrag Noveski, Gjorgji Bozinovski, Milena Jakimovska
EPI_ISL_961486, EPI_ISL_961509, EPI_ISL_961513, EPI_ISL_961518, EPI_ISL_961527, EPI_ISL_961554	Michigan Department of Health and Human Services, Bureau of Laboratories	Michigan Department of Health and Human Services, Bureau of Laboratories	Blankenship HM, Riner D, Soehnlen MK
EPI_ISL_961561, EPI_ISL_961562, EPI_ISL_961563, EPI_ISL_961564, EPI_ISL_961565, EPI_ISL_961566, EPI_ISL_961567, EPI_ISL_961568, EPI_ISL_961569, EPI_ISL_961604, EPI_ISL_961605, EPI_ISL_961606, EPI_ISL_961653, EPI_ISL_961654, EPI_ISL_961655, EPI_ISL_961656, EPI_ISL_961657, EPI_ISL_961658, EPI_ISL_961659, EPI_ISL_961660			
see above	Hôpital Georges L. Dumont	National Microbiology Laboratory (NML)	Anna Majer, Shari Tyson, Grace Seo, Philip Mabon, Elsie Grudeski, Rhiannon Huzarewich, Russell Mandes, Anneliese Landgraff, Jennifer Tanner, Natalie Knox, Morag Graham, Gary Van Domselaar, Richard Garceau, Guillaume Desnoyers, Nathalie Bastien, Yan Li, Timothy Booth, Darian Hole, Madison Chapel, Kirsten Biggar, CanCOGeN's metadata curation team, Public Health Agency of Canada CanCOGeN team
EPI_ISL_961977, EPI_ISL_961978, EPI_ISL_961992, EPI_ISL_962121, EPI_ISL_962129	Illinois Department of Public Health	Gagnon Lab, Southern Illinois University	Keith Gagnon
EPI_ISL_962509	UCLA Clinical Micro Lab	Los Angeles County PHL	P. Hemarajata et al.
EPI_ISL_962818, EPI_ISL_962819, EPI_ISL_962820, EPI_ISL_962821, EPI_ISL_962822	Microbiological Diagnostic Unit - Public Health Laboratory (MDU-PHL)	MDU-PHL	Seemann T., Sait, M.L., Sherry, N.L.
EPI_ISL_962823, EPI_ISL_962824	Victorian Infectious Diseases Reference Laboratory (VIDRL)	VIDRL and MDU-PHL	Caly L., Seemann T., Sait, M.L., Druce J., Sherry, N.L.
EPI_ISL_962895	Akershus University Hospital, Department for Microbiology and Infectious Disease Control	Norwegian Institute of Public Health, Department of Virology	Kathrine Stene-Johansen, Kamilla Heddeland Instefjord, Hilde Elshaug, Ignacio Garcia Llorente, Serina B Engebretsen, Atiya R Ali,Marie Paulsen Madsen, Rasmus Riis Kopperud, Hilde Vollan, Karoline Bragstad, Olav Hungnes
EPI_ISL_964916	Hopital	National Reference Center for Viruses of Respiratory Infections, Institut Pasteur, Paris	Marion Barbet, Sylvie Behillil, Méline Bizard, Angela Brisebarre, Camille Capel, Etienne Simon-Lorière, Vincent Enouf, Maud Vanpeene, Sylvie van der Werf,Smati-Lafarge Mounira
EPI_ISL_964949	Laboratorio de Virologia del HUCA	Laboratorio de Virologia del HUCA	Castelló C, Gómez de Oña J, Boga JA, Rojo S, Alvarez-Arguelles ME, Abreu F, Costales I, Sandoval M, Perez-Martinez Z, Martín-Rodriguez G, Coto E, Melon S
EPI_ISL_965211, EPI_ISL_965212, EPI_ISL_965213	Virginia Division of Consolidated Laboratory Services	Virginia Division of Consolidated Laboratory Services	Virginia DCLS
EPI_ISL_965548, EPI_ISL_965568, EPI_ISL_965608, EPI_ISL_965627, EPI_ISL_965633, EPI_ISL_965653, EPI_ISL_965661, EPI_ISL_965674, EPI_ISL_965802	Dutch COVID-19 response team	Medical Microbiology, Maastricht University Medical Centre	Jozef Dingemans*, Brian van der Veer*, Erik Beuken, Carmen Reumkens, Lieke van Alphen, Christian Hoebe, Paul Savelkoul
EPI_ISL_965967, EPI_ISL_965989, EPI_ISL_966020, EPI_ISL_966044, EPI_ISL_966062, EPI_ISL_966084, EPI_ISL_966108, EPI_ISL_966160, EPI_ISL_966184, EPI_ISL_966204, EPI_ISL_966227, EPI_ISL_966264, EPI_ISL_966289, EPI_ISL_966308, EPI_ISL_966311, EPI_ISL_966314, EPI_ISL_966333, EPI_ISL_966338,			

EPI_ISL_966357, EPI_ISL_966375, EPI_ISL_966376, EPI_ISL_966377, EPI_ISL_966378, EPI_ISL_966380, EPI_ISL_966406, EPI_ISL_966428, EPI_ISL_966452, EPI_ISL_966472, EPI_ISL_966489, EPI_ISL_966496, EPI_ISL_966549, EPI_ISL_966551, EPI_ISL_966552, EPI_ISL_966565, EPI_ISL_966591, EPI_ISL_966605, EPI_ISL_966623, EPI_ISL_966646, EPI_ISL_966663, EPI_ISL_966682, EPI_ISL_966707			
see above	NYU Langone Health	Departments of Pathology and Medicine, New York University School of Medicine	Adriana Heguy, Dacia Dimartino, Emily Guzman, Christian Marier, Peter Meyn, Sitharam Ramaswami, Gael Westby, Paul Zappile, Yutong Zhang, Paolo Cotzia, Guiqing Wang
EPI_ISL_966766, EPI_ISL_966767, EPI_ISL_966768	Maine HETL	Tewhey Lab, The Jackson Laboratory	Matluk,N., Dewey,H., Iosue,F., Barter,M., Lynch,R., Munger,H. and Tewhey,R.
EPI_ISL_967592, EPI_ISL_967594, EPI_ISL_967595, EPI_ISL_967596, EPI_ISL_967597, EPI_ISL_967598, EPI_ISL_967641, EPI_ISL_967644, EPI_ISL_967647, EPI_ISL_967648, EPI_ISL_967650, EPI_ISL_967651, EPI_ISL_967652, EPI_ISL_967665, EPI_ISL_967676, EPI_ISL_967698, EPI_ISL_967699			
see above	State Laboratories Division, Hawaii State Department of Health	State Laboratories Division, Hawaii State Department of Health	Pamela O'Brien, Drew Kuwazaki, Ayana Garnet, Razvan Sultana, Edward Desmond
EPI_ISL_967892, EPI_ISL_967903, EPI_ISL_967915, EPI_ISL_967920, EPI_ISL_967940, EPI_ISL_967958, EPI_ISL_967972, EPI_ISL_967981, EPI_ISL_967984, EPI_ISL_968006, EPI_ISL_968010, EPI_ISL_968018, EPI_ISL_968021, EPI_ISL_968022, EPI_ISL_968026, EPI_ISL_968029, EPI_ISL_968040, EPI_ISL_968042, EPI_ISL_968047, EPI_ISL_968048, EPI_ISL_968054			
see above	TGen North	Sonora Quest Laboratories	"Jolene Bowers, Megan Folkerts, Chris French, Hayley Yaglom, Ashlyn Pfeiffer, Darrin Lemmer, Dave Engelthaler, The Arizona COVID Genomics Union (ACGU)"
EPI_ISL_968855	KEMRI-Wellcome Trust Research Programme/KEMRI-CGMR-C Kilifi	KEMRI-Wellcome Trust Research Programme/KEMRI-CGMR-C Kilifi	Githinji et al
EPI_ISL_976883, EPI_ISL_976884, EPI_ISL_976885, EPI_ISL_976886, EPI_ISL_976887, EPI_ISL_976888, EPI_ISL_976889, EPI_ISL_976890, EPI_ISL_976891, EPI_ISL_976892	BCCDC Public Health Laboratory	BCCDC Public Health Laboratory	Prystajecky Natalie, Linda Hoang, Dan Fornika, John Tyson, Shannon Russell, Kim Macdonald, Kimia Kamelian, Ana Pacagnella, Corrinne Ng, Loretta Janz, Robert Azana Terry Snutch, Mel Krajden
EPI_ISL_977211	Microbiologia e Virologia	Istituto Zooprofilattico Sperimentale delle Venezie	Adelaide Milani, Alessia Schivo, Annalisa Salviato, Erika Giorgia Quaranta, Ambra Pastori, Bianca Zecchin, Alice Fusaro, Isabella Monne, Calogero Terregino, Antonia Ricci
EPI_ISL_977240, EPI_ISL_977241	ULSS 5 Polesana	Istituto Zooprofilattico Sperimentale delle Venezie	Adelaide Milani, Alessia Schivo, Annalisa Salviato, Erika Giorgia Quaranta, Ambra Pastori, Bianca Zecchin, Alice Fusaro, Isabella Monne, Calogero Terregino, Antonia Ricci
EPI_ISL_977659	Caribbean Public Health Agency	Carrington Lab, Department of PreClinical Sciences, Building 36, First Floor Biochemistry Unit, Faculty of Medical Sciences, The University of the West Indies	Nikita S. D. Sahadeo, Arianne Brown-Jordan, Vernie Ramkissoon, Sarah Hill, Naresh Nandram, Avery Hinds, Kenneth George, Jerome Foster, Stanley Giddings, Karla Georges, Marsha Ivey, Rahul Naidu, Risha Singh, SueMin Nathaniel, Rajini Haraksingh, Jaya Jayaraman, Chinna Chinnadurai, Adesh Ramsubhag, Nuno Faria, Oliver Pybus, Christopher Oura, Gabriel Escobar, Christine V. F. Carrington
EPI_ISL_978089, EPI_ISL_978090, EPI_ISL_978091, EPI_ISL_978092, EPI_ISL_978093, EPI_ISL_978094, EPI_ISL_978095, EPI_ISL_978096, EPI_ISL_978097, EPI_ISL_978098, EPI_ISL_978099, EPI_ISL_978100, EPI_ISL_978101, EPI_ISL_978102, EPI_ISL_978103, EPI_ISL_978104, EPI_ISL_978105, EPI_ISL_978106, EPI_ISL_978107, EPI_ISL_978108, EPI_ISL_978109, EPI_ISL_978110, EPI_ISL_978111, EPI_ISL_978112, EPI_ISL_978113, EPI_ISL_978114, EPI_ISL_978115, EPI_ISL_978116, EPI_ISL_978117, EPI_ISL_978118			
see above	Chiu Laboratory, University of California, San Francisco	Chiu Laboratory, University of California, San Francisco	Charles Chiu, Xianding (Wayne) Deng, Candace Wang, Venice Servellita, Jill Hacker, Debra Wadford
EPI_ISL_979136, EPI_ISL_979138, EPI_ISL_979139, EPI_ISL_979140, EPI_ISL_979142	Santa Clara County Public Health Laboratory	Chan-Zuckerberg Biohub	CZB Cliahub Consortium
EPI_ISL_979339, EPI_ISL_979340, EPI_ISL_979341, EPI_ISL_979342	Laboratorio Estatal de Salud Pública de Nuevo León	Laboratorio de Infectología Molecular, Departamento de Bioquímica y Medicina Molecular,Facultad de Medicina - Universidad Autónoma de Nuevo León	Kame A. Galán-Huerta, María F. Herrera-Saldivar, Natalia Martínez-Acuña, Sonia A. Lozano-Sepúlveda, Daniel Arellanos-Soto, Ana M. Rivas-Estilla, Samuel Buentello-Wong, Else del Carmen García-García, Gloria A. Jasso-de-la-Peña, Roberto Montes-de-Oca, Consuelo Treviño-Garza, Manuel E. de-la-O-Cavazos
EPI_ISL_979634, EPI_ISL_979635, EPI_ISL_979636, EPI_ISL_979637	Santa Clara County Public Health Laboratory	Chan-Zuckerberg Biohub	CZB Cliahub Consortium
EPI_ISL_979692, EPI_ISL_979693, EPI_ISL_979694, EPI_ISL_979696, EPI_ISL_979697, EPI_ISL_979705, EPI_ISL_979706, EPI_ISL_979708, EPI_ISL_979709	Orange County Public Health Lab	Chan-Zuckerberg Biohub	CZB Cliahub Consortium
EPI_ISL_981058	Johns Hopkins Hospital Department of Pathology	Johns Hopkins Hospital Department of Pathology	C. Paul Morris, Chun Huai Luo, Adannaya Amadi, Matthew Schwartz, Nicholas Gallagher, Heba H. Mostafa
EPI_ISL_981956, EPI_ISL_981958, EPI_ISL_981959, EPI_ISL_981960	Microbiology Service, Hospital Universitario Clinico San Cecilio, Granada	Microbiology Service, Hospital Universitario Clinico San Cecilio, Granada	Adolfo de Salazar, Natalia Chueca, Laura Viñuela, Ana Fuentes, Federico García
EPI_ISL_981997, EPI_ISL_982034, EPI_ISL_982075, EPI_ISL_982080, EPI_ISL_982103, EPI_ISL_982110	TGen North	Sonora Quest Laboratories	"Jolene Bowers, Megan Folkerts, Chris French, Hayley Yaglom, Ashlyn Pfeiffer, Darrin Lemmer, Dave Engelthaler, The Arizona COVID Genomics Union (ACGU)"
EPI_ISL_982243	Lab voor klinische biologie	Lab voor klinische biologie	Hannelore Hamerlinck, Marija Janevska, Bruno Verhasselt
EPI_ISL_982348, EPI_ISL_982350, EPI_ISL_982351, EPI_ISL_982356, EPI_ISL_982357, EPI_ISL_982358, EPI_ISL_982359, EPI_ISL_982360, EPI_ISL_982361, EPI_ISL_982362, EPI_ISL_982363, EPI_ISL_982364, EPI_ISL_982365, EPI_ISL_982366, EPI_ISL_982367, EPI_ISL_982368, EPI_ISL_982370, EPI_ISL_982374, EPI_ISL_982384			
see above	M Health Fairview	Minnesota Department of Health, Public Health Laboratory	Alexandra Lorentz, Jacob Garfin, Matt Plumb, and Xiong Wang
EPI_ISL_982426, EPI_ISL_982437, EPI_ISL_982438, EPI_ISL_982439, EPI_ISL_982443, EPI_ISL_982444, EPI_ISL_982447, EPI_ISL_982451, EPI_ISL_982452, EPI_ISL_982454, EPI_ISL_982455, EPI_ISL_982458, EPI_ISL_982461, EPI_ISL_982469, EPI_ISL_982474, EPI_ISL_982475, EPI_ISL_982482, EPI_ISL_982483, EPI_ISL_982484, EPI_ISL_982485, EPI_ISL_982488, EPI_ISL_982491, EPI_ISL_982496, EPI_ISL_982497, EPI_ISL_982498			
see above	MONTEFIORE MEDICAL CENTER LABORATORIES	Wadsworth Center, New York State Department of Health	Kirsten St. George, Daryl M. Lamson, Alexis Russel, Matthew Shudt, Melissa A Leisner, Jonathan Plitnick, Navjot Singh, John Kelly, Erasmus Schneider, Erica Lasek-Nesselquist
EPI_ISL_982530, EPI_ISL_982608, EPI_ISL_982653	US Air Force School of Aerospace Medicine	US Air Force School of Aerospace Medicine	Anthony Fries, Jennifer Meyer, William Gruner, William Buggele, Amanda Javorina, Sarah Purves, Clarise Starr, Elizabeth Macias
EPI_ISL_983101, EPI_ISL_983103, EPI_ISL_983104, EPI_ISL_983105, EPI_ISL_983106, EPI_ISL_983108, EPI_ISL_983113, EPI_ISL_983115, EPI_ISL_983134, EPI_ISL_983138, EPI_ISL_983142, EPI_ISL_983144, EPI_ISL_983146, EPI_ISL_983150, EPI_ISL_983152, EPI_ISL_983154, EPI_ISL_983157, EPI_ISL_983166, EPI_ISL_983168, EPI_ISL_983169			
see above	MONTEFIORE MEDICAL CENTER LABORATORIES	Wadsworth Center, New York State Department of Health	Kirsten St. George, Daryl M. Lamson, Alexis Russel, Matthew Shudt, Melissa A Leisner, Jonathan Plitnick, Navjot Singh, John Kelly, Erasmus Schneider, Erica Lasek-Nesselquist
