# Supplementary material for: Detection and characterization of the SARS-CoV-2 lineage B.1.526 in New York
Source: Nat Commun. 2021 Aug 9;12:4886. doi: 10.1038/s41467-021-25168-4 (PMC8352861; doi:10.1038/s41467-021-25168-4)
Supplement: Supplementary file 8 — Supplementary Data 4 [file 41467_2021_25168_MOESM8_ESM.zip › GISAID_acknowledements_tables/gisaid_hcov-19_acknowledgement_table_2021_02_13_00-16.pdf]

We gratefully acknowledge the following Authors from the Originating laboratories responsible for obtaining the specimens, as well as the Submitting laboratories where the genome data were generated and shared via GISAID, on which this research is based.

All Submitters of data may be contacted directly via www.gisaid.org

Authors are sorted alphabetically.

Accession ID	Originating Laboratory	Submitting Laboratory	Authors
EPI_ISL_708798	Sydney South West Pathology Service (SSWPS) - Royal Prince Alfred Hospital - NSW Health Pathology	NSW Health Pathology - Institute of Clinical Pathology and Medical Research; Westmead Hospital; University of Sydney	CIDM-PH et al.
EPI_ISL_708799	Pathology North - Royal North Shore Hospital - NSW Health Pathology	NSW Health Pathology - Institute of Clinical Pathology and Medical Research; Westmead Hospital; University of Sydney	CIDM-PH et al.
EPI_ISL_717701, EPI_ISL_717702	Area of Virology, Serology and Virology Division (SAViD), New South Wales Health Pathology Randwick	Virology Research Laboratory; Area of Virology, Serology and Virology Division (SAViD), New South Wales Health Pathology Randwick	Foster, C.; Au, J.; Ruiz Silva, M.; Deveson, I.; Bull, R.; Van Hal, S.; Rawlinson, W.
EPI_ISL_717978	Army Medical Center, Scientific Department, Virology Laboratory	Army Medical Center, Scientific Department, Virology Laboratory	Silvia Fillo, Giovanni Faggioni, Riccardo De Santis, Antonella Fortunato, Anna Anselmo, Vanessa Vera Fain, Francesco Giordani, Nino D'Amore, Anella Monte, Marzia Cavalli, Alessandra Amoroso, Stella Lia, Roberta Sorrentino, Rossella Tirelli, Federica Galeano, Annalisa Pelo, Margherita De Santis, Giulia Campoli, Andrea Ciammaruconi, Florigio Lista.
EPI_ISL_722357, EPI_ISL_722778, EPI_ISL_722779, EPI_ISL_722780, EPI_ISL_722781, EPI_ISL_722782, EPI_ISL_722783	Dutch COVID-19 response team	Erasmus Medical Center	Bas Oude Munnink, Reina Sikkema, David Nieuwenhuijse, Irina Chestakova, Anne van der Linden, Marjan Boter, Emmanuelle Munger, Corine GeurtsvanKessel, Annemiek van der Eijk, Richard Molenkamp, Marion Koopmans, on behalf of the Dutch national COVID-19 response team.
EPI_ISL_724608, EPI_ISL_724609	University College London, Great Ormond Street Hospital for Children NHS Foundation Trust, Imperial College Healthcare NHS Trust	COVID-19 Genomics UK (COG-UK) Consortium	Sergi Castellano, Rachel Williams, Mark Kristiansen, Paola Resende Silva, Sunando Roy, Tony Brooks, Helena Tutill, Paola Niola, Patricia Dyal, Charlotte Williams, Leysa Forrest, Yasmin Panchbhaya, Jacqueline Findlay, Samuel Weeks, Julianne Brown, Kathryn Harris, Paul Randell, James Price, Alison Holmes, Judith Breuer
EPI_ISL_727865, EPI_ISL_727870, EPI_ISL_727884, EPI_ISL_727891, EPI_ISL_727919, EPI_ISL_727938, EPI_ISL_727953, EPI_ISL_727972, EPI_ISL_727974	Virology Department, Sheffield Teaching Hospitals NHS Foundation Trust/Department of Infection, Immunity and Cardiovascular Disease, The Medical School, University of Sheffield	COVID-19 Genomics UK (COG-UK) Consortium	Thushan de Silva, Matthew Parker, Nikki Smith, Adri Angyal, Rebecca Brown, Luke Green, Rachel Tucker, Paul Parsons, Danielle Groves, Katie Johnson, Laura Carrilero, Alex Keeley, Dave Partridge, Matthew Wyles, Benjamin Lindsey, Mehmet Yavuz, Mohammad Raza, Cariad Evans
EPI_ISL_728642, EPI_ISL_728644, EPI_ISL_728660, EPI_ISL_728661, EPI_ISL_728747	Dutch COVID-19 response team	National Institute for Public Health and the Environment (RIVM)	Adam Meijer, Harry Vennema, Jeroen Cremer, Sharon van den Brink, Bas van der Veer, AnneMarie van den Brandt, Florian Zwagemaker, Dennis Schmitz, Chantal Reusken, on behalf of the national COVID-19 response team
EPI_ISL_732965, EPI_ISL_732967	LabPLUS	Institute of Environmental Science and Research (ESR)	Xiaoyun Ren, Matt Storey, Nikki Freed, Muhammad Faisal, Jing Wang, Hermes Perez, Anja Werno, Antje van der Linden, Arlo Upton, Chris Mansell, David Hammer, Dragana Drinkovic, Gary McAuliffe, Hana Sofia Andersson, James Ussher, Jill Sherwood, Josh Freeman, Julia Howard, Juliet Elvy, Mary DeAlmeida, Matt Blakiston, Matthew Rogers, Max Bloomfield, Michael Addidle, Michelle Balm, Sally Roberts, Sarah Jefferies, Sharmini Muttaiyah, Susan Morpeth, Susan Taylor, Timothy Blackmore, Vani Sathyendran, Veronica Playle, Virginia Hope, Erasmus Smit, Lauren Jelly, Olin Silander, Joep de Ligt
EPI_ISL_733497	University Hospital Zürich	Institute of Medical Virology, University of Zurich	Stefan Schmutz, Verena Kufner, Maryam Zaheri, Gabriela Ziltener, Thomas Scheier, Jürg Böni, Michael Huber, Alexandra Trkola
EPI_ISL_735861, EPI_ISL_735864	Lighthouse Lab in Glasgow	Wellcome Sanger Institute for the COVID-19 Genomics UK (COG-UK) Consortium	Harper VanSteenhouse, Yumi Kasai, David Gray, Carol Clugston, Anna Dominiczak and Alex Alderton, Roberto Amato, Sonia Goncalves, Ewan Harrison, David K. Jackson, Ian Johnston, Dominic Kwiatkowski, Cordelia Langford, John Sillitoe on behalf of the Wellcome Sanger Institute COVID-19 Surveillance Team
EPI_ISL_735867, EPI_ISL_735868, EPI_ISL_735877	Lighthouse Lab in Alderley Park	Wellcome Sanger Institute for the COVID-19 Genomics UK (COG-UK) Consortium	Jacquelyn Wynn, Mairead Hyland, The Lighthouse Lab in Alderley Park and Alex Alderton, Roberto Amato, Sonia Goncalves, Ewan Harrison, David K. Jackson, Ian Johnston, Dominic Kwiatkowski, Cordelia Langford, John Sillitoe on behalf of the Wellcome Sanger Institute COVID-19 Surveillance Team
EPI_ISL_735878, EPI_ISL_735880	Lighthouse Lab in Glasgow	Wellcome Sanger Institute for the COVID-19 Genomics UK (COG-UK) Consortium	Harper VanSteenhouse, Yumi Kasai, David Gray, Carol Clugston, Anna Dominiczak and Alex Alderton, Roberto Amato, Sonia Goncalves, Ewan Harrison, David K. Jackson, Ian Johnston, Dominic Kwiatkowski, Cordelia Langford, John Sillitoe on behalf of the Wellcome Sanger Institute COVID-19 Surveillance Team
EPI_ISL_735881, EPI_ISL_735882	Lighthouse Lab in Alderley Park	Wellcome Sanger Institute for the COVID-19 Genomics UK (COG-UK) Consortium	Jacquelyn Wynn, Mairead Hyland, The Lighthouse Lab in Alderley Park and Alex Alderton, Roberto Amato, Sonia Goncalves, Ewan Harrison, David K. Jackson, Ian Johnston, Dominic Kwiatkowski, Cordelia Langford, John Sillitoe on behalf of the Wellcome Sanger Institute COVID-19 Surveillance Team
EPI_ISL_735886	Lighthouse Lab in Glasgow	Wellcome Sanger Institute for the COVID-19 Genomics UK (COG-UK) Consortium	Harper VanSteenhouse, Yumi Kasai, David Gray, Carol Clugston, Anna Dominiczak and Alex Alderton, Roberto Amato, Sonia Goncalves, Ewan Harrison, David K. Jackson, Ian Johnston, Dominic Kwiatkowski, Cordelia Langford, John Sillitoe on behalf of the Wellcome Sanger Institute COVID-19 Surveillance Team
EPI_ISL_735887, EPI_ISL_735888, EPI_ISL_735889, EPI_ISL_735891	Lighthouse Lab in Alderley Park	Wellcome Sanger Institute for the COVID-19 Genomics UK (COG-UK) Consortium	Jacquelyn Wynn, Mairead Hyland, The Lighthouse Lab in Alderley Park and Alex Alderton, Roberto Amato, Sonia Goncalves, Ewan Harrison, David K. Jackson, Ian Johnston, Dominic Kwiatkowski, Cordelia Langford, John Sillitoe on behalf of the Wellcome Sanger Institute COVID-19 Surveillance Team
EPI_ISL_735894	Lighthouse Lab in Glasgow	Wellcome Sanger Institute for the COVID-19 Genomics UK (COG-UK) Consortium	Harper VanSteenhouse, Yumi Kasai, David Gray, Carol Clugston, Anna Dominiczak and Alex Alderton, Roberto Amato, Sonia Goncalves, Ewan Harrison, David K. Jackson, Ian Johnston, Dominic Kwiatkowski, Cordelia Langford, John Sillitoe on behalf of the Wellcome Sanger Institute COVID-19 Surveillance Team
EPI_ISL_735897, EPI_ISL_735899, EPI_ISL_735900, EPI_ISL_735901, EPI_ISL_735902, EPI_ISL_735904, EPI_ISL_735905, EPI_ISL_735907, EPI_ISL_735908	Lighthouse Lab in Alderley Park	Wellcome Sanger Institute for the COVID-19 Genomics UK (COG-UK) Consortium	Jacquelyn Wynn, Mairead Hyland, The Lighthouse Lab in Alderley Park and Alex Alderton, Roberto Amato, Sonia Goncalves, Ewan Harrison, David K. Jackson, Ian Johnston, Dominic Kwiatkowski, Cordelia Langford, John Sillitoe on behalf of the Wellcome Sanger Institute COVID-19 Surveillance Team
EPI_ISL_735909	Lighthouse Lab in Glasgow	Wellcome Sanger Institute for the COVID-19 Genomics UK (COG-UK) Consortium	Harper VanSteenhouse, Yumi Kasai, David Gray, Carol Clugston, Anna Dominiczak and Alex Alderton, Roberto Amato, Sonia Goncalves, Ewan Harrison, David K. Jackson, Ian Johnston, Dominic Kwiatkowski, Cordelia Langford, John Sillitoe on behalf of the Wellcome Sanger Institute COVID-19 Surveillance Team
EPI_ISL_735912, EPI_ISL_735917	Lighthouse Lab in Alderley Park	Wellcome Sanger Institute for the COVID-19 Genomics UK (COG-UK) Consortium	Jacquelyn Wynn, Mairead Hyland, The Lighthouse Lab in Alderley Park and Alex Alderton, Roberto Amato, Sonia Goncalves, Ewan Harrison, David K. Jackson, Ian Johnston, Dominic Kwiatkowski, Cordelia Langford, John Sillitoe on behalf of the Wellcome Sanger Institute COVID-19 Surveillance Team
EPI_ISL_735920	Lighthouse Lab in Glasgow	Wellcome Sanger Institute for the COVID-19 Genomics UK (COG-UK) Consortium	Harper VanSteenhouse, Yumi Kasai, David Gray, Carol Clugston, Anna Dominiczak and Alex Alderton, Roberto Amato, Sonia Goncalves, Ewan Harrison, David K. Jackson, Ian Johnston, Dominic Kwiatkowski, Cordelia Langford, John Sillitoe on behalf of the Wellcome Sanger Institute COVID-19 Surveillance Team
EPI_ISL_735923, EPI_ISL_735924, EPI_ISL_735926	Lighthouse Lab in Alderley Park	Wellcome Sanger Institute for the COVID-19 Genomics UK (COG-UK) Consortium	Jacquelyn Wynn, Mairead Hyland, The Lighthouse Lab in Alderley Park and Alex Alderton, Roberto Amato, Sonia Goncalves, Ewan Harrison, David K. Jackson, Ian Johnston, Dominic Kwiatkowski, Cordelia Langford, John Sillitoe on behalf of the Wellcome Sanger Institute COVID-19 Surveillance Team
EPI_ISL_735927	Lighthouse Lab in Glasgow	Wellcome Sanger Institute for the COVID-19 Genomics UK	Harper VanSteenhouse, Yumi Kasai, David Gray, Carol Clugston, Anna Dominiczak and Alex Alderton, Roberto Amato, Sonia Goncalves, Ewan Harrison,

		(COG-UK) Consortium	David K. Jackson, Ian Johnston, Dominic Kwiatkowski, Cordelia Langford, John Sillitoe on behalf of the Wellcome Sanger Institute COVID-19 Surveillance Team
EPI_ISL_735930, EPI_ISL_735931, EPI_ISL_735932, EPI_ISL_735936	Lighthouse Lab in Alderley Park	Wellcome Sanger Institute for the COVID-19 Genomics UK (COG-UK) Consortium	Jacquelyn Wynn, Mairead Hyland, The Lighthouse Lab in Alderley Park and Alex Alderton, Roberto Amato, Sonia Goncalves, Ewan Harrison, David K. Jackson, Ian Johnston, Dominic Kwiatkowski, Cordelia Langford, John Sillitoe on behalf of the Wellcome Sanger Institute COVID-19 Surveillance Team
EPI_ISL_735937	Lighthouse Lab in Glasgow	Wellcome Sanger Institute for the COVID-19 Genomics UK (COG-UK) Consortium	Harper VanSteenhouse, Yumi Kasai, David Gray, Carol Clugston, Anna Dominiczak and Alex Alderton, Roberto Amato, Sonia Goncalves, Ewan Harrison, David K. Jackson, Ian Johnston, Dominic Kwiatkowski, Cordelia Langford, John Sillitoe on behalf of the Wellcome Sanger Institute COVID-19 Surveillance Team
EPI_ISL_735938	Lighthouse Lab in Alderley Park	Wellcome Sanger Institute for the COVID-19 Genomics UK (COG-UK) Consortium	Jacquelyn Wynn, Mairead Hyland, The Lighthouse Lab in Alderley Park and Alex Alderton, Roberto Amato, Sonia Goncalves, Ewan Harrison, David K. Jackson, Ian Johnston, Dominic Kwiatkowski, Cordelia Langford, John Sillitoe on behalf of the Wellcome Sanger Institute COVID-19 Surveillance Team
EPI_ISL_735939	Lighthouse Lab in Glasgow	Wellcome Sanger Institute for the COVID-19 Genomics UK (COG-UK) Consortium	Harper VanSteenhouse, Yumi Kasai, David Gray, Carol Clugston, Anna Dominiczak and Alex Alderton, Roberto Amato, Sonia Goncalves, Ewan Harrison, David K. Jackson, Ian Johnston, Dominic Kwiatkowski, Cordelia Langford, John Sillitoe on behalf of the Wellcome Sanger Institute COVID-19 Surveillance Team
EPI_ISL_735941, EPI_ISL_735942, EPI_ISL_735943	Lighthouse Lab in Alderley Park	Wellcome Sanger Institute for the COVID-19 Genomics UK (COG-UK) Consortium	Jacquelyn Wynn, Mairead Hyland, The Lighthouse Lab in Alderley Park and Alex Alderton, Roberto Amato, Sonia Goncalves, Ewan Harrison, David K. Jackson, Ian Johnston, Dominic Kwiatkowski, Cordelia Langford, John Sillitoe on behalf of the Wellcome Sanger Institute COVID-19 Surveillance Team
EPI_ISL_735945, EPI_ISL_735949	Lighthouse Lab in Glasgow	Wellcome Sanger Institute for the COVID-19 Genomics UK (COG-UK) Consortium	Harper VanSteenhouse, Yumi Kasai, David Gray, Carol Clugston, Anna Dominiczak and Alex Alderton, Roberto Amato, Sonia Goncalves, Ewan Harrison, David K. Jackson, Ian Johnston, Dominic Kwiatkowski, Cordelia Langford, John Sillitoe on behalf of the Wellcome Sanger Institute COVID-19 Surveillance Team
EPI_ISL_735951	Lighthouse Lab in Alderley Park	Wellcome Sanger Institute for the COVID-19 Genomics UK (COG-UK) Consortium	Jacquelyn Wynn, Mairead Hyland, The Lighthouse Lab in Alderley Park and Alex Alderton, Roberto Amato, Sonia Goncalves, Ewan Harrison, David K. Jackson, Ian Johnston, Dominic Kwiatkowski, Cordelia Langford, John Sillitoe on behalf of the Wellcome Sanger Institute COVID-19 Surveillance Team
EPI_ISL_735952, EPI_ISL_735956	Lighthouse Lab in Glasgow	Wellcome Sanger Institute for the COVID-19 Genomics UK (COG-UK) Consortium	Harper VanSteenhouse, Yumi Kasai, David Gray, Carol Clugston, Anna Dominiczak and Alex Alderton, Roberto Amato, Sonia Goncalves, Ewan Harrison, David K. Jackson, Ian Johnston, Dominic Kwiatkowski, Cordelia Langford, John Sillitoe on behalf of the Wellcome Sanger Institute COVID-19 Surveillance Team
EPI_ISL_735958	Lighthouse Lab in Alderley Park	Wellcome Sanger Institute for the COVID-19 Genomics UK (COG-UK) Consortium	Jacquelyn Wynn, Mairead Hyland, The Lighthouse Lab in Alderley Park and Alex Alderton, Roberto Amato, Sonia Goncalves, Ewan Harrison, David K. Jackson, Ian Johnston, Dominic Kwiatkowski, Cordelia Langford, John Sillitoe on behalf of the Wellcome Sanger Institute COVID-19 Surveillance Team
EPI_ISL_735961	Lighthouse Lab in Glasgow	Wellcome Sanger Institute for the COVID-19 Genomics UK (COG-UK) Consortium	Harper VanSteenhouse, Yumi Kasai, David Gray, Carol Clugston, Anna Dominiczak and Alex Alderton, Roberto Amato, Sonia Goncalves, Ewan Harrison, David K. Jackson, Ian Johnston, Dominic Kwiatkowski, Cordelia Langford, John Sillitoe on behalf of the Wellcome Sanger Institute COVID-19 Surveillance Team
EPI_ISL_735963, EPI_ISL_735964	Lighthouse Lab in Alderley Park	Wellcome Sanger Institute for the COVID-19 Genomics UK (COG-UK) Consortium	Jacquelyn Wynn, Mairead Hyland, The Lighthouse Lab in Alderley Park and Alex Alderton, Roberto Amato, Sonia Goncalves, Ewan Harrison, David K. Jackson, Ian Johnston, Dominic Kwiatkowski, Cordelia Langford, John Sillitoe on behalf of the Wellcome Sanger Institute COVID-19 Surveillance Team
EPI_ISL_735965	Lighthouse Lab in Glasgow	Wellcome Sanger Institute for the COVID-19 Genomics UK (COG-UK) Consortium	Harper VanSteenhouse, Yumi Kasai, David Gray, Carol Clugston, Anna Dominiczak and Alex Alderton, Roberto Amato, Sonia Goncalves, Ewan Harrison, David K. Jackson, Ian Johnston, Dominic Kwiatkowski, Cordelia Langford, John Sillitoe on behalf of the Wellcome Sanger Institute COVID-19 Surveillance Team
EPI_ISL_735966, EPI_ISL_735967	Lighthouse Lab in Alderley Park	Wellcome Sanger Institute for the COVID-19 Genomics UK (COG-UK) Consortium	Jacquelyn Wynn, Mairead Hyland, The Lighthouse Lab in Alderley Park and Alex Alderton, Roberto Amato, Sonia Goncalves, Ewan Harrison, David K. Jackson, Ian Johnston, Dominic Kwiatkowski, Cordelia Langford, John Sillitoe on behalf of the Wellcome Sanger Institute COVID-19 Surveillance Team
EPI_ISL_735968, EPI_ISL_735972, EPI_ISL_735973	Lighthouse Lab in Glasgow	Wellcome Sanger Institute for the COVID-19 Genomics UK (COG-UK) Consortium	Harper VanSteenhouse, Yumi Kasai, David Gray, Carol Clugston, Anna Dominiczak and Alex Alderton, Roberto Amato, Sonia Goncalves, Ewan Harrison, David K. Jackson, Ian Johnston, Dominic Kwiatkowski, Cordelia Langford, John Sillitoe on behalf of the Wellcome Sanger Institute COVID-19 Surveillance Team
EPI_ISL_735974, EPI_ISL_735975, EPI_ISL_735976	Lighthouse Lab in Alderley Park	Wellcome Sanger Institute for the COVID-19 Genomics UK (COG-UK) Consortium	Jacquelyn Wynn, Mairead Hyland, The Lighthouse Lab in Alderley Park and Alex Alderton, Roberto Amato, Sonia Goncalves, Ewan Harrison, David K. Jackson, Ian Johnston, Dominic Kwiatkowski, Cordelia Langford, John Sillitoe on behalf of the Wellcome Sanger Institute COVID-19 Surveillance Team
EPI_ISL_735978	Lighthouse Lab in Glasgow	Wellcome Sanger Institute for the COVID-19 Genomics UK (COG-UK) Consortium	Harper VanSteenhouse, Yumi Kasai, David Gray, Carol Clugston, Anna Dominiczak and Alex Alderton, Roberto Amato, Sonia Goncalves, Ewan Harrison, David K. Jackson, Ian Johnston, Dominic Kwiatkowski, Cordelia Langford, John Sillitoe on behalf of the Wellcome Sanger Institute COVID-19 Surveillance Team
EPI_ISL_735980, EPI_ISL_735981	Lighthouse Lab in Alderley Park	Wellcome Sanger Institute for the COVID-19 Genomics UK (COG-UK) Consortium	Jacquelyn Wynn, Mairead Hyland, The Lighthouse Lab in Alderley Park and Alex Alderton, Roberto Amato, Sonia Goncalves, Ewan Harrison, David K. Jackson, Ian Johnston, Dominic Kwiatkowski, Cordelia Langford, John Sillitoe on behalf of the Wellcome Sanger Institute COVID-19 Surveillance Team
EPI_ISL_735982	Lighthouse Lab in Glasgow	Wellcome Sanger Institute for the COVID-19 Genomics UK (COG-UK) Consortium	Harper VanSteenhouse, Yumi Kasai, David Gray, Carol Clugston, Anna Dominiczak and Alex Alderton, Roberto Amato, Sonia Goncalves, Ewan Harrison, David K. Jackson, Ian Johnston, Dominic Kwiatkowski, Cordelia Langford, John Sillitoe on behalf of the Wellcome Sanger Institute COVID-19 Surveillance Team
EPI_ISL_735983, EPI_ISL_735986	Lighthouse Lab in Alderley Park	Wellcome Sanger Institute for the COVID-19 Genomics UK (COG-UK) Consortium	Jacquelyn Wynn, Mairead Hyland, The Lighthouse Lab in Alderley Park and Alex Alderton, Roberto Amato, Sonia Goncalves, Ewan Harrison, David K. Jackson, Ian Johnston, Dominic Kwiatkowski, Cordelia Langford, John Sillitoe on behalf of the Wellcome Sanger Institute COVID-19 Surveillance Team
EPI_ISL_735987	Lighthouse Lab in Glasgow	Wellcome Sanger Institute for the COVID-19 Genomics UK (COG-UK) Consortium	Harper VanSteenhouse, Yumi Kasai, David Gray, Carol Clugston, Anna Dominiczak and Alex Alderton, Roberto Amato, Sonia Goncalves, Ewan Harrison, David K. Jackson, Ian Johnston, Dominic Kwiatkowski, Cordelia Langford, John Sillitoe on behalf of the Wellcome Sanger Institute COVID-19 Surveillance Team
EPI_ISL_735992	Lighthouse Lab in Alderley Park	Wellcome Sanger Institute for the COVID-19 Genomics UK (COG-UK) Consortium	Jacquelyn Wynn, Mairead Hyland, The Lighthouse Lab in Alderley Park and Alex Alderton, Roberto Amato, Sonia Goncalves, Ewan Harrison, David K. Jackson, Ian Johnston, Dominic Kwiatkowski, Cordelia Langford, John Sillitoe on behalf of the Wellcome Sanger Institute COVID-19 Surveillance Team
EPI_ISL_735993, EPI_ISL_735995, EPI_ISL_735996	Lighthouse Lab in Glasgow	Wellcome Sanger Institute for the COVID-19 Genomics UK (COG-UK) Consortium	Harper VanSteenhouse, Yumi Kasai, David Gray, Carol Clugston, Anna Dominiczak and Alex Alderton, Roberto Amato, Sonia Goncalves, Ewan Harrison, David K. Jackson, Ian Johnston, Dominic Kwiatkowski, Cordelia Langford, John Sillitoe on behalf of the Wellcome Sanger Institute COVID-19 Surveillance Team
EPI_ISL_735997, EPI_ISL_735998, EPI_ISL_736000	Lighthouse Lab in Alderley Park	Wellcome Sanger Institute for the COVID-19 Genomics UK (COG-UK) Consortium	Jacquelyn Wynn, Mairead Hyland, The Lighthouse Lab in Alderley Park and Alex Alderton, Roberto Amato, Sonia Goncalves, Ewan Harrison, David K. Jackson, Ian Johnston, Dominic Kwiatkowski, Cordelia Langford, John Sillitoe on behalf of the Wellcome Sanger Institute COVID-19 Surveillance Team
EPI_ISL_736001, EPI_ISL_736003, EPI_ISL_736007	Lighthouse Lab in Glasgow	Wellcome Sanger Institute for the COVID-19 Genomics UK (COG-UK) Consortium	Harper VanSteenhouse, Yumi Kasai, David Gray, Carol Clugston, Anna Dominiczak and Alex Alderton, Roberto Amato, Sonia Goncalves, Ewan Harrison, David K. Jackson, Ian Johnston, Dominic Kwiatkowski, Cordelia Langford, John Sillitoe on behalf of the Wellcome Sanger Institute COVID-19 Surveillance Team
EPI_ISL_736008, EPI_ISL_736010, EPI_ISL_736011, EPI_ISL_736012, EPI_ISL_736013	Lighthouse Lab in Alderley Park	Wellcome Sanger Institute for the COVID-19 Genomics UK (COG-UK) Consortium	Jacquelyn Wynn, Mairead Hyland, The Lighthouse Lab in Alderley Park and Alex Alderton, Roberto Amato, Sonia Goncalves, Ewan Harrison, David K. Jackson, Ian Johnston, Dominic Kwiatkowski, Cordelia Langford, John Sillitoe on behalf of the Wellcome Sanger Institute COVID-19 Surveillance Team
EPI_ISL_736015	Lighthouse Lab in Glasgow	Wellcome Sanger Institute for the COVID-19 Genomics UK (COG-UK) Consortium	Harper VanSteenhouse, Yumi Kasai, David Gray, Carol Clugston, Anna Dominiczak and Alex Alderton, Roberto Amato, Sonia Goncalves, Ewan Harrison, David K. Jackson, Ian Johnston, Dominic Kwiatkowski, Cordelia Langford, John Sillitoe on behalf of the Wellcome Sanger Institute COVID-19 Surveillance Team
EPI_ISL_736016	Lighthouse Lab in Alderley Park	Wellcome Sanger Institute for the COVID-19 Genomics UK (COG-UK) Consortium	Jacquelyn Wynn, Mairead Hyland, The Lighthouse Lab in Alderley Park and Alex Alderton, Roberto Amato, Sonia Goncalves, Ewan Harrison, David K. Jackson, Ian Johnston, Dominic Kwiatkowski, Cordelia Langford, John Sillitoe on behalf of the Wellcome Sanger Institute COVID-19 Surveillance Team
EPI_ISL_736017, EPI_ISL_736018, EPI_ISL_736019	Lighthouse Lab in Glasgow	Wellcome Sanger Institute for the COVID-19 Genomics UK (COG-UK) Consortium	Harper VanSteenhouse, Yumi Kasai, David Gray, Carol Clugston, Anna Dominiczak and Alex Alderton, Roberto Amato, Sonia Goncalves, Ewan Harrison, David K. Jackson, Ian Johnston, Dominic Kwiatkowski, Cordelia Langford, John Sillitoe on behalf of the Wellcome Sanger Institute COVID-19 Surveillance Team

EPI_ISL_736021, EPI_ISL_736023	Lighthouse Lab in Alderley Park	Wellcome Sanger Institute for the COVID-19 Genomics UK (COG-UK) Consortium	Jacquelyn Wynn, Mairead Hyland, The Lighthouse Lab in Alderley Park and Alex Alderton, Roberto Amato, Sonia Goncalves, Ewan Harrison, David K. Jackson, Ian Johnston, Dominic Kwiatkowski, Cordelia Langford, John Sillitoe on behalf of the Wellcome Sanger Institute COVID-19 Surveillance Team
EPI_ISL_736024, EPI_ISL_736025, EPI_ISL_736027	Lighthouse Lab in Glasgow	Wellcome Sanger Institute for the COVID-19 Genomics UK (COG-UK) Consortium	Harper VanSteenhouse, Yumi Kasai, David Gray, Carol Clugston, Anna Dominiczak and Alex Alderton, Roberto Amato, Sonia Goncalves, Ewan Harrison, David K. Jackson, Ian Johnston, Dominic Kwiatkowski, Cordelia Langford, John Sillitoe on behalf of the Wellcome Sanger Institute COVID-19 Surveillance Team
EPI_ISL_736028, EPI_ISL_736029, EPI_ISL_736030	Lighthouse Lab in Alderley Park	Wellcome Sanger Institute for the COVID-19 Genomics UK (COG-UK) Consortium	Jacquelyn Wynn, Mairead Hyland, The Lighthouse Lab in Alderley Park and Alex Alderton, Roberto Amato, Sonia Goncalves, Ewan Harrison, David K. Jackson, Ian Johnston, Dominic Kwiatkowski, Cordelia Langford, John Sillitoe on behalf of the Wellcome Sanger Institute COVID-19 Surveillance Team
EPI_ISL_736033	Lighthouse Lab in Glasgow	Wellcome Sanger Institute for the COVID-19 Genomics UK (COG-UK) Consortium	Harper VanSteenhouse, Yumi Kasai, David Gray, Carol Clugston, Anna Dominiczak and Alex Alderton, Roberto Amato, Sonia Goncalves, Ewan Harrison, David K. Jackson, Ian Johnston, Dominic Kwiatkowski, Cordelia Langford, John Sillitoe on behalf of the Wellcome Sanger Institute COVID-19 Surveillance Team
EPI_ISL_736034, EPI_ISL_736037	Lighthouse Lab in Alderley Park	Wellcome Sanger Institute for the COVID-19 Genomics UK (COG-UK) Consortium	Jacquelyn Wynn, Mairead Hyland, The Lighthouse Lab in Alderley Park and Alex Alderton, Roberto Amato, Sonia Goncalves, Ewan Harrison, David K. Jackson, Ian Johnston, Dominic Kwiatkowski, Cordelia Langford, John Sillitoe on behalf of the Wellcome Sanger Institute COVID-19 Surveillance Team
EPI_ISL_736038	Lighthouse Lab in Glasgow	Wellcome Sanger Institute for the COVID-19 Genomics UK (COG-UK) Consortium	Harper VanSteenhouse, Yumi Kasai, David Gray, Carol Clugston, Anna Dominiczak and Alex Alderton, Roberto Amato, Sonia Goncalves, Ewan Harrison, David K. Jackson, Ian Johnston, Dominic Kwiatkowski, Cordelia Langford, John Sillitoe on behalf of the Wellcome Sanger Institute COVID-19 Surveillance Team
EPI_ISL_736039	Lighthouse Lab in Alderley Park	Wellcome Sanger Institute for the COVID-19 Genomics UK (COG-UK) Consortium	Jacquelyn Wynn, Mairead Hyland, The Lighthouse Lab in Alderley Park and Alex Alderton, Roberto Amato, Sonia Goncalves, Ewan Harrison, David K. Jackson, Ian Johnston, Dominic Kwiatkowski, Cordelia Langford, John Sillitoe on behalf of the Wellcome Sanger Institute COVID-19 Surveillance Team
EPI_ISL_736041	Lighthouse Lab in Glasgow	Wellcome Sanger Institute for the COVID-19 Genomics UK (COG-UK) Consortium	Harper VanSteenhouse, Yumi Kasai, David Gray, Carol Clugston, Anna Dominiczak and Alex Alderton, Roberto Amato, Sonia Goncalves, Ewan Harrison, David K. Jackson, Ian Johnston, Dominic Kwiatkowski, Cordelia Langford, John Sillitoe on behalf of the Wellcome Sanger Institute COVID-19 Surveillance Team
EPI_ISL_736046, EPI_ISL_736047, EPI_ISL_736048, EPI_ISL_736050, EPI_ISL_736051	Lighthouse Lab in Alderley Park	Wellcome Sanger Institute for the COVID-19 Genomics UK (COG-UK) Consortium	Jacquelyn Wynn, Mairead Hyland, The Lighthouse Lab in Alderley Park and Alex Alderton, Roberto Amato, Sonia Goncalves, Ewan Harrison, David K. Jackson, Ian Johnston, Dominic Kwiatkowski, Cordelia Langford, John Sillitoe on behalf of the Wellcome Sanger Institute COVID-19 Surveillance Team
EPI_ISL_736052	Lighthouse Lab in Glasgow	Wellcome Sanger Institute for the COVID-19 Genomics UK (COG-UK) Consortium	Harper VanSteenhouse, Yumi Kasai, David Gray, Carol Clugston, Anna Dominiczak and Alex Alderton, Roberto Amato, Sonia Goncalves, Ewan Harrison, David K. Jackson, Ian Johnston, Dominic Kwiatkowski, Cordelia Langford, John Sillitoe on behalf of the Wellcome Sanger Institute COVID-19 Surveillance Team
EPI_ISL_736055	Lighthouse Lab in Alderley Park	Wellcome Sanger Institute for the COVID-19 Genomics UK (COG-UK) Consortium	Jacquelyn Wynn, Mairead Hyland, The Lighthouse Lab in Alderley Park and Alex Alderton, Roberto Amato, Sonia Goncalves, Ewan Harrison, David K. Jackson, Ian Johnston, Dominic Kwiatkowski, Cordelia Langford, John Sillitoe on behalf of the Wellcome Sanger Institute COVID-19 Surveillance Team
EPI_ISL_736057, EPI_ISL_736059	Lighthouse Lab in Glasgow	Wellcome Sanger Institute for the COVID-19 Genomics UK (COG-UK) Consortium	Harper VanSteenhouse, Yumi Kasai, David Gray, Carol Clugston, Anna Dominiczak and Alex Alderton, Roberto Amato, Sonia Goncalves, Ewan Harrison, David K. Jackson, Ian Johnston, Dominic Kwiatkowski, Cordelia Langford, John Sillitoe on behalf of the Wellcome Sanger Institute COVID-19 Surveillance Team
EPI_ISL_736063	Lighthouse Lab in Alderley Park	Wellcome Sanger Institute for the COVID-19 Genomics UK (COG-UK) Consortium	Jacquelyn Wynn, Mairead Hyland, The Lighthouse Lab in Alderley Park and Alex Alderton, Roberto Amato, Sonia Goncalves, Ewan Harrison, David K. Jackson, Ian Johnston, Dominic Kwiatkowski, Cordelia Langford, John Sillitoe on behalf of the Wellcome Sanger Institute COVID-19 Surveillance Team
EPI_ISL_736064	Lighthouse Lab in Glasgow	Wellcome Sanger Institute for the COVID-19 Genomics UK (COG-UK) Consortium	Harper VanSteenhouse, Yumi Kasai, David Gray, Carol Clugston, Anna Dominiczak and Alex Alderton, Roberto Amato, Sonia Goncalves, Ewan Harrison, David K. Jackson, Ian Johnston, Dominic Kwiatkowski, Cordelia Langford, John Sillitoe on behalf of the Wellcome Sanger Institute COVID-19 Surveillance Team
EPI_ISL_736065	Lighthouse Lab in Alderley Park	Wellcome Sanger Institute for the COVID-19 Genomics UK (COG-UK) Consortium	Jacquelyn Wynn, Mairead Hyland, The Lighthouse Lab in Alderley Park and Alex Alderton, Roberto Amato, Sonia Goncalves, Ewan Harrison, David K. Jackson, Ian Johnston, Dominic Kwiatkowski, Cordelia Langford, John Sillitoe on behalf of the Wellcome Sanger Institute COVID-19 Surveillance Team
EPI_ISL_736066, EPI_ISL_736072	Lighthouse Lab in Glasgow	Wellcome Sanger Institute for the COVID-19 Genomics UK (COG-UK) Consortium	Harper VanSteenhouse, Yumi Kasai, David Gray, Carol Clugston, Anna Dominiczak and Alex Alderton, Roberto Amato, Sonia Goncalves, Ewan Harrison, David K. Jackson, Ian Johnston, Dominic Kwiatkowski, Cordelia Langford, John Sillitoe on behalf of the Wellcome Sanger Institute COVID-19 Surveillance Team
EPI_ISL_736078, EPI_ISL_736079, EPI_ISL_736080, EPI_ISL_736083, EPI_ISL_736087	Lighthouse Lab in Alderley Park	Wellcome Sanger Institute for the COVID-19 Genomics UK (COG-UK) Consortium	Jacquelyn Wynn, Mairead Hyland, The Lighthouse Lab in Alderley Park and Alex Alderton, Roberto Amato, Sonia Goncalves, Ewan Harrison, David K. Jackson, Ian Johnston, Dominic Kwiatkowski, Cordelia Langford, John Sillitoe on behalf of the Wellcome Sanger Institute COVID-19 Surveillance Team
EPI_ISL_736090, EPI_ISL_736091, EPI_ISL_736092	Lighthouse Lab in Glasgow	Wellcome Sanger Institute for the COVID-19 Genomics UK (COG-UK) Consortium	Harper VanSteenhouse, Yumi Kasai, David Gray, Carol Clugston, Anna Dominiczak and Alex Alderton, Roberto Amato, Sonia Goncalves, Ewan Harrison, David K. Jackson, Ian Johnston, Dominic Kwiatkowski, Cordelia Langford, John Sillitoe on behalf of the Wellcome Sanger Institute COVID-19 Surveillance Team
EPI_ISL_736093, EPI_ISL_736095	Lighthouse Lab in Alderley Park	Wellcome Sanger Institute for the COVID-19 Genomics UK (COG-UK) Consortium	Jacquelyn Wynn, Mairead Hyland, The Lighthouse Lab in Alderley Park and Alex Alderton, Roberto Amato, Sonia Goncalves, Ewan Harrison, David K. Jackson, Ian Johnston, Dominic Kwiatkowski, Cordelia Langford, John Sillitoe on behalf of the Wellcome Sanger Institute COVID-19 Surveillance Team
EPI_ISL_736096	Lighthouse Lab in Glasgow	Wellcome Sanger Institute for the COVID-19 Genomics UK (COG-UK) Consortium	Harper VanSteenhouse, Yumi Kasai, David Gray, Carol Clugston, Anna Dominiczak and Alex Alderton, Roberto Amato, Sonia Goncalves, Ewan Harrison, David K. Jackson, Ian Johnston, Dominic Kwiatkowski, Cordelia Langford, John Sillitoe on behalf of the Wellcome Sanger Institute COVID-19 Surveillance Team
EPI_ISL_736097	Lighthouse Lab in Alderley Park	Wellcome Sanger Institute for the COVID-19 Genomics UK (COG-UK) Consortium	Jacquelyn Wynn, Mairead Hyland, The Lighthouse Lab in Alderley Park and Alex Alderton, Roberto Amato, Sonia Goncalves, Ewan Harrison, David K. Jackson, Ian Johnston, Dominic Kwiatkowski, Cordelia Langford, John Sillitoe on behalf of the Wellcome Sanger Institute COVID-19 Surveillance Team
EPI_ISL_736099, EPI_ISL_736100, EPI_ISL_736101, EPI_ISL_736102, EPI_ISL_736104	Lighthouse Lab in Glasgow	Wellcome Sanger Institute for the COVID-19 Genomics UK (COG-UK) Consortium	Harper VanSteenhouse, Yumi Kasai, David Gray, Carol Clugston, Anna Dominiczak and Alex Alderton, Roberto Amato, Sonia Goncalves, Ewan Harrison, David K. Jackson, Ian Johnston, Dominic Kwiatkowski, Cordelia Langford, John Sillitoe on behalf of the Wellcome Sanger Institute COVID-19 Surveillance Team
EPI_ISL_736105	Lighthouse Lab in Alderley Park	Wellcome Sanger Institute for the COVID-19 Genomics UK (COG-UK) Consortium	Jacquelyn Wynn, Mairead Hyland, The Lighthouse Lab in Alderley Park and Alex Alderton, Roberto Amato, Sonia Goncalves, Ewan Harrison, David K. Jackson, Ian Johnston, Dominic Kwiatkowski, Cordelia Langford, John Sillitoe on behalf of the Wellcome Sanger Institute COVID-19 Surveillance Team
EPI_ISL_736107	Lighthouse Lab in Glasgow	Wellcome Sanger Institute for the COVID-19 Genomics UK (COG-UK) Consortium	Harper VanSteenhouse, Yumi Kasai, David Gray, Carol Clugston, Anna Dominiczak and Alex Alderton, Roberto Amato, Sonia Goncalves, Ewan Harrison, David K. Jackson, Ian Johnston, Dominic Kwiatkowski, Cordelia Langford, John Sillitoe on behalf of the Wellcome Sanger Institute COVID-19 Surveillance Team
EPI_ISL_736108, EPI_ISL_736110	Lighthouse Lab in Alderley Park	Wellcome Sanger Institute for the COVID-19 Genomics UK (COG-UK) Consortium	Jacquelyn Wynn, Mairead Hyland, The Lighthouse Lab in Alderley Park and Alex Alderton, Roberto Amato, Sonia Goncalves, Ewan Harrison, David K. Jackson, Ian Johnston, Dominic Kwiatkowski, Cordelia Langford, John Sillitoe on behalf of the Wellcome Sanger Institute COVID-19 Surveillance Team
EPI_ISL_736115	Lighthouse Lab in Glasgow	Wellcome Sanger Institute for the COVID-19 Genomics UK (COG-UK) Consortium	Harper VanSteenhouse, Yumi Kasai, David Gray, Carol Clugston, Anna Dominiczak and Alex Alderton, Roberto Amato, Sonia Goncalves, Ewan Harrison, David K. Jackson, Ian Johnston, Dominic Kwiatkowski, Cordelia Langford, John Sillitoe on behalf of the Wellcome Sanger Institute COVID-19 Surveillance Team
EPI_ISL_736118	Lighthouse Lab in Alderley Park	Wellcome Sanger Institute for the COVID-19 Genomics UK (COG-UK) Consortium	Jacquelyn Wynn, Mairead Hyland, The Lighthouse Lab in Alderley Park and Alex Alderton, Roberto Amato, Sonia Goncalves, Ewan Harrison, David K. Jackson, Ian Johnston, Dominic Kwiatkowski, Cordelia Langford, John Sillitoe on behalf of the Wellcome Sanger Institute COVID-19 Surveillance Team
EPI_ISL_736124	Lighthouse Lab in Glasgow	Wellcome Sanger Institute for the COVID-19 Genomics UK (COG-UK) Consortium	Harper VanSteenhouse, Yumi Kasai, David Gray, Carol Clugston, Anna Dominiczak and Alex Alderton, Roberto Amato, Sonia Goncalves, Ewan Harrison, David K. Jackson, Ian Johnston, Dominic Kwiatkowski, Cordelia Langford, John Sillitoe on behalf of the Wellcome Sanger Institute COVID-19 Surveillance Team
EPI_ISL_736125, EPI_ISL_736127	Lighthouse Lab in Alderley Park	Wellcome Sanger Institute for the COVID-19 Genomics UK	Jacquelyn Wynn, Mairead Hyland, The Lighthouse Lab in Alderley Park and Alex Alderton, Roberto Amato, Sonia Goncalves, Ewan Harrison, David K.

		(COG-UK) Consortium	Jackson, Ian Johnston, Dominic Kwiatkowski, Cordelia Langford, John Sillitoe on behalf of the Wellcome Sanger Institute COVID-19 Surveillance Team
EPI_ISL_736130, EPI_ISL_736131, EPI_ISL_736132, EPI_ISL_736135	Lighthouse Lab in Glasgow	Wellcome Sanger Institute for the COVID-19 Genomics UK (COG-UK) Consortium	Harper VanSteenhouse, Yumi Kasai, David Gray, Carol Clugston, Anna Dominiczak and Alex Alderton, Roberto Amato, Sonia Goncalves, Ewan Harrison, David K. Jackson, Ian Johnston, Dominic Kwiatkowski, Cordelia Langford, John Sillitoe on behalf of the Wellcome Sanger Institute COVID-19 Surveillance Team
EPI_ISL_736141, EPI_ISL_736142	Lighthouse Lab in Alderley Park	Wellcome Sanger Institute for the COVID-19 Genomics UK (COG-UK) Consortium	Jacquelyn Wynn, Mairead Hyland, The Lighthouse Lab in Alderley Park and Alex Alderton, Roberto Amato, Sonia Goncalves, Ewan Harrison, David K. Jackson, Ian Johnston, Dominic Kwiatkowski, Cordelia Langford, John Sillitoe on behalf of the Wellcome Sanger Institute COVID-19 Surveillance Team
EPI_ISL_736143, EPI_ISL_736146, EPI_ISL_736149, EPI_ISL_736151	Lighthouse Lab in Glasgow	Wellcome Sanger Institute for the COVID-19 Genomics UK (COG-UK) Consortium	Harper VanSteenhouse, Yumi Kasai, David Gray, Carol Clugston, Anna Dominiczak and Alex Alderton, Roberto Amato, Sonia Goncalves, Ewan Harrison, David K. Jackson, Ian Johnston, Dominic Kwiatkowski, Cordelia Langford, John Sillitoe on behalf of the Wellcome Sanger Institute COVID-19 Surveillance Team
EPI_ISL_736153, EPI_ISL_736158	Lighthouse Lab in Alderley Park	Wellcome Sanger Institute for the COVID-19 Genomics UK (COG-UK) Consortium	Jacquelyn Wynn, Mairead Hyland, The Lighthouse Lab in Alderley Park and Alex Alderton, Roberto Amato, Sonia Goncalves, Ewan Harrison, David K. Jackson, Ian Johnston, Dominic Kwiatkowski, Cordelia Langford, John Sillitoe on behalf of the Wellcome Sanger Institute COVID-19 Surveillance Team
EPI_ISL_736159, EPI_ISL_736162, EPI_ISL_736167, EPI_ISL_736168, EPI_ISL_736169	Lighthouse Lab in Glasgow	Wellcome Sanger Institute for the COVID-19 Genomics UK (COG-UK) Consortium	Harper VanSteenhouse, Yumi Kasai, David Gray, Carol Clugston, Anna Dominiczak and Alex Alderton, Roberto Amato, Sonia Goncalves, Ewan Harrison, David K. Jackson, Ian Johnston, Dominic Kwiatkowski, Cordelia Langford, John Sillitoe on behalf of the Wellcome Sanger Institute COVID-19 Surveillance Team
EPI_ISL_736172	Lighthouse Lab in Alderley Park	Wellcome Sanger Institute for the COVID-19 Genomics UK (COG-UK) Consortium	Jacquelyn Wynn, Mairead Hyland, The Lighthouse Lab in Alderley Park and Alex Alderton, Roberto Amato, Sonia Goncalves, Ewan Harrison, David K. Jackson, Ian Johnston, Dominic Kwiatkowski, Cordelia Langford, John Sillitoe on behalf of the Wellcome Sanger Institute COVID-19 Surveillance Team
EPI_ISL_736175	Lighthouse Lab in Glasgow	Wellcome Sanger Institute for the COVID-19 Genomics UK (COG-UK) Consortium	Harper VanSteenhouse, Yumi Kasai, David Gray, Carol Clugston, Anna Dominiczak and Alex Alderton, Roberto Amato, Sonia Goncalves, Ewan Harrison, David K. Jackson, Ian Johnston, Dominic Kwiatkowski, Cordelia Langford, John Sillitoe on behalf of the Wellcome Sanger Institute COVID-19 Surveillance Team
EPI_ISL_736178	Lighthouse Lab in Alderley Park	Wellcome Sanger Institute for the COVID-19 Genomics UK (COG-UK) Consortium	Jacquelyn Wynn, Mairead Hyland, The Lighthouse Lab in Alderley Park and Alex Alderton, Roberto Amato, Sonia Goncalves, Ewan Harrison, David K. Jackson, Ian Johnston, Dominic Kwiatkowski, Cordelia Langford, John Sillitoe on behalf of the Wellcome Sanger Institute COVID-19 Surveillance Team
EPI_ISL_736182, EPI_ISL_736183, EPI_ISL_736184	Lighthouse Lab in Glasgow	Wellcome Sanger Institute for the COVID-19 Genomics UK (COG-UK) Consortium	Harper VanSteenhouse, Yumi Kasai, David Gray, Carol Clugston, Anna Dominiczak and Alex Alderton, Roberto Amato, Sonia Goncalves, Ewan Harrison, David K. Jackson, Ian Johnston, Dominic Kwiatkowski, Cordelia Langford, John Sillitoe on behalf of the Wellcome Sanger Institute COVID-19 Surveillance Team
EPI_ISL_736187, EPI_ISL_736189	Lighthouse Lab in Alderley Park	Wellcome Sanger Institute for the COVID-19 Genomics UK (COG-UK) Consortium	Jacquelyn Wynn, Mairead Hyland, The Lighthouse Lab in Alderley Park and Alex Alderton, Roberto Amato, Sonia Goncalves, Ewan Harrison, David K. Jackson, Ian Johnston, Dominic Kwiatkowski, Cordelia Langford, John Sillitoe on behalf of the Wellcome Sanger Institute COVID-19 Surveillance Team
EPI_ISL_736190, EPI_ISL_736191	Lighthouse Lab in Glasgow	Wellcome Sanger Institute for the COVID-19 Genomics UK (COG-UK) Consortium	Harper VanSteenhouse, Yumi Kasai, David Gray, Carol Clugston, Anna Dominiczak and Alex Alderton, Roberto Amato, Sonia Goncalves, Ewan Harrison, David K. Jackson, Ian Johnston, Dominic Kwiatkowski, Cordelia Langford, John Sillitoe on behalf of the Wellcome Sanger Institute COVID-19 Surveillance Team
EPI_ISL_736193, EPI_ISL_736199	Lighthouse Lab in Alderley Park	Wellcome Sanger Institute for the COVID-19 Genomics UK (COG-UK) Consortium	Jacquelyn Wynn, Mairead Hyland, The Lighthouse Lab in Alderley Park and Alex Alderton, Roberto Amato, Sonia Goncalves, Ewan Harrison, David K. Jackson, Ian Johnston, Dominic Kwiatkowski, Cordelia Langford, John Sillitoe on behalf of the Wellcome Sanger Institute COVID-19 Surveillance Team
EPI_ISL_736483, EPI_ISL_736488, EPI_ISL_736489, EPI_ISL_736490, EPI_ISL_736491, EPI_ISL_736492, EPI_ISL_736512, EPI_ISL_736519, EPI_ISL_736529, EPI_ISL_736532, EPI_ISL_736533, EPI_ISL_736556, EPI_ISL_736567, EPI_ISL_736573, EPI_ISL_736580, EPI_ISL_736582, EPI_ISL_736605, EPI_ISL_736620, EPI_ISL_736624, EPI_ISL_736627, EPI_ISL_736628, EPI_ISL_736631, EPI_ISL_736633, EPI_ISL_736646, EPI_ISL_736650, EPI_ISL_736657, EPI_ISL_736659, EPI_ISL_736668, EPI_ISL_736672, EPI_ISL_736678, EPI_ISL_736689, EPI_ISL_736691, EPI_ISL_736695, EPI_ISL_736700, EPI_ISL_736734, EPI_ISL_736736, EPI_ISL_736737, EPI_ISL_736743, EPI_ISL_736745, EPI_ISL_736752, EPI_ISL_736754			
see above	Lighthouse Lab in Glasgow	Wellcome Sanger Institute for the COVID-19 Genomics UK (COG-UK) Consortium	Harper VanSteenhouse, Yumi Kasai, David Gray, Carol Clugston, Anna Dominiczak and Alex Alderton, Roberto Amato, Sonia Goncalves, Ewan Harrison, David K. Jackson, Ian Johnston, Dominic Kwiatkowski, Cordelia Langford, John Sillitoe on behalf of the Wellcome Sanger Institute COVID-19 Surveillance Team
EPI_ISL_737245, EPI_ISL_737246, EPI_ISL_737247, EPI_ISL_737248, EPI_ISL_737249, EPI_ISL_737250, EPI_ISL_737251, EPI_ISL_737252, EPI_ISL_737253	Los Angeles County PHL	Los Angeles County PHL	P. Hemarajata et al.
EPI_ISL_737310, EPI_ISL_737311, EPI_ISL_737312, EPI_ISL_737313, EPI_ISL_737314, EPI_ISL_737315, EPI_ISL_737316, EPI_ISL_737317, EPI_ISL_737318, EPI_ISL_737319, EPI_ISL_737320, EPI_ISL_737321, EPI_ISL_737322, EPI_ISL_737323, EPI_ISL_737324, EPI_ISL_737325, EPI_ISL_737326, EPI_ISL_737327, EPI_ISL_737328, EPI_ISL_737329, EPI_ISL_737330, EPI_ISL_737331, EPI_ISL_737332, EPI_ISL_737333, EPI_ISL_737334, EPI_ISL_737335, EPI_ISL_737336, EPI_ISL_737337, EPI_ISL_737338, EPI_ISL_737339, EPI_ISL_737340, EPI_ISL_737341, EPI_ISL_737342, EPI_ISL_737343, EPI_ISL_737344			
see above	Department of Clinical Microbiology	GIGA Medical Genomics	Keith Durkin, Maria Artesi, Sébastien Bontems, Raphaël Boreux, Bouchra Boujemla, Cécile Meex, Pierrette Melin, Marie-Pierre Hayette, Vincent Bours
EPI_ISL_737395, EPI_ISL_737396, EPI_ISL_737399, EPI_ISL_737405, EPI_ISL_737406, EPI_ISL_737407, EPI_ISL_737413, EPI_ISL_737416, EPI_ISL_737418, EPI_ISL_737422, EPI_ISL_737424, EPI_ISL_737425, EPI_ISL_737428, EPI_ISL_737430, EPI_ISL_737431, EPI_ISL_737432, EPI_ISL_737435, EPI_ISL_737436, EPI_ISL_737437, EPI_ISL_737441, EPI_ISL_737442, EPI_ISL_737443, EPI_ISL_737445, EPI_ISL_737446, EPI_ISL_737448, EPI_ISL_737450, EPI_ISL_737451, EPI_ISL_737454, EPI_ISL_737455, EPI_ISL_737457, EPI_ISL_737460, EPI_ISL_737461, EPI_ISL_737462, EPI_ISL_737463, EPI_ISL_737466, EPI_ISL_737467, EPI_ISL_737468, EPI_ISL_737470, EPI_ISL_737472, EPI_ISL_737473, EPI_ISL_737475, EPI_ISL_737477, EPI_ISL_737478, EPI_ISL_737479, EPI_ISL_737485, EPI_ISL_737486, EPI_ISL_737488, EPI_ISL_737491, EPI_ISL_737495, EPI_ISL_737496, EPI_ISL_737501, EPI_ISL_737502, EPI_ISL_737503, EPI_ISL_737504, EPI_ISL_737506, EPI_ISL_737507, EPI_ISL_737508, EPI_ISL_737509, EPI_ISL_737510, EPI_ISL_737511, EPI_ISL_737514, EPI_ISL_737517, EPI_ISL_737519, EPI_ISL_737520, EPI_ISL_737521, EPI_ISL_737522, EPI_ISL_737523, EPI_ISL_737524, EPI_ISL_737526, EPI_ISL_737531, EPI_ISL_737532, EPI_ISL_737534, EPI_ISL_737536, EPI_ISL_737537, EPI_ISL_737539, EPI_ISL_737541, EPI_ISL_737542, EPI_ISL_737543, EPI_ISL_737544, EPI_ISL_737545, EPI_ISL_737546, EPI_ISL_737549, EPI_ISL_737554, EPI_ISL_737556, EPI_ISL_737557, EPI_ISL_737558, EPI_ISL_737559, EPI_ISL_737564, EPI_ISL_737565, EPI_ISL_737566, EPI_ISL_737567, EPI_ISL_737570, EPI_ISL_737571, EPI_ISL_737574, EPI_ISL_737579, EPI_ISL_737580, EPI_ISL_737584, EPI_ISL_737586, EPI_ISL_737587, EPI_ISL_737590, EPI_ISL_737594, EPI_ISL_737596, EPI_ISL_737601, EPI_ISL_737604, EPI_ISL_737606, EPI_ISL_737611, EPI_ISL_737616, EPI_ISL_737617, EPI_ISL_737624, EPI_ISL_737627, EPI_ISL_737642, EPI_ISL_737652, EPI_ISL_737658, EPI_ISL_737659, EPI_ISL_737670, EPI_ISL_737671, EPI_ISL_737672, EPI_ISL_737673, EPI_ISL_737674, EPI_ISL_737675, EPI_ISL_737676, EPI_ISL_737677, EPI_ISL_737678, EPI_ISL_737708, EPI_ISL_737725, EPI_ISL_737726, EPI_ISL_737727, EPI_ISL_737731, EPI_ISL_737740, EPI_ISL_737741, EPI_ISL_737742, EPI_ISL_737743, EPI_ISL_737744, EPI_ISL_737745, EPI_ISL_737767, EPI_ISL_737768, EPI_ISL_737769, EPI_ISL_737791, EPI_ISL_737795, EPI_ISL_737796, EPI_ISL_737797, EPI_ISL_737843, EPI_ISL_737845, EPI_ISL_737846, EPI_ISL_737867, EPI_ISL_737869, EPI_ISL_737870, EPI_ISL_737908, EPI_ISL_737909, EPI_ISL_737913, EPI_ISL_737918			
see above	Viollier AG	Department of Biosystems Science and Engineering, ETH Zürich	Chaoran Chen, Sarah Nadeau, Catharine Aquino, Ivan Topolsky, Philipp Jablonski, Lara Fuhrmann, David Dreifuss, Katharina Jahn, Andreia Cabral de Gouvea, Maria Domenica Moccia, Simon Grüter, Timothy Sykes, Lennart Opitz, Griffin White, Laura Neff, Doris Popovic, Andrea Patrignani, Jay Tracy, Ralph Schlapbach, Christiane Beckmann, Maurice Redondo, Olivier Kobel, Christoph Noppen, Sophie Seidel, Noemie Santamaria de Souza, Niko Beerenwinkel, Tanja Stadler
EPI_ISL_738106	Instituto Nacional de Saude (INSA)	Instituto Nacional de Saude (INSA)	Borges et al
EPI_ISL_738350, EPI_ISL_738351, EPI_ISL_738352, EPI_ISL_738355, EPI_ISL_738357	Utah Public Health Laboratory	Utah Public Health Laboratory	Erin Young, Kelly Oakeson, Tara Gallagher
EPI_ISL_738363, EPI_ISL_738365, EPI_ISL_738369, EPI_ISL_738374, EPI_ISL_738376, EPI_ISL_738380, EPI_ISL_738381, EPI_ISL_738382, EPI_ISL_738386, EPI_ISL_738387, EPI_ISL_738388, EPI_ISL_738392, EPI_ISL_738394, EPI_ISL_738398, EPI_ISL_738400, EPI_ISL_738406, EPI_ISL_738409, EPI_ISL_738412, EPI_ISL_738415, EPI_ISL_738417, EPI_ISL_738420, EPI_ISL_738424, EPI_ISL_738427, EPI_ISL_738430, EPI_ISL_738432, EPI_ISL_738435, EPI_ISL_738439, EPI_ISL_738441, EPI_ISL_738450, EPI_ISL_738452, EPI_ISL_738454, EPI_ISL_738455, EPI_ISL_738458, EPI_ISL_738459, EPI_ISL_738460, EPI_ISL_738461, EPI_ISL_738464, EPI_ISL_738466, EPI_ISL_738467, EPI_ISL_738471, EPI_ISL_738472, EPI_ISL_738474, EPI_ISL_738476, EPI_ISL_738477, EPI_ISL_738479, EPI_ISL_738482, EPI_ISL_738483, EPI_ISL_738485, EPI_ISL_738488, EPI_ISL_738489, EPI_ISL_738491, EPI_ISL_738493, EPI_ISL_738494, EPI_ISL_738497, EPI_ISL_738499			
see above	UZ Leuven, National Reference Laboratory for Coronaviruses, Laboratory Medicine, Leuven, Belgium	KU Leuven, Rega Institute, Clinical and Epidemiological Virology	Tony Wawina-Bokalanga, Joan Marti-Carerras, Bert Vanmechelen, Piet Maes
EPI_ISL_740025, EPI_ISL_740203, EPI_ISL_740345, EPI_ISL_740489, EPI_ISL_740532	Laboratoire national de santé, Microbiology, Virology	Laboratoire national de santé, Microbiology, Microbial Genomics Platform	Anke Wienecke-Baldacchino, Catherine Ragimbeau,Jessica Tapp, Fatu Djabi, Lise Pignon, Raoul Salmon, Tamir Abdelrahman
EPI_ISL_740867, EPI_ISL_740868	South Eastern Area Laboratory Services (SEALS)	NSW Health Pathology - Institute of Clinical Pathology and Medical Research; Westmead Hospital; University of Sydney	CIDM-PH et al.
EPI_ISL_740894, EPI_ISL_740895	Sydney South West Pathology Service (SSWPS) - Royal	NSW Health Pathology - Institute of Clinical Pathology and	CIDM-PH et al.

	Prince Alfred Hospital - NSW Health Pathology	Medical Research; Westmead Hospital; University of Sydney	
EPI_ISL_740999, EPI_ISL_741005, EPI_ISL_741006, EPI_ISL_741007, EPI_ISL_741008, EPI_ISL_741010, EPI_ISL_741013, EPI_ISL_741014, EPI_ISL_741015, EPI_ISL_741016, EPI_ISL_741017			
see above	Department of Pathology, University of Cambridge	COVID-19 Genomics UK (COG-UK) Consortium	Aminu S. Jahun, Yasmin Chaudhry, Grant Hall, Iliana Georgana, Myra Hosmillo, Martin D. Curran, Malte Pinckert, Surendra Parmar, Ian Goodfellow
EPI_ISL_741309, EPI_ISL_741310, EPI_ISL_741311, EPI_ISL_741317, EPI_ISL_741318, EPI_ISL_741319, EPI_ISL_741320, EPI_ISL_741322, EPI_ISL_741323, EPI_ISL_741324, EPI_ISL_741325, EPI_ISL_741326, EPI_ISL_741327, EPI_ISL_741328, EPI_ISL_741329, EPI_ISL_741330, EPI_ISL_741331, EPI_ISL_741332, EPI_ISL_741333, EPI_ISL_741334, EPI_ISL_741335, EPI_ISL_741336, EPI_ISL_741340, EPI_ISL_741346			
see above	University College London, Great Ormond Street Hospital for Children NHS Foundation Trust, Imperial College Healthcare NHS Trust	COVID-19 Genomics UK (COG-UK) Consortium	Sergi Castellano, Rachel Williams, Mark Kristiansen, Paola Resende Silva, Sunando Roy, Tony Brooks, Helena Tutill, Paola Niola, Patricia Dyal, Charlotte Williams, Leysa Forrest, Yasmin Panchbhaya, Jacqueline Findlay, Samuel Weeks, Julianne Brown, Kathryn Harris, Paul Randell, James Price, Alison Holmes, Judith Breuer
EPI_ISL_741453, EPI_ISL_741454, EPI_ISL_741455, EPI_ISL_741456, EPI_ISL_741457, EPI_ISL_741458, EPI_ISL_741459, EPI_ISL_741460, EPI_ISL_741461, EPI_ISL_741462, EPI_ISL_741463, EPI_ISL_741464, EPI_ISL_741465, EPI_ISL_741466, EPI_ISL_741467, EPI_ISL_741468, EPI_ISL_741469, EPI_ISL_741470, EPI_ISL_741471, EPI_ISL_741472, EPI_ISL_741473, EPI_ISL_741474, EPI_ISL_741475, EPI_ISL_741476, EPI_ISL_741477, EPI_ISL_741478, EPI_ISL_741479, EPI_ISL_741480, EPI_ISL_741481, EPI_ISL_741482, EPI_ISL_741483, EPI_ISL_741484, EPI_ISL_741492, EPI_ISL_741583, EPI_ISL_741584, EPI_ISL_741585, EPI_ISL_741586, EPI_ISL_741587, EPI_ISL_741588, EPI_ISL_741589, EPI_ISL_741590, EPI_ISL_741591, EPI_ISL_741592, EPI_ISL_741593, EPI_ISL_741594, EPI_ISL_741595, EPI_ISL_741596, EPI_ISL_741597, EPI_ISL_741598, EPI_ISL_741599, EPI_ISL_741600, EPI_ISL_741601, EPI_ISL_741602, EPI_ISL_741603, EPI_ISL_741604, EPI_ISL_741605, EPI_ISL_741606, EPI_ISL_741607, EPI_ISL_741608			
see above	Quadram Institute Bioscience	COVID-19 Genomics UK (COG-UK) Consortium	Dave J. Baker, Gemma L. Kay, Alp Aydin, Thanh Le-Viet, Steven Rudder, Ana P. Tedim, Anastasia Kolyva, Maria Diaz, Leonardo de Oliveira Martins, Nabil-Fareed Alikhan, Lizzie Meadows, Rachael Stanley, Ngozi Elumogo, Muhammed Yasir, Nicholas M. Thomson, Alexander J Trotter, Rachel Gilroy, Samuel Bloomfield, Claire Stuart, Andrew Bell, Reenesh Prakash, Samir Dervisevic, Alison E. Mather, John Wain, Mark Webber, Andrew J. Page, Justin O'Grady
EPI_ISL_741620, EPI_ISL_741621, EPI_ISL_741622, EPI_ISL_741623, EPI_ISL_741624, EPI_ISL_741625, EPI_ISL_741626, EPI_ISL_741627, EPI_ISL_741628, EPI_ISL_741629, EPI_ISL_741630			
see above	Queens Medical Centre, Clinical Microbiology Department / DeepSeq Nottingham	COVID-19 Genomics UK (COG-UK) Consortium	Gemma Clark, Wendy Smith, Manjinder Khakh, Vicki M Fleming, Michelle M Lister, Hannah Howson-Wells, Jonathan Ball, Patrick McClure, Joseph Chappell, Theocharis Tsoleridis, Nadine Holmes, Matthew Carlisle, Christopher Moore, Fei Sang, Johnny Debebe, Victoria Wright, Matthew Loose
EPI_ISL_741675, EPI_ISL_741693, EPI_ISL_741697, EPI_ISL_741730, EPI_ISL_741747	Centre for Enzyme Innovation, University of Portsmouth / Translational Research Laboratory, Portsmouth Hospitals NHS Trust	COVID-19 Genomics UK (COG-UK) Consortium	Angela Beckett,Yann Bourgeois,Garry Scarlett,Sharon Glaysher,Scott Elliott,Kelly Bicknell,Robert Impey,Allyson Lloyd,Sarah Wyllie,Ethan Butcher,Anoop Chauhan,Samuel Robson
EPI_ISL_741966, EPI_ISL_741974, EPI_ISL_742007, EPI_ISL_742017, EPI_ISL_742050, EPI_ISL_742052, EPI_ISL_742064, EPI_ISL_742082, EPI_ISL_742100, EPI_ISL_742105, EPI_ISL_742114			
see above	Virology Department, Sheffield Teaching Hospitals NHS Foundation Trust/Department of Infection, Immunity and Cardiovascular Disease, The Medical School, University of Sheffield	COVID-19 Genomics UK (COG-UK) Consortium	Thushan de Silva, Matthew Parker, Nikki Smith, Adri Angyal, Rebecca Brown, Luke Green, Rachel Tucker, Paul Parsons, Danielle Groves, Katie Johnson, Laura Carrilero, Alex Keeley, Dave Partridge, Matthew Wyles, Benjamin Lindsey, Mehmet Yavuz, Mohammad Raza, Cariad Evans
EPI_ISL_742194, EPI_ISL_742195, EPI_ISL_742196, EPI_ISL_742197, EPI_ISL_742198, EPI_ISL_742199, EPI_ISL_742200, EPI_ISL_742201	Virology Department, Royal Infirmary of Edinburgh, NHS Lothian / School of Biological Sciences, University of Edinburgh / Institute of Genetics and Molecular Medicine, University of Edinburgh	COVID-19 Genomics UK (COG-UK) Consortium	McHugh M, Dewar R, Rooke S, Gallagher M, Balcaza C, O'Toole Á, Scher E, Hill V, McCrone JT, Colquhoun R, Yu X, Jackson B, Rambaut A, Williams TC, Templeton K
EPI_ISL_743372, EPI_ISL_743603, EPI_ISL_743604, EPI_ISL_743605, EPI_ISL_743782, EPI_ISL_743785, EPI_ISL_743805, EPI_ISL_743806, EPI_ISL_743820, EPI_ISL_743821, EPI_ISL_743822, EPI_ISL_743825, EPI_ISL_743826, EPI_ISL_743827, EPI_ISL_743828, EPI_ISL_743831, EPI_ISL_743832, EPI_ISL_743833, EPI_ISL_743835, EPI_ISL_743836, EPI_ISL_743838, EPI_ISL_743839, EPI_ISL_743840, EPI_ISL_743841, EPI_ISL_743842, EPI_ISL_743843, EPI_ISL_743844, EPI_ISL_743845, EPI_ISL_743846, EPI_ISL_743847, EPI_ISL_743848, EPI_ISL_743849, EPI_ISL_743850, EPI_ISL_743851, EPI_ISL_743852, EPI_ISL_743853, EPI_ISL_743854, EPI_ISL_743855, EPI_ISL_743856, EPI_ISL_743857, EPI_ISL_743858, EPI_ISL_743859, EPI_ISL_743860, EPI_ISL_743861, EPI_ISL_743862, EPI_ISL_743863, EPI_ISL_743864, EPI_ISL_743865, EPI_ISL_743866, EPI_ISL_743867, EPI_ISL_743868, EPI_ISL_743869, EPI_ISL_743870, EPI_ISL_743871, EPI_ISL_743872, EPI_ISL_743873, EPI_ISL_743877, EPI_ISL_743886, EPI_ISL_743887, EPI_ISL_743888, EPI_ISL_743889, EPI_ISL_743898, EPI_ISL_743899, EPI_ISL_743900, EPI_ISL_743903, EPI_ISL_743904, EPI_ISL_743905, EPI_ISL_743906, EPI_ISL_743907, EPI_ISL_743908, EPI_ISL_743909, EPI_ISL_743910, EPI_ISL_743911, EPI_ISL_743912, EPI_ISL_743913, EPI_ISL_743914, EPI_ISL_743915, EPI_ISL_743916, EPI_ISL_743917, EPI_ISL_743918, EPI_ISL_743919, EPI_ISL_743920, EPI_ISL_743921, EPI_ISL_743922, EPI_ISL_743923, EPI_ISL_743924, EPI_ISL_743925, EPI_ISL_743926, EPI_ISL_743927, EPI_ISL_743938, EPI_ISL_743939, EPI_ISL_743940, EPI_ISL_743941, EPI_ISL_743987, EPI_ISL_744000, EPI_ISL_744001, EPI_ISL_744002, EPI_ISL_744003, EPI_ISL_744004, EPI_ISL_744005, EPI_ISL_744006, EPI_ISL_744007, EPI_ISL_744008, EPI_ISL_744009, EPI_ISL_744010, EPI_ISL_744011, EPI_ISL_744012, EPI_ISL_744013, EPI_ISL_744014, EPI_ISL_744015, EPI_ISL_744016, EPI_ISL_744017, EPI_ISL_744018, EPI_ISL_744019, EPI_ISL_744020, EPI_ISL_744021, EPI_ISL_744022, EPI_ISL_744023, EPI_ISL_744024, EPI_ISL_744025, EPI_ISL_744026, EPI_ISL_744027, EPI_ISL_744028, EPI_ISL_744029, EPI_ISL_744030, EPI_ISL_744031, EPI_ISL_744032, EPI_ISL_744033, EPI_ISL_744034, EPI_ISL_744035, EPI_ISL_744036, EPI_ISL_744037, EPI_ISL_744038, EPI_ISL_744039, EPI_ISL_744040, EPI_ISL_744041, EPI_ISL_744042, EPI_ISL_744043, EPI_ISL_744044, EPI_ISL_744045, EPI_ISL_744046, EPI_ISL_744047, EPI_ISL_744048, EPI_ISL_744049, EPI_ISL_744050, EPI_ISL_744051, EPI_ISL_744052, EPI_ISL_744053, EPI_ISL_744054, EPI_ISL_744055, EPI_ISL_744056, EPI_ISL_744057, EPI_ISL_744058, EPI_ISL_744059, EPI_ISL_744060, EPI_ISL_744061, EPI_ISL_744062, EPI_ISL_744063, EPI_ISL_744064, EPI_ISL_744065, EPI_ISL_744066, EPI_ISL_744068, EPI_ISL_744069, EPI_ISL_744070, EPI_ISL_744071, EPI_ISL_744072, EPI_ISL_744073, EPI_ISL_744074, EPI_ISL_744075, EPI_ISL_744076, EPI_ISL_744077, EPI_ISL_744078, EPI_ISL_744079, EPI_ISL_744080, EPI_ISL_744081, EPI_ISL_744082, EPI_ISL_744083, EPI_ISL_744084, EPI_ISL_744085, EPI_ISL_744086, EPI_ISL_744087, EPI_ISL_744088, EPI_ISL_744089, EPI_ISL_744090, EPI_ISL_744091, EPI_ISL_744092, EPI_ISL_744093, EPI_ISL_744094, EPI_ISL_744095, EPI_ISL_744096, EPI_ISL_744097, EPI_ISL_744098, EPI_ISL_744099, EPI_ISL_744100, EPI_ISL_744101, EPI_ISL_744102, EPI_ISL_744103, EPI_ISL_744104, EPI_ISL_744105, EPI_ISL_744106, EPI_ISL_744107, EPI_ISL_744108, EPI_ISL_744109, EPI_ISL_744110, EPI_ISL_744111, EPI_ISL_744112, EPI_ISL_744113, EPI_ISL_744114, EPI_ISL_744115, EPI_ISL_744116, EPI_ISL_744117, EPI_ISL_744118, EPI_ISL_744119, EPI_ISL_744120, EPI_ISL_744121, EPI_ISL_744122, EPI_ISL_744123, EPI_ISL_744124, EPI_ISL_744125, EPI_ISL_744126, EPI_ISL_744127, EPI_ISL_744128, EPI_ISL_744129, EPI_ISL_744130, EPI_ISL_744131, EPI_ISL_744132, EPI_ISL_744133			
see above	Wales Specialist Virology Centre Sequencing lab: Pathogen Genomics Unit	COVID-19 Genomics UK (COG-UK) Consortium	Catherine Moore, Johnathan Evans, Laura Gifford, Malorie Perry, Simon Cottrell, Angela Marchbank, Alec Birchley, Alexander Adams, Amy Gaskin, Bree Gatica-Wilcox, Jason Coombes, Joel Southgate, Lauren Gilbert, Lee Graham, Nicole Pacchiarini, Sara Kumziene-Summerhayes, Sarah Taylor, Sophie Jones, Sara Rey, Matthew Bull, Joanne Watkins, Sally Corden, Tom Connor
EPI_ISL_744158, EPI_ISL_744206, EPI_ISL_744438, EPI_ISL_744456, EPI_ISL_744769, EPI_ISL_744805, EPI_ISL_744894	Laboratoire national de santé, Microbiology, Virology	Laboratoire national de santé, Microbiology, Microbial Genomics Platform	Anke Wienecke-Baldacchino, Catherine Ragimbeau,Jessica Tapp, Fatu Djabi, Lise Pignon, Raoul Salmon, Tamir Abdelrahman
EPI_ISL_745070, EPI_ISL_745071, EPI_ISL_745072, EPI_ISL_745073, EPI_ISL_745074, EPI_ISL_745075, EPI_ISL_745076, EPI_ISL_745077	Israel Central Virology laboratory	Israel Central Virology laboratory	Neta Zuckerman, Efrat Dahan Bucris, Oran Erster, Michal Mandelboim, Orna Mor, Ella Mendelson
EPI_ISL_745212, EPI_ISL_745218, EPI_ISL_745219	Lab voor klinische biologie	Onderzoeksgroep Virologie	Nick Vereecke, Laurens Lambrechts, Marthe Pauwels, Bruno Verhasselt, Linos Vandekerckhove, Hans Nauwynck, Sebastiaan Theuns
EPI_ISL_745222	UZ Leuven, National Reference Laboratory for Coronaviruses, Laboratory Medicine, Leuven, Belgium	KU Leuven, Rega Institute, Clinical and Epidemiological Virology	Tony Wawina-Bokalanga, Joan Marti-Carerras, Bert Vanmechelen, Piet Maes
EPI_ISL_745260	CHUM-Site Glen-LAB Microbiologie	Laboratoire de santé publique du Québec	Sandrine Moreira, Ioannis Ragoussis, Guillaume Bourque, Jesse Shapiro, Mark Lathrop and Michel Roger on behalf of the CoVSeQ research group (http://covseq.ca/researchgroup)
EPI_ISL_745318, EPI_ISL_745324, EPI_ISL_745332, EPI_ISL_745389	CNR Virus des Infections Respiratoires - France SUD	CNR Virus des Infections Respiratoires - France SUD	Antonin Bal, Gregory Destras, Claudia Gonzalez, Gwendolyne Burfin, Quentin Semanas, Martine Valette, Bruno Lina, Laurence Josset
EPI_ISL_746826	National Institute for Infectious Diseases, INMI, "L. Spallanzani" IRCCS	National Institute for Infectious Diseases, INMI, "L. Spallanzani" IRCCS	C.E.M Gruber, B Bartolini, M Rueca, F Messina, E Giombini, A Di Caro, MR Capobianchi
EPI_ISL_747194	Clinical Microbiology Laboratory, Faculty of Medicine, Universitas Indonesia	Clinical Microbiology Laboratory, Faculty of Medicine, Universitas Indonesia	Fera Ibrahim, Augustine Natasha, Andi Yasmon, Pratiwi Sudarmono, Yulia Rosa Saharman, Anis Karuniawati, Fithriyah
EPI_ISL_749156, EPI_ISL_749157, EPI_ISL_749159, EPI_ISL_749160, EPI_ISL_749161, EPI_ISL_749162, EPI_ISL_749163, EPI_ISL_749164, EPI_ISL_749165, EPI_ISL_749166, EPI_ISL_749168, EPI_ISL_749172, EPI_ISL_749173, EPI_ISL_749174, EPI_ISL_749175, EPI_ISL_749178, EPI_ISL_749179, EPI_ISL_749180, EPI_ISL_749182, EPI_ISL_749183, EPI_ISL_749184, EPI_ISL_749185, EPI_ISL_749186, EPI_ISL_749187, EPI_ISL_749188, EPI_ISL_749189, EPI_ISL_749190, EPI_ISL_749191, EPI_ISL_749192, EPI_ISL_749193, EPI_ISL_749194, EPI_ISL_749195, EPI_ISL_749196, EPI_ISL_749197, EPI_ISL_749198, EPI_ISL_749199, EPI_ISL_749200, EPI_ISL_749201, EPI_ISL_749202, EPI_ISL_749203, EPI_ISL_749204, EPI_ISL_749205, EPI_ISL_749206, EPI_ISL_749207, EPI_ISL_749208, EPI_ISL_749209, EPI_ISL_749210, EPI_ISL_749211, EPI_ISL_749212, EPI_ISL_749213, EPI_ISL_749214, EPI_ISL_749215, EPI_ISL_749216, EPI_ISL_749217, EPI_ISL_749218, EPI_ISL_749219, EPI_ISL_749220, EPI_ISL_749221, EPI_ISL_749222, EPI_ISL_749263, EPI_ISL_749264, EPI_ISL_749265, EPI_ISL_749266, EPI_ISL_749269, EPI_ISL_749270, EPI_ISL_749272, EPI_ISL_749275, EPI_ISL_749277, EPI_ISL_749278, EPI_ISL_749280, EPI_ISL_749281, EPI_ISL_749282, EPI_ISL_749283, EPI_ISL_749284, EPI_ISL_749285, EPI_ISL_749286, EPI_ISL_749288, EPI_ISL_749289, EPI_ISL_749296, EPI_ISL_749297, EPI_ISL_749298, EPI_ISL_749299, EPI_ISL_749300, EPI_ISL_749301, EPI_ISL_749302, EPI_ISL_749303, EPI_ISL_749304, EPI_ISL_749305, EPI_ISL_749306, EPI_ISL_749307, EPI_ISL_749308, EPI_ISL_749309, EPI_ISL_749310, EPI_ISL_749311, EPI_ISL_749312, EPI_ISL_749313, EPI_ISL_749314, EPI_ISL_749315, EPI_ISL_749316, EPI_ISL_749317, EPI_ISL_749318, EPI_ISL_749319, EPI_ISL_749320, EPI_ISL_749355, EPI_ISL_749356, EPI_ISL_749358, EPI_ISL_749359, EPI_ISL_749360, EPI_ISL_749362, EPI_ISL_749363, EPI_ISL_749364, EPI_ISL_749365, EPI_ISL_749366, EPI_ISL_749367, EPI_ISL_749368, EPI_ISL_749370, EPI_ISL_749371, EPI_ISL_749372, EPI_ISL_749373, EPI_ISL_749376, EPI_ISL_749377, EPI_ISL_749378, EPI_ISL_749379, EPI_ISL_749380, EPI_ISL_749381, EPI_ISL_749382, EPI_ISL_749383, EPI_ISL_749384, EPI_ISL_749385, EPI_ISL_749386, EPI_ISL_749387, EPI_ISL_749388, EPI_ISL_749389, EPI_ISL_749407, EPI_ISL_749408, EPI_ISL_749409, EPI_ISL_749410, EPI_ISL_749411, EPI_ISL_749412, EPI_ISL_749413, EPI_ISL_749414, EPI_ISL_749415, EPI_ISL_749416, EPI_ISL_749417, EPI_ISL_749418, EPI_ISL_749419, EPI_ISL_749420, EPI_ISL_749421, EPI_ISL_749422, EPI_ISL_749423, EPI_ISL_749424, EPI_ISL_749425, EPI_ISL_749426, EPI_ISL_749427, EPI_ISL_749428, EPI_ISL_749429, EPI_ISL_749430, EPI_ISL_749431, EPI_ISL_749432, EPI_ISL_749433, EPI_ISL_749434, EPI_ISL_749435,			

EPI_ISL_749436, EPI_ISL_749437, EPI_ISL_749438, EPI_ISL_749439, EPI_ISL_749440, EPI_ISL_749441, EPI_ISL_749442, EPI_ISL_749443, EPI_ISL_749444, EPI_ISL_749445, EPI_ISL_749446, EPI_ISL_749447, EPI_ISL_749448, EPI_ISL_749449, EPI_ISL_749450, EPI_ISL_749451, EPI_ISL_749484, EPI_ISL_749485, EPI_ISL_749486, EPI_ISL_749487, EPI_ISL_749488, EPI_ISL_749489, EPI_ISL_749490, EPI_ISL_749493, EPI_ISL_749498, EPI_ISL_749500, EPI_ISL_749501, EPI_ISL_749502, EPI_ISL_749503, EPI_ISL_749504, EPI_ISL_749505, EPI_ISL_749507, EPI_ISL_749511, EPI_ISL_749512, EPI_ISL_749515, EPI_ISL_749516, EPI_ISL_749517, EPI_ISL_749518, EPI_ISL_749519, EPI_ISL_749523, EPI_ISL_749524, EPI_ISL_749528, EPI_ISL_749529, EPI_ISL_749530, EPI_ISL_749531, EPI_ISL_749532, EPI_ISL_749533, EPI_ISL_749534, EPI_ISL_749535, EPI_ISL_749536, EPI_ISL_749537, EPI_ISL_749538, EPI_ISL_749539, EPI_ISL_749542, EPI_ISL_749543, EPI_ISL_749544, EPI_ISL_749545, EPI_ISL_749546, EPI_ISL_749547, EPI_ISL_749548, EPI_ISL_749549, EPI_ISL_749550, EPI_ISL_749551, EPI_ISL_749552, EPI_ISL_749553, EPI_ISL_749554, EPI_ISL_749555, EPI_ISL_749556, EPI_ISL_749557, EPI_ISL_749558, EPI_ISL_749559, EPI_ISL_749560, EPI_ISL_749561, EPI_ISL_749562, EPI_ISL_749563, EPI_ISL_749564, EPI_ISL_749565, EPI_ISL_749566, EPI_ISL_749567, EPI_ISL_749568, EPI_ISL_749569, EPI_ISL_749570, EPI_ISL_749571, EPI_ISL_749572, EPI_ISL_749573, EPI_ISL_749574, EPI_ISL_749575, EPI_ISL_749576, EPI_ISL_749577, EPI_ISL_749578, EPI_ISL_749579, EPI_ISL_749580, EPI_ISL_749581, EPI_ISL_749582, EPI_ISL_749583, EPI_ISL_749584, EPI_ISL_749585, EPI_ISL_749586, EPI_ISL_749587, EPI_ISL_749588, EPI_ISL_749589, EPI_ISL_749590, EPI_ISL_749591, EPI_ISL_749592, EPI_ISL_749593, EPI_ISL_749594, EPI_ISL_749595, EPI_ISL_749596, EPI_ISL_749597, EPI_ISL_749598, EPI_ISL_749599, EPI_ISL_749600, EPI_ISL_749601, EPI_ISL_749602, EPI_ISL_749603, EPI_ISL_749604, EPI_ISL_749605, EPI_ISL_749606, EPI_ISL_749607, EPI_ISL_749608, EPI_ISL_749609, EPI_ISL_749610, EPI_ISL_749611, EPI_ISL_749612, EPI_ISL_749613, EPI_ISL_749614, EPI_ISL_749615, EPI_ISL_749616, EPI_ISL_749617, EPI_ISL_749618, EPI_ISL_749619, EPI_ISL_749620, EPI_ISL_749621, EPI_ISL_749622, EPI_ISL_749623, EPI_ISL_749624, EPI_ISL_749625, EPI_ISL_749626, EPI_ISL_749627, EPI_ISL_749628, EPI_ISL_749629, EPI_ISL_749630, EPI_ISL_749631, EPI_ISL_749632, EPI_ISL_749633, EPI_ISL_749634, EPI_ISL_749635, EPI_ISL_749636, EPI_ISL_749637, EPI_ISL_749638, EPI_ISL_749639, EPI_ISL_749640, EPI_ISL_749641, EPI_ISL_749642, EPI_ISL_749643, EPI_ISL_749644, EPI_ISL_749645, EPI_ISL_749646, EPI_ISL_749647, EPI_ISL_749648, EPI_ISL_749649, EPI_ISL_749650, EPI_ISL_749651, EPI_ISL_749652, EPI_ISL_749653, EPI_ISL_749654, EPI_ISL_749655, EPI_ISL_749656, EPI_ISL_749657, EPI_ISL_749658, EPI_ISL_749659, EPI_ISL_749660, EPI_ISL_749661, EPI_ISL_749662, EPI_ISL_749663, EPI_ISL_749664, EPI_ISL_749665, EPI_ISL_749666, EPI_ISL_749667, EPI_ISL_749668, EPI_ISL_749669, EPI_ISL_749670, EPI_ISL_749671, EPI_ISL_749672, EPI_ISL_749673, EPI_ISL_749674, EPI_ISL_749675, EPI_ISL_749676, EPI_ISL_749677, EPI_ISL_749678, EPI_ISL_749679, EPI_ISL_749680, EPI_ISL_749681, EPI_ISL_749682, EPI_ISL_749683, EPI_ISL_749684, EPI_ISL_749685, EPI_ISL_749686, EPI_ISL_749687, EPI_ISL_749688, EPI_ISL_749689, EPI_ISL_749690, EPI_ISL_749691, EPI_ISL_749692, EPI_ISL_749693, EPI_ISL_749694, EPI_ISL_749695, EPI_ISL_749696, EPI_ISL_749697, EPI_ISL_749698, EPI_ISL_749699, EPI_ISL_749700, EPI_ISL_749701, EPI_ISL_749702, EPI_ISL_749703, EPI_ISL_749704, EPI_ISL_749705, EPI_ISL_749707, EPI_ISL_749708, EPI_ISL_749709, EPI_ISL_749710, EPI_ISL_749711, EPI_ISL_749712, EPI_ISL_749713, EPI_ISL_749714, EPI_ISL_749715, EPI_ISL_749716, EPI_ISL_749717, EPI_ISL_749718, EPI_ISL_749719, EPI_ISL_749720, EPI_ISL_749721, EPI_ISL_749722, EPI_ISL_749723, EPI_ISL_749724, EPI_ISL_749725, EPI_ISL_749726, EPI_ISL_749727, EPI_ISL_749728, EPI_ISL_749729, EPI_ISL_749730, EPI_ISL_749731, EPI_ISL_749732, EPI_ISL_749733, EPI_ISL_749734, EPI_ISL_749735, EPI_ISL_749736, EPI_ISL_749737, EPI_ISL_749738, EPI_ISL_749739, EPI_ISL_749740, EPI_ISL_749741, EPI_ISL_749742, EPI_ISL_749743, EPI_ISL_749744, EPI_ISL_749745, EPI_ISL_749746, EPI_ISL_749747, EPI_ISL_749748, EPI_ISL_749749, EPI_ISL_749750, EPI_ISL_749751, EPI_ISL_749752, EPI_ISL_749753, EPI_ISL_749754, EPI_ISL_749755, EPI_ISL_749756, EPI_ISL_749757, EPI_ISL_749758, EPI_ISL_749759, EPI_ISL_749760, EPI_ISL_749761, EPI_ISL_749762, EPI_ISL_749763, EPI_ISL_749764, EPI_ISL_749765, EPI_ISL_749766, EPI_ISL_749767, EPI_ISL_749768, EPI_ISL_749769, EPI_ISL_749770, EPI_ISL_749771, EPI_ISL_749772, EPI_ISL_749773, EPI_ISL_749774, EPI_ISL_749775, EPI_ISL_749776, EPI_ISL_749777, EPI_ISL_749778, EPI_ISL_749779, EPI_ISL_749780, EPI_ISL_749781, EPI_ISL_749782, EPI_ISL_749783, EPI_ISL_749784, EPI_ISL_749785, EPI_ISL_749786, EPI_ISL_749787, EPI_ISL_749788, EPI_ISL_749789, EPI_ISL_749790, EPI_ISL_749791, EPI_ISL_749792, EPI_ISL_749793, EPI_ISL_749794, EPI_ISL_749795, EPI_ISL_749796, EPI_ISL_749797, EPI_ISL_749798, EPI_ISL_749799, EPI_ISL_749800, EPI_ISL_749801, EPI_ISL_749802, EPI_ISL_749803, EPI_ISL_749804, EPI_ISL_749805, EPI_ISL_749806, EPI_ISL_749807, EPI_ISL_749808, EPI_ISL_749809, EPI_ISL_749810, EPI_ISL_749811, EPI_ISL_749812, EPI_ISL_749813, EPI_ISL_749814, EPI_ISL_749815, EPI_ISL_749816, EPI_ISL_749817, EPI_ISL_749818, EPI_ISL_749819, EPI_ISL_749820, EPI_ISL_749821, EPI_ISL_749822, EPI_ISL_749823, EPI_ISL_749824, EPI_ISL_749825, EPI_ISL_749826, EPI_ISL_749827, EPI_ISL_749828, EPI_ISL_749829, EPI_ISL_749830, EPI_ISL_749831, EPI_ISL_749832, EPI_ISL_749833, EPI_ISL_749834, EPI_ISL_749835, EPI_ISL_749836, EPI_ISL_749837, EPI_ISL_749838, EPI_ISL_749839, EPI_ISL_749840, EPI_ISL_749841, EPI_ISL_749842, EPI_ISL_749843, EPI_ISL_749844, EPI_ISL_749845, EPI_ISL_749846, EPI_ISL_749847, EPI_ISL_749848, EPI_ISL_749849, EPI_ISL_749850, EPI_ISL_749851, EPI_ISL_749852, EPI_ISL_749853, EPI_ISL_749854, EPI_ISL_749855, EPI_ISL_749856, EPI_ISL_749857, EPI_ISL_749858, EPI_ISL_750180, EPI_ISL_750181, EPI_ISL_750182, EPI_ISL_750183, EPI_ISL_750184, EPI_ISL_750185, EPI_ISL_750186, EPI_ISL_750187, EPI_ISL_750188, EPI_ISL_750189, EPI_ISL_750190, EPI_ISL_750191, EPI_ISL_750192, EPI_ISL_750193, EPI_ISL_750194, EPI_ISL_750195, EPI_ISL_750196, EPI_ISL_750197, EPI_ISL_750198, EPI_ISL_750199, EPI_ISL_750200, EPI_ISL_750201, EPI_ISL_750202, EPI_ISL_750203, EPI_ISL_750204, EPI_ISL_750205, EPI_ISL_750206, EPI_ISL_750207, EPI_ISL_750208, EPI_ISL_750209, EPI_ISL_750210, EPI_ISL_750211, EPI_ISL_750212, EPI_ISL_750213, EPI_ISL_750214, EPI_ISL_750215, EPI_ISL_750216, EPI_ISL_750217, EPI_ISL_750218, EPI_ISL_750219, EPI_ISL_750220, EPI_ISL_750221, EPI_ISL_750222, EPI_ISL_750223, EPI_ISL_750224, EPI_ISL_750225, EPI_ISL_750226, EPI_ISL_750227, EPI_ISL_750228, EPI_ISL_750229, EPI_ISL_750230, EPI_ISL_750231, EPI_ISL_750232, EPI_ISL_750233, EPI_ISL_750234, EPI_ISL_750235, EPI_ISL_750236, EPI_ISL_750237, EPI_ISL_750238, EPI_ISL_750239, EPI_ISL_750240, EPI_ISL_750241, EPI_ISL_750242, EPI_ISL_750243, EPI_ISL_750244, EPI_ISL_750245, EPI_ISL_750246, EPI_ISL_750247, EPI_ISL_750248, EPI_ISL_750249, EPI_ISL_750250, EPI_ISL_750251, EPI_ISL_750252, EPI_ISL_750253, EPI_ISL_750254, EPI_ISL_750255, EPI_ISL_750257, EPI_ISL_750258, EPI_ISL_750259, EPI_ISL_750260, EPI_ISL_750261, EPI_ISL_750262, EPI_ISL_750263, EPI_ISL_750264, EPI_ISL_750265, EPI_ISL_750266, EPI_ISL_750267, EPI_ISL_750268, EPI_ISL_750269, EPI_ISL_750270, EPI_ISL_750271, EPI_ISL_750272, EPI_ISL_750273, EPI_ISL_750274, EPI_ISL_750275, EPI_ISL_750276, EPI_ISL_750277, EPI_ISL_750278, EPI_ISL_750279, EPI_ISL_750280, EPI_ISL_750281, EPI_ISL_750282, EPI_ISL_750283, EPI_ISL_750284, EPI_ISL_750285, EPI_ISL_750286, EPI_ISL_750287, EPI_ISL_750288, EPI_ISL_750289, EPI_ISL_750290, EPI_ISL_750291, EPI_ISL_750292, EPI_ISL_750293, EPI_ISL_750294, EPI_ISL_750295, EPI_ISL_750296, EPI_ISL_750297, EPI_ISL_750298, EPI_ISL_750299, EPI_ISL_750300, EPI_ISL_750301, EPI_ISL_750302, EPI_ISL_750303, EPI_ISL_750304, EPI_ISL_750305, EPI_ISL_750306, EPI_ISL_750307, EPI_ISL_750308, EPI_ISL_750309, EPI_ISL_750310, EPI_ISL_750311, EPI_ISL_750312, EPI_ISL_750313, EPI_ISL_750314, EPI_ISL_750315, EPI_ISL_750316, EPI_ISL_750317, EPI_ISL_750318, EPI_ISL_750319, EPI_ISL_750320, EPI_ISL_750321, EPI_ISL_750322, EPI_ISL_750323, EPI_ISL_750324, EPI_ISL_750325, EPI_ISL_750326, EPI_ISL_750327, EPI_ISL_750328, EPI_ISL_750329, EPI_ISL_750330, EPI_ISL_750331, EPI_ISL_750332, EPI_ISL_750333, EPI_ISL_750334, EPI_ISL_750335, EPI_ISL_750336, EPI_ISL_750337, EPI_ISL_750338, EPI_ISL_750339, EPI_ISL_750340, EPI_ISL_750341, EPI_ISL_750342, EPI_ISL_750343, EPI_ISL_750344, EPI_ISL_750345, EPI_ISL_750346, EPI_ISL_750347, EPI_ISL_750348, EPI_ISL_750349, EPI_ISL_750350, EPI_ISL_750351, EPI_ISL_750352, EPI_ISL_750353, EPI_ISL_750354, EPI_ISL_750355, EPI_ISL_750356, EPI_ISL_750357, EPI_ISL_750358, EPI_ISL_750359, EPI_ISL_750360, EPI_ISL_750361, EPI_ISL_750362, EPI_ISL_750363, EPI_ISL_750364, EPI_ISL_750365, EPI_ISL_750366, EPI_ISL_750367, EPI_ISL_750368, EPI_ISL_750369, EPI_ISL_750370, EPI_ISL_750371, EPI_ISL_750372, EPI_ISL_750373, EPI_ISL_750374, EPI_ISL_750375, EPI_ISL_750376, EPI_ISL_750377, EPI_ISL_750378, EPI_ISL_750379, EPI_ISL_750380, EPI_ISL_750381, EPI_ISL_750382, EPI_ISL_750383, EPI_ISL_750384, EPI_ISL_750385, EPI_ISL_750386, EPI_ISL_750387, EPI_ISL_750388, EPI_ISL_750389, EPI_ISL_750390, EPI_ISL_750391, EPI_ISL_750392, EPI_ISL_750393, EPI_ISL_750394, EPI_ISL_750395, EPI_ISL_750396, EPI_ISL_750397, EPI_ISL_750398, EPI_ISL_750399, EPI_ISL_750400, EPI_ISL_750401, EPI_ISL_750402, EPI_ISL_750403, EPI_ISL_750404, EPI_ISL_750405, EPI_ISL_750406, EPI_ISL_750407, EPI_ISL_750408, EPI_ISL_750409, EPI_ISL_750410, EPI_ISL_750411, EPI_ISL_750412, EPI_ISL_750413, EPI_ISL_750414, EPI_ISL_750415, EPI_ISL_750416, EPI_ISL_750417, EPI_ISL_750418, EPI_ISL_750419, EPI_ISL_750420, EPI_ISL_750421, EPI_ISL_750422, EPI_ISL_750423, EPI_ISL_750424, EPI_ISL_750425, EPI_ISL_750426, EPI_ISL_750427, EPI_ISL_750428, EPI_ISL_750429, EPI_ISL_750431, EPI_ISL_750432, EPI_ISL_750433, EPI_ISL_750434, EPI_ISL_750435, EPI_ISL_750436, EPI_ISL_750437, EPI_ISL_750438, EPI_ISL_750439, EPI_ISL_750440, EPI_ISL_750441, EPI_ISL_750442, EPI_ISL_750443, EPI_ISL_750444, EPI_ISL_750445, EPI_ISL_750446, EPI_ISL_750447, EPI_ISL_750448, EPI_ISL_750449, EPI_ISL_750450, EPI_ISL_750451, EPI_ISL_750452, EPI_ISL_750453, EPI_ISL_750454, EPI_ISL_750455, EPI_ISL_750456, EPI_ISL_750457, EPI_ISL_750458, EPI_ISL_750459, EPI_ISL_750460, EPI_ISL_750461, EPI_ISL_750462, EPI_ISL_750463, EPI_ISL_750464, EPI_ISL_750465, EPI_ISL_750466, EPI_ISL_750467, EPI_ISL_750468, EPI_ISL_750469, EPI_ISL_750470, EPI_ISL_750471, EPI_ISL_750472, EPI_ISL_750473, EPI_ISL_750474, EPI_ISL_750475, EPI_ISL_750476, EPI_ISL_750477, EPI_ISL_750478, EPI_ISL_750479, EPI_ISL_750480, EPI_ISL_750481, EPI_ISL_750482, EPI_ISL_750483, EPI_ISL_750484, EPI_ISL_750485, EPI_ISL_750486, EPI_ISL_750487, EPI_ISL_750488, EPI_ISL_750489, EPI_ISL_750490, EPI_ISL_750491, EPI_ISL_750492, EPI_ISL_750493, EPI_ISL_750494, EPI_ISL_750495, EPI_ISL_750496, EPI_ISL_750497, EPI_ISL_750498, EPI_ISL_750499, EPI_ISL_750500, EPI_ISL_750501, EPI_ISL_750502, EPI_ISL_750503, EPI_ISL_750504, EPI_ISL_750505, EPI_ISL_750506, EPI_ISL_750507, EPI_ISL_750508, EPI_ISL_750509, EPI_ISL_750510, EPI_ISL_750511, EPI_ISL_750512, EPI_ISL_750513, EPI_ISL_750514, EPI_ISL_750515, EPI_ISL_750516, EPI_ISL_750517, EPI_ISL_750518, EPI_ISL_750519, EPI_ISL_750520, EPI_ISL_750521, EPI_ISL_750522, EPI_ISL_750523, EPI_ISL_750524, EPI_ISL_750525, EPI_ISL_750526, EPI_ISL_750527, EPI_ISL_750528, EPI_ISL_750529, EPI_ISL_750530, EPI_ISL_750531, EPI_ISL_750532, EPI_ISL_750533, EPI_ISL_750534, EPI_ISL_750535, EPI_ISL_750536, EPI_ISL_750537, EPI_ISL_750538, EPI_ISL_750539, EPI_ISL_750540, EPI_ISL_750541, EPI_ISL_750542, EPI_ISL_750543, EPI_ISL_750544, EPI_ISL_750545, EPI_ISL_750546, EPI_ISL_750547, EPI_ISL_750548, EPI_ISL_750549, EPI_ISL_750550, EPI_ISL_750551, EPI_ISL_750552, EPI_ISL_750553, EPI_ISL_750554, EPI_ISL_750555, EPI_ISL_750556, EPI_ISL_750557, EPI_ISL_750558, EPI_ISL_750559, EPI_ISL_750560, EPI_ISL_750561, EPI_ISL_750562, EPI_ISL_750563, EPI_ISL_750564, EPI_ISL_750565, EPI_ISL_750566, EPI_ISL_750567, EPI_ISL_750568, EPI_ISL_750569, EPI_ISL_750570, EPI_ISL_750571, EPI_ISL_750572, EPI_ISL_750573, EPI_ISL_750574, EPI_ISL_750575, EPI_ISL_750576, EPI_ISL_750577, EPI_ISL_750578, EPI_ISL_750579, EPI_ISL_750580, EPI_ISL_750581, EPI_ISL_750582, EPI_ISL_750583, EPI_ISL_750584, EPI_ISL_750585, EPI_ISL_750586, EPI_ISL_750587, EPI_ISL_750588, EPI_ISL_750589, EPI_ISL_750590, EPI_ISL_750591, EPI_ISL_750592, EPI_ISL_750593, EPI_ISL_750594, EPI_ISL_750595, EPI_ISL_750596, EPI_ISL_750597, EPI_ISL_750598, EPI_ISL_750599, EPI_ISL_750600, EPI_ISL_750601, EPI_ISL_750602, EPI_ISL_750603, EPI_ISL_750604, EPI_ISL_750605, EPI_ISL_750606, EPI_ISL_750607, EPI_ISL_750608, EPI_ISL_750609, EPI_ISL_750610, EPI_ISL_750611, EPI_ISL_750612, EPI_ISL_750613, EPI_ISL_750614, EPI_ISL_750615, EPI_ISL_750616, EPI_ISL_750617, EPI_ISL_750618, EPI_ISL_750619, EPI_ISL_750620, EPI_ISL_750621, EPI_ISL_750622, EPI_ISL_750623, EPI_ISL_750624, EPI_ISL_750625, EPI_ISL_750626, EPI_ISL_750627, EPI_ISL_750628, EPI_ISL_750629, EPI_ISL_750630, EPI_ISL_750631, EPI_ISL_750632, EPI_ISL_750633, EPI_ISL_750634, EPI_ISL_750635, EPI_ISL_750636, EPI_ISL_750637, EPI_ISL_750638, EPI_ISL_750639, EPI_ISL_750640, EPI_ISL_750641, EPI_ISL_750642, EPI_ISL_750643, EPI_ISL_750644, EPI_ISL_750645, EPI_ISL_750646, EPI_ISL_750647, EPI_ISL_750648, EPI_ISL_750649, EPI_ISL_750650, EPI_ISL_750651, EPI_ISL_750652, EPI_ISL_750653, EPI_ISL_750654, EPI_ISL_750655, EPI_ISL_750656, EPI_ISL_750657, EPI_ISL_750658, EPI_ISL_750659, EPI_ISL_750660, EPI_ISL_750661, EPI_ISL_750662, EPI_ISL_750663, EPI_ISL_750664, EPI_ISL_750665, EPI_ISL_750666, EPI_ISL_750667, EPI_ISL_750668, EPI_ISL_750669, EPI_ISL_750670, EPI_ISL_750671, EPI_ISL_750672, EPI_ISL_750673, EPI_ISL_750674, EPI_ISL_750675, EPI_ISL_750676, EPI_ISL_750677, EPI_ISL_750678, EPI_ISL_750679, EPI_ISL_750680, EPI_ISL_750681, EPI_ISL_750682, EPI_ISL_750683, EPI_ISL_750684, EPI_ISL_750685, EPI_ISL_750686, EPI_ISL_750687, EPI_ISL_750688, EPI_ISL_750689, EPI_ISL_750690, EPI_ISL_750691, EPI_ISL_750692, EPI_ISL_750693, EPI_ISL_750694, EPI_ISL_750695, EPI_ISL_750696, EPI_ISL_750697, EPI_ISL_750698, EPI_ISL_750699, EPI_ISL_750700, EPI_ISL_750701, EPI_ISL_750702, EPI_ISL_750703, EPI_ISL_750704, EPI_ISL_750705, EPI_ISL_750706, EPI_ISL_750707, EPI_ISL_750708, EPI_ISL_750709, EPI_ISL_750710, EPI_ISL_750711, EPI_ISL_750712, EPI_ISL_750713, EPI_ISL_750714, EPI_ISL_750715, EPI_ISL_750716, EPI_ISL_750717, EPI_ISL_750718, EPI_ISL_750719, EPI_ISL_750720, EPI_ISL_750721, EPI_ISL_750722, EPI_ISL_750723, EPI_ISL_750724, EPI_ISL_750725, EPI_ISL_750726, EPI_ISL_750727, EPI_ISL_750728, EPI_ISL_750729, EPI_ISL_750730, EPI_ISL_750731, EPI_ISL_750732, EPI_ISL_750733, EPI_ISL_750734, EPI_ISL_750735, EPI_ISL_750736, EPI_ISL_750737, EPI_ISL_750738, EPI_ISL_750739, EPI_ISL_750740, EPI_ISL_750741, EPI_ISL_750742, EPI_ISL_750743, EPI_ISL_750744, EPI_ISL_750745, EPI_ISL_750746, EPI_ISL_750747, EPI_ISL_750748, EPI_ISL_750749, EPI_ISL_750750, EPI_ISL_750751, EPI_ISL_750752, EPI_ISL_750753, EPI_ISL_750754, EPI_ISL_750755, EPI_ISL_750756, EPI_ISL_750757, EPI_ISL_750758, EPI_ISL_750759, EPI_ISL_750760, EPI_ISL_750761, EPI_ISL_750762, EPI_ISL_750763, EPI_ISL_750764, EPI_ISL_750765, EPI_ISL_750766, EPI_ISL_750767, EPI_ISL_750768, EPI_ISL_750769, EPI_ISL_750770, EPI_ISL_750771, EPI_ISL_750772, EPI_ISL_750773, EPI_ISL_750774, EPI_ISL_750775, EPI_ISL_750776, EPI_ISL_750777, EPI_ISL_750778, EPI_ISL_750779, EPI_ISL_750780, EPI_ISL_750781, EPI_ISL_750782, EPI_ISL_750783, EPI_ISL_750784, EPI_ISL_750785, EPI_ISL_750786, EPI_ISL_750787, EPI_ISL_750788, EPI_ISL_750789, EPI_ISL_750790, EPI_ISL_750791, EPI_ISL_750792, EPI_ISL_750793, EPI_ISL_750794, EPI_ISL_750795, EPI_ISL_750796, EPI_ISL_750797, EPI_ISL_750798, EPI_ISL_750799, EPI_ISL_750800, EPI_ISL_750801, EPI_ISL_750802, EPI_ISL_750803, EPI_ISL_750804, EPI_ISL_750805, EPI_ISL_750806, EPI_ISL_750807, EPI_ISL_750808, EPI_ISL_750809, EPI_ISL_750810, EPI_ISL_750811, EPI_ISL_750812, EPI_ISL_750813, EPI_ISL_750814, EPI_ISL_750815, EPI_ISL_750816, EPI_ISL_750817, EPI_ISL_750818, EPI_ISL_750819, EPI_ISL_750821, EPI_ISL_750822, EPI_ISL_750823, EPI_ISL_750824, EPI_ISL_750825, EPI_ISL_750826, EPI_ISL_750827, EPI_ISL_750828, EPI_ISL_750829, EPI_ISL_750830, EPI_ISL_750831, EPI_ISL_750832, EPI_ISL_750833, EPI_ISL_750834, EPI_ISL_750835, EPI_ISL_750836, EPI_ISL_750837, EPI_ISL_750838, EPI_ISL_750839, EPI_ISL_750840, EPI_ISL_750841, EPI_ISL_750842, EPI_ISL_750843, EPI_ISL_750844, EPI_ISL_750845, EPI_ISL_750846, EPI_ISL_750847, EPI_ISL_750848, EPI_ISL_750849, EPI_ISL_750850, EPI_ISL_750851, EPI_ISL_750852, EPI_ISL_750853, EPI_ISL_750854, EPI_ISL_750855, EPI_ISL_750856, EPI_ISL_750857, EPI_ISL_750858, EPI_ISL_750859, EPI_ISL_750860, EPI_ISL_750861, EPI_ISL_750862, EPI_ISL_750863, EPI_ISL_750864, EPI_ISL_750865, EPI_ISL_750866, EPI_ISL_750867, EPI_ISL_750868, EPI_ISL_750869, EPI_ISL_750870, EPI_ISL_750871, EPI_ISL_750872, EPI_ISL_750873, EPI_ISL_750874, EPI_ISL_750875, EPI_ISL_750876, EPI_ISL_750877, EPI_ISL_750878, EPI_ISL_750879, EPI_ISL_750880, EPI_ISL_750881, EPI_ISL_750882, EPI_ISL_750883, EPI_ISL_750884, EPI_ISL_750885, EPI_ISL_750886, EPI_ISL_750887, EPI_ISL_750888, EPI_ISL_750889, EPI_ISL_750890, EPI_ISL_750891, EPI_ISL_750892, EPI_ISL_750893, EPI_ISL_750894, EPI_ISL_750895, EPI_ISL_750896, EPI_ISL_750897, EPI_ISL_750898, EPI_ISL_750899, EPI_ISL_750900, EPI_ISL_750901, EPI_ISL_750902, EPI_ISL_750903, EPI_ISL_750904, EPI_ISL_750905, EPI_ISL_750906, EPI_ISL_750907, EPI_ISL_750908, EPI_ISL_750909, EPI_ISL_750910, EPI_ISL_750911, EPI_ISL_750912, EPI_ISL_750913, EPI_ISL_750914, EPI_ISL_750915, EPI_ISL_750916, EPI_ISL_750917, EPI_ISL_750918, EPI_ISL_750919, EPI_ISL_750920, EPI_ISL_750921, EPI_ISL_750922, EPI_ISL_750923, EPI_ISL_750924, EPI_ISL_750925, EPI_ISL_750926, EPI_ISL_750927, EPI_ISL_750928, EPI_ISL_750929, EPI_ISL_750930, EPI_ISL_750931, EPI_ISL_750932, EPI_ISL_750933, EPI_ISL_750934, EPI_ISL_750935, EPI_ISL_750936, EPI_ISL_750937, EPI_ISL_750938, EPI_ISL_750939, EPI_ISL_750940, EPI_ISL_750941, EPI_ISL_750942, EPI_ISL_750943, EPI_ISL_750944, EPI_ISL_750945, EPI_ISL_750946, EPI_ISL_750947, EPI_ISL_750948, EPI_ISL_750949, EPI_ISL_750950, EPI_ISL_750951, EPI_ISL_750952, EPI_ISL_750953, EPI_ISL_750954, EPI_ISL_750955, EPI_ISL_750956, EPI_ISL_750957, EPI_ISL_750958, EPI_ISL_750959, EPI_ISL_750960, EPI_ISL_750961, EPI_ISL_750962, EPI_ISL_750963, EPI_ISL_750964, EPI_ISL_750965, EPI_ISL_750966, EPI_ISL_750967, EPI_ISL_750968, EPI_ISL_750969, EPI_ISL_750970, EPI_ISL_750971, EPI_ISL_750972, EPI_ISL_750973, EPI_ISL_750974, EPI_ISL_750975, EPI_ISL_750976, EPI_ISL_750977, EPI_ISL_750978, EPI_ISL_750979, EPI_ISL_750980, EPI_ISL_750981, EPI_ISL_750982, EPI_ISL_750983, EPI_ISL_750984, EPI_ISL_750985, EPI_ISL_750986, EPI_ISL_750987, EPI_ISL_750988, EPI_ISL_750989, EPI_ISL_750990, EPI_ISL_750991, EPI_ISL_750992, EPI_ISL_750993, EPI_ISL_750994, EPI_ISL_750995, EPI_ISL_750996, EPI_ISL_750997, EPI_ISL_750998, EPI_ISL_750999, EPI_ISL_751000, EPI_ISL_751001, EPI_ISL_751002, EPI_ISL_751003, EPI_ISL_751004, EPI_ISL_751005, EPI_ISL_751006, EPI_ISL_751007, EPI_ISL_751008, EPI_ISL_751009, EPI_ISL_751010, EPI_ISL_751012, EPI_ISL_751013, EPI_ISL_751014, EPI_ISL_751015, EPI_ISL_751016, EPI_ISL_751017, EPI_ISL_751018, EPI_ISL_751019, EPI_ISL_751020, EPI_ISL_751021, EPI_ISL_751022, EPI_ISL_751023, EPI_ISL_751024, EPI_ISL_751025, EPI_ISL_751026, EPI_ISL_751027, EPI_ISL_751028, EPI_ISL_751029, EPI_ISL_751030, EPI_ISL_751031, EPI_ISL_751032, EPI_ISL_751033, EPI_ISL_751034, EPI_ISL_751035, EPI_ISL_751036, EPI_ISL_751037, EPI_ISL_751038, EPI_ISL_751039, EPI_ISL_751040, EPI_ISL_751041, EPI_ISL_751042, EPI_ISL_751043, EPI_ISL_751044, EPI_ISL_751045, EPI_ISL_751046, EPI_ISL_751047, EPI_ISL_751048, EPI_ISL_751049, EPI_ISL_751050, EPI_ISL_751051, EPI_ISL_751052, EPI_ISL_751053, EPI_ISL_751054, EPI_ISL_751055, EPI_ISL_751056, EPI_ISL_751057, EPI_ISL_751058, EPI_ISL_751059, EPI_ISL_751060, EPI_ISL_751061, EPI_ISL_751062, EPI_ISL_751063, EPI_ISL_751064, EPI_ISL_751065, EPI_ISL_751066, EPI_ISL_751067, EPI_ISL_751068, EPI_ISL_751069, EPI_ISL_751070, EPI_ISL_751071, EPI_ISL_751072, EPI_ISL_751073, EPI_ISL_751074, EPI_ISL_751075, EPI_ISL_751076, EPI_ISL_751077, EPI_ISL_751078, EPI_ISL_751079, EPI_ISL_751080, EPI_ISL_751081, EPI_ISL_751082, EPI_ISL_751083, EPI_ISL_751084, EPI_ISL_751085, EPI_ISL_751086, EPI_ISL_751087, EPI_ISL_751088, EPI_ISL_751089, EPI_ISL_751090, EPI_ISL_751091, EPI_ISL_751092, EPI_ISL_751093, EPI_ISL_751094, EPI_ISL_751095, EPI_ISL_751096, EPI_ISL_751097, EPI_ISL_751098, EPI_ISL_751099, EPI_ISL_751100, EPI_ISL_751101, EPI_ISL_751102, EPI_ISL_751103, EPI_ISL_751104, EPI_ISL_751105, EPI_ISL_751106, EPI_ISL_751107, EPI_ISL_751108, EPI_ISL_751109, EPI_ISL_751110, EPI_ISL_751111, EPI_ISL_751112, EPI_ISL_751113, EPI_ISL_751114, EPI_ISL_751115, EPI_ISL_751116, EPI_ISL_751117, EPI_ISL_751118, EPI_ISL_751119, EPI_ISL_751120, EPI_ISL_751121, EPI_ISL_751122, EPI_ISL_751123, EPI_ISL_751124, EPI_ISL_751125, EPI_ISL_751126, EPI_ISL_751127, EPI_ISL_751128, EPI_ISL_751129, EPI_ISL_751130, EPI_ISL_751131, EPI_ISL_751132, EPI_ISL_751133, EPI_ISL_751134, EPI_ISL_751135, EPI_ISL_751136, EPI_ISL_751137, EPI_ISL_751138, EPI_ISL_751139, EPI_ISL_751140, EPI_ISL_751141, EPI_ISL_751142, EPI_ISL_751143, EPI_ISL_751144, EPI_ISL_751145, EPI_ISL_751146, EPI_ISL_751147, EPI_ISL_751148, EPI_ISL_751149, EPI_ISL_751150, EPI_ISL_751151, EPI_ISL_751152, EPI_ISL_751153, EPI_ISL_751154, EPI_ISL_751155, EPI_ISL_751156, EPI_ISL_751157, EPI_ISL_751158, EPI_ISL_751159, EPI_ISL_751160, EPI_ISL_751161, EPI_ISL_751162, EPI_ISL_751163, EPI_ISL_751164, EPI_ISL_751165, EPI_ISL_751166, EPI_ISL_751167, EPI_ISL_751168, EPI_ISL_751169, EPI_ISL_751170, EPI_ISL_751171, EPI_ISL_751172, EPI_ISL_751173, EPI_ISL_751174, EPI_ISL_751175, EPI_ISL_751176, EPI_ISL_751177, EPI_ISL_751178, EPI_ISL_751179, EPI_ISL_751180, EPI_ISL_751181, EPI_ISL_751182, EPI_ISL_751183

see above	Department of Virus and Microbiological Special Diagnostics, Statens Serum Institut, Copenhagen, Denmark	Albertsen Lab, Department of Chemistry and Bioscience, Aalborg University, Denmark	Danish Covid-19 Genome Consortium
EPI_ISL_753671, EPI_ISL_753672, EPI_ISL_753673, EPI_ISL_753674, EPI_ISL_753689, EPI_ISL_753691	Clinical virology Laboratory, Children's Hospital Los Angeles	Center for Personalized Medicine, Children's Hospital Los Angeles	Gai et al
EPI_ISL_754079, EPI_ISL_754081, EPI_ISL_754085, EPI_ISL_754087, EPI_ISL_754093, EPI_ISL_754096, EPI_ISL_754098, EPI_ISL_754105	National Public Health Laboratory, National Centre for Infectious Diseases	National Public Health Laboratory, National Centre for Infectious Diseases	Tze Minn Mak, Sophie Octavia, Zhenyang Zhou, Lin Cui, Raymond Tzer Pin Lin

EPI_ISL_754312, EPI_ISL_754367	Respiratory Virus Unit, National Infection Service, Public Health England	COVID-19 Genomics UK (COG-UK) Consortium	PHE Covid Sequencing Team
EPI_ISL_754620, EPI_ISL_754621, EPI_ISL_754622, EPI_ISL_754636, EPI_ISL_754637, EPI_ISL_754638, EPI_ISL_754639, EPI_ISL_754640, EPI_ISL_754641, EPI_ISL_754642, EPI_ISL_754643, EPI_ISL_754644, EPI_ISL_754645, EPI_ISL_754646, EPI_ISL_754647			
see above	University of Wisconsin-Madison AIDS Vaccine Research Laboratories	University of Wisconsin-Madison AIDS Vaccine Research Laboratories	Gage Moreno, Katarina Braun, et al. AIDS Vaccine Research Laboratories
EPI_ISL_755171, EPI_ISL_755172, EPI_ISL_755173, EPI_ISL_755175, EPI_ISL_755176, EPI_ISL_755177, EPI_ISL_755178, EPI_ISL_755179, EPI_ISL_755180, EPI_ISL_755183	UCSD EXCITE lab	Andersen lab at Scripps Research	SEARCH Alliance San Diego
EPI_ISL_755500, EPI_ISL_755501, EPI_ISL_755502, EPI_ISL_755503, EPI_ISL_755504, EPI_ISL_755505, EPI_ISL_755506, EPI_ISL_755507, EPI_ISL_755508, EPI_ISL_755509, EPI_ISL_755510, EPI_ISL_755554, EPI_ISL_755555, EPI_ISL_755556, EPI_ISL_755557, EPI_ISL_755558, EPI_ISL_755559, EPI_ISL_755566, EPI_ISL_755567			
see above	Maine Health and Environmental Testing Laboratory	Tewhey Lab, The Jackson Laboratory	Matluk,N., Dewey,H., Iosue,F., Barter,M., Lynch,R., Munger,H. and Tewhey,R.
EPI_ISL_756216, EPI_ISL_756218, EPI_ISL_756219, EPI_ISL_756220, EPI_ISL_756221, EPI_ISL_756222, EPI_ISL_756223, EPI_ISL_756224, EPI_ISL_756225, EPI_ISL_756226, EPI_ISL_756227, EPI_ISL_756231, EPI_ISL_756232, EPI_ISL_756233, EPI_ISL_756300, EPI_ISL_756301, EPI_ISL_756303, EPI_ISL_756304, EPI_ISL_756305			
see above	UW Virology Lab	UW Virology Lab	Pavitra Roychoudhury, Hong Xie, Lasata Shrestha, Meei-Li Huang, Keith R Jerome, Alexander Greninger
EPI_ISL_756689, EPI_ISL_756691, EPI_ISL_756692, EPI_ISL_756693, EPI_ISL_756694, EPI_ISL_756697, EPI_ISL_756698, EPI_ISL_756699, EPI_ISL_756700, EPI_ISL_756701, EPI_ISL_756703, EPI_ISL_756704, EPI_ISL_756707, EPI_ISL_756708, EPI_ISL_756710, EPI_ISL_756712, EPI_ISL_756713, EPI_ISL_756714, EPI_ISL_756715, EPI_ISL_756717, EPI_ISL_756718, EPI_ISL_756719, EPI_ISL_756720, EPI_ISL_756721, EPI_ISL_756722, EPI_ISL_756724, EPI_ISL_756725, EPI_ISL_756726, EPI_ISL_756727, EPI_ISL_756728, EPI_ISL_756730, EPI_ISL_756731, EPI_ISL_756732, EPI_ISL_756733, EPI_ISL_756734, EPI_ISL_756735, EPI_ISL_756736, EPI_ISL_756738, EPI_ISL_756739, EPI_ISL_756740, EPI_ISL_756741, EPI_ISL_756742, EPI_ISL_756743, EPI_ISL_756746, EPI_ISL_756747, EPI_ISL_756749, EPI_ISL_756751, EPI_ISL_756752, EPI_ISL_756753, EPI_ISL_756754, EPI_ISL_756755, EPI_ISL_756756, EPI_ISL_756757, EPI_ISL_756758, EPI_ISL_756759, EPI_ISL_756760, EPI_ISL_756762, EPI_ISL_756763, EPI_ISL_756764, EPI_ISL_756766, EPI_ISL_756767, EPI_ISL_756768, EPI_ISL_756769, EPI_ISL_756770, EPI_ISL_756771, EPI_ISL_756772, EPI_ISL_756774, EPI_ISL_756776, EPI_ISL_756777, EPI_ISL_756778, EPI_ISL_756779, EPI_ISL_756781, EPI_ISL_756782, EPI_ISL_756783, EPI_ISL_756785, EPI_ISL_756786, EPI_ISL_756787, EPI_ISL_756788, EPI_ISL_756790, EPI_ISL_756791, EPI_ISL_756792, EPI_ISL_756793, EPI_ISL_756794, EPI_ISL_756796, EPI_ISL_756797, EPI_ISL_756798, EPI_ISL_756799, EPI_ISL_756801, EPI_ISL_756802, EPI_ISL_756804, EPI_ISL_756806, EPI_ISL_756807, EPI_ISL_756808, EPI_ISL_756809, EPI_ISL_756810, EPI_ISL_756811, EPI_ISL_756812, EPI_ISL_756813, EPI_ISL_756814, EPI_ISL_756815, EPI_ISL_756816, EPI_ISL_756817, EPI_ISL_756818, EPI_ISL_756819, EPI_ISL_756820, EPI_ISL_756821, EPI_ISL_756822, EPI_ISL_756823, EPI_ISL_756825, EPI_ISL_756826, EPI_ISL_756827, EPI_ISL_756828, EPI_ISL_756829, EPI_ISL_756830, EPI_ISL_756831, EPI_ISL_756832, EPI_ISL_756833, EPI_ISL_756834, EPI_ISL_756836, EPI_ISL_756837, EPI_ISL_756838, EPI_ISL_756839, EPI_ISL_756841, EPI_ISL_756842, EPI_ISL_756843, EPI_ISL_756844, EPI_ISL_756845, EPI_ISL_756847, EPI_ISL_756848, EPI_ISL_756851, EPI_ISL_756853, EPI_ISL_756854, EPI_ISL_756855, EPI_ISL_756856, EPI_ISL_756857, EPI_ISL_756858, EPI_ISL_756859, EPI_ISL_756862, EPI_ISL_756863, EPI_ISL_756864, EPI_ISL_756866, EPI_ISL_756868, EPI_ISL_756869, EPI_ISL_756870, EPI_ISL_756871, EPI_ISL_756872, EPI_ISL_756873, EPI_ISL_756874, EPI_ISL_756875, EPI_ISL_756880, EPI_ISL_756882, EPI_ISL_756883, EPI_ISL_756884, EPI_ISL_756890, EPI_ISL_756891, EPI_ISL_756892, EPI_ISL_756896, EPI_ISL_756898, EPI_ISL_756899, EPI_ISL_756901, EPI_ISL_756902, EPI_ISL_756904, EPI_ISL_756905, EPI_ISL_756906, EPI_ISL_756907, EPI_ISL_756908, EPI_ISL_756911, EPI_ISL_756913, EPI_ISL_756914, EPI_ISL_756915, EPI_ISL_756916, EPI_ISL_756917, EPI_ISL_756919, EPI_ISL_756921, EPI_ISL_756922, EPI_ISL_756924, EPI_ISL_756926, EPI_ISL_756927, EPI_ISL_756929, EPI_ISL_756930, EPI_ISL_756932, EPI_ISL_756933, EPI_ISL_756934, EPI_ISL_756936, EPI_ISL_756937, EPI_ISL_756938, EPI_ISL_756939, EPI_ISL_756941, EPI_ISL_756942, EPI_ISL_756943, EPI_ISL_756944, EPI_ISL_756945, EPI_ISL_756948, EPI_ISL_756949, EPI_ISL_756950, EPI_ISL_756954, EPI_ISL_756956, EPI_ISL_756957, EPI_ISL_756958, EPI_ISL_756960, EPI_ISL_756961, EPI_ISL_756962, EPI_ISL_756963, EPI_ISL_756964, EPI_ISL_756965, EPI_ISL_756966, EPI_ISL_756967, EPI_ISL_756968, EPI_ISL_756972, EPI_ISL_756974, EPI_ISL_756975, EPI_ISL_756976, EPI_ISL_756978, EPI_ISL_756979, EPI_ISL_756980, EPI_ISL_756981, EPI_ISL_756983, EPI_ISL_756984, EPI_ISL_756985, EPI_ISL_756986, EPI_ISL_756987, EPI_ISL_756988, EPI_ISL_756989, EPI_ISL_756990, EPI_ISL_756991, EPI_ISL_756992, EPI_ISL_756993, EPI_ISL_756994, EPI_ISL_756995, EPI_ISL_756996, EPI_ISL_756997, EPI_ISL_756999, EPI_ISL_757000, EPI_ISL_757001, EPI_ISL_757002, EPI_ISL_757003, EPI_ISL_757004, EPI_ISL_757006, EPI_ISL_757010, EPI_ISL_757013, EPI_ISL_757015, EPI_ISL_757016, EPI_ISL_757019, EPI_ISL_757022, EPI_ISL_757023, EPI_ISL_757024, EPI_ISL_757025, EPI_ISL_757026, EPI_ISL_757029, EPI_ISL_757030, EPI_ISL_757031, EPI_ISL_757033			
see above	Lighthouse Lab in Milton Keynes	Wellcome Sanger Institute for the COVID-19 Genomics UK (COG-UK) Consortium	The Lighthouse Lab in Milton Keynes and Alex Alderton, Roberto Amato, Sonia Goncalves, Ewan Harrison, David K. Jackson, Ian Johnston, Dominic Kwiatkowski, Cordelia Langford, John Sillitoe on behalf of the Wellcome Sanger Institute COVID-19 Surveillance Team
EPI_ISL_757034, EPI_ISL_757035, EPI_ISL_757036, EPI_ISL_757037, EPI_ISL_757038, EPI_ISL_757039, EPI_ISL_757040, EPI_ISL_757041, EPI_ISL_757083, EPI_ISL_757084, EPI_ISL_757085, EPI_ISL_757086, EPI_ISL_757087, EPI_ISL_757088, EPI_ISL_757089, EPI_ISL_757090, EPI_ISL_757091, EPI_ISL_757092, EPI_ISL_757093, EPI_ISL_757094, EPI_ISL_757095, EPI_ISL_757096, EPI_ISL_757097, EPI_ISL_757098, EPI_ISL_757099, EPI_ISL_757100, EPI_ISL_757101, EPI_ISL_757102, EPI_ISL_757103, EPI_ISL_757104, EPI_ISL_757105, EPI_ISL_757106, EPI_ISL_757107, EPI_ISL_757108, EPI_ISL_757109, EPI_ISL_757110, EPI_ISL_757111, EPI_ISL_757112, EPI_ISL_757113, EPI_ISL_757114, EPI_ISL_757115, EPI_ISL_757116, EPI_ISL_757117, EPI_ISL_757118			
see above	Lighthouse Lab in Glasgow	Wellcome Sanger Institute for the COVID-19 Genomics UK (COG-UK) Consortium	Harper VanSteenhouse, Yumi Kasai, David Gray, Carol Clugston, Anna Dominiczak and Alex Alderton, Roberto Amato, Sonia Goncalves, Ewan Harrison, David K. Jackson, Ian Johnston, Dominic Kwiatkowski, Cordelia Langford, John Sillitoe on behalf of the Wellcome Sanger Institute COVID-19 Surveillance Team
EPI_ISL_757395, EPI_ISL_757396, EPI_ISL_757397, EPI_ISL_757398, EPI_ISL_757399, EPI_ISL_757400, EPI_ISL_757401, EPI_ISL_757402, EPI_ISL_757403, EPI_ISL_757404, EPI_ISL_757405, EPI_ISL_757406, EPI_ISL_757407, EPI_ISL_757408, EPI_ISL_757409, EPI_ISL_757410, EPI_ISL_757411, EPI_ISL_757412, EPI_ISL_757413, EPI_ISL_757414, EPI_ISL_757415, EPI_ISL_757416, EPI_ISL_757417, EPI_ISL_757418, EPI_ISL_757419, EPI_ISL_757420, EPI_ISL_757421, EPI_ISL_757422, EPI_ISL_757423, EPI_ISL_757424, EPI_ISL_757425, EPI_ISL_757426, EPI_ISL_757427, EPI_ISL_757428, EPI_ISL_757429, EPI_ISL_757430, EPI_ISL_757431, EPI_ISL_757432, EPI_ISL_757433, EPI_ISL_757434, EPI_ISL_757435, EPI_ISL_757436, EPI_ISL_757437, EPI_ISL_757438, EPI_ISL_757439, EPI_ISL_757440, EPI_ISL_757441, EPI_ISL_757442, EPI_ISL_757444, EPI_ISL_757445, EPI_ISL_757446, EPI_ISL_757447, EPI_ISL_757449, EPI_ISL_757450, EPI_ISL_757451, EPI_ISL_757452, EPI_ISL_757453, EPI_ISL_757454, EPI_ISL_757455, EPI_ISL_757456, EPI_ISL_757457, EPI_ISL_757458, EPI_ISL_757459, EPI_ISL_757460, EPI_ISL_757461, EPI_ISL_757462, EPI_ISL_757463, EPI_ISL_757464, EPI_ISL_757465, EPI_ISL_757466, EPI_ISL_757467, EPI_ISL_757468, EPI_ISL_757469, EPI_ISL_757470, EPI_ISL_757471, EPI_ISL_757472, EPI_ISL_757473, EPI_ISL_757474, EPI_ISL_757475, EPI_ISL_757476, EPI_ISL_757477, EPI_ISL_757478, EPI_ISL_757479, EPI_ISL_757480, EPI_ISL_757481, EPI_ISL_757482, EPI_ISL_757483, EPI_ISL_757484, EPI_ISL_757485, EPI_ISL_757486, EPI_ISL_757487, EPI_ISL_757488, EPI_ISL_757489, EPI_ISL_757490, EPI_ISL_757492, EPI_ISL_757493, EPI_ISL_757494, EPI_ISL_757495, EPI_ISL_757496, EPI_ISL_757497, EPI_ISL_757499, EPI_ISL_757500, EPI_ISL_757501, EPI_ISL_757502, EPI_ISL_757503, EPI_ISL_757504, EPI_ISL_757505, EPI_ISL_757506, EPI_ISL_757507, EPI_ISL_757508, EPI_ISL_757509, EPI_ISL_757510, EPI_ISL_757511, EPI_ISL_757512, EPI_ISL_757513, EPI_ISL_757514, EPI_ISL_757515, EPI_ISL_757516, EPI_ISL_757517, EPI_ISL_757518, EPI_ISL_757519, EPI_ISL_757520, EPI_ISL_757521, EPI_ISL_757522, EPI_ISL_757523, EPI_ISL_757524, EPI_ISL_757525, EPI_ISL_757526, EPI_ISL_757527, EPI_ISL_757528, EPI_ISL_757529, EPI_ISL_757530, EPI_ISL_757531, EPI_ISL_757532, EPI_ISL_757533, EPI_ISL_757534, EPI_ISL_757535, EPI_ISL_757536, EPI_ISL_757537, EPI_ISL_757538, EPI_ISL_757539, EPI_ISL_757540, EPI_ISL_757541, EPI_ISL_757542, EPI_ISL_757543, EPI_ISL_757544, EPI_ISL_757545, EPI_ISL_757546, EPI_ISL_757547, EPI_ISL_757548, EPI_ISL_757549, EPI_ISL_757550, EPI_ISL_757551, EPI_ISL_757552, EPI_ISL_757553, EPI_ISL_757554, EPI_ISL_757555, EPI_ISL_757556, EPI_ISL_757557, EPI_ISL_757558, EPI_ISL_757559, EPI_ISL_757560, EPI_ISL_757561, EPI_ISL_757562, EPI_ISL_757563, EPI_ISL_757564, EPI_ISL_757565, EPI_ISL_757566, EPI_ISL_757567, EPI_ISL_757568, EPI_ISL_757569, EPI_ISL_757570, EPI_ISL_757571, EPI_ISL_757572, EPI_ISL_757573, EPI_ISL_757574, EPI_ISL_757575, EPI_ISL_757576, EPI_ISL_757577, EPI_ISL_757578, EPI_ISL_757579, EPI_ISL_757580, EPI_ISL_757581, EPI_ISL_757582, EPI_ISL_757583, EPI_ISL_757584, EPI_ISL_757585, EPI_ISL_757586, EPI_ISL_757587, EPI_ISL_757589, EPI_ISL_757590, EPI_ISL_757591, EPI_ISL_757592, EPI_ISL_757593, EPI_ISL_757594, EPI_ISL_757595, EPI_ISL_757596, EPI_ISL_757597, EPI_ISL_757598, EPI_ISL_757599, EPI_ISL_757600, EPI_ISL_757601, EPI_ISL_757602, EPI_ISL_757603, EPI_ISL_757604, EPI_ISL_757605, EPI_ISL_757606, EPI_ISL_757607, EPI_ISL_757608, EPI_ISL_757609, EPI_ISL_757610, EPI_ISL_757611, EPI_ISL_757612, EPI_ISL_757613, EPI_ISL_757614, EPI_ISL_757615, EPI_ISL_757616, EPI_ISL_757617, EPI_ISL_757618, EPI_ISL_757619, EPI_ISL_757620, EPI_ISL_757621, EPI_ISL_757622, EPI_ISL_757623, EPI_ISL_757624, EPI_ISL_757625, EPI_ISL_757626, EPI_ISL_757627, EPI_ISL_757628, EPI_ISL_757629, EPI_ISL_757630, EPI_ISL_757631, EPI_ISL_757632, EPI_ISL_757633, EPI_ISL_757634, EPI_ISL_757635, EPI_ISL_757636, EPI_ISL_757637, EPI_ISL_757638, EPI_ISL_757639, EPI_ISL_757640, EPI_ISL_757641, EPI_ISL_757642, EPI_ISL_757643, EPI_ISL_757644, EPI_ISL_757645, EPI_ISL_757646, EPI_ISL_757647, EPI_ISL_757648, EPI_ISL_757649, EPI_ISL_757650, EPI_ISL_757651, EPI_ISL_757652, EPI_ISL_757653, EPI_ISL_757654, EPI_ISL_757655, EPI_ISL_757656, EPI_ISL_757657, EPI_ISL_757658, EPI_ISL_757659, EPI_ISL_757660, EPI_ISL_757661, EPI_ISL_757662, EPI_ISL_757663, EPI_ISL_757664, EPI_ISL_757665, EPI_ISL_757666, EPI_ISL_757667, EPI_ISL_757668, EPI_ISL_757669, EPI_ISL_757670, EPI_ISL_757671, EPI_ISL_757672, EPI_ISL_757673, EPI_ISL_757674, EPI_ISL_757675, EPI_ISL_757676, EPI_ISL_757677, EPI_ISL_757678, EPI_ISL_757679, EPI_ISL_757680, EPI_ISL_757681, EPI_ISL_757682, EPI_ISL_757683, EPI_ISL_757684, EPI_ISL_757685, EPI_ISL_757686, EPI_ISL_757687, EPI_ISL_757703, EPI_ISL_757704, EPI_ISL_757705, EPI_ISL_757710, EPI_ISL_757712, EPI_ISL_757802, EPI_ISL_757803, EPI_ISL_757804, EPI_ISL_757805, EPI_ISL_757806, EPI_ISL_757807, EPI_ISL_757808, EPI_ISL_757809, EPI_ISL_757810, EPI_ISL_757811, EPI_ISL_757812, EPI_ISL_757813, EPI_ISL_757814, EPI_ISL_757815, EPI_ISL_757816, EPI_ISL_757817, EPI_ISL_757818, EPI_ISL_757819, EPI_ISL_757820, EPI_ISL_757821, EPI_ISL_757822, EPI_ISL_757823, EPI_ISL_757824, EPI_ISL_757825, EPI_ISL_757826, EPI_ISL_757827, EPI_ISL_757828, EPI_ISL_757829, EPI_ISL_757830, EPI_ISL_757831, EPI_ISL_757832, EPI_ISL_757833, EPI_ISL_757834, EPI_ISL_757835, EPI_ISL_757836, EPI_ISL_757837, EPI_ISL_757838, EPI_ISL_757839, EPI_ISL_757840, EPI_ISL_757841, EPI_ISL_757842, EPI_ISL_757843, EPI_ISL_757844, EPI_ISL_757845, EPI_ISL_757846, EPI_ISL_757847, EPI_ISL_757848, EPI_ISL_757849, EPI_ISL_757850, EPI_ISL_757851, EPI_ISL_757852, EPI_ISL_757853, EPI_ISL_757854, EPI_ISL_757859, EPI_ISL_757861, EPI_ISL_757867, EPI_ISL_757872, EPI_ISL_757874, EPI_ISL_757875, EPI_ISL_757876, EPI_ISL_757887, EPI_ISL_757888, EPI_ISL_757889, EPI_ISL_757890, EPI_ISL_757893, EPI_ISL_757894, EPI_ISL_757895, EPI_ISL_757927, EPI_ISL_757928, EPI_ISL_757929, EPI_ISL_757930, EPI_ISL_757931, EPI_ISL_757932, EPI_ISL_757933, EPI_ISL_757934, EPI_ISL_757936, EPI_ISL_757937, EPI_ISL_757938, EPI_ISL_757939, EPI_ISL_757940, EPI_ISL_757941, EPI_ISL_757942, EPI_ISL_757943, EPI_ISL_757944, EPI_ISL_757947, EPI_ISL_757948, EPI_ISL_757949, EPI_ISL_757950, EPI_ISL_757951, EPI_ISL_757953, EPI_ISL_757954, EPI_ISL_757955, EPI_ISL_757956, EPI_ISL_757957, EPI_ISL_757958, EPI_ISL_757967, EPI_ISL_757968, EPI_ISL_757969, EPI_ISL_757970, EPI_ISL_757971, EPI_ISL_757972, EPI_ISL_757973, EPI_ISL_757974, EPI_ISL_758026, EPI_ISL_758027, EPI_ISL_758028, EPI_ISL_758029, EPI_ISL_758030, EPI_ISL_758031, EPI_ISL_758032, EPI_ISL_758033, EPI_ISL_758034, EPI_ISL_758035, EPI_ISL_758036, EPI_ISL_758037, EPI_ISL_758038, EPI_ISL_758039, EPI_ISL_758040, EPI_ISL_758041, EPI_ISL_758042, EPI_ISL_758043, EPI_ISL_758044, EPI_ISL_758045, EPI_ISL_758046, EPI_ISL_758047, EPI_ISL_758048, EPI_ISL_758049, EPI_ISL_758050, EPI_ISL_758051, EPI_ISL_758052, EPI_ISL_758053, EPI_ISL_758054, EPI_ISL_758055, EPI_ISL_758056, EPI_ISL_758057, EPI_ISL_758058, EPI_ISL_758059, EPI_ISL_758060, EPI_ISL_758061, EPI_ISL_758062, EPI_ISL_758063, EPI_ISL_758064, EPI_ISL_758065, EPI_ISL_758066, EPI_ISL_758071, EPI_ISL_758072, EPI_ISL_758082, EPI_ISL_758084, EPI_ISL_758093, EPI_ISL_758101, EPI_ISL_758106, EPI_ISL_758107, EPI_ISL_758108, EPI_ISL_758109, EPI_ISL_758110, EPI_ISL_758111, EPI_ISL_758115, EPI_ISL_758116, EPI_ISL_758122, EPI_ISL_758123, EPI_ISL_758124, EPI_ISL_758125, EPI_ISL_758126, EPI_ISL_758127, EPI_ISL_758484, EPI_ISL_758485, EPI_ISL_758486, EPI_ISL_758487, EPI_ISL_758488, EPI_ISL_758489, EPI_ISL_758490, EPI_ISL_758491, EPI_ISL_758492, EPI_ISL_758493, EPI_ISL_758494, EPI_ISL_758495, EPI_ISL_758496, EPI_ISL_758497, EPI_ISL_758498, EPI_ISL_758499, EPI_ISL_758500, EPI_ISL_758501, EPI_ISL_758502, EPI_ISL_758503, EPI_ISL_758504, EPI_ISL_758505, EPI_ISL_758506, EPI_ISL_758507, EPI_ISL_758508, EPI_ISL_758509, EPI_ISL_758510, EPI_ISL_758511, EPI_ISL_758512, EPI_ISL_758513, EPI_ISL_758514, EPI_ISL_758515, EPI_ISL_758516, EPI_ISL_758517, EPI_ISL_758518, EPI_ISL_758519, EPI_ISL_758520, EPI_ISL_758521, EPI_ISL_758522, EPI_ISL_758523, EPI_ISL_758524, EPI_ISL_758525, EPI_ISL_758526, EPI_ISL_758527, EPI_ISL_758528, EPI_ISL_758529, EPI_ISL_758530, EPI_ISL_758531, EPI_ISL_758532, EPI_ISL_758533, EPI_ISL_758534, EPI_ISL_758535, EPI_ISL_758536, EPI_ISL_758537, EPI_ISL_758538, EPI_ISL_758539, EPI_ISL_758540, EPI_ISL_758541, EPI_ISL_758542, EPI_ISL_758543, EPI_ISL_758544, EPI_ISL_758545, EPI_ISL_758546, EPI_ISL_758547, EPI_ISL_758548, EPI_ISL_758549, EPI_ISL_758550, EPI_ISL_758551, EPI_ISL_758552, EPI_ISL_758553, EPI_ISL_758554, EPI_ISL_758555, EPI_ISL_758556, EPI_ISL_758557, EPI_ISL_758558, EPI_ISL_758559, EPI_ISL_758560, EPI_ISL_758561, EPI_ISL_758562, EPI_ISL_758563, EPI_ISL_758564, EPI_ISL_758565, EPI_ISL_758566, EPI_ISL_758567, EPI_ISL_758568, EPI_ISL_758569, EPI_ISL_758570, EPI_ISL_758571, EPI_ISL_758572, EPI_ISL_758573, EPI_ISL_758574, EPI_ISL_758575, EPI_ISL_758576, EPI_ISL_758577, EPI_ISL_758578, EPI_ISL_758579, EPI_ISL_758580, EPI_ISL_758581, EPI_ISL_758582, EPI_ISL_758583, EPI_ISL_758584, EPI_ISL_758585, EPI_ISL_758586, EPI_ISL_758587, EPI_ISL_758588, EPI_ISL_758589, EPI_ISL_758590, EPI_ISL_758591, EPI_ISL_758592, EPI_ISL_758593, EPI_ISL_758594, EPI_ISL_758595, EPI_ISL_758596, EPI_ISL_758597, EPI_ISL_758598, EPI_ISL_758599, EPI_ISL_758600, EPI_ISL_758601, EPI_ISL_758602, EPI_ISL_758603, EPI_ISL_758604, EPI_ISL_758605, EPI_ISL_758606, EPI_ISL_758607, EPI_ISL_758608, EPI_ISL_758609, EPI_ISL_758610, EPI_ISL_758611, EPI_ISL_758612, EPI_ISL_758613, EPI_ISL_758614, EPI_ISL_758615, EPI_ISL_758616, EPI_ISL_758617, EPI_ISL_758618, EPI_ISL_758619, EPI_ISL_758620, EPI_ISL_758621, EPI_ISL_758622, EPI_ISL_758623, EPI_ISL_758624, EPI_ISL_758625, EPI_ISL_758626, EPI_ISL_758627, EPI_ISL_758628, EPI_ISL_758629, EPI_ISL_758630, EPI_ISL_758631, EPI_ISL_758632, EPI_ISL_758633, EPI_ISL_758634, EPI_ISL_758635, EPI_ISL_758636, EPI_ISL_758637, EPI_ISL_758638, EPI_ISL_758639, EPI_ISL_758640, EPI_ISL_758641, EPI_ISL_758642, EPI_ISL_758643, EPI_ISL_758644, EPI_ISL_758645, EPI_ISL_758646, EPI_ISL_758647, EPI_ISL_758648, EPI_ISL_758649, EPI_ISL_758650, EPI_ISL_758651, EPI_ISL_758652, EPI_ISL_758653, EPI_ISL_758654, EPI_ISL_758655, EPI_ISL_758656, EPI_ISL_758657, EPI_ISL_758658, EPI_ISL_758659, EPI_ISL_758660, EPI_ISL_758661, EPI_ISL_758663, EPI_ISL_758664, EPI_ISL_758665, EPI_ISL_758666, EPI_ISL_758667, EPI_ISL_758668, EPI_ISL_758669, EPI_ISL_758670, EPI_ISL_758671, EPI_ISL_758672, EPI_ISL_758673, EPI_ISL_758674, EPI_ISL_758675, EPI_ISL_758676, EPI_ISL_758677, EPI_ISL_758678, EPI_ISL_758679, EPI_ISL_758680, EPI_ISL_758681, EPI_ISL_758682, EPI_ISL_758683, EPI_ISL_758684, EPI_ISL_758685, EPI_ISL_758686, EPI_ISL_758687, EPI_ISL_758688, EPI_ISL_758689, EPI_ISL_758690, EPI_ISL_758691, EPI_ISL_758692, EPI_ISL_758693, EPI_ISL_758694, EPI_ISL_758695, EPI_ISL_758696, EPI_ISL_758697, EPI_ISL_758698, EPI_ISL_758699, EPI_ISL_758700			
see above	Department of Virus and Microbiological Special Diagnostics, Statens Serum Institut, Copenhagen, Denmark	Albertsen Lab, Department of Chemistry and Bioscience, Aalborg University, Denmark	Danish Covid-19 Genome Consortium
EPI_ISL_760542, EPI_ISL_760560	Lighthouse Lab in Glasgow	Wellcome Sanger Institute for the COVID-19 Genomics UK (COG-UK) Consortium	Harper VanSteenhouse, Yumi Kasai, David Gray, Carol Clugston, Anna Dominiczak and Alex Alderton, Roberto Amato, Sonia Goncalves, Ewan Harrison, David K. Jackson, Ian Johnston, Dominic Kwiatkowski, Cordelia Langford, John Sillitoe on behalf of the Wellcome Sanger Institute COVID-19 Surveillance

			Team
EPI_ISL_760567, EPI_ISL_760600, EPI_ISL_760649	Lighthouse Lab in Alderley Park	Wellcome Sanger Institute for the COVID-19 Genomics UK (COG-UK) Consortium	Jacquelyn Wynn, Mairead Hyland, The Lighthouse Lab in Alderley Park and Alex Alderton, Roberto Amato, Sonia Goncalves, Ewan Harrison, David K. Jackson, Ian Johnston, Dominic Kwiatkowski, Cordelia Langford, John Sillitoe on behalf of the Wellcome Sanger Institute COVID-19 Surveillance Team
EPI_ISL_760658, EPI_ISL_760669, EPI_ISL_760673, EPI_ISL_760739, EPI_ISL_760789	Lighthouse Lab in Glasgow	Wellcome Sanger Institute for the COVID-19 Genomics UK (COG-UK) Consortium	Harper VanSteenhouse, Yumi Kasai, David Gray, Carol Clugston, Anna Dominiczak and Alex Alderton, Roberto Amato, Sonia Goncalves, Ewan Harrison, David K. Jackson, Ian Johnston, Dominic Kwiatkowski, Cordelia Langford, John Sillitoe on behalf of the Wellcome Sanger Institute COVID-19 Surveillance Team
EPI_ISL_760883	Lighthouse Lab in Cambridge	Wellcome Sanger Institute for the COVID-19 Genomics UK (COG-UK) Consortium	Rob Howes, The Lighthouse Lab in Cambridge and Alex Alderton, Roberto Amato, Sonia Goncalves, Ewan Harrison, David K. Jackson, Ian Johnston, Dominic Kwiatkowski, Cordelia Langford, John Sillitoe on behalf of the Wellcome Sanger Institute COVID-19 Surveillance Team
EPI_ISL_760924	Lighthouse Lab in Alderley Park	Wellcome Sanger Institute for the COVID-19 Genomics UK (COG-UK) Consortium	Jacquelyn Wynn, Mairead Hyland, The Lighthouse Lab in Alderley Park and Alex Alderton, Roberto Amato, Sonia Goncalves, Ewan Harrison, David K. Jackson, Ian Johnston, Dominic Kwiatkowski, Cordelia Langford, John Sillitoe on behalf of the Wellcome Sanger Institute COVID-19 Surveillance Team
EPI_ISL_760984	Lighthouse Lab in Glasgow	Wellcome Sanger Institute for the COVID-19 Genomics UK (COG-UK) Consortium	Harper VanSteenhouse, Yumi Kasai, David Gray, Carol Clugston, Anna Dominiczak and Alex Alderton, Roberto Amato, Sonia Goncalves, Ewan Harrison, David K. Jackson, Ian Johnston, Dominic Kwiatkowski, Cordelia Langford, John Sillitoe on behalf of the Wellcome Sanger Institute COVID-19 Surveillance Team
EPI_ISL_760989, EPI_ISL_761036, EPI_ISL_761140, EPI_ISL_761258	Lighthouse Lab in Alderley Park	Wellcome Sanger Institute for the COVID-19 Genomics UK (COG-UK) Consortium	Jacquelyn Wynn, Mairead Hyland, The Lighthouse Lab in Alderley Park and Alex Alderton, Roberto Amato, Sonia Goncalves, Ewan Harrison, David K. Jackson, Ian Johnston, Dominic Kwiatkowski, Cordelia Langford, John Sillitoe on behalf of the Wellcome Sanger Institute COVID-19 Surveillance Team
EPI_ISL_761616	Lighthouse Lab in Milton Keynes	Wellcome Sanger Institute for the COVID-19 Genomics UK (COG-UK) Consortium	The Lighthouse Lab in Milton Keynes and Alex Alderton, Roberto Amato, Sonia Goncalves, Ewan Harrison, David K. Jackson, Ian Johnston, Dominic Kwiatkowski, Cordelia Langford, John Sillitoe on behalf of the Wellcome Sanger Institute COVID-19 Surveillance Team
EPI_ISL_761693, EPI_ISL_761755	Lighthouse Lab in Alderley Park	Wellcome Sanger Institute for the COVID-19 Genomics UK (COG-UK) Consortium	Jacquelyn Wynn, Mairead Hyland, The Lighthouse Lab in Alderley Park and Alex Alderton, Roberto Amato, Sonia Goncalves, Ewan Harrison, David K. Jackson, Ian Johnston, Dominic Kwiatkowski, Cordelia Langford, John Sillitoe on behalf of the Wellcome Sanger Institute COVID-19 Surveillance Team
EPI_ISL_761985, EPI_ISL_761998, EPI_ISL_762013, EPI_ISL_762062, EPI_ISL_762083, EPI_ISL_762110, EPI_ISL_762115, EPI_ISL_762156, EPI_ISL_762169, EPI_ISL_762171	Lighthouse Lab in Glasgow	Wellcome Sanger Institute for the COVID-19 Genomics UK (COG-UK) Consortium	Harper VanSteenhouse, Yumi Kasai, David Gray, Carol Clugston, Anna Dominiczak and Alex Alderton, Roberto Amato, Sonia Goncalves, Ewan Harrison, David K. Jackson, Ian Johnston, Dominic Kwiatkowski, Cordelia Langford, John Sillitoe on behalf of the Wellcome Sanger Institute COVID-19 Surveillance Team
EPI_ISL_762198, EPI_ISL_762204, EPI_ISL_762205, EPI_ISL_762208, EPI_ISL_762227, EPI_ISL_762231, EPI_ISL_762238, EPI_ISL_762244, EPI_ISL_762254, EPI_ISL_762258, EPI_ISL_762271, EPI_ISL_762273, EPI_ISL_762278, EPI_ISL_762281, EPI_ISL_762285, EPI_ISL_762287, EPI_ISL_762293, EPI_ISL_762305, EPI_ISL_762309, EPI_ISL_762324, EPI_ISL_762328, EPI_ISL_762333, EPI_ISL_762340, EPI_ISL_762351, EPI_ISL_762377, EPI_ISL_762378, EPI_ISL_762381, EPI_ISL_762392, EPI_ISL_762395, EPI_ISL_762399, EPI_ISL_762400, EPI_ISL_762408, EPI_ISL_762419, EPI_ISL_762423, EPI_ISL_762434, EPI_ISL_762436, EPI_ISL_762438, EPI_ISL_762440, EPI_ISL_762456, EPI_ISL_762462, EPI_ISL_762463, EPI_ISL_762480, EPI_ISL_762493, EPI_ISL_762494, EPI_ISL_762498, EPI_ISL_762502, EPI_ISL_762505, EPI_ISL_762509, EPI_ISL_762516, EPI_ISL_762519, EPI_ISL_762520, EPI_ISL_762523, EPI_ISL_762864, EPI_ISL_762867, EPI_ISL_762875, EPI_ISL_762880, EPI_ISL_762884, EPI_ISL_762891, EPI_ISL_762895, EPI_ISL_762896, EPI_ISL_762903, EPI_ISL_762908, EPI_ISL_762912, EPI_ISL_762914, EPI_ISL_762917, EPI_ISL_762920, EPI_ISL_762937, EPI_ISL_762943, EPI_ISL_762945, EPI_ISL_762963, EPI_ISL_762965			
see above	Lighthouse Lab in Milton Keynes	Wellcome Sanger Institute for the COVID-19 Genomics UK (COG-UK) Consortium	The Lighthouse Lab in Milton Keynes and Alex Alderton, Roberto Amato, Sonia Goncalves, Ewan Harrison, David K. Jackson, Ian Johnston, Dominic Kwiatkowski, Cordelia Langford, John Sillitoe on behalf of the Wellcome Sanger Institute COVID-19 Surveillance Team
EPI_ISL_762966	Lighthouse Lab in Glasgow	Wellcome Sanger Institute for the COVID-19 Genomics UK (COG-UK) Consortium	Harper VanSteenhouse, Yumi Kasai, David Gray, Carol Clugston, Anna Dominiczak and Alex Alderton, Roberto Amato, Sonia Goncalves, Ewan Harrison, David K. Jackson, Ian Johnston, Dominic Kwiatkowski, Cordelia Langford, John Sillitoe on behalf of the Wellcome Sanger Institute COVID-19 Surveillance Team
EPI_ISL_762967, EPI_ISL_762968, EPI_ISL_762976, EPI_ISL_762978	Lighthouse Lab in Milton Keynes	Wellcome Sanger Institute for the COVID-19 Genomics UK (COG-UK) Consortium	The Lighthouse Lab in Milton Keynes and Alex Alderton, Roberto Amato, Sonia Goncalves, Ewan Harrison, David K. Jackson, Ian Johnston, Dominic Kwiatkowski, Cordelia Langford, John Sillitoe on behalf of the Wellcome Sanger Institute COVID-19 Surveillance Team
EPI_ISL_762979	Lighthouse Lab in Glasgow	Wellcome Sanger Institute for the COVID-19 Genomics UK (COG-UK) Consortium	Harper VanSteenhouse, Yumi Kasai, David Gray, Carol Clugston, Anna Dominiczak and Alex Alderton, Roberto Amato, Sonia Goncalves, Ewan Harrison, David K. Jackson, Ian Johnston, Dominic Kwiatkowski, Cordelia Langford, John Sillitoe on behalf of the Wellcome Sanger Institute COVID-19 Surveillance Team
EPI_ISL_762981	Lighthouse Lab in Milton Keynes	Wellcome Sanger Institute for the COVID-19 Genomics UK (COG-UK) Consortium	The Lighthouse Lab in Milton Keynes and Alex Alderton, Roberto Amato, Sonia Goncalves, Ewan Harrison, David K. Jackson, Ian Johnston, Dominic Kwiatkowski, Cordelia Langford, John Sillitoe on behalf of the Wellcome Sanger Institute COVID-19 Surveillance Team
EPI_ISL_762986	Lighthouse Lab in Glasgow	Wellcome Sanger Institute for the COVID-19 Genomics UK (COG-UK) Consortium	Harper VanSteenhouse, Yumi Kasai, David Gray, Carol Clugston, Anna Dominiczak and Alex Alderton, Roberto Amato, Sonia Goncalves, Ewan Harrison, David K. Jackson, Ian Johnston, Dominic Kwiatkowski, Cordelia Langford, John Sillitoe on behalf of the Wellcome Sanger Institute COVID-19 Surveillance Team
EPI_ISL_763002, EPI_ISL_763012, EPI_ISL_763013, EPI_ISL_763036, EPI_ISL_763037, EPI_ISL_763039, EPI_ISL_763055, EPI_ISL_763056, EPI_ISL_763057, EPI_ISL_763058	Unit 17: Influenza & Other Respiratory Viruses, German National Influenza Center	Project group Epidemiology of Highly Pathogenic Microorganisms, Robert Koch-Institute	Ariane Düx, Andreas Sachse, Grit Schubert, Sébastien Calvignac-Spencer, Fabian Leendertz, Thorsten Wolff, Ralf Dürrwald, Djin-Ye Oh, Marianne Wedde
EPI_ISL_763078, EPI_ISL_763081, EPI_ISL_763082, EPI_ISL_763083	Microbiologia e Virologia	Istituto Zooprofilattico Sperimentale delle Venezie	Adelaide Milani, Alessia Schivo, Annalisa Salviato, Erika Giorgia Quaranta, Ambra Pastori, Bianca Zecchin, Alice Fusaro, Isabella Monne, Calogero Terregino, Antonia Ricci
EPI_ISL_763181, EPI_ISL_763199, EPI_ISL_763201, EPI_ISL_763206, EPI_ISL_763242, EPI_ISL_763245, EPI_ISL_763246, EPI_ISL_763287, EPI_ISL_763289, EPI_ISL_763290	Dutch COVID-19 response team	Erasmus Medical Center	Bas Oude Munnink, Reina Sikkema, David Nieuwenhuijse, Irina Chestakova, Anne van der Linden, Marjan Boter, Emmanuelle Munger, Corine GeurtsvanKessel, Annemiek van der Eijk, Richard Molenkamp, Marion Koopmans, on behalf of the Dutch national COVID-19 response team.
EPI_ISL_763374	Wales Specialist Virology Centre Sequencing lab: Pathogen Genomics Unit	COVID-19 Genomics UK (COG-UK) Consortium	Catherine Moore, Johnathan Evans, Laura Gifford, Malorie Perry, Simon Cottrell, Angela Marchbank, Alec Birchley, Alexander Adams, Amy Gaskin, Bree Gatica-Wilcox, Jason Coombes, Joel Southgate, Lauren Gilbert, Lee Graham, Nicole Pacchiarini, Sara Kumziene-Summerhayes, Sarah Taylor, Sophie Jones, Sara Rey, Matthew Bull, Joanne Watkins, Sally Corden, Tom Connor
EPI_ISL_763381	University College London, Great Ormond Street Hospital for Children NHS Foundation Trust, Imperial College Healthcare NHS Trust	COVID-19 Genomics UK (COG-UK) Consortium	Sergi Castellano, Rachel Williams, Mark Kristiansen, Paola Resende Silva, Sunando Roy, Tony Brooks, Helena Tutill, Paola Niola, Patricia Dyal, Charlotte Williams, Leysa Forrest, Yasmin Panchbhaya, Jacqueline Findlay, Samuel Weeks, Julianne Brown, Kathryn Harris, Paul Randell, James Price, Alison Holmes, Judith Breuer
EPI_ISL_763382, EPI_ISL_763383, EPI_ISL_763384, EPI_ISL_763385, EPI_ISL_763391	University of Exeter	COVID-19 Genomics UK (COG-UK) Consortium	Ben Temperton,Aaron Jeffries,Michelle Michelsen,Joanna Warwick-Dugdale,Audrey Farbos,Robyn Manley,Stephen Michell,Jane Masoli
EPI_ISL_763399	University College London, Great Ormond Street Hospital for Children NHS Foundation Trust, Imperial College Healthcare NHS Trust	COVID-19 Genomics UK (COG-UK) Consortium	Sergi Castellano, Rachel Williams, Mark Kristiansen, Paola Resende Silva, Sunando Roy, Tony Brooks, Helena Tutill, Paola Niola, Patricia Dyal, Charlotte Williams, Leysa Forrest, Yasmin Panchbhaya, Jacqueline Findlay, Samuel Weeks, Julianne Brown, Kathryn Harris, Paul Randell, James Price, Alison Holmes, Judith Breuer
EPI_ISL_763413	Wales Specialist Virology Centre Sequencing lab: Pathogen Genomics Unit	COVID-19 Genomics UK (COG-UK) Consortium	Catherine Moore, Johnathan Evans, Laura Gifford, Malorie Perry, Simon Cottrell, Angela Marchbank, Alec Birchley, Alexander Adams, Amy Gaskin, Bree Gatica-Wilcox, Jason Coombes, Joel Southgate, Lauren Gilbert, Lee Graham, Nicole Pacchiarini, Sara Kumziene-Summerhayes, Sarah Taylor, Sophie Jones, Sara Rey, Matthew Bull, Joanne Watkins, Sally Corden, Tom Connor
EPI_ISL_763414	University of Exeter	COVID-19 Genomics UK (COG-UK) Consortium	Ben Temperton,Aaron Jeffries,Michelle Michelsen,Joanna Warwick-Dugdale,Audrey Farbos,Robyn Manley,Stephen Michell,Jane Masoli
EPI_ISL_763418	Wales Specialist Virology Centre Sequencing lab: Pathogen Genomics Unit	COVID-19 Genomics UK (COG-UK) Consortium	Catherine Moore, Johnathan Evans, Laura Gifford, Malorie Perry, Simon Cottrell, Angela Marchbank, Alec Birchley, Alexander Adams, Amy Gaskin, Bree Gatica-Wilcox, Jason Coombes, Joel Southgate, Lauren Gilbert, Lee Graham, Nicole Pacchiarini, Sara Kumziene-Summerhayes, Sarah Taylor, Sophie Jones, Sara Rey, Matthew Bull, Joanne Watkins, Sally Corden, Tom Connor

EPI_ISL_763425	Centre for Enzyme Innovation, University of Portsmouth / Translational Research Laboratory, Portsmouth Hospitals NHS Trust	COVID-19 Genomics UK (COG-UK) Consortium	Angela Beckett,Yann Bourgeois,Garry Scarlett,Sharon Glaysher,Scott Elliott,Kelly Bicknell,Robert Impey,Allyson Lloyd,Sarah Wyllie,Ethan Butcher,Anoop Chauhan,Samuel Robson
EPI_ISL_763432, EPI_ISL_763433	University of Exeter	COVID-19 Genomics UK (COG-UK) Consortium	Ben Temperton,Aaron Jeffries,Michelle Michelsen,Joanna Warwick-Dugdale,Audrey Farbos,Robyn Manley,Stephen Michell,Jane Masoli
EPI_ISL_763441	Wales Specialist Virology Centre Sequencing lab: Pathogen Genomics Unit	COVID-19 Genomics UK (COG-UK) Consortium	Catherine Moore, Johnathan Evans, Laura Gifford, Malorie Perry, Simon Cottrell, Angela Marchbank, Alec Birchley, Alexander Adams, Amy Gaskin, Bree Gatica-Wilcox, Jason Coombes, Joel Southgate, Lauren Gilbert, Lee Graham, Nicole Pacchiarini, Sara Kumziene-Summerhayes, Sarah Taylor, Sophie Jones, Sara Rey, Matthew Bull, Joanne Watkins, Sally Corden, Tom Connor
EPI_ISL_763452	University of Exeter	COVID-19 Genomics UK (COG-UK) Consortium	Ben Temperton,Aaron Jeffries,Michelle Michelsen,Joanna Warwick-Dugdale,Audrey Farbos,Robyn Manley,Stephen Michell,Jane Masoli
EPI_ISL_763455	University College London, Great Ormond Street Hospital for Children NHS Foundation Trust, Imperial College Healthcare NHS Trust	COVID-19 Genomics UK (COG-UK) Consortium	Sergi Castellano, Rachel Williams, Mark Kristiansen, Paola Resende Silva, Sunando Roy, Tony Brooks, Helena Tutill, Paola Niola, Patricia Dyal, Charlotte Williams, Leysa Forrest, Yasmin Panchbhaya, Jacqueline Findlay, Samuel Weeks, Julianne Brown, Kathryn Harris, Paul Randell, James Price, Alison Holmes, Judith Breuer
EPI_ISL_763458	Wales Specialist Virology Centre Sequencing lab: Pathogen Genomics Unit	COVID-19 Genomics UK (COG-UK) Consortium	Catherine Moore, Johnathan Evans, Laura Gifford, Malorie Perry, Simon Cottrell, Angela Marchbank, Alec Birchley, Alexander Adams, Amy Gaskin, Bree Gatica-Wilcox, Jason Coombes, Joel Southgate, Lauren Gilbert, Lee Graham, Nicole Pacchiarini, Sara Kumziene-Summerhayes, Sarah Taylor, Sophie Jones, Sara Rey, Matthew Bull, Joanne Watkins, Sally Corden, Tom Connor
EPI_ISL_763459, EPI_ISL_763460	University of Exeter	COVID-19 Genomics UK (COG-UK) Consortium	Ben Temperton,Aaron Jeffries,Michelle Michelsen,Joanna Warwick-Dugdale,Audrey Farbos,Robyn Manley,Stephen Michell,Jane Masoli
EPI_ISL_763472, EPI_ISL_763493	Wales Specialist Virology Centre Sequencing lab: Pathogen Genomics Unit	COVID-19 Genomics UK (COG-UK) Consortium	Catherine Moore, Johnathan Evans, Laura Gifford, Malorie Perry, Simon Cottrell, Angela Marchbank, Alec Birchley, Alexander Adams, Amy Gaskin, Bree Gatica-Wilcox, Jason Coombes, Joel Southgate, Lauren Gilbert, Lee Graham, Nicole Pacchiarini, Sara Kumziene-Summerhayes, Sarah Taylor, Sophie Jones, Sara Rey, Matthew Bull, Joanne Watkins, Sally Corden, Tom Connor
EPI_ISL_763496	University of Exeter	COVID-19 Genomics UK (COG-UK) Consortium	Ben Temperton,Aaron Jeffries,Michelle Michelsen,Joanna Warwick-Dugdale,Audrey Farbos,Robyn Manley,Stephen Michell,Jane Masoli
EPI_ISL_763497	Wales Specialist Virology Centre Sequencing lab: Pathogen Genomics Unit	COVID-19 Genomics UK (COG-UK) Consortium	Catherine Moore, Johnathan Evans, Laura Gifford, Malorie Perry, Simon Cottrell, Angela Marchbank, Alec Birchley, Alexander Adams, Amy Gaskin, Bree Gatica-Wilcox, Jason Coombes, Joel Southgate, Lauren Gilbert, Lee Graham, Nicole Pacchiarini, Sara Kumziene-Summerhayes, Sarah Taylor, Sophie Jones, Sara Rey, Matthew Bull, Joanne Watkins, Sally Corden, Tom Connor
EPI_ISL_763520	University College London, Great Ormond Street Hospital for Children NHS Foundation Trust, Imperial College Healthcare NHS Trust	COVID-19 Genomics UK (COG-UK) Consortium	Sergi Castellano, Rachel Williams, Mark Kristiansen, Paola Resende Silva, Sunando Roy, Tony Brooks, Helena Tutill, Paola Niola, Patricia Dyal, Charlotte Williams, Leysa Forrest, Yasmin Panchbhaya, Jacqueline Findlay, Samuel Weeks, Julianne Brown, Kathryn Harris, Paul Randell, James Price, Alison Holmes, Judith Breuer
EPI_ISL_763526	Department of Pathology, University of Cambridge	COVID-19 Genomics UK (COG-UK) Consortium	Aminu S. Jahun, Yasmin Chaudhry, Grant Hall, Iliana Georgana, Myra Hosmillo, Martin D. Curran, Malte Pinckert, Surendra Parmar, Ian Goodfellow
EPI_ISL_763527, EPI_ISL_763528, EPI_ISL_763529, EPI_ISL_763530, EPI_ISL_763531, EPI_ISL_763532	University of Exeter	COVID-19 Genomics UK (COG-UK) Consortium	Ben Temperton,Aaron Jeffries,Michelle Michelsen,Joanna Warwick-Dugdale,Audrey Farbos,Robyn Manley,Stephen Michell,Jane Masoli
EPI_ISL_763539	Wales Specialist Virology Centre Sequencing lab: Pathogen Genomics Unit	COVID-19 Genomics UK (COG-UK) Consortium	Catherine Moore, Johnathan Evans, Laura Gifford, Malorie Perry, Simon Cottrell, Angela Marchbank, Alec Birchley, Alexander Adams, Amy Gaskin, Bree Gatica-Wilcox, Jason Coombes, Joel Southgate, Lauren Gilbert, Lee Graham, Nicole Pacchiarini, Sara Kumziene-Summerhayes, Sarah Taylor, Sophie Jones, Sara Rey, Matthew Bull, Joanne Watkins, Sally Corden, Tom Connor
EPI_ISL_763543	University of Exeter	COVID-19 Genomics UK (COG-UK) Consortium	Ben Temperton,Aaron Jeffries,Michelle Michelsen,Joanna Warwick-Dugdale,Audrey Farbos,Robyn Manley,Stephen Michell,Jane Masoli
EPI_ISL_763544	University College London, Great Ormond Street Hospital for Children NHS Foundation Trust, Imperial College Healthcare NHS Trust	COVID-19 Genomics UK (COG-UK) Consortium	Sergi Castellano, Rachel Williams, Mark Kristiansen, Paola Resende Silva, Sunando Roy, Tony Brooks, Helena Tutill, Paola Niola, Patricia Dyal, Charlotte Williams, Leysa Forrest, Yasmin Panchbhaya, Jacqueline Findlay, Samuel Weeks, Julianne Brown, Kathryn Harris, Paul Randell, James Price, Alison Holmes, Judith Breuer
EPI_ISL_763546	Wales Specialist Virology Centre Sequencing lab: Pathogen Genomics Unit	COVID-19 Genomics UK (COG-UK) Consortium	Catherine Moore, Johnathan Evans, Laura Gifford, Malorie Perry, Simon Cottrell, Angela Marchbank, Alec Birchley, Alexander Adams, Amy Gaskin, Bree Gatica-Wilcox, Jason Coombes, Joel Southgate, Lauren Gilbert, Lee Graham, Nicole Pacchiarini, Sara Kumziene-Summerhayes, Sarah Taylor, Sophie Jones, Sara Rey, Matthew Bull, Joanne Watkins, Sally Corden, Tom Connor
EPI_ISL_763553, EPI_ISL_763554, EPI_ISL_763555, EPI_ISL_763556, EPI_ISL_763563, EPI_ISL_763564	University of Exeter	COVID-19 Genomics UK (COG-UK) Consortium	Ben Temperton,Aaron Jeffries,Michelle Michelsen,Joanna Warwick-Dugdale,Audrey Farbos,Robyn Manley,Stephen Michell,Jane Masoli
EPI_ISL_763567	University College London, Great Ormond Street Hospital for Children NHS Foundation Trust, Imperial College Healthcare NHS Trust	COVID-19 Genomics UK (COG-UK) Consortium	Sergi Castellano, Rachel Williams, Mark Kristiansen, Paola Resende Silva, Sunando Roy, Tony Brooks, Helena Tutill, Paola Niola, Patricia Dyal, Charlotte Williams, Leysa Forrest, Yasmin Panchbhaya, Jacqueline Findlay, Samuel Weeks, Julianne Brown, Kathryn Harris, Paul Randell, James Price, Alison Holmes, Judith Breuer
EPI_ISL_763568, EPI_ISL_763574, EPI_ISL_763575, EPI_ISL_763576	Wales Specialist Virology Centre Sequencing lab: Pathogen Genomics Unit	COVID-19 Genomics UK (COG-UK) Consortium	Catherine Moore, Johnathan Evans, Laura Gifford, Malorie Perry, Simon Cottrell, Angela Marchbank, Alec Birchley, Alexander Adams, Amy Gaskin, Bree Gatica-Wilcox, Jason Coombes, Joel Southgate, Lauren Gilbert, Lee Graham, Nicole Pacchiarini, Sara Kumziene-Summerhayes, Sarah Taylor, Sophie Jones, Sara Rey, Matthew Bull, Joanne Watkins, Sally Corden, Tom Connor
EPI_ISL_763578	University College London, Great Ormond Street Hospital for Children NHS Foundation Trust, Imperial College Healthcare NHS Trust	COVID-19 Genomics UK (COG-UK) Consortium	Sergi Castellano, Rachel Williams, Mark Kristiansen, Paola Resende Silva, Sunando Roy, Tony Brooks, Helena Tutill, Paola Niola, Patricia Dyal, Charlotte Williams, Leysa Forrest, Yasmin Panchbhaya, Jacqueline Findlay, Samuel Weeks, Julianne Brown, Kathryn Harris, Paul Randell, James Price, Alison Holmes, Judith Breuer
EPI_ISL_763584, EPI_ISL_763591, EPI_ISL_763594	Wales Specialist Virology Centre Sequencing lab: Pathogen Genomics Unit	COVID-19 Genomics UK (COG-UK) Consortium	Catherine Moore, Johnathan Evans, Laura Gifford, Malorie Perry, Simon Cottrell, Angela Marchbank, Alec Birchley, Alexander Adams, Amy Gaskin, Bree Gatica-Wilcox, Jason Coombes, Joel Southgate, Lauren Gilbert, Lee Graham, Nicole Pacchiarini, Sara Kumziene-Summerhayes, Sarah Taylor, Sophie Jones, Sara Rey, Matthew Bull, Joanne Watkins, Sally Corden, Tom Connor
EPI_ISL_763607, EPI_ISL_763610	Centre for Enzyme Innovation, University of Portsmouth / Translational Research Laboratory, Portsmouth Hospitals NHS Trust	COVID-19 Genomics UK (COG-UK) Consortium	Angela Beckett,Yann Bourgeois,Garry Scarlett,Sharon Glaysher,Scott Elliott,Kelly Bicknell,Robert Impey,Allyson Lloyd,Sarah Wyllie,Ethan Butcher,Anoop Chauhan,Samuel Robson
EPI_ISL_763629, EPI_ISL_763630, EPI_ISL_763631	University of Exeter	COVID-19 Genomics UK (COG-UK) Consortium	Ben Temperton,Aaron Jeffries,Michelle Michelsen,Joanna Warwick-Dugdale,Audrey Farbos,Robyn Manley,Stephen Michell,Jane Masoli
EPI_ISL_763638	Department of Pathology, University of Cambridge	COVID-19 Genomics UK (COG-UK) Consortium	Aminu S. Jahun, Yasmin Chaudhry, Grant Hall, Iliana Georgana, Myra Hosmillo, Martin D. Curran, Malte Pinckert, Surendra Parmar, Ian Goodfellow
EPI_ISL_763668	Wales Specialist Virology Centre Sequencing lab: Pathogen Genomics Unit	COVID-19 Genomics UK (COG-UK) Consortium	Catherine Moore, Johnathan Evans, Laura Gifford, Malorie Perry, Simon Cottrell, Angela Marchbank, Alec Birchley, Alexander Adams, Amy Gaskin, Bree Gatica-Wilcox, Jason Coombes, Joel Southgate, Lauren Gilbert, Lee Graham, Nicole Pacchiarini, Sara Kumziene-Summerhayes, Sarah Taylor, Sophie Jones, Sara Rey, Matthew Bull, Joanne Watkins, Sally Corden, Tom Connor
EPI_ISL_763669	University of Exeter	COVID-19 Genomics UK (COG-UK) Consortium	Ben Temperton,Aaron Jeffries,Michelle Michelsen,Joanna Warwick-Dugdale,Audrey Farbos,Robyn Manley,Stephen Michell,Jane Masoli
EPI_ISL_763679	Wales Specialist Virology Centre Sequencing lab: Pathogen Genomics Unit	COVID-19 Genomics UK (COG-UK) Consortium	Catherine Moore, Johnathan Evans, Laura Gifford, Malorie Perry, Simon Cottrell, Angela Marchbank, Alec Birchley, Alexander Adams, Amy Gaskin, Bree Gatica-Wilcox, Jason Coombes, Joel Southgate, Lauren Gilbert, Lee Graham, Nicole Pacchiarini, Sara Kumziene-Summerhayes, Sarah Taylor, Sophie Jones, Sara Rey, Matthew Bull, Joanne Watkins, Sally Corden, Tom Connor
EPI_ISL_763684	Quadram Institute Bioscience	COVID-19 Genomics UK (COG-UK) Consortium	Dave J. Baker, Gemma L. Kay, Alp Aydin, Thanh Le-Viet, Steven Rudder, Ana P. Tedim, Anastasia Kolyva, Maria Diaz, Leonardo de Oliveira Martins, Nabil-Fareed Alikhan, Lizzie Meadows, Rachael Stanley, Ngozi Elumogo, Muhammed Yasir, Nicholas M. Thomson, Alexander J Trotter, Rachel Gilroy, Samuel Bloomfield, Claire Stuart, Andrew Bell, Reenesh Prakash, Samir Dervisevic, Alison E. Mather, John Wain, Mark Webber, Andrew J. Page, Justin O'Grady
EPI_ISL_763697, EPI_ISL_763698, EPI_ISL_763699	University of Exeter	COVID-19 Genomics UK (COG-UK) Consortium	Ben Temperton,Aaron Jeffries,Michelle Michelsen,Joanna Warwick-Dugdale,Audrey Farbos,Robyn Manley,Stephen Michell,Jane Masoli

EPI_ISL_763700	University College London, Great Ormond Street Hospital for Children NHS Foundation Trust, Imperial College Healthcare NHS Trust	COVID-19 Genomics UK (COG-UK) Consortium	Sergi Castellano, Rachel Williams, Mark Kristiansen, Paola Resende Silva, Sunando Roy, Tony Brooks, Helena Tutill, Paola Niola, Patricia Dyal, Charlotte Williams, Leysa Forrest, Yasmin Panchbhaya, Jacqueline Findlay, Samuel Weeks, Julianne Brown, Kathryn Harris, Paul Randell, James Price, Alison Holmes, Judith Breuer
EPI_ISL_763709, EPI_ISL_763710, EPI_ISL_763711, EPI_ISL_763712	University of Exeter	COVID-19 Genomics UK (COG-UK) Consortium	Ben Temperton,Aaron Jeffries,Michelle Michelsen,Joanna Warwick-Dugdale,Audrey Farbos,Robyn Manley,Stephen Michell,Jane Masoli
EPI_ISL_763724, EPI_ISL_763733, EPI_ISL_763739	Wales Specialist Virology Centre Sequencing lab: Pathogen Genomics Unit	COVID-19 Genomics UK (COG-UK) Consortium	Catherine Moore, Johnathan Evans, Laura Gifford, Malorie Perry, Simon Cottrell, Angela Marchbank, Alec Birchley, Alexander Adams, Amy Gaskin, Bree Gatica-Wilcox, Jason Coombes, Joel Southgate, Lauren Gilbert, Lee Graham, Nicole Pacchiarini, Sara Kumziene-Summerhayes, Sarah Taylor, Sophie Jones, Sara Rey, Matthew Bull, Joanne Watkins, Sally Corden, Tom Connor
EPI_ISL_763740	Virology Department, Sheffield Teaching Hospitals NHS Foundation Trust/Department of Infection, Immunity and Cardiovascular Disease, The Medical School, University of Sheffield	COVID-19 Genomics UK (COG-UK) Consortium	Thushan de Silva, Matthew Parker, Nikki Smith, Adri Angyal, Rebecca Brown, Luke Green, Rachel Tucker, Paul Parsons, Danielle Groves, Katie Johnson, Laura Carrilero, Alex Keeley, Dave Partridge, Matthew Wyles, Benjamin Lindsey, Mehmet Yavuz, Mohammad Raza, Cariad Evans
EPI_ISL_763751, EPI_ISL_763754	Wales Specialist Virology Centre Sequencing lab: Pathogen Genomics Unit	COVID-19 Genomics UK (COG-UK) Consortium	Catherine Moore, Johnathan Evans, Laura Gifford, Malorie Perry, Simon Cottrell, Angela Marchbank, Alec Birchley, Alexander Adams, Amy Gaskin, Bree Gatica-Wilcox, Jason Coombes, Joel Southgate, Lauren Gilbert, Lee Graham, Nicole Pacchiarini, Sara Kumziene-Summerhayes, Sarah Taylor, Sophie Jones, Sara Rey, Matthew Bull, Joanne Watkins, Sally Corden, Tom Connor
EPI_ISL_763757	University College London, Great Ormond Street Hospital for Children NHS Foundation Trust, Imperial College Healthcare NHS Trust	COVID-19 Genomics UK (COG-UK) Consortium	Sergi Castellano, Rachel Williams, Mark Kristiansen, Paola Resende Silva, Sunando Roy, Tony Brooks, Helena Tutill, Paola Niola, Patricia Dyal, Charlotte Williams, Leysa Forrest, Yasmin Panchbhaya, Jacqueline Findlay, Samuel Weeks, Julianne Brown, Kathryn Harris, Paul Randell, James Price, Alison Holmes, Judith Breuer
EPI_ISL_763762	Wales Specialist Virology Centre Sequencing lab: Pathogen Genomics Unit	COVID-19 Genomics UK (COG-UK) Consortium	Catherine Moore, Johnathan Evans, Laura Gifford, Malorie Perry, Simon Cottrell, Angela Marchbank, Alec Birchley, Alexander Adams, Amy Gaskin, Bree Gatica-Wilcox, Jason Coombes, Joel Southgate, Lauren Gilbert, Lee Graham, Nicole Pacchiarini, Sara Kumziene-Summerhayes, Sarah Taylor, Sophie Jones, Sara Rey, Matthew Bull, Joanne Watkins, Sally Corden, Tom Connor
EPI_ISL_763763	University of Exeter	COVID-19 Genomics UK (COG-UK) Consortium	Ben Temperton,Aaron Jeffries,Michelle Michelsen,Joanna Warwick-Dugdale,Audrey Farbos,Robyn Manley,Stephen Michell,Jane Masoli
EPI_ISL_763764, EPI_ISL_763770, EPI_ISL_763778, EPI_ISL_763788	Wales Specialist Virology Centre Sequencing lab: Pathogen Genomics Unit	COVID-19 Genomics UK (COG-UK) Consortium	Catherine Moore, Johnathan Evans, Laura Gifford, Malorie Perry, Simon Cottrell, Angela Marchbank, Alec Birchley, Alexander Adams, Amy Gaskin, Bree Gatica-Wilcox, Jason Coombes, Joel Southgate, Lauren Gilbert, Lee Graham, Nicole Pacchiarini, Sara Kumziene-Summerhayes, Sarah Taylor, Sophie Jones, Sara Rey, Matthew Bull, Joanne Watkins, Sally Corden, Tom Connor
EPI_ISL_763838, EPI_ISL_763844	Oxford Viromics, NDM, University of Oxford; Oxford University Hospitals; Basingstoke and North Hampshire Hospital	COVID-19 Genomics UK (COG-UK) Consortium	Tanya Golubchik, David Bonsall, George Macintyre, Amy Trebes, Mariateresa de Cesare, Catrin Moore, Alex Mobbs, Anita Justice, Robert Shaw, Monique Andersson, Timothy Peto, Emma Wise, Nathan Moore, Jessica Lynch, Nick Cortes, Matilde Mori, Stephen Kidd, David Buck, John Todd, Christophe Fraser
EPI_ISL_763849	Department of Pathology, University of Cambridge	COVID-19 Genomics UK (COG-UK) Consortium	Aminu S. Jahun, Yasmin Chaudhry, Grant Hall, Iliana Georgana, Myra Hosmillo, Martin D. Curran, Malte Pinckert, Surendra Parmar, Ian Goodfellow
EPI_ISL_763860, EPI_ISL_763862, EPI_ISL_763863	Oxford Viromics, NDM, University of Oxford; Oxford University Hospitals; Basingstoke and North Hampshire Hospital	COVID-19 Genomics UK (COG-UK) Consortium	Tanya Golubchik, David Bonsall, George Macintyre, Amy Trebes, Mariateresa de Cesare, Catrin Moore, Alex Mobbs, Anita Justice, Robert Shaw, Monique Andersson, Timothy Peto, Emma Wise, Nathan Moore, Jessica Lynch, Nick Cortes, Matilde Mori, Stephen Kidd, David Buck, John Todd, Christophe Fraser
EPI_ISL_763874, EPI_ISL_763875, EPI_ISL_763876, EPI_ISL_763877, EPI_ISL_763878, EPI_ISL_763879, EPI_ISL_763880, EPI_ISL_763881	Department of Pathology, University of Cambridge	COVID-19 Genomics UK (COG-UK) Consortium	Aminu S. Jahun, Yasmin Chaudhry, Grant Hall, Iliana Georgana, Myra Hosmillo, Martin D. Curran, Malte Pinckert, Surendra Parmar, Ian Goodfellow
EPI_ISL_763892, EPI_ISL_763902, EPI_ISL_763904	Virology Department, Royal Infirmary of Edinburgh, NHS Lothian / School of Biological Sciences, University of Edinburgh / Institute of Genetics and Molecular Medicine, University of Edinburgh	COVID-19 Genomics UK (COG-UK) Consortium	McHugh M, Dewar R, Rooke S, Gallagher M, Balcaza C, O'Toole Á, Scher E, Hill V, McCrone JT, Colquhoun R, Yu X, Jackson B, Rambaut A, Williams TC, Templeton K
EPI_ISL_763928, EPI_ISL_763929, EPI_ISL_763930, EPI_ISL_763931, EPI_ISL_763936, EPI_ISL_763937, EPI_ISL_763938, EPI_ISL_763939, EPI_ISL_763943, EPI_ISL_763944, EPI_ISL_763949, EPI_ISL_763950, EPI_ISL_763951, EPI_ISL_763952, EPI_ISL_763953, EPI_ISL_763954, EPI_ISL_763955, EPI_ISL_763956, EPI_ISL_763957, EPI_ISL_763958, EPI_ISL_763959, EPI_ISL_763960, EPI_ISL_763961, EPI_ISL_763962, EPI_ISL_763963, EPI_ISL_763964, EPI_ISL_763965, EPI_ISL_763966, EPI_ISL_763967, EPI_ISL_763968, EPI_ISL_763969, EPI_ISL_763970, EPI_ISL_763971, EPI_ISL_763972, EPI_ISL_763973, EPI_ISL_763974			
see above	Oxford Viromics, NDM, University of Oxford; Oxford University Hospitals; Basingstoke and North Hampshire Hospital	COVID-19 Genomics UK (COG-UK) Consortium	Tanya Golubchik, David Bonsall, George Macintyre, Amy Trebes, Mariateresa de Cesare, Catrin Moore, Alex Mobbs, Anita Justice, Robert Shaw, Monique Andersson, Timothy Peto, Emma Wise, Nathan Moore, Jessica Lynch, Nick Cortes, Matilde Mori, Stephen Kidd, David Buck, John Todd, Christophe Fraser
EPI_ISL_763986, EPI_ISL_763990	Wales Specialist Virology Centre Sequencing lab: Pathogen Genomics Unit	COVID-19 Genomics UK (COG-UK) Consortium	Catherine Moore, Johnathan Evans, Laura Gifford, Malorie Perry, Simon Cottrell, Angela Marchbank, Alec Birchley, Alexander Adams, Amy Gaskin, Bree Gatica-Wilcox, Jason Coombes, Joel Southgate, Lauren Gilbert, Lee Graham, Nicole Pacchiarini, Sara Kumziene-Summerhayes, Sarah Taylor, Sophie Jones, Sara Rey, Matthew Bull, Joanne Watkins, Sally Corden, Tom Connor
EPI_ISL_764046, EPI_ISL_764049	University College London, Great Ormond Street Hospital for Children NHS Foundation Trust, Imperial College Healthcare NHS Trust	COVID-19 Genomics UK (COG-UK) Consortium	Sergi Castellano, Rachel Williams, Mark Kristiansen, Paola Resende Silva, Sunando Roy, Tony Brooks, Helena Tutill, Paola Niola, Patricia Dyal, Charlotte Williams, Leysa Forrest, Yasmin Panchbhaya, Jacqueline Findlay, Samuel Weeks, Julianne Brown, Kathryn Harris, Paul Randell, James Price, Alison Holmes, Judith Breuer
EPI_ISL_764061	Virology Department, Royal Infirmary of Edinburgh, NHS Lothian / School of Biological Sciences, University of Edinburgh / Institute of Genetics and Molecular Medicine, University of Edinburgh	COVID-19 Genomics UK (COG-UK) Consortium	McHugh M, Dewar R, Rooke S, Gallagher M, Balcaza C, O'Toole Á, Scher E, Hill V, McCrone JT, Colquhoun R, Yu X, Jackson B, Rambaut A, Williams TC, Templeton K
EPI_ISL_764062	Wales Specialist Virology Centre Sequencing lab: Pathogen Genomics Unit	COVID-19 Genomics UK (COG-UK) Consortium	Catherine Moore, Johnathan Evans, Laura Gifford, Malorie Perry, Simon Cottrell, Angela Marchbank, Alec Birchley, Alexander Adams, Amy Gaskin, Bree Gatica-Wilcox, Jason Coombes, Joel Southgate, Lauren Gilbert, Lee Graham, Nicole Pacchiarini, Sara Kumziene-Summerhayes, Sarah Taylor, Sophie Jones, Sara Rey, Matthew Bull, Joanne Watkins, Sally Corden, Tom Connor
EPI_ISL_764067, EPI_ISL_764068	Oxford Viromics, NDM, University of Oxford; Oxford University Hospitals; Basingstoke and North Hampshire Hospital	COVID-19 Genomics UK (COG-UK) Consortium	Tanya Golubchik, David Bonsall, George Macintyre, Amy Trebes, Mariateresa de Cesare, Catrin Moore, Alex Mobbs, Anita Justice, Robert Shaw, Monique Andersson, Timothy Peto, Emma Wise, Nathan Moore, Jessica Lynch, Nick Cortes, Matilde Mori, Stephen Kidd, David Buck, John Todd, Christophe Fraser
EPI_ISL_764070, EPI_ISL_764071, EPI_ISL_764072, EPI_ISL_764073, EPI_ISL_764074, EPI_ISL_764075	Virology Department, Royal Infirmary of Edinburgh, NHS Lothian / School of Biological Sciences, University of Edinburgh / Institute of Genetics and Molecular Medicine, University of Edinburgh	COVID-19 Genomics UK (COG-UK) Consortium	McHugh M, Dewar R, Rooke S, Gallagher M, Balcaza C, O'Toole Á, Scher E, Hill V, McCrone JT, Colquhoun R, Yu X, Jackson B, Rambaut A, Williams TC, Templeton K
EPI_ISL_764084	University of Exeter	COVID-19 Genomics UK (COG-UK) Consortium	Ben Temperton,Aaron Jeffries,Michelle Michelsen,Joanna Warwick-Dugdale,Audrey Farbos,Robyn Manley,Stephen Michell,Jane Masoli
EPI_ISL_764085, EPI_ISL_764089, EPI_ISL_764090, EPI_ISL_764091, EPI_ISL_764092, EPI_ISL_764093	Oxford Viromics, NDM, University of Oxford; Oxford University Hospitals; Basingstoke and North Hampshire Hospital	COVID-19 Genomics UK (COG-UK) Consortium	Tanya Golubchik, David Bonsall, George Macintyre, Amy Trebes, Mariateresa de Cesare, Catrin Moore, Alex Mobbs, Anita Justice, Robert Shaw, Monique Andersson, Timothy Peto, Emma Wise, Nathan Moore, Jessica Lynch, Nick Cortes, Matilde Mori, Stephen Kidd, David Buck, John Todd, Christophe Fraser
EPI_ISL_764104, EPI_ISL_764108, EPI_ISL_764109	Virology Department, Sheffield Teaching Hospitals NHS Foundation Trust/Department of Infection, Immunity and Cardiovascular Disease, The Medical School, University of Sheffield	COVID-19 Genomics UK (COG-UK) Consortium	Thushan de Silva, Matthew Parker, Nikki Smith, Adri Angyal, Rebecca Brown, Luke Green, Rachel Tucker, Paul Parsons, Danielle Groves, Katie Johnson, Laura Carrilero, Alex Keeley, Dave Partridge, Matthew Wyles, Benjamin Lindsey, Mehmet Yavuz, Mohammad Raza, Cariad Evans
EPI_ISL_764158	University of Exeter	COVID-19 Genomics UK (COG-UK) Consortium	Ben Temperton,Aaron Jeffries,Michelle Michelsen,Joanna Warwick-Dugdale,Audrey Farbos,Robyn Manley,Stephen Michell,Jane Masoli
EPI_ISL_764161	Wales Specialist Virology Centre Sequencing lab: Pathogen Genomics Unit	COVID-19 Genomics UK (COG-UK) Consortium	Catherine Moore, Johnathan Evans, Laura Gifford, Malorie Perry, Simon Cottrell, Angela Marchbank, Alec Birchley, Alexander Adams, Amy Gaskin, Bree Gatica-Wilcox, Jason Coombes, Joel Southgate, Lauren Gilbert, Lee Graham, Nicole Pacchiarini, Sara Kumziene-Summerhayes, Sarah Taylor, Sophie Jones, Sara Rey, Matthew Bull, Joanne Watkins, Sally Corden, Tom Connor
EPI_ISL_764162	Department of Pathology, University of Cambridge	COVID-19 Genomics UK (COG-UK) Consortium	Aminu S. Jahun, Yasmin Chaudhry, Grant Hall, Iliana Georgana, Myra Hosmillo, Martin D. Curran, Malte Pinckert, Surendra Parmar, Ian Goodfellow

EPI_ISL_764163, EPI_ISL_764166	University of Exeter	COVID-19 Genomics UK (COG-UK) Consortium	Ben Temperton,Aaron Jeffries,Michelle Michelsen,Joanna Warwick-Dugdale,Audrey Farbos,Robyn Manley,Stephen Michell,Jane Masoli
EPI_ISL_764175	University College London, Great Ormond Street Hospital for Children NHS Foundation Trust, Imperial College Healthcare NHS Trust	COVID-19 Genomics UK (COG-UK) Consortium	Sergi Castellano, Rachel Williams, Mark Kristiansen, Paola Resende Silva, Sunando Roy, Tony Brooks, Helena Tutill, Paola Niola, Patricia Dyal, Charlotte Williams, Leysa Forrest, Yasmin Panchbhaya, Jacqueline Findlay, Samuel Weeks, Julianne Brown, Kathryn Harris, Paul Randell, James Price, Alison Holmes, Judith Breuer
EPI_ISL_764179	University of Exeter	COVID-19 Genomics UK (COG-UK) Consortium	Ben Temperton,Aaron Jeffries,Michelle Michelsen,Joanna Warwick-Dugdale,Audrey Farbos,Robyn Manley,Stephen Michell,Jane Masoli
EPI_ISL_764180	Wales Specialist Virology Centre Sequencing lab: Pathogen Genomics Unit	COVID-19 Genomics UK (COG-UK) Consortium	Catherine Moore, Johnathan Evans, Laura Gifford, Malorie Perry, Simon Cottrell, Angela Marchbank, Alec Birchley, Alexander Adams, Amy Gaskin, Bree Gatica-Wilcox, Jason Coombes, Joel Southgate, Lauren Gilbert, Lee Graham, Nicole Pacchiarini, Sara Kumziene-Summerhayes, Sarah Taylor, Sophie Jones, Sara Rey, Matthew Bull, Joanne Watkins, Sally Corden, Tom Connor
EPI_ISL_764181, EPI_ISL_764183, EPI_ISL_764184, EPI_ISL_764197	University of Exeter	COVID-19 Genomics UK (COG-UK) Consortium	Ben Temperton,Aaron Jeffries,Michelle Michelsen,Joanna Warwick-Dugdale,Audrey Farbos,Robyn Manley,Stephen Michell,Jane Masoli
EPI_ISL_764202	Department of Pathology, University of Cambridge	COVID-19 Genomics UK (COG-UK) Consortium	Aminu S. Jahun, Yasmin Chaudhry, Grant Hall, Iliana Georgana, Myra Hosmillo, Martin D. Curran, Malte Pinckert, Surendra Parmar, Ian Goodfellow
EPI_ISL_764207	Quadram Institute Bioscience	COVID-19 Genomics UK (COG-UK) Consortium	Dave J. Baker, Gemma L. Kay, Alp Aydin, Thanh Le-Viet, Steven Rudder, Ana P. Tedim, Anastasia Kolyva, Maria Diaz, Leonardo de Oliveira Martins, Nabil-Fareed Alikhan, Lizzie Meadows, Rachael Stanley, Ngozi Elumogo, Muhammed Yasir, Nicholas M. Thomson, Alexander J Trotter, Rachel Gilroy, Samuel Bloomfield, Claire Stuart, Andrew Bell, Reenesh Prakash, Samir Dervisevic, Alison E. Mather, John Wain, Mark Webber, Andrew J. Page, Justin O'Grady
EPI_ISL_764211, EPI_ISL_764217, EPI_ISL_764218, EPI_ISL_764219, EPI_ISL_764220, EPI_ISL_764223, EPI_ISL_764240	University of Exeter	COVID-19 Genomics UK (COG-UK) Consortium	Ben Temperton,Aaron Jeffries,Michelle Michelsen,Joanna Warwick-Dugdale,Audrey Farbos,Robyn Manley,Stephen Michell,Jane Masoli
EPI_ISL_764242	Wales Specialist Virology Centre Sequencing lab: Pathogen Genomics Unit	COVID-19 Genomics UK (COG-UK) Consortium	Catherine Moore, Johnathan Evans, Laura Gifford, Malorie Perry, Simon Cottrell, Angela Marchbank, Alec Birchley, Alexander Adams, Amy Gaskin, Bree Gatica-Wilcox, Jason Coombes, Joel Southgate, Lauren Gilbert, Lee Graham, Nicole Pacchiarini, Sara Kumziene-Summerhayes, Sarah Taylor, Sophie Jones, Sara Rey, Matthew Bull, Joanne Watkins, Sally Corden, Tom Connor
EPI_ISL_764243	Oxford Viromics, NDM, University of Oxford; Oxford University Hospitals; Basingstoke and North Hampshire Hospital	COVID-19 Genomics UK (COG-UK) Consortium	Tanya Golubchik, David Bonsall, George Macintyre, Amy Trebes, Mariateresa de Cesare, Catrin Moore, Alex Mobbs, Anita Justice, Robert Shaw, Monique Andersson, Timothy Peto, Emma Wise, Nathan Moore, Jessica Lynch, Nick Cortes, Matilde Mori, Stephen Kidd, David Buck, John Todd, Christophe Fraser
EPI_ISL_764245, EPI_ISL_764247	Wales Specialist Virology Centre Sequencing lab: Pathogen Genomics Unit	COVID-19 Genomics UK (COG-UK) Consortium	Catherine Moore, Johnathan Evans, Laura Gifford, Malorie Perry, Simon Cottrell, Angela Marchbank, Alec Birchley, Alexander Adams, Amy Gaskin, Bree Gatica-Wilcox, Jason Coombes, Joel Southgate, Lauren Gilbert, Lee Graham, Nicole Pacchiarini, Sara Kumziene-Summerhayes, Sarah Taylor, Sophie Jones, Sara Rey, Matthew Bull, Joanne Watkins, Sally Corden, Tom Connor
EPI_ISL_764250	Oxford Viromics, NDM, University of Oxford; Oxford University Hospitals; Basingstoke and North Hampshire Hospital	COVID-19 Genomics UK (COG-UK) Consortium	Tanya Golubchik, David Bonsall, George Macintyre, Amy Trebes, Mariateresa de Cesare, Catrin Moore, Alex Mobbs, Anita Justice, Robert Shaw, Monique Andersson, Timothy Peto, Emma Wise, Nathan Moore, Jessica Lynch, Nick Cortes, Matilde Mori, Stephen Kidd, David Buck, John Todd, Christophe Fraser
EPI_ISL_764254, EPI_ISL_764258	Wales Specialist Virology Centre Sequencing lab: Pathogen Genomics Unit	COVID-19 Genomics UK (COG-UK) Consortium	Catherine Moore, Johnathan Evans, Laura Gifford, Malorie Perry, Simon Cottrell, Angela Marchbank, Alec Birchley, Alexander Adams, Amy Gaskin, Bree Gatica-Wilcox, Jason Coombes, Joel Southgate, Lauren Gilbert, Lee Graham, Nicole Pacchiarini, Sara Kumziene-Summerhayes, Sarah Taylor, Sophie Jones, Sara Rey, Matthew Bull, Joanne Watkins, Sally Corden, Tom Connor
EPI_ISL_764264, EPI_ISL_764265	Oxford Viromics, NDM, University of Oxford; Oxford University Hospitals; Basingstoke and North Hampshire Hospital	COVID-19 Genomics UK (COG-UK) Consortium	Tanya Golubchik, David Bonsall, George Macintyre, Amy Trebes, Mariateresa de Cesare, Catrin Moore, Alex Mobbs, Anita Justice, Robert Shaw, Monique Andersson, Timothy Peto, Emma Wise, Nathan Moore, Jessica Lynch, Nick Cortes, Matilde Mori, Stephen Kidd, David Buck, John Todd, Christophe Fraser
EPI_ISL_764267, EPI_ISL_764275, EPI_ISL_764282	Wales Specialist Virology Centre Sequencing lab: Pathogen Genomics Unit	COVID-19 Genomics UK (COG-UK) Consortium	Catherine Moore, Johnathan Evans, Laura Gifford, Malorie Perry, Simon Cottrell, Angela Marchbank, Alec Birchley, Alexander Adams, Amy Gaskin, Bree Gatica-Wilcox, Jason Coombes, Joel Southgate, Lauren Gilbert, Lee Graham, Nicole Pacchiarini, Sara Kumziene-Summerhayes, Sarah Taylor, Sophie Jones, Sara Rey, Matthew Bull, Joanne Watkins, Sally Corden, Tom Connor
EPI_ISL_764331, EPI_ISL_764335, EPI_ISL_764336, EPI_ISL_764337, EPI_ISL_764338, EPI_ISL_764339, EPI_ISL_764340, EPI_ISL_764341, EPI_ISL_764342	Department of Pathology, University of Cambridge	COVID-19 Genomics UK (COG-UK) Consortium	Aminu S. Jahun, Yasmin Chaudhry, Grant Hall, Iliana Georgana, Myra Hosmillo, Martin D. Curran, Malte Pinckert, Surendra Parmar, Ian Goodfellow
EPI_ISL_764374, EPI_ISL_764375, EPI_ISL_764376, EPI_ISL_764377	University of Exeter	COVID-19 Genomics UK (COG-UK) Consortium	Ben Temperton,Aaron Jeffries,Michelle Michelsen,Joanna Warwick-Dugdale,Audrey Farbos,Robyn Manley,Stephen Michell,Jane Masoli
EPI_ISL_764385	University College London, Great Ormond Street Hospital for Children NHS Foundation Trust, Imperial College Healthcare NHS Trust	COVID-19 Genomics UK (COG-UK) Consortium	Sergi Castellano, Rachel Williams, Mark Kristiansen, Paola Resende Silva, Sunando Roy, Tony Brooks, Helena Tutill, Paola Niola, Patricia Dyal, Charlotte Williams, Leysa Forrest, Yasmin Panchbhaya, Jacqueline Findlay, Samuel Weeks, Julianne Brown, Kathryn Harris, Paul Randell, James Price, Alison Holmes, Judith Breuer
EPI_ISL_764449, EPI_ISL_764451, EPI_ISL_764453, EPI_ISL_764457, EPI_ISL_764477	Quadram Institute Bioscience	COVID-19 Genomics UK (COG-UK) Consortium	Dave J. Baker, Gemma L. Kay, Alp Aydin, Thanh Le-Viet, Steven Rudder, Ana P. Tedim, Anastasia Kolyva, Maria Diaz, Leonardo de Oliveira Martins, Nabil-Fareed Alikhan, Lizzie Meadows, Rachael Stanley, Ngozi Elumogo, Muhammed Yasir, Nicholas M. Thomson, Alexander J Trotter, Rachel Gilroy, Samuel Bloomfield, Claire Stuart, Andrew Bell, Reenesh Prakash, Samir Dervisevic, Alison E. Mather, John Wain, Mark Webber, Andrew J. Page, Justin O'Grady
EPI_ISL_764525, EPI_ISL_764526, EPI_ISL_764527, EPI_ISL_764528, EPI_ISL_764529, EPI_ISL_764530, EPI_ISL_764531, EPI_ISL_764535, EPI_ISL_764536, EPI_ISL_764537, EPI_ISL_764543, EPI_ISL_764544, EPI_ISL_764545, EPI_ISL_764546, EPI_ISL_764548, EPI_ISL_764549, EPI_ISL_764550, EPI_ISL_764551, EPI_ISL_764552, EPI_ISL_764553, EPI_ISL_764554, EPI_ISL_764555, EPI_ISL_764556, EPI_ISL_764557, EPI_ISL_764559, EPI_ISL_764561, EPI_ISL_764564, EPI_ISL_764565, EPI_ISL_764567, EPI_ISL_764568, EPI_ISL_764569, EPI_ISL_764570, EPI_ISL_764572			
see above	Oxford Viromics, NDM, University of Oxford; Oxford University Hospitals; Basingstoke and North Hampshire Hospital	COVID-19 Genomics UK (COG-UK) Consortium	Tanya Golubchik, David Bonsall, George Macintyre, Amy Trebes, Mariateresa de Cesare, Catrin Moore, Alex Mobbs, Anita Justice, Robert Shaw, Monique Andersson, Timothy Peto, Emma Wise, Nathan Moore, Jessica Lynch, Nick Cortes, Matilde Mori, Stephen Kidd, David Buck, John Todd, Christophe Fraser
EPI_ISL_764574, EPI_ISL_764575, EPI_ISL_764577, EPI_ISL_764583, EPI_ISL_764584, EPI_ISL_764586, EPI_ISL_764587, EPI_ISL_764598, EPI_ISL_764624, EPI_ISL_764653, EPI_ISL_764654, EPI_ISL_764655, EPI_ISL_764656, EPI_ISL_764657, EPI_ISL_764658, EPI_ISL_764660, EPI_ISL_764661, EPI_ISL_764678, EPI_ISL_764679, EPI_ISL_764741, EPI_ISL_764742, EPI_ISL_764743, EPI_ISL_764748, EPI_ISL_764749, EPI_ISL_764750, EPI_ISL_764751, EPI_ISL_764752, EPI_ISL_764756, EPI_ISL_764757, EPI_ISL_764758, EPI_ISL_764759, EPI_ISL_764760, EPI_ISL_764761, EPI_ISL_764762, EPI_ISL_764763, EPI_ISL_764764, EPI_ISL_764768, EPI_ISL_764816, EPI_ISL_764817, EPI_ISL_764818, EPI_ISL_764821, EPI_ISL_764824, EPI_ISL_764826, EPI_ISL_764827, EPI_ISL_764828, EPI_ISL_764829, EPI_ISL_764830, EPI_ISL_764831, EPI_ISL_764832, EPI_ISL_764833, EPI_ISL_764834, EPI_ISL_764835, EPI_ISL_764836, EPI_ISL_764837, EPI_ISL_764838, EPI_ISL_764839, EPI_ISL_764840, EPI_ISL_764841, EPI_ISL_764842, EPI_ISL_764843, EPI_ISL_764844, EPI_ISL_764845, EPI_ISL_764846, EPI_ISL_764847, EPI_ISL_764848, EPI_ISL_764849, EPI_ISL_764850, EPI_ISL_764851, EPI_ISL_764852, EPI_ISL_764853, EPI_ISL_764854, EPI_ISL_764855, EPI_ISL_764856, EPI_ISL_764857, EPI_ISL_764858, EPI_ISL_764859, EPI_ISL_764860, EPI_ISL_764861, EPI_ISL_764862, EPI_ISL_764863, EPI_ISL_764864, EPI_ISL_764865, EPI_ISL_764866, EPI_ISL_764867, EPI_ISL_764868, EPI_ISL_764869, EPI_ISL_764870, EPI_ISL_764871, EPI_ISL_764874, EPI_ISL_764875, EPI_ISL_764879, EPI_ISL_764881, EPI_ISL_764883, EPI_ISL_764884, EPI_ISL_764886, EPI_ISL_764887, EPI_ISL_764897, EPI_ISL_764898, EPI_ISL_764899, EPI_ISL_764900, EPI_ISL_764901, EPI_ISL_764902, EPI_ISL_764903, EPI_ISL_764904, EPI_ISL_764905, EPI_ISL_764906, EPI_ISL_764907, EPI_ISL_764939, EPI_ISL_764940, EPI_ISL_764941, EPI_ISL_764942, EPI_ISL_764943, EPI_ISL_764944, EPI_ISL_764945, EPI_ISL_764946, EPI_ISL_764965, EPI_ISL_764966, EPI_ISL_764967, EPI_ISL_764968, EPI_ISL_764970, EPI_ISL_764971, EPI_ISL_764972, EPI_ISL_764973, EPI_ISL_764974, EPI_ISL_764976, EPI_ISL_764977, EPI_ISL_764978, EPI_ISL_764979, EPI_ISL_764980, EPI_ISL_764981, EPI_ISL_764982, EPI_ISL_764983, EPI_ISL_764984, EPI_ISL_764985, EPI_ISL_764986, EPI_ISL_764987, EPI_ISL_764988, EPI_ISL_764991, EPI_ISL_764992, EPI_ISL_764993, EPI_ISL_764994, EPI_ISL_764995, EPI_ISL_764997, EPI_ISL_765002			
see above	Wales Specialist Virology Centre Sequencing lab: Pathogen Genomics Unit	COVID-19 Genomics UK (COG-UK) Consortium	Catherine Moore, Johnathan Evans, Laura Gifford, Malorie Perry, Simon Cottrell, Angela Marchbank, Alec Birchley, Alexander Adams, Amy Gaskin, Bree Gatica-Wilcox, Jason Coombes, Joel Southgate, Lauren Gilbert, Lee Graham, Nicole Pacchiarini, Sara Kumziene-Summerhayes, Sarah Taylor, Sophie Jones, Sara Rey, Matthew Bull, Joanne Watkins, Sally Corden, Tom Connor
EPI_ISL_765149, EPI_ISL_765153, EPI_ISL_765165, EPI_ISL_765179	Virology Department, Sheffield Teaching Hospitals NHS Foundation Trust/Department of Infection, Immunity and Cardiovascular Disease, The Medical School, University of Sheffield	COVID-19 Genomics UK (COG-UK) Consortium	Thushan de Silva, Matthew Parker, Nikki Smith, Adri Angyal, Rebecca Brown, Luke Green, Rachel Tucker, Paul Parsons, Danielle Groves, Katie Johnson, Laura Carrilero, Alex Keeley, Dave Partridge, Matthew Wyles, Benjamin Lindsey, Mehmet Yavuz, Mohammad Raza, Cariad Evans
EPI_ISL_765481	SARATOGA HOSPITAL LABORATORY	Wadsworth Center, New York State Department.of Health	Kirsten St. George, Daryl M. Lamson, Alexis Russel, Matthew Shudt, Melissa A Leisner, Jonathan Plitnick, Navjot Singh, John Kelly, Sara Griesemer, Erasmus Schneider, Erica Lasek-Nesselquist
EPI_ISL_765489, EPI_ISL_765490	MONTEFIORE MEDICAL CENTER LABORATORIES	Wadsworth Center, New York State Department.of Health	Kirsten St. George, Daryl M. Lamson, Alexis Russel, Matthew Shudt, Melissa A Leisner, Jonathan Plitnick, Navjot Singh, John Kelly, Sara Griesemer, Erasmus Schneider, Erica Lasek-Nesselquist

EPI_ISL_765518, EPI_ISL_765519, EPI_ISL_765523, EPI_ISL_765528, EPI_ISL_765529	SARATOGA HOSPITAL LABORATORY	Wadsworth Center, New York State Department.of Health	Kirsten St. George, Daryl M. Lamson, Alexis Russel, Matthew Shudt, Melissa A Leisner, Jonathan Plitnick, Navjot Singh, John Kelly, Sara Griesemer, Erasmus Schneider, Erica Lasek-Nesselquist
EPI_ISL_765530, EPI_ISL_765541, EPI_ISL_765546, EPI_ISL_765548, EPI_ISL_765552, EPI_ISL_765558, EPI_ISL_765563, EPI_ISL_765564	MONTEFIORE MEDICAL CENTER LABORATORIES	Wadsworth Center, New York State Department.of Health	Kirsten St. George, Daryl M. Lamson, Alexis Russel, Matthew Shudt, Melissa A Leisner, Jonathan Plitnick, Navjot Singh, John Kelly, Sara Griesemer, Erasmus Schneider, Erica Lasek-Nesselquist
EPI_ISL_765986	Massachusetts General Hospital	Infectious Disease Program, Broad Institute of Harvard and MIT	Lemieux,J.E., Siddle,K.J., Shaw,B., Adams,G., Pierce,V., Turbett,S., Anahtar,M., Branda,J., Slater,D., Harris,J., Lin,A.E., Gladden-Young,A., Lagerborg,K., Rudy,M., DeRuff,K., Carter,A., Normandin,E., Bauer,M., Reilly,S., Tomkins-Tinch,C., Loreth,C., Chaluvadi,S., Neumann,A., Cusick,C., Chapman,S.B., Gnirke,A., Flowers,K., Cerrato,F., Birren,B.W., Gallagher,G., Smole,S., Park,D.J., MacInnis,B.L., Ryan,E., LaRocque,R., Rosenberg,E. and Sabeti,P.C.
EPI_ISL_766061, EPI_ISL_766072, EPI_ISL_766073, EPI_ISL_766078, EPI_ISL_766081, EPI_ISL_766084, EPI_ISL_766097, EPI_ISL_766102, EPI_ISL_766105, EPI_ISL_766110, EPI_ISL_766121, EPI_ISL_766132, EPI_ISL_766135, EPI_ISL_766136, EPI_ISL_766137, EPI_ISL_766138, EPI_ISL_766139, EPI_ISL_766140, EPI_ISL_766141, EPI_ISL_766142, EPI_ISL_766149, EPI_ISL_766150, EPI_ISL_766151, EPI_ISL_766152, EPI_ISL_766153, EPI_ISL_766202, EPI_ISL_766203, EPI_ISL_766204, EPI_ISL_766223, EPI_ISL_766224, EPI_ISL_766227, EPI_ISL_766237, EPI_ISL_766258, EPI_ISL_766301, EPI_ISL_766303, EPI_ISL_766310, EPI_ISL_766314, EPI_ISL_766316, EPI_ISL_766317, EPI_ISL_766397, EPI_ISL_766400, EPI_ISL_766403, EPI_ISL_766407, EPI_ISL_766410, EPI_ISL_766411, EPI_ISL_766412, EPI_ISL_766415, EPI_ISL_766421, EPI_ISL_766427, EPI_ISL_766437, EPI_ISL_766438, EPI_ISL_766439, EPI_ISL_766555, EPI_ISL_766564			
see above	Respiratory Virus Unit, National Infection Service, Public Health England	COVID-19 Genomics UK (COG-UK) Consortium	PHE Covid Sequencing Team
EPI_ISL_766575	ULSS 8 Berica	Istituto Zooprofilattico Sperimentale delle Venezie	Adelaide Milani, Alessia Schivo, Annalisa Salviato, Erika Giorgia Quaranta, Ambra Pastori, Bianca Zecchin, Alice Fusaro, Isabella Monne, Calogero Terregino, Antonia Ricci
EPI_ISL_766609, EPI_ISL_766610, EPI_ISL_766637, EPI_ISL_766638, EPI_ISL_766639	Klinisk mikrobiologi	The Public Health Agency of Sweden	Department of Microbiology, The Public Health Agency of Sweden
EPI_ISL_766640, EPI_ISL_766641	Laboratoriemedicin, Klinisk mikrobiologi	The Public Health Agency of Sweden	Department of Microbiology, The Public Health Agency of Sweden
EPI_ISL_766644	Vardcentralen Brinken	The Public Health Agency of Sweden	Department of Microbiology, The Public Health Agency of Sweden
EPI_ISL_766707, EPI_ISL_766711, EPI_ISL_766712, EPI_ISL_766725, EPI_ISL_766726, EPI_ISL_766727, EPI_ISL_766728, EPI_ISL_766729	Klinisk mikrobiologi	The Public Health Agency of Sweden	Department of Microbiology, The Public Health Agency of Sweden
EPI_ISL_767109	Lighthouse Lab in Milton Keynes	Wellcome Sanger Institute for the COVID-19 Genomics UK (COG-UK) Consortium	The Lighthouse Lab in Milton Keynes and Alex Alderton, Roberto Amato, Sonia Goncalves, Ewan Harrison, David K. Jackson, Ian Johnston, Dominic Kwiatkowski, Cordelia Langford, John Sillitoe on behalf of the Wellcome Sanger Institute COVID-19 Surveillance Team
EPI_ISL_767120, EPI_ISL_767122, EPI_ISL_767124, EPI_ISL_767126, EPI_ISL_767127, EPI_ISL_767129, EPI_ISL_767131	Lighthouse Lab in Alderley Park	Wellcome Sanger Institute for the COVID-19 Genomics UK (COG-UK) Consortium	Jacquelyn Wynn, Mairead Hyland, The Lighthouse Lab in Alderley Park and Alex Alderton, Roberto Amato, Sonia Goncalves, Ewan Harrison, David K. Jackson, Ian Johnston, Dominic Kwiatkowski, Cordelia Langford, John Sillitoe on behalf of the Wellcome Sanger Institute COVID-19 Surveillance Team
EPI_ISL_767420, EPI_ISL_767450, EPI_ISL_767451, EPI_ISL_767453, EPI_ISL_767454, EPI_ISL_767455, EPI_ISL_767456, EPI_ISL_767457, EPI_ISL_767458, EPI_ISL_767459, EPI_ISL_767460, EPI_ISL_767461, EPI_ISL_767462, EPI_ISL_767463, EPI_ISL_767464, EPI_ISL_767465, EPI_ISL_767466, EPI_ISL_767467, EPI_ISL_767468, EPI_ISL_767469, EPI_ISL_767470, EPI_ISL_767471, EPI_ISL_767472, EPI_ISL_767473, EPI_ISL_767474, EPI_ISL_767475, EPI_ISL_767476, EPI_ISL_767477, EPI_ISL_767478			
see above	Wadsworth Center, New York State Department.of Health	Wadsworth Center, New York State Department.of Health	Kirsten St. George, Daryl M. Lamson, Alexis Russel, Matthew Shudt, Melissa A Leisner, Jonathan Plitnick, Navjot Singh, John Kelly, Sara Griesemer, Erasmus Schneider, Erica Lasek-Nesselquist
EPI_ISL_767656	MEMORIAL SLOAN KETTERING CANCER CENTER	Wadsworth Center, New York State Department.of Health	Kirsten St. George, Daryl M. Lamson, Alexis Russel, Matthew Shudt, Melissa A Leisner, Jonathan Plitnick, Navjot Singh, John Kelly, Sara Griesemer, Erasmus Schneider, Erica Lasek-Nesselquist
EPI_ISL_767886	South Eastern Area Laboratory Services (SEALS)	NSW Health Pathology - Institute of Clinical Pathology and Medical Research; Westmead Hospital; University of Sydney	CIDM-PH et al.
EPI_ISL_768475, EPI_ISL_768476, EPI_ISL_768477, EPI_ISL_768478, EPI_ISL_768479, EPI_ISL_768480, EPI_ISL_768481, EPI_ISL_768482, EPI_ISL_768483, EPI_ISL_768484, EPI_ISL_768485, EPI_ISL_768486, EPI_ISL_768487, EPI_ISL_768488, EPI_ISL_768489, EPI_ISL_768490, EPI_ISL_768491, EPI_ISL_768492, EPI_ISL_768493, EPI_ISL_768494, EPI_ISL_768495			
see above	LSUHS Emerging Viral Threat Laboratory	Microbial Genome Sequencing Center	Jeremy P. Kamil, Jennifer L. Carroll, Camille F. Abshire, Maarten Van Diest, Andrew D. Yurochko, Martin J. Sapp, Rona S. Scott, Christopher G. Kevil, Daniel J. Snyder, Vaughn S. Cooper, John A. Vanchiere
EPI_ISL_768498	LSUHS Emerging Viral Threat Laboratory	Microbial Genome Sequencing Center	Jennifer L. Carroll, Jeremy P. Kamil, Camille F. Abshire, Maarten Van Diest, Andrew D. Yurochko, Martin J. Sapp, Rona S. Scott, Christopher G. Kevil, Daniel J. Snyder, Vaughn S. Cooper, John A. Vanchiere
EPI_ISL_768902, EPI_ISL_768903, EPI_ISL_768904, EPI_ISL_768905, EPI_ISL_768906, EPI_ISL_768907, EPI_ISL_768908, EPI_ISL_768909, EPI_ISL_768910, EPI_ISL_768912, EPI_ISL_768913, EPI_ISL_768914, EPI_ISL_768915, EPI_ISL_768917, EPI_ISL_768918, EPI_ISL_768920, EPI_ISL_768921, EPI_ISL_768922, EPI_ISL_768923, EPI_ISL_768924, EPI_ISL_768926, EPI_ISL_768927, EPI_ISL_768929, EPI_ISL_768930, EPI_ISL_768933, EPI_ISL_768934, EPI_ISL_768935, EPI_ISL_768938, EPI_ISL_768940, EPI_ISL_768941, EPI_ISL_768942, EPI_ISL_768943, EPI_ISL_768944, EPI_ISL_768947, EPI_ISL_768950, EPI_ISL_768951, EPI_ISL_768954, EPI_ISL_768955, EPI_ISL_768956, EPI_ISL_768958, EPI_ISL_768960, EPI_ISL_768961, EPI_ISL_768962, EPI_ISL_768964, EPI_ISL_768967, EPI_ISL_768968, EPI_ISL_768969, EPI_ISL_768970, EPI_ISL_768971, EPI_ISL_768973, EPI_ISL_768974, EPI_ISL_768975, EPI_ISL_768978, EPI_ISL_768979, EPI_ISL_768980, EPI_ISL_768981, EPI_ISL_768982, EPI_ISL_768983, EPI_ISL_768984, EPI_ISL_768985, EPI_ISL_768986, EPI_ISL_768987, EPI_ISL_768988, EPI_ISL_768989, EPI_ISL_768990, EPI_ISL_768991, EPI_ISL_768994, EPI_ISL_768995, EPI_ISL_768996, EPI_ISL_768997, EPI_ISL_768998, EPI_ISL_768999, EPI_ISL_769000, EPI_ISL_769001, EPI_ISL_769003, EPI_ISL_769004, EPI_ISL_769006, EPI_ISL_769007, EPI_ISL_769008, EPI_ISL_769009, EPI_ISL_769010, EPI_ISL_769011, EPI_ISL_769012, EPI_ISL_769013, EPI_ISL_769014, EPI_ISL_769015, EPI_ISL_769018, EPI_ISL_769019, EPI_ISL_769020, EPI_ISL_769021, EPI_ISL_769022, EPI_ISL_769023, EPI_ISL_769024, EPI_ISL_769025, EPI_ISL_769026, EPI_ISL_769028, EPI_ISL_769030, EPI_ISL_769031, EPI_ISL_769032, EPI_ISL_769034, EPI_ISL_769035, EPI_ISL_769037, EPI_ISL_769039, EPI_ISL_769040, EPI_ISL_769041, EPI_ISL_769042, EPI_ISL_769044, EPI_ISL_769046, EPI_ISL_769047, EPI_ISL_769048, EPI_ISL_769050, EPI_ISL_769051, EPI_ISL_769052, EPI_ISL_769053, EPI_ISL_769054, EPI_ISL_769055, EPI_ISL_769056, EPI_ISL_769058, EPI_ISL_769059, EPI_ISL_769061, EPI_ISL_769063, EPI_ISL_769064, EPI_ISL_769065, EPI_ISL_769066, EPI_ISL_769067, EPI_ISL_769068, EPI_ISL_769069, EPI_ISL_769070, EPI_ISL_769071, EPI_ISL_769072, EPI_ISL_769073, EPI_ISL_769074, EPI_ISL_769075, EPI_ISL_769077, EPI_ISL_769078, EPI_ISL_769079, EPI_ISL_769081, EPI_ISL_769082, EPI_ISL_769083, EPI_ISL_769085, EPI_ISL_769086, EPI_ISL_769088, EPI_ISL_769090, EPI_ISL_769091, EPI_ISL_769092, EPI_ISL_769095, EPI_ISL_769096, EPI_ISL_769097, EPI_ISL_769099, EPI_ISL_769100, EPI_ISL_769101, EPI_ISL_769102, EPI_ISL_769103, EPI_ISL_769104, EPI_ISL_769106, EPI_ISL_769107, EPI_ISL_769108, EPI_ISL_769111, EPI_ISL_769112, EPI_ISL_769113, EPI_ISL_769114, EPI_ISL_769115, EPI_ISL_769116, EPI_ISL_769118, EPI_ISL_769120, EPI_ISL_769121, EPI_ISL_769123, EPI_ISL_769124, EPI_ISL_769125, EPI_ISL_769126, EPI_ISL_769127, EPI_ISL_769128, EPI_ISL_769129, EPI_ISL_769130, EPI_ISL_769131, EPI_ISL_769132, EPI_ISL_769133, EPI_ISL_769134, EPI_ISL_769135, EPI_ISL_769136, EPI_ISL_769137, EPI_ISL_769139, EPI_ISL_769140, EPI_ISL_769142, EPI_ISL_769143, EPI_ISL_769144, EPI_ISL_769145, EPI_ISL_769146, EPI_ISL_769147, EPI_ISL_769148, EPI_ISL_769149, EPI_ISL_769150, EPI_ISL_769151, EPI_ISL_769153, EPI_ISL_769154, EPI_ISL_769155, EPI_ISL_769156, EPI_ISL_769158, EPI_ISL_769159, EPI_ISL_769160, EPI_ISL_769161, EPI_ISL_769162, EPI_ISL_769163, EPI_ISL_769164, EPI_ISL_769165, EPI_ISL_769166, EPI_ISL_769167, EPI_ISL_769170, EPI_ISL_769172, EPI_ISL_769174, EPI_ISL_769175, EPI_ISL_769177, EPI_ISL_769178, EPI_ISL_769179, EPI_ISL_769180, EPI_ISL_769181, EPI_ISL_769182, EPI_ISL_769183, EPI_ISL_769184, EPI_ISL_769185, EPI_ISL_769186, EPI_ISL_769187, EPI_ISL_769188, EPI_ISL_769189, EPI_ISL_769190, EPI_ISL_769191, EPI_ISL_769192, EPI_ISL_769197, EPI_ISL_769199, EPI_ISL_769200, EPI_ISL_769201, EPI_ISL_769202, EPI_ISL_769203, EPI_ISL_769204, EPI_ISL_769205, EPI_ISL_769208, EPI_ISL_769209, EPI_ISL_769210, EPI_ISL_769212, EPI_ISL_769214, EPI_ISL_769216, EPI_ISL_769217, EPI_ISL_769220, EPI_ISL_769221, EPI_ISL_769222			
see above	Lighthouse Lab in Cambridge	Wellcome Sanger Institute for the COVID-19 Genomics UK (COG-UK) Consortium	Rob Howes, The Lighthouse Lab in Cambridge and Alex Alderton, Roberto Amato, Sonia Goncalves, Ewan Harrison, David K. Jackson, Ian Johnston, Dominic Kwiatkowski, Cordelia Langford, John Sillitoe on behalf of the Wellcome Sanger Institute COVID-19 Surveillance Team
EPI_ISL_769223, EPI_ISL_769224, EPI_ISL_769225, EPI_ISL_769229, EPI_ISL_769233, EPI_ISL_769236, EPI_ISL_769243, EPI_ISL_769244, EPI_ISL_769245, EPI_ISL_769246, EPI_ISL_769249, EPI_ISL_769250, EPI_ISL_769252, EPI_ISL_769254, EPI_ISL_769255, EPI_ISL_769257, EPI_ISL_769259, EPI_ISL_769260, EPI_ISL_769261, EPI_ISL_769266, EPI_ISL_769267, EPI_ISL_769273, EPI_ISL_769275, EPI_ISL_769276, EPI_ISL_769280, EPI_ISL_769281, EPI_ISL_769282, EPI_ISL_769284, EPI_ISL_769286, EPI_ISL_769288, EPI_ISL_769289, EPI_ISL_769290, EPI_ISL_769291, EPI_ISL_769294, EPI_ISL_769297, EPI_ISL_769306, EPI_ISL_769308, EPI_ISL_769310, EPI_ISL_769312, EPI_ISL_769314, EPI_ISL_769316, EPI_ISL_769319, EPI_ISL_769320, EPI_ISL_769321, EPI_ISL_769322, EPI_ISL_769323, EPI_ISL_769325, EPI_ISL_769327, EPI_ISL_769329, EPI_ISL_769330, EPI_ISL_769334, EPI_ISL_769335, EPI_ISL_769341, EPI_ISL_769343, EPI_ISL_769345, EPI_ISL_769348, EPI_ISL_769349, EPI_ISL_769352, EPI_ISL_769353, EPI_ISL_769355, EPI_ISL_769360, EPI_ISL_769361, EPI_ISL_769365, EPI_ISL_769367, EPI_ISL_769370, EPI_ISL_769374, EPI_ISL_769380, EPI_ISL_769381, EPI_ISL_769383, EPI_ISL_769384, EPI_ISL_769385, EPI_ISL_769387, EPI_ISL_769388, EPI_ISL_769389, EPI_ISL_769390, EPI_ISL_769391, EPI_ISL_769392, EPI_ISL_769394, EPI_ISL_769396, EPI_ISL_769399, EPI_ISL_769401, EPI_ISL_769402, EPI_ISL_769404, EPI_ISL_769405, EPI_ISL_769406, EPI_ISL_769412, EPI_ISL_769413, EPI_ISL_769416, EPI_ISL_769417, EPI_ISL_769418, EPI_ISL_769419, EPI_ISL_769420, EPI_ISL_769421, EPI_ISL_769422, EPI_ISL_769427, EPI_ISL_769428, EPI_ISL_769430, EPI_ISL_769431, EPI_ISL_769434, EPI_ISL_769440, EPI_ISL_769441, EPI_ISL_769442, EPI_ISL_769445, EPI_ISL_769446, EPI_ISL_769449, EPI_ISL_769450, EPI_ISL_769452, EPI_ISL_769454, EPI_ISL_769457, EPI_ISL_769458, EPI_ISL_769459, EPI_ISL_769460, EPI_ISL_769462, EPI_ISL_769466, EPI_ISL_769468, EPI_ISL_769469, EPI_ISL_769470, EPI_ISL_769471, EPI_ISL_769473, EPI_ISL_769474, EPI_ISL_769475, EPI_ISL_769476, EPI_ISL_769481, EPI_ISL_769482, EPI_ISL_769485, EPI_ISL_769489, EPI_ISL_769497, EPI_ISL_769499, EPI_ISL_769500, EPI_ISL_769501, EPI_ISL_769502, EPI_ISL_769503, EPI_ISL_769504, EPI_ISL_769506, EPI_ISL_769511, EPI_ISL_769512, EPI_ISL_769513, EPI_ISL_769517, EPI_ISL_769518, EPI_ISL_769519, EPI_ISL_769522, EPI_ISL_769524, EPI_ISL_769525, EPI_ISL_769528, EPI_ISL_769530, EPI_ISL_769533, EPI_ISL_769535, EPI_ISL_769539, EPI_ISL_769540, EPI_ISL_769542, EPI_ISL_769543, EPI_ISL_769577, EPI_ISL_769594, EPI_ISL_769641, EPI_ISL_769655, EPI_ISL_769661, EPI_ISL_769802, EPI_ISL_769861			
see above	Lighthouse Lab in Glasgow	Wellcome Sanger Institute for the COVID-19 Genomics UK (COG-UK) Consortium	Harper VanSteenhouse, Yumi Kasai, David Gray, Carol Clugston, Anna Dominiczak and Alex Alderton, Roberto Amato, Sonia Goncalves, Ewan Harrison, David K. Jackson, Ian Johnston, Dominic Kwiatkowski, Cordelia Langford, John Sillitoe on behalf of the Wellcome Sanger Institute COVID-19 Surveillance Team
EPI_ISL_769863	Laboratoire Biolife	Laboratoire de Biotechnologie	Mouna Ouadghiri, Tarik Aanniz, Mohammed Walid Chemao Elfihri, Mohamed Chenaoui, Hanae Dakka, Afaf Alaoui, Otmane Touzani, Amina Benouda, Bouchra Belfquih, Lahcen belyamani, Saaid Amzazi and Azeddine Ibrahimi

EPI_ISL_769877	Respiratory Virus Unit, National Infection Service, Public Health England	COVID-19 Genomics UK (COG-UK) Consortium	PHE Covid Sequencing Team
EPI_ISL_770799	Minnesota Department of Health, Public Health Laboratory	Minnesota Department of Health, Public Health Laboratory	Alexandra Lorentz, Jacob Garfin, Matt Plumb, and Xiong Wang
EPI_ISL_775218	Gonoshasthya-RNA Molecular Research Center	Gonoshasthya-RNA Molecular Research Center	Mohd. Raeed Jamiruddin, Nihad Adnan, Md. Ahsanul Haq, Mohib Ullah Khondoker, Nafisa Azmuda, Firoz Ahmed, Shahana Sharmin, Salma Akter, Taslin Jahan Mou, Mahfuza Marzan, Sayeda Moriam Liza, Nowshin Jahan, Tamanna Ali, Shahad Saif Khandker, Maha Jamiruddin, Mousumi Chaity, Mumtarin Jannat Oishee
EPI_ISL_775219, EPI_ISL_775220, EPI_ISL_775221, EPI_ISL_775225	Laboratoire Biolife	Laboratoire de Biotechnologie	Mouna Ouadghiri, Tarik Aanniz, Mohammed Walid Chemao Elfihri, Mohamed Chenaoui, Hanae Dakka, Afaf Alaoui, Otmane Touzani, Amina Benouda, Bouchra Belfquih, Lahcen belyamani, Saaid Amzazi and Azeddine Ibrahimi
EPI_ISL_775268	National Laboratory for Health, Environment and Food	National Laboratory for Health, Environment and Food	Aleksander Mahnic, Maja Rupnik
EPI_ISL_775272	Dept. of Medical Microbiology, Stavanger University Hospital, Helse Stavanger HF	Norwegian Institute of Public Health, Department of Virology	Kathrine Stene-Johansen, Kamilla Heddeland Instefjord, Hilde Elshaug, Atiya R Ali,Marie Paulsen Madsen, Rasmus Riis Kopperud, Hilde Vollan, Karoline Bragstad, Olav Hungnes
EPI_ISL_775529	Akershus University Hospital, Department for Microbiology and Infectious Disease Control	Norwegian Institute of Public Health, Department of Virology	Kathrine Stene-Johansen, Kamilla Heddeland Instefjord, Hilde Elshaug, Atiya R Ali,Marie Paulsen Madsen, Rasmus Riis Kopperud, Hilde Vollan, Karoline Bragstad, Olav Hungnes
EPI_ISL_775583	Synlab Medilab, Mikrobiologi	The Public Health Agency of Sweden	Department of Microbiology, The Public Health Agency of Sweden
EPI_ISL_775584	Klinisk mikrobiologi	The Public Health Agency of Sweden	Department of Microbiology, The Public Health Agency of Sweden
EPI_ISL_775587	Synlab Medilab, Mikrobiologi	The Public Health Agency of Sweden	Department of Microbiology, The Public Health Agency of Sweden
EPI_ISL_775588, EPI_ISL_775590	Klinisk mikrobiologi	The Public Health Agency of Sweden	Department of Microbiology, The Public Health Agency of Sweden
EPI_ISL_776662, EPI_ISL_776667, EPI_ISL_776706, EPI_ISL_776707, EPI_ISL_776710, EPI_ISL_776712, EPI_ISL_776714, EPI_ISL_776741, EPI_ISL_776745	UW Virology Lab	UW Virology Lab	Pavitra Roychoudhury, Hong Xie, Lasata Shrestha, Meei-Li Huang, Keith R Jerome, Alexander Greninger
EPI_ISL_777011, EPI_ISL_777012, EPI_ISL_777014, EPI_ISL_777015, EPI_ISL_777017, EPI_ISL_777018, EPI_ISL_777020	Lighthouse Lab in Alderley Park	Wellcome Sanger Institute for the COVID-19 Genomics UK (COG-UK) Consortium	Jacquelyn Wynn, Mairead Hyland, The Lighthouse Lab in Alderley Park and Alex Alderton, Roberto Amato, Sonia Goncalves, Ewan Harrison, David K. Jackson, Ian Johnston, Dominic Kwiatkowski, Cordelia Langford, John Sillitoe on behalf of the Wellcome Sanger Institute COVID-19 Surveillance Team
EPI_ISL_777022, EPI_ISL_777023	Lighthouse Lab in Glasgow	Wellcome Sanger Institute for the COVID-19 Genomics UK (COG-UK) Consortium	Harper VanSteenhouse, Yumi Kasai, David Gray, Carol Clugston, Anna Dominiczak and Alex Alderton, Roberto Amato, Sonia Goncalves, Ewan Harrison, David K. Jackson, Ian Johnston, Dominic Kwiatkowski, Cordelia Langford, John Sillitoe on behalf of the Wellcome Sanger Institute COVID-19 Surveillance Team
EPI_ISL_777024, EPI_ISL_777025, EPI_ISL_777026, EPI_ISL_777027, EPI_ISL_777028, EPI_ISL_777030, EPI_ISL_777032	Lighthouse Lab in Alderley Park	Wellcome Sanger Institute for the COVID-19 Genomics UK (COG-UK) Consortium	Jacquelyn Wynn, Mairead Hyland, The Lighthouse Lab in Alderley Park and Alex Alderton, Roberto Amato, Sonia Goncalves, Ewan Harrison, David K. Jackson, Ian Johnston, Dominic Kwiatkowski, Cordelia Langford, John Sillitoe on behalf of the Wellcome Sanger Institute COVID-19 Surveillance Team
EPI_ISL_777034	Lighthouse Lab in Glasgow	Wellcome Sanger Institute for the COVID-19 Genomics UK (COG-UK) Consortium	Harper VanSteenhouse, Yumi Kasai, David Gray, Carol Clugston, Anna Dominiczak and Alex Alderton, Roberto Amato, Sonia Goncalves, Ewan Harrison, David K. Jackson, Ian Johnston, Dominic Kwiatkowski, Cordelia Langford, John Sillitoe on behalf of the Wellcome Sanger Institute COVID-19 Surveillance Team
EPI_ISL_777035, EPI_ISL_777036, EPI_ISL_777041, EPI_ISL_777043, EPI_ISL_777044, EPI_ISL_777045, EPI_ISL_777046, EPI_ISL_777048, EPI_ISL_777049, EPI_ISL_777050, EPI_ISL_777051, EPI_ISL_777052, EPI_ISL_777054			
see above	Lighthouse Lab in Alderley Park	Wellcome Sanger Institute for the COVID-19 Genomics UK (COG-UK) Consortium	Jacquelyn Wynn, Mairead Hyland, The Lighthouse Lab in Alderley Park and Alex Alderton, Roberto Amato, Sonia Goncalves, Ewan Harrison, David K. Jackson, Ian Johnston, Dominic Kwiatkowski, Cordelia Langford, John Sillitoe on behalf of the Wellcome Sanger Institute COVID-19 Surveillance Team
EPI_ISL_777055	Lighthouse Lab in Glasgow	Wellcome Sanger Institute for the COVID-19 Genomics UK (COG-UK) Consortium	Harper VanSteenhouse, Yumi Kasai, David Gray, Carol Clugston, Anna Dominiczak and Alex Alderton, Roberto Amato, Sonia Goncalves, Ewan Harrison, David K. Jackson, Ian Johnston, Dominic Kwiatkowski, Cordelia Langford, John Sillitoe on behalf of the Wellcome Sanger Institute COVID-19 Surveillance Team
EPI_ISL_777057	Lighthouse Lab in Alderley Park	Wellcome Sanger Institute for the COVID-19 Genomics UK (COG-UK) Consortium	Jacquelyn Wynn, Mairead Hyland, The Lighthouse Lab in Alderley Park and Alex Alderton, Roberto Amato, Sonia Goncalves, Ewan Harrison, David K. Jackson, Ian Johnston, Dominic Kwiatkowski, Cordelia Langford, John Sillitoe on behalf of the Wellcome Sanger Institute COVID-19 Surveillance Team
EPI_ISL_777058	Lighthouse Lab in Glasgow	Wellcome Sanger Institute for the COVID-19 Genomics UK (COG-UK) Consortium	Harper VanSteenhouse, Yumi Kasai, David Gray, Carol Clugston, Anna Dominiczak and Alex Alderton, Roberto Amato, Sonia Goncalves, Ewan Harrison, David K. Jackson, Ian Johnston, Dominic Kwiatkowski, Cordelia Langford, John Sillitoe on behalf of the Wellcome Sanger Institute COVID-19 Surveillance Team
EPI_ISL_777060	Lighthouse Lab in Alderley Park	Wellcome Sanger Institute for the COVID-19 Genomics UK (COG-UK) Consortium	Jacquelyn Wynn, Mairead Hyland, The Lighthouse Lab in Alderley Park and Alex Alderton, Roberto Amato, Sonia Goncalves, Ewan Harrison, David K. Jackson, Ian Johnston, Dominic Kwiatkowski, Cordelia Langford, John Sillitoe on behalf of the Wellcome Sanger Institute COVID-19 Surveillance Team
EPI_ISL_777065	Lighthouse Lab in Glasgow	Wellcome Sanger Institute for the COVID-19 Genomics UK (COG-UK) Consortium	Harper VanSteenhouse, Yumi Kasai, David Gray, Carol Clugston, Anna Dominiczak and Alex Alderton, Roberto Amato, Sonia Goncalves, Ewan Harrison, David K. Jackson, Ian Johnston, Dominic Kwiatkowski, Cordelia Langford, John Sillitoe on behalf of the Wellcome Sanger Institute COVID-19 Surveillance Team
EPI_ISL_777069, EPI_ISL_777070, EPI_ISL_777071, EPI_ISL_777072, EPI_ISL_777074, EPI_ISL_777075, EPI_ISL_777076, EPI_ISL_777077, EPI_ISL_777078, EPI_ISL_777079, EPI_ISL_777086, EPI_ISL_777087, EPI_ISL_777088, EPI_ISL_777090, EPI_ISL_777091, EPI_ISL_777092, EPI_ISL_777093, EPI_ISL_777094			
see above	Lighthouse Lab in Alderley Park	Wellcome Sanger Institute for the COVID-19 Genomics UK (COG-UK) Consortium	Jacquelyn Wynn, Mairead Hyland, The Lighthouse Lab in Alderley Park and Alex Alderton, Roberto Amato, Sonia Goncalves, Ewan Harrison, David K. Jackson, Ian Johnston, Dominic Kwiatkowski, Cordelia Langford, John Sillitoe on behalf of the Wellcome Sanger Institute COVID-19 Surveillance Team
EPI_ISL_777095	Lighthouse Lab in Glasgow	Wellcome Sanger Institute for the COVID-19 Genomics UK (COG-UK) Consortium	Harper VanSteenhouse, Yumi Kasai, David Gray, Carol Clugston, Anna Dominiczak and Alex Alderton, Roberto Amato, Sonia Goncalves, Ewan Harrison, David K. Jackson, Ian Johnston, Dominic Kwiatkowski, Cordelia Langford, John Sillitoe on behalf of the Wellcome Sanger Institute COVID-19 Surveillance Team
EPI_ISL_777096, EPI_ISL_777097	Lighthouse Lab in Alderley Park	Wellcome Sanger Institute for the COVID-19 Genomics UK (COG-UK) Consortium	Jacquelyn Wynn, Mairead Hyland, The Lighthouse Lab in Alderley Park and Alex Alderton, Roberto Amato, Sonia Goncalves, Ewan Harrison, David K. Jackson, Ian Johnston, Dominic Kwiatkowski, Cordelia Langford, John Sillitoe on behalf of the Wellcome Sanger Institute COVID-19 Surveillance Team
EPI_ISL_777098	Lighthouse Lab in Glasgow	Wellcome Sanger Institute for the COVID-19 Genomics UK (COG-UK) Consortium	Harper VanSteenhouse, Yumi Kasai, David Gray, Carol Clugston, Anna Dominiczak and Alex Alderton, Roberto Amato, Sonia Goncalves, Ewan Harrison, David K. Jackson, Ian Johnston, Dominic Kwiatkowski, Cordelia Langford, John Sillitoe on behalf of the Wellcome Sanger Institute COVID-19 Surveillance Team
EPI_ISL_777103, EPI_ISL_777106, EPI_ISL_777107, EPI_ISL_777109, EPI_ISL_777110, EPI_ISL_777111, EPI_ISL_777113, EPI_ISL_777114	Lighthouse Lab in Alderley Park	Wellcome Sanger Institute for the COVID-19 Genomics UK (COG-UK) Consortium	Jacquelyn Wynn, Mairead Hyland, The Lighthouse Lab in Alderley Park and Alex Alderton, Roberto Amato, Sonia Goncalves, Ewan Harrison, David K. Jackson, Ian Johnston, Dominic Kwiatkowski, Cordelia Langford, John Sillitoe on behalf of the Wellcome Sanger Institute COVID-19 Surveillance Team
EPI_ISL_777115	Lighthouse Lab in Glasgow	Wellcome Sanger Institute for the COVID-19 Genomics UK (COG-UK) Consortium	Harper VanSteenhouse, Yumi Kasai, David Gray, Carol Clugston, Anna Dominiczak and Alex Alderton, Roberto Amato, Sonia Goncalves, Ewan Harrison, David K. Jackson, Ian Johnston, Dominic Kwiatkowski, Cordelia Langford, John Sillitoe on behalf of the Wellcome Sanger Institute COVID-19 Surveillance Team
EPI_ISL_777116, EPI_ISL_777119, EPI_ISL_777123, EPI_ISL_777125, EPI_ISL_777126, EPI_ISL_777129, EPI_ISL_777132, EPI_ISL_777134, EPI_ISL_777135, EPI_ISL_777136, EPI_ISL_777137, EPI_ISL_777138, EPI_ISL_777139, EPI_ISL_777143, EPI_ISL_777144, EPI_ISL_777147			
see above	Lighthouse Lab in Alderley Park	Wellcome Sanger Institute for the COVID-19 Genomics UK	Jacquelyn Wynn, Mairead Hyland, The Lighthouse Lab in Alderley Park and Alex Alderton, Roberto Amato, Sonia Goncalves, Ewan Harrison, David K.

		(COG-UK) Consortium	Jackson, Ian Johnston, Dominic Kwiatkowski, Cordelia Langford, John Sillitoe on behalf of the Wellcome Sanger Institute COVID-19 Surveillance Team
EPI_ISL_777148, EPI_ISL_777149	Lighthouse Lab in Glasgow	Wellcome Sanger Institute for the COVID-19 Genomics UK (COG-UK) Consortium	Harper VanSteenhouse, Yumi Kasai, David Gray, Carol Clugston, Anna Dominiczak and Alex Alderton, Roberto Amato, Sonia Goncalves, Ewan Harrison, David K. Jackson, Ian Johnston, Dominic Kwiatkowski, Cordelia Langford, John Sillitoe on behalf of the Wellcome Sanger Institute COVID-19 Surveillance Team
EPI_ISL_777150, EPI_ISL_777156, EPI_ISL_777157, EPI_ISL_777159, EPI_ISL_777163, EPI_ISL_777165, EPI_ISL_777166, EPI_ISL_777167, EPI_ISL_777168, EPI_ISL_777170, EPI_ISL_777171, EPI_ISL_777172, EPI_ISL_777174, EPI_ISL_777175, EPI_ISL_777177, EPI_ISL_777178, EPI_ISL_777179, EPI_ISL_777182, EPI_ISL_777185, EPI_ISL_777186			
see above	Lighthouse Lab in Alderley Park	Wellcome Sanger Institute for the COVID-19 Genomics UK (COG-UK) Consortium	Jacquelyn Wynn, Mairead Hyland, The Lighthouse Lab in Alderley Park and Alex Alderton, Roberto Amato, Sonia Goncalves, Ewan Harrison, David K. Jackson, Ian Johnston, Dominic Kwiatkowski, Cordelia Langford, John Sillitoe on behalf of the Wellcome Sanger Institute COVID-19 Surveillance Team
EPI_ISL_777187	Lighthouse Lab in Milton Keynes	Wellcome Sanger Institute for the COVID-19 Genomics UK (COG-UK) Consortium	The Lighthouse Lab in Milton Keynes and Alex Alderton, Roberto Amato, Sonia Goncalves, Ewan Harrison, David K. Jackson, Ian Johnston, Dominic Kwiatkowski, Cordelia Langford, John Sillitoe on behalf of the Wellcome Sanger Institute COVID-19 Surveillance Team
EPI_ISL_777188, EPI_ISL_777190, EPI_ISL_777193, EPI_ISL_777194, EPI_ISL_777195, EPI_ISL_777197, EPI_ISL_777198, EPI_ISL_777199, EPI_ISL_777202, EPI_ISL_777203, EPI_ISL_777204, EPI_ISL_777206, EPI_ISL_777210, EPI_ISL_777212, EPI_ISL_777213, EPI_ISL_777214, EPI_ISL_777216, EPI_ISL_777217, EPI_ISL_777220, EPI_ISL_777221, EPI_ISL_777226, EPI_ISL_777228, EPI_ISL_777231, EPI_ISL_777234, EPI_ISL_777237, EPI_ISL_777239, EPI_ISL_777240, EPI_ISL_777243, EPI_ISL_777247, EPI_ISL_777248, EPI_ISL_777249			
see above	Lighthouse Lab in Alderley Park	Wellcome Sanger Institute for the COVID-19 Genomics UK (COG-UK) Consortium	Jacquelyn Wynn, Mairead Hyland, The Lighthouse Lab in Alderley Park and Alex Alderton, Roberto Amato, Sonia Goncalves, Ewan Harrison, David K. Jackson, Ian Johnston, Dominic Kwiatkowski, Cordelia Langford, John Sillitoe on behalf of the Wellcome Sanger Institute COVID-19 Surveillance Team
EPI_ISL_777250	Lighthouse Lab in Glasgow	Wellcome Sanger Institute for the COVID-19 Genomics UK (COG-UK) Consortium	Harper VanSteenhouse, Yumi Kasai, David Gray, Carol Clugston, Anna Dominiczak and Alex Alderton, Roberto Amato, Sonia Goncalves, Ewan Harrison, David K. Jackson, Ian Johnston, Dominic Kwiatkowski, Cordelia Langford, John Sillitoe on behalf of the Wellcome Sanger Institute COVID-19 Surveillance Team
EPI_ISL_777251, EPI_ISL_777254	Lighthouse Lab in Alderley Park	Wellcome Sanger Institute for the COVID-19 Genomics UK (COG-UK) Consortium	Jacquelyn Wynn, Mairead Hyland, The Lighthouse Lab in Alderley Park and Alex Alderton, Roberto Amato, Sonia Goncalves, Ewan Harrison, David K. Jackson, Ian Johnston, Dominic Kwiatkowski, Cordelia Langford, John Sillitoe on behalf of the Wellcome Sanger Institute COVID-19 Surveillance Team
EPI_ISL_777255	Lighthouse Lab in Glasgow	Wellcome Sanger Institute for the COVID-19 Genomics UK (COG-UK) Consortium	Harper VanSteenhouse, Yumi Kasai, David Gray, Carol Clugston, Anna Dominiczak and Alex Alderton, Roberto Amato, Sonia Goncalves, Ewan Harrison, David K. Jackson, Ian Johnston, Dominic Kwiatkowski, Cordelia Langford, John Sillitoe on behalf of the Wellcome Sanger Institute COVID-19 Surveillance Team
EPI_ISL_777256, EPI_ISL_777257, EPI_ISL_777258, EPI_ISL_777259, EPI_ISL_777260	Lighthouse Lab in Alderley Park	Wellcome Sanger Institute for the COVID-19 Genomics UK (COG-UK) Consortium	Jacquelyn Wynn, Mairead Hyland, The Lighthouse Lab in Alderley Park and Alex Alderton, Roberto Amato, Sonia Goncalves, Ewan Harrison, David K. Jackson, Ian Johnston, Dominic Kwiatkowski, Cordelia Langford, John Sillitoe on behalf of the Wellcome Sanger Institute COVID-19 Surveillance Team
EPI_ISL_777261	Lighthouse Lab in Glasgow	Wellcome Sanger Institute for the COVID-19 Genomics UK (COG-UK) Consortium	Harper VanSteenhouse, Yumi Kasai, David Gray, Carol Clugston, Anna Dominiczak and Alex Alderton, Roberto Amato, Sonia Goncalves, Ewan Harrison, David K. Jackson, Ian Johnston, Dominic Kwiatkowski, Cordelia Langford, John Sillitoe on behalf of the Wellcome Sanger Institute COVID-19 Surveillance Team
EPI_ISL_777262, EPI_ISL_777263, EPI_ISL_777264, EPI_ISL_777265, EPI_ISL_777266, EPI_ISL_777267, EPI_ISL_777271, EPI_ISL_777272, EPI_ISL_777273, EPI_ISL_777274, EPI_ISL_777276, EPI_ISL_777277, EPI_ISL_777278, EPI_ISL_777279, EPI_ISL_777280, EPI_ISL_777281, EPI_ISL_777282			
see above	Lighthouse Lab in Alderley Park	Wellcome Sanger Institute for the COVID-19 Genomics UK (COG-UK) Consortium	Jacquelyn Wynn, Mairead Hyland, The Lighthouse Lab in Alderley Park and Alex Alderton, Roberto Amato, Sonia Goncalves, Ewan Harrison, David K. Jackson, Ian Johnston, Dominic Kwiatkowski, Cordelia Langford, John Sillitoe on behalf of the Wellcome Sanger Institute COVID-19 Surveillance Team
EPI_ISL_777284	Lighthouse Lab in Glasgow	Wellcome Sanger Institute for the COVID-19 Genomics UK (COG-UK) Consortium	Harper VanSteenhouse, Yumi Kasai, David Gray, Carol Clugston, Anna Dominiczak and Alex Alderton, Roberto Amato, Sonia Goncalves, Ewan Harrison, David K. Jackson, Ian Johnston, Dominic Kwiatkowski, Cordelia Langford, John Sillitoe on behalf of the Wellcome Sanger Institute COVID-19 Surveillance Team
EPI_ISL_777285, EPI_ISL_777287, EPI_ISL_777288, EPI_ISL_777291, EPI_ISL_777294, EPI_ISL_777296, EPI_ISL_777297, EPI_ISL_777299, EPI_ISL_777307, EPI_ISL_777308, EPI_ISL_777309, EPI_ISL_777311, EPI_ISL_777312, EPI_ISL_777315, EPI_ISL_777317, EPI_ISL_777318, EPI_ISL_777319, EPI_ISL_777326, EPI_ISL_777327, EPI_ISL_777333, EPI_ISL_777336, EPI_ISL_777338, EPI_ISL_777349, EPI_ISL_777375, EPI_ISL_777380, EPI_ISL_777386, EPI_ISL_777389, EPI_ISL_777394, EPI_ISL_777411, EPI_ISL_777412, EPI_ISL_777416, EPI_ISL_777421, EPI_ISL_777424, EPI_ISL_777425, EPI_ISL_777427, EPI_ISL_777430, EPI_ISL_777435, EPI_ISL_777437, EPI_ISL_777440, EPI_ISL_777448, EPI_ISL_777451, EPI_ISL_777455, EPI_ISL_777458, EPI_ISL_777465, EPI_ISL_777467, EPI_ISL_777470, EPI_ISL_777477, EPI_ISL_777479, EPI_ISL_777481, EPI_ISL_777489, EPI_ISL_777491, EPI_ISL_777495, EPI_ISL_777502, EPI_ISL_777509, EPI_ISL_777515, EPI_ISL_777520, EPI_ISL_777522, EPI_ISL_777524, EPI_ISL_777525, EPI_ISL_777529, EPI_ISL_777530, EPI_ISL_777532, EPI_ISL_777534, EPI_ISL_777535, EPI_ISL_777537, EPI_ISL_777538, EPI_ISL_777539, EPI_ISL_777543, EPI_ISL_777548, EPI_ISL_777550, EPI_ISL_777552, EPI_ISL_777558, EPI_ISL_777561, EPI_ISL_777563, EPI_ISL_777567, EPI_ISL_777568, EPI_ISL_777572, EPI_ISL_777573, EPI_ISL_777576, EPI_ISL_777580, EPI_ISL_777582, EPI_ISL_777584, EPI_ISL_777587, EPI_ISL_777589, EPI_ISL_777591, EPI_ISL_777592, EPI_ISL_777593, EPI_ISL_777595, EPI_ISL_777597, EPI_ISL_777605, EPI_ISL_777621, EPI_ISL_777627, EPI_ISL_777629, EPI_ISL_777633, EPI_ISL_777634, EPI_ISL_777638, EPI_ISL_777639, EPI_ISL_777644, EPI_ISL_777649, EPI_ISL_777650, EPI_ISL_777651, EPI_ISL_777655, EPI_ISL_777659, EPI_ISL_777669, EPI_ISL_777672			
see above	Lighthouse Lab in Alderley Park	Wellcome Sanger Institute for the COVID-19 Genomics UK (COG-UK) Consortium	Jacquelyn Wynn, Mairead Hyland, The Lighthouse Lab in Alderley Park and Alex Alderton, Roberto Amato, Sonia Goncalves, Ewan Harrison, David K. Jackson, Ian Johnston, Dominic Kwiatkowski, Cordelia Langford, John Sillitoe on behalf of the Wellcome Sanger Institute COVID-19 Surveillance Team
EPI_ISL_778297, EPI_ISL_778299, EPI_ISL_778301, EPI_ISL_778302, EPI_ISL_778303	Lighthouse Lab in Glasgow	Wellcome Sanger Institute for the COVID-19 Genomics UK (COG-UK) Consortium	Harper VanSteenhouse, Yumi Kasai, David Gray, Carol Clugston, Anna Dominiczak and Alex Alderton, Roberto Amato, Sonia Goncalves, Ewan Harrison, David K. Jackson, Ian Johnston, Dominic Kwiatkowski, Cordelia Langford, John Sillitoe on behalf of the Wellcome Sanger Institute COVID-19 Surveillance Team
EPI_ISL_778305, EPI_ISL_778309, EPI_ISL_778311, EPI_ISL_778315	Lighthouse Lab in Alderley Park	Wellcome Sanger Institute for the COVID-19 Genomics UK (COG-UK) Consortium	Jacquelyn Wynn, Mairead Hyland, The Lighthouse Lab in Alderley Park and Alex Alderton, Roberto Amato, Sonia Goncalves, Ewan Harrison, David K. Jackson, Ian Johnston, Dominic Kwiatkowski, Cordelia Langford, John Sillitoe on behalf of the Wellcome Sanger Institute COVID-19 Surveillance Team
EPI_ISL_778322, EPI_ISL_778324, EPI_ISL_778325	Lighthouse Lab in Glasgow	Wellcome Sanger Institute for the COVID-19 Genomics UK (COG-UK) Consortium	Harper VanSteenhouse, Yumi Kasai, David Gray, Carol Clugston, Anna Dominiczak and Alex Alderton, Roberto Amato, Sonia Goncalves, Ewan Harrison, David K. Jackson, Ian Johnston, Dominic Kwiatkowski, Cordelia Langford, John Sillitoe on behalf of the Wellcome Sanger Institute COVID-19 Surveillance Team
EPI_ISL_778327, EPI_ISL_778328	Lighthouse Lab in Alderley Park	Wellcome Sanger Institute for the COVID-19 Genomics UK (COG-UK) Consortium	Jacquelyn Wynn, Mairead Hyland, The Lighthouse Lab in Alderley Park and Alex Alderton, Roberto Amato, Sonia Goncalves, Ewan Harrison, David K. Jackson, Ian Johnston, Dominic Kwiatkowski, Cordelia Langford, John Sillitoe on behalf of the Wellcome Sanger Institute COVID-19 Surveillance Team
EPI_ISL_778330	Lighthouse Lab in Glasgow	Wellcome Sanger Institute for the COVID-19 Genomics UK (COG-UK) Consortium	Harper VanSteenhouse, Yumi Kasai, David Gray, Carol Clugston, Anna Dominiczak and Alex Alderton, Roberto Amato, Sonia Goncalves, Ewan Harrison, David K. Jackson, Ian Johnston, Dominic Kwiatkowski, Cordelia Langford, John Sillitoe on behalf of the Wellcome Sanger Institute COVID-19 Surveillance Team
EPI_ISL_778331	Lighthouse Lab in Alderley Park	Wellcome Sanger Institute for the COVID-19 Genomics UK (COG-UK) Consortium	Jacquelyn Wynn, Mairead Hyland, The Lighthouse Lab in Alderley Park and Alex Alderton, Roberto Amato, Sonia Goncalves, Ewan Harrison, David K. Jackson, Ian Johnston, Dominic Kwiatkowski, Cordelia Langford, John Sillitoe on behalf of the Wellcome Sanger Institute COVID-19 Surveillance Team
EPI_ISL_778332	Lighthouse Lab in Glasgow	Wellcome Sanger Institute for the COVID-19 Genomics UK (COG-UK) Consortium	Harper VanSteenhouse, Yumi Kasai, David Gray, Carol Clugston, Anna Dominiczak and Alex Alderton, Roberto Amato, Sonia Goncalves, Ewan Harrison, David K. Jackson, Ian Johnston, Dominic Kwiatkowski, Cordelia Langford, John Sillitoe on behalf of the Wellcome Sanger Institute COVID-19 Surveillance Team
EPI_ISL_778333	Lighthouse Lab in Alderley Park	Wellcome Sanger Institute for the COVID-19 Genomics UK (COG-UK) Consortium	Jacquelyn Wynn, Mairead Hyland, The Lighthouse Lab in Alderley Park and Alex Alderton, Roberto Amato, Sonia Goncalves, Ewan Harrison, David K. Jackson, Ian Johnston, Dominic Kwiatkowski, Cordelia Langford, John Sillitoe on behalf of the Wellcome Sanger Institute COVID-19 Surveillance Team
EPI_ISL_778334	Lighthouse Lab in Glasgow	Wellcome Sanger Institute for the COVID-19 Genomics UK (COG-UK) Consortium	Harper VanSteenhouse, Yumi Kasai, David Gray, Carol Clugston, Anna Dominiczak and Alex Alderton, Roberto Amato, Sonia Goncalves, Ewan Harrison, David K. Jackson, Ian Johnston, Dominic Kwiatkowski, Cordelia Langford, John Sillitoe on behalf of the Wellcome Sanger Institute COVID-19 Surveillance Team
EPI_ISL_778335	Lighthouse Lab in Alderley Park	Wellcome Sanger Institute for the COVID-19 Genomics UK (COG-UK) Consortium	Jacquelyn Wynn, Mairead Hyland, The Lighthouse Lab in Alderley Park and Alex Alderton, Roberto Amato, Sonia Goncalves, Ewan Harrison, David K. Jackson, Ian Johnston, Dominic Kwiatkowski, Cordelia Langford, John Sillitoe on behalf of the Wellcome Sanger Institute COVID-19 Surveillance Team
EPI_ISL_778336, EPI_ISL_778337	Lighthouse Lab in Glasgow	Wellcome Sanger Institute for the COVID-19 Genomics UK (COG-UK) Consortium	Harper VanSteenhouse, Yumi Kasai, David Gray, Carol Clugston, Anna Dominiczak and Alex Alderton, Roberto Amato, Sonia Goncalves, Ewan Harrison, David K. Jackson, Ian Johnston, Dominic Kwiatkowski, Cordelia Langford, John Sillitoe on behalf of the Wellcome Sanger Institute COVID-19 Surveillance Team
EPI_ISL_778342	Lighthouse Lab in Alderley Park	Wellcome Sanger Institute for the COVID-19 Genomics UK (COG-UK) Consortium	Jacquelyn Wynn, Mairead Hyland, The Lighthouse Lab in Alderley Park and Alex Alderton, Roberto Amato, Sonia Goncalves, Ewan Harrison, David K. Jackson, Ian Johnston, Dominic Kwiatkowski, Cordelia Langford, John Sillitoe on behalf of the Wellcome Sanger Institute COVID-19 Surveillance Team
EPI_ISL_778345	Lighthouse Lab in Glasgow	Wellcome Sanger Institute for the COVID-19 Genomics UK	Harper VanSteenhouse, Yumi Kasai, David Gray, Carol Clugston, Anna Dominiczak and Alex Alderton, Roberto Amato, Sonia Goncalves, Ewan Harrison,

		(COG-UK) Consortium	David K. Jackson, Ian Johnston, Dominic Kwiatkowski, Cordelia Langford, John Sillitoe on behalf of the Wellcome Sanger Institute COVID-19 Surveillance Team
EPI_ISL_778346	Lighthouse Lab in Alderley Park	Wellcome Sanger Institute for the COVID-19 Genomics UK (COG-UK) Consortium	Jacquelyn Wynn, Mairead Hyland, The Lighthouse Lab in Alderley Park and Alex Alderton, Roberto Amato, Sonia Goncalves, Ewan Harrison, David K. Jackson, Ian Johnston, Dominic Kwiatkowski, Cordelia Langford, John Sillitoe on behalf of the Wellcome Sanger Institute COVID-19 Surveillance Team
EPI_ISL_778352	Lighthouse Lab in Glasgow	Wellcome Sanger Institute for the COVID-19 Genomics UK (COG-UK) Consortium	Harper VanSteenhouse, Yumi Kasai, David Gray, Carol Clugston, Anna Dominiczak and Alex Alderton, Roberto Amato, Sonia Goncalves, Ewan Harrison, David K. Jackson, Ian Johnston, Dominic Kwiatkowski, Cordelia Langford, John Sillitoe on behalf of the Wellcome Sanger Institute COVID-19 Surveillance Team
EPI_ISL_778353, EPI_ISL_778355	Lighthouse Lab in Alderley Park	Wellcome Sanger Institute for the COVID-19 Genomics UK (COG-UK) Consortium	Jacquelyn Wynn, Mairead Hyland, The Lighthouse Lab in Alderley Park and Alex Alderton, Roberto Amato, Sonia Goncalves, Ewan Harrison, David K. Jackson, Ian Johnston, Dominic Kwiatkowski, Cordelia Langford, John Sillitoe on behalf of the Wellcome Sanger Institute COVID-19 Surveillance Team
EPI_ISL_778356	Lighthouse Lab in Glasgow	Wellcome Sanger Institute for the COVID-19 Genomics UK (COG-UK) Consortium	Harper VanSteenhouse, Yumi Kasai, David Gray, Carol Clugston, Anna Dominiczak and Alex Alderton, Roberto Amato, Sonia Goncalves, Ewan Harrison, David K. Jackson, Ian Johnston, Dominic Kwiatkowski, Cordelia Langford, John Sillitoe on behalf of the Wellcome Sanger Institute COVID-19 Surveillance Team
EPI_ISL_778357, EPI_ISL_778358	Lighthouse Lab in Alderley Park	Wellcome Sanger Institute for the COVID-19 Genomics UK (COG-UK) Consortium	Jacquelyn Wynn, Mairead Hyland, The Lighthouse Lab in Alderley Park and Alex Alderton, Roberto Amato, Sonia Goncalves, Ewan Harrison, David K. Jackson, Ian Johnston, Dominic Kwiatkowski, Cordelia Langford, John Sillitoe on behalf of the Wellcome Sanger Institute COVID-19 Surveillance Team
EPI_ISL_778359	Lighthouse Lab in Glasgow	Wellcome Sanger Institute for the COVID-19 Genomics UK (COG-UK) Consortium	Harper VanSteenhouse, Yumi Kasai, David Gray, Carol Clugston, Anna Dominiczak and Alex Alderton, Roberto Amato, Sonia Goncalves, Ewan Harrison, David K. Jackson, Ian Johnston, Dominic Kwiatkowski, Cordelia Langford, John Sillitoe on behalf of the Wellcome Sanger Institute COVID-19 Surveillance Team
EPI_ISL_778360	Lighthouse Lab in Alderley Park	Wellcome Sanger Institute for the COVID-19 Genomics UK (COG-UK) Consortium	Jacquelyn Wynn, Mairead Hyland, The Lighthouse Lab in Alderley Park and Alex Alderton, Roberto Amato, Sonia Goncalves, Ewan Harrison, David K. Jackson, Ian Johnston, Dominic Kwiatkowski, Cordelia Langford, John Sillitoe on behalf of the Wellcome Sanger Institute COVID-19 Surveillance Team
EPI_ISL_778361, EPI_ISL_778362, EPI_ISL_778364	Lighthouse Lab in Glasgow	Wellcome Sanger Institute for the COVID-19 Genomics UK (COG-UK) Consortium	Harper VanSteenhouse, Yumi Kasai, David Gray, Carol Clugston, Anna Dominiczak and Alex Alderton, Roberto Amato, Sonia Goncalves, Ewan Harrison, David K. Jackson, Ian Johnston, Dominic Kwiatkowski, Cordelia Langford, John Sillitoe on behalf of the Wellcome Sanger Institute COVID-19 Surveillance Team
EPI_ISL_778365, EPI_ISL_778368	Lighthouse Lab in Alderley Park	Wellcome Sanger Institute for the COVID-19 Genomics UK (COG-UK) Consortium	Jacquelyn Wynn, Mairead Hyland, The Lighthouse Lab in Alderley Park and Alex Alderton, Roberto Amato, Sonia Goncalves, Ewan Harrison, David K. Jackson, Ian Johnston, Dominic Kwiatkowski, Cordelia Langford, John Sillitoe on behalf of the Wellcome Sanger Institute COVID-19 Surveillance Team
EPI_ISL_778372, EPI_ISL_778373	Lighthouse Lab in Glasgow	Wellcome Sanger Institute for the COVID-19 Genomics UK (COG-UK) Consortium	Harper VanSteenhouse, Yumi Kasai, David Gray, Carol Clugston, Anna Dominiczak and Alex Alderton, Roberto Amato, Sonia Goncalves, Ewan Harrison, David K. Jackson, Ian Johnston, Dominic Kwiatkowski, Cordelia Langford, John Sillitoe on behalf of the Wellcome Sanger Institute COVID-19 Surveillance Team
EPI_ISL_778374	Lighthouse Lab in Alderley Park	Wellcome Sanger Institute for the COVID-19 Genomics UK (COG-UK) Consortium	Jacquelyn Wynn, Mairead Hyland, The Lighthouse Lab in Alderley Park and Alex Alderton, Roberto Amato, Sonia Goncalves, Ewan Harrison, David K. Jackson, Ian Johnston, Dominic Kwiatkowski, Cordelia Langford, John Sillitoe on behalf of the Wellcome Sanger Institute COVID-19 Surveillance Team
EPI_ISL_778375, EPI_ISL_778376, EPI_ISL_778377, EPI_ISL_778378	Lighthouse Lab in Glasgow	Wellcome Sanger Institute for the COVID-19 Genomics UK (COG-UK) Consortium	Harper VanSteenhouse, Yumi Kasai, David Gray, Carol Clugston, Anna Dominiczak and Alex Alderton, Roberto Amato, Sonia Goncalves, Ewan Harrison, David K. Jackson, Ian Johnston, Dominic Kwiatkowski, Cordelia Langford, John Sillitoe on behalf of the Wellcome Sanger Institute COVID-19 Surveillance Team
EPI_ISL_778380, EPI_ISL_778382, EPI_ISL_778385, EPI_ISL_778390, EPI_ISL_778392, EPI_ISL_778396	Lighthouse Lab in Alderley Park	Wellcome Sanger Institute for the COVID-19 Genomics UK (COG-UK) Consortium	Jacquelyn Wynn, Mairead Hyland, The Lighthouse Lab in Alderley Park and Alex Alderton, Roberto Amato, Sonia Goncalves, Ewan Harrison, David K. Jackson, Ian Johnston, Dominic Kwiatkowski, Cordelia Langford, John Sillitoe on behalf of the Wellcome Sanger Institute COVID-19 Surveillance Team
EPI_ISL_778397	Lighthouse Lab in Glasgow	Wellcome Sanger Institute for the COVID-19 Genomics UK (COG-UK) Consortium	Harper VanSteenhouse, Yumi Kasai, David Gray, Carol Clugston, Anna Dominiczak and Alex Alderton, Roberto Amato, Sonia Goncalves, Ewan Harrison, David K. Jackson, Ian Johnston, Dominic Kwiatkowski, Cordelia Langford, John Sillitoe on behalf of the Wellcome Sanger Institute COVID-19 Surveillance Team
EPI_ISL_778399	Lighthouse Lab in Alderley Park	Wellcome Sanger Institute for the COVID-19 Genomics UK (COG-UK) Consortium	Jacquelyn Wynn, Mairead Hyland, The Lighthouse Lab in Alderley Park and Alex Alderton, Roberto Amato, Sonia Goncalves, Ewan Harrison, David K. Jackson, Ian Johnston, Dominic Kwiatkowski, Cordelia Langford, John Sillitoe on behalf of the Wellcome Sanger Institute COVID-19 Surveillance Team
EPI_ISL_778400	Lighthouse Lab in Glasgow	Wellcome Sanger Institute for the COVID-19 Genomics UK (COG-UK) Consortium	Harper VanSteenhouse, Yumi Kasai, David Gray, Carol Clugston, Anna Dominiczak and Alex Alderton, Roberto Amato, Sonia Goncalves, Ewan Harrison, David K. Jackson, Ian Johnston, Dominic Kwiatkowski, Cordelia Langford, John Sillitoe on behalf of the Wellcome Sanger Institute COVID-19 Surveillance Team
EPI_ISL_778401	Lighthouse Lab in Alderley Park	Wellcome Sanger Institute for the COVID-19 Genomics UK (COG-UK) Consortium	Jacquelyn Wynn, Mairead Hyland, The Lighthouse Lab in Alderley Park and Alex Alderton, Roberto Amato, Sonia Goncalves, Ewan Harrison, David K. Jackson, Ian Johnston, Dominic Kwiatkowski, Cordelia Langford, John Sillitoe on behalf of the Wellcome Sanger Institute COVID-19 Surveillance Team
EPI_ISL_778403	Lighthouse Lab in Glasgow	Wellcome Sanger Institute for the COVID-19 Genomics UK (COG-UK) Consortium	Harper VanSteenhouse, Yumi Kasai, David Gray, Carol Clugston, Anna Dominiczak and Alex Alderton, Roberto Amato, Sonia Goncalves, Ewan Harrison, David K. Jackson, Ian Johnston, Dominic Kwiatkowski, Cordelia Langford, John Sillitoe on behalf of the Wellcome Sanger Institute COVID-19 Surveillance Team
EPI_ISL_778404	Lighthouse Lab in Alderley Park	Wellcome Sanger Institute for the COVID-19 Genomics UK (COG-UK) Consortium	Jacquelyn Wynn, Mairead Hyland, The Lighthouse Lab in Alderley Park and Alex Alderton, Roberto Amato, Sonia Goncalves, Ewan Harrison, David K. Jackson, Ian Johnston, Dominic Kwiatkowski, Cordelia Langford, John Sillitoe on behalf of the Wellcome Sanger Institute COVID-19 Surveillance Team
EPI_ISL_778407	Lighthouse Lab in Glasgow	Wellcome Sanger Institute for the COVID-19 Genomics UK (COG-UK) Consortium	Harper VanSteenhouse, Yumi Kasai, David Gray, Carol Clugston, Anna Dominiczak and Alex Alderton, Roberto Amato, Sonia Goncalves, Ewan Harrison, David K. Jackson, Ian Johnston, Dominic Kwiatkowski, Cordelia Langford, John Sillitoe on behalf of the Wellcome Sanger Institute COVID-19 Surveillance Team
EPI_ISL_778408, EPI_ISL_778410	Lighthouse Lab in Alderley Park	Wellcome Sanger Institute for the COVID-19 Genomics UK (COG-UK) Consortium	Jacquelyn Wynn, Mairead Hyland, The Lighthouse Lab in Alderley Park and Alex Alderton, Roberto Amato, Sonia Goncalves, Ewan Harrison, David K. Jackson, Ian Johnston, Dominic Kwiatkowski, Cordelia Langford, John Sillitoe on behalf of the Wellcome Sanger Institute COVID-19 Surveillance Team
EPI_ISL_778411	Lighthouse Lab in Glasgow	Wellcome Sanger Institute for the COVID-19 Genomics UK (COG-UK) Consortium	Harper VanSteenhouse, Yumi Kasai, David Gray, Carol Clugston, Anna Dominiczak and Alex Alderton, Roberto Amato, Sonia Goncalves, Ewan Harrison, David K. Jackson, Ian Johnston, Dominic Kwiatkowski, Cordelia Langford, John Sillitoe on behalf of the Wellcome Sanger Institute COVID-19 Surveillance Team
EPI_ISL_778412, EPI_ISL_778415, EPI_ISL_778416, EPI_ISL_778417	Lighthouse Lab in Alderley Park	Wellcome Sanger Institute for the COVID-19 Genomics UK (COG-UK) Consortium	Jacquelyn Wynn, Mairead Hyland, The Lighthouse Lab in Alderley Park and Alex Alderton, Roberto Amato, Sonia Goncalves, Ewan Harrison, David K. Jackson, Ian Johnston, Dominic Kwiatkowski, Cordelia Langford, John Sillitoe on behalf of the Wellcome Sanger Institute COVID-19 Surveillance Team
EPI_ISL_778419, EPI_ISL_778420, EPI_ISL_778421, EPI_ISL_778422, EPI_ISL_778423, EPI_ISL_778424	Lighthouse Lab in Glasgow	Wellcome Sanger Institute for the COVID-19 Genomics UK (COG-UK) Consortium	Harper VanSteenhouse, Yumi Kasai, David Gray, Carol Clugston, Anna Dominiczak and Alex Alderton, Roberto Amato, Sonia Goncalves, Ewan Harrison, David K. Jackson, Ian Johnston, Dominic Kwiatkowski, Cordelia Langford, John Sillitoe on behalf of the Wellcome Sanger Institute COVID-19 Surveillance Team
EPI_ISL_778426	Lighthouse Lab in Alderley Park	Wellcome Sanger Institute for the COVID-19 Genomics UK (COG-UK) Consortium	Jacquelyn Wynn, Mairead Hyland, The Lighthouse Lab in Alderley Park and Alex Alderton, Roberto Amato, Sonia Goncalves, Ewan Harrison, David K. Jackson, Ian Johnston, Dominic Kwiatkowski, Cordelia Langford, John Sillitoe on behalf of the Wellcome Sanger Institute COVID-19 Surveillance Team
EPI_ISL_778428	Lighthouse Lab in Glasgow	Wellcome Sanger Institute for the COVID-19 Genomics UK (COG-UK) Consortium	Harper VanSteenhouse, Yumi Kasai, David Gray, Carol Clugston, Anna Dominiczak and Alex Alderton, Roberto Amato, Sonia Goncalves, Ewan Harrison, David K. Jackson, Ian Johnston, Dominic Kwiatkowski, Cordelia Langford, John Sillitoe on behalf of the Wellcome Sanger Institute COVID-19 Surveillance Team
EPI_ISL_778430, EPI_ISL_778432	Lighthouse Lab in Alderley Park	Wellcome Sanger Institute for the COVID-19 Genomics UK (COG-UK) Consortium	Jacquelyn Wynn, Mairead Hyland, The Lighthouse Lab in Alderley Park and Alex Alderton, Roberto Amato, Sonia Goncalves, Ewan Harrison, David K. Jackson, Ian Johnston, Dominic Kwiatkowski, Cordelia Langford, John Sillitoe on behalf of the Wellcome Sanger Institute COVID-19 Surveillance Team
EPI_ISL_778433	Lighthouse Lab in Glasgow	Wellcome Sanger Institute for the COVID-19 Genomics UK (COG-UK) Consortium	Harper VanSteenhouse, Yumi Kasai, David Gray, Carol Clugston, Anna Dominiczak and Alex Alderton, Roberto Amato, Sonia Goncalves, Ewan Harrison, David K. Jackson, Ian Johnston, Dominic Kwiatkowski, Cordelia Langford, John Sillitoe on behalf of the Wellcome Sanger Institute COVID-19 Surveillance Team

EPI_ISL_778444	Lighthouse Lab in Alderley Park	Wellcome Sanger Institute for the COVID-19 Genomics UK (COG-UK) Consortium	Jacquelyn Wynn, Mairead Hyland, The Lighthouse Lab in Alderley Park and Alex Alderton, Roberto Amato, Sonia Goncalves, Ewan Harrison, David K. Jackson, Ian Johnston, Dominic Kwiatkowski, Cordelia Langford, John Sillitoe on behalf of the Wellcome Sanger Institute COVID-19 Surveillance Team
EPI_ISL_778447, EPI_ISL_778450, EPI_ISL_778451	Lighthouse Lab in Glasgow	Wellcome Sanger Institute for the COVID-19 Genomics UK (COG-UK) Consortium	Harper VanSteenhouse, Yumi Kasai, David Gray, Carol Clugston, Anna Dominiczak and Alex Alderton, Roberto Amato, Sonia Goncalves, Ewan Harrison, David K. Jackson, Ian Johnston, Dominic Kwiatkowski, Cordelia Langford, John Sillitoe on behalf of the Wellcome Sanger Institute COVID-19 Surveillance Team
EPI_ISL_778454, EPI_ISL_778455	Lighthouse Lab in Alderley Park	Wellcome Sanger Institute for the COVID-19 Genomics UK (COG-UK) Consortium	Jacquelyn Wynn, Mairead Hyland, The Lighthouse Lab in Alderley Park and Alex Alderton, Roberto Amato, Sonia Goncalves, Ewan Harrison, David K. Jackson, Ian Johnston, Dominic Kwiatkowski, Cordelia Langford, John Sillitoe on behalf of the Wellcome Sanger Institute COVID-19 Surveillance Team
EPI_ISL_778457, EPI_ISL_778459, EPI_ISL_778461, EPI_ISL_778462, EPI_ISL_778463, EPI_ISL_778465	Lighthouse Lab in Glasgow	Wellcome Sanger Institute for the COVID-19 Genomics UK (COG-UK) Consortium	Harper VanSteenhouse, Yumi Kasai, David Gray, Carol Clugston, Anna Dominiczak and Alex Alderton, Roberto Amato, Sonia Goncalves, Ewan Harrison, David K. Jackson, Ian Johnston, Dominic Kwiatkowski, Cordelia Langford, John Sillitoe on behalf of the Wellcome Sanger Institute COVID-19 Surveillance Team
EPI_ISL_778471, EPI_ISL_778472, EPI_ISL_778473, EPI_ISL_778478	Lighthouse Lab in Alderley Park	Wellcome Sanger Institute for the COVID-19 Genomics UK (COG-UK) Consortium	Jacquelyn Wynn, Mairead Hyland, The Lighthouse Lab in Alderley Park and Alex Alderton, Roberto Amato, Sonia Goncalves, Ewan Harrison, David K. Jackson, Ian Johnston, Dominic Kwiatkowski, Cordelia Langford, John Sillitoe on behalf of the Wellcome Sanger Institute COVID-19 Surveillance Team
EPI_ISL_778482	Lighthouse Lab in Glasgow	Wellcome Sanger Institute for the COVID-19 Genomics UK (COG-UK) Consortium	Harper VanSteenhouse, Yumi Kasai, David Gray, Carol Clugston, Anna Dominiczak and Alex Alderton, Roberto Amato, Sonia Goncalves, Ewan Harrison, David K. Jackson, Ian Johnston, Dominic Kwiatkowski, Cordelia Langford, John Sillitoe on behalf of the Wellcome Sanger Institute COVID-19 Surveillance Team
EPI_ISL_778484, EPI_ISL_778487, EPI_ISL_778488, EPI_ISL_778490, EPI_ISL_778491, EPI_ISL_778494	Lighthouse Lab in Alderley Park	Wellcome Sanger Institute for the COVID-19 Genomics UK (COG-UK) Consortium	Jacquelyn Wynn, Mairead Hyland, The Lighthouse Lab in Alderley Park and Alex Alderton, Roberto Amato, Sonia Goncalves, Ewan Harrison, David K. Jackson, Ian Johnston, Dominic Kwiatkowski, Cordelia Langford, John Sillitoe on behalf of the Wellcome Sanger Institute COVID-19 Surveillance Team
EPI_ISL_778496, EPI_ISL_778497, EPI_ISL_778499	Lighthouse Lab in Glasgow	Wellcome Sanger Institute for the COVID-19 Genomics UK (COG-UK) Consortium	Harper VanSteenhouse, Yumi Kasai, David Gray, Carol Clugston, Anna Dominiczak and Alex Alderton, Roberto Amato, Sonia Goncalves, Ewan Harrison, David K. Jackson, Ian Johnston, Dominic Kwiatkowski, Cordelia Langford, John Sillitoe on behalf of the Wellcome Sanger Institute COVID-19 Surveillance Team
EPI_ISL_778500	Lighthouse Lab in Alderley Park	Wellcome Sanger Institute for the COVID-19 Genomics UK (COG-UK) Consortium	Jacquelyn Wynn, Mairead Hyland, The Lighthouse Lab in Alderley Park and Alex Alderton, Roberto Amato, Sonia Goncalves, Ewan Harrison, David K. Jackson, Ian Johnston, Dominic Kwiatkowski, Cordelia Langford, John Sillitoe on behalf of the Wellcome Sanger Institute COVID-19 Surveillance Team
EPI_ISL_778504, EPI_ISL_778506	Lighthouse Lab in Glasgow	Wellcome Sanger Institute for the COVID-19 Genomics UK (COG-UK) Consortium	Harper VanSteenhouse, Yumi Kasai, David Gray, Carol Clugston, Anna Dominiczak and Alex Alderton, Roberto Amato, Sonia Goncalves, Ewan Harrison, David K. Jackson, Ian Johnston, Dominic Kwiatkowski, Cordelia Langford, John Sillitoe on behalf of the Wellcome Sanger Institute COVID-19 Surveillance Team
EPI_ISL_778510, EPI_ISL_778512	Lighthouse Lab in Alderley Park	Wellcome Sanger Institute for the COVID-19 Genomics UK (COG-UK) Consortium	Jacquelyn Wynn, Mairead Hyland, The Lighthouse Lab in Alderley Park and Alex Alderton, Roberto Amato, Sonia Goncalves, Ewan Harrison, David K. Jackson, Ian Johnston, Dominic Kwiatkowski, Cordelia Langford, John Sillitoe on behalf of the Wellcome Sanger Institute COVID-19 Surveillance Team
EPI_ISL_778516	Lighthouse Lab in Glasgow	Wellcome Sanger Institute for the COVID-19 Genomics UK (COG-UK) Consortium	Harper VanSteenhouse, Yumi Kasai, David Gray, Carol Clugston, Anna Dominiczak and Alex Alderton, Roberto Amato, Sonia Goncalves, Ewan Harrison, David K. Jackson, Ian Johnston, Dominic Kwiatkowski, Cordelia Langford, John Sillitoe on behalf of the Wellcome Sanger Institute COVID-19 Surveillance Team
EPI_ISL_778517	Lighthouse Lab in Alderley Park	Wellcome Sanger Institute for the COVID-19 Genomics UK (COG-UK) Consortium	Jacquelyn Wynn, Mairead Hyland, The Lighthouse Lab in Alderley Park and Alex Alderton, Roberto Amato, Sonia Goncalves, Ewan Harrison, David K. Jackson, Ian Johnston, Dominic Kwiatkowski, Cordelia Langford, John Sillitoe on behalf of the Wellcome Sanger Institute COVID-19 Surveillance Team
EPI_ISL_778522	Lighthouse Lab in Glasgow	Wellcome Sanger Institute for the COVID-19 Genomics UK (COG-UK) Consortium	Harper VanSteenhouse, Yumi Kasai, David Gray, Carol Clugston, Anna Dominiczak and Alex Alderton, Roberto Amato, Sonia Goncalves, Ewan Harrison, David K. Jackson, Ian Johnston, Dominic Kwiatkowski, Cordelia Langford, John Sillitoe on behalf of the Wellcome Sanger Institute COVID-19 Surveillance Team
EPI_ISL_778524	Lighthouse Lab in Alderley Park	Wellcome Sanger Institute for the COVID-19 Genomics UK (COG-UK) Consortium	Jacquelyn Wynn, Mairead Hyland, The Lighthouse Lab in Alderley Park and Alex Alderton, Roberto Amato, Sonia Goncalves, Ewan Harrison, David K. Jackson, Ian Johnston, Dominic Kwiatkowski, Cordelia Langford, John Sillitoe on behalf of the Wellcome Sanger Institute COVID-19 Surveillance Team
EPI_ISL_778525, EPI_ISL_778527	Lighthouse Lab in Glasgow	Wellcome Sanger Institute for the COVID-19 Genomics UK (COG-UK) Consortium	Harper VanSteenhouse, Yumi Kasai, David Gray, Carol Clugston, Anna Dominiczak and Alex Alderton, Roberto Amato, Sonia Goncalves, Ewan Harrison, David K. Jackson, Ian Johnston, Dominic Kwiatkowski, Cordelia Langford, John Sillitoe on behalf of the Wellcome Sanger Institute COVID-19 Surveillance Team
EPI_ISL_778528, EPI_ISL_778529	Lighthouse Lab in Alderley Park	Wellcome Sanger Institute for the COVID-19 Genomics UK (COG-UK) Consortium	Jacquelyn Wynn, Mairead Hyland, The Lighthouse Lab in Alderley Park and Alex Alderton, Roberto Amato, Sonia Goncalves, Ewan Harrison, David K. Jackson, Ian Johnston, Dominic Kwiatkowski, Cordelia Langford, John Sillitoe on behalf of the Wellcome Sanger Institute COVID-19 Surveillance Team
EPI_ISL_778530	Lighthouse Lab in Glasgow	Wellcome Sanger Institute for the COVID-19 Genomics UK (COG-UK) Consortium	Harper VanSteenhouse, Yumi Kasai, David Gray, Carol Clugston, Anna Dominiczak and Alex Alderton, Roberto Amato, Sonia Goncalves, Ewan Harrison, David K. Jackson, Ian Johnston, Dominic Kwiatkowski, Cordelia Langford, John Sillitoe on behalf of the Wellcome Sanger Institute COVID-19 Surveillance Team
EPI_ISL_778531	Lighthouse Lab in Alderley Park	Wellcome Sanger Institute for the COVID-19 Genomics UK (COG-UK) Consortium	Jacquelyn Wynn, Mairead Hyland, The Lighthouse Lab in Alderley Park and Alex Alderton, Roberto Amato, Sonia Goncalves, Ewan Harrison, David K. Jackson, Ian Johnston, Dominic Kwiatkowski, Cordelia Langford, John Sillitoe on behalf of the Wellcome Sanger Institute COVID-19 Surveillance Team
EPI_ISL_778534	Lighthouse Lab in Glasgow	Wellcome Sanger Institute for the COVID-19 Genomics UK (COG-UK) Consortium	Harper VanSteenhouse, Yumi Kasai, David Gray, Carol Clugston, Anna Dominiczak and Alex Alderton, Roberto Amato, Sonia Goncalves, Ewan Harrison, David K. Jackson, Ian Johnston, Dominic Kwiatkowski, Cordelia Langford, John Sillitoe on behalf of the Wellcome Sanger Institute COVID-19 Surveillance Team
EPI_ISL_778539	Lighthouse Lab in Alderley Park	Wellcome Sanger Institute for the COVID-19 Genomics UK (COG-UK) Consortium	Jacquelyn Wynn, Mairead Hyland, The Lighthouse Lab in Alderley Park and Alex Alderton, Roberto Amato, Sonia Goncalves, Ewan Harrison, David K. Jackson, Ian Johnston, Dominic Kwiatkowski, Cordelia Langford, John Sillitoe on behalf of the Wellcome Sanger Institute COVID-19 Surveillance Team
EPI_ISL_778540, EPI_ISL_778541	Lighthouse Lab in Glasgow	Wellcome Sanger Institute for the COVID-19 Genomics UK (COG-UK) Consortium	Harper VanSteenhouse, Yumi Kasai, David Gray, Carol Clugston, Anna Dominiczak and Alex Alderton, Roberto Amato, Sonia Goncalves, Ewan Harrison, David K. Jackson, Ian Johnston, Dominic Kwiatkowski, Cordelia Langford, John Sillitoe on behalf of the Wellcome Sanger Institute COVID-19 Surveillance Team
EPI_ISL_778543, EPI_ISL_778547	Lighthouse Lab in Alderley Park	Wellcome Sanger Institute for the COVID-19 Genomics UK (COG-UK) Consortium	Jacquelyn Wynn, Mairead Hyland, The Lighthouse Lab in Alderley Park and Alex Alderton, Roberto Amato, Sonia Goncalves, Ewan Harrison, David K. Jackson, Ian Johnston, Dominic Kwiatkowski, Cordelia Langford, John Sillitoe on behalf of the Wellcome Sanger Institute COVID-19 Surveillance Team
EPI_ISL_778548, EPI_ISL_778551, EPI_ISL_778553	Lighthouse Lab in Glasgow	Wellcome Sanger Institute for the COVID-19 Genomics UK (COG-UK) Consortium	Harper VanSteenhouse, Yumi Kasai, David Gray, Carol Clugston, Anna Dominiczak and Alex Alderton, Roberto Amato, Sonia Goncalves, Ewan Harrison, David K. Jackson, Ian Johnston, Dominic Kwiatkowski, Cordelia Langford, John Sillitoe on behalf of the Wellcome Sanger Institute COVID-19 Surveillance Team
EPI_ISL_778556, EPI_ISL_778558	Lighthouse Lab in Alderley Park	Wellcome Sanger Institute for the COVID-19 Genomics UK (COG-UK) Consortium	Jacquelyn Wynn, Mairead Hyland, The Lighthouse Lab in Alderley Park and Alex Alderton, Roberto Amato, Sonia Goncalves, Ewan Harrison, David K. Jackson, Ian Johnston, Dominic Kwiatkowski, Cordelia Langford, John Sillitoe on behalf of the Wellcome Sanger Institute COVID-19 Surveillance Team
EPI_ISL_778560	Lighthouse Lab in Glasgow	Wellcome Sanger Institute for the COVID-19 Genomics UK (COG-UK) Consortium	Harper VanSteenhouse, Yumi Kasai, David Gray, Carol Clugston, Anna Dominiczak and Alex Alderton, Roberto Amato, Sonia Goncalves, Ewan Harrison, David K. Jackson, Ian Johnston, Dominic Kwiatkowski, Cordelia Langford, John Sillitoe on behalf of the Wellcome Sanger Institute COVID-19 Surveillance Team
EPI_ISL_778562	Lighthouse Lab in Alderley Park	Wellcome Sanger Institute for the COVID-19 Genomics UK (COG-UK) Consortium	Jacquelyn Wynn, Mairead Hyland, The Lighthouse Lab in Alderley Park and Alex Alderton, Roberto Amato, Sonia Goncalves, Ewan Harrison, David K. Jackson, Ian Johnston, Dominic Kwiatkowski, Cordelia Langford, John Sillitoe on behalf of the Wellcome Sanger Institute COVID-19 Surveillance Team
EPI_ISL_778568	Lighthouse Lab in Glasgow	Wellcome Sanger Institute for the COVID-19 Genomics UK (COG-UK) Consortium	Harper VanSteenhouse, Yumi Kasai, David Gray, Carol Clugston, Anna Dominiczak and Alex Alderton, Roberto Amato, Sonia Goncalves, Ewan Harrison, David K. Jackson, Ian Johnston, Dominic Kwiatkowski, Cordelia Langford, John Sillitoe on behalf of the Wellcome Sanger Institute COVID-19 Surveillance Team
EPI_ISL_778569	Lighthouse Lab in Alderley Park	Wellcome Sanger Institute for the COVID-19 Genomics UK (COG-UK) Consortium	Jacquelyn Wynn, Mairead Hyland, The Lighthouse Lab in Alderley Park and Alex Alderton, Roberto Amato, Sonia Goncalves, Ewan Harrison, David K. Jackson, Ian Johnston, Dominic Kwiatkowski, Cordelia Langford, John Sillitoe on behalf of the Wellcome Sanger Institute COVID-19 Surveillance Team

EPI_ISL_778570	Lighthouse Lab in Glasgow	Wellcome Sanger Institute for the COVID-19 Genomics UK (COG-UK) Consortium	Harper VanSteenhouse, Yumi Kasai, David Gray, Carol Clugston, Anna Dominiczak and Alex Alderton, Roberto Amato, Sonia Goncalves, Ewan Harrison, David K. Jackson, Ian Johnston, Dominic Kwiatkowski, Cordelia Langford, John Sillitoe on behalf of the Wellcome Sanger Institute COVID-19 Surveillance Team
EPI_ISL_778571	Lighthouse Lab in Alderley Park	Wellcome Sanger Institute for the COVID-19 Genomics UK (COG-UK) Consortium	Jacquelyn Wynn, Mairead Hyland, The Lighthouse Lab in Alderley Park and Alex Alderton, Roberto Amato, Sonia Goncalves, Ewan Harrison, David K. Jackson, Ian Johnston, Dominic Kwiatkowski, Cordelia Langford, John Sillitoe on behalf of the Wellcome Sanger Institute COVID-19 Surveillance Team
EPI_ISL_778577	Lighthouse Lab in Glasgow	Wellcome Sanger Institute for the COVID-19 Genomics UK (COG-UK) Consortium	Harper VanSteenhouse, Yumi Kasai, David Gray, Carol Clugston, Anna Dominiczak and Alex Alderton, Roberto Amato, Sonia Goncalves, Ewan Harrison, David K. Jackson, Ian Johnston, Dominic Kwiatkowski, Cordelia Langford, John Sillitoe on behalf of the Wellcome Sanger Institute COVID-19 Surveillance Team
EPI_ISL_778578	Lighthouse Lab in Alderley Park	Wellcome Sanger Institute for the COVID-19 Genomics UK (COG-UK) Consortium	Jacquelyn Wynn, Mairead Hyland, The Lighthouse Lab in Alderley Park and Alex Alderton, Roberto Amato, Sonia Goncalves, Ewan Harrison, David K. Jackson, Ian Johnston, Dominic Kwiatkowski, Cordelia Langford, John Sillitoe on behalf of the Wellcome Sanger Institute COVID-19 Surveillance Team
EPI_ISL_778579	Lighthouse Lab in Glasgow	Wellcome Sanger Institute for the COVID-19 Genomics UK (COG-UK) Consortium	Harper VanSteenhouse, Yumi Kasai, David Gray, Carol Clugston, Anna Dominiczak and Alex Alderton, Roberto Amato, Sonia Goncalves, Ewan Harrison, David K. Jackson, Ian Johnston, Dominic Kwiatkowski, Cordelia Langford, John Sillitoe on behalf of the Wellcome Sanger Institute COVID-19 Surveillance Team
EPI_ISL_778584	Lighthouse Lab in Alderley Park	Wellcome Sanger Institute for the COVID-19 Genomics UK (COG-UK) Consortium	Jacquelyn Wynn, Mairead Hyland, The Lighthouse Lab in Alderley Park and Alex Alderton, Roberto Amato, Sonia Goncalves, Ewan Harrison, David K. Jackson, Ian Johnston, Dominic Kwiatkowski, Cordelia Langford, John Sillitoe on behalf of the Wellcome Sanger Institute COVID-19 Surveillance Team
EPI_ISL_778586	Lighthouse Lab in Glasgow	Wellcome Sanger Institute for the COVID-19 Genomics UK (COG-UK) Consortium	Harper VanSteenhouse, Yumi Kasai, David Gray, Carol Clugston, Anna Dominiczak and Alex Alderton, Roberto Amato, Sonia Goncalves, Ewan Harrison, David K. Jackson, Ian Johnston, Dominic Kwiatkowski, Cordelia Langford, John Sillitoe on behalf of the Wellcome Sanger Institute COVID-19 Surveillance Team
EPI_ISL_778593	Lighthouse Lab in Alderley Park	Wellcome Sanger Institute for the COVID-19 Genomics UK (COG-UK) Consortium	Jacquelyn Wynn, Mairead Hyland, The Lighthouse Lab in Alderley Park and Alex Alderton, Roberto Amato, Sonia Goncalves, Ewan Harrison, David K. Jackson, Ian Johnston, Dominic Kwiatkowski, Cordelia Langford, John Sillitoe on behalf of the Wellcome Sanger Institute COVID-19 Surveillance Team
EPI_ISL_778594	Lighthouse Lab in Glasgow	Wellcome Sanger Institute for the COVID-19 Genomics UK (COG-UK) Consortium	Harper VanSteenhouse, Yumi Kasai, David Gray, Carol Clugston, Anna Dominiczak and Alex Alderton, Roberto Amato, Sonia Goncalves, Ewan Harrison, David K. Jackson, Ian Johnston, Dominic Kwiatkowski, Cordelia Langford, John Sillitoe on behalf of the Wellcome Sanger Institute COVID-19 Surveillance Team
EPI_ISL_778598, EPI_ISL_778599, EPI_ISL_778601, EPI_ISL_778605, EPI_ISL_778607, EPI_ISL_778612, EPI_ISL_778614, EPI_ISL_778616	Lighthouse Lab in Alderley Park	Wellcome Sanger Institute for the COVID-19 Genomics UK (COG-UK) Consortium	Jacquelyn Wynn, Mairead Hyland, The Lighthouse Lab in Alderley Park and Alex Alderton, Roberto Amato, Sonia Goncalves, Ewan Harrison, David K. Jackson, Ian Johnston, Dominic Kwiatkowski, Cordelia Langford, John Sillitoe on behalf of the Wellcome Sanger Institute COVID-19 Surveillance Team
EPI_ISL_778617	Lighthouse Lab in Glasgow	Wellcome Sanger Institute for the COVID-19 Genomics UK (COG-UK) Consortium	Harper VanSteenhouse, Yumi Kasai, David Gray, Carol Clugston, Anna Dominiczak and Alex Alderton, Roberto Amato, Sonia Goncalves, Ewan Harrison, David K. Jackson, Ian Johnston, Dominic Kwiatkowski, Cordelia Langford, John Sillitoe on behalf of the Wellcome Sanger Institute COVID-19 Surveillance Team
EPI_ISL_778618	Lighthouse Lab in Alderley Park	Wellcome Sanger Institute for the COVID-19 Genomics UK (COG-UK) Consortium	Jacquelyn Wynn, Mairead Hyland, The Lighthouse Lab in Alderley Park and Alex Alderton, Roberto Amato, Sonia Goncalves, Ewan Harrison, David K. Jackson, Ian Johnston, Dominic Kwiatkowski, Cordelia Langford, John Sillitoe on behalf of the Wellcome Sanger Institute COVID-19 Surveillance Team
EPI_ISL_778619, EPI_ISL_778620	Lighthouse Lab in Glasgow	Wellcome Sanger Institute for the COVID-19 Genomics UK (COG-UK) Consortium	Harper VanSteenhouse, Yumi Kasai, David Gray, Carol Clugston, Anna Dominiczak and Alex Alderton, Roberto Amato, Sonia Goncalves, Ewan Harrison, David K. Jackson, Ian Johnston, Dominic Kwiatkowski, Cordelia Langford, John Sillitoe on behalf of the Wellcome Sanger Institute COVID-19 Surveillance Team
EPI_ISL_778621, EPI_ISL_778623, EPI_ISL_778627, EPI_ISL_778628	Lighthouse Lab in Alderley Park	Wellcome Sanger Institute for the COVID-19 Genomics UK (COG-UK) Consortium	Jacquelyn Wynn, Mairead Hyland, The Lighthouse Lab in Alderley Park and Alex Alderton, Roberto Amato, Sonia Goncalves, Ewan Harrison, David K. Jackson, Ian Johnston, Dominic Kwiatkowski, Cordelia Langford, John Sillitoe on behalf of the Wellcome Sanger Institute COVID-19 Surveillance Team
EPI_ISL_778629	Lighthouse Lab in Glasgow	Wellcome Sanger Institute for the COVID-19 Genomics UK (COG-UK) Consortium	Harper VanSteenhouse, Yumi Kasai, David Gray, Carol Clugston, Anna Dominiczak and Alex Alderton, Roberto Amato, Sonia Goncalves, Ewan Harrison, David K. Jackson, Ian Johnston, Dominic Kwiatkowski, Cordelia Langford, John Sillitoe on behalf of the Wellcome Sanger Institute COVID-19 Surveillance Team
EPI_ISL_778630	Lighthouse Lab in Alderley Park	Wellcome Sanger Institute for the COVID-19 Genomics UK (COG-UK) Consortium	Jacquelyn Wynn, Mairead Hyland, The Lighthouse Lab in Alderley Park and Alex Alderton, Roberto Amato, Sonia Goncalves, Ewan Harrison, David K. Jackson, Ian Johnston, Dominic Kwiatkowski, Cordelia Langford, John Sillitoe on behalf of the Wellcome Sanger Institute COVID-19 Surveillance Team
EPI_ISL_778631	Lighthouse Lab in Glasgow	Wellcome Sanger Institute for the COVID-19 Genomics UK (COG-UK) Consortium	Harper VanSteenhouse, Yumi Kasai, David Gray, Carol Clugston, Anna Dominiczak and Alex Alderton, Roberto Amato, Sonia Goncalves, Ewan Harrison, David K. Jackson, Ian Johnston, Dominic Kwiatkowski, Cordelia Langford, John Sillitoe on behalf of the Wellcome Sanger Institute COVID-19 Surveillance Team
EPI_ISL_778635, EPI_ISL_778638	Lighthouse Lab in Alderley Park	Wellcome Sanger Institute for the COVID-19 Genomics UK (COG-UK) Consortium	Jacquelyn Wynn, Mairead Hyland, The Lighthouse Lab in Alderley Park and Alex Alderton, Roberto Amato, Sonia Goncalves, Ewan Harrison, David K. Jackson, Ian Johnston, Dominic Kwiatkowski, Cordelia Langford, John Sillitoe on behalf of the Wellcome Sanger Institute COVID-19 Surveillance Team
EPI_ISL_778822	AIID	Irish Coronavirus Sequencing Consortium-Teagasc Grange	Matthew McCabe, Aljandro Abner Garcia Leon, Fiona Crispie, Calum Walsh, Michael Carr, John Kenny, Paul Cotter, Patrick Mallon, Gabriel Gonzalez
EPI_ISL_779082, EPI_ISL_779083, EPI_ISL_779084, EPI_ISL_779085, EPI_ISL_779086, EPI_ISL_779087, EPI_ISL_779088, EPI_ISL_779089, EPI_ISL_779090, EPI_ISL_779091, EPI_ISL_779092, EPI_ISL_779093, EPI_ISL_779094, EPI_ISL_779095, EPI_ISL_779096, EPI_ISL_779097, EPI_ISL_779098, EPI_ISL_779099, EPI_ISL_779100, EPI_ISL_779101, EPI_ISL_779102, EPI_ISL_779103, EPI_ISL_779104			
see above	LSUHS Emerging Viral Threat Laboratory	Microbial Genome Sequencing Center	Jeremy P. Kamil, Jennifer L. Carroll, Camille F. Abshire, Maarten Van Diest, Andrew D. Yurochko, Martin J. Sapp, Rona S. Scott, Christopher G. Kevil, Daniel J. Snyder, Vaughn S. Cooper, John A. Vanchiere
EPI_ISL_779105, EPI_ISL_779106, EPI_ISL_779107, EPI_ISL_779108	LSUHS Emerging Viral Threat Laboratory	Microbial Genome Sequencing Center	Jennifer L. Carroll, Jeremy P. Kamil, Camille F. Abshire, Maarten Van Diest, Andrew D. Yurochko, Martin J. Sapp, Rona S. Scott, Christopher G. Kevil, Daniel J. Snyder, Vaughn S. Cooper, John A. Vanchiere
EPI_ISL_779847	Centre Hospotalier Cannes	CNR Virus des Infections Respiratoires - France SUD	Antonin Bal, Gregory Destras, Gwendolyne Burfin, Hadrien Règue, Quentin Semanas, Martine Valette, Bruno Lina, Laurence Josset
EPI_ISL_779855, EPI_ISL_779858, EPI_ISL_779859, EPI_ISL_779860, EPI_ISL_779869, EPI_ISL_779880, EPI_ISL_779884, EPI_ISL_779896, EPI_ISL_779897, EPI_ISL_779907, EPI_ISL_779912, EPI_ISL_779913			
see above	Servicio de Microbiología, Hospital Universitario Son Espases	SeqCOVID-SPAIN consortium/IBV(CSIC)	Carla López-Causapé, Jordi Reina, Antonio Oliver and SeqCOVID-SPAIN consortium
EPI_ISL_779922, EPI_ISL_779924, EPI_ISL_779927, EPI_ISL_779932, EPI_ISL_779939, EPI_ISL_779940, EPI_ISL_779941, EPI_ISL_779942, EPI_ISL_779943, EPI_ISL_779944, EPI_ISL_779945, EPI_ISL_779946, EPI_ISL_779947, EPI_ISL_779948, EPI_ISL_779949, EPI_ISL_779950, EPI_ISL_779951			
see above	Center of Medical Microbiology, Virology, and Hospital Hygiene, University of Duesseldorf	Center of Medical Microbiology, Virology, and Hospital Hygiene, University of Duesseldorf	Maximilian Damagnez, Alexander Dilthey, Ashley-Jane Duplessis, Torsten Houwaart, Lisanna Hülse, Malte Kohns Vasconcelos, Nadine Lübke, Jessica Nicolai, Klaus Pfeffer, Daniel Strelow, Teresa Tamayo, Jörg Timm, Andreas Walker, Tobias Wienemann
EPI_ISL_781551, EPI_ISL_781571, EPI_ISL_781625, EPI_ISL_781671, EPI_ISL_781690, EPI_ISL_781710, EPI_ISL_781824, EPI_ISL_781863	Lighthouse Lab in Cambridge	Wellcome Sanger Institute for the COVID-19 Genomics UK (COG-UK) Consortium	Rob Howes, The Lighthouse Lab in Cambridge and Alex Alderton, Roberto Amato, Sonia Goncalves, Ewan Harrison, David K. Jackson, Ian Johnston, Dominic Kwiatkowski, Cordelia Langford, John Sillitoe on behalf of the Wellcome Sanger Institute COVID-19 Surveillance Team
EPI_ISL_781957, EPI_ISL_782123	Lighthouse Lab in Alderley Park	Wellcome Sanger Institute for the COVID-19 Genomics UK (COG-UK) Consortium	Jacquelyn Wynn, Mairead Hyland, The Lighthouse Lab in Alderley Park and Alex Alderton, Roberto Amato, Sonia Goncalves, Ewan Harrison, David K. Jackson, Ian Johnston, Dominic Kwiatkowski, Cordelia Langford, John Sillitoe on behalf of the Wellcome Sanger Institute COVID-19 Surveillance Team
EPI_ISL_789038, EPI_ISL_789040, EPI_ISL_789041, EPI_ISL_789042, EPI_ISL_789043	Klinisk mikrobiologi	The Public Health Agency of Sweden	Department of Microbiology, The Public Health Agency of Sweden
EPI_ISL_789046	unknown	The Public Health Agency of Sweden	Department of Microbiology, The Public Health Agency of Sweden
EPI_ISL_789047	Klinisk mikrobiologi	The Public Health Agency of Sweden	Department of Microbiology, The Public Health Agency of Sweden
EPI_ISL_790585, EPI_ISL_790592, EPI_ISL_790598, EPI_ISL_790611, EPI_ISL_790624, EPI_ISL_790625, EPI_ISL_790629, EPI_ISL_790633, EPI_ISL_790635, EPI_ISL_790636, EPI_ISL_790637, EPI_ISL_790638, EPI_ISL_790639, EPI_ISL_790640, EPI_ISL_790641, EPI_ISL_790642, EPI_ISL_790643, EPI_ISL_790644,			

EPI_ISL_790645, EPI_ISL_790646, EPI_ISL_790650, EPI_ISL_790667, EPI_ISL_790672, EPI_ISL_790673, EPI_ISL_790703, EPI_ISL_790709, EPI_ISL_790714, EPI_ISL_790715, EPI_ISL_790716, EPI_ISL_790717, EPI_ISL_790718, EPI_ISL_790719, EPI_ISL_790720, EPI_ISL_790721, EPI_ISL_790729, EPI_ISL_790734, EPI_ISL_790735, EPI_ISL_790736, EPI_ISL_790737, EPI_ISL_790742, EPI_ISL_790765, EPI_ISL_790770, EPI_ISL_790772, EPI_ISL_790773, EPI_ISL_790777, EPI_ISL_790784, EPI_ISL_790788, EPI_ISL_790794, EPI_ISL_790795, EPI_ISL_790796, EPI_ISL_790797, EPI_ISL_790807, EPI_ISL_790813, EPI_ISL_790816, EPI_ISL_790832, EPI_ISL_790837, EPI_ISL_790850, EPI_ISL_790876, EPI_ISL_790877, EPI_ISL_790878, EPI_ISL_790889, EPI_ISL_790890, EPI_ISL_790891, EPI_ISL_790901, EPI_ISL_790920, EPI_ISL_790935, EPI_ISL_790936, EPI_ISL_790937, EPI_ISL_790955, EPI_ISL_791081, EPI_ISL_791083			
see above	Dutch COVID-19 response team	National Institute for Public Health and the Environment (RIVM)	Adam Meijer, Harry Vennema, Jeroen Cremer, Sharon van den Brink, Bas van der Veer, AnneMarie van den Brandt, Florian Zwagemaker, Dennis Schmitz, Chantal Reusken, on behalf of the national COVID-19 response team
EPI_ISL_791085	Instituto Nacional de Salud - Unidad de Secuenciación y Análisis Genómico	Instituto Nacional de Salud - Dirección de Investigación en Salud Pública	Katherine Laiton-Donato, Diego A. Álvarez-Díaz, Carlos Franco-Muñoz, Mauricio Pacheco-Montealegre, Jeadran Malagon-Rojas, Jesith Toloza, Julia Almentero, Ronald Lopez, Jonathan Reales, Diego Andrés Prada, Magdalena Wiesner, Martha Lucia Ospina Martinez, Marcela Mercado-Reyes
EPI_ISL_791184, EPI_ISL_791185, EPI_ISL_791186	Respiratory Virus Unit, National Infection Service, Public Health England	COVID-19 Genomics UK (COG-UK) Consortium	PHE Covid Sequencing Team
EPI_ISL_791287, EPI_ISL_791301, EPI_ISL_791306, EPI_ISL_791321	National Virus Reference Laboratory	Irish Coronavirus Sequencing Consortium - Teagasc Moorepark	Alejandro Abner Garcia Leon, Paul Cotter, Fiona Crispie, John Kenny, Paddy Mallon, Calum Walsh
EPI_ISL_791417, EPI_ISL_791484	Johns Hopkins Hospital Department of Pathology	Johns Hopkins Hospital Department of Pathology	C. Paul Morris, Chun Huai Luo, Adannaya Amadi, Nicholas Gallagher, Heba H. Mostafa
EPI_ISL_792522	Laboratorio de Virología del Hospital de Niños Dr. Ricardo Gutierrez	Grupo de Genómica y Bioinformática del Instituto de Investigación de la Cadena Láctea CONICET-INTA on behalf of 'Proyecto Argentino Interinstitucional de genomica de SARS-CoV-2' (PAIS Consortium)	Amadio, AF, Eberhardt, MF; Irazoqui, M; Torres, C; Aulicino, P; König, G; Acevedo, ME; Alvarez Lopez, C; Alexay, S; Jacques, O; Mistchenko, AS, Goya, S; Nabaes Jodar, MS; Viegas, M.
EPI_ISL_792557	Connecticut State Department of Public Health	Grubaugh Lab - Yale School of Public Health	Joseph Fauver, Tara Alpert, Chantal Vogels, Anderson Brito, Nagarjuna Cheemarla, Ellen Foxman, Anthony Muyombwe, Jafar Razeq, Nathan Grubaugh
EPI_ISL_792708, EPI_ISL_792709, EPI_ISL_792711, EPI_ISL_792712, EPI_ISL_792714, EPI_ISL_792715, EPI_ISL_792716, EPI_ISL_792717, EPI_ISL_792718, EPI_ISL_792719, EPI_ISL_792720, EPI_ISL_792721, EPI_ISL_792722, EPI_ISL_792723, EPI_ISL_792724, EPI_ISL_792725, EPI_ISL_792726, EPI_ISL_792727, EPI_ISL_792728, EPI_ISL_792729, EPI_ISL_792730, EPI_ISL_792731, EPI_ISL_792732, EPI_ISL_792733, EPI_ISL_792734, EPI_ISL_792735, EPI_ISL_792736, EPI_ISL_792737, EPI_ISL_792738, EPI_ISL_792739, EPI_ISL_792749, EPI_ISL_792750, EPI_ISL_792751, EPI_ISL_792752, EPI_ISL_792753, EPI_ISL_792755, EPI_ISL_792756, EPI_ISL_792757, EPI_ISL_792758, EPI_ISL_792760, EPI_ISL_792761, EPI_ISL_792762, EPI_ISL_792763, EPI_ISL_792764, EPI_ISL_792765, EPI_ISL_792766, EPI_ISL_792767, EPI_ISL_792768, EPI_ISL_792769, EPI_ISL_792770, EPI_ISL_792771, EPI_ISL_792772, EPI_ISL_792773, EPI_ISL_792774, EPI_ISL_792775, EPI_ISL_792776, EPI_ISL_792777, EPI_ISL_792778, EPI_ISL_792779, EPI_ISL_792781, EPI_ISL_792782, EPI_ISL_792785, EPI_ISL_792786, EPI_ISL_792787, EPI_ISL_792788, EPI_ISL_792789, EPI_ISL_792790, EPI_ISL_792791, EPI_ISL_792792, EPI_ISL_792794, EPI_ISL_792795, EPI_ISL_792796, EPI_ISL_792797, EPI_ISL_792798, EPI_ISL_792799, EPI_ISL_792800, EPI_ISL_792801, EPI_ISL_792802, EPI_ISL_792803, EPI_ISL_792804, EPI_ISL_792805, EPI_ISL_792806, EPI_ISL_792807, EPI_ISL_792808, EPI_ISL_792809, EPI_ISL_792810, EPI_ISL_792811, EPI_ISL_792812, EPI_ISL_792813, EPI_ISL_792814, EPI_ISL_792815, EPI_ISL_792816, EPI_ISL_792817, EPI_ISL_792818, EPI_ISL_792819, EPI_ISL_792820, EPI_ISL_792821, EPI_ISL_792822, EPI_ISL_792823, EPI_ISL_792824, EPI_ISL_792829, EPI_ISL_792830, EPI_ISL_792834, EPI_ISL_792835, EPI_ISL_792840, EPI_ISL_792841, EPI_ISL_792842, EPI_ISL_792843, EPI_ISL_792844, EPI_ISL_792845, EPI_ISL_792846, EPI_ISL_792847, EPI_ISL_792848, EPI_ISL_792849, EPI_ISL_792850, EPI_ISL_792851, EPI_ISL_792852, EPI_ISL_792853, EPI_ISL_792854, EPI_ISL_792855, EPI_ISL_792856, EPI_ISL_792857, EPI_ISL_792858, EPI_ISL_792859, EPI_ISL_792860, EPI_ISL_792861, EPI_ISL_792862, EPI_ISL_792863, EPI_ISL_792864, EPI_ISL_792865, EPI_ISL_792866, EPI_ISL_792867, EPI_ISL_792868, EPI_ISL_792869, EPI_ISL_792870, EPI_ISL_792871, EPI_ISL_792872, EPI_ISL_792873, EPI_ISL_792874, EPI_ISL_792875, EPI_ISL_792876, EPI_ISL_792877, EPI_ISL_792878, EPI_ISL_792879, EPI_ISL_792880, EPI_ISL_792881, EPI_ISL_792882, EPI_ISL_792883, EPI_ISL_792884, EPI_ISL_792885, EPI_ISL_792886, EPI_ISL_792887, EPI_ISL_792888, EPI_ISL_792889, EPI_ISL_792890, EPI_ISL_792891, EPI_ISL_792892, EPI_ISL_792893, EPI_ISL_792894, EPI_ISL_792895, EPI_ISL_792896, EPI_ISL_792897, EPI_ISL_792898, EPI_ISL_792899, EPI_ISL_792900, EPI_ISL_792901, EPI_ISL_792902, EPI_ISL_792903, EPI_ISL_792904, EPI_ISL_792905, EPI_ISL_792906, EPI_ISL_792907, EPI_ISL_792908, EPI_ISL_792909, EPI_ISL_792910, EPI_ISL_792911, EPI_ISL_792912, EPI_ISL_792913, EPI_ISL_792914, EPI_ISL_792915, EPI_ISL_792916, EPI_ISL_792917, EPI_ISL_792918, EPI_ISL_792919, EPI_ISL_792920, EPI_ISL_792921, EPI_ISL_792922, EPI_ISL_792923, EPI_ISL_792924, EPI_ISL_792925, EPI_ISL_792926, EPI_ISL_792927, EPI_ISL_792928, EPI_ISL_792929, EPI_ISL_792930, EPI_ISL_792931, EPI_ISL_792932, EPI_ISL_792933, EPI_ISL_792934, EPI_ISL_792935, EPI_ISL_792936, EPI_ISL_792937, EPI_ISL_792938, EPI_ISL_792939, EPI_ISL_792940, EPI_ISL_792941, EPI_ISL_792942, EPI_ISL_792943, EPI_ISL_792944, EPI_ISL_792945, EPI_ISL_792946, EPI_ISL_792947, EPI_ISL_792948, EPI_ISL_792949, EPI_ISL_792950, EPI_ISL_792951, EPI_ISL_792952, EPI_ISL_792953, EPI_ISL_792954, EPI_ISL_792955, EPI_ISL_792956, EPI_ISL_792957, EPI_ISL_792958, EPI_ISL_792959, EPI_ISL_792960, EPI_ISL_792961, EPI_ISL_792962, EPI_ISL_792963, EPI_ISL_792964, EPI_ISL_792965, EPI_ISL_792966, EPI_ISL_792967, EPI_ISL_792968, EPI_ISL_792969, EPI_ISL_792970, EPI_ISL_792971, EPI_ISL_792972, EPI_ISL_792973, EPI_ISL_792974, EPI_ISL_792975, EPI_ISL_792976, EPI_ISL_792977, EPI_ISL_792978, EPI_ISL_792979, EPI_ISL_793111, EPI_ISL_793112, EPI_ISL_793113, EPI_ISL_793114, EPI_ISL_793146, EPI_ISL_793155, EPI_ISL_793156, EPI_ISL_793157, EPI_ISL_793162, EPI_ISL_793178, EPI_ISL_793201, EPI_ISL_793248, EPI_ISL_793259, EPI_ISL_793268, EPI_ISL_793278, EPI_ISL_793292, EPI_ISL_793315, EPI_ISL_793368, EPI_ISL_793369, EPI_ISL_793380, EPI_ISL_793990, EPI_ISL_793991, EPI_ISL_793992, EPI_ISL_793993, EPI_ISL_793994, EPI_ISL_793995, EPI_ISL_793996, EPI_ISL_793997, EPI_ISL_793998, EPI_ISL_793999, EPI_ISL_794000, EPI_ISL_794001, EPI_ISL_794002, EPI_ISL_794003, EPI_ISL_794004, EPI_ISL_794005, EPI_ISL_794006, EPI_ISL_794007, EPI_ISL_794008, EPI_ISL_794009, EPI_ISL_794010, EPI_ISL_794011, EPI_ISL_794012, EPI_ISL_794013			
see above	Department of Virus and Microbiological Special Diagnostics, Statens Serum Institut, Copenhagen, Denmark	Albertsen Lab, Department of Chemistry and Bioscience, Aalborg University, Denmark	Danish Covid-19 Genome Consortium
EPI_ISL_794297	Columbia University Irving Medical Center	Wadsworth Center, New York State Department.of Health	Kirsten St. George, Daryl M. Lamson, Alexis Russel, Matthew Shudt, Melissa A Leisner, Jonathan Plitnick, Navjot Singh, John Kelly, Sara Griesemer, Erasmus Schneider, Erica Lasek-Nesselquist
EPI_ISL_794326, EPI_ISL_794327, EPI_ISL_794328, EPI_ISL_794339, EPI_ISL_794348, EPI_ISL_794354, EPI_ISL_794355, EPI_ISL_794356, EPI_ISL_794357, EPI_ISL_794358, EPI_ISL_794359, EPI_ISL_794360, EPI_ISL_794368, EPI_ISL_794379, EPI_ISL_794380, EPI_ISL_794381, EPI_ISL_794382, EPI_ISL_794390, EPI_ISL_794391, EPI_ISL_794392, EPI_ISL_794393, EPI_ISL_794394, EPI_ISL_794395, EPI_ISL_794396, EPI_ISL_794397, EPI_ISL_794422, EPI_ISL_794428, EPI_ISL_794429, EPI_ISL_794432, EPI_ISL_794433, EPI_ISL_794436, EPI_ISL_794438, EPI_ISL_794439, EPI_ISL_794449, EPI_ISL_794450, EPI_ISL_794451, EPI_ISL_794452, EPI_ISL_794453, EPI_ISL_794454, EPI_ISL_794455, EPI_ISL_794456, EPI_ISL_794457, EPI_ISL_794458, EPI_ISL_794459, EPI_ISL_794460, EPI_ISL_794461, EPI_ISL_794462, EPI_ISL_794463, EPI_ISL_794464, EPI_ISL_794465, EPI_ISL_794466, EPI_ISL_794467, EPI_ISL_794468, EPI_ISL_794469, EPI_ISL_794470, EPI_ISL_794471, EPI_ISL_794472, EPI_ISL_794473, EPI_ISL_794474, EPI_ISL_794475, EPI_ISL_794476, EPI_ISL_794477, EPI_ISL_794478, EPI_ISL_794479, EPI_ISL_794480, EPI_ISL_794481, EPI_ISL_794482, EPI_ISL_794483, EPI_ISL_794484, EPI_ISL_794485, EPI_ISL_794486, EPI_ISL_794487, EPI_ISL_794488, EPI_ISL_794489, EPI_ISL_794490, EPI_ISL_794491, EPI_ISL_794492, EPI_ISL_794493, EPI_ISL_794494, EPI_ISL_794495			
see above	Department of Virus and Microbiological Special Diagnostics, Statens Serum Institut, Copenhagen, Denmark	Albertsen Lab, Department of Chemistry and Bioscience, Aalborg University, Denmark	Danish Covid-19 Genome Consortium
EPI_ISL_794726, EPI_ISL_794734	PathWest Laboratory Medicine WA	PathWest Laboratory Medicine WA Microbial Surveillance Unit	PathWest Laboratory Medicine WA Microbial Surveillance Unit
EPI_ISL_794788, EPI_ISL_794789, EPI_ISL_794790, EPI_ISL_794793, EPI_ISL_794803, EPI_ISL_794804, EPI_ISL_794805, EPI_ISL_794806, EPI_ISL_794807, EPI_ISL_794808	Istituto Zooprofilattico Sperimentale della Puglia e della Basilicata	Istituto Zooprofilattico Sperimentale della Puglia e della Basilicata	Parisi A., Bianco A., Capozzi L., Del Sambro L., Manzulli V, Rondinone V., Pace L., Cipolletta D., Galante D.
EPI_ISL_794831, EPI_ISL_794832, EPI_ISL_794837, EPI_ISL_794838, EPI_ISL_794839, EPI_ISL_794840, EPI_ISL_794841, EPI_ISL_794842, EPI_ISL_794843, EPI_ISL_794844, EPI_ISL_794845, EPI_ISL_794846, EPI_ISL_794847, EPI_ISL_794848, EPI_ISL_794849, EPI_ISL_794861, EPI_ISL_794863, EPI_ISL_794865, EPI_ISL_794866, EPI_ISL_794867, EPI_ISL_794869, EPI_ISL_794873, EPI_ISL_794875, EPI_ISL_794877, EPI_ISL_794879, EPI_ISL_794881, EPI_ISL_794882, EPI_ISL_794884, EPI_ISL_794889, EPI_ISL_794890, EPI_ISL_794891, EPI_ISL_794892, EPI_ISL_794893, EPI_ISL_794894, EPI_ISL_794904, EPI_ISL_794930, EPI_ISL_794931, EPI_ISL_794932, EPI_ISL_794933, EPI_ISL_794934, EPI_ISL_794935, EPI_ISL_794936, EPI_ISL_794937, EPI_ISL_794938, EPI_ISL_794939, EPI_ISL_794940, EPI_ISL_794941, EPI_ISL_794942, EPI_ISL_794943, EPI_ISL_794944, EPI_ISL_794945, EPI_ISL_794946, EPI_ISL_794947, EPI_ISL_794948, EPI_ISL_794949, EPI_ISL_794950, EPI_ISL_794951, EPI_ISL_794952, EPI_ISL_794953, EPI_ISL_794954, EPI_ISL_794955, EPI_ISL_794956, EPI_ISL_794957, EPI_ISL_794958, EPI_ISL_794959, EPI_ISL_794960, EPI_ISL_794961, EPI_ISL_794962, EPI_ISL_794963, EPI_ISL_794964, EPI_ISL_794965, EPI_ISL_794966, EPI_ISL_794967, EPI_ISL_794968, EPI_ISL_794969, EPI_ISL_794970, EPI_ISL_794971, EPI_ISL_794972, EPI_ISL_794973, EPI_ISL_794974, EPI_ISL_794975, EPI_ISL_794976, EPI_ISL_794977, EPI_ISL_794978, EPI_ISL_794979, EPI_ISL_794980, EPI_ISL_794981, EPI_ISL_794982			
see above	Department of Virus and Microbiological Special Diagnostics, Statens Serum Institut, Copenhagen, Denmark	Albertsen Lab, Department of Chemistry and Bioscience, Aalborg University, Denmark	Danish Covid-19 Genome Consortium
EPI_ISL_796651	Medical Microbiology Unit, Department for Laboratory Medicine, Drammen Hospital, Vestre Viken Health Trust,	Norwegian Institute of Public Health, Department of Virology	Kathrine Stene-Johansen, Kamilla Heddeland Instefjord, Hilde Elshaug, Atiya R Ali,Marie Paulsen Madsen, Rasmus Riis Kopperud, Hilde Vollan, Karoline Bragstad, Olav Hungnes
EPI_ISL_796683	Department of Medical Microbiology - section Molde, Molde Hospital	Norwegian Institute of Public Health, Department of Virology	Kathrine Stene-Johansen, Kamilla Heddeland Instefjord, Hilde Elshaug, Atiya R Ali,Marie Paulsen Madsen, Rasmus Riis Kopperud, Hilde Vollan, Karoline Bragstad, Olav Hungnes
EPI_ISL_796686	Unilabs Laboratory Medicine	Norwegian Institute of Public Health, Department of Virology	Kathrine Stene-Johansen, Kamilla Heddeland Instefjord, Hilde Elshaug, Atiya R Ali,Marie Paulsen Madsen, Rasmus Riis Kopperud, Hilde Vollan, Karoline Bragstad, Olav Hungnes
EPI_ISL_796688	Hospital of Southern Norway - Kristiansand, Department of Medical Microbiology	Norwegian Institute of Public Health, Department of Virology	Kathrine Stene-Johansen, Kamilla Heddeland Instefjord, Hilde Elshaug, Atiya R Ali,Marie Paulsen Madsen, Rasmus Riis Kopperud, Hilde Vollan, Karoline Bragstad, Olav Hungnes
EPI_ISL_796700, EPI_ISL_796701	Unilabs Laboratory Medicine	Norwegian Institute of Public Health, Department of Virology	Kathrine Stene-Johansen, Kamilla Heddeland Instefjord, Hilde Elshaug, Atiya R Ali,Marie Paulsen Madsen, Rasmus Riis Kopperud, Hilde Vollan, Karoline Bragstad, Olav Hungnes
EPI_ISL_796715	Hospital of Southern Norway - Kristiansand, Department of Medical Microbiology	Norwegian Institute of Public Health, Department of Virology	Kathrine Stene-Johansen, Kamilla Heddeland Instefjord, Hilde Elshaug, Atiya R Ali,Marie Paulsen Madsen, Rasmus Riis Kopperud, Hilde Vollan, Karoline Bragstad, Olav Hungnes
EPI_ISL_796717, EPI_ISL_796718	Department of Medical Microbiology, St. Olavs hospital	Norwegian Institute of Public Health, Department of Virology	Kathrine Stene-Johansen, Kamilla Heddeland Instefjord, Hilde Elshaug, Atiya R Ali,Marie Paulsen Madsen, Rasmus Riis Kopperud, Hilde Vollan, Karoline Bragstad, Olav Hungnes
EPI_ISL_796722, EPI_ISL_796723	Furst Medical Laboratory	Norwegian Institute of Public Health, Department of Virology	Kathrine Stene-Johansen, Kamilla Heddeland Instefjord, Hilde Elshaug, Atiya R Ali,Marie Paulsen Madsen, Rasmus Riis Kopperud, Hilde Vollan, Karoline Bragstad, Olav Hungnes
EPI_ISL_796729	Hospital of Southern Norway - Kristiansand, Department of Medical Microbiology	Norwegian Institute of Public Health, Department of Virology	Kathrine Stene-Johansen, Kamilla Heddeland Instefjord, Hilde Elshaug, Atiya R Ali,Marie Paulsen Madsen, Rasmus Riis Kopperud, Hilde Vollan, Karoline Bragstad, Olav Hungnes

EPI_ISL_796780	Instituto Nacional de Saude (INSA)	Instituto Nacional de Saude (INSA)	Borges et al
EPI_ISL_799710, EPI_ISL_799900	Lighthouse Lab in Milton Keynes	Wellcome Sanger Institute for the COVID-19 Genomics UK (COG-UK) Consortium	The Lighthouse Lab in Milton Keynes and Alex Alderton, Roberto Amato, Sonia Goncalves, Ewan Harrison, David K. Jackson, Ian Johnston, Dominic Kwiatkowski, Cordelia Langford, John Sillitoe on behalf of the Wellcome Sanger Institute COVID-19 Surveillance Team
EPI_ISL_799936, EPI_ISL_799971, EPI_ISL_799972, EPI_ISL_799993, EPI_ISL_799994, EPI_ISL_800054, EPI_ISL_800100, EPI_ISL_800139, EPI_ISL_800192, EPI_ISL_800225, EPI_ISL_800244			
see above	Lighthouse Lab in Cambridge	Wellcome Sanger Institute for the COVID-19 Genomics UK (COG-UK) Consortium	Rob Howes, The Lighthouse Lab in Cambridge and Alex Alderton, Roberto Amato, Sonia Goncalves, Ewan Harrison, David K. Jackson, Ian Johnston, Dominic Kwiatkowski, Cordelia Langford, John Sillitoe on behalf of the Wellcome Sanger Institute COVID-19 Surveillance Team
EPI_ISL_801443, EPI_ISL_801448	Dutch COVID-19 response team	Erasmus Medical Center	Bas Oude Munnink, Reina Sikkema, David Nieuwenhuijse, Irina Chestakova, Anne van der Linden, Marjan Boter, Emmanuelle Munger, Corine GeurtsvanKessel, Annemiek van der Eijk, Richard Molenkamp, Marion Koopmans, on behalf of the Dutch national COVID-19 response team.
EPI_ISL_801524, EPI_ISL_801525, EPI_ISL_801526, EPI_ISL_801527, EPI_ISL_801528	Sonic Reference Laboratory	Pathogen Discovery, Respiratory Viruses Branch, Division of Viral Diseases, Centers for Disease Control and Prevention	Ying Tao, Yan Li, Jing Zhang, Krista Queen, Anna Uehara, Peter Cook, Clinton R. Paden, Haibin Wang, Suxiang Tong
EPI_ISL_802512	Dutch COVID-19 response team	Erasmus Medical Center	Bas Oude Munnink, Reina Sikkema, David Nieuwenhuijse, Irina Chestakova, Anne van der Linden, Marjan Boter, Emmanuelle Munger, Corine GeurtsvanKessel, Annemiek van der Eijk, Richard Molenkamp, Marion Koopmans, on behalf of the Dutch national COVID-19 response team.
EPI_ISL_802921, EPI_ISL_802923, EPI_ISL_802924, EPI_ISL_802925, EPI_ISL_802927, EPI_ISL_802929, EPI_ISL_802931, EPI_ISL_802932, EPI_ISL_802934, EPI_ISL_802936, EPI_ISL_802938, EPI_ISL_802941, EPI_ISL_802944, EPI_ISL_802945, EPI_ISL_802946, EPI_ISL_802948, EPI_ISL_802949, EPI_ISL_802953, EPI_ISL_802954, EPI_ISL_802956, EPI_ISL_802959, EPI_ISL_802960, EPI_ISL_802961, EPI_ISL_802964, EPI_ISL_802965, EPI_ISL_802967, EPI_ISL_802968, EPI_ISL_802970, EPI_ISL_802972, EPI_ISL_802973, EPI_ISL_802974, EPI_ISL_802975, EPI_ISL_802976, EPI_ISL_802978, EPI_ISL_802979, EPI_ISL_802980, EPI_ISL_802987, EPI_ISL_802991			
see above	Utah Public Health Laboratory	Utah Public Health Laboratory	Erin Young, Kelly Oakeson, Tara Gallagher
EPI_ISL_803899	Genomic Medicine Laboratory, IRCCS Santa Lucia Foundation	National Institute for Infectious Diseases, INMI, "L. Spallanzani" IRCCS	E Giombini, M. Rueca, B Bartolini, O Butera, C.E.M Gruber, F Messina, E Giardina, MR Capobianchi, A Di Caro
EPI_ISL_804214, EPI_ISL_804215	Respiratory Virus Unit, National Infection Service, Public Health England	COVID-19 Genomics UK (COG-UK) Consortium	PHE Covid Sequencing Team
EPI_ISL_804604, EPI_ISL_804636, EPI_ISL_804642	Michigan Department of Health and Human Services, Bureau of Laboratories	Michigan Department of Health and Human Services, Bureau of Laboratories	Blankenship HM, Riner D, Soehnlen MK
EPI_ISL_804825, EPI_ISL_804827	DB Diagnosticos do Brasil	Laboratório de Parasitologia Médica - Instituto de Medicina Tropical - Universidade de São Paulo	Nuno Faria, Ingra Morales Claro, Darlan Candido, Lucas A. Moyses Franco, Pamela dos Santos Andrade, Thais de Moura Coletti, Camila A. Maia da Silva, Flavia Cristina Sales, Erika Regina Manuli, Renato A. Santana, Nelson Gaburo, Cecília da Cunha Camilo, Nelson Abrahim Fraiji, Myuki Alfaia Esashika Crispim, Maria do Perpétuo Socorro Sampaio Carvalho, Andrew Rambaut, Nick Loman, Oliver G. Pybus, Ester C. Sabino; DB; HEMOAM; CDL; CADDE Genomic Network.
EPI_ISL_804847, EPI_ISL_804870, EPI_ISL_804883, EPI_ISL_804889, EPI_ISL_804935, EPI_ISL_804936, EPI_ISL_804937, EPI_ISL_804938, EPI_ISL_804939, EPI_ISL_804940	DC Public Health Lab/ Dept. of Forensic Sciences	DC Public Health Lab/ Dept. of Forensic Sciences	Scott Nguyen, Elizabeth Zelaya, Connie Maza, Monica Mann, Brittany Hamilton, David Payne, Jocelyn Hauser
EPI_ISL_806851	Alaska State Virology Laboratory	Alaska State Virology Laboratory	Stephanie DeRonde, Lisa Smith, Ph.D., Devin M. Drown, Ph.D., Jack Chen, Ph.D.
EPI_ISL_812360, EPI_ISL_812361, EPI_ISL_812367, EPI_ISL_812368, EPI_ISL_812369, EPI_ISL_812370, EPI_ISL_812371, EPI_ISL_812372, EPI_ISL_812373, EPI_ISL_812374, EPI_ISL_812375, EPI_ISL_812376, EPI_ISL_812377, EPI_ISL_812378, EPI_ISL_812379, EPI_ISL_812380, EPI_ISL_812381, EPI_ISL_812384, EPI_ISL_812385, EPI_ISL_812386, EPI_ISL_812387, EPI_ISL_812388, EPI_ISL_812389, EPI_ISL_812390, EPI_ISL_812391, EPI_ISL_812392, EPI_ISL_812397, EPI_ISL_812398, EPI_ISL_812399, EPI_ISL_812400, EPI_ISL_812401, EPI_ISL_812407, EPI_ISL_812408, EPI_ISL_812409, EPI_ISL_812410, EPI_ISL_812411, EPI_ISL_812412, EPI_ISL_812413, EPI_ISL_812414, EPI_ISL_812415, EPI_ISL_812416, EPI_ISL_812417, EPI_ISL_812418, EPI_ISL_812419, EPI_ISL_812420			
see above	Utah Public Health Laboratory	Utah Public Health Laboratory	Erin L. Young, Kelly F. Oakeson, Tara Gallagher
EPI_ISL_812722, EPI_ISL_812723, EPI_ISL_812724	DOHMH PHL	New York City Public Health Laboratory	Jade Wang, et al.
EPI_ISL_812725	DOHMH Crown Heights	New York City Public Health Laboratory	Jade Wang, et al.
EPI_ISL_812726	DOHMH Corona	New York City Public Health Laboratory	Jade Wang, et al.
EPI_ISL_812727	DOHMH Jamaica	New York City Public Health Laboratory	Jade Wang, et al.
EPI_ISL_812728	DOHMH Riverside	New York City Public Health Laboratory	Jade Wang, et al.
EPI_ISL_812729	DOHMH Central Harlem	New York City Public Health Laboratory	Jade Wang, et al.
EPI_ISL_812730	DOHMH Crown Heights	New York City Public Health Laboratory	Jade Wang, et al.
EPI_ISL_812731	DOHMH Jamaica	New York City Public Health Laboratory	Jade Wang, et al.
EPI_ISL_812732, EPI_ISL_812733	DOHMH Corona	New York City Public Health Laboratory	Jade Wang, et al.
EPI_ISL_812734	DOHMH Crown Heights	New York City Public Health Laboratory	Jade Wang, et al.
EPI_ISL_812735, EPI_ISL_812736, EPI_ISL_812737	DOHMH Morrisania	New York City Public Health Laboratory	Jade Wang, et al.
EPI_ISL_812738	DOHMH Crown Heights	New York City Public Health Laboratory	Jade Wang, et al.
EPI_ISL_812739	DOHMH Central Harlem	New York City Public Health Laboratory	Jade Wang, et al.
EPI_ISL_812740	DOHMH Corona	New York City Public Health Laboratory	Jade Wang, et al.
EPI_ISL_812741	DOHMH Central Harlem	New York City Public Health Laboratory	Jade Wang, et al.
EPI_ISL_812742, EPI_ISL_812743, EPI_ISL_812744	DOHMH Corona	New York City Public Health Laboratory	Jade Wang, et al.
EPI_ISL_813060, EPI_ISL_813061, EPI_ISL_813063, EPI_ISL_813082, EPI_ISL_813084, EPI_ISL_813088, EPI_ISL_813089, EPI_ISL_813090, EPI_ISL_813153	University of Birmingham	COVID-19 Genomics UK (COG-UK) Consortium	Institute of Microbiology, University of Birmingham: Claire McMurray, Joanne Stockton, Samuel Nicholls, Radoslaw Poplawski, Will Rowe, Josh Quick, Nicholas Loman. University of Birmingham Testing Laboratory: Celina M Whalley, Andrew Bosworth, Charlotte Poxon, Kasun Wanigasooriya, Oliver Pickles, Mike Kidd, Alex Richter, Andrew D Beggs PHE Heartlands Lab: Husam Osman, Andrew Bosworth. Queen Elizabeth Hospital: Anna Casey
EPI_ISL_813225, EPI_ISL_813226, EPI_ISL_813239, EPI_ISL_813243, EPI_ISL_813246, EPI_ISL_813252, EPI_ISL_813254, EPI_ISL_813255, EPI_ISL_813262, EPI_ISL_813264, EPI_ISL_813273, EPI_ISL_813274, EPI_ISL_813275, EPI_ISL_813276, EPI_ISL_813277, EPI_ISL_813279, EPI_ISL_813286, EPI_ISL_813288, EPI_ISL_813290, EPI_ISL_813292			
see above	Department of Pathology, University of Cambridge	COVID-19 Genomics UK (COG-UK) Consortium	Aminu S. Jahun, Yasmin Chaudhry, Grant Hall, Iliana Georgana, Myra Hosmillo, Martin D. Curran, Malte Pinckert, Surendra Parmar, Ian Goodfellow
EPI_ISL_813771, EPI_ISL_813772, EPI_ISL_813809	Liverpool Clinical Laboratories	COVID-19 Genomics UK (COG-UK) Consortium	Sam Haldenby, Anita Lucaci, Steve Paterson, Julian Hiscox, Alistair Darby, M Almsaud, A Alrezaihi, Muhannad Alruwaili, Stuart D Armstrong, Jones Benjamin, Eleanor G Bentley, Anu Chawla, Jordan J Clark, Angela Cowell, Richard Eccles, Isabel García-Dorival, Matthew Gemmell, Alessandro Gerada, PKF Gilmore, Richard Gregory, Ximeng Han, Catherine Hartley, Margaret Hughes, Miren Iturriza-Gomara, James Johnson, L Luu, Jenifer Manson, Charlotte Nelson, Elaine O'Toole, Cassie Olateju, Rebekah Penrice-Randal , Lucille Rainbow, N.P Randle, Trevor Ian Robinson, Parul Sharma, Ghada T Shawli, James P Stewart, Neil Swainston, Ecaterina Vamos, Joanne Watts, Mark Whitehead

EPI_ISL_814274, EPI_ISL_814277, EPI_ISL_814278, EPI_ISL_814279, EPI_ISL_814282	Wales Specialist Virology Centre Sequencing lab: Pathogen Genomics Unit	COVID-19 Genomics UK (COG-UK) Consortium	Catherine Moore, Johnathan Evans, Laura Gifford, Malorie Perry, Simon Cottrell, Angela Marchbank, Alec Birchley, Alexander Adams, Amy Gaskin, Bree Gatica-Wilcox, Jason Coombes, Joel Southgate, Lauren Gilbert, Lee Graham, Nicole Pacchiarini, Sara Kumziene-Summerhayes, Sarah Taylor, Sophie Jones, Sara Rey, Matthew Bull, Joanne Watkins, Sally Corden, Tom Connor
EPI_ISL_814293	Liverpool Clinical Laboratories	COVID-19 Genomics UK (COG-UK) Consortium	Sam Haldenby, Anita Lucaci, Steve Paterson, Julian Hiscox, Alistair Darby, M Almsaud, A Alrezaihi, Muhannad Alruwaili, Stuart D Armstrong, Jones Benjamin, Eleanor G Bentley, Anu Chawla, Jordan J Clark, Angela Cowell, Richard Eccles, Isabel García-Dorival, Matthew Gemmell, Alessandro Gerada, PKF Gilmore, Richard Gregory, Ximeng Han, Catherine Hartley, Margaret Hughes, Miren Iturriza-Gomara, James Johnson, L Luu, Jenifer Manson, Charlotte Nelson, Elaine O'Toole, Cassie Olateju, Rebekah Penrice-Randal , Lucille Rainbow, N.P Randle, Trevor Ian Robinson, Parul Sharma, Ghada T Shawli, James P Stewart, Neil Swainston, Ecaterina Vamos, Joanne Watts, Mark Whitehead
EPI_ISL_814294, EPI_ISL_814295, EPI_ISL_814296, EPI_ISL_814300, EPI_ISL_814304, EPI_ISL_814306, EPI_ISL_814309	Wales Specialist Virology Centre Sequencing lab: Pathogen Genomics Unit	COVID-19 Genomics UK (COG-UK) Consortium	Catherine Moore, Johnathan Evans, Laura Gifford, Malorie Perry, Simon Cottrell, Angela Marchbank, Alec Birchley, Alexander Adams, Amy Gaskin, Bree Gatica-Wilcox, Jason Coombes, Joel Southgate, Lauren Gilbert, Lee Graham, Nicole Pacchiarini, Sara Kumziene-Summerhayes, Sarah Taylor, Sophie Jones, Sara Rey, Matthew Bull, Joanne Watkins, Sally Corden, Tom Connor
EPI_ISL_814312	West of Scotland Specialist Virology Centre, NHSGGC / MRC-University of Glasgow Centre for Virus Research	COVID-19 Genomics UK (COG-UK) Consortium	Ana da Silva Filipe, Natasha Johnson, Kathy Smollett, Daniel Mair, Stephen Carmichael, Alice Broos, Lily Tong, Jenna Nichols, Kyriaki Nomikou; Sarah McDonald; Richard Orton, Joseph Hughes, Sreenu Vattipally, David L Robertson; Alasdair MacLean, Rory Gunson; Sharif Shaaban, Matthew Holden; Rachel Blacow, Guy Mollett, Kathy Li, James Shepherd, Antonia Ho, Emma Thomson
EPI_ISL_814319, EPI_ISL_814328, EPI_ISL_814337, EPI_ISL_814356, EPI_ISL_814363, EPI_ISL_814367, EPI_ISL_814369, EPI_ISL_814371, EPI_ISL_814372, EPI_ISL_814373, EPI_ISL_814377, EPI_ISL_814383, EPI_ISL_814386, EPI_ISL_814387, EPI_ISL_814389, EPI_ISL_814394, EPI_ISL_814395, EPI_ISL_814400, EPI_ISL_814411, EPI_ISL_814413, EPI_ISL_814415			
see above	Wales Specialist Virology Centre Sequencing lab: Pathogen Genomics Unit	COVID-19 Genomics UK (COG-UK) Consortium	Catherine Moore, Johnathan Evans, Laura Gifford, Malorie Perry, Simon Cottrell, Angela Marchbank, Alec Birchley, Alexander Adams, Amy Gaskin, Bree Gatica-Wilcox, Jason Coombes, Joel Southgate, Lauren Gilbert, Lee Graham, Nicole Pacchiarini, Sara Kumziene-Summerhayes, Sarah Taylor, Sophie Jones, Sara Rey, Matthew Bull, Joanne Watkins, Sally Corden, Tom Connor
EPI_ISL_814449, EPI_ISL_814457	West of Scotland Specialist Virology Centre, NHSGGC / MRC-University of Glasgow Centre for Virus Research	COVID-19 Genomics UK (COG-UK) Consortium	Ana da Silva Filipe, Natasha Johnson, Kathy Smollett, Daniel Mair, Stephen Carmichael, Alice Broos, Lily Tong, Jenna Nichols, Kyriaki Nomikou; Sarah McDonald; Richard Orton, Joseph Hughes, Sreenu Vattipally, David L Robertson; Alasdair MacLean, Rory Gunson; Sharif Shaaban, Matthew Holden; Rachel Blacow, Guy Mollett, Kathy Li, James Shepherd, Antonia Ho, Emma Thomson
EPI_ISL_814473, EPI_ISL_814474, EPI_ISL_814475, EPI_ISL_814476, EPI_ISL_814477, EPI_ISL_814478, EPI_ISL_814479, EPI_ISL_814511, EPI_ISL_814523, EPI_ISL_814529	Wales Specialist Virology Centre Sequencing lab: Pathogen Genomics Unit	COVID-19 Genomics UK (COG-UK) Consortium	Catherine Moore, Johnathan Evans, Laura Gifford, Malorie Perry, Simon Cottrell, Angela Marchbank, Alec Birchley, Alexander Adams, Amy Gaskin, Bree Gatica-Wilcox, Jason Coombes, Joel Southgate, Lauren Gilbert, Lee Graham, Nicole Pacchiarini, Sara Kumziene-Summerhayes, Sarah Taylor, Sophie Jones, Sara Rey, Matthew Bull, Joanne Watkins, Sally Corden, Tom Connor
EPI_ISL_814530	Bioinformatics and Biostatistics Lab, Advanced Sequencing Facility	COVID-19 Genomics UK (COG-UK) Consortium	Aengus Stewart,Jerome Nicod,Chelsea Sawyer,Laura Cubitt,Harshil Patel,Margaret Crawford
EPI_ISL_814537	Wales Specialist Virology Centre Sequencing lab: Pathogen Genomics Unit	COVID-19 Genomics UK (COG-UK) Consortium	Catherine Moore, Johnathan Evans, Laura Gifford, Malorie Perry, Simon Cottrell, Angela Marchbank, Alec Birchley, Alexander Adams, Amy Gaskin, Bree Gatica-Wilcox, Jason Coombes, Joel Southgate, Lauren Gilbert, Lee Graham, Nicole Pacchiarini, Sara Kumziene-Summerhayes, Sarah Taylor, Sophie Jones, Sara Rey, Matthew Bull, Joanne Watkins, Sally Corden, Tom Connor
EPI_ISL_814547	West of Scotland Specialist Virology Centre, NHSGGC / MRC-University of Glasgow Centre for Virus Research	COVID-19 Genomics UK (COG-UK) Consortium	Ana da Silva Filipe, Natasha Johnson, Kathy Smollett, Daniel Mair, Stephen Carmichael, Alice Broos, Lily Tong, Jenna Nichols, Kyriaki Nomikou; Sarah McDonald; Richard Orton, Joseph Hughes, Sreenu Vattipally, David L Robertson; Alasdair MacLean, Rory Gunson; Sharif Shaaban, Matthew Holden; Rachel Blacow, Guy Mollett, Kathy Li, James Shepherd, Antonia Ho, Emma Thomson
EPI_ISL_814551, EPI_ISL_814556, EPI_ISL_814561, EPI_ISL_814573, EPI_ISL_814578, EPI_ISL_814584, EPI_ISL_814595, EPI_ISL_814606	Wales Specialist Virology Centre Sequencing lab: Pathogen Genomics Unit	COVID-19 Genomics UK (COG-UK) Consortium	Catherine Moore, Johnathan Evans, Laura Gifford, Malorie Perry, Simon Cottrell, Angela Marchbank, Alec Birchley, Alexander Adams, Amy Gaskin, Bree Gatica-Wilcox, Jason Coombes, Joel Southgate, Lauren Gilbert, Lee Graham, Nicole Pacchiarini, Sara Kumziene-Summerhayes, Sarah Taylor, Sophie Jones, Sara Rey, Matthew Bull, Joanne Watkins, Sally Corden, Tom Connor
EPI_ISL_814629, EPI_ISL_814630, EPI_ISL_814648, EPI_ISL_814652, EPI_ISL_814659, EPI_ISL_814660, EPI_ISL_814662	West of Scotland Specialist Virology Centre, NHSGGC / MRC-University of Glasgow Centre for Virus Research	COVID-19 Genomics UK (COG-UK) Consortium	Ana da Silva Filipe, Natasha Johnson, Kathy Smollett, Daniel Mair, Stephen Carmichael, Alice Broos, Lily Tong, Jenna Nichols, Kyriaki Nomikou; Sarah McDonald; Richard Orton, Joseph Hughes, Sreenu Vattipally, David L Robertson; Alasdair MacLean, Rory Gunson; Sharif Shaaban, Matthew Holden; Rachel Blacow, Guy Mollett, Kathy Li, James Shepherd, Antonia Ho, Emma Thomson
EPI_ISL_814689	Oxford Viromics, NDM, University of Oxford; Oxford University Hospitals; Basingstoke and North Hampshire Hospital	COVID-19 Genomics UK (COG-UK) Consortium	Tanya Golubchik, David Bonsall, George Macintyre, Amy Trebes, Mariateresa de Cesare, Catrin Moore, Alex Mobbs, Anita Justice, Robert Shaw, Monique Andersson, Timothy Peto, Emma Wise, Nathan Moore, Jessica Lynch, Nick Cortes, Matilde Mori, Stephen Kidd, David Buck, John Todd, Christophe Fraser
EPI_ISL_814724, EPI_ISL_814727, EPI_ISL_814728, EPI_ISL_814729, EPI_ISL_814730, EPI_ISL_814731, EPI_ISL_814732, EPI_ISL_814733, EPI_ISL_814734, EPI_ISL_814735, EPI_ISL_814736, EPI_ISL_814737, EPI_ISL_814738, EPI_ISL_814739, EPI_ISL_814740, EPI_ISL_814741, EPI_ISL_814742, EPI_ISL_814743, EPI_ISL_814744, EPI_ISL_814745, EPI_ISL_814746, EPI_ISL_814747, EPI_ISL_814748, EPI_ISL_814749, EPI_ISL_814750, EPI_ISL_814751, EPI_ISL_814752, EPI_ISL_814798, EPI_ISL_814799, EPI_ISL_814871, EPI_ISL_814872, EPI_ISL_814873, EPI_ISL_814874, EPI_ISL_814875, EPI_ISL_814876, EPI_ISL_814877, EPI_ISL_814878, EPI_ISL_814879, EPI_ISL_814880, EPI_ISL_814881, EPI_ISL_814882, EPI_ISL_814883, EPI_ISL_814884, EPI_ISL_814885, EPI_ISL_814886, EPI_ISL_814887, EPI_ISL_814888, EPI_ISL_814889, EPI_ISL_814890, EPI_ISL_814891, EPI_ISL_814892, EPI_ISL_814893, EPI_ISL_814894, EPI_ISL_814895, EPI_ISL_814896, EPI_ISL_814897, EPI_ISL_814898, EPI_ISL_814899, EPI_ISL_814900, EPI_ISL_814901, EPI_ISL_814902, EPI_ISL_814903, EPI_ISL_814904, EPI_ISL_814905, EPI_ISL_814906, EPI_ISL_814907, EPI_ISL_814908, EPI_ISL_814909, EPI_ISL_814910, EPI_ISL_814911, EPI_ISL_814912, EPI_ISL_814913, EPI_ISL_814914, EPI_ISL_814915, EPI_ISL_814916, EPI_ISL_814917, EPI_ISL_814918, EPI_ISL_814919, EPI_ISL_814920, EPI_ISL_814921, EPI_ISL_814922, EPI_ISL_814923, EPI_ISL_814924, EPI_ISL_814925, EPI_ISL_814926, EPI_ISL_814927, EPI_ISL_814928, EPI_ISL_814929, EPI_ISL_814930, EPI_ISL_814931, EPI_ISL_814932, EPI_ISL_814933, EPI_ISL_814934, EPI_ISL_814935, EPI_ISL_814936, EPI_ISL_814937, EPI_ISL_814938, EPI_ISL_814939, EPI_ISL_814940, EPI_ISL_814942, EPI_ISL_814943, EPI_ISL_814944, EPI_ISL_814945, EPI_ISL_814946, EPI_ISL_814947, EPI_ISL_814948, EPI_ISL_814949, EPI_ISL_814950, EPI_ISL_814951, EPI_ISL_814952, EPI_ISL_814953, EPI_ISL_815035, EPI_ISL_815037, EPI_ISL_815048, EPI_ISL_815049, EPI_ISL_815050, EPI_ISL_815052, EPI_ISL_815053, EPI_ISL_815054, EPI_ISL_815055, EPI_ISL_815056, EPI_ISL_815057, EPI_ISL_815058, EPI_ISL_815059, EPI_ISL_815060, EPI_ISL_815061, EPI_ISL_815072, EPI_ISL_815073, EPI_ISL_815074, EPI_ISL_815075, EPI_ISL_815106, EPI_ISL_815107, EPI_ISL_815108, EPI_ISL_815109, EPI_ISL_815110, EPI_ISL_815111, EPI_ISL_815112, EPI_ISL_815113, EPI_ISL_815114, EPI_ISL_815115, EPI_ISL_815116, EPI_ISL_815117, EPI_ISL_815118, EPI_ISL_815119, EPI_ISL_815120, EPI_ISL_815121, EPI_ISL_815122, EPI_ISL_815123, EPI_ISL_815124, EPI_ISL_815125, EPI_ISL_815126, EPI_ISL_815127, EPI_ISL_815128, EPI_ISL_815129, EPI_ISL_815130, EPI_ISL_815131, EPI_ISL_815132, EPI_ISL_815133, EPI_ISL_815134, EPI_ISL_815135, EPI_ISL_815136, EPI_ISL_815247, EPI_ISL_815248, EPI_ISL_815249, EPI_ISL_815250, EPI_ISL_815251, EPI_ISL_815252, EPI_ISL_815253, EPI_ISL_815254			
see above	Wales Specialist Virology Centre Sequencing lab: Pathogen Genomics Unit	COVID-19 Genomics UK (COG-UK) Consortium	Catherine Moore, Johnathan Evans, Laura Gifford, Malorie Perry, Simon Cottrell, Angela Marchbank, Alec Birchley, Alexander Adams, Amy Gaskin, Bree Gatica-Wilcox, Jason Coombes, Joel Southgate, Lauren Gilbert, Lee Graham, Nicole Pacchiarini, Sara Kumziene-Summerhayes, Sarah Taylor, Sophie Jones, Sara Rey, Matthew Bull, Joanne Watkins, Sally Corden, Tom Connor
EPI_ISL_815401, EPI_ISL_815407, EPI_ISL_815410, EPI_ISL_815441, EPI_ISL_815442, EPI_ISL_815443, EPI_ISL_815444, EPI_ISL_815445, EPI_ISL_815446, EPI_ISL_815447, EPI_ISL_815448, EPI_ISL_815449, EPI_ISL_815450, EPI_ISL_815451, EPI_ISL_815452, EPI_ISL_815453, EPI_ISL_815468, EPI_ISL_815469, EPI_ISL_815471, EPI_ISL_815475, EPI_ISL_815501, EPI_ISL_815523, EPI_ISL_815524, EPI_ISL_815526, EPI_ISL_815534, EPI_ISL_815535, EPI_ISL_815539, EPI_ISL_815544, EPI_ISL_815581, EPI_ISL_815582, EPI_ISL_815583, EPI_ISL_815584, EPI_ISL_815585, EPI_ISL_815586, EPI_ISL_815587, EPI_ISL_815588, EPI_ISL_815589, EPI_ISL_815590, EPI_ISL_815591, EPI_ISL_815606, EPI_ISL_815650, EPI_ISL_815656, EPI_ISL_815659, EPI_ISL_815962, EPI_ISL_815963, EPI_ISL_815964, EPI_ISL_815965, EPI_ISL_815966, EPI_ISL_815967, EPI_ISL_815968, EPI_ISL_815969, EPI_ISL_815970, EPI_ISL_815971, EPI_ISL_815972, EPI_ISL_815973, EPI_ISL_815974, EPI_ISL_815975, EPI_ISL_815976, EPI_ISL_815977, EPI_ISL_815978, EPI_ISL_815979, EPI_ISL_815980, EPI_ISL_815981, EPI_ISL_815982, EPI_ISL_815983, EPI_ISL_815984, EPI_ISL_815985, EPI_ISL_815986, EPI_ISL_815987, EPI_ISL_815988, EPI_ISL_815989, EPI_ISL_815990, EPI_ISL_815991, EPI_ISL_815992, EPI_ISL_815993, EPI_ISL_815994, EPI_ISL_815995, EPI_ISL_815996, EPI_ISL_815997, EPI_ISL_815998, EPI_ISL_815999, EPI_ISL_816000, EPI_ISL_816001, EPI_ISL_816002, EPI_ISL_816003, EPI_ISL_816004, EPI_ISL_816005, EPI_ISL_816006, EPI_ISL_816007, EPI_ISL_816008, EPI_ISL_816009, EPI_ISL_816010, EPI_ISL_816011, EPI_ISL_816012, EPI_ISL_816013, EPI_ISL_816014, EPI_ISL_816015, EPI_ISL_816016, EPI_ISL_816017, EPI_ISL_816018, EPI_ISL_816019, EPI_ISL_816020, EPI_ISL_816021, EPI_ISL_816022, EPI_ISL_816023, EPI_ISL_816024, EPI_ISL_816025, EPI_ISL_816026, EPI_ISL_816027, EPI_ISL_816028, EPI_ISL_816029, EPI_ISL_816030, EPI_ISL_816031, EPI_ISL_816032, EPI_ISL_816033, EPI_ISL_816034			
see above	Department of Virus and Microbiological Special Diagnostics, Statens Serum Institut, Copenhagen, Denmark	Albertsen Lab, Department of Chemistry and Bioscience, Aalborg University, Denmark	Danish Covid-19 Genome Consortium
EPI_ISL_816234, EPI_ISL_816240, EPI_ISL_816245, EPI_ISL_816259, EPI_ISL_816265, EPI_ISL_816269, EPI_ISL_816274, EPI_ISL_816282, EPI_ISL_816300, EPI_ISL_816301, EPI_ISL_816304, EPI_ISL_816325, EPI_ISL_816329, EPI_ISL_816344, EPI_ISL_816347, EPI_ISL_816375, EPI_ISL_816385, EPI_ISL_816388, EPI_ISL_816400, EPI_ISL_816404, EPI_ISL_816410, EPI_ISL_816418, EPI_ISL_816460, EPI_ISL_816482, EPI_ISL_816486, EPI_ISL_816487, EPI_ISL_816504, EPI_ISL_816514, EPI_ISL_816528, EPI_ISL_816529, EPI_ISL_816535, EPI_ISL_816538, EPI_ISL_816541, EPI_ISL_816544, EPI_ISL_816547, EPI_ISL_816554, EPI_ISL_816556, EPI_ISL_816568, EPI_ISL_816595, EPI_ISL_816617, EPI_ISL_816626, EPI_ISL_816630, EPI_ISL_816640, EPI_ISL_816644, EPI_ISL_816650, EPI_ISL_816654, EPI_ISL_816656			
see above	Virology Department, Sheffield Teaching Hospitals NHS Foundation Trust/Department of Infection, Immunity and Cardiovascular Disease, The Medical School, University of Sheffield	COVID-19 Genomics UK (COG-UK) Consortium	Thushan de Silva, Matthew Parker, Nikki Smith, Adri Angyal, Rebecca Brown, Luke Green, Rachel Tucker, Paul Parsons, Danielle Groves, Katie Johnson, Laura Carrilero, Alex Keeley, Dave Partridge, Matthew Wyles, Benjamin Lindsey, Mehmet Yavuz, Mohammad Raza, Cariad Evans
EPI_ISL_817025, EPI_ISL_817026, EPI_ISL_817027, EPI_ISL_817028, EPI_ISL_817029, EPI_ISL_817030, EPI_ISL_817031, EPI_ISL_817032, EPI_ISL_817033, EPI_ISL_817034, EPI_ISL_817035, EPI_ISL_817036, EPI_ISL_817037, EPI_ISL_817038, EPI_ISL_817040, EPI_ISL_817041, EPI_ISL_817042, EPI_ISL_817043, EPI_ISL_817045, EPI_ISL_817046, EPI_ISL_817047, EPI_ISL_817048, EPI_ISL_817052, EPI_ISL_817055, EPI_ISL_817056, EPI_ISL_817058, EPI_ISL_817059, EPI_ISL_817061, EPI_ISL_817064, EPI_ISL_817065, EPI_ISL_817122			

see above	Bioinformatics and Biostatistics Lab, Advanced Sequencing Facility	COVID-19 Genomics UK (COG-UK) Consortium	Aengus Stewart,Jerome Nicod,Chelsea Sawyer,Laura Cubitt,Harshil Patel,Margaret Crawford
EPI_ISL_819578	Oxford Viromics, NDM, University of Oxford; Oxford University Hospitals; Basingstoke and North Hampshire Hospital	COVID-19 Genomics UK (COG-UK) Consortium	Tanya Golubchik, David Bonsall, George Macintyre, Amy Trebes, Mariateresa de Cesare, Catrin Moore, Alex Mobbs, Anita Justice, Robert Shaw, Monique Andersson, Timothy Peto, Emma Wise, Nathan Moore, Jessica Lynch, Nick Cortes, Matilde Mori, Stephen Kidd, David Buck, John Todd, Christophe Fraser
EPI_ISL_819579, EPI_ISL_819580, EPI_ISL_819581, EPI_ISL_819582, EPI_ISL_819583, EPI_ISL_819584	Queens Medical Centre, Clinical Microbiology Department / DeepSeq Nottingham	COVID-19 Genomics UK (COG-UK) Consortium	Gemma Clark, Wendy Smith, Manjinder Khakh, Vicki M Fleming, Michelle M Lister, Hannah Howson-Wells, Jonathan Ball, Patrick McClure, Joseph Chappell, Theocharis Tsoleridis, Nadine Holmes, Matthew Carlisle, Christopher Moore, Fei Sang, Johnny Debebe, Victoria Wright, Matthew Loose
EPI_ISL_819585, EPI_ISL_819586, EPI_ISL_819587, EPI_ISL_819588, EPI_ISL_819589, EPI_ISL_819590, EPI_ISL_819591, EPI_ISL_819592, EPI_ISL_819593	Oxford Viromics, NDM, University of Oxford; Oxford University Hospitals; Basingstoke and North Hampshire Hospital	COVID-19 Genomics UK (COG-UK) Consortium	Tanya Golubchik, David Bonsall, George Macintyre, Amy Trebes, Mariateresa de Cesare, Catrin Moore, Alex Mobbs, Anita Justice, Robert Shaw, Monique Andersson, Timothy Peto, Emma Wise, Nathan Moore, Jessica Lynch, Nick Cortes, Matilde Mori, Stephen Kidd, David Buck, John Todd, Christophe Fraser
EPI_ISL_819594, EPI_ISL_819595, EPI_ISL_819596, EPI_ISL_819597, EPI_ISL_819598, EPI_ISL_819599, EPI_ISL_819600, EPI_ISL_819601, EPI_ISL_819602, EPI_ISL_819603, EPI_ISL_819604			
see above	Queens Medical Centre, Clinical Microbiology Department / DeepSeq Nottingham	COVID-19 Genomics UK (COG-UK) Consortium	Gemma Clark, Wendy Smith, Manjinder Khakh, Vicki M Fleming, Michelle M Lister, Hannah Howson-Wells, Jonathan Ball, Patrick McClure, Joseph Chappell, Theocharis Tsoleridis, Nadine Holmes, Matthew Carlisle, Christopher Moore, Fei Sang, Johnny Debebe, Victoria Wright, Matthew Loose
EPI_ISL_819605, EPI_ISL_819606, EPI_ISL_819607, EPI_ISL_819608	Oxford Viromics, NDM, University of Oxford; Oxford University Hospitals; Basingstoke and North Hampshire Hospital	COVID-19 Genomics UK (COG-UK) Consortium	Tanya Golubchik, David Bonsall, George Macintyre, Amy Trebes, Mariateresa de Cesare, Catrin Moore, Alex Mobbs, Anita Justice, Robert Shaw, Monique Andersson, Timothy Peto, Emma Wise, Nathan Moore, Jessica Lynch, Nick Cortes, Matilde Mori, Stephen Kidd, David Buck, John Todd, Christophe Fraser
EPI_ISL_820129, EPI_ISL_820130, EPI_ISL_820132, EPI_ISL_820133, EPI_ISL_820135, EPI_ISL_820137, EPI_ISL_820139, EPI_ISL_820140, EPI_ISL_820142, EPI_ISL_820143, EPI_ISL_820145			
see above	Lighthouse Lab in Alderley Park	Wellcome Sanger Institute for the COVID-19 Genomics UK (COG-UK) Consortium	Jacquelyn Wynn, Mairead Hyland, The Lighthouse Lab in Alderley Park and Alex Alderton, Roberto Amato, Sonia Goncalves, Ewan Harrison, David K. Jackson, Ian Johnston, Dominic Kwiatkowski, Cordelia Langford, John Sillitoe on behalf of the Wellcome Sanger Institute COVID-19 Surveillance Team
EPI_ISL_820147	Lighthouse Lab in Glasgow	Wellcome Sanger Institute for the COVID-19 Genomics UK (COG-UK) Consortium	Harper VanSteenhouse, Yumi Kasai, David Gray, Carol Clugston, Anna Dominiczak and Alex Alderton, Roberto Amato, Sonia Goncalves, Ewan Harrison, David K. Jackson, Ian Johnston, Dominic Kwiatkowski, Cordelia Langford, John Sillitoe on behalf of the Wellcome Sanger Institute COVID-19 Surveillance Team
EPI_ISL_820148	Lighthouse Lab in Alderley Park	Wellcome Sanger Institute for the COVID-19 Genomics UK (COG-UK) Consortium	Jacquelyn Wynn, Mairead Hyland, The Lighthouse Lab in Alderley Park and Alex Alderton, Roberto Amato, Sonia Goncalves, Ewan Harrison, David K. Jackson, Ian Johnston, Dominic Kwiatkowski, Cordelia Langford, John Sillitoe on behalf of the Wellcome Sanger Institute COVID-19 Surveillance Team
EPI_ISL_820150, EPI_ISL_820152	Lighthouse Lab in Glasgow	Wellcome Sanger Institute for the COVID-19 Genomics UK (COG-UK) Consortium	Harper VanSteenhouse, Yumi Kasai, David Gray, Carol Clugston, Anna Dominiczak and Alex Alderton, Roberto Amato, Sonia Goncalves, Ewan Harrison, David K. Jackson, Ian Johnston, Dominic Kwiatkowski, Cordelia Langford, John Sillitoe on behalf of the Wellcome Sanger Institute COVID-19 Surveillance Team
EPI_ISL_820153, EPI_ISL_820155, EPI_ISL_820157, EPI_ISL_820158, EPI_ISL_820160, EPI_ISL_820162, EPI_ISL_820163, EPI_ISL_820165, EPI_ISL_820166, EPI_ISL_820168, EPI_ISL_820170			
see above	Lighthouse Lab in Alderley Park	Wellcome Sanger Institute for the COVID-19 Genomics UK (COG-UK) Consortium	Jacquelyn Wynn, Mairead Hyland, The Lighthouse Lab in Alderley Park and Alex Alderton, Roberto Amato, Sonia Goncalves, Ewan Harrison, David K. Jackson, Ian Johnston, Dominic Kwiatkowski, Cordelia Langford, John Sillitoe on behalf of the Wellcome Sanger Institute COVID-19 Surveillance Team
EPI_ISL_820171	Lighthouse Lab in Glasgow	Wellcome Sanger Institute for the COVID-19 Genomics UK (COG-UK) Consortium	Harper VanSteenhouse, Yumi Kasai, David Gray, Carol Clugston, Anna Dominiczak and Alex Alderton, Roberto Amato, Sonia Goncalves, Ewan Harrison, David K. Jackson, Ian Johnston, Dominic Kwiatkowski, Cordelia Langford, John Sillitoe on behalf of the Wellcome Sanger Institute COVID-19 Surveillance Team
EPI_ISL_820173	Lighthouse Lab in Alderley Park	Wellcome Sanger Institute for the COVID-19 Genomics UK (COG-UK) Consortium	Jacquelyn Wynn, Mairead Hyland, The Lighthouse Lab in Alderley Park and Alex Alderton, Roberto Amato, Sonia Goncalves, Ewan Harrison, David K. Jackson, Ian Johnston, Dominic Kwiatkowski, Cordelia Langford, John Sillitoe on behalf of the Wellcome Sanger Institute COVID-19 Surveillance Team
EPI_ISL_820176, EPI_ISL_820177	Lighthouse Lab in Glasgow	Wellcome Sanger Institute for the COVID-19 Genomics UK (COG-UK) Consortium	Harper VanSteenhouse, Yumi Kasai, David Gray, Carol Clugston, Anna Dominiczak and Alex Alderton, Roberto Amato, Sonia Goncalves, Ewan Harrison, David K. Jackson, Ian Johnston, Dominic Kwiatkowski, Cordelia Langford, John Sillitoe on behalf of the Wellcome Sanger Institute COVID-19 Surveillance Team
EPI_ISL_820179, EPI_ISL_820181	Lighthouse Lab in Alderley Park	Wellcome Sanger Institute for the COVID-19 Genomics UK (COG-UK) Consortium	Jacquelyn Wynn, Mairead Hyland, The Lighthouse Lab in Alderley Park and Alex Alderton, Roberto Amato, Sonia Goncalves, Ewan Harrison, David K. Jackson, Ian Johnston, Dominic Kwiatkowski, Cordelia Langford, John Sillitoe on behalf of the Wellcome Sanger Institute COVID-19 Surveillance Team
EPI_ISL_820182, EPI_ISL_820184	Lighthouse Lab in Glasgow	Wellcome Sanger Institute for the COVID-19 Genomics UK (COG-UK) Consortium	Harper VanSteenhouse, Yumi Kasai, David Gray, Carol Clugston, Anna Dominiczak and Alex Alderton, Roberto Amato, Sonia Goncalves, Ewan Harrison, David K. Jackson, Ian Johnston, Dominic Kwiatkowski, Cordelia Langford, John Sillitoe on behalf of the Wellcome Sanger Institute COVID-19 Surveillance Team
EPI_ISL_820185, EPI_ISL_820187, EPI_ISL_820189	Lighthouse Lab in Alderley Park	Wellcome Sanger Institute for the COVID-19 Genomics UK (COG-UK) Consortium	Jacquelyn Wynn, Mairead Hyland, The Lighthouse Lab in Alderley Park and Alex Alderton, Roberto Amato, Sonia Goncalves, Ewan Harrison, David K. Jackson, Ian Johnston, Dominic Kwiatkowski, Cordelia Langford, John Sillitoe on behalf of the Wellcome Sanger Institute COVID-19 Surveillance Team
EPI_ISL_820190	Lighthouse Lab in Glasgow	Wellcome Sanger Institute for the COVID-19 Genomics UK (COG-UK) Consortium	Harper VanSteenhouse, Yumi Kasai, David Gray, Carol Clugston, Anna Dominiczak and Alex Alderton, Roberto Amato, Sonia Goncalves, Ewan Harrison, David K. Jackson, Ian Johnston, Dominic Kwiatkowski, Cordelia Langford, John Sillitoe on behalf of the Wellcome Sanger Institute COVID-19 Surveillance Team
EPI_ISL_820192, EPI_ISL_820194, EPI_ISL_820195	Lighthouse Lab in Alderley Park	Wellcome Sanger Institute for the COVID-19 Genomics UK (COG-UK) Consortium	Jacquelyn Wynn, Mairead Hyland, The Lighthouse Lab in Alderley Park and Alex Alderton, Roberto Amato, Sonia Goncalves, Ewan Harrison, David K. Jackson, Ian Johnston, Dominic Kwiatkowski, Cordelia Langford, John Sillitoe on behalf of the Wellcome Sanger Institute COVID-19 Surveillance Team
EPI_ISL_820197	Lighthouse Lab in Glasgow	Wellcome Sanger Institute for the COVID-19 Genomics UK (COG-UK) Consortium	Harper VanSteenhouse, Yumi Kasai, David Gray, Carol Clugston, Anna Dominiczak and Alex Alderton, Roberto Amato, Sonia Goncalves, Ewan Harrison, David K. Jackson, Ian Johnston, Dominic Kwiatkowski, Cordelia Langford, John Sillitoe on behalf of the Wellcome Sanger Institute COVID-19 Surveillance Team
EPI_ISL_820198	Lighthouse Lab in Alderley Park	Wellcome Sanger Institute for the COVID-19 Genomics UK (COG-UK) Consortium	Jacquelyn Wynn, Mairead Hyland, The Lighthouse Lab in Alderley Park and Alex Alderton, Roberto Amato, Sonia Goncalves, Ewan Harrison, David K. Jackson, Ian Johnston, Dominic Kwiatkowski, Cordelia Langford, John Sillitoe on behalf of the Wellcome Sanger Institute COVID-19 Surveillance Team
EPI_ISL_820200	Lighthouse Lab in Glasgow	Wellcome Sanger Institute for the COVID-19 Genomics UK (COG-UK) Consortium	Harper VanSteenhouse, Yumi Kasai, David Gray, Carol Clugston, Anna Dominiczak and Alex Alderton, Roberto Amato, Sonia Goncalves, Ewan Harrison, David K. Jackson, Ian Johnston, Dominic Kwiatkowski, Cordelia Langford, John Sillitoe on behalf of the Wellcome Sanger Institute COVID-19 Surveillance Team
EPI_ISL_820201, EPI_ISL_820203, EPI_ISL_820205, EPI_ISL_820207, EPI_ISL_820208, EPI_ISL_820210	Lighthouse Lab in Alderley Park	Wellcome Sanger Institute for the COVID-19 Genomics UK (COG-UK) Consortium	Jacquelyn Wynn, Mairead Hyland, The Lighthouse Lab in Alderley Park and Alex Alderton, Roberto Amato, Sonia Goncalves, Ewan Harrison, David K. Jackson, Ian Johnston, Dominic Kwiatkowski, Cordelia Langford, John Sillitoe on behalf of the Wellcome Sanger Institute COVID-19 Surveillance Team
EPI_ISL_820212	Lighthouse Lab in Glasgow	Wellcome Sanger Institute for the COVID-19 Genomics UK (COG-UK) Consortium	Harper VanSteenhouse, Yumi Kasai, David Gray, Carol Clugston, Anna Dominiczak and Alex Alderton, Roberto Amato, Sonia Goncalves, Ewan Harrison, David K. Jackson, Ian Johnston, Dominic Kwiatkowski, Cordelia Langford, John Sillitoe on behalf of the Wellcome Sanger Institute COVID-19 Surveillance Team
EPI_ISL_820214	Lighthouse Lab in Alderley Park	Wellcome Sanger Institute for the COVID-19 Genomics UK (COG-UK) Consortium	Jacquelyn Wynn, Mairead Hyland, The Lighthouse Lab in Alderley Park and Alex Alderton, Roberto Amato, Sonia Goncalves, Ewan Harrison, David K. Jackson, Ian Johnston, Dominic Kwiatkowski, Cordelia Langford, John Sillitoe on behalf of the Wellcome Sanger Institute COVID-19 Surveillance Team
EPI_ISL_820215	Lighthouse Lab in Glasgow	Wellcome Sanger Institute for the COVID-19 Genomics UK (COG-UK) Consortium	Harper VanSteenhouse, Yumi Kasai, David Gray, Carol Clugston, Anna Dominiczak and Alex Alderton, Roberto Amato, Sonia Goncalves, Ewan Harrison, David K. Jackson, Ian Johnston, Dominic Kwiatkowski, Cordelia Langford, John Sillitoe on behalf of the Wellcome Sanger Institute COVID-19 Surveillance Team
EPI_ISL_820217, EPI_ISL_820219	Lighthouse Lab in Alderley Park	Wellcome Sanger Institute for the COVID-19 Genomics UK (COG-UK) Consortium	Jacquelyn Wynn, Mairead Hyland, The Lighthouse Lab in Alderley Park and Alex Alderton, Roberto Amato, Sonia Goncalves, Ewan Harrison, David K. Jackson, Ian Johnston, Dominic Kwiatkowski, Cordelia Langford, John Sillitoe on behalf of the Wellcome Sanger Institute COVID-19 Surveillance Team
EPI_ISL_820220	Lighthouse Lab in Glasgow	Wellcome Sanger Institute for the COVID-19 Genomics UK	Harper VanSteenhouse, Yumi Kasai, David Gray, Carol Clugston, Anna Dominiczak and Alex Alderton, Roberto Amato, Sonia Goncalves, Ewan Harrison,

		(COG-UK) Consortium	David K. Jackson, Ian Johnston, Dominic Kwiatkowski, Cordelia Langford, John Sillitoe on behalf of the Wellcome Sanger Institute COVID-19 Surveillance Team
EPI_ISL_820222, EPI_ISL_820224	Lighthouse Lab in Alderley Park	Wellcome Sanger Institute for the COVID-19 Genomics UK (COG-UK) Consortium	Jacquelyn Wynn, Mairead Hyland, The Lighthouse Lab in Alderley Park and Alex Alderton, Roberto Amato, Sonia Goncalves, Ewan Harrison, David K. Jackson, Ian Johnston, Dominic Kwiatkowski, Cordelia Langford, John Sillitoe on behalf of the Wellcome Sanger Institute COVID-19 Surveillance Team
EPI_ISL_820225, EPI_ISL_820227	Lighthouse Lab in Glasgow	Wellcome Sanger Institute for the COVID-19 Genomics UK (COG-UK) Consortium	Harper VanSteenhouse, Yumi Kasai, David Gray, Carol Clugston, Anna Dominiczak and Alex Alderton, Roberto Amato, Sonia Goncalves, Ewan Harrison, David K. Jackson, Ian Johnston, Dominic Kwiatkowski, Cordelia Langford, John Sillitoe on behalf of the Wellcome Sanger Institute COVID-19 Surveillance Team
EPI_ISL_820228, EPI_ISL_820230, EPI_ISL_820232	Lighthouse Lab in Alderley Park	Wellcome Sanger Institute for the COVID-19 Genomics UK (COG-UK) Consortium	Jacquelyn Wynn, Mairead Hyland, The Lighthouse Lab in Alderley Park and Alex Alderton, Roberto Amato, Sonia Goncalves, Ewan Harrison, David K. Jackson, Ian Johnston, Dominic Kwiatkowski, Cordelia Langford, John Sillitoe on behalf of the Wellcome Sanger Institute COVID-19 Surveillance Team
EPI_ISL_820233	Lighthouse Lab in Glasgow	Wellcome Sanger Institute for the COVID-19 Genomics UK (COG-UK) Consortium	Harper VanSteenhouse, Yumi Kasai, David Gray, Carol Clugston, Anna Dominiczak and Alex Alderton, Roberto Amato, Sonia Goncalves, Ewan Harrison, David K. Jackson, Ian Johnston, Dominic Kwiatkowski, Cordelia Langford, John Sillitoe on behalf of the Wellcome Sanger Institute COVID-19 Surveillance Team
EPI_ISL_820235, EPI_ISL_820237, EPI_ISL_820239, EPI_ISL_820240, EPI_ISL_820242	Lighthouse Lab in Alderley Park	Wellcome Sanger Institute for the COVID-19 Genomics UK (COG-UK) Consortium	Jacquelyn Wynn, Mairead Hyland, The Lighthouse Lab in Alderley Park and Alex Alderton, Roberto Amato, Sonia Goncalves, Ewan Harrison, David K. Jackson, Ian Johnston, Dominic Kwiatkowski, Cordelia Langford, John Sillitoe on behalf of the Wellcome Sanger Institute COVID-19 Surveillance Team
EPI_ISL_820243	Lighthouse Lab in Glasgow	Wellcome Sanger Institute for the COVID-19 Genomics UK (COG-UK) Consortium	Harper VanSteenhouse, Yumi Kasai, David Gray, Carol Clugston, Anna Dominiczak and Alex Alderton, Roberto Amato, Sonia Goncalves, Ewan Harrison, David K. Jackson, Ian Johnston, Dominic Kwiatkowski, Cordelia Langford, John Sillitoe on behalf of the Wellcome Sanger Institute COVID-19 Surveillance Team
EPI_ISL_820245, EPI_ISL_820247, EPI_ISL_820248, EPI_ISL_820250, EPI_ISL_820252, EPI_ISL_820254, EPI_ISL_820255, EPI_ISL_820257, EPI_ISL_820258	Lighthouse Lab in Alderley Park	Wellcome Sanger Institute for the COVID-19 Genomics UK (COG-UK) Consortium	Jacquelyn Wynn, Mairead Hyland, The Lighthouse Lab in Alderley Park and Alex Alderton, Roberto Amato, Sonia Goncalves, Ewan Harrison, David K. Jackson, Ian Johnston, Dominic Kwiatkowski, Cordelia Langford, John Sillitoe on behalf of the Wellcome Sanger Institute COVID-19 Surveillance Team
EPI_ISL_820260, EPI_ISL_820262	Lighthouse Lab in Glasgow	Wellcome Sanger Institute for the COVID-19 Genomics UK (COG-UK) Consortium	Harper VanSteenhouse, Yumi Kasai, David Gray, Carol Clugston, Anna Dominiczak and Alex Alderton, Roberto Amato, Sonia Goncalves, Ewan Harrison, David K. Jackson, Ian Johnston, Dominic Kwiatkowski, Cordelia Langford, John Sillitoe on behalf of the Wellcome Sanger Institute COVID-19 Surveillance Team
EPI_ISL_820263, EPI_ISL_820265, EPI_ISL_820267, EPI_ISL_820269	Lighthouse Lab in Alderley Park	Wellcome Sanger Institute for the COVID-19 Genomics UK (COG-UK) Consortium	Jacquelyn Wynn, Mairead Hyland, The Lighthouse Lab in Alderley Park and Alex Alderton, Roberto Amato, Sonia Goncalves, Ewan Harrison, David K. Jackson, Ian Johnston, Dominic Kwiatkowski, Cordelia Langford, John Sillitoe on behalf of the Wellcome Sanger Institute COVID-19 Surveillance Team
EPI_ISL_820270, EPI_ISL_820272	Lighthouse Lab in Glasgow	Wellcome Sanger Institute for the COVID-19 Genomics UK (COG-UK) Consortium	Harper VanSteenhouse, Yumi Kasai, David Gray, Carol Clugston, Anna Dominiczak and Alex Alderton, Roberto Amato, Sonia Goncalves, Ewan Harrison, David K. Jackson, Ian Johnston, Dominic Kwiatkowski, Cordelia Langford, John Sillitoe on behalf of the Wellcome Sanger Institute COVID-19 Surveillance Team
EPI_ISL_820274, EPI_ISL_820275, EPI_ISL_820277, EPI_ISL_820278, EPI_ISL_820280, EPI_ISL_820281	Lighthouse Lab in Alderley Park	Wellcome Sanger Institute for the COVID-19 Genomics UK (COG-UK) Consortium	Jacquelyn Wynn, Mairead Hyland, The Lighthouse Lab in Alderley Park and Alex Alderton, Roberto Amato, Sonia Goncalves, Ewan Harrison, David K. Jackson, Ian Johnston, Dominic Kwiatkowski, Cordelia Langford, John Sillitoe on behalf of the Wellcome Sanger Institute COVID-19 Surveillance Team
EPI_ISL_820283	Lighthouse Lab in Glasgow	Wellcome Sanger Institute for the COVID-19 Genomics UK (COG-UK) Consortium	Harper VanSteenhouse, Yumi Kasai, David Gray, Carol Clugston, Anna Dominiczak and Alex Alderton, Roberto Amato, Sonia Goncalves, Ewan Harrison, David K. Jackson, Ian Johnston, Dominic Kwiatkowski, Cordelia Langford, John Sillitoe on behalf of the Wellcome Sanger Institute COVID-19 Surveillance Team
EPI_ISL_820285, EPI_ISL_820286, EPI_ISL_820288, EPI_ISL_820290, EPI_ISL_820291, EPI_ISL_820293	Lighthouse Lab in Alderley Park	Wellcome Sanger Institute for the COVID-19 Genomics UK (COG-UK) Consortium	Jacquelyn Wynn, Mairead Hyland, The Lighthouse Lab in Alderley Park and Alex Alderton, Roberto Amato, Sonia Goncalves, Ewan Harrison, David K. Jackson, Ian Johnston, Dominic Kwiatkowski, Cordelia Langford, John Sillitoe on behalf of the Wellcome Sanger Institute COVID-19 Surveillance Team
EPI_ISL_820295	Lighthouse Lab in Glasgow	Wellcome Sanger Institute for the COVID-19 Genomics UK (COG-UK) Consortium	Harper VanSteenhouse, Yumi Kasai, David Gray, Carol Clugston, Anna Dominiczak and Alex Alderton, Roberto Amato, Sonia Goncalves, Ewan Harrison, David K. Jackson, Ian Johnston, Dominic Kwiatkowski, Cordelia Langford, John Sillitoe on behalf of the Wellcome Sanger Institute COVID-19 Surveillance Team
EPI_ISL_820296, EPI_ISL_820298, EPI_ISL_820299, EPI_ISL_820301	Lighthouse Lab in Alderley Park	Wellcome Sanger Institute for the COVID-19 Genomics UK (COG-UK) Consortium	Jacquelyn Wynn, Mairead Hyland, The Lighthouse Lab in Alderley Park and Alex Alderton, Roberto Amato, Sonia Goncalves, Ewan Harrison, David K. Jackson, Ian Johnston, Dominic Kwiatkowski, Cordelia Langford, John Sillitoe on behalf of the Wellcome Sanger Institute COVID-19 Surveillance Team
EPI_ISL_820303	Lighthouse Lab in Glasgow	Wellcome Sanger Institute for the COVID-19 Genomics UK (COG-UK) Consortium	Harper VanSteenhouse, Yumi Kasai, David Gray, Carol Clugston, Anna Dominiczak and Alex Alderton, Roberto Amato, Sonia Goncalves, Ewan Harrison, David K. Jackson, Ian Johnston, Dominic Kwiatkowski, Cordelia Langford, John Sillitoe on behalf of the Wellcome Sanger Institute COVID-19 Surveillance Team
EPI_ISL_820305, EPI_ISL_820307, EPI_ISL_820308, EPI_ISL_820310	Lighthouse Lab in Alderley Park	Wellcome Sanger Institute for the COVID-19 Genomics UK (COG-UK) Consortium	Jacquelyn Wynn, Mairead Hyland, The Lighthouse Lab in Alderley Park and Alex Alderton, Roberto Amato, Sonia Goncalves, Ewan Harrison, David K. Jackson, Ian Johnston, Dominic Kwiatkowski, Cordelia Langford, John Sillitoe on behalf of the Wellcome Sanger Institute COVID-19 Surveillance Team
EPI_ISL_820312	Lighthouse Lab in Glasgow	Wellcome Sanger Institute for the COVID-19 Genomics UK (COG-UK) Consortium	Harper VanSteenhouse, Yumi Kasai, David Gray, Carol Clugston, Anna Dominiczak and Alex Alderton, Roberto Amato, Sonia Goncalves, Ewan Harrison, David K. Jackson, Ian Johnston, Dominic Kwiatkowski, Cordelia Langford, John Sillitoe on behalf of the Wellcome Sanger Institute COVID-19 Surveillance Team
EPI_ISL_820313, EPI_ISL_820315, EPI_ISL_820317	Lighthouse Lab in Alderley Park	Wellcome Sanger Institute for the COVID-19 Genomics UK (COG-UK) Consortium	Jacquelyn Wynn, Mairead Hyland, The Lighthouse Lab in Alderley Park and Alex Alderton, Roberto Amato, Sonia Goncalves, Ewan Harrison, David K. Jackson, Ian Johnston, Dominic Kwiatkowski, Cordelia Langford, John Sillitoe on behalf of the Wellcome Sanger Institute COVID-19 Surveillance Team
EPI_ISL_820319, EPI_ISL_820320	Lighthouse Lab in Glasgow	Wellcome Sanger Institute for the COVID-19 Genomics UK (COG-UK) Consortium	Harper VanSteenhouse, Yumi Kasai, David Gray, Carol Clugston, Anna Dominiczak and Alex Alderton, Roberto Amato, Sonia Goncalves, Ewan Harrison, David K. Jackson, Ian Johnston, Dominic Kwiatkowski, Cordelia Langford, John Sillitoe on behalf of the Wellcome Sanger Institute COVID-19 Surveillance Team
EPI_ISL_820322, EPI_ISL_820324, EPI_ISL_820325, EPI_ISL_820327, EPI_ISL_820329, EPI_ISL_820330, EPI_ISL_820332, EPI_ISL_820334, EPI_ISL_820336, EPI_ISL_820338, EPI_ISL_820339, EPI_ISL_820341, EPI_ISL_820342, EPI_ISL_820344, EPI_ISL_820348			
see above	Lighthouse Lab in Alderley Park	Wellcome Sanger Institute for the COVID-19 Genomics UK (COG-UK) Consortium	Jacquelyn Wynn, Mairead Hyland, The Lighthouse Lab in Alderley Park and Alex Alderton, Roberto Amato, Sonia Goncalves, Ewan Harrison, David K. Jackson, Ian Johnston, Dominic Kwiatkowski, Cordelia Langford, John Sillitoe on behalf of the Wellcome Sanger Institute COVID-19 Surveillance Team
EPI_ISL_820349	Lighthouse Lab in Glasgow	Wellcome Sanger Institute for the COVID-19 Genomics UK (COG-UK) Consortium	Harper VanSteenhouse, Yumi Kasai, David Gray, Carol Clugston, Anna Dominiczak and Alex Alderton, Roberto Amato, Sonia Goncalves, Ewan Harrison, David K. Jackson, Ian Johnston, Dominic Kwiatkowski, Cordelia Langford, John Sillitoe on behalf of the Wellcome Sanger Institute COVID-19 Surveillance Team
EPI_ISL_820352, EPI_ISL_820354	Lighthouse Lab in Alderley Park	Wellcome Sanger Institute for the COVID-19 Genomics UK (COG-UK) Consortium	Jacquelyn Wynn, Mairead Hyland, The Lighthouse Lab in Alderley Park and Alex Alderton, Roberto Amato, Sonia Goncalves, Ewan Harrison, David K. Jackson, Ian Johnston, Dominic Kwiatkowski, Cordelia Langford, John Sillitoe on behalf of the Wellcome Sanger Institute COVID-19 Surveillance Team
EPI_ISL_820356	Lighthouse Lab in Glasgow	Wellcome Sanger Institute for the COVID-19 Genomics UK (COG-UK) Consortium	Harper VanSteenhouse, Yumi Kasai, David Gray, Carol Clugston, Anna Dominiczak and Alex Alderton, Roberto Amato, Sonia Goncalves, Ewan Harrison, David K. Jackson, Ian Johnston, Dominic Kwiatkowski, Cordelia Langford, John Sillitoe on behalf of the Wellcome Sanger Institute COVID-19 Surveillance Team
EPI_ISL_820357, EPI_ISL_820359, EPI_ISL_820361, EPI_ISL_820362, EPI_ISL_820364, EPI_ISL_820366, EPI_ISL_820367, EPI_ISL_820369, EPI_ISL_820370, EPI_ISL_820372, EPI_ISL_820374, EPI_ISL_820375, EPI_ISL_820377, EPI_ISL_820379, EPI_ISL_820380, EPI_ISL_820382			
see above	Lighthouse Lab in Alderley Park	Wellcome Sanger Institute for the COVID-19 Genomics UK (COG-UK) Consortium	Jacquelyn Wynn, Mairead Hyland, The Lighthouse Lab in Alderley Park and Alex Alderton, Roberto Amato, Sonia Goncalves, Ewan Harrison, David K. Jackson, Ian Johnston, Dominic Kwiatkowski, Cordelia Langford, John Sillitoe on behalf of the Wellcome Sanger Institute COVID-19 Surveillance Team
EPI_ISL_820384, EPI_ISL_820386,	Lighthouse Lab in Glasgow	Wellcome Sanger Institute for the COVID-19 Genomics UK	Harper VanSteenhouse, Yumi Kasai, David Gray, Carol Clugston, Anna Dominiczak and Alex Alderton, Roberto Amato, Sonia Goncalves, Ewan Harrison,

EPI_ISL_820387		(COG-UK) Consortium	David K. Jackson, Ian Johnston, Dominic Kwiatkowski, Cordelia Langford, John Sillitoe on behalf of the Wellcome Sanger Institute COVID-19 Surveillance Team
EPI_ISL_820389, EPI_ISL_820390, EPI_ISL_820392, EPI_ISL_820393, EPI_ISL_820395	Lighthouse Lab in Alderley Park	Wellcome Sanger Institute for the COVID-19 Genomics UK (COG-UK) Consortium	Jacquelyn Wynn, Mairead Hyland, The Lighthouse Lab in Alderley Park and Alex Alderton, Roberto Amato, Sonia Goncalves, Ewan Harrison, David K. Jackson, Ian Johnston, Dominic Kwiatkowski, Cordelia Langford, John Sillitoe on behalf of the Wellcome Sanger Institute COVID-19 Surveillance Team
EPI_ISL_820397	Lighthouse Lab in Glasgow	Wellcome Sanger Institute for the COVID-19 Genomics UK (COG-UK) Consortium	Harper VanSteenhouse, Yumi Kasai, David Gray, Carol Clugston, Anna Dominiczak and Alex Alderton, Roberto Amato, Sonia Goncalves, Ewan Harrison, David K. Jackson, Ian Johnston, Dominic Kwiatkowski, Cordelia Langford, John Sillitoe on behalf of the Wellcome Sanger Institute COVID-19 Surveillance Team
EPI_ISL_820398, EPI_ISL_820400, EPI_ISL_820401, EPI_ISL_820403, EPI_ISL_820405, EPI_ISL_820406, EPI_ISL_820408	Lighthouse Lab in Alderley Park	Wellcome Sanger Institute for the COVID-19 Genomics UK (COG-UK) Consortium	Jacquelyn Wynn, Mairead Hyland, The Lighthouse Lab in Alderley Park and Alex Alderton, Roberto Amato, Sonia Goncalves, Ewan Harrison, David K. Jackson, Ian Johnston, Dominic Kwiatkowski, Cordelia Langford, John Sillitoe on behalf of the Wellcome Sanger Institute COVID-19 Surveillance Team
EPI_ISL_820409	Lighthouse Lab in Glasgow	Wellcome Sanger Institute for the COVID-19 Genomics UK (COG-UK) Consortium	Harper VanSteenhouse, Yumi Kasai, David Gray, Carol Clugston, Anna Dominiczak and Alex Alderton, Roberto Amato, Sonia Goncalves, Ewan Harrison, David K. Jackson, Ian Johnston, Dominic Kwiatkowski, Cordelia Langford, John Sillitoe on behalf of the Wellcome Sanger Institute COVID-19 Surveillance Team
EPI_ISL_820411, EPI_ISL_820414, EPI_ISL_820416	Lighthouse Lab in Alderley Park	Wellcome Sanger Institute for the COVID-19 Genomics UK (COG-UK) Consortium	Jacquelyn Wynn, Mairead Hyland, The Lighthouse Lab in Alderley Park and Alex Alderton, Roberto Amato, Sonia Goncalves, Ewan Harrison, David K. Jackson, Ian Johnston, Dominic Kwiatkowski, Cordelia Langford, John Sillitoe on behalf of the Wellcome Sanger Institute COVID-19 Surveillance Team
EPI_ISL_820417	Lighthouse Lab in Glasgow	Wellcome Sanger Institute for the COVID-19 Genomics UK (COG-UK) Consortium	Harper VanSteenhouse, Yumi Kasai, David Gray, Carol Clugston, Anna Dominiczak and Alex Alderton, Roberto Amato, Sonia Goncalves, Ewan Harrison, David K. Jackson, Ian Johnston, Dominic Kwiatkowski, Cordelia Langford, John Sillitoe on behalf of the Wellcome Sanger Institute COVID-19 Surveillance Team
EPI_ISL_820419	Lighthouse Lab in Alderley Park	Wellcome Sanger Institute for the COVID-19 Genomics UK (COG-UK) Consortium	Jacquelyn Wynn, Mairead Hyland, The Lighthouse Lab in Alderley Park and Alex Alderton, Roberto Amato, Sonia Goncalves, Ewan Harrison, David K. Jackson, Ian Johnston, Dominic Kwiatkowski, Cordelia Langford, John Sillitoe on behalf of the Wellcome Sanger Institute COVID-19 Surveillance Team
EPI_ISL_820421	Lighthouse Lab in Glasgow	Wellcome Sanger Institute for the COVID-19 Genomics UK (COG-UK) Consortium	Harper VanSteenhouse, Yumi Kasai, David Gray, Carol Clugston, Anna Dominiczak and Alex Alderton, Roberto Amato, Sonia Goncalves, Ewan Harrison, David K. Jackson, Ian Johnston, Dominic Kwiatkowski, Cordelia Langford, John Sillitoe on behalf of the Wellcome Sanger Institute COVID-19 Surveillance Team
EPI_ISL_820422, EPI_ISL_820424, EPI_ISL_820425, EPI_ISL_820427, EPI_ISL_820429, EPI_ISL_820430, EPI_ISL_820432, EPI_ISL_820434, EPI_ISL_820435	Lighthouse Lab in Alderley Park	Wellcome Sanger Institute for the COVID-19 Genomics UK (COG-UK) Consortium	Jacquelyn Wynn, Mairead Hyland, The Lighthouse Lab in Alderley Park and Alex Alderton, Roberto Amato, Sonia Goncalves, Ewan Harrison, David K. Jackson, Ian Johnston, Dominic Kwiatkowski, Cordelia Langford, John Sillitoe on behalf of the Wellcome Sanger Institute COVID-19 Surveillance Team
EPI_ISL_820437	Lighthouse Lab in Glasgow	Wellcome Sanger Institute for the COVID-19 Genomics UK (COG-UK) Consortium	Harper VanSteenhouse, Yumi Kasai, David Gray, Carol Clugston, Anna Dominiczak and Alex Alderton, Roberto Amato, Sonia Goncalves, Ewan Harrison, David K. Jackson, Ian Johnston, Dominic Kwiatkowski, Cordelia Langford, John Sillitoe on behalf of the Wellcome Sanger Institute COVID-19 Surveillance Team
EPI_ISL_820438, EPI_ISL_820440, EPI_ISL_820442, EPI_ISL_820443, EPI_ISL_820445, EPI_ISL_820447	Lighthouse Lab in Alderley Park	Wellcome Sanger Institute for the COVID-19 Genomics UK (COG-UK) Consortium	Jacquelyn Wynn, Mairead Hyland, The Lighthouse Lab in Alderley Park and Alex Alderton, Roberto Amato, Sonia Goncalves, Ewan Harrison, David K. Jackson, Ian Johnston, Dominic Kwiatkowski, Cordelia Langford, John Sillitoe on behalf of the Wellcome Sanger Institute COVID-19 Surveillance Team
EPI_ISL_820448	Lighthouse Lab in Glasgow	Wellcome Sanger Institute for the COVID-19 Genomics UK (COG-UK) Consortium	Harper VanSteenhouse, Yumi Kasai, David Gray, Carol Clugston, Anna Dominiczak and Alex Alderton, Roberto Amato, Sonia Goncalves, Ewan Harrison, David K. Jackson, Ian Johnston, Dominic Kwiatkowski, Cordelia Langford, John Sillitoe on behalf of the Wellcome Sanger Institute COVID-19 Surveillance Team
EPI_ISL_820450, EPI_ISL_820452, EPI_ISL_820453, EPI_ISL_820455, EPI_ISL_820457, EPI_ISL_820458, EPI_ISL_820460	Lighthouse Lab in Alderley Park	Wellcome Sanger Institute for the COVID-19 Genomics UK (COG-UK) Consortium	Jacquelyn Wynn, Mairead Hyland, The Lighthouse Lab in Alderley Park and Alex Alderton, Roberto Amato, Sonia Goncalves, Ewan Harrison, David K. Jackson, Ian Johnston, Dominic Kwiatkowski, Cordelia Langford, John Sillitoe on behalf of the Wellcome Sanger Institute COVID-19 Surveillance Team
EPI_ISL_820461	Lighthouse Lab in Glasgow	Wellcome Sanger Institute for the COVID-19 Genomics UK (COG-UK) Consortium	Harper VanSteenhouse, Yumi Kasai, David Gray, Carol Clugston, Anna Dominiczak and Alex Alderton, Roberto Amato, Sonia Goncalves, Ewan Harrison, David K. Jackson, Ian Johnston, Dominic Kwiatkowski, Cordelia Langford, John Sillitoe on behalf of the Wellcome Sanger Institute COVID-19 Surveillance Team
EPI_ISL_820463	Lighthouse Lab in Alderley Park	Wellcome Sanger Institute for the COVID-19 Genomics UK (COG-UK) Consortium	Jacquelyn Wynn, Mairead Hyland, The Lighthouse Lab in Alderley Park and Alex Alderton, Roberto Amato, Sonia Goncalves, Ewan Harrison, David K. Jackson, Ian Johnston, Dominic Kwiatkowski, Cordelia Langford, John Sillitoe on behalf of the Wellcome Sanger Institute COVID-19 Surveillance Team
EPI_ISL_820465	Lighthouse Lab in Glasgow	Wellcome Sanger Institute for the COVID-19 Genomics UK (COG-UK) Consortium	Harper VanSteenhouse, Yumi Kasai, David Gray, Carol Clugston, Anna Dominiczak and Alex Alderton, Roberto Amato, Sonia Goncalves, Ewan Harrison, David K. Jackson, Ian Johnston, Dominic Kwiatkowski, Cordelia Langford, John Sillitoe on behalf of the Wellcome Sanger Institute COVID-19 Surveillance Team
EPI_ISL_820466	Lighthouse Lab in Alderley Park	Wellcome Sanger Institute for the COVID-19 Genomics UK (COG-UK) Consortium	Jacquelyn Wynn, Mairead Hyland, The Lighthouse Lab in Alderley Park and Alex Alderton, Roberto Amato, Sonia Goncalves, Ewan Harrison, David K. Jackson, Ian Johnston, Dominic Kwiatkowski, Cordelia Langford, John Sillitoe on behalf of the Wellcome Sanger Institute COVID-19 Surveillance Team
EPI_ISL_820468	Lighthouse Lab in Glasgow	Wellcome Sanger Institute for the COVID-19 Genomics UK (COG-UK) Consortium	Harper VanSteenhouse, Yumi Kasai, David Gray, Carol Clugston, Anna Dominiczak and Alex Alderton, Roberto Amato, Sonia Goncalves, Ewan Harrison, David K. Jackson, Ian Johnston, Dominic Kwiatkowski, Cordelia Langford, John Sillitoe on behalf of the Wellcome Sanger Institute COVID-19 Surveillance Team
EPI_ISL_820469, EPI_ISL_820471, EPI_ISL_820473, EPI_ISL_820474, EPI_ISL_820476	Lighthouse Lab in Alderley Park	Wellcome Sanger Institute for the COVID-19 Genomics UK (COG-UK) Consortium	Jacquelyn Wynn, Mairead Hyland, The Lighthouse Lab in Alderley Park and Alex Alderton, Roberto Amato, Sonia Goncalves, Ewan Harrison, David K. Jackson, Ian Johnston, Dominic Kwiatkowski, Cordelia Langford, John Sillitoe on behalf of the Wellcome Sanger Institute COVID-19 Surveillance Team
EPI_ISL_820477, EPI_ISL_820479, EPI_ISL_820481	Lighthouse Lab in Glasgow	Wellcome Sanger Institute for the COVID-19 Genomics UK (COG-UK) Consortium	Harper VanSteenhouse, Yumi Kasai, David Gray, Carol Clugston, Anna Dominiczak and Alex Alderton, Roberto Amato, Sonia Goncalves, Ewan Harrison, David K. Jackson, Ian Johnston, Dominic Kwiatkowski, Cordelia Langford, John Sillitoe on behalf of the Wellcome Sanger Institute COVID-19 Surveillance Team
EPI_ISL_820482	Lighthouse Lab in Alderley Park	Wellcome Sanger Institute for the COVID-19 Genomics UK (COG-UK) Consortium	Jacquelyn Wynn, Mairead Hyland, The Lighthouse Lab in Alderley Park and Alex Alderton, Roberto Amato, Sonia Goncalves, Ewan Harrison, David K. Jackson, Ian Johnston, Dominic Kwiatkowski, Cordelia Langford, John Sillitoe on behalf of the Wellcome Sanger Institute COVID-19 Surveillance Team
EPI_ISL_820484	Lighthouse Lab in Glasgow	Wellcome Sanger Institute for the COVID-19 Genomics UK (COG-UK) Consortium	Harper VanSteenhouse, Yumi Kasai, David Gray, Carol Clugston, Anna Dominiczak and Alex Alderton, Roberto Amato, Sonia Goncalves, Ewan Harrison, David K. Jackson, Ian Johnston, Dominic Kwiatkowski, Cordelia Langford, John Sillitoe on behalf of the Wellcome Sanger Institute COVID-19 Surveillance Team
EPI_ISL_820486, EPI_ISL_820487	Lighthouse Lab in Alderley Park	Wellcome Sanger Institute for the COVID-19 Genomics UK (COG-UK) Consortium	Jacquelyn Wynn, Mairead Hyland, The Lighthouse Lab in Alderley Park and Alex Alderton, Roberto Amato, Sonia Goncalves, Ewan Harrison, David K. Jackson, Ian Johnston, Dominic Kwiatkowski, Cordelia Langford, John Sillitoe on behalf of the Wellcome Sanger Institute COVID-19 Surveillance Team
EPI_ISL_820489	Lighthouse Lab in Glasgow	Wellcome Sanger Institute for the COVID-19 Genomics UK (COG-UK) Consortium	Harper VanSteenhouse, Yumi Kasai, David Gray, Carol Clugston, Anna Dominiczak and Alex Alderton, Roberto Amato, Sonia Goncalves, Ewan Harrison, David K. Jackson, Ian Johnston, Dominic Kwiatkowski, Cordelia Langford, John Sillitoe on behalf of the Wellcome Sanger Institute COVID-19 Surveillance Team
EPI_ISL_820491, EPI_ISL_820492, EPI_ISL_820494, EPI_ISL_820495, EPI_ISL_820497, EPI_ISL_820499,	Lighthouse Lab in Alderley Park	Wellcome Sanger Institute for the COVID-19 Genomics UK (COG-UK) Consortium	Jacquelyn Wynn, Mairead Hyland, The Lighthouse Lab in Alderley Park and Alex Alderton, Roberto Amato, Sonia Goncalves, Ewan Harrison, David K. Jackson, Ian Johnston, Dominic Kwiatkowski, Cordelia Langford, John Sillitoe on behalf of the Wellcome Sanger Institute COVID-19 Surveillance Team

EPI_ISL_820500, EPI_ISL_820502, EPI_ISL_820503			
EPI_ISL_820505, EPI_ISL_820507	Lighthouse Lab in Glasgow	Wellcome Sanger Institute for the COVID-19 Genomics UK (COG-UK) Consortium	Harper VanSteenhouse, Yumi Kasai, David Gray, Carol Clugston, Anna Dominiczak and Alex Alderton, Roberto Amato, Sonia Goncalves, Ewan Harrison, David K. Jackson, Ian Johnston, Dominic Kwiatkowski, Cordelia Langford, John Sillitoe on behalf of the Wellcome Sanger Institute COVID-19 Surveillance Team
EPI_ISL_820508	Lighthouse Lab in Alderley Park	Wellcome Sanger Institute for the COVID-19 Genomics UK (COG-UK) Consortium	Jacquelyn Wynn, Mairead Hyland, The Lighthouse Lab in Alderley Park and Alex Alderton, Roberto Amato, Sonia Goncalves, Ewan Harrison, David K. Jackson, Ian Johnston, Dominic Kwiatkowski, Cordelia Langford, John Sillitoe on behalf of the Wellcome Sanger Institute COVID-19 Surveillance Team
EPI_ISL_820510, EPI_ISL_820512, EPI_ISL_820513	Lighthouse Lab in Glasgow	Wellcome Sanger Institute for the COVID-19 Genomics UK (COG-UK) Consortium	Harper VanSteenhouse, Yumi Kasai, David Gray, Carol Clugston, Anna Dominiczak and Alex Alderton, Roberto Amato, Sonia Goncalves, Ewan Harrison, David K. Jackson, Ian Johnston, Dominic Kwiatkowski, Cordelia Langford, John Sillitoe on behalf of the Wellcome Sanger Institute COVID-19 Surveillance Team
EPI_ISL_820515, EPI_ISL_820517	Lighthouse Lab in Alderley Park	Wellcome Sanger Institute for the COVID-19 Genomics UK (COG-UK) Consortium	Jacquelyn Wynn, Mairead Hyland, The Lighthouse Lab in Alderley Park and Alex Alderton, Roberto Amato, Sonia Goncalves, Ewan Harrison, David K. Jackson, Ian Johnston, Dominic Kwiatkowski, Cordelia Langford, John Sillitoe on behalf of the Wellcome Sanger Institute COVID-19 Surveillance Team
EPI_ISL_820518	Lighthouse Lab in Glasgow	Wellcome Sanger Institute for the COVID-19 Genomics UK (COG-UK) Consortium	Harper VanSteenhouse, Yumi Kasai, David Gray, Carol Clugston, Anna Dominiczak and Alex Alderton, Roberto Amato, Sonia Goncalves, Ewan Harrison, David K. Jackson, Ian Johnston, Dominic Kwiatkowski, Cordelia Langford, John Sillitoe on behalf of the Wellcome Sanger Institute COVID-19 Surveillance Team
EPI_ISL_820520, EPI_ISL_820522, EPI_ISL_820523, EPI_ISL_820525, EPI_ISL_820527, EPI_ISL_820529, EPI_ISL_820530, EPI_ISL_820532, EPI_ISL_820534, EPI_ISL_820535, EPI_ISL_820537, EPI_ISL_820538, EPI_ISL_820540, EPI_ISL_820542, EPI_ISL_820544, EPI_ISL_820545			
see above	Lighthouse Lab in Alderley Park	Wellcome Sanger Institute for the COVID-19 Genomics UK (COG-UK) Consortium	Jacquelyn Wynn, Mairead Hyland, The Lighthouse Lab in Alderley Park and Alex Alderton, Roberto Amato, Sonia Goncalves, Ewan Harrison, David K. Jackson, Ian Johnston, Dominic Kwiatkowski, Cordelia Langford, John Sillitoe on behalf of the Wellcome Sanger Institute COVID-19 Surveillance Team
EPI_ISL_820547	Lighthouse Lab in Glasgow	Wellcome Sanger Institute for the COVID-19 Genomics UK (COG-UK) Consortium	Harper VanSteenhouse, Yumi Kasai, David Gray, Carol Clugston, Anna Dominiczak and Alex Alderton, Roberto Amato, Sonia Goncalves, Ewan Harrison, David K. Jackson, Ian Johnston, Dominic Kwiatkowski, Cordelia Langford, John Sillitoe on behalf of the Wellcome Sanger Institute COVID-19 Surveillance Team
EPI_ISL_820548, EPI_ISL_820550, EPI_ISL_820551, EPI_ISL_820553, EPI_ISL_820555, EPI_ISL_820556	Lighthouse Lab in Alderley Park	Wellcome Sanger Institute for the COVID-19 Genomics UK (COG-UK) Consortium	Jacquelyn Wynn, Mairead Hyland, The Lighthouse Lab in Alderley Park and Alex Alderton, Roberto Amato, Sonia Goncalves, Ewan Harrison, David K. Jackson, Ian Johnston, Dominic Kwiatkowski, Cordelia Langford, John Sillitoe on behalf of the Wellcome Sanger Institute COVID-19 Surveillance Team
EPI_ISL_820558	Lighthouse Lab in Glasgow	Wellcome Sanger Institute for the COVID-19 Genomics UK (COG-UK) Consortium	Harper VanSteenhouse, Yumi Kasai, David Gray, Carol Clugston, Anna Dominiczak and Alex Alderton, Roberto Amato, Sonia Goncalves, Ewan Harrison, David K. Jackson, Ian Johnston, Dominic Kwiatkowski, Cordelia Langford, John Sillitoe on behalf of the Wellcome Sanger Institute COVID-19 Surveillance Team
EPI_ISL_820559, EPI_ISL_820561, EPI_ISL_820562, EPI_ISL_820564, EPI_ISL_820566, EPI_ISL_820567, EPI_ISL_820569, EPI_ISL_820571, EPI_ISL_820572, EPI_ISL_820574, EPI_ISL_820576			
see above	Lighthouse Lab in Alderley Park	Wellcome Sanger Institute for the COVID-19 Genomics UK (COG-UK) Consortium	Jacquelyn Wynn, Mairead Hyland, The Lighthouse Lab in Alderley Park and Alex Alderton, Roberto Amato, Sonia Goncalves, Ewan Harrison, David K. Jackson, Ian Johnston, Dominic Kwiatkowski, Cordelia Langford, John Sillitoe on behalf of the Wellcome Sanger Institute COVID-19 Surveillance Team
EPI_ISL_820577	Lighthouse Lab in Glasgow	Wellcome Sanger Institute for the COVID-19 Genomics UK (COG-UK) Consortium	Harper VanSteenhouse, Yumi Kasai, David Gray, Carol Clugston, Anna Dominiczak and Alex Alderton, Roberto Amato, Sonia Goncalves, Ewan Harrison, David K. Jackson, Ian Johnston, Dominic Kwiatkowski, Cordelia Langford, John Sillitoe on behalf of the Wellcome Sanger Institute COVID-19 Surveillance Team
EPI_ISL_820579, EPI_ISL_820581, EPI_ISL_820582, EPI_ISL_820584, EPI_ISL_820585, EPI_ISL_820587, EPI_ISL_820589	Lighthouse Lab in Alderley Park	Wellcome Sanger Institute for the COVID-19 Genomics UK (COG-UK) Consortium	Jacquelyn Wynn, Mairead Hyland, The Lighthouse Lab in Alderley Park and Alex Alderton, Roberto Amato, Sonia Goncalves, Ewan Harrison, David K. Jackson, Ian Johnston, Dominic Kwiatkowski, Cordelia Langford, John Sillitoe on behalf of the Wellcome Sanger Institute COVID-19 Surveillance Team
EPI_ISL_820590	Lighthouse Lab in Glasgow	Wellcome Sanger Institute for the COVID-19 Genomics UK (COG-UK) Consortium	Harper VanSteenhouse, Yumi Kasai, David Gray, Carol Clugston, Anna Dominiczak and Alex Alderton, Roberto Amato, Sonia Goncalves, Ewan Harrison, David K. Jackson, Ian Johnston, Dominic Kwiatkowski, Cordelia Langford, John Sillitoe on behalf of the Wellcome Sanger Institute COVID-19 Surveillance Team
EPI_ISL_820592, EPI_ISL_820594, EPI_ISL_820595	Lighthouse Lab in Alderley Park	Wellcome Sanger Institute for the COVID-19 Genomics UK (COG-UK) Consortium	Jacquelyn Wynn, Mairead Hyland, The Lighthouse Lab in Alderley Park and Alex Alderton, Roberto Amato, Sonia Goncalves, Ewan Harrison, David K. Jackson, Ian Johnston, Dominic Kwiatkowski, Cordelia Langford, John Sillitoe on behalf of the Wellcome Sanger Institute COVID-19 Surveillance Team
EPI_ISL_820597	Lighthouse Lab in Glasgow	Wellcome Sanger Institute for the COVID-19 Genomics UK (COG-UK) Consortium	Harper VanSteenhouse, Yumi Kasai, David Gray, Carol Clugston, Anna Dominiczak and Alex Alderton, Roberto Amato, Sonia Goncalves, Ewan Harrison, David K. Jackson, Ian Johnston, Dominic Kwiatkowski, Cordelia Langford, John Sillitoe on behalf of the Wellcome Sanger Institute COVID-19 Surveillance Team
EPI_ISL_820599, EPI_ISL_820600, EPI_ISL_820602, EPI_ISL_820604, EPI_ISL_820606, EPI_ISL_820608	Lighthouse Lab in Alderley Park	Wellcome Sanger Institute for the COVID-19 Genomics UK (COG-UK) Consortium	Jacquelyn Wynn, Mairead Hyland, The Lighthouse Lab in Alderley Park and Alex Alderton, Roberto Amato, Sonia Goncalves, Ewan Harrison, David K. Jackson, Ian Johnston, Dominic Kwiatkowski, Cordelia Langford, John Sillitoe on behalf of the Wellcome Sanger Institute COVID-19 Surveillance Team
EPI_ISL_820609	Lighthouse Lab in Glasgow	Wellcome Sanger Institute for the COVID-19 Genomics UK (COG-UK) Consortium	Harper VanSteenhouse, Yumi Kasai, David Gray, Carol Clugston, Anna Dominiczak and Alex Alderton, Roberto Amato, Sonia Goncalves, Ewan Harrison, David K. Jackson, Ian Johnston, Dominic Kwiatkowski, Cordelia Langford, John Sillitoe on behalf of the Wellcome Sanger Institute COVID-19 Surveillance Team
EPI_ISL_820611, EPI_ISL_820612	Lighthouse Lab in Alderley Park	Wellcome Sanger Institute for the COVID-19 Genomics UK (COG-UK) Consortium	Jacquelyn Wynn, Mairead Hyland, The Lighthouse Lab in Alderley Park and Alex Alderton, Roberto Amato, Sonia Goncalves, Ewan Harrison, David K. Jackson, Ian Johnston, Dominic Kwiatkowski, Cordelia Langford, John Sillitoe on behalf of the Wellcome Sanger Institute COVID-19 Surveillance Team
EPI_ISL_820613	Lighthouse Lab in Glasgow	Wellcome Sanger Institute for the COVID-19 Genomics UK (COG-UK) Consortium	Harper VanSteenhouse, Yumi Kasai, David Gray, Carol Clugston, Anna Dominiczak and Alex Alderton, Roberto Amato, Sonia Goncalves, Ewan Harrison, David K. Jackson, Ian Johnston, Dominic Kwiatkowski, Cordelia Langford, John Sillitoe on behalf of the Wellcome Sanger Institute COVID-19 Surveillance Team
EPI_ISL_820614, EPI_ISL_820615, EPI_ISL_820616	Lighthouse Lab in Alderley Park	Wellcome Sanger Institute for the COVID-19 Genomics UK (COG-UK) Consortium	Jacquelyn Wynn, Mairead Hyland, The Lighthouse Lab in Alderley Park and Alex Alderton, Roberto Amato, Sonia Goncalves, Ewan Harrison, David K. Jackson, Ian Johnston, Dominic Kwiatkowski, Cordelia Langford, John Sillitoe on behalf of the Wellcome Sanger Institute COVID-19 Surveillance Team
EPI_ISL_820617, EPI_ISL_820618	Lighthouse Lab in Glasgow	Wellcome Sanger Institute for the COVID-19 Genomics UK (COG-UK) Consortium	Harper VanSteenhouse, Yumi Kasai, David Gray, Carol Clugston, Anna Dominiczak and Alex Alderton, Roberto Amato, Sonia Goncalves, Ewan Harrison, David K. Jackson, Ian Johnston, Dominic Kwiatkowski, Cordelia Langford, John Sillitoe on behalf of the Wellcome Sanger Institute COVID-19 Surveillance Team
EPI_ISL_820619	Lighthouse Lab in Alderley Park	Wellcome Sanger Institute for the COVID-19 Genomics UK (COG-UK) Consortium	Jacquelyn Wynn, Mairead Hyland, The Lighthouse Lab in Alderley Park and Alex Alderton, Roberto Amato, Sonia Goncalves, Ewan Harrison, David K. Jackson, Ian Johnston, Dominic Kwiatkowski, Cordelia Langford, John Sillitoe on behalf of the Wellcome Sanger Institute COVID-19 Surveillance Team
EPI_ISL_820620	Lighthouse Lab in Glasgow	Wellcome Sanger Institute for the COVID-19 Genomics UK (COG-UK) Consortium	Harper VanSteenhouse, Yumi Kasai, David Gray, Carol Clugston, Anna Dominiczak and Alex Alderton, Roberto Amato, Sonia Goncalves, Ewan Harrison, David K. Jackson, Ian Johnston, Dominic Kwiatkowski, Cordelia Langford, John Sillitoe on behalf of the Wellcome Sanger Institute COVID-19 Surveillance Team
EPI_ISL_820621, EPI_ISL_820622, EPI_ISL_820623, EPI_ISL_820624, EPI_ISL_820625, EPI_ISL_820626, EPI_ISL_820627	Lighthouse Lab in Alderley Park	Wellcome Sanger Institute for the COVID-19 Genomics UK (COG-UK) Consortium	Jacquelyn Wynn, Mairead Hyland, The Lighthouse Lab in Alderley Park and Alex Alderton, Roberto Amato, Sonia Goncalves, Ewan Harrison, David K. Jackson, Ian Johnston, Dominic Kwiatkowski, Cordelia Langford, John Sillitoe on behalf of the Wellcome Sanger Institute COVID-19 Surveillance Team
EPI_ISL_820628	Lighthouse Lab in Glasgow	Wellcome Sanger Institute for the COVID-19 Genomics UK (COG-UK) Consortium	Harper VanSteenhouse, Yumi Kasai, David Gray, Carol Clugston, Anna Dominiczak and Alex Alderton, Roberto Amato, Sonia Goncalves, Ewan Harrison, David K. Jackson, Ian Johnston, Dominic Kwiatkowski, Cordelia Langford, John Sillitoe on behalf of the Wellcome Sanger Institute COVID-19 Surveillance Team

EPI_ISL_820629, EPI_ISL_820630, EPI_ISL_820631, EPI_ISL_820632	Lighthouse Lab in Alderley Park	Wellcome Sanger Institute for the COVID-19 Genomics UK (COG-UK) Consortium	Jacquelyn Wynn, Mairead Hyland, The Lighthouse Lab in Alderley Park and Alex Alderton, Roberto Amato, Sonia Goncalves, Ewan Harrison, David K. Jackson, Ian Johnston, Dominic Kwiatkowski, Cordelia Langford, John Sillitoe on behalf of the Wellcome Sanger Institute COVID-19 Surveillance Team
EPI_ISL_820633, EPI_ISL_820634	Lighthouse Lab in Glasgow	Wellcome Sanger Institute for the COVID-19 Genomics UK (COG-UK) Consortium	Harper VanSteenhouse, Yumi Kasai, David Gray, Carol Clugston, Anna Dominiczak and Alex Alderton, Roberto Amato, Sonia Goncalves, Ewan Harrison, David K. Jackson, Ian Johnston, Dominic Kwiatkowski, Cordelia Langford, John Sillitoe on behalf of the Wellcome Sanger Institute COVID-19 Surveillance Team
EPI_ISL_820635, EPI_ISL_820636, EPI_ISL_820637, EPI_ISL_820638	Lighthouse Lab in Alderley Park	Wellcome Sanger Institute for the COVID-19 Genomics UK (COG-UK) Consortium	Jacquelyn Wynn, Mairead Hyland, The Lighthouse Lab in Alderley Park and Alex Alderton, Roberto Amato, Sonia Goncalves, Ewan Harrison, David K. Jackson, Ian Johnston, Dominic Kwiatkowski, Cordelia Langford, John Sillitoe on behalf of the Wellcome Sanger Institute COVID-19 Surveillance Team
EPI_ISL_820639	Lighthouse Lab in Glasgow	Wellcome Sanger Institute for the COVID-19 Genomics UK (COG-UK) Consortium	Harper VanSteenhouse, Yumi Kasai, David Gray, Carol Clugston, Anna Dominiczak and Alex Alderton, Roberto Amato, Sonia Goncalves, Ewan Harrison, David K. Jackson, Ian Johnston, Dominic Kwiatkowski, Cordelia Langford, John Sillitoe on behalf of the Wellcome Sanger Institute COVID-19 Surveillance Team
EPI_ISL_820640	Lighthouse Lab in Alderley Park	Wellcome Sanger Institute for the COVID-19 Genomics UK (COG-UK) Consortium	Jacquelyn Wynn, Mairead Hyland, The Lighthouse Lab in Alderley Park and Alex Alderton, Roberto Amato, Sonia Goncalves, Ewan Harrison, David K. Jackson, Ian Johnston, Dominic Kwiatkowski, Cordelia Langford, John Sillitoe on behalf of the Wellcome Sanger Institute COVID-19 Surveillance Team
EPI_ISL_820641	Lighthouse Lab in Glasgow	Wellcome Sanger Institute for the COVID-19 Genomics UK (COG-UK) Consortium	Harper VanSteenhouse, Yumi Kasai, David Gray, Carol Clugston, Anna Dominiczak and Alex Alderton, Roberto Amato, Sonia Goncalves, Ewan Harrison, David K. Jackson, Ian Johnston, Dominic Kwiatkowski, Cordelia Langford, John Sillitoe on behalf of the Wellcome Sanger Institute COVID-19 Surveillance Team
EPI_ISL_820642, EPI_ISL_820643, EPI_ISL_820644	Lighthouse Lab in Alderley Park	Wellcome Sanger Institute for the COVID-19 Genomics UK (COG-UK) Consortium	Jacquelyn Wynn, Mairead Hyland, The Lighthouse Lab in Alderley Park and Alex Alderton, Roberto Amato, Sonia Goncalves, Ewan Harrison, David K. Jackson, Ian Johnston, Dominic Kwiatkowski, Cordelia Langford, John Sillitoe on behalf of the Wellcome Sanger Institute COVID-19 Surveillance Team
EPI_ISL_820645	Lighthouse Lab in Glasgow	Wellcome Sanger Institute for the COVID-19 Genomics UK (COG-UK) Consortium	Harper VanSteenhouse, Yumi Kasai, David Gray, Carol Clugston, Anna Dominiczak and Alex Alderton, Roberto Amato, Sonia Goncalves, Ewan Harrison, David K. Jackson, Ian Johnston, Dominic Kwiatkowski, Cordelia Langford, John Sillitoe on behalf of the Wellcome Sanger Institute COVID-19 Surveillance Team
EPI_ISL_820646	Lighthouse Lab in Alderley Park	Wellcome Sanger Institute for the COVID-19 Genomics UK (COG-UK) Consortium	Jacquelyn Wynn, Mairead Hyland, The Lighthouse Lab in Alderley Park and Alex Alderton, Roberto Amato, Sonia Goncalves, Ewan Harrison, David K. Jackson, Ian Johnston, Dominic Kwiatkowski, Cordelia Langford, John Sillitoe on behalf of the Wellcome Sanger Institute COVID-19 Surveillance Team
EPI_ISL_820647	Lighthouse Lab in Glasgow	Wellcome Sanger Institute for the COVID-19 Genomics UK (COG-UK) Consortium	Harper VanSteenhouse, Yumi Kasai, David Gray, Carol Clugston, Anna Dominiczak and Alex Alderton, Roberto Amato, Sonia Goncalves, Ewan Harrison, David K. Jackson, Ian Johnston, Dominic Kwiatkowski, Cordelia Langford, John Sillitoe on behalf of the Wellcome Sanger Institute COVID-19 Surveillance Team
EPI_ISL_822346, EPI_ISL_822853, EPI_ISL_822864, EPI_ISL_822868, EPI_ISL_822891, EPI_ISL_822896, EPI_ISL_822908, EPI_ISL_822942, EPI_ISL_822988, EPI_ISL_823007, EPI_ISL_823008, EPI_ISL_823012, EPI_ISL_823015, EPI_ISL_823021, EPI_ISL_823022, EPI_ISL_823023, EPI_ISL_823024, EPI_ISL_823056, EPI_ISL_823059, EPI_ISL_823060, EPI_ISL_823061, EPI_ISL_823062, EPI_ISL_823063, EPI_ISL_823116, EPI_ISL_823131, EPI_ISL_823137, EPI_ISL_823143, EPI_ISL_823156, EPI_ISL_823170, EPI_ISL_823171, EPI_ISL_823176, EPI_ISL_823177, EPI_ISL_823218, EPI_ISL_823219, EPI_ISL_823220, EPI_ISL_823221, EPI_ISL_823222, EPI_ISL_823223, EPI_ISL_823224, EPI_ISL_823225, EPI_ISL_823226, EPI_ISL_823227, EPI_ISL_823228, EPI_ISL_823229, EPI_ISL_823230, EPI_ISL_823231, EPI_ISL_823232, EPI_ISL_823233, EPI_ISL_823236, EPI_ISL_823237, EPI_ISL_823238, EPI_ISL_823239, EPI_ISL_823240, EPI_ISL_823241, EPI_ISL_823242, EPI_ISL_823243, EPI_ISL_823244, EPI_ISL_823245, EPI_ISL_823397, EPI_ISL_823398, EPI_ISL_823399, EPI_ISL_823400, EPI_ISL_823401, EPI_ISL_823402, EPI_ISL_823403, EPI_ISL_823582, EPI_ISL_823586, EPI_ISL_823608, EPI_ISL_823609, EPI_ISL_823610, EPI_ISL_823611, EPI_ISL_823612, EPI_ISL_823613, EPI_ISL_823614, EPI_ISL_823615, EPI_ISL_823616, EPI_ISL_823617, EPI_ISL_823618, EPI_ISL_823619, EPI_ISL_823620, EPI_ISL_823621, EPI_ISL_823622, EPI_ISL_823623, EPI_ISL_823624, EPI_ISL_823626, EPI_ISL_823627, EPI_ISL_823628, EPI_ISL_823629, EPI_ISL_823630, EPI_ISL_823631, EPI_ISL_823632, EPI_ISL_823633			
see above	Wales Specialist Virology Centre Sequencing lab: Pathogen Genomics Unit	COVID-19 Genomics UK (COG-UK) Consortium	Catherine Moore, Johnathan Evans, Laura Gifford, Malorie Perry, Simon Cottrell, Angela Marchbank, Alec Birchley, Alexander Adams, Amy Gaskin, Bree Gatica-Wilcox, Jason Coombes, Joel Southgate, Lauren Gilbert, Lee Graham, Nicole Pacchiarini, Sara Kumziene-Summerhayes, Sarah Taylor, Sophie Jones, Sara Rey, Matthew Bull, Joanne Watkins, Sally Corden, Tom Connor
EPI_ISL_824284, EPI_ISL_824285	Institute of Microbiology, Universidad San Francisco de Quito	Institute of Microbiology, Universidad San Francisco de Quito	Belén Prado-Vivar, Sully Márquez, Juan José Guadalupe, Monica Becerra-Wong, Bernardo Gutiérrez, Tania Guayasamin, Patricio Reyes, Verónica Barragán, Patricio Rojas-Silva, Gabriel Trueba, Michelle Grunauer, Paúl Cárdenas
EPI_ISL_824295	DOHMH Morrisania	New York City Public Health Laboratory	Jade Wang, et al.
EPI_ISL_824311, EPI_ISL_824312	DOHMH Fort Greene	New York City Public Health Laboratory	Jade Wang, et al.
EPI_ISL_824313	DOHMH Jamaica	New York City Public Health Laboratory	Jade Wang, et al.
EPI_ISL_824314, EPI_ISL_824315	DOHMH Corona	New York City Public Health Laboratory	Jade Wang, et al.
EPI_ISL_824316	DOHMH Crown Heights	New York City Public Health Laboratory	Jade Wang, et al.
EPI_ISL_824317, EPI_ISL_824318	DOHMH Central Harlem	New York City Public Health Laboratory	Jade Wang, et al.
EPI_ISL_824319	DOHMH Chelsea	New York City Public Health Laboratory	Jade Wang, et al.
EPI_ISL_824320	DOHMH Corona	New York City Public Health Laboratory	Jade Wang, et al.
EPI_ISL_824321, EPI_ISL_824322	DOHMH Fort Greene	New York City Public Health Laboratory	Jade Wang, et al.
EPI_ISL_824323, EPI_ISL_824324, EPI_ISL_824325, EPI_ISL_824326, EPI_ISL_824327	DOHMH Morrisania	New York City Public Health Laboratory	Jade Wang, et al.
EPI_ISL_824328	DOHMH Corona	New York City Public Health Laboratory	Jade Wang, et al.
EPI_ISL_824329, EPI_ISL_824330	DOHMH Jamaica	New York City Public Health Laboratory	Jade Wang, et al.
EPI_ISL_824331	DOHMH Corona	New York City Public Health Laboratory	Jade Wang, et al.
EPI_ISL_824343, EPI_ISL_824345, EPI_ISL_824358, EPI_ISL_824359, EPI_ISL_824360, EPI_ISL_824361, EPI_ISL_824363, EPI_ISL_824377, EPI_ISL_824378	Michigan Department of Health and Human Services, Bureau of Laboratories	Michigan Department of Health and Human Services, Bureau of Laboratories	Blankenship HM, Riner D, Soehnlen MK
EPI_ISL_824402	California Department of Public Health	California Department of Public Health	CDPH IDLB COVIDNet
EPI_ISL_824983, EPI_ISL_824984	Maryland Public Health Laboratory	Maryland Public Health Laboratory	Maryland Department of Health Laboratories Administration
EPI_ISL_825026, EPI_ISL_825027	Utah Public Health Laboratory, Utah Public Health Laboratory Infectious Disease submission group	Utah Public Health Laboratory, Utah Public Health Laboratory Infectious Disease submission group	Young,E.L., Oakeson,K.F., Gallagher,T.
EPI_ISL_825378	Hospital Universitari Vall d'Hebron - Vall d'Hebron Institut de Rercerca	Hospital Universitari Vall d'Hebron	Cristina Andrés, Maria Piñana, Josep F Abril, Damir Garcia-Cehic, Ariadna Rando, Juliana Esperalba, Maria Gema Codina, Carla Castillo, Maria Carmen Martin, Tomàs Pumarola, Josep Quer, Andrés Antón
EPI_ISL_825385	Nigeria Centre For Disease Control	National reference Laboratory, NCDC, Gaduwa, Abuja	Dr Ndodo Nnaemeka, Olusola Anuoluwapo Akanbi, Chimaobi Chukwu, Dr Omoare Adesuyi, Kingsley Madubuike, Anthony Ahumibe, Naidoo Dhamari, Nwando Mba, Dr Chikwe Ihekweazu
EPI_ISL_825446, EPI_ISL_825447, EPI_ISL_825448, EPI_ISL_825449, EPI_ISL_825450, EPI_ISL_825451, EPI_ISL_825452, EPI_ISL_825453, EPI_ISL_825454, EPI_ISL_825455, EPI_ISL_825456, EPI_ISL_825457, EPI_ISL_825458, EPI_ISL_825459, EPI_ISL_825460, EPI_ISL_825465, EPI_ISL_825466, EPI_ISL_825467, EPI_ISL_825468, EPI_ISL_825469, EPI_ISL_825470, EPI_ISL_825471, EPI_ISL_825472, EPI_ISL_825473, EPI_ISL_825474, EPI_ISL_825475, EPI_ISL_825476, EPI_ISL_825477, EPI_ISL_825478, EPI_ISL_825479, EPI_ISL_825480, EPI_ISL_825481, EPI_ISL_825482, EPI_ISL_825483, EPI_ISL_825484, EPI_ISL_825485, EPI_ISL_825486, EPI_ISL_825487, EPI_ISL_825488, EPI_ISL_825489			
see above	NHLS-IALCH	KRISP, KZN Research Innovation and Sequencing Platform	Giandhari J, Pillay S, Lessells R, Mdlalose K, York D, Khan S, Tegally H, Wilkinson E, de Oliveira T
EPI_ISL_825617	Nigeria Centre For Disease Control	National reference Laboratory, NCDC, Gaduwa, Abuja	Dr Ndodo Nnaemeka, Olusola Anuoluwapo Akanbi, Chimaobi Chukwu, Dr Omoare Adesuyi, Esebanmen Grace, Anthony Ahumibe, Naidoo Dhamari, Nwando Mba, Dr Chikwe Ihekweazu

EPI_ISL_826464	Lighthouse Lab in Alderley Park	Wellcome Sanger Institute for the COVID-19 Genomics UK (COG-UK) Consortium	Jacquelyn Wynn, Mairead Hyland, The Lighthouse Lab in Alderley Park and Alex Alderton, Roberto Amato, Sonia Goncalves, Ewan Harrison, David K. Jackson, Ian Johnston, Dominic Kwiatkowski, Cordelia Langford, John Sillitoe on behalf of the Wellcome Sanger Institute COVID-19 Surveillance Team
EPI_ISL_827039, EPI_ISL_827778, EPI_ISL_827779, EPI_ISL_828069, EPI_ISL_828071, EPI_ISL_828073, EPI_ISL_829954, EPI_ISL_830219, EPI_ISL_830222, EPI_ISL_830226, EPI_ISL_830228			
see above	deCODE genetics	deCODE genetics	Daniel F Gudbjartsson; Agnar Helgason; Hakon Jonsson; Olafur T Magnusson; Pall Melsted; Gudmundur L Norddahl; Jona Saemundsdottir; Asgeir Sigurdsson; Patrick Sulem; Arna B Agustsdottir; Hannes Eggertsson; Berglind Eiriksdottir; Run Fridriksdottir; Elisabet E Gardarsdottir; Gudmundur Georgsson; Olafia S Gretarsdottir; Kjartan R Gudmundsson; Thora R Gunnarsdottir; Arnaldur Gylfason; Hilma Holm; Brynjar O Jensson; Aslaug Jonasdottir; Kamilla S Josefsdottir; Thordur Kristjansson; Droplaug N Magnusdottir; Solvi Rognvaldsson; Louise le Roux; Gudrun Sigmundsdottir; Gardar Sveinbjornsson; Kristin E Sveinsdottir; Maney Sveinsdottir; Emil A Thorarensen; Bjarni Thorbjornsson; Gisli Masson; Ingileif Jonsdottir; Alma Moller; Thorolfur Gudnason; Karl G Kristinsson; Unnur Thorsteinsdottir; Kari Stefansson
EPI_ISL_830519	The National University Hospital of Iceland	deCODE genetics	Daniel F Gudbjartsson; Agnar Helgason; Hakon Jonsson; Olafur T Magnusson; Pall Melsted; Gudmundur L Norddahl; Jona Saemundsdottir; Asgeir Sigurdsson; Patrick Sulem; Arna B Agustsdottir; Hannes Eggertsson; Berglind Eiriksdottir; Run Fridriksdottir; Elisabet E Gardarsdottir; Gudmundur Georgsson; Olafia S Gretarsdottir; Kjartan R Gudmundsson; Thora R Gunnarsdottir; Arnaldur Gylfason; Hilma Holm; Brynjar O Jensson; Aslaug Jonasdottir; Kamilla S Josefsdottir; Thordur Kristjansson; Droplaug N Magnusdottir; Solvi Rognvaldsson; Louise le Roux; Gudrun Sigmundsdottir; Gardar Sveinbjornsson; Kristin E Sveinsdottir; Maney Sveinsdottir; Emil A Thorarensen; Bjarni Thorbjornsson; Gisli Masson; Ingileif Jonsdottir; Alma Moller; Thorolfur Gudnason; Karl G Kristinsson; Unnur Thorsteinsdottir; Kari Stefansson
EPI_ISL_831027, EPI_ISL_831030, EPI_ISL_831275, EPI_ISL_831276, EPI_ISL_831277, EPI_ISL_831278, EPI_ISL_831279, EPI_ISL_831285, EPI_ISL_831287, EPI_ISL_831288	Hospital Universitario La Paz (Madrid)	SeqCOVID-SPAIN consortium/IBV(CSIC)	Fernando Lázaro-Perona, María Rodríguez-Tejedor, Elias Dahdouh, Jesús Mingorance and SeqCOVID-SPAIN consortium
EPI_ISL_831379	Labor Krone	Robert Koch Institute, Influenza and respiratory viruses FG17 & Bioinformatics MF1, Berlin, Germany	Dr. Münstermann. Prof. Tiemann , Stephan Fuchs, Stefan Kroeger, Marianne Wedde, Oliver Drechsel, Aleksandar Radonic, Rene Kmiecinski, Ralf Duerrwald, Thorsten Wolff
EPI_ISL_831380, EPI_ISL_831381	Limbach - MVZ Labor Dr. Limbach & Kollegen	Robert Koch Institute, Influenza and respiratory viruses FG17 & Bioinformatics MF1, Berlin, Germany	Dr. Konrad Bode, Stephan Fuchs, Stefan Kroeger, Marianne Wedde, Oliver Drechsel, Aleksandar Radonic, Rene Kmiecinski, Ralf Duerrwald, Thorsten Wolff
EPI_ISL_831382, EPI_ISL_831383, EPI_ISL_831384, EPI_ISL_831385	Labor Krone	Robert Koch Institute, Influenza and respiratory viruses FG17 & Bioinformatics MF1, Berlin, Germany	Dr. Münstermann. Prof. Tiemann , Stephan Fuchs, Stefan Kroeger, Marianne Wedde, Oliver Drechsel, Aleksandar Radonic, Rene Kmiecinski, Ralf Duerrwald, Thorsten Wolff
EPI_ISL_831386, EPI_ISL_831387, EPI_ISL_831388	Limbach - MVZ Labor Dr. Limbach & Kollegen	Robert Koch Institute, Influenza and respiratory viruses FG17 & Bioinformatics MF1, Berlin, Germany	Dr. Konrad Bode, Stephan Fuchs, Stefan Kroeger, Marianne Wedde, Oliver Drechsel, Aleksandar Radonic, Rene Kmiecinski, Ralf Duerrwald, Thorsten Wolff
EPI_ISL_831651	Institute for Infectious Diseases, University of Bern, Switzerland	Institute for Infectious Diseases, University of Bern, Switzerland	Michel C Koch, Christian Baumann, Miguel A Terrazos Miani, Cora Sägesser, Pascal Bittel, Stephen L Leib, Peter Keller, Franziska Suter-Riniker, Alban Ramette
EPI_ISL_831695, EPI_ISL_831703, EPI_ISL_831705, EPI_ISL_831712, EPI_ISL_831716, EPI_ISL_831718, EPI_ISL_831737, EPI_ISL_831739, EPI_ISL_831742, EPI_ISL_831749, EPI_ISL_831750, EPI_ISL_831757, EPI_ISL_831758, EPI_ISL_831870, EPI_ISL_831871, EPI_ISL_831872, EPI_ISL_831873, EPI_ISL_831877, EPI_ISL_831878, EPI_ISL_831879, EPI_ISL_831880, EPI_ISL_831881			
see above	United States Air Force School of Aerospace Medicine	United States Air Force School of Aerospace Medicine	Anthony Fries, Jennifer Meyer, William Gruner, Amanda Javorina, Sarah Purves, Clarise Starr, Elizabeth Macias
EPI_ISL_831986, EPI_ISL_831987, EPI_ISL_831988	Synlab Medilab, Mikrobiologi	The Public Health Agency of Sweden	Department of Microbiology, The Public Health Agency of Sweden
EPI_ISL_831989	Laboratorium for klinisk mikrobiologi	The Public Health Agency of Sweden	Department of Microbiology, The Public Health Agency of Sweden
EPI_ISL_831990, EPI_ISL_831992, EPI_ISL_831996, EPI_ISL_831997	Klinisk mikrobiologi	The Public Health Agency of Sweden	Department of Microbiology, The Public Health Agency of Sweden
EPI_ISL_832068	Santa Clara County Public Health Laboratory	Santa Clara County Public Health Laboratory	Santa Clara County Public Health Department
EPI_ISL_832106	MD Laboratories	Los Angeles County PHL	P. Hemarajata et al.
EPI_ISL_832392, EPI_ISL_832394, EPI_ISL_832395, EPI_ISL_832396	Santa Clara County Public Health Laboratory	Santa Clara County Public Health Laboratory	Santa Clara County Public Health Department
EPI_ISL_833134	Laboratorio de Ecologia de Doencas Transmissiveis na Amazonia, Instituto Leonidas e Maria Deane - Fiocruz Amazonia	Laboratorio de Ecologia de Doencas Transmissiveis na Amazonia, Instituto Leonidas e Maria Deane - Fiocruz Amazonia	Valdinete Nascimento, Victor Souza, André Corado, Fernanda Nascimento, George Silva, Ágatha Costa, Debora Duarte, Karina Pessoa, Matilde Mejía, Luciana Gonçalves, Maria Júlia Brandão, Michele Jesus, Felipe Naveca
EPI_ISL_833456	UZA/UAntwerpen (BIS lab), University Hospital Antwerp, Edegem, Belgium	Laboratory of Medical Microbiology, University of Antwerp, Campus Drie Eiken, S6.26, Universiteitsplein 1, 2610, Wilrijk, Antwerp, Belgium	Basil Britto Xavier, Jasmine Coppens, Christine Lammens, Veerle Matheeussen, Herman Goossens
EPI_ISL_836980, EPI_ISL_837113, EPI_ISL_837114, EPI_ISL_837115, EPI_ISL_837200, EPI_ISL_837201, EPI_ISL_837202	Respiratory Virus Unit, National Infection Service, Public Health England	COVID-19 Genomics UK (COG-UK) Consortium	PHE Covid Sequencing Team
EPI_ISL_837342, EPI_ISL_837350	National Virus Reference Laboratory	National Virus Reference Laboratory	Michael Carr, Gabriel Gonzalez, Jonathan Dean, Cillian F De Gascun
EPI_ISL_837444	Istituto Zooprofilattico Sperimentale del Mezzogiorno	TIGEM	Patrizia Annunziata, Andrea Ballabio, Valentina Bouche, Davide Cacchiarelli (CorrespAuthor), Pellegrino Cerino, Chiara Colantuono, Lucio Di Filippo, Antonio Grimaldi, Antonio Limone, Gabriella Loconte, Anna Manfredi, Francesco Panariello, Biancamaria Pierri, Marcello Salvi, Lucia Vassallo
EPI_ISL_838117, EPI_ISL_838119, EPI_ISL_838120, EPI_ISL_838123, EPI_ISL_838127, EPI_ISL_838129, EPI_ISL_838130, EPI_ISL_838131	West of Scotland Specialist Virology Centre, NHSGGC / MRC-University of Glasgow Centre for Virus Research	COVID-19 Genomics UK (COG-UK) Consortium	Ana da Silva Filipe, Natasha Johnson, Kathy Smollett, Daniel Mair, Stephen Carmichael, Alice Broos, Lily Tong, Jenna Nichols, Kyriaki Nomikou; Sarah McDonald; Richard Orton, Joseph Hughes, Sreenu Vattipally, David L Robertson; Alasdair MacLean, Rory Gunson; Sharif Shaaban, Matthew Holden; Rachel Blacow, Guy Mollett, Kathy Li, James Shepherd, Antonia Ho, Emma Thomson
EPI_ISL_838222, EPI_ISL_838223, EPI_ISL_838224, EPI_ISL_838225, EPI_ISL_838226, EPI_ISL_838234, EPI_ISL_838288, EPI_ISL_838289	Virology Department, Royal Infirmary of Edinburgh, NHS Lothian / School of Biological Sciences, University of Edinburgh / Institute of Genetics and Molecular Medicine, University of Edinburgh	COVID-19 Genomics UK (COG-UK) Consortium	McHugh M, Dewar R, Rooke S, Gallagher M, Balcaza C, O'Toole Á, Scher E, Hill V, McCrone JT, Colquhoun R, Yu X, Jackson B, Rambaut A, Williams TC, Templeton K
EPI_ISL_838458, EPI_ISL_838459, EPI_ISL_838460, EPI_ISL_838461, EPI_ISL_838462, EPI_ISL_838463, EPI_ISL_838466, EPI_ISL_838467, EPI_ISL_838468, EPI_ISL_838469, EPI_ISL_838470, EPI_ISL_838473, EPI_ISL_838475, EPI_ISL_838476, EPI_ISL_838477, EPI_ISL_838478, EPI_ISL_838479, EPI_ISL_838480, EPI_ISL_838482, EPI_ISL_838483, EPI_ISL_838484, EPI_ISL_838485, EPI_ISL_838487, EPI_ISL_838489, EPI_ISL_838491, EPI_ISL_838493, EPI_ISL_838494, EPI_ISL_838495, EPI_ISL_838497, EPI_ISL_838498, EPI_ISL_838499, EPI_ISL_838500, EPI_ISL_838501, EPI_ISL_838502, EPI_ISL_838503, EPI_ISL_838504, EPI_ISL_838505, EPI_ISL_838506, EPI_ISL_838507, EPI_ISL_838508, EPI_ISL_838509, EPI_ISL_838510, EPI_ISL_838511, EPI_ISL_838512, EPI_ISL_838513, EPI_ISL_838514, EPI_ISL_838516, EPI_ISL_838517, EPI_ISL_838518, EPI_ISL_838519, EPI_ISL_838520, EPI_ISL_838521, EPI_ISL_838522, EPI_ISL_838523, EPI_ISL_838524, EPI_ISL_838525, EPI_ISL_838526, EPI_ISL_838527, EPI_ISL_838528, EPI_ISL_838530, EPI_ISL_838538, EPI_ISL_838539, EPI_ISL_838540, EPI_ISL_838543, EPI_ISL_838544, EPI_ISL_838546, EPI_ISL_838548, EPI_ISL_838550, EPI_ISL_838557, EPI_ISL_838560, EPI_ISL_838561, EPI_ISL_838562, EPI_ISL_838563, EPI_ISL_838565, EPI_ISL_838566, EPI_ISL_838567			
see above	Liverpool Clinical Laboratories	COVID-19 Genomics UK (COG-UK) Consortium	Sam Haldenby, Anita Lucaci, Steve Paterson, Julian Hiscox, Alistair Darby, M Almsaud, A Alrezaihi, Muhannad Alruwaili, Stuart D Armstrong, Jones Benjamin, Eleanor G Bentley, Anu Chawla, Jordan J Clark, Angela Cowell, Richard Eccles, Isabel García-Dorival, Matthew Gemmell, Alessandro Gerada, PKF Gilmore, Richard Gregory, Ximeng Han, Catherine Hartley, Margaret Hughes, Miren Iturriza-Gomara, James Johnson, L Luu, Jenifer Manson, Charlotte Nelson, Elaine O'Toole, Cassie Olateju, Rebekah Penrice-Randal , Lucille Rainbow, N.P Randle, Trevor Ian Robinson, Parul Sharma, Ghada T Shawli, James P Stewart, Neil Swainston, Ecaterina Vamos, Joanne Watts, Mark Whitehead
EPI_ISL_838687, EPI_ISL_838688, EPI_ISL_838689, EPI_ISL_838690, EPI_ISL_838691, EPI_ISL_838692, EPI_ISL_838693, EPI_ISL_838694, EPI_ISL_838695, EPI_ISL_838696, EPI_ISL_838697, EPI_ISL_838698, EPI_ISL_838699, EPI_ISL_838700, EPI_ISL_838701, EPI_ISL_838702, EPI_ISL_838703, EPI_ISL_838704,			

EPI_ISL_838706, EPI_ISL_838707, EPI_ISL_838716, EPI_ISL_838720, EPI_ISL_838722, EPI_ISL_838728, EPI_ISL_838729			
see above	University College London, Great Ormond Street Hospital for Children NHS Foundation Trust, Imperial College Healthcare NHS Trust	COVID-19 Genomics UK (COG-UK) Consortium	Sergi Castellano, Rachel Williams, Mark Kristiansen, Paola Resende Silva, Sunando Roy, Tony Brooks, Helena Tutill, Paola Niola, Patricia Dyal, Charlotte Williams, Leysa Forrest, Yasmin Panchbhaya, Jacqueline Findlay, Samuel Weeks, Julianne Brown, Kathryn Harris, Paul Randell, James Price, Alison Holmes, Judith Breuer
EPI_ISL_839485, EPI_ISL_839488, EPI_ISL_839490, EPI_ISL_839491, EPI_ISL_839493, EPI_ISL_839494, EPI_ISL_839497, EPI_ISL_839500, EPI_ISL_839507, EPI_ISL_839544, EPI_ISL_839545, EPI_ISL_839546, EPI_ISL_839547, EPI_ISL_839548, EPI_ISL_839549, EPI_ISL_839550, EPI_ISL_839551, EPI_ISL_839552, EPI_ISL_839553, EPI_ISL_839554, EPI_ISL_839555, EPI_ISL_839556, EPI_ISL_839558, EPI_ISL_839559, EPI_ISL_839560, EPI_ISL_839561, EPI_ISL_839562, EPI_ISL_839563, EPI_ISL_839564, EPI_ISL_839565, EPI_ISL_839566, EPI_ISL_839567, EPI_ISL_839568, EPI_ISL_839569, EPI_ISL_839570, EPI_ISL_839571, EPI_ISL_839572, EPI_ISL_839573, EPI_ISL_839574, EPI_ISL_839575, EPI_ISL_839576, EPI_ISL_839577, EPI_ISL_839578, EPI_ISL_839579, EPI_ISL_839580, EPI_ISL_839581, EPI_ISL_839590, EPI_ISL_839632, EPI_ISL_839634, EPI_ISL_839635, EPI_ISL_839637, EPI_ISL_839642, EPI_ISL_839661, EPI_ISL_839662, EPI_ISL_839665			
see above	Northumbria University / South Tees Hospitals NHS Foundation Trust / North Cumbria Integrated Care NHS Foundation Trust / North Tees and Hartlepool NHS Foundation Trust / Newcastle Hospitals NHS Foundation Trust	COVID-19 Genomics UK (COG-UK) Consortium	Darren L Smith,Andrew Nelson,Matthew Bashton,Greg R Young,Joshua Loh,John Allan,Mohammad A Tariq,Giles S Holt,Gary Black,Wen C Yew,Lynn Dover,Paul Baker,Steve Liggett,Sarah Essex,Jane Greenaway,Debra Padgett,Clive Graham,Garren Scott,Edward Barton,Emma Swindells,Brendan Payne,Jennifer Collins,Yusri Taha,Gary Eltringham
EPI_ISL_840048, EPI_ISL_840049, EPI_ISL_840050, EPI_ISL_840146, EPI_ISL_840147, EPI_ISL_840148, EPI_ISL_840149, EPI_ISL_840150, EPI_ISL_840151, EPI_ISL_840152, EPI_ISL_840153, EPI_ISL_840154, EPI_ISL_840155, EPI_ISL_840156, EPI_ISL_840157, EPI_ISL_840158, EPI_ISL_840159, EPI_ISL_840160, EPI_ISL_840161, EPI_ISL_840162, EPI_ISL_840163, EPI_ISL_840164, EPI_ISL_840165, EPI_ISL_840166, EPI_ISL_840167, EPI_ISL_840168, EPI_ISL_840169, EPI_ISL_840170, EPI_ISL_840171, EPI_ISL_840172, EPI_ISL_840173, EPI_ISL_840174, EPI_ISL_840175, EPI_ISL_840176, EPI_ISL_840177, EPI_ISL_840178, EPI_ISL_840179			
see above	Lincolnshire Hospitals and DeepSeq Nottingham	COVID-19 Genomics UK (COG-UK) Consortium	Nichola Duckworth, Tim Sloan, Sarah Walsh, Jonathan Ball, Patrick McClure, Joeseph Chappell, Nadine Holmes, Matthew Carlisle, Christopher Moore, Fei Sang, Johnny Debebe, Victoria Wright, Matthew Loose
EPI_ISL_840259, EPI_ISL_840265, EPI_ISL_840266, EPI_ISL_840281, EPI_ISL_840282, EPI_ISL_840283, EPI_ISL_840284, EPI_ISL_840286, EPI_ISL_840351, EPI_ISL_840358	Oxford Viromics, NDM, University of Oxford; Oxford University Hospitals; Basingstoke and North Hampshire Hospital	COVID-19 Genomics UK (COG-UK) Consortium	Tanya Golubchik, David Bonsall, George Macintyre, Amy Trebes, Mariateresa de Cesare, Catrin Moore, Alex Mobbs, Anita Justice, Robert Shaw, Monique Andersson, Timothy Peto, Emma Wise, Nathan Moore, Jessica Lynch, Nick Cortes, Matilde Mori, Stephen Kidd, David Buck, John Todd, Christophe Fraser
EPI_ISL_840835, EPI_ISL_840885, EPI_ISL_840886	Wales Specialist Virology Centre Sequencing lab: Pathogen Genomics Unit	Public Health Wales Microbiology Cardiff Wales Specialist Virology Centre	Catherine Moore, Johnathan Evans, Laura Gifford, Malorie Perry, Simon Cottrell, Angela Marchbank, Alec Birchley, Alexander Adams, Amy Gaskin, Bree Gatica-Wilcox, Jason Coombes, Joel Southgate, Lauren Gilbert, Lee Graham, Nicole Pacchiarini, Sara Kumziene-Summerhayes, Sarah Taylor, Sophie Jones, Sara Rey, Matthew Bull, Joanne Watkins, Sally Corden, Tom Connor
EPI_ISL_841769, EPI_ISL_841774, EPI_ISL_841780, EPI_ISL_841782, EPI_ISL_841786, EPI_ISL_841788, EPI_ISL_841794, EPI_ISL_841796, EPI_ISL_841812, EPI_ISL_841814, EPI_ISL_841815, EPI_ISL_841816, EPI_ISL_841817, EPI_ISL_841818, EPI_ISL_841819, EPI_ISL_841821, EPI_ISL_841822, EPI_ISL_841823, EPI_ISL_841824, EPI_ISL_841825, EPI_ISL_841826, EPI_ISL_841827, EPI_ISL_841828, EPI_ISL_841829, EPI_ISL_841848, EPI_ISL_841850, EPI_ISL_841852, EPI_ISL_841853, EPI_ISL_841854, EPI_ISL_841861			
see above	Centre for Enzyme Innovation, University of Portsmouth / Translational Research Laboratory, Portsmouth Hospitals NHS Trust	COVID-19 Genomics UK (COG-UK) Consortium	Angela Beckett,Yann Bourgeois,Garry Scarlett,Sharon Glaysher,Scott Elliott,Kelly Bicknell,Robert Impey,Allyson Lloyd,Sarah Wyllie,Ethan Butcher,Anoop Chauhan,Samuel Robson
EPI_ISL_842670, EPI_ISL_842671, EPI_ISL_842672, EPI_ISL_842674, EPI_ISL_842675, EPI_ISL_842676, EPI_ISL_842677, EPI_ISL_842678, EPI_ISL_842679, EPI_ISL_842682, EPI_ISL_842683, EPI_ISL_842684, EPI_ISL_842685, EPI_ISL_842688, EPI_ISL_842691, EPI_ISL_842693, EPI_ISL_842694, EPI_ISL_842695, EPI_ISL_842705, EPI_ISL_842706, EPI_ISL_842707, EPI_ISL_842708, EPI_ISL_842710, EPI_ISL_842711, EPI_ISL_842712, EPI_ISL_842713, EPI_ISL_842714, EPI_ISL_842715, EPI_ISL_842716, EPI_ISL_842726, EPI_ISL_842727, EPI_ISL_842728, EPI_ISL_842729, EPI_ISL_842730, EPI_ISL_842731, EPI_ISL_842732, EPI_ISL_842734			
see above	University College London Hospital	COVID-19 Genomics UK (COG-UK) Consortium	Judith Heaney, Matthew Byott, Catherine Houlihan, Dan Frampton, Stuart Kirk, Moira Spyer and Eleni Nastouli
EPI_ISL_843078, EPI_ISL_843089, EPI_ISL_843090	Barts Health NHS Trust	COVID-19 Genomics UK (COG-UK) Consortium	CUTINO-MOGUEL, Maria-Teresa; HARRINGTON, David; OWOYEMI, Dola; SHYLINI, Raghavendran; BROAD, Claire; KELE, Beatrix
EPI_ISL_844202, EPI_ISL_844203, EPI_ISL_844206, EPI_ISL_844207, EPI_ISL_844208, EPI_ISL_844209, EPI_ISL_844210, EPI_ISL_844211, EPI_ISL_844212, EPI_ISL_844213, EPI_ISL_844226, EPI_ISL_844235, EPI_ISL_844242, EPI_ISL_844243, EPI_ISL_844244, EPI_ISL_844245, EPI_ISL_844246, EPI_ISL_844247, EPI_ISL_844248, EPI_ISL_844253, EPI_ISL_844256, EPI_ISL_844257, EPI_ISL_844259, EPI_ISL_844260, EPI_ISL_844265, EPI_ISL_844268, EPI_ISL_844270, EPI_ISL_844271, EPI_ISL_844272, EPI_ISL_844273, EPI_ISL_844274, EPI_ISL_844275, EPI_ISL_844276, EPI_ISL_844277, EPI_ISL_844278, EPI_ISL_844279, EPI_ISL_844280, EPI_ISL_844281, EPI_ISL_844301, EPI_ISL_844306, EPI_ISL_844307, EPI_ISL_844313, EPI_ISL_844314, EPI_ISL_844315, EPI_ISL_844316, EPI_ISL_844317, EPI_ISL_844318, EPI_ISL_844319, EPI_ISL_844320, EPI_ISL_844321, EPI_ISL_844322, EPI_ISL_844323, EPI_ISL_844324, EPI_ISL_844325, EPI_ISL_844326, EPI_ISL_844327, EPI_ISL_844328, EPI_ISL_844329, EPI_ISL_844330, EPI_ISL_844331, EPI_ISL_844332, EPI_ISL_844333, EPI_ISL_844334, EPI_ISL_844335, EPI_ISL_844336, EPI_ISL_844337, EPI_ISL_844338, EPI_ISL_844339, EPI_ISL_844340, EPI_ISL_844341, EPI_ISL_844342, EPI_ISL_844343, EPI_ISL_844344, EPI_ISL_844345, EPI_ISL_844346, EPI_ISL_844347, EPI_ISL_844348, EPI_ISL_844349, EPI_ISL_844350, EPI_ISL_844351, EPI_ISL_844352, EPI_ISL_844353, EPI_ISL_844354, EPI_ISL_844355, EPI_ISL_844356, EPI_ISL_844357, EPI_ISL_844358, EPI_ISL_844359, EPI_ISL_844360, EPI_ISL_844361, EPI_ISL_844362, EPI_ISL_844363, EPI_ISL_844364, EPI_ISL_844365, EPI_ISL_844366, EPI_ISL_844367, EPI_ISL_844368, EPI_ISL_844369, EPI_ISL_844370, EPI_ISL_844371, EPI_ISL_844372, EPI_ISL_844373, EPI_ISL_844374, EPI_ISL_844375, EPI_ISL_844376, EPI_ISL_844377, EPI_ISL_844378, EPI_ISL_844379, EPI_ISL_844380, EPI_ISL_844381, EPI_ISL_844382, EPI_ISL_844383, EPI_ISL_844384, EPI_ISL_844385, EPI_ISL_844386, EPI_ISL_844387, EPI_ISL_844388, EPI_ISL_844389, EPI_ISL_844390			
see above	Department of Virus and Microbiological Special Diagnostics, Statens Serum Institut, Copenhagen, Denmark	Albertsen Lab, Department of Chemistry and Bioscience, Aalborg University, Denmark	Danish Covid-19 Genome Consortium
EPI_ISL_846582	National Laboratory for Health, Environment and Food	National Laboratory for Health, Environment and Food	Aleksander Mahnic, Sandra Janezic, Maja Rupnik
EPI_ISL_847260, EPI_ISL_847514	Department of Virus and Microbiological Special Diagnostics, Statens Serum Institut, Copenhagen, Denmark	Albertsen Lab, Department of Chemistry and Bioscience, Aalborg University, Denmark	Danish Covid-19 Genome Consortium
EPI_ISL_847517	Chiu Laboratory, University of California, San Francisco	Chiu Laboratory, University of California, San Francisco	Charles Chiu, Xianding (Wayne) Deng, Candace Wang, Brian Bushnell, Scot Federman, Jill Hacker, Debra Wadford
EPI_ISL_847528	California Department of Public Health	Chiu Laboratory, University of California, San Francisco	Charles Chiu, Xianding (Wayne) Deng, Candace Wang, Brian Bushnell, Scot Federman, Jill Hacker, Debra Wadford
EPI_ISL_847540, EPI_ISL_847553	Chiu Laboratory, University of California, San Francisco	Chiu Laboratory, University of California, San Francisco	Charles Chiu, Xianding (Wayne) Deng, Candace Wang, Brian Bushnell, Scot Federman, Jill Hacker, Debra Wadford
EPI_ISL_847555, EPI_ISL_847559, EPI_ISL_847569	California Department of Public Health	Chiu Laboratory, University of California, San Francisco	Charles Chiu, Xianding (Wayne) Deng, Candace Wang, Brian Bushnell, Scot Federman, Jill Hacker, Debra Wadford
EPI_ISL_847579	Chiu Laboratory, University of California, San Francisco	Chiu Laboratory, University of California, San Francisco	Charles Chiu, Xianding (Wayne) Deng, Candace Wang, Brian Bushnell, Scot Federman, Jill Hacker, Debra Wadford
EPI_ISL_847638, EPI_ISL_847639	California Department of Public Health	Chiu Laboratory, University of California, San Francisco	Charles Chiu, Xianding (Wayne) Deng, Candace Wang, Brian Bushnell, Scot Federman, Jill Hacker, Debra Wadford
EPI_ISL_847640, EPI_ISL_847641, EPI_ISL_847642, EPI_ISL_847643, EPI_ISL_847644	Chiu Laboratory, University of California, San Francisco	Chiu Laboratory, University of California, San Francisco	Charles Chiu, Xianding (Wayne) Deng, Candace Wang, Brian Bushnell, Scot Federman, Jill Hacker, Debra Wadford
EPI_ISL_847713	California Department of Public Health	Chiu Laboratory, University of California, San Francisco	Charles Chiu, Xianding (Wayne) Deng, Candace Wang, Brian Bushnell, Scot Federman, Jill Hacker, Debra Wadford
EPI_ISL_847716, EPI_ISL_847720	Chiu Laboratory, University of California, San Francisco	Chiu Laboratory, University of California, San Francisco	Charles Chiu, Xianding (Wayne) Deng, Candace Wang, Brian Bushnell, Scot Federman, Jill Hacker, Debra Wadford
EPI_ISL_847724, EPI_ISL_847740, EPI_ISL_847781	California Department of Public Health	Chiu Laboratory, University of California, San Francisco	Charles Chiu, Xianding (Wayne) Deng, Candace Wang, Brian Bushnell, Scot Federman, Jill Hacker, Debra Wadford
EPI_ISL_847782	Chiu Laboratory, University of California, San Francisco	Chiu Laboratory, University of California, San Francisco	Charles Chiu, Xianding (Wayne) Deng, Candace Wang, Brian Bushnell, Scot Federman, Jill Hacker, Debra Wadford
EPI_ISL_847979, EPI_ISL_847982, EPI_ISL_847990, EPI_ISL_848009, EPI_ISL_848016, EPI_ISL_848021, EPI_ISL_848049, EPI_ISL_848051, EPI_ISL_848053, EPI_ISL_848057	Michigan Department of Health and Human Services, Bureau of Laboratories	Michigan Department of Health and Human Services, Bureau of Laboratories	Blankenship HM, Riner D, Soehnlen MK
EPI_ISL_848260, EPI_ISL_848268, EPI_ISL_848427, EPI_ISL_848428, EPI_ISL_848429, EPI_ISL_848430, EPI_ISL_848441, EPI_ISL_848458, EPI_ISL_848535, EPI_ISL_848536, EPI_ISL_848537			
see above	Illinois Department of Public Health	Gagnon Lab, Southern Illinois University	Keith Gagnon
EPI_ISL_849208, EPI_ISL_849209, EPI_ISL_849212, EPI_ISL_849213, EPI_ISL_849214, EPI_ISL_849216, EPI_ISL_849218, EPI_ISL_849225, EPI_ISL_849226, EPI_ISL_849227, EPI_ISL_849231, EPI_ISL_849233, EPI_ISL_849234, EPI_ISL_849235, EPI_ISL_849236, EPI_ISL_849239, EPI_ISL_849247, EPI_ISL_849248, EPI_ISL_849249, EPI_ISL_849250, EPI_ISL_849251, EPI_ISL_849252, EPI_ISL_849253, EPI_ISL_849254, EPI_ISL_849255, EPI_ISL_849258, EPI_ISL_849260, EPI_ISL_849263, EPI_ISL_849266, EPI_ISL_849270, EPI_ISL_849273, EPI_ISL_849274, EPI_ISL_849277, EPI_ISL_849278			

see above	Utah Public Health Laboratory	Utah Public Health Laboratory	Erin L. Young, Kelly F. Oakeson, Tara Gallagher
EPI_ISL_850658	Division of Emerging Infectious Diseases, Bureau of Infectious Diseases Diagnosis Control, Korea Disease Control and Prevention Agency	Division of Emerging Infectious Diseases, Bureau of Infectious Diseases Diagnosis Control, Korea Disease Control and Prevention Agency	Ae Kyung Park, Il-Hwan Kim, Heui Man Kim, Jeong-Min Kim, Namjoo Lee, Chaeyoung Lee, Sang Hee Woo, Eun-Jin Kim
EPI_ISL_850667, EPI_ISL_850674	The National Institute of Public Health	State Veterinary Institute Prague	Nagy,A;Jirincova,H;Trnka,D;Vecerova,J;Trinklova,M
EPI_ISL_852571, EPI_ISL_852572	Max von Pettenkofer Institute, Virology, National Reference Center for Retroviruses, LMU München	Laboratory for Functional Genome Analysis, Dept. Genomics, Gene Center of the LMU Munich	Max Muenchhoff, Stefan Krebs, Alexander Graf, Oliver Keppler, Helmut Blum
EPI_ISL_852616	National Laboratory for Health, Environment and Food	National Laboratory for Health, Environment and Food	Aleksander Mahnic, Sandra Janezic, Maja Rupnik
EPI_ISL_852817	CHU Purpan - Laboratoire de Virologie - Institut Fédératif de Biologie	CHU Purpan - Laboratoire de Virologie - Institut Fédératif de Biologie	Latour J., Ranger N., Dubois M., Carcenac R., Harter A., Boyer P., Tremeaux P., Izopet J.
EPI_ISL_852958, EPI_ISL_852989	Hospital General Universitario Gregorio Marañón	SeqCOVID-SPAIN consortium/IBV(CSIC)	Darío García de Viedma, Laura Pérez-Lago, Pedro J Sola-Campoy, Sergio Buenestado-Serrano, Marta Herranz, Victor Manuel de la Cueva, Julia Suárez, Pilar Catalán, Patricia Muñoz and SeqCOVID-SPAIN consortium
EPI_ISL_853345	UPMC Clinical Microbiology Laboratory	Microbial Genome Sequencing Center; Microbial Genomic Epidemiology Laboratory	Mustapha M. Mustapha, Jane W. Marsh, Dan Snyder, Marissa P. Griffith, Stephanie L. Mitchell, Vatsala R. Srinivasa, Kady D. Waggle, Chinelo Ezeonwuku, Vaughn S. Cooper, Lee H. Harrison
EPI_ISL_853725, EPI_ISL_853739, EPI_ISL_853744, EPI_ISL_853747, EPI_ISL_853765	Department of Microbiology, University Innsbruck	Bergthaler laboratory, CeMM Research Center for Molecular Medicine of the Austrian Academy of Sciences	Lukas Endler, Alexandra Popa, Benedikt Agerer, Jakob-Wendelin Genger, Alexander Lercher, Anna Schedl, Thomas Penz, Michael Schuster, Jan Laine, Martin Senekowitsch, Christoph Bock, Andreas Bergthaler
EPI_ISL_855409	Servicio de Microbiología, Laboratori Clínic Metropolitana Nord. Hospital Universitari Germans Trias i Pujol. Institut d'Investigació en Ciències de la Salut Germans Trias i Pujol (IGTP)	SeqCOVID-SPAIN consortium/IBV(CSIC)	Elisa Martró, Antoni E. Bordoy, Anna Not, Adrián Antuori, Anabel Fernández, Nona Romaní, Verónica Saludes, Cristina Casañ and SeqCOVID-SPAIN consortium
EPI_ISL_855517, EPI_ISL_855519, EPI_ISL_855531	KEMRI-Wellcome Trust Research Programme/KEMRI-CGMR-C Kilifi	KEMRI-Wellcome Trust Research Programme/KEMRI-CGMR-C Kilifi	Githinji et al
EPI_ISL_856663, EPI_ISL_856664, EPI_ISL_856665, EPI_ISL_856666, EPI_ISL_856667, EPI_ISL_856668, EPI_ISL_856669, EPI_ISL_856670, EPI_ISL_856671, EPI_ISL_856672	Department of Virus and Microbiological Special Diagnostics, Statens Serum Institut, Copenhagen, Denmark	Aalborg University	Danish Covid-19 Genome Consortium
EPI_ISL_856696, EPI_ISL_856697, EPI_ISL_856699, EPI_ISL_856701	Ohio Department of Health Laboratory	Ohio Department of Health Laboratory	Holmes, Jennifer; Eric Brandt, Keoni Omura, Glen McGillivary, Caitlin McDonnell, Kirtana Ramadugu, Erica Leasure, Kelsey Florek, Heather Blankenship, Quanta Brown, and Tammy Bannerman
EPI_ISL_856785, EPI_ISL_856787	Servicio Virosis Respiratorias-Departamento Virología-INEI	Instituto Nacional Enfermedades Infecciosas C.G.Malbran	Baumeister E., Avaro M., Benedetti E., Russo M., Dattero ME, Pontoriero A., Cisterna D., Molina V., Perandones C., Tuduri E., Lorenzo F., Poklepovich T., Campos J.
EPI_ISL_856810, EPI_ISL_856811, EPI_ISL_856814, EPI_ISL_856821, EPI_ISL_856823, EPI_ISL_856827, EPI_ISL_856831, EPI_ISL_856832, EPI_ISL_856835, EPI_ISL_856836, EPI_ISL_856840, EPI_ISL_856843, EPI_ISL_856846, EPI_ISL_856847, EPI_ISL_856851, EPI_ISL_856854, EPI_ISL_856857			
see above	Utah Public Health Laboratory	Utah Public Health Laboratory	Erin L. Young, Kelly F. Oakeson, Tara Gallagher
EPI_ISL_857514	Swiss National Reference Centre for Influenza	Swiss National Reference Centre for Influenza	Ana Rita Goncalves,Samuel Cordey, Laurent Kaiser, Lorenzo Cerutti, Henri Pegeot, Melyssa Elies, Keith Harshman, Ioannis Xenarios, Emmanouil Dermitzakis
EPI_ISL_857537, EPI_ISL_857538, EPI_ISL_857539	Swiss National Reference Centre for Influenza	Swiss National Reference Centre for Influenza	Tim Roloff, Ana Rita Gonçalves, Madlen Stange, Helena MB Seth-Smith, Alfredo Mari, Karoline Leuzinger, Julia Bielicki, Manuel Battegay, Hans Hirsch, Laurent Kaiser, Adrian Egli
EPI_ISL_858408	Lighthouse Lab in Alderley Park	Wellcome Sanger Institute for the COVID-19 Genomics UK (COG-UK) Consortium	Jacquelyn Wynn, Mairead Hyland, The Lighthouse Lab in Alderley Park and Alex Alderton, Roberto Amato, Sonia Goncalves, Ewan Harrison, David K. Jackson, Ian Johnston, Dominic Kwiatkowski, Cordelia Langford, John Sillitoe on behalf of the Wellcome Sanger Institute COVID-19 Surveillance Team
EPI_ISL_858410	Lighthouse Lab in Glasgow	Wellcome Sanger Institute for the COVID-19 Genomics UK (COG-UK) Consortium	Harper VanSteenhouse, Yumi Kasai, David Gray, Carol Clugston, Anna Dominiczak and Alex Alderton, Roberto Amato, Sonia Goncalves, Ewan Harrison, David K. Jackson, Ian Johnston, Dominic Kwiatkowski, Cordelia Langford, John Sillitoe on behalf of the Wellcome Sanger Institute COVID-19 Surveillance Team
EPI_ISL_858411	Lighthouse Lab in Alderley Park	Wellcome Sanger Institute for the COVID-19 Genomics UK (COG-UK) Consortium	Jacquelyn Wynn, Mairead Hyland, The Lighthouse Lab in Alderley Park and Alex Alderton, Roberto Amato, Sonia Goncalves, Ewan Harrison, David K. Jackson, Ian Johnston, Dominic Kwiatkowski, Cordelia Langford, John Sillitoe on behalf of the Wellcome Sanger Institute COVID-19 Surveillance Team
EPI_ISL_858412	Lighthouse Lab in Glasgow	Wellcome Sanger Institute for the COVID-19 Genomics UK (COG-UK) Consortium	Harper VanSteenhouse, Yumi Kasai, David Gray, Carol Clugston, Anna Dominiczak and Alex Alderton, Roberto Amato, Sonia Goncalves, Ewan Harrison, David K. Jackson, Ian Johnston, Dominic Kwiatkowski, Cordelia Langford, John Sillitoe on behalf of the Wellcome Sanger Institute COVID-19 Surveillance Team
EPI_ISL_858413, EPI_ISL_858414	Lighthouse Lab in Alderley Park	Wellcome Sanger Institute for the COVID-19 Genomics UK (COG-UK) Consortium	Jacquelyn Wynn, Mairead Hyland, The Lighthouse Lab in Alderley Park and Alex Alderton, Roberto Amato, Sonia Goncalves, Ewan Harrison, David K. Jackson, Ian Johnston, Dominic Kwiatkowski, Cordelia Langford, John Sillitoe on behalf of the Wellcome Sanger Institute COVID-19 Surveillance Team
EPI_ISL_858415, EPI_ISL_858417	Lighthouse Lab in Glasgow	Wellcome Sanger Institute for the COVID-19 Genomics UK (COG-UK) Consortium	Harper VanSteenhouse, Yumi Kasai, David Gray, Carol Clugston, Anna Dominiczak and Alex Alderton, Roberto Amato, Sonia Goncalves, Ewan Harrison, David K. Jackson, Ian Johnston, Dominic Kwiatkowski, Cordelia Langford, John Sillitoe on behalf of the Wellcome Sanger Institute COVID-19 Surveillance Team
EPI_ISL_858418	Lighthouse Lab in Alderley Park	Wellcome Sanger Institute for the COVID-19 Genomics UK (COG-UK) Consortium	Jacquelyn Wynn, Mairead Hyland, The Lighthouse Lab in Alderley Park and Alex Alderton, Roberto Amato, Sonia Goncalves, Ewan Harrison, David K. Jackson, Ian Johnston, Dominic Kwiatkowski, Cordelia Langford, John Sillitoe on behalf of the Wellcome Sanger Institute COVID-19 Surveillance Team
EPI_ISL_858419, EPI_ISL_858420, EPI_ISL_858421	Lighthouse Lab in Glasgow	Wellcome Sanger Institute for the COVID-19 Genomics UK (COG-UK) Consortium	Harper VanSteenhouse, Yumi Kasai, David Gray, Carol Clugston, Anna Dominiczak and Alex Alderton, Roberto Amato, Sonia Goncalves, Ewan Harrison, David K. Jackson, Ian Johnston, Dominic Kwiatkowski, Cordelia Langford, John Sillitoe on behalf of the Wellcome Sanger Institute COVID-19 Surveillance Team
EPI_ISL_858422	Lighthouse Lab in Alderley Park	Wellcome Sanger Institute for the COVID-19 Genomics UK (COG-UK) Consortium	Jacquelyn Wynn, Mairead Hyland, The Lighthouse Lab in Alderley Park and Alex Alderton, Roberto Amato, Sonia Goncalves, Ewan Harrison, David K. Jackson, Ian Johnston, Dominic Kwiatkowski, Cordelia Langford, John Sillitoe on behalf of the Wellcome Sanger Institute COVID-19 Surveillance Team
EPI_ISL_858423, EPI_ISL_858424	Lighthouse Lab in Glasgow	Wellcome Sanger Institute for the COVID-19 Genomics UK (COG-UK) Consortium	Harper VanSteenhouse, Yumi Kasai, David Gray, Carol Clugston, Anna Dominiczak and Alex Alderton, Roberto Amato, Sonia Goncalves, Ewan Harrison, David K. Jackson, Ian Johnston, Dominic Kwiatkowski, Cordelia Langford, John Sillitoe on behalf of the Wellcome Sanger Institute COVID-19 Surveillance Team
EPI_ISL_858929, EPI_ISL_858973, EPI_ISL_858992, EPI_ISL_858993, EPI_ISL_858995, EPI_ISL_858996, EPI_ISL_858998, EPI_ISL_858999, EPI_ISL_859000, EPI_ISL_859002, EPI_ISL_859004, EPI_ISL_859006, EPI_ISL_859007, EPI_ISL_859009, EPI_ISL_859010, EPI_ISL_859011, EPI_ISL_859013, EPI_ISL_859014, EPI_ISL_859015, EPI_ISL_859016, EPI_ISL_859017, EPI_ISL_859019, EPI_ISL_859021, EPI_ISL_859022, EPI_ISL_859023, EPI_ISL_859025, EPI_ISL_859026, EPI_ISL_859027			
see above	Lighthouse Lab in Alderley Park	Wellcome Sanger Institute for the COVID-19 Genomics UK (COG-UK) Consortium	Jacquelyn Wynn, Mairead Hyland, The Lighthouse Lab in Alderley Park and Alex Alderton, Roberto Amato, Sonia Goncalves, Ewan Harrison, David K. Jackson, Ian Johnston, Dominic Kwiatkowski, Cordelia Langford, John Sillitoe on behalf of the Wellcome Sanger Institute COVID-19 Surveillance Team
EPI_ISL_859326	Lighthouse Lab in Glasgow	Wellcome Sanger Institute for the COVID-19 Genomics UK (COG-UK) Consortium	Harper VanSteenhouse, Yumi Kasai, David Gray, Carol Clugston, Anna Dominiczak and Alex Alderton, Roberto Amato, Sonia Goncalves, Ewan Harrison, David K. Jackson, Ian Johnston, Dominic Kwiatkowski, Cordelia Langford, John Sillitoe on behalf of the Wellcome Sanger Institute COVID-19 Surveillance Team
EPI_ISL_859401, EPI_ISL_859427, EPI_ISL_859432, EPI_ISL_859433	Lighthouse Lab in Alderley Park	Wellcome Sanger Institute for the COVID-19 Genomics UK (COG-UK) Consortium	Jacquelyn Wynn, Mairead Hyland, The Lighthouse Lab in Alderley Park and Alex Alderton, Roberto Amato, Sonia Goncalves, Ewan Harrison, David K. Jackson, Ian Johnston, Dominic Kwiatkowski, Cordelia Langford, John Sillitoe on behalf of the Wellcome Sanger Institute COVID-19 Surveillance Team

EPI_ISL_859456, EPI_ISL_859457, EPI_ISL_859458, EPI_ISL_859459, EPI_ISL_859460, EPI_ISL_859461, EPI_ISL_859464, EPI_ISL_859470, EPI_ISL_859473, EPI_ISL_859474, EPI_ISL_859475, EPI_ISL_859476, EPI_ISL_859481			
see above	Lighthouse Lab in Glasgow	Wellcome Sanger Institute for the COVID-19 Genomics UK (COG-UK) Consortium	Harper VanSteenhouse, Yumi Kasai, David Gray, Carol Clugston, Anna Dominiczak and Alex Alderton, Roberto Amato, Sonia Goncalves, Ewan Harrison, David K. Jackson, Ian Johnston, Dominic Kwiatkowski, Cordelia Langford, John Sillitoe on behalf of the Wellcome Sanger Institute COVID-19 Surveillance Team
EPI_ISL_860114, EPI_ISL_860115	Medical Microbiology Unit, Department for Laboratory Medicine, Drammen Hospital, Vestre Viken Health Trust,	Norwegian Institute of Public Health, Department of Virology	Kathrine Stene-Johansen, Kamilla Heddeland Instefjord, Hilde Elshaug, Atiya R Ali,Marie Paulsen Madsen, Rasmus Riis Kopperud, Hilde Vollan, Karoline Bragstad, Olav Hungnes
EPI_ISL_860218	Ostfold Hospital Trust - Kalnes, Centre for Laboratory Medicine, Section for gene technology and infection serology	Norwegian Institute of Public Health, Department of Virology	Kathrine Stene-Johansen, Kamilla Heddeland Instefjord, Hilde Elshaug, Atiya R Ali,Marie Paulsen Madsen, Rasmus Riis Kopperud, Hilde Vollan, Karoline Bragstad, Olav Hungnes
EPI_ISL_860292	Innlandet Hospital Trust, Division Lillehammer, Department for Medical Microbiology	Norwegian Institute of Public Health, Department of Virology	Kathrine Stene-Johansen, Kamilla Heddeland Instefjord, Hilde Elshaug, Atiya R Ali,Marie Paulsen Madsen, Rasmus Riis Kopperud, Hilde Vollan, Karoline Bragstad, Olav Hungnes
EPI_ISL_860932	Johns Hopkins Hospital Department of Pathology	Johns Hopkins Hospital Department of Pathology	C. Paul Morris, Chun Huai Luo, Adannaya Amadi, Nicholas Gallagher, Heba H. Mostafa
EPI_ISL_861465, EPI_ISL_861469, EPI_ISL_861471	UHAS COVID-19 Lab	UHAS COVID-19 Lab	Kwabena O. Duedu, Jones Gyamfi, Reuben Ayivor-Djanie, John O. Gyapong and the UHAS COVID-19 Lab Team
EPI_ISL_861531, EPI_ISL_861590	Instituto Nacional de Saude (INSA)	Instituto Nacional de Saude (INSA)	Borges et al
EPI_ISL_861775, EPI_ISL_861776, EPI_ISL_861783, EPI_ISL_861786, EPI_ISL_861805, EPI_ISL_861825, EPI_ISL_861826	Hospital General Universitario Gregorio Marañón	SeqCOVID-SPAIN consortium/IBV(CSIC)	Darío García de Viedma, Laura Pérez-Lago, Pedro J Sola-Campoy, Sergio Buenestado-Serrano, Marta Herranz, Victor Manuel de la Cueva, Julia Suárez, Pilar Catalán, Patricia Muñoz and SeqCOVID-SPAIN consortium
EPI_ISL_862190	Respiratory Virus Unit, National Infection Service, Public Health England	COVID-19 Genomics UK (COG-UK) Consortium	PHE Covid Sequencing Team
EPI_ISL_862581, EPI_ISL_862582, EPI_ISL_862583, EPI_ISL_862584	Hospital Clínic	Instituto de Salud Carlos III	Iglesias-Caballero, M.Camarero, S. Molinero Calamita, M. González-Esguevillas, M. Pozo, F. Casas, I. Jiménez, P. Jiménez, M. Zaballos, A. Monzón, S. Varona, S. Juliá, M. Cuesta, I. Marcos, M.A.
EPI_ISL_862780, EPI_ISL_862782	Utah Public Health Laboratory, Utah Public Health Laboratory Infectious Disease submission group	Utah Public Health Laboratory, Utah Public Health Laboratory Infectious Disease submission group	Young,E.L., Oakeson,K.F., Gallagher,T.
EPI_ISL_864574, EPI_ISL_864576	Institute of Medical Microbiology and Hospital Hygiene	Institute of Medical Microbiology and Hospital Hygiene	Prof. Dr. Achim Kaasch, Aljoscha Tersteegen
EPI_ISL_864580	National Institute of Public Health - National Institute of Hygiene	National Institute of Public Health - National Institute of Hygiene, DNA Sequencing and Synthesis Facility (oligo.pl) - Institute of Biochemistry and Biophysics PAS	Wokowicz Tomasz, Zacharczuk Katarzyna, Gawor Jan
EPI_ISL_864796, EPI_ISL_864847, EPI_ISL_864849, EPI_ISL_864851, EPI_ISL_864852	Department of Pathology, University of Cambridge	COVID-19 Genomics UK (COG-UK) Consortium	Aminu S. Jahun, Yasmin Chaudhry, Grant Hall, Iliana Georgana, Myra Hosmillo, Martin D. Curran, Malte Pinckert, Surendra Parmar, Ian Goodfellow
EPI_ISL_865411, EPI_ISL_865412, EPI_ISL_865413, EPI_ISL_865414, EPI_ISL_865415, EPI_ISL_865416, EPI_ISL_865417, EPI_ISL_865418, EPI_ISL_865419, EPI_ISL_865420, EPI_ISL_865422, EPI_ISL_865427, EPI_ISL_865428, EPI_ISL_865433, EPI_ISL_865436, EPI_ISL_865437			
see above	Liverpool Clinical Laboratories	COVID-19 Genomics UK (COG-UK) Consortium	Sam Haldenby, Anita Lucaci, Steve Paterson, Julian Hiscox, Alistair Darby, M Almsaud, A Alrezaihi, Muhannad Alruwaili, Stuart D Armstrong, Jones Benjamin, Eleanor G Bentley, Anu Chawla, Jordan J Clark, Angela Cowell, Richard Eccles, Isabel García-Dorival, Matthew Gemmell, Alessandro Gerada, PKF Gilmore, Richard Gregory, Ximeng Han, Catherine Hartley, Margaret Hughes, Miren Iturriza-Gomara, James Johnson, L Luu, Jenifer Manson, Charlotte Nelson, Elaine O'Toole, Cassie Olateju, Rebekah Penrice-Randal , Lucille Rainbow, N.P Randle, Trevor Ian Robinson, Parul Sharma, Ghada T Shawli, James P Stewart, Neil Swainston, Ecaterina Vamos, Joanne Watts, Mark Whitehead
EPI_ISL_865720	University College London, Great Ormond Street Hospital for Children NHS Foundation Trust, Imperial College Healthcare NHS Trust	COVID-19 Genomics UK (COG-UK) Consortium	Sergi Castellano, Rachel Williams, Mark Kristiansen, Paola Resende Silva, Sunando Roy, Tony Brooks, Helena Tutill, Paola Niola, Patricia Dyal, Charlotte Williams, Leysa Forrest, Yasmin Panchbhaya, Jacqueline Findlay, Samuel Weeks, Julianne Brown, Kathryn Harris, Paul Randell, James Price, Alison Holmes, Judith Breuer
EPI_ISL_866049, EPI_ISL_866106, EPI_ISL_866121	University College London Hospital	COVID-19 Genomics UK (COG-UK) Consortium	Judith Heaney, Matthew Byott, Catherine Houlihan, Dan Frampton, Stuart Kirk, Moira Spyer and Eleni Nastouli
EPI_ISL_866414, EPI_ISL_866415, EPI_ISL_866416, EPI_ISL_866417, EPI_ISL_866418, EPI_ISL_866419, EPI_ISL_866420, EPI_ISL_866421, EPI_ISL_866422, EPI_ISL_866463, EPI_ISL_866464, EPI_ISL_866465, EPI_ISL_866466, EPI_ISL_866467, EPI_ISL_866468, EPI_ISL_866476, EPI_ISL_866477, EPI_ISL_866480, EPI_ISL_866481, EPI_ISL_866482, EPI_ISL_866483, EPI_ISL_866485			
see above	Northumbria University / South Tees Hospitals NHS Foundation Trust / North Cumbria Integrated Care NHS Foundation Trust / North Tees and Hartlepool NHS Foundation Trust / Newcastle Hospitals NHS Foundation Trust	COVID-19 Genomics UK (COG-UK) Consortium	Darren L Smith,Andrew Nelson,Matthew Bashton,Greg R Young,Joshua Loh,John Allan,Mohammad A Tariq,Giles S Holt,Gary Black,Wen C Yew,Lynn Dover,Paul Baker,Steve Liggett,Sarah Essex,Jane Greenaway,Debra Padgett,Clive Graham,Garren Scott,Edward Barton,Emma Swindells,Brendan Payne,Jennifer Collins,Yusri Taha,Gary Eltringham
EPI_ISL_866584, EPI_ISL_866828, EPI_ISL_866830	Quadram Institute Bioscience	COVID-19 Genomics UK (COG-UK) Consortium	Dave J. Baker, Gemma L. Kay, Alp Aydin, Thanh Le-Viet, Steven Rudder, Ana P. Tedim, Anastasia Kolyva, Maria Diaz, Leonardo de Oliveira Martins, Nabil-Fareed Alikhan, Lizzie Meadows, Rachael Stanley, Ngozi Elumogo, Muhammed Yasir, Nicholas M. Thomson, Alexander J Trotter, Rachel Gilroy, Samuel Bloomfield, Claire Stuart, Andrew Bell, Reenesh Prakash, Samir Dervisevic, Alison E. Mather, John Wain, Mark Webber, Andrew J. Page, Justin O'Grady
EPI_ISL_866990, EPI_ISL_866991	Queens Medical Centre, Clinical Microbiology Department / DeepSeq Nottingham	COVID-19 Genomics UK (COG-UK) Consortium	Gemma Clark, Wendy Smith, Manjinder Khakh, Vicki M Fleming, Michelle M Lister, Hannah Howson-Wells, Jonathan Ball, Patrick McClure, Joseph Chappell, Theocharis Tsoleridis, Nadine Holmes, Matthew Carlisle, Christopher Moore, Fei Sang, Johnny Debebe, Victoria Wright, Matthew Loose
EPI_ISL_871716, EPI_ISL_871717, EPI_ISL_871718, EPI_ISL_871719, EPI_ISL_871720, EPI_ISL_871721, EPI_ISL_871722, EPI_ISL_871723, EPI_ISL_871724, EPI_ISL_871725, EPI_ISL_871726, EPI_ISL_871727, EPI_ISL_871728, EPI_ISL_871729, EPI_ISL_871730, EPI_ISL_871731, EPI_ISL_871732, EPI_ISL_871733, EPI_ISL_871734, EPI_ISL_871735, EPI_ISL_871736, EPI_ISL_871737, EPI_ISL_871738, EPI_ISL_871739, EPI_ISL_871740, EPI_ISL_871741, EPI_ISL_871742, EPI_ISL_871743, EPI_ISL_871744, EPI_ISL_871745, EPI_ISL_871746, EPI_ISL_871747, EPI_ISL_871748, EPI_ISL_871749, EPI_ISL_871750, EPI_ISL_871751, EPI_ISL_871752, EPI_ISL_871753, EPI_ISL_871754, EPI_ISL_871755, EPI_ISL_871756, EPI_ISL_871757, EPI_ISL_871758			
see above	Department of Virus and Microbiological Special Diagnostics, Statens Serum Institut, Copenhagen, Denmark	Aalborg University	Danish Covid-19 Genome Consortium
EPI_ISL_871924, EPI_ISL_871932, EPI_ISL_871936	Servicio de Microbiología, Laboratori Clínic Metropolitana Nord. Hospital Universitari Germans Trias i Pujol. Institut d'Investigació en Ciències de la Salut Germans Trias i Pujol (IGTP)	SeqCOVID-SPAIN consortium/IBV(CSIC)	Elisa Martró, Antoni E. Bordoy, Anna Not, Adrián Antuori, Anabel Fernández, Nona Romaní, Verónica Saludes, Cristina Casañ and SeqCOVID-SPAIN consortium
EPI_ISL_872363, EPI_ISL_872364, EPI_ISL_872365, EPI_ISL_872366, EPI_ISL_872367, EPI_ISL_872368	Texas Department of State Health Services (TXDSHS)	Texas Department of State Health Services (TXDSHS)	Bonnie Oh, Anita Pokharel, James Daniel Bonser, Myong Koag, Chung Wang, Rachel Lee, Grace Kubin, Rashmi Tuladhar, Mayela Pedrueza, Maliha Rahman, Jenny Zhang
EPI_ISL_872927, EPI_ISL_872928, EPI_ISL_872929, EPI_ISL_872930, EPI_ISL_872931, EPI_ISL_872932, EPI_ISL_872933, EPI_ISL_872934, EPI_ISL_872935, EPI_ISL_872936, EPI_ISL_872938, EPI_ISL_872939, EPI_ISL_872940, EPI_ISL_872941, EPI_ISL_872942, EPI_ISL_872944, EPI_ISL_872945, EPI_ISL_872947, EPI_ISL_872948, EPI_ISL_872951, EPI_ISL_872959, EPI_ISL_872960, EPI_ISL_872961, EPI_ISL_872962, EPI_ISL_872963, EPI_ISL_872964, EPI_ISL_872965, EPI_ISL_872966, EPI_ISL_872967, EPI_ISL_872968, EPI_ISL_872969, EPI_ISL_872971			
see above	WHO National Influenza Centre Russian Federation	WHO National Influenza Centre Russian Federation	Andrey Komissarov, Artem Fadeev, Anna Ivanova, Kseniya Komissarova, Dmitry Bazhenov, Mikhail Bakaev, Daria Danilenko, Ksenia Safina, Elena Nabieva, Georgii Bazykin, Dmitry Lioznov
EPI_ISL_873219, EPI_ISL_873220	M Health Fairview	Minnesota Department of Health, Public Health Laboratory	Alexandra Lorentz, Jacob Garfin, Matt Plumb, and Xiong Wang
EPI_ISL_876075, EPI_ISL_876100, EPI_ISL_876108, EPI_ISL_876152, EPI_ISL_876166, EPI_ISL_876183, EPI_ISL_876211, EPI_ISL_876212, EPI_ISL_876217, EPI_ISL_876297, EPI_ISL_876298, EPI_ISL_876299			

see above	Massachusetts State Public Health Laboratory	Massachusetts State Public Health Laboratory	Andrew Lang, Timelia Fink, Glen Gallagher, Sandra Smole
EPI_ISL_876520, EPI_ISL_876531, EPI_ISL_876534, EPI_ISL_876535, EPI_ISL_876536, EPI_ISL_876537, EPI_ISL_876538, EPI_ISL_876539, EPI_ISL_876540	Florida Bureau of Public Health Laboratories	Florida Bureau of Public Health Laboratories	Sarah Schmedes, Jason Blanton
EPI_ISL_876784, EPI_ISL_876785, EPI_ISL_876786, EPI_ISL_876787, EPI_ISL_876788, EPI_ISL_876789, EPI_ISL_876790, EPI_ISL_876791, EPI_ISL_876792, EPI_ISL_876793, EPI_ISL_876794			
see above	Istituto Zooprofilattico Sperimentale della Puglia e della Basilicata	Istituto Zooprofilattico Sperimentale della Puglia e della Basilicata	Parisi A., Bianco A., Capozzi L., Del Sambro L., Manzulli V, Rondinone V., Pace L., Cipolletta D., Galante D.
EPI_ISL_877422	Citra Medika Hospital	Institute of Tropical Disease, Universitas Airlangga	Rima R Prasetya, Krisnoadi Rahardjo, Aldise M Nastri, Jezzy R Dewantari, Wahidah Rachmaniyah, Gatot Soegiarto, Laksmi Wulandari, Resti Yudhawati, Soetjipto, Yasuko Mori, Maria I Lusida, Kazufumi Shimizu
EPI_ISL_877437, EPI_ISL_877438, EPI_ISL_877439, EPI_ISL_877440, EPI_ISL_877441, EPI_ISL_877442, EPI_ISL_877443, EPI_ISL_877444, EPI_ISL_877445, EPI_ISL_877446, EPI_ISL_877447, EPI_ISL_877448, EPI_ISL_877449, EPI_ISL_877450			
see above	Institute of Microbiology and Immunology, Faculty of Medicine, University of Ljubljana	Institute of Microbiology and Immunology, Faculty of Medicine, University of Ljubljana	Samo Zakotnik, Tomaž Mark Zorec, Matic Brvar, Miša Korva, Mario Poljak, Tatjana Avši - Županc
EPI_ISL_878721, EPI_ISL_880180, EPI_ISL_880199, EPI_ISL_880200	Rady's Childrens Hospital	Andersen lab at Scripps Research	SEARCH Alliance San Diego with Nanda Radamchar, David Dimmock, Linda Luo, Christina Clarke, Kathryn Bouic, Teresa Mueller, Denise Malicki
EPI_ISL_882958, EPI_ISL_882959	National Institute of Public Health - National Institute of Hygiene	National Institute of Public Health - National Institute of Hygiene	Wokowicz Tomasz, Zacharczuk Katarzyna, Gawor Jan
EPI_ISL_884874, EPI_ISL_884875, EPI_ISL_884876, EPI_ISL_884877, EPI_ISL_884878, EPI_ISL_884879, EPI_ISL_884880, EPI_ISL_884881, EPI_ISL_884882, EPI_ISL_884883, EPI_ISL_884918, EPI_ISL_884919, EPI_ISL_884938, EPI_ISL_884960, EPI_ISL_884962, EPI_ISL_884964, EPI_ISL_884965, EPI_ISL_884966, EPI_ISL_884967, EPI_ISL_884968, EPI_ISL_884969, EPI_ISL_884970, EPI_ISL_884971, EPI_ISL_884973, EPI_ISL_884974, EPI_ISL_884975, EPI_ISL_884976, EPI_ISL_884977, EPI_ISL_884979, EPI_ISL_884980			
see above	Santa Clara County Public Health Laboratory	Chan-Zuckerberg Biohub	CZB Cliahub Consortium
EPI_ISL_887445, EPI_ISL_887469, EPI_ISL_887470, EPI_ISL_887471, EPI_ISL_887474, EPI_ISL_887475	Instituto Nacional de Saude (INS), Mozambique	KRISP, KZN Research Innovation and Sequencing Platform	Nalia Ismael, Nadia Sitoe, Paulo Arnaldo, Nedio Mabunda, Giandhari J, Pillay S, Tegally H, Wilkinson E, de Oliveira T
EPI_ISL_888677	Laboratory for Respiratory Viruses, Cantacuzino National Military-Medical Institute for Research and Development	Cantacuzino Institute Virology	Mihaela Lazar, Luiza Ustea, Nicoleta Paraschiv, Tim Durfee
EPI_ISL_888686, EPI_ISL_888687, EPI_ISL_888688, EPI_ISL_888689, EPI_ISL_888690, EPI_ISL_888693, EPI_ISL_888694, EPI_ISL_888695, EPI_ISL_888696, EPI_ISL_888697, EPI_ISL_888698, EPI_ISL_888699, EPI_ISL_888748, EPI_ISL_888751, EPI_ISL_888772, EPI_ISL_888774, EPI_ISL_888775, EPI_ISL_888777, EPI_ISL_888780, EPI_ISL_888781, EPI_ISL_888782, EPI_ISL_888783, EPI_ISL_888784, EPI_ISL_888787			
see above	KU Leuven, Rega Institute, Clinical and Epidemiological Virology	KU Leuven, Rega Institute, Clinical and Epidemiological Virology	Tony Wawina-Bokalanga, Bert Vanmechelen, Joan Marti-Carerras, Piet Maes
EPI_ISL_888857	Michigan Department of Health and Human Services, Bureau of Laboratories	Michigan Department of Health and Human Services, Bureau of Laboratories	Blankenship HM, Riner D, Soehnlen MK
EPI_ISL_889013	RS Rawa Lumbu	Eijkman Institute for Molecular Biology, Ministry of Research and Technology/National Agency for Research and Innovation	Iskandar Adnan, Lydia V. Panggalo, Sukma Oktavianthi, Willy Agustine, Edison Johar, Hidayat Trimarsanto, Frilasita A Yudhaputri, Safarina G Malik, Khin Saw Myint, Amin Soebandrio
EPI_ISL_890194	Gonoshasthaya-RNA Research Center, Gonoshasthaya-RNA Molecular Diagnostics and Research Center	Gonoshasthaya-RNA Research Center, Gonoshasthaya-RNA Molecular Diagnostics and Research Center	Jamiruddin,M.R., Khondoker,M.U., Sharif,N., Azmuda,N., Ahmed,M.F., Sharmin,S., Akter,S., Mou,T.J., Marzan,M., Liza,S.M., Nahar,S., Jahan,N., Ali,T., Khandker,S.S., Jamiruddin,M., Haq,M.A., Adnan,N., Chaity,M., Oishee,M.
EPI_ISL_892274, EPI_ISL_892357, EPI_ISL_892358, EPI_ISL_892359, EPI_ISL_892360, EPI_ISL_892361, EPI_ISL_892362	Servicio de Microbiología Clínica (Complejo Hospitalario de Navarra, Pamplona), Instituto de Investigación Sanitaria de Navarra (IdiSNA)	SeqCOVID-SPAIN consortium/IBV(CSIC)	Carmen Ezpeleta Baquedano, Ana Navascués, Ana Miqueleiz and SeqCOVID-SPAIN consortium
EPI_ISL_896094	Viollier AG	University Hospital Basel, Clinical Bacteriology	Tim Roloff, Madlen Stange, Helena MB Seth-Smith, Alfredo Mari, Karoline Leuzinger, Julia Bielicki, Christiane Beckmann, Manuel Battegay, Hans Hirsch, Adrian Egli
EPI_ISL_896128, EPI_ISL_896160, EPI_ISL_896187, EPI_ISL_896188, EPI_ISL_896193	MEPHI, Aix Marseille University	MEPHI, Aix Marseille University	Anthony LEVASSEUR
EPI_ISL_896299, EPI_ISL_896310, EPI_ISL_896311, EPI_ISL_896312, EPI_ISL_896313, EPI_ISL_896314, EPI_ISL_896315, EPI_ISL_896316, EPI_ISL_896317, EPI_ISL_896318, EPI_ISL_896319, EPI_ISL_896320, EPI_ISL_896321, EPI_ISL_896322, EPI_ISL_896323, EPI_ISL_896324, EPI_ISL_896325			
see above	MEMORIAL SLOAN KETTERING CANCER CENTER	Wadsworth Center, New York State Department of Health	Kirsten St. George, Daryl M. Lamson, Alexis Russel, Matthew Shudt, Melissa A Leisner, Jonathan Plitnick, Navjot Singh, John Kelly, Erasmus Schneider, Erica Lasek-Nesselquist
EPI_ISL_900086, EPI_ISL_900092, EPI_ISL_900162, EPI_ISL_900236, EPI_ISL_900342, EPI_ISL_900394	MEPHI, Aix Marseille University	MEPHI, Aix Marseille University	Anthony LEVASSEUR
EPI_ISL_900503, EPI_ISL_900571	Hôpital Paris Saint-Joseph	CNR Virus des Infections Respiratoires - France SUD	Antonin Bal, Gregory Destras, Gwendolyne Burfin, Hadrien Règue, Quentin Semanas, Martine Valette, Bruno Lina, Pantxika Bellecave, Camille Ciccone, Isabelle Garrigue, Marie-Edith Lafon, Pascale Trimoulet, Laurence Josset
EPI_ISL_900574, EPI_ISL_900576, EPI_ISL_900579, EPI_ISL_900585, EPI_ISL_900593, EPI_ISL_900610, EPI_ISL_900611, EPI_ISL_900612, EPI_ISL_900613, EPI_ISL_900614, EPI_ISL_900615, EPI_ISL_900616, EPI_ISL_900664, EPI_ISL_900665, EPI_ISL_900666, EPI_ISL_900667, EPI_ISL_900668, EPI_ISL_900669, EPI_ISL_900670			
see above	IZSM	TIGEM	Patrizia Annunziata, Andrea Ballabio, Valentina Bouche, Davide Cacchiarelli (CorrespAuthor), Pellegrino Cerino, Chiara Colantuono, Maria Concetta Cuomo, Denise Di Concilio, Lucio Di Filippo, Antonio Grimaldi, Antonio Limone, Anna Manfredi, Francesco Panariello, Biancamaria Pierri, Marcello Salvi
EPI_ISL_903251, EPI_ISL_903252	M Health Fairview	Minnesota Department of Health, Public Health Laboratory	Alexandra Lorentz, Jacob Garfin, Matt Plumb, and Xiong Wang
EPI_ISL_903566	MO State Public Health Laboratory	Genomics and Discovery, Respiratory Viruses Branch, Division of Viral Diseases, Centers for Disease Control and Prevention	Krista Queen, Yan Li, Ying Tao, Jing Zhang, Anna Uehara, Anna Montmayeur, Clinton R. Paden, Peter W. Cook, Rachel Marine, Mili Sheth, Jasmine Padilla, Sarah Nobles, Mark Burroughs, Lori Rowe, Haibin Wang, Ben L. Rambo-Martin, Dhwani Batra, Justin Lee, Suxiang Tong
EPI_ISL_903641, EPI_ISL_903647	RI State Health Laboratories	Genomics and Discovery, Respiratory Viruses Branch, Division of Viral Diseases, Centers for Disease Control and Prevention	Krista Queen, Yan Li, Ying Tao, Jing Zhang, Anna Uehara, Anna Montmayeur, Clinton R. Paden, Peter W. Cook, Rachel Marine, Mili Sheth, Jasmine Padilla, Sarah Nobles, Mark Burroughs, Lori Rowe, Haibin Wang, Ben L. Rambo-Martin, Dhwani Batra, Justin Lee, Suxiang Tong
EPI_ISL_903652	NC State Laboratory of Public Health	Genomics and Discovery, Respiratory Viruses Branch, Division of Viral Diseases, Centers for Disease Control and Prevention	Krista Queen, Yan Li, Ying Tao, Jing Zhang, Anna Uehara, Anna Montmayeur, Clinton R. Paden, Peter W. Cook, Rachel Marine, Mili Sheth, Jasmine Padilla, Sarah Nobles, Mark Burroughs, Lori Rowe, Haibin Wang, Ben L. Rambo-Martin, Dhwani Batra, Justin Lee, Suxiang Tong
EPI_ISL_903701	MN PHL Division, Minnesota Department of Health	Genomics and Discovery, Respiratory Viruses Branch, Division of Viral Diseases, Centers for Disease Control and Prevention	Krista Queen, Yan Li, Ying Tao, Jing Zhang, Anna Uehara, Anna Montmayeur, Clinton R. Paden, Peter W. Cook, Rachel Marine, Mili Sheth, Jasmine Padilla, Sarah Nobles, Mark Burroughs, Lori Rowe, Haibin Wang, Ben L. Rambo-Martin, Dhwani Batra, Justin Lee, Suxiang Tong
EPI_ISL_903725	SD Public Health Laboratory	Genomics and Discovery, Respiratory Viruses Branch, Division of Viral Diseases, Centers for Disease Control and	Krista Queen, Yan Li, Ying Tao, Jing Zhang, Anna Uehara, Anna Montmayeur, Clinton R. Paden, Peter W. Cook, Rachel Marine, Mili Sheth, Jasmine Padilla, Sarah Nobles, Mark Burroughs, Lori Rowe, Haibin Wang, Ben L. Rambo-Martin, Dhwani Batra, Justin Lee, Suxiang Tong

		Prevention	
EPI_ISL_903731, EPI_ISL_903732, EPI_ISL_903735	NV State Public Health Laboratory	Genomics and Discovery, Respiratory Viruses Branch, Division of Viral Diseases, Centers for Disease Control and Prevention	Krista Queen, Yan Li, Ying Tao, Jing Zhang, Anna Uehara, Anna Montmayeur, Clinton R. Paden, Peter W. Cook, Rachel Marine, Mili Sheth, Jasmine Padilla, Sarah Nobles, Mark Burroughs, Lori Rowe, Haibin Wang, Ben L. Rambo-Martin, Dhwani Batra, Justin Lee, Suxiang Tong
EPI_ISL_903738	MN PHL Division, Minnesota Department of Health	Genomics and Discovery, Respiratory Viruses Branch, Division of Viral Diseases, Centers for Disease Control and Prevention	Krista Queen, Yan Li, Ying Tao, Jing Zhang, Anna Uehara, Anna Montmayeur, Clinton R. Paden, Peter W. Cook, Rachel Marine, Mili Sheth, Jasmine Padilla, Sarah Nobles, Mark Burroughs, Lori Rowe, Haibin Wang, Ben L. Rambo-Martin, Dhwani Batra, Justin Lee, Suxiang Tong
EPI_ISL_903744	SD Public Health Laboratory	Genomics and Discovery, Respiratory Viruses Branch, Division of Viral Diseases, Centers for Disease Control and Prevention	Krista Queen, Yan Li, Ying Tao, Jing Zhang, Anna Uehara, Anna Montmayeur, Clinton R. Paden, Peter W. Cook, Rachel Marine, Mili Sheth, Jasmine Padilla, Sarah Nobles, Mark Burroughs, Lori Rowe, Haibin Wang, Ben L. Rambo-Martin, Dhwani Batra, Justin Lee, Suxiang Tong
EPI_ISL_903772	IA State Hygienic Laboratory	Genomics and Discovery, Respiratory Viruses Branch, Division of Viral Diseases, Centers for Disease Control and Prevention	Krista Queen, Yan Li, Ying Tao, Jing Zhang, Anna Uehara, Anna Montmayeur, Clinton R. Paden, Peter W. Cook, Rachel Marine, Mili Sheth, Jasmine Padilla, Sarah Nobles, Mark Burroughs, Lori Rowe, Haibin Wang, Ben L. Rambo-Martin, Dhwani Batra, Justin Lee, Suxiang Tong
EPI_ISL_903794	AZ SPHL, Arizona Department of Health Services	Genomics and Discovery, Respiratory Viruses Branch, Division of Viral Diseases, Centers for Disease Control and Prevention	Krista Queen, Yan Li, Ying Tao, Jing Zhang, Anna Uehara, Anna Montmayeur, Clinton R. Paden, Peter W. Cook, Rachel Marine, Mili Sheth, Jasmine Padilla, Sarah Nobles, Mark Burroughs, Lori Rowe, Haibin Wang, Ben L. Rambo-Martin, Dhwani Batra, Justin Lee, Suxiang Tong
EPI_ISL_903815	IA State Hygienic Laboratory	Genomics and Discovery, Respiratory Viruses Branch, Division of Viral Diseases, Centers for Disease Control and Prevention	Krista Queen, Yan Li, Ying Tao, Jing Zhang, Anna Uehara, Anna Montmayeur, Clinton R. Paden, Peter W. Cook, Rachel Marine, Mili Sheth, Jasmine Padilla, Sarah Nobles, Mark Burroughs, Lori Rowe, Haibin Wang, Ben L. Rambo-Martin, Dhwani Batra, Justin Lee, Suxiang Tong
EPI_ISL_903831	MO State Public Health Laboratory	Genomics and Discovery, Respiratory Viruses Branch, Division of Viral Diseases, Centers for Disease Control and Prevention	Krista Queen, Yan Li, Ying Tao, Jing Zhang, Anna Uehara, Anna Montmayeur, Clinton R. Paden, Peter W. Cook, Rachel Marine, Mili Sheth, Jasmine Padilla, Sarah Nobles, Mark Burroughs, Lori Rowe, Haibin Wang, Ben L. Rambo-Martin, Dhwani Batra, Justin Lee, Suxiang Tong
EPI_ISL_903836	SD Public Health Laboratory	Genomics and Discovery, Respiratory Viruses Branch, Division of Viral Diseases, Centers for Disease Control and Prevention	Krista Queen, Yan Li, Ying Tao, Jing Zhang, Anna Uehara, Anna Montmayeur, Clinton R. Paden, Peter W. Cook, Rachel Marine, Mili Sheth, Jasmine Padilla, Sarah Nobles, Mark Burroughs, Lori Rowe, Haibin Wang, Ben L. Rambo-Martin, Dhwani Batra, Justin Lee, Suxiang Tong
EPI_ISL_903842	AZ SPHL, Arizona Department of Health Services	Genomics and Discovery, Respiratory Viruses Branch, Division of Viral Diseases, Centers for Disease Control and Prevention	Krista Queen, Yan Li, Ying Tao, Jing Zhang, Anna Uehara, Anna Montmayeur, Clinton R. Paden, Peter W. Cook, Rachel Marine, Mili Sheth, Jasmine Padilla, Sarah Nobles, Mark Burroughs, Lori Rowe, Haibin Wang, Ben L. Rambo-Martin, Dhwani Batra, Justin Lee, Suxiang Tong
EPI_ISL_903861	CO Dept. of Public Health and Environment, Lab Services Division	Genomics and Discovery, Respiratory Viruses Branch, Division of Viral Diseases, Centers for Disease Control and Prevention	Krista Queen, Yan Li, Ying Tao, Jing Zhang, Anna Uehara, Anna Montmayeur, Clinton R. Paden, Peter W. Cook, Rachel Marine, Mili Sheth, Jasmine Padilla, Sarah Nobles, Mark Burroughs, Lori Rowe, Haibin Wang, Ben L. Rambo-Martin, Dhwani Batra, Justin Lee, Suxiang Tong
EPI_ISL_903873	AZ SPHL, Arizona Department of Health Services	Genomics and Discovery, Respiratory Viruses Branch, Division of Viral Diseases, Centers for Disease Control and Prevention	Krista Queen, Yan Li, Ying Tao, Jing Zhang, Anna Uehara, Anna Montmayeur, Clinton R. Paden, Peter W. Cook, Rachel Marine, Mili Sheth, Jasmine Padilla, Sarah Nobles, Mark Burroughs, Lori Rowe, Haibin Wang, Ben L. Rambo-Martin, Dhwani Batra, Justin Lee, Suxiang Tong
EPI_ISL_903884	TX DSHS, Lab Services Section MC 1947	Genomics and Discovery, Respiratory Viruses Branch, Division of Viral Diseases, Centers for Disease Control and Prevention	Krista Queen, Yan Li, Ying Tao, Jing Zhang, Anna Uehara, Anna Montmayeur, Clinton R. Paden, Peter W. Cook, Rachel Marine, Mili Sheth, Jasmine Padilla, Sarah Nobles, Mark Burroughs, Lori Rowe, Haibin Wang, Ben L. Rambo-Martin, Dhwani Batra, Justin Lee, Suxiang Tong
EPI_ISL_903894	SD Public Health Laboratory	Genomics and Discovery, Respiratory Viruses Branch, Division of Viral Diseases, Centers for Disease Control and Prevention	Krista Queen, Yan Li, Ying Tao, Jing Zhang, Anna Uehara, Anna Montmayeur, Clinton R. Paden, Peter W. Cook, Rachel Marine, Mili Sheth, Jasmine Padilla, Sarah Nobles, Mark Burroughs, Lori Rowe, Haibin Wang, Ben L. Rambo-Martin, Dhwani Batra, Justin Lee, Suxiang Tong
EPI_ISL_903897	TX DSHS, Lab Services Section MC 1947	Genomics and Discovery, Respiratory Viruses Branch, Division of Viral Diseases, Centers for Disease Control and Prevention	Krista Queen, Yan Li, Ying Tao, Jing Zhang, Anna Uehara, Anna Montmayeur, Clinton R. Paden, Peter W. Cook, Rachel Marine, Mili Sheth, Jasmine Padilla, Sarah Nobles, Mark Burroughs, Lori Rowe, Haibin Wang, Ben L. Rambo-Martin, Dhwani Batra, Justin Lee, Suxiang Tong
EPI_ISL_903934	SD Public Health Laboratory	Genomics and Discovery, Respiratory Viruses Branch, Division of Viral Diseases, Centers for Disease Control and Prevention	Krista Queen, Yan Li, Ying Tao, Jing Zhang, Anna Uehara, Anna Montmayeur, Clinton R. Paden, Peter W. Cook, Rachel Marine, Mili Sheth, Jasmine Padilla, Sarah Nobles, Mark Burroughs, Lori Rowe, Haibin Wang, Ben L. Rambo-Martin, Dhwani Batra, Justin Lee, Suxiang Tong
EPI_ISL_903950	NYSDOH Wadsworth Center, Virology Lab	Genomics and Discovery, Respiratory Viruses Branch, Division of Viral Diseases, Centers for Disease Control and Prevention	Krista Queen, Yan Li, Ying Tao, Jing Zhang, Anna Uehara, Anna Montmayeur, Clinton R. Paden, Peter W. Cook, Rachel Marine, Mili Sheth, Jasmine Padilla, Sarah Nobles, Mark Burroughs, Lori Rowe, Haibin Wang, Ben L. Rambo-Martin, Dhwani Batra, Justin Lee, Suxiang Tong
EPI_ISL_903962	WA State Department of Health	Genomics and Discovery, Respiratory Viruses Branch, Division of Viral Diseases, Centers for Disease Control and Prevention	Krista Queen, Yan Li, Ying Tao, Jing Zhang, Anna Uehara, Anna Montmayeur, Clinton R. Paden, Peter W. Cook, Rachel Marine, Mili Sheth, Jasmine Padilla, Sarah Nobles, Mark Burroughs, Lori Rowe, Haibin Wang, Ben L. Rambo-Martin, Dhwani Batra, Justin Lee, Suxiang Tong
EPI_ISL_904174, EPI_ISL_904234, EPI_ISL_904244, EPI_ISL_904491, EPI_ISL_904492, EPI_ISL_904493, EPI_ISL_904494, EPI_ISL_904612, EPI_ISL_904613	Dutch COVID-19 response team	Erasmus Medical Center	Bas Oude Munnink, Reina Sikkema, David Nieuwenhuijse, Irina Chestakova, Anne van der Linden, Marjan Boter, Emmanuelle Munger, Corine GeurtsvanKessel, Annemiek van der Eijk, Richard Molenkamp, Marion Koopmans, on behalf of the Dutch national COVID-19 response team.
EPI_ISL_905489, EPI_ISL_905532	Dutch COVID-19 response team	National Institute for Public Health and the Environment (RIVM)	Adam Meijer, Harry Vennema, Dirk Eggink, Jeroen Cremer, Sharon van den Brink, Bas van der Veer, AnneMarie van den Brandt, Florian Zwagemaker, Dennis Schmitz, Chantal Reusken, on behalf of the national COVID-19 response team
EPI_ISL_905799, EPI_ISL_905811, EPI_ISL_905812, EPI_ISL_905813, EPI_ISL_905814, EPI_ISL_905815, EPI_ISL_905816, EPI_ISL_905817, EPI_ISL_905818, EPI_ISL_905819, EPI_ISL_905820			
see above	OHSU Lab Services Molecular Microbiology Lab	Oregon SARS-CoV-2 Genome Sequencing Center	Brendan L. O'Connell, Sally Grindstaff, Kayla Carter, Ruth V. Nichols, Alec J. Hirsch, Donna Hansel, Guang Fan, Xuan, Qin, Daniel N. Streblow, William B. Messer, Andrew C. Adey, Benjamin N. Bimber, Brian J. O'Roak
EPI_ISL_906057	Tilia Laboratories s.r.o.	Tilia Laboratories s.r.o.	Sona Pekova, MD, PhD.
EPI_ISL_906120, EPI_ISL_906121, EPI_ISL_906122, EPI_ISL_906123, EPI_ISL_906124, EPI_ISL_906125, EPI_ISL_906126, EPI_ISL_906127	Institute of Microbiology and Immunology, Faculty of Medicine, University of Ljubljana	Institute of Microbiology and Immunology, Faculty of Medicine, University of Ljubljana	Samo Zakotnik, Tomaž Mark Zorec, Matic Brvar, Miša Korva, Mario Poljak, Tatjana Avši - Županc
EPI_ISL_906294	Nigeria Centre for Disease Control (NCDC)	African Centre of Excellence for Genomics of Infectious Diseases (ACEGID), Redeemer's University	Oluniyi P.E. et al
EPI_ISL_909951	CUSL/UCLouvain COVID testing federal platform	UCLouvain/IREC/MBLG	Jean Ruelle, Lysa Pinsmaye, Benoit Kabamba Mukadi
EPI_ISL_910995, EPI_ISL_910996, EPI_ISL_910997, EPI_ISL_910998, EPI_ISL_910999, EPI_ISL_911000, EPI_ISL_911004, EPI_ISL_911005, EPI_ISL_911006, EPI_ISL_911007, EPI_ISL_911008, EPI_ISL_911009, EPI_ISL_911010, EPI_ISL_911011, EPI_ISL_911012, EPI_ISL_911013, EPI_ISL_911014, EPI_ISL_911015, EPI_ISL_911016, EPI_ISL_911017, EPI_ISL_911018, EPI_ISL_911019, EPI_ISL_911020, EPI_ISL_911021, EPI_ISL_911022, EPI_ISL_911023, EPI_ISL_911024, EPI_ISL_911025, EPI_ISL_911026, EPI_ISL_911027, EPI_ISL_911028, EPI_ISL_911029, EPI_ISL_911030, EPI_ISL_911031, EPI_ISL_911032, EPI_ISL_911033, EPI_ISL_911034, EPI_ISL_911035, EPI_ISL_911036, EPI_ISL_911037, EPI_ISL_911038, EPI_ISL_911039, EPI_ISL_911040, EPI_ISL_911041, EPI_ISL_911042, EPI_ISL_911043, EPI_ISL_911044, EPI_ISL_911045, EPI_ISL_911046, EPI_ISL_911047, EPI_ISL_911048, EPI_ISL_911049, EPI_ISL_911050, EPI_ISL_911051, EPI_ISL_911052, EPI_ISL_911053, EPI_ISL_911054, EPI_ISL_911055, EPI_ISL_911056, EPI_ISL_911057, EPI_ISL_911058, EPI_ISL_911059, EPI_ISL_911060, EPI_ISL_911061, EPI_ISL_911062, EPI_ISL_911063, EPI_ISL_911064, EPI_ISL_911065, EPI_ISL_911066			

see above	Laboratoire national de sante, Microbiology, Virology	Laboratoire national de sante, Microbiology, Microbial Genomics Platform	Anke Wienecke-Baldacchino, Catherine Ragimbeau,Jessica Tapp, Fatu Djabi, Lise Pignon, Raoul Salmon, Tamir Abdelrahman
EPI_ISL_911684, EPI_ISL_911693, EPI_ISL_911695, EPI_ISL_911699	Alaska State Virology Laboratory	Alaska State Virology Laboratory	Stephanie DeRonde, Lisa Smith, Ph.D., Jack Chen, Ph.D.
EPI_ISL_912369, EPI_ISL_912392, EPI_ISL_912393	Fondation Congolaise pour la recherche medicale (FCRM), Francine Ntoumi	NGS Competence Center Tuebingen, Institut für Medizinische Mikrobiologie und Hygiene, Universitaetsklinikum Tübingen	Angel Angelov
EPI_ISL_912399, EPI_ISL_912400	Institute for Medical Research, Infectious Disease Research Centre, National Institutes of Health, Ministry of Health Malaysia	Institute for Medical Research, Infectious Disease Research Centre, National Institutes of Health, Ministry of Health Malaysia	Suppiah J, Kamel K, Azizan MA, Thayan R
EPI_ISL_912459, EPI_ISL_912468, EPI_ISL_912501, EPI_ISL_912520	NHLS Universitas Academic	UFS Virology	PA Bester, MM Nyaga, P Nthiga, MT Mogotsi, D Goedhals, T de Oliveira
EPI_ISL_913113	CHU Purpan - Laboratoire de Virologie - Institut Fédératif de Biologie	CHU Purpan - Laboratoire de Virologie - Institut Fédératif de Biologie	Latour J., Ranger N., Dubois M., Carcenac R., Harter A., Boyer P., Tremeaux P., Izopet J.
EPI_ISL_913310, EPI_ISL_913349, EPI_ISL_913350, EPI_ISL_913481, EPI_ISL_913482, EPI_ISL_913483	Klinisk mikrobiologi	The Public Health Agency of Sweden	Anna-Malin Linde, Maria Lind Karlberg, Carlo Berg, Oskar Karlsson Lindsjo, Sofia Stamouli, Reza Advani, Mattias Haukland, Petra Holmstrom, Noura Walai, Petra Edquist, Mia Brytting, Anna Risberg, Karin Tegmark-Wisell
EPI_ISL_913950, EPI_ISL_913952, EPI_ISL_913962, EPI_ISL_913965, EPI_ISL_913966, EPI_ISL_913979, EPI_ISL_913980, EPI_ISL_913982, EPI_ISL_913984	Instituto de Diagnostico y Referencia Epidemiologicos INDRE_RNLSP	Instituto de Diagnostico y Referencia Epidemiologicos (INDRE)	Claudia Wong-Arambula, Abril Rodriguez-Maldonado, Fabiola Garces-Ayala, Adnan Araiza-Rodriguez, David Fragoso-Fonseca, Sergio Rangel-Guerrero, Mayra Jimenez-Morales, Nancy Munoz-Hernandez, Natividad Cruz-Ortiz, Tatiana Nunez-Garcia, Gisela Barrera-Badillo, Lucia Hernandez-Rivas, Irma Lopez-Martinez, Ernesto Ramirez-Gonzalez.
EPI_ISL_914801	HOSPITAL DR. TOMAS CASAS CASAJUS	Inciensa, Instituto Costarricense de Investigación y Enseñanza en Nutrición y Salud	Francisco Duarte, Hebleen Porras, Claudio Soto-Garita, Estela Cordero, Adriana Godínez, Melany Calderón & Mariel López
EPI_ISL_914804	AREA DE SALUD TURRIALBA-JIMENEZ	Inciensa, Instituto Costarricense de Investigación y Enseñanza en Nutrición y Salud	Francisco Duarte, Hebleen Porras, Claudio Soto-Garita, Estela Cordero, Adriana Godínez, Melany Calderón & Mónica Charpentier-Artavia
EPI_ISL_914880	Instituto de Diagnostico y Referencia Epidemiologicos INDRE_RNLSP	Instituto de Diagnostico y Referencia Epidemiologicos (INDRE)	Claudia Wong-Arambula, Abril Rodriguez-Maldonado, Fabiola Garces-Ayala, Adnan Araiza-Rodriguez, David Fragoso-Fonseca, Sergio Rangel-Guerrero, Mayra Jimenez-Morales, Nancy Munoz-Hernandez, Natividad Cruz-Ortiz, Tatiana Nunez-Garcia, Gisela Barrera-Badillo, Lucia Hernandez-Rivas, Irma Lopez-Martinez, Ernesto Ramirez-Gonzalez.
EPI_ISL_915358, EPI_ISL_915380, EPI_ISL_915382, EPI_ISL_915383, EPI_ISL_915417, EPI_ISL_915418, EPI_ISL_915419, EPI_ISL_915420	Keio University School of Medicine	Keio University School of Medicine	Kenjiro Kosaki, Yuka Iwasaki, Hirotsugu Ishizu, Haruhiko Siomi, Kodai Abe
EPI_ISL_918485, EPI_ISL_918491, EPI_ISL_918494, EPI_ISL_918498	Ohio Department of Health Laboratory	Ohio Department of Health Laboratory	Holmes, Jennifer; Eric Brandt, Keoni Omura, Glen McGillivary, Caitlin McDonnell, Jade Mowery, Stephanie Mccracken, Tyler Payne, Kirtana Ramadugu, Erica Leasure, Brent Lee, Kelsey Florek, Heather Blankenship, Quanta Brown, and Tammy Bannerman
EPI_ISL_918518, EPI_ISL_918519, EPI_ISL_918520	Evandro Chagas Institute	Evandro Chagas Institute	Santos, M.C.; Silva, A.M.; Junior, W.D.C.; Barbagelata, L.S.; Ferreira, J.A.; Sousa, E.M.A.; da Silva, P.S.; Pinheiro, K.C.; L.C.; Sousa Junior, E.C.
EPI_ISL_918540	LACEN - Laboratório Central de Saúde Pública do Ceara	Evandro Chagas Institute	Santos, M.C.; Silva, A.M.; Junior, W.D.C.; Barbagelata, L.S.; Ferreira, J.A.; Sousa, E.M.A.; da Silva, P.S.; Pinheiro, K.C.; L.C.; Sousa Junior, E.C.
EPI_ISL_918562	Ohio Department of Health Laboratory	Ohio Department of Health Laboratory	Holmes, Jennifer; Eric Brandt, Keoni Omura, Glen McGillivary, Caitlin McDonnell, Jade Mowery, Stephanie Mccracken, Tyler Payne, Kirtana Ramadugu, Erica Leasure, Brent Lee, Kelsey Florek, Heather Blankenship, Quanta Brown, and Tammy Bannerman
EPI_ISL_918841, EPI_ISL_918957	University of Birmingham	COVID-19 Genomics UK (COG-UK) Consortium	Institute of Microbiology, University of Birmingham: Claire McMurray, Joanne Stockton, Samuel Nicholls, Radoslaw Poplawski, Will Rowe, Josh Quick, Nicholas Loman. University of Birmingham Testing Laboratory: Celina M Whalley, Andrew Bosworth, Charlotte Poxon, Kasun Wanigasooriya, Oliver Pickles, Mike Kidd, Alex Richter, Andrew D Beggs PHE Heartlands Lab: Husam Osman, Andrew Bosworth. Queen Elizabeth Hospital: Anna Casey
EPI_ISL_919232, EPI_ISL_919236, EPI_ISL_919238, EPI_ISL_919240, EPI_ISL_919244, EPI_ISL_919249, EPI_ISL_919259, EPI_ISL_919263, EPI_ISL_919268	West of Scotland Specialist Virology Centre, NHSGGC / MRC-University of Glasgow Centre for Virus Research	COVID-19 Genomics UK (COG-UK) Consortium	Ana da Silva Filipe, Natasha Johnson, Kathy Smollett, Daniel Mair, Stephen Carmichael, Alice Broos, Lily Tong, Jenna Nichols, Kyriaki Nomikou; Sarah McDonald; Richard Orton, Joseph Hughes, Sreenu Vattipally, David L Robertson; Alasdair MacLean, Rory Gunson; Sharif Shaaban, Matthew Holden; Rachel Blacow, Guy Mollett, Kathy Li, James Shepherd, Antonia Ho, Emma Thomson
EPI_ISL_919313, EPI_ISL_919365, EPI_ISL_919366, EPI_ISL_919368, EPI_ISL_919369, EPI_ISL_919370, EPI_ISL_919394, EPI_ISL_919395, EPI_ISL_919397	Virology Department, Royal Infirmary of Edinburgh, NHS Lothian / School of Biological Sciences, University of Edinburgh / Institute of Genetics and Molecular Medicine, University of Edinburgh	COVID-19 Genomics UK (COG-UK) Consortium	McHugh M, Dewar R, Rooke S, Gallagher M, Balcaza C, O'Toole Á, Scher E, Hill V, McCrone JT, Colquhoun R, Yu X, Jackson B, Rambaut A, Williams TC, Templeton K
EPI_ISL_920199, EPI_ISL_920216, EPI_ISL_920220, EPI_ISL_920256, EPI_ISL_920257, EPI_ISL_920290, EPI_ISL_920331, EPI_ISL_920356, EPI_ISL_920373, EPI_ISL_920378, EPI_ISL_920494, EPI_ISL_920500, EPI_ISL_920503, EPI_ISL_920510, EPI_ISL_920511			
see above	University College London Hospital	COVID-19 Genomics UK (COG-UK) Consortium	Judith Heaney, Matthew Byott, Catherine Houlihan, Dan Frampton, Stuart Kirk, Moira Spyer and Eleni Nastouli
EPI_ISL_925141, EPI_ISL_925167, EPI_ISL_925168, EPI_ISL_925169, EPI_ISL_925170, EPI_ISL_925171, EPI_ISL_925172	Virginia DCLS	Virginia DCLS	Virginia DCLS
EPI_ISL_925387, EPI_ISL_925388, EPI_ISL_925389	Department of Clinical Microbiology	GIGA Medical Genomics	Keith Durkin, Maria Artesi, Sébastien Bontems, Raphaël Boreux, Bouchra Boujemla, Cécile Meex, Pierrette Melin, Marie-Pierre Hayette, Vincent Bours
EPI_ISL_925848, EPI_ISL_925851, EPI_ISL_925852, EPI_ISL_925854, EPI_ISL_925858, EPI_ISL_925859, EPI_ISL_925894, EPI_ISL_925895, EPI_ISL_925896	Nucleic Acid Testing, National Reference Laboratory	GIGA Medical Genomics	Yvan Butera, Keith Durkin, Maria Artesi, Bouchra Boujemla, Robert Rutayisire, Patrick Tuyisenge, Esperence Umumararungu, Sébastien Bontems, Marie-Pierre Hayette, Nathalie Renotte, Swaibu Gatare, Jacob Souopgui, Sabin Nsanzimana, Vincent Bours, Léon Mutesa
EPI_ISL_925952, EPI_ISL_925969, EPI_ISL_925971, EPI_ISL_925983, EPI_ISL_925988, EPI_ISL_926002, EPI_ISL_926005, EPI_ISL_926007, EPI_ISL_926050, EPI_ISL_926053, EPI_ISL_926055, EPI_ISL_926070, EPI_ISL_926093, EPI_ISL_926095, EPI_ISL_926124, EPI_ISL_926128, EPI_ISL_926178, EPI_ISL_926201, EPI_ISL_926209, EPI_ISL_926233, EPI_ISL_926235, EPI_ISL_926255, EPI_ISL_926275, EPI_ISL_926283, EPI_ISL_926315, EPI_ISL_926316, EPI_ISL_926324, EPI_ISL_926345, EPI_ISL_926404, EPI_ISL_926441, EPI_ISL_926469, EPI_ISL_926484, EPI_ISL_926485, EPI_ISL_926528, EPI_ISL_926537, EPI_ISL_926541, EPI_ISL_926547, EPI_ISL_926566, EPI_ISL_926573, EPI_ISL_926576, EPI_ISL_926607, EPI_ISL_926615, EPI_ISL_926617, EPI_ISL_926648, EPI_ISL_926671, EPI_ISL_926679, EPI_ISL_926681, EPI_ISL_926727, EPI_ISL_926773, EPI_ISL_926775, EPI_ISL_926826, EPI_ISL_926838, EPI_ISL_926851, EPI_ISL_926852, EPI_ISL_926859, EPI_ISL_926862, EPI_ISL_926866, EPI_ISL_926870, EPI_ISL_926881, EPI_ISL_926932, EPI_ISL_926941, EPI_ISL_926949, EPI_ISL_926965, EPI_ISL_926970, EPI_ISL_926980, EPI_ISL_926981, EPI_ISL_926990, EPI_ISL_927003, EPI_ISL_927039, EPI_ISL_927047, EPI_ISL_927060, EPI_ISL_927072, EPI_ISL_927089, EPI_ISL_927092, EPI_ISL_927095, EPI_ISL_927119, EPI_ISL_927123, EPI_ISL_927145, EPI_ISL_927151, EPI_ISL_927161, EPI_ISL_927162, EPI_ISL_927164, EPI_ISL_927186, EPI_ISL_927247, EPI_ISL_927276, EPI_ISL_927283, EPI_ISL_927299, EPI_ISL_927311, EPI_ISL_927348, EPI_ISL_927351, EPI_ISL_927353, EPI_ISL_927364, EPI_ISL_927371, EPI_ISL_927375, EPI_ISL_927411, EPI_ISL_927422, EPI_ISL_927428, EPI_ISL_927439, EPI_ISL_927444, EPI_ISL_927474, EPI_ISL_927476, EPI_ISL_927483, EPI_ISL_927501, EPI_ISL_927504, EPI_ISL_927519, EPI_ISL_927558, EPI_ISL_927593, EPI_ISL_927624, EPI_ISL_927627, EPI_ISL_927637, EPI_ISL_927660, EPI_ISL_927665, EPI_ISL_927673, EPI_ISL_927685, EPI_ISL_927689, EPI_ISL_927695, EPI_ISL_927705, EPI_ISL_927707, EPI_ISL_927708, EPI_ISL_927730, EPI_ISL_927744, EPI_ISL_927748, EPI_ISL_927756, EPI_ISL_927771, EPI_ISL_927781, EPI_ISL_927787, EPI_ISL_927789, EPI_ISL_927840, EPI_ISL_927855, EPI_ISL_927859, EPI_ISL_927860, EPI_ISL_927863, EPI_ISL_927871, EPI_ISL_927881, EPI_ISL_927888, EPI_ISL_927891, EPI_ISL_927946, EPI_ISL_927955, EPI_ISL_927969, EPI_ISL_927996, EPI_ISL_928012, EPI_ISL_928032, EPI_ISL_928051, EPI_ISL_928061, EPI_ISL_928066, EPI_ISL_928067, EPI_ISL_928074, EPI_ISL_928076, EPI_ISL_928155, EPI_ISL_928171, EPI_ISL_928221, EPI_ISL_928224, EPI_ISL_928229, EPI_ISL_928231, EPI_ISL_928250, EPI_ISL_928262, EPI_ISL_928307, EPI_ISL_928327, EPI_ISL_928333, EPI_ISL_928344, EPI_ISL_928356, EPI_ISL_928386, EPI_ISL_928389, EPI_ISL_928397, EPI_ISL_928403, EPI_ISL_928420, EPI_ISL_928427, EPI_ISL_928464, EPI_ISL_928490, EPI_ISL_928493, EPI_ISL_928503, EPI_ISL_928522, EPI_ISL_928527, EPI_ISL_928539, EPI_ISL_928563, EPI_ISL_928581, EPI_ISL_928617, EPI_ISL_928631, EPI_ISL_928638, EPI_ISL_928665,			

EPI_ISL_928683, EPI_ISL_928751, EPI_ISL_928777, EPI_ISL_928778, EPI_ISL_928791, EPI_ISL_928821, EPI_ISL_928850, EPI_ISL_928864, EPI_ISL_928876, EPI_ISL_928916, EPI_ISL_928919, EPI_ISL_928924, EPI_ISL_928934, EPI_ISL_928942, EPI_ISL_928967, EPI_ISL_928976, EPI_ISL_929005, EPI_ISL_929057, EPI_ISL_929063, EPI_ISL_929082, EPI_ISL_929136, EPI_ISL_929157, EPI_ISL_929171, EPI_ISL_929185, EPI_ISL_929199, EPI_ISL_929215, EPI_ISL_929219, EPI_ISL_929228, EPI_ISL_929241, EPI_ISL_929253, EPI_ISL_929265, EPI_ISL_929280, EPI_ISL_929281, EPI_ISL_929292, EPI_ISL_929307, EPI_ISL_929339, EPI_ISL_929353, EPI_ISL_929366, EPI_ISL_929383, EPI_ISL_929388, EPI_ISL_929422, EPI_ISL_929438, EPI_ISL_929446, EPI_ISL_929464, EPI_ISL_929466, EPI_ISL_929488, EPI_ISL_929494, EPI_ISL_929501, EPI_ISL_929519, EPI_ISL_929523, EPI_ISL_929534, EPI_ISL_929535, EPI_ISL_929559, EPI_ISL_929583, EPI_ISL_929619, EPI_ISL_929628, EPI_ISL_929655, EPI_ISL_929659, EPI_ISL_929676, EPI_ISL_929694, EPI_ISL_929702, EPI_ISL_929730, EPI_ISL_929740, EPI_ISL_929746, EPI_ISL_929773, EPI_ISL_929782, EPI_ISL_929796, EPI_ISL_929811, EPI_ISL_929832, EPI_ISL_929833, EPI_ISL_929845, EPI_ISL_929854, EPI_ISL_929864, EPI_ISL_929870, EPI_ISL_929880, EPI_ISL_929882, EPI_ISL_929913, EPI_ISL_929926, EPI_ISL_929939, EPI_ISL_930003, EPI_ISL_930007, EPI_ISL_930017, EPI_ISL_930029, EPI_ISL_930030, EPI_ISL_930031, EPI_ISL_930045, EPI_ISL_930058, EPI_ISL_930063, EPI_ISL_930081, EPI_ISL_930082, EPI_ISL_930093, EPI_ISL_930104, EPI_ISL_930129, EPI_ISL_930154, EPI_ISL_930161, EPI_ISL_930170, EPI_ISL_930174, EPI_ISL_930200, EPI_ISL_930206, EPI_ISL_930223, EPI_ISL_930227, EPI_ISL_930252, EPI_ISL_930253, EPI_ISL_930273, EPI_ISL_930275, EPI_ISL_930284, EPI_ISL_930285, EPI_ISL_930307, EPI_ISL_930338, EPI_ISL_930392, EPI_ISL_930394, EPI_ISL_930399, EPI_ISL_930414, EPI_ISL_930430, EPI_ISL_930432, EPI_ISL_930457, EPI_ISL_930464, EPI_ISL_930476, EPI_ISL_930486, EPI_ISL_930503, EPI_ISL_930516, EPI_ISL_930559			
see above	Department of Virus and Microbiological Special Diagnostics, Statens Serum Institut, Copenhagen, Denmark	Aalborg University	Danish Covid-19 Genome Consortium
EPI_ISL_931448	University Hospital Basel, Clinical Virology	University Hospital Basel, Clinical Bacteriology	Tim Roloff, Madlen Stange, Helena MB Seth-Smith, Alfredo Mari, Karoline Leuzinger, Julia Bielicki, Manuel Battegay, Hans Hirsch, Adrian Egli
EPI_ISL_933693, EPI_ISL_933703	Instituto de Diagnostico y Referencia Epidemiologicos INDRE_RNLSP	Instituto de Diagnostico y Referencia Epidemiologicos (INDRE)	Claudia Wong-Arambula, Abril Rodriguez-Maldonado, Fabiola Garces-Ayala, Adnan Araiza-Rodriguez, David Fragoso-Fonseca, Sergio Rangel-Guerrero, Mayra Jimenez-Morales, Nancy Munoz-Hernandez, Natividad Cruz-Ortiz, Tatiana Nunez-Garcia, Gisela Barrera-Badillo, Lucia Hernandez-Rivas, Irma Lopez-Martinez, Ernesto Ramirez-Gonzalez.
EPI_ISL_934096, EPI_ISL_934097, EPI_ISL_934099, EPI_ISL_934100, EPI_ISL_934101, EPI_ISL_934102, EPI_ISL_934103, EPI_ISL_934104, EPI_ISL_934105, EPI_ISL_934106, EPI_ISL_934107, EPI_ISL_934108, EPI_ISL_934109, EPI_ISL_934112, EPI_ISL_934113, EPI_ISL_934114, EPI_ISL_934115, EPI_ISL_934116, EPI_ISL_934117, EPI_ISL_934119, EPI_ISL_934120, EPI_ISL_934121, EPI_ISL_934122, EPI_ISL_934123, EPI_ISL_934124, EPI_ISL_934127, EPI_ISL_934128, EPI_ISL_934129, EPI_ISL_934130, EPI_ISL_934131, EPI_ISL_934132, EPI_ISL_934133, EPI_ISL_934134, EPI_ISL_934135, EPI_ISL_934136, EPI_ISL_934138, EPI_ISL_934139, EPI_ISL_934140, EPI_ISL_934141, EPI_ISL_934142, EPI_ISL_934143, EPI_ISL_934144, EPI_ISL_934145, EPI_ISL_934146, EPI_ISL_934147, EPI_ISL_934148, EPI_ISL_934149, EPI_ISL_934150, EPI_ISL_934151, EPI_ISL_934152, EPI_ISL_934153, EPI_ISL_934154, EPI_ISL_934155, EPI_ISL_934156, EPI_ISL_934157, EPI_ISL_934158, EPI_ISL_934159, EPI_ISL_934160, EPI_ISL_934161, EPI_ISL_934162, EPI_ISL_934163, EPI_ISL_934164, EPI_ISL_934165, EPI_ISL_934166, EPI_ISL_934168, EPI_ISL_934169, EPI_ISL_934170			
see above	Vilnius university hospital Santaros Klinikos, Center of Laboratory Medicine	Vilnius university hospital Santaros Klinikos, Center of Laboratory Medicine	Ingrida Olendraite, Daniel Naumovas, Rimvydas Norvilas, Dovile Ezerskyte, Justinas Slikas, Gytis Dudas
EPI_ISL_934316, EPI_ISL_934317, EPI_ISL_934318, EPI_ISL_934319, EPI_ISL_934320, EPI_ISL_934321	Laboratoriemedicin, Klinisk mikrobiologi	The Public Health Agency of Sweden	Anna-Malin Linde, Maria Lind Karlberg, Carlo Berg, Oskar Karlsson Lindsjo, Sofia Stamouli, Reza Advani, Mattias Haukland, Petra Holmstrom, Noura Walai, Petra Edquist, Mia Brytting, Anna Risberg, Karin Tegmark-Wisell
EPI_ISL_934327, EPI_ISL_934328, EPI_ISL_934329, EPI_ISL_934354	Klinisk mikrobiologi	The Public Health Agency of Sweden	Anna-Malin Linde, Maria Lind Karlberg, Carlo Berg, Oskar Karlsson Lindsjo, Sofia Stamouli, Reza Advani, Mattias Haukland, Petra Holmstrom, Noura Walai, Petra Edquist, Mia Brytting, Anna Risberg, Karin Tegmark-Wisell
EPI_ISL_934414, EPI_ISL_934417	Ohio Department of Health Laboratory	Ohio Department of Health Laboratory	Holmes, Jennifer; Eric Brandt, Keoni Omura, Glen McGillivary, Caitlin McDonnell, Jade Mowery, Stephanie Mccracken, Tyler Payne, Kirtana Ramadugu, Erica Leasure, Brent Lee, Kelsey Florek, Heather Blankenship, Quanta Brown, and Tammy Bannerman
EPI_ISL_934570, EPI_ISL_934571	Department of Laboratory Medicine, Division of Clinical Virology, University of Medicine, Vienna	Bergthaler laboratory, CeMM Research Center for Molecular Medicine of the Austrian Academy of Sciences	Lukas Endler, Anna Schedl, Thomas Penz, Benedikt Agerer, Maelle Le Moing, Michael Schuster, Bekir Erguner, Jan Laine, Martin Senekowitsch, Christoph Bock, Andreas Bergthaler
EPI_ISL_934973, EPI_ISL_934975, EPI_ISL_935020, EPI_ISL_935023, EPI_ISL_935024	Ohio Department of Health Laboratory	Ohio Department of Health Laboratory	Holmes, Jennifer; Eric Brandt, Keoni Omura, Glen McGillivary, Caitlin McDonnell, Jade Mowery, Stephanie Mccracken, Tyler Payne, Kirtana Ramadugu, Erica Leasure, Brent Lee, Kelsey Florek, Heather Blankenship, Quanta Brown, and Tammy Bannerman
EPI_ISL_936491	Institute for Medical Research, Infectious Disease Research Centre, National Institutes of Health, Ministry of Health Malaysia	Institute for Medical Research, Infectious Disease Research Centre, National Institutes of Health, Ministry of Health Malaysia	Suppiah J, Kamel K, Azizan MA, Thayan R
EPI_ISL_936644, EPI_ISL_936645, EPI_ISL_936653, EPI_ISL_936654, EPI_ISL_936655, EPI_ISL_936656, EPI_ISL_936657, EPI_ISL_936658, EPI_ISL_936659	Northwestern Memorial Hospital	Ozer Lab	Ramon Lorenzo-Redondo, Lacy M. Simons, Chad J. Achenbach, Lawrence J. Jennings, Michael G. Ison, Judd F. Hultquist, Egon A. Ozer
EPI_ISL_940078, EPI_ISL_940079, EPI_ISL_940080, EPI_ISL_940083, EPI_ISL_940084, EPI_ISL_940094, EPI_ISL_940095, EPI_ISL_940098, EPI_ISL_940099, EPI_ISL_940101, EPI_ISL_940102, EPI_ISL_940104, EPI_ISL_940105, EPI_ISL_940106, EPI_ISL_940107, EPI_ISL_940114, EPI_ISL_940115, EPI_ISL_940117, EPI_ISL_940121, EPI_ISL_940125, EPI_ISL_940129, EPI_ISL_940132, EPI_ISL_940133			
see above	Charlotte Maxeke Johannesburg Academic Hospital, National Health Laboratory Services, Gauteng, South Africa	National Institute for Communicable Diseases of the National Health Laboratory Service	Amoako DG, Mohale T, Ntuli N, Mahlangu B, Allam M, Ismail A, Bhiman JN
EPI_ISL_940823, EPI_ISL_940824	Virginia DCLS	Virginia DCLS	Virginia DCLS
EPI_ISL_940859	Vaccines and Infectious Diseases Analytics Research Unit (VIDA)	KRISP, KZN Research Innovation and Sequencing Platform	Baillie Vicky, du Plessis Jeanine, Giandhari Jennifer, Pillay Sureshnee, Naidoo Yeshnee, Tegally Houriiyah, de Oliveira Tulio, Madhi Shabir
EPI_ISL_941255, EPI_ISL_941256, EPI_ISL_941257, EPI_ISL_941258, EPI_ISL_941259, EPI_ISL_941260, EPI_ISL_941261, EPI_ISL_941262, EPI_ISL_941263, EPI_ISL_941264, EPI_ISL_941265, EPI_ISL_941266, EPI_ISL_941267, EPI_ISL_941268			
see above	Virginia DCLS	Virginia DCLS	Virginia DCLS
EPI_ISL_942070, EPI_ISL_942071, EPI_ISL_942072, EPI_ISL_942230, EPI_ISL_942232, EPI_ISL_942233, EPI_ISL_942234, EPI_ISL_942235, EPI_ISL_942248, EPI_ISL_942249, EPI_ISL_942250, EPI_ISL_942251, EPI_ISL_942252, EPI_ISL_942253, EPI_ISL_942254, EPI_ISL_942255, EPI_ISL_942256, EPI_ISL_942257, EPI_ISL_942258, EPI_ISL_942259, EPI_ISL_942260, EPI_ISL_942261, EPI_ISL_942264, EPI_ISL_942265, EPI_ISL_942287, EPI_ISL_942288, EPI_ISL_942289, EPI_ISL_942290, EPI_ISL_942291, EPI_ISL_942292, EPI_ISL_942293, EPI_ISL_942294, EPI_ISL_942295, EPI_ISL_942296, EPI_ISL_942297, EPI_ISL_942298, EPI_ISL_942299, EPI_ISL_942300, EPI_ISL_942301, EPI_ISL_942302, EPI_ISL_942303, EPI_ISL_942304, EPI_ISL_942305, EPI_ISL_942306, EPI_ISL_942307, EPI_ISL_942313			
see above	Wisconsin State Laboratory of Hygiene Communicable Disease Division	Wisconsin State Laboratory of Hygiene Communicable Disease Division	Kelsey R. Florek, Abigail C. Shockey
EPI_ISL_942690, EPI_ISL_942691, EPI_ISL_942692, EPI_ISL_942693, EPI_ISL_942694, EPI_ISL_942695, EPI_ISL_942696, EPI_ISL_942697, EPI_ISL_942698, EPI_ISL_942699, EPI_ISL_942700, EPI_ISL_942701, EPI_ISL_942702, EPI_ISL_942703, EPI_ISL_942704, EPI_ISL_942705, EPI_ISL_942706, EPI_ISL_942943			
see above	Gundersen Molecular Diagnostics Laboratory	Kabara Cancer Research Institute	Craig S. Richmond, Paraic A. Kenny
EPI_ISL_942968	General Hospital - Kumanovo	Research Center for Genetic Engineering and Biotechnology "Georgi D. Efremov" , Macedonian Academy of Sciences and Arts	Aleksandar J. Dimovski, Dijana Plasheska-Karanfilska, Predrag Noveski, Gjorgji Bozinovski, Milena Jakimovska
EPI_ISL_942969	General Hospital - Veles	Research Center for Genetic Engineering and Biotechnology "Georgi D. Efremov" , Macedonian Academy of Sciences and Arts	Aleksandar J. Dimovski, Dijana Plasheska-Karanfilska, Predrag Noveski, Gjorgji Bozinovski, Milena Jakimovska
EPI_ISL_942970	General Hospital - Bitola	Research Center for Genetic Engineering and Biotechnology "Georgi D. Efremov" , Macedonian Academy of Sciences and Arts	Aleksandar J. Dimovski, Dijana Plasheska-Karanfilska, Predrag Noveski, Gjorgji Bozinovski, Milena Jakimovska
EPI_ISL_943993	General Hospital - Strumica	Research Center for Genetic Engineering and Biotechnology "Georgi D. Efremov" , Macedon	Aleksandar J. Dimovski, Dijana Plasheska-Karanfilska, Predrag Noveski, Gjorgji Bozinovski
EPI_ISL_943994	General Hospital - Strumica	Research Center for Genetic Engineering and Biotechnology "Georgi D. Efremov" , Macedonian Academy of Sciences and Arts	Aleksandar J. Dimovski, Dijana Plasheska-Karanfilska, Predrag Noveski, Gjorgji Bozinovski, Mi
EPI_ISL_944094, EPI_ISL_944095, EPI_ISL_944096	Institute for Medical Research, Infectious Disease Research Centre, National Institutes of Health, Ministry of Health Malaysia	Institute for Medical Research, Infectious Disease Research Centre, National Institutes of Health, Ministry of Health Malaysia	Suppiah J, Kamel K, Azizan MA, Thayan R
EPI_ISL_945073	Lighthouse Lab in Glasgow	Wellcome Sanger Institute for the COVID-19 Genomics UK	Harper VanSteenhouse, Yumi Kasai, David Gray, Carol Clugston, Anna Dominiczak and Alex Alderton, Roberto Amato, Sonia Goncalves, Ewan Harrison,

		(COG-UK) Consortium	David K. Jackson, Ian Johnston, Dominic Kwiatkowski, Cordelia Langford, John Sillitoe on behalf of the Wellcome Sanger Institute COVID-19 Surveillance Team
EPI_ISL_945140, EPI_ISL_945184	Lighthouse Lab in Milton Keynes	Wellcome Sanger Institute for the COVID-19 Genomics UK (COG-UK) Consortium	The Lighthouse Lab in Milton Keynes and Alex Alderton, Roberto Amato, Sonia Goncalves, Ewan Harrison, David K. Jackson, Ian Johnston, Dominic Kwiatkowski, Cordelia Langford, John Sillitoe on behalf of the Wellcome Sanger Institute COVID-19 Surveillance Team
EPI_ISL_945240, EPI_ISL_945252, EPI_ISL_945289	Lighthouse Lab in Alderley Park	Wellcome Sanger Institute for the COVID-19 Genomics UK (COG-UK) Consortium	Jacquelyn Wynn, Mairead Hyland, The Lighthouse Lab in Alderley Park and Alex Alderton, Roberto Amato, Sonia Goncalves, Ewan Harrison, David K. Jackson, Ian Johnston, Dominic Kwiatkowski, Cordelia Langford, John Sillitoe on behalf of the Wellcome Sanger Institute COVID-19 Surveillance Team
EPI_ISL_945307, EPI_ISL_945338	Lighthouse Lab in Milton Keynes	Wellcome Sanger Institute for the COVID-19 Genomics UK (COG-UK) Consortium	The Lighthouse Lab in Milton Keynes and Alex Alderton, Roberto Amato, Sonia Goncalves, Ewan Harrison, David K. Jackson, Ian Johnston, Dominic Kwiatkowski, Cordelia Langford, John Sillitoe on behalf of the Wellcome Sanger Institute COVID-19 Surveillance Team
EPI_ISL_945357	Lighthouse Lab in Glasgow	Wellcome Sanger Institute for the COVID-19 Genomics UK (COG-UK) Consortium	Harper VanSteenhouse, Yumi Kasai, David Gray, Carol Clugston, Anna Dominiczak and Alex Alderton, Roberto Amato, Sonia Goncalves, Ewan Harrison, David K. Jackson, Ian Johnston, Dominic Kwiatkowski, Cordelia Langford, John Sillitoe on behalf of the Wellcome Sanger Institute COVID-19 Surveillance Team
EPI_ISL_947307, EPI_ISL_947309	RS Sentra Medika Cikarang	Eijkman Institute for Molecular Biology, Ministry of Research and Technology/National Agency for Research and Innovation	Lydia V. Panggalo, Sukma Oktavianthi, Willy Agustine, Edison Johar, Hidayat Trimarsanto, Iskandar Adnan, Frilasita A Yudhaputri, Safarina G Malik, Khin Saw Myint, Amin Soebandrio
EPI_ISL_949403, EPI_ISL_949404	University of Birmingham	COVID-19 Genomics UK (COG-UK) Consortium	Institute of Microbiology, University of Birmingham: Claire McMurray, Joanne Stockton, Sam Nicholls, Radoslaw Poplawski, Will Rowe, Josh Quick, Nicholas Loman. University of Birmingham Testing Laboratory: Celina M Whalley, Andrew Bosworth, Charlotte Poxon, Kasun Wanigasooriya, Oliver Pickles, Mike Kidd, Alex Richter, Andrew D Beggs PHE Heartlands Lab: Husam Osman, Andrew Bosworth. Queen Elizabeth Hospital: Anna Casey
EPI_ISL_949651	Queens Medical Centre, Clinical Microbiology Department / DeepSeq Nottingham	COVID-19 Genomics UK (COG-UK) Consortium	Gemma Clark, Wendy Smith, Manjinder Khakh, Vicki M Fleming, Michelle M Lister, Hannah Howson-Wells, Jonathan Ball, Patrick McClure, Joseph Chappell, Theocharis Tsoleridis, Nadine Holmes, Matthew Carlisle, Christopher Moore, Fei Sang, Johnny Debebe, Victoria Wright, Matthew Loose
EPI_ISL_950166	University College London, Great Ormond Street Hospital for Children NHS Foundation Trust, Imperial College Healthcare NHS Trust	COVID-19 Genomics UK (COG-UK) Consortium	Sergi Castellano, Rachel Williams, Mark Kristiansen, Paola Resende Silva, Sunando Roy, Tony Brooks, Helena Tutill, Paola Niola, Patricia Dyal, Charlotte Williams, Leysa Forrest, Yasmin Panchbhaya, Jacqueline Findlay, Samuel Weeks, Julianne Brown, Kathryn Harris, Paul Randell, James Price, Alison Holmes, Judith Breuer
EPI_ISL_950698, EPI_ISL_950700, EPI_ISL_950701, EPI_ISL_950702, EPI_ISL_950703, EPI_ISL_950704	Lincolnshire Hospitals and DeepSeq Nottingham	COVID-19 Genomics UK (COG-UK) Consortium	Nichola Duckworth, Tim Sloan, Sarah Walsh, Jonathan Ball, Patrick McClure, Joeseph Chappell, Nadine Holmes, Matthew Carlisle, Christopher Moore, Fei Sang, Johnny Debebe, Victoria Wright, Matthew Loose
EPI_ISL_950903, EPI_ISL_950904, EPI_ISL_950911, EPI_ISL_950914, EPI_ISL_950915, EPI_ISL_950916	Oxford Viromics, NDM, University of Oxford; Oxford University Hospitals; Basingstoke and North Hampshire Hospital	COVID-19 Genomics UK (COG-UK) Consortium	Tanya Golubchik, David Bonsall, George Macintyre, Amy Trebes, Mariateresa de Cesare, Catrin Moore, Alex Mobbs, Anita Justice, Robert Shaw, Monique Andersson, Timothy Peto, Emma Wise, Nathan Moore, Jessica Lynch, Nick Cortes, Matilde Mori, Stephen Kidd, David Buck, John Todd, Christophe Fraser
EPI_ISL_959857, EPI_ISL_959859	National Virus Reference Laboratory	National Virus Reference Laboratory	Michael Carr, Gabriel Gonzalez, Jonathan Dean, Cillian F De Gascun
EPI_ISL_960189	Virginia Division of Consolidated Laboratory Services	Virginia Division of Consolidated Laboratory Services	Virginia DCLS
EPI_ISL_960442, EPI_ISL_960472, EPI_ISL_960478, EPI_ISL_960526, EPI_ISL_960527, EPI_ISL_960528, EPI_ISL_960529, EPI_ISL_960530, EPI_ISL_960614, EPI_ISL_960615, EPI_ISL_960616, EPI_ISL_960617, EPI_ISL_960618			
see above	Istituto Zooprofilattico Sperimentale del Mezzogiorno	TIGEM	Patrizia Annunziata, Andrea Ballabio, Valentina Bouche, Davide Cacchiarelli, Pellegrino Cerino, Chiara Colantuono, Maria Concetta Cuomo, Denise Di Concilio, Lucio Di Filippo, Antonio Grimaldi, Antonio Limone, Anna Manfredi, Francesco Panariello, Biancamaria Pierri, Marcello Salvi
EPI_ISL_962123	Illinois Department of Public Health	Gagnon Lab, Southern Illinois University	Keith Gagnon
EPI_ISL_962800, EPI_ISL_962807	San Diego County Public Health Laboratory	Andersen lab at Scripps Research	SEARCH Alliance San Diego with Tracy Basler, Jovan Shephard, Brett Austin
EPI_ISL_965220	Virginia Division of Consolidated Laboratory Services	Virginia Division of Consolidated Laboratory Services	Virginia DCLS
EPI_ISL_965388	University of Liège COVID-19 testing center	GIGA Medical Genomics	Keith Durkin, Maria Artesi, Bouchra Boujemla, Emmanuel André, Marc Van Ranst, Fabrice Bureau, Laurent Gillet, Wouter Coppieters, Vincent Bours
EPI_ISL_965551, EPI_ISL_965569, EPI_ISL_965683, EPI_ISL_965751, EPI_ISL_965804	Dutch COVID-19 response team	Medical Microbiology, Maastricht University Medical Centre	Jozef Dingemans*, Brian van der Veer*, Erik Beuken, Carmen Reumkens, Lieke van Alphen, Christian Hoebe, Paul Savelkoul
EPI_ISL_966757, EPI_ISL_966758, EPI_ISL_966759, EPI_ISL_966760	Maine HETL	Tewhey Lab, The Jackson Laboratory	Matluk,N., Dewey,H., Iosue,F., Barter,M., Lynch,R., Munger,H. and Tewhey,R.
EPI_ISL_967523, EPI_ISL_967526, EPI_ISL_967538	State Laboratories Division, Hawaii State Department of Health	State Laboratories Division, Hawaii State Department of Health	Pamela O'Brien, Drew Kuwazaki, Ayana Garnet, Razvan Sultana, Edward Desmond
EPI_ISL_968832, EPI_ISL_968850	KEMRI-Wellcome Trust Research Programme/KEMRI-CGMR-C Kilifi	KEMRI-Wellcome Trust Research Programme/KEMRI-CGMR-C Kilifi	Githinji et al
EPI_ISL_970834, EPI_ISL_970982, EPI_ISL_971085, EPI_ISL_971151, EPI_ISL_971395, EPI_ISL_971550, EPI_ISL_971608, EPI_ISL_971643, EPI_ISL_971675, EPI_ISL_971749, EPI_ISL_971767, EPI_ISL_971798, EPI_ISL_971846, EPI_ISL_971875, EPI_ISL_971936, EPI_ISL_972025, EPI_ISL_972050, EPI_ISL_972070, EPI_ISL_972082, EPI_ISL_972089, EPI_ISL_972111, EPI_ISL_972135, EPI_ISL_972176, EPI_ISL_972282, EPI_ISL_972398, EPI_ISL_972541, EPI_ISL_972566, EPI_ISL_972585, EPI_ISL_972634, EPI_ISL_972816, EPI_ISL_972877, EPI_ISL_972908, EPI_ISL_972916, EPI_ISL_972917, EPI_ISL_972963, EPI_ISL_972970, EPI_ISL_972973, EPI_ISL_973002, EPI_ISL_973022, EPI_ISL_973088, EPI_ISL_973101, EPI_ISL_973113, EPI_ISL_973178, EPI_ISL_973194, EPI_ISL_973233, EPI_ISL_973357, EPI_ISL_973478, EPI_ISL_973644, EPI_ISL_973701, EPI_ISL_973774			
see above	Department of Virus and Microbiological Special Diagnostics, Statens Serum Institut, Copenhagen, Denmark	Aalborg University	Danish Covid-19 Genome Consortium
EPI_ISL_976424, EPI_ISL_976425, EPI_ISL_976426, EPI_ISL_976427, EPI_ISL_976428, EPI_ISL_976429, EPI_ISL_976430, EPI_ISL_976431, EPI_ISL_976432, EPI_ISL_976433, EPI_ISL_976434, EPI_ISL_976435, EPI_ISL_976436, EPI_ISL_976437, EPI_ISL_976438, EPI_ISL_976439, EPI_ISL_976440, EPI_ISL_976441, EPI_ISL_976442, EPI_ISL_976443, EPI_ISL_976444, EPI_ISL_976445, EPI_ISL_976446, EPI_ISL_976447, EPI_ISL_976448, EPI_ISL_976449, EPI_ISL_976450, EPI_ISL_976451, EPI_ISL_976452, EPI_ISL_976453, EPI_ISL_976454, EPI_ISL_976455, EPI_ISL_976456, EPI_ISL_976457, EPI_ISL_976458, EPI_ISL_976459, EPI_ISL_976460, EPI_ISL_976461, EPI_ISL_976462, EPI_ISL_976463, EPI_ISL_976464, EPI_ISL_976465, EPI_ISL_976466, EPI_ISL_976467, EPI_ISL_976468, EPI_ISL_976469, EPI_ISL_976470, EPI_ISL_976471, EPI_ISL_976472, EPI_ISL_976473, EPI_ISL_976474, EPI_ISL_976475, EPI_ISL_976476, EPI_ISL_976477, EPI_ISL_976478, EPI_ISL_976479, EPI_ISL_976480, EPI_ISL_976481, EPI_ISL_976482, EPI_ISL_976483, EPI_ISL_976484, EPI_ISL_976485, EPI_ISL_976486, EPI_ISL_976487, EPI_ISL_976488, EPI_ISL_976489, EPI_ISL_976490, EPI_ISL_976491, EPI_ISL_976492, EPI_ISL_976493, EPI_ISL_976494, EPI_ISL_976495, EPI_ISL_976496, EPI_ISL_976497, EPI_ISL_976498, EPI_ISL_976499, EPI_ISL_976500, EPI_ISL_976501, EPI_ISL_976502, EPI_ISL_976503, EPI_ISL_976504, EPI_ISL_976505, EPI_ISL_976506, EPI_ISL_976507, EPI_ISL_976508, EPI_ISL_976509, EPI_ISL_976510, EPI_ISL_976511, EPI_ISL_976512, EPI_ISL_976513, EPI_ISL_976514, EPI_ISL_976515, EPI_ISL_976516, EPI_ISL_976517, EPI_ISL_976518, EPI_ISL_976519, EPI_ISL_976520			
see above	BCCDC Public Health Laboratory	BCCDC Public Health Laboratory	Prystajecky Natalie, Linda Hoang, Dan Fornika, John Tyson, Shannon Russell, Kim Macdonald, Kimia Kamelian, Ana Pacagnella, Corrinne Ng, Loretta Janz, Robert Azana Terry Snutch, Mel Krajden
EPI_ISL_977029, EPI_ISL_977030, EPI_ISL_977031, EPI_ISL_977032	Rhode Island Department of Health	Infectious Disease Program, Broad Institute of Harvard and MIT	Lemieux,J.E., Siddle,K.J., Huard,R., King,E., Azevedo,K., Miller,A., Adams,G., Gladden-Young,A., Lagerborg,K., Rudy,M., DeRuff,K., Carter,A., Normandin,E., Bauer,M., Reilly,S., Tomkins-Tinch,C., Loreth,C., Chaluvadi,S., Birren,B.W., Gallagher,G., Smole,S., Park,D.J., MacInnis,B.L., and Sabeti,P.C.
EPI_ISL_977164, EPI_ISL_977170	Microbiologia e Virologia	Istituto Zooprofilattico Sperimentale delle Venezie	Adelaide Milani, Alessia Schivo, Annalisa Salviato, Erika Giorgia Quaranta, Ambra Pastori, Bianca Zecchin, Alice Fusaro, Isabella Monne, Calogero Terregino, Antonia Ricci
EPI_ISL_977336	University of Zambia, School of Veterinary Medicine	UNZAVET and PATH	Mulenga Mwenda-Chimfwembe, Ngonda Saasa, Daniel Bridges
EPI_ISL_978010	Chiu Laboratory, University of California, San Francisco	Chiu Laboratory, University of California, San Francisco	Charles Chiu, Xianding (Wayne) Deng, Candace Wang, Venice Servellita, Jill Hacker, Debra Wadford
EPI_ISL_978198, EPI_ISL_978199, EPI_ISL_978200, EPI_ISL_978213	Virginia Division of Consolidated Laboratory Services	Virginia Division of Consolidated Laboratory Services	Virginia DCLS
EPI_ISL_979173, EPI_ISL_979174, EPI_ISL_979175, EPI_ISL_979176, EPI_ISL_979177, EPI_ISL_979178, EPI_ISL_979179, EPI_ISL_979180, EPI_ISL_979181, EPI_ISL_979182, EPI_ISL_979185, EPI_ISL_979186, EPI_ISL_979192, EPI_ISL_979193, EPI_ISL_979194			
see above	Humboldt County Public Health Laboratory	Chan-Zuckerberg Biohub	CZB Cliahub Consortium
EPI_ISL_979248	Institute of Microbiology and Immunology, Faculty of Medicine, University of Ljubljana	Institute of Microbiology and Immunology, Faculty of Medicine, University of Ljubljana	Samo Zakotnik, Tomaž Mark Zorec, Matic Brvar, Doroteja Vlaj, Patricija Pozvek,Špela Pleh, Miša Korva, Mario Poljak, Tatjana Avši - Županc

EPI_ISL_979295, EPI_ISL_979296, EPI_ISL_979297, EPI_ISL_979298, EPI_ISL_979303	Cadham Provincial laboratory	National Microbiology Laboratory (NML)	Anna Majer, Shari Tyson, Grace Seo, Philip Mabon, Elsie Grudeski, Rhiannon Huzarewich, Russell Mandes, Anneliese Landgraff, Jennifer Tanner, Natalie Knox, Morag Graham, Gary Van Domselaar, Paul Van Caeseele, Jared Bullard, David Alexander, Kerry Dust, Nathalie Bastien, Yan Li, Timothy Booth, Darian Hole, Madison Chapel, Kirsten Biggar, CanCOGeN's metadata curation team, Public Health Agency of Canada CanCOGeN team
EPI_ISL_981903, EPI_ISL_981904, EPI_ISL_981905, EPI_ISL_981906, EPI_ISL_981907, EPI_ISL_981908, EPI_ISL_981909, EPI_ISL_981910, EPI_ISL_981911, EPI_ISL_981912, EPI_ISL_981913, EPI_ISL_981914, EPI_ISL_981915, EPI_ISL_981916, EPI_ISL_981917, EPI_ISL_981918, EPI_ISL_981919			
see above	Microbiology Service, Hospital Universitario Clinico San Cecilio, Granada	Microbiology Service, Hospital Universitario Clinico San Cecilio, Granada	Adolfo de Salazar, Natalia Chueca, Laura Viñuela, Ana Fuentes, Federico García
EPI_ISL_982847, EPI_ISL_982848, EPI_ISL_982849, EPI_ISL_982850	UK Healthcare Clinical Microbiology	Kentucky State Public Health Lab	Stephanie Lunn, Karim George, Joshua Tobias, William Grooms, Vaneet Arora, Matthew Johnson, Rachel Zinner, Rhonda Lucas
