# Supplementary material for: Detection and characterization of the SARS-CoV-2 lineage B.1.526 in New York
Source: Nat Commun. 2021 Aug 9;12:4886. doi: 10.1038/s41467-021-25168-4 (PMC8352861; doi:10.1038/s41467-021-25168-4)
Supplement: Supplementary file 8 — Supplementary Data 4 [file 41467_2021_25168_MOESM8_ESM.zip › GISAID_acknowledements_tables/gisaid_hcov-19_acknowledgement_table_2021_02_13_00-14.pdf]

We gratefully acknowledge the following Authors from the Originating laboratories responsible for obtaining the specimens, as well as the Submitting laboratories where the genome data were generated and shared via GISAID, on which this research is based.

All Submitters of data may be contacted directly via www.gisaid.org

Authors are sorted alphabetically.

Accession ID	Originating Laboratory	Submitting Laboratory	Authors
EPI_ISL_702633	Virology Department, Sheffield Teaching Hospitals NHS Foundation Trust/Department of Infection, Immunity and Cardiovascular Disease, The Medical School, University of Sheffield	COVID-19 Genomics UK (COG-UK) Consortium	Thushan de Silva, Matthew Parker, Nikki Smith, Adri Angyal, Rebecca Brown, Luke Green, Rachel Tucker, Paul Parsons, Danielle Groves, Katie Johnson, Laura Carrilero, Alex Keeley, Dave Partridge, Matthew Wyles, Benjamin Lindsey, Mehmet Yavuz, Mohammad Raza, Cariad Evans
EPI_ISL_704583, EPI_ISL_704780	Queens Medical Centre, Clinical Microbiology Department / DeepSeq Nottingham	COVID-19 Genomics UK (COG-UK) Consortium	Gemma Clark, Wendy Smith, Manjinder Khakh, Vicki M Fleming, Michelle M Lister, Hannah Howson-Wells, Jonathan Ball, Patrick McClure, Joseph Chappell, Theocharis Tsoleridis, Nadine Holmes, Matthew Carlisle, Christopher Moore, Fei Sang, Johnny Debebe, Victoria Wright, Matthew Loose
EPI_ISL_705204, EPI_ISL_705421	Virology Department, Sheffield Teaching Hospitals NHS Foundation Trust/Department of Infection, Immunity and Cardiovascular Disease, The Medical School, University of Sheffield	COVID-19 Genomics UK (COG-UK) Consortium	Thushan de Silva, Matthew Parker, Nikki Smith, Adri Angyal, Rebecca Brown, Luke Green, Rachel Tucker, Paul Parsons, Danielle Groves, Katie Johnson, Laura Carrilero, Alex Keeley, Dave Partridge, Matthew Wyles, Benjamin Lindsey, Mehmet Yavuz, Mohammad Raza, Cariad Evans
EPI_ISL_706435, EPI_ISL_706436, EPI_ISL_706437	Queens Medical Centre, Clinical Microbiology Department / DeepSeq Nottingham	COVID-19 Genomics UK (COG-UK) Consortium	Gemma Clark, Wendy Smith, Manjinder Khakh, Vicki M Fleming, Michelle M Lister, Hannah Howson-Wells, Jonathan Ball, Patrick McClure, Joseph Chappell, Theocharis Tsoleridis, Nadine Holmes, Matthew Carlisle, Christopher Moore, Fei Sang, Johnny Debebe, Victoria Wright, Matthew Loose
EPI_ISL_706976, EPI_ISL_706984, EPI_ISL_706985, EPI_ISL_706986	Virology Department, Sheffield Teaching Hospitals NHS Foundation Trust/Department of Infection, Immunity and Cardiovascular Disease, The Medical School, University of Sheffield	COVID-19 Genomics UK (COG-UK) Consortium	Thushan de Silva, Matthew Parker, Nikki Smith, Adri Angyal, Rebecca Brown, Luke Green, Rachel Tucker, Paul Parsons, Danielle Groves, Katie Johnson, Laura Carrilero, Alex Keeley, Dave Partridge, Matthew Wyles, Benjamin Lindsey, Mehmet Yavuz, Mohammad Raza, Cariad Evans
EPI_ISL_712099, EPI_ISL_712100, EPI_ISL_712119, EPI_ISL_712120, EPI_ISL_712121, EPI_ISL_712122, EPI_ISL_712123, EPI_ISL_712124, EPI_ISL_712125, EPI_ISL_712126, EPI_ISL_712127, EPI_ISL_712128, EPI_ISL_712250, EPI_ISL_712251, EPI_ISL_712252, EPI_ISL_712253, EPI_ISL_712295, EPI_ISL_712321, EPI_ISL_712340, EPI_ISL_712341, EPI_ISL_712342, EPI_ISL_712343, EPI_ISL_712344, EPI_ISL_712345, EPI_ISL_712346, EPI_ISL_712417, EPI_ISL_712431, EPI_ISL_712439, EPI_ISL_712446, EPI_ISL_712447, EPI_ISL_712448, EPI_ISL_712455, EPI_ISL_712456, EPI_ISL_712457, EPI_ISL_712458, EPI_ISL_712459, EPI_ISL_712460, EPI_ISL_712461, EPI_ISL_712462, EPI_ISL_712463, EPI_ISL_712464, EPI_ISL_712465, EPI_ISL_712466, EPI_ISL_712467, EPI_ISL_712468, EPI_ISL_712469, EPI_ISL_712470, EPI_ISL_712471, EPI_ISL_712472, EPI_ISL_712473, EPI_ISL_712474, EPI_ISL_712475, EPI_ISL_712476, EPI_ISL_712477, EPI_ISL_712478, EPI_ISL_712479, EPI_ISL_712480, EPI_ISL_712481, EPI_ISL_712482, EPI_ISL_712483, EPI_ISL_712484, EPI_ISL_712485, EPI_ISL_712486, EPI_ISL_712487, EPI_ISL_712488, EPI_ISL_712489, EPI_ISL_712490, EPI_ISL_712491, EPI_ISL_712492, EPI_ISL_712493, EPI_ISL_712494, EPI_ISL_712495, EPI_ISL_712496, EPI_ISL_712497, EPI_ISL_712498, EPI_ISL_712499, EPI_ISL_712500, EPI_ISL_712501, EPI_ISL_712502, EPI_ISL_712503, EPI_ISL_712504, EPI_ISL_712505, EPI_ISL_712506, EPI_ISL_712507, EPI_ISL_712508, EPI_ISL_712509, EPI_ISL_712510, EPI_ISL_712511, EPI_ISL_712512, EPI_ISL_712513, EPI_ISL_712514, EPI_ISL_712515, EPI_ISL_712516, EPI_ISL_712517, EPI_ISL_712518, EPI_ISL_712519, EPI_ISL_712520, EPI_ISL_712521, EPI_ISL_712522, EPI_ISL_712523, EPI_ISL_712524, EPI_ISL_712525, EPI_ISL_712526, EPI_ISL_712527, EPI_ISL_712528, EPI_ISL_712529, EPI_ISL_712530			
see above	Department of Virus and Microbiological Special Diagnostics, Statens Serum Institut, Copenhagen, Denmark	Albertsen Lab, Department of Chemistry and Bioscience, Aalborg University, Denmark	Danish Covid-19 Genome Consortium
EPI_ISL_717703, EPI_ISL_717708, EPI_ISL_717709, EPI_ISL_717710, EPI_ISL_717711, EPI_ISL_717712, EPI_ISL_717713	Area of Virology, Serology and Virology Division (SAViD), New South Wales Health Pathology Randwick	Virology Research Laboratory; Area of Virology, Serology and Virology Division (SAViD), New South Wales Health Pathology Randwick	Foster, C.; Au, J.; Ruiz Silva, M.; Deveson, I.; Bull, R.; Van Hal, S.; Rawlinson, W.
EPI_ISL_722299, EPI_ISL_722315, EPI_ISL_722402, EPI_ISL_722461, EPI_ISL_722462, EPI_ISL_722796, EPI_ISL_722797, EPI_ISL_722798, EPI_ISL_722799	Dutch COVID-19 response team	Erasmus Medical Center	Bas Oude Munnink, Reina Sikkema, David Nieuwenhuijse, Irina Chestakova, Anne van der Linden, Marjan Boter, Emmanuelle Munger, Corine GeurtsvanKessel, Annemiek van der Eijk, Richard Molenkamp, Marion Koopmans, on behalf of the Dutch national COVID-19 response team.
EPI_ISL_722974, EPI_ISL_722996, EPI_ISL_722997, EPI_ISL_722998, EPI_ISL_723001, EPI_ISL_723002, EPI_ISL_723003, EPI_ISL_723004, EPI_ISL_723005, EPI_ISL_723038, EPI_ISL_723039, EPI_ISL_723040, EPI_ISL_723041, EPI_ISL_723042, EPI_ISL_723043			
see above	Respiratory Virus Unit, National Infection Service, Public Health England	COVID-19 Genomics UK (COG-UK) Consortium	PHE Covid Sequencing Team
EPI_ISL_723139, EPI_ISL_723140, EPI_ISL_723146	Minnesota Department of Health, Public Health Laboratory	Minnesota Department of Health, Public Health Laboratory	Alexandra Lorentz, Jacob Garfin, Matt Plumb, and Xiong Wang
EPI_ISL_723933, EPI_ISL_723935, EPI_ISL_723936, EPI_ISL_723947, EPI_ISL_723948	Department of Pathology, University of Cambridge	COVID-19 Genomics UK (COG-UK) Consortium	Aminu S. Jahun, Yasmin Chaudhry, Grant Hall, Iliana Georgana, Myra Hosmillo, Martin D. Curran, Malte Pinckert, Surendra Parmar, Ian Goodfellow
EPI_ISL_724217, EPI_ISL_724219, EPI_ISL_724220	West of Scotland Specialist Virology Centre, NHSGGC / MRC-University of Glasgow Centre for Virus Research	COVID-19 Genomics UK (COG-UK) Consortium	Ana da Silva Filipe, Natasha Johnson, Kathy Smollett, Daniel Mair, Stephen Carmichael, Alice Broos, Lily Tong, Jenna Nichols, Kyriaki Nomikou; Sarah McDonald; Richard Orton, Joseph Hughes, Sreenu Vattipally, David L Robertson; Alasdair MacLean, Rory Gunson; Sharif Shaaban, Matthew Holden; Rachel Blacow, Guy Mollett, Kathy Li, James Shepherd, Antonia Ho, Emma Thomson
EPI_ISL_724228, EPI_ISL_724229, EPI_ISL_724231, EPI_ISL_724232, EPI_ISL_724233, EPI_ISL_724234, EPI_ISL_724235, EPI_ISL_724236, EPI_ISL_724237, EPI_ISL_724238, EPI_ISL_724239, EPI_ISL_724240, EPI_ISL_724241, EPI_ISL_724242, EPI_ISL_724243, EPI_ISL_724244, EPI_ISL_724246, EPI_ISL_724247			
see above	Virology Department, Royal Infirmary of Edinburgh, NHS Lothian / School of Biological Sciences, University of Edinburgh / Institute of Genetics and Molecular Medicine, University of Edinburgh	COVID-19 Genomics UK (COG-UK) Consortium	McHugh M, Dewar R, Rooke S, Gallagher M, Balcaza C, O'Toole Á, Scher E, Hill V, McCrone JT, Colquhoun R, Yu X, Jackson B, Rambaut A, Williams TC, Templeton K
EPI_ISL_724357, EPI_ISL_724358, EPI_ISL_724361, EPI_ISL_724363, EPI_ISL_724367, EPI_ISL_724368, EPI_ISL_724373, EPI_ISL_724374, EPI_ISL_724376, EPI_ISL_724379, EPI_ISL_724381, EPI_ISL_724385, EPI_ISL_724386, EPI_ISL_724387, EPI_ISL_724388, EPI_ISL_724389, EPI_ISL_724390, EPI_ISL_724391, EPI_ISL_724392, EPI_ISL_724393, EPI_ISL_724394, EPI_ISL_724395			
see above	Liverpool Clinical Laboratories	COVID-19 Genomics UK (COG-UK) Consortium	Sam Haldenby, Anita Lucaci, Steve Paterson, Julian Hiscox, Alistair Darby, M Almsaud, A Alrezaihi, Muhannad Alruwaili, Stuart D Armstrong, Jones Benjamin, Eleanor G Bentley, Anu Chawla, Jordan J Clark, Angela Cowell, Richard Eccles, Isabel García-Dorival, Matthew Gemmell, Alessandro Gerada, PKF Gilmore, Richard Gregory, Ximeng Han, Catherine Hartley, Margaret Hughes, Miren Iturriza-Gomara, James Johnson, L Luu, Jenifer Manson, Charlotte Nelson, Elaine O'Toole, Cassie Olateju, Rebekah Penrice-Randal , Lucille Rainbow, N.P Randle, Trevor Ian Robinson, Parul Sharma, Ghada T Shawli, James P Stewart, Neil Swainston, Ecaterina Vamos, Joanne Watts, Mark Whitehead
EPI_ISL_724598, EPI_ISL_724599, EPI_ISL_724600, EPI_ISL_724603, EPI_ISL_724604, EPI_ISL_724605	University College London, Great Ormond Street Hospital for Children NHS Foundation Trust, Imperial College Healthcare NHS Trust	COVID-19 Genomics UK (COG-UK) Consortium	Sergi Castellano, Rachel Williams, Mark Kristiansen, Paola Resende Silva, Sunando Roy, Tony Brooks, Helena Tutill, Paola Niola, Patricia Dyal, Charlotte Williams, Leysa Forrest, Yasmin Panchbhaya, Jacqueline Findlay, Samuel Weeks, Julianne Brown, Kathryn Harris, Paul Randell, James Price, Alison Holmes, Judith Breuer
EPI_ISL_724908, EPI_ISL_724912, EPI_ISL_724914, EPI_ISL_724917, EPI_ISL_724918, EPI_ISL_724919, EPI_ISL_724920, EPI_ISL_724921, EPI_ISL_724922, EPI_ISL_724923	Northumbria University / South Tees Hospitals NHS Foundation Trust / North Cumbria Integrated Care NHS Foundation Trust / North Tees and Hartlepool NHS Foundation Trust / Newcastle Hospitals NHS Foundation Trust	COVID-19 Genomics UK (COG-UK) Consortium	Darren L Smith,Andrew Nelson,Matthew Bashton,Greg R Young,Joshua Loh,John Allan,Mohammad A Tariq,Giles S Holt,Gary Black,Wen C Yew,Lynn Dover,Paul Baker,Steve Liggett,Sarah Essex,Jane Greenaway,Debra Padgett,Clive Graham,Garren Scott,Edward Barton,Emma Swindells,Brendan Payne,Jennifer Collins,Yusri Taha,Gary Eltringham
EPI_ISL_725029, EPI_ISL_725030, EPI_ISL_725031, EPI_ISL_725032, EPI_ISL_725036, EPI_ISL_725037, EPI_ISL_725039, EPI_ISL_725040, EPI_ISL_725042, EPI_ISL_725043, EPI_ISL_725044, EPI_ISL_725045, EPI_ISL_725046, EPI_ISL_725047, EPI_ISL_725048, EPI_ISL_725049, EPI_ISL_725050, EPI_ISL_725051,			

EPI_ISL_725052, EPI_ISL_725053, EPI_ISL_725054, EPI_ISL_725056, EPI_ISL_725057, EPI_ISL_725058, EPI_ISL_725059, EPI_ISL_725060, EPI_ISL_725061, EPI_ISL_725139, EPI_ISL_725140, EPI_ISL_725142, EPI_ISL_725145, EPI_ISL_725154, EPI_ISL_725158, EPI_ISL_725302, EPI_ISL_725303, EPI_ISL_725307, EPI_ISL_725309, EPI_ISL_725310, EPI_ISL_725311, EPI_ISL_725312, EPI_ISL_725313, EPI_ISL_725314, EPI_ISL_725321, EPI_ISL_725324, EPI_ISL_725325, EPI_ISL_725327, EPI_ISL_725328, EPI_ISL_725331, EPI_ISL_725365, EPI_ISL_725415, EPI_ISL_725416, EPI_ISL_725418, EPI_ISL_725421, EPI_ISL_725426, EPI_ISL_725427, EPI_ISL_725429, EPI_ISL_725431, EPI_ISL_725432, EPI_ISL_725434, EPI_ISL_725435, EPI_ISL_725436, EPI_ISL_725437, EPI_ISL_725439, EPI_ISL_725440, EPI_ISL_725441, EPI_ISL_725442, EPI_ISL_725443, EPI_ISL_725444, EPI_ISL_725445, EPI_ISL_725446, EPI_ISL_725447, EPI_ISL_725448, EPI_ISL_725449, EPI_ISL_725450, EPI_ISL_725451, EPI_ISL_725452, EPI_ISL_725453, EPI_ISL_725454, EPI_ISL_725455, EPI_ISL_725456, EPI_ISL_725457, EPI_ISL_725460, EPI_ISL_725461, EPI_ISL_725462, EPI_ISL_725465, EPI_ISL_725470, EPI_ISL_725473, EPI_ISL_725474, EPI_ISL_725477, EPI_ISL_725478, EPI_ISL_725479, EPI_ISL_725482, EPI_ISL_725483, EPI_ISL_725484, EPI_ISL_725485, EPI_ISL_725486, EPI_ISL_725488, EPI_ISL_725489, EPI_ISL_725490, EPI_ISL_725491, EPI_ISL_725511, EPI_ISL_725514			
see above	Quadram Institute Bioscience	COVID-19 Genomics UK (COG-UK) Consortium	Dave J. Baker, Gemma L. Kay, Alp Aydin, Thanh Le-Viet, Steven Rudder, Ana P. Tedim, Anastasia Kolyva, Maria Diaz, Leonardo de Oliveira Martins, Nabil-Fareed Alikhan, Lizzie Meadows, Rachael Stanley, Ngozi Elumogo, Muhammed Yasir, Nicholas M. Thomson, Alexander J Trotter, Rachel Gilroy, Samuel Bloomfield, Claire Stuart, Andrew Bell, Reenesh Prakash, Samir Dervisevic, Alison E. Mather, John Wain, Mark Webber, Andrew J. Page, Justin O'Grady
EPI_ISL_725606, EPI_ISL_725607, EPI_ISL_725608, EPI_ISL_725609, EPI_ISL_725610, EPI_ISL_725611, EPI_ISL_725612, EPI_ISL_725613, EPI_ISL_725614, EPI_ISL_725615, EPI_ISL_725616, EPI_ISL_725617, EPI_ISL_725618, EPI_ISL_725619, EPI_ISL_725620, EPI_ISL_725621, EPI_ISL_725622, EPI_ISL_725623, EPI_ISL_725624, EPI_ISL_725625, EPI_ISL_725626, EPI_ISL_725627, EPI_ISL_725628, EPI_ISL_725629, EPI_ISL_725630, EPI_ISL_725631, EPI_ISL_725632, EPI_ISL_725633, EPI_ISL_725634, EPI_ISL_725635, EPI_ISL_725636, EPI_ISL_725637, EPI_ISL_725638, EPI_ISL_725639, EPI_ISL_725640, EPI_ISL_725641, EPI_ISL_725642			
see above	Queens Medical Centre, Clinical Microbiology Department / DeepSeq Nottingham	COVID-19 Genomics UK (COG-UK) Consortium	Gemma Clark, Wendy Smith, Manjinder Khakh, Vicki M Fleming, Michelle M Lister, Hannah Howson-Wells, Jonathan Ball, Patrick McClure, Joseph Chappell, Theocharis Tsoleridis, Nadine Holmes, Matthew Carlisle, Christopher Moore, Fei Sang, Johnny Debebe, Victoria Wright, Matthew Loose
EPI_ISL_727871, EPI_ISL_727885, EPI_ISL_727905, EPI_ISL_727906, EPI_ISL_727920, EPI_ISL_727922, EPI_ISL_727923, EPI_ISL_727964, EPI_ISL_727966, EPI_ISL_727975, EPI_ISL_727987			
see above	Virology Department, Sheffield Teaching Hospitals NHS Foundation Trust/Department of Infection, Immunity and Cardiovascular Disease, The Medical School, University of Sheffield	COVID-19 Genomics UK (COG-UK) Consortium	Thushan de Silva, Matthew Parker, Nikki Smith, Adri Angyal, Rebecca Brown, Luke Green, Rachel Tucker, Paul Parsons, Danielle Groves, Katie Johnson, Laura Carrilero, Alex Keeley, Dave Partridge, Matthew Wyles, Benjamin Lindsey, Mehmet Yavuz, Mohammad Raza, Cariad Evans
EPI_ISL_728180, EPI_ISL_728183, EPI_ISL_728184, EPI_ISL_728189, EPI_ISL_728191, EPI_ISL_728201	National Public Health Laboratory, National Centre for Infectious Diseases	National Public Health Laboratory, National Centre for Infectious Diseases	Tze Minn Mak, Sophie Octavia, Zhenyang Zhou, Lin Cui, Raymond Tzer Pin Lin
EPI_ISL_728205	Institute of Microbiology, Universidad San Francisco de Quito	Institute of Microbiology, Universidad San Francisco de Quito	Belén Prado-Vivar, Sully Márquez, Juan José Guadalupe, Monica Becerra-Wong, Diana Zambrano, Fredy Loor, Juan Zuñiga, Edison Chavez, Bernardo Gutiérrez, Verónica Barragán, Patricio Rojas-Silva, Gabriel Trueba, Michelle Grunauer, Paúl Cárdenas
EPI_ISL_728557, EPI_ISL_728558, EPI_ISL_728559, EPI_ISL_728560, EPI_ISL_728561, EPI_ISL_728562, EPI_ISL_728563	Institute for Urban Disease Control and Prevention	COVID-19 Network Investigations (CONI) Alliance	Kamolthip Atsawawaranunt, Elizabeth Batty, Wasun Chantratita, Thanat Chookajorn, Stefan Fernandez, Angkana Huang, Anthony R. Jones, Khajohn Joonsalak, Chonticha Klungtong, Theerarat Kochakarn, Prayuth Kaewmalang, Amornmas Kongklieng, Namfon Kotanan, Krittikorn Kumpornsin, Wudtichai Manasatienkij, Anek Mungaomklang, Bhakbhoom Panthan, Pukkaporn Parnwijitkul, Ekawat Pasomsub, Kingkan Rakmanee, Insee Sensorn, Janjira Thaipadungpanit, Arporn Wangwiwatsin, Treewat Watthanachockchai
EPI_ISL_728570, EPI_ISL_728571, EPI_ISL_728572, EPI_ISL_728573, EPI_ISL_728574, EPI_ISL_728575, EPI_ISL_728576, EPI_ISL_728578, EPI_ISL_728579, EPI_ISL_728581, EPI_ISL_728582, EPI_ISL_728583, EPI_ISL_728584, EPI_ISL_728585, EPI_ISL_728588, EPI_ISL_728589, EPI_ISL_728590, EPI_ISL_728591, EPI_ISL_728595, EPI_ISL_728596, EPI_ISL_728600, EPI_ISL_728601, EPI_ISL_728602, EPI_ISL_728603, EPI_ISL_728606, EPI_ISL_728607, EPI_ISL_728608, EPI_ISL_728609, EPI_ISL_728610, EPI_ISL_728611, EPI_ISL_728612, EPI_ISL_728613, EPI_ISL_728618, EPI_ISL_728619, EPI_ISL_728622, EPI_ISL_728623, EPI_ISL_728624, EPI_ISL_728625, EPI_ISL_728626, EPI_ISL_728627, EPI_ISL_728628, EPI_ISL_728629, EPI_ISL_728630, EPI_ISL_728631, EPI_ISL_728649, EPI_ISL_728650, EPI_ISL_728652, EPI_ISL_728653, EPI_ISL_728654, EPI_ISL_728655, EPI_ISL_728656, EPI_ISL_728657, EPI_ISL_728658, EPI_ISL_728659, EPI_ISL_728662, EPI_ISL_728663, EPI_ISL_728664, EPI_ISL_728665, EPI_ISL_728666, EPI_ISL_728667, EPI_ISL_728695, EPI_ISL_728698, EPI_ISL_728699, EPI_ISL_728700, EPI_ISL_728702, EPI_ISL_728703, EPI_ISL_728721, EPI_ISL_728722, EPI_ISL_728746			
see above	Dutch COVID-19 response team	National Institute for Public Health and the Environment (RIVM)	Adam Meijer, Harry Vennema, Jeroen Cremer, Sharon van den Brink, Bas van der Veer, AnneMarie van den Brandt, Florian Zwagemaker, Dennis Schmitz, Chantal Reusken, on behalf of the national COVID-19 response team
EPI_ISL_728764, EPI_ISL_728769, EPI_ISL_728771, EPI_ISL_728772	Viollier AG	Department of Biosystems Science and Engineering, ETH Zürich	Chaoran Chen, Sarah Nadeau, Catharine Aquino, Ivan Topolsky, Pedro Ferreira, Philipp Jablonski, Susana Posada-Céspedes, Andreia Cabral de Gouvea, Maria Domenica Moccia, Simon Grüter, Timothy Sykes, Lennart Opitz, Ralph Schlapbach, Christiane Beckmann, Maurice Redondo, Olivier Kobel, Christoph Noppen, Sophie Seidel, Noemie Santamaria de Souza, Niko Beerenwinkel, Tanja Stadler
EPI_ISL_728776	Viollier AG	Department of Biosystems Science and Engineering, ETH Zürich	Christian Beisel, Sarah Nadeau, Chaoran Chen, Ivan Topolsky, Pedro Ferreira, Philipp Jablonski, Susana Posada-Céspedes, Tobias Schär, Ina Nissen, Natascha Santacroce, Elodie Burcklen, Christiane Beckmann, Maurice Redondo, Olivier Kobel, Christoph Noppen, Sophie Seidel, Noemie Santamaria de Souza, Niko Beerenwinkel, Tanja Stadler
EPI_ISL_728779	Viollier AG	Department of Biosystems Science and Engineering, ETH Zürich	Chaoran Chen, Sarah Nadeau, Catharine Aquino, Ivan Topolsky, Pedro Ferreira, Philipp Jablonski, Susana Posada-Céspedes, Andreia Cabral de Gouvea, Maria Domenica Moccia, Simon Grüter, Timothy Sykes, Lennart Opitz, Ralph Schlapbach, Christiane Beckmann, Maurice Redondo, Olivier Kobel, Christoph Noppen, Sophie Seidel, Noemie Santamaria de Souza, Niko Beerenwinkel, Tanja Stadler
EPI_ISL_728808	Viollier AG	Department of Biosystems Science and Engineering, ETH Zürich	Christian Beisel, Sarah Nadeau, Chaoran Chen, Ivan Topolsky, Pedro Ferreira, Philipp Jablonski, Susana Posada-Céspedes, Tobias Schär, Ina Nissen, Natascha Santacroce, Elodie Burcklen, Christiane Beckmann, Maurice Redondo, Olivier Kobel, Christoph Noppen, Sophie Seidel, Noemie Santamaria de Souza, Niko Beerenwinkel, Tanja Stadler
EPI_ISL_728809	Viollier AG	Department of Biosystems Science and Engineering, ETH Zürich	Chaoran Chen, Sarah Nadeau, Catharine Aquino, Ivan Topolsky, Pedro Ferreira, Philipp Jablonski, Susana Posada-Céspedes, Andreia Cabral de Gouvea, Maria Domenica Moccia, Simon Grüter, Timothy Sykes, Lennart Opitz, Ralph Schlapbach, Christiane Beckmann, Maurice Redondo, Olivier Kobel, Christoph Noppen, Sophie Seidel, Noemie Santamaria de Souza, Niko Beerenwinkel, Tanja Stadler
EPI_ISL_728828, EPI_ISL_728882	Viollier AG	Department of Biosystems Science and Engineering, ETH Zürich	Christian Beisel, Sarah Nadeau, Chaoran Chen, Ivan Topolsky, Pedro Ferreira, Philipp Jablonski, Susana Posada-Céspedes, Tobias Schär, Ina Nissen, Natascha Santacroce, Elodie Burcklen, Christiane Beckmann, Maurice Redondo, Olivier Kobel, Christoph Noppen, Sophie Seidel, Noemie Santamaria de Souza, Niko Beerenwinkel, Tanja Stadler
EPI_ISL_728886, EPI_ISL_728887, EPI_ISL_728888, EPI_ISL_728889, EPI_ISL_728890, EPI_ISL_728891, EPI_ISL_728894, EPI_ISL_728895, EPI_ISL_728898, EPI_ISL_728899, EPI_ISL_728901, EPI_ISL_728902, EPI_ISL_728903, EPI_ISL_728904, EPI_ISL_728905, EPI_ISL_728910, EPI_ISL_728917, EPI_ISL_728920, EPI_ISL_728925, EPI_ISL_728926, EPI_ISL_728927, EPI_ISL_728933, EPI_ISL_728936, EPI_ISL_728937, EPI_ISL_728949, EPI_ISL_728952, EPI_ISL_728953, EPI_ISL_728954, EPI_ISL_728958, EPI_ISL_728962, EPI_ISL_728964, EPI_ISL_728969, EPI_ISL_728980, EPI_ISL_728988, EPI_ISL_728990, EPI_ISL_728997, EPI_ISL_728999, EPI_ISL_729001, EPI_ISL_729003, EPI_ISL_729005, EPI_ISL_729009, EPI_ISL_729010, EPI_ISL_729012, EPI_ISL_729013, EPI_ISL_729014, EPI_ISL_729016, EPI_ISL_729017, EPI_ISL_729020, EPI_ISL_729023, EPI_ISL_729024, EPI_ISL_729026, EPI_ISL_729027, EPI_ISL_729028, EPI_ISL_729031, EPI_ISL_729033, EPI_ISL_729043, EPI_ISL_729044			
see above	Viollier AG	Department of Biosystems Science and Engineering, ETH Zürich	Chaoran Chen, Sarah Nadeau, Catharine Aquino, Ivan Topolsky, Pedro Ferreira, Philipp Jablonski, Susana Posada-Céspedes, Andreia Cabral de Gouvea, Maria Domenica Moccia, Simon Grüter, Timothy Sykes, Lennart Opitz, Ralph Schlapbach, Christiane Beckmann, Maurice Redondo, Olivier Kobel, Christoph Noppen, Sophie Seidel, Noemie Santamaria de Souza, Niko Beerenwinkel, Tanja Stadler
EPI_ISL_729050, EPI_ISL_729057, EPI_ISL_729059, EPI_ISL_729060, EPI_ISL_729061, EPI_ISL_729062, EPI_ISL_729063, EPI_ISL_729064, EPI_ISL_729065, EPI_ISL_729066	Viollier AG	Department of Biosystems Science and Engineering, ETH Zürich	Christian Beisel, Sarah Nadeau, Chaoran Chen, Ivan Topolsky, Pedro Ferreira, Philipp Jablonski, Susana Posada-Céspedes, Tobias Schär, Ina Nissen, Natascha Santacroce, Elodie Burcklen, Christiane Beckmann, Maurice Redondo, Olivier Kobel, Christoph Noppen, Sophie Seidel, Noemie Santamaria de Souza, Niko Beerenwinkel, Tanja Stadler
EPI_ISL_729139, EPI_ISL_729140, EPI_ISL_729141, EPI_ISL_729142, EPI_ISL_729143, EPI_ISL_729144, EPI_ISL_729145, EPI_ISL_729146, EPI_ISL_729147, EPI_ISL_729148, EPI_ISL_729149, EPI_ISL_729150, EPI_ISL_729151			
see above	Viollier AG	Department of Biosystems Science and Engineering, ETH Zürich	Chaoran Chen, Sarah Nadeau, Catharine Aquino, Ivan Topolsky, Pedro Ferreira, Philipp Jablonski, Susana Posada-Céspedes, Andreia Cabral de Gouvea, Maria Domenica Moccia, Simon Grüter, Timothy Sykes, Lennart Opitz, Ralph Schlapbach, Christiane Beckmann, Maurice Redondo, Olivier Kobel, Christoph Noppen, Sophie Seidel, Noemie Santamaria de Souza, Niko Beerenwinkel, Tanja Stadler
EPI_ISL_729156, EPI_ISL_729157, EPI_ISL_729158, EPI_ISL_729159, EPI_ISL_729160, EPI_ISL_729161, EPI_ISL_729162, EPI_ISL_729163, EPI_ISL_729164, EPI_ISL_729165, EPI_ISL_729166, EPI_ISL_729167, EPI_ISL_729168, EPI_ISL_729169, EPI_ISL_729170, EPI_ISL_729171, EPI_ISL_729172, EPI_ISL_729173, EPI_ISL_729174, EPI_ISL_729175, EPI_ISL_729176, EPI_ISL_729177, EPI_ISL_729178, EPI_ISL_729179, EPI_ISL_729180, EPI_ISL_729181, EPI_ISL_729182, EPI_ISL_729183, EPI_ISL_729184, EPI_ISL_729185, EPI_ISL_729186			
see above	Viollier AG	Department of Biosystems Science and Engineering, ETH Zürich	Christian Beisel, Sarah Nadeau, Chaoran Chen, Ivan Topolsky, Pedro Ferreira, Philipp Jablonski, Susana Posada-Céspedes, Tobias Schär, Ina Nissen, Natascha Santacroce, Elodie Burcklen, Christiane Beckmann, Maurice Redondo, Olivier Kobel, Christoph Noppen, Sophie Seidel, Noemie Santamaria de Souza, Niko Beerenwinkel, Tanja Stadler
EPI_ISL_730654, EPI_ISL_730660, EPI_ISL_730661, EPI_ISL_730662, EPI_ISL_730663, EPI_ISL_730665, EPI_ISL_730666, EPI_ISL_730669, EPI_ISL_730670, EPI_ISL_730671, EPI_ISL_730672, EPI_ISL_730674, EPI_ISL_730677, EPI_ISL_730679, EPI_ISL_730681, EPI_ISL_730684, EPI_ISL_730687, EPI_ISL_730688, EPI_ISL_730689, EPI_ISL_730690, EPI_ISL_730691, EPI_ISL_730693, EPI_ISL_730694, EPI_ISL_730698, EPI_ISL_730700, EPI_ISL_730701, EPI_ISL_730703, EPI_ISL_730706, EPI_ISL_730707, EPI_ISL_730708, EPI_ISL_730713, EPI_ISL_730714, EPI_ISL_730715, EPI_ISL_730716, EPI_ISL_730717, EPI_ISL_730719,			

EPI_ISL_730720, EPI_ISL_730721			
see above	Lighthouse Lab in Alderley Park	Wellcome Sanger Institute for the COVID-19 Genomics UK (COG-UK) Consortium	Jacquelyn Wynn, Mairead Hyland, The Lighthouse Lab in Alderley Park and Alex Alderton, Roberto Amato, Sonia Goncalves, Ewan Harrison, David K. Jackson, Ian Johnston, Dominic Kwiatkowski, Cordelia Langford, John Sillitoe on behalf of the Wellcome Sanger Institute COVID-19 Surveillance Team
EPI_ISL_730723	Lighthouse Lab in Glasgow	Wellcome Sanger Institute for the COVID-19 Genomics UK (COG-UK) Consortium	Harper VanSteenhouse, Yumi Kasai, David Gray, Carol Clugston, Anna Dominiczak and Alex Alderton, Roberto Amato, Sonia Goncalves, Ewan Harrison, David K. Jackson, Ian Johnston, Dominic Kwiatkowski, Cordelia Langford, John Sillitoe on behalf of the Wellcome Sanger Institute COVID-19 Surveillance Team
EPI_ISL_730724, EPI_ISL_730725, EPI_ISL_730726, EPI_ISL_730727, EPI_ISL_730728, EPI_ISL_730730, EPI_ISL_730735, EPI_ISL_730736, EPI_ISL_730738, EPI_ISL_730739, EPI_ISL_730740, EPI_ISL_730741, EPI_ISL_730744, EPI_ISL_730745, EPI_ISL_730746, EPI_ISL_730747, EPI_ISL_730748, EPI_ISL_730749, EPI_ISL_730755, EPI_ISL_730756, EPI_ISL_730757, EPI_ISL_730760, EPI_ISL_730761, EPI_ISL_730762, EPI_ISL_730763, EPI_ISL_730764, EPI_ISL_730766, EPI_ISL_730767, EPI_ISL_730768, EPI_ISL_730769, EPI_ISL_730770, EPI_ISL_730772, EPI_ISL_730775, EPI_ISL_730776, EPI_ISL_730779, EPI_ISL_730781, EPI_ISL_730782, EPI_ISL_730783, EPI_ISL_730787, EPI_ISL_730790, EPI_ISL_730791, EPI_ISL_730792, EPI_ISL_730797, EPI_ISL_730798, EPI_ISL_730799, EPI_ISL_730800, EPI_ISL_730807			
see above	Lighthouse Lab in Alderley Park	Wellcome Sanger Institute for the COVID-19 Genomics UK (COG-UK) Consortium	Jacquelyn Wynn, Mairead Hyland, The Lighthouse Lab in Alderley Park and Alex Alderton, Roberto Amato, Sonia Goncalves, Ewan Harrison, David K. Jackson, Ian Johnston, Dominic Kwiatkowski, Cordelia Langford, John Sillitoe on behalf of the Wellcome Sanger Institute COVID-19 Surveillance Team
EPI_ISL_730808	Lighthouse Lab in Glasgow	Wellcome Sanger Institute for the COVID-19 Genomics UK (COG-UK) Consortium	Harper VanSteenhouse, Yumi Kasai, David Gray, Carol Clugston, Anna Dominiczak and Alex Alderton, Roberto Amato, Sonia Goncalves, Ewan Harrison, David K. Jackson, Ian Johnston, Dominic Kwiatkowski, Cordelia Langford, John Sillitoe on behalf of the Wellcome Sanger Institute COVID-19 Surveillance Team
EPI_ISL_730810, EPI_ISL_730811, EPI_ISL_730812, EPI_ISL_730816, EPI_ISL_730817, EPI_ISL_730820, EPI_ISL_730821, EPI_ISL_730822, EPI_ISL_730827, EPI_ISL_730835, EPI_ISL_730836, EPI_ISL_730837, EPI_ISL_730838, EPI_ISL_730841, EPI_ISL_730842, EPI_ISL_730843, EPI_ISL_730851, EPI_ISL_730853, EPI_ISL_730856, EPI_ISL_730857, EPI_ISL_730863, EPI_ISL_730866, EPI_ISL_730868, EPI_ISL_730871, EPI_ISL_730872, EPI_ISL_730874, EPI_ISL_730879, EPI_ISL_730880, EPI_ISL_730882, EPI_ISL_730883, EPI_ISL_730884, EPI_ISL_730885, EPI_ISL_730888, EPI_ISL_730891, EPI_ISL_730892, EPI_ISL_730895, EPI_ISL_730896, EPI_ISL_730897, EPI_ISL_730899, EPI_ISL_730901, EPI_ISL_730903, EPI_ISL_730905, EPI_ISL_730906, EPI_ISL_730907, EPI_ISL_730909, EPI_ISL_730912, EPI_ISL_730915, EPI_ISL_730916, EPI_ISL_730921, EPI_ISL_730924, EPI_ISL_730929, EPI_ISL_730935, EPI_ISL_730939, EPI_ISL_730948, EPI_ISL_730959, EPI_ISL_730966, EPI_ISL_730967, EPI_ISL_730969, EPI_ISL_730970, EPI_ISL_730971, EPI_ISL_730973, EPI_ISL_730980, EPI_ISL_730987, EPI_ISL_730989, EPI_ISL_730994, EPI_ISL_731000, EPI_ISL_731006, EPI_ISL_731017, EPI_ISL_731019, EPI_ISL_731020, EPI_ISL_731024, EPI_ISL_731026, EPI_ISL_731027, EPI_ISL_731033, EPI_ISL_731035, EPI_ISL_731040, EPI_ISL_731045, EPI_ISL_731050, EPI_ISL_731052, EPI_ISL_731054, EPI_ISL_731055, EPI_ISL_731057, EPI_ISL_731059, EPI_ISL_731065, EPI_ISL_731066, EPI_ISL_731074, EPI_ISL_731076, EPI_ISL_731079, EPI_ISL_731080, EPI_ISL_731082, EPI_ISL_731083, EPI_ISL_731086, EPI_ISL_731087, EPI_ISL_731088, EPI_ISL_731094, EPI_ISL_731096, EPI_ISL_731104, EPI_ISL_731106, EPI_ISL_731107, EPI_ISL_731110, EPI_ISL_731116, EPI_ISL_731119, EPI_ISL_731120, EPI_ISL_731123, EPI_ISL_731126, EPI_ISL_731129, EPI_ISL_731130, EPI_ISL_731131, EPI_ISL_731133, EPI_ISL_731135, EPI_ISL_731137, EPI_ISL_731141, EPI_ISL_731143, EPI_ISL_731147, EPI_ISL_731153, EPI_ISL_731154, EPI_ISL_731157, EPI_ISL_731158, EPI_ISL_731160, EPI_ISL_731161, EPI_ISL_731169, EPI_ISL_731171, EPI_ISL_731181, EPI_ISL_731183, EPI_ISL_731188, EPI_ISL_731196, EPI_ISL_731199, EPI_ISL_731200, EPI_ISL_731204, EPI_ISL_731206, EPI_ISL_731214, EPI_ISL_731215, EPI_ISL_731233, EPI_ISL_731235, EPI_ISL_731240, EPI_ISL_731247, EPI_ISL_731248, EPI_ISL_731254, EPI_ISL_731260			
see above	Lighthouse Lab in Alderley Park	Wellcome Sanger Institute for the COVID-19 Genomics UK (COG-UK) Consortium	Jacquelyn Wynn, Mairead Hyland, The Lighthouse Lab in Alderley Park and Alex Alderton, Roberto Amato, Sonia Goncalves, Ewan Harrison, David K. Jackson, Ian Johnston, Dominic Kwiatkowski, Cordelia Langford, John Sillitoe on behalf of the Wellcome Sanger Institute COVID-19 Surveillance Team
EPI_ISL_731261, EPI_ISL_731262	Lighthouse Lab in Glasgow	Wellcome Sanger Institute for the COVID-19 Genomics UK (COG-UK) Consortium	Harper VanSteenhouse, Yumi Kasai, David Gray, Carol Clugston, Anna Dominiczak and Alex Alderton, Roberto Amato, Sonia Goncalves, Ewan Harrison, David K. Jackson, Ian Johnston, Dominic Kwiatkowski, Cordelia Langford, John Sillitoe on behalf of the Wellcome Sanger Institute COVID-19 Surveillance Team
EPI_ISL_731263	Lighthouse Lab in Alderley Park	Wellcome Sanger Institute for the COVID-19 Genomics UK (COG-UK) Consortium	Jacquelyn Wynn, Mairead Hyland, The Lighthouse Lab in Alderley Park and Alex Alderton, Roberto Amato, Sonia Goncalves, Ewan Harrison, David K. Jackson, Ian Johnston, Dominic Kwiatkowski, Cordelia Langford, John Sillitoe on behalf of the Wellcome Sanger Institute COVID-19 Surveillance Team
EPI_ISL_731264	Lighthouse Lab in Glasgow	Wellcome Sanger Institute for the COVID-19 Genomics UK (COG-UK) Consortium	Harper VanSteenhouse, Yumi Kasai, David Gray, Carol Clugston, Anna Dominiczak and Alex Alderton, Roberto Amato, Sonia Goncalves, Ewan Harrison, David K. Jackson, Ian Johnston, Dominic Kwiatkowski, Cordelia Langford, John Sillitoe on behalf of the Wellcome Sanger Institute COVID-19 Surveillance Team
EPI_ISL_731265, EPI_ISL_731266, EPI_ISL_731268, EPI_ISL_731269, EPI_ISL_731270, EPI_ISL_731272, EPI_ISL_731274, EPI_ISL_731275, EPI_ISL_731276, EPI_ISL_731277, EPI_ISL_731278, EPI_ISL_731279			
see above	Lighthouse Lab in Alderley Park	Wellcome Sanger Institute for the COVID-19 Genomics UK (COG-UK) Consortium	Jacquelyn Wynn, Mairead Hyland, The Lighthouse Lab in Alderley Park and Alex Alderton, Roberto Amato, Sonia Goncalves, Ewan Harrison, David K. Jackson, Ian Johnston, Dominic Kwiatkowski, Cordelia Langford, John Sillitoe on behalf of the Wellcome Sanger Institute COVID-19 Surveillance Team
EPI_ISL_731283, EPI_ISL_731284	Lighthouse Lab in Glasgow	Wellcome Sanger Institute for the COVID-19 Genomics UK (COG-UK) Consortium	Harper VanSteenhouse, Yumi Kasai, David Gray, Carol Clugston, Anna Dominiczak and Alex Alderton, Roberto Amato, Sonia Goncalves, Ewan Harrison, David K. Jackson, Ian Johnston, Dominic Kwiatkowski, Cordelia Langford, John Sillitoe on behalf of the Wellcome Sanger Institute COVID-19 Surveillance Team
EPI_ISL_731285, EPI_ISL_731288, EPI_ISL_731289	Lighthouse Lab in Alderley Park	Wellcome Sanger Institute for the COVID-19 Genomics UK (COG-UK) Consortium	Jacquelyn Wynn, Mairead Hyland, The Lighthouse Lab in Alderley Park and Alex Alderton, Roberto Amato, Sonia Goncalves, Ewan Harrison, David K. Jackson, Ian Johnston, Dominic Kwiatkowski, Cordelia Langford, John Sillitoe on behalf of the Wellcome Sanger Institute COVID-19 Surveillance Team
EPI_ISL_731290	Lighthouse Lab in Glasgow	Wellcome Sanger Institute for the COVID-19 Genomics UK (COG-UK) Consortium	Harper VanSteenhouse, Yumi Kasai, David Gray, Carol Clugston, Anna Dominiczak and Alex Alderton, Roberto Amato, Sonia Goncalves, Ewan Harrison, David K. Jackson, Ian Johnston, Dominic Kwiatkowski, Cordelia Langford, John Sillitoe on behalf of the Wellcome Sanger Institute COVID-19 Surveillance Team
EPI_ISL_731292	Lighthouse Lab in Alderley Park	Wellcome Sanger Institute for the COVID-19 Genomics UK (COG-UK) Consortium	Jacquelyn Wynn, Mairead Hyland, The Lighthouse Lab in Alderley Park and Alex Alderton, Roberto Amato, Sonia Goncalves, Ewan Harrison, David K. Jackson, Ian Johnston, Dominic Kwiatkowski, Cordelia Langford, John Sillitoe on behalf of the Wellcome Sanger Institute COVID-19 Surveillance Team
EPI_ISL_731293	Lighthouse Lab in Glasgow	Wellcome Sanger Institute for the COVID-19 Genomics UK (COG-UK) Consortium	Harper VanSteenhouse, Yumi Kasai, David Gray, Carol Clugston, Anna Dominiczak and Alex Alderton, Roberto Amato, Sonia Goncalves, Ewan Harrison, David K. Jackson, Ian Johnston, Dominic Kwiatkowski, Cordelia Langford, John Sillitoe on behalf of the Wellcome Sanger Institute COVID-19 Surveillance Team
EPI_ISL_731294, EPI_ISL_731296, EPI_ISL_731298	Lighthouse Lab in Alderley Park	Wellcome Sanger Institute for the COVID-19 Genomics UK (COG-UK) Consortium	Jacquelyn Wynn, Mairead Hyland, The Lighthouse Lab in Alderley Park and Alex Alderton, Roberto Amato, Sonia Goncalves, Ewan Harrison, David K. Jackson, Ian Johnston, Dominic Kwiatkowski, Cordelia Langford, John Sillitoe on behalf of the Wellcome Sanger Institute COVID-19 Surveillance Team
EPI_ISL_731299	Lighthouse Lab in Glasgow	Wellcome Sanger Institute for the COVID-19 Genomics UK (COG-UK) Consortium	Harper VanSteenhouse, Yumi Kasai, David Gray, Carol Clugston, Anna Dominiczak and Alex Alderton, Roberto Amato, Sonia Goncalves, Ewan Harrison, David K. Jackson, Ian Johnston, Dominic Kwiatkowski, Cordelia Langford, John Sillitoe on behalf of the Wellcome Sanger Institute COVID-19 Surveillance Team
EPI_ISL_731300, EPI_ISL_731301, EPI_ISL_731302, EPI_ISL_731303, EPI_ISL_731304	Lighthouse Lab in Alderley Park	Wellcome Sanger Institute for the COVID-19 Genomics UK (COG-UK) Consortium	Jacquelyn Wynn, Mairead Hyland, The Lighthouse Lab in Alderley Park and Alex Alderton, Roberto Amato, Sonia Goncalves, Ewan Harrison, David K. Jackson, Ian Johnston, Dominic Kwiatkowski, Cordelia Langford, John Sillitoe on behalf of the Wellcome Sanger Institute COVID-19 Surveillance Team
EPI_ISL_731306	Lighthouse Lab in Glasgow	Wellcome Sanger Institute for the COVID-19 Genomics UK (COG-UK) Consortium	Harper VanSteenhouse, Yumi Kasai, David Gray, Carol Clugston, Anna Dominiczak and Alex Alderton, Roberto Amato, Sonia Goncalves, Ewan Harrison, David K. Jackson, Ian Johnston, Dominic Kwiatkowski, Cordelia Langford, John Sillitoe on behalf of the Wellcome Sanger Institute COVID-19 Surveillance Team
EPI_ISL_731307, EPI_ISL_731311, EPI_ISL_731312, EPI_ISL_731313, EPI_ISL_731315, EPI_ISL_731316, EPI_ISL_731317, EPI_ISL_731318, EPI_ISL_731323, EPI_ISL_731324, EPI_ISL_731325, EPI_ISL_731328, EPI_ISL_731329, EPI_ISL_731331			
see above	Lighthouse Lab in Alderley Park	Wellcome Sanger Institute for the COVID-19 Genomics UK (COG-UK) Consortium	Jacquelyn Wynn, Mairead Hyland, The Lighthouse Lab in Alderley Park and Alex Alderton, Roberto Amato, Sonia Goncalves, Ewan Harrison, David K. Jackson, Ian Johnston, Dominic Kwiatkowski, Cordelia Langford, John Sillitoe on behalf of the Wellcome Sanger Institute COVID-19 Surveillance Team
EPI_ISL_731333, EPI_ISL_731334	Lighthouse Lab in Glasgow	Wellcome Sanger Institute for the COVID-19 Genomics UK (COG-UK) Consortium	Harper VanSteenhouse, Yumi Kasai, David Gray, Carol Clugston, Anna Dominiczak and Alex Alderton, Roberto Amato, Sonia Goncalves, Ewan Harrison, David K. Jackson, Ian Johnston, Dominic Kwiatkowski, Cordelia Langford, John Sillitoe on behalf of the Wellcome Sanger Institute COVID-19 Surveillance Team
EPI_ISL_731335, EPI_ISL_731336, EPI_ISL_731339, EPI_ISL_731342, EPI_ISL_731343, EPI_ISL_731344, EPI_ISL_731348	Lighthouse Lab in Alderley Park	Wellcome Sanger Institute for the COVID-19 Genomics UK (COG-UK) Consortium	Jacquelyn Wynn, Mairead Hyland, The Lighthouse Lab in Alderley Park and Alex Alderton, Roberto Amato, Sonia Goncalves, Ewan Harrison, David K. Jackson, Ian Johnston, Dominic Kwiatkowski, Cordelia Langford, John Sillitoe on behalf of the Wellcome Sanger Institute COVID-19 Surveillance Team
EPI_ISL_731349	Lighthouse Lab in Glasgow	Wellcome Sanger Institute for the COVID-19 Genomics UK (COG-UK) Consortium	Harper VanSteenhouse, Yumi Kasai, David Gray, Carol Clugston, Anna Dominiczak and Alex Alderton, Roberto Amato, Sonia Goncalves, Ewan Harrison, David K. Jackson, Ian Johnston, Dominic Kwiatkowski, Cordelia Langford, John Sillitoe on behalf of the Wellcome Sanger Institute COVID-19 Surveillance Team
EPI_ISL_731350, EPI_ISL_731351, EPI_ISL_731352	Lighthouse Lab in Alderley Park	Wellcome Sanger Institute for the COVID-19 Genomics UK (COG-UK) Consortium	Jacquelyn Wynn, Mairead Hyland, The Lighthouse Lab in Alderley Park and Alex Alderton, Roberto Amato, Sonia Goncalves, Ewan Harrison, David K. Jackson, Ian Johnston, Dominic Kwiatkowski, Cordelia Langford, John Sillitoe on behalf of the Wellcome Sanger Institute COVID-19 Surveillance Team

EPI_ISL_731353	Lighthouse Lab in Glasgow	Wellcome Sanger Institute for the COVID-19 Genomics UK (COG-UK) Consortium	Harper VanSteenhouse, Yumi Kasai, David Gray, Carol Clugston, Anna Dominiczak and Alex Alderton, Roberto Amato, Sonia Goncalves, Ewan Harrison, David K. Jackson, Ian Johnston, Dominic Kwiatkowski, Cordelia Langford, John Sillitoe on behalf of the Wellcome Sanger Institute COVID-19 Surveillance Team
EPI_ISL_731356, EPI_ISL_731358, EPI_ISL_731359, EPI_ISL_731360, EPI_ISL_731362, EPI_ISL_731363, EPI_ISL_731364, EPI_ISL_731365, EPI_ISL_731366, EPI_ISL_731369, EPI_ISL_731370, EPI_ISL_731371, EPI_ISL_731374, EPI_ISL_731375, EPI_ISL_731377			
see above	Lighthouse Lab in Alderley Park	Wellcome Sanger Institute for the COVID-19 Genomics UK (COG-UK) Consortium	Jacquelyn Wynn, Mairead Hyland, The Lighthouse Lab in Alderley Park and Alex Alderton, Roberto Amato, Sonia Goncalves, Ewan Harrison, David K. Jackson, Ian Johnston, Dominic Kwiatkowski, Cordelia Langford, John Sillitoe on behalf of the Wellcome Sanger Institute COVID-19 Surveillance Team
EPI_ISL_731378	Lighthouse Lab in Glasgow	Wellcome Sanger Institute for the COVID-19 Genomics UK (COG-UK) Consortium	Harper VanSteenhouse, Yumi Kasai, David Gray, Carol Clugston, Anna Dominiczak and Alex Alderton, Roberto Amato, Sonia Goncalves, Ewan Harrison, David K. Jackson, Ian Johnston, Dominic Kwiatkowski, Cordelia Langford, John Sillitoe on behalf of the Wellcome Sanger Institute COVID-19 Surveillance Team
EPI_ISL_731381, EPI_ISL_731384, EPI_ISL_731385, EPI_ISL_731387, EPI_ISL_731388, EPI_ISL_731389, EPI_ISL_731391	Lighthouse Lab in Alderley Park	Wellcome Sanger Institute for the COVID-19 Genomics UK (COG-UK) Consortium	Jacquelyn Wynn, Mairead Hyland, The Lighthouse Lab in Alderley Park and Alex Alderton, Roberto Amato, Sonia Goncalves, Ewan Harrison, David K. Jackson, Ian Johnston, Dominic Kwiatkowski, Cordelia Langford, John Sillitoe on behalf of the Wellcome Sanger Institute COVID-19 Surveillance Team
EPI_ISL_731393	Lighthouse Lab in Glasgow	Wellcome Sanger Institute for the COVID-19 Genomics UK (COG-UK) Consortium	Harper VanSteenhouse, Yumi Kasai, David Gray, Carol Clugston, Anna Dominiczak and Alex Alderton, Roberto Amato, Sonia Goncalves, Ewan Harrison, David K. Jackson, Ian Johnston, Dominic Kwiatkowski, Cordelia Langford, John Sillitoe on behalf of the Wellcome Sanger Institute COVID-19 Surveillance Team
EPI_ISL_731394, EPI_ISL_731395	Lighthouse Lab in Alderley Park	Wellcome Sanger Institute for the COVID-19 Genomics UK (COG-UK) Consortium	Jacquelyn Wynn, Mairead Hyland, The Lighthouse Lab in Alderley Park and Alex Alderton, Roberto Amato, Sonia Goncalves, Ewan Harrison, David K. Jackson, Ian Johnston, Dominic Kwiatkowski, Cordelia Langford, John Sillitoe on behalf of the Wellcome Sanger Institute COVID-19 Surveillance Team
EPI_ISL_731396, EPI_ISL_731398	Lighthouse Lab in Glasgow	Wellcome Sanger Institute for the COVID-19 Genomics UK (COG-UK) Consortium	Harper VanSteenhouse, Yumi Kasai, David Gray, Carol Clugston, Anna Dominiczak and Alex Alderton, Roberto Amato, Sonia Goncalves, Ewan Harrison, David K. Jackson, Ian Johnston, Dominic Kwiatkowski, Cordelia Langford, John Sillitoe on behalf of the Wellcome Sanger Institute COVID-19 Surveillance Team
EPI_ISL_731399	Lighthouse Lab in Alderley Park	Wellcome Sanger Institute for the COVID-19 Genomics UK (COG-UK) Consortium	Jacquelyn Wynn, Mairead Hyland, The Lighthouse Lab in Alderley Park and Alex Alderton, Roberto Amato, Sonia Goncalves, Ewan Harrison, David K. Jackson, Ian Johnston, Dominic Kwiatkowski, Cordelia Langford, John Sillitoe on behalf of the Wellcome Sanger Institute COVID-19 Surveillance Team
EPI_ISL_731400, EPI_ISL_731405	Lighthouse Lab in Glasgow	Wellcome Sanger Institute for the COVID-19 Genomics UK (COG-UK) Consortium	Harper VanSteenhouse, Yumi Kasai, David Gray, Carol Clugston, Anna Dominiczak and Alex Alderton, Roberto Amato, Sonia Goncalves, Ewan Harrison, David K. Jackson, Ian Johnston, Dominic Kwiatkowski, Cordelia Langford, John Sillitoe on behalf of the Wellcome Sanger Institute COVID-19 Surveillance Team
EPI_ISL_731409, EPI_ISL_731410, EPI_ISL_731412, EPI_ISL_731413, EPI_ISL_731415, EPI_ISL_731418, EPI_ISL_731419	Lighthouse Lab in Alderley Park	Wellcome Sanger Institute for the COVID-19 Genomics UK (COG-UK) Consortium	Jacquelyn Wynn, Mairead Hyland, The Lighthouse Lab in Alderley Park and Alex Alderton, Roberto Amato, Sonia Goncalves, Ewan Harrison, David K. Jackson, Ian Johnston, Dominic Kwiatkowski, Cordelia Langford, John Sillitoe on behalf of the Wellcome Sanger Institute COVID-19 Surveillance Team
EPI_ISL_731421	Lighthouse Lab in Glasgow	Wellcome Sanger Institute for the COVID-19 Genomics UK (COG-UK) Consortium	Harper VanSteenhouse, Yumi Kasai, David Gray, Carol Clugston, Anna Dominiczak and Alex Alderton, Roberto Amato, Sonia Goncalves, Ewan Harrison, David K. Jackson, Ian Johnston, Dominic Kwiatkowski, Cordelia Langford, John Sillitoe on behalf of the Wellcome Sanger Institute COVID-19 Surveillance Team
EPI_ISL_731422, EPI_ISL_731423, EPI_ISL_731424, EPI_ISL_731427, EPI_ISL_731428, EPI_ISL_731430	Lighthouse Lab in Alderley Park	Wellcome Sanger Institute for the COVID-19 Genomics UK (COG-UK) Consortium	Jacquelyn Wynn, Mairead Hyland, The Lighthouse Lab in Alderley Park and Alex Alderton, Roberto Amato, Sonia Goncalves, Ewan Harrison, David K. Jackson, Ian Johnston, Dominic Kwiatkowski, Cordelia Langford, John Sillitoe on behalf of the Wellcome Sanger Institute COVID-19 Surveillance Team
EPI_ISL_731433	Lighthouse Lab in Glasgow	Wellcome Sanger Institute for the COVID-19 Genomics UK (COG-UK) Consortium	Harper VanSteenhouse, Yumi Kasai, David Gray, Carol Clugston, Anna Dominiczak and Alex Alderton, Roberto Amato, Sonia Goncalves, Ewan Harrison, David K. Jackson, Ian Johnston, Dominic Kwiatkowski, Cordelia Langford, John Sillitoe on behalf of the Wellcome Sanger Institute COVID-19 Surveillance Team
EPI_ISL_731434, EPI_ISL_731436	Lighthouse Lab in Alderley Park	Wellcome Sanger Institute for the COVID-19 Genomics UK (COG-UK) Consortium	Jacquelyn Wynn, Mairead Hyland, The Lighthouse Lab in Alderley Park and Alex Alderton, Roberto Amato, Sonia Goncalves, Ewan Harrison, David K. Jackson, Ian Johnston, Dominic Kwiatkowski, Cordelia Langford, John Sillitoe on behalf of the Wellcome Sanger Institute COVID-19 Surveillance Team
EPI_ISL_731438	Lighthouse Lab in Glasgow	Wellcome Sanger Institute for the COVID-19 Genomics UK (COG-UK) Consortium	Harper VanSteenhouse, Yumi Kasai, David Gray, Carol Clugston, Anna Dominiczak and Alex Alderton, Roberto Amato, Sonia Goncalves, Ewan Harrison, David K. Jackson, Ian Johnston, Dominic Kwiatkowski, Cordelia Langford, John Sillitoe on behalf of the Wellcome Sanger Institute COVID-19 Surveillance Team
EPI_ISL_731439, EPI_ISL_731440, EPI_ISL_731441, EPI_ISL_731442, EPI_ISL_731443, EPI_ISL_731445, EPI_ISL_731446, EPI_ISL_731448, EPI_ISL_731451, EPI_ISL_731455	Lighthouse Lab in Alderley Park	Wellcome Sanger Institute for the COVID-19 Genomics UK (COG-UK) Consortium	Jacquelyn Wynn, Mairead Hyland, The Lighthouse Lab in Alderley Park and Alex Alderton, Roberto Amato, Sonia Goncalves, Ewan Harrison, David K. Jackson, Ian Johnston, Dominic Kwiatkowski, Cordelia Langford, John Sillitoe on behalf of the Wellcome Sanger Institute COVID-19 Surveillance Team
EPI_ISL_731456	Lighthouse Lab in Glasgow	Wellcome Sanger Institute for the COVID-19 Genomics UK (COG-UK) Consortium	Harper VanSteenhouse, Yumi Kasai, David Gray, Carol Clugston, Anna Dominiczak and Alex Alderton, Roberto Amato, Sonia Goncalves, Ewan Harrison, David K. Jackson, Ian Johnston, Dominic Kwiatkowski, Cordelia Langford, John Sillitoe on behalf of the Wellcome Sanger Institute COVID-19 Surveillance Team
EPI_ISL_731457	Lighthouse Lab in Alderley Park	Wellcome Sanger Institute for the COVID-19 Genomics UK (COG-UK) Consortium	Jacquelyn Wynn, Mairead Hyland, The Lighthouse Lab in Alderley Park and Alex Alderton, Roberto Amato, Sonia Goncalves, Ewan Harrison, David K. Jackson, Ian Johnston, Dominic Kwiatkowski, Cordelia Langford, John Sillitoe on behalf of the Wellcome Sanger Institute COVID-19 Surveillance Team
EPI_ISL_731459	Lighthouse Lab in Glasgow	Wellcome Sanger Institute for the COVID-19 Genomics UK (COG-UK) Consortium	Harper VanSteenhouse, Yumi Kasai, David Gray, Carol Clugston, Anna Dominiczak and Alex Alderton, Roberto Amato, Sonia Goncalves, Ewan Harrison, David K. Jackson, Ian Johnston, Dominic Kwiatkowski, Cordelia Langford, John Sillitoe on behalf of the Wellcome Sanger Institute COVID-19 Surveillance Team
EPI_ISL_731460, EPI_ISL_731461, EPI_ISL_731462, EPI_ISL_731463, EPI_ISL_731464, EPI_ISL_731465	Lighthouse Lab in Alderley Park	Wellcome Sanger Institute for the COVID-19 Genomics UK (COG-UK) Consortium	Jacquelyn Wynn, Mairead Hyland, The Lighthouse Lab in Alderley Park and Alex Alderton, Roberto Amato, Sonia Goncalves, Ewan Harrison, David K. Jackson, Ian Johnston, Dominic Kwiatkowski, Cordelia Langford, John Sillitoe on behalf of the Wellcome Sanger Institute COVID-19 Surveillance Team
EPI_ISL_731466	Lighthouse Lab in Glasgow	Wellcome Sanger Institute for the COVID-19 Genomics UK (COG-UK) Consortium	Harper VanSteenhouse, Yumi Kasai, David Gray, Carol Clugston, Anna Dominiczak and Alex Alderton, Roberto Amato, Sonia Goncalves, Ewan Harrison, David K. Jackson, Ian Johnston, Dominic Kwiatkowski, Cordelia Langford, John Sillitoe on behalf of the Wellcome Sanger Institute COVID-19 Surveillance Team
EPI_ISL_731467, EPI_ISL_731468, EPI_ISL_731470, EPI_ISL_731473, EPI_ISL_731474, EPI_ISL_731475, EPI_ISL_731478	Lighthouse Lab in Alderley Park	Wellcome Sanger Institute for the COVID-19 Genomics UK (COG-UK) Consortium	Jacquelyn Wynn, Mairead Hyland, The Lighthouse Lab in Alderley Park and Alex Alderton, Roberto Amato, Sonia Goncalves, Ewan Harrison, David K. Jackson, Ian Johnston, Dominic Kwiatkowski, Cordelia Langford, John Sillitoe on behalf of the Wellcome Sanger Institute COVID-19 Surveillance Team
EPI_ISL_731479	Lighthouse Lab in Glasgow	Wellcome Sanger Institute for the COVID-19 Genomics UK (COG-UK) Consortium	Harper VanSteenhouse, Yumi Kasai, David Gray, Carol Clugston, Anna Dominiczak and Alex Alderton, Roberto Amato, Sonia Goncalves, Ewan Harrison, David K. Jackson, Ian Johnston, Dominic Kwiatkowski, Cordelia Langford, John Sillitoe on behalf of the Wellcome Sanger Institute COVID-19 Surveillance Team
EPI_ISL_731480, EPI_ISL_731481, EPI_ISL_731483, EPI_ISL_731487, EPI_ISL_731489, EPI_ISL_731490, EPI_ISL_731492, EPI_ISL_731495, EPI_ISL_731498, EPI_ISL_731500, EPI_ISL_731502, EPI_ISL_731503, EPI_ISL_731504, EPI_ISL_731506, EPI_ISL_731509, EPI_ISL_731510, EPI_ISL_731511, EPI_ISL_731512, EPI_ISL_731514, EPI_ISL_731515, EPI_ISL_731516, EPI_ISL_731518, EPI_ISL_731520, EPI_ISL_731521			
see above	Lighthouse Lab in Alderley Park	Wellcome Sanger Institute for the COVID-19 Genomics UK (COG-UK) Consortium	Jacquelyn Wynn, Mairead Hyland, The Lighthouse Lab in Alderley Park and Alex Alderton, Roberto Amato, Sonia Goncalves, Ewan Harrison, David K. Jackson, Ian Johnston, Dominic Kwiatkowski, Cordelia Langford, John Sillitoe on behalf of the Wellcome Sanger Institute COVID-19 Surveillance Team

EPI_ISL_731522	Lighthouse Lab in Glasgow	Wellcome Sanger Institute for the COVID-19 Genomics UK (COG-UK) Consortium	Harper VanSteenhouse, Yumi Kasai, David Gray, Carol Clugston, Anna Dominiczak and Alex Alderton, Roberto Amato, Sonia Goncalves, Ewan Harrison, David K. Jackson, Ian Johnston, Dominic Kwiatkowski, Cordelia Langford, John Sillitoe on behalf of the Wellcome Sanger Institute COVID-19 Surveillance Team
EPI_ISL_731524, EPI_ISL_731525, EPI_ISL_731526, EPI_ISL_731527, EPI_ISL_731528, EPI_ISL_731529, EPI_ISL_731530, EPI_ISL_731531, EPI_ISL_731532, EPI_ISL_731533, EPI_ISL_731534			
see above	Lighthouse Lab in Alderley Park	Wellcome Sanger Institute for the COVID-19 Genomics UK (COG-UK) Consortium	Jacquelyn Wynn, Mairead Hyland, The Lighthouse Lab in Alderley Park and Alex Alderton, Roberto Amato, Sonia Goncalves, Ewan Harrison, David K. Jackson, Ian Johnston, Dominic Kwiatkowski, Cordelia Langford, John Sillitoe on behalf of the Wellcome Sanger Institute COVID-19 Surveillance Team
EPI_ISL_731535	Lighthouse Lab in Glasgow	Wellcome Sanger Institute for the COVID-19 Genomics UK (COG-UK) Consortium	Harper VanSteenhouse, Yumi Kasai, David Gray, Carol Clugston, Anna Dominiczak and Alex Alderton, Roberto Amato, Sonia Goncalves, Ewan Harrison, David K. Jackson, Ian Johnston, Dominic Kwiatkowski, Cordelia Langford, John Sillitoe on behalf of the Wellcome Sanger Institute COVID-19 Surveillance Team
EPI_ISL_731538, EPI_ISL_731539, EPI_ISL_731540	Lighthouse Lab in Alderley Park	Wellcome Sanger Institute for the COVID-19 Genomics UK (COG-UK) Consortium	Jacquelyn Wynn, Mairead Hyland, The Lighthouse Lab in Alderley Park and Alex Alderton, Roberto Amato, Sonia Goncalves, Ewan Harrison, David K. Jackson, Ian Johnston, Dominic Kwiatkowski, Cordelia Langford, John Sillitoe on behalf of the Wellcome Sanger Institute COVID-19 Surveillance Team
EPI_ISL_731541	Lighthouse Lab in Glasgow	Wellcome Sanger Institute for the COVID-19 Genomics UK (COG-UK) Consortium	Harper VanSteenhouse, Yumi Kasai, David Gray, Carol Clugston, Anna Dominiczak and Alex Alderton, Roberto Amato, Sonia Goncalves, Ewan Harrison, David K. Jackson, Ian Johnston, Dominic Kwiatkowski, Cordelia Langford, John Sillitoe on behalf of the Wellcome Sanger Institute COVID-19 Surveillance Team
EPI_ISL_731542, EPI_ISL_731543, EPI_ISL_731545, EPI_ISL_731546, EPI_ISL_731547, EPI_ISL_731549, EPI_ISL_731555, EPI_ISL_731565, EPI_ISL_731570, EPI_ISL_731571, EPI_ISL_731576, EPI_ISL_731580, EPI_ISL_731584, EPI_ISL_731585, EPI_ISL_731588, EPI_ISL_731592, EPI_ISL_731599, EPI_ISL_731611, EPI_ISL_731624, EPI_ISL_731626, EPI_ISL_731629, EPI_ISL_731632, EPI_ISL_731635, EPI_ISL_731639, EPI_ISL_731640, EPI_ISL_731641, EPI_ISL_731644, EPI_ISL_731647, EPI_ISL_731648, EPI_ISL_731649, EPI_ISL_731655, EPI_ISL_731656, EPI_ISL_731664, EPI_ISL_731665, EPI_ISL_731667, EPI_ISL_731671, EPI_ISL_731680, EPI_ISL_731684, EPI_ISL_731686, EPI_ISL_731688, EPI_ISL_731690, EPI_ISL_731691, EPI_ISL_731692, EPI_ISL_731693, EPI_ISL_731697, EPI_ISL_731701, EPI_ISL_731705, EPI_ISL_731707, EPI_ISL_731709, EPI_ISL_731711, EPI_ISL_731714, EPI_ISL_731719, EPI_ISL_731721, EPI_ISL_731722, EPI_ISL_731724, EPI_ISL_731731, EPI_ISL_731736, EPI_ISL_731737, EPI_ISL_731763, EPI_ISL_731767, EPI_ISL_731770, EPI_ISL_731775, EPI_ISL_731785, EPI_ISL_731787, EPI_ISL_731788, EPI_ISL_731792, EPI_ISL_731795, EPI_ISL_731796, EPI_ISL_731799, EPI_ISL_731800, EPI_ISL_731806, EPI_ISL_731808, EPI_ISL_731810, EPI_ISL_731819, EPI_ISL_731824, EPI_ISL_731827, EPI_ISL_731836, EPI_ISL_731838, EPI_ISL_731840, EPI_ISL_731841, EPI_ISL_731842, EPI_ISL_731846, EPI_ISL_731850, EPI_ISL_731854, EPI_ISL_731858, EPI_ISL_731863, EPI_ISL_731868, EPI_ISL_731874, EPI_ISL_731875, EPI_ISL_731877, EPI_ISL_731881, EPI_ISL_731884, EPI_ISL_731885, EPI_ISL_731887, EPI_ISL_731888, EPI_ISL_731889			
see above	Lighthouse Lab in Alderley Park	Wellcome Sanger Institute for the COVID-19 Genomics UK (COG-UK) Consortium	Jacquelyn Wynn, Mairead Hyland, The Lighthouse Lab in Alderley Park and Alex Alderton, Roberto Amato, Sonia Goncalves, Ewan Harrison, David K. Jackson, Ian Johnston, Dominic Kwiatkowski, Cordelia Langford, John Sillitoe on behalf of the Wellcome Sanger Institute COVID-19 Surveillance Team
EPI_ISL_732657	Barnakuten	The Public Health Agency of Sweden	Department of Microbiology, The Public Health Agency of Sweden
EPI_ISL_732962	LabPLUS	Institute of Environmental Science and Research (ESR)	Xiaoyun Ren, Matt Storey, Nikki Freed, Muhammad Faisal, Jing Wang, Hermes Perez, Anja Werno, Antje van der Linden, Arlo Upton, Chris Mansell, David Hammer, Dragana Drinkovic, Gary McAuliffe, Hana Sofia Andersson, James Ussher, Jill Sherwood, Josh Freeman, Julia Howard, Juliet Elvy, Mary DeAlmeida, Matt Blakiston, Matthew Rogers, Max Bloomfield, Michael Addidle, Michelle Balm, Sally Roberts, Sarah Jefferies, Sharmini Muttaiyah, Susan Morpeth, Susan Taylor, Timothy Blackmore, Vani Sathyendran, Veronica Playle, Virginia Hope, Erasmus Smit, Lauren Jelly, Olin Silander, Joep de Ligt
EPI_ISL_732968, EPI_ISL_732969, EPI_ISL_732970	Middlemore Hospital	Institute of Environmental Science and Research (ESR)	Xiaoyun Ren, Matt Storey, Nikki Freed, Muhammad Faisal, Jing Wang, Hermes Perez, Anja Werno, Antje van der Linden, Arlo Upton, Chris Mansell, David Hammer, Dragana Drinkovic, Gary McAuliffe, Hana Sofia Andersson, James Ussher, Jill Sherwood, Josh Freeman, Julia Howard, Juliet Elvy, Mary DeAlmeida, Matt Blakiston, Matthew Rogers, Max Bloomfield, Michael Addidle, Michelle Balm, Sally Roberts, Sarah Jefferies, Sharmini Muttaiyah, Susan Morpeth, Susan Taylor, Timothy Blackmore, Vani Sathyendran, Veronica Playle, Virginia Hope, Erasmus Smit, Lauren Jelly, Olin Silander, Joep de Ligt
EPI_ISL_733573, EPI_ISL_733578, EPI_ISL_733579	Temporary Specimen Collection Centre	Hong Kong Department of Health	Alan K.L. Tsang, Peter C.W. Yip, Edman T.K. Lam, Rickjason C.W. Chan, Dominic N.C. Tsang
EPI_ISL_733589, EPI_ISL_733592	Respiratory Virus Unit, National Infection Service, Public Health England	COVID-19 Genomics UK (COG-UK) Consortium	PHE Covid Sequencing Team
EPI_ISL_733596, EPI_ISL_733597, EPI_ISL_733599	Lighthouse Lab in Cambridge	Wellcome Sanger Institute for the COVID-19 Genomics UK (COG-UK) Consortium	Rob Howes, The Lighthouse Lab in Cambridge and Alex Alderton, Roberto Amato, Sonia Goncalves, Ewan Harrison, David K. Jackson, Ian Johnston, Dominic Kwiatkowski, Cordelia Langford, John Sillitoe on behalf of the Wellcome Sanger Institute COVID-19 Surveillance Team
EPI_ISL_733600	Lighthouse Lab in Milton Keynes	Wellcome Sanger Institute for the COVID-19 Genomics UK (COG-UK) Consortium	The Lighthouse Lab in Milton Keynes and Alex Alderton, Roberto Amato, Sonia Goncalves, Ewan Harrison, David K. Jackson, Ian Johnston, Dominic Kwiatkowski, Cordelia Langford, John Sillitoe on behalf of the Wellcome Sanger Institute COVID-19 Surveillance Team
EPI_ISL_733601, EPI_ISL_733602, EPI_ISL_733604	Lighthouse Lab in Cambridge	Wellcome Sanger Institute for the COVID-19 Genomics UK (COG-UK) Consortium	Rob Howes, The Lighthouse Lab in Cambridge and Alex Alderton, Roberto Amato, Sonia Goncalves, Ewan Harrison, David K. Jackson, Ian Johnston, Dominic Kwiatkowski, Cordelia Langford, John Sillitoe on behalf of the Wellcome Sanger Institute COVID-19 Surveillance Team
EPI_ISL_733607	Lighthouse Lab in Milton Keynes	Wellcome Sanger Institute for the COVID-19 Genomics UK (COG-UK) Consortium	The Lighthouse Lab in Milton Keynes and Alex Alderton, Roberto Amato, Sonia Goncalves, Ewan Harrison, David K. Jackson, Ian Johnston, Dominic Kwiatkowski, Cordelia Langford, John Sillitoe on behalf of the Wellcome Sanger Institute COVID-19 Surveillance Team
EPI_ISL_733610, EPI_ISL_733612, EPI_ISL_733614, EPI_ISL_733615	Lighthouse Lab in Cambridge	Wellcome Sanger Institute for the COVID-19 Genomics UK (COG-UK) Consortium	Rob Howes, The Lighthouse Lab in Cambridge and Alex Alderton, Roberto Amato, Sonia Goncalves, Ewan Harrison, David K. Jackson, Ian Johnston, Dominic Kwiatkowski, Cordelia Langford, John Sillitoe on behalf of the Wellcome Sanger Institute COVID-19 Surveillance Team
EPI_ISL_733616, EPI_ISL_733617	Lighthouse Lab in Milton Keynes	Wellcome Sanger Institute for the COVID-19 Genomics UK (COG-UK) Consortium	The Lighthouse Lab in Milton Keynes and Alex Alderton, Roberto Amato, Sonia Goncalves, Ewan Harrison, David K. Jackson, Ian Johnston, Dominic Kwiatkowski, Cordelia Langford, John Sillitoe on behalf of the Wellcome Sanger Institute COVID-19 Surveillance Team
EPI_ISL_733619, EPI_ISL_733620, EPI_ISL_733622, EPI_ISL_733624, EPI_ISL_733626, EPI_ISL_733627	Lighthouse Lab in Cambridge	Wellcome Sanger Institute for the COVID-19 Genomics UK (COG-UK) Consortium	Rob Howes, The Lighthouse Lab in Cambridge and Alex Alderton, Roberto Amato, Sonia Goncalves, Ewan Harrison, David K. Jackson, Ian Johnston, Dominic Kwiatkowski, Cordelia Langford, John Sillitoe on behalf of the Wellcome Sanger Institute COVID-19 Surveillance Team
EPI_ISL_733628	Lighthouse Lab in Milton Keynes	Wellcome Sanger Institute for the COVID-19 Genomics UK (COG-UK) Consortium	The Lighthouse Lab in Milton Keynes and Alex Alderton, Roberto Amato, Sonia Goncalves, Ewan Harrison, David K. Jackson, Ian Johnston, Dominic Kwiatkowski, Cordelia Langford, John Sillitoe on behalf of the Wellcome Sanger Institute COVID-19 Surveillance Team
EPI_ISL_733629	Lighthouse Lab in Cambridge	Wellcome Sanger Institute for the COVID-19 Genomics UK (COG-UK) Consortium	Rob Howes, The Lighthouse Lab in Cambridge and Alex Alderton, Roberto Amato, Sonia Goncalves, Ewan Harrison, David K. Jackson, Ian Johnston, Dominic Kwiatkowski, Cordelia Langford, John Sillitoe on behalf of the Wellcome Sanger Institute COVID-19 Surveillance Team
EPI_ISL_733630	Lighthouse Lab in Milton Keynes	Wellcome Sanger Institute for the COVID-19 Genomics UK (COG-UK) Consortium	The Lighthouse Lab in Milton Keynes and Alex Alderton, Roberto Amato, Sonia Goncalves, Ewan Harrison, David K. Jackson, Ian Johnston, Dominic Kwiatkowski, Cordelia Langford, John Sillitoe on behalf of the Wellcome Sanger Institute COVID-19 Surveillance Team
EPI_ISL_733631, EPI_ISL_733632, EPI_ISL_733633, EPI_ISL_733634	Lighthouse Lab in Cambridge	Wellcome Sanger Institute for the COVID-19 Genomics UK (COG-UK) Consortium	Rob Howes, The Lighthouse Lab in Cambridge and Alex Alderton, Roberto Amato, Sonia Goncalves, Ewan Harrison, David K. Jackson, Ian Johnston, Dominic Kwiatkowski, Cordelia Langford, John Sillitoe on behalf of the Wellcome Sanger Institute COVID-19 Surveillance Team
EPI_ISL_733636	Lighthouse Lab in Milton Keynes	Wellcome Sanger Institute for the COVID-19 Genomics UK (COG-UK) Consortium	The Lighthouse Lab in Milton Keynes and Alex Alderton, Roberto Amato, Sonia Goncalves, Ewan Harrison, David K. Jackson, Ian Johnston, Dominic Kwiatkowski, Cordelia Langford, John Sillitoe on behalf of the Wellcome Sanger Institute COVID-19 Surveillance Team
EPI_ISL_733637, EPI_ISL_733640	Lighthouse Lab in Cambridge	Wellcome Sanger Institute for the COVID-19 Genomics UK (COG-UK) Consortium	Rob Howes, The Lighthouse Lab in Cambridge and Alex Alderton, Roberto Amato, Sonia Goncalves, Ewan Harrison, David K. Jackson, Ian Johnston, Dominic Kwiatkowski, Cordelia Langford, John Sillitoe on behalf of the Wellcome Sanger Institute COVID-19 Surveillance Team
EPI_ISL_733644, EPI_ISL_733645	Lighthouse Lab in Milton Keynes	Wellcome Sanger Institute for the COVID-19 Genomics UK (COG-UK) Consortium	The Lighthouse Lab in Milton Keynes and Alex Alderton, Roberto Amato, Sonia Goncalves, Ewan Harrison, David K. Jackson, Ian Johnston, Dominic Kwiatkowski, Cordelia Langford, John Sillitoe on behalf of the Wellcome Sanger Institute COVID-19 Surveillance Team
EPI_ISL_733646	Lighthouse Lab in Cambridge	Wellcome Sanger Institute for the COVID-19 Genomics UK (COG-UK) Consortium	Rob Howes, The Lighthouse Lab in Cambridge and Alex Alderton, Roberto Amato, Sonia Goncalves, Ewan Harrison, David K. Jackson, Ian Johnston, Dominic Kwiatkowski, Cordelia Langford, John Sillitoe on behalf of the Wellcome Sanger Institute COVID-19 Surveillance Team
EPI_ISL_733648	Lighthouse Lab in Milton Keynes	Wellcome Sanger Institute for the COVID-19 Genomics UK (COG-UK) Consortium	The Lighthouse Lab in Milton Keynes and Alex Alderton, Roberto Amato, Sonia Goncalves, Ewan Harrison, David K. Jackson, Ian Johnston, Dominic Kwiatkowski, Cordelia Langford, John Sillitoe on behalf of the Wellcome Sanger Institute COVID-19 Surveillance Team
EPI_ISL_733649, EPI_ISL_733650, EPI_ISL_733651	Lighthouse Lab in Cambridge	Wellcome Sanger Institute for the COVID-19 Genomics UK (COG-UK) Consortium	Rob Howes, The Lighthouse Lab in Cambridge and Alex Alderton, Roberto Amato, Sonia Goncalves, Ewan Harrison, David K. Jackson, Ian Johnston, Dominic Kwiatkowski, Cordelia Langford, John Sillitoe on behalf of the Wellcome Sanger Institute COVID-19 Surveillance Team
EPI_ISL_733652	Lighthouse Lab in Milton Keynes	Wellcome Sanger Institute for the COVID-19 Genomics UK (COG-UK) Consortium	The Lighthouse Lab in Milton Keynes and Alex Alderton, Roberto Amato, Sonia Goncalves, Ewan Harrison, David K. Jackson, Ian Johnston, Dominic Kwiatkowski, Cordelia Langford, John Sillitoe on behalf of the Wellcome Sanger Institute COVID-19 Surveillance Team

EPI_ISL_733655, EPI_ISL_733656	Lighthouse Lab in Cambridge	Wellcome Sanger Institute for the COVID-19 Genomics UK (COG-UK) Consortium	Rob Howes, The Lighthouse Lab in Cambridge and Alex Alderton, Roberto Amato, Sonia Goncalves, Ewan Harrison, David K. Jackson, Ian Johnston, Dominic Kwiatkowski, Cordelia Langford, John Sillitoe on behalf of the Wellcome Sanger Institute COVID-19 Surveillance Team
EPI_ISL_733657, EPI_ISL_733658	Lighthouse Lab in Milton Keynes	Wellcome Sanger Institute for the COVID-19 Genomics UK (COG-UK) Consortium	The Lighthouse Lab in Milton Keynes and Alex Alderton, Roberto Amato, Sonia Goncalves, Ewan Harrison, David K. Jackson, Ian Johnston, Dominic Kwiatkowski, Cordelia Langford, John Sillitoe on behalf of the Wellcome Sanger Institute COVID-19 Surveillance Team
EPI_ISL_733660, EPI_ISL_733661, EPI_ISL_733662, EPI_ISL_733663	Lighthouse Lab in Cambridge	Wellcome Sanger Institute for the COVID-19 Genomics UK (COG-UK) Consortium	Rob Howes, The Lighthouse Lab in Cambridge and Alex Alderton, Roberto Amato, Sonia Goncalves, Ewan Harrison, David K. Jackson, Ian Johnston, Dominic Kwiatkowski, Cordelia Langford, John Sillitoe on behalf of the Wellcome Sanger Institute COVID-19 Surveillance Team
EPI_ISL_733664	Lighthouse Lab in Milton Keynes	Wellcome Sanger Institute for the COVID-19 Genomics UK (COG-UK) Consortium	The Lighthouse Lab in Milton Keynes and Alex Alderton, Roberto Amato, Sonia Goncalves, Ewan Harrison, David K. Jackson, Ian Johnston, Dominic Kwiatkowski, Cordelia Langford, John Sillitoe on behalf of the Wellcome Sanger Institute COVID-19 Surveillance Team
EPI_ISL_733665	Lighthouse Lab in Cambridge	Wellcome Sanger Institute for the COVID-19 Genomics UK (COG-UK) Consortium	Rob Howes, The Lighthouse Lab in Cambridge and Alex Alderton, Roberto Amato, Sonia Goncalves, Ewan Harrison, David K. Jackson, Ian Johnston, Dominic Kwiatkowski, Cordelia Langford, John Sillitoe on behalf of the Wellcome Sanger Institute COVID-19 Surveillance Team
EPI_ISL_733666	Lighthouse Lab in Milton Keynes	Wellcome Sanger Institute for the COVID-19 Genomics UK (COG-UK) Consortium	The Lighthouse Lab in Milton Keynes and Alex Alderton, Roberto Amato, Sonia Goncalves, Ewan Harrison, David K. Jackson, Ian Johnston, Dominic Kwiatkowski, Cordelia Langford, John Sillitoe on behalf of the Wellcome Sanger Institute COVID-19 Surveillance Team
EPI_ISL_733667, EPI_ISL_733668	Lighthouse Lab in Cambridge	Wellcome Sanger Institute for the COVID-19 Genomics UK (COG-UK) Consortium	Rob Howes, The Lighthouse Lab in Cambridge and Alex Alderton, Roberto Amato, Sonia Goncalves, Ewan Harrison, David K. Jackson, Ian Johnston, Dominic Kwiatkowski, Cordelia Langford, John Sillitoe on behalf of the Wellcome Sanger Institute COVID-19 Surveillance Team
EPI_ISL_733669	Lighthouse Lab in Milton Keynes	Wellcome Sanger Institute for the COVID-19 Genomics UK (COG-UK) Consortium	The Lighthouse Lab in Milton Keynes and Alex Alderton, Roberto Amato, Sonia Goncalves, Ewan Harrison, David K. Jackson, Ian Johnston, Dominic Kwiatkowski, Cordelia Langford, John Sillitoe on behalf of the Wellcome Sanger Institute COVID-19 Surveillance Team
EPI_ISL_733670, EPI_ISL_733671, EPI_ISL_733672	Lighthouse Lab in Cambridge	Wellcome Sanger Institute for the COVID-19 Genomics UK (COG-UK) Consortium	Rob Howes, The Lighthouse Lab in Cambridge and Alex Alderton, Roberto Amato, Sonia Goncalves, Ewan Harrison, David K. Jackson, Ian Johnston, Dominic Kwiatkowski, Cordelia Langford, John Sillitoe on behalf of the Wellcome Sanger Institute COVID-19 Surveillance Team
EPI_ISL_733673	Lighthouse Lab in Milton Keynes	Wellcome Sanger Institute for the COVID-19 Genomics UK (COG-UK) Consortium	The Lighthouse Lab in Milton Keynes and Alex Alderton, Roberto Amato, Sonia Goncalves, Ewan Harrison, David K. Jackson, Ian Johnston, Dominic Kwiatkowski, Cordelia Langford, John Sillitoe on behalf of the Wellcome Sanger Institute COVID-19 Surveillance Team
EPI_ISL_733674, EPI_ISL_733675, EPI_ISL_733676, EPI_ISL_733677	Lighthouse Lab in Cambridge	Wellcome Sanger Institute for the COVID-19 Genomics UK (COG-UK) Consortium	Rob Howes, The Lighthouse Lab in Cambridge and Alex Alderton, Roberto Amato, Sonia Goncalves, Ewan Harrison, David K. Jackson, Ian Johnston, Dominic Kwiatkowski, Cordelia Langford, John Sillitoe on behalf of the Wellcome Sanger Institute COVID-19 Surveillance Team
EPI_ISL_733678, EPI_ISL_733679	Lighthouse Lab in Milton Keynes	Wellcome Sanger Institute for the COVID-19 Genomics UK (COG-UK) Consortium	The Lighthouse Lab in Milton Keynes and Alex Alderton, Roberto Amato, Sonia Goncalves, Ewan Harrison, David K. Jackson, Ian Johnston, Dominic Kwiatkowski, Cordelia Langford, John Sillitoe on behalf of the Wellcome Sanger Institute COVID-19 Surveillance Team
EPI_ISL_733680, EPI_ISL_733681, EPI_ISL_733684	Lighthouse Lab in Cambridge	Wellcome Sanger Institute for the COVID-19 Genomics UK (COG-UK) Consortium	Rob Howes, The Lighthouse Lab in Cambridge and Alex Alderton, Roberto Amato, Sonia Goncalves, Ewan Harrison, David K. Jackson, Ian Johnston, Dominic Kwiatkowski, Cordelia Langford, John Sillitoe on behalf of the Wellcome Sanger Institute COVID-19 Surveillance Team
EPI_ISL_733686, EPI_ISL_733687	Lighthouse Lab in Milton Keynes	Wellcome Sanger Institute for the COVID-19 Genomics UK (COG-UK) Consortium	The Lighthouse Lab in Milton Keynes and Alex Alderton, Roberto Amato, Sonia Goncalves, Ewan Harrison, David K. Jackson, Ian Johnston, Dominic Kwiatkowski, Cordelia Langford, John Sillitoe on behalf of the Wellcome Sanger Institute COVID-19 Surveillance Team
EPI_ISL_733688, EPI_ISL_733689, EPI_ISL_733690, EPI_ISL_733691	Lighthouse Lab in Cambridge	Wellcome Sanger Institute for the COVID-19 Genomics UK (COG-UK) Consortium	Rob Howes, The Lighthouse Lab in Cambridge and Alex Alderton, Roberto Amato, Sonia Goncalves, Ewan Harrison, David K. Jackson, Ian Johnston, Dominic Kwiatkowski, Cordelia Langford, John Sillitoe on behalf of the Wellcome Sanger Institute COVID-19 Surveillance Team
EPI_ISL_733692	Lighthouse Lab in Milton Keynes	Wellcome Sanger Institute for the COVID-19 Genomics UK (COG-UK) Consortium	The Lighthouse Lab in Milton Keynes and Alex Alderton, Roberto Amato, Sonia Goncalves, Ewan Harrison, David K. Jackson, Ian Johnston, Dominic Kwiatkowski, Cordelia Langford, John Sillitoe on behalf of the Wellcome Sanger Institute COVID-19 Surveillance Team
EPI_ISL_733694, EPI_ISL_733695, EPI_ISL_733696, EPI_ISL_733698, EPI_ISL_733700, EPI_ISL_733702, EPI_ISL_733704	Lighthouse Lab in Cambridge	Wellcome Sanger Institute for the COVID-19 Genomics UK (COG-UK) Consortium	Rob Howes, The Lighthouse Lab in Cambridge and Alex Alderton, Roberto Amato, Sonia Goncalves, Ewan Harrison, David K. Jackson, Ian Johnston, Dominic Kwiatkowski, Cordelia Langford, John Sillitoe on behalf of the Wellcome Sanger Institute COVID-19 Surveillance Team
EPI_ISL_733707	Lighthouse Lab in Milton Keynes	Wellcome Sanger Institute for the COVID-19 Genomics UK (COG-UK) Consortium	The Lighthouse Lab in Milton Keynes and Alex Alderton, Roberto Amato, Sonia Goncalves, Ewan Harrison, David K. Jackson, Ian Johnston, Dominic Kwiatkowski, Cordelia Langford, John Sillitoe on behalf of the Wellcome Sanger Institute COVID-19 Surveillance Team
EPI_ISL_733708, EPI_ISL_733709	Lighthouse Lab in Cambridge	Wellcome Sanger Institute for the COVID-19 Genomics UK (COG-UK) Consortium	Rob Howes, The Lighthouse Lab in Cambridge and Alex Alderton, Roberto Amato, Sonia Goncalves, Ewan Harrison, David K. Jackson, Ian Johnston, Dominic Kwiatkowski, Cordelia Langford, John Sillitoe on behalf of the Wellcome Sanger Institute COVID-19 Surveillance Team
EPI_ISL_733711, EPI_ISL_733713	Lighthouse Lab in Milton Keynes	Wellcome Sanger Institute for the COVID-19 Genomics UK (COG-UK) Consortium	The Lighthouse Lab in Milton Keynes and Alex Alderton, Roberto Amato, Sonia Goncalves, Ewan Harrison, David K. Jackson, Ian Johnston, Dominic Kwiatkowski, Cordelia Langford, John Sillitoe on behalf of the Wellcome Sanger Institute COVID-19 Surveillance Team
EPI_ISL_733714, EPI_ISL_733715, EPI_ISL_733716, EPI_ISL_733717, EPI_ISL_733719, EPI_ISL_733720, EPI_ISL_733721, EPI_ISL_733722, EPI_ISL_733723, EPI_ISL_733724, EPI_ISL_733726, EPI_ISL_733727, EPI_ISL_733728, EPI_ISL_733729, EPI_ISL_733730, EPI_ISL_733732, EPI_ISL_733733			
see above	Lighthouse Lab in Cambridge	Wellcome Sanger Institute for the COVID-19 Genomics UK (COG-UK) Consortium	Rob Howes, The Lighthouse Lab in Cambridge and Alex Alderton, Roberto Amato, Sonia Goncalves, Ewan Harrison, David K. Jackson, Ian Johnston, Dominic Kwiatkowski, Cordelia Langford, John Sillitoe on behalf of the Wellcome Sanger Institute COVID-19 Surveillance Team
EPI_ISL_733735	Lighthouse Lab in Milton Keynes	Wellcome Sanger Institute for the COVID-19 Genomics UK (COG-UK) Consortium	The Lighthouse Lab in Milton Keynes and Alex Alderton, Roberto Amato, Sonia Goncalves, Ewan Harrison, David K. Jackson, Ian Johnston, Dominic Kwiatkowski, Cordelia Langford, John Sillitoe on behalf of the Wellcome Sanger Institute COVID-19 Surveillance Team
EPI_ISL_733736, EPI_ISL_733738	Lighthouse Lab in Cambridge	Wellcome Sanger Institute for the COVID-19 Genomics UK (COG-UK) Consortium	Rob Howes, The Lighthouse Lab in Cambridge and Alex Alderton, Roberto Amato, Sonia Goncalves, Ewan Harrison, David K. Jackson, Ian Johnston, Dominic Kwiatkowski, Cordelia Langford, John Sillitoe on behalf of the Wellcome Sanger Institute COVID-19 Surveillance Team
EPI_ISL_733739	Lighthouse Lab in Milton Keynes	Wellcome Sanger Institute for the COVID-19 Genomics UK (COG-UK) Consortium	The Lighthouse Lab in Milton Keynes and Alex Alderton, Roberto Amato, Sonia Goncalves, Ewan Harrison, David K. Jackson, Ian Johnston, Dominic Kwiatkowski, Cordelia Langford, John Sillitoe on behalf of the Wellcome Sanger Institute COVID-19 Surveillance Team
EPI_ISL_733740, EPI_ISL_733741	Lighthouse Lab in Cambridge	Wellcome Sanger Institute for the COVID-19 Genomics UK (COG-UK) Consortium	Rob Howes, The Lighthouse Lab in Cambridge and Alex Alderton, Roberto Amato, Sonia Goncalves, Ewan Harrison, David K. Jackson, Ian Johnston, Dominic Kwiatkowski, Cordelia Langford, John Sillitoe on behalf of the Wellcome Sanger Institute COVID-19 Surveillance Team
EPI_ISL_733743	Lighthouse Lab in Milton Keynes	Wellcome Sanger Institute for the COVID-19 Genomics UK (COG-UK) Consortium	The Lighthouse Lab in Milton Keynes and Alex Alderton, Roberto Amato, Sonia Goncalves, Ewan Harrison, David K. Jackson, Ian Johnston, Dominic Kwiatkowski, Cordelia Langford, John Sillitoe on behalf of the Wellcome Sanger Institute COVID-19 Surveillance Team
EPI_ISL_733744, EPI_ISL_733745	Lighthouse Lab in Cambridge	Wellcome Sanger Institute for the COVID-19 Genomics UK (COG-UK) Consortium	Rob Howes, The Lighthouse Lab in Cambridge and Alex Alderton, Roberto Amato, Sonia Goncalves, Ewan Harrison, David K. Jackson, Ian Johnston, Dominic Kwiatkowski, Cordelia Langford, John Sillitoe on behalf of the Wellcome Sanger Institute COVID-19 Surveillance Team
EPI_ISL_733746, EPI_ISL_733747, EPI_ISL_733748, EPI_ISL_733749	Lighthouse Lab in Milton Keynes	Wellcome Sanger Institute for the COVID-19 Genomics UK (COG-UK) Consortium	The Lighthouse Lab in Milton Keynes and Alex Alderton, Roberto Amato, Sonia Goncalves, Ewan Harrison, David K. Jackson, Ian Johnston, Dominic Kwiatkowski, Cordelia Langford, John Sillitoe on behalf of the Wellcome Sanger Institute COVID-19 Surveillance Team
EPI_ISL_733750, EPI_ISL_733751, EPI_ISL_733752	Lighthouse Lab in Cambridge	Wellcome Sanger Institute for the COVID-19 Genomics UK (COG-UK) Consortium	Rob Howes, The Lighthouse Lab in Cambridge and Alex Alderton, Roberto Amato, Sonia Goncalves, Ewan Harrison, David K. Jackson, Ian Johnston, Dominic Kwiatkowski, Cordelia Langford, John Sillitoe on behalf of the Wellcome Sanger Institute COVID-19 Surveillance Team
EPI_ISL_733756, EPI_ISL_733757	Lighthouse Lab in Milton Keynes	Wellcome Sanger Institute for the COVID-19 Genomics UK (COG-UK) Consortium	The Lighthouse Lab in Milton Keynes and Alex Alderton, Roberto Amato, Sonia Goncalves, Ewan Harrison, David K. Jackson, Ian Johnston, Dominic Kwiatkowski, Cordelia Langford, John Sillitoe on behalf of the Wellcome Sanger Institute COVID-19 Surveillance Team
EPI_ISL_733758	Lighthouse Lab in Cambridge	Wellcome Sanger Institute for the COVID-19 Genomics UK (COG-UK) Consortium	Rob Howes, The Lighthouse Lab in Cambridge and Alex Alderton, Roberto Amato, Sonia Goncalves, Ewan Harrison, David K. Jackson, Ian Johnston, Dominic Kwiatkowski, Cordelia Langford, John Sillitoe on behalf of the Wellcome Sanger Institute COVID-19 Surveillance Team
EPI_ISL_733760, EPI_ISL_733761	Lighthouse Lab in Milton Keynes	Wellcome Sanger Institute for the COVID-19 Genomics UK (COG-UK) Consortium	The Lighthouse Lab in Milton Keynes and Alex Alderton, Roberto Amato, Sonia Goncalves, Ewan Harrison, David K. Jackson, Ian Johnston, Dominic Kwiatkowski, Cordelia Langford, John Sillitoe on behalf of the Wellcome Sanger Institute COVID-19 Surveillance Team
EPI_ISL_733763	Lighthouse Lab in Cambridge	Wellcome Sanger Institute for the COVID-19 Genomics UK (COG-UK) Consortium	Rob Howes, The Lighthouse Lab in Cambridge and Alex Alderton, Roberto Amato, Sonia Goncalves, Ewan Harrison, David K. Jackson, Ian Johnston, Dominic Kwiatkowski, Cordelia Langford, John Sillitoe on behalf of the Wellcome Sanger Institute COVID-19 Surveillance Team
EPI_ISL_733764	Lighthouse Lab in Milton Keynes	Wellcome Sanger Institute for the COVID-19 Genomics UK (COG-UK) Consortium	The Lighthouse Lab in Milton Keynes and Alex Alderton, Roberto Amato, Sonia Goncalves, Ewan Harrison, David K. Jackson, Ian Johnston, Dominic Kwiatkowski, Cordelia Langford, John Sillitoe on behalf of the Wellcome Sanger Institute COVID-19 Surveillance Team

EPI_ISL_733765, EPI_ISL_733767	Lighthouse Lab in Cambridge	Wellcome Sanger Institute for the COVID-19 Genomics UK (COG-UK) Consortium	Rob Howes, The Lighthouse Lab in Cambridge and Alex Alderton, Roberto Amato, Sonia Goncalves, Ewan Harrison, David K. Jackson, Ian Johnston, Dominic Kwiatkowski, Cordelia Langford, John Sillitoe on behalf of the Wellcome Sanger Institute COVID-19 Surveillance Team
EPI_ISL_733768	Lighthouse Lab in Milton Keynes	Wellcome Sanger Institute for the COVID-19 Genomics UK (COG-UK) Consortium	The Lighthouse Lab in Milton Keynes and Alex Alderton, Roberto Amato, Sonia Goncalves, Ewan Harrison, David K. Jackson, Ian Johnston, Dominic Kwiatkowski, Cordelia Langford, John Sillitoe on behalf of the Wellcome Sanger Institute COVID-19 Surveillance Team
EPI_ISL_733770	Lighthouse Lab in Cambridge	Wellcome Sanger Institute for the COVID-19 Genomics UK (COG-UK) Consortium	Rob Howes, The Lighthouse Lab in Cambridge and Alex Alderton, Roberto Amato, Sonia Goncalves, Ewan Harrison, David K. Jackson, Ian Johnston, Dominic Kwiatkowski, Cordelia Langford, John Sillitoe on behalf of the Wellcome Sanger Institute COVID-19 Surveillance Team
EPI_ISL_733771	Lighthouse Lab in Milton Keynes	Wellcome Sanger Institute for the COVID-19 Genomics UK (COG-UK) Consortium	The Lighthouse Lab in Milton Keynes and Alex Alderton, Roberto Amato, Sonia Goncalves, Ewan Harrison, David K. Jackson, Ian Johnston, Dominic Kwiatkowski, Cordelia Langford, John Sillitoe on behalf of the Wellcome Sanger Institute COVID-19 Surveillance Team
EPI_ISL_733772, EPI_ISL_733773, EPI_ISL_733774, EPI_ISL_733775	Lighthouse Lab in Cambridge	Wellcome Sanger Institute for the COVID-19 Genomics UK (COG-UK) Consortium	Rob Howes, The Lighthouse Lab in Cambridge and Alex Alderton, Roberto Amato, Sonia Goncalves, Ewan Harrison, David K. Jackson, Ian Johnston, Dominic Kwiatkowski, Cordelia Langford, John Sillitoe on behalf of the Wellcome Sanger Institute COVID-19 Surveillance Team
EPI_ISL_733776	Lighthouse Lab in Milton Keynes	Wellcome Sanger Institute for the COVID-19 Genomics UK (COG-UK) Consortium	The Lighthouse Lab in Milton Keynes and Alex Alderton, Roberto Amato, Sonia Goncalves, Ewan Harrison, David K. Jackson, Ian Johnston, Dominic Kwiatkowski, Cordelia Langford, John Sillitoe on behalf of the Wellcome Sanger Institute COVID-19 Surveillance Team
EPI_ISL_733778, EPI_ISL_733779	Lighthouse Lab in Cambridge	Wellcome Sanger Institute for the COVID-19 Genomics UK (COG-UK) Consortium	Rob Howes, The Lighthouse Lab in Cambridge and Alex Alderton, Roberto Amato, Sonia Goncalves, Ewan Harrison, David K. Jackson, Ian Johnston, Dominic Kwiatkowski, Cordelia Langford, John Sillitoe on behalf of the Wellcome Sanger Institute COVID-19 Surveillance Team
EPI_ISL_733780, EPI_ISL_733781	Lighthouse Lab in Milton Keynes	Wellcome Sanger Institute for the COVID-19 Genomics UK (COG-UK) Consortium	The Lighthouse Lab in Milton Keynes and Alex Alderton, Roberto Amato, Sonia Goncalves, Ewan Harrison, David K. Jackson, Ian Johnston, Dominic Kwiatkowski, Cordelia Langford, John Sillitoe on behalf of the Wellcome Sanger Institute COVID-19 Surveillance Team
EPI_ISL_733782, EPI_ISL_733784, EPI_ISL_733786, EPI_ISL_733787, EPI_ISL_733788	Lighthouse Lab in Cambridge	Wellcome Sanger Institute for the COVID-19 Genomics UK (COG-UK) Consortium	Rob Howes, The Lighthouse Lab in Cambridge and Alex Alderton, Roberto Amato, Sonia Goncalves, Ewan Harrison, David K. Jackson, Ian Johnston, Dominic Kwiatkowski, Cordelia Langford, John Sillitoe on behalf of the Wellcome Sanger Institute COVID-19 Surveillance Team
EPI_ISL_733789	Lighthouse Lab in Milton Keynes	Wellcome Sanger Institute for the COVID-19 Genomics UK (COG-UK) Consortium	The Lighthouse Lab in Milton Keynes and Alex Alderton, Roberto Amato, Sonia Goncalves, Ewan Harrison, David K. Jackson, Ian Johnston, Dominic Kwiatkowski, Cordelia Langford, John Sillitoe on behalf of the Wellcome Sanger Institute COVID-19 Surveillance Team
EPI_ISL_733790, EPI_ISL_733791, EPI_ISL_733792, EPI_ISL_733793	Lighthouse Lab in Cambridge	Wellcome Sanger Institute for the COVID-19 Genomics UK (COG-UK) Consortium	Rob Howes, The Lighthouse Lab in Cambridge and Alex Alderton, Roberto Amato, Sonia Goncalves, Ewan Harrison, David K. Jackson, Ian Johnston, Dominic Kwiatkowski, Cordelia Langford, John Sillitoe on behalf of the Wellcome Sanger Institute COVID-19 Surveillance Team
EPI_ISL_733796, EPI_ISL_733798	Lighthouse Lab in Milton Keynes	Wellcome Sanger Institute for the COVID-19 Genomics UK (COG-UK) Consortium	The Lighthouse Lab in Milton Keynes and Alex Alderton, Roberto Amato, Sonia Goncalves, Ewan Harrison, David K. Jackson, Ian Johnston, Dominic Kwiatkowski, Cordelia Langford, John Sillitoe on behalf of the Wellcome Sanger Institute COVID-19 Surveillance Team
EPI_ISL_733799, EPI_ISL_733800	Lighthouse Lab in Cambridge	Wellcome Sanger Institute for the COVID-19 Genomics UK (COG-UK) Consortium	Rob Howes, The Lighthouse Lab in Cambridge and Alex Alderton, Roberto Amato, Sonia Goncalves, Ewan Harrison, David K. Jackson, Ian Johnston, Dominic Kwiatkowski, Cordelia Langford, John Sillitoe on behalf of the Wellcome Sanger Institute COVID-19 Surveillance Team
EPI_ISL_733801	Lighthouse Lab in Milton Keynes	Wellcome Sanger Institute for the COVID-19 Genomics UK (COG-UK) Consortium	The Lighthouse Lab in Milton Keynes and Alex Alderton, Roberto Amato, Sonia Goncalves, Ewan Harrison, David K. Jackson, Ian Johnston, Dominic Kwiatkowski, Cordelia Langford, John Sillitoe on behalf of the Wellcome Sanger Institute COVID-19 Surveillance Team
EPI_ISL_733802, EPI_ISL_733804, EPI_ISL_733805, EPI_ISL_733809, EPI_ISL_733810	Lighthouse Lab in Cambridge	Wellcome Sanger Institute for the COVID-19 Genomics UK (COG-UK) Consortium	Rob Howes, The Lighthouse Lab in Cambridge and Alex Alderton, Roberto Amato, Sonia Goncalves, Ewan Harrison, David K. Jackson, Ian Johnston, Dominic Kwiatkowski, Cordelia Langford, John Sillitoe on behalf of the Wellcome Sanger Institute COVID-19 Surveillance Team
EPI_ISL_733811	Lighthouse Lab in Milton Keynes	Wellcome Sanger Institute for the COVID-19 Genomics UK (COG-UK) Consortium	The Lighthouse Lab in Milton Keynes and Alex Alderton, Roberto Amato, Sonia Goncalves, Ewan Harrison, David K. Jackson, Ian Johnston, Dominic Kwiatkowski, Cordelia Langford, John Sillitoe on behalf of the Wellcome Sanger Institute COVID-19 Surveillance Team
EPI_ISL_733812	Lighthouse Lab in Cambridge	Wellcome Sanger Institute for the COVID-19 Genomics UK (COG-UK) Consortium	Rob Howes, The Lighthouse Lab in Cambridge and Alex Alderton, Roberto Amato, Sonia Goncalves, Ewan Harrison, David K. Jackson, Ian Johnston, Dominic Kwiatkowski, Cordelia Langford, John Sillitoe on behalf of the Wellcome Sanger Institute COVID-19 Surveillance Team
EPI_ISL_733813	Lighthouse Lab in Milton Keynes	Wellcome Sanger Institute for the COVID-19 Genomics UK (COG-UK) Consortium	The Lighthouse Lab in Milton Keynes and Alex Alderton, Roberto Amato, Sonia Goncalves, Ewan Harrison, David K. Jackson, Ian Johnston, Dominic Kwiatkowski, Cordelia Langford, John Sillitoe on behalf of the Wellcome Sanger Institute COVID-19 Surveillance Team
EPI_ISL_733814, EPI_ISL_733816, EPI_ISL_733817	Lighthouse Lab in Cambridge	Wellcome Sanger Institute for the COVID-19 Genomics UK (COG-UK) Consortium	Rob Howes, The Lighthouse Lab in Cambridge and Alex Alderton, Roberto Amato, Sonia Goncalves, Ewan Harrison, David K. Jackson, Ian Johnston, Dominic Kwiatkowski, Cordelia Langford, John Sillitoe on behalf of the Wellcome Sanger Institute COVID-19 Surveillance Team
EPI_ISL_733820	Lighthouse Lab in Milton Keynes	Wellcome Sanger Institute for the COVID-19 Genomics UK (COG-UK) Consortium	The Lighthouse Lab in Milton Keynes and Alex Alderton, Roberto Amato, Sonia Goncalves, Ewan Harrison, David K. Jackson, Ian Johnston, Dominic Kwiatkowski, Cordelia Langford, John Sillitoe on behalf of the Wellcome Sanger Institute COVID-19 Surveillance Team
EPI_ISL_733821, EPI_ISL_733822, EPI_ISL_733823, EPI_ISL_733824	Lighthouse Lab in Cambridge	Wellcome Sanger Institute for the COVID-19 Genomics UK (COG-UK) Consortium	Rob Howes, The Lighthouse Lab in Cambridge and Alex Alderton, Roberto Amato, Sonia Goncalves, Ewan Harrison, David K. Jackson, Ian Johnston, Dominic Kwiatkowski, Cordelia Langford, John Sillitoe on behalf of the Wellcome Sanger Institute COVID-19 Surveillance Team
EPI_ISL_733825, EPI_ISL_733826	Lighthouse Lab in Milton Keynes	Wellcome Sanger Institute for the COVID-19 Genomics UK (COG-UK) Consortium	The Lighthouse Lab in Milton Keynes and Alex Alderton, Roberto Amato, Sonia Goncalves, Ewan Harrison, David K. Jackson, Ian Johnston, Dominic Kwiatkowski, Cordelia Langford, John Sillitoe on behalf of the Wellcome Sanger Institute COVID-19 Surveillance Team
EPI_ISL_733827	Lighthouse Lab in Cambridge	Wellcome Sanger Institute for the COVID-19 Genomics UK (COG-UK) Consortium	Rob Howes, The Lighthouse Lab in Cambridge and Alex Alderton, Roberto Amato, Sonia Goncalves, Ewan Harrison, David K. Jackson, Ian Johnston, Dominic Kwiatkowski, Cordelia Langford, John Sillitoe on behalf of the Wellcome Sanger Institute COVID-19 Surveillance Team
EPI_ISL_733828, EPI_ISL_733829	Lighthouse Lab in Milton Keynes	Wellcome Sanger Institute for the COVID-19 Genomics UK (COG-UK) Consortium	The Lighthouse Lab in Milton Keynes and Alex Alderton, Roberto Amato, Sonia Goncalves, Ewan Harrison, David K. Jackson, Ian Johnston, Dominic Kwiatkowski, Cordelia Langford, John Sillitoe on behalf of the Wellcome Sanger Institute COVID-19 Surveillance Team
EPI_ISL_733831	Lighthouse Lab in Cambridge	Wellcome Sanger Institute for the COVID-19 Genomics UK (COG-UK) Consortium	Rob Howes, The Lighthouse Lab in Cambridge and Alex Alderton, Roberto Amato, Sonia Goncalves, Ewan Harrison, David K. Jackson, Ian Johnston, Dominic Kwiatkowski, Cordelia Langford, John Sillitoe on behalf of the Wellcome Sanger Institute COVID-19 Surveillance Team
EPI_ISL_733832	Lighthouse Lab in Milton Keynes	Wellcome Sanger Institute for the COVID-19 Genomics UK (COG-UK) Consortium	The Lighthouse Lab in Milton Keynes and Alex Alderton, Roberto Amato, Sonia Goncalves, Ewan Harrison, David K. Jackson, Ian Johnston, Dominic Kwiatkowski, Cordelia Langford, John Sillitoe on behalf of the Wellcome Sanger Institute COVID-19 Surveillance Team
EPI_ISL_733834, EPI_ISL_733835	Lighthouse Lab in Cambridge	Wellcome Sanger Institute for the COVID-19 Genomics UK (COG-UK) Consortium	Rob Howes, The Lighthouse Lab in Cambridge and Alex Alderton, Roberto Amato, Sonia Goncalves, Ewan Harrison, David K. Jackson, Ian Johnston, Dominic Kwiatkowski, Cordelia Langford, John Sillitoe on behalf of the Wellcome Sanger Institute COVID-19 Surveillance Team
EPI_ISL_733836, EPI_ISL_733838	Lighthouse Lab in Milton Keynes	Wellcome Sanger Institute for the COVID-19 Genomics UK (COG-UK) Consortium	The Lighthouse Lab in Milton Keynes and Alex Alderton, Roberto Amato, Sonia Goncalves, Ewan Harrison, David K. Jackson, Ian Johnston, Dominic Kwiatkowski, Cordelia Langford, John Sillitoe on behalf of the Wellcome Sanger Institute COVID-19 Surveillance Team
EPI_ISL_733839, EPI_ISL_733840, EPI_ISL_733841	Lighthouse Lab in Cambridge	Wellcome Sanger Institute for the COVID-19 Genomics UK (COG-UK) Consortium	Rob Howes, The Lighthouse Lab in Cambridge and Alex Alderton, Roberto Amato, Sonia Goncalves, Ewan Harrison, David K. Jackson, Ian Johnston, Dominic Kwiatkowski, Cordelia Langford, John Sillitoe on behalf of the Wellcome Sanger Institute COVID-19 Surveillance Team
EPI_ISL_733842, EPI_ISL_733845	Lighthouse Lab in Milton Keynes	Wellcome Sanger Institute for the COVID-19 Genomics UK (COG-UK) Consortium	The Lighthouse Lab in Milton Keynes and Alex Alderton, Roberto Amato, Sonia Goncalves, Ewan Harrison, David K. Jackson, Ian Johnston, Dominic Kwiatkowski, Cordelia Langford, John Sillitoe on behalf of the Wellcome Sanger Institute COVID-19 Surveillance Team
EPI_ISL_733846, EPI_ISL_733847, EPI_ISL_733849	Lighthouse Lab in Cambridge	Wellcome Sanger Institute for the COVID-19 Genomics UK (COG-UK) Consortium	Rob Howes, The Lighthouse Lab in Cambridge and Alex Alderton, Roberto Amato, Sonia Goncalves, Ewan Harrison, David K. Jackson, Ian Johnston, Dominic Kwiatkowski, Cordelia Langford, John Sillitoe on behalf of the Wellcome Sanger Institute COVID-19 Surveillance Team
EPI_ISL_733851	Lighthouse Lab in Milton Keynes	Wellcome Sanger Institute for the COVID-19 Genomics UK (COG-UK) Consortium	The Lighthouse Lab in Milton Keynes and Alex Alderton, Roberto Amato, Sonia Goncalves, Ewan Harrison, David K. Jackson, Ian Johnston, Dominic Kwiatkowski, Cordelia Langford, John Sillitoe on behalf of the Wellcome Sanger Institute COVID-19 Surveillance Team
EPI_ISL_733852, EPI_ISL_733853, EPI_ISL_733854	Lighthouse Lab in Cambridge	Wellcome Sanger Institute for the COVID-19 Genomics UK (COG-UK) Consortium	Rob Howes, The Lighthouse Lab in Cambridge and Alex Alderton, Roberto Amato, Sonia Goncalves, Ewan Harrison, David K. Jackson, Ian Johnston, Dominic Kwiatkowski, Cordelia Langford, John Sillitoe on behalf of the Wellcome Sanger Institute COVID-19 Surveillance Team
EPI_ISL_733855	Lighthouse Lab in Milton Keynes	Wellcome Sanger Institute for the COVID-19 Genomics UK (COG-UK) Consortium	The Lighthouse Lab in Milton Keynes and Alex Alderton, Roberto Amato, Sonia Goncalves, Ewan Harrison, David K. Jackson, Ian Johnston, Dominic Kwiatkowski, Cordelia Langford, John Sillitoe on behalf of the Wellcome Sanger Institute COVID-19 Surveillance Team

EPI_ISL_733856, EPI_ISL_733857	Lighthouse Lab in Cambridge	Wellcome Sanger Institute for the COVID-19 Genomics UK (COG-UK) Consortium	Rob Howes, The Lighthouse Lab in Cambridge and Alex Alderton, Roberto Amato, Sonia Goncalves, Ewan Harrison, David K. Jackson, Ian Johnston, Dominic Kwiatkowski, Cordelia Langford, John Sillitoe on behalf of the Wellcome Sanger Institute COVID-19 Surveillance Team
EPI_ISL_733858	Lighthouse Lab in Milton Keynes	Wellcome Sanger Institute for the COVID-19 Genomics UK (COG-UK) Consortium	The Lighthouse Lab in Milton Keynes and Alex Alderton, Roberto Amato, Sonia Goncalves, Ewan Harrison, David K. Jackson, Ian Johnston, Dominic Kwiatkowski, Cordelia Langford, John Sillitoe on behalf of the Wellcome Sanger Institute COVID-19 Surveillance Team
EPI_ISL_733860	Lighthouse Lab in Cambridge	Wellcome Sanger Institute for the COVID-19 Genomics UK (COG-UK) Consortium	Rob Howes, The Lighthouse Lab in Cambridge and Alex Alderton, Roberto Amato, Sonia Goncalves, Ewan Harrison, David K. Jackson, Ian Johnston, Dominic Kwiatkowski, Cordelia Langford, John Sillitoe on behalf of the Wellcome Sanger Institute COVID-19 Surveillance Team
EPI_ISL_733861	Lighthouse Lab in Milton Keynes	Wellcome Sanger Institute for the COVID-19 Genomics UK (COG-UK) Consortium	The Lighthouse Lab in Milton Keynes and Alex Alderton, Roberto Amato, Sonia Goncalves, Ewan Harrison, David K. Jackson, Ian Johnston, Dominic Kwiatkowski, Cordelia Langford, John Sillitoe on behalf of the Wellcome Sanger Institute COVID-19 Surveillance Team
EPI_ISL_733862	Lighthouse Lab in Cambridge	Wellcome Sanger Institute for the COVID-19 Genomics UK (COG-UK) Consortium	Rob Howes, The Lighthouse Lab in Cambridge and Alex Alderton, Roberto Amato, Sonia Goncalves, Ewan Harrison, David K. Jackson, Ian Johnston, Dominic Kwiatkowski, Cordelia Langford, John Sillitoe on behalf of the Wellcome Sanger Institute COVID-19 Surveillance Team
EPI_ISL_733863, EPI_ISL_733864, EPI_ISL_733865	Lighthouse Lab in Milton Keynes	Wellcome Sanger Institute for the COVID-19 Genomics UK (COG-UK) Consortium	The Lighthouse Lab in Milton Keynes and Alex Alderton, Roberto Amato, Sonia Goncalves, Ewan Harrison, David K. Jackson, Ian Johnston, Dominic Kwiatkowski, Cordelia Langford, John Sillitoe on behalf of the Wellcome Sanger Institute COVID-19 Surveillance Team
EPI_ISL_733866, EPI_ISL_733868, EPI_ISL_733869, EPI_ISL_733870, EPI_ISL_733871	Lighthouse Lab in Cambridge	Wellcome Sanger Institute for the COVID-19 Genomics UK (COG-UK) Consortium	Rob Howes, The Lighthouse Lab in Cambridge and Alex Alderton, Roberto Amato, Sonia Goncalves, Ewan Harrison, David K. Jackson, Ian Johnston, Dominic Kwiatkowski, Cordelia Langford, John Sillitoe on behalf of the Wellcome Sanger Institute COVID-19 Surveillance Team
EPI_ISL_733872, EPI_ISL_733873	Lighthouse Lab in Milton Keynes	Wellcome Sanger Institute for the COVID-19 Genomics UK (COG-UK) Consortium	The Lighthouse Lab in Milton Keynes and Alex Alderton, Roberto Amato, Sonia Goncalves, Ewan Harrison, David K. Jackson, Ian Johnston, Dominic Kwiatkowski, Cordelia Langford, John Sillitoe on behalf of the Wellcome Sanger Institute COVID-19 Surveillance Team
EPI_ISL_733874	Lighthouse Lab in Cambridge	Wellcome Sanger Institute for the COVID-19 Genomics UK (COG-UK) Consortium	Rob Howes, The Lighthouse Lab in Cambridge and Alex Alderton, Roberto Amato, Sonia Goncalves, Ewan Harrison, David K. Jackson, Ian Johnston, Dominic Kwiatkowski, Cordelia Langford, John Sillitoe on behalf of the Wellcome Sanger Institute COVID-19 Surveillance Team
EPI_ISL_733875	Lighthouse Lab in Milton Keynes	Wellcome Sanger Institute for the COVID-19 Genomics UK (COG-UK) Consortium	The Lighthouse Lab in Milton Keynes and Alex Alderton, Roberto Amato, Sonia Goncalves, Ewan Harrison, David K. Jackson, Ian Johnston, Dominic Kwiatkowski, Cordelia Langford, John Sillitoe on behalf of the Wellcome Sanger Institute COVID-19 Surveillance Team
EPI_ISL_733876, EPI_ISL_733878, EPI_ISL_733881	Lighthouse Lab in Cambridge	Wellcome Sanger Institute for the COVID-19 Genomics UK (COG-UK) Consortium	Rob Howes, The Lighthouse Lab in Cambridge and Alex Alderton, Roberto Amato, Sonia Goncalves, Ewan Harrison, David K. Jackson, Ian Johnston, Dominic Kwiatkowski, Cordelia Langford, John Sillitoe on behalf of the Wellcome Sanger Institute COVID-19 Surveillance Team
EPI_ISL_733884	Lighthouse Lab in Milton Keynes	Wellcome Sanger Institute for the COVID-19 Genomics UK (COG-UK) Consortium	The Lighthouse Lab in Milton Keynes and Alex Alderton, Roberto Amato, Sonia Goncalves, Ewan Harrison, David K. Jackson, Ian Johnston, Dominic Kwiatkowski, Cordelia Langford, John Sillitoe on behalf of the Wellcome Sanger Institute COVID-19 Surveillance Team
EPI_ISL_733886	Lighthouse Lab in Cambridge	Wellcome Sanger Institute for the COVID-19 Genomics UK (COG-UK) Consortium	Rob Howes, The Lighthouse Lab in Cambridge and Alex Alderton, Roberto Amato, Sonia Goncalves, Ewan Harrison, David K. Jackson, Ian Johnston, Dominic Kwiatkowski, Cordelia Langford, John Sillitoe on behalf of the Wellcome Sanger Institute COVID-19 Surveillance Team
EPI_ISL_733887	Lighthouse Lab in Milton Keynes	Wellcome Sanger Institute for the COVID-19 Genomics UK (COG-UK) Consortium	The Lighthouse Lab in Milton Keynes and Alex Alderton, Roberto Amato, Sonia Goncalves, Ewan Harrison, David K. Jackson, Ian Johnston, Dominic Kwiatkowski, Cordelia Langford, John Sillitoe on behalf of the Wellcome Sanger Institute COVID-19 Surveillance Team
EPI_ISL_733888	Lighthouse Lab in Cambridge	Wellcome Sanger Institute for the COVID-19 Genomics UK (COG-UK) Consortium	Rob Howes, The Lighthouse Lab in Cambridge and Alex Alderton, Roberto Amato, Sonia Goncalves, Ewan Harrison, David K. Jackson, Ian Johnston, Dominic Kwiatkowski, Cordelia Langford, John Sillitoe on behalf of the Wellcome Sanger Institute COVID-19 Surveillance Team
EPI_ISL_733889, EPI_ISL_733890, EPI_ISL_733891	Lighthouse Lab in Milton Keynes	Wellcome Sanger Institute for the COVID-19 Genomics UK (COG-UK) Consortium	The Lighthouse Lab in Milton Keynes and Alex Alderton, Roberto Amato, Sonia Goncalves, Ewan Harrison, David K. Jackson, Ian Johnston, Dominic Kwiatkowski, Cordelia Langford, John Sillitoe on behalf of the Wellcome Sanger Institute COVID-19 Surveillance Team
EPI_ISL_733892	Lighthouse Lab in Cambridge	Wellcome Sanger Institute for the COVID-19 Genomics UK (COG-UK) Consortium	Rob Howes, The Lighthouse Lab in Cambridge and Alex Alderton, Roberto Amato, Sonia Goncalves, Ewan Harrison, David K. Jackson, Ian Johnston, Dominic Kwiatkowski, Cordelia Langford, John Sillitoe on behalf of the Wellcome Sanger Institute COVID-19 Surveillance Team
EPI_ISL_733893, EPI_ISL_733894	Lighthouse Lab in Milton Keynes	Wellcome Sanger Institute for the COVID-19 Genomics UK (COG-UK) Consortium	The Lighthouse Lab in Milton Keynes and Alex Alderton, Roberto Amato, Sonia Goncalves, Ewan Harrison, David K. Jackson, Ian Johnston, Dominic Kwiatkowski, Cordelia Langford, John Sillitoe on behalf of the Wellcome Sanger Institute COVID-19 Surveillance Team
EPI_ISL_733895, EPI_ISL_733896, EPI_ISL_733898	Lighthouse Lab in Cambridge	Wellcome Sanger Institute for the COVID-19 Genomics UK (COG-UK) Consortium	Rob Howes, The Lighthouse Lab in Cambridge and Alex Alderton, Roberto Amato, Sonia Goncalves, Ewan Harrison, David K. Jackson, Ian Johnston, Dominic Kwiatkowski, Cordelia Langford, John Sillitoe on behalf of the Wellcome Sanger Institute COVID-19 Surveillance Team
EPI_ISL_733900, EPI_ISL_733902, EPI_ISL_733903, EPI_ISL_733904, EPI_ISL_733905	Lighthouse Lab in Milton Keynes	Wellcome Sanger Institute for the COVID-19 Genomics UK (COG-UK) Consortium	The Lighthouse Lab in Milton Keynes and Alex Alderton, Roberto Amato, Sonia Goncalves, Ewan Harrison, David K. Jackson, Ian Johnston, Dominic Kwiatkowski, Cordelia Langford, John Sillitoe on behalf of the Wellcome Sanger Institute COVID-19 Surveillance Team
EPI_ISL_733908, EPI_ISL_733909, EPI_ISL_733911	Lighthouse Lab in Cambridge	Wellcome Sanger Institute for the COVID-19 Genomics UK (COG-UK) Consortium	Rob Howes, The Lighthouse Lab in Cambridge and Alex Alderton, Roberto Amato, Sonia Goncalves, Ewan Harrison, David K. Jackson, Ian Johnston, Dominic Kwiatkowski, Cordelia Langford, John Sillitoe on behalf of the Wellcome Sanger Institute COVID-19 Surveillance Team
EPI_ISL_733914	Lighthouse Lab in Milton Keynes	Wellcome Sanger Institute for the COVID-19 Genomics UK (COG-UK) Consortium	The Lighthouse Lab in Milton Keynes and Alex Alderton, Roberto Amato, Sonia Goncalves, Ewan Harrison, David K. Jackson, Ian Johnston, Dominic Kwiatkowski, Cordelia Langford, John Sillitoe on behalf of the Wellcome Sanger Institute COVID-19 Surveillance Team
EPI_ISL_733915	Lighthouse Lab in Cambridge	Wellcome Sanger Institute for the COVID-19 Genomics UK (COG-UK) Consortium	Rob Howes, The Lighthouse Lab in Cambridge and Alex Alderton, Roberto Amato, Sonia Goncalves, Ewan Harrison, David K. Jackson, Ian Johnston, Dominic Kwiatkowski, Cordelia Langford, John Sillitoe on behalf of the Wellcome Sanger Institute COVID-19 Surveillance Team
EPI_ISL_733928, EPI_ISL_733950, EPI_ISL_734041, EPI_ISL_734044, EPI_ISL_734093, EPI_ISL_734117, EPI_ISL_734131	Lighthouse Lab in Glasgow	Wellcome Sanger Institute for the COVID-19 Genomics UK (COG-UK) Consortium	Harper VanSteenhouse, Yumi Kasai, David Gray, Carol Clugston, Anna Dominiczak and Alex Alderton, Roberto Amato, Sonia Goncalves, Ewan Harrison, David K. Jackson, Ian Johnston, Dominic Kwiatkowski, Cordelia Langford, John Sillitoe on behalf of the Wellcome Sanger Institute COVID-19 Surveillance Team
EPI_ISL_736202, EPI_ISL_736240, EPI_ISL_736252, EPI_ISL_736264, EPI_ISL_736274, EPI_ISL_736289, EPI_ISL_736292, EPI_ISL_736293, EPI_ISL_736303, EPI_ISL_736349, EPI_ISL_736369, EPI_ISL_736380, EPI_ISL_736392, EPI_ISL_736408, EPI_ISL_736449			
see above	Lighthouse Lab in Milton Keynes	Wellcome Sanger Institute for the COVID-19 Genomics UK (COG-UK) Consortium	The Lighthouse Lab in Milton Keynes and Alex Alderton, Roberto Amato, Sonia Goncalves, Ewan Harrison, David K. Jackson, Ian Johnston, Dominic Kwiatkowski, Cordelia Langford, John Sillitoe on behalf of the Wellcome Sanger Institute COVID-19 Surveillance Team
EPI_ISL_736458	Lighthouse Lab in Glasgow	Wellcome Sanger Institute for the COVID-19 Genomics UK (COG-UK) Consortium	Harper VanSteenhouse, Yumi Kasai, David Gray, Carol Clugston, Anna Dominiczak and Alex Alderton, Roberto Amato, Sonia Goncalves, Ewan Harrison, David K. Jackson, Ian Johnston, Dominic Kwiatkowski, Cordelia Langford, John Sillitoe on behalf of the Wellcome Sanger Institute COVID-19 Surveillance Team
EPI_ISL_736469	Lighthouse Lab in Milton Keynes	Wellcome Sanger Institute for the COVID-19 Genomics UK (COG-UK) Consortium	The Lighthouse Lab in Milton Keynes and Alex Alderton, Roberto Amato, Sonia Goncalves, Ewan Harrison, David K. Jackson, Ian Johnston, Dominic Kwiatkowski, Cordelia Langford, John Sillitoe on behalf of the Wellcome Sanger Institute COVID-19 Surveillance Team
EPI_ISL_736477	Lighthouse Lab in Glasgow	Wellcome Sanger Institute for the COVID-19 Genomics UK (COG-UK) Consortium	Harper VanSteenhouse, Yumi Kasai, David Gray, Carol Clugston, Anna Dominiczak and Alex Alderton, Roberto Amato, Sonia Goncalves, Ewan Harrison, David K. Jackson, Ian Johnston, Dominic Kwiatkowski, Cordelia Langford, John Sillitoe on behalf of the Wellcome Sanger Institute COVID-19 Surveillance Team
EPI_ISL_736547, EPI_ISL_736686, EPI_ISL_736694, EPI_ISL_736749	Lighthouse Lab in Milton Keynes	Wellcome Sanger Institute for the COVID-19 Genomics UK (COG-UK) Consortium	The Lighthouse Lab in Milton Keynes and Alex Alderton, Roberto Amato, Sonia Goncalves, Ewan Harrison, David K. Jackson, Ian Johnston, Dominic Kwiatkowski, Cordelia Langford, John Sillitoe on behalf of the Wellcome Sanger Institute COVID-19 Surveillance Team
EPI_ISL_736758	Lighthouse Lab in Alderley Park	Wellcome Sanger Institute for the COVID-19 Genomics UK (COG-UK) Consortium	Jacquelyn Wynn, Mairead Hyland, The Lighthouse Lab in Alderley Park and Alex Alderton, Roberto Amato, Sonia Goncalves, Ewan Harrison, David K. Jackson, Ian Johnston, Dominic Kwiatkowski, Cordelia Langford, John Sillitoe on behalf of the Wellcome Sanger Institute COVID-19 Surveillance Team
EPI_ISL_736927	MDS	KRISP, KZN Research Innovation and Sequencing Platform	Giandhari J, Pillay S, Lessells R, ChimukangaraB, Mdlalose K, York D, Khan S, Tegally H, Wilkinson E, de Oliveira T
EPI_ISL_736938, EPI_ISL_736970, EPI_ISL_736971, EPI_ISL_736973, EPI_ISL_736974, EPI_ISL_736975, EPI_ISL_736976, EPI_ISL_736977, EPI_ISL_736978, EPI_ISL_736979, EPI_ISL_736980			
see above	NHLS-IALCH	KRISP, KZN Research Innovation and Sequencing Platform	Giandhari J, Pillay S, Lessells R, ChimukangaraB, Mdlalose K, York D, Khan S, Tegally H, Wilkinson E, de Oliveira T
EPI_ISL_736995	MDS	KRISP, KZN Research Innovation and Sequencing Platform	Giandhari J, Pillay S, Lessells R, ChimukangaraB, Mdlalose K, York D, Khan S, Tegally H, Wilkinson E, de Oliveira T

EPI_ISL_737105, EPI_ISL_737282, EPI_ISL_737301	Michigan Department of Health and Human Services, Bureau of Laboratories	Michigan Department of Health and Human Services, Bureau of Laboratories	Blankenship HM, Riner D, Soehnlen MK
EPI_ISL_737630, EPI_ISL_737857, EPI_ISL_737859, EPI_ISL_737860, EPI_ISL_737861, EPI_ISL_737862	Viollier AG	Department of Biosystems Science and Engineering, ETH Zürich	Chaoran Chen, Sarah Nadeau, Catharine Aquino, Ivan Topolsky, Philipp Jablonski, Lara Fuhrmann, David Dreifuss, Katharina Jahn, Andreia Cabral de Gouvea, Maria Domenica Moccia, Simon Grüter, Timothy Sykes, Lennart Opitz, Griffin White, Laura Neff, Doris Popovic, Andrea Patrignani, Jay Tracy, Ralph Schlapbach, Christiane Beckmann, Maurice Redondo, Olivier Kobel, Christoph Noppen, Sophie Seidel, Noemie Santamaria de Souza, Niko Beerenwinkel, Tanja Stadler
EPI_ISL_738108, EPI_ISL_738112	Instituto Nacional de Saude (INSA)	Instituto Nacional de Saude (INSA)	Borges et al
EPI_ISL_738361, EPI_ISL_738371, EPI_ISL_738389, EPI_ISL_738414, EPI_ISL_738431, EPI_ISL_738437	UZ Leuven, National Reference Laboratory for Coronaviruses, Laboratory Medicine, Leuven, Belgium	KU Leuven, Rega Institute, Clinical and Epidemiological Virology	Tony Wawina-Bokalanga, Joan Marti-Carerras, Bert Vanmechelen, Piet Maes
EPI_ISL_738658	Monterey County Public Health Lab	Chan-Zuckerberg Biohub	CZB Cliahub Consortium
EPI_ISL_740871, EPI_ISL_740872, EPI_ISL_740873, EPI_ISL_740874	South Eastern Area Laboratory Services (SEALS)	NSW Health Pathology - Institute of Clinical Pathology and Medical Research; Westmead Hospital; University of Sydney	CIDM-PH et al.
EPI_ISL_740958, EPI_ISL_740963, EPI_ISL_740964, EPI_ISL_740965, EPI_ISL_740966, EPI_ISL_740967, EPI_ISL_740968, EPI_ISL_740969, EPI_ISL_740970, EPI_ISL_740972, EPI_ISL_740973, EPI_ISL_740974, EPI_ISL_740975, EPI_ISL_740976, EPI_ISL_740977, EPI_ISL_740978, EPI_ISL_740979, EPI_ISL_740980, EPI_ISL_740981, EPI_ISL_740982, EPI_ISL_740983, EPI_ISL_740984, EPI_ISL_740985, EPI_ISL_740990, EPI_ISL_740991, EPI_ISL_740993			
see above	Department of Pathology, University of Cambridge	COVID-19 Genomics UK (COG-UK) Consortium	Aminu S. Jahun, Yasmin Chaudhry, Grant Hall, Iliana Georgana, Myra Hosmillo, Martin D. Curran, Malte Pinckert, Surendra Parmar, Ian Goodfellow
EPI_ISL_741187, EPI_ISL_741207, EPI_ISL_741255, EPI_ISL_741264, EPI_ISL_741265, EPI_ISL_741266, EPI_ISL_741267, EPI_ISL_741268, EPI_ISL_741269, EPI_ISL_741271, EPI_ISL_741272, EPI_ISL_741273, EPI_ISL_741274, EPI_ISL_741276, EPI_ISL_741278, EPI_ISL_741279, EPI_ISL_741280, EPI_ISL_741281, EPI_ISL_741282, EPI_ISL_741283, EPI_ISL_741284, EPI_ISL_741285, EPI_ISL_741286, EPI_ISL_741288, EPI_ISL_741289, EPI_ISL_741290, EPI_ISL_741291, EPI_ISL_741292, EPI_ISL_741293, EPI_ISL_741295, EPI_ISL_741300, EPI_ISL_741303, EPI_ISL_741308			
see above	University College London, Great Ormond Street Hospital for Children NHS Foundation Trust, Imperial College Healthcare NHS Trust	COVID-19 Genomics UK (COG-UK) Consortium	Sergi Castellano, Rachel Williams, Mark Kristiansen, Paola Resende Silva, Sunando Roy, Tony Brooks, Helena Tutill, Paola Niola, Patricia Dyal, Charlotte Williams, Leysa Forrest, Yasmin Panchbhaya, Jacqueline Findlay, Samuel Weeks, Julianne Brown, Kathryn Harris, Paul Randell, James Price, Alison Holmes, Judith Breuer
EPI_ISL_741613	Queens Medical Centre, Clinical Microbiology Department / DeepSeq Nottingham	COVID-19 Genomics UK (COG-UK) Consortium	Gemma Clark, Wendy Smith, Manjinder Khakh, Vicki M Fleming, Michelle M Lister, Hannah Howson-Wells, Jonathan Ball, Patrick McClure, Joseph Chappell, Theocharis Tsoleridis, Nadine Holmes, Matthew Carlisle, Christopher Moore, Fei Sang, Johnny Debebe, Victoria Wright, Matthew Loose
EPI_ISL_741734, EPI_ISL_741750, EPI_ISL_741751, EPI_ISL_741752	Centre for Enzyme Innovation, University of Portsmouth / Translational Research Laboratory, Portsmouth Hospitals NHS Trust	COVID-19 Genomics UK (COG-UK) Consortium	Angela Beckett,Yann Bourgeois,Garry Scarlett,Sharon Glaysher,Scott Elliott,Kelly Bicknell,Robert Impey,Allyson Lloyd,Sarah Wyllie,Ethan Butcher,Anoop Chauhan,Samuel Robson
EPI_ISL_741939, EPI_ISL_741940, EPI_ISL_741941, EPI_ISL_741949, EPI_ISL_741955, EPI_ISL_741959, EPI_ISL_741973, EPI_ISL_741979, EPI_ISL_741987, EPI_ISL_741988, EPI_ISL_741993, EPI_ISL_742001, EPI_ISL_742006, EPI_ISL_742008, EPI_ISL_742011, EPI_ISL_742021, EPI_ISL_742027, EPI_ISL_742029, EPI_ISL_742036, EPI_ISL_742038, EPI_ISL_742042, EPI_ISL_742043, EPI_ISL_742045, EPI_ISL_742053, EPI_ISL_742057, EPI_ISL_742058, EPI_ISL_742085, EPI_ISL_742088, EPI_ISL_742091, EPI_ISL_742093, EPI_ISL_742095, EPI_ISL_742110			
see above	Virology Department, Sheffield Teaching Hospitals NHS Foundation Trust/Department of Infection, Immunity and Cardiovascular Disease, The Medical School, University of Sheffield	COVID-19 Genomics UK (COG-UK) Consortium	Thushan de Silva, Matthew Parker, Nikki Smith, Adri Angyal, Rebecca Brown, Luke Green, Rachel Tucker, Paul Parsons, Danielle Groves, Katie Johnson, Laura Carrilero, Alex Keeley, Dave Partridge, Matthew Wyles, Benjamin Lindsey, Mehmet Yavuz, Mohammad Raza, Cariad Evans
EPI_ISL_742180	Virology Department, Royal Infirmary of Edinburgh, NHS Lothian / School of Biological Sciences, University of Edinburgh / Institute of Genetics and Molecular Medicine, University of Edinburgh	COVID-19 Genomics UK (COG-UK) Consortium	McHugh M, Dewar R, Rooke S, Gallagher M, Balcaza C, O'Toole Á, Scher E, Hill V, McCrone JT, Colquhoun R, Yu X, Jackson B, Rambaut A, Williams TC, Templeton K
EPI_ISL_742263, EPI_ISL_742266, EPI_ISL_742288, EPI_ISL_742289, EPI_ISL_742290, EPI_ISL_742291, EPI_ISL_742292, EPI_ISL_742293, EPI_ISL_742294, EPI_ISL_742295, EPI_ISL_742296, EPI_ISL_742297, EPI_ISL_742356, EPI_ISL_742359, EPI_ISL_742360, EPI_ISL_742361, EPI_ISL_742362, EPI_ISL_742363, EPI_ISL_742488, EPI_ISL_742489, EPI_ISL_742490, EPI_ISL_742491, EPI_ISL_742492, EPI_ISL_742493, EPI_ISL_742494, EPI_ISL_742495, EPI_ISL_742496, EPI_ISL_742497, EPI_ISL_742498, EPI_ISL_742499, EPI_ISL_742500, EPI_ISL_742501, EPI_ISL_742502, EPI_ISL_742503, EPI_ISL_742504, EPI_ISL_742505, EPI_ISL_742506, EPI_ISL_742507, EPI_ISL_742508, EPI_ISL_742509, EPI_ISL_742510, EPI_ISL_742511, EPI_ISL_742512, EPI_ISL_742513, EPI_ISL_742514, EPI_ISL_742515, EPI_ISL_742516, EPI_ISL_742517, EPI_ISL_742518, EPI_ISL_742519, EPI_ISL_742520, EPI_ISL_742521, EPI_ISL_742522, EPI_ISL_742523, EPI_ISL_742524, EPI_ISL_742525, EPI_ISL_742526, EPI_ISL_742527, EPI_ISL_742528, EPI_ISL_742529, EPI_ISL_742531, EPI_ISL_742532, EPI_ISL_742533, EPI_ISL_742534, EPI_ISL_742535, EPI_ISL_742536, EPI_ISL_742537, EPI_ISL_742538, EPI_ISL_742539, EPI_ISL_742540, EPI_ISL_742545, EPI_ISL_742550, EPI_ISL_742551, EPI_ISL_742552, EPI_ISL_742553, EPI_ISL_742554, EPI_ISL_742565, EPI_ISL_742637, EPI_ISL_742638, EPI_ISL_742639, EPI_ISL_742640, EPI_ISL_742646, EPI_ISL_742649, EPI_ISL_742651, EPI_ISL_742653, EPI_ISL_742654, EPI_ISL_742655, EPI_ISL_742656, EPI_ISL_742657, EPI_ISL_742658, EPI_ISL_742659, EPI_ISL_742660, EPI_ISL_742661, EPI_ISL_742662, EPI_ISL_742663, EPI_ISL_742664, EPI_ISL_742665, EPI_ISL_742666, EPI_ISL_742667, EPI_ISL_742668, EPI_ISL_742669, EPI_ISL_742670, EPI_ISL_742671, EPI_ISL_742672, EPI_ISL_742673, EPI_ISL_742674, EPI_ISL_742675, EPI_ISL_742676, EPI_ISL_742677, EPI_ISL_742678, EPI_ISL_742679, EPI_ISL_742683, EPI_ISL_742684, EPI_ISL_742685, EPI_ISL_742686, EPI_ISL_742687, EPI_ISL_742688, EPI_ISL_742689, EPI_ISL_742690, EPI_ISL_742691, EPI_ISL_742694, EPI_ISL_742695, EPI_ISL_742696, EPI_ISL_742697, EPI_ISL_742700, EPI_ISL_742701, EPI_ISL_742702, EPI_ISL_742704, EPI_ISL_742705, EPI_ISL_742707, EPI_ISL_742709, EPI_ISL_742710, EPI_ISL_742713, EPI_ISL_742714, EPI_ISL_742716, EPI_ISL_742717, EPI_ISL_742718, EPI_ISL_742719, EPI_ISL_742720, EPI_ISL_742849, EPI_ISL_742850, EPI_ISL_742851, EPI_ISL_742852, EPI_ISL_742853, EPI_ISL_742854, EPI_ISL_742855, EPI_ISL_742858, EPI_ISL_742859, EPI_ISL_742860, EPI_ISL_742861, EPI_ISL_742862, EPI_ISL_742863, EPI_ISL_742864, EPI_ISL_742865, EPI_ISL_742866, EPI_ISL_742867, EPI_ISL_742868, EPI_ISL_742869, EPI_ISL_742870, EPI_ISL_742871, EPI_ISL_742872, EPI_ISL_742873, EPI_ISL_742874, EPI_ISL_742875, EPI_ISL_742876, EPI_ISL_742877, EPI_ISL_742878, EPI_ISL_742879, EPI_ISL_742911, EPI_ISL_742912, EPI_ISL_742915, EPI_ISL_742924, EPI_ISL_742925, EPI_ISL_742939, EPI_ISL_742946, EPI_ISL_742947, EPI_ISL_742948, EPI_ISL_742949, EPI_ISL_742950, EPI_ISL_742951, EPI_ISL_742952, EPI_ISL_742953, EPI_ISL_742954, EPI_ISL_742955, EPI_ISL_742956, EPI_ISL_742957, EPI_ISL_742958, EPI_ISL_742959, EPI_ISL_742960, EPI_ISL_742961, EPI_ISL_742962, EPI_ISL_742963, EPI_ISL_742964, EPI_ISL_742965, EPI_ISL_742966, EPI_ISL_742967, EPI_ISL_742968, EPI_ISL_742969, EPI_ISL_742970, EPI_ISL_742971, EPI_ISL_742972, EPI_ISL_742973, EPI_ISL_742974, EPI_ISL_742975, EPI_ISL_742976, EPI_ISL_742977, EPI_ISL_742978, EPI_ISL_742979, EPI_ISL_742980, EPI_ISL_742981, EPI_ISL_742982, EPI_ISL_742983, EPI_ISL_742984, EPI_ISL_742985, EPI_ISL_742986, EPI_ISL_742987, EPI_ISL_742988, EPI_ISL_742989, EPI_ISL_742990, EPI_ISL_742991, EPI_ISL_742992, EPI_ISL_742993, EPI_ISL_742994, EPI_ISL_742995, EPI_ISL_742996, EPI_ISL_742997, EPI_ISL_742998, EPI_ISL_742999, EPI_ISL_743000, EPI_ISL_743001, EPI_ISL_743002, EPI_ISL_743003, EPI_ISL_743004, EPI_ISL_743005, EPI_ISL_743006, EPI_ISL_743007, EPI_ISL_743008, EPI_ISL_743009, EPI_ISL_743010, EPI_ISL_743011, EPI_ISL_743012, EPI_ISL_743013, EPI_ISL_743014, EPI_ISL_743015, EPI_ISL_743016, EPI_ISL_743017, EPI_ISL_743018, EPI_ISL_743019, EPI_ISL_743020, EPI_ISL_743021, EPI_ISL_743022, EPI_ISL_743023, EPI_ISL_743024, EPI_ISL_743025, EPI_ISL_743026, EPI_ISL_743027, EPI_ISL_743028, EPI_ISL_743029, EPI_ISL_743030, EPI_ISL_743031, EPI_ISL_743032, EPI_ISL_743033, EPI_ISL_743034, EPI_ISL_743035, EPI_ISL_743036, EPI_ISL_743037, EPI_ISL_743038, EPI_ISL_743039, EPI_ISL_743040, EPI_ISL_743041, EPI_ISL_743042, EPI_ISL_743043, EPI_ISL_743044, EPI_ISL_743045, EPI_ISL_743046, EPI_ISL_743047, EPI_ISL_743048, EPI_ISL_743049, EPI_ISL_743050, EPI_ISL_743051, EPI_ISL_743052, EPI_ISL_743053, EPI_ISL_743054, EPI_ISL_743055, EPI_ISL_743056, EPI_ISL_743057, EPI_ISL_743058, EPI_ISL_743059, EPI_ISL_743060, EPI_ISL_743061, EPI_ISL_743062, EPI_ISL_743063, EPI_ISL_743064, EPI_ISL_743065, EPI_ISL_743066, EPI_ISL_743067, EPI_ISL_743068, EPI_ISL_743069, EPI_ISL_743070, EPI_ISL_743071, EPI_ISL_743072, EPI_ISL_743073, EPI_ISL_743074, EPI_ISL_743075, EPI_ISL_743076, EPI_ISL_743077, EPI_ISL_743078, EPI_ISL_743079, EPI_ISL_743080, EPI_ISL_743081, EPI_ISL_743082, EPI_ISL_743083, EPI_ISL_743084, EPI_ISL_743085, EPI_ISL_743086, EPI_ISL_743087, EPI_ISL_743088, EPI_ISL_743089, EPI_ISL_743090, EPI_ISL_743091, EPI_ISL_743092, EPI_ISL_743093, EPI_ISL_743094, EPI_ISL_743095, EPI_ISL_743096, EPI_ISL_743097, EPI_ISL_743098, EPI_ISL_743099, EPI_ISL_743100, EPI_ISL_743101, EPI_ISL_743102, EPI_ISL_743103, EPI_ISL_743104, EPI_ISL_743105, EPI_ISL_743106, EPI_ISL_743107, EPI_ISL_743108, EPI_ISL_743109, EPI_ISL_743110, EPI_ISL_743111, EPI_ISL_743112, EPI_ISL_743113, EPI_ISL_743114, EPI_ISL_743115, EPI_ISL_743116, EPI_ISL_743121, EPI_ISL_743122, EPI_ISL_743123, EPI_ISL_743128, EPI_ISL_743137, EPI_ISL_743138, EPI_ISL_743139, EPI_ISL_743140, EPI_ISL_743141, EPI_ISL_743142, EPI_ISL_743143, EPI_ISL_743144, EPI_ISL_743145, EPI_ISL_743159, EPI_ISL_743160, EPI_ISL_743163, EPI_ISL_743164, EPI_ISL_743165, EPI_ISL_743175, EPI_ISL_743176, EPI_ISL_743177, EPI_ISL_743178, EPI_ISL_743179, EPI_ISL_743180, EPI_ISL_743181, EPI_ISL_743182, EPI_ISL_743190, EPI_ISL_743192, EPI_ISL_743193, EPI_ISL_743194, EPI_ISL_743195, EPI_ISL_743196, EPI_ISL_743197, EPI_ISL_743198, EPI_ISL_743199, EPI_ISL_743200, EPI_ISL_743201, EPI_ISL_743202, EPI_ISL_743203, EPI_ISL_743204, EPI_ISL_743205, EPI_ISL_743206, EPI_ISL_743207, EPI_ISL_743208, EPI_ISL_743209, EPI_ISL_743210, EPI_ISL_743211, EPI_ISL_743212, EPI_ISL_743213, EPI_ISL_743214, EPI_ISL_743215, EPI_ISL_743216, EPI_ISL_743217, EPI_ISL_743218, EPI_ISL_743219, EPI_ISL_743220, EPI_ISL_743221, EPI_ISL_743222, EPI_ISL_743223, EPI_ISL_743224, EPI_ISL_743225, EPI_ISL_743226, EPI_ISL_743227, EPI_ISL_743228, EPI_ISL_743229, EPI_ISL_743230, EPI_ISL_743231, EPI_ISL_743232, EPI_ISL_743233, EPI_ISL_743234, EPI_ISL_743235, EPI_ISL_743236, EPI_ISL_743237, EPI_ISL_743238, EPI_ISL_743239, EPI_ISL_743240, EPI_ISL_743241, EPI_ISL_743242, EPI_ISL_743243, EPI_ISL_743244, EPI_ISL_743245, EPI_ISL_743246, EPI_ISL_743247, EPI_ISL_743248, EPI_ISL_743249, EPI_ISL_743250, EPI_ISL_743251, EPI_ISL_743252, EPI_ISL_743253, EPI_ISL_743254, EPI_ISL_743255, EPI_ISL_743256, EPI_ISL_743257, EPI_ISL_743258, EPI_ISL_743259, EPI_ISL_743260, EPI_ISL_743261, EPI_ISL_743262, EPI_ISL_743263, EPI_ISL_743264, EPI_ISL_743265, EPI_ISL_743266, EPI_ISL_743267, EPI_ISL_743268, EPI_ISL_743269, EPI_ISL_743270, EPI_ISL_743271, EPI_ISL_743272, EPI_ISL_743273, EPI_ISL_743274, EPI_ISL_743275, EPI_ISL_743276, EPI_ISL_743277, EPI_ISL_743278, EPI_ISL_743279, EPI_ISL_743280, EPI_ISL_743281, EPI_ISL_743282, EPI_ISL_743283, EPI_ISL_743284, EPI_ISL_743285, EPI_ISL_743286, EPI_ISL_743287, EPI_ISL_743288, EPI_ISL_743289, EPI_ISL_743290, EPI_ISL_743291, EPI_ISL_743292, EPI_ISL_743293, EPI_ISL_743294, EPI_ISL_743295, EPI_ISL_743296, EPI_ISL_743297, EPI_ISL_743298, EPI_ISL_743299, EPI_ISL_743300, EPI_ISL_743301, EPI_ISL_743302, EPI_ISL_743303, EPI_ISL_743304, EPI_ISL_743305, EPI_ISL_743306, EPI_ISL_743307, EPI_ISL_743308, EPI_ISL_743309, EPI_ISL_743310, EPI_ISL_743311, EPI_ISL_743312, EPI_ISL_743313, EPI_ISL_743314, EPI_ISL_743315, EPI_ISL_743316, EPI_ISL_743317, EPI_ISL_743318, EPI_ISL_743319, EPI_ISL_743320, EPI_ISL_743323, EPI_ISL_743324, EPI_ISL_743325, EPI_ISL_743327, EPI_ISL_743328, EPI_ISL_743329, EPI_ISL_743330, EPI_ISL_743331, EPI_ISL_743332, EPI_ISL_743333, EPI_ISL_743334, EPI_ISL_743335, EPI_ISL_743336, EPI_ISL_743337, EPI_ISL_743338, EPI_ISL_743339, EPI_ISL_743340, EPI_ISL_743346, EPI_ISL_743347, EPI_ISL_743348, EPI_ISL_743349, EPI_ISL_743350, EPI_ISL_743351, EPI_ISL_743356, EPI_ISL_743357, EPI_ISL_743411, EPI_ISL_743454, EPI_ISL_743455, EPI_ISL_743456, EPI_ISL_743457, EPI_ISL_743458, EPI_ISL_743459, EPI_ISL_743460, EPI_ISL_743461, EPI_ISL_743462, EPI_ISL_743463, EPI_ISL_743464, EPI_ISL_743465, EPI_ISL_743466, EPI_ISL_743467, EPI_ISL_743468, EPI_ISL_743469, EPI_ISL_743470, EPI_ISL_743471, EPI_ISL_743472, EPI_ISL_743473, EPI_ISL_743474, EPI_ISL_743475, EPI_ISL_743476, EPI_ISL_743477, EPI_ISL_743478, EPI_ISL_743479, EPI_ISL_743480, EPI_ISL_743481, EPI_ISL_743482, EPI_ISL_743483, EPI_ISL_743484, EPI_ISL_743485, EPI_ISL_743486, EPI_ISL_743487, EPI_ISL_743488, EPI_ISL_743489, EPI_ISL_743490, EPI_ISL_743491, EPI_ISL_743492, EPI_ISL_743493, EPI_ISL_743494, EPI_ISL_743495, EPI_ISL_743496, EPI_ISL_743497, EPI_ISL_743498, EPI_ISL_743499, EPI_ISL_743500, EPI_ISL_743501, EPI_ISL_743502, EPI_ISL_743503, EPI_ISL_743504, EPI_ISL_743505, EPI_ISL_743506, EPI_ISL_743507, EPI_ISL_743508, EPI_ISL_743510, EPI_ISL_743568, EPI_ISL_743580, EPI_ISL_743640, EPI_ISL_743642, EPI_ISL_743643, EPI_ISL_743644, EPI_ISL_743645, EPI_ISL_743646, EPI_ISL_743647, EPI_ISL_743648, EPI_ISL_743649, EPI_ISL_743650, EPI_ISL_743651, EPI_ISL_743652, EPI_ISL_743653, EPI_ISL_743654, EPI_ISL_743655, EPI_ISL_743656, EPI_ISL_743657, EPI_ISL_743658, EPI_ISL_743659, EPI_ISL_743660, EPI_ISL_743661, EPI_ISL_743665, EPI_ISL_743666, EPI_ISL_743667, EPI_ISL_743668, EPI_ISL_743669, EPI_ISL_743670, EPI_ISL_743671, EPI_ISL_743672, EPI_ISL_743673, EPI_ISL_743674, EPI_ISL_743676, EPI_ISL_743677, EPI_ISL_743679, EPI_ISL_743901, EPI_ISL_743943, EPI_ISL_743945, EPI_ISL_743946, EPI_ISL_743947, EPI_ISL_743948, EPI_ISL_743949, EPI_ISL_743950, EPI_ISL_743951, EPI_ISL_743953, EPI_ISL_743954, EPI_ISL_743955, EPI_ISL_743956, EPI_ISL_743957			
see above	Wales Specialist Virology Centre Sequencing lab: Pathogen Genomics Unit	COVID-19 Genomics UK (COG-UK) Consortium	Catherine Moore, Johnathan Evans, Laura Gifford, Malorie Perry, Simon Cottrell, Angela Marchbank, Alec Birchley, Alexander Adams, Amy Gaskin, Bree Gatica-Wilcox, Jason Coombes, Joel Southgate, Lauren Gilbert, Lee Graham, Nicole Pacchiarini, Sara Kumziene-Summerhayes, Sarah Taylor, Sophie Jones, Sara Rey, Matthew Bull, Joanne Watkins, Sally Corden, Tom Connor
EPI_ISL_745043, EPI_ISL_745044, EPI_ISL_745045, EPI_ISL_745046, EPI_ISL_745047, EPI_ISL_745048, EPI_ISL_745049, EPI_ISL_745050, EPI_ISL_745051, EPI_ISL_745052, EPI_ISL_745053, EPI_ISL_745054, EPI_ISL_745055, EPI_ISL_745057			
see above	Israel Central Virology laboratory	Israel Central Virology laboratory	Neta Zuckerman, Efrat Dahan Bucris, Oran Erster, Michal Mandelboim, Orna Mor, Ella Mendelson

EPI_ISL_745127	Khayelitsha (Site B) CHC wc KHC	National Health Laboratory Service (NHLS), Tygerberg	Susan Engelbrecht, Kayla Delaney, Bronwyn Kleinhans, Houriiyah Tegally, Eduan Wilkindon, Gert van Zyl, Wolfgang Preiser, Tulio de Oliveira
EPI_ISL_745131, EPI_ISL_745133, EPI_ISL_745136	Kraaifontein CHC wc KFP	National Health Laboratory Service (NHLS), Tygerberg	Susan Engelbrecht, Kayla Delaney, Bronwyn Kleinhans, Houriiyah Tegally, Eduan Wilkindon, Gert van Zyl, Wolfgang Preiser, Tulio de Oliveira
EPI_ISL_745140, EPI_ISL_745141, EPI_ISL_745147, EPI_ISL_745148, EPI_ISL_745149, EPI_ISL_745150	Tygerberg Hospital wc TBH	National Health Laboratory Service (NHLS), Tygerberg	Susan Engelbrecht, Kayla Delaney, Bronwyn Kleinhans, Houriiyah Tegally, Eduan Wilkindon, Gert van Zyl, Wolfgang Preiser, Tulio de Oliveira
EPI_ISL_745168, EPI_ISL_745169	Bishop Lavis CDC wc BLP	National Health Laboratory Service (NHLS), Tygerberg	Susan Engelbrecht, Kayla Delaney, Bronwyn Kleinhans, Houriiyah Tegally, Eduan Wilkindon, Gert van Zyl, Wolfgang Preiser, Tulio de Oliveira
EPI_ISL_745170	Brackengate Field Hospital COVID-19 wc BRG	National Health Laboratory Service (NHLS), Tygerberg	Susan Engelbrecht, Kayla Delaney, Bronwyn Kleinhans, Houriiyah Tegally, Eduan Wilkindon, Gert van Zyl, Wolfgang Preiser, Tulio de Oliveira
EPI_ISL_745171, EPI_ISL_745172	Delft CHC wc DFP	National Health Laboratory Service (NHLS), Tygerberg	Susan Engelbrecht, Kayla Delaney, Bronwyn Kleinhans, Houriiyah Tegally, Eduan Wilkindon, Gert van Zyl, Wolfgang Preiser, Tulio de Oliveira
EPI_ISL_745173, EPI_ISL_745174, EPI_ISL_745175, EPI_ISL_745176, EPI_ISL_745177, EPI_ISL_745178	Eerste River Hospital wc ERH	National Health Laboratory Service (NHLS), Tygerberg	Susan Engelbrecht, Kayla Delaney, Bronwyn Kleinhans, Houriiyah Tegally, Eduan Wilkindon, Gert van Zyl, Wolfgang Preiser, Tulio de Oliveira
EPI_ISL_745179	Kraaifontein CHC wc KFP	National Health Laboratory Service (NHLS), Tygerberg	Susan Engelbrecht, Kayla Delaney, Bronwyn Kleinhans, Houriiyah Tegally, Eduan Wilkindon, Gert van Zyl, Wolfgang Preiser, Tulio de Oliveira
EPI_ISL_745180, EPI_ISL_745181, EPI_ISL_745182, EPI_ISL_745183, EPI_ISL_745184, EPI_ISL_745185	Tygerberg Hospital wc TBH	National Health Laboratory Service (NHLS), Tygerberg	Susan Engelbrecht, Kayla Delaney, Bronwyn Kleinhans, Houriiyah Tegally, Eduan Wilkindon, Gert van Zyl, Wolfgang Preiser, Tulio de Oliveira
EPI_ISL_745186	Wallacedene Clinic wc WAL	National Health Laboratory Service (NHLS), Tygerberg	Susan Engelbrecht, Kayla Delaney, Bronwyn Kleinhans, Houriiyah Tegally, Eduan Wilkindon, Gert van Zyl, Wolfgang Preiser, Tulio de Oliveira
EPI_ISL_745190	Brackengate Field Hospital COVID-19 wc BRG	National Health Laboratory Service (NHLS), Tygerberg	Susan Engelbrecht, Kayla Delaney, Bronwyn Kleinhans, Houriiyah Tegally, Eduan Wilkindon, Gert van Zyl, Wolfgang Preiser, Tulio de Oliveira
EPI_ISL_745208, EPI_ISL_745209, EPI_ISL_745210	Lab voor klinische biologie	Onderzoeksgroep Virologie	Laurens Lambrechts, Nick Vereecke, Marthe Pauwels, Bruno Verhasselt, Linos Vandekerckhove, Hans Nauwynck, Sebastiaan Theuns
EPI_ISL_745309, EPI_ISL_745330, EPI_ISL_745368, EPI_ISL_745369, EPI_ISL_745370, EPI_ISL_745371, EPI_ISL_745372, EPI_ISL_745373, EPI_ISL_745374, EPI_ISL_745375, EPI_ISL_745376, EPI_ISL_745377, EPI_ISL_745397			
see above	CNR Virus des Infections Respiratoires - France SUD	CNR Virus des Infections Respiratoires - France SUD	Antonin Bal, Gregory Destras, Claudia Gonzalez, Gwendolyne Burfin, Quentin Semanas, Martine Valette, Bruno Lina, Laurence Josset
EPI_ISL_746966	Utah Public Health Laboratory	Utah Public Health Laboratory	Erin Young, Kelly Oakeson, Tara Gallagher
EPI_ISL_747195, EPI_ISL_747196, EPI_ISL_747197, EPI_ISL_747198, EPI_ISL_747199, EPI_ISL_747200, EPI_ISL_747201, EPI_ISL_747202, EPI_ISL_747203, EPI_ISL_747204, EPI_ISL_747205, EPI_ISL_747206, EPI_ISL_747207, EPI_ISL_747208, EPI_ISL_747209, EPI_ISL_747210, EPI_ISL_747211, EPI_ISL_747212, EPI_ISL_747213, EPI_ISL_747214, EPI_ISL_747215, EPI_ISL_747216, EPI_ISL_747217, EPI_ISL_747218, EPI_ISL_747219, EPI_ISL_747220, EPI_ISL_747221, EPI_ISL_747222, EPI_ISL_747223, EPI_ISL_747224, EPI_ISL_747225, EPI_ISL_747226, EPI_ISL_747227, EPI_ISL_747228, EPI_ISL_747229, EPI_ISL_747230, EPI_ISL_747231, EPI_ISL_747232			
see above	UW Virology Lab	UW Virology Lab	Pavitra Roychoudhury, Hong Xie, Lasata Shrestha, Michelle Lin, Meei-Li Huang, Keith R Jerome, Alexander Greninger
EPI_ISL_747527, EPI_ISL_747528, EPI_ISL_747529, EPI_ISL_747533, EPI_ISL_747535, EPI_ISL_747536, EPI_ISL_747540, EPI_ISL_747541, EPI_ISL_747542, EPI_ISL_747543, EPI_ISL_747544, EPI_ISL_747545, EPI_ISL_747546, EPI_ISL_747547, EPI_ISL_747548, EPI_ISL_747549, EPI_ISL_747550, EPI_ISL_747551, EPI_ISL_747552, EPI_ISL_747553, EPI_ISL_747554, EPI_ISL_747555, EPI_ISL_747556, EPI_ISL_747557, EPI_ISL_747558, EPI_ISL_747559, EPI_ISL_747560, EPI_ISL_747561, EPI_ISL_747562, EPI_ISL_747563, EPI_ISL_747564, EPI_ISL_747565, EPI_ISL_747566, EPI_ISL_747567, EPI_ISL_747568, EPI_ISL_747569, EPI_ISL_747570, EPI_ISL_747571, EPI_ISL_747572, EPI_ISL_747573, EPI_ISL_747574, EPI_ISL_747575, EPI_ISL_747576, EPI_ISL_747589, EPI_ISL_747590, EPI_ISL_747591, EPI_ISL_747592, EPI_ISL_747593, EPI_ISL_747594, EPI_ISL_747595, EPI_ISL_747599, EPI_ISL_747601, EPI_ISL_747602, EPI_ISL_747607, EPI_ISL_747608, EPI_ISL_747610, EPI_ISL_747611, EPI_ISL_747612, EPI_ISL_747613, EPI_ISL_747614, EPI_ISL_747616, EPI_ISL_747617, EPI_ISL_747618, EPI_ISL_747619, EPI_ISL_747620, EPI_ISL_747621, EPI_ISL_747622, EPI_ISL_747623, EPI_ISL_747632, EPI_ISL_747636, EPI_ISL_747637, EPI_ISL_747638, EPI_ISL_747639, EPI_ISL_747640, EPI_ISL_747642, EPI_ISL_747643, EPI_ISL_747644, EPI_ISL_747645, EPI_ISL_747648, EPI_ISL_747652, EPI_ISL_747655, EPI_ISL_747656, EPI_ISL_747658, EPI_ISL_747659, EPI_ISL_747660, EPI_ISL_747661, EPI_ISL_747662, EPI_ISL_747663, EPI_ISL_747664, EPI_ISL_747665, EPI_ISL_747666, EPI_ISL_747667, EPI_ISL_747668, EPI_ISL_747669, EPI_ISL_747670, EPI_ISL_747671, EPI_ISL_747672, EPI_ISL_747673, EPI_ISL_747674, EPI_ISL_747681, EPI_ISL_747682, EPI_ISL_747683, EPI_ISL_747684, EPI_ISL_747685, EPI_ISL_747686, EPI_ISL_747687, EPI_ISL_747688, EPI_ISL_747689, EPI_ISL_747690, EPI_ISL_747691, EPI_ISL_747692, EPI_ISL_747693, EPI_ISL_747694, EPI_ISL_747695, EPI_ISL_747696, EPI_ISL_747697, EPI_ISL_747698, EPI_ISL_747699, EPI_ISL_747700, EPI_ISL_747701, EPI_ISL_747702, EPI_ISL_747703, EPI_ISL_747704, EPI_ISL_747705, EPI_ISL_747706, EPI_ISL_747707, EPI_ISL_747708, EPI_ISL_747721, EPI_ISL_747722, EPI_ISL_747723, EPI_ISL_747728, EPI_ISL_747729, EPI_ISL_747731, EPI_ISL_747732, EPI_ISL_747733, EPI_ISL_747734, EPI_ISL_747739, EPI_ISL_747740, EPI_ISL_747741, EPI_ISL_747742, EPI_ISL_747743, EPI_ISL_747745, EPI_ISL_747746, EPI_ISL_747748, EPI_ISL_747750, EPI_ISL_747752, EPI_ISL_747753, EPI_ISL_747754, EPI_ISL_747756, EPI_ISL_747757, EPI_ISL_747758, EPI_ISL_747759, EPI_ISL_747760, EPI_ISL_747761, EPI_ISL_747762, EPI_ISL_747763, EPI_ISL_747764, EPI_ISL_747765, EPI_ISL_747766, EPI_ISL_747767, EPI_ISL_747768, EPI_ISL_747769, EPI_ISL_747770, EPI_ISL_747771, EPI_ISL_747772, EPI_ISL_747773, EPI_ISL_747774, EPI_ISL_747775, EPI_ISL_747776, EPI_ISL_747777, EPI_ISL_747778, EPI_ISL_747779, EPI_ISL_747780, EPI_ISL_747781, EPI_ISL_747782, EPI_ISL_747783, EPI_ISL_747784, EPI_ISL_747785, EPI_ISL_747786, EPI_ISL_747787, EPI_ISL_747788, EPI_ISL_747789, EPI_ISL_747790, EPI_ISL_747791, EPI_ISL_747792, EPI_ISL_747793, EPI_ISL_747794, EPI_ISL_747795, EPI_ISL_747796, EPI_ISL_747797, EPI_ISL_747798, EPI_ISL_747799, EPI_ISL_747800, EPI_ISL_747801, EPI_ISL_747802, EPI_ISL_747803, EPI_ISL_747804, EPI_ISL_747805, EPI_ISL_747806, EPI_ISL_747807, EPI_ISL_747808, EPI_ISL_747809, EPI_ISL_747810, EPI_ISL_747811, EPI_ISL_747812, EPI_ISL_747813, EPI_ISL_747814, EPI_ISL_747815, EPI_ISL_747816, EPI_ISL_747817, EPI_ISL_747818, EPI_ISL_747819, EPI_ISL_747820, EPI_ISL_747821, EPI_ISL_747822, EPI_ISL_747823, EPI_ISL_747824, EPI_ISL_747825, EPI_ISL_747826, EPI_ISL_747827, EPI_ISL_747828, EPI_ISL_747829, EPI_ISL_747830, EPI_ISL_747831, EPI_ISL_747832, EPI_ISL_747833, EPI_ISL_747834, EPI_ISL_747835, EPI_ISL_747836, EPI_ISL_747837, EPI_ISL_747838, EPI_ISL_747839, EPI_ISL_747840, EPI_ISL_747841, EPI_ISL_747842, EPI_ISL_747843, EPI_ISL_747844, EPI_ISL_747845, EPI_ISL_747846, EPI_ISL_747847, EPI_ISL_747848, EPI_ISL_747849, EPI_ISL_747850, EPI_ISL_747851, EPI_ISL_747852, EPI_ISL_747853, EPI_ISL_747854, EPI_ISL_747855, EPI_ISL_747856, EPI_ISL_747857, EPI_ISL_747858, EPI_ISL_747859, EPI_ISL_747860, EPI_ISL_747861, EPI_ISL_747862, EPI_ISL_747863, EPI_ISL_747864, EPI_ISL_747865, EPI_ISL_747866, EPI_ISL_747867, EPI_ISL_747868, EPI_ISL_747869, EPI_ISL_747870, EPI_ISL_747871, EPI_ISL_747872, EPI_ISL_747873, EPI_ISL_747874, EPI_ISL_747875, EPI_ISL_747876, EPI_ISL_747877, EPI_ISL_747878, EPI_ISL_747879, EPI_ISL_747880, EPI_ISL_747881, EPI_ISL_747882, EPI_ISL_747883, EPI_ISL_747884, EPI_ISL_747885, EPI_ISL_747886, EPI_ISL_747887, EPI_ISL_747888, EPI_ISL_747889, EPI_ISL_747890, EPI_ISL_747891, EPI_ISL_747892, EPI_ISL_747893, EPI_ISL_747894, EPI_ISL_747895, EPI_ISL_747896, EPI_ISL_747897, EPI_ISL_747898, EPI_ISL_747899, EPI_ISL_747900, EPI_ISL_747901, EPI_ISL_747902, EPI_ISL_747903, EPI_ISL_747904, EPI_ISL_747905, EPI_ISL_747906, EPI_ISL_747907, EPI_ISL_747908, EPI_ISL_747909, EPI_ISL_747910, EPI_ISL_747911, EPI_ISL_747912, EPI_ISL_747913, EPI_ISL_747914, EPI_ISL_747915, EPI_ISL_747916, EPI_ISL_747917, EPI_ISL_747918, EPI_ISL_747919, EPI_ISL_747920, EPI_ISL_747921, EPI_ISL_747922, EPI_ISL_747923, EPI_ISL_747924, EPI_ISL_747925, EPI_ISL_747926, EPI_ISL_747927, EPI_ISL_747928, EPI_ISL_747929, EPI_ISL_747930, EPI_ISL_747931, EPI_ISL_747932, EPI_ISL_747933, EPI_ISL_747934, EPI_ISL_747935, EPI_ISL_747936, EPI_ISL_747937, EPI_ISL_747938, EPI_ISL_747939, EPI_ISL_747940, EPI_ISL_747941, EPI_ISL_747942, EPI_ISL_747943, EPI_ISL_747944, EPI_ISL_747945, EPI_ISL_747946, EPI_ISL_747947, EPI_ISL_747948, EPI_ISL_747949, EPI_ISL_747950, EPI_ISL_747951, EPI_ISL_747952, EPI_ISL_747953, EPI_ISL_747954, EPI_ISL_747955, EPI_ISL_747956, EPI_ISL_747957, EPI_ISL_747958, EPI_ISL_747959, EPI_ISL_747960, EPI_ISL_747961, EPI_ISL_747962, EPI_ISL_747963, EPI_ISL_747964, EPI_ISL_747965, EPI_ISL_747966, EPI_ISL_747967, EPI_ISL_747968, EPI_ISL_747969, EPI_ISL_747970, EPI_ISL_747971, EPI_ISL_747972, EPI_ISL_747973, EPI_ISL_747974, EPI_ISL_747975, EPI_ISL_747976, EPI_ISL_747977, EPI_ISL_747978, EPI_ISL_747979, EPI_ISL_747980, EPI_ISL_747981, EPI_ISL_747982, EPI_ISL_747983, EPI_ISL_747984, EPI_ISL_747985, EPI_ISL_747986, EPI_ISL_747987, EPI_ISL_747988, EPI_ISL_747989, EPI_ISL_747990, EPI_ISL_747991, EPI_ISL_747992, EPI_ISL_747993, EPI_ISL_747994, EPI_ISL_747995, EPI_ISL_747996, EPI_ISL_747997, EPI_ISL_747998, EPI_ISL_747999, EPI_ISL_748000, EPI_ISL_748001, EPI_ISL_748002, EPI_ISL_748003, EPI_ISL_748004, EPI_ISL_748005, EPI_ISL_748006, EPI_ISL_748007, EPI_ISL_748008, EPI_ISL_748009, EPI_ISL_748010, EPI_ISL_748011, EPI_ISL_748012, EPI_ISL_748013, EPI_ISL_748014, EPI_ISL_748015, EPI_ISL_748016, EPI_ISL_748017, EPI_ISL_748018, EPI_ISL_748019, EPI_ISL_748020, EPI_ISL_748146, EPI_ISL_748147, EPI_ISL_748148, EPI_ISL_748149, EPI_ISL_748150, EPI_ISL_748151, EPI_ISL_748152, EPI_ISL_748153, EPI_ISL_748154, EPI_ISL_748155, EPI_ISL_748156, EPI_ISL_748157, EPI_ISL_748158, EPI_ISL_748159, EPI_ISL_748160, EPI_ISL_748161, EPI_ISL_748162, EPI_ISL_748163, EPI_ISL_748164, EPI_ISL_748165, EPI_ISL_748166, EPI_ISL_748167, EPI_ISL_748168, EPI_ISL_748169, EPI_ISL_748170, EPI_ISL_748171, EPI_ISL_748172, EPI_ISL_748173, EPI_ISL_748174, EPI_ISL_748175, EPI_ISL_748176, EPI_ISL_748177, EPI_ISL_748178, EPI_ISL_748179, EPI_ISL_748180, EPI_ISL_748181, EPI_ISL_748182, EPI_ISL_748183, EPI_ISL_748184, EPI_ISL_748185, EPI_ISL_748186, EPI_ISL_748187, EPI_ISL_748188, EPI_ISL_748189, EPI_ISL_748190, EPI_ISL_748191, EPI_ISL_748192, EPI_ISL_748193, EPI_ISL_748194, EPI_ISL_748195, EPI_ISL_748196, EPI_ISL_748197, EPI_ISL_748198, EPI_ISL_748199, EPI_ISL_748200, EPI_ISL_748201, EPI_ISL_748202, EPI_ISL_748203, EPI_ISL_748204, EPI_ISL_748205, EPI_ISL_748206, EPI_ISL_748207, EPI_ISL_748208, EPI_ISL_748209, EPI_ISL_748210, EPI_ISL_748211, EPI_ISL_748212, EPI_ISL_748213, EPI_ISL_748214, EPI_ISL_748215, EPI_ISL_748216, EPI_ISL_748217, EPI_ISL_748218, EPI_ISL_748219, EPI_ISL_748220, EPI_ISL_748221, EPI_ISL_748222, EPI_ISL_748223, EPI_ISL_748224, EPI_ISL_748225, EPI_ISL_748226, EPI_ISL_748227, EPI_ISL_748228, EPI_ISL_748229, EPI_ISL_748230, EPI_ISL_748231, EPI_ISL_748232, EPI_ISL_748233, EPI_ISL_748234, EPI_ISL_748235, EPI_ISL_748236, EPI_ISL_748237, EPI_ISL_748238, EPI_ISL_748239, EPI_ISL_748240, EPI_ISL_748241, EPI_ISL_748242, EPI_ISL_748243, EPI_ISL_748244, EPI_ISL_748245, EPI_ISL_748246, EPI_ISL_748247, EPI_ISL_748248, EPI_ISL_748249, EPI_ISL_748250, EPI_ISL_748251, EPI_ISL_748252, EPI_ISL_748253, EPI_ISL_748254, EPI_ISL_748255, EPI_ISL_748256, EPI_ISL_748257, EPI_ISL_748258, EPI_ISL_748259, EPI_ISL_748260, EPI_ISL_748261, EPI_ISL_748262, EPI_ISL_748263, EPI_ISL_748264, EPI_ISL_748265, EPI_ISL_748266, EPI_ISL_748267, EPI_ISL_748268, EPI_ISL_748269, EPI_ISL_748270, EPI_ISL_748271, EPI_ISL_748272, EPI_ISL_748273, EPI_ISL_748274, EPI_ISL_748275, EPI_ISL_748276, EPI_ISL_748277, EPI_ISL_748278, EPI_ISL_748279, EPI_ISL_748280, EPI_ISL_748281, EPI_ISL_748282, EPI_ISL_748283, EPI_ISL_748284, EPI_ISL_748285, EPI_ISL_748286, EPI_ISL_748287, EPI_ISL_748288, EPI_ISL_748289, EPI_ISL_748290, EPI_ISL_748291, EPI_ISL_748292, EPI_ISL_748293, EPI_ISL_748294, EPI_ISL_748295, EPI_ISL_748296, EPI_ISL_748297, EPI_ISL_748298, EPI_ISL_748299, EPI_ISL_748300, EPI_ISL_748301, EPI_ISL_748302, EPI_ISL_748303, EPI_ISL_748304, EPI_ISL_748305, EPI_ISL_748306, EPI_ISL_748307, EPI_ISL_748308, EPI_ISL_748309, EPI_ISL_748310, EPI_ISL_748311, EPI_ISL_748312, EPI_ISL_748313, EPI_ISL_748314, EPI_ISL_748315, EPI_ISL_748316, EPI_ISL_748317, EPI_ISL_748318, EPI_ISL_748319, EPI_ISL_748320, EPI_ISL_748321, EPI_ISL_748322, EPI_ISL_748323, EPI_ISL_748324, EPI_ISL_748325, EPI_ISL_748326, EPI_ISL_748327, EPI_ISL_748328, EPI_ISL_748329, EPI_ISL_748330, EPI_ISL_748331, EPI_ISL_748332, EPI_ISL_748333, EPI_ISL_748334, EPI_ISL_748335, EPI_ISL_748336, EPI_ISL_748337, EPI_ISL_748338, EPI_ISL_748339, EPI_ISL_748340, EPI_ISL_748341, EPI_ISL_748342, EPI_ISL_748343, EPI_ISL_748344, EPI_ISL_748345, EPI_ISL_748346, EPI_ISL_748347, EPI_ISL_748348, EPI_ISL_748349, EPI_ISL_748350, EPI_ISL_748351, EPI_ISL_748352, EPI_ISL_748353, EPI_ISL_748354, EPI_ISL_748355, EPI_ISL_748356, EPI_ISL_748357, EPI_ISL_748358, EPI_ISL_748359, EPI_ISL_748360, EPI_ISL_748361, EPI_ISL_748362, EPI_ISL_748363, EPI_ISL_748364, EPI_ISL_748365, EPI_ISL_748366, EPI_ISL_748367, EPI_ISL_748368, EPI_ISL_748369, EPI_ISL_748370, EPI_ISL_748371, EPI_ISL_748372, EPI_ISL_748373, EPI_ISL_748374, EPI_ISL_748375, EPI_ISL_748376, EPI_ISL_748377, EPI_ISL_748378, EPI_ISL_748379, EPI_ISL_748380, EPI_ISL_748381, EPI_ISL_748382, EPI_ISL_748383, EPI_ISL_748384, EPI_ISL_748385, EPI_ISL_748386, EPI_ISL_748387, EPI_ISL_748388, EPI_ISL_748389, EPI_ISL_748390, EPI_ISL_748391, EPI_ISL_748392, EPI_ISL_748393, EPI_ISL_748394, EPI_ISL_748395, EPI_ISL_748396, EPI_ISL_748397, EPI_ISL_748398, EPI_ISL_748399, EPI_ISL_748400, EPI_ISL_748401, EPI_ISL_748402, EPI_ISL_748403, EPI_ISL_748404, EPI_ISL_748405, EPI_ISL_748406, EPI_ISL_748407, EPI_ISL_748408, EPI_ISL_748409, EPI_ISL_748410, EPI_ISL_748411, EPI_ISL_748412, EPI_ISL_748413, EPI_ISL_748414, EPI_ISL_748415, EPI_ISL_748416, EPI_ISL_748417, EPI_ISL_748418, EPI_ISL_748419, EPI_ISL_748420, EPI_ISL_748421, EPI_ISL_748422, EPI_ISL_748423, EPI_ISL_748424, EPI_ISL_748425, EPI_ISL_748426, EPI_ISL_748427, EPI_ISL_748428, EPI_ISL_748429, EPI_ISL_748430, EPI_ISL_748431, EPI_ISL_748432, EPI_ISL_748433, EPI_ISL_748434, EPI_ISL_748435, EPI_ISL_748436, EPI_ISL_748437, EPI_ISL_748438, EPI_ISL_748439, EPI_ISL_748440, EPI_ISL_748441, EPI_ISL_748442, EPI_ISL_748443, EPI_ISL_748444, EPI_ISL_748445, EPI_ISL_748446, EPI_ISL_748447, EPI_ISL_748448, EPI_ISL_748449, EPI_ISL_748450, EPI_ISL_748451, EPI_ISL_748452, EPI_ISL_748453, EPI_ISL_748454, EPI_ISL_748455, EPI_ISL_748456, EPI_ISL_748457, EPI_ISL_748458, EPI_ISL_748459, EPI_ISL_748460, EPI_ISL_748461, EPI_ISL_748462, EPI_ISL_748463, EPI_ISL_748464, EPI_ISL_748465, EPI_ISL_748466, EPI_ISL_748467, EPI_ISL_748468, EPI_ISL_748469, EPI_ISL_748470, EPI_ISL_748471, EPI_ISL_748472, EPI_ISL_748473, EPI_ISL_748474, EPI_ISL_748475, EPI_ISL_748476, EPI_ISL_748477, EPI_ISL_748478, EPI_ISL_748479, EPI_ISL_748480, EPI_ISL_748481, EPI_ISL_748482, EPI_ISL_748483, EPI_ISL_748484, EPI_ISL_748485, EPI_ISL_748486, EPI_ISL_748487, EPI_ISL_748488, EPI_ISL_748489, EPI_ISL_748490, EPI_ISL_748491, EPI_ISL_748492, EPI_ISL_748493, EPI_ISL_748494, EPI_ISL_748495, EPI_ISL_748496, EPI_ISL_748497, EPI_ISL_748498, EPI_ISL_748499, EPI_ISL_748500, EPI_ISL_748501, EPI_ISL_748502, EPI_ISL_748503, EPI_ISL_748504, EPI_ISL_748505, EPI_ISL_748506, EPI_ISL_748507, EPI_ISL_748508, EPI_ISL_748509, EPI_ISL_748510, EPI_ISL_748511, EPI_ISL_748512, EPI_ISL_748513, EPI_ISL_748514, EPI_ISL_748515, EPI_ISL_748516, EPI_ISL_748517, EPI_ISL_748518, EPI_ISL_748519, EPI_ISL_748520, EPI_ISL_748521, EPI_ISL_748522, EPI_ISL_748523, EPI_ISL_748524, EPI_ISL_748525, EPI_ISL_748526, EPI_ISL_748527, EPI_ISL_748528, EPI_ISL_748529, EPI_ISL_748530, EPI_ISL_748531, EPI_ISL_748532, EPI_ISL_748533, EPI_ISL_748534, EPI_ISL_748535, EPI_ISL_748536, EPI_ISL_748537, EPI_ISL_748538, EPI_ISL_748539, EPI_ISL_748540, EPI_ISL_748541, EPI_ISL_748542, EPI_ISL_748543, EPI_ISL_748544, EPI_ISL_748545, EPI_ISL_748546, EPI_ISL_748547, EPI_ISL_748548, EPI_ISL_748549, EPI_ISL_748550, EPI_ISL_748551, EPI_ISL_748552, EPI_ISL_748553, EPI_ISL_748554, EPI_ISL_748555, EPI_ISL_748556, EPI_ISL_748557, EPI_ISL_748558, EPI_ISL_748559, EPI_ISL_748560, EPI_ISL_748561, EPI_ISL_748562, EPI_ISL_748563, EPI_ISL_748564, EPI_ISL_748565, EPI_ISL_748566, EPI_ISL_748567, EPI_ISL_748568, EPI_ISL_748569, EPI_ISL_748570, EPI_ISL_748571, EPI_ISL_748572, EPI_ISL_748573, EPI_ISL_748574, EPI_ISL_748575, EPI_ISL_748576, EPI_ISL_748577, EPI_ISL_748578, EPI_ISL_748579, EPI_ISL_748580, EPI_ISL_748581, EPI_ISL_748582, EPI_ISL_748583, EPI_ISL_748584, EPI_ISL_748585, EPI_ISL_748586, EPI_ISL_748587, EPI_ISL_748588, EPI_ISL_748589, EPI_ISL_748590, EPI_ISL_748591, EPI_ISL_748592, EPI_ISL_748593, EPI_ISL_748594, EPI_ISL_748595, EPI_ISL_748596, EPI_ISL_748597, EPI_ISL_748598, EPI_ISL_748599, EPI_ISL_748600, EPI_ISL_748601, EPI_ISL_748602, EPI_ISL_748603, EPI_ISL_748604, EPI_ISL_748605, EPI_ISL_748606, EPI_ISL_748607, EPI_ISL_748608, EPI_ISL_748609, EPI_ISL_748610, EPI_ISL_748611, EPI_ISL_748612, EPI_ISL_748613, EPI_ISL_748614, EPI_ISL_748615, EPI_ISL_748616, EPI_ISL_748617, EPI_ISL_748618, EPI_ISL_748619, EPI_ISL_748620, EPI_ISL_748621, EPI_ISL_748622, EPI_ISL_748623, EPI_ISL_748624, EPI_ISL_748625, EPI_ISL_748626, EPI_ISL_748627, EPI_ISL_748628, EPI_ISL_748629, EPI_ISL_748630, EPI_ISL_748631, EPI_ISL_748632, EPI_ISL_748633, EPI_ISL_748634, EPI_ISL_748635, EPI_ISL_748636, EPI_ISL_748637, EPI_ISL_748638, EPI_ISL_748639, EPI_ISL_748640, EPI_ISL_748641, EPI_ISL_748642, EPI_ISL_748643, EPI_ISL_748644, EPI_ISL_748645, EPI_ISL_748646, EPI_ISL_748647, EPI_ISL_748648, EPI_ISL_748649, EPI_ISL_748650, EPI_ISL_748651, EPI_ISL_748652, EPI_ISL_748653, EPI_ISL_748654, EPI_ISL_748655, EPI_ISL_748656, EPI_ISL_748657, EPI_ISL_748658, EPI_ISL_748659, EPI_ISL_748660, EPI_ISL_748661, EPI_ISL_748662, EPI_ISL_748663, EPI_ISL_748664, EPI_ISL_748665, EPI_ISL_748666, EPI_ISL_748668, EPI_ISL_748669,			

EPI_ISL_748670, EPI_ISL_748671, EPI_ISL_748672, EPI_ISL_748673, EPI_ISL_748674, EPI_ISL_748675, EPI_ISL_748676, EPI_ISL_748677, EPI_ISL_748678, EPI_ISL_748679, EPI_ISL_748680, EPI_ISL_748681, EPI_ISL_748682, EPI_ISL_748683, EPI_ISL_748684, EPI_ISL_748685, EPI_ISL_748686, EPI_ISL_748687, EPI_ISL_748688, EPI_ISL_748689, EPI_ISL_748690, EPI_ISL_748691, EPI_ISL_748692, EPI_ISL_748693, EPI_ISL_748694, EPI_ISL_748695, EPI_ISL_748696, EPI_ISL_748697, EPI_ISL_748698, EPI_ISL_748699, EPI_ISL_748700, EPI_ISL_748701, EPI_ISL_748702, EPI_ISL_748703, EPI_ISL_748704, EPI_ISL_748705, EPI_ISL_748706, EPI_ISL_748707, EPI_ISL_748708, EPI_ISL_748709, EPI_ISL_748710, EPI_ISL_748711, EPI_ISL_748712, EPI_ISL_748713, EPI_ISL_748714, EPI_ISL_748715, EPI_ISL_748716, EPI_ISL_748717, EPI_ISL_748718, EPI_ISL_748719, EPI_ISL_748720, EPI_ISL_748721, EPI_ISL_748722, EPI_ISL_748723, EPI_ISL_748724, EPI_ISL_748725, EPI_ISL_748726, EPI_ISL_748727, EPI_ISL_748728, EPI_ISL_748729, EPI_ISL_748730, EPI_ISL_748731, EPI_ISL_748732, EPI_ISL_748733, EPI_ISL_748734, EPI_ISL_748735, EPI_ISL_748736, EPI_ISL_748737, EPI_ISL_748738, EPI_ISL_748739, EPI_ISL_748740, EPI_ISL_748741, EPI_ISL_748742, EPI_ISL_748743, EPI_ISL_748744, EPI_ISL_748745, EPI_ISL_748746, EPI_ISL_748747, EPI_ISL_748748, EPI_ISL_748749, EPI_ISL_748750, EPI_ISL_748751, EPI_ISL_748752, EPI_ISL_748753, EPI_ISL_748754, EPI_ISL_748755, EPI_ISL_748756, EPI_ISL_748757, EPI_ISL_748758, EPI_ISL_748759, EPI_ISL_748760, EPI_ISL_748761, EPI_ISL_748762, EPI_ISL_748763, EPI_ISL_748764, EPI_ISL_748765, EPI_ISL_748766, EPI_ISL_748767, EPI_ISL_748768, EPI_ISL_748769, EPI_ISL_748770, EPI_ISL_748771, EPI_ISL_748772, EPI_ISL_748773, EPI_ISL_748774, EPI_ISL_748775, EPI_ISL_748776, EPI_ISL_748777, EPI_ISL_748778, EPI_ISL_748779, EPI_ISL_748780, EPI_ISL_748781, EPI_ISL_748782, EPI_ISL_748783, EPI_ISL_748784, EPI_ISL_748785, EPI_ISL_748786, EPI_ISL_748787, EPI_ISL_748788, EPI_ISL_748789, EPI_ISL_748790, EPI_ISL_748791, EPI_ISL_748792, EPI_ISL_748793, EPI_ISL_748794, EPI_ISL_748795, EPI_ISL_748796, EPI_ISL_748797, EPI_ISL_748798, EPI_ISL_748799, EPI_ISL_748800, EPI_ISL_748801, EPI_ISL_748802, EPI_ISL_748803, EPI_ISL_748804, EPI_ISL_748805, EPI_ISL_748806, EPI_ISL_748807, EPI_ISL_748808, EPI_ISL_748809, EPI_ISL_748810, EPI_ISL_748811, EPI_ISL_748812, EPI_ISL_748813, EPI_ISL_748814, EPI_ISL_748815, EPI_ISL_748816, EPI_ISL_748817, EPI_ISL_748818, EPI_ISL_748819, EPI_ISL_748820, EPI_ISL_748821, EPI_ISL_748822, EPI_ISL_748823, EPI_ISL_748824, EPI_ISL_748825, EPI_ISL_748826, EPI_ISL_748827, EPI_ISL_748828, EPI_ISL_748829, EPI_ISL_748830, EPI_ISL_748831, EPI_ISL_748832, EPI_ISL_748833, EPI_ISL_748834, EPI_ISL_748835, EPI_ISL_748836, EPI_ISL_748837, EPI_ISL_748838, EPI_ISL_748839, EPI_ISL_748840, EPI_ISL_748841, EPI_ISL_748842, EPI_ISL_748843, EPI_ISL_748844, EPI_ISL_748845, EPI_ISL_748846, EPI_ISL_748847, EPI_ISL_748848, EPI_ISL_748849, EPI_ISL_748850, EPI_ISL_748851, EPI_ISL_748852, EPI_ISL_748853, EPI_ISL_748854, EPI_ISL_748855, EPI_ISL_748856, EPI_ISL_748857, EPI_ISL_748858, EPI_ISL_748859, EPI_ISL_748860, EPI_ISL_748861, EPI_ISL_748862, EPI_ISL_748863, EPI_ISL_748864, EPI_ISL_748865, EPI_ISL_748866, EPI_ISL_748867, EPI_ISL_748868, EPI_ISL_748869, EPI_ISL_748870, EPI_ISL_748871, EPI_ISL_748872, EPI_ISL_748873, EPI_ISL_748874, EPI_ISL_748875, EPI_ISL_748876, EPI_ISL_748877, EPI_ISL_748878, EPI_ISL_748879, EPI_ISL_748880, EPI_ISL_748881, EPI_ISL_748882, EPI_ISL_748883, EPI_ISL_748884, EPI_ISL_748885, EPI_ISL_748886, EPI_ISL_748887, EPI_ISL_748888, EPI_ISL_748889, EPI_ISL_748890, EPI_ISL_748891, EPI_ISL_748892, EPI_ISL_748893, EPI_ISL_748894, EPI_ISL_748895, EPI_ISL_748896, EPI_ISL_748897, EPI_ISL_748898, EPI_ISL_748899, EPI_ISL_748900, EPI_ISL_748901, EPI_ISL_748902, EPI_ISL_748903, EPI_ISL_748904, EPI_ISL_748905, EPI_ISL_748906, EPI_ISL_748907, EPI_ISL_748908, EPI_ISL_748909, EPI_ISL_748910, EPI_ISL_748911, EPI_ISL_748912, EPI_ISL_748913, EPI_ISL_748914, EPI_ISL_748915, EPI_ISL_748916, EPI_ISL_748917, EPI_ISL_748918, EPI_ISL_748919, EPI_ISL_748920, EPI_ISL_748921, EPI_ISL_748922, EPI_ISL_748923, EPI_ISL_748924, EPI_ISL_748925, EPI_ISL_748926, EPI_ISL_748927, EPI_ISL_748928, EPI_ISL_748929, EPI_ISL_748930, EPI_ISL_748931, EPI_ISL_748932, EPI_ISL_748933, EPI_ISL_748934, EPI_ISL_748935, EPI_ISL_748936, EPI_ISL_748937, EPI_ISL_748938, EPI_ISL_748939, EPI_ISL_748940, EPI_ISL_748941, EPI_ISL_748942, EPI_ISL_748943, EPI_ISL_748944, EPI_ISL_748945, EPI_ISL_748946, EPI_ISL_748947, EPI_ISL_748948, EPI_ISL_748949, EPI_ISL_748950, EPI_ISL_748951, EPI_ISL_748952, EPI_ISL_748953, EPI_ISL_748954, EPI_ISL_748955, EPI_ISL_748956, EPI_ISL_748957, EPI_ISL_748958, EPI_ISL_748959, EPI_ISL_748960, EPI_ISL_748961, EPI_ISL_748962, EPI_ISL_748963, EPI_ISL_748964, EPI_ISL_748965, EPI_ISL_748966, EPI_ISL_748967, EPI_ISL_748968, EPI_ISL_748969, EPI_ISL_748970, EPI_ISL_748971, EPI_ISL_748972, EPI_ISL_748973, EPI_ISL_748974, EPI_ISL_748975, EPI_ISL_748976, EPI_ISL_748977, EPI_ISL_748978, EPI_ISL_748979, EPI_ISL_748980, EPI_ISL_748981, EPI_ISL_748982, EPI_ISL_748983, EPI_ISL_748984, EPI_ISL_748985, EPI_ISL_748986, EPI_ISL_748987, EPI_ISL_748988, EPI_ISL_748989, EPI_ISL_748990, EPI_ISL_748991, EPI_ISL_748992, EPI_ISL_748993, EPI_ISL_748994, EPI_ISL_748995, EPI_ISL_748996, EPI_ISL_748997, EPI_ISL_748998, EPI_ISL_748999, EPI_ISL_749000, EPI_ISL_749001, EPI_ISL_749002, EPI_ISL_749003, EPI_ISL_749004, EPI_ISL_749005, EPI_ISL_749006, EPI_ISL_749007, EPI_ISL_749008, EPI_ISL_749009, EPI_ISL_749010, EPI_ISL_749011, EPI_ISL_749012, EPI_ISL_749013, EPI_ISL_749014, EPI_ISL_749015, EPI_ISL_749016, EPI_ISL_749017, EPI_ISL_749018, EPI_ISL_749019, EPI_ISL_749020, EPI_ISL_749021, EPI_ISL_749022, EPI_ISL_749023, EPI_ISL_749024, EPI_ISL_749025, EPI_ISL_749026, EPI_ISL_749027, EPI_ISL_749028, EPI_ISL_749029, EPI_ISL_749030, EPI_ISL_749031, EPI_ISL_749032, EPI_ISL_749033, EPI_ISL_749034, EPI_ISL_749035, EPI_ISL_749037, EPI_ISL_749038, EPI_ISL_749039, EPI_ISL_749040, EPI_ISL_749041, EPI_ISL_749042, EPI_ISL_749043, EPI_ISL_749044, EPI_ISL_749045, EPI_ISL_749046, EPI_ISL_749047, EPI_ISL_749048, EPI_ISL_749049, EPI_ISL_749050, EPI_ISL_749051, EPI_ISL_749052, EPI_ISL_749053, EPI_ISL_749054, EPI_ISL_749055, EPI_ISL_749056, EPI_ISL_749057, EPI_ISL_749058, EPI_ISL_749059, EPI_ISL_749060, EPI_ISL_749061, EPI_ISL_749062, EPI_ISL_749063, EPI_ISL_749064, EPI_ISL_749065, EPI_ISL_749066, EPI_ISL_749067, EPI_ISL_749068, EPI_ISL_749069, EPI_ISL_749070, EPI_ISL_749071, EPI_ISL_749072, EPI_ISL_749073, EPI_ISL_749074, EPI_ISL_749075, EPI_ISL_749076, EPI_ISL_749077, EPI_ISL_749078, EPI_ISL_749079, EPI_ISL_749080, EPI_ISL_749081, EPI_ISL_749082, EPI_ISL_749083, EPI_ISL_749084, EPI_ISL_749085, EPI_ISL_749086, EPI_ISL_749087, EPI_ISL_749088, EPI_ISL_749089, EPI_ISL_749090, EPI_ISL_749091, EPI_ISL_749092, EPI_ISL_749093, EPI_ISL_749094, EPI_ISL_749095, EPI_ISL_749096, EPI_ISL_749097, EPI_ISL_749098, EPI_ISL_749099, EPI_ISL_749100, EPI_ISL_749101, EPI_ISL_749102, EPI_ISL_749103, EPI_ISL_749104, EPI_ISL_749105, EPI_ISL_749106, EPI_ISL_749107, EPI_ISL_749108, EPI_ISL_749109, EPI_ISL_749110, EPI_ISL_749111, EPI_ISL_749112, EPI_ISL_749113, EPI_ISL_749114, EPI_ISL_749115, EPI_ISL_749116, EPI_ISL_749117, EPI_ISL_749118, EPI_ISL_749119, EPI_ISL_749120, EPI_ISL_749121, EPI_ISL_749122, EPI_ISL_749123, EPI_ISL_749124, EPI_ISL_749125, EPI_ISL_749126, EPI_ISL_749127, EPI_ISL_749128, EPI_ISL_749129, EPI_ISL_749130, EPI_ISL_749131, EPI_ISL_749132, EPI_ISL_749133, EPI_ISL_749134, EPI_ISL_749135, EPI_ISL_749136, EPI_ISL_749137, EPI_ISL_749138, EPI_ISL_749139, EPI_ISL_749140, EPI_ISL_749141, EPI_ISL_749142, EPI_ISL_749143, EPI_ISL_749144, EPI_ISL_749145, EPI_ISL_749146, EPI_ISL_749147, EPI_ISL_749158, EPI_ISL_749167, EPI_ISL_749169, EPI_ISL_749170, EPI_ISL_749171, EPI_ISL_749176, EPI_ISL_749177, EPI_ISL_749181, EPI_ISL_749223, EPI_ISL_749224, EPI_ISL_749225, EPI_ISL_749226, EPI_ISL_749227, EPI_ISL_749228, EPI_ISL_749229, EPI_ISL_749230, EPI_ISL_749231, EPI_ISL_749232, EPI_ISL_749233, EPI_ISL_749234, EPI_ISL_749235, EPI_ISL_749236, EPI_ISL_749237, EPI_ISL_749239, EPI_ISL_749240, EPI_ISL_749241, EPI_ISL_749242, EPI_ISL_749243, EPI_ISL_749244, EPI_ISL_749245, EPI_ISL_749246, EPI_ISL_749247, EPI_ISL_749248, EPI_ISL_749249, EPI_ISL_749250, EPI_ISL_749251, EPI_ISL_749252, EPI_ISL_749253, EPI_ISL_749254, EPI_ISL_749255, EPI_ISL_749256, EPI_ISL_749257, EPI_ISL_749258, EPI_ISL_749259, EPI_ISL_749260, EPI_ISL_749261, EPI_ISL_749262, EPI_ISL_749267, EPI_ISL_749268, EPI_ISL_749271, EPI_ISL_749273, EPI_ISL_749274, EPI_ISL_749276, EPI_ISL_749279, EPI_ISL_749287, EPI_ISL_749290, EPI_ISL_749291, EPI_ISL_749292, EPI_ISL_749293, EPI_ISL_749294, EPI_ISL_749295, EPI_ISL_749321, EPI_ISL_749322, EPI_ISL_749323, EPI_ISL_749324, EPI_ISL_749325, EPI_ISL_749326, EPI_ISL_749327, EPI_ISL_749328, EPI_ISL_749329, EPI_ISL_749330, EPI_ISL_749331, EPI_ISL_749332, EPI_ISL_749333, EPI_ISL_749334, EPI_ISL_749335, EPI_ISL_749336, EPI_ISL_749337, EPI_ISL_749338, EPI_ISL_749339, EPI_ISL_749340, EPI_ISL_749341, EPI_ISL_749342, EPI_ISL_749343, EPI_ISL_749344, EPI_ISL_749345, EPI_ISL_749346, EPI_ISL_749347, EPI_ISL_749348, EPI_ISL_749349, EPI_ISL_749350, EPI_ISL_749351, EPI_ISL_749352, EPI_ISL_749353, EPI_ISL_749354, EPI_ISL_749357, EPI_ISL_749361, EPI_ISL_749369, EPI_ISL_749374, EPI_ISL_749375, EPI_ISL_749390, EPI_ISL_749391, EPI_ISL_749392, EPI_ISL_749393, EPI_ISL_749394, EPI_ISL_749395, EPI_ISL_749396, EPI_ISL_749397, EPI_ISL_749398, EPI_ISL_749399, EPI_ISL_749400, EPI_ISL_749401, EPI_ISL_749402, EPI_ISL_749403, EPI_ISL_749404, EPI_ISL_749405, EPI_ISL_749406, EPI_ISL_749452, EPI_ISL_749453, EPI_ISL_749454, EPI_ISL_749455, EPI_ISL_749456, EPI_ISL_749457, EPI_ISL_749458, EPI_ISL_749459, EPI_ISL_749460, EPI_ISL_749461, EPI_ISL_749462, EPI_ISL_749463, EPI_ISL_749464, EPI_ISL_749465, EPI_ISL_749466, EPI_ISL_749467, EPI_ISL_749468, EPI_ISL_749469, EPI_ISL_749470, EPI_ISL_749471, EPI_ISL_749472, EPI_ISL_749473, EPI_ISL_749475, EPI_ISL_749476, EPI_ISL_749477, EPI_ISL_749478, EPI_ISL_749479, EPI_ISL_749480, EPI_ISL_749481, EPI_ISL_749482, EPI_ISL_749483, EPI_ISL_749491, EPI_ISL_749492, EPI_ISL_749494, EPI_ISL_749495, EPI_ISL_749496, EPI_ISL_749497, EPI_ISL_749499, EPI_ISL_749506, EPI_ISL_749508, EPI_ISL_749509, EPI_ISL_749510, EPI_ISL_749513, EPI_ISL_749514, EPI_ISL_749520, EPI_ISL_749521, EPI_ISL_749522, EPI_ISL_749525, EPI_ISL_749526, EPI_ISL_749527, EPI_ISL_749540, EPI_ISL_749541, EPI_ISL_749859, EPI_ISL_749860, EPI_ISL_749861, EPI_ISL_749862, EPI_ISL_749863, EPI_ISL_749864, EPI_ISL_749865, EPI_ISL_749866, EPI_ISL_749867, EPI_ISL_749868, EPI_ISL_749869, EPI_ISL_749870, EPI_ISL_749871, EPI_ISL_749872, EPI_ISL_749873, EPI_ISL_749874, EPI_ISL_749875, EPI_ISL_749876, EPI_ISL_749877, EPI_ISL_749878, EPI_ISL_749879, EPI_ISL_749880, EPI_ISL_749881, EPI_ISL_749882, EPI_ISL_749883, EPI_ISL_749884, EPI_ISL_749885, EPI_ISL_749886, EPI_ISL_749887, EPI_ISL_749888, EPI_ISL_749889, EPI_ISL_749890, EPI_ISL_749891, EPI_ISL_749892, EPI_ISL_749893, EPI_ISL_749894, EPI_ISL_749895, EPI_ISL_749896, EPI_ISL_749897, EPI_ISL_749898, EPI_ISL_749899, EPI_ISL_749900, EPI_ISL_749901, EPI_ISL_749902, EPI_ISL_749903, EPI_ISL_749904, EPI_ISL_749905, EPI_ISL_749907, EPI_ISL_749908, EPI_ISL_749909, EPI_ISL_749910, EPI_ISL_749911, EPI_ISL_749912, EPI_ISL_749913, EPI_ISL_749914, EPI_ISL_749915, EPI_ISL_749916, EPI_ISL_749917, EPI_ISL_749918, EPI_ISL_749919, EPI_ISL_749920, EPI_ISL_749921, EPI_ISL_749922, EPI_ISL_749923, EPI_ISL_749924, EPI_ISL_749925, EPI_ISL_749926, EPI_ISL_749927, EPI_ISL_749928, EPI_ISL_749929, EPI_ISL_749930, EPI_ISL_749931, EPI_ISL_749932, EPI_ISL_749933, EPI_ISL_749934, EPI_ISL_749935, EPI_ISL_749936, EPI_ISL_749937, EPI_ISL_749938, EPI_ISL_749939, EPI_ISL_749940, EPI_ISL_749941, EPI_ISL_749942, EPI_ISL_749943, EPI_ISL_749944, EPI_ISL_749945, EPI_ISL_749946, EPI_ISL_749947, EPI_ISL_749948, EPI_ISL_749949, EPI_ISL_749950, EPI_ISL_749951, EPI_ISL_749952, EPI_ISL_749953, EPI_ISL_749954, EPI_ISL_749955, EPI_ISL_749956, EPI_ISL_749957, EPI_ISL_749958, EPI_ISL_749959, EPI_ISL_749960, EPI_ISL_749961, EPI_ISL_749962, EPI_ISL_749963, EPI_ISL_749964, EPI_ISL_749965, EPI_ISL_749966, EPI_ISL_749967, EPI_ISL_749968, EPI_ISL_749969, EPI_ISL_749970, EPI_ISL_749971, EPI_ISL_749972, EPI_ISL_749973, EPI_ISL_749974, EPI_ISL_749975, EPI_ISL_749976, EPI_ISL_749977, EPI_ISL_749978, EPI_ISL_749979, EPI_ISL_749980, EPI_ISL_749981, EPI_ISL_749982, EPI_ISL_749983, EPI_ISL_749984, EPI_ISL_749985, EPI_ISL_749986, EPI_ISL_749987, EPI_ISL_749988, EPI_ISL_749989, EPI_ISL_749990, EPI_ISL_749991, EPI_ISL_749992, EPI_ISL_749993, EPI_ISL_749994, EPI_ISL_749995, EPI_ISL_749996, EPI_ISL_749997, EPI_ISL_749998, EPI_ISL_749999, EPI_ISL_750000, EPI_ISL_750001, EPI_ISL_750002, EPI_ISL_750003, EPI_ISL_750004, EPI_ISL_750005, EPI_ISL_750006, EPI_ISL_750007, EPI_ISL_750008, EPI_ISL_750009, EPI_ISL_750010, EPI_ISL_750011, EPI_ISL_750012, EPI_ISL_750013, EPI_ISL_750014, EPI_ISL_750015, EPI_ISL_750016, EPI_ISL_750017, EPI_ISL_750018, EPI_ISL_750019, EPI_ISL_750020, EPI_ISL_750021, EPI_ISL_750022, EPI_ISL_750023, EPI_ISL_750024, EPI_ISL_750025, EPI_ISL_750026, EPI_ISL_750027, EPI_ISL_750028, EPI_ISL_750029, EPI_ISL_750030, EPI_ISL_750031, EPI_ISL_750032, EPI_ISL_750033, EPI_ISL_750034, EPI_ISL_750035, EPI_ISL_750036, EPI_ISL_750037, EPI_ISL_750038, EPI_ISL_750039, EPI_ISL_750040, EPI_ISL_750041, EPI_ISL_750042, EPI_ISL_750043, EPI_ISL_750044, EPI_ISL_750045, EPI_ISL_750046, EPI_ISL_750047, EPI_ISL_750048, EPI_ISL_750049, EPI_ISL_750050, EPI_ISL_750051, EPI_ISL_750052, EPI_ISL_750053, EPI_ISL_750054, EPI_ISL_750055, EPI_ISL_750056, EPI_ISL_750057, EPI_ISL_750058, EPI_ISL_750059, EPI_ISL_750060, EPI_ISL_750061, EPI_ISL_750062, EPI_ISL_750063, EPI_ISL_750064, EPI_ISL_750065, EPI_ISL_750066, EPI_ISL_750067, EPI_ISL_750068, EPI_ISL_750069, EPI_ISL_750070, EPI_ISL_750071, EPI_ISL_750072, EPI_ISL_750073, EPI_ISL_750074, EPI_ISL_750075, EPI_ISL_750076, EPI_ISL_750077, EPI_ISL_750078, EPI_ISL_750079, EPI_ISL_750080, EPI_ISL_750081, EPI_ISL_750082, EPI_ISL_750083, EPI_ISL_750084, EPI_ISL_750085, EPI_ISL_750086, EPI_ISL_750087, EPI_ISL_750088, EPI_ISL_750089, EPI_ISL_750090, EPI_ISL_750091, EPI_ISL_750092, EPI_ISL_750093, EPI_ISL_750094, EPI_ISL_750095, EPI_ISL_750096, EPI_ISL_750097, EPI_ISL_750098, EPI_ISL_750099, EPI_ISL_750100, EPI_ISL_750101, EPI_ISL_750102, EPI_ISL_750103, EPI_ISL_750104, EPI_ISL_750105, EPI_ISL_750106, EPI_ISL_750107, EPI_ISL_750109, EPI_ISL_750110, EPI_ISL_750111, EPI_ISL_750112, EPI_ISL_750113, EPI_ISL_750114, EPI_ISL_750115, EPI_ISL_750116, EPI_ISL_750117, EPI_ISL_750118, EPI_ISL_750119, EPI_ISL_750120, EPI_ISL_750121, EPI_ISL_750122, EPI_ISL_750123, EPI_ISL_750124, EPI_ISL_750125, EPI_ISL_750126, EPI_ISL_750127, EPI_ISL_750128, EPI_ISL_750129, EPI_ISL_750130, EPI_ISL_750131, EPI_ISL_750132, EPI_ISL_750133, EPI_ISL_750134, EPI_ISL_750135, EPI_ISL_750136, EPI_ISL_750137, EPI_ISL_750138, EPI_ISL_750139, EPI_ISL_750140, EPI_ISL_750141, EPI_ISL_750142, EPI_ISL_750143, EPI_ISL_750144, EPI_ISL_750145, EPI_ISL_750146, EPI_ISL_750147, EPI_ISL_750148, EPI_ISL_750149, EPI_ISL_750150, EPI_ISL_750151, EPI_ISL_750152, EPI_ISL_750153, EPI_ISL_750154, EPI_ISL_750155, EPI_ISL_750156, EPI_ISL_750157, EPI_ISL_750158, EPI_ISL_750159, EPI_ISL_750160			
see above	Department of Virus and Microbiological Special Diagnostics, Statens Serum Institut, Copenhagen, Denmark	Albertsen Lab, Department of Chemistry and Bioscience, Aalborg University, Denmark	Danish Covid-19 Genome Consortium
EPI_ISL_751191	Limmattal Hospital	Institute of Medical Virology, University of Zurich	Stefan Schmutz, Kevin Steiner, Verena Kufner, Maryam Zaheri, Gabriela Ziltener, Jürg Böni, Michael Huber, Alexandra Trkola, Roberto Buonomano
EPI_ISL_751799	Labor Dr. Wisplinghoff - Köln	Robert Koch Institute, Bioinformatics MF1, Berlin, Germany	Dr. R. Grosser, Stephan Fuchs, Stefan Kroeger, Marianne Wedde, Oliver Drechsel, Aleksandar Radonic, Rene Kmiecinski, Ralf Duerrwald, Thorsten Wolff
EPI_ISL_753225, EPI_ISL_753226, EPI_ISL_753227, EPI_ISL_753228, EPI_ISL_753229, EPI_ISL_753230, EPI_ISL_753231, EPI_ISL_753232, EPI_ISL_753233, EPI_ISL_753234, EPI_ISL_753235, EPI_ISL_753236, EPI_ISL_753237, EPI_ISL_753241, EPI_ISL_753242, EPI_ISL_753243, EPI_ISL_753244, EPI_ISL_753245, EPI_ISL_753246, EPI_ISL_753247, EPI_ISL_753248, EPI_ISL_753249, EPI_ISL_753250, EPI_ISL_753251, EPI_ISL_753252, EPI_ISL_753253, EPI_ISL_753254, EPI_ISL_753255, EPI_ISL_753256, EPI_ISL_753257, EPI_ISL_753258, EPI_ISL_753259, EPI_ISL_753260, EPI_ISL_753261			
see above	State Laboratories Division, Hawaii State Department of Health	State Laboratories Division, Hawaii State Department of Health	Pamela O'Brien, Sabrina Diemert, Drew Kuwazaki, Razvan Sultana, Edward Desmond
EPI_ISL_753661, EPI_ISL_753662, EPI_ISL_753664, EPI_ISL_753665, EPI_ISL_753666, EPI_ISL_753667, EPI_ISL_753668, EPI_ISL_753670	Clinical virology Laboratory, Children's Hospital Los Angeles	Center for Personalized Medicine, Children's Hospital Los Angeles	Gai et al
EPI_ISL_754149, EPI_ISL_754150, EPI_ISL_754163, EPI_ISL_754165, EPI_ISL_754166	Laboratoire de Microbiologie	National Reference Center for Viruses of Respiratory Infections, Institut Pasteur, Paris	Marion Barbet, Sylvie Behillil, Méline Bizard, Angela Brisebarre, Camille Capel, Etienne Simon-Lorière, Vincent Enouf, Maud Vanpeene, Sylvie van der Werf, Marie-Sarah Fangous
EPI_ISL_754275	Respiratory Virus Unit, National Infection Service, Public Health England	COVID-19 Genomics UK (COG-UK) Consortium	PHE Covid Sequencing Team
EPI_ISL_754394, EPI_ISL_754399, EPI_ISL_754448, EPI_ISL_754449, EPI_ISL_754450, EPI_ISL_754451, EPI_ISL_754452, EPI_ISL_754453, EPI_ISL_754454, EPI_ISL_754455, EPI_ISL_754456, EPI_ISL_754457, EPI_ISL_754458, EPI_ISL_754459, EPI_ISL_754460, EPI_ISL_754461, EPI_ISL_754462, EPI_ISL_754463, EPI_ISL_754464, EPI_ISL_754465, EPI_ISL_754466, EPI_ISL_754467, EPI_ISL_754468, EPI_ISL_754469, EPI_ISL_754470, EPI_ISL_754471, EPI_ISL_754472, EPI_ISL_754473, EPI_ISL_754474, EPI_ISL_754475, EPI_ISL_754476, EPI_ISL_754547, EPI_ISL_754548, EPI_ISL_754549, EPI_ISL_754572			
see above	Wadsworth Center, New York State Department.of Health	Wadsworth Center, New York State Department.of Health	Kirsten St. George, Daryl M. Lamson, Alexis Russel, Matthew Shudt, Melissa A Leisner, Jonathan Plitnick, Navjot Singh, John Kelly, Sara Griesemer, Erasmus Schneider, Erica Lasek-Nesselquist
EPI_ISL_754624, EPI_ISL_754673, EPI_ISL_754674, EPI_ISL_754675	University of Wisconsin-Madison AIDS Vaccine Research Laboratories	University of Wisconsin-Madison AIDS Vaccine Research Laboratories	Gage Moreno, Katarina Braun, et al. AIDS Vaccine Research Laboratories
EPI_ISL_755133, EPI_ISL_755140, EPI_ISL_755147, EPI_ISL_755148, EPI_ISL_755149, EPI_ISL_755150, EPI_ISL_755151, EPI_ISL_755152, EPI_ISL_755153, EPI_ISL_755154, EPI_ISL_755155, EPI_ISL_755156, EPI_ISL_755157, EPI_ISL_755158, EPI_ISL_755159, EPI_ISL_755160, EPI_ISL_755161, EPI_ISL_755162			
see above	UCSD EXCITE lab	Andersen lab at Scripps Research	SEARCH Alliance San Diego
EPI_ISL_755489, EPI_ISL_755490, EPI_ISL_755491, EPI_ISL_755492,	Maine Health and Environmental Testing Laboratory	Tewhey Lab, The Jackson Laboratory	Matluk,N., Dewey,H., Iosue,F., Barter,M., Lynch,R., Munger,H. and Tewhey,R.

EPI_ISL_755493, EPI_ISL_755496			
EPI_ISL_755643	Instituto Adolfo Lutz - Central	Instituto Adolfo Lutz, Interdiciplinary Procedures Center, Strategic Laboratory	Claudio Tavares Sacchi, Claudia Regina Gonçalves, Erica Valessa Ramos Gomes, Karoline Rodrigues Campos
EPI_ISL_755644, EPI_ISL_755645	Lab LOC - Itapecerica da Serra	Instituto Adolfo Lutz, Interdiciplinary Procedures Center, Strategic Laboratory	Claudio Tavares Sacchi, Claudia Regina Gonçalves, Erica Valessa Ramos Gomes, Karoline Rodrigues Campos
EPI_ISL_755646, EPI_ISL_755647	Instituto Adolfo Lutz - Regional de Santo Andre	Instituto Adolfo Lutz, Interdiciplinary Procedures Center, Strategic Laboratory	Claudio Tavares Sacchi, Claudia Regina Gonçalves, Erica Valessa Ramos Gomes, Karoline Rodrigues Campos
EPI_ISL_755648, EPI_ISL_755650	Instituto Adolfo Lutz - Regional de Taubate	Instituto Adolfo Lutz, Interdiciplinary Procedures Center, Strategic Laboratory	Claudio Tavares Sacchi, Claudia Regina Gonçalves, Erica Valessa Ramos Gomes, Karoline Rodrigues Campos
EPI_ISL_755652	Lab LOC - Itapecerica da Serra	Instituto Adolfo Lutz, Interdiciplinary Procedures Center, Strategic Laboratory	Claudio Tavares Sacchi, Claudia Regina Gonçalves, Erica Valessa Ramos Gomes, Karoline Rodrigues Campos
EPI_ISL_756202, EPI_ISL_756203, EPI_ISL_756204, EPI_ISL_756205, EPI_ISL_756207, EPI_ISL_756298	UW Virology Lab	UW Virology Lab	Pavitra Roychoudhury, Hong Xie, Lasata Shrestha, Meei-Li Huang, Keith R Jerome, Alexander Greninger
EPI_ISL_757226, EPI_ISL_757227, EPI_ISL_757231	Lighthouse Lab in Glasgow	Wellcome Sanger Institute for the COVID-19 Genomics UK (COG-UK) Consortium	Harper VanSteenhouse, Yumi Kasai, David Gray, Carol Clugston, Anna Dominiczak and Alex Alderton, Roberto Amato, Sonia Goncalves, Ewan Harrison, David K. Jackson, Ian Johnston, Dominic Kwiatkowski, Cordelia Langford, John Sillitoe on behalf of the Wellcome Sanger Institute COVID-19 Surveillance Team
EPI_ISL_757443, EPI_ISL_757448, EPI_ISL_757491, EPI_ISL_757688, EPI_ISL_757689, EPI_ISL_757690, EPI_ISL_757691, EPI_ISL_757692, EPI_ISL_757693, EPI_ISL_757694, EPI_ISL_757695, EPI_ISL_757696, EPI_ISL_757697, EPI_ISL_757698			
see above	Department of Virus and Microbiological Special Diagnostics, Statens Serum Institut, Copenhagen, Denmark	Albertsen Lab, Department of Chemistry and Bioscience, Aalborg University, Denmark	Danish Covid-19 Genome Consortium
EPI_ISL_759989	Norwegian Institute of Public Health, Department of Virology	Norwegian Institute of Public Health, Department of Virology	Kathrine Stene-Johansen, Kamilla Heddeland Instefjord, Hilde Elshaug, Marie Paulsen Madsen, Rasmus Riis Kopperud, Hilde Vollan, Karoline Bragstad, Olav Hungnes
EPI_ISL_760822	Lighthouse Lab in Cambridge	Wellcome Sanger Institute for the COVID-19 Genomics UK (COG-UK) Consortium	Rob Howes, The Lighthouse Lab in Cambridge and Alex Alderton, Roberto Amato, Sonia Goncalves, Ewan Harrison, David K. Jackson, Ian Johnston, Dominic Kwiatkowski, Cordelia Langford, John Sillitoe on behalf of the Wellcome Sanger Institute COVID-19 Surveillance Team
EPI_ISL_760844, EPI_ISL_760871	Lighthouse Lab in Milton Keynes	Wellcome Sanger Institute for the COVID-19 Genomics UK (COG-UK) Consortium	The Lighthouse Lab in Milton Keynes and Alex Alderton, Roberto Amato, Sonia Goncalves, Ewan Harrison, David K. Jackson, Ian Johnston, Dominic Kwiatkowski, Cordelia Langford, John Sillitoe on behalf of the Wellcome Sanger Institute COVID-19 Surveillance Team
EPI_ISL_760891, EPI_ISL_760929	Lighthouse Lab in Alderley Park	Wellcome Sanger Institute for the COVID-19 Genomics UK (COG-UK) Consortium	Jacquelyn Wynn, Mairead Hyland, The Lighthouse Lab in Alderley Park and Alex Alderton, Roberto Amato, Sonia Goncalves, Ewan Harrison, David K. Jackson, Ian Johnston, Dominic Kwiatkowski, Cordelia Langford, John Sillitoe on behalf of the Wellcome Sanger Institute COVID-19 Surveillance Team
EPI_ISL_760939	Lighthouse Lab in Cambridge	Wellcome Sanger Institute for the COVID-19 Genomics UK (COG-UK) Consortium	Rob Howes, The Lighthouse Lab in Cambridge and Alex Alderton, Roberto Amato, Sonia Goncalves, Ewan Harrison, David K. Jackson, Ian Johnston, Dominic Kwiatkowski, Cordelia Langford, John Sillitoe on behalf of the Wellcome Sanger Institute COVID-19 Surveillance Team
EPI_ISL_760982	Lighthouse Lab in Alderley Park	Wellcome Sanger Institute for the COVID-19 Genomics UK (COG-UK) Consortium	Jacquelyn Wynn, Mairead Hyland, The Lighthouse Lab in Alderley Park and Alex Alderton, Roberto Amato, Sonia Goncalves, Ewan Harrison, David K. Jackson, Ian Johnston, Dominic Kwiatkowski, Cordelia Langford, John Sillitoe on behalf of the Wellcome Sanger Institute COVID-19 Surveillance Team
EPI_ISL_760986	Lighthouse Lab in Glasgow	Wellcome Sanger Institute for the COVID-19 Genomics UK (COG-UK) Consortium	Harper VanSteenhouse, Yumi Kasai, David Gray, Carol Clugston, Anna Dominiczak and Alex Alderton, Roberto Amato, Sonia Goncalves, Ewan Harrison, David K. Jackson, Ian Johnston, Dominic Kwiatkowski, Cordelia Langford, John Sillitoe on behalf of the Wellcome Sanger Institute COVID-19 Surveillance Team
EPI_ISL_760988	Lighthouse Lab in Cambridge	Wellcome Sanger Institute for the COVID-19 Genomics UK (COG-UK) Consortium	Rob Howes, The Lighthouse Lab in Cambridge and Alex Alderton, Roberto Amato, Sonia Goncalves, Ewan Harrison, David K. Jackson, Ian Johnston, Dominic Kwiatkowski, Cordelia Langford, John Sillitoe on behalf of the Wellcome Sanger Institute COVID-19 Surveillance Team
EPI_ISL_761013, EPI_ISL_761046	Lighthouse Lab in Alderley Park	Wellcome Sanger Institute for the COVID-19 Genomics UK (COG-UK) Consortium	Jacquelyn Wynn, Mairead Hyland, The Lighthouse Lab in Alderley Park and Alex Alderton, Roberto Amato, Sonia Goncalves, Ewan Harrison, David K. Jackson, Ian Johnston, Dominic Kwiatkowski, Cordelia Langford, John Sillitoe on behalf of the Wellcome Sanger Institute COVID-19 Surveillance Team
EPI_ISL_761047	Lighthouse Lab in Glasgow	Wellcome Sanger Institute for the COVID-19 Genomics UK (COG-UK) Consortium	Harper VanSteenhouse, Yumi Kasai, David Gray, Carol Clugston, Anna Dominiczak and Alex Alderton, Roberto Amato, Sonia Goncalves, Ewan Harrison, David K. Jackson, Ian Johnston, Dominic Kwiatkowski, Cordelia Langford, John Sillitoe on behalf of the Wellcome Sanger Institute COVID-19 Surveillance Team
EPI_ISL_761050	Lighthouse Lab in Milton Keynes	Wellcome Sanger Institute for the COVID-19 Genomics UK (COG-UK) Consortium	The Lighthouse Lab in Milton Keynes and Alex Alderton, Roberto Amato, Sonia Goncalves, Ewan Harrison, David K. Jackson, Ian Johnston, Dominic Kwiatkowski, Cordelia Langford, John Sillitoe on behalf of the Wellcome Sanger Institute COVID-19 Surveillance Team
EPI_ISL_763004, EPI_ISL_763005, EPI_ISL_763006, EPI_ISL_763014, EPI_ISL_763015, EPI_ISL_763016, EPI_ISL_763017, EPI_ISL_763018, EPI_ISL_763019, EPI_ISL_763031, EPI_ISL_763032, EPI_ISL_763043, EPI_ISL_763044, EPI_ISL_763046, EPI_ISL_763060, EPI_ISL_763061			
see above	Unit 17: Influenza & Other Respiratory Viruses, German National Influenza Center	Project group Epidemiology of Highly Pathogenic Microorganisms, Robert Koch-Institute	Ariane Düx, Andreas Sachse, Grit Schubert, Sébastien Calvignac-Spencer, Fabian Leendertz, Thorsten Wolff, Ralf Dürrwald, Djin-Ye Oh, Marianne Wedde
EPI_ISL_763126, EPI_ISL_763174, EPI_ISL_763176, EPI_ISL_763191, EPI_ISL_763192, EPI_ISL_763193, EPI_ISL_763200	Dutch COVID-19 response team	Erasmus Medical Center	Bas Oude Munnink, Reina Sikkema, David Nieuwenhuijse, Irina Chestakova, Anne van der Linden, Marjan Boter, Emmanuelle Munger, Corine GeurtsvanKessel, Annemiek van der Eijk, Richard Molenkamp, Marion Koopmans, on behalf of the Dutch national COVID-19 response team.
EPI_ISL_763370	Department of Pathology, University of Cambridge	COVID-19 Genomics UK (COG-UK) Consortium	Aminu S. Jahun, Yasmin Chaudhry, Grant Hall, Iliana Georgana, Myra Hosmillo, Martin D. Curran, Malte Pinckert, Surendra Parmar, Ian Goodfellow
EPI_ISL_763392, EPI_ISL_763409, EPI_ISL_763416, EPI_ISL_763420, EPI_ISL_763422, EPI_ISL_763423, EPI_ISL_763454, EPI_ISL_763462, EPI_ISL_763467, EPI_ISL_763470, EPI_ISL_763479, EPI_ISL_763480, EPI_ISL_763481, EPI_ISL_763489, EPI_ISL_763492, EPI_ISL_763565, EPI_ISL_763572, EPI_ISL_763590, EPI_ISL_763658, EPI_ISL_763721, EPI_ISL_763725, EPI_ISL_763750, EPI_ISL_763760, EPI_ISL_763782, EPI_ISL_763821			
see above	Wales Specialist Virology Centre Sequencing lab: Pathogen Genomics Unit	COVID-19 Genomics UK (COG-UK) Consortium	Catherine Moore, Johnathan Evans, Laura Gifford, Malorie Perry, Simon Cottrell, Angela Marchbank, Alec Birchley, Alexander Adams, Amy Gaskin, Bree Gatica-Wilcox, Jason Coombes, Joel Southgate, Lauren Gilbert, Lee Graham, Nicole Pacchiarini, Sara Kumziene-Summerhayes, Sarah Taylor, Sophie Jones, Sara Rey, Matthew Bull, Joanne Watkins, Sally Corden, Tom Connor
EPI_ISL_763903	Virology Department, Royal Infirmary of Edinburgh, NHS Lothian / School of Biological Sciences, University of Edinburgh / Institute of Genetics and Molecular Medicine, University of Edinburgh	COVID-19 Genomics UK (COG-UK) Consortium	McHugh M, Dewar R, Rooke S, Gallagher M, Balcaza C, O'Toole Á, Scher E, Hill V, McCrone JT, Colquhoun R, Yu X, Jackson B, Rambaut A, Williams TC, Templeton K
EPI_ISL_763934, EPI_ISL_763935, EPI_ISL_763940, EPI_ISL_763941, EPI_ISL_763945	Oxford Viromics, NDM, University of Oxford; Oxford University Hospitals; Basingstoke and North Hampshire Hospital	COVID-19 Genomics UK (COG-UK) Consortium	Tanya Golubchik, David Bonsall, George Macintyre, Amy Trebes, Mariateresa de Cesare, Catrin Moore, Alex Mobbs, Anita Justice, Robert Shaw, Monique Andersson, Timothy Peto, Emma Wise, Nathan Moore, Jessica Lynch, Nick Cortes, Matilde Mori, Stephen Kidd, David Buck, John Todd, Christophe Fraser
EPI_ISL_764064	Wales Specialist Virology Centre Sequencing lab: Pathogen Genomics Unit	COVID-19 Genomics UK (COG-UK) Consortium	Catherine Moore, Johnathan Evans, Laura Gifford, Malorie Perry, Simon Cottrell, Angela Marchbank, Alec Birchley, Alexander Adams, Amy Gaskin, Bree Gatica-Wilcox, Jason Coombes, Joel Southgate, Lauren Gilbert, Lee Graham, Nicole Pacchiarini, Sara Kumziene-Summerhayes, Sarah Taylor, Sophie Jones, Sara Rey, Matthew Bull, Joanne Watkins, Sally Corden, Tom Connor
EPI_ISL_764066, EPI_ISL_764087	Oxford Viromics, NDM, University of Oxford; Oxford University Hospitals; Basingstoke and North Hampshire Hospital	COVID-19 Genomics UK (COG-UK) Consortium	Tanya Golubchik, David Bonsall, George Macintyre, Amy Trebes, Mariateresa de Cesare, Catrin Moore, Alex Mobbs, Anita Justice, Robert Shaw, Monique Andersson, Timothy Peto, Emma Wise, Nathan Moore, Jessica Lynch, Nick Cortes, Matilde Mori, Stephen Kidd, David Buck, John Todd, Christophe Fraser
EPI_ISL_764133, EPI_ISL_764138, EPI_ISL_764155, EPI_ISL_764157, EPI_ISL_764192, EPI_ISL_764200,	Wales Specialist Virology Centre Sequencing lab: Pathogen Genomics Unit	COVID-19 Genomics UK (COG-UK) Consortium	Catherine Moore, Johnathan Evans, Laura Gifford, Malorie Perry, Simon Cottrell, Angela Marchbank, Alec Birchley, Alexander Adams, Amy Gaskin, Bree Gatica-Wilcox, Jason Coombes, Joel Southgate, Lauren Gilbert, Lee Graham, Nicole Pacchiarini, Sara Kumziene-Summerhayes, Sarah Taylor, Sophie Jones, Sara Rey, Matthew Bull, Joanne Watkins, Sally Corden, Tom Connor

EPI_ISL_764201, EPI_ISL_764235			
EPI_ISL_764238	University College London, Great Ormond Street Hospital for Children NHS Foundation Trust, Imperial College Healthcare NHS Trust	COVID-19 Genomics UK (COG-UK) Consortium	Sergi Castellano, Rachel Williams, Mark Kristiansen, Paola Resende Silva, Sunando Roy, Tony Brooks, Helena Tutill, Paola Niola, Patricia Dyal, Charlotte Williams, Leysa Forrest, Yasmin Panchbhaya, Jacqueline Findlay, Samuel Weeks, Julianne Brown, Kathryn Harris, Paul Randell, James Price, Alison Holmes, Judith Breuer
EPI_ISL_764255	Wales Specialist Virology Centre Sequencing lab: Pathogen Genomics Unit	COVID-19 Genomics UK (COG-UK) Consortium	Catherine Moore, Johnathan Evans, Laura Gifford, Malorie Perry, Simon Cottrell, Angela Marchbank, Alec Birchley, Alexander Adams, Amy Gaskin, Bree Gatica-Wilcox, Jason Coombes, Joel Southgate, Lauren Gilbert, Lee Graham, Nicole Pacchiarini, Sara Kumziene-Summerhayes, Sarah Taylor, Sophie Jones, Sara Rey, Matthew Bull, Joanne Watkins, Sally Corden, Tom Connor
EPI_ISL_764303, EPI_ISL_764304	Department of Pathology, University of Cambridge	COVID-19 Genomics UK (COG-UK) Consortium	Aminu S. Jahun, Yasmin Chaudhry, Grant Hall, Iliana Georgana, Myra Hosmillo, Martin D. Curran, Malte Pinckert, Surendra Parmar, Ian Goodfellow
EPI_ISL_764532, EPI_ISL_764533, EPI_ISL_764538, EPI_ISL_764539	Oxford Viromics, NDM, University of Oxford; Oxford University Hospitals; Basingstoke and North Hampshire Hospital	COVID-19 Genomics UK (COG-UK) Consortium	Tanya Golubchik, David Bonsall, George Macintyre, Amy Trebes, Mariateresa de Cesare, Catrin Moore, Alex Mobbs, Anita Justice, Robert Shaw, Monique Andersson, Timothy Peto, Emma Wise, Nathan Moore, Jessica Lynch, Nick Cortes, Matilde Mori, Stephen Kidd, David Buck, John Todd, Christophe Fraser
EPI_ISL_764582, EPI_ISL_764746, EPI_ISL_764747, EPI_ISL_764753, EPI_ISL_764754, EPI_ISL_764755, EPI_ISL_764769, EPI_ISL_764770, EPI_ISL_764771, EPI_ISL_764772, EPI_ISL_764773, EPI_ISL_764774, EPI_ISL_764775, EPI_ISL_764776, EPI_ISL_764777, EPI_ISL_764778, EPI_ISL_764779, EPI_ISL_764780, EPI_ISL_764781, EPI_ISL_764782, EPI_ISL_764783, EPI_ISL_764784, EPI_ISL_764785, EPI_ISL_764786, EPI_ISL_764787, EPI_ISL_764788, EPI_ISL_764789, EPI_ISL_764790, EPI_ISL_764791, EPI_ISL_764792, EPI_ISL_764793, EPI_ISL_764794, EPI_ISL_764795, EPI_ISL_764796, EPI_ISL_764797, EPI_ISL_764798, EPI_ISL_764799, EPI_ISL_764800, EPI_ISL_764801, EPI_ISL_764802, EPI_ISL_764803, EPI_ISL_764804			
see above	Wales Specialist Virology Centre Sequencing lab: Pathogen Genomics Unit	COVID-19 Genomics UK (COG-UK) Consortium	Catherine Moore, Johnathan Evans, Laura Gifford, Malorie Perry, Simon Cottrell, Angela Marchbank, Alec Birchley, Alexander Adams, Amy Gaskin, Bree Gatica-Wilcox, Jason Coombes, Joel Southgate, Lauren Gilbert, Lee Graham, Nicole Pacchiarini, Sara Kumziene-Summerhayes, Sarah Taylor, Sophie Jones, Sara Rey, Matthew Bull, Joanne Watkins, Sally Corden, Tom Connor
EPI_ISL_765818, EPI_ISL_765819, EPI_ISL_765820, EPI_ISL_765821, EPI_ISL_765822, EPI_ISL_765823	North Shore Medical Center	Infectious Disease Program, Broad Institute of Harvard and MIT	Lemieux,J.E., Siddle,K.J., Shaw,B., Adams,G., Pierce,V., Turbett,S., Anahtar,M., Branda,J., Slater,D., Harris,J., Lin,A.E., Gladden-Young,A., Lagerborg,K., Rudy,M., DeRuff,K., Carter,A., Normandin,E., Bauer,M., Reilly,S., Tomkins-Tinch,C., Loreth,C., Chaluvadi,S., Neumann,A., Cusick,C., Chapman,S.B., Gnirke,A., Flowers,K., Cerrato,F., Birren,B.W., Gallagher,G., Smole,S., Park,D.J., MacInnis,B.L., Ryan,E., LaRocque,R., Rosenberg,E. and Sabeti,P.C.
EPI_ISL_765893, EPI_ISL_765894	Unit 17: Influenza & Other Respiratory Viruses, German National Influenza Center	Project group Epidemiology of Highly Pathogenic Microorganisms, Robert Koch-Institute	Ariane Düx, Andreas Sachse, Grit Schubert, Sébastien Calvignac-Spencer, Fabian Leendertz, Thorsten Wolff, Ralf Dürrwald, Djin-Ye Oh, Marianne Wedde
EPI_ISL_765960, EPI_ISL_765961, EPI_ISL_765962, EPI_ISL_765963, EPI_ISL_765964, EPI_ISL_765965, EPI_ISL_765966, EPI_ISL_765967, EPI_ISL_765968, EPI_ISL_765969, EPI_ISL_765970, EPI_ISL_765971, EPI_ISL_765972, EPI_ISL_765973, EPI_ISL_765974, EPI_ISL_765975, EPI_ISL_765976, EPI_ISL_765977, EPI_ISL_765978			
see above	Wyoming Public Health Laboratory	Wyoming Public Health Laboratory	Noah Hull, Taylor Fearing, Channing Weber, Ashley Norberg, Bailey Bowcutt, and Wanda Manley
EPI_ISL_766096, EPI_ISL_766125, EPI_ISL_766129, EPI_ISL_766130, EPI_ISL_766213, EPI_ISL_766246, EPI_ISL_766247, EPI_ISL_766281, EPI_ISL_766282, EPI_ISL_766283, EPI_ISL_766285, EPI_ISL_766289, EPI_ISL_766291, EPI_ISL_766292, EPI_ISL_766506, EPI_ISL_766507, EPI_ISL_766510, EPI_ISL_766511, EPI_ISL_766512, EPI_ISL_766513, EPI_ISL_766514, EPI_ISL_766515, EPI_ISL_766516, EPI_ISL_766558, EPI_ISL_766559, EPI_ISL_766561			
see above	Respiratory Virus Unit, National Infection Service, Public Health England	COVID-19 Genomics UK (COG-UK) Consortium	PHE Covid Sequencing Team
EPI_ISL_766630	Klinisk mikrobiologi, Viruslab	The Public Health Agency of Sweden	Department of Microbiology, The Public Health Agency of Sweden
EPI_ISL_766651, EPI_ISL_766658	Texas Department of State Health Services	Texas Department of State Health Services	Rashmi Tuladhar, Bonnie Oh, Jenny Zhang, Maliha Rahman, Anita Pokharel, Myong Koag, Chung Wang, Rachel Lee, Grace Kubin, Mayela Pedrueza, James Daniel Bonser
EPI_ISL_766853	Respiratory Virus Unit, National Infection Service, Public Health England	COVID-19 Genomics UK (COG-UK) Consortium	PHE Covid Sequencing Team
EPI_ISL_767134	Lighthouse Lab in Milton Keynes	Wellcome Sanger Institute for the COVID-19 Genomics UK (COG-UK) Consortium	The Lighthouse Lab in Milton Keynes and Alex Alderton, Roberto Amato, Sonia Goncalves, Ewan Harrison, David K. Jackson, Ian Johnston, Dominic Kwiatkowski, Cordelia Langford, John Sillitoe on behalf of the Wellcome Sanger Institute COVID-19 Surveillance Team
EPI_ISL_767151, EPI_ISL_767152, EPI_ISL_767153, EPI_ISL_767154, EPI_ISL_767155, EPI_ISL_767156, EPI_ISL_767157, EPI_ISL_767158, EPI_ISL_767159, EPI_ISL_767160, EPI_ISL_767161, EPI_ISL_767162, EPI_ISL_767163, EPI_ISL_767164, EPI_ISL_767165, EPI_ISL_767166, EPI_ISL_767167, EPI_ISL_767168, EPI_ISL_767169, EPI_ISL_767170, EPI_ISL_767171, EPI_ISL_767172, EPI_ISL_767173, EPI_ISL_767174, EPI_ISL_767175, EPI_ISL_767176, EPI_ISL_767177, EPI_ISL_767179, EPI_ISL_767180, EPI_ISL_767181, EPI_ISL_767182, EPI_ISL_767183, EPI_ISL_767184, EPI_ISL_767185, EPI_ISL_767186, EPI_ISL_767187, EPI_ISL_767188, EPI_ISL_767189, EPI_ISL_767190, EPI_ISL_767191, EPI_ISL_767192, EPI_ISL_767194, EPI_ISL_767195, EPI_ISL_767196, EPI_ISL_767197, EPI_ISL_767198, EPI_ISL_767199, EPI_ISL_767200, EPI_ISL_767201, EPI_ISL_767202, EPI_ISL_767203, EPI_ISL_767204, EPI_ISL_767206, EPI_ISL_767207, EPI_ISL_767208, EPI_ISL_767209, EPI_ISL_767210, EPI_ISL_767211, EPI_ISL_767212, EPI_ISL_767213, EPI_ISL_767214, EPI_ISL_767215, EPI_ISL_767221, EPI_ISL_767230, EPI_ISL_767251, EPI_ISL_767252, EPI_ISL_767253, EPI_ISL_767254, EPI_ISL_767255, EPI_ISL_767256, EPI_ISL_767257, EPI_ISL_767258, EPI_ISL_767259, EPI_ISL_767260, EPI_ISL_767261, EPI_ISL_767262, EPI_ISL_767263, EPI_ISL_767264, EPI_ISL_767265, EPI_ISL_767266, EPI_ISL_767267, EPI_ISL_767268, EPI_ISL_767269, EPI_ISL_767270, EPI_ISL_767271, EPI_ISL_767272, EPI_ISL_767273, EPI_ISL_767274, EPI_ISL_767275, EPI_ISL_767276, EPI_ISL_767278, EPI_ISL_767284			
see above	Lighthouse Lab in Alderley Park	Wellcome Sanger Institute for the COVID-19 Genomics UK (COG-UK) Consortium	Jacquelyn Wynn, Mairead Hyland, The Lighthouse Lab in Alderley Park and Alex Alderton, Roberto Amato, Sonia Goncalves, Ewan Harrison, David K. Jackson, Ian Johnston, Dominic Kwiatkowski, Cordelia Langford, John Sillitoe on behalf of the Wellcome Sanger Institute COVID-19 Surveillance Team
EPI_ISL_767344, EPI_ISL_767345, EPI_ISL_767347, EPI_ISL_767349, EPI_ISL_767350, EPI_ISL_767354, EPI_ISL_767358, EPI_ISL_767359, EPI_ISL_767361	Michigan Department of Health and Human Services, Bureau of Laboratories	Michigan Department of Health and Human Services, Bureau of Laboratories	Blankenship HM, Riner D, Soehnlen MK
EPI_ISL_767655, EPI_ISL_767658, EPI_ISL_767690, EPI_ISL_767691, EPI_ISL_767692, EPI_ISL_767693, EPI_ISL_767694, EPI_ISL_767695, EPI_ISL_767696, EPI_ISL_767697, EPI_ISL_767698, EPI_ISL_767699, EPI_ISL_767700, EPI_ISL_767701, EPI_ISL_767702, EPI_ISL_767703, EPI_ISL_767704, EPI_ISL_767705, EPI_ISL_767706, EPI_ISL_767707, EPI_ISL_767708, EPI_ISL_767709			
see above	MEMORIAL SLOAN KETTERING CANCER CENTER	Wadsworth Center, New York State Department.of Health	Kirsten St. George, Daryl M. Lamson, Alexis Russel, Matthew Shudt, Melissa A Leisner, Jonathan Plitnick, Navjot Singh, John Kelly, Sara Griesemer, Erasmus Schneider, Erica Lasek-Nesselquist
EPI_ISL_768398, EPI_ISL_768399, EPI_ISL_768400, EPI_ISL_768401, EPI_ISL_768402, EPI_ISL_768403, EPI_ISL_768404, EPI_ISL_768405, EPI_ISL_768406, EPI_ISL_768407, EPI_ISL_768408, EPI_ISL_768409, EPI_ISL_768410, EPI_ISL_768411, EPI_ISL_768412, EPI_ISL_768413, EPI_ISL_768414, EPI_ISL_768415, EPI_ISL_768416, EPI_ISL_768417, EPI_ISL_768418			
see above	LSUHS Emerging Viral Threat Laboratory	Microbial Genome Sequencing Center	Jeremy P. Kamil, Jennifer L. Carroll, Camille F. Abshire, Maarten Van Diest, Andrew D. Yurochko, Martin J. Sapp, Rona S. Scott, Christopher G. Kevil, Daniel J. Snyder, Vaughn S. Cooper, John A. Vanchiere
EPI_ISL_768633	Jena University Hospital, Institute for Infectious Diseases and Infection Control	Institute of infectious medicine & hospital hygiene, CaSe-Group	Spott, Riccardo; Marquet, Mike; Pletz, Matthias W.; Brandt, Christian
EPI_ISL_768801, EPI_ISL_768802, EPI_ISL_768803	Respiratory Virus Unit, National Infection Service, Public Health England	COVID-19 Genomics UK (COG-UK) Consortium	PHE Covid Sequencing Team
EPI_ISL_769545, EPI_ISL_769584, EPI_ISL_769590, EPI_ISL_769597, EPI_ISL_769646, EPI_ISL_769715, EPI_ISL_769744, EPI_ISL_769764, EPI_ISL_769776, EPI_ISL_769845, EPI_ISL_769852, EPI_ISL_769857			
see above	Lighthouse Lab in Milton Keynes	Wellcome Sanger Institute for the COVID-19 Genomics UK (COG-UK) Consortium	The Lighthouse Lab in Milton Keynes and Alex Alderton, Roberto Amato, Sonia Goncalves, Ewan Harrison, David K. Jackson, Ian Johnston, Dominic Kwiatkowski, Cordelia Langford, John Sillitoe on behalf of the Wellcome Sanger Institute COVID-19 Surveillance Team
EPI_ISL_769868, EPI_ISL_769870, EPI_ISL_769878	Respiratory Virus Unit, National Infection Service, Public Health England	COVID-19 Genomics UK (COG-UK) Consortium	PHE Covid Sequencing Team
EPI_ISL_770791, EPI_ISL_770792	Minnesota Department of Health, Public Health Laboratory	Minnesota Department of Health, Public Health Laboratory	Alexandra Lorentz, Jacob Garfin, Matt Plumb, and Xiong Wang
EPI_ISL_775020	Gonoshasthya-RNA Molecular Research Center	Gonoshasthya-RNA Molecular Research Center	Mohd. Raeed Jamiruddin, Nihad Adnan, Md. Ahsanul Haq, Mohib Ullah Khondoker, Nafisa Azmuda, Firoz Ahmed, Shahana Sharmin, Salma Akter, Taslin Jahan Mou, Mahfuza Marzan, Sayeda Moriam Liza, Nowshin Jahan, Tamanna Ali, Shahad Saif Khandker, Maha Jamiruddin, Mousumi Chaity, Mumtarin Jannat Oishee
EPI_ISL_775213	Gonoshasthya-RNA Molecular Research Center	Gonoshasthya-RNA Molecular Research Center	Nihad Adnan, Mohd. Raeed Jamiruddin, Md. Ahsanul Haq, Mohib Ullah Khondoker, Nafisa Azmuda, Firoz Ahmed, Shahana Sharmin, Salma Akter, Taslin Jahan Mou, Mahfuza Marzan, Sayeda Moriam Liza, Nowshin Jahan, Tamanna Ali, Maha Jamiruddin, Mousumi Chaity, Shahad Saif Khandker, Mumtarin Jannat Oishee

EPI_ISL_775293	Oslo University Hospital, Department of Medical Microbiology	Norwegian Institute of Public Health, Department of Virology	Kathrine Stene-Johansen, Kamilla Heddeland Instefjord, Hilde Elshaug, Atiya R Ali,Marie Paulsen Madsen, Rasmus Riis Kopperud, Hilde Vollan, Karoline Bragstad, Olav Hungnes
EPI_ISL_775403	Nordland Hospital - Bodo, Laboratory Department, Molecular Biology Unit	Norwegian Institute of Public Health, Department of Virology	Kathrine Stene-Johansen, Kamilla Heddeland Instefjord, Hilde Elshaug, Atiya R Ali,Marie Paulsen Madsen, Rasmus Riis Kopperud, Hilde Vollan, Karoline Bragstad, Olav Hungnes
EPI_ISL_775406, EPI_ISL_775463	Unilabs Laboratory Medicine	Norwegian Institute of Public Health, Department of Virology	Kathrine Stene-Johansen, Kamilla Heddeland Instefjord, Hilde Elshaug, Atiya R Ali,Marie Paulsen Madsen, Rasmus Riis Kopperud, Hilde Vollan, Karoline Bragstad, Olav Hungnes
EPI_ISL_775505, EPI_ISL_775506, EPI_ISL_775507, EPI_ISL_775508	Department of Medical Microbiology, St. Olavs hospital	Norwegian Institute of Public Health, Department of Virology	Kathrine Stene-Johansen, Kamilla Heddeland Instefjord, Hilde Elshaug, Atiya R Ali,Marie Paulsen Madsen, Rasmus Riis Kopperud, Hilde Vollan, Karoline Bragstad, Olav Hungnes
EPI_ISL_775516, EPI_ISL_775517	Unilabs Laboratory Medicine	Norwegian Institute of Public Health, Department of Virology	Kathrine Stene-Johansen, Kamilla Heddeland Instefjord, Hilde Elshaug, Atiya R Ali,Marie Paulsen Madsen, Rasmus Riis Kopperud, Hilde Vollan, Karoline Bragstad, Olav Hungnes
EPI_ISL_775520	Hospital of Southern Norway - Kristiansand, Department of Medical Microbiology	Norwegian Institute of Public Health, Department of Virology	Kathrine Stene-Johansen, Kamilla Heddeland Instefjord, Hilde Elshaug, Atiya R Ali,Marie Paulsen Madsen, Rasmus Riis Kopperud, Hilde Vollan, Karoline Bragstad, Olav Hungnes
EPI_ISL_775531, EPI_ISL_775544, EPI_ISL_775545	Klinisk mikrobiologi, Viruslab	The Public Health Agency of Sweden	Department of Microbiology, The Public Health Agency of Sweden
EPI_ISL_775549, EPI_ISL_775592	Klinisk mikrobiologi	The Public Health Agency of Sweden	Department of Microbiology, The Public Health Agency of Sweden
EPI_ISL_776696, EPI_ISL_776697, EPI_ISL_776698, EPI_ISL_776699, EPI_ISL_776700, EPI_ISL_776701, EPI_ISL_776702, EPI_ISL_776703, EPI_ISL_776704, EPI_ISL_776705, EPI_ISL_776730, EPI_ISL_776746			
see above	UW Virology Lab	UW Virology Lab	Pavitra Roychoudhury, Hong Xie, Lasata Shrestha, Meei-Li Huang, Keith R Jerome, Alexander Greninger
EPI_ISL_777013	Lighthouse Lab in Glasgow	Wellcome Sanger Institute for the COVID-19 Genomics UK (COG-UK) Consortium	Harper VanSteenhouse, Yumi Kasai, David Gray, Carol Clugston, Anna Dominiczak and Alex Alderton, Roberto Amato, Sonia Goncalves, Ewan Harrison, David K. Jackson, Ian Johnston, Dominic Kwiatkowski, Cordelia Langford, John Sillitoe on behalf of the Wellcome Sanger Institute COVID-19 Surveillance Team
EPI_ISL_777039	Lighthouse Lab in Milton Keynes	Wellcome Sanger Institute for the COVID-19 Genomics UK (COG-UK) Consortium	The Lighthouse Lab in Milton Keynes and Alex Alderton, Roberto Amato, Sonia Goncalves, Ewan Harrison, David K. Jackson, Ian Johnston, Dominic Kwiatkowski, Cordelia Langford, John Sillitoe on behalf of the Wellcome Sanger Institute COVID-19 Surveillance Team
EPI_ISL_777081	Lighthouse Lab in Glasgow	Wellcome Sanger Institute for the COVID-19 Genomics UK (COG-UK) Consortium	Harper VanSteenhouse, Yumi Kasai, David Gray, Carol Clugston, Anna Dominiczak and Alex Alderton, Roberto Amato, Sonia Goncalves, Ewan Harrison, David K. Jackson, Ian Johnston, Dominic Kwiatkowski, Cordelia Langford, John Sillitoe on behalf of the Wellcome Sanger Institute COVID-19 Surveillance Team
EPI_ISL_777085	Lighthouse Lab in Milton Keynes	Wellcome Sanger Institute for the COVID-19 Genomics UK (COG-UK) Consortium	The Lighthouse Lab in Milton Keynes and Alex Alderton, Roberto Amato, Sonia Goncalves, Ewan Harrison, David K. Jackson, Ian Johnston, Dominic Kwiatkowski, Cordelia Langford, John Sillitoe on behalf of the Wellcome Sanger Institute COVID-19 Surveillance Team
EPI_ISL_777101	Lighthouse Lab in Glasgow	Wellcome Sanger Institute for the COVID-19 Genomics UK (COG-UK) Consortium	Harper VanSteenhouse, Yumi Kasai, David Gray, Carol Clugston, Anna Dominiczak and Alex Alderton, Roberto Amato, Sonia Goncalves, Ewan Harrison, David K. Jackson, Ian Johnston, Dominic Kwiatkowski, Cordelia Langford, John Sillitoe on behalf of the Wellcome Sanger Institute COVID-19 Surveillance Team
EPI_ISL_777102, EPI_ISL_777108	Lighthouse Lab in Milton Keynes	Wellcome Sanger Institute for the COVID-19 Genomics UK (COG-UK) Consortium	The Lighthouse Lab in Milton Keynes and Alex Alderton, Roberto Amato, Sonia Goncalves, Ewan Harrison, David K. Jackson, Ian Johnston, Dominic Kwiatkowski, Cordelia Langford, John Sillitoe on behalf of the Wellcome Sanger Institute COVID-19 Surveillance Team
EPI_ISL_777142	Lighthouse Lab in Glasgow	Wellcome Sanger Institute for the COVID-19 Genomics UK (COG-UK) Consortium	Harper VanSteenhouse, Yumi Kasai, David Gray, Carol Clugston, Anna Dominiczak and Alex Alderton, Roberto Amato, Sonia Goncalves, Ewan Harrison, David K. Jackson, Ian Johnston, Dominic Kwiatkowski, Cordelia Langford, John Sillitoe on behalf of the Wellcome Sanger Institute COVID-19 Surveillance Team
EPI_ISL_777145	Lighthouse Lab in Milton Keynes	Wellcome Sanger Institute for the COVID-19 Genomics UK (COG-UK) Consortium	The Lighthouse Lab in Milton Keynes and Alex Alderton, Roberto Amato, Sonia Goncalves, Ewan Harrison, David K. Jackson, Ian Johnston, Dominic Kwiatkowski, Cordelia Langford, John Sillitoe on behalf of the Wellcome Sanger Institute COVID-19 Surveillance Team
EPI_ISL_777169	Lighthouse Lab in Alderley Park	Wellcome Sanger Institute for the COVID-19 Genomics UK (COG-UK) Consortium	Jacquelyn Wynn, Mairead Hyland, The Lighthouse Lab in Alderley Park and Alex Alderton, Roberto Amato, Sonia Goncalves, Ewan Harrison, David K. Jackson, Ian Johnston, Dominic Kwiatkowski, Cordelia Langford, John Sillitoe on behalf of the Wellcome Sanger Institute COVID-19 Surveillance Team
EPI_ISL_777192	Lighthouse Lab in Glasgow	Wellcome Sanger Institute for the COVID-19 Genomics UK (COG-UK) Consortium	Harper VanSteenhouse, Yumi Kasai, David Gray, Carol Clugston, Anna Dominiczak and Alex Alderton, Roberto Amato, Sonia Goncalves, Ewan Harrison, David K. Jackson, Ian Johnston, Dominic Kwiatkowski, Cordelia Langford, John Sillitoe on behalf of the Wellcome Sanger Institute COVID-19 Surveillance Team
EPI_ISL_777201	Lighthouse Lab in Cambridge	Wellcome Sanger Institute for the COVID-19 Genomics UK (COG-UK) Consortium	Rob Howes, The Lighthouse Lab in Cambridge and Alex Alderton, Roberto Amato, Sonia Goncalves, Ewan Harrison, David K. Jackson, Ian Johnston, Dominic Kwiatkowski, Cordelia Langford, John Sillitoe on behalf of the Wellcome Sanger Institute COVID-19 Surveillance Team
EPI_ISL_777295, EPI_ISL_777313	Lighthouse Lab in Milton Keynes	Wellcome Sanger Institute for the COVID-19 Genomics UK (COG-UK) Consortium	The Lighthouse Lab in Milton Keynes and Alex Alderton, Roberto Amato, Sonia Goncalves, Ewan Harrison, David K. Jackson, Ian Johnston, Dominic Kwiatkowski, Cordelia Langford, John Sillitoe on behalf of the Wellcome Sanger Institute COVID-19 Surveillance Team
EPI_ISL_777323	Lighthouse Lab in Cambridge	Wellcome Sanger Institute for the COVID-19 Genomics UK (COG-UK) Consortium	Rob Howes, The Lighthouse Lab in Cambridge and Alex Alderton, Roberto Amato, Sonia Goncalves, Ewan Harrison, David K. Jackson, Ian Johnston, Dominic Kwiatkowski, Cordelia Langford, John Sillitoe on behalf of the Wellcome Sanger Institute COVID-19 Surveillance Team
EPI_ISL_778837, EPI_ISL_778838	AIID	Irish Coronavirus Sequencing Consortium-Teagasc Grange	Matthew McCabe, Aljandro Abner Garcia Leon, Fiona Crispie, Calum Walsh, Michael Carr, John Kenny, Paul Cotter, Patrick Mallon, Gabriel Gonzalez
EPI_ISL_779813	CNR Virus des Infections Respiratoires - France SUD	CNR Virus des Infections Respiratoires - France SUD	Antonin Bal, Gregory Destras, Gwendolyne Burfin, Hadrien Règue, Quentin Semanas, Martine Valette, Bruno Lina, Laurence Josset
EPI_ISL_779837	CHU Bordeaux	CNR Virus des Infections Respiratoires - France SUD	Antonin Bal, Gregory Destras, Gwendolyne Burfin, Hadrien Règue, Quentin Semanas, Martine Valette, Bruno Lina, Pantxika Bellecave, Camille Ciccone, Isabelle Garrigue, Marie-Edith Lafon, Pascale Trimoulet, Laurence Josset
EPI_ISL_779845	CNR Virus des Infections Respiratoires - France SUD	CNR Virus des Infections Respiratoires - France SUD	Antonin Bal, Gregory Destras, Gwendolyne Burfin, Hadrien Règue, Quentin Semanas, Martine Valette, Bruno Lina, Laurence Josset
EPI_ISL_779862, EPI_ISL_779867, EPI_ISL_779870, EPI_ISL_779873, EPI_ISL_779876, EPI_ISL_779877, EPI_ISL_779887, EPI_ISL_779900, EPI_ISL_779903, EPI_ISL_779904, EPI_ISL_779920			
see above	Servicio de Microbiología, Hospital Universitario Son Espases	SeqCOVID-SPAIN consortium/IBV(CSIC)	Carla López-Causapé, Jordi Reina, Antonio Oliver and SeqCOVID-SPAIN consortium
EPI_ISL_782486, EPI_ISL_782494, EPI_ISL_782500, EPI_ISL_782504, EPI_ISL_782512, EPI_ISL_782536, EPI_ISL_782538, EPI_ISL_782568, EPI_ISL_782580, EPI_ISL_782584, EPI_ISL_782596, EPI_ISL_782605, EPI_ISL_782618, EPI_ISL_782624, EPI_ISL_782631, EPI_ISL_782633, EPI_ISL_782674, EPI_ISL_782679, EPI_ISL_782689, EPI_ISL_782710			
see above	Lighthouse Lab in Glasgow	Wellcome Sanger Institute for the COVID-19 Genomics UK (COG-UK) Consortium	Harper VanSteenhouse, Yumi Kasai, David Gray, Carol Clugston, Anna Dominiczak and Alex Alderton, Roberto Amato, Sonia Goncalves, Ewan Harrison, David K. Jackson, Ian Johnston, Dominic Kwiatkowski, Cordelia Langford, John Sillitoe on behalf of the Wellcome Sanger Institute COVID-19 Surveillance Team
EPI_ISL_782734, EPI_ISL_782735, EPI_ISL_782736, EPI_ISL_782738	Lighthouse Lab in Milton Keynes	Wellcome Sanger Institute for the COVID-19 Genomics UK (COG-UK) Consortium	The Lighthouse Lab in Milton Keynes and Alex Alderton, Roberto Amato, Sonia Goncalves, Ewan Harrison, David K. Jackson, Ian Johnston, Dominic Kwiatkowski, Cordelia Langford, John Sillitoe on behalf of the Wellcome Sanger Institute COVID-19 Surveillance Team
EPI_ISL_782739	Lighthouse Lab in Cambridge	Wellcome Sanger Institute for the COVID-19 Genomics UK (COG-UK) Consortium	Rob Howes, The Lighthouse Lab in Cambridge and Alex Alderton, Roberto Amato, Sonia Goncalves, Ewan Harrison, David K. Jackson, Ian Johnston, Dominic Kwiatkowski, Cordelia Langford, John Sillitoe on behalf of the Wellcome Sanger Institute COVID-19 Surveillance Team
EPI_ISL_782740, EPI_ISL_782741, EPI_ISL_782742, EPI_ISL_782743, EPI_ISL_782744	Lighthouse Lab in Milton Keynes	Wellcome Sanger Institute for the COVID-19 Genomics UK (COG-UK) Consortium	The Lighthouse Lab in Milton Keynes and Alex Alderton, Roberto Amato, Sonia Goncalves, Ewan Harrison, David K. Jackson, Ian Johnston, Dominic Kwiatkowski, Cordelia Langford, John Sillitoe on behalf of the Wellcome Sanger Institute COVID-19 Surveillance Team
EPI_ISL_782746	Lighthouse Lab in Cambridge	Wellcome Sanger Institute for the COVID-19 Genomics UK (COG-UK) Consortium	Rob Howes, The Lighthouse Lab in Cambridge and Alex Alderton, Roberto Amato, Sonia Goncalves, Ewan Harrison, David K. Jackson, Ian Johnston, Dominic Kwiatkowski, Cordelia Langford, John Sillitoe on behalf of the Wellcome Sanger Institute COVID-19 Surveillance Team
EPI_ISL_782749, EPI_ISL_782750,	Lighthouse Lab in Milton Keynes	Wellcome Sanger Institute for the COVID-19 Genomics UK	The Lighthouse Lab in Milton Keynes and Alex Alderton, Roberto Amato, Sonia Goncalves, Ewan Harrison, David K. Jackson, Ian Johnston, Dominic

EPI_ISL_782751, EPI_ISL_782752, EPI_ISL_782753, EPI_ISL_782754, EPI_ISL_782755, EPI_ISL_782756		(COG-UK) Consortium	Kwiatkowski, Cordelia Langford, John Sillitoe on behalf of the Wellcome Sanger Institute COVID-19 Surveillance Team
EPI_ISL_782757, EPI_ISL_782759	Lighthouse Lab in Cambridge	Wellcome Sanger Institute for the COVID-19 Genomics UK (COG-UK) Consortium	Rob Howes, The Lighthouse Lab in Cambridge and Alex Alderton, Roberto Amato, Sonia Goncalves, Ewan Harrison, David K. Jackson, Ian Johnston, Dominic Kwiatkowski, Cordelia Langford, John Sillitoe on behalf of the Wellcome Sanger Institute COVID-19 Surveillance Team
EPI_ISL_782760	Lighthouse Lab in Milton Keynes	Wellcome Sanger Institute for the COVID-19 Genomics UK (COG-UK) Consortium	The Lighthouse Lab in Milton Keynes and Alex Alderton, Roberto Amato, Sonia Goncalves, Ewan Harrison, David K. Jackson, Ian Johnston, Dominic Kwiatkowski, Cordelia Langford, John Sillitoe on behalf of the Wellcome Sanger Institute COVID-19 Surveillance Team
EPI_ISL_782761	Lighthouse Lab in Cambridge	Wellcome Sanger Institute for the COVID-19 Genomics UK (COG-UK) Consortium	Rob Howes, The Lighthouse Lab in Cambridge and Alex Alderton, Roberto Amato, Sonia Goncalves, Ewan Harrison, David K. Jackson, Ian Johnston, Dominic Kwiatkowski, Cordelia Langford, John Sillitoe on behalf of the Wellcome Sanger Institute COVID-19 Surveillance Team
EPI_ISL_782762	Lighthouse Lab in Milton Keynes	Wellcome Sanger Institute for the COVID-19 Genomics UK (COG-UK) Consortium	The Lighthouse Lab in Milton Keynes and Alex Alderton, Roberto Amato, Sonia Goncalves, Ewan Harrison, David K. Jackson, Ian Johnston, Dominic Kwiatkowski, Cordelia Langford, John Sillitoe on behalf of the Wellcome Sanger Institute COVID-19 Surveillance Team
EPI_ISL_782763, EPI_ISL_782764	Lighthouse Lab in Cambridge	Wellcome Sanger Institute for the COVID-19 Genomics UK (COG-UK) Consortium	Rob Howes, The Lighthouse Lab in Cambridge and Alex Alderton, Roberto Amato, Sonia Goncalves, Ewan Harrison, David K. Jackson, Ian Johnston, Dominic Kwiatkowski, Cordelia Langford, John Sillitoe on behalf of the Wellcome Sanger Institute COVID-19 Surveillance Team
EPI_ISL_782765, EPI_ISL_782766, EPI_ISL_782767	Lighthouse Lab in Milton Keynes	Wellcome Sanger Institute for the COVID-19 Genomics UK (COG-UK) Consortium	The Lighthouse Lab in Milton Keynes and Alex Alderton, Roberto Amato, Sonia Goncalves, Ewan Harrison, David K. Jackson, Ian Johnston, Dominic Kwiatkowski, Cordelia Langford, John Sillitoe on behalf of the Wellcome Sanger Institute COVID-19 Surveillance Team
EPI_ISL_782769	Lighthouse Lab in Cambridge	Wellcome Sanger Institute for the COVID-19 Genomics UK (COG-UK) Consortium	Rob Howes, The Lighthouse Lab in Cambridge and Alex Alderton, Roberto Amato, Sonia Goncalves, Ewan Harrison, David K. Jackson, Ian Johnston, Dominic Kwiatkowski, Cordelia Langford, John Sillitoe on behalf of the Wellcome Sanger Institute COVID-19 Surveillance Team
EPI_ISL_782770, EPI_ISL_782772, EPI_ISL_782773, EPI_ISL_782775	Lighthouse Lab in Milton Keynes	Wellcome Sanger Institute for the COVID-19 Genomics UK (COG-UK) Consortium	The Lighthouse Lab in Milton Keynes and Alex Alderton, Roberto Amato, Sonia Goncalves, Ewan Harrison, David K. Jackson, Ian Johnston, Dominic Kwiatkowski, Cordelia Langford, John Sillitoe on behalf of the Wellcome Sanger Institute COVID-19 Surveillance Team
EPI_ISL_782776, EPI_ISL_782777	Lighthouse Lab in Cambridge	Wellcome Sanger Institute for the COVID-19 Genomics UK (COG-UK) Consortium	Rob Howes, The Lighthouse Lab in Cambridge and Alex Alderton, Roberto Amato, Sonia Goncalves, Ewan Harrison, David K. Jackson, Ian Johnston, Dominic Kwiatkowski, Cordelia Langford, John Sillitoe on behalf of the Wellcome Sanger Institute COVID-19 Surveillance Team
EPI_ISL_782778, EPI_ISL_782779	Lighthouse Lab in Milton Keynes	Wellcome Sanger Institute for the COVID-19 Genomics UK (COG-UK) Consortium	The Lighthouse Lab in Milton Keynes and Alex Alderton, Roberto Amato, Sonia Goncalves, Ewan Harrison, David K. Jackson, Ian Johnston, Dominic Kwiatkowski, Cordelia Langford, John Sillitoe on behalf of the Wellcome Sanger Institute COVID-19 Surveillance Team
EPI_ISL_782780	Lighthouse Lab in Cambridge	Wellcome Sanger Institute for the COVID-19 Genomics UK (COG-UK) Consortium	Rob Howes, The Lighthouse Lab in Cambridge and Alex Alderton, Roberto Amato, Sonia Goncalves, Ewan Harrison, David K. Jackson, Ian Johnston, Dominic Kwiatkowski, Cordelia Langford, John Sillitoe on behalf of the Wellcome Sanger Institute COVID-19 Surveillance Team
EPI_ISL_782781, EPI_ISL_782782, EPI_ISL_782785, EPI_ISL_782786	Lighthouse Lab in Milton Keynes	Wellcome Sanger Institute for the COVID-19 Genomics UK (COG-UK) Consortium	The Lighthouse Lab in Milton Keynes and Alex Alderton, Roberto Amato, Sonia Goncalves, Ewan Harrison, David K. Jackson, Ian Johnston, Dominic Kwiatkowski, Cordelia Langford, John Sillitoe on behalf of the Wellcome Sanger Institute COVID-19 Surveillance Team
EPI_ISL_782788	Lighthouse Lab in Cambridge	Wellcome Sanger Institute for the COVID-19 Genomics UK (COG-UK) Consortium	Rob Howes, The Lighthouse Lab in Cambridge and Alex Alderton, Roberto Amato, Sonia Goncalves, Ewan Harrison, David K. Jackson, Ian Johnston, Dominic Kwiatkowski, Cordelia Langford, John Sillitoe on behalf of the Wellcome Sanger Institute COVID-19 Surveillance Team
EPI_ISL_782789, EPI_ISL_782790, EPI_ISL_782793, EPI_ISL_782794	Lighthouse Lab in Milton Keynes	Wellcome Sanger Institute for the COVID-19 Genomics UK (COG-UK) Consortium	The Lighthouse Lab in Milton Keynes and Alex Alderton, Roberto Amato, Sonia Goncalves, Ewan Harrison, David K. Jackson, Ian Johnston, Dominic Kwiatkowski, Cordelia Langford, John Sillitoe on behalf of the Wellcome Sanger Institute COVID-19 Surveillance Team
EPI_ISL_782797	Lighthouse Lab in Cambridge	Wellcome Sanger Institute for the COVID-19 Genomics UK (COG-UK) Consortium	Rob Howes, The Lighthouse Lab in Cambridge and Alex Alderton, Roberto Amato, Sonia Goncalves, Ewan Harrison, David K. Jackson, Ian Johnston, Dominic Kwiatkowski, Cordelia Langford, John Sillitoe on behalf of the Wellcome Sanger Institute COVID-19 Surveillance Team
EPI_ISL_782798, EPI_ISL_782799	Lighthouse Lab in Milton Keynes	Wellcome Sanger Institute for the COVID-19 Genomics UK (COG-UK) Consortium	The Lighthouse Lab in Milton Keynes and Alex Alderton, Roberto Amato, Sonia Goncalves, Ewan Harrison, David K. Jackson, Ian Johnston, Dominic Kwiatkowski, Cordelia Langford, John Sillitoe on behalf of the Wellcome Sanger Institute COVID-19 Surveillance Team
EPI_ISL_782800	Lighthouse Lab in Cambridge	Wellcome Sanger Institute for the COVID-19 Genomics UK (COG-UK) Consortium	Rob Howes, The Lighthouse Lab in Cambridge and Alex Alderton, Roberto Amato, Sonia Goncalves, Ewan Harrison, David K. Jackson, Ian Johnston, Dominic Kwiatkowski, Cordelia Langford, John Sillitoe on behalf of the Wellcome Sanger Institute COVID-19 Surveillance Team
EPI_ISL_782802	Lighthouse Lab in Milton Keynes	Wellcome Sanger Institute for the COVID-19 Genomics UK (COG-UK) Consortium	The Lighthouse Lab in Milton Keynes and Alex Alderton, Roberto Amato, Sonia Goncalves, Ewan Harrison, David K. Jackson, Ian Johnston, Dominic Kwiatkowski, Cordelia Langford, John Sillitoe on behalf of the Wellcome Sanger Institute COVID-19 Surveillance Team
EPI_ISL_782803	Lighthouse Lab in Cambridge	Wellcome Sanger Institute for the COVID-19 Genomics UK (COG-UK) Consortium	Rob Howes, The Lighthouse Lab in Cambridge and Alex Alderton, Roberto Amato, Sonia Goncalves, Ewan Harrison, David K. Jackson, Ian Johnston, Dominic Kwiatkowski, Cordelia Langford, John Sillitoe on behalf of the Wellcome Sanger Institute COVID-19 Surveillance Team
EPI_ISL_782804, EPI_ISL_782807	Lighthouse Lab in Milton Keynes	Wellcome Sanger Institute for the COVID-19 Genomics UK (COG-UK) Consortium	The Lighthouse Lab in Milton Keynes and Alex Alderton, Roberto Amato, Sonia Goncalves, Ewan Harrison, David K. Jackson, Ian Johnston, Dominic Kwiatkowski, Cordelia Langford, John Sillitoe on behalf of the Wellcome Sanger Institute COVID-19 Surveillance Team
EPI_ISL_782809, EPI_ISL_782810	Lighthouse Lab in Cambridge	Wellcome Sanger Institute for the COVID-19 Genomics UK (COG-UK) Consortium	Rob Howes, The Lighthouse Lab in Cambridge and Alex Alderton, Roberto Amato, Sonia Goncalves, Ewan Harrison, David K. Jackson, Ian Johnston, Dominic Kwiatkowski, Cordelia Langford, John Sillitoe on behalf of the Wellcome Sanger Institute COVID-19 Surveillance Team
EPI_ISL_782811, EPI_ISL_782812	Lighthouse Lab in Milton Keynes	Wellcome Sanger Institute for the COVID-19 Genomics UK (COG-UK) Consortium	The Lighthouse Lab in Milton Keynes and Alex Alderton, Roberto Amato, Sonia Goncalves, Ewan Harrison, David K. Jackson, Ian Johnston, Dominic Kwiatkowski, Cordelia Langford, John Sillitoe on behalf of the Wellcome Sanger Institute COVID-19 Surveillance Team
EPI_ISL_782814	Lighthouse Lab in Cambridge	Wellcome Sanger Institute for the COVID-19 Genomics UK (COG-UK) Consortium	Rob Howes, The Lighthouse Lab in Cambridge and Alex Alderton, Roberto Amato, Sonia Goncalves, Ewan Harrison, David K. Jackson, Ian Johnston, Dominic Kwiatkowski, Cordelia Langford, John Sillitoe on behalf of the Wellcome Sanger Institute COVID-19 Surveillance Team
EPI_ISL_782815	Lighthouse Lab in Milton Keynes	Wellcome Sanger Institute for the COVID-19 Genomics UK (COG-UK) Consortium	The Lighthouse Lab in Milton Keynes and Alex Alderton, Roberto Amato, Sonia Goncalves, Ewan Harrison, David K. Jackson, Ian Johnston, Dominic Kwiatkowski, Cordelia Langford, John Sillitoe on behalf of the Wellcome Sanger Institute COVID-19 Surveillance Team
EPI_ISL_782816, EPI_ISL_782817, EPI_ISL_782818	Lighthouse Lab in Cambridge	Wellcome Sanger Institute for the COVID-19 Genomics UK (COG-UK) Consortium	Rob Howes, The Lighthouse Lab in Cambridge and Alex Alderton, Roberto Amato, Sonia Goncalves, Ewan Harrison, David K. Jackson, Ian Johnston, Dominic Kwiatkowski, Cordelia Langford, John Sillitoe on behalf of the Wellcome Sanger Institute COVID-19 Surveillance Team
EPI_ISL_782819, EPI_ISL_782821	Lighthouse Lab in Milton Keynes	Wellcome Sanger Institute for the COVID-19 Genomics UK (COG-UK) Consortium	The Lighthouse Lab in Milton Keynes and Alex Alderton, Roberto Amato, Sonia Goncalves, Ewan Harrison, David K. Jackson, Ian Johnston, Dominic Kwiatkowski, Cordelia Langford, John Sillitoe on behalf of the Wellcome Sanger Institute COVID-19 Surveillance Team
EPI_ISL_782822	Lighthouse Lab in Cambridge	Wellcome Sanger Institute for the COVID-19 Genomics UK (COG-UK) Consortium	Rob Howes, The Lighthouse Lab in Cambridge and Alex Alderton, Roberto Amato, Sonia Goncalves, Ewan Harrison, David K. Jackson, Ian Johnston, Dominic Kwiatkowski, Cordelia Langford, John Sillitoe on behalf of the Wellcome Sanger Institute COVID-19 Surveillance Team
EPI_ISL_782823, EPI_ISL_782824, EPI_ISL_782826, EPI_ISL_782828, EPI_ISL_782829	Lighthouse Lab in Milton Keynes	Wellcome Sanger Institute for the COVID-19 Genomics UK (COG-UK) Consortium	The Lighthouse Lab in Milton Keynes and Alex Alderton, Roberto Amato, Sonia Goncalves, Ewan Harrison, David K. Jackson, Ian Johnston, Dominic Kwiatkowski, Cordelia Langford, John Sillitoe on behalf of the Wellcome Sanger Institute COVID-19 Surveillance Team
EPI_ISL_782830	Lighthouse Lab in Cambridge	Wellcome Sanger Institute for the COVID-19 Genomics UK (COG-UK) Consortium	Rob Howes, The Lighthouse Lab in Cambridge and Alex Alderton, Roberto Amato, Sonia Goncalves, Ewan Harrison, David K. Jackson, Ian Johnston, Dominic Kwiatkowski, Cordelia Langford, John Sillitoe on behalf of the Wellcome Sanger Institute COVID-19 Surveillance Team
EPI_ISL_782831, EPI_ISL_782833	Lighthouse Lab in Milton Keynes	Wellcome Sanger Institute for the COVID-19 Genomics UK (COG-UK) Consortium	The Lighthouse Lab in Milton Keynes and Alex Alderton, Roberto Amato, Sonia Goncalves, Ewan Harrison, David K. Jackson, Ian Johnston, Dominic Kwiatkowski, Cordelia Langford, John Sillitoe on behalf of the Wellcome Sanger Institute COVID-19 Surveillance Team
EPI_ISL_782834	Lighthouse Lab in Cambridge	Wellcome Sanger Institute for the COVID-19 Genomics UK (COG-UK) Consortium	Rob Howes, The Lighthouse Lab in Cambridge and Alex Alderton, Roberto Amato, Sonia Goncalves, Ewan Harrison, David K. Jackson, Ian Johnston, Dominic Kwiatkowski, Cordelia Langford, John Sillitoe on behalf of the Wellcome Sanger Institute COVID-19 Surveillance Team
EPI_ISL_782835, EPI_ISL_782836, EPI_ISL_782837, EPI_ISL_782839	Lighthouse Lab in Milton Keynes	Wellcome Sanger Institute for the COVID-19 Genomics UK (COG-UK) Consortium	The Lighthouse Lab in Milton Keynes and Alex Alderton, Roberto Amato, Sonia Goncalves, Ewan Harrison, David K. Jackson, Ian Johnston, Dominic Kwiatkowski, Cordelia Langford, John Sillitoe on behalf of the Wellcome Sanger Institute COVID-19 Surveillance Team
EPI_ISL_782840, EPI_ISL_782842	Lighthouse Lab in Cambridge	Wellcome Sanger Institute for the COVID-19 Genomics UK (COG-UK) Consortium	Rob Howes, The Lighthouse Lab in Cambridge and Alex Alderton, Roberto Amato, Sonia Goncalves, Ewan Harrison, David K. Jackson, Ian Johnston, Dominic Kwiatkowski, Cordelia Langford, John Sillitoe on behalf of the Wellcome Sanger Institute COVID-19 Surveillance Team
EPI_ISL_782845	Lighthouse Lab in Milton Keynes	Wellcome Sanger Institute for the COVID-19 Genomics UK	The Lighthouse Lab in Milton Keynes and Alex Alderton, Roberto Amato, Sonia Goncalves, Ewan Harrison, David K. Jackson, Ian Johnston, Dominic

		(COG-UK) Consortium	Kwiatkowski, Cordelia Langford, John Sillitoe on behalf of the Wellcome Sanger Institute COVID-19 Surveillance Team
EPI_ISL_782846	Lighthouse Lab in Cambridge	Wellcome Sanger Institute for the COVID-19 Genomics UK (COG-UK) Consortium	Rob Howes, The Lighthouse Lab in Cambridge and Alex Alderton, Roberto Amato, Sonia Goncalves, Ewan Harrison, David K. Jackson, Ian Johnston, Dominic Kwiatkowski, Cordelia Langford, John Sillitoe on behalf of the Wellcome Sanger Institute COVID-19 Surveillance Team
EPI_ISL_782847, EPI_ISL_782848	Lighthouse Lab in Milton Keynes	Wellcome Sanger Institute for the COVID-19 Genomics UK (COG-UK) Consortium	The Lighthouse Lab in Milton Keynes and Alex Alderton, Roberto Amato, Sonia Goncalves, Ewan Harrison, David K. Jackson, Ian Johnston, Dominic Kwiatkowski, Cordelia Langford, John Sillitoe on behalf of the Wellcome Sanger Institute COVID-19 Surveillance Team
EPI_ISL_782850	Lighthouse Lab in Cambridge	Wellcome Sanger Institute for the COVID-19 Genomics UK (COG-UK) Consortium	Rob Howes, The Lighthouse Lab in Cambridge and Alex Alderton, Roberto Amato, Sonia Goncalves, Ewan Harrison, David K. Jackson, Ian Johnston, Dominic Kwiatkowski, Cordelia Langford, John Sillitoe on behalf of the Wellcome Sanger Institute COVID-19 Surveillance Team
EPI_ISL_782851, EPI_ISL_782852	Lighthouse Lab in Milton Keynes	Wellcome Sanger Institute for the COVID-19 Genomics UK (COG-UK) Consortium	The Lighthouse Lab in Milton Keynes and Alex Alderton, Roberto Amato, Sonia Goncalves, Ewan Harrison, David K. Jackson, Ian Johnston, Dominic Kwiatkowski, Cordelia Langford, John Sillitoe on behalf of the Wellcome Sanger Institute COVID-19 Surveillance Team
EPI_ISL_782853, EPI_ISL_782854, EPI_ISL_782857	Lighthouse Lab in Cambridge	Wellcome Sanger Institute for the COVID-19 Genomics UK (COG-UK) Consortium	Rob Howes, The Lighthouse Lab in Cambridge and Alex Alderton, Roberto Amato, Sonia Goncalves, Ewan Harrison, David K. Jackson, Ian Johnston, Dominic Kwiatkowski, Cordelia Langford, John Sillitoe on behalf of the Wellcome Sanger Institute COVID-19 Surveillance Team
EPI_ISL_782858, EPI_ISL_782859	Lighthouse Lab in Milton Keynes	Wellcome Sanger Institute for the COVID-19 Genomics UK (COG-UK) Consortium	The Lighthouse Lab in Milton Keynes and Alex Alderton, Roberto Amato, Sonia Goncalves, Ewan Harrison, David K. Jackson, Ian Johnston, Dominic Kwiatkowski, Cordelia Langford, John Sillitoe on behalf of the Wellcome Sanger Institute COVID-19 Surveillance Team
EPI_ISL_782860	Lighthouse Lab in Cambridge	Wellcome Sanger Institute for the COVID-19 Genomics UK (COG-UK) Consortium	Rob Howes, The Lighthouse Lab in Cambridge and Alex Alderton, Roberto Amato, Sonia Goncalves, Ewan Harrison, David K. Jackson, Ian Johnston, Dominic Kwiatkowski, Cordelia Langford, John Sillitoe on behalf of the Wellcome Sanger Institute COVID-19 Surveillance Team
EPI_ISL_782861	Lighthouse Lab in Milton Keynes	Wellcome Sanger Institute for the COVID-19 Genomics UK (COG-UK) Consortium	The Lighthouse Lab in Milton Keynes and Alex Alderton, Roberto Amato, Sonia Goncalves, Ewan Harrison, David K. Jackson, Ian Johnston, Dominic Kwiatkowski, Cordelia Langford, John Sillitoe on behalf of the Wellcome Sanger Institute COVID-19 Surveillance Team
EPI_ISL_782862	Lighthouse Lab in Cambridge	Wellcome Sanger Institute for the COVID-19 Genomics UK (COG-UK) Consortium	Rob Howes, The Lighthouse Lab in Cambridge and Alex Alderton, Roberto Amato, Sonia Goncalves, Ewan Harrison, David K. Jackson, Ian Johnston, Dominic Kwiatkowski, Cordelia Langford, John Sillitoe on behalf of the Wellcome Sanger Institute COVID-19 Surveillance Team
EPI_ISL_782863, EPI_ISL_782864, EPI_ISL_782865	Lighthouse Lab in Milton Keynes	Wellcome Sanger Institute for the COVID-19 Genomics UK (COG-UK) Consortium	The Lighthouse Lab in Milton Keynes and Alex Alderton, Roberto Amato, Sonia Goncalves, Ewan Harrison, David K. Jackson, Ian Johnston, Dominic Kwiatkowski, Cordelia Langford, John Sillitoe on behalf of the Wellcome Sanger Institute COVID-19 Surveillance Team
EPI_ISL_782866, EPI_ISL_782867, EPI_ISL_782868	Lighthouse Lab in Cambridge	Wellcome Sanger Institute for the COVID-19 Genomics UK (COG-UK) Consortium	Rob Howes, The Lighthouse Lab in Cambridge and Alex Alderton, Roberto Amato, Sonia Goncalves, Ewan Harrison, David K. Jackson, Ian Johnston, Dominic Kwiatkowski, Cordelia Langford, John Sillitoe on behalf of the Wellcome Sanger Institute COVID-19 Surveillance Team
EPI_ISL_782870, EPI_ISL_782871, EPI_ISL_782874, EPI_ISL_782875, EPI_ISL_782876, EPI_ISL_782879	Lighthouse Lab in Milton Keynes	Wellcome Sanger Institute for the COVID-19 Genomics UK (COG-UK) Consortium	The Lighthouse Lab in Milton Keynes and Alex Alderton, Roberto Amato, Sonia Goncalves, Ewan Harrison, David K. Jackson, Ian Johnston, Dominic Kwiatkowski, Cordelia Langford, John Sillitoe on behalf of the Wellcome Sanger Institute COVID-19 Surveillance Team
EPI_ISL_782880	Lighthouse Lab in Cambridge	Wellcome Sanger Institute for the COVID-19 Genomics UK (COG-UK) Consortium	Rob Howes, The Lighthouse Lab in Cambridge and Alex Alderton, Roberto Amato, Sonia Goncalves, Ewan Harrison, David K. Jackson, Ian Johnston, Dominic Kwiatkowski, Cordelia Langford, John Sillitoe on behalf of the Wellcome Sanger Institute COVID-19 Surveillance Team
EPI_ISL_782881	Lighthouse Lab in Milton Keynes	Wellcome Sanger Institute for the COVID-19 Genomics UK (COG-UK) Consortium	The Lighthouse Lab in Milton Keynes and Alex Alderton, Roberto Amato, Sonia Goncalves, Ewan Harrison, David K. Jackson, Ian Johnston, Dominic Kwiatkowski, Cordelia Langford, John Sillitoe on behalf of the Wellcome Sanger Institute COVID-19 Surveillance Team
EPI_ISL_782882, EPI_ISL_782883	Lighthouse Lab in Cambridge	Wellcome Sanger Institute for the COVID-19 Genomics UK (COG-UK) Consortium	Rob Howes, The Lighthouse Lab in Cambridge and Alex Alderton, Roberto Amato, Sonia Goncalves, Ewan Harrison, David K. Jackson, Ian Johnston, Dominic Kwiatkowski, Cordelia Langford, John Sillitoe on behalf of the Wellcome Sanger Institute COVID-19 Surveillance Team
EPI_ISL_782884	Lighthouse Lab in Milton Keynes	Wellcome Sanger Institute for the COVID-19 Genomics UK (COG-UK) Consortium	The Lighthouse Lab in Milton Keynes and Alex Alderton, Roberto Amato, Sonia Goncalves, Ewan Harrison, David K. Jackson, Ian Johnston, Dominic Kwiatkowski, Cordelia Langford, John Sillitoe on behalf of the Wellcome Sanger Institute COVID-19 Surveillance Team
EPI_ISL_782885, EPI_ISL_782886, EPI_ISL_782887	Lighthouse Lab in Cambridge	Wellcome Sanger Institute for the COVID-19 Genomics UK (COG-UK) Consortium	Rob Howes, The Lighthouse Lab in Cambridge and Alex Alderton, Roberto Amato, Sonia Goncalves, Ewan Harrison, David K. Jackson, Ian Johnston, Dominic Kwiatkowski, Cordelia Langford, John Sillitoe on behalf of the Wellcome Sanger Institute COVID-19 Surveillance Team
EPI_ISL_782888	Lighthouse Lab in Milton Keynes	Wellcome Sanger Institute for the COVID-19 Genomics UK (COG-UK) Consortium	The Lighthouse Lab in Milton Keynes and Alex Alderton, Roberto Amato, Sonia Goncalves, Ewan Harrison, David K. Jackson, Ian Johnston, Dominic Kwiatkowski, Cordelia Langford, John Sillitoe on behalf of the Wellcome Sanger Institute COVID-19 Surveillance Team
EPI_ISL_782889, EPI_ISL_782890	Lighthouse Lab in Cambridge	Wellcome Sanger Institute for the COVID-19 Genomics UK (COG-UK) Consortium	Rob Howes, The Lighthouse Lab in Cambridge and Alex Alderton, Roberto Amato, Sonia Goncalves, Ewan Harrison, David K. Jackson, Ian Johnston, Dominic Kwiatkowski, Cordelia Langford, John Sillitoe on behalf of the Wellcome Sanger Institute COVID-19 Surveillance Team
EPI_ISL_782891, EPI_ISL_782895, EPI_ISL_782896, EPI_ISL_782898, EPI_ISL_782900, EPI_ISL_782901, EPI_ISL_782906	Lighthouse Lab in Milton Keynes	Wellcome Sanger Institute for the COVID-19 Genomics UK (COG-UK) Consortium	The Lighthouse Lab in Milton Keynes and Alex Alderton, Roberto Amato, Sonia Goncalves, Ewan Harrison, David K. Jackson, Ian Johnston, Dominic Kwiatkowski, Cordelia Langford, John Sillitoe on behalf of the Wellcome Sanger Institute COVID-19 Surveillance Team
EPI_ISL_782907, EPI_ISL_782908, EPI_ISL_782909, EPI_ISL_782910, EPI_ISL_782912, EPI_ISL_782913	Lighthouse Lab in Cambridge	Wellcome Sanger Institute for the COVID-19 Genomics UK (COG-UK) Consortium	Rob Howes, The Lighthouse Lab in Cambridge and Alex Alderton, Roberto Amato, Sonia Goncalves, Ewan Harrison, David K. Jackson, Ian Johnston, Dominic Kwiatkowski, Cordelia Langford, John Sillitoe on behalf of the Wellcome Sanger Institute COVID-19 Surveillance Team
EPI_ISL_782914, EPI_ISL_782915, EPI_ISL_782916, EPI_ISL_782917	Lighthouse Lab in Milton Keynes	Wellcome Sanger Institute for the COVID-19 Genomics UK (COG-UK) Consortium	The Lighthouse Lab in Milton Keynes and Alex Alderton, Roberto Amato, Sonia Goncalves, Ewan Harrison, David K. Jackson, Ian Johnston, Dominic Kwiatkowski, Cordelia Langford, John Sillitoe on behalf of the Wellcome Sanger Institute COVID-19 Surveillance Team
EPI_ISL_782920	Lighthouse Lab in Cambridge	Wellcome Sanger Institute for the COVID-19 Genomics UK (COG-UK) Consortium	Rob Howes, The Lighthouse Lab in Cambridge and Alex Alderton, Roberto Amato, Sonia Goncalves, Ewan Harrison, David K. Jackson, Ian Johnston, Dominic Kwiatkowski, Cordelia Langford, John Sillitoe on behalf of the Wellcome Sanger Institute COVID-19 Surveillance Team
EPI_ISL_782921, EPI_ISL_782922	Lighthouse Lab in Milton Keynes	Wellcome Sanger Institute for the COVID-19 Genomics UK (COG-UK) Consortium	The Lighthouse Lab in Milton Keynes and Alex Alderton, Roberto Amato, Sonia Goncalves, Ewan Harrison, David K. Jackson, Ian Johnston, Dominic Kwiatkowski, Cordelia Langford, John Sillitoe on behalf of the Wellcome Sanger Institute COVID-19 Surveillance Team
EPI_ISL_782923	Lighthouse Lab in Cambridge	Wellcome Sanger Institute for the COVID-19 Genomics UK (COG-UK) Consortium	Rob Howes, The Lighthouse Lab in Cambridge and Alex Alderton, Roberto Amato, Sonia Goncalves, Ewan Harrison, David K. Jackson, Ian Johnston, Dominic Kwiatkowski, Cordelia Langford, John Sillitoe on behalf of the Wellcome Sanger Institute COVID-19 Surveillance Team
EPI_ISL_782925, EPI_ISL_782927	Lighthouse Lab in Milton Keynes	Wellcome Sanger Institute for the COVID-19 Genomics UK (COG-UK) Consortium	The Lighthouse Lab in Milton Keynes and Alex Alderton, Roberto Amato, Sonia Goncalves, Ewan Harrison, David K. Jackson, Ian Johnston, Dominic Kwiatkowski, Cordelia Langford, John Sillitoe on behalf of the Wellcome Sanger Institute COVID-19 Surveillance Team
EPI_ISL_782928, EPI_ISL_782930, EPI_ISL_782932	Lighthouse Lab in Cambridge	Wellcome Sanger Institute for the COVID-19 Genomics UK (COG-UK) Consortium	Rob Howes, The Lighthouse Lab in Cambridge and Alex Alderton, Roberto Amato, Sonia Goncalves, Ewan Harrison, David K. Jackson, Ian Johnston, Dominic Kwiatkowski, Cordelia Langford, John Sillitoe on behalf of the Wellcome Sanger Institute COVID-19 Surveillance Team
EPI_ISL_782933, EPI_ISL_782934, EPI_ISL_782935	Lighthouse Lab in Milton Keynes	Wellcome Sanger Institute for the COVID-19 Genomics UK (COG-UK) Consortium	The Lighthouse Lab in Milton Keynes and Alex Alderton, Roberto Amato, Sonia Goncalves, Ewan Harrison, David K. Jackson, Ian Johnston, Dominic Kwiatkowski, Cordelia Langford, John Sillitoe on behalf of the Wellcome Sanger Institute COVID-19 Surveillance Team
EPI_ISL_782937	Lighthouse Lab in Cambridge	Wellcome Sanger Institute for the COVID-19 Genomics UK (COG-UK) Consortium	Rob Howes, The Lighthouse Lab in Cambridge and Alex Alderton, Roberto Amato, Sonia Goncalves, Ewan Harrison, David K. Jackson, Ian Johnston, Dominic Kwiatkowski, Cordelia Langford, John Sillitoe on behalf of the Wellcome Sanger Institute COVID-19 Surveillance Team
EPI_ISL_782938, EPI_ISL_782939, EPI_ISL_782940	Lighthouse Lab in Milton Keynes	Wellcome Sanger Institute for the COVID-19 Genomics UK (COG-UK) Consortium	The Lighthouse Lab in Milton Keynes and Alex Alderton, Roberto Amato, Sonia Goncalves, Ewan Harrison, David K. Jackson, Ian Johnston, Dominic Kwiatkowski, Cordelia Langford, John Sillitoe on behalf of the Wellcome Sanger Institute COVID-19 Surveillance Team
EPI_ISL_782941, EPI_ISL_782942	Lighthouse Lab in Cambridge	Wellcome Sanger Institute for the COVID-19 Genomics UK (COG-UK) Consortium	Rob Howes, The Lighthouse Lab in Cambridge and Alex Alderton, Roberto Amato, Sonia Goncalves, Ewan Harrison, David K. Jackson, Ian Johnston, Dominic Kwiatkowski, Cordelia Langford, John Sillitoe on behalf of the Wellcome Sanger Institute COVID-19 Surveillance Team
EPI_ISL_782943, EPI_ISL_782944	Lighthouse Lab in Milton Keynes	Wellcome Sanger Institute for the COVID-19 Genomics UK (COG-UK) Consortium	The Lighthouse Lab in Milton Keynes and Alex Alderton, Roberto Amato, Sonia Goncalves, Ewan Harrison, David K. Jackson, Ian Johnston, Dominic Kwiatkowski, Cordelia Langford, John Sillitoe on behalf of the Wellcome Sanger Institute COVID-19 Surveillance Team
EPI_ISL_782947	Lighthouse Lab in Cambridge	Wellcome Sanger Institute for the COVID-19 Genomics UK (COG-UK) Consortium	Rob Howes, The Lighthouse Lab in Cambridge and Alex Alderton, Roberto Amato, Sonia Goncalves, Ewan Harrison, David K. Jackson, Ian Johnston, Dominic Kwiatkowski, Cordelia Langford, John Sillitoe on behalf of the Wellcome Sanger Institute COVID-19 Surveillance Team

EPI_ISL_782948, EPI_ISL_782949, EPI_ISL_782950, EPI_ISL_782951, EPI_ISL_782952, EPI_ISL_782953	Lighthouse Lab in Milton Keynes	Wellcome Sanger Institute for the COVID-19 Genomics UK (COG-UK) Consortium	The Lighthouse Lab in Milton Keynes and Alex Alderton, Roberto Amato, Sonia Goncalves, Ewan Harrison, David K. Jackson, Ian Johnston, Dominic Kwiatkowski, Cordelia Langford, John Sillitoe on behalf of the Wellcome Sanger Institute COVID-19 Surveillance Team
EPI_ISL_782954	Lighthouse Lab in Cambridge	Wellcome Sanger Institute for the COVID-19 Genomics UK (COG-UK) Consortium	Rob Howes, The Lighthouse Lab in Cambridge and Alex Alderton, Roberto Amato, Sonia Goncalves, Ewan Harrison, David K. Jackson, Ian Johnston, Dominic Kwiatkowski, Cordelia Langford, John Sillitoe on behalf of the Wellcome Sanger Institute COVID-19 Surveillance Team
EPI_ISL_782955, EPI_ISL_782957, EPI_ISL_782958	Lighthouse Lab in Milton Keynes	Wellcome Sanger Institute for the COVID-19 Genomics UK (COG-UK) Consortium	The Lighthouse Lab in Milton Keynes and Alex Alderton, Roberto Amato, Sonia Goncalves, Ewan Harrison, David K. Jackson, Ian Johnston, Dominic Kwiatkowski, Cordelia Langford, John Sillitoe on behalf of the Wellcome Sanger Institute COVID-19 Surveillance Team
EPI_ISL_782959, EPI_ISL_782960	Lighthouse Lab in Cambridge	Wellcome Sanger Institute for the COVID-19 Genomics UK (COG-UK) Consortium	Rob Howes, The Lighthouse Lab in Cambridge and Alex Alderton, Roberto Amato, Sonia Goncalves, Ewan Harrison, David K. Jackson, Ian Johnston, Dominic Kwiatkowski, Cordelia Langford, John Sillitoe on behalf of the Wellcome Sanger Institute COVID-19 Surveillance Team
EPI_ISL_782961, EPI_ISL_782962, EPI_ISL_782965	Lighthouse Lab in Milton Keynes	Wellcome Sanger Institute for the COVID-19 Genomics UK (COG-UK) Consortium	The Lighthouse Lab in Milton Keynes and Alex Alderton, Roberto Amato, Sonia Goncalves, Ewan Harrison, David K. Jackson, Ian Johnston, Dominic Kwiatkowski, Cordelia Langford, John Sillitoe on behalf of the Wellcome Sanger Institute COVID-19 Surveillance Team
EPI_ISL_782966	Lighthouse Lab in Cambridge	Wellcome Sanger Institute for the COVID-19 Genomics UK (COG-UK) Consortium	Rob Howes, The Lighthouse Lab in Cambridge and Alex Alderton, Roberto Amato, Sonia Goncalves, Ewan Harrison, David K. Jackson, Ian Johnston, Dominic Kwiatkowski, Cordelia Langford, John Sillitoe on behalf of the Wellcome Sanger Institute COVID-19 Surveillance Team
EPI_ISL_782968	Lighthouse Lab in Milton Keynes	Wellcome Sanger Institute for the COVID-19 Genomics UK (COG-UK) Consortium	The Lighthouse Lab in Milton Keynes and Alex Alderton, Roberto Amato, Sonia Goncalves, Ewan Harrison, David K. Jackson, Ian Johnston, Dominic Kwiatkowski, Cordelia Langford, John Sillitoe on behalf of the Wellcome Sanger Institute COVID-19 Surveillance Team
EPI_ISL_782971	Lighthouse Lab in Cambridge	Wellcome Sanger Institute for the COVID-19 Genomics UK (COG-UK) Consortium	Rob Howes, The Lighthouse Lab in Cambridge and Alex Alderton, Roberto Amato, Sonia Goncalves, Ewan Harrison, David K. Jackson, Ian Johnston, Dominic Kwiatkowski, Cordelia Langford, John Sillitoe on behalf of the Wellcome Sanger Institute COVID-19 Surveillance Team
EPI_ISL_782972, EPI_ISL_782973	Lighthouse Lab in Milton Keynes	Wellcome Sanger Institute for the COVID-19 Genomics UK (COG-UK) Consortium	The Lighthouse Lab in Milton Keynes and Alex Alderton, Roberto Amato, Sonia Goncalves, Ewan Harrison, David K. Jackson, Ian Johnston, Dominic Kwiatkowski, Cordelia Langford, John Sillitoe on behalf of the Wellcome Sanger Institute COVID-19 Surveillance Team
EPI_ISL_782974	Lighthouse Lab in Cambridge	Wellcome Sanger Institute for the COVID-19 Genomics UK (COG-UK) Consortium	Rob Howes, The Lighthouse Lab in Cambridge and Alex Alderton, Roberto Amato, Sonia Goncalves, Ewan Harrison, David K. Jackson, Ian Johnston, Dominic Kwiatkowski, Cordelia Langford, John Sillitoe on behalf of the Wellcome Sanger Institute COVID-19 Surveillance Team
EPI_ISL_782975, EPI_ISL_782977, EPI_ISL_782978	Lighthouse Lab in Milton Keynes	Wellcome Sanger Institute for the COVID-19 Genomics UK (COG-UK) Consortium	The Lighthouse Lab in Milton Keynes and Alex Alderton, Roberto Amato, Sonia Goncalves, Ewan Harrison, David K. Jackson, Ian Johnston, Dominic Kwiatkowski, Cordelia Langford, John Sillitoe on behalf of the Wellcome Sanger Institute COVID-19 Surveillance Team
EPI_ISL_782979	Lighthouse Lab in Cambridge	Wellcome Sanger Institute for the COVID-19 Genomics UK (COG-UK) Consortium	Rob Howes, The Lighthouse Lab in Cambridge and Alex Alderton, Roberto Amato, Sonia Goncalves, Ewan Harrison, David K. Jackson, Ian Johnston, Dominic Kwiatkowski, Cordelia Langford, John Sillitoe on behalf of the Wellcome Sanger Institute COVID-19 Surveillance Team
EPI_ISL_782980, EPI_ISL_782981, EPI_ISL_782982	Lighthouse Lab in Milton Keynes	Wellcome Sanger Institute for the COVID-19 Genomics UK (COG-UK) Consortium	The Lighthouse Lab in Milton Keynes and Alex Alderton, Roberto Amato, Sonia Goncalves, Ewan Harrison, David K. Jackson, Ian Johnston, Dominic Kwiatkowski, Cordelia Langford, John Sillitoe on behalf of the Wellcome Sanger Institute COVID-19 Surveillance Team
EPI_ISL_782983, EPI_ISL_782984, EPI_ISL_782985	Lighthouse Lab in Cambridge	Wellcome Sanger Institute for the COVID-19 Genomics UK (COG-UK) Consortium	Rob Howes, The Lighthouse Lab in Cambridge and Alex Alderton, Roberto Amato, Sonia Goncalves, Ewan Harrison, David K. Jackson, Ian Johnston, Dominic Kwiatkowski, Cordelia Langford, John Sillitoe on behalf of the Wellcome Sanger Institute COVID-19 Surveillance Team
EPI_ISL_782986	Lighthouse Lab in Milton Keynes	Wellcome Sanger Institute for the COVID-19 Genomics UK (COG-UK) Consortium	The Lighthouse Lab in Milton Keynes and Alex Alderton, Roberto Amato, Sonia Goncalves, Ewan Harrison, David K. Jackson, Ian Johnston, Dominic Kwiatkowski, Cordelia Langford, John Sillitoe on behalf of the Wellcome Sanger Institute COVID-19 Surveillance Team
EPI_ISL_782987	Lighthouse Lab in Cambridge	Wellcome Sanger Institute for the COVID-19 Genomics UK (COG-UK) Consortium	Rob Howes, The Lighthouse Lab in Cambridge and Alex Alderton, Roberto Amato, Sonia Goncalves, Ewan Harrison, David K. Jackson, Ian Johnston, Dominic Kwiatkowski, Cordelia Langford, John Sillitoe on behalf of the Wellcome Sanger Institute COVID-19 Surveillance Team
EPI_ISL_782989, EPI_ISL_782990	Lighthouse Lab in Milton Keynes	Wellcome Sanger Institute for the COVID-19 Genomics UK (COG-UK) Consortium	The Lighthouse Lab in Milton Keynes and Alex Alderton, Roberto Amato, Sonia Goncalves, Ewan Harrison, David K. Jackson, Ian Johnston, Dominic Kwiatkowski, Cordelia Langford, John Sillitoe on behalf of the Wellcome Sanger Institute COVID-19 Surveillance Team
EPI_ISL_782991	Lighthouse Lab in Cambridge	Wellcome Sanger Institute for the COVID-19 Genomics UK (COG-UK) Consortium	Rob Howes, The Lighthouse Lab in Cambridge and Alex Alderton, Roberto Amato, Sonia Goncalves, Ewan Harrison, David K. Jackson, Ian Johnston, Dominic Kwiatkowski, Cordelia Langford, John Sillitoe on behalf of the Wellcome Sanger Institute COVID-19 Surveillance Team
EPI_ISL_782992, EPI_ISL_782995	Lighthouse Lab in Milton Keynes	Wellcome Sanger Institute for the COVID-19 Genomics UK (COG-UK) Consortium	The Lighthouse Lab in Milton Keynes and Alex Alderton, Roberto Amato, Sonia Goncalves, Ewan Harrison, David K. Jackson, Ian Johnston, Dominic Kwiatkowski, Cordelia Langford, John Sillitoe on behalf of the Wellcome Sanger Institute COVID-19 Surveillance Team
EPI_ISL_782996, EPI_ISL_782998	Lighthouse Lab in Cambridge	Wellcome Sanger Institute for the COVID-19 Genomics UK (COG-UK) Consortium	Rob Howes, The Lighthouse Lab in Cambridge and Alex Alderton, Roberto Amato, Sonia Goncalves, Ewan Harrison, David K. Jackson, Ian Johnston, Dominic Kwiatkowski, Cordelia Langford, John Sillitoe on behalf of the Wellcome Sanger Institute COVID-19 Surveillance Team
EPI_ISL_782999, EPI_ISL_783001, EPI_ISL_783003, EPI_ISL_783004, EPI_ISL_783005, EPI_ISL_783006, EPI_ISL_783009, EPI_ISL_783010, EPI_ISL_783011	Lighthouse Lab in Milton Keynes	Wellcome Sanger Institute for the COVID-19 Genomics UK (COG-UK) Consortium	The Lighthouse Lab in Milton Keynes and Alex Alderton, Roberto Amato, Sonia Goncalves, Ewan Harrison, David K. Jackson, Ian Johnston, Dominic Kwiatkowski, Cordelia Langford, John Sillitoe on behalf of the Wellcome Sanger Institute COVID-19 Surveillance Team
EPI_ISL_783012	Lighthouse Lab in Cambridge	Wellcome Sanger Institute for the COVID-19 Genomics UK (COG-UK) Consortium	Rob Howes, The Lighthouse Lab in Cambridge and Alex Alderton, Roberto Amato, Sonia Goncalves, Ewan Harrison, David K. Jackson, Ian Johnston, Dominic Kwiatkowski, Cordelia Langford, John Sillitoe on behalf of the Wellcome Sanger Institute COVID-19 Surveillance Team
EPI_ISL_783013, EPI_ISL_783016, EPI_ISL_783017, EPI_ISL_783018	Lighthouse Lab in Milton Keynes	Wellcome Sanger Institute for the COVID-19 Genomics UK (COG-UK) Consortium	The Lighthouse Lab in Milton Keynes and Alex Alderton, Roberto Amato, Sonia Goncalves, Ewan Harrison, David K. Jackson, Ian Johnston, Dominic Kwiatkowski, Cordelia Langford, John Sillitoe on behalf of the Wellcome Sanger Institute COVID-19 Surveillance Team
EPI_ISL_783019	Lighthouse Lab in Cambridge	Wellcome Sanger Institute for the COVID-19 Genomics UK (COG-UK) Consortium	Rob Howes, The Lighthouse Lab in Cambridge and Alex Alderton, Roberto Amato, Sonia Goncalves, Ewan Harrison, David K. Jackson, Ian Johnston, Dominic Kwiatkowski, Cordelia Langford, John Sillitoe on behalf of the Wellcome Sanger Institute COVID-19 Surveillance Team
EPI_ISL_783020, EPI_ISL_783021, EPI_ISL_783022	Lighthouse Lab in Milton Keynes	Wellcome Sanger Institute for the COVID-19 Genomics UK (COG-UK) Consortium	The Lighthouse Lab in Milton Keynes and Alex Alderton, Roberto Amato, Sonia Goncalves, Ewan Harrison, David K. Jackson, Ian Johnston, Dominic Kwiatkowski, Cordelia Langford, John Sillitoe on behalf of the Wellcome Sanger Institute COVID-19 Surveillance Team
EPI_ISL_783023	Lighthouse Lab in Cambridge	Wellcome Sanger Institute for the COVID-19 Genomics UK (COG-UK) Consortium	Rob Howes, The Lighthouse Lab in Cambridge and Alex Alderton, Roberto Amato, Sonia Goncalves, Ewan Harrison, David K. Jackson, Ian Johnston, Dominic Kwiatkowski, Cordelia Langford, John Sillitoe on behalf of the Wellcome Sanger Institute COVID-19 Surveillance Team
EPI_ISL_783024, EPI_ISL_783025, EPI_ISL_783027	Lighthouse Lab in Milton Keynes	Wellcome Sanger Institute for the COVID-19 Genomics UK (COG-UK) Consortium	The Lighthouse Lab in Milton Keynes and Alex Alderton, Roberto Amato, Sonia Goncalves, Ewan Harrison, David K. Jackson, Ian Johnston, Dominic Kwiatkowski, Cordelia Langford, John Sillitoe on behalf of the Wellcome Sanger Institute COVID-19 Surveillance Team
EPI_ISL_783028	Lighthouse Lab in Cambridge	Wellcome Sanger Institute for the COVID-19 Genomics UK (COG-UK) Consortium	Rob Howes, The Lighthouse Lab in Cambridge and Alex Alderton, Roberto Amato, Sonia Goncalves, Ewan Harrison, David K. Jackson, Ian Johnston, Dominic Kwiatkowski, Cordelia Langford, John Sillitoe on behalf of the Wellcome Sanger Institute COVID-19 Surveillance Team
EPI_ISL_783029	Lighthouse Lab in Milton Keynes	Wellcome Sanger Institute for the COVID-19 Genomics UK (COG-UK) Consortium	The Lighthouse Lab in Milton Keynes and Alex Alderton, Roberto Amato, Sonia Goncalves, Ewan Harrison, David K. Jackson, Ian Johnston, Dominic Kwiatkowski, Cordelia Langford, John Sillitoe on behalf of the Wellcome Sanger Institute COVID-19 Surveillance Team
EPI_ISL_783031	Lighthouse Lab in Cambridge	Wellcome Sanger Institute for the COVID-19 Genomics UK (COG-UK) Consortium	Rob Howes, The Lighthouse Lab in Cambridge and Alex Alderton, Roberto Amato, Sonia Goncalves, Ewan Harrison, David K. Jackson, Ian Johnston, Dominic Kwiatkowski, Cordelia Langford, John Sillitoe on behalf of the Wellcome Sanger Institute COVID-19 Surveillance Team
EPI_ISL_783035	Lighthouse Lab in Milton Keynes	Wellcome Sanger Institute for the COVID-19 Genomics UK (COG-UK) Consortium	The Lighthouse Lab in Milton Keynes and Alex Alderton, Roberto Amato, Sonia Goncalves, Ewan Harrison, David K. Jackson, Ian Johnston, Dominic Kwiatkowski, Cordelia Langford, John Sillitoe on behalf of the Wellcome Sanger Institute COVID-19 Surveillance Team
EPI_ISL_783036, EPI_ISL_783037, EPI_ISL_783038	Lighthouse Lab in Cambridge	Wellcome Sanger Institute for the COVID-19 Genomics UK (COG-UK) Consortium	Rob Howes, The Lighthouse Lab in Cambridge and Alex Alderton, Roberto Amato, Sonia Goncalves, Ewan Harrison, David K. Jackson, Ian Johnston, Dominic Kwiatkowski, Cordelia Langford, John Sillitoe on behalf of the Wellcome Sanger Institute COVID-19 Surveillance Team
EPI_ISL_783039, EPI_ISL_783040, EPI_ISL_783041	Lighthouse Lab in Milton Keynes	Wellcome Sanger Institute for the COVID-19 Genomics UK (COG-UK) Consortium	The Lighthouse Lab in Milton Keynes and Alex Alderton, Roberto Amato, Sonia Goncalves, Ewan Harrison, David K. Jackson, Ian Johnston, Dominic Kwiatkowski, Cordelia Langford, John Sillitoe on behalf of the Wellcome Sanger Institute COVID-19 Surveillance Team
EPI_ISL_783042, EPI_ISL_783044	Lighthouse Lab in Cambridge	Wellcome Sanger Institute for the COVID-19 Genomics UK	Rob Howes, The Lighthouse Lab in Cambridge and Alex Alderton, Roberto Amato, Sonia Goncalves, Ewan Harrison, David K. Jackson, Ian Johnston,

		(COG-UK) Consortium	Dominic Kwiatkowski, Cordelia Langford, John Sillitoe on behalf of the Wellcome Sanger Institute COVID-19 Surveillance Team
EPI_ISL_783048, EPI_ISL_783049, EPI_ISL_783050	Lighthouse Lab in Milton Keynes	Wellcome Sanger Institute for the COVID-19 Genomics UK (COG-UK) Consortium	The Lighthouse Lab in Milton Keynes and Alex Alderton, Roberto Amato, Sonia Goncalves, Ewan Harrison, David K. Jackson, Ian Johnston, Dominic Kwiatkowski, Cordelia Langford, John Sillitoe on behalf of the Wellcome Sanger Institute COVID-19 Surveillance Team
EPI_ISL_783051	Lighthouse Lab in Cambridge	Wellcome Sanger Institute for the COVID-19 Genomics UK (COG-UK) Consortium	Rob Howes, The Lighthouse Lab in Cambridge and Alex Alderton, Roberto Amato, Sonia Goncalves, Ewan Harrison, David K. Jackson, Ian Johnston, Dominic Kwiatkowski, Cordelia Langford, John Sillitoe on behalf of the Wellcome Sanger Institute COVID-19 Surveillance Team
EPI_ISL_783053, EPI_ISL_783054	Lighthouse Lab in Milton Keynes	Wellcome Sanger Institute for the COVID-19 Genomics UK (COG-UK) Consortium	The Lighthouse Lab in Milton Keynes and Alex Alderton, Roberto Amato, Sonia Goncalves, Ewan Harrison, David K. Jackson, Ian Johnston, Dominic Kwiatkowski, Cordelia Langford, John Sillitoe on behalf of the Wellcome Sanger Institute COVID-19 Surveillance Team
EPI_ISL_783055, EPI_ISL_783057	Lighthouse Lab in Cambridge	Wellcome Sanger Institute for the COVID-19 Genomics UK (COG-UK) Consortium	Rob Howes, The Lighthouse Lab in Cambridge and Alex Alderton, Roberto Amato, Sonia Goncalves, Ewan Harrison, David K. Jackson, Ian Johnston, Dominic Kwiatkowski, Cordelia Langford, John Sillitoe on behalf of the Wellcome Sanger Institute COVID-19 Surveillance Team
EPI_ISL_783058, EPI_ISL_783059, EPI_ISL_783060, EPI_ISL_783061, EPI_ISL_783062, EPI_ISL_783063, EPI_ISL_783064, EPI_ISL_783065	Lighthouse Lab in Milton Keynes	Wellcome Sanger Institute for the COVID-19 Genomics UK (COG-UK) Consortium	The Lighthouse Lab in Milton Keynes and Alex Alderton, Roberto Amato, Sonia Goncalves, Ewan Harrison, David K. Jackson, Ian Johnston, Dominic Kwiatkowski, Cordelia Langford, John Sillitoe on behalf of the Wellcome Sanger Institute COVID-19 Surveillance Team
EPI_ISL_783066	Lighthouse Lab in Cambridge	Wellcome Sanger Institute for the COVID-19 Genomics UK (COG-UK) Consortium	Rob Howes, The Lighthouse Lab in Cambridge and Alex Alderton, Roberto Amato, Sonia Goncalves, Ewan Harrison, David K. Jackson, Ian Johnston, Dominic Kwiatkowski, Cordelia Langford, John Sillitoe on behalf of the Wellcome Sanger Institute COVID-19 Surveillance Team
EPI_ISL_783067	Lighthouse Lab in Milton Keynes	Wellcome Sanger Institute for the COVID-19 Genomics UK (COG-UK) Consortium	The Lighthouse Lab in Milton Keynes and Alex Alderton, Roberto Amato, Sonia Goncalves, Ewan Harrison, David K. Jackson, Ian Johnston, Dominic Kwiatkowski, Cordelia Langford, John Sillitoe on behalf of the Wellcome Sanger Institute COVID-19 Surveillance Team
EPI_ISL_789037, EPI_ISL_789039	Klinisk mikrobiologi	The Public Health Agency of Sweden	Department of Microbiology, The Public Health Agency of Sweden
EPI_ISL_790626, EPI_ISL_790647, EPI_ISL_790651, EPI_ISL_790652, EPI_ISL_790653, EPI_ISL_790665, EPI_ISL_790675, EPI_ISL_790676, EPI_ISL_790677, EPI_ISL_790678, EPI_ISL_790686, EPI_ISL_790688, EPI_ISL_790689, EPI_ISL_790693, EPI_ISL_790745, EPI_ISL_790746, EPI_ISL_790747, EPI_ISL_790814, EPI_ISL_790823, EPI_ISL_791034			
see above	Dutch COVID-19 response team	National Institute for Public Health and the Environment (RIVM)	Adam Meijer, Harry Vennema, Jeroen Cremer, Sharon van den Brink, Bas van der Veer, AnneMarie van den Brandt, Florian Zwagemaker, Dennis Schmitz, Chantal Reusken, on behalf of the national COVID-19 response team
EPI_ISL_791093, EPI_ISL_791094, EPI_ISL_791095	Instituto Nacional de Salud - Unidad de Secuenciación y Análisis Genómico	Instituto Nacional de Salud - Dirección de Investigación en Salud Pública	Katherine Laiton-Donato, Diego A. Álvarez-Díaz, Carlos Franco-Muñoz, Mauricio Pacheco-Montealegre, Jonathan Reales, Sheryl Corchuelo, Maria T. Herrera, Julian Naizaque, Gerardo Santamaría, Paola Muñoz-Laiton, Diego Andrés Prada, Magdalena Wiesner, Martha Lucia Ospina Martinez, Marcela Mercado-Reyes
EPI_ISL_791314, EPI_ISL_791322	National Virus Reference Laboratory	Irish Coronavirus Sequencing Consortium - Teagasc Moorepark	Alejandro Abner Garcia Leon, Paul Cotter, Fiona Crispie, John Kenny, Paddy Mallon, Calum Walsh
EPI_ISL_791994, EPI_ISL_791995	CHU - Hôpital Cavale Blanche	National Reference Center for Viruses of Respiratory Infections, Institut Pasteur, Paris	Marion Barbet, Sylvie Behillil, Méline Bizard, Angela Brisebarre, Camille Capel, Etienne Simon-Lorière, Vincent Enouf, Maud Vanpeene, Sylvie van der Werf, Léa Pilorgé
EPI_ISL_792556	Centre for Dengue Research and AICBU, Department of Immunology and Molecular Medicine	Centre for Dengue Research and AICBU, Department of Immunology and Molecular Medicine	Chandima Jeewandara, Deshni Jayathilaka, Dinuka Ariyaratne, Diyanath Ranasinghe, Laksiri Gomes, Gathsaurie Neelika Malavige
EPI_ISL_792699, EPI_ISL_792704	The National Institute of Public Health	State Veterinary Institute Prague	Nagy,A;Jirincova,H;Trnka,D;Vecerova,J
EPI_ISL_792710, EPI_ISL_792713, EPI_ISL_792740, EPI_ISL_792741, EPI_ISL_792742, EPI_ISL_792743, EPI_ISL_792744, EPI_ISL_792745, EPI_ISL_792746, EPI_ISL_792747, EPI_ISL_792748, EPI_ISL_792754, EPI_ISL_792759, EPI_ISL_792780, EPI_ISL_792783, EPI_ISL_792784, EPI_ISL_792793, EPI_ISL_792825, EPI_ISL_792826, EPI_ISL_792827, EPI_ISL_792831, EPI_ISL_792832, EPI_ISL_792833, EPI_ISL_792836, EPI_ISL_792837, EPI_ISL_792980, EPI_ISL_792981, EPI_ISL_792982, EPI_ISL_792983, EPI_ISL_792984, EPI_ISL_792985, EPI_ISL_792986, EPI_ISL_792987, EPI_ISL_792988, EPI_ISL_792989, EPI_ISL_792990, EPI_ISL_792991, EPI_ISL_792992, EPI_ISL_792993, EPI_ISL_792994, EPI_ISL_792995, EPI_ISL_792996, EPI_ISL_792997, EPI_ISL_792998, EPI_ISL_792999, EPI_ISL_793000, EPI_ISL_793001, EPI_ISL_793002, EPI_ISL_793003, EPI_ISL_793004, EPI_ISL_793005, EPI_ISL_793006, EPI_ISL_793007, EPI_ISL_793008, EPI_ISL_793009, EPI_ISL_794324, EPI_ISL_794329, EPI_ISL_794330, EPI_ISL_794331, EPI_ISL_794332, EPI_ISL_794333, EPI_ISL_794334, EPI_ISL_794335, EPI_ISL_794336, EPI_ISL_794341, EPI_ISL_794343, EPI_ISL_794344, EPI_ISL_794345, EPI_ISL_794346, EPI_ISL_794347, EPI_ISL_794349, EPI_ISL_794350, EPI_ISL_794351, EPI_ISL_794361, EPI_ISL_794362, EPI_ISL_794363, EPI_ISL_794364, EPI_ISL_794365, EPI_ISL_794366, EPI_ISL_794367, EPI_ISL_794369, EPI_ISL_794371, EPI_ISL_794375, EPI_ISL_794376, EPI_ISL_794377, EPI_ISL_794383, EPI_ISL_794384, EPI_ISL_794385, EPI_ISL_794386, EPI_ISL_794387, EPI_ISL_794388, EPI_ISL_794398, EPI_ISL_794399, EPI_ISL_794400, EPI_ISL_794401, EPI_ISL_794402, EPI_ISL_794403, EPI_ISL_794404, EPI_ISL_794405, EPI_ISL_794406, EPI_ISL_794407, EPI_ISL_794408, EPI_ISL_794409, EPI_ISL_794410, EPI_ISL_794411, EPI_ISL_794420, EPI_ISL_794421, EPI_ISL_794423, EPI_ISL_794425, EPI_ISL_794426, EPI_ISL_794430, EPI_ISL_794434, EPI_ISL_794496, EPI_ISL_794497, EPI_ISL_794498, EPI_ISL_794499, EPI_ISL_794500, EPI_ISL_794501, EPI_ISL_794502, EPI_ISL_794503, EPI_ISL_794504, EPI_ISL_794505, EPI_ISL_794506, EPI_ISL_794507, EPI_ISL_794508, EPI_ISL_794509, EPI_ISL_794510, EPI_ISL_794511, EPI_ISL_794512, EPI_ISL_794513, EPI_ISL_794514, EPI_ISL_794515, EPI_ISL_794516, EPI_ISL_794517, EPI_ISL_794518, EPI_ISL_794519, EPI_ISL_794520, EPI_ISL_794521, EPI_ISL_794522, EPI_ISL_794523, EPI_ISL_794524, EPI_ISL_794525, EPI_ISL_794526, EPI_ISL_794527, EPI_ISL_794528, EPI_ISL_794529, EPI_ISL_794530, EPI_ISL_794531, EPI_ISL_794532, EPI_ISL_794533, EPI_ISL_794534, EPI_ISL_794535, EPI_ISL_794536, EPI_ISL_794537, EPI_ISL_794538, EPI_ISL_794539, EPI_ISL_794540, EPI_ISL_794541, EPI_ISL_794542, EPI_ISL_794543, EPI_ISL_794544, EPI_ISL_794545, EPI_ISL_794546, EPI_ISL_794547, EPI_ISL_794548, EPI_ISL_794549, EPI_ISL_794550, EPI_ISL_794551, EPI_ISL_794552, EPI_ISL_794553, EPI_ISL_794554			
see above	Department of Virus and Microbiological Special Diagnostics, Statens Serum Institut, Copenhagen, Denmark	Albertsen Lab, Department of Chemistry and Bioscience, Aalborg University, Denmark	Danish Covid-19 Genome Consortium
EPI_ISL_794716, EPI_ISL_794730	PathWest Laboratory Medicine WA	PathWest Laboratory Medicine WA Microbial Surveillance Unit	PathWest Laboratory Medicine WA Microbial Surveillance Unit
EPI_ISL_794850, EPI_ISL_794851, EPI_ISL_794852, EPI_ISL_794853, EPI_ISL_794854, EPI_ISL_794855, EPI_ISL_794856, EPI_ISL_794857, EPI_ISL_794860, EPI_ISL_794862, EPI_ISL_794864, EPI_ISL_794871, EPI_ISL_794876, EPI_ISL_794883, EPI_ISL_794895, EPI_ISL_794896, EPI_ISL_794897, EPI_ISL_794898, EPI_ISL_794899, EPI_ISL_794901, EPI_ISL_794905, EPI_ISL_794983, EPI_ISL_794984, EPI_ISL_794985, EPI_ISL_794986, EPI_ISL_794987, EPI_ISL_794988, EPI_ISL_794989, EPI_ISL_794990, EPI_ISL_794991, EPI_ISL_794992, EPI_ISL_794993, EPI_ISL_794994, EPI_ISL_794995, EPI_ISL_794996, EPI_ISL_794997, EPI_ISL_794998, EPI_ISL_794999, EPI_ISL_795000, EPI_ISL_795001, EPI_ISL_795002, EPI_ISL_795003, EPI_ISL_795004, EPI_ISL_795005, EPI_ISL_795006, EPI_ISL_795007			
see above	Department of Virus and Microbiological Special Diagnostics, Statens Serum Institut, Copenhagen, Denmark	Albertsen Lab, Department of Chemistry and Bioscience, Aalborg University, Denmark	Danish Covid-19 Genome Consortium
EPI_ISL_796650	Nordland Hospital - Bodo, Laboratory Department, Molecular Biology Unit	Norwegian Institute of Public Health, Department of Virology	Kathrine Stene-Johansen, Kamilla Heddeland Instefjord, Hilde Elshaug, Atiya R Ali,Marie Paulsen Madsen, Rasmus Riis Kopperud, Hilde Vollan, Karoline Bragstad, Olav Hungnes
EPI_ISL_796663	Dept. of Medical Microbiology, Stavanger University Hospital, Helse Stavanger HF	Norwegian Institute of Public Health, Department of Virology	Kathrine Stene-Johansen, Kamilla Heddeland Instefjord, Hilde Elshaug, Atiya R Ali,Marie Paulsen Madsen, Rasmus Riis Kopperud, Hilde Vollan, Karoline Bragstad, Olav Hungnes
EPI_ISL_796667, EPI_ISL_796668, EPI_ISL_796669, EPI_ISL_796672, EPI_ISL_796691, EPI_ISL_796692, EPI_ISL_796693, EPI_ISL_796694	Nordland Hospital - Bodo, Laboratory Department, Molecular Biology Unit	Norwegian Institute of Public Health, Department of Virology	Kathrine Stene-Johansen, Kamilla Heddeland Instefjord, Hilde Elshaug, Atiya R Ali,Marie Paulsen Madsen, Rasmus Riis Kopperud, Hilde Vollan, Karoline Bragstad, Olav Hungnes
EPI_ISL_796695, EPI_ISL_796696, EPI_ISL_796697	Ostfold Hospital Trust - Kalnes, Centre for Laboratory Medicine, Section for gene technology and infection serology	Norwegian Institute of Public Health, Department of Virology	Kathrine Stene-Johansen, Kamilla Heddeland Instefjord, Hilde Elshaug, Atiya R Ali,Marie Paulsen Madsen, Rasmus Riis Kopperud, Hilde Vollan, Karoline Bragstad, Olav Hungnes
EPI_ISL_796698	Dept. of Medical Microbiology, Stavanger University Hospital, Helse Stavanger HF	Norwegian Institute of Public Health, Department of Virology	Kathrine Stene-Johansen, Kamilla Heddeland Instefjord, Hilde Elshaug, Atiya R Ali,Marie Paulsen Madsen, Rasmus Riis Kopperud, Hilde Vollan, Karoline Bragstad, Olav Hungnes
EPI_ISL_796702	Unilabs Laboratory Medicine	Norwegian Institute of Public Health, Department of Virology	Kathrine Stene-Johansen, Kamilla Heddeland Instefjord, Hilde Elshaug, Atiya R Ali,Marie Paulsen Madsen, Rasmus Riis Kopperud, Hilde Vollan, Karoline Bragstad, Olav Hungnes
EPI_ISL_796730, EPI_ISL_796731, EPI_ISL_796732	Furst Medical Laboratory	Norwegian Institute of Public Health, Department of Virology	Kathrine Stene-Johansen, Kamilla Heddeland Instefjord, Hilde Elshaug, Atiya R Ali,Marie Paulsen Madsen, Rasmus Riis Kopperud, Hilde Vollan, Karoline Bragstad, Olav Hungnes
EPI_ISL_800089, EPI_ISL_800157, EPI_ISL_800203, EPI_ISL_800586, EPI_ISL_800587, EPI_ISL_800589, EPI_ISL_800590	Lighthouse Lab in Cambridge	Wellcome Sanger Institute for the COVID-19 Genomics UK (COG-UK) Consortium	Rob Howes, The Lighthouse Lab in Cambridge and Alex Alderton, Roberto Amato, Sonia Goncalves, Ewan Harrison, David K. Jackson, Ian Johnston, Dominic Kwiatkowski, Cordelia Langford, John Sillitoe on behalf of the Wellcome Sanger Institute COVID-19 Surveillance Team
EPI_ISL_800591	Lighthouse Lab in Glasgow	Wellcome Sanger Institute for the COVID-19 Genomics UK (COG-UK) Consortium	Harper VanSteenhouse, Yumi Kasai, David Gray, Carol Clugston, Anna Dominiczak and Alex Alderton, Roberto Amato, Sonia Goncalves, Ewan Harrison, David K. Jackson, Ian Johnston, Dominic Kwiatkowski, Cordelia Langford, John Sillitoe on behalf of the Wellcome Sanger Institute COVID-19 Surveillance Team

EPI_ISL_800592	Lighthouse Lab in Cambridge	Wellcome Sanger Institute for the COVID-19 Genomics UK (COG-UK) Consortium	Rob Howes, The Lighthouse Lab in Cambridge and Alex Alderton, Roberto Amato, Sonia Goncalves, Ewan Harrison, David K. Jackson, Ian Johnston, Dominic Kwiatkowski, Cordelia Langford, John Sillitoe on behalf of the Wellcome Sanger Institute COVID-19 Surveillance Team
EPI_ISL_800594, EPI_ISL_800596	Lighthouse Lab in Glasgow	Wellcome Sanger Institute for the COVID-19 Genomics UK (COG-UK) Consortium	Harper VanSteenhouse, Yumi Kasai, David Gray, Carol Clugston, Anna Dominiczak and Alex Alderton, Roberto Amato, Sonia Goncalves, Ewan Harrison, David K. Jackson, Ian Johnston, Dominic Kwiatkowski, Cordelia Langford, John Sillitoe on behalf of the Wellcome Sanger Institute COVID-19 Surveillance Team
EPI_ISL_800597, EPI_ISL_800600	Lighthouse Lab in Cambridge	Wellcome Sanger Institute for the COVID-19 Genomics UK (COG-UK) Consortium	Rob Howes, The Lighthouse Lab in Cambridge and Alex Alderton, Roberto Amato, Sonia Goncalves, Ewan Harrison, David K. Jackson, Ian Johnston, Dominic Kwiatkowski, Cordelia Langford, John Sillitoe on behalf of the Wellcome Sanger Institute COVID-19 Surveillance Team
EPI_ISL_800601, EPI_ISL_800602, EPI_ISL_800603, EPI_ISL_800604, EPI_ISL_800605, EPI_ISL_800607, EPI_ISL_800608	Lighthouse Lab in Glasgow	Wellcome Sanger Institute for the COVID-19 Genomics UK (COG-UK) Consortium	Harper VanSteenhouse, Yumi Kasai, David Gray, Carol Clugston, Anna Dominiczak and Alex Alderton, Roberto Amato, Sonia Goncalves, Ewan Harrison, David K. Jackson, Ian Johnston, Dominic Kwiatkowski, Cordelia Langford, John Sillitoe on behalf of the Wellcome Sanger Institute COVID-19 Surveillance Team
EPI_ISL_800609, EPI_ISL_800610	Lighthouse Lab in Cambridge	Wellcome Sanger Institute for the COVID-19 Genomics UK (COG-UK) Consortium	Rob Howes, The Lighthouse Lab in Cambridge and Alex Alderton, Roberto Amato, Sonia Goncalves, Ewan Harrison, David K. Jackson, Ian Johnston, Dominic Kwiatkowski, Cordelia Langford, John Sillitoe on behalf of the Wellcome Sanger Institute COVID-19 Surveillance Team
EPI_ISL_800611, EPI_ISL_800612, EPI_ISL_800614	Lighthouse Lab in Glasgow	Wellcome Sanger Institute for the COVID-19 Genomics UK (COG-UK) Consortium	Harper VanSteenhouse, Yumi Kasai, David Gray, Carol Clugston, Anna Dominiczak and Alex Alderton, Roberto Amato, Sonia Goncalves, Ewan Harrison, David K. Jackson, Ian Johnston, Dominic Kwiatkowski, Cordelia Langford, John Sillitoe on behalf of the Wellcome Sanger Institute COVID-19 Surveillance Team
EPI_ISL_800615	Lighthouse Lab in Cambridge	Wellcome Sanger Institute for the COVID-19 Genomics UK (COG-UK) Consortium	Rob Howes, The Lighthouse Lab in Cambridge and Alex Alderton, Roberto Amato, Sonia Goncalves, Ewan Harrison, David K. Jackson, Ian Johnston, Dominic Kwiatkowski, Cordelia Langford, John Sillitoe on behalf of the Wellcome Sanger Institute COVID-19 Surveillance Team
EPI_ISL_800616, EPI_ISL_800618, EPI_ISL_800620, EPI_ISL_800621	Lighthouse Lab in Glasgow	Wellcome Sanger Institute for the COVID-19 Genomics UK (COG-UK) Consortium	Harper VanSteenhouse, Yumi Kasai, David Gray, Carol Clugston, Anna Dominiczak and Alex Alderton, Roberto Amato, Sonia Goncalves, Ewan Harrison, David K. Jackson, Ian Johnston, Dominic Kwiatkowski, Cordelia Langford, John Sillitoe on behalf of the Wellcome Sanger Institute COVID-19 Surveillance Team
EPI_ISL_800622	Lighthouse Lab in Cambridge	Wellcome Sanger Institute for the COVID-19 Genomics UK (COG-UK) Consortium	Rob Howes, The Lighthouse Lab in Cambridge and Alex Alderton, Roberto Amato, Sonia Goncalves, Ewan Harrison, David K. Jackson, Ian Johnston, Dominic Kwiatkowski, Cordelia Langford, John Sillitoe on behalf of the Wellcome Sanger Institute COVID-19 Surveillance Team
EPI_ISL_800625	Lighthouse Lab in Glasgow	Wellcome Sanger Institute for the COVID-19 Genomics UK (COG-UK) Consortium	Harper VanSteenhouse, Yumi Kasai, David Gray, Carol Clugston, Anna Dominiczak and Alex Alderton, Roberto Amato, Sonia Goncalves, Ewan Harrison, David K. Jackson, Ian Johnston, Dominic Kwiatkowski, Cordelia Langford, John Sillitoe on behalf of the Wellcome Sanger Institute COVID-19 Surveillance Team
EPI_ISL_800626	Lighthouse Lab in Cambridge	Wellcome Sanger Institute for the COVID-19 Genomics UK (COG-UK) Consortium	Rob Howes, The Lighthouse Lab in Cambridge and Alex Alderton, Roberto Amato, Sonia Goncalves, Ewan Harrison, David K. Jackson, Ian Johnston, Dominic Kwiatkowski, Cordelia Langford, John Sillitoe on behalf of the Wellcome Sanger Institute COVID-19 Surveillance Team
EPI_ISL_800627, EPI_ISL_800628, EPI_ISL_800629, EPI_ISL_800630, EPI_ISL_800631, EPI_ISL_800633, EPI_ISL_800634, EPI_ISL_800635	Lighthouse Lab in Glasgow	Wellcome Sanger Institute for the COVID-19 Genomics UK (COG-UK) Consortium	Harper VanSteenhouse, Yumi Kasai, David Gray, Carol Clugston, Anna Dominiczak and Alex Alderton, Roberto Amato, Sonia Goncalves, Ewan Harrison, David K. Jackson, Ian Johnston, Dominic Kwiatkowski, Cordelia Langford, John Sillitoe on behalf of the Wellcome Sanger Institute COVID-19 Surveillance Team
EPI_ISL_800637	Lighthouse Lab in Cambridge	Wellcome Sanger Institute for the COVID-19 Genomics UK (COG-UK) Consortium	Rob Howes, The Lighthouse Lab in Cambridge and Alex Alderton, Roberto Amato, Sonia Goncalves, Ewan Harrison, David K. Jackson, Ian Johnston, Dominic Kwiatkowski, Cordelia Langford, John Sillitoe on behalf of the Wellcome Sanger Institute COVID-19 Surveillance Team
EPI_ISL_800638, EPI_ISL_800639, EPI_ISL_800640	Lighthouse Lab in Glasgow	Wellcome Sanger Institute for the COVID-19 Genomics UK (COG-UK) Consortium	Harper VanSteenhouse, Yumi Kasai, David Gray, Carol Clugston, Anna Dominiczak and Alex Alderton, Roberto Amato, Sonia Goncalves, Ewan Harrison, David K. Jackson, Ian Johnston, Dominic Kwiatkowski, Cordelia Langford, John Sillitoe on behalf of the Wellcome Sanger Institute COVID-19 Surveillance Team
EPI_ISL_800641	Lighthouse Lab in Cambridge	Wellcome Sanger Institute for the COVID-19 Genomics UK (COG-UK) Consortium	Rob Howes, The Lighthouse Lab in Cambridge and Alex Alderton, Roberto Amato, Sonia Goncalves, Ewan Harrison, David K. Jackson, Ian Johnston, Dominic Kwiatkowski, Cordelia Langford, John Sillitoe on behalf of the Wellcome Sanger Institute COVID-19 Surveillance Team
EPI_ISL_800643, EPI_ISL_800646	Lighthouse Lab in Glasgow	Wellcome Sanger Institute for the COVID-19 Genomics UK (COG-UK) Consortium	Harper VanSteenhouse, Yumi Kasai, David Gray, Carol Clugston, Anna Dominiczak and Alex Alderton, Roberto Amato, Sonia Goncalves, Ewan Harrison, David K. Jackson, Ian Johnston, Dominic Kwiatkowski, Cordelia Langford, John Sillitoe on behalf of the Wellcome Sanger Institute COVID-19 Surveillance Team
EPI_ISL_800647, EPI_ISL_800648, EPI_ISL_800649	Lighthouse Lab in Cambridge	Wellcome Sanger Institute for the COVID-19 Genomics UK (COG-UK) Consortium	Rob Howes, The Lighthouse Lab in Cambridge and Alex Alderton, Roberto Amato, Sonia Goncalves, Ewan Harrison, David K. Jackson, Ian Johnston, Dominic Kwiatkowski, Cordelia Langford, John Sillitoe on behalf of the Wellcome Sanger Institute COVID-19 Surveillance Team
EPI_ISL_800651, EPI_ISL_800653, EPI_ISL_800654, EPI_ISL_800655, EPI_ISL_800656	Lighthouse Lab in Glasgow	Wellcome Sanger Institute for the COVID-19 Genomics UK (COG-UK) Consortium	Harper VanSteenhouse, Yumi Kasai, David Gray, Carol Clugston, Anna Dominiczak and Alex Alderton, Roberto Amato, Sonia Goncalves, Ewan Harrison, David K. Jackson, Ian Johnston, Dominic Kwiatkowski, Cordelia Langford, John Sillitoe on behalf of the Wellcome Sanger Institute COVID-19 Surveillance Team
EPI_ISL_800658	Lighthouse Lab in Cambridge	Wellcome Sanger Institute for the COVID-19 Genomics UK (COG-UK) Consortium	Rob Howes, The Lighthouse Lab in Cambridge and Alex Alderton, Roberto Amato, Sonia Goncalves, Ewan Harrison, David K. Jackson, Ian Johnston, Dominic Kwiatkowski, Cordelia Langford, John Sillitoe on behalf of the Wellcome Sanger Institute COVID-19 Surveillance Team
EPI_ISL_800659, EPI_ISL_800662	Lighthouse Lab in Glasgow	Wellcome Sanger Institute for the COVID-19 Genomics UK (COG-UK) Consortium	Harper VanSteenhouse, Yumi Kasai, David Gray, Carol Clugston, Anna Dominiczak and Alex Alderton, Roberto Amato, Sonia Goncalves, Ewan Harrison, David K. Jackson, Ian Johnston, Dominic Kwiatkowski, Cordelia Langford, John Sillitoe on behalf of the Wellcome Sanger Institute COVID-19 Surveillance Team
EPI_ISL_800663, EPI_ISL_800665	Lighthouse Lab in Cambridge	Wellcome Sanger Institute for the COVID-19 Genomics UK (COG-UK) Consortium	Rob Howes, The Lighthouse Lab in Cambridge and Alex Alderton, Roberto Amato, Sonia Goncalves, Ewan Harrison, David K. Jackson, Ian Johnston, Dominic Kwiatkowski, Cordelia Langford, John Sillitoe on behalf of the Wellcome Sanger Institute COVID-19 Surveillance Team
EPI_ISL_800666, EPI_ISL_800667, EPI_ISL_800668	Lighthouse Lab in Glasgow	Wellcome Sanger Institute for the COVID-19 Genomics UK (COG-UK) Consortium	Harper VanSteenhouse, Yumi Kasai, David Gray, Carol Clugston, Anna Dominiczak and Alex Alderton, Roberto Amato, Sonia Goncalves, Ewan Harrison, David K. Jackson, Ian Johnston, Dominic Kwiatkowski, Cordelia Langford, John Sillitoe on behalf of the Wellcome Sanger Institute COVID-19 Surveillance Team
EPI_ISL_800669	Lighthouse Lab in Cambridge	Wellcome Sanger Institute for the COVID-19 Genomics UK (COG-UK) Consortium	Rob Howes, The Lighthouse Lab in Cambridge and Alex Alderton, Roberto Amato, Sonia Goncalves, Ewan Harrison, David K. Jackson, Ian Johnston, Dominic Kwiatkowski, Cordelia Langford, John Sillitoe on behalf of the Wellcome Sanger Institute COVID-19 Surveillance Team
EPI_ISL_800670, EPI_ISL_800671	Lighthouse Lab in Glasgow	Wellcome Sanger Institute for the COVID-19 Genomics UK (COG-UK) Consortium	Harper VanSteenhouse, Yumi Kasai, David Gray, Carol Clugston, Anna Dominiczak and Alex Alderton, Roberto Amato, Sonia Goncalves, Ewan Harrison, David K. Jackson, Ian Johnston, Dominic Kwiatkowski, Cordelia Langford, John Sillitoe on behalf of the Wellcome Sanger Institute COVID-19 Surveillance Team
EPI_ISL_800672	Lighthouse Lab in Cambridge	Wellcome Sanger Institute for the COVID-19 Genomics UK (COG-UK) Consortium	Rob Howes, The Lighthouse Lab in Cambridge and Alex Alderton, Roberto Amato, Sonia Goncalves, Ewan Harrison, David K. Jackson, Ian Johnston, Dominic Kwiatkowski, Cordelia Langford, John Sillitoe on behalf of the Wellcome Sanger Institute COVID-19 Surveillance Team
EPI_ISL_800675	Lighthouse Lab in Glasgow	Wellcome Sanger Institute for the COVID-19 Genomics UK (COG-UK) Consortium	Harper VanSteenhouse, Yumi Kasai, David Gray, Carol Clugston, Anna Dominiczak and Alex Alderton, Roberto Amato, Sonia Goncalves, Ewan Harrison, David K. Jackson, Ian Johnston, Dominic Kwiatkowski, Cordelia Langford, John Sillitoe on behalf of the Wellcome Sanger Institute COVID-19 Surveillance Team
EPI_ISL_800679, EPI_ISL_800680	Lighthouse Lab in Cambridge	Wellcome Sanger Institute for the COVID-19 Genomics UK (COG-UK) Consortium	Rob Howes, The Lighthouse Lab in Cambridge and Alex Alderton, Roberto Amato, Sonia Goncalves, Ewan Harrison, David K. Jackson, Ian Johnston, Dominic Kwiatkowski, Cordelia Langford, John Sillitoe on behalf of the Wellcome Sanger Institute COVID-19 Surveillance Team
EPI_ISL_800681	Lighthouse Lab in Milton Keynes	Wellcome Sanger Institute for the COVID-19 Genomics UK (COG-UK) Consortium	The Lighthouse Lab in Milton Keynes and Alex Alderton, Roberto Amato, Sonia Goncalves, Ewan Harrison, David K. Jackson, Ian Johnston, Dominic Kwiatkowski, Cordelia Langford, John Sillitoe on behalf of the Wellcome Sanger Institute COVID-19 Surveillance Team
EPI_ISL_800682	Lighthouse Lab in Cambridge	Wellcome Sanger Institute for the COVID-19 Genomics UK (COG-UK) Consortium	Rob Howes, The Lighthouse Lab in Cambridge and Alex Alderton, Roberto Amato, Sonia Goncalves, Ewan Harrison, David K. Jackson, Ian Johnston, Dominic Kwiatkowski, Cordelia Langford, John Sillitoe on behalf of the Wellcome Sanger Institute COVID-19 Surveillance Team

EPI_ISL_800685	Lighthouse Lab in Glasgow	Wellcome Sanger Institute for the COVID-19 Genomics UK (COG-UK) Consortium	Harper VanSteenhouse, Yumi Kasai, David Gray, Carol Clugston, Anna Dominiczak and Alex Alderton, Roberto Amato, Sonia Goncalves, Ewan Harrison, David K. Jackson, Ian Johnston, Dominic Kwiatkowski, Cordelia Langford, John Sillitoe on behalf of the Wellcome Sanger Institute COVID-19 Surveillance Team
EPI_ISL_800686, EPI_ISL_800687	Lighthouse Lab in Cambridge	Wellcome Sanger Institute for the COVID-19 Genomics UK (COG-UK) Consortium	Rob Howes, The Lighthouse Lab in Cambridge and Alex Alderton, Roberto Amato, Sonia Goncalves, Ewan Harrison, David K. Jackson, Ian Johnston, Dominic Kwiatkowski, Cordelia Langford, John Sillitoe on behalf of the Wellcome Sanger Institute COVID-19 Surveillance Team
EPI_ISL_800688, EPI_ISL_800689	Lighthouse Lab in Glasgow	Wellcome Sanger Institute for the COVID-19 Genomics UK (COG-UK) Consortium	Harper VanSteenhouse, Yumi Kasai, David Gray, Carol Clugston, Anna Dominiczak and Alex Alderton, Roberto Amato, Sonia Goncalves, Ewan Harrison, David K. Jackson, Ian Johnston, Dominic Kwiatkowski, Cordelia Langford, John Sillitoe on behalf of the Wellcome Sanger Institute COVID-19 Surveillance Team
EPI_ISL_800690	Lighthouse Lab in Cambridge	Wellcome Sanger Institute for the COVID-19 Genomics UK (COG-UK) Consortium	Rob Howes, The Lighthouse Lab in Cambridge and Alex Alderton, Roberto Amato, Sonia Goncalves, Ewan Harrison, David K. Jackson, Ian Johnston, Dominic Kwiatkowski, Cordelia Langford, John Sillitoe on behalf of the Wellcome Sanger Institute COVID-19 Surveillance Team
EPI_ISL_800691, EPI_ISL_800692, EPI_ISL_800693, EPI_ISL_800694, EPI_ISL_800695, EPI_ISL_800696, EPI_ISL_800697, EPI_ISL_800699, EPI_ISL_800700	Lighthouse Lab in Glasgow	Wellcome Sanger Institute for the COVID-19 Genomics UK (COG-UK) Consortium	Harper VanSteenhouse, Yumi Kasai, David Gray, Carol Clugston, Anna Dominiczak and Alex Alderton, Roberto Amato, Sonia Goncalves, Ewan Harrison, David K. Jackson, Ian Johnston, Dominic Kwiatkowski, Cordelia Langford, John Sillitoe on behalf of the Wellcome Sanger Institute COVID-19 Surveillance Team
EPI_ISL_800702	Lighthouse Lab in Cambridge	Wellcome Sanger Institute for the COVID-19 Genomics UK (COG-UK) Consortium	Rob Howes, The Lighthouse Lab in Cambridge and Alex Alderton, Roberto Amato, Sonia Goncalves, Ewan Harrison, David K. Jackson, Ian Johnston, Dominic Kwiatkowski, Cordelia Langford, John Sillitoe on behalf of the Wellcome Sanger Institute COVID-19 Surveillance Team
EPI_ISL_800703, EPI_ISL_800704, EPI_ISL_800705, EPI_ISL_800706	Lighthouse Lab in Glasgow	Wellcome Sanger Institute for the COVID-19 Genomics UK (COG-UK) Consortium	Harper VanSteenhouse, Yumi Kasai, David Gray, Carol Clugston, Anna Dominiczak and Alex Alderton, Roberto Amato, Sonia Goncalves, Ewan Harrison, David K. Jackson, Ian Johnston, Dominic Kwiatkowski, Cordelia Langford, John Sillitoe on behalf of the Wellcome Sanger Institute COVID-19 Surveillance Team
EPI_ISL_800707, EPI_ISL_800708, EPI_ISL_800709, EPI_ISL_800712, EPI_ISL_800714	Lighthouse Lab in Cambridge	Wellcome Sanger Institute for the COVID-19 Genomics UK (COG-UK) Consortium	Rob Howes, The Lighthouse Lab in Cambridge and Alex Alderton, Roberto Amato, Sonia Goncalves, Ewan Harrison, David K. Jackson, Ian Johnston, Dominic Kwiatkowski, Cordelia Langford, John Sillitoe on behalf of the Wellcome Sanger Institute COVID-19 Surveillance Team
EPI_ISL_800715	Lighthouse Lab in Glasgow	Wellcome Sanger Institute for the COVID-19 Genomics UK (COG-UK) Consortium	Harper VanSteenhouse, Yumi Kasai, David Gray, Carol Clugston, Anna Dominiczak and Alex Alderton, Roberto Amato, Sonia Goncalves, Ewan Harrison, David K. Jackson, Ian Johnston, Dominic Kwiatkowski, Cordelia Langford, John Sillitoe on behalf of the Wellcome Sanger Institute COVID-19 Surveillance Team
EPI_ISL_800716, EPI_ISL_800717	Lighthouse Lab in Cambridge	Wellcome Sanger Institute for the COVID-19 Genomics UK (COG-UK) Consortium	Rob Howes, The Lighthouse Lab in Cambridge and Alex Alderton, Roberto Amato, Sonia Goncalves, Ewan Harrison, David K. Jackson, Ian Johnston, Dominic Kwiatkowski, Cordelia Langford, John Sillitoe on behalf of the Wellcome Sanger Institute COVID-19 Surveillance Team
EPI_ISL_800719	Lighthouse Lab in Glasgow	Wellcome Sanger Institute for the COVID-19 Genomics UK (COG-UK) Consortium	Harper VanSteenhouse, Yumi Kasai, David Gray, Carol Clugston, Anna Dominiczak and Alex Alderton, Roberto Amato, Sonia Goncalves, Ewan Harrison, David K. Jackson, Ian Johnston, Dominic Kwiatkowski, Cordelia Langford, John Sillitoe on behalf of the Wellcome Sanger Institute COVID-19 Surveillance Team
EPI_ISL_800720, EPI_ISL_800721	Lighthouse Lab in Cambridge	Wellcome Sanger Institute for the COVID-19 Genomics UK (COG-UK) Consortium	Rob Howes, The Lighthouse Lab in Cambridge and Alex Alderton, Roberto Amato, Sonia Goncalves, Ewan Harrison, David K. Jackson, Ian Johnston, Dominic Kwiatkowski, Cordelia Langford, John Sillitoe on behalf of the Wellcome Sanger Institute COVID-19 Surveillance Team
EPI_ISL_800722, EPI_ISL_800723	Lighthouse Lab in Glasgow	Wellcome Sanger Institute for the COVID-19 Genomics UK (COG-UK) Consortium	Harper VanSteenhouse, Yumi Kasai, David Gray, Carol Clugston, Anna Dominiczak and Alex Alderton, Roberto Amato, Sonia Goncalves, Ewan Harrison, David K. Jackson, Ian Johnston, Dominic Kwiatkowski, Cordelia Langford, John Sillitoe on behalf of the Wellcome Sanger Institute COVID-19 Surveillance Team
EPI_ISL_800724	Lighthouse Lab in Cambridge	Wellcome Sanger Institute for the COVID-19 Genomics UK (COG-UK) Consortium	Rob Howes, The Lighthouse Lab in Cambridge and Alex Alderton, Roberto Amato, Sonia Goncalves, Ewan Harrison, David K. Jackson, Ian Johnston, Dominic Kwiatkowski, Cordelia Langford, John Sillitoe on behalf of the Wellcome Sanger Institute COVID-19 Surveillance Team
EPI_ISL_800725, EPI_ISL_800726, EPI_ISL_800727, EPI_ISL_800728	Lighthouse Lab in Glasgow	Wellcome Sanger Institute for the COVID-19 Genomics UK (COG-UK) Consortium	Harper VanSteenhouse, Yumi Kasai, David Gray, Carol Clugston, Anna Dominiczak and Alex Alderton, Roberto Amato, Sonia Goncalves, Ewan Harrison, David K. Jackson, Ian Johnston, Dominic Kwiatkowski, Cordelia Langford, John Sillitoe on behalf of the Wellcome Sanger Institute COVID-19 Surveillance Team
EPI_ISL_800730	Lighthouse Lab in Cambridge	Wellcome Sanger Institute for the COVID-19 Genomics UK (COG-UK) Consortium	Rob Howes, The Lighthouse Lab in Cambridge and Alex Alderton, Roberto Amato, Sonia Goncalves, Ewan Harrison, David K. Jackson, Ian Johnston, Dominic Kwiatkowski, Cordelia Langford, John Sillitoe on behalf of the Wellcome Sanger Institute COVID-19 Surveillance Team
EPI_ISL_800731	Lighthouse Lab in Glasgow	Wellcome Sanger Institute for the COVID-19 Genomics UK (COG-UK) Consortium	Harper VanSteenhouse, Yumi Kasai, David Gray, Carol Clugston, Anna Dominiczak and Alex Alderton, Roberto Amato, Sonia Goncalves, Ewan Harrison, David K. Jackson, Ian Johnston, Dominic Kwiatkowski, Cordelia Langford, John Sillitoe on behalf of the Wellcome Sanger Institute COVID-19 Surveillance Team
EPI_ISL_800733, EPI_ISL_800734	Lighthouse Lab in Cambridge	Wellcome Sanger Institute for the COVID-19 Genomics UK (COG-UK) Consortium	Rob Howes, The Lighthouse Lab in Cambridge and Alex Alderton, Roberto Amato, Sonia Goncalves, Ewan Harrison, David K. Jackson, Ian Johnston, Dominic Kwiatkowski, Cordelia Langford, John Sillitoe on behalf of the Wellcome Sanger Institute COVID-19 Surveillance Team
EPI_ISL_800735, EPI_ISL_800736	Lighthouse Lab in Glasgow	Wellcome Sanger Institute for the COVID-19 Genomics UK (COG-UK) Consortium	Harper VanSteenhouse, Yumi Kasai, David Gray, Carol Clugston, Anna Dominiczak and Alex Alderton, Roberto Amato, Sonia Goncalves, Ewan Harrison, David K. Jackson, Ian Johnston, Dominic Kwiatkowski, Cordelia Langford, John Sillitoe on behalf of the Wellcome Sanger Institute COVID-19 Surveillance Team
EPI_ISL_800737	Lighthouse Lab in Cambridge	Wellcome Sanger Institute for the COVID-19 Genomics UK (COG-UK) Consortium	Rob Howes, The Lighthouse Lab in Cambridge and Alex Alderton, Roberto Amato, Sonia Goncalves, Ewan Harrison, David K. Jackson, Ian Johnston, Dominic Kwiatkowski, Cordelia Langford, John Sillitoe on behalf of the Wellcome Sanger Institute COVID-19 Surveillance Team
EPI_ISL_800739, EPI_ISL_800742	Lighthouse Lab in Glasgow	Wellcome Sanger Institute for the COVID-19 Genomics UK (COG-UK) Consortium	Harper VanSteenhouse, Yumi Kasai, David Gray, Carol Clugston, Anna Dominiczak and Alex Alderton, Roberto Amato, Sonia Goncalves, Ewan Harrison, David K. Jackson, Ian Johnston, Dominic Kwiatkowski, Cordelia Langford, John Sillitoe on behalf of the Wellcome Sanger Institute COVID-19 Surveillance Team
EPI_ISL_800744	Lighthouse Lab in Cambridge	Wellcome Sanger Institute for the COVID-19 Genomics UK (COG-UK) Consortium	Rob Howes, The Lighthouse Lab in Cambridge and Alex Alderton, Roberto Amato, Sonia Goncalves, Ewan Harrison, David K. Jackson, Ian Johnston, Dominic Kwiatkowski, Cordelia Langford, John Sillitoe on behalf of the Wellcome Sanger Institute COVID-19 Surveillance Team
EPI_ISL_800745	Lighthouse Lab in Glasgow	Wellcome Sanger Institute for the COVID-19 Genomics UK (COG-UK) Consortium	Harper VanSteenhouse, Yumi Kasai, David Gray, Carol Clugston, Anna Dominiczak and Alex Alderton, Roberto Amato, Sonia Goncalves, Ewan Harrison, David K. Jackson, Ian Johnston, Dominic Kwiatkowski, Cordelia Langford, John Sillitoe on behalf of the Wellcome Sanger Institute COVID-19 Surveillance Team
EPI_ISL_800746	Lighthouse Lab in Cambridge	Wellcome Sanger Institute for the COVID-19 Genomics UK (COG-UK) Consortium	Rob Howes, The Lighthouse Lab in Cambridge and Alex Alderton, Roberto Amato, Sonia Goncalves, Ewan Harrison, David K. Jackson, Ian Johnston, Dominic Kwiatkowski, Cordelia Langford, John Sillitoe on behalf of the Wellcome Sanger Institute COVID-19 Surveillance Team
EPI_ISL_800748, EPI_ISL_800749, EPI_ISL_800753, EPI_ISL_800754	Lighthouse Lab in Glasgow	Wellcome Sanger Institute for the COVID-19 Genomics UK (COG-UK) Consortium	Harper VanSteenhouse, Yumi Kasai, David Gray, Carol Clugston, Anna Dominiczak and Alex Alderton, Roberto Amato, Sonia Goncalves, Ewan Harrison, David K. Jackson, Ian Johnston, Dominic Kwiatkowski, Cordelia Langford, John Sillitoe on behalf of the Wellcome Sanger Institute COVID-19 Surveillance Team
EPI_ISL_800755	Lighthouse Lab in Cambridge	Wellcome Sanger Institute for the COVID-19 Genomics UK (COG-UK) Consortium	Rob Howes, The Lighthouse Lab in Cambridge and Alex Alderton, Roberto Amato, Sonia Goncalves, Ewan Harrison, David K. Jackson, Ian Johnston, Dominic Kwiatkowski, Cordelia Langford, John Sillitoe on behalf of the Wellcome Sanger Institute COVID-19 Surveillance Team
EPI_ISL_800759, EPI_ISL_800760	Lighthouse Lab in Glasgow	Wellcome Sanger Institute for the COVID-19 Genomics UK (COG-UK) Consortium	Harper VanSteenhouse, Yumi Kasai, David Gray, Carol Clugston, Anna Dominiczak and Alex Alderton, Roberto Amato, Sonia Goncalves, Ewan Harrison, David K. Jackson, Ian Johnston, Dominic Kwiatkowski, Cordelia Langford, John Sillitoe on behalf of the Wellcome Sanger Institute COVID-19 Surveillance Team
EPI_ISL_800763	Lighthouse Lab in Cambridge	Wellcome Sanger Institute for the COVID-19 Genomics UK (COG-UK) Consortium	Rob Howes, The Lighthouse Lab in Cambridge and Alex Alderton, Roberto Amato, Sonia Goncalves, Ewan Harrison, David K. Jackson, Ian Johnston, Dominic Kwiatkowski, Cordelia Langford, John Sillitoe on behalf of the Wellcome Sanger Institute COVID-19 Surveillance Team

EPI_ISL_800765, EPI_ISL_800766, EPI_ISL_800767	Lighthouse Lab in Glasgow	Wellcome Sanger Institute for the COVID-19 Genomics UK (COG-UK) Consortium	Harper VanSteenhouse, Yumi Kasai, David Gray, Carol Clugston, Anna Dominiczak and Alex Alderton, Roberto Amato, Sonia Goncalves, Ewan Harrison, David K. Jackson, Ian Johnston, Dominic Kwiatkowski, Cordelia Langford, John Sillitoe on behalf of the Wellcome Sanger Institute COVID-19 Surveillance Team
EPI_ISL_800768, EPI_ISL_800770, EPI_ISL_800771	Lighthouse Lab in Cambridge	Wellcome Sanger Institute for the COVID-19 Genomics UK (COG-UK) Consortium	Rob Howes, The Lighthouse Lab in Cambridge and Alex Alderton, Roberto Amato, Sonia Goncalves, Ewan Harrison, David K. Jackson, Ian Johnston, Dominic Kwiatkowski, Cordelia Langford, John Sillitoe on behalf of the Wellcome Sanger Institute COVID-19 Surveillance Team
EPI_ISL_800772, EPI_ISL_800775	Lighthouse Lab in Glasgow	Wellcome Sanger Institute for the COVID-19 Genomics UK (COG-UK) Consortium	Harper VanSteenhouse, Yumi Kasai, David Gray, Carol Clugston, Anna Dominiczak and Alex Alderton, Roberto Amato, Sonia Goncalves, Ewan Harrison, David K. Jackson, Ian Johnston, Dominic Kwiatkowski, Cordelia Langford, John Sillitoe on behalf of the Wellcome Sanger Institute COVID-19 Surveillance Team
EPI_ISL_800776	Lighthouse Lab in Cambridge	Wellcome Sanger Institute for the COVID-19 Genomics UK (COG-UK) Consortium	Rob Howes, The Lighthouse Lab in Cambridge and Alex Alderton, Roberto Amato, Sonia Goncalves, Ewan Harrison, David K. Jackson, Ian Johnston, Dominic Kwiatkowski, Cordelia Langford, John Sillitoe on behalf of the Wellcome Sanger Institute COVID-19 Surveillance Team
EPI_ISL_800779, EPI_ISL_800780, EPI_ISL_800781	Lighthouse Lab in Glasgow	Wellcome Sanger Institute for the COVID-19 Genomics UK (COG-UK) Consortium	Harper VanSteenhouse, Yumi Kasai, David Gray, Carol Clugston, Anna Dominiczak and Alex Alderton, Roberto Amato, Sonia Goncalves, Ewan Harrison, David K. Jackson, Ian Johnston, Dominic Kwiatkowski, Cordelia Langford, John Sillitoe on behalf of the Wellcome Sanger Institute COVID-19 Surveillance Team
EPI_ISL_800784	Lighthouse Lab in Cambridge	Wellcome Sanger Institute for the COVID-19 Genomics UK (COG-UK) Consortium	Rob Howes, The Lighthouse Lab in Cambridge and Alex Alderton, Roberto Amato, Sonia Goncalves, Ewan Harrison, David K. Jackson, Ian Johnston, Dominic Kwiatkowski, Cordelia Langford, John Sillitoe on behalf of the Wellcome Sanger Institute COVID-19 Surveillance Team
EPI_ISL_800785, EPI_ISL_800786, EPI_ISL_800790	Lighthouse Lab in Glasgow	Wellcome Sanger Institute for the COVID-19 Genomics UK (COG-UK) Consortium	Harper VanSteenhouse, Yumi Kasai, David Gray, Carol Clugston, Anna Dominiczak and Alex Alderton, Roberto Amato, Sonia Goncalves, Ewan Harrison, David K. Jackson, Ian Johnston, Dominic Kwiatkowski, Cordelia Langford, John Sillitoe on behalf of the Wellcome Sanger Institute COVID-19 Surveillance Team
EPI_ISL_800791, EPI_ISL_800792, EPI_ISL_800793	Lighthouse Lab in Cambridge	Wellcome Sanger Institute for the COVID-19 Genomics UK (COG-UK) Consortium	Rob Howes, The Lighthouse Lab in Cambridge and Alex Alderton, Roberto Amato, Sonia Goncalves, Ewan Harrison, David K. Jackson, Ian Johnston, Dominic Kwiatkowski, Cordelia Langford, John Sillitoe on behalf of the Wellcome Sanger Institute COVID-19 Surveillance Team
EPI_ISL_800794	Lighthouse Lab in Glasgow	Wellcome Sanger Institute for the COVID-19 Genomics UK (COG-UK) Consortium	Harper VanSteenhouse, Yumi Kasai, David Gray, Carol Clugston, Anna Dominiczak and Alex Alderton, Roberto Amato, Sonia Goncalves, Ewan Harrison, David K. Jackson, Ian Johnston, Dominic Kwiatkowski, Cordelia Langford, John Sillitoe on behalf of the Wellcome Sanger Institute COVID-19 Surveillance Team
EPI_ISL_800795	Lighthouse Lab in Cambridge	Wellcome Sanger Institute for the COVID-19 Genomics UK (COG-UK) Consortium	Rob Howes, The Lighthouse Lab in Cambridge and Alex Alderton, Roberto Amato, Sonia Goncalves, Ewan Harrison, David K. Jackson, Ian Johnston, Dominic Kwiatkowski, Cordelia Langford, John Sillitoe on behalf of the Wellcome Sanger Institute COVID-19 Surveillance Team
EPI_ISL_800796, EPI_ISL_800797	Lighthouse Lab in Glasgow	Wellcome Sanger Institute for the COVID-19 Genomics UK (COG-UK) Consortium	Harper VanSteenhouse, Yumi Kasai, David Gray, Carol Clugston, Anna Dominiczak and Alex Alderton, Roberto Amato, Sonia Goncalves, Ewan Harrison, David K. Jackson, Ian Johnston, Dominic Kwiatkowski, Cordelia Langford, John Sillitoe on behalf of the Wellcome Sanger Institute COVID-19 Surveillance Team
EPI_ISL_800798	Lighthouse Lab in Cambridge	Wellcome Sanger Institute for the COVID-19 Genomics UK (COG-UK) Consortium	Rob Howes, The Lighthouse Lab in Cambridge and Alex Alderton, Roberto Amato, Sonia Goncalves, Ewan Harrison, David K. Jackson, Ian Johnston, Dominic Kwiatkowski, Cordelia Langford, John Sillitoe on behalf of the Wellcome Sanger Institute COVID-19 Surveillance Team
EPI_ISL_800799, EPI_ISL_800800, EPI_ISL_800801	Lighthouse Lab in Glasgow	Wellcome Sanger Institute for the COVID-19 Genomics UK (COG-UK) Consortium	Harper VanSteenhouse, Yumi Kasai, David Gray, Carol Clugston, Anna Dominiczak and Alex Alderton, Roberto Amato, Sonia Goncalves, Ewan Harrison, David K. Jackson, Ian Johnston, Dominic Kwiatkowski, Cordelia Langford, John Sillitoe on behalf of the Wellcome Sanger Institute COVID-19 Surveillance Team
EPI_ISL_800802, EPI_ISL_800803	Lighthouse Lab in Cambridge	Wellcome Sanger Institute for the COVID-19 Genomics UK (COG-UK) Consortium	Rob Howes, The Lighthouse Lab in Cambridge and Alex Alderton, Roberto Amato, Sonia Goncalves, Ewan Harrison, David K. Jackson, Ian Johnston, Dominic Kwiatkowski, Cordelia Langford, John Sillitoe on behalf of the Wellcome Sanger Institute COVID-19 Surveillance Team
EPI_ISL_800804, EPI_ISL_800805	Lighthouse Lab in Glasgow	Wellcome Sanger Institute for the COVID-19 Genomics UK (COG-UK) Consortium	Harper VanSteenhouse, Yumi Kasai, David Gray, Carol Clugston, Anna Dominiczak and Alex Alderton, Roberto Amato, Sonia Goncalves, Ewan Harrison, David K. Jackson, Ian Johnston, Dominic Kwiatkowski, Cordelia Langford, John Sillitoe on behalf of the Wellcome Sanger Institute COVID-19 Surveillance Team
EPI_ISL_800806	Lighthouse Lab in Cambridge	Wellcome Sanger Institute for the COVID-19 Genomics UK (COG-UK) Consortium	Rob Howes, The Lighthouse Lab in Cambridge and Alex Alderton, Roberto Amato, Sonia Goncalves, Ewan Harrison, David K. Jackson, Ian Johnston, Dominic Kwiatkowski, Cordelia Langford, John Sillitoe on behalf of the Wellcome Sanger Institute COVID-19 Surveillance Team
EPI_ISL_800809	Lighthouse Lab in Glasgow	Wellcome Sanger Institute for the COVID-19 Genomics UK (COG-UK) Consortium	Harper VanSteenhouse, Yumi Kasai, David Gray, Carol Clugston, Anna Dominiczak and Alex Alderton, Roberto Amato, Sonia Goncalves, Ewan Harrison, David K. Jackson, Ian Johnston, Dominic Kwiatkowski, Cordelia Langford, John Sillitoe on behalf of the Wellcome Sanger Institute COVID-19 Surveillance Team
EPI_ISL_800810	Lighthouse Lab in Cambridge	Wellcome Sanger Institute for the COVID-19 Genomics UK (COG-UK) Consortium	Rob Howes, The Lighthouse Lab in Cambridge and Alex Alderton, Roberto Amato, Sonia Goncalves, Ewan Harrison, David K. Jackson, Ian Johnston, Dominic Kwiatkowski, Cordelia Langford, John Sillitoe on behalf of the Wellcome Sanger Institute COVID-19 Surveillance Team
EPI_ISL_800812, EPI_ISL_800813, EPI_ISL_800815	Lighthouse Lab in Glasgow	Wellcome Sanger Institute for the COVID-19 Genomics UK (COG-UK) Consortium	Harper VanSteenhouse, Yumi Kasai, David Gray, Carol Clugston, Anna Dominiczak and Alex Alderton, Roberto Amato, Sonia Goncalves, Ewan Harrison, David K. Jackson, Ian Johnston, Dominic Kwiatkowski, Cordelia Langford, John Sillitoe on behalf of the Wellcome Sanger Institute COVID-19 Surveillance Team
EPI_ISL_800816, EPI_ISL_800817	Lighthouse Lab in Cambridge	Wellcome Sanger Institute for the COVID-19 Genomics UK (COG-UK) Consortium	Rob Howes, The Lighthouse Lab in Cambridge and Alex Alderton, Roberto Amato, Sonia Goncalves, Ewan Harrison, David K. Jackson, Ian Johnston, Dominic Kwiatkowski, Cordelia Langford, John Sillitoe on behalf of the Wellcome Sanger Institute COVID-19 Surveillance Team
EPI_ISL_800818	Lighthouse Lab in Glasgow	Wellcome Sanger Institute for the COVID-19 Genomics UK (COG-UK) Consortium	Harper VanSteenhouse, Yumi Kasai, David Gray, Carol Clugston, Anna Dominiczak and Alex Alderton, Roberto Amato, Sonia Goncalves, Ewan Harrison, David K. Jackson, Ian Johnston, Dominic Kwiatkowski, Cordelia Langford, John Sillitoe on behalf of the Wellcome Sanger Institute COVID-19 Surveillance Team
EPI_ISL_800819	Lighthouse Lab in Cambridge	Wellcome Sanger Institute for the COVID-19 Genomics UK (COG-UK) Consortium	Rob Howes, The Lighthouse Lab in Cambridge and Alex Alderton, Roberto Amato, Sonia Goncalves, Ewan Harrison, David K. Jackson, Ian Johnston, Dominic Kwiatkowski, Cordelia Langford, John Sillitoe on behalf of the Wellcome Sanger Institute COVID-19 Surveillance Team
EPI_ISL_800823, EPI_ISL_800825, EPI_ISL_800826, EPI_ISL_800827	Lighthouse Lab in Glasgow	Wellcome Sanger Institute for the COVID-19 Genomics UK (COG-UK) Consortium	Harper VanSteenhouse, Yumi Kasai, David Gray, Carol Clugston, Anna Dominiczak and Alex Alderton, Roberto Amato, Sonia Goncalves, Ewan Harrison, David K. Jackson, Ian Johnston, Dominic Kwiatkowski, Cordelia Langford, John Sillitoe on behalf of the Wellcome Sanger Institute COVID-19 Surveillance Team
EPI_ISL_800828	Lighthouse Lab in Cambridge	Wellcome Sanger Institute for the COVID-19 Genomics UK (COG-UK) Consortium	Rob Howes, The Lighthouse Lab in Cambridge and Alex Alderton, Roberto Amato, Sonia Goncalves, Ewan Harrison, David K. Jackson, Ian Johnston, Dominic Kwiatkowski, Cordelia Langford, John Sillitoe on behalf of the Wellcome Sanger Institute COVID-19 Surveillance Team
EPI_ISL_800829	Lighthouse Lab in Glasgow	Wellcome Sanger Institute for the COVID-19 Genomics UK (COG-UK) Consortium	Harper VanSteenhouse, Yumi Kasai, David Gray, Carol Clugston, Anna Dominiczak and Alex Alderton, Roberto Amato, Sonia Goncalves, Ewan Harrison, David K. Jackson, Ian Johnston, Dominic Kwiatkowski, Cordelia Langford, John Sillitoe on behalf of the Wellcome Sanger Institute COVID-19 Surveillance Team
EPI_ISL_800832	Lighthouse Lab in Cambridge	Wellcome Sanger Institute for the COVID-19 Genomics UK (COG-UK) Consortium	Rob Howes, The Lighthouse Lab in Cambridge and Alex Alderton, Roberto Amato, Sonia Goncalves, Ewan Harrison, David K. Jackson, Ian Johnston, Dominic Kwiatkowski, Cordelia Langford, John Sillitoe on behalf of the Wellcome Sanger Institute COVID-19 Surveillance Team
EPI_ISL_800833, EPI_ISL_800835, EPI_ISL_800838	Lighthouse Lab in Glasgow	Wellcome Sanger Institute for the COVID-19 Genomics UK (COG-UK) Consortium	Harper VanSteenhouse, Yumi Kasai, David Gray, Carol Clugston, Anna Dominiczak and Alex Alderton, Roberto Amato, Sonia Goncalves, Ewan Harrison, David K. Jackson, Ian Johnston, Dominic Kwiatkowski, Cordelia Langford, John Sillitoe on behalf of the Wellcome Sanger Institute COVID-19 Surveillance Team
EPI_ISL_800839	Lighthouse Lab in Cambridge	Wellcome Sanger Institute for the COVID-19 Genomics UK (COG-UK) Consortium	Rob Howes, The Lighthouse Lab in Cambridge and Alex Alderton, Roberto Amato, Sonia Goncalves, Ewan Harrison, David K. Jackson, Ian Johnston, Dominic Kwiatkowski, Cordelia Langford, John Sillitoe on behalf of the Wellcome Sanger Institute COVID-19 Surveillance Team
EPI_ISL_800841	Lighthouse Lab in Glasgow	Wellcome Sanger Institute for the COVID-19 Genomics UK (COG-UK) Consortium	Harper VanSteenhouse, Yumi Kasai, David Gray, Carol Clugston, Anna Dominiczak and Alex Alderton, Roberto Amato, Sonia Goncalves, Ewan Harrison, David K. Jackson, Ian Johnston, Dominic Kwiatkowski, Cordelia Langford, John Sillitoe on behalf of the Wellcome Sanger Institute COVID-19 Surveillance Team

EPI_ISL_800842	Lighthouse Lab in Cambridge	Wellcome Sanger Institute for the COVID-19 Genomics UK (COG-UK) Consortium	Rob Howes, The Lighthouse Lab in Cambridge and Alex Alderton, Roberto Amato, Sonia Goncalves, Ewan Harrison, David K. Jackson, Ian Johnston, Dominic Kwiatkowski, Cordelia Langford, John Sillitoe on behalf of the Wellcome Sanger Institute COVID-19 Surveillance Team
EPI_ISL_800843, EPI_ISL_800847, EPI_ISL_800848, EPI_ISL_800850, EPI_ISL_800851, EPI_ISL_800852	Lighthouse Lab in Glasgow	Wellcome Sanger Institute for the COVID-19 Genomics UK (COG-UK) Consortium	Harper VanSteenhouse, Yumi Kasai, David Gray, Carol Clugston, Anna Dominiczak and Alex Alderton, Roberto Amato, Sonia Goncalves, Ewan Harrison, David K. Jackson, Ian Johnston, Dominic Kwiatkowski, Cordelia Langford, John Sillitoe on behalf of the Wellcome Sanger Institute COVID-19 Surveillance Team
EPI_ISL_800853, EPI_ISL_800854, EPI_ISL_800855	Lighthouse Lab in Cambridge	Wellcome Sanger Institute for the COVID-19 Genomics UK (COG-UK) Consortium	Rob Howes, The Lighthouse Lab in Cambridge and Alex Alderton, Roberto Amato, Sonia Goncalves, Ewan Harrison, David K. Jackson, Ian Johnston, Dominic Kwiatkowski, Cordelia Langford, John Sillitoe on behalf of the Wellcome Sanger Institute COVID-19 Surveillance Team
EPI_ISL_800856, EPI_ISL_800858	Lighthouse Lab in Glasgow	Wellcome Sanger Institute for the COVID-19 Genomics UK (COG-UK) Consortium	Harper VanSteenhouse, Yumi Kasai, David Gray, Carol Clugston, Anna Dominiczak and Alex Alderton, Roberto Amato, Sonia Goncalves, Ewan Harrison, David K. Jackson, Ian Johnston, Dominic Kwiatkowski, Cordelia Langford, John Sillitoe on behalf of the Wellcome Sanger Institute COVID-19 Surveillance Team
EPI_ISL_800859	Lighthouse Lab in Cambridge	Wellcome Sanger Institute for the COVID-19 Genomics UK (COG-UK) Consortium	Rob Howes, The Lighthouse Lab in Cambridge and Alex Alderton, Roberto Amato, Sonia Goncalves, Ewan Harrison, David K. Jackson, Ian Johnston, Dominic Kwiatkowski, Cordelia Langford, John Sillitoe on behalf of the Wellcome Sanger Institute COVID-19 Surveillance Team
EPI_ISL_800860, EPI_ISL_800861, EPI_ISL_800862	Lighthouse Lab in Glasgow	Wellcome Sanger Institute for the COVID-19 Genomics UK (COG-UK) Consortium	Harper VanSteenhouse, Yumi Kasai, David Gray, Carol Clugston, Anna Dominiczak and Alex Alderton, Roberto Amato, Sonia Goncalves, Ewan Harrison, David K. Jackson, Ian Johnston, Dominic Kwiatkowski, Cordelia Langford, John Sillitoe on behalf of the Wellcome Sanger Institute COVID-19 Surveillance Team
EPI_ISL_800865, EPI_ISL_800870, EPI_ISL_800872, EPI_ISL_800873	Lighthouse Lab in Cambridge	Wellcome Sanger Institute for the COVID-19 Genomics UK (COG-UK) Consortium	Rob Howes, The Lighthouse Lab in Cambridge and Alex Alderton, Roberto Amato, Sonia Goncalves, Ewan Harrison, David K. Jackson, Ian Johnston, Dominic Kwiatkowski, Cordelia Langford, John Sillitoe on behalf of the Wellcome Sanger Institute COVID-19 Surveillance Team
EPI_ISL_800874, EPI_ISL_800875	Lighthouse Lab in Glasgow	Wellcome Sanger Institute for the COVID-19 Genomics UK (COG-UK) Consortium	Harper VanSteenhouse, Yumi Kasai, David Gray, Carol Clugston, Anna Dominiczak and Alex Alderton, Roberto Amato, Sonia Goncalves, Ewan Harrison, David K. Jackson, Ian Johnston, Dominic Kwiatkowski, Cordelia Langford, John Sillitoe on behalf of the Wellcome Sanger Institute COVID-19 Surveillance Team
EPI_ISL_800876	Lighthouse Lab in Cambridge	Wellcome Sanger Institute for the COVID-19 Genomics UK (COG-UK) Consortium	Rob Howes, The Lighthouse Lab in Cambridge and Alex Alderton, Roberto Amato, Sonia Goncalves, Ewan Harrison, David K. Jackson, Ian Johnston, Dominic Kwiatkowski, Cordelia Langford, John Sillitoe on behalf of the Wellcome Sanger Institute COVID-19 Surveillance Team
EPI_ISL_800880, EPI_ISL_800882	Lighthouse Lab in Glasgow	Wellcome Sanger Institute for the COVID-19 Genomics UK (COG-UK) Consortium	Harper VanSteenhouse, Yumi Kasai, David Gray, Carol Clugston, Anna Dominiczak and Alex Alderton, Roberto Amato, Sonia Goncalves, Ewan Harrison, David K. Jackson, Ian Johnston, Dominic Kwiatkowski, Cordelia Langford, John Sillitoe on behalf of the Wellcome Sanger Institute COVID-19 Surveillance Team
EPI_ISL_800883	Lighthouse Lab in Cambridge	Wellcome Sanger Institute for the COVID-19 Genomics UK (COG-UK) Consortium	Rob Howes, The Lighthouse Lab in Cambridge and Alex Alderton, Roberto Amato, Sonia Goncalves, Ewan Harrison, David K. Jackson, Ian Johnston, Dominic Kwiatkowski, Cordelia Langford, John Sillitoe on behalf of the Wellcome Sanger Institute COVID-19 Surveillance Team
EPI_ISL_800884	Lighthouse Lab in Glasgow	Wellcome Sanger Institute for the COVID-19 Genomics UK (COG-UK) Consortium	Harper VanSteenhouse, Yumi Kasai, David Gray, Carol Clugston, Anna Dominiczak and Alex Alderton, Roberto Amato, Sonia Goncalves, Ewan Harrison, David K. Jackson, Ian Johnston, Dominic Kwiatkowski, Cordelia Langford, John Sillitoe on behalf of the Wellcome Sanger Institute COVID-19 Surveillance Team
EPI_ISL_801180, EPI_ISL_801181	Lighthouse Lab in Milton Keynes	Wellcome Sanger Institute for the COVID-19 Genomics UK (COG-UK) Consortium	The Lighthouse Lab in Milton Keynes and Alex Alderton, Roberto Amato, Sonia Goncalves, Ewan Harrison, David K. Jackson, Ian Johnston, Dominic Kwiatkowski, Cordelia Langford, John Sillitoe on behalf of the Wellcome Sanger Institute COVID-19 Surveillance Team
EPI_ISL_801405, EPI_ISL_801409, EPI_ISL_801426, EPI_ISL_801471, EPI_ISL_801473, EPI_ISL_801474, EPI_ISL_801475, EPI_ISL_801476, EPI_ISL_801481	Dutch COVID-19 response team	Erasmus Medical Center	Bas Oude Munnink, Reina Sikkema, David Nieuwenhuijse, Irina Chestakova, Anne van der Linden, Marjan Boter, Emmanuelle Munger, Corine GeurtsvanKessel, Annemiek van der Eijk, Richard Molenkamp, Marion Koopmans, on behalf of the Dutch national COVID-19 response team.
EPI_ISL_802865, EPI_ISL_802866, EPI_ISL_802869, EPI_ISL_802872, EPI_ISL_802876, EPI_ISL_802879, EPI_ISL_802880, EPI_ISL_802886, EPI_ISL_802887, EPI_ISL_802893, EPI_ISL_802894, EPI_ISL_802898, EPI_ISL_802902, EPI_ISL_802906, EPI_ISL_802908, EPI_ISL_802909, EPI_ISL_802911, EPI_ISL_802914, EPI_ISL_802917			
see above	Utah Public Health Laboratory	Utah Public Health Laboratory	Erin Young, Kelly Oakeson, Tara Gallagher
EPI_ISL_803874, EPI_ISL_803875	Institute of Medical Microbiology and Hospital Hygiene	Institute of Medical Microbiology and Hospital Hygiene	Prof. Dr. Achim Kaasch
EPI_ISL_803911	BBMP Urban PHC	Department of Neurovirology, National Institute of Mental Health and Neurosciences (NIMHANS)	Chitra Pattabiraman, Pramada Prasad, Risha Rasheed, Anita S Desai, V Ravi
EPI_ISL_804609, EPI_ISL_804694, EPI_ISL_804707	Michigan Department of Health and Human Services, Bureau of Laboratories	Michigan Department of Health and Human Services, Bureau of Laboratories	Blankenship HM, Riner D, Soehnlen MK
EPI_ISL_804934	DC Public Health Lab/ Dept. of Forensic Sciences	DC Public Health Lab/ Dept. of Forensic Sciences	Scott Nguyen, Elizabeth Zelaya, Connie Maza, Monica Mann, Brittany Hamilton, David Payne, Jocelyn Hauser
EPI_ISL_806843, EPI_ISL_806844	Alaska State Virology Laboratory	Alaska State Virology Laboratory	Stephanie DeRonde, Lisa Smith, Ph.D., Devin M. Drown, Ph.D., Jack Chen, Ph.D.
EPI_ISL_812402	Utah Public Health Laboratory	Utah Public Health Laboratory	Erin L. Young, Kelly F. Oakeson, Tara Gallagher
EPI_ISL_813030, EPI_ISL_813031, EPI_ISL_813032, EPI_ISL_813033, EPI_ISL_813034, EPI_ISL_813035, EPI_ISL_813036, EPI_ISL_813071	University of Birmingham	COVID-19 Genomics UK (COG-UK) Consortium	Institute of Microbiology, University of Birmingham: Claire McMurray, Joanne Stockton, Samuel Nicholls, Radoslaw Poplawski, Will Rowe, Josh Quick, Nicholas Loman. University of Birmingham Testing Laboratory: Celina M Whalley, Andrew Bosworth, Charlotte Poxon, Kasun Wanigasooriya, Oliver Pickles, Mike Kidd, Alex Richter, Andrew D Beggs PHE Heartlands Lab: Husam Osman, Andrew Bosworth. Queen Elizabeth Hospital: Anna Casey
EPI_ISL_813678, EPI_ISL_813679, EPI_ISL_813681, EPI_ISL_813682, EPI_ISL_813683, EPI_ISL_813684, EPI_ISL_813685, EPI_ISL_813686, EPI_ISL_813688, EPI_ISL_813689, EPI_ISL_813690, EPI_ISL_813691, EPI_ISL_813692, EPI_ISL_813693, EPI_ISL_813694, EPI_ISL_813695, EPI_ISL_813696, EPI_ISL_813697, EPI_ISL_813698, EPI_ISL_813699, EPI_ISL_813700, EPI_ISL_813701, EPI_ISL_813702, EPI_ISL_813703, EPI_ISL_813704, EPI_ISL_813723, EPI_ISL_813724, EPI_ISL_813725, EPI_ISL_813726, EPI_ISL_813727, EPI_ISL_813728, EPI_ISL_813729, EPI_ISL_813730, EPI_ISL_813731			
see above	Liverpool Clinical Laboratories	COVID-19 Genomics UK (COG-UK) Consortium	Sam Haldenby, Anita Lucaci, Steve Paterson, Julian Hiscox, Alistair Darby, M Almsaud, A Alrezaihi, Muhannad Alruwaili, Stuart D Armstrong, Jones Benjamin, Eleanor G Bentley, Anu Chawla, Jordan J Clark, Angela Cowell, Richard Eccles, Isabel García-Dorival, Matthew Gemmell, Alessandro Gerada, PKF Gilmore, Richard Gregory, Ximeng Han, Catherine Hartley, Margaret Hughes, Miren Iturriza-Gomara, James Johnson, L Luu, Jenifer Manson, Charlotte Nelson, Elaine O'Toole, Cassie Olateju, Rebekah Penrice-Randal , Lucille Rainbow, N.P Randle, Trevor Ian Robinson, Parul Sharma, Ghada T Shawli, James P Stewart, Neil Swainston, Ecaterina Vamos, Joanne Watts, Mark Whitehead
EPI_ISL_814299, EPI_ISL_814307, EPI_ISL_814316, EPI_ISL_814322, EPI_ISL_814327	Wales Specialist Virology Centre Sequencing lab: Pathogen Genomics Unit	COVID-19 Genomics UK (COG-UK) Consortium	Catherine Moore, Johnathan Evans, Laura Gifford, Malorie Perry, Simon Cottrell, Angela Marchbank, Alec Birchley, Alexander Adams, Amy Gaskin, Bree Gatica-Wilcox, Jason Coombes, Joel Southgate, Lauren Gilbert, Lee Graham, Nicole Pacchiarini, Sara Kumziene-Summerhayes, Sarah Taylor, Sophie Jones, Sara Rey, Matthew Bull, Joanne Watkins, Sally Corden, Tom Connor
EPI_ISL_814365	Virology Department, Royal Infirmary of Edinburgh, NHS Lothian / School of Biological Sciences, University of Edinburgh / Institute of Genetics and Molecular Medicine, University of Edinburgh	COVID-19 Genomics UK (COG-UK) Consortium	McHugh M, Dewar R, Rooke S, Gallagher M, Balcaza C, O'Toole Á, Scher E, Hill V, McCrone JT, Colquhoun R, Yu X, Jackson B, Rambaut A, Williams TC, Templeton K
EPI_ISL_814391, EPI_ISL_814409, EPI_ISL_814545, EPI_ISL_814559	Wales Specialist Virology Centre Sequencing lab: Pathogen Genomics Unit	COVID-19 Genomics UK (COG-UK) Consortium	Catherine Moore, Johnathan Evans, Laura Gifford, Malorie Perry, Simon Cottrell, Angela Marchbank, Alec Birchley, Alexander Adams, Amy Gaskin, Bree Gatica-Wilcox, Jason Coombes, Joel Southgate, Lauren Gilbert, Lee Graham, Nicole Pacchiarini, Sara Kumziene-Summerhayes, Sarah Taylor, Sophie Jones, Sara Rey, Matthew Bull, Joanne Watkins, Sally Corden, Tom Connor
EPI_ISL_814590, EPI_ISL_814591, EPI_ISL_814592, EPI_ISL_814593	West of Scotland Specialist Virology Centre, NHSGGC / MRC-University of Glasgow Centre for Virus Research	COVID-19 Genomics UK (COG-UK) Consortium	Ana da Silva Filipe, Natasha Johnson, Kathy Smollett, Daniel Mair, Stephen Carmichael, Alice Broos, Lily Tong, Jenna Nichols, Kyriaki Nomikou; Sarah McDonald; Richard Orton, Joseph Hughes, Sreenu Vattipally, David L Robertson; Alasdair MacLean, Rory Gunson; Sharif Shaaban, Matthew Holden; Rachel Blacow, Guy Mollett, Kathy Li, James Shepherd, Antonia Ho, Emma Thomson
EPI_ISL_814607	Wales Specialist Virology Centre Sequencing lab: Pathogen	COVID-19 Genomics UK (COG-UK) Consortium	Catherine Moore, Johnathan Evans, Laura Gifford, Malorie Perry, Simon Cottrell, Angela Marchbank, Alec Birchley, Alexander Adams, Amy Gaskin, Bree

	Genomics Unit		Gatica-Wilcox, Jason Coombes, Joel Southgate, Lauren Gilbert, Lee Graham, Nicole Pacchiarini, Sara Kumziene-Summerhayes, Sarah Taylor, Sophie Jones, Sara Rey, Matthew Bull, Joanne Watkins, Sally Corden, Tom Connor
EPI_ISL_814671, EPI_ISL_814672, EPI_ISL_814673, EPI_ISL_814674, EPI_ISL_814676, EPI_ISL_814677	Virology Department, Royal Infirmary of Edinburgh, NHS Lothian / School of Biological Sciences, University of Edinburgh / Institute of Genetics and Molecular Medicine, University of Edinburgh	COVID-19 Genomics UK (COG-UK) Consortium	McHugh M, Dewar R, Rooke S, Gallagher M, Balcaza C, O'Toole Á, Scher E, Hill V, McCrone JT, Colquhoun R, Yu X, Jackson B, Rambaut A, Williams TC, Templeton K
EPI_ISL_814705, EPI_ISL_814706, EPI_ISL_814707, EPI_ISL_814708, EPI_ISL_814709, EPI_ISL_814710, EPI_ISL_814711, EPI_ISL_814712, EPI_ISL_814713, EPI_ISL_814714, EPI_ISL_814715, EPI_ISL_814716, EPI_ISL_815088, EPI_ISL_815089, EPI_ISL_815091, EPI_ISL_815092			
see above	Wales Specialist Virology Centre Sequencing lab: Pathogen Genomics Unit	COVID-19 Genomics UK (COG-UK) Consortium	Catherine Moore, Johnathan Evans, Laura Gifford, Malorie Perry, Simon Cottrell, Angela Marchbank, Alec Birchley, Alexander Adams, Amy Gaskin, Bree Gatica-Wilcox, Jason Coombes, Joel Southgate, Lauren Gilbert, Lee Graham, Nicole Pacchiarini, Sara Kumziene-Summerhayes, Sarah Taylor, Sophie Jones, Sara Rey, Matthew Bull, Joanne Watkins, Sally Corden, Tom Connor
EPI_ISL_815411, EPI_ISL_815454, EPI_ISL_815455, EPI_ISL_815456, EPI_ISL_815457, EPI_ISL_815458, EPI_ISL_815459, EPI_ISL_815460, EPI_ISL_815461, EPI_ISL_815462, EPI_ISL_815474, EPI_ISL_815480, EPI_ISL_815527, EPI_ISL_815536, EPI_ISL_815540, EPI_ISL_815592, EPI_ISL_815593, EPI_ISL_815594, EPI_ISL_815595, EPI_ISL_815596, EPI_ISL_815607, EPI_ISL_815621, EPI_ISL_816035, EPI_ISL_816036, EPI_ISL_816037, EPI_ISL_816038, EPI_ISL_816039, EPI_ISL_816040, EPI_ISL_816041, EPI_ISL_816042, EPI_ISL_816043, EPI_ISL_816044, EPI_ISL_816045, EPI_ISL_816046, EPI_ISL_816047, EPI_ISL_816048, EPI_ISL_816049, EPI_ISL_816050, EPI_ISL_816051, EPI_ISL_816052, EPI_ISL_816053, EPI_ISL_816054, EPI_ISL_816055, EPI_ISL_816056, EPI_ISL_816057, EPI_ISL_816058, EPI_ISL_816059, EPI_ISL_816060, EPI_ISL_816061, EPI_ISL_816062, EPI_ISL_816063, EPI_ISL_816064, EPI_ISL_816065, EPI_ISL_816066			
see above	Department of Virus and Microbiological Special Diagnostics, Statens Serum Institut, Copenhagen, Denmark	Albertsen Lab, Department of Chemistry and Bioscience, Aalborg University, Denmark	Danish Covid-19 Genome Consortium
EPI_ISL_816263, EPI_ISL_816270, EPI_ISL_816342, EPI_ISL_816356, EPI_ISL_816397, EPI_ISL_816445, EPI_ISL_816476, EPI_ISL_816485, EPI_ISL_816489, EPI_ISL_816499, EPI_ISL_816516, EPI_ISL_816530, EPI_ISL_816545, EPI_ISL_816570, EPI_ISL_816602			
see above	Virology Department, Sheffield Teaching Hospitals NHS Foundation Trust/Department of Infection, Immunity and Cardiovascular Disease, The Medical School, University of Sheffield	COVID-19 Genomics UK (COG-UK) Consortium	Thushan de Silva, Matthew Parker, Nikki Smith, Adri Angyal, Rebecca Brown, Luke Green, Rachel Tucker, Paul Parsons, Danielle Groves, Katie Johnson, Laura Carrilero, Alex Keeley, Dave Partridge, Matthew Wyles, Benjamin Lindsey, Mehmet Yavuz, Mohammad Raza, Cariad Evans
EPI_ISL_816861, EPI_ISL_816862, EPI_ISL_816863, EPI_ISL_816864, EPI_ISL_816865, EPI_ISL_816867, EPI_ISL_816868, EPI_ISL_816869, EPI_ISL_816870, EPI_ISL_816871, EPI_ISL_816872, EPI_ISL_816873, EPI_ISL_816874, EPI_ISL_816875, EPI_ISL_816876, EPI_ISL_816878, EPI_ISL_816879, EPI_ISL_816880, EPI_ISL_816881, EPI_ISL_816882, EPI_ISL_816883, EPI_ISL_816884, EPI_ISL_816885, EPI_ISL_816886, EPI_ISL_816887, EPI_ISL_816888, EPI_ISL_816889, EPI_ISL_816890, EPI_ISL_816891, EPI_ISL_816893, EPI_ISL_816895, EPI_ISL_816900, EPI_ISL_816901, EPI_ISL_816902, EPI_ISL_816904, EPI_ISL_816906, EPI_ISL_816909, EPI_ISL_816910, EPI_ISL_816911, EPI_ISL_816913, EPI_ISL_816915, EPI_ISL_816916, EPI_ISL_816918, EPI_ISL_816920, EPI_ISL_816923, EPI_ISL_816924, EPI_ISL_816925, EPI_ISL_816958, EPI_ISL_816960, EPI_ISL_816962, EPI_ISL_816967, EPI_ISL_816968, EPI_ISL_816969, EPI_ISL_816971, EPI_ISL_816973, EPI_ISL_816976, EPI_ISL_816977, EPI_ISL_816978, EPI_ISL_816980, EPI_ISL_816982, EPI_ISL_816983, EPI_ISL_816985, EPI_ISL_816987, EPI_ISL_816990, EPI_ISL_816991, EPI_ISL_816992			
see above	Bioinformatics and Biostatistics Lab, Advanced Sequencing Facility	COVID-19 Genomics UK (COG-UK) Consortium	Aengus Stewart,Jerome Nicod,Chelsea Sawyer,Laura Cubitt,Harshil Patel,Margaret Crawford
EPI_ISL_819569, EPI_ISL_819570, EPI_ISL_819571, EPI_ISL_819572, EPI_ISL_819573, EPI_ISL_819574	Northumbria University / South Tees Hospitals NHS Foundation Trust / North Cumbria Integrated Care NHS Foundation Trust / North Tees and Hartlepool NHS Foundation Trust / Newcastle Hospitals NHS Foundation Trust	COVID-19 Genomics UK (COG-UK) Consortium	Darren L Smith,Andrew Nelson,Matthew Bashton,Greg R Young,Joshua Loh,John Allan,Mohammad A Tariq,Giles S Holt,Gary Black,Wen C Yew,Lynn Dover,Paul Baker,Steve Liggett,Sarah Essex,Jane Greenaway,Debra Padgett,Clive Graham,Garren Scott,Edward Barton,Emma Swindells,Brendan Payne,Jennifer Collins,Yusri Taha,Gary Eltringham
EPI_ISL_822900, EPI_ISL_823206, EPI_ISL_823207	Wales Specialist Virology Centre Sequencing lab: Pathogen Genomics Unit	COVID-19 Genomics UK (COG-UK) Consortium	Catherine Moore, Johnathan Evans, Laura Gifford, Malorie Perry, Simon Cottrell, Angela Marchbank, Alec Birchley, Alexander Adams, Amy Gaskin, Bree Gatica-Wilcox, Jason Coombes, Joel Southgate, Lauren Gilbert, Lee Graham, Nicole Pacchiarini, Sara Kumziene-Summerhayes, Sarah Taylor, Sophie Jones, Sara Rey, Matthew Bull, Joanne Watkins, Sally Corden, Tom Connor
EPI_ISL_823838, EPI_ISL_823839, EPI_ISL_823840	DOHMH Central Harlem	New York City Public Health Laboratory	Jade Wang, et al.
EPI_ISL_823841	DOHMH Riverside	New York City Public Health Laboratory	Jade Wang, et al.
EPI_ISL_823842	DOHMH PHL	New York City Public Health Laboratory	Jade Wang, et al.
EPI_ISL_823843, EPI_ISL_823844	DOHMH Central Harlem	New York City Public Health Laboratory	Jade Wang, et al.
EPI_ISL_823845	DOHMH Morrisania	New York City Public Health Laboratory	Jade Wang, et al.
EPI_ISL_823846, EPI_ISL_823847, EPI_ISL_823848	DOHMH Jamaica	New York City Public Health Laboratory	Jade Wang, et al.
EPI_ISL_823849	DOHMH Fort Greene	New York City Public Health Laboratory	Jade Wang, et al.
EPI_ISL_823850	DOHMH Corona	New York City Public Health Laboratory	Jade Wang, et al.
EPI_ISL_823851	DOHMH Fort Greene	New York City Public Health Laboratory	Jade Wang, et al.
EPI_ISL_823852, EPI_ISL_823853	DOHMH Crown Heights	New York City Public Health Laboratory	Jade Wang, et al.
EPI_ISL_823854, EPI_ISL_823855, EPI_ISL_823856, EPI_ISL_823857, EPI_ISL_823858, EPI_ISL_823859, EPI_ISL_823860	DOHMH Morrisania	New York City Public Health Laboratory	Jade Wang, et al.
EPI_ISL_823861, EPI_ISL_823862, EPI_ISL_823863	DOHMH Corona	New York City Public Health Laboratory	Jade Wang, et al.
EPI_ISL_823864	DOHMH Chelsea	New York City Public Health Laboratory	Jade Wang, et al.
EPI_ISL_823865	DOHMH Central Harlem	New York City Public Health Laboratory	Jade Wang, et al.
EPI_ISL_823866, EPI_ISL_823867	DOHMH Fort Greene	New York City Public Health Laboratory	Jade Wang, et al.
EPI_ISL_823868	DOHMH Corona	New York City Public Health Laboratory	Jade Wang, et al.
EPI_ISL_823869	DOHMH Central Harlem	New York City Public Health Laboratory	Jade Wang, et al.
EPI_ISL_823870, EPI_ISL_823871	DOHMH Jamaica	New York City Public Health Laboratory	Jade Wang, et al.
EPI_ISL_823872, EPI_ISL_823873, EPI_ISL_823874	DOHMH Morrisania	New York City Public Health Laboratory	Jade Wang, et al.
EPI_ISL_823875	DOHMH PHL	New York City Public Health Laboratory	Jade Wang, et al.
EPI_ISL_823876, EPI_ISL_823877	DOHMH Jamaica	New York City Public Health Laboratory	Jade Wang, et al.
EPI_ISL_823878, EPI_ISL_823879, EPI_ISL_823880, EPI_ISL_823881, EPI_ISL_823882	DOHMH Corona	New York City Public Health Laboratory	Jade Wang, et al.
EPI_ISL_823883	DOHMH Riverside	New York City Public Health Laboratory	Jade Wang, et al.
EPI_ISL_823884, EPI_ISL_823885, EPI_ISL_823886, EPI_ISL_823887	DOHMH Central Harlem	New York City Public Health Laboratory	Jade Wang, et al.
EPI_ISL_823888	DOHMH Riverside	New York City Public Health Laboratory	Jade Wang, et al.
EPI_ISL_823889, EPI_ISL_823890,	DOHMH PHL	New York City Public Health Laboratory	Jade Wang, et al.

EPI_ISL_823891			
EPI_ISL_823892, EPI_ISL_823893	DOHMH Morrisania	New York City Public Health Laboratory	Jade Wang, et al.
EPI_ISL_823894, EPI_ISL_823895	DOHMH Jamaica	New York City Public Health Laboratory	Jade Wang, et al.
EPI_ISL_823896, EPI_ISL_823897	DOHMH PHL	New York City Public Health Laboratory	Jade Wang, et al.
EPI_ISL_823898, EPI_ISL_823899	DOHMH Corona	New York City Public Health Laboratory	Jade Wang, et al.
EPI_ISL_823900	DOHMH Riverside	New York City Public Health Laboratory	Jade Wang, et al.
EPI_ISL_823901, EPI_ISL_823902	DOHMH Central Harlem	New York City Public Health Laboratory	Jade Wang, et al.
EPI_ISL_823903	DOHMH Morrisania	New York City Public Health Laboratory	Jade Wang, et al.
EPI_ISL_823904	DOHMH Fort Greene	New York City Public Health Laboratory	Jade Wang, et al.
EPI_ISL_823905, EPI_ISL_823906	DOHMH Corona	New York City Public Health Laboratory	Jade Wang, et al.
EPI_ISL_823907, EPI_ISL_823908	DOHMH Jamaica	New York City Public Health Laboratory	Jade Wang, et al.
EPI_ISL_823909	DOHMH PHL	New York City Public Health Laboratory	Jade Wang, et al.
EPI_ISL_823910, EPI_ISL_823911	DOHMH Fort Greene	New York City Public Health Laboratory	Jade Wang, et al.
EPI_ISL_823912	DOHMH Central Harlem	New York City Public Health Laboratory	Jade Wang, et al.
EPI_ISL_823913, EPI_ISL_823914	DOHMH PHL	New York City Public Health Laboratory	Jade Wang, et al.
EPI_ISL_823915, EPI_ISL_823916, EPI_ISL_823917	DOHMH Jamaica	New York City Public Health Laboratory	Jade Wang, et al.
EPI_ISL_823918, EPI_ISL_823919, EPI_ISL_823920, EPI_ISL_823921, EPI_ISL_823922	DOHMH Corona	New York City Public Health Laboratory	Jade Wang, et al.
EPI_ISL_823923	DOHMH Fort Greene	New York City Public Health Laboratory	Jade Wang, et al.
EPI_ISL_823924	DOHMH Chelsea	New York City Public Health Laboratory	Jade Wang, et al.
EPI_ISL_823925	DOHMH Crown Heights	New York City Public Health Laboratory	Jade Wang, et al.
EPI_ISL_824234, EPI_ISL_824235, EPI_ISL_824236, EPI_ISL_824237, EPI_ISL_824238, EPI_ISL_824239, EPI_ISL_824240	Dutch COVID-19 response team	National Institute for Public Health and the Environment (RIVM)	Adam Meijer, Harry Vennema, Jeroen Cremer, Sharon van den Brink, Bas van der Veer, AnneMarie van den Brandt, Florian Zwagemaker, Dennis Schmitz, Chantal Reusken, on behalf of the national COVID-19 response team
EPI_ISL_824346, EPI_ISL_824362, EPI_ISL_824372, EPI_ISL_824373	Michigan Department of Health and Human Services, Bureau of Laboratories	Michigan Department of Health and Human Services, Bureau of Laboratories	Blankenship HM, Riner D, Soehnlen MK
EPI_ISL_824400	California Department of Public Health	California Department of Public Health	CDPH IDLB COVIDNet
EPI_ISL_824656, EPI_ISL_824666, EPI_ISL_824667, EPI_ISL_824678, EPI_ISL_824679, EPI_ISL_824690, EPI_ISL_824691, EPI_ISL_824702, EPI_ISL_824714, EPI_ISL_824725, EPI_ISL_824726, EPI_ISL_824736, EPI_ISL_824737			
see above	Cedars-Sinai Medical Center, Department of Pathology & Laboratory Medicine, Molecular Pathology Laboratory	Cedars-Sinai Medical Center, Molecular Pathology Laboratory of Department of Pathology & Laboratory Medicine and Genomic Core	Wenjuan Zhang, Brian Davis, Stephanie Chen, Jorge Mario Sincuir Martinez, Jasmine T Plummer, Eric Vail
EPI_ISL_824765, EPI_ISL_824766, EPI_ISL_824767, EPI_ISL_824768, EPI_ISL_824769, EPI_ISL_824770, EPI_ISL_824771, EPI_ISL_824772, EPI_ISL_824773, EPI_ISL_824774, EPI_ISL_824775, EPI_ISL_824776, EPI_ISL_824777, EPI_ISL_824778, EPI_ISL_824779, EPI_ISL_824780, EPI_ISL_824781, EPI_ISL_824782, EPI_ISL_824783, EPI_ISL_824784, EPI_ISL_824785, EPI_ISL_824786			
see above	A. Krumbholz, Labor Dr. Krause und Kollegen MVZ GmbH, Kiel	Charité Universitätsmedizin Berlin, Institut für Virologie	Victor M Corman, Jörn Beheim-Schwarzbach, Julia Schneider, Talitha Veith, Barbara Mühlemann, Terry Jones, Christian Drosten
EPI_ISL_825020, EPI_ISL_825022, EPI_ISL_825024, EPI_ISL_825025	Utah Public Health Laboratory, Utah Public Health Laboratory Infectious Disease submission group	Utah Public Health Laboratory, Utah Public Health Laboratory Infectious Disease submission group	Young,E.L., Oakeson,K.F., Gallagher,T.
EPI_ISL_825374	Hospital Universitari Vall d'Hebron - Vall d'Hebron Institut de Rercerca	Hospital Universitari Vall d'Hebron	Cristina Andrés, Maria Piñana, Josep F Abril, Damir Garcia-Cehic, Ariadna Rando, Juliana Esperalba, Maria Gema Codina, Carla Castillo, Maria Carmen Martin, Tomàs Pumarola, Josep Quer, Andrés Antón
EPI_ISL_826539, EPI_ISL_826541, EPI_ISL_826550, EPI_ISL_826551, EPI_ISL_826557, EPI_ISL_826561, EPI_ISL_826566, EPI_ISL_826570, EPI_ISL_826575	Texas Department of State Health Services	Texas Department of State Health Services	Rashmi Tuladhar, Bonnie Oh, Jenny Zhang, Maliha Rahman, Anita Pokharel, Myong Koag, Chung Wang, Rachel Lee, Grace Kubin, Mayela Pedrueza, James Daniel Bonser
EPI_ISL_826806, EPI_ISL_826808, EPI_ISL_826895	deCODE genetics	deCODE genetics	Daniel F Gudbjartsson; Agnar Helgason; Hakon Jonsson; Olafur T Magnusson; Pall Melsted; Gudmundur L Norddahl; Jona Saemundsdottir; Asgeir Sigurdsson; Patrick Sulem; Arna B Agustsdottir; Hannes Eggertsson; Berglind Eiriksdottir; Run Fridriksdottir; Elisabet E Gardarsdottir; Gudmundur Georgsson; Olafia S Gretarsdottir; Kjartan R Gudmundsson; Thora R Gunnarsdottir; Arnaldur Gylfason; Hilma Holm; Brynjar O Jensson; Aslaug Jonasdottir; Kamilla S Josefsdottir; Thordur Kristjansson; Droplaug N Magnusdottir; Solvi Rognvaldsson; Louise le Roux; Gudrun Sigmundsdottir; Gardar Sveinbjornsson; Kristin E Sveinsdottir; Maney Sveinsdottir; Emil A Thorarensen; Bjarni Thorbjornsson; Gisli Masson; Ingileif Jonsdottir; Alma Moller; Thorolfur Gudnason; Karl G Kristinsson; Unnur Thorsteinsdottir; Kari Stefansson
EPI_ISL_826980	The National University Hospital of Iceland	deCODE genetics	Daniel F Gudbjartsson; Agnar Helgason; Hakon Jonsson; Olafur T Magnusson; Pall Melsted; Gudmundur L Norddahl; Jona Saemundsdottir; Asgeir Sigurdsson; Patrick Sulem; Arna B Agustsdottir; Hannes Eggertsson; Berglind Eiriksdottir; Run Fridriksdottir; Elisabet E Gardarsdottir; Gudmundur Georgsson; Olafia S Gretarsdottir; Kjartan R Gudmundsson; Thora R Gunnarsdottir; Arnaldur Gylfason; Hilma Holm; Brynjar O Jensson; Aslaug Jonasdottir; Kamilla S Josefsdottir; Thordur Kristjansson; Droplaug N Magnusdottir; Solvi Rognvaldsson; Louise le Roux; Gudrun Sigmundsdottir; Gardar Sveinbjornsson; Kristin E Sveinsdottir; Maney Sveinsdottir; Emil A Thorarensen; Bjarni Thorbjornsson; Gisli Masson; Ingileif Jonsdottir; Alma Moller; Thorolfur Gudnason; Karl G Kristinsson; Unnur Thorsteinsdottir; Kari Stefansson
EPI_ISL_827089, EPI_ISL_827420, EPI_ISL_827431, EPI_ISL_828088	deCODE genetics	deCODE genetics	Daniel F Gudbjartsson; Agnar Helgason; Hakon Jonsson; Olafur T Magnusson; Pall Melsted; Gudmundur L Norddahl; Jona Saemundsdottir; Asgeir Sigurdsson; Patrick Sulem; Arna B Agustsdottir; Hannes Eggertsson; Berglind Eiriksdottir; Run Fridriksdottir; Elisabet E Gardarsdottir; Gudmundur Georgsson; Olafia S Gretarsdottir; Kjartan R Gudmundsson; Thora R Gunnarsdottir; Arnaldur Gylfason; Hilma Holm; Brynjar O Jensson; Aslaug Jonasdottir; Kamilla S Josefsdottir; Thordur Kristjansson; Droplaug N Magnusdottir; Solvi Rognvaldsson; Louise le Roux; Gudrun Sigmundsdottir; Gardar Sveinbjornsson; Kristin E Sveinsdottir; Maney Sveinsdottir; Emil A Thorarensen; Bjarni Thorbjornsson; Gisli Masson; Ingileif Jonsdottir; Alma Moller; Thorolfur Gudnason; Karl G Kristinsson; Unnur Thorsteinsdottir; Kari Stefansson
EPI_ISL_828363, EPI_ISL_828383	The National University Hospital of Iceland	deCODE genetics	Daniel F Gudbjartsson; Agnar Helgason; Hakon Jonsson; Olafur T Magnusson; Pall Melsted; Gudmundur L Norddahl; Jona Saemundsdottir; Asgeir Sigurdsson; Patrick Sulem; Arna B Agustsdottir; Hannes Eggertsson; Berglind Eiriksdottir; Run Fridriksdottir; Elisabet E Gardarsdottir; Gudmundur Georgsson; Olafia S Gretarsdottir; Kjartan R Gudmundsson; Thora R Gunnarsdottir; Arnaldur Gylfason; Hilma Holm; Brynjar O Jensson; Aslaug Jonasdottir; Kamilla S Josefsdottir; Thordur Kristjansson; Droplaug N Magnusdottir; Solvi Rognvaldsson; Louise le Roux; Gudrun Sigmundsdottir; Gardar Sveinbjornsson; Kristin E Sveinsdottir; Maney Sveinsdottir; Emil A Thorarensen; Bjarni Thorbjornsson; Gisli Masson; Ingileif Jonsdottir; Alma Moller; Thorolfur Gudnason; Karl G Kristinsson; Unnur Thorsteinsdottir; Kari Stefansson

EPI_ISL_828410, EPI_ISL_829054, EPI_ISL_829058, EPI_ISL_829071, EPI_ISL_829527, EPI_ISL_829528, EPI_ISL_829701, EPI_ISL_829704, EPI_ISL_829794, EPI_ISL_829795, EPI_ISL_829796, EPI_ISL_829797, EPI_ISL_829903, EPI_ISL_830164, EPI_ISL_830165			
see above	deCODE genetics	deCODE genetics	Daniel F Gudbjartsson; Agnar Helgason; Hakon Jonsson; Olafur T Magnusson; Pall Melsted; Gudmundur L Norddahl; Jona Saemundsdottir; Asgeir Sigurdsson; Patrick Sulem; Arna B Agustsdottir; Hannes Eggertsson; Berglind Eiriksdottir; Run Fridriksdottir; Elisabet E Gardarsdottir; Gudmundur Georgsson; Olafia S Gretarsdottir; Kjartan R Gudmundsson; Thora R Gunnarsdottir; Arnaldur Gylfason; Hilma Holm; Brynjar O Jensson; Aslaug Jonasdottir; Kamilla S Josefsdottir; Thordur Kristjansson; Droplaug N Magnusdottir; Solvi Rognvaldsson; Louise le Roux; Gudrun Sigmundsdottir; Gardar Sveinbjornsson; Kristin E Sveinsdottir; Maney Sveinsdottir; Emil A Thorarensen; Bjarni Thorbjornsson; Gisli Masson; Ingileif Jonsdottir; Alma Moller; Thorolfur Gudnason; Karl G Kristinsson; Unnur Thorsteinsdottir; Kari Stefansson
EPI_ISL_830168, EPI_ISL_830169	The National University Hospital of Iceland	deCODE genetics	Daniel F Gudbjartsson; Agnar Helgason; Hakon Jonsson; Olafur T Magnusson; Pall Melsted; Gudmundur L Norddahl; Jona Saemundsdottir; Asgeir Sigurdsson; Patrick Sulem; Arna B Agustsdottir; Hannes Eggertsson; Berglind Eiriksdottir; Run Fridriksdottir; Elisabet E Gardarsdottir; Gudmundur Georgsson; Olafia S Gretarsdottir; Kjartan R Gudmundsson; Thora R Gunnarsdottir; Arnaldur Gylfason; Hilma Holm; Brynjar O Jensson; Aslaug Jonasdottir; Kamilla S Josefsdottir; Thordur Kristjansson; Droplaug N Magnusdottir; Solvi Rognvaldsson; Louise le Roux; Gudrun Sigmundsdottir; Gardar Sveinbjornsson; Kristin E Sveinsdottir; Maney Sveinsdottir; Emil A Thorarensen; Bjarni Thorbjornsson; Gisli Masson; Ingileif Jonsdottir; Alma Moller; Thorolfur Gudnason; Karl G Kristinsson; Unnur Thorsteinsdottir; Kari Stefansson
EPI_ISL_830499	deCODE genetics	deCODE genetics	Daniel F Gudbjartsson; Agnar Helgason; Hakon Jonsson; Olafur T Magnusson; Pall Melsted; Gudmundur L Norddahl; Jona Saemundsdottir; Asgeir Sigurdsson; Patrick Sulem; Arna B Agustsdottir; Hannes Eggertsson; Berglind Eiriksdottir; Run Fridriksdottir; Elisabet E Gardarsdottir; Gudmundur Georgsson; Olafia S Gretarsdottir; Kjartan R Gudmundsson; Thora R Gunnarsdottir; Arnaldur Gylfason; Hilma Holm; Brynjar O Jensson; Aslaug Jonasdottir; Kamilla S Josefsdottir; Thordur Kristjansson; Droplaug N Magnusdottir; Solvi Rognvaldsson; Louise le Roux; Gudrun Sigmundsdottir; Gardar Sveinbjornsson; Kristin E Sveinsdottir; Maney Sveinsdottir; Emil A Thorarensen; Bjarni Thorbjornsson; Gisli Masson; Ingileif Jonsdottir; Alma Moller; Thorolfur Gudnason; Karl G Kristinsson; Unnur Thorsteinsdottir; Kari Stefansson
EPI_ISL_831247, EPI_ISL_831248, EPI_ISL_831249, EPI_ISL_831250, EPI_ISL_831251, EPI_ISL_831252, EPI_ISL_831253, EPI_ISL_831254, EPI_ISL_831255, EPI_ISL_831257, EPI_ISL_831258			
see above	Hospital Universitario La Paz (Madrid)	SeqCOVID-SPAIN consortium/IBV(CSIC)	Fernando Lázaro-Perona, María Rodríguez-Tejedor, Elias Dahdouh, Jesús Mingorance and SeqCOVID-SPAIN consortium
EPI_ISL_831389	Limbach - MVZ Labor Dr. Limbach & Kollegen	Robert Koch Institute, Influenza and respiratory viruses FG17 & Bioinformatics MF1, Berlin, Germany	Dr. Konrad Bode, Stephan Fuchs, Stefan Kroeger, Marianne Wedde, Oliver Drechsel, Aleksandar Radonic, Rene Kmiecinski, Ralf Duerrwald, Thorsten Wolff
EPI_ISL_831390	Labor Krone	Robert Koch Institute, Influenza and respiratory viruses FG17 & Bioinformatics MF1, Berlin, Germany	Dr. Münstermann. Prof. Tiemann , Stephan Fuchs, Stefan Kroeger, Marianne Wedde, Oliver Drechsel, Aleksandar Radonic, Rene Kmiecinski, Ralf Duerrwald, Thorsten Wolff
EPI_ISL_831391	Limbach - MVZ Labor Dr. Limbach & Kollegen	Robert Koch Institute, Influenza and respiratory viruses FG17 & Bioinformatics MF1, Berlin, Germany	Dr. Konrad Bode, Stephan Fuchs, Stefan Kroeger, Marianne Wedde, Oliver Drechsel, Aleksandar Radonic, Rene Kmiecinski, Ralf Duerrwald, Thorsten Wolff
EPI_ISL_831392	Labor Krone	Robert Koch Institute, Influenza and respiratory viruses FG17 & Bioinformatics MF1, Berlin, Germany	Dr. Münstermann. Prof. Tiemann , Stephan Fuchs, Stefan Kroeger, Marianne Wedde, Oliver Drechsel, Aleksandar Radonic, Rene Kmiecinski, Ralf Duerrwald, Thorsten Wolff
EPI_ISL_831393	Limbach - MVZ Labor Dr. Limbach & Kollegen	Robert Koch Institute, Influenza and respiratory viruses FG17 & Bioinformatics MF1, Berlin, Germany	Dr. Konrad Bode, Stephan Fuchs, Stefan Kroeger, Marianne Wedde, Oliver Drechsel, Aleksandar Radonic, Rene Kmiecinski, Ralf Duerrwald, Thorsten Wolff
EPI_ISL_831394, EPI_ISL_831395	Labor Krone	Robert Koch Institute, Influenza and respiratory viruses FG17 & Bioinformatics MF1, Berlin, Germany	Dr. Münstermann. Prof. Tiemann , Stephan Fuchs, Stefan Kroeger, Marianne Wedde, Oliver Drechsel, Aleksandar Radonic, Rene Kmiecinski, Ralf Duerrwald, Thorsten Wolff
EPI_ISL_831396	Limbach - MVZ Labor Dr. Limbach & Kollegen	Robert Koch Institute, Influenza and respiratory viruses FG17 & Bioinformatics MF1, Berlin, Germany	Dr. Konrad Bode, Stephan Fuchs, Stefan Kroeger, Marianne Wedde, Oliver Drechsel, Aleksandar Radonic, Rene Kmiecinski, Ralf Duerrwald, Thorsten Wolff
EPI_ISL_831397, EPI_ISL_831398, EPI_ISL_831399	Labor Krone	Robert Koch Institute, Influenza and respiratory viruses FG17 & Bioinformatics MF1, Berlin, Germany	Dr. Münstermann. Prof. Tiemann , Stephan Fuchs, Stefan Kroeger, Marianne Wedde, Oliver Drechsel, Aleksandar Radonic, Rene Kmiecinski, Ralf Duerrwald, Thorsten Wolff
EPI_ISL_831400	Limbach - MVZ Labor Dr. Limbach & Kollegen	Robert Koch Institute, Influenza and respiratory viruses FG17 & Bioinformatics MF1, Berlin, Germany	Dr. Konrad Bode, Stephan Fuchs, Stefan Kroeger, Marianne Wedde, Oliver Drechsel, Aleksandar Radonic, Rene Kmiecinski, Ralf Duerrwald, Thorsten Wolff
EPI_ISL_831401	Labor Krone	Robert Koch Institute, Influenza and respiratory viruses FG17 & Bioinformatics MF1, Berlin, Germany	Dr. Münstermann. Prof. Tiemann , Stephan Fuchs, Stefan Kroeger, Marianne Wedde, Oliver Drechsel, Aleksandar Radonic, Rene Kmiecinski, Ralf Duerrwald, Thorsten Wolff
EPI_ISL_831402	Labor Dr. Wisplinghoff - Köln	Robert Koch Institute, Influenza and respiratory viruses FG17 & Bioinformatics MF1, Berlin, Germany	Dr. R. Grosser, Stephan Fuchs, Stefan Kroeger, Marianne Wedde, Oliver Drechsel, Aleksandar Radonic, Rene Kmiecinski, Ralf Duerrwald, Thorsten Wolff
EPI_ISL_831403	MVZ Düsseldorf Zentrum GbR	Robert Koch Institute, Influenza and respiratory viruses FG17 & Bioinformatics MF1, Berlin, Germany	Dr. Finzer, Stephan Fuchs, Stefan Kroeger, Marianne Wedde, Oliver Drechsel, Aleksandar Radonic, Rene Kmiecinski, Ralf Duerrwald, Thorsten Wolff
EPI_ISL_831404, EPI_ISL_831405	Labor Dr. Krause & Kollegen MVZ GmbH	Robert Koch Institute, Influenza and respiratory viruses FG17 & Bioinformatics MF1, Berlin, Germany	Dr. Lorentz, Stephan Fuchs, Stefan Kroeger, Marianne Wedde, Oliver Drechsel, Aleksandar Radonic, Rene Kmiecinski, Ralf Duerrwald, Thorsten Wolff
EPI_ISL_831406	MVZ Düsseldorf Zentrum GbR	Robert Koch Institute, Influenza and respiratory viruses FG17 & Bioinformatics MF1, Berlin, Germany	Dr. Finzer, Stephan Fuchs, Stefan Kroeger, Marianne Wedde, Oliver Drechsel, Aleksandar Radonic, Rene Kmiecinski, Ralf Duerrwald, Thorsten Wolff
EPI_ISL_831407	Labor Dr. Krause & Kollegen MVZ GmbH	Robert Koch Institute, Influenza and respiratory viruses FG17 & Bioinformatics MF1, Berlin, Germany	Dr. Lorentz, Stephan Fuchs, Stefan Kroeger, Marianne Wedde, Oliver Drechsel, Aleksandar Radonic, Rene Kmiecinski, Ralf Duerrwald, Thorsten Wolff
EPI_ISL_831408	MVZ Düsseldorf Zentrum GbR	Robert Koch Institute, Influenza and respiratory viruses FG17 & Bioinformatics MF1, Berlin, Germany	Dr. Finzer, Stephan Fuchs, Stefan Kroeger, Marianne Wedde, Oliver Drechsel, Aleksandar Radonic, Rene Kmiecinski, Ralf Duerrwald, Thorsten Wolff
EPI_ISL_831409	Labor Dr. Wisplinghoff - Köln	Robert Koch Institute, Influenza and respiratory viruses FG17 & Bioinformatics MF1, Berlin, Germany	Dr. R. Grosser, Stephan Fuchs, Stefan Kroeger, Marianne Wedde, Oliver Drechsel, Aleksandar Radonic, Rene Kmiecinski, Ralf Duerrwald, Thorsten Wolff
EPI_ISL_831410	MVZ Düsseldorf Zentrum GbR	Robert Koch Institute, Influenza and respiratory viruses FG17 & Bioinformatics MF1, Berlin, Germany	Dr. Finzer, Stephan Fuchs, Stefan Kroeger, Marianne Wedde, Oliver Drechsel, Aleksandar Radonic, Rene Kmiecinski, Ralf Duerrwald, Thorsten Wolff
EPI_ISL_831411	Labor Dr. Wisplinghoff - Köln	Robert Koch Institute, Influenza and respiratory viruses FG17 & Bioinformatics MF1, Berlin, Germany	Dr. R. Grosser, Stephan Fuchs, Stefan Kroeger, Marianne Wedde, Oliver Drechsel, Aleksandar Radonic, Rene Kmiecinski, Ralf Duerrwald, Thorsten Wolff
EPI_ISL_831412, EPI_ISL_831413	Labor Dr. Krause & Kollegen MVZ GmbH	Robert Koch Institute, Influenza and respiratory viruses FG17 & Bioinformatics MF1, Berlin, Germany	Dr. Lorentz, Stephan Fuchs, Stefan Kroeger, Marianne Wedde, Oliver Drechsel, Aleksandar Radonic, Rene Kmiecinski, Ralf Duerrwald, Thorsten Wolff
EPI_ISL_831654, EPI_ISL_831655, EPI_ISL_831656	Institute for Infectious Diseases, University of Bern, Switzerland	Institute for Infectious Diseases, University of Bern, Switzerland	Michel C Koch, Christian Baumann, Miguel A Terrazos Miani, Cora Sägesser, Pascal Bittel, Stephen L Leib, Peter Keller, Franziska Suter-Riniker, Alban Ramette
EPI_ISL_831687	Santa Clara County Public Health Laboratory	Santa Clara County Public Health Laboratory	Santa Clara County Public Health Department
EPI_ISL_831698, EPI_ISL_831704, EPI_ISL_831732, EPI_ISL_831762, EPI_ISL_831850, EPI_ISL_831865	United States Air Force School of Aerospace Medicine	United States Air Force School of Aerospace Medicine	Anthony Fries, Jennifer Meyer, William Gruner, Amanda Javorina, Sarah Purves, Clarise Starr, Elizabeth Macias
EPI_ISL_831893, EPI_ISL_831896, EPI_ISL_831897, EPI_ISL_831899	Santa Clara County Public Health Laboratory	Santa Clara County Public Health Laboratory	Santa Clara County Public Health Department
EPI_ISL_831946	Klinisk mikrobiologi, virus F68	The Public Health Agency of Sweden	Department of Microbiology, The Public Health Agency of Sweden
EPI_ISL_831979	Klinisk mikrobiologi, Viruslab	The Public Health Agency of Sweden	Department of Microbiology, The Public Health Agency of Sweden
EPI_ISL_831991	Klinisk mikrobiologi	The Public Health Agency of Sweden	Department of Microbiology, The Public Health Agency of Sweden

EPI_ISL_832066	Santa Clara County Public Health Laboratory	Santa Clara County Public Health Laboratory	Santa Clara County Public Health Department
EPI_ISL_832128	Sentinelles IDF	National Reference Center for Viruses of Respiratory Infections, Institut Pasteur, Paris	Marion Barbet, Sylvie Behillil, Méline Bizard, Angela Brisebarre, Camille Capel, Etienne Simon-Lorière, Vincent Enouf, Maud Vanpeene, Sylvie van der Werf, Patricia Lefebure
EPI_ISL_832129	Sentinelles IDF	National Reference Center for Viruses of Respiratory Infections, Institut Pasteur, Paris	Marion Barbet, Sylvie Behillil, Méline Bizard, Angela Brisebarre, Camille Capel, Etienne Simon-Lorière, Vincent Enouf, Maud Vanpeene, Sylvie van der Werf, Claire Watermez
EPI_ISL_832164, EPI_ISL_832165, EPI_ISL_832166	Hospital	National Reference Center for Viruses of Respiratory Infections, Institut Pasteur, Paris	Marion Barbet, Sylvie Behillil, Méline Bizard, Angela Brisebarre, Camille Capel, Etienne Simon-Lorière, Vincent Enouf, Maud Vanpeene, Sylvie van der Werf, Léa Pilorge
EPI_ISL_832369, EPI_ISL_832370, EPI_ISL_832371, EPI_ISL_832372, EPI_ISL_832373, EPI_ISL_832374	Center of Medical Microbiology, Virology, and Hospital Hygiene, University of Duesseldorf	Center of Medical Microbiology, Virology, and Hospital Hygiene, University of Duesseldorf	Maximilian Damagnez, Alexander Dilthey, Ashley-Jane Duplessis, Torsten Houwaart, Lisanna Hülse, Malte Kohns Vasconcelos, Nadine Lübke, Jessica Nicolai, Klaus Pfeffer, Daniel Strelow, Teresa Tamayo, Jörg Timm, Andreas Walker, Tobias Wienemann
EPI_ISL_832402, EPI_ISL_832403	Santa Clara County Public Health Laboratory	Santa Clara County Public Health Laboratory	Santa Clara County Public Health Department
EPI_ISL_833459, EPI_ISL_833464, EPI_ISL_833465, EPI_ISL_833474, EPI_ISL_833480	CHU Purpan - Laboratoire de Virologie - Institut Fédératif de Biologie	CHU Purpan - Laboratoire de Virologie - Institut Fédératif de Biologie	Latour J., Ranger N., Dubois M., Carcenac R., Harter A., Boyer P., Tremeaux P., Izopet J.
EPI_ISL_836143	Hospital de Campanha COVID-19 de Mairipora	Instituto Adolfo Lutz, Interdiciplinary Procedures Center, Strategic Laboratory	Claudio Tavares Sacchi, Claudia Regina Gonçalves, Erica Valessa Ramos Gomes, Karoline Rodrigues Campos
EPI_ISL_836977	Hospital Municiplal Dr. Jose de Carvalho Florence	Instituto Adolfo Lutz, Interdiciplinary Procedures Center, Strategic Laboratory	Claudio Tavares Sacchi, Claudia Regina Gonçalves, Erica Valessa Ramos Gomes, Karoline Rodrigues Campos
EPI_ISL_836978	Irmandade da Santa Casa de Misericordia de Lorena	Instituto Adolfo Lutz, Interdiciplinary Procedures Center, Strategic Laboratory	Claudio Tavares Sacchi, Claudia Regina Gonçalves, Erica Valessa Ramos Gomes, Karoline Rodrigues Campos
EPI_ISL_836979	Respiratory Virus Unit, National Infection Service, Public Health England	COVID-19 Genomics UK (COG-UK) Consortium	PHE Covid Sequencing Team
EPI_ISL_837053	UBS Darcy Alves e Robalinho	Instituto Adolfo Lutz, Interdiciplinary Procedures Center, Strategic Laboratory	Claudio Tavares Sacchi, Claudia Regina Gonçalves, Erica Valessa Ramos Gomes, Karoline Rodrigues Campos
EPI_ISL_837054	UBS Jose Sabino Ferreira	Instituto Adolfo Lutz, Interdiciplinary Procedures Center, Strategic Laboratory	Claudio Tavares Sacchi, Claudia Regina Gonçalves, Erica Valessa Ramos Gomes, Karoline Rodrigues Campos
EPI_ISL_837083, EPI_ISL_837100, EPI_ISL_837112, EPI_ISL_837185, EPI_ISL_837199	Respiratory Virus Unit, National Infection Service, Public Health England	COVID-19 Genomics UK (COG-UK) Consortium	PHE Covid Sequencing Team
EPI_ISL_838072, EPI_ISL_838106, EPI_ISL_838108, EPI_ISL_838109, EPI_ISL_838111, EPI_ISL_838112, EPI_ISL_838113, EPI_ISL_838114, EPI_ISL_838115, EPI_ISL_838209, EPI_ISL_838210, EPI_ISL_838211, EPI_ISL_838212			
see above	West of Scotland Specialist Virology Centre, NHSGGC / MRC-University of Glasgow Centre for Virus Research	COVID-19 Genomics UK (COG-UK) Consortium	Ana da Silva Filipe, Natasha Johnson, Kathy Smollett, Daniel Mair, Stephen Carmichael, Alice Broos, Lily Tong, Jenna Nichols, Kyriaki Nomikou; Sarah McDonald; Richard Orton, Joseph Hughes, Sreenu Vattipally, David L Robertson; Alasdair MacLean, Rory Gunson; Sharif Shaaban, Matthew Holden; Rachel Blacow, Guy Mollett, Kathy Li, James Shepherd, Antonia Ho, Emma Thomson
EPI_ISL_838216, EPI_ISL_838217, EPI_ISL_838218, EPI_ISL_838219, EPI_ISL_838283	Virology Department, Royal Infirmary of Edinburgh, NHS Lothian / School of Biological Sciences, University of Edinburgh / Institute of Genetics and Molecular Medicine, University of Edinburgh	COVID-19 Genomics UK (COG-UK) Consortium	McHugh M, Dewar R, Rooke S, Gallagher M, Balcaza C, O'Toole Á, Scher E, Hill V, McCrone JT, Colquhoun R, Yu X, Jackson B, Rambaut A, Williams TC, Templeton K
EPI_ISL_838585, EPI_ISL_838664	Liverpool Clinical Laboratories	COVID-19 Genomics UK (COG-UK) Consortium	Sam Haldenby, Anita Lucaci, Steve Paterson, Julian Hiscox, Alistair Darby, M Almsaud, A Alrezaihi, Muhannad Alruwaili, Stuart D Armstrong, Jones Benjamin, Eleanor G Bentley, Anu Chawla, Jordan J Clark, Angela Cowell, Richard Eccles, Isabel García-Dorival, Matthew Gemmell, Alessandro Gerada, PKF Gilmore, Richard Gregory, Ximeng Han, Catherine Hartley, Margaret Hughes, Miren Iturriza-Gomara, James Johnson, L Luu, Jenifer Manson, Charlotte Nelson, Elaine O'Toole, Cassie Olateju, Rebekah Penrice-Randal , Lucille Rainbow, N.P Randle, Trevor Ian Robinson, Parul Sharma, Ghada T Shawli, James P Stewart, Neil Swainston, Ecaterina Vamos, Joanne Watts, Mark Whitehead
EPI_ISL_839331, EPI_ISL_839332, EPI_ISL_839333	University College London, Great Ormond Street Hospital for Children NHS Foundation Trust, Imperial College Healthcare NHS Trust	COVID-19 Genomics UK (COG-UK) Consortium	Sergi Castellano, Rachel Williams, Mark Kristiansen, Paola Resende Silva, Sunando Roy, Tony Brooks, Helena Tutill, Paola Niola, Patricia Dyal, Charlotte Williams, Leysa Forrest, Yasmin Panchbhaya, Jacqueline Findlay, Samuel Weeks, Julianne Brown, Kathryn Harris, Paul Randell, James Price, Alison Holmes, Judith Breuer
EPI_ISL_839520, EPI_ISL_839521, EPI_ISL_839522, EPI_ISL_839523, EPI_ISL_839524, EPI_ISL_839525, EPI_ISL_839526, EPI_ISL_839527, EPI_ISL_839528, EPI_ISL_839529, EPI_ISL_839530, EPI_ISL_839531, EPI_ISL_839532, EPI_ISL_839533, EPI_ISL_839739, EPI_ISL_839740, EPI_ISL_839741, EPI_ISL_839742, EPI_ISL_839743, EPI_ISL_839744, EPI_ISL_839745, EPI_ISL_839746, EPI_ISL_839747, EPI_ISL_839748			
see above	Northumbria University / South Tees Hospitals NHS Foundation Trust / North Cumbria Integrated Care NHS Foundation Trust / North Tees and Hartlepool NHS Foundation Trust / Newcastle Hospitals NHS Foundation Trust	COVID-19 Genomics UK (COG-UK) Consortium	Darren L Smith,Andrew Nelson,Matthew Bashton,Greg R Young,Joshua Loh,John Allan,Mohammad A Tariq,Giles S Holt,Gary Black,Wen C Yew,Lynn Dover,Paul Baker,Steve Liggett,Sarah Essex,Jane Greenaway,Debra Padgett,Clive Graham,Garren Scott,Edward Barton,Emma Swindells,Brendan Payne,Jennifer Collins,Yusri Taha,Gary Eltringham
EPI_ISL_840037, EPI_ISL_840038, EPI_ISL_840039, EPI_ISL_840040	Lincolnshire Hospitals and DeepSeq Nottingham	COVID-19 Genomics UK (COG-UK) Consortium	Nichola Duckworth, Tim Sloan, Sarah Walsh, Jonathan Ball, Patrick McClure, Joeseph Chappell, Nadine Holmes, Matthew Carlisle, Christopher Moore, Fei Sang, Johnny Debebe, Victoria Wright, Matthew Loose
EPI_ISL_841579	Originating lab: Wales Specialist Virology Centre Sequencing lab: Pathogen Genomics Unit	Public Health Wales Microbiology Cardiff Wales Specialist Virology Centre	Catherine Moore, Johnathan Evans, Laura Gifford, Malorie Perry, Simon Cottrell, Angela Marchbank, Alec Birchley, Alexander Adams, Amy Gaskin, Bree Gatica-Wilcox, Jason Coombes, Joel Southgate, Lauren Gilbert, Lee Graham, Nicole Pacchiarini, Sara Kumziene-Summerhayes, Sarah Taylor, Sophie Jones, Sara Rey, Matthew Bull, Joanne Watkins, Sally Corden, Tom Connor
EPI_ISL_841767	Centre for Enzyme Innovation, University of Portsmouth / Translational Research Laboratory, Portsmouth Hospitals NHS Trust	COVID-19 Genomics UK (COG-UK) Consortium	Angela Beckett,Yann Bourgeois,Garry Scarlett,Sharon Glaysher,Scott Elliott,Kelly Bicknell,Robert Impey,Allyson Lloyd,Sarah Wyllie,Ethan Butcher,Anoop Chauhan,Samuel Robson
EPI_ISL_842355, EPI_ISL_842357, EPI_ISL_842359, EPI_ISL_842364, EPI_ISL_842365, EPI_ISL_842366, EPI_ISL_842368, EPI_ISL_842370, EPI_ISL_842373, EPI_ISL_842374, EPI_ISL_842375, EPI_ISL_842377, EPI_ISL_842379, EPI_ISL_842380, EPI_ISL_842382, EPI_ISL_842384, EPI_ISL_842387, EPI_ISL_842388, EPI_ISL_842389			
see above	Bioinformatics and Biostatistics Lab, Advanced Sequencing Facility	COVID-19 Genomics UK (COG-UK) Consortium	Aengus Stewart,Jerome Nicod,Chelsea Sawyer,Laura Cubitt,Harshil Patel,Margaret Crawford
EPI_ISL_842653, EPI_ISL_842654, EPI_ISL_842656, EPI_ISL_842657, EPI_ISL_842659, EPI_ISL_842660, EPI_ISL_842661, EPI_ISL_842663, EPI_ISL_842666, EPI_ISL_842667, EPI_ISL_842668, EPI_ISL_842669, EPI_ISL_842697, EPI_ISL_842766, EPI_ISL_842767, EPI_ISL_842783, EPI_ISL_842790			
see above	University College London Hospital	COVID-19 Genomics UK (COG-UK) Consortium	Judith Heaney, Matthew Byott, Catherine Houlihan, Dan Frampton, Stuart Kirk, Moira Spyer and Eleni Nastouli
EPI_ISL_843006, EPI_ISL_843056, EPI_ISL_843058, EPI_ISL_843059, EPI_ISL_843062, EPI_ISL_843063, EPI_ISL_843088	Barts Health NHS Trust	COVID-19 Genomics UK (COG-UK) Consortium	CUTINO-MOGUEL, Maria-Teresa; HARRINGTON, David; OWOYEMI, Dola; SHYLINI, Raghavendran; BROAD, Claire; KELE, Beatrix
EPI_ISL_843189, EPI_ISL_843190	Maryland Public Health Laboratory	Maryland Public Health Laboratory	Maryland Department of Health Laboratories Administration
EPI_ISL_844205, EPI_ISL_844214, EPI_ISL_844215, EPI_ISL_844216, EPI_ISL_844217, EPI_ISL_844218, EPI_ISL_844219, EPI_ISL_844224, EPI_ISL_844225, EPI_ISL_844227, EPI_ISL_844230, EPI_ISL_844231, EPI_ISL_844237, EPI_ISL_844240, EPI_ISL_844249, EPI_ISL_844250, EPI_ISL_844255, EPI_ISL_844258, EPI_ISL_844261, EPI_ISL_844267, EPI_ISL_844282, EPI_ISL_844283, EPI_ISL_844284, EPI_ISL_844285, EPI_ISL_844286, EPI_ISL_844295, EPI_ISL_844298, EPI_ISL_844308, EPI_ISL_844391, EPI_ISL_844392, EPI_ISL_844393, EPI_ISL_844394, EPI_ISL_844395, EPI_ISL_844396, EPI_ISL_844397, EPI_ISL_844398,			

EPI_ISL_844399, EPI_ISL_844400, EPI_ISL_844401, EPI_ISL_844402, EPI_ISL_844403, EPI_ISL_844404, EPI_ISL_844405, EPI_ISL_844406, EPI_ISL_844407, EPI_ISL_844408, EPI_ISL_844409, EPI_ISL_844410, EPI_ISL_844411, EPI_ISL_844412, EPI_ISL_844413, EPI_ISL_844414, EPI_ISL_844415, EPI_ISL_844416, EPI_ISL_844417, EPI_ISL_844418, EPI_ISL_844419, EPI_ISL_844420, EPI_ISL_844421, EPI_ISL_844422, EPI_ISL_844423, EPI_ISL_844424, EPI_ISL_844425, EPI_ISL_844426, EPI_ISL_844427, EPI_ISL_844428, EPI_ISL_844429, EPI_ISL_844430, EPI_ISL_844431, EPI_ISL_844432, EPI_ISL_844433, EPI_ISL_844434, EPI_ISL_844435, EPI_ISL_844436, EPI_ISL_844437, EPI_ISL_844438, EPI_ISL_844439, EPI_ISL_844440, EPI_ISL_844441, EPI_ISL_844442, EPI_ISL_844443, EPI_ISL_844444, EPI_ISL_844445, EPI_ISL_844446, EPI_ISL_844447, EPI_ISL_844448, EPI_ISL_844449, EPI_ISL_844450, EPI_ISL_844451, EPI_ISL_844452, EPI_ISL_844453, EPI_ISL_844454, EPI_ISL_844455, EPI_ISL_844456, EPI_ISL_844457, EPI_ISL_844458, EPI_ISL_844459, EPI_ISL_844460, EPI_ISL_844461			
see above	Department of Virus and Microbiological Special Diagnostics, Statens Serum Institut, Copenhagen, Denmark	Albertsen Lab, Department of Chemistry and Bioscience, Aalborg University, Denmark	Danish Covid-19 Genome Consortium
EPI_ISL_847525, EPI_ISL_847533, EPI_ISL_847537, EPI_ISL_847542, EPI_ISL_847544, EPI_ISL_847547, EPI_ISL_847562, EPI_ISL_847565, EPI_ISL_847567, EPI_ISL_847581, EPI_ISL_847585, EPI_ISL_847587, EPI_ISL_847649, EPI_ISL_847650, EPI_ISL_847651, EPI_ISL_847652, EPI_ISL_847653, EPI_ISL_847654, EPI_ISL_847655, EPI_ISL_847656, EPI_ISL_847657, EPI_ISL_847658, EPI_ISL_847705, EPI_ISL_847728, EPI_ISL_847733, EPI_ISL_847739, EPI_ISL_847745, EPI_ISL_847752, EPI_ISL_847784, EPI_ISL_847785, EPI_ISL_847786, EPI_ISL_847787, EPI_ISL_847788, EPI_ISL_847789, EPI_ISL_847790, EPI_ISL_847791, EPI_ISL_847792, EPI_ISL_847793			
see above	California Department of Public Health	Chiu Laboratory, University of California, San Francisco	Charles Chiu, Xianding (Wayne) Deng, Candace Wang, Brian Bushnell, Scot Federman, Jill Hacker, Debra Wadford
EPI_ISL_848006, EPI_ISL_848023, EPI_ISL_848058	Michigan Department of Health and Human Services, Bureau of Laboratories	Michigan Department of Health and Human Services, Bureau of Laboratories	Blankenship HM, Riner D, Soehnlen MK
EPI_ISL_848425	Illinois Department of Public Health	Gagnon Lab, Southern Illinois University	Keith Gagnon
EPI_ISL_848644, EPI_ISL_848645, EPI_ISL_848646, EPI_ISL_848647	Florida Bureau of Public Health Laboratories	Florida Bureau of Public Health Laboratories	Sarah Schmedes, Jason Blanton
EPI_ISL_849385, EPI_ISL_849394, EPI_ISL_849398, EPI_ISL_849403, EPI_ISL_849412, EPI_ISL_849413, EPI_ISL_849415, EPI_ISL_849426, EPI_ISL_849429, EPI_ISL_849436, EPI_ISL_849449, EPI_ISL_849455, EPI_ISL_849460, EPI_ISL_849465, EPI_ISL_849521, EPI_ISL_849524, EPI_ISL_849541, EPI_ISL_849542, EPI_ISL_849543, EPI_ISL_849544, EPI_ISL_849545, EPI_ISL_849546, EPI_ISL_849547, EPI_ISL_849548, EPI_ISL_849549, EPI_ISL_849550, EPI_ISL_849551, EPI_ISL_849559, EPI_ISL_849562, EPI_ISL_849565, EPI_ISL_849566, EPI_ISL_849572, EPI_ISL_849576, EPI_ISL_849578, EPI_ISL_849579, EPI_ISL_849580, EPI_ISL_849581, EPI_ISL_849585, EPI_ISL_849588, EPI_ISL_849616, EPI_ISL_849617, EPI_ISL_849619, EPI_ISL_849628, EPI_ISL_849629, EPI_ISL_849630, EPI_ISL_849631, EPI_ISL_849632, EPI_ISL_849633			
see above	Washington State Department of Health	Seattle Flu Study	Deborah A. Nickerson, Chris D. Frazar, Jover Lee, Benjamin Pelle, Matthew Richardson, Amanda Adler, Elisabeth Brandstetter, Peter D. Han, Kairsten Fay, Misja Ilcisin, Kirsten Lacombe, Thomas R. Sibley, Melissa Truong, Caitlin R. Wolf, Romesh Gautom, Geoff Melly, Brian Hiatt, Philip Dykema, Scott Lindquist, Michael Boeckh, Janet A. Englund, Michael Famulare, Barry R. Lutz, Mark J. Rieder, Lea M. Starita, Matthew Thompson, Helen Y. Chu, Jay Shendure, Trevor Bedford
EPI_ISL_849731	unknown	PHV-FSS	Son Nguyen et al.
EPI_ISL_849985, EPI_ISL_849986, EPI_ISL_849987, EPI_ISL_849988, EPI_ISL_849989, EPI_ISL_849990, EPI_ISL_849991, EPI_ISL_849992, EPI_ISL_849993, EPI_ISL_849994, EPI_ISL_849995, EPI_ISL_849996, EPI_ISL_849997, EPI_ISL_849998, EPI_ISL_849999, EPI_ISL_850000, EPI_ISL_850001, EPI_ISL_850002, EPI_ISL_850003, EPI_ISL_850004, EPI_ISL_850005, EPI_ISL_850006, EPI_ISL_850007, EPI_ISL_850008, EPI_ISL_850009, EPI_ISL_850010, EPI_ISL_850011			
see above	Santa Clara County Public Health Laboratory	Chan-Zuckerberg Biohub	CZB Cliahub Consortium
EPI_ISL_850664, EPI_ISL_850665	The National Institute of Public Health	State Veterinary Institute Prague	Nagy,A;Jirincova,H;Trnka,D;Vecerova,J;Trinklova,M
EPI_ISL_852597	Max von Pettenkofer Institute, Virology, National Reference Center for Retroviruses, LMU München	Laboratory for Functional Genome Analysis, Dept. Genomics, Gene Center of the LMU Munich	Max Muenchhoff, Stefan Krebs, Alexander Graf, Oliver Keppler, Helmut Blum
EPI_ISL_853341, EPI_ISL_853342, EPI_ISL_853375, EPI_ISL_853376, EPI_ISL_853381, EPI_ISL_853383, EPI_ISL_853384, EPI_ISL_853385, EPI_ISL_853387, EPI_ISL_853392, EPI_ISL_853393			
see above	UPMC Clinical Microbiology Laboratory	Microbial Genome Sequencing Center; Microbial Genomic Epidemiology Laboratory	Mustapha M. Mustapha, Jane W. Marsh, Dan Snyder, Marissa P. Griffith, Stephanie L. Mitchell, Vatsala R. Srinivasa, Kady D. Waggle, Chinelo Ezeonwuku, Vaughn S. Cooper, Lee H. Harrison
EPI_ISL_853931, EPI_ISL_853955	Department of Microbiology, University Innsbruck	Bergthaler laboratory, CeMM Research Center for Molecular Medicine of the Austrian Academy of Sciences	Lukas Endler, Alexandra Popa, Benedikt Agerer, Jakob-Wendelin Genger, Alexander Lercher, Anna Schedl, Thomas Penz, Michael Schuster, Jan Laine, Martin Senekowitsch, Christoph Bock, Andreas Bergthaler
EPI_ISL_854438	SARATOGA HOSPITAL LABORATORY	Wadsworth Center, New York State Department of Health	Kirsten St. George, Daryl M. Lamson, Alexis Russel, Matthew Shudt, Melissa A Leisner, Jonathan Plitnick, Navjot Singh, John Kelly, Erasmus Schneider, Erica Lasek-Nesselquist
EPI_ISL_855354	Hospital	National Reference Center for Viruses of Respiratory Infections, Institut Pasteur, Paris	Marion Barbet, Sylvie Behillil, Méline Bizard, Angela Brisebarre, Camille Capel, Etienne Simon-Lorière, Vincent Enouf, Maud Vanpeene, Sylvie van der Werf
EPI_ISL_855410, EPI_ISL_855411, EPI_ISL_855412, EPI_ISL_855413, EPI_ISL_855414, EPI_ISL_855415, EPI_ISL_855416, EPI_ISL_855417, EPI_ISL_855418, EPI_ISL_855419, EPI_ISL_855427			
see above	Servicio de Microbiología, Laboratori Clínic Metropolitana Nord. Hospital Universitari Germans Trias i Pujol. Institut d'Investigació en Ciències de la Salut Germans Trias i Pujol (IGTP)	SeqCOVID-SPAIN consortium/IBV(CSIC)	Elisa Martró, Antoni E. Bordoy, Anna Not, Adrián Antuori, Anabel Fernández, Nona Romaní, Verónica Saludes, Cristina Casañ and SeqCOVID-SPAIN consortium
EPI_ISL_855515, EPI_ISL_855518, EPI_ISL_855522, EPI_ISL_855535	KEMRI-Wellcome Trust Research Programme/KEMRI-CGMR-C Kilifi	KEMRI-Wellcome Trust Research Programme/KEMRI-CGMR-C Kilifi	Githinji et al
EPI_ISL_856673, EPI_ISL_856674, EPI_ISL_856675, EPI_ISL_856676	Department of Virus and Microbiological Special Diagnostics, Statens Serum Institut, Copenhagen, Denmark	Aalborg University	Danish Covid-19 Genome Consortium
EPI_ISL_856776, EPI_ISL_856780, EPI_ISL_856781, EPI_ISL_856782	Servicio Virosis Respiratorias-Departamento Virología-INEI	Instituto Nacional Enfermedades Infecciosas C.G.Malbran	Baumeister E., Avaro M., Benedetti E., Russo M., Dattero ME, Pontoriero A., Cisterna D., Molina V., Perandones C., Tuduri E., Lorenzo F., Poklepovich T., Campos J.
EPI_ISL_857508, EPI_ISL_857509, EPI_ISL_857510, EPI_ISL_857511, EPI_ISL_857512	Swiss National Reference Centre for Influenza	Swiss National Reference Centre for Influenza	Ana Rita Goncalves,Samuel Cordey, Laurent Kaiser, Lorenzo Cerutti, Henri Pegeot, Melyssa Elies, Keith Harshman, Ioannis Xenarios, Emmanouil Dermitzakis
EPI_ISL_857531, EPI_ISL_857532, EPI_ISL_857533, EPI_ISL_857534, EPI_ISL_857535	Swiss National Reference Centre for Influenza	Swiss National Reference Centre for Influenza	Tim Roloff, Ana Rita Gonçalves, Madlen Stange, Helena MB Seth-Smith, Alfredo Mari, Karoline Leuzinger, Julia Bielicki, Manuel Battegay, Hans Hirsch, Laurent Kaiser, Adrian Egli
EPI_ISL_859469	Lighthouse Lab in Milton Keynes	Wellcome Sanger Institute for the COVID-19 Genomics UK (COG-UK) Consortium	The Lighthouse Lab in Milton Keynes and Alex Alderton, Roberto Amato, Sonia Goncalves, Ewan Harrison, David K. Jackson, Ian Johnston, Dominic Kwiatkowski, Cordelia Langford, John Sillitoe on behalf of the Wellcome Sanger Institute COVID-19 Surveillance Team
EPI_ISL_859541, EPI_ISL_859542, EPI_ISL_859543, EPI_ISL_859544, EPI_ISL_859545, EPI_ISL_859546, EPI_ISL_859547, EPI_ISL_859548, EPI_ISL_859549, EPI_ISL_859550, EPI_ISL_859551, EPI_ISL_859552			
see above	Lighthouse Lab in Glasgow	Wellcome Sanger Institute for the COVID-19 Genomics UK (COG-UK) Consortium	Harper VanSteenhouse, Yumi Kasai, David Gray, Carol Clugston, Anna Dominiczak and Alex Alderton, Roberto Amato, Sonia Goncalves, Ewan Harrison, David K. Jackson, Ian Johnston, Dominic Kwiatkowski, Cordelia Langford, John Sillitoe on behalf of the Wellcome Sanger Institute COVID-19 Surveillance Team
EPI_ISL_860181	Keio University School of Medicine	Keio University School of Medicine	Kenjiro Kosaki, Yuka Iwasaki, Hirotsugu Ishizu, Haruhiko Siomi, Kodai Abe
EPI_ISL_861470	UHAS COVID-19 Lab	UHAS COVID-19 Lab	Kwabena O. Duedu, Jones Gyamfi, Reuben Ayivor-Djanie, John O. Gyapong and the UHAS COVID-19 Lab Team
EPI_ISL_861584, EPI_ISL_861585	Instituto Nacional de Saude (INSA)	Instituto Nacional de Saude (INSA)	Borges et al
EPI_ISL_861669	Lab LOC - Itapecerica da Serra	Instituto Adolfo Lutz, Interdiciplinary Procedures Center, Strategic Laboratory	Claudio Tavares Sacchi, Claudia Regina Gonçalves, Erica Valessa Ramos Gomes, Karoline Rodrigues Campos
EPI_ISL_861670	Instituto Adolfo Lutz - Regional de Taubate	Instituto Adolfo Lutz, Interdiciplinary Procedures Center, Strategic Laboratory	Claudio Tavares Sacchi, Claudia Regina Gonçalves, Erica Valessa Ramos Gomes, Karoline Rodrigues Campos
EPI_ISL_861773, EPI_ISL_861774, EPI_ISL_861777, EPI_ISL_861789, EPI_ISL_861792, EPI_ISL_861795, EPI_ISL_861797, EPI_ISL_861798, EPI_ISL_861800, EPI_ISL_861801, EPI_ISL_861802, EPI_ISL_861803, EPI_ISL_861808, EPI_ISL_861809, EPI_ISL_861812, EPI_ISL_861815, EPI_ISL_861816, EPI_ISL_861819, EPI_ISL_861820, EPI_ISL_861821, EPI_ISL_861823, EPI_ISL_861830, EPI_ISL_861831, EPI_ISL_861832, EPI_ISL_861833, EPI_ISL_861834, EPI_ISL_861835, EPI_ISL_861836, EPI_ISL_861837, EPI_ISL_861838, EPI_ISL_861839, EPI_ISL_861840, EPI_ISL_861841, EPI_ISL_861842, EPI_ISL_861843, EPI_ISL_861844			

see above	Hospital General Universitario Gregorio Marañón	SeqCOVID-SPAIN consortium/IBV(CSIC)	Darío García de Viedma, Laura Pérez-Lago, Pedro J Sola-Campoy, Sergio Buenestado-Serrano, Marta Herranz, Victor Manuel de la Cueva, Julia Suárez, Pilar Catalán, Patricia Muñoz and SeqCOVID-SPAIN consortium
EPI_ISL_862578	Hospital Clínic	Instituto de Salud Carlos III	Iglesias-Caballero, M.Camarero, S. Molinero Calamita, M. González-Esguevillas, M. Pozo, F. Casas, I. Jiménez, P. Jiménez, M. Zaballos, A. Monzón, S. Varona, S. Juliá, M. Cuesta, I. Marcos, M.A.
EPI_ISL_864868, EPI_ISL_864870, EPI_ISL_864888, EPI_ISL_864890, EPI_ISL_864892, EPI_ISL_864894	Department of Pathology, University of Cambridge	COVID-19 Genomics UK (COG-UK) Consortium	Aminu S. Jahun, Yasmin Chaudhry, Grant Hall, Iliana Georgana, Myra Hosmillo, Martin D. Curran, Malte Pinckert, Surendra Parmar, Ian Goodfellow
EPI_ISL_865069, EPI_ISL_865070, EPI_ISL_865071, EPI_ISL_865155, EPI_ISL_865157, EPI_ISL_865161	Virology Department, Royal Infirmary of Edinburgh, NHS Lothian / School of Biological Sciences, University of Edinburgh / Institute of Genetics and Molecular Medicine, University of Edinburgh	COVID-19 Genomics UK (COG-UK) Consortium	McHugh M, Dewar R, Rooke S, Gallagher M, Balcaza C, O'Toole Á, Scher E, Hill V, McCrone JT, Colquhoun R, Yu X, Jackson B, Rambaut A, Williams TC, Templeton K
EPI_ISL_865207, EPI_ISL_865209, EPI_ISL_865211, EPI_ISL_865212, EPI_ISL_865213, EPI_ISL_865214, EPI_ISL_865215, EPI_ISL_865216, EPI_ISL_865261, EPI_ISL_865264, EPI_ISL_865265, EPI_ISL_865268			
see above	Liverpool Clinical Laboratories	COVID-19 Genomics UK (COG-UK) Consortium	Sam Haldenby, Anita Lucaci, Steve Paterson, Julian Hiscox, Alistair Darby, M Almsaud, A Alrezaihi, Muhannad Alruwaili, Stuart D Armstrong, Jones Benjamin, Eleanor G Bentley, Anu Chawla, Jordan J Clark, Angela Cowell, Richard Eccles, Isabel García-Dorival, Matthew Gemmell, Alessandro Gerada, PKF Gilmore, Richard Gregory, Ximeng Han, Catherine Hartley, Margaret Hughes, Miren Iturriza-Gomara, James Johnson, L Luu, Jenifer Manson, Charlotte Nelson, Elaine O'Toole, Cassie Olateju, Rebekah Penrice-Randal , Lucille Rainbow, N.P Randle, Trevor Ian Robinson, Parul Sharma, Ghada T Shawli, James P Stewart, Neil Swainston, Ecaterina Vamos, Joanne Watts, Mark Whitehead
EPI_ISL_865717	University College London, Great Ormond Street Hospital for Children NHS Foundation Trust, Imperial College Healthcare NHS Trust	COVID-19 Genomics UK (COG-UK) Consortium	Sergi Castellano, Rachel Williams, Mark Kristiansen, Paola Resende Silva, Sunando Roy, Tony Brooks, Helena Tutill, Paola Niola, Patricia Dyal, Charlotte Williams, Leysa Forrest, Yasmin Panchbhaya, Jacqueline Findlay, Samuel Weeks, Julianne Brown, Kathryn Harris, Paul Randell, James Price, Alison Holmes, Judith Breuer
EPI_ISL_866059, EPI_ISL_866060, EPI_ISL_866061, EPI_ISL_866062, EPI_ISL_866063, EPI_ISL_866064	University College London Hospital	COVID-19 Genomics UK (COG-UK) Consortium	Judith Heaney, Matthew Byott, Catherine Houlihan, Dan Frampton, Stuart Kirk, Moira Spyer and Eleni Nastouli
EPI_ISL_866335, EPI_ISL_866341, EPI_ISL_866345	University College London, Great Ormond Street Hospital for Children NHS Foundation Trust, Imperial College Healthcare NHS Trust	COVID-19 Genomics UK (COG-UK) Consortium	Sergi Castellano, Rachel Williams, Mark Kristiansen, Paola Resende Silva, Sunando Roy, Tony Brooks, Helena Tutill, Paola Niola, Patricia Dyal, Charlotte Williams, Leysa Forrest, Yasmin Panchbhaya, Jacqueline Findlay, Samuel Weeks, Julianne Brown, Kathryn Harris, Paul Randell, James Price, Alison Holmes, Judith Breuer
EPI_ISL_866427, EPI_ISL_866430, EPI_ISL_866433, EPI_ISL_866434, EPI_ISL_866436, EPI_ISL_866438, EPI_ISL_866440	Northumbria University / South Tees Hospitals NHS Foundation Trust / North Cumbria Integrated Care NHS Foundation Trust / North Tees and Hartlepool NHS Foundation Trust / Newcastle Hospitals NHS Foundation Trust	COVID-19 Genomics UK (COG-UK) Consortium	Darren L Smith,Andrew Nelson,Matthew Bashton,Greg R Young,Joshua Loh,John Allan,Mohammad A Tariq,Giles S Holt,Gary Black,Wen C Yew,Lynn Dover,Paul Baker,Steve Liggett,Sarah Essex,Jane Greenaway,Debra Padgett,Clive Graham,Garren Scott,Edward Barton,Emma Swindells,Brendan Payne,Jennifer Collins,Yusri Taha,Gary Eltringham
EPI_ISL_866579, EPI_ISL_866809, EPI_ISL_866810, EPI_ISL_866811, EPI_ISL_866816, EPI_ISL_866823	Quadram Institute Bioscience	COVID-19 Genomics UK (COG-UK) Consortium	Dave J. Baker, Gemma L. Kay, Alp Aydin, Thanh Le-Viet, Steven Rudder, Ana P. Tedim, Anastasia Kolyva, Maria Diaz, Leonardo de Oliveira Martins, Nabil-Fareed Alikhan, Lizzie Meadows, Rachael Stanley, Ngozi Elumogo, Muhammed Yasir, Nicholas M. Thomson, Alexander J Trotter, Rachel Gilroy, Samuel Bloomfield, Claire Stuart, Andrew Bell, Reenesh Prakash, Samir Dervisevic, Alison E. Mather, John Wain, Mark Webber, Andrew J. Page, Justin O'Grady
EPI_ISL_867285	Originating lab: Wales Specialist Virology Centre Sequencing lab: Pathogen Genomics Unit	Public Health Wales Microbiology Cardiff Wales Specialist Virology Centre	Catherine Moore, Johnathan Evans, Laura Gifford, Malorie Perry, Simon Cottrell, Angela Marchbank, Alec Birchley, Alexander Adams, Amy Gaskin, Bree Gatica-Wilcox, Jason Coombes, Joel Southgate, Lauren Gilbert, Lee Graham, Nicole Pacchiarini, Sara Kumziene-Summerhayes, Sarah Taylor, Sophie Jones, Sara Rey, Matthew Bull, Joanne Watkins, Sally Corden, Tom Connor
EPI_ISL_867952, EPI_ISL_867953	Centre for Enzyme Innovation, University of Portsmouth / Translational Research Laboratory, Portsmouth Hospitals NHS Trust	COVID-19 Genomics UK (COG-UK) Consortium	Angela Beckett,Yann Bourgeois,Garry Scarlett,Sharon Glaysher,Scott Elliott,Kelly Bicknell,Robert Impey,Allyson Lloyd,Sarah Wyllie,Ethan Butcher,Anoop Chauhan,Samuel Robson
EPI_ISL_868950, EPI_ISL_868951, EPI_ISL_868952, EPI_ISL_868954, EPI_ISL_868955, EPI_ISL_868956, EPI_ISL_868957, EPI_ISL_868958, EPI_ISL_868960, EPI_ISL_868961, EPI_ISL_868962, EPI_ISL_868969, EPI_ISL_868973, EPI_ISL_868979, EPI_ISL_868980			
see above	Bioinformatics and Biostatistics Lab, Advanced Sequencing Facility	COVID-19 Genomics UK (COG-UK) Consortium	Aengus Stewart,Jerome Nicod,Chelsea Sawyer,Laura Cubitt,Harshil Patel,Margaret Crawford
EPI_ISL_871759, EPI_ISL_871760, EPI_ISL_871761, EPI_ISL_871762, EPI_ISL_871763, EPI_ISL_871764, EPI_ISL_871765, EPI_ISL_871766, EPI_ISL_871767, EPI_ISL_871768, EPI_ISL_871769, EPI_ISL_871770, EPI_ISL_871771, EPI_ISL_871772, EPI_ISL_871773, EPI_ISL_871774, EPI_ISL_871775, EPI_ISL_871776, EPI_ISL_871777, EPI_ISL_871778, EPI_ISL_871779, EPI_ISL_871780			
see above	Department of Virus and Microbiological Special Diagnostics, Statens Serum Institut, Copenhagen, Denmark	Aalborg University	Danish Covid-19 Genome Consortium
EPI_ISL_872616	Nigeria Centre for Disease Control (NCDC)	African Centre of Excellence for Genomics of Infectious Diseases (ACEGID), Redeemer's University	Oluniyi P.E. et al
EPI_ISL_872732, EPI_ISL_872733, EPI_ISL_872734, EPI_ISL_872735, EPI_ISL_872736, EPI_ISL_872737, EPI_ISL_872738, EPI_ISL_872739, EPI_ISL_872740	Rhode Island Department of Health	Infectious Disease Program, Broad Institute of Harvard and MIT	Lemieux,J.E., Siddle,K.J., Huard,R., King,E., Azevedo,K., Miller,A., Adams,G., Gladden-Young,A., Lagerborg,K., Rudy,M., DeRuff,K., Carter,A., Normandin,E., Bauer,M., Reilly,S., Tomkins-Tinch,C., Loreth,C., Chaluvadi,S., Birren,B.W., Gallagher,G., Smole,S., Park,D.J., MacInnis,B.L., and Sabeti,P.C.
EPI_ISL_872904, EPI_ISL_872906, EPI_ISL_872907, EPI_ISL_872909, EPI_ISL_872912	WHO National Influenza Centre Russian Federation	WHO National Influenza Centre Russian Federation	Andrey Komissarov, Artem Fadeev, Anna Ivanova, Kseniya Komissarova, Dmitry Bazhenov, Mikhail Bakaev, Daria Danilenko, Ksenia Safina, Elena Nabieva, Georgii Bazykin, Dmitry Lioznov
EPI_ISL_873125, EPI_ISL_873161, EPI_ISL_873163	University of Michigan Clinical Microbiology Laboratory	Lauring Lab, University of Michigan, Department of Microbiology and Immunology	Valesano
EPI_ISL_873215	M Health Fairview	Minnesota Department of Health, Public Health Laboratory	Alexandra Lorentz, Jacob Garfin, Matt Plumb, and Xiong Wang
EPI_ISL_876109, EPI_ISL_876126, EPI_ISL_876130, EPI_ISL_876154, EPI_ISL_876156, EPI_ISL_876168, EPI_ISL_876179, EPI_ISL_876201, EPI_ISL_876206, EPI_ISL_876213, EPI_ISL_876305, EPI_ISL_876306, EPI_ISL_876307, EPI_ISL_876308, EPI_ISL_876309, EPI_ISL_876310, EPI_ISL_876311			
see above	Massachusetts State Public Health Laboratory	Massachusetts State Public Health Laboratory	Andrew Lang, Timelia Fink, Glen Gallagher, Sandra Smole
EPI_ISL_876541, EPI_ISL_876542, EPI_ISL_876543, EPI_ISL_876544, EPI_ISL_876545, EPI_ISL_876546, EPI_ISL_876547, EPI_ISL_876548	Florida Bureau of Public Health Laboratories	Florida Bureau of Public Health Laboratories	Sarah Schmedes, Jason Blanton
EPI_ISL_876590, EPI_ISL_876591, EPI_ISL_876593	TN Division of Laboratory Services	Pathogen Discovery, Respiratory Viruses Branch, Division of Viral Diseases, Centers for Disease Control and Prevention	Ying Tao, Yan Li, Jing Zhang, Krista Queen, Anna Uehara, Peter Cook, Clinton R. Paden, Haibin Wang, Suxiang Tong
EPI_ISL_878152, EPI_ISL_878155, EPI_ISL_878159, EPI_ISL_878162, EPI_ISL_878165, EPI_ISL_878172, EPI_ISL_878175, EPI_ISL_878178, EPI_ISL_878186, EPI_ISL_878189, EPI_ISL_878194, EPI_ISL_878197, EPI_ISL_878199, EPI_ISL_878207, EPI_ISL_878209, EPI_ISL_878217, EPI_ISL_878221, EPI_ISL_878223, EPI_ISL_878234, EPI_ISL_878237, EPI_ISL_878242, EPI_ISL_878245, EPI_ISL_878256, EPI_ISL_878266, EPI_ISL_878268, EPI_ISL_878271, EPI_ISL_878384, EPI_ISL_878389			
see above	San Diego County Public Health Laboratory	Andersen lab at Scripps Research	SEARCH Alliance San Diego with Tracy Basler, Jovan Shephard, Brett Austin
EPI_ISL_878707	Rady's Childrens Hospital	Andersen lab at Scripps Research	SEARCH Alliance San Diego with Nanda Radamchar, David Dimmock, Linda Luo, Christina Clarke, Kathryn Bouic, Teresa Mueller, Denise Malicki
EPI_ISL_879929, EPI_ISL_879939,	San Diego County Public Health Laboratory	Andersen lab at Scripps Research	SEARCH Alliance San Diego with Tracy Basler, Jovan Shephard, Brett Austin

EPI_ISL_879945, EPI_ISL_880006, EPI_ISL_880024			
EPI_ISL_880174, EPI_ISL_880186, EPI_ISL_880187, EPI_ISL_880190, EPI_ISL_880191, EPI_ISL_880194, EPI_ISL_880196	Rady's Childrens Hospital	Andersen lab at Scripps Research	SEARCH Alliance San Diego with Nanda Radamchar, David Dimmock, Linda Luo, Christina Clarke, Kathryn Bouic, Teresa Mueller, Denise Malicki
EPI_ISL_882679, EPI_ISL_882765, EPI_ISL_882766, EPI_ISL_882767	1.AO Universitaria 'S. Giovanni di Dio e Ruggi D'Aragona, Scuola Medica Salernitana' Hospital / 2.UOC di Virologia e Microbiologia, Università della Campania 'L. Vanvitelli' / 3.AO Universitaria 'Federico II' Napoli Hospital / 4.AORN 'San Giuseppe Moscati' Avellino Hospital / 5.AO 'San Pio - presidio G. Rummo' Benevento Hospital / 6.AO 'Sant'Anna e San Sebastiano' Caserta Hospital / 7.PO 'Maria Santissima Addolorata' Eboli Hospital / 8.Biogem Istituto di Ricerche Genetiche	1. Genome Research Center for Health (CRGS) / 2. Laboratory of Molecular Medicine and Genomics(LMMGe) / 3. Center for Research in Pure and Applied Mathematics (CRMPA)	Giorgio Giurato, Francesca Rizzo, Alessandro Weisz, Gianluigi Franci, Giovanni Nassa, Pasquale Pagliano, Roberta Tarallo, Elena Alexandrova, Ylenia D'Agostino, Carlo Ferravante, Jessica Lamberti, Viola Melone, Domenico Memoli, Valeria Mirici Cappa, Domenico Palumbo, Giovanni Pecoraro, Assunta Sellitto, Oriana Strianese, Ilaria Terenzi, Giuseppe Fenza, Aniello Gentile, Antonello Saccomanno, Sonia Amabile, Teresa Rocco, Annamaria Salvati, Emilia Vaccaro, Massimiliano Galdiero, Michele Cennamo, Giuseppe Portella, Maria Grazia Foti, Mariarosaria Ingino, Maria Landi, Maurizio Fumi, Vincenzo Rocco, Rita Greco, Vittoria Letizia, Arnolfo Petruzziello, Maddalena Schioppa, Gregorio Goffredi, Francesca Marciano, Michele Caraglia, Alessia Cossu, Marianna Scrima
EPI_ISL_883006, EPI_ISL_883007, EPI_ISL_883008	Maryland Public Health Laboratory	Maryland Public Health Laboratory	Maryland Department of Health Laboratories Administration
EPI_ISL_883867, EPI_ISL_883868, EPI_ISL_883869, EPI_ISL_883870, EPI_ISL_883871, EPI_ISL_883872, EPI_ISL_883873, EPI_ISL_883874, EPI_ISL_883875, EPI_ISL_883876, EPI_ISL_883877, EPI_ISL_883879, EPI_ISL_883880, EPI_ISL_883881, EPI_ISL_883882, EPI_ISL_883883, EPI_ISL_883884, EPI_ISL_883885, EPI_ISL_883886, EPI_ISL_883887, EPI_ISL_883888, EPI_ISL_883889, EPI_ISL_883890, EPI_ISL_883891, EPI_ISL_883892, EPI_ISL_883893, EPI_ISL_883894, EPI_ISL_883895, EPI_ISL_883896, EPI_ISL_883897, EPI_ISL_883898, EPI_ISL_883899, EPI_ISL_883900, EPI_ISL_883901, EPI_ISL_883902, EPI_ISL_883903, EPI_ISL_883904, EPI_ISL_883905, EPI_ISL_883906, EPI_ISL_883907, EPI_ISL_883908, EPI_ISL_883909, EPI_ISL_883910, EPI_ISL_883911, EPI_ISL_883912, EPI_ISL_883913, EPI_ISL_883914, EPI_ISL_883915, EPI_ISL_883916, EPI_ISL_883917, EPI_ISL_883918, EPI_ISL_883919, EPI_ISL_883920, EPI_ISL_883922, EPI_ISL_883923, EPI_ISL_883924, EPI_ISL_883925, EPI_ISL_883926, EPI_ISL_883927, EPI_ISL_883928, EPI_ISL_883929, EPI_ISL_883930, EPI_ISL_883931, EPI_ISL_883932, EPI_ISL_883933, EPI_ISL_883934, EPI_ISL_883935, EPI_ISL_883936, EPI_ISL_883939, EPI_ISL_883940, EPI_ISL_883941, EPI_ISL_883942, EPI_ISL_883943, EPI_ISL_883944, EPI_ISL_883945, EPI_ISL_883946, EPI_ISL_883947, EPI_ISL_883948, EPI_ISL_883949, EPI_ISL_883950, EPI_ISL_883951, EPI_ISL_883952, EPI_ISL_883953, EPI_ISL_883954, EPI_ISL_883955, EPI_ISL_883956			
see above	Eurofins Diatherix	Hudsonalpha Genome Sequencing Center	Jane Grimwood, Melissa Williams, Lori H. Handley, Joshua Stough, Leslie Malone, Stefan Brzezinski, Ada Stewart, Teresa Jones, Jenell Webber, John Lovell, Jennifer Cart, and Jeremy Schmutz
EPI_ISL_884217, EPI_ISL_884218	Alaska State Virology Laboratory (Alaska DHHS)	Alaska State Virology Laboratory (Alaska DHHS)	Stephanie DeRonde, Lisa Smith, PhD. Jack Chen, PhD.
EPI_ISL_884251, EPI_ISL_884252, EPI_ISL_884253	Maryland Public Health Laboratory	Maryland Public Health Laboratory	Maryland Department of Health Laboratories Administration
EPI_ISL_884890, EPI_ISL_884893, EPI_ISL_884894, EPI_ISL_884895, EPI_ISL_884896, EPI_ISL_884897, EPI_ISL_884902, EPI_ISL_884921, EPI_ISL_884922, EPI_ISL_884957	Santa Clara County Public Health Laboratory	Chan-Zuckerberg Biohub	CZB Cliahub Consortium
EPI_ISL_885131, EPI_ISL_885132	California Institute of Technology	Chan-Zuckerberg Biohub	CZB Cliahub Consortium
EPI_ISL_888844	Michigan Department of Health and Human Services, Bureau of Laboratories	Michigan Department of Health and Human Services, Bureau of Laboratories	Blankenship HM, Riner D, Soehnlen MK
EPI_ISL_888980	RS Anna	Eijkman Institute for Molecular Biology, Ministry of Research and Technology/National Agency for Research and Innovation	Hidayat Trimarsanto, Iskandar Adnan, Lydia V. Panggalo, Sukma Oktavianthi, Willy Agustine, Edison Johar, Frilasita A Yudhaputri, Safarina G Malik, Khin Saw Myint, Amin Soebandrio
EPI_ISL_890189	Gonoshasthaya-RNA Research Center, Gonoshasthaya-RNA Molecular Diagnostics and Research Center	Gonoshasthaya-RNA Research Center, Gonoshasthaya-RNA Molecular Diagnostics and Research Center	Jamiruddin,M.R., Khondoker,M.U., Sharif,N., Azmuda,N., Ahmed,M.F., Sharmin,S., Akter,S., Mou,T.J., Marzan,M., Liza,S.M., Nahar,S., Jahan,N., Ali,T., Khandker,S.S., Jamiruddin,M., Haq,M.A., Adnan,N., Chaity,M., Oishee,M.
EPI_ISL_890949	Seattle Flu Study	Seattle Flu Study	Deborah A. Nickerson, Chris D. Frazar, Jover Lee, Benjamin Pelle, Erica Ryke, Matthew Richardson, Amanda Adler, Elisabeth Brandstetter, Peter D. Han, Kairsten Fay, Misja Ilcisin, Kirsten Lacombe, Thomas R. Sibley, Melissa Truong, Caitlin R. Wolf, Karen Cowgill, Stephanie Schrag, Jeff Duchin, Michael Boeckh, Janet A. Englund, Michael Famulare, Barry R. Lutz, Mark J. Rieder, Lea M. Starita, Matthew Thompson, Helen Y. Chu, Trevor Bedford, Jay Shendure
EPI_ISL_890950	Seattle Flu Study	Seattle Flu Study	Deborah A. Nickerson, Chris D. Frazar, Jover Lee, Benjamin Pelle, Erica Ryke, Matthew Richardson, Amanda Adler, Elisabeth Brandstetter, Peter D. Han, Kairsten Fay, Misja Ilcisin, Kirsten Lacombe, Thomas R. Sibley, Melissa Truong, Caitlin R. Wolf, Michael Boeckh, Janet A. Englund, Michael Famulare, Barry R. Lutz, Mark J. Rieder, Lea M. Starita, Matthew Thompson, Jay Shendure, Trevor Bedford, Helen Y. Chu
EPI_ISL_890958, EPI_ISL_890959	Seattle Flu Study	Seattle Flu Study	Deborah A. Nickerson, Chris D. Frazar, Jover Lee, Benjamin Pelle, Erica Ryke, Matthew Richardson, Amanda Adler, Elisabeth Brandstetter, Peter D. Han, Kairsten Fay, Misja Ilcisin, Kirsten Lacombe, Thomas R. Sibley, Melissa Truong, Caitlin R. Wolf, Karen Cowgill, Stephanie Schrag, Jeff Duchin, Michael Boeckh, Janet A. Englund, Michael Famulare, Barry R. Lutz, Mark J. Rieder, Lea M. Starita, Matthew Thompson, Helen Y. Chu, Trevor Bedford, Jay Shendure
EPI_ISL_892276	Servicio de Microbiología Clínica (Complejo Hospitalario de Navarra, Pamplona), Instituto de Investigación Sanitaria de Navarra (IdiSNA)	SeqCOVID-SPAIN consortium/IBV(CSIC)	Carmen Ezpeleta Baquedano, Ana Navascués, Ana Miqueleiz and SeqCOVID-SPAIN consortium
EPI_ISL_892278, EPI_ISL_892303, EPI_ISL_892304, EPI_ISL_892305, EPI_ISL_892306, EPI_ISL_892307, EPI_ISL_892308, EPI_ISL_892309, EPI_ISL_892310, EPI_ISL_892311, EPI_ISL_892312, EPI_ISL_892313			
see above	Servicio de Microbiología. Hospital Universitario Donostia. OSI Donostialdea. Área de Enfermedades Infecciosas, Grupo de Infección Respiratoria y Resistencia Antimicrobiana. Instituto de Investigación Sanitaria Biodonostia	SeqCOVID-SPAIN consortium/IBV(CSIC)	Gustavo Cilla Eguiluz, Milagrosa Montes Ros, Luis Piñeiro Vázquez, Ane Sorrarain, Jose Maria Marimón and SeqCOVID-SPAIN consortium
EPI_ISL_892339, EPI_ISL_892340, EPI_ISL_892341, EPI_ISL_892342, EPI_ISL_892343, EPI_ISL_892344	Servicio de Microbiología Clínica (Complejo Hospitalario de Navarra, Pamplona), Instituto de Investigación Sanitaria de Navarra (IdiSNA)	SeqCOVID-SPAIN consortium/IBV(CSIC)	Carmen Ezpeleta Baquedano, Ana Navascués, Ana Miqueleiz and SeqCOVID-SPAIN consortium
EPI_ISL_892364, EPI_ISL_892368, EPI_ISL_893367, EPI_ISL_893740, EPI_ISL_893741, EPI_ISL_893742	Ohio Department of Health Laboratory	Ohio Department of Health Laboratory	Holmes, Jennifer; Eric Brandt, Keoni Omura, Glen McGillivary, Caitlin McDonnell, Jade Mowery, Stephanie Mccracken, Tyler Payne, Kirtana Ramadugu, Erica Leasure, Brent Lee, Kelsey Florek, Heather Blankenship, Quanta Brown, and Tammy Bannerman
EPI_ISL_893785	Institute of Virology, Medical Center, University of Freiburg, Freiburg, Germany	Institute of Virology, Clinial Virus Genomics, Medical Center, University of Freiburg, Freiburg, Germany	Jonas Fuchs, Lisa Kern, Sandra Reuter, Hajo Grundmann, Marcus Panning
EPI_ISL_894170, EPI_ISL_894947, EPI_ISL_895289, EPI_ISL_895727, EPI_ISL_900742	Ohio Department of Health Laboratory	Ohio Department of Health Laboratory	Holmes, Jennifer; Eric Brandt, Keoni Omura, Glen McGillivary, Caitlin McDonnell, Jade Mowery, Stephanie Mccracken, Tyler Payne, Kirtana Ramadugu, Erica Leasure, Brent Lee, Kelsey Florek, Heather Blankenship, Quanta Brown, and Tammy Bannerman
EPI_ISL_903235, EPI_ISL_903253	M Health Fairview	Minnesota Department of Health, Public Health Laboratory	Alexandra Lorentz, Jacob Garfin, Matt Plumb, and Xiong Wang
EPI_ISL_903570	WVDHHR - Office of Laboratory Services	Genomics and Discovery, Respiratory Viruses Branch, Division of Viral Diseases, Centers for Disease Control and Prevention	Krista Queen, Yan Li, Ying Tao, Jing Zhang, Anna Uehara, Anna Montmayeur, Clinton R. Paden, Peter W. Cook, Rachel Marine, Mili Sheth, Jasmine Padilla, Sarah Nobles, Mark Burroughs, Lori Rowe, Haibin Wang, Ben L. Rambo-Martin, Dhwani Batra, Justin Lee, Suxiang Tong
EPI_ISL_903573	WA State Department of Health	Genomics and Discovery, Respiratory Viruses Branch, Division of Viral Diseases, Centers for Disease Control and	Krista Queen, Yan Li, Ying Tao, Jing Zhang, Anna Uehara, Anna Montmayeur, Clinton R. Paden, Peter W. Cook, Rachel Marine, Mili Sheth, Jasmine Padilla, Sarah Nobles, Mark Burroughs, Lori Rowe, Haibin Wang, Ben L. Rambo-Martin, Dhwani Batra, Justin Lee, Suxiang Tong

		Prevention	
EPI_ISL_903576	WVDHHR - Office of Laboratory Services	Genomics and Discovery, Respiratory Viruses Branch, Division of Viral Diseases, Centers for Disease Control and Prevention	Krista Queen, Yan Li, Ying Tao, Jing Zhang, Anna Uehara, Anna Montmayeur, Clinton R. Paden, Peter W. Cook, Rachel Marine, Mili Sheth, Jasmine Padilla, Sarah Nobles, Mark Burroughs, Lori Rowe, Haibin Wang, Ben L. Rambo-Martin, Dhwani Batra, Justin Lee, Suxiang Tong
EPI_ISL_903590	PA Department of Health, Bureau of Laboratories	Genomics and Discovery, Respiratory Viruses Branch, Division of Viral Diseases, Centers for Disease Control and Prevention	Krista Queen, Yan Li, Ying Tao, Jing Zhang, Anna Uehara, Anna Montmayeur, Clinton R. Paden, Peter W. Cook, Rachel Marine, Mili Sheth, Jasmine Padilla, Sarah Nobles, Mark Burroughs, Lori Rowe, Haibin Wang, Ben L. Rambo-Martin, Dhwani Batra, Justin Lee, Suxiang Tong
EPI_ISL_903601, EPI_ISL_903602, EPI_ISL_903612	OR State PHL-Virology/Immunology Section	Genomics and Discovery, Respiratory Viruses Branch, Division of Viral Diseases, Centers for Disease Control and Prevention	Krista Queen, Yan Li, Ying Tao, Jing Zhang, Anna Uehara, Anna Montmayeur, Clinton R. Paden, Peter W. Cook, Rachel Marine, Mili Sheth, Jasmine Padilla, Sarah Nobles, Mark Burroughs, Lori Rowe, Haibin Wang, Ben L. Rambo-Martin, Dhwani Batra, Justin Lee, Suxiang Tong
EPI_ISL_903643, EPI_ISL_903644	RI State Health Laboratories	Genomics and Discovery, Respiratory Viruses Branch, Division of Viral Diseases, Centers for Disease Control and Prevention	Krista Queen, Yan Li, Ying Tao, Jing Zhang, Anna Uehara, Anna Montmayeur, Clinton R. Paden, Peter W. Cook, Rachel Marine, Mili Sheth, Jasmine Padilla, Sarah Nobles, Mark Burroughs, Lori Rowe, Haibin Wang, Ben L. Rambo-Martin, Dhwani Batra, Justin Lee, Suxiang Tong
EPI_ISL_903653	WA State Department of Health	Genomics and Discovery, Respiratory Viruses Branch, Division of Viral Diseases, Centers for Disease Control and Prevention	Krista Queen, Yan Li, Ying Tao, Jing Zhang, Anna Uehara, Anna Montmayeur, Clinton R. Paden, Peter W. Cook, Rachel Marine, Mili Sheth, Jasmine Padilla, Sarah Nobles, Mark Burroughs, Lori Rowe, Haibin Wang, Ben L. Rambo-Martin, Dhwani Batra, Justin Lee, Suxiang Tong
EPI_ISL_903662	PA Department of Health, Bureau of Laboratories	Genomics and Discovery, Respiratory Viruses Branch, Division of Viral Diseases, Centers for Disease Control and Prevention	Krista Queen, Yan Li, Ying Tao, Jing Zhang, Anna Uehara, Anna Montmayeur, Clinton R. Paden, Peter W. Cook, Rachel Marine, Mili Sheth, Jasmine Padilla, Sarah Nobles, Mark Burroughs, Lori Rowe, Haibin Wang, Ben L. Rambo-Martin, Dhwani Batra, Justin Lee, Suxiang Tong
EPI_ISL_903707, EPI_ISL_903746	MN PHL Division, Minnesota Department of Health	Genomics and Discovery, Respiratory Viruses Branch, Division of Viral Diseases, Centers for Disease Control and Prevention	Krista Queen, Yan Li, Ying Tao, Jing Zhang, Anna Uehara, Anna Montmayeur, Clinton R. Paden, Peter W. Cook, Rachel Marine, Mili Sheth, Jasmine Padilla, Sarah Nobles, Mark Burroughs, Lori Rowe, Haibin Wang, Ben L. Rambo-Martin, Dhwani Batra, Justin Lee, Suxiang Tong
EPI_ISL_903784, EPI_ISL_903786	WVDHHR - Office of Laboratory Services	Genomics and Discovery, Respiratory Viruses Branch, Division of Viral Diseases, Centers for Disease Control and Prevention	Krista Queen, Yan Li, Ying Tao, Jing Zhang, Anna Uehara, Anna Montmayeur, Clinton R. Paden, Peter W. Cook, Rachel Marine, Mili Sheth, Jasmine Padilla, Sarah Nobles, Mark Burroughs, Lori Rowe, Haibin Wang, Ben L. Rambo-Martin, Dhwani Batra, Justin Lee, Suxiang Tong
EPI_ISL_903788	TX DSHS, Lab Services Section MC 1947	Genomics and Discovery, Respiratory Viruses Branch, Division of Viral Diseases, Centers for Disease Control and Prevention	Krista Queen, Yan Li, Ying Tao, Jing Zhang, Anna Uehara, Anna Montmayeur, Clinton R. Paden, Peter W. Cook, Rachel Marine, Mili Sheth, Jasmine Padilla, Sarah Nobles, Mark Burroughs, Lori Rowe, Haibin Wang, Ben L. Rambo-Martin, Dhwani Batra, Justin Lee, Suxiang Tong
EPI_ISL_903791	NE Public Health Laboratory	Genomics and Discovery, Respiratory Viruses Branch, Division of Viral Diseases, Centers for Disease Control and Prevention	Krista Queen, Yan Li, Ying Tao, Jing Zhang, Anna Uehara, Anna Montmayeur, Clinton R. Paden, Peter W. Cook, Rachel Marine, Mili Sheth, Jasmine Padilla, Sarah Nobles, Mark Burroughs, Lori Rowe, Haibin Wang, Ben L. Rambo-Martin, Dhwani Batra, Justin Lee, Suxiang Tong
EPI_ISL_903795	WVDHHR - Office of Laboratory Services	Genomics and Discovery, Respiratory Viruses Branch, Division of Viral Diseases, Centers for Disease Control and Prevention	Krista Queen, Yan Li, Ying Tao, Jing Zhang, Anna Uehara, Anna Montmayeur, Clinton R. Paden, Peter W. Cook, Rachel Marine, Mili Sheth, Jasmine Padilla, Sarah Nobles, Mark Burroughs, Lori Rowe, Haibin Wang, Ben L. Rambo-Martin, Dhwani Batra, Justin Lee, Suxiang Tong
EPI_ISL_903843	OR State PHL-Virology/Immunology Section	Genomics and Discovery, Respiratory Viruses Branch, Division of Viral Diseases, Centers for Disease Control and Prevention	Krista Queen, Yan Li, Ying Tao, Jing Zhang, Anna Uehara, Anna Montmayeur, Clinton R. Paden, Peter W. Cook, Rachel Marine, Mili Sheth, Jasmine Padilla, Sarah Nobles, Mark Burroughs, Lori Rowe, Haibin Wang, Ben L. Rambo-Martin, Dhwani Batra, Justin Lee, Suxiang Tong
EPI_ISL_903867	NC State Laboratory of Public Health	Genomics and Discovery, Respiratory Viruses Branch, Division of Viral Diseases, Centers for Disease Control and Prevention	Krista Queen, Yan Li, Ying Tao, Jing Zhang, Anna Uehara, Anna Montmayeur, Clinton R. Paden, Peter W. Cook, Rachel Marine, Mili Sheth, Jasmine Padilla, Sarah Nobles, Mark Burroughs, Lori Rowe, Haibin Wang, Ben L. Rambo-Martin, Dhwani Batra, Justin Lee, Suxiang Tong
EPI_ISL_903868	MN PHL Division, Minnesota Department of Health	Genomics and Discovery, Respiratory Viruses Branch, Division of Viral Diseases, Centers for Disease Control and Prevention	Krista Queen, Yan Li, Ying Tao, Jing Zhang, Anna Uehara, Anna Montmayeur, Clinton R. Paden, Peter W. Cook, Rachel Marine, Mili Sheth, Jasmine Padilla, Sarah Nobles, Mark Burroughs, Lori Rowe, Haibin Wang, Ben L. Rambo-Martin, Dhwani Batra, Justin Lee, Suxiang Tong
EPI_ISL_903869	VA-Division of Consolidated Laboratory Services	Genomics and Discovery, Respiratory Viruses Branch, Division of Viral Diseases, Centers for Disease Control and Prevention	Krista Queen, Yan Li, Ying Tao, Jing Zhang, Anna Uehara, Anna Montmayeur, Clinton R. Paden, Peter W. Cook, Rachel Marine, Mili Sheth, Jasmine Padilla, Sarah Nobles, Mark Burroughs, Lori Rowe, Haibin Wang, Ben L. Rambo-Martin, Dhwani Batra, Justin Lee, Suxiang Tong
EPI_ISL_903881	WVDHHR - Office of Laboratory Services	Genomics and Discovery, Respiratory Viruses Branch, Division of Viral Diseases, Centers for Disease Control and Prevention	Krista Queen, Yan Li, Ying Tao, Jing Zhang, Anna Uehara, Anna Montmayeur, Clinton R. Paden, Peter W. Cook, Rachel Marine, Mili Sheth, Jasmine Padilla, Sarah Nobles, Mark Burroughs, Lori Rowe, Haibin Wang, Ben L. Rambo-Martin, Dhwani Batra, Justin Lee, Suxiang Tong
EPI_ISL_903898, EPI_ISL_903916	MO State Public Health Laboratory	Genomics and Discovery, Respiratory Viruses Branch, Division of Viral Diseases, Centers for Disease Control and Prevention	Krista Queen, Yan Li, Ying Tao, Jing Zhang, Anna Uehara, Anna Montmayeur, Clinton R. Paden, Peter W. Cook, Rachel Marine, Mili Sheth, Jasmine Padilla, Sarah Nobles, Mark Burroughs, Lori Rowe, Haibin Wang, Ben L. Rambo-Martin, Dhwani Batra, Justin Lee, Suxiang Tong
EPI_ISL_903917	PA Department of Health, Bureau of Laboratories	Genomics and Discovery, Respiratory Viruses Branch, Division of Viral Diseases, Centers for Disease Control and Prevention	Krista Queen, Yan Li, Ying Tao, Jing Zhang, Anna Uehara, Anna Montmayeur, Clinton R. Paden, Peter W. Cook, Rachel Marine, Mili Sheth, Jasmine Padilla, Sarah Nobles, Mark Burroughs, Lori Rowe, Haibin Wang, Ben L. Rambo-Martin, Dhwani Batra, Justin Lee, Suxiang Tong
EPI_ISL_903932	AZ SPHL, Arizona Department of Health Services	Genomics and Discovery, Respiratory Viruses Branch, Division of Viral Diseases, Centers for Disease Control and Prevention	Krista Queen, Yan Li, Ying Tao, Jing Zhang, Anna Uehara, Anna Montmayeur, Clinton R. Paden, Peter W. Cook, Rachel Marine, Mili Sheth, Jasmine Padilla, Sarah Nobles, Mark Burroughs, Lori Rowe, Haibin Wang, Ben L. Rambo-Martin, Dhwani Batra, Justin Lee, Suxiang Tong
EPI_ISL_903956, EPI_ISL_903974	WA State Department of Health	Genomics and Discovery, Respiratory Viruses Branch, Division of Viral Diseases, Centers for Disease Control and Prevention	Krista Queen, Yan Li, Ying Tao, Jing Zhang, Anna Uehara, Anna Montmayeur, Clinton R. Paden, Peter W. Cook, Rachel Marine, Mili Sheth, Jasmine Padilla, Sarah Nobles, Mark Burroughs, Lori Rowe, Haibin Wang, Ben L. Rambo-Martin, Dhwani Batra, Justin Lee, Suxiang Tong
EPI_ISL_904007, EPI_ISL_904008	Veterinary Specialized Institute Kraljevo	Veterinary Specialized Institute "Kraljevo", Serbia	Vidanovic,D., Tesovic,B., Knezevic,A., Jovanovic,T., Jankovic,M., Sekler,M., Banovic Djeri,B., Petrovic,T., Volkening,J., Afonso,C.
EPI_ISL_904178, EPI_ISL_904242, EPI_ISL_904262, EPI_ISL_904481, EPI_ISL_904482	Dutch COVID-19 response team	Erasmus Medical Center	Bas Oude Munnink, Reina Sikkema, David Nieuwenhuijse, Irina Chestakova, Anne van der Linden, Marjan Boter, Emmanuelle Munger, Corine GeurtsvanKessel, Annemiek van der Eijk, Richard Molenkamp, Marion Koopmans, on behalf of the Dutch national COVID-19 response team.
EPI_ISL_904862, EPI_ISL_904863, EPI_ISL_904865, EPI_ISL_904888, EPI_ISL_904901, EPI_ISL_905085, EPI_ISL_905088, EPI_ISL_905090, EPI_ISL_905535	Dutch COVID-19 response team	National Institute for Public Health and the Environment (RIVM)	Adam Meijer, Harry Vennema, Dirk Eggink, Jeroen Cremer, Sharon van den Brink, Bas van der Veer, AnneMarie van den Brandt, Florian Zwagemaker, Dennis Schmitz, Chantal Reusken, on behalf of the national COVID-19 response team
EPI_ISL_905801, EPI_ISL_905802, EPI_ISL_905803, EPI_ISL_905804, EPI_ISL_905805, EPI_ISL_905806	OHSU Lab Services Molecular Microbiology Lab	Oregon SARS-CoV-2 Genome Sequencing Center	Brendan L. O'Connell, Sally Grindstaff, Kayla Carter, Ruth V. Nichols, Alec J. Hirsch, Donna Hansel, Guang Fan, Xuan, Qin, Daniel N. Streblow, William B. Messer, Andrew C. Adey, Benjamin N. Bimber, Brian J. O'Roak
EPI_ISL_906100	Child Health Research Foundation	Child Health Research Foundation	Senjuti Saha, Sharmistha Goswami, Afroza Akter Tanni, Syed Muktadir Al Sium, Arif Mohammad Tanmoy, Roly Malaker, Md Hafizur Rahman, Samir K Saha

EPI_ISL_906112	Child Health Research Foundation	Child Health Research Foundation	Senjuti Saha, Afroza Akter Tanni, Sharmistha Goswami, Syed Muktadir Al Sium, Arif Mohammad Tanmoy, Roly Malaker, Md Hafizur Rahman, Samir K Saha
EPI_ISL_906305	Nigeria Centre for Disease Control (NCDC)	African Centre of Excellence for Genomics of Infectious Diseases (ACEGID), Redeemer's University	Oluniyi P.E. et al
EPI_ISL_906549	Laboratorio de Virologia-Instituto Nacional de Salud	Instituto Nacional de Salud- Dirección de Investigación en Salud Pública, Universidad de los Andes- Applied genomics research group, Vicerrectoria de Investigación y Creación, Universidad de los Andes- Systems and Computing Engineering Department	Katherine Laiton-Donato, Diego A. Álvarez-Díaz, Carlos Franco-Muñoz, Mauricio Pacheco-Montealegre, Héctor Alejandro Ruiz-Moreno, Maria T. Herrera-Sepúlveda, Diego Andrés Prada, Jhonnatan Reales-González, Sheryll Corchuelo, Julian Naizaque, Gerardo Santamaría Jorge Duitama, Laura Natalia Gonzalez, Jorge Ivan Diaz, Silvia Restrepo-Restrepo, Magdalena Wiesner, Martha Lucia Ospina Martinez, Marcela Mercado-Reyes
EPI_ISL_909741, EPI_ISL_909743	Ohio Department of Health Laboratory	Ohio Department of Health Laboratory	Holmes, Jennifer; Eric Brandt, Keoni Omura, Glen McGillivary, Caitlin McDonnell, Jade Mowery, Stephanie Mccracken, Tyler Payne, Kirtana Ramadugu, Erica Leasure, Brent Lee, Kelsey Florek, Heather Blankenship, Quanta Brown, and Tammy Bannerman
EPI_ISL_909952	CUSL/UCLouvain COVID testing federal platform	UCLouvain/IREC/MBLG	Jean Ruelle, Lysa Pinsmaye, Benoit Kabamba Mukadi
EPI_ISL_910490, EPI_ISL_910491, EPI_ISL_910492, EPI_ISL_910493, EPI_ISL_910494, EPI_ISL_910495, EPI_ISL_910644, EPI_ISL_910645, EPI_ISL_910646, EPI_ISL_910648, EPI_ISL_910649, EPI_ISL_910650, EPI_ISL_910651, EPI_ISL_910652, EPI_ISL_910653, EPI_ISL_910654, EPI_ISL_910655, EPI_ISL_910656, EPI_ISL_910657, EPI_ISL_910658, EPI_ISL_910659, EPI_ISL_910660, EPI_ISL_910661, EPI_ISL_910662, EPI_ISL_910663, EPI_ISL_910664, EPI_ISL_910665, EPI_ISL_910666, EPI_ISL_910667, EPI_ISL_910668, EPI_ISL_910669, EPI_ISL_910670, EPI_ISL_910671, EPI_ISL_910672, EPI_ISL_910673, EPI_ISL_910674, EPI_ISL_910675, EPI_ISL_910676, EPI_ISL_910677, EPI_ISL_910678, EPI_ISL_910679, EPI_ISL_910680, EPI_ISL_910681, EPI_ISL_910682, EPI_ISL_910683, EPI_ISL_910684, EPI_ISL_910685, EPI_ISL_910686, EPI_ISL_910687, EPI_ISL_910688, EPI_ISL_910689, EPI_ISL_910690, EPI_ISL_910691, EPI_ISL_910692, EPI_ISL_910693, EPI_ISL_910694, EPI_ISL_910695, EPI_ISL_910696, EPI_ISL_910697, EPI_ISL_910698, EPI_ISL_910699, EPI_ISL_910704, EPI_ISL_910706, EPI_ISL_910707, EPI_ISL_910708, EPI_ISL_910709, EPI_ISL_910710, EPI_ISL_910711, EPI_ISL_910712, EPI_ISL_910713, EPI_ISL_910721, EPI_ISL_910722, EPI_ISL_910724, EPI_ISL_910725, EPI_ISL_910726, EPI_ISL_910727, EPI_ISL_910728, EPI_ISL_910729, EPI_ISL_910730, EPI_ISL_910731, EPI_ISL_910732, EPI_ISL_910733, EPI_ISL_910734, EPI_ISL_910735, EPI_ISL_910736, EPI_ISL_910737, EPI_ISL_910738, EPI_ISL_910739, EPI_ISL_910740, EPI_ISL_910741, EPI_ISL_910742, EPI_ISL_910743, EPI_ISL_910744, EPI_ISL_910745, EPI_ISL_910746, EPI_ISL_910747			
see above	Laboratoire national de sante, Microbiology, Virology	Laboratoire national de sante, Microbiology, Microbial Genomics Platform	Anke Wienecke-Baldacchino, Catherine Ragimbeau,Jessica Tapp, Fatu Djabi, Lise Pignon, Raoul Salmon, Tamir Abdelrahman
EPI_ISL_911514	Ohio Department of Health Laboratory	Ohio Department of Health Laboratory	Holmes, Jennifer; Eric Brandt, Keoni Omura, Glen McGillivary, Caitlin McDonnell, Jade Mowery, Stephanie Mccracken, Tyler Payne, Kirtana Ramadugu, Erica Leasure, Brent Lee, Kelsey Florek, Heather Blankenship, Quanta Brown, and Tammy Bannerman
EPI_ISL_911681, EPI_ISL_911688, EPI_ISL_911691, EPI_ISL_911692	Alaska State Virology Laboratory	Alaska State Virology Laboratory	Stephanie DeRonde, Lisa Smith, Ph.D., Jack Chen, Ph.D.
EPI_ISL_912355, EPI_ISL_912356	Fondation Congolaise pour la recherche medicale (FCRM), Francine Ntoumi	NGS Competence Center Tuebingen, Institut für Medizinische Mikrobiologie und Hygiene, Universitaetsklinikum Tübingen	Angel Angelov
EPI_ISL_913105, EPI_ISL_913106, EPI_ISL_913111	CHU Purpan - Laboratoire de Virologie - Institut Fédératif de Biologie	CHU Purpan - Laboratoire de Virologie - Institut Fédératif de Biologie	Latour J., Ranger N., Dubois M., Carcenac R., Harter A., Boyer P., Tremeaux P., Izopet J.
EPI_ISL_913668, EPI_ISL_913670, EPI_ISL_913672	Vault Health	Minnesota Department of Health, Public Health Laboratory	Alexandra Lorentz, Jacob Garfin, Matt Plumb, and Xiong Wang
EPI_ISL_913940, EPI_ISL_913941, EPI_ISL_913942, EPI_ISL_913943, EPI_ISL_913945, EPI_ISL_913960, EPI_ISL_913961, EPI_ISL_913972, EPI_ISL_913973, EPI_ISL_913974	Instituto de Diagnostico y Referencia Epidemiologicos INDRE_RNLSP	Instituto de Diagnostico y Referencia Epidemiologicos (INDRE)	Claudia Wong-Arambula, Abril Rodriguez-Maldonado, Fabiola Garces-Ayala, Adnan Araiza-Rodriguez, David Fragoso-Fonseca, Sergio Rangel-Guerrero, Mayra Jimenez-Morales, Nancy Munoz-Hernandez, Natividad Cruz-Ortiz, Tatiana Nunez-Garcia, Gisela Barrera-Badillo, Lucia Hernandez-Rivas, Irma Lopez-Martinez, Ernesto Ramirez-Gonzalez.
EPI_ISL_914797	TAMIZAJE COMUNITARIO - PASO CANOAS	Inciensa, Instituto Costarricense de Investigación y Enseñanza en Nutrición y Salud	Francisco Duarte, Hebleen Porras, Claudio Soto-Garita, Estela Cordero, Adriana Godínez, Melany Calderón & Mariel López
EPI_ISL_914883	Vilnius university hospital Santaros Klinikos, Center of Laboratory Medicine	Vilnius University Hospital Santaros Klinikos	Ingrida Olendraite, Daniel Naumovas, Rimvydas Norvilas, Dovil Ežerskyt, Justinas Šlikas
EPI_ISL_915360	Keio University School of Medicine	Keio University School of Medicine	Kenjiro Kosaki, Yuka Iwasaki, Hirotsugu Ishizu, Haruhiko Siomi, Kodai Abe
EPI_ISL_918168	Department of Infectious Diseases and Immunology, National Hospital Organization Nagoya Medical Center	Clinical Research Center, National Hospital Organization Nagoya Medical Center	Yoshihiro Nakata, Hirotaka Ode, Mai Kubota, Masakazu Matsuda, Kazuhiro Matsuoka, Miho Nakasuji, Mikiko Mori, Mayumi Imahashi, Yoshiyuki Yokomaku, Yasumasa Iwatani
EPI_ISL_918186, EPI_ISL_918208	Innovative Genomics Institute, UC Berkeley	Innovative Genomics Institute, UC Berkeley	Stacia Wyman, Haridha Shivram, Phil Frankino, Liana Lareau, Shana McDevitt, Justin Choi
EPI_ISL_918524, EPI_ISL_918525, EPI_ISL_918527, EPI_ISL_918528	LACEN - Laboratório Central de Saúde Pública do Para	Evandro Chagas Institute	Santos, M.C.; Silva, A.M.; Junior, W.D.C.; Barbagelata, L.S.; Ferreira, J.A.; Sousa, E.M.A.; da Silva, P.S.; Pinheiro, K.C.; L.C.; Sousa Junior, E.C.
EPI_ISL_918553	LACEN - Laboratório Central de Saúde Pública do Amapa	Evandro Chagas Institute	Santos, M.C.; Silva, A.M.; Junior, W.D.C.; Barbagelata, L.S.; Ferreira, J.A.; Sousa, E.M.A.; da Silva, P.S.; Pinheiro, K.C.; L.C.; Sousa Junior, E.C.
EPI_ISL_918600, EPI_ISL_918601, EPI_ISL_918604, EPI_ISL_918605, EPI_ISL_918606	University of Birmingham	COVID-19 Genomics UK (COG-UK) Consortium	Institute of Microbiology, University of Birmingham: Claire McMurray, Joanne Stockton, Samuel Nicholls, Radoslaw Poplawski, Will Rowe, Josh Quick, Nicholas Loman. University of Birmingham Testing Laboratory: Celina M Whalley, Andrew Bosworth, Charlotte Poxon, Kasun Wanigasooriya, Oliver Pickles, Mike Kidd, Alex Richter, Andrew D Beggs PHE Heartlands Lab: Husam Osman, Andrew Bosworth. Queen Elizabeth Hospital: Anna Casey
EPI_ISL_919234	West of Scotland Specialist Virology Centre, NHSGGC / MRC-University of Glasgow Centre for Virus Research	COVID-19 Genomics UK (COG-UK) Consortium	Ana da Silva Filipe, Natasha Johnson, Kathy Smollett, Daniel Mair, Stephen Carmichael, Alice Broos, Lily Tong, Jenna Nichols, Kyriaki Nomikou; Sarah McDonald; Richard Orton, Joseph Hughes, Sreenu Vattipally, David L Robertson; Alasdair MacLean, Rory Gunson; Sharif Shaaban, Matthew Holden; Rachel Blacow, Guy Mollett, Kathy Li, James Shepherd, Antonia Ho, Emma Thomson
EPI_ISL_919310, EPI_ISL_919349, EPI_ISL_919350, EPI_ISL_919351, EPI_ISL_919352, EPI_ISL_919353, EPI_ISL_919354, EPI_ISL_919355, EPI_ISL_919356, EPI_ISL_919357, EPI_ISL_919358, EPI_ISL_919382, EPI_ISL_919383, EPI_ISL_919384, EPI_ISL_919385, EPI_ISL_919386, EPI_ISL_919387, EPI_ISL_919402, EPI_ISL_919403			
see above	Virology Department, Royal Infirmary of Edinburgh, NHS Lothian / School of Biological Sciences, University of Edinburgh / Institute of Genetics and Molecular Medicine, University of Edinburgh	COVID-19 Genomics UK (COG-UK) Consortium	McHugh M, Dewar R, Rooke S, Gallagher M, Balcaza C, O'Toole Á, Scher E, Hill V, McCrone JT, Colquhoun R, Yu X, Jackson B, Rambaut A, Williams TC, Templeton K
EPI_ISL_920377, EPI_ISL_920504	University College London Hospital	COVID-19 Genomics UK (COG-UK) Consortium	Judith Heaney, Matthew Byott, Catherine Houlihan, Dan Frampton, Stuart Kirk, Moira Spyer and Eleni Nastouli
EPI_ISL_922892, EPI_ISL_922893, EPI_ISL_922967, EPI_ISL_922968, EPI_ISL_922969	Wales Specialist Virology Centre Sequencing lab: Pathogen Genomics Unit	Public Health Wales Microbiology Cardiff Wales Specialist Virology Centre	Catherine Moore, Johnathan Evans, Laura Gifford, Malorie Perry, Simon Cottrell, Angela Marchbank, Alec Birchley, Alexander Adams, Amy Gaskin, Bree Gatica-Wilcox, Jason Coombes, Joel Southgate, Lauren Gilbert, Lee Graham, Nicole Pacchiarini, Sara Kumziene-Summerhayes, Sarah Taylor, Sophie Jones, Sara Rey, Matthew Bull, Joanne Watkins, Sally Corden, Tom Connor
EPI_ISL_925942, EPI_ISL_926015, EPI_ISL_926153, EPI_ISL_926187, EPI_ISL_926357, EPI_ISL_926381, EPI_ISL_926396, EPI_ISL_926402, EPI_ISL_926459, EPI_ISL_926517, EPI_ISL_926525, EPI_ISL_926555, EPI_ISL_926564, EPI_ISL_926587, EPI_ISL_926588, EPI_ISL_926594, EPI_ISL_926627, EPI_ISL_926698, EPI_ISL_926717, EPI_ISL_926751, EPI_ISL_926767, EPI_ISL_926873, EPI_ISL_926903, EPI_ISL_926916, EPI_ISL_926964, EPI_ISL_926972, EPI_ISL_926976, EPI_ISL_927101, EPI_ISL_927117, EPI_ISL_927149, EPI_ISL_927215, EPI_ISL_927228, EPI_ISL_927249, EPI_ISL_927262, EPI_ISL_927277, EPI_ISL_927321, EPI_ISL_927342, EPI_ISL_927346, EPI_ISL_927399, EPI_ISL_927456, EPI_ISL_927459, EPI_ISL_927460, EPI_ISL_927569, EPI_ISL_927571, EPI_ISL_927595, EPI_ISL_927651, EPI_ISL_927679, EPI_ISL_927703, EPI_ISL_927818, EPI_ISL_927869, EPI_ISL_927879, EPI_ISL_927882, EPI_ISL_927904, EPI_ISL_928005, EPI_ISL_928068, EPI_ISL_928080, EPI_ISL_928192, EPI_ISL_928211, EPI_ISL_928236, EPI_ISL_928258, EPI_ISL_928320, EPI_ISL_928355, EPI_ISL_928363, EPI_ISL_928372, EPI_ISL_928432, EPI_ISL_928467, EPI_ISL_928509, EPI_ISL_928523, EPI_ISL_928580, EPI_ISL_928585, EPI_ISL_928600, EPI_ISL_928664, EPI_ISL_928676, EPI_ISL_928708, EPI_ISL_928730, EPI_ISL_928794, EPI_ISL_928804, EPI_ISL_928817, EPI_ISL_928869, EPI_ISL_928886, EPI_ISL_928899, EPI_ISL_928909, EPI_ISL_928915, EPI_ISL_928951, EPI_ISL_929018, EPI_ISL_929035, EPI_ISL_929040, EPI_ISL_929047, EPI_ISL_929096, EPI_ISL_929158, EPI_ISL_929269, EPI_ISL_929409, EPI_ISL_929428, EPI_ISL_929433, EPI_ISL_929468, EPI_ISL_929497, EPI_ISL_929598, EPI_ISL_929608, EPI_ISL_929639, EPI_ISL_929647, EPI_ISL_929672, EPI_ISL_929683, EPI_ISL_929701, EPI_ISL_929735, EPI_ISL_929750, EPI_ISL_929757, EPI_ISL_929758, EPI_ISL_929772, EPI_ISL_929803, EPI_ISL_929829, EPI_ISL_929886, EPI_ISL_929911, EPI_ISL_929919, EPI_ISL_929954, EPI_ISL_930068, EPI_ISL_930126, EPI_ISL_930134, EPI_ISL_930151, EPI_ISL_930235, EPI_ISL_930321, EPI_ISL_930375, EPI_ISL_930407, EPI_ISL_930410, EPI_ISL_930444			
see above	Department of Virus and Microbiological Special Diagnostics, Statens Serum Institut, Copenhagen, Denmark	Aalborg University	Danish Covid-19 Genome Consortium
EPI_ISL_931392, EPI_ISL_931449	University Hospital Basel, Clinical Virology	University Hospital Basel, Clinical Bacteriology	Tim Roloff, Madlen Stange, Helena MB Seth-Smith, Alfredo Mari, Karoline Leuzinger, Julia Bielicki, Manuel Battegay, Hans Hirsch, Adrian Egli
EPI_ISL_933493, EPI_ISL_933494	Lighthouse Lab in Glasgow	Wellcome Sanger Institute for the COVID-19 Genomics UK	Harper VanSteenhouse, Yumi Kasai, David Gray, Carol Clugston, Anna Dominiczak and Alex Alderton, Roberto Amato, Sonia Goncalves, Ewan Harrison,

		(COG-UK) Consortium	David K. Jackson, Ian Johnston, Dominic Kwiatkowski, Cordelia Langford, John Sillitoe on behalf of the Wellcome Sanger Institute COVID-19 Surveillance Team
EPI_ISL_933533	Laboratory for HIV and opportunistic infections diagnosis The Republican Research and Practical Center for Epidemiology and Microbiology (RRPCEM)	Laboratory for HIV and opportunistic infections diagnosis The Republican Research and Practical Center for Epidemiology and Microbiology (RRPCEM)	Elena Gasich, Kirill Bulda, Artur Akhremchuk, Leonid Valentovich, Vladimir Gorbunov
EPI_ISL_933958, EPI_ISL_933984, EPI_ISL_933985, EPI_ISL_933986, EPI_ISL_933987, EPI_ISL_933988, EPI_ISL_933989, EPI_ISL_933990, EPI_ISL_933992, EPI_ISL_933993, EPI_ISL_933994, EPI_ISL_933995, EPI_ISL_933996, EPI_ISL_933997, EPI_ISL_933998, EPI_ISL_933999, EPI_ISL_934000, EPI_ISL_934001, EPI_ISL_934002, EPI_ISL_934003, EPI_ISL_934004, EPI_ISL_934005, EPI_ISL_934006, EPI_ISL_934007, EPI_ISL_934008, EPI_ISL_934009, EPI_ISL_934010, EPI_ISL_934011, EPI_ISL_934012, EPI_ISL_934013, EPI_ISL_934015, EPI_ISL_934016, EPI_ISL_934017, EPI_ISL_934018, EPI_ISL_934019, EPI_ISL_934020, EPI_ISL_934021, EPI_ISL_934022, EPI_ISL_934023, EPI_ISL_934024, EPI_ISL_934025, EPI_ISL_934026, EPI_ISL_934027, EPI_ISL_934028, EPI_ISL_934029, EPI_ISL_934030, EPI_ISL_934031, EPI_ISL_934032, EPI_ISL_934033, EPI_ISL_934034, EPI_ISL_934035, EPI_ISL_934036, EPI_ISL_934037, EPI_ISL_934038, EPI_ISL_934039, EPI_ISL_934040, EPI_ISL_934041, EPI_ISL_934042, EPI_ISL_934043, EPI_ISL_934044, EPI_ISL_934045, EPI_ISL_934046, EPI_ISL_934047, EPI_ISL_934048, EPI_ISL_934049, EPI_ISL_934050, EPI_ISL_934051, EPI_ISL_934052, EPI_ISL_934053, EPI_ISL_934054, EPI_ISL_934055, EPI_ISL_934056, EPI_ISL_934057, EPI_ISL_934058, EPI_ISL_934059, EPI_ISL_934060			
see above	Vilnius university hospital Santaros Klinikos, Center of Laboratory Medicine	Vilnius university hospital Santaros Klinikos, Center of Laboratory Medicine	Ingrida Olendraite, Daniel Naumovas, Rimvydas Norvilas, Dovile Ezerskyte, Justinas Slikas, Gytis Dudas
EPI_ISL_935241	KU Leuven, Rega Institute, Clinical and Epidemiological Virology	KU Leuven, Rega Institute, Clinical and Epidemiological Virology	Tony Wawina-Bokalanga, Bert Vanmechelen, Joan Marti-Carerras, Piet Maes
EPI_ISL_936639, EPI_ISL_936640, EPI_ISL_936641	Northwestern Memorial Hospital	Ozer Lab	Ramon Lorenzo-Redondo, Lacy M. Simons, Chad J. Achenbach, Lawrence J. Jennings, Michael G. Ison, Judd F. Hultquist, Egon A. Ozer
EPI_ISL_937338, EPI_ISL_937341, EPI_ISL_937342	Utah Public Health Laboratory	Utah Public Health Laboratory	Erin L. Young, Kelly F. Oakeson, Tara Gallagher
EPI_ISL_940122, EPI_ISL_940123, EPI_ISL_940128, EPI_ISL_940134	Charlotte Maxeke Johannesburg Academic Hospital, National Health Laboratory Services, Gauteng, South Africa	National Institute for Communicable Diseases of the National Health Laboratory Service	Amoako DG, Mohale T, Ntuli N, Mahlangu B, Allam M, Ismail A, Bhiman JN
EPI_ISL_940825, EPI_ISL_940826, EPI_ISL_940832	Virginia DCLS	Virginia DCLS	Virginia DCLS
EPI_ISL_940858	Vaccines and Infectious Diseases Analytics Research Unit (VIDA)	KRISP, KZN Research Innovation and Sequencing Platform	Baillie Vicky, du Plessis Jeanine, Giandhari Jennifer, Pillay Sureshnee, Naidoo Yeshnee, Tegally Houriiyah, de Oliveira Tulio, Madhi Shabir
EPI_ISL_941240, EPI_ISL_941241, EPI_ISL_941242, EPI_ISL_941243, EPI_ISL_941244, EPI_ISL_941249, EPI_ISL_941250	Virginia DCLS	Virginia DCLS	Virginia DCLS
EPI_ISL_942024, EPI_ISL_942025, EPI_ISL_942069, EPI_ISL_942170, EPI_ISL_942171, EPI_ISL_942172, EPI_ISL_942173, EPI_ISL_942174, EPI_ISL_942175, EPI_ISL_942176, EPI_ISL_942177, EPI_ISL_942178, EPI_ISL_942179, EPI_ISL_942180, EPI_ISL_942181, EPI_ISL_942182, EPI_ISL_942183, EPI_ISL_942184, EPI_ISL_942193, EPI_ISL_942194, EPI_ISL_942195, EPI_ISL_942196, EPI_ISL_942197, EPI_ISL_942198, EPI_ISL_942199, EPI_ISL_942200, EPI_ISL_942201, EPI_ISL_942202, EPI_ISL_942203, EPI_ISL_942206, EPI_ISL_942207, EPI_ISL_942208, EPI_ISL_942210, EPI_ISL_942212, EPI_ISL_942214, EPI_ISL_942316			
see above	Wisconsin State Laboratory of Hygiene Communicable Disease Division	Wisconsin State Laboratory of Hygiene Communicable Disease Division	Kelsey R. Florek, Abigail C. Shockey
EPI_ISL_942629, EPI_ISL_942630, EPI_ISL_942631, EPI_ISL_942632, EPI_ISL_942633, EPI_ISL_942634, EPI_ISL_942635, EPI_ISL_942636, EPI_ISL_942637, EPI_ISL_942638, EPI_ISL_942639, EPI_ISL_942640, EPI_ISL_942641, EPI_ISL_942642, EPI_ISL_942643, EPI_ISL_942644, EPI_ISL_942645, EPI_ISL_942646, EPI_ISL_942647, EPI_ISL_942648, EPI_ISL_942649, EPI_ISL_942650, EPI_ISL_942651, EPI_ISL_942652, EPI_ISL_942653, EPI_ISL_942654, EPI_ISL_942655, EPI_ISL_942656, EPI_ISL_942657, EPI_ISL_942660, EPI_ISL_942661			
see above	Gundersen Molecular Diagnostics Laboratory	Kabara Cancer Research Institute	Craig S. Richmond, Paraic A. Kenny
EPI_ISL_942963	General Hospital - Bitola	Research Center for Genetic Engineering and Biotechnology "Georgi D. Efremov" , Macedonian Academy of Sciences and Arts	Aleksandar J.Dimovski, Dijana Plasheska-Karanfilska, Predrag Noveski, Gjorgji Bozinovski, Milena Jakimovska
EPI_ISL_942964	General Hospital - Kumanovo	Research Center for Genetic Engineering and Biotechnology "Georgi D. Efremov" , Macedonian Academy of Sciences and Arts	Aleksandar J.Dimovski, Dijana Plasheska-Karanfilska, Predrag Noveski, Gjorgji Bozinovski, Milen
EPI_ISL_942965	General Hospital - Veles	Research Center for Genetic Engineering and Biotechnology "Georgi D. Efremov" , Macedonian Academy of Sciences and Arts	Aleksandar J.Dimovski, Dijana Plasheska-Karanfilska, Predrag Noveski, Gjorgji Bozinovski, Milena Jakimovska
EPI_ISL_943803, EPI_ISL_943804	Utah Public Health Laboratory	Utah Public Health Laboratory	Erin L. Young, Kelly F. Oakeson, Tara Gallagher
EPI_ISL_947293	Eijkman Institute for Molecular Biology, Ministry of Research and Technology/National Agency for Research and Innovation	Eijkman Institute for Molecular Biology, Ministry of Research and Technology/National Agency for Research and Innovation	Edison Johar, Frilasita A Yudhaputri, Hidayat Trimarsanto, Iskandar Adnan, Lydia V. Panggalo, Sukma Oktavianthi, Willy Agustine, Safarina G Malik, Khin Saw Myint, Amin Soebandrio
EPI_ISL_949392, EPI_ISL_949393, EPI_ISL_949394, EPI_ISL_949395, EPI_ISL_949399, EPI_ISL_949400, EPI_ISL_949401	University of Birmingham	COVID-19 Genomics UK (COG-UK) Consortium	Institute of Microbiology, University of Birmingham: Claire McMurray, Joanne Stockton, Samuel Nicholls, Radoslaw Poplawski, Will Rowe, Josh Quick, Nicholas Loman. University of Birmingham Testing Laboratory: Celina M Whalley, Andrew Bosworth, Charlotte Poxon, Kasun Wanigasooriya, Oliver Pickles, Mike Kidd, Alex Richter, Andrew D Beggs PHE Heartlands Lab: Husam Osman, Andrew Bosworth. Queen Elizabeth Hospital: Anna Casey
EPI_ISL_949650	Queens Medical Centre, Clinical Microbiology Department / DeepSeq Nottingham	COVID-19 Genomics UK (COG-UK) Consortium	Gemma Clark, Wendy Smith, Manjinder Khakh, Vicki M Fleming, Michelle M Lister, Hannah Howson-Wells, Jonathan Ball, Patrick McClure, Joseph Chappell, Theocharis Tsoleridis, Nadine Holmes, Matthew Carlisle, Christopher Moore, Fei Sang, Johnny Debebe, Victoria Wright, Matthew Loose
EPI_ISL_949787	University College London, Great Ormond Street Hospital for Children NHS Foundation Trust, Imperial College Healthcare NHS Trust	COVID-19 Genomics UK (COG-UK) Consortium	Sergi Castellano, Rachel Williams, Mark Kristiansen, Paola Resende Silva, Sunando Roy, Tony Brooks, Helena Tutill, Paola Niola, Patricia Dyal, Charlotte Williams, Leysa Forrest, Yasmin Panchbhaya, Jacqueline Findlay, Samuel Weeks, Julianne Brown, Kathryn Harris, Paul Randell, James Price, Alison Holmes, Judith Breuer
EPI_ISL_950577, EPI_ISL_950587	Quadram Institute Bioscience	COVID-19 Genomics UK (COG-UK) Consortium	Dave J. Baker, Gemma L. Kay, Alp Aydin, Thanh Le-Viet, Steven Rudder, Ana P. Tedim, Anastasia Kolyva, Maria Diaz, Leonardo de Oliveira Martins, Nabil-Fareed Alikhan, Lizzie Meadows, Rachael Stanley, Ngozi Elumogo, Muhammed Yasir, Nicholas M. Thomson, Alexander J Trotter, Rachel Gilroy, Samuel Bloomfield, Claire Stuart, Andrew Bell, Reenesh Prakash, Samir Dervisevic, Alison E. Mather, John Wain, Mark Webber, Andrew J. Page, Justin O'Grady
EPI_ISL_954197	1.AO Universitaria 'S. Giovanni di Dio e Ruggi D'Aragona, Scuola Medica Salernitana' Hospital / 2.UOC di Virologia e Microbiologia, Università della Campania 'L. Vanvitelli' / 3.AO Universitaria 'Federico II' Napoli Hospital / 4.AORN 'San Giuseppe Moscati' Avellino Hospital / 5.AO 'San Pio - presidio G. Rummo' Benevento Hospital / 6.AO 'Sant'Anna e San Sebastiano' Caserta Hospital / 7.PO 'Maria Santissima Addolorata' Eboli Hospital / 8.Biogem Istituto di Ricerche Genetiche	1. Genome Research Center for Health (CRGS) / 2. Laboratory of Molecular Medicine and Genomics(LMMGe) / 3. Center for Research in Pure and Applied Mathematics (CRMPA)	Giorgio Giurato, Francesca Rizzo, Alessandro Weisz, Gianluigi Franci, Giovanni Nassa, Pasquale Pagliano, Roberta Tarallo, Elena Alexandrova, Ylenia D'Agostino, Carlo Ferravante, Jessica Lamberti, Viola Melone, Domenico Memoli, Valeria Mirici Cappa, Domenico Palumbo, Giovanni Pecoraro, Assunta Sellitto, Oriana Strianese, Ilaria Terenzi, Giuseppe Fenza, Aniello Gentile, Antonello Saccomanno, Sonia Amabile, Teresa Rocco, Annamaria Salvati, Emilia Vaccaro, Massimiliano Galdiero, Michele Cennamo, Giuseppe Portella, Maria Grazia Foti, Mariarosaria Ingino, Maria Landi, Maurizio Fumi, Vincenzo Rocco, Rita Greco, Vittoria Letizia, Arnolfo Petruzziello, Maddalena Schioppa, Gregorio Goffredi, Francesca Marciano, Michele Caraglia, Alessia Cossu, Marianna Scrima, Edmondo Adorisio, Morena D'Avenia, Michela Iacobellis, Rosanna Piluscio, Giorgio Dirani, Vittorio Sambri, Simona Semprini, Silvia Zanoli, Francesco Curcio, Stefania Marzinotto, Andreina Baj, Fausto Sessa.
EPI_ISL_955336	GA Department of Public Health Laboratory	Pathogen Discovery, Respiratory Viruses Branch, Division of Viral Diseases, Centers for Disease Control and Prevention	Ying Tao, Jing Zhang, Yan Li, Krista Queen, Anna Uehara, Peter Cook, Clinton R. Paden, Haibin Wang, Suxiang Tong
EPI_ISL_955353, EPI_ISL_955354, EPI_ISL_955355, EPI_ISL_955356, EPI_ISL_955357, EPI_ISL_955358, EPI_ISL_955359, EPI_ISL_955360, EPI_ISL_955361, EPI_ISL_955371, EPI_ISL_955372, EPI_ISL_955375, EPI_ISL_955376, EPI_ISL_955377			
see above	Santa Clara County Public Health Laboratory	Chan-Zuckerberg Biohub	CZB Cliahub Consortium

EPI_ISL_955633, EPI_ISL_955634, EPI_ISL_955635, EPI_ISL_955636, EPI_ISL_955637, EPI_ISL_955638, EPI_ISL_955639, EPI_ISL_955640, EPI_ISL_955641, EPI_ISL_955642, EPI_ISL_955643, EPI_ISL_955644, EPI_ISL_955645, EPI_ISL_955646, EPI_ISL_955647, EPI_ISL_955648, EPI_ISL_955649, EPI_ISL_955650, EPI_ISL_955651, EPI_ISL_955652, EPI_ISL_955653, EPI_ISL_955654, EPI_ISL_955655, EPI_ISL_955656, EPI_ISL_955657, EPI_ISL_955658, EPI_ISL_955659, EPI_ISL_955660, EPI_ISL_955661, EPI_ISL_955662, EPI_ISL_955663, EPI_ISL_955664, EPI_ISL_955666, EPI_ISL_955670			
see above	Orange County Public Health Lab	Chan-Zuckerberg Biohub	CZB Cliahub Consortium
EPI_ISL_960394	University of Wisconsin-Madison AIDS Vaccine Research Laboratories	University of Wisconsin-Madison AIDS Vaccine Research Laboratories	Gage Moreno, Katarina Braun, et al. AIDS Vaccine Research Laboratories
EPI_ISL_960417	The National Institute of Public Health	State Veterinary Institute Prague	Nagy,A;Vecerova,J;Cernikova,L;Stara,M;Jirincova,H;Trnka,D
EPI_ISL_960446, EPI_ISL_960453, EPI_ISL_960474, EPI_ISL_960503, EPI_ISL_960504, EPI_ISL_960505, EPI_ISL_960506, EPI_ISL_960507, EPI_ISL_960508, EPI_ISL_960509, EPI_ISL_960510, EPI_ISL_960511, EPI_ISL_960512, EPI_ISL_960513, EPI_ISL_960514, EPI_ISL_960515, EPI_ISL_960570, EPI_ISL_960590, EPI_ISL_960593, EPI_ISL_960594, EPI_ISL_960595, EPI_ISL_960596, EPI_ISL_960597, EPI_ISL_960598, EPI_ISL_960599, EPI_ISL_960600, EPI_ISL_960601, EPI_ISL_960602, EPI_ISL_960603, EPI_ISL_960604, EPI_ISL_960605, EPI_ISL_960606			
see above	Istituto Zooprofilattico Sperimentale del Mezzogiorno	TIGEM	Patrizia Annunziata, Andrea Ballabio, Valentina Bouche, Davide Cacchiarelli, Pellegrino Cerino, Chiara Colantuono, Maria Concetta Cuomo, Denise Di Concilio, Lucio Di Filippo, Antonio Grimaldi, Antonio Limone, Anna Manfredi, Francesco Panariello, Biancamaria Pierri, Marcello Salvi
EPI_ISL_961666	Hôpital Georges L. Dumont	National Microbiology Laboratory (NML)	Anna Majer, Shari Tyson, Grace Seo, Philip Mabon, Elsie Grudeski, Rhiannon Huzarewich, Russell Mandes, Anneliese Landgraff, Jennifer Tanner, Natalie Knox, Morag Graham, Gary Van Domselaar, Richard Garceau, Guillaume Desnoyers, Nathalie Bastien, Yan Li, Timothy Booth, Darian Hole, Madison Chapel, Kirsten Biggar, CanCOGeN's metadata curation team, Public Health Agency of Canada CanCOGeN team
EPI_ISL_961893, EPI_ISL_961928, EPI_ISL_961929, EPI_ISL_961973, EPI_ISL_961984, EPI_ISL_962015, EPI_ISL_962067, EPI_ISL_962068, EPI_ISL_962069, EPI_ISL_962070, EPI_ISL_962071, EPI_ISL_962072, EPI_ISL_962073, EPI_ISL_962074, EPI_ISL_962075, EPI_ISL_962076, EPI_ISL_962077, EPI_ISL_962078, EPI_ISL_962079, EPI_ISL_962082, EPI_ISL_962084, EPI_ISL_962091, EPI_ISL_962158, EPI_ISL_962159			
see above	Illinois Department of Public Health	Gagnon Lab, Southern Illinois University	Keith Gagnon
EPI_ISL_962506	UCLA Clinical Micro Lab	Los Angeles County PHL	P. Hemarajata et al.
EPI_ISL_962610, EPI_ISL_962768, EPI_ISL_962770, EPI_ISL_962776, EPI_ISL_962777, EPI_ISL_962783, EPI_ISL_962784, EPI_ISL_962787, EPI_ISL_962790, EPI_ISL_962795, EPI_ISL_962799, EPI_ISL_962806			
see above	San Diego County Public Health Laboratory	Andersen lab at Scripps Research	SEARCH Alliance San Diego with Tracy Basler, Jovan Shephard, Brett Austin
EPI_ISL_962970	Hospital Universitario de Gran Canaria Dr. Negrín	SeqCOVID-SPAIN consortium/IBV(CSIC)	M. Carmen Pérez González, Francisco J. Chamizo López, Ana Bordes Benítez and SeqCOVID-SPAIN consortium
EPI_ISL_965544, EPI_ISL_965574, EPI_ISL_965586, EPI_ISL_965610, EPI_ISL_965616, EPI_ISL_965626, EPI_ISL_965636, EPI_ISL_965647, EPI_ISL_965681, EPI_ISL_965696, EPI_ISL_965725, EPI_ISL_965730, EPI_ISL_965782, EPI_ISL_965807, EPI_ISL_965808			
see above	Dutch COVID-19 response team	Medical Microbiology, Maastricht University Medical Centre	Jozef Dingemans*, Brian van der Veer*, Erik Beuken, Carmen Reumkens, Lieke van Alphen, Christian Hoebe, Paul Savelkoul
EPI_ISL_967551, EPI_ISL_967552, EPI_ISL_967553, EPI_ISL_967555, EPI_ISL_967556	State Laboratories Division, Hawaii State Department of Health	State Laboratories Division, Hawaii State Department of Health	Pamela O'Brien, Drew Kuwazaki, Ayana Garnet, Razvan Sultana, Edward Desmond
EPI_ISL_968807, EPI_ISL_968813, EPI_ISL_968817, EPI_ISL_968819, EPI_ISL_968820, EPI_ISL_968821, EPI_ISL_968831	KEMRI-Wellcome Trust Research Programme/KEMRI-CGMR-C Kilifi	KEMRI-Wellcome Trust Research Programme/KEMRI-CGMR-C Kilifi	Githinji et al
EPI_ISL_970879, EPI_ISL_971006, EPI_ISL_971143, EPI_ISL_971345, EPI_ISL_971811, EPI_ISL_972238, EPI_ISL_972410, EPI_ISL_972624, EPI_ISL_973276, EPI_ISL_973514	Department of Virus and Microbiological Special Diagnostics, Statens Serum Institut, Copenhagen, Denmark	Aalborg University	Danish Covid-19 Genome Consortium
EPI_ISL_976176, EPI_ISL_976177, EPI_ISL_976178, EPI_ISL_976179, EPI_ISL_976180, EPI_ISL_976181, EPI_ISL_976182, EPI_ISL_976183, EPI_ISL_976184, EPI_ISL_976185, EPI_ISL_976186, EPI_ISL_976187, EPI_ISL_976188, EPI_ISL_976189, EPI_ISL_976190, EPI_ISL_976191, EPI_ISL_976192, EPI_ISL_976193, EPI_ISL_976194, EPI_ISL_976195, EPI_ISL_976196, EPI_ISL_976197, EPI_ISL_976198, EPI_ISL_976199, EPI_ISL_976200, EPI_ISL_976201, EPI_ISL_976202, EPI_ISL_976203, EPI_ISL_976204, EPI_ISL_976205, EPI_ISL_976206, EPI_ISL_976207, EPI_ISL_976208, EPI_ISL_976209, EPI_ISL_976210, EPI_ISL_976211, EPI_ISL_976212, EPI_ISL_976213, EPI_ISL_976214, EPI_ISL_976215, EPI_ISL_976216, EPI_ISL_976217, EPI_ISL_976218, EPI_ISL_976219, EPI_ISL_976220, EPI_ISL_976221, EPI_ISL_976222, EPI_ISL_976223, EPI_ISL_976224, EPI_ISL_976225, EPI_ISL_976226, EPI_ISL_976227, EPI_ISL_976228, EPI_ISL_976229, EPI_ISL_976230, EPI_ISL_976231, EPI_ISL_976232, EPI_ISL_976233, EPI_ISL_976234, EPI_ISL_976235, EPI_ISL_976236, EPI_ISL_976237, EPI_ISL_976238, EPI_ISL_976239, EPI_ISL_976240, EPI_ISL_976241, EPI_ISL_976242, EPI_ISL_976243, EPI_ISL_976244, EPI_ISL_976245, EPI_ISL_976246, EPI_ISL_976247, EPI_ISL_976248, EPI_ISL_976249, EPI_ISL_976250, EPI_ISL_976251, EPI_ISL_976252, EPI_ISL_976253, EPI_ISL_976254, EPI_ISL_976255, EPI_ISL_976256, EPI_ISL_976257, EPI_ISL_976258, EPI_ISL_976259, EPI_ISL_976260, EPI_ISL_976261, EPI_ISL_976262, EPI_ISL_976263, EPI_ISL_976264, EPI_ISL_976265, EPI_ISL_976266, EPI_ISL_976267, EPI_ISL_976268, EPI_ISL_976269, EPI_ISL_976270, EPI_ISL_976271, EPI_ISL_976272, EPI_ISL_976273, EPI_ISL_976274, EPI_ISL_976275, EPI_ISL_976276, EPI_ISL_976277, EPI_ISL_976278, EPI_ISL_976279, EPI_ISL_976280, EPI_ISL_976281, EPI_ISL_976282, EPI_ISL_976283, EPI_ISL_976284, EPI_ISL_976285, EPI_ISL_976286, EPI_ISL_976287, EPI_ISL_976288, EPI_ISL_976289, EPI_ISL_976290, EPI_ISL_976291, EPI_ISL_976292, EPI_ISL_976293, EPI_ISL_976294, EPI_ISL_976295, EPI_ISL_976296, EPI_ISL_976297, EPI_ISL_976298, EPI_ISL_976299, EPI_ISL_976300			
see above	BCCDC Public Health Laboratory	BCCDC Public Health Laboratory	Prystajecky Natalie, Linda Hoang, Dan Fornika, John Tyson, Shannon Russell, Kim Macdonald, Kimia Kamelian, Ana Pacagnella, Corrinne Ng, Loretta Janz, Robert Azana Terry Snutch, Mel Krajden
EPI_ISL_977024, EPI_ISL_977025, EPI_ISL_977026, EPI_ISL_977027	Rhode Island Department of Health	Infectious Disease Program, Broad Institute of Harvard and MIT	Lemieux,J.E., Siddle,K.J., Huard,R., King,E., Azevedo,K., Miller,A., Adams,G., Gladden-Young,A., Lagerborg,K., Rudy,M., DeRuff,K., Carter,A., Normandin,E., Bauer,M., Reilly,S., Tomkins-Tinch,C., Loreth,C., Chaluvadi,S., Birren,B.W., Gallagher,G., Smole,S., Park,D.J., MacInnis,B.L., and Sabeti,P.C.
EPI_ISL_977200, EPI_ISL_977201, EPI_ISL_977202	ULSS 7 Pedemontana - Distretto 2	Istituto Zooprofilattico Sperimentale delle Venezie	Adelaide Milani, Alessia Schivo, Annalisa Salviato, Erika Giorgia Quaranta, Ambra Pastori, Bianca Zecchin, Alice Fusaro, Isabella Monne, Calogero Terregino, Antonia Ricci
EPI_ISL_977213, EPI_ISL_977214, EPI_ISL_977215, EPI_ISL_977216, EPI_ISL_977217, EPI_ISL_977218, EPI_ISL_977219, EPI_ISL_977220, EPI_ISL_977221	ULSS 1 Dolomiti	Istituto Zooprofilattico Sperimentale delle Venezie	Adelaide Milani, Alessia Schivo, Annalisa Salviato, Erika Giorgia Quaranta, Ambra Pastori, Bianca Zecchin, Alice Fusaro, Isabella Monne, Calogero Terregino, Antonia Ricci
EPI_ISL_978194, EPI_ISL_978195, EPI_ISL_978196, EPI_ISL_978197, EPI_ISL_978214	Virginia Division of Consolidated Laboratory Services	Virginia Division of Consolidated Laboratory Services	Virginia DCLS
EPI_ISL_978989, EPI_ISL_978990, EPI_ISL_978991, EPI_ISL_978992, EPI_ISL_978993, EPI_ISL_978994, EPI_ISL_978995, EPI_ISL_978996, EPI_ISL_978997, EPI_ISL_978998, EPI_ISL_978999, EPI_ISL_979000, EPI_ISL_979001, EPI_ISL_979002, EPI_ISL_979003, EPI_ISL_979004, EPI_ISL_979005, EPI_ISL_979006, EPI_ISL_979007, EPI_ISL_979008, EPI_ISL_979009, EPI_ISL_979010, EPI_ISL_979011, EPI_ISL_979012, EPI_ISL_979013, EPI_ISL_979014, EPI_ISL_979015, EPI_ISL_979016, EPI_ISL_979019, EPI_ISL_979020, EPI_ISL_979021, EPI_ISL_979022, EPI_ISL_979023, EPI_ISL_979024, EPI_ISL_979025, EPI_ISL_979026, EPI_ISL_979027, EPI_ISL_979028, EPI_ISL_979029, EPI_ISL_979030, EPI_ISL_979031, EPI_ISL_979032, EPI_ISL_979033, EPI_ISL_979034, EPI_ISL_979035, EPI_ISL_979036, EPI_ISL_979037, EPI_ISL_979038, EPI_ISL_979039, EPI_ISL_979040, EPI_ISL_979041, EPI_ISL_979042, EPI_ISL_979043, EPI_ISL_979044, EPI_ISL_979045, EPI_ISL_979046, EPI_ISL_979047, EPI_ISL_979048, EPI_ISL_979049, EPI_ISL_979050, EPI_ISL_979051, EPI_ISL_979052, EPI_ISL_979053, EPI_ISL_979054, EPI_ISL_979055			
see above	Santa Clara County Public Health Laboratory	Chan-Zuckerberg Biohub	CZB Cliahub Consortium
EPI_ISL_979310	Cadham Provincial laboratory	National Microbiology Laboratory (NML)	Anna Majer, Shari Tyson, Grace Seo, Philip Mabon, Elsie Grudeski, Rhiannon Huzarewich, Russell Mandes, Anneliese Landgraff, Jennifer Tanner, Natalie Knox, Morag Graham, Gary Van Domselaar, Paul Van Caeseele, Jared Bullard, David Alexander, Kerry Dust, Nathalie Bastien, Yan Li, Timothy Booth, Darian Hole, Madison Chapel, Kirsten Biggar, CanCOGeN's metadata curation team, Public Health Agency of Canada CanCOGeN team
EPI_ISL_979545, EPI_ISL_979546, EPI_ISL_979547, EPI_ISL_979548, EPI_ISL_979549, EPI_ISL_979550, EPI_ISL_979551, EPI_ISL_979552, EPI_ISL_979553, EPI_ISL_979554, EPI_ISL_979555, EPI_ISL_979557, EPI_ISL_979558, EPI_ISL_979559, EPI_ISL_979560, EPI_ISL_979561, EPI_ISL_979562, EPI_ISL_979563, EPI_ISL_979564, EPI_ISL_979565, EPI_ISL_979566, EPI_ISL_979567, EPI_ISL_979568, EPI_ISL_979569, EPI_ISL_979570, EPI_ISL_979571, EPI_ISL_979572, EPI_ISL_979573, EPI_ISL_979574, EPI_ISL_979575, EPI_ISL_979576, EPI_ISL_979577, EPI_ISL_979578, EPI_ISL_979579, EPI_ISL_979580, EPI_ISL_979581, EPI_ISL_979582, EPI_ISL_979583, EPI_ISL_979584, EPI_ISL_979585, EPI_ISL_979586, EPI_ISL_979587, EPI_ISL_979588, EPI_ISL_979589, EPI_ISL_979590, EPI_ISL_979591, EPI_ISL_979592, EPI_ISL_979593			
see above	Santa Clara County Public Health Laboratory	Chan-Zuckerberg Biohub	CZB Cliahub Consortium
EPI_ISL_979756, EPI_ISL_979757, EPI_ISL_979758, EPI_ISL_979759, EPI_ISL_979760, EPI_ISL_979761, EPI_ISL_979762, EPI_ISL_979763, EPI_ISL_979764, EPI_ISL_979765, EPI_ISL_979766, EPI_ISL_979767, EPI_ISL_979768, EPI_ISL_979769, EPI_ISL_979770, EPI_ISL_979771, EPI_ISL_979772, EPI_ISL_979773, EPI_ISL_979774, EPI_ISL_979775, EPI_ISL_979776, EPI_ISL_979777, EPI_ISL_979778, EPI_ISL_979779, EPI_ISL_979780, EPI_ISL_979781, EPI_ISL_979782, EPI_ISL_979783, EPI_ISL_979784, EPI_ISL_979785, EPI_ISL_979786, EPI_ISL_979787, EPI_ISL_979788, EPI_ISL_979789, EPI_ISL_979790, EPI_ISL_979791, EPI_ISL_979792, EPI_ISL_979793, EPI_ISL_979794, EPI_ISL_979795, EPI_ISL_979796			
see above	Humboldt County Public Health Laboratory	Chan-Zuckerberg Biohub	CZB Cliahub Consortium

EPI_ISL_981006	AZ Klina	AZ Klina	Dr. C. Vael
EPI_ISL_981882, EPI_ISL_981888, EPI_ISL_981889, EPI_ISL_981890, EPI_ISL_981891, EPI_ISL_981892, EPI_ISL_981893, EPI_ISL_981894, EPI_ISL_981895	Microbiology Service, Hospital Universitario Clinico San Cecilio, Granada	Microbiology Service, Hospital Universitario Clinico San Cecilio, Granada	Adolfo de Salazar, Natalia Chueca, Laura Viñuela, Ana Fuentes, Federico García
EPI_ISL_982585	Landstuhl Regional Medical Center	US Air Force School of Aerospace Medicine	Anthony Fries, Jennifer Meyer, William Gruner, William Buggele, Amanda Javorina, Sarah Purves, Fritz Castillo, Cole Anderson, Clarise Starr, Elizabeth Macias
EPI_ISL_982843	Kentucky State Public Health Lab	Kentucky State Public Health Lab	Stephanie Lunn, Karim George, Joshua Tobias, William Grooms, Vaneet Arora, Matthew Johnson, Rachel Zinner, Rhonda Lucas
