# Supplementary material for: Detection and characterization of the SARS-CoV-2 lineage B.1.526 in New York
Source: Nat Commun. 2021 Aug 9;12:4886. doi: 10.1038/s41467-021-25168-4 (PMC8352861; doi:10.1038/s41467-021-25168-4)
Supplement: Supplementary file 8 — Supplementary Data 4 [file 41467_2021_25168_MOESM8_ESM.zip › GISAID_acknowledements_tables/gisaid_hcov-19_acknowledgement_table_2021_02_13_010-4.pdf]

We gratefully acknowledge the following Authors from the Originating laboratories responsible for obtaining the specimens, as well as the Submitting laboratories where the genome data were generated and shared via GISAID, on which this research is based.

All Submitters of data may be contacted directly via www.gisaid.org

Authors are sorted alphabetically.

Accession ID	Originating Laboratory	Submitting Laboratory	Authors
EPI_ISL_745029, EPI_ISL_745030, EPI_ISL_745031	Area of Virology, Serology and Virology Division (SAViD), New South Wales Health Pathology Randwick	Area of Virology, Serology and Virology Division (SAViD), New South Wales Health Pathology Randwick	Rawlinson, W.
EPI_ISL_752599	SA Pathology	SA Pathology	Lex Leong, Julien Soubrier, Chuan Kok Lim, Song Gao, Mark Turra, Karin Kassahn, Ivan Bastian, Geoff Higgins
EPI_ISL_754302	Respiratory Virus Unit, National Infection Service, Public Health England	COVID-19 Genomics UK (COG-UK) Consortium	PHE Covid Sequencing Team
EPI_ISL_755574	Center of Advanced Studies and Technology, CAST	Center of Advanced Studies and Technology, CAST	Ferrante,R., Mandatori,D., De Fabritiis,S.
EPI_ISL_755632, EPI_ISL_755633	LabPLUS	Institute of Environmental Science and Research (ESR)	Xiaoyun Ren, Matt Storey, Nikki Freed, Muhammad Faisal, Jing Wang, Hermes Perez, Anja Werno, Antje van der Linden, Arlo Upton, Chris Mansell, David Hammer, Dragana Drinkovic, Gary McAuliffe, Hana Sofia Andersson, James Ussher, Jill Sherwood, Josh Freeman, Julia Howard, Juliet Elvy, Mary DeAlmeida, Matt Blakiston, Matthew Rogers, Max Bloomfield, Michael Addidle, Michelle Balm, Sally Roberts, Sarah Jefferies, Sharmini Muttaiyah, Susan Morpeth, Susan Taylor, Timothy Blackmore, Vani Sathyendran, Veronica Playle, Virginia Hope, Erasmus Smit, Lauren Jelly, Olin Silander, Joep de Ligt
EPI_ISL_755635, EPI_ISL_755636	Middlemore Hospital	Institute of Environmental Science and Research (ESR)	Xiaoyun Ren, Matt Storey, Nikki Freed, Muhammad Faisal, Jing Wang, Hermes Perez, Anja Werno, Antje van der Linden, Arlo Upton, Chris Mansell, David Hammer, Dragana Drinkovic, Gary McAuliffe, Hana Sofia Andersson, James Ussher, Jill Sherwood, Josh Freeman, Julia Howard, Juliet Elvy, Mary DeAlmeida, Matt Blakiston, Matthew Rogers, Max Bloomfield, Michael Addidle, Michelle Balm, Sally Roberts, Sarah Jefferies, Sharmini Muttaiyah, Susan Morpeth, Susan Taylor, Timothy Blackmore, Vani Sathyendran, Veronica Playle, Virginia Hope, Erasmus Smit, Lauren Jelly, Olin Silander, Joep de Ligt
EPI_ISL_756274	Hospital General Universitario Gregorio Marañón	Hospital General Universitario Gregorio Marañón	Sergio Buenestado Serrano, Pedro Sola Campoy, Pilar Catalán, Patricia Muñoz, Darío García de Viedma.
EPI_ISL_756375	Siriraj Hospital, Mahidol University	National Institute of Health, Department of Medical Sciences, Ministry of Public Health, Thailand	Pilailuk Okada; Siripaporn Phuygun; Sittiporn Parnmen; Navin Horthongkham; Ratana Tacharoenmuang; Pakorn Piromtong; Natchaya Khiadsang; Thanutsapa Thanadachakul; Warawan Wongboot; Sirikanda wimol; Sunthareeya Waicharoen; Niracha Athipanyasilp; Archiraya Pattama; Chutikarn Chaimayo; Ruengpung Sutthent; Wannee Kantakamalakul.
EPI_ISL_757286	Department of Virology, Public Health Laboratories Division	Department of Virology, Public Health Laboratories Division	Massab Umair, Aamer Ikram, Muhammad Salman
EPI_ISL_759723	Instituto Nacional de Saude (INSA)	Instituto Nacional de Saude (INSA)	Borges et al
EPI_ISL_759724, EPI_ISL_759741, EPI_ISL_759743, EPI_ISL_759745, EPI_ISL_759746, EPI_ISL_759747, EPI_ISL_759748, EPI_ISL_759749, EPI_ISL_759750, EPI_ISL_759751	University of Wisconsin-Madison AIDS Vaccine Research Laboratories	University of Wisconsin-Madison AIDS Vaccine Research Laboratories	Gage Moreno, Katarina Braun, et al. AIDS Vaccine Research Laboratories
EPI_ISL_763310	Dutch COVID-19 response team	Erasmus Medical Center	Bas Oude Munnink, Reina Sikkema, David Nieuwenhuijse, Irina Chestakova, Anne van der Linden, Marjan Boter, Emmanuelle Munger, Corine GeurtsvanKessel, Annemiek van der Eijk, Richard Molenkamp, Marion Koopmans, on behalf of the Dutch national COVID-19 response team.
EPI_ISL_763888, EPI_ISL_764141	Department of Pathology, University of Cambridge	COVID-19 Genomics UK (COG-UK) Consortium	Aminu S. Jahun, Yasmin Chaudhry, Grant Hall, Iliana Georgana, Myra Hosmillo, Martin D. Curran, Malte Pinckert, Surendra Parmar, Ian Goodfellow
EPI_ISL_764231	Virology Department, Sheffield Teaching Hospitals NHS Foundation Trust/Department of Infection, Immunity and Cardiovascular Disease, The Medical School, University of Sheffield	COVID-19 Genomics UK (COG-UK) Consortium	Thushan de Silva, Matthew Parker, Nikki Smith, Adri Angyal, Rebecca Brown, Luke Green, Rachel Tucker, Paul Parsons, Danielle Groves, Katie Johnson, Laura Carrilero, Alex Keeley, Dave Partridge, Matthew Wyles, Benjamin Lindsey, Mehmet Yavuz, Mohammad Raza, Cariad Evans
EPI_ISL_764354	Department of Pathology, University of Cambridge	COVID-19 Genomics UK (COG-UK) Consortium	Aminu S. Jahun, Yasmin Chaudhry, Grant Hall, Iliana Georgana, Myra Hosmillo, Martin D. Curran, Malte Pinckert, Surendra Parmar, Ian Goodfellow
EPI_ISL_765571	National Institute for Infectious Diseases, INMI, "L. Spallanzani" IRCCS	National Institute for Infectious Diseases, INMI, "L. Spallanzani" IRCCS	E Giombini, B Bartolini, O Butera, C.E.M Gruber, M Rueca, F Messina, MR Capobianchi, A Di Caro
EPI_ISL_766022, EPI_ISL_766023, EPI_ISL_766027	Department of Virology and Immunology, University of Helsinki and Helsinki University Hospital, Huslab Finland	Department of Virology, Faculty of Medicine, University of Helsinki, Helsinki, Finland	Teemu Smura, Olli Vapalahti, Maija Lappalainen, Satu Kurkela
EPI_ISL_766587	Analytica Medizinische Laboratorien AG	Institute of Medical Virology, University of Zurich	Stefan Schmutz, Maryam Zaheri, Verena Kufner, Annette Audigé, Maria Grünberg, Kevin Steiner, Jon Huder, Cyril Shah, Riccarda Capaul, Jürg Böni, Michael Huber, Alexandra Trkola
EPI_ISL_766697	Klinisk mikrobiologi, bakteriologi	The Public Health Agency of Sweden	Department of Microbiology, The Public Health Agency of Sweden
EPI_ISL_766699	A05 Biomedicum	The Public Health Agency of Sweden	Department of Microbiology, The Public Health Agency of Sweden
EPI_ISL_766714	Klinisk mikrobiologi	The Public Health Agency of Sweden	Department of Microbiology, The Public Health Agency of Sweden
EPI_ISL_766718, EPI_ISL_766719	A05 Biomedicum	The Public Health Agency of Sweden	Department of Microbiology, The Public Health Agency of Sweden
EPI_ISL_766938	Nevada State Health Lab	Nevada State Health Lab	Andrew Gorzalski
EPI_ISL_767013	University of Bari Biomedical Sciences and Human Oncology	University of Bari Biomedical Sciences and Human Oncology	Maria Chironna, Anna Sallustio, Daniela Loconsole, Marisa Accogli
EPI_ISL_767044, EPI_ISL_767047, EPI_ISL_767048, EPI_ISL_767050, EPI_ISL_767064, EPI_ISL_767065, EPI_ISL_767066, EPI_ISL_767068, EPI_ISL_767069, EPI_ISL_767073, EPI_ISL_767074, EPI_ISL_767075, EPI_ISL_767076, EPI_ISL_767077, EPI_ISL_767078, EPI_ISL_767079, EPI_ISL_767080, EPI_ISL_767081, EPI_ISL_767082, EPI_ISL_767083, EPI_ISL_767084, EPI_ISL_767085, EPI_ISL_767086, EPI_ISL_767087, EPI_ISL_767088			
see above	New Mexico Department of Health Scientific Laboratory	New Mexico Department of Health Scientific Laboratory	D'eldra Malone, Ellie Johnson, Anastacia Griego-Fisher
EPI_ISL_767613	SARATOGA HOSPITAL LABORATORY	Wadsworth Center, New York State Department.of Health	Kirsten St. George, Daryl M. Lamson, Alexis Russel, Matthew Shudt, Melissa A Leisner, Jonathan Plitnick, Navjot Singh, John Kelly, Sara Griesemer, Erasmus Schneider, Erica Lasek-Nesselquist
EPI_ISL_767902	Australian Clinical Labs	NSW Health Pathology - Institute of Clinical Pathology and Medical Research; Westmead Hospital; University of Sydney	CIDM-PH et al.
EPI_ISL_767903	Laverty Pathology	NSW Health Pathology - Institute of Clinical Pathology and Medical Research; Westmead Hospital; University of Sydney	CIDM-PH et al.
EPI_ISL_767905	Sydney South West Pathology Service (SSWPS) - Liverpool Hospital - NSW Health Pathology	NSW Health Pathology - Institute of Clinical Pathology and Medical Research; Westmead Hospital; University of Sydney	CIDM-PH et al.
EPI_ISL_767907	Sydney South West Pathology Service (SSWPS) - Royal Prince Alfred Hospital - NSW Health Pathology	NSW Health Pathology - Institute of Clinical Pathology and Medical Research; Westmead Hospital; University of Sydney	CIDM-PH et al.
EPI_ISL_767916, EPI_ISL_767917, EPI_ISL_767918	Australian Clinical Labs	NSW Health Pathology - Institute of Clinical Pathology and Medical Research; Westmead Hospital; University of Sydney	CIDM-PH et al.

EPI_ISL_767919	Laverty Pathology	NSW Health Pathology - Institute of Clinical Pathology and Medical Research; Westmead Hospital; University of Sydney	CIDM-PH et al.
EPI_ISL_767920	Pathology North - Hunter - NSW Health Pathology	NSW Health Pathology - Institute of Clinical Pathology and Medical Research; Westmead Hospital; University of Sydney	CIDM-PH et al.
EPI_ISL_767923, EPI_ISL_767924	South Eastern Area Laboratory Services (SEALS)	NSW Health Pathology - Institute of Clinical Pathology and Medical Research; Westmead Hospital; University of Sydney	CIDM-PH et al.
EPI_ISL_767925, EPI_ISL_767926	Histopath	NSW Health Pathology - Institute of Clinical Pathology and Medical Research; Westmead Hospital; University of Sydney	CIDM-PH et al.
EPI_ISL_767929	Southern. IML Pathology	NSW Health Pathology - Institute of Clinical Pathology and Medical Research; Westmead Hospital; University of Sydney	CIDM-PH et al.
EPI_ISL_767930	Sydney South West Pathology Service (SSWPS) - Royal Prince Alfred Hospital - NSW Health Pathology	NSW Health Pathology - Institute of Clinical Pathology and Medical Research; Westmead Hospital; University of Sydney	CIDM-PH et al.
EPI_ISL_768602	South Eastern Area Laboratory Services (SEALS)	NSW Health Pathology - Institute of Clinical Pathology and Medical Research; Westmead Hospital; University of Sydney	CIDM-PH et al.
EPI_ISL_768619, EPI_ISL_768622, EPI_ISL_768625, EPI_ISL_768626, EPI_ISL_768627	National Public Health Laboratory, National Centre for Infectious Diseases	National Public Health Laboratory, National Centre for Infectious Diseases	Tze Minn Mak, Sophie Octavia, Zhenyang Zhou, Lin Cui, Raymond Tzer Pin Lin
EPI_ISL_768824	Laboratoire BIOLAB33	CNR Virus des Infections Respiratoires - France SUD	Antonin Bal, Gregory Destras, Gwendolyne Burfin, Hadrien Règue, Quentin Semanas, Martine Valette, Bruno Lina, Laurence Josset
EPI_ISL_768829	Hôpital d'Instruction des Armées Sainte-Anne	CNR Virus des Infections Respiratoires - France SUD	Antonin Bal, Gregory Destras, Gwendolyne Burfin, Hadrien Règue, Quentin Semanas, Martine Valette, Bruno Lina, Laurence Josset
EPI_ISL_768830	Laboratoire BIOMED 05	CNR Virus des Infections Respiratoires - France SUD	Antonin Bal, Gregory Destras, Gwendolyne Burfin, Hadrien Règue, Quentin Semanas, Martine Valette, Bruno Lina, Laurence Josset
EPI_ISL_769917, EPI_ISL_769918, EPI_ISL_769941, EPI_ISL_769946, EPI_ISL_769950, EPI_ISL_769951, EPI_ISL_769952, EPI_ISL_769953, EPI_ISL_769954, EPI_ISL_769956, EPI_ISL_769957, EPI_ISL_769958, EPI_ISL_769971, EPI_ISL_769972, EPI_ISL_769973, EPI_ISL_769974, EPI_ISL_769975, EPI_ISL_769976, EPI_ISL_769977, EPI_ISL_769978, EPI_ISL_769979, EPI_ISL_769980, EPI_ISL_769981, EPI_ISL_769982, EPI_ISL_769983, EPI_ISL_769984, EPI_ISL_769985			
see above	Albany Medical Center Hospital Clinical Laboratories	Wadsworth Center, New York State Department.of Health	Kirsten St. George, Daryl M. Lamson, Alexis Russel, Matthew Shudt, Melissa A Leisner, Jonathan Plitnick, Navjot Singh, John Kelly, Sara Griesemer, Erasmus Schneider, Erica Lasek-Nesselquist
EPI_ISL_770442, EPI_ISL_770443, EPI_ISL_770444	Wyoming Public Health Laboratory	Wyoming Public Health Laboratory	Noah Hull, Taylor Fearing, Lynette Gumbleton, Channing Weber, Ashley Norberg, Bailey Bowcutt, and Wanda Manley
EPI_ISL_770462, EPI_ISL_770463, EPI_ISL_770464, EPI_ISL_770465, EPI_ISL_770466, EPI_ISL_770467, EPI_ISL_770468, EPI_ISL_770469	Respiratory Virus Unit, National Infection Service, Public Health England	COVID-19 Genomics UK (COG-UK) Consortium	PHE Covid Sequencing Team
EPI_ISL_770850, EPI_ISL_770851, EPI_ISL_770852, EPI_ISL_770855, EPI_ISL_770856, EPI_ISL_770858, EPI_ISL_770860, EPI_ISL_770868, EPI_ISL_770872, EPI_ISL_770881, EPI_ISL_770883, EPI_ISL_770889, EPI_ISL_770895, EPI_ISL_771051, EPI_ISL_771052, EPI_ISL_771060, EPI_ISL_771090, EPI_ISL_771092, EPI_ISL_771093, EPI_ISL_771095, EPI_ISL_771096, EPI_ISL_771112, EPI_ISL_771113, EPI_ISL_771114, EPI_ISL_771115, EPI_ISL_771116, EPI_ISL_771117, EPI_ISL_771118, EPI_ISL_771119, EPI_ISL_771120, EPI_ISL_771121, EPI_ISL_771122, EPI_ISL_771123, EPI_ISL_771124, EPI_ISL_771125, EPI_ISL_771126, EPI_ISL_771127, EPI_ISL_771133, EPI_ISL_771134, EPI_ISL_771141, EPI_ISL_771142, EPI_ISL_771143, EPI_ISL_771144, EPI_ISL_771145, EPI_ISL_771146			
see above	Laboratoire national de santé, Microbiology, Virology	Laboratoire national de santé, Microbiology, Microbial Genomics Platform	Anke Wienecke-Baldacchino, Catherine Ragimbeau,Jessica Tapp, Fatu Djabi, Lise Pignon, Raoul Salmon, Tamir Abdelrahman
EPI_ISL_775428, EPI_ISL_775429, EPI_ISL_775432, EPI_ISL_775433, EPI_ISL_775436	Department of Medical Microbiology - section Molde, Molde Hospital	Norwegian Institute of Public Health, Department of Virology	Kathrine Stene-Johansen, Kamilla Heddeland Instefjord, Hilde Elshaug, Atiya R Ali,Marie Paulsen Madsen, Rasmus Riis Kopperud, Hilde Vollan, Karoline Bragstad, Olav Hungnes
EPI_ISL_776569, EPI_ISL_776575, EPI_ISL_776590, EPI_ISL_776604, EPI_ISL_776609, EPI_ISL_776653, EPI_ISL_776654, EPI_ISL_776655, EPI_ISL_776656, EPI_ISL_776775, EPI_ISL_776776, EPI_ISL_776777, EPI_ISL_776778, EPI_ISL_776779, EPI_ISL_776780, EPI_ISL_776781, EPI_ISL_776782, EPI_ISL_776783, EPI_ISL_776784, EPI_ISL_776785, EPI_ISL_776786, EPI_ISL_776787, EPI_ISL_776788, EPI_ISL_776789, EPI_ISL_776790, EPI_ISL_776791, EPI_ISL_776792, EPI_ISL_776793, EPI_ISL_776794, EPI_ISL_776795, EPI_ISL_776796, EPI_ISL_776797, EPI_ISL_776798, EPI_ISL_776799, EPI_ISL_776800, EPI_ISL_776801, EPI_ISL_776802, EPI_ISL_776803, EPI_ISL_776804, EPI_ISL_776805, EPI_ISL_776806, EPI_ISL_776807, EPI_ISL_776808, EPI_ISL_776809, EPI_ISL_776810, EPI_ISL_776811, EPI_ISL_776812, EPI_ISL_776813, EPI_ISL_776814, EPI_ISL_776815, EPI_ISL_776816, EPI_ISL_776817, EPI_ISL_776818, EPI_ISL_776819, EPI_ISL_776820, EPI_ISL_776821, EPI_ISL_776822, EPI_ISL_776823, EPI_ISL_776824, EPI_ISL_776825, EPI_ISL_776826, EPI_ISL_776827, EPI_ISL_776828, EPI_ISL_776829, EPI_ISL_776830, EPI_ISL_776831, EPI_ISL_776832, EPI_ISL_776833, EPI_ISL_776834, EPI_ISL_776835, EPI_ISL_776836, EPI_ISL_776837, EPI_ISL_776838, EPI_ISL_776839, EPI_ISL_776840, EPI_ISL_776841, EPI_ISL_776842, EPI_ISL_776843, EPI_ISL_776844, EPI_ISL_776845, EPI_ISL_776846, EPI_ISL_776847, EPI_ISL_776848, EPI_ISL_776849, EPI_ISL_776850, EPI_ISL_776851, EPI_ISL_776852, EPI_ISL_776853, EPI_ISL_776854, EPI_ISL_776855, EPI_ISL_776856, EPI_ISL_776857, EPI_ISL_776858, EPI_ISL_776859, EPI_ISL_776860, EPI_ISL_776861, EPI_ISL_776862, EPI_ISL_776863, EPI_ISL_776864, EPI_ISL_776865, EPI_ISL_776866, EPI_ISL_776867, EPI_ISL_776868			
see above	Israel Central Virology laboratory	Israel Central Virology laboratory	Neta Zuckerman, Efrat Dahan Bucris, Oran Erster, Michal Mandelboim, Orna Mor, Ella Mendelson
EPI_ISL_778821	AIID	Irish Coronavirus Sequencing Consortium-Teagasc Grange	Matthew McCabe, Aljandro Abner Garcia Leon, Fiona Crispie, Calum Walsh, Michael Carr, John Kenny, Paul Cotter, Patrick Mallon, Gabriel Gonzalez
EPI_ISL_778842	Clinical Pathology Labs	Centers for Disease Control and Prevention Division of Viral Diseases, Pathogen Discovery	Ying Tao Yan Li Jing Zhang Krista Queen Anna Uehara Peter Cook Clinton R. Paden Haibin Wang Suxiang Tong
EPI_ISL_779137, EPI_ISL_779141, EPI_ISL_779142, EPI_ISL_779143, EPI_ISL_779144, EPI_ISL_779145, EPI_ISL_779146	Yale Clinical Virology Laboratory	Grubaugh Lab - Yale School of Public Health	Tara Alpert, Joseph Fauver, Anderson Brito, Mallery Breban, Anne Wyllie, Chantal Vogels, Mary Petrone, Chaney Kalinich, Isabel Ott, Arnau Casanovas, Catherine Muenker, Adam Moore, Alice Lu, Maria Tokuyama, Patrick Wong, Peiwen Lu, Saad Omer, Richard Martinello, Allison Nelson, Shelli Farhadian, Akiko Iwasaki, Charlese Dela Cruz, Albert Ko, Nathan Grubaugh
EPI_ISL_779356, EPI_ISL_779364, EPI_ISL_779369, EPI_ISL_779371	University of Wisconsin-Madison AIDS Vaccine Research Laboratories	University of Wisconsin-Madison AIDS Vaccine Research Laboratories	Gage Moreno, Katarina Braun, et al. AIDS Vaccine Research Laboratories
EPI_ISL_779621	Victorian Infectious Diseases Reference Laboratory (VIDRL)	VIDRL and MDU-PHL	Caly L., Seemann T., Sait, M.L., Druce J., Sherry, N.L.
EPI_ISL_779651	Institute of Virology, Biomedical Research Center of the Slovak Academy of Sciences, Bratislava	Faculty of Natural Sciences, Comenius University, Bratislava	Viktória abanová, Kristína Boršová, Broa Brejová, Viktória Hodorová, Sabina Fumaová Havlíková, Juraj Kopáek, Martina Liková, ubomíra Lukáiková, Martina Neboháová, Monika Sláviková, Andrej Belák, Tomáš Vina, Jozef Nosek, Boris Klempa
EPI_ISL_779652	Institute of Virology, Biomedical Research Center of the Slovak Academy of Sciences, Bratislava	Faculty of Natural Sciences, Comenius University, Bratislava	Kristína Boršová, Viktória abanová, Broa Brejová, Viktória Hodorová, Sabina Fumaová Havlíková, Juraj Kopáek, Martina Liková, ubomíra Lukáiková, Martina Neboháová, Monika Sláviková, Andrej Belák, Tomáš Vina, Boris Klempa, Jozef Nosek
EPI_ISL_779653	Institute of Virology, Biomedical Research Center of the Slovak Academy of Sciences, Bratislava	Faculty of Natural Sciences, Comenius University, Bratislava	Viktória abanová, Kristína Boršová, Broa Brejová, Viktória Hodorová, Sabina Fumaová Havlíková, Juraj Kopáek, Martina Liková, ubomíra Lukáiková, Martina Neboháová, Monika Sláviková, Andrej Belák, Tomáš Vina, Jozef Nosek, Boris Klempa
EPI_ISL_779654	Institute of Virology, Biomedical Research Center of the Slovak Academy of Sciences, Bratislava	Faculty of Natural Sciences, Comenius University, Bratislava	Kristína Boršová, Viktória abanová, Broa Brejová, Viktória Hodorová, Sabina Fumaová Havlíková, Juraj Kopáek, Martina Liková, ubomíra Lukáiková, Martina Neboháová, Monika Sláviková, Andrej Belák, Tomáš Vina, Boris Klempa, Jozef Nosek
EPI_ISL_779655	Institute of Virology, Biomedical Research Center of the Slovak Academy of Sciences, Bratislava	Faculty of Natural Sciences, Comenius University, Bratislava	Viktória abanová, Kristína Boršová, Broa Brejová, Viktória Hodorová, Sabina Fumaová Havlíková, Juraj Kopáek, Martina Liková, ubomíra Lukáiková, Martina Neboháová, Monika Sláviková, Andrej Belák, Tomáš Vina, Jozef Nosek, Boris Klempa
EPI_ISL_779656	Institute of Virology, Biomedical Research Center of the Slovak Academy of Sciences, Bratislava	Faculty of Natural Sciences, Comenius University, Bratislava	Kristína Boršová, Viktória abanová, Broa Brejová, Viktória Hodorová, Sabina Fumaová Havlíková, Juraj Kopáek, Martina Liková, ubomíra Lukáiková, Martina Neboháová, Monika Sláviková, Andrej Belák, Tomáš Vina, Boris Klempa, Jozef Nosek
EPI_ISL_779657, EPI_ISL_779658, EPI_ISL_779659	Institute of Virology, Biomedical Research Center of the Slovak Academy of Sciences, Bratislava	Faculty of Natural Sciences, Comenius University, Bratislava	Broa Brejová, Viktória abanová, Kristína Boršová, Viktória Hodorová, Sabina Fumaová Havlíková, Juraj Kopáek, Martina Liková, ubomíra Lukáiková, Martina Neboháová, Monika Sláviková, Andrej Belák, Tomáš Vina, Jozef Nosek, Boris Klempa
EPI_ISL_779660	Institute of Virology, Biomedical Research Center of the Slovak Academy of Sciences, Bratislava	Faculty of Natural Sciences, Comenius University, Bratislava	Viktória abanová, Kristína Boršová, Broa Brejová, Viktória Hodorová, Sabina Fumaová Havlíková, Juraj Kopáek, Martina Liková, ubomíra Lukáiková, Martina Neboháová, Monika Sláviková, Andrej Belák, Tomáš Vina, Jozef Nosek, Boris Klempa

EPI_ISL_779810, EPI_ISL_779811, EPI_ISL_779812	CNR Virus des Infections Respiratoires - France SUD	CNR Virus des Infections Respiratoires - France SUD	Antonin Bal, Gregory Destras, Gwendolyne Burfin, Hadrien Règue, Quentin Semanas, Martine Valette, Bruno Lina, Laurence Josset
EPI_ISL_779836	Laboratoire Mirialis	CNR Virus des Infections Respiratoires - France SUD	Antonin Bal, Gregory Destras, Gwendolyne Burfin, Hadrien Règue, Quentin Semanas, Martine Valette, Bruno Lina, Laurence Josset
EPI_ISL_788920	University of Wisconsin-Madison AIDS Vaccine Research Laboratories	University of Wisconsin-Madison AIDS Vaccine Research Laboratories	Gage Moreno, Katarina Braun, et al. AIDS Vaccine Research Laboratories
EPI_ISL_789044	Klinisk mikrobiologi	The Public Health Agency of Sweden	Department of Microbiology, The Public Health Agency of Sweden
EPI_ISL_789054	Unilabs AB	The Public Health Agency of Sweden	Department of Microbiology, The Public Health Agency of Sweden
EPI_ISL_790588, EPI_ISL_790984, EPI_ISL_790985, EPI_ISL_791032	Dutch COVID-19 response team	National Institute for Public Health and the Environment (RIVM)	Adam Meijer, Harry Vennema, Jeroen Cremer, Sharon van den Brink, Bas van der Veer, AnneMarie van den Brandt, Florian Zwagemaker, Dennis Schmitz, Chantal Reusken, on behalf of the national COVID-19 response team
EPI_ISL_791087, EPI_ISL_791088	Instituto Nacional de Salud - Unidad de Secuenciación y Análisis Genómico	Instituto Nacional de Salud - Dirección de Investigación en Salud Pública	Katherine Laiton-Donato, Diego A. Álvarez-Díaz, Carlos Franco-Muñoz, Mauricio Pacheco-Montealegre, Jonathan Reales, Sheryl Corchuelo, Maria T. Herrera, Julian Naizaque, Gerardo Santamaría, Paola Muñoz-Laiton, Diego Andrés Prada, Magdalena Wiesner, Martha Lucia Ospina Martinez, Marcela Mercado-Reyes
EPI_ISL_791139	University of Wisconsin-Madison AIDS Vaccine Research Laboratories	University of Wisconsin-Madison AIDS Vaccine Research Laboratories	Gage Moreno, Katarina Braun, et al. AIDS Vaccine Research Laboratories
EPI_ISL_791335, EPI_ISL_791337, EPI_ISL_791339, EPI_ISL_791424, EPI_ISL_791435, EPI_ISL_791436, EPI_ISL_791443, EPI_ISL_791444, EPI_ISL_791451, EPI_ISL_791463, EPI_ISL_791474, EPI_ISL_791477, EPI_ISL_791485, EPI_ISL_791486			
see above	Johns Hopkins Hospital Department of Pathology	Johns Hopkins Hospital Department of Pathology	C. Paul Morris, Chun Huai Luo, Adannaya Amadi, Nicholas Gallagher, Heba H. Mostafa
EPI_ISL_792039	hospital	National Reference Center for Viruses of Respiratory Infections, Institut Pasteur, Paris	Marion Barbet, Sylvie Behillil, Méline Bizard, Angela Brisebarre, Camille Capel, Etienne Simon-Lorière, Vincent Enouf, Maud Vanpeene, Sylvie van der Werf,Laurent Roudière
EPI_ISL_792054	hospital	National Reference Center for Viruses of Respiratory Infections, Institut Pasteur, Paris	Marion Barbet, Sylvie Behillil, Méline Bizard, Angela Brisebarre, Camille Capel, Etienne Simon-Lorière, Vincent Enouf, Maud Vanpeene, Sylvie van der Werf,Esther Gyde
EPI_ISL_792055	hospital	National Reference Center for Viruses of Respiratory Infections, Institut Pasteur, Paris	Marion Barbet, Sylvie Behillil, Méline Bizard, Angela Brisebarre, Camille Capel, Etienne Simon-Lorière, Vincent Enouf, Maud Vanpeene, Sylvie van der Werf,Patricia Stoessel
EPI_ISL_792084, EPI_ISL_792085	Toronto Invasive Bacterial Diseases Network	McMaster University	Allison McGeer, Patryk Aftanas, Hooman Derakhshani, Angel Li, Kuganya Nirmalarajah, Emily Panousis, Ahmed Draia, Jalees Nasir, Michael Surette, Samira Mubareka, Andrew G. McArthur
EPI_ISL_792674, EPI_ISL_792675	Los Angeles County PHL	Los Angeles County PHL	P. Hemarajata et al.
EPI_ISL_793030, EPI_ISL_793031, EPI_ISL_793032, EPI_ISL_793033, EPI_ISL_793034, EPI_ISL_793035, EPI_ISL_793036, EPI_ISL_793037, EPI_ISL_793038, EPI_ISL_793039, EPI_ISL_793040, EPI_ISL_793041, EPI_ISL_793042, EPI_ISL_793043, EPI_ISL_793044, EPI_ISL_793045, EPI_ISL_793046, EPI_ISL_793047, EPI_ISL_793048, EPI_ISL_793049, EPI_ISL_793050, EPI_ISL_793116, EPI_ISL_793117, EPI_ISL_793120, EPI_ISL_793130, EPI_ISL_793134, EPI_ISL_793142, EPI_ISL_793145, EPI_ISL_793150, EPI_ISL_793151, EPI_ISL_793159, EPI_ISL_793163, EPI_ISL_793164, EPI_ISL_793171, EPI_ISL_793179, EPI_ISL_793180, EPI_ISL_793181, EPI_ISL_793182, EPI_ISL_793183, EPI_ISL_793184, EPI_ISL_793185, EPI_ISL_793186, EPI_ISL_793187, EPI_ISL_793188, EPI_ISL_793189, EPI_ISL_793190, EPI_ISL_793191, EPI_ISL_793192, EPI_ISL_793193, EPI_ISL_793205, EPI_ISL_793206, EPI_ISL_793207, EPI_ISL_793208, EPI_ISL_793209, EPI_ISL_793210, EPI_ISL_793211, EPI_ISL_793212, EPI_ISL_793213, EPI_ISL_793214, EPI_ISL_793215, EPI_ISL_793216, EPI_ISL_793217, EPI_ISL_793296, EPI_ISL_793299, EPI_ISL_793301, EPI_ISL_793316, EPI_ISL_793317, EPI_ISL_793318, EPI_ISL_793319, EPI_ISL_793320, EPI_ISL_793321, EPI_ISL_793322, EPI_ISL_793323, EPI_ISL_793324, EPI_ISL_793325, EPI_ISL_793326, EPI_ISL_793327, EPI_ISL_793328, EPI_ISL_793329, EPI_ISL_793330, EPI_ISL_793331, EPI_ISL_793371, EPI_ISL_793374, EPI_ISL_793394, EPI_ISL_793396, EPI_ISL_793399, EPI_ISL_793400, EPI_ISL_793401, EPI_ISL_793402, EPI_ISL_793403, EPI_ISL_793406, EPI_ISL_793417, EPI_ISL_793421, EPI_ISL_793422, EPI_ISL_793423, EPI_ISL_793424, EPI_ISL_793425, EPI_ISL_793426, EPI_ISL_793427, EPI_ISL_793428, EPI_ISL_793429, EPI_ISL_793430, EPI_ISL_793431, EPI_ISL_793432, EPI_ISL_793433, EPI_ISL_793434, EPI_ISL_793435, EPI_ISL_793436, EPI_ISL_793437, EPI_ISL_793438, EPI_ISL_793439, EPI_ISL_793440, EPI_ISL_793441, EPI_ISL_793442, EPI_ISL_793443, EPI_ISL_793444, EPI_ISL_793445, EPI_ISL_793446, EPI_ISL_793447, EPI_ISL_793448, EPI_ISL_793449, EPI_ISL_793450, EPI_ISL_793451, EPI_ISL_793452, EPI_ISL_793453, EPI_ISL_793454, EPI_ISL_793455, EPI_ISL_793456, EPI_ISL_793457, EPI_ISL_793458, EPI_ISL_793459, EPI_ISL_793460, EPI_ISL_793461, EPI_ISL_793462, EPI_ISL_793463, EPI_ISL_793464, EPI_ISL_793465, EPI_ISL_793466, EPI_ISL_793467, EPI_ISL_793468, EPI_ISL_793469, EPI_ISL_793470, EPI_ISL_793471, EPI_ISL_793472, EPI_ISL_793473, EPI_ISL_793474, EPI_ISL_793475, EPI_ISL_793476, EPI_ISL_793477, EPI_ISL_793478, EPI_ISL_793479, EPI_ISL_793480, EPI_ISL_793481, EPI_ISL_793482, EPI_ISL_793483, EPI_ISL_793484, EPI_ISL_793485, EPI_ISL_793486, EPI_ISL_793487, EPI_ISL_793488, EPI_ISL_793489, EPI_ISL_793490, EPI_ISL_793491, EPI_ISL_793492, EPI_ISL_793493, EPI_ISL_793494, EPI_ISL_793495, EPI_ISL_793496, EPI_ISL_793497, EPI_ISL_793498, EPI_ISL_793499, EPI_ISL_793500, EPI_ISL_793501, EPI_ISL_793502, EPI_ISL_793503, EPI_ISL_793504, EPI_ISL_793505, EPI_ISL_793506, EPI_ISL_793507, EPI_ISL_793508, EPI_ISL_793509, EPI_ISL_793510, EPI_ISL_793511, EPI_ISL_793512, EPI_ISL_793513, EPI_ISL_793514, EPI_ISL_793515, EPI_ISL_793516, EPI_ISL_793517, EPI_ISL_793518, EPI_ISL_793519, EPI_ISL_793520, EPI_ISL_793521, EPI_ISL_793522, EPI_ISL_793523, EPI_ISL_793524, EPI_ISL_793525, EPI_ISL_793526, EPI_ISL_793527, EPI_ISL_793528, EPI_ISL_793529, EPI_ISL_793530, EPI_ISL_793531, EPI_ISL_793532, EPI_ISL_793533, EPI_ISL_793534, EPI_ISL_793535, EPI_ISL_793536, EPI_ISL_793537, EPI_ISL_793538, EPI_ISL_793539, EPI_ISL_793540, EPI_ISL_793541, EPI_ISL_793542, EPI_ISL_793543, EPI_ISL_793544, EPI_ISL_793545, EPI_ISL_793546, EPI_ISL_793547, EPI_ISL_793548, EPI_ISL_793549, EPI_ISL_793550, EPI_ISL_793551, EPI_ISL_793552, EPI_ISL_793553, EPI_ISL_793554, EPI_ISL_793555, EPI_ISL_793556, EPI_ISL_793557, EPI_ISL_793558, EPI_ISL_793559, EPI_ISL_793560, EPI_ISL_793561, EPI_ISL_793562, EPI_ISL_793563, EPI_ISL_793564, EPI_ISL_793565, EPI_ISL_793566, EPI_ISL_793567, EPI_ISL_793568, EPI_ISL_793569, EPI_ISL_793570, EPI_ISL_793571, EPI_ISL_793572, EPI_ISL_793573, EPI_ISL_793574, EPI_ISL_793575, EPI_ISL_793576, EPI_ISL_793577, EPI_ISL_793578, EPI_ISL_793579, EPI_ISL_793580, EPI_ISL_793581, EPI_ISL_793582, EPI_ISL_793583, EPI_ISL_793584, EPI_ISL_793585, EPI_ISL_793586, EPI_ISL_793587, EPI_ISL_793588, EPI_ISL_793589, EPI_ISL_793590, EPI_ISL_793591, EPI_ISL_793592, EPI_ISL_793593, EPI_ISL_793594, EPI_ISL_793595, EPI_ISL_793596, EPI_ISL_793597, EPI_ISL_793598, EPI_ISL_793599, EPI_ISL_793600, EPI_ISL_793601, EPI_ISL_793602, EPI_ISL_793603, EPI_ISL_793604, EPI_ISL_793605, EPI_ISL_793606, EPI_ISL_793607, EPI_ISL_793608, EPI_ISL_793609, EPI_ISL_793610, EPI_ISL_793611, EPI_ISL_793612, EPI_ISL_793613, EPI_ISL_793614, EPI_ISL_793615, EPI_ISL_793616, EPI_ISL_793617, EPI_ISL_793618, EPI_ISL_793619, EPI_ISL_793620, EPI_ISL_793621, EPI_ISL_793622, EPI_ISL_793623, EPI_ISL_793624, EPI_ISL_793625, EPI_ISL_793626, EPI_ISL_793627, EPI_ISL_793628, EPI_ISL_793629, EPI_ISL_793630, EPI_ISL_793631, EPI_ISL_793632, EPI_ISL_793633, EPI_ISL_793634, EPI_ISL_793635, EPI_ISL_793636, EPI_ISL_793637, EPI_ISL_793638, EPI_ISL_793639, EPI_ISL_793640, EPI_ISL_793641, EPI_ISL_793642, EPI_ISL_793643, EPI_ISL_793644, EPI_ISL_793645, EPI_ISL_793646, EPI_ISL_793647, EPI_ISL_793648, EPI_ISL_793649			
see above	Department of Virus and Microbiological Special Diagnostics, Statens Serum Institut, Copenhagen, Denmark	Albertsen Lab, Department of Chemistry and Bioscience, Aalborg University, Denmark	Danish Covid-19 Genome Consortium
EPI_ISL_794083, EPI_ISL_794084, EPI_ISL_794085, EPI_ISL_794086, EPI_ISL_794087, EPI_ISL_794088, EPI_ISL_794089, EPI_ISL_794090, EPI_ISL_794091, EPI_ISL_794093, EPI_ISL_794094, EPI_ISL_794095, EPI_ISL_794096, EPI_ISL_794097, EPI_ISL_794098, EPI_ISL_794099, EPI_ISL_794100, EPI_ISL_794101, EPI_ISL_794102, EPI_ISL_794103, EPI_ISL_794105, EPI_ISL_794106			
see above	URMC LABS	Wadsworth Center, New York State Department.of Health	Kirsten St. George, Daryl M. Lamson, Alexis Russel, Matthew Shudt, Melissa A Leisner, Jonathan Plitnick, Navjot Singh, John Kelly, Sara Griesemer, Erasmus Schneider, Erica Lasek-Nesselquist
EPI_ISL_794112, EPI_ISL_794113, EPI_ISL_794115, EPI_ISL_794118	GLENS FALLS HOSPITAL LABORATORY	Wadsworth Center, New York State Department.of Health	Kirsten St. George, Daryl M. Lamson, Alexis Russel, Matthew Shudt, Melissa A Leisner, Jonathan Plitnick, Navjot Singh, John Kelly, Sara Griesemer, Erasmus Schneider, Erica Lasek-Nesselquist
EPI_ISL_794138, EPI_ISL_794139, EPI_ISL_794141, EPI_ISL_794143, EPI_ISL_794144, EPI_ISL_794145, EPI_ISL_794148, EPI_ISL_794149, EPI_ISL_794150, EPI_ISL_794151, EPI_ISL_794152, EPI_ISL_794153, EPI_ISL_794154, EPI_ISL_794155, EPI_ISL_794156, EPI_ISL_794157, EPI_ISL_794158			
see above	NORTHWELL HEALTH LABORATORIES	Wadsworth Center, New York State Department.of Health	Kirsten St. George, Daryl M. Lamson, Alexis Russel, Matthew Shudt, Melissa A Leisner, Jonathan Plitnick, Navjot Singh, John Kelly, Sara Griesemer, Erasmus Schneider, Erica Lasek-Nesselquist
EPI_ISL_794166, EPI_ISL_794167	Wadsworth Center, New York State Department.of Health	Wadsworth Center, New York State Department.of Health	Kirsten St. George, Daryl M. Lamson, Alexis Russel, Matthew Shudt, Melissa A Leisner, Jonathan Plitnick, Navjot Singh, John Kelly, Sara Griesemer, Erasmus Schneider, Erica Lasek-Nesselquist
EPI_ISL_794182, EPI_ISL_794183, EPI_ISL_794184, EPI_ISL_794185, EPI_ISL_794186, EPI_ISL_794187, EPI_ISL_794188, EPI_ISL_794189, EPI_ISL_794190, EPI_ISL_794191, EPI_ISL_794192, EPI_ISL_794193, EPI_ISL_794194, EPI_ISL_794195, EPI_ISL_794196, EPI_ISL_794197, EPI_ISL_794198, EPI_ISL_794199, EPI_ISL_794200, EPI_ISL_794201, EPI_ISL_794202, EPI_ISL_794203, EPI_ISL_794204, EPI_ISL_794205			
see above	URMC LABS	Wadsworth Center, New York State Department.of Health	Kirsten St. George, Daryl M. Lamson, Alexis Russel, Matthew Shudt, Melissa A Leisner, Jonathan Plitnick, Navjot Singh, John Kelly, Sara Griesemer, Erasmus Schneider, Erica Lasek-Nesselquist
EPI_ISL_794219, EPI_ISL_794220, EPI_ISL_794221, EPI_ISL_794222, EPI_ISL_794223, EPI_ISL_794224, EPI_ISL_794225, EPI_ISL_794226, EPI_ISL_794227, EPI_ISL_794228, EPI_ISL_794229, EPI_ISL_794230, EPI_ISL_794231, EPI_ISL_794232, EPI_ISL_794233, EPI_ISL_794235, EPI_ISL_794238, EPI_ISL_794241, EPI_ISL_794242, EPI_ISL_794243			
see above	WESTCHESTER MEDICAL CENTER	Wadsworth Center, New York State Department.of Health	Kirsten St. George, Daryl M. Lamson, Alexis Russel, Matthew Shudt, Melissa A Leisner, Jonathan Plitnick, Navjot Singh, John Kelly, Sara Griesemer, Erasmus Schneider, Erica Lasek-Nesselquist
EPI_ISL_794661	DRECCION DE SANIDAD POLICIA NACIONAL	Instituto Nacional de Salud - Dirección de Investigación en Salud Pública	Katherine Laiton-Donato, Diego A. Álvarez-Díaz, Carlos Franco-Muñoz, Mauricio Pacheco-Montealegre, Jonathan Reales, Sheryl Corchuelo, Maria T. Herrera, Julian Naizaque, Gerardo Santamaría, Paola Muñoz-Laiton, Diego Andrés Prada, Magdalena Wiesner, Martha Lucia Ospina Martinez, Marcela Mercado-Reyes
EPI_ISL_795011, EPI_ISL_795012, EPI_ISL_795013, EPI_ISL_795014, EPI_ISL_795015, EPI_ISL_795016, EPI_ISL_795017, EPI_ISL_795018, EPI_ISL_795019, EPI_ISL_795021, EPI_ISL_795022, EPI_ISL_795023, EPI_ISL_795024, EPI_ISL_795025, EPI_ISL_795026, EPI_ISL_795027, EPI_ISL_795028, EPI_ISL_795029, EPI_ISL_795030, EPI_ISL_795031, EPI_ISL_795032, EPI_ISL_795033, EPI_ISL_795034, EPI_ISL_795035, EPI_ISL_795036, EPI_ISL_795037, EPI_ISL_795038, EPI_ISL_795040, EPI_ISL_795041, EPI_ISL_795042, EPI_ISL_795043, EPI_ISL_795044, EPI_ISL_795045, EPI_ISL_795046, EPI_ISL_795047, EPI_ISL_795048, EPI_ISL_795049, EPI_ISL_795050, EPI_ISL_795051, EPI_ISL_795052, EPI_ISL_795053, EPI_ISL_795054, EPI_ISL_795055, EPI_ISL_795056, EPI_ISL_795057, EPI_ISL_795058, EPI_ISL_795059, EPI_ISL_795060, EPI_ISL_795061, EPI_ISL_795062, EPI_ISL_795063, EPI_ISL_795064, EPI_ISL_795065, EPI_ISL_795066, EPI_ISL_795067, EPI_ISL_795068, EPI_ISL_795069, EPI_ISL_795070, EPI_ISL_795071, EPI_ISL_795072, EPI_ISL_795073, EPI_ISL_795074, EPI_ISL_795075, EPI_ISL_795076, EPI_ISL_795077, EPI_ISL_795078, EPI_ISL_795079, EPI_ISL_795080, EPI_ISL_795081, EPI_ISL_795082, EPI_ISL_795083, EPI_ISL_795084, EPI_ISL_795085, EPI_ISL_795086, EPI_ISL_795087, EPI_ISL_795088, EPI_ISL_795089, EPI_ISL_795090, EPI_ISL_795091, EPI_ISL_795092, EPI_ISL_795093, EPI_ISL_795094, EPI_ISL_795095, EPI_ISL_795096, EPI_ISL_795097, EPI_ISL_795098, EPI_ISL_795099, EPI_ISL_795100, EPI_ISL_795101, EPI_ISL_795102, EPI_ISL_795103, EPI_ISL_795104, EPI_ISL_795105, EPI_ISL_795106, EPI_ISL_795107, EPI_ISL_795108, EPI_ISL_795109, EPI_ISL_795110, EPI_ISL_795111, EPI_ISL_795112, EPI_ISL_795113, EPI_ISL_795114, EPI_ISL_795115, EPI_ISL_795116, EPI_ISL_795117, EPI_ISL_795118, EPI_ISL_795119, EPI_ISL_795120,			

EPI_ISL_795121, EPI_ISL_795122, EPI_ISL_795123, EPI_ISL_795124, EPI_ISL_795125, EPI_ISL_795126, EPI_ISL_795127, EPI_ISL_795128, EPI_ISL_795129, EPI_ISL_795130, EPI_ISL_795131, EPI_ISL_795132, EPI_ISL_795133, EPI_ISL_795134, EPI_ISL_795135, EPI_ISL_795136, EPI_ISL_795137, EPI_ISL_795138, EPI_ISL_795139, EPI_ISL_795140, EPI_ISL_795141, EPI_ISL_795142, EPI_ISL_795143, EPI_ISL_795144, EPI_ISL_795145, EPI_ISL_795146, EPI_ISL_795147, EPI_ISL_795148, EPI_ISL_795154, EPI_ISL_795155, EPI_ISL_795156, EPI_ISL_795157, EPI_ISL_795158, EPI_ISL_795159, EPI_ISL_795160, EPI_ISL_795161, EPI_ISL_795162, EPI_ISL_795163, EPI_ISL_795164, EPI_ISL_795165, EPI_ISL_795166, EPI_ISL_795167, EPI_ISL_795168, EPI_ISL_795169, EPI_ISL_795171, EPI_ISL_795172, EPI_ISL_795173, EPI_ISL_795175, EPI_ISL_795176, EPI_ISL_795177, EPI_ISL_795178, EPI_ISL_795179, EPI_ISL_795180, EPI_ISL_795181, EPI_ISL_795182, EPI_ISL_795183, EPI_ISL_795184, EPI_ISL_795185, EPI_ISL_795186, EPI_ISL_795187, EPI_ISL_795188, EPI_ISL_795189, EPI_ISL_795190, EPI_ISL_795191, EPI_ISL_795192, EPI_ISL_795193, EPI_ISL_795194, EPI_ISL_795195, EPI_ISL_795196, EPI_ISL_795197, EPI_ISL_795198, EPI_ISL_795199, EPI_ISL_795200, EPI_ISL_795201, EPI_ISL_795202, EPI_ISL_795203, EPI_ISL_795204, EPI_ISL_795205, EPI_ISL_795206, EPI_ISL_795207, EPI_ISL_795208, EPI_ISL_795209, EPI_ISL_795210, EPI_ISL_795211, EPI_ISL_795212, EPI_ISL_795213, EPI_ISL_795214, EPI_ISL_795215, EPI_ISL_795216, EPI_ISL_795217, EPI_ISL_795218, EPI_ISL_795219, EPI_ISL_795220, EPI_ISL_795221, EPI_ISL_795222, EPI_ISL_795223, EPI_ISL_795224, EPI_ISL_795225, EPI_ISL_795226, EPI_ISL_795227, EPI_ISL_795228, EPI_ISL_795229, EPI_ISL_795230, EPI_ISL_795231, EPI_ISL_795232, EPI_ISL_795233, EPI_ISL_795234, EPI_ISL_795235, EPI_ISL_795236, EPI_ISL_795237, EPI_ISL_795238, EPI_ISL_795242, EPI_ISL_795244, EPI_ISL_795245, EPI_ISL_795246, EPI_ISL_795247, EPI_ISL_795248, EPI_ISL_795249, EPI_ISL_795250, EPI_ISL_795252, EPI_ISL_795253, EPI_ISL_795254, EPI_ISL_795255, EPI_ISL_795256, EPI_ISL_795257, EPI_ISL_795258, EPI_ISL_795259, EPI_ISL_795260, EPI_ISL_795261, EPI_ISL_795262, EPI_ISL_795263, EPI_ISL_795264, EPI_ISL_795265, EPI_ISL_795266, EPI_ISL_795267, EPI_ISL_795268, EPI_ISL_795269, EPI_ISL_795270, EPI_ISL_795271, EPI_ISL_795272, EPI_ISL_795273, EPI_ISL_795274, EPI_ISL_795275, EPI_ISL_795276, EPI_ISL_795277, EPI_ISL_795281, EPI_ISL_795282, EPI_ISL_795283, EPI_ISL_795284, EPI_ISL_795285, EPI_ISL_795286, EPI_ISL_795287, EPI_ISL_795288, EPI_ISL_795289, EPI_ISL_795290, EPI_ISL_795291, EPI_ISL_795292, EPI_ISL_795293, EPI_ISL_795294, EPI_ISL_795295, EPI_ISL_795296, EPI_ISL_795297, EPI_ISL_795298, EPI_ISL_795299, EPI_ISL_795300, EPI_ISL_795301, EPI_ISL_795302, EPI_ISL_795303, EPI_ISL_795304, EPI_ISL_795305, EPI_ISL_795306, EPI_ISL_795307, EPI_ISL_795308, EPI_ISL_795309, EPI_ISL_795310, EPI_ISL_795311, EPI_ISL_795312, EPI_ISL_795313, EPI_ISL_795314, EPI_ISL_795315, EPI_ISL_795316, EPI_ISL_795317, EPI_ISL_795318, EPI_ISL_795319, EPI_ISL_795320, EPI_ISL_795321, EPI_ISL_795322, EPI_ISL_795323, EPI_ISL_795324, EPI_ISL_795325, EPI_ISL_795326, EPI_ISL_795327, EPI_ISL_795328, EPI_ISL_795329, EPI_ISL_795330, EPI_ISL_795331, EPI_ISL_795332, EPI_ISL_795333, EPI_ISL_795334, EPI_ISL_795335, EPI_ISL_795336, EPI_ISL_795337, EPI_ISL_795338, EPI_ISL_795339, EPI_ISL_795340, EPI_ISL_795341, EPI_ISL_795342, EPI_ISL_795343, EPI_ISL_795344, EPI_ISL_795345, EPI_ISL_795346, EPI_ISL_795347, EPI_ISL_795348, EPI_ISL_795349, EPI_ISL_795350, EPI_ISL_795351, EPI_ISL_795352, EPI_ISL_795353, EPI_ISL_795354, EPI_ISL_795355, EPI_ISL_795356, EPI_ISL_795357, EPI_ISL_795358, EPI_ISL_795359, EPI_ISL_795360, EPI_ISL_795361, EPI_ISL_795368, EPI_ISL_795369, EPI_ISL_795370, EPI_ISL_795371, EPI_ISL_795372, EPI_ISL_795373, EPI_ISL_795374, EPI_ISL_795375, EPI_ISL_795376, EPI_ISL_795377, EPI_ISL_795378, EPI_ISL_795379, EPI_ISL_795380, EPI_ISL_795381, EPI_ISL_795382, EPI_ISL_795384, EPI_ISL_795385, EPI_ISL_795386, EPI_ISL_795387, EPI_ISL_795388, EPI_ISL_795389, EPI_ISL_795390, EPI_ISL_795391, EPI_ISL_795392, EPI_ISL_795393, EPI_ISL_795394, EPI_ISL_795395, EPI_ISL_795396, EPI_ISL_795397, EPI_ISL_795398, EPI_ISL_795399, EPI_ISL_795400, EPI_ISL_795401, EPI_ISL_795402, EPI_ISL_795403, EPI_ISL_795404, EPI_ISL_795405, EPI_ISL_795406, EPI_ISL_795407, EPI_ISL_795408, EPI_ISL_795409, EPI_ISL_795410, EPI_ISL_795411, EPI_ISL_795412, EPI_ISL_795414, EPI_ISL_795415, EPI_ISL_795416, EPI_ISL_795417, EPI_ISL_795418, EPI_ISL_795419, EPI_ISL_795420, EPI_ISL_795421, EPI_ISL_795422, EPI_ISL_795423, EPI_ISL_795424, EPI_ISL_795426, EPI_ISL_795427, EPI_ISL_795428, EPI_ISL_795429, EPI_ISL_795430, EPI_ISL_795431, EPI_ISL_795432, EPI_ISL_795433, EPI_ISL_795434, EPI_ISL_795435, EPI_ISL_795436, EPI_ISL_795437, EPI_ISL_795438, EPI_ISL_795439, EPI_ISL_795440, EPI_ISL_795441, EPI_ISL_795442, EPI_ISL_795443, EPI_ISL_795444, EPI_ISL_795445, EPI_ISL_795446, EPI_ISL_795447, EPI_ISL_795448, EPI_ISL_795449, EPI_ISL_795450, EPI_ISL_795451, EPI_ISL_795452, EPI_ISL_795453, EPI_ISL_795454, EPI_ISL_795455, EPI_ISL_795456, EPI_ISL_795457, EPI_ISL_795458, EPI_ISL_795459, EPI_ISL_795460, EPI_ISL_795461, EPI_ISL_795462, EPI_ISL_795463, EPI_ISL_795464, EPI_ISL_795465, EPI_ISL_795466, EPI_ISL_795467, EPI_ISL_795468, EPI_ISL_795469, EPI_ISL_795470, EPI_ISL_795471, EPI_ISL_795472, EPI_ISL_795473, EPI_ISL_795474, EPI_ISL_795475, EPI_ISL_795476, EPI_ISL_795477, EPI_ISL_795478, EPI_ISL_795479, EPI_ISL_795480, EPI_ISL_795481, EPI_ISL_795482, EPI_ISL_795483, EPI_ISL_795484, EPI_ISL_795485, EPI_ISL_795486, EPI_ISL_795487, EPI_ISL_795488, EPI_ISL_795489, EPI_ISL_795490, EPI_ISL_795491, EPI_ISL_795492, EPI_ISL_795493, EPI_ISL_795494, EPI_ISL_795495, EPI_ISL_795496, EPI_ISL_795497, EPI_ISL_795498, EPI_ISL_795499, EPI_ISL_795500, EPI_ISL_795501, EPI_ISL_795502, EPI_ISL_795503, EPI_ISL_795504, EPI_ISL_795505, EPI_ISL_795506, EPI_ISL_795507, EPI_ISL_795508, EPI_ISL_795509, EPI_ISL_795510, EPI_ISL_795511, EPI_ISL_795512, EPI_ISL_795513, EPI_ISL_795514, EPI_ISL_795515, EPI_ISL_795516, EPI_ISL_795517, EPI_ISL_795518, EPI_ISL_795519, EPI_ISL_795520, EPI_ISL_795521, EPI_ISL_795522, EPI_ISL_795523, EPI_ISL_795524, EPI_ISL_795525, EPI_ISL_795526, EPI_ISL_795527, EPI_ISL_795528, EPI_ISL_795529, EPI_ISL_795530, EPI_ISL_795531, EPI_ISL_795532, EPI_ISL_795533, EPI_ISL_795534, EPI_ISL_795535, EPI_ISL_795536, EPI_ISL_795537, EPI_ISL_795538, EPI_ISL_795539, EPI_ISL_795540, EPI_ISL_795541, EPI_ISL_795542, EPI_ISL_795543, EPI_ISL_795544, EPI_ISL_795545, EPI_ISL_795546, EPI_ISL_795547, EPI_ISL_795548, EPI_ISL_795549, EPI_ISL_795550, EPI_ISL_795551, EPI_ISL_795552, EPI_ISL_795553, EPI_ISL_795554, EPI_ISL_795555, EPI_ISL_795556, EPI_ISL_795557, EPI_ISL_795558, EPI_ISL_795559, EPI_ISL_795560, EPI_ISL_795561, EPI_ISL_795562, EPI_ISL_795563, EPI_ISL_795564, EPI_ISL_795565, EPI_ISL_795566, EPI_ISL_795567, EPI_ISL_795568, EPI_ISL_795569, EPI_ISL_795570, EPI_ISL_795571, EPI_ISL_795572, EPI_ISL_795573, EPI_ISL_795574, EPI_ISL_795575, EPI_ISL_795576, EPI_ISL_795577, EPI_ISL_795578, EPI_ISL_795579, EPI_ISL_795580, EPI_ISL_795581, EPI_ISL_795582, EPI_ISL_795583, EPI_ISL_795584, EPI_ISL_795585, EPI_ISL_795586, EPI_ISL_795587, EPI_ISL_795588, EPI_ISL_795589, EPI_ISL_795590, EPI_ISL_795591, EPI_ISL_795592, EPI_ISL_795593, EPI_ISL_795594, EPI_ISL_795595, EPI_ISL_795596, EPI_ISL_795597, EPI_ISL_795598, EPI_ISL_795599, EPI_ISL_795600, EPI_ISL_795601, EPI_ISL_795602, EPI_ISL_795603, EPI_ISL_795604, EPI_ISL_795605, EPI_ISL_795606, EPI_ISL_795607, EPI_ISL_795608, EPI_ISL_795609, EPI_ISL_795610, EPI_ISL_795611, EPI_ISL_795612, EPI_ISL_795613, EPI_ISL_795614, EPI_ISL_795615, EPI_ISL_795616, EPI_ISL_795617, EPI_ISL_795618, EPI_ISL_795619, EPI_ISL_795620, EPI_ISL_795621, EPI_ISL_795622, EPI_ISL_795623, EPI_ISL_795624, EPI_ISL_795625, EPI_ISL_795626, EPI_ISL_795627, EPI_ISL_795628, EPI_ISL_795629, EPI_ISL_795630, EPI_ISL_795631, EPI_ISL_795632, EPI_ISL_795633, EPI_ISL_795634, EPI_ISL_795635, EPI_ISL_795636, EPI_ISL_795637, EPI_ISL_795638, EPI_ISL_795639, EPI_ISL_795640, EPI_ISL_795641, EPI_ISL_795642, EPI_ISL_795643, EPI_ISL_795644, EPI_ISL_795645, EPI_ISL_795646, EPI_ISL_795647, EPI_ISL_795648, EPI_ISL_795649, EPI_ISL_795650, EPI_ISL_795651, EPI_ISL_795652, EPI_ISL_795653, EPI_ISL_795654, EPI_ISL_795655, EPI_ISL_795656, EPI_ISL_795657, EPI_ISL_795658, EPI_ISL_795659, EPI_ISL_795660, EPI_ISL_795661, EPI_ISL_795662, EPI_ISL_795663, EPI_ISL_795664, EPI_ISL_795665, EPI_ISL_795666, EPI_ISL_795667, EPI_ISL_795668, EPI_ISL_795669, EPI_ISL_795670, EPI_ISL_795671, EPI_ISL_795672, EPI_ISL_795673, EPI_ISL_795674, EPI_ISL_795675, EPI_ISL_795676, EPI_ISL_795677, EPI_ISL_795678, EPI_ISL_795679, EPI_ISL_795680, EPI_ISL_795681, EPI_ISL_795682, EPI_ISL_795683, EPI_ISL_795684, EPI_ISL_795685, EPI_ISL_795686, EPI_ISL_795687, EPI_ISL_795688, EPI_ISL_795689, EPI_ISL_795690, EPI_ISL_795691, EPI_ISL_795692, EPI_ISL_795693, EPI_ISL_795694, EPI_ISL_795695, EPI_ISL_795696, EPI_ISL_795697, EPI_ISL_795698, EPI_ISL_795699, EPI_ISL_795700, EPI_ISL_795701, EPI_ISL_795702, EPI_ISL_795703, EPI_ISL_795704, EPI_ISL_795705, EPI_ISL_795706, EPI_ISL_795707, EPI_ISL_795708, EPI_ISL_795709, EPI_ISL_795710, EPI_ISL_795711, EPI_ISL_795712, EPI_ISL_795713, EPI_ISL_795714, EPI_ISL_795715, EPI_ISL_795716, EPI_ISL_795717, EPI_ISL_795718, EPI_ISL_795719, EPI_ISL_795720, EPI_ISL_795721, EPI_ISL_795722, EPI_ISL_795723, EPI_ISL_795724, EPI_ISL_795725, EPI_ISL_795726, EPI_ISL_795727, EPI_ISL_795728, EPI_ISL_795729, EPI_ISL_795730, EPI_ISL_795731, EPI_ISL_795732, EPI_ISL_795733, EPI_ISL_795734, EPI_ISL_795735, EPI_ISL_795736, EPI_ISL_795737, EPI_ISL_795738, EPI_ISL_795739, EPI_ISL_795740, EPI_ISL_795741, EPI_ISL_795742, EPI_ISL_795743, EPI_ISL_795744, EPI_ISL_795745, EPI_ISL_795746, EPI_ISL_795747, EPI_ISL_795748, EPI_ISL_795749, EPI_ISL_795750, EPI_ISL_795751, EPI_ISL_795752, EPI_ISL_795753, EPI_ISL_795754, EPI_ISL_795755, EPI_ISL_795756, EPI_ISL_795757, EPI_ISL_795758, EPI_ISL_795759, EPI_ISL_795760, EPI_ISL_795761, EPI_ISL_795762, EPI_ISL_795763, EPI_ISL_795764, EPI_ISL_795765, EPI_ISL_795766, EPI_ISL_795767, EPI_ISL_795768, EPI_ISL_795769, EPI_ISL_795770, EPI_ISL_795771, EPI_ISL_795772, EPI_ISL_795773, EPI_ISL_795774, EPI_ISL_795775, EPI_ISL_795776, EPI_ISL_795777, EPI_ISL_795778, EPI_ISL_795779, EPI_ISL_795780, EPI_ISL_795781, EPI_ISL_795782, EPI_ISL_795783, EPI_ISL_795784, EPI_ISL_795785, EPI_ISL_795786, EPI_ISL_795787, EPI_ISL_795788, EPI_ISL_795789, EPI_ISL_795790, EPI_ISL_795791, EPI_ISL_795792, EPI_ISL_795793, EPI_ISL_795794, EPI_ISL_795795, EPI_ISL_795796, EPI_ISL_795797, EPI_ISL_795798, EPI_ISL_795799, EPI_ISL_795800, EPI_ISL_795801, EPI_ISL_795802, EPI_ISL_795803, EPI_ISL_795804, EPI_ISL_795805, EPI_ISL_795806, EPI_ISL_795807, EPI_ISL_795808, EPI_ISL_795809, EPI_ISL_795810, EPI_ISL_795811, EPI_ISL_795812, EPI_ISL_795813, EPI_ISL_795814, EPI_ISL_795815, EPI_ISL_795816, EPI_ISL_795817, EPI_ISL_795818, EPI_ISL_795819, EPI_ISL_795820, EPI_ISL_795821, EPI_ISL_795822, EPI_ISL_795823, EPI_ISL_795824, EPI_ISL_795825, EPI_ISL_795826, EPI_ISL_795827, EPI_ISL_795828, EPI_ISL_795829, EPI_ISL_795830, EPI_ISL_795831, EPI_ISL_795832, EPI_ISL_795833, EPI_ISL_795834, EPI_ISL_795835, EPI_ISL_795836, EPI_ISL_795837, EPI_ISL_795838, EPI_ISL_795839, EPI_ISL_795840, EPI_ISL_795841, EPI_ISL_795842, EPI_ISL_795843, EPI_ISL_795844, EPI_ISL_795845, EPI_ISL_795846, EPI_ISL_795847, EPI_ISL_795848, EPI_ISL_795849, EPI_ISL_795850, EPI_ISL_795851, EPI_ISL_795852, EPI_ISL_795853, EPI_ISL_795854, EPI_ISL_795855, EPI_ISL_795856, EPI_ISL_795857, EPI_ISL_795858, EPI_ISL_795859, EPI_ISL_795860, EPI_ISL_795861, EPI_ISL_795862, EPI_ISL_795863, EPI_ISL_795864, EPI_ISL_795865, EPI_ISL_795866, EPI_ISL_795867, EPI_ISL_795868, EPI_ISL_795869, EPI_ISL_795870, EPI_ISL_795871, EPI_ISL_795872, EPI_ISL_795873, EPI_ISL_795874, EPI_ISL_795875, EPI_ISL_795876, EPI_ISL_795877, EPI_ISL_795878, EPI_ISL_795879, EPI_ISL_795880, EPI_ISL_795881, EPI_ISL_795882, EPI_ISL_795883, EPI_ISL_795884, EPI_ISL_795885, EPI_ISL_795886, EPI_ISL_795887, EPI_ISL_795888, EPI_ISL_795889, EPI_ISL_795890, EPI_ISL_795891, EPI_ISL_795892, EPI_ISL_795893, EPI_ISL_795894, EPI_ISL_795895, EPI_ISL_795896, EPI_ISL_795897, EPI_ISL_795898, EPI_ISL_795899, EPI_ISL_795900, EPI_ISL_795901, EPI_ISL_795902, EPI_ISL_795903, EPI_ISL_795904, EPI_ISL_795905, EPI_ISL_795906, EPI_ISL_795907, EPI_ISL_795908, EPI_ISL_795909, EPI_ISL_795910, EPI_ISL_795911, EPI_ISL_795912, EPI_ISL_795913, EPI_ISL_795914, EPI_ISL_795915, EPI_ISL_795916, EPI_ISL_795917, EPI_ISL_795918, EPI_ISL_795919, EPI_ISL_795920, EPI_ISL_795921, EPI_ISL_795922, EPI_ISL_795923, EPI_ISL_795924, EPI_ISL_795925, EPI_ISL_795926, EPI_ISL_795927, EPI_ISL_795928, EPI_ISL_795929, EPI_ISL_795930, EPI_ISL_795931, EPI_ISL_795932, EPI_ISL_795933, EPI_ISL_795934, EPI_ISL_795935, EPI_ISL_795936, EPI_ISL_795937, EPI_ISL_795938, EPI_ISL_795939, EPI_ISL_795940, EPI_ISL_795941, EPI_ISL_795942, EPI_ISL_795943, EPI_ISL_795944, EPI_ISL_795945, EPI_ISL_795946, EPI_ISL_795947, EPI_ISL_795948, EPI_ISL_795949, EPI_ISL_795950, EPI_ISL_795951, EPI_ISL_795952, EPI_ISL_795953, EPI_ISL_795954, EPI_ISL_795955, EPI_ISL_795956, EPI_ISL_795957, EPI_ISL_795958, EPI_ISL_795959, EPI_ISL_795960, EPI_ISL_795961, EPI_ISL_795962, EPI_ISL_795963, EPI_ISL_795964, EPI_ISL_795965, EPI_ISL_795966, EPI_ISL_795967, EPI_ISL_795968, EPI_ISL_795969, EPI_ISL_795970, EPI_ISL_795971, EPI_ISL_795972, EPI_ISL_795973, EPI_ISL_795974, EPI_ISL_795975, EPI_ISL_795976, EPI_ISL_795977, EPI_ISL_795978, EPI_ISL_795979, EPI_ISL_795980, EPI_ISL_795981, EPI_ISL_795982, EPI_ISL_795983			
see above	Department of Virus and Microbiological Special Diagnostics, Statens Serum Institut, Copenhagen, Denmark	Albertsen Lab, Department of Chemistry and Bioscience, Aalborg University, Denmark	Danish Covid-19 Genome Consortium
EPI_ISL_796159, EPI_ISL_796160, EPI_ISL_796167, EPI_ISL_796171, EPI_ISL_796172, EPI_ISL_796173, EPI_ISL_796174, EPI_ISL_796177, EPI_ISL_796179, EPI_ISL_796180, EPI_ISL_796184, EPI_ISL_796188, EPI_ISL_796189, EPI_ISL_796190, EPI_ISL_796192, EPI_ISL_796194, EPI_ISL_796198, EPI_ISL_796199, EPI_ISL_796202, EPI_ISL_796204, EPI_ISL_796208, EPI_ISL_796218, EPI_ISL_796222, EPI_ISL_796223, EPI_ISL_796225, EPI_ISL_796226, EPI_ISL_796227, EPI_ISL_796229, EPI_ISL_796233, EPI_ISL_796242, EPI_ISL_796245, EPI_ISL_796246, EPI_ISL_796247, EPI_ISL_796249, EPI_ISL_796250, EPI_ISL_796252, EPI_ISL_796253, EPI_ISL_796256, EPI_ISL_796266, EPI_ISL_796268, EPI_ISL_796270, EPI_ISL_796271, EPI_ISL_796273, EPI_ISL_796275, EPI_ISL_796276, EPI_ISL_796279, EPI_ISL_796280, EPI_ISL_796282, EPI_ISL_796283, EPI_ISL_796284, EPI_ISL_796290, EPI_ISL_796293, EPI_ISL_796297, EPI_ISL_796298, EPI_ISL_796302, EPI_ISL_796303, EPI_ISL_796312, EPI_ISL_796316, EPI_ISL_796318, EPI_ISL_796319, EPI_ISL_796326, EPI_ISL_796333, EPI_ISL_796337, EPI_ISL_796340, EPI_ISL_796342, EPI_ISL_796348, EPI_ISL_796355, EPI_ISL_796359, EPI_ISL_796361, EPI_ISL_796362, EPI_ISL_796363, EPI_ISL_796364, EPI_ISL_796368, EPI_ISL_796371, EPI_ISL_796372, EPI_ISL_796374, EPI_ISL_796382, EPI_ISL_796396, EPI_ISL_796416, EPI_ISL_796418, EPI_ISL_796420, EPI_ISL_796421, EPI_ISL_796422, EPI_ISL_796423, EPI_ISL_796427, EPI_ISL_796428, EPI_ISL_796431, EPI_ISL_796436, EPI_ISL_796438, EPI_ISL_796443, EPI_ISL_796444, EPI_ISL_796446, EPI_ISL_796447, EPI_ISL_796449, EPI_ISL_796450, EPI_ISL_796452, EPI_ISL_796453, EPI_ISL_796454, EPI_ISL_796455, EPI_ISL_796458, EPI_ISL_796459, EPI_ISL_796463, EPI_ISL_796466, EPI_ISL_796467, EPI_ISL_796468, EPI_ISL_796469, EPI_ISL_796472, EPI_ISL_796473, EPI_ISL_796474, EPI_ISL_796475, EPI_ISL_796478, EPI_ISL_796484, EPI_ISL_796488, EPI_ISL_796491, EPI_ISL_796497, EPI_ISL_796501, EPI_ISL_796506, EPI_ISL_796515, EPI_ISL_796516, EPI_ISL_796528, EPI_ISL_796531, EPI_ISL_796536, EPI_ISL_796537, EPI_ISL_796549, EPI_ISL_796551, EPI_ISL_796553, EPI_ISL_796558, EPI_ISL_796564, EPI_ISL_796565, EPI_ISL_796575, EPI_ISL_796576, EPI_ISL_796581, EPI_ISL_796584, EPI_ISL_796595, EPI_ISL_796598, EPI_ISL_796599, EPI_ISL_796602, EPI_ISL_796604, EPI_ISL_796606, EPI_ISL_796608, EPI_ISL_796612, EPI_ISL_796622, EPI_ISL_796627, EPI_ISL_796628, EPI_ISL_796630, EPI_ISL_796633, EPI_ISL_796635			
see above	Viollier AG	Department of Biosystems Science and Engineering, ETH Zürich	Chaoran Chen, Sarah Nadeau, Catharine Aquino, Ivan Topolsky, Philipp Jablonski, Lara Fuhrmann, David Dreifuss, Katharina Jahn, Andreia Cabral de Gouvea, Maria Domenica Moccia, Simon Grüter, Timothy Sykes, Lennart Opitz, Griffin White, Laura Neff, Doris Popovic, Andrea Patrignani, Jay Tracy, Ralph Schlapbach, Christiane Beckmann, Maurice Redondo, Olivier Kobel, Christoph Noppen, Sophie Seidel, Noemie Santamaria de Souza, Niko Beerenwinkel, Tanja Stadler
EPI_ISL_796733, EPI_ISL_796734, EPI_ISL_796752, EPI_ISL_796753, EPI_ISL_796764, EPI_ISL_796774, EPI_ISL_796775	Instituto Nacional de Saude (INSA)	Instituto Nacional de Saude (INSA)	Borges et al
EPI_ISL_796795, EPI_ISL_796997	Lighthouse Lab in Alderley Park	Wellcome Sanger Institute for the COVID-19 Genomics UK (COG-UK) Consortium	Jacquelyn Wynn, Mairead Hyland, The Lighthouse Lab in Alderley Park and Alex Alderton, Roberto Amato, Sonia Goncalves, Ewan Harrison, David K. Jackson, Ian Johnston, Dominic Kwiatkowski, Cordelia Langford, John Sillitoe on behalf of the Wellcome Sanger Institute COVID-19 Surveillance Team
EPI_ISL_797119, EPI_ISL_797122, EPI_ISL_797123, EPI_ISL_797124, EPI_ISL_797127, EPI_ISL_797130, EPI_ISL_797131, EPI_ISL_797132, EPI_ISL_797135, EPI_ISL_797136, EPI_ISL_797137, EPI_ISL_797140, EPI_ISL_797145, EPI_ISL_797147, EPI_ISL_797150, EPI_ISL_797152, EPI_ISL_797157, EPI_ISL_797158, EPI_ISL_797159, EPI_ISL_797162, EPI_ISL_797166, EPI_ISL_797167, EPI_ISL_797169, EPI_ISL_797170, EPI_ISL_797172, EPI_ISL_797174, EPI_ISL_797180, EPI_ISL_797181, EPI_ISL_797182, EPI_ISL_797183, EPI_ISL_797184, EPI_ISL_797186, EPI_ISL_797188, EPI_ISL_797189, EPI_ISL_797191, EPI_ISL_797192, EPI_ISL_797193, EPI_ISL_797195, EPI_ISL_797196, EPI_ISL_797197, EPI_ISL_797199, EPI_ISL_797200, EPI_ISL_797201, EPI_ISL_797206, EPI_ISL_797208, EPI_ISL_797209, EPI_ISL_797211, EPI_ISL_797213, EPI_ISL_797214, EPI_ISL_797215, EPI_ISL_797217, EPI_ISL_797219, EPI_ISL_797221, EPI_ISL_797222, EPI_ISL_797223, EPI_ISL_797224, EPI_ISL_797227, EPI_ISL_797230, EPI_ISL_797233, EPI_ISL_797235, EPI_ISL_797236, EPI_ISL_797237, EPI_ISL_797238, EPI_ISL_797241, EPI_ISL_797244, EPI_ISL_797245, EPI_ISL_797249, EPI_ISL_797250, EPI_ISL_797251, EPI_ISL_797255, EPI_ISL_797257, EPI_ISL_797260, EPI_ISL_797262, EPI_ISL_797263, EPI_ISL_797265, EPI_ISL_797267, EPI_ISL_797268, EPI_ISL_797270, EPI_ISL_797271, EPI_ISL_797273, EPI_ISL_797274, EPI_ISL_797275, EPI_ISL_797276, EPI_ISL_797278, EPI_ISL_797279, EPI_ISL_797280, EPI_ISL_797282, EPI_ISL_797283, EPI_ISL_797284, EPI_ISL_797285, EPI_ISL_797287, EPI_ISL_797288, EPI_ISL_797291, EPI_ISL_797292, EPI_ISL_797293, EPI_ISL_797294, EPI_ISL_797297, EPI_ISL_797298, EPI_ISL_797299, EPI_ISL_797300, EPI_ISL_797302, EPI_ISL_797303, EPI_ISL_797304, EPI_ISL_797306, EPI_ISL_797307, EPI_ISL_797308, EPI_ISL_797309, EPI_ISL_797310, EPI_ISL_797311, EPI_ISL_797312, EPI_ISL_797314, EPI_ISL_797316, EPI_ISL_797320, EPI_ISL_797321, EPI_ISL_797322, EPI_ISL_797324, EPI_ISL_797328, EPI_ISL_797329, EPI_ISL_797330, EPI_ISL_797331, EPI_ISL_797333, EPI_ISL_797334, EPI_ISL_797336, EPI_ISL_797340, EPI_ISL_797341, EPI_ISL_797343, EPI_ISL_797344, EPI_ISL_797347, EPI_ISL_797348, EPI_ISL_797350, EPI_ISL_797351, EPI_ISL_797352, EPI_ISL_797353, EPI_ISL_797355, EPI_ISL_797356, EPI_ISL_797358, EPI_ISL_797359, EPI_ISL_797360, EPI_ISL_797361, EPI_ISL_797364, EPI_ISL_797368, EPI_ISL_797369, EPI_ISL_797370, EPI_ISL_797371, EPI_ISL_797372, EPI_ISL_797373, EPI_ISL_797374, EPI_ISL_797375, EPI_ISL_797377, EPI_ISL_797378, EPI_ISL_797381, EPI_ISL_797382, EPI_ISL_797383, EPI_ISL_797384, EPI_ISL_797386, EPI_ISL_797387, EPI_ISL_797388, EPI_ISL_797392, EPI_ISL_797393, EPI_ISL_797394, EPI_ISL_797396			
see above	Lighthouse Lab in Milton Keynes	Wellcome Sanger Institute for the COVID-19 Genomics UK (COG-UK) Consortium	The Lighthouse Lab in Milton Keynes and Alex Alderton, Roberto Amato, Sonia Goncalves, Ewan Harrison, David K. Jackson, Ian Johnston, Dominic Kwiatkowski, Cordelia Langford, John Sillitoe on behalf of the Wellcome Sanger Institute COVID-19 Surveillance Team
EPI_ISL_797400, EPI_ISL_797403, EPI_ISL_797434, EPI_ISL_797446, EPI_ISL_797465, EPI_ISL_797481, EPI_ISL_797483, EPI_ISL_797492, EPI_ISL_797497, EPI_ISL_797498, EPI_ISL_797502, EPI_ISL_797505, EPI_ISL_797510, EPI_ISL_797511, EPI_ISL_797532, EPI_ISL_797537, EPI_ISL_797548, EPI_ISL_797566, EPI_ISL_797578, EPI_ISL_797579, EPI_ISL_797580, EPI_ISL_797590, EPI_ISL_797593, EPI_ISL_797595, EPI_ISL_797597, EPI_ISL_797600, EPI_ISL_797625, EPI_ISL_797631, EPI_ISL_797643, EPI_ISL_797645, EPI_ISL_797646, EPI_ISL_797647, EPI_ISL_797648, EPI_ISL_797652, EPI_ISL_797657, EPI_ISL_797659, EPI_ISL_797664, EPI_ISL_797668, EPI_ISL_797676, EPI_ISL_797688, EPI_ISL_797690, EPI_ISL_797692, EPI_ISL_797694, EPI_ISL_797705			
see above	Lighthouse Lab in Cambridge	Wellcome Sanger Institute for the COVID-19 Genomics UK	Rob Howes, The Lighthouse Lab in Cambridge and Alex Alderton, Roberto Amato, Sonia Goncalves, Ewan Harrison, David K. Jackson, Ian Johnston,

		(COG-UK) Consortium	Dominic Kwiatkowski, Cordelia Langford, John Sillitoe on behalf of the Wellcome Sanger Institute COVID-19 Surveillance Team
EPI_ISL_797739, EPI_ISL_797775, EPI_ISL_797789, EPI_ISL_797797, EPI_ISL_797817, EPI_ISL_797831, EPI_ISL_797838, EPI_ISL_797851, EPI_ISL_797868, EPI_ISL_797884, EPI_ISL_797903, EPI_ISL_797916, EPI_ISL_797921, EPI_ISL_797937, EPI_ISL_797952, EPI_ISL_797965, EPI_ISL_797985			
see above	Lighthouse Lab in Glasgow	Wellcome Sanger Institute for the COVID-19 Genomics UK (COG-UK) Consortium	Harper VanSteenhouse, Yumi Kasai, David Gray, Carol Clugston, Anna Dominiczak and Alex Alderton, Roberto Amato, Sonia Goncalves, Ewan Harrison, David K. Jackson, Ian Johnston, Dominic Kwiatkowski, Cordelia Langford, John Sillitoe on behalf of the Wellcome Sanger Institute COVID-19 Surveillance Team
EPI_ISL_797999, EPI_ISL_798002, EPI_ISL_798007, EPI_ISL_798009, EPI_ISL_798011, EPI_ISL_798013, EPI_ISL_798015	Lighthouse Lab in Alderley Park	Wellcome Sanger Institute for the COVID-19 Genomics UK (COG-UK) Consortium	Jacquelyn Wynn, Mairead Hyland, The Lighthouse Lab in Alderley Park and Alex Alderton, Roberto Amato, Sonia Goncalves, Ewan Harrison, David K. Jackson, Ian Johnston, Dominic Kwiatkowski, Cordelia Langford, John Sillitoe on behalf of the Wellcome Sanger Institute COVID-19 Surveillance Team
EPI_ISL_798017, EPI_ISL_798022	Lighthouse Lab in Glasgow	Wellcome Sanger Institute for the COVID-19 Genomics UK (COG-UK) Consortium	Harper VanSteenhouse, Yumi Kasai, David Gray, Carol Clugston, Anna Dominiczak and Alex Alderton, Roberto Amato, Sonia Goncalves, Ewan Harrison, David K. Jackson, Ian Johnston, Dominic Kwiatkowski, Cordelia Langford, John Sillitoe on behalf of the Wellcome Sanger Institute COVID-19 Surveillance Team
EPI_ISL_798025, EPI_ISL_798027, EPI_ISL_798029, EPI_ISL_798035	Lighthouse Lab in Alderley Park	Wellcome Sanger Institute for the COVID-19 Genomics UK (COG-UK) Consortium	Jacquelyn Wynn, Mairead Hyland, The Lighthouse Lab in Alderley Park and Alex Alderton, Roberto Amato, Sonia Goncalves, Ewan Harrison, David K. Jackson, Ian Johnston, Dominic Kwiatkowski, Cordelia Langford, John Sillitoe on behalf of the Wellcome Sanger Institute COVID-19 Surveillance Team
EPI_ISL_798043, EPI_ISL_798051	Lighthouse Lab in Glasgow	Wellcome Sanger Institute for the COVID-19 Genomics UK (COG-UK) Consortium	Harper VanSteenhouse, Yumi Kasai, David Gray, Carol Clugston, Anna Dominiczak and Alex Alderton, Roberto Amato, Sonia Goncalves, Ewan Harrison, David K. Jackson, Ian Johnston, Dominic Kwiatkowski, Cordelia Langford, John Sillitoe on behalf of the Wellcome Sanger Institute COVID-19 Surveillance Team
EPI_ISL_798054, EPI_ISL_798061, EPI_ISL_798062, EPI_ISL_798064, EPI_ISL_798066, EPI_ISL_798068, EPI_ISL_798073	Lighthouse Lab in Alderley Park	Wellcome Sanger Institute for the COVID-19 Genomics UK (COG-UK) Consortium	Jacquelyn Wynn, Mairead Hyland, The Lighthouse Lab in Alderley Park and Alex Alderton, Roberto Amato, Sonia Goncalves, Ewan Harrison, David K. Jackson, Ian Johnston, Dominic Kwiatkowski, Cordelia Langford, John Sillitoe on behalf of the Wellcome Sanger Institute COVID-19 Surveillance Team
EPI_ISL_798080	Lighthouse Lab in Glasgow	Wellcome Sanger Institute for the COVID-19 Genomics UK (COG-UK) Consortium	Harper VanSteenhouse, Yumi Kasai, David Gray, Carol Clugston, Anna Dominiczak and Alex Alderton, Roberto Amato, Sonia Goncalves, Ewan Harrison, David K. Jackson, Ian Johnston, Dominic Kwiatkowski, Cordelia Langford, John Sillitoe on behalf of the Wellcome Sanger Institute COVID-19 Surveillance Team
EPI_ISL_798083, EPI_ISL_798084, EPI_ISL_798096, EPI_ISL_798101, EPI_ISL_798103, EPI_ISL_798106	Lighthouse Lab in Alderley Park	Wellcome Sanger Institute for the COVID-19 Genomics UK (COG-UK) Consortium	Jacquelyn Wynn, Mairead Hyland, The Lighthouse Lab in Alderley Park and Alex Alderton, Roberto Amato, Sonia Goncalves, Ewan Harrison, David K. Jackson, Ian Johnston, Dominic Kwiatkowski, Cordelia Langford, John Sillitoe on behalf of the Wellcome Sanger Institute COVID-19 Surveillance Team
EPI_ISL_798107	Lighthouse Lab in Glasgow	Wellcome Sanger Institute for the COVID-19 Genomics UK (COG-UK) Consortium	Harper VanSteenhouse, Yumi Kasai, David Gray, Carol Clugston, Anna Dominiczak and Alex Alderton, Roberto Amato, Sonia Goncalves, Ewan Harrison, David K. Jackson, Ian Johnston, Dominic Kwiatkowski, Cordelia Langford, John Sillitoe on behalf of the Wellcome Sanger Institute COVID-19 Surveillance Team
EPI_ISL_798108, EPI_ISL_798119, EPI_ISL_798122, EPI_ISL_798123, EPI_ISL_798124, EPI_ISL_798125, EPI_ISL_798127, EPI_ISL_798131, EPI_ISL_798133, EPI_ISL_798134, EPI_ISL_798139			
see above	Lighthouse Lab in Alderley Park	Wellcome Sanger Institute for the COVID-19 Genomics UK (COG-UK) Consortium	Jacquelyn Wynn, Mairead Hyland, The Lighthouse Lab in Alderley Park and Alex Alderton, Roberto Amato, Sonia Goncalves, Ewan Harrison, David K. Jackson, Ian Johnston, Dominic Kwiatkowski, Cordelia Langford, John Sillitoe on behalf of the Wellcome Sanger Institute COVID-19 Surveillance Team
EPI_ISL_798141	Lighthouse Lab in Glasgow	Wellcome Sanger Institute for the COVID-19 Genomics UK (COG-UK) Consortium	Harper VanSteenhouse, Yumi Kasai, David Gray, Carol Clugston, Anna Dominiczak and Alex Alderton, Roberto Amato, Sonia Goncalves, Ewan Harrison, David K. Jackson, Ian Johnston, Dominic Kwiatkowski, Cordelia Langford, John Sillitoe on behalf of the Wellcome Sanger Institute COVID-19 Surveillance Team
EPI_ISL_798152, EPI_ISL_798154	Lighthouse Lab in Alderley Park	Wellcome Sanger Institute for the COVID-19 Genomics UK (COG-UK) Consortium	Jacquelyn Wynn, Mairead Hyland, The Lighthouse Lab in Alderley Park and Alex Alderton, Roberto Amato, Sonia Goncalves, Ewan Harrison, David K. Jackson, Ian Johnston, Dominic Kwiatkowski, Cordelia Langford, John Sillitoe on behalf of the Wellcome Sanger Institute COVID-19 Surveillance Team
EPI_ISL_798159	Lighthouse Lab in Glasgow	Wellcome Sanger Institute for the COVID-19 Genomics UK (COG-UK) Consortium	Harper VanSteenhouse, Yumi Kasai, David Gray, Carol Clugston, Anna Dominiczak and Alex Alderton, Roberto Amato, Sonia Goncalves, Ewan Harrison, David K. Jackson, Ian Johnston, Dominic Kwiatkowski, Cordelia Langford, John Sillitoe on behalf of the Wellcome Sanger Institute COVID-19 Surveillance Team
EPI_ISL_798169, EPI_ISL_798170	Lighthouse Lab in Alderley Park	Wellcome Sanger Institute for the COVID-19 Genomics UK (COG-UK) Consortium	Jacquelyn Wynn, Mairead Hyland, The Lighthouse Lab in Alderley Park and Alex Alderton, Roberto Amato, Sonia Goncalves, Ewan Harrison, David K. Jackson, Ian Johnston, Dominic Kwiatkowski, Cordelia Langford, John Sillitoe on behalf of the Wellcome Sanger Institute COVID-19 Surveillance Team
EPI_ISL_798171	Lighthouse Lab in Glasgow	Wellcome Sanger Institute for the COVID-19 Genomics UK (COG-UK) Consortium	Harper VanSteenhouse, Yumi Kasai, David Gray, Carol Clugston, Anna Dominiczak and Alex Alderton, Roberto Amato, Sonia Goncalves, Ewan Harrison, David K. Jackson, Ian Johnston, Dominic Kwiatkowski, Cordelia Langford, John Sillitoe on behalf of the Wellcome Sanger Institute COVID-19 Surveillance Team
EPI_ISL_798173, EPI_ISL_798174, EPI_ISL_798175, EPI_ISL_798176	Lighthouse Lab in Alderley Park	Wellcome Sanger Institute for the COVID-19 Genomics UK (COG-UK) Consortium	Jacquelyn Wynn, Mairead Hyland, The Lighthouse Lab in Alderley Park and Alex Alderton, Roberto Amato, Sonia Goncalves, Ewan Harrison, David K. Jackson, Ian Johnston, Dominic Kwiatkowski, Cordelia Langford, John Sillitoe on behalf of the Wellcome Sanger Institute COVID-19 Surveillance Team
EPI_ISL_798178	Lighthouse Lab in Glasgow	Wellcome Sanger Institute for the COVID-19 Genomics UK (COG-UK) Consortium	Harper VanSteenhouse, Yumi Kasai, David Gray, Carol Clugston, Anna Dominiczak and Alex Alderton, Roberto Amato, Sonia Goncalves, Ewan Harrison, David K. Jackson, Ian Johnston, Dominic Kwiatkowski, Cordelia Langford, John Sillitoe on behalf of the Wellcome Sanger Institute COVID-19 Surveillance Team
EPI_ISL_798181, EPI_ISL_798182	Lighthouse Lab in Alderley Park	Wellcome Sanger Institute for the COVID-19 Genomics UK (COG-UK) Consortium	Jacquelyn Wynn, Mairead Hyland, The Lighthouse Lab in Alderley Park and Alex Alderton, Roberto Amato, Sonia Goncalves, Ewan Harrison, David K. Jackson, Ian Johnston, Dominic Kwiatkowski, Cordelia Langford, John Sillitoe on behalf of the Wellcome Sanger Institute COVID-19 Surveillance Team
EPI_ISL_798184, EPI_ISL_798192	Lighthouse Lab in Glasgow	Wellcome Sanger Institute for the COVID-19 Genomics UK (COG-UK) Consortium	Harper VanSteenhouse, Yumi Kasai, David Gray, Carol Clugston, Anna Dominiczak and Alex Alderton, Roberto Amato, Sonia Goncalves, Ewan Harrison, David K. Jackson, Ian Johnston, Dominic Kwiatkowski, Cordelia Langford, John Sillitoe on behalf of the Wellcome Sanger Institute COVID-19 Surveillance Team
EPI_ISL_798202, EPI_ISL_798204, EPI_ISL_798208, EPI_ISL_798220, EPI_ISL_798221, EPI_ISL_798222	Lighthouse Lab in Alderley Park	Wellcome Sanger Institute for the COVID-19 Genomics UK (COG-UK) Consortium	Jacquelyn Wynn, Mairead Hyland, The Lighthouse Lab in Alderley Park and Alex Alderton, Roberto Amato, Sonia Goncalves, Ewan Harrison, David K. Jackson, Ian Johnston, Dominic Kwiatkowski, Cordelia Langford, John Sillitoe on behalf of the Wellcome Sanger Institute COVID-19 Surveillance Team
EPI_ISL_798223	Lighthouse Lab in Glasgow	Wellcome Sanger Institute for the COVID-19 Genomics UK (COG-UK) Consortium	Harper VanSteenhouse, Yumi Kasai, David Gray, Carol Clugston, Anna Dominiczak and Alex Alderton, Roberto Amato, Sonia Goncalves, Ewan Harrison, David K. Jackson, Ian Johnston, Dominic Kwiatkowski, Cordelia Langford, John Sillitoe on behalf of the Wellcome Sanger Institute COVID-19 Surveillance Team
EPI_ISL_798225, EPI_ISL_798229	Lighthouse Lab in Alderley Park	Wellcome Sanger Institute for the COVID-19 Genomics UK (COG-UK) Consortium	Jacquelyn Wynn, Mairead Hyland, The Lighthouse Lab in Alderley Park and Alex Alderton, Roberto Amato, Sonia Goncalves, Ewan Harrison, David K. Jackson, Ian Johnston, Dominic Kwiatkowski, Cordelia Langford, John Sillitoe on behalf of the Wellcome Sanger Institute COVID-19 Surveillance Team
EPI_ISL_798231	Lighthouse Lab in Glasgow	Wellcome Sanger Institute for the COVID-19 Genomics UK (COG-UK) Consortium	Harper VanSteenhouse, Yumi Kasai, David Gray, Carol Clugston, Anna Dominiczak and Alex Alderton, Roberto Amato, Sonia Goncalves, Ewan Harrison, David K. Jackson, Ian Johnston, Dominic Kwiatkowski, Cordelia Langford, John Sillitoe on behalf of the Wellcome Sanger Institute COVID-19 Surveillance Team
EPI_ISL_798234, EPI_ISL_798235, EPI_ISL_798236, EPI_ISL_798239, EPI_ISL_798241, EPI_ISL_798242, EPI_ISL_798245, EPI_ISL_798246, EPI_ISL_798248	Lighthouse Lab in Alderley Park	Wellcome Sanger Institute for the COVID-19 Genomics UK (COG-UK) Consortium	Jacquelyn Wynn, Mairead Hyland, The Lighthouse Lab in Alderley Park and Alex Alderton, Roberto Amato, Sonia Goncalves, Ewan Harrison, David K. Jackson, Ian Johnston, Dominic Kwiatkowski, Cordelia Langford, John Sillitoe on behalf of the Wellcome Sanger Institute COVID-19 Surveillance Team
EPI_ISL_798249	Lighthouse Lab in Glasgow	Wellcome Sanger Institute for the COVID-19 Genomics UK (COG-UK) Consortium	Harper VanSteenhouse, Yumi Kasai, David Gray, Carol Clugston, Anna Dominiczak and Alex Alderton, Roberto Amato, Sonia Goncalves, Ewan Harrison, David K. Jackson, Ian Johnston, Dominic Kwiatkowski, Cordelia Langford, John Sillitoe on behalf of the Wellcome Sanger Institute COVID-19 Surveillance

			Team
EPI_ISL_798250, EPI_ISL_798255	Lighthouse Lab in Alderley Park	Wellcome Sanger Institute for the COVID-19 Genomics UK (COG-UK) Consortium	Jacquelyn Wynn, Mairead Hyland, The Lighthouse Lab in Alderley Park and Alex Alderton, Roberto Amato, Sonia Goncalves, Ewan Harrison, David K. Jackson, Ian Johnston, Dominic Kwiatkowski, Cordelia Langford, John Sillitoe on behalf of the Wellcome Sanger Institute COVID-19 Surveillance Team
EPI_ISL_798261	Lighthouse Lab in Glasgow	Wellcome Sanger Institute for the COVID-19 Genomics UK (COG-UK) Consortium	Harper VanSteenhouse, Yumi Kasai, David Gray, Carol Clugston, Anna Dominiczak and Alex Alderton, Roberto Amato, Sonia Goncalves, Ewan Harrison, David K. Jackson, Ian Johnston, Dominic Kwiatkowski, Cordelia Langford, John Sillitoe on behalf of the Wellcome Sanger Institute COVID-19 Surveillance Team
EPI_ISL_798268	Lighthouse Lab in Alderley Park	Wellcome Sanger Institute for the COVID-19 Genomics UK (COG-UK) Consortium	Jacquelyn Wynn, Mairead Hyland, The Lighthouse Lab in Alderley Park and Alex Alderton, Roberto Amato, Sonia Goncalves, Ewan Harrison, David K. Jackson, Ian Johnston, Dominic Kwiatkowski, Cordelia Langford, John Sillitoe on behalf of the Wellcome Sanger Institute COVID-19 Surveillance Team
EPI_ISL_798271	Lighthouse Lab in Glasgow	Wellcome Sanger Institute for the COVID-19 Genomics UK (COG-UK) Consortium	Harper VanSteenhouse, Yumi Kasai, David Gray, Carol Clugston, Anna Dominiczak and Alex Alderton, Roberto Amato, Sonia Goncalves, Ewan Harrison, David K. Jackson, Ian Johnston, Dominic Kwiatkowski, Cordelia Langford, John Sillitoe on behalf of the Wellcome Sanger Institute COVID-19 Surveillance Team
EPI_ISL_798274, EPI_ISL_798275, EPI_ISL_798277	Lighthouse Lab in Alderley Park	Wellcome Sanger Institute for the COVID-19 Genomics UK (COG-UK) Consortium	Jacquelyn Wynn, Mairead Hyland, The Lighthouse Lab in Alderley Park and Alex Alderton, Roberto Amato, Sonia Goncalves, Ewan Harrison, David K. Jackson, Ian Johnston, Dominic Kwiatkowski, Cordelia Langford, John Sillitoe on behalf of the Wellcome Sanger Institute COVID-19 Surveillance Team
EPI_ISL_798278	Lighthouse Lab in Glasgow	Wellcome Sanger Institute for the COVID-19 Genomics UK (COG-UK) Consortium	Harper VanSteenhouse, Yumi Kasai, David Gray, Carol Clugston, Anna Dominiczak and Alex Alderton, Roberto Amato, Sonia Goncalves, Ewan Harrison, David K. Jackson, Ian Johnston, Dominic Kwiatkowski, Cordelia Langford, John Sillitoe on behalf of the Wellcome Sanger Institute COVID-19 Surveillance Team
EPI_ISL_798280, EPI_ISL_798282, EPI_ISL_798283, EPI_ISL_798284, EPI_ISL_798285	Lighthouse Lab in Alderley Park	Wellcome Sanger Institute for the COVID-19 Genomics UK (COG-UK) Consortium	Jacquelyn Wynn, Mairead Hyland, The Lighthouse Lab in Alderley Park and Alex Alderton, Roberto Amato, Sonia Goncalves, Ewan Harrison, David K. Jackson, Ian Johnston, Dominic Kwiatkowski, Cordelia Langford, John Sillitoe on behalf of the Wellcome Sanger Institute COVID-19 Surveillance Team
EPI_ISL_798287	Lighthouse Lab in Glasgow	Wellcome Sanger Institute for the COVID-19 Genomics UK (COG-UK) Consortium	Harper VanSteenhouse, Yumi Kasai, David Gray, Carol Clugston, Anna Dominiczak and Alex Alderton, Roberto Amato, Sonia Goncalves, Ewan Harrison, David K. Jackson, Ian Johnston, Dominic Kwiatkowski, Cordelia Langford, John Sillitoe on behalf of the Wellcome Sanger Institute COVID-19 Surveillance Team
EPI_ISL_798292, EPI_ISL_798295, EPI_ISL_798296, EPI_ISL_798299, EPI_ISL_798305	Lighthouse Lab in Alderley Park	Wellcome Sanger Institute for the COVID-19 Genomics UK (COG-UK) Consortium	Jacquelyn Wynn, Mairead Hyland, The Lighthouse Lab in Alderley Park and Alex Alderton, Roberto Amato, Sonia Goncalves, Ewan Harrison, David K. Jackson, Ian Johnston, Dominic Kwiatkowski, Cordelia Langford, John Sillitoe on behalf of the Wellcome Sanger Institute COVID-19 Surveillance Team
EPI_ISL_798308	Lighthouse Lab in Glasgow	Wellcome Sanger Institute for the COVID-19 Genomics UK (COG-UK) Consortium	Harper VanSteenhouse, Yumi Kasai, David Gray, Carol Clugston, Anna Dominiczak and Alex Alderton, Roberto Amato, Sonia Goncalves, Ewan Harrison, David K. Jackson, Ian Johnston, Dominic Kwiatkowski, Cordelia Langford, John Sillitoe on behalf of the Wellcome Sanger Institute COVID-19 Surveillance Team
EPI_ISL_798310, EPI_ISL_798314, EPI_ISL_798321, EPI_ISL_798323, EPI_ISL_798324	Lighthouse Lab in Alderley Park	Wellcome Sanger Institute for the COVID-19 Genomics UK (COG-UK) Consortium	Jacquelyn Wynn, Mairead Hyland, The Lighthouse Lab in Alderley Park and Alex Alderton, Roberto Amato, Sonia Goncalves, Ewan Harrison, David K. Jackson, Ian Johnston, Dominic Kwiatkowski, Cordelia Langford, John Sillitoe on behalf of the Wellcome Sanger Institute COVID-19 Surveillance Team
EPI_ISL_798325	Lighthouse Lab in Glasgow	Wellcome Sanger Institute for the COVID-19 Genomics UK (COG-UK) Consortium	Harper VanSteenhouse, Yumi Kasai, David Gray, Carol Clugston, Anna Dominiczak and Alex Alderton, Roberto Amato, Sonia Goncalves, Ewan Harrison, David K. Jackson, Ian Johnston, Dominic Kwiatkowski, Cordelia Langford, John Sillitoe on behalf of the Wellcome Sanger Institute COVID-19 Surveillance Team
EPI_ISL_798328, EPI_ISL_798329	Lighthouse Lab in Alderley Park	Wellcome Sanger Institute for the COVID-19 Genomics UK (COG-UK) Consortium	Jacquelyn Wynn, Mairead Hyland, The Lighthouse Lab in Alderley Park and Alex Alderton, Roberto Amato, Sonia Goncalves, Ewan Harrison, David K. Jackson, Ian Johnston, Dominic Kwiatkowski, Cordelia Langford, John Sillitoe on behalf of the Wellcome Sanger Institute COVID-19 Surveillance Team
EPI_ISL_798331, EPI_ISL_798332	Lighthouse Lab in Glasgow	Wellcome Sanger Institute for the COVID-19 Genomics UK (COG-UK) Consortium	Harper VanSteenhouse, Yumi Kasai, David Gray, Carol Clugston, Anna Dominiczak and Alex Alderton, Roberto Amato, Sonia Goncalves, Ewan Harrison, David K. Jackson, Ian Johnston, Dominic Kwiatkowski, Cordelia Langford, John Sillitoe on behalf of the Wellcome Sanger Institute COVID-19 Surveillance Team

EPI_ISL_798334, EPI_ISL_798336, EPI_ISL_798341, EPI_ISL_798345, EPI_ISL_798346, EPI_ISL_798349, EPI_ISL_798356, EPI_ISL_798362, EPI_ISL_798368, EPI_ISL_798369, EPI_ISL_798370, EPI_ISL_798378, EPI_ISL_798379, EPI_ISL_798384, EPI_ISL_798386, EPI_ISL_798388, EPI_ISL_798389, EPI_ISL_798396, EPI_ISL_798399, EPI_ISL_798404, EPI_ISL_798405, EPI_ISL_798413, EPI_ISL_798416, EPI_ISL_798417, EPI_ISL_798431, EPI_ISL_798436, EPI_ISL_798444, EPI_ISL_798455, EPI_ISL_798465, EPI_ISL_798468, EPI_ISL_798475, EPI_ISL_798479, EPI_ISL_798483, EPI_ISL_798487, EPI_ISL_798492, EPI_ISL_798493, EPI_ISL_798500, EPI_ISL_798502, EPI_ISL_798508, EPI_ISL_798510, EPI_ISL_798513, EPI_ISL_798516, EPI_ISL_798517, EPI_ISL_798519, EPI_ISL_798524, EPI_ISL_798531, EPI_ISL_798534, EPI_ISL_798539, EPI_ISL_798541, EPI_ISL_798542, EPI_ISL_798548, EPI_ISL_798550, EPI_ISL_798555, EPI_ISL_798561, EPI_ISL_798564, EPI_ISL_798579, EPI_ISL_798591, EPI_ISL_798593, EPI_ISL_798598, EPI_ISL_798601, EPI_ISL_798607, EPI_ISL_798609, EPI_ISL_798612, EPI_ISL_798622, EPI_ISL_798631, EPI_ISL_798632, EPI_ISL_798633, EPI_ISL_798635, EPI_ISL_798637, EPI_ISL_798639, EPI_ISL_798640, EPI_ISL_798641, EPI_ISL_798643, EPI_ISL_798652, EPI_ISL_798659, EPI_ISL_798661, EPI_ISL_798665, EPI_ISL_798667, EPI_ISL_798668, EPI_ISL_798669, EPI_ISL_798671

see above	Lighthouse Lab in Alderley Park	Wellcome Sanger Institute for the COVID-19 Genomics UK (COG-UK) Consortium	Jacquelyn Wynn, Mairead Hyland, The Lighthouse Lab in Alderley Park and Alex Alderton, Roberto Amato, Sonia Goncalves, Ewan Harrison, David K. Jackson, Ian Johnston, Dominic Kwiatkowski, Cordelia Langford, John Sillitoe on behalf of the Wellcome Sanger Institute COVID-19 Surveillance Team
EPI_ISL_798674	Lighthouse Lab in Cambridge	Wellcome Sanger Institute for the COVID-19 Genomics UK (COG-UK) Consortium	Rob Howes, The Lighthouse Lab in Cambridge and Alex Alderton, Roberto Amato, Sonia Goncalves, Ewan Harrison, David K. Jackson, Ian Johnston, Dominic Kwiatkowski, Cordelia Langford, John Sillitoe on behalf of the Wellcome Sanger Institute COVID-19 Surveillance Team
EPI_ISL_798676	Lighthouse Lab in Alderley Park	Wellcome Sanger Institute for the COVID-19 Genomics UK (COG-UK) Consortium	Jacquelyn Wynn, Mairead Hyland, The Lighthouse Lab in Alderley Park and Alex Alderton, Roberto Amato, Sonia Goncalves, Ewan Harrison, David K. Jackson, Ian Johnston, Dominic Kwiatkowski, Cordelia Langford, John Sillitoe on behalf of the Wellcome Sanger Institute COVID-19 Surveillance Team
EPI_ISL_798678, EPI_ISL_798687	Lighthouse Lab in Cambridge	Wellcome Sanger Institute for the COVID-19 Genomics UK (COG-UK) Consortium	Rob Howes, The Lighthouse Lab in Cambridge and Alex Alderton, Roberto Amato, Sonia Goncalves, Ewan Harrison, David K. Jackson, Ian Johnston, Dominic Kwiatkowski, Cordelia Langford, John Sillitoe on behalf of the Wellcome Sanger Institute COVID-19 Surveillance Team
EPI_ISL_798688, EPI_ISL_798689, EPI_ISL_798690, EPI_ISL_798692, EPI_ISL_798695	Lighthouse Lab in Alderley Park	Wellcome Sanger Institute for the COVID-19 Genomics UK (COG-UK) Consortium	Jacquelyn Wynn, Mairead Hyland, The Lighthouse Lab in Alderley Park and Alex Alderton, Roberto Amato, Sonia Goncalves, Ewan Harrison, David K. Jackson, Ian Johnston, Dominic Kwiatkowski, Cordelia Langford, John Sillitoe on behalf of the Wellcome Sanger Institute COVID-19 Surveillance Team
EPI_ISL_798700	Lighthouse Lab in Cambridge	Wellcome Sanger Institute for the COVID-19 Genomics UK (COG-UK) Consortium	Rob Howes, The Lighthouse Lab in Cambridge and Alex Alderton, Roberto Amato, Sonia Goncalves, Ewan Harrison, David K. Jackson, Ian Johnston, Dominic Kwiatkowski, Cordelia Langford, John Sillitoe on behalf of the Wellcome Sanger Institute COVID-19 Surveillance Team
EPI_ISL_798704, EPI_ISL_798705, EPI_ISL_798722, EPI_ISL_798727, EPI_ISL_798735, EPI_ISL_798744, EPI_ISL_798749, EPI_ISL_798768, EPI_ISL_798771	Lighthouse Lab in Alderley Park	Wellcome Sanger Institute for the COVID-19 Genomics UK (COG-UK) Consortium	Jacquelyn Wynn, Mairead Hyland, The Lighthouse Lab in Alderley Park and Alex Alderton, Roberto Amato, Sonia Goncalves, Ewan Harrison, David K. Jackson, Ian Johnston, Dominic Kwiatkowski, Cordelia Langford, John Sillitoe on behalf of the Wellcome Sanger Institute COVID-19 Surveillance Team
EPI_ISL_798778	Lighthouse Lab in Cambridge	Wellcome Sanger Institute for the COVID-19 Genomics UK (COG-UK) Consortium	Rob Howes, The Lighthouse Lab in Cambridge and Alex Alderton, Roberto Amato, Sonia Goncalves, Ewan Harrison, David K. Jackson, Ian Johnston, Dominic Kwiatkowski, Cordelia Langford, John Sillitoe on behalf of the Wellcome Sanger Institute COVID-19 Surveillance Team
EPI_ISL_798784	Lighthouse Lab in Alderley Park	Wellcome Sanger Institute for the COVID-19 Genomics UK (COG-UK) Consortium	Jacquelyn Wynn, Mairead Hyland, The Lighthouse Lab in Alderley Park and Alex Alderton, Roberto Amato, Sonia Goncalves, Ewan Harrison, David K. Jackson, Ian Johnston, Dominic Kwiatkowski, Cordelia Langford, John Sillitoe on behalf of the Wellcome Sanger Institute COVID-19 Surveillance Team
EPI_ISL_798786, EPI_ISL_798790	Lighthouse Lab in Cambridge	Wellcome Sanger Institute for the COVID-19 Genomics UK (COG-UK) Consortium	Rob Howes, The Lighthouse Lab in Cambridge and Alex Alderton, Roberto Amato, Sonia Goncalves, Ewan Harrison, David K. Jackson, Ian Johnston, Dominic Kwiatkowski, Cordelia Langford, John Sillitoe on behalf of the Wellcome Sanger Institute COVID-19 Surveillance Team
EPI_ISL_798791, EPI_ISL_798793, EPI_ISL_798798, EPI_ISL_798804, EPI_ISL_798811, EPI_ISL_798814, EPI_ISL_798819, EPI_ISL_798824	Lighthouse Lab in Alderley Park	Wellcome Sanger Institute for the COVID-19 Genomics UK (COG-UK) Consortium	Jacquelyn Wynn, Mairead Hyland, The Lighthouse Lab in Alderley Park and Alex Alderton, Roberto Amato, Sonia Goncalves, Ewan Harrison, David K. Jackson, Ian Johnston, Dominic Kwiatkowski, Cordelia Langford, John Sillitoe on behalf of the Wellcome Sanger Institute COVID-19 Surveillance Team
EPI_ISL_798825	Lighthouse Lab in Cambridge	Wellcome Sanger Institute for the COVID-19 Genomics UK (COG-UK) Consortium	Rob Howes, The Lighthouse Lab in Cambridge and Alex Alderton, Roberto Amato, Sonia Goncalves, Ewan Harrison, David K. Jackson, Ian Johnston, Dominic Kwiatkowski, Cordelia Langford, John Sillitoe on behalf of the Wellcome Sanger Institute COVID-19 Surveillance Team

EPI_ISL_798826, EPI_ISL_798833	Lighthouse Lab in Alderley Park	Wellcome Sanger Institute for the COVID-19 Genomics UK (COG-UK) Consortium	Jacquelyn Wynn, Mairead Hyland, The Lighthouse Lab in Alderley Park and Alex Alderton, Roberto Amato, Sonia Goncalves, Ewan Harrison, David K. Jackson, Ian Johnston, Dominic Kwiatkowski, Cordelia Langford, John Sillitoe on behalf of the Wellcome Sanger Institute COVID-19 Surveillance Team
EPI_ISL_798834, EPI_ISL_798841	Lighthouse Lab in Cambridge	Wellcome Sanger Institute for the COVID-19 Genomics UK (COG-UK) Consortium	Rob Howes, The Lighthouse Lab in Cambridge and Alex Alderton, Roberto Amato, Sonia Goncalves, Ewan Harrison, David K. Jackson, Ian Johnston, Dominic Kwiatkowski, Cordelia Langford, John Sillitoe on behalf of the Wellcome Sanger Institute COVID-19 Surveillance Team
EPI_ISL_798845	Lighthouse Lab in Alderley Park	Wellcome Sanger Institute for the COVID-19 Genomics UK (COG-UK) Consortium	Jacquelyn Wynn, Mairead Hyland, The Lighthouse Lab in Alderley Park and Alex Alderton, Roberto Amato, Sonia Goncalves, Ewan Harrison, David K. Jackson, Ian Johnston, Dominic Kwiatkowski, Cordelia Langford, John Sillitoe on behalf of the Wellcome Sanger Institute COVID-19 Surveillance Team
EPI_ISL_798848	Lighthouse Lab in Cambridge	Wellcome Sanger Institute for the COVID-19 Genomics UK (COG-UK) Consortium	Rob Howes, The Lighthouse Lab in Cambridge and Alex Alderton, Roberto Amato, Sonia Goncalves, Ewan Harrison, David K. Jackson, Ian Johnston, Dominic Kwiatkowski, Cordelia Langford, John Sillitoe on behalf of the Wellcome Sanger Institute COVID-19 Surveillance Team
EPI_ISL_798850, EPI_ISL_798852, EPI_ISL_798869, EPI_ISL_798872, EPI_ISL_798878, EPI_ISL_798884, EPI_ISL_798888, EPI_ISL_798891, EPI_ISL_798898, EPI_ISL_798902, EPI_ISL_798906			
see above	Lighthouse Lab in Alderley Park	Wellcome Sanger Institute for the COVID-19 Genomics UK (COG-UK) Consortium	Jacquelyn Wynn, Mairead Hyland, The Lighthouse Lab in Alderley Park and Alex Alderton, Roberto Amato, Sonia Goncalves, Ewan Harrison, David K. Jackson, Ian Johnston, Dominic Kwiatkowski, Cordelia Langford, John Sillitoe on behalf of the Wellcome Sanger Institute COVID-19 Surveillance Team
EPI_ISL_798907	Lighthouse Lab in Cambridge	Wellcome Sanger Institute for the COVID-19 Genomics UK (COG-UK) Consortium	Rob Howes, The Lighthouse Lab in Cambridge and Alex Alderton, Roberto Amato, Sonia Goncalves, Ewan Harrison, David K. Jackson, Ian Johnston, Dominic Kwiatkowski, Cordelia Langford, John Sillitoe on behalf of the Wellcome Sanger Institute COVID-19 Surveillance Team
EPI_ISL_798914, EPI_ISL_798921, EPI_ISL_798924, EPI_ISL_798926, EPI_ISL_798928	Lighthouse Lab in Alderley Park	Wellcome Sanger Institute for the COVID-19 Genomics UK (COG-UK) Consortium	Jacquelyn Wynn, Mairead Hyland, The Lighthouse Lab in Alderley Park and Alex Alderton, Roberto Amato, Sonia Goncalves, Ewan Harrison, David K. Jackson, Ian Johnston, Dominic Kwiatkowski, Cordelia Langford, John Sillitoe on behalf of the Wellcome Sanger Institute COVID-19 Surveillance Team
EPI_ISL_798931	Lighthouse Lab in Cambridge	Wellcome Sanger Institute for the COVID-19 Genomics UK (COG-UK) Consortium	Rob Howes, The Lighthouse Lab in Cambridge and Alex Alderton, Roberto Amato, Sonia Goncalves, Ewan Harrison, David K. Jackson, Ian Johnston, Dominic Kwiatkowski, Cordelia Langford, John Sillitoe on behalf of the Wellcome Sanger Institute COVID-19 Surveillance Team
EPI_ISL_798934, EPI_ISL_798939	Lighthouse Lab in Alderley Park	Wellcome Sanger Institute for the COVID-19 Genomics UK (COG-UK) Consortium	Jacquelyn Wynn, Mairead Hyland, The Lighthouse Lab in Alderley Park and Alex Alderton, Roberto Amato, Sonia Goncalves, Ewan Harrison, David K. Jackson, Ian Johnston, Dominic Kwiatkowski, Cordelia Langford, John Sillitoe on behalf of the Wellcome Sanger Institute COVID-19 Surveillance Team
EPI_ISL_798944	Lighthouse Lab in Cambridge	Wellcome Sanger Institute for the COVID-19 Genomics UK (COG-UK) Consortium	Rob Howes, The Lighthouse Lab in Cambridge and Alex Alderton, Roberto Amato, Sonia Goncalves, Ewan Harrison, David K. Jackson, Ian Johnston, Dominic Kwiatkowski, Cordelia Langford, John Sillitoe on behalf of the Wellcome Sanger Institute COVID-19 Surveillance Team
EPI_ISL_798951, EPI_ISL_798952, EPI_ISL_798962, EPI_ISL_798963, EPI_ISL_798964, EPI_ISL_798968, EPI_ISL_798969, EPI_ISL_798984	Lighthouse Lab in Alderley Park	Wellcome Sanger Institute for the COVID-19 Genomics UK (COG-UK) Consortium	Jacquelyn Wynn, Mairead Hyland, The Lighthouse Lab in Alderley Park and Alex Alderton, Roberto Amato, Sonia Goncalves, Ewan Harrison, David K. Jackson, Ian Johnston, Dominic Kwiatkowski, Cordelia Langford, John Sillitoe on behalf of the Wellcome Sanger Institute COVID-19 Surveillance Team
EPI_ISL_798985	Lighthouse Lab in Cambridge	Wellcome Sanger Institute for the COVID-19 Genomics UK (COG-UK) Consortium	Rob Howes, The Lighthouse Lab in Cambridge and Alex Alderton, Roberto Amato, Sonia Goncalves, Ewan Harrison, David K. Jackson, Ian Johnston, Dominic Kwiatkowski, Cordelia Langford, John Sillitoe on behalf of the Wellcome Sanger Institute COVID-19 Surveillance Team
EPI_ISL_798986, EPI_ISL_798988, EPI_ISL_798991, EPI_ISL_799000, EPI_ISL_799003, EPI_ISL_799005, EPI_ISL_799010	Lighthouse Lab in Alderley Park	Wellcome Sanger Institute for the COVID-19 Genomics UK (COG-UK) Consortium	Jacquelyn Wynn, Mairead Hyland, The Lighthouse Lab in Alderley Park and Alex Alderton, Roberto Amato, Sonia Goncalves, Ewan Harrison, David K. Jackson, Ian Johnston, Dominic Kwiatkowski, Cordelia Langford, John Sillitoe on behalf of the Wellcome Sanger Institute COVID-19 Surveillance Team
EPI_ISL_799020	Lighthouse Lab in Cambridge	Wellcome Sanger Institute for the COVID-19 Genomics UK (COG-UK) Consortium	Rob Howes, The Lighthouse Lab in Cambridge and Alex Alderton, Roberto Amato, Sonia Goncalves, Ewan Harrison, David K. Jackson, Ian Johnston, Dominic Kwiatkowski, Cordelia Langford, John Sillitoe on behalf of the Wellcome Sanger Institute COVID-19 Surveillance Team
EPI_ISL_799021, EPI_ISL_799022, EPI_ISL_799023, EPI_ISL_799025, EPI_ISL_799026, EPI_ISL_799029	Lighthouse Lab in Alderley Park	Wellcome Sanger Institute for the COVID-19 Genomics UK (COG-UK) Consortium	Jacquelyn Wynn, Mairead Hyland, The Lighthouse Lab in Alderley Park and Alex Alderton, Roberto Amato, Sonia Goncalves, Ewan Harrison, David K. Jackson, Ian Johnston, Dominic Kwiatkowski, Cordelia Langford, John Sillitoe on behalf of the Wellcome Sanger Institute COVID-19 Surveillance Team
EPI_ISL_799031	Lighthouse Lab in Cambridge	Wellcome Sanger Institute for the COVID-19 Genomics UK (COG-UK) Consortium	Rob Howes, The Lighthouse Lab in Cambridge and Alex Alderton, Roberto Amato, Sonia Goncalves, Ewan Harrison, David K. Jackson, Ian Johnston, Dominic Kwiatkowski, Cordelia Langford, John Sillitoe on behalf of the Wellcome Sanger Institute COVID-19 Surveillance Team
EPI_ISL_799040, EPI_ISL_799045, EPI_ISL_799047, EPI_ISL_799053, EPI_ISL_799055, EPI_ISL_799057, EPI_ISL_799059, EPI_ISL_799069, EPI_ISL_799070	Lighthouse Lab in Alderley Park	Wellcome Sanger Institute for the COVID-19 Genomics UK (COG-UK) Consortium	Jacquelyn Wynn, Mairead Hyland, The Lighthouse Lab in Alderley Park and Alex Alderton, Roberto Amato, Sonia Goncalves, Ewan Harrison, David K. Jackson, Ian Johnston, Dominic Kwiatkowski, Cordelia Langford, John Sillitoe on behalf of the Wellcome Sanger Institute COVID-19 Surveillance Team
EPI_ISL_799074	Lighthouse Lab in Cambridge	Wellcome Sanger Institute for the COVID-19 Genomics UK (COG-UK) Consortium	Rob Howes, The Lighthouse Lab in Cambridge and Alex Alderton, Roberto Amato, Sonia Goncalves, Ewan Harrison, David K. Jackson, Ian Johnston, Dominic Kwiatkowski, Cordelia Langford, John Sillitoe on behalf of the Wellcome Sanger Institute COVID-19 Surveillance Team
EPI_ISL_799079	Lighthouse Lab in Alderley Park	Wellcome Sanger Institute for the COVID-19 Genomics UK (COG-UK) Consortium	Jacquelyn Wynn, Mairead Hyland, The Lighthouse Lab in Alderley Park and Alex Alderton, Roberto Amato, Sonia Goncalves, Ewan Harrison, David K. Jackson, Ian Johnston, Dominic Kwiatkowski, Cordelia Langford, John Sillitoe on behalf of the Wellcome Sanger Institute COVID-19 Surveillance Team
EPI_ISL_799083	Lighthouse Lab in Cambridge	Wellcome Sanger Institute for the COVID-19 Genomics UK (COG-UK) Consortium	Rob Howes, The Lighthouse Lab in Cambridge and Alex Alderton, Roberto Amato, Sonia Goncalves, Ewan Harrison, David K. Jackson, Ian Johnston, Dominic Kwiatkowski, Cordelia Langford, John Sillitoe on behalf of the Wellcome Sanger Institute COVID-19 Surveillance Team
EPI_ISL_799085, EPI_ISL_799086	Lighthouse Lab in Alderley Park	Wellcome Sanger Institute for the COVID-19 Genomics UK (COG-UK) Consortium	Jacquelyn Wynn, Mairead Hyland, The Lighthouse Lab in Alderley Park and Alex Alderton, Roberto Amato, Sonia Goncalves, Ewan Harrison, David K. Jackson, Ian Johnston, Dominic Kwiatkowski, Cordelia Langford, John Sillitoe on behalf of the Wellcome Sanger Institute COVID-19 Surveillance Team
EPI_ISL_799087	Lighthouse Lab in Cambridge	Wellcome Sanger Institute for the COVID-19 Genomics UK (COG-UK) Consortium	Rob Howes, The Lighthouse Lab in Cambridge and Alex Alderton, Roberto Amato, Sonia Goncalves, Ewan Harrison, David K. Jackson, Ian Johnston, Dominic Kwiatkowski, Cordelia Langford, John Sillitoe on behalf of the Wellcome Sanger Institute COVID-19 Surveillance Team
EPI_ISL_799099, EPI_ISL_799103, EPI_ISL_799104, EPI_ISL_799105, EPI_ISL_799106, EPI_ISL_799111, EPI_ISL_799113, EPI_ISL_799114, EPI_ISL_799122, EPI_ISL_799124, EPI_ISL_799137, EPI_ISL_799138, EPI_ISL_799139, EPI_ISL_799143, EPI_ISL_799145, EPI_ISL_799149			
see above	Lighthouse Lab in Alderley Park	Wellcome Sanger Institute for the COVID-19 Genomics UK (COG-UK) Consortium	Jacquelyn Wynn, Mairead Hyland, The Lighthouse Lab in Alderley Park and Alex Alderton, Roberto Amato, Sonia Goncalves, Ewan Harrison, David K. Jackson, Ian Johnston, Dominic Kwiatkowski, Cordelia Langford, John Sillitoe on behalf of the Wellcome Sanger Institute COVID-19 Surveillance Team
EPI_ISL_799152, EPI_ISL_799153	Lighthouse Lab in Cambridge	Wellcome Sanger Institute for the COVID-19 Genomics UK (COG-UK) Consortium	Rob Howes, The Lighthouse Lab in Cambridge and Alex Alderton, Roberto Amato, Sonia Goncalves, Ewan Harrison, David K. Jackson, Ian Johnston, Dominic Kwiatkowski, Cordelia Langford, John Sillitoe on behalf of the Wellcome Sanger Institute COVID-19 Surveillance Team
EPI_ISL_799154, EPI_ISL_799160, EPI_ISL_799161, EPI_ISL_799165, EPI_ISL_799166, EPI_ISL_799170, EPI_ISL_799171, EPI_ISL_799177, EPI_ISL_799178, EPI_ISL_799179, EPI_ISL_799182			
see above	Lighthouse Lab in Alderley Park	Wellcome Sanger Institute for the COVID-19 Genomics UK (COG-UK) Consortium	Jacquelyn Wynn, Mairead Hyland, The Lighthouse Lab in Alderley Park and Alex Alderton, Roberto Amato, Sonia Goncalves, Ewan Harrison, David K. Jackson, Ian Johnston, Dominic Kwiatkowski, Cordelia Langford, John Sillitoe on behalf of the Wellcome Sanger Institute COVID-19 Surveillance Team
EPI_ISL_799186	Lighthouse Lab in Cambridge	Wellcome Sanger Institute for the COVID-19 Genomics UK (COG-UK) Consortium	Rob Howes, The Lighthouse Lab in Cambridge and Alex Alderton, Roberto Amato, Sonia Goncalves, Ewan Harrison, David K. Jackson, Ian Johnston, Dominic Kwiatkowski, Cordelia Langford, John Sillitoe on behalf of the Wellcome Sanger Institute COVID-19 Surveillance Team
EPI_ISL_799188, EPI_ISL_799191, EPI_ISL_799198, EPI_ISL_799200, EPI_ISL_799202	Lighthouse Lab in Alderley Park	Wellcome Sanger Institute for the COVID-19 Genomics UK (COG-UK) Consortium	Jacquelyn Wynn, Mairead Hyland, The Lighthouse Lab in Alderley Park and Alex Alderton, Roberto Amato, Sonia Goncalves, Ewan Harrison, David K. Jackson, Ian Johnston, Dominic Kwiatkowski, Cordelia Langford, John Sillitoe on behalf of the Wellcome Sanger Institute COVID-19 Surveillance Team
EPI_ISL_799208, EPI_ISL_799210	Lighthouse Lab in Cambridge	Wellcome Sanger Institute for the COVID-19 Genomics UK (COG-UK) Consortium	Rob Howes, The Lighthouse Lab in Cambridge and Alex Alderton, Roberto Amato, Sonia Goncalves, Ewan Harrison, David K. Jackson, Ian Johnston, Dominic Kwiatkowski, Cordelia Langford, John Sillitoe on behalf of the Wellcome Sanger Institute COVID-19 Surveillance Team
EPI_ISL_799212, EPI_ISL_799213, EPI_ISL_799214, EPI_ISL_799219	Lighthouse Lab in Alderley Park	Wellcome Sanger Institute for the COVID-19 Genomics UK (COG-UK) Consortium	Jacquelyn Wynn, Mairead Hyland, The Lighthouse Lab in Alderley Park and Alex Alderton, Roberto Amato, Sonia Goncalves, Ewan Harrison, David K. Jackson, Ian Johnston, Dominic Kwiatkowski, Cordelia Langford, John Sillitoe on behalf of the Wellcome Sanger Institute COVID-19 Surveillance Team
EPI_ISL_799226	Lighthouse Lab in Cambridge	Wellcome Sanger Institute for the COVID-19 Genomics UK	Rob Howes, The Lighthouse Lab in Cambridge and Alex Alderton, Roberto Amato, Sonia Goncalves, Ewan Harrison, David K. Jackson, Ian Johnston,

		(COG-UK) Consortium	Dominic Kwiatkowski, Cordelia Langford, John Sillitoe on behalf of the Wellcome Sanger Institute COVID-19 Surveillance Team
EPI_ISL_799227, EPI_ISL_799228, EPI_ISL_799229, EPI_ISL_799231, EPI_ISL_799233, EPI_ISL_799234, EPI_ISL_799235, EPI_ISL_799237, EPI_ISL_799244, EPI_ISL_799248, EPI_ISL_799252, EPI_ISL_799257, EPI_ISL_799261, EPI_ISL_799265, EPI_ISL_799269, EPI_ISL_799271, EPI_ISL_799276, EPI_ISL_799278, EPI_ISL_799281			
see above	Lighthouse Lab in Alderley Park	Wellcome Sanger Institute for the COVID-19 Genomics UK (COG-UK) Consortium	Jacquelyn Wynn, Mairead Hyland, The Lighthouse Lab in Alderley Park and Alex Alderton, Roberto Amato, Sonia Goncalves, Ewan Harrison, David K. Jackson, Ian Johnston, Dominic Kwiatkowski, Cordelia Langford, John Sillitoe on behalf of the Wellcome Sanger Institute COVID-19 Surveillance Team
EPI_ISL_799291, EPI_ISL_799292	Lighthouse Lab in Cambridge	Wellcome Sanger Institute for the COVID-19 Genomics UK (COG-UK) Consortium	Rob Howes, The Lighthouse Lab in Cambridge and Alex Alderton, Roberto Amato, Sonia Goncalves, Ewan Harrison, David K. Jackson, Ian Johnston, Dominic Kwiatkowski, Cordelia Langford, John Sillitoe on behalf of the Wellcome Sanger Institute COVID-19 Surveillance Team
EPI_ISL_799297, EPI_ISL_799300, EPI_ISL_799301, EPI_ISL_799304, EPI_ISL_799307, EPI_ISL_799309, EPI_ISL_799312, EPI_ISL_799313, EPI_ISL_799316, EPI_ISL_799321, EPI_ISL_799324, EPI_ISL_799328, EPI_ISL_799329, EPI_ISL_799330, EPI_ISL_799331, EPI_ISL_799332, EPI_ISL_799335, EPI_ISL_799336, EPI_ISL_799337, EPI_ISL_799338, EPI_ISL_799339, EPI_ISL_799340, EPI_ISL_799341, EPI_ISL_799342, EPI_ISL_799343, EPI_ISL_799344, EPI_ISL_799349, EPI_ISL_799358, EPI_ISL_799359, EPI_ISL_799361, EPI_ISL_799365, EPI_ISL_799367, EPI_ISL_799371, EPI_ISL_799374, EPI_ISL_799379, EPI_ISL_799381, EPI_ISL_799382, EPI_ISL_799383, EPI_ISL_799385, EPI_ISL_799389, EPI_ISL_799391, EPI_ISL_799393, EPI_ISL_799396, EPI_ISL_799402, EPI_ISL_799407, EPI_ISL_799408, EPI_ISL_799409, EPI_ISL_799414, EPI_ISL_799421, EPI_ISL_799423, EPI_ISL_799426, EPI_ISL_799428, EPI_ISL_799429, EPI_ISL_799431, EPI_ISL_799435, EPI_ISL_799442, EPI_ISL_799444, EPI_ISL_799449, EPI_ISL_799450, EPI_ISL_799451, EPI_ISL_799453, EPI_ISL_799455, EPI_ISL_799457, EPI_ISL_799459, EPI_ISL_799460, EPI_ISL_799462, EPI_ISL_799463, EPI_ISL_799464, EPI_ISL_799471, EPI_ISL_799472, EPI_ISL_799475, EPI_ISL_799478, EPI_ISL_799480, EPI_ISL_799481, EPI_ISL_799483, EPI_ISL_799484, EPI_ISL_799486, EPI_ISL_799489, EPI_ISL_799491, EPI_ISL_799493, EPI_ISL_799495, EPI_ISL_799499, EPI_ISL_799503, EPI_ISL_799504, EPI_ISL_799507, EPI_ISL_799511, EPI_ISL_799513, EPI_ISL_799516, EPI_ISL_799517, EPI_ISL_799518, EPI_ISL_799521, EPI_ISL_799524, EPI_ISL_799527, EPI_ISL_799532, EPI_ISL_799539, EPI_ISL_799544, EPI_ISL_799548, EPI_ISL_799552, EPI_ISL_799553, EPI_ISL_799554, EPI_ISL_799558, EPI_ISL_799561, EPI_ISL_799564, EPI_ISL_799568, EPI_ISL_799569, EPI_ISL_799571, EPI_ISL_799574, EPI_ISL_799576, EPI_ISL_799581, EPI_ISL_799584, EPI_ISL_799585, EPI_ISL_799589, EPI_ISL_799591, EPI_ISL_799592, EPI_ISL_799593, EPI_ISL_799594, EPI_ISL_799596, EPI_ISL_799599, EPI_ISL_799601, EPI_ISL_799605, EPI_ISL_799606, EPI_ISL_799609, EPI_ISL_799612, EPI_ISL_799613, EPI_ISL_799615, EPI_ISL_799634, EPI_ISL_799659, EPI_ISL_799669, EPI_ISL_799681, EPI_ISL_799682, EPI_ISL_799686			
see above	Lighthouse Lab in Glasgow	Wellcome Sanger Institute for the COVID-19 Genomics UK (COG-UK) Consortium	Harper VanSteenhouse, Yumi Kasai, David Gray, Carol Clugston, Anna Dominiczak and Alex Alderton, Roberto Amato, Sonia Goncalves, Ewan Harrison, David K. Jackson, Ian Johnston, Dominic Kwiatkowski, Cordelia Langford, John Sillitoe on behalf of the Wellcome Sanger Institute COVID-19 Surveillance Team
EPI_ISL_799687, EPI_ISL_799693	Lighthouse Lab in Alderley Park	Wellcome Sanger Institute for the COVID-19 Genomics UK (COG-UK) Consortium	Jacquelyn Wynn, Mairead Hyland, The Lighthouse Lab in Alderley Park and Alex Alderton, Roberto Amato, Sonia Goncalves, Ewan Harrison, David K. Jackson, Ian Johnston, Dominic Kwiatkowski, Cordelia Langford, John Sillitoe on behalf of the Wellcome Sanger Institute COVID-19 Surveillance Team
EPI_ISL_799699, EPI_ISL_799707, EPI_ISL_799726	Lighthouse Lab in Glasgow	Wellcome Sanger Institute for the COVID-19 Genomics UK (COG-UK) Consortium	Harper VanSteenhouse, Yumi Kasai, David Gray, Carol Clugston, Anna Dominiczak and Alex Alderton, Roberto Amato, Sonia Goncalves, Ewan Harrison, David K. Jackson, Ian Johnston, Dominic Kwiatkowski, Cordelia Langford, John Sillitoe on behalf of the Wellcome Sanger Institute COVID-19 Surveillance Team
EPI_ISL_799734, EPI_ISL_799744, EPI_ISL_799747	Lighthouse Lab in Alderley Park	Wellcome Sanger Institute for the COVID-19 Genomics UK (COG-UK) Consortium	Jacquelyn Wynn, Mairead Hyland, The Lighthouse Lab in Alderley Park and Alex Alderton, Roberto Amato, Sonia Goncalves, Ewan Harrison, David K. Jackson, Ian Johnston, Dominic Kwiatkowski, Cordelia Langford, John Sillitoe on behalf of the Wellcome Sanger Institute COVID-19 Surveillance Team
EPI_ISL_799748, EPI_ISL_799758, EPI_ISL_799763, EPI_ISL_799765	Lighthouse Lab in Glasgow	Wellcome Sanger Institute for the COVID-19 Genomics UK (COG-UK) Consortium	Harper VanSteenhouse, Yumi Kasai, David Gray, Carol Clugston, Anna Dominiczak and Alex Alderton, Roberto Amato, Sonia Goncalves, Ewan Harrison, David K. Jackson, Ian Johnston, Dominic Kwiatkowski, Cordelia Langford, John Sillitoe on behalf of the Wellcome Sanger Institute COVID-19 Surveillance Team
EPI_ISL_799767	Lighthouse Lab in Alderley Park	Wellcome Sanger Institute for the COVID-19 Genomics UK (COG-UK) Consortium	Jacquelyn Wynn, Mairead Hyland, The Lighthouse Lab in Alderley Park and Alex Alderton, Roberto Amato, Sonia Goncalves, Ewan Harrison, David K. Jackson, Ian Johnston, Dominic Kwiatkowski, Cordelia Langford, John Sillitoe on behalf of the Wellcome Sanger Institute COVID-19 Surveillance Team
EPI_ISL_799768	Lighthouse Lab in Glasgow	Wellcome Sanger Institute for the COVID-19 Genomics UK (COG-UK) Consortium	Harper VanSteenhouse, Yumi Kasai, David Gray, Carol Clugston, Anna Dominiczak and Alex Alderton, Roberto Amato, Sonia Goncalves, Ewan Harrison, David K. Jackson, Ian Johnston, Dominic Kwiatkowski, Cordelia Langford, John Sillitoe on behalf of the Wellcome Sanger Institute COVID-19 Surveillance Team
EPI_ISL_799784, EPI_ISL_799787, EPI_ISL_799799	Lighthouse Lab in Alderley Park	Wellcome Sanger Institute for the COVID-19 Genomics UK (COG-UK) Consortium	Jacquelyn Wynn, Mairead Hyland, The Lighthouse Lab in Alderley Park and Alex Alderton, Roberto Amato, Sonia Goncalves, Ewan Harrison, David K. Jackson, Ian Johnston, Dominic Kwiatkowski, Cordelia Langford, John Sillitoe on behalf of the Wellcome Sanger Institute COVID-19 Surveillance Team
EPI_ISL_799801, EPI_ISL_799805	Lighthouse Lab in Glasgow	Wellcome Sanger Institute for the COVID-19 Genomics UK (COG-UK) Consortium	Harper VanSteenhouse, Yumi Kasai, David Gray, Carol Clugston, Anna Dominiczak and Alex Alderton, Roberto Amato, Sonia Goncalves, Ewan Harrison, David K. Jackson, Ian Johnston, Dominic Kwiatkowski, Cordelia Langford, John Sillitoe on behalf of the Wellcome Sanger Institute COVID-19 Surveillance Team
EPI_ISL_799807, EPI_ISL_799812	Lighthouse Lab in Alderley Park	Wellcome Sanger Institute for the COVID-19 Genomics UK (COG-UK) Consortium	Jacquelyn Wynn, Mairead Hyland, The Lighthouse Lab in Alderley Park and Alex Alderton, Roberto Amato, Sonia Goncalves, Ewan Harrison, David K. Jackson, Ian Johnston, Dominic Kwiatkowski, Cordelia Langford, John Sillitoe on behalf of the Wellcome Sanger Institute COVID-19 Surveillance Team
EPI_ISL_799819	Lighthouse Lab in Glasgow	Wellcome Sanger Institute for the COVID-19 Genomics UK (COG-UK) Consortium	Harper VanSteenhouse, Yumi Kasai, David Gray, Carol Clugston, Anna Dominiczak and Alex Alderton, Roberto Amato, Sonia Goncalves, Ewan Harrison, David K. Jackson, Ian Johnston, Dominic Kwiatkowski, Cordelia Langford, John Sillitoe on behalf of the Wellcome Sanger Institute COVID-19 Surveillance Team
EPI_ISL_799821, EPI_ISL_799822, EPI_ISL_799823	Lighthouse Lab in Alderley Park	Wellcome Sanger Institute for the COVID-19 Genomics UK (COG-UK) Consortium	Jacquelyn Wynn, Mairead Hyland, The Lighthouse Lab in Alderley Park and Alex Alderton, Roberto Amato, Sonia Goncalves, Ewan Harrison, David K. Jackson, Ian Johnston, Dominic Kwiatkowski, Cordelia Langford, John Sillitoe on behalf of the Wellcome Sanger Institute COVID-19 Surveillance Team
EPI_ISL_799827	Lighthouse Lab in Glasgow	Wellcome Sanger Institute for the COVID-19 Genomics UK (COG-UK) Consortium	Harper VanSteenhouse, Yumi Kasai, David Gray, Carol Clugston, Anna Dominiczak and Alex Alderton, Roberto Amato, Sonia Goncalves, Ewan Harrison, David K. Jackson, Ian Johnston, Dominic Kwiatkowski, Cordelia Langford, John Sillitoe on behalf of the Wellcome Sanger Institute COVID-19 Surveillance Team
EPI_ISL_799831, EPI_ISL_799832	Lighthouse Lab in Alderley Park	Wellcome Sanger Institute for the COVID-19 Genomics UK (COG-UK) Consortium	Jacquelyn Wynn, Mairead Hyland, The Lighthouse Lab in Alderley Park and Alex Alderton, Roberto Amato, Sonia Goncalves, Ewan Harrison, David K. Jackson, Ian Johnston, Dominic Kwiatkowski, Cordelia Langford, John Sillitoe on behalf of the Wellcome Sanger Institute COVID-19 Surveillance Team
EPI_ISL_799838, EPI_ISL_799839	Lighthouse Lab in Glasgow	Wellcome Sanger Institute for the COVID-19 Genomics UK (COG-UK) Consortium	Harper VanSteenhouse, Yumi Kasai, David Gray, Carol Clugston, Anna Dominiczak and Alex Alderton, Roberto Amato, Sonia Goncalves, Ewan Harrison, David K. Jackson, Ian Johnston, Dominic Kwiatkowski, Cordelia Langford, John Sillitoe on behalf of the Wellcome Sanger Institute COVID-19 Surveillance Team
EPI_ISL_799841, EPI_ISL_799844, EPI_ISL_799846	Lighthouse Lab in Alderley Park	Wellcome Sanger Institute for the COVID-19 Genomics UK (COG-UK) Consortium	Jacquelyn Wynn, Mairead Hyland, The Lighthouse Lab in Alderley Park and Alex Alderton, Roberto Amato, Sonia Goncalves, Ewan Harrison, David K. Jackson, Ian Johnston, Dominic Kwiatkowski, Cordelia Langford, John Sillitoe on behalf of the Wellcome Sanger Institute COVID-19 Surveillance Team
EPI_ISL_799855	Lighthouse Lab in Glasgow	Wellcome Sanger Institute for the COVID-19 Genomics UK (COG-UK) Consortium	Harper VanSteenhouse, Yumi Kasai, David Gray, Carol Clugston, Anna Dominiczak and Alex Alderton, Roberto Amato, Sonia Goncalves, Ewan Harrison, David K. Jackson, Ian Johnston, Dominic Kwiatkowski, Cordelia Langford, John Sillitoe on behalf of the Wellcome Sanger Institute COVID-19 Surveillance Team
EPI_ISL_799859	Lighthouse Lab in Alderley Park	Wellcome Sanger Institute for the COVID-19 Genomics UK (COG-UK) Consortium	Jacquelyn Wynn, Mairead Hyland, The Lighthouse Lab in Alderley Park and Alex Alderton, Roberto Amato, Sonia Goncalves, Ewan Harrison, David K. Jackson, Ian Johnston, Dominic Kwiatkowski, Cordelia Langford, John Sillitoe on behalf of the Wellcome Sanger Institute COVID-19 Surveillance Team
EPI_ISL_799865, EPI_ISL_799888, EPI_ISL_799892, EPI_ISL_799894, EPI_ISL_799903, EPI_ISL_799906	Lighthouse Lab in Glasgow	Wellcome Sanger Institute for the COVID-19 Genomics UK (COG-UK) Consortium	Harper VanSteenhouse, Yumi Kasai, David Gray, Carol Clugston, Anna Dominiczak and Alex Alderton, Roberto Amato, Sonia Goncalves, Ewan Harrison, David K. Jackson, Ian Johnston, Dominic Kwiatkowski, Cordelia Langford, John Sillitoe on behalf of the Wellcome Sanger Institute COVID-19 Surveillance Team
EPI_ISL_799920, EPI_ISL_799924, EPI_ISL_799930	Lighthouse Lab in Alderley Park	Wellcome Sanger Institute for the COVID-19 Genomics UK (COG-UK) Consortium	Jacquelyn Wynn, Mairead Hyland, The Lighthouse Lab in Alderley Park and Alex Alderton, Roberto Amato, Sonia Goncalves, Ewan Harrison, David K. Jackson, Ian Johnston, Dominic Kwiatkowski, Cordelia Langford, John Sillitoe on behalf of the Wellcome Sanger Institute COVID-19 Surveillance Team
EPI_ISL_799932	Lighthouse Lab in Glasgow	Wellcome Sanger Institute for the COVID-19 Genomics UK (COG-UK) Consortium	Harper VanSteenhouse, Yumi Kasai, David Gray, Carol Clugston, Anna Dominiczak and Alex Alderton, Roberto Amato, Sonia Goncalves, Ewan Harrison, David K. Jackson, Ian Johnston, Dominic Kwiatkowski, Cordelia Langford, John Sillitoe on behalf of the Wellcome Sanger Institute COVID-19 Surveillance Team
EPI_ISL_799933	Lighthouse Lab in Alderley Park	Wellcome Sanger Institute for the COVID-19 Genomics UK (COG-UK) Consortium	Jacquelyn Wynn, Mairead Hyland, The Lighthouse Lab in Alderley Park and Alex Alderton, Roberto Amato, Sonia Goncalves, Ewan Harrison, David K. Jackson, Ian Johnston, Dominic Kwiatkowski, Cordelia Langford, John Sillitoe on behalf of the Wellcome Sanger Institute COVID-19 Surveillance Team
EPI_ISL_799937	Lighthouse Lab in Glasgow	Wellcome Sanger Institute for the COVID-19 Genomics UK (COG-UK) Consortium	Harper VanSteenhouse, Yumi Kasai, David Gray, Carol Clugston, Anna Dominiczak and Alex Alderton, Roberto Amato, Sonia Goncalves, Ewan Harrison, David K. Jackson, Ian Johnston, Dominic Kwiatkowski, Cordelia Langford, John Sillitoe on behalf of the Wellcome Sanger Institute COVID-19 Surveillance Team

EPI_ISL_799940, EPI_ISL_799941, EPI_ISL_799943	Lighthouse Lab in Alderley Park	Wellcome Sanger Institute for the COVID-19 Genomics UK (COG-UK) Consortium	Jacquelyn Wynn, Mairead Hyland, The Lighthouse Lab in Alderley Park and Alex Alderton, Roberto Amato, Sonia Goncalves, Ewan Harrison, David K. Jackson, Ian Johnston, Dominic Kwiatkowski, Cordelia Langford, John Sillitoe on behalf of the Wellcome Sanger Institute COVID-19 Surveillance Team
EPI_ISL_799946	Lighthouse Lab in Glasgow	Wellcome Sanger Institute for the COVID-19 Genomics UK (COG-UK) Consortium	Harper VanSteenhouse, Yumi Kasai, David Gray, Carol Clugston, Anna Dominiczak and Alex Alderton, Roberto Amato, Sonia Goncalves, Ewan Harrison, David K. Jackson, Ian Johnston, Dominic Kwiatkowski, Cordelia Langford, John Sillitoe on behalf of the Wellcome Sanger Institute COVID-19 Surveillance Team
EPI_ISL_799948	Lighthouse Lab in Alderley Park	Wellcome Sanger Institute for the COVID-19 Genomics UK (COG-UK) Consortium	Jacquelyn Wynn, Mairead Hyland, The Lighthouse Lab in Alderley Park and Alex Alderton, Roberto Amato, Sonia Goncalves, Ewan Harrison, David K. Jackson, Ian Johnston, Dominic Kwiatkowski, Cordelia Langford, John Sillitoe on behalf of the Wellcome Sanger Institute COVID-19 Surveillance Team
EPI_ISL_799949, EPI_ISL_799954	Lighthouse Lab in Glasgow	Wellcome Sanger Institute for the COVID-19 Genomics UK (COG-UK) Consortium	Harper VanSteenhouse, Yumi Kasai, David Gray, Carol Clugston, Anna Dominiczak and Alex Alderton, Roberto Amato, Sonia Goncalves, Ewan Harrison, David K. Jackson, Ian Johnston, Dominic Kwiatkowski, Cordelia Langford, John Sillitoe on behalf of the Wellcome Sanger Institute COVID-19 Surveillance Team
EPI_ISL_799956, EPI_ISL_799961, EPI_ISL_799964	Lighthouse Lab in Alderley Park	Wellcome Sanger Institute for the COVID-19 Genomics UK (COG-UK) Consortium	Jacquelyn Wynn, Mairead Hyland, The Lighthouse Lab in Alderley Park and Alex Alderton, Roberto Amato, Sonia Goncalves, Ewan Harrison, David K. Jackson, Ian Johnston, Dominic Kwiatkowski, Cordelia Langford, John Sillitoe on behalf of the Wellcome Sanger Institute COVID-19 Surveillance Team
EPI_ISL_799965	Lighthouse Lab in Glasgow	Wellcome Sanger Institute for the COVID-19 Genomics UK (COG-UK) Consortium	Harper VanSteenhouse, Yumi Kasai, David Gray, Carol Clugston, Anna Dominiczak and Alex Alderton, Roberto Amato, Sonia Goncalves, Ewan Harrison, David K. Jackson, Ian Johnston, Dominic Kwiatkowski, Cordelia Langford, John Sillitoe on behalf of the Wellcome Sanger Institute COVID-19 Surveillance Team
EPI_ISL_799967	Lighthouse Lab in Alderley Park	Wellcome Sanger Institute for the COVID-19 Genomics UK (COG-UK) Consortium	Jacquelyn Wynn, Mairead Hyland, The Lighthouse Lab in Alderley Park and Alex Alderton, Roberto Amato, Sonia Goncalves, Ewan Harrison, David K. Jackson, Ian Johnston, Dominic Kwiatkowski, Cordelia Langford, John Sillitoe on behalf of the Wellcome Sanger Institute COVID-19 Surveillance Team
EPI_ISL_799968, EPI_ISL_799969	Lighthouse Lab in Glasgow	Wellcome Sanger Institute for the COVID-19 Genomics UK (COG-UK) Consortium	Harper VanSteenhouse, Yumi Kasai, David Gray, Carol Clugston, Anna Dominiczak and Alex Alderton, Roberto Amato, Sonia Goncalves, Ewan Harrison, David K. Jackson, Ian Johnston, Dominic Kwiatkowski, Cordelia Langford, John Sillitoe on behalf of the Wellcome Sanger Institute COVID-19 Surveillance Team
EPI_ISL_799970, EPI_ISL_799975, EPI_ISL_799977, EPI_ISL_799979	Lighthouse Lab in Alderley Park	Wellcome Sanger Institute for the COVID-19 Genomics UK (COG-UK) Consortium	Jacquelyn Wynn, Mairead Hyland, The Lighthouse Lab in Alderley Park and Alex Alderton, Roberto Amato, Sonia Goncalves, Ewan Harrison, David K. Jackson, Ian Johnston, Dominic Kwiatkowski, Cordelia Langford, John Sillitoe on behalf of the Wellcome Sanger Institute COVID-19 Surveillance Team
EPI_ISL_799980	Lighthouse Lab in Glasgow	Wellcome Sanger Institute for the COVID-19 Genomics UK (COG-UK) Consortium	Harper VanSteenhouse, Yumi Kasai, David Gray, Carol Clugston, Anna Dominiczak and Alex Alderton, Roberto Amato, Sonia Goncalves, Ewan Harrison, David K. Jackson, Ian Johnston, Dominic Kwiatkowski, Cordelia Langford, John Sillitoe on behalf of the Wellcome Sanger Institute COVID-19 Surveillance Team
EPI_ISL_799984	Lighthouse Lab in Alderley Park	Wellcome Sanger Institute for the COVID-19 Genomics UK (COG-UK) Consortium	Jacquelyn Wynn, Mairead Hyland, The Lighthouse Lab in Alderley Park and Alex Alderton, Roberto Amato, Sonia Goncalves, Ewan Harrison, David K. Jackson, Ian Johnston, Dominic Kwiatkowski, Cordelia Langford, John Sillitoe on behalf of the Wellcome Sanger Institute COVID-19 Surveillance Team
EPI_ISL_799986, EPI_ISL_799987, EPI_ISL_799992	Lighthouse Lab in Glasgow	Wellcome Sanger Institute for the COVID-19 Genomics UK (COG-UK) Consortium	Harper VanSteenhouse, Yumi Kasai, David Gray, Carol Clugston, Anna Dominiczak and Alex Alderton, Roberto Amato, Sonia Goncalves, Ewan Harrison, David K. Jackson, Ian Johnston, Dominic Kwiatkowski, Cordelia Langford, John Sillitoe on behalf of the Wellcome Sanger Institute COVID-19 Surveillance Team
EPI_ISL_799995, EPI_ISL_799999	Lighthouse Lab in Alderley Park	Wellcome Sanger Institute for the COVID-19 Genomics UK (COG-UK) Consortium	Jacquelyn Wynn, Mairead Hyland, The Lighthouse Lab in Alderley Park and Alex Alderton, Roberto Amato, Sonia Goncalves, Ewan Harrison, David K. Jackson, Ian Johnston, Dominic Kwiatkowski, Cordelia Langford, John Sillitoe on behalf of the Wellcome Sanger Institute COVID-19 Surveillance Team
EPI_ISL_800000	Lighthouse Lab in Glasgow	Wellcome Sanger Institute for the COVID-19 Genomics UK (COG-UK) Consortium	Harper VanSteenhouse, Yumi Kasai, David Gray, Carol Clugston, Anna Dominiczak and Alex Alderton, Roberto Amato, Sonia Goncalves, Ewan Harrison, David K. Jackson, Ian Johnston, Dominic Kwiatkowski, Cordelia Langford, John Sillitoe on behalf of the Wellcome Sanger Institute COVID-19 Surveillance Team
EPI_ISL_800002, EPI_ISL_800009, EPI_ISL_800011, EPI_ISL_800013, EPI_ISL_800014	Lighthouse Lab in Alderley Park	Wellcome Sanger Institute for the COVID-19 Genomics UK (COG-UK) Consortium	Jacquelyn Wynn, Mairead Hyland, The Lighthouse Lab in Alderley Park and Alex Alderton, Roberto Amato, Sonia Goncalves, Ewan Harrison, David K. Jackson, Ian Johnston, Dominic Kwiatkowski, Cordelia Langford, John Sillitoe on behalf of the Wellcome Sanger Institute COVID-19 Surveillance Team
EPI_ISL_800015	Lighthouse Lab in Glasgow	Wellcome Sanger Institute for the COVID-19 Genomics UK (COG-UK) Consortium	Harper VanSteenhouse, Yumi Kasai, David Gray, Carol Clugston, Anna Dominiczak and Alex Alderton, Roberto Amato, Sonia Goncalves, Ewan Harrison, David K. Jackson, Ian Johnston, Dominic Kwiatkowski, Cordelia Langford, John Sillitoe on behalf of the Wellcome Sanger Institute COVID-19 Surveillance Team
EPI_ISL_800016, EPI_ISL_800019, EPI_ISL_800020, EPI_ISL_800021, EPI_ISL_800023, EPI_ISL_800031	Lighthouse Lab in Alderley Park	Wellcome Sanger Institute for the COVID-19 Genomics UK (COG-UK) Consortium	Jacquelyn Wynn, Mairead Hyland, The Lighthouse Lab in Alderley Park and Alex Alderton, Roberto Amato, Sonia Goncalves, Ewan Harrison, David K. Jackson, Ian Johnston, Dominic Kwiatkowski, Cordelia Langford, John Sillitoe on behalf of the Wellcome Sanger Institute COVID-19 Surveillance Team
EPI_ISL_800032	Lighthouse Lab in Glasgow	Wellcome Sanger Institute for the COVID-19 Genomics UK (COG-UK) Consortium	Harper VanSteenhouse, Yumi Kasai, David Gray, Carol Clugston, Anna Dominiczak and Alex Alderton, Roberto Amato, Sonia Goncalves, Ewan Harrison, David K. Jackson, Ian Johnston, Dominic Kwiatkowski, Cordelia Langford, John Sillitoe on behalf of the Wellcome Sanger Institute COVID-19 Surveillance Team
EPI_ISL_800033, EPI_ISL_800035, EPI_ISL_800041, EPI_ISL_800042, EPI_ISL_800046	Lighthouse Lab in Alderley Park	Wellcome Sanger Institute for the COVID-19 Genomics UK (COG-UK) Consortium	Jacquelyn Wynn, Mairead Hyland, The Lighthouse Lab in Alderley Park and Alex Alderton, Roberto Amato, Sonia Goncalves, Ewan Harrison, David K. Jackson, Ian Johnston, Dominic Kwiatkowski, Cordelia Langford, John Sillitoe on behalf of the Wellcome Sanger Institute COVID-19 Surveillance Team
EPI_ISL_800048	Lighthouse Lab in Glasgow	Wellcome Sanger Institute for the COVID-19 Genomics UK (COG-UK) Consortium	Harper VanSteenhouse, Yumi Kasai, David Gray, Carol Clugston, Anna Dominiczak and Alex Alderton, Roberto Amato, Sonia Goncalves, Ewan Harrison, David K. Jackson, Ian Johnston, Dominic Kwiatkowski, Cordelia Langford, John Sillitoe on behalf of the Wellcome Sanger Institute COVID-19 Surveillance Team
EPI_ISL_800049	Lighthouse Lab in Alderley Park	Wellcome Sanger Institute for the COVID-19 Genomics UK (COG-UK) Consortium	Jacquelyn Wynn, Mairead Hyland, The Lighthouse Lab in Alderley Park and Alex Alderton, Roberto Amato, Sonia Goncalves, Ewan Harrison, David K. Jackson, Ian Johnston, Dominic Kwiatkowski, Cordelia Langford, John Sillitoe on behalf of the Wellcome Sanger Institute COVID-19 Surveillance Team
EPI_ISL_800052	Lighthouse Lab in Glasgow	Wellcome Sanger Institute for the COVID-19 Genomics UK (COG-UK) Consortium	Harper VanSteenhouse, Yumi Kasai, David Gray, Carol Clugston, Anna Dominiczak and Alex Alderton, Roberto Amato, Sonia Goncalves, Ewan Harrison, David K. Jackson, Ian Johnston, Dominic Kwiatkowski, Cordelia Langford, John Sillitoe on behalf of the Wellcome Sanger Institute COVID-19 Surveillance Team
EPI_ISL_800053	Lighthouse Lab in Alderley Park	Wellcome Sanger Institute for the COVID-19 Genomics UK (COG-UK) Consortium	Jacquelyn Wynn, Mairead Hyland, The Lighthouse Lab in Alderley Park and Alex Alderton, Roberto Amato, Sonia Goncalves, Ewan Harrison, David K. Jackson, Ian Johnston, Dominic Kwiatkowski, Cordelia Langford, John Sillitoe on behalf of the Wellcome Sanger Institute COVID-19 Surveillance Team
EPI_ISL_800057	Lighthouse Lab in Glasgow	Wellcome Sanger Institute for the COVID-19 Genomics UK (COG-UK) Consortium	Harper VanSteenhouse, Yumi Kasai, David Gray, Carol Clugston, Anna Dominiczak and Alex Alderton, Roberto Amato, Sonia Goncalves, Ewan Harrison, David K. Jackson, Ian Johnston, Dominic Kwiatkowski, Cordelia Langford, John Sillitoe on behalf of the Wellcome Sanger Institute COVID-19 Surveillance Team
EPI_ISL_800058, EPI_ISL_800060, EPI_ISL_800062, EPI_ISL_800063	Lighthouse Lab in Alderley Park	Wellcome Sanger Institute for the COVID-19 Genomics UK (COG-UK) Consortium	Jacquelyn Wynn, Mairead Hyland, The Lighthouse Lab in Alderley Park and Alex Alderton, Roberto Amato, Sonia Goncalves, Ewan Harrison, David K. Jackson, Ian Johnston, Dominic Kwiatkowski, Cordelia Langford, John Sillitoe on behalf of the Wellcome Sanger Institute COVID-19 Surveillance Team
EPI_ISL_800064	Lighthouse Lab in Glasgow	Wellcome Sanger Institute for the COVID-19 Genomics UK (COG-UK) Consortium	Harper VanSteenhouse, Yumi Kasai, David Gray, Carol Clugston, Anna Dominiczak and Alex Alderton, Roberto Amato, Sonia Goncalves, Ewan Harrison, David K. Jackson, Ian Johnston, Dominic Kwiatkowski, Cordelia Langford, John Sillitoe on behalf of the Wellcome Sanger Institute COVID-19 Surveillance Team
EPI_ISL_800066, EPI_ISL_800069	Lighthouse Lab in Alderley Park	Wellcome Sanger Institute for the COVID-19 Genomics UK (COG-UK) Consortium	Jacquelyn Wynn, Mairead Hyland, The Lighthouse Lab in Alderley Park and Alex Alderton, Roberto Amato, Sonia Goncalves, Ewan Harrison, David K. Jackson, Ian Johnston, Dominic Kwiatkowski, Cordelia Langford, John Sillitoe on behalf of the Wellcome Sanger Institute COVID-19 Surveillance Team
EPI_ISL_800071	Lighthouse Lab in Glasgow	Wellcome Sanger Institute for the COVID-19 Genomics UK (COG-UK) Consortium	Harper VanSteenhouse, Yumi Kasai, David Gray, Carol Clugston, Anna Dominiczak and Alex Alderton, Roberto Amato, Sonia Goncalves, Ewan Harrison, David K. Jackson, Ian Johnston, Dominic Kwiatkowski, Cordelia Langford, John Sillitoe on behalf of the Wellcome Sanger Institute COVID-19 Surveillance Team

EPI_ISL_800073, EPI_ISL_800075, EPI_ISL_800076, EPI_ISL_800080, EPI_ISL_800081, EPI_ISL_800087, EPI_ISL_800090, EPI_ISL_800091, EPI_ISL_800097, EPI_ISL_800099, EPI_ISL_800104			
see above	Lighthouse Lab in Alderley Park	Wellcome Sanger Institute for the COVID-19 Genomics UK (COG-UK) Consortium	Jacquelyn Wynn, Mairead Hyland, The Lighthouse Lab in Alderley Park and Alex Alderton, Roberto Amato, Sonia Goncalves, Ewan Harrison, David K. Jackson, Ian Johnston, Dominic Kwiatkowski, Cordelia Langford, John Sillitoe on behalf of the Wellcome Sanger Institute COVID-19 Surveillance Team
EPI_ISL_800106	Lighthouse Lab in Glasgow	Wellcome Sanger Institute for the COVID-19 Genomics UK (COG-UK) Consortium	Harper VanSteenhouse, Yumi Kasai, David Gray, Carol Clugston, Anna Dominiczak and Alex Alderton, Roberto Amato, Sonia Goncalves, Ewan Harrison, David K. Jackson, Ian Johnston, Dominic Kwiatkowski, Cordelia Langford, John Sillitoe on behalf of the Wellcome Sanger Institute COVID-19 Surveillance Team
EPI_ISL_800107, EPI_ISL_800108, EPI_ISL_800109	Lighthouse Lab in Alderley Park	Wellcome Sanger Institute for the COVID-19 Genomics UK (COG-UK) Consortium	Jacquelyn Wynn, Mairead Hyland, The Lighthouse Lab in Alderley Park and Alex Alderton, Roberto Amato, Sonia Goncalves, Ewan Harrison, David K. Jackson, Ian Johnston, Dominic Kwiatkowski, Cordelia Langford, John Sillitoe on behalf of the Wellcome Sanger Institute COVID-19 Surveillance Team
EPI_ISL_800111, EPI_ISL_800113, EPI_ISL_800117, EPI_ISL_800120, EPI_ISL_800122	Lighthouse Lab in Glasgow	Wellcome Sanger Institute for the COVID-19 Genomics UK (COG-UK) Consortium	Harper VanSteenhouse, Yumi Kasai, David Gray, Carol Clugston, Anna Dominiczak and Alex Alderton, Roberto Amato, Sonia Goncalves, Ewan Harrison, David K. Jackson, Ian Johnston, Dominic Kwiatkowski, Cordelia Langford, John Sillitoe on behalf of the Wellcome Sanger Institute COVID-19 Surveillance Team
EPI_ISL_800126, EPI_ISL_800129	Lighthouse Lab in Alderley Park	Wellcome Sanger Institute for the COVID-19 Genomics UK (COG-UK) Consortium	Jacquelyn Wynn, Mairead Hyland, The Lighthouse Lab in Alderley Park and Alex Alderton, Roberto Amato, Sonia Goncalves, Ewan Harrison, David K. Jackson, Ian Johnston, Dominic Kwiatkowski, Cordelia Langford, John Sillitoe on behalf of the Wellcome Sanger Institute COVID-19 Surveillance Team
EPI_ISL_800131	Lighthouse Lab in Glasgow	Wellcome Sanger Institute for the COVID-19 Genomics UK (COG-UK) Consortium	Harper VanSteenhouse, Yumi Kasai, David Gray, Carol Clugston, Anna Dominiczak and Alex Alderton, Roberto Amato, Sonia Goncalves, Ewan Harrison, David K. Jackson, Ian Johnston, Dominic Kwiatkowski, Cordelia Langford, John Sillitoe on behalf of the Wellcome Sanger Institute COVID-19 Surveillance Team
EPI_ISL_800135, EPI_ISL_800136	Lighthouse Lab in Alderley Park	Wellcome Sanger Institute for the COVID-19 Genomics UK (COG-UK) Consortium	Jacquelyn Wynn, Mairead Hyland, The Lighthouse Lab in Alderley Park and Alex Alderton, Roberto Amato, Sonia Goncalves, Ewan Harrison, David K. Jackson, Ian Johnston, Dominic Kwiatkowski, Cordelia Langford, John Sillitoe on behalf of the Wellcome Sanger Institute COVID-19 Surveillance Team
EPI_ISL_800138	Lighthouse Lab in Glasgow	Wellcome Sanger Institute for the COVID-19 Genomics UK (COG-UK) Consortium	Harper VanSteenhouse, Yumi Kasai, David Gray, Carol Clugston, Anna Dominiczak and Alex Alderton, Roberto Amato, Sonia Goncalves, Ewan Harrison, David K. Jackson, Ian Johnston, Dominic Kwiatkowski, Cordelia Langford, John Sillitoe on behalf of the Wellcome Sanger Institute COVID-19 Surveillance Team
EPI_ISL_800140	Lighthouse Lab in Alderley Park	Wellcome Sanger Institute for the COVID-19 Genomics UK (COG-UK) Consortium	Jacquelyn Wynn, Mairead Hyland, The Lighthouse Lab in Alderley Park and Alex Alderton, Roberto Amato, Sonia Goncalves, Ewan Harrison, David K. Jackson, Ian Johnston, Dominic Kwiatkowski, Cordelia Langford, John Sillitoe on behalf of the Wellcome Sanger Institute COVID-19 Surveillance Team
EPI_ISL_800142	Lighthouse Lab in Glasgow	Wellcome Sanger Institute for the COVID-19 Genomics UK (COG-UK) Consortium	Harper VanSteenhouse, Yumi Kasai, David Gray, Carol Clugston, Anna Dominiczak and Alex Alderton, Roberto Amato, Sonia Goncalves, Ewan Harrison, David K. Jackson, Ian Johnston, Dominic Kwiatkowski, Cordelia Langford, John Sillitoe on behalf of the Wellcome Sanger Institute COVID-19 Surveillance Team
EPI_ISL_800144, EPI_ISL_800145, EPI_ISL_800146	Lighthouse Lab in Alderley Park	Wellcome Sanger Institute for the COVID-19 Genomics UK (COG-UK) Consortium	Jacquelyn Wynn, Mairead Hyland, The Lighthouse Lab in Alderley Park and Alex Alderton, Roberto Amato, Sonia Goncalves, Ewan Harrison, David K. Jackson, Ian Johnston, Dominic Kwiatkowski, Cordelia Langford, John Sillitoe on behalf of the Wellcome Sanger Institute COVID-19 Surveillance Team
EPI_ISL_800147, EPI_ISL_800158	Lighthouse Lab in Glasgow	Wellcome Sanger Institute for the COVID-19 Genomics UK (COG-UK) Consortium	Harper VanSteenhouse, Yumi Kasai, David Gray, Carol Clugston, Anna Dominiczak and Alex Alderton, Roberto Amato, Sonia Goncalves, Ewan Harrison, David K. Jackson, Ian Johnston, Dominic Kwiatkowski, Cordelia Langford, John Sillitoe on behalf of the Wellcome Sanger Institute COVID-19 Surveillance Team
EPI_ISL_800159, EPI_ISL_800160, EPI_ISL_800162, EPI_ISL_800165, EPI_ISL_800171	Lighthouse Lab in Alderley Park	Wellcome Sanger Institute for the COVID-19 Genomics UK (COG-UK) Consortium	Jacquelyn Wynn, Mairead Hyland, The Lighthouse Lab in Alderley Park and Alex Alderton, Roberto Amato, Sonia Goncalves, Ewan Harrison, David K. Jackson, Ian Johnston, Dominic Kwiatkowski, Cordelia Langford, John Sillitoe on behalf of the Wellcome Sanger Institute COVID-19 Surveillance Team
EPI_ISL_800174, EPI_ISL_800183	Lighthouse Lab in Glasgow	Wellcome Sanger Institute for the COVID-19 Genomics UK (COG-UK) Consortium	Harper VanSteenhouse, Yumi Kasai, David Gray, Carol Clugston, Anna Dominiczak and Alex Alderton, Roberto Amato, Sonia Goncalves, Ewan Harrison, David K. Jackson, Ian Johnston, Dominic Kwiatkowski, Cordelia Langford, John Sillitoe on behalf of the Wellcome Sanger Institute COVID-19 Surveillance Team
EPI_ISL_800185	Lighthouse Lab in Alderley Park	Wellcome Sanger Institute for the COVID-19 Genomics UK (COG-UK) Consortium	Jacquelyn Wynn, Mairead Hyland, The Lighthouse Lab in Alderley Park and Alex Alderton, Roberto Amato, Sonia Goncalves, Ewan Harrison, David K. Jackson, Ian Johnston, Dominic Kwiatkowski, Cordelia Langford, John Sillitoe on behalf of the Wellcome Sanger Institute COVID-19 Surveillance Team
EPI_ISL_800186, EPI_ISL_800189	Lighthouse Lab in Glasgow	Wellcome Sanger Institute for the COVID-19 Genomics UK (COG-UK) Consortium	Harper VanSteenhouse, Yumi Kasai, David Gray, Carol Clugston, Anna Dominiczak and Alex Alderton, Roberto Amato, Sonia Goncalves, Ewan Harrison, David K. Jackson, Ian Johnston, Dominic Kwiatkowski, Cordelia Langford, John Sillitoe on behalf of the Wellcome Sanger Institute COVID-19 Surveillance Team
EPI_ISL_800193, EPI_ISL_800194, EPI_ISL_800197, EPI_ISL_800198, EPI_ISL_800199, EPI_ISL_800202	Lighthouse Lab in Alderley Park	Wellcome Sanger Institute for the COVID-19 Genomics UK (COG-UK) Consortium	Jacquelyn Wynn, Mairead Hyland, The Lighthouse Lab in Alderley Park and Alex Alderton, Roberto Amato, Sonia Goncalves, Ewan Harrison, David K. Jackson, Ian Johnston, Dominic Kwiatkowski, Cordelia Langford, John Sillitoe on behalf of the Wellcome Sanger Institute COVID-19 Surveillance Team
EPI_ISL_800204	Lighthouse Lab in Glasgow	Wellcome Sanger Institute for the COVID-19 Genomics UK (COG-UK) Consortium	Harper VanSteenhouse, Yumi Kasai, David Gray, Carol Clugston, Anna Dominiczak and Alex Alderton, Roberto Amato, Sonia Goncalves, Ewan Harrison, David K. Jackson, Ian Johnston, Dominic Kwiatkowski, Cordelia Langford, John Sillitoe on behalf of the Wellcome Sanger Institute COVID-19 Surveillance Team
EPI_ISL_800205, EPI_ISL_800206, EPI_ISL_800207, EPI_ISL_800208, EPI_ISL_800209	Lighthouse Lab in Alderley Park	Wellcome Sanger Institute for the COVID-19 Genomics UK (COG-UK) Consortium	Jacquelyn Wynn, Mairead Hyland, The Lighthouse Lab in Alderley Park and Alex Alderton, Roberto Amato, Sonia Goncalves, Ewan Harrison, David K. Jackson, Ian Johnston, Dominic Kwiatkowski, Cordelia Langford, John Sillitoe on behalf of the Wellcome Sanger Institute COVID-19 Surveillance Team
EPI_ISL_800210	Lighthouse Lab in Glasgow	Wellcome Sanger Institute for the COVID-19 Genomics UK (COG-UK) Consortium	Harper VanSteenhouse, Yumi Kasai, David Gray, Carol Clugston, Anna Dominiczak and Alex Alderton, Roberto Amato, Sonia Goncalves, Ewan Harrison, David K. Jackson, Ian Johnston, Dominic Kwiatkowski, Cordelia Langford, John Sillitoe on behalf of the Wellcome Sanger Institute COVID-19 Surveillance Team
EPI_ISL_800212, EPI_ISL_800213, EPI_ISL_800215, EPI_ISL_800216	Lighthouse Lab in Alderley Park	Wellcome Sanger Institute for the COVID-19 Genomics UK (COG-UK) Consortium	Jacquelyn Wynn, Mairead Hyland, The Lighthouse Lab in Alderley Park and Alex Alderton, Roberto Amato, Sonia Goncalves, Ewan Harrison, David K. Jackson, Ian Johnston, Dominic Kwiatkowski, Cordelia Langford, John Sillitoe on behalf of the Wellcome Sanger Institute COVID-19 Surveillance Team
EPI_ISL_800217	Lighthouse Lab in Glasgow	Wellcome Sanger Institute for the COVID-19 Genomics UK (COG-UK) Consortium	Harper VanSteenhouse, Yumi Kasai, David Gray, Carol Clugston, Anna Dominiczak and Alex Alderton, Roberto Amato, Sonia Goncalves, Ewan Harrison, David K. Jackson, Ian Johnston, Dominic Kwiatkowski, Cordelia Langford, John Sillitoe on behalf of the Wellcome Sanger Institute COVID-19 Surveillance Team
EPI_ISL_800219	Lighthouse Lab in Alderley Park	Wellcome Sanger Institute for the COVID-19 Genomics UK (COG-UK) Consortium	Jacquelyn Wynn, Mairead Hyland, The Lighthouse Lab in Alderley Park and Alex Alderton, Roberto Amato, Sonia Goncalves, Ewan Harrison, David K. Jackson, Ian Johnston, Dominic Kwiatkowski, Cordelia Langford, John Sillitoe on behalf of the Wellcome Sanger Institute COVID-19 Surveillance Team
EPI_ISL_800221	Lighthouse Lab in Glasgow	Wellcome Sanger Institute for the COVID-19 Genomics UK (COG-UK) Consortium	Harper VanSteenhouse, Yumi Kasai, David Gray, Carol Clugston, Anna Dominiczak and Alex Alderton, Roberto Amato, Sonia Goncalves, Ewan Harrison, David K. Jackson, Ian Johnston, Dominic Kwiatkowski, Cordelia Langford, John Sillitoe on behalf of the Wellcome Sanger Institute COVID-19 Surveillance Team
EPI_ISL_800223, EPI_ISL_800226, EPI_ISL_800229, EPI_ISL_800230, EPI_ISL_800231, EPI_ISL_800232	Lighthouse Lab in Alderley Park	Wellcome Sanger Institute for the COVID-19 Genomics UK (COG-UK) Consortium	Jacquelyn Wynn, Mairead Hyland, The Lighthouse Lab in Alderley Park and Alex Alderton, Roberto Amato, Sonia Goncalves, Ewan Harrison, David K. Jackson, Ian Johnston, Dominic Kwiatkowski, Cordelia Langford, John Sillitoe on behalf of the Wellcome Sanger Institute COVID-19 Surveillance Team
EPI_ISL_800237	Lighthouse Lab in Glasgow	Wellcome Sanger Institute for the COVID-19 Genomics UK (COG-UK) Consortium	Harper VanSteenhouse, Yumi Kasai, David Gray, Carol Clugston, Anna Dominiczak and Alex Alderton, Roberto Amato, Sonia Goncalves, Ewan Harrison, David K. Jackson, Ian Johnston, Dominic Kwiatkowski, Cordelia Langford, John Sillitoe on behalf of the Wellcome Sanger Institute COVID-19 Surveillance Team
EPI_ISL_800239, EPI_ISL_800242	Lighthouse Lab in Alderley Park	Wellcome Sanger Institute for the COVID-19 Genomics UK (COG-UK) Consortium	Jacquelyn Wynn, Mairead Hyland, The Lighthouse Lab in Alderley Park and Alex Alderton, Roberto Amato, Sonia Goncalves, Ewan Harrison, David K. Jackson, Ian Johnston, Dominic Kwiatkowski, Cordelia Langford, John Sillitoe on behalf of the Wellcome Sanger Institute COVID-19 Surveillance Team
EPI_ISL_800245	Lighthouse Lab in Glasgow	Wellcome Sanger Institute for the COVID-19 Genomics UK	Harper VanSteenhouse, Yumi Kasai, David Gray, Carol Clugston, Anna Dominiczak and Alex Alderton, Roberto Amato, Sonia Goncalves, Ewan Harrison,

		(COG-UK) Consortium	David K. Jackson, Ian Johnston, Dominic Kwiatkowski, Cordelia Langford, John Sillitoe on behalf of the Wellcome Sanger Institute COVID-19 Surveillance Team
EPI_ISL_800247, EPI_ISL_800249	Lighthouse Lab in Alderley Park	Wellcome Sanger Institute for the COVID-19 Genomics UK (COG-UK) Consortium	Jacquelyn Wynn, Mairead Hyland, The Lighthouse Lab in Alderley Park and Alex Alderton, Roberto Amato, Sonia Goncalves, Ewan Harrison, David K. Jackson, Ian Johnston, Dominic Kwiatkowski, Cordelia Langford, John Sillitoe on behalf of the Wellcome Sanger Institute COVID-19 Surveillance Team
EPI_ISL_800251, EPI_ISL_800253	Lighthouse Lab in Glasgow	Wellcome Sanger Institute for the COVID-19 Genomics UK (COG-UK) Consortium	Harper VanSteenhouse, Yumi Kasai, David Gray, Carol Clugston, Anna Dominiczak and Alex Alderton, Roberto Amato, Sonia Goncalves, Ewan Harrison, David K. Jackson, Ian Johnston, Dominic Kwiatkowski, Cordelia Langford, John Sillitoe on behalf of the Wellcome Sanger Institute COVID-19 Surveillance Team
EPI_ISL_800267, EPI_ISL_800271, EPI_ISL_800274, EPI_ISL_800280, EPI_ISL_800281, EPI_ISL_800282, EPI_ISL_800286	Lighthouse Lab in Alderley Park	Wellcome Sanger Institute for the COVID-19 Genomics UK (COG-UK) Consortium	Jacquelyn Wynn, Mairead Hyland, The Lighthouse Lab in Alderley Park and Alex Alderton, Roberto Amato, Sonia Goncalves, Ewan Harrison, David K. Jackson, Ian Johnston, Dominic Kwiatkowski, Cordelia Langford, John Sillitoe on behalf of the Wellcome Sanger Institute COVID-19 Surveillance Team
EPI_ISL_800297	Lighthouse Lab in Cambridge	Wellcome Sanger Institute for the COVID-19 Genomics UK (COG-UK) Consortium	Rob Howes, The Lighthouse Lab in Cambridge and Alex Alderton, Roberto Amato, Sonia Goncalves, Ewan Harrison, David K. Jackson, Ian Johnston, Dominic Kwiatkowski, Cordelia Langford, John Sillitoe on behalf of the Wellcome Sanger Institute COVID-19 Surveillance Team
EPI_ISL_800299, EPI_ISL_800301, EPI_ISL_800303, EPI_ISL_800308, EPI_ISL_800317, EPI_ISL_800338, EPI_ISL_800340, EPI_ISL_800342, EPI_ISL_800347, EPI_ISL_800348, EPI_ISL_800350, EPI_ISL_800351, EPI_ISL_800354, EPI_ISL_800359, EPI_ISL_800360, EPI_ISL_800365, EPI_ISL_800367, EPI_ISL_800370			
see above	Lighthouse Lab in Alderley Park	Wellcome Sanger Institute for the COVID-19 Genomics UK (COG-UK) Consortium	Jacquelyn Wynn, Mairead Hyland, The Lighthouse Lab in Alderley Park and Alex Alderton, Roberto Amato, Sonia Goncalves, Ewan Harrison, David K. Jackson, Ian Johnston, Dominic Kwiatkowski, Cordelia Langford, John Sillitoe on behalf of the Wellcome Sanger Institute COVID-19 Surveillance Team
EPI_ISL_800372	Lighthouse Lab in Cambridge	Wellcome Sanger Institute for the COVID-19 Genomics UK (COG-UK) Consortium	Rob Howes, The Lighthouse Lab in Cambridge and Alex Alderton, Roberto Amato, Sonia Goncalves, Ewan Harrison, David K. Jackson, Ian Johnston, Dominic Kwiatkowski, Cordelia Langford, John Sillitoe on behalf of the Wellcome Sanger Institute COVID-19 Surveillance Team
EPI_ISL_800376, EPI_ISL_800384	Lighthouse Lab in Alderley Park	Wellcome Sanger Institute for the COVID-19 Genomics UK (COG-UK) Consortium	Jacquelyn Wynn, Mairead Hyland, The Lighthouse Lab in Alderley Park and Alex Alderton, Roberto Amato, Sonia Goncalves, Ewan Harrison, David K. Jackson, Ian Johnston, Dominic Kwiatkowski, Cordelia Langford, John Sillitoe on behalf of the Wellcome Sanger Institute COVID-19 Surveillance Team
EPI_ISL_800389	Lighthouse Lab in Cambridge	Wellcome Sanger Institute for the COVID-19 Genomics UK (COG-UK) Consortium	Rob Howes, The Lighthouse Lab in Cambridge and Alex Alderton, Roberto Amato, Sonia Goncalves, Ewan Harrison, David K. Jackson, Ian Johnston, Dominic Kwiatkowski, Cordelia Langford, John Sillitoe on behalf of the Wellcome Sanger Institute COVID-19 Surveillance Team
EPI_ISL_800392, EPI_ISL_800407, EPI_ISL_800420, EPI_ISL_800428, EPI_ISL_800441	Lighthouse Lab in Alderley Park	Wellcome Sanger Institute for the COVID-19 Genomics UK (COG-UK) Consortium	Jacquelyn Wynn, Mairead Hyland, The Lighthouse Lab in Alderley Park and Alex Alderton, Roberto Amato, Sonia Goncalves, Ewan Harrison, David K. Jackson, Ian Johnston, Dominic Kwiatkowski, Cordelia Langford, John Sillitoe on behalf of the Wellcome Sanger Institute COVID-19 Surveillance Team
EPI_ISL_800442	Lighthouse Lab in Cambridge	Wellcome Sanger Institute for the COVID-19 Genomics UK (COG-UK) Consortium	Rob Howes, The Lighthouse Lab in Cambridge and Alex Alderton, Roberto Amato, Sonia Goncalves, Ewan Harrison, David K. Jackson, Ian Johnston, Dominic Kwiatkowski, Cordelia Langford, John Sillitoe on behalf of the Wellcome Sanger Institute COVID-19 Surveillance Team
EPI_ISL_800444, EPI_ISL_800446, EPI_ISL_800452, EPI_ISL_800453, EPI_ISL_800454, EPI_ISL_800456, EPI_ISL_800457, EPI_ISL_800468, EPI_ISL_800474, EPI_ISL_800481, EPI_ISL_800485, EPI_ISL_800494, EPI_ISL_800495, EPI_ISL_800501, EPI_ISL_800502, EPI_ISL_800509, EPI_ISL_800516, EPI_ISL_800518, EPI_ISL_800524, EPI_ISL_800525, EPI_ISL_800528, EPI_ISL_800531, EPI_ISL_800558, EPI_ISL_800566			
see above	Lighthouse Lab in Alderley Park	Wellcome Sanger Institute for the COVID-19 Genomics UK (COG-UK) Consortium	Jacquelyn Wynn, Mairead Hyland, The Lighthouse Lab in Alderley Park and Alex Alderton, Roberto Amato, Sonia Goncalves, Ewan Harrison, David K. Jackson, Ian Johnston, Dominic Kwiatkowski, Cordelia Langford, John Sillitoe on behalf of the Wellcome Sanger Institute COVID-19 Surveillance Team
EPI_ISL_801432, EPI_ISL_801433, EPI_ISL_801434, EPI_ISL_801435, EPI_ISL_801436, EPI_ISL_801439, EPI_ISL_801440, EPI_ISL_801442, EPI_ISL_801458, EPI_ISL_801496, EPI_ISL_801498, EPI_ISL_801499, EPI_ISL_801500, EPI_ISL_801501, EPI_ISL_801502, EPI_ISL_801503, EPI_ISL_801504, EPI_ISL_801505, EPI_ISL_801506, EPI_ISL_801507, EPI_ISL_801508, EPI_ISL_801509, EPI_ISL_801515			
see above	Dutch COVID-19 response team	Erasmus Medical Center	Bas Oude Munnink, Reina Sikkema, David Nieuwenhuijse, Irina Chestakova, Anne van der Linden, Marjan Boter, Emmanuelle Munger, Corine GeurtsvanKessel, Annemiek van der Eijk, Richard Molenkamp, Marion Koopmans, on behalf of the Dutch national COVID-19 response team.
EPI_ISL_801521	Instituto Nacional de Saude (INSA)	Instituto Nacional de Saude (INSA)	Borges et al
EPI_ISL_802430, EPI_ISL_802445, EPI_ISL_802446	BIO-REFERENCE LABORATORIES	Wadsworth Center, New York State Department.of Health	Kirsten St. George, Daryl M. Lamson, Alexis Russel, Matthew Shudt, Melissa A Leisner, Jonathan Plitnick, Navjot Singh, John Kelly, Sara Griesemer, Erasmus Schneider, Erica Lasek-Nesselquist
EPI_ISL_802449, EPI_ISL_802450, EPI_ISL_802451, EPI_ISL_802452, EPI_ISL_802453, EPI_ISL_802454, EPI_ISL_802455, EPI_ISL_802456, EPI_ISL_802457, EPI_ISL_802458, EPI_ISL_802459, EPI_ISL_802460, EPI_ISL_802461, EPI_ISL_802462			
see above	Wadsworth Center, New York State Department.of Health	Wadsworth Center, New York State Department.of Health	Kirsten St. George, Daryl M. Lamson, Alexis Russel, Matthew Shudt, Melissa A Leisner, Jonathan Plitnick, Navjot Singh, John Kelly, Sara Griesemer, Erasmus Schneider, Erica Lasek-Nesselquist
EPI_ISL_802575, EPI_ISL_802577, EPI_ISL_802579, EPI_ISL_802580, EPI_ISL_802585, EPI_ISL_802586, EPI_ISL_802587, EPI_ISL_802589, EPI_ISL_802590, EPI_ISL_802591, EPI_ISL_802592, EPI_ISL_802593, EPI_ISL_802594, EPI_ISL_802597			
see above	Vault Health	Minnesota Department of Health, Public Health Laboratory	Alexandra Lorentz, Jacob Garfin, Matt Plumb, and Xiong Wang
EPI_ISL_802600, EPI_ISL_802605, EPI_ISL_802608, EPI_ISL_802609, EPI_ISL_802610, EPI_ISL_802611, EPI_ISL_802629, EPI_ISL_802630, EPI_ISL_802631, EPI_ISL_802637, EPI_ISL_802638, EPI_ISL_802639, EPI_ISL_802640, EPI_ISL_802648, EPI_ISL_802650, EPI_ISL_802654, EPI_ISL_802656, EPI_ISL_802657, EPI_ISL_802658, EPI_ISL_802659, EPI_ISL_802660, EPI_ISL_802669, EPI_ISL_802670, EPI_ISL_802671, EPI_ISL_802672, EPI_ISL_802673, EPI_ISL_802674, EPI_ISL_802679, EPI_ISL_802680, EPI_ISL_802681, EPI_ISL_802684, EPI_ISL_802685, EPI_ISL_802686, EPI_ISL_802688, EPI_ISL_802690, EPI_ISL_802699, EPI_ISL_802700, EPI_ISL_802706, EPI_ISL_802707, EPI_ISL_802708, EPI_ISL_802709, EPI_ISL_802710, EPI_ISL_802719, EPI_ISL_802722			
see above	Helix/Illumina	Genomics and Discovery, Respiratory Viruses Branch, Division of Viral Diseases, Centers for Disease Control and Prevention	Peter W. Cook, Dhwani Batra, Ben L. Rambo-Martin Eileen de Feo, Jan Antico, Christine Tran, Matthew Tolentino, Shannon Wickline, Kim Gietzen, Brad Sickler, Jingtao Liu, Eric Allen, Phil Febbo, Summer Galloway, Nicole L. Washington, Simon White, Geraint Levan, Kelly Schiabor Barrett, Elizabeth Cirulli, Alexandre Bolze, Ary Ascencio, Charlotte Rivera-Garcia, Ryan Cho, Jason Nguyen, Sherry Wang, Jimmy Ramirez, Tyler Cassens, Efren Sandoval, Magnus Isaksson, William Lee, David Becker, Marc Laurent, James Lu, Clinton R. Paden, Suxiang Tong, Duncan MacCannell
EPI_ISL_802788, EPI_ISL_802789	Columbia University Irving Medical Center	Wadsworth Center, New York State Department.of Health	Kirsten St. George, Daryl M. Lamson, Alexis Russel, Matthew Shudt, Melissa A Leisner, Jonathan Plitnick, Navjot Singh, John Kelly, Sara Griesemer, Erasmus Schneider, Erica Lasek-Nesselquist
EPI_ISL_803079	Quest Diagnostics	Quest Diagnostics	Rosenthal,S.H., Gerasimova,A., Kagan,R.M., Anderson, B., Livingston, K.E., Hua, M., Liu Y., Shalhout, D.F., Owen, R., Lacbawan, F.
EPI_ISL_803894	Robert Koch Institut	Robert Koch Institut	Annika Brinkmann, Janine Michel, Livia Schrick, Steven Uddin, Dominique Seifert, Alexander Dalpke, Leo Büttner, Kristina Hochauf-Stange, Dirk Lindemann, Lars Schaade, Andreas Nitsche
EPI_ISL_803900	National Institute for Infectious Diseases, INMI, "L. Spallanzani" IRCCS	National Institute for Infectious Diseases, INMI, "L. Spallanzani" IRCCS	B Bartolini, O Butera, C.E.M Gruber, M Rueca, F Messina, E Giombini, MR Capobianchi, A Di Caro
EPI_ISL_803955, EPI_ISL_803956, EPI_ISL_803957	Labor Dr. Wisplinghoff - Köln	Robert Koch Institute, Influenza and respiratory viruses FG17 & Bioinformatics MF1, Berlin, Germany	Dr. R. Grosser, Stephan Fuchs, Stefan Kroeger, Marianne Wedde, Oliver Drechsel, Aleksandar Radonic, Rene Kmiecinski, Ralf Duerrwald, Thorsten Wolff
EPI_ISL_803984, EPI_ISL_803985, EPI_ISL_803986, EPI_ISL_803987, EPI_ISL_803988	National Public Health Laboratory, National Centre for Infectious Diseases	National Public Health Laboratory, National Centre for Infectious Diseases	Tze Minn Mak, Sophie Octavia, Zhenyang Zhou, Lin Cui, Raymond Tzer Pin Lin
EPI_ISL_804020, EPI_ISL_804021, EPI_ISL_804022, EPI_ISL_804023, EPI_ISL_804024, EPI_ISL_804025, EPI_ISL_804027, EPI_ISL_804028	Maryland Public Health Laboratory	Maryland Public Health Laboratory	Maryland Department of Health Laboratories Administration
EPI_ISL_804056, EPI_ISL_804071, EPI_ISL_804072, EPI_ISL_804073, EPI_ISL_804074, EPI_ISL_804075, EPI_ISL_804076, EPI_ISL_804077, EPI_ISL_804078, EPI_ISL_804079, EPI_ISL_804080, EPI_ISL_804081, EPI_ISL_804082, EPI_ISL_804083, EPI_ISL_804092, EPI_ISL_804093, EPI_ISL_804094, EPI_ISL_804096, EPI_ISL_804098, EPI_ISL_804102, EPI_ISL_804103, EPI_ISL_804104, EPI_ISL_804106, EPI_ISL_804142, EPI_ISL_804143, EPI_ISL_804144, EPI_ISL_804145, EPI_ISL_804146, EPI_ISL_804147, EPI_ISL_804148, EPI_ISL_804149, EPI_ISL_804150, EPI_ISL_804151, EPI_ISL_804152, EPI_ISL_804153, EPI_ISL_804154, EPI_ISL_804155, EPI_ISL_804156, EPI_ISL_804157, EPI_ISL_804158, EPI_ISL_804159, EPI_ISL_804160, EPI_ISL_804161, EPI_ISL_804162, EPI_ISL_804163, EPI_ISL_804164, EPI_ISL_804165, EPI_ISL_804166, EPI_ISL_804167, EPI_ISL_804168, EPI_ISL_804169, EPI_ISL_804170, EPI_ISL_804171, EPI_ISL_804172, EPI_ISL_804173, EPI_ISL_804174, EPI_ISL_804175, EPI_ISL_804176, EPI_ISL_804177, EPI_ISL_804178, EPI_ISL_804179, EPI_ISL_804180, EPI_ISL_804181, EPI_ISL_804182, EPI_ISL_804183, EPI_ISL_804184, EPI_ISL_804185, EPI_ISL_804186, EPI_ISL_804187, EPI_ISL_804188, EPI_ISL_804191, EPI_ISL_804209, EPI_ISL_804210, EPI_ISL_804211, EPI_ISL_804212			
see above	Israel Central Virology laboratory	Israel National Consortium for SARS-CoV-2 sequencing	Neta Zuckerman, Efrat Dahan Bucris, Michal Mandelboim, Dana Bar-Ilan, Oran Erster, Tzvia Mann, Omer Murik, David A. Zeevi, Assaf Rokney, Joseph

			Jaffe, Eva Nachum, Maya Davidovich Cohen, Ephraim Fass, Gal Zizelski Valenci, Mor Rubinstein, Efrat Rorman, Israel Nissan, Efrat Glick-Saar, Omri Nayshool, Gideon Rechavi, Ella Mendelson, Orna Mor
EPI_ISL_804223, EPI_ISL_804253, EPI_ISL_804274, EPI_ISL_804275, EPI_ISL_804277, EPI_ISL_804278, EPI_ISL_804279	Respiratory Virus Unit, National Infection Service, Public Health England	COVID-19 Genomics UK (COG-UK) Consortium	PHE Covid Sequencing Team
EPI_ISL_804946	BIO-REFERENCE LABORATORIES	Wadsworth Center, New York State Department.of Health	Kirsten St. George, Daryl M. Lamson, Alexis Russel, Matthew Shudt, Melissa A Leisner, Jonathan Plitnick, Navjot Singh, John Kelly, Sara Griesemer, Erasmus Schneider, Erica Lasek-Nesselquist
EPI_ISL_806721	Histopath	NSW Health Pathology - Institute of Clinical Pathology and Medical Research; Westmead Hospital; University of Sydney	CIDM-PH et al.
EPI_ISL_806722	South Eastern Area Laboratory Services (SEALS)	NSW Health Pathology - Institute of Clinical Pathology and Medical Research; Westmead Hospital; University of Sydney	CIDM-PH et al.
EPI_ISL_807156	Deva County Emergency Hospital	National Institute of Infectious Diseases-Prof. Dr. Matei Bals Molecular Diagnostics Laboratory	Leontina Banica, Marius Surleac, Corina Casangiu, Petre Milu, Andreea Tudor, Simona Paraschiv, Dan Otelea
EPI_ISL_810804	PathWest Laboratory Medicine WA	PathWest Laboratory Medicine WA Microbial Surveillance Unit	PathWest Laboratory Medicine WA Microbial Surveillance Unit
EPI_ISL_811122	Respiratory Virus Unit, National Infection Service, Public Health England	COVID-19 Genomics UK (COG-UK) Consortium	PHE Covid Sequencing Team
EPI_ISL_811809, EPI_ISL_811810, EPI_ISL_811813, EPI_ISL_811815, EPI_ISL_811816, EPI_ISL_811820, EPI_ISL_811822, EPI_ISL_811829, EPI_ISL_811838, EPI_ISL_811842, EPI_ISL_811844, EPI_ISL_811863, EPI_ISL_811864, EPI_ISL_811866, EPI_ISL_811872, EPI_ISL_811876, EPI_ISL_811882, EPI_ISL_811895, EPI_ISL_811901, EPI_ISL_811903, EPI_ISL_811904, EPI_ISL_811906, EPI_ISL_811908, EPI_ISL_811911, EPI_ISL_811920, EPI_ISL_811926, EPI_ISL_811933, EPI_ISL_811934, EPI_ISL_811940, EPI_ISL_811947, EPI_ISL_811961, EPI_ISL_811963, EPI_ISL_811964, EPI_ISL_811966, EPI_ISL_811980, EPI_ISL_811982, EPI_ISL_811992, EPI_ISL_811995, EPI_ISL_812001, EPI_ISL_812004, EPI_ISL_812005, EPI_ISL_812006, EPI_ISL_812008, EPI_ISL_812011, EPI_ISL_812013, EPI_ISL_812016, EPI_ISL_812023, EPI_ISL_812029, EPI_ISL_812031, EPI_ISL_812032, EPI_ISL_812034, EPI_ISL_812039, EPI_ISL_812043, EPI_ISL_812044, EPI_ISL_812053, EPI_ISL_812056, EPI_ISL_812057, EPI_ISL_812060, EPI_ISL_812065, EPI_ISL_812071, EPI_ISL_812072, EPI_ISL_812081, EPI_ISL_812082, EPI_ISL_812087, EPI_ISL_812089, EPI_ISL_812095, EPI_ISL_812107, EPI_ISL_812108, EPI_ISL_812110, EPI_ISL_812111, EPI_ISL_812112			
see above	Lighthouse Lab in Milton Keynes	Wellcome Sanger Institute for the COVID-19 Genomics UK (COG-UK) Consortium	The Lighthouse Lab in Milton Keynes and Alex Alderton, Roberto Amato, Sonia Goncalves, Ewan Harrison, David K. Jackson, Ian Johnston, Dominic Kwiatkowski, Cordelia Langford, John Sillitoe on behalf of the Wellcome Sanger Institute COVID-19 Surveillance Team
EPI_ISL_812114	Arizona State University	Arizona State University	LaRinda A. Holland, Peter T. Skidmore, Emily A. Kaelin, Nicholas J. Mellor, Kristina Buss, Joy M. Blain, Valerie Harris, Joshua LaBaer, Vel Murugan, Efrem S. Lim
EPI_ISL_812286, EPI_ISL_812287, EPI_ISL_812288, EPI_ISL_812289, EPI_ISL_812297, EPI_ISL_812298, EPI_ISL_812299, EPI_ISL_812300, EPI_ISL_812301, EPI_ISL_812302, EPI_ISL_812303, EPI_ISL_812304, EPI_ISL_812305, EPI_ISL_812306, EPI_ISL_812307			
see above	Delaware Public Health Lab	Delaware Public Health Lab	Gregory Hovan
EPI_ISL_812763, EPI_ISL_812764, EPI_ISL_812765, EPI_ISL_812766, EPI_ISL_812767	Ministry of Health Turkey	Ministry of Health Turkey	Fatma Bayrakdar, Yasemin Cogun, Süleyman Yalcin, Aye Baak Alta, Gülay Korukluolu
EPI_ISL_813094, EPI_ISL_813095, EPI_ISL_813096, EPI_ISL_813097, EPI_ISL_813098, EPI_ISL_813099, EPI_ISL_813100, EPI_ISL_813101	University of Birmingham	COVID-19 Genomics UK (COG-UK) Consortium	Institute of Microbiology, University of Birmingham: Claire McMurray, Joanne Stockton, Samuel Nicholls, Radoslaw Poplawski, Will Rowe, Josh Quick, Nicholas Loman. University of Birmingham Testing Laboratory: Celina M Whalley, Andrew Bosworth, Charlotte Poxon, Kasun Wanigasooriya, Oliver Pickles, Mike Kidd, Alex Richter, Andrew D Beggs PHE Heartlands Lab: Husam Osman, Andrew Bosworth. Queen Elizabeth Hospital: Anna Casey
EPI_ISL_813167, EPI_ISL_813171, EPI_ISL_813173, EPI_ISL_813175, EPI_ISL_813183, EPI_ISL_813227, EPI_ISL_813230, EPI_ISL_813250, EPI_ISL_813256, EPI_ISL_813265, EPI_ISL_813266, EPI_ISL_813267, EPI_ISL_813268, EPI_ISL_813269, EPI_ISL_813278, EPI_ISL_813281, EPI_ISL_813283, EPI_ISL_813285, EPI_ISL_813294			
see above	Department of Pathology, University of Cambridge	COVID-19 Genomics UK (COG-UK) Consortium	Aminu S. Jahun, Yasmin Chaudhry, Grant Hall, Iliana Georgana, Myra Hosmillo, Martin D. Curran, Malte Pinckert, Surendra Parmar, Ian Goodfellow
EPI_ISL_813581, EPI_ISL_813582, EPI_ISL_813583, EPI_ISL_813587, EPI_ISL_813588, EPI_ISL_813589, EPI_ISL_813590, EPI_ISL_813591, EPI_ISL_813592, EPI_ISL_813593, EPI_ISL_813594, EPI_ISL_813595, EPI_ISL_813596, EPI_ISL_813597, EPI_ISL_813598, EPI_ISL_813600, EPI_ISL_813601, EPI_ISL_813602, EPI_ISL_813603, EPI_ISL_813604, EPI_ISL_813605, EPI_ISL_813606, EPI_ISL_813607, EPI_ISL_813609, EPI_ISL_813610, EPI_ISL_813611, EPI_ISL_813612, EPI_ISL_813613, EPI_ISL_813614, EPI_ISL_813615, EPI_ISL_813616, EPI_ISL_813617, EPI_ISL_813618, EPI_ISL_813619, EPI_ISL_813620, EPI_ISL_813621, EPI_ISL_813622, EPI_ISL_813624, EPI_ISL_813637			
see above	University of Exeter	COVID-19 Genomics UK (COG-UK) Consortium	Ben Temperton,Aaron Jeffries,Michelle Michelsen,Joanna Warwick-Dugdale,Audrey Farbos,Robyn Manley,Stephen Michell,Jane Masoli
EPI_ISL_813773, EPI_ISL_813774, EPI_ISL_813775, EPI_ISL_813776, EPI_ISL_813777, EPI_ISL_813778, EPI_ISL_813779, EPI_ISL_813780, EPI_ISL_813781, EPI_ISL_813782, EPI_ISL_813784			
see above	Liverpool Clinical Laboratories	COVID-19 Genomics UK (COG-UK) Consortium	Sam Haldenby, Anita Lucaci, Steve Paterson, Julian Hiscox, Alistair Darby, M Almsaud, A Alrezaihi, Muhannad Alruwaili, Stuart D Armstrong, Jones Benjamin, Eleanor G Bentley, Anu Chawla, Jordan J Clark, Angela Cowell, Richard Eccles, Isabel García-Dorival, Matthew Gemmell, Alessandro Gerada, PKF Gilmore, Richard Gregory, Ximeng Han, Catherine Hartley, Margaret Hughes, Miren Iturriza-Gomara, James Johnson, L Luu, Jenifer Manson, Charlotte Nelson, Elaine O'Toole, Cassie Olateju, Rebekah Penrice-Randal , Lucille Rainbow, N.P Randle, Trevor Ian Robinson, Parul Sharma, Ghada T Shawli, James P Stewart, Neil Swainston, Ecaterina Vamos, Joanne Watts, Mark Whitehead
EPI_ISL_813900, EPI_ISL_813901, EPI_ISL_813902, EPI_ISL_813903, EPI_ISL_813904, EPI_ISL_813905, EPI_ISL_813906, EPI_ISL_813956, EPI_ISL_813959, EPI_ISL_813962, EPI_ISL_813963, EPI_ISL_813964, EPI_ISL_813966			
see above	University College London, Great Ormond Street Hospital for Children NHS Foundation Trust, Imperial College Healthcare NHS Trust	COVID-19 Genomics UK (COG-UK) Consortium	Sergi Castellano, Rachel Williams, Mark Kristiansen, Paola Resende Silva, Sunando Roy, Tony Brooks, Helena Tutill, Paola Niola, Patricia Dyal, Charlotte Williams, Leysa Forrest, Yasmin Panchbhaya, Jacqueline Findlay, Samuel Weeks, Julianne Brown, Kathryn Harris, Paul Randell, James Price, Alison Holmes, Judith Breuer
EPI_ISL_813975	Akershus University Hospital, Department for Microbiology and Infectious Disease Control	Norwegian Institute of Public Health, Department of Virology	Kathrine Stene-Johansen, Kamilla Heddeland Instefjord, Hilde Elshaug, Atiya R Ali,Marie Paulsen Madsen, Rasmus Riis Kopperud, Hilde Vollan, Karoline Bragstad, Olav Hungnes
EPI_ISL_814068	Ministry of Health Turkey	Ministry of Health Turkey	Fatma Bayrakdar, Yasemin Cogun, Süleyman Yalcin, Aye Baak Alta, Gülay Korukluolu
EPI_ISL_814092, EPI_ISL_814093, EPI_ISL_814094, EPI_ISL_814095, EPI_ISL_814096, EPI_ISL_814098, EPI_ISL_814099, EPI_ISL_814100, EPI_ISL_814101, EPI_ISL_814180, EPI_ISL_814181, EPI_ISL_814182, EPI_ISL_814183, EPI_ISL_814184, EPI_ISL_814185, EPI_ISL_814186, EPI_ISL_814187, EPI_ISL_814188, EPI_ISL_814189, EPI_ISL_814190, EPI_ISL_814191, EPI_ISL_814192, EPI_ISL_814193, EPI_ISL_814194, EPI_ISL_814195, EPI_ISL_814196, EPI_ISL_814197, EPI_ISL_814198, EPI_ISL_814199, EPI_ISL_814200, EPI_ISL_814201, EPI_ISL_814202, EPI_ISL_814203, EPI_ISL_814204, EPI_ISL_814205, EPI_ISL_814206, EPI_ISL_814207, EPI_ISL_814208, EPI_ISL_814209, EPI_ISL_814210, EPI_ISL_814211, EPI_ISL_814212, EPI_ISL_814213, EPI_ISL_814214, EPI_ISL_814215, EPI_ISL_814216, EPI_ISL_814217, EPI_ISL_814218, EPI_ISL_814219, EPI_ISL_814220, EPI_ISL_814221, EPI_ISL_814222, EPI_ISL_814223, EPI_ISL_814224, EPI_ISL_814225, EPI_ISL_814226, EPI_ISL_814227, EPI_ISL_814228, EPI_ISL_814229, EPI_ISL_814230, EPI_ISL_814231, EPI_ISL_814232, EPI_ISL_814233, EPI_ISL_814234, EPI_ISL_814235, EPI_ISL_814236, EPI_ISL_814237, EPI_ISL_814238, EPI_ISL_814239, EPI_ISL_814240, EPI_ISL_814241, EPI_ISL_814242, EPI_ISL_814243, EPI_ISL_814244, EPI_ISL_814245, EPI_ISL_814246, EPI_ISL_814247, EPI_ISL_814248, EPI_ISL_814249, EPI_ISL_814250, EPI_ISL_814251, EPI_ISL_814252, EPI_ISL_814253, EPI_ISL_814254, EPI_ISL_814255, EPI_ISL_814256, EPI_ISL_814257, EPI_ISL_814258, EPI_ISL_814259, EPI_ISL_814260, EPI_ISL_814261, EPI_ISL_814262, EPI_ISL_814263, EPI_ISL_814264, EPI_ISL_814265, EPI_ISL_814266, EPI_ISL_814267, EPI_ISL_814268, EPI_ISL_814269, EPI_ISL_814270, EPI_ISL_814271, EPI_ISL_814272, EPI_ISL_814273			
see above	Israel Central Virology laboratory	Israel National Consortium for SARS-CoV-2 sequencing	Neta Zuckerman, Efrat Dahan Bucris, Michal Mandelboim, Dana Bar-Ilan, Oran Erster, Tzvia Mann, Omer Murik, David A. Zeevi, Assaf Rokney, Joseph Jaffe, Eva Nachum, Maya Davidovich Cohen, Ephraim Fass, Gal Zizelski Valenci, Mor Rubinstein, Efrat Rorman, Israel Nissan, Efrat Glick-Saar, Omri Nayshool, Gideon Rechavi, Ella Mendelson, Orna Mor
EPI_ISL_814350, EPI_ISL_814487, EPI_ISL_814504, EPI_ISL_814505, EPI_ISL_814506, EPI_ISL_814508, EPI_ISL_814509, EPI_ISL_814510, EPI_ISL_814541, EPI_ISL_814602, EPI_ISL_815045, EPI_ISL_815046, EPI_ISL_815068, EPI_ISL_815069, EPI_ISL_815070, EPI_ISL_815071, EPI_ISL_815233, EPI_ISL_815234, EPI_ISL_815235, EPI_ISL_815236, EPI_ISL_815237, EPI_ISL_815238, EPI_ISL_815239, EPI_ISL_815240, EPI_ISL_815241, EPI_ISL_815242, EPI_ISL_815243			
see above	Wales Specialist Virology Centre Sequencing lab: Pathogen Genomics Unit	COVID-19 Genomics UK (COG-UK) Consortium	Catherine Moore, Johnathan Evans, Laura Gifford, Malorie Perry, Simon Cottrell, Angela Marchbank, Alec Birchley, Alexander Adams, Amy Gaskin, Bree Gatica-Wilcox, Jason Coombes, Joel Southgate, Lauren Gilbert, Lee Graham, Nicole Pacchiarini, Sara Kumziene-Summerhayes, Sarah Taylor, Sophie Jones, Sara Rey, Matthew Bull, Joanne Watkins, Sally Corden, Tom Connor
EPI_ISL_815402, EPI_ISL_815404, EPI_ISL_815405, EPI_ISL_815406, EPI_ISL_815408, EPI_ISL_815409, EPI_ISL_815412, EPI_ISL_815413, EPI_ISL_815414, EPI_ISL_815415, EPI_ISL_815470, EPI_ISL_815472, EPI_ISL_815476, EPI_ISL_815485, EPI_ISL_815488, EPI_ISL_815492, EPI_ISL_815502, EPI_ISL_815503, EPI_ISL_815504, EPI_ISL_815505, EPI_ISL_815506, EPI_ISL_815507, EPI_ISL_815537, EPI_ISL_815538, EPI_ISL_815547, EPI_ISL_815548, EPI_ISL_815549, EPI_ISL_815550, EPI_ISL_815559, EPI_ISL_815560, EPI_ISL_815561, EPI_ISL_815562, EPI_ISL_815605, EPI_ISL_815608, EPI_ISL_815609, EPI_ISL_815622, EPI_ISL_815627, EPI_ISL_815631, EPI_ISL_815632, EPI_ISL_815633, EPI_ISL_815637, EPI_ISL_815646, EPI_ISL_815661, EPI_ISL_815662, EPI_ISL_815663, EPI_ISL_815664, EPI_ISL_815665, EPI_ISL_815666, EPI_ISL_815667, EPI_ISL_815668, EPI_ISL_815669, EPI_ISL_815670, EPI_ISL_815671, EPI_ISL_815672,			

EPI_ISL_815673, EPI_ISL_815674, EPI_ISL_815675, EPI_ISL_815676, EPI_ISL_815677, EPI_ISL_815678, EPI_ISL_815679, EPI_ISL_815680, EPI_ISL_815681, EPI_ISL_815682, EPI_ISL_815683, EPI_ISL_815684, EPI_ISL_815685, EPI_ISL_815686, EPI_ISL_815687, EPI_ISL_815688, EPI_ISL_815689, EPI_ISL_815690, EPI_ISL_815691, EPI_ISL_815692, EPI_ISL_815693, EPI_ISL_815694, EPI_ISL_815695, EPI_ISL_815696, EPI_ISL_815697, EPI_ISL_815698, EPI_ISL_815699, EPI_ISL_815700, EPI_ISL_815701, EPI_ISL_815702, EPI_ISL_815703, EPI_ISL_815704, EPI_ISL_815705, EPI_ISL_815706, EPI_ISL_815707, EPI_ISL_815708, EPI_ISL_815709, EPI_ISL_815710, EPI_ISL_815711, EPI_ISL_815712, EPI_ISL_815713, EPI_ISL_815714, EPI_ISL_815715, EPI_ISL_815716, EPI_ISL_815717, EPI_ISL_815718, EPI_ISL_815719, EPI_ISL_815720, EPI_ISL_815721			
see above	Department of Virus and Microbiological Special Diagnostics, Statens Serum Institut, Copenhagen, Denmark	Albertsen Lab, Department of Chemistry and Bioscience, Aalborg University, Denmark	Danish Covid-19 Genome Consortium
EPI_ISL_816194, EPI_ISL_816195, EPI_ISL_816196, EPI_ISL_816203, EPI_ISL_816224, EPI_ISL_816225	Centre for Enzyme Innovation, University of Portsmouth / Translational Research Laboratory, Portsmouth Hospitals NHS Trust	COVID-19 Genomics UK (COG-UK) Consortium	Angela Beckett,Yann Bourgeois,Garry Scarlett,Sharon Glaysher,Scott Elliott,Kelly Bicknell,Robert Impey,Allyson Lloyd,Sarah Wyllie,Ethan Butcher,Anoop Chauhan,Samuel Robson
EPI_ISL_816230, EPI_ISL_816236, EPI_ISL_816239, EPI_ISL_816255, EPI_ISL_816262, EPI_ISL_816268, EPI_ISL_816290, EPI_ISL_816292, EPI_ISL_816302, EPI_ISL_816310, EPI_ISL_816313, EPI_ISL_816316, EPI_ISL_816320, EPI_ISL_816350, EPI_ISL_816357, EPI_ISL_816361, EPI_ISL_816365, EPI_ISL_816380, EPI_ISL_816382, EPI_ISL_816392, EPI_ISL_816421, EPI_ISL_816425, EPI_ISL_816430, EPI_ISL_816435, EPI_ISL_816447, EPI_ISL_816474, EPI_ISL_816480, EPI_ISL_816503, EPI_ISL_816505, EPI_ISL_816507, EPI_ISL_816513, EPI_ISL_816531, EPI_ISL_816533, EPI_ISL_816537, EPI_ISL_816542, EPI_ISL_816548, EPI_ISL_816560, EPI_ISL_816562, EPI_ISL_816577, EPI_ISL_816585, EPI_ISL_816589, EPI_ISL_816597, EPI_ISL_816599, EPI_ISL_816600, EPI_ISL_816605, EPI_ISL_816606, EPI_ISL_816607, EPI_ISL_816616, EPI_ISL_816619, EPI_ISL_816620, EPI_ISL_816621, EPI_ISL_816625, EPI_ISL_816635			
see above	Virology Department, Sheffield Teaching Hospitals NHS Foundation Trust/Department of Infection, Immunity and Cardiovascular Disease, The Medical School, University of Sheffield	COVID-19 Genomics UK (COG-UK) Consortium	Thushan de Silva, Matthew Parker, Nikki Smith, Adri Angyal, Rebecca Brown, Luke Green, Rachel Tucker, Paul Parsons, Danielle Groves, Katie Johnson, Laura Carrilero, Alex Keeley, Dave Partridge, Matthew Wyles, Benjamin Lindsey, Mehmet Yavuz, Mohammad Raza, Cariad Evans
EPI_ISL_817123, EPI_ISL_817124, EPI_ISL_817125, EPI_ISL_817126, EPI_ISL_817127, EPI_ISL_817128, EPI_ISL_817129, EPI_ISL_817130, EPI_ISL_817131, EPI_ISL_817132, EPI_ISL_817133, EPI_ISL_817134, EPI_ISL_817136, EPI_ISL_817139, EPI_ISL_817140, EPI_ISL_817141, EPI_ISL_817142, EPI_ISL_817143, EPI_ISL_817144, EPI_ISL_817145, EPI_ISL_817148, EPI_ISL_817150, EPI_ISL_817151, EPI_ISL_817152, EPI_ISL_817153, EPI_ISL_817154, EPI_ISL_817155, EPI_ISL_817156, EPI_ISL_817157, EPI_ISL_817158, EPI_ISL_817177, EPI_ISL_817178, EPI_ISL_817179, EPI_ISL_817180, EPI_ISL_817181, EPI_ISL_817182, EPI_ISL_817183, EPI_ISL_817184, EPI_ISL_817185, EPI_ISL_817186, EPI_ISL_817187, EPI_ISL_817188, EPI_ISL_817189, EPI_ISL_817190, EPI_ISL_817191, EPI_ISL_817192, EPI_ISL_817193, EPI_ISL_817194, EPI_ISL_817195, EPI_ISL_817196, EPI_ISL_817197, EPI_ISL_817198, EPI_ISL_817199, EPI_ISL_817200, EPI_ISL_817201, EPI_ISL_817202, EPI_ISL_817203, EPI_ISL_817204, EPI_ISL_817205, EPI_ISL_817206, EPI_ISL_817207, EPI_ISL_817208, EPI_ISL_817209, EPI_ISL_817210, EPI_ISL_817211, EPI_ISL_817212, EPI_ISL_817213, EPI_ISL_817214, EPI_ISL_817215, EPI_ISL_817216, EPI_ISL_817217, EPI_ISL_817218, EPI_ISL_817219, EPI_ISL_817220, EPI_ISL_817221, EPI_ISL_817222, EPI_ISL_817223, EPI_ISL_817224, EPI_ISL_817225, EPI_ISL_817226, EPI_ISL_817227, EPI_ISL_817228, EPI_ISL_817229, EPI_ISL_817230, EPI_ISL_817231, EPI_ISL_817232, EPI_ISL_817233, EPI_ISL_817234, EPI_ISL_817235, EPI_ISL_817236, EPI_ISL_817237, EPI_ISL_817238, EPI_ISL_817239, EPI_ISL_817240, EPI_ISL_817241, EPI_ISL_817242, EPI_ISL_817243, EPI_ISL_817244, EPI_ISL_817245, EPI_ISL_817246, EPI_ISL_817247, EPI_ISL_817248, EPI_ISL_817249, EPI_ISL_817250, EPI_ISL_817251, EPI_ISL_817252, EPI_ISL_817253, EPI_ISL_817254, EPI_ISL_817255, EPI_ISL_817256, EPI_ISL_817257, EPI_ISL_817258, EPI_ISL_817259, EPI_ISL_817260, EPI_ISL_817261, EPI_ISL_817262, EPI_ISL_817263, EPI_ISL_817265, EPI_ISL_817266, EPI_ISL_817267, EPI_ISL_817268, EPI_ISL_817270, EPI_ISL_817271, EPI_ISL_817274, EPI_ISL_817275, EPI_ISL_817276, EPI_ISL_817277, EPI_ISL_817280, EPI_ISL_817283, EPI_ISL_817284, EPI_ISL_817285, EPI_ISL_817286, EPI_ISL_817287, EPI_ISL_817289, EPI_ISL_817293, EPI_ISL_817294, EPI_ISL_817295, EPI_ISL_817298, EPI_ISL_817300, EPI_ISL_817302, EPI_ISL_817304, EPI_ISL_817305, EPI_ISL_817307, EPI_ISL_817309, EPI_ISL_817310, EPI_ISL_817311, EPI_ISL_817313, EPI_ISL_817314, EPI_ISL_817318, EPI_ISL_817319, EPI_ISL_817320, EPI_ISL_817321, EPI_ISL_817322, EPI_ISL_817323, EPI_ISL_817324, EPI_ISL_817325, EPI_ISL_817326, EPI_ISL_817327, EPI_ISL_817328, EPI_ISL_817329, EPI_ISL_817330, EPI_ISL_817331, EPI_ISL_817332, EPI_ISL_817336, EPI_ISL_817337, EPI_ISL_817338, EPI_ISL_817339, EPI_ISL_817340, EPI_ISL_817341, EPI_ISL_817342, EPI_ISL_817343, EPI_ISL_817344, EPI_ISL_817345, EPI_ISL_817346, EPI_ISL_817347, EPI_ISL_817348, EPI_ISL_817349, EPI_ISL_817350, EPI_ISL_817351, EPI_ISL_817352, EPI_ISL_817353, EPI_ISL_817354, EPI_ISL_817355, EPI_ISL_817356, EPI_ISL_817357, EPI_ISL_817358, EPI_ISL_817359, EPI_ISL_817360, EPI_ISL_817361, EPI_ISL_817362, EPI_ISL_817363, EPI_ISL_817364, EPI_ISL_817365, EPI_ISL_817366, EPI_ISL_817367, EPI_ISL_817368, EPI_ISL_817369, EPI_ISL_817370, EPI_ISL_817371, EPI_ISL_817372, EPI_ISL_817373, EPI_ISL_817374, EPI_ISL_817377, EPI_ISL_817378, EPI_ISL_817379, EPI_ISL_817380, EPI_ISL_817381, EPI_ISL_817382, EPI_ISL_817387, EPI_ISL_817388, EPI_ISL_817389, EPI_ISL_817390, EPI_ISL_817391, EPI_ISL_817393, EPI_ISL_817394, EPI_ISL_817395, EPI_ISL_817396, EPI_ISL_817397, EPI_ISL_817400, EPI_ISL_817402, EPI_ISL_817403, EPI_ISL_817406, EPI_ISL_817407, EPI_ISL_817408, EPI_ISL_817409, EPI_ISL_817410, EPI_ISL_817411, EPI_ISL_817412, EPI_ISL_817420, EPI_ISL_817421, EPI_ISL_817422, EPI_ISL_817423, EPI_ISL_817424, EPI_ISL_817425, EPI_ISL_817426, EPI_ISL_817427, EPI_ISL_817428, EPI_ISL_817429, EPI_ISL_817430, EPI_ISL_817431, EPI_ISL_817432, EPI_ISL_817433, EPI_ISL_817434, EPI_ISL_817435, EPI_ISL_817436, EPI_ISL_817437, EPI_ISL_817438, EPI_ISL_817439, EPI_ISL_817440, EPI_ISL_817441, EPI_ISL_817442, EPI_ISL_817443, EPI_ISL_817444, EPI_ISL_817445, EPI_ISL_817446, EPI_ISL_817447, EPI_ISL_817448, EPI_ISL_817449, EPI_ISL_817450, EPI_ISL_817451, EPI_ISL_817452, EPI_ISL_817453, EPI_ISL_817454, EPI_ISL_817455, EPI_ISL_817456, EPI_ISL_817457, EPI_ISL_817458, EPI_ISL_817459, EPI_ISL_817460, EPI_ISL_817461, EPI_ISL_817462, EPI_ISL_817463, EPI_ISL_817464, EPI_ISL_817465, EPI_ISL_817466, EPI_ISL_817467, EPI_ISL_817468, EPI_ISL_817469, EPI_ISL_817470, EPI_ISL_817471, EPI_ISL_817472, EPI_ISL_817473, EPI_ISL_817474, EPI_ISL_817475, EPI_ISL_817476, EPI_ISL_817477, EPI_ISL_817478, EPI_ISL_817479, EPI_ISL_817480, EPI_ISL_817481, EPI_ISL_817482, EPI_ISL_817483, EPI_ISL_817484, EPI_ISL_817485, EPI_ISL_817486, EPI_ISL_817488, EPI_ISL_817489, EPI_ISL_817490, EPI_ISL_817491, EPI_ISL_817492, EPI_ISL_817493, EPI_ISL_817494, EPI_ISL_817495, EPI_ISL_817498, EPI_ISL_817500, EPI_ISL_817502, EPI_ISL_817503, EPI_ISL_817504, EPI_ISL_817505, EPI_ISL_817506, EPI_ISL_817507, EPI_ISL_817512, EPI_ISL_817513, EPI_ISL_817514, EPI_ISL_817515, EPI_ISL_817516, EPI_ISL_817517, EPI_ISL_817518, EPI_ISL_817519, EPI_ISL_817520, EPI_ISL_817521, EPI_ISL_817522, EPI_ISL_817523, EPI_ISL_817524, EPI_ISL_817525, EPI_ISL_817526, EPI_ISL_817527, EPI_ISL_817528, EPI_ISL_817529, EPI_ISL_817531, EPI_ISL_817533, EPI_ISL_817534, EPI_ISL_817535, EPI_ISL_817536, EPI_ISL_817539, EPI_ISL_817540, EPI_ISL_817541, EPI_ISL_817542, EPI_ISL_817543, EPI_ISL_817544, EPI_ISL_817545, EPI_ISL_817546, EPI_ISL_817547, EPI_ISL_817548, EPI_ISL_817549, EPI_ISL_817550, EPI_ISL_817551, EPI_ISL_817552, EPI_ISL_817553, EPI_ISL_817554, EPI_ISL_817653, EPI_ISL_817654, EPI_ISL_817655, EPI_ISL_817656, EPI_ISL_817657, EPI_ISL_817658, EPI_ISL_817659, EPI_ISL_817660, EPI_ISL_817661, EPI_ISL_817662, EPI_ISL_817663, EPI_ISL_817664, EPI_ISL_817665, EPI_ISL_817666, EPI_ISL_817667, EPI_ISL_817668, EPI_ISL_817669, EPI_ISL_817670, EPI_ISL_817671, EPI_ISL_817672, EPI_ISL_817673, EPI_ISL_817674, EPI_ISL_817675, EPI_ISL_817676, EPI_ISL_817677, EPI_ISL_817678, EPI_ISL_817679, EPI_ISL_817680, EPI_ISL_817681, EPI_ISL_817682, EPI_ISL_817683, EPI_ISL_817684, EPI_ISL_817685, EPI_ISL_817686, EPI_ISL_817687, EPI_ISL_817688, EPI_ISL_817689, EPI_ISL_817690, EPI_ISL_817691, EPI_ISL_817692, EPI_ISL_817693, EPI_ISL_817694, EPI_ISL_817695, EPI_ISL_817696, EPI_ISL_817697, EPI_ISL_817698, EPI_ISL_817699, EPI_ISL_817700, EPI_ISL_817701, EPI_ISL_817702, EPI_ISL_817703, EPI_ISL_817704, EPI_ISL_817705, EPI_ISL_817706, EPI_ISL_817707, EPI_ISL_817708, EPI_ISL_817709, EPI_ISL_817710, EPI_ISL_817711, EPI_ISL_817712, EPI_ISL_817713, EPI_ISL_817714, EPI_ISL_817715, EPI_ISL_817716, EPI_ISL_817717, EPI_ISL_817718, EPI_ISL_817719, EPI_ISL_817720, EPI_ISL_817721, EPI_ISL_817722, EPI_ISL_817723, EPI_ISL_817724, EPI_ISL_817725, EPI_ISL_817726, EPI_ISL_817727, EPI_ISL_817728, EPI_ISL_817729, EPI_ISL_817730, EPI_ISL_817731, EPI_ISL_817732, EPI_ISL_817733, EPI_ISL_817734, EPI_ISL_817735, EPI_ISL_817736, EPI_ISL_817737, EPI_ISL_817738, EPI_ISL_817739, EPI_ISL_817740, EPI_ISL_817741, EPI_ISL_817742, EPI_ISL_817743, EPI_ISL_817744, EPI_ISL_817745, EPI_ISL_817746, EPI_ISL_817747, EPI_ISL_817748, EPI_ISL_817749, EPI_ISL_817750, EPI_ISL_817751, EPI_ISL_817752, EPI_ISL_817753, EPI_ISL_817754, EPI_ISL_817755, EPI_ISL_817756, EPI_ISL_817757, EPI_ISL_817758, EPI_ISL_817759, EPI_ISL_817760, EPI_ISL_817761, EPI_ISL_817762, EPI_ISL_817763, EPI_ISL_817764, EPI_ISL_817765, EPI_ISL_817766, EPI_ISL_817767, EPI_ISL_817768, EPI_ISL_817769, EPI_ISL_817770, EPI_ISL_817771, EPI_ISL_817772, EPI_ISL_817773, EPI_ISL_817774, EPI_ISL_817775, EPI_ISL_817776, EPI_ISL_817777, EPI_ISL_817778, EPI_ISL_817779, EPI_ISL_817780, EPI_ISL_817781, EPI_ISL_817782, EPI_ISL_817783, EPI_ISL_817784, EPI_ISL_817785, EPI_ISL_817786, EPI_ISL_817787, EPI_ISL_817788, EPI_ISL_817789, EPI_ISL_817790, EPI_ISL_817791, EPI_ISL_817792, EPI_ISL_817793, EPI_ISL_817794, EPI_ISL_817795, EPI_ISL_817796, EPI_ISL_817797, EPI_ISL_817798, EPI_ISL_817799, EPI_ISL_817800, EPI_ISL_817801, EPI_ISL_817802, EPI_ISL_817803, EPI_ISL_817804, EPI_ISL_817805, EPI_ISL_817806, EPI_ISL_817807, EPI_ISL_817808, EPI_ISL_817809, EPI_ISL_817810, EPI_ISL_817811, EPI_ISL_817812, EPI_ISL_817813, EPI_ISL_817814, EPI_ISL_817815, EPI_ISL_817816, EPI_ISL_817817, EPI_ISL_817818, EPI_ISL_817819, EPI_ISL_817820, EPI_ISL_817821, EPI_ISL_817822, EPI_ISL_817823, EPI_ISL_817824, EPI_ISL_817825, EPI_ISL_817826, EPI_ISL_817827, EPI_ISL_817828, EPI_ISL_817829, EPI_ISL_817830, EPI_ISL_817831, EPI_ISL_817832, EPI_ISL_817833, EPI_ISL_817834, EPI_ISL_817835, EPI_ISL_817836, EPI_ISL_817837, EPI_ISL_817838, EPI_ISL_817839, EPI_ISL_817840, EPI_ISL_817841, EPI_ISL_817842, EPI_ISL_817843, EPI_ISL_817844, EPI_ISL_817845, EPI_ISL_817846, EPI_ISL_817847, EPI_ISL_817848, EPI_ISL_817849, EPI_ISL_817850, EPI_ISL_817851, EPI_ISL_817852, EPI_ISL_817853, EPI_ISL_817854, EPI_ISL_817855, EPI_ISL_817856, EPI_ISL_817857, EPI_ISL_817858, EPI_ISL_817859, EPI_ISL_817860, EPI_ISL_817861, EPI_ISL_817862, EPI_ISL_817863, EPI_ISL_817864, EPI_ISL_817865, EPI_ISL_817866, EPI_ISL_817867, EPI_ISL_817868, EPI_ISL_817869, EPI_ISL_817870, EPI_ISL_817871, EPI_ISL_817872, EPI_ISL_817873, EPI_ISL_817874, EPI_ISL_817875, EPI_ISL_817876, EPI_ISL_817877, EPI_ISL_817878, EPI_ISL_817879, EPI_ISL_817880, EPI_ISL_817881, EPI_ISL_817882, EPI_ISL_817883, EPI_ISL_817884, EPI_ISL_817885, EPI_ISL_817886, EPI_ISL_817887, EPI_ISL_817888, EPI_ISL_817889, EPI_ISL_817890, EPI_ISL_817891, EPI_ISL_817892, EPI_ISL_817893, EPI_ISL_817894, EPI_ISL_817895, EPI_ISL_817896, EPI_ISL_817897, EPI_ISL_817898, EPI_ISL_817899, EPI_ISL_817900, EPI_ISL_817901, EPI_ISL_817902, EPI_ISL_817903, EPI_ISL_817904, EPI_ISL_817905, EPI_ISL_817906, EPI_ISL_817907, EPI_ISL_817908, EPI_ISL_817909, EPI_ISL_817910, EPI_ISL_817911, EPI_ISL_817912, EPI_ISL_817913, EPI_ISL_817914, EPI_ISL_817915, EPI_ISL_817916, EPI_ISL_817917, EPI_ISL_817918, EPI_ISL_817919, EPI_ISL_817920, EPI_ISL_817921, EPI_ISL_817922, EPI_ISL_817923, EPI_ISL_817924, EPI_ISL_817925, EPI_ISL_817926, EPI_ISL_817927, EPI_ISL_817928, EPI_ISL_817929, EPI_ISL_817930, EPI_ISL_817931, EPI_ISL_817932, EPI_ISL_817933, EPI_ISL_817934, EPI_ISL_817935, EPI_ISL_817936, EPI_ISL_817937, EPI_ISL_817938, EPI_ISL_817939, EPI_ISL_817940, EPI_ISL_817941, EPI_ISL_817942, EPI_ISL_817943, EPI_ISL_817944, EPI_ISL_817945, EPI_ISL_817946, EPI_ISL_817947, EPI_ISL_817948, EPI_ISL_817949, EPI_ISL_817950, EPI_ISL_817951, EPI_ISL_817952, EPI_ISL_817953, EPI_ISL_817954, EPI_ISL_817955, EPI_ISL_817956, EPI_ISL_817957, EPI_ISL_817958, EPI_ISL_817959, EPI_ISL_817960, EPI_ISL_817961, EPI_ISL_817962, EPI_ISL_817963, EPI_ISL_817964, EPI_ISL_817965, EPI_ISL_817966, EPI_ISL_817967, EPI_ISL_817968, EPI_ISL_817969, EPI_ISL_817970, EPI_ISL_817971, EPI_ISL_817972, EPI_ISL_817973, EPI_ISL_817974, EPI_ISL_817975, EPI_ISL_817976, EPI_ISL_817977, EPI_ISL_817978, EPI_ISL_817979, EPI_ISL_817980, EPI_ISL_817981, EPI_ISL_817982, EPI_ISL_817983, EPI_ISL_817984, EPI_ISL_817985, EPI_ISL_817986, EPI_ISL_817987, EPI_ISL_817988, EPI_ISL_817989, EPI_ISL_817990, EPI_ISL_817991, EPI_ISL_817992, EPI_ISL_817993, EPI_ISL_817994, EPI_ISL_817995, EPI_ISL_817996, EPI_ISL_817997, EPI_ISL_817998, EPI_ISL_817999, EPI_ISL_818000, EPI_ISL_818001, EPI_ISL_818002, EPI_ISL_818003, EPI_ISL_818004, EPI_ISL_818005, EPI_ISL_818006, EPI_ISL_818007, EPI_ISL_818008, EPI_ISL_818009, EPI_ISL_818010, EPI_ISL_818011, EPI_ISL_818012, EPI_ISL_818013, EPI_ISL_818014, EPI_ISL_818015, EPI_ISL_818016, EPI_ISL_818017, EPI_ISL_818018, EPI_ISL_818019, EPI_ISL_818020, EPI_ISL_818021, EPI_ISL_818022, EPI_ISL_818023, EPI_ISL_818024, EPI_ISL_818025, EPI_ISL_818026, EPI_ISL_818027, EPI_ISL_818028, EPI_ISL_818029, EPI_ISL_818030, EPI_ISL_818031, EPI_ISL_818032, EPI_ISL_818033, EPI_ISL_818034, EPI_ISL_818035, EPI_ISL_818036, EPI_ISL_818037, EPI_ISL_818038, EPI_ISL_818039, EPI_ISL_818040, EPI_ISL_818041, EPI_ISL_818042, EPI_ISL_818043, EPI_ISL_818044, EPI_ISL_818045, EPI_ISL_818046, EPI_ISL_818047, EPI_ISL_818048, EPI_ISL_818049, EPI_ISL_818050, EPI_ISL_818051, EPI_ISL_818052, EPI_ISL_818053, EPI_ISL_818054, EPI_ISL_818055, EPI_ISL_818056, EPI_ISL_818057, EPI_ISL_818058, EPI_ISL_818059, EPI_ISL_818060, EPI_ISL_818061, EPI_ISL_818062, EPI_ISL_818063, EPI_ISL_818064, EPI_ISL_818065, EPI_ISL_818066, EPI_ISL_818067, EPI_ISL_818068, EPI_ISL_818069, EPI_ISL_818070, EPI_ISL_818071, EPI_ISL_818072, EPI_ISL_818073, EPI_ISL_818074, EPI_ISL_818075, EPI_ISL_818076, EPI_ISL_818077, EPI_ISL_818078, EPI_ISL_818079, EPI_ISL_818080, EPI_ISL_818081, EPI_ISL_818082, EPI_ISL_818083, EPI_ISL_818084, EPI_ISL_818085, EPI_ISL_818086, EPI_ISL_818087, EPI_ISL_818088, EPI_ISL_818089, EPI_ISL_818090, EPI_ISL_818091, EPI_ISL_818092, EPI_ISL_818093, EPI_ISL_818094, EPI_ISL_818095, EPI_ISL_818096, EPI_ISL_818097, EPI_ISL_818098, EPI_ISL_818099, EPI_ISL_818100, EPI_ISL_818101, EPI_ISL_818102, EPI_ISL_818103, EPI_ISL_818104, EPI_ISL_818105, EPI_ISL_818106, EPI_ISL_818107, EPI_ISL_818108, EPI_ISL_818109, EPI_ISL_818110, EPI_ISL_818111, EPI_ISL_818112, EPI_ISL_818113, EPI_ISL_818114, EPI_ISL_818115, EPI_ISL_818116, EPI_ISL_818117, EPI_ISL_818118, EPI_ISL_818119, EPI_ISL_818120, EPI_ISL_818121, EPI_ISL_818122			
see above	Department of Virus and Microbiological Special Diagnostics, Statens Serum Institut, Copenhagen, Denmark	Albertsen Lab, Department of Chemistry and Bioscience, Aalborg University, Denmark	Danish Covid-19 Genome Consortium
EPI_ISL_819140, EPI_ISL_819144, EPI_ISL_819149, EPI_ISL_819180, EPI_ISL_819201	Servicio de Microbiología, Hospital Universitario Son Espases	SeqCOVID-SPAIN consortium/IBV(CSIC)	Carla López-Causapé, Jordi Reina, Antonio Oliver and SeqCOVID-SPAIN consortium
EPI_ISL_819370, EPI_ISL_819437, EPI_ISL_819439	Quadram Institute Bioscience	COVID-19 Genomics UK (COG-UK) Consortium	Dave J. Baker, Gemma L. Kay, Alp Aydin, Thanh Le-Viet, Steven Rudder, Ana P. Tedim, Anastasia Kolyva, Maria Diaz, Leonardo de Oliveira Martins, Nabil-Fareed Alikhan, Lizzie Meadows, Rachael Stanley, Ngozi Elumogo, Muhammed Yasir, Nicholas M. Thomson, Alexander J Trotter, Rachel Gilroy, Samuel Bloomfield, Claire Stuart, Andrew Bell, Reenesh Prakash, Samir Dervisevic, Alison E. Mather, John Wain, Mark Webber, Andrew J. Page, Justin O'Grady
EPI_ISL_820109	University College London, Great Ormond Street Hospital for Children NHS Foundation Trust, Imperial College Healthcare NHS Trust	COVID-19 Genomics UK (COG-UK) Consortium	Sergi Castellano, Rachel Williams, Mark Kristiansen, Paola Resende Silva, Sunando Roy, Tony Brooks, Helena Tutill, Paola Niola, Patricia Dyal, Charlotte Williams, Leysa Forrest, Yasmin Panchbhaya, Jacqueline Findlay, Samuel Weeks, Julianne Brown, Kathryn Harris, Paul Randell, James Price, Alison Holmes, Judith Breuer
EPI_ISL_820110	Quadram Institute Bioscience	COVID-19 Genomics UK (COG-UK) Consortium	Dave J. Baker, Gemma L. Kay, Alp Aydin, Thanh Le-Viet, Steven Rudder, Ana P. Tedim, Anastasia Kolyva, Maria Diaz, Leonardo de Oliveira Martins, Nabil-Fareed Alikhan, Lizzie Meadows, Rachael Stanley, Ngozi Elumogo, Muhammed Yasir, Nicholas M. Thomson, Alexander J Trotter, Rachel Gilroy, Samuel Bloomfield, Claire Stuart, Andrew Bell, Reenesh Prakash, Samir Dervisevic, Alison E. Mather, John Wain, Mark Webber, Andrew J. Page, Justin O'Grady
EPI_ISL_820111	University College London, Great Ormond Street Hospital for Children NHS Foundation Trust, Imperial College Healthcare NHS Trust	COVID-19 Genomics UK (COG-UK) Consortium	Sergi Castellano, Rachel Williams, Mark Kristiansen, Paola Resende Silva, Sunando Roy, Tony Brooks, Helena Tutill, Paola Niola, Patricia Dyal, Charlotte Williams, Leysa Forrest, Yasmin Panchbhaya, Jacqueline Findlay, Samuel Weeks, Julianne Brown, Kathryn Harris, Paul Randell, James Price, Alison Holmes, Judith Breuer
EPI_ISL_820112	Quadram Institute Bioscience	COVID-19 Genomics UK (COG-UK) Consortium	Dave J. Baker, Gemma L. Kay, Alp Aydin, Thanh Le-Viet, Steven Rudder, Ana P. Tedim, Anastasia Kolyva, Maria Diaz, Leonardo de Oliveira Martins,

			Nabil-Fareed Alikhan, Lizzie Meadows, Rachael Stanley, Ngozi Elumogo, Muhammed Yasir, Nicholas M. Thomson, Alexander J Trotter, Rachel Gilroy, Samuel Bloomfield, Claire Stuart, Andrew Bell, Reenesh Prakash, Samir Dervisevic, Alison E. Mather, John Wain, Mark Webber, Andrew J. Page, Justin O'Grady
EPI_ISL_820113, EPI_ISL_820114, EPI_ISL_820115, EPI_ISL_820116, EPI_ISL_820117, EPI_ISL_820118	University College London, Great Ormond Street Hospital for Children NHS Foundation Trust, Imperial College Healthcare NHS Trust	COVID-19 Genomics UK (COG-UK) Consortium	Sergi Castellano, Rachel Williams, Mark Kristiansen, Paola Resende Silva, Sunando Roy, Tony Brooks, Helena Tutill, Paola Niola, Patricia Dyal, Charlotte Williams, Leysa Forrest, Yasmin Panchbhaya, Jacqueline Findlay, Samuel Weeks, Julianne Brown, Kathryn Harris, Paul Randell, James Price, Alison Holmes, Judith Breuer
EPI_ISL_820119	Quadram Institute Bioscience	COVID-19 Genomics UK (COG-UK) Consortium	Dave J. Baker, Gemma L. Kay, Alp Aydin, Thanh Le-Viet, Steven Rudder, Ana P. Tedim, Anastasia Kolyva, Maria Diaz, Leonardo de Oliveira Martins, Nabil-Fareed Alikhan, Lizzie Meadows, Rachael Stanley, Ngozi Elumogo, Muhammed Yasir, Nicholas M. Thomson, Alexander J Trotter, Rachel Gilroy, Samuel Bloomfield, Claire Stuart, Andrew Bell, Reenesh Prakash, Samir Dervisevic, Alison E. Mather, John Wain, Mark Webber, Andrew J. Page, Justin O'Grady
EPI_ISL_820120, EPI_ISL_820121, EPI_ISL_820122, EPI_ISL_820123	University College London, Great Ormond Street Hospital for Children NHS Foundation Trust, Imperial College Healthcare NHS Trust	COVID-19 Genomics UK (COG-UK) Consortium	Sergi Castellano, Rachel Williams, Mark Kristiansen, Paola Resende Silva, Sunando Roy, Tony Brooks, Helena Tutill, Paola Niola, Patricia Dyal, Charlotte Williams, Leysa Forrest, Yasmin Panchbhaya, Jacqueline Findlay, Samuel Weeks, Julianne Brown, Kathryn Harris, Paul Randell, James Price, Alison Holmes, Judith Breuer
EPI_ISL_820124	Quadram Institute Bioscience	COVID-19 Genomics UK (COG-UK) Consortium	Dave J. Baker, Gemma L. Kay, Alp Aydin, Thanh Le-Viet, Steven Rudder, Ana P. Tedim, Anastasia Kolyva, Maria Diaz, Leonardo de Oliveira Martins, Nabil-Fareed Alikhan, Lizzie Meadows, Rachael Stanley, Ngozi Elumogo, Muhammed Yasir, Nicholas M. Thomson, Alexander J Trotter, Rachel Gilroy, Samuel Bloomfield, Claire Stuart, Andrew Bell, Reenesh Prakash, Samir Dervisevic, Alison E. Mather, John Wain, Mark Webber, Andrew J. Page, Justin O'Grady
EPI_ISL_820125	University College London, Great Ormond Street Hospital for Children NHS Foundation Trust, Imperial College Healthcare NHS Trust	COVID-19 Genomics UK (COG-UK) Consortium	Sergi Castellano, Rachel Williams, Mark Kristiansen, Paola Resende Silva, Sunando Roy, Tony Brooks, Helena Tutill, Paola Niola, Patricia Dyal, Charlotte Williams, Leysa Forrest, Yasmin Panchbhaya, Jacqueline Findlay, Samuel Weeks, Julianne Brown, Kathryn Harris, Paul Randell, James Price, Alison Holmes, Judith Breuer
EPI_ISL_820126	Quadram Institute Bioscience	COVID-19 Genomics UK (COG-UK) Consortium	Dave J. Baker, Gemma L. Kay, Alp Aydin, Thanh Le-Viet, Steven Rudder, Ana P. Tedim, Anastasia Kolyva, Maria Diaz, Leonardo de Oliveira Martins, Nabil-Fareed Alikhan, Lizzie Meadows, Rachael Stanley, Ngozi Elumogo, Muhammed Yasir, Nicholas M. Thomson, Alexander J Trotter, Rachel Gilroy, Samuel Bloomfield, Claire Stuart, Andrew Bell, Reenesh Prakash, Samir Dervisevic, Alison E. Mather, John Wain, Mark Webber, Andrew J. Page, Justin O'Grady
EPI_ISL_820127, EPI_ISL_820128	University College London, Great Ormond Street Hospital for Children NHS Foundation Trust, Imperial College Healthcare NHS Trust	COVID-19 Genomics UK (COG-UK) Consortium	Sergi Castellano, Rachel Williams, Mark Kristiansen, Paola Resende Silva, Sunando Roy, Tony Brooks, Helena Tutill, Paola Niola, Patricia Dyal, Charlotte Williams, Leysa Forrest, Yasmin Panchbhaya, Jacqueline Findlay, Samuel Weeks, Julianne Brown, Kathryn Harris, Paul Randell, James Price, Alison Holmes, Judith Breuer
EPI_ISL_820131, EPI_ISL_820134, EPI_ISL_820136	Quadram Institute Bioscience	COVID-19 Genomics UK (COG-UK) Consortium	Dave J. Baker, Gemma L. Kay, Alp Aydin, Thanh Le-Viet, Steven Rudder, Ana P. Tedim, Anastasia Kolyva, Maria Diaz, Leonardo de Oliveira Martins, Nabil-Fareed Alikhan, Lizzie Meadows, Rachael Stanley, Ngozi Elumogo, Muhammed Yasir, Nicholas M. Thomson, Alexander J Trotter, Rachel Gilroy, Samuel Bloomfield, Claire Stuart, Andrew Bell, Reenesh Prakash, Samir Dervisevic, Alison E. Mather, John Wain, Mark Webber, Andrew J. Page, Justin O'Grady
EPI_ISL_820138	University College London, Great Ormond Street Hospital for Children NHS Foundation Trust, Imperial College Healthcare NHS Trust	COVID-19 Genomics UK (COG-UK) Consortium	Sergi Castellano, Rachel Williams, Mark Kristiansen, Paola Resende Silva, Sunando Roy, Tony Brooks, Helena Tutill, Paola Niola, Patricia Dyal, Charlotte Williams, Leysa Forrest, Yasmin Panchbhaya, Jacqueline Findlay, Samuel Weeks, Julianne Brown, Kathryn Harris, Paul Randell, James Price, Alison Holmes, Judith Breuer
EPI_ISL_820141, EPI_ISL_820144, EPI_ISL_820146, EPI_ISL_820149, EPI_ISL_820151, EPI_ISL_820154, EPI_ISL_820156, EPI_ISL_820159, EPI_ISL_820161, EPI_ISL_820164, EPI_ISL_820167, EPI_ISL_820169, EPI_ISL_820172, EPI_ISL_820175, EPI_ISL_820178, EPI_ISL_820180, EPI_ISL_820183, EPI_ISL_820186, EPI_ISL_820188, EPI_ISL_820191, EPI_ISL_820193, EPI_ISL_820196, EPI_ISL_820199, EPI_ISL_820202			
see above	Quadram Institute Bioscience	COVID-19 Genomics UK (COG-UK) Consortium	Dave J. Baker, Gemma L. Kay, Alp Aydin, Thanh Le-Viet, Steven Rudder, Ana P. Tedim, Anastasia Kolyva, Maria Diaz, Leonardo de Oliveira Martins, Nabil-Fareed Alikhan, Lizzie Meadows, Rachael Stanley, Ngozi Elumogo, Muhammed Yasir, Nicholas M. Thomson, Alexander J Trotter, Rachel Gilroy, Samuel Bloomfield, Claire Stuart, Andrew Bell, Reenesh Prakash, Samir Dervisevic, Alison E. Mather, John Wain, Mark Webber, Andrew J. Page, Justin O'Grady
EPI_ISL_820204, EPI_ISL_820206, EPI_ISL_820209, EPI_ISL_820211, EPI_ISL_820213, EPI_ISL_820216, EPI_ISL_820218	University College London, Great Ormond Street Hospital for Children NHS Foundation Trust, Imperial College Healthcare NHS Trust	COVID-19 Genomics UK (COG-UK) Consortium	Sergi Castellano, Rachel Williams, Mark Kristiansen, Paola Resende Silva, Sunando Roy, Tony Brooks, Helena Tutill, Paola Niola, Patricia Dyal, Charlotte Williams, Leysa Forrest, Yasmin Panchbhaya, Jacqueline Findlay, Samuel Weeks, Julianne Brown, Kathryn Harris, Paul Randell, James Price, Alison Holmes, Judith Breuer
EPI_ISL_820221, EPI_ISL_820223	Quadram Institute Bioscience	COVID-19 Genomics UK (COG-UK) Consortium	Dave J. Baker, Gemma L. Kay, Alp Aydin, Thanh Le-Viet, Steven Rudder, Ana P. Tedim, Anastasia Kolyva, Maria Diaz, Leonardo de Oliveira Martins, Nabil-Fareed Alikhan, Lizzie Meadows, Rachael Stanley, Ngozi Elumogo, Muhammed Yasir, Nicholas M. Thomson, Alexander J Trotter, Rachel Gilroy, Samuel Bloomfield, Claire Stuart, Andrew Bell, Reenesh Prakash, Samir Dervisevic, Alison E. Mather, John Wain, Mark Webber, Andrew J. Page, Justin O'Grady
EPI_ISL_820226, EPI_ISL_820229	University College London, Great Ormond Street Hospital for Children NHS Foundation Trust, Imperial College Healthcare NHS Trust	COVID-19 Genomics UK (COG-UK) Consortium	Sergi Castellano, Rachel Williams, Mark Kristiansen, Paola Resende Silva, Sunando Roy, Tony Brooks, Helena Tutill, Paola Niola, Patricia Dyal, Charlotte Williams, Leysa Forrest, Yasmin Panchbhaya, Jacqueline Findlay, Samuel Weeks, Julianne Brown, Kathryn Harris, Paul Randell, James Price, Alison Holmes, Judith Breuer
EPI_ISL_820231, EPI_ISL_820234	Quadram Institute Bioscience	COVID-19 Genomics UK (COG-UK) Consortium	Dave J. Baker, Gemma L. Kay, Alp Aydin, Thanh Le-Viet, Steven Rudder, Ana P. Tedim, Anastasia Kolyva, Maria Diaz, Leonardo de Oliveira Martins, Nabil-Fareed Alikhan, Lizzie Meadows, Rachael Stanley, Ngozi Elumogo, Muhammed Yasir, Nicholas M. Thomson, Alexander J Trotter, Rachel Gilroy, Samuel Bloomfield, Claire Stuart, Andrew Bell, Reenesh Prakash, Samir Dervisevic, Alison E. Mather, John Wain, Mark Webber, Andrew J. Page, Justin O'Grady
EPI_ISL_820236	University College London, Great Ormond Street Hospital for Children NHS Foundation Trust, Imperial College Healthcare NHS Trust	COVID-19 Genomics UK (COG-UK) Consortium	Sergi Castellano, Rachel Williams, Mark Kristiansen, Paola Resende Silva, Sunando Roy, Tony Brooks, Helena Tutill, Paola Niola, Patricia Dyal, Charlotte Williams, Leysa Forrest, Yasmin Panchbhaya, Jacqueline Findlay, Samuel Weeks, Julianne Brown, Kathryn Harris, Paul Randell, James Price, Alison Holmes, Judith Breuer
EPI_ISL_820238, EPI_ISL_820241, EPI_ISL_820244	Quadram Institute Bioscience	COVID-19 Genomics UK (COG-UK) Consortium	Dave J. Baker, Gemma L. Kay, Alp Aydin, Thanh Le-Viet, Steven Rudder, Ana P. Tedim, Anastasia Kolyva, Maria Diaz, Leonardo de Oliveira Martins, Nabil-Fareed Alikhan, Lizzie Meadows, Rachael Stanley, Ngozi Elumogo, Muhammed Yasir, Nicholas M. Thomson, Alexander J Trotter, Rachel Gilroy, Samuel Bloomfield, Claire Stuart, Andrew Bell, Reenesh Prakash, Samir Dervisevic, Alison E. Mather, John Wain, Mark Webber, Andrew J. Page, Justin O'Grady
EPI_ISL_820246, EPI_ISL_820249, EPI_ISL_820251	University College London, Great Ormond Street Hospital for Children NHS Foundation Trust, Imperial College Healthcare NHS Trust	COVID-19 Genomics UK (COG-UK) Consortium	Sergi Castellano, Rachel Williams, Mark Kristiansen, Paola Resende Silva, Sunando Roy, Tony Brooks, Helena Tutill, Paola Niola, Patricia Dyal, Charlotte Williams, Leysa Forrest, Yasmin Panchbhaya, Jacqueline Findlay, Samuel Weeks, Julianne Brown, Kathryn Harris, Paul Randell, James Price, Alison Holmes, Judith Breuer
EPI_ISL_820253	Quadram Institute Bioscience	COVID-19 Genomics UK (COG-UK) Consortium	Dave J. Baker, Gemma L. Kay, Alp Aydin, Thanh Le-Viet, Steven Rudder, Ana P. Tedim, Anastasia Kolyva, Maria Diaz, Leonardo de Oliveira Martins, Nabil-Fareed Alikhan, Lizzie Meadows, Rachael Stanley, Ngozi Elumogo, Muhammed Yasir, Nicholas M. Thomson, Alexander J Trotter, Rachel Gilroy, Samuel Bloomfield, Claire Stuart, Andrew Bell, Reenesh Prakash, Samir Dervisevic, Alison E. Mather, John Wain, Mark Webber, Andrew J. Page, Justin O'Grady
EPI_ISL_820256, EPI_ISL_820259	University College London, Great Ormond Street Hospital for Children NHS Foundation Trust, Imperial College Healthcare NHS Trust	COVID-19 Genomics UK (COG-UK) Consortium	Sergi Castellano, Rachel Williams, Mark Kristiansen, Paola Resende Silva, Sunando Roy, Tony Brooks, Helena Tutill, Paola Niola, Patricia Dyal, Charlotte Williams, Leysa Forrest, Yasmin Panchbhaya, Jacqueline Findlay, Samuel Weeks, Julianne Brown, Kathryn Harris, Paul Randell, James Price, Alison Holmes, Judith Breuer
EPI_ISL_820261	Quadram Institute Bioscience	COVID-19 Genomics UK (COG-UK) Consortium	Dave J. Baker, Gemma L. Kay, Alp Aydin, Thanh Le-Viet, Steven Rudder, Ana P. Tedim, Anastasia Kolyva, Maria Diaz, Leonardo de Oliveira Martins, Nabil-Fareed Alikhan, Lizzie Meadows, Rachael Stanley, Ngozi Elumogo, Muhammed Yasir, Nicholas M. Thomson, Alexander J Trotter, Rachel Gilroy, Samuel Bloomfield, Claire Stuart, Andrew Bell, Reenesh Prakash, Samir Dervisevic, Alison E. Mather, John Wain, Mark Webber, Andrew J. Page, Justin O'Grady

EPI_ISL_820264, EPI_ISL_820266, EPI_ISL_820268, EPI_ISL_820271, EPI_ISL_820273, EPI_ISL_820276	University College London, Great Ormond Street Hospital for Children NHS Foundation Trust, Imperial College Healthcare NHS Trust	COVID-19 Genomics UK (COG-UK) Consortium	Sergi Castellano, Rachel Williams, Mark Kristiansen, Paola Resende Silva, Sunando Roy, Tony Brooks, Helena Tutill, Paola Niola, Patricia Dyal, Charlotte Williams, Leysa Forrest, Yasmin Panchbhaya, Jacqueline Findlay, Samuel Weeks, Julianne Brown, Kathryn Harris, Paul Randell, James Price, Alison Holmes, Judith Breuer
EPI_ISL_820279	Quadram Institute Bioscience	COVID-19 Genomics UK (COG-UK) Consortium	Dave J. Baker, Gemma L. Kay, Alp Aydin, Thanh Le-Viet, Steven Rudder, Ana P. Tedim, Anastasia Kolyva, Maria Diaz, Leonardo de Oliveira Martins, Nabil-Fareed Alikhan, Lizzie Meadows, Rachael Stanley, Ngozi Elumogo, Muhammed Yasir, Nicholas M. Thomson, Alexander J Trotter, Rachel Gilroy, Samuel Bloomfield, Claire Stuart, Andrew Bell, Reenesh Prakash, Samir Dervisevic, Alison E. Mather, John Wain, Mark Webber, Andrew J. Page, Justin O'Grady
EPI_ISL_820282	University College London, Great Ormond Street Hospital for Children NHS Foundation Trust, Imperial College Healthcare NHS Trust	COVID-19 Genomics UK (COG-UK) Consortium	Sergi Castellano, Rachel Williams, Mark Kristiansen, Paola Resende Silva, Sunando Roy, Tony Brooks, Helena Tutill, Paola Niola, Patricia Dyal, Charlotte Williams, Leysa Forrest, Yasmin Panchbhaya, Jacqueline Findlay, Samuel Weeks, Julianne Brown, Kathryn Harris, Paul Randell, James Price, Alison Holmes, Judith Breuer
EPI_ISL_820284, EPI_ISL_820287, EPI_ISL_820289, EPI_ISL_820292, EPI_ISL_820294	Quadram Institute Bioscience	COVID-19 Genomics UK (COG-UK) Consortium	Dave J. Baker, Gemma L. Kay, Alp Aydin, Thanh Le-Viet, Steven Rudder, Ana P. Tedim, Anastasia Kolyva, Maria Diaz, Leonardo de Oliveira Martins, Nabil-Fareed Alikhan, Lizzie Meadows, Rachael Stanley, Ngozi Elumogo, Muhammed Yasir, Nicholas M. Thomson, Alexander J Trotter, Rachel Gilroy, Samuel Bloomfield, Claire Stuart, Andrew Bell, Reenesh Prakash, Samir Dervisevic, Alison E. Mather, John Wain, Mark Webber, Andrew J. Page, Justin O'Grady
EPI_ISL_820297	Queens Medical Centre, Clinical Microbiology Department / DeepSeq Nottingham	COVID-19 Genomics UK (COG-UK) Consortium	Gemma Clark, Wendy Smith, Manjinder Khakh, Vicki M Fleming, Michelle M Lister, Hannah Howson-Wells, Jonathan Ball, Patrick McClure, Joseph Chappell, Theocharis Tsoleridis, Nadine Holmes, Matthew Carlisle, Christopher Moore, Fei Sang, Johnny Debebe, Victoria Wright, Matthew Loose
EPI_ISL_820300, EPI_ISL_820302, EPI_ISL_820304, EPI_ISL_820306	Quadram Institute Bioscience	COVID-19 Genomics UK (COG-UK) Consortium	Dave J. Baker, Gemma L. Kay, Alp Aydin, Thanh Le-Viet, Steven Rudder, Ana P. Tedim, Anastasia Kolyva, Maria Diaz, Leonardo de Oliveira Martins, Nabil-Fareed Alikhan, Lizzie Meadows, Rachael Stanley, Ngozi Elumogo, Muhammed Yasir, Nicholas M. Thomson, Alexander J Trotter, Rachel Gilroy, Samuel Bloomfield, Claire Stuart, Andrew Bell, Reenesh Prakash, Samir Dervisevic, Alison E. Mather, John Wain, Mark Webber, Andrew J. Page, Justin O'Grady
EPI_ISL_820309	University College London, Great Ormond Street Hospital for Children NHS Foundation Trust, Imperial College Healthcare NHS Trust	COVID-19 Genomics UK (COG-UK) Consortium	Sergi Castellano, Rachel Williams, Mark Kristiansen, Paola Resende Silva, Sunando Roy, Tony Brooks, Helena Tutill, Paola Niola, Patricia Dyal, Charlotte Williams, Leysa Forrest, Yasmin Panchbhaya, Jacqueline Findlay, Samuel Weeks, Julianne Brown, Kathryn Harris, Paul Randell, James Price, Alison Holmes, Judith Breuer
EPI_ISL_820311	Quadram Institute Bioscience	COVID-19 Genomics UK (COG-UK) Consortium	Dave J. Baker, Gemma L. Kay, Alp Aydin, Thanh Le-Viet, Steven Rudder, Ana P. Tedim, Anastasia Kolyva, Maria Diaz, Leonardo de Oliveira Martins, Nabil-Fareed Alikhan, Lizzie Meadows, Rachael Stanley, Ngozi Elumogo, Muhammed Yasir, Nicholas M. Thomson, Alexander J Trotter, Rachel Gilroy, Samuel Bloomfield, Claire Stuart, Andrew Bell, Reenesh Prakash, Samir Dervisevic, Alison E. Mather, John Wain, Mark Webber, Andrew J. Page, Justin O'Grady
EPI_ISL_820314, EPI_ISL_820316, EPI_ISL_820318, EPI_ISL_820321	University College London, Great Ormond Street Hospital for Children NHS Foundation Trust, Imperial College Healthcare NHS Trust	COVID-19 Genomics UK (COG-UK) Consortium	Sergi Castellano, Rachel Williams, Mark Kristiansen, Paola Resende Silva, Sunando Roy, Tony Brooks, Helena Tutill, Paola Niola, Patricia Dyal, Charlotte Williams, Leysa Forrest, Yasmin Panchbhaya, Jacqueline Findlay, Samuel Weeks, Julianne Brown, Kathryn Harris, Paul Randell, James Price, Alison Holmes, Judith Breuer
EPI_ISL_820323	Quadram Institute Bioscience	COVID-19 Genomics UK (COG-UK) Consortium	Dave J. Baker, Gemma L. Kay, Alp Aydin, Thanh Le-Viet, Steven Rudder, Ana P. Tedim, Anastasia Kolyva, Maria Diaz, Leonardo de Oliveira Martins, Nabil-Fareed Alikhan, Lizzie Meadows, Rachael Stanley, Ngozi Elumogo, Muhammed Yasir, Nicholas M. Thomson, Alexander J Trotter, Rachel Gilroy, Samuel Bloomfield, Claire Stuart, Andrew Bell, Reenesh Prakash, Samir Dervisevic, Alison E. Mather, John Wain, Mark Webber, Andrew J. Page, Justin O'Grady
EPI_ISL_821629, EPI_ISL_821630, EPI_ISL_821631, EPI_ISL_821633, EPI_ISL_821634, EPI_ISL_821635, EPI_ISL_821636, EPI_ISL_821637, EPI_ISL_821641, EPI_ISL_821648, EPI_ISL_821653			
see above	Lighthouse Lab in Milton Keynes	Wellcome Sanger Institute for the COVID-19 Genomics UK (COG-UK) Consortium	The Lighthouse Lab in Milton Keynes and Alex Alderton, Roberto Amato, Sonia Goncalves, Ewan Harrison, David K. Jackson, Ian Johnston, Dominic Kwiatkowski, Cordelia Langford, John Sillitoe on behalf of the Wellcome Sanger Institute COVID-19 Surveillance Team
EPI_ISL_821667, EPI_ISL_821672, EPI_ISL_821677, EPI_ISL_821733, EPI_ISL_821787, EPI_ISL_821790, EPI_ISL_821827, EPI_ISL_821877, EPI_ISL_821889, EPI_ISL_821934, EPI_ISL_821943, EPI_ISL_821959			
see above	Lighthouse Lab in Cambridge	Wellcome Sanger Institute for the COVID-19 Genomics UK (COG-UK) Consortium	Rob Howes, The Lighthouse Lab in Cambridge and Alex Alderton, Roberto Amato, Sonia Goncalves, Ewan Harrison, David K. Jackson, Ian Johnston, Dominic Kwiatkowski, Cordelia Langford, John Sillitoe on behalf of the Wellcome Sanger Institute COVID-19 Surveillance Team
EPI_ISL_822291, EPI_ISL_822294, EPI_ISL_822297, EPI_ISL_822298, EPI_ISL_822300, EPI_ISL_822303	Lighthouse Lab in Alderley Park	Wellcome Sanger Institute for the COVID-19 Genomics UK (COG-UK) Consortium	Jacquelyn Wynn, Mairead Hyland, The Lighthouse Lab in Alderley Park and Alex Alderton, Roberto Amato, Sonia Goncalves, Ewan Harrison, David K. Jackson, Ian Johnston, Dominic Kwiatkowski, Cordelia Langford, John Sillitoe on behalf of the Wellcome Sanger Institute COVID-19 Surveillance Team
EPI_ISL_822307	Lighthouse Lab in Glasgow	Wellcome Sanger Institute for the COVID-19 Genomics UK (COG-UK) Consortium	Harper VanSteenhouse, Yumi Kasai, David Gray, Carol Clugston, Anna Dominiczak and Alex Alderton, Roberto Amato, Sonia Goncalves, Ewan Harrison, David K. Jackson, Ian Johnston, Dominic Kwiatkowski, Cordelia Langford, John Sillitoe on behalf of the Wellcome Sanger Institute COVID-19 Surveillance Team
EPI_ISL_822529, EPI_ISL_822530, EPI_ISL_822531, EPI_ISL_822532, EPI_ISL_822533, EPI_ISL_822534, EPI_ISL_822535, EPI_ISL_822536, EPI_ISL_822537, EPI_ISL_822538, EPI_ISL_822539, EPI_ISL_822540, EPI_ISL_822541, EPI_ISL_822542, EPI_ISL_822543, EPI_ISL_822544, EPI_ISL_822545, EPI_ISL_822546, EPI_ISL_822547, EPI_ISL_822548, EPI_ISL_822549, EPI_ISL_822550, EPI_ISL_822551, EPI_ISL_822552, EPI_ISL_822553, EPI_ISL_822554, EPI_ISL_822555, EPI_ISL_822556, EPI_ISL_822557, EPI_ISL_822558, EPI_ISL_822559, EPI_ISL_822560, EPI_ISL_822561, EPI_ISL_822562, EPI_ISL_822563, EPI_ISL_822564, EPI_ISL_822565, EPI_ISL_822566, EPI_ISL_822567, EPI_ISL_822568, EPI_ISL_822569, EPI_ISL_822570, EPI_ISL_822571, EPI_ISL_822572, EPI_ISL_822573, EPI_ISL_822574, EPI_ISL_822575, EPI_ISL_822576, EPI_ISL_822577, EPI_ISL_822578, EPI_ISL_822579, EPI_ISL_822580, EPI_ISL_822581, EPI_ISL_822582, EPI_ISL_822583, EPI_ISL_822584, EPI_ISL_822585, EPI_ISL_822586, EPI_ISL_822587, EPI_ISL_822588, EPI_ISL_822589, EPI_ISL_822590, EPI_ISL_822591, EPI_ISL_822592, EPI_ISL_822593, EPI_ISL_822594, EPI_ISL_822595, EPI_ISL_822596, EPI_ISL_822597, EPI_ISL_822598, EPI_ISL_822599, EPI_ISL_822600, EPI_ISL_822601, EPI_ISL_822602, EPI_ISL_822603, EPI_ISL_822604, EPI_ISL_822605, EPI_ISL_822606, EPI_ISL_822607, EPI_ISL_822608, EPI_ISL_822609, EPI_ISL_822610, EPI_ISL_822611, EPI_ISL_822612, EPI_ISL_822613, EPI_ISL_822614, EPI_ISL_822615, EPI_ISL_822616, EPI_ISL_822617, EPI_ISL_822618, EPI_ISL_822619, EPI_ISL_822620, EPI_ISL_822621, EPI_ISL_822622, EPI_ISL_822623, EPI_ISL_822624, EPI_ISL_822625, EPI_ISL_822626, EPI_ISL_822627, EPI_ISL_822628, EPI_ISL_822629, EPI_ISL_822630, EPI_ISL_822631, EPI_ISL_822632, EPI_ISL_822633, EPI_ISL_822634, EPI_ISL_822635, EPI_ISL_822636, EPI_ISL_822637, EPI_ISL_822638, EPI_ISL_822639, EPI_ISL_822640, EPI_ISL_822641, EPI_ISL_822642, EPI_ISL_822643, EPI_ISL_822644, EPI_ISL_822645, EPI_ISL_822646, EPI_ISL_822647, EPI_ISL_822648, EPI_ISL_822649, EPI_ISL_822650, EPI_ISL_822651, EPI_ISL_822652, EPI_ISL_822653, EPI_ISL_822654, EPI_ISL_822655, EPI_ISL_822656, EPI_ISL_822657, EPI_ISL_822658, EPI_ISL_822839, EPI_ISL_822865, EPI_ISL_822885, EPI_ISL_822887, EPI_ISL_822921, EPI_ISL_822932, EPI_ISL_822949, EPI_ISL_822954, EPI_ISL_822963, EPI_ISL_822978, EPI_ISL_823019, EPI_ISL_823065, EPI_ISL_823093, EPI_ISL_823094, EPI_ISL_823095, EPI_ISL_823096, EPI_ISL_823097, EPI_ISL_823104, EPI_ISL_823106, EPI_ISL_823107, EPI_ISL_823108, EPI_ISL_823115, EPI_ISL_823141, EPI_ISL_823153, EPI_ISL_823643, EPI_ISL_823644, EPI_ISL_823645, EPI_ISL_823769, EPI_ISL_823770, EPI_ISL_823771, EPI_ISL_823772, EPI_ISL_823773, EPI_ISL_823774, EPI_ISL_823775, EPI_ISL_823776, EPI_ISL_823777, EPI_ISL_823778, EPI_ISL_823779, EPI_ISL_823800, EPI_ISL_823801, EPI_ISL_823803, EPI_ISL_823804, EPI_ISL_823808, EPI_ISL_823809, EPI_ISL_823810			
see above	Wales Specialist Virology Centre Sequencing lab: Pathogen Genomics Unit	COVID-19 Genomics UK (COG-UK) Consortium	Catherine Moore, Johnathan Evans, Laura Gifford, Malorie Perry, Simon Cottrell, Angela Marchbank, Alec Birchley, Alexander Adams, Amy Gaskin, Bree Gatica-Wilcox, Jason Coombes, Joel Southgate, Lauren Gilbert, Lee Graham, Nicole Pacchiarini, Sara Kumziene-Summerhayes, Sarah Taylor, Sophie Jones, Sara Rey, Matthew Bull, Joanne Watkins, Sally Corden, Tom Connor
EPI_ISL_823979, EPI_ISL_823980, EPI_ISL_823992, EPI_ISL_824002, EPI_ISL_824041, EPI_ISL_824042, EPI_ISL_824043, EPI_ISL_824044, EPI_ISL_824045, EPI_ISL_824046, EPI_ISL_824056, EPI_ISL_824057, EPI_ISL_824058, EPI_ISL_824059, EPI_ISL_824069, EPI_ISL_824070, EPI_ISL_824072, EPI_ISL_824094, EPI_ISL_824110, EPI_ISL_824115, EPI_ISL_824117, EPI_ISL_824118, EPI_ISL_824119, EPI_ISL_824121, EPI_ISL_824122, EPI_ISL_824140, EPI_ISL_824163, EPI_ISL_824170, EPI_ISL_824171, EPI_ISL_824172, EPI_ISL_824173, EPI_ISL_824174, EPI_ISL_824176, EPI_ISL_824177, EPI_ISL_824178, EPI_ISL_824179, EPI_ISL_824180, EPI_ISL_824195, EPI_ISL_824196, EPI_ISL_824210, EPI_ISL_824211, EPI_ISL_824212, EPI_ISL_824213, EPI_ISL_824230, EPI_ISL_824231, EPI_ISL_824248, EPI_ISL_824265, EPI_ISL_824269, EPI_ISL_824271, EPI_ISL_824274, EPI_ISL_824275, EPI_ISL_824277, EPI_ISL_824281, EPI_ISL_824282, EPI_ISL_824283			
see above	Dutch COVID-19 response team	National Institute for Public Health and the Environment (RIVM)	Adam Meijer, Harry Vennema, Jeroen Cremer, Sharon van den Brink, Bas van der Veer, AnneMarie van den Brandt, Florian Zwagemaker, Dennis Schmitz, Chantal Reusken, on behalf of the national COVID-19 response team
EPI_ISL_824286, EPI_ISL_824287	Institute of Microbiology, Universidad San Francisco de Quito	Institute of Microbiology, Universidad San Francisco de Quito	Belén Prado-Vivar, Sully Márquez, Juan José Guadalupe, Monica Becerra-Wong, Bernardo Gutiérrez, Andrea Cunguan, Nabih Dahik, Verónica Barragán, Patricio Rojas-Silva, Gabriel Trueba, Michelle Grunauer, Paúl Cárdenas
EPI_ISL_824288, EPI_ISL_824289	Institute of Microbiology, Universidad San Francisco de Quito	Institute of Microbiology, Universidad San Francisco de Quito	Belén Prado-Vivar, Sully Márquez, Juan José Guadalupe, Monica Becerra-Wong, Bernardo Gutiérrez, Tania Guayasamin, Patricio Reyes, Verónica Barragán, Patricio Rojas-Silva, Gabriel Trueba, Michelle Grunauer, Paúl Cárdenas
EPI_ISL_824519, EPI_ISL_824520, EPI_ISL_824521, EPI_ISL_824522, EPI_ISL_824523, EPI_ISL_824536, EPI_ISL_824537	New Mexico Department of Health Scientific Laboratory	New Mexico Department of Health Scientific Laboratory	Ellie Johnson, Anastacia Griego-Fisher, D'eldra Malone
EPI_ISL_824556, EPI_ISL_824557, EPI_ISL_824562, EPI_ISL_824563, EPI_ISL_824564, EPI_ISL_824566, EPI_ISL_824567, EPI_ISL_824568, EPI_ISL_824569, EPI_ISL_824574, EPI_ISL_824575, EPI_ISL_824576, EPI_ISL_824577, EPI_ISL_824578, EPI_ISL_824579, EPI_ISL_824584, EPI_ISL_824585, EPI_ISL_824586, EPI_ISL_824587, EPI_ISL_824588, EPI_ISL_824589, EPI_ISL_824596, EPI_ISL_824597, EPI_ISL_824598, EPI_ISL_824599, EPI_ISL_824600, EPI_ISL_824601, EPI_ISL_824607, EPI_ISL_824608, EPI_ISL_824609, EPI_ISL_824610, EPI_ISL_824611, EPI_ISL_824612, EPI_ISL_824618, EPI_ISL_824619, EPI_ISL_824620, EPI_ISL_824621, EPI_ISL_824622, EPI_ISL_824623, EPI_ISL_824625, EPI_ISL_824629, EPI_ISL_824630, EPI_ISL_824631, EPI_ISL_824632, EPI_ISL_824633, EPI_ISL_824634, EPI_ISL_824635, EPI_ISL_824637, EPI_ISL_824641, EPI_ISL_824642, EPI_ISL_824643, EPI_ISL_824644, EPI_ISL_824645, EPI_ISL_824646,			

EPI_ISL_824647, EPI_ISL_824663, EPI_ISL_824675, EPI_ISL_824695, EPI_ISL_824719			
see above	Cedars-Sinai Medical Center, Department of Pathology & Laboratory Medicine, Molecular Pathology Laboratory	Cedars-Sinai Medical Center, Molecular Pathology Laboratory of Department of Pathology & Laboratory Medicine and Genomic Core	Wenjuan Zhang, Brian Davis, Stephanie Chen, Jorge Mario Sincuir Martinez, Jasmine T Plummer, Eric Vail
EPI_ISL_824800, EPI_ISL_824801, EPI_ISL_824802, EPI_ISL_824803, EPI_ISL_824804, EPI_ISL_824805, EPI_ISL_824806, EPI_ISL_824807, EPI_ISL_824808	Department of Clinical Microbiology	GIGA Medical Genomics	Keith Durkin, Maria Artesi, Sébastien Bontems, Raphaël Boreux, Bouchra Boujemla, Cécile Meex, Pierrette Melin, Marie-Pierre Hayette, Vincent Bours
EPI_ISL_825011, EPI_ISL_825012	Arizona State Public Health Laboratory	Arizona State Public Health Laboratory	Trung Huynh, Jessica Escobar, Katherine Fullerton, Nobuko Fukushima, Stacy White, Linda Getsinger, Victor Waddell
EPI_ISL_825152	National Institute of Mental Health and Neurosciences (NIMHANS)	Department of Neurovirology, National Institute of Mental Health and Neurosciences (NIMHANS)	Chitra Pattabiraman, Pramada Prasad, Anita S Desai, V Ravi
EPI_ISL_825504, EPI_ISL_825532, EPI_ISL_825533, EPI_ISL_825556	Respiratory Virus Unit, National Infection Service, Public Health England	COVID-19 Genomics UK (COG-UK) Consortium	PHE Covid Sequencing Team
EPI_ISL_826536	Dutch COVID-19 response team	National Institute for Public Health and the Environment (RIVM)	Adam Meijer, Harry Vennema, Jeroen Cremer, Sharon van den Brink, Bas van der Veer, AnneMarie van den Brandt, Florian Zwagemaker, Dennis Schmitz, Chantal Reusken, on behalf of the national COVID-19 response team
EPI_ISL_827820, EPI_ISL_828111, EPI_ISL_828140, EPI_ISL_828809, EPI_ISL_828998, EPI_ISL_829136, EPI_ISL_829139, EPI_ISL_829310, EPI_ISL_829733, EPI_ISL_829805, EPI_ISL_830121, EPI_ISL_830122, EPI_ISL_830123, EPI_ISL_830124, EPI_ISL_830126			
see above	deCODE genetics	deCODE genetics	Daniel F Gudbjartsson; Agnar Helgason; Hakon Jonsson; Olafur T Magnusson; Pall Melsted; Gudmundur L Norddahl; Jona Saemundsdottir; Asgeir Sigurdsson; Patrick Sulem; Arna B Agustsdottir; Hannes Eggertsson; Berglind Eiriksdottir; Run Fridriksdottir; Elisabet E Gardarsdottir; Gudmundur Georgsson; Olafia S Gretarsdottir; Kjartan R Gudmundsson; Thora R Gunnarsdottir; Arnaldur Gylfason; Hilma Holm; Brynjar O Jensson; Aslaug Jonasdottir; Kamilla S Josefsdottir; Thordur Kristjansson; Droplaug N Magnusdottir; Solvi Rognvaldsson; Louise le Roux; Gudrun Sigmundsdottir; Gardar Sveinbjornsson; Kristin E Sveinsdottir; Maney Sveinsdottir; Emil A Thorarensen; Bjarni Thorbjornsson; Gisli Masson; Ingileif Jonsdottir; Alma Moller; Thorolfur Gudnason; Karl G Kristinsson; Unnur Thorsteinsdottir; Kari Stefansson
EPI_ISL_830212, EPI_ISL_830243, EPI_ISL_830571, EPI_ISL_830573, EPI_ISL_830583, EPI_ISL_830584, EPI_ISL_830585, EPI_ISL_830586, EPI_ISL_830587, EPI_ISL_830588, EPI_ISL_830589, EPI_ISL_830590, EPI_ISL_830591, EPI_ISL_830592, EPI_ISL_830593, EPI_ISL_830594, EPI_ISL_830595, EPI_ISL_830596, EPI_ISL_830597, EPI_ISL_830598, EPI_ISL_830599, EPI_ISL_830600, EPI_ISL_830601, EPI_ISL_830602, EPI_ISL_830603, EPI_ISL_830604, EPI_ISL_830605, EPI_ISL_830606, EPI_ISL_830607			
see above	KALEIDA CENTER FOR LABORATORY MEDICINE	Wadsworth Center, New York State Department of Health	Kirsten St. George, Daryl M. Lamson, Alexis Russel, Matthew Shudt, Melissa A Leisner, Jonathan Plitnick, Navjot Singh, John Kelly, Erasmus Schneider, Erica Lasek-Nesselquist
EPI_ISL_830720, EPI_ISL_830721	BIO-REFERENCE LABORATORIES	Wadsworth Center, New York State Department of Health	Kirsten St. George, Daryl M. Lamson, Alexis Russel, Matthew Shudt, Melissa A Leisner, Jonathan Plitnick, Navjot Singh, John Kelly, Erasmus Schneider, Erica Lasek-Nesselquist
EPI_ISL_831021, EPI_ISL_831022, EPI_ISL_831023, EPI_ISL_831024, EPI_ISL_831025, EPI_ISL_831026	Hospital Universitario La Paz (Madrid)	SeqCOVID-SPAIN consortium/IBV(CSIC)	Fernando Lázaro-Perona, María Rodríguez-Tejedor, Elias Dahdouh, Jesús Mingorance and SeqCOVID-SPAIN consortium
EPI_ISL_832019	Southern Nevada Public Health Laboratory	Southern Nevada Public Health Laboratory	Michael Picker, Holly Hansen, Erin Buttery, Joseph Benson
EPI_ISL_832047, EPI_ISL_832048, EPI_ISL_832049, EPI_ISL_832050, EPI_ISL_832051, EPI_ISL_832052, EPI_ISL_832053, EPI_ISL_832054, EPI_ISL_832055, EPI_ISL_832056, EPI_ISL_832057, EPI_ISL_832058, EPI_ISL_832059, EPI_ISL_832060, EPI_ISL_832061, EPI_ISL_832062, EPI_ISL_832063, EPI_ISL_832064			
see above	Wyoming Public Health Laboratory	Wyoming Public Health Laboratory	Noah Hull, Taylor Fearing, Lynette Gumbleton, Channing Weber, Ashley Norberg, Bailey Bowcutt, and Wanda Manley
EPI_ISL_832078	Santa Clara County Public Health Laboratory	Santa Clara County Public Health Laboratory	Santa Clara County Public Health Department
EPI_ISL_832116	MD Laboratories	Los Angeles County PHL	P. Hemarajata et al.
EPI_ISL_832197	Hospital	National Reference Center for Viruses of Respiratory Infections, Institut Pasteur, Paris	Marion Barbet, Sylvie Behillil, Méline Bizard, Angela Brisebarre, Camille Capel, Etienne Simon-Lorière, Vincent Enouf, Maud Vanpeene, Sylvie van der Werf, Esther Gyde
EPI_ISL_832199	Hospital	National Reference Center for Viruses of Respiratory Infections, Institut Pasteur, Paris	Marion Barbet, Sylvie Behillil, Méline Bizard, Angela Brisebarre, Camille Capel, Etienne Simon-Lorière, Vincent Enouf, Maud Vanpeene, Sylvie van der Werf, Patricia Stoessel-Thouvenin
EPI_ISL_832265	DOHMH Jamaica	New York City Public Health Laboratory	Jade Wang, et al.
EPI_ISL_832355, EPI_ISL_832356	DOHMH Corona	New York City Public Health Laboratory	Jade Wang, et al.
EPI_ISL_832357, EPI_ISL_832358	DOHMH Jamaica	New York City Public Health Laboratory	Jade Wang, et al.
EPI_ISL_832359, EPI_ISL_832360	DOHMH Corona	New York City Public Health Laboratory	Jade Wang, et al.
EPI_ISL_832361	DOHMH Jamaica	New York City Public Health Laboratory	Jade Wang, et al.
EPI_ISL_832362, EPI_ISL_832363	DOHMH Chelsea	New York City Public Health Laboratory	Jade Wang, et al.
EPI_ISL_832835, EPI_ISL_832836, EPI_ISL_832837, EPI_ISL_832838, EPI_ISL_832839, EPI_ISL_832840, EPI_ISL_832841, EPI_ISL_832842, EPI_ISL_832843, EPI_ISL_832844, EPI_ISL_832845, EPI_ISL_832846, EPI_ISL_832847, EPI_ISL_832848, EPI_ISL_832849, EPI_ISL_832850, EPI_ISL_832875, EPI_ISL_832911, EPI_ISL_832912, EPI_ISL_832914, EPI_ISL_832915, EPI_ISL_832916, EPI_ISL_832917, EPI_ISL_832918, EPI_ISL_832927, EPI_ISL_832928, EPI_ISL_832929, EPI_ISL_832933, EPI_ISL_832934			
see above	Maine HETL	Tewhey Lab, The Jackson Laboratory	Matluk,N., Dewey,H., Iosue,F., Barter,M., Lynch,R., Munger,H. and Tewhey,R.
EPI_ISL_833149	Platform BIS UZA/UAntwerpen, University Hospital Antwerp, Edegem, Belgium	UAntwerp, Laboratory of Medical Microbiology, Campus Drie Eiken S6.26, Universiteitsplein 1, 2610, Wilrijk, Belgium	Basil Britto Xavier, Jasmine Coppens, Sahaya Glingston Rajakani, Christine Lammens, Veerle Matheeussen, Herman Goossens
EPI_ISL_833183	Microbiology Department. Complexo Hospitalario Universitario de Vigo	Microbiology Department. Complexo Hospitalario Universitario de Vigo	Microbiology Department, Complexo Hospitalario Universitario de Vigo. EPICOVIGAL.
EPI_ISL_833186	Department of Clinical Microbiology	GIGA Medical Genomics	Keith Durkin, Maria Artesi, Sébastien Bontems, Raphaël Boreux, Bouchra Boujemla, Cécile Meex, Pierrette Melin, Marie-Pierre Hayette, Vincent Bours
EPI_ISL_833210, EPI_ISL_833227	Department of Virology and Immunology, University of Helsinki and Helsinki University Hospital, Huslab Finland	Department of Virology, Faculty of Medicine, University of Helsinki, Helsinki, Finland	Teemu Smura, Ravi Kant, Phuoc Truong, Hussein Alburkat, Hannimari Kallio-Kokko, Jenni Virtanen, Maija Suvanto, Fathiah Zakham, Essi Korhonen, Sari Hannula, Harri Kangas, Pekka Ellonen, Olli Vapalahti
EPI_ISL_833251	Ospedale Civile Atri-Medicina Interna	Istituto Zooprofilattico Sperimentale dell'Abruzzo e Molise "G. Caporale"	Lorusso A, Marcacci M, Di Domenico M, Ancora M, Curini V, Mangone I, Rinaldi A, Di Pasquale A, Cammà C, Puglia I, Calistri P, Savini G
EPI_ISL_833252	Ospedale Civile Giulianova-Pronto Soccorso	Istituto Zooprofilattico Sperimentale dell'Abruzzo e Molise "G. Caporale"	Lorusso A, Marcacci M, Di Domenico M, Ancora M, Curini V, Mangone I, Rinaldi A, Di Pasquale A, Cammà C, Puglia I, Calistri P, Savini G
EPI_ISL_833253	USCA-Avezzano	Istituto Zooprofilattico Sperimentale dell'Abruzzo e Molise "G. Caporale"	Lorusso A, Marcacci M, Di Domenico M, Ancora M, Curini V, Mangone I, Rinaldi A, Di Pasquale A, Cammà C, Puglia I, Calistri P, Savini G
EPI_ISL_833254	USCA-Pescina	Istituto Zooprofilattico Sperimentale dell'Abruzzo e Molise "G. Caporale"	Lorusso A, Marcacci M, Di Domenico M, Ancora M, Curini V, Mangone I, Rinaldi A, Di Pasquale A, Cammà C, Puglia I, Calistri P, Savini G
EPI_ISL_833255, EPI_ISL_833256, EPI_ISL_833257	Ospedale Civile Atri	Istituto Zooprofilattico Sperimentale dell'Abruzzo e Molise "G. Caporale"	Lorusso A, Marcacci M, Di Domenico M, Ancora M, Curini V, Mangone I, Rinaldi A, Di Pasquale A, Cammà C, Puglia I, Calistri P, Savini G
EPI_ISL_833258, EPI_ISL_833259	SIESP DIPARTIMENTO DI PREVENZIONE TERAMO	Istituto Zooprofilattico Sperimentale dell'Abruzzo e Molise "G. Caporale"	Lorusso A, Marcacci M, Di Domenico M, Ancora M, Curini V, Mangone I, Rinaldi A, Di Pasquale A, Cammà C, Puglia I, Calistri P, Savini G

EPI_ISL_833260, EPI_ISL_833261	Giulianova	Istituto Zooprofilattico Sperimentale dell'Abruzzo e Molise "G. Caporale"	Lorusso A, Marcacci M, Di Domenico M, Ancora M, Curini V, Mangone I, Rinaldi A, Di Pasquale A, Cammà C, Puglia I, Calistri P, Savini G
EPI_ISL_833262, EPI_ISL_833263, EPI_ISL_833264	SIESP DIPARTIMENTO DI PREVENZIONE CHIETI	Istituto Zooprofilattico Sperimentale dell'Abruzzo e Molise "G. Caporale"	Lorusso A, Marcacci M, Di Domenico M, Ancora M, Curini V, Mangone I, Rinaldi A, Di Pasquale A, Cammà C, Puglia I, Calistri P, Savini G
EPI_ISL_833265, EPI_ISL_833266, EPI_ISL_833267, EPI_ISL_833268, EPI_ISL_833269	Ospedale SS Annunziata Chieti	Istituto Zooprofilattico Sperimentale dell'Abruzzo e Molise "G. Caporale"	Lorusso A, Marcacci M, Di Domenico M, Ancora M, Curini V, Mangone I, Rinaldi A, Di Pasquale A, Cammà C, Puglia I, Calistri P, Savini G
EPI_ISL_833270, EPI_ISL_833272, EPI_ISL_833273, EPI_ISL_833274, EPI_ISL_833275	SIESP CHIETI - DRIVE IN ORTONA	Istituto Zooprofilattico Sperimentale dell'Abruzzo e Molise "G. Caporale"	Lorusso A, Marcacci M, Di Domenico M, Ancora M, Curini V, Mangone I, Rinaldi A, Di Pasquale A, Cammà C, Puglia I, Calistri P, Savini G
EPI_ISL_833276	Ospedale Civile G.Mazzini-Teramo	Istituto Zooprofilattico Sperimentale dell'Abruzzo e Molise "G. Caporale"	Lorusso A, Marcacci M, Di Domenico M, Ancora M, Curini V, Mangone I, Rinaldi A, Di Pasquale A, Cammà C, Puglia I, Calistri P, Savini G
EPI_ISL_833278, EPI_ISL_833279, EPI_ISL_833280	SIESP CHIETI- DRIVE IN CHIETI	Istituto Zooprofilattico Sperimentale dell'Abruzzo e Molise "G. Caporale"	Lorusso A, Marcacci M, Di Domenico M, Ancora M, Curini V, Mangone I, Rinaldi A, Di Pasquale A, Cammà C, Puglia I, Calistri P, Savini G
EPI_ISL_833281, EPI_ISL_833282	SIESP DIPARTIMENTO DI PREVENZIONE TERAMO	Istituto Zooprofilattico Sperimentale dell'Abruzzo e Molise "G. Caporale"	Lorusso A, Marcacci M, Di Domenico M, Ancora M, Curini V, Mangone I, Rinaldi A, Di Pasquale A, Cammà C, Puglia I, Calistri P, Savini G
EPI_ISL_833283	DIPARTIMENTO PREVENZIONE AVEZZANO-SERVIZIO DI IGIENE EPIDEMIOLOGIA E SANITA' PUBBLICA	Istituto Zooprofilattico Sperimentale dell'Abruzzo e Molise "G. Caporale"	Lorusso A, Marcacci M, Di Domenico M, Ancora M, Curini V, Mangone I, Rinaldi A, Di Pasquale A, Cammà C, Puglia I, Calistri P, Savini G
EPI_ISL_833284, EPI_ISL_833285, EPI_ISL_833286, EPI_ISL_833287	SIESP CHIETI - DRIVE IN ORTONA	Istituto Zooprofilattico Sperimentale dell'Abruzzo e Molise "G. Caporale"	Lorusso A, Marcacci M, Di Domenico M, Ancora M, Curini V, Mangone I, Rinaldi A, Di Pasquale A, Cammà C, Puglia I, Calistri P, Savini G
EPI_ISL_833288, EPI_ISL_833290	PRESIDIO TERRITORIALE CASOLI CH	Istituto Zooprofilattico Sperimentale dell'Abruzzo e Molise "G. Caporale"	Lorusso A, Marcacci M, Di Domenico M, Ancora M, Curini V, Mangone I, Rinaldi A, Di Pasquale A, Cammà C, Puglia I, Calistri P, Savini G
EPI_ISL_833291	SIESP DIPARTIMENTO DI PREVENZIONE DELL'AQUILA	Istituto Zooprofilattico Sperimentale dell'Abruzzo e Molise "G. Caporale"	Lorusso A, Marcacci M, Di Domenico M, Ancora M, Curini V, Mangone I, Rinaldi A, Di Pasquale A, Cammà C, Puglia I, Calistri P, Savini G
EPI_ISL_833292, EPI_ISL_833293, EPI_ISL_833294	SIESP CHIETI - DRIVE IN LANCIANO	Istituto Zooprofilattico Sperimentale dell'Abruzzo e Molise "G. Caporale"	Lorusso A, Marcacci M, Di Domenico M, Ancora M, Curini V, Mangone I, Rinaldi A, Di Pasquale A, Cammà C, Puglia I, Calistri P, Savini G
EPI_ISL_833295, EPI_ISL_833296	SIESP DIPARTIMENTO DI PREVENZIONE TERAMO	Istituto Zooprofilattico Sperimentale dell'Abruzzo e Molise "G. Caporale"	Lorusso A, Marcacci M, Di Domenico M, Ancora M, Curini V, Mangone I, Rinaldi A, Di Pasquale A, Cammà C, Puglia I, Calistri P, Savini G
EPI_ISL_833298, EPI_ISL_833300	SIESP CHIETI - DRIVE IN LANCIANO	Istituto Zooprofilattico Sperimentale dell'Abruzzo e Molise "G. Caporale"	Lorusso A, Marcacci M, Di Domenico M, Ancora M, Curini V, Mangone I, Rinaldi A, Di Pasquale A, Cammà C, Puglia I, Calistri P, Savini G
EPI_ISL_833301	DIPARTIMENTO PREVENZIONE AVEZZANO-SERVIZIO DI IGIENE EPIDEMIOLOGIA E SANITA' PUBBLICA	Istituto Zooprofilattico Sperimentale dell'Abruzzo e Molise "G. Caporale"	Lorusso A, Marcacci M, Di Domenico M, Ancora M, Curini V, Mangone I, Rinaldi A, Di Pasquale A, Cammà C, Puglia I, Calistri P, Savini G
EPI_ISL_833302, EPI_ISL_833303, EPI_ISL_833304, EPI_ISL_833305	SIESP DIPARTIMENTO DI PREVENZIONE TERAMO	Istituto Zooprofilattico Sperimentale dell'Abruzzo e Molise "G. Caporale"	Lorusso A, Marcacci M, Di Domenico M, Ancora M, Curini V, Mangone I, Rinaldi A, Di Pasquale A, Cammà C, Puglia I, Calistri P, Savini G
EPI_ISL_833306, EPI_ISL_833307, EPI_ISL_833308, EPI_ISL_833309	SIESP CHIETI - DRIVE IN ORTONA	Istituto Zooprofilattico Sperimentale dell'Abruzzo e Molise "G. Caporale"	Lorusso A, Marcacci M, Di Domenico M, Ancora M, Curini V, Mangone I, Rinaldi A, Di Pasquale A, Cammà C, Puglia I, Calistri P, Savini G
EPI_ISL_833311, EPI_ISL_833312	SIESP CHIETI- DRIVE IN CHIETI	Istituto Zooprofilattico Sperimentale dell'Abruzzo e Molise "G. Caporale"	Lorusso A, Marcacci M, Di Domenico M, Ancora M, Curini V, Mangone I, Rinaldi A, Di Pasquale A, Cammà C, Puglia I, Calistri P, Savini G
EPI_ISL_833313, EPI_ISL_833314, EPI_ISL_833315	SIESP CHIETI - DRIVE IN LANCIANO	Istituto Zooprofilattico Sperimentale dell'Abruzzo e Molise "G. Caporale"	Lorusso A, Marcacci M, Di Domenico M, Ancora M, Curini V, Mangone I, Rinaldi A, Di Pasquale A, Cammà C, Puglia I, Calistri P, Savini G
EPI_ISL_833316	SIESP CHIETI - DRIVE IN GISSI	Istituto Zooprofilattico Sperimentale dell'Abruzzo e Molise "G. Caporale"	Lorusso A, Marcacci M, Di Domenico M, Ancora M, Curini V, Mangone I, Rinaldi A, Di Pasquale A, Cammà C, Puglia I, Calistri P, Savini G
EPI_ISL_833317, EPI_ISL_833318	SIESP CHIETI - DRIVE IN CHIETI	Istituto Zooprofilattico Sperimentale dell'Abruzzo e Molise "G. Caporale"	Lorusso A, Marcacci M, Di Domenico M, Ancora M, Curini V, Mangone I, Rinaldi A, Di Pasquale A, Cammà C, Puglia I, Calistri P, Savini G
EPI_ISL_833319, EPI_ISL_833320, EPI_ISL_833322	SIESP DIPARTIMENTO DI PREVENZIONE SULMONA	Istituto Zooprofilattico Sperimentale dell'Abruzzo e Molise "G. Caporale"	Lorusso A, Marcacci M, Di Domenico M, Ancora M, Curini V, Mangone I, Rinaldi A, Di Pasquale A, Cammà C, Puglia I, Calistri P, Savini G
EPI_ISL_833323	SIESP CHIETI - DRIVE IN CHIETI	Istituto Zooprofilattico Sperimentale dell'Abruzzo e Molise "G. Caporale"	Lorusso A, Marcacci M, Di Domenico M, Ancora M, Curini V, Mangone I, Rinaldi A, Di Pasquale A, Cammà C, Puglia I, Calistri P, Savini G
EPI_ISL_833324	SIESP DIPARTIMENTO DI PREVENZIONE CHIETI	Istituto Zooprofilattico Sperimentale dell'Abruzzo e Molise "G. Caporale"	Lorusso A, Marcacci M, Di Domenico M, Ancora M, Curini V, Mangone I, Rinaldi A, Di Pasquale A, Cammà C, Puglia I, Calistri P, Savini G
EPI_ISL_833325	Ospedale SS Annunziata Chieti	Istituto Zooprofilattico Sperimentale dell'Abruzzo e Molise "G. Caporale"	Lorusso A, Marcacci M, Di Domenico M, Ancora M, Curini V, Mangone I, Rinaldi A, Di Pasquale A, Cammà C, Puglia I, Calistri P, Savini G
EPI_ISL_833326	SIESP DIPARTIMENTO DI PREVENZIONE CHIETI	Istituto Zooprofilattico Sperimentale dell'Abruzzo e Molise "G. Caporale"	Lorusso A, Marcacci M, Di Domenico M, Ancora M, Curini V, Mangone I, Rinaldi A, Di Pasquale A, Cammà C, Puglia I, Calistri P, Savini G
EPI_ISL_833327	Ospedale SS Annunziata Chieti	Istituto Zooprofilattico Sperimentale dell'Abruzzo e Molise "G. Caporale"	Lorusso A, Marcacci M, Di Domenico M, Ancora M, Curini V, Mangone I, Rinaldi A, Di Pasquale A, Cammà C, Puglia I, Calistri P, Savini G
EPI_ISL_833328, EPI_ISL_833329, EPI_ISL_833330	SIESP CHIETI - DRIVE IN ORTONA	Istituto Zooprofilattico Sperimentale dell'Abruzzo e Molise "G. Caporale"	Lorusso A, Marcacci M, Di Domenico M, Ancora M, Curini V, Mangone I, Rinaldi A, Di Pasquale A, Cammà C, Puglia I, Calistri P, Savini G
EPI_ISL_833331	Ospedale Civile Atri	Istituto Zooprofilattico Sperimentale dell'Abruzzo e Molise "G. Caporale"	Lorusso A, Marcacci M, Di Domenico M, Ancora M, Curini V, Mangone I, Rinaldi A, Di Pasquale A, Cammà C, Puglia I, Calistri P, Savini G
EPI_ISL_834578	Lighthouse Lab in Glasgow	Wellcome Sanger Institute for the COVID-19 Genomics UK (COG-UK) Consortium	Harper VanSteenhouse, Yumi Kasai, David Gray, Carol Clugston, Anna Dominiczak and Alex Alderton, Roberto Amato, Sonia Goncalves, Ewan Harrison, David K. Jackson, Ian Johnston, Dominic Kwiatkowski, Cordelia Langford, John Sillitoe on behalf of the Wellcome Sanger Institute COVID-19 Surveillance Team
EPI_ISL_837225	Respiratory Virus Unit, National Infection Service, Public Health England	COVID-19 Genomics UK (COG-UK) Consortium	PHE Covid Sequencing Team
EPI_ISL_837254, EPI_ISL_837326, EPI_ISL_837327, EPI_ISL_837328, EPI_ISL_837329, EPI_ISL_837330	Istituto Zooprofilattico Sperimentale del Mezzogiorno	TIGEM	Patrizia Annunziata, Andrea Ballabio, Valentina Bouche, Davide Cacchiarelli (CorrespAuthor), Pellegrino Cerino, Chiara Colantuono, Lucio Di Filippo, Antonio Grimaldi, Antonio Limone, Gabriella Loconte, Anna Manfredi, Francesco Panariello, Biancamaria Pierri, Marcello Salvi, Lucia Vassallo

EPI_ISL_837341, EPI_ISL_837343, EPI_ISL_837344, EPI_ISL_837346, EPI_ISL_837347, EPI_ISL_837349, EPI_ISL_837351, EPI_ISL_837352, EPI_ISL_837354, EPI_ISL_837355, EPI_ISL_837356, EPI_ISL_837357, EPI_ISL_837358, EPI_ISL_837359, EPI_ISL_837360, EPI_ISL_837361, EPI_ISL_837362, EPI_ISL_837363, EPI_ISL_837364, EPI_ISL_837365, EPI_ISL_837366, EPI_ISL_837367, EPI_ISL_837368, EPI_ISL_837369, EPI_ISL_837370, EPI_ISL_837371, EPI_ISL_837372, EPI_ISL_837373, EPI_ISL_837374, EPI_ISL_837375, EPI_ISL_837376, EPI_ISL_837377, EPI_ISL_837378, EPI_ISL_837379, EPI_ISL_837380, EPI_ISL_837381,

EPI_ISL_837384, EPI_ISL_837385, EPI_ISL_837386, EPI_ISL_837388, EPI_ISL_837389, EPI_ISL_837390, EPI_ISL_837391, EPI_ISL_837392, EPI_ISL_837393, EPI_ISL_837396, EPI_ISL_837397, EPI_ISL_837398, EPI_ISL_837399, EPI_ISL_837400, EPI_ISL_837401, EPI_ISL_837402, EPI_ISL_837403, EPI_ISL_837404, EPI_ISL_837405, EPI_ISL_837406, EPI_ISL_837407, EPI_ISL_837408, EPI_ISL_837409, EPI_ISL_837410, EPI_ISL_837411, EPI_ISL_837412, EPI_ISL_837413, EPI_ISL_837414, EPI_ISL_837415, EPI_ISL_837417, EPI_ISL_837418, EPI_ISL_837419, EPI_ISL_837420, EPI_ISL_837421, EPI_ISL_837422, EPI_ISL_837423, EPI_ISL_837427, EPI_ISL_837428, EPI_ISL_837429, EPI_ISL_837431			
see above	National Virus Reference Laboratory	National Virus Reference Laboratory	Michael Carr, Gabriel Gonzalez, Jonathan Dean, Cillian F De Gascun
EPI_ISL_837448, EPI_ISL_837483, EPI_ISL_837484, EPI_ISL_837485	Istituto Zooprofilattico Sperimentale del Mezzogiorno	TIGEM	Patrizia Annunziata, Andrea Ballabio, Valentina Bouche, Davide Cacchiarelli (CorrespAuthor), Pellegrino Cerino, Chiara Colantuono, Lucio Di Filippo, Antonio Grimaldi, Antonio Limone, Gabriella Loconte, Anna Manfredi, Francesco Panariello, Biancamaria Pierri, Marcello Salvi, Lucia Vassallo
EPI_ISL_837518, EPI_ISL_837521, EPI_ISL_837522, EPI_ISL_837546	UW Virology Lab	UW Virology Lab	Pavitra Roychoudhury, Hong Xie, Lasata Shrestha, Meei-Li Huang, Keith R Jerome, Alexander Greninger
EPI_ISL_837829, EPI_ISL_837830, EPI_ISL_837832	Wyoming Public Health Laboratory	Wyoming Public Health Laboratory	Noah Hull, Taylor Fearing, Lynette Gumbleton, Channing Weber, Ashley Norberg, Bailey Bowcutt, and Wanda Manley
EPI_ISL_837879, EPI_ISL_837889, EPI_ISL_837891, EPI_ISL_837892, EPI_ISL_837893, EPI_ISL_837895, EPI_ISL_837898, EPI_ISL_837901	Department of Pathology, University of Cambridge	COVID-19 Genomics UK (COG-UK) Consortium	Aminu S. Jahun, Yasmin Chaudhry, Grant Hall, Iliana Georgana, Myra Hosmillo, Martin D. Curran, Malte Pinckert, Surendra Parmar, Ian Goodfellow
EPI_ISL_838185, EPI_ISL_838188, EPI_ISL_838189, EPI_ISL_838190, EPI_ISL_838191, EPI_ISL_838192, EPI_ISL_838193, EPI_ISL_838194, EPI_ISL_838195, EPI_ISL_838196, EPI_ISL_838197			
see above	West of Scotland Specialist Virology Centre, NHSGGC / MRC-University of Glasgow Centre for Virus Research	COVID-19 Genomics UK (COG-UK) Consortium	Ana da Silva Filipe, Natasha Johnson, Kathy Smollett, Daniel Mair, Stephen Carmichael, Alice Broos, Lily Tong, Jenna Nichols, Kyriaki Nomikou; Sarah McDonald; Richard Orton, Joseph Hughes, Sreenu Vattipally, David L Robertson; Alasdair MacLean, Rory Gunson; Sharif Shaaban, Matthew Holden; Rachel Blacow, Guy Mollett, Kathy Li, James Shepherd, Antonia Ho, Emma Thomson
EPI_ISL_838765, EPI_ISL_838766, EPI_ISL_838772, EPI_ISL_838774, EPI_ISL_838779, EPI_ISL_838780, EPI_ISL_838781, EPI_ISL_838782, EPI_ISL_838784, EPI_ISL_838785, EPI_ISL_838786, EPI_ISL_838787, EPI_ISL_838788, EPI_ISL_838789, EPI_ISL_838790, EPI_ISL_838791, EPI_ISL_838792, EPI_ISL_838793, EPI_ISL_838794, EPI_ISL_838795, EPI_ISL_838796, EPI_ISL_838797, EPI_ISL_839311, EPI_ISL_839313, EPI_ISL_839314, EPI_ISL_839324, EPI_ISL_839325, EPI_ISL_839326, EPI_ISL_839327, EPI_ISL_839328			
see above	University College London, Great Ormond Street Hospital for Children NHS Foundation Trust, Imperial College Healthcare NHS Trust	COVID-19 Genomics UK (COG-UK) Consortium	Sergi Castellano, Rachel Williams, Mark Kristiansen, Paola Resende Silva, Sunando Roy, Tony Brooks, Helena Tutill, Paola Niola, Patricia Dyal, Charlotte Williams, Leysa Forrest, Yasmin Panchbhaya, Jacqueline Findlay, Samuel Weeks, Julianne Brown, Kathryn Harris, Paul Randell, James Price, Alison Holmes, Judith Breuer
EPI_ISL_839668, EPI_ISL_839669, EPI_ISL_839670, EPI_ISL_839671, EPI_ISL_839673, EPI_ISL_839674, EPI_ISL_839675, EPI_ISL_839677, EPI_ISL_839678, EPI_ISL_839679, EPI_ISL_839680, EPI_ISL_839681, EPI_ISL_839683, EPI_ISL_839684, EPI_ISL_839686, EPI_ISL_839687, EPI_ISL_839688, EPI_ISL_839689, EPI_ISL_839690, EPI_ISL_839691, EPI_ISL_839692, EPI_ISL_839693, EPI_ISL_839694, EPI_ISL_839695, EPI_ISL_839696, EPI_ISL_839697, EPI_ISL_839698, EPI_ISL_839701, EPI_ISL_839702, EPI_ISL_839705, EPI_ISL_839706, EPI_ISL_839707, EPI_ISL_839708			
see above	Northumbria University / South Tees Hospitals NHS Foundation Trust / North Cumbria Integrated Care NHS Foundation Trust / North Tees and Hartlepool NHS Foundation Trust / Newcastle Hospitals NHS Foundation Trust	COVID-19 Genomics UK (COG-UK) Consortium	Darren L Smith,Andrew Nelson,Matthew Bashton,Greg R Young,Joshua Loh,John Allan,Mohammad A Tariq,Giles S Holt,Gary Black,Wen C Yew,Lynn Dover,Paul Baker,Steve Liggett,Sarah Essex,Jane Greenaway,Debra Padgett,Clive Graham,Garren Scott,Edward Barton,Emma Swindells,Brendan Payne,Jennifer Collins,Yusri Taha,Gary Eltringham
EPI_ISL_839992, EPI_ISL_839994	Queens Medical Centre, Clinical Microbiology Department / DeepSeq Nottingham	COVID-19 Genomics UK (COG-UK) Consortium	Gemma Clark, Wendy Smith, Manjinder Khakh, Vicki M Fleming, Michelle M Lister, Hannah Howson-Wells, Jonathan Ball, Patrick McClure, Joseph Chappell, Theocharis Tsoleridis, Nadine Holmes, Matthew Carlisle, Christopher Moore, Fei Sang, Johnny Debebe, Victoria Wright, Matthew Loose
EPI_ISL_840104, EPI_ISL_840105, EPI_ISL_840106, EPI_ISL_840107, EPI_ISL_840111	Lincolnshire Hospitals and DeepSeq Nottingham	COVID-19 Genomics UK (COG-UK) Consortium	Nichola Duckworth, Tim Sloan, Sarah Walsh, Jonathan Ball, Patrick McClure, Joeseph Chappell, Nadine Holmes, Matthew Carlisle, Christopher Moore, Fei Sang, Johnny Debebe, Victoria Wright, Matthew Loose
EPI_ISL_840332, EPI_ISL_840333, EPI_ISL_840334, EPI_ISL_840335, EPI_ISL_840336, EPI_ISL_840338, EPI_ISL_840343, EPI_ISL_840344, EPI_ISL_840345, EPI_ISL_840348	Oxford Viromics, NDM, University of Oxford; Oxford University Hospitals; Basingstoke and North Hampshire Hospital	COVID-19 Genomics UK (COG-UK) Consortium	Tanya Golubchik, David Bonsall, George Macintyre, Amy Trebes, Mariateresa de Cesare, Catrin Moore, Alex Mobbs, Anita Justice, Robert Shaw, Monique Andersson, Timothy Peto, Emma Wise, Nathan Moore, Jessica Lynch, Nick Cortes, Matilde Mori, Stephen Kidd, David Buck, John Todd, Christophe Fraser
EPI_ISL_840370, EPI_ISL_840387, EPI_ISL_840388, EPI_ISL_840389, EPI_ISL_840513, EPI_ISL_840514, EPI_ISL_840519, EPI_ISL_840520, EPI_ISL_840521, EPI_ISL_840522, EPI_ISL_840523, EPI_ISL_840524, EPI_ISL_840525, EPI_ISL_840526, EPI_ISL_840527, EPI_ISL_840528, EPI_ISL_840529, EPI_ISL_840530, EPI_ISL_840531, EPI_ISL_840532, EPI_ISL_840533, EPI_ISL_840534, EPI_ISL_840535, EPI_ISL_840536, EPI_ISL_840537, EPI_ISL_840538, EPI_ISL_840539, EPI_ISL_840540, EPI_ISL_840541, EPI_ISL_840542, EPI_ISL_840543, EPI_ISL_840544, EPI_ISL_840545, EPI_ISL_840546, EPI_ISL_840547, EPI_ISL_840548, EPI_ISL_840549, EPI_ISL_840550, EPI_ISL_840551, EPI_ISL_840553, EPI_ISL_840555, EPI_ISL_840556, EPI_ISL_840557, EPI_ISL_840558, EPI_ISL_840559, EPI_ISL_840560, EPI_ISL_840561, EPI_ISL_840562, EPI_ISL_840563, EPI_ISL_840564, EPI_ISL_840565, EPI_ISL_840566, EPI_ISL_840567, EPI_ISL_840568, EPI_ISL_840569, EPI_ISL_840571, EPI_ISL_840572, EPI_ISL_840573, EPI_ISL_840574, EPI_ISL_840575, EPI_ISL_840576, EPI_ISL_840577, EPI_ISL_840578, EPI_ISL_840581, EPI_ISL_840582, EPI_ISL_840583, EPI_ISL_840585, EPI_ISL_840587, EPI_ISL_840588, EPI_ISL_840589, EPI_ISL_840591, EPI_ISL_840592, EPI_ISL_840593, EPI_ISL_840594, EPI_ISL_840595, EPI_ISL_840599			
see above	Originating lab: Wales Specialist Virology Centre Sequencing lab: Pathogen Genomics Unit	Public Health Wales Microbiology Cardiff Wales Specialist Virology Centre	Catherine Moore, Johnathan Evans, Laura Gifford, Malorie Perry, Simon Cottrell, Angela Marchbank, Alec Birchley, Alexander Adams, Amy Gaskin, Bree Gatica-Wilcox, Jason Coombes, Joel Southgate, Lauren Gilbert, Lee Graham, Nicole Pacchiarini, Sara Kumziene-Summerhayes, Sarah Taylor, Sophie Jones, Sara Rey, Matthew Bull, Joanne Watkins, Sally Corden, Tom Connor
EPI_ISL_842010, EPI_ISL_842013	Centre for Enzyme Innovation, University of Portsmouth / Translational Research Laboratory, Portsmouth Hospitals NHS Trust	COVID-19 Genomics UK (COG-UK) Consortium	Angela Beckett,Yann Bourgeois,Garry Scarlett,Sharon Glaysher,Scott Elliott,Kelly Bicknell,Robert Impey,Allyson Lloyd,Sarah Wyllie,Ethan Butcher,Anoop Chauhan,Samuel Robson
EPI_ISL_842230, EPI_ISL_842270, EPI_ISL_842297, EPI_ISL_842325	Virology Department, Sheffield Teaching Hospitals NHS Foundation Trust/Department of Infection, Immunity and Cardiovascular Disease, The Medical School, University of Sheffield	COVID-19 Genomics UK (COG-UK) Consortium	Thushan de Silva, Matthew Parker, Nikki Smith, Adri Angyal, Rebecca Brown, Luke Green, Rachel Tucker, Paul Parsons, Danielle Groves, Katie Johnson, Laura Carrilero, Alex Keeley, Dave Partridge, Matthew Wyles, Benjamin Lindsey, Mehmet Yavuz, Mohammad Raza, Cariad Evans
EPI_ISL_842609, EPI_ISL_842613, EPI_ISL_842616, EPI_ISL_842620, EPI_ISL_842621, EPI_ISL_842622, EPI_ISL_842629, EPI_ISL_842630	Wyoming Public Health Laboratory	Wyoming Public Health Laboratory	Noah Hull, Taylor Fearing, Lynette Gumbleton, Channing Weber, Ashley Norberg, Bailey Bowcutt, and Wanda Manley
EPI_ISL_842643	Department of Medical Microbiology, St. Olavs hospital	Norwegian Institute of Public Health, Department of Virology	Kathrine Stene-Johansen, Kamilla Heddeland Instefjord, Hilde Elshaug, Atiya R Ali,Marie Paulsen Madsen, Rasmus Riis Kopperud, Hilde Vollan, Karoline Bragstad, Olav Hungnes
EPI_ISL_843144, EPI_ISL_843145	Barts Health NHS Trust	COVID-19 Genomics UK (COG-UK) Consortium	CUTINO-MOGUEL, Maria-Teresa; HARRINGTON, David; OWOYEMI, Dola; SHYLINI, Raghavendran; BROAD, Claire; KELE, Beatrix
EPI_ISL_843151, EPI_ISL_843152, EPI_ISL_843153, EPI_ISL_843154, EPI_ISL_843163	Regional Virus Laboratory, Belfast Health and Social Care Trust	COVID-19 Genomics UK (COG-UK) Consortium	Conall McCaughey, James McKenna, Tanya Curran, Susan Feeney, Alison Watt, Ciara Cox, Mairead Connor, Zoltan Molnar, David Simpson, Derek Fairley
EPI_ISL_843213, EPI_ISL_843216, EPI_ISL_843270, EPI_ISL_843302, EPI_ISL_843316, EPI_ISL_843341, EPI_ISL_843360, EPI_ISL_843370, EPI_ISL_843384, EPI_ISL_843547, EPI_ISL_843574, EPI_ISL_843591, EPI_ISL_843595, EPI_ISL_843598, EPI_ISL_843608, EPI_ISL_843615, EPI_ISL_843616, EPI_ISL_843617, EPI_ISL_843618, EPI_ISL_843619, EPI_ISL_843620, EPI_ISL_843621, EPI_ISL_843622, EPI_ISL_843623, EPI_ISL_843624, EPI_ISL_843625, EPI_ISL_843626, EPI_ISL_843627, EPI_ISL_843628, EPI_ISL_843629, EPI_ISL_843630, EPI_ISL_843631, EPI_ISL_843632, EPI_ISL_843633, EPI_ISL_843634, EPI_ISL_843635, EPI_ISL_843636, EPI_ISL_843637, EPI_ISL_843638, EPI_ISL_843639, EPI_ISL_843640, EPI_ISL_843641, EPI_ISL_843642, EPI_ISL_843643, EPI_ISL_843644, EPI_ISL_843645, EPI_ISL_843646, EPI_ISL_843647, EPI_ISL_843648, EPI_ISL_843649, EPI_ISL_843650, EPI_ISL_843651, EPI_ISL_843652, EPI_ISL_843653, EPI_ISL_843654, EPI_ISL_843655, EPI_ISL_843656, EPI_ISL_843657, EPI_ISL_843658, EPI_ISL_843659, EPI_ISL_843660, EPI_ISL_843661, EPI_ISL_843662, EPI_ISL_843663, EPI_ISL_843664, EPI_ISL_843665, EPI_ISL_843666, EPI_ISL_843667, EPI_ISL_843668, EPI_ISL_843669, EPI_ISL_843670, EPI_ISL_843671, EPI_ISL_843674, EPI_ISL_843675, EPI_ISL_843676, EPI_ISL_843677, EPI_ISL_843678, EPI_ISL_843679, EPI_ISL_843680, EPI_ISL_843681, EPI_ISL_843682, EPI_ISL_843683, EPI_ISL_843684, EPI_ISL_843685, EPI_ISL_843686, EPI_ISL_843687, EPI_ISL_843688, EPI_ISL_843689, EPI_ISL_843690, EPI_ISL_843691, EPI_ISL_843692, EPI_ISL_843693, EPI_ISL_843694, EPI_ISL_843695, EPI_ISL_843696, EPI_ISL_843697, EPI_ISL_843698, EPI_ISL_843699, EPI_ISL_843701, EPI_ISL_843702, EPI_ISL_843703, EPI_ISL_843704, EPI_ISL_843705, EPI_ISL_843706, EPI_ISL_843707, EPI_ISL_843708, EPI_ISL_843709, EPI_ISL_843710, EPI_ISL_843711, EPI_ISL_843712, EPI_ISL_843713, EPI_ISL_843714, EPI_ISL_843715, EPI_ISL_843716, EPI_ISL_843717, EPI_ISL_843718, EPI_ISL_843719, EPI_ISL_843720, EPI_ISL_843721, EPI_ISL_843722, EPI_ISL_843723, EPI_ISL_843724, EPI_ISL_843725, EPI_ISL_843726, EPI_ISL_843727, EPI_ISL_843728, EPI_ISL_843729, EPI_ISL_843730, EPI_ISL_843731, EPI_ISL_843732, EPI_ISL_843733, EPI_ISL_843734, EPI_ISL_843735, EPI_ISL_843736, EPI_ISL_843737, EPI_ISL_843738, EPI_ISL_843739, EPI_ISL_843740, EPI_ISL_843741, EPI_ISL_843742, EPI_ISL_843743, EPI_ISL_843744, EPI_ISL_843745, EPI_ISL_843746, EPI_ISL_843747, EPI_ISL_843748, EPI_ISL_843749, EPI_ISL_843750, EPI_ISL_843751, EPI_ISL_843752, EPI_ISL_843753, EPI_ISL_843754, EPI_ISL_843755, EPI_ISL_843756, EPI_ISL_843757, EPI_ISL_843758, EPI_ISL_843759, EPI_ISL_843760, EPI_ISL_843761, EPI_ISL_843762, EPI_ISL_843763, EPI_ISL_843764, EPI_ISL_843765, EPI_ISL_843766, EPI_ISL_843767, EPI_ISL_843768, EPI_ISL_843769, EPI_ISL_843770, EPI_ISL_843771, EPI_ISL_843772, EPI_ISL_843773, EPI_ISL_843774, EPI_ISL_843775, EPI_ISL_843776, EPI_ISL_843777, EPI_ISL_843778, EPI_ISL_843779, EPI_ISL_843780, EPI_ISL_843781, EPI_ISL_843782, EPI_ISL_843783, EPI_ISL_843784, EPI_ISL_843785, EPI_ISL_843786, EPI_ISL_843787, EPI_ISL_843788, EPI_ISL_843790, EPI_ISL_843791, EPI_ISL_843792, EPI_ISL_843793, EPI_ISL_843794, EPI_ISL_843795, EPI_ISL_843796, EPI_ISL_843797, EPI_ISL_843798, EPI_ISL_843799, EPI_ISL_843800, EPI_ISL_843801, EPI_ISL_843802, EPI_ISL_843804, EPI_ISL_843805, EPI_ISL_843806, EPI_ISL_843807, EPI_ISL_843808, EPI_ISL_843809, EPI_ISL_843810, EPI_ISL_843811, EPI_ISL_843812, EPI_ISL_843813, EPI_ISL_843814, EPI_ISL_843815, EPI_ISL_843816, EPI_ISL_843817, EPI_ISL_843818, EPI_ISL_843819, EPI_ISL_843820,			

EPI_ISL_843821, EPI_ISL_843822, EPI_ISL_843823, EPI_ISL_843824, EPI_ISL_843825, EPI_ISL_843826, EPI_ISL_843827, EPI_ISL_843828, EPI_ISL_843829, EPI_ISL_843830, EPI_ISL_843831, EPI_ISL_843832, EPI_ISL_843833, EPI_ISL_843834, EPI_ISL_843835, EPI_ISL_843836, EPI_ISL_843837, EPI_ISL_843838, EPI_ISL_843839, EPI_ISL_843840, EPI_ISL_843841, EPI_ISL_843842, EPI_ISL_843843, EPI_ISL_843844, EPI_ISL_843845, EPI_ISL_843846, EPI_ISL_843847, EPI_ISL_843848, EPI_ISL_843849, EPI_ISL_843850, EPI_ISL_843851, EPI_ISL_843852, EPI_ISL_843853, EPI_ISL_843854, EPI_ISL_843855, EPI_ISL_843856, EPI_ISL_843857, EPI_ISL_843858, EPI_ISL_843859, EPI_ISL_843860, EPI_ISL_843861, EPI_ISL_843862, EPI_ISL_843863, EPI_ISL_843864, EPI_ISL_843865, EPI_ISL_843866, EPI_ISL_843867, EPI_ISL_843868, EPI_ISL_843869, EPI_ISL_843870, EPI_ISL_843871, EPI_ISL_843872, EPI_ISL_843873, EPI_ISL_843874, EPI_ISL_843875, EPI_ISL_843876, EPI_ISL_843877, EPI_ISL_843878, EPI_ISL_843879, EPI_ISL_843880, EPI_ISL_843881, EPI_ISL_843882, EPI_ISL_843883, EPI_ISL_843884, EPI_ISL_843885, EPI_ISL_843886, EPI_ISL_843887, EPI_ISL_843888, EPI_ISL_843889, EPI_ISL_843890, EPI_ISL_843891, EPI_ISL_843892, EPI_ISL_843893, EPI_ISL_843894, EPI_ISL_843895, EPI_ISL_843896, EPI_ISL_843897, EPI_ISL_843898, EPI_ISL_843899, EPI_ISL_843900, EPI_ISL_843901, EPI_ISL_843902, EPI_ISL_843903, EPI_ISL_843904, EPI_ISL_843905, EPI_ISL_843906, EPI_ISL_843907, EPI_ISL_843908, EPI_ISL_843909, EPI_ISL_843910, EPI_ISL_843911, EPI_ISL_843913, EPI_ISL_843914, EPI_ISL_843915, EPI_ISL_843916, EPI_ISL_843917, EPI_ISL_843918, EPI_ISL_843919, EPI_ISL_843920, EPI_ISL_843921, EPI_ISL_843922, EPI_ISL_843923, EPI_ISL_843924, EPI_ISL_843926, EPI_ISL_843927, EPI_ISL_843928, EPI_ISL_843929, EPI_ISL_843930, EPI_ISL_843931, EPI_ISL_843932, EPI_ISL_843933, EPI_ISL_843934, EPI_ISL_843935, EPI_ISL_843936, EPI_ISL_843937, EPI_ISL_843938, EPI_ISL_843939, EPI_ISL_843940, EPI_ISL_843941, EPI_ISL_843942, EPI_ISL_843943, EPI_ISL_843944, EPI_ISL_843945, EPI_ISL_843946, EPI_ISL_843947, EPI_ISL_843948, EPI_ISL_843949, EPI_ISL_843950, EPI_ISL_843951, EPI_ISL_843952, EPI_ISL_843953, EPI_ISL_843954, EPI_ISL_843955, EPI_ISL_843956, EPI_ISL_843957, EPI_ISL_843958, EPI_ISL_843959, EPI_ISL_843960, EPI_ISL_843961, EPI_ISL_843962, EPI_ISL_843963, EPI_ISL_843964, EPI_ISL_843965, EPI_ISL_843966, EPI_ISL_843967, EPI_ISL_843968, EPI_ISL_843969, EPI_ISL_843970, EPI_ISL_843971, EPI_ISL_843972, EPI_ISL_843974, EPI_ISL_843975, EPI_ISL_843976, EPI_ISL_843977, EPI_ISL_843978, EPI_ISL_843979, EPI_ISL_843980, EPI_ISL_843981, EPI_ISL_843982, EPI_ISL_843983, EPI_ISL_843984, EPI_ISL_843985, EPI_ISL_843986, EPI_ISL_843987, EPI_ISL_843988, EPI_ISL_843989, EPI_ISL_843990, EPI_ISL_843991, EPI_ISL_843992, EPI_ISL_843993, EPI_ISL_843994, EPI_ISL_843995, EPI_ISL_843996, EPI_ISL_843997, EPI_ISL_843998, EPI_ISL_843999, EPI_ISL_844001, EPI_ISL_844002, EPI_ISL_844003, EPI_ISL_844004, EPI_ISL_844005, EPI_ISL_844006, EPI_ISL_844007, EPI_ISL_844009, EPI_ISL_844010, EPI_ISL_844011, EPI_ISL_844012, EPI_ISL_844013, EPI_ISL_844014, EPI_ISL_844016, EPI_ISL_844017, EPI_ISL_844018, EPI_ISL_844019, EPI_ISL_844020, EPI_ISL_844021, EPI_ISL_844022, EPI_ISL_844023, EPI_ISL_844024, EPI_ISL_844025, EPI_ISL_844027, EPI_ISL_844028, EPI_ISL_844029, EPI_ISL_844030, EPI_ISL_844031, EPI_ISL_844032, EPI_ISL_844033, EPI_ISL_844034, EPI_ISL_844035, EPI_ISL_844036, EPI_ISL_844037, EPI_ISL_844038, EPI_ISL_844039, EPI_ISL_844040, EPI_ISL_844041, EPI_ISL_844042, EPI_ISL_844043, EPI_ISL_844044, EPI_ISL_844045, EPI_ISL_844046, EPI_ISL_844047, EPI_ISL_844048, EPI_ISL_844049, EPI_ISL_844050, EPI_ISL_844051, EPI_ISL_844052, EPI_ISL_844053, EPI_ISL_844054, EPI_ISL_844055, EPI_ISL_844056, EPI_ISL_844057, EPI_ISL_844058, EPI_ISL_844059, EPI_ISL_844060, EPI_ISL_844061, EPI_ISL_844062, EPI_ISL_844063, EPI_ISL_844064, EPI_ISL_844065, EPI_ISL_844066, EPI_ISL_844068, EPI_ISL_844069, EPI_ISL_844070, EPI_ISL_844071, EPI_ISL_844072, EPI_ISL_844073, EPI_ISL_844074, EPI_ISL_844075, EPI_ISL_844076, EPI_ISL_844077, EPI_ISL_844078, EPI_ISL_844079, EPI_ISL_844080, EPI_ISL_844081, EPI_ISL_844082, EPI_ISL_844083, EPI_ISL_844084, EPI_ISL_844085, EPI_ISL_844086, EPI_ISL_844087, EPI_ISL_844088, EPI_ISL_844089, EPI_ISL_844090, EPI_ISL_844091, EPI_ISL_844092, EPI_ISL_844093, EPI_ISL_844094, EPI_ISL_844095, EPI_ISL_844096, EPI_ISL_844097, EPI_ISL_844098, EPI_ISL_844099, EPI_ISL_844100, EPI_ISL_844101, EPI_ISL_844102, EPI_ISL_844103, EPI_ISL_844104, EPI_ISL_844105, EPI_ISL_844106, EPI_ISL_844107, EPI_ISL_844109, EPI_ISL_844110, EPI_ISL_844111, EPI_ISL_844112, EPI_ISL_844114, EPI_ISL_844115, EPI_ISL_844116, EPI_ISL_844117, EPI_ISL_844118, EPI_ISL_844119, EPI_ISL_844121, EPI_ISL_844122, EPI_ISL_844123, EPI_ISL_844124, EPI_ISL_844125, EPI_ISL_844126, EPI_ISL_844127, EPI_ISL_844128, EPI_ISL_844130, EPI_ISL_844131, EPI_ISL_844132, EPI_ISL_844133, EPI_ISL_844134, EPI_ISL_844135, EPI_ISL_844136, EPI_ISL_844137, EPI_ISL_844138, EPI_ISL_844139, EPI_ISL_844140, EPI_ISL_844141, EPI_ISL_844142, EPI_ISL_844143, EPI_ISL_844144, EPI_ISL_844190, EPI_ISL_844196			
see above	Department of Virus and Microbiological Special Diagnostics, Statens Serum Institut, Copenhagen, Denmark	Albertsen Lab, Department of Chemistry and Bioscience, Aalborg University, Denmark	Danish Covid-19 Genome Consortium
EPI_ISL_845592, EPI_ISL_845594, EPI_ISL_845596, EPI_ISL_845604	KU Leuven, Rega Institute, Clinical and Epidemiological Virology	KU Leuven, Rega Institute, Clinical and Epidemiological Virology	Tony Wawina-Bokalanga, Bert Vanmechelen, Joan Marti-Carerras, Piet Maes
EPI_ISL_845620	compensar calle 63	Instituto Nacional de Salud - Dirección de Investigación en Salud Pública	Katherine Laiton-Donato, Diego A. Álvarez-Díaz, Carlos Franco-Muñoz, Mauricio Pacheco-Montealegre, Maria T. Herrera-Sepúlveda, Jonathan Reales, Sheryll Corchuelo, Julian Naizaque, Gerardo Santamaría, Paola Muñoz-Laiton, Diego Andrés Prada, Magdalena Wiesner, Martha Lucia Ospina Martinez, Marcela Mercado-Reyes
EPI_ISL_845622	Dirección de Sanidad Ejército	Instituto Nacional de Salud - Dirección de Investigación en Salud Pública	Katherine Laiton-Donato, Diego A. Álvarez-Díaz, Carlos Franco-Muñoz, Mauricio Pacheco-Montealegre, Maria T. Herrera-Sepúlveda, Jonathan Reales, Sheryll Corchuelo, Julian Naizaque, Gerardo Santamaría, Paola Muñoz-Laiton, Diego Andrés Prada, Magdalena Wiesner, Martha Lucia Ospina Martinez, Marcela Mercado-Reyes
EPI_ISL_845630, EPI_ISL_845631, EPI_ISL_845632, EPI_ISL_845633	IDIME S.A	Instituto Nacional de Salud - Dirección de Investigación en Salud Pública	Katherine Laiton-Donato, Diego A. Álvarez-Díaz, Carlos Franco-Muñoz, Mauricio Pacheco-Montealegre, Maria T. Herrera-Sepúlveda, Jonathan Reales, Sheryll Corchuelo, Julian Naizaque, Gerardo Santamaría, Paola Muñoz-Laiton, Diego Andrés Prada, Magdalena Wiesner, Martha Lucia Ospina Martinez, Marcela Mercado-Reyes
EPI_ISL_845650	Laboratorio de Salud Pública - Secretaría Distrital de Salud	Instituto Nacional de Salud - Dirección de Investigación en Salud Pública	Katherine Laiton-Donato, Diego A. Álvarez-Díaz, Carlos Franco-Muñoz, Mauricio Pacheco-Montealegre, Maria T. Herrera-Sepúlveda, Jonathan Reales, Sheryll Corchuelo, Julian Naizaque, Gerardo Santamaría, Paola Muñoz-Laiton, Diego Andrés Prada, Magdalena Wiesner, Martha Lucia Ospina Martinez, Marcela Mercado-Reyes
EPI_ISL_845850, EPI_ISL_845851, EPI_ISL_845852, EPI_ISL_845854, EPI_ISL_845855, EPI_ISL_845856, EPI_ISL_845858, EPI_ISL_845859, EPI_ISL_845860	TGen North	TGen North	Jolene Bowers, Megan Folkerts, Chris French, Hayley Yaglom, Ashlyn Pfeiffer, Darrin Lemmer, Dave Engelthaler, The Arizona COVID Genomics Union (ACGU)
EPI_ISL_845874, EPI_ISL_845875, EPI_ISL_845876, EPI_ISL_845883, EPI_ISL_845884, EPI_ISL_845889	BBMP Urban PHC	Department of Neurovirology, National Institute of Mental Health and Neurosciences (NIMHANS)	Chitra Pattabiraman, Pramada Prasad, Risha Rasheed, Darshan Sreenivas, Nakka Vijay Kiran Reddy, Anita S Desai, V Ravi
EPI_ISL_846818, EPI_ISL_846819, EPI_ISL_846851, EPI_ISL_846852, EPI_ISL_846861, EPI_ISL_846866, EPI_ISL_846894, EPI_ISL_846895, EPI_ISL_846913, EPI_ISL_847168, EPI_ISL_847169, EPI_ISL_847170, EPI_ISL_847171, EPI_ISL_847172, EPI_ISL_847173, EPI_ISL_847174, EPI_ISL_847175, EPI_ISL_847176, EPI_ISL_847320, EPI_ISL_847348, EPI_ISL_847349, EPI_ISL_847368, EPI_ISL_847369, EPI_ISL_847370, EPI_ISL_847510, EPI_ISL_847511, EPI_ISL_847512, EPI_ISL_847513			
see above	Department of Virus and Microbiological Special Diagnostics, Statens Serum Institut, Copenhagen, Denmark	Albertsen Lab, Department of Chemistry and Bioscience, Aalborg University, Denmark	Danish Covid-19 Genome Consortium
EPI_ISL_847532	California Department of Public Health	Chiu Laboratory, University of California, San Francisco	Charles Chiu, Xianding (Wayne) Deng, Candace Wang, Brian Bushnell, Scot Federman, Jill Hacker, Debra Wadford
EPI_ISL_847536	Chiu Laboratory, University of California, San Francisco	Chiu Laboratory, University of California, San Francisco	Charles Chiu, Xianding (Wayne) Deng, Candace Wang, Brian Bushnell, Scot Federman, Jill Hacker, Debra Wadford
EPI_ISL_847572, EPI_ISL_847574, EPI_ISL_847601, EPI_ISL_847602, EPI_ISL_847603, EPI_ISL_847604, EPI_ISL_847605, EPI_ISL_847606, EPI_ISL_847607	California Department of Public Health	Chiu Laboratory, University of California, San Francisco	Charles Chiu, Xianding (Wayne) Deng, Candace Wang, Brian Bushnell, Scot Federman, Jill Hacker, Debra Wadford
EPI_ISL_847736, EPI_ISL_847741	Chiu Laboratory, University of California, San Francisco	Chiu Laboratory, University of California, San Francisco	Charles Chiu, Xianding (Wayne) Deng, Candace Wang, Brian Bushnell, Scot Federman, Jill Hacker, Debra Wadford
EPI_ISL_847766, EPI_ISL_847767, EPI_ISL_847768, EPI_ISL_847769, EPI_ISL_847770, EPI_ISL_847771	California Department of Public Health	Chiu Laboratory, University of California, San Francisco	Charles Chiu, Xianding (Wayne) Deng, Candace Wang, Brian Bushnell, Scot Federman, Jill Hacker, Debra Wadford
EPI_ISL_847830	Tempus	Grubaugh Lab - Yale School of Public Health	Tara Alpert, Joseph Fauver, Anderson Brito, Mallery Breban, Anne Wyllie, Chantal Vogels, Mary Petrone, Chaney Kalinich, Isabel Ott, Nathan Grubaugh
EPI_ISL_847845, EPI_ISL_847848, EPI_ISL_847849, EPI_ISL_847890, EPI_ISL_847891, EPI_ISL_847892, EPI_ISL_847893, EPI_ISL_847894, EPI_ISL_847895, EPI_ISL_847896, EPI_ISL_847897, EPI_ISL_847898, EPI_ISL_847899, EPI_ISL_847900, EPI_ISL_847901, EPI_ISL_847925, EPI_ISL_847960, EPI_ISL_847961, EPI_ISL_847962, EPI_ISL_847963, EPI_ISL_847964, EPI_ISL_847965, EPI_ISL_847966, EPI_ISL_847967			
see above	University Hospitals of Geneva, Laboratory of Virology	HUG, Laboratory of Virology and the Health2030 Genome Center	Samuel Cordey, Ana Rita Goncalves, Laurent Kaiser, Lorenzo Cerutti, Henri Pegeot, Melyssa Elies, Keith Harshman, Ioannis Xenarios, Emmanouil Dermitzakis
EPI_ISL_847989, EPI_ISL_847993, EPI_ISL_848000, EPI_ISL_848002, EPI_ISL_848012	Michigan Department of Health and Human Services, Bureau of Laboratories	Michigan Department of Health and Human Services, Bureau of Laboratories	Blankenship HM, Riner D, Soehnlen MK
EPI_ISL_848063, EPI_ISL_848064	CHU Purpan - Laboratoire de Virologie - Institut Fédératif de Biologie	CHU Purpan - Laboratoire de Virologie - Institut Fédératif de Biologie	Latour J., Ranger N., Dubois M., Carcenac R., Harter A., Boyer P., Tremeaux P., Izopet J.
EPI_ISL_849783, EPI_ISL_849787, EPI_ISL_849793, EPI_ISL_849881, EPI_ISL_849889, EPI_ISL_849890, EPI_ISL_849891, EPI_ISL_849892, EPI_ISL_849893, EPI_ISL_849894, EPI_ISL_849896, EPI_ISL_849897, EPI_ISL_849898, EPI_ISL_849899			
see above	Utah Public Health Laboratory	Utah Public Health Laboratory	Erin L. Young, Kelly F. Oakeson, Tara Gallagher
EPI_ISL_849926	UCSF Clinical Microbiology Laboratory	Chan-Zuckerberg Biohub	CZB Cliahub Consortium
EPI_ISL_850657, EPI_ISL_850662	Division of Emerging Infectious Diseases, Bureau of Infectious Diseases Diagnosis Control, Korea Disease Control and Prevention Agency	Division of Emerging Infectious Diseases, Bureau of Infectious Diseases Diagnosis Control, Korea Disease Control and Prevention Agency	Ae Kyung Park, Il-Hwan Kim, Heui Man Kim, Jeong-Min Kim, Namjoo Lee, Chaeyoung Lee, Sang Hee Woo, Eun-Jin Kim
EPI_ISL_852961	Hospital General Universitario Gregorio Marañón	SeqCOVID-SPAIN consortium/IBV(CSIC)	Darío García de Viedma, Laura Pérez-Lago, Pedro J Sola-Campoy, Sergio Buenestado-Serrano, Marta Herranz, Victor Manuel de la Cueva, Julia Suárez, Pilar Catalán, Patricia Muñoz and SeqCOVID-SPAIN consortium

EPI_ISL_853373, EPI_ISL_853374	UPMC Clinical Microbiology Laboratory	Microbial Genome Sequencing Center; Microbial Genomic Epidemiology Laboratory	Mustapha M. Mustapha, Jane W. Marsh, Dan Snyder, Marissa P. Griffith, Stephanie L. Mitchell, Vatsala R. Srinivasa, Kady D. Waggle, Chinelo Ezeonwuku, Vaughn S. Cooper, Lee H. Harrison
EPI_ISL_853722, EPI_ISL_853743, EPI_ISL_853745, EPI_ISL_853956, EPI_ISL_854226	Department of Microbiology, University Innsbruck	Bergthaler laboratory, CeMM Research Center for Molecular Medicine of the Austrian Academy of Sciences	Lukas Endler, Alexandra Popa, Benedikt Agerer, Jakob-Wendelin Genger, Alexander Lercher, Anna Schedl, Thomas Penz, Michael Schuster, Jan Laine, Martin Senekowitsch, Christoph Bock, Andreas Bergthaler
EPI_ISL_854416, EPI_ISL_854417, EPI_ISL_854418, EPI_ISL_854419, EPI_ISL_854420	MONTEFIORE MEDICAL CENTER LABORATORIES	Wadsworth Center, New York State Department of Health	Kirsten St. George, Daryl M. Lamson, Alexis Russel, Matthew Shudt, Melissa A Leisner, Jonathan Plitnick, Navjot Singh, John Kelly, Erasmus Schneider, Erica Lasek-Nesselquist
EPI_ISL_854421	SARATOGA HOSPITAL LABORATORY	Wadsworth Center, New York State Department of Health	Kirsten St. George, Daryl M. Lamson, Alexis Russel, Matthew Shudt, Melissa A Leisner, Jonathan Plitnick, Navjot Singh, John Kelly, Erasmus Schneider, Erica Lasek-Nesselquist
EPI_ISL_854422, EPI_ISL_854423, EPI_ISL_854424, EPI_ISL_854425, EPI_ISL_854426, EPI_ISL_854453, EPI_ISL_854456, EPI_ISL_854459	MONTEFIORE MEDICAL CENTER LABORATORIES	Wadsworth Center, New York State Department of Health	Kirsten St. George, Daryl M. Lamson, Alexis Russel, Matthew Shudt, Melissa A Leisner, Jonathan Plitnick, Navjot Singh, John Kelly, Erasmus Schneider, Erica Lasek-Nesselquist
EPI_ISL_855016, EPI_ISL_855019, EPI_ISL_855023, EPI_ISL_855027, EPI_ISL_855030, EPI_ISL_855031, EPI_ISL_855033, EPI_ISL_855034, EPI_ISL_855037, EPI_ISL_855038, EPI_ISL_855039, EPI_ISL_855041, EPI_ISL_855046, EPI_ISL_855047, EPI_ISL_855049, EPI_ISL_855050, EPI_ISL_855052, EPI_ISL_855053, EPI_ISL_855054, EPI_ISL_855057, EPI_ISL_855059, EPI_ISL_855060, EPI_ISL_855061, EPI_ISL_855062, EPI_ISL_855063			
see above	Quest Diagnostics	Quest Diagnostics	Rosenthal,S.H., Gerasimova,A., Kagan,R.M., Anderson, B., Hua, M., Liu Y., Bernstein, L.E., Livingston, K.E., Perez, A., Shalhout, D.F., Shlyakhter, I.A., Owen, R., Tanpaiboon, P., Lacbawan, F.
EPI_ISL_855401	Chiu Laboratory, University of California, San Francisco	Chiu Laboratory, University of California, San Francisco	Charles Chiu, Xianding (Wayne) Deng, Candace Wang, Brian Bushnell, Scot Federman, Jill Hacker, Debra Wadford
EPI_ISL_855910, EPI_ISL_855912	Lab voor klinische biologie	Onderzoeksgroep Virologie	Laurens Lambrechts, Nick Vereecke, Marthe Pauwels, Bruno Verhasselt, Linos Vandekerckhove, Hans Nauwynck, Sebastiaan Theuns
EPI_ISL_856612, EPI_ISL_856613, EPI_ISL_856614, EPI_ISL_856615, EPI_ISL_856616, EPI_ISL_856617, EPI_ISL_856618, EPI_ISL_856619, EPI_ISL_856620, EPI_ISL_856621, EPI_ISL_856622, EPI_ISL_856623, EPI_ISL_856624, EPI_ISL_856625, EPI_ISL_856626, EPI_ISL_856627, EPI_ISL_856628, EPI_ISL_856629, EPI_ISL_856630, EPI_ISL_856631, EPI_ISL_856632, EPI_ISL_856633, EPI_ISL_856634, EPI_ISL_856635, EPI_ISL_856636, EPI_ISL_856637, EPI_ISL_856638, EPI_ISL_856639, EPI_ISL_856640, EPI_ISL_856641, EPI_ISL_856642, EPI_ISL_856643, EPI_ISL_856644, EPI_ISL_856645, EPI_ISL_856646			
see above	Department of Virus and Microbiological Special Diagnostics, Statens Serum Institut, Copenhagen, Denmark	Aalborg University	Danish Covid-19 Genome Consortium
EPI_ISL_856973, EPI_ISL_857040	Platform BIS UZA/UAntwerpen, University Hospital Antwerp, Edegem, Belgium	UAntwerp, Laboratory of Medical Microbiology, Campus Drie Eiken S6.26, Universiteitsplein 1, 2610, Wilrijk, Antwerp, Belgium	Basil Britto Xavier, Jasmine Coppens, Christine Lammens, Veerle Matheeussen, Herman Goossens
EPI_ISL_857047	Colorado Department of Public Health and Environment	Colorado Department of Puplic Health and Environment	Laura Bankers, Molly C. Hetherington-Rauth, Diana Ir, Shannon Ely, Shannon R. Matzinger, Sarah Elizabeth Totten, Emily A. Travanty
EPI_ISL_857187, EPI_ISL_857192	DOHMH PHL	New York City Public Health Laboratory	Jade Wang, et al.
EPI_ISL_857198	Department of Homeless Services	New York City Public Health Laboratory	Jade Wang, et al.
EPI_ISL_857279, EPI_ISL_857280	DOHMH Fort Greene	New York City Public Health Laboratory	Jade Wang, et al.
EPI_ISL_857281	DOHMH Riverside	New York City Public Health Laboratory	Jade Wang, et al.
EPI_ISL_857282, EPI_ISL_857283	DOHMH Morrisania	New York City Public Health Laboratory	Jade Wang, et al.
EPI_ISL_857284	DOHMH PHL	New York City Public Health Laboratory	Jade Wang, et al.
EPI_ISL_857285, EPI_ISL_857286	DOHMH Central Harlem	New York City Public Health Laboratory	Jade Wang, et al.
EPI_ISL_857287, EPI_ISL_857288	Department of Homeless Services	New York City Public Health Laboratory	Jade Wang, et al.
EPI_ISL_857289	OCME Office Of Chief Medical Examiner	New York City Public Health Laboratory	Jade Wang, et al.
EPI_ISL_857290	Department of Homeless Services	New York City Public Health Laboratory	Jade Wang, et al.
EPI_ISL_857515	Swiss National Reference Centre for Influenza	Swiss National Reference Centre for Influenza	Ana Rita Goncalves,Samuel Cordey, Laurent Kaiser, Lorenzo Cerutti, Henri Pegeot, Melyssa Elies, Keith Harshman, Ioannis Xenarios, Emmanouil Dermitzakis
EPI_ISL_858590, EPI_ISL_858597, EPI_ISL_858619	Lighthouse Lab in Glasgow	Wellcome Sanger Institute for the COVID-19 Genomics UK (COG-UK) Consortium	Harper VanSteenhouse, Yumi Kasai, David Gray, Carol Clugston, Anna Dominiczak and Alex Alderton, Roberto Amato, Sonia Goncalves, Ewan Harrison, David K. Jackson, Ian Johnston, Dominic Kwiatkowski, Cordelia Langford, John Sillitoe on behalf of the Wellcome Sanger Institute COVID-19 Surveillance Team
EPI_ISL_858701, EPI_ISL_858702, EPI_ISL_858704, EPI_ISL_858706, EPI_ISL_858707, EPI_ISL_858708, EPI_ISL_858709, EPI_ISL_858713, EPI_ISL_858714, EPI_ISL_858719, EPI_ISL_858720, EPI_ISL_858725, EPI_ISL_858732, EPI_ISL_858737, EPI_ISL_858739, EPI_ISL_858748, EPI_ISL_858752, EPI_ISL_858753			
see above	Lighthouse Lab in Alderley Park	Wellcome Sanger Institute for the COVID-19 Genomics UK (COG-UK) Consortium	Jacquelyn Wynn, Mairead Hyland, The Lighthouse Lab in Alderley Park and Alex Alderton, Roberto Amato, Sonia Goncalves, Ewan Harrison, David K. Jackson, Ian Johnston, Dominic Kwiatkowski, Cordelia Langford, John Sillitoe on behalf of the Wellcome Sanger Institute COVID-19 Surveillance Team
EPI_ISL_858778	Lighthouse Lab in Glasgow	Wellcome Sanger Institute for the COVID-19 Genomics UK (COG-UK) Consortium	Harper VanSteenhouse, Yumi Kasai, David Gray, Carol Clugston, Anna Dominiczak and Alex Alderton, Roberto Amato, Sonia Goncalves, Ewan Harrison, David K. Jackson, Ian Johnston, Dominic Kwiatkowski, Cordelia Langford, John Sillitoe on behalf of the Wellcome Sanger Institute COVID-19 Surveillance Team
EPI_ISL_858779, EPI_ISL_858780	Lighthouse Lab in Alderley Park	Wellcome Sanger Institute for the COVID-19 Genomics UK (COG-UK) Consortium	Jacquelyn Wynn, Mairead Hyland, The Lighthouse Lab in Alderley Park and Alex Alderton, Roberto Amato, Sonia Goncalves, Ewan Harrison, David K. Jackson, Ian Johnston, Dominic Kwiatkowski, Cordelia Langford, John Sillitoe on behalf of the Wellcome Sanger Institute COVID-19 Surveillance Team
EPI_ISL_858782	Lighthouse Lab in Glasgow	Wellcome Sanger Institute for the COVID-19 Genomics UK (COG-UK) Consortium	Harper VanSteenhouse, Yumi Kasai, David Gray, Carol Clugston, Anna Dominiczak and Alex Alderton, Roberto Amato, Sonia Goncalves, Ewan Harrison, David K. Jackson, Ian Johnston, Dominic Kwiatkowski, Cordelia Langford, John Sillitoe on behalf of the Wellcome Sanger Institute COVID-19 Surveillance Team
EPI_ISL_858784, EPI_ISL_858785, EPI_ISL_858786, EPI_ISL_858788, EPI_ISL_858789, EPI_ISL_858790, EPI_ISL_858791	Lighthouse Lab in Alderley Park	Wellcome Sanger Institute for the COVID-19 Genomics UK (COG-UK) Consortium	Jacquelyn Wynn, Mairead Hyland, The Lighthouse Lab in Alderley Park and Alex Alderton, Roberto Amato, Sonia Goncalves, Ewan Harrison, David K. Jackson, Ian Johnston, Dominic Kwiatkowski, Cordelia Langford, John Sillitoe on behalf of the Wellcome Sanger Institute COVID-19 Surveillance Team
EPI_ISL_858793, EPI_ISL_858794, EPI_ISL_858796, EPI_ISL_858797, EPI_ISL_858801, EPI_ISL_858802, EPI_ISL_858806	Lighthouse Lab in Glasgow	Wellcome Sanger Institute for the COVID-19 Genomics UK (COG-UK) Consortium	Harper VanSteenhouse, Yumi Kasai, David Gray, Carol Clugston, Anna Dominiczak and Alex Alderton, Roberto Amato, Sonia Goncalves, Ewan Harrison, David K. Jackson, Ian Johnston, Dominic Kwiatkowski, Cordelia Langford, John Sillitoe on behalf of the Wellcome Sanger Institute COVID-19 Surveillance Team
EPI_ISL_858809	Lighthouse Lab in Alderley Park	Wellcome Sanger Institute for the COVID-19 Genomics UK (COG-UK) Consortium	Jacquelyn Wynn, Mairead Hyland, The Lighthouse Lab in Alderley Park and Alex Alderton, Roberto Amato, Sonia Goncalves, Ewan Harrison, David K. Jackson, Ian Johnston, Dominic Kwiatkowski, Cordelia Langford, John Sillitoe on behalf of the Wellcome Sanger Institute COVID-19 Surveillance Team
EPI_ISL_858810	Lighthouse Lab in Glasgow	Wellcome Sanger Institute for the COVID-19 Genomics UK (COG-UK) Consortium	Harper VanSteenhouse, Yumi Kasai, David Gray, Carol Clugston, Anna Dominiczak and Alex Alderton, Roberto Amato, Sonia Goncalves, Ewan Harrison, David K. Jackson, Ian Johnston, Dominic Kwiatkowski, Cordelia Langford, John Sillitoe on behalf of the Wellcome Sanger Institute COVID-19 Surveillance Team
EPI_ISL_858811, EPI_ISL_858813, EPI_ISL_858814	Lighthouse Lab in Alderley Park	Wellcome Sanger Institute for the COVID-19 Genomics UK (COG-UK) Consortium	Jacquelyn Wynn, Mairead Hyland, The Lighthouse Lab in Alderley Park and Alex Alderton, Roberto Amato, Sonia Goncalves, Ewan Harrison, David K. Jackson, Ian Johnston, Dominic Kwiatkowski, Cordelia Langford, John Sillitoe on behalf of the Wellcome Sanger Institute COVID-19 Surveillance Team
EPI_ISL_858815	Lighthouse Lab in Glasgow	Wellcome Sanger Institute for the COVID-19 Genomics UK	Harper VanSteenhouse, Yumi Kasai, David Gray, Carol Clugston, Anna Dominiczak and Alex Alderton, Roberto Amato, Sonia Goncalves, Ewan Harrison,

		(COG-UK) Consortium	David K. Jackson, Ian Johnston, Dominic Kwiatkowski, Cordelia Langford, John Sillitoe on behalf of the Wellcome Sanger Institute COVID-19 Surveillance Team
EPI_ISL_858816, EPI_ISL_858817, EPI_ISL_858821, EPI_ISL_858822, EPI_ISL_858824, EPI_ISL_858825, EPI_ISL_858827, EPI_ISL_858828	Lighthouse Lab in Alderley Park	Wellcome Sanger Institute for the COVID-19 Genomics UK (COG-UK) Consortium	Jacquelyn Wynn, Mairead Hyland, The Lighthouse Lab in Alderley Park and Alex Alderton, Roberto Amato, Sonia Goncalves, Ewan Harrison, David K. Jackson, Ian Johnston, Dominic Kwiatkowski, Cordelia Langford, John Sillitoe on behalf of the Wellcome Sanger Institute COVID-19 Surveillance Team
EPI_ISL_858830	Lighthouse Lab in Glasgow	Wellcome Sanger Institute for the COVID-19 Genomics UK (COG-UK) Consortium	Harper VanSteenhouse, Yumi Kasai, David Gray, Carol Clugston, Anna Dominiczak and Alex Alderton, Roberto Amato, Sonia Goncalves, Ewan Harrison, David K. Jackson, Ian Johnston, Dominic Kwiatkowski, Cordelia Langford, John Sillitoe on behalf of the Wellcome Sanger Institute COVID-19 Surveillance Team
EPI_ISL_858831, EPI_ISL_858832, EPI_ISL_858834, EPI_ISL_858835	Lighthouse Lab in Alderley Park	Wellcome Sanger Institute for the COVID-19 Genomics UK (COG-UK) Consortium	Jacquelyn Wynn, Mairead Hyland, The Lighthouse Lab in Alderley Park and Alex Alderton, Roberto Amato, Sonia Goncalves, Ewan Harrison, David K. Jackson, Ian Johnston, Dominic Kwiatkowski, Cordelia Langford, John Sillitoe on behalf of the Wellcome Sanger Institute COVID-19 Surveillance Team
EPI_ISL_858842, EPI_ISL_858843, EPI_ISL_858844	Lighthouse Lab in Glasgow	Wellcome Sanger Institute for the COVID-19 Genomics UK (COG-UK) Consortium	Harper VanSteenhouse, Yumi Kasai, David Gray, Carol Clugston, Anna Dominiczak and Alex Alderton, Roberto Amato, Sonia Goncalves, Ewan Harrison, David K. Jackson, Ian Johnston, Dominic Kwiatkowski, Cordelia Langford, John Sillitoe on behalf of the Wellcome Sanger Institute COVID-19 Surveillance Team
EPI_ISL_858847, EPI_ISL_858851, EPI_ISL_858854, EPI_ISL_858856	Lighthouse Lab in Alderley Park	Wellcome Sanger Institute for the COVID-19 Genomics UK (COG-UK) Consortium	Jacquelyn Wynn, Mairead Hyland, The Lighthouse Lab in Alderley Park and Alex Alderton, Roberto Amato, Sonia Goncalves, Ewan Harrison, David K. Jackson, Ian Johnston, Dominic Kwiatkowski, Cordelia Langford, John Sillitoe on behalf of the Wellcome Sanger Institute COVID-19 Surveillance Team
EPI_ISL_858859, EPI_ISL_858860, EPI_ISL_858863, EPI_ISL_858865, EPI_ISL_858867	Lighthouse Lab in Glasgow	Wellcome Sanger Institute for the COVID-19 Genomics UK (COG-UK) Consortium	Harper VanSteenhouse, Yumi Kasai, David Gray, Carol Clugston, Anna Dominiczak and Alex Alderton, Roberto Amato, Sonia Goncalves, Ewan Harrison, David K. Jackson, Ian Johnston, Dominic Kwiatkowski, Cordelia Langford, John Sillitoe on behalf of the Wellcome Sanger Institute COVID-19 Surveillance Team
EPI_ISL_858873	Lighthouse Lab in Alderley Park	Wellcome Sanger Institute for the COVID-19 Genomics UK (COG-UK) Consortium	Jacquelyn Wynn, Mairead Hyland, The Lighthouse Lab in Alderley Park and Alex Alderton, Roberto Amato, Sonia Goncalves, Ewan Harrison, David K. Jackson, Ian Johnston, Dominic Kwiatkowski, Cordelia Langford, John Sillitoe on behalf of the Wellcome Sanger Institute COVID-19 Surveillance Team
EPI_ISL_858874	Lighthouse Lab in Glasgow	Wellcome Sanger Institute for the COVID-19 Genomics UK (COG-UK) Consortium	Harper VanSteenhouse, Yumi Kasai, David Gray, Carol Clugston, Anna Dominiczak and Alex Alderton, Roberto Amato, Sonia Goncalves, Ewan Harrison, David K. Jackson, Ian Johnston, Dominic Kwiatkowski, Cordelia Langford, John Sillitoe on behalf of the Wellcome Sanger Institute COVID-19 Surveillance Team
EPI_ISL_858879, EPI_ISL_858887, EPI_ISL_858891, EPI_ISL_858895	Lighthouse Lab in Alderley Park	Wellcome Sanger Institute for the COVID-19 Genomics UK (COG-UK) Consortium	Jacquelyn Wynn, Mairead Hyland, The Lighthouse Lab in Alderley Park and Alex Alderton, Roberto Amato, Sonia Goncalves, Ewan Harrison, David K. Jackson, Ian Johnston, Dominic Kwiatkowski, Cordelia Langford, John Sillitoe on behalf of the Wellcome Sanger Institute COVID-19 Surveillance Team
EPI_ISL_860027, EPI_ISL_860042, EPI_ISL_860043, EPI_ISL_860048, EPI_ISL_860049, EPI_ISL_860051, EPI_ISL_860052, EPI_ISL_860053, EPI_ISL_860054, EPI_ISL_860055, EPI_ISL_860056, EPI_ISL_860057, EPI_ISL_860063, EPI_ISL_860064, EPI_ISL_860066, EPI_ISL_860067, EPI_ISL_860068, EPI_ISL_860069, EPI_ISL_860071, EPI_ISL_860073, EPI_ISL_860075, EPI_ISL_860076, EPI_ISL_860079, EPI_ISL_860080, EPI_ISL_860089			
see above	BTC, Khalifa University	BTC, Khalifa University	Al Safar et al
EPI_ISL_860203, EPI_ISL_860204, EPI_ISL_860205	Lab. Microbiologia e Virologia, Cotugno, A.O. dei Colli	Lab. Microbiologia e Virologia, Cotugno, A.O. dei Colli	Luigi Atripaldi, Claudia Tiberio, Anna Perfetti
EPI_ISL_860229	Ostfold Hospital Trust - Kalnes, Centre for Laboratory Medicine, Section for gene technology and infection serology	Norwegian Institute of Public Health, Department of Virology	Kathrine Stene-Johansen, Kamilla Heddeland Instefjord, Hilde Elshaug, Atiya R Ali,Marie Paulsen Madsen, Rasmus Riis Kopperud, Hilde Vollan, Karoline Bragstad, Olav Hungnes
EPI_ISL_860231	Vestfold Hospital, Toensberg Department of Microbiology	Norwegian Institute of Public Health, Department of Virology	Kathrine Stene-Johansen, Kamilla Heddeland Instefjord, Hilde Elshaug, Atiya R Ali,Marie Paulsen Madsen, Rasmus Riis Kopperud, Hilde Vollan, Karoline Bragstad, Olav Hungnes
EPI_ISL_860247	University Hospitals of Geneva, Laboratory of Virology	HUG, Laboratory of Virology and Universitätsspital Basel	Samuel Cordey, Ana Rita Goncalves, Laurent Kaiser, Tim Roloff, Madlen Stange, Helena MB Seth-Smith, Alfredo Mari, Karoline Leuzinger, Julia Bielicki, Manuel Battegay, Hans Hirsch, Adrian Egli
EPI_ISL_860257	Lab. Microbiologia e Virologia, Cotugno, A.O. dei Colli	Lab. Microbiologia e Virologia, Cotugno, A.O. dei Colli	Luigi Atripaldi, Claudia Tiberio, Anna Perfetti
EPI_ISL_860298, EPI_ISL_860301	Unit 17: Influenza & Other Respiratory Viruses, German National Influenza Center	Project group Epidemiology of Highly Pathogenic Microorganisms, Robert Koch-Institute	Andreas Sachse, Grit Schubert, Essia Belarbi, Sébastien Calvignac-Spencer, Thorsten Wolff, Ralf Dürrwald, Djin-Ye Oh, Marianne Wedde
EPI_ISL_860619	NHLS-IALCH	KRISP, KZn Research Innovation and Sequencing Platform	Giandhari J, Pillay S, Lessells R, Mdlalose K, York D, Khan S, Tegally H, Wilkinson E, de Oliveira T
EPI_ISL_860641	Respiratory Virus Unit, National Infection Service, Public Health England	COVID-19 Genomics UK (COG-UK) Consortium	PHE Covid Sequencing Team
EPI_ISL_860942, EPI_ISL_860945, EPI_ISL_861101, EPI_ISL_861103	Johns Hopkins Hospital Department of Pathology	Johns Hopkins Hospital Department of Pathology	C. Paul Morris, Chun Huai Luo, Adannaya Amadi, Nicholas Gallagher, Heba H. Mostafa
EPI_ISL_861119	New York Presbyterian Hospital	Wadsworth Center, New York State Department of Health	Kirsten St. George, Daryl M. Lamson, Alexis Russel, Matthew Shudt, Melissa A Leisner, Jonathan Plitnick, Navjot Singh, John Kelly, Erasmus Schneider, Erica Lasek-Nesselquist
EPI_ISL_861175, EPI_ISL_861176, EPI_ISL_861179, EPI_ISL_861185, EPI_ISL_861187, EPI_ISL_861210, EPI_ISL_861211, EPI_ISL_861212, EPI_ISL_861213, EPI_ISL_861214, EPI_ISL_861215, EPI_ISL_861216, EPI_ISL_861217, EPI_ISL_861218, EPI_ISL_861219, EPI_ISL_861220, EPI_ISL_861221, EPI_ISL_861222, EPI_ISL_861223, EPI_ISL_861224, EPI_ISL_861225, EPI_ISL_861226, EPI_ISL_861227, EPI_ISL_861228, EPI_ISL_861229, EPI_ISL_861230, EPI_ISL_861231, EPI_ISL_861232			
see above	BIO-REFERENCE LABORATORIES	Wadsworth Center, New York State Department of Health	Kirsten St. George, Daryl M. Lamson, Alexis Russel, Matthew Shudt, Melissa A Leisner, Jonathan Plitnick, Navjot Singh, John Kelly, Erasmus Schneider, Erica Lasek-Nesselquist
EPI_ISL_861243	Ohio Department of Health Laboratory	Ohio Department of Health Laboratory	Holmes, Jennifer; Eric Brandt, Keoni Omura, Glen McGillivary, Caitlin McDonnell, Kirtana Ramadugu, Erica Leasure, Kelsey Florek, Heather Blankenship, Quanta Brown, and Tammy Bannerman
EPI_ISL_861245, EPI_ISL_861246, EPI_ISL_861247, EPI_ISL_861249, EPI_ISL_861253, EPI_ISL_861256, EPI_ISL_861261, EPI_ISL_861267, EPI_ISL_861289, EPI_ISL_861291, EPI_ISL_861293, EPI_ISL_861294, EPI_ISL_861299, EPI_ISL_861301, EPI_ISL_861302, EPI_ISL_861308, EPI_ISL_861309, EPI_ISL_861311, EPI_ISL_861313, EPI_ISL_861315, EPI_ISL_861318, EPI_ISL_861319, EPI_ISL_861320, EPI_ISL_861321, EPI_ISL_861324, EPI_ISL_861326			
see above	MONTEFIORE MEDICAL CENTER LABORATORIES	Wadsworth Center, New York State Department of Health	Kirsten St. George, Daryl M. Lamson, Alexis Russel, Matthew Shudt, Melissa A Leisner, Jonathan Plitnick, Navjot Singh, John Kelly, Erasmus Schneider, Erica Lasek-Nesselquist
EPI_ISL_861534, EPI_ISL_861537, EPI_ISL_861573, EPI_ISL_861574, EPI_ISL_861588	Instituto Nacional de Saude (INSA)	Instituto Nacional de Saude (INSA)	Borges et al
EPI_ISL_861679	Instituto Adolfo Lutz - Regional de Taubate	Instituto Adolfo Lutz, Interdiciplinary Procedures Center, Strategic Laboratory	Claudio Tavares Sacchi, Claudia Regina Gonçalves, Erica Valessa Ramos Gomes, Karoline Rodrigues Campos
EPI_ISL_861862	Labormedizinisches Zentrum Dr Risch	University Hospital Basel, Clinical Bacteriology	Tim Roloff, Madlen Stange, Helena MB Seth-Smith, Alfredo Mari, Karoline Leuzinger, Julia Bielicki, Nadia Wohlwend,Martin Risch, Lorenz Risch, Manuel Battegay, Hans Hirsch, Adrian Egli
EPI_ISL_861865	Bioanalytika AG	University Hospital Basel, Clinical Bacteriology	Tim Roloff, Madlen Stange, Helena MB Seth-Smith, Alfredo Mari, Karoline Leuzinger, Julia Bielicki, Adrian Härri, Manuel Battegay, Hans Hirsch, Adrian Egli
EPI_ISL_861997, EPI_ISL_861998, EPI_ISL_861999	OHSU Lab Services Molecular Microbiology Lab	Oregon SARS-CoV-2 Genome Sequencing Center	Brendan L. O'Connell, Sally Grindstaff, Kayla Carter, Ruth V. Nichols, Alec J. Hirsch, Donna Hansel, Guang Fan, Xuan Qin, Daniel N. Streblow, William B. Messer, Andrew C. Adey, Benjamin N. Bimber, Brian J. O'Roak
EPI_ISL_862039	RSUP Dr. Sardjito	Genetics Working Group (Pokja Genetik) Faculty of Medicine, Public Health and Nursing Universitas Gadjah Mada (FK-KMK UGM); Disease Investigation Center Wates Ministry of Agriculture Indonesia; Department of Microbiology FK-KMK	Gunadi, Hendra Wibawa, . Marcellus, Mohamad S. Hakim, Edwin W. Daniwijaya, Ludhang P. Rizki, Endah Supriyati, Eggi Arguni, Titik Nuryastuti, Tri Wibawa, Dwi AA Nugrahaningsih, Afiahayati, Siswanto, Kristy Iskandar, Nungki Anggorowati, Ika Trisnawati, Sumardi, Eko Budiono, Bambang Sigit Riyanto, Heni Retnowulan, Munawar Gani, Satria Maulana, Nur Rahmi Ananda, Riat El Khair, Yunika Puspadewi, Osman Sianipar, Umi Solekhah Intansari, Elizabeth Henny Herningtiyas, Ira Puspitawati, Nur Imma Fatimah Harahap,William Widitjiarso, Maria Patricia Inggriani

		UGM; Laboratorium Diagnostik Yayasan Tahija World Mosquito Program (WMP) Yogyakarta Center for Tropical Medicine FK-KMK UGM; Integrated Research Center FK-KMK UGM; Department of Computer Science and Electronics FMIPA UGM; RSUP Dr. Sardjito	
EPI_ISL_862040	RSUP Dr. Sardjito	Genetics Working Group (Pokja Genetik) Faculty of Medicine, Public Health and Nursing Universitas Gadjah Mada (FK-KMK UGM); Disease Investigation Center Wates Ministry of Agriculture Indonesia; Department of Microbiology FK-KMK UGM; Laboratorium Diagnostik Yayasan Tahija World Mosquito Program (WMP) Yogyakarta Center for Tropical Medicine FK-KMK UGM; Integrated Research Center FK-KMK UGM; Department of Computer Science and Electronics FMIPA UGM; RSUP Dr. Sardjito	Gunadi, Hendra Wibawa, . Marcellus, Mohamad S. Hakim, Edwin W. Daniwijaya, Ludhang P. Rizki, Endah Supriyati, Eggi Arguni, Titik Nuryastuti, Tri Wibawa, Dwi AA Nugrahaningsih, Afiahayati, Siswanto, Kristy Iskandar, Nungki Anggorowati, Ika Trisnawati, Sumardi, Eko Budiono, Bambang Sigit Riyanto, Heni Retnowulan, Munawar Gani, Satria Maulana, Nur Rahmi Ananda, Riat El Khair, Yunika Puspadewi, Osman Sianipar, Umi Solekhah Intansari, Elizabeth Henny Herningtiyas, Ira Puspitawati, Nur Imma Fatimah Harahap,Alvin Santoso Kalim, Untung Riawan
EPI_ISL_862041	RSUP Dr. Sardjito	Genetics Working Group (Pokja Genetik) Faculty of Medicine, Public Health and Nursing Universitas Gadjah Mada (FK-KMK UGM); Disease Investigation Center Wates Ministry of Agriculture Indonesia; Department of Microbiology FK-KMK UGM; Laboratorium Diagnostik Yayasan Tahija World Mosquito Program (WMP) Yogyakarta Center for Tropical Medicine FK-KMK UGM; Integrated Research Center FK-KMK UGM; Department of Computer Science and Electronics FMIPA UGM; RSUP Dr. Sardjito	Gunadi, Hendra Wibawa, . Marcellus, Mohamad S. Hakim, Edwin W. Daniwijaya, Ludhang P. Rizki, Endah Supriyati, Eggi Arguni, Titik Nuryastuti, Tri Wibawa, Dwi AA Nugrahaningsih, Afiahayati, Siswanto, Kristy Iskandar, Nungki Anggorowati, Ika Trisnawati, Sumardi, Eko Budiono, Bambang Sigit Riyanto, Heni Retnowulan, Munawar Gani, Satria Maulana, Nur Rahmi Ananda, Riat El Khair, Yunika Puspadewi, Osman Sianipar, Umi Solekhah Intansari, Elizabeth Henny Herningtiyas, Ira Puspitawati, Nur Imma Fatimah Harahap, Dyah Ayu Puspitarani, Audric Kenny Tedja
EPI_ISL_862548	Hospital Clínico San Carlos	Instituto de Salud Carlos III	Iglesias-Caballero, M. Camarero, S. Molinero Calamita, M. González-Esguevillas, M. Pozo, F. Casas, I. Jiménez, P. Jiménez, M. Zaballos, A. Monzón, S. Varona, S. Juliá, M. Cuesta, I. Rodríguez, I.
EPI_ISL_862551	Hospital J.M. Morales Meseguer	Instituto de Salud Carlos III	Iglesias-Caballero, M. Camarero, S. Molinero Calamita, M. González-Esguevillas, M. . Pozo, F. Casas, I. Jiménez, P. Jiménez, M. Zaballos, A. Monzón, S. Varona, S. Juliá, M. Cuesta, I. Guerrero, C.
EPI_ISL_862552	Complejo Hospitalario de Navarra	Instituto de Salud Carlos III	Iglesias-Caballero, M.Camarero, S. Molinero Calamita, M. González-Esguevillas, M. Pozo, F. Casas, I. Jiménez, P. Jiménez, M. Zaballos, A. Monzón, S. Varona, S. Juliá, M. Cuesta, I. Ezpeleta, C.
EPI_ISL_862557, EPI_ISL_862559, EPI_ISL_862560, EPI_ISL_862561, EPI_ISL_862566	Consejería de Sanidad y Asuntos Sociales	Instituto de Salud Carlos III	Iglesias-Caballero, M. Camarero, S. Molinero Calamita, M. González-Esguevillas, M. Pozo, F. Casas, I. Jiménez, P. Jiménez, M. Zaballos, A. Monzón, S. Varona, S. Juliá, M. Cuesta, I. Gutiérrez, G.
EPI_ISL_862569	Hospital Clínico San Carlos	Instituto de Salud Carlos III	Iglesias-Caballero, M. Camarero, S. Molinero Calamita, M. González-Esguevillas, M. Pozo, F. Casas, I. Jiménez, P. Jiménez, M. Zaballos, A. Monzón, S. Varona, S. Juliá, M. Cuesta, I. Rodríguez, I.
EPI_ISL_862573, EPI_ISL_862576, EPI_ISL_862577, EPI_ISL_862580, EPI_ISL_862585, EPI_ISL_862587	Hospital Clínic	Instituto de Salud Carlos III	Iglesias-Caballero, M.Camarero, S. Molinero Calamita, M. González-Esguevillas, M. Pozo, F. Casas, I. Jiménez, P. Jiménez, M. Zaballos, A. Monzón, S. Varona, S. Juliá, M. Cuesta, I. Marcos, M.A.
EPI_ISL_862728, EPI_ISL_862748, EPI_ISL_862749, EPI_ISL_862754, EPI_ISL_862756, EPI_ISL_862758, EPI_ISL_862765, EPI_ISL_862775	Utah Public Health Laboratory, Utah Public Health Laboratory Infectious Disease submission group	Utah Public Health Laboratory, Utah Public Health Laboratory Infectious Disease submission group	Young,E.L., Oakeson,K.F., Gallagher,T.
EPI_ISL_865079, EPI_ISL_865083, EPI_ISL_865084, EPI_ISL_865085, EPI_ISL_865086, EPI_ISL_865088, EPI_ISL_865142	Virology Department, Royal Infirmary of Edinburgh, NHS Lothian / School of Biological Sciences, University of Edinburgh / Institute of Genetics and Molecular Medicine, University of Edinburgh	COVID-19 Genomics UK (COG-UK) Consortium	McHugh M, Dewar R, Rooke S, Gallagher M, Balcaza C, O'Toole Á, Scher E, Hill V, McCrone JT, Colquhoun R, Yu X, Jackson B, Rambaut A, Williams TC, Templeton K
EPI_ISL_865373, EPI_ISL_865374, EPI_ISL_865376, EPI_ISL_865398, EPI_ISL_865400, EPI_ISL_865405	Liverpool Clinical Laboratories	COVID-19 Genomics UK (COG-UK) Consortium	Sam Haldenby, Anita Lucaci, Steve Paterson, Julian Hiscox, Alistair Darby, M Almsaud, A Alrezaihi, Muhannad Alruwaili, Stuart D Armstrong, Jones Benjamin, Eleanor G Bentley, Anu Chawla, Jordan J Clark, Angela Cowell, Richard Eccles, Isabel García-Dorival, Matthew Gemmell, Alessandro Gerada, PKF Gilmore, Richard Gregory, Ximeng Han, Catherine Hartley, Margaret Hughes, Miren Iturriza-Gomara, James Johnson, L Luu, Jenifer Manson, Charlotte Nelson, Elaine O'Toole, Cassie Olateju, Rebekah Penrice-Randal , Lucille Rainbow, N.P Randle, Trevor Ian Robinson, Parul Sharma, Ghada T Shawli, James P Stewart, Neil Swainston, Ecaterina Vamos, Joanne Watts, Mark Whitehead
EPI_ISL_865488, EPI_ISL_865489, EPI_ISL_865494, EPI_ISL_865495, EPI_ISL_865496, EPI_ISL_865497, EPI_ISL_865501, EPI_ISL_865514, EPI_ISL_865515, EPI_ISL_865516, EPI_ISL_865517, EPI_ISL_865518, EPI_ISL_865519, EPI_ISL_865520, EPI_ISL_865521, EPI_ISL_865522, EPI_ISL_865523, EPI_ISL_865524, EPI_ISL_865525, EPI_ISL_865526, EPI_ISL_865563, EPI_ISL_865564, EPI_ISL_865565, EPI_ISL_865566, EPI_ISL_865576, EPI_ISL_865577, EPI_ISL_865578			
see above	Barts Health NHS Trust	COVID-19 Genomics UK (COG-UK) Consortium	CUTINO-MOGUEL, Maria-Teresa; HARRINGTON, David; OWOYEMI, Dola; KULASEGARAN-SHYLINI, Raghavendran; BROAD, Claire; KELE, Beatrix
EPI_ISL_865689, EPI_ISL_865797, EPI_ISL_865798, EPI_ISL_865799, EPI_ISL_865800, EPI_ISL_865801, EPI_ISL_865802, EPI_ISL_865803, EPI_ISL_865804, EPI_ISL_865805, EPI_ISL_865806, EPI_ISL_865807, EPI_ISL_865808, EPI_ISL_865809, EPI_ISL_865810, EPI_ISL_865811, EPI_ISL_865814, EPI_ISL_865815, EPI_ISL_865818, EPI_ISL_865837			
see above	University College London, Great Ormond Street Hospital for Children NHS Foundation Trust, Imperial College Healthcare NHS Trust	COVID-19 Genomics UK (COG-UK) Consortium	Sergi Castellano, Rachel Williams, Mark Kristiansen, Paola Resende Silva, Sunando Roy, Tony Brooks, Helena Tutill, Paola Niola, Patricia Dyal, Charlotte Williams, Leysa Forrest, Yasmin Panchbhaya, Jacqueline Findlay, Samuel Weeks, Julianne Brown, Kathryn Harris, Paul Randell, James Price, Alison Holmes, Judith Breuer
EPI_ISL_866131, EPI_ISL_866183	University College London Hospital	COVID-19 Genomics UK (COG-UK) Consortium	Judith Heaney, Matthew Byott, Catherine Houlihan, Dan Frampton, Stuart Kirk, Moira Spyer and Eleni Nastouli
EPI_ISL_867037, EPI_ISL_867038, EPI_ISL_867047, EPI_ISL_867051, EPI_ISL_867055	Oxford Viromics, NDM, University of Oxford; Oxford University Hospitals; Basingstoke and North Hampshire Hospital	COVID-19 Genomics UK (COG-UK) Consortium	Tanya Golubchik, David Bonsall, George Macintyre, Amy Trebes, Mariateresa de Cesare, Catrin Moore, Alex Mobbs, Anita Justice, Robert Shaw, Monique Andersson, Timothy Peto, Emma Wise, Nathan Moore, Jessica Lynch, Nick Cortes, Matilde Mori, Stephen Kidd, David Buck, John Todd, Christophe Fraser
EPI_ISL_867218	Originating lab: Wales Specialist Virology Centre Sequencing lab: Pathogen Genomics Unit	Public Health Wales Microbiology Cardiff Wales Specialist Virology Centre	Catherine Moore, Johnathan Evans, Laura Gifford, Malorie Perry, Simon Cottrell, Angela Marchbank, Alec Birchley, Alexander Adams, Amy Gaskin, Bree Gatica-Wilcox, Jason Coombes, Joel Southgate, Lauren Gilbert, Lee Graham, Nicole Pacchiarini, Sara Kumziene-Summerhayes, Sarah Taylor, Sophie Jones, Sara Rey, Matthew Bull, Joanne Watkins, Sally Corden, Tom Connor
EPI_ISL_868372, EPI_ISL_868438, EPI_ISL_868559, EPI_ISL_868570, EPI_ISL_868576	Virology Department, Sheffield Teaching Hospitals NHS Foundation Trust/Department of Infection, Immunity and Cardiovascular Disease, The Medical School, University of Sheffield	COVID-19 Genomics UK (COG-UK) Consortium	Thushan de Silva, Matthew Parker, Nikki Smith, Adri Angyal, Rebecca Brown, Luke Green, Rachel Tucker, Paul Parsons, Danielle Groves, Katie Johnson, Laura Carrilero, Alex Keeley, Dave Partridge, Matthew Wyles, Benjamin Lindsey, Mehmet Yavuz, Mohammad Raza, Cariad Evans
EPI_ISL_869079, EPI_ISL_869080, EPI_ISL_869081, EPI_ISL_871325	Ohio Department of Health Laboratory	Ohio Department of Health Laboratory	Holmes, Jennifer; Eric Brandt, Keoni Omura, Glen McGillivary, Caitlin McDonnell, Kirtana Ramadugu, Erica Leasure, Kelsey Florek, Heather Blankenship, Quanta Brown, and Tammy Bannerman
EPI_ISL_871458, EPI_ISL_871459, EPI_ISL_871460, EPI_ISL_871461, EPI_ISL_871462, EPI_ISL_871463, EPI_ISL_871464, EPI_ISL_871465, EPI_ISL_871466, EPI_ISL_871467, EPI_ISL_871468, EPI_ISL_871469, EPI_ISL_871470, EPI_ISL_871471, EPI_ISL_871472, EPI_ISL_871473, EPI_ISL_871474, EPI_ISL_871475, EPI_ISL_871476, EPI_ISL_871477, EPI_ISL_871478, EPI_ISL_871479, EPI_ISL_871480, EPI_ISL_871481, EPI_ISL_871482, EPI_ISL_871483, EPI_ISL_871484, EPI_ISL_871485, EPI_ISL_871486, EPI_ISL_871487, EPI_ISL_871488, EPI_ISL_871489, EPI_ISL_871490, EPI_ISL_871491, EPI_ISL_871492, EPI_ISL_871493, EPI_ISL_871494, EPI_ISL_871495, EPI_ISL_871496, EPI_ISL_871497, EPI_ISL_871498, EPI_ISL_871499, EPI_ISL_871500, EPI_ISL_871501, EPI_ISL_871502, EPI_ISL_871503, EPI_ISL_871504, EPI_ISL_871505, EPI_ISL_871506, EPI_ISL_871507, EPI_ISL_871508, EPI_ISL_871509, EPI_ISL_871510, EPI_ISL_871511, EPI_ISL_871512, EPI_ISL_871513, EPI_ISL_871514, EPI_ISL_871515, EPI_ISL_871516, EPI_ISL_871517, EPI_ISL_871518, EPI_ISL_871519, EPI_ISL_871520, EPI_ISL_871521, EPI_ISL_871522, EPI_ISL_871523, EPI_ISL_871524, EPI_ISL_871525, EPI_ISL_871526, EPI_ISL_871527, EPI_ISL_871528, EPI_ISL_871529,			

EPI_ISL_871530, EPI_ISL_871531, EPI_ISL_871532, EPI_ISL_871533, EPI_ISL_871534, EPI_ISL_871535, EPI_ISL_871536, EPI_ISL_871537, EPI_ISL_871538, EPI_ISL_871539, EPI_ISL_871540, EPI_ISL_871541, EPI_ISL_871542, EPI_ISL_871543, EPI_ISL_871544, EPI_ISL_871545, EPI_ISL_871546, EPI_ISL_871547, EPI_ISL_871548, EPI_ISL_871549, EPI_ISL_871550, EPI_ISL_871551, EPI_ISL_871552, EPI_ISL_871553, EPI_ISL_871554, EPI_ISL_871555, EPI_ISL_871556, EPI_ISL_871557, EPI_ISL_871558, EPI_ISL_871559, EPI_ISL_871560, EPI_ISL_871561, EPI_ISL_871562, EPI_ISL_871563, EPI_ISL_871564, EPI_ISL_871565, EPI_ISL_871566, EPI_ISL_871567, EPI_ISL_871568, EPI_ISL_871569, EPI_ISL_871570, EPI_ISL_871571, EPI_ISL_871572, EPI_ISL_871573, EPI_ISL_871574, EPI_ISL_871575, EPI_ISL_871576, EPI_ISL_871577			
see above	Department of Virus and Microbiological Special Diagnostics, Statens Serum Institut, Copenhagen, Denmark	Aalborg University	Danish Covid-19 Genome Consortium
EPI_ISL_871788, EPI_ISL_871791	Ohio Department of Health Laboratory	Ohio Department of Health Laboratory	Holmes, Jennifer; Eric Brandt, Keoni Omura, Glen McGillivary, Caitlin McDonnell, Kirtana Ramadugu, Erica Leasure, Kelsey Florek, Heather Blankenship, Quanta Brown, and Tammy Bannerman
EPI_ISL_871806, EPI_ISL_871810	AIID	Irish Coronavirus Sequencing Consortium-Teagasc Grange	Matthew McCabe, Aljandro Abner Garcia Leon, Fiona Crispie, Calum Walsh, Michael Carr, John Kenny, Paul Cotter, Patrick Mallon, Gabriel Gonzalez
EPI_ISL_871893	Hospital Universitario Marqués de Valdecilla - IDIVAL (Santander, Cantabria)	SeqCOVID-SPAIN consortium/IBV(CSIC)	Mónica Gozalo Margüello, María Eliecer Cano García, Jose Manuel Méndez Legaza, Daniel Pablo Marcos, Jesús Rodríguez Rodríguez, María Siller Ruiz and SeqCOVID-SPAIN consortium
EPI_ISL_871910	Servicio de Microbiología Clínica (Complejo Hospitalario de Navarra, Pamplona), Instituto de Investigación Sanitaria de Navarra (IdiSNA)	SeqCOVID-SPAIN consortium/IBV(CSIC)	Carmen Ezpeleta Baquedano, Ana Navascués, Ana Miqueleiz and SeqCOVID-SPAIN consortium
EPI_ISL_872040, EPI_ISL_872058, EPI_ISL_872059, EPI_ISL_872061, EPI_ISL_872062	Department of Clinical Microbiology	GIGA Medical Genomics	Keith Durkin, Maria Artesi, Sébastien Bontems, Raphaël Boreux, Bouchra Boujemla, Cécile Meex, Pierrette Melin, Marie-Pierre Hayette, Vincent Bours
EPI_ISL_872188	RSUP Dr. Sardjito	Genetics Working Group (Pokja Genetik) Faculty of Medicine, Public Health and Nursing Universitas Gadjah Mada (FK-KMK UGM); Disease Investigation Center Wates Ministry of Agriculture Indonesia; Department of Microbiology FK-KMK UGM; Laboratorium Diagnostik Yayasan Tahija World Mosquito Program (WMP) Yogyakarta Center for Tropical Medicine FK-KMK UGM; Integrated Research Center FK-KMK UGM; Department of Computer Science and Electronics FMIPA UGM; RSUP Dr. Sardjito	Gunadi, Hendra Wibawa, . Marcellus, Mohamad S. Hakim, Edwin W. Daniwijaya, Ludhang P. Rizki, Endah Supriyati, Eggi Arguni, Titik Nuryastuti, Tri Wibawa, Dwi AA Nugrahaningsih, Afiahayati, Siswanto, Kristy Iskandar, Nungki Anggorowati, Ika Trisnawati, Sumardi, Eko Budiono, Bambang Sigit Riyanto, Heni Retnowulan, Munawar Gani, Satria Maulana, Nur Rahmi Ananda, Riat El Khair, Yunika Puspadewi, Osman Sianipar, Umi Solekhah Intansari, Elizabeth Henny Herningtiyas, Ira Puspitawati, Nur Imma Fatimah Harahap, Aditya Rifqi Fauzi, Fadil Fahri
EPI_ISL_872189	RSUP Dr. Sardjito	Genetics Working Group (Pokja Genetik) Faculty of Medicine, Public Health and Nursing Universitas Gadjah Mada (FK-KMK UGM); Disease Investigation Center Wates Ministry of Agriculture Indonesia; Department of Microbiology FK-KMK UGM; Laboratorium Diagnostik Yayasan Tahija World Mosquito Program (WMP) Yogyakarta Center for Tropical Medicine FK-KMK UGM; Integrated Research Center FK-KMK UGM; Department of Computer Science and Electronics FMIPA UGM; RSUP Dr. Sardjito	Gunadi, Hendra Wibawa, . Marcellus, Mohamad S. Hakim, Edwin W. Daniwijaya, Ludhang P. Rizki, Endah Supriyati, Eggi Arguni, Titik Nuryastuti, Tri Wibawa, Dwi AA Nugrahaningsih, Afiahayati, Siswanto, Kristy Iskandar, Nungki Anggorowati, Ika Trisnawati, Sumardi, Eko Budiono, Bambang Sigit Riyanto, Heni Retnowulan, Munawar Gani, Satria Maulana, Nur Rahmi Ananda, Riat El Khair, Yunika Puspadewi, Osman Sianipar, Umi Solekhah Intansari, Elizabeth Henny Herningtiyas, Ira Puspitawati, Nur Imma Fatimah Harahap, Alvin S. Kalim, Dwiki afandy
EPI_ISL_872190	RSUP Dr. Sardjito	Genetics Working Group (Pokja Genetik) Faculty of Medicine, Public Health and Nursing Universitas Gadjah Mada (FK-KMK UGM); Disease Investigation Center Wates Ministry of Agriculture Indonesia; Department of Microbiology FK-KMK UGM; Laboratorium Diagnostik Yayasan Tahija World Mosquito Program (WMP) Yogyakarta Center for Tropical Medicine FK-KMK UGM; Integrated Research Center FK-KMK UGM; Department of Computer Science and Electronics FMIPA UGM; RSUP Dr. Sardjito	Gunadi, Hendra Wibawa, . Marcellus, Mohamad S. Hakim, Edwin W. Daniwijaya, Ludhang P. Rizki, Endah Supriyati, Eggi Arguni, Titik Nuryastuti, Tri Wibawa, Dwi AA Nugrahaningsih, Afiahayati, Siswanto, Kristy Iskandar, Nungki Anggorowati, Ika Trisnawati, Sumardi, Eko Budiono, Bambang Sigit Riyanto, Heni Retnowulan, Munawar Gani, Satria Maulana, Nur Rahmi Ananda, Riat El Khair, Yunika Puspadewi, Osman Sianipar, Umi Solekhah Intansari, Elizabeth Henny Herningtiyas, Ira Puspitawati, Nur Imma Fatimah Harahap, Dyah Ayu Puspitarani, Kemala Athollah
EPI_ISL_872380, EPI_ISL_872381, EPI_ISL_872386, EPI_ISL_872387, EPI_ISL_872388, EPI_ISL_872389, EPI_ISL_872390, EPI_ISL_872391, EPI_ISL_872392, EPI_ISL_872393	Texas Department of State Health Services (TXDSHS)	Texas Department of State Health Services (TXDSHS)	Bonnie Oh, Anita Pokharel, James Daniel Bonser, Myong Koag, Chung Wang, Rachel Lee, Grace Kubin, Rashmi Tuladhar, Mayela Pedrueza, Maliha Rahman, Jenny Zhang
EPI_ISL_872623, EPI_ISL_872624	Nigeria Centre for Disease Control (NCDC)	African Centre of Excellence for Genomics of Infectious Diseases (ACEGID), Redeemer's University	Oluniyi P.E. et al
EPI_ISL_872741	Rhode Island Department of Health	Infectious Disease Program, Broad Institute of Harvard and MIT	Lemieux,J.E., Siddle,K.J., Huard,R., King,E., Azevedo,K., Miller,A., Adams,G., Gladden-Young,A., Lagerborg,K., Rudy,M., DeRuff,K., Carter,A., Normandin,E., Bauer,M., Reilly,S., Tomkins-Tinch,C., Loreth,C., Chaluvadi,S., Birren,B.W., Gallagher,G., Smole,S., Park,D.J., MacInnis,B.L., and Sabeti,P.C.
EPI_ISL_873021, EPI_ISL_873022, EPI_ISL_873023, EPI_ISL_873024, EPI_ISL_873025, EPI_ISL_873026, EPI_ISL_873034	HELIX LLC	WHO National Influenza Centre Russian Federation	Andrey Komissarov, Artem Fadeev, Anna Ivanova, Kseniya Komissarova, Dmitry Bazhenov, Mikhail Bakaev, Daria Danilenko, Ksenia Safina, Elena Nabieva, Georgii Bazykin, Dmitry Lioznov
EPI_ISL_873234	M Health Fairview	Minnesota Department of Health, Public Health Laboratory	Alexandra Lorentz, Jacob Garfin, Matt Plumb, and Xiong Wang
EPI_ISL_875513	National Virus Reference Laboratory	National Virus Reference Laboratory	Michael Carr, Gabriel Gonzalez, Jonathan Dean, Cillian F De Gascun
EPI_ISL_876058, EPI_ISL_876072, EPI_ISL_876088, EPI_ISL_876097, EPI_ISL_876132, EPI_ISL_876150, EPI_ISL_876151, EPI_ISL_876169, EPI_ISL_876182, EPI_ISL_876261, EPI_ISL_876262, EPI_ISL_876263, EPI_ISL_876264, EPI_ISL_876265, EPI_ISL_876266, EPI_ISL_876267, EPI_ISL_876268, EPI_ISL_876269, EPI_ISL_876270, EPI_ISL_876271, EPI_ISL_876272			
see above	Massachusetts State Public Health Laboratory	Massachusetts State Public Health Laboratory	Andrew Lang, Timelia Fink, Glen Gallagher, Sandra Smole
EPI_ISL_876744, EPI_ISL_876745, EPI_ISL_876746, EPI_ISL_876747	Istituto Zooprofilattico Sperimentale della Puglia e della Basilicata	Istituto Zooprofilattico Sperimentale della Puglia e della Basilicata	Parisi A., Bianco A., Capozzi L., Del Sambro L., Manzulli V, Rondinone V., Pace L., Cipolletta D., Galante D.
EPI_ISL_877186, EPI_ISL_877188	Quest Diagnostics	Quest Diagnostics	Rosenthal,S.H., Gerasimova,A., Kagan,R.M., Anderson, B., Hua, M., Liu Y., Bernstein, L.E., Livingston, K.E., Perez, A., Shalhout, D.F., Shlyakhter, I.A., Owen, R., Tanpaiboon, P., Lacbawan, F.
EPI_ISL_877228	Institute for Medical Research, Infectious Disease Research Centre, National Institutes of Health, Ministry of Health Malaysia	Institute for Medical Research, Infectious Disease Research Centre, National Institutes of Health, Ministry of Health Malaysia	Suppiah J, Kamel K, Azizan MA, Thayan R
EPI_ISL_877451, EPI_ISL_877452	Institute of Microbiology and Immunology, Faculty of Medicine, University of Ljubljana	Institute of Microbiology and Immunology, Faculty of Medicine, University of Ljubljana	Samo Zakotnik, Tomaž Mark Zorec, Matic Brvar, Miša Korva, Mario Poljak, Tatjana Avši - Županc
EPI_ISL_877538	Siti Khodijah Hospital	Institute of Tropical Disease, Universitas Airlangga	Krisnoadi Rahardjo, Aldise M Nastri, Jezzy R Dewantari, Rima R Prasetya, Muhammad Hamdan, Gatot Soegiarto, Laksmi Wulandari, Resti Yudhawati, Soetjipto, Yasuko Mori, Maria I Lusida, Kazufumi Shimizu
EPI_ISL_878779, EPI_ISL_880173, EPI_ISL_880195	Rady's Childrens Hospital	Andersen lab at Scripps Research	SEARCH Alliance San Diego with Nanda Radamchar, David Dimmock, Linda Luo, Christina Clarke, Kathryn Bouic, Teresa Mueller, Denise Malicki

EPI_ISL_882314	Lighthouse Lab in Alderley Park	Wellcome Sanger Institute for the COVID-19 Genomics UK (COG-UK) Consortium	Jacquelyn Wynn, Mairead Hyland, The Lighthouse Lab in Alderley Park and Alex Alderton, Roberto Amato, Sonia Goncalves, Ewan Harrison, David K. Jackson, Ian Johnston, Dominic Kwiatkowski, Cordelia Langford, John Sillitoe on behalf of the Wellcome Sanger Institute COVID-19 Surveillance Team
EPI_ISL_882771	Siti Khodijah Hospital	Institute of Tropical Disease, Universitas Airlangga	Kazufumi Shimizu, Krisnoadi Rahardjo, Aldise M Nastri, Jezzy R Dewantari, Rima R Prasetya, Muhammad Hamdan, Gatot Soegiarto, Laksmi Wulandari, Resti Yudhawati, Yasuko Mori, Soetjipto, Maria I Lusida
EPI_ISL_882921	Genomic Medicine Laboratory, IRCCS Santa Lucia Foundation	INMI Lazzaro Spallanzani IRCCS	M Rueca, E Giombini, C.E.M Gruber, B Bartolini, O Butera, F Messina, E Giardina, A Di Caro, MR Capobianchi
EPI_ISL_882924	INMI Lazzaro Spallanzani IRCCS	INMI Lazzaro Spallanzani IRCCS	F Messina, O Butera, E Giombini, M Rueca, B Bartolini, C.E.M Gruber, MR Capobianchi, A Di Caro
EPI_ISL_882925	INMI Lazzaro Spallanzani IRCCS	INMI Lazzaro Spallanzani IRCCS	C.E.M Gruber, B Bartolini, E Giombini, M Rueca, O Butera, F Messina, A Di Caro, MR Capobianchi
EPI_ISL_884260, EPI_ISL_884264, EPI_ISL_884272, EPI_ISL_884273, EPI_ISL_884275, EPI_ISL_884276, EPI_ISL_884288, EPI_ISL_884289, EPI_ISL_884290	Institute of Medical Microbiology and Hospital Hygiene	Institute of Medical Microbiology and Hospital Hygiene	Prof. Dr. Achim Kaasch, Aljoscha Tersteegen
EPI_ISL_884983, EPI_ISL_884987, EPI_ISL_884988, EPI_ISL_884989, EPI_ISL_884990, EPI_ISL_884991, EPI_ISL_884992, EPI_ISL_884993, EPI_ISL_884994, EPI_ISL_885009, EPI_ISL_885010, EPI_ISL_885011			
see above	Santa Clara County Public Health Laboratory	Chan-Zuckerberg Biohub	CZB Cliahub Consortium
EPI_ISL_885108, EPI_ISL_885109, EPI_ISL_885110, EPI_ISL_885111, EPI_ISL_885112, EPI_ISL_885113, EPI_ISL_885114, EPI_ISL_885115, EPI_ISL_885116, EPI_ISL_885117, EPI_ISL_885118, EPI_ISL_885119, EPI_ISL_885120, EPI_ISL_885121, EPI_ISL_885122, EPI_ISL_885123, EPI_ISL_885124, EPI_ISL_885125, EPI_ISL_885126, EPI_ISL_885127			
see above	Orange County Public Health Lab	Chan-Zuckerberg Biohub	CZB Cliahub Consortium
EPI_ISL_885142	RSUP Dr. Sardjito	Genetics Working Group (Pokja Genetik) Faculty of Medicine, Public Health and Nursing Universitas Gadjah Mada (FK-KMK UGM); Disease Investigation Center Wates Ministry of Agriculture Indonesia; Department of Microbiology FK-KMK UGM; Laboratorium Diagnostik Yayasan Tahija World Mosquito Program (WMP) Yogyakarta Center for Tropical Medicine FK-KMK UGM; Integrated Research Center FK-KMK UGM; Department of Computer Science and Electronics FMIPA UGM; RSUP Dr. Sardjito	Gunadi, Hendra Wibawa, . Marcellus, Mohamad S. Hakim, Edwin W. Daniwijaya, Ludhang P. Rizki, Endah Supriyati, Eggi Arguni, Titik Nuryastuti, Tri Wibawa, Dwi AA Nugrahaningsih, Afiahayati, Siswanto, Kristy Iskandar, Nungki Anggorowati, Ika Trisnawati, Sumardi, Eko Budiono, Bambang Sigit Riyanto, Heni Retnowulan, Munawar Gani, Satria Maulana, Nur Rahmi Ananda, Riat El Khair, Yunika Puspadewi, Osman Sianipar, Umi Solekhah Intansari, Elizabeth Henny Herningtiyas, Ira Puspitawati, Nur Imma Fatimah Harahap, Alvin Santoso Kalim, Susan Simanjaya
EPI_ISL_886825	Labcorp	Genomics and Discovery, Respiratory Viruses Branch, Division of Viral Diseases, Centers for Disease Control and Prevention	Peter W. Cook,Dhwani Batra,Ben L. Rambo-Martin,Summer Galloway,Brian Krueger,Minoo Agarwal,Eyad Almasri,Debbie Boles,Ayla Burns,Nuthawin Charoensri,Oren Cohen,Susan Countryman,Mary Ann Cristobal,Bobbi Croy,Suzanne Dale,Hrushikesh Deshmukh,Amanda Douglas,Vincent Drouillon,Marcia Eisenberg,Howard Engler,Rama Ghatti,Prashant Gupta,Susan Hicks,Jake Humphrey,Lax Iyer,Manoj Jain,Mohan Kolli,Tim Kuphal,Stanley Letovsky,Michael Levandoski,Craig Lukasik,Jonathan Meltzer,Brian Norvell,Mindy Nye,Scott Parker,Christos Petropoulos,John Pruitt,Steven Ragan,Scott Ryan,Mike Sapeta,Jana Schroth,Suresh Babu Selvaraju,Goran Stevovic,Amanda Suchanek,Andrea Throop,Lyndon Tilson,Thomas Urban,Joe Voshell,Kimberly Wagner,Jonathan Williams,Mary Williamson,Qian Zeng,Tricia Zwiefelhofer,Clinton R. Paden,Suxiang Tong,Duncan MacCannell,
EPI_ISL_887440, EPI_ISL_887486, EPI_ISL_887487, EPI_ISL_887488, EPI_ISL_887489, EPI_ISL_887490, EPI_ISL_887491, EPI_ISL_887492	Instituto Nacional de Saude (INS), Mozambique	KRISP, KZN Research Innovation and Sequencing Platform	Nalia Ismael, Nadia Sitoe, Paulo Arnaldo, Nedio Mabunda, Giandhari J, Pillay S, Tegally H, Wilkinson E, de Oliveira T
EPI_ISL_887593, EPI_ISL_887594, EPI_ISL_887596, EPI_ISL_887597, EPI_ISL_887601, EPI_ISL_887602, EPI_ISL_887603, EPI_ISL_887605, EPI_ISL_887607, EPI_ISL_887608, EPI_ISL_887609, EPI_ISL_887613, EPI_ISL_887615, EPI_ISL_887622, EPI_ISL_887627, EPI_ISL_887630, EPI_ISL_887631, EPI_ISL_887634, EPI_ISL_887640, EPI_ISL_887646, EPI_ISL_887649, EPI_ISL_887652, EPI_ISL_887661, EPI_ISL_887664, EPI_ISL_887666, EPI_ISL_887667, EPI_ISL_887675, EPI_ISL_887689, EPI_ISL_887690, EPI_ISL_887691, EPI_ISL_887693, EPI_ISL_887695, EPI_ISL_887704, EPI_ISL_887705, EPI_ISL_887707, EPI_ISL_887710, EPI_ISL_887712, EPI_ISL_887713, EPI_ISL_887717, EPI_ISL_887718, EPI_ISL_887720, EPI_ISL_887727, EPI_ISL_887734, EPI_ISL_887735, EPI_ISL_887736, EPI_ISL_887737, EPI_ISL_887741, EPI_ISL_887749, EPI_ISL_887750, EPI_ISL_887764, EPI_ISL_887769, EPI_ISL_887772, EPI_ISL_887774, EPI_ISL_887775, EPI_ISL_887783, EPI_ISL_887794, EPI_ISL_887795, EPI_ISL_887796, EPI_ISL_887799, EPI_ISL_887800, EPI_ISL_887801, EPI_ISL_887802, EPI_ISL_887803, EPI_ISL_887804, EPI_ISL_887805, EPI_ISL_887806, EPI_ISL_887809, EPI_ISL_887815, EPI_ISL_887818, EPI_ISL_887819, EPI_ISL_887820, EPI_ISL_887825, EPI_ISL_887827, EPI_ISL_887833, EPI_ISL_887835, EPI_ISL_887839, EPI_ISL_887843, EPI_ISL_887844, EPI_ISL_887846, EPI_ISL_887847, EPI_ISL_887848, EPI_ISL_887850, EPI_ISL_887854, EPI_ISL_887860, EPI_ISL_887863, EPI_ISL_887875, EPI_ISL_887876, EPI_ISL_887878, EPI_ISL_887881, EPI_ISL_887882, EPI_ISL_887888, EPI_ISL_887895, EPI_ISL_887896, EPI_ISL_887897, EPI_ISL_887899, EPI_ISL_887900, EPI_ISL_887904, EPI_ISL_887911, EPI_ISL_887914, EPI_ISL_887926, EPI_ISL_887929, EPI_ISL_887933, EPI_ISL_887941, EPI_ISL_887942, EPI_ISL_887943, EPI_ISL_887948, EPI_ISL_887952, EPI_ISL_887955, EPI_ISL_887958, EPI_ISL_887967, EPI_ISL_887969, EPI_ISL_887982, EPI_ISL_887983, EPI_ISL_887990, EPI_ISL_887996, EPI_ISL_887999, EPI_ISL_888005, EPI_ISL_888006, EPI_ISL_888011, EPI_ISL_888016, EPI_ISL_888017, EPI_ISL_888019, EPI_ISL_888020, EPI_ISL_888021, EPI_ISL_888025, EPI_ISL_888031, EPI_ISL_888034, EPI_ISL_888040, EPI_ISL_888046, EPI_ISL_888048, EPI_ISL_888052, EPI_ISL_888055, EPI_ISL_888056, EPI_ISL_888061, EPI_ISL_888067, EPI_ISL_888070, EPI_ISL_888072, EPI_ISL_888073, EPI_ISL_888077, EPI_ISL_888078, EPI_ISL_888079, EPI_ISL_888082, EPI_ISL_888086, EPI_ISL_888087, EPI_ISL_888089, EPI_ISL_888093, EPI_ISL_888098, EPI_ISL_888099, EPI_ISL_888106, EPI_ISL_888108, EPI_ISL_888115, EPI_ISL_888118, EPI_ISL_888119, EPI_ISL_888121, EPI_ISL_888124, EPI_ISL_888126, EPI_ISL_888129, EPI_ISL_888130, EPI_ISL_888131, EPI_ISL_888135, EPI_ISL_888136, EPI_ISL_888137, EPI_ISL_888138, EPI_ISL_888141, EPI_ISL_888142, EPI_ISL_888145, EPI_ISL_888146, EPI_ISL_888148, EPI_ISL_888163, EPI_ISL_888167, EPI_ISL_888168, EPI_ISL_888169, EPI_ISL_888170, EPI_ISL_888171, EPI_ISL_888172, EPI_ISL_888177, EPI_ISL_888178, EPI_ISL_888180, EPI_ISL_888181, EPI_ISL_888182, EPI_ISL_888190, EPI_ISL_888193, EPI_ISL_888194, EPI_ISL_888197, EPI_ISL_888198, EPI_ISL_888200, EPI_ISL_888203, EPI_ISL_888206, EPI_ISL_888212, EPI_ISL_888213, EPI_ISL_888214, EPI_ISL_888216, EPI_ISL_888224, EPI_ISL_888227, EPI_ISL_888230, EPI_ISL_888231, EPI_ISL_888233, EPI_ISL_888236, EPI_ISL_888239, EPI_ISL_888240, EPI_ISL_888244, EPI_ISL_888246, EPI_ISL_888264, EPI_ISL_888267, EPI_ISL_888269, EPI_ISL_888270, EPI_ISL_888279, EPI_ISL_888288, EPI_ISL_888289, EPI_ISL_888294, EPI_ISL_888295, EPI_ISL_888298, EPI_ISL_888303, EPI_ISL_888313, EPI_ISL_888317, EPI_ISL_888319, EPI_ISL_888321, EPI_ISL_888322, EPI_ISL_888331, EPI_ISL_888336, EPI_ISL_888338, EPI_ISL_888340, EPI_ISL_888343, EPI_ISL_888345, EPI_ISL_888346, EPI_ISL_888349, EPI_ISL_888355, EPI_ISL_888357, EPI_ISL_888358, EPI_ISL_888359, EPI_ISL_888361, EPI_ISL_888362, EPI_ISL_888364, EPI_ISL_888371, EPI_ISL_888374, EPI_ISL_888376, EPI_ISL_888383, EPI_ISL_888390, EPI_ISL_888398, EPI_ISL_888403, EPI_ISL_888412, EPI_ISL_888416, EPI_ISL_888418, EPI_ISL_888423, EPI_ISL_888425, EPI_ISL_888428, EPI_ISL_888430, EPI_ISL_888431, EPI_ISL_888433, EPI_ISL_888434, EPI_ISL_888438, EPI_ISL_888455, EPI_ISL_888456, EPI_ISL_888457, EPI_ISL_888459, EPI_ISL_888461, EPI_ISL_888465, EPI_ISL_888469, EPI_ISL_888470, EPI_ISL_888472, EPI_ISL_888473, EPI_ISL_888474, EPI_ISL_888477, EPI_ISL_888482, EPI_ISL_888485, EPI_ISL_888487, EPI_ISL_888489, EPI_ISL_888494, EPI_ISL_888495, EPI_ISL_888497, EPI_ISL_888499, EPI_ISL_888502, EPI_ISL_888503, EPI_ISL_888505, EPI_ISL_888506, EPI_ISL_888507, EPI_ISL_888508, EPI_ISL_888517, EPI_ISL_888518, EPI_ISL_888519, EPI_ISL_888520, EPI_ISL_888530, EPI_ISL_888531, EPI_ISL_888533, EPI_ISL_888542, EPI_ISL_888549, EPI_ISL_888550, EPI_ISL_888551, EPI_ISL_888554, EPI_ISL_888565, EPI_ISL_888569, EPI_ISL_888570, EPI_ISL_888575, EPI_ISL_888576, EPI_ISL_888577, EPI_ISL_888578, EPI_ISL_888579, EPI_ISL_888580, EPI_ISL_888584, EPI_ISL_888585, EPI_ISL_888586			
see above	Labcorp	Genomics and Discovery, Respiratory Viruses Branch, Division of Viral Diseases, Centers for Disease Control and Prevention	Peter W. Cook,Dhwani Batra,Ben L. Rambo-Martin,Summer Galloway,Brian Krueger,Minoo Agarwal,Eyad Almasri,Debbie Boles,Ayla Burns,Nuthawin Charoensri,Oren Cohen,Susan Countryman,Mary Ann Cristobal,Bobbi Croy,Suzanne Dale,Hrushikesh Deshmukh,Amanda Douglas,Vincent Drouillon,Marcia Eisenberg,Howard Engler,Rama Ghatti,Prashant Gupta,Susan Hicks,Jake Humphrey,Lax Iyer,Manoj Jain,Mohan Kolli,Tim Kuphal,Stanley Letovsky,Michael Levandoski,Craig Lukasik,Jonathan Meltzer,Brian Norvell,Mindy Nye,Scott Parker,Christos Petropoulos,John Pruitt,Steven Ragan,Scott Ryan,Mike Sapeta,Jana Schroth,Suresh Babu Selvaraju,Goran Stevovic,Amanda Suchanek,Andrea Throop,Lyndon Tilson,Thomas Urban,Joe Voshell,Kimberly Wagner,Jonathan Williams,Mary Williamson,Qian Zeng,Tricia Zwiefelhofer,Clinton R. Paden,Suxiang Tong,Duncan MacCannell,
EPI_ISL_888676	Center of Advanced Studies and Technology, Molecular Genetics Laboratory	Center of Advanced Studies and Technology, Molecular Genetics Laboratory	De Fabritiis Simone, Mandatori Domitilla, Ferrante Rossella
EPI_ISL_888786	KU Leuven, Rega Institute, Clinical and Epidemiological Virology	KU Leuven, Rega Institute, Clinical and Epidemiological Virology	Tony Wawina-Bokalanga, Bert Vanmechelen, Joan Marti-Carerras, Piet Maes
EPI_ISL_888869	Michigan Department of Health and Human Services, Bureau of Laboratories	Michigan Department of Health and Human Services, Bureau of Laboratories	Blankenship HM, Riner D, Soehnlen MK
EPI_ISL_889028, EPI_ISL_889029, EPI_ISL_889030, EPI_ISL_889031, EPI_ISL_889032, EPI_ISL_889033, EPI_ISL_889034, EPI_ISL_889035, EPI_ISL_889036, EPI_ISL_889037, EPI_ISL_889038, EPI_ISL_889039, EPI_ISL_889040, EPI_ISL_889041, EPI_ISL_889042, EPI_ISL_889043, EPI_ISL_889044, EPI_ISL_889045, EPI_ISL_889046, EPI_ISL_889047, EPI_ISL_889048, EPI_ISL_889049, EPI_ISL_889050, EPI_ISL_889051, EPI_ISL_889052, EPI_ISL_889053, EPI_ISL_889054, EPI_ISL_889055, EPI_ISL_889056, EPI_ISL_889057, EPI_ISL_889058, EPI_ISL_889059, EPI_ISL_889060, EPI_ISL_889061, EPI_ISL_889062, EPI_ISL_889063, EPI_ISL_889064, EPI_ISL_889065, EPI_ISL_889066, EPI_ISL_889067, EPI_ISL_889068, EPI_ISL_889069, EPI_ISL_889070, EPI_ISL_889154, EPI_ISL_889155, EPI_ISL_889156, EPI_ISL_889157, EPI_ISL_889162, EPI_ISL_889163, EPI_ISL_889164, EPI_ISL_889165, EPI_ISL_889166, EPI_ISL_889167, EPI_ISL_889168, EPI_ISL_889169, EPI_ISL_889170, EPI_ISL_889171, EPI_ISL_889172, EPI_ISL_889173, EPI_ISL_889174, EPI_ISL_889175			
see above	Israel Central Virology laboratory	Israel National Consortium for SARS-CoV-2 sequencing	Neta Zuckerman, Efrat Dahan Bucris, Michal Mandelboim, Dana Bar-Ilan, Oran Erster, Tzvia Mann, Omer Murik, David A. Zeevi, Assaf Rokney, Joseph Jaffe, Eva Nachum, Maya Davidovich Cohen, Ephraim Fass, Gal Zizelski Valenci, Mor Rubinstein, Efrat Rorman, Israel Nissan, Efrat Glick-Saar, Omri Nayshool, Gideon Rechavi, Ella Mendelson, Orna Mor
EPI_ISL_889461, EPI_ISL_889462, EPI_ISL_889463, EPI_ISL_889464, EPI_ISL_889465, EPI_ISL_889466, EPI_ISL_889467, EPI_ISL_889468, EPI_ISL_889469, EPI_ISL_889470, EPI_ISL_889471, EPI_ISL_889472, EPI_ISL_889473, EPI_ISL_889474, EPI_ISL_889475, EPI_ISL_889476, EPI_ISL_889477, EPI_ISL_889478, EPI_ISL_889479, EPI_ISL_889480, EPI_ISL_889481, EPI_ISL_889483, EPI_ISL_889484, EPI_ISL_889485, EPI_ISL_889497			
see above	LSUHS Emerging Viral Threat Laboratory	Microbial Genome Sequencing Center	Jeremy P. Kamil, Jennifer L. Carroll, Camille F. Abshire, Maarten Van Diest, Mohammed N.A. Siddiquey, Andrew D. Yurochko, Martin J. Sapp, Rona S. Scott, Christopher G. Kevil, Daniel J. Snyder, Vaughn S. Cooper, John A. Vanchiere

EPI_ISL_890236	Center for Genome Sciences, US Army Medical Research Institute of Infectious Disease (USAMRIID)	Center for Genome Sciences, US Army Medical Research Institute of Infectious Disease (USAMRIID)	Kugelman,J., Di Paola,N., Richardson,J., Gibson,W., Bateman,S. and Jerke,K.
EPI_ISL_890248, EPI_ISL_890252, EPI_ISL_890257, EPI_ISL_890277, EPI_ISL_890328, EPI_ISL_890332	KU Leuven, Rega Institute, Clinical and Epidemiological Virology	KU Leuven, Rega Institute, Clinical and Epidemiological Virology	Tony Wawina-Bokalanga, Bert Vanmechelen, Joan Marti-Carerras, Piet Maes
EPI_ISL_890894	Hospital	National Reference Center for Viruses of Respiratory Infections, Institut Pasteur, Paris	Marion Barbet, Sylvie Behillil, Méline Bizard, Angela Brisebarre, Camille Capel, Etienne Simon-Lorière, Vincent Enouf, Maud Vanpeene, Sylvie van der Werf, Patricia Stoessel-Thouvenin
EPI_ISL_890998, EPI_ISL_890999, EPI_ISL_891000, EPI_ISL_891001, EPI_ISL_891002, EPI_ISL_891003, EPI_ISL_891004, EPI_ISL_891005, EPI_ISL_891006, EPI_ISL_891007, EPI_ISL_891008, EPI_ISL_891009, EPI_ISL_891010, EPI_ISL_891011, EPI_ISL_891020, EPI_ISL_891021, EPI_ISL_891022, EPI_ISL_891023, EPI_ISL_891024, EPI_ISL_891025, EPI_ISL_891026, EPI_ISL_891027, EPI_ISL_891028, EPI_ISL_891029, EPI_ISL_891030, EPI_ISL_891031, EPI_ISL_891032, EPI_ISL_891033, EPI_ISL_891034, EPI_ISL_891036, EPI_ISL_891037, EPI_ISL_891038, EPI_ISL_891039, EPI_ISL_891040, EPI_ISL_891041, EPI_ISL_891042, EPI_ISL_891043, EPI_ISL_891045, EPI_ISL_891048			
see above	Washington State Department of Health	Seattle Flu Study	Deborah A. Nickerson, Chris D. Frazar, Jover Lee, Benjamin Pelle, Erica Ryke, Matthew Richardson, Amanda Adler, Elisabeth Brandstetter, Peter D. Han, Kairsten Fay, Misja Ilcisin, Kirsten Lacombe, Thomas R. Sibley, Melissa Truong, Caitlin R. Wolf, Romesh Gautom, Geoff Melly, Brian Hiatt, Philip Dykema, Scott Lindquist, Michael Boeckh, Janet A. Englund, Michael Famulare, Barry R. Lutz, Mark J. Rieder, Lea M. Starita, Matthew Thompson, Helen Y. Chu, Jay Shendure, Trevor Bedford
EPI_ISL_891211	SMS Medical College Jaipur	SMS Medical College jaipur	Bharti Malhotra, Swati Gautam, Himanshu Sharma, Pratibha Sharma, Neha Bhomia, Nivedita Gupta, Pragya Yadav, Varsha Potdar
EPI_ISL_891213	SMS Medical College Jaipur	SMS Medical College Jaipur	Bharti Malhotra, Swati Gautam, Himanshu Sharma, Pratibha Sharma, Neha Bhomia, Nivedita Gupta, Pragya Yadav, Varsha Potdar
EPI_ISL_891214	SMS Medical College Jaipur	SMS Medical College Jaipur	Bharti Malhotra, Swati Gautam, Himanshu Sharma, Pratibha Sharma, Neha Bhomia, Nivedita Gupta, Pragya Yadav,Varsha Potdar
EPI_ISL_891219	SMS Medical College Jaipur	SMS Medical College Jaipur	Bharti Malhotra, Swati Gautam, Himanshu Sharma, Pratibha Sharma, Neha Bhomia, Nivedita Gupta, Pragya Yadav, Varsha Potdar
EPI_ISL_892212	Lighthouse Lab in Alderley Park	Wellcome Sanger Institute for the COVID-19 Genomics UK (COG-UK) Consortium	Jacquelyn Wynn, Mairead Hyland, The Lighthouse Lab in Alderley Park and Alex Alderton, Roberto Amato, Sonia Goncalves, Ewan Harrison, David K. Jackson, Ian Johnston, Dominic Kwiatkowski, Cordelia Langford, John Sillitoe on behalf of the Wellcome Sanger Institute COVID-19 Surveillance Team
EPI_ISL_893771, EPI_ISL_893773	Institute of Virology, Medical Center, University of Freiburg, Freiburg, Germany	Institute of Virology, Clinial Virus Genomics, Medical Center, University of Freiburg, Freiburg, Germany	Jonas Fuchs, Lisa Kern, Sandra Reuter, Hajo Grundmann, Marcus Panning
EPI_ISL_894169	Institute of Medical Microbiology and Hospital Hygiene	Institute of Medical Microbiology and Hospital Hygiene	Prof. Dr. Achim Kaasch, Aljoscha Tersteegen
EPI_ISL_896114	Viollier AG	University Hospital Basel, Clinical Bacteriology	Tim Roloff, Madlen Stange, Helena MB Seth-Smith, Alfredo Mari, Karoline Leuzinger, Julia Bielicki, Christiane Beckmann, Manuel Battegay, Hans Hirsch, Adrian Egli
EPI_ISL_896204	MEPHI, Aix Marseille University	MEPHI, Aix Marseille University	Anthony LEVASSEUR
EPI_ISL_899015, EPI_ISL_899019, EPI_ISL_899020, EPI_ISL_899033, EPI_ISL_899109, EPI_ISL_899118, EPI_ISL_899119, EPI_ISL_899120, EPI_ISL_899134, EPI_ISL_899161, EPI_ISL_899179, EPI_ISL_899185, EPI_ISL_899186, EPI_ISL_899187, EPI_ISL_899285, EPI_ISL_899286, EPI_ISL_899287, EPI_ISL_899288, EPI_ISL_899289, EPI_ISL_899290, EPI_ISL_899291, EPI_ISL_899292, EPI_ISL_899293, EPI_ISL_899294, EPI_ISL_899295, EPI_ISL_899296, EPI_ISL_899297, EPI_ISL_899405, EPI_ISL_899406, EPI_ISL_899407, EPI_ISL_899408, EPI_ISL_899409, EPI_ISL_899410, EPI_ISL_899411, EPI_ISL_899412, EPI_ISL_899413, EPI_ISL_899414, EPI_ISL_899415, EPI_ISL_899416, EPI_ISL_899417, EPI_ISL_899418, EPI_ISL_899419, EPI_ISL_899420, EPI_ISL_899517, EPI_ISL_899518, EPI_ISL_899519, EPI_ISL_899520, EPI_ISL_899521, EPI_ISL_899559, EPI_ISL_899597, EPI_ISL_899621, EPI_ISL_899623, EPI_ISL_899624, EPI_ISL_899625, EPI_ISL_899626, EPI_ISL_899664, EPI_ISL_899768, EPI_ISL_899769, EPI_ISL_899770, EPI_ISL_899771, EPI_ISL_899772, EPI_ISL_899774, EPI_ISL_899775, EPI_ISL_899776, EPI_ISL_899777, EPI_ISL_899778, EPI_ISL_899779, EPI_ISL_899780, EPI_ISL_899811, EPI_ISL_899812, EPI_ISL_899813, EPI_ISL_899814, EPI_ISL_899815, EPI_ISL_899816, EPI_ISL_899817, EPI_ISL_899818, EPI_ISL_899819, EPI_ISL_899820, EPI_ISL_899821, EPI_ISL_899822, EPI_ISL_899823, EPI_ISL_899824, EPI_ISL_899825, EPI_ISL_899826, EPI_ISL_899827, EPI_ISL_899863, EPI_ISL_899875, EPI_ISL_899876, EPI_ISL_899877, EPI_ISL_899878, EPI_ISL_899879, EPI_ISL_899880, EPI_ISL_899881, EPI_ISL_899933, EPI_ISL_899937, EPI_ISL_899938, EPI_ISL_899939, EPI_ISL_899940, EPI_ISL_899941, EPI_ISL_899944, EPI_ISL_899945, EPI_ISL_899946, EPI_ISL_899947, EPI_ISL_899948, EPI_ISL_899949, EPI_ISL_899950, EPI_ISL_899951			
see above	Viollier AG	Department of Biosystems Science and Engineering, ETH Zürich	Christian Beisel, Sarah Nadeau, Chaoran Chen, Ivan Topolsky, Philipp Jablonski, Lara Fuhrmann, David Dreifuss, Katharina Jahn, Tobias Schär, Ina Nissen, Natascha Santacroce, Elodie Burcklen, Christiane Beckmann, Maurice Redondo, Olivier Kobel, Christoph Noppen, Sophie Seidel, Noemie Santamaria de Souza, Niko Beerenwinkel, Tanja Stadler
EPI_ISL_900512	Centre hospitalier Emile Roux	CNR Virus des Infections Respiratoires - France SUD	Antonin Bal, Gregory Destras, Gwendolyne Burfin, Hadrien Règue, Quentin Semanas, Martine Valette, Bruno Lina, Laurence Josset
EPI_ISL_900520	Biosterel	CNR Virus des Infections Respiratoires - France SUD	Antonin Bal, Gregory Destras, Gwendolyne Burfin, Hadrien Règue, Quentin Semanas, Martine Valette, Bruno Lina, Laurence Josset
EPI_ISL_900533, EPI_ISL_900544	CNR Virus des Infections Respiratoires - France SUD	CNR Virus des Infections Respiratoires - France SUD	Antonin Bal, Gregory Destras, Gwendolyne Burfin, Hadrien Règue, Quentin Semanas, Martine Valette, Bruno Lina, Laurence Josset
EPI_ISL_900583, EPI_ISL_900633, EPI_ISL_900634, EPI_ISL_900635, EPI_ISL_900636, EPI_ISL_900637, EPI_ISL_900638, EPI_ISL_900639, EPI_ISL_900640, EPI_ISL_900641, EPI_ISL_900642, EPI_ISL_900643, EPI_ISL_900644, EPI_ISL_900645, EPI_ISL_900646, EPI_ISL_900648, EPI_ISL_900649, EPI_ISL_900650, EPI_ISL_900685, EPI_ISL_900686, EPI_ISL_900687, EPI_ISL_900688, EPI_ISL_900689, EPI_ISL_900690			
see above	IZSM	TIGEM	Patrizia Annunziata, Andrea Ballabio, Valentina Bouche, Davide Cacchiarelli (CorrespAuthor), Pellegrino Cerino, Chiara Colantuono, Maria Concetta Cuomo, Denise Di Concilio, Lucio Di Filippo, Antonio Grimaldi, Antonio Limone, Anna Manfredi, Francesco Panariello, Biancamaria Pierri, Marcello Salvi
EPI_ISL_903037, EPI_ISL_903038, EPI_ISL_903039, EPI_ISL_903040, EPI_ISL_903041, EPI_ISL_903042, EPI_ISL_903045, EPI_ISL_903046, EPI_ISL_903047, EPI_ISL_903082, EPI_ISL_903083			
see above	Seattle Flu Study	Seattle Flu Study	Deborah A. Nickerson, Chris D. Frazar, Jover Lee, Benjamin Pelle, Erica Ryke, Matthew Richardson, Amanda Adler, Elisabeth Brandstetter, Peter D. Han, Kairsten Fay, Misja Ilcisin, Kirsten Lacombe, Thomas R. Sibley, Melissa Truong, Caitlin R. Wolf, Karen Cowgill, Stephanie Schrag, Jeff Duchin, Michael Boeckh, Janet A. Englund, Michael Famulare, Barry R. Lutz, Mark J. Rieder, Lea M. Starita, Matthew Thompson, Helen Y. Chu, Trevor Bedford, Jay Shendure
EPI_ISL_903106	Washington State Department of Health	Seattle Flu Study	Deborah A. Nickerson, Chris D. Frazar, Jover Lee, Benjamin Pelle, Erica Ryke, Matthew Richardson, Amanda Adler, Elisabeth Brandstetter, Peter D. Han, Kairsten Fay, Misja Ilcisin, Kirsten Lacombe, Thomas R. Sibley, Melissa Truong, Caitlin R. Wolf, Romesh Gautom, Geoff Melly, Brian Hiatt, Philip Dykema, Scott Lindquist, Michael Boeckh, Janet A. Englund, Michael Famulare, Barry R. Lutz, Mark J. Rieder, Lea M. Starita, Matthew Thompson, Helen Y. Chu, Jay Shendure, Trevor Bedford
EPI_ISL_903264, EPI_ISL_903273	M Health Fairview	Minnesota Department of Health, Public Health Laboratory	Alexandra Lorentz, Jacob Garfin, Matt Plumb, and Xiong Wang
EPI_ISL_903375, EPI_ISL_903377, EPI_ISL_903379, EPI_ISL_903380, EPI_ISL_903381, EPI_ISL_903388, EPI_ISL_903389	MOH - Jaber Al-Ahmad Hospital (Innovation Research Laboratory)	MOH - Jaber Al-Ahmad Hospital (Innovation Research Laboratory)	Salman Al-Sabah , Mohammad Alghounaim
EPI_ISL_903574	HI Dept. of Health, State Laboratories Division	Genomics and Discovery, Respiratory Viruses Branch, Division of Viral Diseases, Centers for Disease Control and Prevention	Krista Queen, Yan Li, Ying Tao, Jing Zhang, Anna Uehara, Anna Montmayeur, Clinton R. Paden, Peter W. Cook, Rachel Marine, Mili Sheth, Jasmine Padilla, Sarah Nobles, Mark Burroughs, Lori Rowe, Haibin Wang, Ben L. Rambo-Martin, Dhwani Batra, Justin Lee, Suxiang Tong
EPI_ISL_903580	AZ SPHL, Arizona Department of Health Services	Genomics and Discovery, Respiratory Viruses Branch, Division of Viral Diseases, Centers for Disease Control and Prevention	Krista Queen, Yan Li, Ying Tao, Jing Zhang, Anna Uehara, Anna Montmayeur, Clinton R. Paden, Peter W. Cook, Rachel Marine, Mili Sheth, Jasmine Padilla, Sarah Nobles, Mark Burroughs, Lori Rowe, Haibin Wang, Ben L. Rambo-Martin, Dhwani Batra, Justin Lee, Suxiang Tong
EPI_ISL_903584	OH Department of Health Laboratory	Genomics and Discovery, Respiratory Viruses Branch, Division of Viral Diseases, Centers for Disease Control and Prevention	Krista Queen, Yan Li, Ying Tao, Jing Zhang, Anna Uehara, Anna Montmayeur, Clinton R. Paden, Peter W. Cook, Rachel Marine, Mili Sheth, Jasmine Padilla, Sarah Nobles, Mark Burroughs, Lori Rowe, Haibin Wang, Ben L. Rambo-Martin, Dhwani Batra, Justin Lee, Suxiang Tong
EPI_ISL_903592	PA Department of Health, Bureau of Laboratories	Genomics and Discovery, Respiratory Viruses Branch, Division of Viral Diseases, Centers for Disease Control and Prevention	Krista Queen, Yan Li, Ying Tao, Jing Zhang, Anna Uehara, Anna Montmayeur, Clinton R. Paden, Peter W. Cook, Rachel Marine, Mili Sheth, Jasmine Padilla, Sarah Nobles, Mark Burroughs, Lori Rowe, Haibin Wang, Ben L. Rambo-Martin, Dhwani Batra, Justin Lee, Suxiang Tong
EPI_ISL_903622	NM Dept. Health, Scientific Laboratory Division	Genomics and Discovery, Respiratory Viruses Branch, Division of Viral Diseases, Centers for Disease Control and Prevention	Krista Queen, Yan Li, Ying Tao, Jing Zhang, Anna Uehara, Anna Montmayeur, Clinton R. Paden, Peter W. Cook, Rachel Marine, Mili Sheth, Jasmine Padilla, Sarah Nobles, Mark Burroughs, Lori Rowe, Haibin Wang, Ben L. Rambo-Martin, Dhwani Batra, Justin Lee, Suxiang Tong
EPI_ISL_903634	AZ SPHL, Arizona Department of Health Services	Genomics and Discovery, Respiratory Viruses Branch,	Krista Queen, Yan Li, Ying Tao, Jing Zhang, Anna Uehara, Anna Montmayeur, Clinton R. Paden, Peter W. Cook, Rachel Marine, Mili Sheth, Jasmine

		Division of Viral Diseases, Centers for Disease Control and Prevention	Padilla, Sarah Nobles, Mark Burroughs, Lori Rowe, Haibin Wang, Ben L. Rambo-Martin, Dhwani Batra, Justin Lee, Suxiang Tong
EPI_ISL_903677	MN PHL Division, Minnesota Department of Health	Genomics and Discovery, Respiratory Viruses Branch, Division of Viral Diseases, Centers for Disease Control and Prevention	Krista Queen, Yan Li, Ying Tao, Jing Zhang, Anna Uehara, Anna Montmayeur, Clinton R. Paden, Peter W. Cook, Rachel Marine, Mili Sheth, Jasmine Padilla, Sarah Nobles, Mark Burroughs, Lori Rowe, Haibin Wang, Ben L. Rambo-Martin, Dhwani Batra, Justin Lee, Suxiang Tong
EPI_ISL_903715	VT Dept. of Health Laboratory	Genomics and Discovery, Respiratory Viruses Branch, Division of Viral Diseases, Centers for Disease Control and Prevention	Krista Queen, Yan Li, Ying Tao, Jing Zhang, Anna Uehara, Anna Montmayeur, Clinton R. Paden, Peter W. Cook, Rachel Marine, Mili Sheth, Jasmine Padilla, Sarah Nobles, Mark Burroughs, Lori Rowe, Haibin Wang, Ben L. Rambo-Martin, Dhwani Batra, Justin Lee, Suxiang Tong
EPI_ISL_903740, EPI_ISL_903755	NM Dept. Health, Scientific Laboratory Division	Genomics and Discovery, Respiratory Viruses Branch, Division of Viral Diseases, Centers for Disease Control and Prevention	Krista Queen, Yan Li, Ying Tao, Jing Zhang, Anna Uehara, Anna Montmayeur, Clinton R. Paden, Peter W. Cook, Rachel Marine, Mili Sheth, Jasmine Padilla, Sarah Nobles, Mark Burroughs, Lori Rowe, Haibin Wang, Ben L. Rambo-Martin, Dhwani Batra, Justin Lee, Suxiang Tong
EPI_ISL_903778	PA Department of Health, Bureau of Laboratories	Genomics and Discovery, Respiratory Viruses Branch, Division of Viral Diseases, Centers for Disease Control and Prevention	Krista Queen, Yan Li, Ying Tao, Jing Zhang, Anna Uehara, Anna Montmayeur, Clinton R. Paden, Peter W. Cook, Rachel Marine, Mili Sheth, Jasmine Padilla, Sarah Nobles, Mark Burroughs, Lori Rowe, Haibin Wang, Ben L. Rambo-Martin, Dhwani Batra, Justin Lee, Suxiang Tong
EPI_ISL_903796, EPI_ISL_903800	NM Dept. Health, Scientific Laboratory Division	Genomics and Discovery, Respiratory Viruses Branch, Division of Viral Diseases, Centers for Disease Control and Prevention	Krista Queen, Yan Li, Ying Tao, Jing Zhang, Anna Uehara, Anna Montmayeur, Clinton R. Paden, Peter W. Cook, Rachel Marine, Mili Sheth, Jasmine Padilla, Sarah Nobles, Mark Burroughs, Lori Rowe, Haibin Wang, Ben L. Rambo-Martin, Dhwani Batra, Justin Lee, Suxiang Tong
EPI_ISL_903808	AZ SPHL, Arizona Department of Health Services	Genomics and Discovery, Respiratory Viruses Branch, Division of Viral Diseases, Centers for Disease Control and Prevention	Krista Queen, Yan Li, Ying Tao, Jing Zhang, Anna Uehara, Anna Montmayeur, Clinton R. Paden, Peter W. Cook, Rachel Marine, Mili Sheth, Jasmine Padilla, Sarah Nobles, Mark Burroughs, Lori Rowe, Haibin Wang, Ben L. Rambo-Martin, Dhwani Batra, Justin Lee, Suxiang Tong
EPI_ISL_903811	NM Dept. Health, Scientific Laboratory Division	Genomics and Discovery, Respiratory Viruses Branch, Division of Viral Diseases, Centers for Disease Control and Prevention	Krista Queen, Yan Li, Ying Tao, Jing Zhang, Anna Uehara, Anna Montmayeur, Clinton R. Paden, Peter W. Cook, Rachel Marine, Mili Sheth, Jasmine Padilla, Sarah Nobles, Mark Burroughs, Lori Rowe, Haibin Wang, Ben L. Rambo-Martin, Dhwani Batra, Justin Lee, Suxiang Tong
EPI_ISL_903834	PA Department of Health, Bureau of Laboratories	Genomics and Discovery, Respiratory Viruses Branch, Division of Viral Diseases, Centers for Disease Control and Prevention	Krista Queen, Yan Li, Ying Tao, Jing Zhang, Anna Uehara, Anna Montmayeur, Clinton R. Paden, Peter W. Cook, Rachel Marine, Mili Sheth, Jasmine Padilla, Sarah Nobles, Mark Burroughs, Lori Rowe, Haibin Wang, Ben L. Rambo-Martin, Dhwani Batra, Justin Lee, Suxiang Tong
EPI_ISL_903838, EPI_ISL_903855	AZ SPHL, Arizona Department of Health Services	Genomics and Discovery, Respiratory Viruses Branch, Division of Viral Diseases, Centers for Disease Control and Prevention	Krista Queen, Yan Li, Ying Tao, Jing Zhang, Anna Uehara, Anna Montmayeur, Clinton R. Paden, Peter W. Cook, Rachel Marine, Mili Sheth, Jasmine Padilla, Sarah Nobles, Mark Burroughs, Lori Rowe, Haibin Wang, Ben L. Rambo-Martin, Dhwani Batra, Justin Lee, Suxiang Tong
EPI_ISL_903864	DC Public Health Lab/ Dept. of Forensic Sciences	Genomics and Discovery, Respiratory Viruses Branch, Division of Viral Diseases, Centers for Disease Control and Prevention	Krista Queen, Yan Li, Ying Tao, Jing Zhang, Anna Uehara, Anna Montmayeur, Clinton R. Paden, Peter W. Cook, Rachel Marine, Mili Sheth, Jasmine Padilla, Sarah Nobles, Mark Burroughs, Lori Rowe, Haibin Wang, Ben L. Rambo-Martin, Dhwani Batra, Justin Lee, Suxiang Tong
EPI_ISL_903866	MD DOH Laboratories Administration	Genomics and Discovery, Respiratory Viruses Branch, Division of Viral Diseases, Centers for Disease Control and Prevention	Krista Queen, Yan Li, Ying Tao, Jing Zhang, Anna Uehara, Anna Montmayeur, Clinton R. Paden, Peter W. Cook, Rachel Marine, Mili Sheth, Jasmine Padilla, Sarah Nobles, Mark Burroughs, Lori Rowe, Haibin Wang, Ben L. Rambo-Martin, Dhwani Batra, Justin Lee, Suxiang Tong
EPI_ISL_903870	NM Dept. Health, Scientific Laboratory Division	Genomics and Discovery, Respiratory Viruses Branch, Division of Viral Diseases, Centers for Disease Control and Prevention	Krista Queen, Yan Li, Ying Tao, Jing Zhang, Anna Uehara, Anna Montmayeur, Clinton R. Paden, Peter W. Cook, Rachel Marine, Mili Sheth, Jasmine Padilla, Sarah Nobles, Mark Burroughs, Lori Rowe, Haibin Wang, Ben L. Rambo-Martin, Dhwani Batra, Justin Lee, Suxiang Tong
EPI_ISL_903876	PA Department of Health, Bureau of Laboratories	Genomics and Discovery, Respiratory Viruses Branch, Division of Viral Diseases, Centers for Disease Control and Prevention	Krista Queen, Yan Li, Ying Tao, Jing Zhang, Anna Uehara, Anna Montmayeur, Clinton R. Paden, Peter W. Cook, Rachel Marine, Mili Sheth, Jasmine Padilla, Sarah Nobles, Mark Burroughs, Lori Rowe, Haibin Wang, Ben L. Rambo-Martin, Dhwani Batra, Justin Lee, Suxiang Tong
EPI_ISL_903885	NE Public Health Laboratory	Genomics and Discovery, Respiratory Viruses Branch, Division of Viral Diseases, Centers for Disease Control and Prevention	Krista Queen, Yan Li, Ying Tao, Jing Zhang, Anna Uehara, Anna Montmayeur, Clinton R. Paden, Peter W. Cook, Rachel Marine, Mili Sheth, Jasmine Padilla, Sarah Nobles, Mark Burroughs, Lori Rowe, Haibin Wang, Ben L. Rambo-Martin, Dhwani Batra, Justin Lee, Suxiang Tong
EPI_ISL_903886	IN State Department of Health Laboratory Services	Genomics and Discovery, Respiratory Viruses Branch, Division of Viral Diseases, Centers for Disease Control and Prevention	Krista Queen, Yan Li, Ying Tao, Jing Zhang, Anna Uehara, Anna Montmayeur, Clinton R. Paden, Peter W. Cook, Rachel Marine, Mili Sheth, Jasmine Padilla, Sarah Nobles, Mark Burroughs, Lori Rowe, Haibin Wang, Ben L. Rambo-Martin, Dhwani Batra, Justin Lee, Suxiang Tong
EPI_ISL_903902, EPI_ISL_903919, EPI_ISL_903935	AZ SPHL, Arizona Department of Health Services	Genomics and Discovery, Respiratory Viruses Branch, Division of Viral Diseases, Centers for Disease Control and Prevention	Krista Queen, Yan Li, Ying Tao, Jing Zhang, Anna Uehara, Anna Montmayeur, Clinton R. Paden, Peter W. Cook, Rachel Marine, Mili Sheth, Jasmine Padilla, Sarah Nobles, Mark Burroughs, Lori Rowe, Haibin Wang, Ben L. Rambo-Martin, Dhwani Batra, Justin Lee, Suxiang Tong
EPI_ISL_903958	MD DOH Laboratories Administration	Genomics and Discovery, Respiratory Viruses Branch, Division of Viral Diseases, Centers for Disease Control and Prevention	Krista Queen, Yan Li, Ying Tao, Jing Zhang, Anna Uehara, Anna Montmayeur, Clinton R. Paden, Peter W. Cook, Rachel Marine, Mili Sheth, Jasmine Padilla, Sarah Nobles, Mark Burroughs, Lori Rowe, Haibin Wang, Ben L. Rambo-Martin, Dhwani Batra, Justin Lee, Suxiang Tong
EPI_ISL_903959, EPI_ISL_903964	DC Public Health Lab/ Dept. of Forensic Sciences	Genomics and Discovery, Respiratory Viruses Branch, Division of Viral Diseases, Centers for Disease Control and Prevention	Krista Queen, Yan Li, Ying Tao, Jing Zhang, Anna Uehara, Anna Montmayeur, Clinton R. Paden, Peter W. Cook, Rachel Marine, Mili Sheth, Jasmine Padilla, Sarah Nobles, Mark Burroughs, Lori Rowe, Haibin Wang, Ben L. Rambo-Martin, Dhwani Batra, Justin Lee, Suxiang Tong
EPI_ISL_903967	MD DOH Laboratories Administration	Genomics and Discovery, Respiratory Viruses Branch, Division of Viral Diseases, Centers for Disease Control and Prevention	Krista Queen, Yan Li, Ying Tao, Jing Zhang, Anna Uehara, Anna Montmayeur, Clinton R. Paden, Peter W. Cook, Rachel Marine, Mili Sheth, Jasmine Padilla, Sarah Nobles, Mark Burroughs, Lori Rowe, Haibin Wang, Ben L. Rambo-Martin, Dhwani Batra, Justin Lee, Suxiang Tong
EPI_ISL_903976	DC Public Health Lab/ Dept. of Forensic Sciences	Genomics and Discovery, Respiratory Viruses Branch, Division of Viral Diseases, Centers for Disease Control and Prevention	Krista Queen, Yan Li, Ying Tao, Jing Zhang, Anna Uehara, Anna Montmayeur, Clinton R. Paden, Peter W. Cook, Rachel Marine, Mili Sheth, Jasmine Padilla, Sarah Nobles, Mark Burroughs, Lori Rowe, Haibin Wang, Ben L. Rambo-Martin, Dhwani Batra, Justin Lee, Suxiang Tong
EPI_ISL_904170, EPI_ISL_904196, EPI_ISL_904197, EPI_ISL_904198, EPI_ISL_904300, EPI_ISL_904301, EPI_ISL_904302, EPI_ISL_904363, EPI_ISL_904506, EPI_ISL_904507, EPI_ISL_904508, EPI_ISL_904509, EPI_ISL_904510, EPI_ISL_904511, EPI_ISL_904512, EPI_ISL_904513, EPI_ISL_904514, EPI_ISL_904515, EPI_ISL_904516, EPI_ISL_904517, EPI_ISL_904518, EPI_ISL_904519, EPI_ISL_904618, EPI_ISL_904619, EPI_ISL_904620			
see above	Dutch COVID-19 response team	Erasmus Medical Center	Bas Oude Munnink, Reina Sikkema, David Nieuwenhuijse, Irina Chestakova, Anne van der Linden, Marjan Boter, Emmanuelle Munger, Corine GeurtsvanKessel, Annemiek van der Eijk, Richard Molenkamp, Marion Koopmans, on behalf of the Dutch national COVID-19 response team.
EPI_ISL_904658, EPI_ISL_904746, EPI_ISL_904785, EPI_ISL_904887, EPI_ISL_904899, EPI_ISL_904903, EPI_ISL_905004, EPI_ISL_905099, EPI_ISL_905100, EPI_ISL_905128, EPI_ISL_905151, EPI_ISL_905156, EPI_ISL_905167, EPI_ISL_905174, EPI_ISL_905215, EPI_ISL_905224, EPI_ISL_905229, EPI_ISL_905232, EPI_ISL_905233, EPI_ISL_905237, EPI_ISL_905298, EPI_ISL_905370, EPI_ISL_905371, EPI_ISL_905377, EPI_ISL_905394, EPI_ISL_905395, EPI_ISL_905396, EPI_ISL_905397, EPI_ISL_905398, EPI_ISL_905399, EPI_ISL_905400, EPI_ISL_905401, EPI_ISL_905402, EPI_ISL_905403, EPI_ISL_905428, EPI_ISL_905433, EPI_ISL_905462, EPI_ISL_905463, EPI_ISL_905464, EPI_ISL_905465, EPI_ISL_905466, EPI_ISL_905467, EPI_ISL_905468, EPI_ISL_905469, EPI_ISL_905470, EPI_ISL_905471, EPI_ISL_905483, EPI_ISL_905491, EPI_ISL_905492, EPI_ISL_905493, EPI_ISL_905495, EPI_ISL_905496, EPI_ISL_905497, EPI_ISL_905498, EPI_ISL_905499, EPI_ISL_905500, EPI_ISL_905504, EPI_ISL_905505, EPI_ISL_905506, EPI_ISL_905508, EPI_ISL_905509, EPI_ISL_905510, EPI_ISL_905514, EPI_ISL_905560, EPI_ISL_905671, EPI_ISL_905672, EPI_ISL_905709, EPI_ISL_905710			
see above	Dutch COVID-19 response team	National Institute for Public Health and the Environment (RIVM)	Adam Meijer, Harry Vennema, Dirk Eggink, Jeroen Cremer, Sharon van den Brink, Bas van der Veer, AnneMarie van den Brandt, Florian Zwagemaker, Dennis Schmitz, Chantal Reusken, on behalf of the national COVID-19 response team
EPI_ISL_906128, EPI_ISL_906129	Institute of Microbiology and Immunology, Faculty of Medicine, University of Ljubljana	Institute of Microbiology and Immunology, Faculty of Medicine, University of Ljubljana	Samo Zakotnik, Tomaž Mark Zorec, Matic Brvar, Miša Korva, Mario Poljak, Tatjana Avši - Županc

EPI_ISL_906543	FUNDACION HOSPITAL SAN PEDRO	Instituto Nacional de Salud- Dirección de Investigación en Salud Pública, Universidad de los Andes- Applied genomics research group, Vicerrectoria de Investigación y Creación, Universidad de los Andes- Systems and Computing Engineering Department	Katherine Laiton-Donato, Diego A. Álvarez-Díaz, Carlos Franco-Muñoz, Mauricio Pacheco-Montealegre, Héctor Alejandro Ruiz-Moreno, Maria T. Herrera-Sepúlveda, Diego Andrés Prada, Jhonnatan Reales-González, Sheryll Corchuelo, Julian Naizaque, Gerardo Santamaría Jorge Duitama, Laura Natalia Gonzalez, Jorge Ivan Diaz, Silvia Restrepo-Restrepo, Magdalena Wiesner, Martha Lucia Ospina Martinez, Marcela Mercado-Reyes
EPI_ISL_906553, EPI_ISL_906555	Laboratorio Bienestar	Instituto Nacional de Salud- Dirección de Investigación en Salud Pública, Universidad de los Andes- Applied genomics research group, Vicerrectoria de Investigación y Creación, Universidad de los Andes- Systems and Computing Engineering Department	Katherine Laiton-Donato, Diego A. Álvarez-Díaz, Carlos Franco-Muñoz, Mauricio Pacheco-Montealegre, Héctor Alejandro Ruiz-Moreno, Maria T. Herrera-Sepúlveda, Diego Andrés Prada, Jhonnatan Reales-González, Sheryll Corchuelo, Julian Naizaque, Gerardo Santamaría Jorge Duitama, Laura Natalia Gonzalez, Jorge Ivan Diaz, Silvia Restrepo-Restrepo, Magdalena Wiesner, Martha Lucia Ospina Martinez, Marcela Mercado-Reyes
EPI_ISL_906571	Maine Health and Environmental Testing Laboratory (Maine HETL)	Tewhey Lab, The Jackson Laboratory	Matluk,N., Dewey,H., Iosue,F., Barter,M., Lynch,R., Munger,H. and Tewhey,R.
EPI_ISL_906848, EPI_ISL_906851	Respiratory Viruses Branch, Centers for Disease Control and Prevention	Respiratory Viruses Branch, Centers for Disease Control and Prevention	Tao,Y., Li,Y., Zhang,J., Queen,K., Uehara,A., Cook,P., Paden,C.R., Wang,H., Tong,S.
EPI_ISL_906918	Bureau of Public Health Laboratories, Florida Department of Health (BPHL, FLDOH)	Bureau of Public Health Laboratories, Florida Department of Health (BPHL, FLDOH)	Schmedes,S., Blanton,J.
EPI_ISL_910547, EPI_ISL_910548, EPI_ISL_910550, EPI_ISL_910551, EPI_ISL_910552, EPI_ISL_910633, EPI_ISL_911241	Laboratoire national de sante, Microbiology, Virology	Laboratoire national de sante, Microbiology, Microbial Genomics Platform	Anke Wienecke-Baldacchino, Catherine Ragimbeau,Jessica Tapp, Fatu Djabi, Lise Pignon, Raoul Salmon, Tamir Abdelrahman
EPI_ISL_912279, EPI_ISL_912297, EPI_ISL_912299, EPI_ISL_912315, EPI_ISL_912324, EPI_ISL_912336, EPI_ISL_912352	Hospital General Universitario Gregorio Marañón	SeqCOVID-SPAIN consortium / IBV (CSIC)	Darío García de Viedma, Laura Pérez-Lago, Pedro J Sola-Campoy, Sergio Buenestado-Serrano, Marta Herranz, Victor Manuel de la Cueva, Julia Suárez, Pilar Catalán, Patricia Muñoz and SeqCOVID-SPAIN consortium
EPI_ISL_912407, EPI_ISL_912409, EPI_ISL_912417, EPI_ISL_912426	KU Leuven, Rega Institute, Clinical and Epidemiological Virology	KU Leuven, Rega Institute, Clinical and Epidemiological Virology	Tony Wawina-Bokalanga, Bert Vanmechelen, Joan Marti-Carerras, Piet Maes
EPI_ISL_912508	NHLS Universitas Academic	UFS Virology	PA Bester, MM Nyaga, P Nthiga, MT Mogotsi, D Goedhals, T de Oliveira
EPI_ISL_912892, EPI_ISL_912896, EPI_ISL_912906, EPI_ISL_912921, EPI_ISL_912929, EPI_ISL_912932, EPI_ISL_913012, EPI_ISL_913013, EPI_ISL_913014, EPI_ISL_913015, EPI_ISL_913016, EPI_ISL_913017, EPI_ISL_913018, EPI_ISL_913019			
see above	Hôpital Henri Mondor	Department of Virology, Henri Mondor University Hospital, Assistance Publique Hôpitaux de Paris, Université Paris-Est Créteil, INSERM U955	Christophe Rodriguez, Slim Fourati, Vanessa Demontant, Guillaume Gricourt, Melissa N'Debi, Alexandre Soulier, Elisabeth Trawinski, Jean-Michel Pawlotsky
EPI_ISL_913051	Hospital Comarcal Sierrallana	Instituto de Salud Carlos III	Iglesias-Caballero, M. Camarero, S. Sandonís,V. Vázquez, S. Pozo, F. Casas, I. Jiménez, P. Zaballos, A. Monzón, S. Varona, S. Cuesta, I. De Benito, I.
EPI_ISL_913052, EPI_ISL_913053	Complejo Asistencial Universitario de Salamanca	Instituto de Salud Carlos III	Iglesias-Caballero, M. Camarero, S. Sandonís,V. Vázquez, S. Pozo, F. Casas, I. Jiménez, P. Zaballos, A. Monzón, S. Varona, S. Cuesta, I. Ávila, A.
EPI_ISL_913276	Synlab Medilab, Mikrobiologi	The Public Health Agency of Sweden	Anna-Malin Linde, Maria Lind Karlberg, Carlo Berg, Oskar Karlsson Lindsjo, Sofia Stamouli, Reza Advani, Mattias Haukland, Petra Holmstrom, Noura Walai, Petra Edquist, Mia Brytting, Anna Risberg, Karin Tegmark-Wisell
EPI_ISL_913280	Klinisk mikrobiologi	The Public Health Agency of Sweden	Anna-Malin Linde, Maria Lind Karlberg, Carlo Berg, Oskar Karlsson Lindsjo, Sofia Stamouli, Reza Advani, Mattias Haukland, Petra Holmstrom, Noura Walai, Petra Edquist, Mia Brytting, Anna Risberg, Karin Tegmark-Wisell
EPI_ISL_913284	Synlab Medilab, Mikrobiologi	The Public Health Agency of Sweden	Anna-Malin Linde, Maria Lind Karlberg, Carlo Berg, Oskar Karlsson Lindsjo, Sofia Stamouli, Reza Advani, Mattias Haukland, Petra Holmstrom, Noura Walai, Petra Edquist, Mia Brytting, Anna Risberg, Karin Tegmark-Wisell
EPI_ISL_913286, EPI_ISL_913314	The Public Health Agency of Sweden	The Public Health Agency of Sweden	Anna-Malin Linde, Maria Lind Karlberg, Carlo Berg, Oskar Karlsson Lindsjo, Sofia Stamouli, Reza Advani, Mattias Haukland, Petra Holmstrom, Noura Walai, Petra Edquist, Mia Brytting, Anna Risberg, Karin Tegmark-Wisell
EPI_ISL_913327, EPI_ISL_913329	Klinisk mikrobiologi	The Public Health Agency of Sweden	Anna-Malin Linde, Maria Lind Karlberg, Carlo Berg, Oskar Karlsson Lindsjo, Sofia Stamouli, Reza Advani, Mattias Haukland, Petra Holmstrom, Noura Walai, Petra Edquist, Mia Brytting, Anna Risberg, Karin Tegmark-Wisell
EPI_ISL_913340	Unilabs, Mikrobiologiska laboratoriet	The Public Health Agency of Sweden	Anna-Malin Linde, Maria Lind Karlberg, Carlo Berg, Oskar Karlsson Lindsjo, Sofia Stamouli, Reza Advani, Mattias Haukland, Petra Holmstrom, Noura Walai, Petra Edquist, Mia Brytting, Anna Risberg, Karin Tegmark-Wisell
EPI_ISL_913378, EPI_ISL_913379	Dynamic Code AB	The Public Health Agency of Sweden	Anna-Malin Linde, Maria Lind Karlberg, Carlo Berg, Oskar Karlsson Lindsjo, Sofia Stamouli, Reza Advani, Mattias Haukland, Petra Holmstrom, Noura Walai, Petra Edquist, Mia Brytting, Anna Risberg, Karin Tegmark-Wisell
EPI_ISL_913463	Klinisk mikrobiologi	The Public Health Agency of Sweden	Anna-Malin Linde, Maria Lind Karlberg, Carlo Berg, Oskar Karlsson Lindsjo, Sofia Stamouli, Reza Advani, Mattias Haukland, Petra Holmstrom, Noura Walai, Petra Edquist, Mia Brytting, Anna Risberg, Karin Tegmark-Wisell
EPI_ISL_913464	The Public Health Agency of Sweden	The Public Health Agency of Sweden	Anna-Malin Linde, Maria Lind Karlberg, Carlo Berg, Oskar Karlsson Lindsjo, Sofia Stamouli, Reza Advani, Mattias Haukland, Petra Holmstrom, Noura Walai, Petra Edquist, Mia Brytting, Anna Risberg, Karin Tegmark-Wisell
EPI_ISL_913485	Klinisk mikrobiologi	The Public Health Agency of Sweden	Anna-Malin Linde, Maria Lind Karlberg, Carlo Berg, Oskar Karlsson Lindsjo, Sofia Stamouli, Reza Advani, Mattias Haukland, Petra Holmstrom, Noura Walai, Petra Edquist, Mia Brytting, Anna Risberg, Karin Tegmark-Wisell
EPI_ISL_913519, EPI_ISL_913520, EPI_ISL_913521, EPI_ISL_913522, EPI_ISL_913523, EPI_ISL_913524, EPI_ISL_913526, EPI_ISL_913527, EPI_ISL_913536	M Health Fairview	Minnesota Department of Health, Public Health Laboratory	Alexandra Lorentz, Jacob Garfin, Matt Plumb, and Xiong Wang
EPI_ISL_913611, EPI_ISL_913612, EPI_ISL_913634, EPI_ISL_913635, EPI_ISL_913637, EPI_ISL_913638, EPI_ISL_913639	Michigan Department of Health and Human Services, Bureau of Laboratories	Michigan Department of Health and Human Services, Bureau of Laboratories	Blankenship HM, Riner D, Soehnlen MK
EPI_ISL_913676, EPI_ISL_913677, EPI_ISL_913678, EPI_ISL_913679, EPI_ISL_913680, EPI_ISL_913681, EPI_ISL_913682, EPI_ISL_913683, EPI_ISL_913684, EPI_ISL_913685, EPI_ISL_913692			
see above	Minnesota Department of Health, Public Health Laboratory	Minnesota Department of Health, Public Health Laboratory	Alexandra Lorentz, Jacob Garfin, Matt Plumb, and Xiong Wang
EPI_ISL_913811, EPI_ISL_913897	TGen North	TGen North	"Jolene Bowers, Megan Folkerts, Chris French, Hayley Yaglom, Ashlyn Pfeiffer, Darrin Lemmer, Dave Engelthaler, The Arizona COVID Genomics Union (ACGU)"
EPI_ISL_914648, EPI_ISL_914649, EPI_ISL_914650, EPI_ISL_914651, EPI_ISL_914652, EPI_ISL_914653, EPI_ISL_914654, EPI_ISL_914655, EPI_ISL_914656, EPI_ISL_914657, EPI_ISL_914658, EPI_ISL_914659, EPI_ISL_914660, EPI_ISL_914661, EPI_ISL_914663, EPI_ISL_914664, EPI_ISL_914665, EPI_ISL_914666, EPI_ISL_914696, EPI_ISL_914697, EPI_ISL_914698, EPI_ISL_914699, EPI_ISL_914700, EPI_ISL_914701			
see above	Utah Public Health Laboratory	Utah Public Health Laboratory	Erin L. Young, Kelly F. Oakeson, Tara Gallagher
EPI_ISL_914814	HOSPITAL METROPOLITANO	Inciensa, Instituto Costarricense de Investigación y Enseñanza en Nutrición y Salud	Francisco Duarte, Hebleen Porras, Claudio Soto-Garita, Estela Cordero, Adriana Godínez, Melany Calderón & Margarita Lee-Lui
EPI_ISL_918172, EPI_ISL_918173	Department of Infectious Diseases and Immunology, National	Clinical Research Center, National Hospital Organization	Yoshihiro Nakata, Hirotaka Ode, Mai Kubota, Masakazu Matsuda, Kazuhiro Matsuoka, Miho Nakasuji, Mikiko Mori, Mayumi Imahashi, Yoshiyuki

	Hospital Organization Nagoya Medical Center	Nagoya Medical Center	Yokomaku, Yasumasa Iwatani
EPI_ISL_918193, EPI_ISL_918241, EPI_ISL_918248, EPI_ISL_918250	Innovative Genomics Institute, UC Berkeley	Innovative Genomics Institute, UC Berkeley	Stacia Wyman, Haridha Shivram, Phil Frankino, Liana Lareau, Shana McDevitt, Justin Choi
EPI_ISL_918349, EPI_ISL_918350	Institute of Virology, Medical Center, University of Freiburg, Freiburg, Germany	Institute of Virology, Clinial Virus Genomics, Medical Center, University of Freiburg, Freiburg, Germany	Jonas Fuchs, Lisa Kern, Sandra Reuter, Hajo Grundmann, Marcus Panning
EPI_ISL_918408	Ospedale Santa Caterina Novella	Istituto Zooprofilattico Sperimentale della Puglia e della Basilicata	Parisi A., Bianco A., Capozzi L., Del Sambro L., Simone D., Manzulli V, Rondinone V., Pace L., Cipolletta D., Galante D.
EPI_ISL_918500, EPI_ISL_918501, EPI_ISL_918503	LACEN - Laboratório Central de Saúde Pública do Amazonas	Evandro Chagas Institute	Santos, M.C.; Silva, A.M.; Junior, W.D.C.; Barbagelata, L.S.; Ferreira, J.A.; Sousa, E.M.A.; da Silva, P.S.; Pinheiro, K.C.; L.C.; Sousa Junior, E.C.
EPI_ISL_918955	University of Birmingham	COVID-19 Genomics UK (COG-UK) Consortium	Institute of Microbiology, University of Birmingham: Claire McMurray, Joanne Stockton, Samuel Nicholls, Radoslaw Poplawski, Will Rowe, Josh Quick, Nicholas Loman. University of Birmingham Testing Laboratory: Celina M Whalley, Andrew Bosworth, Charlotte Poxon, Kasun Wanigasooriya, Oliver Pickles, Mike Kidd, Alex Richter, Andrew D Beggs PHE Heartlands Lab: Husam Osman, Andrew Bosworth. Queen Elizabeth Hospital: Anna Casey
EPI_ISL_919406	Virology Department, Royal Infirmary of Edinburgh, NHS Lothian / School of Biological Sciences, University of Edinburgh / Institute of Genetics and Molecular Medicine, University of Edinburgh	COVID-19 Genomics UK (COG-UK) Consortium	McHugh M, Dewar R, Rooke S, Gallagher M, Balcaza C, O'Toole Á, Scher E, Hill V, McCrone JT, Colquhoun R, Yu X, Jackson B, Rambaut A, Williams TC, Templeton K
EPI_ISL_919456	Liverpool Clinical Laboratories	COVID-19 Genomics UK (COG-UK) Consortium	Sam Haldenby, Anita Lucaci, Steve Paterson, Julian Hiscox, Alistair Darby, M Almsaud, A Alrezaihi, Muhannad Alruwaili, Stuart D Armstrong, Jones Benjamin, Eleanor G Bentley, Anu Chawla, Jordan J Clark, Angela Cowell, Richard Eccles, Isabel García-Dorival, Matthew Gemmell, Alessandro Gerada, PKF Gilmore, Richard Gregory, Ximeng Han, Catherine Hartley, Margaret Hughes, Miren Iturriza-Gomara, James Johnson, L Luu, Jenifer Manson, Charlotte Nelson, Elaine O'Toole, Cassie Olateju, Rebekah Penrice-Randal , Lucille Rainbow, N.P Randle, Trevor Ian Robinson, Parul Sharma, Ghada T Shawli, James P Stewart, Neil Swainston, Ecaterina Vamos, Joanne Watts, Mark Whitehead
EPI_ISL_919760, EPI_ISL_919761, EPI_ISL_919762, EPI_ISL_919763, EPI_ISL_919764, EPI_ISL_919765, EPI_ISL_919766, EPI_ISL_919767	University College London, Great Ormond Street Hospital for Children NHS Foundation Trust, Imperial College Healthcare NHS Trust	COVID-19 Genomics UK (COG-UK) Consortium	Sergi Castellano, Rachel Williams, Mark Kristiansen, Paola Resende Silva, Sunando Roy, Tony Brooks, Helena Tutill, Paola Niola, Patricia Dyal, Charlotte Williams, Leysa Forrest, Yasmin Panchbhaya, Jacqueline Findlay, Samuel Weeks, Julianne Brown, Kathryn Harris, Paul Randell, James Price, Alison Holmes, Judith Breuer
EPI_ISL_920189, EPI_ISL_920319, EPI_ISL_920320, EPI_ISL_920354, EPI_ISL_920370, EPI_ISL_920569, EPI_ISL_920629, EPI_ISL_920653, EPI_ISL_920697	University College London Hospital	COVID-19 Genomics UK (COG-UK) Consortium	Judith Heaney, Matthew Byott, Catherine Houlihan, Dan Frampton, Stuart Kirk, Moira Spyer and Eleni Nastouli
EPI_ISL_920841, EPI_ISL_920854, EPI_ISL_920855	University College London, Great Ormond Street Hospital for Children NHS Foundation Trust, Imperial College Healthcare NHS Trust	COVID-19 Genomics UK (COG-UK) Consortium	Sergi Castellano, Rachel Williams, Mark Kristiansen, Paola Resende Silva, Sunando Roy, Tony Brooks, Helena Tutill, Paola Niola, Patricia Dyal, Charlotte Williams, Leysa Forrest, Yasmin Panchbhaya, Jacqueline Findlay, Samuel Weeks, Julianne Brown, Kathryn Harris, Paul Randell, James Price, Alison Holmes, Judith Breuer
EPI_ISL_921090	Regional Virus Laboratory, Belfast Health and Social Care Trust	COVID-19 Genomics UK (COG-UK) Consortium	Conall McCaughey, James McKenna, Tanya Curran, Susan Feeney, Alison Watt, Ciara Cox, Mairead Connor, Zoltan Molnar, David Simpson, Derek Fairley
EPI_ISL_921227, EPI_ISL_921230, EPI_ISL_921231, EPI_ISL_921232, EPI_ISL_921233, EPI_ISL_921234, EPI_ISL_921235, EPI_ISL_921237, EPI_ISL_921240, EPI_ISL_921364, EPI_ISL_921365, EPI_ISL_921366, EPI_ISL_921367, EPI_ISL_921368, EPI_ISL_921369, EPI_ISL_921370, EPI_ISL_921372, EPI_ISL_921374, EPI_ISL_921375, EPI_ISL_921376, EPI_ISL_921377, EPI_ISL_921670, EPI_ISL_921671, EPI_ISL_921771, EPI_ISL_921774, EPI_ISL_921778, EPI_ISL_921779			
see above	Northumbria University / South Tees Hospitals NHS Foundation Trust / North Cumbria Integrated Care NHS Foundation Trust / North Tees and Hartlepool NHS Foundation Trust / Newcastle Hospitals NHS Foundation Trust	COVID-19 Genomics UK (COG-UK) Consortium	Darren L Smith,Andrew Nelson,Matthew Bashton,Greg R Young,Joshua Loh,John Allan,Mohammad A Tariq,Giles S Holt,Gary Black,Wen C Yew,Lynn Dover,Paul Baker,Steve Liggett,Sarah Essex,Jane Greenaway,Debra Padgett,Clive Graham,Garren Scott,Edward Barton,Emma Swindells,Brendan Payne,Jennifer Collins,Yusri Taha,Gary Eltringham
EPI_ISL_921789, EPI_ISL_921790, EPI_ISL_921791, EPI_ISL_921792, EPI_ISL_921796, EPI_ISL_921798, EPI_ISL_921799, EPI_ISL_921801, EPI_ISL_921802, EPI_ISL_921811, EPI_ISL_921815, EPI_ISL_921820, EPI_ISL_921821, EPI_ISL_921824, EPI_ISL_921826, EPI_ISL_921828, EPI_ISL_921839, EPI_ISL_921841, EPI_ISL_921842, EPI_ISL_921846, EPI_ISL_921847, EPI_ISL_921848, EPI_ISL_921849			
see above	Quadram Institute Bioscience	COVID-19 Genomics UK (COG-UK) Consortium	Dave J. Baker, Gemma L. Kay, Alp Aydin, Thanh Le-Viet, Steven Rudder, Ana P. Tedim, Anastasia Kolyva, Maria Diaz, Leonardo de Oliveira Martins, Nabil-Fareed Alikhan, Lizzie Meadows, Rachael Stanley, Ngozi Elumogo, Muhammed Yasir, Nicholas M. Thomson, Alexander J Trotter, Rachel Gilroy, Samuel Bloomfield, Claire Stuart, Andrew Bell, Reenesh Prakash, Samir Dervisevic, Alison E. Mather, John Wain, Mark Webber, Andrew J. Page, Justin O'Grady
EPI_ISL_922268, EPI_ISL_922270, EPI_ISL_922276, EPI_ISL_922281, EPI_ISL_922282, EPI_ISL_922307, EPI_ISL_922313, EPI_ISL_922314	Oxford Viromics, NDM, University of Oxford; Oxford University Hospitals; Basingstoke and North Hampshire Hospital	COVID-19 Genomics UK (COG-UK) Consortium	Tanya Golubchik, David Bonsall, George Macintyre, Amy Trebes, Mariateresa de Cesare, Catrin Moore, Alex Mobbs, Anita Justice, Robert Shaw, Monique Andersson, Timothy Peto, Emma Wise, Nathan Moore, Jessica Lynch, Nick Cortes, Matilde Mori, Stephen Kidd, David Buck, John Todd, Christophe Fraser
EPI_ISL_923258, EPI_ISL_923267, EPI_ISL_923268	Centre for Enzyme Innovation, University of Portsmouth / Translational Research Laboratory, Portsmouth Hospitals NHS Trust	COVID-19 Genomics UK (COG-UK) Consortium	Angela Beckett,Salman Goudarzi,Christopher Fearn,Kate Cook,Katie Loveson,Sharon Glaysher,Scott Elliott,Samuel Robson
EPI_ISL_925923, EPI_ISL_925937, EPI_ISL_925953, EPI_ISL_926020, EPI_ISL_926024, EPI_ISL_926046, EPI_ISL_926059, EPI_ISL_926177, EPI_ISL_926192, EPI_ISL_926218, EPI_ISL_926224, EPI_ISL_926286, EPI_ISL_926289, EPI_ISL_926361, EPI_ISL_926438, EPI_ISL_926487, EPI_ISL_926539, EPI_ISL_926551, EPI_ISL_926575, EPI_ISL_926631, EPI_ISL_926661, EPI_ISL_926708, EPI_ISL_926770, EPI_ISL_926793, EPI_ISL_926809, EPI_ISL_926914, EPI_ISL_926937, EPI_ISL_926940, EPI_ISL_926942, EPI_ISL_926959, EPI_ISL_926960, EPI_ISL_926987, EPI_ISL_927099, EPI_ISL_927192, EPI_ISL_927194, EPI_ISL_927216, EPI_ISL_927314, EPI_ISL_927493, EPI_ISL_927505, EPI_ISL_927544, EPI_ISL_927565, EPI_ISL_927602, EPI_ISL_927626, EPI_ISL_927652, EPI_ISL_927657, EPI_ISL_927671, EPI_ISL_927721, EPI_ISL_927845, EPI_ISL_927981, EPI_ISL_928047, EPI_ISL_928048, EPI_ISL_928092, EPI_ISL_928149, EPI_ISL_928274, EPI_ISL_928395, EPI_ISL_928404, EPI_ISL_928446, EPI_ISL_928468, EPI_ISL_928532, EPI_ISL_928546, EPI_ISL_928560, EPI_ISL_928609, EPI_ISL_928636, EPI_ISL_928675, EPI_ISL_928750, EPI_ISL_928772, EPI_ISL_928796, EPI_ISL_928812, EPI_ISL_928814, EPI_ISL_928816, EPI_ISL_928843, EPI_ISL_928849, EPI_ISL_928935, EPI_ISL_928966, EPI_ISL_928984, EPI_ISL_929093, EPI_ISL_929141, EPI_ISL_929147, EPI_ISL_929169, EPI_ISL_929173, EPI_ISL_929175, EPI_ISL_929227, EPI_ISL_929319, EPI_ISL_929334, EPI_ISL_929387, EPI_ISL_929452, EPI_ISL_929458, EPI_ISL_929552, EPI_ISL_929570, EPI_ISL_929588, EPI_ISL_929605, EPI_ISL_929627, EPI_ISL_929706, EPI_ISL_929712, EPI_ISL_929745, EPI_ISL_929771, EPI_ISL_929802, EPI_ISL_929972, EPI_ISL_930001, EPI_ISL_930019, EPI_ISL_930044, EPI_ISL_930053, EPI_ISL_930062, EPI_ISL_930064, EPI_ISL_930208, EPI_ISL_930238, EPI_ISL_930257, EPI_ISL_930272, EPI_ISL_930300, EPI_ISL_930327, EPI_ISL_930343, EPI_ISL_930385, EPI_ISL_930465, EPI_ISL_930496			
see above	Department of Virus and Microbiological Special Diagnostics, Statens Serum Institut, Copenhagen, Denmark	Aalborg University	Danish Covid-19 Genome Consortium
EPI_ISL_930665, EPI_ISL_930666, EPI_ISL_930667, EPI_ISL_930671, EPI_ISL_930672, EPI_ISL_930673, EPI_ISL_930674, EPI_ISL_930720, EPI_ISL_930722, EPI_ISL_930723, EPI_ISL_930724, EPI_ISL_930725, EPI_ISL_930726, EPI_ISL_930727, EPI_ISL_930728, EPI_ISL_930730, EPI_ISL_930731, EPI_ISL_930732, EPI_ISL_930733, EPI_ISL_930735, EPI_ISL_930736, EPI_ISL_930737, EPI_ISL_930738, EPI_ISL_930739, EPI_ISL_930740, EPI_ISL_930741, EPI_ISL_930742, EPI_ISL_930743, EPI_ISL_930744, EPI_ISL_930745, EPI_ISL_930746, EPI_ISL_930747, EPI_ISL_930748, EPI_ISL_930749, EPI_ISL_930750, EPI_ISL_930751, EPI_ISL_930752, EPI_ISL_930753, EPI_ISL_930754, EPI_ISL_930755, EPI_ISL_930756, EPI_ISL_930757, EPI_ISL_930758, EPI_ISL_930759, EPI_ISL_930761, EPI_ISL_930762, EPI_ISL_930763, EPI_ISL_930765, EPI_ISL_930767, EPI_ISL_930768, EPI_ISL_930777, EPI_ISL_930779, EPI_ISL_930781			
see above	Utah Public Health Laboratory	Utah Public Health Laboratory	Erin L. Young, Kelly F. Oakeson, Tara Gallagher
EPI_ISL_931471	Maryland Public Health Laboratory (MD PHL)	Maryland Public Health Laboratory (MD PHL)	Maryland Department of Health Laboratories Administration
EPI_ISL_931507, EPI_ISL_931516, EPI_ISL_931537, EPI_ISL_931539, EPI_ISL_931540	Utah Public Health Laboratory	Utah Public Health Laboratory	Erin L. Young, Kelly F. Oakeson, Tara Gallagher
EPI_ISL_933426, EPI_ISL_933427, EPI_ISL_933428, EPI_ISL_933429, EPI_ISL_933431, EPI_ISL_933432, EPI_ISL_933433, EPI_ISL_933434, EPI_ISL_933436, EPI_ISL_933437, EPI_ISL_933438, EPI_ISL_933439, EPI_ISL_933440, EPI_ISL_933447, EPI_ISL_933453, EPI_ISL_933456, EPI_ISL_933463, EPI_ISL_933467, EPI_ISL_933469, EPI_ISL_933472, EPI_ISL_933474, EPI_ISL_933475			
see above	Lighthouse Lab in Milton Keynes	Wellcome Sanger Institute for the COVID-19 Genomics UK (COG-UK) Consortium	The Lighthouse Lab in Milton Keynes and Alex Alderton, Roberto Amato, Sonia Goncalves, Ewan Harrison, David K. Jackson, Ian Johnston, Dominic Kwiatkowski, Cordelia Langford, John Sillitoe on behalf of the Wellcome Sanger Institute COVID-19 Surveillance Team

EPI_ISL_934349	Klinisk mikrobiologi	The Public Health Agency of Sweden	Anna-Malin Linde, Maria Lind Karlberg, Carlo Berg, Oskar Karlsson Lindsjo, Sofia Stamouli, Reza Advani, Mattias Haukland, Petra Holmstrom, Noura Walai, Petra Edquist, Mia Brytting, Anna Risberg, Karin Tegmark-Wisell
EPI_ISL_934388	Klinisk Mikrobiologi	The Public Health Agency of Sweden	Anna-Malin Linde, Maria Lind Karlberg, Carlo Berg, Oskar Karlsson Lindsjo, Sofia Stamouli, Reza Advani, Mattias Haukland, Petra Holmstrom, Noura Walai, Petra Edquist, Mia Brytting, Anna Risberg, Karin Tegmark-Wisell
EPI_ISL_934639, EPI_ISL_934642, EPI_ISL_934646	Department of Microbiology, University Innsbruck	Bergthaler laboratory, CeMM Research Center for Molecular Medicine of the Austrian Academy of Sciences	Lukas Endler, Anna Schedl, Thomas Penz, Benedikt Agerer, Maelle Le Moing, Michael Schuster, Bekir Erguner, Jan Laine, Martin Senekowitsch, Christoph Bock, Andreas Bergthaler
EPI_ISL_935209	KU Leuven, Rega Institute, Clinical and Epidemiological Virology	KU Leuven, Rega Institute, Clinical and Epidemiological Virology	Tony Wawina-Bokalanga, Bert Vanmechelen, Joan Marti-Carerras, Piet Maes
EPI_ISL_935708, EPI_ISL_935709, EPI_ISL_935710, EPI_ISL_935750, EPI_ISL_935751, EPI_ISL_935752	Houston Health Dept.	Houston Health Dept.	Ryker Penn, Pamela Brown, Adolpho Lara
EPI_ISL_936036, EPI_ISL_936037, EPI_ISL_936038, EPI_ISL_936039, EPI_ISL_936040, EPI_ISL_936041, EPI_ISL_936042, EPI_ISL_936043	Columbia University Irving Medical Center	Wadsworth Center, New York State Department of Health	Kirsten St. George, Daryl M. Lamson, Alexis Russel, Matthew Shudt, Melissa A Leisner, Jonathan Plitnick, Navjot Singh, John Kelly, Erasmus Schneider, Erica Lasek-Nesselquist
EPI_ISL_936180	ALBANY MEDICAL CENTER	Wadsworth Center, New York State Department of Health	Kirsten St. George, Daryl M. Lamson, Alexis Russel, Matthew Shudt, Melissa A Leisner, Jonathan Plitnick, Navjot Singh, John Kelly, Erasmus Schneider, Erica Lasek-Nesselquist
EPI_ISL_936424	TGen North	TGen North	Jolene Bowers, Megan Folkerts, Chris French, Hayley Yaglom, Ashlyn Pfeiffer, Darrin Lemmer, Dave Engelthaler, The Arizona COVID Genomics Union (ACGU)
EPI_ISL_936813, EPI_ISL_936814, EPI_ISL_936815, EPI_ISL_936816	Northwestern Memorial Hospital	Ozer Lab	Ramon Lorenzo-Redondo, Lacy M. Simons, Chad J. Achenbach, Lawrence J. Jennings, Michael G. Ison, Judd F. Hultquist, Egon A. Ozer
EPI_ISL_940149	NHLS Universitas Academic	UFS Virology	PA Bester, MM Nyaga, P Nthiga, MT Mogotsi, D Goedhals, T de Oliveira
EPI_ISL_940802, EPI_ISL_940819, EPI_ISL_940820	Houston Health Dept.	Houston Health Dept.	Ryker Penn, Pamela Brown, Adolpho Lara
EPI_ISL_940861, EPI_ISL_940883	Vaccines and Infectious Diseases Analytics Research Unit (VIDA)	KRISP, KZN Research Innovation and Sequencing Platform	Baillie Vicky, du Plessis Jeanine, Giandhari Jennifer, Pillay Sureshnee, Naidoo Yeshnee, Tegally Houriiyah, de Oliveira Tulio, Madhi Shabir
EPI_ISL_941233, EPI_ISL_941234, EPI_ISL_941235, EPI_ISL_941236, EPI_ISL_941237, EPI_ISL_941238, EPI_ISL_941239	Hospital Universitario de La Ribera (Alzira, València)	SeqCOVID-SPAIN consortium/IBV(CSIC)	Olalla Martínez Macias, Julia González Cantó and SeqCOVID-SPAIN consortium
EPI_ISL_941269	Virginia DCLS	Virginia DCLS	Virginia DCLS
EPI_ISL_941624	Instituto Nacional de Saude (INSA)	Instituto Nacional de Saude (INSA)	Borges et al
EPI_ISL_941932	Florida Bureau of Public Health Laboratories	Florida Bureau of Public Health Laboratories	Sarah Schmedes, Jason Blanton
EPI_ISL_942096, EPI_ISL_942142	Wisconsin State Laboratory of Hygiene Communicable Disease Division	Wisconsin State Laboratory of Hygiene Communicable Disease Division	Kelsey R. Florek, Abigail C. Shockey
EPI_ISL_942753, EPI_ISL_942754, EPI_ISL_942755, EPI_ISL_942756, EPI_ISL_942757, EPI_ISL_942758, EPI_ISL_942759, EPI_ISL_942760, EPI_ISL_942761, EPI_ISL_942762	Gundersen Molecular Diagnostics Laboratory	Kabara Cancer Research Institute	Craig S. Richmond, Paraic A. Kenny
EPI_ISL_943568	Servizo de Microbioloxía. Complexo Hospitalario Universitario de Santiago de Compostela	Servizo de Microbioloxía. Complexo Hospitalario Universitario de Santiago de Compostela	Antonio Aguilera, Gema Barbeito, Amparo Coira, José Costa, Rocio Trastoy, María Luisa Pérez del Molino.
EPI_ISL_943818	Utah Public Health Laboratory	Utah Public Health Laboratory	Erin L. Young, Kelly F. Oakeson, Tara Gallagher
EPI_ISL_945126, EPI_ISL_945136	Lighthouse Lab in Glasgow	Wellcome Sanger Institute for the COVID-19 Genomics UK (COG-UK) Consortium	Harper VanSteenhouse, Yumi Kasai, David Gray, Carol Clugston, Anna Dominiczak and Alex Alderton, Roberto Amato, Sonia Goncalves, Ewan Harrison, David K. Jackson, Ian Johnston, Dominic Kwiatkowski, Cordelia Langford, John Sillitoe on behalf of the Wellcome Sanger Institute COVID-19 Surveillance Team
EPI_ISL_945231	Lighthouse Lab in Milton Keynes	Wellcome Sanger Institute for the COVID-19 Genomics UK (COG-UK) Consortium	The Lighthouse Lab in Milton Keynes and Alex Alderton, Roberto Amato, Sonia Goncalves, Ewan Harrison, David K. Jackson, Ian Johnston, Dominic Kwiatkowski, Cordelia Langford, John Sillitoe on behalf of the Wellcome Sanger Institute COVID-19 Surveillance Team
EPI_ISL_945335	Lighthouse Lab in Alderley Park	Wellcome Sanger Institute for the COVID-19 Genomics UK (COG-UK) Consortium	Jacquelyn Wynn, Mairead Hyland, The Lighthouse Lab in Alderley Park and Alex Alderton, Roberto Amato, Sonia Goncalves, Ewan Harrison, David K. Jackson, Ian Johnston, Dominic Kwiatkowski, Cordelia Langford, John Sillitoe on behalf of the Wellcome Sanger Institute COVID-19 Surveillance Team
EPI_ISL_949761	Barts Health NHS Trust	COVID-19 Genomics UK (COG-UK) Consortium	CUTINO-MOGUEL, Maria-Teresa; HARRINGTON, David; OWOYEMI, Dola; KULASEGARAN-SHYLINI, Raghavendran; BROAD, Claire; KELE, Beatrix
EPI_ISL_950105, EPI_ISL_950138, EPI_ISL_950145	University College London, Great Ormond Street Hospital for Children NHS Foundation Trust, Imperial College Healthcare NHS Trust	COVID-19 Genomics UK (COG-UK) Consortium	Sergi Castellano, Rachel Williams, Mark Kristiansen, Paola Resende Silva, Sunando Roy, Tony Brooks, Helena Tutill, Paola Niola, Patricia Dyal, Charlotte Williams, Leysa Forrest, Yasmin Panchbhaya, Jacqueline Findlay, Samuel Weeks, Julianne Brown, Kathryn Harris, Paul Randell, James Price, Alison Holmes, Judith Breuer
EPI_ISL_950240, EPI_ISL_950436, EPI_ISL_950438, EPI_ISL_950439, EPI_ISL_950440, EPI_ISL_950441, EPI_ISL_950442, EPI_ISL_950443	Northumbria University / South Tees Hospitals NHS Foundation Trust / North Cumbria Integrated Care NHS Foundation Trust / North Tees and Hartlepool NHS Foundation Trust / Newcastle Hospitals NHS Foundation Trust	COVID-19 Genomics UK (COG-UK) Consortium	Darren L Smith,Andrew Nelson,Matthew Bashton,Greg R Young,Joshua Loh,John Allan,Mohammad A Tariq,Giles S Holt,Gary Black,Wen C Yew,Lynn Dover,Paul Baker,Steve Liggett,Sarah Essex,Jane Greenaway,Debra Padgett,Clive Graham,Garren Scott,Edward Barton,Emma Swindells,Brendan Payne,Jennifer Collins,Yusri Taha,Gary Eltringham
EPI_ISL_950544, EPI_ISL_950560, EPI_ISL_950561, EPI_ISL_950562, EPI_ISL_950572, EPI_ISL_950578, EPI_ISL_950579, EPI_ISL_950583, EPI_ISL_950584, EPI_ISL_950589, EPI_ISL_950595			
see above	Quadram Institute Bioscience	COVID-19 Genomics UK (COG-UK) Consortium	Dave J. Baker, Gemma L. Kay, Alp Aydin, Thanh Le-Viet, Steven Rudder, Ana P. Tedim, Anastasia Kolyva, Maria Diaz, Leonardo de Oliveira Martins, Nabil-Fareed Alikhan, Lizzie Meadows, Rachael Stanley, Ngozi Elumogo, Muhammed Yasir, Nicholas M. Thomson, Alexander J Trotter, Rachel Gilroy, Samuel Bloomfield, Claire Stuart, Andrew Bell, Reenesh Prakash, Samir Dervisevic, Alison E. Mather, John Wain, Mark Webber, Andrew J. Page, Justin O'Grady
EPI_ISL_950849, EPI_ISL_950850, EPI_ISL_950851, EPI_ISL_950852, EPI_ISL_950853, EPI_ISL_950854, EPI_ISL_950855, EPI_ISL_950856, EPI_ISL_950857, EPI_ISL_950858, EPI_ISL_950859, EPI_ISL_950861, EPI_ISL_950862, EPI_ISL_950863, EPI_ISL_950864, EPI_ISL_950865, EPI_ISL_950868, EPI_ISL_950871, EPI_ISL_950879, EPI_ISL_950880, EPI_ISL_950881, EPI_ISL_950882, EPI_ISL_950884, EPI_ISL_950885, EPI_ISL_950886, EPI_ISL_950887, EPI_ISL_950889, EPI_ISL_950890, EPI_ISL_950891, EPI_ISL_950892, EPI_ISL_950893, EPI_ISL_950894, EPI_ISL_950895, EPI_ISL_950896, EPI_ISL_950897, EPI_ISL_950898, EPI_ISL_950899, EPI_ISL_950955, EPI_ISL_950961, EPI_ISL_950963, EPI_ISL_950964, EPI_ISL_950966, EPI_ISL_950968, EPI_ISL_950970, EPI_ISL_950971, EPI_ISL_950972, EPI_ISL_950973, EPI_ISL_950974, EPI_ISL_950975, EPI_ISL_950976, EPI_ISL_950977, EPI_ISL_950984, EPI_ISL_950997, EPI_ISL_951005, EPI_ISL_951006, EPI_ISL_951007, EPI_ISL_951012, EPI_ISL_951015, EPI_ISL_951343			
see above	Oxford Viromics, NDM, University of Oxford; Oxford University Hospitals; Basingstoke and North Hampshire Hospital	COVID-19 Genomics UK (COG-UK) Consortium	Tanya Golubchik, David Bonsall, George Macintyre, Amy Trebes, Mariateresa de Cesare, Catrin Moore, Alex Mobbs, Anita Justice, Robert Shaw, Monique Andersson, Timothy Peto, Emma Wise, Nathan Moore, Jessica Lynch, Nick Cortes, Matilde Mori, Stephen Kidd, David Buck, John Todd, Christophe Fraser
EPI_ISL_954210	1.AO Universitaria 'S. Giovanni di Dio e Ruggi D'Aragona, Scuola Medica Salernitana' Hospital / 2.UOC di Virologia e Microbiologia, Università della Campania 'L. Vanvitelli' / 3.AO	1. Genome Research Center for Health (CRGS) / 2. Laboratory of Molecular Medicine and Genomics(LMMGe) / 3. Center for Research in Pure and Applied Mathematics	Giorgio Giurato, Francesca Rizzo, Alessandro Weisz, Gianluigi Franci, Giovanni Nassa, Pasquale Pagliano, Roberta Tarallo, Elena Alexandrova, Ylenia D'Agostino, Carlo Ferravante, Jessica Lamberti, Viola Melone, Domenico Memoli, Valeria Mirici Cappa, Domenico Palumbo, Giovanni Pecoraro, Assunta Sellitto, Oriana Strianese, Ilaria Terenzi, Giuseppe Fenza, Aniello Gentile, Antonello Saccomanno, Sonia Amabile, Teresa Rocco, Annamaria Salvati,

	Universitaria 'Federico II' Napoli Hospital / 4.AORN 'San Giuseppe Moscati' Avellino Hospital / 5.AO 'San Pio - presidio G. Rummo' Benevento Hospital / 6.AO 'Sant'Anna e San Sebastiano' Caserta Hospital / 7.PO 'Maria Santissima Addolorata' Eboli Hospital / 8.Biogem Istituto di Ricerche Genetiche	(CRMPA)	Emilia Vaccaro, Massimiliano Galdiero, Michele Cennamo, Giuseppe Portella, Maria Grazia Foti, Mariarosaria Ingino, Maria Landi, Maurizio Fumi, Vincenzo Rocco, Rita Greco, Vittoria Letizia, Arnolfo Petruzziello, Maddalena Schioppa, Gregorio Goffredi, Francesca Marciano, Michele Caraglia, Alessia Cossu, Marianna Scrima, Edmondo Adorisio, Morena D'Avenia, Michela Iacobellis, Rosanna Piluscio, Giorgio Dirani, Vittorio Sambri, Simona Semprini, Silvia Zanoli, Francesco Curcio, Stefania Marzinotto, Andreina Baj, Fausto Sessa.
EPI_ISL_954227, EPI_ISL_954230, EPI_ISL_954256, EPI_ISL_954257, EPI_ISL_954258, EPI_ISL_954259, EPI_ISL_954260, EPI_ISL_954261, EPI_ISL_954262, EPI_ISL_954263, EPI_ISL_954264, EPI_ISL_954266, EPI_ISL_954267, EPI_ISL_954268, EPI_ISL_954269, EPI_ISL_954270, EPI_ISL_954271, EPI_ISL_954272, EPI_ISL_954273, EPI_ISL_954274, EPI_ISL_954275, EPI_ISL_954276, EPI_ISL_954277, EPI_ISL_954278, EPI_ISL_954279, EPI_ISL_954280			
see above	MRC/UVRI & LSHTM Uganda Research Unit	Where sequence data have been generated and submitted to GISAID	Matthew Cotten, Dan Lule Bugembe, My V.T. Phan, Isaac Sseeewanyana, Patrick Semanda, Susan Nabadda, Pontiano Kaleebu
EPI_ISL_954869	Colorado Department of Public Health and Environment	Colorado Department of Puplic Health and Environment	Laura Bankers, Molly C. Hetherington-Rauth, Diana Ir, Shannon Ely, Shannon R. Matzinger, Sarah Elizabeth Totten, Emily A. Travanty
EPI_ISL_955128	Innovative Genomics Institute, UC Berkeley	Innovative Genomics Institute, UC Berkeley	Stacia Wyman, Haridha Shivram, Phil Frankino, Liana Lareau, Shana McDevitt, Justin Choi
EPI_ISL_955261, EPI_ISL_955263	Fulgent Genetics	Fulgent Genetics	Harry Gao, Mickey Li, John Gao, Joseph Fierro, Benafsh Sapra, Becky Tsai, Yan Meng, Doreen Ng, James Xie
EPI_ISL_955389	Alameda County Public Health Lab	Chan-Zuckerberg Biohub	CZB Cliahub Consortium
EPI_ISL_955697, EPI_ISL_955698, EPI_ISL_955700, EPI_ISL_955704, EPI_ISL_955706, EPI_ISL_955707, EPI_ISL_955708, EPI_ISL_955709, EPI_ISL_955710, EPI_ISL_955711, EPI_ISL_955712, EPI_ISL_955713, EPI_ISL_955714, EPI_ISL_955715, EPI_ISL_955716, EPI_ISL_955717, EPI_ISL_955718, EPI_ISL_955719, EPI_ISL_955720, EPI_ISL_955721, EPI_ISL_955722, EPI_ISL_955723, EPI_ISL_955724, EPI_ISL_955725, EPI_ISL_955726, EPI_ISL_955732, EPI_ISL_955733			
see above	Humboldt County Public Health Laboratory	Chan-Zuckerberg Biohub	CZB Cliahub Consortium
EPI_ISL_956352, EPI_ISL_956353, EPI_ISL_956354	Houston Health Department, Disease Prevention and Control	Houston Health Department, Disease Prevention and Control	Penn,R., Brown,P., Lara,A.
EPI_ISL_956402	General Hospital - Ohrid	Research Center for Genetic Engineering and Biotechnology "Georgi D. Efremov" , Macedonian Academy of Sciences and Arts	Aleksandar J. Dimovski, Dijana Plasheska-Karanfilska, Predrag Noveski, Gjorgji Bozinovski, Milena Jakimovska
EPI_ISL_956403	Institute for prevention, treatment and rehabilitation of cardiovascular diseases - Ohrid	Research Center for Genetic Engineering and Biotechnology "Georgi D. Efremov" , Macedonian Academy of Sciences and Arts	Aleksandar J. Dimovski, Dijana Plasheska-Karanfilska, Predrag Noveski, Gjorgji Bozinovski, Milena Jakimovska
EPI_ISL_960463, EPI_ISL_960464, EPI_ISL_960471, EPI_ISL_960560, EPI_ISL_960561, EPI_ISL_960562, EPI_ISL_960563, EPI_ISL_960564, EPI_ISL_960565, EPI_ISL_960566, EPI_ISL_960567, EPI_ISL_960649, EPI_ISL_960650, EPI_ISL_960651, EPI_ISL_960652, EPI_ISL_960653, EPI_ISL_960654, EPI_ISL_960655, EPI_ISL_960656, EPI_ISL_960657, EPI_ISL_960659			
see above	Istituto Zooprofilattico Sperimentale del Mezzogiorno	TIGEM	Patrizia Annunziata, Andrea Ballabio, Valentina Bouche, Davide Cacchiarelli, Pellegrino Cerino, Chiara Colantuono, Maria Concetta Cuomo, Denise Di Concilio, Lucio Di Filippo, Antonio Grimaldi, Antonio Limone, Anna Manfredi, Francesco Panariello, Biancamaria Pierri, Marcello Salvi
EPI_ISL_960669, EPI_ISL_960672, EPI_ISL_960675, EPI_ISL_960681, EPI_ISL_960682, EPI_ISL_960692, EPI_ISL_960693, EPI_ISL_960708, EPI_ISL_960718, EPI_ISL_960719, EPI_ISL_960723, EPI_ISL_960748, EPI_ISL_960749, EPI_ISL_960750, EPI_ISL_960762, EPI_ISL_960783, EPI_ISL_960823, EPI_ISL_960824			
see above	Germano de sousa	Instituto Gulbenkian de Ciencia	Susana Ladeiro, Cathy Paulino, João Costa, João Sobral, Maria Costa, Ricardo Leite
EPI_ISL_962013	Illinois Department of Public Health	Gagnon Lab, Southern Illinois University	Keith Gagnon
EPI_ISL_962876	UCLA Clinical Micro Lab	Los Angeles County PHL	P. Hemarajata et al.
EPI_ISL_962877, EPI_ISL_962878	National Virology Reference Laboratory	National Public Health Laboratory, National Centre for Infectious Diseases	Tze Minn Mak, Zhenyang Zhou, Zaini Zainun, Taib Surita, Lin Cui, Raymond Tzer Pin Lin
EPI_ISL_962930, EPI_ISL_962931, EPI_ISL_962932, EPI_ISL_962942	Hospital Universitario de Gran Canaria Dr. Negrín	SeqCOVID-SPAIN consortium/IBV(CSIC)	M. Carmen Pérez González, Francisco J. Chamizo López, Ana Bordes Benítez and SeqCOVID-SPAIN consortium
EPI_ISL_965217	Virginia Division of Consolidated Laboratory Services	Virginia Division of Consolidated Laboratory Services	Virginia DCLS
EPI_ISL_965531, EPI_ISL_965534, EPI_ISL_965555, EPI_ISL_965588, EPI_ISL_965591, EPI_ISL_965642, EPI_ISL_965669, EPI_ISL_965701, EPI_ISL_965708, EPI_ISL_965726, EPI_ISL_965739, EPI_ISL_965742, EPI_ISL_965743, EPI_ISL_965744, EPI_ISL_965753, EPI_ISL_965757, EPI_ISL_965760, EPI_ISL_965768, EPI_ISL_965777			
see above	Dutch COVID-19 response team	Medical Microbiology, Maastricht University Medical Centre	Jozef Dingemans*, Brian van der Veer*, Erik Beuken, Carmen Reumkens, Lieke van Alphen, Christian Hoebe, Paul Savelkoul
EPI_ISL_967591, EPI_ISL_967615, EPI_ISL_967616, EPI_ISL_967619, EPI_ISL_967622, EPI_ISL_967623, EPI_ISL_967626, EPI_ISL_967627, EPI_ISL_967633, EPI_ISL_967636, EPI_ISL_967640, EPI_ISL_967645, EPI_ISL_967646, EPI_ISL_967679, EPI_ISL_967687, EPI_ISL_967688, EPI_ISL_967725, EPI_ISL_967728, EPI_ISL_967730, EPI_ISL_967752			
see above	State Laboratories Division, Hawaii State Department of Health	State Laboratories Division, Hawaii State Department of Health	Pamela O'Brien, Drew Kuwazaki, Ayana Garnet, Razvan Sultana, Edward Desmond
EPI_ISL_968110, EPI_ISL_968111, EPI_ISL_968137, EPI_ISL_968138, EPI_ISL_968139	Houston Health Department, Disease Prevention and Control	Houston Health Department, Disease Prevention and Control	Penn,R., Brown,P., Lara,A.
EPI_ISL_970811, EPI_ISL_970821, EPI_ISL_970942, EPI_ISL_971140, EPI_ISL_971279, EPI_ISL_971532, EPI_ISL_971617, EPI_ISL_971844, EPI_ISL_971862, EPI_ISL_971872, EPI_ISL_971921, EPI_ISL_971945, EPI_ISL_972051, EPI_ISL_972263, EPI_ISL_972342, EPI_ISL_972479, EPI_ISL_972653, EPI_ISL_972714, EPI_ISL_972752, EPI_ISL_972805, EPI_ISL_972811, EPI_ISL_972871, EPI_ISL_972882, EPI_ISL_972929, EPI_ISL_972972, EPI_ISL_972996, EPI_ISL_973116, EPI_ISL_973213, EPI_ISL_973234, EPI_ISL_973342, EPI_ISL_973369, EPI_ISL_973415, EPI_ISL_973417, EPI_ISL_973463, EPI_ISL_973582			
see above	Department of Virus and Microbiological Special Diagnostics, Statens Serum Institut, Copenhagen, Denmark	Aalborg University	Danish Covid-19 Genome Consortium
EPI_ISL_976790, EPI_ISL_976791, EPI_ISL_976792, EPI_ISL_976793, EPI_ISL_976794, EPI_ISL_976795, EPI_ISL_976796, EPI_ISL_976797, EPI_ISL_976798, EPI_ISL_976799, EPI_ISL_976800, EPI_ISL_976801, EPI_ISL_976802, EPI_ISL_976803, EPI_ISL_976804, EPI_ISL_976805, EPI_ISL_976806, EPI_ISL_976807, EPI_ISL_976808, EPI_ISL_976809, EPI_ISL_976810, EPI_ISL_976811, EPI_ISL_976812, EPI_ISL_976813, EPI_ISL_976814, EPI_ISL_976815, EPI_ISL_976817, EPI_ISL_976818, EPI_ISL_976819, EPI_ISL_976820, EPI_ISL_976821, EPI_ISL_976822, EPI_ISL_976823, EPI_ISL_976824, EPI_ISL_976825, EPI_ISL_976826, EPI_ISL_976827, EPI_ISL_976828, EPI_ISL_976829, EPI_ISL_976830, EPI_ISL_976831, EPI_ISL_976832, EPI_ISL_976833, EPI_ISL_976834, EPI_ISL_976835, EPI_ISL_976836, EPI_ISL_976837, EPI_ISL_976838, EPI_ISL_976839, EPI_ISL_976840, EPI_ISL_976841, EPI_ISL_976842, EPI_ISL_976843, EPI_ISL_976844, EPI_ISL_976845, EPI_ISL_976846, EPI_ISL_976847, EPI_ISL_976848, EPI_ISL_976849, EPI_ISL_976850			
see above	BCCDC Public Health Laboratory	BCCDC Public Health Laboratory	Prystajecky Natalie, Linda Hoang, Dan Fornika, John Tyson, Shannon Russell, Kim Macdonald, Kimia Kamelian, Ana Pacagnella, Corrinne Ng, Loretta Janz, Robert Azana Terry Snutch, Mel Krajden
EPI_ISL_977082	Massachusetts General Hospital	Infectious Disease Program, Broad Institute of Harvard and MIT	Lemieux,J.E., Siddle,K.J., Shaw,B., Adams,G., Pierce,V., Turbett,S., Anahtar,M., Branda,J., Slater,D., Harris,J., Lin,A.E., Gladden-Young,A., Lagerborg,K., Rudy,M., DeRuff,K., Carter,A., Normandin,E., Bauer,M., Reilly,S., Tomkins-Tinch,C., Loreth,C., Chaluvadi,S., Neumann,A., Cusick,C., Chapman,S.B., Gnirke,A., Flowers,K., Cerrato,F., Birren,B.W., Gallagher,G., Smole,S., Park,D.J., MacInnis,B.L., Ryan,E., LaRocque,R., Rosenberg,E. and Sabeti,P.C.
EPI_ISL_977156, EPI_ISL_977249, EPI_ISL_977250	ULSS 03 Venezia	Istituto Zooprofilattico Sperimentale delle Venezie	Adelaide Milani, Alessia Schivo, Annalisa Salviato, Erika Giorgia Quaranta, Ambra Pastori, Bianca Zecchin, Alice Fusaro, Isabella Monne, Calogero Terregino, Antonia Ricci
EPI_ISL_977721, EPI_ISL_977722, EPI_ISL_977723, EPI_ISL_977726, EPI_ISL_977727, EPI_ISL_977728, EPI_ISL_977729, EPI_ISL_977730, EPI_ISL_977733, EPI_ISL_977734, EPI_ISL_977740			
see above	California Department of Public Health	Chiu Laboratory, University of California, San Francisco	Charles Chiu, Xianding (Wayne) Deng, Candace Wang, Venice Servellita, Jill Hacker, Debra Wadford
EPI_ISL_978044, EPI_ISL_978048, EPI_ISL_978051, EPI_ISL_978052, EPI_ISL_978053, EPI_ISL_978054, EPI_ISL_978055, EPI_ISL_978056, EPI_ISL_978070, EPI_ISL_978071, EPI_ISL_978072, EPI_ISL_978073, EPI_ISL_978074			
see above	Chiu Laboratory, University of California, San Francisco	Chiu Laboratory, University of California, San Francisco	Charles Chiu, Xianding (Wayne) Deng, Candace Wang, Venice Servellita, Jill Hacker, Debra Wadford
EPI_ISL_978417, EPI_ISL_978418, EPI_ISL_978419, EPI_ISL_978420, EPI_ISL_978421, EPI_ISL_978422,	Arizona State Public Health Laboratory	Arizona State Public Health Laboratory	Trung Huynh, Jessica Escobar, Katherine Fullerton, Nobuko Fukushima, Stacy White, Linda Getsinger, Victor Waddell

EPI_ISL_978423			
EPI_ISL_978960	Chiu Laboratory, University of California, San Francisco	Chiu Laboratory, University of California, San Francisco	Charles Chiu, Xianding (Wayne) Deng, Candace Wang, Venice Servellita, Jill Hacker, Debra Wadford
EPI_ISL_979060	Santa Clara County Public Health Laboratory	Chan-Zuckerberg Biohub	CZB Cliahub Consortium
EPI_ISL_979246	Institute of Microbiology and Immunology, Faculty of Medicine, University of Ljubljana	Institute of Microbiology and Immunology, Faculty of Medicine, University of Ljubljana	Samo Zakotnik, Tomaž Mark Zorec, Matic Brvar, Doroteja Vlaj, Patricija Pozvek,Špela Pleh, Miša Korva, Mario Poljak, Tatjana Avši - Županc
EPI_ISL_979335, EPI_ISL_979336, EPI_ISL_979337	Laboratorio Estatal de Salud Pública de Nuevo León	Laboratorio de Infectología Molecular, Departamento de Bioquímica y Medicina Molecular,Facultad de Medicina - Universidad Autónoma de Nuevo León	Kame A. Galán-Huerta, María F. Herrera-Saldivar, Natalia Martínez-Acuña, Sonia A. Lozano-Sepúlveda, Daniel Arellanos-Soto, Ana M. Rivas-Estilla, Samuel Buentello-Wong, Else del Carmen García-García, Gloria A. Jasso-de-la-Peña, Roberto Montes-de-Oca, Consuelo Treviño-Garza, Manuel E. de-la-O-Cavazos
EPI_ISL_979596, EPI_ISL_979597, EPI_ISL_979598, EPI_ISL_979599, EPI_ISL_979600, EPI_ISL_979601, EPI_ISL_979602, EPI_ISL_979603, EPI_ISL_979604, EPI_ISL_979605, EPI_ISL_979606, EPI_ISL_979612, EPI_ISL_979613, EPI_ISL_979629, EPI_ISL_979630			
see above	Santa Clara County Public Health Laboratory	Chan-Zuckerberg Biohub	CZB Cliahub Consortium
EPI_ISL_979656, EPI_ISL_979657	Orange County Public Health Lab	Chan-Zuckerberg Biohub	CZB Cliahub Consortium
EPI_ISL_981062, EPI_ISL_981068	Johns Hopkins Hospital Department of Pathology	Johns Hopkins Hospital Department of Pathology	C. Paul Morris, Chun Huai Luo, Adannaya Amadi, Matthew Schwartz, Nicholas Gallagher, Heba H. Mostafa
EPI_ISL_983323	INMI Lazzaro Spallanzani IRCCS	INMI Lazzaro Spallanzani IRCCS	CEM Gruber, B Bartolini, E Giombini, M Rueca, O Butera, F Messina, A Di Caro, MR Capobianchi
EPI_ISL_983853, EPI_ISL_983854, EPI_ISL_983855	Colorado Department of Public Health and Environment	Colorado Department of Puplic Health and Environment	Laura Bankers, Molly C. Hetherington-Rauth, Diana Ir, Shannon Ely, Shannon R. Matzinger, Sarah Elizabeth Totten, Emily A. Travanty
