# Supplementary material for: Detection and characterization of the SARS-CoV-2 lineage B.1.526 in New York
Source: Nat Commun. 2021 Aug 9;12:4886. doi: 10.1038/s41467-021-25168-4 (PMC8352861; doi:10.1038/s41467-021-25168-4)
Supplement: Supplementary file 8 — Supplementary Data 4 [file 41467_2021_25168_MOESM8_ESM.zip › GISAID_acknowledements_tables/gisaid_hcov-19_acknowledgement_table_2021_02_12_23-9.pdf]

We gratefully acknowledge the following Authors from the Originating laboratories responsible for obtaining the specimens, as well as the Submitting laboratories where the genome data were generated and shared via GISAID, on which this research is based.

All Submitters of data may be contacted directly via www.gisaid.org

Authors are sorted alphabetically.

Accession ID	Originating Laboratory	Submitting Laboratory	Authors
EPI_ISL_572405	Wales Specialist Virology Centre Sequencing lab: Pathogen Genomics Unit	COVID-19 Genomics UK (COG-UK) Consortium	Catherine Moore, Johnathan Evans, Laura Gifford, Malorie Perry, Simon Cottrell, Angela Marchbank, Alec Birchley, Alexander Adams, Amy Gaskin, Bree Gatica-Wilcox, Jason Coombes, Joel Southgate, Lauren Gilbert, Lee Graham, Nicole Pacchiarini, Sara Kumziene-Summerhayes, Sarah Taylor, Sophie Jones, Sara Rey, Matthew Bull, Joanne Watkins, Sally Corden, Tom Connor
EPI_ISL_572419	Quadram Institute Bioscience	COVID-19 Genomics UK (COG-UK) Consortium	Dave J. Baker, Gemma L. Kay, Alp Aydin, Thanh Le-Viet, Steven Rudder, Ana P. Tedim, Anastasia Kolyva, Maria Diaz, Leonardo de Oliveira Martins, Nabil-Fareed Alikhan, Lizzie Meadows, Rachael Stanley, Ngozi Elumogo, Muhammed Yasir, Nicholas M. Thomson, Alexander J Trotter, Rachel Gilroy, Samuel Bloomfield, Claire Stuart, Andrew Bell, Reenesh Prakash, Samir Dervisevic, Alison E. Mather, John Wain, Mark Webber, Andrew J. Page, Justin O'Grady
EPI_ISL_572444, EPI_ISL_572460	Queens Medical Centre, Clinical Microbiology Department / DeepSeq Nottingham	COVID-19 Genomics UK (COG-UK) Consortium	Gemma Clark, Wendy Smith, Manjinder Khakh, Vicki M Fleming, Michelle M Lister, Hannah Howson-Wells, Jonathan Ball, Patrick McClure, Joseph Chappell, Theocharis Tsoleridis, Nadine Holmes, Matthew Carlisle, Christopher Moore, Fei Sang, Johnny Debebe, Victoria Wright, Matthew Loose
EPI_ISL_572462	Quadram Institute Bioscience	COVID-19 Genomics UK (COG-UK) Consortium	Dave J. Baker, Gemma L. Kay, Alp Aydin, Thanh Le-Viet, Steven Rudder, Ana P. Tedim, Anastasia Kolyva, Maria Diaz, Leonardo de Oliveira Martins, Nabil-Fareed Alikhan, Lizzie Meadows, Rachael Stanley, Ngozi Elumogo, Muhammed Yasir, Nicholas M. Thomson, Alexander J Trotter, Rachel Gilroy, Samuel Bloomfield, Claire Stuart, Andrew Bell, Reenesh Prakash, Samir Dervisevic, Alison E. Mather, John Wain, Mark Webber, Andrew J. Page, Justin O'Grady
EPI_ISL_572474	Wales Specialist Virology Centre Sequencing lab: Pathogen Genomics Unit	COVID-19 Genomics UK (COG-UK) Consortium	Catherine Moore, Johnathan Evans, Laura Gifford, Malorie Perry, Simon Cottrell, Angela Marchbank, Alec Birchley, Alexander Adams, Amy Gaskin, Bree Gatica-Wilcox, Jason Coombes, Joel Southgate, Lauren Gilbert, Lee Graham, Nicole Pacchiarini, Sara Kumziene-Summerhayes, Sarah Taylor, Sophie Jones, Sara Rey, Matthew Bull, Joanne Watkins, Sally Corden, Tom Connor
EPI_ISL_572475, EPI_ISL_572476	Queens Medical Centre, Clinical Microbiology Department / DeepSeq Nottingham	COVID-19 Genomics UK (COG-UK) Consortium	Gemma Clark, Wendy Smith, Manjinder Khakh, Vicki M Fleming, Michelle M Lister, Hannah Howson-Wells, Jonathan Ball, Patrick McClure, Joseph Chappell, Theocharis Tsoleridis, Nadine Holmes, Matthew Carlisle, Christopher Moore, Fei Sang, Johnny Debebe, Victoria Wright, Matthew Loose
EPI_ISL_572481	Virology Department, Sheffield Teaching Hospitals NHS Foundation Trust/Department of Infection, Immunity and Cardiovascular Disease, The Medical School, University of Sheffield	COVID-19 Genomics UK (COG-UK) Consortium	Thushan de Silva, Matthew Parker, Nikki Smith, Adri Angyal, Rebecca Brown, Luke Green, Rachel Tucker, Paul Parsons, Danielle Groves, Katie Johnson, Laura Carrilero, Alex Keeley, Dave Partridge, Matthew Wyles, Benjamin Lindsey, Mehmet Yavuz, Mohammad Raza, Cariad Evans
EPI_ISL_572501	Wales Specialist Virology Centre Sequencing lab: Pathogen Genomics Unit	COVID-19 Genomics UK (COG-UK) Consortium	Catherine Moore, Johnathan Evans, Laura Gifford, Malorie Perry, Simon Cottrell, Angela Marchbank, Alec Birchley, Alexander Adams, Amy Gaskin, Bree Gatica-Wilcox, Jason Coombes, Joel Southgate, Lauren Gilbert, Lee Graham, Nicole Pacchiarini, Sara Kumziene-Summerhayes, Sarah Taylor, Sophie Jones, Sara Rey, Matthew Bull, Joanne Watkins, Sally Corden, Tom Connor
EPI_ISL_572522	Virology Department, Sheffield Teaching Hospitals NHS Foundation Trust/Department of Infection, Immunity and Cardiovascular Disease, The Medical School, University of Sheffield	COVID-19 Genomics UK (COG-UK) Consortium	Thushan de Silva, Matthew Parker, Nikki Smith, Adri Angyal, Rebecca Brown, Luke Green, Rachel Tucker, Paul Parsons, Danielle Groves, Katie Johnson, Laura Carrilero, Alex Keeley, Dave Partridge, Matthew Wyles, Benjamin Lindsey, Mehmet Yavuz, Mohammad Raza, Cariad Evans
EPI_ISL_572555, EPI_ISL_572560, EPI_ISL_572565, EPI_ISL_572575	Wales Specialist Virology Centre Sequencing lab: Pathogen Genomics Unit	COVID-19 Genomics UK (COG-UK) Consortium	Catherine Moore, Johnathan Evans, Laura Gifford, Malorie Perry, Simon Cottrell, Angela Marchbank, Alec Birchley, Alexander Adams, Amy Gaskin, Bree Gatica-Wilcox, Jason Coombes, Joel Southgate, Lauren Gilbert, Lee Graham, Nicole Pacchiarini, Sara Kumziene-Summerhayes, Sarah Taylor, Sophie Jones, Sara Rey, Matthew Bull, Joanne Watkins, Sally Corden, Tom Connor
EPI_ISL_572582	Queens Medical Centre, Clinical Microbiology Department / DeepSeq Nottingham	COVID-19 Genomics UK (COG-UK) Consortium	Gemma Clark, Wendy Smith, Manjinder Khakh, Vicki M Fleming, Michelle M Lister, Hannah Howson-Wells, Jonathan Ball, Patrick McClure, Joseph Chappell, Theocharis Tsoleridis, Nadine Holmes, Matthew Carlisle, Christopher Moore, Fei Sang, Johnny Debebe, Victoria Wright, Matthew Loose
EPI_ISL_572589	Virology Department, Sheffield Teaching Hospitals NHS Foundation Trust/Department of Infection, Immunity and Cardiovascular Disease, The Medical School, University of Sheffield	COVID-19 Genomics UK (COG-UK) Consortium	Thushan de Silva, Matthew Parker, Nikki Smith, Adri Angyal, Rebecca Brown, Luke Green, Rachel Tucker, Paul Parsons, Danielle Groves, Katie Johnson, Laura Carrilero, Alex Keeley, Dave Partridge, Matthew Wyles, Benjamin Lindsey, Mehmet Yavuz, Mohammad Raza, Cariad Evans
EPI_ISL_572598, EPI_ISL_572603	Queens Medical Centre, Clinical Microbiology Department / DeepSeq Nottingham	COVID-19 Genomics UK (COG-UK) Consortium	Gemma Clark, Wendy Smith, Manjinder Khakh, Vicki M Fleming, Michelle M Lister, Hannah Howson-Wells, Jonathan Ball, Patrick McClure, Joseph Chappell, Theocharis Tsoleridis, Nadine Holmes, Matthew Carlisle, Christopher Moore, Fei Sang, Johnny Debebe, Victoria Wright, Matthew Loose
EPI_ISL_572605	Wales Specialist Virology Centre Sequencing lab: Pathogen Genomics Unit	COVID-19 Genomics UK (COG-UK) Consortium	Catherine Moore, Johnathan Evans, Laura Gifford, Malorie Perry, Simon Cottrell, Angela Marchbank, Alec Birchley, Alexander Adams, Amy Gaskin, Bree Gatica-Wilcox, Jason Coombes, Joel Southgate, Lauren Gilbert, Lee Graham, Nicole Pacchiarini, Sara Kumziene-Summerhayes, Sarah Taylor, Sophie Jones, Sara Rey, Matthew Bull, Joanne Watkins, Sally Corden, Tom Connor
EPI_ISL_572613, EPI_ISL_572618	Virology Department, Sheffield Teaching Hospitals NHS Foundation Trust/Department of Infection, Immunity and Cardiovascular Disease, The Medical School, University of Sheffield	COVID-19 Genomics UK (COG-UK) Consortium	Thushan de Silva, Matthew Parker, Nikki Smith, Adri Angyal, Rebecca Brown, Luke Green, Rachel Tucker, Paul Parsons, Danielle Groves, Katie Johnson, Laura Carrilero, Alex Keeley, Dave Partridge, Matthew Wyles, Benjamin Lindsey, Mehmet Yavuz, Mohammad Raza, Cariad Evans
EPI_ISL_572651	Quadram Institute Bioscience	COVID-19 Genomics UK (COG-UK) Consortium	Dave J. Baker, Gemma L. Kay, Alp Aydin, Thanh Le-Viet, Steven Rudder, Ana P. Tedim, Anastasia Kolyva, Maria Diaz, Leonardo de Oliveira Martins, Nabil-Fareed Alikhan, Lizzie Meadows, Rachael Stanley, Ngozi Elumogo, Muhammed Yasir, Nicholas M. Thomson, Alexander J Trotter, Rachel Gilroy, Samuel Bloomfield, Claire Stuart, Andrew Bell, Reenesh Prakash, Samir Dervisevic, Alison E. Mather, John Wain, Mark Webber, Andrew J. Page, Justin O'Grady
EPI_ISL_572652, EPI_ISL_572654, EPI_ISL_572668	Wales Specialist Virology Centre Sequencing lab: Pathogen Genomics Unit	COVID-19 Genomics UK (COG-UK) Consortium	Catherine Moore, Johnathan Evans, Laura Gifford, Malorie Perry, Simon Cottrell, Angela Marchbank, Alec Birchley, Alexander Adams, Amy Gaskin, Bree Gatica-Wilcox, Jason Coombes, Joel Southgate, Lauren Gilbert, Lee Graham, Nicole Pacchiarini, Sara Kumziene-Summerhayes, Sarah Taylor, Sophie Jones, Sara Rey, Matthew Bull, Joanne Watkins, Sally Corden, Tom Connor
EPI_ISL_572706	Queens Medical Centre, Clinical Microbiology Department / DeepSeq Nottingham	COVID-19 Genomics UK (COG-UK) Consortium	Gemma Clark, Wendy Smith, Manjinder Khakh, Vicki M Fleming, Michelle M Lister, Hannah Howson-Wells, Jonathan Ball, Patrick McClure, Joseph Chappell, Theocharis Tsoleridis, Nadine Holmes, Matthew Carlisle, Christopher Moore, Fei Sang, Johnny Debebe, Victoria Wright, Matthew Loose
EPI_ISL_572732	Quadram Institute Bioscience	COVID-19 Genomics UK (COG-UK) Consortium	Dave J. Baker, Gemma L. Kay, Alp Aydin, Thanh Le-Viet, Steven Rudder, Ana P. Tedim, Anastasia Kolyva, Maria Diaz, Leonardo de Oliveira Martins, Nabil-Fareed Alikhan, Lizzie Meadows, Rachael Stanley, Ngozi Elumogo, Muhammed Yasir, Nicholas M. Thomson, Alexander J Trotter, Rachel Gilroy, Samuel Bloomfield, Claire Stuart, Andrew Bell, Reenesh Prakash, Samir Dervisevic, Alison E. Mather, John Wain, Mark Webber, Andrew J. Page, Justin O'Grady
EPI_ISL_572754	Wales Specialist Virology Centre Sequencing lab: Pathogen Genomics Unit	COVID-19 Genomics UK (COG-UK) Consortium	Catherine Moore, Johnathan Evans, Laura Gifford, Malorie Perry, Simon Cottrell, Angela Marchbank, Alec Birchley, Alexander Adams, Amy Gaskin, Bree Gatica-Wilcox, Jason Coombes, Joel Southgate, Lauren Gilbert, Lee Graham, Nicole Pacchiarini, Sara Kumziene-Summerhayes, Sarah Taylor, Sophie Jones, Sara Rey, Matthew Bull, Joanne Watkins, Sally Corden, Tom Connor
EPI_ISL_572764	Quadram Institute Bioscience	COVID-19 Genomics UK (COG-UK) Consortium	Dave J. Baker, Gemma L. Kay, Alp Aydin, Thanh Le-Viet, Steven Rudder, Ana P. Tedim, Anastasia Kolyva, Maria Diaz, Leonardo de Oliveira Martins, Nabil-Fareed Alikhan, Lizzie Meadows, Rachael Stanley, Ngozi Elumogo, Muhammed Yasir, Nicholas M. Thomson, Alexander J Trotter, Rachel Gilroy, Samuel Bloomfield, Claire Stuart, Andrew Bell, Reenesh Prakash, Samir Dervisevic, Alison E. Mather, John Wain, Mark Webber, Andrew J. Page, Justin O'Grady
EPI_ISL_572778	Wales Specialist Virology Centre Sequencing lab: Pathogen Genomics Unit	COVID-19 Genomics UK (COG-UK) Consortium	Catherine Moore, Johnathan Evans, Laura Gifford, Malorie Perry, Simon Cottrell, Angela Marchbank, Alec Birchley, Alexander Adams, Amy Gaskin, Bree Gatica-Wilcox, Jason Coombes, Joel Southgate, Lauren Gilbert, Lee Graham, Nicole Pacchiarini, Sara Kumziene-Summerhayes, Sarah Taylor, Sophie Jones, Sara Rey, Matthew Bull, Joanne Watkins, Sally Corden, Tom Connor

EPI_ISL_572799	Queens Medical Centre, Clinical Microbiology Department / DeepSeq Nottingham	COVID-19 Genomics UK (COG-UK) Consortium	Gemma Clark, Wendy Smith, Manjinder Khakh, Vicki M Fleming, Michelle M Lister, Hannah Howson-Wells, Jonathan Ball, Patrick McClure, Joseph Chappell, Theocharis Tsoleridis, Nadine Holmes, Matthew Carlisle, Christopher Moore, Fei Sang, Johnny Debebe, Victoria Wright, Matthew Loose
EPI_ISL_572805, EPI_ISL_572810	Wales Specialist Virology Centre Sequencing lab: Pathogen Genomics Unit	COVID-19 Genomics UK (COG-UK) Consortium	Catherine Moore, Johnathan Evans, Laura Gifford, Malorie Perry, Simon Cottrell, Angela Marchbank, Alec Birchley, Alexander Adams, Amy Gaskin, Bree Gatica-Wilcox, Jason Coombes, Joel Southgate, Lauren Gilbert, Lee Graham, Nicole Pacchiarini, Sara Kumziene-Summerhayes, Sarah Taylor, Sophie Jones, Sara Rey, Matthew Bull, Joanne Watkins, Sally Corden, Tom Connor
EPI_ISL_572814	Quadram Institute Bioscience	COVID-19 Genomics UK (COG-UK) Consortium	Dave J. Baker, Gemma L. Kay, Alp Aydin, Thanh Le-Viet, Steven Rudder, Ana P. Tedim, Anastasia Kolyva, Maria Diaz, Leonardo de Oliveira Martins, Nabil-Fareed Alikhan, Lizzie Meadows, Rachael Stanley, Ngozi Elumogo, Muhammed Yasir, Nicholas M. Thomson, Alexander J Trotter, Rachel Gilroy, Samuel Bloomfield, Claire Stuart, Andrew Bell, Reenesh Prakash, Samir Dervisevic, Alison E. Mather, John Wain, Mark Webber, Andrew J. Page, Justin O'Grady
EPI_ISL_572833	Queens Medical Centre, Clinical Microbiology Department / DeepSeq Nottingham	COVID-19 Genomics UK (COG-UK) Consortium	Gemma Clark, Wendy Smith, Manjinder Khakh, Vicki M Fleming, Michelle M Lister, Hannah Howson-Wells, Jonathan Ball, Patrick McClure, Joseph Chappell, Theocharis Tsoleridis, Nadine Holmes, Matthew Carlisle, Christopher Moore, Fei Sang, Johnny Debebe, Victoria Wright, Matthew Loose
EPI_ISL_572842	Quadram Institute Bioscience	COVID-19 Genomics UK (COG-UK) Consortium	Dave J. Baker, Gemma L. Kay, Alp Aydin, Thanh Le-Viet, Steven Rudder, Ana P. Tedim, Anastasia Kolyva, Maria Diaz, Leonardo de Oliveira Martins, Nabil-Fareed Alikhan, Lizzie Meadows, Rachael Stanley, Ngozi Elumogo, Muhammed Yasir, Nicholas M. Thomson, Alexander J Trotter, Rachel Gilroy, Samuel Bloomfield, Claire Stuart, Andrew Bell, Reenesh Prakash, Samir Dervisevic, Alison E. Mather, John Wain, Mark Webber, Andrew J. Page, Justin O'Grady
EPI_ISL_572881, EPI_ISL_572882, EPI_ISL_572886, EPI_ISL_572888	Wales Specialist Virology Centre Sequencing lab: Pathogen Genomics Unit	COVID-19 Genomics UK (COG-UK) Consortium	Catherine Moore, Johnathan Evans, Laura Gifford, Malorie Perry, Simon Cottrell, Angela Marchbank, Alec Birchley, Alexander Adams, Amy Gaskin, Bree Gatica-Wilcox, Jason Coombes, Joel Southgate, Lauren Gilbert, Lee Graham, Nicole Pacchiarini, Sara Kumziene-Summerhayes, Sarah Taylor, Sophie Jones, Sara Rey, Matthew Bull, Joanne Watkins, Sally Corden, Tom Connor
EPI_ISL_572929, EPI_ISL_572948	Virology Department, Sheffield Teaching Hospitals NHS Foundation Trust/Department of Infection, Immunity and Cardiovascular Disease, The Medical School, University of Sheffield	COVID-19 Genomics UK (COG-UK) Consortium	Thushan de Silva, Matthew Parker, Nikki Smith, Adri Angyal, Rebecca Brown, Luke Green, Rachel Tucker, Paul Parsons, Danielle Groves, Katie Johnson, Laura Carrilero, Alex Keeley, Dave Partridge, Matthew Wyles, Benjamin Lindsey, Mehmet Yavuz, Mohammad Raza, Cariad Evans
EPI_ISL_573450, EPI_ISL_573451, EPI_ISL_573452, EPI_ISL_573453, EPI_ISL_573454, EPI_ISL_573455, EPI_ISL_573456, EPI_ISL_573457, EPI_ISL_573458, EPI_ISL_573459, EPI_ISL_573460, EPI_ISL_573461, EPI_ISL_573462, EPI_ISL_573463, EPI_ISL_573464, EPI_ISL_573465, EPI_ISL_573466, EPI_ISL_573467, EPI_ISL_573468, EPI_ISL_573469, EPI_ISL_573470, EPI_ISL_573471, EPI_ISL_573472, EPI_ISL_573473, EPI_ISL_573474, EPI_ISL_573475, EPI_ISL_573476, EPI_ISL_573477			
see above	Queens Medical Centre, Clinical Microbiology Department / DeepSeq Nottingham	COVID-19 Genomics UK (COG-UK) Consortium	Gemma Clark, Wendy Smith, Manjinder Khakh, Vicki M Fleming, Michelle M Lister, Hannah Howson-Wells, Jonathan Ball, Patrick McClure, Joseph Chappell, Theocharis Tsoleridis, Nadine Holmes, Matthew Carlisle, Christopher Moore, Fei Sang, Johnny Debebe, Victoria Wright, Matthew Loose
EPI_ISL_573687, EPI_ISL_573691, EPI_ISL_573697, EPI_ISL_573703, EPI_ISL_573707, EPI_ISL_573710, EPI_ISL_573712, EPI_ISL_573724, EPI_ISL_573730, EPI_ISL_573731, EPI_ISL_573738, EPI_ISL_573744, EPI_ISL_573751, EPI_ISL_573752			
see above	Virology Department, Sheffield Teaching Hospitals NHS Foundation Trust/Department of Infection, Immunity and Cardiovascular Disease, The Medical School, University of Sheffield	COVID-19 Genomics UK (COG-UK) Consortium	Thushan de Silva, Matthew Parker, Nikki Smith, Adri Angyal, Rebecca Brown, Luke Green, Rachel Tucker, Paul Parsons, Danielle Groves, Katie Johnson, Laura Carrilero, Alex Keeley, Dave Partridge, Matthew Wyles, Benjamin Lindsey, Mehmet Yavuz, Mohammad Raza, Cariad Evans
EPI_ISL_574018, EPI_ISL_574019, EPI_ISL_574030, EPI_ISL_574032, EPI_ISL_574034, EPI_ISL_574036, EPI_ISL_574039, EPI_ISL_574050, EPI_ISL_574054, EPI_ISL_574055, EPI_ISL_574061, EPI_ISL_574062, EPI_ISL_574064, EPI_ISL_574067, EPI_ISL_574069, EPI_ISL_574070, EPI_ISL_574087, EPI_ISL_574090, EPI_ISL_574094, EPI_ISL_574102, EPI_ISL_574108, EPI_ISL_574112, EPI_ISL_574113, EPI_ISL_574118, EPI_ISL_574121, EPI_ISL_574125, EPI_ISL_574129, EPI_ISL_574143, EPI_ISL_574146, EPI_ISL_574150, EPI_ISL_574151, EPI_ISL_574155, EPI_ISL_574171, EPI_ISL_574179, EPI_ISL_574183, EPI_ISL_574185, EPI_ISL_574187, EPI_ISL_574190, EPI_ISL_574192, EPI_ISL_574199, EPI_ISL_574201, EPI_ISL_574206, EPI_ISL_574208, EPI_ISL_574211, EPI_ISL_574213, EPI_ISL_574222, EPI_ISL_574229, EPI_ISL_574234, EPI_ISL_574241, EPI_ISL_574246, EPI_ISL_574249, EPI_ISL_574253, EPI_ISL_574256			
see above	Wales Specialist Virology Centre Sequencing lab: Pathogen Genomics Unit	COVID-19 Genomics UK (COG-UK) Consortium	Catherine Moore, Johnathan Evans, Laura Gifford, Malorie Perry, Simon Cottrell, Angela Marchbank, Alec Birchley, Alexander Adams, Amy Gaskin, Bree Gatica-Wilcox, Jason Coombes, Joel Southgate, Lauren Gilbert, Lee Graham, Nicole Pacchiarini, Sara Kumziene-Summerhayes, Sarah Taylor, Sophie Jones, Sara Rey, Matthew Bull, Joanne Watkins, Sally Corden, Tom Connor
EPI_ISL_574494, EPI_ISL_574519	National Public Health Laboratory, National Centre for Infectious Diseases	National Public Health Laboratory, National Centre for Infectious Diseases	Tze Minn Mak, Sophie Octavia, Zhenyang Zhou, Lin Cui, Raymond Tzer Pin Lin
EPI_ISL_576198, EPI_ISL_576199, EPI_ISL_576200, EPI_ISL_576201, EPI_ISL_576202, EPI_ISL_576203, EPI_ISL_576204, EPI_ISL_576205, EPI_ISL_576206, EPI_ISL_576207, EPI_ISL_576208, EPI_ISL_576209, EPI_ISL_576210, EPI_ISL_576211, EPI_ISL_576212, EPI_ISL_576213, EPI_ISL_576214, EPI_ISL_576215, EPI_ISL_576216, EPI_ISL_576217, EPI_ISL_576218, EPI_ISL_576219, EPI_ISL_576220, EPI_ISL_576221			
see above	Michigan Department of Health and Human Services, Bureau of Laboratories	Michigan Department of Health and Human Services, Bureau of Laboratories	Blankenship HM, Riner D, Soehnlen MK
EPI_ISL_576396	Genome Centre	Genome Centre	Selina Akter Pravas Chandra Roy Amina Ferdaus manami Habiba Ibnat A. S. M. Rubayet Ul Alam Shireen Nigar Iqbal Kabir Jahid and M. Anwar Hossain
EPI_ISL_576487, EPI_ISL_576488, EPI_ISL_576557	UW Virology Lab	UW Virology Lab	Pavitra Roychoudhury, Hong Xie, Lasata Shrestha, Amin Addetia, Victoria M Rachleff, Meei-Li Huang, Keith R Jerome, Alexander Greninger
EPI_ISL_576902	Department of Pathology, University of Cambridge	COVID-19 Genomics UK (COG-UK) Consortium	Aminu S. Jahun, Yasmin Chaudhry, Grant Hall, Iliana Georgana, Myra Hosmillo, Martin D. Curran, Malte Pinckert, Surendra Parmar, Ian Goodfellow
EPI_ISL_576981, EPI_ISL_576982, EPI_ISL_576983	Liverpool Clinical Laboratories	COVID-19 Genomics UK (COG-UK) Consortium	Sam Haldenby, Anita Lucaci, Steve Paterson, Julian Hiscox, Alistair Darby, M Almsaud, A Alrezaihi, Muhannad Alruwaili, Stuart D Armstrong, Jones Benjamin, Eleanor G Bentley, Anu Chawla, Jordan J Clark, Angela Cowell, Richard Eccles, Isabel García-Dorival, Matthew Gemmell, Alessandro Gerada, PKF Gilmore, Richard Gregory, Ximeng Han, Catherine Hartley, Margaret Hughes, Miren Iturriza-Gomara, James Johnson, L Luu, Jenifer Manson, Charlotte Nelson, Elaine O'Toole, Cassie Olateju, Rebekah Penrice-Randal , Lucille Rainbow, N.P Randle, Trevor Ian Robinson, Parul Sharma, Ghada T Shawli, James P Stewart, Neil Swainston, Ecaterina Vamos, Joanne Watts, Mark Whitehead
EPI_ISL_577096, EPI_ISL_577097, EPI_ISL_577100, EPI_ISL_577101, EPI_ISL_577102, EPI_ISL_577105, EPI_ISL_577106, EPI_ISL_577107, EPI_ISL_577108, EPI_ISL_577109	Quadram Institute Bioscience	COVID-19 Genomics UK (COG-UK) Consortium	Dave J. Baker, Gemma L. Kay, Alp Aydin, Thanh Le-Viet, Steven Rudder, Ana P. Tedim, Anastasia Kolyva, Maria Diaz, Leonardo de Oliveira Martins, Nabil-Fareed Alikhan, Lizzie Meadows, Rachael Stanley, Ngozi Elumogo, Muhammed Yasir, Nicholas M. Thomson, Alexander J Trotter, Rachel Gilroy, Samuel Bloomfield, Claire Stuart, Andrew Bell, Reenesh Prakash, Samir Dervisevic, Alison E. Mather, John Wain, Mark Webber, Andrew J. Page, Justin O'Grady
EPI_ISL_577110, EPI_ISL_577111, EPI_ISL_577112, EPI_ISL_577113, EPI_ISL_577114, EPI_ISL_577115, EPI_ISL_577116, EPI_ISL_577117, EPI_ISL_577118, EPI_ISL_577119, EPI_ISL_577120, EPI_ISL_577121, EPI_ISL_577122, EPI_ISL_577123			
see above	Queens Medical Centre, Clinical Microbiology Department / DeepSeq Nottingham	COVID-19 Genomics UK (COG-UK) Consortium	Gemma Clark, Wendy Smith, Manjinder Khakh, Vicki M Fleming, Michelle M Lister, Hannah Howson-Wells, Jonathan Ball, Patrick McClure, Joseph Chappell, Theocharis Tsoleridis, Nadine Holmes, Matthew Carlisle, Christopher Moore, Fei Sang, Johnny Debebe, Victoria Wright, Matthew Loose
EPI_ISL_577418, EPI_ISL_577421, EPI_ISL_577423, EPI_ISL_577424, EPI_ISL_577425, EPI_ISL_577426, EPI_ISL_577428, EPI_ISL_577429, EPI_ISL_577431, EPI_ISL_577434, EPI_ISL_577435, EPI_ISL_577436, EPI_ISL_577438, EPI_ISL_577442, EPI_ISL_577443, EPI_ISL_577444, EPI_ISL_577445, EPI_ISL_577446, EPI_ISL_577447, EPI_ISL_577448, EPI_ISL_577450, EPI_ISL_577451, EPI_ISL_577452, EPI_ISL_577453, EPI_ISL_577454, EPI_ISL_577458, EPI_ISL_577459, EPI_ISL_577462, EPI_ISL_577463, EPI_ISL_577465, EPI_ISL_577466, EPI_ISL_577469, EPI_ISL_577472, EPI_ISL_577475, EPI_ISL_577476, EPI_ISL_577479, EPI_ISL_577481, EPI_ISL_577492, EPI_ISL_577493, EPI_ISL_577494, EPI_ISL_577495, EPI_ISL_577497, EPI_ISL_577499, EPI_ISL_577500, EPI_ISL_577501, EPI_ISL_577503, EPI_ISL_577504, EPI_ISL_577505, EPI_ISL_577507, EPI_ISL_577508, EPI_ISL_577509, EPI_ISL_577511, EPI_ISL_577512, EPI_ISL_577513, EPI_ISL_577515, EPI_ISL_577517, EPI_ISL_577521, EPI_ISL_577522, EPI_ISL_577523, EPI_ISL_577526, EPI_ISL_577527, EPI_ISL_577528, EPI_ISL_577529, EPI_ISL_577530, EPI_ISL_577531, EPI_ISL_577532, EPI_ISL_577534, EPI_ISL_577535, EPI_ISL_577536, EPI_ISL_577537, EPI_ISL_577539, EPI_ISL_577540, EPI_ISL_577541, EPI_ISL_577542, EPI_ISL_577543			
see above	Wales Specialist Virology Centre Sequencing lab: Pathogen Genomics Unit	COVID-19 Genomics UK (COG-UK) Consortium	Catherine Moore, Johnathan Evans, Laura Gifford, Malorie Perry, Simon Cottrell, Angela Marchbank, Alec Birchley, Alexander Adams, Amy Gaskin, Bree Gatica-Wilcox, Jason Coombes, Joel Southgate, Lauren Gilbert, Lee Graham, Nicole Pacchiarini, Sara Kumziene-Summerhayes, Sarah Taylor, Sophie Jones, Sara Rey, Matthew Bull, Joanne Watkins, Sally Corden, Tom Connor
EPI_ISL_577640, EPI_ISL_577677, EPI_ISL_577679, EPI_ISL_577680, EPI_ISL_577681, EPI_ISL_577682, EPI_ISL_577683, EPI_ISL_577684, EPI_ISL_577685, EPI_ISL_577686, EPI_ISL_577687, EPI_ISL_577688, EPI_ISL_577689, EPI_ISL_577690, EPI_ISL_577691, EPI_ISL_577692, EPI_ISL_577693, EPI_ISL_577694, EPI_ISL_577695, EPI_ISL_577696, EPI_ISL_577697, EPI_ISL_577698, EPI_ISL_577699, EPI_ISL_577700, EPI_ISL_577701, EPI_ISL_577702, EPI_ISL_577706, EPI_ISL_577709, EPI_ISL_577710, EPI_ISL_577711, EPI_ISL_577712, EPI_ISL_577713, EPI_ISL_577717, EPI_ISL_577720, EPI_ISL_577721, EPI_ISL_577722, EPI_ISL_577723			
see above	NIV Influenza	NIV Influenza	Potdar V
EPI_ISL_577834, EPI_ISL_577835, EPI_ISL_577836, EPI_ISL_578052, EPI_ISL_578073, EPI_ISL_578074, EPI_ISL_578075, EPI_ISL_578076, EPI_ISL_578077, EPI_ISL_578078, EPI_ISL_578079			
see above	Dutch COVID-19 response team	Erasmus Medical Center	Bas Oude Munnink, Reina Sikkema, David Nieuwenhuijse, Irina Chestakova, Anne van der Linden, Marjan Boter, Emmanuelle Munger, Corine GeurtsvanKessel, Annemiek van der Eijk, Richard Molenkamp, Marion Koopmans, on behalf of the Dutch national COVID-19 response team.

EPI_ISL_579113, EPI_ISL_579114, EPI_ISL_579115	LabPLUS	Institute of Environmental Science and Research (ESR)	Xiaoyun Ren, Matt Storey, Nikki Freed, Muhammad Faisal, Jing Wang, Hermes Perez, Anja Werno, Antje van der Linden, Arlo Upton, Chris Mansell, David Hammer, Dragana Drinkovic, Gary McAuliffe, Hana Sofia Andersson, James Ussher, Jill Sherwood, Josh Freeman, Julia Howard, Juliet Elvy, Mary DeAlmeida, Matt Blakiston, Matthew Rogers, Max Bloomfield, Michael Addidle, Michelle Balm, Sally Roberts, Sarah Jefferies, Sharmini Muttaiyah, Susan Morpeth, Susan Taylor, Timothy Blackmore, Vani Sathyendran, Veronica Playle, Virginia Hope, Erasmus Smit, Lauren Jelly, Olin Silander, Joep de Ligt
EPI_ISL_580302, EPI_ISL_580337, EPI_ISL_580405, EPI_ISL_580411, EPI_ISL_580424, EPI_ISL_580443, EPI_ISL_580501, EPI_ISL_580514, EPI_ISL_580526, EPI_ISL_580602	Lighthouse Lab in Alderley Park	Wellcome Sanger Institute for the COVID-19 Genomics UK (COG-UK) consortium	Jacquelyn Wynn, Mairead Hyland, The Lighthouse Lab in Alderley Park and Alex Alderton, Roberto Amato, Sonia Goncalves, Ewan Harrison, David K. Jackson, Ian Johnston, Dominic Kwiatkowski, Cordelia Langford, John Sillitoe on behalf of the Wellcome Sanger Institute COVID-19 Surveillance Team
EPI_ISL_580654, EPI_ISL_580661, EPI_ISL_580665, EPI_ISL_580707, EPI_ISL_580711, EPI_ISL_580717, EPI_ISL_580725, EPI_ISL_580729, EPI_ISL_580739, EPI_ISL_580748, EPI_ISL_580760, EPI_ISL_580773, EPI_ISL_580777, EPI_ISL_580786, EPI_ISL_580812, EPI_ISL_580818, EPI_ISL_580826, EPI_ISL_580830, EPI_ISL_580831, EPI_ISL_580839			
see above	Lighthouse Lab in Milton Keynes	Wellcome Sanger Institute for the COVID-19 Genomics UK (COG-UK) consortium	The Lighthouse Lab in Milton Keynes and Alex Alderton, Roberto Amato, Sonia Goncalves, Ewan Harrison, David K. Jackson, Ian Johnston, Dominic Kwiatkowski, Cordelia Langford, John Sillitoe on behalf of the Wellcome Sanger Institute COVID-19 Surveillance Team
EPI_ISL_581664, EPI_ISL_581665, EPI_ISL_581666	Department of Clinical Microbiology	GIGA Medical Genomics	Keith Durkin, Maria Artesi, Sébastien Bontems, Raphaël Boreux, Bouchra Boujemla, Cécile Meex, Pierrette Melin, Marie-Pierre Hayette, Vincent Bours
EPI_ISL_582241	NIV Influenza	NIV Influenza	Potdar V
EPI_ISL_582689, EPI_ISL_582690, EPI_ISL_582691	Sheikh Khalifa Medical City	Molecular/Surveillance lab Sheikh Khalifa Medical City	Amirtharaj Francis, Sajeed Abdul, Hala Imambaccus, Sahar Almarzooqi, Hiba Saud, Stefan Weber
EPI_ISL_583421, EPI_ISL_583422, EPI_ISL_583423, EPI_ISL_583425	University of Michigan Clinical Microbiology Laboratory	Lauring Lab, University of Michigan, Department of Microbiology and Immunology	Valesano
EPI_ISL_583514, EPI_ISL_583515, EPI_ISL_583516, EPI_ISL_583517, EPI_ISL_583518, EPI_ISL_583519, EPI_ISL_583520, EPI_ISL_583521, EPI_ISL_583525, EPI_ISL_583526, EPI_ISL_583527, EPI_ISL_583529, EPI_ISL_583532			
see above	Michigan Department of Health and Human Services, Bureau of Laboratories	Michigan Department of Health and Human Services, Bureau of Laboratories	Blankenship HM, Riner D, Soehnlen MK
EPI_ISL_583554, EPI_ISL_583555, EPI_ISL_583556	Genome Centre	Genome Centre	Selina Akter, Pravas Chandra Roy, Amina Ferdaus manami, Habiba Ibnat, A. S. M. Rubayet Ul Alam, Shireen Nigar, Iqbal Kabir Jahid, M.Anwar Hossain
EPI_ISL_583893	Singapore General Hospital	Department of Microbiology	Nurdyana Abdul Rahman, Kun Lee Lim, Chenhao Li, Sui Sin Goh, Kenneth Xin Long Chan, Kian Sing Chan, Lynette Oon, Kern Rei Chng, Niranjan Nagarajan, Karrie Ko
EPI_ISL_583968, EPI_ISL_583970, EPI_ISL_583971, EPI_ISL_583972, EPI_ISL_583973, EPI_ISL_583974, EPI_ISL_583982, EPI_ISL_583983, EPI_ISL_583984, EPI_ISL_583985, EPI_ISL_583986, EPI_ISL_583987, EPI_ISL_583990, EPI_ISL_583991, EPI_ISL_584066			
see above	Respiratory Virus Unit, Microbiology Services Colindale, Public Health England	Respiratory Virus Unit, Microbiology Services Colindale, Public Health England	PHE Covid Sequencing Team
EPI_ISL_584069, EPI_ISL_584071	IZSM	IZSM	Maurizio Viscardi, Lorena Cardillo, Giovanna Fusco
EPI_ISL_584286, EPI_ISL_584287, EPI_ISL_584288, EPI_ISL_584289, EPI_ISL_584290, EPI_ISL_584294, EPI_ISL_584295, EPI_ISL_584296, EPI_ISL_584297, EPI_ISL_584298	Department of Pathology, University of Cambridge	COVID-19 Genomics UK (COG-UK) Consortium	Aminu S. Jahun, Yasmin Chaudhry, Grant Hall, Iliana Georgana, Myra Hosmillo, Martin D. Curran, Malte Pinckert, Surendra Parmar, Ian Goodfellow
EPI_ISL_584592, EPI_ISL_584595, EPI_ISL_584596, EPI_ISL_584598, EPI_ISL_584599, EPI_ISL_584600, EPI_ISL_584601, EPI_ISL_584602, EPI_ISL_584603, EPI_ISL_584604, EPI_ISL_584605			
see above	Liverpool Clinical Laboratories	COVID-19 Genomics UK (COG-UK) Consortium	Sam Haldenby, Anita Lucaci, Steve Paterson, Julian Hiscox, Alistair Darby, M Almsaud, A Alrezaihi, Muhannad Alruwaili, Stuart D Armstrong, Jones Benjamin, Eleanor G Bentley, Anu Chawla, Jordan J Clark, Angela Cowell, Richard Eccles, Isabel García-Dorival, Matthew Gemmell, Alessandro Gerada, PKF Gilmore, Richard Gregory, Ximeng Han, Catherine Hartley, Margaret Hughes, Miren Iturriza-Gomara, James Johnson, L Luu, Jenifer Manson, Charlotte Nelson, Elaine O'Toole, Cassie Olateju, Rebekah Penrice-Randal , Lucille Rainbow, N.P Randle, Trevor Ian Robinson, Parul Sharma, Ghada T Shawli, James P Stewart, Neil Swainston, Ecaterina Vamos, Joanne Watts, Mark Whitehead
EPI_ISL_584672	University College London, Great Ormond Street Hospital for Children NHS Foundation Trust, Imperial College Healthcare NHS Trust	COVID-19 Genomics UK (COG-UK) Consortium	Sergi Castellano, Rachel Williams, Mark Kristiansen, Paola Resende Silva, Sunando Roy, Tony Brooks, Helena Tutill, Paola Niola, Patricia Dyal, Charlotte Williams, Leysa Forrest, Yasmin Panchbhaya, Jacqueline Findlay, Samuel Weeks, Julianne Brown, Kathryn Harris, Paul Randell, James Price, Alison Holmes, Judith Breuer
EPI_ISL_584690, EPI_ISL_584691, EPI_ISL_584692, EPI_ISL_584693, EPI_ISL_584694, EPI_ISL_584695, EPI_ISL_584696, EPI_ISL_584697, EPI_ISL_584698, EPI_ISL_584699, EPI_ISL_584700, EPI_ISL_584701, EPI_ISL_584702, EPI_ISL_584703, EPI_ISL_584704, EPI_ISL_584705			
see above	Northumbria University / South Tees Hospitals NHS Foundation Trust / North Cumbria Integrated Care NHS Foundation Trust / North Tees and Hartlepool NHS Foundation Trust / Newcastle Hospitals NHS Foundation Trust	COVID-19 Genomics UK (COG-UK) Consortium	Darren L Smith,Andrew Nelson,Matthew Bashton,Greg R Young,Joshua Loh,John Allan,Mohammad A Tariq,Giles S Holt,Gary Black,Wen C Yew,Lynn Dover,Paul Baker,Steve Liggett,Sarah Essex,Jane Greenaway,Debra Padgett,Clive Graham,Garren Scott,Edward Barton,Emma Swindells,Brendan Payne,Jennifer Collins,Yusri Taha,Gary Eltringham
EPI_ISL_584716, EPI_ISL_584719, EPI_ISL_584722, EPI_ISL_584723	Quadram Institute Bioscience	COVID-19 Genomics UK (COG-UK) Consortium	Dave J. Baker, Gemma L. Kay, Alp Aydin, Thanh Le-Viet, Steven Rudder, Ana P. Tedim, Anastasia Kolyva, Maria Diaz, Leonardo de Oliveira Martins, Nabil-Fareed Alikhan, Lizzie Meadows, Rachael Stanley, Ngozi Elumogo, Muhammed Yasir, Nicholas M. Thomson, Alexander J Trotter, Rachel Gilroy, Samuel Bloomfield, Claire Stuart, Andrew Bell, Reenesh Prakash, Samir Dervisevic, Alison E. Mather, John Wain, Mark Webber, Andrew J. Page, Justin O'Grady
EPI_ISL_584898, EPI_ISL_584899, EPI_ISL_584901, EPI_ISL_584902	University of Exeter	COVID-19 Genomics UK (COG-UK) Consortium	Ben Temperton,Aaron Jeffries,Michelle Michelsen,Joanna Warwick-Dugdale,Audrey Farbos,Robyn Manley,Stephen Michell,Jane Masoli
EPI_ISL_584906, EPI_ISL_584907, EPI_ISL_584908, EPI_ISL_584909, EPI_ISL_584910, EPI_ISL_584911, EPI_ISL_584912, EPI_ISL_584913, EPI_ISL_584914, EPI_ISL_584915, EPI_ISL_584916, EPI_ISL_584917, EPI_ISL_584918, EPI_ISL_584919, EPI_ISL_584920, EPI_ISL_584921, EPI_ISL_584922, EPI_ISL_584923, EPI_ISL_584924, EPI_ISL_584925, EPI_ISL_584926, EPI_ISL_584927, EPI_ISL_584928, EPI_ISL_584929, EPI_ISL_584930, EPI_ISL_584931, EPI_ISL_584932, EPI_ISL_584933, EPI_ISL_584934, EPI_ISL_584935, EPI_ISL_584936, EPI_ISL_584937, EPI_ISL_584938, EPI_ISL_584939, EPI_ISL_584940, EPI_ISL_584941, EPI_ISL_584942, EPI_ISL_584943, EPI_ISL_584944, EPI_ISL_584945, EPI_ISL_584946, EPI_ISL_584947, EPI_ISL_584948, EPI_ISL_584949, EPI_ISL_584950, EPI_ISL_584951, EPI_ISL_584952, EPI_ISL_584956, EPI_ISL_584961, EPI_ISL_584962, EPI_ISL_584963, EPI_ISL_584964, EPI_ISL_584965, EPI_ISL_584966, EPI_ISL_584967, EPI_ISL_584968, EPI_ISL_584969, EPI_ISL_584970, EPI_ISL_584971, EPI_ISL_584972, EPI_ISL_584973, EPI_ISL_584981, EPI_ISL_584982, EPI_ISL_584983, EPI_ISL_584984, EPI_ISL_584985, EPI_ISL_584986, EPI_ISL_584987, EPI_ISL_584988, EPI_ISL_584989, EPI_ISL_584990, EPI_ISL_584991, EPI_ISL_584992, EPI_ISL_584993, EPI_ISL_584994, EPI_ISL_584995, EPI_ISL_584996, EPI_ISL_584997, EPI_ISL_584998, EPI_ISL_584999, EPI_ISL_585001, EPI_ISL_585006, EPI_ISL_585007, EPI_ISL_585008			
see above	Quadram Institute Bioscience	COVID-19 Genomics UK (COG-UK) Consortium	Dave J. Baker, Gemma L. Kay, Alp Aydin, Thanh Le-Viet, Steven Rudder, Ana P. Tedim, Anastasia Kolyva, Maria Diaz, Leonardo de Oliveira Martins, Nabil-Fareed Alikhan, Lizzie Meadows, Rachael Stanley, Ngozi Elumogo, Muhammed Yasir, Nicholas M. Thomson, Alexander J Trotter, Rachel Gilroy, Samuel Bloomfield, Claire Stuart, Andrew Bell, Reenesh Prakash, Samir Dervisevic, Alison E. Mather, John Wain, Mark Webber, Andrew J. Page, Justin O'Grady
EPI_ISL_585011, EPI_ISL_585013, EPI_ISL_585014, EPI_ISL_585017, EPI_ISL_585019, EPI_ISL_585020, EPI_ISL_585021, EPI_ISL_585022, EPI_ISL_585030, EPI_ISL_585031, EPI_ISL_585032, EPI_ISL_585034, EPI_ISL_585039, EPI_ISL_585040, EPI_ISL_585043, EPI_ISL_585049, EPI_ISL_585051, EPI_ISL_585052, EPI_ISL_585053, EPI_ISL_585055, EPI_ISL_585057, EPI_ISL_585058, EPI_ISL_585059, EPI_ISL_585060, EPI_ISL_585061, EPI_ISL_585062, EPI_ISL_585064, EPI_ISL_585066, EPI_ISL_585068, EPI_ISL_585070, EPI_ISL_585076, EPI_ISL_585078, EPI_ISL_585080, EPI_ISL_585082, EPI_ISL_585083, EPI_ISL_585087, EPI_ISL_585089			
see above	Virology Department, Sheffield Teaching Hospitals NHS Foundation Trust/Department of Infection, Immunity and Cardiovascular Disease, The Medical School, University of Sheffield	COVID-19 Genomics UK (COG-UK) Consortium	Thushan de Silva, Matthew Parker, Nikki Smith, Adri Angyal, Rebecca Brown, Luke Green, Rachel Tucker, Paul Parsons, Danielle Groves, Katie Johnson, Laura Carrilero, Alex Keeley, Dave Partridge, Matthew Wyles, Benjamin Lindsey, Mehmet Yavuz, Mohammad Raza, Cariad Evans
EPI_ISL_585260	Quadram Institute Bioscience	COVID-19 Genomics UK (COG-UK) Consortium	Dave J. Baker, Gemma L. Kay, Alp Aydin, Thanh Le-Viet, Steven Rudder, Ana P. Tedim, Anastasia Kolyva, Maria Diaz, Leonardo de Oliveira Martins, Nabil-Fareed Alikhan, Lizzie Meadows, Rachael Stanley, Ngozi Elumogo, Muhammed Yasir, Nicholas M. Thomson, Alexander J Trotter, Rachel Gilroy, Samuel Bloomfield, Claire Stuart, Andrew Bell, Reenesh Prakash, Samir Dervisevic, Alison E. Mather, John Wain, Mark Webber, Andrew J. Page, Justin O'Grady

EPI_ISL_585425	West of Scotland Specialist Virology Centre, NHSGGC / MRC-University of Glasgow Centre for Virus Research	COVID-19 Genomics UK (COG-UK) Consortium	Ana da Silva Filipe, Natasha Johnson, Kathy Smollett, Daniel Mair, Stephen Carmichael, Lily Tong, Jenna Nichols, Elihu Aranday-Cortes, Kyriaki Nomikou; Sarah McDonald, Marc Niebel, Patawee Asamaphan; Richard Orton, Joseph Hughes, Sreenu Vattipally, David L Robertson; Alasdair MacLean, Rory Gunson; Kathy Li, Igor Starinskij, Natasha Jesudason, Rajiv Shah, James Shepherd, Antonia Ho, Emma Thomson
EPI_ISL_585451, EPI_ISL_585452, EPI_ISL_585453, EPI_ISL_585454, EPI_ISL_585455, EPI_ISL_585456, EPI_ISL_585458, EPI_ISL_585459, EPI_ISL_585460, EPI_ISL_585461, EPI_ISL_585462, EPI_ISL_585463, EPI_ISL_585464, EPI_ISL_585465, EPI_ISL_585466, EPI_ISL_585467, EPI_ISL_585468, EPI_ISL_585469, EPI_ISL_585470, EPI_ISL_585471, EPI_ISL_585472, EPI_ISL_585473, EPI_ISL_585488, EPI_ISL_585492, EPI_ISL_585493, EPI_ISL_585496, EPI_ISL_585497			
see above	Virology Department, Royal Infirmary of Edinburgh, NHS Lothian / School of Biological Sciences, University of Edinburgh / Institute of Genetics and Molecular Medicine, University of Edinburgh	COVID-19 Genomics UK (COG-UK) Consortium	McHugh M, Dewar R, Rooke S, Gallagher M, Balcaza C, O'Toole Á, Scher E, Hill V, McCrone JT, Colquhoun R, Yu X, Jackson B, Rambaut A, Williams TC, Templeton K
EPI_ISL_585583, EPI_ISL_585584, EPI_ISL_585585, EPI_ISL_585586, EPI_ISL_585587, EPI_ISL_585588, EPI_ISL_585589, EPI_ISL_585590, EPI_ISL_585591, EPI_ISL_585592, EPI_ISL_585593, EPI_ISL_585594, EPI_ISL_585595, EPI_ISL_585596, EPI_ISL_585597, EPI_ISL_585606, EPI_ISL_585607, EPI_ISL_585608, EPI_ISL_585619			
see above	Quadram Institute Bioscience	COVID-19 Genomics UK (COG-UK) Consortium	Dave J. Baker, Gemma L. Kay, Alp Aydin, Thanh Le-Viet, Steven Rudder, Ana P. Tedim, Anastasia Kolyva, Maria Diaz, Leonardo de Oliveira Martins, Nabil-Fareed Alikhan, Lizzie Meadows, Rachael Stanley, Ngozi Elumogo, Muhammed Yasir, Nicholas M. Thomson, Alexander J Trotter, Rachel Gilroy, Samuel Bloomfield, Claire Stuart, Andrew Bell, Reenesh Prakash, Samir Dervisevic, Alison E. Mather, John Wain, Mark Webber, Andrew J. Page, Justin O'Grady
EPI_ISL_585631, EPI_ISL_585633, EPI_ISL_585634, EPI_ISL_585635, EPI_ISL_585637, EPI_ISL_585638, EPI_ISL_585639, EPI_ISL_585640, EPI_ISL_585641, EPI_ISL_585642, EPI_ISL_585643, EPI_ISL_585645, EPI_ISL_585646, EPI_ISL_585647, EPI_ISL_585649, EPI_ISL_585650, EPI_ISL_585651, EPI_ISL_585654, EPI_ISL_585655, EPI_ISL_585657, EPI_ISL_585658, EPI_ISL_585660, EPI_ISL_585661, EPI_ISL_585663, EPI_ISL_585664, EPI_ISL_585665, EPI_ISL_585666, EPI_ISL_585667, EPI_ISL_585668, EPI_ISL_585669, EPI_ISL_585672, EPI_ISL_585674, EPI_ISL_585675, EPI_ISL_585676, EPI_ISL_585677, EPI_ISL_585678, EPI_ISL_585679, EPI_ISL_585681, EPI_ISL_585683, EPI_ISL_585684, EPI_ISL_585685, EPI_ISL_585686, EPI_ISL_585687, EPI_ISL_585689, EPI_ISL_585690, EPI_ISL_585691, EPI_ISL_585692, EPI_ISL_585694, EPI_ISL_585698, EPI_ISL_585699, EPI_ISL_585700, EPI_ISL_585702, EPI_ISL_585703, EPI_ISL_585705, EPI_ISL_585707, EPI_ISL_585708, EPI_ISL_585709, EPI_ISL_585710, EPI_ISL_585711, EPI_ISL_585712, EPI_ISL_585713, EPI_ISL_585714, EPI_ISL_585715, EPI_ISL_585716, EPI_ISL_585718, EPI_ISL_585719, EPI_ISL_585721, EPI_ISL_585722, EPI_ISL_585723, EPI_ISL_585725, EPI_ISL_585726, EPI_ISL_585728, EPI_ISL_585729, EPI_ISL_585730, EPI_ISL_585732, EPI_ISL_585733, EPI_ISL_585735, EPI_ISL_585736, EPI_ISL_585738, EPI_ISL_585739, EPI_ISL_585741, EPI_ISL_585746, EPI_ISL_585747, EPI_ISL_585749, EPI_ISL_585750, EPI_ISL_585751, EPI_ISL_585752, EPI_ISL_585754, EPI_ISL_585755, EPI_ISL_585756, EPI_ISL_585757, EPI_ISL_585758, EPI_ISL_585759, EPI_ISL_585760, EPI_ISL_585762, EPI_ISL_585763, EPI_ISL_585765, EPI_ISL_585779, EPI_ISL_585781, EPI_ISL_585782, EPI_ISL_585783, EPI_ISL_585791, EPI_ISL_585794, EPI_ISL_585796, EPI_ISL_585798, EPI_ISL_585800, EPI_ISL_585801, EPI_ISL_585804, EPI_ISL_585806, EPI_ISL_585807, EPI_ISL_585825, EPI_ISL_585830, EPI_ISL_585833, EPI_ISL_585834, EPI_ISL_585835, EPI_ISL_585857, EPI_ISL_585862, EPI_ISL_585864, EPI_ISL_585867, EPI_ISL_585876, EPI_ISL_585878, EPI_ISL_585879, EPI_ISL_585886, EPI_ISL_585887, EPI_ISL_585888, EPI_ISL_585892, EPI_ISL_585893, EPI_ISL_585895, EPI_ISL_585899, EPI_ISL_585903, EPI_ISL_585904, EPI_ISL_585908, EPI_ISL_585918, EPI_ISL_585919, EPI_ISL_585920, EPI_ISL_585926, EPI_ISL_585929, EPI_ISL_585930, EPI_ISL_585931, EPI_ISL_585937, EPI_ISL_585938, EPI_ISL_585940, EPI_ISL_585942, EPI_ISL_585955, EPI_ISL_585962, EPI_ISL_585964, EPI_ISL_585975, EPI_ISL_585977, EPI_ISL_585978, EPI_ISL_585980, EPI_ISL_585985, EPI_ISL_585986, EPI_ISL_585988, EPI_ISL_585990, EPI_ISL_585998, EPI_ISL_586005, EPI_ISL_586011, EPI_ISL_586012, EPI_ISL_586013, EPI_ISL_586017, EPI_ISL_586020, EPI_ISL_586023, EPI_ISL_586024, EPI_ISL_586057, EPI_ISL_586074, EPI_ISL_586078, EPI_ISL_586080, EPI_ISL_586092, EPI_ISL_586128, EPI_ISL_586135, EPI_ISL_586162, EPI_ISL_586164, EPI_ISL_586167, EPI_ISL_586213, EPI_ISL_586225			
see above	Wales Specialist Virology Centre Sequencing lab: Pathogen Genomics Unit	COVID-19 Genomics UK (COG-UK) Consortium	Catherine Moore, Johnathan Evans, Laura Gifford, Malorie Perry, Simon Cottrell, Angela Marchbank, Alec Birchley, Alexander Adams, Amy Gaskin, Bree Gatica-Wilcox, Jason Coombes, Joel Southgate, Lauren Gilbert, Lee Graham, Nicole Pacchiarini, Sara Kumziene-Summerhayes, Sarah Taylor, Sophie Jones, Sara Rey, Matthew Bull, Joanne Watkins, Sally Corden, Tom Connor
EPI_ISL_586240, EPI_ISL_586241, EPI_ISL_586242	Quadram Institute Bioscience	COVID-19 Genomics UK (COG-UK) Consortium	Dave J. Baker, Gemma L. Kay, Alp Aydin, Thanh Le-Viet, Steven Rudder, Ana P. Tedim, Anastasia Kolyva, Maria Diaz, Leonardo de Oliveira Martins, Nabil-Fareed Alikhan, Lizzie Meadows, Rachael Stanley, Ngozi Elumogo, Muhammed Yasir, Nicholas M. Thomson, Alexander J Trotter, Rachel Gilroy, Samuel Bloomfield, Claire Stuart, Andrew Bell, Reenesh Prakash, Samir Dervisevic, Alison E. Mather, John Wain, Mark Webber, Andrew J. Page, Justin O'Grady
EPI_ISL_587303	Lighthouse Lab in Glasgow	Wellcome Sanger Institute for the COVID-19 Genomics UK (COG-UK) Consortium	Harper VanSteenhouse, Yumi Kasai, David Gray, Carol Clugston, Anna Dominiczak and Alex Alderton, Roberto Amato, Sonia Goncalves, Ewan Harrison, David K. Jackson, Ian Johnston, Dominic Kwiatkowski, Cordelia Langford, John Sillitoe on behalf of the Wellcome Sanger Institute COVID-19 Surveillance Team
EPI_ISL_587304, EPI_ISL_587305, EPI_ISL_587306, EPI_ISL_587307, EPI_ISL_587308, EPI_ISL_587309, EPI_ISL_587310, EPI_ISL_587311, EPI_ISL_587312, EPI_ISL_587313, EPI_ISL_587314, EPI_ISL_587315, EPI_ISL_587316, EPI_ISL_587317, EPI_ISL_587318, EPI_ISL_587319, EPI_ISL_587320, EPI_ISL_587321, EPI_ISL_587322, EPI_ISL_587323, EPI_ISL_587324, EPI_ISL_587325, EPI_ISL_587326, EPI_ISL_587327, EPI_ISL_587328, EPI_ISL_587329, EPI_ISL_587330, EPI_ISL_587331, EPI_ISL_587332, EPI_ISL_587333, EPI_ISL_587334, EPI_ISL_587335, EPI_ISL_587336, EPI_ISL_587337, EPI_ISL_587338, EPI_ISL_587339, EPI_ISL_587340, EPI_ISL_587341, EPI_ISL_587342, EPI_ISL_587343, EPI_ISL_587344, EPI_ISL_587345, EPI_ISL_587346, EPI_ISL_587347, EPI_ISL_587348, EPI_ISL_587349, EPI_ISL_587350, EPI_ISL_587351, EPI_ISL_587352, EPI_ISL_587353, EPI_ISL_587354, EPI_ISL_587355, EPI_ISL_587356, EPI_ISL_587357, EPI_ISL_587358, EPI_ISL_587359, EPI_ISL_587360, EPI_ISL_587361, EPI_ISL_587362, EPI_ISL_587363, EPI_ISL_587364, EPI_ISL_587365, EPI_ISL_587366, EPI_ISL_587367, EPI_ISL_587368, EPI_ISL_587369, EPI_ISL_587370, EPI_ISL_587371, EPI_ISL_587372, EPI_ISL_587373, EPI_ISL_587374, EPI_ISL_587375, EPI_ISL_587376, EPI_ISL_587377, EPI_ISL_587378, EPI_ISL_587379, EPI_ISL_587380, EPI_ISL_587381, EPI_ISL_587382, EPI_ISL_587383, EPI_ISL_587384, EPI_ISL_587385			
see above	Lighthouse Lab in Glasgow	Wellcome Sanger Institute for the COVID-19 Genomics UK (COG-UK) consortium	Harper VanSteenhouse, Yumi Kasai, David Gray, Carol Clugston, Anna Dominiczak and Alex Alderton, Roberto Amato, Sonia Goncalves, Ewan Harrison, David K. Jackson, Ian Johnston, Dominic Kwiatkowski, Cordelia Langford, John Sillitoe on behalf of the Wellcome Sanger Institute COVID-19 Surveillance Team
EPI_ISL_587386	Lighthouse Lab in Glasgow	Wellcome Sanger Institute for the COVID-19 Genomics UK (COG-UK) Consortium	Harper VanSteenhouse, Yumi Kasai, David Gray, Carol Clugston, Anna Dominiczak and Alex Alderton, Roberto Amato, Sonia Goncalves, Ewan Harrison, David K. Jackson, Ian Johnston, Dominic Kwiatkowski, Cordelia Langford, John Sillitoe on behalf of the Wellcome Sanger Institute COVID-19 Surveillance Team
EPI_ISL_587387, EPI_ISL_587388, EPI_ISL_587389, EPI_ISL_587390, EPI_ISL_587391, EPI_ISL_587392, EPI_ISL_587393, EPI_ISL_587394, EPI_ISL_587395, EPI_ISL_587396, EPI_ISL_587397, EPI_ISL_587398, EPI_ISL_587399, EPI_ISL_587400, EPI_ISL_587401, EPI_ISL_587402, EPI_ISL_587403, EPI_ISL_587404, EPI_ISL_587405, EPI_ISL_587406, EPI_ISL_587407, EPI_ISL_587408, EPI_ISL_587409, EPI_ISL_587410, EPI_ISL_587411, EPI_ISL_587412, EPI_ISL_587413, EPI_ISL_587414, EPI_ISL_587415, EPI_ISL_587416, EPI_ISL_587417, EPI_ISL_587418, EPI_ISL_587419, EPI_ISL_587420, EPI_ISL_587421, EPI_ISL_587422, EPI_ISL_587423, EPI_ISL_587424, EPI_ISL_587425, EPI_ISL_587426, EPI_ISL_587427, EPI_ISL_587428, EPI_ISL_587429, EPI_ISL_587430, EPI_ISL_587431, EPI_ISL_587432, EPI_ISL_587433, EPI_ISL_587434, EPI_ISL_587435, EPI_ISL_587436, EPI_ISL_587437, EPI_ISL_587438, EPI_ISL_587439, EPI_ISL_587440, EPI_ISL_587441, EPI_ISL_587442, EPI_ISL_587443, EPI_ISL_587444, EPI_ISL_587445, EPI_ISL_587446, EPI_ISL_587447, EPI_ISL_587448, EPI_ISL_587449, EPI_ISL_587450, EPI_ISL_587451, EPI_ISL_587452, EPI_ISL_587453, EPI_ISL_587454, EPI_ISL_587455, EPI_ISL_587456, EPI_ISL_587457, EPI_ISL_587458, EPI_ISL_587459, EPI_ISL_587460, EPI_ISL_587461, EPI_ISL_587462, EPI_ISL_587463, EPI_ISL_587464, EPI_ISL_587465, EPI_ISL_587466, EPI_ISL_587467, EPI_ISL_587468, EPI_ISL_587469, EPI_ISL_587470, EPI_ISL_587471, EPI_ISL_587472, EPI_ISL_587473, EPI_ISL_587474, EPI_ISL_587475, EPI_ISL_587476			
see above	Lighthouse Lab in Glasgow	Wellcome Sanger Institute for the COVID-19 Genomics UK (COG-UK) consortium	Harper VanSteenhouse, Yumi Kasai, David Gray, Carol Clugston, Anna Dominiczak and Alex Alderton, Roberto Amato, Sonia Goncalves, Ewan Harrison, David K. Jackson, Ian Johnston, Dominic Kwiatkowski, Cordelia Langford, John Sillitoe on behalf of the Wellcome Sanger Institute COVID-19 Surveillance Team
EPI_ISL_587477	Lighthouse Lab in Glasgow	Wellcome Sanger Institute for the COVID-19 Genomics UK (COG-UK) Consortium	Harper VanSteenhouse, Yumi Kasai, David Gray, Carol Clugston, Anna Dominiczak and Alex Alderton, Roberto Amato, Sonia Goncalves, Ewan Harrison, David K. Jackson, Ian Johnston, Dominic Kwiatkowski, Cordelia Langford, John Sillitoe on behalf of the Wellcome Sanger Institute COVID-19 Surveillance Team
EPI_ISL_587478, EPI_ISL_587479, EPI_ISL_587480, EPI_ISL_587481, EPI_ISL_587482, EPI_ISL_587483, EPI_ISL_587484, EPI_ISL_587485, EPI_ISL_587486, EPI_ISL_587487, EPI_ISL_587488, EPI_ISL_587489, EPI_ISL_587490, EPI_ISL_587491, EPI_ISL_587492, EPI_ISL_587493, EPI_ISL_587494, EPI_ISL_587495, EPI_ISL_587496, EPI_ISL_587497, EPI_ISL_587498, EPI_ISL_587499, EPI_ISL_587500, EPI_ISL_587501, EPI_ISL_587502, EPI_ISL_587503, EPI_ISL_587504, EPI_ISL_587505, EPI_ISL_587506, EPI_ISL_587507, EPI_ISL_587508, EPI_ISL_587509, EPI_ISL_587510, EPI_ISL_587511, EPI_ISL_587512, EPI_ISL_587513, EPI_ISL_587514, EPI_ISL_587515, EPI_ISL_587516, EPI_ISL_587517, EPI_ISL_587518, EPI_ISL_587519, EPI_ISL_587520, EPI_ISL_587521, EPI_ISL_587522, EPI_ISL_587523, EPI_ISL_587524, EPI_ISL_587525, EPI_ISL_587526, EPI_ISL_587527, EPI_ISL_587528, EPI_ISL_587529, EPI_ISL_587530, EPI_ISL_587531, EPI_ISL_587532, EPI_ISL_587533, EPI_ISL_587534, EPI_ISL_587535, EPI_ISL_587536, EPI_ISL_587537, EPI_ISL_587538, EPI_ISL_587539, EPI_ISL_587540, EPI_ISL_587541, EPI_ISL_587542, EPI_ISL_587543, EPI_ISL_587544, EPI_ISL_587545, EPI_ISL_587546, EPI_ISL_587547, EPI_ISL_587548, EPI_ISL_587549, EPI_ISL_587550, EPI_ISL_587551, EPI_ISL_587552, EPI_ISL_587553, EPI_ISL_587554, EPI_ISL_587555, EPI_ISL_587556, EPI_ISL_587557, EPI_ISL_587558, EPI_ISL_587559, EPI_ISL_587560, EPI_ISL_587561, EPI_ISL_587562, EPI_ISL_587563, EPI_ISL_587564, EPI_ISL_587565, EPI_ISL_587566, EPI_ISL_587567, EPI_ISL_587568, EPI_ISL_587569, EPI_ISL_587570, EPI_ISL_587571, EPI_ISL_587572, EPI_ISL_587573, EPI_ISL_587574, EPI_ISL_587575, EPI_ISL_587576, EPI_ISL_587577, EPI_ISL_587578, EPI_ISL_587579, EPI_ISL_587580, EPI_ISL_587581, EPI_ISL_587582, EPI_ISL_587583, EPI_ISL_587584, EPI_ISL_587585, EPI_ISL_587586, EPI_ISL_587587, EPI_ISL_587588, EPI_ISL_587589, EPI_ISL_587590, EPI_ISL_587591, EPI_ISL_587592, EPI_ISL_587593, EPI_ISL_587594, EPI_ISL_587595, EPI_ISL_587596, EPI_ISL_587597, EPI_ISL_587598, EPI_ISL_587599, EPI_ISL_587600, EPI_ISL_587601, EPI_ISL_587602, EPI_ISL_587603, EPI_ISL_587604, EPI_ISL_587605, EPI_ISL_587606, EPI_ISL_587607, EPI_ISL_587608, EPI_ISL_587609, EPI_ISL_587610, EPI_ISL_587611, EPI_ISL_587612, EPI_ISL_587613, EPI_ISL_587614, EPI_ISL_587615, EPI_ISL_587616, EPI_ISL_587617, EPI_ISL_587618, EPI_ISL_587619, EPI_ISL_587620, EPI_ISL_587621, EPI_ISL_587622, EPI_ISL_587623, EPI_ISL_587624, EPI_ISL_587625, EPI_ISL_587626, EPI_ISL_587627, EPI_ISL_587628, EPI_ISL_587629, EPI_ISL_587630, EPI_ISL_587631, EPI_ISL_587632, EPI_ISL_587633, EPI_ISL_587634, EPI_ISL_587635, EPI_ISL_587636, EPI_ISL_587637, EPI_ISL_587638, EPI_ISL_587639, EPI_ISL_587640, EPI_ISL_587641, EPI_ISL_587642, EPI_ISL_587643, EPI_ISL_587644, EPI_ISL_587645, EPI_ISL_587646, EPI_ISL_587647, EPI_ISL_587648, EPI_ISL_587649, EPI_ISL_587650, EPI_ISL_587651, EPI_ISL_587652, EPI_ISL_587653, EPI_ISL_587654, EPI_ISL_587655, EPI_ISL_587656, EPI_ISL_587657, EPI_ISL_587658, EPI_ISL_587659, EPI_ISL_587660, EPI_ISL_587661, EPI_ISL_587662, EPI_ISL_587663, EPI_ISL_587664, EPI_ISL_587665, EPI_ISL_587666, EPI_ISL_587667, EPI_ISL_587668, EPI_ISL_587669, EPI_ISL_587670, EPI_ISL_587671, EPI_ISL_587672, EPI_ISL_587673, EPI_ISL_587674, EPI_ISL_587675, EPI_ISL_587676, EPI_ISL_587677, EPI_ISL_587678, EPI_ISL_587679, EPI_ISL_587680, EPI_ISL_587681, EPI_ISL_587682, EPI_ISL_587683, EPI_ISL_587684, EPI_ISL_587685, EPI_ISL_587686, EPI_ISL_587687, EPI_ISL_587688, EPI_ISL_587689, EPI_ISL_587690, EPI_ISL_587691, EPI_ISL_587692, EPI_ISL_587693, EPI_ISL_587694, EPI_ISL_587695, EPI_ISL_587696, EPI_ISL_587697, EPI_ISL_587698, EPI_ISL_587699, EPI_ISL_587700, EPI_ISL_587701, EPI_ISL_587702, EPI_ISL_587703, EPI_ISL_587704, EPI_ISL_587705, EPI_ISL_587706, EPI_ISL_587707, EPI_ISL_587708, EPI_ISL_587709, EPI_ISL_587710			
see above	Lighthouse Lab in Glasgow	Wellcome Sanger Institute for the COVID-19 Genomics UK (COG-UK) consortium	Harper VanSteenhouse, Yumi Kasai, David Gray, Carol Clugston, Anna Dominiczak and Alex Alderton, Roberto Amato, Sonia Goncalves, Ewan Harrison, David K. Jackson, Ian Johnston, Dominic Kwiatkowski, Cordelia Langford, John Sillitoe on behalf of the Wellcome Sanger Institute COVID-19 Surveillance Team
EPI_ISL_587711, EPI_ISL_587712, EPI_ISL_587713, EPI_ISL_587714, EPI_ISL_587718, EPI_ISL_587720, EPI_ISL_587721, EPI_ISL_587722, EPI_ISL_587724, EPI_ISL_587726, EPI_ISL_587727, EPI_ISL_587729, EPI_ISL_587730, EPI_ISL_587731, EPI_ISL_587734, EPI_ISL_587738, EPI_ISL_587741, EPI_ISL_587744, EPI_ISL_587746, EPI_ISL_587748, EPI_ISL_587750, EPI_ISL_587751, EPI_ISL_587753, EPI_ISL_587754, EPI_ISL_587755, EPI_ISL_587758, EPI_ISL_587760, EPI_ISL_587761, EPI_ISL_587762, EPI_ISL_587763, EPI_ISL_587764, EPI_ISL_587765, EPI_ISL_587766, EPI_ISL_587768, EPI_ISL_587769, EPI_ISL_587770, EPI_ISL_587772, EPI_ISL_587773, EPI_ISL_587774, EPI_ISL_587775, EPI_ISL_587776, EPI_ISL_587777, EPI_ISL_587778, EPI_ISL_587779, EPI_ISL_587781, EPI_ISL_587782, EPI_ISL_587784, EPI_ISL_587785, EPI_ISL_587786, EPI_ISL_587787, EPI_ISL_587788, EPI_ISL_587789, EPI_ISL_587791, EPI_ISL_587792, EPI_ISL_587793, EPI_ISL_587794, EPI_ISL_587796, EPI_ISL_587797, EPI_ISL_587799, EPI_ISL_587800, EPI_ISL_587801, EPI_ISL_587803, EPI_ISL_587806, EPI_ISL_587807, EPI_ISL_587808, EPI_ISL_587809, EPI_ISL_587814, EPI_ISL_587816, EPI_ISL_587818, EPI_ISL_587819, EPI_ISL_587820, EPI_ISL_587822, EPI_ISL_587824, EPI_ISL_587825, EPI_ISL_587828, EPI_ISL_587829, EPI_ISL_587830, EPI_ISL_587833, EPI_ISL_587834, EPI_ISL_587835, EPI_ISL_587837, EPI_ISL_587838, EPI_ISL_587840, EPI_ISL_587843, EPI_ISL_587844, EPI_ISL_587846, EPI_ISL_587847, EPI_ISL_587849, EPI_ISL_587852, EPI_ISL_587853, EPI_ISL_587856, EPI_ISL_587859, EPI_ISL_587860, EPI_ISL_587861, EPI_ISL_587862, EPI_ISL_587864, EPI_ISL_587865, EPI_ISL_587866, EPI_ISL_587867, EPI_ISL_587869, EPI_ISL_587870, EPI_ISL_587873, EPI_ISL_587874, EPI_ISL_587875, EPI_ISL_587876, EPI_ISL_587883, EPI_ISL_587884, EPI_ISL_587885, EPI_ISL_587886, EPI_ISL_587887, EPI_ISL_587888, EPI_ISL_587890, EPI_ISL_587891, EPI_ISL_587897, EPI_ISL_587898, EPI_ISL_587899, EPI_ISL_587900, EPI_ISL_587901, EPI_ISL_587902, EPI_ISL_587904, EPI_ISL_587905, EPI_ISL_587906, EPI_ISL_587907, EPI_ISL_587908, EPI_ISL_587910, EPI_ISL_587911, EPI_ISL_587912, EPI_ISL_587913, EPI_ISL_587916, EPI_ISL_587917, EPI_ISL_587920, EPI_ISL_587922, EPI_ISL_587923, EPI_ISL_587924, EPI_ISL_587927, EPI_ISL_587928, EPI_ISL_587929, EPI_ISL_587930, EPI_ISL_587931, EPI_ISL_587932, EPI_ISL_587933, EPI_ISL_587934, EPI_ISL_587935, EPI_ISL_587937,			

EPI_ISL_587938, EPI_ISL_587941, EPI_ISL_587944, EPI_ISL_587946, EPI_ISL_587947, EPI_ISL_587950, EPI_ISL_587951, EPI_ISL_587952, EPI_ISL_587953, EPI_ISL_587957, EPI_ISL_587958, EPI_ISL_587960, EPI_ISL_587961, EPI_ISL_587965, EPI_ISL_587966, EPI_ISL_587967, EPI_ISL_587971, EPI_ISL_587972, EPI_ISL_587973, EPI_ISL_587976			
see above	Lighthouse Lab in Alderley Park	Wellcome Sanger Institute for the COVID-19 Genomics UK (COG-UK) consortium	Jacquelyn Wynn, Mairead Hyland, The Lighthouse Lab in Alderley Park and Alex Alderton, Roberto Amato, Sonia Goncalves, Ewan Harrison, David K. Jackson, Ian Johnston, Dominic Kwiatkowski, Cordelia Langford, John Sillitoe on behalf of the Wellcome Sanger Institute COVID-19 Surveillance Team
EPI_ISL_587977	Lighthouse Lab in Alderley Park	Wellcome Sanger Institute for the COVID-19 Genomics UK (COG-UK) Consortium	Jacquelyn Wynn, Mairead Hyland, The Lighthouse Lab in Alderley Park and Alex Alderton, Roberto Amato, Sonia Goncalves, Ewan Harrison, David K. Jackson, Ian Johnston, Dominic Kwiatkowski, Cordelia Langford, John Sillitoe on behalf of the Wellcome Sanger Institute COVID-19 Surveillance Team
EPI_ISL_587978, EPI_ISL_587981, EPI_ISL_587984, EPI_ISL_587986, EPI_ISL_587987, EPI_ISL_587990, EPI_ISL_587992, EPI_ISL_587993, EPI_ISL_587996, EPI_ISL_587997, EPI_ISL_587998, EPI_ISL_587999, EPI_ISL_588000, EPI_ISL_588001, EPI_ISL_588002, EPI_ISL_588004			
see above	Lighthouse Lab in Alderley Park	Wellcome Sanger Institute for the COVID-19 Genomics UK (COG-UK) consortium	Jacquelyn Wynn, Mairead Hyland, The Lighthouse Lab in Alderley Park and Alex Alderton, Roberto Amato, Sonia Goncalves, Ewan Harrison, David K. Jackson, Ian Johnston, Dominic Kwiatkowski, Cordelia Langford, John Sillitoe on behalf of the Wellcome Sanger Institute COVID-19 Surveillance Team
EPI_ISL_588007	Lighthouse Lab in Alderley Park	Wellcome Sanger Institute for the COVID-19 Genomics UK (COG-UK) Consortium	Jacquelyn Wynn, Mairead Hyland, The Lighthouse Lab in Alderley Park and Alex Alderton, Roberto Amato, Sonia Goncalves, Ewan Harrison, David K. Jackson, Ian Johnston, Dominic Kwiatkowski, Cordelia Langford, John Sillitoe on behalf of the Wellcome Sanger Institute COVID-19 Surveillance Team
EPI_ISL_588009, EPI_ISL_588010, EPI_ISL_588011, EPI_ISL_588012, EPI_ISL_588013, EPI_ISL_588014, EPI_ISL_588015, EPI_ISL_588017, EPI_ISL_588018, EPI_ISL_588019, EPI_ISL_588021, EPI_ISL_588022, EPI_ISL_588024, EPI_ISL_588025			
see above	Lighthouse Lab in Alderley Park	Wellcome Sanger Institute for the COVID-19 Genomics UK (COG-UK) consortium	Jacquelyn Wynn, Mairead Hyland, The Lighthouse Lab in Alderley Park and Alex Alderton, Roberto Amato, Sonia Goncalves, Ewan Harrison, David K. Jackson, Ian Johnston, Dominic Kwiatkowski, Cordelia Langford, John Sillitoe on behalf of the Wellcome Sanger Institute COVID-19 Surveillance Team
EPI_ISL_588026	Lighthouse Lab in Alderley Park	Wellcome Sanger Institute for the COVID-19 Genomics UK (COG-UK) Consortium	Jacquelyn Wynn, Mairead Hyland, The Lighthouse Lab in Alderley Park and Alex Alderton, Roberto Amato, Sonia Goncalves, Ewan Harrison, David K. Jackson, Ian Johnston, Dominic Kwiatkowski, Cordelia Langford, John Sillitoe on behalf of the Wellcome Sanger Institute COVID-19 Surveillance Team
EPI_ISL_588028, EPI_ISL_588029, EPI_ISL_588031, EPI_ISL_588032, EPI_ISL_588034, EPI_ISL_588036, EPI_ISL_588037, EPI_ISL_588038, EPI_ISL_588040, EPI_ISL_588042, EPI_ISL_588044, EPI_ISL_588045, EPI_ISL_588046, EPI_ISL_588048, EPI_ISL_588049, EPI_ISL_588051, EPI_ISL_588054, EPI_ISL_588055, EPI_ISL_588057, EPI_ISL_588059			
see above	Lighthouse Lab in Alderley Park	Wellcome Sanger Institute for the COVID-19 Genomics UK (COG-UK) consortium	Jacquelyn Wynn, Mairead Hyland, The Lighthouse Lab in Alderley Park and Alex Alderton, Roberto Amato, Sonia Goncalves, Ewan Harrison, David K. Jackson, Ian Johnston, Dominic Kwiatkowski, Cordelia Langford, John Sillitoe on behalf of the Wellcome Sanger Institute COVID-19 Surveillance Team
EPI_ISL_588060, EPI_ISL_588061, EPI_ISL_588062, EPI_ISL_588063, EPI_ISL_588064, EPI_ISL_588065, EPI_ISL_588066, EPI_ISL_588067, EPI_ISL_588068, EPI_ISL_588069, EPI_ISL_588070, EPI_ISL_588071, EPI_ISL_588072, EPI_ISL_588073, EPI_ISL_588074			
see above	Lighthouse Lab in Glasgow	Wellcome Sanger Institute for the COVID-19 Genomics UK (COG-UK) consortium	Harper VanSteenhouse, Yumi Kasai, David Gray, Carol Clugston, Anna Dominiczak and Alex Alderton, Roberto Amato, Sonia Goncalves, Ewan Harrison, David K. Jackson, Ian Johnston, Dominic Kwiatkowski, Cordelia Langford, John Sillitoe on behalf of the Wellcome Sanger Institute COVID-19 Surveillance Team
EPI_ISL_588075	Lighthouse Lab in Glasgow	Wellcome Sanger Institute for the COVID-19 Genomics UK (COG-UK) Consortium	Harper VanSteenhouse, Yumi Kasai, David Gray, Carol Clugston, Anna Dominiczak and Alex Alderton, Roberto Amato, Sonia Goncalves, Ewan Harrison, David K. Jackson, Ian Johnston, Dominic Kwiatkowski, Cordelia Langford, John Sillitoe on behalf of the Wellcome Sanger Institute COVID-19 Surveillance Team
EPI_ISL_588076, EPI_ISL_588077, EPI_ISL_588078, EPI_ISL_588079, EPI_ISL_588080, EPI_ISL_588081, EPI_ISL_588082, EPI_ISL_588083, EPI_ISL_588084, EPI_ISL_588085, EPI_ISL_588086, EPI_ISL_588087, EPI_ISL_588088, EPI_ISL_588089, EPI_ISL_588090, EPI_ISL_588091, EPI_ISL_588092, EPI_ISL_588093, EPI_ISL_588094, EPI_ISL_588095, EPI_ISL_588096, EPI_ISL_588097, EPI_ISL_588098, EPI_ISL_588099, EPI_ISL_588100, EPI_ISL_588101, EPI_ISL_588102, EPI_ISL_588103, EPI_ISL_588104, EPI_ISL_588105, EPI_ISL_588106, EPI_ISL_588107, EPI_ISL_588108, EPI_ISL_588109, EPI_ISL_588110, EPI_ISL_588111, EPI_ISL_588112, EPI_ISL_588113, EPI_ISL_588114, EPI_ISL_588115, EPI_ISL_588116, EPI_ISL_588117, EPI_ISL_588118, EPI_ISL_588119, EPI_ISL_588120, EPI_ISL_588121, EPI_ISL_588122, EPI_ISL_588123, EPI_ISL_588124			
see above	Lighthouse Lab in Glasgow	Wellcome Sanger Institute for the COVID-19 Genomics UK (COG-UK) consortium	Harper VanSteenhouse, Yumi Kasai, David Gray, Carol Clugston, Anna Dominiczak and Alex Alderton, Roberto Amato, Sonia Goncalves, Ewan Harrison, David K. Jackson, Ian Johnston, Dominic Kwiatkowski, Cordelia Langford, John Sillitoe on behalf of the Wellcome Sanger Institute COVID-19 Surveillance Team
EPI_ISL_588125	Lighthouse Lab in Glasgow	Wellcome Sanger Institute for the COVID-19 Genomics UK (COG-UK) Consortium	Harper VanSteenhouse, Yumi Kasai, David Gray, Carol Clugston, Anna Dominiczak and Alex Alderton, Roberto Amato, Sonia Goncalves, Ewan Harrison, David K. Jackson, Ian Johnston, Dominic Kwiatkowski, Cordelia Langford, John Sillitoe on behalf of the Wellcome Sanger Institute COVID-19 Surveillance Team
EPI_ISL_588126, EPI_ISL_588127	Lighthouse Lab in Glasgow	Wellcome Sanger Institute for the COVID-19 Genomics UK (COG-UK) consortium	Harper VanSteenhouse, Yumi Kasai, David Gray, Carol Clugston, Anna Dominiczak and Alex Alderton, Roberto Amato, Sonia Goncalves, Ewan Harrison, David K. Jackson, Ian Johnston, Dominic Kwiatkowski, Cordelia Langford, John Sillitoe on behalf of the Wellcome Sanger Institute COVID-19 Surveillance Team
EPI_ISL_588128	Lighthouse Lab in Glasgow	Wellcome Sanger Institute for the COVID-19 Genomics UK (COG-UK) Consortium	Harper VanSteenhouse, Yumi Kasai, David Gray, Carol Clugston, Anna Dominiczak and Alex Alderton, Roberto Amato, Sonia Goncalves, Ewan Harrison, David K. Jackson, Ian Johnston, Dominic Kwiatkowski, Cordelia Langford, John Sillitoe on behalf of the Wellcome Sanger Institute COVID-19 Surveillance Team
EPI_ISL_588129, EPI_ISL_588130, EPI_ISL_588131, EPI_ISL_588132, EPI_ISL_588133, EPI_ISL_588134, EPI_ISL_588135, EPI_ISL_588136, EPI_ISL_588137, EPI_ISL_588138, EPI_ISL_588139, EPI_ISL_588140, EPI_ISL_588141, EPI_ISL_588142, EPI_ISL_588143, EPI_ISL_588144, EPI_ISL_588145, EPI_ISL_588146, EPI_ISL_588147, EPI_ISL_588148, EPI_ISL_588149, EPI_ISL_588150, EPI_ISL_588151, EPI_ISL_588152, EPI_ISL_588153, EPI_ISL_588154, EPI_ISL_588155, EPI_ISL_588156, EPI_ISL_588157, EPI_ISL_588158, EPI_ISL_588159, EPI_ISL_588160, EPI_ISL_588161, EPI_ISL_588162, EPI_ISL_588163, EPI_ISL_588164, EPI_ISL_588165, EPI_ISL_588166, EPI_ISL_588167, EPI_ISL_588168, EPI_ISL_588169, EPI_ISL_588170, EPI_ISL_588171, EPI_ISL_588172, EPI_ISL_588173, EPI_ISL_588174			
see above	Lighthouse Lab in Glasgow	Wellcome Sanger Institute for the COVID-19 Genomics UK (COG-UK) consortium	Harper VanSteenhouse, Yumi Kasai, David Gray, Carol Clugston, Anna Dominiczak and Alex Alderton, Roberto Amato, Sonia Goncalves, Ewan Harrison, David K. Jackson, Ian Johnston, Dominic Kwiatkowski, Cordelia Langford, John Sillitoe on behalf of the Wellcome Sanger Institute COVID-19 Surveillance Team
EPI_ISL_588175	Lighthouse Lab in Glasgow	Wellcome Sanger Institute for the COVID-19 Genomics UK (COG-UK) Consortium	Harper VanSteenhouse, Yumi Kasai, David Gray, Carol Clugston, Anna Dominiczak and Alex Alderton, Roberto Amato, Sonia Goncalves, Ewan Harrison, David K. Jackson, Ian Johnston, Dominic Kwiatkowski, Cordelia Langford, John Sillitoe on behalf of the Wellcome Sanger Institute COVID-19 Surveillance Team
EPI_ISL_588176, EPI_ISL_588177, EPI_ISL_588178, EPI_ISL_588179, EPI_ISL_588180, EPI_ISL_588181, EPI_ISL_588182, EPI_ISL_588183, EPI_ISL_588184, EPI_ISL_588185, EPI_ISL_588186, EPI_ISL_588187, EPI_ISL_588188, EPI_ISL_588189, EPI_ISL_588190, EPI_ISL_588191, EPI_ISL_588192, EPI_ISL_588193, EPI_ISL_588194, EPI_ISL_588195, EPI_ISL_588196, EPI_ISL_588197, EPI_ISL_588198, EPI_ISL_588199, EPI_ISL_588200, EPI_ISL_588201, EPI_ISL_588202, EPI_ISL_588203, EPI_ISL_588204, EPI_ISL_588205, EPI_ISL_588207, EPI_ISL_588208, EPI_ISL_588209, EPI_ISL_588210, EPI_ISL_588211, EPI_ISL_588212, EPI_ISL_588213, EPI_ISL_588214, EPI_ISL_588215, EPI_ISL_588216, EPI_ISL_588217, EPI_ISL_588218, EPI_ISL_588219, EPI_ISL_588220, EPI_ISL_588221, EPI_ISL_588222, EPI_ISL_588223, EPI_ISL_588224, EPI_ISL_588225, EPI_ISL_588226, EPI_ISL_588227, EPI_ISL_588228, EPI_ISL_588229, EPI_ISL_588230, EPI_ISL_588231, EPI_ISL_588232, EPI_ISL_588233, EPI_ISL_588234, EPI_ISL_588235, EPI_ISL_588236, EPI_ISL_588237, EPI_ISL_588238, EPI_ISL_588239, EPI_ISL_588240, EPI_ISL_588241, EPI_ISL_588242, EPI_ISL_588243, EPI_ISL_588244, EPI_ISL_588245, EPI_ISL_588246, EPI_ISL_588247, EPI_ISL_588248, EPI_ISL_588249, EPI_ISL_588250, EPI_ISL_588251, EPI_ISL_588252, EPI_ISL_588253, EPI_ISL_588254, EPI_ISL_588255, EPI_ISL_588256, EPI_ISL_588257, EPI_ISL_588258, EPI_ISL_588259, EPI_ISL_588260, EPI_ISL_588261, EPI_ISL_588262, EPI_ISL_588263, EPI_ISL_588264, EPI_ISL_588265, EPI_ISL_588266, EPI_ISL_588267, EPI_ISL_588268, EPI_ISL_588269, EPI_ISL_588270, EPI_ISL_588271, EPI_ISL_588272, EPI_ISL_588273, EPI_ISL_588274, EPI_ISL_588275, EPI_ISL_588276, EPI_ISL_588278, EPI_ISL_588279, EPI_ISL_588280, EPI_ISL_588281, EPI_ISL_588282, EPI_ISL_588283, EPI_ISL_588285, EPI_ISL_588286, EPI_ISL_588287, EPI_ISL_588288, EPI_ISL_588289, EPI_ISL_588290, EPI_ISL_588291, EPI_ISL_588292, EPI_ISL_588293, EPI_ISL_588294, EPI_ISL_588295, EPI_ISL_588296, EPI_ISL_588297, EPI_ISL_588298, EPI_ISL_588299, EPI_ISL_588300, EPI_ISL_588301, EPI_ISL_588302, EPI_ISL_588303, EPI_ISL_588304, EPI_ISL_588305, EPI_ISL_588306, EPI_ISL_588307, EPI_ISL_588309, EPI_ISL_588310, EPI_ISL_588311, EPI_ISL_588312, EPI_ISL_588315, EPI_ISL_588316, EPI_ISL_588317, EPI_ISL_588318, EPI_ISL_588319, EPI_ISL_588320, EPI_ISL_588321, EPI_ISL_588322, EPI_ISL_588323, EPI_ISL_588324, EPI_ISL_588325, EPI_ISL_588326, EPI_ISL_588327, EPI_ISL_588328, EPI_ISL_588329, EPI_ISL_588331, EPI_ISL_588332, EPI_ISL_588333, EPI_ISL_588334, EPI_ISL_588335, EPI_ISL_588336, EPI_ISL_588337, EPI_ISL_588338, EPI_ISL_588339, EPI_ISL_588340, EPI_ISL_588341, EPI_ISL_588342, EPI_ISL_588343, EPI_ISL_588344, EPI_ISL_588345, EPI_ISL_588348, EPI_ISL_588349, EPI_ISL_588350, EPI_ISL_588351, EPI_ISL_588353, EPI_ISL_588354, EPI_ISL_588355, EPI_ISL_588356, EPI_ISL_588357, EPI_ISL_588358, EPI_ISL_588359, EPI_ISL_588360, EPI_ISL_588361, EPI_ISL_588362, EPI_ISL_588363, EPI_ISL_588364, EPI_ISL_588365, EPI_ISL_588366, EPI_ISL_588367, EPI_ISL_588368, EPI_ISL_588369, EPI_ISL_588370, EPI_ISL_588371, EPI_ISL_588372, EPI_ISL_588373, EPI_ISL_588374, EPI_ISL_588375, EPI_ISL_588376, EPI_ISL_588377, EPI_ISL_588379, EPI_ISL_588380, EPI_ISL_588382, EPI_ISL_588383, EPI_ISL_588384, EPI_ISL_588385, EPI_ISL_588386, EPI_ISL_588388, EPI_ISL_588390, EPI_ISL_588391, EPI_ISL_588392, EPI_ISL_588395, EPI_ISL_588396, EPI_ISL_588397, EPI_ISL_588398, EPI_ISL_588399, EPI_ISL_588400, EPI_ISL_588401, EPI_ISL_588402, EPI_ISL_588403, EPI_ISL_588404, EPI_ISL_588405, EPI_ISL_588406, EPI_ISL_588407, EPI_ISL_588408, EPI_ISL_588409, EPI_ISL_588410, EPI_ISL_588411, EPI_ISL_588412, EPI_ISL_588413, EPI_ISL_588414, EPI_ISL_588415, EPI_ISL_588417, EPI_ISL_588418, EPI_ISL_588419, EPI_ISL_588420, EPI_ISL_588421, EPI_ISL_588423, EPI_ISL_588425, EPI_ISL_588426, EPI_ISL_588427, EPI_ISL_588429, EPI_ISL_588431, EPI_ISL_588432, EPI_ISL_588433, EPI_ISL_588434, EPI_ISL_588435, EPI_ISL_588436, EPI_ISL_588440, EPI_ISL_588441, EPI_ISL_588442, EPI_ISL_588444, EPI_ISL_588445, EPI_ISL_588446, EPI_ISL_588447, EPI_ISL_588448, EPI_ISL_588449, EPI_ISL_588450, EPI_ISL_588451, EPI_ISL_588452, EPI_ISL_588453, EPI_ISL_588454, EPI_ISL_588455, EPI_ISL_588457, EPI_ISL_588458, EPI_ISL_588459, EPI_ISL_588460, EPI_ISL_588461, EPI_ISL_588462, EPI_ISL_588464, EPI_ISL_588465, EPI_ISL_588466, EPI_ISL_588467, EPI_ISL_588468, EPI_ISL_588469, EPI_ISL_588470, EPI_ISL_588471, EPI_ISL_588472, EPI_ISL_588473, EPI_ISL_588474, EPI_ISL_588475, EPI_ISL_588476, EPI_ISL_588479, EPI_ISL_588488, EPI_ISL_588490			
see above	Lighthouse Lab in Glasgow	Wellcome Sanger Institute for the COVID-19 Genomics UK (COG-UK) consortium	Harper VanSteenhouse, Yumi Kasai, David Gray, Carol Clugston, Anna Dominiczak and Alex Alderton, Roberto Amato, Sonia Goncalves, Ewan Harrison, David K. Jackson, Ian Johnston, Dominic Kwiatkowski, Cordelia Langford, John Sillitoe on behalf of the Wellcome Sanger Institute COVID-19 Surveillance Team
EPI_ISL_588496	Lighthouse Lab in Glasgow	Wellcome Sanger Institute for the COVID-19 Genomics UK (COG-UK) Consortium	Harper VanSteenhouse, Yumi Kasai, David Gray, Carol Clugston, Anna Dominiczak and Alex Alderton, Roberto Amato, Sonia Goncalves, Ewan Harrison, David K. Jackson, Ian Johnston, Dominic Kwiatkowski, Cordelia Langford, John Sillitoe on behalf of the Wellcome Sanger Institute COVID-19 Surveillance Team
EPI_ISL_588523, EPI_ISL_588533, EPI_ISL_588537, EPI_ISL_588542, EPI_ISL_588546, EPI_ISL_588548, EPI_ISL_588552, EPI_ISL_588553, EPI_ISL_588556, EPI_ISL_588558, EPI_ISL_588563, EPI_ISL_588585, EPI_ISL_588589, EPI_ISL_588592, EPI_ISL_588598, EPI_ISL_588601, EPI_ISL_588608, EPI_ISL_588609, EPI_ISL_588614, EPI_ISL_588619, EPI_ISL_588626, EPI_ISL_588628, EPI_ISL_588633, EPI_ISL_588643, EPI_ISL_588656, EPI_ISL_588659, EPI_ISL_588661, EPI_ISL_588668, EPI_ISL_588672, EPI_ISL_588675, EPI_ISL_588682, EPI_ISL_588684, EPI_ISL_588691, EPI_ISL_588698, EPI_ISL_588699, EPI_ISL_588702,			

EPI_ISL_588706, EPI_ISL_588712			
see above	Lighthouse Lab in Glasgow	Wellcome Sanger Institute for the COVID-19 Genomics UK (COG-UK) consortium	Harper VanSteenhouse, Yumi Kasai, David Gray, Carol Clugston, Anna Dominiczak and Alex Alderton, Roberto Amato, Sonia Goncalves, Ewan Harrison, David K. Jackson, Ian Johnston, Dominic Kwiatkowski, Cordelia Langford, John Sillitoe on behalf of the Wellcome Sanger Institute COVID-19 Surveillance Team
EPI_ISL_588717	Lighthouse Lab in Glasgow	Wellcome Sanger Institute for the COVID-19 Genomics UK (COG-UK) Consortium	Harper VanSteenhouse, Yumi Kasai, David Gray, Carol Clugston, Anna Dominiczak and Alex Alderton, Roberto Amato, Sonia Goncalves, Ewan Harrison, David K. Jackson, Ian Johnston, Dominic Kwiatkowski, Cordelia Langford, John Sillitoe on behalf of the Wellcome Sanger Institute COVID-19 Surveillance Team
EPI_ISL_588725, EPI_ISL_588726, EPI_ISL_588727, EPI_ISL_588728, EPI_ISL_588729, EPI_ISL_588732, EPI_ISL_588733, EPI_ISL_588734, EPI_ISL_588735, EPI_ISL_588736, EPI_ISL_588737, EPI_ISL_588738, EPI_ISL_588739, EPI_ISL_588740, EPI_ISL_588741, EPI_ISL_588742, EPI_ISL_588743, EPI_ISL_588744, EPI_ISL_588745, EPI_ISL_588746, EPI_ISL_588747, EPI_ISL_588748, EPI_ISL_588749, EPI_ISL_588750, EPI_ISL_588751, EPI_ISL_588753, EPI_ISL_588754, EPI_ISL_588755, EPI_ISL_588756, EPI_ISL_588757, EPI_ISL_588758, EPI_ISL_588759, EPI_ISL_588760, EPI_ISL_588761, EPI_ISL_588762, EPI_ISL_588764, EPI_ISL_588765, EPI_ISL_588766, EPI_ISL_588767, EPI_ISL_588768, EPI_ISL_588769, EPI_ISL_588770, EPI_ISL_588771, EPI_ISL_588772, EPI_ISL_588773, EPI_ISL_588774, EPI_ISL_588775, EPI_ISL_588776, EPI_ISL_588777, EPI_ISL_588778, EPI_ISL_588779, EPI_ISL_588780, EPI_ISL_588781, EPI_ISL_588782, EPI_ISL_588783, EPI_ISL_588784, EPI_ISL_588785, EPI_ISL_588786, EPI_ISL_588787, EPI_ISL_588788, EPI_ISL_588789, EPI_ISL_588790, EPI_ISL_588791, EPI_ISL_588792, EPI_ISL_588793, EPI_ISL_588794, EPI_ISL_588795, EPI_ISL_588796, EPI_ISL_588797, EPI_ISL_588798, EPI_ISL_588799, EPI_ISL_588800, EPI_ISL_588801, EPI_ISL_588802, EPI_ISL_588804, EPI_ISL_588805, EPI_ISL_588807, EPI_ISL_588808, EPI_ISL_588809, EPI_ISL_588810, EPI_ISL_588811, EPI_ISL_588812, EPI_ISL_588813, EPI_ISL_588814, EPI_ISL_588815, EPI_ISL_588816, EPI_ISL_588817, EPI_ISL_588818, EPI_ISL_588819, EPI_ISL_588820, EPI_ISL_588821, EPI_ISL_588822, EPI_ISL_588823, EPI_ISL_588824, EPI_ISL_588825, EPI_ISL_588826, EPI_ISL_588827, EPI_ISL_588828, EPI_ISL_588829, EPI_ISL_588830, EPI_ISL_588831, EPI_ISL_588832, EPI_ISL_588833, EPI_ISL_588834, EPI_ISL_588835, EPI_ISL_588836, EPI_ISL_588837, EPI_ISL_588838, EPI_ISL_588839, EPI_ISL_588840, EPI_ISL_588841, EPI_ISL_588842, EPI_ISL_588843			
see above	Lighthouse Lab in Glasgow	Wellcome Sanger Institute for the COVID-19 Genomics UK (COG-UK) consortium	Harper VanSteenhouse, Yumi Kasai, David Gray, Carol Clugston, Anna Dominiczak and Alex Alderton, Roberto Amato, Sonia Goncalves, Ewan Harrison, David K. Jackson, Ian Johnston, Dominic Kwiatkowski, Cordelia Langford, John Sillitoe on behalf of the Wellcome Sanger Institute COVID-19 Surveillance Team
EPI_ISL_588844, EPI_ISL_588845	Lighthouse Lab in Glasgow	Wellcome Sanger Institute for the COVID-19 Genomics UK (COG-UK) Consortium	Harper VanSteenhouse, Yumi Kasai, David Gray, Carol Clugston, Anna Dominiczak and Alex Alderton, Roberto Amato, Sonia Goncalves, Ewan Harrison, David K. Jackson, Ian Johnston, Dominic Kwiatkowski, Cordelia Langford, John Sillitoe on behalf of the Wellcome Sanger Institute COVID-19 Surveillance Team
EPI_ISL_588846, EPI_ISL_588847, EPI_ISL_588848, EPI_ISL_588849, EPI_ISL_588850, EPI_ISL_588851, EPI_ISL_588852, EPI_ISL_588853, EPI_ISL_588854, EPI_ISL_588855, EPI_ISL_588856, EPI_ISL_588857, EPI_ISL_588858, EPI_ISL_588859, EPI_ISL_588860, EPI_ISL_588861, EPI_ISL_588862, EPI_ISL_588863, EPI_ISL_588864, EPI_ISL_588865, EPI_ISL_588866, EPI_ISL_588867, EPI_ISL_588868, EPI_ISL_588869, EPI_ISL_588870, EPI_ISL_588871, EPI_ISL_588872, EPI_ISL_588873, EPI_ISL_588874, EPI_ISL_588875, EPI_ISL_588876, EPI_ISL_588877, EPI_ISL_588878, EPI_ISL_588879, EPI_ISL_588880, EPI_ISL_588881, EPI_ISL_588882, EPI_ISL_588883, EPI_ISL_588884, EPI_ISL_588885, EPI_ISL_588886, EPI_ISL_588887, EPI_ISL_588888, EPI_ISL_588890, EPI_ISL_588891, EPI_ISL_588892, EPI_ISL_588893, EPI_ISL_588894, EPI_ISL_588895, EPI_ISL_588896, EPI_ISL_588897, EPI_ISL_588898, EPI_ISL_588899, EPI_ISL_588900, EPI_ISL_588901, EPI_ISL_588902, EPI_ISL_588903, EPI_ISL_588904, EPI_ISL_588905, EPI_ISL_588906, EPI_ISL_588907, EPI_ISL_588908, EPI_ISL_588909, EPI_ISL_588910, EPI_ISL_588911, EPI_ISL_588913, EPI_ISL_588914, EPI_ISL_588915, EPI_ISL_588916, EPI_ISL_588917, EPI_ISL_588918, EPI_ISL_588919, EPI_ISL_588920, EPI_ISL_588921, EPI_ISL_588922, EPI_ISL_588923, EPI_ISL_588924, EPI_ISL_588925, EPI_ISL_588926			
see above	Lighthouse Lab in Glasgow	Wellcome Sanger Institute for the COVID-19 Genomics UK (COG-UK) consortium	Harper VanSteenhouse, Yumi Kasai, David Gray, Carol Clugston, Anna Dominiczak and Alex Alderton, Roberto Amato, Sonia Goncalves, Ewan Harrison, David K. Jackson, Ian Johnston, Dominic Kwiatkowski, Cordelia Langford, John Sillitoe on behalf of the Wellcome Sanger Institute COVID-19 Surveillance Team
EPI_ISL_588927	Lighthouse Lab in Glasgow	Wellcome Sanger Institute for the COVID-19 Genomics UK (COG-UK) Consortium	Harper VanSteenhouse, Yumi Kasai, David Gray, Carol Clugston, Anna Dominiczak and Alex Alderton, Roberto Amato, Sonia Goncalves, Ewan Harrison, David K. Jackson, Ian Johnston, Dominic Kwiatkowski, Cordelia Langford, John Sillitoe on behalf of the Wellcome Sanger Institute COVID-19 Surveillance Team
EPI_ISL_588928, EPI_ISL_588929, EPI_ISL_588930, EPI_ISL_588931, EPI_ISL_588932, EPI_ISL_588933, EPI_ISL_588934, EPI_ISL_588935, EPI_ISL_588936, EPI_ISL_588937, EPI_ISL_588938, EPI_ISL_588939			
see above	Lighthouse Lab in Glasgow	Wellcome Sanger Institute for the COVID-19 Genomics UK (COG-UK) consortium	Harper VanSteenhouse, Yumi Kasai, David Gray, Carol Clugston, Anna Dominiczak and Alex Alderton, Roberto Amato, Sonia Goncalves, Ewan Harrison, David K. Jackson, Ian Johnston, Dominic Kwiatkowski, Cordelia Langford, John Sillitoe on behalf of the Wellcome Sanger Institute COVID-19 Surveillance Team
EPI_ISL_588940	Lighthouse Lab in Glasgow	Wellcome Sanger Institute for the COVID-19 Genomics UK (COG-UK) Consortium	Harper VanSteenhouse, Yumi Kasai, David Gray, Carol Clugston, Anna Dominiczak and Alex Alderton, Roberto Amato, Sonia Goncalves, Ewan Harrison, David K. Jackson, Ian Johnston, Dominic Kwiatkowski, Cordelia Langford, John Sillitoe on behalf of the Wellcome Sanger Institute COVID-19 Surveillance Team
EPI_ISL_588941, EPI_ISL_588942, EPI_ISL_588943, EPI_ISL_588944, EPI_ISL_588945, EPI_ISL_588946, EPI_ISL_588947, EPI_ISL_588948, EPI_ISL_588949, EPI_ISL_588950, EPI_ISL_588951, EPI_ISL_588952, EPI_ISL_588953, EPI_ISL_588954, EPI_ISL_588955, EPI_ISL_588956, EPI_ISL_588957, EPI_ISL_588958, EPI_ISL_588959, EPI_ISL_588960, EPI_ISL_588961			
see above	Lighthouse Lab in Glasgow	Wellcome Sanger Institute for the COVID-19 Genomics UK (COG-UK) consortium	Harper VanSteenhouse, Yumi Kasai, David Gray, Carol Clugston, Anna Dominiczak and Alex Alderton, Roberto Amato, Sonia Goncalves, Ewan Harrison, David K. Jackson, Ian Johnston, Dominic Kwiatkowski, Cordelia Langford, John Sillitoe on behalf of the Wellcome Sanger Institute COVID-19 Surveillance Team
EPI_ISL_588962	Lighthouse Lab in Glasgow	Wellcome Sanger Institute for the COVID-19 Genomics UK (COG-UK) Consortium	Harper VanSteenhouse, Yumi Kasai, David Gray, Carol Clugston, Anna Dominiczak and Alex Alderton, Roberto Amato, Sonia Goncalves, Ewan Harrison, David K. Jackson, Ian Johnston, Dominic Kwiatkowski, Cordelia Langford, John Sillitoe on behalf of the Wellcome Sanger Institute COVID-19 Surveillance Team
EPI_ISL_588963, EPI_ISL_588964, EPI_ISL_588965, EPI_ISL_588966, EPI_ISL_588967, EPI_ISL_588968, EPI_ISL_588969, EPI_ISL_588970, EPI_ISL_588971, EPI_ISL_588972, EPI_ISL_588974, EPI_ISL_588975, EPI_ISL_588976, EPI_ISL_588977, EPI_ISL_588978, EPI_ISL_588979, EPI_ISL_588980, EPI_ISL_588981, EPI_ISL_588982, EPI_ISL_588983, EPI_ISL_588984, EPI_ISL_588985, EPI_ISL_588986, EPI_ISL_588987, EPI_ISL_588988, EPI_ISL_588989, EPI_ISL_588990, EPI_ISL_588991, EPI_ISL_588992, EPI_ISL_588993, EPI_ISL_588994, EPI_ISL_588996, EPI_ISL_588997, EPI_ISL_588998, EPI_ISL_588999, EPI_ISL_589000, EPI_ISL_589001, EPI_ISL_589003, EPI_ISL_589004, EPI_ISL_589005, EPI_ISL_589006, EPI_ISL_589007, EPI_ISL_589008, EPI_ISL_589009, EPI_ISL_589010, EPI_ISL_589011, EPI_ISL_589012, EPI_ISL_589013, EPI_ISL_589014, EPI_ISL_589015, EPI_ISL_589016			
see above	Lighthouse Lab in Glasgow	Wellcome Sanger Institute for the COVID-19 Genomics UK (COG-UK) consortium	Harper VanSteenhouse, Yumi Kasai, David Gray, Carol Clugston, Anna Dominiczak and Alex Alderton, Roberto Amato, Sonia Goncalves, Ewan Harrison, David K. Jackson, Ian Johnston, Dominic Kwiatkowski, Cordelia Langford, John Sillitoe on behalf of the Wellcome Sanger Institute COVID-19 Surveillance Team
EPI_ISL_589017, EPI_ISL_589018	Lighthouse Lab in Milton Keynes	Wellcome Sanger Institute for the COVID-19 Genomics UK (COG-UK) consortium	The Lighthouse Lab in Milton Keynes and Alex Alderton, Roberto Amato, Sonia Goncalves, Ewan Harrison, David K. Jackson, Ian Johnston, Dominic Kwiatkowski, Cordelia Langford, John Sillitoe on behalf of the Wellcome Sanger Institute COVID-19 Surveillance Team
EPI_ISL_589020, EPI_ISL_589022, EPI_ISL_589023, EPI_ISL_589025	Lighthouse Lab in Glasgow	Wellcome Sanger Institute for the COVID-19 Genomics UK (COG-UK) consortium	Harper VanSteenhouse, Yumi Kasai, David Gray, Carol Clugston, Anna Dominiczak and Alex Alderton, Roberto Amato, Sonia Goncalves, Ewan Harrison, David K. Jackson, Ian Johnston, Dominic Kwiatkowski, Cordelia Langford, John Sillitoe on behalf of the Wellcome Sanger Institute COVID-19 Surveillance Team
EPI_ISL_589026	Lighthouse Lab in Milton Keynes	Wellcome Sanger Institute for the COVID-19 Genomics UK (COG-UK) consortium	The Lighthouse Lab in Milton Keynes and Alex Alderton, Roberto Amato, Sonia Goncalves, Ewan Harrison, David K. Jackson, Ian Johnston, Dominic Kwiatkowski, Cordelia Langford, John Sillitoe on behalf of the Wellcome Sanger Institute COVID-19 Surveillance Team
EPI_ISL_589027, EPI_ISL_589031, EPI_ISL_589032, EPI_ISL_589033, EPI_ISL_589035, EPI_ISL_589036, EPI_ISL_589037, EPI_ISL_589038	Lighthouse Lab in Glasgow	Wellcome Sanger Institute for the COVID-19 Genomics UK (COG-UK) consortium	Harper VanSteenhouse, Yumi Kasai, David Gray, Carol Clugston, Anna Dominiczak and Alex Alderton, Roberto Amato, Sonia Goncalves, Ewan Harrison, David K. Jackson, Ian Johnston, Dominic Kwiatkowski, Cordelia Langford, John Sillitoe on behalf of the Wellcome Sanger Institute COVID-19 Surveillance Team
EPI_ISL_589041	Lighthouse Lab in Milton Keynes	Wellcome Sanger Institute for the COVID-19 Genomics UK (COG-UK) consortium	The Lighthouse Lab in Milton Keynes and Alex Alderton, Roberto Amato, Sonia Goncalves, Ewan Harrison, David K. Jackson, Ian Johnston, Dominic Kwiatkowski, Cordelia Langford, John Sillitoe on behalf of the Wellcome Sanger Institute COVID-19 Surveillance Team
EPI_ISL_589042	Lighthouse Lab in Glasgow	Wellcome Sanger Institute for the COVID-19 Genomics UK (COG-UK) consortium	Harper VanSteenhouse, Yumi Kasai, David Gray, Carol Clugston, Anna Dominiczak and Alex Alderton, Roberto Amato, Sonia Goncalves, Ewan Harrison, David K. Jackson, Ian Johnston, Dominic Kwiatkowski, Cordelia Langford, John Sillitoe on behalf of the Wellcome Sanger Institute COVID-19 Surveillance Team
EPI_ISL_589043	Lighthouse Lab in Milton Keynes	Wellcome Sanger Institute for the COVID-19 Genomics UK (COG-UK) consortium	The Lighthouse Lab in Milton Keynes and Alex Alderton, Roberto Amato, Sonia Goncalves, Ewan Harrison, David K. Jackson, Ian Johnston, Dominic Kwiatkowski, Cordelia Langford, John Sillitoe on behalf of the Wellcome Sanger Institute COVID-19 Surveillance Team
EPI_ISL_589044, EPI_ISL_589045, EPI_ISL_589046, EPI_ISL_589047, EPI_ISL_589048, EPI_ISL_589049	Lighthouse Lab in Glasgow	Wellcome Sanger Institute for the COVID-19 Genomics UK (COG-UK) consortium	Harper VanSteenhouse, Yumi Kasai, David Gray, Carol Clugston, Anna Dominiczak and Alex Alderton, Roberto Amato, Sonia Goncalves, Ewan Harrison, David K. Jackson, Ian Johnston, Dominic Kwiatkowski, Cordelia Langford, John Sillitoe on behalf of the Wellcome Sanger Institute COVID-19 Surveillance Team
EPI_ISL_589050	Lighthouse Lab in Milton Keynes	Wellcome Sanger Institute for the COVID-19 Genomics UK (COG-UK) Consortium	The Lighthouse Lab in Milton Keynes and Alex Alderton, Roberto Amato, Sonia Goncalves, Ewan Harrison, David K. Jackson, Ian Johnston, Dominic Kwiatkowski, Cordelia Langford, John Sillitoe on behalf of the Wellcome Sanger Institute COVID-19 Surveillance Team

EPI_ISL_589053, EPI_ISL_589054, EPI_ISL_589055, EPI_ISL_589056	Lighthouse Lab in Glasgow	Wellcome Sanger Institute for the COVID-19 Genomics UK (COG-UK) consortium	Harper VanSteenhouse, Yumi Kasai, David Gray, Carol Clugston, Anna Dominiczak and Alex Alderton, Roberto Amato, Sonia Goncalves, Ewan Harrison, David K. Jackson, Ian Johnston, Dominic Kwiatkowski, Cordelia Langford, John Sillitoe on behalf of the Wellcome Sanger Institute COVID-19 Surveillance Team
EPI_ISL_589057	Lighthouse Lab in Milton Keynes	Wellcome Sanger Institute for the COVID-19 Genomics UK (COG-UK) consortium	The Lighthouse Lab in Milton Keynes and Alex Alderton, Roberto Amato, Sonia Goncalves, Ewan Harrison, David K. Jackson, Ian Johnston, Dominic Kwiatkowski, Cordelia Langford, John Sillitoe on behalf of the Wellcome Sanger Institute COVID-19 Surveillance Team
EPI_ISL_589058, EPI_ISL_589059, EPI_ISL_589060, EPI_ISL_589061, EPI_ISL_589062, EPI_ISL_589064, EPI_ISL_589065, EPI_ISL_589066, EPI_ISL_589068, EPI_ISL_589069	Lighthouse Lab in Glasgow	Wellcome Sanger Institute for the COVID-19 Genomics UK (COG-UK) consortium	Harper VanSteenhouse, Yumi Kasai, David Gray, Carol Clugston, Anna Dominiczak and Alex Alderton, Roberto Amato, Sonia Goncalves, Ewan Harrison, David K. Jackson, Ian Johnston, Dominic Kwiatkowski, Cordelia Langford, John Sillitoe on behalf of the Wellcome Sanger Institute COVID-19 Surveillance Team
EPI_ISL_589070	Lighthouse Lab in Milton Keynes	Wellcome Sanger Institute for the COVID-19 Genomics UK (COG-UK) consortium	The Lighthouse Lab in Milton Keynes and Alex Alderton, Roberto Amato, Sonia Goncalves, Ewan Harrison, David K. Jackson, Ian Johnston, Dominic Kwiatkowski, Cordelia Langford, John Sillitoe on behalf of the Wellcome Sanger Institute COVID-19 Surveillance Team
EPI_ISL_589071, EPI_ISL_589072, EPI_ISL_589073	Lighthouse Lab in Glasgow	Wellcome Sanger Institute for the COVID-19 Genomics UK (COG-UK) consortium	Harper VanSteenhouse, Yumi Kasai, David Gray, Carol Clugston, Anna Dominiczak and Alex Alderton, Roberto Amato, Sonia Goncalves, Ewan Harrison, David K. Jackson, Ian Johnston, Dominic Kwiatkowski, Cordelia Langford, John Sillitoe on behalf of the Wellcome Sanger Institute COVID-19 Surveillance Team
EPI_ISL_589075	Lighthouse Lab in Milton Keynes	Wellcome Sanger Institute for the COVID-19 Genomics UK (COG-UK) consortium	The Lighthouse Lab in Milton Keynes and Alex Alderton, Roberto Amato, Sonia Goncalves, Ewan Harrison, David K. Jackson, Ian Johnston, Dominic Kwiatkowski, Cordelia Langford, John Sillitoe on behalf of the Wellcome Sanger Institute COVID-19 Surveillance Team
EPI_ISL_589076, EPI_ISL_589078, EPI_ISL_589082	Lighthouse Lab in Glasgow	Wellcome Sanger Institute for the COVID-19 Genomics UK (COG-UK) consortium	Harper VanSteenhouse, Yumi Kasai, David Gray, Carol Clugston, Anna Dominiczak and Alex Alderton, Roberto Amato, Sonia Goncalves, Ewan Harrison, David K. Jackson, Ian Johnston, Dominic Kwiatkowski, Cordelia Langford, John Sillitoe on behalf of the Wellcome Sanger Institute COVID-19 Surveillance Team
EPI_ISL_589083	Lighthouse Lab in Milton Keynes	Wellcome Sanger Institute for the COVID-19 Genomics UK (COG-UK) consortium	The Lighthouse Lab in Milton Keynes and Alex Alderton, Roberto Amato, Sonia Goncalves, Ewan Harrison, David K. Jackson, Ian Johnston, Dominic Kwiatkowski, Cordelia Langford, John Sillitoe on behalf of the Wellcome Sanger Institute COVID-19 Surveillance Team
EPI_ISL_589084, EPI_ISL_589085, EPI_ISL_589087, EPI_ISL_589088, EPI_ISL_589089, EPI_ISL_589092, EPI_ISL_589093, EPI_ISL_589094	Lighthouse Lab in Glasgow	Wellcome Sanger Institute for the COVID-19 Genomics UK (COG-UK) consortium	Harper VanSteenhouse, Yumi Kasai, David Gray, Carol Clugston, Anna Dominiczak and Alex Alderton, Roberto Amato, Sonia Goncalves, Ewan Harrison, David K. Jackson, Ian Johnston, Dominic Kwiatkowski, Cordelia Langford, John Sillitoe on behalf of the Wellcome Sanger Institute COVID-19 Surveillance Team
EPI_ISL_589095	Lighthouse Lab in Milton Keynes	Wellcome Sanger Institute for the COVID-19 Genomics UK (COG-UK) consortium	The Lighthouse Lab in Milton Keynes and Alex Alderton, Roberto Amato, Sonia Goncalves, Ewan Harrison, David K. Jackson, Ian Johnston, Dominic Kwiatkowski, Cordelia Langford, John Sillitoe on behalf of the Wellcome Sanger Institute COVID-19 Surveillance Team
EPI_ISL_589096, EPI_ISL_589100, EPI_ISL_589102, EPI_ISL_589105, EPI_ISL_589106, EPI_ISL_589107, EPI_ISL_589112, EPI_ISL_589113, EPI_ISL_589116	Lighthouse Lab in Glasgow	Wellcome Sanger Institute for the COVID-19 Genomics UK (COG-UK) consortium	Harper VanSteenhouse, Yumi Kasai, David Gray, Carol Clugston, Anna Dominiczak and Alex Alderton, Roberto Amato, Sonia Goncalves, Ewan Harrison, David K. Jackson, Ian Johnston, Dominic Kwiatkowski, Cordelia Langford, John Sillitoe on behalf of the Wellcome Sanger Institute COVID-19 Surveillance Team
EPI_ISL_589118	Lighthouse Lab in Milton Keynes	Wellcome Sanger Institute for the COVID-19 Genomics UK (COG-UK) consortium	The Lighthouse Lab in Milton Keynes and Alex Alderton, Roberto Amato, Sonia Goncalves, Ewan Harrison, David K. Jackson, Ian Johnston, Dominic Kwiatkowski, Cordelia Langford, John Sillitoe on behalf of the Wellcome Sanger Institute COVID-19 Surveillance Team
EPI_ISL_589119, EPI_ISL_589120	Lighthouse Lab in Glasgow	Wellcome Sanger Institute for the COVID-19 Genomics UK (COG-UK) consortium	Harper VanSteenhouse, Yumi Kasai, David Gray, Carol Clugston, Anna Dominiczak and Alex Alderton, Roberto Amato, Sonia Goncalves, Ewan Harrison, David K. Jackson, Ian Johnston, Dominic Kwiatkowski, Cordelia Langford, John Sillitoe on behalf of the Wellcome Sanger Institute COVID-19 Surveillance Team
EPI_ISL_589123	Lighthouse Lab in Milton Keynes	Wellcome Sanger Institute for the COVID-19 Genomics UK (COG-UK) consortium	The Lighthouse Lab in Milton Keynes and Alex Alderton, Roberto Amato, Sonia Goncalves, Ewan Harrison, David K. Jackson, Ian Johnston, Dominic Kwiatkowski, Cordelia Langford, John Sillitoe on behalf of the Wellcome Sanger Institute COVID-19 Surveillance Team
EPI_ISL_589124, EPI_ISL_589125, EPI_ISL_589126	Lighthouse Lab in Glasgow	Wellcome Sanger Institute for the COVID-19 Genomics UK (COG-UK) consortium	Harper VanSteenhouse, Yumi Kasai, David Gray, Carol Clugston, Anna Dominiczak and Alex Alderton, Roberto Amato, Sonia Goncalves, Ewan Harrison, David K. Jackson, Ian Johnston, Dominic Kwiatkowski, Cordelia Langford, John Sillitoe on behalf of the Wellcome Sanger Institute COVID-19 Surveillance Team
EPI_ISL_589127	Lighthouse Lab in Milton Keynes	Wellcome Sanger Institute for the COVID-19 Genomics UK (COG-UK) consortium	The Lighthouse Lab in Milton Keynes and Alex Alderton, Roberto Amato, Sonia Goncalves, Ewan Harrison, David K. Jackson, Ian Johnston, Dominic Kwiatkowski, Cordelia Langford, John Sillitoe on behalf of the Wellcome Sanger Institute COVID-19 Surveillance Team
EPI_ISL_589128, EPI_ISL_589129	Lighthouse Lab in Glasgow	Wellcome Sanger Institute for the COVID-19 Genomics UK (COG-UK) consortium	Harper VanSteenhouse, Yumi Kasai, David Gray, Carol Clugston, Anna Dominiczak and Alex Alderton, Roberto Amato, Sonia Goncalves, Ewan Harrison, David K. Jackson, Ian Johnston, Dominic Kwiatkowski, Cordelia Langford, John Sillitoe on behalf of the Wellcome Sanger Institute COVID-19 Surveillance Team
EPI_ISL_589130	Lighthouse Lab in Milton Keynes	Wellcome Sanger Institute for the COVID-19 Genomics UK (COG-UK) Consortium	The Lighthouse Lab in Milton Keynes and Alex Alderton, Roberto Amato, Sonia Goncalves, Ewan Harrison, David K. Jackson, Ian Johnston, Dominic Kwiatkowski, Cordelia Langford, John Sillitoe on behalf of the Wellcome Sanger Institute COVID-19 Surveillance Team
EPI_ISL_589131, EPI_ISL_589132	Lighthouse Lab in Glasgow	Wellcome Sanger Institute for the COVID-19 Genomics UK (COG-UK) consortium	Harper VanSteenhouse, Yumi Kasai, David Gray, Carol Clugston, Anna Dominiczak and Alex Alderton, Roberto Amato, Sonia Goncalves, Ewan Harrison, David K. Jackson, Ian Johnston, Dominic Kwiatkowski, Cordelia Langford, John Sillitoe on behalf of the Wellcome Sanger Institute COVID-19 Surveillance Team
EPI_ISL_589133	Lighthouse Lab in Milton Keynes	Wellcome Sanger Institute for the COVID-19 Genomics UK (COG-UK) consortium	The Lighthouse Lab in Milton Keynes and Alex Alderton, Roberto Amato, Sonia Goncalves, Ewan Harrison, David K. Jackson, Ian Johnston, Dominic Kwiatkowski, Cordelia Langford, John Sillitoe on behalf of the Wellcome Sanger Institute COVID-19 Surveillance Team
EPI_ISL_589134	Lighthouse Lab in Glasgow	Wellcome Sanger Institute for the COVID-19 Genomics UK (COG-UK) consortium	Harper VanSteenhouse, Yumi Kasai, David Gray, Carol Clugston, Anna Dominiczak and Alex Alderton, Roberto Amato, Sonia Goncalves, Ewan Harrison, David K. Jackson, Ian Johnston, Dominic Kwiatkowski, Cordelia Langford, John Sillitoe on behalf of the Wellcome Sanger Institute COVID-19 Surveillance Team
EPI_ISL_589135	Lighthouse Lab in Milton Keynes	Wellcome Sanger Institute for the COVID-19 Genomics UK (COG-UK) consortium	The Lighthouse Lab in Milton Keynes and Alex Alderton, Roberto Amato, Sonia Goncalves, Ewan Harrison, David K. Jackson, Ian Johnston, Dominic Kwiatkowski, Cordelia Langford, John Sillitoe on behalf of the Wellcome Sanger Institute COVID-19 Surveillance Team
EPI_ISL_589136, EPI_ISL_589138, EPI_ISL_589139, EPI_ISL_589140, EPI_ISL_589141, EPI_ISL_589142, EPI_ISL_589143, EPI_ISL_589144, EPI_ISL_589145, EPI_ISL_589146	Lighthouse Lab in Glasgow	Wellcome Sanger Institute for the COVID-19 Genomics UK (COG-UK) consortium	Harper VanSteenhouse, Yumi Kasai, David Gray, Carol Clugston, Anna Dominiczak and Alex Alderton, Roberto Amato, Sonia Goncalves, Ewan Harrison, David K. Jackson, Ian Johnston, Dominic Kwiatkowski, Cordelia Langford, John Sillitoe on behalf of the Wellcome Sanger Institute COVID-19 Surveillance Team
EPI_ISL_589147	Lighthouse Lab in Milton Keynes	Wellcome Sanger Institute for the COVID-19 Genomics UK (COG-UK) consortium	The Lighthouse Lab in Milton Keynes and Alex Alderton, Roberto Amato, Sonia Goncalves, Ewan Harrison, David K. Jackson, Ian Johnston, Dominic Kwiatkowski, Cordelia Langford, John Sillitoe on behalf of the Wellcome Sanger Institute COVID-19 Surveillance Team
EPI_ISL_589148, EPI_ISL_589149, EPI_ISL_589150, EPI_ISL_589151, EPI_ISL_589152, EPI_ISL_589154, EPI_ISL_589155, EPI_ISL_589156	Lighthouse Lab in Glasgow	Wellcome Sanger Institute for the COVID-19 Genomics UK (COG-UK) consortium	Harper VanSteenhouse, Yumi Kasai, David Gray, Carol Clugston, Anna Dominiczak and Alex Alderton, Roberto Amato, Sonia Goncalves, Ewan Harrison, David K. Jackson, Ian Johnston, Dominic Kwiatkowski, Cordelia Langford, John Sillitoe on behalf of the Wellcome Sanger Institute COVID-19 Surveillance Team
EPI_ISL_589157	Lighthouse Lab in Milton Keynes	Wellcome Sanger Institute for the COVID-19 Genomics UK (COG-UK) consortium	The Lighthouse Lab in Milton Keynes and Alex Alderton, Roberto Amato, Sonia Goncalves, Ewan Harrison, David K. Jackson, Ian Johnston, Dominic Kwiatkowski, Cordelia Langford, John Sillitoe on behalf of the Wellcome Sanger Institute COVID-19 Surveillance Team
EPI_ISL_589158, EPI_ISL_589159	Lighthouse Lab in Glasgow	Wellcome Sanger Institute for the COVID-19 Genomics UK	Harper VanSteenhouse, Yumi Kasai, David Gray, Carol Clugston, Anna Dominiczak and Alex Alderton, Roberto Amato, Sonia Goncalves, Ewan Harrison,

		(COG-UK) consortium	David K. Jackson, Ian Johnston, Dominic Kwiatkowski, Cordelia Langford, John Sillitoe on behalf of the Wellcome Sanger Institute COVID-19 Surveillance Team
EPI_ISL_589160, EPI_ISL_589161	Lighthouse Lab in Milton Keynes	Wellcome Sanger Institute for the COVID-19 Genomics UK (COG-UK) consortium	The Lighthouse Lab in Milton Keynes and Alex Alderton, Roberto Amato, Sonia Goncalves, Ewan Harrison, David K. Jackson, Ian Johnston, Dominic Kwiatkowski, Cordelia Langford, John Sillitoe on behalf of the Wellcome Sanger Institute COVID-19 Surveillance Team
EPI_ISL_589162, EPI_ISL_589163, EPI_ISL_589164, EPI_ISL_589165	Lighthouse Lab in Glasgow	Wellcome Sanger Institute for the COVID-19 Genomics UK (COG-UK) consortium	Harper VanSteenhouse, Yumi Kasai, David Gray, Carol Clugston, Anna Dominiczak and Alex Alderton, Roberto Amato, Sonia Goncalves, Ewan Harrison, David K. Jackson, Ian Johnston, Dominic Kwiatkowski, Cordelia Langford, John Sillitoe on behalf of the Wellcome Sanger Institute COVID-19 Surveillance Team
EPI_ISL_589168	Lighthouse Lab in Milton Keynes	Wellcome Sanger Institute for the COVID-19 Genomics UK (COG-UK) consortium	The Lighthouse Lab in Milton Keynes and Alex Alderton, Roberto Amato, Sonia Goncalves, Ewan Harrison, David K. Jackson, Ian Johnston, Dominic Kwiatkowski, Cordelia Langford, John Sillitoe on behalf of the Wellcome Sanger Institute COVID-19 Surveillance Team
EPI_ISL_589175, EPI_ISL_589177, EPI_ISL_589178	Lighthouse Lab in Glasgow	Wellcome Sanger Institute for the COVID-19 Genomics UK (COG-UK) consortium	Harper VanSteenhouse, Yumi Kasai, David Gray, Carol Clugston, Anna Dominiczak and Alex Alderton, Roberto Amato, Sonia Goncalves, Ewan Harrison, David K. Jackson, Ian Johnston, Dominic Kwiatkowski, Cordelia Langford, John Sillitoe on behalf of the Wellcome Sanger Institute COVID-19 Surveillance Team
EPI_ISL_589179	Lighthouse Lab in Milton Keynes	Wellcome Sanger Institute for the COVID-19 Genomics UK (COG-UK) consortium	The Lighthouse Lab in Milton Keynes and Alex Alderton, Roberto Amato, Sonia Goncalves, Ewan Harrison, David K. Jackson, Ian Johnston, Dominic Kwiatkowski, Cordelia Langford, John Sillitoe on behalf of the Wellcome Sanger Institute COVID-19 Surveillance Team
EPI_ISL_589180, EPI_ISL_589181	Lighthouse Lab in Glasgow	Wellcome Sanger Institute for the COVID-19 Genomics UK (COG-UK) consortium	Harper VanSteenhouse, Yumi Kasai, David Gray, Carol Clugston, Anna Dominiczak and Alex Alderton, Roberto Amato, Sonia Goncalves, Ewan Harrison, David K. Jackson, Ian Johnston, Dominic Kwiatkowski, Cordelia Langford, John Sillitoe on behalf of the Wellcome Sanger Institute COVID-19 Surveillance Team
EPI_ISL_589182	Lighthouse Lab in Milton Keynes	Wellcome Sanger Institute for the COVID-19 Genomics UK (COG-UK) consortium	The Lighthouse Lab in Milton Keynes and Alex Alderton, Roberto Amato, Sonia Goncalves, Ewan Harrison, David K. Jackson, Ian Johnston, Dominic Kwiatkowski, Cordelia Langford, John Sillitoe on behalf of the Wellcome Sanger Institute COVID-19 Surveillance Team
EPI_ISL_589184	Lighthouse Lab in Milton Keynes	Wellcome Sanger Institute for the COVID-19 Genomics UK (COG-UK) Consortium	The Lighthouse Lab in Milton Keynes and Alex Alderton, Roberto Amato, Sonia Goncalves, Ewan Harrison, David K. Jackson, Ian Johnston, Dominic Kwiatkowski, Cordelia Langford, John Sillitoe on behalf of the Wellcome Sanger Institute COVID-19 Surveillance Team
EPI_ISL_589185	Lighthouse Lab in Milton Keynes	Wellcome Sanger Institute for the COVID-19 Genomics UK (COG-UK) consortium	The Lighthouse Lab in Milton Keynes and Alex Alderton, Roberto Amato, Sonia Goncalves, Ewan Harrison, David K. Jackson, Ian Johnston, Dominic Kwiatkowski, Cordelia Langford, John Sillitoe on behalf of the Wellcome Sanger Institute COVID-19 Surveillance Team
EPI_ISL_589186	Lighthouse Lab in Glasgow	Wellcome Sanger Institute for the COVID-19 Genomics UK (COG-UK) consortium	Harper VanSteenhouse, Yumi Kasai, David Gray, Carol Clugston, Anna Dominiczak and Alex Alderton, Roberto Amato, Sonia Goncalves, Ewan Harrison, David K. Jackson, Ian Johnston, Dominic Kwiatkowski, Cordelia Langford, John Sillitoe on behalf of the Wellcome Sanger Institute COVID-19 Surveillance Team
EPI_ISL_589187	Lighthouse Lab in Milton Keynes	Wellcome Sanger Institute for the COVID-19 Genomics UK (COG-UK) consortium	The Lighthouse Lab in Milton Keynes and Alex Alderton, Roberto Amato, Sonia Goncalves, Ewan Harrison, David K. Jackson, Ian Johnston, Dominic Kwiatkowski, Cordelia Langford, John Sillitoe on behalf of the Wellcome Sanger Institute COVID-19 Surveillance Team
EPI_ISL_589188, EPI_ISL_589189, EPI_ISL_589190, EPI_ISL_589191, EPI_ISL_589192, EPI_ISL_589193, EPI_ISL_589194	Lighthouse Lab in Glasgow	Wellcome Sanger Institute for the COVID-19 Genomics UK (COG-UK) consortium	Harper VanSteenhouse, Yumi Kasai, David Gray, Carol Clugston, Anna Dominiczak and Alex Alderton, Roberto Amato, Sonia Goncalves, Ewan Harrison, David K. Jackson, Ian Johnston, Dominic Kwiatkowski, Cordelia Langford, John Sillitoe on behalf of the Wellcome Sanger Institute COVID-19 Surveillance Team
EPI_ISL_589195, EPI_ISL_589196	Lighthouse Lab in Milton Keynes	Wellcome Sanger Institute for the COVID-19 Genomics UK (COG-UK) consortium	The Lighthouse Lab in Milton Keynes and Alex Alderton, Roberto Amato, Sonia Goncalves, Ewan Harrison, David K. Jackson, Ian Johnston, Dominic Kwiatkowski, Cordelia Langford, John Sillitoe on behalf of the Wellcome Sanger Institute COVID-19 Surveillance Team
EPI_ISL_589197, EPI_ISL_589201, EPI_ISL_589202, EPI_ISL_589203, EPI_ISL_589204, EPI_ISL_589206, EPI_ISL_589208, EPI_ISL_589209, EPI_ISL_589210	Lighthouse Lab in Glasgow	Wellcome Sanger Institute for the COVID-19 Genomics UK (COG-UK) consortium	Harper VanSteenhouse, Yumi Kasai, David Gray, Carol Clugston, Anna Dominiczak and Alex Alderton, Roberto Amato, Sonia Goncalves, Ewan Harrison, David K. Jackson, Ian Johnston, Dominic Kwiatkowski, Cordelia Langford, John Sillitoe on behalf of the Wellcome Sanger Institute COVID-19 Surveillance Team
EPI_ISL_589211	Lighthouse Lab in Milton Keynes	Wellcome Sanger Institute for the COVID-19 Genomics UK (COG-UK) consortium	The Lighthouse Lab in Milton Keynes and Alex Alderton, Roberto Amato, Sonia Goncalves, Ewan Harrison, David K. Jackson, Ian Johnston, Dominic Kwiatkowski, Cordelia Langford, John Sillitoe on behalf of the Wellcome Sanger Institute COVID-19 Surveillance Team
EPI_ISL_589213, EPI_ISL_589214, EPI_ISL_589215, EPI_ISL_589216, EPI_ISL_589217, EPI_ISL_589218, EPI_ISL_589219, EPI_ISL_589220	Lighthouse Lab in Glasgow	Wellcome Sanger Institute for the COVID-19 Genomics UK (COG-UK) consortium	Harper VanSteenhouse, Yumi Kasai, David Gray, Carol Clugston, Anna Dominiczak and Alex Alderton, Roberto Amato, Sonia Goncalves, Ewan Harrison, David K. Jackson, Ian Johnston, Dominic Kwiatkowski, Cordelia Langford, John Sillitoe on behalf of the Wellcome Sanger Institute COVID-19 Surveillance Team
EPI_ISL_589221	Lighthouse Lab in Milton Keynes	Wellcome Sanger Institute for the COVID-19 Genomics UK (COG-UK) consortium	The Lighthouse Lab in Milton Keynes and Alex Alderton, Roberto Amato, Sonia Goncalves, Ewan Harrison, David K. Jackson, Ian Johnston, Dominic Kwiatkowski, Cordelia Langford, John Sillitoe on behalf of the Wellcome Sanger Institute COVID-19 Surveillance Team
EPI_ISL_589222, EPI_ISL_589223	Lighthouse Lab in Glasgow	Wellcome Sanger Institute for the COVID-19 Genomics UK (COG-UK) consortium	Harper VanSteenhouse, Yumi Kasai, David Gray, Carol Clugston, Anna Dominiczak and Alex Alderton, Roberto Amato, Sonia Goncalves, Ewan Harrison, David K. Jackson, Ian Johnston, Dominic Kwiatkowski, Cordelia Langford, John Sillitoe on behalf of the Wellcome Sanger Institute COVID-19 Surveillance Team
EPI_ISL_589226	Lighthouse Lab in Milton Keynes	Wellcome Sanger Institute for the COVID-19 Genomics UK (COG-UK) consortium	The Lighthouse Lab in Milton Keynes and Alex Alderton, Roberto Amato, Sonia Goncalves, Ewan Harrison, David K. Jackson, Ian Johnston, Dominic Kwiatkowski, Cordelia Langford, John Sillitoe on behalf of the Wellcome Sanger Institute COVID-19 Surveillance Team
EPI_ISL_589228	Lighthouse Lab in Glasgow	Wellcome Sanger Institute for the COVID-19 Genomics UK (COG-UK) consortium	Harper VanSteenhouse, Yumi Kasai, David Gray, Carol Clugston, Anna Dominiczak and Alex Alderton, Roberto Amato, Sonia Goncalves, Ewan Harrison, David K. Jackson, Ian Johnston, Dominic Kwiatkowski, Cordelia Langford, John Sillitoe on behalf of the Wellcome Sanger Institute COVID-19 Surveillance Team
EPI_ISL_589229	Lighthouse Lab in Milton Keynes	Wellcome Sanger Institute for the COVID-19 Genomics UK (COG-UK) consortium	The Lighthouse Lab in Milton Keynes and Alex Alderton, Roberto Amato, Sonia Goncalves, Ewan Harrison, David K. Jackson, Ian Johnston, Dominic Kwiatkowski, Cordelia Langford, John Sillitoe on behalf of the Wellcome Sanger Institute COVID-19 Surveillance Team
EPI_ISL_589230, EPI_ISL_589232	Lighthouse Lab in Glasgow	Wellcome Sanger Institute for the COVID-19 Genomics UK (COG-UK) consortium	Harper VanSteenhouse, Yumi Kasai, David Gray, Carol Clugston, Anna Dominiczak and Alex Alderton, Roberto Amato, Sonia Goncalves, Ewan Harrison, David K. Jackson, Ian Johnston, Dominic Kwiatkowski, Cordelia Langford, John Sillitoe on behalf of the Wellcome Sanger Institute COVID-19 Surveillance Team
EPI_ISL_589233	Lighthouse Lab in Milton Keynes	Wellcome Sanger Institute for the COVID-19 Genomics UK (COG-UK) consortium	The Lighthouse Lab in Milton Keynes and Alex Alderton, Roberto Amato, Sonia Goncalves, Ewan Harrison, David K. Jackson, Ian Johnston, Dominic Kwiatkowski, Cordelia Langford, John Sillitoe on behalf of the Wellcome Sanger Institute COVID-19 Surveillance Team
EPI_ISL_589234, EPI_ISL_589236, EPI_ISL_589238, EPI_ISL_589239, EPI_ISL_589240, EPI_ISL_589241	Lighthouse Lab in Glasgow	Wellcome Sanger Institute for the COVID-19 Genomics UK (COG-UK) consortium	Harper VanSteenhouse, Yumi Kasai, David Gray, Carol Clugston, Anna Dominiczak and Alex Alderton, Roberto Amato, Sonia Goncalves, Ewan Harrison, David K. Jackson, Ian Johnston, Dominic Kwiatkowski, Cordelia Langford, John Sillitoe on behalf of the Wellcome Sanger Institute COVID-19 Surveillance Team
EPI_ISL_589242, EPI_ISL_589243	Lighthouse Lab in Milton Keynes	Wellcome Sanger Institute for the COVID-19 Genomics UK (COG-UK) consortium	The Lighthouse Lab in Milton Keynes and Alex Alderton, Roberto Amato, Sonia Goncalves, Ewan Harrison, David K. Jackson, Ian Johnston, Dominic Kwiatkowski, Cordelia Langford, John Sillitoe on behalf of the Wellcome Sanger Institute COVID-19 Surveillance Team
EPI_ISL_589244, EPI_ISL_589245, EPI_ISL_589246	Lighthouse Lab in Glasgow	Wellcome Sanger Institute for the COVID-19 Genomics UK (COG-UK) consortium	Harper VanSteenhouse, Yumi Kasai, David Gray, Carol Clugston, Anna Dominiczak and Alex Alderton, Roberto Amato, Sonia Goncalves, Ewan Harrison, David K. Jackson, Ian Johnston, Dominic Kwiatkowski, Cordelia Langford, John Sillitoe on behalf of the Wellcome Sanger Institute COVID-19 Surveillance Team
EPI_ISL_589248	Lighthouse Lab in Glasgow	Wellcome Sanger Institute for the COVID-19 Genomics UK (COG-UK) Consortium	Harper VanSteenhouse, Yumi Kasai, David Gray, Carol Clugston, Anna Dominiczak and Alex Alderton, Roberto Amato, Sonia Goncalves, Ewan Harrison, David K. Jackson, Ian Johnston, Dominic Kwiatkowski, Cordelia Langford, John Sillitoe on behalf of the Wellcome Sanger Institute COVID-19 Surveillance Team

EPI_ISL_589249, EPI_ISL_589250	Lighthouse Lab in Glasgow	Wellcome Sanger Institute for the COVID-19 Genomics UK (COG-UK) consortium	Harper VanSteenhouse, Yumi Kasai, David Gray, Carol Clugston, Anna Dominiczak and Alex Alderton, Roberto Amato, Sonia Goncalves, Ewan Harrison, David K. Jackson, Ian Johnston, Dominic Kwiatkowski, Cordelia Langford, John Sillitoe on behalf of the Wellcome Sanger Institute COVID-19 Surveillance Team
EPI_ISL_589251	Lighthouse Lab in Milton Keynes	Wellcome Sanger Institute for the COVID-19 Genomics UK (COG-UK) consortium	The Lighthouse Lab in Milton Keynes and Alex Alderton, Roberto Amato, Sonia Goncalves, Ewan Harrison, David K. Jackson, Ian Johnston, Dominic Kwiatkowski, Cordelia Langford, John Sillitoe on behalf of the Wellcome Sanger Institute COVID-19 Surveillance Team
EPI_ISL_589252	Lighthouse Lab in Glasgow	Wellcome Sanger Institute for the COVID-19 Genomics UK (COG-UK) consortium	Harper VanSteenhouse, Yumi Kasai, David Gray, Carol Clugston, Anna Dominiczak and Alex Alderton, Roberto Amato, Sonia Goncalves, Ewan Harrison, David K. Jackson, Ian Johnston, Dominic Kwiatkowski, Cordelia Langford, John Sillitoe on behalf of the Wellcome Sanger Institute COVID-19 Surveillance Team
EPI_ISL_589253	Lighthouse Lab in Milton Keynes	Wellcome Sanger Institute for the COVID-19 Genomics UK (COG-UK) consortium	The Lighthouse Lab in Milton Keynes and Alex Alderton, Roberto Amato, Sonia Goncalves, Ewan Harrison, David K. Jackson, Ian Johnston, Dominic Kwiatkowski, Cordelia Langford, John Sillitoe on behalf of the Wellcome Sanger Institute COVID-19 Surveillance Team
EPI_ISL_589254, EPI_ISL_589255, EPI_ISL_589258	Lighthouse Lab in Glasgow	Wellcome Sanger Institute for the COVID-19 Genomics UK (COG-UK) consortium	Harper VanSteenhouse, Yumi Kasai, David Gray, Carol Clugston, Anna Dominiczak and Alex Alderton, Roberto Amato, Sonia Goncalves, Ewan Harrison, David K. Jackson, Ian Johnston, Dominic Kwiatkowski, Cordelia Langford, John Sillitoe on behalf of the Wellcome Sanger Institute COVID-19 Surveillance Team
EPI_ISL_589259, EPI_ISL_589261	Lighthouse Lab in Milton Keynes	Wellcome Sanger Institute for the COVID-19 Genomics UK (COG-UK) consortium	The Lighthouse Lab in Milton Keynes and Alex Alderton, Roberto Amato, Sonia Goncalves, Ewan Harrison, David K. Jackson, Ian Johnston, Dominic Kwiatkowski, Cordelia Langford, John Sillitoe on behalf of the Wellcome Sanger Institute COVID-19 Surveillance Team
EPI_ISL_589263	Lighthouse Lab in Glasgow	Wellcome Sanger Institute for the COVID-19 Genomics UK (COG-UK) consortium	Harper VanSteenhouse, Yumi Kasai, David Gray, Carol Clugston, Anna Dominiczak and Alex Alderton, Roberto Amato, Sonia Goncalves, Ewan Harrison, David K. Jackson, Ian Johnston, Dominic Kwiatkowski, Cordelia Langford, John Sillitoe on behalf of the Wellcome Sanger Institute COVID-19 Surveillance Team
EPI_ISL_589266	Lighthouse Lab in Milton Keynes	Wellcome Sanger Institute for the COVID-19 Genomics UK (COG-UK) consortium	The Lighthouse Lab in Milton Keynes and Alex Alderton, Roberto Amato, Sonia Goncalves, Ewan Harrison, David K. Jackson, Ian Johnston, Dominic Kwiatkowski, Cordelia Langford, John Sillitoe on behalf of the Wellcome Sanger Institute COVID-19 Surveillance Team
EPI_ISL_589577, EPI_ISL_589585, EPI_ISL_589586, EPI_ISL_589588, EPI_ISL_589591, EPI_ISL_589592, EPI_ISL_589594, EPI_ISL_589600, EPI_ISL_589602, EPI_ISL_589603, EPI_ISL_589608, EPI_ISL_589613, EPI_ISL_589614, EPI_ISL_589618, EPI_ISL_589621, EPI_ISL_589625, EPI_ISL_589626, EPI_ISL_589627, EPI_ISL_589628, EPI_ISL_589629, EPI_ISL_589636, EPI_ISL_589639, EPI_ISL_589642, EPI_ISL_589647, EPI_ISL_589649, EPI_ISL_589654, EPI_ISL_589655, EPI_ISL_589656, EPI_ISL_589657, EPI_ISL_589658, EPI_ISL_589664, EPI_ISL_589668, EPI_ISL_589669, EPI_ISL_589670, EPI_ISL_589671, EPI_ISL_589673, EPI_ISL_589674, EPI_ISL_589675, EPI_ISL_589678, EPI_ISL_589679, EPI_ISL_589681, EPI_ISL_589685, EPI_ISL_589688, EPI_ISL_589695, EPI_ISL_589698, EPI_ISL_589700, EPI_ISL_589709, EPI_ISL_589710, EPI_ISL_589713, EPI_ISL_589716, EPI_ISL_589717, EPI_ISL_589726, EPI_ISL_589730, EPI_ISL_589733, EPI_ISL_589734, EPI_ISL_589735, EPI_ISL_589739, EPI_ISL_589742, EPI_ISL_589743, EPI_ISL_589745, EPI_ISL_589746, EPI_ISL_589748, EPI_ISL_589754, EPI_ISL_589755, EPI_ISL_589757, EPI_ISL_589758, EPI_ISL_589759, EPI_ISL_589766			
see above	Lighthouse Lab in Milton Keynes	Wellcome Sanger Institute for the COVID-19 Genomics UK (COG-UK) consortium	The Lighthouse Lab in Milton Keynes and Alex Alderton, Roberto Amato, Sonia Goncalves, Ewan Harrison, David K. Jackson, Ian Johnston, Dominic Kwiatkowski, Cordelia Langford, John Sillitoe on behalf of the Wellcome Sanger Institute COVID-19 Surveillance Team (http://www.sanger.ac.uk/covid-team)
EPI_ISL_590699, EPI_ISL_590702, EPI_ISL_590704, EPI_ISL_590705, EPI_ISL_590707, EPI_ISL_590709, EPI_ISL_590710, EPI_ISL_590711, EPI_ISL_590715, EPI_ISL_590716, EPI_ISL_590719, EPI_ISL_590720, EPI_ISL_590722, EPI_ISL_590723, EPI_ISL_590725, EPI_ISL_590726, EPI_ISL_590727, EPI_ISL_590728, EPI_ISL_590729, EPI_ISL_590730, EPI_ISL_590731, EPI_ISL_590732, EPI_ISL_590733, EPI_ISL_590734, EPI_ISL_590735, EPI_ISL_590736, EPI_ISL_590737, EPI_ISL_590738, EPI_ISL_590739, EPI_ISL_590740, EPI_ISL_590742, EPI_ISL_590743, EPI_ISL_590744, EPI_ISL_590745, EPI_ISL_590746			
see above	University of Michigan Clinical Microbiology Laboratory	Lauring Lab, University of Michigan, Department of Microbiology and Immunology	Valesano
EPI_ISL_590877	Dept. of Medical Microbiology, Stavanger University Hospital, Helse Stavanger HF	Norwegian Institute of Public Health, Department of Virology	Kathrine Stene-Johansen, Kamilla Heddeland Instefjord, Hilde Elshaug, Marie Paulsen Madsen, Rasmus Riis Kopperud, Hilde Vollan, Karoline Bragstad, Olav Hungnes
EPI_ISL_590955	Dept. of Medical Microbiology, Stavanger University Hospital, Helse Stavanger HF	Norwegian Institute of Public Health, Department of Virology	Kathrine Stene-Johansen, Iren Löhr, Kamilla Heddeland Instefjord, Hilde Elshaug, Rasmus Riis Kopperud, Hilde Vollan, Karoline Bragstad, Olav Hungnes
EPI_ISL_590979, EPI_ISL_590980	Oslo University Hospital, Department of Medical Microbiology	Norwegian Institute of Public Health, Department of Virology	Kathrine Stene-Johansen, Kamilla Heddeland Instefjord, Hilde Elshaug, Rasmus Riis Kopperud, Hilde Vollan, Karoline Bragstad, Olav Hungnes
EPI_ISL_590981, EPI_ISL_591008	Foerde Hospital, Department of Microbiology	Norwegian Institute of Public Health, Department of Virology	Kathrine Stene-Johansen, Kamilla Heddeland Instefjord, Hilde Elshaug, Rasmus Riis Kopperud, Hilde Vollan, Karoline Bragstad, Olav Hungnes
EPI_ISL_591009	Ostfold Hospital Trust - Kalnes, Centre for Laboratory Medicine, Section for gene technology and infection serology	Norwegian Institute of Public Health, Department of Virology	Kathrine Stene-Johansen, Kamilla Heddeland Instefjord, Hilde Elshaug, Rasmus Riis Kopperud, Hilde Vollan, Karoline Bragstad, Olav Hungnes
EPI_ISL_591011	Oslo University Hospital, Department of Medical Microbiology	Norwegian Institute of Public Health, Department of Virology	Kathrine Stene-Johansen, Kamilla Heddeland Instefjord, Hilde Elshaug, Rasmus Riis Kopperud, Hilde Vollan, Karoline Bragstad, Olav Hungnes
EPI_ISL_591012	Department of Medical Microbiology - section Molde, Molde Hospital	Norwegian Institute of Public Health, Department of Virology	Kathrine Stene-Johansen, Kamilla Heddeland Instefjord, Hilde Elshaug, Rasmus Riis Kopperud, Hilde Vollan, Karoline Bragstad, Olav Hungnes
EPI_ISL_591014	Department of Medical Microbiology, St. Olavs hospital	Norwegian Institute of Public Health, Department of Virology	Kathrine Stene-Johansen, Kamilla Heddeland Instefjord, Hilde Elshaug, Rasmus Riis Kopperud, Hilde Vollan, Karoline Bragstad, Olav Hungnes
EPI_ISL_591018, EPI_ISL_591019	Department of Medical Microbiology - section Molde, Molde Hospital	Norwegian Institute of Public Health, Department of Virology	Kathrine Stene-Johansen, Kamilla Heddeland Instefjord, Hilde Elshaug, Rasmus Riis Kopperud, Hilde Vollan, Karoline Bragstad, Olav Hungnes
EPI_ISL_591033, EPI_ISL_591034, EPI_ISL_591035, EPI_ISL_591036	MD PHL	MD PHL	Maryland Department of Health Laboratories Administration
EPI_ISL_591279	National Institute for Viral Disease Control and Prevention, China CDC	National Institute for Viral Disease Control and Prevention, China CDC	Huilai Ma, Zhaoguo Wang, Xiang Zhao, Jun Han, Yong Zhang, Hong Wang, Cao Chen, Ji Wang, Jingdong Song, Yao Meng, Yuchao Wu, Zhixiao Chen, Dayan Wang, Ruqin Gao, George F.Gao, Wenbo Xu
EPI_ISL_591517	Sydney South West Pathology Service (SSWPS) - Royal Prince Alfred Hospital - NSW Health Pathology	NSW Health Pathology - Institute of Clinical Pathology and Medical Research; Westmead Hospital; University of Sydney	CIDM-PH et al.
EPI_ISL_592584, EPI_ISL_592588, EPI_ISL_592609, EPI_ISL_592749	Microbiological Diagnostic Unit - Public Health Laboratory (MDU-PHL)	MDU-PHL	Seemann T., Schultz, M. B., Sait, M., Sherry, N.
EPI_ISL_592795	Victorian Infectious Diseases Reference Laboratory (VIDRL)	VIDRL and MDU-PHL	Caly L., Seemann T., Sait, M., Schultz, M. B., Druce J., Sherry, N.
EPI_ISL_592801, EPI_ISL_592802, EPI_ISL_592817, EPI_ISL_593013, EPI_ISL_593014, EPI_ISL_593016, EPI_ISL_593099, EPI_ISL_593218, EPI_ISL_593220, EPI_ISL_593222, EPI_ISL_593238, EPI_ISL_593239, EPI_ISL_593284, EPI_ISL_593347, EPI_ISL_593348			
see above	Microbiological Diagnostic Unit - Public Health Laboratory (MDU-PHL)	MDU-PHL	Seemann T., Schultz, M. B., Sait, M., Sherry, N.
EPI_ISL_593744, EPI_ISL_593753	South Eastern Area Laboratory Services (SEALS)	NSW Health Pathology - Institute of Clinical Pathology and Medical Research; Westmead Hospital; University of Sydney	CIDM-PH et al.
EPI_ISL_593804	Respiratory Virus Unit, Microbiology Services Colindale, Public Health England	Respiratory Virus Unit, Microbiology Services Colindale, Public Health England	PHE Covid Sequencing Team
EPI_ISL_593893, EPI_ISL_593894, EPI_ISL_593901	CHU Purpan - Laboratoire de Virologie - Institut Fédératif de Biologie	CHU Purpan - Laboratoire de Virologie - Institut Fédératif de Biologie	Latour J., Ranger N., Dubois M., Carcenac R., Harter A., Boyer P., Tremeaux P., Izopet J.
EPI_ISL_593935	Sentinelles, Plessis-Trevise	National Reference Center for Viruses of Respiratory Infections, Institut Pasteur, Paris	Sylvie Behillil, Fabiana Gambaro, Etienne Simon-Lorière, Vincent Enouf, Maud Vanpeene, Sylvie van der Werf
EPI_ISL_593936	Sentinelles, Fondettes	National Reference Center for Viruses of Respiratory Infections, Institut Pasteur, Paris	Sylvie Behillil, Fabiana Gambaro, Etienne Simon-Lorière, Vincent Enouf, Maud Vanpeene, Sylvie van der Werf
EPI_ISL_593980	Delaware Public Health Lab	Delaware Public Health Lab	Gregory Hovan
EPI_ISL_594062	Utah Public Health Laboratory	Utah Public Health Laboratory	Erin Young, Kelly Oakeson
EPI_ISL_594175, EPI_ISL_594176, EPI_ISL_594177, EPI_ISL_594178,	PathWest Laboratory Medicine WA	PathWest Laboratory Medicine WA Microbial Surveillance Unit	PathWest Laboratory Medicine WA Microbial Surveillance Unit

EPI_ISL_594179			
EPI_ISL_594478, EPI_ISL_594479, EPI_ISL_594480, EPI_ISL_594481, EPI_ISL_594482, EPI_ISL_594483	Oxford Viromics, NDM, University of Oxford; Oxford University Hospitals; Basingstoke and North Hampshire Hospital	COVID-19 Genomics UK (COG-UK) Consortium	Tanya Golubchik, David Bonsall, George Macintyre, Amy Trebes, Mariateresa de Cesare, Catrin Moore, Alex Mobbs, Anita Justice, Robert Shaw, Monique Andersson, Timothy Peto, Emma Wise, Nathan Moore, Jessica Lynch, Nick Cortes, Matilde Mori, Stephen Kidd, David Buck, John Todd, Christophe Fraser
EPI_ISL_594484, EPI_ISL_594532, EPI_ISL_594533, EPI_ISL_594534, EPI_ISL_594535, EPI_ISL_594536, EPI_ISL_594537, EPI_ISL_594538, EPI_ISL_594539, EPI_ISL_594540, EPI_ISL_594541, EPI_ISL_594542, EPI_ISL_594543, EPI_ISL_594544, EPI_ISL_594545, EPI_ISL_594546, EPI_ISL_594547, EPI_ISL_594548, EPI_ISL_594549, EPI_ISL_594550, EPI_ISL_594551, EPI_ISL_594552, EPI_ISL_594553, EPI_ISL_594554, EPI_ISL_594555, EPI_ISL_594556, EPI_ISL_594557, EPI_ISL_594558, EPI_ISL_594597, EPI_ISL_594598, EPI_ISL_594601, EPI_ISL_594602, EPI_ISL_594603, EPI_ISL_594604, EPI_ISL_594605, EPI_ISL_594606, EPI_ISL_594608			
see above	University of Birmingham	COVID-19 Genomics UK (COG-UK) Consortium	Institute of Microbiology, University of Birmingham: Claire McMurray, Joanne Stockton, Samuel Nicholls, Radoslaw Poplawski, Will Rowe, Josh Quick, Nicholas Loman. University of Birmingham Testing Laboratory: Celina M Whalley, Andrew Bosworth, Charlotte Poxon, Kasun Wanigasooriya, Oliver Pickles, Mike Kidd, Alex Richter, Andrew D Beggs PHE Heartlands Lab: Husam Osman, Andrew Bosworth. Queen Elizabeth Hospital: Anna Casey
EPI_ISL_594655, EPI_ISL_594659, EPI_ISL_594667, EPI_ISL_594671, EPI_ISL_594681, EPI_ISL_594692, EPI_ISL_594700, EPI_ISL_594710, EPI_ISL_594713, EPI_ISL_594714, EPI_ISL_594721, EPI_ISL_594733			
see above	West of Scotland Specialist Virology Centre, NHSGGC / MRC-University of Glasgow Centre for Virus Research	COVID-19 Genomics UK (COG-UK) Consortium	Ana da Silva Filipe, Natasha Johnson, Kathy Smollett, Daniel Mair, Stephen Carmichael, Lily Tong, Jenna Nichols, Elihu Aranday-Cortes, Kyriaki Nomikou; Sarah McDonald, Marc Niebel, Patawee Asamaphan; Richard Orton, Joseph Hughes, Sreenu Vattipally, David L Robertson; Alasdair MacLean, Rory Gunson; Kathy Li, Igor Starinskij, Natasha Jesudason, Rajiv Shah, James Shepherd, Antonia Ho, Emma Thomson
EPI_ISL_594816, EPI_ISL_594817, EPI_ISL_594818, EPI_ISL_594819, EPI_ISL_594820, EPI_ISL_594821, EPI_ISL_594822, EPI_ISL_594823, EPI_ISL_594824, EPI_ISL_594825, EPI_ISL_594826, EPI_ISL_594827			
see above	Virology Department, Royal Infirmary of Edinburgh, NHS Lothian / School of Biological Sciences, University of Edinburgh / Institute of Genetics and Molecular Medicine, University of Edinburgh	COVID-19 Genomics UK (COG-UK) Consortium	McHugh M, Dewar R, Rooke S, Gallagher M, Balcaza C, O'Toole Á, Scher E, Hill V, McCrone JT, Colquhoun R, Yu X, Jackson B, Rambaut A, Williams TC, Templeton K
EPI_ISL_594853, EPI_ISL_594854, EPI_ISL_594855, EPI_ISL_594856, EPI_ISL_594859, EPI_ISL_594860	Liverpool Clinical Laboratories	COVID-19 Genomics UK (COG-UK) Consortium	Sam Haldenby, Anita Lucaci, Steve Paterson, Julian Hiscox, Alistair Darby, M Almsaud, A Alrezaihi, Muhannad Alruwaili, Stuart D Armstrong, Jones Benjamin, Eleanor G Bentley, Anu Chawla, Jordan J Clark, Angela Cowell, Richard Eccles, Isabel García-Dorival, Matthew Gemmell, Alessandro Gerada, PKF Gilmore, Richard Gregory, Ximeng Han, Catherine Hartley, Margaret Hughes, Miren Iturriza-Gomara, James Johnson, L Luu, Jenifer Manson, Charlotte Nelson, Elaine O'Toole, Cassie Olateju, Rebekah Penrice-Randal , Lucille Rainbow, N.P Randle, Trevor Ian Robinson, Parul Sharma, Ghada T Shawli, James P Stewart, Neil Swainston, Ecaterina Vamos, Joanne Watts, Mark Whitehead
EPI_ISL_594861, EPI_ISL_594862, EPI_ISL_594863, EPI_ISL_594864, EPI_ISL_594867, EPI_ISL_594868, EPI_ISL_594871	University College London, Great Ormond Street Hospital for Children NHS Foundation Trust, Imperial College Healthcare NHS Trust	COVID-19 Genomics UK (COG-UK) Consortium	Sergi Castellano, Rachel Williams, Mark Kristiansen, Paola Resende Silva, Sunando Roy, Tony Brooks, Helena Tutill, Paola Niola, Patricia Dyal, Charlotte Williams, Leysa Forrest, Yasmin Panchbhaya, Jacqueline Findlay, Samuel Weeks, Julianne Brown, Kathryn Harris, Paul Randell, James Price, Alison Holmes, Judith Breuer
EPI_ISL_594900, EPI_ISL_594901, EPI_ISL_594913, EPI_ISL_594914, EPI_ISL_594915, EPI_ISL_594918, EPI_ISL_594919, EPI_ISL_594920, EPI_ISL_594921, EPI_ISL_594922	Oxford Viromics, NDM, University of Oxford; Oxford University Hospitals; Basingstoke and North Hampshire Hospital	COVID-19 Genomics UK (COG-UK) Consortium	Tanya Golubchik, David Bonsall, George Macintyre, Amy Trebes, Mariateresa de Cesare, Catrin Moore, Alex Mobbs, Anita Justice, Robert Shaw, Monique Andersson, Timothy Peto, Emma Wise, Nathan Moore, Jessica Lynch, Nick Cortes, Matilde Mori, Stephen Kidd, David Buck, John Todd, Christophe Fraser
EPI_ISL_594987, EPI_ISL_594988, EPI_ISL_594989, EPI_ISL_594990, EPI_ISL_594991, EPI_ISL_594992, EPI_ISL_594993, EPI_ISL_594994, EPI_ISL_594995, EPI_ISL_594996, EPI_ISL_594997, EPI_ISL_594998, EPI_ISL_594999, EPI_ISL_595000, EPI_ISL_595001, EPI_ISL_595002, EPI_ISL_595003, EPI_ISL_595004, EPI_ISL_595005, EPI_ISL_595006, EPI_ISL_595007, EPI_ISL_595008, EPI_ISL_595009, EPI_ISL_595010, EPI_ISL_595011, EPI_ISL_595012, EPI_ISL_595013, EPI_ISL_595014, EPI_ISL_595015, EPI_ISL_595016, EPI_ISL_595017, EPI_ISL_595018, EPI_ISL_595019, EPI_ISL_595020, EPI_ISL_595021, EPI_ISL_595022, EPI_ISL_595023, EPI_ISL_595024, EPI_ISL_595025, EPI_ISL_595026, EPI_ISL_595027, EPI_ISL_595028, EPI_ISL_595029, EPI_ISL_595030, EPI_ISL_595031, EPI_ISL_595032, EPI_ISL_595033, EPI_ISL_595034, EPI_ISL_595035, EPI_ISL_595036, EPI_ISL_595037, EPI_ISL_595038, EPI_ISL_595039, EPI_ISL_595040, EPI_ISL_595041, EPI_ISL_595042, EPI_ISL_595043, EPI_ISL_595044, EPI_ISL_595045			
see above	Northumbria University / South Tees Hospitals NHS Foundation Trust / North Cumbria Integrated Care NHS Foundation Trust / North Tees and Hartlepool NHS Foundation Trust / Newcastle Hospitals NHS Foundation Trust	COVID-19 Genomics UK (COG-UK) Consortium	Darren L Smith,Andrew Nelson,Matthew Bashton,Greg R Young,Joshua Loh,John Allan,Mohammad A Tariq,Giles S Holt,Gary Black,Wen C Yew,Lynn Dover,Paul Baker,Steve Liggett,Sarah Essex,Jane Greenaway,Debra Padgett,Clive Graham,Garren Scott,Edward Barton,Emma Swindells,Brendan Payne,Jennifer Collins,Yusri Taha,Gary Eltringham
EPI_ISL_595095, EPI_ISL_595096, EPI_ISL_595097, EPI_ISL_595098	Queens Medical Centre, Clinical Microbiology Department / DeepSeq Nottingham	COVID-19 Genomics UK (COG-UK) Consortium	Gemma Clark, Wendy Smith, Manjinder Khakh, Vicki M Fleming, Michelle M Lister, Hannah Howson-Wells, Jonathan Ball, Patrick McClure, Joseph Chappell, Theocharis Tsoleridis, Nadine Holmes, Matthew Carlisle, Christopher Moore, Fei Sang, Johnny Debebe, Victoria Wright, Matthew Loose
EPI_ISL_595326, EPI_ISL_595327, EPI_ISL_595328	Oxford Viromics, NDM, University of Oxford; Oxford University Hospitals; Basingstoke and North Hampshire Hospital	COVID-19 Genomics UK (COG-UK) Consortium	Tanya Golubchik, David Bonsall, George Macintyre, Amy Trebes, Mariateresa de Cesare, Catrin Moore, Alex Mobbs, Anita Justice, Robert Shaw, Monique Andersson, Timothy Peto, Emma Wise, Nathan Moore, Jessica Lynch, Nick Cortes, Matilde Mori, Stephen Kidd, David Buck, John Todd, Christophe Fraser
EPI_ISL_595369, EPI_ISL_595377, EPI_ISL_595380, EPI_ISL_595381, EPI_ISL_595382, EPI_ISL_595388, EPI_ISL_595391, EPI_ISL_595397, EPI_ISL_595402, EPI_ISL_595403, EPI_ISL_595404, EPI_ISL_595405, EPI_ISL_595407, EPI_ISL_595408, EPI_ISL_595412, EPI_ISL_595413, EPI_ISL_595414, EPI_ISL_595417, EPI_ISL_595424, EPI_ISL_595426, EPI_ISL_595427, EPI_ISL_595430, EPI_ISL_595435, EPI_ISL_595441, EPI_ISL_595444, EPI_ISL_595448, EPI_ISL_595452, EPI_ISL_595455, EPI_ISL_595457, EPI_ISL_595459, EPI_ISL_595468, EPI_ISL_595471, EPI_ISL_595473, EPI_ISL_595476, EPI_ISL_595478, EPI_ISL_595483, EPI_ISL_595491, EPI_ISL_595493, EPI_ISL_595494, EPI_ISL_595495, EPI_ISL_595496, EPI_ISL_595497, EPI_ISL_595498, EPI_ISL_595500, EPI_ISL_595501, EPI_ISL_595504, EPI_ISL_595505, EPI_ISL_595514, EPI_ISL_595520, EPI_ISL_595525, EPI_ISL_595527, EPI_ISL_595530, EPI_ISL_595532, EPI_ISL_595534, EPI_ISL_595538, EPI_ISL_595550, EPI_ISL_595554, EPI_ISL_595555, EPI_ISL_595556, EPI_ISL_595559, EPI_ISL_595562, EPI_ISL_595564, EPI_ISL_595565, EPI_ISL_595571, EPI_ISL_595572, EPI_ISL_595573, EPI_ISL_595579, EPI_ISL_595580, EPI_ISL_595586, EPI_ISL_595591, EPI_ISL_595592, EPI_ISL_595607, EPI_ISL_595610, EPI_ISL_595612			
see above	Wales Specialist Virology Centre Sequencing lab: Pathogen Genomics Unit	COVID-19 Genomics UK (COG-UK) Consortium	Catherine Moore, Johnathan Evans, Laura Gifford, Malorie Perry, Simon Cottrell, Angela Marchbank, Alec Birchley, Alexander Adams, Amy Gaskin, Bree Gatica-Wilcox, Jason Coombes, Joel Southgate, Lauren Gilbert, Lee Graham, Nicole Pacchiarini, Sara Kumziene-Summerhayes, Sarah Taylor, Sophie Jones, Sara Rey, Matthew Bull, Joanne Watkins, Sally Corden, Tom Connor
EPI_ISL_595617, EPI_ISL_595618, EPI_ISL_595619, EPI_ISL_595620, EPI_ISL_595621, EPI_ISL_595622, EPI_ISL_595623, EPI_ISL_595624, EPI_ISL_595625, EPI_ISL_595626, EPI_ISL_595627, EPI_ISL_595628, EPI_ISL_595629, EPI_ISL_595630, EPI_ISL_595631, EPI_ISL_595632, EPI_ISL_595633, EPI_ISL_595634, EPI_ISL_595635, EPI_ISL_595636, EPI_ISL_595637, EPI_ISL_595638, EPI_ISL_595639, EPI_ISL_595640, EPI_ISL_595641, EPI_ISL_595642, EPI_ISL_595643, EPI_ISL_595644, EPI_ISL_595645, EPI_ISL_595646, EPI_ISL_595647, EPI_ISL_595648, EPI_ISL_595649, EPI_ISL_595650, EPI_ISL_595651, EPI_ISL_595652, EPI_ISL_595653, EPI_ISL_595654, EPI_ISL_595655, EPI_ISL_595656, EPI_ISL_595657, EPI_ISL_595658, EPI_ISL_595659, EPI_ISL_595660, EPI_ISL_595661, EPI_ISL_595662, EPI_ISL_595663, EPI_ISL_595664, EPI_ISL_595665, EPI_ISL_595666, EPI_ISL_595667, EPI_ISL_595668, EPI_ISL_595669, EPI_ISL_595670, EPI_ISL_595671, EPI_ISL_595672, EPI_ISL_595673, EPI_ISL_595674, EPI_ISL_595675, EPI_ISL_595676, EPI_ISL_595677, EPI_ISL_595678, EPI_ISL_595679, EPI_ISL_595680, EPI_ISL_595681, EPI_ISL_595682, EPI_ISL_595683, EPI_ISL_595684, EPI_ISL_595685, EPI_ISL_595686, EPI_ISL_595687, EPI_ISL_595688, EPI_ISL_595689, EPI_ISL_595690, EPI_ISL_595691, EPI_ISL_595692, EPI_ISL_595693, EPI_ISL_595694, EPI_ISL_595695, EPI_ISL_595696, EPI_ISL_595697, EPI_ISL_595698, EPI_ISL_595699, EPI_ISL_595700, EPI_ISL_595701, EPI_ISL_595702, EPI_ISL_595703, EPI_ISL_595704, EPI_ISL_595705, EPI_ISL_595706, EPI_ISL_595707, EPI_ISL_595708, EPI_ISL_595709, EPI_ISL_595710, EPI_ISL_595711, EPI_ISL_595712, EPI_ISL_595713, EPI_ISL_595714, EPI_ISL_595715, EPI_ISL_595716, EPI_ISL_595717, EPI_ISL_595718, EPI_ISL_595719, EPI_ISL_595720, EPI_ISL_595721, EPI_ISL_595722, EPI_ISL_595723, EPI_ISL_595724, EPI_ISL_595725, EPI_ISL_595726, EPI_ISL_595727, EPI_ISL_595728, EPI_ISL_595729, EPI_ISL_595730, EPI_ISL_595731, EPI_ISL_595732, EPI_ISL_595733, EPI_ISL_595734, EPI_ISL_595735, EPI_ISL_595736, EPI_ISL_595737, EPI_ISL_595738, EPI_ISL_595740, EPI_ISL_595741, EPI_ISL_595742, EPI_ISL_595743, EPI_ISL_595744, EPI_ISL_595745, EPI_ISL_595746, EPI_ISL_595747, EPI_ISL_595748, EPI_ISL_595749, EPI_ISL_595750, EPI_ISL_595751, EPI_ISL_595752, EPI_ISL_595753, EPI_ISL_595754, EPI_ISL_595755, EPI_ISL_595756, EPI_ISL_595757, EPI_ISL_595758, EPI_ISL_595759, EPI_ISL_595760, EPI_ISL_595761, EPI_ISL_595762, EPI_ISL_595763, EPI_ISL_595764, EPI_ISL_595765, EPI_ISL_595766, EPI_ISL_595767, EPI_ISL_595768, EPI_ISL_595769, EPI_ISL_595770, EPI_ISL_595771, EPI_ISL_595772, EPI_ISL_595773, EPI_ISL_595774, EPI_ISL_595775, EPI_ISL_595776, EPI_ISL_595777, EPI_ISL_595778, EPI_ISL_595779, EPI_ISL_595780, EPI_ISL_595781, EPI_ISL_595782, EPI_ISL_595783, EPI_ISL_595784, EPI_ISL_595785, EPI_ISL_595786, EPI_ISL_595787, EPI_ISL_595788, EPI_ISL_595789, EPI_ISL_595790, EPI_ISL_595791, EPI_ISL_595792, EPI_ISL_595793, EPI_ISL_595794, EPI_ISL_595795, EPI_ISL_595796, EPI_ISL_595797, EPI_ISL_595798			
see above	Oxford Viromics, NDM, University of Oxford; Oxford University Hospitals; Basingstoke and North Hampshire Hospital	COVID-19 Genomics UK (COG-UK) Consortium	Tanya Golubchik, David Bonsall, George Macintyre, Amy Trebes, Mariateresa de Cesare, Catrin Moore, Alex Mobbs, Anita Justice, Robert Shaw, Monique Andersson, Timothy Peto, Emma Wise, Nathan Moore, Jessica Lynch, Nick Cortes, Matilde Mori, Stephen Kidd, David Buck, John Todd, Christophe Fraser
EPI_ISL_595799	Liverpool Clinical Laboratories	COVID-19 Genomics UK (COG-UK) Consortium	Sam Haldenby, Anita Lucaci, Steve Paterson, Julian Hiscox, Alistair Darby, M Almsaud, A Alrezaihi, Muhannad Alruwaili, Stuart D Armstrong, Jones Benjamin, Eleanor G Bentley, Anu Chawla, Jordan J Clark, Angela Cowell, Richard Eccles, Isabel García-Dorival, Matthew Gemmell, Alessandro Gerada, PKF Gilmore, Richard Gregory, Ximeng Han, Catherine Hartley, Margaret Hughes, Miren Iturriza-Gomara, James Johnson, L Luu, Jenifer Manson, Charlotte Nelson, Elaine O'Toole, Cassie Olateju, Rebekah Penrice-Randal , Lucille Rainbow, N.P Randle, Trevor Ian Robinson, Parul Sharma, Ghada T Shawli, James P Stewart, Neil Swainston, Ecaterina Vamos, Joanne Watts, Mark Whitehead
EPI_ISL_595803, EPI_ISL_595813, EPI_ISL_595814, EPI_ISL_595815, EPI_ISL_595816, EPI_ISL_595817, EPI_ISL_595818, EPI_ISL_595823, EPI_ISL_595824, EPI_ISL_595825, EPI_ISL_595826, EPI_ISL_595827, EPI_ISL_595831			
see above	Virology Department, Sheffield Teaching Hospitals NHS Foundation Trust/Department of Infection, Immunity and	COVID-19 Genomics UK (COG-UK) Consortium	Thushan de Silva, Matthew Parker, Nikki Smith, Adri Angyal, Rebecca Brown, Luke Green, Rachel Tucker, Paul Parsons, Danielle Groves, Katie Johnson, Laura Carrilero, Alex Keeley, Dave Partridge, Matthew Wyles, Benjamin Lindsey, Mehmet Yavuz, Mohammad Raza, Cariad Evans

	Cardiovascular Disease, The Medical School, University of Sheffield		
EPI_ISL_595837, EPI_ISL_595838, EPI_ISL_595839, EPI_ISL_595840, EPI_ISL_595841, EPI_ISL_595842, EPI_ISL_595843, EPI_ISL_595844, EPI_ISL_595845, EPI_ISL_595846, EPI_ISL_595847			
see above	Quadram Institute Bioscience	COVID-19 Genomics UK (COG-UK) Consortium	Dave J. Baker, Gemma L. Kay, Alp Aydin, Thanh Le-Viet, Steven Rudder, Ana P. Tedim, Anastasia Kolyva, Maria Diaz, Leonardo de Oliveira Martins, Nabil-Fareed Alikhan, Lizzie Meadows, Rachel Stanley, Ngozi Elumogo, Muhammed Yasir, Nicholas M. Thomson, Alexander J Trotter, Rachel Gilroy, Samuel Bloomfield, Claire Stuart, Andrew Bell, Reenesh Prakash, Samir Dervisevic, Alison E. Mather, John Wain, Mark Webber, Andrew J. Page, Justin O'Grady
EPI_ISL_596201, EPI_ISL_596202, EPI_ISL_596205, EPI_ISL_596209, EPI_ISL_596213, EPI_ISL_596215, EPI_ISL_596218, EPI_ISL_596220	Virology Department, Sheffield Teaching Hospitals NHS Foundation Trust/Department of Infection, Immunity and Cardiovascular Disease, The Medical School, University of Sheffield	COVID-19 Genomics UK (COG-UK) Consortium	Thushan de Silva, Matthew Parker, Nikki Smith, Adri Angyal, Rebecca Brown, Luke Green, Rachel Tucker, Paul Parsons, Danielle Groves, Katie Johnson, Laura Carrilero, Alex Keeley, Dave Partridge, Matthew Wyles, Benjamin Lindsey, Mehmet Yavuz, Mohammad Raza, Cariad Evans
EPI_ISL_596246, EPI_ISL_596247, EPI_ISL_596248, EPI_ISL_596249	WHO National Influenza Centre Russian Federation	WHO National Influenza Centre Russian Federation	Andrey Komissarov, Artem Fadeev, Anna Ivanova, Kseniya Komissarova, Dmitry Bazhenov, Daria Danilenko
EPI_ISL_596409	Seattle Flu Study	Seattle Flu Study	Deborah A. Nickerson, Chris D. Frazar, Jover Lee, Benjamin Pelle, Matthew Richardson, Amanda Adler, Elisabeth Brandstetter, Peter D. Han, Kairsten Fay, Misja Ilcisin, Kirsten Lacombe, Thomas R. Sibley, Melissa Truong, Caitlin R. Wolf, Karen Cowgill, Stephanie Schrag, Jeff Duchin, Michael Boeckh, Janet A. Englund, Michael Famulare, Barry R. Lutz, Mark J. Rieder, Lea M. Starita, Matthew Thompson, Helen Y. Chu, Trevor Bedford, Jay Shendure
EPI_ISL_596410	Seattle Flu Study	Seattle Flu Study	Deborah A. Nickerson, Chris D. Frazar, Jover Lee, Benjamin Pelle, Matthew Richardson, Amanda Adler, Elisabeth Brandstetter, Peter D. Han, Kairsten Fay, Misja Ilcisin, Kirsten Lacombe, Thomas R. Sibley, Melissa Truong, Caitlin R. Wolf, Michael Boeckh, Janet A. Englund, Michael Famulare, Barry R. Lutz, Mark J. Rieder, Lea M. Starita, Matthew Thompson, Jay Shendure, Trevor Bedford, Helen Y. Chu
EPI_ISL_596411	Seattle Flu Study	Seattle Flu Study	Deborah A. Nickerson, Chris D. Frazar, Jover Lee, Benjamin Pelle, Matthew Richardson, Amanda Adler, Elisabeth Brandstetter, Peter D. Han, Kairsten Fay, Misja Ilcisin, Kirsten Lacombe, Thomas R. Sibley, Melissa Truong, Caitlin R. Wolf, Karen Cowgill, Stephanie Schrag, Jeff Duchin, Michael Boeckh, Janet A. Englund, Michael Famulare, Barry R. Lutz, Mark J. Rieder, Lea M. Starita, Matthew Thompson, Helen Y. Chu, Trevor Bedford, Jay Shendure
EPI_ISL_596412, EPI_ISL_596413, EPI_ISL_596414	Seattle Flu Study	Seattle Flu Study	Deborah A. Nickerson, Chris D. Frazar, Jover Lee, Benjamin Pelle, Matthew Richardson, Amanda Adler, Elisabeth Brandstetter, Peter D. Han, Kairsten Fay, Misja Ilcisin, Kirsten Lacombe, Thomas R. Sibley, Melissa Truong, Caitlin R. Wolf, Michael Boeckh, Janet A. Englund, Michael Famulare, Barry R. Lutz, Mark J. Rieder, Lea M. Starita, Matthew Thompson, Jay Shendure, Trevor Bedford, Helen Y. Chu
EPI_ISL_596416	Seattle Flu Study	Seattle Flu Study	Deborah A. Nickerson, Chris D. Frazar, Jover Lee, Benjamin Pelle, Matthew Richardson, Amanda Adler, Elisabeth Brandstetter, Peter D. Han, Kairsten Fay, Misja Ilcisin, Kirsten Lacombe, Thomas R. Sibley, Melissa Truong, Caitlin R. Wolf, Karen Cowgill, Stephanie Schrag, Jeff Duchin, Michael Boeckh, Janet A. Englund, Michael Famulare, Barry R. Lutz, Mark J. Rieder, Lea M. Starita, Matthew Thompson, Helen Y. Chu, Trevor Bedford, Jay Shendure
EPI_ISL_596417, EPI_ISL_596418, EPI_ISL_596419, EPI_ISL_596420	Seattle Flu Study	Seattle Flu Study	Deborah A. Nickerson, Chris D. Frazar, Jover Lee, Benjamin Pelle, Matthew Richardson, Amanda Adler, Elisabeth Brandstetter, Peter D. Han, Kairsten Fay, Misja Ilcisin, Kirsten Lacombe, Thomas R. Sibley, Melissa Truong, Caitlin R. Wolf, Michael Boeckh, Janet A. Englund, Michael Famulare, Barry R. Lutz, Mark J. Rieder, Lea M. Starita, Matthew Thompson, Jay Shendure, Trevor Bedford, Helen Y. Chu
EPI_ISL_596421, EPI_ISL_596422, EPI_ISL_596423, EPI_ISL_596424, EPI_ISL_596425	Seattle Flu Study	Seattle Flu Study	Deborah A. Nickerson, Chris D. Frazar, Jover Lee, Benjamin Pelle, Matthew Richardson, Amanda Adler, Elisabeth Brandstetter, Peter D. Han, Kairsten Fay, Misja Ilcisin, Kirsten Lacombe, Thomas R. Sibley, Melissa Truong, Caitlin R. Wolf, Karen Cowgill, Stephanie Schrag, Jeff Duchin, Michael Boeckh, Janet A. Englund, Michael Famulare, Barry R. Lutz, Mark J. Rieder, Lea M. Starita, Matthew Thompson, Helen Y. Chu, Trevor Bedford, Jay Shendure
EPI_ISL_596426, EPI_ISL_596427	Seattle Flu Study	Seattle Flu Study	Deborah A. Nickerson, Chris D. Frazar, Jover Lee, Benjamin Pelle, Matthew Richardson, Amanda Adler, Elisabeth Brandstetter, Peter D. Han, Kairsten Fay, Misja Ilcisin, Kirsten Lacombe, Thomas R. Sibley, Melissa Truong, Caitlin R. Wolf, Michael Boeckh, Janet A. Englund, Michael Famulare, Barry R. Lutz, Mark J. Rieder, Lea M. Starita, Matthew Thompson, Jay Shendure, Trevor Bedford, Helen Y. Chu
EPI_ISL_596463, EPI_ISL_596468, EPI_ISL_596477, EPI_ISL_596481	National Public Health Laboratory, National Centre for Infectious Diseases	National Public Health Laboratory, National Centre for Infectious Diseases	Tze Minn Mak, Sophie Octavia, Zhenyang Zhou, Lin Cui, Raymond Tzer Pin Lin
EPI_ISL_596572, EPI_ISL_596581	University of Michigan Clinical Microbiology Laboratory	Lauring Lab, University of Michigan, Department of Microbiology and Immunology	Valesano
EPI_ISL_596883	PathWest Laboratory Medicine WA	PathWest Laboratory Medicine WA Microbial Surveillance Unit	PathWest Laboratory Medicine WA Microbial Surveillance Unit
EPI_ISL_598831, EPI_ISL_598832, EPI_ISL_598833, EPI_ISL_598834, EPI_ISL_598835, EPI_ISL_598836, EPI_ISL_598837, EPI_ISL_598838, EPI_ISL_598839, EPI_ISL_598840, EPI_ISL_598841, EPI_ISL_598842, EPI_ISL_598843, EPI_ISL_598844, EPI_ISL_598845, EPI_ISL_598846, EPI_ISL_598847, EPI_ISL_598848, EPI_ISL_598849, EPI_ISL_598850, EPI_ISL_598851, EPI_ISL_598852, EPI_ISL_598853, EPI_ISL_598854, EPI_ISL_598855, EPI_ISL_598856, EPI_ISL_598857, EPI_ISL_598858, EPI_ISL_598859, EPI_ISL_598860, EPI_ISL_598861, EPI_ISL_598862, EPI_ISL_598863, EPI_ISL_598864, EPI_ISL_598865, EPI_ISL_598866, EPI_ISL_598867, EPI_ISL_598868, EPI_ISL_598869, EPI_ISL_598870, EPI_ISL_598871, EPI_ISL_598872, EPI_ISL_598873, EPI_ISL_598874, EPI_ISL_598875, EPI_ISL_598876, EPI_ISL_598877, EPI_ISL_598878, EPI_ISL_598879, EPI_ISL_598880, EPI_ISL_598881, EPI_ISL_598882, EPI_ISL_598883, EPI_ISL_598884, EPI_ISL_598885, EPI_ISL_598886, EPI_ISL_598887, EPI_ISL_598888, EPI_ISL_598889, EPI_ISL_598890, EPI_ISL_598891, EPI_ISL_598892, EPI_ISL_598893, EPI_ISL_598894, EPI_ISL_598895, EPI_ISL_598896, EPI_ISL_598897, EPI_ISL_598898, EPI_ISL_598899, EPI_ISL_598900, EPI_ISL_598901, EPI_ISL_598902, EPI_ISL_598903, EPI_ISL_598904, EPI_ISL_598905, EPI_ISL_598906, EPI_ISL_598907, EPI_ISL_598908, EPI_ISL_598909, EPI_ISL_598910, EPI_ISL_598911, EPI_ISL_598912, EPI_ISL_598913, EPI_ISL_598914, EPI_ISL_598915, EPI_ISL_598916, EPI_ISL_598917, EPI_ISL_598918, EPI_ISL_598919, EPI_ISL_598920, EPI_ISL_598921, EPI_ISL_598922, EPI_ISL_598923, EPI_ISL_598924, EPI_ISL_598925, EPI_ISL_598926, EPI_ISL_598927, EPI_ISL_598928, EPI_ISL_598929, EPI_ISL_598930, EPI_ISL_598931, EPI_ISL_598932, EPI_ISL_598933, EPI_ISL_598934, EPI_ISL_598935, EPI_ISL_598936, EPI_ISL_598937, EPI_ISL_598938, EPI_ISL_598939, EPI_ISL_598940, EPI_ISL_598941, EPI_ISL_598942, EPI_ISL_598943, EPI_ISL_598944, EPI_ISL_598945, EPI_ISL_598946, EPI_ISL_598947, EPI_ISL_598948, EPI_ISL_598949, EPI_ISL_598950, EPI_ISL_598951, EPI_ISL_598952, EPI_ISL_598953, EPI_ISL_598954, EPI_ISL_598955, EPI_ISL_598956, EPI_ISL_598957, EPI_ISL_598958, EPI_ISL_598959, EPI_ISL_598960, EPI_ISL_598961, EPI_ISL_598962, EPI_ISL_598963, EPI_ISL_598964, EPI_ISL_598965, EPI_ISL_598966, EPI_ISL_598967, EPI_ISL_598968, EPI_ISL_598969			
see above	Lighthouse Lab in Glasgow	Wellcome Sanger Institute for the COVID-19 Genomics UK (COG-UK) consortium	Harper VanSteenhouse, Yumi Kasai, David Gray, Carol Clugston, Anna Dominiczak and Alex Alderton, Roberto Amato, Sonia Goncalves, Ewan Harrison, David K. Jackson, Ian Johnston, Dominic Kwiatkowski, Cordelia Langford, John Sillitoe on behalf of the Wellcome Sanger Institute COVID-19 Surveillance Team (http://www.sanger.ac.uk/covid-team)
EPI_ISL_598970	Lighthouse Lab in Glasgow	Wellcome Sanger Institute for the COVID-19 Genomics UK (COG-UK) Consortium	Harper VanSteenhouse, Yumi Kasai, David Gray, Carol Clugston, Anna Dominiczak and Alex Alderton, Roberto Amato, Sonia Goncalves, Ewan Harrison, David K. Jackson, Ian Johnston, Dominic Kwiatkowski, Cordelia Langford, John Sillitoe on behalf of the Wellcome Sanger Institute COVID-19 Surveillance Team
EPI_ISL_598971, EPI_ISL_598972, EPI_ISL_598973, EPI_ISL_598974, EPI_ISL_598975, EPI_ISL_598976, EPI_ISL_598977, EPI_ISL_598978, EPI_ISL_598979, EPI_ISL_598980, EPI_ISL_598981, EPI_ISL_598982, EPI_ISL_598983, EPI_ISL_598984, EPI_ISL_598985, EPI_ISL_598986, EPI_ISL_598987, EPI_ISL_598988, EPI_ISL_598989, EPI_ISL_598990, EPI_ISL_598991, EPI_ISL_598992, EPI_ISL_598993, EPI_ISL_598994, EPI_ISL_598995, EPI_ISL_598996, EPI_ISL_598997, EPI_ISL_598998, EPI_ISL_598999, EPI_ISL_599000, EPI_ISL_599001, EPI_ISL_599002, EPI_ISL_599003, EPI_ISL_599004, EPI_ISL_599005, EPI_ISL_599006, EPI_ISL_599007, EPI_ISL_599008, EPI_ISL_599009			
see above	Lighthouse Lab in Glasgow	Wellcome Sanger Institute for the COVID-19 Genomics UK (COG-UK) consortium	Harper VanSteenhouse, Yumi Kasai, David Gray, Carol Clugston, Anna Dominiczak and Alex Alderton, Roberto Amato, Sonia Goncalves, Ewan Harrison, David K. Jackson, Ian Johnston, Dominic Kwiatkowski, Cordelia Langford, John Sillitoe on behalf of the Wellcome Sanger Institute COVID-19 Surveillance Team (http://www.sanger.ac.uk/covid-team)
EPI_ISL_599010	Lighthouse Lab in Glasgow	Wellcome Sanger Institute for the COVID-19 Genomics UK (COG-UK) Consortium	Harper VanSteenhouse, Yumi Kasai, David Gray, Carol Clugston, Anna Dominiczak and Alex Alderton, Roberto Amato, Sonia Goncalves, Ewan Harrison, David K. Jackson, Ian Johnston, Dominic Kwiatkowski, Cordelia Langford, John Sillitoe on behalf of the Wellcome Sanger Institute COVID-19 Surveillance Team
EPI_ISL_599011, EPI_ISL_599012, EPI_ISL_599013, EPI_ISL_599014, EPI_ISL_599015, EPI_ISL_599016, EPI_ISL_599017, EPI_ISL_599018, EPI_ISL_599019, EPI_ISL_599020, EPI_ISL_599021, EPI_ISL_599022, EPI_ISL_599023, EPI_ISL_599024, EPI_ISL_599025, EPI_ISL_599026, EPI_ISL_599027, EPI_ISL_599028, EPI_ISL_599029, EPI_ISL_599030, EPI_ISL_599031, EPI_ISL_599032, EPI_ISL_599033, EPI_ISL_599034, EPI_ISL_599035, EPI_ISL_599036, EPI_ISL_599037, EPI_ISL_599038, EPI_ISL_599039, EPI_ISL_599040, EPI_ISL_599041, EPI_ISL_599042, EPI_ISL_599043, EPI_ISL_599044, EPI_ISL_599045, EPI_ISL_599046, EPI_ISL_599047, EPI_ISL_599048, EPI_ISL_599049, EPI_ISL_599050, EPI_ISL_599051, EPI_ISL_599052, EPI_ISL_599053, EPI_ISL_599054, EPI_ISL_599055, EPI_ISL_599056, EPI_ISL_599057, EPI_ISL_599058, EPI_ISL_599059, EPI_ISL_599060, EPI_ISL_599061, EPI_ISL_599062, EPI_ISL_599063, EPI_ISL_599064, EPI_ISL_599065, EPI_ISL_599066, EPI_ISL_599067, EPI_ISL_599068, EPI_ISL_599069, EPI_ISL_599070, EPI_ISL_599071, EPI_ISL_599072, EPI_ISL_599073, EPI_ISL_599074, EPI_ISL_599075, EPI_ISL_599076			
see above	Lighthouse Lab in Glasgow	Wellcome Sanger Institute for the COVID-19 Genomics UK (COG-UK) consortium	Harper VanSteenhouse, Yumi Kasai, David Gray, Carol Clugston, Anna Dominiczak and Alex Alderton, Roberto Amato, Sonia Goncalves, Ewan Harrison, David K. Jackson, Ian Johnston, Dominic Kwiatkowski, Cordelia Langford, John Sillitoe on behalf of the Wellcome Sanger Institute COVID-19 Surveillance Team (http://www.sanger.ac.uk/covid-team)
EPI_ISL_599077	Lighthouse Lab in Glasgow	Wellcome Sanger Institute for the COVID-19 Genomics UK (COG-UK) Consortium	Harper VanSteenhouse, Yumi Kasai, David Gray, Carol Clugston, Anna Dominiczak and Alex Alderton, Roberto Amato, Sonia Goncalves, Ewan Harrison, David K. Jackson, Ian Johnston, Dominic Kwiatkowski, Cordelia Langford, John Sillitoe on behalf of the Wellcome Sanger Institute COVID-19 Surveillance Team

EPI_ISL_599078, EPI_ISL_599079, EPI_ISL_599080, EPI_ISL_599081, EPI_ISL_599082, EPI_ISL_599083, EPI_ISL_599084, EPI_ISL_599085, EPI_ISL_599086, EPI_ISL_599087, EPI_ISL_599088, EPI_ISL_599089, EPI_ISL_599090, EPI_ISL_599091, EPI_ISL_599092, EPI_ISL_599093, EPI_ISL_599094, EPI_ISL_599095, EPI_ISL_599096, EPI_ISL_599097, EPI_ISL_599098, EPI_ISL_599099, EPI_ISL_599100, EPI_ISL_599101, EPI_ISL_599102, EPI_ISL_599103, EPI_ISL_599104, EPI_ISL_599105, EPI_ISL_599106, EPI_ISL_599107, EPI_ISL_599108, EPI_ISL_599109, EPI_ISL_599110, EPI_ISL_599111, EPI_ISL_599112, EPI_ISL_599113, EPI_ISL_599114, EPI_ISL_599115, EPI_ISL_599116, EPI_ISL_599117, EPI_ISL_599118, EPI_ISL_599119, EPI_ISL_599120, EPI_ISL_599121, EPI_ISL_599122, EPI_ISL_599123, EPI_ISL_599124, EPI_ISL_599125, EPI_ISL_599126, EPI_ISL_599127, EPI_ISL_599128, EPI_ISL_599129, EPI_ISL_599130, EPI_ISL_599131, EPI_ISL_599132, EPI_ISL_599133, EPI_ISL_599134, EPI_ISL_599135, EPI_ISL_599136, EPI_ISL_599137, EPI_ISL_599139, EPI_ISL_599141, EPI_ISL_599142, EPI_ISL_599143, EPI_ISL_599144, EPI_ISL_599147, EPI_ISL_599150, EPI_ISL_599151, EPI_ISL_599152, EPI_ISL_599153, EPI_ISL_599155, EPI_ISL_599156, EPI_ISL_599157, EPI_ISL_599158, EPI_ISL_599159, EPI_ISL_599163, EPI_ISL_599164, EPI_ISL_599167, EPI_ISL_599168, EPI_ISL_599172, EPI_ISL_599174, EPI_ISL_599180, EPI_ISL_599182			
see above	Lighthouse Lab in Glasgow	Wellcome Sanger Institute for the COVID-19 Genomics UK (COG-UK) consortium	Harper VanSteenhouse, Yumi Kasai, David Gray, Carol Clugston, Anna Dominiczak and Alex Alderton, Roberto Amato, Sonia Goncalves, Ewan Harrison, David K. Jackson, Ian Johnston, Dominic Kwiatkowski, Cordelia Langford, John Sillitoe on behalf of the Wellcome Sanger Institute COVID-19 Surveillance Team (http://www.sanger.ac.uk/covid-team)
EPI_ISL_599183	Lighthouse Lab in Glasgow	Wellcome Sanger Institute for the COVID-19 Genomics UK (COG-UK) Consortium	Harper VanSteenhouse, Yumi Kasai, David Gray, Carol Clugston, Anna Dominiczak and Alex Alderton, Roberto Amato, Sonia Goncalves, Ewan Harrison, David K. Jackson, Ian Johnston, Dominic Kwiatkowski, Cordelia Langford, John Sillitoe on behalf of the Wellcome Sanger Institute COVID-19 Surveillance Team
EPI_ISL_599184, EPI_ISL_599185, EPI_ISL_599186, EPI_ISL_599190, EPI_ISL_599192, EPI_ISL_599193, EPI_ISL_599195, EPI_ISL_599198, EPI_ISL_599200, EPI_ISL_599205, EPI_ISL_599208, EPI_ISL_599213, EPI_ISL_599217, EPI_ISL_599218, EPI_ISL_599221, EPI_ISL_599224, EPI_ISL_599226, EPI_ISL_599229, EPI_ISL_599230, EPI_ISL_599232, EPI_ISL_599233, EPI_ISL_599240, EPI_ISL_599242, EPI_ISL_599243, EPI_ISL_599244, EPI_ISL_599245, EPI_ISL_599247, EPI_ISL_599251, EPI_ISL_599252, EPI_ISL_599254, EPI_ISL_599258, EPI_ISL_599259, EPI_ISL_599260, EPI_ISL_599261, EPI_ISL_599263, EPI_ISL_599264, EPI_ISL_599265			
see above	Lighthouse Lab in Glasgow	Wellcome Sanger Institute for the COVID-19 Genomics UK (COG-UK) consortium	Harper VanSteenhouse, Yumi Kasai, David Gray, Carol Clugston, Anna Dominiczak and Alex Alderton, Roberto Amato, Sonia Goncalves, Ewan Harrison, David K. Jackson, Ian Johnston, Dominic Kwiatkowski, Cordelia Langford, John Sillitoe on behalf of the Wellcome Sanger Institute COVID-19 Surveillance Team (http://www.sanger.ac.uk/covid-team)
EPI_ISL_599270	Lighthouse Lab in Glasgow	Wellcome Sanger Institute for the COVID-19 Genomics UK (COG-UK) Consortium	Harper VanSteenhouse, Yumi Kasai, David Gray, Carol Clugston, Anna Dominiczak and Alex Alderton, Roberto Amato, Sonia Goncalves, Ewan Harrison, David K. Jackson, Ian Johnston, Dominic Kwiatkowski, Cordelia Langford, John Sillitoe on behalf of the Wellcome Sanger Institute COVID-19 Surveillance Team
EPI_ISL_599272, EPI_ISL_599273, EPI_ISL_599274, EPI_ISL_599275, EPI_ISL_599278, EPI_ISL_599281, EPI_ISL_599282, EPI_ISL_599283, EPI_ISL_599284, EPI_ISL_599285, EPI_ISL_599287, EPI_ISL_599289, EPI_ISL_599290, EPI_ISL_599292, EPI_ISL_599296, EPI_ISL_599297, EPI_ISL_599298, EPI_ISL_599300, EPI_ISL_599301, EPI_ISL_599302, EPI_ISL_599303, EPI_ISL_599304, EPI_ISL_599305, EPI_ISL_599308, EPI_ISL_599309, EPI_ISL_599312, EPI_ISL_599313			
see above	Lighthouse Lab in Glasgow	Wellcome Sanger Institute for the COVID-19 Genomics UK (COG-UK) consortium	Harper VanSteenhouse, Yumi Kasai, David Gray, Carol Clugston, Anna Dominiczak and Alex Alderton, Roberto Amato, Sonia Goncalves, Ewan Harrison, David K. Jackson, Ian Johnston, Dominic Kwiatkowski, Cordelia Langford, John Sillitoe on behalf of the Wellcome Sanger Institute COVID-19 Surveillance Team (http://www.sanger.ac.uk/covid-team)
EPI_ISL_599314	Lighthouse Lab in Glasgow	Wellcome Sanger Institute for the COVID-19 Genomics UK (COG-UK) Consortium	Harper VanSteenhouse, Yumi Kasai, David Gray, Carol Clugston, Anna Dominiczak and Alex Alderton, Roberto Amato, Sonia Goncalves, Ewan Harrison, David K. Jackson, Ian Johnston, Dominic Kwiatkowski, Cordelia Langford, John Sillitoe on behalf of the Wellcome Sanger Institute COVID-19 Surveillance Team
EPI_ISL_599315, EPI_ISL_599317, EPI_ISL_599319, EPI_ISL_599322, EPI_ISL_599324, EPI_ISL_599327, EPI_ISL_599329, EPI_ISL_599334, EPI_ISL_599335, EPI_ISL_599336, EPI_ISL_599337, EPI_ISL_599338, EPI_ISL_599346, EPI_ISL_599348, EPI_ISL_599349, EPI_ISL_599350, EPI_ISL_599351, EPI_ISL_599355, EPI_ISL_599356, EPI_ISL_599357, EPI_ISL_599358, EPI_ISL_599359, EPI_ISL_599360, EPI_ISL_599363, EPI_ISL_599364, EPI_ISL_599365, EPI_ISL_599367, EPI_ISL_599368, EPI_ISL_599370, EPI_ISL_599371, EPI_ISL_599373, EPI_ISL_599375, EPI_ISL_599380, EPI_ISL_599381, EPI_ISL_599382, EPI_ISL_599383, EPI_ISL_599386, EPI_ISL_599387, EPI_ISL_599388, EPI_ISL_599389, EPI_ISL_599391, EPI_ISL_599392, EPI_ISL_599395, EPI_ISL_599396, EPI_ISL_599400, EPI_ISL_599402, EPI_ISL_599405, EPI_ISL_599406, EPI_ISL_599407, EPI_ISL_599415, EPI_ISL_599416, EPI_ISL_599417, EPI_ISL_599421, EPI_ISL_599422, EPI_ISL_599423, EPI_ISL_599424, EPI_ISL_599425, EPI_ISL_599427, EPI_ISL_599428, EPI_ISL_599430, EPI_ISL_599432, EPI_ISL_599433, EPI_ISL_599434, EPI_ISL_599439, EPI_ISL_599441, EPI_ISL_599442, EPI_ISL_599443, EPI_ISL_599446, EPI_ISL_599448, EPI_ISL_599451, EPI_ISL_599453, EPI_ISL_599454, EPI_ISL_599455, EPI_ISL_599456, EPI_ISL_599458, EPI_ISL_599460, EPI_ISL_599461, EPI_ISL_599462, EPI_ISL_599464, EPI_ISL_599467, EPI_ISL_599469, EPI_ISL_599473, EPI_ISL_599476, EPI_ISL_599478, EPI_ISL_599479, EPI_ISL_599483			
see above	Lighthouse Lab in Glasgow	Wellcome Sanger Institute for the COVID-19 Genomics UK (COG-UK) consortium	Harper VanSteenhouse, Yumi Kasai, David Gray, Carol Clugston, Anna Dominiczak and Alex Alderton, Roberto Amato, Sonia Goncalves, Ewan Harrison, David K. Jackson, Ian Johnston, Dominic Kwiatkowski, Cordelia Langford, John Sillitoe on behalf of the Wellcome Sanger Institute COVID-19 Surveillance Team (http://www.sanger.ac.uk/covid-team)
EPI_ISL_599818, EPI_ISL_599819, EPI_ISL_599820, EPI_ISL_599821, EPI_ISL_599822, EPI_ISL_599823, EPI_ISL_599824, EPI_ISL_599825, EPI_ISL_599826, EPI_ISL_599827, EPI_ISL_599828, EPI_ISL_599829, EPI_ISL_599830, EPI_ISL_599831, EPI_ISL_599832, EPI_ISL_599833, EPI_ISL_599834, EPI_ISL_599835, EPI_ISL_599836, EPI_ISL_599837, EPI_ISL_599838, EPI_ISL_599839, EPI_ISL_599840, EPI_ISL_599841, EPI_ISL_599842, EPI_ISL_599843, EPI_ISL_599844			
see above	Lighthouse Lab in Milton Keynes	Wellcome Sanger Institute for the COVID-19 Genomics UK (COG-UK) consortium	The Lighthouse Lab in Milton Keynes and Alex Alderton, Roberto Amato, Sonia Goncalves, Ewan Harrison, David K. Jackson, Ian Johnston, Dominic Kwiatkowski, Cordelia Langford, John Sillitoe on behalf of the Wellcome Sanger Institute COVID-19 Surveillance Team (http://www.sanger.ac.uk/covid-team)
EPI_ISL_599845	Lighthouse Lab in Milton Keynes	Wellcome Sanger Institute for the COVID-19 Genomics UK (COG-UK) Consortium	The Lighthouse Lab in Milton Keynes and Alex Alderton, Roberto Amato, Sonia Goncalves, Ewan Harrison, David K. Jackson, Ian Johnston, Dominic Kwiatkowski, Cordelia Langford, John Sillitoe on behalf of the Wellcome Sanger Institute COVID-19 Surveillance Team
EPI_ISL_599846, EPI_ISL_599847, EPI_ISL_599848, EPI_ISL_599849, EPI_ISL_599850, EPI_ISL_599851, EPI_ISL_599852, EPI_ISL_599853, EPI_ISL_599854, EPI_ISL_599855, EPI_ISL_599856, EPI_ISL_599857, EPI_ISL_599858, EPI_ISL_599859, EPI_ISL_599860, EPI_ISL_599861, EPI_ISL_599862, EPI_ISL_599863, EPI_ISL_599864, EPI_ISL_599865, EPI_ISL_599866, EPI_ISL_599867, EPI_ISL_599868, EPI_ISL_599869, EPI_ISL_599870, EPI_ISL_599871, EPI_ISL_599872, EPI_ISL_599873, EPI_ISL_599874, EPI_ISL_599875, EPI_ISL_599876, EPI_ISL_599877, EPI_ISL_599878, EPI_ISL_599879, EPI_ISL_599880, EPI_ISL_599881, EPI_ISL_599882, EPI_ISL_599883, EPI_ISL_599884, EPI_ISL_599885, EPI_ISL_599886, EPI_ISL_599887			
see above	Lighthouse Lab in Milton Keynes	Wellcome Sanger Institute for the COVID-19 Genomics UK (COG-UK) consortium	The Lighthouse Lab in Milton Keynes and Alex Alderton, Roberto Amato, Sonia Goncalves, Ewan Harrison, David K. Jackson, Ian Johnston, Dominic Kwiatkowski, Cordelia Langford, John Sillitoe on behalf of the Wellcome Sanger Institute COVID-19 Surveillance Team (http://www.sanger.ac.uk/covid-team)
EPI_ISL_599888	Lighthouse Lab in Milton Keynes	Wellcome Sanger Institute for the COVID-19 Genomics UK (COG-UK) Consortium	The Lighthouse Lab in Milton Keynes and Alex Alderton, Roberto Amato, Sonia Goncalves, Ewan Harrison, David K. Jackson, Ian Johnston, Dominic Kwiatkowski, Cordelia Langford, John Sillitoe on behalf of the Wellcome Sanger Institute COVID-19 Surveillance Team
EPI_ISL_599889, EPI_ISL_599890, EPI_ISL_599891, EPI_ISL_599892, EPI_ISL_599893, EPI_ISL_599894, EPI_ISL_599895, EPI_ISL_599896, EPI_ISL_599897, EPI_ISL_599898, EPI_ISL_599899, EPI_ISL_599900, EPI_ISL_599901, EPI_ISL_599902, EPI_ISL_599903, EPI_ISL_599904, EPI_ISL_599905, EPI_ISL_599906, EPI_ISL_599907, EPI_ISL_599908, EPI_ISL_599909, EPI_ISL_599910, EPI_ISL_599911, EPI_ISL_599912, EPI_ISL_599913, EPI_ISL_599914, EPI_ISL_599915, EPI_ISL_599916, EPI_ISL_599917, EPI_ISL_599918, EPI_ISL_599919, EPI_ISL_599920, EPI_ISL_599921, EPI_ISL_599922, EPI_ISL_599923, EPI_ISL_599924, EPI_ISL_599925, EPI_ISL_599926, EPI_ISL_599927, EPI_ISL_599928, EPI_ISL_599929, EPI_ISL_599930, EPI_ISL_599931, EPI_ISL_599932, EPI_ISL_599933, EPI_ISL_599934, EPI_ISL_599935, EPI_ISL_599936, EPI_ISL_599937, EPI_ISL_599938, EPI_ISL_599939, EPI_ISL_599940, EPI_ISL_599941, EPI_ISL_599942, EPI_ISL_599943, EPI_ISL_599944, EPI_ISL_599945, EPI_ISL_599946, EPI_ISL_599947			
see above	Lighthouse Lab in Milton Keynes	Wellcome Sanger Institute for the COVID-19 Genomics UK (COG-UK) consortium	The Lighthouse Lab in Milton Keynes and Alex Alderton, Roberto Amato, Sonia Goncalves, Ewan Harrison, David K. Jackson, Ian Johnston, Dominic Kwiatkowski, Cordelia Langford, John Sillitoe on behalf of the Wellcome Sanger Institute COVID-19 Surveillance Team (http://www.sanger.ac.uk/covid-team)
EPI_ISL_599948	Lighthouse Lab in Milton Keynes	Wellcome Sanger Institute for the COVID-19 Genomics UK (COG-UK) Consortium	The Lighthouse Lab in Milton Keynes and Alex Alderton, Roberto Amato, Sonia Goncalves, Ewan Harrison, David K. Jackson, Ian Johnston, Dominic Kwiatkowski, Cordelia Langford, John Sillitoe on behalf of the Wellcome Sanger Institute COVID-19 Surveillance Team
EPI_ISL_599949, EPI_ISL_599950, EPI_ISL_599951, EPI_ISL_599952, EPI_ISL_599953, EPI_ISL_599954, EPI_ISL_599955, EPI_ISL_599956, EPI_ISL_599957, EPI_ISL_599958, EPI_ISL_599959			
see above	Lighthouse Lab in Milton Keynes	Wellcome Sanger Institute for the COVID-19 Genomics UK (COG-UK) consortium	The Lighthouse Lab in Milton Keynes and Alex Alderton, Roberto Amato, Sonia Goncalves, Ewan Harrison, David K. Jackson, Ian Johnston, Dominic Kwiatkowski, Cordelia Langford, John Sillitoe on behalf of the Wellcome Sanger Institute COVID-19 Surveillance Team (http://www.sanger.ac.uk/covid-team)
EPI_ISL_599960	Lighthouse Lab in Milton Keynes	Wellcome Sanger Institute for the COVID-19 Genomics UK (COG-UK) Consortium	The Lighthouse Lab in Milton Keynes and Alex Alderton, Roberto Amato, Sonia Goncalves, Ewan Harrison, David K. Jackson, Ian Johnston, Dominic Kwiatkowski, Cordelia Langford, John Sillitoe on behalf of the Wellcome Sanger Institute COVID-19 Surveillance Team
EPI_ISL_599961, EPI_ISL_599962, EPI_ISL_599963, EPI_ISL_599964, EPI_ISL_599965, EPI_ISL_599966, EPI_ISL_599967, EPI_ISL_599968, EPI_ISL_599969, EPI_ISL_599970, EPI_ISL_599971, EPI_ISL_599972, EPI_ISL_599973, EPI_ISL_599974, EPI_ISL_599975, EPI_ISL_599976, EPI_ISL_599977, EPI_ISL_599978, EPI_ISL_599979, EPI_ISL_599980, EPI_ISL_599981, EPI_ISL_599982, EPI_ISL_599983, EPI_ISL_599984, EPI_ISL_599985, EPI_ISL_599986, EPI_ISL_599987, EPI_ISL_599988, EPI_ISL_599989, EPI_ISL_599990, EPI_ISL_599991, EPI_ISL_599992, EPI_ISL_599993, EPI_ISL_599994, EPI_ISL_599995, EPI_ISL_599996, EPI_ISL_599997, EPI_ISL_599998, EPI_ISL_599999, EPI_ISL_600000, EPI_ISL_600001, EPI_ISL_600002, EPI_ISL_600003, EPI_ISL_600004, EPI_ISL_600005, EPI_ISL_600006, EPI_ISL_600007, EPI_ISL_600008, EPI_ISL_600009, EPI_ISL_600010, EPI_ISL_600011, EPI_ISL_600012, EPI_ISL_600013, EPI_ISL_600014, EPI_ISL_600015, EPI_ISL_600016, EPI_ISL_600017, EPI_ISL_600018, EPI_ISL_600019, EPI_ISL_600020, EPI_ISL_600021, EPI_ISL_600022, EPI_ISL_600023, EPI_ISL_600024, EPI_ISL_600025, EPI_ISL_600026, EPI_ISL_600027, EPI_ISL_600028, EPI_ISL_600029, EPI_ISL_600030, EPI_ISL_600031, EPI_ISL_600032, EPI_ISL_600033, EPI_ISL_600034, EPI_ISL_600035, EPI_ISL_600036, EPI_ISL_600037, EPI_ISL_600038, EPI_ISL_600039, EPI_ISL_600040, EPI_ISL_600041, EPI_ISL_600042, EPI_ISL_600043, EPI_ISL_600044, EPI_ISL_600045, EPI_ISL_600046, EPI_ISL_600047, EPI_ISL_600048, EPI_ISL_600049, EPI_ISL_600050, EPI_ISL_600051, EPI_ISL_600052, EPI_ISL_600053, EPI_ISL_600054, EPI_ISL_600055			
see above	Lighthouse Lab in Milton Keynes	Wellcome Sanger Institute for the COVID-19 Genomics UK (COG-UK) consortium	The Lighthouse Lab in Milton Keynes and Alex Alderton, Roberto Amato, Sonia Goncalves, Ewan Harrison, David K. Jackson, Ian Johnston, Dominic Kwiatkowski, Cordelia Langford, John Sillitoe on behalf of the Wellcome Sanger Institute COVID-19 Surveillance Team (http://www.sanger.ac.uk/covid-team)
EPI_ISL_600056	Lighthouse Lab in Milton Keynes	Wellcome Sanger Institute for the COVID-19 Genomics UK (COG-UK) Consortium	The Lighthouse Lab in Milton Keynes and Alex Alderton, Roberto Amato, Sonia Goncalves, Ewan Harrison, David K. Jackson, Ian Johnston, Dominic Kwiatkowski, Cordelia Langford, John Sillitoe on behalf of the Wellcome Sanger Institute COVID-19 Surveillance Team
EPI_ISL_600057, EPI_ISL_600058, EPI_ISL_600059, EPI_ISL_600060, EPI_ISL_600061, EPI_ISL_600062, EPI_ISL_600063, EPI_ISL_600064, EPI_ISL_600065, EPI_ISL_600066, EPI_ISL_600067, EPI_ISL_600068, EPI_ISL_600069, EPI_ISL_600070, EPI_ISL_600071, EPI_ISL_600072, EPI_ISL_600073, EPI_ISL_600074, EPI_ISL_600075, EPI_ISL_600076, EPI_ISL_600077, EPI_ISL_600078, EPI_ISL_600079, EPI_ISL_600080, EPI_ISL_600081, EPI_ISL_600082, EPI_ISL_600083, EPI_ISL_600084, EPI_ISL_600085, EPI_ISL_600086, EPI_ISL_600087, EPI_ISL_600088, EPI_ISL_600089, EPI_ISL_600090, EPI_ISL_600091, EPI_ISL_600092,			

EPI_ISL_600093, EPI_ISL_600094, EPI_ISL_600095, EPI_ISL_600096, EPI_ISL_600097, EPI_ISL_600098, EPI_ISL_600099, EPI_ISL_600100, EPI_ISL_600101, EPI_ISL_600102, EPI_ISL_600103, EPI_ISL_600104, EPI_ISL_600105, EPI_ISL_600106, EPI_ISL_600107, EPI_ISL_600108, EPI_ISL_600109, EPI_ISL_600110, EPI_ISL_600111, EPI_ISL_600112, EPI_ISL_600113, EPI_ISL_600114, EPI_ISL_600115, EPI_ISL_600116, EPI_ISL_600117, EPI_ISL_600118, EPI_ISL_600119, EPI_ISL_600120, EPI_ISL_600121, EPI_ISL_600122, EPI_ISL_600123, EPI_ISL_600124, EPI_ISL_600125, EPI_ISL_600126, EPI_ISL_600127			
see above	Lighthouse Lab in Milton Keynes	Wellcome Sanger Institute for the COVID-19 Genomics UK (COG-UK) consortium	The Lighthouse Lab in Milton Keynes and Alex Alderton, Roberto Amato, Sonia Goncalves, Ewan Harrison, David K. Jackson, Ian Johnston, Dominic Kwiatkowski, Cordelia Langford, John Sillitoe on behalf of the Wellcome Sanger Institute COVID-19 Surveillance Team (http://www.sanger.ac.uk/covid-team)
EPI_ISL_600128, EPI_ISL_600130, EPI_ISL_600133, EPI_ISL_600134, EPI_ISL_600135, EPI_ISL_600136	Lighthouse Lab in Glasgow	Wellcome Sanger Institute for the COVID-19 Genomics UK (COG-UK) consortium	Harper VanSteenhouse, Yumi Kasai, David Gray, Carol Clugston, Anna Dominiczak and Alex Alderton, Roberto Amato, Sonia Goncalves, Ewan Harrison, David K. Jackson, Ian Johnston, Dominic Kwiatkowski, Cordelia Langford, John Sillitoe on behalf of the Wellcome Sanger Institute COVID-19 Surveillance Team (http://www.sanger.ac.uk/covid-team)
EPI_ISL_600139	Lighthouse Lab in Glasgow	Wellcome Sanger Institute for the COVID-19 Genomics UK (COG-UK) Consortium	Harper VanSteenhouse, Yumi Kasai, David Gray, Carol Clugston, Anna Dominiczak and Alex Alderton, Roberto Amato, Sonia Goncalves, Ewan Harrison, David K. Jackson, Ian Johnston, Dominic Kwiatkowski, Cordelia Langford, John Sillitoe on behalf of the Wellcome Sanger Institute COVID-19 Surveillance Team
EPI_ISL_600144, EPI_ISL_600147, EPI_ISL_600148, EPI_ISL_600150, EPI_ISL_600151, EPI_ISL_600152, EPI_ISL_600157, EPI_ISL_600158, EPI_ISL_600159, EPI_ISL_600160, EPI_ISL_600161, EPI_ISL_600163, EPI_ISL_600166, EPI_ISL_600167, EPI_ISL_600172, EPI_ISL_600177, EPI_ISL_600179, EPI_ISL_600180, EPI_ISL_600182, EPI_ISL_600185, EPI_ISL_600192, EPI_ISL_600193, EPI_ISL_600194, EPI_ISL_600196, EPI_ISL_600197, EPI_ISL_600200, EPI_ISL_600201, EPI_ISL_600204, EPI_ISL_600205, EPI_ISL_600209, EPI_ISL_600210, EPI_ISL_600212, EPI_ISL_600215, EPI_ISL_600217, EPI_ISL_600223, EPI_ISL_600224, EPI_ISL_600225, EPI_ISL_600231, EPI_ISL_600232, EPI_ISL_600233, EPI_ISL_600238, EPI_ISL_600239, EPI_ISL_600240, EPI_ISL_600241, EPI_ISL_600243, EPI_ISL_600249, EPI_ISL_600250, EPI_ISL_600252, EPI_ISL_600254, EPI_ISL_600257, EPI_ISL_600258, EPI_ISL_600262, EPI_ISL_600263, EPI_ISL_600266, EPI_ISL_600267, EPI_ISL_600269, EPI_ISL_600271, EPI_ISL_600272, EPI_ISL_600273, EPI_ISL_600279, EPI_ISL_600281, EPI_ISL_600283, EPI_ISL_600285, EPI_ISL_600286, EPI_ISL_600287, EPI_ISL_600290, EPI_ISL_600292, EPI_ISL_600293, EPI_ISL_600298, EPI_ISL_600299, EPI_ISL_600303, EPI_ISL_600304, EPI_ISL_600306, EPI_ISL_600309, EPI_ISL_600311, EPI_ISL_600313, EPI_ISL_600314, EPI_ISL_600315, EPI_ISL_600317, EPI_ISL_600325, EPI_ISL_600332, EPI_ISL_600334, EPI_ISL_600338, EPI_ISL_600342, EPI_ISL_600344, EPI_ISL_600345, EPI_ISL_600352, EPI_ISL_600355, EPI_ISL_600356, EPI_ISL_600357, EPI_ISL_600359, EPI_ISL_600361, EPI_ISL_600366, EPI_ISL_600369, EPI_ISL_600375, EPI_ISL_600378, EPI_ISL_600380, EPI_ISL_600381, EPI_ISL_600389, EPI_ISL_600391, EPI_ISL_600393, EPI_ISL_600396, EPI_ISL_600402, EPI_ISL_600404, EPI_ISL_600406, EPI_ISL_600407, EPI_ISL_600413, EPI_ISL_600414, EPI_ISL_600415, EPI_ISL_600416, EPI_ISL_600417, EPI_ISL_600419, EPI_ISL_600421, EPI_ISL_600422, EPI_ISL_600424, EPI_ISL_600427, EPI_ISL_600447, EPI_ISL_600448, EPI_ISL_600452, EPI_ISL_600455, EPI_ISL_600472, EPI_ISL_600475, EPI_ISL_600477, EPI_ISL_600479, EPI_ISL_600485, EPI_ISL_600496, EPI_ISL_600501, EPI_ISL_600503, EPI_ISL_600504, EPI_ISL_600509, EPI_ISL_600520			
see above	Lighthouse Lab in Glasgow	Wellcome Sanger Institute for the COVID-19 Genomics UK (COG-UK) consortium	Harper VanSteenhouse, Yumi Kasai, David Gray, Carol Clugston, Anna Dominiczak and Alex Alderton, Roberto Amato, Sonia Goncalves, Ewan Harrison, David K. Jackson, Ian Johnston, Dominic Kwiatkowski, Cordelia Langford, John Sillitoe on behalf of the Wellcome Sanger Institute COVID-19 Surveillance Team (http://www.sanger.ac.uk/covid-team)
EPI_ISL_600521, EPI_ISL_600523, EPI_ISL_600525, EPI_ISL_600528, EPI_ISL_600529, EPI_ISL_600532, EPI_ISL_600536, EPI_ISL_600537	Lighthouse Lab in Alderley Park	Wellcome Sanger Institute for the COVID-19 Genomics UK (COG-UK) consortium	Jacquelyn Wynn, Mairead Hyland, The Lighthouse Lab in Alderley Park and Alex Alderton, Roberto Amato, Sonia Goncalves, Ewan Harrison, David K. Jackson, Ian Johnston, Dominic Kwiatkowski, Cordelia Langford, John Sillitoe on behalf of the Wellcome Sanger Institute COVID-19 Surveillance Team (http://www.sanger.ac.uk/covid-team)
EPI_ISL_600539, EPI_ISL_600540, EPI_ISL_600542, EPI_ISL_600544, EPI_ISL_600545, EPI_ISL_600547, EPI_ISL_600548, EPI_ISL_600550, EPI_ISL_600551, EPI_ISL_600553, EPI_ISL_600555, EPI_ISL_600556, EPI_ISL_600558, EPI_ISL_600560, EPI_ISL_600561, EPI_ISL_600563, EPI_ISL_600565, EPI_ISL_600566, EPI_ISL_600568, EPI_ISL_600569, EPI_ISL_600571, EPI_ISL_600572, EPI_ISL_600573, EPI_ISL_600574, EPI_ISL_600575, EPI_ISL_600576, EPI_ISL_600577, EPI_ISL_600578, EPI_ISL_600579, EPI_ISL_600580, EPI_ISL_600581, EPI_ISL_600582, EPI_ISL_600583, EPI_ISL_600584, EPI_ISL_600585, EPI_ISL_600586, EPI_ISL_600587, EPI_ISL_600588, EPI_ISL_600589, EPI_ISL_600590, EPI_ISL_600591, EPI_ISL_600592, EPI_ISL_600593, EPI_ISL_600594, EPI_ISL_600595, EPI_ISL_600596, EPI_ISL_600597, EPI_ISL_600598, EPI_ISL_600599, EPI_ISL_600600, EPI_ISL_600601, EPI_ISL_600602, EPI_ISL_600603, EPI_ISL_600604, EPI_ISL_600605, EPI_ISL_600606, EPI_ISL_600607, EPI_ISL_600608, EPI_ISL_600609, EPI_ISL_600610, EPI_ISL_600611, EPI_ISL_600612, EPI_ISL_600613, EPI_ISL_600614, EPI_ISL_600615, EPI_ISL_600616, EPI_ISL_600617, EPI_ISL_600618, EPI_ISL_600619, EPI_ISL_600620, EPI_ISL_600621, EPI_ISL_600622, EPI_ISL_600623, EPI_ISL_600624, EPI_ISL_600625, EPI_ISL_600626, EPI_ISL_600627, EPI_ISL_600628, EPI_ISL_600629, EPI_ISL_600630, EPI_ISL_600631, EPI_ISL_600632, EPI_ISL_600633, EPI_ISL_600634, EPI_ISL_600635, EPI_ISL_600636, EPI_ISL_600637, EPI_ISL_600638, EPI_ISL_600639, EPI_ISL_600640, EPI_ISL_600641, EPI_ISL_600642			
see above	Lighthouse Lab in Glasgow	Wellcome Sanger Institute for the COVID-19 Genomics UK (COG-UK) consortium	Harper VanSteenhouse, Yumi Kasai, David Gray, Carol Clugston, Anna Dominiczak and Alex Alderton, Roberto Amato, Sonia Goncalves, Ewan Harrison, David K. Jackson, Ian Johnston, Dominic Kwiatkowski, Cordelia Langford, John Sillitoe on behalf of the Wellcome Sanger Institute COVID-19 Surveillance Team (http://www.sanger.ac.uk/covid-team)
EPI_ISL_600643	Lighthouse Lab in Glasgow	Wellcome Sanger Institute for the COVID-19 Genomics UK (COG-UK) Consortium	Harper VanSteenhouse, Yumi Kasai, David Gray, Carol Clugston, Anna Dominiczak and Alex Alderton, Roberto Amato, Sonia Goncalves, Ewan Harrison, David K. Jackson, Ian Johnston, Dominic Kwiatkowski, Cordelia Langford, John Sillitoe on behalf of the Wellcome Sanger Institute COVID-19 Surveillance Team
EPI_ISL_600644, EPI_ISL_600645, EPI_ISL_600646, EPI_ISL_600647, EPI_ISL_600648, EPI_ISL_600649, EPI_ISL_600650, EPI_ISL_600651, EPI_ISL_600652, EPI_ISL_600653, EPI_ISL_600654, EPI_ISL_600655, EPI_ISL_600656, EPI_ISL_600657, EPI_ISL_600658, EPI_ISL_600659, EPI_ISL_600660, EPI_ISL_600661, EPI_ISL_600662, EPI_ISL_600663, EPI_ISL_600664, EPI_ISL_600665, EPI_ISL_600666, EPI_ISL_600667, EPI_ISL_600668, EPI_ISL_600669, EPI_ISL_600670, EPI_ISL_600671, EPI_ISL_600672, EPI_ISL_600673, EPI_ISL_600674, EPI_ISL_600675, EPI_ISL_600676, EPI_ISL_600677, EPI_ISL_600678, EPI_ISL_600679, EPI_ISL_600680, EPI_ISL_600681, EPI_ISL_600682, EPI_ISL_600683, EPI_ISL_600684, EPI_ISL_600685, EPI_ISL_600686, EPI_ISL_600687, EPI_ISL_600688, EPI_ISL_600689, EPI_ISL_600690, EPI_ISL_600691, EPI_ISL_600692, EPI_ISL_600693, EPI_ISL_600694, EPI_ISL_600695, EPI_ISL_600696, EPI_ISL_600697, EPI_ISL_600698, EPI_ISL_600699, EPI_ISL_600700, EPI_ISL_600701, EPI_ISL_600702, EPI_ISL_600703, EPI_ISL_600704, EPI_ISL_600705, EPI_ISL_600706, EPI_ISL_600707, EPI_ISL_600708, EPI_ISL_600709, EPI_ISL_600710, EPI_ISL_600711, EPI_ISL_600712, EPI_ISL_600713, EPI_ISL_600714, EPI_ISL_600715, EPI_ISL_600716, EPI_ISL_600717, EPI_ISL_600718, EPI_ISL_600719, EPI_ISL_600720, EPI_ISL_600721, EPI_ISL_600722, EPI_ISL_600723, EPI_ISL_600724, EPI_ISL_600725, EPI_ISL_600726, EPI_ISL_600727, EPI_ISL_600728, EPI_ISL_600729, EPI_ISL_600730, EPI_ISL_600731, EPI_ISL_600732, EPI_ISL_600733, EPI_ISL_600734, EPI_ISL_600735, EPI_ISL_600736, EPI_ISL_600737, EPI_ISL_600738, EPI_ISL_600739, EPI_ISL_600740, EPI_ISL_600741, EPI_ISL_600742, EPI_ISL_600743, EPI_ISL_600744, EPI_ISL_600745, EPI_ISL_600746, EPI_ISL_600747, EPI_ISL_600748, EPI_ISL_600749, EPI_ISL_600750, EPI_ISL_600751, EPI_ISL_600752, EPI_ISL_600753, EPI_ISL_600754, EPI_ISL_600755, EPI_ISL_600756, EPI_ISL_600757, EPI_ISL_600758, EPI_ISL_600759, EPI_ISL_600761, EPI_ISL_600766, EPI_ISL_600768, EPI_ISL_600770, EPI_ISL_600773, EPI_ISL_600778, EPI_ISL_600780, EPI_ISL_600782, EPI_ISL_600783, EPI_ISL_600784, EPI_ISL_600785, EPI_ISL_600787, EPI_ISL_600788, EPI_ISL_600789, EPI_ISL_600790, EPI_ISL_600791, EPI_ISL_600792, EPI_ISL_600793, EPI_ISL_600794, EPI_ISL_600796, EPI_ISL_600797, EPI_ISL_600798, EPI_ISL_600803, EPI_ISL_600805, EPI_ISL_600807, EPI_ISL_600808, EPI_ISL_600809, EPI_ISL_600810, EPI_ISL_600812, EPI_ISL_600817, EPI_ISL_600818, EPI_ISL_600819, EPI_ISL_600820, EPI_ISL_600821, EPI_ISL_600823			
see above	Lighthouse Lab in Glasgow	Wellcome Sanger Institute for the COVID-19 Genomics UK (COG-UK) consortium	Harper VanSteenhouse, Yumi Kasai, David Gray, Carol Clugston, Anna Dominiczak and Alex Alderton, Roberto Amato, Sonia Goncalves, Ewan Harrison, David K. Jackson, Ian Johnston, Dominic Kwiatkowski, Cordelia Langford, John Sillitoe on behalf of the Wellcome Sanger Institute COVID-19 Surveillance Team (http://www.sanger.ac.uk/covid-team)
EPI_ISL_600824	Lighthouse Lab in Glasgow	Wellcome Sanger Institute for the COVID-19 Genomics UK (COG-UK) Consortium	Harper VanSteenhouse, Yumi Kasai, David Gray, Carol Clugston, Anna Dominiczak and Alex Alderton, Roberto Amato, Sonia Goncalves, Ewan Harrison, David K. Jackson, Ian Johnston, Dominic Kwiatkowski, Cordelia Langford, John Sillitoe on behalf of the Wellcome Sanger Institute COVID-19 Surveillance Team
EPI_ISL_600825, EPI_ISL_600826, EPI_ISL_600829, EPI_ISL_600830, EPI_ISL_600831, EPI_ISL_600832, EPI_ISL_600833, EPI_ISL_600834, EPI_ISL_600835, EPI_ISL_600836, EPI_ISL_600837, EPI_ISL_600838, EPI_ISL_600839, EPI_ISL_600840, EPI_ISL_600841, EPI_ISL_600842, EPI_ISL_600843, EPI_ISL_600844, EPI_ISL_600845, EPI_ISL_600846, EPI_ISL_600847, EPI_ISL_600848, EPI_ISL_600849, EPI_ISL_600850, EPI_ISL_600851, EPI_ISL_600852, EPI_ISL_600853, EPI_ISL_600854, EPI_ISL_600855, EPI_ISL_600856, EPI_ISL_600857, EPI_ISL_600858, EPI_ISL_600859, EPI_ISL_600860, EPI_ISL_600861, EPI_ISL_600862, EPI_ISL_600863, EPI_ISL_600864, EPI_ISL_600865, EPI_ISL_600866, EPI_ISL_600867, EPI_ISL_600868, EPI_ISL_600869, EPI_ISL_600870, EPI_ISL_600871, EPI_ISL_600872, EPI_ISL_600873, EPI_ISL_600874, EPI_ISL_600875, EPI_ISL_600876, EPI_ISL_600877, EPI_ISL_600878, EPI_ISL_600879, EPI_ISL_600880, EPI_ISL_600881, EPI_ISL_600882, EPI_ISL_600883, EPI_ISL_600884, EPI_ISL_600885, EPI_ISL_600886, EPI_ISL_600887, EPI_ISL_600888, EPI_ISL_600889, EPI_ISL_600890, EPI_ISL_600891, EPI_ISL_600893, EPI_ISL_600894, EPI_ISL_600895, EPI_ISL_600896, EPI_ISL_600897, EPI_ISL_600898, EPI_ISL_600899, EPI_ISL_600900, EPI_ISL_600902, EPI_ISL_600903			
see above	Lighthouse Lab in Glasgow	Wellcome Sanger Institute for the COVID-19 Genomics UK (COG-UK) consortium	Harper VanSteenhouse, Yumi Kasai, David Gray, Carol Clugston, Anna Dominiczak and Alex Alderton, Roberto Amato, Sonia Goncalves, Ewan Harrison, David K. Jackson, Ian Johnston, Dominic Kwiatkowski, Cordelia Langford, John Sillitoe on behalf of the Wellcome Sanger Institute COVID-19 Surveillance Team (http://www.sanger.ac.uk/covid-team)
EPI_ISL_600904	Lighthouse Lab in Glasgow	Wellcome Sanger Institute for the COVID-19 Genomics UK (COG-UK) Consortium	Harper VanSteenhouse, Yumi Kasai, David Gray, Carol Clugston, Anna Dominiczak and Alex Alderton, Roberto Amato, Sonia Goncalves, Ewan Harrison, David K. Jackson, Ian Johnston, Dominic Kwiatkowski, Cordelia Langford, John Sillitoe on behalf of the Wellcome Sanger Institute COVID-19 Surveillance Team
EPI_ISL_600905, EPI_ISL_600906, EPI_ISL_600907, EPI_ISL_600908, EPI_ISL_600909, EPI_ISL_600910, EPI_ISL_600911, EPI_ISL_600914, EPI_ISL_600916, EPI_ISL_600917, EPI_ISL_600918, EPI_ISL_600919, EPI_ISL_600920, EPI_ISL_600921, EPI_ISL_600922, EPI_ISL_600924			
see above	Lighthouse Lab in Glasgow	Wellcome Sanger Institute for the COVID-19 Genomics UK (COG-UK) consortium	Harper VanSteenhouse, Yumi Kasai, David Gray, Carol Clugston, Anna Dominiczak and Alex Alderton, Roberto Amato, Sonia Goncalves, Ewan Harrison, David K. Jackson, Ian Johnston, Dominic Kwiatkowski, Cordelia Langford, John Sillitoe on behalf of the Wellcome Sanger Institute COVID-19 Surveillance Team (http://www.sanger.ac.uk/covid-team)
EPI_ISL_600925, EPI_ISL_600928, EPI_ISL_600929	Lighthouse Lab in Milton Keynes	Wellcome Sanger Institute for the COVID-19 Genomics UK (COG-UK) consortium	The Lighthouse Lab in Milton Keynes and Alex Alderton, Roberto Amato, Sonia Goncalves, Ewan Harrison, David K. Jackson, Ian Johnston, Dominic Kwiatkowski, Cordelia Langford, John Sillitoe on behalf of the Wellcome Sanger Institute COVID-19 Surveillance Team (http://www.sanger.ac.uk/covid-team)
EPI_ISL_602258, EPI_ISL_602259, EPI_ISL_602260, EPI_ISL_602261, EPI_ISL_602262, EPI_ISL_602263	MD PHL	MD PHL	Maryland Department of Health Laboratories Administration
EPI_ISL_602304	Istituto Zooprofilattico Sperimentale del Mezzogiorno	U.O. Diagnostica Virologica Dip. Sanità Animale IZSM	Maurizio Viscardi, Lorena Cardillo , e Giovanna Fusco
EPI_ISL_602379, EPI_ISL_602390, EPI_ISL_602394, EPI_ISL_602395, EPI_ISL_602396, EPI_ISL_602405, EPI_ISL_602407, EPI_ISL_602408, EPI_ISL_602419, EPI_ISL_602421, EPI_ISL_602422, EPI_ISL_602431, EPI_ISL_602432, EPI_ISL_602435, EPI_ISL_602436, EPI_ISL_602437, EPI_ISL_602448, EPI_ISL_602454, EPI_ISL_602455, EPI_ISL_602458			
see above	HELIX LLC	WHO National Influenza Centre Russian Federation	Andrey Komissarov, Artem Fadeev, Kseniya Komissarova, Anna Ivanova, Dmitry Bazhenov, Daria Danilenko

EPI_ISL_602830, EPI_ISL_602831, EPI_ISL_602832, EPI_ISL_602833, EPI_ISL_602834, EPI_ISL_602835, EPI_ISL_602836, EPI_ISL_602837, EPI_ISL_602838, EPI_ISL_602839, EPI_ISL_602840, EPI_ISL_602841, EPI_ISL_602842, EPI_ISL_602843, EPI_ISL_602844, EPI_ISL_602852, EPI_ISL_602853, EPI_ISL_602854, EPI_ISL_602855, EPI_ISL_602856, EPI_ISL_602857, EPI_ISL_602858, EPI_ISL_602859, EPI_ISL_602860, EPI_ISL_602861, EPI_ISL_602862, EPI_ISL_602863, EPI_ISL_602864, EPI_ISL_602866, EPI_ISL_602912, EPI_ISL_602913, EPI_ISL_602914, EPI_ISL_602915, EPI_ISL_602916, EPI_ISL_602917, EPI_ISL_602918, EPI_ISL_602919, EPI_ISL_602920, EPI_ISL_602921, EPI_ISL_602922, EPI_ISL_602923, EPI_ISL_602924, EPI_ISL_602925, EPI_ISL_602926, EPI_ISL_602927, EPI_ISL_602928, EPI_ISL_602929, EPI_ISL_602930			
see above	NHLS-IALCH	KRISP, KZN Research Innovation and Sequencing Platform	Giandhari J, Pillay S, Lessells R, Mdlalose K, York D, Khan S, Tegally H, Wilkinson E, de Oliveira T
EPI_ISL_602977, EPI_ISL_602978, EPI_ISL_602979, EPI_ISL_602980, EPI_ISL_602981, EPI_ISL_602982, EPI_ISL_602983, EPI_ISL_602984	Minnesota Department of Health, Public Health Laboratory	Minnesota Department of Health, Public Health Laboratory	Matt Plumb, Jacob Garfin, Alexandra Lorentz, and Xiong Wang
EPI_ISL_603002, EPI_ISL_603003	Sanford South University Medical Center	Minnesota Department of Health, Public Health Laboratory	Matt Plumb, Jacob Garfin, Alexandra Lorentz, and Xiong Wang
EPI_ISL_603004	Essentia Health-St. Mary's Medical Center	Minnesota Department of Health, Public Health Laboratory	Matt Plumb, Jacob Garfin, Alexandra Lorentz, and Xiong Wang
EPI_ISL_603011	Mayo Clinic & Mayo Clinic Laboratories	Minnesota Department of Health, Public Health Laboratory	Matt Plumb, Jacob Garfin, Alexandra Lorentz, and Xiong Wang
EPI_ISL_603218, EPI_ISL_603219, EPI_ISL_603220	CHU Purpan - Laboratoire de Virologie - Institut Fédératif de Biologie	CHU Purpan - Laboratoire de Virologie - Institut Fédératif de Biologie	Latour J., Ranger N., Dubois M., Carcenac R., Harter A., Boyer P., Tremeaux P., Izopet J.
EPI_ISL_603252, EPI_ISL_603254, EPI_ISL_603255	UW Virology Lab	UW Virology Lab	Pavitra Roychoudhury, Hong Xie, Lasata Shrestha, Meei-Li Huang, Keith R Jerome, Alexander Greninger
EPI_ISL_603267, EPI_ISL_603268, EPI_ISL_603269, EPI_ISL_603270, EPI_ISL_603271, EPI_ISL_603272, EPI_ISL_603273, EPI_ISL_603274, EPI_ISL_603275, EPI_ISL_603276, EPI_ISL_603277, EPI_ISL_603278, EPI_ISL_603279, EPI_ISL_603280, EPI_ISL_603281, EPI_ISL_603282, EPI_ISL_603283, EPI_ISL_603284, EPI_ISL_603285, EPI_ISL_603286, EPI_ISL_603287, EPI_ISL_603288, EPI_ISL_603289, EPI_ISL_603290, EPI_ISL_603291, EPI_ISL_603292, EPI_ISL_603293, EPI_ISL_603294, EPI_ISL_603295, EPI_ISL_603296, EPI_ISL_603297, EPI_ISL_603298, EPI_ISL_603299, EPI_ISL_603300, EPI_ISL_603301, EPI_ISL_603302, EPI_ISL_603303, EPI_ISL_603304, EPI_ISL_603305, EPI_ISL_603306, EPI_ISL_603307, EPI_ISL_603308, EPI_ISL_603309, EPI_ISL_603310, EPI_ISL_603311, EPI_ISL_603312, EPI_ISL_603313, EPI_ISL_603314, EPI_ISL_603315, EPI_ISL_603316, EPI_ISL_603317, EPI_ISL_603318, EPI_ISL_603319, EPI_ISL_603320, EPI_ISL_603321, EPI_ISL_603322, EPI_ISL_603323, EPI_ISL_603324, EPI_ISL_603325, EPI_ISL_603326, EPI_ISL_603327, EPI_ISL_603328, EPI_ISL_603329, EPI_ISL_603330, EPI_ISL_603331, EPI_ISL_603332, EPI_ISL_603333, EPI_ISL_603334, EPI_ISL_603335, EPI_ISL_603336, EPI_ISL_603337, EPI_ISL_603338, EPI_ISL_603339, EPI_ISL_603340, EPI_ISL_603341, EPI_ISL_603342, EPI_ISL_603343, EPI_ISL_603344, EPI_ISL_603345, EPI_ISL_603346, EPI_ISL_603347, EPI_ISL_603348, EPI_ISL_603349, EPI_ISL_603350, EPI_ISL_603351, EPI_ISL_603352, EPI_ISL_603353, EPI_ISL_603354, EPI_ISL_603355, EPI_ISL_603356, EPI_ISL_603357, EPI_ISL_603358, EPI_ISL_603359, EPI_ISL_603360, EPI_ISL_603361, EPI_ISL_603362, EPI_ISL_603363, EPI_ISL_603364, EPI_ISL_603365, EPI_ISL_603366, EPI_ISL_603367, EPI_ISL_603368, EPI_ISL_603369, EPI_ISL_603370, EPI_ISL_603371, EPI_ISL_603372, EPI_ISL_603373, EPI_ISL_603374, EPI_ISL_603375, EPI_ISL_603376, EPI_ISL_603377, EPI_ISL_603378, EPI_ISL_603379, EPI_ISL_603380, EPI_ISL_603381, EPI_ISL_603382, EPI_ISL_603383, EPI_ISL_603384, EPI_ISL_603385, EPI_ISL_603386, EPI_ISL_603387, EPI_ISL_603388, EPI_ISL_603389, EPI_ISL_603390, EPI_ISL_603391, EPI_ISL_603392, EPI_ISL_603393, EPI_ISL_603394, EPI_ISL_603395, EPI_ISL_603396, EPI_ISL_603397, EPI_ISL_603398, EPI_ISL_603399, EPI_ISL_603400, EPI_ISL_603401, EPI_ISL_603402, EPI_ISL_603403, EPI_ISL_603404, EPI_ISL_603405, EPI_ISL_603406, EPI_ISL_603407, EPI_ISL_603408, EPI_ISL_603409, EPI_ISL_603410, EPI_ISL_603411, EPI_ISL_603412, EPI_ISL_603413, EPI_ISL_603414, EPI_ISL_603415, EPI_ISL_603416, EPI_ISL_603417, EPI_ISL_603418, EPI_ISL_603419, EPI_ISL_603420, EPI_ISL_603421, EPI_ISL_603422			
see above	Viollier AG	Department of Biosystems Science and Engineering, ETH Zürich	Christian Beisel, Sarah Nadeau, Ivan Topolsky, Pedro Ferreira, Philipp Jablonski, Susana Posada-Céspedes, Tobias Schär, Ina Nissen, Natascha Santacroce, Elodie Burcklen, Christiane Beckmann, Maurice Redondo, Olivier Kobel, Christoph Noppen, Sophie Seidel, Noemie Santamaria de Souza, Niko Beerenwinkel, Tanja Stadler
EPI_ISL_605483, EPI_ISL_605484, EPI_ISL_605495, EPI_ISL_605496, EPI_ISL_605498, EPI_ISL_605499, EPI_ISL_605500, EPI_ISL_605501, EPI_ISL_605763, EPI_ISL_605767	University of Wisconsin-Madison AIDS Vaccine Research Laboratories	University of Wisconsin-Madison AIDS Vaccine Research Laboratories	Gage Moreno, Katarina Braun, et al. AIDS Vaccine Research Laboratories
EPI_ISL_605786, EPI_ISL_605789, EPI_ISL_605790	NHLS-IALCH	KRISP, KZN Research Innovation and Sequencing Platform	Giandhari J, Pillay S, Lessells R, Mdlalose K, York D, Khan S, Tegally H, Wilkinson E, de Oliveira T
EPI_ISL_609539	Lighthouse Lab in Cambridge	Wellcome Sanger Institute for the COVID-19 Genomics UK (COG-UK) consortium	Rob Howes, The Lighthouse Lab in Cambridge and Alex Alderton, Roberto Amato, Sonia Goncalves, Ewan Harrison, David K. Jackson, Ian Johnston, Dominic Kwiatkowski, Cordelia Langford, John Sillitoe on behalf of the Wellcome Sanger Institute COVID-19 Surveillance Team
EPI_ISL_609827, EPI_ISL_609828, EPI_ISL_609829, EPI_ISL_609839, EPI_ISL_609857	Respiratory Virus Unit, Microbiology Services Colindale, Public Health England	Respiratory Virus Unit, Microbiology Services Colindale, Public Health England	PHE Covid Sequencing Team
EPI_ISL_610153	Singapore General Hospital	Department of Microbiology	Nurdyana Abdul Rahman, Kun Lee Lim, Chenhao Li, Sui Sin Goh, Kenneth Xin Long Chan, Kian Sing Chan, Lynette Oon, Kern Rei Chng, Niranjan Nagarajan, Karrie Ko
EPI_ISL_610196, EPI_ISL_610197, EPI_ISL_610198, EPI_ISL_610204	Department of Health Technology and Informatics, The Hong Kong Polytechnic University	Department of Health Technology and Informatics, The Hong Kong Polytechnic University	Siu,G.K.-H., Lee,L.-K., Leung,K.S.-S., Leung,J.S.-L., Ng,T.T.-L., Chan,C.T.-M., Tam,K.K.-G., Lao,H.-Y., Wu,A.K.-L., Yau,M.C.-Y., Lai,Y.W.-M., Fung,K.S.-C., Chau,S.K.-Y., Wong,B.K.-C., To,W.-K., Luk,K., Ho,A.Y.-M., Que,T.-L., Yip,K.-T., Yam,W.C., Shum,D.H.-K., Yip,S.P.
EPI_ISL_611517	Liverpool Clinical Laboratories	COVID-19 Genomics UK (COG-UK) Consortium	Sam Haldenby, Anita Lucaci, Steve Paterson, Julian Hiscox, Alistair Darby, M Almsaud, A Alrezaihi, Muhannad Alruwaili, Stuart D Armstrong, Jones Benjamin, Eleanor G Bentley, Anu Chawla, Jordan J Clark, Angela Cowell, Richard Eccles, Isabel García-Dorival, Matthew Gemmell, Alessandro Gerada, PKF Gilmore, Richard Gregory, Ximeng Han, Catherine Hartley, Margaret Hughes, Miren Iturriza-Gomara, James Johnson, L Luu, Jenifer Manson, Charlotte Nelson, Elaine O'Toole, Cassie Olateju, Rebekah Penrice-Randal , Lucille Rainbow, N.P Randle, Trevor Ian Robinson, Parul Sharma, Ghada T Shawli, James P Stewart, Neil Swainston, Ecaterina Vamos, Joanne Watts, Mark Whitehead
EPI_ISL_611533, EPI_ISL_611547, EPI_ISL_611548	West of Scotland Specialist Virology Centre, NHSGGC / MRC-University of Glasgow Centre for Virus Research	COVID-19 Genomics UK (COG-UK) Consortium	Ana da Silva Filipe, Natasha Johnson, Kathy Smollett, Daniel Mair, Stephen Carmichael, Lily Tong, Jenna Nichols, Elihu Aranday-Cortes, Kyriaki Nomikou; Sarah McDonald, Marc Niebel, Patawee Asamaphan; Richard Orton, Joseph Hughes, Sreenu Vattipally, David L Robertson; Alasdair MacLean, Rory Gunson; Kathy Li, Igor Starinskij, Natasha Jesudason, Rajiv Shah, James Shepherd, Antonia Ho, Emma Thomson
EPI_ISL_611572	Northumbria University / South Tees Hospitals NHS Foundation Trust / North Cumbria Integrated Care NHS Foundation Trust / North Tees and Hartlepool NHS Foundation Trust / Newcastle Hospitals NHS Foundation Trust	COVID-19 Genomics UK (COG-UK) Consortium	Darren L Smith,Andrew Nelson,Matthew Bashton,Greg R Young,Joshua Loh,John Allan,Mohammad A Tariq,Giles S Holt,Gary Black,Wen C Yew,Lynn Dover,Paul Baker,Steve Liggett,Sarah Essex,Jane Greenaway,Debra Padgett,Clive Graham,Garren Scott,Edward Barton,Emma Swindells,Brendan Payne,Jennifer Collins,Yusri Taha,Gary Eltringham
EPI_ISL_611603	Liverpool Clinical Laboratories	COVID-19 Genomics UK (COG-UK) Consortium	Sam Haldenby, Anita Lucaci, Steve Paterson, Julian Hiscox, Alistair Darby, M Almsaud, A Alrezaihi, Muhannad Alruwaili, Stuart D Armstrong, Jones Benjamin, Eleanor G Bentley, Anu Chawla, Jordan J Clark, Angela Cowell, Richard Eccles, Isabel García-Dorival, Matthew Gemmell, Alessandro Gerada, PKF Gilmore, Richard Gregory, Ximeng Han, Catherine Hartley, Margaret Hughes, Miren Iturriza-Gomara, James Johnson, L Luu, Jenifer Manson, Charlotte Nelson, Elaine O'Toole, Cassie Olateju, Rebekah Penrice-Randal , Lucille Rainbow, N.P Randle, Trevor Ian Robinson, Parul Sharma, Ghada T Shawli, James P Stewart, Neil Swainston, Ecaterina Vamos, Joanne Watts, Mark Whitehead
EPI_ISL_611610, EPI_ISL_611611, EPI_ISL_611614	West of Scotland Specialist Virology Centre, NHSGGC / MRC-University of Glasgow Centre for Virus Research	COVID-19 Genomics UK (COG-UK) Consortium	Ana da Silva Filipe, Natasha Johnson, Kathy Smollett, Daniel Mair, Stephen Carmichael, Lily Tong, Jenna Nichols, Elihu Aranday-Cortes, Kyriaki Nomikou; Sarah McDonald, Marc Niebel, Patawee Asamaphan; Richard Orton, Joseph Hughes, Sreenu Vattipally, David L Robertson; Alasdair MacLean, Rory Gunson; Kathy Li, Igor Starinskij, Natasha Jesudason, Rajiv Shah, James Shepherd, Antonia Ho, Emma Thomson
EPI_ISL_611631	Wales Specialist Virology Centre Sequencing lab: Pathogen Genomics Unit	COVID-19 Genomics UK (COG-UK) Consortium	Catherine Moore, Johnathan Evans, Laura Gifford, Malorie Perry, Simon Cottrell, Angela Marchbank, Alec Birchley, Alexander Adams, Amy Gaskin, Bree Gatica-Wilcox, Jason Coombes, Joel Southgate, Lauren Gilbert, Lee Graham, Nicole Pacchiarini, Sara Kumziene-Summerhayes, Sarah Taylor, Sophie Jones, Sara Rey, Matthew Bull, Joanne Watkins, Sally Corden, Tom Connor
EPI_ISL_611632	Liverpool Clinical Laboratories	COVID-19 Genomics UK (COG-UK) Consortium	Sam Haldenby, Anita Lucaci, Steve Paterson, Julian Hiscox, Alistair Darby, M Almsaud, A Alrezaihi, Muhannad Alruwaili, Stuart D Armstrong, Jones Benjamin, Eleanor G Bentley, Anu Chawla, Jordan J Clark, Angela Cowell, Richard Eccles, Isabel García-Dorival, Matthew Gemmell, Alessandro Gerada, PKF Gilmore, Richard Gregory, Ximeng Han, Catherine Hartley, Margaret Hughes, Miren Iturriza-Gomara, James Johnson, L Luu, Jenifer Manson, Charlotte Nelson, Elaine O'Toole, Cassie Olateju, Rebekah Penrice-Randal , Lucille Rainbow, N.P Randle, Trevor Ian Robinson, Parul Sharma, Ghada T Shawli, James P Stewart, Neil Swainston, Ecaterina Vamos, Joanne Watts, Mark Whitehead
EPI_ISL_611636	Wales Specialist Virology Centre Sequencing lab: Pathogen Genomics Unit	COVID-19 Genomics UK (COG-UK) Consortium	Catherine Moore, Johnathan Evans, Laura Gifford, Malorie Perry, Simon Cottrell, Angela Marchbank, Alec Birchley, Alexander Adams, Amy Gaskin, Bree Gatica-Wilcox, Jason Coombes, Joel Southgate, Lauren Gilbert, Lee Graham, Nicole Pacchiarini, Sara Kumziene-Summerhayes, Sarah Taylor, Sophie Jones, Sara Rey, Matthew Bull, Joanne Watkins, Sally Corden, Tom Connor

EPI_ISL_611642	Liverpool Clinical Laboratories	COVID-19 Genomics UK (COG-UK) Consortium	Sam Haldenby, Anita Lucaci, Steve Paterson, Julian Hiscox, Alistair Darby, M Almsaud, A Alrezaihi, Muhannad Alruwaili, Stuart D Armstrong, Jones Benjamin, Eleanor G Bentley, Anu Chawla, Jordan J Clark, Angela Cowell, Richard Eccles, Isabel García-Dorival, Matthew Gemmell, Alessandro Gerada, PKF Gilmore, Richard Gregory, Ximeng Han, Catherine Hartley, Margaret Hughes, Miren Iturriza-Gomara, James Johnson, L Luu, Jenifer Manson, Charlotte Nelson, Elaine O'Toole, Cassie Olateju, Rebekah Penrice-Randal , Lucille Rainbow, N.P Randle, Trevor Ian Robinson, Parul Sharma, Ghada T Shawli, James P Stewart, Neil Swainston, Ecaterina Vamos, Joanne Watts, Mark Whitehead
EPI_ISL_611644	Northumbria University / South Tees Hospitals NHS Foundation Trust / North Cumbria Integrated Care NHS Foundation Trust / North Tees and Hartlepool NHS Foundation Trust / Newcastle Hospitals NHS Foundation Trust	COVID-19 Genomics UK (COG-UK) Consortium	Darren L Smith,Andrew Nelson,Matthew Bashton,Greg R Young,Joshua Loh,John Allan,Mohammad A Tariq,Giles S Holt,Gary Black,Wen C Yew,Lynn Dover,Paul Baker,Steve Liggett,Sarah Essex,Jane Greenaway,Debra Padgett,Clive Graham,Garren Scott,Edward Barton,Emma Swindells,Brendan Payne,Jennifer Collins,Yusri Taha,Gary Eltringham
EPI_ISL_611660	West of Scotland Specialist Virology Centre, NHSGGC / MRC-University of Glasgow Centre for Virus Research	COVID-19 Genomics UK (COG-UK) Consortium	Ana da Silva Filipe, Natasha Johnson, Kathy Smollett, Daniel Mair, Stephen Carmichael, Lily Tong, Jenna Nichols, Elihu Aranday-Cortes, Kyriaki Nomikou; Sarah McDonald, Marc Niebel, Patawee Asamaphan; Richard Orton, Joseph Hughes, Sreenu Vattipally, David L Robertson; Alasdair MacLean, Rory Gunson; Kathy Li, Igor Starinskij, Natasha Jesudason, Rajiv Shah, James Shepherd, Antonia Ho, Emma Thomson
EPI_ISL_611664	Liverpool Clinical Laboratories	COVID-19 Genomics UK (COG-UK) Consortium	Sam Haldenby, Anita Lucaci, Steve Paterson, Julian Hiscox, Alistair Darby, M Almsaud, A Alrezaihi, Muhannad Alruwaili, Stuart D Armstrong, Jones Benjamin, Eleanor G Bentley, Anu Chawla, Jordan J Clark, Angela Cowell, Richard Eccles, Isabel García-Dorival, Matthew Gemmell, Alessandro Gerada, PKF Gilmore, Richard Gregory, Ximeng Han, Catherine Hartley, Margaret Hughes, Miren Iturriza-Gomara, James Johnson, L Luu, Jenifer Manson, Charlotte Nelson, Elaine O'Toole, Cassie Olateju, Rebekah Penrice-Randal , Lucille Rainbow, N.P Randle, Trevor Ian Robinson, Parul Sharma, Ghada T Shawli, James P Stewart, Neil Swainston, Ecaterina Vamos, Joanne Watts, Mark Whitehead
EPI_ISL_611679	Virology Department, Sheffield Teaching Hospitals NHS Foundation Trust/Department of Infection, Immunity and Cardiovascular Disease, The Medical School, University of Sheffield	COVID-19 Genomics UK (COG-UK) Consortium	Thushan de Silva, Matthew Parker, Nikki Smith, Adri Angyal, Rebecca Brown, Luke Green, Rachel Tucker, Paul Parsons, Danielle Groves, Katie Johnson, Laura Carrilero, Alex Keeley, Dave Partridge, Matthew Wyles, Benjamin Lindsey, Mehmet Yavuz, Mohammad Raza, Cariad Evans
EPI_ISL_611695	Liverpool Clinical Laboratories	COVID-19 Genomics UK (COG-UK) Consortium	Sam Haldenby, Anita Lucaci, Steve Paterson, Julian Hiscox, Alistair Darby, M Almsaud, A Alrezaihi, Muhannad Alruwaili, Stuart D Armstrong, Jones Benjamin, Eleanor G Bentley, Anu Chawla, Jordan J Clark, Angela Cowell, Richard Eccles, Isabel García-Dorival, Matthew Gemmell, Alessandro Gerada, PKF Gilmore, Richard Gregory, Ximeng Han, Catherine Hartley, Margaret Hughes, Miren Iturriza-Gomara, James Johnson, L Luu, Jenifer Manson, Charlotte Nelson, Elaine O'Toole, Cassie Olateju, Rebekah Penrice-Randal , Lucille Rainbow, N.P Randle, Trevor Ian Robinson, Parul Sharma, Ghada T Shawli, James P Stewart, Neil Swainston, Ecaterina Vamos, Joanne Watts, Mark Whitehead
EPI_ISL_611709, EPI_ISL_611712, EPI_ISL_611713	West of Scotland Specialist Virology Centre, NHSGGC / MRC-University of Glasgow Centre for Virus Research	COVID-19 Genomics UK (COG-UK) Consortium	Ana da Silva Filipe, Natasha Johnson, Kathy Smollett, Daniel Mair, Stephen Carmichael, Lily Tong, Jenna Nichols, Elihu Aranday-Cortes, Kyriaki Nomikou; Sarah McDonald, Marc Niebel, Patawee Asamaphan; Richard Orton, Joseph Hughes, Sreenu Vattipally, David L Robertson; Alasdair MacLean, Rory Gunson; Kathy Li, Igor Starinskij, Natasha Jesudason, Rajiv Shah, James Shepherd, Antonia Ho, Emma Thomson
EPI_ISL_611721, EPI_ISL_611728, EPI_ISL_611730, EPI_ISL_611736, EPI_ISL_611743	Liverpool Clinical Laboratories	COVID-19 Genomics UK (COG-UK) Consortium	Sam Haldenby, Anita Lucaci, Steve Paterson, Julian Hiscox, Alistair Darby, M Almsaud, A Alrezaihi, Muhannad Alruwaili, Stuart D Armstrong, Jones Benjamin, Eleanor G Bentley, Anu Chawla, Jordan J Clark, Angela Cowell, Richard Eccles, Isabel García-Dorival, Matthew Gemmell, Alessandro Gerada, PKF Gilmore, Richard Gregory, Ximeng Han, Catherine Hartley, Margaret Hughes, Miren Iturriza-Gomara, James Johnson, L Luu, Jenifer Manson, Charlotte Nelson, Elaine O'Toole, Cassie Olateju, Rebekah Penrice-Randal , Lucille Rainbow, N.P Randle, Trevor Ian Robinson, Parul Sharma, Ghada T Shawli, James P Stewart, Neil Swainston, Ecaterina Vamos, Joanne Watts, Mark Whitehead
EPI_ISL_611746, EPI_ISL_611747	Northumbria University / South Tees Hospitals NHS Foundation Trust / North Cumbria Integrated Care NHS Foundation Trust / North Tees and Hartlepool NHS Foundation Trust / Newcastle Hospitals NHS Foundation Trust	COVID-19 Genomics UK (COG-UK) Consortium	Darren L Smith,Andrew Nelson,Matthew Bashton,Greg R Young,Joshua Loh,John Allan,Mohammad A Tariq,Giles S Holt,Gary Black,Wen C Yew,Lynn Dover,Paul Baker,Steve Liggett,Sarah Essex,Jane Greenaway,Debra Padgett,Clive Graham,Garren Scott,Edward Barton,Emma Swindells,Brendan Payne,Jennifer Collins,Yusri Taha,Gary Eltringham
EPI_ISL_611784, EPI_ISL_611785, EPI_ISL_611790, EPI_ISL_611815	Liverpool Clinical Laboratories	COVID-19 Genomics UK (COG-UK) Consortium	Sam Haldenby, Anita Lucaci, Steve Paterson, Julian Hiscox, Alistair Darby, M Almsaud, A Alrezaihi, Muhannad Alruwaili, Stuart D Armstrong, Jones Benjamin, Eleanor G Bentley, Anu Chawla, Jordan J Clark, Angela Cowell, Richard Eccles, Isabel García-Dorival, Matthew Gemmell, Alessandro Gerada, PKF Gilmore, Richard Gregory, Ximeng Han, Catherine Hartley, Margaret Hughes, Miren Iturriza-Gomara, James Johnson, L Luu, Jenifer Manson, Charlotte Nelson, Elaine O'Toole, Cassie Olateju, Rebekah Penrice-Randal , Lucille Rainbow, N.P Randle, Trevor Ian Robinson, Parul Sharma, Ghada T Shawli, James P Stewart, Neil Swainston, Ecaterina Vamos, Joanne Watts, Mark Whitehead
EPI_ISL_611818, EPI_ISL_611820, EPI_ISL_611826	West of Scotland Specialist Virology Centre, NHSGGC / MRC-University of Glasgow Centre for Virus Research	COVID-19 Genomics UK (COG-UK) Consortium	Ana da Silva Filipe, Natasha Johnson, Kathy Smollett, Daniel Mair, Stephen Carmichael, Lily Tong, Jenna Nichols, Elihu Aranday-Cortes, Kyriaki Nomikou; Sarah McDonald, Marc Niebel, Patawee Asamaphan; Richard Orton, Joseph Hughes, Sreenu Vattipally, David L Robertson; Alasdair MacLean, Rory Gunson; Kathy Li, Igor Starinskij, Natasha Jesudason, Rajiv Shah, James Shepherd, Antonia Ho, Emma Thomson
EPI_ISL_611827	Liverpool Clinical Laboratories	COVID-19 Genomics UK (COG-UK) Consortium	Sam Haldenby, Anita Lucaci, Steve Paterson, Julian Hiscox, Alistair Darby, M Almsaud, A Alrezaihi, Muhannad Alruwaili, Stuart D Armstrong, Jones Benjamin, Eleanor G Bentley, Anu Chawla, Jordan J Clark, Angela Cowell, Richard Eccles, Isabel García-Dorival, Matthew Gemmell, Alessandro Gerada, PKF Gilmore, Richard Gregory, Ximeng Han, Catherine Hartley, Margaret Hughes, Miren Iturriza-Gomara, James Johnson, L Luu, Jenifer Manson, Charlotte Nelson, Elaine O'Toole, Cassie Olateju, Rebekah Penrice-Randal , Lucille Rainbow, N.P Randle, Trevor Ian Robinson, Parul Sharma, Ghada T Shawli, James P Stewart, Neil Swainston, Ecaterina Vamos, Joanne Watts, Mark Whitehead
EPI_ISL_611844	University of Exeter	COVID-19 Genomics UK (COG-UK) Consortium	Ben Temperton,Aaron Jeffries,Michelle Michelsen,Joanna Warwick-Dugdale,Audrey Farbos,Robyn Manley,Stephen Michell,Jane Masoli
EPI_ISL_611848	West of Scotland Specialist Virology Centre, NHSGGC / MRC-University of Glasgow Centre for Virus Research	COVID-19 Genomics UK (COG-UK) Consortium	Ana da Silva Filipe, Natasha Johnson, Kathy Smollett, Daniel Mair, Stephen Carmichael, Lily Tong, Jenna Nichols, Elihu Aranday-Cortes, Kyriaki Nomikou; Sarah McDonald, Marc Niebel, Patawee Asamaphan; Richard Orton, Joseph Hughes, Sreenu Vattipally, David L Robertson; Alasdair MacLean, Rory Gunson; Kathy Li, Igor Starinskij, Natasha Jesudason, Rajiv Shah, James Shepherd, Antonia Ho, Emma Thomson
EPI_ISL_611849	Wales Specialist Virology Centre Sequencing lab: Pathogen Genomics Unit	COVID-19 Genomics UK (COG-UK) Consortium	Catherine Moore, Johnathan Evans, Laura Gifford, Malorie Perry, Simon Cottrell, Angela Marchbank, Alec Birchley, Alexander Adams, Amy Gaskin, Bree Gatica-Wilcox, Jason Coombes, Joel Southgate, Lauren Gilbert, Lee Graham, Nicole Pacchiarini, Sara Kumziene-Summerhayes, Sarah Taylor, Sophie Jones, Sara Rey, Matthew Bull, Joanne Watkins, Sally Corden, Tom Connor
EPI_ISL_611851	Liverpool Clinical Laboratories	COVID-19 Genomics UK (COG-UK) Consortium	Sam Haldenby, Anita Lucaci, Steve Paterson, Julian Hiscox, Alistair Darby, M Almsaud, A Alrezaihi, Muhannad Alruwaili, Stuart D Armstrong, Jones Benjamin, Eleanor G Bentley, Anu Chawla, Jordan J Clark, Angela Cowell, Richard Eccles, Isabel García-Dorival, Matthew Gemmell, Alessandro Gerada, PKF Gilmore, Richard Gregory, Ximeng Han, Catherine Hartley, Margaret Hughes, Miren Iturriza-Gomara, James Johnson, L Luu, Jenifer Manson, Charlotte Nelson, Elaine O'Toole, Cassie Olateju, Rebekah Penrice-Randal , Lucille Rainbow, N.P Randle, Trevor Ian Robinson, Parul Sharma, Ghada T Shawli, James P Stewart, Neil Swainston, Ecaterina Vamos, Joanne Watts, Mark Whitehead
EPI_ISL_611852	West of Scotland Specialist Virology Centre, NHSGGC / MRC-University of Glasgow Centre for Virus Research	COVID-19 Genomics UK (COG-UK) Consortium	Ana da Silva Filipe, Natasha Johnson, Kathy Smollett, Daniel Mair, Stephen Carmichael, Lily Tong, Jenna Nichols, Elihu Aranday-Cortes, Kyriaki Nomikou; Sarah McDonald, Marc Niebel, Patawee Asamaphan; Richard Orton, Joseph Hughes, Sreenu Vattipally, David L Robertson; Alasdair MacLean, Rory Gunson; Kathy Li, Igor Starinskij, Natasha Jesudason, Rajiv Shah, James Shepherd, Antonia Ho, Emma Thomson
EPI_ISL_611857	Liverpool Clinical Laboratories	COVID-19 Genomics UK (COG-UK) Consortium	Sam Haldenby, Anita Lucaci, Steve Paterson, Julian Hiscox, Alistair Darby, M Almsaud, A Alrezaihi, Muhannad Alruwaili, Stuart D Armstrong, Jones Benjamin, Eleanor G Bentley, Anu Chawla, Jordan J Clark, Angela Cowell, Richard Eccles, Isabel García-Dorival, Matthew Gemmell, Alessandro Gerada, PKF Gilmore, Richard Gregory, Ximeng Han, Catherine Hartley, Margaret Hughes, Miren Iturriza-Gomara, James Johnson, L Luu, Jenifer Manson, Charlotte Nelson, Elaine O'Toole, Cassie Olateju, Rebekah Penrice-Randal , Lucille Rainbow, N.P Randle, Trevor Ian Robinson, Parul Sharma, Ghada T Shawli, James P Stewart, Neil Swainston, Ecaterina Vamos, Joanne Watts, Mark Whitehead
EPI_ISL_611867, EPI_ISL_611872, EPI_ISL_611873, EPI_ISL_611877, EPI_ISL_611878, EPI_ISL_611879, EPI_ISL_611880, EPI_ISL_611881, EPI_ISL_611882, EPI_ISL_611883, EPI_ISL_611884, EPI_ISL_611890			
see above	West of Scotland Specialist Virology Centre, NHSGGC / MRC-University of Glasgow Centre for Virus Research	COVID-19 Genomics UK (COG-UK) Consortium	Ana da Silva Filipe, Natasha Johnson, Kathy Smollett, Daniel Mair, Stephen Carmichael, Lily Tong, Jenna Nichols, Elihu Aranday-Cortes, Kyriaki Nomikou; Sarah McDonald, Marc Niebel, Patawee Asamaphan; Richard Orton, Joseph Hughes, Sreenu Vattipally, David L Robertson; Alasdair MacLean, Rory Gunson; Kathy Li, Igor Starinskij, Natasha Jesudason, Rajiv Shah, James Shepherd, Antonia Ho, Emma Thomson

EPI_ISL_611892	Liverpool Clinical Laboratories	COVID-19 Genomics UK (COG-UK) Consortium	Sam Haldenby, Anita Lucaci, Steve Paterson, Julian Hiscox, Alistair Darby, M Almsaud, A Alrezaihi, Muhannad Alruwaili, Stuart D Armstrong, Jones Benjamin, Eleanor G Bentley, Anu Chawla, Jordan J Clark, Angela Cowell, Richard Eccles, Isabel García-Dorival, Matthew Gemmell, Alessandro Gerada, PKF Gilmore, Richard Gregory, Ximeng Han, Catherine Hartley, Margaret Hughes, Miren Iturriza-Gomara, James Johnson, L Luu, Jenifer Manson, Charlotte Nelson, Elaine O'Toole, Cassie Olateju, Rebekah Penrice-Randal , Lucille Rainbow, N.P Randle, Trevor Ian Robinson, Parul Sharma, Ghada T Shawli, James P Stewart, Neil Swainston, Ecaterina Vamos, Joanne Watts, Mark Whitehead
EPI_ISL_611917, EPI_ISL_611952	Northumbria University / South Tees Hospitals NHS Foundation Trust / North Cumbria Integrated Care NHS Foundation Trust / North Tees and Hartlepool NHS Foundation Trust / Newcastle Hospitals NHS Foundation Trust	COVID-19 Genomics UK (COG-UK) Consortium	Darren L Smith,Andrew Nelson,Matthew Bashton,Greg R Young,Joshua Loh,John Allan,Mohammad A Tariq,Giles S Holt,Gary Black,Wen C Yew,Lynn Dover,Paul Baker,Steve Liggett,Sarah Essex,Jane Greenaway,Debra Padgett,Clive Graham,Garren Scott,Edward Barton,Emma Swindells,Brendan Payne,Jennifer Collins,Yusri Taha,Gary Eltringham
EPI_ISL_611969, EPI_ISL_611970	Wales Specialist Virology Centre Sequencing lab: Pathogen Genomics Unit	COVID-19 Genomics UK (COG-UK) Consortium	Catherine Moore, Johnathan Evans, Laura Gifford, Malorie Perry, Simon Cottrell, Angela Marchbank, Alec Birchley, Alexander Adams, Amy Gaskin, Bree Gatica-Wilcox, Jason Coombes, Joel Southgate, Lauren Gilbert, Lee Graham, Nicole Pacchiarini, Sara Kumziene-Summerhayes, Sarah Taylor, Sophie Jones, Sara Rey, Matthew Bull, Joanne Watkins, Sally Corden, Tom Connor
EPI_ISL_611978, EPI_ISL_611984, EPI_ISL_612001, EPI_ISL_612009, EPI_ISL_612010, EPI_ISL_612039, EPI_ISL_612071, EPI_ISL_612101	Liverpool Clinical Laboratories	COVID-19 Genomics UK (COG-UK) Consortium	Sam Haldenby, Anita Lucaci, Steve Paterson, Julian Hiscox, Alistair Darby, M Almsaud, A Alrezaihi, Muhannad Alruwaili, Stuart D Armstrong, Jones Benjamin, Eleanor G Bentley, Anu Chawla, Jordan J Clark, Angela Cowell, Richard Eccles, Isabel García-Dorival, Matthew Gemmell, Alessandro Gerada, PKF Gilmore, Richard Gregory, Ximeng Han, Catherine Hartley, Margaret Hughes, Miren Iturriza-Gomara, James Johnson, L Luu, Jenifer Manson, Charlotte Nelson, Elaine O'Toole, Cassie Olateju, Rebekah Penrice-Randal , Lucille Rainbow, N.P Randle, Trevor Ian Robinson, Parul Sharma, Ghada T Shawli, James P Stewart, Neil Swainston, Ecaterina Vamos, Joanne Watts, Mark Whitehead
EPI_ISL_612121, EPI_ISL_612132, EPI_ISL_612133	Wales Specialist Virology Centre Sequencing lab: Pathogen Genomics Unit	COVID-19 Genomics UK (COG-UK) Consortium	Catherine Moore, Johnathan Evans, Laura Gifford, Malorie Perry, Simon Cottrell, Angela Marchbank, Alec Birchley, Alexander Adams, Amy Gaskin, Bree Gatica-Wilcox, Jason Coombes, Joel Southgate, Lauren Gilbert, Lee Graham, Nicole Pacchiarini, Sara Kumziene-Summerhayes, Sarah Taylor, Sophie Jones, Sara Rey, Matthew Bull, Joanne Watkins, Sally Corden, Tom Connor
EPI_ISL_612262, EPI_ISL_612263, EPI_ISL_612264	Department of Pathology, University of Cambridge	COVID-19 Genomics UK (COG-UK) Consortium	Aminu S. Jahun, Yasmin Chaudhry, Grant Hall, Iliana Georgana, Myra Hosmillo, Martin D. Curran, Malte Pinckert, Surendra Parmar, Ian Goodfellow
EPI_ISL_612288, EPI_ISL_612289, EPI_ISL_612290, EPI_ISL_612291, EPI_ISL_612292, EPI_ISL_612293, EPI_ISL_612294, EPI_ISL_612297, EPI_ISL_612298, EPI_ISL_612299, EPI_ISL_612300, EPI_ISL_612301, EPI_ISL_612302, EPI_ISL_612303, EPI_ISL_612304, EPI_ISL_612305, EPI_ISL_612306, EPI_ISL_612307, EPI_ISL_612308, EPI_ISL_612309, EPI_ISL_612310, EPI_ISL_612311, EPI_ISL_612312, EPI_ISL_612313			
see above	West of Scotland Specialist Virology Centre, NHSGGC / MRC-University of Glasgow Centre for Virus Research	COVID-19 Genomics UK (COG-UK) Consortium	Ana da Silva Filipe, Natasha Johnson, Kathy Smollett, Daniel Mair, Stephen Carmichael, Lily Tong, Jenna Nichols, Elihu Aranday-Cortes, Kyriaki Nomikou; Sarah McDonald, Marc Niebel, Patawee Asamaphan; Richard Orton, Joseph Hughes, Sreenu Vattipally, David L Robertson; Alasdair MacLean, Rory Gunson; Kathy Li, Igor Starinskij, Natasha Jesudason, Rajiv Shah, James Shepherd, Antonia Ho, Emma Thomson
EPI_ISL_612320, EPI_ISL_612321, EPI_ISL_612322, EPI_ISL_612323, EPI_ISL_612324	Virology Department, Royal Infirmary of Edinburgh, NHS Lothian / School of Biological Sciences, University of Edinburgh / Institute of Genetics and Molecular Medicine, University of Edinburgh	COVID-19 Genomics UK (COG-UK) Consortium	McHugh M, Dewar R, Rooke S, Gallagher M, Balcaza C, O'Toole Á, Scher E, Hill V, McCrone JT, Colquhoun R, Yu X, Jackson B, Rambaut A, Williams TC, Templeton K
EPI_ISL_612375, EPI_ISL_612376, EPI_ISL_612377, EPI_ISL_612378, EPI_ISL_612379	University of Exeter	COVID-19 Genomics UK (COG-UK) Consortium	Ben Temperton,Aaron Jeffries,Michelle Michelsen,Joanna Warwick-Dugdale,Audrey Farbos,Robyn Manley,Stephen Michell,Jane Masoli
EPI_ISL_612433, EPI_ISL_612435, EPI_ISL_612436	Liverpool Clinical Laboratories	COVID-19 Genomics UK (COG-UK) Consortium	Sam Haldenby, Anita Lucaci, Steve Paterson, Julian Hiscox, Alistair Darby, M Almsaud, A Alrezaihi, Muhannad Alruwaili, Stuart D Armstrong, Jones Benjamin, Eleanor G Bentley, Anu Chawla, Jordan J Clark, Angela Cowell, Richard Eccles, Isabel García-Dorival, Matthew Gemmell, Alessandro Gerada, PKF Gilmore, Richard Gregory, Ximeng Han, Catherine Hartley, Margaret Hughes, Miren Iturriza-Gomara, James Johnson, L Luu, Jenifer Manson, Charlotte Nelson, Elaine O'Toole, Cassie Olateju, Rebekah Penrice-Randal , Lucille Rainbow, N.P Randle, Trevor Ian Robinson, Parul Sharma, Ghada T Shawli, James P Stewart, Neil Swainston, Ecaterina Vamos, Joanne Watts, Mark Whitehead
EPI_ISL_612458, EPI_ISL_612459, EPI_ISL_612460, EPI_ISL_612461, EPI_ISL_612462, EPI_ISL_612463, EPI_ISL_612464, EPI_ISL_612465, EPI_ISL_612466, EPI_ISL_612467, EPI_ISL_612468, EPI_ISL_612469, EPI_ISL_612470, EPI_ISL_612471, EPI_ISL_612472, EPI_ISL_612473, EPI_ISL_612474, EPI_ISL_612475, EPI_ISL_612476, EPI_ISL_612477, EPI_ISL_612478, EPI_ISL_612479, EPI_ISL_612480, EPI_ISL_612481, EPI_ISL_612482, EPI_ISL_612483, EPI_ISL_612484, EPI_ISL_612485, EPI_ISL_612486, EPI_ISL_612487, EPI_ISL_612493, EPI_ISL_612494, EPI_ISL_612495, EPI_ISL_612496, EPI_ISL_612497, EPI_ISL_612498, EPI_ISL_612499, EPI_ISL_612500			
see above	Wales Specialist Virology Centre Sequencing lab: Pathogen Genomics Unit	COVID-19 Genomics UK (COG-UK) Consortium	Catherine Moore, Johnathan Evans, Laura Gifford, Malorie Perry, Simon Cottrell, Angela Marchbank, Alec Birchley, Alexander Adams, Amy Gaskin, Bree Gatica-Wilcox, Jason Coombes, Joel Southgate, Lauren Gilbert, Lee Graham, Nicole Pacchiarini, Sara Kumziene-Summerhayes, Sarah Taylor, Sophie Jones, Sara Rey, Matthew Bull, Joanne Watkins, Sally Corden, Tom Connor
EPI_ISL_612554, EPI_ISL_612555, EPI_ISL_612556, EPI_ISL_612558, EPI_ISL_612559, EPI_ISL_612560, EPI_ISL_612561, EPI_ISL_612562, EPI_ISL_612563	Northumbria University / South Tees Hospitals NHS Foundation Trust / North Cumbria Integrated Care NHS Foundation Trust / North Tees and Hartlepool NHS Foundation Trust / Newcastle Hospitals NHS Foundation Trust	COVID-19 Genomics UK (COG-UK) Consortium	Darren L Smith,Andrew Nelson,Matthew Bashton,Greg R Young,Joshua Loh,John Allan,Mohammad A Tariq,Giles S Holt,Gary Black,Wen C Yew,Lynn Dover,Paul Baker,Steve Liggett,Sarah Essex,Jane Greenaway,Debra Padgett,Clive Graham,Garren Scott,Edward Barton,Emma Swindells,Brendan Payne,Jennifer Collins,Yusri Taha,Gary Eltringham
EPI_ISL_612880, EPI_ISL_612978, EPI_ISL_613003, EPI_ISL_613010, EPI_ISL_613014, EPI_ISL_613020, EPI_ISL_613045, EPI_ISL_613061, EPI_ISL_613078, EPI_ISL_613098, EPI_ISL_613105, EPI_ISL_613149, EPI_ISL_613151			
see above	Wales Specialist Virology Centre Sequencing lab: Pathogen Genomics Unit	COVID-19 Genomics UK (COG-UK) Consortium	Catherine Moore, Johnathan Evans, Laura Gifford, Malorie Perry, Simon Cottrell, Angela Marchbank, Alec Birchley, Alexander Adams, Amy Gaskin, Bree Gatica-Wilcox, Jason Coombes, Joel Southgate, Lauren Gilbert, Lee Graham, Nicole Pacchiarini, Sara Kumziene-Summerhayes, Sarah Taylor, Sophie Jones, Sara Rey, Matthew Bull, Joanne Watkins, Sally Corden, Tom Connor
EPI_ISL_613379	Virology Department, Sheffield Teaching Hospitals NHS Foundation Trust/Department of Infection, Immunity and Cardiovascular Disease, The Medical School, University of Sheffield	COVID-19 Genomics UK (COG-UK) Consortium	Thushan de Silva, Matthew Parker, Nikki Smith, Adri Angyal, Rebecca Brown, Luke Green, Rachel Tucker, Paul Parsons, Danielle Groves, Katie Johnson, Laura Carrilero, Alex Keeley, Dave Partridge, Matthew Wyles, Benjamin Lindsey, Mehmet Yavuz, Mohammad Raza, Cariad Evans
EPI_ISL_613489, EPI_ISL_613490, EPI_ISL_613491, EPI_ISL_613492	Public Health Laboratory - Infectious Disease Lab, Minnesota Department of Health Infectious Disease Laboratory Submission Group	Minnesota Department of Health, Public Health Laboratory	Plumb,M., Garfin,J., Lorentz,A., Wang,X.
EPI_ISL_613718, EPI_ISL_613721, EPI_ISL_613722, EPI_ISL_613733, EPI_ISL_613737, EPI_ISL_613741, EPI_ISL_613752, EPI_ISL_613754, EPI_ISL_613756, EPI_ISL_613760, EPI_ISL_613778, EPI_ISL_613790, EPI_ISL_613796, EPI_ISL_613822, EPI_ISL_613843, EPI_ISL_613844, EPI_ISL_613845, EPI_ISL_613846, EPI_ISL_613847, EPI_ISL_613848, EPI_ISL_613849, EPI_ISL_613850, EPI_ISL_613851, EPI_ISL_613852, EPI_ISL_613853, EPI_ISL_613854, EPI_ISL_613855			
see above	Florida Bureau of Public Health Laboratories	Florida Bureau of Public Health Laboratories	Sarah Schmedes, Jason Blanton
EPI_ISL_614049, EPI_ISL_614065, EPI_ISL_614071, EPI_ISL_614072, EPI_ISL_614145, EPI_ISL_614146, EPI_ISL_614147, EPI_ISL_614148	Virginia DCLS	Virginia DCLS	Virginia DCLS
EPI_ISL_614160, EPI_ISL_614161, EPI_ISL_614165, EPI_ISL_614166, EPI_ISL_614168, EPI_ISL_614171, EPI_ISL_614175, EPI_ISL_614178	Michigan Department of Health and Human Services, Bureau of Laboratories	Michigan Department of Health and Human Services, Bureau of Laboratories	Blankenship HM, Riner D, Soehnlen MK
EPI_ISL_614295, EPI_ISL_614304, EPI_ISL_614316, EPI_ISL_614317	Faroese National Reference Laboratory for Fish and Animal Diseases	Faroese National Reference Laboratory for Fish and Animal Diseases	Maria Marjunardóttir Dahl, Petra Elisabeth Petersen, Debes Hammershaimb Christiansen
EPI_ISL_615117, EPI_ISL_615118,	Klinisk mikrobiologi Linkoping	The Public Health Agency of Sweden	Anna-Malin Linde, Maria Lind Karlberg, Mattias Haukland, Reza Advani, Olov Svartstrom, Oskar Karlsson Lindsjo, Sandra Broddesson, Petra Edquist, Mia

EPI_ISL_615119			Brytting, Anna Risberg, Karin Tegmark-Wisell
EPI_ISL_615120	Orebro klinisk mikrobiologi	The Public Health Agency of Sweden	Anna-Malin Linde, Maria Lind Karlberg, Mattias Haukland, Reza Advani, Olov Svartstrom, Oskar Karlsson Lindsjo, Sandra Broddesson, Petra Edquist, Mia Brytting, Anna Risberg, Karin Tegmark-Wisell
EPI_ISL_619906, EPI_ISL_619907, EPI_ISL_619908, EPI_ISL_619909, EPI_ISL_619910, EPI_ISL_619911, EPI_ISL_619912, EPI_ISL_619913, EPI_ISL_619914, EPI_ISL_619915, EPI_ISL_619916, EPI_ISL_619917, EPI_ISL_619918, EPI_ISL_619919, EPI_ISL_619920, EPI_ISL_619921, EPI_ISL_619922, EPI_ISL_619923, EPI_ISL_619924, EPI_ISL_619925, EPI_ISL_619926, EPI_ISL_619927, EPI_ISL_619928, EPI_ISL_619929, EPI_ISL_619930, EPI_ISL_619931, EPI_ISL_619932, EPI_ISL_619933, EPI_ISL_619934, EPI_ISL_619935, EPI_ISL_619936, EPI_ISL_619937, EPI_ISL_619938, EPI_ISL_619939, EPI_ISL_619940, EPI_ISL_619941, EPI_ISL_619942, EPI_ISL_619943, EPI_ISL_619944, EPI_ISL_619945, EPI_ISL_619946, EPI_ISL_619947, EPI_ISL_619948, EPI_ISL_619949, EPI_ISL_619950, EPI_ISL_619951, EPI_ISL_619952, EPI_ISL_619953, EPI_ISL_619954, EPI_ISL_619955, EPI_ISL_619956, EPI_ISL_619957, EPI_ISL_619958, EPI_ISL_619959, EPI_ISL_619960, EPI_ISL_619961, EPI_ISL_619962, EPI_ISL_619963, EPI_ISL_619964, EPI_ISL_619965, EPI_ISL_619966, EPI_ISL_619967, EPI_ISL_619968, EPI_ISL_619969, EPI_ISL_619970, EPI_ISL_619971, EPI_ISL_619972, EPI_ISL_619973, EPI_ISL_619974, EPI_ISL_619975, EPI_ISL_619976, EPI_ISL_620783, EPI_ISL_620800, EPI_ISL_620804, EPI_ISL_620805, EPI_ISL_620806, EPI_ISL_620807, EPI_ISL_620808, EPI_ISL_620809, EPI_ISL_620810, EPI_ISL_620811, EPI_ISL_620812, EPI_ISL_620813, EPI_ISL_620829, EPI_ISL_620850, EPI_ISL_620851, EPI_ISL_620852, EPI_ISL_620854, EPI_ISL_620855, EPI_ISL_620856, EPI_ISL_620857, EPI_ISL_620858, EPI_ISL_620859, EPI_ISL_620860, EPI_ISL_620861, EPI_ISL_620862, EPI_ISL_620863, EPI_ISL_620864, EPI_ISL_620865, EPI_ISL_620866, EPI_ISL_620867, EPI_ISL_620868, EPI_ISL_620869, EPI_ISL_620870, EPI_ISL_620871, EPI_ISL_620872, EPI_ISL_620873, EPI_ISL_620874, EPI_ISL_620875, EPI_ISL_620876, EPI_ISL_620877, EPI_ISL_620878, EPI_ISL_620879, EPI_ISL_620880, EPI_ISL_620881, EPI_ISL_620882, EPI_ISL_620883, EPI_ISL_620884, EPI_ISL_620885, EPI_ISL_620886, EPI_ISL_620887, EPI_ISL_620888, EPI_ISL_620889, EPI_ISL_620890, EPI_ISL_620891, EPI_ISL_620892, EPI_ISL_620893, EPI_ISL_620894, EPI_ISL_620895, EPI_ISL_620896, EPI_ISL_620897, EPI_ISL_620898, EPI_ISL_620899, EPI_ISL_620900, EPI_ISL_620901, EPI_ISL_620902, EPI_ISL_620903, EPI_ISL_620904, EPI_ISL_620905, EPI_ISL_620906, EPI_ISL_620907, EPI_ISL_620908, EPI_ISL_620909, EPI_ISL_620910, EPI_ISL_620911, EPI_ISL_620912, EPI_ISL_620913, EPI_ISL_620914, EPI_ISL_620915, EPI_ISL_620916, EPI_ISL_620917, EPI_ISL_620918, EPI_ISL_620919, EPI_ISL_620920, EPI_ISL_620922, EPI_ISL_620923, EPI_ISL_620924, EPI_ISL_620925, EPI_ISL_620927, EPI_ISL_620928, EPI_ISL_620929, EPI_ISL_620930, EPI_ISL_620931, EPI_ISL_620932, EPI_ISL_620933, EPI_ISL_620934, EPI_ISL_620935, EPI_ISL_620936, EPI_ISL_620937, EPI_ISL_620938, EPI_ISL_620939, EPI_ISL_620940, EPI_ISL_620941, EPI_ISL_620942, EPI_ISL_620943, EPI_ISL_620944, EPI_ISL_620945, EPI_ISL_620946, EPI_ISL_620947, EPI_ISL_620948, EPI_ISL_620949, EPI_ISL_620950, EPI_ISL_620951, EPI_ISL_620952, EPI_ISL_620953, EPI_ISL_620954, EPI_ISL_620955, EPI_ISL_620956, EPI_ISL_620957, EPI_ISL_620958, EPI_ISL_620959, EPI_ISL_620960, EPI_ISL_620961, EPI_ISL_620962, EPI_ISL_620963, EPI_ISL_620964, EPI_ISL_620965, EPI_ISL_620966, EPI_ISL_620967, EPI_ISL_620968, EPI_ISL_620969, EPI_ISL_620970, EPI_ISL_620971, EPI_ISL_620972, EPI_ISL_620973, EPI_ISL_620974, EPI_ISL_620975, EPI_ISL_620976, EPI_ISL_620977, EPI_ISL_620978, EPI_ISL_620979, EPI_ISL_620980, EPI_ISL_620981, EPI_ISL_620982, EPI_ISL_620983, EPI_ISL_620984, EPI_ISL_620985, EPI_ISL_620986, EPI_ISL_620987, EPI_ISL_620988, EPI_ISL_620989, EPI_ISL_620990, EPI_ISL_620991, EPI_ISL_620992, EPI_ISL_620993, EPI_ISL_620994, EPI_ISL_620995, EPI_ISL_620996, EPI_ISL_620997, EPI_ISL_620998, EPI_ISL_620999, EPI_ISL_621000, EPI_ISL_621001, EPI_ISL_621002, EPI_ISL_621003, EPI_ISL_621004, EPI_ISL_621005, EPI_ISL_621006, EPI_ISL_621007, EPI_ISL_621008, EPI_ISL_621009, EPI_ISL_621010, EPI_ISL_621011, EPI_ISL_621012, EPI_ISL_621013, EPI_ISL_621014, EPI_ISL_621015, EPI_ISL_621016, EPI_ISL_621017, EPI_ISL_621018, EPI_ISL_621019, EPI_ISL_621020, EPI_ISL_621021, EPI_ISL_621022, EPI_ISL_621023, EPI_ISL_621024, EPI_ISL_621025, EPI_ISL_621026, EPI_ISL_621027, EPI_ISL_621028, EPI_ISL_621029, EPI_ISL_621030, EPI_ISL_621031, EPI_ISL_621032, EPI_ISL_621033, EPI_ISL_621034, EPI_ISL_621035, EPI_ISL_621036, EPI_ISL_621037, EPI_ISL_621038, EPI_ISL_621039, EPI_ISL_621040, EPI_ISL_621041, EPI_ISL_621042, EPI_ISL_621043, EPI_ISL_621044, EPI_ISL_621045, EPI_ISL_621046, EPI_ISL_621047, EPI_ISL_621048, EPI_ISL_621049, EPI_ISL_621050, EPI_ISL_621051, EPI_ISL_621052, EPI_ISL_621053, EPI_ISL_621054, EPI_ISL_621055, EPI_ISL_621056, EPI_ISL_621057, EPI_ISL_621058, EPI_ISL_621059, EPI_ISL_621060, EPI_ISL_621061, EPI_ISL_621062, EPI_ISL_621063, EPI_ISL_621064, EPI_ISL_621065, EPI_ISL_621066, EPI_ISL_621067, EPI_ISL_621068, EPI_ISL_621069, EPI_ISL_621070, EPI_ISL_621071, EPI_ISL_621072, EPI_ISL_621073, EPI_ISL_621074, EPI_ISL_621075, EPI_ISL_621076, EPI_ISL_621077, EPI_ISL_621078, EPI_ISL_621079, EPI_ISL_621080, EPI_ISL_621081, EPI_ISL_621082, EPI_ISL_621090, EPI_ISL_621095, EPI_ISL_621096, EPI_ISL_621097, EPI_ISL_621098, EPI_ISL_621099, EPI_ISL_621100, EPI_ISL_621101, EPI_ISL_621102, EPI_ISL_621103, EPI_ISL_621104, EPI_ISL_621105, EPI_ISL_621106, EPI_ISL_621107, EPI_ISL_621108, EPI_ISL_621109, EPI_ISL_621110, EPI_ISL_621111, EPI_ISL_621112, EPI_ISL_621113, EPI_ISL_621118, EPI_ISL_621119, EPI_ISL_621120, EPI_ISL_621121, EPI_ISL_621122, EPI_ISL_621123, EPI_ISL_621124, EPI_ISL_621125, EPI_ISL_621150, EPI_ISL_621211, EPI_ISL_621212, EPI_ISL_621213, EPI_ISL_621214, EPI_ISL_621215, EPI_ISL_621216, EPI_ISL_621217, EPI_ISL_621218, EPI_ISL_621219, EPI_ISL_621220, EPI_ISL_621233, EPI_ISL_621234, EPI_ISL_621235, EPI_ISL_621236, EPI_ISL_621237, EPI_ISL_621238, EPI_ISL_621239, EPI_ISL_621240, EPI_ISL_621241, EPI_ISL_621242, EPI_ISL_621243, EPI_ISL_621244, EPI_ISL_621245, EPI_ISL_621246, EPI_ISL_621247, EPI_ISL_621248, EPI_ISL_621249, EPI_ISL_621250, EPI_ISL_621251, EPI_ISL_621252, EPI_ISL_621253, EPI_ISL_621254, EPI_ISL_621255, EPI_ISL_621256, EPI_ISL_621257, EPI_ISL_621258, EPI_ISL_621259, EPI_ISL_621260, EPI_ISL_621261, EPI_ISL_622302, EPI_ISL_622306, EPI_ISL_622307, EPI_ISL_622308, EPI_ISL_622309, EPI_ISL_622310, EPI_ISL_622311, EPI_ISL_622312, EPI_ISL_622313, EPI_ISL_622314, EPI_ISL_622315, EPI_ISL_622316, EPI_ISL_622317, EPI_ISL_622318, EPI_ISL_622319, EPI_ISL_622320, EPI_ISL_622321, EPI_ISL_622322, EPI_ISL_622323, EPI_ISL_622324, EPI_ISL_622325, EPI_ISL_622326, EPI_ISL_622327, EPI_ISL_622328, EPI_ISL_622329, EPI_ISL_622330, EPI_ISL_622331, EPI_ISL_622333, EPI_ISL_622339, EPI_ISL_622340			
see above	Department of Virus and Microbiological Special Diagnostics, Statens Serum Institut, Denmark	Albertsen lab, Department of Chemistry and Bioscience, Aalborg University, Denmark	Danish Covid-19 Genome Consortia
EPI_ISL_622833, EPI_ISL_622834, EPI_ISL_622835, EPI_ISL_622836, EPI_ISL_622837, EPI_ISL_622838, EPI_ISL_622839, EPI_ISL_622840, EPI_ISL_622841, EPI_ISL_622842, EPI_ISL_622892, EPI_ISL_622893			
see above	Respiratory Virus Unit, Microbiology Services Colindale, Public Health England	Respiratory Virus Unit, Microbiology Services Colindale, Public Health England	PHE Covid Sequencing Team
EPI_ISL_623179	Utah Public Health Laboratory	Utah Public Health Laboratory	Erin Young, Kelly Oakeson
EPI_ISL_625485, EPI_ISL_625492, EPI_ISL_625495, EPI_ISL_625496, EPI_ISL_625497, EPI_ISL_625498, EPI_ISL_625499	Santa Clara County Public Health Laboratory	Chan-Zuckerberg Biohub	CZB Cliahub Consortium
EPI_ISL_625546	Alameda County Public Health Lab	Chan-Zuckerberg Biohub	CZB Cliahub Consortium
EPI_ISL_625601	Santa Clara County Public Health Laboratory	Chan-Zuckerberg Biohub	CZB Cliahub Consortium
EPI_ISL_625611, EPI_ISL_625612, EPI_ISL_625613	Alameda County Public Health Lab	Chan-Zuckerberg Biohub	CZB Cliahub Consortium
EPI_ISL_625647, EPI_ISL_625648, EPI_ISL_625649, EPI_ISL_625650, EPI_ISL_625651, EPI_ISL_625652, EPI_ISL_625653, EPI_ISL_625654, EPI_ISL_625655, EPI_ISL_625656, EPI_ISL_625657			
see above	Orange County Public Health Lab	Chan-Zuckerberg Biohub	CZB Cliahub Consortium
EPI_ISL_625668	UCSF Clinical Microbiology Laboratory	Chan-Zuckerberg Biohub	CZB Cliahub Consortium
EPI_ISL_625674	Laboratory of Molecular Medicine, University of Magallanes	Centro Asistencial Docente y de Investigacion, Universidad de Magallanes	Jorge Gonzalez, Jacqueline Aldridge, Diego Alvarez, Marcelo Navarrete
EPI_ISL_626586, EPI_ISL_626587, EPI_ISL_626588, EPI_ISL_626589, EPI_ISL_626590, EPI_ISL_626591, EPI_ISL_626599, EPI_ISL_626600, EPI_ISL_626601	The National Institute of Public Health	State Veterinary Institute Prague	Nagy,A;Jirincova,H;Novakova,L;Trnka,D;Vecerova,J
EPI_ISL_626794	University of Exeter	COVID-19 Genomics UK (COG-UK) Consortium	Ben Temperton,Aaron Jeffries,Michelle Michelsen,Joanna Warwick-Dugdale,Audrey Farbos,Robyn Manley,Stephen Michell,Jane Masoli
EPI_ISL_626833, EPI_ISL_627026, EPI_ISL_627146, EPI_ISL_627203, EPI_ISL_627212, EPI_ISL_627220	Wales Specialist Virology Centre Sequencing lab: Pathogen Genomics Unit	COVID-19 Genomics UK (COG-UK) Consortium	Catherine Moore, Johnathan Evans, Laura Gifford, Malorie Perry, Simon Cottrell, Angela Marchbank, Alec Birchley, Alexander Adams, Amy Gaskin, Bree Gatica-Wilcox, Jason Coombes, Joel Southgate, Lauren Gilbert, Lee Graham, Nicole Pacchiarini, Sara Kumziene-Summerhayes, Sarah Taylor, Sophie Jones, Sara Rey, Matthew Bull, Joanne Watkins, Sally Corden, Tom Connor
EPI_ISL_627436, EPI_ISL_627438, EPI_ISL_627439	University of Exeter	COVID-19 Genomics UK (COG-UK) Consortium	Ben Temperton,Aaron Jeffries,Michelle Michelsen,Joanna Warwick-Dugdale,Audrey Farbos,Robyn Manley,Stephen Michell,Jane Masoli
EPI_ISL_627482, EPI_ISL_627483, EPI_ISL_627484, EPI_ISL_627485, EPI_ISL_627486, EPI_ISL_627487	Wales Specialist Virology Centre Sequencing lab: Pathogen Genomics Unit	COVID-19 Genomics UK (COG-UK) Consortium	Catherine Moore, Johnathan Evans, Laura Gifford, Malorie Perry, Simon Cottrell, Angela Marchbank, Alec Birchley, Alexander Adams, Amy Gaskin, Bree Gatica-Wilcox, Jason Coombes, Joel Southgate, Lauren Gilbert, Lee Graham, Nicole Pacchiarini, Sara Kumziene-Summerhayes, Sarah Taylor, Sophie Jones, Sara Rey, Matthew Bull, Joanne Watkins, Sally Corden, Tom Connor
EPI_ISL_627491	University College London, Great Ormond Street Hospital for Children NHS Foundation Trust, Imperial College Healthcare NHS Trust	COVID-19 Genomics UK (COG-UK) Consortium	Sergi Castellano, Rachel Williams, Mark Kristiansen, Paola Resende Silva, Sunando Roy, Tony Brooks, Helena Tutill, Paola Niola, Patricia Dyal, Charlotte Williams, Leysa Forrest, Yasmin Panchbhaya, Jacqueline Findlay, Samuel Weeks, Julianne Brown, Kathryn Harris, Paul Randell, James Price, Alison Holmes, Judith Breuer
EPI_ISL_627730, EPI_ISL_627731, EPI_ISL_627871, EPI_ISL_627899	Wales Specialist Virology Centre Sequencing lab: Pathogen Genomics Unit	COVID-19 Genomics UK (COG-UK) Consortium	Catherine Moore, Johnathan Evans, Laura Gifford, Malorie Perry, Simon Cottrell, Angela Marchbank, Alec Birchley, Alexander Adams, Amy Gaskin, Bree Gatica-Wilcox, Jason Coombes, Joel Southgate, Lauren Gilbert, Lee Graham, Nicole Pacchiarini, Sara Kumziene-Summerhayes, Sarah Taylor, Sophie Jones, Sara Rey, Matthew Bull, Joanne Watkins, Sally Corden, Tom Connor
EPI_ISL_628374	Virology Department, Sheffield Teaching Hospitals NHS Foundation Trust/Department of Infection, Immunity and Cardiovascular Disease, The Medical School, University of Sheffield	COVID-19 Genomics UK (COG-UK) Consortium	Thushan de Silva, Matthew Parker, Nikki Smith, Adri Angyal, Rebecca Brown, Luke Green, Rachel Tucker, Paul Parsons, Danielle Groves, Katie Johnson, Laura Carrilero, Alex Keeley, Dave Partridge, Matthew Wyles, Benjamin Lindsey, Mehmet Yavuz, Mohammad Raza, Cariad Evans
EPI_ISL_628939, EPI_ISL_628940, EPI_ISL_628941, EPI_ISL_628946, EPI_ISL_628947, EPI_ISL_628948, EPI_ISL_628949, EPI_ISL_628956, EPI_ISL_628960	Utah Public Health Laboratory	Utah Public Health Laboratory	Erin Young, Kelly Oakeson
EPI_ISL_629030	South Eastern Area Laboratory Services (SEALS)	NSW Health Pathology - Institute of Clinical Pathology and	CIDM-PH et al.

		Medical Research; Westmead Hospital; University of Sydney	
EPI_ISL_631362, EPI_ISL_631363, EPI_ISL_631364	ZOTZ KLIMAS MVZ Düsseldorf-Centrum GbR ÜBAG für Labormedizin, Genetik, Zytologie, Pathologie	Center of Medical Microbiology, Virology, and Hospital Hygiene, University of Duesseldorf	Maximilian Damagnez, Alexander Dilthey, Ashley-Jane Duplessis, Patrick Finzer, Katrin Hoffmann, Torsten Houwaart, Lisanna Hülse, Malte Kohns Vasconcelos, Marek Korencak, Nadine Lübke, Jessica Nicolai, Klaus Pfeffer, Daniel Strelow, Jörg Timm, Andreas Walker, Tobias Wienemann, Rainer Zotz
EPI_ISL_631377, EPI_ISL_631382, EPI_ISL_631386, EPI_ISL_631388	University Hospital Cologne	Center of Medical Microbiology, Virology, and Hospital Hygiene, University of Duesseldorf	Maximilian Damagnez, Alexander Dilthey, Ashley-Jane Duplessis, Eva Heger, Torsten Houwaart, Rolf Kaiser, Florian Klein, Elena Knops, Malte Kohns Vasconcelos, Jessica Nicolai, Klaus Pfeffer, Gibran Rubio Quintanares, Saleta Sierra-Aragón, Daniel Strelow, Jörg Timm, Andreas Walker, Tobias Wienemann
EPI_ISL_632323, EPI_ISL_632345, EPI_ISL_632352, EPI_ISL_632355, EPI_ISL_632356, EPI_ISL_632360, EPI_ISL_632363, EPI_ISL_632367, EPI_ISL_632378	Dutch COVID-19 response team	Erasmus Medical Center	Bas Oude Munnink, David Nieuwenhuijse, Reina Sikkema, Claudia Schapendonk, Irina Chestakova, Anne van der Linden, Theo Bestebroer, Stefan van Nieuwkoop, Mark Pronk, Pascal Lexmond, Corien Swaan, Manon Haverkate, Madelief Mollers, Mart Stein, Sandra Kengne Kamga Mobou, Jeroen van Kampen, Jolanda Voermans, Aura Timen, Corine GeurtsvanKessel, Annemiek van der Eijk, Richard Molenkamp, Marion Koopmans, on behalf of the Dutch national COVID-19 response team.
EPI_ISL_632385	Dutch COVID-19 response team	Erasmus Medical Center	OH consortium
EPI_ISL_632389, EPI_ISL_632390, EPI_ISL_632399, EPI_ISL_632403, EPI_ISL_632410, EPI_ISL_632421	Dutch COVID-19 response team	Erasmus Medical Center	Bas Oude Munnink, David Nieuwenhuijse, Reina Sikkema, Claudia Schapendonk, Irina Chestakova, Anne van der Linden, Theo Bestebroer, Stefan van Nieuwkoop, Mark Pronk, Pascal Lexmond, Corien Swaan, Manon Haverkate, Madelief Mollers, Mart Stein, Sandra Kengne Kamga Mobou, Jeroen van Kampen, Jolanda Voermans, Aura Timen, Corine GeurtsvanKessel, Annemiek van der Eijk, Richard Molenkamp, Marion Koopmans, on behalf of the Dutch national COVID-19 response team.
EPI_ISL_632456, EPI_ISL_632457, EPI_ISL_632458, EPI_ISL_632459	Dutch COVID-19 response team	Erasmus Medical Center	OH consortium
EPI_ISL_632461, EPI_ISL_632493, EPI_ISL_632494, EPI_ISL_632502, EPI_ISL_632506, EPI_ISL_632510, EPI_ISL_632511, EPI_ISL_632513, EPI_ISL_632530, EPI_ISL_632531, EPI_ISL_632535, EPI_ISL_632544, EPI_ISL_632545, EPI_ISL_632546, EPI_ISL_632551, EPI_ISL_632552, EPI_ISL_632553, EPI_ISL_632554, EPI_ISL_632555, EPI_ISL_632556, EPI_ISL_632557, EPI_ISL_632558, EPI_ISL_632568, EPI_ISL_632569, EPI_ISL_632570, EPI_ISL_632571, EPI_ISL_632572, EPI_ISL_632592, EPI_ISL_632593, EPI_ISL_632596, EPI_ISL_632598, EPI_ISL_632599, EPI_ISL_632612, EPI_ISL_632613, EPI_ISL_632614, EPI_ISL_632615, EPI_ISL_632616, EPI_ISL_632617, EPI_ISL_632618, EPI_ISL_632619, EPI_ISL_632620, EPI_ISL_632621, EPI_ISL_632622, EPI_ISL_632665, EPI_ISL_632670, EPI_ISL_632671, EPI_ISL_632697, EPI_ISL_632716, EPI_ISL_632717, EPI_ISL_632718, EPI_ISL_632733, EPI_ISL_632754, EPI_ISL_632785			
see above	Dutch COVID-19 response team	Erasmus Medical Center	Bas Oude Munnink, David Nieuwenhuijse, Reina Sikkema, Claudia Schapendonk, Irina Chestakova, Anne van der Linden, Theo Bestebroer, Stefan van Nieuwkoop, Mark Pronk, Pascal Lexmond, Corien Swaan, Manon Haverkate, Madelief Mollers, Mart Stein, Sandra Kengne Kamga Mobou, Jeroen van Kampen, Jolanda Voermans, Aura Timen, Corine GeurtsvanKessel, Annemiek van der Eijk, Richard Molenkamp, Marion Koopmans, on behalf of the Dutch national COVID-19 response team.
EPI_ISL_632983, EPI_ISL_632984, EPI_ISL_632985, EPI_ISL_632986, EPI_ISL_632987, EPI_ISL_632988, EPI_ISL_632989, EPI_ISL_632990, EPI_ISL_632991	DOHMH Corona	New York City Public Health Laboratory	Jade Wang, et al.
EPI_ISL_632995	DOHMH Crown Heights	New York City Public Health Laboratory	Jade Wang, et al.
EPI_ISL_633004, EPI_ISL_633005, EPI_ISL_633006, EPI_ISL_633007	DOHMH Jamaica	New York City Public Health Laboratory	Jade Wang, et al.
EPI_ISL_633009, EPI_ISL_633010, EPI_ISL_633018	DOHMH Central Harlem	New York City Public Health Laboratory	Jade Wang, et al.
EPI_ISL_633019, EPI_ISL_633020, EPI_ISL_633021, EPI_ISL_633022	DOHMH Morrisania	New York City Public Health Laboratory	Jade Wang, et al.
EPI_ISL_633023	DOHMH Chelsea	New York City Public Health Laboratory	Jade Wang, et al.
EPI_ISL_633024	DOHMH Morrisania	New York City Public Health Laboratory	Jade Wang, et al.
EPI_ISL_633043	DOHMH Riverside	New York City Public Health Laboratory	Jade Wang, et al.
EPI_ISL_633044	DOHMH Morrisania	New York City Public Health Laboratory	Jade Wang, et al.
EPI_ISL_633045	DOHMH PHL	New York City Public Health Laboratory	Jade Wang, et al.
EPI_ISL_633046, EPI_ISL_633047	DOHMH Chelsea	New York City Public Health Laboratory	Jade Wang, et al.
EPI_ISL_633048, EPI_ISL_633049	DOHMH Morrisania	New York City Public Health Laboratory	Jade Wang, et al.
EPI_ISL_633050, EPI_ISL_633051	DOHMH PHL	New York City Public Health Laboratory	Jade Wang, et al.
EPI_ISL_633052	DOHMH Chelsea	New York City Public Health Laboratory	Jade Wang, et al.
EPI_ISL_634884	Lab voor klinische biologie	Onderzoeksgroep Virologie	Laurens Lambrechts, Nick Vereecke, Marthe Pauwels, Bruno Verhasselt, Linos Vandekerckhove, Hans Nauwynck, Sebastiaan Theuns
EPI_ISL_634923	Utah Public Health Laboratory	Utah Public Health Laboratory	Erin L. Young, Kelly F. Oakeson
EPI_ISL_634978, EPI_ISL_634979, EPI_ISL_634980, EPI_ISL_634981, EPI_ISL_634982, EPI_ISL_634983, EPI_ISL_634984, EPI_ISL_634985, EPI_ISL_634986, EPI_ISL_634988, EPI_ISL_634989, EPI_ISL_634990, EPI_ISL_634991, EPI_ISL_634992			
see above	National Health Laboratory Service - Inkosi Albert Luthuli Central Hospital (NHLS-IALCH)	KRISP, KZN Research Innovation and Sequencing Platform	Giandhari J, Pillay S, Lessells R, Mdlalose K, York D, Khan S, Tegally H, Wilkinson E, de Oliveira T
EPI_ISL_635064, EPI_ISL_635065	Foerde Hospital, Department of Microbiology	Norwegian Institute of Public Health, Department of Virology	Kathrine Stene-Johansen, Kamilla Heddeland Instefjord, Hilde Elshaug, Marie Paulsen Madsen, Rasmus Riis Kopperud, Hilde Vollan, Karoline Bragstad, Olav Hungnes
EPI_ISL_635092	Medical Microbiology Unit, Department for Laboratory Medicine, Drammen Hospital, Vestre Viken Health Trust,	Norwegian Institute of Public Health, Department of Virology	Kathrine Stene-Johansen, Kamilla Heddeland Instefjord, Hilde Elshaug, Marie Paulsen Madsen, Rasmus Riis Kopperud, Hilde Vollan, Karoline Bragstad, Olav Hungnes
EPI_ISL_635094	Oslo University Hospital, Department of Medical Microbiology	Norwegian Institute of Public Health, Department of Virology	Kathrine Stene-Johansen, Kamilla Heddeland Instefjord, Hilde Elshaug, Marie Paulsen Madsen, Rasmus Riis Kopperud, Hilde Vollan, Karoline Bragstad, Olav Hungnes
EPI_ISL_635098	Foerde Hospital, Department of Microbiology	Norwegian Institute of Public Health, Department of Virology	Kathrine Stene-Johansen, Kamilla Heddeland Instefjord, Hilde Elshaug, Marie Paulsen Madsen, Rasmus Riis Kopperud, Hilde Vollan, Karoline Bragstad, Olav Hungnes
EPI_ISL_635110, EPI_ISL_635111	Department of Medical Microbiology, St. Olavs hospital	Norwegian Institute of Public Health, Department of Virology	Kathrine Stene-Johansen, Kamilla Heddeland Instefjord, Hilde Elshaug, Marie Paulsen Madsen, Rasmus Riis Kopperud, Hilde Vollan, Karoline Bragstad, Olav Hungnes
EPI_ISL_635120	Dept. of Medical Microbiology, Stavanger University Hospital, Helse Stavanger HF	Norwegian Institute of Public Health, Department of Virology	Kathrine Stene-Johansen, Kamilla Heddeland Instefjord, Hilde Elshaug, Marie Paulsen Madsen, Rasmus Riis Kopperud, Hilde Vollan, Karoline Bragstad, Olav Hungnes
EPI_ISL_635121, EPI_ISL_635122, EPI_ISL_635123	Hospital of Southern Norway - Kristiansand, Department of Medical Microbiology	Norwegian Institute of Public Health, Department of Virology	Kathrine Stene-Johansen, Kamilla Heddeland Instefjord, Hilde Elshaug, Marie Paulsen Madsen, Rasmus Riis Kopperud, Hilde Vollan, Karoline Bragstad, Olav Hungnes
EPI_ISL_635126	Department of Medical Microbiology, St. Olavs hospital	Norwegian Institute of Public Health, Department of Virology	Kathrine Stene-Johansen, Kamilla Heddeland Instefjord, Hilde Elshaug, Marie Paulsen Madsen, Rasmus Riis Kopperud, Hilde Vollan, Karoline Bragstad, Olav Hungnes
EPI_ISL_635127	Foerde Hospital, Department of Microbiology	Norwegian Institute of Public Health, Department of Virology	Kathrine Stene-Johansen, Kamilla Heddeland Instefjord, Hilde Elshaug, Marie Paulsen Madsen, Rasmus Riis Kopperud, Hilde Vollan, Karoline Bragstad, Olav Hungnes
EPI_ISL_635132	Unilabs Laboratory Medicine	Norwegian Institute of Public Health, Department of Virology	Kathrine Stene-Johansen, Kamilla Heddeland Instefjord, Hilde Elshaug, Marie Paulsen Madsen, Rasmus Riis Kopperud, Hilde Vollan, Karoline Bragstad, Olav Hungnes

EPI_ISL_635135, EPI_ISL_635136	Department of Medical Microbiology, St. Olavs hospital	Norwegian Institute of Public Health, Department of Virology	Kathrine Stene-Johansen, Kamilla Heddeland Instefjord, Hilde Elshaug, Marie Paulsen Madsen, Rasmus Riis Kopperud, Hilde Vollan, Karoline Bragstad, Olav Hungnes
EPI_ISL_635137	University Hospital of Northern Norway, Department for Microbiology and Infectious Disease Control	Norwegian Institute of Public Health, Department of Virology	Kathrine Stene-Johansen, Kamilla Heddeland Instefjord, Hilde Elshaug, Marie Paulsen Madsen, Rasmus Riis Kopperud, Hilde Vollan, Karoline Bragstad, Olav Hungnes
EPI_ISL_635155	Oslo University Hospital, Department of Medical Microbiology	Norwegian Institute of Public Health, Department of Virology	Kathrine Stene-Johansen, Kamilla Heddeland Instefjord, Hilde Elshaug, Marie Paulsen Madsen, Rasmus Riis Kopperud, Hilde Vollan, Karoline Bragstad, Olav Hungnes
EPI_ISL_635158	Unilabs Laboratory Medicine	Norwegian Institute of Public Health, Department of Virology	Kathrine Stene-Johansen, Kamilla Heddeland Instefjord, Hilde Elshaug, Marie Paulsen Madsen, Rasmus Riis Kopperud, Hilde Vollan, Karoline Bragstad, Olav Hungnes
EPI_ISL_635188	Norwegian Institute of Public Health, Department of Virology	Norwegian Institute of Public Health, Department of Virology	Kathrine Stene-Johansen, Kamilla Heddeland Instefjord, Hilde Elshaug, Marie Paulsen Madsen, Rasmus Riis Kopperud, Hilde Vollan, Karoline Bragstad, Olav Hungnes
EPI_ISL_636121, EPI_ISL_636122, EPI_ISL_636123, EPI_ISL_636126, EPI_ISL_636127, EPI_ISL_636130, EPI_ISL_636134, EPI_ISL_636140, EPI_ISL_636141, EPI_ISL_636144, EPI_ISL_636146, EPI_ISL_636147, EPI_ISL_636150, EPI_ISL_636153, EPI_ISL_636156, EPI_ISL_636157, EPI_ISL_636158, EPI_ISL_636160, EPI_ISL_636161			
see above	San Diego County Public Health Laboratory	Andersen lab at Scripps Research	SEARCH Alliance San Diego with Tracy Basler, Jovan Shephard, Brett Austin
EPI_ISL_636267, EPI_ISL_636268, EPI_ISL_636269, EPI_ISL_636270, EPI_ISL_636271, EPI_ISL_636272, EPI_ISL_636273, EPI_ISL_636274, EPI_ISL_636275, EPI_ISL_636276, EPI_ISL_636277, EPI_ISL_636278, EPI_ISL_636279, EPI_ISL_636280, EPI_ISL_636281, EPI_ISL_636282, EPI_ISL_636283, EPI_ISL_636284, EPI_ISL_636285, EPI_ISL_636286, EPI_ISL_636287, EPI_ISL_636288, EPI_ISL_636289, EPI_ISL_636290, EPI_ISL_636291, EPI_ISL_636292, EPI_ISL_636293, EPI_ISL_636294, EPI_ISL_636295, EPI_ISL_636296, EPI_ISL_636297, EPI_ISL_636298, EPI_ISL_636299, EPI_ISL_636300, EPI_ISL_636301, EPI_ISL_636302, EPI_ISL_636303, EPI_ISL_636304, EPI_ISL_636305, EPI_ISL_636306, EPI_ISL_636307, EPI_ISL_636308, EPI_ISL_636309, EPI_ISL_636310, EPI_ISL_636311, EPI_ISL_636312, EPI_ISL_636313, EPI_ISL_636314, EPI_ISL_636315, EPI_ISL_636316, EPI_ISL_636317, EPI_ISL_636318, EPI_ISL_636319, EPI_ISL_636320, EPI_ISL_636321, EPI_ISL_636322, EPI_ISL_636323, EPI_ISL_636324, EPI_ISL_636325, EPI_ISL_636326, EPI_ISL_636327, EPI_ISL_636328			
see above	Biolab Diagnostic Laboratories	Andersen lab at Scripps Research	Issa Abu-Dayyeh, Ahmad Tibi, Lama Hussein, Lina Mohammad, Zein Naber, Amid Abdelnour with SEARCH Alliance San Diego
EPI_ISL_636534, EPI_ISL_636535, EPI_ISL_636579	Dutch COVID-19 response team	National Institute for Public Health and the Environment (RIVM)	Adam Meijer, Harry Vennema, Jeroen Cremer, Sharon van den Brink, Bas van der Veer, AnneMarie van den Brandt, Florian Zwagemaker, Dennis Schmitz, Chantal Reusken, on behalf of the national COVID-19 response team
EPI_ISL_636688, EPI_ISL_636689	Respiratory Virus Unit, Microbiology Services Colindale, Public Health England	Respiratory Virus Unit, Microbiology Services Colindale, Public Health England	PHE Covid Sequencing Team
EPI_ISL_637017	Department of Infectious Diseases and Immunology, National Hospital Organization Nagoya Medical Center	Clinical Research Center, National Hospital Organization Nagoya Medical Center	Yoshihiro Nakata, Hirotaka Ode, Mai Kubota, Masakazu Matsuda, Kazuhiro Matsuoka, Miho Nakasuji, Mikiko Mori, Mayumi Imahashi, Yoshiyuki Yokomaku, Yasumasa Iwatani
EPI_ISL_637307, EPI_ISL_637354, EPI_ISL_637554, EPI_ISL_637598, EPI_ISL_637666	Department of Pathology, University of Cambridge	COVID-19 Genomics UK (COG-UK) Consortium	Aminu S. Jahun, Yasmin Chaudhry, Grant Hall, Iliana Georgana, Myra Hosmillo, Martin D. Curran, Malte Pinckert, Surendra Parmar, Ian Goodfellow
EPI_ISL_637793	Northumbria University / South Tees Hospitals NHS Foundation Trust / North Cumbria Integrated Care NHS Foundation Trust / North Tees and Hartlepool NHS Foundation Trust / Newcastle Hospitals NHS Foundation Trust	COVID-19 Genomics UK (COG-UK) Consortium	Darren L Smith,Andrew Nelson,Matthew Bashton,Greg R Young,Joshua Loh,John Allan,Mohammad A Tariq,Giles S Holt,Gary Black,Wen C Yew,Lynn Dover,Paul Baker,Steve Liggett,Sarah Essex,Jane Greenaway,Debra Padgett,Clive Graham,Garren Scott,Edward Barton,Emma Swindells,Brendan Payne,Jennifer Collins,Yusri Taha,Gary Eltringham
EPI_ISL_637803, EPI_ISL_637804, EPI_ISL_637805, EPI_ISL_637806, EPI_ISL_637807, EPI_ISL_637808, EPI_ISL_637809, EPI_ISL_637856, EPI_ISL_637880, EPI_ISL_637888, EPI_ISL_637916			
see above	Department of Pathology, University of Cambridge	COVID-19 Genomics UK (COG-UK) Consortium	Aminu S. Jahun, Yasmin Chaudhry, Grant Hall, Iliana Georgana, Myra Hosmillo, Martin D. Curran, Malte Pinckert, Surendra Parmar, Ian Goodfellow
EPI_ISL_637995	University College London, Great Ormond Street Hospital for Children NHS Foundation Trust, Imperial College Healthcare NHS Trust	COVID-19 Genomics UK (COG-UK) Consortium	Sergi Castellano, Rachel Williams, Mark Kristiansen, Paola Resende Silva, Sunando Roy, Tony Brooks, Helena Tutill, Paola Niola, Patricia Dyal, Charlotte Williams, Leysa Forrest, Yasmin Panchbhaya, Jacqueline Findlay, Samuel Weeks, Julianne Brown, Kathryn Harris, Paul Randell, James Price, Alison Holmes, Judith Breuer
EPI_ISL_638080, EPI_ISL_638081, EPI_ISL_638082, EPI_ISL_638083, EPI_ISL_638174	Department of Pathology, University of Cambridge	COVID-19 Genomics UK (COG-UK) Consortium	Aminu S. Jahun, Yasmin Chaudhry, Grant Hall, Iliana Georgana, Myra Hosmillo, Martin D. Curran, Malte Pinckert, Surendra Parmar, Ian Goodfellow
EPI_ISL_638562	Northumbria University / South Tees Hospitals NHS Foundation Trust / North Cumbria Integrated Care NHS Foundation Trust / North Tees and Hartlepool NHS Foundation Trust / Newcastle Hospitals NHS Foundation Trust	COVID-19 Genomics UK (COG-UK) Consortium	Darren L Smith,Andrew Nelson,Matthew Bashton,Greg R Young,Joshua Loh,John Allan,Mohammad A Tariq,Giles S Holt,Gary Black,Wen C Yew,Lynn Dover,Paul Baker,Steve Liggett,Sarah Essex,Jane Greenaway,Debra Padgett,Clive Graham,Garren Scott,Edward Barton,Emma Swindells,Brendan Payne,Jennifer Collins,Yusri Taha,Gary Eltringham
EPI_ISL_638642, EPI_ISL_638643, EPI_ISL_638645, EPI_ISL_638646, EPI_ISL_638647, EPI_ISL_638648, EPI_ISL_638649, EPI_ISL_638650, EPI_ISL_638651, EPI_ISL_638652, EPI_ISL_638653, EPI_ISL_638654, EPI_ISL_638655, EPI_ISL_638656, EPI_ISL_638657, EPI_ISL_638658, EPI_ISL_638659, EPI_ISL_638660, EPI_ISL_638661, EPI_ISL_638662, EPI_ISL_638663, EPI_ISL_638664, EPI_ISL_638665, EPI_ISL_638666, EPI_ISL_638667, EPI_ISL_638668, EPI_ISL_638669, EPI_ISL_638670, EPI_ISL_638671, EPI_ISL_638672, EPI_ISL_638673, EPI_ISL_638674, EPI_ISL_638675, EPI_ISL_638676, EPI_ISL_638677, EPI_ISL_638678, EPI_ISL_638679, EPI_ISL_638681, EPI_ISL_638914, EPI_ISL_638915, EPI_ISL_638916, EPI_ISL_638917, EPI_ISL_638918, EPI_ISL_638919, EPI_ISL_638920, EPI_ISL_638921, EPI_ISL_638922, EPI_ISL_638923, EPI_ISL_638924, EPI_ISL_638925, EPI_ISL_638926, EPI_ISL_638927			
see above	Department of Pathology, University of Cambridge	COVID-19 Genomics UK (COG-UK) Consortium	Aminu S. Jahun, Yasmin Chaudhry, Grant Hall, Iliana Georgana, Myra Hosmillo, Martin D. Curran, Malte Pinckert, Surendra Parmar, Ian Goodfellow
EPI_ISL_639668, EPI_ISL_639670, EPI_ISL_639673, EPI_ISL_639675	E. Gulbja Laboratorija	Latvian Biomedical Research and Study Centre	Ivars Silamielis, Kaspars Megnis, Monta Ustinova, ikita Zrelovs, Vita Rovte, Mikus Gavars, Dmitrijs Perminovs, Uga Dumpis, Jnis Kloviš
EPI_ISL_639676, EPI_ISL_639677, EPI_ISL_639678, EPI_ISL_639679, EPI_ISL_639680	Centrl Laboratorija	Latvian Biomedical Research and Study Centre	Ivars Silamielis, Kaspars Megnis, Monta Ustinova, ikita Zrelovs, Vita Rovte, Stella Lapia, Jana Oste, Marta Priedte, Uga Dumpis, Jnis Kloviš
EPI_ISL_639682	E. Gulbja Laboratorija	Latvian Biomedical Research and Study Centre	Ivars Silamielis, Kaspars Megnis, Monta Ustinova, ikita Zrelovs, Vita Rovte, Mikus Gavars, Dmitrijs Perminovs, Uga Dumpis, Jnis Kloviš
EPI_ISL_639892, EPI_ISL_639893, EPI_ISL_639894, EPI_ISL_639895, EPI_ISL_639896, EPI_ISL_639897, EPI_ISL_639898, EPI_ISL_639899, EPI_ISL_639900, EPI_ISL_639901, EPI_ISL_639902, EPI_ISL_639903, EPI_ISL_639905, EPI_ISL_639906, EPI_ISL_639907, EPI_ISL_639908			
see above	National Virus Reference Laboratory	National Virus Reference Laboratory	Michael Carr, Gabriel Gonzalez, Jonathan Dean, Daniel Hare, Cillian F De Gascun
EPI_ISL_639953, EPI_ISL_639954, EPI_ISL_639955, EPI_ISL_639956, EPI_ISL_639957, EPI_ISL_639958, EPI_ISL_639959, EPI_ISL_639962, EPI_ISL_639968, EPI_ISL_639972	HELIX LLC	WHO National Influenza Centre Russian Federation	Andrey Komissarov, Artem Fadeev, Kseniya Komissarova, Anna Ivanova, Dmitry Bazhenov, Daria Danilenko
EPI_ISL_639991, EPI_ISL_639993, EPI_ISL_639995	CNR Virus des Infections Respiratoires - France SUD	CNR Virus des Infections Respiratoires - France SUD	Antonin Bal, Gregory Destras, Gwendolyne Burfin, Hadrien Règue, Alexandre Gaymard, Maude Bouscambert-Duchamp, Florence Morfin-Sherpa, Martine Valette, Bruno Lina, Laurence Josset
EPI_ISL_640084	Groote Schuur Hospital wc GSH	NHLS/UCT	Arash Iranzadeh, Deelan Doolabh, Lynn Tyers, Bruna Galvao, Innocent Mudau, Marvin Hsiao, Kruger Marais, Diana Hardie, Stephen Korsman, Carolyn Williamson
EPI_ISL_640085	Victoria Hospital wc VHW	NHLS/UCT	Arash Iranzadeh, Deelan Doolabh, Lynn Tyers, Bruna Galvao, Innocent Mudau, Marvin Hsiao, Kruger Marais, Diana Hardie, Stephen Korsman, Carolyn Williamson
EPI_ISL_640086	Red Cross Children's Hospital wc RXH	NHLS/UCT	Arash Iranzadeh, Deelan Doolabh, Lynn Tyers, Bruna Galvao, Innocent Mudau, Marvin Hsiao, Kruger Marais, Diana Hardie, Stephen Korsman, Carolyn Williamson
EPI_ISL_640131, EPI_ISL_640132, EPI_ISL_640133, EPI_ISL_640134,	Groote Schuur Hospital wc GSH	NHLS/UCT	Arash Iranzadeh, Deelan Doolabh, Lynn Tyers, Bruna Galvao, Innocent Mudau, Marvin Hsiao, Kruger Marais, Diana Hardie, Stephen Korsman, Carolyn Williamson

EPI_ISL_640135			
EPI_ISL_640136	Red Cross Children's Hospital wc RXH	NHLS/UCT	Arash Iranzadeh, Deelan Doolabh, Lynn Tyers, Bruna Galvao, Innocent Mudau, Marvin Hsiao, Kruger Marais, Diana Hardie, Stephen Korsman, Carolyn Williamson
EPI_ISL_641424, EPI_ISL_641425, EPI_ISL_641426, EPI_ISL_641427, EPI_ISL_641428, EPI_ISL_641429, EPI_ISL_641430, EPI_ISL_641431, EPI_ISL_641432, EPI_ISL_641433, EPI_ISL_641434, EPI_ISL_641435, EPI_ISL_641436, EPI_ISL_641437, EPI_ISL_641438, EPI_ISL_641439, EPI_ISL_641440, EPI_ISL_641441, EPI_ISL_641442, EPI_ISL_641443			
see above	Department of Virus and Microbiological Special Diagnostics, Statens Serum Institut, Copenhagen, Denmark	Albertsen lab, Department of Chemistry and Bioscience, Aalborg University, Denmark	Thomas Bruun Rasmussen, Jannik Fonager, Morten Rasmussen
EPI_ISL_644383, EPI_ISL_644386, EPI_ISL_644389, EPI_ISL_644495, EPI_ISL_644497, EPI_ISL_644498, EPI_ISL_644499, EPI_ISL_644500, EPI_ISL_644501, EPI_ISL_644503, EPI_ISL_644504, EPI_ISL_644505, EPI_ISL_644507, EPI_ISL_644509, EPI_ISL_644510, EPI_ISL_644512, EPI_ISL_644514, EPI_ISL_644515, EPI_ISL_644516, EPI_ISL_644517, EPI_ISL_644518, EPI_ISL_644519, EPI_ISL_644520, EPI_ISL_644521, EPI_ISL_644522, EPI_ISL_644523, EPI_ISL_644524, EPI_ISL_644525, EPI_ISL_644526, EPI_ISL_644527			
see above	MEPHI, Aix Marseille University	MEPHI, Aix Marseille University	Anthony LEVASSEUR
EPI_ISL_644900, EPI_ISL_644901, EPI_ISL_644921, EPI_ISL_644922, EPI_ISL_644923, EPI_ISL_644924, EPI_ISL_644925, EPI_ISL_644926, EPI_ISL_644927, EPI_ISL_644928, EPI_ISL_644929, EPI_ISL_644930, EPI_ISL_644931			
see above	Virginia DCLS	Virginia DCLS	Virginia DCLS
EPI_ISL_644936, EPI_ISL_644938, EPI_ISL_644939	Mayo Clinic & Mayo Clinic Laboratories	Minnesota Department of Health, Public Health Laboratory	Matt Plumb, Jacob Garfin, Alexandra Lorentz, and Xiong Wang
EPI_ISL_645240, EPI_ISL_645473, EPI_ISL_645499, EPI_ISL_645508	Lighthouse Lab in Alderley Park	Wellcome Sanger Institute for the COVID-19 Genomics UK (COG-UK) Consortium	Jacquelyn Wynn, Mairead Hyland, The Lighthouse Lab in Alderley Park and Alex Alderton, Roberto Amato, Sonia Goncalves, Ewan Harrison, David K. Jackson, Ian Johnston, Dominic Kwiatkowski, Cordelia Langford, John Sillitoe on behalf of the Wellcome Sanger Institute COVID-19 Surveillance Team
EPI_ISL_648141	Gavle klinisk mikrobiologi	The Public Health Agency of Sweden	Anna-Malin Linde, Maria Lind Karlberg, Mattias Haukland, Reza Advani, Olov Svartstrom, Oskar Karlsson Lindsjo, Sandra Broddesson, Petra Edquist, Mia Brytting, Anna Risberg, Karin Tegmark-Wisell
EPI_ISL_648146	The Public Health Agency of Sweden	The Public Health Agency of Sweden	Anna-Malin Linde, Maria Lind Karlberg, Mattias Haukland, Reza Advani, Olov Svartstrom, Oskar Karlsson Lindsjo, Sandra Broddesson, Petra Edquist, Mia Brytting, Anna Risberg, Karin Tegmark-Wisell
EPI_ISL_648162	Klinisk mikrobiologi Vasternorrland	The Public Health Agency of Sweden	Anna-Malin Linde, Maria Lind Karlberg, Mattias Haukland, Reza Advani, Olov Svartstrom, Oskar Karlsson Lindsjo, Sandra Broddesson, Petra Edquist, Mia Brytting, Anna Risberg, Karin Tegmark-Wisell
EPI_ISL_648164	Kalmar klinisk mikrobiologi	The Public Health Agency of Sweden	Anna-Malin Linde, Maria Lind Karlberg, Mattias Haukland, Reza Advani, Olov Svartstrom, Oskar Karlsson Lindsjo, Sandra Broddesson, Petra Edquist, Mia Brytting, Anna Risberg, Karin Tegmark-Wisell
EPI_ISL_648174, EPI_ISL_648175, EPI_ISL_648197	The Public Health Agency of Sweden	The Public Health Agency of Sweden	Anna-Malin Linde, Maria Lind Karlberg, Mattias Haukland, Reza Advani, Olov Svartstrom, Oskar Karlsson Lindsjo, Sandra Broddesson, Petra Edquist, Mia Brytting, Anna Risberg, Karin Tegmark-Wisell
EPI_ISL_648205, EPI_ISL_648206	Orebro klinisk mikrobiologi	The Public Health Agency of Sweden	Anna-Malin Linde, Maria Lind Karlberg, Mattias Haukland, Reza Advani, Olov Svartstrom, Oskar Karlsson Lindsjo, Sandra Broddesson, Petra Edquist, Mia Brytting, Anna Risberg, Karin Tegmark-Wisell
EPI_ISL_648468, EPI_ISL_648469, EPI_ISL_648470, EPI_ISL_648471, EPI_ISL_648472, EPI_ISL_648473	Santa Clara County Public Health Laboratory	Chan-Zuckerberg Biohub	CZB Cliahub Consortium
EPI_ISL_648537, EPI_ISL_648538	Tulare County Public Health Lab	Chan-Zuckerberg Biohub	CZB Cliahub Consortium
EPI_ISL_648922, EPI_ISL_648931, EPI_ISL_648933, EPI_ISL_648938, EPI_ISL_648945, EPI_ISL_648946, EPI_ISL_648952, EPI_ISL_648958, EPI_ISL_648959, EPI_ISL_648963, EPI_ISL_649026, EPI_ISL_649028, EPI_ISL_649032, EPI_ISL_649034, EPI_ISL_649035, EPI_ISL_649039, EPI_ISL_649045, EPI_ISL_649054			
see above	San Diego County Public Health Laboratory	Andersen lab at Scripps Research	SEARCH Alliance San Diego with Tracy Basler, Jovan Shephard, Brett Austin
EPI_ISL_650503	West of Scotland Specialist Virology Centre, NHSGGC / MRC-University of Glasgow Centre for Virus Research	COVID-19 Genomics UK (COG-UK) Consortium	Ana da Silva Filipe, Natasha Johnson, Kathy Smollett, Daniel Mair, Stephen Carmichael, Alice Broos, Lily Tong, Jenna Nichols, Kyriaki Nomikou; Sarah McDonald; Richard Orton, Joseph Hughes, Sreenu Vattipally, David L Robertson; Alasdair MacLean, Rory Gunson; Sharif Shaaban, Matthew Holden; Rachel Blacow, Guy Mollett, Kathy Li, James Shepherd, Antonia Ho, Emma Thomson
EPI_ISL_650684	Northumbria University / South Tees Hospitals NHS Foundation Trust / North Cumbria Integrated Care NHS Foundation Trust / North Tees and Hartlepool NHS Foundation Trust / Newcastle Hospitals NHS Foundation Trust	COVID-19 Genomics UK (COG-UK) Consortium	Darren L Smith,Andrew Nelson,Matthew Bashton,Greg R Young,Joshua Loh,John Allan,Mohammad A Tariq,Giles S Holt,Gary Black,Wen C Yew,Lynn Dover,Paul Baker,Steve Liggett,Sarah Essex,Jane Greenaway,Debra Padgett,Clive Graham,Garren Scott,Edward Barton,Emma Swindells,Brendan Payne,Jennifer Collins,Yusri Taha,Gary Eltringham
EPI_ISL_650762	West of Scotland Specialist Virology Centre, NHSGGC / MRC-University of Glasgow Centre for Virus Research	COVID-19 Genomics UK (COG-UK) Consortium	Ana da Silva Filipe, Natasha Johnson, Kathy Smollett, Daniel Mair, Stephen Carmichael, Alice Broos, Lily Tong, Jenna Nichols, Kyriaki Nomikou; Sarah McDonald; Richard Orton, Joseph Hughes, Sreenu Vattipally, David L Robertson; Alasdair MacLean, Rory Gunson; Sharif Shaaban, Matthew Holden; Rachel Blacow, Guy Mollett, Kathy Li, James Shepherd, Antonia Ho, Emma Thomson
EPI_ISL_651126, EPI_ISL_651356	Northumbria University / South Tees Hospitals NHS Foundation Trust / North Cumbria Integrated Care NHS Foundation Trust / North Tees and Hartlepool NHS Foundation Trust / Newcastle Hospitals NHS Foundation Trust	COVID-19 Genomics UK (COG-UK) Consortium	Darren L Smith,Andrew Nelson,Matthew Bashton,Greg R Young,Joshua Loh,John Allan,Mohammad A Tariq,Giles S Holt,Gary Black,Wen C Yew,Lynn Dover,Paul Baker,Steve Liggett,Sarah Essex,Jane Greenaway,Debra Padgett,Clive Graham,Garren Scott,Edward Barton,Emma Swindells,Brendan Payne,Jennifer Collins,Yusri Taha,Gary Eltringham
EPI_ISL_651498, EPI_ISL_651583	West of Scotland Specialist Virology Centre, NHSGGC / MRC-University of Glasgow Centre for Virus Research	COVID-19 Genomics UK (COG-UK) Consortium	Ana da Silva Filipe, Natasha Johnson, Kathy Smollett, Daniel Mair, Stephen Carmichael, Alice Broos, Lily Tong, Jenna Nichols, Kyriaki Nomikou; Sarah McDonald; Richard Orton, Joseph Hughes, Sreenu Vattipally, David L Robertson; Alasdair MacLean, Rory Gunson; Sharif Shaaban, Matthew Holden; Rachel Blacow, Guy Mollett, Kathy Li, James Shepherd, Antonia Ho, Emma Thomson
EPI_ISL_652344	Northumbria University / South Tees Hospitals NHS Foundation Trust / North Cumbria Integrated Care NHS Foundation Trust / North Tees and Hartlepool NHS Foundation Trust / Newcastle Hospitals NHS Foundation Trust	COVID-19 Genomics UK (COG-UK) Consortium	Darren L Smith,Andrew Nelson,Matthew Bashton,Greg R Young,Joshua Loh,John Allan,Mohammad A Tariq,Giles S Holt,Gary Black,Wen C Yew,Lynn Dover,Paul Baker,Steve Liggett,Sarah Essex,Jane Greenaway,Debra Padgett,Clive Graham,Garren Scott,Edward Barton,Emma Swindells,Brendan Payne,Jennifer Collins,Yusri Taha,Gary Eltringham
EPI_ISL_653785, EPI_ISL_653786	Istituto Zooprofilattico Sperimentale della Puglia e della Basilicata	Istituto Zooprofilattico Sperimentale della Puglia e della Basilicata	Parisi A., Bianco A., Capozzi L., Del Sambro L., Manzulli V, Rondinone V., Pace L., Cipolletta D., Galante D.
EPI_ISL_654162, EPI_ISL_654163, EPI_ISL_654164, EPI_ISL_654165, EPI_ISL_654169, EPI_ISL_654171, EPI_ISL_654174, EPI_ISL_654175, EPI_ISL_654176, EPI_ISL_654177, EPI_ISL_654178, EPI_ISL_654180, EPI_ISL_654183, EPI_ISL_654185, EPI_ISL_654194, EPI_ISL_654205, EPI_ISL_654206, EPI_ISL_654207, EPI_ISL_654208, EPI_ISL_654209, EPI_ISL_654210, EPI_ISL_654211, EPI_ISL_654212, EPI_ISL_654213, EPI_ISL_654214, EPI_ISL_654215, EPI_ISL_654216, EPI_ISL_654217, EPI_ISL_654218, EPI_ISL_654219, EPI_ISL_654220, EPI_ISL_654221, EPI_ISL_654222, EPI_ISL_654223, EPI_ISL_654224, EPI_ISL_654225, EPI_ISL_654226, EPI_ISL_654227, EPI_ISL_654228, EPI_ISL_654229, EPI_ISL_654230, EPI_ISL_654283			
see above	Hospital General Universitario Gregorio Marañón	SeqCOVID-SPAIN consortium/IBV(CSIC)	Darío García de Viedma, Laura Pérez-Lago, Marta Herranz, Jon Sicilia, Julia Suárez, Pilar Catalán, Patricia Muñoz and SeqCOVID-SPAIN consortium
EPI_ISL_654401, EPI_ISL_654404, EPI_ISL_654406, EPI_ISL_654412, EPI_ISL_654414, EPI_ISL_654416, EPI_ISL_654421, EPI_ISL_654422, EPI_ISL_654423, EPI_ISL_654424, EPI_ISL_654425, EPI_ISL_654433, EPI_ISL_654435, EPI_ISL_654437, EPI_ISL_654438, EPI_ISL_654439, EPI_ISL_654441, EPI_ISL_654442, EPI_ISL_654443, EPI_ISL_654444, EPI_ISL_654445, EPI_ISL_654446, EPI_ISL_654447, EPI_ISL_654448, EPI_ISL_654449, EPI_ISL_654450, EPI_ISL_654451, EPI_ISL_654452, EPI_ISL_654453, EPI_ISL_654454			
see above	Servicio de Microbiología, Hospital Miguel Servet, Zaragoza	SeqCOVID-SPAIN consortium/IBV(CSIC)	Antonio Rezusta López, Alexander Tristancho Baró, Ana Milagro, Yolanda Gracia Grataloup, Nieves Martínez Cameo and SeqCOVID-SPAIN consortium
EPI_ISL_654510, EPI_ISL_654521	Servicio de Microbiología, Laboratori Clínic Metropolitana Nord. Hospital Universitari Germans Trias i Pujol. Institut d'Investigació en Ciències de la Salut Germans Trias i Pujol (IGTP)	SeqCOVID-SPAIN consortium/IBV(CSIC)	Elisa Martró, Antoni E. Bordoy, Anna Not, Adrián Antuori, Anabel Fernández, Nona Romaní and SeqCOVID-SPAIN consortium
EPI_ISL_654553, EPI_ISL_654554	Servicio de Microbiología. Hospital Universitario Donostia.	SeqCOVID-SPAIN consortium/IBV(CSIC)	Gustavo Cilla Eguiluz, Milagrosa Montes Ros, Luis Piñeiro Vázquez, Ane Sorrarain, Jose Maria Marimón and SeqCOVID-SPAIN consortium

	OSI Donostialdea. Área de Enfermedades Infecciosas, Grupo de Infección Respiratoria y Resistencia Antimicrobiana. Instituto de Investigación Sanitaria Biodonostia		
EPI_ISL_654696, EPI_ISL_654697	Essentia Health-St. Mary's Medical Center	Minnesota Department of Health, Public Health Laboratory	Matt Plumb, Jacob Garfin, Alexandra Lorentz, and Xiong Wang
EPI_ISL_660466, EPI_ISL_660467, EPI_ISL_660504	Laboratoire de Microbiologie CHU Souro Sanou	Centre Muraz	Abdoul-Salam Ouedraogo, Yacouba Sawadogo, Essia Belarbi, Grit Schubert, Fabian Leendertz, Arsène Zongo, Soumeya Ouangraoua, Zekiba Tarnagda, Lassana Sangaré, Halidou Tinto
EPI_ISL_660529, EPI_ISL_660530, EPI_ISL_660531	Institute of Microbiology, Universidad San Francisco de Quito	Institute of Microbiology, Universidad San Francisco de Quito	Sully Márquez, Belén Prado-Vivar, Juan José Guadalupe, Monica Becerra-Wong, Bernardo Gutiérrez, Manuel Jaramillo, Verónica Barragán, Patricio Rojas-Silva, Gabriel Trueba, Michelle Grunauer, Paúl Cárdenas
EPI_ISL_660558	The National Institute of Public Health	State Veterinary Institute Prague	Nagy,A;Jirincova,H;Novakova,L;Trnka,D;Vecerova,J
EPI_ISL_660854, EPI_ISL_660855, EPI_ISL_660856, EPI_ISL_660857, EPI_ISL_660858, EPI_ISL_660859, EPI_ISL_660860, EPI_ISL_660861, EPI_ISL_660862, EPI_ISL_660863, EPI_ISL_660864, EPI_ISL_660865, EPI_ISL_660866, EPI_ISL_660867, EPI_ISL_660868			
see above	Gundersen Molecular Diagnostics Laboratory	Kabara Cancer Research Institute	Craig S. Richmond, Paraic A. Kenny
EPI_ISL_660869, EPI_ISL_660870, EPI_ISL_660871, EPI_ISL_660872, EPI_ISL_660873, EPI_ISL_660874	Gundersen Clinical Microbiology Laboratory	Kabara Cancer Research Institute	Craig S. Richmond, Paraic A. Kenny
EPI_ISL_661284, EPI_ISL_661285	Klinisk mikrobiologi Linkoping	The Public Health Agency of Sweden	Department of Microbiology, The Public Health Agency of Sweden
EPI_ISL_664281, EPI_ISL_665158, EPI_ISL_665159, EPI_ISL_665160, EPI_ISL_665183, EPI_ISL_665184, EPI_ISL_665185, EPI_ISL_665223	University College London Hospital	COVID-19 Genomics UK (COG-UK) Consortium	Judith Heaney, Matthew Byott, Catherine Houlihan, Dan Frampton, Stuart Kirk, Moira Spyer and Eleni Nastouli
EPI_ISL_665278	Northumbria University / South Tees Hospitals NHS Foundation Trust / North Cumbria Integrated Care NHS Foundation Trust / North Tees and Hartlepool NHS Foundation Trust / Newcastle Hospitals NHS Foundation Trust	COVID-19 Genomics UK (COG-UK) Consortium	Darren L Smith,Andrew Nelson,Matthew Bashton,Greg R Young,Joshua Loh,John Allan,Mohammad A Tariq,Giles S Holt,Gary Black,Wen C Yew,Lynn Dover,Paul Baker,Steve Liggett,Sarah Essex,Jane Greenaway,Debra Padgett,Clive Graham,Garren Scott,Edward Barton,Emma Swindells,Brendan Payne,Jennifer Collins,Yusri Taha,Gary Eltringham
EPI_ISL_667454, EPI_ISL_667455, EPI_ISL_667456, EPI_ISL_667457, EPI_ISL_667458, EPI_ISL_667459, EPI_ISL_667460, EPI_ISL_667461, EPI_ISL_667462, EPI_ISL_667463, EPI_ISL_667464, EPI_ISL_667465, EPI_ISL_667466, EPI_ISL_667467, EPI_ISL_667468, EPI_ISL_667469, EPI_ISL_667470, EPI_ISL_667471, EPI_ISL_667472, EPI_ISL_667473, EPI_ISL_667474, EPI_ISL_667475, EPI_ISL_667476, EPI_ISL_667477, EPI_ISL_667478, EPI_ISL_667479, EPI_ISL_667480, EPI_ISL_667481, EPI_ISL_667482, EPI_ISL_667484			
see above	OHSU Lab Services Molecular Microbiology Lab	Oregon SARS-CoV-2 Genome Sequencing Center	Brendan L. O'Connell, Ruth V. Nichols, Sally Grindstaff, Alec J. Hirsch, Donna Hansel, Guang Fan, Daniel N. Streblow, William B. Messer, Andrew C. Adey, Benjamin N. Bimber, Brian J. O'Roak
EPI_ISL_667611, EPI_ISL_667612, EPI_ISL_667613, EPI_ISL_667614, EPI_ISL_667615, EPI_ISL_667616, EPI_ISL_667617, EPI_ISL_667618, EPI_ISL_667619, EPI_ISL_667620, EPI_ISL_667637, EPI_ISL_667638, EPI_ISL_667667, EPI_ISL_667668, EPI_ISL_667669, EPI_ISL_667670, EPI_ISL_667671, EPI_ISL_667672, EPI_ISL_667673, EPI_ISL_667674, EPI_ISL_667675, EPI_ISL_667676, EPI_ISL_667677, EPI_ISL_667678, EPI_ISL_667679, EPI_ISL_667680, EPI_ISL_667681, EPI_ISL_667682, EPI_ISL_667683, EPI_ISL_667684, EPI_ISL_667685, EPI_ISL_667686, EPI_ISL_667687, EPI_ISL_667688, EPI_ISL_667689, EPI_ISL_667690, EPI_ISL_667691, EPI_ISL_667692, EPI_ISL_667693, EPI_ISL_667737, EPI_ISL_667738, EPI_ISL_667739			
see above	Pathogen Genomics Center, National Institute of Infectious Diseases	Pathogen Genomics Center, National Institute of Infectious Diseases	Tsuyoshi Sekizuka, Kentaro Itokawa, Rina Tanaka, Masanori Hashino, Makoto Kuroda
EPI_ISL_668390	Vestfold Hospital, Toensberg Department of Microbiology	Norwegian Institute of Public Health, Department of Virology	Kathrine Stene-Johansen, Kamilla Heddeland Instefjord, Hilde Elshaug, Marie Paulsen Madsen, Rasmus Riis Kopperud, Hilde Vollan, Karoline Bragstad, Olav Hungnes
EPI_ISL_668393	Akershus University Hospital, Department for Microbiology and Infectious Disease Control	Norwegian Institute of Public Health, Department of Virology	Kathrine Stene-Johansen, Kamilla Heddeland Instefjord, Hilde Elshaug, Marie Paulsen Madsen, Rasmus Riis Kopperud, Hilde Vollan, Karoline Bragstad, Olav Hungnes
EPI_ISL_668439	Ostfold Hospital Trust - Kalnes, Centre for Laboratory Medicine, Section for gene technology and infection serology	Norwegian Institute of Public Health, Department of Virology	Kathrine Stene-Johansen, Kamilla Heddeland Instefjord, Hilde Elshaug, Marie Paulsen Madsen, Rasmus Riis Kopperud, Hilde Vollan, Karoline Bragstad, Olav Hungnes
EPI_ISL_671652, EPI_ISL_671655, EPI_ISL_671660, EPI_ISL_671661, EPI_ISL_671662, EPI_ISL_671663, EPI_ISL_671664, EPI_ISL_671668, EPI_ISL_671670, EPI_ISL_671671	Unity Health Toronto	Ontario Institute for Cancer Research	Ramzi Fattouh, Larissa M. Matukas, Yan Chen,Mark Downing, Trina Otterman, Karel Boissinot, Wai Sum Siu, Zhi Cui, Le Luu, Samira Mubareka, TIBDN, Ilinca Lungu, Bernard Lam, Jeremy Johns, Paul Krzyzanowski, Richard de Borja, Felicia Vincelli, Philip Zuzarte, Jared T. Simpson
EPI_ISL_671978	Laboratorio de Virología y Microbiología Molecular, Depto. de Microbiología, Facultad de Medicina, Universidad de El Salvador/INS-laboratorio de Ref. Ministerio de Salud	Laboratorio de Virología y Microbiología Molecular, Depto. de Microbiología, Facultad de Medicina, Universidad de El Salvador/INS-laboratorio de Ref. Ministerio de Salud	Rivera NR et al
EPI_ISL_672012	Laboratorio de Virología y Microbiología Molecular, Depto. de Microbiología, Facultad de Medicina, Universidad de El Salvador/INS-laboratorio de Ref. Ministerio de Salud. 1*: Dr. Noé Rigoberto Rivera, profesor del Departamento de Bioquímica e Investigador adjunto de la Sección de Virología y Microbiología Molecular; director de la Unidad de Investigaciones Científicas (UNICA), Facultad de Medicina Universidad de El Salvador, El Salvador C.A 1**: Dr. Carlos Alexander Ortega Pérez, Profesor del Departamento de Microbiología; investigador y Jefe de la Sección de Virología, Director Metodológico de la Unidad de Investigaciones Científicas (UNICA), Facultad de Medicina, Universidad de El Salvador, El Salvador C.A 2*: Dra. Xochitl Sandoval López: Directora e investigadora del Instituto Nacional de Salud (INS) El Salvador C.A 2** Dr. Hernandez Avila Carlos E Director de Gobernanza e investigador del Instituto Nacional de Salud (INS) El Salvador C.A	Laboratorio de Virología y Microbiología Molecular, Depto. de Microbiología, Facultad de Medicina, Universidad de El Salvador/INS-laboratorio de Ref. Ministerio de Salud.	Ortega Pérez CA et al
EPI_ISL_672033, EPI_ISL_672089	Santa Clara County Public Health Laboratory	Chan-Zuckerberg Biohub	CZB Cliahub Consortium
EPI_ISL_672109	Orange County Public Health Lab	Chan-Zuckerberg Biohub	CZB Cliahub Consortium
EPI_ISL_672111, EPI_ISL_672277, EPI_ISL_672278, EPI_ISL_672279, EPI_ISL_672280, EPI_ISL_672281, EPI_ISL_672282, EPI_ISL_672283	Santa Clara County Public Health Laboratory	Chan-Zuckerberg Biohub	CZB Cliahub Consortium
EPI_ISL_672570	Laboratorio de Virología y Microbiología Molecular, Depto. de Microbiología, Facultad de Medicina, Universidad de El Salvador/INS-laboratorio de Ref. Ministerio de Salud	Laboratorio de Virología y Microbiología Molecular, Depto. de Microbiología, Facultad de Medicina, Universidad de El Salvador/INS-laboratorio de Ref. Ministerio de Salud	Ortega Pérez CA et al
EPI_ISL_672572	Laboratorio de Virología y Microbiología Molecular, Depto. de	Laboratorio de Virología y Microbiología Molecular, Depto. de	Rivera NR et al

	Microbiología, Facultad de Medicina, Universidad de El Salvador/INS-laboratorio de Ref. Ministerio de Salud. 1*: Dr. Noé Rigoberto Rivera, profesor del Departamento de Bioquímica e Investigador adjunto de la Sección de Virología y Microbiología Molecular; director de la Unidad de Investigaciones Científicas (UNICA), Facultad de Medicina Universidad de El Salvador, El Salvador C.A 1**: Dr. Carlos Alexander Ortega Pérez, Profesor del Departamento de Microbiología; investigador y Jefe de la Sección de Virología, Director Metodológico de la Unidad de Investigaciones Científicas (UNICA), Facultad de Medicina, Universidad de El Salvador, El Salvador C.A 2*: Dra. Xochitl Sandoval López: Directora e investigadora del Instituto Nacional de Salud (INS) El Salvador C.A 2** Dr. Hernandez Avila Carlos E Director de Gobernanza e investigador del Instituto Nacional de Salud (INS) El Salvador C.A	Microbiología, Facultad de Medicina, Universidad de El Salvador/INS-laboratorio de Ref. Ministerio de Salud.	
EPI_ISL_672644, EPI_ISL_672645, EPI_ISL_672646, EPI_ISL_672647, EPI_ISL_672648, EPI_ISL_672649, EPI_ISL_672650, EPI_ISL_672651, EPI_ISL_672652, EPI_ISL_672653	PathWest Laboratory Medicine WA	PathWest Laboratory Medicine WA Microbial Surveillance Unit	PathWest Laboratory Medicine WA Microbial Surveillance Unit
EPI_ISL_676493	Orebro klinisk mikrobiologi	The Public Health Agency of Sweden	Department of Microbiology, The Public Health Agency of Sweden
EPI_ISL_676506	Klinisk mikrobiologi	The Public Health Agency of Sweden	Department of Microbiology, The Public Health Agency of Sweden
EPI_ISL_677131, EPI_ISL_677132, EPI_ISL_677133	Wadsworth Center, New York State Department.of Health	Wadsworth Center, New York State Department.of Health	Kirsten St. George, Daryl M. Lamson, Alexis Russel, Jonathan Plitnick, Navjot Singh, John Kelly, Sara Griesemer, Erasmus Schneider, Erica Lasek-Nesselquist
EPI_ISL_677344, EPI_ISL_677346, EPI_ISL_677353, EPI_ISL_677354, EPI_ISL_677355, EPI_ISL_677357, EPI_ISL_677358, EPI_ISL_677359, EPI_ISL_677362, EPI_ISL_677366, EPI_ISL_677368, EPI_ISL_677370, EPI_ISL_677373, EPI_ISL_677375, EPI_ISL_677380, EPI_ISL_677384, EPI_ISL_677385, EPI_ISL_677388, EPI_ISL_677390, EPI_ISL_677394, EPI_ISL_677395, EPI_ISL_677396, EPI_ISL_677397, EPI_ISL_677399, EPI_ISL_677400, EPI_ISL_677401, EPI_ISL_677402, EPI_ISL_677403, EPI_ISL_677404, EPI_ISL_677405, EPI_ISL_677406, EPI_ISL_677407, EPI_ISL_677408, EPI_ISL_677427, EPI_ISL_677429, EPI_ISL_677432, EPI_ISL_677437, EPI_ISL_677438, EPI_ISL_677443, EPI_ISL_677444, EPI_ISL_677446, EPI_ISL_677451, EPI_ISL_677453, EPI_ISL_677465, EPI_ISL_677474, EPI_ISL_677493, EPI_ISL_677494, EPI_ISL_677495, EPI_ISL_677497, EPI_ISL_677509, EPI_ISL_677510, EPI_ISL_677511, EPI_ISL_677512, EPI_ISL_677514, EPI_ISL_677516, EPI_ISL_677517, EPI_ISL_677518, EPI_ISL_677520, EPI_ISL_677525, EPI_ISL_677529, EPI_ISL_677530, EPI_ISL_677534, EPI_ISL_677535, EPI_ISL_677536, EPI_ISL_677620, EPI_ISL_677621, EPI_ISL_677622, EPI_ISL_677623, EPI_ISL_677625, EPI_ISL_677660			
see above	University of Wisconsin-Madison AIDS Vaccine Research Laboratories	University of Wisconsin-Madison AIDS Vaccine Research Laboratories	Gage Moreno, Katarina Braun, et al. AIDS Vaccine Research Laboratories
EPI_ISL_677727, EPI_ISL_677769, EPI_ISL_677772, EPI_ISL_677773, EPI_ISL_677774, EPI_ISL_677777, EPI_ISL_677782, EPI_ISL_677789, EPI_ISL_677790, EPI_ISL_677795, EPI_ISL_677797, EPI_ISL_677798, EPI_ISL_677799, EPI_ISL_677801, EPI_ISL_677802, EPI_ISL_677803, EPI_ISL_677804, EPI_ISL_677805, EPI_ISL_677815			
see above	University of Szeged, Institute of Clinical Microbiology	National Laboratory of Virology, Szentágothai Research Centre	Endre Gábor Tóth, Balázs Somogyi, Brigitta, Gabriella Terhes, Ferenc Jakab, Gábor Kemenesi
EPI_ISL_678351	Area of Virology, Serology and Virology Division (SAViD), New South Wales Health Pathology Randwick	Virology Research Laboratory; Area of Virology, Serology and Virology Division (SAViD), New South Wales Health Pathology Randwick	Foster, C.; Au, J.; Ruiz Silva, M.; Deveson, I.; Bull, R.; Van Hal, S.; Rawlinson, W.
EPI_ISL_679627, EPI_ISL_679628	University College London, Great Ormond Street Hospital for Children NHS Foundation Trust, Imperial College Healthcare NHS Trust	COVID-19 Genomics UK (COG-UK) Consortium	Sergi Castellano, Rachel Williams, Mark Kristiansen, Paola Resende Silva, Sunando Roy, Tony Brooks, Helena Tutill, Paola Niola, Patricia Dyal, Charlotte Williams, Leysa Forrest, Yasmin Panchbhaya, Jacqueline Findlay, Samuel Weeks, Julianne Brown, Kathryn Harris, Paul Randell, James Price, Alison Holmes, Judith Breuer
EPI_ISL_679986, EPI_ISL_679987, EPI_ISL_679988, EPI_ISL_679989, EPI_ISL_679990, EPI_ISL_679991, EPI_ISL_679992, EPI_ISL_679993, EPI_ISL_679994, EPI_ISL_679995, EPI_ISL_679996, EPI_ISL_679997, EPI_ISL_679998, EPI_ISL_679999, EPI_ISL_680000, EPI_ISL_680001, EPI_ISL_680002, EPI_ISL_680003, EPI_ISL_680004, EPI_ISL_680005, EPI_ISL_680006, EPI_ISL_680007, EPI_ISL_680008, EPI_ISL_680009, EPI_ISL_680010, EPI_ISL_680011, EPI_ISL_680012, EPI_ISL_680013, EPI_ISL_680014, EPI_ISL_680015			
see above	Virology Department, Sheffield Teaching Hospitals NHS Foundation Trust/Department of Infection, Immunity and Cardiovascular Disease, The Medical School, University of Sheffield	COVID-19 Genomics UK (COG-UK) Consortium	Thushan de Silva, Matthew Parker, Nikki Smith, Adri Angyal, Rebecca Brown, Luke Green, Rachel Tucker, Paul Parsons, Danielle Groves, Katie Johnson, Laura Carrilero, Alex Keeley, Dave Partridge, Matthew Wyles, Benjamin Lindsey, Mehmet Yavuz, Mohammad Raza, Cariad Evans
EPI_ISL_680231, EPI_ISL_680232, EPI_ISL_680233, EPI_ISL_680234, EPI_ISL_680235, EPI_ISL_680236, EPI_ISL_680237, EPI_ISL_680238, EPI_ISL_680239, EPI_ISL_680240, EPI_ISL_680241, EPI_ISL_680242, EPI_ISL_680243, EPI_ISL_680244, EPI_ISL_680245, EPI_ISL_680246, EPI_ISL_680247, EPI_ISL_680248, EPI_ISL_680249, EPI_ISL_680250, EPI_ISL_680251, EPI_ISL_680252, EPI_ISL_680253, EPI_ISL_680254, EPI_ISL_680255, EPI_ISL_680256, EPI_ISL_680257, EPI_ISL_680259, EPI_ISL_680260, EPI_ISL_680261, EPI_ISL_680262, EPI_ISL_680263, EPI_ISL_680264, EPI_ISL_680274, EPI_ISL_680275, EPI_ISL_680276, EPI_ISL_680277, EPI_ISL_680278, EPI_ISL_680282, EPI_ISL_680292, EPI_ISL_680293, EPI_ISL_680294, EPI_ISL_680295, EPI_ISL_680298, EPI_ISL_680299, EPI_ISL_680305, EPI_ISL_680306, EPI_ISL_680307, EPI_ISL_680308, EPI_ISL_680309, EPI_ISL_680310, EPI_ISL_680311, EPI_ISL_680312, EPI_ISL_680313, EPI_ISL_680314, EPI_ISL_680315, EPI_ISL_680441			
see above	Regional Virus Laboratory, Belfast Health and Social Care Trust	COVID-19 Genomics UK (COG-UK) Consortium	Conall McCaughey, James McKenna, Tanya Curran, Susan Feeney, Alison Watt, Ciara Cox, Mairead Connor, Zoltan Molnar, David Simpson, Derek Fairley
EPI_ISL_681321	Pathology and Laboratory Medicine, UW-Madison	Pathology and Laboratory Medicine, UW-Madison	Moreno,G., Braun,K., Baczenas,J.J., Baker,D.
EPI_ISL_682330, EPI_ISL_682332, EPI_ISL_682334, EPI_ISL_682335, EPI_ISL_682337, EPI_ISL_682342, EPI_ISL_682344	NHLS Universitas Academic	UFS Virology	PA Bester, MM Nyaga, P Nthiga, MT Mogotsi, D Goedhals, T de Oliveira
EPI_ISL_684014	Utah Public Health Laboratory	Utah Public Health Laboratory	Erin Young, Kelly Oakeson
EPI_ISL_686535, EPI_ISL_686536, EPI_ISL_686537, EPI_ISL_686538, EPI_ISL_686539, EPI_ISL_686540, EPI_ISL_686597	Respiratory Virus Unit, Microbiology Services Colindale, Public Health England	COVID-19 Genomics UK (COG-UK) Consortium	PHE Covid Sequencing Team
EPI_ISL_691616, EPI_ISL_691624, EPI_ISL_691644, EPI_ISL_691651, EPI_ISL_691667, EPI_ISL_691668, EPI_ISL_691678	Servicio de Microbiología, Hospital Universitario Son Espases	SeqCOVID-SPAIN consortium/IBV(CSIC)	Carla López-Causapé, Jordi Reina, Antonio Oliver and SeqCOVID-SPAIN consortium
EPI_ISL_692866, EPI_ISL_692867, EPI_ISL_692901, EPI_ISL_692903, EPI_ISL_692904, EPI_ISL_692906, EPI_ISL_692907, EPI_ISL_692908, EPI_ISL_692909, EPI_ISL_692911, EPI_ISL_692912, EPI_ISL_692913, EPI_ISL_692914, EPI_ISL_692915, EPI_ISL_692916, EPI_ISL_692917, EPI_ISL_692918, EPI_ISL_692919, EPI_ISL_692920, EPI_ISL_692921, EPI_ISL_692922, EPI_ISL_692923, EPI_ISL_692924, EPI_ISL_692925			
see above	Massachusetts State Public Health Laboratory	Massachusetts State Public Health Laboratory	Andrew Lang, Timelia Fink, Glen Gallagher, Sandra Smole
EPI_ISL_693480	Central Public Health Laboratory	National Public Health Laboratory, National Centre for Infectious Diseases	Tze Minn Mak, Sophie Octavia, Zhenyang Zhou, Esorom Daoni, Theresa Palou, Lin Cui, Raymond Tzer Pin Lin
EPI_ISL_693512	CNR Virus des Infections Respiratoires - France SUD	CNR Virus des Infections Respiratoires - France SUD	Antonin Bal, Gregory Destras, Gwendolyne Burfin, Quentin Semanas, Martine Valette, Bruno Lina, Laurence Josset
EPI_ISL_693692, EPI_ISL_693721, EPI_ISL_693746	Delaware Public Health Laboratory	Delaware Public Health Laboratory	Gregory Hovan

EPI_ISL_694046	Los Angeles County Public Health Laboratory	Los Angeles County Public Health Laboratory	Peera Hemarajata et al.
EPI_ISL_697794, EPI_ISL_697795	Institute of Microbiology, Universidad San Francisco de Quito	Institute of Microbiology, Universidad San Francisco de Quito	Andrea Macias, Belén Prado-Vivar, Sully Márquez, Juan José Guadalupe, Monica Becerra-Wong, Bernardo Gutiérrez, Verónica Barragán, Patricio Rojas-Silva, Gabriel Trueba, Michelle Grunauer, Paúl Cárdenas
EPI_ISL_700319, EPI_ISL_700320, EPI_ISL_700321, EPI_ISL_700322, EPI_ISL_700323, EPI_ISL_700324, EPI_ISL_700325	Hematopathology Laboratory, ACTREC, TMC	Hematopathology Laboratory, ACTREC, TMC	Hematopathology Laboratory, ACTREC
EPI_ISL_700347	Child Health Research Foundation	Child Health Research Foundation	Senjuti Saha, Afroza Akter Tanni, Syed Muktadir Al Sium, Roly Malaker, Sharmistha Goswami, Arif Mohammad Tanmoy, Md Hafizur Rahman, Samir K Saha
EPI_ISL_700727, EPI_ISL_700731, EPI_ISL_700739	Texas Department of State Health Services	Texas Department of State Health Services	Rashmi Tuladhar, Bonnie Oh, Jenny Zhang, Maliha Rahman, Anita Pokharel, Myong Koag, Chung Wang, Rachel Lee, Grace Kubin, Mayela Pedrueza, James Daniel Bonser
EPI_ISL_705476	Oxford Viromics, NDM, University of Oxford; Oxford University Hospitals; Basingstoke and North Hampshire Hospital	COVID-19 Genomics UK (COG-UK) Consortium	Tanya Golubchik, David Bonsall, George Macintyre, Amy Trebes, Mariateresa de Cesare, Catrin Moore, Alex Mobbs, Anita Justice, Robert Shaw, Monique Andersson, Timothy Peto, Emma Wise, Nathan Moore, Jessica Lynch, Nick Cortes, Matilde Mori, Stephen Kidd, David Buck, John Todd, Christophe Fraser
EPI_ISL_707694	Los Angeles County Public Health Laboratory	Los Angeles County Public Health Laboratory	P. Hemarajata et al.
EPI_ISL_707778	Medical Research Center, Faculty of Medicine, Syarif Hidayatullah State Islamic University Jakarta	Medical Research Center, Faculty of Medicine, Syarif Hidayatullah State Islamic University Jakarta	Erike A Suwarsono, Chris Adhiyanto, Laifa Hendarmin, Zeti Harriyati, Rini Puspitaningrum, Ferania Mela, Dennis Nurjadi
EPI_ISL_708395, EPI_ISL_708429, EPI_ISL_708430	Delaware Public Health Lab	Delaware Public Health Lab	Gregory Hovan
EPI_ISL_709947	Lighthouse Lab in Milton Keynes	Wellcome Sanger Institute for the COVID-19 Genomics UK (COG-UK) Consortium	The Lighthouse Lab in Milton Keynes and Alex Alderton, Roberto Amato, Sonia Goncalves, Ewan Harrison, David K. Jackson, Ian Johnston, Dominic Kwiatkowski, Cordelia Langford, John Sillitoe on behalf of the Wellcome Sanger Institute COVID-19 Surveillance Team
EPI_ISL_710265, EPI_ISL_710278, EPI_ISL_710348	Colorado Department of Public Health and Environment	Colorado Department of Puplic Health and Environment	Laura Bankers, Molly C. Hetherington-Rauth, Shannon Ely, Shannon R. Matzinger, Sarah Elizabeth Totten, Emily A. Travanty
EPI_ISL_710552, EPI_ISL_710553, EPI_ISL_710565, EPI_ISL_710566, EPI_ISL_710567, EPI_ISL_710568, EPI_ISL_710569, EPI_ISL_710570	University Hospital Dubrava	Ruer Boškovic Institute; Forensic Science Centre Ivan Vueti; University of Zagreb Faculty of Science	Robert Beluži, Marina Korolija, Ana Livun, Vjekoslav Tomai, Dunja Glavaš, Maja Kuzman, Paula Štancl, Lucija Markulin, Lucija Basi, Antonela Blažekovi, Fran Boroveki, Lidija Cvetko-Krajinovi, Ivana elap, Fuad osovi, Mirjana Domazet-Lošo, Tomislav Domazet-Lošo, Valentina umlijan-Combaj, Kristina Gotovac Jerei, Jasna Kašman, Vladimir Krajinovi, Danilo Licastro, Boris Maek, Željka Maak Šafranko, Gordana Maravi Vlahoviek, Senica Pejša, Josipa Skelin, Ivan Šamija, Mario Štefanovi, Sanja Tadinac, Katarina Marija Tupek, Petra Vrabec, Rosa Karli, Kristian Vlahoviek
EPI_ISL_712077, EPI_ISL_712078, EPI_ISL_712092	East London NHLS Laboratory, Eastern Cape, South Africa	National Institute for Communicable Diseases of the National Health Laboratory Service	Mohale T, Ntuli N, Mahlangu B, Allam M, Ismail A, Bhiman JN
EPI_ISL_712568	Laboratoire de Microbiologie- CHU Habib Bourguiba - Sfax adresse	Laboratoire des Procédés de Criblage Moléculaire et Cellulaire-Centre de Biotechnologie de Sfax	Souissi,A., Abid,N., Ben Ayed,I., Gargouri,S., Abdelmoulah,F.,Elargoubi,A., Smeti,I., Bensaid,M., Stambouli,N., Kharat,N., Ajili,F., Fki-berrajah,L., Mhalla,S., Chtourou,A., Gaaloul,I., Nabli,A., Turki,M., Aouni,M., Hammami,A., Mastouri,M., Karray Hakim,H., Kamoun,S., Rebai,A. and Masmoudi,S.
EPI_ISL_717777, EPI_ISL_717778, EPI_ISL_717779, EPI_ISL_717780, EPI_ISL_717781	UW Virology Lab	UW Virology Lab	Pavitra Roychoudhury, Hong Xie, Lasata Shrestha, Michelle Lin, Meei-Li Huang, Keith R Jerome, Alexander Greninger
EPI_ISL_717809	LACEN Dr. Francisco Rimolo Neto	Bioinformatics Laboratory / LNCC	Carolina M Voloch, Ronaldo da Silva F Jr, Luiz G P de Almeida, Cynthia C Cardoso, Otavio Bustrolini, Alexandra L Gerber, Ana Paula de C Guimarães, Diana Mariani, Andréa Cony Cavalcanti, Claudia dos Santos Rodrigues, Terezinha M P P Castiñeira, Amilcar Tanuri, Ana Tereza R de Vasconcelos
EPI_ISL_718214, EPI_ISL_718215	Ministry of Health Hospitals	Institute of Health and Community Medicine	David Perera, Ooi Mong How, Chua Hock Hin, Tonnii Sia Loong Loong, Wong Jyn Shan, Wong Kiing Aik, Chan Chia Jui
EPI_ISL_718230	Hospital	National Reference Center for Viruses of Respiratory Infections, Institut Pasteur, Paris	Marion Barbet, Sylvie Behillil, Méline Bizard, Angela Brisebarre, Camille Capel, Etienne Simon-Lorière, Vincent Enouf, Maud Vanpeene, Sylvie van der Werf, Gisèle Lagathu
EPI_ISL_718285, EPI_ISL_718286, EPI_ISL_718310, EPI_ISL_718312, EPI_ISL_718313	Institute for Medical Research, Infectious Disease Research Centre, National Institutes of Health, Ministry of Health Malaysia	Institute for Medical Research, Infectious Disease Research Centre, National Institutes of Health, Ministry of Health Malaysia	Suppiah J, Kamel K, Mohd-Zawawi Z, Thayan R
EPI_ISL_722224, EPI_ISL_722225, EPI_ISL_722226, EPI_ISL_722227, EPI_ISL_722255, EPI_ISL_722256, EPI_ISL_722257, EPI_ISL_722260, EPI_ISL_722261	Servicio de Microbiología, Hospital Miguel Servet, Zaragoza	SeqCOVID-SPAIN consortium/IBV(CSIC)	Antonio Rezusta López, Alexander Tristancho Baró, Ana Milagro, Yolanda Gracia Grataloup, Nieves Martínez Cameo and SeqCOVID-SPAIN consortium
EPI_ISL_722274, EPI_ISL_722278, EPI_ISL_722294, EPI_ISL_722297, EPI_ISL_722310, EPI_ISL_722353, EPI_ISL_722403, EPI_ISL_722413, EPI_ISL_722492, EPI_ISL_722493, EPI_ISL_722494, EPI_ISL_722495, EPI_ISL_722829, EPI_ISL_722830, EPI_ISL_722831, EPI_ISL_722832, EPI_ISL_722833, EPI_ISL_722834, EPI_ISL_722835, EPI_ISL_722836, EPI_ISL_722837, EPI_ISL_722838, EPI_ISL_722839, EPI_ISL_722840, EPI_ISL_722841			
see above	Dutch COVID-19 response team	Erasmus Medical Center	Bas Oude Munnink, Reina Sikkema, David Nieuwenhuijse, Irina Chestakova, Anne van der Linden, Marjan Boter, Emmanuelle Munger, Corine GeurtsvanKessel, Annemiek van der Eijk, Richard Molenkamp, Marion Koopmans, on behalf of the Dutch national COVID-19 response team.
EPI_ISL_724967, EPI_ISL_724968, EPI_ISL_724969, EPI_ISL_724978	Northumbria University / South Tees Hospitals NHS Foundation Trust / North Cumbria Integrated Care NHS Foundation Trust / North Tees and Hartlepool NHS Foundation Trust / Newcastle Hospitals NHS Foundation Trust	COVID-19 Genomics UK (COG-UK) Consortium	Darren L Smith,Andrew Nelson,Matthew Bashton,Greg R Young,Joshua Loh,John Allan,Mohammad A Tariq,Giles S Holt,Gary Black,Wen C Yew,Lynn Dover,Paul Baker,Steve Liggett,Sarah Essex,Jane Greenaway,Debra Padgett,Clive Graham,Garren Scott,Edward Barton,Emma Swindells,Brendan Payne,Jennifer Collins,Yusri Taha,Gary Eltringham
EPI_ISL_727705, EPI_ISL_727707, EPI_ISL_727712	Centre for Enzyme Innovation, University of Portsmouth / Translational Research Laboratory, Portsmouth Hospitals NHS Trust	COVID-19 Genomics UK (COG-UK) Consortium	Angela Beckett,Yann Bourgeois,Garry Scarlett,Sharon Glaysher,Scott Elliott,Kelly Bicknell,Robert Impey,Allyson Lloyd,Sarah Wyllie,Ethan Butcher,Anoop Chauhan,Samuel Robson
EPI_ISL_728013, EPI_ISL_728018, EPI_ISL_728019, EPI_ISL_728024, EPI_ISL_728025, EPI_ISL_728026, EPI_ISL_728093	University of Wisconsin-Madison AIDS Vaccine Research Laboratories	University of Wisconsin-Madison AIDS Vaccine Research Laboratories	Gage Moreno, Katarina Braun, et al. AIDS Vaccine Research Laboratories
EPI_ISL_728157, EPI_ISL_728158, EPI_ISL_728161, EPI_ISL_728162, EPI_ISL_728163	Institute for Medical Research, Infectious Disease Research Centre, National Institutes of Health, Ministry of Health Malaysia	Institute for Medical Research, Infectious Disease Research Centre, National Institutes of Health, Ministry of Health Malaysia	Suppiah J, Kamel K, Mohd-Zawawi Z, Thayan R
EPI_ISL_729374, EPI_ISL_729403, EPI_ISL_729419, EPI_ISL_729610, EPI_ISL_729611, EPI_ISL_729615, EPI_ISL_729616	A. Krumbholz, Labor Dr. Krause und Kollegen MVZ GmbH, Kiel	Charité Universitätsmedizin Berlin, Institut für Virologie	Victor M Corman, Barbara Mühlemann, Jörn Beheim-Schwarzbach, Talitha Veith, Julia Schneider, Terry Jones, Christian Drosten
EPI_ISL_729870, EPI_ISL_729903, EPI_ISL_729907	Instituto de Medicina Tropical, Universidad Nacional Toribio Rodríguez de Mendoza de Amazonas	Laboratorio de Genómica Microbiana, Universidad Peruana Cayetano Heredia	Pablo Tsukayama, Alejandra Dávila-Barclay, Luis González, Pedro E. Romero, Brenda Ayzanoa, Janet Huancachoque, Pool Marcos, Stella Chenet, Rafael Tapia, Cecilia Pajuelo, Carla Montenegro
EPI_ISL_730085, EPI_ISL_730092, EPI_ISL_730097, EPI_ISL_730098,	San Diego County Public Health Laboratory	Andersen lab at Scripps Research	SEARCH Alliance San Diego with Tracy Basler, Jovan Shephard, Brett Austin

EPI_ISL_730105, EPI_ISL_730106, EPI_ISL_730107, EPI_ISL_730120, EPI_ISL_730345			
EPI_ISL_730567	Gazi University Faculty of Medicine, Medical Virology Laboratory	Gazi University Faculty of Medicine, Medical Virology Laboratory	Erdem ahin, Hager Muftah, Selin Yiit, Shaknoza Sarzhanova, Özlem Güzel Tunçcan, Murat Dizbay, Il Fidan, Kayhan Çalar, Gülendam Bozday
EPI_ISL_730580	Home Quarantine Taskforce	Hong Kong Department of Health	Mak Gannon C.K., Lam Edman T.K., Chan Rickjason C.W., Tsang Dominic N.C.
EPI_ISL_730587	Pamela Youde Nethersole Eastern Hospital	Hong Kong Department of Health	Mak Gannon C.K., Lam Edman T.K., Chan Rickjason C.W., Tsang Dominic N.C.
EPI_ISL_730588, EPI_ISL_730590	Prince of Wales Hospital	Hong Kong Department of Health	Mak Gannon C.K., Lam Edman T.K., Chan Rickjason C.W., Tsang Dominic N.C.
EPI_ISL_730592	Princess Margaret Hospital	Hong Kong Department of Health	Mak Gannon C.K., Lam Edman T.K., Chan Rickjason C.W., Tsang Dominic N.C.
EPI_ISL_730597	Queen Mary Hospital	Hong Kong Department of Health	Mak Gannon C.K., Lam Edman T.K., Chan Rickjason C.W., Tsang Dominic N.C.
EPI_ISL_730614	United Christian Hospital	Hong Kong Department of Health	Mak Gannon C.K., Lam Edman T.K., Chan Rickjason C.W., Tsang Dominic N.C.
EPI_ISL_732517	National Virus Reference Laboratory	National Virus Reference Laboratory	Michael Carr, Gabriel Gonzalez, Jonathan Dean, Daniel Hare, Cillian F De Gascun
EPI_ISL_732694, EPI_ISL_732695	CNR Virus des Infections Respiratoires - France SUD	CNR Virus des Infections Respiratoires - France SUD	Antonin Bal, Gregory Destras, Claudia Gonzalez, Gwendolyne Burfin, Quentin Semanas, Martine Valette, Bruno Lina, Laurence Josset
EPI_ISL_732784, EPI_ISL_732794, EPI_ISL_732804	Centro de Investigación Biomédica de La Rioja - Hospital San Pedro Logroño	SeqCOVID-SPAIN consortium/IBV(CSIC)	María de Toro, José Manuel Azcona Gutiérrez, María Pilar Bea Escudero, Miriam Blasco Alberdi and SeqCOVID-SPAIN consortium
EPI_ISL_732820, EPI_ISL_732821, EPI_ISL_732822, EPI_ISL_732823, EPI_ISL_732824, EPI_ISL_732825, EPI_ISL_732826	Nebraska Public Health Laboratory	UNMC COVID-19 Response Team	UNMC COVID-19 Response Team
EPI_ISL_733026, EPI_ISL_733028, EPI_ISL_733029, EPI_ISL_733030, EPI_ISL_733031, EPI_ISL_733033, EPI_ISL_733038, EPI_ISL_733040, EPI_ISL_733042, EPI_ISL_733047, EPI_ISL_733051, EPI_ISL_733052, EPI_ISL_733056, EPI_ISL_733059, EPI_ISL_733060, EPI_ISL_733064, EPI_ISL_733065, EPI_ISL_733066, EPI_ISL_733068, EPI_ISL_733074, EPI_ISL_733079, EPI_ISL_733080, EPI_ISL_733081, EPI_ISL_733082, EPI_ISL_733093, EPI_ISL_733094, EPI_ISL_733095, EPI_ISL_733096, EPI_ISL_733097			
see above	HELIX LLC	WHO National Influenza Centre Russian Federation	Andrey Komissarov, Artem Fadeev, Anna Ivanova, Kseniya Komissarova, Dmitry Bazhenov, Daria Danilenko, Ksenia Safina, Elena Nabieva, Georgii Bazykin, Dmitry Lioznov
EPI_ISL_733190, EPI_ISL_733192, EPI_ISL_733208, EPI_ISL_733220	Pathogenic Microorganisms Variability Laboratory	WHO National Influenza Centre Russian Federation	Andrey Komissarov, Artem Fadeev, Anna Ivanova, Kseniya Komissarova, Dmitry Bazhenov, Daria Danilenko, Ksenia Safina, Elena Nabieva, Georgii Bazykin, Nadezhda Kuznetsova, Elena Shidlovskaya, Sergey Alkhovsky, Tatyana Vishnevskaya, Elizaveta Divisenko, Alexey Shchetinin, Maria Nikiforova, Andrey Pochtovyy, Evgeny Usachev, Elena Vokalova, Maxim Rubalsky, Oleg Rubalsky, Artem Tkachuk, Vladimir Gushchin, Alexander Gintsburg, Dmitry Lioznov
EPI_ISL_733233, EPI_ISL_733235, EPI_ISL_733236	UMMC-Health	WHO National Influenza Centre Russian Federation	Andrey Komissarov, Artem Fadeev, Anna Ivanova, Kseniya Komissarova, Dmitry Bazhenov, Tatiana Platonova, Daria Danilenko, Ksenia Safina, Elena Nabieva, Georgii Bazykin, Dmitry Lioznov
EPI_ISL_733242, EPI_ISL_733261, EPI_ISL_733262, EPI_ISL_733263, EPI_ISL_733296, EPI_ISL_733297, EPI_ISL_733394, EPI_ISL_733403	WHO National Influenza Centre Russian Federation	WHO National Influenza Centre Russian Federation	Andrey Komissarov, Artem Fadeev, Anna Ivanova, Kseniya Komissarova, Dmitry Bazhenov, Daria Danilenko, Ksenia Safina, Elena Nabieva, Georgii Bazykin, Dmitry Lioznov
EPI_ISL_733405, EPI_ISL_733406, EPI_ISL_733407, EPI_ISL_733408, EPI_ISL_733409, EPI_ISL_733410, EPI_ISL_733411, EPI_ISL_733412	UMMC-Health	WHO National Influenza Centre Russian Federation	Andrey Komissarov, Artem Fadeev, Anna Ivanova, Kseniya Komissarova, Dmitry Bazhenov, Tatiana Platonova, Daria Danilenko, Ksenia Safina, Elena Nabieva, Georgii Bazykin, Dmitry Lioznov
EPI_ISL_734309, EPI_ISL_734310, EPI_ISL_734311, EPI_ISL_734312, EPI_ISL_734313	Wadsworth Center, New York State Department.of Health	Wadsworth Center, New York State Department.of Health	Kirsten St. George, Daryl M. Lamson, Alexis Russel, Jonathan Plitnick, Navjot Singh, John Kelly, Sara Griesemer, Erasmus Schneider, Erica Lasek-Nesselquist
EPI_ISL_735451	UW Virology Lab	UW Virology Lab	Pavitra Roychoudhury, Hong Xie, Lasata Shrestha, Meei-Li Huang, Keith R Jerome, Alexander Greninger
EPI_ISL_738038, EPI_ISL_738039, EPI_ISL_738040, EPI_ISL_738041, EPI_ISL_738042, EPI_ISL_738043	Uganda Central Public Health Lab and Uganda Virus Research Institute	MRC/UVRI & LSHTM Uganda Research Unit	Matthew Cotten, Dan Lule Bugembe, My V.T. Phan, Pontiano Kaleebu et al.
EPI_ISL_739086, EPI_ISL_739264, EPI_ISL_739297, EPI_ISL_739489	Humboldt County Public Health Laboratory	Chan-Zuckerberg Biohub	CZB Cliahub Consortium
EPI_ISL_739795, EPI_ISL_740027, EPI_ISL_740222, EPI_ISL_740226, EPI_ISL_740291	Laboratoire national de santé, Microbiology, Virology	Laboratoire national de santé, Microbiology, Microbial Genomics Platform	Anke Wienecke-Baldacchino, Catherine Ragimbeau,Jessica Tapp, Fatu Djabi, Lise Pignon, Raoul Salmon, Tamir Abdelrahman
EPI_ISL_740548, EPI_ISL_740549, EPI_ISL_740550, EPI_ISL_740551, EPI_ISL_740552, EPI_ISL_740553, EPI_ISL_740554, EPI_ISL_740555, EPI_ISL_740556, EPI_ISL_740557, EPI_ISL_740558, EPI_ISL_740559, EPI_ISL_740560, EPI_ISL_740561, EPI_ISL_740562, EPI_ISL_740563, EPI_ISL_740564, EPI_ISL_740565, EPI_ISL_740566, EPI_ISL_740567, EPI_ISL_740568, EPI_ISL_740569, EPI_ISL_740570, EPI_ISL_740571, EPI_ISL_740572, EPI_ISL_740573, EPI_ISL_740574, EPI_ISL_740575, EPI_ISL_740576, EPI_ISL_740577, EPI_ISL_740578, EPI_ISL_740579, EPI_ISL_740580, EPI_ISL_740581, EPI_ISL_740582, EPI_ISL_740583, EPI_ISL_740584, EPI_ISL_740585, EPI_ISL_740586, EPI_ISL_740587, EPI_ISL_740588, EPI_ISL_740589, EPI_ISL_740590, EPI_ISL_740591, EPI_ISL_740592, EPI_ISL_740593, EPI_ISL_740594, EPI_ISL_740595, EPI_ISL_740596, EPI_ISL_740597, EPI_ISL_740598, EPI_ISL_740599, EPI_ISL_740600, EPI_ISL_740601, EPI_ISL_740602, EPI_ISL_740603, EPI_ISL_740604, EPI_ISL_740605, EPI_ISL_740606, EPI_ISL_740607, EPI_ISL_740608, EPI_ISL_740609, EPI_ISL_740610, EPI_ISL_740611, EPI_ISL_740612, EPI_ISL_740613, EPI_ISL_740614, EPI_ISL_740615, EPI_ISL_740616, EPI_ISL_740617, EPI_ISL_740618, EPI_ISL_740619, EPI_ISL_740620, EPI_ISL_740621, EPI_ISL_740622, EPI_ISL_740655, EPI_ISL_740677, EPI_ISL_740681, EPI_ISL_740682, EPI_ISL_740684, EPI_ISL_740767, EPI_ISL_740777, EPI_ISL_740778, EPI_ISL_740779, EPI_ISL_740780, EPI_ISL_740781, EPI_ISL_740784, EPI_ISL_740785, EPI_ISL_740786, EPI_ISL_740787, EPI_ISL_740788, EPI_ISL_740789			
see above	BCCDC Public Health Laboratory	BCCDC Public Health Laboratory	Prystajecky Natalie, Linda Hoang, Dan Fornika, Shannon Russell, Kim Macdonald, Kimia Kamelian, John Tyson, Inna Sekirov, Mel Krajden
EPI_ISL_741347	Wales Specialist Virology Centre Sequencing lab: Pathogen Genomics Unit	COVID-19 Genomics UK (COG-UK) Consortium	Catherine Moore, Johnathan Evans, Laura Gifford, Malorie Perry, Simon Cottrell, Angela Marchbank, Alec Birchley, Alexander Adams, Amy Gaskin, Bree Gatica-Wilcox, Jason Coombes, Joel Southgate, Lauren Gilbert, Lee Graham, Nicole Pacchiarini, Sara Kumziene-Summerhayes, Sarah Taylor, Sophie Jones, Sara Rey, Matthew Bull, Joanne Watkins, Sally Corden, Tom Connor
EPI_ISL_744484, EPI_ISL_744521, EPI_ISL_744761, EPI_ISL_744861, EPI_ISL_744994, EPI_ISL_745023	Laboratoire national de santé, Microbiology, Virology	Laboratoire national de santé, Microbiology, Microbial Genomics Platform	Anke Wienecke-Baldacchino, Catherine Ragimbeau,Jessica Tapp, Fatu Djabi, Lise Pignon, Raoul Salmon, Tamir Abdelrahman
EPI_ISL_745639, EPI_ISL_746121, EPI_ISL_746124	Ginkgo Bioworks Clinical Laboratory	Utah Public Health Laboratory	Erin L. Young, Kelly Oakeson, Tara Gallagher, Michael T. Pyne, E. Susan Slechta, Melanie A. Mallory, Jeffrey B. Stevenson, Salika M. Shakir, David R. Hillyard, Malaika McKenzie-Bennett, James McGann, Jim Griffin, Keith Robison, Alex Plocik, Becky Schilling, Martha Pierson, Rebecca Littlefield, Michelle Spencer, Birgitte Simen
EPI_ISL_746480, EPI_ISL_746483, EPI_ISL_746491, EPI_ISL_746492, EPI_ISL_746498, EPI_ISL_746501, EPI_ISL_746502, EPI_ISL_746511, EPI_ISL_746512, EPI_ISL_746521, EPI_ISL_746522, EPI_ISL_746523, EPI_ISL_746524, EPI_ISL_746525, EPI_ISL_746526, EPI_ISL_746527, EPI_ISL_746528, EPI_ISL_746529, EPI_ISL_746742, EPI_ISL_746743, EPI_ISL_746744, EPI_ISL_746745, EPI_ISL_746746, EPI_ISL_746747, EPI_ISL_746748, EPI_ISL_746749, EPI_ISL_746750, EPI_ISL_746751, EPI_ISL_746752, EPI_ISL_746753, EPI_ISL_746754, EPI_ISL_746755, EPI_ISL_746756			
see above	Genetica Molecular and Subdepartamento de Virologia ISP Chile	Instituto de Salud Publica de Chile	Javier Tognarelli, Barbara Parra, Loredana Arata, Jaime Lagos, Gisselle Barra, Patricia Bustos, Rodrigo Fasce, Andres Castillo, Jorge Fernandez
EPI_ISL_747039, EPI_ISL_747040	Respiratory Viruses Branch, Centers for Disease Control and Prevention	Respiratory Viruses Branch, Centers for Disease Control and Prevention	Queen,K., Li,Y., Tao,Y., Uehara,A., Montmayeur,A., Paden,C.R., Cook,P.W., Marine,R., Sheth,M., Wang,H., Lee,J., Tong,S.
EPI_ISL_747237	National Institute of Health Research and Development	National Institute of Health Research and Development	Subangkit,Nafiandi,Estaria,V;Pawestri,HA;Puspa,KD;Nugraha,AA;Ikawati,HD;Pangesti,KNA;Soekarso,T;Paisal;Puspandari,N;Setiawaty,V
EPI_ISL_747307, EPI_ISL_747315, EPI_ISL_747316, EPI_ISL_747328, EPI_ISL_747331, EPI_ISL_747343, EPI_ISL_747344, EPI_ISL_747345, EPI_ISL_747346, EPI_ISL_747347, EPI_ISL_747348, EPI_ISL_747349, EPI_ISL_747350, EPI_ISL_747351, EPI_ISL_747352, EPI_ISL_747353, EPI_ISL_747354, EPI_ISL_747355			

see above	Division of Emerging Infectious Diseases, Bureau of Infectious Diseases Diagnosis Control, Korea Disease Control and Prevention Agency	Division of Emerging Infectious Diseases, Bureau of Infectious Diseases Diagnosis Control, Korea Disease Control and Prevention Agency	Ae Kyung Park, Il-Hwan Kim, Heui Man Kim, Jeong-Min Kim, Namjoo Lee, Chaeyoung Lee, Sang Hee Woo, Eun-Jin Kim
EPI_ISL_747597, EPI_ISL_748132, EPI_ISL_748133, EPI_ISL_748134, EPI_ISL_748135	Department of Virus and Microbiological Special Diagnostics, Statens Serum Institut, Copenhagen, Denmark	Albertsen Lab, Department of Chemistry and Bioscience, Aalborg University, Denmark	Danish Covid-19 Genome Consortium
EPI_ISL_751499	CHU Purpan - Laboratoire de Virologie - Institut Fédératif de Biologie	CHU Purpan - Laboratoire de Virologie - Institut Fédératif de Biologie	Latour J., Ranger N., Dubois M., Carcenac R., Harter A., Boyer P., Tremeaux P., Izopet J.
EPI_ISL_752912, EPI_ISL_752913, EPI_ISL_752914, EPI_ISL_752915, EPI_ISL_752916, EPI_ISL_752917, EPI_ISL_752918, EPI_ISL_752919, EPI_ISL_752920, EPI_ISL_752921, EPI_ISL_752922, EPI_ISL_752933, EPI_ISL_752934, EPI_ISL_752935, EPI_ISL_752936, EPI_ISL_752937, EPI_ISL_752938, EPI_ISL_752939, EPI_ISL_752940, EPI_ISL_752972, EPI_ISL_752973, EPI_ISL_752974, EPI_ISL_752975, EPI_ISL_752976, EPI_ISL_752977, EPI_ISL_753029, EPI_ISL_753030, EPI_ISL_753123, EPI_ISL_753149			
see above	State Laboratories Division, Hawaii State Department of Health	State Laboratories Division, Hawaii State Department of Health	Pamela O'Brien, Sabrina Diemert, Drew Kuwazaki, Razvan Sultana, Edward Desmond
EPI_ISL_753731, EPI_ISL_753743, EPI_ISL_753751, EPI_ISL_753777, EPI_ISL_753786, EPI_ISL_753787, EPI_ISL_753788, EPI_ISL_753819, EPI_ISL_753820, EPI_ISL_753821, EPI_ISL_753837, EPI_ISL_753860, EPI_ISL_753876, EPI_ISL_753879, EPI_ISL_753884, EPI_ISL_753885, EPI_ISL_753886, EPI_ISL_753907, EPI_ISL_754021			
see above	Charité Universitätsmedizin Berlin, Institut für Virologie/Labor Berlin	Charité Universitätsmedizin Berlin, Institut für Virologie	Victor M Corman, Jörn Beheim-Schwarzbach, Barbara Mühlemann, Julia Schneider, Talitha Veith, Terry Jones, Christian Drosten
EPI_ISL_754182	Wyoming Public Health Laboratory	Wyoming Public Health Laboratory	Noah Hull, Taylor Fearing, Channing Weber, Ashley Norberg, Bailey Bowcutt, and Wanda Manley
EPI_ISL_754185	Charité Universitätsmedizin Berlin, Institut für Virologie/Labor Berlin	Charité Universitätsmedizin Berlin, Institut für Virologie	Victor M Corman, Jörn Beheim-Schwarzbach, Barbara Mühlemann, Julia Schneider, Talitha Veith, Terry Jones, Christian Drosten
EPI_ISL_754393	Genetica Molecular and Subdepartamento de Virologia ISP Chile	Insituto de Salud Publica de Chile	Javier Tognarelli, Barbara Parra, Loredana Arata, Jaime Lagos, Gisselle Barra, Patricia Bustos, Rodrigo Fasce, Andres Castillo, Jorge Fernandez
EPI_ISL_754526, EPI_ISL_754527, EPI_ISL_754528, EPI_ISL_754529, EPI_ISL_754530, EPI_ISL_754531, EPI_ISL_754532	Wadsworth Center, New York State Department.of Health	Wadsworth Center, New York State Department.of Health	Kirsten St. George, Daryl M. Lamson, Alexis Russel, Matthew Shudt, Melissa A Leisner, Jonathan Plitnick, Navjot Singh, John Kelly, Sara Griesemer, Erasmus Schneider, Erica Lasek-Nesselquist
EPI_ISL_754902	Laboratory Diagnostics and Clinical Immunology of Developmental Age, Medical University of Warsaw	genXone SA, Research & Development Laboratory; The Faculty of Mathematics, Informatics and Mechanics of the University of Warsaw	Maciej Sykulski, Grzegorz Nowicki, Monika Makowska-Woniak, Jakub Grabowski, Natalia Drwska-Matelska, ukasz Krych, Micha Kaszuba, Anna Gambin, Urszula Demkow
EPI_ISL_754915, EPI_ISL_754917, EPI_ISL_754921, EPI_ISL_754923, EPI_ISL_754931, EPI_ISL_754934, EPI_ISL_754952, EPI_ISL_754974, EPI_ISL_754975, EPI_ISL_754979, EPI_ISL_754981, EPI_ISL_754995, EPI_ISL_755016, EPI_ISL_755027, EPI_ISL_755031, EPI_ISL_755032, EPI_ISL_755057, EPI_ISL_755058, EPI_ISL_755059			
see above	California Department of Public Health	California Department of Public Health	CDPH IDLB COVIDNet
EPI_ISL_755386, EPI_ISL_755387, EPI_ISL_755388, EPI_ISL_755389, EPI_ISL_755390, EPI_ISL_755391, EPI_ISL_755392, EPI_ISL_755393, EPI_ISL_755394, EPI_ISL_755395, EPI_ISL_755396, EPI_ISL_755397, EPI_ISL_755398, EPI_ISL_755399, EPI_ISL_755400, EPI_ISL_755401, EPI_ISL_755402, EPI_ISL_755403, EPI_ISL_755404, EPI_ISL_755405, EPI_ISL_755406, EPI_ISL_755407, EPI_ISL_755408, EPI_ISL_755409, EPI_ISL_755410, EPI_ISL_755411, EPI_ISL_755412, EPI_ISL_755413, EPI_ISL_755414, EPI_ISL_755415, EPI_ISL_755416, EPI_ISL_755417, EPI_ISL_755418, EPI_ISL_755419			
see above	Maine Health and Environmental Testing Laboratory	Tewhey Lab, The Jackson Laboratory	Matluk,N., Dewey,H., Iosue,F., Barter,M., Lynch,R., Munger,H. and Tewhey,R.
EPI_ISL_755746	Toronto Invasive Bacterial Diseases Network	McMaster University	Allison McGeer, Patryk Aftanas, Hooman Derakhshani, Angel Li, Kuganya Nirmalarajah, Emily Panousis, Ahmed Draia, Jalees Nasir, Michael Surette, Samira Mubareka, Andrew G. McArthur
EPI_ISL_756278	State Laboratories Division, Hawaii State Department of Health	State Laboratories Division, Hawaii State Department of Health	Pamela O'Brien, Sabrina Diemert, Drew Kuwazaki, Razvan Sultana, Edward Desmond
EPI_ISL_757343, EPI_ISL_757344, EPI_ISL_757345, EPI_ISL_757346, EPI_ISL_757347, EPI_ISL_757348, EPI_ISL_757349, EPI_ISL_757350, EPI_ISL_757351, EPI_ISL_757352, EPI_ISL_757353, EPI_ISL_757354, EPI_ISL_757355, EPI_ISL_757356, EPI_ISL_757357, EPI_ISL_757358, EPI_ISL_757359, EPI_ISL_757360, EPI_ISL_757361, EPI_ISL_757362, EPI_ISL_757363, EPI_ISL_757364, EPI_ISL_759916, EPI_ISL_759917, EPI_ISL_759918, EPI_ISL_759919, EPI_ISL_759920, EPI_ISL_759921, EPI_ISL_759922, EPI_ISL_759923, EPI_ISL_759924, EPI_ISL_759925, EPI_ISL_759926, EPI_ISL_759927, EPI_ISL_759928, EPI_ISL_759945, EPI_ISL_759946, EPI_ISL_759947, EPI_ISL_759948, EPI_ISL_759949, EPI_ISL_759950, EPI_ISL_759951, EPI_ISL_759952, EPI_ISL_759953, EPI_ISL_759954			
see above	Department of Virology and Immunology, University of Helsinki and Helsinki University Hospital, Huslab Finland	Department of Virology, Faculty of Medicine, University of Helsinki, Helsinki, Finland	Teemu Smura, Ravi Kant, Phuoc Truong, Hussein Alburkat, Hannimari Kallio-Kokko, Jenni Virtanen, Maija Suvanto, Sari Hannula, Harri Kangas, Pekka Ellonen, Olli Vapalahti
EPI_ISL_760037, EPI_ISL_760038, EPI_ISL_760043, EPI_ISL_760044, EPI_ISL_760048	Hong Kong Department of Health	School of Public Health, The University of Hong Kong	Daniel Chu, Haogao Gu, Pavithra Krishnan, Daisy Ng, Gigi Liu, Carrie Wan, Malik Peiris, Leo Poon
EPI_ISL_760117	Division of Emerging Infectious Diseases, Bureau of Infectious Diseases Diagnosis Control, Korea Disease Control and Prevention Agency	Division of Emerging Infectious Diseases, Bureau of Infectious Diseases Diagnosis Control, Korea Disease Control and Prevention Agency	Ae Kyung Park, Il-Hwan Kim, Heui Man Kim, Jeong-Min Kim, Namjoo Lee, Chaeyoung Lee, Sang Hee Woo, Eun-Jin Kim
EPI_ISL_765586, EPI_ISL_765589, EPI_ISL_765592, EPI_ISL_765595, EPI_ISL_765596, EPI_ISL_765597, EPI_ISL_765598, EPI_ISL_765599, EPI_ISL_765600, EPI_ISL_765601, EPI_ISL_765602, EPI_ISL_765603, EPI_ISL_765604, EPI_ISL_765605, EPI_ISL_765606, EPI_ISL_765607, EPI_ISL_765608, EPI_ISL_765609, EPI_ISL_765610, EPI_ISL_765612			
see above	Brigham and Womens Hospital	Infectious Disease Program, Broad Institute of Harvard and MIT	Lemieux,J.E., Siddle,K.J., Shaw,B., Adams,G., Pierce,V., Turbett,S., Anahtar,M., Branda,J., Slater,D., Harris,J., Lin,A.E., Gladden-Young,A., Lagerborg,K., Rudy,M., DeRuff,K., Carter,A., Normandin,E., Bauer,M., Reilly,S., Tomkins-Tinch,C., Loreth,C., Chaluvadi,S., Neumann,A., Cusick,C., Chapman,S.B., Gnirke,A., Flowers,K., Cerrato,F., Birren,B.W., Gallagher,G., Smole,S., Park,D.J., MacInnis,B.L., Ryan,E., LaRocque,R., Rosenberg,E. and Sabeti,P.C.
EPI_ISL_765613, EPI_ISL_765615, EPI_ISL_765620, EPI_ISL_765624, EPI_ISL_765641, EPI_ISL_765642, EPI_ISL_765735, EPI_ISL_765737, EPI_ISL_765738, EPI_ISL_765739, EPI_ISL_765740, EPI_ISL_765741, EPI_ISL_765742, EPI_ISL_765743, EPI_ISL_765744, EPI_ISL_765745, EPI_ISL_765746, EPI_ISL_765747			
see above	Massachusetts General Hospital	Infectious Disease Program, Broad Institute of Harvard and MIT	Lemieux,J.E., Siddle,K.J., Shaw,B., Adams,G., Pierce,V., Turbett,S., Anahtar,M., Branda,J., Slater,D., Harris,J., Lin,A.E., Gladden-Young,A., Lagerborg,K., Rudy,M., DeRuff,K., Carter,A., Normandin,E., Bauer,M., Reilly,S., Tomkins-Tinch,C., Loreth,C., Chaluvadi,S., Neumann,A., Cusick,C., Chapman,S.B., Gnirke,A., Flowers,K., Cerrato,F., Birren,B.W., Gallagher,G., Smole,S., Park,D.J., MacInnis,B.L., Ryan,E., LaRocque,R., Rosenberg,E. and Sabeti,P.C.
EPI_ISL_765945, EPI_ISL_765946, EPI_ISL_765947, EPI_ISL_765948, EPI_ISL_765949, EPI_ISL_765950, EPI_ISL_765951	Worobey Lab, Department of Ecology and Evolutionary Biology, University of Arizona	Worobey Lab, Department of Ecology and Evolutionary Biology, University of Arizona	Brendan Larsen, Grace Quirk, Thomas Watts, David Baltrus, Michael Worobey
EPI_ISL_767439, EPI_ISL_767440, EPI_ISL_767441, EPI_ISL_767442, EPI_ISL_767443, EPI_ISL_767444, EPI_ISL_767445, EPI_ISL_767446, EPI_ISL_767447, EPI_ISL_767448, EPI_ISL_767449, EPI_ISL_767578			
see above	Wadsworth Center, New York State Department.of Health	Wadsworth Center, New York State Department.of Health	Kirsten St. George, Daryl M. Lamson, Alexis Russel, Matthew Shudt, Melissa A Leisner, Jonathan Plitnick, Navjot Singh, John Kelly, Sara Griesemer, Erasmus Schneider, Erica Lasek-Nesselquist
EPI_ISL_768646, EPI_ISL_768647, EPI_ISL_768648, EPI_ISL_768649, EPI_ISL_768650, EPI_ISL_768659, EPI_ISL_768660	Pathogen Genomics Center, National Institute of Infectious Diseases	Pathogen Genomics Center, National Institute of Infectious Diseases	Tsuyoshi Sekizuka, Kentaro Itokawa, Rina Tanaka, Masanori Hashino, Makoto Kuroda
EPI_ISL_768773	AIID	Irish Coronavirus Sequencing Consortium - National Virus Reference Laboratory	Michael Carr, Gabriel Gonzalez, Alejandro Abner Garcia Leon, Patrick Mallon
EPI_ISL_769992, EPI_ISL_769995	Hospital Metropolitano	Inciensa, Instituto Costarricense de Investigación y	Francisco Duarte, Hebleen Porras, Claudio Soto-Garita, Estela Cordero, Adriana Godínez, Melany Calderón & Margarita Lee Lui

		Enseñanza en Nutrición y Salud	
EPI_ISL_770007	Area De Salud San Rafael	Inciensa, Instituto Costarricense de Investigación y Enseñanza en Nutrición y Salud	Francisco Duarte, Hebleen Porras, Claudio Soto-Garita, Estela Cordero, Adriana Godínez, Melany Calderón & Mariel López
EPI_ISL_770027	Area De Salud La Cruz	Inciensa, Instituto Costarricense de Investigación y Enseñanza en Nutrición y Salud	Francisco Duarte, Hebleen Porras, Claudio Soto-Garita, Estela Cordero, Adriana Godínez, Melany Calderón & Mariel López
EPI_ISL_770031, EPI_ISL_770032	Hle-Asociacion De Atencion Integral Del Anciano San Cayetano	Inciensa, Instituto Costarricense de Investigación y Enseñanza en Nutrición y Salud	Francisco Duarte, Hebleen Porras, Claudio Soto-Garita, Estela Cordero, Adriana Godínez, Melany Calderón & Mariel López
EPI_ISL_770638, EPI_ISL_770642, EPI_ISL_770647, EPI_ISL_770648, EPI_ISL_770649, EPI_ISL_770650, EPI_ISL_770651, EPI_ISL_770652, EPI_ISL_770654, EPI_ISL_770655, EPI_ISL_770657, EPI_ISL_770662, EPI_ISL_770663, EPI_ISL_770665, EPI_ISL_770667, EPI_ISL_770668, EPI_ISL_770671, EPI_ISL_770672			
see above	UMMC-Health	WHO National Influenza Centre Russian Federation	Andrey Komissarov, Artem Fadeev, Anna Ivanova, Kseniya Komissarova, Dmitry Bazhenov, Tatiana Platonova, Daria Danilenko, Ksenia Safina, Elena Nabieva, Georgii Bazykin, Dmitry Lioznov
EPI_ISL_770752, EPI_ISL_770753, EPI_ISL_770754, EPI_ISL_770755, EPI_ISL_770756, EPI_ISL_770757, EPI_ISL_770758, EPI_ISL_770759, EPI_ISL_770760, EPI_ISL_770761, EPI_ISL_770762, EPI_ISL_770763, EPI_ISL_770764, EPI_ISL_770765, EPI_ISL_770766, EPI_ISL_770767, EPI_ISL_770768, EPI_ISL_770769, EPI_ISL_770770, EPI_ISL_770771			
see above	ZOTZ KLIMAS MVZ Düsseldorf-Centrum GbR ÜBAG für Labormedizin, Genetik, Zytologie, Pathologie	Center of Medical Microbiology, Virology, and Hospital Hygiene, University of Duesseldorf	Maximilian Damagnez, Alexander Dilthey, Ashley-Jane Duplessis, Patrick Finzer, Katrin Hoffmann, Torsten Houwaart, Lisanna Hülse, Malte Kohns Vasconcelos, Marek Korencak, Nadine Lübke, Jessica Nicolai, Klaus Pfeffer, Daniel Strelow, Jörg Timm, Andreas Walker, Tobias Wienemann, Rainer Zotz
EPI_ISL_770876	Laboratoire national de santé, Microbiology, Virology	Laboratoire national de santé, Microbiology, Microbial Genomics Platform	Anke Wienecke-Baldacchino, Catherine Ragimbeau,Jessica Tapp, Fatu Djabi, Lise Pignon, Raoul Salmon, Tamir Abdelrahman
EPI_ISL_775307, EPI_ISL_775308	Oslo University Hospital, Department of Medical Microbiology	Norwegian Institute of Public Health, Department of Virology	Kathrine Stene-Johansen, Kamilla Heddeland Instefjord, Hilde Elshaug, Atiya R Ali,Marie Paulsen Madsen, Rasmus Riis Kopperud, Hilde Vollan, Karoline Bragstad, Olav Hungnes
EPI_ISL_775310	Medical Microbiology Unit, Department for Laboratory Medicine, Drammen Hospital, Vestre Viken Health Trust,	Norwegian Institute of Public Health, Department of Virology	Kathrine Stene-Johansen, Kamilla Heddeland Instefjord, Hilde Elshaug, Atiya R Ali,Marie Paulsen Madsen, Rasmus Riis Kopperud, Hilde Vollan, Karoline Bragstad, Olav Hungnes
EPI_ISL_775464, EPI_ISL_775465, EPI_ISL_775466	Department of Medical Microbiology, St. Olavs hospital	Norwegian Institute of Public Health, Department of Virology	Kathrine Stene-Johansen, Kamilla Heddeland Instefjord, Hilde Elshaug, Atiya R Ali,Marie Paulsen Madsen, Rasmus Riis Kopperud, Hilde Vollan, Karoline Bragstad, Olav Hungnes
EPI_ISL_775467	Department of Medical Microbiology - section Molde, Molde Hospital	Norwegian Institute of Public Health, Department of Virology	Kathrine Stene-Johansen, Kamilla Heddeland Instefjord, Hilde Elshaug, Atiya R Ali,Marie Paulsen Madsen, Rasmus Riis Kopperud, Hilde Vollan, Karoline Bragstad, Olav Hungnes
EPI_ISL_775468	Unilabs Laboratory Medicine	Norwegian Institute of Public Health, Department of Virology	Kathrine Stene-Johansen, Kamilla Heddeland Instefjord, Hilde Elshaug, Atiya R Ali,Marie Paulsen Madsen, Rasmus Riis Kopperud, Hilde Vollan, Karoline Bragstad, Olav Hungnes
EPI_ISL_775479	Akershus University Hospital, Department for Microbiology and Infectious Disease Control	Norwegian Institute of Public Health, Department of Virology	Kathrine Stene-Johansen, Kamilla Heddeland Instefjord, Hilde Elshaug, Atiya R Ali,Marie Paulsen Madsen, Rasmus Riis Kopperud, Hilde Vollan, Karoline Bragstad, Olav Hungnes
EPI_ISL_775481	Department of Medical Microbiology, St. Olavs hospital	Norwegian Institute of Public Health, Department of Virology	Kathrine Stene-Johansen, Kamilla Heddeland Instefjord, Hilde Elshaug, Atiya R Ali,Marie Paulsen Madsen, Rasmus Riis Kopperud, Hilde Vollan, Karoline Bragstad, Olav Hungnes
EPI_ISL_775523	Department of Medical Microbiology - section Molde, Molde Hospital	Norwegian Institute of Public Health, Department of Virology	Kathrine Stene-Johansen, Kamilla Heddeland Instefjord, Hilde Elshaug, Atiya R Ali,Marie Paulsen Madsen, Rasmus Riis Kopperud, Hilde Vollan, Karoline Bragstad, Olav Hungnes
EPI_ISL_775597	RS Pertamina Sorong	National Institute of Health Research and Development	Puspa,KD;Subangkit;Pawestri,HA;Ikawati,HD;Nugraha,AA;Yosepin;Pangesti,KNA;Soekarso,T;Puspandari,N;Setiawaty,V
EPI_ISL_776960, EPI_ISL_776961, EPI_ISL_776962, EPI_ISL_776963, EPI_ISL_776964, EPI_ISL_776965, EPI_ISL_776966, EPI_ISL_776967, EPI_ISL_778691, EPI_ISL_778692, EPI_ISL_778693, EPI_ISL_778694, EPI_ISL_778695			
see above	Istituto Zooprofilattico Sperimentale del Mezzogiorno	TIGEM	Patrizia Annunziata, Andrea Ballabio, Valentina Bouche, Davide Cacchiarelli (CorrespAuthor), Pellegrino Cerino, Chiara Colantuono, Lucio Di Filippo, Antonio Grimaldi, Antonio Limone, Gabriella Loconte, Anna Manfredi, Francesco Panariello, Biancamaria Pierri, Marcello Salvi, Lucia Vassallo
EPI_ISL_779221, EPI_ISL_779222, EPI_ISL_779223, EPI_ISL_779224, EPI_ISL_779225, EPI_ISL_779226, EPI_ISL_779227, EPI_ISL_779251, EPI_ISL_779252, EPI_ISL_779253, EPI_ISL_779666, EPI_ISL_779667, EPI_ISL_779668, EPI_ISL_779669			
see above	Pathogen Genomics Center, National Institute of Infectious Diseases	Pathogen Genomics Center, National Institute of Infectious Diseases	Tsuyoshi Sekizuka, Kentaro Itokawa, Rina Tanaka, Masanori Hashino, Makoto Kuroda
EPI_ISL_786200, EPI_ISL_786201, EPI_ISL_786202, EPI_ISL_786203, EPI_ISL_786212, EPI_ISL_786213, EPI_ISL_786214, EPI_ISL_786215, EPI_ISL_786224, EPI_ISL_786225, EPI_ISL_786226, EPI_ISL_786227, EPI_ISL_786236, EPI_ISL_786237, EPI_ISL_786238, EPI_ISL_786239, EPI_ISL_786248, EPI_ISL_786249, EPI_ISL_786250, EPI_ISL_787389, EPI_ISL_787390, EPI_ISL_787391, EPI_ISL_787401, EPI_ISL_787402, EPI_ISL_787403, EPI_ISL_787412, EPI_ISL_787413, EPI_ISL_787414, EPI_ISL_787424, EPI_ISL_787425, EPI_ISL_787426, EPI_ISL_787436, EPI_ISL_787437, EPI_ISL_787438, EPI_ISL_787448, EPI_ISL_787449, EPI_ISL_787450, EPI_ISL_787460, EPI_ISL_787461, EPI_ISL_787462, EPI_ISL_787472, EPI_ISL_787473, EPI_ISL_787474, EPI_ISL_787964, EPI_ISL_787965, EPI_ISL_787966, EPI_ISL_787967, EPI_ISL_787976, EPI_ISL_787977, EPI_ISL_787978, EPI_ISL_787979, EPI_ISL_787988, EPI_ISL_787989, EPI_ISL_787990, EPI_ISL_787991, EPI_ISL_789789, EPI_ISL_789799, EPI_ISL_789806, EPI_ISL_789808, EPI_ISL_789814, EPI_ISL_789823, EPI_ISL_789834, EPI_ISL_789838, EPI_ISL_789874, EPI_ISL_789883, EPI_ISL_789888, EPI_ISL_789890, EPI_ISL_789894, EPI_ISL_789895, EPI_ISL_789903, EPI_ISL_789916, EPI_ISL_789934, EPI_ISL_789935, EPI_ISL_789936, EPI_ISL_789937, EPI_ISL_789941, EPI_ISL_789954, EPI_ISL_789961, EPI_ISL_789962, EPI_ISL_789963, EPI_ISL_789965, EPI_ISL_789966, EPI_ISL_789969, EPI_ISL_789970, EPI_ISL_789977, EPI_ISL_789988, EPI_ISL_790208, EPI_ISL_790220, EPI_ISL_790222, EPI_ISL_790229, EPI_ISL_790231, EPI_ISL_790268, EPI_ISL_790290, EPI_ISL_790291, EPI_ISL_790292, EPI_ISL_790293, EPI_ISL_790294, EPI_ISL_790295, EPI_ISL_790296, EPI_ISL_790297, EPI_ISL_790298, EPI_ISL_790299, EPI_ISL_790300, EPI_ISL_790301, EPI_ISL_790302, EPI_ISL_790303, EPI_ISL_790304, EPI_ISL_790305, EPI_ISL_790307, EPI_ISL_790308, EPI_ISL_790309, EPI_ISL_790310, EPI_ISL_790311, EPI_ISL_790312, EPI_ISL_790313, EPI_ISL_790314, EPI_ISL_790315, EPI_ISL_790316, EPI_ISL_790317, EPI_ISL_790318, EPI_ISL_790319, EPI_ISL_790320, EPI_ISL_790321, EPI_ISL_790322, EPI_ISL_790323, EPI_ISL_790324, EPI_ISL_790325, EPI_ISL_790326, EPI_ISL_790327, EPI_ISL_790328, EPI_ISL_790329, EPI_ISL_790330, EPI_ISL_790331, EPI_ISL_790332, EPI_ISL_790333, EPI_ISL_790334, EPI_ISL_790335, EPI_ISL_790336, EPI_ISL_790337, EPI_ISL_790338, EPI_ISL_790339, EPI_ISL_790340, EPI_ISL_790341, EPI_ISL_790342, EPI_ISL_790343, EPI_ISL_790344, EPI_ISL_790345, EPI_ISL_790346, EPI_ISL_790347, EPI_ISL_790348, EPI_ISL_790349, EPI_ISL_790350, EPI_ISL_790351, EPI_ISL_790352, EPI_ISL_790353, EPI_ISL_790354, EPI_ISL_790355, EPI_ISL_790356, EPI_ISL_790357, EPI_ISL_790358, EPI_ISL_790359, EPI_ISL_790360, EPI_ISL_790361, EPI_ISL_790362, EPI_ISL_790363, EPI_ISL_790364, EPI_ISL_790365, EPI_ISL_790366, EPI_ISL_790367, EPI_ISL_790368, EPI_ISL_790369, EPI_ISL_790370, EPI_ISL_790371, EPI_ISL_790372, EPI_ISL_790373, EPI_ISL_790374, EPI_ISL_790375, EPI_ISL_790376, EPI_ISL_790377, EPI_ISL_790378, EPI_ISL_790379, EPI_ISL_790380, EPI_ISL_790381, EPI_ISL_790382, EPI_ISL_790383, EPI_ISL_790384, EPI_ISL_790385, EPI_ISL_790386, EPI_ISL_790387, EPI_ISL_790388, EPI_ISL_790389, EPI_ISL_790390, EPI_ISL_790391, EPI_ISL_790392, EPI_ISL_790393, EPI_ISL_790394, EPI_ISL_790395			
see above	Houston Methodist Hospital	Houston Methodist Hospital	S. Wesley Long, Randall J. Olsen, Paul A. Christensen, David W. Bernard, James J. Davis, Maulik Shukla, Marcus Nguyen, Matthew Ojeda Saavedra, Prasanti Yerramilli, Layne Pruitt, Sishir Subedi, Heather Hendrickson, and James M. Musser
EPI_ISL_790927, EPI_ISL_790942, EPI_ISL_790953	Dutch COVID-19 response team	National Institute for Public Health and the Environment (RIVM)	Adam Meijer, Harry Vennema, Jeroen Cremer, Sharon van den Brink, Bas van der Veer, AnneMarie van den Brandt, Florian Zwagemaker, Dennis Schmitz, Chantal Reusken, on behalf of the national COVID-19 response team
EPI_ISL_791316	National Virus Reference Laboratory	Irish Coronavirus Sequencing Consortium - Teagasc Moorepark	Alejandro Abner Garcia Leon, Paul Cotter, Fiona Crispie, John Kenny, Paddy Mallon, Calum Walsh
EPI_ISL_792602, EPI_ISL_792620, EPI_ISL_792621	LACEN-PB	Laboratory of Respiratory Viruses and Measles, Oswaldo Cruz Institute, FIOCRUZ	Paola Resende, Luciana Appolinario, Fernando Motta, Anna Carolina Paixao, Ana Carolina Mendonca, João Felipe Bezerra, Romero Henrique Teixeira de Vasconcelos, Dalane Loudal Florentino Teixeira, Thiago Franco de Oliveira Carneiro, Marilda Siqueira
EPI_ISL_794035, EPI_ISL_794038, EPI_ISL_794039, EPI_ISL_794040, EPI_ISL_794041	Wadsworth Center, New York State Department.of Health	Wadsworth Center, New York State Department.of Health	Kirsten St. George, Daryl M. Lamson, Alexis Russel, Matthew Shudt, Melissa A Leisner, Jonathan Plitnick, Navjot Singh, John Kelly, Sara Griesemer, Erasmus Schneider, Erica Lasek-Nesselquist
EPI_ISL_796032, EPI_ISL_796036, EPI_ISL_796037, EPI_ISL_796038, EPI_ISL_796048, EPI_ISL_796049, EPI_ISL_796050, EPI_ISL_796056, EPI_ISL_796057, EPI_ISL_796058	Institute of Virology, University of Cologne	Institute of Virology, University of Cologne	Saleta Sierra, Gibran Rubio, Zevanya Tessalonica, Dominik Aschenmeier, Eva Heger, Elena Knops, Rolf Kaiser, Maximilian Damagnez, Andreas Walker, Jörg Timm, Alexander Dilthey, Martin Däumer, Alex Thielen
EPI_ISL_801885, EPI_ISL_801916, EPI_ISL_801918, EPI_ISL_801933, EPI_ISL_801942, EPI_ISL_801943, EPI_ISL_801947, EPI_ISL_801954, EPI_ISL_801959, EPI_ISL_801971, EPI_ISL_801972, EPI_ISL_802270, EPI_ISL_802271, EPI_ISL_802272, EPI_ISL_802273, EPI_ISL_802274, EPI_ISL_802275, EPI_ISL_802276, EPI_ISL_802277, EPI_ISL_802278, EPI_ISL_802279, EPI_ISL_802280, EPI_ISL_802281, EPI_ISL_802282, EPI_ISL_802283, EPI_ISL_802284, EPI_ISL_802285, EPI_ISL_802286, EPI_ISL_802287, EPI_ISL_802288, EPI_ISL_802289, EPI_ISL_802290, EPI_ISL_802291, EPI_ISL_802292, EPI_ISL_802293, EPI_ISL_802294, EPI_ISL_802295, EPI_ISL_802296, EPI_ISL_802297, EPI_ISL_802298, EPI_ISL_802299, EPI_ISL_802300, EPI_ISL_802301, EPI_ISL_802302, EPI_ISL_802303, EPI_ISL_802304, EPI_ISL_802305, EPI_ISL_802306, EPI_ISL_802307, EPI_ISL_802308, EPI_ISL_802309, EPI_ISL_802310, EPI_ISL_802311, EPI_ISL_802312, EPI_ISL_802313, EPI_ISL_802314, EPI_ISL_802315, EPI_ISL_802316, EPI_ISL_802317, EPI_ISL_802318, EPI_ISL_802319, EPI_ISL_802320, EPI_ISL_802321, EPI_ISL_802322, EPI_ISL_802323, EPI_ISL_802324, EPI_ISL_802325			
see above	MSHS Clinical Microbiology Laboratories	MSHS Pathogen Surveillance Program	Ana S. Gonzalez-Reiche, Hala Alshammary, Mitchell J. Sullivan, Brianne Ciferri, Ajay Obla, Angela Amoako, Mahmoud Awawda, Elena Hirsch, Ashley S. Salimbangon, Levy Sominsky, Katherine Beach, Kayla Russo, Charles Gleason, Shelcie Fabre, Giulio Kleiner, Zenab Khan, Bremy Alburquerque, Adriana van de Guchte, Komal Srivastava, Matthew M. Hernandez, Jayeeta Dutta, Denise Jurczyszak, Emily Ferreri, Rachel Chernet, Nancy Francoeur, Betsaida Salom

			Melo, Irina Oussenko, Gintaras Deikus, Juan Soto, Shwetha Hara Sridhar, Ying-Chih Wang, Kathryn Twyman, Andrew Kasarskis, Deena R. Altman, Robert Sebra, Adolfo García-Sastre, Marta Luksza, Gopi Patel, Sarah Schaefer, Melissa Gitman, Michael D. Nowak, Alberto Paniz-Mondolfi, Emilia Mia Sordillo, Viviana Simon, Harm van Bakel
EPI_ISL_802860	Vilnius University Hospital Santaros Klinikos, Vilnius University	Institute of Biotechnology, Life Sciences Center, Vilnius University	Emilija Vasiliunaite, Milda Norkiene, Albertas Timinskas, Alma Gedvilaite, Aurelija Zvirbliene, Daniel Naumovas, Laimonas Griskevicius
EPI_ISL_803308, EPI_ISL_803319, EPI_ISL_803320, EPI_ISL_803321, EPI_ISL_803323, EPI_ISL_803324, EPI_ISL_803325, EPI_ISL_803327, EPI_ISL_803328, EPI_ISL_803329, EPI_ISL_803330, EPI_ISL_803331, EPI_ISL_803332, EPI_ISL_803353, EPI_ISL_803559, EPI_ISL_803560, EPI_ISL_803561, EPI_ISL_803562, EPI_ISL_803563, EPI_ISL_803564, EPI_ISL_803565, EPI_ISL_803566, EPI_ISL_803567, EPI_ISL_803568, EPI_ISL_803569, EPI_ISL_803570, EPI_ISL_803571, EPI_ISL_803572, EPI_ISL_803573, EPI_ISL_803574, EPI_ISL_803575, EPI_ISL_803576, EPI_ISL_803587, EPI_ISL_803588, EPI_ISL_803589, EPI_ISL_803590, EPI_ISL_803591, EPI_ISL_803592, EPI_ISL_803593, EPI_ISL_803594, EPI_ISL_803595, EPI_ISL_803596, EPI_ISL_803597, EPI_ISL_803601, EPI_ISL_803605, EPI_ISL_803606, EPI_ISL_803607, EPI_ISL_803608, EPI_ISL_803609, EPI_ISL_803610, EPI_ISL_803611, EPI_ISL_803612, EPI_ISL_803613, EPI_ISL_803614, EPI_ISL_803615, EPI_ISL_803616, EPI_ISL_803617, EPI_ISL_803618, EPI_ISL_803619, EPI_ISL_803620, EPI_ISL_803621, EPI_ISL_803622, EPI_ISL_803623, EPI_ISL_803624, EPI_ISL_803625, EPI_ISL_803626, EPI_ISL_803628, EPI_ISL_803629, EPI_ISL_803725, EPI_ISL_803726, EPI_ISL_803750, EPI_ISL_803751, EPI_ISL_803752, EPI_ISL_803753, EPI_ISL_803754, EPI_ISL_803755			
see above	Wisconsin State Laboratory of Hygiene Communicable Disease Division	Wisconsin State Laboratory of Hygiene Communicable Disease Division	Kelsey R. Florek, Abigail C. Shockey
EPI_ISL_804261, EPI_ISL_804262, EPI_ISL_804263	Respiratory Virus Unit, National Infection Service, Public Health England	COVID-19 Genomics UK (COG-UK) Consortium	PHE Covid Sequencing Team
EPI_ISL_804963	Hospital Comarcal de Melilla	Instituto de Salud Carlos III	Iglesias-Caballero, M. Molinero Calamita, M. González-Esguevillas, M. Camarero, S. Pozo, F. Casas, I. Jiménez, P. Jiménez, M. Zaballos, A. Monzón, S. Varona, S. Juliá, M. Cuesta, I, J. López
EPI_ISL_810981, EPI_ISL_810982, EPI_ISL_810986, EPI_ISL_811011, EPI_ISL_811037	MRCG at LSHTM Genomics lab	MRCG at LSHTM Genomics lab	Abdul Karim sesay, Abdoulie Kanteh, Jarra Manneh, Mariama Kujabi, Bakary Sanyang
EPI_ISL_812121	Santa Clara County Public Health Laboratory	Santa Clara County Public Health Laboratory	Santa Clara County Public Health Department
EPI_ISL_812134, EPI_ISL_812232	UT-Unified State Labs: Public Health Utah DOH	Pathogen Discovery, Respiratory Viruses Branch, Division of Viral Diseases, Centers for Disease Control and Prevention	Yan Li, Ying Tao, Anna Montmayeur, Jing Zhang, Brian Lynch, Krista Queen, Anna Uehara, Rachel Marine, Peter Cook, Clinton R. Paden, Haibin Wang, Suxiang Tong
EPI_ISL_813661	Liverpool Clinical Laboratories	COVID-19 Genomics UK (COG-UK) Consortium	Sam Haldenby, Anita Lucaci, Steve Paterson, Julian Hiscox, Alistair Darby, M Almsaud, A Alrezaihi, Muhannad Alruwaili, Stuart D Armstrong, Jones Benjamin, Eleanor G Bentley, Anu Chawla, Jordan J Clark, Angela Cowell, Richard Eccles, Isabel García-Dorival, Matthew Gemmell, Alessandro Gerada, PKF Gilmore, Richard Gregory, Ximeng Han, Catherine Hartley, Margaret Hughes, Miren Iturriza-Gomara, James Johnson, L Luu, Jenifer Manson, Charlotte Nelson, Elaine O'Toole, Cassie Olateju, Rebekah Penrice-Randal , Lucille Rainbow, N.P Randle, Trevor Ian Robinson, Parul Sharma, Ghada T Shawli, James P Stewart, Neil Swainston, Ecaterina Vamos, Joanne Watts, Mark Whitehead
EPI_ISL_813993	Hospital General Universitario Gregorio Marañón	SeqCOVID-SPAIN consortium/IBV(CSIC)	Darío García de Viedma, Laura Pérez-Lago, Marta Herranz, Jon Sicilia, Julia Suárez, Pilar Catalán, Patricia Muñoz and SeqCOVID-SPAIN consortium
EPI_ISL_815258, EPI_ISL_815290, EPI_ISL_815294, EPI_ISL_815295, EPI_ISL_815298, EPI_ISL_815312, EPI_ISL_815332, EPI_ISL_815340, EPI_ISL_815344, EPI_ISL_815348, EPI_ISL_815349, EPI_ISL_815354, EPI_ISL_815359, EPI_ISL_815361, EPI_ISL_815362, EPI_ISL_815363, EPI_ISL_815368, EPI_ISL_815369, EPI_ISL_815373, EPI_ISL_815376, EPI_ISL_815382, EPI_ISL_815383, EPI_ISL_815389			
see above	Centogene	Centogene	Peter Bauer, Krishna Kumar Kandaswamy, Vivi Hue-Trang Lieu
EPI_ISL_822318, EPI_ISL_822319, EPI_ISL_822320, EPI_ISL_822321	Lighthouse Lab in Glasgow	Wellcome Sanger Institute for the COVID-19 Genomics UK (COG-UK) Consortium	Harper VanSteenhouse, Yumi Kasai, David Gray, Carol Clugston, Anna Dominiczak and Alex Alderton, Roberto Amato, Sonia Goncalves, Ewan Harrison, David K. Jackson, Ian Johnston, Dominic Kwiatkowski, Cordelia Langford, John Sillitoe on behalf of the Wellcome Sanger Institute COVID-19 Surveillance Team
EPI_ISL_824003, EPI_ISL_824157	Dutch COVID-19 response team	National Institute for Public Health and the Environment (RIVM)	Adam Meijer, Harry Vennema, Jeroen Cremer, Sharon van den Brink, Bas van der Veer, AnneMarie van den Brandt, Florian Zwagemaker, Dennis Schmitz, Chantal Reusken, on behalf of the national COVID-19 response team
EPI_ISL_824495, EPI_ISL_824496, EPI_ISL_824497, EPI_ISL_824498, EPI_ISL_824499, EPI_ISL_824500, EPI_ISL_824501, EPI_ISL_824502	Hospital Universitari Vall d'Hebron - Vall d'Hebron Institut de Rercerca	Hospital Universitari Vall d'Hebron	Cristina Andrés, Maria Piñana, Josep F Abril, Damir Garcia-Cehic, Ariadna Rando, Juliana Esperalba, Maria Gema Codina, Carla Castillo, Maria Carmen Martin, Tomàs Pumarola, Josep Quer, Andrés Antón
EPI_ISL_824513	New Mexico Department of Health Scientific Laboratory	New Mexico Department of Health Scientific Laboratory	Ellie Johnson, Anastacia Griego-Fisher, D'eldra Malone
EPI_ISL_825493, EPI_ISL_825575	Respiratory Virus Unit, National Infection Service, Public Health England	COVID-19 Genomics UK (COG-UK) Consortium	PHE Covid Sequencing Team
EPI_ISL_826934, EPI_ISL_826935, EPI_ISL_826936, EPI_ISL_826938, EPI_ISL_826939, EPI_ISL_826940, EPI_ISL_826941, EPI_ISL_826942, EPI_ISL_826943	deCODE genetics	deCODE genetics	Daniel F Gudbjartsson; Agnar Helgason; Hakon Jonsson; Olafur T Magnusson; Pall Melsted; Gudmundur L Norddahl; Jona Saemundsdottir; Asgeir Sigurdsson; Patrick Sulem; Arna B Agustsdottir; Hannes Eggertsson; Berglind Eiriksdottir; Run Fridriksdottir; Elisabet E Gardarsdottir; Gudmundur Georgsson; Olafia S Gretarsdottir; Kjartan R Gudmundsson; Thora R Gunnarsdottir; Arnaldur Gylfason; Hilma Holm; Brynjar O Jensson; Aslaug Jonasdottir; Kamilla S Josefsdottir; Thordur Kristjansson; Droplaug N Magnusdottir; Solvi Rognvaldsson; Louise le Roux; Gudrun Sigmundsdottir; Gardar Sveinbjornsson; Kristin E Sveinsdottir; Maney Sveinsdottir; Emil A Thorarensen; Bjarni Thorbjornsson; Gisli Masson; Ingileif Jonsdottir; Alma Moller; Thorolfur Gudnason; Karl G Kristinsson; Unnur Thorsteinsdottir; Kari Stefansson
EPI_ISL_826955, EPI_ISL_827045, EPI_ISL_827069, EPI_ISL_827076, EPI_ISL_827082, EPI_ISL_827110, EPI_ISL_827126, EPI_ISL_827142	The National University Hospital of Iceland	deCODE genetics	Daniel F Gudbjartsson; Agnar Helgason; Hakon Jonsson; Olafur T Magnusson; Pall Melsted; Gudmundur L Norddahl; Jona Saemundsdottir; Asgeir Sigurdsson; Patrick Sulem; Arna B Agustsdottir; Hannes Eggertsson; Berglind Eiriksdottir; Run Fridriksdottir; Elisabet E Gardarsdottir; Gudmundur Georgsson; Olafia S Gretarsdottir; Kjartan R Gudmundsson; Thora R Gunnarsdottir; Arnaldur Gylfason; Hilma Holm; Brynjar O Jensson; Aslaug Jonasdottir; Kamilla S Josefsdottir; Thordur Kristjansson; Droplaug N Magnusdottir; Solvi Rognvaldsson; Louise le Roux; Gudrun Sigmundsdottir; Gardar Sveinbjornsson; Kristin E Sveinsdottir; Maney Sveinsdottir; Emil A Thorarensen; Bjarni Thorbjornsson; Gisli Masson; Ingileif Jonsdottir; Alma Moller; Thorolfur Gudnason; Karl G Kristinsson; Unnur Thorsteinsdottir; Kari Stefansson
EPI_ISL_827322, EPI_ISL_827323, EPI_ISL_827325, EPI_ISL_827326, EPI_ISL_827327, EPI_ISL_827328, EPI_ISL_827329, EPI_ISL_827332	deCODE genetics	deCODE genetics	Daniel F Gudbjartsson; Agnar Helgason; Hakon Jonsson; Olafur T Magnusson; Pall Melsted; Gudmundur L Norddahl; Jona Saemundsdottir; Asgeir Sigurdsson; Patrick Sulem; Arna B Agustsdottir; Hannes Eggertsson; Berglind Eiriksdottir; Run Fridriksdottir; Elisabet E Gardarsdottir; Gudmundur Georgsson; Olafia S Gretarsdottir; Kjartan R Gudmundsson; Thora R Gunnarsdottir; Arnaldur Gylfason; Hilma Holm; Brynjar O Jensson; Aslaug Jonasdottir; Kamilla S Josefsdottir; Thordur Kristjansson; Droplaug N Magnusdottir; Solvi Rognvaldsson; Louise le Roux; Gudrun Sigmundsdottir; Gardar Sveinbjornsson; Kristin E Sveinsdottir; Maney Sveinsdottir; Emil A Thorarensen; Bjarni Thorbjornsson; Gisli Masson; Ingileif Jonsdottir; Alma Moller; Thorolfur Gudnason; Karl G Kristinsson; Unnur Thorsteinsdottir; Kari Stefansson
EPI_ISL_827354	The National University Hospital of Iceland	deCODE genetics	Daniel F Gudbjartsson; Agnar Helgason; Hakon Jonsson; Olafur T Magnusson; Pall Melsted; Gudmundur L Norddahl; Jona Saemundsdottir; Asgeir Sigurdsson; Patrick Sulem; Arna B Agustsdottir; Hannes Eggertsson; Berglind Eiriksdottir; Run Fridriksdottir; Elisabet E Gardarsdottir; Gudmundur Georgsson; Olafia S Gretarsdottir; Kjartan R Gudmundsson; Thora R Gunnarsdottir; Arnaldur Gylfason; Hilma Holm; Brynjar O Jensson; Aslaug Jonasdottir; Kamilla S Josefsdottir; Thordur Kristjansson; Droplaug N Magnusdottir; Solvi Rognvaldsson; Louise le Roux; Gudrun Sigmundsdottir; Gardar Sveinbjornsson; Kristin E Sveinsdottir; Maney Sveinsdottir; Emil A Thorarensen; Bjarni Thorbjornsson; Gisli Masson; Ingileif Jonsdottir; Alma Moller; Thorolfur Gudnason; Karl G Kristinsson; Unnur Thorsteinsdottir; Kari Stefansson
EPI_ISL_827610, EPI_ISL_827611, EPI_ISL_827612, EPI_ISL_827613, EPI_ISL_827614, EPI_ISL_827615, EPI_ISL_827616, EPI_ISL_827617, EPI_ISL_827618, EPI_ISL_827619, EPI_ISL_827630, EPI_ISL_827631, EPI_ISL_827632, EPI_ISL_827634, EPI_ISL_827635, EPI_ISL_827636, EPI_ISL_827738, EPI_ISL_827740, EPI_ISL_827741, EPI_ISL_827742, EPI_ISL_827743			
see above	deCODE genetics	deCODE genetics	Daniel F Gudbjartsson; Agnar Helgason; Hakon Jonsson; Olafur T Magnusson; Pall Melsted; Gudmundur L Norddahl; Jona Saemundsdottir; Asgeir Sigurdsson; Patrick Sulem; Arna B Agustsdottir; Hannes Eggertsson; Berglind Eiriksdottir; Run Fridriksdottir; Elisabet E Gardarsdottir; Gudmundur Georgsson; Olafia S Gretarsdottir; Kjartan R Gudmundsson; Thora R Gunnarsdottir; Arnaldur Gylfason; Hilma Holm; Brynjar O Jensson; Aslaug Jonasdottir; Kamilla S Josefsdottir; Thordur Kristjansson; Droplaug N Magnusdottir; Solvi Rognvaldsson; Louise le Roux; Gudrun Sigmundsdottir; Gardar Sveinbjornsson; Kristin E Sveinsdottir; Maney Sveinsdottir; Emil A Thorarensen; Bjarni Thorbjornsson; Gisli Masson; Ingileif Jonsdottir; Alma Moller; Thorolfur Gudnason; Karl G Kristinsson; Unnur Thorsteinsdottir; Kari Stefansson

EPI_ISL_827746	The National University Hospital of Iceland	deCODE genetics	Daniel F Gudbjartsson; Agnar Helgason; Hakon Jonsson; Olafur T Magnusson; Pall Melsted; Gudmundur L Norddahl; Jona Saemundsdottir; Asgeir Sigurdsson; Patrick Sulem; Arna B Agustsdottir; Hannes Eggertsson; Berglind Eiriksdottir; Run Fridriksdottir; Elisabet E Gardarsdottir; Gudmundur Georgsson; Olafia S Gretarsdottir; Kjartan R Gudmundsson; Thora R Gunnarsdottir; Arnaldur Gylfason; Hilma Holm; Brynjar O Jensson; Aslaug Jonasdottir; Kamilla S Josefsdottir; Thordur Kristjansson; Droplaug N Magnusdottir; Solvi Rognvaldsson; Louise le Roux; Gudrun Sigmundsdottir; Gardar Sveinbjornsson; Kristin E Sveinsdottir; Maney Sveinsdottir; Emil A Thorarensen; Bjarni Thorbjornsson; Gisli Masson; Ingileif Jonsdottir; Alma Moller; Thorolfur Gudnason; Karl G Kristinsson; Unnur Thorsteinsdottir; Kari Stefansson
EPI_ISL_827755, EPI_ISL_827756	deCODE genetics	deCODE genetics	Daniel F Gudbjartsson; Agnar Helgason; Hakon Jonsson; Olafur T Magnusson; Pall Melsted; Gudmundur L Norddahl; Jona Saemundsdottir; Asgeir Sigurdsson; Patrick Sulem; Arna B Agustsdottir; Hannes Eggertsson; Berglind Eiriksdottir; Run Fridriksdottir; Elisabet E Gardarsdottir; Gudmundur Georgsson; Olafia S Gretarsdottir; Kjartan R Gudmundsson; Thora R Gunnarsdottir; Arnaldur Gylfason; Hilma Holm; Brynjar O Jensson; Aslaug Jonasdottir; Kamilla S Josefsdottir; Thordur Kristjansson; Droplaug N Magnusdottir; Solvi Rognvaldsson; Louise le Roux; Gudrun Sigmundsdottir; Gardar Sveinbjornsson; Kristin E Sveinsdottir; Maney Sveinsdottir; Emil A Thorarensen; Bjarni Thorbjornsson; Gisli Masson; Ingileif Jonsdottir; Alma Moller; Thorolfur Gudnason; Karl G Kristinsson; Unnur Thorsteinsdottir; Kari Stefansson
EPI_ISL_827800, EPI_ISL_827802, EPI_ISL_827803, EPI_ISL_827804, EPI_ISL_827805	The National University Hospital of Iceland	deCODE genetics	Daniel F Gudbjartsson; Agnar Helgason; Hakon Jonsson; Olafur T Magnusson; Pall Melsted; Gudmundur L Norddahl; Jona Saemundsdottir; Asgeir Sigurdsson; Patrick Sulem; Arna B Agustsdottir; Hannes Eggertsson; Berglind Eiriksdottir; Run Fridriksdottir; Elisabet E Gardarsdottir; Gudmundur Georgsson; Olafia S Gretarsdottir; Kjartan R Gudmundsson; Thora R Gunnarsdottir; Arnaldur Gylfason; Hilma Holm; Brynjar O Jensson; Aslaug Jonasdottir; Kamilla S Josefsdottir; Thordur Kristjansson; Droplaug N Magnusdottir; Solvi Rognvaldsson; Louise le Roux; Gudrun Sigmundsdottir; Gardar Sveinbjornsson; Kristin E Sveinsdottir; Maney Sveinsdottir; Emil A Thorarensen; Bjarni Thorbjornsson; Gisli Masson; Ingileif Jonsdottir; Alma Moller; Thorolfur Gudnason; Karl G Kristinsson; Unnur Thorsteinsdottir; Kari Stefansson
EPI_ISL_827883, EPI_ISL_827914, EPI_ISL_827915, EPI_ISL_827916, EPI_ISL_827917, EPI_ISL_827918, EPI_ISL_827919	deCODE genetics	deCODE genetics	Daniel F Gudbjartsson; Agnar Helgason; Hakon Jonsson; Olafur T Magnusson; Pall Melsted; Gudmundur L Norddahl; Jona Saemundsdottir; Asgeir Sigurdsson; Patrick Sulem; Arna B Agustsdottir; Hannes Eggertsson; Berglind Eiriksdottir; Run Fridriksdottir; Elisabet E Gardarsdottir; Gudmundur Georgsson; Olafia S Gretarsdottir; Kjartan R Gudmundsson; Thora R Gunnarsdottir; Arnaldur Gylfason; Hilma Holm; Brynjar O Jensson; Aslaug Jonasdottir; Kamilla S Josefsdottir; Thordur Kristjansson; Droplaug N Magnusdottir; Solvi Rognvaldsson; Louise le Roux; Gudrun Sigmundsdottir; Gardar Sveinbjornsson; Kristin E Sveinsdottir; Maney Sveinsdottir; Emil A Thorarensen; Bjarni Thorbjornsson; Gisli Masson; Ingileif Jonsdottir; Alma Moller; Thorolfur Gudnason; Karl G Kristinsson; Unnur Thorsteinsdottir; Kari Stefansson
EPI_ISL_827942, EPI_ISL_827943, EPI_ISL_828031, EPI_ISL_828032, EPI_ISL_828035, EPI_ISL_828036	The National University Hospital of Iceland	deCODE genetics	Daniel F Gudbjartsson; Agnar Helgason; Hakon Jonsson; Olafur T Magnusson; Pall Melsted; Gudmundur L Norddahl; Jona Saemundsdottir; Asgeir Sigurdsson; Patrick Sulem; Arna B Agustsdottir; Hannes Eggertsson; Berglind Eiriksdottir; Run Fridriksdottir; Elisabet E Gardarsdottir; Gudmundur Georgsson; Olafia S Gretarsdottir; Kjartan R Gudmundsson; Thora R Gunnarsdottir; Arnaldur Gylfason; Hilma Holm; Brynjar O Jensson; Aslaug Jonasdottir; Kamilla S Josefsdottir; Thordur Kristjansson; Droplaug N Magnusdottir; Solvi Rognvaldsson; Louise le Roux; Gudrun Sigmundsdottir; Gardar Sveinbjornsson; Kristin E Sveinsdottir; Maney Sveinsdottir; Emil A Thorarensen; Bjarni Thorbjornsson; Gisli Masson; Ingileif Jonsdottir; Alma Moller; Thorolfur Gudnason; Karl G Kristinsson; Unnur Thorsteinsdottir; Kari Stefansson
EPI_ISL_828040, EPI_ISL_828041, EPI_ISL_828042, EPI_ISL_828043, EPI_ISL_828044, EPI_ISL_828045, EPI_ISL_828046, EPI_ISL_828048, EPI_ISL_828049	deCODE genetics	deCODE genetics	Daniel F Gudbjartsson; Agnar Helgason; Hakon Jonsson; Olafur T Magnusson; Pall Melsted; Gudmundur L Norddahl; Jona Saemundsdottir; Asgeir Sigurdsson; Patrick Sulem; Arna B Agustsdottir; Hannes Eggertsson; Berglind Eiriksdottir; Run Fridriksdottir; Elisabet E Gardarsdottir; Gudmundur Georgsson; Olafia S Gretarsdottir; Kjartan R Gudmundsson; Thora R Gunnarsdottir; Arnaldur Gylfason; Hilma Holm; Brynjar O Jensson; Aslaug Jonasdottir; Kamilla S Josefsdottir; Thordur Kristjansson; Droplaug N Magnusdottir; Solvi Rognvaldsson; Louise le Roux; Gudrun Sigmundsdottir; Gardar Sveinbjornsson; Kristin E Sveinsdottir; Maney Sveinsdottir; Emil A Thorarensen; Bjarni Thorbjornsson; Gisli Masson; Ingileif Jonsdottir; Alma Moller; Thorolfur Gudnason; Karl G Kristinsson; Unnur Thorsteinsdottir; Kari Stefansson
EPI_ISL_828053, EPI_ISL_828064, EPI_ISL_828070, EPI_ISL_828099	The National University Hospital of Iceland	deCODE genetics	Daniel F Gudbjartsson; Agnar Helgason; Hakon Jonsson; Olafur T Magnusson; Pall Melsted; Gudmundur L Norddahl; Jona Saemundsdottir; Asgeir Sigurdsson; Patrick Sulem; Arna B Agustsdottir; Hannes Eggertsson; Berglind Eiriksdottir; Run Fridriksdottir; Elisabet E Gardarsdottir; Gudmundur Georgsson; Olafia S Gretarsdottir; Kjartan R Gudmundsson; Thora R Gunnarsdottir; Arnaldur Gylfason; Hilma Holm; Brynjar O Jensson; Aslaug Jonasdottir; Kamilla S Josefsdottir; Thordur Kristjansson; Droplaug N Magnusdottir; Solvi Rognvaldsson; Louise le Roux; Gudrun Sigmundsdottir; Gardar Sveinbjornsson; Kristin E Sveinsdottir; Maney Sveinsdottir; Emil A Thorarensen; Bjarni Thorbjornsson; Gisli Masson; Ingileif Jonsdottir; Alma Moller; Thorolfur Gudnason; Karl G Kristinsson; Unnur Thorsteinsdottir; Kari Stefansson
EPI_ISL_828104	deCODE genetics	deCODE genetics	Daniel F Gudbjartsson; Agnar Helgason; Hakon Jonsson; Olafur T Magnusson; Pall Melsted; Gudmundur L Norddahl; Jona Saemundsdottir; Asgeir Sigurdsson; Patrick Sulem; Arna B Agustsdottir; Hannes Eggertsson; Berglind Eiriksdottir; Run Fridriksdottir; Elisabet E Gardarsdottir; Gudmundur Georgsson; Olafia S Gretarsdottir; Kjartan R Gudmundsson; Thora R Gunnarsdottir; Arnaldur Gylfason; Hilma Holm; Brynjar O Jensson; Aslaug Jonasdottir; Kamilla S Josefsdottir; Thordur Kristjansson; Droplaug N Magnusdottir; Solvi Rognvaldsson; Louise le Roux; Gudrun Sigmundsdottir; Gardar Sveinbjornsson; Kristin E Sveinsdottir; Maney Sveinsdottir; Emil A Thorarensen; Bjarni Thorbjornsson; Gisli Masson; Ingileif Jonsdottir; Alma Moller; Thorolfur Gudnason; Karl G Kristinsson; Unnur Thorsteinsdottir; Kari Stefansson
EPI_ISL_828142, EPI_ISL_828143, EPI_ISL_828144	The National University Hospital of Iceland	deCODE genetics	Daniel F Gudbjartsson; Agnar Helgason; Hakon Jonsson; Olafur T Magnusson; Pall Melsted; Gudmundur L Norddahl; Jona Saemundsdottir; Asgeir Sigurdsson; Patrick Sulem; Arna B Agustsdottir; Hannes Eggertsson; Berglind Eiriksdottir; Run Fridriksdottir; Elisabet E Gardarsdottir; Gudmundur Georgsson; Olafia S Gretarsdottir; Kjartan R Gudmundsson; Thora R Gunnarsdottir; Arnaldur Gylfason; Hilma Holm; Brynjar O Jensson; Aslaug Jonasdottir; Kamilla S Josefsdottir; Thordur Kristjansson; Droplaug N Magnusdottir; Solvi Rognvaldsson; Louise le Roux; Gudrun Sigmundsdottir; Gardar Sveinbjornsson; Kristin E Sveinsdottir; Maney Sveinsdottir; Emil A Thorarensen; Bjarni Thorbjornsson; Gisli Masson; Ingileif Jonsdottir; Alma Moller; Thorolfur Gudnason; Karl G Kristinsson; Unnur Thorsteinsdottir; Kari Stefansson
EPI_ISL_828153, EPI_ISL_828155, EPI_ISL_828156	deCODE genetics	deCODE genetics	Daniel F Gudbjartsson; Agnar Helgason; Hakon Jonsson; Olafur T Magnusson; Pall Melsted; Gudmundur L Norddahl; Jona Saemundsdottir; Asgeir Sigurdsson; Patrick Sulem; Arna B Agustsdottir; Hannes Eggertsson; Berglind Eiriksdottir; Run Fridriksdottir; Elisabet E Gardarsdottir; Gudmundur Georgsson; Olafia S Gretarsdottir; Kjartan R Gudmundsson; Thora R Gunnarsdottir; Arnaldur Gylfason; Hilma Holm; Brynjar O Jensson; Aslaug Jonasdottir; Kamilla S Josefsdottir; Thordur Kristjansson; Droplaug N Magnusdottir; Solvi Rognvaldsson; Louise le Roux; Gudrun Sigmundsdottir; Gardar Sveinbjornsson; Kristin E Sveinsdottir; Maney Sveinsdottir; Emil A Thorarensen; Bjarni Thorbjornsson; Gisli Masson; Ingileif Jonsdottir; Alma Moller; Thorolfur Gudnason; Karl G Kristinsson; Unnur Thorsteinsdottir; Kari Stefansson
EPI_ISL_828157, EPI_ISL_828158, EPI_ISL_828159, EPI_ISL_828160, EPI_ISL_828162	The National University Hospital of Iceland	deCODE genetics	Daniel F Gudbjartsson; Agnar Helgason; Hakon Jonsson; Olafur T Magnusson; Pall Melsted; Gudmundur L Norddahl; Jona Saemundsdottir; Asgeir Sigurdsson; Patrick Sulem; Arna B Agustsdottir; Hannes Eggertsson; Berglind Eiriksdottir; Run Fridriksdottir; Elisabet E Gardarsdottir; Gudmundur Georgsson; Olafia S Gretarsdottir; Kjartan R Gudmundsson; Thora R Gunnarsdottir; Arnaldur Gylfason; Hilma Holm; Brynjar O Jensson; Aslaug Jonasdottir; Kamilla S Josefsdottir; Thordur Kristjansson; Droplaug N Magnusdottir; Solvi Rognvaldsson; Louise le Roux; Gudrun Sigmundsdottir; Gardar Sveinbjornsson; Kristin E Sveinsdottir; Maney Sveinsdottir; Emil A Thorarensen; Bjarni Thorbjornsson; Gisli Masson; Ingileif Jonsdottir; Alma Moller; Thorolfur Gudnason; Karl G Kristinsson; Unnur Thorsteinsdottir; Kari Stefansson
EPI_ISL_828176, EPI_ISL_828180, EPI_ISL_828181, EPI_ISL_828189, EPI_ISL_828193, EPI_ISL_828207, EPI_ISL_828237, EPI_ISL_828238, EPI_ISL_828239, EPI_ISL_828240, EPI_ISL_828280, EPI_ISL_828284			
see above	deCODE genetics	deCODE genetics	Daniel F Gudbjartsson; Agnar Helgason; Hakon Jonsson; Olafur T Magnusson; Pall Melsted; Gudmundur L Norddahl; Jona Saemundsdottir; Asgeir Sigurdsson; Patrick Sulem; Arna B Agustsdottir; Hannes Eggertsson; Berglind Eiriksdottir; Run Fridriksdottir; Elisabet E Gardarsdottir; Gudmundur Georgsson; Olafia S Gretarsdottir; Kjartan R Gudmundsson; Thora R Gunnarsdottir; Arnaldur Gylfason; Hilma Holm; Brynjar O Jensson; Aslaug Jonasdottir; Kamilla S Josefsdottir; Thordur Kristjansson; Droplaug N Magnusdottir; Solvi Rognvaldsson; Louise le Roux; Gudrun Sigmundsdottir; Gardar Sveinbjornsson; Kristin E Sveinsdottir; Maney Sveinsdottir; Emil A Thorarensen; Bjarni Thorbjornsson; Gisli Masson; Ingileif Jonsdottir; Alma Moller; Thorolfur Gudnason; Karl G Kristinsson; Unnur Thorsteinsdottir; Kari Stefansson
EPI_ISL_828287	The National University Hospital of Iceland	deCODE genetics	Daniel F Gudbjartsson; Agnar Helgason; Hakon Jonsson; Olafur T Magnusson; Pall Melsted; Gudmundur L Norddahl; Jona Saemundsdottir; Asgeir Sigurdsson; Patrick Sulem; Arna B Agustsdottir; Hannes Eggertsson; Berglind Eiriksdottir; Run Fridriksdottir; Elisabet E Gardarsdottir; Gudmundur Georgsson; Olafia S Gretarsdottir; Kjartan R Gudmundsson; Thora R Gunnarsdottir; Arnaldur Gylfason; Hilma Holm; Brynjar O Jensson; Aslaug Jonasdottir; Kamilla S Josefsdottir; Thordur Kristjansson; Droplaug N Magnusdottir; Solvi Rognvaldsson; Louise le Roux; Gudrun Sigmundsdottir; Gardar Sveinbjornsson; Kristin E Sveinsdottir; Maney Sveinsdottir; Emil A Thorarensen; Bjarni Thorbjornsson; Gisli Masson; Ingileif Jonsdottir; Alma Moller; Thorolfur Gudnason; Karl G Kristinsson; Unnur Thorsteinsdottir; Kari Stefansson
EPI_ISL_828289, EPI_ISL_828293,	deCODE genetics	deCODE genetics	Daniel F Gudbjartsson; Agnar Helgason; Hakon Jonsson; Olafur T Magnusson; Pall Melsted; Gudmundur L Norddahl; Jona Saemundsdottir; Asgeir

EPI_ISL_828297, EPI_ISL_828302, EPI_ISL_828307, EPI_ISL_828311, EPI_ISL_828316, EPI_ISL_828318, EPI_ISL_828319			Sigurdsson; Patrick Sulem; Arna B Agustsdottir; Hannes Eggertsson; Berglind Eiriksdottir; Run Fridriksdottir; Elisabet E Gardarsdottir; Gudmundur Georgsson; Olafia S Gretarsdottir; Kjartan R Gudmundsson; Thora R Gunnarsdottir; Arnaldur Gylfason; Hilma Holm; Brynjar O Jensson; Aslaug Jonasdottir; Kamilla S Josefsdottir; Thordur Kristjansson; Droplaug N Magnusdottir; Solvi Rognvaldsson; Louise le Roux; Gudrun Sigmundsdottir; Gardar Sveinbjornsson; Kristin E Sveinsdottir; Maney Sveinsdottir; Emil A Thorarensen; Bjarni Thorbjornsson; Gisli Masson; Ingileif Jonsdottir; Alma Moller; Thorolfur Gudnason; Karl G Kristinsson; Unnur Thorsteinsdottir; Kari Stefansson
EPI_ISL_828323	The National University Hospital of Iceland	deCODE genetics	Daniel F Gudbjartsson; Agnar Helgason; Hakon Jonsson; Olafur T Magnusson; Pall Melsted; Gudmundur L Norddahl; Jona Saemundsdottir; Asgeir Sigurdsson; Patrick Sulem; Arna B Agustsdottir; Hannes Eggertsson; Berglind Eiriksdottir; Run Fridriksdottir; Elisabet E Gardarsdottir; Gudmundur Georgsson; Olafia S Gretarsdottir; Kjartan R Gudmundsson; Thora R Gunnarsdottir; Arnaldur Gylfason; Hilma Holm; Brynjar O Jensson; Aslaug Jonasdottir; Kamilla S Josefsdottir; Thordur Kristjansson; Droplaug N Magnusdottir; Solvi Rognvaldsson; Louise le Roux; Gudrun Sigmundsdottir; Gardar Sveinbjornsson; Kristin E Sveinsdottir; Maney Sveinsdottir; Emil A Thorarensen; Bjarni Thorbjornsson; Gisli Masson; Ingileif Jonsdottir; Alma Moller; Thorolfur Gudnason; Karl G Kristinsson; Unnur Thorsteinsdottir; Kari Stefansson
EPI_ISL_828417, EPI_ISL_828419, EPI_ISL_828420, EPI_ISL_828421, EPI_ISL_828422, EPI_ISL_828423, EPI_ISL_828424, EPI_ISL_828425, EPI_ISL_828426, EPI_ISL_828559, EPI_ISL_828560, EPI_ISL_828561, EPI_ISL_828563, EPI_ISL_828564, EPI_ISL_828579, EPI_ISL_828580, EPI_ISL_828581, EPI_ISL_828582, EPI_ISL_828583, EPI_ISL_828584, EPI_ISL_828585, EPI_ISL_828586			
see above	deCODE genetics	deCODE genetics	Daniel F Gudbjartsson; Agnar Helgason; Hakon Jonsson; Olafur T Magnusson; Pall Melsted; Gudmundur L Norddahl; Jona Saemundsdottir; Asgeir Sigurdsson; Patrick Sulem; Arna B Agustsdottir; Hannes Eggertsson; Berglind Eiriksdottir; Run Fridriksdottir; Elisabet E Gardarsdottir; Gudmundur Georgsson; Olafia S Gretarsdottir; Kjartan R Gudmundsson; Thora R Gunnarsdottir; Arnaldur Gylfason; Hilma Holm; Brynjar O Jensson; Aslaug Jonasdottir; Kamilla S Josefsdottir; Thordur Kristjansson; Droplaug N Magnusdottir; Solvi Rognvaldsson; Louise le Roux; Gudrun Sigmundsdottir; Gardar Sveinbjornsson; Kristin E Sveinsdottir; Maney Sveinsdottir; Emil A Thorarensen; Bjarni Thorbjornsson; Gisli Masson; Ingileif Jonsdottir; Alma Moller; Thorolfur Gudnason; Karl G Kristinsson; Unnur Thorsteinsdottir; Kari Stefansson
EPI_ISL_828589, EPI_ISL_828591, EPI_ISL_828593, EPI_ISL_828594	The National University Hospital of Iceland	deCODE genetics	Daniel F Gudbjartsson; Agnar Helgason; Hakon Jonsson; Olafur T Magnusson; Pall Melsted; Gudmundur L Norddahl; Jona Saemundsdottir; Asgeir Sigurdsson; Patrick Sulem; Arna B Agustsdottir; Hannes Eggertsson; Berglind Eiriksdottir; Run Fridriksdottir; Elisabet E Gardarsdottir; Gudmundur Georgsson; Olafia S Gretarsdottir; Kjartan R Gudmundsson; Thora R Gunnarsdottir; Arnaldur Gylfason; Hilma Holm; Brynjar O Jensson; Aslaug Jonasdottir; Kamilla S Josefsdottir; Thordur Kristjansson; Droplaug N Magnusdottir; Solvi Rognvaldsson; Louise le Roux; Gudrun Sigmundsdottir; Gardar Sveinbjornsson; Kristin E Sveinsdottir; Maney Sveinsdottir; Emil A Thorarensen; Bjarni Thorbjornsson; Gisli Masson; Ingileif Jonsdottir; Alma Moller; Thorolfur Gudnason; Karl G Kristinsson; Unnur Thorsteinsdottir; Kari Stefansson
EPI_ISL_828695, EPI_ISL_828703, EPI_ISL_828716, EPI_ISL_828799, EPI_ISL_828803, EPI_ISL_828805, EPI_ISL_828827, EPI_ISL_828830, EPI_ISL_828831, EPI_ISL_828832, EPI_ISL_828833, EPI_ISL_828939, EPI_ISL_828941, EPI_ISL_828942, EPI_ISL_828943			
see above	deCODE genetics	deCODE genetics	Daniel F Gudbjartsson; Agnar Helgason; Hakon Jonsson; Olafur T Magnusson; Pall Melsted; Gudmundur L Norddahl; Jona Saemundsdottir; Asgeir Sigurdsson; Patrick Sulem; Arna B Agustsdottir; Hannes Eggertsson; Berglind Eiriksdottir; Run Fridriksdottir; Elisabet E Gardarsdottir; Gudmundur Georgsson; Olafia S Gretarsdottir; Kjartan R Gudmundsson; Thora R Gunnarsdottir; Arnaldur Gylfason; Hilma Holm; Brynjar O Jensson; Aslaug Jonasdottir; Kamilla S Josefsdottir; Thordur Kristjansson; Droplaug N Magnusdottir; Solvi Rognvaldsson; Louise le Roux; Gudrun Sigmundsdottir; Gardar Sveinbjornsson; Kristin E Sveinsdottir; Maney Sveinsdottir; Emil A Thorarensen; Bjarni Thorbjornsson; Gisli Masson; Ingileif Jonsdottir; Alma Moller; Thorolfur Gudnason; Karl G Kristinsson; Unnur Thorsteinsdottir; Kari Stefansson
EPI_ISL_828970, EPI_ISL_828971, EPI_ISL_828976, EPI_ISL_828977, EPI_ISL_829010, EPI_ISL_829011	The National University Hospital of Iceland	deCODE genetics	Daniel F Gudbjartsson; Agnar Helgason; Hakon Jonsson; Olafur T Magnusson; Pall Melsted; Gudmundur L Norddahl; Jona Saemundsdottir; Asgeir Sigurdsson; Patrick Sulem; Arna B Agustsdottir; Hannes Eggertsson; Berglind Eiriksdottir; Run Fridriksdottir; Elisabet E Gardarsdottir; Gudmundur Georgsson; Olafia S Gretarsdottir; Kjartan R Gudmundsson; Thora R Gunnarsdottir; Arnaldur Gylfason; Hilma Holm; Brynjar O Jensson; Aslaug Jonasdottir; Kamilla S Josefsdottir; Thordur Kristjansson; Droplaug N Magnusdottir; Solvi Rognvaldsson; Louise le Roux; Gudrun Sigmundsdottir; Gardar Sveinbjornsson; Kristin E Sveinsdottir; Maney Sveinsdottir; Emil A Thorarensen; Bjarni Thorbjornsson; Gisli Masson; Ingileif Jonsdottir; Alma Moller; Thorolfur Gudnason; Karl G Kristinsson; Unnur Thorsteinsdottir; Kari Stefansson
EPI_ISL_829096, EPI_ISL_829097, EPI_ISL_829098, EPI_ISL_829099, EPI_ISL_829101, EPI_ISL_829102, EPI_ISL_829200, EPI_ISL_829201, EPI_ISL_829202, EPI_ISL_829203, EPI_ISL_829204, EPI_ISL_829205, EPI_ISL_829206, EPI_ISL_829207, EPI_ISL_829208			
see above	deCODE genetics	deCODE genetics	Daniel F Gudbjartsson; Agnar Helgason; Hakon Jonsson; Olafur T Magnusson; Pall Melsted; Gudmundur L Norddahl; Jona Saemundsdottir; Asgeir Sigurdsson; Patrick Sulem; Arna B Agustsdottir; Hannes Eggertsson; Berglind Eiriksdottir; Run Fridriksdottir; Elisabet E Gardarsdottir; Gudmundur Georgsson; Olafia S Gretarsdottir; Kjartan R Gudmundsson; Thora R Gunnarsdottir; Arnaldur Gylfason; Hilma Holm; Brynjar O Jensson; Aslaug Jonasdottir; Kamilla S Josefsdottir; Thordur Kristjansson; Droplaug N Magnusdottir; Solvi Rognvaldsson; Louise le Roux; Gudrun Sigmundsdottir; Gardar Sveinbjornsson; Kristin E Sveinsdottir; Maney Sveinsdottir; Emil A Thorarensen; Bjarni Thorbjornsson; Gisli Masson; Ingileif Jonsdottir; Alma Moller; Thorolfur Gudnason; Karl G Kristinsson; Unnur Thorsteinsdottir; Kari Stefansson
EPI_ISL_829239, EPI_ISL_829290, EPI_ISL_829291, EPI_ISL_829292, EPI_ISL_829293	The National University Hospital of Iceland	deCODE genetics	Daniel F Gudbjartsson; Agnar Helgason; Hakon Jonsson; Olafur T Magnusson; Pall Melsted; Gudmundur L Norddahl; Jona Saemundsdottir; Asgeir Sigurdsson; Patrick Sulem; Arna B Agustsdottir; Hannes Eggertsson; Berglind Eiriksdottir; Run Fridriksdottir; Elisabet E Gardarsdottir; Gudmundur Georgsson; Olafia S Gretarsdottir; Kjartan R Gudmundsson; Thora R Gunnarsdottir; Arnaldur Gylfason; Hilma Holm; Brynjar O Jensson; Aslaug Jonasdottir; Kamilla S Josefsdottir; Thordur Kristjansson; Droplaug N Magnusdottir; Solvi Rognvaldsson; Louise le Roux; Gudrun Sigmundsdottir; Gardar Sveinbjornsson; Kristin E Sveinsdottir; Maney Sveinsdottir; Emil A Thorarensen; Bjarni Thorbjornsson; Gisli Masson; Ingileif Jonsdottir; Alma Moller; Thorolfur Gudnason; Karl G Kristinsson; Unnur Thorsteinsdottir; Kari Stefansson
EPI_ISL_829466, EPI_ISL_829467, EPI_ISL_829468, EPI_ISL_829469, EPI_ISL_829472, EPI_ISL_829473, EPI_ISL_829474, EPI_ISL_829475	deCODE genetics	deCODE genetics	Daniel F Gudbjartsson; Agnar Helgason; Hakon Jonsson; Olafur T Magnusson; Pall Melsted; Gudmundur L Norddahl; Jona Saemundsdottir; Asgeir Sigurdsson; Patrick Sulem; Arna B Agustsdottir; Hannes Eggertsson; Berglind Eiriksdottir; Run Fridriksdottir; Elisabet E Gardarsdottir; Gudmundur Georgsson; Olafia S Gretarsdottir; Kjartan R Gudmundsson; Thora R Gunnarsdottir; Arnaldur Gylfason; Hilma Holm; Brynjar O Jensson; Aslaug Jonasdottir; Kamilla S Josefsdottir; Thordur Kristjansson; Droplaug N Magnusdottir; Solvi Rognvaldsson; Louise le Roux; Gudrun Sigmundsdottir; Gardar Sveinbjornsson; Kristin E Sveinsdottir; Maney Sveinsdottir; Emil A Thorarensen; Bjarni Thorbjornsson; Gisli Masson; Ingileif Jonsdottir; Alma Moller; Thorolfur Gudnason; Karl G Kristinsson; Unnur Thorsteinsdottir; Kari Stefansson
EPI_ISL_829725, EPI_ISL_829735	The National University Hospital of Iceland	deCODE genetics	Daniel F Gudbjartsson; Agnar Helgason; Hakon Jonsson; Olafur T Magnusson; Pall Melsted; Gudmundur L Norddahl; Jona Saemundsdottir; Asgeir Sigurdsson; Patrick Sulem; Arna B Agustsdottir; Hannes Eggertsson; Berglind Eiriksdottir; Run Fridriksdottir; Elisabet E Gardarsdottir; Gudmundur Georgsson; Olafia S Gretarsdottir; Kjartan R Gudmundsson; Thora R Gunnarsdottir; Arnaldur Gylfason; Hilma Holm; Brynjar O Jensson; Aslaug Jonasdottir; Kamilla S Josefsdottir; Thordur Kristjansson; Droplaug N Magnusdottir; Solvi Rognvaldsson; Louise le Roux; Gudrun Sigmundsdottir; Gardar Sveinbjornsson; Kristin E Sveinsdottir; Maney Sveinsdottir; Emil A Thorarensen; Bjarni Thorbjornsson; Gisli Masson; Ingileif Jonsdottir; Alma Moller; Thorolfur Gudnason; Karl G Kristinsson; Unnur Thorsteinsdottir; Kari Stefansson
EPI_ISL_829831, EPI_ISL_829832, EPI_ISL_829833, EPI_ISL_829834, EPI_ISL_829836, EPI_ISL_829837	deCODE genetics	deCODE genetics	Daniel F Gudbjartsson; Agnar Helgason; Hakon Jonsson; Olafur T Magnusson; Pall Melsted; Gudmundur L Norddahl; Jona Saemundsdottir; Asgeir Sigurdsson; Patrick Sulem; Arna B Agustsdottir; Hannes Eggertsson; Berglind Eiriksdottir; Run Fridriksdottir; Elisabet E Gardarsdottir; Gudmundur Georgsson; Olafia S Gretarsdottir; Kjartan R Gudmundsson; Thora R Gunnarsdottir; Arnaldur Gylfason; Hilma Holm; Brynjar O Jensson; Aslaug Jonasdottir; Kamilla S Josefsdottir; Thordur Kristjansson; Droplaug N Magnusdottir; Solvi Rognvaldsson; Louise le Roux; Gudrun Sigmundsdottir; Gardar Sveinbjornsson; Kristin E Sveinsdottir; Maney Sveinsdottir; Emil A Thorarensen; Bjarni Thorbjornsson; Gisli Masson; Ingileif Jonsdottir; Alma Moller; Thorolfur Gudnason; Karl G Kristinsson; Unnur Thorsteinsdottir; Kari Stefansson
EPI_ISL_829916	The National University Hospital of Iceland	deCODE genetics	Daniel F Gudbjartsson; Agnar Helgason; Hakon Jonsson; Olafur T Magnusson; Pall Melsted; Gudmundur L Norddahl; Jona Saemundsdottir; Asgeir Sigurdsson; Patrick Sulem; Arna B Agustsdottir; Hannes Eggertsson; Berglind Eiriksdottir; Run Fridriksdottir; Elisabet E Gardarsdottir; Gudmundur Georgsson; Olafia S Gretarsdottir; Kjartan R Gudmundsson; Thora R Gunnarsdottir; Arnaldur Gylfason; Hilma Holm; Brynjar O Jensson; Aslaug Jonasdottir; Kamilla S Josefsdottir; Thordur Kristjansson; Droplaug N Magnusdottir; Solvi Rognvaldsson; Louise le Roux; Gudrun Sigmundsdottir; Gardar Sveinbjornsson; Kristin E Sveinsdottir; Maney Sveinsdottir; Emil A Thorarensen; Bjarni Thorbjornsson; Gisli Masson; Ingileif Jonsdottir; Alma Moller; Thorolfur Gudnason; Karl G Kristinsson; Unnur Thorsteinsdottir; Kari Stefansson
EPI_ISL_830087, EPI_ISL_830089, EPI_ISL_830090, EPI_ISL_830091, EPI_ISL_830092	deCODE genetics	deCODE genetics	Daniel F Gudbjartsson; Agnar Helgason; Hakon Jonsson; Olafur T Magnusson; Pall Melsted; Gudmundur L Norddahl; Jona Saemundsdottir; Asgeir Sigurdsson; Patrick Sulem; Arna B Agustsdottir; Hannes Eggertsson; Berglind Eiriksdottir; Run Fridriksdottir; Elisabet E Gardarsdottir; Gudmundur Georgsson; Olafia S Gretarsdottir; Kjartan R Gudmundsson; Thora R Gunnarsdottir; Arnaldur Gylfason; Hilma Holm; Brynjar O Jensson; Aslaug Jonasdottir; Kamilla S

			Josefsdottir; Thordur Kristjansson; Droplaug N Magnusdottir; Solvi Rognvaldsson; Louise le Roux; Gudrun Sigmundsdottir; Gardar Sveinbjornsson; Kristin E Sveinsdottir; Maney Sveinsdottir; Emil A Thorarensen; Bjarni Thorbjornsson; Gisli Masson; Ingileif Jonsdottir; Alma Moller; Thorolfur Gudnason; Karl G Kristinsson; Unnur Thorsteinsdottir; Kari Stefansson
EPI_ISL_830097, EPI_ISL_830098, EPI_ISL_830100	The National University Hospital of Iceland	deCODE genetics	Daniel F Gudbjartsson; Agnar Helgason; Hakon Jonsson; Olafur T Magnusson; Pall Melsted; Gudmundur L Norddahl; Jona Saemundsdottir; Asgeir Sigurdsson; Patrick Sulem; Arna B Agustsdottir; Hannes Eggertsson; Berglind Eiriksdottir; Run Fridriksdottir; Elisabet E Gardarsdottir; Gudmundur Georgsson; Olafia S Gretarsdottir; Kjartan R Gudmundsson; Thora R Gunnarsdottir; Arnaldur Gylfason; Hilma Holm; Brynjar O Jensson; Aslaug Jonasdottir; Kamilla S Josefsdottir; Thordur Kristjansson; Droplaug N Magnusdottir; Solvi Rognvaldsson; Louise le Roux; Gudrun Sigmundsdottir; Gardar Sveinbjornsson; Kristin E Sveinsdottir; Maney Sveinsdottir; Emil A Thorarensen; Bjarni Thorbjornsson; Gisli Masson; Ingileif Jonsdottir; Alma Moller; Thorolfur Gudnason; Karl G Kristinsson; Unnur Thorsteinsdottir; Kari Stefansson
EPI_ISL_830106, EPI_ISL_830107, EPI_ISL_830108	deCODE genetics	deCODE genetics	Daniel F Gudbjartsson; Agnar Helgason; Hakon Jonsson; Olafur T Magnusson; Pall Melsted; Gudmundur L Norddahl; Jona Saemundsdottir; Asgeir Sigurdsson; Patrick Sulem; Arna B Agustsdottir; Hannes Eggertsson; Berglind Eiriksdottir; Run Fridriksdottir; Elisabet E Gardarsdottir; Gudmundur Georgsson; Olafia S Gretarsdottir; Kjartan R Gudmundsson; Thora R Gunnarsdottir; Arnaldur Gylfason; Hilma Holm; Brynjar O Jensson; Aslaug Jonasdottir; Kamilla S Josefsdottir; Thordur Kristjansson; Droplaug N Magnusdottir; Solvi Rognvaldsson; Louise le Roux; Gudrun Sigmundsdottir; Gardar Sveinbjornsson; Kristin E Sveinsdottir; Maney Sveinsdottir; Emil A Thorarensen; Bjarni Thorbjornsson; Gisli Masson; Ingileif Jonsdottir; Alma Moller; Thorolfur Gudnason; Karl G Kristinsson; Unnur Thorsteinsdottir; Kari Stefansson
EPI_ISL_830184, EPI_ISL_830266, EPI_ISL_830309, EPI_ISL_830314, EPI_ISL_830368, EPI_ISL_830445	The National University Hospital of Iceland	deCODE genetics	Daniel F Gudbjartsson; Agnar Helgason; Hakon Jonsson; Olafur T Magnusson; Pall Melsted; Gudmundur L Norddahl; Jona Saemundsdottir; Asgeir Sigurdsson; Patrick Sulem; Arna B Agustsdottir; Hannes Eggertsson; Berglind Eiriksdottir; Run Fridriksdottir; Elisabet E Gardarsdottir; Gudmundur Georgsson; Olafia S Gretarsdottir; Kjartan R Gudmundsson; Thora R Gunnarsdottir; Arnaldur Gylfason; Hilma Holm; Brynjar O Jensson; Aslaug Jonasdottir; Kamilla S Josefsdottir; Thordur Kristjansson; Droplaug N Magnusdottir; Solvi Rognvaldsson; Louise le Roux; Gudrun Sigmundsdottir; Gardar Sveinbjornsson; Kristin E Sveinsdottir; Maney Sveinsdottir; Emil A Thorarensen; Bjarni Thorbjornsson; Gisli Masson; Ingileif Jonsdottir; Alma Moller; Thorolfur Gudnason; Karl G Kristinsson; Unnur Thorsteinsdottir; Kari Stefansson
EPI_ISL_830448, EPI_ISL_830450, EPI_ISL_830496, EPI_ISL_830550	deCODE genetics	deCODE genetics	Daniel F Gudbjartsson; Agnar Helgason; Hakon Jonsson; Olafur T Magnusson; Pall Melsted; Gudmundur L Norddahl; Jona Saemundsdottir; Asgeir Sigurdsson; Patrick Sulem; Arna B Agustsdottir; Hannes Eggertsson; Berglind Eiriksdottir; Run Fridriksdottir; Elisabet E Gardarsdottir; Gudmundur Georgsson; Olafia S Gretarsdottir; Kjartan R Gudmundsson; Thora R Gunnarsdottir; Arnaldur Gylfason; Hilma Holm; Brynjar O Jensson; Aslaug Jonasdottir; Kamilla S Josefsdottir; Thordur Kristjansson; Droplaug N Magnusdottir; Solvi Rognvaldsson; Louise le Roux; Gudrun Sigmundsdottir; Gardar Sveinbjornsson; Kristin E Sveinsdottir; Maney Sveinsdottir; Emil A Thorarensen; Bjarni Thorbjornsson; Gisli Masson; Ingileif Jonsdottir; Alma Moller; Thorolfur Gudnason; Karl G Kristinsson; Unnur Thorsteinsdottir; Kari Stefansson
EPI_ISL_830739, EPI_ISL_830764, EPI_ISL_830765, EPI_ISL_830773, EPI_ISL_830815, EPI_ISL_830816, EPI_ISL_830817, EPI_ISL_830818, EPI_ISL_830819, EPI_ISL_830820, EPI_ISL_830821, EPI_ISL_830822, EPI_ISL_830823, EPI_ISL_830824, EPI_ISL_830825, EPI_ISL_830826, EPI_ISL_830827, EPI_ISL_831003, EPI_ISL_831004			
see above	University Hospital Basel, Clinical Virology	University Hospital Basel, Clinical Bacteriology	Tim Roloff, Madlen Stange, Helena MB Seth-Smith, Alfredo Mari, Karoline Leuzinger, Julia Bielicki, Manuel Battegay, Hans Hirsch, Adrian Egli
EPI_ISL_831180, EPI_ISL_831181, EPI_ISL_831182, EPI_ISL_831183, EPI_ISL_831184, EPI_ISL_831185, EPI_ISL_831186, EPI_ISL_831187, EPI_ISL_831188, EPI_ISL_831189	Hospital Universitario La Paz (Madrid)	SeqCOVID-SPAIN consortium/IBV(CSIC)	María Rodríguez-Tejedor, Elias Dahdouh, Fernando Lázaro-Perona, Jesús Mingorance and SeqCOVID-SPAIN consortium
EPI_ISL_831976	Orebro klinisk mikrobiologi	The Public Health Agency of Sweden	Department of Microbiology, The Public Health Agency of Sweden
EPI_ISL_831977, EPI_ISL_831978	Gavle klinisk mikrobiologi	The Public Health Agency of Sweden	Department of Microbiology, The Public Health Agency of Sweden
EPI_ISL_833333	Batangas City Health Office	Research Institute for Tropical Medicine	Hannah Leah Morito, Othoniel Jan Onza, John Leonard Chan, Ma Angelica Tujan, Francisco Gerardo Polotan, Inez Andrea Medado, Kirstyn Brunker, Edelwisa Mercado, Daria Manalo, Catalino Demetria
EPI_ISL_833339	St. Pascual De Baylon Hospital	Research Institute for Tropical Medicine	Hannah Leah Morito, Othoniel Jan Onza, John Leonard Chan, Ma Angelica Tujan, Francisco Gerardo Polotan, Inez Andrea Medado, Kirstyn Brunker, Edelwisa Mercado, Daria Manalo, Catalino Demetria
EPI_ISL_833341	Batangas City Health Office	Research Institute for Tropical Medicine	Hannah Leah Morito, Othoniel Jan Onza, John Leonard Chan, Ma Angelica Tujan, Francisco Gerardo Polotan, Inez Andrea Medado, Kirstyn Brunker, Edelwisa Mercado, Daria Manalo, Catalino Demetria
EPI_ISL_839732	Northumbria University / South Tees Hospitals NHS Foundation Trust / North Cumbria Integrated Care NHS Foundation Trust / North Tees and Hartlepool NHS Foundation Trust / Newcastle Hospitals NHS Foundation Trust	COVID-19 Genomics UK (COG-UK) Consortium	Darren L Smith,Andrew Nelson,Matthew Bashton,Greg R Young,Joshua Loh,John Allan,Mohammad A Tariq,Giles S Holt,Gary Black,Wen C Yew,Lynn Dover,Paul Baker,Steve Liggett,Sarah Essex,Jane Greenaway,Debra Padgett,Clive Graham,Garren Scott,Edward Barton,Emma Swindells,Brendan Payne,Jennifer Collins,Yusri Taha,Gary Eltringham
EPI_ISL_842100	Quadram Institute Bioscience	COVID-19 Genomics UK (COG-UK) Consortium	Dave J. Baker, Gemma L. Kay, Alp Aydin, Thanh Le-Viet, Steven Rudder, Ana P. Tedim, Anastasia Kolyva, Maria Diaz, Leonardo de Oliveira Martins, Nabil-Fareed Alikhan, Lizzie Meadows, Rachael Stanley, Ngozi Elumogo, Muhammed Yasir, Nicholas M. Thomson, Alexander J Trotter, Rachel Gilroy, Samuel Bloomfield, Claire Stuart, Andrew Bell, Reenesh Prakash, Samir Dervisevic, Alison E. Mather, John Wain, Mark Webber, Andrew J. Page, Justin O'Grady
EPI_ISL_842651	University Hospital Basel, Clinical Virology	University Hospital Basel, Clinical Bacteriology	Tim Roloff, Madlen Stange, Helena MB Seth-Smith, Alfredo Mari, Karoline Leuzinger, Julia Bielicki, Manuel Battegay, Hans Hirsch, Adrian Egli
EPI_ISL_842847, EPI_ISL_842848, EPI_ISL_842849, EPI_ISL_842850, EPI_ISL_842851, EPI_ISL_842852, EPI_ISL_842853, EPI_ISL_842854, EPI_ISL_842855, EPI_ISL_842856, EPI_ISL_842857			
see above	Barts Health NHS Trust	COVID-19 Genomics UK (COG-UK) Consortium	CUTINO-MOGUEL, Maria-Teresa; HARRINGTON, David; OWOYEMI, Dola; SHYLINI, Raghavendran; BROAD, Claire; KELE, Beatrix
EPI_ISL_848078, EPI_ISL_848081	Laboratory of Clinical Research on Dermatozoonoses in Domestic Animals, Evandro Chagas National Institute of Infectious Diseases, Oswaldo Cruz Foundation (Fiocruz), Rio de Janeiro, RJ, Brazil.	Laboratory of Respiratory Viruses and Measles, Oswaldo Cruz Institute, FIOCRUZ	Guilherme Amaral Calvet, Michelle Fernanda Borges da Silva, Anielle de Pina Costa, Ezequias Batista Martins, Isabella Campos Vargas de Moraes, Lusiele Guaraldo, Patrícia Brasil, Sandro Antônio Pereira, Rodrigo Caldas Menezes, Isabella Dib Ferreira Gremião, Lucas Oliveira Keidel, Shanna Araujo dos Santos, Artur Augusto Velho Mendes Junior, Renato Orsini Ornellas, Maria Ogrzewalska, PaolaCristina Resende, Alex Pauvolid-Corrêa, Fernando do Couto Motta, Alice Sampaio Barreto da Rocha, Thiago C. Souza, Marilda Mendonça Siqueira
EPI_ISL_848319	Illinois Department of Public Health	Gagnon Lab, Southern Illinois University	Keith Gagnon
EPI_ISL_849011, EPI_ISL_849012, EPI_ISL_849013, EPI_ISL_849014, EPI_ISL_849015, EPI_ISL_849016, EPI_ISL_849017, EPI_ISL_849018, EPI_ISL_849019, EPI_ISL_849020, EPI_ISL_849021, EPI_ISL_849022, EPI_ISL_849023, EPI_ISL_849024, EPI_ISL_849025, EPI_ISL_849026			
see above	Florida Bureau of Public Health Laboratories	Florida Bureau of Public Health Laboratories	Sarah Schmedes, Jason Blanton
EPI_ISL_849483	Seattle Flu Study	Seattle Flu Study	Deborah A. Nickerson, Chris D. Frazar, Jover Lee, Benjamin Pelle, Matthew Richardson, Amanda Adler, Elisabeth Brandstetter, Peter D. Han, Kairsten Fay, Misja Ilcisin, Kirsten Lacombe, Thomas R. Sibley, Melissa Truong, Caitlin R. Wolf, Michael Boeckh, Janet A. Englund, Michael Famulare, Barry R. Lutz, Mark J. Rieder, Lea M. Starita, Matthew Thompson, Jay Shendure, Trevor Bedford, Helen Y. Chu
EPI_ISL_853294, EPI_ISL_853306, EPI_ISL_853347, EPI_ISL_853348, EPI_ISL_853349, EPI_ISL_853350, EPI_ISL_853351, EPI_ISL_853390	UPMC Clinical Microbiology Laboratory	Microbial Genome Sequencing Center; Microbial Genomic Epidemiology Laboratory	Mustapha M. Mustapha, Jane W. Marsh, Dan Snyder, Marissa P. Griffith, Stephanie L. Mitchell, Vatsala R. Srinivasa, Kady D. Waggle, Chinelo Ezeonwuku, Vaughn S. Cooper, Lee H. Harrison
EPI_ISL_854234	Department of Microbiology, University Innsbruck	Bergthaler laboratory, CeMM Research Center for Molecular Medicine of the Austrian Academy of Sciences	Lukas Endler, Alexandra Popa, Benedikt Agerer, Jakob-Wendelin Genger, Alexander Lercher, Anna Schedl, Thomas Penz, Michael Schuster, Jan Laine, Martin Senekowitsch, Christoph Bock, Andreas Bergthaler
EPI_ISL_854714	Public Health Ontario Laboratory	Public Health Ontario Laboratory	Vanessa G Allen, Philip Banh, Yao Chen, Richard de Borja, Alireza Eshaghi, Nahuel Fittipaldi, Christine Frantz, Jonathan B Gubbay, Jennifer L Guthrie, Lawrence Heisler, Esha Joshi, Michael Laszloffy, Aimin Li, Michael CY Li, Dean Maxwell, Sandeep Nagra, Samir N Patel, Jared Simpson, Karthikeyan Sivaraman, Ashleigh Sullivan, Yogi Sundaravadanam, Sarah Teatero, Matthew Watson, Andre Villegas, Sandra Zittermann
EPI_ISL_855356	Hospital	National Reference Center for Viruses of Respiratory	Marion Barbet, Sylvie Behillil, Méline Bizard, Angela Brisebarre, Camille Capel, Etienne Simon-Lorière, Vincent Enouf, Maud Vanpeene, Sylvie van der Werf

		Infections, Institut Pasteur, Paris	
EPI_ISL_855527	KEMRI-Wellcome Trust Research Programme/KEMRI-CGMR-C Kilifi	KEMRI-Wellcome Trust Research Programme/KEMRI-CGMR-C Kilifi	Githinji et al
EPI_ISL_859685, EPI_ISL_859686, EPI_ISL_859688, EPI_ISL_859690, EPI_ISL_859691, EPI_ISL_859698, EPI_ISL_859699, EPI_ISL_859700, EPI_ISL_859701, EPI_ISL_859702, EPI_ISL_859703, EPI_ISL_859704, EPI_ISL_859705, EPI_ISL_859706, EPI_ISL_859707, EPI_ISL_859708, EPI_ISL_859709, EPI_ISL_859710, EPI_ISL_859711, EPI_ISL_859712, EPI_ISL_859713, EPI_ISL_859714, EPI_ISL_859715, EPI_ISL_859716, EPI_ISL_859717, EPI_ISL_859718, EPI_ISL_859719, EPI_ISL_859720, EPI_ISL_859721, EPI_ISL_859722, EPI_ISL_859723, EPI_ISL_859724, EPI_ISL_859725, EPI_ISL_859726, EPI_ISL_859727, EPI_ISL_859728, EPI_ISL_859729, EPI_ISL_859730, EPI_ISL_859731, EPI_ISL_859732, EPI_ISL_859733, EPI_ISL_859734, EPI_ISL_859735, EPI_ISL_859736, EPI_ISL_859737, EPI_ISL_859738, EPI_ISL_859739, EPI_ISL_859740, EPI_ISL_859741, EPI_ISL_859742, EPI_ISL_859743, EPI_ISL_859744, EPI_ISL_859745, EPI_ISL_859746, EPI_ISL_859747, EPI_ISL_859748, EPI_ISL_859749, EPI_ISL_859750, EPI_ISL_859751, EPI_ISL_859752, EPI_ISL_859753, EPI_ISL_859754			
see above	BTC, Khalifa University	BTC, Khalifa University	Al Safar et al
EPI_ISL_860145	Keio University School of Medicine	Keio University School of Medicine	Kenjiro Kosaki, Yuka Iwasaki, Hirotsugu Ishizu, Haruhiko Siomi, Kodai Abe
EPI_ISL_860548	St.Olavs hospital/NTNU	IKOM, NTNU	Aleksandr Ianevski, Svein Arne Nordbo, Denis Kainov
EPI_ISL_860685	Respiratory Virus Unit, National Infection Service, Public Health England	COVID-19 Genomics UK (COG-UK) Consortium	PHE Covid Sequencing Team
EPI_ISL_860745	Swiss National Reference Centre for Influenza	Swiss National Reference Centre for Influenza	Ana Rita Gonçalves Cabecinhas, Samuel Cordey, Florian Laubscher,Christoph Grünig, Laurent Kaiser
EPI_ISL_862056, EPI_ISL_862057, EPI_ISL_862058, EPI_ISL_862059, EPI_ISL_862060, EPI_ISL_862061, EPI_ISL_862062, EPI_ISL_862063, EPI_ISL_862064, EPI_ISL_862065, EPI_ISL_862066, EPI_ISL_862067, EPI_ISL_862068, EPI_ISL_862069, EPI_ISL_862070, EPI_ISL_862071, EPI_ISL_862072, EPI_ISL_862073, EPI_ISL_862074, EPI_ISL_862082, EPI_ISL_862083, EPI_ISL_862084, EPI_ISL_862085, EPI_ISL_862086, EPI_ISL_862087, EPI_ISL_862088, EPI_ISL_862089, EPI_ISL_862090, EPI_ISL_862091, EPI_ISL_862092, EPI_ISL_862093, EPI_ISL_862094, EPI_ISL_862095, EPI_ISL_862096, EPI_ISL_862097, EPI_ISL_862098, EPI_ISL_862099, EPI_ISL_862100, EPI_ISL_862101, EPI_ISL_862102, EPI_ISL_862103, EPI_ISL_862104, EPI_ISL_862105, EPI_ISL_862106, EPI_ISL_862107, EPI_ISL_862108, EPI_ISL_862109, EPI_ISL_862110, EPI_ISL_862111, EPI_ISL_862112, EPI_ISL_862113, EPI_ISL_862114, EPI_ISL_862115, EPI_ISL_862116, EPI_ISL_862117, EPI_ISL_862118, EPI_ISL_862119, EPI_ISL_862120			
see above	Department of Virology and Immunology, University of Helsinki and Helsinki University Hospital, Huslab Finland	Department of Virology, Faculty of Medicine, University of Helsinki, Helsinki, Finland	Teemu Smura, Ravi Kant, Phuoc Truong, Hussein Alburkat, Hanna Liimatainen, Hannimari Kallio-Kokko, Jenni Virtanen, Maija Suvanto, Sari Hannula, Harri Kangas, Pekka Ellonen, Olli Vapalahti
EPI_ISL_869242, EPI_ISL_869243, EPI_ISL_869246, EPI_ISL_869248, EPI_ISL_869249, EPI_ISL_869251, EPI_ISL_869253, EPI_ISL_869254, EPI_ISL_869255, EPI_ISL_869256, EPI_ISL_869257, EPI_ISL_869258, EPI_ISL_869259, EPI_ISL_869260, EPI_ISL_872569			
see above	UMMC-Health	WHO National Influenza Centre Russian Federation	Andrey Komissarov, Artem Fadeev, Anna Ivanova, Kseniya Komissarova, Dmitry Bazhenov, Mikhail Bakaev,Tatiana Platonova, Daria Danilenko, Ksenia Safina, Elena Nabieva, Georgii Bazykin, Dmitry Lioznov
EPI_ISL_876814	Quest Diagnostics	Quest Diagnostics	Rosenthal,S.H., Gerasimova,A., Kagan,R.M., Anderson, B., Hua, M., Liu Y., Bernstein, L.E., Livingston, K.E., Perez, A., Shalhout, D.F., Shlyakhter, I.A., Owen, R., Tanpaiboon, P., Lacbawan, F.
EPI_ISL_878653	Rady's Childrens Hospital	Andersen lab at Scripps Research	SEARCH Alliance San Diego with Nanda Radamchar, David Dimmock, Linda Luo, Christina Clarke, Kathryn Bouic, Teresa Mueller, Denise Malicki
EPI_ISL_882626	COVID lab, Mymensingh Medical College	Department of Pathology, Bangladesh Agricultural University & Department of Microbiology, Mymensingh Medical College	Afrin, S. Z. Paul, S. K. Parvin, R.
EPI_ISL_884306, EPI_ISL_884314, EPI_ISL_884332, EPI_ISL_884347, EPI_ISL_884348, EPI_ISL_884350, EPI_ISL_884351, EPI_ISL_884352, EPI_ISL_884360, EPI_ISL_884362, EPI_ISL_884365, EPI_ISL_884388			
see above	Infectious Diseases, Quest Diagnostics	Infectious Diseases, Quest Diagnostics	Rosenthal,S.H., Gerasimova,A., Kagan,R.M., Anderson,B., Bernstein,L.E., Livingston,K.E., Hua,M., Liu,Y., Shalhout,D.F., Owen,R., Lacbawan,F.
EPI_ISL_887110	Institute of Medical Microbiology and Virology, University Hospital Carl Gustav Carus, TU Dresden	DRESDEN-concept Genome Center, CMCB, TU Dresden	Beil J., Brown T., Büttner L., Gscheidel N., Hochauf-Stange K., Klemroth S., Lindemann D., Mehnert G., Petzold A., Reinhardt S., Rost F., Sameith K., Winkler S.
EPI_ISL_887153	Massachusetts General Hospital	Infectious Disease Program, Broad Institute of Harvard and MIT	Lemieux,J.E., Siddle,K.J., Shaw,B., Adams,G., Pierce,V., Turbett,S., Anahtar,M., Branda,J., Slater,D., Harris,J., Lin,A.E., Gladden-Young,A., Lagerborg,K., Rudy,M., DeRuff,K., Carter,A., Normandin,E., Bauer,M., Reilly,S., Tomkins-Tinch,C., Loreth,C., Chaluvadi,S., Neumann,A., Cusick,C., Chapman,S.B., Gnirke,A., Flowers,K., Cerrato,F., Birren,B.W., Gallagher,G., Smole,S., Park,D.J., MacInnis,B.L., Ryan,E., LaRocque,R., Rosenberg,E. and Sabeti,P.C.
EPI_ISL_887434	Instituto Nacional de Saude (INS), Mozambique	KRISP, KZN Research Innovation and Sequencing Platform	Nalia Ismael, Nadia Sitoe, Paulo Arnaldo, Nedio Mabunda, Giandhari J, Pillay S, Tegally H, Wilkinson E, de Oliveira T
EPI_ISL_888826	Hospital	National Reference Center for Viruses of Respiratory Infections, Institut Pasteur, Paris	Marion Barbet, Sylvie Behillil, Méline Bizard, Angela Brisebarre, Camille Capel, Etienne Simon-Lorière, Vincent Enouf, Maud Vanpeene, Sylvie van der Werf, Gisèle Lagathu
EPI_ISL_890184	Seattle Flu Study	Seattle Flu Study	Deborah A. Nickerson, Chris D. Frazar, Jover Lee, Benjamin Pelle, Erica Ryke, Matthew Richardson, Amanda Adler, Elisabeth Brandstetter, Peter D. Han, Kairsten Fay, Misja Ilcisin, Kirsten Lacombe, Thomas R. Sibley, Melissa Truong, Caitlin R. Wolf, Karen Cowgill, Stephanie Schrag, Jeff Duchin, Michael Boeckh, Janet A. Englund, Michael Famulare, Barry R. Lutz, Mark J. Rieder, Lea M. Starita, Matthew Thompson, Helen Y. Chu, Trevor Bedford, Jay Shendure
EPI_ISL_891179, EPI_ISL_891191	DPH, Massachusetts State Public Health Lab	DPH, Massachusetts State Public Health Lab	Lang,A.S., Fink,T., Gallagher,G.R., Smole,S.C.
EPI_ISL_891271	Institute of Biocides and Medical Ecology, Belgarde, Serbia	Virology department Institute of microbiology and immunology Faculty of Medicine University of Belgrade	Banko Ana, Miljanovic Danijela, Milicevic Ognjen, Loncar Ana, Abazovic Dzihan, Despot Dragana
EPI_ISL_892604, EPI_ISL_892605, EPI_ISL_892606, EPI_ISL_892607, EPI_ISL_892608, EPI_ISL_892609, EPI_ISL_892610	Saitama Prefectural Institute of Public Health	Pathogen Genomics Center, National Institute of Infectious Diseases	Tsuyoshi Sekizuka, Kentaro Itokawa, Rina Tanaka, Masanori Hashino, Makoto Kuroda
EPI_ISL_893269, EPI_ISL_893270, EPI_ISL_893271	Pathogen Genomics Center, National Institute of Infectious Diseases	Pathogen Genomics Center, National Institute of Infectious Diseases	Tsuyoshi Sekizuka, Kentaro Itokawa, Rina Tanaka, Masanori Hashino, Makoto Kuroda
EPI_ISL_893368, EPI_ISL_893375, EPI_ISL_893376, EPI_ISL_893377, EPI_ISL_893378, EPI_ISL_893379, EPI_ISL_893380, EPI_ISL_893381, EPI_ISL_893382, EPI_ISL_893383, EPI_ISL_893384, EPI_ISL_893385, EPI_ISL_893386, EPI_ISL_893387, EPI_ISL_893388, EPI_ISL_893389, EPI_ISL_893390, EPI_ISL_893391, EPI_ISL_893392, EPI_ISL_893393, EPI_ISL_893394, EPI_ISL_893395, EPI_ISL_893396, EPI_ISL_893397, EPI_ISL_893398, EPI_ISL_893399, EPI_ISL_893400, EPI_ISL_893401, EPI_ISL_893402, EPI_ISL_893403, EPI_ISL_893404, EPI_ISL_893405, EPI_ISL_893406, EPI_ISL_893407, EPI_ISL_893408, EPI_ISL_893420, EPI_ISL_893421, EPI_ISL_893422, EPI_ISL_893423, EPI_ISL_893424, EPI_ISL_893425, EPI_ISL_893426, EPI_ISL_893427, EPI_ISL_893428, EPI_ISL_893429, EPI_ISL_893430, EPI_ISL_893431, EPI_ISL_893432, EPI_ISL_893433, EPI_ISL_893434, EPI_ISL_893435, EPI_ISL_893436, EPI_ISL_893437, EPI_ISL_893438, EPI_ISL_893439, EPI_ISL_893440, EPI_ISL_893441, EPI_ISL_893442, EPI_ISL_893673			
see above	Department of Infectious Diseases, Kobe Institute of Health	Department of Infectious Diseases, Kobe Institute of Health	Ryohei Nomoto, Tomotada Iwamoto, Tsuyoshi Sekizuka, Kentaro Itokawa, Rina Tanaka, Masanori Hashino, Makoto Kuroda
EPI_ISL_894277, EPI_ISL_894302, EPI_ISL_894465, EPI_ISL_894466, EPI_ISL_894467, EPI_ISL_894468, EPI_ISL_894469, EPI_ISL_894470, EPI_ISL_894471, EPI_ISL_894472, EPI_ISL_894473, EPI_ISL_894474, EPI_ISL_894475, EPI_ISL_894476, EPI_ISL_894477, EPI_ISL_894478, EPI_ISL_894479, EPI_ISL_894480, EPI_ISL_894481, EPI_ISL_894482, EPI_ISL_894483, EPI_ISL_894484, EPI_ISL_894485, EPI_ISL_894486, EPI_ISL_894487, EPI_ISL_894488, EPI_ISL_894489, EPI_ISL_894490, EPI_ISL_894491, EPI_ISL_894492, EPI_ISL_894493, EPI_ISL_894494, EPI_ISL_894495, EPI_ISL_894496, EPI_ISL_894497, EPI_ISL_894498, EPI_ISL_894499, EPI_ISL_894500, EPI_ISL_894501, EPI_ISL_894502, EPI_ISL_894503, EPI_ISL_894504, EPI_ISL_894505, EPI_ISL_894506, EPI_ISL_894507, EPI_ISL_894508, EPI_ISL_894509, EPI_ISL_894510, EPI_ISL_894511, EPI_ISL_894512, EPI_ISL_894513, EPI_ISL_894514, EPI_ISL_894515, EPI_ISL_894516, EPI_ISL_894517, EPI_ISL_894518, EPI_ISL_894519, EPI_ISL_894520, EPI_ISL_894521, EPI_ISL_894522, EPI_ISL_894523, EPI_ISL_894524, EPI_ISL_894525, EPI_ISL_894526, EPI_ISL_894527, EPI_ISL_894528, EPI_ISL_894529, EPI_ISL_894530, EPI_ISL_895096, EPI_ISL_895097, EPI_ISL_895098, EPI_ISL_895099, EPI_ISL_895100, EPI_ISL_895101, EPI_ISL_895102, EPI_ISL_895103, EPI_ISL_895104, EPI_ISL_895105, EPI_ISL_895160, EPI_ISL_895161, EPI_ISL_895162, EPI_ISL_895163, EPI_ISL_895164, EPI_ISL_895165, EPI_ISL_895166, EPI_ISL_895167, EPI_ISL_895168, EPI_ISL_895169, EPI_ISL_895170, EPI_ISL_895171, EPI_ISL_895172, EPI_ISL_895173, EPI_ISL_895174, EPI_ISL_895175, EPI_ISL_895176, EPI_ISL_895177, EPI_ISL_895178, EPI_ISL_895616, EPI_ISL_895617, EPI_ISL_895618, EPI_ISL_895619, EPI_ISL_895620, EPI_ISL_895621, EPI_ISL_895622, EPI_ISL_895623, EPI_ISL_895624, EPI_ISL_895625, EPI_ISL_895626, EPI_ISL_895627, EPI_ISL_895628, EPI_ISL_895629, EPI_ISL_895630, EPI_ISL_895631, EPI_ISL_895632, EPI_ISL_895633, EPI_ISL_895634, EPI_ISL_895635, EPI_ISL_895636, EPI_ISL_895637, EPI_ISL_895638, EPI_ISL_896019			
see above	Pathogen Genomics Center, National Institute of Infectious Diseases	Pathogen Genomics Center, National Institute of Infectious Diseases	Tsuyoshi Sekizuka, Kentaro Itokawa, Rina Tanaka, Masanori Hashino, Makoto Kuroda
EPI_ISL_896142, EPI_ISL_896147, EPI_ISL_896153, EPI_ISL_896176	MEPHI, Aix Marseille University	MEPHI, Aix Marseille University	Anthony LEVASSEUR
EPI_ISL_896612, EPI_ISL_896613, EPI_ISL_896614, EPI_ISL_896615, EPI_ISL_896616, EPI_ISL_896617, EPI_ISL_896618, EPI_ISL_896619, EPI_ISL_896620, EPI_ISL_896621, EPI_ISL_896622, EPI_ISL_896623, EPI_ISL_896624, EPI_ISL_896625, EPI_ISL_896626, EPI_ISL_896627, EPI_ISL_896628, EPI_ISL_896629, EPI_ISL_896630, EPI_ISL_896631, EPI_ISL_896632, EPI_ISL_896633, EPI_ISL_896634, EPI_ISL_896635, EPI_ISL_896636, EPI_ISL_896637, EPI_ISL_896638, EPI_ISL_896639, EPI_ISL_896640, EPI_ISL_896641, EPI_ISL_896658, EPI_ISL_896698, EPI_ISL_896909, EPI_ISL_896910, EPI_ISL_896911, EPI_ISL_896912, EPI_ISL_896913, EPI_ISL_896914, EPI_ISL_896915, EPI_ISL_896916, EPI_ISL_896917, EPI_ISL_896918, EPI_ISL_896919, EPI_ISL_896920, EPI_ISL_896921, EPI_ISL_896922, EPI_ISL_896923, EPI_ISL_896924, EPI_ISL_896925, EPI_ISL_896926, EPI_ISL_896927, EPI_ISL_896928, EPI_ISL_896929, EPI_ISL_896930, EPI_ISL_896931, EPI_ISL_896932, EPI_ISL_896933, EPI_ISL_896934, EPI_ISL_896935, EPI_ISL_896936, EPI_ISL_896937, EPI_ISL_896938, EPI_ISL_896939, EPI_ISL_897024, EPI_ISL_897025, EPI_ISL_897026, EPI_ISL_897027, EPI_ISL_897028, EPI_ISL_897029, EPI_ISL_897030, EPI_ISL_897031, EPI_ISL_897032, EPI_ISL_897033			
see above	Pathogen Genomics Center, National Institute of Infectious Diseases	Pathogen Genomics Center, National Institute of Infectious Diseases	Tsuyoshi Sekizuka, Kentaro Itokawa, Rina Tanaka, Masanori Hashino, Makoto Kuroda

EPI_ISL_897059, EPI_ISL_897060, EPI_ISL_897066	Niigata Prefectural Institute of Public Health and Environmental Sciences	Pathogen Genomics Center, National Institute of Infectious Diseases	Tsuyoshi Sekizuka, Kentaro Itokawa, Rina Tanaka, Masanori Hashino, Makoto Kuroda
EPI_ISL_897081, EPI_ISL_897082, EPI_ISL_897083, EPI_ISL_897084, EPI_ISL_897085, EPI_ISL_897086, EPI_ISL_897087, EPI_ISL_897149, EPI_ISL_897150, EPI_ISL_897273, EPI_ISL_897274, EPI_ISL_897275, EPI_ISL_897276, EPI_ISL_897277, EPI_ISL_897278, EPI_ISL_897279, EPI_ISL_897280, EPI_ISL_897281, EPI_ISL_897282, EPI_ISL_897316, EPI_ISL_897317, EPI_ISL_897318, EPI_ISL_897319, EPI_ISL_897320, EPI_ISL_897321, EPI_ISL_897322, EPI_ISL_897323, EPI_ISL_897324, EPI_ISL_897325, EPI_ISL_897326, EPI_ISL_897327, EPI_ISL_897328, EPI_ISL_897329, EPI_ISL_897330, EPI_ISL_897331, EPI_ISL_897332, EPI_ISL_897333, EPI_ISL_897334, EPI_ISL_897335, EPI_ISL_897336, EPI_ISL_897337, EPI_ISL_897338, EPI_ISL_897339, EPI_ISL_897447, EPI_ISL_897448, EPI_ISL_897449, EPI_ISL_897450, EPI_ISL_897451, EPI_ISL_897452, EPI_ISL_897593, EPI_ISL_897594, EPI_ISL_898033, EPI_ISL_898034, EPI_ISL_898035, EPI_ISL_898036			
see above	Pathogen Genomics Center, National Institute of Infectious Diseases	Pathogen Genomics Center, National Institute of Infectious Diseases	Tsuyoshi Sekizuka, Kentaro Itokawa, Rina Tanaka, Masanori Hashino, Makoto Kuroda
EPI_ISL_898037, EPI_ISL_898038, EPI_ISL_898039, EPI_ISL_898040, EPI_ISL_898041, EPI_ISL_898042, EPI_ISL_898043, EPI_ISL_898044, EPI_ISL_898045, EPI_ISL_898046, EPI_ISL_898047, EPI_ISL_898048, EPI_ISL_898049, EPI_ISL_898050, EPI_ISL_898051, EPI_ISL_898052, EPI_ISL_898053			
see above	Sakai City Institute of Public Health	Pathogen Genomics Center, National Institute of Infectious Diseases	Tsuyoshi Sekizuka, Kentaro Itokawa, Rina Tanaka, Masanori Hashino, Makoto Kuroda
EPI_ISL_898057, EPI_ISL_898058, EPI_ISL_898059, EPI_ISL_898060, EPI_ISL_898061, EPI_ISL_898062, EPI_ISL_898063, EPI_ISL_898064, EPI_ISL_898065, EPI_ISL_898066, EPI_ISL_898067, EPI_ISL_898068, EPI_ISL_898069, EPI_ISL_898070, EPI_ISL_898071, EPI_ISL_898072, EPI_ISL_898073, EPI_ISL_898077, EPI_ISL_898078, EPI_ISL_898079, EPI_ISL_898080, EPI_ISL_898081, EPI_ISL_898082, EPI_ISL_898083, EPI_ISL_898084, EPI_ISL_898085, EPI_ISL_898088, EPI_ISL_898134, EPI_ISL_898143, EPI_ISL_898144, EPI_ISL_898145, EPI_ISL_898269, EPI_ISL_898299, EPI_ISL_898300, EPI_ISL_898301, EPI_ISL_898302, EPI_ISL_898303, EPI_ISL_898304, EPI_ISL_898305, EPI_ISL_898306, EPI_ISL_898313, EPI_ISL_898314			
see above	Pathogen Genomics Center, National Institute of Infectious Diseases	Pathogen Genomics Center, National Institute of Infectious Diseases	Tsuyoshi Sekizuka, Kentaro Itokawa, Rina Tanaka, Masanori Hashino, Makoto Kuroda
EPI_ISL_898321	Toyama Institute of Health	Pathogen Genomics Center, National Institute of Infectious Diseases	Tsuyoshi Sekizuka, Kentaro Itokawa, Rina Tanaka, Masanori Hashino, Makoto Kuroda
EPI_ISL_898326, EPI_ISL_898327, EPI_ISL_898328	Pathogen Genomics Center, National Institute of Infectious Diseases	Pathogen Genomics Center, National Institute of Infectious Diseases	Tsuyoshi Sekizuka, Kentaro Itokawa, Rina Tanaka, Masanori Hashino, Makoto Kuroda
EPI_ISL_898331	Yamagata Prefectural Institute of Public Health	Pathogen Genomics Center, National Institute of Infectious Diseases	Tsuyoshi Sekizuka, Kentaro Itokawa, Rina Tanaka, Masanori Hashino, Makoto Kuroda
EPI_ISL_898539, EPI_ISL_898540, EPI_ISL_898541, EPI_ISL_898542, EPI_ISL_898543, EPI_ISL_898544, EPI_ISL_898545, EPI_ISL_898546, EPI_ISL_898547, EPI_ISL_898548, EPI_ISL_898549, EPI_ISL_898550			
see above	Kanagawa Prefectural Institute of Public Health	Pathogen Genomics Center, National Institute of Infectious Diseases	Tsuyoshi Sekizuka, Kentaro Itokawa, Rina Tanaka, Masanori Hashino, Makoto Kuroda
EPI_ISL_898552, EPI_ISL_898553, EPI_ISL_898607, EPI_ISL_898608, EPI_ISL_898619, EPI_ISL_898620, EPI_ISL_898621, EPI_ISL_898622, EPI_ISL_898623, EPI_ISL_898624, EPI_ISL_898629, EPI_ISL_898630, EPI_ISL_898631, EPI_ISL_898632, EPI_ISL_898633, EPI_ISL_898634, EPI_ISL_898635, EPI_ISL_898636, EPI_ISL_898696, EPI_ISL_898697, EPI_ISL_898698, EPI_ISL_898699, EPI_ISL_898700, EPI_ISL_898701, EPI_ISL_898702, EPI_ISL_898703, EPI_ISL_898704, EPI_ISL_898705, EPI_ISL_898706, EPI_ISL_898707, EPI_ISL_898708, EPI_ISL_898859, EPI_ISL_898860, EPI_ISL_898861, EPI_ISL_898862, EPI_ISL_898863, EPI_ISL_898864, EPI_ISL_898865, EPI_ISL_898866, EPI_ISL_898867, EPI_ISL_898868, EPI_ISL_898869, EPI_ISL_898870, EPI_ISL_898871, EPI_ISL_898894, EPI_ISL_898895, EPI_ISL_898896, EPI_ISL_898897, EPI_ISL_898898, EPI_ISL_898899, EPI_ISL_898900, EPI_ISL_898901, EPI_ISL_898902, EPI_ISL_898903, EPI_ISL_898904, EPI_ISL_898905, EPI_ISL_898906, EPI_ISL_898907, EPI_ISL_898908, EPI_ISL_898909, EPI_ISL_898910, EPI_ISL_898911, EPI_ISL_898912, EPI_ISL_898913, EPI_ISL_898914			
see above	Pathogen Genomics Center, National Institute of Infectious Diseases	Pathogen Genomics Center, National Institute of Infectious Diseases	Tsuyoshi Sekizuka, Kentaro Itokawa, Rina Tanaka, Masanori Hashino, Makoto Kuroda
EPI_ISL_900059, EPI_ISL_900074, EPI_ISL_900075, EPI_ISL_900105, EPI_ISL_900127, EPI_ISL_900174, EPI_ISL_900178, EPI_ISL_900188, EPI_ISL_900199, EPI_ISL_900223, EPI_ISL_900225, EPI_ISL_900252, EPI_ISL_900276, EPI_ISL_900326, EPI_ISL_900353, EPI_ISL_900391, EPI_ISL_900406, EPI_ISL_900449, EPI_ISL_900464			
see above	MEPHI, Aix Marseille University	MEPHI, Aix Marseille University	Anthony LEVASSEUR
EPI_ISL_900760, EPI_ISL_900761, EPI_ISL_900762, EPI_ISL_900763, EPI_ISL_900764, EPI_ISL_900765, EPI_ISL_900766, EPI_ISL_900767, EPI_ISL_900768, EPI_ISL_900769, EPI_ISL_900770, EPI_ISL_900771, EPI_ISL_900776, EPI_ISL_900777, EPI_ISL_900778, EPI_ISL_900779, EPI_ISL_900780, EPI_ISL_900781, EPI_ISL_900782, EPI_ISL_900783, EPI_ISL_900784, EPI_ISL_900785, EPI_ISL_900786, EPI_ISL_900787, EPI_ISL_900788, EPI_ISL_900789, EPI_ISL_900790, EPI_ISL_900791, EPI_ISL_900792, EPI_ISL_900793, EPI_ISL_900794, EPI_ISL_900795, EPI_ISL_900796, EPI_ISL_900797, EPI_ISL_900798, EPI_ISL_900799, EPI_ISL_900800, EPI_ISL_900801, EPI_ISL_900802, EPI_ISL_900803, EPI_ISL_900804, EPI_ISL_900805, EPI_ISL_900806, EPI_ISL_900807, EPI_ISL_900808, EPI_ISL_900809, EPI_ISL_900810, EPI_ISL_900811, EPI_ISL_900812, EPI_ISL_900813, EPI_ISL_900814, EPI_ISL_900815, EPI_ISL_900816, EPI_ISL_900817, EPI_ISL_900818, EPI_ISL_900819, EPI_ISL_900820, EPI_ISL_900821, EPI_ISL_900822, EPI_ISL_900823, EPI_ISL_900824, EPI_ISL_900825, EPI_ISL_900826, EPI_ISL_900827, EPI_ISL_900828, EPI_ISL_900829, EPI_ISL_900830, EPI_ISL_900831, EPI_ISL_900832, EPI_ISL_900833, EPI_ISL_900834, EPI_ISL_900835, EPI_ISL_900836, EPI_ISL_900837, EPI_ISL_900838, EPI_ISL_900839, EPI_ISL_900840, EPI_ISL_900841, EPI_ISL_900842, EPI_ISL_900843, EPI_ISL_900844, EPI_ISL_900845, EPI_ISL_900846, EPI_ISL_900847, EPI_ISL_900848, EPI_ISL_900985, EPI_ISL_900986, EPI_ISL_900987, EPI_ISL_900988, EPI_ISL_900989, EPI_ISL_900990, EPI_ISL_900991, EPI_ISL_900992, EPI_ISL_900993, EPI_ISL_900994, EPI_ISL_900995, EPI_ISL_900996, EPI_ISL_900997, EPI_ISL_900998, EPI_ISL_900999, EPI_ISL_901000, EPI_ISL_901001, EPI_ISL_901002, EPI_ISL_901003, EPI_ISL_901004, EPI_ISL_901005, EPI_ISL_901006, EPI_ISL_901007, EPI_ISL_901008, EPI_ISL_901009, EPI_ISL_901010, EPI_ISL_901011, EPI_ISL_901012, EPI_ISL_901013, EPI_ISL_901014, EPI_ISL_901015, EPI_ISL_901016, EPI_ISL_901017, EPI_ISL_901018, EPI_ISL_901019, EPI_ISL_901020, EPI_ISL_901021, EPI_ISL_901022, EPI_ISL_901023, EPI_ISL_901024, EPI_ISL_901025, EPI_ISL_901026, EPI_ISL_901027, EPI_ISL_901028, EPI_ISL_901029, EPI_ISL_901030, EPI_ISL_901031, EPI_ISL_901032, EPI_ISL_901033, EPI_ISL_901034, EPI_ISL_901035, EPI_ISL_901036, EPI_ISL_901037, EPI_ISL_901038, EPI_ISL_901039, EPI_ISL_901040, EPI_ISL_901041, EPI_ISL_901042, EPI_ISL_901043, EPI_ISL_901044, EPI_ISL_901045, EPI_ISL_901046, EPI_ISL_901047, EPI_ISL_901048, EPI_ISL_901049, EPI_ISL_901050, EPI_ISL_901051, EPI_ISL_901052, EPI_ISL_901053, EPI_ISL_901252, EPI_ISL_901253, EPI_ISL_901254, EPI_ISL_901255, EPI_ISL_901256, EPI_ISL_901257, EPI_ISL_901258, EPI_ISL_901259, EPI_ISL_901260, EPI_ISL_901261, EPI_ISL_901262, EPI_ISL_901263, EPI_ISL_901264, EPI_ISL_901265, EPI_ISL_901266, EPI_ISL_901267, EPI_ISL_901268, EPI_ISL_901269, EPI_ISL_901270, EPI_ISL_901271, EPI_ISL_901306, EPI_ISL_901307, EPI_ISL_901308, EPI_ISL_901309, EPI_ISL_901310, EPI_ISL_901311, EPI_ISL_901312, EPI_ISL_901313, EPI_ISL_901314, EPI_ISL_901315, EPI_ISL_901316, EPI_ISL_901317, EPI_ISL_901318, EPI_ISL_901319, EPI_ISL_901320, EPI_ISL_901321, EPI_ISL_901360, EPI_ISL_901361, EPI_ISL_901362, EPI_ISL_901363, EPI_ISL_901368, EPI_ISL_901369, EPI_ISL_901370, EPI_ISL_901371, EPI_ISL_901372, EPI_ISL_901373, EPI_ISL_901374, EPI_ISL_901375, EPI_ISL_901376, EPI_ISL_901377, EPI_ISL_901378, EPI_ISL_901379, EPI_ISL_901380, EPI_ISL_901381, EPI_ISL_901382, EPI_ISL_901383, EPI_ISL_901384, EPI_ISL_901385, EPI_ISL_901386, EPI_ISL_901387, EPI_ISL_901388, EPI_ISL_901389, EPI_ISL_901390, EPI_ISL_901396, EPI_ISL_901397, EPI_ISL_901398, EPI_ISL_901399, EPI_ISL_901400, EPI_ISL_901401, EPI_ISL_901402, EPI_ISL_901403, EPI_ISL_901404, EPI_ISL_901405, EPI_ISL_901406, EPI_ISL_901407, EPI_ISL_901408, EPI_ISL_901409, EPI_ISL_901410, EPI_ISL_901411, EPI_ISL_901412, EPI_ISL_901413, EPI_ISL_901414, EPI_ISL_901415, EPI_ISL_901416, EPI_ISL_901417, EPI_ISL_901418, EPI_ISL_901419, EPI_ISL_901420, EPI_ISL_901421, EPI_ISL_901422, EPI_ISL_901423, EPI_ISL_901424, EPI_ISL_901425, EPI_ISL_901426, EPI_ISL_901427, EPI_ISL_901428, EPI_ISL_901429, EPI_ISL_901430, EPI_ISL_901431, EPI_ISL_901432, EPI_ISL_901450, EPI_ISL_901451, EPI_ISL_901452, EPI_ISL_901453, EPI_ISL_901454, EPI_ISL_901455, EPI_ISL_901456, EPI_ISL_901457			
see above	Pathogen Genomics Center, National Institute of Infectious Diseases	Pathogen Genomics Center, National Institute of Infectious Diseases	Tsuyoshi Sekizuka, Kentaro Itokawa, Rina Tanaka, Masanori Hashino, Makoto Kuroda
EPI_ISL_901458, EPI_ISL_901459, EPI_ISL_901460, EPI_ISL_901461, EPI_ISL_901462, EPI_ISL_901463, EPI_ISL_901464, EPI_ISL_901465, EPI_ISL_901466, EPI_ISL_901467, EPI_ISL_901468, EPI_ISL_901469, EPI_ISL_901470, EPI_ISL_901471, EPI_ISL_901472, EPI_ISL_901473, EPI_ISL_901474, EPI_ISL_901475, EPI_ISL_901476, EPI_ISL_901477, EPI_ISL_901478, EPI_ISL_901479, EPI_ISL_901480, EPI_ISL_901481, EPI_ISL_901482, EPI_ISL_901483, EPI_ISL_901484, EPI_ISL_901485, EPI_ISL_901486, EPI_ISL_901487, EPI_ISL_901488, EPI_ISL_901489, EPI_ISL_901490			
see above	Gunma Prefectural Institute of Public Health and Environmental Sciences	Pathogen Genomics Center, National Institute of Infectious Diseases	Tsuyoshi Sekizuka, Kentaro Itokawa, Rina Tanaka, Masanori Hashino, Makoto Kuroda
EPI_ISL_901491, EPI_ISL_901492, EPI_ISL_901493, EPI_ISL_901494, EPI_ISL_901495, EPI_ISL_901496, EPI_ISL_901497, EPI_ISL_901501, EPI_ISL_901502, EPI_ISL_901503, EPI_ISL_901504, EPI_ISL_901505, EPI_ISL_901506, EPI_ISL_901507, EPI_ISL_901508, EPI_ISL_901509, EPI_ISL_901510, EPI_ISL_901511, EPI_ISL_901512, EPI_ISL_901513, EPI_ISL_901514, EPI_ISL_901515, EPI_ISL_901516, EPI_ISL_901644, EPI_ISL_901645, EPI_ISL_901646, EPI_ISL_901647, EPI_ISL_901648			
see above	Pathogen Genomics Center, National Institute of Infectious Diseases	Pathogen Genomics Center, National Institute of Infectious Diseases	Tsuyoshi Sekizuka, Kentaro Itokawa, Rina Tanaka, Masanori Hashino, Makoto Kuroda
EPI_ISL_901717, EPI_ISL_901718, EPI_ISL_901719, EPI_ISL_901720, EPI_ISL_901721	Chiba Prefectural Institute of Public Health	Pathogen Genomics Center, National Institute of Infectious Diseases	Tsuyoshi Sekizuka, Kentaro Itokawa, Rina Tanaka, Masanori Hashino, Makoto Kuroda
EPI_ISL_901722, EPI_ISL_901723, EPI_ISL_901724, EPI_ISL_901725, EPI_ISL_901726, EPI_ISL_901727, EPI_ISL_901728, EPI_ISL_901729, EPI_ISL_901730, EPI_ISL_901731, EPI_ISL_901732, EPI_ISL_901733, EPI_ISL_901807, EPI_ISL_901808			
see above	Pathogen Genomics Center, National Institute of Infectious Diseases	Pathogen Genomics Center, National Institute of Infectious Diseases	Tsuyoshi Sekizuka, Kentaro Itokawa, Rina Tanaka, Masanori Hashino, Makoto Kuroda
EPI_ISL_902024, EPI_ISL_902025, EPI_ISL_902026, EPI_ISL_902027, EPI_ISL_902028, EPI_ISL_902029, EPI_ISL_902030, EPI_ISL_902031, EPI_ISL_902032, EPI_ISL_902033, EPI_ISL_902034, EPI_ISL_902045, EPI_ISL_902046, EPI_ISL_902047, EPI_ISL_902049, EPI_ISL_902050, EPI_ISL_902051, EPI_ISL_902052, EPI_ISL_902053			
see above	Department of Infectious Diseases, Kobe Institute of Health	Pathogen Genomics Center, National Institute of Infectious Diseases	Tsuyoshi Sekizuka, Kentaro Itokawa, Rina Tanaka, Masanori Hashino, Makoto Kuroda
EPI_ISL_902197, EPI_ISL_902198, EPI_ISL_902199, EPI_ISL_902200, EPI_ISL_902321, EPI_ISL_902322, EPI_ISL_902323, EPI_ISL_902324, EPI_ISL_902325, EPI_ISL_902326, EPI_ISL_902327, EPI_ISL_902328, EPI_ISL_902329, EPI_ISL_902330, EPI_ISL_902331, EPI_ISL_902332, EPI_ISL_902333			
see above	Pathogen Genomics Center, National Institute of Infectious Diseases	Pathogen Genomics Center, National Institute of Infectious Diseases	Tsuyoshi Sekizuka, Kentaro Itokawa, Rina Tanaka, Masanori Hashino, Makoto Kuroda
EPI_ISL_902344	Toyama Institute of Health	Pathogen Genomics Center, National Institute of Infectious	Tsuyoshi Sekizuka, Kentaro Itokawa, Rina Tanaka, Masanori Hashino, Makoto Kuroda

		Diseases	
EPI_ISL_902415, EPI_ISL_902416, EPI_ISL_902417, EPI_ISL_902418, EPI_ISL_902419, EPI_ISL_902420, EPI_ISL_902421, EPI_ISL_902422, EPI_ISL_902423, EPI_ISL_902424, EPI_ISL_902425, EPI_ISL_902426, EPI_ISL_902427, EPI_ISL_902428, EPI_ISL_902429, EPI_ISL_902430, EPI_ISL_902431, EPI_ISL_902432, EPI_ISL_902433, EPI_ISL_902434, EPI_ISL_902435, EPI_ISL_902436, EPI_ISL_902437, EPI_ISL_902438, EPI_ISL_902439, EPI_ISL_902440, EPI_ISL_902441, EPI_ISL_902442, EPI_ISL_902444, EPI_ISL_902445, EPI_ISL_902446, EPI_ISL_902447, EPI_ISL_902448, EPI_ISL_902449, EPI_ISL_902450, EPI_ISL_902451, EPI_ISL_902452, EPI_ISL_902453, EPI_ISL_902454, EPI_ISL_902455, EPI_ISL_902456, EPI_ISL_902457, EPI_ISL_902458, EPI_ISL_902459, EPI_ISL_902460, EPI_ISL_902461, EPI_ISL_902462, EPI_ISL_902463, EPI_ISL_902464, EPI_ISL_902465, EPI_ISL_902466, EPI_ISL_902467, EPI_ISL_902468, EPI_ISL_902469, EPI_ISL_902470, EPI_ISL_902471, EPI_ISL_902472, EPI_ISL_902474, EPI_ISL_902500			
see above	Pathogen Genomics Center, National Institute of Infectious Diseases	Pathogen Genomics Center, National Institute of Infectious Diseases	Tsuyoshi Sekizuka, Kentaro Itokawa, Rina Tanaka, Masanori Hashino, Makoto Kuroda
EPI_ISL_902504, EPI_ISL_902505, EPI_ISL_902506, EPI_ISL_902507, EPI_ISL_902508, EPI_ISL_902509, EPI_ISL_902510, EPI_ISL_902511, EPI_ISL_902512, EPI_ISL_902513, EPI_ISL_902514, EPI_ISL_902515, EPI_ISL_902516, EPI_ISL_902517, EPI_ISL_902518, EPI_ISL_902519, EPI_ISL_902520, EPI_ISL_902521, EPI_ISL_902522, EPI_ISL_902523, EPI_ISL_902524, EPI_ISL_902525, EPI_ISL_902526, EPI_ISL_902527, EPI_ISL_902528, EPI_ISL_902529, EPI_ISL_902530, EPI_ISL_902531, EPI_ISL_902532, EPI_ISL_902533, EPI_ISL_902534, EPI_ISL_902535, EPI_ISL_902536, EPI_ISL_902537, EPI_ISL_902538, EPI_ISL_902539, EPI_ISL_902540, EPI_ISL_902541, EPI_ISL_902542, EPI_ISL_902543, EPI_ISL_902544, EPI_ISL_902545, EPI_ISL_902546, EPI_ISL_902547, EPI_ISL_902548, EPI_ISL_902549, EPI_ISL_902550, EPI_ISL_902551, EPI_ISL_902552			
see above	Department of Infectious Diseases, Kobe Institute of Health	Pathogen Genomics Center, National Institute of Infectious Diseases	Tsuyoshi Sekizuka, Kentaro Itokawa, Rina Tanaka, Masanori Hashino, Makoto Kuroda
EPI_ISL_902560, EPI_ISL_902561, EPI_ISL_902562, EPI_ISL_902563, EPI_ISL_902564, EPI_ISL_902565, EPI_ISL_902566, EPI_ISL_902567, EPI_ISL_902568, EPI_ISL_902569, EPI_ISL_902570, EPI_ISL_902571, EPI_ISL_902572, EPI_ISL_902573, EPI_ISL_902574, EPI_ISL_902578, EPI_ISL_902682			
see above	Pathogen Genomics Center, National Institute of Infectious Diseases	Pathogen Genomics Center, National Institute of Infectious Diseases	Tsuyoshi Sekizuka, Kentaro Itokawa, Rina Tanaka, Masanori Hashino, Makoto Kuroda
EPI_ISL_902919	Tanjungpura University Hospital	Tanjungpura University Hospital	Andriani; Mahyarudin; Virhan Novianry ; Delima Fajar Liana; Sofi Siti Shofiyah; Puji Astuti P; Muhammad Ibnu Kahtan; Ambar Rialita; Eka Ardiani Putri EA; Wiwik Windarti ; Helmi Sastriawan; Arif Wicaksono
EPI_ISL_904936, EPI_ISL_904938, EPI_ISL_904943, EPI_ISL_904944	Vilnius University Hospital Santaros Klinikos, Vilnius University	Institute of Biotechnology, Life Sciences Center, Vilnius University	Emilija Vasiliunaite, Milda Norkiene, Albertas Timinskas, Alma Gedvilaite, Aurelija Zvirbliene, Daniel Naumovas, Laimonas Griskevicius
EPI_ISL_906054	Tilia Laboratories s.r.o.	Tilia Laboratories s.r.o.	Sona Pekova, MD, PhD.
EPI_ISL_906055	Tilia Laboratories s.r.o.	Tilia Laboratories s.r.o.	Sona Pekova MD, PhD.
EPI_ISL_906059	Tilia Laboratories s.r.o.	Tilia Laboratories s.r.o.	Sona Pekova, MD, PhD.
EPI_ISL_906469, EPI_ISL_906470, EPI_ISL_906471, EPI_ISL_906472, EPI_ISL_906473	Pathogen Genomics Center, National Institute of Infectious Diseases	Pathogen Genomics Center, National Institute of Infectious Diseases	Tsuyoshi Sekizuka, Kentaro Itokawa, Rina Tanaka, Masanori Hashino, Makoto Kuroda
EPI_ISL_906479, EPI_ISL_906480, EPI_ISL_906481, EPI_ISL_906482, EPI_ISL_906483, EPI_ISL_906484, EPI_ISL_906485, EPI_ISL_906486, EPI_ISL_906487, EPI_ISL_906488, EPI_ISL_906489, EPI_ISL_906490, EPI_ISL_906491, EPI_ISL_906492, EPI_ISL_906493, EPI_ISL_906494, EPI_ISL_906495, EPI_ISL_906496, EPI_ISL_906497, EPI_ISL_906498, EPI_ISL_906499, EPI_ISL_906500, EPI_ISL_906501, EPI_ISL_906502, EPI_ISL_906503, EPI_ISL_906504, EPI_ISL_906505			
see above	Sakai City Institute of Public Health	Pathogen Genomics Center, National Institute of Infectious Diseases	Tsuyoshi Sekizuka, Kentaro Itokawa, Rina Tanaka, Masanori Hashino, Makoto Kuroda
EPI_ISL_906506, EPI_ISL_906520, EPI_ISL_906521	Pathogen Genomics Center, National Institute of Infectious Diseases	Pathogen Genomics Center, National Institute of Infectious Diseases	Tsuyoshi Sekizuka, Kentaro Itokawa, Rina Tanaka, Masanori Hashino, Makoto Kuroda
EPI_ISL_906711	Hematology Laboratory, Section of Molecular Diagnostics, University Clinical Centre, Medical University of Gdansk	Laboratory of Recombinant Vaccines	Lukasz Rabalski, Maciej Kosinski, Anna Piotrowska-Mietelska,Izabela Szczygielska, Boguslaw Szewczyk, Krystyna Bienkowska-Szewczyk
EPI_ISL_907075	Department of Biology, University of Basrah	Department of Biology, University of Basrah	Abu-Ali,H.M. and Al-Badran,I.F.
EPI_ISL_910252, EPI_ISL_910253, EPI_ISL_910254, EPI_ISL_910255, EPI_ISL_910256, EPI_ISL_910257, EPI_ISL_910258, EPI_ISL_910259, EPI_ISL_910260, EPI_ISL_910261, EPI_ISL_910262, EPI_ISL_910263, EPI_ISL_910264, EPI_ISL_910265, EPI_ISL_910266, EPI_ISL_910267, EPI_ISL_910268, EPI_ISL_910269, EPI_ISL_910270, EPI_ISL_910271, EPI_ISL_910272, EPI_ISL_910273, EPI_ISL_910274, EPI_ISL_910275, EPI_ISL_910276, EPI_ISL_910277, EPI_ISL_910278, EPI_ISL_910279, EPI_ISL_910280, EPI_ISL_910281, EPI_ISL_910282, EPI_ISL_910283, EPI_ISL_910284, EPI_ISL_910285, EPI_ISL_910286, EPI_ISL_910287, EPI_ISL_910288, EPI_ISL_910289, EPI_ISL_910290, EPI_ISL_910291, EPI_ISL_910292, EPI_ISL_910293			
see above	CSIR-Centre for Cellular and Molecular Biology	CSIR-Centre for Cellular and Molecular Biology	Payel Mukherjee,Pratheusa Maccha,Namami Gaur,Lamuk Zaveri,Tulasi Nagabandi,Purushotham Vodnala,Blessy B John,Viswagithe S L,B Himasri,Sofia Banu,Priya Singh,Archana Bharadwaj Siva,Karthik Bharadwaj Tallapaka,Rakesh K Mishra,Divya Tej Sowpati
EPI_ISL_912193, EPI_ISL_912194	California Institute of Technology	Chan-Zuckerberg Biohub	CZB Cliahub Consortium
EPI_ISL_913092	Labor Mustafa Wien	Center for Virology	Jeremy V. Camp, Irene Goerzer, Monika Redlberger-Fritz, Stephan W. Aberle
EPI_ISL_913094	Center for Virology	Center for Virology	Jeremy V. Camp, Irene Goerzer, Monika Redlberger-Fritz, Stephan W. Aberle
EPI_ISL_915399, EPI_ISL_915400, EPI_ISL_915402	Keio University School of Medicine	Keio University School of Medicine	Kenjiro Kosaki, Yuka Iwasaki, Hirotsugu Ishizu, Haruhiko Siomi, Kodai Abe
EPI_ISL_918378	Ibaraki Prefectural Institute of Public Health	Ibaraki Prefectural Institute of Public Health	Keiko Goto, Tsuyoshi Sekizuka, Kentaro Itokawa, Rina Tanaka, Masanori Hashino, Makoto Kuroda
EPI_ISL_924077	Quadram Institute Bioscience	COVID-19 Genomics UK (COG-UK) Consortium	Dave J. Baker, Gemma L. Kay, Alp Aydin, Thanh Le-Viet, Steven Rudder, Ana P. Tedim, Anastasia Kolyva, Maria Diaz, Leonardo de Oliveira Martins, Nabil-Fareed Alikhan, Lizzie Meadows, Rachael Stanley, Ngozi Elumogo, Muhammed Yasir, Nicholas M. Thomson, Alexander J Trotter, Rachel Gilroy, Samuel Bloomfield, Claire Stuart, Andrew Bell, Reenesh Prakash, Samir Dervisevic, Alison E. Mather, John Wain, Mark Webber, Andrew J. Page, Justin O'Grady
EPI_ISL_925585	Public Health Ontario Laboratory	Public Health Ontario Laboratory	Vanessa G Allen, Philip Banh, Yao Chen, Richard de Borja, Alireza Eshaghi, Nahuel Fittipaldi, Christine Frantz, Jonathan B Gubbay, Jennifer L Guthrie, Lawrence Heisler, Esha Joshi, Michael Laszloffy, Aimin Li, Michael CY Li, Dean Maxwell, Sandeep Nagra, Samir N Patel, Jared Simpson, Karthikeyan Sivaraman, Ashleigh Sullivan, Yogi Sundaravadanam, Sarah Teatero, Matthew Watson, Andre Villegas, Sandra Zittermann
EPI_ISL_931473, EPI_ISL_931474, EPI_ISL_931475, EPI_ISL_931476, EPI_ISL_931477, EPI_ISL_931478, EPI_ISL_931479, EPI_ISL_931480, EPI_ISL_931481, EPI_ISL_931482, EPI_ISL_931483, EPI_ISL_931484, EPI_ISL_931485			
see above	City of Milwaukee Health Department Laboratory	City of Milwaukee Health Department Laboratory	Sanjib Bhattacharyya
EPI_ISL_935862, EPI_ISL_935864, EPI_ISL_935865, EPI_ISL_935871, EPI_ISL_935872, EPI_ISL_935873	Cadham Provincial laboratory	National Microbiology Laboratory (NML)	Anna Majer, Shari Tyson, Grace Seo, Philip Mabon, Elsie Grudeski, Rhiannon Huzarewich, Russell Mandes, Anneliese Landgraff, Jennifer Tanner, Natalie Knox, Morag Graham, Gary Van Domselaar, Paul Van Caeseele, Jared Bullard, David Alexander, Kerry Dust, Nathalie Bastien, Yan Li, Timothy Booth, Darian Hole, Madison Chapel, Kirsten Biggar, CanCOGeN's metadata curation team, Public Health Agency of Canada CanCOGeN team
EPI_ISL_936849, EPI_ISL_936850, EPI_ISL_936851	Northwestern Memorial Hospital	Ozer Lab	Ramon Lorenzo-Redondo, Lacy M. Simons, Chad J. Achenbach, Lawrence J. Jennings, Michael G. Ison, Judd F. Hultquist, Egon A. Ozer
EPI_ISL_937037, EPI_ISL_937098	Quest Diagnostics	Quest Diagnostics	Rosenthal,S.H., Gerasimova,A., Kagan,R.M., Anderson, B., Livingston, K.E., Hua, M., Liu Y., Shalhout, D.F., Owen, R., Lacbawan, F.
EPI_ISL_940174, EPI_ISL_940188, EPI_ISL_940189, EPI_ISL_940241, EPI_ISL_940245, EPI_ISL_940266, EPI_ISL_940267, EPI_ISL_940271	Hôpital Bichat Claude Bernard, Laboratoire de Virologie	IAME UMR1137 Inserm, Université de Paris, Hôpital Bichat	Antoine Bridier-Nahmias, Amélie Recoing, Quentin Le Hingrat, Lena Daniel, Siham Hamri, Gilles Collin, Alexandre Storto, Mélanie Bertine, Charlotte Charpentier, Nadhira Houhou-Fidouh, Diane Descamps, Benoit Visseaux
EPI_ISL_940960, EPI_ISL_940961, EPI_ISL_940983, EPI_ISL_940986, EPI_ISL_940987, EPI_ISL_942008	Centers for Disease Control and Prevention, Dengue Branch	Centers for Disease Control and Prevention, Dengue Branch	Gilberto A. Santiago, Glenda Gonzalez, Betzabel Flores, Keyla Charriez, Gabriela Paz-Bailey, Jorge L. Munoz-Jordan
EPI_ISL_943974, EPI_ISL_943975, EPI_ISL_943976, EPI_ISL_943977	LACEN do Estado de Tocantins	Instituto Adolfo Lutz, Interdiciplinary Procedures Center, Strategic Laboratory	Claudio Tavares Sacchi, Claudia Regina Gonçalves, Erica Valessa Ramos Gomes, Karoline Rodrigues Campos
EPI_ISL_949231	Departamento de Microbiología, CDB, Hospital Clínic, Barcelona	SeqCOVID-SPAIN consortium/IBV(CSIC)	Andrea Vergara, Mikel Martínez, Elisa Rubio, Jéssica Navero, Aida Peiró and SeqCOVID-SPAIN consortium

EPI_ISL_956332	Department of Biology, University of Basrah	Department of Biology, University of Basrah	Abu-Ali,H.F. and Al-Badran,A.I.
EPI_ISL_959897, EPI_ISL_959899, EPI_ISL_959904, EPI_ISL_959908, EPI_ISL_959909, EPI_ISL_959920, EPI_ISL_959921, EPI_ISL_959922, EPI_ISL_959923, EPI_ISL_959924, EPI_ISL_959925, EPI_ISL_959926, EPI_ISL_959927, EPI_ISL_959928, EPI_ISL_959929, EPI_ISL_959930, EPI_ISL_959931, EPI_ISL_959932, EPI_ISL_959933, EPI_ISL_959934, EPI_ISL_959935, EPI_ISL_959936, EPI_ISL_959937, EPI_ISL_959938, EPI_ISL_959939, EPI_ISL_959940, EPI_ISL_959941, EPI_ISL_959942, EPI_ISL_959943, EPI_ISL_959944, EPI_ISL_959945, EPI_ISL_959946, EPI_ISL_959947, EPI_ISL_959948, EPI_ISL_959949, EPI_ISL_959950, EPI_ISL_959951, EPI_ISL_959952, EPI_ISL_959953, EPI_ISL_959954, EPI_ISL_959955, EPI_ISL_959956, EPI_ISL_959957			
see above	University Medical Center Hamburg Eppendorf	Heinrich Pette Institute, Leibniz Institute for Experimental Virology	Alexis Robitaille, Thomas Günther, Johannes Knobloch, Martin Aepfelbacher, Nicole Fischer, Adam Grundhoff
EPI_ISL_960148	Mitchells Plain Hospital wc MPH	National Health Laboratory Service/UCT	Arash Iranzadeh, Deelan Doolabh, Lynn Tyers, Bruna Galvao, Innocent Mudau, Marvin Hsiao, Kruger Marais, Diana Hardie, Stephen Korsman, Carolyn Williamson
EPI_ISL_960149	Great Brak River Clinic wc GBC	National Health Laboratory Service/UCT	Arash Iranzadeh, Deelan Doolabh, Lynn Tyers, Bruna Galvao, Innocent Mudau, Marvin Hsiao, Kruger Marais, Diana Hardie, Stephen Korsman, Carolyn Williamson
EPI_ISL_973760, EPI_ISL_973764, EPI_ISL_973767, EPI_ISL_973769, EPI_ISL_973771, EPI_ISL_973775, EPI_ISL_973776, EPI_ISL_973779, EPI_ISL_973781, EPI_ISL_973783, EPI_ISL_973785, EPI_ISL_973788, EPI_ISL_973791, EPI_ISL_973794, EPI_ISL_973795, EPI_ISL_973797, EPI_ISL_973800, EPI_ISL_973802, EPI_ISL_973805, EPI_ISL_973807, EPI_ISL_973810, EPI_ISL_973813, EPI_ISL_973815, EPI_ISL_973818, EPI_ISL_973821, EPI_ISL_973823, EPI_ISL_973826, EPI_ISL_973828, EPI_ISL_973830, EPI_ISL_973832, EPI_ISL_973834, EPI_ISL_973837, EPI_ISL_973838, EPI_ISL_973839, EPI_ISL_973840, EPI_ISL_973841, EPI_ISL_973842, EPI_ISL_973843, EPI_ISL_973844, EPI_ISL_973845, EPI_ISL_973846, EPI_ISL_973847, EPI_ISL_973848, EPI_ISL_973849, EPI_ISL_973850, EPI_ISL_973851, EPI_ISL_973852, EPI_ISL_973853, EPI_ISL_973854, EPI_ISL_973855, EPI_ISL_973856, EPI_ISL_973857, EPI_ISL_973858, EPI_ISL_973859, EPI_ISL_973860, EPI_ISL_973861, EPI_ISL_973862, EPI_ISL_973863, EPI_ISL_973864, EPI_ISL_973865, EPI_ISL_973866, EPI_ISL_973867, EPI_ISL_973868, EPI_ISL_973869, EPI_ISL_973870, EPI_ISL_973871, EPI_ISL_973872, EPI_ISL_973873, EPI_ISL_973874, EPI_ISL_973875, EPI_ISL_973876, EPI_ISL_973877, EPI_ISL_973878, EPI_ISL_973879, EPI_ISL_973880, EPI_ISL_973881, EPI_ISL_973882, EPI_ISL_973883, EPI_ISL_973884, EPI_ISL_973885, EPI_ISL_973886, EPI_ISL_973887, EPI_ISL_973888, EPI_ISL_973889, EPI_ISL_973890, EPI_ISL_973891, EPI_ISL_973892, EPI_ISL_973893, EPI_ISL_973894, EPI_ISL_973895, EPI_ISL_973896, EPI_ISL_973897, EPI_ISL_973898, EPI_ISL_973899, EPI_ISL_973900, EPI_ISL_973901, EPI_ISL_973902, EPI_ISL_973903, EPI_ISL_973904, EPI_ISL_973905, EPI_ISL_973906, EPI_ISL_973907, EPI_ISL_973908, EPI_ISL_973909, EPI_ISL_973910, EPI_ISL_973911, EPI_ISL_973912, EPI_ISL_973913, EPI_ISL_973914, EPI_ISL_973915, EPI_ISL_973916, EPI_ISL_973917, EPI_ISL_973918, EPI_ISL_973919, EPI_ISL_973920, EPI_ISL_973921, EPI_ISL_973922, EPI_ISL_973923, EPI_ISL_973924, EPI_ISL_973925, EPI_ISL_973926, EPI_ISL_973927, EPI_ISL_973928, EPI_ISL_973929, EPI_ISL_973930, EPI_ISL_973931, EPI_ISL_973932, EPI_ISL_973933, EPI_ISL_973934, EPI_ISL_973935, EPI_ISL_973936, EPI_ISL_973937, EPI_ISL_973938, EPI_ISL_973939, EPI_ISL_973940, EPI_ISL_973941, EPI_ISL_973942, EPI_ISL_973943, EPI_ISL_973944, EPI_ISL_973945, EPI_ISL_973946, EPI_ISL_973947, EPI_ISL_973948, EPI_ISL_973949, EPI_ISL_973950, EPI_ISL_973951, EPI_ISL_973952, EPI_ISL_973953, EPI_ISL_973954, EPI_ISL_973955, EPI_ISL_973956, EPI_ISL_973957, EPI_ISL_973958, EPI_ISL_973959, EPI_ISL_973960, EPI_ISL_973961, EPI_ISL_973962, EPI_ISL_973963, EPI_ISL_973964, EPI_ISL_973965, EPI_ISL_973966, EPI_ISL_973967, EPI_ISL_973968, EPI_ISL_973969, EPI_ISL_973970, EPI_ISL_973971, EPI_ISL_973972, EPI_ISL_973973, EPI_ISL_973974, EPI_ISL_973975, EPI_ISL_973976, EPI_ISL_973977, EPI_ISL_973978, EPI_ISL_973979, EPI_ISL_973980, EPI_ISL_973981, EPI_ISL_973982, EPI_ISL_973983, EPI_ISL_973984, EPI_ISL_973985, EPI_ISL_973986, EPI_ISL_973987, EPI_ISL_973988, EPI_ISL_973989, EPI_ISL_973990, EPI_ISL_973991, EPI_ISL_973992, EPI_ISL_973993, EPI_ISL_973994, EPI_ISL_973995, EPI_ISL_973996, EPI_ISL_973997, EPI_ISL_973998, EPI_ISL_973999, EPI_ISL_974000, EPI_ISL_974001, EPI_ISL_974002, EPI_ISL_974003, EPI_ISL_974004, EPI_ISL_974005, EPI_ISL_974006, EPI_ISL_974007, EPI_ISL_974008, EPI_ISL_974009, EPI_ISL_974010, EPI_ISL_974011, EPI_ISL_974012, EPI_ISL_974013, EPI_ISL_974014, EPI_ISL_974015, EPI_ISL_974016, EPI_ISL_974017			
see above	BCCDC Public Health Laboratory	BCCDC Public Health Laboratory	Prystajecky Natalie, Linda Hoang, Dan Fornika, John Tyson, Shannon Russell, Kim Macdonald, Kimia Kamelian, Ana Pacagnella, Corrinne Ng, Loretta Janz, Robert Azana Terry Snutch, Mel Krajden
EPI_ISL_977476, EPI_ISL_977477	Instituto Adolfo Lutz Central	Instituto Adolfo Lutz, Interdiciplinary Procedures Center, Strategic Laboratory	Claudio Tavares Sacchi, Claudia Regina Gonçalves, Erica Valessa Ramos Gomes, Karoline Rodrigues Campos
