# Supplementary material for: Detection and characterization of the SARS-CoV-2 lineage B.1.526 in New York
Source: Nat Commun. 2021 Aug 9;12:4886. doi: 10.1038/s41467-021-25168-4 (PMC8352861; doi:10.1038/s41467-021-25168-4)
Supplement: Supplementary file 8 — Supplementary Data 4 [file 41467_2021_25168_MOESM8_ESM.zip › GISAID_acknowledements_tables/gisaid_hcov-19_acknowledgement_table_2021_02_13_010-5.pdf]

We gratefully acknowledge the following Authors from the Originating laboratories responsible for obtaining the specimens, as well as the Submitting laboratories where the genome data were generated and shared via GISAID, on which this research is based.

All Submitters of data may be contacted directly via www.gisaid.org

Authors are sorted alphabetically.

Accession ID	Originating Laboratory	Submitting Laboratory	Authors
EPI_ISL_751801	UCSD EXCITE	Andersen lab at Scripps Research	SEARCH Alliance San Diego
EPI_ISL_754238	Laboratory for Respiratory Viruses, Cantacuzino National Military-Medical Institute for Research and Development	Cantacuzino Institute Virology	Luiza Ustea, Mühlemann Barbara, Mihaela Lazar
EPI_ISL_755568, EPI_ISL_755569, EPI_ISL_755570, EPI_ISL_755571	SA Pathology	SA Pathology	Lex Leong, Julien Soubrier, Chuan Kok Lim, Song Gao, Mark Turra, Karin Kassahn, Ivan Bastian, Geoff Higgins
EPI_ISL_763769	Quadram Institute Bioscience	COVID-19 Genomics UK (COG-UK) Consortium	Dave J. Baker, Gemma L. Kay, Alp Aydin, Thanh Le-Viet, Steven Rudder, Ana P. Tedim, Anastasia Kolyva, Maria Diaz, Leonardo de Oliveira Martins, Nabil-Fareed Alikhan, Lizzie Meadows, Rachael Stanley, Ngozi Elumogo, Muhammed Yasir, Nicholas M. Thomson, Alexander J Trotter, Rachel Gilroy, Samuel Bloomfield, Claire Stuart, Andrew Bell, Reenesh Prakash, Samir Dervisevic, Alison E. Mather, John Wain, Mark Webber, Andrew J. Page, Justin O'Grady
EPI_ISL_766221	Respiratory Virus Unit, National Infection Service, Public Health England	COVID-19 Genomics UK (COG-UK) Consortium	PHE Covid Sequencing Team
EPI_ISL_766585	Triemli Hospital	Institute of Medical Virology, University of Zurich	Stefan Schmutz, Maryam Zaheri, Verena Kufner, Annette Audigé, Maria Grünberg, Kevin Steiner, Jon Huder, Cyril Shah, Riccarda Capaul, Jürg Böni, Michael Huber, Alexandra Trkola
EPI_ISL_766694	Klinisk mikrobiologi	The Public Health Agency of Sweden	Department of Microbiology, The Public Health Agency of Sweden
EPI_ISL_767906, EPI_ISL_767908, EPI_ISL_767909, EPI_ISL_767910	Sydney South West Pathology Service (SSWPS) - Royal Prince Alfred Hospital - NSW Health Pathology	NSW Health Pathology - Institute of Clinical Pathology and Medical Research; Westmead Hospital; University of Sydney	CIDM-PH et al.
EPI_ISL_767911, EPI_ISL_767912, EPI_ISL_767913	Sydney South West Pathology Service (SSWPS) - Liverpool Hospital - NSW Health Pathology	NSW Health Pathology - Institute of Clinical Pathology and Medical Research; Westmead Hospital; University of Sydney	CIDM-PH et al.
EPI_ISL_767914	Sydney South West Pathology Service (SSWPS) - Royal Prince Alfred Hospital - NSW Health Pathology	NSW Health Pathology - Institute of Clinical Pathology and Medical Research; Westmead Hospital; University of Sydney	CIDM-PH et al.
EPI_ISL_767921	South Eastern Area Laboratory Services (SEALS)	NSW Health Pathology - Institute of Clinical Pathology and Medical Research; Westmead Hospital; University of Sydney	CIDM-PH et al.
EPI_ISL_767927	St Vincent's Pathology (SydPath)	NSW Health Pathology - Institute of Clinical Pathology and Medical Research; Westmead Hospital; University of Sydney	CIDM-PH et al.
EPI_ISL_767928	South Eastern Area Laboratory Services (SEALS)	NSW Health Pathology - Institute of Clinical Pathology and Medical Research; Westmead Hospital; University of Sydney	CIDM-PH et al.
EPI_ISL_767933, EPI_ISL_767934, EPI_ISL_767935, EPI_ISL_767936	Pathology West - NSW Health Pathology	NSW Health Pathology - Institute of Clinical Pathology and Medical Research; Westmead Hospital; University of Sydney	CIDM-PH et al.
EPI_ISL_768324	Singapore General Hospital	Department of Microbiology	Nurdyana Abdul Rahman, Kun Lee Lim, Chenhao Li, Sui Sin Goh, Kenneth Xin Long Chan, Kian Sing Chan, Lynette Oon, Kern Rei Chng, Niranjan Nagarajan, Karrie Ko
EPI_ISL_769955	Albany Medical Center Hospital Clinical Laboratories	Wadsworth Center, New York State Department.of Health	Kirsten St. George, Daryl M. Lamson, Alexis Russel, Matthew Shudt, Melissa A Leisner, Jonathan Plitnick, Navjot Singh, John Kelly, Sara Griesemer, Erasmus Schneider, Erica Lasek-Nesselquist
EPI_ISL_770445, EPI_ISL_770446, EPI_ISL_770447, EPI_ISL_770448, EPI_ISL_770449, EPI_ISL_770450, EPI_ISL_770451, EPI_ISL_770452, EPI_ISL_770453, EPI_ISL_770454, EPI_ISL_770455, EPI_ISL_770456, EPI_ISL_770457, EPI_ISL_770458, EPI_ISL_770459, EPI_ISL_770460, EPI_ISL_770461, EPI_ISL_770476, EPI_ISL_770478, EPI_ISL_770479, EPI_ISL_770480, EPI_ISL_770481, EPI_ISL_770482, EPI_ISL_770483, EPI_ISL_770484, EPI_ISL_770485, EPI_ISL_770486, EPI_ISL_770487, EPI_ISL_770488, EPI_ISL_770489, EPI_ISL_770490, EPI_ISL_770491, EPI_ISL_770492, EPI_ISL_770493, EPI_ISL_770494, EPI_ISL_770495			
see above	Wyoming Public Health Laboratory	Wyoming Public Health Laboratory	Noah Hull, Taylor Fearing, Lynette Gumbleton, Channing Weber, Ashley Norberg, Bailey Bowcutt, and Wanda Manley
EPI_ISL_770820, EPI_ISL_770821	South East Regional Hospital	NSW Health Pathology - Institute of Clinical Pathology and Medical Research; Westmead Hospital; University of Sydney	CIDM-PH et al.
EPI_ISL_770871, EPI_ISL_770880, EPI_ISL_770886, EPI_ISL_770887, EPI_ISL_770905, EPI_ISL_771097, EPI_ISL_771098, EPI_ISL_771099, EPI_ISL_771128, EPI_ISL_771129, EPI_ISL_771130, EPI_ISL_771131, EPI_ISL_771132, EPI_ISL_771135, EPI_ISL_771136, EPI_ISL_771137, EPI_ISL_771138, EPI_ISL_771139, EPI_ISL_771140, EPI_ISL_771147, EPI_ISL_771148, EPI_ISL_771149, EPI_ISL_771150			
see above	Laboratoire national de santé, Microbiology, Virology	Laboratoire national de santé, Microbiology, Microbial Genomics Platform	Anke Wienecke-Baldacchino, Catherine Ragimbeau,Jessica Tapp, Fatu Djabi, Lise Pignon, Raoul Salmon, Tamir Abdelrahman
EPI_ISL_771367	SA Pathology	SA Pathology	Lex Leong, Julien Soubrier, Chuan Kok Lim, Song Gao, Mark Turra, Karin Kassahn, Ivan Bastian, Geoff Higgins
EPI_ISL_775416	Oslo University Hospital, Department of Medical Microbiology	Norwegian Institute of Public Health, Department of Virology	Kathrine Stene-Johansen, Kamilla Heddeland Instefjord, Hilde Elshaug, Atiya R Ali,Marie Paulsen Madsen, Rasmus Riis Kopperud, Hilde Vollan, Karoline Bragstad, Olav Hungnes
EPI_ISL_775550, EPI_ISL_775551, EPI_ISL_775552, EPI_ISL_775553, EPI_ISL_775554, EPI_ISL_775555, EPI_ISL_775556, EPI_ISL_775557, EPI_ISL_775558, EPI_ISL_775559, EPI_ISL_775560, EPI_ISL_775561, EPI_ISL_775562, EPI_ISL_775563, EPI_ISL_775564, EPI_ISL_775565, EPI_ISL_775566, EPI_ISL_775567, EPI_ISL_775568, EPI_ISL_775569, EPI_ISL_775570, EPI_ISL_775571			
see above	ABC	The Public Health Agency of Sweden	Department of Microbiology, The Public Health Agency of Sweden
EPI_ISL_775572, EPI_ISL_775573, EPI_ISL_775574	Klinisk mikrobiologi	The Public Health Agency of Sweden	Department of Microbiology, The Public Health Agency of Sweden
EPI_ISL_775575	ABC	The Public Health Agency of Sweden	Department of Microbiology, The Public Health Agency of Sweden
EPI_ISL_779138, EPI_ISL_779139, EPI_ISL_779140, EPI_ISL_779147, EPI_ISL_779148, EPI_ISL_779149, EPI_ISL_779150, EPI_ISL_779152	Yale Clinical Virology Laboratory	Grubaugh Lab - Yale School of Public Health	Tara Alpert, Joseph Fauver, Anderson Brito, Mallery Breban, Anne Wyllie, Chantal Vogels, Mary Petrone, Chaney Kalinich, Isabel Ott, Arnau Casanovas, Catherine Muenker, Adam Moore, Alice Lu, Maria Tokuyama, Patrick Wong, Peiwen Lu, Saad Omer, Richard Martinello, Allison Nelson, Shelli Farhadian, Akiko Iwasaki, Charlese Dela Cruz, Albert Ko, Nathan Grubaugh
EPI_ISL_779319, EPI_ISL_779320, EPI_ISL_779321, EPI_ISL_779322, EPI_ISL_779323, EPI_ISL_779324, EPI_ISL_779325, EPI_ISL_779326, EPI_ISL_779327, EPI_ISL_779328, EPI_ISL_779329, EPI_ISL_779330, EPI_ISL_779331, EPI_ISL_779332, EPI_ISL_779333, EPI_ISL_779334, EPI_ISL_779335, EPI_ISL_779336, EPI_ISL_779337, EPI_ISL_779338, EPI_ISL_779339, EPI_ISL_779340, EPI_ISL_779341, EPI_ISL_779342, EPI_ISL_779343, EPI_ISL_779344, EPI_ISL_779345, EPI_ISL_779346, EPI_ISL_779347, EPI_ISL_779348, EPI_ISL_779349, EPI_ISL_779350, EPI_ISL_779351, EPI_ISL_779352, EPI_ISL_779353, EPI_ISL_779354, EPI_ISL_779355, EPI_ISL_779357, EPI_ISL_779358, EPI_ISL_779359, EPI_ISL_779360, EPI_ISL_779361, EPI_ISL_779362, EPI_ISL_779363, EPI_ISL_779365, EPI_ISL_779366, EPI_ISL_779367, EPI_ISL_779368, EPI_ISL_779370, EPI_ISL_779372, EPI_ISL_779373, EPI_ISL_779374, EPI_ISL_779375, EPI_ISL_779376, EPI_ISL_779377, EPI_ISL_779378, EPI_ISL_779379, EPI_ISL_779380, EPI_ISL_779381, EPI_ISL_779382			
see above	University of Wisconsin-Madison AIDS Vaccine Research Laboratories	University of Wisconsin-Madison AIDS Vaccine Research Laboratories	Gage Moreno, Katarina Braun, et al. AIDS Vaccine Research Laboratories

EPI_ISL_779399, EPI_ISL_779400	South Eastern Area Laboratory Services (SEALS)	NSW Health Pathology - Institute of Clinical Pathology and Medical Research; Westmead Hospital; University of Sydney	CIDM-PH et al.
EPI_ISL_779413	Royal Darwin Hospital Pathology	MDU-PHL	Meumann, E., Caly L., Seemann T., Sait, M.L., Druce J., Sherry, N.L.
EPI_ISL_779624	Microbiological Diagnostic Unit - Public Health Laboratory (MDU-PHL)	MDU-PHL	Seemann T., Sait, M.L., Sherry, N.L.
EPI_ISL_779626	Victorian Infectious Diseases Reference Laboratory (VIDRL)	VIDRL and MDU-PHL	Caly L., Seemann T., Sait, M.L., Druce J., Sherry, N.L.
EPI_ISL_779628, EPI_ISL_779630	Microbiological Diagnostic Unit - Public Health Laboratory (MDU-PHL)	MDU-PHL	Seemann T., Sait, M.L., Sherry, N.L.
EPI_ISL_779631, EPI_ISL_779632, EPI_ISL_779633	Victorian Infectious Diseases Reference Laboratory (VIDRL)	VIDRL and MDU-PHL	Caly L., Seemann T., Sait, M.L., Druce J., Sherry, N.L.
EPI_ISL_779641, EPI_ISL_779642, EPI_ISL_779643, EPI_ISL_779644, EPI_ISL_779645, EPI_ISL_779646	Microbiological Diagnostic Unit - Public Health Laboratory (MDU-PHL)	MDU-PHL	Seemann T., Sait, M.L., Sherry, N.L.
EPI_ISL_779784	CNR Virus des Infections Respiratoires - France SUD	CNR Virus des Infections Respiratoires - France SUD	Antonin Bal, Gregory Destras, Gwendolyne Burfin, Hadrien Règue, Quentin Semanas, Martine Valette, Bruno Lina, Laurence Josset
EPI_ISL_779848, EPI_ISL_779849	Laboratoire BIOMED 05	CNR Virus des Infections Respiratoires - France SUD	Antonin Bal, Gregory Destras, Gwendolyne Burfin, Hadrien Règue, Quentin Semanas, Martine Valette, Bruno Lina, Laurence Josset
EPI_ISL_788898, EPI_ISL_788899, EPI_ISL_788900, EPI_ISL_788901, EPI_ISL_788902, EPI_ISL_788903, EPI_ISL_788904, EPI_ISL_788905, EPI_ISL_788906, EPI_ISL_788907, EPI_ISL_788908, EPI_ISL_788919, EPI_ISL_788939, EPI_ISL_788940, EPI_ISL_788941			
see above	University of Wisconsin-Madison AIDS Vaccine Research Laboratories	University of Wisconsin-Madison AIDS Vaccine Research Laboratories	Gage Moreno, Katarina Braun, et al. AIDS Vaccine Research Laboratories
EPI_ISL_788993, EPI_ISL_789000, EPI_ISL_789001, EPI_ISL_789002, EPI_ISL_789003, EPI_ISL_789004, EPI_ISL_789005, EPI_ISL_789006, EPI_ISL_789007, EPI_ISL_789008, EPI_ISL_789009, EPI_ISL_789010, EPI_ISL_789011, EPI_ISL_789018, EPI_ISL_789019, EPI_ISL_789020, EPI_ISL_789021, EPI_ISL_789022, EPI_ISL_789023, EPI_ISL_789024			
see above	ABC	The Public Health Agency of Sweden	Department of Microbiology, The Public Health Agency of Sweden
EPI_ISL_789062, EPI_ISL_789063, EPI_ISL_789064, EPI_ISL_789065, EPI_ISL_789066, EPI_ISL_789073, EPI_ISL_789076, EPI_ISL_789079, EPI_ISL_789083, EPI_ISL_789086, EPI_ISL_789092, EPI_ISL_789095, EPI_ISL_789098			
see above	National Virus Reference Laboratory	National Virus Reference Laboratory	Michael Carr, Gabriel Gonzalez, Dana Alalwan, Jonathan Dean, Daniel Hare, Cillian F De Gascun
EPI_ISL_790573, EPI_ISL_790574, EPI_ISL_790617, EPI_ISL_790618, EPI_ISL_790986, EPI_ISL_790987, EPI_ISL_790988, EPI_ISL_791002, EPI_ISL_791003, EPI_ISL_791004, EPI_ISL_791033, EPI_ISL_791038, EPI_ISL_791039, EPI_ISL_791040, EPI_ISL_791041, EPI_ISL_791054, EPI_ISL_791055, EPI_ISL_791056, EPI_ISL_791057, EPI_ISL_791058, EPI_ISL_791059, EPI_ISL_791060, EPI_ISL_791061, EPI_ISL_791062, EPI_ISL_791063			
see above	Dutch COVID-19 response team	National Institute for Public Health and the Environment (RIVM)	Adam Meijer, Harry Vennema, Jeroen Cremer, Sharon van den Brink, Bas van der Veer, AnneMarie van den Brandt, Florian Zwagemaker, Dennis Schmitz, Chantal Reusken, on behalf of the national COVID-19 response team
EPI_ISL_791144, EPI_ISL_791150, EPI_ISL_791152, EPI_ISL_791153, EPI_ISL_791154, EPI_ISL_791155, EPI_ISL_791157, EPI_ISL_791158, EPI_ISL_791159, EPI_ISL_791160, EPI_ISL_791161, EPI_ISL_791163, EPI_ISL_791164, EPI_ISL_791165, EPI_ISL_791166, EPI_ISL_791167, EPI_ISL_791168, EPI_ISL_791169, EPI_ISL_791170, EPI_ISL_791171, EPI_ISL_791172, EPI_ISL_791173, EPI_ISL_791174, EPI_ISL_791175, EPI_ISL_791176, EPI_ISL_791177, EPI_ISL_791178, EPI_ISL_791179, EPI_ISL_791180, EPI_ISL_791181, EPI_ISL_791182			
see above	University of Wisconsin-Madison AIDS Vaccine Research Laboratories	University of Wisconsin-Madison AIDS Vaccine Research Laboratories	Gage Moreno, Katarina Braun, et al. AIDS Vaccine Research Laboratories
EPI_ISL_791193	Respiratory Virus Unit, National Infection Service, Public Health England	COVID-19 Genomics UK (COG-UK) Consortium	PHE Covid Sequencing Team
EPI_ISL_791330, EPI_ISL_791331	University of Wisconsin-Madison AIDS Vaccine Research Laboratories	University of Wisconsin-Madison AIDS Vaccine Research Laboratories	Gage Moreno, Katarina Braun, et al. AIDS Vaccine Research Laboratories
EPI_ISL_791340, EPI_ISL_791346, EPI_ISL_791415, EPI_ISL_791421, EPI_ISL_791426, EPI_ISL_791437, EPI_ISL_791441, EPI_ISL_791442, EPI_ISL_791445, EPI_ISL_791446, EPI_ISL_791452, EPI_ISL_791453, EPI_ISL_791455, EPI_ISL_791456, EPI_ISL_791457, EPI_ISL_791461, EPI_ISL_791465, EPI_ISL_791466, EPI_ISL_791468, EPI_ISL_791469, EPI_ISL_791471, EPI_ISL_791472, EPI_ISL_791480, EPI_ISL_791487			
see above	Johns Hopkins Hospital Department of Pathology	Johns Hopkins Hospital Department of Pathology	C. Paul Morris, Chun Huai Luo, Adannaya Amadi, Nicholas Gallagher, Heba H. Mostafa
EPI_ISL_792058	Hopital	National Reference Center for Viruses of Respiratory Infections, Institut Pasteur, Paris	Marion Barbet, Sylvie Behillil, Méline Bizard, Angela Brisebarre, Camille Capel, Etienne Simon-Lorière, Vincent Enouf, Maud Vanpeene, Sylvie van der Werf,Patricia Stoessel
EPI_ISL_792088	Toronto Invasive Bacterial Diseases Network	McMaster University	Allison McGeer, Patryk Aftanas, Hooman Derakhshani, Angel Li, Kuganya Nirmalarajah, Emily Panousis, Ahmed Draia, Jalees Nasir, Michael Surette, Samira Mubareka, Andrew G. McArthur
EPI_ISL_792550	Centre for Dengue Research and AICBU, Department of Immunology and Molecular Medicine	Centre for Dengue Research and AICBU, Department of Immunology and Molecular Medicine	Chandima Jeewandara, Deshni Jayathilaka, Dinuka Ariyaratne, Diyanath Ranasinghe, Laksiri Gomes, Gathsaurie Neelika Malavige
EPI_ISL_792668, EPI_ISL_792669, EPI_ISL_792670, EPI_ISL_792671, EPI_ISL_792672, EPI_ISL_792673	Los Angeles County PHL	Los Angeles County PHL	P. Hemarajata et al.
EPI_ISL_794092	URMC LABS	Wadsworth Center, New York State Department.of Health	Kirsten St. George, Daryl M. Lamson, Alexis Russel, Matthew Shudt, Melissa A Leisner, Jonathan Plitnick, Navjot Singh, John Kelly, Sara Griesemer, Erasmus Schneider, Erica Lasek-Nesselquist
EPI_ISL_794116, EPI_ISL_794117	GLENS FALLS HOSPITAL LABORATORY	Wadsworth Center, New York State Department.of Health	Kirsten St. George, Daryl M. Lamson, Alexis Russel, Matthew Shudt, Melissa A Leisner, Jonathan Plitnick, Navjot Singh, John Kelly, Sara Griesemer, Erasmus Schneider, Erica Lasek-Nesselquist
EPI_ISL_794159, EPI_ISL_794160, EPI_ISL_794161, EPI_ISL_794162, EPI_ISL_794163, EPI_ISL_794164, EPI_ISL_794165, EPI_ISL_794168, EPI_ISL_794169, EPI_ISL_794170, EPI_ISL_794171, EPI_ISL_794172, EPI_ISL_794173, EPI_ISL_794174, EPI_ISL_794175, EPI_ISL_794176, EPI_ISL_794177, EPI_ISL_794178, EPI_ISL_794179, EPI_ISL_794180, EPI_ISL_794181			
see above	Wadsworth Center, New York State Department.of Health	Wadsworth Center, New York State Department.of Health	Kirsten St. George, Daryl M. Lamson, Alexis Russel, Matthew Shudt, Melissa A Leisner, Jonathan Plitnick, Navjot Singh, John Kelly, Sara Griesemer, Erasmus Schneider, Erica Lasek-Nesselquist
EPI_ISL_794234, EPI_ISL_794236, EPI_ISL_794237, EPI_ISL_794244, EPI_ISL_794245, EPI_ISL_794246, EPI_ISL_794247, EPI_ISL_794248, EPI_ISL_794249, EPI_ISL_794250, EPI_ISL_794251, EPI_ISL_794252, EPI_ISL_794253, EPI_ISL_794254, EPI_ISL_794255, EPI_ISL_794256, EPI_ISL_794257, EPI_ISL_794258, EPI_ISL_794259, EPI_ISL_794260, EPI_ISL_794281, EPI_ISL_794282			
see above	WESTCHESTER MEDICAL CENTER	Wadsworth Center, New York State Department.of Health	Kirsten St. George, Daryl M. Lamson, Alexis Russel, Matthew Shudt, Melissa A Leisner, Jonathan Plitnick, Navjot Singh, John Kelly, Sara Griesemer, Erasmus Schneider, Erica Lasek-Nesselquist
EPI_ISL_794592	Laboratorio Estatal de Salud Pública de Tamaulipas	Instituto de diagnóstico y Referencia Epidemiologicos (INDRE)	Abril Rodriguez-Maldonado, Claudia Wong-Arambula, Fabiola Garces-Ayala, Gisela Barrera-Badillo, Ana Maria Cortez-Calderon, Bernardita Reyes-Berrones, Hilda del Carmen-Selvera, Gloria Molina-Gamboa, Lucia Hernandez-Rivas, Irma Lopez-Martinez, Celia Alpuche-Aranda, Jose Luis Alomia-Zegarra, Hugo Lopez Gatell-Ramirez, Ernesto Ramirez-Gonzalez.
EPI_ISL_794610, EPI_ISL_794620, EPI_ISL_794621, EPI_ISL_794622	LabPLUS	Institute of Environmental Science and Research (ESR)	Xiaoyun Ren, Matt Storey, Nikki Freed, Muhammad Faisal, Jing Wang, Hermes Perez, Anja Werno, Antje van der Linden, Arlo Upton, Chris Mansell, David Hammer, Dragana Drinkovic, Gary McAuliffe, Hana Sofia Andersson, James Ussher, Jill Sherwood, Josh Freeman, Julia Howard, Juliet Elvy, Mary DeAlmeida, Matt Blakiston, Matthew Rogers, Max Bloomfield, Michael Addidle, Michelle Balm, Sally Roberts, Sarah Jefferies, Sharmini Muttaiyah, Susan Morpeth, Susan Taylor, Timothy Blackmore, Vani Sathyendran, Veronica Playle, Virginia Hope, Erasmus Smit, Lauren Jelly, Olin Silander, Joep de Ligt
EPI_ISL_794624	Middlemore Hospital	Institute of Environmental Science and Research (ESR)	Xiaoyun Ren, Matt Storey, Nikki Freed, Muhammad Faisal, Jing Wang, Hermes Perez, Anja Werno, Antje van der Linden, Arlo Upton, Chris Mansell, David Hammer, Dragana Drinkovic, Gary McAuliffe, Hana Sofia Andersson, James Ussher, Jill Sherwood, Josh Freeman, Julia Howard, Juliet Elvy, Mary DeAlmeida, Matt Blakiston, Matthew Rogers, Max Bloomfield, Michael Addidle, Michelle Balm, Sally Roberts, Sarah Jefferies, Sharmini Muttaiyah, Susan Morpeth, Susan Taylor, Timothy Blackmore, Vani Sathyendran, Veronica Playle, Virginia Hope, Erasmus Smit, Lauren Jelly, Olin Silander, Joep de Ligt

EPI_ISL_794627	Canterbury Health Laboratories	Institute of Environmental Science and Research (ESR)	Xiaoyun Ren, Matt Storey, Nikki Freed, Muhammad Faisal, Jing Wang, Hermes Perez, Anja Werno, Antje van der Linden, Arlo Upton, Chris Mansell, David Hammer, Dragana Drinkovic, Gary McAuliffe, Hana Sofia Andersson, James Ussher, Jill Sherwood, Josh Freeman, Julia Howard, Juliet Elvy, Mary DeAlmeida, Matt Blakiston, Matthew Rogers, Max Bloomfield, Michael Addidle, Michelle Balm, Sally Roberts, Sarah Jefferies, Sharmini Muttaiyah, Susan Morpeth, Susan Taylor, Timothy Blackmore, Vani Sathyendran, Veronica Playle, Virginia Hope, Erasmus Smit, Lauren Jelly, Olin Silander, Joep de Ligt
EPI_ISL_794649, EPI_ISL_794650, EPI_ISL_794651	Fundación Cardio Infantil	Instituto Nacional de Salud - Dirección de Investigación en Salud Pública	Katherine Laiton-Donato, Diego A. Álvarez-Díaz, Carlos Franco-Muñoz, Mauricio Pacheco-Montealegre, Jonathan Reales, Sheryl Corchuelo, Maria T. Herrera, Julian Naizaque, Gerardo Santamaría, Paola Muñoz-Laiton, Diego Andrés Prada, Magdalena Wiesner, Martha Lucia Ospina Martinez, Marcela Mercado-Reyes
EPI_ISL_794652, EPI_ISL_794653	LSP DEL TOLIMA	Instituto Nacional de Salud - Dirección de Investigación en Salud Pública	Katherine Laiton-Donato, Diego A. Álvarez-Díaz, Carlos Franco-Muñoz, Mauricio Pacheco-Montealegre, Jonathan Reales, Sheryl Corchuelo, Maria T. Herrera, Julian Naizaque, Gerardo Santamaría, Paola Muñoz-Laiton, Diego Andrés Prada, Magdalena Wiesner, Martha Lucia Ospina Martinez, Marcela Mercado-Reyes
EPI_ISL_794654, EPI_ISL_794655	Fundación Valle del Lili	Instituto Nacional de Salud - Dirección de Investigación en Salud Pública	Katherine Laiton-Donato, Diego A. Álvarez-Díaz, Carlos Franco-Muñoz, Mauricio Pacheco-Montealegre, Jonathan Reales, Sheryl Corchuelo, Maria T. Herrera, Julian Naizaque, Gerardo Santamaría, Paola Muñoz-Laiton, Diego Andrés Prada, Magdalena Wiesner, Martha Lucia Ospina Martinez, Marcela Mercado-Reyes
EPI_ISL_794656, EPI_ISL_794657	UNIDAD HEMATOLOGICA ESPECIALIZADA	Instituto Nacional de Salud - Dirección de Investigación en Salud Pública	Katherine Laiton-Donato, Diego A. Álvarez-Díaz, Carlos Franco-Muñoz, Mauricio Pacheco-Montealegre, Jonathan Reales, Sheryl Corchuelo, Maria T. Herrera, Julian Naizaque, Gerardo Santamaría, Paola Muñoz-Laiton, Diego Andrés Prada, Magdalena Wiesner, Martha Lucia Ospina Martinez, Marcela Mercado-Reyes
EPI_ISL_794658	DIRECCION DE SANIDAD POLICIA NACIONAL	Instituto Nacional de Salud - Dirección de Investigación en Salud Pública	Katherine Laiton-Donato, Diego A. Álvarez-Díaz, Carlos Franco-Muñoz, Mauricio Pacheco-Montealegre, Jonathan Reales, Sheryl Corchuelo, Maria T. Herrera, Julian Naizaque, Gerardo Santamaría, Paola Muñoz-Laiton, Diego Andrés Prada, Magdalena Wiesner, Martha Lucia Ospina Martinez, Marcela Mercado-Reyes
EPI_ISL_794659, EPI_ISL_794660	HOSPITAL UNIVERSITARIO SAN IGNACIO	Instituto Nacional de Salud - Dirección de Investigación en Salud Pública	Katherine Laiton-Donato, Diego A. Álvarez-Díaz, Carlos Franco-Muñoz, Mauricio Pacheco-Montealegre, Jonathan Reales, Sheryl Corchuelo, Maria T. Herrera, Julian Naizaque, Gerardo Santamaría, Paola Muñoz-Laiton, Diego Andrés Prada, Magdalena Wiesner, Martha Lucia Ospina Martinez, Marcela Mercado-Reyes
EPI_ISL_794662	Fundación Cardio Infantil	Instituto Nacional de Salud - Dirección de Investigación en Salud Pública	Katherine Laiton-Donato, Diego A. Álvarez-Díaz, Carlos Franco-Muñoz, Mauricio Pacheco-Montealegre, Jonathan Reales, Sheryl Corchuelo, Maria T. Herrera, Julian Naizaque, Gerardo Santamaría, Paola Muñoz-Laiton, Diego Andrés Prada, Magdalena Wiesner, Martha Lucia Ospina Martinez, Marcela Mercado-Reyes
EPI_ISL_794663	Carvajal Laboratorios	Instituto Nacional de Salud - Dirección de Investigación en Salud Pública	Katherine Laiton-Donato, Diego A. Álvarez-Díaz, Carlos Franco-Muñoz, Mauricio Pacheco-Montealegre, Jonathan Reales, Sheryl Corchuelo, Maria T. Herrera, Julian Naizaque, Gerardo Santamaría, Paola Muñoz-Laiton, Diego Andrés Prada, Magdalena Wiesner, Martha Lucia Ospina Martinez, Marcela Mercado-Reyes
EPI_ISL_794664, EPI_ISL_794665	Fundación Valle del Lili	Instituto Nacional de Salud - Dirección de Investigación en Salud Pública	Katherine Laiton-Donato, Diego A. Álvarez-Díaz, Carlos Franco-Muñoz, Mauricio Pacheco-Montealegre, Jonathan Reales, Sheryl Corchuelo, Maria T. Herrera, Julian Naizaque, Gerardo Santamaría, Paola Muñoz-Laiton, Diego Andrés Prada, Magdalena Wiesner, Martha Lucia Ospina Martinez, Marcela Mercado-Reyes
EPI_ISL_794672, EPI_ISL_794673, EPI_ISL_794674	PathWest Laboratory Medicine WA	PathWest Laboratory Medicine WA Microbial Surveillance Unit	PathWest Laboratory Medicine WA Microbial Surveillance Unit
EPI_ISL_794818, EPI_ISL_794819, EPI_ISL_794820, EPI_ISL_794822, EPI_ISL_794823, EPI_ISL_794824	Greek Genome Center, Biomedical Research Foundation of the Academy of Athens (BRFAA)	Greek Genome Center, Biomedical Research Foundation of the Academy of Athens (BRFAA)	Emmanouil Athanasiadis, Ioannis Vatsellas, Thodoris Loupis, Christina Maria Kravvari, Katerina Zoi, Dimitrios Thanos
EPI_ISL_796161, EPI_ISL_796162, EPI_ISL_796166, EPI_ISL_796168, EPI_ISL_796169, EPI_ISL_796175, EPI_ISL_796176, EPI_ISL_796182, EPI_ISL_796183, EPI_ISL_796185, EPI_ISL_796191, EPI_ISL_796195, EPI_ISL_796196, EPI_ISL_796197, EPI_ISL_796201, EPI_ISL_796203, EPI_ISL_796206, EPI_ISL_796209, EPI_ISL_796210, EPI_ISL_796211, EPI_ISL_796212, EPI_ISL_796214, EPI_ISL_796215, EPI_ISL_796216, EPI_ISL_796217, EPI_ISL_796219, EPI_ISL_796220, EPI_ISL_796224, EPI_ISL_796228, EPI_ISL_796234, EPI_ISL_796235, EPI_ISL_796236, EPI_ISL_796237, EPI_ISL_796240, EPI_ISL_796243, EPI_ISL_796251, EPI_ISL_796255, EPI_ISL_796259, EPI_ISL_796264, EPI_ISL_796265, EPI_ISL_796267, EPI_ISL_796269, EPI_ISL_796285, EPI_ISL_796286, EPI_ISL_796287, EPI_ISL_796288, EPI_ISL_796289, EPI_ISL_796291, EPI_ISL_796294, EPI_ISL_796295, EPI_ISL_796300, EPI_ISL_796301, EPI_ISL_796304, EPI_ISL_796305, EPI_ISL_796306, EPI_ISL_796307, EPI_ISL_796309, EPI_ISL_796310, EPI_ISL_796317, EPI_ISL_796320, EPI_ISL_796321, EPI_ISL_796322, EPI_ISL_796324, EPI_ISL_796325, EPI_ISL_796327, EPI_ISL_796334, EPI_ISL_796351, EPI_ISL_796353, EPI_ISL_796356, EPI_ISL_796369, EPI_ISL_796373, EPI_ISL_796376, EPI_ISL_796377, EPI_ISL_796378, EPI_ISL_796384, EPI_ISL_796385, EPI_ISL_796386, EPI_ISL_796390, EPI_ISL_796391, EPI_ISL_796393, EPI_ISL_796395, EPI_ISL_796398, EPI_ISL_796399, EPI_ISL_796400, EPI_ISL_796401, EPI_ISL_796403, EPI_ISL_796407, EPI_ISL_796411, EPI_ISL_796413, EPI_ISL_796417, EPI_ISL_796426, EPI_ISL_796437, EPI_ISL_796439, EPI_ISL_796440, EPI_ISL_796441, EPI_ISL_796448, EPI_ISL_796451, EPI_ISL_796456, EPI_ISL_796457, EPI_ISL_796461, EPI_ISL_796462, EPI_ISL_796464, EPI_ISL_796465, EPI_ISL_796470, EPI_ISL_796471, EPI_ISL_796480, EPI_ISL_796481, EPI_ISL_796482, EPI_ISL_796486, EPI_ISL_796489, EPI_ISL_796493, EPI_ISL_796498, EPI_ISL_796504, EPI_ISL_796505, EPI_ISL_796511, EPI_ISL_796514, EPI_ISL_796517, EPI_ISL_796518, EPI_ISL_796523, EPI_ISL_796535, EPI_ISL_796538, EPI_ISL_796539, EPI_ISL_796540, EPI_ISL_796541, EPI_ISL_796542, EPI_ISL_796543, EPI_ISL_796544, EPI_ISL_796545, EPI_ISL_796546, EPI_ISL_796547, EPI_ISL_796548, EPI_ISL_796550, EPI_ISL_796561, EPI_ISL_796567, EPI_ISL_796571, EPI_ISL_796572, EPI_ISL_796573, EPI_ISL_796574, EPI_ISL_796577, EPI_ISL_796578, EPI_ISL_796579, EPI_ISL_796582, EPI_ISL_796585, EPI_ISL_796586, EPI_ISL_796587, EPI_ISL_796589, EPI_ISL_796591, EPI_ISL_796592, EPI_ISL_796600, EPI_ISL_796601, EPI_ISL_796607, EPI_ISL_796609, EPI_ISL_796610, EPI_ISL_796611, EPI_ISL_796613, EPI_ISL_796614, EPI_ISL_796615, EPI_ISL_796616, EPI_ISL_796617, EPI_ISL_796619, EPI_ISL_796620, EPI_ISL_796621, EPI_ISL_796623, EPI_ISL_796624, EPI_ISL_796625, EPI_ISL_796626, EPI_ISL_796629, EPI_ISL_796631, EPI_ISL_796632, EPI_ISL_796634, EPI_ISL_796636, EPI_ISL_796637, EPI_ISL_796638, EPI_ISL_796639, EPI_ISL_796640, EPI_ISL_796643			
see above	Viollier AG	Department of Biosystems Science and Engineering, ETH Zürich	Chaoran Chen, Sarah Nadeau, Catharine Aquino, Ivan Topolsky, Philipp Jablonski, Lara Fuhrmann, David Dreifuss, Katharina Jahn, Andreia Cabral de Gouvea, Maria Domenica Moccia, Simon Grüter, Timothy Sykes, Lennart Opitz, Griffin White, Laura Neff, Doris Popovic, Andrea Patrignani, Jay Tracy, Ralph Schlapbach, Christiane Beckmann, Maurice Redondo, Olivier Kobel, Christoph Noppen, Sophie Seidel, Noemie Santamaria de Souza, Niko Beerenwinkel, Tanja Stadler
EPI_ISL_796661, EPI_ISL_796675	Norwegian Institute of Public Health, Department of Virology	Norwegian Institute of Public Health, Department of Virology	Kathrine Stene-Johansen, Kamilla Heddeland Instefjord, Hilde Elshaug, Atiya R Ali,Marie Paulsen Madsen, Rasmus Riis Kopperud, Hilde Vollan, Karoline Bragstad, Olav Hungnes
EPI_ISL_796737, EPI_ISL_796738, EPI_ISL_796754, EPI_ISL_796761, EPI_ISL_796768, EPI_ISL_796771, EPI_ISL_796773	Instituto Nacional de Saude (INSA)	Instituto Nacional de Saude (INSA)	Borges et al
EPI_ISL_796784, EPI_ISL_796785, EPI_ISL_796786, EPI_ISL_796787, EPI_ISL_796788, EPI_ISL_796789, EPI_ISL_796790, EPI_ISL_796791, EPI_ISL_796792, EPI_ISL_796793, EPI_ISL_796794, EPI_ISL_796796, EPI_ISL_796797, EPI_ISL_796798, EPI_ISL_796799, EPI_ISL_796800, EPI_ISL_796801, EPI_ISL_796802, EPI_ISL_796803, EPI_ISL_796804, EPI_ISL_796805, EPI_ISL_796806, EPI_ISL_796807, EPI_ISL_796808, EPI_ISL_796809, EPI_ISL_796810, EPI_ISL_796811, EPI_ISL_796812, EPI_ISL_796813, EPI_ISL_796814, EPI_ISL_796815, EPI_ISL_796816, EPI_ISL_796817, EPI_ISL_796818, EPI_ISL_796819, EPI_ISL_796820, EPI_ISL_796821, EPI_ISL_796822, EPI_ISL_796823, EPI_ISL_796824, EPI_ISL_796825, EPI_ISL_796826, EPI_ISL_796827, EPI_ISL_796828, EPI_ISL_796829, EPI_ISL_796830, EPI_ISL_796831, EPI_ISL_796832, EPI_ISL_796833, EPI_ISL_796834, EPI_ISL_796835, EPI_ISL_796836, EPI_ISL_796837, EPI_ISL_796838, EPI_ISL_796839, EPI_ISL_796840, EPI_ISL_796841, EPI_ISL_796842, EPI_ISL_796843, EPI_ISL_796844, EPI_ISL_796845, EPI_ISL_796846, EPI_ISL_796847, EPI_ISL_796848, EPI_ISL_796849, EPI_ISL_796850, EPI_ISL_796851, EPI_ISL_796852, EPI_ISL_796853, EPI_ISL_796854, EPI_ISL_796855, EPI_ISL_796856, EPI_ISL_796857, EPI_ISL_796858, EPI_ISL_796859, EPI_ISL_796860, EPI_ISL_796861, EPI_ISL_796862, EPI_ISL_796863, EPI_ISL_796864, EPI_ISL_796865, EPI_ISL_796866, EPI_ISL_796867, EPI_ISL_796868, EPI_ISL_796869, EPI_ISL_796870, EPI_ISL_796871, EPI_ISL_796872, EPI_ISL_796873, EPI_ISL_796874, EPI_ISL_796875, EPI_ISL_796876, EPI_ISL_796877, EPI_ISL_796878, EPI_ISL_796879, EPI_ISL_796880, EPI_ISL_796881, EPI_ISL_796882, EPI_ISL_796883, EPI_ISL_796884, EPI_ISL_796885, EPI_ISL_796886, EPI_ISL_796887, EPI_ISL_796888, EPI_ISL_796889, EPI_ISL_796890, EPI_ISL_796891, EPI_ISL_796892, EPI_ISL_796893, EPI_ISL_796894, EPI_ISL_796895, EPI_ISL_796896, EPI_ISL_796897, EPI_ISL_796898, EPI_ISL_796899, EPI_ISL_796900, EPI_ISL_796901, EPI_ISL_796902, EPI_ISL_796903, EPI_ISL_796904, EPI_ISL_796905, EPI_ISL_796906, EPI_ISL_796907, EPI_ISL_796908, EPI_ISL_796909, EPI_ISL_796910, EPI_ISL_796911, EPI_ISL_796912, EPI_ISL_796913, EPI_ISL_796914, EPI_ISL_796915, EPI_ISL_796916, EPI_ISL_796917, EPI_ISL_796918, EPI_ISL_796919, EPI_ISL_796920, EPI_ISL_796921, EPI_ISL_796922, EPI_ISL_796923, EPI_ISL_796924, EPI_ISL_796925, EPI_ISL_796926, EPI_ISL_796927, EPI_ISL_796928, EPI_ISL_796929, EPI_ISL_796930, EPI_ISL_796931, EPI_ISL_796932, EPI_ISL_796933, EPI_ISL_796934, EPI_ISL_796935, EPI_ISL_796936, EPI_ISL_796937, EPI_ISL_796938, EPI_ISL_796939, EPI_ISL_796940, EPI_ISL_796941, EPI_ISL_796942, EPI_ISL_796943, EPI_ISL_796944, EPI_ISL_796945, EPI_ISL_796946, EPI_ISL_796947, EPI_ISL_796948, EPI_ISL_796949, EPI_ISL_796950, EPI_ISL_796951, EPI_ISL_796952, EPI_ISL_796953, EPI_ISL_796954, EPI_ISL_796955, EPI_ISL_796956, EPI_ISL_796957, EPI_ISL_796958, EPI_ISL_796959, EPI_ISL_796960, EPI_ISL_796961, EPI_ISL_796962, EPI_ISL_796963, EPI_ISL_796964, EPI_ISL_796965, EPI_ISL_796966, EPI_ISL_796967, EPI_ISL_796968, EPI_ISL_796969, EPI_ISL_796970, EPI_ISL_796971, EPI_ISL_796972, EPI_ISL_796973, EPI_ISL_796974, EPI_ISL_796975, EPI_ISL_796976, EPI_ISL_796977, EPI_ISL_796978, EPI_ISL_796979, EPI_ISL_796980, EPI_ISL_796981, EPI_ISL_796982, EPI_ISL_796983, EPI_ISL_796984, EPI_ISL_796985, EPI_ISL_796986, EPI_ISL_796987, EPI_ISL_796988, EPI_ISL_796989, EPI_ISL_796990, EPI_ISL_796991, EPI_ISL_796992, EPI_ISL_796993, EPI_ISL_796994, EPI_ISL_796995, EPI_ISL_796996, EPI_ISL_796998, EPI_ISL_796999, EPI_ISL_797000, EPI_ISL_797001, EPI_ISL_797002, EPI_ISL_797003, EPI_ISL_797004, EPI_ISL_797005, EPI_ISL_797006, EPI_ISL_797007, EPI_ISL_797008, EPI_ISL_797009, EPI_ISL_797010, EPI_ISL_797011, EPI_ISL_797012, EPI_ISL_797013, EPI_ISL_797014, EPI_ISL_797015, EPI_ISL_797016, EPI_ISL_797017, EPI_ISL_797018, EPI_ISL_797019, EPI_ISL_797020, EPI_ISL_797021, EPI_ISL_797022, EPI_ISL_797023, EPI_ISL_797024, EPI_ISL_797025, EPI_ISL_797026, EPI_ISL_797027, EPI_ISL_797028, EPI_ISL_797029, EPI_ISL_797030, EPI_ISL_797031, EPI_ISL_797032, EPI_ISL_797033, EPI_ISL_797034, EPI_ISL_797035, EPI_ISL_797036, EPI_ISL_797037, EPI_ISL_797038, EPI_ISL_797039, EPI_ISL_797040, EPI_ISL_797041, EPI_ISL_797042, EPI_ISL_797043, EPI_ISL_797044, EPI_ISL_797045, EPI_ISL_797046, EPI_ISL_797047, EPI_ISL_797048, EPI_ISL_797049, EPI_ISL_797050, EPI_ISL_797051, EPI_ISL_797052, EPI_ISL_797053, EPI_ISL_797054, EPI_ISL_797055, EPI_ISL_797056, EPI_ISL_797057, EPI_ISL_797058, EPI_ISL_797059, EPI_ISL_797060, EPI_ISL_797061, EPI_ISL_797062, EPI_ISL_797063, EPI_ISL_797064, EPI_ISL_797065, EPI_ISL_797066, EPI_ISL_797067, EPI_ISL_797068, EPI_ISL_797069, EPI_ISL_797070, EPI_ISL_797071, EPI_ISL_797072, EPI_ISL_797073, EPI_ISL_797074, EPI_ISL_797075, EPI_ISL_797076, EPI_ISL_797077, EPI_ISL_797078, EPI_ISL_797079, EPI_ISL_797080, EPI_ISL_797081, EPI_ISL_797082, EPI_ISL_797083, EPI_ISL_797084, EPI_ISL_797085, EPI_ISL_797086, EPI_ISL_797088, EPI_ISL_797089, EPI_ISL_797090, EPI_ISL_797091, EPI_ISL_797092, EPI_ISL_797093, EPI_ISL_797094, EPI_ISL_797095, EPI_ISL_797096, EPI_ISL_797097, EPI_ISL_797098, EPI_ISL_797099, EPI_ISL_797100, EPI_ISL_797101, EPI_ISL_797102, EPI_ISL_797103, EPI_ISL_797104, EPI_ISL_797105, EPI_ISL_797106, EPI_ISL_797107, EPI_ISL_797108, EPI_ISL_797109, EPI_ISL_797110, EPI_ISL_797111, EPI_ISL_797112, EPI_ISL_797113, EPI_ISL_797114, EPI_ISL_797115, EPI_ISL_797116, EPI_ISL_797117			
see above	Lighthouse Lab in Alderley Park	Wellcome Sanger Institute for the COVID-19 Genomics UK (COG-UK) Consortium	Jacquelyn Wynn, Mairead Hyland, The Lighthouse Lab in Alderley Park and Alex Alderton, Roberto Amato, Sonia Goncalves, Ewan Harrison, David K. Jackson, Ian Johnston, Dominic Kwiatkowski, Cordelia Langford, John Sillitoe on behalf of the Wellcome Sanger Institute COVID-19 Surveillance Team
EPI_ISL_797118, EPI_ISL_797120, EPI_ISL_797121, EPI_ISL_797125, EPI_ISL_797126, EPI_ISL_797128, EPI_ISL_797129, EPI_ISL_797133, EPI_ISL_797134, EPI_ISL_797138, EPI_ISL_797139, EPI_ISL_797141, EPI_ISL_797142, EPI_ISL_797143, EPI_ISL_797144, EPI_ISL_797146, EPI_ISL_797148, EPI_ISL_797149,			

EPI_ISL_797151, EPI_ISL_797153, EPI_ISL_797154, EPI_ISL_797155, EPI_ISL_797156, EPI_ISL_797160, EPI_ISL_797161, EPI_ISL_797163, EPI_ISL_797164, EPI_ISL_797165, EPI_ISL_797168, EPI_ISL_797171, EPI_ISL_797173, EPI_ISL_797175, EPI_ISL_797176, EPI_ISL_797177, EPI_ISL_797178, EPI_ISL_797179, EPI_ISL_797185, EPI_ISL_797187, EPI_ISL_797190, EPI_ISL_797194, EPI_ISL_797198, EPI_ISL_797202, EPI_ISL_797203, EPI_ISL_797204, EPI_ISL_797205, EPI_ISL_797207, EPI_ISL_797210, EPI_ISL_797212, EPI_ISL_797216, EPI_ISL_797218, EPI_ISL_797220, EPI_ISL_797225, EPI_ISL_797226, EPI_ISL_797228, EPI_ISL_797229, EPI_ISL_797231, EPI_ISL_797232, EPI_ISL_797234, EPI_ISL_797239, EPI_ISL_797240, EPI_ISL_797242, EPI_ISL_797243, EPI_ISL_797246, EPI_ISL_797247, EPI_ISL_797248, EPI_ISL_797252, EPI_ISL_797253, EPI_ISL_797254, EPI_ISL_797256, EPI_ISL_797258, EPI_ISL_797259, EPI_ISL_797261, EPI_ISL_797264, EPI_ISL_797266, EPI_ISL_797269, EPI_ISL_797272, EPI_ISL_797277, EPI_ISL_797281, EPI_ISL_797286, EPI_ISL_797289, EPI_ISL_797290, EPI_ISL_797295, EPI_ISL_797296, EPI_ISL_797301, EPI_ISL_797305, EPI_ISL_797313, EPI_ISL_797315, EPI_ISL_797317, EPI_ISL_797318, EPI_ISL_797319, EPI_ISL_797323, EPI_ISL_797325, EPI_ISL_797326, EPI_ISL_797327, EPI_ISL_797332, EPI_ISL_797335, EPI_ISL_797337, EPI_ISL_797338, EPI_ISL_797339, EPI_ISL_797342, EPI_ISL_797345, EPI_ISL_797346, EPI_ISL_797349, EPI_ISL_797354, EPI_ISL_797357, EPI_ISL_797362, EPI_ISL_797363, EPI_ISL_797365, EPI_ISL_797366, EPI_ISL_797367, EPI_ISL_797376, EPI_ISL_797379, EPI_ISL_797380, EPI_ISL_797385, EPI_ISL_797389, EPI_ISL_797390, EPI_ISL_797391, EPI_ISL_797395			
see above	Lighthouse Lab in Milton Keynes	Wellcome Sanger Institute for the COVID-19 Genomics UK (COG-UK) Consortium	The Lighthouse Lab in Milton Keynes and Alex Alderton, Roberto Amato, Sonia Goncalves, Ewan Harrison, David K. Jackson, Ian Johnston, Dominic Kwiatkowski, Cordelia Langford, John Sillitoe on behalf of the Wellcome Sanger Institute COVID-19 Surveillance Team
EPI_ISL_797397, EPI_ISL_797398	Lighthouse Lab in Cambridge	Wellcome Sanger Institute for the COVID-19 Genomics UK (COG-UK) Consortium	Rob Howes, The Lighthouse Lab in Cambridge and Alex Alderton, Roberto Amato, Sonia Goncalves, Ewan Harrison, David K. Jackson, Ian Johnston, Dominic Kwiatkowski, Cordelia Langford, John Sillitoe on behalf of the Wellcome Sanger Institute COVID-19 Surveillance Team
EPI_ISL_797399	Lighthouse Lab in Alderley Park	Wellcome Sanger Institute for the COVID-19 Genomics UK (COG-UK) Consortium	Jacquelyn Wynn, Mairead Hyland, The Lighthouse Lab in Alderley Park and Alex Alderton, Roberto Amato, Sonia Goncalves, Ewan Harrison, David K. Jackson, Ian Johnston, Dominic Kwiatkowski, Cordelia Langford, John Sillitoe on behalf of the Wellcome Sanger Institute COVID-19 Surveillance Team
EPI_ISL_797401	Lighthouse Lab in Cambridge	Wellcome Sanger Institute for the COVID-19 Genomics UK (COG-UK) Consortium	Rob Howes, The Lighthouse Lab in Cambridge and Alex Alderton, Roberto Amato, Sonia Goncalves, Ewan Harrison, David K. Jackson, Ian Johnston, Dominic Kwiatkowski, Cordelia Langford, John Sillitoe on behalf of the Wellcome Sanger Institute COVID-19 Surveillance Team
EPI_ISL_797402	Lighthouse Lab in Alderley Park	Wellcome Sanger Institute for the COVID-19 Genomics UK (COG-UK) Consortium	Jacquelyn Wynn, Mairead Hyland, The Lighthouse Lab in Alderley Park and Alex Alderton, Roberto Amato, Sonia Goncalves, Ewan Harrison, David K. Jackson, Ian Johnston, Dominic Kwiatkowski, Cordelia Langford, John Sillitoe on behalf of the Wellcome Sanger Institute COVID-19 Surveillance Team
EPI_ISL_797404, EPI_ISL_797406, EPI_ISL_797407	Lighthouse Lab in Cambridge	Wellcome Sanger Institute for the COVID-19 Genomics UK (COG-UK) Consortium	Rob Howes, The Lighthouse Lab in Cambridge and Alex Alderton, Roberto Amato, Sonia Goncalves, Ewan Harrison, David K. Jackson, Ian Johnston, Dominic Kwiatkowski, Cordelia Langford, John Sillitoe on behalf of the Wellcome Sanger Institute COVID-19 Surveillance Team
EPI_ISL_797409	Lighthouse Lab in Alderley Park	Wellcome Sanger Institute for the COVID-19 Genomics UK (COG-UK) Consortium	Jacquelyn Wynn, Mairead Hyland, The Lighthouse Lab in Alderley Park and Alex Alderton, Roberto Amato, Sonia Goncalves, Ewan Harrison, David K. Jackson, Ian Johnston, Dominic Kwiatkowski, Cordelia Langford, John Sillitoe on behalf of the Wellcome Sanger Institute COVID-19 Surveillance Team
EPI_ISL_797410, EPI_ISL_797412	Lighthouse Lab in Cambridge	Wellcome Sanger Institute for the COVID-19 Genomics UK (COG-UK) Consortium	Rob Howes, The Lighthouse Lab in Cambridge and Alex Alderton, Roberto Amato, Sonia Goncalves, Ewan Harrison, David K. Jackson, Ian Johnston, Dominic Kwiatkowski, Cordelia Langford, John Sillitoe on behalf of the Wellcome Sanger Institute COVID-19 Surveillance Team
EPI_ISL_797413	Lighthouse Lab in Alderley Park	Wellcome Sanger Institute for the COVID-19 Genomics UK (COG-UK) Consortium	Jacquelyn Wynn, Mairead Hyland, The Lighthouse Lab in Alderley Park and Alex Alderton, Roberto Amato, Sonia Goncalves, Ewan Harrison, David K. Jackson, Ian Johnston, Dominic Kwiatkowski, Cordelia Langford, John Sillitoe on behalf of the Wellcome Sanger Institute COVID-19 Surveillance Team
EPI_ISL_797414, EPI_ISL_797415	Lighthouse Lab in Cambridge	Wellcome Sanger Institute for the COVID-19 Genomics UK (COG-UK) Consortium	Rob Howes, The Lighthouse Lab in Cambridge and Alex Alderton, Roberto Amato, Sonia Goncalves, Ewan Harrison, David K. Jackson, Ian Johnston, Dominic Kwiatkowski, Cordelia Langford, John Sillitoe on behalf of the Wellcome Sanger Institute COVID-19 Surveillance Team
EPI_ISL_797416	Lighthouse Lab in Alderley Park	Wellcome Sanger Institute for the COVID-19 Genomics UK (COG-UK) Consortium	Jacquelyn Wynn, Mairead Hyland, The Lighthouse Lab in Alderley Park and Alex Alderton, Roberto Amato, Sonia Goncalves, Ewan Harrison, David K. Jackson, Ian Johnston, Dominic Kwiatkowski, Cordelia Langford, John Sillitoe on behalf of the Wellcome Sanger Institute COVID-19 Surveillance Team
EPI_ISL_797417, EPI_ISL_797418, EPI_ISL_797419	Lighthouse Lab in Cambridge	Wellcome Sanger Institute for the COVID-19 Genomics UK (COG-UK) Consortium	Rob Howes, The Lighthouse Lab in Cambridge and Alex Alderton, Roberto Amato, Sonia Goncalves, Ewan Harrison, David K. Jackson, Ian Johnston, Dominic Kwiatkowski, Cordelia Langford, John Sillitoe on behalf of the Wellcome Sanger Institute COVID-19 Surveillance Team
EPI_ISL_797420, EPI_ISL_797421	Lighthouse Lab in Alderley Park	Wellcome Sanger Institute for the COVID-19 Genomics UK (COG-UK) Consortium	Jacquelyn Wynn, Mairead Hyland, The Lighthouse Lab in Alderley Park and Alex Alderton, Roberto Amato, Sonia Goncalves, Ewan Harrison, David K. Jackson, Ian Johnston, Dominic Kwiatkowski, Cordelia Langford, John Sillitoe on behalf of the Wellcome Sanger Institute COVID-19 Surveillance Team
EPI_ISL_797422, EPI_ISL_797423	Lighthouse Lab in Cambridge	Wellcome Sanger Institute for the COVID-19 Genomics UK (COG-UK) Consortium	Rob Howes, The Lighthouse Lab in Cambridge and Alex Alderton, Roberto Amato, Sonia Goncalves, Ewan Harrison, David K. Jackson, Ian Johnston, Dominic Kwiatkowski, Cordelia Langford, John Sillitoe on behalf of the Wellcome Sanger Institute COVID-19 Surveillance Team
EPI_ISL_797424	Lighthouse Lab in Alderley Park	Wellcome Sanger Institute for the COVID-19 Genomics UK (COG-UK) Consortium	Jacquelyn Wynn, Mairead Hyland, The Lighthouse Lab in Alderley Park and Alex Alderton, Roberto Amato, Sonia Goncalves, Ewan Harrison, David K. Jackson, Ian Johnston, Dominic Kwiatkowski, Cordelia Langford, John Sillitoe on behalf of the Wellcome Sanger Institute COVID-19 Surveillance Team
EPI_ISL_797425, EPI_ISL_797426, EPI_ISL_797427	Lighthouse Lab in Cambridge	Wellcome Sanger Institute for the COVID-19 Genomics UK (COG-UK) Consortium	Rob Howes, The Lighthouse Lab in Cambridge and Alex Alderton, Roberto Amato, Sonia Goncalves, Ewan Harrison, David K. Jackson, Ian Johnston, Dominic Kwiatkowski, Cordelia Langford, John Sillitoe on behalf of the Wellcome Sanger Institute COVID-19 Surveillance Team
EPI_ISL_797428	Lighthouse Lab in Alderley Park	Wellcome Sanger Institute for the COVID-19 Genomics UK (COG-UK) Consortium	Jacquelyn Wynn, Mairead Hyland, The Lighthouse Lab in Alderley Park and Alex Alderton, Roberto Amato, Sonia Goncalves, Ewan Harrison, David K. Jackson, Ian Johnston, Dominic Kwiatkowski, Cordelia Langford, John Sillitoe on behalf of the Wellcome Sanger Institute COVID-19 Surveillance Team
EPI_ISL_797429	Lighthouse Lab in Cambridge	Wellcome Sanger Institute for the COVID-19 Genomics UK (COG-UK) Consortium	Rob Howes, The Lighthouse Lab in Cambridge and Alex Alderton, Roberto Amato, Sonia Goncalves, Ewan Harrison, David K. Jackson, Ian Johnston, Dominic Kwiatkowski, Cordelia Langford, John Sillitoe on behalf of the Wellcome Sanger Institute COVID-19 Surveillance Team
EPI_ISL_797430	Lighthouse Lab in Alderley Park	Wellcome Sanger Institute for the COVID-19 Genomics UK (COG-UK) Consortium	Jacquelyn Wynn, Mairead Hyland, The Lighthouse Lab in Alderley Park and Alex Alderton, Roberto Amato, Sonia Goncalves, Ewan Harrison, David K. Jackson, Ian Johnston, Dominic Kwiatkowski, Cordelia Langford, John Sillitoe on behalf of the Wellcome Sanger Institute COVID-19 Surveillance Team
EPI_ISL_797431, EPI_ISL_797433	Lighthouse Lab in Cambridge	Wellcome Sanger Institute for the COVID-19 Genomics UK (COG-UK) Consortium	Rob Howes, The Lighthouse Lab in Cambridge and Alex Alderton, Roberto Amato, Sonia Goncalves, Ewan Harrison, David K. Jackson, Ian Johnston, Dominic Kwiatkowski, Cordelia Langford, John Sillitoe on behalf of the Wellcome Sanger Institute COVID-19 Surveillance Team
EPI_ISL_797435	Lighthouse Lab in Alderley Park	Wellcome Sanger Institute for the COVID-19 Genomics UK (COG-UK) Consortium	Jacquelyn Wynn, Mairead Hyland, The Lighthouse Lab in Alderley Park and Alex Alderton, Roberto Amato, Sonia Goncalves, Ewan Harrison, David K. Jackson, Ian Johnston, Dominic Kwiatkowski, Cordelia Langford, John Sillitoe on behalf of the Wellcome Sanger Institute COVID-19 Surveillance Team
EPI_ISL_797436, EPI_ISL_797437, EPI_ISL_797439, EPI_ISL_797440, EPI_ISL_797442, EPI_ISL_797443, EPI_ISL_797444, EPI_ISL_797447, EPI_ISL_797448, EPI_ISL_797449, EPI_ISL_797450, EPI_ISL_797452, EPI_ISL_797453, EPI_ISL_797454, EPI_ISL_797455			
see above	Lighthouse Lab in Cambridge	Wellcome Sanger Institute for the COVID-19 Genomics UK (COG-UK) Consortium	Rob Howes, The Lighthouse Lab in Cambridge and Alex Alderton, Roberto Amato, Sonia Goncalves, Ewan Harrison, David K. Jackson, Ian Johnston, Dominic Kwiatkowski, Cordelia Langford, John Sillitoe on behalf of the Wellcome Sanger Institute COVID-19 Surveillance Team
EPI_ISL_797456	Lighthouse Lab in Alderley Park	Wellcome Sanger Institute for the COVID-19 Genomics UK (COG-UK) Consortium	Jacquelyn Wynn, Mairead Hyland, The Lighthouse Lab in Alderley Park and Alex Alderton, Roberto Amato, Sonia Goncalves, Ewan Harrison, David K. Jackson, Ian Johnston, Dominic Kwiatkowski, Cordelia Langford, John Sillitoe on behalf of the Wellcome Sanger Institute COVID-19 Surveillance Team
EPI_ISL_797457, EPI_ISL_797458, EPI_ISL_797460, EPI_ISL_797463, EPI_ISL_797464, EPI_ISL_797466, EPI_ISL_797467	Lighthouse Lab in Cambridge	Wellcome Sanger Institute for the COVID-19 Genomics UK (COG-UK) Consortium	Rob Howes, The Lighthouse Lab in Cambridge and Alex Alderton, Roberto Amato, Sonia Goncalves, Ewan Harrison, David K. Jackson, Ian Johnston, Dominic Kwiatkowski, Cordelia Langford, John Sillitoe on behalf of the Wellcome Sanger Institute COVID-19 Surveillance Team
EPI_ISL_797468	Lighthouse Lab in Alderley Park	Wellcome Sanger Institute for the COVID-19 Genomics UK (COG-UK) Consortium	Jacquelyn Wynn, Mairead Hyland, The Lighthouse Lab in Alderley Park and Alex Alderton, Roberto Amato, Sonia Goncalves, Ewan Harrison, David K. Jackson, Ian Johnston, Dominic Kwiatkowski, Cordelia Langford, John Sillitoe on behalf of the Wellcome Sanger Institute COVID-19 Surveillance Team
EPI_ISL_797469, EPI_ISL_797470, EPI_ISL_797471, EPI_ISL_797472, EPI_ISL_797473, EPI_ISL_797474, EPI_ISL_797476, EPI_ISL_797477, EPI_ISL_797478, EPI_ISL_797479	Lighthouse Lab in Cambridge	Wellcome Sanger Institute for the COVID-19 Genomics UK (COG-UK) Consortium	Rob Howes, The Lighthouse Lab in Cambridge and Alex Alderton, Roberto Amato, Sonia Goncalves, Ewan Harrison, David K. Jackson, Ian Johnston, Dominic Kwiatkowski, Cordelia Langford, John Sillitoe on behalf of the Wellcome Sanger Institute COVID-19 Surveillance Team
EPI_ISL_797480	Lighthouse Lab in Alderley Park	Wellcome Sanger Institute for the COVID-19 Genomics UK (COG-UK) Consortium	Jacquelyn Wynn, Mairead Hyland, The Lighthouse Lab in Alderley Park and Alex Alderton, Roberto Amato, Sonia Goncalves, Ewan Harrison, David K. Jackson, Ian Johnston, Dominic Kwiatkowski, Cordelia Langford, John Sillitoe on behalf of the Wellcome Sanger Institute COVID-19 Surveillance Team
EPI_ISL_797482, EPI_ISL_797485, EPI_ISL_797486, EPI_ISL_797487, EPI_ISL_797488	Lighthouse Lab in Cambridge	Wellcome Sanger Institute for the COVID-19 Genomics UK (COG-UK) Consortium	Rob Howes, The Lighthouse Lab in Cambridge and Alex Alderton, Roberto Amato, Sonia Goncalves, Ewan Harrison, David K. Jackson, Ian Johnston, Dominic Kwiatkowski, Cordelia Langford, John Sillitoe on behalf of the Wellcome Sanger Institute COVID-19 Surveillance Team
EPI_ISL_797489	Lighthouse Lab in Alderley Park	Wellcome Sanger Institute for the COVID-19 Genomics UK (COG-UK) Consortium	Jacquelyn Wynn, Mairead Hyland, The Lighthouse Lab in Alderley Park and Alex Alderton, Roberto Amato, Sonia Goncalves, Ewan Harrison, David K. Jackson, Ian Johnston, Dominic Kwiatkowski, Cordelia Langford, John Sillitoe on behalf of the Wellcome Sanger Institute COVID-19 Surveillance Team
EPI_ISL_797490	Lighthouse Lab in Cambridge	Wellcome Sanger Institute for the COVID-19 Genomics UK	Rob Howes, The Lighthouse Lab in Cambridge and Alex Alderton, Roberto Amato, Sonia Goncalves, Ewan Harrison, David K. Jackson, Ian Johnston,

		(COG-UK) Consortium	Dominic Kwiatkowski, Cordelia Langford, John Sillitoe on behalf of the Wellcome Sanger Institute COVID-19 Surveillance Team
EPI_ISL_797491	Lighthouse Lab in Alderley Park	Wellcome Sanger Institute for the COVID-19 Genomics UK (COG-UK) Consortium	Jacquelyn Wynn, Mairead Hyland, The Lighthouse Lab in Alderley Park and Alex Alderton, Roberto Amato, Sonia Goncalves, Ewan Harrison, David K. Jackson, Ian Johnston, Dominic Kwiatkowski, Cordelia Langford, John Sillitoe on behalf of the Wellcome Sanger Institute COVID-19 Surveillance Team
EPI_ISL_797493, EPI_ISL_797494, EPI_ISL_797495, EPI_ISL_797496	Lighthouse Lab in Cambridge	Wellcome Sanger Institute for the COVID-19 Genomics UK (COG-UK) Consortium	Rob Howes, The Lighthouse Lab in Cambridge and Alex Alderton, Roberto Amato, Sonia Goncalves, Ewan Harrison, David K. Jackson, Ian Johnston, Dominic Kwiatkowski, Cordelia Langford, John Sillitoe on behalf of the Wellcome Sanger Institute COVID-19 Surveillance Team
EPI_ISL_797499	Lighthouse Lab in Alderley Park	Wellcome Sanger Institute for the COVID-19 Genomics UK (COG-UK) Consortium	Jacquelyn Wynn, Mairead Hyland, The Lighthouse Lab in Alderley Park and Alex Alderton, Roberto Amato, Sonia Goncalves, Ewan Harrison, David K. Jackson, Ian Johnston, Dominic Kwiatkowski, Cordelia Langford, John Sillitoe on behalf of the Wellcome Sanger Institute COVID-19 Surveillance Team
EPI_ISL_797501, EPI_ISL_797503, EPI_ISL_797506, EPI_ISL_797507, EPI_ISL_797508	Lighthouse Lab in Cambridge	Wellcome Sanger Institute for the COVID-19 Genomics UK (COG-UK) Consortium	Rob Howes, The Lighthouse Lab in Cambridge and Alex Alderton, Roberto Amato, Sonia Goncalves, Ewan Harrison, David K. Jackson, Ian Johnston, Dominic Kwiatkowski, Cordelia Langford, John Sillitoe on behalf of the Wellcome Sanger Institute COVID-19 Surveillance Team
EPI_ISL_797512	Lighthouse Lab in Alderley Park	Wellcome Sanger Institute for the COVID-19 Genomics UK (COG-UK) Consortium	Jacquelyn Wynn, Mairead Hyland, The Lighthouse Lab in Alderley Park and Alex Alderton, Roberto Amato, Sonia Goncalves, Ewan Harrison, David K. Jackson, Ian Johnston, Dominic Kwiatkowski, Cordelia Langford, John Sillitoe on behalf of the Wellcome Sanger Institute COVID-19 Surveillance Team
EPI_ISL_797513, EPI_ISL_797515, EPI_ISL_797516, EPI_ISL_797517, EPI_ISL_797518, EPI_ISL_797519, EPI_ISL_797520, EPI_ISL_797521, EPI_ISL_797522, EPI_ISL_797523, EPI_ISL_797524, EPI_ISL_797525, EPI_ISL_797526, EPI_ISL_797527, EPI_ISL_797528, EPI_ISL_797529, EPI_ISL_797530			
see above	Lighthouse Lab in Cambridge	Wellcome Sanger Institute for the COVID-19 Genomics UK (COG-UK) Consortium	Rob Howes, The Lighthouse Lab in Cambridge and Alex Alderton, Roberto Amato, Sonia Goncalves, Ewan Harrison, David K. Jackson, Ian Johnston, Dominic Kwiatkowski, Cordelia Langford, John Sillitoe on behalf of the Wellcome Sanger Institute COVID-19 Surveillance Team
EPI_ISL_797533	Lighthouse Lab in Alderley Park	Wellcome Sanger Institute for the COVID-19 Genomics UK (COG-UK) Consortium	Jacquelyn Wynn, Mairead Hyland, The Lighthouse Lab in Alderley Park and Alex Alderton, Roberto Amato, Sonia Goncalves, Ewan Harrison, David K. Jackson, Ian Johnston, Dominic Kwiatkowski, Cordelia Langford, John Sillitoe on behalf of the Wellcome Sanger Institute COVID-19 Surveillance Team
EPI_ISL_797534	Lighthouse Lab in Cambridge	Wellcome Sanger Institute for the COVID-19 Genomics UK (COG-UK) Consortium	Rob Howes, The Lighthouse Lab in Cambridge and Alex Alderton, Roberto Amato, Sonia Goncalves, Ewan Harrison, David K. Jackson, Ian Johnston, Dominic Kwiatkowski, Cordelia Langford, John Sillitoe on behalf of the Wellcome Sanger Institute COVID-19 Surveillance Team
EPI_ISL_797535	Lighthouse Lab in Alderley Park	Wellcome Sanger Institute for the COVID-19 Genomics UK (COG-UK) Consortium	Jacquelyn Wynn, Mairead Hyland, The Lighthouse Lab in Alderley Park and Alex Alderton, Roberto Amato, Sonia Goncalves, Ewan Harrison, David K. Jackson, Ian Johnston, Dominic Kwiatkowski, Cordelia Langford, John Sillitoe on behalf of the Wellcome Sanger Institute COVID-19 Surveillance Team
EPI_ISL_797536, EPI_ISL_797538, EPI_ISL_797539	Lighthouse Lab in Cambridge	Wellcome Sanger Institute for the COVID-19 Genomics UK (COG-UK) Consortium	Rob Howes, The Lighthouse Lab in Cambridge and Alex Alderton, Roberto Amato, Sonia Goncalves, Ewan Harrison, David K. Jackson, Ian Johnston, Dominic Kwiatkowski, Cordelia Langford, John Sillitoe on behalf of the Wellcome Sanger Institute COVID-19 Surveillance Team
EPI_ISL_797540, EPI_ISL_797541	Lighthouse Lab in Alderley Park	Wellcome Sanger Institute for the COVID-19 Genomics UK (COG-UK) Consortium	Jacquelyn Wynn, Mairead Hyland, The Lighthouse Lab in Alderley Park and Alex Alderton, Roberto Amato, Sonia Goncalves, Ewan Harrison, David K. Jackson, Ian Johnston, Dominic Kwiatkowski, Cordelia Langford, John Sillitoe on behalf of the Wellcome Sanger Institute COVID-19 Surveillance Team
EPI_ISL_797542	Lighthouse Lab in Cambridge	Wellcome Sanger Institute for the COVID-19 Genomics UK (COG-UK) Consortium	Rob Howes, The Lighthouse Lab in Cambridge and Alex Alderton, Roberto Amato, Sonia Goncalves, Ewan Harrison, David K. Jackson, Ian Johnston, Dominic Kwiatkowski, Cordelia Langford, John Sillitoe on behalf of the Wellcome Sanger Institute COVID-19 Surveillance Team
EPI_ISL_797543	Lighthouse Lab in Alderley Park	Wellcome Sanger Institute for the COVID-19 Genomics UK (COG-UK) Consortium	Jacquelyn Wynn, Mairead Hyland, The Lighthouse Lab in Alderley Park and Alex Alderton, Roberto Amato, Sonia Goncalves, Ewan Harrison, David K. Jackson, Ian Johnston, Dominic Kwiatkowski, Cordelia Langford, John Sillitoe on behalf of the Wellcome Sanger Institute COVID-19 Surveillance Team
EPI_ISL_797544, EPI_ISL_797545, EPI_ISL_797546	Lighthouse Lab in Cambridge	Wellcome Sanger Institute for the COVID-19 Genomics UK (COG-UK) Consortium	Rob Howes, The Lighthouse Lab in Cambridge and Alex Alderton, Roberto Amato, Sonia Goncalves, Ewan Harrison, David K. Jackson, Ian Johnston, Dominic Kwiatkowski, Cordelia Langford, John Sillitoe on behalf of the Wellcome Sanger Institute COVID-19 Surveillance Team
EPI_ISL_797547, EPI_ISL_797549	Lighthouse Lab in Alderley Park	Wellcome Sanger Institute for the COVID-19 Genomics UK (COG-UK) Consortium	Jacquelyn Wynn, Mairead Hyland, The Lighthouse Lab in Alderley Park and Alex Alderton, Roberto Amato, Sonia Goncalves, Ewan Harrison, David K. Jackson, Ian Johnston, Dominic Kwiatkowski, Cordelia Langford, John Sillitoe on behalf of the Wellcome Sanger Institute COVID-19 Surveillance Team
EPI_ISL_797550, EPI_ISL_797551, EPI_ISL_797552, EPI_ISL_797555, EPI_ISL_797556, EPI_ISL_797557, EPI_ISL_797558, EPI_ISL_797561, EPI_ISL_797562, EPI_ISL_797565, EPI_ISL_797567			
see above	Lighthouse Lab in Cambridge	Wellcome Sanger Institute for the COVID-19 Genomics UK (COG-UK) Consortium	Rob Howes, The Lighthouse Lab in Cambridge and Alex Alderton, Roberto Amato, Sonia Goncalves, Ewan Harrison, David K. Jackson, Ian Johnston, Dominic Kwiatkowski, Cordelia Langford, John Sillitoe on behalf of the Wellcome Sanger Institute COVID-19 Surveillance Team
EPI_ISL_797568	Lighthouse Lab in Alderley Park	Wellcome Sanger Institute for the COVID-19 Genomics UK (COG-UK) Consortium	Jacquelyn Wynn, Mairead Hyland, The Lighthouse Lab in Alderley Park and Alex Alderton, Roberto Amato, Sonia Goncalves, Ewan Harrison, David K. Jackson, Ian Johnston, Dominic Kwiatkowski, Cordelia Langford, John Sillitoe on behalf of the Wellcome Sanger Institute COVID-19 Surveillance Team
EPI_ISL_797569, EPI_ISL_797570, EPI_ISL_797571, EPI_ISL_797572, EPI_ISL_797573, EPI_ISL_797574, EPI_ISL_797577, EPI_ISL_797581, EPI_ISL_797582, EPI_ISL_797583, EPI_ISL_797584, EPI_ISL_797585			
see above	Lighthouse Lab in Cambridge	Wellcome Sanger Institute for the COVID-19 Genomics UK (COG-UK) Consortium	Rob Howes, The Lighthouse Lab in Cambridge and Alex Alderton, Roberto Amato, Sonia Goncalves, Ewan Harrison, David K. Jackson, Ian Johnston, Dominic Kwiatkowski, Cordelia Langford, John Sillitoe on behalf of the Wellcome Sanger Institute COVID-19 Surveillance Team
EPI_ISL_797587	Lighthouse Lab in Alderley Park	Wellcome Sanger Institute for the COVID-19 Genomics UK (COG-UK) Consortium	Jacquelyn Wynn, Mairead Hyland, The Lighthouse Lab in Alderley Park and Alex Alderton, Roberto Amato, Sonia Goncalves, Ewan Harrison, David K. Jackson, Ian Johnston, Dominic Kwiatkowski, Cordelia Langford, John Sillitoe on behalf of the Wellcome Sanger Institute COVID-19 Surveillance Team
EPI_ISL_797588, EPI_ISL_797589, EPI_ISL_797591, EPI_ISL_797592, EPI_ISL_797594, EPI_ISL_797596, EPI_ISL_797598	Lighthouse Lab in Cambridge	Wellcome Sanger Institute for the COVID-19 Genomics UK (COG-UK) Consortium	Rob Howes, The Lighthouse Lab in Cambridge and Alex Alderton, Roberto Amato, Sonia Goncalves, Ewan Harrison, David K. Jackson, Ian Johnston, Dominic Kwiatkowski, Cordelia Langford, John Sillitoe on behalf of the Wellcome Sanger Institute COVID-19 Surveillance Team
EPI_ISL_797599	Lighthouse Lab in Alderley Park	Wellcome Sanger Institute for the COVID-19 Genomics UK (COG-UK) Consortium	Jacquelyn Wynn, Mairead Hyland, The Lighthouse Lab in Alderley Park and Alex Alderton, Roberto Amato, Sonia Goncalves, Ewan Harrison, David K. Jackson, Ian Johnston, Dominic Kwiatkowski, Cordelia Langford, John Sillitoe on behalf of the Wellcome Sanger Institute COVID-19 Surveillance Team
EPI_ISL_797601, EPI_ISL_797602, EPI_ISL_797603, EPI_ISL_797604	Lighthouse Lab in Cambridge	Wellcome Sanger Institute for the COVID-19 Genomics UK (COG-UK) Consortium	Rob Howes, The Lighthouse Lab in Cambridge and Alex Alderton, Roberto Amato, Sonia Goncalves, Ewan Harrison, David K. Jackson, Ian Johnston, Dominic Kwiatkowski, Cordelia Langford, John Sillitoe on behalf of the Wellcome Sanger Institute COVID-19 Surveillance Team
EPI_ISL_797606	Lighthouse Lab in Alderley Park	Wellcome Sanger Institute for the COVID-19 Genomics UK (COG-UK) Consortium	Jacquelyn Wynn, Mairead Hyland, The Lighthouse Lab in Alderley Park and Alex Alderton, Roberto Amato, Sonia Goncalves, Ewan Harrison, David K. Jackson, Ian Johnston, Dominic Kwiatkowski, Cordelia Langford, John Sillitoe on behalf of the Wellcome Sanger Institute COVID-19 Surveillance Team
EPI_ISL_797607, EPI_ISL_797608, EPI_ISL_797609	Lighthouse Lab in Cambridge	Wellcome Sanger Institute for the COVID-19 Genomics UK (COG-UK) Consortium	Rob Howes, The Lighthouse Lab in Cambridge and Alex Alderton, Roberto Amato, Sonia Goncalves, Ewan Harrison, David K. Jackson, Ian Johnston, Dominic Kwiatkowski, Cordelia Langford, John Sillitoe on behalf of the Wellcome Sanger Institute COVID-19 Surveillance Team
EPI_ISL_797610	Lighthouse Lab in Alderley Park	Wellcome Sanger Institute for the COVID-19 Genomics UK (COG-UK) Consortium	Jacquelyn Wynn, Mairead Hyland, The Lighthouse Lab in Alderley Park and Alex Alderton, Roberto Amato, Sonia Goncalves, Ewan Harrison, David K. Jackson, Ian Johnston, Dominic Kwiatkowski, Cordelia Langford, John Sillitoe on behalf of the Wellcome Sanger Institute COVID-19 Surveillance Team
EPI_ISL_797611, EPI_ISL_797612	Lighthouse Lab in Cambridge	Wellcome Sanger Institute for the COVID-19 Genomics UK (COG-UK) Consortium	Rob Howes, The Lighthouse Lab in Cambridge and Alex Alderton, Roberto Amato, Sonia Goncalves, Ewan Harrison, David K. Jackson, Ian Johnston, Dominic Kwiatkowski, Cordelia Langford, John Sillitoe on behalf of the Wellcome Sanger Institute COVID-19 Surveillance Team
EPI_ISL_797613	Lighthouse Lab in Alderley Park	Wellcome Sanger Institute for the COVID-19 Genomics UK (COG-UK) Consortium	Jacquelyn Wynn, Mairead Hyland, The Lighthouse Lab in Alderley Park and Alex Alderton, Roberto Amato, Sonia Goncalves, Ewan Harrison, David K. Jackson, Ian Johnston, Dominic Kwiatkowski, Cordelia Langford, John Sillitoe on behalf of the Wellcome Sanger Institute COVID-19 Surveillance Team
EPI_ISL_797614	Lighthouse Lab in Cambridge	Wellcome Sanger Institute for the COVID-19 Genomics UK (COG-UK) Consortium	Rob Howes, The Lighthouse Lab in Cambridge and Alex Alderton, Roberto Amato, Sonia Goncalves, Ewan Harrison, David K. Jackson, Ian Johnston, Dominic Kwiatkowski, Cordelia Langford, John Sillitoe on behalf of the Wellcome Sanger Institute COVID-19 Surveillance Team
EPI_ISL_797616	Lighthouse Lab in Alderley Park	Wellcome Sanger Institute for the COVID-19 Genomics UK (COG-UK) Consortium	Jacquelyn Wynn, Mairead Hyland, The Lighthouse Lab in Alderley Park and Alex Alderton, Roberto Amato, Sonia Goncalves, Ewan Harrison, David K. Jackson, Ian Johnston, Dominic Kwiatkowski, Cordelia Langford, John Sillitoe on behalf of the Wellcome Sanger Institute COVID-19 Surveillance Team
EPI_ISL_797617, EPI_ISL_797618	Lighthouse Lab in Cambridge	Wellcome Sanger Institute for the COVID-19 Genomics UK (COG-UK) Consortium	Rob Howes, The Lighthouse Lab in Cambridge and Alex Alderton, Roberto Amato, Sonia Goncalves, Ewan Harrison, David K. Jackson, Ian Johnston, Dominic Kwiatkowski, Cordelia Langford, John Sillitoe on behalf of the Wellcome Sanger Institute COVID-19 Surveillance Team
EPI_ISL_797619	Lighthouse Lab in Alderley Park	Wellcome Sanger Institute for the COVID-19 Genomics UK (COG-UK) Consortium	Jacquelyn Wynn, Mairead Hyland, The Lighthouse Lab in Alderley Park and Alex Alderton, Roberto Amato, Sonia Goncalves, Ewan Harrison, David K. Jackson, Ian Johnston, Dominic Kwiatkowski, Cordelia Langford, John Sillitoe on behalf of the Wellcome Sanger Institute COVID-19 Surveillance Team
EPI_ISL_797621, EPI_ISL_797622, EPI_ISL_797623, EPI_ISL_797624, EPI_ISL_797626, EPI_ISL_797628, EPI_ISL_797629, EPI_ISL_797630, EPI_ISL_797633, EPI_ISL_797634, EPI_ISL_797636			

see above	Lighthouse Lab in Cambridge	Wellcome Sanger Institute for the COVID-19 Genomics UK (COG-UK) Consortium	Rob Howes, The Lighthouse Lab in Cambridge and Alex Alderton, Roberto Amato, Sonia Goncalves, Ewan Harrison, David K. Jackson, Ian Johnston, Dominic Kwiatkowski, Cordelia Langford, John Sillitoe on behalf of the Wellcome Sanger Institute COVID-19 Surveillance Team
EPI_ISL_797637	Lighthouse Lab in Alderley Park	Wellcome Sanger Institute for the COVID-19 Genomics UK (COG-UK) Consortium	Jacquelyn Wynn, Mairead Hyland, The Lighthouse Lab in Alderley Park and Alex Alderton, Roberto Amato, Sonia Goncalves, Ewan Harrison, David K. Jackson, Ian Johnston, Dominic Kwiatkowski, Cordelia Langford, John Sillitoe on behalf of the Wellcome Sanger Institute COVID-19 Surveillance Team
EPI_ISL_797638, EPI_ISL_797639, EPI_ISL_797640	Lighthouse Lab in Cambridge	Wellcome Sanger Institute for the COVID-19 Genomics UK (COG-UK) Consortium	Rob Howes, The Lighthouse Lab in Cambridge and Alex Alderton, Roberto Amato, Sonia Goncalves, Ewan Harrison, David K. Jackson, Ian Johnston, Dominic Kwiatkowski, Cordelia Langford, John Sillitoe on behalf of the Wellcome Sanger Institute COVID-19 Surveillance Team
EPI_ISL_797641, EPI_ISL_797642, EPI_ISL_797644	Lighthouse Lab in Alderley Park	Wellcome Sanger Institute for the COVID-19 Genomics UK (COG-UK) Consortium	Jacquelyn Wynn, Mairead Hyland, The Lighthouse Lab in Alderley Park and Alex Alderton, Roberto Amato, Sonia Goncalves, Ewan Harrison, David K. Jackson, Ian Johnston, Dominic Kwiatkowski, Cordelia Langford, John Sillitoe on behalf of the Wellcome Sanger Institute COVID-19 Surveillance Team
EPI_ISL_797649, EPI_ISL_797650, EPI_ISL_797651, EPI_ISL_797653	Lighthouse Lab in Cambridge	Wellcome Sanger Institute for the COVID-19 Genomics UK (COG-UK) Consortium	Rob Howes, The Lighthouse Lab in Cambridge and Alex Alderton, Roberto Amato, Sonia Goncalves, Ewan Harrison, David K. Jackson, Ian Johnston, Dominic Kwiatkowski, Cordelia Langford, John Sillitoe on behalf of the Wellcome Sanger Institute COVID-19 Surveillance Team
EPI_ISL_797654	Lighthouse Lab in Alderley Park	Wellcome Sanger Institute for the COVID-19 Genomics UK (COG-UK) Consortium	Jacquelyn Wynn, Mairead Hyland, The Lighthouse Lab in Alderley Park and Alex Alderton, Roberto Amato, Sonia Goncalves, Ewan Harrison, David K. Jackson, Ian Johnston, Dominic Kwiatkowski, Cordelia Langford, John Sillitoe on behalf of the Wellcome Sanger Institute COVID-19 Surveillance Team
EPI_ISL_797655, EPI_ISL_797656, EPI_ISL_797658	Lighthouse Lab in Cambridge	Wellcome Sanger Institute for the COVID-19 Genomics UK (COG-UK) Consortium	Rob Howes, The Lighthouse Lab in Cambridge and Alex Alderton, Roberto Amato, Sonia Goncalves, Ewan Harrison, David K. Jackson, Ian Johnston, Dominic Kwiatkowski, Cordelia Langford, John Sillitoe on behalf of the Wellcome Sanger Institute COVID-19 Surveillance Team
EPI_ISL_797660	Lighthouse Lab in Alderley Park	Wellcome Sanger Institute for the COVID-19 Genomics UK (COG-UK) Consortium	Jacquelyn Wynn, Mairead Hyland, The Lighthouse Lab in Alderley Park and Alex Alderton, Roberto Amato, Sonia Goncalves, Ewan Harrison, David K. Jackson, Ian Johnston, Dominic Kwiatkowski, Cordelia Langford, John Sillitoe on behalf of the Wellcome Sanger Institute COVID-19 Surveillance Team
EPI_ISL_797661, EPI_ISL_797662	Lighthouse Lab in Cambridge	Wellcome Sanger Institute for the COVID-19 Genomics UK (COG-UK) Consortium	Rob Howes, The Lighthouse Lab in Cambridge and Alex Alderton, Roberto Amato, Sonia Goncalves, Ewan Harrison, David K. Jackson, Ian Johnston, Dominic Kwiatkowski, Cordelia Langford, John Sillitoe on behalf of the Wellcome Sanger Institute COVID-19 Surveillance Team
EPI_ISL_797663	Lighthouse Lab in Alderley Park	Wellcome Sanger Institute for the COVID-19 Genomics UK (COG-UK) Consortium	Jacquelyn Wynn, Mairead Hyland, The Lighthouse Lab in Alderley Park and Alex Alderton, Roberto Amato, Sonia Goncalves, Ewan Harrison, David K. Jackson, Ian Johnston, Dominic Kwiatkowski, Cordelia Langford, John Sillitoe on behalf of the Wellcome Sanger Institute COVID-19 Surveillance Team
EPI_ISL_797665	Lighthouse Lab in Cambridge	Wellcome Sanger Institute for the COVID-19 Genomics UK (COG-UK) Consortium	Rob Howes, The Lighthouse Lab in Cambridge and Alex Alderton, Roberto Amato, Sonia Goncalves, Ewan Harrison, David K. Jackson, Ian Johnston, Dominic Kwiatkowski, Cordelia Langford, John Sillitoe on behalf of the Wellcome Sanger Institute COVID-19 Surveillance Team
EPI_ISL_797666	Lighthouse Lab in Alderley Park	Wellcome Sanger Institute for the COVID-19 Genomics UK (COG-UK) Consortium	Jacquelyn Wynn, Mairead Hyland, The Lighthouse Lab in Alderley Park and Alex Alderton, Roberto Amato, Sonia Goncalves, Ewan Harrison, David K. Jackson, Ian Johnston, Dominic Kwiatkowski, Cordelia Langford, John Sillitoe on behalf of the Wellcome Sanger Institute COVID-19 Surveillance Team
EPI_ISL_797667, EPI_ISL_797669, EPI_ISL_797670, EPI_ISL_797671, EPI_ISL_797672, EPI_ISL_797674, EPI_ISL_797675, EPI_ISL_797679, EPI_ISL_797680, EPI_ISL_797681, EPI_ISL_797682, EPI_ISL_797683, EPI_ISL_797684, EPI_ISL_797687, EPI_ISL_797689, EPI_ISL_797691, EPI_ISL_797693, EPI_ISL_797695, EPI_ISL_797696, EPI_ISL_797697, EPI_ISL_797698			
see above	Lighthouse Lab in Cambridge	Wellcome Sanger Institute for the COVID-19 Genomics UK (COG-UK) Consortium	Rob Howes, The Lighthouse Lab in Cambridge and Alex Alderton, Roberto Amato, Sonia Goncalves, Ewan Harrison, David K. Jackson, Ian Johnston, Dominic Kwiatkowski, Cordelia Langford, John Sillitoe on behalf of the Wellcome Sanger Institute COVID-19 Surveillance Team
EPI_ISL_797699	Lighthouse Lab in Alderley Park	Wellcome Sanger Institute for the COVID-19 Genomics UK (COG-UK) Consortium	Jacquelyn Wynn, Mairead Hyland, The Lighthouse Lab in Alderley Park and Alex Alderton, Roberto Amato, Sonia Goncalves, Ewan Harrison, David K. Jackson, Ian Johnston, Dominic Kwiatkowski, Cordelia Langford, John Sillitoe on behalf of the Wellcome Sanger Institute COVID-19 Surveillance Team
EPI_ISL_797700	Lighthouse Lab in Cambridge	Wellcome Sanger Institute for the COVID-19 Genomics UK (COG-UK) Consortium	Rob Howes, The Lighthouse Lab in Cambridge and Alex Alderton, Roberto Amato, Sonia Goncalves, Ewan Harrison, David K. Jackson, Ian Johnston, Dominic Kwiatkowski, Cordelia Langford, John Sillitoe on behalf of the Wellcome Sanger Institute COVID-19 Surveillance Team
EPI_ISL_797701	Lighthouse Lab in Alderley Park	Wellcome Sanger Institute for the COVID-19 Genomics UK (COG-UK) Consortium	Jacquelyn Wynn, Mairead Hyland, The Lighthouse Lab in Alderley Park and Alex Alderton, Roberto Amato, Sonia Goncalves, Ewan Harrison, David K. Jackson, Ian Johnston, Dominic Kwiatkowski, Cordelia Langford, John Sillitoe on behalf of the Wellcome Sanger Institute COVID-19 Surveillance Team
EPI_ISL_797702, EPI_ISL_797703, EPI_ISL_797706, EPI_ISL_797707, EPI_ISL_797708	Lighthouse Lab in Cambridge	Wellcome Sanger Institute for the COVID-19 Genomics UK (COG-UK) Consortium	Rob Howes, The Lighthouse Lab in Cambridge and Alex Alderton, Roberto Amato, Sonia Goncalves, Ewan Harrison, David K. Jackson, Ian Johnston, Dominic Kwiatkowski, Cordelia Langford, John Sillitoe on behalf of the Wellcome Sanger Institute COVID-19 Surveillance Team
EPI_ISL_797709	Lighthouse Lab in Alderley Park	Wellcome Sanger Institute for the COVID-19 Genomics UK (COG-UK) Consortium	Jacquelyn Wynn, Mairead Hyland, The Lighthouse Lab in Alderley Park and Alex Alderton, Roberto Amato, Sonia Goncalves, Ewan Harrison, David K. Jackson, Ian Johnston, Dominic Kwiatkowski, Cordelia Langford, John Sillitoe on behalf of the Wellcome Sanger Institute COVID-19 Surveillance Team
EPI_ISL_797710, EPI_ISL_797711	Lighthouse Lab in Cambridge	Wellcome Sanger Institute for the COVID-19 Genomics UK (COG-UK) Consortium	Rob Howes, The Lighthouse Lab in Cambridge and Alex Alderton, Roberto Amato, Sonia Goncalves, Ewan Harrison, David K. Jackson, Ian Johnston, Dominic Kwiatkowski, Cordelia Langford, John Sillitoe on behalf of the Wellcome Sanger Institute COVID-19 Surveillance Team
EPI_ISL_797712, EPI_ISL_797714, EPI_ISL_797715, EPI_ISL_797717	Lighthouse Lab in Alderley Park	Wellcome Sanger Institute for the COVID-19 Genomics UK (COG-UK) Consortium	Jacquelyn Wynn, Mairead Hyland, The Lighthouse Lab in Alderley Park and Alex Alderton, Roberto Amato, Sonia Goncalves, Ewan Harrison, David K. Jackson, Ian Johnston, Dominic Kwiatkowski, Cordelia Langford, John Sillitoe on behalf of the Wellcome Sanger Institute COVID-19 Surveillance Team
EPI_ISL_797718	Lighthouse Lab in Cambridge	Wellcome Sanger Institute for the COVID-19 Genomics UK (COG-UK) Consortium	Rob Howes, The Lighthouse Lab in Cambridge and Alex Alderton, Roberto Amato, Sonia Goncalves, Ewan Harrison, David K. Jackson, Ian Johnston, Dominic Kwiatkowski, Cordelia Langford, John Sillitoe on behalf of the Wellcome Sanger Institute COVID-19 Surveillance Team
EPI_ISL_797719, EPI_ISL_797720, EPI_ISL_797721, EPI_ISL_797722, EPI_ISL_797723, EPI_ISL_797724, EPI_ISL_797725, EPI_ISL_797726, EPI_ISL_797727, EPI_ISL_797728, EPI_ISL_797729, EPI_ISL_797730, EPI_ISL_797731, EPI_ISL_797732, EPI_ISL_797733, EPI_ISL_797734, EPI_ISL_797735, EPI_ISL_797736, EPI_ISL_797737, EPI_ISL_797738, EPI_ISL_797740, EPI_ISL_797741, EPI_ISL_797742, EPI_ISL_797743, EPI_ISL_797744, EPI_ISL_797745, EPI_ISL_797746, EPI_ISL_797747, EPI_ISL_797748, EPI_ISL_797749, EPI_ISL_797750, EPI_ISL_797751, EPI_ISL_797752, EPI_ISL_797753, EPI_ISL_797754, EPI_ISL_797755, EPI_ISL_797756, EPI_ISL_797757, EPI_ISL_797758, EPI_ISL_797759, EPI_ISL_797760, EPI_ISL_797761, EPI_ISL_797762, EPI_ISL_797763, EPI_ISL_797764, EPI_ISL_797765, EPI_ISL_797767, EPI_ISL_797768, EPI_ISL_797769, EPI_ISL_797770, EPI_ISL_797771, EPI_ISL_797772, EPI_ISL_797773, EPI_ISL_797774, EPI_ISL_797776, EPI_ISL_797777, EPI_ISL_797778, EPI_ISL_797779, EPI_ISL_797780, EPI_ISL_797781, EPI_ISL_797782, EPI_ISL_797783, EPI_ISL_797784, EPI_ISL_797785, EPI_ISL_797786, EPI_ISL_797787, EPI_ISL_797788, EPI_ISL_797790, EPI_ISL_797791, EPI_ISL_797792, EPI_ISL_797793, EPI_ISL_797794, EPI_ISL_797795, EPI_ISL_797796, EPI_ISL_797798, EPI_ISL_797799, EPI_ISL_797800, EPI_ISL_797801, EPI_ISL_797802, EPI_ISL_797803, EPI_ISL_797804, EPI_ISL_797805, EPI_ISL_797806, EPI_ISL_797807, EPI_ISL_797808, EPI_ISL_797809, EPI_ISL_797810, EPI_ISL_797811, EPI_ISL_797812, EPI_ISL_797813, EPI_ISL_797814, EPI_ISL_797815, EPI_ISL_797816, EPI_ISL_797818, EPI_ISL_797819, EPI_ISL_797820, EPI_ISL_797821, EPI_ISL_797822, EPI_ISL_797823, EPI_ISL_797824, EPI_ISL_797826, EPI_ISL_797827, EPI_ISL_797828, EPI_ISL_797832, EPI_ISL_797833, EPI_ISL_797834, EPI_ISL_797835, EPI_ISL_797836, EPI_ISL_797837, EPI_ISL_797839, EPI_ISL_797840, EPI_ISL_797841, EPI_ISL_797842, EPI_ISL_797843, EPI_ISL_797844, EPI_ISL_797845, EPI_ISL_797846, EPI_ISL_797848, EPI_ISL_797849, EPI_ISL_797852, EPI_ISL_797854, EPI_ISL_797855, EPI_ISL_797856, EPI_ISL_797857, EPI_ISL_797858, EPI_ISL_797859, EPI_ISL_797860, EPI_ISL_797861, EPI_ISL_797862, EPI_ISL_797863, EPI_ISL_797864, EPI_ISL_797865, EPI_ISL_797866, EPI_ISL_797869, EPI_ISL_797871, EPI_ISL_797872, EPI_ISL_797873, EPI_ISL_797874, EPI_ISL_797875, EPI_ISL_797876, EPI_ISL_797877, EPI_ISL_797878, EPI_ISL_797879, EPI_ISL_797880, EPI_ISL_797881, EPI_ISL_797882, EPI_ISL_797883, EPI_ISL_797886, EPI_ISL_797887, EPI_ISL_797888, EPI_ISL_797889, EPI_ISL_797890, EPI_ISL_797891, EPI_ISL_797892, EPI_ISL_797893, EPI_ISL_797894, EPI_ISL_797895, EPI_ISL_797896, EPI_ISL_797897, EPI_ISL_797898, EPI_ISL_797899, EPI_ISL_797900, EPI_ISL_797901, EPI_ISL_797902, EPI_ISL_797904, EPI_ISL_797905, EPI_ISL_797906, EPI_ISL_797907, EPI_ISL_797908, EPI_ISL_797909, EPI_ISL_797910, EPI_ISL_797911, EPI_ISL_797912, EPI_ISL_797913, EPI_ISL_797915, EPI_ISL_797917, EPI_ISL_797918, EPI_ISL_797919, EPI_ISL_797920, EPI_ISL_797922, EPI_ISL_797923, EPI_ISL_797924, EPI_ISL_797925, EPI_ISL_797926, EPI_ISL_797927, EPI_ISL_797928, EPI_ISL_797930, EPI_ISL_797931, EPI_ISL_797932, EPI_ISL_797933, EPI_ISL_797934, EPI_ISL_797935, EPI_ISL_797936, EPI_ISL_797938, EPI_ISL_797939, EPI_ISL_797940, EPI_ISL_797941, EPI_ISL_797942, EPI_ISL_797943, EPI_ISL_797944, EPI_ISL_797946, EPI_ISL_797947, EPI_ISL_797948, EPI_ISL_797949, EPI_ISL_797950, EPI_ISL_797951, EPI_ISL_797953, EPI_ISL_797954, EPI_ISL_797955, EPI_ISL_797956, EPI_ISL_797957, EPI_ISL_797958, EPI_ISL_797959, EPI_ISL_797960, EPI_ISL_797962, EPI_ISL_797963, EPI_ISL_797964, EPI_ISL_797966, EPI_ISL_797967, EPI_ISL_797968, EPI_ISL_797969, EPI_ISL_797971, EPI_ISL_797972, EPI_ISL_797973, EPI_ISL_797974, EPI_ISL_797975, EPI_ISL_797976, EPI_ISL_797977, EPI_ISL_797978, EPI_ISL_797979, EPI_ISL_797981, EPI_ISL_797982, EPI_ISL_797983, EPI_ISL_797984, EPI_ISL_797986, EPI_ISL_797987, EPI_ISL_797988, EPI_ISL_797989, EPI_ISL_797990, EPI_ISL_797991, EPI_ISL_797992, EPI_ISL_797993, EPI_ISL_797995, EPI_ISL_797997, EPI_ISL_797998, EPI_ISL_798001, EPI_ISL_798004, EPI_ISL_798005, EPI_ISL_798006, EPI_ISL_798008, EPI_ISL_798010, EPI_ISL_798012, EPI_ISL_798014, EPI_ISL_798016, EPI_ISL_798019, EPI_ISL_798020, EPI_ISL_798021, EPI_ISL_798023, EPI_ISL_798024, EPI_ISL_798026, EPI_ISL_798028, EPI_ISL_798030, EPI_ISL_798031, EPI_ISL_798033, EPI_ISL_798034, EPI_ISL_798036, EPI_ISL_798037, EPI_ISL_798039, EPI_ISL_798041, EPI_ISL_798042, EPI_ISL_798044, EPI_ISL_798045, EPI_ISL_798047, EPI_ISL_798049, EPI_ISL_798050, EPI_ISL_798052, EPI_ISL_798056, EPI_ISL_798057, EPI_ISL_798059, EPI_ISL_798063, EPI_ISL_798065, EPI_ISL_798067, EPI_ISL_798070, EPI_ISL_798071, EPI_ISL_798074, EPI_ISL_798075, EPI_ISL_798081, EPI_ISL_798082, EPI_ISL_798085, EPI_ISL_798087, EPI_ISL_798089, EPI_ISL_798090, EPI_ISL_798091, EPI_ISL_798092, EPI_ISL_798098, EPI_ISL_798099, EPI_ISL_798100, EPI_ISL_798102, EPI_ISL_798104, EPI_ISL_798109, EPI_ISL_798114, EPI_ISL_798116, EPI_ISL_798118, EPI_ISL_798120, EPI_ISL_798129, EPI_ISL_798135, EPI_ISL_798136, EPI_ISL_798138, EPI_ISL_798142, EPI_ISL_798144, EPI_ISL_798146, EPI_ISL_798147, EPI_ISL_798150, EPI_ISL_798155, EPI_ISL_798156, EPI_ISL_798157, EPI_ISL_798158, EPI_ISL_798162, EPI_ISL_798163, EPI_ISL_798164, EPI_ISL_798165, EPI_ISL_798172, EPI_ISL_798177, EPI_ISL_798179, EPI_ISL_798180, EPI_ISL_798187, EPI_ISL_798188, EPI_ISL_798189, EPI_ISL_798190, EPI_ISL_798191, EPI_ISL_798194, EPI_ISL_798195, EPI_ISL_798196, EPI_ISL_798198, EPI_ISL_798200, EPI_ISL_798205, EPI_ISL_798206, EPI_ISL_798207, EPI_ISL_798209, EPI_ISL_798210, EPI_ISL_798212, EPI_ISL_798215, EPI_ISL_798219, EPI_ISL_798226, EPI_ISL_798227, EPI_ISL_798228, EPI_ISL_798232, EPI_ISL_798233, EPI_ISL_798237, EPI_ISL_798238, EPI_ISL_798240, EPI_ISL_798247, EPI_ISL_798257, EPI_ISL_798259, EPI_ISL_798260, EPI_ISL_798262, EPI_ISL_798263, EPI_ISL_798264, EPI_ISL_798265, EPI_ISL_798267, EPI_ISL_798269, EPI_ISL_798276, EPI_ISL_798279, EPI_ISL_798286, EPI_ISL_798290, EPI_ISL_798291, EPI_ISL_798293, EPI_ISL_798297, EPI_ISL_798301, EPI_ISL_798302, EPI_ISL_798303, EPI_ISL_798304, EPI_ISL_798306, EPI_ISL_798309, EPI_ISL_798313, EPI_ISL_798317, EPI_ISL_798318, EPI_ISL_798320, EPI_ISL_798330, EPI_ISL_799006, EPI_ISL_799009, EPI_ISL_799011, EPI_ISL_799012, EPI_ISL_799014, EPI_ISL_799015, EPI_ISL_799016, EPI_ISL_799019, EPI_ISL_799024, EPI_ISL_799027, EPI_ISL_799028, EPI_ISL_799030, EPI_ISL_799033, EPI_ISL_799034, EPI_ISL_799038, EPI_ISL_799041, EPI_ISL_799042, EPI_ISL_799043, EPI_ISL_799044, EPI_ISL_799046, EPI_ISL_799049, EPI_ISL_799050, EPI_ISL_799051, EPI_ISL_799054, EPI_ISL_799056, EPI_ISL_799060, EPI_ISL_799062, EPI_ISL_799063, EPI_ISL_799064, EPI_ISL_799065, EPI_ISL_799066, EPI_ISL_799067, EPI_ISL_799068, EPI_ISL_799071, EPI_ISL_799072, EPI_ISL_799073, EPI_ISL_799075, EPI_ISL_799077, EPI_ISL_799080, EPI_ISL_799081, EPI_ISL_799082, EPI_ISL_799084, EPI_ISL_799089, EPI_ISL_799090, EPI_ISL_799096, EPI_ISL_799098, EPI_ISL_799100, EPI_ISL_799101, EPI_ISL_799102, EPI_ISL_799107, EPI_ISL_799108, EPI_ISL_799112, EPI_ISL_799115, EPI_ISL_799118, EPI_ISL_799120, EPI_ISL_799123, EPI_ISL_799125, EPI_ISL_799126, EPI_ISL_799127, EPI_ISL_799129, EPI_ISL_799130, EPI_ISL_799131, EPI_ISL_799132, EPI_ISL_799134, EPI_ISL_799140, EPI_ISL_799144, EPI_ISL_799147, EPI_ISL_799150, EPI_ISL_799151, EPI_ISL_799155, EPI_ISL_799156, EPI_ISL_799157, EPI_ISL_799158, EPI_ISL_799162, EPI_ISL_799163, EPI_ISL_799164, EPI_ISL_799167, EPI_ISL_799169, EPI_ISL_799172, EPI_ISL_799175, EPI_ISL_799176, EPI_ISL_799180, EPI_ISL_799183, EPI_ISL_799189, EPI_ISL_799190, EPI_ISL_799192, EPI_ISL_799193, EPI_ISL_799194, EPI_ISL_799196, EPI_ISL_799197, EPI_ISL_799201, EPI_ISL_799203, EPI_ISL_799204, EPI_ISL_799205, EPI_ISL_799207, EPI_ISL_799209, EPI_ISL_799215, EPI_ISL_799216, EPI_ISL_799218, EPI_ISL_799221, EPI_ISL_799222, EPI_ISL_799224, EPI_ISL_799230, EPI_ISL_799232, EPI_ISL_799236, EPI_ISL_799240, EPI_ISL_799243, EPI_ISL_799245, EPI_ISL_799246, EPI_ISL_799249, EPI_ISL_799250, EPI_ISL_799255, EPI_ISL_799256, EPI_ISL_799258, EPI_ISL_799260, EPI_ISL_799262, EPI_ISL_799263, EPI_ISL_799264, EPI_ISL_799267, EPI_ISL_799268, EPI_ISL_799270, EPI_ISL_799272, EPI_ISL_799273, EPI_ISL_799274, EPI_ISL_799275, EPI_ISL_799277, EPI_ISL_799279, EPI_ISL_799280, EPI_ISL_799282, EPI_ISL_799283, EPI_ISL_799285, EPI_ISL_799286, EPI_ISL_799287, EPI_ISL_799288, EPI_ISL_799289, EPI_ISL_799290, EPI_ISL_799294			
see above	Lighthouse Lab in Glasgow	Wellcome Sanger Institute for the COVID-19 Genomics UK (COG-UK) Consortium	Harper VanSteenhouse, Yumi Kasai, David Gray, Carol Clugston, Anna Dominiczak and Alex Alderton, Roberto Amato, Sonia Goncalves, Ewan Harrison, David K. Jackson, Ian Johnston, Dominic Kwiatkowski, Cordelia Langford, John Sillitoe on behalf of the Wellcome Sanger Institute COVID-19 Surveillance

			Team
EPI_ISL_801412, EPI_ISL_801437, EPI_ISL_801511	Dutch COVID-19 response team	Erasmus Medical Center	Bas Oude Munnink, Reina Sikkema, David Nieuwenhuijse, Irina Chestakova, Anne van der Linden, Marjan Boter, Emmanuelle Munger, Corine GeurtsvanKessel, Annemiek van der Eijk, Richard Molenkamp, Marion Koopmans, on behalf of the Dutch national COVID-19 response team.
EPI_ISL_801517, EPI_ISL_801518	Instituto Nacional de Saude (INSA)	Instituto Nacional de Saude (INSA)	Borges et al
EPI_ISL_802429, EPI_ISL_802431	Wadsworth Center, New York State Department.of Health	Wadsworth Center, New York State Department.of Health	Kirsten St. George, Daryl M. Lamson, Alexis Russel, Matthew Shudt, Melissa A Leisner, Jonathan Plitnick, Navjot Singh, John Kelly, Sara Griesemer, Erasmus Schneider, Erica Lasek-Nesselquist
EPI_ISL_802434, EPI_ISL_802435	WESTCHESTER MEDICAL CENTER	Wadsworth Center, New York State Department.of Health	Kirsten St. George, Daryl M. Lamson, Alexis Russel, Matthew Shudt, Melissa A Leisner, Jonathan Plitnick, Navjot Singh, John Kelly, Sara Griesemer, Erasmus Schneider, Erica Lasek-Nesselquist
EPI_ISL_802439	SARATOGA HOSPITAL LABORATORY	Wadsworth Center, New York State Department.of Health	Kirsten St. George, Daryl M. Lamson, Alexis Russel, Matthew Shudt, Melissa A Leisner, Jonathan Plitnick, Navjot Singh, John Kelly, Sara Griesemer, Erasmus Schneider, Erica Lasek-Nesselquist
EPI_ISL_802440, EPI_ISL_802441	WESTCHESTER MEDICAL CENTER	Wadsworth Center, New York State Department.of Health	Kirsten St. George, Daryl M. Lamson, Alexis Russel, Matthew Shudt, Melissa A Leisner, Jonathan Plitnick, Navjot Singh, John Kelly, Sara Griesemer, Erasmus Schneider, Erica Lasek-Nesselquist
EPI_ISL_802442	Wadsworth Center, New York State Department.of Health	Wadsworth Center, New York State Department.of Health	Kirsten St. George, Daryl M. Lamson, Alexis Russel, Matthew Shudt, Melissa A Leisner, Jonathan Plitnick, Navjot Singh, John Kelly, Sara Griesemer, Erasmus Schneider, Erica Lasek-Nesselquist
EPI_ISL_802443	WESTCHESTER MEDICAL CENTER	Wadsworth Center, New York State Department.of Health	Kirsten St. George, Daryl M. Lamson, Alexis Russel, Matthew Shudt, Melissa A Leisner, Jonathan Plitnick, Navjot Singh, John Kelly, Sara Griesemer, Erasmus Schneider, Erica Lasek-Nesselquist
EPI_ISL_802447	BIO-REFERENCE LABORATORIES	Wadsworth Center, New York State Department.of Health	Kirsten St. George, Daryl M. Lamson, Alexis Russel, Matthew Shudt, Melissa A Leisner, Jonathan Plitnick, Navjot Singh, John Kelly, Sara Griesemer, Erasmus Schneider, Erica Lasek-Nesselquist
EPI_ISL_802448	WESTCHESTER MEDICAL CENTER	Wadsworth Center, New York State Department.of Health	Kirsten St. George, Daryl M. Lamson, Alexis Russel, Matthew Shudt, Melissa A Leisner, Jonathan Plitnick, Navjot Singh, John Kelly, Sara Griesemer, Erasmus Schneider, Erica Lasek-Nesselquist
EPI_ISL_802463, EPI_ISL_802464, EPI_ISL_802465, EPI_ISL_802466	Wadsworth Center, New York State Department.of Health	Wadsworth Center, New York State Department.of Health	Kirsten St. George, Daryl M. Lamson, Alexis Russel, Matthew Shudt, Melissa A Leisner, Jonathan Plitnick, Navjot Singh, John Kelly, Sara Griesemer, Erasmus Schneider, Erica Lasek-Nesselquist
EPI_ISL_802467, EPI_ISL_802468	SARATOGA HOSPITAL LABORATORY	Wadsworth Center, New York State Department.of Health	Kirsten St. George, Daryl M. Lamson, Alexis Russel, Matthew Shudt, Melissa A Leisner, Jonathan Plitnick, Navjot Singh, John Kelly, Sara Griesemer, Erasmus Schneider, Erica Lasek-Nesselquist
EPI_ISL_802469, EPI_ISL_802470, EPI_ISL_802471, EPI_ISL_802472, EPI_ISL_802473, EPI_ISL_802474, EPI_ISL_802475, EPI_ISL_802476, EPI_ISL_802477, EPI_ISL_802478, EPI_ISL_802479, EPI_ISL_802480, EPI_ISL_802481, EPI_ISL_802482, EPI_ISL_802483, EPI_ISL_802484, EPI_ISL_802485, EPI_ISL_802486, EPI_ISL_802487, EPI_ISL_802488, EPI_ISL_802489, EPI_ISL_802490			
see above	WESTCHESTER MEDICAL CENTER	Wadsworth Center, New York State Department.of Health	Kirsten St. George, Daryl M. Lamson, Alexis Russel, Matthew Shudt, Melissa A Leisner, Jonathan Plitnick, Navjot Singh, John Kelly, Sara Griesemer, Erasmus Schneider, Erica Lasek-Nesselquist
EPI_ISL_802506, EPI_ISL_802509, EPI_ISL_802510, EPI_ISL_802511, EPI_ISL_802517, EPI_ISL_802518, EPI_ISL_802519, EPI_ISL_802526, EPI_ISL_802527, EPI_ISL_802528, EPI_ISL_802529, EPI_ISL_802530, EPI_ISL_802536, EPI_ISL_802542			
see above	Dutch COVID-19 response team	Erasmus Medical Center	Bas Oude Munnink, Reina Sikkema, David Nieuwenhuijse, Irina Chestakova, Anne van der Linden, Marjan Boter, Emmanuelle Munger, Corine GeurtsvanKessel, Annemiek van der Eijk, Richard Molenkamp, Marion Koopmans, on behalf of the Dutch national COVID-19 response team.
EPI_ISL_802599, EPI_ISL_802601, EPI_ISL_802604, EPI_ISL_802606, EPI_ISL_802607, EPI_ISL_802618, EPI_ISL_802619, EPI_ISL_802620, EPI_ISL_802621, EPI_ISL_802622, EPI_ISL_802623, EPI_ISL_802624, EPI_ISL_802625, EPI_ISL_802626, EPI_ISL_802627, EPI_ISL_802628, EPI_ISL_802641, EPI_ISL_802642, EPI_ISL_802643, EPI_ISL_802644, EPI_ISL_802645, EPI_ISL_802646, EPI_ISL_802649, EPI_ISL_802652, EPI_ISL_802662, EPI_ISL_802663, EPI_ISL_802664, EPI_ISL_802665, EPI_ISL_802666, EPI_ISL_802667, EPI_ISL_802668, EPI_ISL_802682, EPI_ISL_802683, EPI_ISL_802687, EPI_ISL_802689, EPI_ISL_802691, EPI_ISL_802692, EPI_ISL_802693, EPI_ISL_802694, EPI_ISL_802695, EPI_ISL_802696, EPI_ISL_802711, EPI_ISL_802712, EPI_ISL_802713, EPI_ISL_802715, EPI_ISL_802716, EPI_ISL_802717			
see above	Helix/Illumina	Genomics and Discovery, Respiratory Viruses Branch, Division of Viral Diseases, Centers for Disease Control and Prevention	Peter W. Cook, Dhwani Batra, Ben L. Rambo-Martin Eileen de Feo, Jan Antico, Christine Tran, Matthew Tolentino, Shannon Wickline, Kim Gietzen, Brad Sickler, Jingtao Liu, Eric Allen, Phil Febbo, Summer Galloway, Nicole L. Washington, Simon White, Geraint Levan, Kelly Schiabor Barrett, Elizabeth Cirulli, Alexandre Bolze, Ary Ascencio, Charlotte Rivera-Garcia, Ryan Cho, Jason Nguyen, Sherry Wang, Jimmy Ramirez, Tyler Cassens, Efren Sandoval, Magnus Isaksson, William Lee, David Becker, Marc Laurent, James Lu, Clinton R. Paden, Suxiang Tong, Duncan MacCannell
EPI_ISL_802725	Florida Bureau of Public Health Laboratories	Florida Bureau of Public Health Laboratories	Sarah Schmedes, Jason Blanton
EPI_ISL_802790, EPI_ISL_802791, EPI_ISL_802792, EPI_ISL_802793	BIO-REFERENCE LABORATORIES	Wadsworth Center, New York State Department.of Health	Kirsten St. George, Daryl M. Lamson, Alexis Russel, Matthew Shudt, Melissa A Leisner, Jonathan Plitnick, Navjot Singh, John Kelly, Sara Griesemer, Erasmus Schneider, Erica Lasek-Nesselquist
EPI_ISL_803855	Hospital General Universitario Gregorio Marañón	Hospital General Universitario Gregorio Marañón	Sergio Buenestado Serrano, Pedro Sola Campoy, Laura Pérez-Lago, Pilar Catalán, Patricia Muñoz, Darío García de Viedma
EPI_ISL_803856, EPI_ISL_803857, EPI_ISL_803858, EPI_ISL_803859, EPI_ISL_803860, EPI_ISL_803861, EPI_ISL_803862, EPI_ISL_803863, EPI_ISL_803864	Hospital General Universitario Gregorio Marañón	Hospital General Universitario Gregorio Marañón	Sergio Buenestado Serrano, Pedro Sola Campoy, Laura Pérez-Lago, Pilar Catalán, Patricia Muñoz, Darío García de Viedma.
EPI_ISL_803959, EPI_ISL_803960, EPI_ISL_803961, EPI_ISL_803966, EPI_ISL_803969, EPI_ISL_803970, EPI_ISL_803971, EPI_ISL_803972, EPI_ISL_803973, EPI_ISL_803974, EPI_ISL_803975, EPI_ISL_803976, EPI_ISL_803977, EPI_ISL_803978, EPI_ISL_803979, EPI_ISL_803980, EPI_ISL_803981, EPI_ISL_803982, EPI_ISL_803983			
see above	National Public Health Laboratory, National Centre for Infectious Diseases	National Public Health Laboratory, National Centre for Infectious Diseases	Tze Minn Mak, Sophie Octavia, Zhenyang Zhou, Lin Cui, Raymond Tzer Pin Lin
EPI_ISL_804026, EPI_ISL_804029, EPI_ISL_804030, EPI_ISL_804031, EPI_ISL_804032, EPI_ISL_804033, EPI_ISL_804034, EPI_ISL_804035, EPI_ISL_804036	Maryland Public Health Laboratory	Maryland Public Health Laboratory	Maryland Department of Health Laboratories Administration
EPI_ISL_804040	SC (UCO) Igiene e Sanità Pubblica (funzione integrata con SC Microbiologia e Virologia) e Laboratory of Molecular Virology of the International Centre for Genetic Engineering and Biotechnology (ICGEB)	ARGO Laboratorio Genomica ed Epigenomica	Licastro D, Dal Monego S, Degasperi M, Marcello A, D'Agaro P
EPI_ISL_804057, EPI_ISL_804058, EPI_ISL_804059, EPI_ISL_804060, EPI_ISL_804061, EPI_ISL_804062, EPI_ISL_804063, EPI_ISL_804064, EPI_ISL_804065, EPI_ISL_804066, EPI_ISL_804067, EPI_ISL_804068, EPI_ISL_804069, EPI_ISL_804070, EPI_ISL_804085, EPI_ISL_804086, EPI_ISL_804087, EPI_ISL_804088, EPI_ISL_804089, EPI_ISL_804090, EPI_ISL_804091, EPI_ISL_804095, EPI_ISL_804097, EPI_ISL_804099, EPI_ISL_804100, EPI_ISL_804101, EPI_ISL_804105, EPI_ISL_804107, EPI_ISL_804108, EPI_ISL_804109, EPI_ISL_804110, EPI_ISL_804111, EPI_ISL_804112, EPI_ISL_804113, EPI_ISL_804114, EPI_ISL_804115, EPI_ISL_804116, EPI_ISL_804117, EPI_ISL_804118, EPI_ISL_804119, EPI_ISL_804120, EPI_ISL_804121, EPI_ISL_804122, EPI_ISL_804123, EPI_ISL_804124, EPI_ISL_804125, EPI_ISL_804126, EPI_ISL_804127, EPI_ISL_804128, EPI_ISL_804129, EPI_ISL_804130, EPI_ISL_804131, EPI_ISL_804132, EPI_ISL_804133, EPI_ISL_804134, EPI_ISL_804135, EPI_ISL_804136, EPI_ISL_804137, EPI_ISL_804138, EPI_ISL_804139, EPI_ISL_804140, EPI_ISL_804141, EPI_ISL_804189, EPI_ISL_804190, EPI_ISL_804192, EPI_ISL_804193, EPI_ISL_804194, EPI_ISL_804195, EPI_ISL_804196, EPI_ISL_804197, EPI_ISL_804198, EPI_ISL_804199, EPI_ISL_804200, EPI_ISL_804201, EPI_ISL_804202, EPI_ISL_804203, EPI_ISL_804204, EPI_ISL_804205, EPI_ISL_804206, EPI_ISL_804207, EPI_ISL_804208, EPI_ISL_804213			
see above	Israel Central Virology laboratory	Israel National Consortium for SARS-CoV-2 sequencing	Neta Zuckerman, Efrat Dahan Bucris, Michal Mandelboim, Dana Bar-Ilan, Oran Erster, Tzvia Mann, Omer Murik, David A. Zeevi, Assaf Rokney, Joseph Jaffe, Eva Nachum, Maya Davidovich Cohen, Ephraim Fass, Gal Zizelski Valenci, Mor Rubinstein, Efrat Rorman, Israel Nissan, Efrat Glick-Saar, Omri Nayshool, Gideon Rechavi, Ella Mendelson, Orna Mor
EPI_ISL_804222, EPI_ISL_804224, EPI_ISL_804242, EPI_ISL_804243, EPI_ISL_804244, EPI_ISL_804245, EPI_ISL_804246, EPI_ISL_804247, EPI_ISL_804248, EPI_ISL_804251, EPI_ISL_804252, EPI_ISL_804260, EPI_ISL_804280, EPI_ISL_804281, EPI_ISL_804282, EPI_ISL_804283, EPI_ISL_804284, EPI_ISL_804285, EPI_ISL_804286, EPI_ISL_804287, EPI_ISL_804288, EPI_ISL_804289, EPI_ISL_804290, EPI_ISL_804291, EPI_ISL_804292, EPI_ISL_804293			
see above	Respiratory Virus Unit, National Infection Service, Public Health England	COVID-19 Genomics UK (COG-UK) Consortium	PHE Covid Sequencing Team

EPI_ISL_804463, EPI_ISL_804464, EPI_ISL_804465	Maryland Public Health Laboratory	Maryland Public Health Laboratory	Maryland Department of Health Laboratories Administration
EPI_ISL_804982, EPI_ISL_804983, EPI_ISL_804984	BIO-REFERENCE LABORATORIES	Wadsworth Center, New York State Department of Health	Kirsten St. George, Daryl M. Lamson, Alexis Russel, Matthew Shudt, Melissa A Leisner, Jonathan Plitnick, Navjot Singh, John Kelly, Erasmus Schneider, Erica Lasek-Nesselquist
EPI_ISL_806723	4Cyte Pathology	NSW Health Pathology - Institute of Clinical Pathology and Medical Research; Westmead Hospital; University of Sydney	CIDM-PH et al.
EPI_ISL_806724	Sydney South West Pathology Service (SSWPS) - Royal Prince Alfred Hospital - NSW Health Pathology	NSW Health Pathology - Institute of Clinical Pathology and Medical Research; Westmead Hospital; University of Sydney	CIDM-PH et al.
EPI_ISL_806727	4Cyte Pathology	NSW Health Pathology - Institute of Clinical Pathology and Medical Research; Westmead Hospital; University of Sydney	CIDM-PH et al.
EPI_ISL_811040, EPI_ISL_811041, EPI_ISL_811042, EPI_ISL_811043, EPI_ISL_811044, EPI_ISL_811045, EPI_ISL_811046, EPI_ISL_811047, EPI_ISL_811048, EPI_ISL_811049, EPI_ISL_811050, EPI_ISL_811051, EPI_ISL_811052, EPI_ISL_811053, EPI_ISL_811054, EPI_ISL_811055, EPI_ISL_811056, EPI_ISL_811057, EPI_ISL_811058, EPI_ISL_811059, EPI_ISL_811060, EPI_ISL_811061, EPI_ISL_811062, EPI_ISL_811063, EPI_ISL_811064, EPI_ISL_811065, EPI_ISL_811066, EPI_ISL_811067, EPI_ISL_811068, EPI_ISL_811069, EPI_ISL_811070, EPI_ISL_811071, EPI_ISL_811073, EPI_ISL_811074, EPI_ISL_811075, EPI_ISL_811076, EPI_ISL_811077, EPI_ISL_811078, EPI_ISL_811079, EPI_ISL_811080, EPI_ISL_811081, EPI_ISL_811082, EPI_ISL_811083, EPI_ISL_811084, EPI_ISL_811085, EPI_ISL_811086, EPI_ISL_811087, EPI_ISL_811088, EPI_ISL_811089, EPI_ISL_811090, EPI_ISL_811091, EPI_ISL_811092, EPI_ISL_811093, EPI_ISL_811094, EPI_ISL_811095, EPI_ISL_811096, EPI_ISL_811097, EPI_ISL_811098, EPI_ISL_811099, EPI_ISL_811100, EPI_ISL_811101, EPI_ISL_811102, EPI_ISL_811103, EPI_ISL_811104, EPI_ISL_811105, EPI_ISL_811115, EPI_ISL_811117, EPI_ISL_811118, EPI_ISL_811119			
see above	Respiratory Virus Unit, National Infection Service, Public Health England	COVID-19 Genomics UK (COG-UK) Consortium	PHE Covid Sequencing Team
EPI_ISL_811137, EPI_ISL_811138, EPI_ISL_811139, EPI_ISL_811143	Ministry of Health Turkey	Ministry of Health Turkey	Fatma Bayrakdar, Yasemin Cogun, Süleyman Yalcin, Aye Baak Alta, Gülay Korukluolu
EPI_ISL_811149	Laboratorio de Ecologia de Doencas Transmissiveis na Amazonia, Instituto Leonidas e Maria Deane - Fiocruz Amazonia	Laboratorio de Ecologia de Doencas Transmissiveis na Amazonia, Instituto Leonidas e Maria Deane - Fiocruz Amazonia	Valdinete Nascimento, Victor Souza, André Corado, Fernanda Nascimento, George Silva, Ágatha Costa, Debora Duarte, Luciana Gonçalves, Matilde Mejía, Karina Pessoa, Maria Júlia Brandão, Michele Jesus, Felipe Naveca
EPI_ISL_811214, EPI_ISL_811234, EPI_ISL_811236, EPI_ISL_811241, EPI_ISL_811247, EPI_ISL_811275, EPI_ISL_811293, EPI_ISL_811310, EPI_ISL_811312, EPI_ISL_811321, EPI_ISL_811345, EPI_ISL_811350, EPI_ISL_811353, EPI_ISL_811366, EPI_ISL_811377, EPI_ISL_811414, EPI_ISL_811422, EPI_ISL_811472, EPI_ISL_811490, EPI_ISL_811494, EPI_ISL_811500, EPI_ISL_811516, EPI_ISL_811519, EPI_ISL_811524, EPI_ISL_811530, EPI_ISL_811548, EPI_ISL_811563, EPI_ISL_811573, EPI_ISL_811580, EPI_ISL_811584, EPI_ISL_811594, EPI_ISL_811617, EPI_ISL_811618, EPI_ISL_811620, EPI_ISL_811621, EPI_ISL_811627, EPI_ISL_811645, EPI_ISL_811650, EPI_ISL_811651, EPI_ISL_811661, EPI_ISL_811669, EPI_ISL_811670, EPI_ISL_811671, EPI_ISL_811677, EPI_ISL_811678, EPI_ISL_811682, EPI_ISL_811688			
see above	Lighthouse Lab in Milton Keynes	Wellcome Sanger Institute for the COVID-19 Genomics UK (COG-UK) Consortium	The Lighthouse Lab in Milton Keynes and Alex Alderton, Roberto Amato, Sonia Goncalves, Ewan Harrison, David K. Jackson, Ian Johnston, Dominic Kwiatkowski, Cordelia Langford, John Sillitoe on behalf of the Wellcome Sanger Institute COVID-19 Surveillance Team
EPI_ISL_811713	Lighthouse Lab in Cambridge	Wellcome Sanger Institute for the COVID-19 Genomics UK (COG-UK) Consortium	Rob Howes, The Lighthouse Lab in Cambridge and Alex Alderton, Roberto Amato, Sonia Goncalves, Ewan Harrison, David K. Jackson, Ian Johnston, Dominic Kwiatkowski, Cordelia Langford, John Sillitoe on behalf of the Wellcome Sanger Institute COVID-19 Surveillance Team
EPI_ISL_811720, EPI_ISL_811727, EPI_ISL_811750, EPI_ISL_811753, EPI_ISL_811756, EPI_ISL_811759, EPI_ISL_811767, EPI_ISL_811785, EPI_ISL_811808, EPI_ISL_811811, EPI_ISL_811812, EPI_ISL_811814, EPI_ISL_811817, EPI_ISL_811818, EPI_ISL_811819, EPI_ISL_811821, EPI_ISL_811823, EPI_ISL_811824, EPI_ISL_811825, EPI_ISL_811826, EPI_ISL_811827, EPI_ISL_811828, EPI_ISL_811830, EPI_ISL_811831, EPI_ISL_811832, EPI_ISL_811833, EPI_ISL_811834, EPI_ISL_811835, EPI_ISL_811836, EPI_ISL_811837, EPI_ISL_811839, EPI_ISL_811840, EPI_ISL_811841, EPI_ISL_811843, EPI_ISL_811845, EPI_ISL_811846, EPI_ISL_811847, EPI_ISL_811848, EPI_ISL_811849, EPI_ISL_811850, EPI_ISL_811851, EPI_ISL_811852, EPI_ISL_811853, EPI_ISL_811854, EPI_ISL_811855, EPI_ISL_811856, EPI_ISL_811857, EPI_ISL_811858, EPI_ISL_811859, EPI_ISL_811860, EPI_ISL_811861, EPI_ISL_811862, EPI_ISL_811865, EPI_ISL_811867, EPI_ISL_811868, EPI_ISL_811869, EPI_ISL_811870, EPI_ISL_811871, EPI_ISL_811873, EPI_ISL_811874, EPI_ISL_811875, EPI_ISL_811877, EPI_ISL_811878, EPI_ISL_811879, EPI_ISL_811880, EPI_ISL_811881, EPI_ISL_811883, EPI_ISL_811884, EPI_ISL_811885, EPI_ISL_811886, EPI_ISL_811887, EPI_ISL_811888, EPI_ISL_811889, EPI_ISL_811890, EPI_ISL_811891, EPI_ISL_811892, EPI_ISL_811893, EPI_ISL_811894, EPI_ISL_811896, EPI_ISL_811897, EPI_ISL_811898, EPI_ISL_811899, EPI_ISL_811900, EPI_ISL_811902, EPI_ISL_811905, EPI_ISL_811907, EPI_ISL_811909, EPI_ISL_811910, EPI_ISL_811912, EPI_ISL_811913, EPI_ISL_811914, EPI_ISL_811915, EPI_ISL_811916, EPI_ISL_811917, EPI_ISL_811918, EPI_ISL_811919, EPI_ISL_811921, EPI_ISL_811922, EPI_ISL_811923, EPI_ISL_811924, EPI_ISL_811925, EPI_ISL_811927, EPI_ISL_811928, EPI_ISL_811929, EPI_ISL_811930, EPI_ISL_811931, EPI_ISL_811932, EPI_ISL_811935, EPI_ISL_811936, EPI_ISL_811937, EPI_ISL_811938, EPI_ISL_811939, EPI_ISL_811941, EPI_ISL_811942, EPI_ISL_811943, EPI_ISL_811944, EPI_ISL_811945, EPI_ISL_811946, EPI_ISL_811948, EPI_ISL_811949, EPI_ISL_811950, EPI_ISL_811951, EPI_ISL_811952, EPI_ISL_811953, EPI_ISL_811954, EPI_ISL_811955, EPI_ISL_811956, EPI_ISL_811957, EPI_ISL_811958, EPI_ISL_811959, EPI_ISL_811960, EPI_ISL_811962, EPI_ISL_811965, EPI_ISL_811967, EPI_ISL_811968, EPI_ISL_811969, EPI_ISL_811970, EPI_ISL_811971, EPI_ISL_811972, EPI_ISL_811973, EPI_ISL_811974, EPI_ISL_811975, EPI_ISL_811976, EPI_ISL_811977, EPI_ISL_811978, EPI_ISL_811979, EPI_ISL_811981, EPI_ISL_811983, EPI_ISL_811984, EPI_ISL_811985, EPI_ISL_811986, EPI_ISL_811987, EPI_ISL_811988, EPI_ISL_811989, EPI_ISL_811990, EPI_ISL_811991, EPI_ISL_811993, EPI_ISL_811994, EPI_ISL_811996, EPI_ISL_811997, EPI_ISL_811998, EPI_ISL_811999, EPI_ISL_812000, EPI_ISL_812002, EPI_ISL_812003, EPI_ISL_812007, EPI_ISL_812009, EPI_ISL_812010, EPI_ISL_812012, EPI_ISL_812014, EPI_ISL_812015, EPI_ISL_812017, EPI_ISL_812018, EPI_ISL_812019, EPI_ISL_812020, EPI_ISL_812021, EPI_ISL_812022, EPI_ISL_812024, EPI_ISL_812025, EPI_ISL_812026, EPI_ISL_812027, EPI_ISL_812028, EPI_ISL_812030, EPI_ISL_812033, EPI_ISL_812035, EPI_ISL_812036, EPI_ISL_812037, EPI_ISL_812038, EPI_ISL_812040, EPI_ISL_812041, EPI_ISL_812042, EPI_ISL_812045, EPI_ISL_812046, EPI_ISL_812047, EPI_ISL_812048, EPI_ISL_812049, EPI_ISL_812050, EPI_ISL_812051, EPI_ISL_812052, EPI_ISL_812054, EPI_ISL_812055, EPI_ISL_812058, EPI_ISL_812059, EPI_ISL_812061, EPI_ISL_812062, EPI_ISL_812063, EPI_ISL_812064, EPI_ISL_812066, EPI_ISL_812067, EPI_ISL_812068, EPI_ISL_812069, EPI_ISL_812070, EPI_ISL_812073, EPI_ISL_812074, EPI_ISL_812075, EPI_ISL_812076, EPI_ISL_812077, EPI_ISL_812078, EPI_ISL_812079, EPI_ISL_812080, EPI_ISL_812083, EPI_ISL_812084, EPI_ISL_812085, EPI_ISL_812086, EPI_ISL_812088, EPI_ISL_812090, EPI_ISL_812091, EPI_ISL_812092, EPI_ISL_812093, EPI_ISL_812094, EPI_ISL_812096, EPI_ISL_812097, EPI_ISL_812098, EPI_ISL_812099, EPI_ISL_812100, EPI_ISL_812101, EPI_ISL_812102, EPI_ISL_812103, EPI_ISL_812104, EPI_ISL_812105, EPI_ISL_812106, EPI_ISL_812109, EPI_ISL_812113			
see above	Lighthouse Lab in Milton Keynes	Wellcome Sanger Institute for the COVID-19 Genomics UK (COG-UK) Consortium	The Lighthouse Lab in Milton Keynes and Alex Alderton, Roberto Amato, Sonia Goncalves, Ewan Harrison, David K. Jackson, Ian Johnston, Dominic Kwiatkowski, Cordelia Langford, John Sillitoe on behalf of the Wellcome Sanger Institute COVID-19 Surveillance Team
EPI_ISL_812250	Invenimus AG	Institute of Medical Virology, University of Zurich	Stefan Schmutz, Maryam Zaheri, Verena Kufner, Annette Audigé, Maria Grünberg, Kevin Steiner, Jon Huder, Cyril Shah, Riccarda Capaul, Guido Bloemberg, Jürg Böni, Michael Huber, Alexandra Trkola
EPI_ISL_812348	University of Wisconsin-Madison AIDS Vaccine Research Laboratories	University of Wisconsin-Madison AIDS Vaccine Research Laboratories	Gage Moreno, Katarina Braun, et al. AIDS Vaccine Research Laboratories
EPI_ISL_812424, EPI_ISL_812425, EPI_ISL_812426, EPI_ISL_812427, EPI_ISL_812428, EPI_ISL_812429, EPI_ISL_812430, EPI_ISL_812431, EPI_ISL_812432, EPI_ISL_812433	Microbiological Diagnostic Unit - Public Health Laboratory (MDU-PHL)	MDU-PHL	Seemann T., Sait, M.L., Sherry, N.L.
EPI_ISL_812437	Victorian Infectious Diseases Reference Laboratory (VIDRL)	VIDRL and MDU-PHL	Caly L., Seemann T., Sait, M.L., Druce J., Sherry, N.L.
EPI_ISL_812768, EPI_ISL_812769, EPI_ISL_812770, EPI_ISL_812771, EPI_ISL_812777, EPI_ISL_812779, EPI_ISL_812780, EPI_ISL_812781, EPI_ISL_812874, EPI_ISL_812875, EPI_ISL_812876			
see above	Ministry of Health Turkey	Ministry of Health Turkey	Fatma Bayrakdar, Yasemin Cogun, Süleyman Yalcin, Aye Baak Alta, Gülay Korukluolu
EPI_ISL_813091, EPI_ISL_813092, EPI_ISL_813093, EPI_ISL_813116	University of Birmingham	COVID-19 Genomics UK (COG-UK) Consortium	Institute of Microbiology, University of Birmingham: Claire McMurray, Joanne Stockton, Samuel Nicholls, Radoslaw Poplawski, Will Rowe, Josh Quick, Nicholas Loman. University of Birmingham Testing Laboratory: Celina M Whalley, Andrew Bosworth, Charlotte Poxon, Kasun Wanigasooriya, Oliver Pickles, Mike Kidd, Alex Richter, Andrew D Beggs PHE Heartlands Lab: Husam Osman, Andrew Bosworth. Queen Elizabeth Hospital: Anna Casey
EPI_ISL_813198, EPI_ISL_813201, EPI_ISL_813202, EPI_ISL_813203, EPI_ISL_813204, EPI_ISL_813205, EPI_ISL_813206, EPI_ISL_813207, EPI_ISL_813209, EPI_ISL_813210, EPI_ISL_813211, EPI_ISL_813212, EPI_ISL_813213, EPI_ISL_813214, EPI_ISL_813215, EPI_ISL_813216, EPI_ISL_813217, EPI_ISL_813219, EPI_ISL_813220, EPI_ISL_813221, EPI_ISL_813222, EPI_ISL_813223, EPI_ISL_813224			
see above	Department of Pathology, University of Cambridge	COVID-19 Genomics UK (COG-UK) Consortium	Aminu S. Jahun, Yasmin Chaudhry, Grant Hall, Iliana Georgana, Myra Hosmillo, Martin D. Curran, Malte Pinckert, Surendra Parmar, Ian Goodfellow
EPI_ISL_813608, EPI_ISL_813623, EPI_ISL_813625, EPI_ISL_813626, EPI_ISL_813627, EPI_ISL_813628, EPI_ISL_813629, EPI_ISL_813630, EPI_ISL_813631, EPI_ISL_813632, EPI_ISL_813633, EPI_ISL_813634, EPI_ISL_813635, EPI_ISL_813636, EPI_ISL_813638, EPI_ISL_813639, EPI_ISL_813640, EPI_ISL_813641, EPI_ISL_813642, EPI_ISL_813643, EPI_ISL_813644, EPI_ISL_813645, EPI_ISL_813646, EPI_ISL_813647, EPI_ISL_813648, EPI_ISL_813649, EPI_ISL_813650, EPI_ISL_813651, EPI_ISL_813652, EPI_ISL_813653, EPI_ISL_813654			
see above	University of Exeter	COVID-19 Genomics UK (COG-UK) Consortium	Ben Temperton,Aaron Jeffries,Michelle Michelsen,Joanna Warwick-Dugdale,Audrey Farbos,Robyn Manley,Stephen Michell,Jane Masoli
EPI_ISL_813810, EPI_ISL_813811, EPI_ISL_813812, EPI_ISL_813813, EPI_ISL_813814, EPI_ISL_813815	Liverpool Clinical Laboratories	COVID-19 Genomics UK (COG-UK) Consortium	Sam Haldenby, Anita Lucaci, Steve Paterson, Julian Hiscox, Alistair Darby, M Almsaud, A Alrezaihi, Muhannad Alruwaili, Stuart D Armstrong, Jones Benjamin, Eleanor G Bentley, Anu Chawla, Jordan J Clark, Angela Cowell, Richard Eccles, Isabel García-Dorival, Matthew Gemmell, Alessandro Gerada, PKF Gilmore, Richard Gregory, Ximeng Han, Catherine Hartley, Margaret Hughes, Miren Iturriza-Gomara, James Johnson, L Luu, Jenifer Manson, Charlotte Nelson, Elaine O'Toole, Cassie Olateju, Rebekah Penrice-Randal , Lucille Rainbow, N.P Randle, Trevor Ian Robinson, Parul Sharma, Ghada T Shawli, James P Stewart, Neil Swainston, Ecaterina Vamos, Joanne Watts, Mark Whitehead
EPI_ISL_813958	University College London, Great Ormond Street Hospital for Children NHS Foundation Trust, Imperial College Healthcare NHS Trust	COVID-19 Genomics UK (COG-UK) Consortium	Sergi Castellano, Rachel Williams, Mark Kristiansen, Paola Resende Silva, Sunando Roy, Tony Brooks, Helena Tutill, Paola Niola, Patricia Dyal, Charlotte Williams, Leysa Forrest, Yasmin Panchbhaya, Jacqueline Findlay, Samuel Weeks, Julianne Brown, Kathryn Harris, Paul Randell, James Price, Alison Holmes, Judith Breuer
EPI_ISL_813980, EPI_ISL_813981, EPI_ISL_813982, EPI_ISL_813984, EPI_ISL_813985, EPI_ISL_813986, EPI_ISL_813989, EPI_ISL_813990, EPI_ISL_813991, EPI_ISL_813992, EPI_ISL_813994, EPI_ISL_813997, EPI_ISL_813998, EPI_ISL_814001, EPI_ISL_814002, EPI_ISL_814003, EPI_ISL_814004, EPI_ISL_814005,			

EPI_ISL_814006, EPI_ISL_814008, EPI_ISL_814009, EPI_ISL_814015, EPI_ISL_814018, EPI_ISL_814019, EPI_ISL_814020, EPI_ISL_814027, EPI_ISL_814028, EPI_ISL_814029, EPI_ISL_814030, EPI_ISL_814031, EPI_ISL_814032, EPI_ISL_814033, EPI_ISL_814037, EPI_ISL_814038, EPI_ISL_814039, EPI_ISL_814040, EPI_ISL_814051, EPI_ISL_814052, EPI_ISL_814053, EPI_ISL_814054, EPI_ISL_814055, EPI_ISL_814056, EPI_ISL_814057, EPI_ISL_814058, EPI_ISL_814059, EPI_ISL_814060, EPI_ISL_814061			
see above	Hospital General Universitario Gregorio Marañón	SeqCOVID-SPAIN consortium/IBV(CSIC)	Darío García de Viedma, Laura Pérez-Lago, Marta Herranz, Jon Sicilia, Julia Suárez, Pilar Catalán, Patricia Muñoz and SeqCOVID-SPAIN consortium
EPI_ISL_814358, EPI_ISL_814429, EPI_ISL_814433, EPI_ISL_814434	Bioinformatics and Biostatistics Lab, Advanced Sequencing Facility	COVID-19 Genomics UK (COG-UK) Consortium	Aengus Stewart,Jerome Nicod,Chelsea Sawyer,Laura Cubitt,Harshil Patel,Margaret Crawford
EPI_ISL_816213, EPI_ISL_816214, EPI_ISL_816215, EPI_ISL_816220, EPI_ISL_816221, EPI_ISL_816222, EPI_ISL_816223	Centre for Enzyme Innovation, University of Portsmouth / Translational Research Laboratory, Portsmouth Hospitals NHS Trust	COVID-19 Genomics UK (COG-UK) Consortium	Angela Beckett,Yann Bourgeois,Garry Scarlett,Sharon Glaysher,Scott Elliott,Kelly Bicknell,Robert Impey,Allyson Lloyd,Sarah Wyllie,Ethan Butcher,Anoop Chauhan,Samuel Robson
EPI_ISL_816226, EPI_ISL_816227, EPI_ISL_816228, EPI_ISL_816229, EPI_ISL_816232, EPI_ISL_816233, EPI_ISL_816235, EPI_ISL_816246, EPI_ISL_816248, EPI_ISL_816256, EPI_ISL_816261, EPI_ISL_816275, EPI_ISL_816276, EPI_ISL_816277, EPI_ISL_816285, EPI_ISL_816291, EPI_ISL_816294, EPI_ISL_816296, EPI_ISL_816297, EPI_ISL_816305, EPI_ISL_816311, EPI_ISL_816314, EPI_ISL_816318, EPI_ISL_816319, EPI_ISL_816323, EPI_ISL_816327, EPI_ISL_816331, EPI_ISL_816348, EPI_ISL_816353, EPI_ISL_816364, EPI_ISL_816368, EPI_ISL_816383, EPI_ISL_816390, EPI_ISL_816396, EPI_ISL_816405, EPI_ISL_816406, EPI_ISL_816407, EPI_ISL_816409, EPI_ISL_816415, EPI_ISL_816423, EPI_ISL_816426, EPI_ISL_816427, EPI_ISL_816431, EPI_ISL_816434, EPI_ISL_816437, EPI_ISL_816438, EPI_ISL_816441, EPI_ISL_816442, EPI_ISL_816444, EPI_ISL_816448, EPI_ISL_816449, EPI_ISL_816462, EPI_ISL_816469, EPI_ISL_816472, EPI_ISL_816475, EPI_ISL_816477, EPI_ISL_816492, EPI_ISL_816494, EPI_ISL_816495, EPI_ISL_816496, EPI_ISL_816501, EPI_ISL_816508, EPI_ISL_816525, EPI_ISL_816536, EPI_ISL_816539, EPI_ISL_816555, EPI_ISL_816561, EPI_ISL_816566, EPI_ISL_816571, EPI_ISL_816576, EPI_ISL_816581, EPI_ISL_816582, EPI_ISL_816584, EPI_ISL_816601, EPI_ISL_816608, EPI_ISL_816611, EPI_ISL_816613, EPI_ISL_816629, EPI_ISL_816633, EPI_ISL_816634, EPI_ISL_816641, EPI_ISL_816642, EPI_ISL_816646			
see above	Virology Department, Sheffield Teaching Hospitals NHS Foundation Trust/Department of Infection, Immunity and Cardiovascular Disease, The Medical School, University of Sheffield	COVID-19 Genomics UK (COG-UK) Consortium	Thushan de Silva, Matthew Parker, Nikki Smith, Adri Angyal, Rebecca Brown, Luke Green, Rachel Tucker, Paul Parsons, Danielle Groves, Katie Johnson, Laura Carrilero, Alex Keeley, Dave Partridge, Matthew Wyles, Benjamin Lindsey, Mehmet Yavuz, Mohammad Raza, Cariad Evans
EPI_ISL_816825, EPI_ISL_816826, EPI_ISL_816827, EPI_ISL_816828, EPI_ISL_816829, EPI_ISL_816830, EPI_ISL_816831, EPI_ISL_816832, EPI_ISL_816833, EPI_ISL_816834, EPI_ISL_816835, EPI_ISL_816836, EPI_ISL_816837, EPI_ISL_816838, EPI_ISL_816839, EPI_ISL_816840, EPI_ISL_816841, EPI_ISL_816842, EPI_ISL_816843, EPI_ISL_816844, EPI_ISL_816845, EPI_ISL_816846, EPI_ISL_816847, EPI_ISL_816848, EPI_ISL_816849, EPI_ISL_816850, EPI_ISL_816851, EPI_ISL_816852, EPI_ISL_816853, EPI_ISL_816854, EPI_ISL_816855, EPI_ISL_816856, EPI_ISL_816857, EPI_ISL_816858, EPI_ISL_816859, EPI_ISL_816860, EPI_ISL_816892, EPI_ISL_816897, EPI_ISL_816898, EPI_ISL_816903, EPI_ISL_816959, EPI_ISL_816964, EPI_ISL_816965, EPI_ISL_816970, EPI_ISL_817039			
see above	Bioinformatics and Biostatistics Lab, Advanced Sequencing Facility	COVID-19 Genomics UK (COG-UK) Consortium	Aengus Stewart,Jerome Nicod,Chelsea Sawyer,Laura Cubitt,Harshil Patel,Margaret Crawford
EPI_ISL_818342	Charité Universitätsmedizin Berlin, Institute of Virology	Charité Universitätsmedizin Berlin, Institute of Virology	Victor M Corman, Julia Schneider, Jörn Beheim-Schwarzbach, Barbara Mühlemann, Talitha Veith, Terry Jones, Christian Drosten
EPI_ISL_819133, EPI_ISL_819135, EPI_ISL_819142, EPI_ISL_819156, EPI_ISL_819157, EPI_ISL_819163, EPI_ISL_819169, EPI_ISL_819171, EPI_ISL_819175, EPI_ISL_819186, EPI_ISL_819187, EPI_ISL_819191, EPI_ISL_819195			
see above	Servicio de Microbiología, Hospital Universitario Son Espases	SeqCOVID-SPAIN consortium/IBV(CSIC)	Carla López-Causapé, Jordi Reina, Antonio Oliver and SeqCOVID-SPAIN consortium
EPI_ISL_819378, EPI_ISL_819387, EPI_ISL_819388, EPI_ISL_819389, EPI_ISL_819390, EPI_ISL_819398, EPI_ISL_819399, EPI_ISL_819400, EPI_ISL_819401, EPI_ISL_819402, EPI_ISL_819403, EPI_ISL_819404, EPI_ISL_819428, EPI_ISL_819431, EPI_ISL_819433, EPI_ISL_819438			
see above	Quadram Institute Bioscience	COVID-19 Genomics UK (COG-UK) Consortium	Dave J. Baker, Gemma L. Kay, Alp Aydin, Thanh Le-Viet, Steven Rudder, Ana P. Tedim, Anastasia Kolyva, Maria Diaz, Leonardo de Oliveira Martins, Nabil-Fareed Alikhan, Lizzie Meadows, Rachael Stanley, Ngozi Elumogo, Muhammed Yasir, Nicholas M. Thomson, Alexander J Trotter, Rachel Gilroy, Samuel Bloomfield, Claire Stuart, Andrew Bell, Reenesh Prakash, Samir Dervisevic, Alison E. Mather, John Wain, Mark Webber, Andrew J. Page, Justin O'Grady
EPI_ISL_820326, EPI_ISL_820328, EPI_ISL_820331, EPI_ISL_820333, EPI_ISL_820335, EPI_ISL_820337, EPI_ISL_820340, EPI_ISL_820343, EPI_ISL_820345, EPI_ISL_820347, EPI_ISL_820350			
see above	University College London, Great Ormond Street Hospital for Children NHS Foundation Trust, Imperial College Healthcare NHS Trust	COVID-19 Genomics UK (COG-UK) Consortium	Sergi Castellano, Rachel Williams, Mark Kristiansen, Paola Resende Silva, Sunando Roy, Tony Brooks, Helena Tutill, Paola Niola, Patricia Dyal, Charlotte Williams, Leysa Forrest, Yasmin Panchbhaya, Jacqueline Findlay, Samuel Weeks, Julianne Brown, Kathryn Harris, Paul Randell, James Price, Alison Holmes, Judith Breuer
EPI_ISL_820353, EPI_ISL_820355, EPI_ISL_820358, EPI_ISL_820360, EPI_ISL_820363, EPI_ISL_820365, EPI_ISL_820368, EPI_ISL_820371	Quadram Institute Bioscience	COVID-19 Genomics UK (COG-UK) Consortium	Dave J. Baker, Gemma L. Kay, Alp Aydin, Thanh Le-Viet, Steven Rudder, Ana P. Tedim, Anastasia Kolyva, Maria Diaz, Leonardo de Oliveira Martins, Nabil-Fareed Alikhan, Lizzie Meadows, Rachael Stanley, Ngozi Elumogo, Muhammed Yasir, Nicholas M. Thomson, Alexander J Trotter, Rachel Gilroy, Samuel Bloomfield, Claire Stuart, Andrew Bell, Reenesh Prakash, Samir Dervisevic, Alison E. Mather, John Wain, Mark Webber, Andrew J. Page, Justin O'Grady
EPI_ISL_820373, EPI_ISL_820376	University College London, Great Ormond Street Hospital for Children NHS Foundation Trust, Imperial College Healthcare NHS Trust	COVID-19 Genomics UK (COG-UK) Consortium	Sergi Castellano, Rachel Williams, Mark Kristiansen, Paola Resende Silva, Sunando Roy, Tony Brooks, Helena Tutill, Paola Niola, Patricia Dyal, Charlotte Williams, Leysa Forrest, Yasmin Panchbhaya, Jacqueline Findlay, Samuel Weeks, Julianne Brown, Kathryn Harris, Paul Randell, James Price, Alison Holmes, Judith Breuer
EPI_ISL_820378	Quadram Institute Bioscience	COVID-19 Genomics UK (COG-UK) Consortium	Dave J. Baker, Gemma L. Kay, Alp Aydin, Thanh Le-Viet, Steven Rudder, Ana P. Tedim, Anastasia Kolyva, Maria Diaz, Leonardo de Oliveira Martins, Nabil-Fareed Alikhan, Lizzie Meadows, Rachael Stanley, Ngozi Elumogo, Muhammed Yasir, Nicholas M. Thomson, Alexander J Trotter, Rachel Gilroy, Samuel Bloomfield, Claire Stuart, Andrew Bell, Reenesh Prakash, Samir Dervisevic, Alison E. Mather, John Wain, Mark Webber, Andrew J. Page, Justin O'Grady
EPI_ISL_820381, EPI_ISL_820383, EPI_ISL_820385	University College London, Great Ormond Street Hospital for Children NHS Foundation Trust, Imperial College Healthcare NHS Trust	COVID-19 Genomics UK (COG-UK) Consortium	Sergi Castellano, Rachel Williams, Mark Kristiansen, Paola Resende Silva, Sunando Roy, Tony Brooks, Helena Tutill, Paola Niola, Patricia Dyal, Charlotte Williams, Leysa Forrest, Yasmin Panchbhaya, Jacqueline Findlay, Samuel Weeks, Julianne Brown, Kathryn Harris, Paul Randell, James Price, Alison Holmes, Judith Breuer
EPI_ISL_820388, EPI_ISL_820391	Quadram Institute Bioscience	COVID-19 Genomics UK (COG-UK) Consortium	Dave J. Baker, Gemma L. Kay, Alp Aydin, Thanh Le-Viet, Steven Rudder, Ana P. Tedim, Anastasia Kolyva, Maria Diaz, Leonardo de Oliveira Martins, Nabil-Fareed Alikhan, Lizzie Meadows, Rachael Stanley, Ngozi Elumogo, Muhammed Yasir, Nicholas M. Thomson, Alexander J Trotter, Rachel Gilroy, Samuel Bloomfield, Claire Stuart, Andrew Bell, Reenesh Prakash, Samir Dervisevic, Alison E. Mather, John Wain, Mark Webber, Andrew J. Page, Justin O'Grady
EPI_ISL_820394	Queens Medical Centre, Clinical Microbiology Department / DeepSeq Nottingham	COVID-19 Genomics UK (COG-UK) Consortium	Gemma Clark, Wendy Smith, Manjinder Khakh, Vicki M Fleming, Michelle M Lister, Hannah Howson-Wells, Jonathan Ball, Patrick McClure, Joseph Chappell, Theocharis Tsoleridis, Nadine Holmes, Matthew Carlisle, Christopher Moore, Fei Sang, Johnny Debebe, Victoria Wright, Matthew Loose
EPI_ISL_820396	University College London, Great Ormond Street Hospital for Children NHS Foundation Trust, Imperial College Healthcare NHS Trust	COVID-19 Genomics UK (COG-UK) Consortium	Sergi Castellano, Rachel Williams, Mark Kristiansen, Paola Resende Silva, Sunando Roy, Tony Brooks, Helena Tutill, Paola Niola, Patricia Dyal, Charlotte Williams, Leysa Forrest, Yasmin Panchbhaya, Jacqueline Findlay, Samuel Weeks, Julianne Brown, Kathryn Harris, Paul Randell, James Price, Alison Holmes, Judith Breuer
EPI_ISL_820399	Quadram Institute Bioscience	COVID-19 Genomics UK (COG-UK) Consortium	Dave J. Baker, Gemma L. Kay, Alp Aydin, Thanh Le-Viet, Steven Rudder, Ana P. Tedim, Anastasia Kolyva, Maria Diaz, Leonardo de Oliveira Martins, Nabil-Fareed Alikhan, Lizzie Meadows, Rachael Stanley, Ngozi Elumogo, Muhammed Yasir, Nicholas M. Thomson, Alexander J Trotter, Rachel Gilroy, Samuel Bloomfield, Claire Stuart, Andrew Bell, Reenesh Prakash, Samir Dervisevic, Alison E. Mather, John Wain, Mark Webber, Andrew J. Page, Justin O'Grady
EPI_ISL_820402	Queens Medical Centre, Clinical Microbiology Department / DeepSeq Nottingham	COVID-19 Genomics UK (COG-UK) Consortium	Gemma Clark, Wendy Smith, Manjinder Khakh, Vicki M Fleming, Michelle M Lister, Hannah Howson-Wells, Jonathan Ball, Patrick McClure, Joseph Chappell, Theocharis Tsoleridis, Nadine Holmes, Matthew Carlisle, Christopher Moore, Fei Sang, Johnny Debebe, Victoria Wright, Matthew Loose
EPI_ISL_820404, EPI_ISL_820407, EPI_ISL_820410, EPI_ISL_820412, EPI_ISL_820415	Quadram Institute Bioscience	COVID-19 Genomics UK (COG-UK) Consortium	Dave J. Baker, Gemma L. Kay, Alp Aydin, Thanh Le-Viet, Steven Rudder, Ana P. Tedim, Anastasia Kolyva, Maria Diaz, Leonardo de Oliveira Martins, Nabil-Fareed Alikhan, Lizzie Meadows, Rachael Stanley, Ngozi Elumogo, Muhammed Yasir, Nicholas M. Thomson, Alexander J Trotter, Rachel Gilroy, Samuel Bloomfield, Claire Stuart, Andrew Bell, Reenesh Prakash, Samir Dervisevic, Alison E. Mather, John Wain, Mark Webber, Andrew J. Page, Justin O'Grady
EPI_ISL_820418	Queens Medical Centre, Clinical Microbiology Department / DeepSeq Nottingham	COVID-19 Genomics UK (COG-UK) Consortium	Gemma Clark, Wendy Smith, Manjinder Khakh, Vicki M Fleming, Michelle M Lister, Hannah Howson-Wells, Jonathan Ball, Patrick McClure, Joseph Chappell, Theocharis Tsoleridis, Nadine Holmes, Matthew Carlisle, Christopher Moore, Fei Sang, Johnny Debebe, Victoria Wright, Matthew Loose
EPI_ISL_820420	University College London, Great Ormond Street Hospital for Children NHS Foundation Trust, Imperial College Healthcare NHS Trust	COVID-19 Genomics UK (COG-UK) Consortium	Sergi Castellano, Rachel Williams, Mark Kristiansen, Paola Resende Silva, Sunando Roy, Tony Brooks, Helena Tutill, Paola Niola, Patricia Dyal, Charlotte Williams, Leysa Forrest, Yasmin Panchbhaya, Jacqueline Findlay, Samuel Weeks, Julianne Brown, Kathryn Harris, Paul Randell, James Price, Alison Holmes, Judith Breuer
EPI_ISL_820423	Queens Medical Centre, Clinical Microbiology Department /	COVID-19 Genomics UK (COG-UK) Consortium	Gemma Clark, Wendy Smith, Manjinder Khakh, Vicki M Fleming, Michelle M Lister, Hannah Howson-Wells, Jonathan Ball, Patrick McClure, Joseph

	DeepSeq Nottingham		Chappell, Theocharis Tsoleridis, Nadine Holmes, Matthew Carlisle, Christopher Moore, Fei Sang, Johnny Debebe, Victoria Wright, Matthew Loose
EPI_ISL_820426	Quadram Institute Bioscience	COVID-19 Genomics UK (COG-UK) Consortium	Dave J. Baker, Gemma L. Kay, Alp Aydin, Thanh Le-Viet, Steven Rudder, Ana P. Tedim, Anastasia Kolyva, Maria Diaz, Leonardo de Oliveira Martins, Nabil-Fareed Alikhan, Lizzie Meadows, Rachael Stanley, Ngozi Elumogo, Muhammed Yasir, Nicholas M. Thomson, Alexander J Trotter, Rachel Gilroy, Samuel Bloomfield, Claire Stuart, Andrew Bell, Reenesh Prakash, Samir Dervisevic, Alison E. Mather, John Wain, Mark Webber, Andrew J. Page, Justin O'Grady
EPI_ISL_820428, EPI_ISL_820431, EPI_ISL_820433	Queens Medical Centre, Clinical Microbiology Department / DeepSeq Nottingham	COVID-19 Genomics UK (COG-UK) Consortium	Gemma Clark, Wendy Smith, Manjinder Khakh, Vicki M Fleming, Michelle M Lister, Rachael Howson-Wells, Jonathan Ball, Patrick McClure, Joseph Chappell, Theocharis Tsoleridis, Nadine Holmes, Matthew Carlisle, Christopher Moore, Fei Sang, Johnny Debebe, Victoria Wright, Matthew Loose
EPI_ISL_820436, EPI_ISL_820439, EPI_ISL_820441, EPI_ISL_820444, EPI_ISL_820446, EPI_ISL_820449, EPI_ISL_820451, EPI_ISL_820454, EPI_ISL_820456, EPI_ISL_820459, EPI_ISL_820462, EPI_ISL_820464, EPI_ISL_820467, EPI_ISL_820470, EPI_ISL_820472, EPI_ISL_820475, EPI_ISL_820478, EPI_ISL_820480, EPI_ISL_820483, EPI_ISL_820485, EPI_ISL_820488, EPI_ISL_820490, EPI_ISL_820493, EPI_ISL_820496, EPI_ISL_820498, EPI_ISL_820501, EPI_ISL_820504, EPI_ISL_820506, EPI_ISL_820509, EPI_ISL_820511, EPI_ISL_820514, EPI_ISL_820516, EPI_ISL_820519, EPI_ISL_820521, EPI_ISL_820524, EPI_ISL_820526, EPI_ISL_820528, EPI_ISL_820531, EPI_ISL_820533, EPI_ISL_820536, EPI_ISL_820539, EPI_ISL_820541, EPI_ISL_820543, EPI_ISL_820546, EPI_ISL_820549, EPI_ISL_820552, EPI_ISL_820554, EPI_ISL_820557			
see above	Quadram Institute Bioscience	COVID-19 Genomics UK (COG-UK) Consortium	Dave J. Baker, Gemma L. Kay, Alp Aydin, Thanh Le-Viet, Steven Rudder, Ana P. Tedim, Anastasia Kolyva, Maria Diaz, Leonardo de Oliveira Martins, Nabil-Fareed Alikhan, Lizzie Meadows, Rachael Stanley, Ngozi Elumogo, Muhammed Yasir, Nicholas M. Thomson, Alexander J Trotter, Rachel Gilroy, Samuel Bloomfield, Claire Stuart, Andrew Bell, Reenesh Prakash, Samir Dervisevic, Alison E. Mather, John Wain, Mark Webber, Andrew J. Page, Justin O'Grady
EPI_ISL_821039, EPI_ISL_821128, EPI_ISL_821140, EPI_ISL_821163, EPI_ISL_821165, EPI_ISL_821172, EPI_ISL_821197, EPI_ISL_821220, EPI_ISL_821235, EPI_ISL_821263, EPI_ISL_821270, EPI_ISL_821271			
see above	Lighthouse Lab in Alderley Park	Wellcome Sanger Institute for the COVID-19 Genomics UK (COG-UK) Consortium	Jacquelyn Wynn, Mairead Hyland, The Lighthouse Lab in Alderley Park and Alex Alderton, Roberto Amato, Sonia Goncalves, Ewan Harrison, David K. Jackson, Ian Johnston, Dominic Kwiatkowski, Cordelia Langford, John Sillitoe on behalf of the Wellcome Sanger Institute COVID-19 Surveillance Team
EPI_ISL_821275, EPI_ISL_821277	Lighthouse Lab in Milton Keynes	Wellcome Sanger Institute for the COVID-19 Genomics UK (COG-UK) Consortium	The Lighthouse Lab in Milton Keynes and Alex Alderton, Roberto Amato, Sonia Goncalves, Ewan Harrison, David K. Jackson, Ian Johnston, Dominic Kwiatkowski, Cordelia Langford, John Sillitoe on behalf of the Wellcome Sanger Institute COVID-19 Surveillance Team
EPI_ISL_821278	Lighthouse Lab in Cambridge	Wellcome Sanger Institute for the COVID-19 Genomics UK (COG-UK) Consortium	Rob Howes, The Lighthouse Lab in Cambridge and Alex Alderton, Roberto Amato, Sonia Goncalves, Ewan Harrison, David K. Jackson, Ian Johnston, Dominic Kwiatkowski, Cordelia Langford, John Sillitoe on behalf of the Wellcome Sanger Institute COVID-19 Surveillance Team
EPI_ISL_821291, EPI_ISL_821299, EPI_ISL_821302, EPI_ISL_821305	Lighthouse Lab in Milton Keynes	Wellcome Sanger Institute for the COVID-19 Genomics UK (COG-UK) Consortium	The Lighthouse Lab in Milton Keynes and Alex Alderton, Roberto Amato, Sonia Goncalves, Ewan Harrison, David K. Jackson, Ian Johnston, Dominic Kwiatkowski, Cordelia Langford, John Sillitoe on behalf of the Wellcome Sanger Institute COVID-19 Surveillance Team
EPI_ISL_821311	Lighthouse Lab in Cambridge	Wellcome Sanger Institute for the COVID-19 Genomics UK (COG-UK) Consortium	Rob Howes, The Lighthouse Lab in Cambridge and Alex Alderton, Roberto Amato, Sonia Goncalves, Ewan Harrison, David K. Jackson, Ian Johnston, Dominic Kwiatkowski, Cordelia Langford, John Sillitoe on behalf of the Wellcome Sanger Institute COVID-19 Surveillance Team
EPI_ISL_821312, EPI_ISL_821313, EPI_ISL_821315	Lighthouse Lab in Milton Keynes	Wellcome Sanger Institute for the COVID-19 Genomics UK (COG-UK) Consortium	The Lighthouse Lab in Milton Keynes and Alex Alderton, Roberto Amato, Sonia Goncalves, Ewan Harrison, David K. Jackson, Ian Johnston, Dominic Kwiatkowski, Cordelia Langford, John Sillitoe on behalf of the Wellcome Sanger Institute COVID-19 Surveillance Team
EPI_ISL_821316	Lighthouse Lab in Cambridge	Wellcome Sanger Institute for the COVID-19 Genomics UK (COG-UK) Consortium	Rob Howes, The Lighthouse Lab in Cambridge and Alex Alderton, Roberto Amato, Sonia Goncalves, Ewan Harrison, David K. Jackson, Ian Johnston, Dominic Kwiatkowski, Cordelia Langford, John Sillitoe on behalf of the Wellcome Sanger Institute COVID-19 Surveillance Team
EPI_ISL_821318	Lighthouse Lab in Alderley Park	Wellcome Sanger Institute for the COVID-19 Genomics UK (COG-UK) Consortium	Jacquelyn Wynn, Mairead Hyland, The Lighthouse Lab in Alderley Park and Alex Alderton, Roberto Amato, Sonia Goncalves, Ewan Harrison, David K. Jackson, Ian Johnston, Dominic Kwiatkowski, Cordelia Langford, John Sillitoe on behalf of the Wellcome Sanger Institute COVID-19 Surveillance Team
EPI_ISL_821320	Lighthouse Lab in Cambridge	Wellcome Sanger Institute for the COVID-19 Genomics UK (COG-UK) Consortium	Rob Howes, The Lighthouse Lab in Cambridge and Alex Alderton, Roberto Amato, Sonia Goncalves, Ewan Harrison, David K. Jackson, Ian Johnston, Dominic Kwiatkowski, Cordelia Langford, John Sillitoe on behalf of the Wellcome Sanger Institute COVID-19 Surveillance Team
EPI_ISL_821321, EPI_ISL_821322, EPI_ISL_821325, EPI_ISL_821326, EPI_ISL_821330, EPI_ISL_821332, EPI_ISL_821337, EPI_ISL_821345, EPI_ISL_821349, EPI_ISL_821355, EPI_ISL_821357, EPI_ISL_821361, EPI_ISL_821364			
see above	Lighthouse Lab in Milton Keynes	Wellcome Sanger Institute for the COVID-19 Genomics UK (COG-UK) Consortium	The Lighthouse Lab in Milton Keynes and Alex Alderton, Roberto Amato, Sonia Goncalves, Ewan Harrison, David K. Jackson, Ian Johnston, Dominic Kwiatkowski, Cordelia Langford, John Sillitoe on behalf of the Wellcome Sanger Institute COVID-19 Surveillance Team
EPI_ISL_821368	Lighthouse Lab in Cambridge	Wellcome Sanger Institute for the COVID-19 Genomics UK (COG-UK) Consortium	Rob Howes, The Lighthouse Lab in Cambridge and Alex Alderton, Roberto Amato, Sonia Goncalves, Ewan Harrison, David K. Jackson, Ian Johnston, Dominic Kwiatkowski, Cordelia Langford, John Sillitoe on behalf of the Wellcome Sanger Institute COVID-19 Surveillance Team
EPI_ISL_821369	Lighthouse Lab in Milton Keynes	Wellcome Sanger Institute for the COVID-19 Genomics UK (COG-UK) Consortium	The Lighthouse Lab in Milton Keynes and Alex Alderton, Roberto Amato, Sonia Goncalves, Ewan Harrison, David K. Jackson, Ian Johnston, Dominic Kwiatkowski, Cordelia Langford, John Sillitoe on behalf of the Wellcome Sanger Institute COVID-19 Surveillance Team
EPI_ISL_821376, EPI_ISL_821393	Lighthouse Lab in Cambridge	Wellcome Sanger Institute for the COVID-19 Genomics UK (COG-UK) Consortium	Rob Howes, The Lighthouse Lab in Cambridge and Alex Alderton, Roberto Amato, Sonia Goncalves, Ewan Harrison, David K. Jackson, Ian Johnston, Dominic Kwiatkowski, Cordelia Langford, John Sillitoe on behalf of the Wellcome Sanger Institute COVID-19 Surveillance Team
EPI_ISL_821396	Lighthouse Lab in Milton Keynes	Wellcome Sanger Institute for the COVID-19 Genomics UK (COG-UK) Consortium	The Lighthouse Lab in Milton Keynes and Alex Alderton, Roberto Amato, Sonia Goncalves, Ewan Harrison, David K. Jackson, Ian Johnston, Dominic Kwiatkowski, Cordelia Langford, John Sillitoe on behalf of the Wellcome Sanger Institute COVID-19 Surveillance Team
EPI_ISL_821399	Lighthouse Lab in Cambridge	Wellcome Sanger Institute for the COVID-19 Genomics UK (COG-UK) Consortium	Rob Howes, The Lighthouse Lab in Cambridge and Alex Alderton, Roberto Amato, Sonia Goncalves, Ewan Harrison, David K. Jackson, Ian Johnston, Dominic Kwiatkowski, Cordelia Langford, John Sillitoe on behalf of the Wellcome Sanger Institute COVID-19 Surveillance Team
EPI_ISL_821413, EPI_ISL_821420	Lighthouse Lab in Milton Keynes	Wellcome Sanger Institute for the COVID-19 Genomics UK (COG-UK) Consortium	The Lighthouse Lab in Milton Keynes and Alex Alderton, Roberto Amato, Sonia Goncalves, Ewan Harrison, David K. Jackson, Ian Johnston, Dominic Kwiatkowski, Cordelia Langford, John Sillitoe on behalf of the Wellcome Sanger Institute COVID-19 Surveillance Team
EPI_ISL_821421, EPI_ISL_821427	Lighthouse Lab in Cambridge	Wellcome Sanger Institute for the COVID-19 Genomics UK (COG-UK) Consortium	Rob Howes, The Lighthouse Lab in Cambridge and Alex Alderton, Roberto Amato, Sonia Goncalves, Ewan Harrison, David K. Jackson, Ian Johnston, Dominic Kwiatkowski, Cordelia Langford, John Sillitoe on behalf of the Wellcome Sanger Institute COVID-19 Surveillance Team
EPI_ISL_821429, EPI_ISL_821430	Lighthouse Lab in Milton Keynes	Wellcome Sanger Institute for the COVID-19 Genomics UK (COG-UK) Consortium	The Lighthouse Lab in Milton Keynes and Alex Alderton, Roberto Amato, Sonia Goncalves, Ewan Harrison, David K. Jackson, Ian Johnston, Dominic Kwiatkowski, Cordelia Langford, John Sillitoe on behalf of the Wellcome Sanger Institute COVID-19 Surveillance Team
EPI_ISL_821442, EPI_ISL_821444	Lighthouse Lab in Cambridge	Wellcome Sanger Institute for the COVID-19 Genomics UK (COG-UK) Consortium	Rob Howes, The Lighthouse Lab in Cambridge and Alex Alderton, Roberto Amato, Sonia Goncalves, Ewan Harrison, David K. Jackson, Ian Johnston, Dominic Kwiatkowski, Cordelia Langford, John Sillitoe on behalf of the Wellcome Sanger Institute COVID-19 Surveillance Team
EPI_ISL_821450	Lighthouse Lab in Milton Keynes	Wellcome Sanger Institute for the COVID-19 Genomics UK (COG-UK) Consortium	The Lighthouse Lab in Milton Keynes and Alex Alderton, Roberto Amato, Sonia Goncalves, Ewan Harrison, David K. Jackson, Ian Johnston, Dominic Kwiatkowski, Cordelia Langford, John Sillitoe on behalf of the Wellcome Sanger Institute COVID-19 Surveillance Team
EPI_ISL_821454	Lighthouse Lab in Cambridge	Wellcome Sanger Institute for the COVID-19 Genomics UK (COG-UK) Consortium	Rob Howes, The Lighthouse Lab in Cambridge and Alex Alderton, Roberto Amato, Sonia Goncalves, Ewan Harrison, David K. Jackson, Ian Johnston, Dominic Kwiatkowski, Cordelia Langford, John Sillitoe on behalf of the Wellcome Sanger Institute COVID-19 Surveillance Team
EPI_ISL_821455	Lighthouse Lab in Milton Keynes	Wellcome Sanger Institute for the COVID-19 Genomics UK (COG-UK) Consortium	The Lighthouse Lab in Milton Keynes and Alex Alderton, Roberto Amato, Sonia Goncalves, Ewan Harrison, David K. Jackson, Ian Johnston, Dominic Kwiatkowski, Cordelia Langford, John Sillitoe on behalf of the Wellcome Sanger Institute COVID-19 Surveillance Team
EPI_ISL_821456	Lighthouse Lab in Alderley Park	Wellcome Sanger Institute for the COVID-19 Genomics UK (COG-UK) Consortium	Jacquelyn Wynn, Mairead Hyland, The Lighthouse Lab in Alderley Park and Alex Alderton, Roberto Amato, Sonia Goncalves, Ewan Harrison, David K. Jackson, Ian Johnston, Dominic Kwiatkowski, Cordelia Langford, John Sillitoe on behalf of the Wellcome Sanger Institute COVID-19 Surveillance Team
EPI_ISL_821463	Lighthouse Lab in Cambridge	Wellcome Sanger Institute for the COVID-19 Genomics UK (COG-UK) Consortium	Rob Howes, The Lighthouse Lab in Cambridge and Alex Alderton, Roberto Amato, Sonia Goncalves, Ewan Harrison, David K. Jackson, Ian Johnston, Dominic Kwiatkowski, Cordelia Langford, John Sillitoe on behalf of the Wellcome Sanger Institute COVID-19 Surveillance Team
EPI_ISL_821464, EPI_ISL_821471	Lighthouse Lab in Milton Keynes	Wellcome Sanger Institute for the COVID-19 Genomics UK (COG-UK) Consortium	The Lighthouse Lab in Milton Keynes and Alex Alderton, Roberto Amato, Sonia Goncalves, Ewan Harrison, David K. Jackson, Ian Johnston, Dominic Kwiatkowski, Cordelia Langford, John Sillitoe on behalf of the Wellcome Sanger Institute COVID-19 Surveillance Team
EPI_ISL_821473	Lighthouse Lab in Cambridge	Wellcome Sanger Institute for the COVID-19 Genomics UK (COG-UK) Consortium	Rob Howes, The Lighthouse Lab in Cambridge and Alex Alderton, Roberto Amato, Sonia Goncalves, Ewan Harrison, David K. Jackson, Ian Johnston, Dominic Kwiatkowski, Cordelia Langford, John Sillitoe on behalf of the Wellcome Sanger Institute COVID-19 Surveillance Team
EPI_ISL_821481	Lighthouse Lab in Milton Keynes	Wellcome Sanger Institute for the COVID-19 Genomics UK (COG-UK) Consortium	The Lighthouse Lab in Milton Keynes and Alex Alderton, Roberto Amato, Sonia Goncalves, Ewan Harrison, David K. Jackson, Ian Johnston, Dominic Kwiatkowski, Cordelia Langford, John Sillitoe on behalf of the Wellcome Sanger Institute COVID-19 Surveillance Team
EPI_ISL_821485	Lighthouse Lab in Cambridge	Wellcome Sanger Institute for the COVID-19 Genomics UK	Rob Howes, The Lighthouse Lab in Cambridge and Alex Alderton, Roberto Amato, Sonia Goncalves, Ewan Harrison, David K. Jackson, Ian Johnston,

		(COG-UK) Consortium	Dominic Kwiatkowski, Cordelia Langford, John Sillitoe on behalf of the Wellcome Sanger Institute COVID-19 Surveillance Team
EPI_ISL_821489, EPI_ISL_821495, EPI_ISL_821499, EPI_ISL_821505, EPI_ISL_821508, EPI_ISL_821512	Lighthouse Lab in Milton Keynes	Wellcome Sanger Institute for the COVID-19 Genomics UK (COG-UK) Consortium	The Lighthouse Lab in Milton Keynes and Alex Alderton, Roberto Amato, Sonia Goncalves, Ewan Harrison, David K. Jackson, Ian Johnston, Dominic Kwiatkowski, Cordelia Langford, John Sillitoe on behalf of the Wellcome Sanger Institute COVID-19 Surveillance Team
EPI_ISL_821513	Lighthouse Lab in Cambridge	Wellcome Sanger Institute for the COVID-19 Genomics UK (COG-UK) Consortium	Rob Howes, The Lighthouse Lab in Cambridge and Alex Alderton, Roberto Amato, Sonia Goncalves, Ewan Harrison, David K. Jackson, Ian Johnston, Dominic Kwiatkowski, Cordelia Langford, John Sillitoe on behalf of the Wellcome Sanger Institute COVID-19 Surveillance Team
EPI_ISL_821525	Lighthouse Lab in Alderley Park	Wellcome Sanger Institute for the COVID-19 Genomics UK (COG-UK) Consortium	Jacquelyn Wynn, Mairead Hyland, The Lighthouse Lab in Alderley Park and Alex Alderton, Roberto Amato, Sonia Goncalves, Ewan Harrison, David K. Jackson, Ian Johnston, Dominic Kwiatkowski, Cordelia Langford, John Sillitoe on behalf of the Wellcome Sanger Institute COVID-19 Surveillance Team
EPI_ISL_821531	Lighthouse Lab in Milton Keynes	Wellcome Sanger Institute for the COVID-19 Genomics UK (COG-UK) Consortium	The Lighthouse Lab in Milton Keynes and Alex Alderton, Roberto Amato, Sonia Goncalves, Ewan Harrison, David K. Jackson, Ian Johnston, Dominic Kwiatkowski, Cordelia Langford, John Sillitoe on behalf of the Wellcome Sanger Institute COVID-19 Surveillance Team
EPI_ISL_821546	Lighthouse Lab in Cambridge	Wellcome Sanger Institute for the COVID-19 Genomics UK (COG-UK) Consortium	Rob Howes, The Lighthouse Lab in Cambridge and Alex Alderton, Roberto Amato, Sonia Goncalves, Ewan Harrison, David K. Jackson, Ian Johnston, Dominic Kwiatkowski, Cordelia Langford, John Sillitoe on behalf of the Wellcome Sanger Institute COVID-19 Surveillance Team
EPI_ISL_821557, EPI_ISL_821578, EPI_ISL_821581, EPI_ISL_821591	Lighthouse Lab in Milton Keynes	Wellcome Sanger Institute for the COVID-19 Genomics UK (COG-UK) Consortium	The Lighthouse Lab in Milton Keynes and Alex Alderton, Roberto Amato, Sonia Goncalves, Ewan Harrison, David K. Jackson, Ian Johnston, Dominic Kwiatkowski, Cordelia Langford, John Sillitoe on behalf of the Wellcome Sanger Institute COVID-19 Surveillance Team
EPI_ISL_821602	Lighthouse Lab in Cambridge	Wellcome Sanger Institute for the COVID-19 Genomics UK (COG-UK) Consortium	Rob Howes, The Lighthouse Lab in Cambridge and Alex Alderton, Roberto Amato, Sonia Goncalves, Ewan Harrison, David K. Jackson, Ian Johnston, Dominic Kwiatkowski, Cordelia Langford, John Sillitoe on behalf of the Wellcome Sanger Institute COVID-19 Surveillance Team
EPI_ISL_821611	Lighthouse Lab in Milton Keynes	Wellcome Sanger Institute for the COVID-19 Genomics UK (COG-UK) Consortium	The Lighthouse Lab in Milton Keynes and Alex Alderton, Roberto Amato, Sonia Goncalves, Ewan Harrison, David K. Jackson, Ian Johnston, Dominic Kwiatkowski, Cordelia Langford, John Sillitoe on behalf of the Wellcome Sanger Institute COVID-19 Surveillance Team
EPI_ISL_821613	Lighthouse Lab in Cambridge	Wellcome Sanger Institute for the COVID-19 Genomics UK (COG-UK) Consortium	Rob Howes, The Lighthouse Lab in Cambridge and Alex Alderton, Roberto Amato, Sonia Goncalves, Ewan Harrison, David K. Jackson, Ian Johnston, Dominic Kwiatkowski, Cordelia Langford, John Sillitoe on behalf of the Wellcome Sanger Institute COVID-19 Surveillance Team
EPI_ISL_821617	Lighthouse Lab in Alderley Park	Wellcome Sanger Institute for the COVID-19 Genomics UK (COG-UK) Consortium	Jacquelyn Wynn, Mairead Hyland, The Lighthouse Lab in Alderley Park and Alex Alderton, Roberto Amato, Sonia Goncalves, Ewan Harrison, David K. Jackson, Ian Johnston, Dominic Kwiatkowski, Cordelia Langford, John Sillitoe on behalf of the Wellcome Sanger Institute COVID-19 Surveillance Team
EPI_ISL_821619	Lighthouse Lab in Milton Keynes	Wellcome Sanger Institute for the COVID-19 Genomics UK (COG-UK) Consortium	The Lighthouse Lab in Milton Keynes and Alex Alderton, Roberto Amato, Sonia Goncalves, Ewan Harrison, David K. Jackson, Ian Johnston, Dominic Kwiatkowski, Cordelia Langford, John Sillitoe on behalf of the Wellcome Sanger Institute COVID-19 Surveillance Team
EPI_ISL_821626, EPI_ISL_821627, EPI_ISL_821628	Lighthouse Lab in Alderley Park	Wellcome Sanger Institute for the COVID-19 Genomics UK (COG-UK) Consortium	Jacquelyn Wynn, Mairead Hyland, The Lighthouse Lab in Alderley Park and Alex Alderton, Roberto Amato, Sonia Goncalves, Ewan Harrison, David K. Jackson, Ian Johnston, Dominic Kwiatkowski, Cordelia Langford, John Sillitoe on behalf of the Wellcome Sanger Institute COVID-19 Surveillance Team
EPI_ISL_821632, EPI_ISL_821638, EPI_ISL_821639, EPI_ISL_821640, EPI_ISL_821642, EPI_ISL_821643, EPI_ISL_821644, EPI_ISL_821645, EPI_ISL_821646, EPI_ISL_821647, EPI_ISL_821649, EPI_ISL_821650, EPI_ISL_821651, EPI_ISL_821652, EPI_ISL_821654, EPI_ISL_821655, EPI_ISL_821656, EPI_ISL_821657, EPI_ISL_821658			
see above	Lighthouse Lab in Milton Keynes	Wellcome Sanger Institute for the COVID-19 Genomics UK (COG-UK) Consortium	The Lighthouse Lab in Milton Keynes and Alex Alderton, Roberto Amato, Sonia Goncalves, Ewan Harrison, David K. Jackson, Ian Johnston, Dominic Kwiatkowski, Cordelia Langford, John Sillitoe on behalf of the Wellcome Sanger Institute COVID-19 Surveillance Team
EPI_ISL_821659, EPI_ISL_821660	Lighthouse Lab in Glasgow	Wellcome Sanger Institute for the COVID-19 Genomics UK (COG-UK) Consortium	Harper VanSteenhouse, Yumi Kasai, David Gray, Carol Clugston, Anna Dominiczak and Alex Alderton, Roberto Amato, Sonia Goncalves, Ewan Harrison, David K. Jackson, Ian Johnston, Dominic Kwiatkowski, Cordelia Langford, John Sillitoe on behalf of the Wellcome Sanger Institute COVID-19 Surveillance Team
EPI_ISL_821661, EPI_ISL_821662, EPI_ISL_821663, EPI_ISL_821664, EPI_ISL_821665, EPI_ISL_821666, EPI_ISL_821668, EPI_ISL_821670, EPI_ISL_821671, EPI_ISL_821673, EPI_ISL_821674, EPI_ISL_821675, EPI_ISL_821676, EPI_ISL_821678, EPI_ISL_821679, EPI_ISL_821680, EPI_ISL_821681, EPI_ISL_821682, EPI_ISL_821683, EPI_ISL_821684, EPI_ISL_821685, EPI_ISL_821686, EPI_ISL_821687, EPI_ISL_821688, EPI_ISL_821689, EPI_ISL_821690, EPI_ISL_821691, EPI_ISL_821692, EPI_ISL_821693, EPI_ISL_821694, EPI_ISL_821695, EPI_ISL_821696, EPI_ISL_821697, EPI_ISL_821698, EPI_ISL_821699, EPI_ISL_821700, EPI_ISL_821701, EPI_ISL_821702, EPI_ISL_821703, EPI_ISL_821704, EPI_ISL_821705, EPI_ISL_821706, EPI_ISL_821707, EPI_ISL_821708, EPI_ISL_821709, EPI_ISL_821710, EPI_ISL_821711, EPI_ISL_821712, EPI_ISL_821713, EPI_ISL_821714, EPI_ISL_821715, EPI_ISL_821716, EPI_ISL_821717, EPI_ISL_821718, EPI_ISL_821719, EPI_ISL_821720, EPI_ISL_821721, EPI_ISL_821722, EPI_ISL_821723, EPI_ISL_821724, EPI_ISL_821725, EPI_ISL_821726, EPI_ISL_821727, EPI_ISL_821728, EPI_ISL_821729, EPI_ISL_821730, EPI_ISL_821731, EPI_ISL_821732, EPI_ISL_821734, EPI_ISL_821735, EPI_ISL_821736, EPI_ISL_821737, EPI_ISL_821738, EPI_ISL_821739, EPI_ISL_821740, EPI_ISL_821741, EPI_ISL_821742, EPI_ISL_821743, EPI_ISL_821744, EPI_ISL_821745, EPI_ISL_821746, EPI_ISL_821747, EPI_ISL_821748, EPI_ISL_821749, EPI_ISL_821750, EPI_ISL_821751, EPI_ISL_821752, EPI_ISL_821753, EPI_ISL_821754, EPI_ISL_821755, EPI_ISL_821756, EPI_ISL_821757, EPI_ISL_821758, EPI_ISL_821759, EPI_ISL_821760, EPI_ISL_821761, EPI_ISL_821762, EPI_ISL_821763, EPI_ISL_821764, EPI_ISL_821765, EPI_ISL_821766, EPI_ISL_821767, EPI_ISL_821768, EPI_ISL_821769, EPI_ISL_821770, EPI_ISL_821771, EPI_ISL_821772, EPI_ISL_821773, EPI_ISL_821774, EPI_ISL_821775, EPI_ISL_821776, EPI_ISL_821777, EPI_ISL_821778, EPI_ISL_821779, EPI_ISL_821780, EPI_ISL_821781, EPI_ISL_821782, EPI_ISL_821783, EPI_ISL_821784, EPI_ISL_821785, EPI_ISL_821786, EPI_ISL_821788, EPI_ISL_821789, EPI_ISL_821791, EPI_ISL_821792, EPI_ISL_821793, EPI_ISL_821794, EPI_ISL_821795, EPI_ISL_821796, EPI_ISL_821797, EPI_ISL_821798, EPI_ISL_821799, EPI_ISL_821800, EPI_ISL_821801, EPI_ISL_821802, EPI_ISL_821803, EPI_ISL_821804, EPI_ISL_821805, EPI_ISL_821806, EPI_ISL_821807, EPI_ISL_821808, EPI_ISL_821809, EPI_ISL_821810, EPI_ISL_821811, EPI_ISL_821812, EPI_ISL_821813, EPI_ISL_821814, EPI_ISL_821815, EPI_ISL_821816, EPI_ISL_821817, EPI_ISL_821818, EPI_ISL_821819, EPI_ISL_821820, EPI_ISL_821821, EPI_ISL_821822, EPI_ISL_821823, EPI_ISL_821824, EPI_ISL_821825, EPI_ISL_821826, EPI_ISL_821828, EPI_ISL_821829, EPI_ISL_821830, EPI_ISL_821831, EPI_ISL_821832, EPI_ISL_821833, EPI_ISL_821835, EPI_ISL_821836, EPI_ISL_821837, EPI_ISL_821838, EPI_ISL_821839, EPI_ISL_821840, EPI_ISL_821841, EPI_ISL_821843, EPI_ISL_821844, EPI_ISL_821845, EPI_ISL_821846, EPI_ISL_821847, EPI_ISL_821848, EPI_ISL_821849, EPI_ISL_821850, EPI_ISL_821851, EPI_ISL_821852, EPI_ISL_821853, EPI_ISL_821854, EPI_ISL_821855, EPI_ISL_821856, EPI_ISL_821857, EPI_ISL_821859, EPI_ISL_821860, EPI_ISL_821862, EPI_ISL_821863, EPI_ISL_821864, EPI_ISL_821865, EPI_ISL_821866, EPI_ISL_821867, EPI_ISL_821868, EPI_ISL_821869, EPI_ISL_821870, EPI_ISL_821871, EPI_ISL_821872, EPI_ISL_821873, EPI_ISL_821874, EPI_ISL_821875, EPI_ISL_821876, EPI_ISL_821878, EPI_ISL_821879, EPI_ISL_821880, EPI_ISL_821881, EPI_ISL_821882, EPI_ISL_821883, EPI_ISL_821884, EPI_ISL_821885, EPI_ISL_821886, EPI_ISL_821887, EPI_ISL_821888, EPI_ISL_821890, EPI_ISL_821891, EPI_ISL_821892, EPI_ISL_821893, EPI_ISL_821894, EPI_ISL_821896, EPI_ISL_821897, EPI_ISL_821898, EPI_ISL_821899, EPI_ISL_821900, EPI_ISL_821901, EPI_ISL_821902, EPI_ISL_821904, EPI_ISL_821905, EPI_ISL_821906, EPI_ISL_821907, EPI_ISL_821908, EPI_ISL_821909, EPI_ISL_821910, EPI_ISL_821911, EPI_ISL_821912, EPI_ISL_821913, EPI_ISL_821914, EPI_ISL_821915, EPI_ISL_821916, EPI_ISL_821917, EPI_ISL_821918, EPI_ISL_821919, EPI_ISL_821920, EPI_ISL_821921, EPI_ISL_821922, EPI_ISL_821923, EPI_ISL_821924, EPI_ISL_821925, EPI_ISL_821926, EPI_ISL_821927, EPI_ISL_821928, EPI_ISL_821929, EPI_ISL_821930, EPI_ISL_821931, EPI_ISL_821932, EPI_ISL_821933, EPI_ISL_821935, EPI_ISL_821936, EPI_ISL_821937, EPI_ISL_821938, EPI_ISL_821939, EPI_ISL_821940, EPI_ISL_821941, EPI_ISL_821942, EPI_ISL_821944, EPI_ISL_821945, EPI_ISL_821946, EPI_ISL_821947, EPI_ISL_821948, EPI_ISL_821949, EPI_ISL_821951, EPI_ISL_821952, EPI_ISL_821953, EPI_ISL_821954, EPI_ISL_821955, EPI_ISL_821956, EPI_ISL_821957, EPI_ISL_821958, EPI_ISL_821960, EPI_ISL_821961, EPI_ISL_821962, EPI_ISL_821963, EPI_ISL_821964, EPI_ISL_821965, EPI_ISL_821966, EPI_ISL_821968, EPI_ISL_821969, EPI_ISL_821970			
see above	Lighthouse Lab in Cambridge	Wellcome Sanger Institute for the COVID-19 Genomics UK (COG-UK) Consortium	Rob Howes, The Lighthouse Lab in Cambridge and Alex Alderton, Roberto Amato, Sonia Goncalves, Ewan Harrison, David K. Jackson, Ian Johnston, Dominic Kwiatkowski, Cordelia Langford, John Sillitoe on behalf of the Wellcome Sanger Institute COVID-19 Surveillance Team
EPI_ISL_821971, EPI_ISL_821974, EPI_ISL_821976, EPI_ISL_821977, EPI_ISL_821978, EPI_ISL_821979, EPI_ISL_821980, EPI_ISL_821981, EPI_ISL_821982, EPI_ISL_821983, EPI_ISL_821984, EPI_ISL_821985, EPI_ISL_821986, EPI_ISL_821989, EPI_ISL_821990, EPI_ISL_821994, EPI_ISL_821998, EPI_ISL_821999, EPI_ISL_822000, EPI_ISL_822002, EPI_ISL_822003, EPI_ISL_822007, EPI_ISL_822008, EPI_ISL_822009, EPI_ISL_822010, EPI_ISL_822012, EPI_ISL_822014, EPI_ISL_822015, EPI_ISL_822016, EPI_ISL_822017, EPI_ISL_822018, EPI_ISL_822020, EPI_ISL_822021, EPI_ISL_822024, EPI_ISL_822025, EPI_ISL_822026, EPI_ISL_822027, EPI_ISL_822028, EPI_ISL_822031, EPI_ISL_822032, EPI_ISL_822034, EPI_ISL_822036, EPI_ISL_822037, EPI_ISL_822038, EPI_ISL_822039, EPI_ISL_822042, EPI_ISL_822045, EPI_ISL_822046, EPI_ISL_822049, EPI_ISL_822050, EPI_ISL_822052, EPI_ISL_822054, EPI_ISL_822055, EPI_ISL_822056, EPI_ISL_822057, EPI_ISL_822058, EPI_ISL_822061, EPI_ISL_822062, EPI_ISL_822064, EPI_ISL_822066, EPI_ISL_822068, EPI_ISL_822070, EPI_ISL_822071, EPI_ISL_822073, EPI_ISL_822074, EPI_ISL_822076, EPI_ISL_822077, EPI_ISL_822079, EPI_ISL_822082, EPI_ISL_822084, EPI_ISL_822085, EPI_ISL_822087, EPI_ISL_822088, EPI_ISL_822089, EPI_ISL_822090, EPI_ISL_822091, EPI_ISL_822092, EPI_ISL_822095, EPI_ISL_822096, EPI_ISL_822100, EPI_ISL_822101, EPI_ISL_822102, EPI_ISL_822103, EPI_ISL_822106, EPI_ISL_822107, EPI_ISL_822108, EPI_ISL_822109, EPI_ISL_822110, EPI_ISL_822111, EPI_ISL_822112, EPI_ISL_822114, EPI_ISL_822115, EPI_ISL_822116, EPI_ISL_822118, EPI_ISL_822120, EPI_ISL_822121, EPI_ISL_822123, EPI_ISL_822124, EPI_ISL_822125, EPI_ISL_822126, EPI_ISL_822128, EPI_ISL_822129, EPI_ISL_822131, EPI_ISL_822132, EPI_ISL_822133, EPI_ISL_822134, EPI_ISL_822136, EPI_ISL_822137, EPI_ISL_822138, EPI_ISL_822143, EPI_ISL_822144, EPI_ISL_822145, EPI_ISL_822146, EPI_ISL_822147, EPI_ISL_822149, EPI_ISL_822151, EPI_ISL_822152, EPI_ISL_822154, EPI_ISL_822155, EPI_ISL_822157, EPI_ISL_822160, EPI_ISL_822161, EPI_ISL_822162, EPI_ISL_822163, EPI_ISL_822164, EPI_ISL_822166, EPI_ISL_822167, EPI_ISL_822168, EPI_ISL_822171, EPI_ISL_822172, EPI_ISL_822174, EPI_ISL_822175, EPI_ISL_822176, EPI_ISL_822177, EPI_ISL_822179, EPI_ISL_822180, EPI_ISL_822181, EPI_ISL_822185, EPI_ISL_822186, EPI_ISL_822187, EPI_ISL_822188, EPI_ISL_822189, EPI_ISL_822190, EPI_ISL_822192, EPI_ISL_822194, EPI_ISL_822195, EPI_ISL_822196, EPI_ISL_822198, EPI_ISL_822200, EPI_ISL_822203, EPI_ISL_822204, EPI_ISL_822205, EPI_ISL_822206, EPI_ISL_822207, EPI_ISL_822208, EPI_ISL_822210, EPI_ISL_822211, EPI_ISL_822212, EPI_ISL_822213, EPI_ISL_822215, EPI_ISL_822216, EPI_ISL_822217, EPI_ISL_822218, EPI_ISL_822219, EPI_ISL_822220, EPI_ISL_822221, EPI_ISL_822222, EPI_ISL_822225, EPI_ISL_822226, EPI_ISL_822227, EPI_ISL_822228, EPI_ISL_822229, EPI_ISL_822231, EPI_ISL_822232, EPI_ISL_822233, EPI_ISL_822234, EPI_ISL_822235, EPI_ISL_822236, EPI_ISL_822238, EPI_ISL_822239, EPI_ISL_822241, EPI_ISL_822246, EPI_ISL_822251, EPI_ISL_822252, EPI_ISL_822254, EPI_ISL_822255, EPI_ISL_822257, EPI_ISL_822258, EPI_ISL_822259, EPI_ISL_822261, EPI_ISL_822262, EPI_ISL_822263, EPI_ISL_822266, EPI_ISL_822269, EPI_ISL_822270, EPI_ISL_822271, EPI_ISL_822273, EPI_ISL_822274, EPI_ISL_822276, EPI_ISL_822277, EPI_ISL_822278, EPI_ISL_822279, EPI_ISL_822280, EPI_ISL_822284, EPI_ISL_822285, EPI_ISL_822286, EPI_ISL_822287, EPI_ISL_822289, EPI_ISL_822290			
see above	Lighthouse Lab in Alderley Park	Wellcome Sanger Institute for the COVID-19 Genomics UK (COG-UK) Consortium	Jacquelyn Wynn, Mairead Hyland, The Lighthouse Lab in Alderley Park and Alex Alderton, Roberto Amato, Sonia Goncalves, Ewan Harrison, David K. Jackson, Ian Johnston, Dominic Kwiatkowski, Cordelia Langford, John Sillitoe on behalf of the Wellcome Sanger Institute COVID-19 Surveillance Team
EPI_ISL_822292, EPI_ISL_822293, EPI_ISL_822295, EPI_ISL_822296, EPI_ISL_822302, EPI_ISL_822304	Lighthouse Lab in Glasgow	Wellcome Sanger Institute for the COVID-19 Genomics UK (COG-UK) Consortium	Harper VanSteenhouse, Yumi Kasai, David Gray, Carol Clugston, Anna Dominiczak and Alex Alderton, Roberto Amato, Sonia Goncalves, Ewan Harrison, David K. Jackson, Ian Johnston, Dominic Kwiatkowski, Cordelia Langford, John Sillitoe on behalf of the Wellcome Sanger Institute COVID-19 Surveillance Team
EPI_ISL_822659, EPI_ISL_822660, EPI_ISL_822661, EPI_ISL_822662, EPI_ISL_822663, EPI_ISL_822664, EPI_ISL_822665, EPI_ISL_822666, EPI_ISL_822667, EPI_ISL_822668, EPI_ISL_822669, EPI_ISL_822670, EPI_ISL_822671, EPI_ISL_822672, EPI_ISL_822673, EPI_ISL_822674, EPI_ISL_822675, EPI_ISL_822676, EPI_ISL_822677, EPI_ISL_822678, EPI_ISL_822679, EPI_ISL_822680, EPI_ISL_822681, EPI_ISL_822682, EPI_ISL_822683, EPI_ISL_822684, EPI_ISL_822685, EPI_ISL_822686, EPI_ISL_822687, EPI_ISL_822688, EPI_ISL_822689, EPI_ISL_822690, EPI_ISL_822691, EPI_ISL_822692, EPI_ISL_822693, EPI_ISL_822694, EPI_ISL_822695, EPI_ISL_822696, EPI_ISL_822697, EPI_ISL_822698, EPI_ISL_822699, EPI_ISL_822700, EPI_ISL_822701, EPI_ISL_822702, EPI_ISL_822703, EPI_ISL_822704, EPI_ISL_822705, EPI_ISL_822706, EPI_ISL_822707, EPI_ISL_822708, EPI_ISL_822709, EPI_ISL_822710, EPI_ISL_822711, EPI_ISL_822712, EPI_ISL_822713, EPI_ISL_822714, EPI_ISL_822715, EPI_ISL_822716, EPI_ISL_822717, EPI_ISL_822718, EPI_ISL_822719, EPI_ISL_822720, EPI_ISL_822721, EPI_ISL_822722, EPI_ISL_822723, EPI_ISL_822724, EPI_ISL_822725, EPI_ISL_822726, EPI_ISL_822727, EPI_ISL_822728, EPI_ISL_822729, EPI_ISL_822730, EPI_ISL_822731, EPI_ISL_822732, EPI_ISL_822733, EPI_ISL_822734, EPI_ISL_822735, EPI_ISL_822736, EPI_ISL_822737, EPI_ISL_822738, EPI_ISL_822739, EPI_ISL_822740, EPI_ISL_822741, EPI_ISL_822742, EPI_ISL_822743, EPI_ISL_822744, EPI_ISL_822745, EPI_ISL_822746, EPI_ISL_822747, EPI_ISL_822748, EPI_ISL_822749, EPI_ISL_822750, EPI_ISL_822751, EPI_ISL_822752, EPI_ISL_822753, EPI_ISL_822754, EPI_ISL_822755, EPI_ISL_822756, EPI_ISL_822757, EPI_ISL_822758, EPI_ISL_822759, EPI_ISL_822760, EPI_ISL_822761, EPI_ISL_822762, EPI_ISL_822763, EPI_ISL_822764, EPI_ISL_822765, EPI_ISL_822766, EPI_ISL_822767, EPI_ISL_822768, EPI_ISL_822769, EPI_ISL_822770, EPI_ISL_822771, EPI_ISL_822772, EPI_ISL_822773, EPI_ISL_822774, EPI_ISL_822775, EPI_ISL_822776, EPI_ISL_822777, EPI_ISL_822778, EPI_ISL_822779, EPI_ISL_822780, EPI_ISL_822781, EPI_ISL_822782, EPI_ISL_822783, EPI_ISL_822784, EPI_ISL_822785, EPI_ISL_822786, EPI_ISL_822787, EPI_ISL_822788, EPI_ISL_822789, EPI_ISL_822790, EPI_ISL_822791, EPI_ISL_822792, EPI_ISL_822793, EPI_ISL_822794, EPI_ISL_822795, EPI_ISL_822796, EPI_ISL_822797, EPI_ISL_822798, EPI_ISL_822799, EPI_ISL_822800, EPI_ISL_822801, EPI_ISL_822802,			

EPI_ISL_822803, EPI_ISL_822804, EPI_ISL_822805, EPI_ISL_822844, EPI_ISL_822880, EPI_ISL_822925, EPI_ISL_822940, EPI_ISL_822947, EPI_ISL_822951, EPI_ISL_822952, EPI_ISL_822955, EPI_ISL_822957, EPI_ISL_822971, EPI_ISL_823003, EPI_ISL_823066, EPI_ISL_823098, EPI_ISL_823099, EPI_ISL_823100, EPI_ISL_823101, EPI_ISL_823102, EPI_ISL_823103, EPI_ISL_823112, EPI_ISL_823149, EPI_ISL_823152, EPI_ISL_823154, EPI_ISL_823155, EPI_ISL_823646, EPI_ISL_823647, EPI_ISL_823648, EPI_ISL_823649, EPI_ISL_823780, EPI_ISL_823781, EPI_ISL_823782, EPI_ISL_823783, EPI_ISL_823784, EPI_ISL_823785, EPI_ISL_823786, EPI_ISL_823787, EPI_ISL_823788, EPI_ISL_823789, EPI_ISL_823790, EPI_ISL_823791, EPI_ISL_823792, EPI_ISL_823793, EPI_ISL_823794, EPI_ISL_823795			
see above	Wales Specialist Virology Centre Sequencing lab: Pathogen Genomics Unit	COVID-19 Genomics UK (COG-UK) Consortium	Catherine Moore, Johnathan Evans, Laura Gifford, Malorie Perry, Simon Cottrell, Angela Marchbank, Alec Birchley, Alexander Adams, Amy Gaskin, Bree Gatica-Wilcox, Jason Coombes, Joel Southgate, Lauren Gilbert, Lee Graham, Nicole Pacchiarini, Sara Kumziene-Summerhayes, Sarah Taylor, Sophie Jones, Sara Rey, Matthew Bull, Joanne Watkins, Sally Corden, Tom Connor
EPI_ISL_823976, EPI_ISL_823981, EPI_ISL_823983, EPI_ISL_823984, EPI_ISL_823985, EPI_ISL_823986, EPI_ISL_823987, EPI_ISL_823988, EPI_ISL_823990, EPI_ISL_823991, EPI_ISL_823994, EPI_ISL_823995, EPI_ISL_823996, EPI_ISL_824020, EPI_ISL_824025, EPI_ISL_824026, EPI_ISL_824038, EPI_ISL_824039, EPI_ISL_824040, EPI_ISL_824047, EPI_ISL_824048, EPI_ISL_824051, EPI_ISL_824052, EPI_ISL_824068, EPI_ISL_824071, EPI_ISL_824088, EPI_ISL_824109, EPI_ISL_824111, EPI_ISL_824112, EPI_ISL_824113, EPI_ISL_824114, EPI_ISL_824116, EPI_ISL_824120, EPI_ISL_824123, EPI_ISL_824164, EPI_ISL_824165, EPI_ISL_824166, EPI_ISL_824167, EPI_ISL_824168, EPI_ISL_824181, EPI_ISL_824182, EPI_ISL_824199, EPI_ISL_824200, EPI_ISL_824201, EPI_ISL_824202, EPI_ISL_824203, EPI_ISL_824204, EPI_ISL_824207, EPI_ISL_824208, EPI_ISL_824214, EPI_ISL_824215, EPI_ISL_824216, EPI_ISL_824217, EPI_ISL_824242, EPI_ISL_824243, EPI_ISL_824244, EPI_ISL_824245, EPI_ISL_824246, EPI_ISL_824247, EPI_ISL_824249, EPI_ISL_824250, EPI_ISL_824251, EPI_ISL_824252, EPI_ISL_824259, EPI_ISL_824260, EPI_ISL_824266, EPI_ISL_824272, EPI_ISL_824273, EPI_ISL_824276, EPI_ISL_824278			
see above	Dutch COVID-19 response team	National Institute for Public Health and the Environment (RIVM)	Adam Meijer, Harry Vennema, Jeroen Cremer, Sharon van den Brink, Bas van der Veer, AnneMarie van den Brandt, Florian Zwagemaker, Dennis Schmitz, Chantal Reusken, on behalf of the national COVID-19 response team
EPI_ISL_824290, EPI_ISL_824291	Institute of Microbiology, Universidad San Francisco de Quito	Institute of Microbiology, Universidad San Francisco de Quito	Belén Prado-Vivar, Sully Márquez, Juan José Guadalupe, Monica Becerra-Wong, Bernardo Gutiérrez, Andrea Cunguan, Nabih Dahik, Verónica Barragán, Patricio Rojas-Silva, Gabriel Trueba, Michelle Grunauer, Paúl Cárdenas
EPI_ISL_824342	Michigan Department of Health and Human Services, Bureau of Laboratories	Michigan Department of Health and Human Services, Bureau of Laboratories	Blankenship HM, Riner D, Soehnlen MK
EPI_ISL_824524, EPI_ISL_824525, EPI_ISL_824526, EPI_ISL_824527, EPI_ISL_824528, EPI_ISL_824529, EPI_ISL_824530, EPI_ISL_824531, EPI_ISL_824532, EPI_ISL_824533, EPI_ISL_824534, EPI_ISL_824535, EPI_ISL_824538, EPI_ISL_824539, EPI_ISL_824540, EPI_ISL_824541, EPI_ISL_824542, EPI_ISL_824543, EPI_ISL_824544, EPI_ISL_824545, EPI_ISL_824546, EPI_ISL_824547, EPI_ISL_824548, EPI_ISL_824549, EPI_ISL_824550, EPI_ISL_824551, EPI_ISL_824552, EPI_ISL_824553			
see above	New Mexico Department of Health Scientific Laboratory	New Mexico Department of Health Scientific Laboratory	Ellie Johnson, Anastacia Griego-Fisher, D'eldra Malone
EPI_ISL_824790, EPI_ISL_824791, EPI_ISL_824792, EPI_ISL_824793, EPI_ISL_824794, EPI_ISL_824795, EPI_ISL_824796, EPI_ISL_824797, EPI_ISL_824798, EPI_ISL_824809, EPI_ISL_824810, EPI_ISL_824811, EPI_ISL_824812, EPI_ISL_824813, EPI_ISL_824814, EPI_ISL_824815, EPI_ISL_824816, EPI_ISL_824817, EPI_ISL_824818, EPI_ISL_824819, EPI_ISL_824820, EPI_ISL_824821, EPI_ISL_824822, EPI_ISL_824823, EPI_ISL_824824, EPI_ISL_824825, EPI_ISL_824826, EPI_ISL_824827, EPI_ISL_824828, EPI_ISL_824829, EPI_ISL_824830, EPI_ISL_824831, EPI_ISL_824834			
see above	Department of Clinical Microbiology	GIGA Medical Genomics	Keith Durkin, Maria Artesi, Sébastien Bontems, Raphaël Boreux, Bouchra Boujemla, Cécile Meex, Pierrette Melin, Marie-Pierre Hayette, Vincent Bours
EPI_ISL_824921, EPI_ISL_824944	Charité Universitätsmedizin Berlin, Institute of Virology, Charitéplatz 1, 10117 Berlin, Germany	Charité Universitätsmedizin Berlin, Institute of Virology, Charitéplatz 1, 10117 Berlin, Germany	Victor M Corman, Jörn Beheim-Schwarzbach, Tobias Bleicker, Julia Tesch, Barbara Mühlemann, Talitha Veith, Julia Schneider, Terry Jones, Christian Drosten
EPI_ISL_824945, EPI_ISL_824946, EPI_ISL_824947, EPI_ISL_824948, EPI_ISL_824949, EPI_ISL_824950, EPI_ISL_824951, EPI_ISL_824952	Maryland Public Health Laboratory	Maryland Public Health Laboratory	Maryland Department of Health Laboratories Administration
EPI_ISL_824987	Arizona State Public Health Laboratory	Arizona State Public Health Laboratory	Trung Huynh, Jessica Escobar, Katherine Fullerton, Nobuko Fukushima, Stacy White, Linda Getsinger, Victor Waddell
EPI_ISL_825014	Northwestern Memorial Hospital	Ozer Lab	Ramon Lorenzo-Redondo, Lacy M. Simons, Lawrence J. Jennings, Michael G. Ison, Judd F. Hultquist, Egon A. Ozer
EPI_ISL_825061	GMERS Medical College and Hospital, Gotri, Vadodara	Gujarat Biotechnology Research Centre	Apurvasinh Puvar, Ramesh Pandit, Janvi Raval, Zarna Patel, Nitin Savaliya, Dinesh Kumar, Zuber Saiyed, Afzal Ansari, Nikha Trivedi, Mitesh Kamothi, Bithika Duttaroy, Kalpesh Mistry, Chaitanya Joshi, Madhvi Joshi
EPI_ISL_825062	GMERS Medical College and Hospital, Gotri, Vadodara	Gujarat Biotechnology Research Centre	Ramesh Pandit, Janvi Raval, Zarna Patel, Nitin Savaliya, Dinesh Kumar, Zuber Saiyed, Afzal Ansari, Nikha Trivedi, Apurvasinh Puvar, Mitesh Kamothi, Bithika Duttaroy, Kalpesh Mistry, Chaitanya Joshi, Madhvi Joshi
EPI_ISL_825063	NHL Municipal Medical College, Ahmedbad	Gujarat Biotechnology Research Centre	Janvi Raval, Zarna Patel, Nitin Savaliya, Dinesh Kumar, Zuber Saiyed, Afzal Ansari, Nikha Trivedi, Apurvasinh Puvar, Ramesh Pandit, Jayshri Pethani, Monila Patel, Atit Shah, NM Shaikh, Bimal Chauhan, Tanmay Mehta, Bhavin Prajapati, Chaitanya Joshi, Madhvi Joshi
EPI_ISL_825156	Microbiology and Virology Unit, Florence Careggi University Hospital	Microbiology and Virology Unit, Florence Careggi University Hospital	Vincenzo Di Pilato, Marco Coppi, Fabio Morecchiato, Alberto Antonelli, Emanuele Gori, Gian Maria Rossolini
EPI_ISL_825165, EPI_ISL_825169, EPI_ISL_825170, EPI_ISL_825171, EPI_ISL_825172	Robert Koch Institute, ZBS1 Highly Pathogenic Viruses, Berlin, Germany	Robert Koch Institute, ZBS1 Highly Pathogenic Viruses & Bioinformatics MF1, Berlin, Germany	Annika Brinkmann, Janine Michel, Livia Schrick, Steven Uddin, Dominique Seifert, Alexander Dalpke, Leo Büttner, Kristina Hochauf-Stange, Dirk Lindemann, Lars Schaade, Andreas Nitsche
EPI_ISL_825499, EPI_ISL_825500, EPI_ISL_825534, EPI_ISL_825535, EPI_ISL_825536, EPI_ISL_825537, EPI_ISL_825538, EPI_ISL_825539, EPI_ISL_825540, EPI_ISL_825541, EPI_ISL_825542, EPI_ISL_825543, EPI_ISL_825544, EPI_ISL_825545, EPI_ISL_825568, EPI_ISL_825569, EPI_ISL_825570, EPI_ISL_825610, EPI_ISL_825611, EPI_ISL_825612, EPI_ISL_825613, EPI_ISL_825614, EPI_ISL_825615			
see above	Respiratory Virus Unit, National Infection Service, Public Health England	COVID-19 Genomics UK (COG-UK) Consortium	PHE Covid Sequencing Team
EPI_ISL_825626, EPI_ISL_825627, EPI_ISL_825628, EPI_ISL_825629, EPI_ISL_825630, EPI_ISL_825631	Hospital Universitari Vall d'Hebron - Vall d'Hebron Institut de Rercerca	Hospital Universitari Vall d'Hebron	Cristina Andrés, Maria Piñana, Josep F Abril, Damir Garcia-Cehic, Ariadna Rando, Juliana Esperalba, Maria Gema Codina, Carla Castillo, Maria Carmen Martin, Tomàs Pumarola, Josep Quer, Andrés Antón
EPI_ISL_826458	University of Bari Biomedical Sciences and Human Oncology	University of Bari Biomedical Sciences and Human Oncology	Chironna Maria, Sallustio Anna, Loconsole Daniela, Accogli Marisa
EPI_ISL_826463	The Ohio State University	OSU Polaris Molecular Laboratory	Huolin Tu, Matthew R Avenarius, Laura Kubatko, Matthew Hunt, Xiaokang Pan, Peng Ru, Jason Garee, Keelie Thomas, Peter Mohler, Preeti Pancholi, Dan Jones
EPI_ISL_826472	Lighthouse Lab in Alderley Park	Wellcome Sanger Institute for the COVID-19 Genomics UK (COG-UK) Consortium	Jacquelyn Wynn, Mairead Hyland, The Lighthouse Lab in Alderley Park and Alex Alderton, Roberto Amato, Sonia Goncalves, Ewan Harrison, David K. Jackson, Ian Johnston, Dominic Kwiatkowski, Cordelia Langford, John Sillitoe on behalf of the Wellcome Sanger Institute COVID-19 Surveillance Team
EPI_ISL_826475	Lighthouse Lab in Cambridge	Wellcome Sanger Institute for the COVID-19 Genomics UK (COG-UK) Consortium	Rob Howes, The Lighthouse Lab in Cambridge and Alex Alderton, Roberto Amato, Sonia Goncalves, Ewan Harrison, David K. Jackson, Ian Johnston, Dominic Kwiatkowski, Cordelia Langford, John Sillitoe on behalf of the Wellcome Sanger Institute COVID-19 Surveillance Team
EPI_ISL_826476	Lighthouse Lab in Alderley Park	Wellcome Sanger Institute for the COVID-19 Genomics UK (COG-UK) Consortium	Jacquelyn Wynn, Mairead Hyland, The Lighthouse Lab in Alderley Park and Alex Alderton, Roberto Amato, Sonia Goncalves, Ewan Harrison, David K. Jackson, Ian Johnston, Dominic Kwiatkowski, Cordelia Langford, John Sillitoe on behalf of the Wellcome Sanger Institute COVID-19 Surveillance Team
EPI_ISL_826521	The Ohio State University	James Molecular Laboratory	Huolin Tu, Matthew R Avenarius, Laura Kubatko, Matthew Hunt, Xiaokang Pan, Peng Ru, Jason Garee, Keelie Thomas, Peter Mohler, Preeti Pancholi, Dan Jones
EPI_ISL_826523, EPI_ISL_826524, EPI_ISL_826525, EPI_ISL_826526, EPI_ISL_826527, EPI_ISL_826528, EPI_ISL_826529, EPI_ISL_826530, EPI_ISL_826531, EPI_ISL_826532, EPI_ISL_826533, EPI_ISL_826534, EPI_ISL_826535			
see above	Dutch COVID-19 response team	National Institute for Public Health and the Environment (RIVM)	Adam Meijer, Harry Vennema, Jeroen Cremer, Sharon van den Brink, Bas van der Veer, AnneMarie van den Brandt, Florian Zwagemaker, Dennis Schmitz, Chantal Reusken, on behalf of the national COVID-19 response team
EPI_ISL_827042, EPI_ISL_827043	Institute of Virology, University of Cologne	Institute of Virology, University of Cologne	Saleta Sierra, Gibran Rubio, Zevanya Tessalonica, Dominik Aschenmeier, Eva Heger, Elena Knops, Rolf Kaiser, Martin Däumer, Alex Thielen
EPI_ISL_827146, EPI_ISL_827927, EPI_ISL_827929, EPI_ISL_827932, EPI_ISL_828221, EPI_ISL_828335	deCODE genetics	deCODE genetics	Daniel F Gudbjartsson; Agnar Helgason; Hakon Jonsson; Olafur T Magnusson; Pall Melsted; Gudmundur L Norddahl; Jona Saemundsdottir; Asgeir Sigurdsson; Patrick Sulem; Arna B Agustsdottir; Hannes Eggertsson; Berglind Eiriksdottir; Run Fridriksdottir; Elisabet E Gardarsdottir; Gudmundur Georgsson; Olafia S Gretarsdottir; Kjartan R Gudmundsson; Thora R Gunnarsdottir; Arnaldur Gylfason; Hilma Holm; Brynjar O Jensson; Aslaug Jonasdottir; Kamilla S Josefsdottir; Thordur Kristjansson; Droplaug N Magnusdottir; Solvi Rognvaldsson; Louise le Roux; Gudrun Sigmundsdottir; Gardar Sveinbjornsson; Kristin E Sveinsdottir; Maney Sveinsdottir; Emil A Thorarensen; Bjarni Thorbjornsson; Gisli Masson; Ingileif Jonsdottir; Alma Moller; Thorolfur Gudnason; Karl G Kristinsson; Unnur Thorsteinsdottir; Kari Stefansson
EPI_ISL_828336	The National University Hospital of Iceland	deCODE genetics	Daniel F Gudbjartsson; Agnar Helgason; Hakon Jonsson; Olafur T Magnusson; Pall Melsted; Gudmundur L Norddahl; Jona Saemundsdottir; Asgeir Sigurdsson; Patrick Sulem; Arna B Agustsdottir; Hannes Eggertsson; Berglind Eiriksdottir; Run Fridriksdottir; Elisabet E Gardarsdottir; Gudmundur Georgsson; Olafia S Gretarsdottir; Kjartan R Gudmundsson; Thora R Gunnarsdottir; Arnaldur Gylfason; Hilma Holm; Brynjar O Jensson; Aslaug

			Jonasdottir; Kamilla S Josefsdottir; Thordur Kristjansson; Droplaug N Magnusdottir; Solvi Rognvaldsson; Louise le Roux; Gudrun Sigmundsdottir; Gardar Sveinbjornsson; Kristin E Sveinsdottir; Maney Sveinsdottir; Emil A Thorarensen; Bjarni Thorbjornsson; Gisli Masson; Ingileif Jonsdottir; Alma Moller; Thorolfur Gudnason; Karl G Kristinsson; Unnur Thorsteinsdottir; Kari Stefansson
EPI_ISL_828414, EPI_ISL_828786, EPI_ISL_829305, EPI_ISL_829306, EPI_ISL_829307, EPI_ISL_829311	deCODE genetics	deCODE genetics	Daniel F Gudbjartsson; Agnar Helgason; Hakon Jonsson; Olafur T Magnusson; Pall Melsted; Gudmundur L Norddahl; Jona Saemundsdottir; Asgeir Sigurdsson; Patrick Sulem; Arna B Agustsdottir; Hannes Eggertsson; Berglind Eiriksdottir; Run Fridriksdottir; Elisabet E Gardarsdottir; Gudmundur Georgsson; Olafia S Gretarsdottir; Kjartan R Gudmundsson; Thora R Gunnarsdottir; Arnaldur Gylfason; Hilma Holm; Brynjar O Jensson; Aslaug Jonasdottir; Kamilla S Josefsdottir; Thordur Kristjansson; Droplaug N Magnusdottir; Solvi Rognvaldsson; Louise le Roux; Gudrun Sigmundsdottir; Gardar Sveinbjornsson; Kristin E Sveinsdottir; Maney Sveinsdottir; Emil A Thorarensen; Bjarni Thorbjornsson; Gisli Masson; Ingileif Jonsdottir; Alma Moller; Thorolfur Gudnason; Karl G Kristinsson; Unnur Thorsteinsdottir; Kari Stefansson
EPI_ISL_829543	The National University Hospital of Iceland	deCODE genetics	Daniel F Gudbjartsson; Agnar Helgason; Hakon Jonsson; Olafur T Magnusson; Pall Melsted; Gudmundur L Norddahl; Jona Saemundsdottir; Asgeir Sigurdsson; Patrick Sulem; Arna B Agustsdottir; Hannes Eggertsson; Berglind Eiriksdottir; Run Fridriksdottir; Elisabet E Gardarsdottir; Gudmundur Georgsson; Olafia S Gretarsdottir; Kjartan R Gudmundsson; Thora R Gunnarsdottir; Arnaldur Gylfason; Hilma Holm; Brynjar O Jensson; Aslaug Jonasdottir; Kamilla S Josefsdottir; Thordur Kristjansson; Droplaug N Magnusdottir; Solvi Rognvaldsson; Louise le Roux; Gudrun Sigmundsdottir; Gardar Sveinbjornsson; Kristin E Sveinsdottir; Maney Sveinsdottir; Emil A Thorarensen; Bjarni Thorbjornsson; Gisli Masson; Ingileif Jonsdottir; Alma Moller; Thorolfur Gudnason; Karl G Kristinsson; Unnur Thorsteinsdottir; Kari Stefansson
EPI_ISL_829545, EPI_ISL_829546, EPI_ISL_829547	deCODE genetics	deCODE genetics	Daniel F Gudbjartsson; Agnar Helgason; Hakon Jonsson; Olafur T Magnusson; Pall Melsted; Gudmundur L Norddahl; Jona Saemundsdottir; Asgeir Sigurdsson; Patrick Sulem; Arna B Agustsdottir; Hannes Eggertsson; Berglind Eiriksdottir; Run Fridriksdottir; Elisabet E Gardarsdottir; Gudmundur Georgsson; Olafia S Gretarsdottir; Kjartan R Gudmundsson; Thora R Gunnarsdottir; Arnaldur Gylfason; Hilma Holm; Brynjar O Jensson; Aslaug Jonasdottir; Kamilla S Josefsdottir; Thordur Kristjansson; Droplaug N Magnusdottir; Solvi Rognvaldsson; Louise le Roux; Gudrun Sigmundsdottir; Gardar Sveinbjornsson; Kristin E Sveinsdottir; Maney Sveinsdottir; Emil A Thorarensen; Bjarni Thorbjornsson; Gisli Masson; Ingileif Jonsdottir; Alma Moller; Thorolfur Gudnason; Karl G Kristinsson; Unnur Thorsteinsdottir; Kari Stefansson
EPI_ISL_830214	BOSTON HEART DIAGNOSTICS CORP	Wadsworth Center, New York State Department of Health	Kirsten St. George, Daryl M. Lamson, Alexis Russel, Matthew Shudt, Melissa A Leisner, Jonathan Plitnick, Navjot Singh, John Kelly, Erasmus Schneider, Erica Lasek-Nesselquist
EPI_ISL_830608, EPI_ISL_830609, EPI_ISL_830610, EPI_ISL_830611, EPI_ISL_830612, EPI_ISL_830613, EPI_ISL_830614, EPI_ISL_830615, EPI_ISL_830616, EPI_ISL_830617, EPI_ISL_830618, EPI_ISL_830619, EPI_ISL_830620, EPI_ISL_830621, EPI_ISL_830622, EPI_ISL_830623, EPI_ISL_830624, EPI_ISL_830625, EPI_ISL_830626, EPI_ISL_830627			
see above	KALEIDA CENTER FOR LABORATORY MEDICINE	Wadsworth Center, New York State Department of Health	Kirsten St. George, Daryl M. Lamson, Alexis Russel, Matthew Shudt, Melissa A Leisner, Jonathan Plitnick, Navjot Singh, John Kelly, Erasmus Schneider, Erica Lasek-Nesselquist
EPI_ISL_830649	Gundersen Molecular Diagnostics Laboratory	Kabara Cancer Research Institute	Craig S. Richmond, Paraic A. Kenny
EPI_ISL_830650, EPI_ISL_830651, EPI_ISL_830652, EPI_ISL_830653, EPI_ISL_830654, EPI_ISL_830655, EPI_ISL_830656, EPI_ISL_830657, EPI_ISL_830658, EPI_ISL_830659, EPI_ISL_830660, EPI_ISL_830661, EPI_ISL_830662, EPI_ISL_830663, EPI_ISL_830665, EPI_ISL_830666, EPI_ISL_830667, EPI_ISL_830668, EPI_ISL_830670, EPI_ISL_830671, EPI_ISL_830672, EPI_ISL_830673, EPI_ISL_830674, EPI_ISL_830675, EPI_ISL_830676, EPI_ISL_830677, EPI_ISL_830678, EPI_ISL_830679, EPI_ISL_830680, EPI_ISL_830681			
see above	SUNY UPSTATE MEDICAL UNIVERSITY	Wadsworth Center, New York State Department of Health	Kirsten St. George, Daryl M. Lamson, Alexis Russel, Matthew Shudt, Melissa A Leisner, Jonathan Plitnick, Navjot Singh, John Kelly, Erasmus Schneider, Erica Lasek-Nesselquist
EPI_ISL_830722, EPI_ISL_830723	BIO-REFERENCE LABORATORIES	Wadsworth Center, New York State Department of Health	Kirsten St. George, Daryl M. Lamson, Alexis Russel, Matthew Shudt, Melissa A Leisner, Jonathan Plitnick, Navjot Singh, John Kelly, Erasmus Schneider, Erica Lasek-Nesselquist
EPI_ISL_831490, EPI_ISL_831498, EPI_ISL_831499, EPI_ISL_831572, EPI_ISL_831573, EPI_ISL_831575, EPI_ISL_831577, EPI_ISL_831578, EPI_ISL_831579, EPI_ISL_831580, EPI_ISL_831581, EPI_ISL_831582, EPI_ISL_831583, EPI_ISL_831584, EPI_ISL_831585, EPI_ISL_831586			
see above	University of Wisconsin-Madison AIDS Vaccine Research Laboratories	University of Wisconsin-Madison AIDS Vaccine Research Laboratories	Gage Moreno, Katarina Braun, et al. AIDS Vaccine Research Laboratories
EPI_ISL_831649	Institute for Infectious Diseases, University of Bern, Switzerland	Institute for Infectious Diseases, University of Bern, Switzerland	Michel C Koch, Christian Baumann, Miguel A Terrazos Miani, Cora Sägesser, Pascal Bittel, Stephen L Leib, Peter Keller, Franziska Suter-Riniker, Alban Ramette
EPI_ISL_831944, EPI_ISL_831947, EPI_ISL_831948, EPI_ISL_831949, EPI_ISL_831950, EPI_ISL_831951, EPI_ISL_831952	ABC	The Public Health Agency of Sweden	Department of Microbiology, The Public Health Agency of Sweden
EPI_ISL_831980, EPI_ISL_831981	Klinisk mikrobiologi	The Public Health Agency of Sweden	Department of Microbiology, The Public Health Agency of Sweden
EPI_ISL_832008	Unilabs, Mikrobiologiska laboratoriet	The Public Health Agency of Sweden	Department of Microbiology, The Public Health Agency of Sweden
EPI_ISL_832014	Utah Public Health Laboratory	Utah Public Health Laboratory	Erin L. Young, Kelly F. Oakeson, Tara Gallagher
EPI_ISL_832017	University of Wisconsin-Madison AIDS Vaccine Research Laboratories	University of Wisconsin-Madison AIDS Vaccine Research Laboratories	Gage Moreno, Katarina Braun, et al. AIDS Vaccine Research Laboratories
EPI_ISL_832079, EPI_ISL_832080, EPI_ISL_832081	Santa Clara County Public Health Laboratory	Santa Clara County Public Health Laboratory	Santa Clara County Public Health Department
EPI_ISL_832207	Department of Clinical Microbiology	GIGA Medical Genomics	Keith Durkin, Maria Artesi, Sébastien Bontems, Raphaël Boreux, Bouchra Boujemla, Cécile Meex, Pierrette Melin, Marie-Pierre Hayette, Vincent Bours
EPI_ISL_832823, EPI_ISL_832824, EPI_ISL_832825, EPI_ISL_832826, EPI_ISL_832827, EPI_ISL_832828, EPI_ISL_832829, EPI_ISL_832830, EPI_ISL_832831, EPI_ISL_832832, EPI_ISL_832833, EPI_ISL_832834, EPI_ISL_832852, EPI_ISL_832853, EPI_ISL_832854, EPI_ISL_832855, EPI_ISL_832856, EPI_ISL_832857, EPI_ISL_832858, EPI_ISL_832859, EPI_ISL_832860, EPI_ISL_832861, EPI_ISL_832862, EPI_ISL_832863, EPI_ISL_832864, EPI_ISL_832865, EPI_ISL_832866, EPI_ISL_832867, EPI_ISL_832868, EPI_ISL_832869, EPI_ISL_832870, EPI_ISL_832873, EPI_ISL_832874, EPI_ISL_832885, EPI_ISL_832886, EPI_ISL_832887, EPI_ISL_832888, EPI_ISL_832889, EPI_ISL_832890, EPI_ISL_832891, EPI_ISL_832892, EPI_ISL_832893, EPI_ISL_832894, EPI_ISL_832895, EPI_ISL_832896, EPI_ISL_832897, EPI_ISL_832926, EPI_ISL_832935, EPI_ISL_832936, EPI_ISL_832937, EPI_ISL_832938, EPI_ISL_832939, EPI_ISL_832940, EPI_ISL_832941, EPI_ISL_832942, EPI_ISL_832943, EPI_ISL_832944, EPI_ISL_832947, EPI_ISL_832948, EPI_ISL_832949, EPI_ISL_832950, EPI_ISL_832951, EPI_ISL_832952, EPI_ISL_832953, EPI_ISL_832954, EPI_ISL_832955, EPI_ISL_832956, EPI_ISL_832957, EPI_ISL_832958, EPI_ISL_832959, EPI_ISL_832960, EPI_ISL_832961, EPI_ISL_832962, EPI_ISL_832963, EPI_ISL_832964, EPI_ISL_832965, EPI_ISL_832966, EPI_ISL_832967			
see above	Maine HETL	Tewhey Lab, The Jackson Laboratory	Matluk,N., Dewey,H., Iosue,F., Barter,M., Lynch,R., Munger,H. and Tewhey,R.
EPI_ISL_833038	Laboratorio de Referencia Nacional de Virus Respiratorio. Instituto Nacional de Salud Perú	Laboratorio de Referencia Nacional de Biotecnología y Biología Molecular. Instituto Nacional de Salud Perú	Carlos Padilla Rojas, Luis Barcena, Karolyn Vega Chozo, Priscila Lope Pari, Omar Caceres Rey, Marco Galarza Perez, Maribel Huaringa Nuñez, Johanna Balbuena Torrez, Henri Bailon Calderon, Nancy Rojas Serrano
EPI_ISL_833136	Laboratorio de Ecologia de Doencas Transmissiveis na Amazonia, Instituto Leonidas e Maria Deane - Fiocruz Amazonia	Laboratorio de Ecologia de Doencas Transmissiveis na Amazonia, Instituto Leonidas e Maria Deane - Fiocruz Amazonia	Valdinete Nascimento, Victor Souza, André Corado, Fernanda Nascimento, George Silva, Ágatha Costa, Debora Duarte, Karina Pessoa, Matilde Mejía, Luciana Gonçalves, Maria Júlia Brandão, Michele Jesus, Felipe Naveca
EPI_ISL_833146	Genomic Laboratory (GLAB) (Conjoint lab of Health Directorate of Istanbul and Istanbul Technical University)	Genomic Laboratory (GLAB), Istanbul Technical University	Ilker Karacan, Tugba Kizilboga Akgun, Payam Zolfagharian, Nisan Denizce Can, Pari Sharifli, Levent Doganay, Gizem Dinler Doganay
EPI_ISL_833187	Department of Clinical Microbiology	GIGA Medical Genomics	Keith Durkin, Maria Artesi, Sébastien Bontems, Raphaël Boreux, Bouchra Boujemla, Cécile Meex, Pierrette Melin, Marie-Pierre Hayette, Vincent Bours
EPI_ISL_833202, EPI_ISL_833204, EPI_ISL_833207, EPI_ISL_833211, EPI_ISL_833212, EPI_ISL_833216, EPI_ISL_833217, EPI_ISL_833218, EPI_ISL_833219	Department of Virology and Immunology, University of Helsinki and Helsinki University Hospital, Huslab Finland	Department of Virology, Faculty of Medicine, University of Helsinki, Helsinki, Finland	Teemu Smura, Ravi Kant, Phuoc Truong, Hussein Alburkat, Hannimari Kallio-Kokko, Jenni Virtanen, Maija Suvanto, Fathiah Zakham, Essi Korhonen, Sari Hannula, Harri Kangas, Pekka Ellonen, Olli Vapalahti
EPI_ISL_833228, EPI_ISL_833229	R.P. GUARDIAGRELE Ospedale di Comunità	Istituto Zooprofilattico Sperimentale dell'Abruzzo e Molise "G.Caporale"	Lorusso A, Marcacci M, Di Domenico M, Curini V, Ancora M, Cammà C, Rinaldi A, Mangone I, Di Pasquale A, Puglia I, Calistri P, Savini G.
EPI_ISL_833230	OSPEDALE S.S. ANNUNZIATA CHIETI - CLINICA MEDICA	Istituto Zooprofilattico Sperimentale dell'Abruzzo e Molise	Lorusso A, Marcacci M, Di Domenico M, Curini V, Ancora M, Cammà C, Rinaldi A, Mangone I, Di Pasquale A, Puglia I, Calistri P, Savini G.

	(MEDICINA GENERALE 1)	"G.Caporale"	
EPI_ISL_833231	SIESP CHIETI - DRIVE IN LANCIANO	Istituto Zooprofilattico Sperimentale dell'Abruzzo e Molise "G.Caporale"	Lorusso A, Marcacci M, Di Domenico M, Curini V, Ancora M, Cammà C, Rinaldi A, Mangone I, Di Pasquale A, Puglia I, Calistri P, Savini G.
EPI_ISL_833232	SIESP CHIETI - DRIVE IN CHIETI	Istituto Zooprofilattico Sperimentale dell'Abruzzo e Molise "G.Caporale"	Lorusso A, Marcacci M, Di Domenico M, Curini V, Ancora M, Cammà C, Rinaldi A, Mangone I, Di Pasquale A, Puglia I, Calistri P, Savini G.
EPI_ISL_833233, EPI_ISL_833234, EPI_ISL_833235, EPI_ISL_833236, EPI_ISL_833237, EPI_ISL_833238	SIESP DIPARTIMENTO DI PREVENZIONE CHIETI	Istituto Zooprofilattico Sperimentale dell'Abruzzo e Molise "G.Caporale"	Lorusso A, Marcacci M, Di Domenico M, Curini V, Ancora M, Cammà C, Rinaldi A, Mangone I, Di Pasquale A, Puglia I, Calistri P, Savini G.
EPI_ISL_833239, EPI_ISL_833240	SIESP DIPARTIMENTO DI PREVENZIONE TERAMO	Istituto Zooprofilattico Sperimentale dell'Abruzzo e Molise "G.Caporale"	Lorusso A, Marcacci M, Di Domenico M, Curini V, Ancora M, Cammà C, Rinaldi A, Mangone I, Di Pasquale A, Puglia I, Calistri P, Savini G.
EPI_ISL_833241	SIESP DIPARTIMENTO DI PREVENZIONE CHIETI	Istituto Zooprofilattico Sperimentale dell'Abruzzo e Molise "G.Caporale"	Lorusso A, Marcacci M, Di Domenico M, Curini V, Ancora M, Cammà C, Rinaldi A, Mangone I, Di Pasquale A, Puglia I, Calistri P, Savini G.
EPI_ISL_833242	SIESP CHIETI - DRIVE IN LANCIANO	Istituto Zooprofilattico Sperimentale dell'Abruzzo e Molise "G.Caporale"	Lorusso A, Marcacci M, Di Domenico M, Curini V, Ancora M, Cammà C, Rinaldi A, Mangone I, Di Pasquale A, Puglia I, Calistri P, Savini G.
EPI_ISL_833243, EPI_ISL_833244, EPI_ISL_833245, EPI_ISL_833246, EPI_ISL_833247	SIESP DIPARTIMENTO DI PREVENZIONE CHIETI	Istituto Zooprofilattico Sperimentale dell'Abruzzo e Molise "G.Caporale"	Lorusso A, Marcacci M, Di Domenico M, Curini V, Ancora M, Cammà C, Rinaldi A, Mangone I, Di Pasquale A, Puglia I, Calistri P, Savini G.
EPI_ISL_833297	Ospedale Civile Giulianova	Istituto Zooprofilattico Sperimentale dell'Abruzzo e Molise "G. Caporale"	Lorusso A, Marcacci M, Di Domenico M, Ancora M, Curini V, Mangone I, Rinaldi A, Di Pasquale A, Cammà C, Puglia I, Calistri P, Savini G
EPI_ISL_833435, EPI_ISL_833436, EPI_ISL_833437, EPI_ISL_833438, EPI_ISL_833439, EPI_ISL_833440, EPI_ISL_833441, EPI_ISL_833442, EPI_ISL_833443, EPI_ISL_833444, EPI_ISL_833445, EPI_ISL_833446			
see above	SC (UCO) Igiene e Sanità Pubblica (funzione integrata con SC Microbiologia e Virologia) e Laboratory of Molecular Virology of the International Centre for Genetic Engineering and Biotechnology (ICGEB)	ARGO Laboratorio Genomica ed Epigenomica	Licastro D, Dal Monego S, Degasperi M, Marcello A, D'Agaro P
EPI_ISL_834146, EPI_ISL_834211, EPI_ISL_834212, EPI_ISL_834223, EPI_ISL_834226, EPI_ISL_834228, EPI_ISL_834231, EPI_ISL_834233, EPI_ISL_834236, EPI_ISL_834237, EPI_ISL_834248, EPI_ISL_834250, EPI_ISL_834256, EPI_ISL_834264, EPI_ISL_834271, EPI_ISL_834280, EPI_ISL_834283, EPI_ISL_834288, EPI_ISL_834290, EPI_ISL_834293, EPI_ISL_834298, EPI_ISL_834299, EPI_ISL_834311, EPI_ISL_834312, EPI_ISL_834313, EPI_ISL_834344, EPI_ISL_834352, EPI_ISL_834353, EPI_ISL_834360, EPI_ISL_834373, EPI_ISL_834376, EPI_ISL_834377, EPI_ISL_834378, EPI_ISL_834380, EPI_ISL_834381, EPI_ISL_834382, EPI_ISL_834388, EPI_ISL_834404, EPI_ISL_834407, EPI_ISL_834409, EPI_ISL_834417, EPI_ISL_834423, EPI_ISL_834427, EPI_ISL_834440, EPI_ISL_834443, EPI_ISL_834445, EPI_ISL_834448, EPI_ISL_834449, EPI_ISL_834459, EPI_ISL_834466, EPI_ISL_834467, EPI_ISL_834468, EPI_ISL_834485, EPI_ISL_834486, EPI_ISL_834488, EPI_ISL_834494, EPI_ISL_834497, EPI_ISL_834499, EPI_ISL_834500, EPI_ISL_834503, EPI_ISL_834511, EPI_ISL_834517, EPI_ISL_834529, EPI_ISL_834541, EPI_ISL_834545, EPI_ISL_834804, EPI_ISL_834814, EPI_ISL_834823, EPI_ISL_834825, EPI_ISL_834826, EPI_ISL_834833, EPI_ISL_834834, EPI_ISL_834837, EPI_ISL_834848, EPI_ISL_834865, EPI_ISL_834870, EPI_ISL_834874, EPI_ISL_834903, EPI_ISL_834909, EPI_ISL_834910, EPI_ISL_834920, EPI_ISL_834925, EPI_ISL_834931, EPI_ISL_834933, EPI_ISL_834949, EPI_ISL_834959, EPI_ISL_834972, EPI_ISL_834979, EPI_ISL_834981, EPI_ISL_834983, EPI_ISL_834989, EPI_ISL_834991, EPI_ISL_835001, EPI_ISL_835004, EPI_ISL_835008, EPI_ISL_835017, EPI_ISL_835038, EPI_ISL_835047, EPI_ISL_835055, EPI_ISL_835058, EPI_ISL_835060, EPI_ISL_835067, EPI_ISL_835112, EPI_ISL_835114, EPI_ISL_835115, EPI_ISL_835127, EPI_ISL_835130, EPI_ISL_835132, EPI_ISL_835140, EPI_ISL_835483, EPI_ISL_835492, EPI_ISL_835495, EPI_ISL_835532, EPI_ISL_835546, EPI_ISL_835550, EPI_ISL_835562, EPI_ISL_835615, EPI_ISL_835639, EPI_ISL_835655, EPI_ISL_835680, EPI_ISL_835698			
see above	Lighthouse Lab in Alderley Park	Wellcome Sanger Institute for the COVID-19 Genomics UK (COG-UK) Consortium	Jacquelyn Wynn, Mairead Hyland, The Lighthouse Lab in Alderley Park and Alex Alderton, Roberto Amato, Sonia Goncalves, Ewan Harrison, David K. Jackson, Ian Johnston, Dominic Kwiatkowski, Cordelia Langford, John Sillitoe on behalf of the Wellcome Sanger Institute COVID-19 Surveillance Team
EPI_ISL_837041, EPI_ISL_837042, EPI_ISL_837043, EPI_ISL_837044, EPI_ISL_837045, EPI_ISL_837046, EPI_ISL_837047, EPI_ISL_837048, EPI_ISL_837049, EPI_ISL_837050, EPI_ISL_837051, EPI_ISL_837064, EPI_ISL_837070, EPI_ISL_837080, EPI_ISL_837086, EPI_ISL_837092, EPI_ISL_837093, EPI_ISL_837094, EPI_ISL_837096, EPI_ISL_837135, EPI_ISL_837136, EPI_ISL_837137, EPI_ISL_837138, EPI_ISL_837139, EPI_ISL_837140, EPI_ISL_837141, EPI_ISL_837142, EPI_ISL_837143, EPI_ISL_837144, EPI_ISL_837145, EPI_ISL_837146, EPI_ISL_837147, EPI_ISL_837148, EPI_ISL_837149, EPI_ISL_837150, EPI_ISL_837151, EPI_ISL_837152, EPI_ISL_837153, EPI_ISL_837154, EPI_ISL_837155, EPI_ISL_837156, EPI_ISL_837157, EPI_ISL_837158, EPI_ISL_837159, EPI_ISL_837160, EPI_ISL_837161, EPI_ISL_837162, EPI_ISL_837163, EPI_ISL_837164, EPI_ISL_837165, EPI_ISL_837166, EPI_ISL_837167, EPI_ISL_837168, EPI_ISL_837169, EPI_ISL_837170, EPI_ISL_837171, EPI_ISL_837184, EPI_ISL_837188, EPI_ISL_837190, EPI_ISL_837191, EPI_ISL_837192, EPI_ISL_837193, EPI_ISL_837194, EPI_ISL_837226, EPI_ISL_837227, EPI_ISL_837228, EPI_ISL_837229, EPI_ISL_837230, EPI_ISL_837231, EPI_ISL_837232, EPI_ISL_837233, EPI_ISL_837234, EPI_ISL_837235, EPI_ISL_837236, EPI_ISL_837237, EPI_ISL_837238			
see above	Respiratory Virus Unit, National Infection Service, Public Health England	COVID-19 Genomics UK (COG-UK) Consortium	PHE Covid Sequencing Team
EPI_ISL_837345, EPI_ISL_837348, EPI_ISL_837353, EPI_ISL_837382, EPI_ISL_837383, EPI_ISL_837394, EPI_ISL_837416, EPI_ISL_837424, EPI_ISL_837425, EPI_ISL_837430, EPI_ISL_837432			
see above	National Virus Reference Laboratory	National Virus Reference Laboratory	Michael Carr, Gabriel Gonzalez, Jonathan Dean, Cillian F De Gascun
EPI_ISL_837525, EPI_ISL_837527	UW Virology Lab	UW Virology Lab	Pavitra Roychoudhury, Hong Xie, Lasata Shrestha, Meei-Li Huang, Keith R Jerome, Alexander Greninger
EPI_ISL_837817, EPI_ISL_837820, EPI_ISL_837821, EPI_ISL_837826, EPI_ISL_837828, EPI_ISL_837831	Wyoming Public Health Laboratory	Wyoming Public Health Laboratory	Noah Hull, Taylor Fearing, Lynette Gumbleton, Channing Weber, Ashley Norberg, Bailey Bowcutt, and Wanda Manley
EPI_ISL_837851, EPI_ISL_837853, EPI_ISL_837858, EPI_ISL_837866, EPI_ISL_837867, EPI_ISL_837868, EPI_ISL_837872, EPI_ISL_837873, EPI_ISL_837874, EPI_ISL_837875, EPI_ISL_837878, EPI_ISL_837880, EPI_ISL_837881, EPI_ISL_837883, EPI_ISL_837884, EPI_ISL_837885, EPI_ISL_837888, EPI_ISL_837890, EPI_ISL_837894, EPI_ISL_837897, EPI_ISL_837899, EPI_ISL_837900			
see above	Department of Pathology, University of Cambridge	COVID-19 Genomics UK (COG-UK) Consortium	Aminu S. Jahun, Yasmin Chaudhry, Grant Hall, Iliana Georgana, Myra Hosmillo, Martin D. Curran, Malte Pinckert, Surendra Parmar, Ian Goodfellow
EPI_ISL_838268, EPI_ISL_838269, EPI_ISL_838270, EPI_ISL_838271, EPI_ISL_838273, EPI_ISL_838277, EPI_ISL_838278, EPI_ISL_838279	Virology Department, Royal Infirmary of Edinburgh, NHS Lothian / School of Biological Sciences, University of Edinburgh / Institute of Genetics and Molecular Medicine, University of Edinburgh	COVID-19 Genomics UK (COG-UK) Consortium	McHugh M, Dewar R, Rooke S, Gallagher M, Balcaza C, O'Toole Á, Scher E, Hill V, McCrone JT, Colquhoun R, Yu X, Jackson B, Rambaut A, Williams TC, Templeton K
EPI_ISL_838330, EPI_ISL_838332, EPI_ISL_838333	University of Exeter	COVID-19 Genomics UK (COG-UK) Consortium	Ben Temperton,Aaron Jeffries,Michelle Michelsen,Joanna Warwick-Dugdale,Audrey Farbos,Robyn Manley,Stephen Michell,Jane Masoli
EPI_ISL_838580, EPI_ISL_838581, EPI_ISL_838582, EPI_ISL_838583, EPI_ISL_838584, EPI_ISL_838589, EPI_ISL_838590, EPI_ISL_838591, EPI_ISL_838594, EPI_ISL_838595, EPI_ISL_838596, EPI_ISL_838601, EPI_ISL_838604, EPI_ISL_838606, EPI_ISL_838607, EPI_ISL_838608, EPI_ISL_838609, EPI_ISL_838611, EPI_ISL_838614, EPI_ISL_838615, EPI_ISL_838616, EPI_ISL_838621, EPI_ISL_838623, EPI_ISL_838624, EPI_ISL_838629, EPI_ISL_838630, EPI_ISL_838631, EPI_ISL_838632, EPI_ISL_838634, EPI_ISL_838635, EPI_ISL_838636, EPI_ISL_838637, EPI_ISL_838639, EPI_ISL_838642, EPI_ISL_838643, EPI_ISL_838644, EPI_ISL_838647, EPI_ISL_838648, EPI_ISL_838649, EPI_ISL_838650, EPI_ISL_838651, EPI_ISL_838654, EPI_ISL_838656, EPI_ISL_838657, EPI_ISL_838658, EPI_ISL_838659, EPI_ISL_838660, EPI_ISL_838661, EPI_ISL_838663			
see above	Liverpool Clinical Laboratories	COVID-19 Genomics UK (COG-UK) Consortium	Sam Haldenby, Anita Lucaci, Steve Paterson, Julian Hiscox, Alistair Darby, M Almsaud, A Alrezaihi, Muhannad Alruwaili, Stuart D Armstrong, Jones Benjamin, Eleanor G Bentley, Anu Chawla, Jordan J Clark, Angela Cowell, Richard Eccles, Isabel García-Dorival, Matthew Gemmell, Alessandro Gerada, PKF Gilmore, Richard Gregory, Ximeng Han, Catherine Hartley, Margaret Hughes, Miren Iturriza-Gomara, James Johnson, L Luu, Jenifer Manson, Charlotte Nelson, Elaine O'Toole, Cassie Olateju, Rebekah Penrice-Randal , Lucille Rainbow, N.P Randle, Trevor Ian Robinson, Parul Sharma, Ghada T Shawli, James P Stewart, Neil Swainston, Ecaterina Vamos, Joanne Watts, Mark Whitehead
EPI_ISL_838767, EPI_ISL_838768, EPI_ISL_838769, EPI_ISL_838770, EPI_ISL_838771, EPI_ISL_838773, EPI_ISL_838775, EPI_ISL_838776, EPI_ISL_838777, EPI_ISL_838799, EPI_ISL_838800, EPI_ISL_838801, EPI_ISL_838802, EPI_ISL_838803, EPI_ISL_838804, EPI_ISL_838805, EPI_ISL_838806, EPI_ISL_838808, EPI_ISL_838809, EPI_ISL_838810, EPI_ISL_838811, EPI_ISL_838812, EPI_ISL_838813, EPI_ISL_838814, EPI_ISL_838815, EPI_ISL_838816, EPI_ISL_838817, EPI_ISL_838818, EPI_ISL_838819, EPI_ISL_838820, EPI_ISL_838821, EPI_ISL_838822, EPI_ISL_838823, EPI_ISL_838824, EPI_ISL_838825, EPI_ISL_838826, EPI_ISL_838827, EPI_ISL_839041, EPI_ISL_839042, EPI_ISL_839043, EPI_ISL_839063, EPI_ISL_839064, EPI_ISL_839065, EPI_ISL_839066, EPI_ISL_839067, EPI_ISL_839068, EPI_ISL_839069, EPI_ISL_839080, EPI_ISL_839081, EPI_ISL_839082, EPI_ISL_839083, EPI_ISL_839084, EPI_ISL_839101, EPI_ISL_839102, EPI_ISL_839103, EPI_ISL_839104, EPI_ISL_839105, EPI_ISL_839106, EPI_ISL_839107, EPI_ISL_839108, EPI_ISL_839109, EPI_ISL_839110, EPI_ISL_839111, EPI_ISL_839112, EPI_ISL_839113, EPI_ISL_839114, EPI_ISL_839116, EPI_ISL_839215, EPI_ISL_839218, EPI_ISL_839219, EPI_ISL_839220, EPI_ISL_839222, EPI_ISL_839223, EPI_ISL_839226, EPI_ISL_839227, EPI_ISL_839305, EPI_ISL_839306, EPI_ISL_839307, EPI_ISL_839308, EPI_ISL_839310, EPI_ISL_839316, EPI_ISL_839317, EPI_ISL_839330			
see above	University College London, Great Ormond Street Hospital for Children NHS Foundation Trust, Imperial College Healthcare NHS Trust	COVID-19 Genomics UK (COG-UK) Consortium	Sergi Castellano, Rachel Williams, Mark Kristiansen, Paola Resende Silva, Sunando Roy, Tony Brooks, Helena Tutill, Paola Niola, Patricia Dyal, Charlotte Williams, Leysa Forrest, Yasmin Panchbhaya, Jacqueline Findlay, Samuel Weeks, Julianne Brown, Kathryn Harris, Paul Randell, James Price, Alison Holmes, Judith Breuer
EPI_ISL_839710, EPI_ISL_839711, EPI_ISL_839712, EPI_ISL_839714, EPI_ISL_839715, EPI_ISL_839716, EPI_ISL_839717, EPI_ISL_839718, EPI_ISL_839719, EPI_ISL_839720, EPI_ISL_839721, EPI_ISL_839722, EPI_ISL_839723, EPI_ISL_839724, EPI_ISL_839725, EPI_ISL_839726, EPI_ISL_839727, EPI_ISL_839728, EPI_ISL_839729, EPI_ISL_839730			

see above	Northumbria University / South Tees Hospitals NHS Foundation Trust / North Cumbria Integrated Care NHS Foundation Trust / North Tees and Hartlepool NHS Foundation Trust / Newcastle Hospitals NHS Foundation Trust	COVID-19 Genomics UK (COG-UK) Consortium	Darren L Smith,Andrew Nelson,Matthew Bashton,Greg R Young,Joshua Loh,John Allan,Mohammad A Tariq,Giles S Holt,Gary Black,Wen C Yew,Lynn Dover,Paul Baker,Steve Liggett,Sarah Essex,Jane Greenaway,Debra Padgett,Clive Graham,Garren Scott,Edward Barton,Emma Swindells,Brendan Payne,Jennifer Collins,Yusri Taha,Gary Eltringham
EPI_ISL_840108, EPI_ISL_840109, EPI_ISL_840110, EPI_ISL_840112, EPI_ISL_840113, EPI_ISL_840114, EPI_ISL_840115, EPI_ISL_840116, EPI_ISL_840117, EPI_ISL_840118, EPI_ISL_840119, EPI_ISL_840120			
see above	Lincolnshire Hospitals and DeepSeq Nottingham	COVID-19 Genomics UK (COG-UK) Consortium	Nichola Duckworth, Tim Sloan, Sarah Walsh, Jonathan Ball, Patrick McClure, Joeseph Chappell, Nadine Holmes, Matthew Carlisle, Christopher Moore, Fei Sang, Johnny Debebe, Victoria Wright, Matthew Loose
EPI_ISL_840371, EPI_ISL_840372, EPI_ISL_840390, EPI_ISL_840391, EPI_ISL_840392, EPI_ISL_840393, EPI_ISL_840394, EPI_ISL_840395, EPI_ISL_840396, EPI_ISL_840397, EPI_ISL_840399, EPI_ISL_840600, EPI_ISL_840603, EPI_ISL_840604, EPI_ISL_840606, EPI_ISL_840609, EPI_ISL_840610, EPI_ISL_840611, EPI_ISL_840612, EPI_ISL_840615, EPI_ISL_840617, EPI_ISL_840618, EPI_ISL_840619, EPI_ISL_840620, EPI_ISL_840621, EPI_ISL_840622, EPI_ISL_840623, EPI_ISL_840624, EPI_ISL_840625, EPI_ISL_840626, EPI_ISL_840627, EPI_ISL_840628, EPI_ISL_840629, EPI_ISL_840630, EPI_ISL_840631, EPI_ISL_840632, EPI_ISL_840633, EPI_ISL_840634, EPI_ISL_840635, EPI_ISL_840636			
see above	Originating lab: Wales Specialist Virology Centre Sequencing lab: Pathogen Genomics Unit	Public Health Wales Microbiology Cardiff Wales Specialist Virology Centre	Catherine Moore, Johnathan Evans, Laura Gifford, Malorie Perry, Simon Cottrell, Angela Marchbank, Alec Birchley, Alexander Adams, Amy Gaskin, Bree Gatica-Wilcox, Jason Coombes, Joel Southgate, Lauren Gilbert, Lee Graham, Nicole Pacchiarini, Sara Kumziene-Summerhayes, Sarah Taylor, Sophie Jones, Sara Rey, Matthew Bull, Joanne Watkins, Sally Corden, Tom Connor
EPI_ISL_840637	Wyoming Public Health Laboratory	Wyoming Public Health Laboratory	Noah Hull, Taylor Fearing, Lynette Gumbleton, Channing Weber, Ashley Norberg, Bailey Bowcutt, and Wanda Manley
EPI_ISL_840638, EPI_ISL_840639, EPI_ISL_840640, EPI_ISL_840641, EPI_ISL_840643, EPI_ISL_840644, EPI_ISL_840645, EPI_ISL_840646, EPI_ISL_840647, EPI_ISL_840648, EPI_ISL_840649, EPI_ISL_840651, EPI_ISL_840653, EPI_ISL_840655, EPI_ISL_840656, EPI_ISL_840657, EPI_ISL_840658, EPI_ISL_840671, EPI_ISL_840672, EPI_ISL_840695, EPI_ISL_840696, EPI_ISL_840697, EPI_ISL_840698, EPI_ISL_840699, EPI_ISL_840700, EPI_ISL_840701, EPI_ISL_840702, EPI_ISL_840703, EPI_ISL_840774, EPI_ISL_840775, EPI_ISL_840776, EPI_ISL_840777, EPI_ISL_840778, EPI_ISL_840779, EPI_ISL_840781, EPI_ISL_840782, EPI_ISL_840783, EPI_ISL_840785, EPI_ISL_840787, EPI_ISL_840790, EPI_ISL_840794, EPI_ISL_840795, EPI_ISL_840796, EPI_ISL_840798, EPI_ISL_840799, EPI_ISL_840801, EPI_ISL_840802, EPI_ISL_840803, EPI_ISL_840804, EPI_ISL_840805, EPI_ISL_840806, EPI_ISL_840808, EPI_ISL_840810			
see above	Originating lab: Wales Specialist Virology Centre Sequencing lab: Pathogen Genomics Unit	Public Health Wales Microbiology Cardiff Wales Specialist Virology Centre	Catherine Moore, Johnathan Evans, Laura Gifford, Malorie Perry, Simon Cottrell, Angela Marchbank, Alec Birchley, Alexander Adams, Amy Gaskin, Bree Gatica-Wilcox, Jason Coombes, Joel Southgate, Lauren Gilbert, Lee Graham, Nicole Pacchiarini, Sara Kumziene-Summerhayes, Sarah Taylor, Sophie Jones, Sara Rey, Matthew Bull, Joanne Watkins, Sally Corden, Tom Connor
EPI_ISL_840837	Wales Specialist Virology Centre Sequencing lab: Pathogen Genomics Unit	Public Health Wales Microbiology Cardiff Wales Specialist Virology Centre	Catherine Moore, Johnathan Evans, Laura Gifford, Malorie Perry, Simon Cottrell, Angela Marchbank, Alec Birchley, Alexander Adams, Amy Gaskin, Bree Gatica-Wilcox, Jason Coombes, Joel Southgate, Lauren Gilbert, Lee Graham, Nicole Pacchiarini, Sara Kumziene-Summerhayes, Sarah Taylor, Sophie Jones, Sara Rey, Matthew Bull, Joanne Watkins, Sally Corden, Tom Connor
EPI_ISL_840850	Wyoming Public Health Laboratory	Wyoming Public Health Laboratory	Noah Hull, Taylor Fearing, Lynette Gumbleton, Channing Weber, Ashley Norberg, Bailey Bowcutt, and Wanda Manley
EPI_ISL_840895, EPI_ISL_840897, EPI_ISL_840898, EPI_ISL_840899, EPI_ISL_840900, EPI_ISL_841057, EPI_ISL_841058, EPI_ISL_841083, EPI_ISL_841087, EPI_ISL_841088, EPI_ISL_841089, EPI_ISL_841090, EPI_ISL_841091, EPI_ISL_841092, EPI_ISL_841093, EPI_ISL_841094, EPI_ISL_841095, EPI_ISL_841096, EPI_ISL_841097, EPI_ISL_841099, EPI_ISL_841100, EPI_ISL_841101, EPI_ISL_841102, EPI_ISL_841103, EPI_ISL_841104, EPI_ISL_841105, EPI_ISL_841106, EPI_ISL_841107, EPI_ISL_841109, EPI_ISL_841110, EPI_ISL_841111, EPI_ISL_841112, EPI_ISL_841113, EPI_ISL_841302, EPI_ISL_841303, EPI_ISL_841304, EPI_ISL_841305			
see above	Wales Specialist Virology Centre Sequencing lab: Pathogen Genomics Unit	Public Health Wales Microbiology Cardiff Wales Specialist Virology Centre	Catherine Moore, Johnathan Evans, Laura Gifford, Malorie Perry, Simon Cottrell, Angela Marchbank, Alec Birchley, Alexander Adams, Amy Gaskin, Bree Gatica-Wilcox, Jason Coombes, Joel Southgate, Lauren Gilbert, Lee Graham, Nicole Pacchiarini, Sara Kumziene-Summerhayes, Sarah Taylor, Sophie Jones, Sara Rey, Matthew Bull, Joanne Watkins, Sally Corden, Tom Connor
EPI_ISL_841321, EPI_ISL_841355, EPI_ISL_841359, EPI_ISL_841360, EPI_ISL_841361, EPI_ISL_841521, EPI_ISL_841522, EPI_ISL_841580, EPI_ISL_841581, EPI_ISL_841584, EPI_ISL_841587			
see above	Originating lab: Wales Specialist Virology Centre Sequencing lab: Pathogen Genomics Unit	Public Health Wales Microbiology Cardiff Wales Specialist Virology Centre	Catherine Moore, Johnathan Evans, Laura Gifford, Malorie Perry, Simon Cottrell, Angela Marchbank, Alec Birchley, Alexander Adams, Amy Gaskin, Bree Gatica-Wilcox, Jason Coombes, Joel Southgate, Lauren Gilbert, Lee Graham, Nicole Pacchiarini, Sara Kumziene-Summerhayes, Sarah Taylor, Sophie Jones, Sara Rey, Matthew Bull, Joanne Watkins, Sally Corden, Tom Connor
EPI_ISL_841978, EPI_ISL_842004, EPI_ISL_842014	Centre for Enzyme Innovation, University of Portsmouth / Translational Research Laboratory, Portsmouth Hospitals NHS Trust	COVID-19 Genomics UK (COG-UK) Consortium	Angela Beckett,Yann Bourgeois,Garry Scarlett,Sharon Glaysher,Scott Elliott,Kelly Bicknell,Robert Impey,Allyson Lloyd,Sarah Wyllie,Ethan Butcher,Anoop Chauhan,Samuel Robson
EPI_ISL_842209, EPI_ISL_842265, EPI_ISL_842273, EPI_ISL_842293, EPI_ISL_842312, EPI_ISL_842338, EPI_ISL_842341	Virology Department, Sheffield Teaching Hospitals NHS Foundation Trust/Department of Infection, Immunity and Cardiovascular Disease, The Medical School, University of Sheffield	COVID-19 Genomics UK (COG-UK) Consortium	Thushan de Silva, Matthew Parker, Nikki Smith, Adri Angyal, Rebecca Brown, Luke Green, Rachel Tucker, Paul Parsons, Danielle Groves, Katie Johnson, Laura Carrilero, Alex Keeley, Dave Partridge, Matthew Wyles, Benjamin Lindsey, Mehmet Yavuz, Mohammad Raza, Cariad Evans
EPI_ISL_842356, EPI_ISL_842361, EPI_ISL_842362, EPI_ISL_842367, EPI_ISL_842422, EPI_ISL_842423, EPI_ISL_842424, EPI_ISL_842425, EPI_ISL_842426, EPI_ISL_842427, EPI_ISL_842428, EPI_ISL_842429, EPI_ISL_842430, EPI_ISL_842431, EPI_ISL_842432, EPI_ISL_842433, EPI_ISL_842434, EPI_ISL_842435, EPI_ISL_842436, EPI_ISL_842437, EPI_ISL_842438, EPI_ISL_842439, EPI_ISL_842440, EPI_ISL_842441, EPI_ISL_842442, EPI_ISL_842443, EPI_ISL_842444, EPI_ISL_842445, EPI_ISL_842447, EPI_ISL_842448, EPI_ISL_842449, EPI_ISL_842450, EPI_ISL_842451, EPI_ISL_842452, EPI_ISL_842453, EPI_ISL_842454, EPI_ISL_842455, EPI_ISL_842456, EPI_ISL_842457, EPI_ISL_842458, EPI_ISL_842459, EPI_ISL_842460, EPI_ISL_842461, EPI_ISL_842462, EPI_ISL_842463, EPI_ISL_842464, EPI_ISL_842465, EPI_ISL_842466, EPI_ISL_842467, EPI_ISL_842468, EPI_ISL_842469, EPI_ISL_842470, EPI_ISL_842472, EPI_ISL_842473, EPI_ISL_842474, EPI_ISL_842475, EPI_ISL_842476, EPI_ISL_842477, EPI_ISL_842478, EPI_ISL_842479, EPI_ISL_842480, EPI_ISL_842481, EPI_ISL_842482, EPI_ISL_842483, EPI_ISL_842484, EPI_ISL_842485, EPI_ISL_842486, EPI_ISL_842487, EPI_ISL_842488, EPI_ISL_842489, EPI_ISL_842490, EPI_ISL_842491, EPI_ISL_842492, EPI_ISL_842493, EPI_ISL_842494, EPI_ISL_842496, EPI_ISL_842497, EPI_ISL_842498, EPI_ISL_842499, EPI_ISL_842500, EPI_ISL_842501, EPI_ISL_842502, EPI_ISL_842503, EPI_ISL_842504, EPI_ISL_842505, EPI_ISL_842506, EPI_ISL_842507, EPI_ISL_842508, EPI_ISL_842509, EPI_ISL_842510, EPI_ISL_842511, EPI_ISL_842513, EPI_ISL_842583, EPI_ISL_842585, EPI_ISL_842586, EPI_ISL_842587, EPI_ISL_842588, EPI_ISL_842589, EPI_ISL_842590, EPI_ISL_842591, EPI_ISL_842592, EPI_ISL_842593, EPI_ISL_842594, EPI_ISL_842596, EPI_ISL_842598, EPI_ISL_842599, EPI_ISL_842600, EPI_ISL_842601, EPI_ISL_842602, EPI_ISL_842603, EPI_ISL_842604, EPI_ISL_842605, EPI_ISL_842606, EPI_ISL_842607, EPI_ISL_842608			
see above	Bioinformatics and Biostatistics Lab, Advanced Sequencing Facility	COVID-19 Genomics UK (COG-UK) Consortium	Aengus Stewart,Jerome Nicod,Chelsea Sawyer,Laura Cubitt,Harshil Patel,Margaret Crawford
EPI_ISL_842610, EPI_ISL_842611, EPI_ISL_842612, EPI_ISL_842614, EPI_ISL_842615, EPI_ISL_842617, EPI_ISL_842618, EPI_ISL_842619, EPI_ISL_842623, EPI_ISL_842624, EPI_ISL_842625, EPI_ISL_842626, EPI_ISL_842627, EPI_ISL_842628, EPI_ISL_842631, EPI_ISL_842632			
see above	Wyoming Public Health Laboratory	Wyoming Public Health Laboratory	Noah Hull, Taylor Fearing, Lynette Gumbleton, Channing Weber, Ashley Norberg, Bailey Bowcutt, and Wanda Manley
EPI_ISL_843147, EPI_ISL_843148, EPI_ISL_843149, EPI_ISL_843150	Barts Health NHS Trust	COVID-19 Genomics UK (COG-UK) Consortium	CUTINO-MOGUEL, Maria-Teresa; HARRINGTON, David; OWOYEMI, Dola; SHYLINI, Raghavendran; BROAD, Claire; KELE, Beatrix
EPI_ISL_843155, EPI_ISL_843156, EPI_ISL_843157, EPI_ISL_843158, EPI_ISL_843159, EPI_ISL_843160, EPI_ISL_843161, EPI_ISL_843164, EPI_ISL_843165, EPI_ISL_843166	Regional Virus Laboratory, Belfast Health and Social Care Trust	COVID-19 Genomics UK (COG-UK) Consortium	Conall McCaughey, James McKenna, Tanya Curran, Susan Feeney, Alison Watt, Ciara Cox, Mairead Connor, Zoltan Molnar, David Simpson, Derek Fairley
EPI_ISL_843196	LabPLUS	Institute of Environmental Science and Research (ESR)	Xiaoyun Ren, Matt Storey, Nikki Freed, Muhammad Faisal, Jing Wang, Hermes Perez, Anja Werno, Antje van der Linden, Arlo Upton, Chris Mansell, David Hammer, Dragana Drinkovic, Gary McAuliffe, Hana Sofia Andersson, James Ussher, Jill Sherwood, Josh Freeman, Julia Howard, Juliet Elvy, Mary DeAlmeida, Matt Blakiston, Matthew Rogers, Max Bloomfield, Michael Addidle, Michelle Balm, Sally Roberts, Sarah Jefferies, Sharmini Muttaiyah, Susan Morpeth, Susan Taylor, Timothy Blackmore, Vani Sathyendran, Veronica Playle, Virginia Hope, Erasmus Smit, Lauren Jelly, Olin Silander, Joep de Ligt
EPI_ISL_845543, EPI_ISL_845544	Santa Clara County Public Health Laboratory	Santa Clara County Public Health Laboratory	Santa Clara County Public Health Department
EPI_ISL_845588, EPI_ISL_845590, EPI_ISL_845593, EPI_ISL_845606, EPI_ISL_845608, EPI_ISL_845610, EPI_ISL_845611, EPI_ISL_845614, EPI_ISL_845615	KU Leuven, Rega Institute, Clinical and Epidemiological Virology	KU Leuven, Rega Institute, Clinical and Epidemiological Virology	Tony Wawina-Bokalanga, Bert Vanmechelen, Joan Marti-Carerras, Piet Maes
EPI_ISL_845626	FUNDACION CARDIOINFANTIL	Instituto Nacional de Salud - Dirección de Investigación en Salud Pública	Katherine Laiton-Donato, Diego A. Álvarez-Díaz, Carlos Franco-Muñoz, Mauricio Pacheco-Montealegre, Maria T. Herrera-Sepúlveda, Jonathan Reales, Sheryll Corchuelo, Julian Naizaque, Gerardo Santamaría, Paola Muñoz-Laiton, Diego Andrés Prada, Magdalena Wiesner, Martha Lucia Ospina Martinez, Marcela Mercado-Reyes
EPI_ISL_845634	Instituto Nacional de Cancerologia	Instituto Nacional de Salud - Dirección de Investigación en Salud Pública	Katherine Laiton-Donato, Diego A. Álvarez-Díaz, Carlos Franco-Muñoz, Mauricio Pacheco-Montealegre, Maria T. Herrera-Sepúlveda, Jonathan Reales, Sheryll Corchuelo, Julian Naizaque, Gerardo Santamaría, Paola Muñoz-Laiton, Diego Andrés Prada, Magdalena Wiesner, Martha Lucia Ospina Martinez,

			Marcela Mercado-Reyes
EPI_ISL_845636	Laboratorio de Salud Pública - Secretaría Distrital de Salud	Instituto Nacional de Salud - Dirección de Investigación en Salud Pública	Katherine Laiton-Donato, Diego A. Álvarez-Díaz, Carlos Franco-Muñoz, Mauricio Pacheco-Montealegre, Maria T. Herrera-Sepúlveda, Jonathan Reales, Sheryll Corchuelo, Julian Naizaque, Gerardo Santamaría, Paola Muñoz-Laiton, Diego Andrés Prada, Magdalena Wiesner, Martha Lucia Ospina Martinez, Marcela Mercado-Reyes
EPI_ISL_845770	Emory Molecular Diagnostics Laboratory, Emory Healthcare	Piantadosi Lab, Emory Department of Pathology	Ahmed Babiker, Anne Piantadosi
EPI_ISL_845825, EPI_ISL_845826, EPI_ISL_845827, EPI_ISL_845828, EPI_ISL_845829, EPI_ISL_845830, EPI_ISL_845831, EPI_ISL_845832, EPI_ISL_845833, EPI_ISL_845834, EPI_ISL_845835, EPI_ISL_845836, EPI_ISL_845837, EPI_ISL_845838, EPI_ISL_845839, EPI_ISL_845840, EPI_ISL_845841, EPI_ISL_845842, EPI_ISL_845843, EPI_ISL_845844, EPI_ISL_845845, EPI_ISL_845846, EPI_ISL_845847, EPI_ISL_845848, EPI_ISL_845849, EPI_ISL_845853, EPI_ISL_845857, EPI_ISL_845872, EPI_ISL_845873			
see above	TGen North	TGen North	Jolene Bowers, Megan Folkerts, Chris French, Hayley Yaglom, Ashlyn Pfeiffer, Darrin Lemmer, Dave Engelthaler, The Arizona COVID Genomics Union (ACGU)
EPI_ISL_845877	National Institute of Mental Health and Neurosciences (NIMHANS)	Department of Neurovirology, National Institute of Mental Health and Neurosciences (NIMHANS)	Chitra Pattabiraman, Pramada Prasad, Risha Rasheed, Darshan Sreenivas, Nakka Vijay Kiran Reddy, Anita S Desai, V Ravi
EPI_ISL_845878, EPI_ISL_845879, EPI_ISL_845880	BBMP Urban PHC	Department of Neurovirology, National Institute of Mental Health and Neurosciences (NIMHANS)	Chitra Pattabiraman, Pramada Prasad, Risha Rasheed, Darshan Sreenivas, Nakka Vijay Kiran Reddy, Anita S Desai, V Ravi
EPI_ISL_845881	Kidwai Memorial Institute of Oncology	Department of Neurovirology, National Institute of Mental Health and Neurosciences (NIMHANS)	Chitra Pattabiraman, Pramada Prasad, Risha Rasheed, Darshan Sreenivas, Nakka Vijay Kiran Reddy, Anita S Desai, V Ravi
EPI_ISL_845882	VRDL, Mysore Medical College	Department of Neurovirology, National Institute of Mental Health and Neurosciences (NIMHANS)	Chitra Pattabiraman, Pramada Prasad, Risha Rasheed, Darshan Sreenivas, Nakka Vijay Kiran Reddy, Anita S Desai, V Ravi
EPI_ISL_845885, EPI_ISL_845886	Railway Hospital	Department of Neurovirology, National Institute of Mental Health and Neurosciences (NIMHANS)	Chitra Pattabiraman, Pramada Prasad, Risha Rasheed, Darshan Sreenivas, Nakka Vijay Kiran Reddy, Anita S Desai, V Ravi
EPI_ISL_845887, EPI_ISL_845888, EPI_ISL_845890	BBMP Urban PHC	Department of Neurovirology, National Institute of Mental Health and Neurosciences (NIMHANS)	Chitra Pattabiraman, Pramada Prasad, Risha Rasheed, Darshan Sreenivas, Nakka Vijay Kiran Reddy, Anita S Desai, V Ravi
EPI_ISL_846526, EPI_ISL_846536	Lighthouse Lab in Alderley Park	Wellcome Sanger Institute for the COVID-19 Genomics UK (COG-UK) Consortium	Jacquelyn Wynn, Mairead Hyland, The Lighthouse Lab in Alderley Park and Alex Alderton, Roberto Amato, Sonia Goncalves, Ewan Harrison, David K. Jackson, Ian Johnston, Dominic Kwiatkowski, Cordelia Langford, John Sillitoe on behalf of the Wellcome Sanger Institute COVID-19 Surveillance Team
EPI_ISL_847594, EPI_ISL_847595, EPI_ISL_847596, EPI_ISL_847597, EPI_ISL_847598, EPI_ISL_847599, EPI_ISL_847600	California Department of Public Health	Chiu Laboratory, University of California, San Francisco	Charles Chiu, Xianding (Wayne) Deng, Candace Wang, Brian Bushnell, Scot Federman, Jill Hacker, Debra Wadford
EPI_ISL_847730, EPI_ISL_847743	Chiu Laboratory, University of California, San Francisco	Chiu Laboratory, University of California, San Francisco	Charles Chiu, Xianding (Wayne) Deng, Candace Wang, Brian Bushnell, Scot Federman, Jill Hacker, Debra Wadford
EPI_ISL_847756, EPI_ISL_847757, EPI_ISL_847760, EPI_ISL_847761, EPI_ISL_847762, EPI_ISL_847763, EPI_ISL_847764, EPI_ISL_847765	California Department of Public Health	Chiu Laboratory, University of California, San Francisco	Charles Chiu, Xianding (Wayne) Deng, Candace Wang, Brian Bushnell, Scot Federman, Jill Hacker, Debra Wadford
EPI_ISL_847836, EPI_ISL_847839	Tempus	Grubaugh Lab - Yale School of Public Health	Tara Alpert, Joseph Fauver, Anderson Brito, Mallery Breban, Anne Wyllie, Chantal Vogels, Mary Petrone, Chaney Kalinich, Isabel Ott, Nathan Grubaugh
EPI_ISL_847841	Yale New Haven Hospital	Grubaugh Lab - Yale School of Public Health	Tara Alpert, Joseph Fauver, Anderson Brito, Mallery Breban, Anne Wyllie, Chantal Vogels, Mary Petrone, Chaney Kalinich, Isabel Ott, Nathan Grubaugh
EPI_ISL_847873, EPI_ISL_847874, EPI_ISL_847875, EPI_ISL_847876, EPI_ISL_847877, EPI_ISL_847878, EPI_ISL_847879, EPI_ISL_847880, EPI_ISL_847881, EPI_ISL_847882, EPI_ISL_847883, EPI_ISL_847884, EPI_ISL_847885, EPI_ISL_847886, EPI_ISL_847887, EPI_ISL_847888, EPI_ISL_847889, EPI_ISL_847921, EPI_ISL_847924, EPI_ISL_847927, EPI_ISL_847928, EPI_ISL_847937, EPI_ISL_847938, EPI_ISL_847953, EPI_ISL_847954, EPI_ISL_847955, EPI_ISL_847956, EPI_ISL_847957, EPI_ISL_847958, EPI_ISL_847959			
see above	University Hospitals of Geneva, Laboratory of Virology	HUG, Laboratory of Virology and the Health2030 Genome Center	Samuel Cordey, Ana Rita Goncalves, Laurent Kaiser, Lorenzo Cerutti, Henri Pegeot, Melyssa Elies, Keith Harshman, Ioannis Xenarios, Emmanouil Dermitzakis
EPI_ISL_847996, EPI_ISL_848013, EPI_ISL_848044	Michigan Department of Health and Human Services, Bureau of Laboratories	Michigan Department of Health and Human Services, Bureau of Laboratories	Blankenship HM, Riner D, Soehnlen MK
EPI_ISL_848633, EPI_ISL_848642	Montana Public Health Laboratory	Wyoming Public Health Laboratory	Noah Hull, Joy Ritter, Taylor Fearing, Lynette Gumbleton, Channing Weber, Ashley Norberg, Bailey Bowcutt, Wanda Manley, Deborah Gibson
EPI_ISL_848643	Florida Bureau of Public Health Laboratories	Florida Bureau of Public Health Laboratories	Sarah Schmedes, Jason Blanton
EPI_ISL_849322, EPI_ISL_849323, EPI_ISL_849324, EPI_ISL_849325	Delaware Public Health Lab	Delaware Public Health Lab	Gregory Hovan
EPI_ISL_849634	SIESP DIPARTIMENTO DI PREVENZIONE CHIETI	Istituto Zooprofilattico Sperimentale dell'Abruzzo e Molise "G.Caporale"	Lorusso A, Marcacci M, Di Domenico M, Curini V, Ancora M, Cammà C, Rinaldi A, Mangone I, Di Pasquale A, Puglia I, Savini G.
EPI_ISL_849635, EPI_ISL_849636	DIPARTIMENTO PREVENZIONE AVEZZANO-SERVIZIO DI IGIENE EPIDEMIOLOGIA E SANITA' PUBBLICA	Istituto Zooprofilattico Sperimentale dell'Abruzzo e Molise "G.Caporale"	Lorusso A, Marcacci M, Di Domenico M, Curini V, Ancora M, Cammà C, Rinaldi A, Mangone I, Di Pasquale A, Puglia I, Savini G.
EPI_ISL_849637, EPI_ISL_849638, EPI_ISL_849639, EPI_ISL_849640, EPI_ISL_849641, EPI_ISL_849642, EPI_ISL_849643, EPI_ISL_849644, EPI_ISL_849645, EPI_ISL_849646	SIESP DIPARTIMENTO DI PREVENZIONE CHIETI	Istituto Zooprofilattico Sperimentale dell'Abruzzo e Molise "G.Caporale"	Lorusso A, Marcacci M, Di Domenico M, Curini V, Ancora M, Cammà C, Rinaldi A, Mangone I, Di Pasquale A, Puglia I, Savini G.
EPI_ISL_849647, EPI_ISL_849648, EPI_ISL_849649	SIESP- DRIVE IN CHIETI	Istituto Zooprofilattico Sperimentale dell'Abruzzo e Molise "G.Caporale"	Lorusso A, Marcacci M, Di Domenico M, Curini V, Ancora M, Cammà C, Rinaldi A, Mangone I, Di Pasquale A, Puglia I, Savini G.
EPI_ISL_849865, EPI_ISL_849866	A. Krumbholz, Labor Dr. Krause und Kollegen MVZ GmbH, Kiel	Charité Universitätsmedizin Berlin, Institut für Virologie	Victor M Corman, Jörn Beheim-Schwarzbach, Talitha Veith, Julia Schneider, Tobias Bleicker, Julia Tesch, Barbara Mühlemann, Terry Jones, Christian Drosten
EPI_ISL_849895	Utah Public Health Laboratory	Utah Public Health Laboratory	Erin L. Young, Kelly F. Oakeson, Tara Gallagher
EPI_ISL_849925, EPI_ISL_849927	UCSF Clinical Microbiology Laboratory	Chan-Zuckerberg Biohub	CZB Cliahub Consortium
EPI_ISL_850508	Hospital de Tortosa Verge de la Cinta	Hospital Universitari Vall d'Hebron - Vall d'Hebron Institut de Rercerca	Cristina Andrés, Maria Piñana, Josep F Abril, Damir Garcia-Cehic, Ariadna Rando, Juliana Esperalba, Maria Gema Codina, Carla Castillo, Maria Carmen Martin, Tomàs Pumarola, Josep Quer, Andrés Antón
EPI_ISL_850601, EPI_ISL_850602, EPI_ISL_850603, EPI_ISL_850604, EPI_ISL_850605, EPI_ISL_850606, EPI_ISL_850607, EPI_ISL_850608, EPI_ISL_850609, EPI_ISL_850610, EPI_ISL_850611, EPI_ISL_850612, EPI_ISL_850613, EPI_ISL_850614, EPI_ISL_850615, EPI_ISL_850616, EPI_ISL_850617, EPI_ISL_850618, EPI_ISL_850619, EPI_ISL_850620, EPI_ISL_850621, EPI_ISL_850622, EPI_ISL_850623, EPI_ISL_850624, EPI_ISL_850625, EPI_ISL_850626, EPI_ISL_850627, EPI_ISL_850628, EPI_ISL_850629, EPI_ISL_850630, EPI_ISL_850631, EPI_ISL_850632, EPI_ISL_850633, EPI_ISL_850634, EPI_ISL_850635, EPI_ISL_850636, EPI_ISL_850637, EPI_ISL_850638, EPI_ISL_850639, EPI_ISL_850640, EPI_ISL_850641, EPI_ISL_850642, EPI_ISL_850643, EPI_ISL_850644, EPI_ISL_850645, EPI_ISL_850646, EPI_ISL_850647, EPI_ISL_850648, EPI_ISL_850649, EPI_ISL_850650, EPI_ISL_850651			
see above	Helix/Illumina	Genomics and Discovery, Respiratory Viruses Branch, Division of Viral Diseases, Centers for Disease Control and Prevention	Peter W. Cook, Dhwani Batra, Ben L. Rambo-Martin Eileen de Feo, Jan Antico, Christine Tran, Matthew Tolentino, Shannon Wickline, Kim Gietzen, Brad Sickler, Jingtao Liu, Eric Allen, Phil Febbo, Summer Galloway, Nicole L. Washington, Simon White, Geraint Levan, Kelly Schiabor Barrett, Elizabeth Cirulli, Alexandre Bolze, Ary Ascencio, Charlotte Rivera-Garcia, Ryan Cho, Jason Nguyen, Sherry Wang, Jimmy Ramirez, Tyler Cassens, Efren Sandoval, Magnus Isaksson, William Lee, David Becker, Marc Laurent, James Lu, Clinton R. Paden, Suxiang Tong, Duncan MacCannell
EPI_ISL_850660, EPI_ISL_850661	Division of Emerging Infectious Diseases, Bureau of Infectious Diseases Diagnosis Control, Korea Disease Control and Prevention Agency	Division of Emerging Infectious Diseases, Bureau of Infectious Diseases Diagnosis Control, Korea Disease Control and Prevention Agency	Ae Kyung Park, Il-Hwan Kim, Heui Man Kim, Jeong-Min Kim, Namjoo Lee, Chaeyoung Lee, Sang Hee Woo, Eun-Jin Kim

EPI_ISL_850942, EPI_ISL_850943, EPI_ISL_850944	Helix / Illumina	Genomics and Discovery, Respiratory Viruses Branch, Division of Viral Diseases, Centers for Disease Control and Prevention	Peter W. Cook, Dhwani Batra, Ben L. Rambo-Martin Eileen de Feo, Jan Antico, Christine Tran, Matthew Tolentino, Shannon Wickline, Kim Gietzen, Brad Sickler, Jingtao Liu, Eric Allen, Phil Febbo, Summer Galloway, Nicole L. Washington, Simon White, Geraint Levan, Kelly Schiabor Barrett, Elizabeth Cirulli, Alexandre Bolze, Ary Ascencio, Charlotte Rivera-Garcia, Ryan Cho, Jason Nguyen, Sherry Wang, Jimmy Ramirez, Tyler Cassens, Efren Sandoval, Magnus Isaksson, William Lee, David Becker, Marc Laurent, James Lu, Clinton R. Paden, Suxiang Tong, Duncan MacCannell
EPI_ISL_851007, EPI_ISL_851008, EPI_ISL_851009, EPI_ISL_851010, EPI_ISL_851011, EPI_ISL_851012, EPI_ISL_851013, EPI_ISL_851014, EPI_ISL_851015, EPI_ISL_851016, EPI_ISL_851017, EPI_ISL_851018, EPI_ISL_851019, EPI_ISL_851020, EPI_ISL_851026, EPI_ISL_851027, EPI_ISL_851028, EPI_ISL_851033, EPI_ISL_851034, EPI_ISL_851035, EPI_ISL_851036, EPI_ISL_851037, EPI_ISL_851038, EPI_ISL_851039, EPI_ISL_851040			
see above	Helix/Illumina	Genomics and Discovery, Respiratory Viruses Branch, Division of Viral Diseases, Centers for Disease Control and Prevention	Peter W. Cook, Dhwani Batra, Ben L. Rambo-Martin Eileen de Feo, Jan Antico, Christine Tran, Matthew Tolentino, Shannon Wickline, Kim Gietzen, Brad Sickler, Jingtao Liu, Eric Allen, Phil Febbo, Summer Galloway, Nicole L. Washington, Simon White, Geraint Levan, Kelly Schiabor Barrett, Elizabeth Cirulli, Alexandre Bolze, Ary Ascencio, Charlotte Rivera-Garcia, Ryan Cho, Jason Nguyen, Sherry Wang, Jimmy Ramirez, Tyler Cassens, Efren Sandoval, Magnus Isaksson, William Lee, David Becker, Marc Laurent, James Lu, Clinton R. Paden, Suxiang Tong, Duncan MacCannell
EPI_ISL_852017	Lighthouse Lab in Milton Keynes	Wellcome Sanger Institute for the COVID-19 Genomics UK (COG-UK) Consortium	The Lighthouse Lab in Milton Keynes and Alex Alderton, Roberto Amato, Sonia Goncalves, Ewan Harrison, David K. Jackson, Ian Johnston, Dominic Kwiatkowski, Cordelia Langford, John Sillitoe on behalf of the Wellcome Sanger Institute COVID-19 Surveillance Team
EPI_ISL_852191	Lighthouse Lab in Alderley Park	Wellcome Sanger Institute for the COVID-19 Genomics UK (COG-UK) Consortium	Jacquelyn Wynn, Mairead Hyland, The Lighthouse Lab in Alderley Park and Alex Alderton, Roberto Amato, Sonia Goncalves, Ewan Harrison, David K. Jackson, Ian Johnston, Dominic Kwiatkowski, Cordelia Langford, John Sillitoe on behalf of the Wellcome Sanger Institute COVID-19 Surveillance Team
EPI_ISL_852565, EPI_ISL_852579	Max von Pettenkofer Institute, Virology, National Reference Center for Retroviruses, LMU München	Laboratory for Functional Genome Analysis, Dept. Genomics, Gene Center of the LMU Munich	Max Muenchhoff, Stefan Krebs, Alexander Graf, Oliver Keppler, Helmut Blum
EPI_ISL_852619, EPI_ISL_852622, EPI_ISL_852628	Lab. Microbiologia e Virologia, Cotugno, A.O. dei Colli	Lab. Microbiologia e Virologia, Cotugno, A.O. dei Colli	Luigi Atripaldi, Claudia Tiberio, Anna Perfetti
EPI_ISL_852818	CHU Purpan - Laboratoire de Virologie - Institut Fédératif de Biologie	CHU Purpan - Laboratoire de Virologie - Institut Fédératif de Biologie	Latour J., Ranger N., Dubois M., Carcenac R., Harter A., Boyer P., Tremeaux P., Izopet J.
EPI_ISL_852974, EPI_ISL_853002	Hospital General Universitario Gregorio Marañón	SeqCOVID-SPAIN consortium/IBV(CSIC)	Darío García de Viedma, Laura Pérez-Lago, Pedro J Sola-Campoy, Sergio Buenestado-Serrano, Marta Herranz, Victor Manuel de la Cueva, Julia Suárez, Pilar Catalán, Patricia Muñoz and SeqCOVID-SPAIN consortium
EPI_ISL_853401, EPI_ISL_853402, EPI_ISL_853403	Charité Universitätsmedizin Berlin, Institut für Virologie/Labor Berlin	Charité Universitätsmedizin Berlin, Institut für Virologie	Victor M Corman, Julia Schneider, Barbara Mühlemann, Jörn Beheim-Schwarzbach, Talitha Veith, Julia Tesch, Tobias Bleicker, Terry Jones, Christian Drosten
EPI_ISL_853688	THE MARY IMOGENE BASSETT HOSPITAL	Wadsworth Center, New York State Department of Health	Kirsten St. George, Daryl M. Lamson, Alexis Russel, Matthew Shudt, Melissa A Leisner, Jonathan Plitnick, Navjot Singh, John Kelly, Erasmus Schneider, Erica Lasek-Nesselquist
EPI_ISL_853689	SUNY UPSTATE MEDICAL UNIVERSITY	Wadsworth Center, New York State Department of Health	Kirsten St. George, Daryl M. Lamson, Alexis Russel, Matthew Shudt, Melissa A Leisner, Jonathan Plitnick, Navjot Singh, John Kelly, Erasmus Schneider, Erica Lasek-Nesselquist
EPI_ISL_853690, EPI_ISL_853691, EPI_ISL_853692, EPI_ISL_853693, EPI_ISL_853694, EPI_ISL_853695, EPI_ISL_853696, EPI_ISL_853697, EPI_ISL_853698, EPI_ISL_853699, EPI_ISL_853700, EPI_ISL_853701, EPI_ISL_853702, EPI_ISL_853703, EPI_ISL_853704			
see above	ALBANY MEDICAL CENTER HOSPITAL CLINICAL LABORATORIES	Wadsworth Center, New York State Department of Health	Kirsten St. George, Daryl M. Lamson, Alexis Russel, Matthew Shudt, Melissa A Leisner, Jonathan Plitnick, Navjot Singh, John Kelly, Erasmus Schneider, Erica Lasek-Nesselquist
EPI_ISL_853705	NORTHWELL HEALTH LABORATORIES	Wadsworth Center, New York State Department of Health	Kirsten St. George, Daryl M. Lamson, Alexis Russel, Matthew Shudt, Melissa A Leisner, Jonathan Plitnick, Navjot Singh, John Kelly, Erasmus Schneider, Erica Lasek-Nesselquist
EPI_ISL_853706, EPI_ISL_853707, EPI_ISL_853708	SUNY UPSTATE MEDICAL UNIVERSITY	Wadsworth Center, New York State Department of Health	Kirsten St. George, Daryl M. Lamson, Alexis Russel, Matthew Shudt, Melissa A Leisner, Jonathan Plitnick, Navjot Singh, John Kelly, Erasmus Schneider, Erica Lasek-Nesselquist
EPI_ISL_853709, EPI_ISL_853710, EPI_ISL_853711	ALBANY MEDICAL CENTER HOSPITAL CLINICAL LABORATORIES	Wadsworth Center, New York State Department of Health	Kirsten St. George, Daryl M. Lamson, Alexis Russel, Matthew Shudt, Melissa A Leisner, Jonathan Plitnick, Navjot Singh, John Kelly, Erasmus Schneider, Erica Lasek-Nesselquist
EPI_ISL_853715	SUNY UPSTATE MEDICAL UNIVERSITY	Wadsworth Center, New York State Department of Health	Kirsten St. George, Daryl M. Lamson, Alexis Russel, Matthew Shudt, Melissa A Leisner, Jonathan Plitnick, Navjot Singh, John Kelly, Erasmus Schneider, Erica Lasek-Nesselquist
EPI_ISL_853742	Department of Microbiology, University Innsbruck	Bergthaler laboratory, CeMM Research Center for Molecular Medicine of the Austrian Academy of Sciences	Lukas Endler, Alexandra Popa, Benedikt Agerer, Jakob-Wendelin Genger, Alexander Lercher, Anna Schedl, Thomas Penz, Michael Schuster, Jan Laine, Martin Senekowitsch, Christoph Bock, Andreas Bergthaler
EPI_ISL_854350, EPI_ISL_854351, EPI_ISL_854352, EPI_ISL_854353, EPI_ISL_854354, EPI_ISL_854355, EPI_ISL_854356, EPI_ISL_854357, EPI_ISL_854358, EPI_ISL_854359, EPI_ISL_854360, EPI_ISL_854361, EPI_ISL_854362, EPI_ISL_854363, EPI_ISL_854364, EPI_ISL_854365, EPI_ISL_854366, EPI_ISL_854367, EPI_ISL_854369, EPI_ISL_854372			
see above	BIO-REFERENCE LABORATORIES	Wadsworth Center, New York State Department of Health	Kirsten St. George, Daryl M. Lamson, Alexis Russel, Matthew Shudt, Melissa A Leisner, Jonathan Plitnick, Navjot Singh, John Kelly, Erasmus Schneider, Erica Lasek-Nesselquist
EPI_ISL_854405, EPI_ISL_854406, EPI_ISL_854407, EPI_ISL_854408, EPI_ISL_854409, EPI_ISL_854410, EPI_ISL_854411, EPI_ISL_854412, EPI_ISL_854413, EPI_ISL_854414, EPI_ISL_854415, EPI_ISL_854450, EPI_ISL_854452, EPI_ISL_854454, EPI_ISL_854455, EPI_ISL_854457, EPI_ISL_854458			
see above	MONTEFIORE MEDICAL CENTER LABORATORIES	Wadsworth Center, New York State Department of Health	Kirsten St. George, Daryl M. Lamson, Alexis Russel, Matthew Shudt, Melissa A Leisner, Jonathan Plitnick, Navjot Singh, John Kelly, Erasmus Schneider, Erica Lasek-Nesselquist
EPI_ISL_854748	Victorian Infectious Diseases Reference Laboratory (VIDRL)	VIDRL and MDU-PHL	Caly L., Seemann T., Sait, M.L., Druce J., Sherry, N.L.
EPI_ISL_855388	Hospital	National Reference Center for Viruses of Respiratory Infections, Institut Pasteur, Paris	Marion Barbet, Sylvie Behillil, Méline Bizard, Angela Brisebarre, Camille Capel, Etienne Simon-Lorière, Vincent Enouf, Maud Vanpeene, Sylvie van der Werf,Ducancelle Alexandra
EPI_ISL_855393, EPI_ISL_855394	California Department of Public Health	Chiu Laboratory, University of California, San Francisco	Charles Chiu, Xianding (Wayne) Deng, Candace Wang, Brian Bushnell, Scot Federman, Jill Hacker, Debra Wadford
EPI_ISL_855556	Lab. Microbiologia e Virologia, Cotugno, A.O. dei Colli	Lab. Microbiologia e Virologia, Cotugno, A.O. dei Colli	Luigi Atripaldi, Claudia Tiberio, Anna Perfetti
EPI_ISL_855908	Lab voor klinische biologie	Onderzoeksgroep Virologie	Nick Vereecke, Laurens Lambrechts, Marthe Pauwels, Bruno Verhasselt, Linos Vandekerckhove, Hans Nauwynck, Sebastiaan Theuns
EPI_ISL_855923, EPI_ISL_855925, EPI_ISL_855927, EPI_ISL_855928, EPI_ISL_855934	Lab voor klinische biologie	Onderzoeksgroep Virologie	Laurens Lambrechts, Nick Vereecke, Marthe Pauwels, Bruno Verhasselt, Linos Vandekerckhove, Hans Nauwynck, Sebastiaan Theuns
EPI_ISL_855940, EPI_ISL_855942	Lab voor klinische biologie	Onderzoeksgroep Virologie	Nick Vereecke, Laurens Lambrechts, Marthe Pauwels, Bruno Verhasselt, Linos Vandekerckhove, Hans Nauwynck, Sebastiaan Theuns
EPI_ISL_856680	Charité Universitätsmedizin Berlin, Institute of Virology, Charitéplatz 1, 10117 Berlin, Germany	Charité Universitätsmedizin Berlin, Institute of Virology, Charitéplatz 1, 10117 Berlin, Germany	Victor M Corman, Julia Schneider, Jörn Beheim-Schwarzbach, Tobias Bleicker, Julia Tesch, Barbara Mühlemann, Talitha Veith, Terry Jones, Christian Drosten
EPI_ISL_857052, EPI_ISL_857055	Platform BIS UZA/UAntwerpen, University Hospital Antwerp, Edegem, Belgium	UAntwerp, Laboratory of Medical Microbiology, Campus Drie Eiken S6.26, Universiteitsplein 1, 2610, Wilrijk, Antwerp, Belgium	Basil Britto Xavier, Jasmine Coppens, Christine Lammens, Veerle Matheeussen, Herman Goossens
EPI_ISL_857056, EPI_ISL_857057, EPI_ISL_857058	OCME Office Of Chief Medical Examiner	New York City Public Health Laboratory	Jade Wang, et al.
EPI_ISL_857189	DOHMH Corona	New York City Public Health Laboratory	Jade Wang, et al.
EPI_ISL_857190	DOHMH PHL	New York City Public Health Laboratory	Jade Wang, et al.
EPI_ISL_857191	DOHMH Morrisania	New York City Public Health Laboratory	Jade Wang, et al.
EPI_ISL_857195	DOHMH Riverside	New York City Public Health Laboratory	Jade Wang, et al.

EPI_ISL_857196	DOHMH Crown Heights	New York City Public Health Laboratory	Jade Wang, et al.
EPI_ISL_857197	DOHMH Jamaica	New York City Public Health Laboratory	Jade Wang, et al.
EPI_ISL_857202	DOHMH PHL	New York City Public Health Laboratory	Jade Wang, et al.
EPI_ISL_857204, EPI_ISL_857205	DOHMH Central Harlem	New York City Public Health Laboratory	Jade Wang, et al.
EPI_ISL_857227, EPI_ISL_857228	DOHMH Riverside	New York City Public Health Laboratory	Jade Wang, et al.
EPI_ISL_857229	DOHMH Morrisania	New York City Public Health Laboratory	Jade Wang, et al.
EPI_ISL_857230	DOHMH Chelsea	New York City Public Health Laboratory	Jade Wang, et al.
EPI_ISL_857231, EPI_ISL_857232, EPI_ISL_857233	DOHMH Central Harlem	New York City Public Health Laboratory	Jade Wang, et al.
EPI_ISL_857234, EPI_ISL_857235, EPI_ISL_857236, EPI_ISL_857237	DOHMH Jamaica	New York City Public Health Laboratory	Jade Wang, et al.
EPI_ISL_857238, EPI_ISL_857239, EPI_ISL_857240	DOHMH Corona	New York City Public Health Laboratory	Jade Wang, et al.
EPI_ISL_857241, EPI_ISL_857242, EPI_ISL_857243, EPI_ISL_857244, EPI_ISL_857245, EPI_ISL_857246, EPI_ISL_857247, EPI_ISL_857248, EPI_ISL_857249, EPI_ISL_857250	DOHMH Jamaica	New York City Public Health Laboratory	Jade Wang, et al.
EPI_ISL_857251, EPI_ISL_857252	DOHMH Morrisania	New York City Public Health Laboratory	Jade Wang, et al.
EPI_ISL_857253, EPI_ISL_857254	DOHMH Chelsea	New York City Public Health Laboratory	Jade Wang, et al.
EPI_ISL_857255	DOHMH Central Harlem	New York City Public Health Laboratory	Jade Wang, et al.
EPI_ISL_857256	DOHMH Fort Greene	New York City Public Health Laboratory	Jade Wang, et al.
EPI_ISL_857257	DOHMH PHL	New York City Public Health Laboratory	Jade Wang, et al.
EPI_ISL_857258, EPI_ISL_857259	DOHMH Central Harlem	New York City Public Health Laboratory	Jade Wang, et al.
EPI_ISL_857260, EPI_ISL_857261, EPI_ISL_857262	DOHMH Chelsea	New York City Public Health Laboratory	Jade Wang, et al.
EPI_ISL_857263, EPI_ISL_857264	DOHMH Corona	New York City Public Health Laboratory	Jade Wang, et al.
EPI_ISL_857265	DOHMH Fort Greene	New York City Public Health Laboratory	Jade Wang, et al.
EPI_ISL_857266	DOHMH PHL	New York City Public Health Laboratory	Jade Wang, et al.
EPI_ISL_857267, EPI_ISL_857268, EPI_ISL_857269, EPI_ISL_857270	DOHMH Jamaica	New York City Public Health Laboratory	Jade Wang, et al.
EPI_ISL_857271, EPI_ISL_857272, EPI_ISL_857273	DOHMH Morrisania	New York City Public Health Laboratory	Jade Wang, et al.
EPI_ISL_857274, EPI_ISL_857275, EPI_ISL_857276	DOHMH Jamaica	New York City Public Health Laboratory	Jade Wang, et al.
EPI_ISL_857277	DOHMH Corona	New York City Public Health Laboratory	Jade Wang, et al.
EPI_ISL_857278	DOHMH PHL	New York City Public Health Laboratory	Jade Wang, et al.
EPI_ISL_858274, EPI_ISL_858313	Lighthouse Lab in Glasgow	Wellcome Sanger Institute for the COVID-19 Genomics UK (COG-UK) Consortium	Harper VanSteenhouse, Yumi Kasai, David Gray, Carol Clugston, Anna Dominiczak and Alex Alderton, Roberto Amato, Sonia Goncalves, Ewan Harrison, David K. Jackson, Ian Johnston, Dominic Kwiatkowski, Cordelia Langford, John Sillitoe on behalf of the Wellcome Sanger Institute COVID-19 Surveillance Team
EPI_ISL_858573, EPI_ISL_858575, EPI_ISL_858577, EPI_ISL_858578, EPI_ISL_858579, EPI_ISL_858582	Lighthouse Lab in Alderley Park	Wellcome Sanger Institute for the COVID-19 Genomics UK (COG-UK) Consortium	Jacquelyn Wynn, Mairead Hyland, The Lighthouse Lab in Alderley Park and Alex Alderton, Roberto Amato, Sonia Goncalves, Ewan Harrison, David K. Jackson, Ian Johnston, Dominic Kwiatkowski, Cordelia Langford, John Sillitoe on behalf of the Wellcome Sanger Institute COVID-19 Surveillance Team
EPI_ISL_858589, EPI_ISL_858591, EPI_ISL_858593, EPI_ISL_858595, EPI_ISL_858596, EPI_ISL_858599, EPI_ISL_858602, EPI_ISL_858603, EPI_ISL_858605, EPI_ISL_858606, EPI_ISL_858611, EPI_ISL_858617, EPI_ISL_858620, EPI_ISL_858622, EPI_ISL_858625, EPI_ISL_858629, EPI_ISL_858631, EPI_ISL_858632, EPI_ISL_858639			
see above	Lighthouse Lab in Glasgow	Wellcome Sanger Institute for the COVID-19 Genomics UK (COG-UK) Consortium	Harper VanSteenhouse, Yumi Kasai, David Gray, Carol Clugston, Anna Dominiczak and Alex Alderton, Roberto Amato, Sonia Goncalves, Ewan Harrison, David K. Jackson, Ian Johnston, Dominic Kwiatkowski, Cordelia Langford, John Sillitoe on behalf of the Wellcome Sanger Institute COVID-19 Surveillance Team
EPI_ISL_858644, EPI_ISL_858647, EPI_ISL_858648, EPI_ISL_858649, EPI_ISL_858653, EPI_ISL_858654, EPI_ISL_858655, EPI_ISL_858662, EPI_ISL_858663, EPI_ISL_858666, EPI_ISL_858667, EPI_ISL_858669, EPI_ISL_858670, EPI_ISL_858671, EPI_ISL_858672, EPI_ISL_858673, EPI_ISL_858674, EPI_ISL_858675, EPI_ISL_858676, EPI_ISL_858677, EPI_ISL_858678, EPI_ISL_858679, EPI_ISL_858680, EPI_ISL_858681, EPI_ISL_858682, EPI_ISL_858683, EPI_ISL_858684, EPI_ISL_858685, EPI_ISL_858686, EPI_ISL_858687, EPI_ISL_858688, EPI_ISL_858689, EPI_ISL_858690, EPI_ISL_858691, EPI_ISL_858692, EPI_ISL_858693, EPI_ISL_858694, EPI_ISL_858695, EPI_ISL_858696, EPI_ISL_858697, EPI_ISL_858698, EPI_ISL_858699, EPI_ISL_858700			
see above	Lighthouse Lab in Alderley Park	Wellcome Sanger Institute for the COVID-19 Genomics UK (COG-UK) Consortium	Jacquelyn Wynn, Mairead Hyland, The Lighthouse Lab in Alderley Park and Alex Alderton, Roberto Amato, Sonia Goncalves, Ewan Harrison, David K. Jackson, Ian Johnston, Dominic Kwiatkowski, Cordelia Langford, John Sillitoe on behalf of the Wellcome Sanger Institute COVID-19 Surveillance Team
EPI_ISL_858711, EPI_ISL_858712, EPI_ISL_858715, EPI_ISL_858716, EPI_ISL_858717, EPI_ISL_858718, EPI_ISL_858721, EPI_ISL_858722, EPI_ISL_858723, EPI_ISL_858724, EPI_ISL_858726, EPI_ISL_858727, EPI_ISL_858728, EPI_ISL_858729, EPI_ISL_858730, EPI_ISL_858731, EPI_ISL_858733, EPI_ISL_858734, EPI_ISL_858735, EPI_ISL_858736, EPI_ISL_858738, EPI_ISL_858740, EPI_ISL_858741, EPI_ISL_858742, EPI_ISL_858743, EPI_ISL_858744, EPI_ISL_858745, EPI_ISL_858746, EPI_ISL_858747, EPI_ISL_858749, EPI_ISL_858750, EPI_ISL_858751, EPI_ISL_858754, EPI_ISL_858755, EPI_ISL_858756, EPI_ISL_858757, EPI_ISL_858758, EPI_ISL_858759, EPI_ISL_858760, EPI_ISL_858761, EPI_ISL_858762, EPI_ISL_858763, EPI_ISL_858764, EPI_ISL_858765, EPI_ISL_858766, EPI_ISL_858767, EPI_ISL_858768, EPI_ISL_858769, EPI_ISL_858770, EPI_ISL_858771, EPI_ISL_858772, EPI_ISL_858773, EPI_ISL_858774, EPI_ISL_858775, EPI_ISL_858776, EPI_ISL_858777, EPI_ISL_858781, EPI_ISL_858783			
see above	Lighthouse Lab in Glasgow	Wellcome Sanger Institute for the COVID-19 Genomics UK (COG-UK) Consortium	Harper VanSteenhouse, Yumi Kasai, David Gray, Carol Clugston, Anna Dominiczak and Alex Alderton, Roberto Amato, Sonia Goncalves, Ewan Harrison, David K. Jackson, Ian Johnston, Dominic Kwiatkowski, Cordelia Langford, John Sillitoe on behalf of the Wellcome Sanger Institute COVID-19 Surveillance Team
EPI_ISL_860026, EPI_ISL_860028, EPI_ISL_860029, EPI_ISL_860030, EPI_ISL_860031, EPI_ISL_860032, EPI_ISL_860033, EPI_ISL_860034, EPI_ISL_860035, EPI_ISL_860036, EPI_ISL_860037, EPI_ISL_860038, EPI_ISL_860039, EPI_ISL_860040, EPI_ISL_860041, EPI_ISL_860044, EPI_ISL_860045, EPI_ISL_860046, EPI_ISL_860047, EPI_ISL_860070, EPI_ISL_860072, EPI_ISL_860074, EPI_ISL_860078			
see above	BTC, Khalifa University	BTC, Khalifa University	Al Safar et al
EPI_ISL_860182, EPI_ISL_860183	Bangalore Medical College and Research Institute	Department of Neurovirology, National Institute of Mental Health and Neurosciences (NIMHANS)	Chitra Pattabiraman, Pramada Prasad, Anson Kunjumon George, Risha Rasheed, Darshan Sreenivas, Nakka Vijay Kiran Reddy, Anita S Desai, V Ravi
EPI_ISL_860208, EPI_ISL_860211	Haukeland University Hospital, Dept. of Microbiology	Norwegian Institute of Public Health, Department of Virology	Kathrine Stene-Johansen, Kamilla Heddeland Instefjord, Hilde Elshaug, Atiya R Ali,Marie Paulsen Madsen, Rasmus Riis Kopperud, Hilde Vollan, Karoline Bragstad, Olav Hungnes
EPI_ISL_860213	Furst Medical Laboratory	Norwegian Institute of Public Health, Department of Virology	Kathrine Stene-Johansen, Kamilla Heddeland Instefjord, Hilde Elshaug, Atiya R Ali,Marie Paulsen Madsen, Rasmus Riis Kopperud, Hilde Vollan, Karoline Bragstad, Olav Hungnes

EPI_ISL_860230	Ostfold Hospital Trust - Kalnes, Centre for Laboratory Medicine, Section for gene technology and infection serology	Norwegian Institute of Public Health, Department of Virology	Kathrine Stene-Johansen, Kamilla Heddeland Instefjord, Hilde Elshaug, Atiya R Ali,Marie Paulsen Madsen, Rasmus Riis Kopperud, Hilde Vollan, Karoline Bragstad, Olav Hungnes
EPI_ISL_860236	Hospital of Southern Norway - Kristiansand, Department of Medical Microbiology	Norwegian Institute of Public Health, Department of Virology	Kathrine Stene-Johansen, Kamilla Heddeland Instefjord, Hilde Elshaug, Atiya R Ali,Marie Paulsen Madsen, Rasmus Riis Kopperud, Hilde Vollan, Karoline Bragstad, Olav Hungnes
EPI_ISL_860289	Dept. of Medical Microbiology, Stavanger University Hospital, Helse Stavanger HF	Norwegian Institute of Public Health, Department of Virology	Kathrine Stene-Johansen, Kamilla Heddeland Instefjord, Hilde Elshaug, Atiya R Ali,Marie Paulsen Madsen, Rasmus Riis Kopperud, Hilde Vollan, Karoline Bragstad, Olav Hungnes
EPI_ISL_860302, EPI_ISL_860303	Unit 17: Influenza & Other Respiratory Viruses, German National Influenza Center	Project group Epidemiology of Highly Pathogenic Microorganisms, Robert Koch-Institute	Andreas Sachse, Grit Schubert, Essia Belarbi, Sébastien Calvignac-Spencer, Thorsten Wolff, Ralf Dürrwald, Djin-Ye Oh, Marianne Wedde
EPI_ISL_860559, EPI_ISL_860576, EPI_ISL_860579, EPI_ISL_860580, EPI_ISL_860581, EPI_ISL_860583, EPI_ISL_860584, EPI_ISL_860586	NHLS-IALCH	KRISP, KZn Research Innovation and Sequencing Platform	Giandhari J, Pillay S, Lessells R, Mdlalose K, York D, Khan S, Tegally H, Wilkinson E, de Oliveira T
EPI_ISL_860642, EPI_ISL_860643, EPI_ISL_860644, EPI_ISL_860645, EPI_ISL_860646, EPI_ISL_860647, EPI_ISL_860648, EPI_ISL_860649, EPI_ISL_860650, EPI_ISL_860651, EPI_ISL_860653, EPI_ISL_860654, EPI_ISL_860655, EPI_ISL_860656, EPI_ISL_860657, EPI_ISL_860658, EPI_ISL_860659, EPI_ISL_860660, EPI_ISL_860661, EPI_ISL_860662, EPI_ISL_860663, EPI_ISL_860664, EPI_ISL_860665, EPI_ISL_860782			
see above	Respiratory Virus Unit, National Infection Service, Public Health England	COVID-19 Genomics UK (COG-UK) Consortium	PHE Covid Sequencing Team
EPI_ISL_860864, EPI_ISL_860869, EPI_ISL_860870, EPI_ISL_860871, EPI_ISL_860882, EPI_ISL_860883, EPI_ISL_860884	Labo Analyses Med	National Reference Center for Viruses of Respiratory Infections, Institut Pasteur, Paris	Marion Barbet, Sylvie Behillil, Méline Bizard, Angela Brisebarre, Camille Capel, Etienne Simon-Lorière, Vincent Enouf, Maud Vanpeene, Sylvie van der Werf,Lefaure Brieuc
EPI_ISL_860898	Centre Hospitalier Compiègne-Noyon - Laboratoire	National Reference Center for Viruses of Respiratory Infections, Institut Pasteur, Paris	Marion Barbet, Sylvie Behillil, Méline Bizard, Angela Brisebarre, Camille Capel, Etienne Simon-Lorière, Vincent Enouf, Maud Vanpeene, Sylvie van der Werf,Emond Jean-Philippe
EPI_ISL_860938, EPI_ISL_860946, EPI_ISL_860950, EPI_ISL_860957, EPI_ISL_860960, EPI_ISL_860969, EPI_ISL_861097, EPI_ISL_861102	Johns Hopkins Hospital Department of Pathology	Johns Hopkins Hospital Department of Pathology	C. Paul Morris, Chun Huai Luo, Adannaya Amadi, Nicholas Gallagher, Heba H. Mostafa
EPI_ISL_861109	New York Presbyterian Hospital	Wadsworth Center, New York State Department of Health	Kirsten St. George, Daryl M. Lamson, Alexis Russel, Matthew Shudt, Melissa A Leisner, Jonathan Plitnick, Navjot Singh, John Kelly, Erasmus Schneider, Erica Lasek-Nesselquist
EPI_ISL_861135, EPI_ISL_861136, EPI_ISL_861137, EPI_ISL_861138, EPI_ISL_861139, EPI_ISL_861140, EPI_ISL_861141, EPI_ISL_861142, EPI_ISL_861143	ADIRONDACK MEDICAL CENTER	Wadsworth Center, New York State Department of Health	Kirsten St. George, Daryl M. Lamson, Alexis Russel, Matthew Shudt, Melissa A Leisner, Jonathan Plitnick, Navjot Singh, John Kelly, Erasmus Schneider, Erica Lasek-Nesselquist
EPI_ISL_861191	NORTH SHORE UNIVERSITY HOSPITAL	Wadsworth Center, New York State Department of Health	Kirsten St. George, Daryl M. Lamson, Alexis Russel, Matthew Shudt, Melissa A Leisner, Jonathan Plitnick, Navjot Singh, John Kelly, Erasmus Schneider, Erica Lasek-Nesselquist
EPI_ISL_861290, EPI_ISL_861292, EPI_ISL_861295, EPI_ISL_861296, EPI_ISL_861297, EPI_ISL_861298, EPI_ISL_861300, EPI_ISL_861305, EPI_ISL_861306, EPI_ISL_861310, EPI_ISL_861312, EPI_ISL_861314, EPI_ISL_861317, EPI_ISL_861323, EPI_ISL_861325, EPI_ISL_861327, EPI_ISL_861329			
see above	MONTEFIORE MEDICAL CENTER LABORATORIES	Wadsworth Center, New York State Department of Health	Kirsten St. George, Daryl M. Lamson, Alexis Russel, Matthew Shudt, Melissa A Leisner, Jonathan Plitnick, Navjot Singh, John Kelly, Erasmus Schneider, Erica Lasek-Nesselquist
EPI_ISL_861413	NORTH SHORE UNIVERSITY HOSPITAL	Wadsworth Center, New York State Department of Health	Kirsten St. George, Daryl M. Lamson, Alexis Russel, Matthew Shudt, Melissa A Leisner, Jonathan Plitnick, Navjot Singh, John Kelly, Erasmus Schneider, Erica Lasek-Nesselquist
EPI_ISL_861434, EPI_ISL_861435	Ohio State University	James Molecular Laboratory	Huolin Tu, Matthew R Avenarius, Laura Kubatko, Matthew Hunt, Xiaokang Pan, Peng Ru, Jason Garee, Keelie Thomas, Peter Mohler, Preeti Pancholi, Dan Jones
EPI_ISL_861439, EPI_ISL_861440, EPI_ISL_861441, EPI_ISL_861442	The Ohio State University	James Molecular Laboratory	Huolin Tu, Matthew R Avenarius, Laura Kubatko, Matthew Hunt, Xiaokang Pan, Peng Ru, Jason Garee, Keelie Thomas, Peter Mohler, Preeti Pancholi, Dan Jones
EPI_ISL_861538, EPI_ISL_861552, EPI_ISL_861568, EPI_ISL_861570, EPI_ISL_861589	Instituto Nacional de Saude (INSA)	Instituto Nacional de Saude (INSA)	Borges et al
EPI_ISL_861699	Los Angeles County PHL	Los Angeles County PHL	P. Hemarajata et al.
EPI_ISL_861866	Bioanalytika AG	University Hospital Basel, Clinical Bacteriology	Tim Roloff, Madlen Stange, Helena MB Seth-Smith, Alfredo Mari, Karoline Leuzinger, Julia Bielicki, Adrian Härri, Manuel Battegay, Hans Hirsch, Adrian Egli
EPI_ISL_862000, EPI_ISL_862001, EPI_ISL_862002, EPI_ISL_862003, EPI_ISL_862004, EPI_ISL_862005, EPI_ISL_862006, EPI_ISL_862007, EPI_ISL_862008, EPI_ISL_862009, EPI_ISL_862010, EPI_ISL_862011, EPI_ISL_862012, EPI_ISL_862013, EPI_ISL_862014, EPI_ISL_862015, EPI_ISL_862016, EPI_ISL_862017, EPI_ISL_862018, EPI_ISL_862019, EPI_ISL_862020, EPI_ISL_862021, EPI_ISL_862022, EPI_ISL_862023, EPI_ISL_862024, EPI_ISL_862025, EPI_ISL_862026, EPI_ISL_862027, EPI_ISL_862028, EPI_ISL_862029, EPI_ISL_862030, EPI_ISL_862031, EPI_ISL_862032, EPI_ISL_862033, EPI_ISL_862034, EPI_ISL_862035			
see above	OHSU Lab Services Molecular Microbiology Lab	Oregon SARS-CoV-2 Genome Sequencing Center	Brendan L. O'Connell, Sally Grindstaff, Kayla Carter, Ruth V. Nichols, Alec J. Hirsch, Donna Hansel, Guang Fan, Xuan Qin, Daniel N. Streblow, William B. Messer, Andrew C. Adey, Benjamin N. Bimber, Brian J. O'Roak
EPI_ISL_862125, EPI_ISL_862132	Charité Universitätsmedizin Berlin, Institut fur Virologie/Labor Berlin	Charité Universitätsmedizin Berlin, Institut für Virologie	Victor M Corman, Barbara Mühlemann, Jörn Beheim-Schwarzbach, Tobias Bleicker, Julia Tesch, Talitha Veith, Julia Schneider, Terry Jones, Christian Drosten
EPI_ISL_862556	Gerencia de Asistencia Sanitaria de Soria	Instituto de Salud Carlos III	Iglesias-Caballero, M.Camarero, S. Molinero Calamita, M. González-Esguevillas, M. Pozo, F. Casas, I. Jiménez, P. Jiménez, M. Zaballos, A. Monzón, S. Varona, S. Juliá, M. Cuesta, I. Aldea, C.
EPI_ISL_862575	Hospital Clínic	Instituto de Salud Carlos III	Iglesias-Caballero, M.Camarero, S. Molinero Calamita, M. González-Esguevillas, M. Pozo, F. Casas, I. Jiménez, P. Jiménez, M. Zaballos, A. Monzón, S. Varona, S. Juliá, M. Cuesta, I. Marcos, M.A.
EPI_ISL_862759	Utah Public Health Laboratory, Utah Public Health Laboratory Infectious Disease submission group	Utah Public Health Laboratory, Utah Public Health Laboratory Infectious Disease submission group	Young,E.L., Oakeson,K.F., Gallagher,T.
EPI_ISL_862832	National Institute of Infectious Diseases-Prof. Dr. Matei Bals Molecular Diagnostics Laboratory	National Institute of Infectious Diseases-Prof. Dr. Matei Bals Molecular Diagnostics Laboratory	Leontina Banica, Marius Surleac, Corina Casangiu, Petre Milu, Andreea Tudor, Simona Paraschiv, Dan Otelea
EPI_ISL_863532, EPI_ISL_863590, EPI_ISL_863600, EPI_ISL_863631, EPI_ISL_863635, EPI_ISL_863639, EPI_ISL_863645, EPI_ISL_863651, EPI_ISL_863707, EPI_ISL_863721, EPI_ISL_863729, EPI_ISL_863746, EPI_ISL_863803, EPI_ISL_863811, EPI_ISL_863830, EPI_ISL_863841, EPI_ISL_863853			
see above	Lighthouse Lab in Glasgow	Wellcome Sanger Institute for the COVID-19 Genomics UK (COG-UK) Consortium	Harper VanSteenhouse, Yumi Kasai, David Gray, Carol Clugston, Anna Dominiczak and Alex Alderton, Roberto Amato, Sonia Goncalves, Ewan Harrison, David K. Jackson, Ian Johnston, Dominic Kwiatkowski, Cordelia Langford, John Sillitoe on behalf of the Wellcome Sanger Institute COVID-19 Surveillance Team
EPI_ISL_864738, EPI_ISL_864739, EPI_ISL_864740, EPI_ISL_864741, EPI_ISL_864742, EPI_ISL_864743, EPI_ISL_864744, EPI_ISL_864745, EPI_ISL_864752, EPI_ISL_864753, EPI_ISL_864787, EPI_ISL_864869, EPI_ISL_864871, EPI_ISL_864873			
see above	Department of Pathology, University of Cambridge	COVID-19 Genomics UK (COG-UK) Consortium	Aminu S. Jahun, Yasmin Chaudhry, Grant Hall, Iliana Georgana, Myra Hosmillo, Martin D. Curran, Malte Pinckert, Surendra Parmar, Ian Goodfellow
EPI_ISL_864961, EPI_ISL_864962, EPI_ISL_864963, EPI_ISL_864964, EPI_ISL_864965, EPI_ISL_864966, EPI_ISL_864967, EPI_ISL_864968, EPI_ISL_864969, EPI_ISL_864970, EPI_ISL_864971, EPI_ISL_864972, EPI_ISL_864973, EPI_ISL_864974, EPI_ISL_864975, EPI_ISL_864976, EPI_ISL_864977, EPI_ISL_864978, EPI_ISL_864979, EPI_ISL_864980, EPI_ISL_864982, EPI_ISL_864983, EPI_ISL_864992, EPI_ISL_864993			

see above	West of Scotland Specialist Virology Centre, NHSGGC / MRC-University of Glasgow Centre for Virus Research	COVID-19 Genomics UK (COG-UK) Consortium	Ana da Silva Filipe, Natasha Johnson, Kathy Smollett, Daniel Mair, Stephen Carmichael, Alice Broos, Lily Tong, Jenna Nichols, Kyriaki Nomikou; Sarah McDonald; Richard Orton, Joseph Hughes, Sreenu Vattipally, David L Robertson; Alasdair MacLean, Rory Gunson; Sharif Shaaban, Matthew Holden; Rachel Blacow, Guy Mollett, Kathy Li, James Shepherd, Antonia Ho, Emma Thomson
EPI_ISL_865000, EPI_ISL_865001, EPI_ISL_865002, EPI_ISL_865003, EPI_ISL_865008	Lighthouse Lab in Glasgow / MRC-University of Glasgow Centre for Virus Research	COVID-19 Genomics UK (COG-UK) Consortium	Ana da Silva Filipe, Natasha Johnson, Kathy Smollett, Daniel Mair, Stephen Carmichael, Alice Broos, Lily Tong, Jenna Nichols, Kyriaki Nomikou; Sarah McDonald; Harper VanSteenhouse, Yumi Kasai, David Gray, Carol Clugston, Anna Dominiczak; Alasdair MacLean, Rory Gunson; Richard Orton, Joseph Hughes, Sreenu Vattipally, David L Robertson; Sharif Shaaban, Matthew Holden; Kathy Li, James Shepherd, Antonia Ho, Emma Thomson
EPI_ISL_865080, EPI_ISL_865081, EPI_ISL_865082, EPI_ISL_865087	Virology Department, Royal Infirmary of Edinburgh, NHS Lothian / School of Biological Sciences, University of Edinburgh / Institute of Genetics and Molecular Medicine, University of Edinburgh	COVID-19 Genomics UK (COG-UK) Consortium	McHugh M, Dewar R, Rooke S, Gallagher M, Balcaza C, O'Toole Á, Scher E, Hill V, McCrone JT, Colquhoun R, Yu X, Jackson B, Rambaut A, Williams TC, Templeton K
EPI_ISL_865177, EPI_ISL_865354, EPI_ISL_865355, EPI_ISL_865356, EPI_ISL_865357, EPI_ISL_865358, EPI_ISL_865359, EPI_ISL_865360, EPI_ISL_865361, EPI_ISL_865362, EPI_ISL_865363, EPI_ISL_865371, EPI_ISL_865372, EPI_ISL_865375, EPI_ISL_865377, EPI_ISL_865378, EPI_ISL_865379, EPI_ISL_865380, EPI_ISL_865381, EPI_ISL_865382, EPI_ISL_865386			
see above	Liverpool Clinical Laboratories	COVID-19 Genomics UK (COG-UK) Consortium	Sam Haldenby, Anita Lucaci, Steve Paterson, Julian Hiscox, Alistair Darby, M Almsaud, A Alrezaihi, Muhannad Alruwaili, Stuart D Armstrong, Jones Benjamin, Eleanor G Bentley, Anu Chawla, Jordan J Clark, Angela Cowell, Richard Eccles, Isabel García-Dorival, Matthew Gemmell, Alessandro Gerada, PKF Gilmore, Richard Gregory, Ximeng Han, Catherine Hartley, Margaret Hughes, Miren Iturriza-Gomara, James Johnson, L Luu, Jenifer Manson, Charlotte Nelson, Elaine O'Toole, Cassie Olateju, Rebekah Penrice-Randal , Lucille Rainbow, N.P Randle, Trevor Ian Robinson, Parul Sharma, Ghada T Shawli, James P Stewart, Neil Swainston, Ecaterina Vamos, Joanne Watts, Mark Whitehead
EPI_ISL_865498, EPI_ISL_865502, EPI_ISL_865527, EPI_ISL_865529, EPI_ISL_865530, EPI_ISL_865531, EPI_ISL_865532, EPI_ISL_865533, EPI_ISL_865534, EPI_ISL_865535, EPI_ISL_865536, EPI_ISL_865537, EPI_ISL_865538, EPI_ISL_865539, EPI_ISL_865540, EPI_ISL_865541, EPI_ISL_865542, EPI_ISL_865546, EPI_ISL_865547, EPI_ISL_865548, EPI_ISL_865549, EPI_ISL_865550, EPI_ISL_865551, EPI_ISL_865552, EPI_ISL_865553, EPI_ISL_865554, EPI_ISL_865555, EPI_ISL_865556, EPI_ISL_865557, EPI_ISL_865558, EPI_ISL_865561, EPI_ISL_865562, EPI_ISL_865567, EPI_ISL_865568, EPI_ISL_865569, EPI_ISL_865570, EPI_ISL_865579, EPI_ISL_865580, EPI_ISL_865582, EPI_ISL_865583			
see above	Barts Health NHS Trust	COVID-19 Genomics UK (COG-UK) Consortium	CUTINO-MOGUEL, Maria-Teresa; HARRINGTON, David; OWOYEMI, Dola; KULASEGARAN-SHYLINI, Raghavendran; BROAD, Claire; KELE, Beatrix
EPI_ISL_865682, EPI_ISL_865691, EPI_ISL_865692, EPI_ISL_865693, EPI_ISL_865694, EPI_ISL_865812, EPI_ISL_865816, EPI_ISL_865817, EPI_ISL_865819, EPI_ISL_865820, EPI_ISL_865821, EPI_ISL_865822, EPI_ISL_865823, EPI_ISL_865824, EPI_ISL_865825, EPI_ISL_865826, EPI_ISL_865827, EPI_ISL_865828, EPI_ISL_865829, EPI_ISL_865830, EPI_ISL_865831, EPI_ISL_865832, EPI_ISL_865833, EPI_ISL_865834, EPI_ISL_865835, EPI_ISL_865836, EPI_ISL_865838, EPI_ISL_865839, EPI_ISL_865840, EPI_ISL_865841, EPI_ISL_865843, EPI_ISL_865845, EPI_ISL_865846			
see above	University College London, Great Ormond Street Hospital for Children NHS Foundation Trust, Imperial College Healthcare NHS Trust	COVID-19 Genomics UK (COG-UK) Consortium	Sergi Castellano, Rachel Williams, Mark Kristiansen, Paola Resende Silva, Sunando Roy, Tony Brooks, Helena Tutill, Paola Niola, Patricia Dyal, Charlotte Williams, Leysa Forrest, Yasmin Panchbhaya, Jacqueline Findlay, Samuel Weeks, Julianne Brown, Kathryn Harris, Paul Randell, James Price, Alison Holmes, Judith Breuer
EPI_ISL_866071, EPI_ISL_866072, EPI_ISL_866073, EPI_ISL_866074, EPI_ISL_866115, EPI_ISL_866122, EPI_ISL_866127, EPI_ISL_866135, EPI_ISL_866140, EPI_ISL_866162, EPI_ISL_866168			
see above	University College London Hospital	COVID-19 Genomics UK (COG-UK) Consortium	Judith Heaney, Matthew Byott, Catherine Houlihan, Dan Frampton, Stuart Kirk, Moira Spyer and Eleni Nastouli
EPI_ISL_866349, EPI_ISL_866350, EPI_ISL_866351, EPI_ISL_866352, EPI_ISL_866364, EPI_ISL_866365, EPI_ISL_866372	Regional Virus Laboratory, Belfast Health and Social Care Trust	COVID-19 Genomics UK (COG-UK) Consortium	Conall McCaughey, James McKenna, Tanya Curran, Susan Feeney, Alison Watt, Ciara Cox, Mairead Connor, Zoltan Molnar, David Simpson, Derek Fairley
EPI_ISL_866486, EPI_ISL_866487, EPI_ISL_866489, EPI_ISL_866490, EPI_ISL_866491, EPI_ISL_866492, EPI_ISL_866493, EPI_ISL_866494, EPI_ISL_866495, EPI_ISL_866496, EPI_ISL_866497, EPI_ISL_866498, EPI_ISL_866499, EPI_ISL_866501, EPI_ISL_866502, EPI_ISL_866503, EPI_ISL_866504, EPI_ISL_866506, EPI_ISL_866507, EPI_ISL_866508, EPI_ISL_866510, EPI_ISL_866511, EPI_ISL_866512, EPI_ISL_866515, EPI_ISL_866516, EPI_ISL_866517, EPI_ISL_866518, EPI_ISL_866519, EPI_ISL_866522, EPI_ISL_866524, EPI_ISL_866525			
see above	Northumbria University / South Tees Hospitals NHS Foundation Trust / North Cumbria Integrated Care NHS Foundation Trust / North Tees and Hartlepool NHS Foundation Trust / Newcastle Hospitals NHS Foundation Trust	COVID-19 Genomics UK (COG-UK) Consortium	Darren L Smith,Andrew Nelson,Matthew Bashton,Greg R Young,Joshua Loh,John Allan,Mohammad A Tariq,Giles S Holt,Gary Black,Wen C Yew,Lynn Dover,Paul Baker,Steve Liggett,Sarah Essex,Jane Greenaway,Debra Padgett,Clive Graham,Garren Scott,Edward Barton,Emma Swindells,Brendan Payne,Jennifer Collins,Yusri Taha,Gary Eltringham
EPI_ISL_866905	Queens Medical Centre, Clinical Microbiology Department / DeepSeq Nottingham	COVID-19 Genomics UK (COG-UK) Consortium	Gemma Clark, Wendy Smith, Manjinder Khakh, Vicki M Fleming, Michelle M Lister, Hannah Howson-Wells, Jonathan Ball, Patrick McClure, Joseph Chappell, Theocharis Tsoleridis, Nadine Holmes, Matthew Carlisle, Christopher Moore, Fei Sang, Johnny Debebe, Victoria Wright, Matthew Loose
EPI_ISL_866992, EPI_ISL_866993, EPI_ISL_866994, EPI_ISL_866995, EPI_ISL_866996, EPI_ISL_866997, EPI_ISL_866998, EPI_ISL_866999, EPI_ISL_867000, EPI_ISL_867001, EPI_ISL_867002, EPI_ISL_867003, EPI_ISL_867004, EPI_ISL_867005, EPI_ISL_867006, EPI_ISL_867007, EPI_ISL_867010			
see above	Lincolnshire Hospitals and DeepSeq Nottingham	COVID-19 Genomics UK (COG-UK) Consortium	Nichola Duckworth, Tim Sloan, Sarah Walsh, Jonathan Ball, Patrick McClure, Joeseph Chappell, Nadine Holmes, Matthew Carlisle, Christopher Moore, Fei Sang, Johnny Debebe, Victoria Wright, Matthew Loose
EPI_ISL_867039, EPI_ISL_867040, EPI_ISL_867041, EPI_ISL_867044, EPI_ISL_867045, EPI_ISL_867046, EPI_ISL_867048, EPI_ISL_867049, EPI_ISL_867050, EPI_ISL_867052, EPI_ISL_867053, EPI_ISL_867054, EPI_ISL_867056, EPI_ISL_867057, EPI_ISL_867058, EPI_ISL_867059, EPI_ISL_867060, EPI_ISL_867061, EPI_ISL_867062, EPI_ISL_867063, EPI_ISL_867064, EPI_ISL_867065, EPI_ISL_867066, EPI_ISL_867067, EPI_ISL_867068, EPI_ISL_867069, EPI_ISL_867071, EPI_ISL_867072, EPI_ISL_867073, EPI_ISL_867074, EPI_ISL_867075, EPI_ISL_867077, EPI_ISL_867078, EPI_ISL_867079, EPI_ISL_867080, EPI_ISL_867081, EPI_ISL_867082, EPI_ISL_867084, EPI_ISL_867087, EPI_ISL_867088, EPI_ISL_867089, EPI_ISL_867090, EPI_ISL_867093, EPI_ISL_867094, EPI_ISL_867095, EPI_ISL_867096, EPI_ISL_867097, EPI_ISL_867098, EPI_ISL_867130, EPI_ISL_867155, EPI_ISL_867159, EPI_ISL_867161, EPI_ISL_867162, EPI_ISL_867168, EPI_ISL_867169, EPI_ISL_867170, EPI_ISL_867171, EPI_ISL_867181			
see above	Oxford Viromics, NDM, University of Oxford; Oxford University Hospitals; Basingstoke and North Hampshire Hospital	COVID-19 Genomics UK (COG-UK) Consortium	Tanya Golubchik, David Bonsall, George Macintyre, Amy Trebes, Mariateresa de Cesare, Catrin Moore, Alex Mobbs, Anita Justice, Robert Shaw, Monique Andersson, Timothy Peto, Emma Wise, Nathan Moore, Jessica Lynch, Nick Cortes, Matilde Mori, Stephen Kidd, David Buck, John Todd, Christophe Fraser
EPI_ISL_867216, EPI_ISL_867217, EPI_ISL_867437, EPI_ISL_867438, EPI_ISL_867439, EPI_ISL_867440, EPI_ISL_867441, EPI_ISL_867442, EPI_ISL_867443	Originating lab: Wales Specialist Virology Centre Sequencing lab: Pathogen Genomics Unit	Public Health Wales Microbiology Cardiff Wales Specialist Virology Centre	Catherine Moore, Johnathan Evans, Laura Gifford, Malorie Perry, Simon Cottrell, Angela Marchbank, Alec Birchley, Alexander Adams, Amy Gaskin, Bree Gatica-Wilcox, Jason Coombes, Joel Southgate, Lauren Gilbert, Lee Graham, Nicole Pacchiarini, Sara Kumziene-Summerhayes, Sarah Taylor, Sophie Jones, Sara Rey, Matthew Bull, Joanne Watkins, Sally Corden, Tom Connor
EPI_ISL_868420, EPI_ISL_868423, EPI_ISL_868485, EPI_ISL_868629	Virology Department, Sheffield Teaching Hospitals NHS Foundation Trust/Department of Infection, Immunity and Cardiovascular Disease, The Medical School, University of Sheffield	COVID-19 Genomics UK (COG-UK) Consortium	Thushan de Silva, Matthew Parker, Nikki Smith, Adri Angyal, Rebecca Brown, Luke Green, Rachel Tucker, Paul Parsons, Danielle Groves, Katie Johnson, Laura Carrilero, Alex Keeley, Dave Partridge, Matthew Wyles, Benjamin Lindsey, Mehmet Yavuz, Mohammad Raza, Cariad Evans
EPI_ISL_868716, EPI_ISL_868717, EPI_ISL_868718, EPI_ISL_868719, EPI_ISL_868720, EPI_ISL_868721, EPI_ISL_868722, EPI_ISL_868723, EPI_ISL_868724, EPI_ISL_868725, EPI_ISL_868726, EPI_ISL_868727, EPI_ISL_868728, EPI_ISL_868729, EPI_ISL_868730, EPI_ISL_868731, EPI_ISL_868732, EPI_ISL_868733, EPI_ISL_868734, EPI_ISL_868735, EPI_ISL_868736, EPI_ISL_868737, EPI_ISL_868738, EPI_ISL_868739, EPI_ISL_868740, EPI_ISL_868741, EPI_ISL_868742, EPI_ISL_868743, EPI_ISL_868744, EPI_ISL_868745, EPI_ISL_868746, EPI_ISL_868747, EPI_ISL_868748, EPI_ISL_868749, EPI_ISL_868750, EPI_ISL_868751, EPI_ISL_868752, EPI_ISL_868753, EPI_ISL_868754, EPI_ISL_868755, EPI_ISL_868756, EPI_ISL_868757, EPI_ISL_868842, EPI_ISL_868846, EPI_ISL_868847, EPI_ISL_868849, EPI_ISL_868852, EPI_ISL_868857, EPI_ISL_868858, EPI_ISL_868862, EPI_ISL_868870, EPI_ISL_868871, EPI_ISL_868874, EPI_ISL_868876, EPI_ISL_868877, EPI_ISL_868901, EPI_ISL_868902, EPI_ISL_868903, EPI_ISL_868904, EPI_ISL_868905, EPI_ISL_868906, EPI_ISL_868907, EPI_ISL_868908, EPI_ISL_868909, EPI_ISL_868910, EPI_ISL_868911, EPI_ISL_868912, EPI_ISL_868913, EPI_ISL_868914, EPI_ISL_868915, EPI_ISL_868916, EPI_ISL_868917, EPI_ISL_868918, EPI_ISL_868919, EPI_ISL_868920, EPI_ISL_868921, EPI_ISL_868922, EPI_ISL_868923, EPI_ISL_868924, EPI_ISL_868925, EPI_ISL_868926, EPI_ISL_868927, EPI_ISL_868928, EPI_ISL_868929, EPI_ISL_868930, EPI_ISL_868931, EPI_ISL_868932, EPI_ISL_868933, EPI_ISL_868934, EPI_ISL_868935, EPI_ISL_868936, EPI_ISL_868937			
see above	Bioinformatics and Biostatistics Lab, Advanced Sequencing Facility	COVID-19 Genomics UK (COG-UK) Consortium	Aengus Stewart,Jerome Nicod,Chelsea Sawyer,Laura Cubitt,Harshil Patel,Margaret Crawford
EPI_ISL_868949	Charité Universitätsmedizin Berlin, Institute of Virology, Charitéplatz 1, 10117 Berlin, Germany	Charité Universitätsmedizin Berlin, Institute of Virology, Charitéplatz 1, 10117 Berlin, Germany	Victor M Corman, Julia Schneider, Jörn Beheim-Schwarzbach, Tobias Bleicker, Julia Tesch, Barbara Mühlemann, Talitha Veith, Terry Jones, Christian Drosten
EPI_ISL_869014, EPI_ISL_869015, EPI_ISL_869016, EPI_ISL_869017, EPI_ISL_869018, EPI_ISL_869019, EPI_ISL_869020, EPI_ISL_869021, EPI_ISL_869022, EPI_ISL_869023, EPI_ISL_869024, EPI_ISL_869025, EPI_ISL_869027, EPI_ISL_869028, EPI_ISL_869029, EPI_ISL_869030, EPI_ISL_869031, EPI_ISL_869032, EPI_ISL_869033, EPI_ISL_869034, EPI_ISL_869035, EPI_ISL_869036, EPI_ISL_869037, EPI_ISL_869038, EPI_ISL_869039, EPI_ISL_869040, EPI_ISL_869041, EPI_ISL_869042, EPI_ISL_869043, EPI_ISL_869044, EPI_ISL_869051, EPI_ISL_869052, EPI_ISL_869053, EPI_ISL_869054, EPI_ISL_869056, EPI_ISL_869058, EPI_ISL_869061, EPI_ISL_869062, EPI_ISL_869067, EPI_ISL_869069, EPI_ISL_869071, EPI_ISL_869072, EPI_ISL_869073, EPI_ISL_869074, EPI_ISL_869075, EPI_ISL_869077, EPI_ISL_869078			
see above	Bioinformatics and Biostatistics Lab, Advanced Sequencing Facility	COVID-19 Genomics UK (COG-UK) Consortium	Aengus Stewart,Jerome Nicod,Chelsea Sawyer,Laura Cubitt,Harshil Patel,Margaret Crawford
EPI_ISL_870065	Ohio Department of Health Laboratory	Ohio Department of Health Laboratory	Holmes, Jennifer; Eric Brandt, Keoni Omura, Glen McGillivary, Caitlin McDonnell, Kirtana Ramadugu, Erica Leasure, Kelsey Florek, Heather Blankenship,

			Quanta Brown, and Tammy Bannerman
EPI_ISL_871794, EPI_ISL_871795, EPI_ISL_871797, EPI_ISL_871800, EPI_ISL_871801, EPI_ISL_871802, EPI_ISL_871803, EPI_ISL_871804, EPI_ISL_871807, EPI_ISL_871808, EPI_ISL_871811, EPI_ISL_871812			
see above	AIID	Irish Coronavirus Sequencing Consortium-Teagasc Grange	Matthew McCabe, Aljandro Abner Garcia Leon, Fiona Crispie, Calum Walsh, Michael Carr, John Kenny, Paul Cotter, Patrick Mallon, Gabriel Gonzalez
EPI_ISL_871816, EPI_ISL_871818, EPI_ISL_871819	Ohio Department of Health Laboratory	Ohio Department of Health Laboratory	Holmes, Jennifer; Eric Brandt, Keoni Omura, Glen McGillivary, Caitlin McDonnell, Kirtana Ramadugu, Erica Leasure, Kelsey Florek, Heather Blankenship, Quanta Brown, and Tammy Bannerman
EPI_ISL_871908, EPI_ISL_871909	Servicio de Microbiología Clínica (Complejo Hospitalario de Navarra, Pamplona), Instituto de Investigación Sanitaria de Navarra (IdiSNA)	SeqCOVID-SPAIN consortium/IBV(CSIC)	Carmen Ezpeleta Baquedano, Ana Navascués, Ana Miqueleiz and SeqCOVID-SPAIN consortium
EPI_ISL_871911	Hospital Universitario Marqués de Valdecilla - IDIVAL (Santander, Cantabria)	SeqCOVID-SPAIN consortium/IBV(CSIC)	Mónica Gozalo Margüello, María Eliecer Cano García, Jose Manuel Méndez Legaza, Daniel Pablo Marcos, Jesús Rodríguez Rodríguez, María Siller Ruiz and SeqCOVID-SPAIN consortium
EPI_ISL_871956, EPI_ISL_871961	Servicio de Microbiología Clínica (Complejo Hospitalario de Navarra, Pamplona), Instituto de Investigación Sanitaria de Navarra (IdiSNA)	SeqCOVID-SPAIN consortium/IBV(CSIC)	Carmen Ezpeleta Baquedano, Ana Navascués, Ana Miqueleiz and SeqCOVID-SPAIN consortium
EPI_ISL_871965, EPI_ISL_871966, EPI_ISL_871967, EPI_ISL_871968, EPI_ISL_871969, EPI_ISL_872002	Ohio Department of Health Laboratory	Ohio Department of Health Laboratory	Holmes, Jennifer; Eric Brandt, Keoni Omura, Glen McGillivary, Caitlin McDonnell, Kirtana Ramadugu, Erica Leasure, Kelsey Florek, Heather Blankenship, Quanta Brown, and Tammy Bannerman
EPI_ISL_872041, EPI_ISL_872063, EPI_ISL_872064, EPI_ISL_872153, EPI_ISL_872154, EPI_ISL_872155	Department of Clinical Microbiology	GIGA Medical Genomics	Keith Durkin, Maria Artesi, Sébastien Bontems, Raphaël Boreux, Bouchra Boujemla, Cécile Meex, Pierrette Melin, Marie-Pierre Hayette, Vincent Bours
EPI_ISL_872209, EPI_ISL_872259, EPI_ISL_872290	Labo Analyses Med	National Reference Center for Viruses of Respiratory Infections, Institut Pasteur, Paris	Marion Barbet, Sylvie Behillil, Méline Bizard, Angela Brisebarre, Camille Capel, Etienne Simon-Lorière, Vincent Enouf, Maud Vanpeene, Sylvie van der Werf
EPI_ISL_872313	Labo analyses med	National Reference Center for Viruses of Respiratory Infections, Institut Pasteur, Paris	Marion Barbet, Sylvie Behillil, Méline Bizard, Angela Brisebarre, Camille Capel, Etienne Simon-Lorière, Vincent Enouf, Maud Vanpeene, Sylvie van der Werf,Amzalag Jonas
EPI_ISL_872323	Labo Analyses Med	National Reference Center for Viruses of Respiratory Infections, Institut Pasteur, Paris	Marion Barbet, Sylvie Behillil, Méline Bizard, Angela Brisebarre, Camille Capel, Etienne Simon-Lorière, Vincent Enouf, Maud Vanpeene, Sylvie van der Werf,Rouah Raquel
EPI_ISL_872382, EPI_ISL_872383, EPI_ISL_872384, EPI_ISL_872385	Texas Department of State Health Services (TXDSHS)	Texas Department of State Health Services (TXDSHS)	Bonnie Oh, Anita Pokharel, James Daniel Bonser, Myong Koag, Chung Wang, Rachel Lee, Grace Kubin, Rashmi Tuladhar, Mayela Pedrueza, Maliha Rahman, Jenny Zhang
EPI_ISL_872572	Sydney South West Pathology Service (SSWPS) - Liverpool Hospital - NSW Health Pathology	NSW Health Pathology - Institute of Clinical Pathology and Medical Research; Westmead Hospital; University of Sydney	CIDM-PH et al.
EPI_ISL_872625, EPI_ISL_872626, EPI_ISL_872627	Nigeria Centre for Disease Control (NCDC)	African Centre of Excellence for Genomics of Infectious Diseases (ACEGID), Redeemer's University	Oluniyi P.E. et al
EPI_ISL_872742, EPI_ISL_872743	Rhode Island Department of Health	Infectious Disease Program, Broad Institute of Harvard and MIT	Lemieux,J.E., Siddle,K.J., Huard,R., King,E., Azevedo,K., Miller,A., Adams,G., Gladden-Young,A., Lagerborg,K., Rudy,M., DeRuff,K., Carter,A., Normandin,E., Bauer,M., Reilly,S., Tomkins-Tinch,C., Loreth,C., Chaluvadi,S., Birren,B.W., Gallagher,G., Smole,S., Park,D.J., MacInnis,B.L., and Sabeti,P.C.
EPI_ISL_875347, EPI_ISL_875348	Ohio Department of Health Laboratory	Ohio Department of Health Laboratory	Holmes, Jennifer; Eric Brandt, Keoni Omura, Glen McGillivary, Caitlin McDonnell, Kirtana Ramadugu, Erica Leasure, Kelsey Florek, Heather Blankenship, Quanta Brown, and Tammy Bannerman
EPI_ISL_875516	Institute of Virology, Biomedical Research Center of the Slovak Academy of Sciences, Bratislava	Faculty of Natural Sciences, Comenius University, Bratislava	Kristína Boršová, Viktória abanová, Broa Brejová, Viktória Hodorová, Sabina Fumaová Havlíková, Juraj Kopáek, Martina Liková, ubomíra Lukáiková, Martina Neboháová, Monika Sláviková, Tomáš Vina, Boris Klempa, Jozef Nosek
EPI_ISL_876054, EPI_ISL_876076, EPI_ISL_876078, EPI_ISL_876084, EPI_ISL_876106, EPI_ISL_876122, EPI_ISL_876141, EPI_ISL_876145, EPI_ISL_876171, EPI_ISL_876204, EPI_ISL_876205, EPI_ISL_876252, EPI_ISL_876253, EPI_ISL_876254, EPI_ISL_876255, EPI_ISL_876256, EPI_ISL_876257, EPI_ISL_876258, EPI_ISL_876259, EPI_ISL_876260			
see above	Massachusetts State Public Health Laboratory	Massachusetts State Public Health Laboratory	Andrew Lang, Timelia Fink, Glen Gallagher, Sandra Smole
EPI_ISL_876738	Istituto Zooprofilattico Sperimentale della Puglia e della Basilicata	Istituto Zooprofilattico Sperimentale della Puglia e della Basilicata	Parisi A., Bianco A., Capozzi L., Del Sambro L., Manzulli V, Rondinone V., Pace L., Cipolletta D., Galante D.
EPI_ISL_876742	Ospedale Santa Caterina Novella	Istituto Zooprofilattico Sperimentale della Puglia e della Basilicata	Parisi A., Bianco A., Capozzi L., Del Sambro L., Manzulli V, Rondinone V., Pace L., Cipolletta D., Galante D.
EPI_ISL_876743	Presidio di Brindisi Di Summa - Perrino	Istituto Zooprofilattico Sperimentale della Puglia e della Basilicata	Parisi A., Bianco A., Capozzi L., Del Sambro L., Manzulli V, Rondinone V., Pace L., Cipolletta D., Galante D.
EPI_ISL_878734, EPI_ISL_878750, EPI_ISL_878752, EPI_ISL_878771, EPI_ISL_878773, EPI_ISL_878789, EPI_ISL_880171	Rady's Childrens Hospital	Andersen lab at Scripps Research	SEARCH Alliance San Diego with Nanda Radamchar, David Dimmock, Linda Luo, Christina Clarke, Kathryn Bouic, Teresa Mueller, Denise Malicki
EPI_ISL_882303, EPI_ISL_882305, EPI_ISL_882307, EPI_ISL_882308, EPI_ISL_882309, EPI_ISL_882316, EPI_ISL_882320, EPI_ISL_882323, EPI_ISL_882327, EPI_ISL_882332, EPI_ISL_882343, EPI_ISL_882352, EPI_ISL_882361, EPI_ISL_882363, EPI_ISL_882364, EPI_ISL_882371, EPI_ISL_882372, EPI_ISL_882375, EPI_ISL_882376, EPI_ISL_882377, EPI_ISL_882385, EPI_ISL_882388, EPI_ISL_882390, EPI_ISL_882393, EPI_ISL_882397, EPI_ISL_882398, EPI_ISL_882402, EPI_ISL_882404, EPI_ISL_882409, EPI_ISL_882413, EPI_ISL_882415, EPI_ISL_882418, EPI_ISL_882420, EPI_ISL_882421, EPI_ISL_882427, EPI_ISL_882437, EPI_ISL_882438, EPI_ISL_882440, EPI_ISL_882441, EPI_ISL_882442, EPI_ISL_882452, EPI_ISL_882471, EPI_ISL_882473, EPI_ISL_882475, EPI_ISL_882476, EPI_ISL_882480, EPI_ISL_882481, EPI_ISL_882484, EPI_ISL_882491, EPI_ISL_882506, EPI_ISL_882512, EPI_ISL_882518, EPI_ISL_882520, EPI_ISL_882525, EPI_ISL_882534, EPI_ISL_882538, EPI_ISL_882539, EPI_ISL_882542, EPI_ISL_882548, EPI_ISL_882550, EPI_ISL_882559, EPI_ISL_882562, EPI_ISL_882563, EPI_ISL_882571, EPI_ISL_882579, EPI_ISL_882580, EPI_ISL_882585, EPI_ISL_882586, EPI_ISL_882589, EPI_ISL_882595			
see above	Lighthouse Lab in Alderley Park	Wellcome Sanger Institute for the COVID-19 Genomics UK (COG-UK) Consortium	Jacquelyn Wynn, Mairead Hyland, The Lighthouse Lab in Alderley Park and Alex Alderton, Roberto Amato, Sonia Goncalves, Ewan Harrison, David K. Jackson, Ian Johnston, Dominic Kwiatkowski, Cordelia Langford, John Sillitoe on behalf of the Wellcome Sanger Institute COVID-19 Surveillance Team
EPI_ISL_882617, EPI_ISL_882621, EPI_ISL_882623	Hospital Ramón y Cajal	Hospital Ramón y Cajal	José M Gonzalez-Alba, Concepción Rodríguez, Melanie Abreu, Laura Martínez, Val F Lanza, Luz Leticia Olavarrieta, Rafael Cantón, JC Galán
EPI_ISL_882635	SC (UCO) Igiene e Sanità Pubblica (funzione integrata con SC Microbiologia e Virologia) e Laboratory of Molecular Virology of the International Centre for Genetic Engineering and Biotechnology (ICGEB)	ARGO Laboratorio Genomica ed EpigenomicA	Licastro D, Dal Monego S, Degasperi M, Marcello A, D'Agaro P
EPI_ISL_882636	SC (UCO) Igiene e Sanità Pubblica (funzione integrata con SC Microbiologia e Virologia) e Laboratory of Molecular Virology of the International Centre for Genetic Engineering and Biotechnology (ICGEB)	ARGO Laboratorio Genomica ed Epigenomica	Licastro D, Dal Monego S, Degasperi M, Marcello A, D'Agaro P
EPI_ISL_882663, EPI_ISL_882664	LACEN do Estado do Piaui, Dr. Costa Alvarenga	Instituto Adolfo Lutz, Interdiciplinary Procedures Center, Strategic Laboratory	Claudio Tavares Sacchi, Claudia Regina Gonçalves, Erica Valessa Ramos Gomes, Karoline Rodrigues Campos
EPI_ISL_882779, EPI_ISL_882781, EPI_ISL_882800, EPI_ISL_882801, EPI_ISL_882802, EPI_ISL_882803, EPI_ISL_882804	Institute for Urban Disease Control and Prevention	COVID-19 Network Investigations (CONI) Alliance	Kamolthip Atsawawaranunt, Elizabeth Batty, Wasun Chantratita, Thanat Chookajorn, Stefan Fernandez, Angkana Huang, Anthony R. Jones, Khajohn Joonsalak, Chonticha Klungtong, Theerarat Kochakarn, Prayuth Kaewmalang, Amornmas Kongklieng, Namfon Kotanan, Krittikorn Kumpornsin, Duangkamon Loesbanluechai, Wudtichai Manasatienkij, Anek Mungaomklang, Bhakbhoom Panthan, Pukkaporn Parnwijitkul, Ekawat Pasomsub, Vichan Pawun, Kingkan Rakmanee, Insee Sensorn, Janjira Thaipadungpanit, Arporn Wangwiwatsin, Treewat Watthanachockchai

EPI_ISL_882955	State Veterinary Institute Prague	State Veterinary Institute Prague	Nagy,A;Cernikova,L;Stara,M
EPI_ISL_883033, EPI_ISL_883034	Maryland Public Health Laboratory	Maryland Public Health Laboratory	Maryland Department of Health Laboratories Administration
EPI_ISL_883350	OCME Office Of Chief Medical Examiner	New York City Public Health Laboratory	Jade Wang, et al.
EPI_ISL_884265, EPI_ISL_884268, EPI_ISL_884269, EPI_ISL_884270, EPI_ISL_884274, EPI_ISL_884277, EPI_ISL_884279, EPI_ISL_884286, EPI_ISL_884287	Institute of Medical Microbiology and Hospital Hygiene	Institute of Medical Microbiology and Hospital Hygiene	Prof. Dr. Achim Kaasch, Aljoscha Tersteegen
EPI_ISL_884984, EPI_ISL_884985, EPI_ISL_884986, EPI_ISL_885013, EPI_ISL_885014, EPI_ISL_885015, EPI_ISL_885016, EPI_ISL_885017, EPI_ISL_885018, EPI_ISL_885019, EPI_ISL_885020, EPI_ISL_885021, EPI_ISL_885023, EPI_ISL_885024, EPI_ISL_885025, EPI_ISL_885033, EPI_ISL_885034, EPI_ISL_885036, EPI_ISL_885039, EPI_ISL_885040, EPI_ISL_885043			
see above	Santa Clara County Public Health Laboratory	Chan-Zuckerberg Biohub	CZB Cliahub Consortium
EPI_ISL_885139	PathWest Laboratory Medicine WA	PathWest Laboratory Medicine WA Microbial Surveillance Unit	PathWest Laboratory Medicine WA Microbial Surveillance Unit
EPI_ISL_886232, EPI_ISL_886279, EPI_ISL_886361, EPI_ISL_886455, EPI_ISL_886472, EPI_ISL_886576, EPI_ISL_886600, EPI_ISL_886610, EPI_ISL_886702, EPI_ISL_886863, EPI_ISL_887054, EPI_ISL_887103, EPI_ISL_887635, EPI_ISL_887648, EPI_ISL_887672, EPI_ISL_887676, EPI_ISL_887682, EPI_ISL_887685, EPI_ISL_887688, EPI_ISL_887714, EPI_ISL_887719, EPI_ISL_887722, EPI_ISL_887730, EPI_ISL_887760, EPI_ISL_887761, EPI_ISL_887781, EPI_ISL_887790, EPI_ISL_887814, EPI_ISL_887836, EPI_ISL_887840, EPI_ISL_887842, EPI_ISL_887853, EPI_ISL_887855, EPI_ISL_887858, EPI_ISL_887865, EPI_ISL_887879, EPI_ISL_887883, EPI_ISL_887884, EPI_ISL_887889, EPI_ISL_887894, EPI_ISL_887906, EPI_ISL_887907, EPI_ISL_887919, EPI_ISL_887954, EPI_ISL_887956, EPI_ISL_887962, EPI_ISL_887963, EPI_ISL_887977, EPI_ISL_888033, EPI_ISL_888035, EPI_ISL_888038, EPI_ISL_888039, EPI_ISL_888045, EPI_ISL_888047, EPI_ISL_888049, EPI_ISL_888090, EPI_ISL_888092, EPI_ISL_888096, EPI_ISL_888134, EPI_ISL_888154, EPI_ISL_888161, EPI_ISL_888282, EPI_ISL_888285, EPI_ISL_888335, EPI_ISL_888504, EPI_ISL_888513, EPI_ISL_888521, EPI_ISL_888529, EPI_ISL_888532, EPI_ISL_888534, EPI_ISL_888535, EPI_ISL_888536, EPI_ISL_888537, EPI_ISL_888539, EPI_ISL_888540, EPI_ISL_888552, EPI_ISL_888555, EPI_ISL_888558, EPI_ISL_888561, EPI_ISL_888573, EPI_ISL_888574, EPI_ISL_888583			
see above	Labcorp	Genomics and Discovery, Respiratory Viruses Branch, Division of Viral Diseases, Centers for Disease Control and Prevention	Peter W. Cook,Dhwani Batra,Ben L. Rambo-Martin,Summer Galloway,Brian Krueger,Minoo Agarwal,Eyad Almasri,Debbie Boles,Ayla Burns,Nuthawin Charoensri,Oren Cohen,Susan Countryman,Mary Ann Cristobal,Bobbi Croy,Suzanne Dale,Hrushikesh Deshmukh,Amanda Douglas,Vincent Drouillon,Marcia Eisenberg,Howard Engler,Rama Ghatti,Prashant Gupta,Susan Hicks,Jake Humphrey,Lax Iyer,Manoj Jain,Mohan Kolli,Tim Kuphal,Stanley Letovsky,Michael Levandoski,Craig Lukasik,Jonathan Meltzer,Brian Norvell,Mindy Nye,Scott Parker,Christos Petropoulos,John Pruitt,Steven Ragan,Scott Ryan,Mike Sapeta,Jana Schroth,Suresh Babu Selvaraju,Goran Stevovic,Amanda Suchanek,Andrea Throop,Lyndon Tilson,Thomas Urban,Joe Voshell,Kimberly Wagner,Jonathan Williams,Mary Williamson,Qian Zeng,Tricia Zwiefelhofer,Clinton R. Paden,Suxiang Tong,Duncan MacCannell,
EPI_ISL_888785, EPI_ISL_888789, EPI_ISL_888790	KU Leuven, Rega Institute, Clinical and Epidemiological Virology	KU Leuven, Rega Institute, Clinical and Epidemiological Virology	Tony Wawina-Bokalanga, Bert Vanmechelen, Joan Marti-Carerras, Piet Maes
EPI_ISL_888832, EPI_ISL_888834	National Virus Reference Laboratory	National Virus Reference Laboratory	Michael Carr, Gabriel Gonzalez, Jonathan Dean, Cillian F De Gascun
EPI_ISL_888845, EPI_ISL_888846, EPI_ISL_888847, EPI_ISL_888852	Michigan Department of Health and Human Services, Bureau of Laboratories	Michigan Department of Health and Human Services, Bureau of Laboratories	Blankenship HM, Riner D, Soehnlen MK
EPI_ISL_888994	RSU MMC	Eijkman Institute for Molecular Biology, Ministry of Research and Technology/National Agency for Research and Innovation	Lydia V. Panggalo, Sukma Oktavianthi, Willy Agustine, Edison Johar, Hidayat Trimarsanto, Iskandar Adnan, Frilasita A Yudhaputri, Safarina G Malik, Khin Saw Myint, Amin Soebandrio
EPI_ISL_888995	RSU Sumber Waras	Eijkman Institute for Molecular Biology, Ministry of Research and Technology/National Agency for Research and Innovation	Lydia V. Panggalo, Sukma Oktavianthi, Willy Agustine, Edison Johar, Hidayat Trimarsanto, Iskandar Adnan, Frilasita A Yudhaputri, Safarina G Malik, Khin Saw Myint, Amin Soebandrio
EPI_ISL_888996	RSU Graha Juanda	Eijkman Institute for Molecular Biology, Ministry of Research and Technology/National Agency for Research and Innovation	Lydia V. Panggalo, Sukma Oktavianthi, Willy Agustine, Edison Johar, Hidayat Trimarsanto, Iskandar Adnan, Frilasita A Yudhaputri, Safarina G Malik, Khin Saw Myint, Amin Soebandrio
EPI_ISL_889003	RS Mitra Keluarga Gading Serpong	Eijkman Institute for Molecular Biology, Ministry of Research and Technology/National Agency for Research and Innovation	Willy Agustine, Edison Johar, Hidayat Trimarsanto, Iskandar Adnan, Lydia V. Panggalo, Sukma Oktavianthi, Frilasita A Yudhaputri, Safarina G Malik, Khin Saw Myint, Amin Soebandrio
EPI_ISL_889017	RS Hermina Tangerang	Eijkman Institute for Molecular Biology, Ministry of Research and Technology/National Agency for Research and Innovation	Lydia V. Panggalo, Sukma Oktavianthi, Willy Agustine, Edison Johar, Hidayat Trimarsanto, Iskandar Adnan, Frilasita A Yudhaputri, Safarina G Malik, Khin Saw Myint, Amin Soebandrio
EPI_ISL_889023, EPI_ISL_889024, EPI_ISL_889025, EPI_ISL_889026, EPI_ISL_889027, EPI_ISL_889071, EPI_ISL_889072, EPI_ISL_889073, EPI_ISL_889074, EPI_ISL_889075, EPI_ISL_889076, EPI_ISL_889077, EPI_ISL_889078, EPI_ISL_889079, EPI_ISL_889080, EPI_ISL_889081, EPI_ISL_889082, EPI_ISL_889083, EPI_ISL_889084, EPI_ISL_889085, EPI_ISL_889086, EPI_ISL_889087, EPI_ISL_889088, EPI_ISL_889089, EPI_ISL_889090, EPI_ISL_889091, EPI_ISL_889092, EPI_ISL_889093, EPI_ISL_889094, EPI_ISL_889095, EPI_ISL_889096, EPI_ISL_889097, EPI_ISL_889098, EPI_ISL_889099, EPI_ISL_889100, EPI_ISL_889101, EPI_ISL_889102, EPI_ISL_889103, EPI_ISL_889104, EPI_ISL_889105, EPI_ISL_889106, EPI_ISL_889107, EPI_ISL_889108, EPI_ISL_889109, EPI_ISL_889110, EPI_ISL_889111, EPI_ISL_889112, EPI_ISL_889113, EPI_ISL_889114, EPI_ISL_889115, EPI_ISL_889116, EPI_ISL_889117, EPI_ISL_889118, EPI_ISL_889119, EPI_ISL_889120, EPI_ISL_889121, EPI_ISL_889122, EPI_ISL_889123, EPI_ISL_889124, EPI_ISL_889125, EPI_ISL_889126, EPI_ISL_889127, EPI_ISL_889128, EPI_ISL_889129, EPI_ISL_889130, EPI_ISL_889131, EPI_ISL_889132, EPI_ISL_889133, EPI_ISL_889134, EPI_ISL_889135, EPI_ISL_889136, EPI_ISL_889137, EPI_ISL_889138, EPI_ISL_889139, EPI_ISL_889140, EPI_ISL_889141, EPI_ISL_889142, EPI_ISL_889143, EPI_ISL_889144, EPI_ISL_889145, EPI_ISL_889146, EPI_ISL_889147, EPI_ISL_889148, EPI_ISL_889149, EPI_ISL_889150, EPI_ISL_889151, EPI_ISL_889152, EPI_ISL_889153, EPI_ISL_889158, EPI_ISL_889159, EPI_ISL_889160, EPI_ISL_889161, EPI_ISL_889176, EPI_ISL_889177, EPI_ISL_889178, EPI_ISL_889179, EPI_ISL_889180, EPI_ISL_889181, EPI_ISL_889182, EPI_ISL_889183, EPI_ISL_889184, EPI_ISL_889185, EPI_ISL_889186, EPI_ISL_889187, EPI_ISL_889188, EPI_ISL_889189, EPI_ISL_889190, EPI_ISL_889191, EPI_ISL_889192, EPI_ISL_889193, EPI_ISL_889194, EPI_ISL_889195, EPI_ISL_889196, EPI_ISL_889197, EPI_ISL_889198, EPI_ISL_889199, EPI_ISL_889200, EPI_ISL_889201, EPI_ISL_889202, EPI_ISL_889203, EPI_ISL_889204, EPI_ISL_889205, EPI_ISL_889206, EPI_ISL_889207, EPI_ISL_889208, EPI_ISL_889209, EPI_ISL_889210, EPI_ISL_889211, EPI_ISL_889212, EPI_ISL_889213, EPI_ISL_889214, EPI_ISL_889215, EPI_ISL_889216, EPI_ISL_889217, EPI_ISL_889218, EPI_ISL_889219, EPI_ISL_889220, EPI_ISL_889221, EPI_ISL_889222, EPI_ISL_889223, EPI_ISL_889224, EPI_ISL_889225, EPI_ISL_889226, EPI_ISL_889227, EPI_ISL_889228, EPI_ISL_889229, EPI_ISL_889230, EPI_ISL_889231, EPI_ISL_889232, EPI_ISL_889233, EPI_ISL_889234, EPI_ISL_889235, EPI_ISL_889236, EPI_ISL_889237, EPI_ISL_889238, EPI_ISL_889239, EPI_ISL_889240, EPI_ISL_889241, EPI_ISL_889242, EPI_ISL_889243, EPI_ISL_889244, EPI_ISL_889245, EPI_ISL_889246, EPI_ISL_889247, EPI_ISL_889248, EPI_ISL_889249, EPI_ISL_889250, EPI_ISL_889251, EPI_ISL_889252, EPI_ISL_889253, EPI_ISL_889254, EPI_ISL_889255, EPI_ISL_889256, EPI_ISL_889257, EPI_ISL_889258, EPI_ISL_889259, EPI_ISL_889260, EPI_ISL_889261, EPI_ISL_889262, EPI_ISL_889263, EPI_ISL_889264, EPI_ISL_889265, EPI_ISL_889266, EPI_ISL_889267, EPI_ISL_889268, EPI_ISL_889269, EPI_ISL_889270, EPI_ISL_889271, EPI_ISL_889272, EPI_ISL_889273, EPI_ISL_889274, EPI_ISL_889275, EPI_ISL_889276, EPI_ISL_889277, EPI_ISL_889278, EPI_ISL_889279, EPI_ISL_889280, EPI_ISL_889281, EPI_ISL_889282, EPI_ISL_889283, EPI_ISL_889284, EPI_ISL_889285, EPI_ISL_889286, EPI_ISL_889287, EPI_ISL_889288, EPI_ISL_889289, EPI_ISL_889290, EPI_ISL_889291, EPI_ISL_889292, EPI_ISL_889293, EPI_ISL_889294, EPI_ISL_889295, EPI_ISL_889296, EPI_ISL_889297, EPI_ISL_889298, EPI_ISL_889299, EPI_ISL_889300, EPI_ISL_889301, EPI_ISL_889302, EPI_ISL_889303, EPI_ISL_889304, EPI_ISL_889305, EPI_ISL_889306			
see above	Israel Central Virology laboratory	Israel National Consortium for SARS-CoV-2 sequencing	Neta Zuckerman, Efrat Dahan Bucris, Michal Mandelboim, Dana Bar-Ilan, Oran Erster, Tzvia Mann, Omer Murik, David A. Zeevi, Assaf Rokney, Joseph Jaffe, Eva Nachum, Maya Davidovich Cohen, Ephraim Fass, Gal Zizelski Valenci, Mor Rubinstein, Efrat Rorman, Israel Nissan, Efrat Glick-Saar, Omri Nayshool, Gideon Rechavi, Ella Mendelson, Orna Mor
EPI_ISL_889482, EPI_ISL_889486, EPI_ISL_889487, EPI_ISL_889488, EPI_ISL_889489, EPI_ISL_889490, EPI_ISL_889491, EPI_ISL_889492, EPI_ISL_889493, EPI_ISL_889494, EPI_ISL_889495, EPI_ISL_889496, EPI_ISL_889498, EPI_ISL_889499, EPI_ISL_889500, EPI_ISL_889501, EPI_ISL_889502, EPI_ISL_889503, EPI_ISL_889504, EPI_ISL_889505, EPI_ISL_889506, EPI_ISL_889507, EPI_ISL_889508, EPI_ISL_889509, EPI_ISL_889510, EPI_ISL_889511, EPI_ISL_889512, EPI_ISL_889513, EPI_ISL_889514, EPI_ISL_889515, EPI_ISL_889516, EPI_ISL_889517, EPI_ISL_889518, EPI_ISL_889519, EPI_ISL_889520, EPI_ISL_889521, EPI_ISL_889522, EPI_ISL_889523, EPI_ISL_889524, EPI_ISL_889525, EPI_ISL_889526, EPI_ISL_889527, EPI_ISL_889528, EPI_ISL_889529, EPI_ISL_889530, EPI_ISL_889531, EPI_ISL_889532, EPI_ISL_889533, EPI_ISL_889534, EPI_ISL_889535, EPI_ISL_889536, EPI_ISL_889537, EPI_ISL_889538, EPI_ISL_889539, EPI_ISL_889540, EPI_ISL_889541, EPI_ISL_889543, EPI_ISL_889544, EPI_ISL_889545, EPI_ISL_889546, EPI_ISL_889547, EPI_ISL_889548, EPI_ISL_889549, EPI_ISL_889550, EPI_ISL_889551			
see above	LSUHS Emerging Viral Threat Laboratory	Microbial Genome Sequencing Center	Jeremy P. Kamil, Jennifer L. Carroll, Camille F. Abshire, Maarten Van Diest, Mohammed N.A. Siddiquey, Andrew D. Yurochko, Martin J. Sapp, Rona S. Scott, Christopher G. Kevil, Daniel J. Snyder, Vaughn S. Cooper, John A. Vanchiere
EPI_ISL_890238, EPI_ISL_890243, EPI_ISL_890295, EPI_ISL_890336, EPI_ISL_890340, EPI_ISL_890343, EPI_ISL_890346, EPI_ISL_890350	KU Leuven, Rega Institute, Clinical and Epidemiological Virology	KU Leuven, Rega Institute, Clinical and Epidemiological Virology	Tony Wawina-Bokalanga, Bert Vanmechelen, Joan Marti-Carerras, Piet Maes
EPI_ISL_890361, EPI_ISL_890362	LSUHS Emerging Viral Threat Laboratory	Microbial Genome Sequencing Center	Jeremy P. Kamil, Jennifer L. Carroll, Camille F. Abshire, Maarten Van Diest, Mohammed N.A. Siddiquey, Andrew D. Yurochko, Martin J. Sapp, Rona S. Scott, Christopher G. Kevil, Daniel J. Snyder, Vaughn S. Cooper, John A. Vanchiere
EPI_ISL_891035, EPI_ISL_891044, EPI_ISL_891046, EPI_ISL_891047, EPI_ISL_891049, EPI_ISL_891050, EPI_ISL_891051, EPI_ISL_891052, EPI_ISL_891053, EPI_ISL_891054, EPI_ISL_891055, EPI_ISL_891056, EPI_ISL_891057			
see above	Washington State Department of Health	Seattle Flu Study	Deborah A. Nickerson, Chris D. Frazar, Jover Lee, Benjamin Pelle, Erica Ryke, Matthew Richardson, Amanda Adler, Elisabeth Brandstetter, Peter D. Han, Kairsten Fay, Misja Ilcisin, Kirsten Lacombe, Thomas R. Sibley, Melissa Truong, Caitlin R. Wolf, Romesh Gautom, Geoff Melly, Brian Hiatt, Philip Dykema, Scott Lindquist, Michael Boeckh, Janet A. Englund, Michael Famulare, Barry R. Lutz, Mark J. Rieder, Lea M. Starita, Matthew Thompson, Helen Y. Chu, Jay Shendure, Trevor Bedford
EPI_ISL_891136	The Jackson Laboratory	The Jackson Laboratory	Lloyd M, Sanderson B, Srivastava A, Maurya R, Renzette N, Omerza G, Kelly K, Li L, Wei C L, Adams M
EPI_ISL_891198, EPI_ISL_891201	DPH, Massachusetts State Public Health Lab	DPH, Massachusetts State Public Health Lab	Lang,A.S., Fink,T., Gallagher,G.R., Smole,S.C.

EPI_ISL_892244	National Laboratory for Health, Environment and Food	National Laboratory for Health, Environment and Food	Aleksander Mahnic, Sandra Janezic, Maja Rupnik
EPI_ISL_893744, EPI_ISL_893774	Institute of Virology, Medical Center, University of Freiburg, Freiburg, Germany	Institute of Virology, Clinial Virus Genomics, Medical Center, University of Freiburg, Freiburg, Germany	Jonas Fuchs, Lisa Kern, Sandra Reuter, Hajo Grundmann, Marcus Panning
EPI_ISL_894168	Institute of Medical Microbiology and Hospital Hygiene	Institute of Medical Microbiology and Hospital Hygiene	Prof. Dr. Achim Kaasch, Aljoscha Tersteegen
EPI_ISL_896072	Viollier AG	University Hospital Basel, Clinical Bacteriology	Tim Roloff, Madlen Stange, Helena MB Seth-Smith, Alfredo Mari, Karoline Leuzinger, Julia Bielicki, Christiane Beckmann, Manuel Battegay, Hans Hirsch, Adrian Egli
EPI_ISL_896087, EPI_ISL_896088	Labormedizinisches Zentrum Dr Risch	University Hospital Basel, Clinical Bacteriology	Tim Roloff, Madlen Stange, Helena MB Seth-Smith, Alfredo Mari, Karoline Leuzinger, Julia Bielicki, Nadia Wohlwend,Martin Risch, Lorenz Risch, Manuel Battegay, Hans Hirsch, Adrian Egli
EPI_ISL_896095	Bioanalytika AG	University Hospital Basel, Clinical Bacteriology	Tim Roloff, Madlen Stange, Helena MB Seth-Smith, Alfredo Mari, Karoline Leuzinger, Julia Bielicki, Adrian Härri, Manuel Battegay, Hans Hirsch, Adrian Egli
EPI_ISL_896101, EPI_ISL_896102	Kantonsspital Aarau, Institut für Labormedizin	University Hospital Basel, Clinical Bacteriology	Tim Roloff, Madlen Stange, Helena MB Seth-Smith, Alfredo Mari, Karoline Leuzinger, Julia Bielicki, Michael Oberle, Manuel Battegay, Hans Hirsch, Adrian Egli
EPI_ISL_896103, EPI_ISL_896104	Labormedizinisches Zentrum Dr Risch	University Hospital Basel, Clinical Bacteriology	Tim Roloff, Madlen Stange, Helena MB Seth-Smith, Alfredo Mari, Karoline Leuzinger, Julia Bielicki, Nadia Wohlwend,Martin Risch, Lorenz Risch, Manuel Battegay, Hans Hirsch, Adrian Egli
EPI_ISL_898010, EPI_ISL_898011, EPI_ISL_898025, EPI_ISL_898026, EPI_ISL_898027, EPI_ISL_898028	KU Leuven, Rega Institute, Clinical and Epidemiological Virology	KU Leuven, Rega Institute, Clinical and Epidemiological Virology	Tony Wawina-Bokalanga, Bert Vanmechelen, Joan Marti-Carerras, Piet Maes
EPI_ISL_899008, EPI_ISL_899021, EPI_ISL_899045, EPI_ISL_899046, EPI_ISL_899049, EPI_ISL_899066, EPI_ISL_899067, EPI_ISL_899068, EPI_ISL_899091, EPI_ISL_899111, EPI_ISL_899121, EPI_ISL_899122, EPI_ISL_899135, EPI_ISL_899143, EPI_ISL_899144, EPI_ISL_899155, EPI_ISL_899168, EPI_ISL_899170, EPI_ISL_899173, EPI_ISL_899175, EPI_ISL_899188, EPI_ISL_899189, EPI_ISL_899190, EPI_ISL_899191, EPI_ISL_899192, EPI_ISL_899193, EPI_ISL_899194, EPI_ISL_899195, EPI_ISL_899196, EPI_ISL_899298, EPI_ISL_899299, EPI_ISL_899300, EPI_ISL_899301, EPI_ISL_899421, EPI_ISL_899422, EPI_ISL_899423, EPI_ISL_899424, EPI_ISL_899425, EPI_ISL_899426, EPI_ISL_899427, EPI_ISL_899428, EPI_ISL_899522, EPI_ISL_899523, EPI_ISL_899537, EPI_ISL_899547, EPI_ISL_899548, EPI_ISL_899578, EPI_ISL_899579, EPI_ISL_899580, EPI_ISL_899581, EPI_ISL_899582, EPI_ISL_899598, EPI_ISL_899622, EPI_ISL_899627, EPI_ISL_899628, EPI_ISL_899629, EPI_ISL_899630, EPI_ISL_899665, EPI_ISL_899666, EPI_ISL_899667, EPI_ISL_899773, EPI_ISL_899828, EPI_ISL_899829, EPI_ISL_899830, EPI_ISL_899831, EPI_ISL_899832, EPI_ISL_899833, EPI_ISL_899834, EPI_ISL_899835, EPI_ISL_899836, EPI_ISL_899837, EPI_ISL_899921, EPI_ISL_899942, EPI_ISL_899943, EPI_ISL_899952, EPI_ISL_899953, EPI_ISL_899954, EPI_ISL_899955, EPI_ISL_899956, EPI_ISL_899957, EPI_ISL_899958, EPI_ISL_899959, EPI_ISL_899960, EPI_ISL_899961, EPI_ISL_899962, EPI_ISL_899963, EPI_ISL_899964, EPI_ISL_899965, EPI_ISL_899966, EPI_ISL_899967, EPI_ISL_899968, EPI_ISL_899969, EPI_ISL_899970, EPI_ISL_899971, EPI_ISL_899972, EPI_ISL_899973			
see above	Viollier AG	Department of Biosystems Science and Engineering, ETH Zürich	Christian Beisel, Sarah Nadeau, Chaoran Chen, Ivan Topolsky, Philipp Jablonski, Lara Fuhrmann, David Dreifuss, Katharina Jahn, Tobias Schär, Ina Nissen, Natascha Santacroce, Elodie Burcklen, Christiane Beckmann, Maurice Redondo, Olivier Kobel, Christoph Noppen, Sophie Seidel, Noemie Santamaria de Souza, Niko Beerenwinkel, Tanja Stadler
EPI_ISL_900502	Gen-Bio	CNR Virus des Infections Respiratoires - France SUD	Antonin Bal, Gregory Destras, Gwendolyne Burfin, Hadrien Règue, Quentin Semanas, Martine Valette, Bruno Lina, Sylvie Larrat, Laurence Josset
EPI_ISL_900504, EPI_ISL_900507, EPI_ISL_900508, EPI_ISL_900509	Mirialis	CNR Virus des Infections Respiratoires - France SUD	Antonin Bal, Gregory Destras, Gwendolyne Burfin, Hadrien Règue, Quentin Semanas, Martine Valette, Bruno Lina, Laurence Josset
EPI_ISL_900510	Laboratoire de virologie, CHU de Grenoble - CS 10217 - 38043 Grenoble cedex 9	CNR Virus des Infections Respiratoires - France SUD	Antonin Bal, Gregory Destras, Gwendolyne Burfin, Hadrien Règue, Quentin Semanas, Martine Valette, Bruno Lina, Sylvie Larrat, Laurence Josset
EPI_ISL_900513	Labosud	CNR Virus des Infections Respiratoires - France SUD	Antonin Bal, Gregory Destras, Gwendolyne Burfin, Hadrien Règue, Quentin Semanas, Martine Valette, Bruno Lina, Laurence Josset
EPI_ISL_900515, EPI_ISL_900516, EPI_ISL_900517	Bioesterel	CNR Virus des Infections Respiratoires - France SUD	Antonin Bal, Gregory Destras, Gwendolyne Burfin, Hadrien Règue, Quentin Semanas, Martine Valette, Bruno Lina, Laurence Josset
EPI_ISL_900521	Novabio	CNR Virus des Infections Respiratoires - France SUD	Antonin Bal, Gregory Destras, Gwendolyne Burfin, Hadrien Règue, Quentin Semanas, Martine Valette, Bruno Lina, Laurence Josset
EPI_ISL_900523	Novabio	CNR Virus des Infections Respiratoires - France SUD	Antonin Bal, Gregory Destras, Gwendolyne Burfin, Hadrien Règue, Quentin Semanas, Martine Valette, Bruno Lina, Sylvie Larrat, Laurence Josset
EPI_ISL_900525	Astralab	CNR Virus des Infections Respiratoires - France SUD	Antonin Bal, Gregory Destras, Gwendolyne Burfin, Hadrien Règue, Quentin Semanas, Martine Valette, Bruno Lina, Laurence Josset
EPI_ISL_900526	CNR Virus des Infections Respiratoires - France SUD	CNR Virus des Infections Respiratoires - France SUD	Antonin Bal, Gregory Destras, Gwendolyne Burfin, Hadrien Règue, Quentin Semanas, Martine Valette, Bruno Lina, Laurence Josset
EPI_ISL_900527	Cerballiance	CNR Virus des Infections Respiratoires - France SUD	Antonin Bal, Gregory Destras, Gwendolyne Burfin, Hadrien Règue, Quentin Semanas, Martine Valette, Bruno Lina, Laurence Josset
EPI_ISL_900529	Bioesterel	CNR Virus des Infections Respiratoires - France SUD	Antonin Bal, Gregory Destras, Gwendolyne Burfin, Hadrien Règue, Quentin Semanas, Martine Valette, Bruno Lina, Laurence Josset
EPI_ISL_900530	Centre Hospitalier de Guéret	CNR Virus des Infections Respiratoires - France SUD	Antonin Bal, Gregory Destras, Gwendolyne Burfin, Hadrien Règue, Quentin Semanas, Martine Valette, Bruno Lina, Sylvie Larrat, Laurence Josset
EPI_ISL_900531	CHU Poitiers	CNR Virus des Infections Respiratoires - France SUD	Antonin Bal, Gregory Destras, Gwendolyne Burfin, Hadrien Règue, Quentin Semanas, Martine Valette, Bruno Lina, Agnès Beby-Defaux, Magali Garcia, Clément Jousselin, Nicolas Lévêque, Laurence Josset
EPI_ISL_900532	CNR Virus des Infections Respiratoires - France SUD	CNR Virus des Infections Respiratoires - France SUD	Antonin Bal, Gregory Destras, Gwendolyne Burfin, Hadrien Règue, Quentin Semanas, Martine Valette, Bruno Lina, Laurence Josset
EPI_ISL_900536, EPI_ISL_900537, EPI_ISL_900538, EPI_ISL_900539	Gen-Bio	CNR Virus des Infections Respiratoires - France SUD	Antonin Bal, Gregory Destras, Gwendolyne Burfin, Hadrien Règue, Quentin Semanas, Martine Valette, Bruno Lina, Laurence Josset
EPI_ISL_900540, EPI_ISL_900541, EPI_ISL_900542	Gen-Bio	CNR Virus des Infections Respiratoires - France SUD	Antonin Bal, Gregory Destras, Gwendolyne Burfin, Hadrien Règue, Quentin Semanas, Martine Valette, Bruno Lina, Sylvie Larrat, Laurence Josset
EPI_ISL_900545, EPI_ISL_900546, EPI_ISL_900547, EPI_ISL_900548, EPI_ISL_900549, EPI_ISL_900550, EPI_ISL_900551, EPI_ISL_900552, EPI_ISL_900553, EPI_ISL_900554, EPI_ISL_900555, EPI_ISL_900556, EPI_ISL_900557, EPI_ISL_900558, EPI_ISL_900559, EPI_ISL_900560, EPI_ISL_900561, EPI_ISL_900562, EPI_ISL_900563, EPI_ISL_900564, EPI_ISL_900566, EPI_ISL_900567			
see above	CNR Virus des Infections Respiratoires - France SUD	CNR Virus des Infections Respiratoires - France SUD	Antonin Bal, Gregory Destras, Gwendolyne Burfin, Hadrien Règue, Quentin Semanas, Martine Valette, Bruno Lina, Laurence Josset
EPI_ISL_902856, EPI_ISL_902857, EPI_ISL_902858, EPI_ISL_902859, EPI_ISL_902860, EPI_ISL_902861, EPI_ISL_902862, EPI_ISL_902863, EPI_ISL_902864, EPI_ISL_902865, EPI_ISL_902866, EPI_ISL_902867, EPI_ISL_902868, EPI_ISL_902869, EPI_ISL_902870, EPI_ISL_902871, EPI_ISL_902872, EPI_ISL_902873, EPI_ISL_902874, EPI_ISL_902875, EPI_ISL_902876, EPI_ISL_902877, EPI_ISL_902878, EPI_ISL_902879, EPI_ISL_902880, EPI_ISL_902881, EPI_ISL_902882, EPI_ISL_902883, EPI_ISL_902884, EPI_ISL_902885, EPI_ISL_902886, EPI_ISL_902887, EPI_ISL_902888			
see above	Department of Virology and Immunology, University of Helsinki and Helsinki University Hospital, Huslab Finland	Department of Virology, Faculty of Medicine, University of Helsinki, Helsinki, Finland	Teemu Smura, Ravi Kant, Phuoc Truong, Hussein Alburkat, Hannimari Kallio-Kokko, Jenni Virtanen, Maija Suvanto, Essi Korhonen, Sari Hannula, Harri Kangas, Hanna Liimatainen, Satu Kurkela, Hanna Jarva, Maija Lappalainen, Pekka Ellonen, Olli Vapalahti
EPI_ISL_903043, EPI_ISL_903044, EPI_ISL_903048	Seattle Flu Study	Seattle Flu Study	Deborah A. Nickerson, Chris D. Frazar, Jover Lee, Benjamin Pelle, Erica Ryke, Matthew Richardson, Amanda Adler, Elisabeth Brandstetter, Peter D. Han, Kairsten Fay, Misja Ilcisin, Kirsten Lacombe, Thomas R. Sibley, Melissa Truong, Caitlin R. Wolf, Michael Boeckh, Janet A. Englund, Michael Famulare, Barry R. Lutz, Mark J. Rieder, Lea M. Starita, Matthew Thompson, Jay Shendure, Trevor Bedford, Helen Y. Chu
EPI_ISL_903049, EPI_ISL_903050, EPI_ISL_903051, EPI_ISL_903052, EPI_ISL_903053	Seattle Flu Study	Seattle Flu Study	Deborah A. Nickerson, Chris D. Frazar, Jover Lee, Benjamin Pelle, Erica Ryke, Matthew Richardson, Amanda Adler, Elisabeth Brandstetter, Peter D. Han, Kairsten Fay, Misja Ilcisin, Kirsten Lacombe, Thomas R. Sibley, Melissa Truong, Caitlin R. Wolf, Karen Cowgill, Stephanie Schrag, Jeff Duchin, Michael Boeckh, Janet A. Englund, Michael Famulare, Barry R. Lutz, Mark J. Rieder, Lea M. Starita, Matthew Thompson, Helen Y. Chu, Trevor Bedford, Jay Shendure
EPI_ISL_903054	Seattle Flu Study	Seattle Flu Study	Deborah A. Nickerson, Chris D. Frazar, Jover Lee, Benjamin Pelle, Erica Ryke, Matthew Richardson, Amanda Adler, Elisabeth Brandstetter, Peter D. Han, Kairsten Fay, Misja Ilcisin, Kirsten Lacombe, Thomas R. Sibley, Melissa Truong, Caitlin R. Wolf, Michael Boeckh, Janet A. Englund, Michael Famulare, Barry R. Lutz, Mark J. Rieder, Lea M. Starita, Matthew Thompson, Jay Shendure, Trevor Bedford, Helen Y. Chu
EPI_ISL_903055	Seattle Flu Study	Seattle Flu Study	Deborah A. Nickerson, Chris D. Frazar, Jover Lee, Benjamin Pelle, Erica Ryke, Matthew Richardson, Amanda Adler, Elisabeth Brandstetter, Peter D. Han, Kairsten Fay, Misja Ilcisin, Kirsten Lacombe, Thomas R. Sibley, Melissa Truong, Caitlin R. Wolf, Karen Cowgill, Stephanie Schrag, Jeff Duchin, Michael Boeckh, Janet A. Englund, Michael Famulare, Barry R. Lutz, Mark J. Rieder, Lea M. Starita, Matthew Thompson, Helen Y. Chu, Trevor Bedford, Jay Shendure
EPI_ISL_903056	Seattle Flu Study	Seattle Flu Study	Deborah A. Nickerson, Chris D. Frazar, Jover Lee, Benjamin Pelle, Erica Ryke, Matthew Richardson, Amanda Adler, Elisabeth Brandstetter, Peter D. Han, Kairsten Fay, Misja Ilcisin, Kirsten Lacombe, Thomas R. Sibley, Melissa Truong, Caitlin R. Wolf, Michael Boeckh, Janet A. Englund, Michael Famulare,

			Barry R. Lutz, Mark J. Rieder, Lea M. Starita, Matthew Thompson, Jay Shendure, Trevor Bedford, Helen Y. Chu
EPI_ISL_903057, EPI_ISL_903058, EPI_ISL_903059, EPI_ISL_903060, EPI_ISL_903087, EPI_ISL_903088, EPI_ISL_903089, EPI_ISL_903090, EPI_ISL_903091	Seattle Flu Study	Seattle Flu Study	Deborah A. Nickerson, Chris D. Frazar, Jover Lee, Benjamin Pelle, Erica Ryke, Matthew Richardson, Amanda Adler, Elisabeth Brandstetter, Peter D. Han, Kairsten Fay, Misja Ilcisin, Kirsten Lacombe, Thomas R. Sibley, Melissa Truong, Caitlin R. Wolf, Karen Cowgill, Stephanie Schrag, Jeff Duchin, Michael Boeckh, Janet A. Englund, Michael Famulare, Barry R. Lutz, Mark J. Rieder, Lea M. Starita, Matthew Thompson, Helen Y. Chu, Trevor Bedford, Jay Shendure
EPI_ISL_903105, EPI_ISL_903107, EPI_ISL_903109, EPI_ISL_903112, EPI_ISL_903114, EPI_ISL_903115, EPI_ISL_903116, EPI_ISL_903117, EPI_ISL_903118, EPI_ISL_903119, EPI_ISL_903124, EPI_ISL_903125, EPI_ISL_903126, EPI_ISL_903127, EPI_ISL_903128			
see above	Washington State Department of Health	Seattle Flu Study	Deborah A. Nickerson, Chris D. Frazar, Jover Lee, Benjamin Pelle, Erica Ryke, Matthew Richardson, Amanda Adler, Elisabeth Brandstetter, Peter D. Han, Kairsten Fay, Misja Ilcisin, Kirsten Lacombe, Thomas R. Sibley, Melissa Truong, Caitlin R. Wolf, Romesh Gautom, Geoff Melly, Brian Hiatt, Philip Dykema, Scott Lindquist, Michael Boeckh, Janet A. Englund, Michael Famulare, Barry R. Lutz, Mark J. Rieder, Lea M. Starita, Matthew Thompson, Helen Y. Chu, Jay Shendure, Trevor Bedford
EPI_ISL_903241, EPI_ISL_903274, EPI_ISL_903275, EPI_ISL_903276, EPI_ISL_903277, EPI_ISL_903278, EPI_ISL_903279, EPI_ISL_903280, EPI_ISL_903282, EPI_ISL_903283, EPI_ISL_903286, EPI_ISL_903288			
see above	M Health Fairview	Minnesota Department of Health, Public Health Laboratory	Alexandra Lorentz, Jacob Garfin, Matt Plumb, and Xiong Wang
EPI_ISL_903378, EPI_ISL_903383	MOH - Jaber Al-Ahmad Hospital (Innovation Research Laboratory)	MOH - Jaber Al-Ahmad Hospital (Innovation Research Laboratory)	Salman Al-Sabah , Mohammad Alghounaim
EPI_ISL_903577	NJ Public Health and Environmental Laboratories	Genomics and Discovery, Respiratory Viruses Branch, Division of Viral Diseases, Centers for Disease Control and Prevention	Krista Queen, Yan Li, Ying Tao, Jing Zhang, Anna Uehara, Anna Montmayeur, Clinton R. Paden, Peter W. Cook, Rachel Marine, Mili Sheth, Jasmine Padilla, Sarah Nobles, Mark Burroughs, Lori Rowe, Haibin Wang, Ben L. Rambo-Martin, Dhwani Batra, Justin Lee, Suxiang Tong
EPI_ISL_903585	MD DOH Laboratories Administration	Genomics and Discovery, Respiratory Viruses Branch, Division of Viral Diseases, Centers for Disease Control and Prevention	Krista Queen, Yan Li, Ying Tao, Jing Zhang, Anna Uehara, Anna Montmayeur, Clinton R. Paden, Peter W. Cook, Rachel Marine, Mili Sheth, Jasmine Padilla, Sarah Nobles, Mark Burroughs, Lori Rowe, Haibin Wang, Ben L. Rambo-Martin, Dhwani Batra, Justin Lee, Suxiang Tong
EPI_ISL_903591	CDPH, Viral and Rickettsial Disease Laboratory	Genomics and Discovery, Respiratory Viruses Branch, Division of Viral Diseases, Centers for Disease Control and Prevention	Krista Queen, Yan Li, Ying Tao, Jing Zhang, Anna Uehara, Anna Montmayeur, Clinton R. Paden, Peter W. Cook, Rachel Marine, Mili Sheth, Jasmine Padilla, Sarah Nobles, Mark Burroughs, Lori Rowe, Haibin Wang, Ben L. Rambo-Martin, Dhwani Batra, Justin Lee, Suxiang Tong
EPI_ISL_903594, EPI_ISL_903595	NJ Public Health and Environmental Laboratories	Genomics and Discovery, Respiratory Viruses Branch, Division of Viral Diseases, Centers for Disease Control and Prevention	Krista Queen, Yan Li, Ying Tao, Jing Zhang, Anna Uehara, Anna Montmayeur, Clinton R. Paden, Peter W. Cook, Rachel Marine, Mili Sheth, Jasmine Padilla, Sarah Nobles, Mark Burroughs, Lori Rowe, Haibin Wang, Ben L. Rambo-Martin, Dhwani Batra, Justin Lee, Suxiang Tong
EPI_ISL_903596	OK Public Health Laboratory, Oklahoma State DOH	Genomics and Discovery, Respiratory Viruses Branch, Division of Viral Diseases, Centers for Disease Control and Prevention	Krista Queen, Yan Li, Ying Tao, Jing Zhang, Anna Uehara, Anna Montmayeur, Clinton R. Paden, Peter W. Cook, Rachel Marine, Mili Sheth, Jasmine Padilla, Sarah Nobles, Mark Burroughs, Lori Rowe, Haibin Wang, Ben L. Rambo-Martin, Dhwani Batra, Justin Lee, Suxiang Tong
EPI_ISL_903607	IL Dept. of Public Health Springfield Laboratory	Genomics and Discovery, Respiratory Viruses Branch, Division of Viral Diseases, Centers for Disease Control and Prevention	Krista Queen, Yan Li, Ying Tao, Jing Zhang, Anna Uehara, Anna Montmayeur, Clinton R. Paden, Peter W. Cook, Rachel Marine, Mili Sheth, Jasmine Padilla, Sarah Nobles, Mark Burroughs, Lori Rowe, Haibin Wang, Ben L. Rambo-Martin, Dhwani Batra, Justin Lee, Suxiang Tong
EPI_ISL_903609	NJ Public Health and Environmental Laboratories	Genomics and Discovery, Respiratory Viruses Branch, Division of Viral Diseases, Centers for Disease Control and Prevention	Krista Queen, Yan Li, Ying Tao, Jing Zhang, Anna Uehara, Anna Montmayeur, Clinton R. Paden, Peter W. Cook, Rachel Marine, Mili Sheth, Jasmine Padilla, Sarah Nobles, Mark Burroughs, Lori Rowe, Haibin Wang, Ben L. Rambo-Martin, Dhwani Batra, Justin Lee, Suxiang Tong
EPI_ISL_903610	NV State Public Health Laboratory	Genomics and Discovery, Respiratory Viruses Branch, Division of Viral Diseases, Centers for Disease Control and Prevention	Krista Queen, Yan Li, Ying Tao, Jing Zhang, Anna Uehara, Anna Montmayeur, Clinton R. Paden, Peter W. Cook, Rachel Marine, Mili Sheth, Jasmine Padilla, Sarah Nobles, Mark Burroughs, Lori Rowe, Haibin Wang, Ben L. Rambo-Martin, Dhwani Batra, Justin Lee, Suxiang Tong
EPI_ISL_903616	IN State Department of Health Laboratory Services	Genomics and Discovery, Respiratory Viruses Branch, Division of Viral Diseases, Centers for Disease Control and Prevention	Krista Queen, Yan Li, Ying Tao, Jing Zhang, Anna Uehara, Anna Montmayeur, Clinton R. Paden, Peter W. Cook, Rachel Marine, Mili Sheth, Jasmine Padilla, Sarah Nobles, Mark Burroughs, Lori Rowe, Haibin Wang, Ben L. Rambo-Martin, Dhwani Batra, Justin Lee, Suxiang Tong
EPI_ISL_903637, EPI_ISL_903638	NJ Public Health and Environmental Laboratories	Genomics and Discovery, Respiratory Viruses Branch, Division of Viral Diseases, Centers for Disease Control and Prevention	Krista Queen, Yan Li, Ying Tao, Jing Zhang, Anna Uehara, Anna Montmayeur, Clinton R. Paden, Peter W. Cook, Rachel Marine, Mili Sheth, Jasmine Padilla, Sarah Nobles, Mark Burroughs, Lori Rowe, Haibin Wang, Ben L. Rambo-Martin, Dhwani Batra, Justin Lee, Suxiang Tong
EPI_ISL_903642	PA Department of Health, Bureau of Laboratories	Genomics and Discovery, Respiratory Viruses Branch, Division of Viral Diseases, Centers for Disease Control and Prevention	Krista Queen, Yan Li, Ying Tao, Jing Zhang, Anna Uehara, Anna Montmayeur, Clinton R. Paden, Peter W. Cook, Rachel Marine, Mili Sheth, Jasmine Padilla, Sarah Nobles, Mark Burroughs, Lori Rowe, Haibin Wang, Ben L. Rambo-Martin, Dhwani Batra, Justin Lee, Suxiang Tong
EPI_ISL_903651	LA Office of Public Health Laboratories	Genomics and Discovery, Respiratory Viruses Branch, Division of Viral Diseases, Centers for Disease Control and Prevention	Krista Queen, Yan Li, Ying Tao, Jing Zhang, Anna Uehara, Anna Montmayeur, Clinton R. Paden, Peter W. Cook, Rachel Marine, Mili Sheth, Jasmine Padilla, Sarah Nobles, Mark Burroughs, Lori Rowe, Haibin Wang, Ben L. Rambo-Martin, Dhwani Batra, Justin Lee, Suxiang Tong
EPI_ISL_903654, EPI_ISL_903655, EPI_ISL_903658	NJ Public Health and Environmental Laboratories	Genomics and Discovery, Respiratory Viruses Branch, Division of Viral Diseases, Centers for Disease Control and Prevention	Krista Queen, Yan Li, Ying Tao, Jing Zhang, Anna Uehara, Anna Montmayeur, Clinton R. Paden, Peter W. Cook, Rachel Marine, Mili Sheth, Jasmine Padilla, Sarah Nobles, Mark Burroughs, Lori Rowe, Haibin Wang, Ben L. Rambo-Martin, Dhwani Batra, Justin Lee, Suxiang Tong
EPI_ISL_903663	IL Dept. of Public Health Springfield Laboratory	Genomics and Discovery, Respiratory Viruses Branch, Division of Viral Diseases, Centers for Disease Control and Prevention	Krista Queen, Yan Li, Ying Tao, Jing Zhang, Anna Uehara, Anna Montmayeur, Clinton R. Paden, Peter W. Cook, Rachel Marine, Mili Sheth, Jasmine Padilla, Sarah Nobles, Mark Burroughs, Lori Rowe, Haibin Wang, Ben L. Rambo-Martin, Dhwani Batra, Justin Lee, Suxiang Tong
EPI_ISL_903664	NV State Public Health Laboratory	Genomics and Discovery, Respiratory Viruses Branch, Division of Viral Diseases, Centers for Disease Control and Prevention	Krista Queen, Yan Li, Ying Tao, Jing Zhang, Anna Uehara, Anna Montmayeur, Clinton R. Paden, Peter W. Cook, Rachel Marine, Mili Sheth, Jasmine Padilla, Sarah Nobles, Mark Burroughs, Lori Rowe, Haibin Wang, Ben L. Rambo-Martin, Dhwani Batra, Justin Lee, Suxiang Tong
EPI_ISL_903673, EPI_ISL_903674, EPI_ISL_903678, EPI_ISL_903680	NJ Public Health and Environmental Laboratories	Genomics and Discovery, Respiratory Viruses Branch, Division of Viral Diseases, Centers for Disease Control and Prevention	Krista Queen, Yan Li, Ying Tao, Jing Zhang, Anna Uehara, Anna Montmayeur, Clinton R. Paden, Peter W. Cook, Rachel Marine, Mili Sheth, Jasmine Padilla, Sarah Nobles, Mark Burroughs, Lori Rowe, Haibin Wang, Ben L. Rambo-Martin, Dhwani Batra, Justin Lee, Suxiang Tong
EPI_ISL_903682	IL Dept. of Public Health Springfield Laboratory	Genomics and Discovery, Respiratory Viruses Branch, Division of Viral Diseases, Centers for Disease Control and Prevention	Krista Queen, Yan Li, Ying Tao, Jing Zhang, Anna Uehara, Anna Montmayeur, Clinton R. Paden, Peter W. Cook, Rachel Marine, Mili Sheth, Jasmine Padilla, Sarah Nobles, Mark Burroughs, Lori Rowe, Haibin Wang, Ben L. Rambo-Martin, Dhwani Batra, Justin Lee, Suxiang Tong
EPI_ISL_903683, EPI_ISL_903686	NJ Public Health and Environmental Laboratories	Genomics and Discovery, Respiratory Viruses Branch, Division of Viral Diseases, Centers for Disease Control and Prevention	Krista Queen, Yan Li, Ying Tao, Jing Zhang, Anna Uehara, Anna Montmayeur, Clinton R. Paden, Peter W. Cook, Rachel Marine, Mili Sheth, Jasmine Padilla, Sarah Nobles, Mark Burroughs, Lori Rowe, Haibin Wang, Ben L. Rambo-Martin, Dhwani Batra, Justin Lee, Suxiang Tong
EPI_ISL_903689	LA Office of Public Health Laboratories	Genomics and Discovery, Respiratory Viruses Branch, Division of Viral Diseases, Centers for Disease Control and Prevention	Krista Queen, Yan Li, Ying Tao, Jing Zhang, Anna Uehara, Anna Montmayeur, Clinton R. Paden, Peter W. Cook, Rachel Marine, Mili Sheth, Jasmine Padilla, Sarah Nobles, Mark Burroughs, Lori Rowe, Haibin Wang, Ben L. Rambo-Martin, Dhwani Batra, Justin Lee, Suxiang Tong
EPI_ISL_903690	NV State Public Health Laboratory	Genomics and Discovery, Respiratory Viruses Branch, Division of Viral Diseases, Centers for Disease Control and	Krista Queen, Yan Li, Ying Tao, Jing Zhang, Anna Uehara, Anna Montmayeur, Clinton R. Paden, Peter W. Cook, Rachel Marine, Mili Sheth, Jasmine Padilla, Sarah Nobles, Mark Burroughs, Lori Rowe, Haibin Wang, Ben L. Rambo-Martin, Dhwani Batra, Justin Lee, Suxiang Tong

		Prevention	
EPI_ISL_903692	NJ Public Health and Environmental Laboratories	Genomics and Discovery, Respiratory Viruses Branch, Division of Viral Diseases, Centers for Disease Control and Prevention	Krista Queen, Yan Li, Ying Tao, Jing Zhang, Anna Uehara, Anna Montmayeur, Clinton R. Paden, Peter W. Cook, Rachel Marine, Mili Sheth, Jasmine Padilla, Sarah Nobles, Mark Burroughs, Lori Rowe, Haibin Wang, Ben L. Rambo-Martin, Dhwani Batra, Justin Lee, Suxiang Tong
EPI_ISL_903695	OK Public Health Laboratory, Oklahoma State DOH	Genomics and Discovery, Respiratory Viruses Branch, Division of Viral Diseases, Centers for Disease Control and Prevention	Krista Queen, Yan Li, Ying Tao, Jing Zhang, Anna Uehara, Anna Montmayeur, Clinton R. Paden, Peter W. Cook, Rachel Marine, Mili Sheth, Jasmine Padilla, Sarah Nobles, Mark Burroughs, Lori Rowe, Haibin Wang, Ben L. Rambo-Martin, Dhwani Batra, Justin Lee, Suxiang Tong
EPI_ISL_903699	NJ Public Health and Environmental Laboratories	Genomics and Discovery, Respiratory Viruses Branch, Division of Viral Diseases, Centers for Disease Control and Prevention	Krista Queen, Yan Li, Ying Tao, Jing Zhang, Anna Uehara, Anna Montmayeur, Clinton R. Paden, Peter W. Cook, Rachel Marine, Mili Sheth, Jasmine Padilla, Sarah Nobles, Mark Burroughs, Lori Rowe, Haibin Wang, Ben L. Rambo-Martin, Dhwani Batra, Justin Lee, Suxiang Tong
EPI_ISL_903700, EPI_ISL_903702	NV State Public Health Laboratory	Genomics and Discovery, Respiratory Viruses Branch, Division of Viral Diseases, Centers for Disease Control and Prevention	Krista Queen, Yan Li, Ying Tao, Jing Zhang, Anna Uehara, Anna Montmayeur, Clinton R. Paden, Peter W. Cook, Rachel Marine, Mili Sheth, Jasmine Padilla, Sarah Nobles, Mark Burroughs, Lori Rowe, Haibin Wang, Ben L. Rambo-Martin, Dhwani Batra, Justin Lee, Suxiang Tong
EPI_ISL_903704	NJ Public Health and Environmental Laboratories	Genomics and Discovery, Respiratory Viruses Branch, Division of Viral Diseases, Centers for Disease Control and Prevention	Krista Queen, Yan Li, Ying Tao, Jing Zhang, Anna Uehara, Anna Montmayeur, Clinton R. Paden, Peter W. Cook, Rachel Marine, Mili Sheth, Jasmine Padilla, Sarah Nobles, Mark Burroughs, Lori Rowe, Haibin Wang, Ben L. Rambo-Martin, Dhwani Batra, Justin Lee, Suxiang Tong
EPI_ISL_903709	LA Office of Public Health Laboratories	Genomics and Discovery, Respiratory Viruses Branch, Division of Viral Diseases, Centers for Disease Control and Prevention	Krista Queen, Yan Li, Ying Tao, Jing Zhang, Anna Uehara, Anna Montmayeur, Clinton R. Paden, Peter W. Cook, Rachel Marine, Mili Sheth, Jasmine Padilla, Sarah Nobles, Mark Burroughs, Lori Rowe, Haibin Wang, Ben L. Rambo-Martin, Dhwani Batra, Justin Lee, Suxiang Tong
EPI_ISL_903711, EPI_ISL_903714	NJ Public Health and Environmental Laboratories	Genomics and Discovery, Respiratory Viruses Branch, Division of Viral Diseases, Centers for Disease Control and Prevention	Krista Queen, Yan Li, Ying Tao, Jing Zhang, Anna Uehara, Anna Montmayeur, Clinton R. Paden, Peter W. Cook, Rachel Marine, Mili Sheth, Jasmine Padilla, Sarah Nobles, Mark Burroughs, Lori Rowe, Haibin Wang, Ben L. Rambo-Martin, Dhwani Batra, Justin Lee, Suxiang Tong
EPI_ISL_903716	OK Public Health Laboratory, Oklahoma State DOH	Genomics and Discovery, Respiratory Viruses Branch, Division of Viral Diseases, Centers for Disease Control and Prevention	Krista Queen, Yan Li, Ying Tao, Jing Zhang, Anna Uehara, Anna Montmayeur, Clinton R. Paden, Peter W. Cook, Rachel Marine, Mili Sheth, Jasmine Padilla, Sarah Nobles, Mark Burroughs, Lori Rowe, Haibin Wang, Ben L. Rambo-Martin, Dhwani Batra, Justin Lee, Suxiang Tong
EPI_ISL_903721, EPI_ISL_903723	NV State Public Health Laboratory	Genomics and Discovery, Respiratory Viruses Branch, Division of Viral Diseases, Centers for Disease Control and Prevention	Krista Queen, Yan Li, Ying Tao, Jing Zhang, Anna Uehara, Anna Montmayeur, Clinton R. Paden, Peter W. Cook, Rachel Marine, Mili Sheth, Jasmine Padilla, Sarah Nobles, Mark Burroughs, Lori Rowe, Haibin Wang, Ben L. Rambo-Martin, Dhwani Batra, Justin Lee, Suxiang Tong
EPI_ISL_903726	NJ Public Health and Environmental Laboratories	Genomics and Discovery, Respiratory Viruses Branch, Division of Viral Diseases, Centers for Disease Control and Prevention	Krista Queen, Yan Li, Ying Tao, Jing Zhang, Anna Uehara, Anna Montmayeur, Clinton R. Paden, Peter W. Cook, Rachel Marine, Mili Sheth, Jasmine Padilla, Sarah Nobles, Mark Burroughs, Lori Rowe, Haibin Wang, Ben L. Rambo-Martin, Dhwani Batra, Justin Lee, Suxiang Tong
EPI_ISL_903727	NV State Public Health Laboratory	Genomics and Discovery, Respiratory Viruses Branch, Division of Viral Diseases, Centers for Disease Control and Prevention	Krista Queen, Yan Li, Ying Tao, Jing Zhang, Anna Uehara, Anna Montmayeur, Clinton R. Paden, Peter W. Cook, Rachel Marine, Mili Sheth, Jasmine Padilla, Sarah Nobles, Mark Burroughs, Lori Rowe, Haibin Wang, Ben L. Rambo-Martin, Dhwani Batra, Justin Lee, Suxiang Tong
EPI_ISL_903728	LA Office of Public Health Laboratories	Genomics and Discovery, Respiratory Viruses Branch, Division of Viral Diseases, Centers for Disease Control and Prevention	Krista Queen, Yan Li, Ying Tao, Jing Zhang, Anna Uehara, Anna Montmayeur, Clinton R. Paden, Peter W. Cook, Rachel Marine, Mili Sheth, Jasmine Padilla, Sarah Nobles, Mark Burroughs, Lori Rowe, Haibin Wang, Ben L. Rambo-Martin, Dhwani Batra, Justin Lee, Suxiang Tong
EPI_ISL_903733	NV State Public Health Laboratory	Genomics and Discovery, Respiratory Viruses Branch, Division of Viral Diseases, Centers for Disease Control and Prevention	Krista Queen, Yan Li, Ying Tao, Jing Zhang, Anna Uehara, Anna Montmayeur, Clinton R. Paden, Peter W. Cook, Rachel Marine, Mili Sheth, Jasmine Padilla, Sarah Nobles, Mark Burroughs, Lori Rowe, Haibin Wang, Ben L. Rambo-Martin, Dhwani Batra, Justin Lee, Suxiang Tong
EPI_ISL_903734	NJ Public Health and Environmental Laboratories	Genomics and Discovery, Respiratory Viruses Branch, Division of Viral Diseases, Centers for Disease Control and Prevention	Krista Queen, Yan Li, Ying Tao, Jing Zhang, Anna Uehara, Anna Montmayeur, Clinton R. Paden, Peter W. Cook, Rachel Marine, Mili Sheth, Jasmine Padilla, Sarah Nobles, Mark Burroughs, Lori Rowe, Haibin Wang, Ben L. Rambo-Martin, Dhwani Batra, Justin Lee, Suxiang Tong
EPI_ISL_903751	PA Department of Health, Bureau of Laboratories	Genomics and Discovery, Respiratory Viruses Branch, Division of Viral Diseases, Centers for Disease Control and Prevention	Krista Queen, Yan Li, Ying Tao, Jing Zhang, Anna Uehara, Anna Montmayeur, Clinton R. Paden, Peter W. Cook, Rachel Marine, Mili Sheth, Jasmine Padilla, Sarah Nobles, Mark Burroughs, Lori Rowe, Haibin Wang, Ben L. Rambo-Martin, Dhwani Batra, Justin Lee, Suxiang Tong
EPI_ISL_903754	IN State Department of Health Laboratory Services	Genomics and Discovery, Respiratory Viruses Branch, Division of Viral Diseases, Centers for Disease Control and Prevention	Krista Queen, Yan Li, Ying Tao, Jing Zhang, Anna Uehara, Anna Montmayeur, Clinton R. Paden, Peter W. Cook, Rachel Marine, Mili Sheth, Jasmine Padilla, Sarah Nobles, Mark Burroughs, Lori Rowe, Haibin Wang, Ben L. Rambo-Martin, Dhwani Batra, Justin Lee, Suxiang Tong
EPI_ISL_903759	NM Dept. Health, Scientific Laboratory Division	Genomics and Discovery, Respiratory Viruses Branch, Division of Viral Diseases, Centers for Disease Control and Prevention	Krista Queen, Yan Li, Ying Tao, Jing Zhang, Anna Uehara, Anna Montmayeur, Clinton R. Paden, Peter W. Cook, Rachel Marine, Mili Sheth, Jasmine Padilla, Sarah Nobles, Mark Burroughs, Lori Rowe, Haibin Wang, Ben L. Rambo-Martin, Dhwani Batra, Justin Lee, Suxiang Tong
EPI_ISL_903761	CDPH, Viral and Rickettsial Disease Laboratory	Genomics and Discovery, Respiratory Viruses Branch, Division of Viral Diseases, Centers for Disease Control and Prevention	Krista Queen, Yan Li, Ying Tao, Jing Zhang, Anna Uehara, Anna Montmayeur, Clinton R. Paden, Peter W. Cook, Rachel Marine, Mili Sheth, Jasmine Padilla, Sarah Nobles, Mark Burroughs, Lori Rowe, Haibin Wang, Ben L. Rambo-Martin, Dhwani Batra, Justin Lee, Suxiang Tong
EPI_ISL_903762	MD DOH Laboratories Administration	Genomics and Discovery, Respiratory Viruses Branch, Division of Viral Diseases, Centers for Disease Control and Prevention	Krista Queen, Yan Li, Ying Tao, Jing Zhang, Anna Uehara, Anna Montmayeur, Clinton R. Paden, Peter W. Cook, Rachel Marine, Mili Sheth, Jasmine Padilla, Sarah Nobles, Mark Burroughs, Lori Rowe, Haibin Wang, Ben L. Rambo-Martin, Dhwani Batra, Justin Lee, Suxiang Tong
EPI_ISL_903773	IN State Department of Health Laboratory Services	Genomics and Discovery, Respiratory Viruses Branch, Division of Viral Diseases, Centers for Disease Control and Prevention	Krista Queen, Yan Li, Ying Tao, Jing Zhang, Anna Uehara, Anna Montmayeur, Clinton R. Paden, Peter W. Cook, Rachel Marine, Mili Sheth, Jasmine Padilla, Sarah Nobles, Mark Burroughs, Lori Rowe, Haibin Wang, Ben L. Rambo-Martin, Dhwani Batra, Justin Lee, Suxiang Tong
EPI_ISL_903779, EPI_ISL_903789	IL Dept. of Public Health Springfield Laboratory	Genomics and Discovery, Respiratory Viruses Branch, Division of Viral Diseases, Centers for Disease Control and Prevention	Krista Queen, Yan Li, Ying Tao, Jing Zhang, Anna Uehara, Anna Montmayeur, Clinton R. Paden, Peter W. Cook, Rachel Marine, Mili Sheth, Jasmine Padilla, Sarah Nobles, Mark Burroughs, Lori Rowe, Haibin Wang, Ben L. Rambo-Martin, Dhwani Batra, Justin Lee, Suxiang Tong
EPI_ISL_903790	PA Department of Health, Bureau of Laboratories	Genomics and Discovery, Respiratory Viruses Branch, Division of Viral Diseases, Centers for Disease Control and Prevention	Krista Queen, Yan Li, Ying Tao, Jing Zhang, Anna Uehara, Anna Montmayeur, Clinton R. Paden, Peter W. Cook, Rachel Marine, Mili Sheth, Jasmine Padilla, Sarah Nobles, Mark Burroughs, Lori Rowe, Haibin Wang, Ben L. Rambo-Martin, Dhwani Batra, Justin Lee, Suxiang Tong
EPI_ISL_903810	CDPH, Viral and Rickettsial Disease Laboratory	Genomics and Discovery, Respiratory Viruses Branch, Division of Viral Diseases, Centers for Disease Control and Prevention	Krista Queen, Yan Li, Ying Tao, Jing Zhang, Anna Uehara, Anna Montmayeur, Clinton R. Paden, Peter W. Cook, Rachel Marine, Mili Sheth, Jasmine Padilla, Sarah Nobles, Mark Burroughs, Lori Rowe, Haibin Wang, Ben L. Rambo-Martin, Dhwani Batra, Justin Lee, Suxiang Tong
EPI_ISL_903823	OK Public Health Laboratory, Oklahoma State DOH	Genomics and Discovery, Respiratory Viruses Branch, Division of Viral Diseases, Centers for Disease Control and Prevention	Krista Queen, Yan Li, Ying Tao, Jing Zhang, Anna Uehara, Anna Montmayeur, Clinton R. Paden, Peter W. Cook, Rachel Marine, Mili Sheth, Jasmine Padilla, Sarah Nobles, Mark Burroughs, Lori Rowe, Haibin Wang, Ben L. Rambo-Martin, Dhwani Batra, Justin Lee, Suxiang Tong
EPI_ISL_903828	IL Dept. of Public Health Springfield Laboratory	Genomics and Discovery, Respiratory Viruses Branch, Division of Viral Diseases, Centers for Disease Control and	Krista Queen, Yan Li, Ying Tao, Jing Zhang, Anna Uehara, Anna Montmayeur, Clinton R. Paden, Peter W. Cook, Rachel Marine, Mili Sheth, Jasmine Padilla, Sarah Nobles, Mark Burroughs, Lori Rowe, Haibin Wang, Ben L. Rambo-Martin, Dhwani Batra, Justin Lee, Suxiang Tong

		Prevention	
EPI_ISL_903832	LA Office of Public Health Laboratories	Genomics and Discovery, Respiratory Viruses Branch, Division of Viral Diseases, Centers for Disease Control and Prevention	Krista Queen, Yan Li, Ying Tao, Jing Zhang, Anna Uehara, Anna Montmayeur, Clinton R. Paden, Peter W. Cook, Rachel Marine, Mili Sheth, Jasmine Padilla, Sarah Nobles, Mark Burroughs, Lori Rowe, Haibin Wang, Ben L. Rambo-Martin, Dhwani Batra, Justin Lee, Suxiang Tong
EPI_ISL_903837	PA Department of Health, Bureau of Laboratories	Genomics and Discovery, Respiratory Viruses Branch, Division of Viral Diseases, Centers for Disease Control and Prevention	Krista Queen, Yan Li, Ying Tao, Jing Zhang, Anna Uehara, Anna Montmayeur, Clinton R. Paden, Peter W. Cook, Rachel Marine, Mili Sheth, Jasmine Padilla, Sarah Nobles, Mark Burroughs, Lori Rowe, Haibin Wang, Ben L. Rambo-Martin, Dhwani Batra, Justin Lee, Suxiang Tong
EPI_ISL_903848	MD DOH Laboratories Administration	Genomics and Discovery, Respiratory Viruses Branch, Division of Viral Diseases, Centers for Disease Control and Prevention	Krista Queen, Yan Li, Ying Tao, Jing Zhang, Anna Uehara, Anna Montmayeur, Clinton R. Paden, Peter W. Cook, Rachel Marine, Mili Sheth, Jasmine Padilla, Sarah Nobles, Mark Burroughs, Lori Rowe, Haibin Wang, Ben L. Rambo-Martin, Dhwani Batra, Justin Lee, Suxiang Tong
EPI_ISL_903852, EPI_ISL_903859	PA Department of Health, Bureau of Laboratories	Genomics and Discovery, Respiratory Viruses Branch, Division of Viral Diseases, Centers for Disease Control and Prevention	Krista Queen, Yan Li, Ying Tao, Jing Zhang, Anna Uehara, Anna Montmayeur, Clinton R. Paden, Peter W. Cook, Rachel Marine, Mili Sheth, Jasmine Padilla, Sarah Nobles, Mark Burroughs, Lori Rowe, Haibin Wang, Ben L. Rambo-Martin, Dhwani Batra, Justin Lee, Suxiang Tong
EPI_ISL_903862	NM Dept. Health, Scientific Laboratory Division	Genomics and Discovery, Respiratory Viruses Branch, Division of Viral Diseases, Centers for Disease Control and Prevention	Krista Queen, Yan Li, Ying Tao, Jing Zhang, Anna Uehara, Anna Montmayeur, Clinton R. Paden, Peter W. Cook, Rachel Marine, Mili Sheth, Jasmine Padilla, Sarah Nobles, Mark Burroughs, Lori Rowe, Haibin Wang, Ben L. Rambo-Martin, Dhwani Batra, Justin Lee, Suxiang Tong
EPI_ISL_903875	IN State Department of Health Laboratory Services	Genomics and Discovery, Respiratory Viruses Branch, Division of Viral Diseases, Centers for Disease Control and Prevention	Krista Queen, Yan Li, Ying Tao, Jing Zhang, Anna Uehara, Anna Montmayeur, Clinton R. Paden, Peter W. Cook, Rachel Marine, Mili Sheth, Jasmine Padilla, Sarah Nobles, Mark Burroughs, Lori Rowe, Haibin Wang, Ben L. Rambo-Martin, Dhwani Batra, Justin Lee, Suxiang Tong
EPI_ISL_903877	LA Office of Public Health Laboratories	Genomics and Discovery, Respiratory Viruses Branch, Division of Viral Diseases, Centers for Disease Control and Prevention	Krista Queen, Yan Li, Ying Tao, Jing Zhang, Anna Uehara, Anna Montmayeur, Clinton R. Paden, Peter W. Cook, Rachel Marine, Mili Sheth, Jasmine Padilla, Sarah Nobles, Mark Burroughs, Lori Rowe, Haibin Wang, Ben L. Rambo-Martin, Dhwani Batra, Justin Lee, Suxiang Tong
EPI_ISL_903883	IN State Department of Health Laboratory Services	Genomics and Discovery, Respiratory Viruses Branch, Division of Viral Diseases, Centers for Disease Control and Prevention	Krista Queen, Yan Li, Ying Tao, Jing Zhang, Anna Uehara, Anna Montmayeur, Clinton R. Paden, Peter W. Cook, Rachel Marine, Mili Sheth, Jasmine Padilla, Sarah Nobles, Mark Burroughs, Lori Rowe, Haibin Wang, Ben L. Rambo-Martin, Dhwani Batra, Justin Lee, Suxiang Tong
EPI_ISL_903892	CDPH, Viral and Rickettsial Disease Laboratory	Genomics and Discovery, Respiratory Viruses Branch, Division of Viral Diseases, Centers for Disease Control and Prevention	Krista Queen, Yan Li, Ying Tao, Jing Zhang, Anna Uehara, Anna Montmayeur, Clinton R. Paden, Peter W. Cook, Rachel Marine, Mili Sheth, Jasmine Padilla, Sarah Nobles, Mark Burroughs, Lori Rowe, Haibin Wang, Ben L. Rambo-Martin, Dhwani Batra, Justin Lee, Suxiang Tong
EPI_ISL_903896	IL Dept. of Public Health Springfield Laboratory	Genomics and Discovery, Respiratory Viruses Branch, Division of Viral Diseases, Centers for Disease Control and Prevention	Krista Queen, Yan Li, Ying Tao, Jing Zhang, Anna Uehara, Anna Montmayeur, Clinton R. Paden, Peter W. Cook, Rachel Marine, Mili Sheth, Jasmine Padilla, Sarah Nobles, Mark Burroughs, Lori Rowe, Haibin Wang, Ben L. Rambo-Martin, Dhwani Batra, Justin Lee, Suxiang Tong
EPI_ISL_903904	IN State Department of Health Laboratory Services	Genomics and Discovery, Respiratory Viruses Branch, Division of Viral Diseases, Centers for Disease Control and Prevention	Krista Queen, Yan Li, Ying Tao, Jing Zhang, Anna Uehara, Anna Montmayeur, Clinton R. Paden, Peter W. Cook, Rachel Marine, Mili Sheth, Jasmine Padilla, Sarah Nobles, Mark Burroughs, Lori Rowe, Haibin Wang, Ben L. Rambo-Martin, Dhwani Batra, Justin Lee, Suxiang Tong
EPI_ISL_903909	PA Department of Health, Bureau of Laboratories	Genomics and Discovery, Respiratory Viruses Branch, Division of Viral Diseases, Centers for Disease Control and Prevention	Krista Queen, Yan Li, Ying Tao, Jing Zhang, Anna Uehara, Anna Montmayeur, Clinton R. Paden, Peter W. Cook, Rachel Marine, Mili Sheth, Jasmine Padilla, Sarah Nobles, Mark Burroughs, Lori Rowe, Haibin Wang, Ben L. Rambo-Martin, Dhwani Batra, Justin Lee, Suxiang Tong
EPI_ISL_903915	CDPH, Viral and Rickettsial Disease Laboratory	Genomics and Discovery, Respiratory Viruses Branch, Division of Viral Diseases, Centers for Disease Control and Prevention	Krista Queen, Yan Li, Ying Tao, Jing Zhang, Anna Uehara, Anna Montmayeur, Clinton R. Paden, Peter W. Cook, Rachel Marine, Mili Sheth, Jasmine Padilla, Sarah Nobles, Mark Burroughs, Lori Rowe, Haibin Wang, Ben L. Rambo-Martin, Dhwani Batra, Justin Lee, Suxiang Tong
EPI_ISL_903933	PA Department of Health, Bureau of Laboratories	Genomics and Discovery, Respiratory Viruses Branch, Division of Viral Diseases, Centers for Disease Control and Prevention	Krista Queen, Yan Li, Ying Tao, Jing Zhang, Anna Uehara, Anna Montmayeur, Clinton R. Paden, Peter W. Cook, Rachel Marine, Mili Sheth, Jasmine Padilla, Sarah Nobles, Mark Burroughs, Lori Rowe, Haibin Wang, Ben L. Rambo-Martin, Dhwani Batra, Justin Lee, Suxiang Tong
EPI_ISL_903963	MD DOH Laboratories Administration	Genomics and Discovery, Respiratory Viruses Branch, Division of Viral Diseases, Centers for Disease Control and Prevention	Krista Queen, Yan Li, Ying Tao, Jing Zhang, Anna Uehara, Anna Montmayeur, Clinton R. Paden, Peter W. Cook, Rachel Marine, Mili Sheth, Jasmine Padilla, Sarah Nobles, Mark Burroughs, Lori Rowe, Haibin Wang, Ben L. Rambo-Martin, Dhwani Batra, Justin Lee, Suxiang Tong
EPI_ISL_904036, EPI_ISL_904037, EPI_ISL_904038, EPI_ISL_904039, EPI_ISL_904040, EPI_ISL_904041, EPI_ISL_904042	New Mexico Department of Health Scientific Laboratory	New Mexico Department of Health Scientific Laboratory	Ellie Johnson, Anastacia Griego-Fisher, D'eldra Malone
EPI_ISL_904148, EPI_ISL_904153, EPI_ISL_904164, EPI_ISL_904186, EPI_ISL_904187, EPI_ISL_904190, EPI_ISL_904199, EPI_ISL_904265, EPI_ISL_904266, EPI_ISL_904267, EPI_ISL_904303, EPI_ISL_904304, EPI_ISL_904364, EPI_ISL_904365, EPI_ISL_904520, EPI_ISL_904521, EPI_ISL_904522, EPI_ISL_904523, EPI_ISL_904524, EPI_ISL_904525, EPI_ISL_904526, EPI_ISL_904527, EPI_ISL_904528, EPI_ISL_904621, EPI_ISL_904622, EPI_ISL_904623, EPI_ISL_904624			
see above	Dutch COVID-19 response team	Erasmus Medical Center	Bas Oude Munnink, Reina Sikkema, David Nieuwenhuijse, Irina Chestakova, Anne van der Linden, Marjan Boter, Emmanuelle Munger, Corine GeurtsvanKessel, Annemiek van der Eijk, Richard Molenkamp, Marion Koopmans, on behalf of the Dutch national COVID-19 response team.
EPI_ISL_904767, EPI_ISL_904784, EPI_ISL_904891, EPI_ISL_904904, EPI_ISL_904972, EPI_ISL_904992, EPI_ISL_904994, EPI_ISL_905011, EPI_ISL_905033, EPI_ISL_905069, EPI_ISL_905101, EPI_ISL_905102, EPI_ISL_905103, EPI_ISL_905104, EPI_ISL_905149, EPI_ISL_905153, EPI_ISL_905157, EPI_ISL_905164, EPI_ISL_905169, EPI_ISL_905187, EPI_ISL_905201, EPI_ISL_905213, EPI_ISL_905214, EPI_ISL_905218, EPI_ISL_905219, EPI_ISL_905225, EPI_ISL_905226, EPI_ISL_905227, EPI_ISL_905352, EPI_ISL_905353, EPI_ISL_905354, EPI_ISL_905372, EPI_ISL_905373, EPI_ISL_905374, EPI_ISL_905375, EPI_ISL_905378, EPI_ISL_905379, EPI_ISL_905380, EPI_ISL_905381, EPI_ISL_905382, EPI_ISL_905404, EPI_ISL_905418, EPI_ISL_905429, EPI_ISL_905430, EPI_ISL_905431, EPI_ISL_905434, EPI_ISL_905435, EPI_ISL_905472, EPI_ISL_905473, EPI_ISL_905486, EPI_ISL_905502, EPI_ISL_905503, EPI_ISL_905507, EPI_ISL_905515, EPI_ISL_905536, EPI_ISL_905538, EPI_ISL_905539, EPI_ISL_905540, EPI_ISL_905551, EPI_ISL_905553, EPI_ISL_905554, EPI_ISL_905555, EPI_ISL_905556, EPI_ISL_905557, EPI_ISL_905558, EPI_ISL_905559, EPI_ISL_905561, EPI_ISL_905562, EPI_ISL_905563, EPI_ISL_905564, EPI_ISL_905565, EPI_ISL_905662, EPI_ISL_905673, EPI_ISL_905674, EPI_ISL_905675, EPI_ISL_905688, EPI_ISL_905711, EPI_ISL_905712, EPI_ISL_905713, EPI_ISL_905725, EPI_ISL_905729			
see above	Dutch COVID-19 response team	National Institute for Public Health and the Environment (RIVM)	Adam Meijer, Harry Vennema, Dirk Eggink, Jeroen Cremer, Sharon van den Brink, Bas van der Veer, AnneMarie van den Brandt, Florian Zwagemaker, Dennis Schmitz, Chantal Reusken, on behalf of the national COVID-19 response team
EPI_ISL_905849, EPI_ISL_905850, EPI_ISL_905851, EPI_ISL_905852, EPI_ISL_905853, EPI_ISL_905854, EPI_ISL_905855, EPI_ISL_905856, EPI_ISL_905857, EPI_ISL_905858, EPI_ISL_905859, EPI_ISL_905860, EPI_ISL_905861, EPI_ISL_905862, EPI_ISL_905863, EPI_ISL_905864, EPI_ISL_905865, EPI_ISL_905900, EPI_ISL_905901			
see above	OHSU Lab Services Molecular Microbiology Lab	Oregon SARS-CoV-2 Genome Sequencing Center	Brendan L. O'Connell, Sally Grindstaff, Kayla Carter, Ruth V. Nichols, Alec J. Hirsch, Donna Hansel, Guang Fan, Xuan, Qin, Daniel N. Streblow, William B. Messer, Andrew C. Adey, Benjamin N. Bimber, Brian J. O'Roak
EPI_ISL_906058, EPI_ISL_906062	Tilia Laboratories s.r.o.	Tilia Laboratories s.r.o.	Sona Pekova, MD, PhD.
EPI_ISL_906091	Shimantik Pathology and Diagnostic Center	Child Health Research Foundation	Senjuti Saha, Syed Muktadir Al Sium, Arif Mohammad Tanmoy, Afroza Akter Tanni, Sharmistha Goswami, Roly Malaker Md Hafizur Rahman, Md. Parvej Alam, Md. Mobarok Karim, Samir K Saha
EPI_ISL_906116	Child Health Research Foundation	Child Health Research Foundation	Senjuti Saha, Afroza Akter Tanni, Sharmistha Goswami, Syed Muktadir Al Sium, Arif Mohammad Tanmoy, Roly Malaker, Md Hafizur Rahman, Samir K Saha
EPI_ISL_906536	CLINICA OCCIDENTE	Instituto Nacional de Salud- Dirección de Investigación en Salud Pública, Universidad de los Andes- Applied genomics	Katherine Laiton-Donato, Diego A. Álvarez-Díaz, Carlos Franco-Muñoz, Mauricio Pacheco-Montealegre, Héctor Alejandro Ruiz-Moreno, Maria T. Herrera-Sepúlveda, Diego Andrés Prada, Jhonnatan Reales-González, Sheryll Corchuelo, Julian Naizaque, Gerardo Santamaría Jorge Duitama, Laura Natalia Gonzalez, Jorge Ivan Diaz, Silvia Restrepo-Restrepo, Magdalena Wiesner, Martha Lucia Ospina Martinez, Marcela Mercado-Reyes

		research group, Vicerrectoria de Investigación y Creación, Universidad de los Andes- Systems and Computing Engineering Department	
EPI_ISL_906540, EPI_ISL_906544	CLINICA DE OCCIDENTE	Instituto Nacional de Salud- Dirección de Investigación en Salud Pública, Universidad de los Andes- Applied genomics research group, Vicerrectoria de Investigación y Creación, Universidad de los Andes- Systems and Computing Engineering Department	Katherine Laiton-Donato, Diego A. Álvarez-Díaz, Carlos Franco-Muñoz, Mauricio Pacheco-Montealegre, Héctor Alejandro Ruiz-Moreno, Maria T. Herrera-Sepúlveda, Diego Andrés Prada, Jhonnatan Reales-González, Sheryll Corchuelo, Julian Naizaque, Gerardo Santamaría Jorge Duitama, Laura Natalia Gonzalez, Jorge Ivan Diaz, Silvia Restrepo-Restrepo, Magdalena Wiesner, Martha Lucia Ospina Martinez, Marcela Mercado-Reyes
EPI_ISL_906572	Maine Health and Environmental Testing Laboratory (Maine HETL)	Tewhey Lab, The Jackson Laboratory	Matluk,N., Dewey,H., Iosue,F., Barter,M., Lynch,R., Munger,H. and Tewhey,R.
EPI_ISL_906777, EPI_ISL_906778	New Mexico Department of Health Scientific Laboratory	New Mexico Department of Health Scientific Laboratory	Ellie Johnson, Anastacia Griego-Fisher, D'eldra Malone
EPI_ISL_909686	GH Cochin-Broca Hôtel Dieu/Lab Virologie	National Reference Center for Viruses of Respiratory Infections, Institut Pasteur, Paris	Marion Barbet, Sylvie Behillil, Méline Bizard, Angela Brisebarre, Camille Capel, Etienne Simon-Lorière, Vincent Enouf, Maud Vanpeene, Sylvie van der Werf,L'Honneur Anne-Sophie
EPI_ISL_910030, EPI_ISL_910222, EPI_ISL_910324	Viollier AG	University Hospital Basel, Clinical Bacteriology	Tim Roloff, Madlen Stange, Helena MB Seth-Smith, Alfredo Mari, Karoline Leuzinger, Julia Bielicki, Christiane Beckmann, Manuel Battegay, Hans Hirsch, Adrian Egli
EPI_ISL_910336	Laboratory for Respiratory Viruses, Cantacuzino National Military-Medical Institute for Research and Development	Cantacuzino Institute Virology	Luiza Ustea, Nicoleta Paraschiv, Mihaela Lazar
EPI_ISL_910553, EPI_ISL_910554, EPI_ISL_910555, EPI_ISL_910556, EPI_ISL_910557, EPI_ISL_910558, EPI_ISL_910559, EPI_ISL_910560, EPI_ISL_910561, EPI_ISL_910562, EPI_ISL_910563, EPI_ISL_910564, EPI_ISL_910565, EPI_ISL_910566, EPI_ISL_910567, EPI_ISL_910568, EPI_ISL_910569, EPI_ISL_910571, EPI_ISL_910572, EPI_ISL_910573, EPI_ISL_910574, EPI_ISL_910575, EPI_ISL_910576, EPI_ISL_910577, EPI_ISL_910580, EPI_ISL_910581, EPI_ISL_910582, EPI_ISL_910583, EPI_ISL_910584, EPI_ISL_911233			
see above	Laboratoire national de sante, Microbiology, Virology	Laboratoire national de sante, Microbiology, Microbial Genomics Platform	Anke Wienecke-Baldacchino, Catherine Ragimbeau,Jessica Tapp, Fatu Djabi, Lise Pignon, Raoul Salmon, Tamir Abdelrahman
EPI_ISL_911331	Servicio de Microbiología, Hospital Universitario Son Espases	SeqCOVID-SPAIN consortium/IBV(CSIC)	Carla López-Causapé, Jordi Reina, Antonio Oliver and SeqCOVID-SPAIN consortium
EPI_ISL_911524	Microbiology and Virology Unit, Florence Careggi University Hospital	Microbiology and Virology Unit, Florence Careggi University Hospital	Vincenzo Di Pilato, Marco Coppi, Fabio Morecchiato, Noemi Aiezza, Ilaria Baccani, Alberto Antonelli, Emanuele Gori, Gian Maria Rossolini
EPI_ISL_911967	LabCorp	Wyoming Public Health Laboratory	Noah Hull, Taylor Fearing, Lynette Gumbleton, Channing Weber, Ashley Norberg, Bailey Bowcutt, and Wanda Manley
EPI_ISL_912165	Connecticut Department of Health	Grubaugh Lab - Yale School of Public Health	Tara Alpert, Joseph Fauver, Anderson Brito, Mallery Breban, Anne Wyllie, Chantal Vogels, Mary Petrone, Annie Watkins, Chaney Kalinich, Isabel Ott, Nathan Grubaugh
EPI_ISL_912269, EPI_ISL_912306	Hospital General Universitario Gregorio Marañón	SeqCOVID-SPAIN consortium / IBV (CSIC)	Darío García de Viedma, Laura Pérez-Lago, Pedro J Sola-Campoy, Sergio Buenestado-Serrano, Marta Herranz, Victor Manuel de la Cueva, Julia Suárez, Pilar Catalán, Patricia Muñoz and SeqCOVID-SPAIN consortium
EPI_ISL_912374, EPI_ISL_912381, EPI_ISL_912382, EPI_ISL_912383, EPI_ISL_912384, EPI_ISL_912385	Fondation Congolaise pour la recherche medicale (FCRM), Francine Ntoumi	NGS Competence Center Tuebingen, Institut für Medizinische Mikrobiologie und Hygiene, Universitaetsklinikum Tübingen	Angel Angelov
EPI_ISL_912405, EPI_ISL_912411, EPI_ISL_912412, EPI_ISL_912419	KU Leuven, Rega Institute, Clinical and Epidemiological Virology	KU Leuven, Rega Institute, Clinical and Epidemiological Virology	Tony Wawina-Bokalanga, Bert Vanmechelen, Joan Marti-Carerras, Piet Maes
EPI_ISL_912458, EPI_ISL_912461, EPI_ISL_912491, EPI_ISL_912519	NHLS Universitas Academic	UFS Virology	PA Bester, MM Nyaga, P Nthiga, MT Mogotsi, D Goedhals, T de Oliveira
EPI_ISL_912900, EPI_ISL_912922, EPI_ISL_912937, EPI_ISL_912962, EPI_ISL_912963, EPI_ISL_912964, EPI_ISL_912965, EPI_ISL_912966, EPI_ISL_912967, EPI_ISL_912968, EPI_ISL_912969, EPI_ISL_912970, EPI_ISL_912971, EPI_ISL_912972, EPI_ISL_912973, EPI_ISL_912974, EPI_ISL_912975, EPI_ISL_912976, EPI_ISL_912977			
see above	Hôpital Henri Mondor	Department of Virology, Henri Mondor University Hospital, Assistance Publique Hôpitaux de Paris, Université Paris-Est Créteil, INSERM U955	Christophe Rodriguez, Slim Fourati, Vanessa Demontant, Guillaume Gricourt, Melissa N'Debi, Alexandre Soulier, Elisabeth Trawinski, Jean-Michel Pawlotsky
EPI_ISL_913021, EPI_ISL_913022, EPI_ISL_913023	Hospital San Pedro de Alcántara	Instituto de Salud Carlos III	Iglesias-Caballero, M. Camarero, S. Sandonís,V. Vázquez, S. Pozo, F. Casas, I. Jiménez, P. Zaballos, A. Monzón, S. Varona, S. Cuesta, I. Rodríguez, G.
EPI_ISL_913024	Complejo Hospitalario Universitario de Santiago	Instituto de Salud Carlos III	Iglesias-Caballero, M. Camarero, S. Sandonís,V. Vázquez, S. Pozo, F. Casas, I. Jiménez, P. Zaballos, A. Monzón, S. Varona, S. Cuesta, I. Peña,F.
EPI_ISL_913033	Hospital San Pedro de Alcántara	Instituto de Salud Carlos III	Iglesias-Caballero, M. Camarero, S. Sandonís,V. Vázquez, S. Pozo, F. Casas, I. Jiménez, P. Zaballos, A. Monzón, S. Varona, S. Cuesta, I. Rodríguez, G.
EPI_ISL_913040	Hospital Universitario de Guadalajara	Instituto de Salud Carlos III	Iglesias-Caballero, M. Camarero, S. Sandonís,V. Vázquez, S. Pozo, F. Casas, I. Jiménez, P. Zaballos, A. Monzón, S. Varona, S. Cuesta, I. González, A.
EPI_ISL_913041	Consejería de Sanidad y Asuntos Sociales	Instituto de Salud Carlos III	Iglesias-Caballero, M. Camarero, S. Sandonís,V. Vázquez, S. Pozo, F. Casas, I. Jiménez, P. Zaballos, A. Monzón, S. Varona, S. Cuesta, I. Gutiérrez, G.
EPI_ISL_913048, EPI_ISL_913049, EPI_ISL_913050	Hospital Comarcal Sierrallana	Instituto de Salud Carlos III	Iglesias-Caballero, M. Camarero, S. Sandonís,V. Vázquez, S. Pozo, F. Casas, I. Jiménez, P. Zaballos, A. Monzón, S. Varona, S. Cuesta, I. De Benito, I.
EPI_ISL_913076, EPI_ISL_913085	Center for Virology	Center for Virology	Jeremy V. Camp, Irene Goerzer, Monika Redlberger-Fritz, Stephan W. Aberle
EPI_ISL_913102	CHU Purpan - Laboratoire de Virologie - Institut Fédératif de Biologie	CHU Purpan - Laboratoire de Virologie - Institut Fédératif de Biologie	Latour J., Ranger N., Dubois M., Carcenac R., Harter A., Boyer P., Tremeaux P., Izopet J.
EPI_ISL_913269, EPI_ISL_913274	Klinisk mikrobiologi	The Public Health Agency of Sweden	Anna-Malin Linde, Maria Lind Karlberg, Carlo Berg, Oskar Karlsson Lindsjo, Sofia Stamouli, Reza Advani, Mattias Haukland, Petra Holmstrom, Noura Walai, Petra Edquist, Mia Brytting, Anna Risberg, Karin Tegmark-Wisell
EPI_ISL_913291, EPI_ISL_913292, EPI_ISL_913293	ABC Labs	The Public Health Agency of Sweden	Anna-Malin Linde, Maria Lind Karlberg, Carlo Berg, Oskar Karlsson Lindsjo, Sofia Stamouli, Reza Advani, Mattias Haukland, Petra Holmstrom, Noura Walai, Petra Edquist, Mia Brytting, Anna Risberg, Karin Tegmark-Wisell
EPI_ISL_913296	Klinisk mikrobiologi	The Public Health Agency of Sweden	Anna-Malin Linde, Maria Lind Karlberg, Carlo Berg, Oskar Karlsson Lindsjo, Sofia Stamouli, Reza Advani, Mattias Haukland, Petra Holmstrom, Noura Walai, Petra Edquist, Mia Brytting, Anna Risberg, Karin Tegmark-Wisell
EPI_ISL_913305	Synlab Medilab, Mikrobiologi	The Public Health Agency of Sweden	Anna-Malin Linde, Maria Lind Karlberg, Carlo Berg, Oskar Karlsson Lindsjo, Sofia Stamouli, Reza Advani, Mattias Haukland, Petra Holmstrom, Noura Walai, Petra Edquist, Mia Brytting, Anna Risberg, Karin Tegmark-Wisell
EPI_ISL_913311	Klinisk mikrobiologi	The Public Health Agency of Sweden	Anna-Malin Linde, Maria Lind Karlberg, Carlo Berg, Oskar Karlsson Lindsjo, Sofia Stamouli, Reza Advani, Mattias Haukland, Petra Holmstrom, Noura Walai, Petra Edquist, Mia Brytting, Anna Risberg, Karin Tegmark-Wisell
EPI_ISL_913339, EPI_ISL_913341, EPI_ISL_913342	Unilabs, Mikrobiologiska laboratoriet	The Public Health Agency of Sweden	Anna-Malin Linde, Maria Lind Karlberg, Carlo Berg, Oskar Karlsson Lindsjo, Sofia Stamouli, Reza Advani, Mattias Haukland, Petra Holmstrom, Noura Walai, Petra Edquist, Mia Brytting, Anna Risberg, Karin Tegmark-Wisell
EPI_ISL_913367, EPI_ISL_913369	Klinisk mikrobiologi	The Public Health Agency of Sweden	Anna-Malin Linde, Maria Lind Karlberg, Carlo Berg, Oskar Karlsson Lindsjo, Sofia Stamouli, Reza Advani, Mattias Haukland, Petra Holmstrom, Noura Walai, Petra Edquist, Mia Brytting, Anna Risberg, Karin Tegmark-Wisell
EPI_ISL_913380, EPI_ISL_913381, EPI_ISL_913382, EPI_ISL_913383, EPI_ISL_913384	Dynamic Code AB	The Public Health Agency of Sweden	Anna-Malin Linde, Maria Lind Karlberg, Carlo Berg, Oskar Karlsson Lindsjo, Sofia Stamouli, Reza Advani, Mattias Haukland, Petra Holmstrom, Noura Walai, Petra Edquist, Mia Brytting, Anna Risberg, Karin Tegmark-Wisell
EPI_ISL_913450	Unilabs AB Skovde	The Public Health Agency of Sweden	Anna-Malin Linde, Maria Lind Karlberg, Carlo Berg, Oskar Karlsson Lindsjo, Sofia Stamouli, Reza Advani, Mattias Haukland, Petra Holmstrom, Noura

			Walai, Petra Edquist, Mia Brytting, Anna Risberg, Karin Tegmark-Wisell
EPI_ISL_913486	ABC Labs	The Public Health Agency of Sweden	Anna-Malin Linde, Maria Lind Karlberg, Carlo Berg, Oskar Karlsson Lindsjo, Sofia Stamouli, Reza Advani, Mattias Haukland, Petra Holmstrom, Noura Walai, Petra Edquist, Mia Brytting, Anna Risberg, Karin Tegmark-Wisell
EPI_ISL_913487, EPI_ISL_913488, EPI_ISL_913489	Klinisk mikrobiologi	The Public Health Agency of Sweden	Anna-Malin Linde, Maria Lind Karlberg, Carlo Berg, Oskar Karlsson Lindsjo, Sofia Stamouli, Reza Advani, Mattias Haukland, Petra Holmstrom, Noura Walai, Petra Edquist, Mia Brytting, Anna Risberg, Karin Tegmark-Wisell
EPI_ISL_913495	Complejo Hospitalario Universitario de Santiago	Instituto de Salud Carlos III	Iglesias-Caballero, M. Camarero, S. Sandonís,V. Vázquez, S. Pozo, F. Casas, I. Jiménez, P. Zaballos, A. Monzón, S. Varona, S. Cuesta, I. Peña,F.
EPI_ISL_913525, EPI_ISL_913528, EPI_ISL_913529, EPI_ISL_913530, EPI_ISL_913531, EPI_ISL_913532, EPI_ISL_913533, EPI_ISL_913534, EPI_ISL_913535, EPI_ISL_913537, EPI_ISL_913538, EPI_ISL_913539, EPI_ISL_913540, EPI_ISL_913541, EPI_ISL_913542, EPI_ISL_913543, EPI_ISL_913544, EPI_ISL_913545, EPI_ISL_913546, EPI_ISL_913547, EPI_ISL_913548, EPI_ISL_913549, EPI_ISL_913550, EPI_ISL_913551, EPI_ISL_913552, EPI_ISL_913553, EPI_ISL_913554, EPI_ISL_913555, EPI_ISL_913556, EPI_ISL_913557, EPI_ISL_913558, EPI_ISL_913559, EPI_ISL_913560, EPI_ISL_913561, EPI_ISL_913562			
see above	M Health Fairview	Minnesota Department of Health, Public Health Laboratory	Alexandra Lorentz, Jacob Garfin, Matt Plumb, and Xiong Wang
EPI_ISL_913613, EPI_ISL_913614, EPI_ISL_913640, EPI_ISL_913641, EPI_ISL_913642, EPI_ISL_913643, EPI_ISL_913644, EPI_ISL_913645, EPI_ISL_913646	Michigan Department of Health and Human Services, Bureau of Laboratories	Michigan Department of Health and Human Services, Bureau of Laboratories	Blankenship HM, Riner D, Soehnlen MK
EPI_ISL_913686, EPI_ISL_913687, EPI_ISL_913688, EPI_ISL_913689, EPI_ISL_913690, EPI_ISL_913691, EPI_ISL_913693, EPI_ISL_913694, EPI_ISL_913695, EPI_ISL_913696, EPI_ISL_913697, EPI_ISL_913698, EPI_ISL_913699, EPI_ISL_913700, EPI_ISL_913701, EPI_ISL_913702, EPI_ISL_913703, EPI_ISL_913704, EPI_ISL_913705, EPI_ISL_913706, EPI_ISL_913707, EPI_ISL_913708, EPI_ISL_913709, EPI_ISL_913710			
see above	Minnesota Department of Health, Public Health Laboratory	Minnesota Department of Health, Public Health Laboratory	Alexandra Lorentz, Jacob Garfin, Matt Plumb, and Xiong Wang
EPI_ISL_913726	Missouri State Public Health Laboratory	Minnesota Department of Health, Public Health Laboratory	Alexandra Lorentz, Jacob Garfin, Matt Plumb, and Xiong Wang
EPI_ISL_913791, EPI_ISL_913808, EPI_ISL_913822, EPI_ISL_913832, EPI_ISL_913839, EPI_ISL_913846, EPI_ISL_913862, EPI_ISL_913877, EPI_ISL_913883, EPI_ISL_913884, EPI_ISL_913891, EPI_ISL_913892, EPI_ISL_913893, EPI_ISL_913898, EPI_ISL_913902, EPI_ISL_913903, EPI_ISL_913904, EPI_ISL_913906, EPI_ISL_913909			
see above	TGen North	TGen North	"Jolene Bowers, Megan Folkerts, Chris French, Hayley Yaglom, Ashlyn Pfeiffer, Darrin Lemmer, Dave Engelthaler, The Arizona COVID Genomics Union (ACGU)"
EPI_ISL_913999	TX DSHS, Lab Services Section MC 1947	Pathogen Discovery, Respiratory Viruses Branch, Division of Viral Diseases, Centers for Disease Control and Prevention	Ying Tao, Yan Li, Jing Zhang, Krista Queen, Anna Uehara, Peter Cook, Clinton R. Paden, Haibin Wang, Suxiang Tong
EPI_ISL_914038	CT-Dr. Katherine A. Kelley State Public Health Lab	Pathogen Discovery, Respiratory Viruses Branch, Division of Viral Diseases, Centers for Disease Control and Prevention	Ying Tao, Yan Li, Jing Zhang, Krista Queen, Anna Uehara, Peter Cook, Clinton R. Paden, Haibin Wang, Suxiang Tong
EPI_ISL_914644	Santa Clara County Public Health Laboratory	Santa Clara County Public Health Laboratory	Santa Clara County Public Health Department
EPI_ISL_914817, EPI_ISL_914819	HOSPITAL DE NIÑOS DR. CARLOS SAENZ HERRERA	Inciensa, Instituto Costarricense de Investigación y Enseñanza en Nutrición y Salud	Francisco Duarte, Hebleen Porras, Claudio Soto-Garita, Estela Cordero, Adriana Godínez, Melany Calderón & Cristian Pérez-Corrales
EPI_ISL_918189, EPI_ISL_918194, EPI_ISL_918224, EPI_ISL_918225, EPI_ISL_918227, EPI_ISL_918228, EPI_ISL_918233, EPI_ISL_918239, EPI_ISL_918240, EPI_ISL_918242, EPI_ISL_918244, EPI_ISL_918261			
see above	Innovative Genomics Institute, UC Berkeley	Innovative Genomics Institute, UC Berkeley	Stacia Wyman, Haridha Shivram, Phil Frankino, Liana Lareau, Shana McDevitt, Justin Choi
EPI_ISL_918351	Institute of Virology, Medical Center, University of Freiburg, Freiburg, Germany	Institute of Virology, Clinial Virus Genomics, Medical Center, University of Freiburg, Freiburg, Germany	Jonas Fuchs, Lisa Kern, Sandra Reuter, Hajo Grundmann, Marcus Panning
EPI_ISL_918409	Ospedale Santa Caterina Novella	Istituto Zooprofilattico Sperimentale della Puglia e della Basilicata	Parisi A., Bianco A., Capozzi L., Del Sambro L., Simone D., Manzulli V, Rondinone V., Pace L., Cipolletta D., Galante D.
EPI_ISL_918444, EPI_ISL_918445, EPI_ISL_918446, EPI_ISL_918447, EPI_ISL_918448, EPI_ISL_918449, EPI_ISL_918450, EPI_ISL_918451, EPI_ISL_918452, EPI_ISL_918453, EPI_ISL_918454, EPI_ISL_918455, EPI_ISL_918456			
see above	AIID	Irish Coronavirus Sequencing Consortium-Teagasc Grange	Matthew McCabe, Aljandro Abner Garcia Leon, Fiona Crispie, Calum Walsh, Michael Carr, John Kenny, Paul Cotter, Patrick Mallon, Gabriel Gonzalez
EPI_ISL_918502, EPI_ISL_918505, EPI_ISL_918506, EPI_ISL_918511	LACEN - Laboratório Central de Saúde Pública do Amazonas	Evandro Chagas Institute	Santos, M.C.; Silva, A.M.; Junior, W.D.C.; Barbagelata, L.S.; Ferreira, J.A.; Sousa, E.M.A.; da Silva, P.S.; Pinheiro, K.C.; L.C.; Sousa Junior, E.C.
EPI_ISL_918748, EPI_ISL_918749, EPI_ISL_918750, EPI_ISL_918751, EPI_ISL_918752, EPI_ISL_918753, EPI_ISL_918754, EPI_ISL_918755, EPI_ISL_918756, EPI_ISL_918757, EPI_ISL_918760, EPI_ISL_918761, EPI_ISL_918762, EPI_ISL_918763, EPI_ISL_918940, EPI_ISL_918941, EPI_ISL_918942, EPI_ISL_918943, EPI_ISL_918944, EPI_ISL_918945, EPI_ISL_918946, EPI_ISL_918947, EPI_ISL_918948, EPI_ISL_918949, EPI_ISL_918950, EPI_ISL_918951, EPI_ISL_918952, EPI_ISL_918953, EPI_ISL_918954, EPI_ISL_918959, EPI_ISL_918961, EPI_ISL_918962, EPI_ISL_918963, EPI_ISL_918965			
see above	University of Birmingham	COVID-19 Genomics UK (COG-UK) Consortium	Institute of Microbiology, University of Birmingham: Claire McMurray, Joanne Stockton, Samuel Nicholls, Radoslaw Poplawski, Will Rowe, Josh Quick, Nicholas Loman. University of Birmingham Testing Laboratory: Celina M Whalley, Andrew Bosworth, Charlotte Poxon, Kasun Wanigasooriya, Oliver Pickles, Mike Kidd, Alex Richter, Andrew D Beggs PHE Heartlands Lab: Husam Osman, Andrew Bosworth. Queen Elizabeth Hospital: Anna Casey
EPI_ISL_919257	West of Scotland Specialist Virology Centre, NHSGGC / MRC-University of Glasgow Centre for Virus Research	COVID-19 Genomics UK (COG-UK) Consortium	Ana da Silva Filipe, Natasha Johnson, Kathy Smollett, Daniel Mair, Stephen Carmichael, Alice Broos, Lily Tong, Jenna Nichols, Kyriaki Nomikou; Sarah McDonald; Richard Orton, Joseph Hughes, Sreenu Vattipally, David L Robertson; Alasdair MacLean, Rory Gunson; Sharif Shaaban, Matthew Holden; Rachel Blacow, Guy Mollett, Kathy Li, James Shepherd, Antonia Ho, Emma Thomson
EPI_ISL_919321, EPI_ISL_919322, EPI_ISL_919323, EPI_ISL_919324, EPI_ISL_919325, EPI_ISL_919326, EPI_ISL_919327, EPI_ISL_919328, EPI_ISL_919329, EPI_ISL_919330, EPI_ISL_919331, EPI_ISL_919332, EPI_ISL_919333, EPI_ISL_919334, EPI_ISL_919335			
see above	Virology Department, Royal Infirmary of Edinburgh, NHS Lothian / School of Biological Sciences, University of Edinburgh / Institute of Genetics and Molecular Medicine, University of Edinburgh	COVID-19 Genomics UK (COG-UK) Consortium	McHugh M, Dewar R, Rooke S, Gallagher M, Balcaza C, O'Toole Á, Scher E, Hill V, McCrone JT, Colquhoun R, Yu X, Jackson B, Rambaut A, Williams TC, Templeton K
EPI_ISL_919452, EPI_ISL_919453, EPI_ISL_919476	Liverpool Clinical Laboratories	COVID-19 Genomics UK (COG-UK) Consortium	Sam Haldenby, Anita Lucaci, Steve Paterson, Julian Hiscox, Alistair Darby, M Almsaud, A Alrezaihi, Muhannad Alruwaili, Stuart D Armstrong, Jones Benjamin, Eleanor G Bentley, Anu Chawla, Jordan J Clark, Angela Cowell, Richard Eccles, Isabel García-Dorival, Matthew Gemmell, Alessandro Gerada, PKF Gilmore, Richard Gregory, Ximeng Han, Catherine Hartley, Margaret Hughes, Miren Iturriza-Gomara, James Johnson, L Luu, Jenifer Manson, Charlotte Nelson, Elaine O'Toole, Cassie Olateju, Rebekah Penrice-Randal , Lucille Rainbow, N.P Randle, Trevor Ian Robinson, Parul Sharma, Ghada T Shawli, James P Stewart, Neil Swainston, Ecaterina Vamos, Joanne Watts, Mark Whitehead
EPI_ISL_919781, EPI_ISL_919782, EPI_ISL_919783, EPI_ISL_919784, EPI_ISL_919785, EPI_ISL_919786, EPI_ISL_919787, EPI_ISL_919788, EPI_ISL_919789, EPI_ISL_919790, EPI_ISL_919791, EPI_ISL_919792, EPI_ISL_919793, EPI_ISL_919794, EPI_ISL_919795, EPI_ISL_919796			
see above	Barts Health NHS Trust	COVID-19 Genomics UK (COG-UK) Consortium	CUTINO-MOGUEL, Maria-Teresa; HARRINGTON, David; OWOYEMI, Dola; KULASEGARAN-SHYLINI, Raghavendran; BROAD, Claire; KELE, Beatrix
EPI_ISL_920499	University College London Hospital	COVID-19 Genomics UK (COG-UK) Consortium	Judith Heaney, Matthew Byott, Catherine Houlihan, Dan Frampton, Stuart Kirk, Moira Spyer and Eleni Nastouli
EPI_ISL_920843, EPI_ISL_920844, EPI_ISL_920845, EPI_ISL_920846, EPI_ISL_920847	University College London, Great Ormond Street Hospital for Children NHS Foundation Trust, Imperial College Healthcare NHS Trust	COVID-19 Genomics UK (COG-UK) Consortium	Sergi Castellano, Rachel Williams, Mark Kristiansen, Paola Resende Silva, Sunando Roy, Tony Brooks, Helena Tutill, Paola Niola, Patricia Dyal, Charlotte Williams, Leysa Forrest, Yasmin Panchbhaya, Jacqueline Findlay, Samuel Weeks, Julianne Brown, Kathryn Harris, Paul Randell, James Price, Alison Holmes, Judith Breuer
EPI_ISL_921022, EPI_ISL_921027, EPI_ISL_921028, EPI_ISL_921029, EPI_ISL_921030, EPI_ISL_921031, EPI_ISL_921032, EPI_ISL_921034, EPI_ISL_921035, EPI_ISL_921036, EPI_ISL_921042, EPI_ISL_921043, EPI_ISL_921044, EPI_ISL_921045, EPI_ISL_921085, EPI_ISL_921087, EPI_ISL_921088, EPI_ISL_921089, EPI_ISL_921118, EPI_ISL_921119, EPI_ISL_921120, EPI_ISL_921133, EPI_ISL_921135, EPI_ISL_921136			
see above	Regional Virus Laboratory, Belfast Health and Social Care Trust	COVID-19 Genomics UK (COG-UK) Consortium	Conall McCaughey, James McKenna, Tanya Curran, Susan Feeney, Alison Watt, Ciara Cox, Mairead Connor, Zoltan Molnar, David Simpson, Derek Fairley
EPI_ISL_921253, EPI_ISL_921255, EPI_ISL_921258, EPI_ISL_921259, EPI_ISL_921261, EPI_ISL_921262, EPI_ISL_921264, EPI_ISL_921265, EPI_ISL_921271, EPI_ISL_921272, EPI_ISL_921275, EPI_ISL_921276, EPI_ISL_921371, EPI_ISL_921379, EPI_ISL_921380, EPI_ISL_921381, EPI_ISL_921382, EPI_ISL_921384, EPI_ISL_921385, EPI_ISL_921386, EPI_ISL_921387, EPI_ISL_921388, EPI_ISL_921389, EPI_ISL_921390, EPI_ISL_921392, EPI_ISL_921393, EPI_ISL_921394, EPI_ISL_921395, EPI_ISL_921396, EPI_ISL_921397, EPI_ISL_921398, EPI_ISL_921399, EPI_ISL_921400, EPI_ISL_921401, EPI_ISL_921402, EPI_ISL_921403, EPI_ISL_921404, EPI_ISL_921405, EPI_ISL_921406, EPI_ISL_921414, EPI_ISL_921415, EPI_ISL_921416, EPI_ISL_921417, EPI_ISL_921418, EPI_ISL_921420, EPI_ISL_921422			

see above	Northumbria University / South Tees Hospitals NHS Foundation Trust / North Cumbria Integrated Care NHS Foundation Trust / North Tees and Hartlepool NHS Foundation Trust / Newcastle Hospitals NHS Foundation Trust	COVID-19 Genomics UK (COG-UK) Consortium	Darren L Smith,Andrew Nelson,Matthew Bashton,Greg R Young,Joshua Loh,John Allan,Mohammad A Tariq,Giles S Holt,Gary Black,Wen C Yew,Lynn Dover,Paul Baker,Steve Liggett,Sarah Essex,Jane Greenaway,Debra Padgett,Clive Graham,Garren Scott,Edward Barton,Emma Swindells,Brendan Payne,Jennifer Collins,Yusri Taha,Gary Eltringham
EPI_ISL_921786, EPI_ISL_921787, EPI_ISL_921788, EPI_ISL_921793, EPI_ISL_921800, EPI_ISL_921805, EPI_ISL_921808, EPI_ISL_921809, EPI_ISL_921810, EPI_ISL_921813, EPI_ISL_921819, EPI_ISL_921825			
see above	Quadram Institute Bioscience	COVID-19 Genomics UK (COG-UK) Consortium	Dave J. Baker, Gemma L. Kay, Alp Aydin, Thanh Le-Viet, Steven Rudder, Ana P. Tedim, Anastasia Kolyva, Maria Diaz, Leonardo de Oliveira Martins, Nabil-Fareed Alikhan, Lizzie Meadows, Rachael Stanley, Ngozi Elumogo, Muhammed Yasir, Nicholas M. Thomson, Alexander J Trotter, Rachel Gilroy, Samuel Bloomfield, Claire Stuart, Andrew Bell, Reenesh Prakash, Samir Dervisevic, Alison E. Mather, John Wain, Mark Webber, Andrew J. Page, Justin O'Grady
EPI_ISL_922241, EPI_ISL_922260, EPI_ISL_922261, EPI_ISL_922262, EPI_ISL_922263, EPI_ISL_922264, EPI_ISL_922267, EPI_ISL_922271, EPI_ISL_922272, EPI_ISL_922274, EPI_ISL_922275, EPI_ISL_922277, EPI_ISL_922279, EPI_ISL_922287, EPI_ISL_922288, EPI_ISL_922290, EPI_ISL_922291, EPI_ISL_922292, EPI_ISL_922293, EPI_ISL_922296, EPI_ISL_922297, EPI_ISL_922299, EPI_ISL_922300, EPI_ISL_922302, EPI_ISL_922305			
see above	Oxford Viromics, NDM, University of Oxford; Oxford University Hospitals; Basingstoke and North Hampshire Hospital	COVID-19 Genomics UK (COG-UK) Consortium	Tanya Golubchik, David Bonsall, George Macintyre, Amy Trebes, Mariateresa de Cesare, Catrin Moore, Alex Mobbs, Anita Justice, Robert Shaw, Monique Andersson, Timothy Peto, Emma Wise, Nathan Moore, Jessica Lynch, Nick Cortes, Matilde Mori, Stephen Kidd, David Buck, John Todd, Christophe Fraser
EPI_ISL_923244, EPI_ISL_923265	Centre for Enzyme Innovation, University of Portsmouth / Translational Research Laboratory, Portsmouth Hospitals NHS Trust	COVID-19 Genomics UK (COG-UK) Consortium	Angela Beckett,Salman Goudarzi,Christopher Fearn,Kate Cook,Katie Loveson,Sharon Glaysher,Scott Elliott,Samuel Robson
EPI_ISL_924094, EPI_ISL_924168, EPI_ISL_924213, EPI_ISL_924237, EPI_ISL_924250, EPI_ISL_924280, EPI_ISL_924283	Virology Department, Sheffield Teaching Hospitals NHS Foundation Trust/Department of Infection, Immunity and Cardiovascular Disease, The Medical School, University of Sheffield	COVID-19 Genomics UK (COG-UK) Consortium	Thushan de Silva, Matthew Parker, Nikki Smith, Adri Angyal, Rebecca Brown, Luke Green, Rachel Tucker, Paul Parsons, Danielle Groves, Katie Johnson, Laura Carrilero, Alex Keeley, Dave Partridge, Matthew Wyles, Benjamin Lindsey, Mehmet Yavuz, Mohammad Raza, Cariad Evans
EPI_ISL_924430, EPI_ISL_924431, EPI_ISL_924432, EPI_ISL_924433, EPI_ISL_924434, EPI_ISL_924435, EPI_ISL_924436, EPI_ISL_924437, EPI_ISL_924438, EPI_ISL_924439, EPI_ISL_924440, EPI_ISL_924441, EPI_ISL_924443, EPI_ISL_924444, EPI_ISL_924445, EPI_ISL_924446, EPI_ISL_924447, EPI_ISL_924448, EPI_ISL_924449, EPI_ISL_924450, EPI_ISL_924451, EPI_ISL_924452, EPI_ISL_924453, EPI_ISL_924454, EPI_ISL_924455, EPI_ISL_924456, EPI_ISL_924457, EPI_ISL_924458, EPI_ISL_924459, EPI_ISL_924460, EPI_ISL_924467, EPI_ISL_924468, EPI_ISL_924469, EPI_ISL_924470, EPI_ISL_924472, EPI_ISL_924474, EPI_ISL_924476, EPI_ISL_924477, EPI_ISL_924478, EPI_ISL_924479, EPI_ISL_924483, EPI_ISL_924485, EPI_ISL_924487, EPI_ISL_924488, EPI_ISL_924489, EPI_ISL_924490, EPI_ISL_924491, EPI_ISL_924493, EPI_ISL_924494, EPI_ISL_924497, EPI_ISL_924498, EPI_ISL_924500, EPI_ISL_924501, EPI_ISL_924502, EPI_ISL_924503, EPI_ISL_924504, EPI_ISL_924505, EPI_ISL_924506, EPI_ISL_924507, EPI_ISL_924508, EPI_ISL_924509, EPI_ISL_924510, EPI_ISL_924511, EPI_ISL_924512, EPI_ISL_924513, EPI_ISL_924514, EPI_ISL_924515, EPI_ISL_924516, EPI_ISL_924519, EPI_ISL_924520, EPI_ISL_924522, EPI_ISL_924523, EPI_ISL_924524, EPI_ISL_924525, EPI_ISL_924526, EPI_ISL_924527, EPI_ISL_924532, EPI_ISL_924534, EPI_ISL_924535, EPI_ISL_924537, EPI_ISL_924538, EPI_ISL_924549, EPI_ISL_924551, EPI_ISL_924554, EPI_ISL_924561, EPI_ISL_924569			
see above	Bioinformatics and Biostatistics Lab, Advanced Sequencing Facility	COVID-19 Genomics UK (COG-UK) Consortium	Aengus Stewart,Jerome Nicod,Chelsea Sawyer,Laura Cubitt,Harshil Patel,Margaret Crawford
EPI_ISL_925395, EPI_ISL_925396	Department of Clinical Microbiology	GIGA Medical Genomics	Keith Durkin, Maria Artesi, Sébastien Bontems, Raphaël Boreux, Bouchra Boujemla, Cécile Meex, Pierrette Melin, Marie-Pierre Hayette, Vincent Bours
EPI_ISL_925528	Arizona State Public Health Laboratory	Arizona State Public Health Laboratory	Trung Huynh, Jessica Escobar, Katherine Fullerton, Nobuko Fukushima, Stacy White, Linda Getsinger, Victor Waddell
EPI_ISL_925913	Nucleic Acid Testing, National Reference Laboratory	GIGA Medical Genomics	Yvan Butera, Keith Durkin, Maria Artesi, Bouchra Boujemla, Robert Rutayisire, Patrick Tuyisenge, Esperence Umumararungu, Sébastien Bontems, Marie-Pierre Hayette, Nathalie Renotte, Swaibu Gatare, Jacob Souopgui, Sabin Nsanzimana, Vincent Bours, Léon Mutesa
EPI_ISL_930668, EPI_ISL_930669, EPI_ISL_930670, EPI_ISL_930675, EPI_ISL_930676, EPI_ISL_930677, EPI_ISL_930678, EPI_ISL_930679, EPI_ISL_930680, EPI_ISL_930681, EPI_ISL_930682, EPI_ISL_930683, EPI_ISL_930684, EPI_ISL_930685, EPI_ISL_930686, EPI_ISL_930687, EPI_ISL_930688, EPI_ISL_930689, EPI_ISL_930690, EPI_ISL_930691, EPI_ISL_930692, EPI_ISL_930693, EPI_ISL_930694, EPI_ISL_930695, EPI_ISL_930696, EPI_ISL_930697, EPI_ISL_930698, EPI_ISL_930699, EPI_ISL_930700, EPI_ISL_930701, EPI_ISL_930702, EPI_ISL_930703, EPI_ISL_930704, EPI_ISL_930705, EPI_ISL_930706, EPI_ISL_930707, EPI_ISL_930708, EPI_ISL_930709, EPI_ISL_930710, EPI_ISL_930711, EPI_ISL_930712, EPI_ISL_930713, EPI_ISL_930714, EPI_ISL_930715, EPI_ISL_930716, EPI_ISL_930717, EPI_ISL_930718, EPI_ISL_930719, EPI_ISL_930721, EPI_ISL_930729, EPI_ISL_930760, EPI_ISL_930764, EPI_ISL_930766, EPI_ISL_930769, EPI_ISL_930770, EPI_ISL_930771, EPI_ISL_930772, EPI_ISL_930773, EPI_ISL_930774, EPI_ISL_930775, EPI_ISL_930776, EPI_ISL_930778, EPI_ISL_930780, EPI_ISL_930782, EPI_ISL_931508, EPI_ISL_931509, EPI_ISL_931510, EPI_ISL_931511, EPI_ISL_931512, EPI_ISL_931513, EPI_ISL_931514, EPI_ISL_931515, EPI_ISL_931517, EPI_ISL_931518, EPI_ISL_931519, EPI_ISL_931520, EPI_ISL_931521, EPI_ISL_931522, EPI_ISL_931523, EPI_ISL_931524, EPI_ISL_931525, EPI_ISL_931526, EPI_ISL_931527, EPI_ISL_931528, EPI_ISL_931529, EPI_ISL_931530, EPI_ISL_931531, EPI_ISL_931532, EPI_ISL_931533, EPI_ISL_931534, EPI_ISL_931535, EPI_ISL_931536, EPI_ISL_931538, EPI_ISL_931541, EPI_ISL_931542, EPI_ISL_931543, EPI_ISL_931544, EPI_ISL_931545, EPI_ISL_931546, EPI_ISL_931547, EPI_ISL_931548, EPI_ISL_931549, EPI_ISL_931550, EPI_ISL_931551, EPI_ISL_931552, EPI_ISL_931553, EPI_ISL_931554, EPI_ISL_931555, EPI_ISL_931556, EPI_ISL_931557, EPI_ISL_931558, EPI_ISL_931559, EPI_ISL_931560, EPI_ISL_931561, EPI_ISL_931562, EPI_ISL_931563, EPI_ISL_931564, EPI_ISL_931565, EPI_ISL_931566, EPI_ISL_931567, EPI_ISL_931568, EPI_ISL_931569, EPI_ISL_931571, EPI_ISL_931572, EPI_ISL_931573			
see above	Utah Public Health Laboratory	Utah Public Health Laboratory	Erin L. Young, Kelly F. Oakeson, Tara Gallagher
EPI_ISL_933441, EPI_ISL_933442, EPI_ISL_933443, EPI_ISL_933444, EPI_ISL_933445, EPI_ISL_933446, EPI_ISL_933448, EPI_ISL_933449, EPI_ISL_933450, EPI_ISL_933451, EPI_ISL_933452, EPI_ISL_933454, EPI_ISL_933455, EPI_ISL_933457, EPI_ISL_933458, EPI_ISL_933459, EPI_ISL_933460, EPI_ISL_933461, EPI_ISL_933462, EPI_ISL_933464, EPI_ISL_933465, EPI_ISL_933466, EPI_ISL_933468, EPI_ISL_933470, EPI_ISL_933471, EPI_ISL_933473, EPI_ISL_933476, EPI_ISL_933477, EPI_ISL_933478			
see above	Lighthouse Lab in Milton Keynes	Wellcome Sanger Institute for the COVID-19 Genomics UK (COG-UK) Consortium	The Lighthouse Lab in Milton Keynes and Alex Alderton, Roberto Amato, Sonia Goncalves, Ewan Harrison, David K. Jackson, Ian Johnston, Dominic Kwiatkowski, Cordelia Langford, John Sillitoe on behalf of the Wellcome Sanger Institute COVID-19 Surveillance Team
EPI_ISL_933679	Instituto de Diagnostico y Referencia Epidemiologicos INDRE_RNLSP	Instituto de Diagnostico y Referencia Epidemiologicos (INDRE)	Claudia Wong-Arambula, Abril Rodriguez-Maldonado, Fabiola Garces-Ayala, Adnan Araiza-Rodriguez, David Fragoso-Fonseca, Sergio Rangel-Guerrero, Mayra Jimenez-Morales, Nancy Munoz-Hernandez, Natividad Cruz-Ortiz, Tatiana Nunez-Garcia, Gisela Barrera-Badillo, Lucia Hernandez-Rivas, Irma Lopez-Martinez, Ernesto Ramirez-Gonzalez.
EPI_ISL_933780, EPI_ISL_933781, EPI_ISL_933782, EPI_ISL_933783, EPI_ISL_933784	Virology Unit, Institut Pasteur du Cambodge	Virology Unit, Institut Pasteur du Cambodge	Sokhoun Yann, Ly Sovann, Kraing Sidonn, Yi Sengdoeurn, Chin Savuth, Chau Darapheak, Etienne Simon-Loriere, Veasna Duong, Erik A Karlsson
EPI_ISL_934348, EPI_ISL_934358, EPI_ISL_934359, EPI_ISL_934361, EPI_ISL_934362, EPI_ISL_934363	Klinisk mikrobiologi	The Public Health Agency of Sweden	Anna-Malin Linde, Maria Lind Karlberg, Carlo Berg, Oskar Karlsson Lindsjo, Sofia Stamouli, Reza Advani, Mattias Haukland, Petra Holmstrom, Noura Walai, Petra Edquist, Mia Brytting, Anna Risberg, Karin Tegmark-Wisell
EPI_ISL_934390, EPI_ISL_934407	Klinisk Mikrobiologi	The Public Health Agency of Sweden	Anna-Malin Linde, Maria Lind Karlberg, Carlo Berg, Oskar Karlsson Lindsjo, Sofia Stamouli, Reza Advani, Mattias Haukland, Petra Holmstrom, Noura Walai, Petra Edquist, Mia Brytting, Anna Risberg, Karin Tegmark-Wisell
EPI_ISL_934569	Department of Laboratory Medicine, Division of Clinical Virology, University of Medicine, Vienna	Bergthaler laboratory, CeMM Research Center for Molecular Medicine of the Austrian Academy of Sciences	Lukas Endler, Anna Schedl, Thomas Penz, Benedikt Agerer, Maelle Le Moing, Michael Schuster, Bekir Erguner, Jan Laine, Martin Senekowitsch, Christoph Bock, Andreas Bergthaler
EPI_ISL_934640	Department of Microbiology, University Innsbruck	Bergthaler laboratory, CeMM Research Center for Molecular Medicine of the Austrian Academy of Sciences	Lukas Endler, Anna Schedl, Thomas Penz, Benedikt Agerer, Maelle Le Moing, Michael Schuster, Bekir Erguner, Jan Laine, Martin Senekowitsch, Christoph Bock, Andreas Bergthaler
EPI_ISL_935214, EPI_ISL_935217, EPI_ISL_935218, EPI_ISL_935219	KU Leuven, Rega Institute, Clinical and Epidemiological Virology	KU Leuven, Rega Institute, Clinical and Epidemiological Virology	Tony Wawina-Bokalanga, Bert Vanmechelen, Joan Marti-Carerras, Piet Maes
EPI_ISL_935982, EPI_ISL_935983, EPI_ISL_935984, EPI_ISL_935985	GLENS FALLS HOSPITAL LABORATORY	Wadsworth Center, New York State Department of Health	Kirsten St. George, Daryl M. Lamson, Alexis Russel, Matthew Shudt, Melissa A Leisner, Jonathan Plitnick, Navjot Singh, John Kelly, Erasmus Schneider, Erica Lasek-Nesselquist
EPI_ISL_936044, EPI_ISL_936045	Columbia University Irving Medical Center	Wadsworth Center, New York State Department of Health	Kirsten St. George, Daryl M. Lamson, Alexis Russel, Matthew Shudt, Melissa A Leisner, Jonathan Plitnick, Navjot Singh, John Kelly, Erasmus Schneider, Erica Lasek-Nesselquist
EPI_ISL_936060	KALEIDA CENTER FOR LABORATORY MEDICINE	Wadsworth Center, New York State Department of Health	Kirsten St. George, Daryl M. Lamson, Alexis Russel, Matthew Shudt, Melissa A Leisner, Jonathan Plitnick, Navjot Singh, John Kelly, Erasmus Schneider, Erica Lasek-Nesselquist
EPI_ISL_936063, EPI_ISL_936064, EPI_ISL_936065, EPI_ISL_936066, EPI_ISL_936069, EPI_ISL_936082, EPI_ISL_936086, EPI_ISL_936089, EPI_ISL_936092, EPI_ISL_936093	Columbia University Irving Medical Center	Wadsworth Center, New York State Department of Health	Kirsten St. George, Daryl M. Lamson, Alexis Russel, Matthew Shudt, Melissa A Leisner, Jonathan Plitnick, Navjot Singh, John Kelly, Erasmus Schneider, Erica Lasek-Nesselquist
EPI_ISL_936147	New York Presbyterian Hospital	Wadsworth Center, New York State Department of Health	Kirsten St. George, Daryl M. Lamson, Alexis Russel, Matthew Shudt, Melissa A Leisner, Jonathan Plitnick, Navjot Singh, John Kelly, Erasmus Schneider, Erica Lasek-Nesselquist

EPI_ISL_936390, EPI_ISL_936396, EPI_ISL_936397, EPI_ISL_936400, EPI_ISL_936401, EPI_ISL_936402, EPI_ISL_936405, EPI_ISL_936406, EPI_ISL_936407, EPI_ISL_936408, EPI_ISL_936410, EPI_ISL_936414, EPI_ISL_936415, EPI_ISL_936419, EPI_ISL_936420, EPI_ISL_936423, EPI_ISL_936437, EPI_ISL_936439, EPI_ISL_936450, EPI_ISL_936451, EPI_ISL_936453, EPI_ISL_936456, EPI_ISL_936458, EPI_ISL_936459, EPI_ISL_936460, EPI_ISL_936463			
see above	TGen North	TGen North	Jolene Bowers, Megan Folkerts, Chris French, Hayley Yaglom, Ashlyn Pfeiffer, Darrin Lemmer, Dave Engelthaler, The Arizona COVID Genomics Union (ACGU)
EPI_ISL_936817, EPI_ISL_936818, EPI_ISL_936819, EPI_ISL_936820, EPI_ISL_936821, EPI_ISL_936822, EPI_ISL_936823, EPI_ISL_936824, EPI_ISL_936825, EPI_ISL_936826, EPI_ISL_936827			
see above	Northwestern Memorial Hospital	Ozer Lab	Ramon Lorenzo-Redondo, Lacy M. Simons, Chad J. Achenbach, Lawrence J. Jennings, Michael G. Ison, Judd F. Hultquist, Egon A. Ozer
EPI_ISL_940148	NHLS Universitas Academic	UFS Virology	PA Bester, MM Nyaga, P Nthiga, MT Mogotsi, D Goedhals, T de Oliveira
EPI_ISL_940769, EPI_ISL_940775	INSPI-CRN de Influenza y otros virus respiratorios	INSPI-Centro de Investigación Multidisciplinaria de la DTIDI	Leandro Patiño, Doménica de Mora, Maritza Olmedo, Andrés Carrazco-Montalvo, Orson Mestanza, Mary Regato-Arrata, Melissa Zambrano, Manuel González, Alfredo Bruno, Alberto Orlando.
EPI_ISL_940863, EPI_ISL_940873, EPI_ISL_940884, EPI_ISL_940885	Vaccines and Infectious Diseases Analytics Research Unit (VIDA)	KRISP, KZN Research Innovation and Sequencing Platform	Baillie Vicky, du Plessis Jeanine, Giandhari Jennifer, Pillay Sureshnee, Naidoo Yeshnee, Tegally Houriiyah, de Oliveira Tulio, Madhi Shabir
EPI_ISL_941223, EPI_ISL_941225	Laboratorio de Microbiología. Hospital General Universitario de Elda, Alicante	SeqCOVID-SPAIN consortium/IBV(CSIC)	Mª Isabel Gascón Ros, Cristina Torregrosa Hetland, Eva Pastor Boix, Paloma Cascales Ramos and SeqCOVID-SPAIN consortium
EPI_ISL_941285, EPI_ISL_941286, EPI_ISL_941287, EPI_ISL_941289, EPI_ISL_941290, EPI_ISL_941291	Nigeria Centre for Disease Control (NCDC)	African Centre of Excellence for Genomics of Infectious Diseases (ACEGID), Redeemer's University	Oluniyi P.E. et al
EPI_ISL_941370, EPI_ISL_941623	Instituto Nacional de Saude (INSA)	Instituto Nacional de Saude (INSA)	Borges et al
EPI_ISL_942763, EPI_ISL_942764, EPI_ISL_942765, EPI_ISL_942766, EPI_ISL_942767, EPI_ISL_942768, EPI_ISL_942769, EPI_ISL_942771	Gundersen Molecular Diagnostics Laboratory	Kabara Cancer Research Institute	Craig S. Richmond, Paraic A. Kenny
EPI_ISL_943564, EPI_ISL_943565, EPI_ISL_943567	Servizo de Microbioloxía. Complexo Hospitalario Universitario de Santiago de Compostela	Servizo de Microbioloxía. Complexo Hospitalario Universitario de Santiago de Compostela	Antonio Aguilera, Gema Barbeito, Amparo Coira, José Costa, Rocio Trastoy, María Luisa Pérez del Molino.
EPI_ISL_943819, EPI_ISL_943820, EPI_ISL_943821, EPI_ISL_943931	Utah Public Health Laboratory	Utah Public Health Laboratory	Erin L. Young, Kelly F. Oakeson, Tara Gallagher
EPI_ISL_944126, EPI_ISL_944129, EPI_ISL_944140, EPI_ISL_944141, EPI_ISL_944142, EPI_ISL_944143, EPI_ISL_944149	National Health Laboratory Service, South Africa	KRISP, KZN Research Innovation and Sequencing Platform	Laguda-Akingba O, Giandhari J, Pillay S, Lessells R, Mdlalose K, York D, Khan S, Emmanuel SJ, Tegally H, Wilkinson E, de Oliveira T
EPI_ISL_944202, EPI_ISL_944225, EPI_ISL_944458	Israel Central Virology laboratory	Israel National Consortium for SARS-CoV-2 sequencing	Neta Zuckerman, Efrat Dahan Bucris, Michal Mandelboim, Dana Bar-Ilan, Oran Erster, Tzvia Mann, Omer Murik, David A. Zeevi, Assaf Rokney, Joseph Jaffe, Eva Nachum, Maya Davidovich Cohen, Ephraim Fass, Gal Zizelski Valenci, Mor Rubinstein, Efrat Rorman, Israel Nissan, Efrat Glick-Saar, Omri Nayshool, Gideon Rechavi, Ella Mendelson, Orna Mor
EPI_ISL_944737	unknown	Public Health Virology-Forensic and Scientific Services (PHV-FSS)	Son Nguyen et al.
EPI_ISL_945065	Lighthouse Lab in Glasgow	Wellcome Sanger Institute for the COVID-19 Genomics UK (COG-UK) Consortium	Harper VanSteenhouse, Yumi Kasai, David Gray, Carol Clugston, Anna Dominiczak and Alex Alderton, Roberto Amato, Sonia Goncalves, Ewan Harrison, David K. Jackson, Ian Johnston, Dominic Kwiatkowski, Cordelia Langford, John Sillitoe on behalf of the Wellcome Sanger Institute COVID-19 Surveillance Team
EPI_ISL_945273	Lighthouse Lab in Milton Keynes	Wellcome Sanger Institute for the COVID-19 Genomics UK (COG-UK) Consortium	The Lighthouse Lab in Milton Keynes and Alex Alderton, Roberto Amato, Sonia Goncalves, Ewan Harrison, David K. Jackson, Ian Johnston, Dominic Kwiatkowski, Cordelia Langford, John Sillitoe on behalf of the Wellcome Sanger Institute COVID-19 Surveillance Team
EPI_ISL_945301	Lighthouse Lab in Glasgow	Wellcome Sanger Institute for the COVID-19 Genomics UK (COG-UK) Consortium	Harper VanSteenhouse, Yumi Kasai, David Gray, Carol Clugston, Anna Dominiczak and Alex Alderton, Roberto Amato, Sonia Goncalves, Ewan Harrison, David K. Jackson, Ian Johnston, Dominic Kwiatkowski, Cordelia Langford, John Sillitoe on behalf of the Wellcome Sanger Institute COVID-19 Surveillance Team
EPI_ISL_945339	Lighthouse Lab in Milton Keynes	Wellcome Sanger Institute for the COVID-19 Genomics UK (COG-UK) Consortium	The Lighthouse Lab in Milton Keynes and Alex Alderton, Roberto Amato, Sonia Goncalves, Ewan Harrison, David K. Jackson, Ian Johnston, Dominic Kwiatkowski, Cordelia Langford, John Sillitoe on behalf of the Wellcome Sanger Institute COVID-19 Surveillance Team
EPI_ISL_949959, EPI_ISL_950131, EPI_ISL_950132, EPI_ISL_950137, EPI_ISL_950139, EPI_ISL_950140, EPI_ISL_950141, EPI_ISL_950143, EPI_ISL_950144, EPI_ISL_950149, EPI_ISL_950167			
see above	University College London, Great Ormond Street Hospital for Children NHS Foundation Trust, Imperial College Healthcare NHS Trust	COVID-19 Genomics UK (COG-UK) Consortium	Sergi Castellano, Rachel Williams, Mark Kristiansen, Paola Resende Silva, Sunando Roy, Tony Brooks, Helena Tutill, Paola Niola, Patricia Dyal, Charlotte Williams, Leysa Forrest, Yasmin Panchbhaya, Jacqueline Findlay, Samuel Weeks, Julianne Brown, Kathryn Harris, Paul Randell, James Price, Alison Holmes, Judith Breuer
EPI_ISL_950234, EPI_ISL_950237, EPI_ISL_950238, EPI_ISL_950249, EPI_ISL_950444, EPI_ISL_950445, EPI_ISL_950446, EPI_ISL_950447, EPI_ISL_950448, EPI_ISL_950449, EPI_ISL_950450, EPI_ISL_950451, EPI_ISL_950452, EPI_ISL_950453, EPI_ISL_950454, EPI_ISL_950455, EPI_ISL_950456, EPI_ISL_950457, EPI_ISL_950458			
see above	Northumbria University / South Tees Hospitals NHS Foundation Trust / North Cumbria Integrated Care NHS Foundation Trust / North Tees and Hartlepool NHS Foundation Trust / Newcastle Hospitals NHS Foundation Trust	COVID-19 Genomics UK (COG-UK) Consortium	Darren L Smith,Andrew Nelson,Matthew Bashton,Greg R Young,Joshua Loh,John Allan,Mohammad A Tariq,Giles S Holt,Gary Black,Wen C Yew,Lynn Dover,Paul Baker,Steve Liggett,Sarah Essex,Jane Greenaway,Debra Padgett,Clive Graham,Garren Scott,Edward Barton,Emma Swindells,Brendan Payne,Jennifer Collins,Yusri Taha,Gary Eltringham
EPI_ISL_950555	Quadram Institute Bioscience	COVID-19 Genomics UK (COG-UK) Consortium	Dave J. Baker, Gemma L. Kay, Alp Aydin, Thanh Le-Viet, Steven Rudder, Ana P. Tedim, Anastasia Kolyva, Maria Diaz, Leonardo de Oliveira Martins, Nabil-Fareed Alikhan, Lizzie Meadows, Rachael Stanley, Ngozi Elumogo, Muhammed Yasir, Nicholas M. Thomson, Alexander J Trotter, Rachel Gilroy, Samuel Bloomfield, Claire Stuart, Andrew Bell, Reenesh Prakash, Samir Dervisevic, Alison E. Mather, John Wain, Mark Webber, Andrew J. Page, Justin O'Grady
EPI_ISL_950710	Lincolnshire Hospitals and DeepSeq Nottingham	COVID-19 Genomics UK (COG-UK) Consortium	Nichola Duckworth, Tim Sloan, Sarah Walsh, Jonathan Ball, Patrick McClure, Joeseph Chappell, Nadine Holmes, Matthew Carlisle, Christopher Moore, Fei Sang, Johnny Debebe, Victoria Wright, Matthew Loose
EPI_ISL_950953, EPI_ISL_950954, EPI_ISL_950956, EPI_ISL_950957, EPI_ISL_950958, EPI_ISL_950959, EPI_ISL_950965, EPI_ISL_950979, EPI_ISL_950980, EPI_ISL_950981, EPI_ISL_950982, EPI_ISL_950983, EPI_ISL_950985, EPI_ISL_950987, EPI_ISL_950988, EPI_ISL_950989, EPI_ISL_950990, EPI_ISL_950991, EPI_ISL_950992, EPI_ISL_950993, EPI_ISL_950994, EPI_ISL_950995, EPI_ISL_950996, EPI_ISL_950999, EPI_ISL_951000, EPI_ISL_951002, EPI_ISL_951003, EPI_ISL_951004, EPI_ISL_951008, EPI_ISL_951009, EPI_ISL_951010, EPI_ISL_951011, EPI_ISL_951013, EPI_ISL_951014, EPI_ISL_951016, EPI_ISL_951018, EPI_ISL_951020, EPI_ISL_951021, EPI_ISL_951023, EPI_ISL_951024, EPI_ISL_951025, EPI_ISL_951026, EPI_ISL_951027, EPI_ISL_951028, EPI_ISL_951029, EPI_ISL_951030, EPI_ISL_951031, EPI_ISL_951032, EPI_ISL_951033, EPI_ISL_951034, EPI_ISL_951036, EPI_ISL_951037, EPI_ISL_951038, EPI_ISL_951042, EPI_ISL_951043, EPI_ISL_951044, EPI_ISL_951045, EPI_ISL_951046, EPI_ISL_951049, EPI_ISL_951050, EPI_ISL_951052, EPI_ISL_951053, EPI_ISL_951054, EPI_ISL_951055, EPI_ISL_951056, EPI_ISL_951057, EPI_ISL_951058, EPI_ISL_951059, EPI_ISL_951061, EPI_ISL_951062, EPI_ISL_951064, EPI_ISL_951066, EPI_ISL_951079, EPI_ISL_951080, EPI_ISL_951081, EPI_ISL_951083, EPI_ISL_951094, EPI_ISL_951097, EPI_ISL_951098, EPI_ISL_951163, EPI_ISL_951164, EPI_ISL_951165, EPI_ISL_951166, EPI_ISL_951169, EPI_ISL_951170, EPI_ISL_951171, EPI_ISL_951249			
see above	Oxford Viromics, NDM, University of Oxford; Oxford University Hospitals; Basingstoke and North Hampshire Hospital	COVID-19 Genomics UK (COG-UK) Consortium	Tanya Golubchik, David Bonsall, George Macintyre, Amy Trebes, Mariateresa de Cesare, Catrin Moore, Alex Mobbs, Anita Justice, Robert Shaw, Monique Andersson, Timothy Peto, Emma Wise, Nathan Moore, Jessica Lynch, Nick Cortes, Matilde Mori, Stephen Kidd, David Buck, John Todd, Christophe Fraser
EPI_ISL_953665	University Hospitals of Geneva, Laboratory of Virology	HUG, Laboratory of Virology and the Health2030 Genome Center	Samuel Cordey, Ana Rita Goncalves, Laurent Kaiser, Lorenzo Cerutti, Henri Pegeot, Melyssa Elies, Deborah Penet, Keith Harshman, Ioannis Xenarios, Emmanouil Dermitzakis
EPI_ISL_954211, EPI_ISL_954212, EPI_ISL_954213, EPI_ISL_954214, EPI_ISL_954215	1.AO Universitaria 'S. Giovanni di Dio e Ruggi D'Aragona, Scuola Medica Salernitana' Hospital / 2.UOC di Virologia e Microbiologia, Università della Campania 'L. Vanvitelli' / 3.AO Universitaria 'Federico II' Napoli Hospital / 4.AORN 'San	1. Genome Research Center for Health (CRGS) / 2. Laboratory of Molecular Medicine and Genomics(LMMGe) / 3. Center for Research in Pure and Applied Mathematics (CRMPA)	Giorgio Giurato, Francesca Rizzo, Alessandro Weisz, Gianluigi Franci, Giovanni Nassa, Pasquale Pagliano, Roberta Tarallo, Elena Alexandrova, Ylenia D'Agostino, Carlo Ferravante, Jessica Lamberti, Viola Melone, Domenico Memoli, Valeria Mirici Cappa, Domenico Palumbo, Giovanni Pecoraro, Assunta Sellitto, Oriana Strianese, Ilaria Terenzi, Giuseppe Fenza, Aniello Gentile, Antonello Saccomanno, Sonia Amabile, Teresa Rocco, Annamaria Salvati, Emilia Vaccaro, Massimiliano Galdiero, Michele Cennamo, Giuseppe Portella, Maria Grazia Foti, Mariarosaria Ingino, Maria Landi, Maurizio Fumi, Vincenzo

	Giuseppe Moscati' Avellino Hospital / 5.AO 'San Pio - presidio G. Rummo' Benevento Hospital / 6.AO 'Sant'Anna e San Sebastiano' Caserta Hospital / 7.PO 'Maria Santissima Addolorata' Eboli Hospital / 8.Biogem Istituto di Ricerche Genetiche		Rocco, Rita Greco, Vittoria Letizia, Arnolfo Petruzziello, Maddalena Schioppa, Gregorio Goffredi, Francesca Marciano, Michele Caraglia, Alessia Cossu, Marianna Scrima, Edmondo Adorisio, Morena D'Avenia, Michela Iacobellis, Rosanna Piluscio, Giorgio Dirani, Vittorio Sambri, Simona Semprini, Silvia Zanoli, Francesco Curcio, Stefania Marzinotto, Andreina Baj, Fausto Sessa.
EPI_ISL_954281, EPI_ISL_954282, EPI_ISL_954283, EPI_ISL_954284, EPI_ISL_954285, EPI_ISL_954286, EPI_ISL_954287, EPI_ISL_954288, EPI_ISL_954289, EPI_ISL_954290, EPI_ISL_954291, EPI_ISL_954292, EPI_ISL_954293, EPI_ISL_954294, EPI_ISL_954295, EPI_ISL_954296			
see above	MRC/UVRI & LSHTM Uganda Research Unit	Where sequence data have been generated and submitted to GISAID	Matthew Cotten, Dan Lule Bugembe, My V.T. Phan, Isaac Sseeewanyana, Patrick Semanda, Susan Nabadda, Pontiano Kaleebu
EPI_ISL_955126	Innovative Genomics Institute, UC Berkeley	Innovative Genomics Institute, UC Berkeley	Stacia Wyman, Haridha Shivram, Phil Frankino, Liana Lareau, Shana McDevitt, Justin Choi
EPI_ISL_955136	MRC/UVRI & LSHTM Uganda Research Unit	Where sequence data have been generated and submitted to GISAID	Matthew Cotten, Dan Lule Bugembe, My V.T. Phan, Isaac Sseeewanyana, Patrick Semanda, Susan Nabadda, Pontiano Kaleebu
EPI_ISL_955387, EPI_ISL_955388, EPI_ISL_955390	Alameda County Public Health Lab	Chan-Zuckerberg Biohub	CZB Cliahub Consortium
EPI_ISL_955727, EPI_ISL_955728, EPI_ISL_955729, EPI_ISL_955730, EPI_ISL_955731, EPI_ISL_955734, EPI_ISL_955735, EPI_ISL_955736, EPI_ISL_955737, EPI_ISL_955738, EPI_ISL_955739, EPI_ISL_955740, EPI_ISL_955741, EPI_ISL_955742, EPI_ISL_955743, EPI_ISL_955744, EPI_ISL_955745, EPI_ISL_955746, EPI_ISL_955747, EPI_ISL_955748, EPI_ISL_955749, EPI_ISL_955750, EPI_ISL_955751, EPI_ISL_955752, EPI_ISL_955753, EPI_ISL_955754, EPI_ISL_955755, EPI_ISL_955756, EPI_ISL_955757, EPI_ISL_955758, EPI_ISL_955759, EPI_ISL_955760, EPI_ISL_955761, EPI_ISL_955762, EPI_ISL_955763, EPI_ISL_955765, EPI_ISL_955766			
see above	Humboldt County Public Health Laboratory	Chan-Zuckerberg Biohub	CZB Cliahub Consortium
EPI_ISL_956279	Siti Khodijah Hospital	Institute of Tropical Disease, Universitas Airlangga	Jezzy R Dewantari, Rima R Prasetya, Krisnoadi Rahardjo, Aldise M Nastri, Muhammad Hamdan, Gatot Soegiarto, Laksmi Wulandari, Resti Yudhawati, Soetjipto, Yasuko Mori, Maria I Lusida, Kazufumi Shimizu
EPI_ISL_956326	Laboratory Medicine	Department of Laboratory Medicine, Lin-Kou Chang Gung Memorial Hospital, Taoyuan, Taiwan	Kuo-Chien Tsao, Yu-Nong Gong, Shu-Li Yang, Yi-Chun Liu, Chung-Guei Huang, Mei-Jen Hsiao, Po-Wei Huang, Cheng-Ta Yang, Cheng-Hsun Chiu, Peng-Nien Huang, Kuo-Ming Lee, Guang-Wu Chen, Shin-Ru Shih
EPI_ISL_960224	QEII Health Sciences Centre	National Microbiology Laboratory (NML)	Anna Majer, Shari Tyson, Grace Seo, Philip Mabon, Elsie Grudeski, Rhiannon Huzarewich, Russell Mandes, Anneliese Landgraff, Jennifer Tanner, Natalie Knox, Morag Graham, Gary Van Domselaar, Todd Hatchette, Jason LeBlanc, Janice Pettipas, Dan Gaston, Nathalie Bastien, Yan Li, Timothy Booth, Darian Hole, Madison Chapel, Kirsten Biggar, CanCOGeN's metadata curation team, Public Health Agency of Canada CanCOGeN team
EPI_ISL_960425, EPI_ISL_960438	The National Institute of Public Health	State Veterinary Institute Prague	Nagy,A;Vecerova,J;Cernikova,L;Stara,M;Jirincova,H;Trnka,D
EPI_ISL_960460, EPI_ISL_960466, EPI_ISL_960467, EPI_ISL_960479, EPI_ISL_960571	Istituto Zooprofilattico Sperimentale del Mezzogiorno	TIGEM	Patrizia Annunziata, Andrea Ballabio, Valentina Bouche, Davide Cacchiarelli, Pellegrino Cerino, Chiara Colantuono, Maria Concetta Cuomo, Denise Di Concilio, Lucio Di Filippo, Antonio Grimaldi, Antonio Limone, Anna Manfredi, Francesco Panariello, Biancamaria Pierri, Marcello Salvi
EPI_ISL_961270, EPI_ISL_961271	Hospital General Universitario de Alicante - Instituto de Investigación Sanitaria y Biomédica de Alicante	SeqCOVID-SPAIN consortium/IBV(CSIC)	Maripaz Ventero Martín, Carmen Molina Pardines and SeqCOVID-SPAIN consortium
EPI_ISL_961570, EPI_ISL_961571, EPI_ISL_961572, EPI_ISL_961573, EPI_ISL_961574, EPI_ISL_961575, EPI_ISL_961576, EPI_ISL_961577, EPI_ISL_961578, EPI_ISL_961579, EPI_ISL_961607, EPI_ISL_961608			
see above	Hôpital Georges L. Dumont	National Microbiology Laboratory (NML)	Anna Majer, Shari Tyson, Grace Seo, Philip Mabon, Elsie Grudeski, Rhiannon Huzarewich, Russell Mandes, Anneliese Landgraff, Jennifer Tanner, Natalie Knox, Morag Graham, Gary Van Domselaar, Richard Garceau, Guillaume Desnoyers, Nathalie Bastien, Yan Li, Timothy Booth, Darian Hole, Madison Chapel, Kirsten Biggar, CanCOGeN's metadata curation team, Public Health Agency of Canada CanCOGeN team
EPI_ISL_961891, EPI_ISL_961936, EPI_ISL_962004, EPI_ISL_962108, EPI_ISL_962109, EPI_ISL_962110, EPI_ISL_962111, EPI_ISL_962112, EPI_ISL_962113, EPI_ISL_962114, EPI_ISL_962115, EPI_ISL_962116, EPI_ISL_962117, EPI_ISL_962118, EPI_ISL_962119, EPI_ISL_962120, EPI_ISL_962128			
see above	Illinois Department of Public Health	Gagnon Lab, Southern Illinois University	Keith Gagnon
EPI_ISL_962611, EPI_ISL_962613	San Diego County Public Health Laboratory	Andersen lab at Scripps Research	SEARCH Alliance San Diego with Tracy Basler, Jovan Shephard, Brett Austin
EPI_ISL_962815	Robert Garry lab	Andersen lab at Scripps Research	Allison Smither, Gilberto Sabino-Santos, Patricia Snarski, Lilia Melnik, Antoinette Bell, Kaylynn Genemaras, Arnaud Drouin, Dahlene Fusco, Robert Garry with SEARCH Alliance San Diego
EPI_ISL_962907	Ostfold Hospital Trust - Kalnes, Centre for Laboratory Medicine, Section for gene technology and infection serology	Norwegian Institute of Public Health, Department of Virology	Kathrine Stene-Johansen, Kamilla Heddeland Instefjord, Hilde Elshaug, Ignacio Garcia Llorente, Serina B Engebretsen, Atiya R Ali,Marie Paulsen Madsen, Rasmus Riis Kopperud, Hilde Vollan, Karoline Bragstad, Olav Hungnes
EPI_ISL_962936, EPI_ISL_962940, EPI_ISL_962941	Hospital Universitario de Gran Canaria Dr. Negrín	SeqCOVID-SPAIN consortium/IBV(CSIC)	M. Carmen Pérez González, Francisco J. Chamizo López, Ana Bordes Benítez and SeqCOVID-SPAIN consortium
EPI_ISL_965053	Wyoming Public Health Laboratory	Wyoming Public Health Laboratory	Noah Hull, Taylor Fearing, Lynette Gumbleton, Channing Weber, Ashley Norberg, Bailey Bowcutt, and Wanda Manley
EPI_ISL_965189, EPI_ISL_965214, EPI_ISL_965215, EPI_ISL_965216	Virginia Division of Consolidated Laboratory Services	Virginia Division of Consolidated Laboratory Services	Virginia DCLS
EPI_ISL_965528	NYU Langone Health	Departments of Pathology and Medicine, New York University School of Medicine	Adriana Heguy, Dacia Dimartino, Emily Guzman, Christian Marier, Peter Meyn, Sitharam Ramaswami, Gael Westby, Paul Zappile, Yutong Zhang, Paolo Cotzia, Guiqing Wang
EPI_ISL_965533, EPI_ISL_965536, EPI_ISL_965547, EPI_ISL_965549, EPI_ISL_965556, EPI_ISL_965560, EPI_ISL_965561, EPI_ISL_965563, EPI_ISL_965589, EPI_ISL_965594, EPI_ISL_965601, EPI_ISL_965606, EPI_ISL_965607, EPI_ISL_965620, EPI_ISL_965667, EPI_ISL_965720, EPI_ISL_965729, EPI_ISL_965731, EPI_ISL_965741, EPI_ISL_965747, EPI_ISL_965748, EPI_ISL_965749, EPI_ISL_965756, EPI_ISL_965759, EPI_ISL_965761, EPI_ISL_965762			
see above	Dutch COVID-19 response team	Medical Microbiology, Maastricht University Medical Centre	Jozef Dingemans*, Brian van der Veer*, Erik Beuken, Carmen Reumkens, Lieke van Alphen, Christian Hoebe, Paul Savelkoul
EPI_ISL_965770	NYU Langone Health	Departments of Pathology and Medicine, New York University School of Medicine	Adriana Heguy, Dacia Dimartino, Emily Guzman, Christian Marier, Peter Meyn, Sitharam Ramaswami, Gael Westby, Paul Zappile, Yutong Zhang, Paolo Cotzia, Guiqing Wang
EPI_ISL_965773, EPI_ISL_965774, EPI_ISL_965776, EPI_ISL_965778, EPI_ISL_965779, EPI_ISL_965785, EPI_ISL_965805	Dutch COVID-19 response team	Medical Microbiology, Maastricht University Medical Centre	Jozef Dingemans*, Brian van der Veer*, Erik Beuken, Carmen Reumkens, Lieke van Alphen, Christian Hoebe, Paul Savelkoul
EPI_ISL_965812, EPI_ISL_965814, EPI_ISL_965826, EPI_ISL_965837, EPI_ISL_965838, EPI_ISL_965839, EPI_ISL_965910, EPI_ISL_965948, EPI_ISL_965962, EPI_ISL_965963, EPI_ISL_965964, EPI_ISL_965965, EPI_ISL_965968, EPI_ISL_965969, EPI_ISL_965970, EPI_ISL_965971, EPI_ISL_965972, EPI_ISL_965973, EPI_ISL_965977, EPI_ISL_965980			
see above	NYU Langone Health	Departments of Pathology and Medicine, New York University School of Medicine	Adriana Heguy, Dacia Dimartino, Emily Guzman, Christian Marier, Peter Meyn, Sitharam Ramaswami, Gael Westby, Paul Zappile, Yutong Zhang, Paolo Cotzia, Guiqing Wang
EPI_ISL_967618, EPI_ISL_967620, EPI_ISL_967621, EPI_ISL_967624, EPI_ISL_967625, EPI_ISL_967628, EPI_ISL_967630, EPI_ISL_967631, EPI_ISL_967632, EPI_ISL_967634, EPI_ISL_967635, EPI_ISL_967637, EPI_ISL_967639, EPI_ISL_967643, EPI_ISL_967689, EPI_ISL_967690, EPI_ISL_967691, EPI_ISL_967692, EPI_ISL_967706, EPI_ISL_967707, EPI_ISL_967708, EPI_ISL_967722, EPI_ISL_967723, EPI_ISL_967724, EPI_ISL_967727, EPI_ISL_967729, EPI_ISL_967750, EPI_ISL_967751, EPI_ISL_967758, EPI_ISL_967759, EPI_ISL_967760, EPI_ISL_967762, EPI_ISL_967766			
see above	State Laboratories Division, Hawaii State Department of Health	State Laboratories Division, Hawaii State Department of Health	Pamela O'Brien, Drew Kuwazaki, Ayana Garnet, Razvan Sultana, Edward Desmond
EPI_ISL_968081	Public Health Virology-Forensic and Scientific Services (PHV-FSS)	Public Health Virology-Forensic and Scientific Services (PHV-FSS)	Alyssa T. Pyke et al.
EPI_ISL_968854, EPI_ISL_968878	KEMRI-Wellcome Trust Research Programme/KEMRI-CGMR-C Kilifi	KEMRI-Wellcome Trust Research Programme/KEMRI-CGMR-C Kilifi	Githinji et al
EPI_ISL_976851, EPI_ISL_976852, EPI_ISL_976853, EPI_ISL_976854, EPI_ISL_976855, EPI_ISL_976856, EPI_ISL_976857, EPI_ISL_976858, EPI_ISL_976859, EPI_ISL_976860, EPI_ISL_976861, EPI_ISL_976862, EPI_ISL_976863, EPI_ISL_976864, EPI_ISL_976865, EPI_ISL_976866, EPI_ISL_976867, EPI_ISL_976868,			

EPI_ISL_976869, EPI_ISL_976870, EPI_ISL_976871, EPI_ISL_976872, EPI_ISL_976873, EPI_ISL_976874, EPI_ISL_976875, EPI_ISL_976876, EPI_ISL_976877, EPI_ISL_976878, EPI_ISL_976879, EPI_ISL_976880, EPI_ISL_976881, EPI_ISL_976882			
see above	BCCDC Public Health Laboratory	BCCDC Public Health Laboratory	Prystajecky Natalie, Linda Hoang, Dan Fornika, John Tyson, Shannon Russell, Kim Macdonald, Kimia Kamelian, Ana Pacagnella, Corrinne Ng, Loretta Janz, Robert Azana Terry Snutch, Mel Krajden
EPI_ISL_977078	Massachusetts General Hospital	Infectious Disease Program, Broad Institute of Harvard and MIT	Lemieux,J.E., Siddle,K.J., Shaw,B., Adams,G., Pierce,V., Turbett,S., Anahtar,M., Branda,J., Slater,D., Harris,J., Lin,A.E., Gladden-Young,A., Lagerborg,K., Rudy,M., DeRuff,K., Carter,A., Normandin,E., Bauer,M., Reilly,S., Tomkins-Tinch,C., Loreth,C., Chaluvadi,S., Neumann,A., Cusick,C., Chapman,S.B., Gnirke,A., Flowers,K., Cerrato,F., Birren,B.W., Gallagher,G., Smole,S., Park,D.J., MacInnis,B.L., Ryan,E., LaRocque,R., Rosenberg,E. and Sabeti,P.C.
EPI_ISL_977210	Microbiologia e Virologia	Istituto Zooprofilattico Sperimentale delle Venezie	Adelaide Milani, Alessia Schivo, Annalisa Salviato, Erika Giorgia Quaranta, Ambra Pastori, Bianca Zecchin, Alice Fusaro, Isabella Monne, Calogero Terregino, Antonia Ricci
EPI_ISL_977656, EPI_ISL_977657, EPI_ISL_977658	Caribbean Public Health Agency	Carrington Lab, Department of PreClinical Sciences, Building 36, First Floor Biochemistry Unit, Faculty of Medical Sciences, The University of the West Indies	Nikita S. D. Sahadeo, Arianne Brown-Jordan, Vernie Ramkissoon, Sarah Hill, Naresh Nandram, Avery Hinds, Kenneth George, Jerome Foster, Stanley Giddings, Karla Georges, Marsha Ivey, Rahul Naidu, Risha Singh, SueMin Nathaniel, Rajini Haraksingh, Jaya Jayaraman, Chinna Chinnadurai, Adesh Ramsubhag, Nuno Faria, Oliver Pybus, Christopher Oura, Gabriel Escobar, Christine V. F. Carrington
EPI_ISL_977686, EPI_ISL_977693, EPI_ISL_977694, EPI_ISL_977697, EPI_ISL_977705, EPI_ISL_977708, EPI_ISL_977711, EPI_ISL_977719, EPI_ISL_977720	California Department of Public Health	Chiu Laboratory, University of California, San Francisco	Charles Chiu, Xianding (Wayne) Deng, Candace Wang, Venice Servellita, Jill Hacker, Debra Wadford
EPI_ISL_978063, EPI_ISL_978064, EPI_ISL_978065, EPI_ISL_978066, EPI_ISL_978067, EPI_ISL_978068, EPI_ISL_978069, EPI_ISL_978075, EPI_ISL_978076, EPI_ISL_978077, EPI_ISL_978078, EPI_ISL_978079, EPI_ISL_978080, EPI_ISL_978081, EPI_ISL_978082, EPI_ISL_978083, EPI_ISL_978084, EPI_ISL_978085, EPI_ISL_978086, EPI_ISL_978087, EPI_ISL_978088, EPI_ISL_978119, EPI_ISL_978120, EPI_ISL_978121, EPI_ISL_978122, EPI_ISL_978123, EPI_ISL_978124, EPI_ISL_978125, EPI_ISL_978126, EPI_ISL_978127, EPI_ISL_978128, EPI_ISL_978129, EPI_ISL_978130			
see above	Chiu Laboratory, University of California, San Francisco	Chiu Laboratory, University of California, San Francisco	Charles Chiu, Xianding (Wayne) Deng, Candace Wang, Venice Servellita, Jill Hacker, Debra Wadford
EPI_ISL_978411, EPI_ISL_978412, EPI_ISL_978413, EPI_ISL_978414, EPI_ISL_978415, EPI_ISL_978416	Arizona State Public Health Laboratory	Arizona State Public Health Laboratory	Trung Huynh, Jessica Escobar, Katherine Fullerton, Nobuko Fukushima, Stacy White, Linda Getsinger, Victor Waddell
EPI_ISL_978920, EPI_ISL_978921, EPI_ISL_978922, EPI_ISL_978923	Centre for Dengue Research and AICBU, Department of Immunology and Molecular Medicine	Centre for Dengue Research and AICBU, Department of Immunology and Molecular Medicine	Chandima Jeewandara, Deshni Jayathilaka, Dinuka Ariyaratne, Tibutius Thanesh Pramanayagam, Diyanath Ranasinghe, Laksiri Gomes, Gathsaurie Neelika Malavige
EPI_ISL_978961	Chiu Laboratory, University of California, San Francisco	Chiu Laboratory, University of California, San Francisco	Charles Chiu, Xianding (Wayne) Deng, Candace Wang, Venice Servellita, Jill Hacker, Debra Wadford
EPI_ISL_979141	Santa Clara County Public Health Laboratory	Chan-Zuckerberg Biohub	CZB Cliahub Consortium
EPI_ISL_979250	Institute of Microbiology and Immunology, Faculty of Medicine, University of Ljubljana	Institute of Microbiology and Immunology, Faculty of Medicine, University of Ljubljana	Samo Zakotnik, Tomaž Mark Zorec, Matic Brvar, Doroteja Vlaj, Patricija Pozvek,Špela Pleh, Miša Korva, Mario Poljak, Tatjana Avši - Županc
EPI_ISL_979338	Laboratorio Estatal de Salud Pública de Nuevo León	Laboratorio de Infectología Molecular, Departamento de Bioquímica y Medicina Molecular,Facultad de Medicina - Universidad Autónoma de Nuevo León	Kame A. Galán-Huerta, María F. Herrera-Saldivar, Natalia Martínez-Acuña, Sonia A. Lozano-Sepúlveda, Daniel Arellanos-Soto, Ana M. Rivas-Estilla, Samuel Buentello-Wong, Else del Carmen García-García, Gloria A. Jasso-de-la-Peña, Roberto Montes-de-Oca, Consuelo Treviño-Garza, Manuel E. de-la-O-Cavazos
EPI_ISL_979623, EPI_ISL_979624, EPI_ISL_979625, EPI_ISL_979626, EPI_ISL_979627, EPI_ISL_979628, EPI_ISL_979631, EPI_ISL_979632, EPI_ISL_979633	Santa Clara County Public Health Laboratory	Chan-Zuckerberg Biohub	CZB Cliahub Consortium
EPI_ISL_979643, EPI_ISL_979644, EPI_ISL_979645, EPI_ISL_979646, EPI_ISL_979647, EPI_ISL_979648, EPI_ISL_979649, EPI_ISL_979650, EPI_ISL_979651, EPI_ISL_979652, EPI_ISL_979653, EPI_ISL_979654, EPI_ISL_979655, EPI_ISL_979658, EPI_ISL_979659, EPI_ISL_979660, EPI_ISL_979661, EPI_ISL_979662, EPI_ISL_979663, EPI_ISL_979664, EPI_ISL_979665, EPI_ISL_979666, EPI_ISL_979667, EPI_ISL_979668, EPI_ISL_979669, EPI_ISL_979670, EPI_ISL_979671, EPI_ISL_979672, EPI_ISL_979673, EPI_ISL_979674, EPI_ISL_979675, EPI_ISL_979676, EPI_ISL_979677, EPI_ISL_979678, EPI_ISL_979679, EPI_ISL_979680, EPI_ISL_979681, EPI_ISL_979682, EPI_ISL_979683, EPI_ISL_979684, EPI_ISL_979685, EPI_ISL_979686, EPI_ISL_979687, EPI_ISL_979688, EPI_ISL_979689, EPI_ISL_979690, EPI_ISL_979691			
see above	Orange County Public Health Lab	Chan-Zuckerberg Biohub	CZB Cliahub Consortium
EPI_ISL_980974	Innovative Genomics Institute, UC Berkeley	Innovative Genomics Institute, UC Berkeley	Stacia Wyman, Haridha Shivram, Phil Frankino, Liana Lareau
EPI_ISL_981069	Johns Hopkins Hospital Department of Pathology	Johns Hopkins Hospital Department of Pathology	C. Paul Morris, Chun Huai Luo, Adannaya Amadi, Matthew Schwartz, Nicholas Gallagher, Heba H. Mostafa
EPI_ISL_981954, EPI_ISL_981955, EPI_ISL_981957	Microbiology Service, Hospital Universitario Clinico San Cecilio, Granada	Microbiology Service, Hospital Universitario Clinico San Cecilio, Granada	Adolfo de Salazar, Natalia Chueca, Laura Viñuela, Ana Fuentes, Federico García
EPI_ISL_982239	Lab voor klinische biologie	Lab voor klinische biologie	Hannelore Hamerlinck, Marija Janevska, Bruno Verhasselt
EPI_ISL_982355	M Health Fairview	Minnesota Department of Health, Public Health Laboratory	Alexandra Lorentz, Jacob Garfin, Matt Plumb, and Xiong Wang
EPI_ISL_982442	MONTEFIORE MEDICAL CENTER LABORATORIES	Wadsworth Center, New York State Department of Health	Kirsten St. George, Daryl M. Lamson, Alexis Russel, Matthew Shudt, Melissa A Leisner, Jonathan Plitnick, Navjot Singh, John Kelly, Erasmus Schneider, Erica Lasek-Nesselquist
EPI_ISL_982613	Landstuhl Regional Medical Center	US Air Force School of Aerospace Medicine	Anthony Fries, Jennifer Meyer, William Gruner, William Buggele, Amanda Javorina, Sarah Purves, Fritz Castillo, Cole Anderson, Clarise Starr, Elizabeth Macias
EPI_ISL_982853	Kentucky State Public Health Lab	Kentucky State Public Health Lab	Stephanie Lunn, Karim George, Joshua Tobias, William Grooms, Vaneet Arora, Matthew Johnson, Rachel Zinner, Rhonda Lucas
EPI_ISL_983408	Utah Public Health Laboratory	Utah Public Health Laboratory	Erin L. Young, Kelly F. Oakeson, Tara Gallagher
EPI_ISL_983836, EPI_ISL_983837, EPI_ISL_983838, EPI_ISL_983839, EPI_ISL_983840, EPI_ISL_983841, EPI_ISL_983842, EPI_ISL_983843, EPI_ISL_983844, EPI_ISL_983845, EPI_ISL_983846, EPI_ISL_983847, EPI_ISL_983848, EPI_ISL_983849, EPI_ISL_983850, EPI_ISL_983851, EPI_ISL_983852			
see above	Colorado Department of Public Health and Environment	Colorado Department of Puplic Health and Environment	Laura Bankers, Molly C. Hetherington-Rauth, Diana Ir, Shannon Ely, Shannon R. Matzinger, Sarah Elizabeth Totten, Emily A. Travanty
