# Supplementary material for: Detection and characterization of the SARS-CoV-2 lineage B.1.526 in New York
Source: Nat Commun. 2021 Aug 9;12:4886. doi: 10.1038/s41467-021-25168-4 (PMC8352861; doi:10.1038/s41467-021-25168-4)
Supplement: Supplementary file 8 — Supplementary Data 4 [file 41467_2021_25168_MOESM8_ESM.zip › GISAID_acknowledements_tables/gisaid_hcov-19_acknowledgement_table_2021_02_13_00-15.pdf]

We gratefully acknowledge the following Authors from the Originating laboratories responsible for obtaining the specimens, as well as the Submitting laboratories where the genome data were generated and shared via GISAID, on which this research is based.

All Submitters of data may be contacted directly via www.gisaid.org

Authors are sorted alphabetically.

Accession ID	Originating Laboratory	Submitting Laboratory	Authors
EPI_ISL_717704, EPI_ISL_717705, EPI_ISL_717706, EPI_ISL_717707	Area of Virology, Serology and Virology Division (SAViD), New South Wales Health Pathology Randwick	Virology Research Laboratory; Area of Virology, Serology and Virology Division (SAViD), New South Wales Health Pathology Randwick	Foster, C.; Au, J.; Ruiz Silva, M.; Deveson, I.; Bull, R.; Van Hal, S.; Rawlinson, W.
EPI_ISL_718318, EPI_ISL_718319, EPI_ISL_718320, EPI_ISL_718321, EPI_ISL_718322, EPI_ISL_718323, EPI_ISL_718324, EPI_ISL_718325, EPI_ISL_718326, EPI_ISL_718327, EPI_ISL_718328, EPI_ISL_718329, EPI_ISL_718330, EPI_ISL_718331, EPI_ISL_718332, EPI_ISL_718333, EPI_ISL_718334, EPI_ISL_718335, EPI_ISL_718336, EPI_ISL_718337, EPI_ISL_718338, EPI_ISL_718339, EPI_ISL_718340, EPI_ISL_718341, EPI_ISL_718342, EPI_ISL_718343, EPI_ISL_718344, EPI_ISL_718345, EPI_ISL_718346, EPI_ISL_718347, EPI_ISL_718348, EPI_ISL_718349, EPI_ISL_718350, EPI_ISL_718351, EPI_ISL_718352, EPI_ISL_718353, EPI_ISL_718354, EPI_ISL_718355, EPI_ISL_718356, EPI_ISL_718357, EPI_ISL_718358, EPI_ISL_718359, EPI_ISL_718360, EPI_ISL_718361, EPI_ISL_718362, EPI_ISL_718363, EPI_ISL_718364, EPI_ISL_718365, EPI_ISL_718366, EPI_ISL_718367, EPI_ISL_718368, EPI_ISL_718369, EPI_ISL_718370, EPI_ISL_718371, EPI_ISL_718372, EPI_ISL_718373, EPI_ISL_718374, EPI_ISL_718375, EPI_ISL_718376, EPI_ISL_718377, EPI_ISL_718378, EPI_ISL_718379, EPI_ISL_718380, EPI_ISL_718381, EPI_ISL_718382, EPI_ISL_718383, EPI_ISL_718384, EPI_ISL_718385, EPI_ISL_718386, EPI_ISL_718387, EPI_ISL_718388, EPI_ISL_718389, EPI_ISL_718390, EPI_ISL_718391, EPI_ISL_718392, EPI_ISL_718393, EPI_ISL_718394, EPI_ISL_718395, EPI_ISL_718396, EPI_ISL_718397, EPI_ISL_718398, EPI_ISL_718399, EPI_ISL_718400, EPI_ISL_718401, EPI_ISL_718402, EPI_ISL_718403, EPI_ISL_718404, EPI_ISL_718405, EPI_ISL_718406, EPI_ISL_718407, EPI_ISL_718408, EPI_ISL_718409, EPI_ISL_718410, EPI_ISL_718411, EPI_ISL_718412, EPI_ISL_718413, EPI_ISL_718414, EPI_ISL_718415, EPI_ISL_718416, EPI_ISL_718417, EPI_ISL_718418, EPI_ISL_718419, EPI_ISL_718420, EPI_ISL_718421, EPI_ISL_718422, EPI_ISL_718423, EPI_ISL_718424, EPI_ISL_718425, EPI_ISL_718426, EPI_ISL_718427, EPI_ISL_718428, EPI_ISL_718429, EPI_ISL_718430, EPI_ISL_718431, EPI_ISL_718432, EPI_ISL_718433, EPI_ISL_718434, EPI_ISL_718435, EPI_ISL_718436, EPI_ISL_718437, EPI_ISL_718438, EPI_ISL_718439, EPI_ISL_718440, EPI_ISL_718441, EPI_ISL_718442, EPI_ISL_718443, EPI_ISL_718444, EPI_ISL_718445, EPI_ISL_718446, EPI_ISL_718447, EPI_ISL_718448, EPI_ISL_718449, EPI_ISL_718450, EPI_ISL_718451, EPI_ISL_718452, EPI_ISL_718453, EPI_ISL_718454, EPI_ISL_718455, EPI_ISL_718456, EPI_ISL_718457, EPI_ISL_718458, EPI_ISL_718459, EPI_ISL_718460, EPI_ISL_718461, EPI_ISL_718462, EPI_ISL_718463, EPI_ISL_718464, EPI_ISL_718465, EPI_ISL_718466, EPI_ISL_718467, EPI_ISL_718468, EPI_ISL_718469, EPI_ISL_718470, EPI_ISL_718471, EPI_ISL_718472, EPI_ISL_718473, EPI_ISL_718474, EPI_ISL_718475, EPI_ISL_718476, EPI_ISL_718477, EPI_ISL_718478, EPI_ISL_718479, EPI_ISL_718480, EPI_ISL_718481, EPI_ISL_718482, EPI_ISL_718483, EPI_ISL_718484, EPI_ISL_718485, EPI_ISL_718486, EPI_ISL_718487, EPI_ISL_718488, EPI_ISL_718489, EPI_ISL_718490, EPI_ISL_718491, EPI_ISL_718492, EPI_ISL_718493, EPI_ISL_718494, EPI_ISL_718495, EPI_ISL_718496, EPI_ISL_718497, EPI_ISL_718498, EPI_ISL_718499, EPI_ISL_718500, EPI_ISL_718501, EPI_ISL_718502, EPI_ISL_718503, EPI_ISL_718504, EPI_ISL_718505, EPI_ISL_718506, EPI_ISL_718507, EPI_ISL_718508, EPI_ISL_718509, EPI_ISL_718510, EPI_ISL_718511, EPI_ISL_718512, EPI_ISL_718513, EPI_ISL_718514, EPI_ISL_718515, EPI_ISL_718516, EPI_ISL_718517, EPI_ISL_718518, EPI_ISL_718519, EPI_ISL_718520, EPI_ISL_718521, EPI_ISL_718522, EPI_ISL_718523, EPI_ISL_718524, EPI_ISL_718525, EPI_ISL_718526, EPI_ISL_718527, EPI_ISL_718528, EPI_ISL_718529, EPI_ISL_718530, EPI_ISL_718531, EPI_ISL_718532, EPI_ISL_718533, EPI_ISL_718534, EPI_ISL_718535, EPI_ISL_718536, EPI_ISL_718537, EPI_ISL_718538, EPI_ISL_718539, EPI_ISL_718540, EPI_ISL_718541, EPI_ISL_718542, EPI_ISL_718543, EPI_ISL_718544, EPI_ISL_718545, EPI_ISL_718546, EPI_ISL_718547, EPI_ISL_718548, EPI_ISL_718549, EPI_ISL_718550, EPI_ISL_718551, EPI_ISL_718552, EPI_ISL_718553, EPI_ISL_718554, EPI_ISL_718555, EPI_ISL_718556, EPI_ISL_718557, EPI_ISL_718558, EPI_ISL_718559, EPI_ISL_718560, EPI_ISL_718561, EPI_ISL_718562, EPI_ISL_718563, EPI_ISL_718564, EPI_ISL_718565, EPI_ISL_718566, EPI_ISL_718567, EPI_ISL_718568, EPI_ISL_718569, EPI_ISL_718570, EPI_ISL_718571, EPI_ISL_718572, EPI_ISL_718573, EPI_ISL_718574, EPI_ISL_718575, EPI_ISL_718576, EPI_ISL_718577, EPI_ISL_718578, EPI_ISL_718579, EPI_ISL_718580, EPI_ISL_718581, EPI_ISL_718582, EPI_ISL_718583, EPI_ISL_718584, EPI_ISL_718585, EPI_ISL_718586, EPI_ISL_718587, EPI_ISL_718588, EPI_ISL_718589, EPI_ISL_718590, EPI_ISL_718591, EPI_ISL_718592, EPI_ISL_718593, EPI_ISL_718594, EPI_ISL_718595, EPI_ISL_718596, EPI_ISL_718597, EPI_ISL_718598, EPI_ISL_718599, EPI_ISL_718600, EPI_ISL_718601, EPI_ISL_718602, EPI_ISL_718603, EPI_ISL_718604, EPI_ISL_718605, EPI_ISL_718606, EPI_ISL_718607, EPI_ISL_718608, EPI_ISL_718609, EPI_ISL_718610, EPI_ISL_718611, EPI_ISL_718612, EPI_ISL_718613, EPI_ISL_718614, EPI_ISL_718615, EPI_ISL_718616, EPI_ISL_718617, EPI_ISL_718618, EPI_ISL_718619, EPI_ISL_718620, EPI_ISL_718621, EPI_ISL_718622, EPI_ISL_718623, EPI_ISL_718624, EPI_ISL_718625, EPI_ISL_718626, EPI_ISL_718627, EPI_ISL_718628			
see above	Lighthouse Lab in Cambridge	Wellcome Sanger Institute for the COVID-19 Genomics UK (COG-UK) Consortium	Rob Howes, The Lighthouse Lab in Cambridge and Alex Alderton, Roberto Amato, Sonia Goncalves, Ewan Harrison, David K. Jackson, Ian Johnston, Dominic Kwiatkowski, Cordelia Langford, John Sillitoe on behalf of the Wellcome Sanger Institute COVID-19 Surveillance Team
EPI_ISL_718629	Lighthouse Lab in Alderley Park	Wellcome Sanger Institute for the COVID-19 Genomics UK (COG-UK) Consortium	Jacquelyn Wynn, Mairead Hyland, The Lighthouse Lab in Alderley Park and Alex Alderton, Roberto Amato, Sonia Goncalves, Ewan Harrison, David K. Jackson, Ian Johnston, Dominic Kwiatkowski, Cordelia Langford, John Sillitoe on behalf of the Wellcome Sanger Institute COVID-19 Surveillance Team
EPI_ISL_718630	Lighthouse Lab in Cambridge	Wellcome Sanger Institute for the COVID-19 Genomics UK (COG-UK) Consortium	Rob Howes, The Lighthouse Lab in Cambridge and Alex Alderton, Roberto Amato, Sonia Goncalves, Ewan Harrison, David K. Jackson, Ian Johnston, Dominic Kwiatkowski, Cordelia Langford, John Sillitoe on behalf of the Wellcome Sanger Institute COVID-19 Surveillance Team
EPI_ISL_718631	Lighthouse Lab in Alderley Park	Wellcome Sanger Institute for the COVID-19 Genomics UK (COG-UK) Consortium	Jacquelyn Wynn, Mairead Hyland, The Lighthouse Lab in Alderley Park and Alex Alderton, Roberto Amato, Sonia Goncalves, Ewan Harrison, David K. Jackson, Ian Johnston, Dominic Kwiatkowski, Cordelia Langford, John Sillitoe on behalf of the Wellcome Sanger Institute COVID-19 Surveillance Team
EPI_ISL_718632	Lighthouse Lab in Cambridge	Wellcome Sanger Institute for the COVID-19 Genomics UK (COG-UK) Consortium	Rob Howes, The Lighthouse Lab in Cambridge and Alex Alderton, Roberto Amato, Sonia Goncalves, Ewan Harrison, David K. Jackson, Ian Johnston, Dominic Kwiatkowski, Cordelia Langford, John Sillitoe on behalf of the Wellcome Sanger Institute COVID-19 Surveillance Team
EPI_ISL_718633	Lighthouse Lab in Alderley Park	Wellcome Sanger Institute for the COVID-19 Genomics UK (COG-UK) Consortium	Jacquelyn Wynn, Mairead Hyland, The Lighthouse Lab in Alderley Park and Alex Alderton, Roberto Amato, Sonia Goncalves, Ewan Harrison, David K. Jackson, Ian Johnston, Dominic Kwiatkowski, Cordelia Langford, John Sillitoe on behalf of the Wellcome Sanger Institute COVID-19 Surveillance Team
EPI_ISL_718634	Lighthouse Lab in Cambridge	Wellcome Sanger Institute for the COVID-19 Genomics UK (COG-UK) Consortium	Rob Howes, The Lighthouse Lab in Cambridge and Alex Alderton, Roberto Amato, Sonia Goncalves, Ewan Harrison, David K. Jackson, Ian Johnston, Dominic Kwiatkowski, Cordelia Langford, John Sillitoe on behalf of the Wellcome Sanger Institute COVID-19 Surveillance Team
EPI_ISL_718635	Lighthouse Lab in Alderley Park	Wellcome Sanger Institute for the COVID-19 Genomics UK (COG-UK) Consortium	Jacquelyn Wynn, Mairead Hyland, The Lighthouse Lab in Alderley Park and Alex Alderton, Roberto Amato, Sonia Goncalves, Ewan Harrison, David K. Jackson, Ian Johnston, Dominic Kwiatkowski, Cordelia Langford, John Sillitoe on behalf of the Wellcome Sanger Institute COVID-19 Surveillance Team
EPI_ISL_718636	Lighthouse Lab in Cambridge	Wellcome Sanger Institute for the COVID-19 Genomics UK (COG-UK) Consortium	Rob Howes, The Lighthouse Lab in Cambridge and Alex Alderton, Roberto Amato, Sonia Goncalves, Ewan Harrison, David K. Jackson, Ian Johnston, Dominic Kwiatkowski, Cordelia Langford, John Sillitoe on behalf of the Wellcome Sanger Institute COVID-19 Surveillance Team
EPI_ISL_718637	Lighthouse Lab in Alderley Park	Wellcome Sanger Institute for the COVID-19 Genomics UK (COG-UK) Consortium	Jacquelyn Wynn, Mairead Hyland, The Lighthouse Lab in Alderley Park and Alex Alderton, Roberto Amato, Sonia Goncalves, Ewan Harrison, David K. Jackson, Ian Johnston, Dominic Kwiatkowski, Cordelia Langford, John Sillitoe on behalf of the Wellcome Sanger Institute COVID-19 Surveillance Team
EPI_ISL_718638	Lighthouse Lab in Cambridge	Wellcome Sanger Institute for the COVID-19 Genomics UK (COG-UK) Consortium	Rob Howes, The Lighthouse Lab in Cambridge and Alex Alderton, Roberto Amato, Sonia Goncalves, Ewan Harrison, David K. Jackson, Ian Johnston, Dominic Kwiatkowski, Cordelia Langford, John Sillitoe on behalf of the Wellcome Sanger Institute COVID-19 Surveillance Team
EPI_ISL_718639, EPI_ISL_718640, EPI_ISL_718641	Lighthouse Lab in Alderley Park	Wellcome Sanger Institute for the COVID-19 Genomics UK (COG-UK) Consortium	Jacquelyn Wynn, Mairead Hyland, The Lighthouse Lab in Alderley Park and Alex Alderton, Roberto Amato, Sonia Goncalves, Ewan Harrison, David K. Jackson, Ian Johnston, Dominic Kwiatkowski, Cordelia Langford, John Sillitoe on behalf of the Wellcome Sanger Institute COVID-19 Surveillance Team
EPI_ISL_718642	Lighthouse Lab in Cambridge	Wellcome Sanger Institute for the COVID-19 Genomics UK (COG-UK) Consortium	Rob Howes, The Lighthouse Lab in Cambridge and Alex Alderton, Roberto Amato, Sonia Goncalves, Ewan Harrison, David K. Jackson, Ian Johnston, Dominic Kwiatkowski, Cordelia Langford, John Sillitoe on behalf of the Wellcome Sanger Institute COVID-19 Surveillance Team
EPI_ISL_718643, EPI_ISL_718644	Lighthouse Lab in Alderley Park	Wellcome Sanger Institute for the COVID-19 Genomics UK (COG-UK) Consortium	Jacquelyn Wynn, Mairead Hyland, The Lighthouse Lab in Alderley Park and Alex Alderton, Roberto Amato, Sonia Goncalves, Ewan Harrison, David K. Jackson, Ian Johnston, Dominic Kwiatkowski, Cordelia Langford, John Sillitoe on behalf of the Wellcome Sanger Institute COVID-19 Surveillance Team
EPI_ISL_718645, EPI_ISL_718646	Lighthouse Lab in Cambridge	Wellcome Sanger Institute for the COVID-19 Genomics UK (COG-UK) Consortium	Rob Howes, The Lighthouse Lab in Cambridge and Alex Alderton, Roberto Amato, Sonia Goncalves, Ewan Harrison, David K. Jackson, Ian Johnston, Dominic Kwiatkowski, Cordelia Langford, John Sillitoe on behalf of the Wellcome Sanger Institute COVID-19 Surveillance Team
EPI_ISL_718647	Lighthouse Lab in Alderley Park	Wellcome Sanger Institute for the COVID-19 Genomics UK (COG-UK) Consortium	Jacquelyn Wynn, Mairead Hyland, The Lighthouse Lab in Alderley Park and Alex Alderton, Roberto Amato, Sonia Goncalves, Ewan Harrison, David K. Jackson, Ian Johnston, Dominic Kwiatkowski, Cordelia Langford, John Sillitoe on behalf of the Wellcome Sanger Institute COVID-19 Surveillance Team
EPI_ISL_718648, EPI_ISL_718649	Lighthouse Lab in Cambridge	Wellcome Sanger Institute for the COVID-19 Genomics UK (COG-UK) Consortium	Rob Howes, The Lighthouse Lab in Cambridge and Alex Alderton, Roberto Amato, Sonia Goncalves, Ewan Harrison, David K. Jackson, Ian Johnston, Dominic Kwiatkowski, Cordelia Langford, John Sillitoe on behalf of the Wellcome Sanger Institute COVID-19 Surveillance Team
EPI_ISL_718650	Lighthouse Lab in Alderley Park	Wellcome Sanger Institute for the COVID-19 Genomics UK (COG-UK) Consortium	Jacquelyn Wynn, Mairead Hyland, The Lighthouse Lab in Alderley Park and Alex Alderton, Roberto Amato, Sonia Goncalves, Ewan Harrison, David K. Jackson, Ian Johnston, Dominic Kwiatkowski, Cordelia Langford, John Sillitoe on behalf of the Wellcome Sanger Institute COVID-19 Surveillance Team
EPI_ISL_718651, EPI_ISL_718652	Lighthouse Lab in Cambridge	Wellcome Sanger Institute for the COVID-19 Genomics UK (COG-UK) Consortium	Rob Howes, The Lighthouse Lab in Cambridge and Alex Alderton, Roberto Amato, Sonia Goncalves, Ewan Harrison, David K. Jackson, Ian Johnston, Dominic Kwiatkowski, Cordelia Langford, John Sillitoe on behalf of the Wellcome Sanger Institute COVID-19 Surveillance Team
EPI_ISL_718653	Lighthouse Lab in Alderley Park	Wellcome Sanger Institute for the COVID-19 Genomics UK (COG-UK) Consortium	Jacquelyn Wynn, Mairead Hyland, The Lighthouse Lab in Alderley Park and Alex Alderton, Roberto Amato, Sonia Goncalves, Ewan Harrison, David K. Jackson, Ian Johnston, Dominic Kwiatkowski, Cordelia Langford, John Sillitoe on behalf of the Wellcome Sanger Institute COVID-19 Surveillance Team
EPI_ISL_718654, EPI_ISL_718655, EPI_ISL_718656	Lighthouse Lab in Cambridge	Wellcome Sanger Institute for the COVID-19 Genomics UK (COG-UK) Consortium	Rob Howes, The Lighthouse Lab in Cambridge and Alex Alderton, Roberto Amato, Sonia Goncalves, Ewan Harrison, David K. Jackson, Ian Johnston, Dominic Kwiatkowski, Cordelia Langford, John Sillitoe on behalf of the Wellcome Sanger Institute COVID-19 Surveillance Team
EPI_ISL_718657, EPI_ISL_718658	Lighthouse Lab in Alderley Park	Wellcome Sanger Institute for the COVID-19 Genomics UK	Jacquelyn Wynn, Mairead Hyland, The Lighthouse Lab in Alderley Park and Alex Alderton, Roberto Amato, Sonia Goncalves, Ewan Harrison, David K.

		(COG-UK) Consortium	Jackson, Ian Johnston, Dominic Kwiatkowski, Cordelia Langford, John Sillitoe on behalf of the Wellcome Sanger Institute COVID-19 Surveillance Team
EPI_ISL_718659, EPI_ISL_718660, EPI_ISL_718661	Lighthouse Lab in Cambridge	Wellcome Sanger Institute for the COVID-19 Genomics UK (COG-UK) Consortium	Rob Howes, The Lighthouse Lab in Cambridge and Alex Alderton, Roberto Amato, Sonia Goncalves, Ewan Harrison, David K. Jackson, Ian Johnston, Dominic Kwiatkowski, Cordelia Langford, John Sillitoe on behalf of the Wellcome Sanger Institute COVID-19 Surveillance Team
EPI_ISL_718662, EPI_ISL_718663, EPI_ISL_718664, EPI_ISL_718665, EPI_ISL_718666	Lighthouse Lab in Alderley Park	Wellcome Sanger Institute for the COVID-19 Genomics UK (COG-UK) Consortium	Jacquelyn Wynn, Mairead Hyland, The Lighthouse Lab in Alderley Park and Alex Alderton, Roberto Amato, Sonia Goncalves, Ewan Harrison, David K. Jackson, Ian Johnston, Dominic Kwiatkowski, Cordelia Langford, John Sillitoe on behalf of the Wellcome Sanger Institute COVID-19 Surveillance Team
EPI_ISL_718667, EPI_ISL_718668, EPI_ISL_718669	Lighthouse Lab in Cambridge	Wellcome Sanger Institute for the COVID-19 Genomics UK (COG-UK) Consortium	Rob Howes, The Lighthouse Lab in Cambridge and Alex Alderton, Roberto Amato, Sonia Goncalves, Ewan Harrison, David K. Jackson, Ian Johnston, Dominic Kwiatkowski, Cordelia Langford, John Sillitoe on behalf of the Wellcome Sanger Institute COVID-19 Surveillance Team
EPI_ISL_718670	Lighthouse Lab in Alderley Park	Wellcome Sanger Institute for the COVID-19 Genomics UK (COG-UK) Consortium	Jacquelyn Wynn, Mairead Hyland, The Lighthouse Lab in Alderley Park and Alex Alderton, Roberto Amato, Sonia Goncalves, Ewan Harrison, David K. Jackson, Ian Johnston, Dominic Kwiatkowski, Cordelia Langford, John Sillitoe on behalf of the Wellcome Sanger Institute COVID-19 Surveillance Team
EPI_ISL_718671	Lighthouse Lab in Cambridge	Wellcome Sanger Institute for the COVID-19 Genomics UK (COG-UK) Consortium	Rob Howes, The Lighthouse Lab in Cambridge and Alex Alderton, Roberto Amato, Sonia Goncalves, Ewan Harrison, David K. Jackson, Ian Johnston, Dominic Kwiatkowski, Cordelia Langford, John Sillitoe on behalf of the Wellcome Sanger Institute COVID-19 Surveillance Team
EPI_ISL_718672	Lighthouse Lab in Alderley Park	Wellcome Sanger Institute for the COVID-19 Genomics UK (COG-UK) Consortium	Jacquelyn Wynn, Mairead Hyland, The Lighthouse Lab in Alderley Park and Alex Alderton, Roberto Amato, Sonia Goncalves, Ewan Harrison, David K. Jackson, Ian Johnston, Dominic Kwiatkowski, Cordelia Langford, John Sillitoe on behalf of the Wellcome Sanger Institute COVID-19 Surveillance Team
EPI_ISL_718673	Lighthouse Lab in Cambridge	Wellcome Sanger Institute for the COVID-19 Genomics UK (COG-UK) Consortium	Rob Howes, The Lighthouse Lab in Cambridge and Alex Alderton, Roberto Amato, Sonia Goncalves, Ewan Harrison, David K. Jackson, Ian Johnston, Dominic Kwiatkowski, Cordelia Langford, John Sillitoe on behalf of the Wellcome Sanger Institute COVID-19 Surveillance Team
EPI_ISL_718674, EPI_ISL_718675, EPI_ISL_718676	Lighthouse Lab in Alderley Park	Wellcome Sanger Institute for the COVID-19 Genomics UK (COG-UK) Consortium	Jacquelyn Wynn, Mairead Hyland, The Lighthouse Lab in Alderley Park and Alex Alderton, Roberto Amato, Sonia Goncalves, Ewan Harrison, David K. Jackson, Ian Johnston, Dominic Kwiatkowski, Cordelia Langford, John Sillitoe on behalf of the Wellcome Sanger Institute COVID-19 Surveillance Team
EPI_ISL_718677	Lighthouse Lab in Cambridge	Wellcome Sanger Institute for the COVID-19 Genomics UK (COG-UK) Consortium	Rob Howes, The Lighthouse Lab in Cambridge and Alex Alderton, Roberto Amato, Sonia Goncalves, Ewan Harrison, David K. Jackson, Ian Johnston, Dominic Kwiatkowski, Cordelia Langford, John Sillitoe on behalf of the Wellcome Sanger Institute COVID-19 Surveillance Team
EPI_ISL_718678	Lighthouse Lab in Alderley Park	Wellcome Sanger Institute for the COVID-19 Genomics UK (COG-UK) Consortium	Jacquelyn Wynn, Mairead Hyland, The Lighthouse Lab in Alderley Park and Alex Alderton, Roberto Amato, Sonia Goncalves, Ewan Harrison, David K. Jackson, Ian Johnston, Dominic Kwiatkowski, Cordelia Langford, John Sillitoe on behalf of the Wellcome Sanger Institute COVID-19 Surveillance Team
EPI_ISL_718679, EPI_ISL_718680, EPI_ISL_718681	Lighthouse Lab in Cambridge	Wellcome Sanger Institute for the COVID-19 Genomics UK (COG-UK) Consortium	Rob Howes, The Lighthouse Lab in Cambridge and Alex Alderton, Roberto Amato, Sonia Goncalves, Ewan Harrison, David K. Jackson, Ian Johnston, Dominic Kwiatkowski, Cordelia Langford, John Sillitoe on behalf of the Wellcome Sanger Institute COVID-19 Surveillance Team
EPI_ISL_718682	Lighthouse Lab in Alderley Park	Wellcome Sanger Institute for the COVID-19 Genomics UK (COG-UK) Consortium	Jacquelyn Wynn, Mairead Hyland, The Lighthouse Lab in Alderley Park and Alex Alderton, Roberto Amato, Sonia Goncalves, Ewan Harrison, David K. Jackson, Ian Johnston, Dominic Kwiatkowski, Cordelia Langford, John Sillitoe on behalf of the Wellcome Sanger Institute COVID-19 Surveillance Team
EPI_ISL_718683, EPI_ISL_718684, EPI_ISL_718685	Lighthouse Lab in Cambridge	Wellcome Sanger Institute for the COVID-19 Genomics UK (COG-UK) Consortium	Rob Howes, The Lighthouse Lab in Cambridge and Alex Alderton, Roberto Amato, Sonia Goncalves, Ewan Harrison, David K. Jackson, Ian Johnston, Dominic Kwiatkowski, Cordelia Langford, John Sillitoe on behalf of the Wellcome Sanger Institute COVID-19 Surveillance Team
EPI_ISL_718686, EPI_ISL_718687	Lighthouse Lab in Alderley Park	Wellcome Sanger Institute for the COVID-19 Genomics UK (COG-UK) Consortium	Jacquelyn Wynn, Mairead Hyland, The Lighthouse Lab in Alderley Park and Alex Alderton, Roberto Amato, Sonia Goncalves, Ewan Harrison, David K. Jackson, Ian Johnston, Dominic Kwiatkowski, Cordelia Langford, John Sillitoe on behalf of the Wellcome Sanger Institute COVID-19 Surveillance Team
EPI_ISL_718688, EPI_ISL_718689, EPI_ISL_718690	Lighthouse Lab in Cambridge	Wellcome Sanger Institute for the COVID-19 Genomics UK (COG-UK) Consortium	Rob Howes, The Lighthouse Lab in Cambridge and Alex Alderton, Roberto Amato, Sonia Goncalves, Ewan Harrison, David K. Jackson, Ian Johnston, Dominic Kwiatkowski, Cordelia Langford, John Sillitoe on behalf of the Wellcome Sanger Institute COVID-19 Surveillance Team
EPI_ISL_718691	Lighthouse Lab in Alderley Park	Wellcome Sanger Institute for the COVID-19 Genomics UK (COG-UK) Consortium	Jacquelyn Wynn, Mairead Hyland, The Lighthouse Lab in Alderley Park and Alex Alderton, Roberto Amato, Sonia Goncalves, Ewan Harrison, David K. Jackson, Ian Johnston, Dominic Kwiatkowski, Cordelia Langford, John Sillitoe on behalf of the Wellcome Sanger Institute COVID-19 Surveillance Team
EPI_ISL_718692, EPI_ISL_718693	Lighthouse Lab in Cambridge	Wellcome Sanger Institute for the COVID-19 Genomics UK (COG-UK) Consortium	Rob Howes, The Lighthouse Lab in Cambridge and Alex Alderton, Roberto Amato, Sonia Goncalves, Ewan Harrison, David K. Jackson, Ian Johnston, Dominic Kwiatkowski, Cordelia Langford, John Sillitoe on behalf of the Wellcome Sanger Institute COVID-19 Surveillance Team
EPI_ISL_718694, EPI_ISL_718695	Lighthouse Lab in Alderley Park	Wellcome Sanger Institute for the COVID-19 Genomics UK (COG-UK) Consortium	Jacquelyn Wynn, Mairead Hyland, The Lighthouse Lab in Alderley Park and Alex Alderton, Roberto Amato, Sonia Goncalves, Ewan Harrison, David K. Jackson, Ian Johnston, Dominic Kwiatkowski, Cordelia Langford, John Sillitoe on behalf of the Wellcome Sanger Institute COVID-19 Surveillance Team
EPI_ISL_718696, EPI_ISL_718697, EPI_ISL_718698, EPI_ISL_718699	Lighthouse Lab in Cambridge	Wellcome Sanger Institute for the COVID-19 Genomics UK (COG-UK) Consortium	Rob Howes, The Lighthouse Lab in Cambridge and Alex Alderton, Roberto Amato, Sonia Goncalves, Ewan Harrison, David K. Jackson, Ian Johnston, Dominic Kwiatkowski, Cordelia Langford, John Sillitoe on behalf of the Wellcome Sanger Institute COVID-19 Surveillance Team
EPI_ISL_718700	Lighthouse Lab in Alderley Park	Wellcome Sanger Institute for the COVID-19 Genomics UK (COG-UK) Consortium	Jacquelyn Wynn, Mairead Hyland, The Lighthouse Lab in Alderley Park and Alex Alderton, Roberto Amato, Sonia Goncalves, Ewan Harrison, David K. Jackson, Ian Johnston, Dominic Kwiatkowski, Cordelia Langford, John Sillitoe on behalf of the Wellcome Sanger Institute COVID-19 Surveillance Team
EPI_ISL_718701, EPI_ISL_718702, EPI_ISL_718703, EPI_ISL_718704	Lighthouse Lab in Cambridge	Wellcome Sanger Institute for the COVID-19 Genomics UK (COG-UK) Consortium	Rob Howes, The Lighthouse Lab in Cambridge and Alex Alderton, Roberto Amato, Sonia Goncalves, Ewan Harrison, David K. Jackson, Ian Johnston, Dominic Kwiatkowski, Cordelia Langford, John Sillitoe on behalf of the Wellcome Sanger Institute COVID-19 Surveillance Team
EPI_ISL_718705	Lighthouse Lab in Alderley Park	Wellcome Sanger Institute for the COVID-19 Genomics UK (COG-UK) Consortium	Jacquelyn Wynn, Mairead Hyland, The Lighthouse Lab in Alderley Park and Alex Alderton, Roberto Amato, Sonia Goncalves, Ewan Harrison, David K. Jackson, Ian Johnston, Dominic Kwiatkowski, Cordelia Langford, John Sillitoe on behalf of the Wellcome Sanger Institute COVID-19 Surveillance Team
EPI_ISL_718706	Lighthouse Lab in Cambridge	Wellcome Sanger Institute for the COVID-19 Genomics UK (COG-UK) Consortium	Rob Howes, The Lighthouse Lab in Cambridge and Alex Alderton, Roberto Amato, Sonia Goncalves, Ewan Harrison, David K. Jackson, Ian Johnston, Dominic Kwiatkowski, Cordelia Langford, John Sillitoe on behalf of the Wellcome Sanger Institute COVID-19 Surveillance Team
EPI_ISL_718707	Lighthouse Lab in Alderley Park	Wellcome Sanger Institute for the COVID-19 Genomics UK (COG-UK) Consortium	Jacquelyn Wynn, Mairead Hyland, The Lighthouse Lab in Alderley Park and Alex Alderton, Roberto Amato, Sonia Goncalves, Ewan Harrison, David K. Jackson, Ian Johnston, Dominic Kwiatkowski, Cordelia Langford, John Sillitoe on behalf of the Wellcome Sanger Institute COVID-19 Surveillance Team
EPI_ISL_718708, EPI_ISL_718709	Lighthouse Lab in Cambridge	Wellcome Sanger Institute for the COVID-19 Genomics UK (COG-UK) Consortium	Rob Howes, The Lighthouse Lab in Cambridge and Alex Alderton, Roberto Amato, Sonia Goncalves, Ewan Harrison, David K. Jackson, Ian Johnston, Dominic Kwiatkowski, Cordelia Langford, John Sillitoe on behalf of the Wellcome Sanger Institute COVID-19 Surveillance Team
EPI_ISL_718710, EPI_ISL_718711, EPI_ISL_718712, EPI_ISL_718713, EPI_ISL_718714, EPI_ISL_718715, EPI_ISL_718716	Lighthouse Lab in Alderley Park	Wellcome Sanger Institute for the COVID-19 Genomics UK (COG-UK) Consortium	Jacquelyn Wynn, Mairead Hyland, The Lighthouse Lab in Alderley Park and Alex Alderton, Roberto Amato, Sonia Goncalves, Ewan Harrison, David K. Jackson, Ian Johnston, Dominic Kwiatkowski, Cordelia Langford, John Sillitoe on behalf of the Wellcome Sanger Institute COVID-19 Surveillance Team
EPI_ISL_718717	Lighthouse Lab in Cambridge	Wellcome Sanger Institute for the COVID-19 Genomics UK (COG-UK) Consortium	Rob Howes, The Lighthouse Lab in Cambridge and Alex Alderton, Roberto Amato, Sonia Goncalves, Ewan Harrison, David K. Jackson, Ian Johnston, Dominic Kwiatkowski, Cordelia Langford, John Sillitoe on behalf of the Wellcome Sanger Institute COVID-19 Surveillance Team
EPI_ISL_718718, EPI_ISL_718719	Lighthouse Lab in Alderley Park	Wellcome Sanger Institute for the COVID-19 Genomics UK (COG-UK) Consortium	Jacquelyn Wynn, Mairead Hyland, The Lighthouse Lab in Alderley Park and Alex Alderton, Roberto Amato, Sonia Goncalves, Ewan Harrison, David K. Jackson, Ian Johnston, Dominic Kwiatkowski, Cordelia Langford, John Sillitoe on behalf of the Wellcome Sanger Institute COVID-19 Surveillance Team
EPI_ISL_718720	Lighthouse Lab in Cambridge	Wellcome Sanger Institute for the COVID-19 Genomics UK (COG-UK) Consortium	Rob Howes, The Lighthouse Lab in Cambridge and Alex Alderton, Roberto Amato, Sonia Goncalves, Ewan Harrison, David K. Jackson, Ian Johnston, Dominic Kwiatkowski, Cordelia Langford, John Sillitoe on behalf of the Wellcome Sanger Institute COVID-19 Surveillance Team
EPI_ISL_718721	Lighthouse Lab in Alderley Park	Wellcome Sanger Institute for the COVID-19 Genomics UK (COG-UK) Consortium	Jacquelyn Wynn, Mairead Hyland, The Lighthouse Lab in Alderley Park and Alex Alderton, Roberto Amato, Sonia Goncalves, Ewan Harrison, David K. Jackson, Ian Johnston, Dominic Kwiatkowski, Cordelia Langford, John Sillitoe on behalf of the Wellcome Sanger Institute COVID-19 Surveillance Team
EPI_ISL_718722	Lighthouse Lab in Cambridge	Wellcome Sanger Institute for the COVID-19 Genomics UK (COG-UK) Consortium	Rob Howes, The Lighthouse Lab in Cambridge and Alex Alderton, Roberto Amato, Sonia Goncalves, Ewan Harrison, David K. Jackson, Ian Johnston, Dominic Kwiatkowski, Cordelia Langford, John Sillitoe on behalf of the Wellcome Sanger Institute COVID-19 Surveillance Team
EPI_ISL_718723, EPI_ISL_718724, EPI_ISL_718725	Lighthouse Lab in Alderley Park	Wellcome Sanger Institute for the COVID-19 Genomics UK (COG-UK) Consortium	Jacquelyn Wynn, Mairead Hyland, The Lighthouse Lab in Alderley Park and Alex Alderton, Roberto Amato, Sonia Goncalves, Ewan Harrison, David K. Jackson, Ian Johnston, Dominic Kwiatkowski, Cordelia Langford, John Sillitoe on behalf of the Wellcome Sanger Institute COVID-19 Surveillance Team
EPI_ISL_718726, EPI_ISL_718727	Lighthouse Lab in Cambridge	Wellcome Sanger Institute for the COVID-19 Genomics UK (COG-UK) Consortium	Rob Howes, The Lighthouse Lab in Cambridge and Alex Alderton, Roberto Amato, Sonia Goncalves, Ewan Harrison, David K. Jackson, Ian Johnston, Dominic Kwiatkowski, Cordelia Langford, John Sillitoe on behalf of the Wellcome Sanger Institute COVID-19 Surveillance Team
EPI_ISL_718728, EPI_ISL_718729	Lighthouse Lab in Alderley Park	Wellcome Sanger Institute for the COVID-19 Genomics UK	Jacquelyn Wynn, Mairead Hyland, The Lighthouse Lab in Alderley Park and Alex Alderton, Roberto Amato, Sonia Goncalves, Ewan Harrison, David K.

		(COG-UK) Consortium	Jackson, Ian Johnston, Dominic Kwiatkowski, Cordelia Langford, John Sillitoe on behalf of the Wellcome Sanger Institute COVID-19 Surveillance Team
EPI_ISL_718730, EPI_ISL_718731, EPI_ISL_718732, EPI_ISL_718733, EPI_ISL_718734	Lighthouse Lab in Cambridge	Wellcome Sanger Institute for the COVID-19 Genomics UK (COG-UK) Consortium	Rob Howes, The Lighthouse Lab in Cambridge and Alex Alderton, Roberto Amato, Sonia Goncalves, Ewan Harrison, David K. Jackson, Ian Johnston, Dominic Kwiatkowski, Cordelia Langford, John Sillitoe on behalf of the Wellcome Sanger Institute COVID-19 Surveillance Team
EPI_ISL_718735	Lighthouse Lab in Alderley Park	Wellcome Sanger Institute for the COVID-19 Genomics UK (COG-UK) Consortium	Jacquelyn Wynn, Mairead Hyland, The Lighthouse Lab in Alderley Park and Alex Alderton, Roberto Amato, Sonia Goncalves, Ewan Harrison, David K. Jackson, Ian Johnston, Dominic Kwiatkowski, Cordelia Langford, John Sillitoe on behalf of the Wellcome Sanger Institute COVID-19 Surveillance Team
EPI_ISL_718736, EPI_ISL_718737	Lighthouse Lab in Cambridge	Wellcome Sanger Institute for the COVID-19 Genomics UK (COG-UK) Consortium	Rob Howes, The Lighthouse Lab in Cambridge and Alex Alderton, Roberto Amato, Sonia Goncalves, Ewan Harrison, David K. Jackson, Ian Johnston, Dominic Kwiatkowski, Cordelia Langford, John Sillitoe on behalf of the Wellcome Sanger Institute COVID-19 Surveillance Team
EPI_ISL_718738, EPI_ISL_718739, EPI_ISL_718740	Lighthouse Lab in Alderley Park	Wellcome Sanger Institute for the COVID-19 Genomics UK (COG-UK) Consortium	Jacquelyn Wynn, Mairead Hyland, The Lighthouse Lab in Alderley Park and Alex Alderton, Roberto Amato, Sonia Goncalves, Ewan Harrison, David K. Jackson, Ian Johnston, Dominic Kwiatkowski, Cordelia Langford, John Sillitoe on behalf of the Wellcome Sanger Institute COVID-19 Surveillance Team
EPI_ISL_718741	Lighthouse Lab in Cambridge	Wellcome Sanger Institute for the COVID-19 Genomics UK (COG-UK) Consortium	Rob Howes, The Lighthouse Lab in Cambridge and Alex Alderton, Roberto Amato, Sonia Goncalves, Ewan Harrison, David K. Jackson, Ian Johnston, Dominic Kwiatkowski, Cordelia Langford, John Sillitoe on behalf of the Wellcome Sanger Institute COVID-19 Surveillance Team
EPI_ISL_718742	Lighthouse Lab in Alderley Park	Wellcome Sanger Institute for the COVID-19 Genomics UK (COG-UK) Consortium	Jacquelyn Wynn, Mairead Hyland, The Lighthouse Lab in Alderley Park and Alex Alderton, Roberto Amato, Sonia Goncalves, Ewan Harrison, David K. Jackson, Ian Johnston, Dominic Kwiatkowski, Cordelia Langford, John Sillitoe on behalf of the Wellcome Sanger Institute COVID-19 Surveillance Team
EPI_ISL_718743	Lighthouse Lab in Cambridge	Wellcome Sanger Institute for the COVID-19 Genomics UK (COG-UK) Consortium	Rob Howes, The Lighthouse Lab in Cambridge and Alex Alderton, Roberto Amato, Sonia Goncalves, Ewan Harrison, David K. Jackson, Ian Johnston, Dominic Kwiatkowski, Cordelia Langford, John Sillitoe on behalf of the Wellcome Sanger Institute COVID-19 Surveillance Team
EPI_ISL_718744, EPI_ISL_718745, EPI_ISL_718746, EPI_ISL_718747	Lighthouse Lab in Alderley Park	Wellcome Sanger Institute for the COVID-19 Genomics UK (COG-UK) Consortium	Jacquelyn Wynn, Mairead Hyland, The Lighthouse Lab in Alderley Park and Alex Alderton, Roberto Amato, Sonia Goncalves, Ewan Harrison, David K. Jackson, Ian Johnston, Dominic Kwiatkowski, Cordelia Langford, John Sillitoe on behalf of the Wellcome Sanger Institute COVID-19 Surveillance Team
EPI_ISL_718748, EPI_ISL_718749	Lighthouse Lab in Cambridge	Wellcome Sanger Institute for the COVID-19 Genomics UK (COG-UK) Consortium	Rob Howes, The Lighthouse Lab in Cambridge and Alex Alderton, Roberto Amato, Sonia Goncalves, Ewan Harrison, David K. Jackson, Ian Johnston, Dominic Kwiatkowski, Cordelia Langford, John Sillitoe on behalf of the Wellcome Sanger Institute COVID-19 Surveillance Team
EPI_ISL_718750	Lighthouse Lab in Alderley Park	Wellcome Sanger Institute for the COVID-19 Genomics UK (COG-UK) Consortium	Jacquelyn Wynn, Mairead Hyland, The Lighthouse Lab in Alderley Park and Alex Alderton, Roberto Amato, Sonia Goncalves, Ewan Harrison, David K. Jackson, Ian Johnston, Dominic Kwiatkowski, Cordelia Langford, John Sillitoe on behalf of the Wellcome Sanger Institute COVID-19 Surveillance Team
EPI_ISL_718751, EPI_ISL_718752, EPI_ISL_718753	Lighthouse Lab in Cambridge	Wellcome Sanger Institute for the COVID-19 Genomics UK (COG-UK) Consortium	Rob Howes, The Lighthouse Lab in Cambridge and Alex Alderton, Roberto Amato, Sonia Goncalves, Ewan Harrison, David K. Jackson, Ian Johnston, Dominic Kwiatkowski, Cordelia Langford, John Sillitoe on behalf of the Wellcome Sanger Institute COVID-19 Surveillance Team
EPI_ISL_718754, EPI_ISL_718755	Lighthouse Lab in Alderley Park	Wellcome Sanger Institute for the COVID-19 Genomics UK (COG-UK) Consortium	Jacquelyn Wynn, Mairead Hyland, The Lighthouse Lab in Alderley Park and Alex Alderton, Roberto Amato, Sonia Goncalves, Ewan Harrison, David K. Jackson, Ian Johnston, Dominic Kwiatkowski, Cordelia Langford, John Sillitoe on behalf of the Wellcome Sanger Institute COVID-19 Surveillance Team
EPI_ISL_718756, EPI_ISL_718757, EPI_ISL_718758	Lighthouse Lab in Cambridge	Wellcome Sanger Institute for the COVID-19 Genomics UK (COG-UK) Consortium	Rob Howes, The Lighthouse Lab in Cambridge and Alex Alderton, Roberto Amato, Sonia Goncalves, Ewan Harrison, David K. Jackson, Ian Johnston, Dominic Kwiatkowski, Cordelia Langford, John Sillitoe on behalf of the Wellcome Sanger Institute COVID-19 Surveillance Team
EPI_ISL_718759	Lighthouse Lab in Alderley Park	Wellcome Sanger Institute for the COVID-19 Genomics UK (COG-UK) Consortium	Jacquelyn Wynn, Mairead Hyland, The Lighthouse Lab in Alderley Park and Alex Alderton, Roberto Amato, Sonia Goncalves, Ewan Harrison, David K. Jackson, Ian Johnston, Dominic Kwiatkowski, Cordelia Langford, John Sillitoe on behalf of the Wellcome Sanger Institute COVID-19 Surveillance Team
EPI_ISL_718760	Lighthouse Lab in Cambridge	Wellcome Sanger Institute for the COVID-19 Genomics UK (COG-UK) Consortium	Rob Howes, The Lighthouse Lab in Cambridge and Alex Alderton, Roberto Amato, Sonia Goncalves, Ewan Harrison, David K. Jackson, Ian Johnston, Dominic Kwiatkowski, Cordelia Langford, John Sillitoe on behalf of the Wellcome Sanger Institute COVID-19 Surveillance Team
EPI_ISL_718761, EPI_ISL_718762	Lighthouse Lab in Alderley Park	Wellcome Sanger Institute for the COVID-19 Genomics UK (COG-UK) Consortium	Jacquelyn Wynn, Mairead Hyland, The Lighthouse Lab in Alderley Park and Alex Alderton, Roberto Amato, Sonia Goncalves, Ewan Harrison, David K. Jackson, Ian Johnston, Dominic Kwiatkowski, Cordelia Langford, John Sillitoe on behalf of the Wellcome Sanger Institute COVID-19 Surveillance Team
EPI_ISL_718763	Lighthouse Lab in Cambridge	Wellcome Sanger Institute for the COVID-19 Genomics UK (COG-UK) Consortium	Rob Howes, The Lighthouse Lab in Cambridge and Alex Alderton, Roberto Amato, Sonia Goncalves, Ewan Harrison, David K. Jackson, Ian Johnston, Dominic Kwiatkowski, Cordelia Langford, John Sillitoe on behalf of the Wellcome Sanger Institute COVID-19 Surveillance Team
EPI_ISL_718764, EPI_ISL_718765, EPI_ISL_718766, EPI_ISL_718767	Lighthouse Lab in Alderley Park	Wellcome Sanger Institute for the COVID-19 Genomics UK (COG-UK) Consortium	Jacquelyn Wynn, Mairead Hyland, The Lighthouse Lab in Alderley Park and Alex Alderton, Roberto Amato, Sonia Goncalves, Ewan Harrison, David K. Jackson, Ian Johnston, Dominic Kwiatkowski, Cordelia Langford, John Sillitoe on behalf of the Wellcome Sanger Institute COVID-19 Surveillance Team
EPI_ISL_718768	Lighthouse Lab in Cambridge	Wellcome Sanger Institute for the COVID-19 Genomics UK (COG-UK) Consortium	Rob Howes, The Lighthouse Lab in Cambridge and Alex Alderton, Roberto Amato, Sonia Goncalves, Ewan Harrison, David K. Jackson, Ian Johnston, Dominic Kwiatkowski, Cordelia Langford, John Sillitoe on behalf of the Wellcome Sanger Institute COVID-19 Surveillance Team
EPI_ISL_718769	Lighthouse Lab in Alderley Park	Wellcome Sanger Institute for the COVID-19 Genomics UK (COG-UK) Consortium	Jacquelyn Wynn, Mairead Hyland, The Lighthouse Lab in Alderley Park and Alex Alderton, Roberto Amato, Sonia Goncalves, Ewan Harrison, David K. Jackson, Ian Johnston, Dominic Kwiatkowski, Cordelia Langford, John Sillitoe on behalf of the Wellcome Sanger Institute COVID-19 Surveillance Team
EPI_ISL_718770, EPI_ISL_718771	Lighthouse Lab in Cambridge	Wellcome Sanger Institute for the COVID-19 Genomics UK (COG-UK) Consortium	Rob Howes, The Lighthouse Lab in Cambridge and Alex Alderton, Roberto Amato, Sonia Goncalves, Ewan Harrison, David K. Jackson, Ian Johnston, Dominic Kwiatkowski, Cordelia Langford, John Sillitoe on behalf of the Wellcome Sanger Institute COVID-19 Surveillance Team
EPI_ISL_718772, EPI_ISL_718773, EPI_ISL_718774	Lighthouse Lab in Alderley Park	Wellcome Sanger Institute for the COVID-19 Genomics UK (COG-UK) Consortium	Jacquelyn Wynn, Mairead Hyland, The Lighthouse Lab in Alderley Park and Alex Alderton, Roberto Amato, Sonia Goncalves, Ewan Harrison, David K. Jackson, Ian Johnston, Dominic Kwiatkowski, Cordelia Langford, John Sillitoe on behalf of the Wellcome Sanger Institute COVID-19 Surveillance Team
EPI_ISL_718775, EPI_ISL_718776	Lighthouse Lab in Cambridge	Wellcome Sanger Institute for the COVID-19 Genomics UK (COG-UK) Consortium	Rob Howes, The Lighthouse Lab in Cambridge and Alex Alderton, Roberto Amato, Sonia Goncalves, Ewan Harrison, David K. Jackson, Ian Johnston, Dominic Kwiatkowski, Cordelia Langford, John Sillitoe on behalf of the Wellcome Sanger Institute COVID-19 Surveillance Team
EPI_ISL_718777	Lighthouse Lab in Alderley Park	Wellcome Sanger Institute for the COVID-19 Genomics UK (COG-UK) Consortium	Jacquelyn Wynn, Mairead Hyland, The Lighthouse Lab in Alderley Park and Alex Alderton, Roberto Amato, Sonia Goncalves, Ewan Harrison, David K. Jackson, Ian Johnston, Dominic Kwiatkowski, Cordelia Langford, John Sillitoe on behalf of the Wellcome Sanger Institute COVID-19 Surveillance Team
EPI_ISL_718778	Lighthouse Lab in Cambridge	Wellcome Sanger Institute for the COVID-19 Genomics UK (COG-UK) Consortium	Rob Howes, The Lighthouse Lab in Cambridge and Alex Alderton, Roberto Amato, Sonia Goncalves, Ewan Harrison, David K. Jackson, Ian Johnston, Dominic Kwiatkowski, Cordelia Langford, John Sillitoe on behalf of the Wellcome Sanger Institute COVID-19 Surveillance Team
EPI_ISL_718779, EPI_ISL_718780, EPI_ISL_718781, EPI_ISL_718782, EPI_ISL_718783	Lighthouse Lab in Alderley Park	Wellcome Sanger Institute for the COVID-19 Genomics UK (COG-UK) Consortium	Jacquelyn Wynn, Mairead Hyland, The Lighthouse Lab in Alderley Park and Alex Alderton, Roberto Amato, Sonia Goncalves, Ewan Harrison, David K. Jackson, Ian Johnston, Dominic Kwiatkowski, Cordelia Langford, John Sillitoe on behalf of the Wellcome Sanger Institute COVID-19 Surveillance Team
EPI_ISL_718784, EPI_ISL_718785, EPI_ISL_718786	Lighthouse Lab in Cambridge	Wellcome Sanger Institute for the COVID-19 Genomics UK (COG-UK) Consortium	Rob Howes, The Lighthouse Lab in Cambridge and Alex Alderton, Roberto Amato, Sonia Goncalves, Ewan Harrison, David K. Jackson, Ian Johnston, Dominic Kwiatkowski, Cordelia Langford, John Sillitoe on behalf of the Wellcome Sanger Institute COVID-19 Surveillance Team
EPI_ISL_718787	Lighthouse Lab in Alderley Park	Wellcome Sanger Institute for the COVID-19 Genomics UK (COG-UK) Consortium	Jacquelyn Wynn, Mairead Hyland, The Lighthouse Lab in Alderley Park and Alex Alderton, Roberto Amato, Sonia Goncalves, Ewan Harrison, David K. Jackson, Ian Johnston, Dominic Kwiatkowski, Cordelia Langford, John Sillitoe on behalf of the Wellcome Sanger Institute COVID-19 Surveillance Team
EPI_ISL_718788	Lighthouse Lab in Cambridge	Wellcome Sanger Institute for the COVID-19 Genomics UK (COG-UK) Consortium	Rob Howes, The Lighthouse Lab in Cambridge and Alex Alderton, Roberto Amato, Sonia Goncalves, Ewan Harrison, David K. Jackson, Ian Johnston, Dominic Kwiatkowski, Cordelia Langford, John Sillitoe on behalf of the Wellcome Sanger Institute COVID-19 Surveillance Team
EPI_ISL_718789	Lighthouse Lab in Alderley Park	Wellcome Sanger Institute for the COVID-19 Genomics UK (COG-UK) Consortium	Jacquelyn Wynn, Mairead Hyland, The Lighthouse Lab in Alderley Park and Alex Alderton, Roberto Amato, Sonia Goncalves, Ewan Harrison, David K. Jackson, Ian Johnston, Dominic Kwiatkowski, Cordelia Langford, John Sillitoe on behalf of the Wellcome Sanger Institute COVID-19 Surveillance Team
EPI_ISL_718790	Lighthouse Lab in Cambridge	Wellcome Sanger Institute for the COVID-19 Genomics UK (COG-UK) Consortium	Rob Howes, The Lighthouse Lab in Cambridge and Alex Alderton, Roberto Amato, Sonia Goncalves, Ewan Harrison, David K. Jackson, Ian Johnston, Dominic Kwiatkowski, Cordelia Langford, John Sillitoe on behalf of the Wellcome Sanger Institute COVID-19 Surveillance Team
EPI_ISL_718791	Lighthouse Lab in Alderley Park	Wellcome Sanger Institute for the COVID-19 Genomics UK (COG-UK) Consortium	Jacquelyn Wynn, Mairead Hyland, The Lighthouse Lab in Alderley Park and Alex Alderton, Roberto Amato, Sonia Goncalves, Ewan Harrison, David K. Jackson, Ian Johnston, Dominic Kwiatkowski, Cordelia Langford, John Sillitoe on behalf of the Wellcome Sanger Institute COVID-19 Surveillance Team
EPI_ISL_718792, EPI_ISL_718793, EPI_ISL_718794	Lighthouse Lab in Cambridge	Wellcome Sanger Institute for the COVID-19 Genomics UK (COG-UK) Consortium	Rob Howes, The Lighthouse Lab in Cambridge and Alex Alderton, Roberto Amato, Sonia Goncalves, Ewan Harrison, David K. Jackson, Ian Johnston, Dominic Kwiatkowski, Cordelia Langford, John Sillitoe on behalf of the Wellcome Sanger Institute COVID-19 Surveillance Team
EPI_ISL_718795, EPI_ISL_718796	Lighthouse Lab in Alderley Park	Wellcome Sanger Institute for the COVID-19 Genomics UK (COG-UK) Consortium	Jacquelyn Wynn, Mairead Hyland, The Lighthouse Lab in Alderley Park and Alex Alderton, Roberto Amato, Sonia Goncalves, Ewan Harrison, David K. Jackson, Ian Johnston, Dominic Kwiatkowski, Cordelia Langford, John Sillitoe on behalf of the Wellcome Sanger Institute COVID-19 Surveillance Team

EPI_ISL_718797	Lighthouse Lab in Cambridge	Wellcome Sanger Institute for the COVID-19 Genomics UK (COG-UK) Consortium	Rob Howes, The Lighthouse Lab in Cambridge and Alex Alderton, Roberto Amato, Sonia Goncalves, Ewan Harrison, David K. Jackson, Ian Johnston, Dominic Kwiatkowski, Cordelia Langford, John Sillitoe on behalf of the Wellcome Sanger Institute COVID-19 Surveillance Team
EPI_ISL_718798, EPI_ISL_718799	Lighthouse Lab in Alderley Park	Wellcome Sanger Institute for the COVID-19 Genomics UK (COG-UK) Consortium	Jacquelyn Wynn, Mairead Hyland, The Lighthouse Lab in Alderley Park and Alex Alderton, Roberto Amato, Sonia Goncalves, Ewan Harrison, David K. Jackson, Ian Johnston, Dominic Kwiatkowski, Cordelia Langford, John Sillitoe on behalf of the Wellcome Sanger Institute COVID-19 Surveillance Team
EPI_ISL_718800	Lighthouse Lab in Cambridge	Wellcome Sanger Institute for the COVID-19 Genomics UK (COG-UK) Consortium	Rob Howes, The Lighthouse Lab in Cambridge and Alex Alderton, Roberto Amato, Sonia Goncalves, Ewan Harrison, David K. Jackson, Ian Johnston, Dominic Kwiatkowski, Cordelia Langford, John Sillitoe on behalf of the Wellcome Sanger Institute COVID-19 Surveillance Team
EPI_ISL_718801, EPI_ISL_718802, EPI_ISL_718803	Lighthouse Lab in Alderley Park	Wellcome Sanger Institute for the COVID-19 Genomics UK (COG-UK) Consortium	Jacquelyn Wynn, Mairead Hyland, The Lighthouse Lab in Alderley Park and Alex Alderton, Roberto Amato, Sonia Goncalves, Ewan Harrison, David K. Jackson, Ian Johnston, Dominic Kwiatkowski, Cordelia Langford, John Sillitoe on behalf of the Wellcome Sanger Institute COVID-19 Surveillance Team
EPI_ISL_718804, EPI_ISL_718805	Lighthouse Lab in Cambridge	Wellcome Sanger Institute for the COVID-19 Genomics UK (COG-UK) Consortium	Rob Howes, The Lighthouse Lab in Cambridge and Alex Alderton, Roberto Amato, Sonia Goncalves, Ewan Harrison, David K. Jackson, Ian Johnston, Dominic Kwiatkowski, Cordelia Langford, John Sillitoe on behalf of the Wellcome Sanger Institute COVID-19 Surveillance Team
EPI_ISL_718806, EPI_ISL_718807, EPI_ISL_718808	Lighthouse Lab in Alderley Park	Wellcome Sanger Institute for the COVID-19 Genomics UK (COG-UK) Consortium	Jacquelyn Wynn, Mairead Hyland, The Lighthouse Lab in Alderley Park and Alex Alderton, Roberto Amato, Sonia Goncalves, Ewan Harrison, David K. Jackson, Ian Johnston, Dominic Kwiatkowski, Cordelia Langford, John Sillitoe on behalf of the Wellcome Sanger Institute COVID-19 Surveillance Team
EPI_ISL_718809, EPI_ISL_718810	Lighthouse Lab in Cambridge	Wellcome Sanger Institute for the COVID-19 Genomics UK (COG-UK) Consortium	Rob Howes, The Lighthouse Lab in Cambridge and Alex Alderton, Roberto Amato, Sonia Goncalves, Ewan Harrison, David K. Jackson, Ian Johnston, Dominic Kwiatkowski, Cordelia Langford, John Sillitoe on behalf of the Wellcome Sanger Institute COVID-19 Surveillance Team
EPI_ISL_718811, EPI_ISL_718812, EPI_ISL_718813	Lighthouse Lab in Alderley Park	Wellcome Sanger Institute for the COVID-19 Genomics UK (COG-UK) Consortium	Jacquelyn Wynn, Mairead Hyland, The Lighthouse Lab in Alderley Park and Alex Alderton, Roberto Amato, Sonia Goncalves, Ewan Harrison, David K. Jackson, Ian Johnston, Dominic Kwiatkowski, Cordelia Langford, John Sillitoe on behalf of the Wellcome Sanger Institute COVID-19 Surveillance Team
EPI_ISL_718814, EPI_ISL_718815, EPI_ISL_718816, EPI_ISL_718817, EPI_ISL_718818	Lighthouse Lab in Cambridge	Wellcome Sanger Institute for the COVID-19 Genomics UK (COG-UK) Consortium	Rob Howes, The Lighthouse Lab in Cambridge and Alex Alderton, Roberto Amato, Sonia Goncalves, Ewan Harrison, David K. Jackson, Ian Johnston, Dominic Kwiatkowski, Cordelia Langford, John Sillitoe on behalf of the Wellcome Sanger Institute COVID-19 Surveillance Team
EPI_ISL_718819	Lighthouse Lab in Alderley Park	Wellcome Sanger Institute for the COVID-19 Genomics UK (COG-UK) Consortium	Jacquelyn Wynn, Mairead Hyland, The Lighthouse Lab in Alderley Park and Alex Alderton, Roberto Amato, Sonia Goncalves, Ewan Harrison, David K. Jackson, Ian Johnston, Dominic Kwiatkowski, Cordelia Langford, John Sillitoe on behalf of the Wellcome Sanger Institute COVID-19 Surveillance Team
EPI_ISL_718820	Lighthouse Lab in Cambridge	Wellcome Sanger Institute for the COVID-19 Genomics UK (COG-UK) Consortium	Rob Howes, The Lighthouse Lab in Cambridge and Alex Alderton, Roberto Amato, Sonia Goncalves, Ewan Harrison, David K. Jackson, Ian Johnston, Dominic Kwiatkowski, Cordelia Langford, John Sillitoe on behalf of the Wellcome Sanger Institute COVID-19 Surveillance Team
EPI_ISL_718821	Lighthouse Lab in Alderley Park	Wellcome Sanger Institute for the COVID-19 Genomics UK (COG-UK) Consortium	Jacquelyn Wynn, Mairead Hyland, The Lighthouse Lab in Alderley Park and Alex Alderton, Roberto Amato, Sonia Goncalves, Ewan Harrison, David K. Jackson, Ian Johnston, Dominic Kwiatkowski, Cordelia Langford, John Sillitoe on behalf of the Wellcome Sanger Institute COVID-19 Surveillance Team
EPI_ISL_718822	Lighthouse Lab in Cambridge	Wellcome Sanger Institute for the COVID-19 Genomics UK (COG-UK) Consortium	Rob Howes, The Lighthouse Lab in Cambridge and Alex Alderton, Roberto Amato, Sonia Goncalves, Ewan Harrison, David K. Jackson, Ian Johnston, Dominic Kwiatkowski, Cordelia Langford, John Sillitoe on behalf of the Wellcome Sanger Institute COVID-19 Surveillance Team
EPI_ISL_718823	Lighthouse Lab in Alderley Park	Wellcome Sanger Institute for the COVID-19 Genomics UK (COG-UK) Consortium	Jacquelyn Wynn, Mairead Hyland, The Lighthouse Lab in Alderley Park and Alex Alderton, Roberto Amato, Sonia Goncalves, Ewan Harrison, David K. Jackson, Ian Johnston, Dominic Kwiatkowski, Cordelia Langford, John Sillitoe on behalf of the Wellcome Sanger Institute COVID-19 Surveillance Team
EPI_ISL_718824, EPI_ISL_718825, EPI_ISL_718826	Lighthouse Lab in Cambridge	Wellcome Sanger Institute for the COVID-19 Genomics UK (COG-UK) Consortium	Rob Howes, The Lighthouse Lab in Cambridge and Alex Alderton, Roberto Amato, Sonia Goncalves, Ewan Harrison, David K. Jackson, Ian Johnston, Dominic Kwiatkowski, Cordelia Langford, John Sillitoe on behalf of the Wellcome Sanger Institute COVID-19 Surveillance Team
EPI_ISL_718827, EPI_ISL_718828, EPI_ISL_718829, EPI_ISL_718830, EPI_ISL_718831, EPI_ISL_718832, EPI_ISL_718833	Lighthouse Lab in Alderley Park	Wellcome Sanger Institute for the COVID-19 Genomics UK (COG-UK) Consortium	Jacquelyn Wynn, Mairead Hyland, The Lighthouse Lab in Alderley Park and Alex Alderton, Roberto Amato, Sonia Goncalves, Ewan Harrison, David K. Jackson, Ian Johnston, Dominic Kwiatkowski, Cordelia Langford, John Sillitoe on behalf of the Wellcome Sanger Institute COVID-19 Surveillance Team
EPI_ISL_718834, EPI_ISL_718835, EPI_ISL_718836	Lighthouse Lab in Cambridge	Wellcome Sanger Institute for the COVID-19 Genomics UK (COG-UK) Consortium	Rob Howes, The Lighthouse Lab in Cambridge and Alex Alderton, Roberto Amato, Sonia Goncalves, Ewan Harrison, David K. Jackson, Ian Johnston, Dominic Kwiatkowski, Cordelia Langford, John Sillitoe on behalf of the Wellcome Sanger Institute COVID-19 Surveillance Team
EPI_ISL_718837, EPI_ISL_718838, EPI_ISL_718839	Lighthouse Lab in Alderley Park	Wellcome Sanger Institute for the COVID-19 Genomics UK (COG-UK) Consortium	Jacquelyn Wynn, Mairead Hyland, The Lighthouse Lab in Alderley Park and Alex Alderton, Roberto Amato, Sonia Goncalves, Ewan Harrison, David K. Jackson, Ian Johnston, Dominic Kwiatkowski, Cordelia Langford, John Sillitoe on behalf of the Wellcome Sanger Institute COVID-19 Surveillance Team
EPI_ISL_718840	Lighthouse Lab in Cambridge	Wellcome Sanger Institute for the COVID-19 Genomics UK (COG-UK) Consortium	Rob Howes, The Lighthouse Lab in Cambridge and Alex Alderton, Roberto Amato, Sonia Goncalves, Ewan Harrison, David K. Jackson, Ian Johnston, Dominic Kwiatkowski, Cordelia Langford, John Sillitoe on behalf of the Wellcome Sanger Institute COVID-19 Surveillance Team
EPI_ISL_718841	Lighthouse Lab in Alderley Park	Wellcome Sanger Institute for the COVID-19 Genomics UK (COG-UK) Consortium	Jacquelyn Wynn, Mairead Hyland, The Lighthouse Lab in Alderley Park and Alex Alderton, Roberto Amato, Sonia Goncalves, Ewan Harrison, David K. Jackson, Ian Johnston, Dominic Kwiatkowski, Cordelia Langford, John Sillitoe on behalf of the Wellcome Sanger Institute COVID-19 Surveillance Team
EPI_ISL_718842, EPI_ISL_718843	Lighthouse Lab in Cambridge	Wellcome Sanger Institute for the COVID-19 Genomics UK (COG-UK) Consortium	Rob Howes, The Lighthouse Lab in Cambridge and Alex Alderton, Roberto Amato, Sonia Goncalves, Ewan Harrison, David K. Jackson, Ian Johnston, Dominic Kwiatkowski, Cordelia Langford, John Sillitoe on behalf of the Wellcome Sanger Institute COVID-19 Surveillance Team
EPI_ISL_718844	Lighthouse Lab in Alderley Park	Wellcome Sanger Institute for the COVID-19 Genomics UK (COG-UK) Consortium	Jacquelyn Wynn, Mairead Hyland, The Lighthouse Lab in Alderley Park and Alex Alderton, Roberto Amato, Sonia Goncalves, Ewan Harrison, David K. Jackson, Ian Johnston, Dominic Kwiatkowski, Cordelia Langford, John Sillitoe on behalf of the Wellcome Sanger Institute COVID-19 Surveillance Team
EPI_ISL_718845, EPI_ISL_718846	Lighthouse Lab in Cambridge	Wellcome Sanger Institute for the COVID-19 Genomics UK (COG-UK) Consortium	Rob Howes, The Lighthouse Lab in Cambridge and Alex Alderton, Roberto Amato, Sonia Goncalves, Ewan Harrison, David K. Jackson, Ian Johnston, Dominic Kwiatkowski, Cordelia Langford, John Sillitoe on behalf of the Wellcome Sanger Institute COVID-19 Surveillance Team
EPI_ISL_718847	Lighthouse Lab in Alderley Park	Wellcome Sanger Institute for the COVID-19 Genomics UK (COG-UK) Consortium	Jacquelyn Wynn, Mairead Hyland, The Lighthouse Lab in Alderley Park and Alex Alderton, Roberto Amato, Sonia Goncalves, Ewan Harrison, David K. Jackson, Ian Johnston, Dominic Kwiatkowski, Cordelia Langford, John Sillitoe on behalf of the Wellcome Sanger Institute COVID-19 Surveillance Team
EPI_ISL_718848	Lighthouse Lab in Cambridge	Wellcome Sanger Institute for the COVID-19 Genomics UK (COG-UK) Consortium	Rob Howes, The Lighthouse Lab in Cambridge and Alex Alderton, Roberto Amato, Sonia Goncalves, Ewan Harrison, David K. Jackson, Ian Johnston, Dominic Kwiatkowski, Cordelia Langford, John Sillitoe on behalf of the Wellcome Sanger Institute COVID-19 Surveillance Team
EPI_ISL_718849, EPI_ISL_718850	Lighthouse Lab in Alderley Park	Wellcome Sanger Institute for the COVID-19 Genomics UK (COG-UK) Consortium	Jacquelyn Wynn, Mairead Hyland, The Lighthouse Lab in Alderley Park and Alex Alderton, Roberto Amato, Sonia Goncalves, Ewan Harrison, David K. Jackson, Ian Johnston, Dominic Kwiatkowski, Cordelia Langford, John Sillitoe on behalf of the Wellcome Sanger Institute COVID-19 Surveillance Team
EPI_ISL_718851, EPI_ISL_718852	Lighthouse Lab in Cambridge	Wellcome Sanger Institute for the COVID-19 Genomics UK (COG-UK) Consortium	Rob Howes, The Lighthouse Lab in Cambridge and Alex Alderton, Roberto Amato, Sonia Goncalves, Ewan Harrison, David K. Jackson, Ian Johnston, Dominic Kwiatkowski, Cordelia Langford, John Sillitoe on behalf of the Wellcome Sanger Institute COVID-19 Surveillance Team
EPI_ISL_718853, EPI_ISL_718854	Lighthouse Lab in Alderley Park	Wellcome Sanger Institute for the COVID-19 Genomics UK (COG-UK) Consortium	Jacquelyn Wynn, Mairead Hyland, The Lighthouse Lab in Alderley Park and Alex Alderton, Roberto Amato, Sonia Goncalves, Ewan Harrison, David K. Jackson, Ian Johnston, Dominic Kwiatkowski, Cordelia Langford, John Sillitoe on behalf of the Wellcome Sanger Institute COVID-19 Surveillance Team
EPI_ISL_718855, EPI_ISL_718856, EPI_ISL_718857	Lighthouse Lab in Cambridge	Wellcome Sanger Institute for the COVID-19 Genomics UK (COG-UK) Consortium	Rob Howes, The Lighthouse Lab in Cambridge and Alex Alderton, Roberto Amato, Sonia Goncalves, Ewan Harrison, David K. Jackson, Ian Johnston, Dominic Kwiatkowski, Cordelia Langford, John Sillitoe on behalf of the Wellcome Sanger Institute COVID-19 Surveillance Team
EPI_ISL_718858, EPI_ISL_718859	Lighthouse Lab in Alderley Park	Wellcome Sanger Institute for the COVID-19 Genomics UK (COG-UK) Consortium	Jacquelyn Wynn, Mairead Hyland, The Lighthouse Lab in Alderley Park and Alex Alderton, Roberto Amato, Sonia Goncalves, Ewan Harrison, David K. Jackson, Ian Johnston, Dominic Kwiatkowski, Cordelia Langford, John Sillitoe on behalf of the Wellcome Sanger Institute COVID-19 Surveillance Team
EPI_ISL_718860, EPI_ISL_718861, EPI_ISL_718862, EPI_ISL_718863	Lighthouse Lab in Cambridge	Wellcome Sanger Institute for the COVID-19 Genomics UK (COG-UK) Consortium	Rob Howes, The Lighthouse Lab in Cambridge and Alex Alderton, Roberto Amato, Sonia Goncalves, Ewan Harrison, David K. Jackson, Ian Johnston, Dominic Kwiatkowski, Cordelia Langford, John Sillitoe on behalf of the Wellcome Sanger Institute COVID-19 Surveillance Team
EPI_ISL_718864, EPI_ISL_718865, EPI_ISL_718866	Lighthouse Lab in Alderley Park	Wellcome Sanger Institute for the COVID-19 Genomics UK (COG-UK) Consortium	Jacquelyn Wynn, Mairead Hyland, The Lighthouse Lab in Alderley Park and Alex Alderton, Roberto Amato, Sonia Goncalves, Ewan Harrison, David K. Jackson, Ian Johnston, Dominic Kwiatkowski, Cordelia Langford, John Sillitoe on behalf of the Wellcome Sanger Institute COVID-19 Surveillance Team
EPI_ISL_718867, EPI_ISL_718868, EPI_ISL_718869	Lighthouse Lab in Cambridge	Wellcome Sanger Institute for the COVID-19 Genomics UK (COG-UK) Consortium	Rob Howes, The Lighthouse Lab in Cambridge and Alex Alderton, Roberto Amato, Sonia Goncalves, Ewan Harrison, David K. Jackson, Ian Johnston, Dominic Kwiatkowski, Cordelia Langford, John Sillitoe on behalf of the Wellcome Sanger Institute COVID-19 Surveillance Team
EPI_ISL_718870, EPI_ISL_718871, EPI_ISL_718872, EPI_ISL_718873	Lighthouse Lab in Alderley Park	Wellcome Sanger Institute for the COVID-19 Genomics UK (COG-UK) Consortium	Jacquelyn Wynn, Mairead Hyland, The Lighthouse Lab in Alderley Park and Alex Alderton, Roberto Amato, Sonia Goncalves, Ewan Harrison, David K. Jackson, Ian Johnston, Dominic Kwiatkowski, Cordelia Langford, John Sillitoe on behalf of the Wellcome Sanger Institute COVID-19 Surveillance Team

EPI_ISL_718874, EPI_ISL_718875, EPI_ISL_718876	Lighthouse Lab in Cambridge	Wellcome Sanger Institute for the COVID-19 Genomics UK (COG-UK) Consortium	Rob Howes, The Lighthouse Lab in Cambridge and Alex Alderton, Roberto Amato, Sonia Goncalves, Ewan Harrison, David K. Jackson, Ian Johnston, Dominic Kwiatkowski, Cordelia Langford, John Sillitoe on behalf of the Wellcome Sanger Institute COVID-19 Surveillance Team
EPI_ISL_718877, EPI_ISL_718878	Lighthouse Lab in Alderley Park	Wellcome Sanger Institute for the COVID-19 Genomics UK (COG-UK) Consortium	Jacquelyn Wynn, Mairead Hyland, The Lighthouse Lab in Alderley Park and Alex Alderton, Roberto Amato, Sonia Goncalves, Ewan Harrison, David K. Jackson, Ian Johnston, Dominic Kwiatkowski, Cordelia Langford, John Sillitoe on behalf of the Wellcome Sanger Institute COVID-19 Surveillance Team
EPI_ISL_718879	Lighthouse Lab in Cambridge	Wellcome Sanger Institute for the COVID-19 Genomics UK (COG-UK) Consortium	Rob Howes, The Lighthouse Lab in Cambridge and Alex Alderton, Roberto Amato, Sonia Goncalves, Ewan Harrison, David K. Jackson, Ian Johnston, Dominic Kwiatkowski, Cordelia Langford, John Sillitoe on behalf of the Wellcome Sanger Institute COVID-19 Surveillance Team
EPI_ISL_718880, EPI_ISL_718881	Lighthouse Lab in Alderley Park	Wellcome Sanger Institute for the COVID-19 Genomics UK (COG-UK) Consortium	Jacquelyn Wynn, Mairead Hyland, The Lighthouse Lab in Alderley Park and Alex Alderton, Roberto Amato, Sonia Goncalves, Ewan Harrison, David K. Jackson, Ian Johnston, Dominic Kwiatkowski, Cordelia Langford, John Sillitoe on behalf of the Wellcome Sanger Institute COVID-19 Surveillance Team
EPI_ISL_718882, EPI_ISL_718883, EPI_ISL_718884	Lighthouse Lab in Cambridge	Wellcome Sanger Institute for the COVID-19 Genomics UK (COG-UK) Consortium	Rob Howes, The Lighthouse Lab in Cambridge and Alex Alderton, Roberto Amato, Sonia Goncalves, Ewan Harrison, David K. Jackson, Ian Johnston, Dominic Kwiatkowski, Cordelia Langford, John Sillitoe on behalf of the Wellcome Sanger Institute COVID-19 Surveillance Team
EPI_ISL_718885, EPI_ISL_718886, EPI_ISL_718887, EPI_ISL_718888, EPI_ISL_718889, EPI_ISL_718890, EPI_ISL_718891	Lighthouse Lab in Alderley Park	Wellcome Sanger Institute for the COVID-19 Genomics UK (COG-UK) Consortium	Jacquelyn Wynn, Mairead Hyland, The Lighthouse Lab in Alderley Park and Alex Alderton, Roberto Amato, Sonia Goncalves, Ewan Harrison, David K. Jackson, Ian Johnston, Dominic Kwiatkowski, Cordelia Langford, John Sillitoe on behalf of the Wellcome Sanger Institute COVID-19 Surveillance Team
EPI_ISL_718892, EPI_ISL_718893	Lighthouse Lab in Cambridge	Wellcome Sanger Institute for the COVID-19 Genomics UK (COG-UK) Consortium	Rob Howes, The Lighthouse Lab in Cambridge and Alex Alderton, Roberto Amato, Sonia Goncalves, Ewan Harrison, David K. Jackson, Ian Johnston, Dominic Kwiatkowski, Cordelia Langford, John Sillitoe on behalf of the Wellcome Sanger Institute COVID-19 Surveillance Team
EPI_ISL_718894, EPI_ISL_718895	Lighthouse Lab in Alderley Park	Wellcome Sanger Institute for the COVID-19 Genomics UK (COG-UK) Consortium	Jacquelyn Wynn, Mairead Hyland, The Lighthouse Lab in Alderley Park and Alex Alderton, Roberto Amato, Sonia Goncalves, Ewan Harrison, David K. Jackson, Ian Johnston, Dominic Kwiatkowski, Cordelia Langford, John Sillitoe on behalf of the Wellcome Sanger Institute COVID-19 Surveillance Team
EPI_ISL_718896, EPI_ISL_718897, EPI_ISL_718898, EPI_ISL_718899, EPI_ISL_718900	Lighthouse Lab in Cambridge	Wellcome Sanger Institute for the COVID-19 Genomics UK (COG-UK) Consortium	Rob Howes, The Lighthouse Lab in Cambridge and Alex Alderton, Roberto Amato, Sonia Goncalves, Ewan Harrison, David K. Jackson, Ian Johnston, Dominic Kwiatkowski, Cordelia Langford, John Sillitoe on behalf of the Wellcome Sanger Institute COVID-19 Surveillance Team
EPI_ISL_718901, EPI_ISL_718902, EPI_ISL_718903	Lighthouse Lab in Alderley Park	Wellcome Sanger Institute for the COVID-19 Genomics UK (COG-UK) Consortium	Jacquelyn Wynn, Mairead Hyland, The Lighthouse Lab in Alderley Park and Alex Alderton, Roberto Amato, Sonia Goncalves, Ewan Harrison, David K. Jackson, Ian Johnston, Dominic Kwiatkowski, Cordelia Langford, John Sillitoe on behalf of the Wellcome Sanger Institute COVID-19 Surveillance Team
EPI_ISL_718904, EPI_ISL_718905, EPI_ISL_718906	Lighthouse Lab in Cambridge	Wellcome Sanger Institute for the COVID-19 Genomics UK (COG-UK) Consortium	Rob Howes, The Lighthouse Lab in Cambridge and Alex Alderton, Roberto Amato, Sonia Goncalves, Ewan Harrison, David K. Jackson, Ian Johnston, Dominic Kwiatkowski, Cordelia Langford, John Sillitoe on behalf of the Wellcome Sanger Institute COVID-19 Surveillance Team
EPI_ISL_718907, EPI_ISL_718908	Lighthouse Lab in Alderley Park	Wellcome Sanger Institute for the COVID-19 Genomics UK (COG-UK) Consortium	Jacquelyn Wynn, Mairead Hyland, The Lighthouse Lab in Alderley Park and Alex Alderton, Roberto Amato, Sonia Goncalves, Ewan Harrison, David K. Jackson, Ian Johnston, Dominic Kwiatkowski, Cordelia Langford, John Sillitoe on behalf of the Wellcome Sanger Institute COVID-19 Surveillance Team
EPI_ISL_718909, EPI_ISL_718910	Lighthouse Lab in Cambridge	Wellcome Sanger Institute for the COVID-19 Genomics UK (COG-UK) Consortium	Rob Howes, The Lighthouse Lab in Cambridge and Alex Alderton, Roberto Amato, Sonia Goncalves, Ewan Harrison, David K. Jackson, Ian Johnston, Dominic Kwiatkowski, Cordelia Langford, John Sillitoe on behalf of the Wellcome Sanger Institute COVID-19 Surveillance Team
EPI_ISL_718911	Lighthouse Lab in Alderley Park	Wellcome Sanger Institute for the COVID-19 Genomics UK (COG-UK) Consortium	Jacquelyn Wynn, Mairead Hyland, The Lighthouse Lab in Alderley Park and Alex Alderton, Roberto Amato, Sonia Goncalves, Ewan Harrison, David K. Jackson, Ian Johnston, Dominic Kwiatkowski, Cordelia Langford, John Sillitoe on behalf of the Wellcome Sanger Institute COVID-19 Surveillance Team
EPI_ISL_718912, EPI_ISL_718913	Lighthouse Lab in Cambridge	Wellcome Sanger Institute for the COVID-19 Genomics UK (COG-UK) Consortium	Rob Howes, The Lighthouse Lab in Cambridge and Alex Alderton, Roberto Amato, Sonia Goncalves, Ewan Harrison, David K. Jackson, Ian Johnston, Dominic Kwiatkowski, Cordelia Langford, John Sillitoe on behalf of the Wellcome Sanger Institute COVID-19 Surveillance Team
EPI_ISL_718914, EPI_ISL_718915	Lighthouse Lab in Alderley Park	Wellcome Sanger Institute for the COVID-19 Genomics UK (COG-UK) Consortium	Jacquelyn Wynn, Mairead Hyland, The Lighthouse Lab in Alderley Park and Alex Alderton, Roberto Amato, Sonia Goncalves, Ewan Harrison, David K. Jackson, Ian Johnston, Dominic Kwiatkowski, Cordelia Langford, John Sillitoe on behalf of the Wellcome Sanger Institute COVID-19 Surveillance Team
EPI_ISL_718916	Lighthouse Lab in Cambridge	Wellcome Sanger Institute for the COVID-19 Genomics UK (COG-UK) Consortium	Rob Howes, The Lighthouse Lab in Cambridge and Alex Alderton, Roberto Amato, Sonia Goncalves, Ewan Harrison, David K. Jackson, Ian Johnston, Dominic Kwiatkowski, Cordelia Langford, John Sillitoe on behalf of the Wellcome Sanger Institute COVID-19 Surveillance Team
EPI_ISL_718917	Lighthouse Lab in Alderley Park	Wellcome Sanger Institute for the COVID-19 Genomics UK (COG-UK) Consortium	Jacquelyn Wynn, Mairead Hyland, The Lighthouse Lab in Alderley Park and Alex Alderton, Roberto Amato, Sonia Goncalves, Ewan Harrison, David K. Jackson, Ian Johnston, Dominic Kwiatkowski, Cordelia Langford, John Sillitoe on behalf of the Wellcome Sanger Institute COVID-19 Surveillance Team
EPI_ISL_718918	Lighthouse Lab in Cambridge	Wellcome Sanger Institute for the COVID-19 Genomics UK (COG-UK) Consortium	Rob Howes, The Lighthouse Lab in Cambridge and Alex Alderton, Roberto Amato, Sonia Goncalves, Ewan Harrison, David K. Jackson, Ian Johnston, Dominic Kwiatkowski, Cordelia Langford, John Sillitoe on behalf of the Wellcome Sanger Institute COVID-19 Surveillance Team
EPI_ISL_718919	Lighthouse Lab in Alderley Park	Wellcome Sanger Institute for the COVID-19 Genomics UK (COG-UK) Consortium	Jacquelyn Wynn, Mairead Hyland, The Lighthouse Lab in Alderley Park and Alex Alderton, Roberto Amato, Sonia Goncalves, Ewan Harrison, David K. Jackson, Ian Johnston, Dominic Kwiatkowski, Cordelia Langford, John Sillitoe on behalf of the Wellcome Sanger Institute COVID-19 Surveillance Team
EPI_ISL_718920, EPI_ISL_718921	Lighthouse Lab in Cambridge	Wellcome Sanger Institute for the COVID-19 Genomics UK (COG-UK) Consortium	Rob Howes, The Lighthouse Lab in Cambridge and Alex Alderton, Roberto Amato, Sonia Goncalves, Ewan Harrison, David K. Jackson, Ian Johnston, Dominic Kwiatkowski, Cordelia Langford, John Sillitoe on behalf of the Wellcome Sanger Institute COVID-19 Surveillance Team
EPI_ISL_718922, EPI_ISL_718923	Lighthouse Lab in Alderley Park	Wellcome Sanger Institute for the COVID-19 Genomics UK (COG-UK) Consortium	Jacquelyn Wynn, Mairead Hyland, The Lighthouse Lab in Alderley Park and Alex Alderton, Roberto Amato, Sonia Goncalves, Ewan Harrison, David K. Jackson, Ian Johnston, Dominic Kwiatkowski, Cordelia Langford, John Sillitoe on behalf of the Wellcome Sanger Institute COVID-19 Surveillance Team
EPI_ISL_718924, EPI_ISL_718925	Lighthouse Lab in Cambridge	Wellcome Sanger Institute for the COVID-19 Genomics UK (COG-UK) Consortium	Rob Howes, The Lighthouse Lab in Cambridge and Alex Alderton, Roberto Amato, Sonia Goncalves, Ewan Harrison, David K. Jackson, Ian Johnston, Dominic Kwiatkowski, Cordelia Langford, John Sillitoe on behalf of the Wellcome Sanger Institute COVID-19 Surveillance Team
EPI_ISL_718926	Lighthouse Lab in Alderley Park	Wellcome Sanger Institute for the COVID-19 Genomics UK (COG-UK) Consortium	Jacquelyn Wynn, Mairead Hyland, The Lighthouse Lab in Alderley Park and Alex Alderton, Roberto Amato, Sonia Goncalves, Ewan Harrison, David K. Jackson, Ian Johnston, Dominic Kwiatkowski, Cordelia Langford, John Sillitoe on behalf of the Wellcome Sanger Institute COVID-19 Surveillance Team
EPI_ISL_718927, EPI_ISL_718928	Lighthouse Lab in Cambridge	Wellcome Sanger Institute for the COVID-19 Genomics UK (COG-UK) Consortium	Rob Howes, The Lighthouse Lab in Cambridge and Alex Alderton, Roberto Amato, Sonia Goncalves, Ewan Harrison, David K. Jackson, Ian Johnston, Dominic Kwiatkowski, Cordelia Langford, John Sillitoe on behalf of the Wellcome Sanger Institute COVID-19 Surveillance Team
EPI_ISL_718929, EPI_ISL_718930	Lighthouse Lab in Alderley Park	Wellcome Sanger Institute for the COVID-19 Genomics UK (COG-UK) Consortium	Jacquelyn Wynn, Mairead Hyland, The Lighthouse Lab in Alderley Park and Alex Alderton, Roberto Amato, Sonia Goncalves, Ewan Harrison, David K. Jackson, Ian Johnston, Dominic Kwiatkowski, Cordelia Langford, John Sillitoe on behalf of the Wellcome Sanger Institute COVID-19 Surveillance Team
EPI_ISL_718931	Lighthouse Lab in Cambridge	Wellcome Sanger Institute for the COVID-19 Genomics UK (COG-UK) Consortium	Rob Howes, The Lighthouse Lab in Cambridge and Alex Alderton, Roberto Amato, Sonia Goncalves, Ewan Harrison, David K. Jackson, Ian Johnston, Dominic Kwiatkowski, Cordelia Langford, John Sillitoe on behalf of the Wellcome Sanger Institute COVID-19 Surveillance Team
EPI_ISL_718932	Lighthouse Lab in Alderley Park	Wellcome Sanger Institute for the COVID-19 Genomics UK (COG-UK) Consortium	Jacquelyn Wynn, Mairead Hyland, The Lighthouse Lab in Alderley Park and Alex Alderton, Roberto Amato, Sonia Goncalves, Ewan Harrison, David K. Jackson, Ian Johnston, Dominic Kwiatkowski, Cordelia Langford, John Sillitoe on behalf of the Wellcome Sanger Institute COVID-19 Surveillance Team
EPI_ISL_718933, EPI_ISL_718934	Lighthouse Lab in Cambridge	Wellcome Sanger Institute for the COVID-19 Genomics UK (COG-UK) Consortium	Rob Howes, The Lighthouse Lab in Cambridge and Alex Alderton, Roberto Amato, Sonia Goncalves, Ewan Harrison, David K. Jackson, Ian Johnston, Dominic Kwiatkowski, Cordelia Langford, John Sillitoe on behalf of the Wellcome Sanger Institute COVID-19 Surveillance Team
EPI_ISL_718935, EPI_ISL_718936	Lighthouse Lab in Alderley Park	Wellcome Sanger Institute for the COVID-19 Genomics UK (COG-UK) Consortium	Jacquelyn Wynn, Mairead Hyland, The Lighthouse Lab in Alderley Park and Alex Alderton, Roberto Amato, Sonia Goncalves, Ewan Harrison, David K. Jackson, Ian Johnston, Dominic Kwiatkowski, Cordelia Langford, John Sillitoe on behalf of the Wellcome Sanger Institute COVID-19 Surveillance Team
EPI_ISL_718937, EPI_ISL_718938	Lighthouse Lab in Cambridge	Wellcome Sanger Institute for the COVID-19 Genomics UK (COG-UK) Consortium	Rob Howes, The Lighthouse Lab in Cambridge and Alex Alderton, Roberto Amato, Sonia Goncalves, Ewan Harrison, David K. Jackson, Ian Johnston, Dominic Kwiatkowski, Cordelia Langford, John Sillitoe on behalf of the Wellcome Sanger Institute COVID-19 Surveillance Team
EPI_ISL_718939	Lighthouse Lab in Alderley Park	Wellcome Sanger Institute for the COVID-19 Genomics UK (COG-UK) Consortium	Jacquelyn Wynn, Mairead Hyland, The Lighthouse Lab in Alderley Park and Alex Alderton, Roberto Amato, Sonia Goncalves, Ewan Harrison, David K. Jackson, Ian Johnston, Dominic Kwiatkowski, Cordelia Langford, John Sillitoe on behalf of the Wellcome Sanger Institute COVID-19 Surveillance Team
EPI_ISL_718940, EPI_ISL_718941, EPI_ISL_718942	Lighthouse Lab in Cambridge	Wellcome Sanger Institute for the COVID-19 Genomics UK (COG-UK) Consortium	Rob Howes, The Lighthouse Lab in Cambridge and Alex Alderton, Roberto Amato, Sonia Goncalves, Ewan Harrison, David K. Jackson, Ian Johnston, Dominic Kwiatkowski, Cordelia Langford, John Sillitoe on behalf of the Wellcome Sanger Institute COVID-19 Surveillance Team
EPI_ISL_718943	Lighthouse Lab in Alderley Park	Wellcome Sanger Institute for the COVID-19 Genomics UK (COG-UK) Consortium	Jacquelyn Wynn, Mairead Hyland, The Lighthouse Lab in Alderley Park and Alex Alderton, Roberto Amato, Sonia Goncalves, Ewan Harrison, David K. Jackson, Ian Johnston, Dominic Kwiatkowski, Cordelia Langford, John Sillitoe on behalf of the Wellcome Sanger Institute COVID-19 Surveillance Team

EPI_ISL_718944	Lighthouse Lab in Cambridge	Wellcome Sanger Institute for the COVID-19 Genomics UK (COG-UK) Consortium	Rob Howes, The Lighthouse Lab in Cambridge and Alex Alderton, Roberto Amato, Sonia Goncalves, Ewan Harrison, David K. Jackson, Ian Johnston, Dominic Kwiatkowski, Cordelia Langford, John Sillitoe on behalf of the Wellcome Sanger Institute COVID-19 Surveillance Team
EPI_ISL_718945	Lighthouse Lab in Alderley Park	Wellcome Sanger Institute for the COVID-19 Genomics UK (COG-UK) Consortium	Jacquelyn Wynn, Mairead Hyland, The Lighthouse Lab in Alderley Park and Alex Alderton, Roberto Amato, Sonia Goncalves, Ewan Harrison, David K. Jackson, Ian Johnston, Dominic Kwiatkowski, Cordelia Langford, John Sillitoe on behalf of the Wellcome Sanger Institute COVID-19 Surveillance Team
EPI_ISL_718946, EPI_ISL_718947, EPI_ISL_718948, EPI_ISL_718949	Lighthouse Lab in Cambridge	Wellcome Sanger Institute for the COVID-19 Genomics UK (COG-UK) Consortium	Rob Howes, The Lighthouse Lab in Cambridge and Alex Alderton, Roberto Amato, Sonia Goncalves, Ewan Harrison, David K. Jackson, Ian Johnston, Dominic Kwiatkowski, Cordelia Langford, John Sillitoe on behalf of the Wellcome Sanger Institute COVID-19 Surveillance Team
EPI_ISL_718950	Lighthouse Lab in Alderley Park	Wellcome Sanger Institute for the COVID-19 Genomics UK (COG-UK) Consortium	Jacquelyn Wynn, Mairead Hyland, The Lighthouse Lab in Alderley Park and Alex Alderton, Roberto Amato, Sonia Goncalves, Ewan Harrison, David K. Jackson, Ian Johnston, Dominic Kwiatkowski, Cordelia Langford, John Sillitoe on behalf of the Wellcome Sanger Institute COVID-19 Surveillance Team
EPI_ISL_718951	Lighthouse Lab in Cambridge	Wellcome Sanger Institute for the COVID-19 Genomics UK (COG-UK) Consortium	Rob Howes, The Lighthouse Lab in Cambridge and Alex Alderton, Roberto Amato, Sonia Goncalves, Ewan Harrison, David K. Jackson, Ian Johnston, Dominic Kwiatkowski, Cordelia Langford, John Sillitoe on behalf of the Wellcome Sanger Institute COVID-19 Surveillance Team
EPI_ISL_718952, EPI_ISL_718953	Lighthouse Lab in Alderley Park	Wellcome Sanger Institute for the COVID-19 Genomics UK (COG-UK) Consortium	Jacquelyn Wynn, Mairead Hyland, The Lighthouse Lab in Alderley Park and Alex Alderton, Roberto Amato, Sonia Goncalves, Ewan Harrison, David K. Jackson, Ian Johnston, Dominic Kwiatkowski, Cordelia Langford, John Sillitoe on behalf of the Wellcome Sanger Institute COVID-19 Surveillance Team
EPI_ISL_718954	Lighthouse Lab in Cambridge	Wellcome Sanger Institute for the COVID-19 Genomics UK (COG-UK) Consortium	Rob Howes, The Lighthouse Lab in Cambridge and Alex Alderton, Roberto Amato, Sonia Goncalves, Ewan Harrison, David K. Jackson, Ian Johnston, Dominic Kwiatkowski, Cordelia Langford, John Sillitoe on behalf of the Wellcome Sanger Institute COVID-19 Surveillance Team
EPI_ISL_718955	Lighthouse Lab in Alderley Park	Wellcome Sanger Institute for the COVID-19 Genomics UK (COG-UK) Consortium	Jacquelyn Wynn, Mairead Hyland, The Lighthouse Lab in Alderley Park and Alex Alderton, Roberto Amato, Sonia Goncalves, Ewan Harrison, David K. Jackson, Ian Johnston, Dominic Kwiatkowski, Cordelia Langford, John Sillitoe on behalf of the Wellcome Sanger Institute COVID-19 Surveillance Team
EPI_ISL_718956	Lighthouse Lab in Cambridge	Wellcome Sanger Institute for the COVID-19 Genomics UK (COG-UK) Consortium	Rob Howes, The Lighthouse Lab in Cambridge and Alex Alderton, Roberto Amato, Sonia Goncalves, Ewan Harrison, David K. Jackson, Ian Johnston, Dominic Kwiatkowski, Cordelia Langford, John Sillitoe on behalf of the Wellcome Sanger Institute COVID-19 Surveillance Team
EPI_ISL_718957	Lighthouse Lab in Alderley Park	Wellcome Sanger Institute for the COVID-19 Genomics UK (COG-UK) Consortium	Jacquelyn Wynn, Mairead Hyland, The Lighthouse Lab in Alderley Park and Alex Alderton, Roberto Amato, Sonia Goncalves, Ewan Harrison, David K. Jackson, Ian Johnston, Dominic Kwiatkowski, Cordelia Langford, John Sillitoe on behalf of the Wellcome Sanger Institute COVID-19 Surveillance Team
EPI_ISL_718958	Lighthouse Lab in Cambridge	Wellcome Sanger Institute for the COVID-19 Genomics UK (COG-UK) Consortium	Rob Howes, The Lighthouse Lab in Cambridge and Alex Alderton, Roberto Amato, Sonia Goncalves, Ewan Harrison, David K. Jackson, Ian Johnston, Dominic Kwiatkowski, Cordelia Langford, John Sillitoe on behalf of the Wellcome Sanger Institute COVID-19 Surveillance Team
EPI_ISL_718959	Lighthouse Lab in Alderley Park	Wellcome Sanger Institute for the COVID-19 Genomics UK (COG-UK) Consortium	Jacquelyn Wynn, Mairead Hyland, The Lighthouse Lab in Alderley Park and Alex Alderton, Roberto Amato, Sonia Goncalves, Ewan Harrison, David K. Jackson, Ian Johnston, Dominic Kwiatkowski, Cordelia Langford, John Sillitoe on behalf of the Wellcome Sanger Institute COVID-19 Surveillance Team
EPI_ISL_718960, EPI_ISL_718961	Lighthouse Lab in Cambridge	Wellcome Sanger Institute for the COVID-19 Genomics UK (COG-UK) Consortium	Rob Howes, The Lighthouse Lab in Cambridge and Alex Alderton, Roberto Amato, Sonia Goncalves, Ewan Harrison, David K. Jackson, Ian Johnston, Dominic Kwiatkowski, Cordelia Langford, John Sillitoe on behalf of the Wellcome Sanger Institute COVID-19 Surveillance Team
EPI_ISL_718962, EPI_ISL_718963, EPI_ISL_718964	Lighthouse Lab in Alderley Park	Wellcome Sanger Institute for the COVID-19 Genomics UK (COG-UK) Consortium	Jacquelyn Wynn, Mairead Hyland, The Lighthouse Lab in Alderley Park and Alex Alderton, Roberto Amato, Sonia Goncalves, Ewan Harrison, David K. Jackson, Ian Johnston, Dominic Kwiatkowski, Cordelia Langford, John Sillitoe on behalf of the Wellcome Sanger Institute COVID-19 Surveillance Team
EPI_ISL_718965	Lighthouse Lab in Cambridge	Wellcome Sanger Institute for the COVID-19 Genomics UK (COG-UK) Consortium	Rob Howes, The Lighthouse Lab in Cambridge and Alex Alderton, Roberto Amato, Sonia Goncalves, Ewan Harrison, David K. Jackson, Ian Johnston, Dominic Kwiatkowski, Cordelia Langford, John Sillitoe on behalf of the Wellcome Sanger Institute COVID-19 Surveillance Team
EPI_ISL_718966, EPI_ISL_718967	Lighthouse Lab in Alderley Park	Wellcome Sanger Institute for the COVID-19 Genomics UK (COG-UK) Consortium	Jacquelyn Wynn, Mairead Hyland, The Lighthouse Lab in Alderley Park and Alex Alderton, Roberto Amato, Sonia Goncalves, Ewan Harrison, David K. Jackson, Ian Johnston, Dominic Kwiatkowski, Cordelia Langford, John Sillitoe on behalf of the Wellcome Sanger Institute COVID-19 Surveillance Team
EPI_ISL_718968, EPI_ISL_718969	Lighthouse Lab in Cambridge	Wellcome Sanger Institute for the COVID-19 Genomics UK (COG-UK) Consortium	Rob Howes, The Lighthouse Lab in Cambridge and Alex Alderton, Roberto Amato, Sonia Goncalves, Ewan Harrison, David K. Jackson, Ian Johnston, Dominic Kwiatkowski, Cordelia Langford, John Sillitoe on behalf of the Wellcome Sanger Institute COVID-19 Surveillance Team
EPI_ISL_718970	Lighthouse Lab in Alderley Park	Wellcome Sanger Institute for the COVID-19 Genomics UK (COG-UK) Consortium	Jacquelyn Wynn, Mairead Hyland, The Lighthouse Lab in Alderley Park and Alex Alderton, Roberto Amato, Sonia Goncalves, Ewan Harrison, David K. Jackson, Ian Johnston, Dominic Kwiatkowski, Cordelia Langford, John Sillitoe on behalf of the Wellcome Sanger Institute COVID-19 Surveillance Team
EPI_ISL_718971, EPI_ISL_718972, EPI_ISL_718973	Lighthouse Lab in Cambridge	Wellcome Sanger Institute for the COVID-19 Genomics UK (COG-UK) Consortium	Rob Howes, The Lighthouse Lab in Cambridge and Alex Alderton, Roberto Amato, Sonia Goncalves, Ewan Harrison, David K. Jackson, Ian Johnston, Dominic Kwiatkowski, Cordelia Langford, John Sillitoe on behalf of the Wellcome Sanger Institute COVID-19 Surveillance Team
EPI_ISL_718974, EPI_ISL_718975, EPI_ISL_718976, EPI_ISL_718977, EPI_ISL_718978, EPI_ISL_718979	Lighthouse Lab in Alderley Park	Wellcome Sanger Institute for the COVID-19 Genomics UK (COG-UK) Consortium	Jacquelyn Wynn, Mairead Hyland, The Lighthouse Lab in Alderley Park and Alex Alderton, Roberto Amato, Sonia Goncalves, Ewan Harrison, David K. Jackson, Ian Johnston, Dominic Kwiatkowski, Cordelia Langford, John Sillitoe on behalf of the Wellcome Sanger Institute COVID-19 Surveillance Team
EPI_ISL_718980, EPI_ISL_718981, EPI_ISL_718982, EPI_ISL_718983, EPI_ISL_718984, EPI_ISL_718985, EPI_ISL_718986, EPI_ISL_718987, EPI_ISL_718988, EPI_ISL_718989, EPI_ISL_718990, EPI_ISL_718991, EPI_ISL_718992, EPI_ISL_718993, EPI_ISL_718994, EPI_ISL_718995, EPI_ISL_718996, EPI_ISL_718997, EPI_ISL_718998, EPI_ISL_718999, EPI_ISL_719000, EPI_ISL_719001, EPI_ISL_719002, EPI_ISL_719003, EPI_ISL_719004, EPI_ISL_719005, EPI_ISL_719006, EPI_ISL_719007, EPI_ISL_719008, EPI_ISL_719009, EPI_ISL_719010, EPI_ISL_719011, EPI_ISL_719012, EPI_ISL_719013, EPI_ISL_719014, EPI_ISL_719015, EPI_ISL_719016, EPI_ISL_719017, EPI_ISL_719018, EPI_ISL_719019, EPI_ISL_719020, EPI_ISL_719021, EPI_ISL_719022, EPI_ISL_719023, EPI_ISL_719024, EPI_ISL_719025, EPI_ISL_719026, EPI_ISL_719027, EPI_ISL_719028, EPI_ISL_719029, EPI_ISL_719030, EPI_ISL_719031, EPI_ISL_719032, EPI_ISL_719033, EPI_ISL_719034, EPI_ISL_719035, EPI_ISL_719036, EPI_ISL_719037, EPI_ISL_719038, EPI_ISL_719039, EPI_ISL_719040, EPI_ISL_719041, EPI_ISL_719042, EPI_ISL_719043, EPI_ISL_719044, EPI_ISL_719045, EPI_ISL_719046, EPI_ISL_719047, EPI_ISL_719048, EPI_ISL_719049, EPI_ISL_719050, EPI_ISL_719051, EPI_ISL_719052, EPI_ISL_719053, EPI_ISL_719054, EPI_ISL_719055, EPI_ISL_719056, EPI_ISL_719057, EPI_ISL_719058, EPI_ISL_719059, EPI_ISL_719060, EPI_ISL_719061, EPI_ISL_719062, EPI_ISL_719063, EPI_ISL_719064, EPI_ISL_719065, EPI_ISL_719066, EPI_ISL_719067, EPI_ISL_719068, EPI_ISL_719069, EPI_ISL_719070, EPI_ISL_719071, EPI_ISL_719072, EPI_ISL_719073, EPI_ISL_719074, EPI_ISL_719075, EPI_ISL_719076, EPI_ISL_719077, EPI_ISL_719078, EPI_ISL_719079, EPI_ISL_719080, EPI_ISL_719081, EPI_ISL_719082, EPI_ISL_719083, EPI_ISL_719084, EPI_ISL_719085, EPI_ISL_719086, EPI_ISL_719087, EPI_ISL_719088, EPI_ISL_719089, EPI_ISL_719090, EPI_ISL_719091, EPI_ISL_719092, EPI_ISL_719093, EPI_ISL_719094, EPI_ISL_719095, EPI_ISL_719096, EPI_ISL_719097, EPI_ISL_719098, EPI_ISL_719099, EPI_ISL_719100, EPI_ISL_719101, EPI_ISL_719102, EPI_ISL_719103, EPI_ISL_719104, EPI_ISL_719105, EPI_ISL_719106, EPI_ISL_719107, EPI_ISL_719108, EPI_ISL_719109, EPI_ISL_719110, EPI_ISL_719111, EPI_ISL_719112, EPI_ISL_719113, EPI_ISL_719114, EPI_ISL_719115, EPI_ISL_719116, EPI_ISL_719117, EPI_ISL_719118, EPI_ISL_719119, EPI_ISL_719120, EPI_ISL_719121, EPI_ISL_719122, EPI_ISL_719123, EPI_ISL_719124, EPI_ISL_719125, EPI_ISL_719126, EPI_ISL_719127, EPI_ISL_719128, EPI_ISL_719129, EPI_ISL_719130, EPI_ISL_719131, EPI_ISL_719132, EPI_ISL_719133, EPI_ISL_719134, EPI_ISL_719135, EPI_ISL_719136, EPI_ISL_719137, EPI_ISL_719138, EPI_ISL_719139, EPI_ISL_719140, EPI_ISL_719141, EPI_ISL_719142, EPI_ISL_719143, EPI_ISL_719144, EPI_ISL_719145, EPI_ISL_719146, EPI_ISL_719147, EPI_ISL_719148, EPI_ISL_719149, EPI_ISL_719150, EPI_ISL_719151, EPI_ISL_719152, EPI_ISL_719153, EPI_ISL_719154, EPI_ISL_719155, EPI_ISL_719156, EPI_ISL_719157, EPI_ISL_719158, EPI_ISL_719159, EPI_ISL_719160, EPI_ISL_719161, EPI_ISL_719162, EPI_ISL_719163, EPI_ISL_719164, EPI_ISL_719165, EPI_ISL_719166, EPI_ISL_719167, EPI_ISL_719168, EPI_ISL_719169, EPI_ISL_719170, EPI_ISL_719171, EPI_ISL_719172, EPI_ISL_719173, EPI_ISL_719174, EPI_ISL_719175, EPI_ISL_719176, EPI_ISL_719177, EPI_ISL_719178, EPI_ISL_719179, EPI_ISL_719180, EPI_ISL_719181, EPI_ISL_719182, EPI_ISL_719183, EPI_ISL_719184, EPI_ISL_719185, EPI_ISL_719186, EPI_ISL_719187, EPI_ISL_719188, EPI_ISL_719189, EPI_ISL_719190, EPI_ISL_719191, EPI_ISL_719192, EPI_ISL_719193, EPI_ISL_719194, EPI_ISL_719195, EPI_ISL_719196, EPI_ISL_719197, EPI_ISL_719198, EPI_ISL_719199, EPI_ISL_719200, EPI_ISL_719201, EPI_ISL_719202, EPI_ISL_719203, EPI_ISL_719204, EPI_ISL_719205, EPI_ISL_719206, EPI_ISL_719207, EPI_ISL_719208, EPI_ISL_719209, EPI_ISL_719210, EPI_ISL_719211, EPI_ISL_719212, EPI_ISL_719213, EPI_ISL_719214, EPI_ISL_719215, EPI_ISL_719216, EPI_ISL_719217, EPI_ISL_719218, EPI_ISL_719219, EPI_ISL_719220, EPI_ISL_719221, EPI_ISL_719222, EPI_ISL_719223, EPI_ISL_719224, EPI_ISL_719225, EPI_ISL_719226, EPI_ISL_719227, EPI_ISL_719228, EPI_ISL_719229, EPI_ISL_719230, EPI_ISL_719231, EPI_ISL_719232, EPI_ISL_719233, EPI_ISL_719234, EPI_ISL_719235, EPI_ISL_719236, EPI_ISL_719237, EPI_ISL_719238, EPI_ISL_719239, EPI_ISL_719240, EPI_ISL_719241, EPI_ISL_719242, EPI_ISL_719243, EPI_ISL_719244, EPI_ISL_719245, EPI_ISL_719246, EPI_ISL_719247, EPI_ISL_719248, EPI_ISL_719249, EPI_ISL_719250, EPI_ISL_719251, EPI_ISL_719252, EPI_ISL_719253, EPI_ISL_719254, EPI_ISL_719255, EPI_ISL_719256, EPI_ISL_719257, EPI_ISL_719258, EPI_ISL_719259, EPI_ISL_719260, EPI_ISL_719261, EPI_ISL_719262, EPI_ISL_719263, EPI_ISL_719264, EPI_ISL_719265, EPI_ISL_719266, EPI_ISL_719267, EPI_ISL_719268, EPI_ISL_719269, EPI_ISL_719270, EPI_ISL_719271, EPI_ISL_719272, EPI_ISL_719273, EPI_ISL_719274, EPI_ISL_719275, EPI_ISL_719276, EPI_ISL_719277, EPI_ISL_719278, EPI_ISL_719279, EPI_ISL_719280, EPI_ISL_719281, EPI_ISL_719282, EPI_ISL_719283, EPI_ISL_719284, EPI_ISL_719285, EPI_ISL_719286, EPI_ISL_719287, EPI_ISL_719288, EPI_ISL_719289, EPI_ISL_719290, EPI_ISL_719291, EPI_ISL_719292, EPI_ISL_719293, EPI_ISL_719294, EPI_ISL_719295, EPI_ISL_719296, EPI_ISL_719297, EPI_ISL_719298, EPI_ISL_719299, EPI_ISL_719300, EPI_ISL_719301, EPI_ISL_719302, EPI_ISL_719303, EPI_ISL_719304, EPI_ISL_719305, EPI_ISL_719306, EPI_ISL_719307, EPI_ISL_719308, EPI_ISL_719309, EPI_ISL_719310, EPI_ISL_719311, EPI_ISL_719312, EPI_ISL_719313, EPI_ISL_719314, EPI_ISL_719315, EPI_ISL_719316, EPI_ISL_719317, EPI_ISL_719318, EPI_ISL_719319, EPI_ISL_719320, EPI_ISL_719321, EPI_ISL_719322, EPI_ISL_719323, EPI_ISL_719324, EPI_ISL_719325, EPI_ISL_719326, EPI_ISL_719327, EPI_ISL_719328, EPI_ISL_719329, EPI_ISL_719330, EPI_ISL_719331, EPI_ISL_719332, EPI_ISL_719333, EPI_ISL_719334, EPI_ISL_719335, EPI_ISL_719336, EPI_ISL_719337, EPI_ISL_719338, EPI_ISL_719339, EPI_ISL_719340, EPI_ISL_719341, EPI_ISL_719342, EPI_ISL_719343, EPI_ISL_719344, EPI_ISL_719345, EPI_ISL_719346, EPI_ISL_719347, EPI_ISL_719348, EPI_ISL_719349, EPI_ISL_719350, EPI_ISL_719351, EPI_ISL_719352, EPI_ISL_719353, EPI_ISL_719354, EPI_ISL_719355, EPI_ISL_719356, EPI_ISL_719357, EPI_ISL_719358, EPI_ISL_719359, EPI_ISL_719360, EPI_ISL_719361, EPI_ISL_719362, EPI_ISL_719363, EPI_ISL_719364, EPI_ISL_719365, EPI_ISL_719366, EPI_ISL_719367, EPI_ISL_719368, EPI_ISL_719369, EPI_ISL_719370, EPI_ISL_719371, EPI_ISL_719372, EPI_ISL_719373, EPI_ISL_719374, EPI_ISL_719375, EPI_ISL_719376, EPI_ISL_719377, EPI_ISL_719378, EPI_ISL_719379, EPI_ISL_719380, EPI_ISL_719381, EPI_ISL_719382, EPI_ISL_719383, EPI_ISL_719384, EPI_ISL_719385, EPI_ISL_719386, EPI_ISL_719387, EPI_ISL_719388, EPI_ISL_719389, EPI_ISL_719390, EPI_ISL_719391, EPI_ISL_719392, EPI_ISL_719393, EPI_ISL_719394, EPI_ISL_719395, EPI_ISL_719396, EPI_ISL_719397, EPI_ISL_719398, EPI_ISL_719399, EPI_ISL_719400, EPI_ISL_719401, EPI_ISL_719402, EPI_ISL_719403, EPI_ISL_719404, EPI_ISL_719405, EPI_ISL_719406, EPI_ISL_719407, EPI_ISL_719408, EPI_ISL_719409, EPI_ISL_719410, EPI_ISL_719411, EPI_ISL_719412, EPI_ISL_719413, EPI_ISL_719414, EPI_ISL_719415, EPI_ISL_719416, EPI_ISL_719417, EPI_ISL_719418, EPI_ISL_719419, EPI_ISL_719420, EPI_ISL_719421, EPI_ISL_719422, EPI_ISL_719423, EPI_ISL_719424, EPI_ISL_719425, EPI_ISL_719426, EPI_ISL_719427, EPI_ISL_719428, EPI_ISL_719429, EPI_ISL_719430, EPI_ISL_719431, EPI_ISL_719432, EPI_ISL_719433, EPI_ISL_719434, EPI_ISL_719435, EPI_ISL_719436, EPI_ISL_719437, EPI_ISL_719438, EPI_ISL_719439, EPI_ISL_719440, EPI_ISL_719441, EPI_ISL_719442, EPI_ISL_719443, EPI_ISL_719444, EPI_ISL_719445, EPI_ISL_719446, EPI_ISL_719447, EPI_ISL_719448, EPI_ISL_719449, EPI_ISL_719450, EPI_ISL_719451, EPI_ISL_719452, EPI_ISL_719453, EPI_ISL_719454, EPI_ISL_719455, EPI_ISL_719456, EPI_ISL_719457, EPI_ISL_719458, EPI_ISL_719459, EPI_ISL_719460, EPI_ISL_719461, EPI_ISL_719462, EPI_ISL_719463, EPI_ISL_719464, EPI_ISL_719465, EPI_ISL_719466, EPI_ISL_719467, EPI_ISL_719468, EPI_ISL_719469, EPI_ISL_719470, EPI_ISL_719471, EPI_ISL_719472, EPI_ISL_719473, EPI_ISL_719474, EPI_ISL_719475, EPI_ISL_719476, EPI_ISL_719477, EPI_ISL_719478, EPI_ISL_719479, EPI_ISL_719480, EPI_ISL_719481, EPI_ISL_719482, EPI_ISL_719483, EPI_ISL_719484, EPI_ISL_719485, EPI_ISL_719486, EPI_ISL_719487, EPI_ISL_719488, EPI_ISL_719489, EPI_ISL_719490, EPI_ISL_719491, EPI_ISL_719492, EPI_ISL_719493, EPI_ISL_719494, EPI_ISL_719495, EPI_ISL_719496, EPI_ISL_719497, EPI_ISL_719498, EPI_ISL_719499, EPI_ISL_719500, EPI_ISL_719501, EPI_ISL_719502, EPI_ISL_719503, EPI_ISL_719504, EPI_ISL_719505, EPI_ISL_719506, EPI_ISL_719507, EPI_ISL_719508, EPI_ISL_719509, EPI_ISL_719510, EPI_ISL_719511, EPI_ISL_719512, EPI_ISL_719513, EPI_ISL_719514, EPI_ISL_719515, EPI_ISL_719516, EPI_ISL_719517, EPI_ISL_719518, EPI_ISL_719519, EPI_ISL_719520, EPI_ISL_719521, EPI_ISL_719522, EPI_ISL_719523, EPI_ISL_719524, EPI_ISL_719525, EPI_ISL_719526, EPI_ISL_719527, EPI_ISL_719528, EPI_ISL_719529, EPI_ISL_719530, EPI_ISL_719531, EPI_ISL_719532, EPI_ISL_719533, EPI_ISL_719534, EPI_ISL_719535, EPI_ISL_719536, EPI_ISL_719537, EPI_ISL_719538, EPI_ISL_719539, EPI_ISL_719540, EPI_ISL_719541, EPI_ISL_719542, EPI_ISL_719543, EPI_ISL_719544, EPI_ISL_719545, EPI_ISL_719546, EPI_ISL_719547, EPI_ISL_719548, EPI_ISL_719549, EPI_ISL_719550, EPI_ISL_719551, EPI_ISL_719552, EPI_ISL_719553, EPI_ISL_719554, EPI_ISL_719555, EPI_ISL_719556, EPI_ISL_719557, EPI_ISL_719558, EPI_ISL_719559, EPI_ISL_719560, EPI_ISL_719561, EPI_ISL_719562, EPI_ISL_719563, EPI_ISL_719564, EPI_ISL_719565, EPI_ISL_719566, EPI_ISL_719567, EPI_ISL_719568, EPI_ISL_719569, EPI_ISL_719570, EPI_ISL_719571, EPI_ISL_719572, EPI_ISL_719573, EPI_ISL_719574, EPI_ISL_719575, EPI_ISL_719576, EPI_ISL_719577, EPI_ISL_719578, EPI_ISL_719579, EPI_ISL_719580, EPI_ISL_719581, EPI_ISL_719582, EPI_ISL_719583, EPI_ISL_719584, EPI_ISL_719585, EPI_ISL_719586, EPI_ISL_719587, EPI_ISL_719588, EPI_ISL_719589, EPI_ISL_719590, EPI_ISL_719591, EPI_ISL_719592, EPI_ISL_719593, EPI_ISL_719594, EPI_ISL_719595, EPI_ISL_719596, EPI_ISL_719597, EPI_ISL_719598, EPI_ISL_719599, EPI_ISL_719600, EPI_ISL_719601, EPI_ISL_719602, EPI_ISL_719603, EPI_ISL_719604			
see above	Lighthouse Lab in Cambridge	Wellcome Sanger Institute for the COVID-19 Genomics UK	Rob Howes, The Lighthouse Lab in Cambridge and Alex Alderton, Roberto Amato, Sonia Goncalves, Ewan Harrison, David K. Jackson, Ian Johnston,

		(COG-UK) Consortium	Dominic Kwiatkowski, Cordelia Langford, John Sillitoe on behalf of the Wellcome Sanger Institute COVID-19 Surveillance Team
EPI_ISL_722342, EPI_ISL_722356, EPI_ISL_722381, EPI_ISL_722386, EPI_ISL_722411, EPI_ISL_722433, EPI_ISL_722455, EPI_ISL_722456, EPI_ISL_722457, EPI_ISL_722458, EPI_ISL_722459, EPI_ISL_722460, EPI_ISL_722763, EPI_ISL_722764, EPI_ISL_722765, EPI_ISL_722766, EPI_ISL_722767, EPI_ISL_722768, EPI_ISL_722769, EPI_ISL_722770, EPI_ISL_722771, EPI_ISL_722772, EPI_ISL_722773, EPI_ISL_722774, EPI_ISL_722775, EPI_ISL_722776, EPI_ISL_722777			
see above	Dutch COVID-19 response team	Erasmus Medical Center	Bas Oude Munnink, Reina Sikkema, David Nieuwenhuijse, Irina Chestakova, Anne van der Linden, Marjan Boter, Emmanuelle Munger, Corine GeurtsvanKessel, Annemiek van der Eijk, Richard Molenkamp, Marion Koopmans, on behalf of the Dutch national COVID-19 response team.
EPI_ISL_722999, EPI_ISL_723000, EPI_ISL_723006, EPI_ISL_723007, EPI_ISL_723015, EPI_ISL_723044	Respiratory Virus Unit, National Infection Service, Public Health England	COVID-19 Genomics UK (COG-UK) Consortium	PHE Covid Sequencing Team
EPI_ISL_723922, EPI_ISL_723923, EPI_ISL_723924, EPI_ISL_723925, EPI_ISL_723926, EPI_ISL_723927, EPI_ISL_723928, EPI_ISL_723929, EPI_ISL_723930, EPI_ISL_723931, EPI_ISL_723932, EPI_ISL_723934, EPI_ISL_723937, EPI_ISL_723938, EPI_ISL_723939, EPI_ISL_723940, EPI_ISL_723941, EPI_ISL_723942, EPI_ISL_723943, EPI_ISL_723944, EPI_ISL_723945, EPI_ISL_723946			
see above	Department of Pathology, University of Cambridge	COVID-19 Genomics UK (COG-UK) Consortium	Aminu S. Jahun, Yasmin Chaudhry, Grant Hall, Iliana Georgana, Myra Hosmillo, Martin D. Curran, Malte Pinckert, Surendra Parmar, Ian Goodfellow
EPI_ISL_724245, EPI_ISL_724248, EPI_ISL_724249, EPI_ISL_724250, EPI_ISL_724251, EPI_ISL_724252	Virology Department, Royal Infirmary of Edinburgh, NHS Lothian / School of Biological Sciences, University of Edinburgh / Institute of Genetics and Molecular Medicine, University of Edinburgh	COVID-19 Genomics UK (COG-UK) Consortium	McHugh M, Dewar R, Rooke S, Gallagher M, Balcaza C, O'Toole Á, Scher E, Hill V, McCrone JT, Colquhoun R, Yu X, Jackson B, Rambaut A, Williams TC, Templeton K
EPI_ISL_724525, EPI_ISL_724526, EPI_ISL_724527, EPI_ISL_724528, EPI_ISL_724529, EPI_ISL_724530, EPI_ISL_724531, EPI_ISL_724532, EPI_ISL_724533, EPI_ISL_724534, EPI_ISL_724535, EPI_ISL_724536, EPI_ISL_724537, EPI_ISL_724538, EPI_ISL_724539			
see above	Liverpool Clinical Laboratories	COVID-19 Genomics UK (COG-UK) Consortium	Sam Haldenby, Anita Lucaci, Steve Paterson, Julian Hiscox, Alistair Darby, M Almsaud, A Alrezaihi, Muhannad Alruwaili, Stuart D Armstrong, Jones Benjamin, Eleanor G Bentley, Anu Chawla, Jordan J Clark, Angela Cowell, Richard Eccles, Isabel García-Dorival, Matthew Gemmell, Alessandro Gerada, PKF Gilmore, Richard Gregory, Ximeng Han, Catherine Hartley, Margaret Hughes, Miren Iturriza-Gomara, James Johnson, L Luu, Jenifer Manson, Charlotte Nelson, Elaine O'Toole, Cassie Olateju, Rebekah Penrice-Randal , Lucille Rainbow, N.P Randle, Trevor Ian Robinson, Parul Sharma, Ghada T Shawli, James P Stewart, Neil Swainston, Ecaterina Vamos, Joanne Watts, Mark Whitehead
EPI_ISL_724593, EPI_ISL_724606, EPI_ISL_724607	University College London, Great Ormond Street Hospital for Children NHS Foundation Trust, Imperial College Healthcare NHS Trust	COVID-19 Genomics UK (COG-UK) Consortium	Sergi Castellano, Rachel Williams, Mark Kristiansen, Paola Resende Silva, Sunando Roy, Tony Brooks, Helena Tutill, Paola Niola, Patricia Dyal, Charlotte Williams, Leysa Forrest, Yasmin Panchbhaya, Jacqueline Findlay, Samuel Weeks, Julianne Brown, Kathryn Harris, Paul Randell, James Price, Alison Holmes, Judith Breuer
EPI_ISL_725033, EPI_ISL_725034, EPI_ISL_725035, EPI_ISL_725038, EPI_ISL_725041, EPI_ISL_725055, EPI_ISL_725062, EPI_ISL_725063, EPI_ISL_725064, EPI_ISL_725065, EPI_ISL_725066, EPI_ISL_725067, EPI_ISL_725068, EPI_ISL_725069, EPI_ISL_725070, EPI_ISL_725071, EPI_ISL_725072, EPI_ISL_725073, EPI_ISL_725074, EPI_ISL_725075, EPI_ISL_725076, EPI_ISL_725077, EPI_ISL_725078, EPI_ISL_725079, EPI_ISL_725080, EPI_ISL_725081, EPI_ISL_725082, EPI_ISL_725083, EPI_ISL_725084, EPI_ISL_725085, EPI_ISL_725086, EPI_ISL_725087, EPI_ISL_725088, EPI_ISL_725089, EPI_ISL_725090, EPI_ISL_725091, EPI_ISL_725092, EPI_ISL_725093, EPI_ISL_725094, EPI_ISL_725095, EPI_ISL_725096, EPI_ISL_725097, EPI_ISL_725098, EPI_ISL_725099, EPI_ISL_725100, EPI_ISL_725101, EPI_ISL_725102, EPI_ISL_725103, EPI_ISL_725104, EPI_ISL_725105, EPI_ISL_725151, EPI_ISL_725155, EPI_ISL_725296, EPI_ISL_725297, EPI_ISL_725298, EPI_ISL_725299, EPI_ISL_725300, EPI_ISL_725301, EPI_ISL_725343, EPI_ISL_725344, EPI_ISL_725346, EPI_ISL_725347, EPI_ISL_725348, EPI_ISL_725349, EPI_ISL_725350, EPI_ISL_725351, EPI_ISL_725352, EPI_ISL_725353, EPI_ISL_725354, EPI_ISL_725355, EPI_ISL_725356, EPI_ISL_725357, EPI_ISL_725358, EPI_ISL_725359, EPI_ISL_725360, EPI_ISL_725361, EPI_ISL_725362, EPI_ISL_725363, EPI_ISL_725364, EPI_ISL_725366, EPI_ISL_725367, EPI_ISL_725368, EPI_ISL_725369, EPI_ISL_725417, EPI_ISL_725419, EPI_ISL_725420, EPI_ISL_725422, EPI_ISL_725423, EPI_ISL_725424, EPI_ISL_725428, EPI_ISL_725430, EPI_ISL_725433, EPI_ISL_725438, EPI_ISL_725458, EPI_ISL_725459, EPI_ISL_725463, EPI_ISL_725464, EPI_ISL_725466, EPI_ISL_725467, EPI_ISL_725468, EPI_ISL_725469, EPI_ISL_725471, EPI_ISL_725472, EPI_ISL_725475, EPI_ISL_725476, EPI_ISL_725480, EPI_ISL_725481, EPI_ISL_725487, EPI_ISL_725492, EPI_ISL_725493, EPI_ISL_725494, EPI_ISL_725495			
see above	Quadram Institute Bioscience	COVID-19 Genomics UK (COG-UK) Consortium	Dave J. Baker, Gemma L. Kay, Alp Aydin, Thanh Le-Viet, Steven Rudder, Ana P. Tedim, Anastasia Kolyva, Maria Diaz, Leonardo de Oliveira Martins, Nabil-Fareed Alikhan, Lizzie Meadows, Rachael Stanley, Ngozi Elumogo, Muhammed Yasir, Nicholas M. Thomson, Alexander J Trotter, Rachel Gilroy, Samuel Bloomfield, Claire Stuart, Andrew Bell, Reenesh Prakash, Samir Dervisevic, Alison E. Mather, John Wain, Mark Webber, Andrew J. Page, Justin O'Grady
EPI_ISL_725643, EPI_ISL_725644, EPI_ISL_725645, EPI_ISL_725646, EPI_ISL_725647, EPI_ISL_725648, EPI_ISL_725649, EPI_ISL_725650, EPI_ISL_725651, EPI_ISL_725652, EPI_ISL_725653, EPI_ISL_725654, EPI_ISL_725655, EPI_ISL_725656, EPI_ISL_725657, EPI_ISL_725658, EPI_ISL_725659, EPI_ISL_725660, EPI_ISL_725661, EPI_ISL_725662, EPI_ISL_725663, EPI_ISL_725664, EPI_ISL_725665, EPI_ISL_725666, EPI_ISL_725667, EPI_ISL_725668, EPI_ISL_725669			
see above	Queens Medical Centre, Clinical Microbiology Department / DeepSeq Nottingham	COVID-19 Genomics UK (COG-UK) Consortium	Gemma Clark, Wendy Smith, Manjinder Khakh, Vicki M Fleming, Michelle M Lister, Hannah Howson-Wells, Jonathan Ball, Patrick McClure, Joseph Chappell, Theocharis Tsoleridis, Nadine Holmes, Matthew Carlisle, Christopher Moore, Fei Sang, Johnny Debebe, Victoria Wright, Matthew Loose
EPI_ISL_727862, EPI_ISL_727864, EPI_ISL_727866, EPI_ISL_727867, EPI_ISL_727872, EPI_ISL_727873, EPI_ISL_727876, EPI_ISL_727878, EPI_ISL_727881, EPI_ISL_727886, EPI_ISL_727889, EPI_ISL_727893, EPI_ISL_727894, EPI_ISL_727898, EPI_ISL_727899, EPI_ISL_727900, EPI_ISL_727902, EPI_ISL_727904, EPI_ISL_727907, EPI_ISL_727910, EPI_ISL_727912, EPI_ISL_727913, EPI_ISL_727916, EPI_ISL_727921, EPI_ISL_727924, EPI_ISL_727925, EPI_ISL_727926, EPI_ISL_727928, EPI_ISL_727930, EPI_ISL_727933, EPI_ISL_727935, EPI_ISL_727937, EPI_ISL_727944, EPI_ISL_727945, EPI_ISL_727946, EPI_ISL_727952, EPI_ISL_727958, EPI_ISL_727959, EPI_ISL_727962, EPI_ISL_727965, EPI_ISL_727977, EPI_ISL_727984, EPI_ISL_727989			
see above	Virology Department, Sheffield Teaching Hospitals NHS Foundation Trust/Department of Infection, Immunity and Cardiovascular Disease, The Medical School, University of Sheffield	COVID-19 Genomics UK (COG-UK) Consortium	Thushan de Silva, Matthew Parker, Nikki Smith, Adri Angyal, Rebecca Brown, Luke Green, Rachel Tucker, Paul Parsons, Danielle Groves, Katie Johnson, Laura Carrilero, Alex Keeley, Dave Partridge, Matthew Wyles, Benjamin Lindsey, Mehmet Yavuz, Mohammad Raza, Cariad Evans
EPI_ISL_728192, EPI_ISL_728193, EPI_ISL_728195, EPI_ISL_728196, EPI_ISL_728197, EPI_ISL_728198, EPI_ISL_728199, EPI_ISL_728200	National Public Health Laboratory, National Centre for Infectious Diseases	National Public Health Laboratory, National Centre for Infectious Diseases	Tze Minn Mak, Sophie Octavia, Zhenyang Zhou, Lin Cui, Raymond Tzer Pin Lin
EPI_ISL_728299	Lighthouse Lab in Cambridge	Wellcome Sanger Institute for the COVID-19 Genomics UK (COG-UK) Consortium	Rob Howes, The Lighthouse Lab in Cambridge and Alex Alderton, Roberto Amato, Sonia Goncalves, Ewan Harrison, David K. Jackson, Ian Johnston, Dominic Kwiatkowski, Cordelia Langford, John Sillitoe on behalf of the Wellcome Sanger Institute COVID-19 Surveillance Team
EPI_ISL_728343	Respiratory Virus Unit, National Infection Service, Public Health England	COVID-19 Genomics UK (COG-UK) Consortium	PHE Covid Sequencing Team
EPI_ISL_728568, EPI_ISL_728569, EPI_ISL_728586, EPI_ISL_728592, EPI_ISL_728593, EPI_ISL_728594, EPI_ISL_728614, EPI_ISL_728615, EPI_ISL_728616, EPI_ISL_728617, EPI_ISL_728632, EPI_ISL_728633, EPI_ISL_728634, EPI_ISL_728651, EPI_ISL_728697			
see above	Dutch COVID-19 response team	National Institute for Public Health and the Environment (RIVM)	Adam Meijer, Harry Vennema, Jeroen Cremer, Sharon van den Brink, Bas van der Veer, AnneMarie van den Brandt, Florian Zwagemaker, Dennis Schmitz, Chantal Reusken, on behalf of the national COVID-19 response team
EPI_ISL_728774	Viollier AG	Department of Biosystems Science and Engineering, ETH Zürich	Chaoran Chen, Sarah Nadeau, Catharine Aquino, Ivan Topolsky, Pedro Ferreira, Philipp Jablonski, Susana Posada-Céspedes, Andreia Cabral de Gouvea, Maria Domenica Moccia, Simon Grüter, Timothy Sykes, Lennart Opitz, Ralph Schlapbach, Christiane Beckmann, Maurice Redondo, Olivier Kobel, Christoph Noppen, Sophie Seidel, Noemie Santamaria de Souza, Niko Beerenwinkel, Tanja Stadler
EPI_ISL_728863, EPI_ISL_728883	Viollier AG	Department of Biosystems Science and Engineering, ETH Zürich	Christian Beisel, Sarah Nadeau, Chaoran Chen, Ivan Topolsky, Pedro Ferreira, Philipp Jablonski, Susana Posada-Céspedes, Tobias Schär, Ina Nissen, Natascha Santacroce, Elodie Burcklen, Christiane Beckmann, Maurice Redondo, Olivier Kobel, Christoph Noppen, Sophie Seidel, Noemie Santamaria de Souza, Niko Beerenwinkel, Tanja Stadler
EPI_ISL_728913, EPI_ISL_728918, EPI_ISL_729007, EPI_ISL_729034, EPI_ISL_729042, EPI_ISL_729047	Viollier AG	Department of Biosystems Science and Engineering, ETH Zürich	Chaoran Chen, Sarah Nadeau, Catharine Aquino, Ivan Topolsky, Pedro Ferreira, Philipp Jablonski, Susana Posada-Céspedes, Andreia Cabral de Gouvea, Maria Domenica Moccia, Simon Grüter, Timothy Sykes, Lennart Opitz, Ralph Schlapbach, Christiane Beckmann, Maurice Redondo, Olivier Kobel, Christoph Noppen, Sophie Seidel, Noemie Santamaria de Souza, Niko Beerenwinkel, Tanja Stadler
EPI_ISL_729049, EPI_ISL_729053, EPI_ISL_729067, EPI_ISL_729088, EPI_ISL_729089, EPI_ISL_729187, EPI_ISL_729188, EPI_ISL_729189	Viollier AG	Department of Biosystems Science and Engineering, ETH Zürich	Christian Beisel, Sarah Nadeau, Chaoran Chen, Ivan Topolsky, Pedro Ferreira, Philipp Jablonski, Susana Posada-Céspedes, Tobias Schär, Ina Nissen, Natascha Santacroce, Elodie Burcklen, Christiane Beckmann, Maurice Redondo, Olivier Kobel, Christoph Noppen, Sophie Seidel, Noemie Santamaria de Souza, Niko Beerenwinkel, Tanja Stadler
EPI_ISL_730655	Lighthouse Lab in Alderley Park	Wellcome Sanger Institute for the COVID-19 Genomics UK (COG-UK) Consortium	Jacquelyn Wynn, Mairead Hyland, The Lighthouse Lab in Alderley Park and Alex Alderton, Roberto Amato, Sonia Goncalves, Ewan Harrison, David K. Jackson, Ian Johnston, Dominic Kwiatkowski, Cordelia Langford, John Sillitoe on behalf of the Wellcome Sanger Institute COVID-19 Surveillance Team
EPI_ISL_730656, EPI_ISL_730657	Lighthouse Lab in Glasgow	Wellcome Sanger Institute for the COVID-19 Genomics UK (COG-UK) Consortium	Harper VanSteenhouse, Yumi Kasai, David Gray, Carol Clugston, Anna Dominiczak and Alex Alderton, Roberto Amato, Sonia Goncalves, Ewan Harrison, David K. Jackson, Ian Johnston, Dominic Kwiatkowski, Cordelia Langford, John Sillitoe on behalf of the Wellcome Sanger Institute COVID-19 Surveillance

			Team
EPI_ISL_730658	Lighthouse Lab in Alderley Park	Wellcome Sanger Institute for the COVID-19 Genomics UK (COG-UK) Consortium	Jacquelyn Wynn, Mairead Hyland, The Lighthouse Lab in Alderley Park and Alex Alderton, Roberto Amato, Sonia Goncalves, Ewan Harrison, David K. Jackson, Ian Johnston, Dominic Kwiatkowski, Cordelia Langford, John Sillitoe on behalf of the Wellcome Sanger Institute COVID-19 Surveillance Team
EPI_ISL_730659, EPI_ISL_730664	Lighthouse Lab in Glasgow	Wellcome Sanger Institute for the COVID-19 Genomics UK (COG-UK) Consortium	Harper VanSteenhouse, Yumi Kasai, David Gray, Carol Clugston, Anna Dominiczak and Alex Alderton, Roberto Amato, Sonia Goncalves, Ewan Harrison, David K. Jackson, Ian Johnston, Dominic Kwiatkowski, Cordelia Langford, John Sillitoe on behalf of the Wellcome Sanger Institute COVID-19 Surveillance Team
EPI_ISL_730667, EPI_ISL_730668	Lighthouse Lab in Alderley Park	Wellcome Sanger Institute for the COVID-19 Genomics UK (COG-UK) Consortium	Jacquelyn Wynn, Mairead Hyland, The Lighthouse Lab in Alderley Park and Alex Alderton, Roberto Amato, Sonia Goncalves, Ewan Harrison, David K. Jackson, Ian Johnston, Dominic Kwiatkowski, Cordelia Langford, John Sillitoe on behalf of the Wellcome Sanger Institute COVID-19 Surveillance Team
EPI_ISL_730673, EPI_ISL_730675, EPI_ISL_730676, EPI_ISL_730678	Lighthouse Lab in Glasgow	Wellcome Sanger Institute for the COVID-19 Genomics UK (COG-UK) Consortium	Harper VanSteenhouse, Yumi Kasai, David Gray, Carol Clugston, Anna Dominiczak and Alex Alderton, Roberto Amato, Sonia Goncalves, Ewan Harrison, David K. Jackson, Ian Johnston, Dominic Kwiatkowski, Cordelia Langford, John Sillitoe on behalf of the Wellcome Sanger Institute COVID-19 Surveillance Team
EPI_ISL_730680, EPI_ISL_730682, EPI_ISL_730683	Lighthouse Lab in Alderley Park	Wellcome Sanger Institute for the COVID-19 Genomics UK (COG-UK) Consortium	Jacquelyn Wynn, Mairead Hyland, The Lighthouse Lab in Alderley Park and Alex Alderton, Roberto Amato, Sonia Goncalves, Ewan Harrison, David K. Jackson, Ian Johnston, Dominic Kwiatkowski, Cordelia Langford, John Sillitoe on behalf of the Wellcome Sanger Institute COVID-19 Surveillance Team
EPI_ISL_730685	Lighthouse Lab in Glasgow	Wellcome Sanger Institute for the COVID-19 Genomics UK (COG-UK) Consortium	Harper VanSteenhouse, Yumi Kasai, David Gray, Carol Clugston, Anna Dominiczak and Alex Alderton, Roberto Amato, Sonia Goncalves, Ewan Harrison, David K. Jackson, Ian Johnston, Dominic Kwiatkowski, Cordelia Langford, John Sillitoe on behalf of the Wellcome Sanger Institute COVID-19 Surveillance Team
EPI_ISL_730686, EPI_ISL_730692, EPI_ISL_730695, EPI_ISL_730696	Lighthouse Lab in Alderley Park	Wellcome Sanger Institute for the COVID-19 Genomics UK (COG-UK) Consortium	Jacquelyn Wynn, Mairead Hyland, The Lighthouse Lab in Alderley Park and Alex Alderton, Roberto Amato, Sonia Goncalves, Ewan Harrison, David K. Jackson, Ian Johnston, Dominic Kwiatkowski, Cordelia Langford, John Sillitoe on behalf of the Wellcome Sanger Institute COVID-19 Surveillance Team
EPI_ISL_730697	Lighthouse Lab in Glasgow	Wellcome Sanger Institute for the COVID-19 Genomics UK (COG-UK) Consortium	Harper VanSteenhouse, Yumi Kasai, David Gray, Carol Clugston, Anna Dominiczak and Alex Alderton, Roberto Amato, Sonia Goncalves, Ewan Harrison, David K. Jackson, Ian Johnston, Dominic Kwiatkowski, Cordelia Langford, John Sillitoe on behalf of the Wellcome Sanger Institute COVID-19 Surveillance Team
EPI_ISL_730699, EPI_ISL_730702, EPI_ISL_730704	Lighthouse Lab in Alderley Park	Wellcome Sanger Institute for the COVID-19 Genomics UK (COG-UK) Consortium	Jacquelyn Wynn, Mairead Hyland, The Lighthouse Lab in Alderley Park and Alex Alderton, Roberto Amato, Sonia Goncalves, Ewan Harrison, David K. Jackson, Ian Johnston, Dominic Kwiatkowski, Cordelia Langford, John Sillitoe on behalf of the Wellcome Sanger Institute COVID-19 Surveillance Team
EPI_ISL_730705, EPI_ISL_730709, EPI_ISL_730710	Lighthouse Lab in Glasgow	Wellcome Sanger Institute for the COVID-19 Genomics UK (COG-UK) Consortium	Harper VanSteenhouse, Yumi Kasai, David Gray, Carol Clugston, Anna Dominiczak and Alex Alderton, Roberto Amato, Sonia Goncalves, Ewan Harrison, David K. Jackson, Ian Johnston, Dominic Kwiatkowski, Cordelia Langford, John Sillitoe on behalf of the Wellcome Sanger Institute COVID-19 Surveillance Team
EPI_ISL_730711, EPI_ISL_730712	Lighthouse Lab in Alderley Park	Wellcome Sanger Institute for the COVID-19 Genomics UK (COG-UK) Consortium	Jacquelyn Wynn, Mairead Hyland, The Lighthouse Lab in Alderley Park and Alex Alderton, Roberto Amato, Sonia Goncalves, Ewan Harrison, David K. Jackson, Ian Johnston, Dominic Kwiatkowski, Cordelia Langford, John Sillitoe on behalf of the Wellcome Sanger Institute COVID-19 Surveillance Team
EPI_ISL_730718	Lighthouse Lab in Glasgow	Wellcome Sanger Institute for the COVID-19 Genomics UK (COG-UK) Consortium	Harper VanSteenhouse, Yumi Kasai, David Gray, Carol Clugston, Anna Dominiczak and Alex Alderton, Roberto Amato, Sonia Goncalves, Ewan Harrison, David K. Jackson, Ian Johnston, Dominic Kwiatkowski, Cordelia Langford, John Sillitoe on behalf of the Wellcome Sanger Institute COVID-19 Surveillance Team
EPI_ISL_730722, EPI_ISL_730729, EPI_ISL_730731, EPI_ISL_730732, EPI_ISL_730733, EPI_ISL_730734	Lighthouse Lab in Alderley Park	Wellcome Sanger Institute for the COVID-19 Genomics UK (COG-UK) Consortium	Jacquelyn Wynn, Mairead Hyland, The Lighthouse Lab in Alderley Park and Alex Alderton, Roberto Amato, Sonia Goncalves, Ewan Harrison, David K. Jackson, Ian Johnston, Dominic Kwiatkowski, Cordelia Langford, John Sillitoe on behalf of the Wellcome Sanger Institute COVID-19 Surveillance Team
EPI_ISL_730737	Lighthouse Lab in Glasgow	Wellcome Sanger Institute for the COVID-19 Genomics UK (COG-UK) Consortium	Harper VanSteenhouse, Yumi Kasai, David Gray, Carol Clugston, Anna Dominiczak and Alex Alderton, Roberto Amato, Sonia Goncalves, Ewan Harrison, David K. Jackson, Ian Johnston, Dominic Kwiatkowski, Cordelia Langford, John Sillitoe on behalf of the Wellcome Sanger Institute COVID-19 Surveillance Team
EPI_ISL_730742, EPI_ISL_730743, EPI_ISL_730750, EPI_ISL_730751	Lighthouse Lab in Alderley Park	Wellcome Sanger Institute for the COVID-19 Genomics UK (COG-UK) Consortium	Jacquelyn Wynn, Mairead Hyland, The Lighthouse Lab in Alderley Park and Alex Alderton, Roberto Amato, Sonia Goncalves, Ewan Harrison, David K. Jackson, Ian Johnston, Dominic Kwiatkowski, Cordelia Langford, John Sillitoe on behalf of the Wellcome Sanger Institute COVID-19 Surveillance Team
EPI_ISL_730752	Lighthouse Lab in Glasgow	Wellcome Sanger Institute for the COVID-19 Genomics UK (COG-UK) Consortium	Harper VanSteenhouse, Yumi Kasai, David Gray, Carol Clugston, Anna Dominiczak and Alex Alderton, Roberto Amato, Sonia Goncalves, Ewan Harrison, David K. Jackson, Ian Johnston, Dominic Kwiatkowski, Cordelia Langford, John Sillitoe on behalf of the Wellcome Sanger Institute COVID-19 Surveillance Team
EPI_ISL_730753, EPI_ISL_730754, EPI_ISL_730758	Lighthouse Lab in Alderley Park	Wellcome Sanger Institute for the COVID-19 Genomics UK (COG-UK) Consortium	Jacquelyn Wynn, Mairead Hyland, The Lighthouse Lab in Alderley Park and Alex Alderton, Roberto Amato, Sonia Goncalves, Ewan Harrison, David K. Jackson, Ian Johnston, Dominic Kwiatkowski, Cordelia Langford, John Sillitoe on behalf of the Wellcome Sanger Institute COVID-19 Surveillance Team
EPI_ISL_730759, EPI_ISL_730765	Lighthouse Lab in Glasgow	Wellcome Sanger Institute for the COVID-19 Genomics UK (COG-UK) Consortium	Harper VanSteenhouse, Yumi Kasai, David Gray, Carol Clugston, Anna Dominiczak and Alex Alderton, Roberto Amato, Sonia Goncalves, Ewan Harrison, David K. Jackson, Ian Johnston, Dominic Kwiatkowski, Cordelia Langford, John Sillitoe on behalf of the Wellcome Sanger Institute COVID-19 Surveillance Team
EPI_ISL_730771, EPI_ISL_730773, EPI_ISL_730774	Lighthouse Lab in Alderley Park	Wellcome Sanger Institute for the COVID-19 Genomics UK (COG-UK) Consortium	Jacquelyn Wynn, Mairead Hyland, The Lighthouse Lab in Alderley Park and Alex Alderton, Roberto Amato, Sonia Goncalves, Ewan Harrison, David K. Jackson, Ian Johnston, Dominic Kwiatkowski, Cordelia Langford, John Sillitoe on behalf of the Wellcome Sanger Institute COVID-19 Surveillance Team
EPI_ISL_730777, EPI_ISL_730778, EPI_ISL_730780	Lighthouse Lab in Glasgow	Wellcome Sanger Institute for the COVID-19 Genomics UK (COG-UK) Consortium	Harper VanSteenhouse, Yumi Kasai, David Gray, Carol Clugston, Anna Dominiczak and Alex Alderton, Roberto Amato, Sonia Goncalves, Ewan Harrison, David K. Jackson, Ian Johnston, Dominic Kwiatkowski, Cordelia Langford, John Sillitoe on behalf of the Wellcome Sanger Institute COVID-19 Surveillance Team
EPI_ISL_730784	Lighthouse Lab in Alderley Park	Wellcome Sanger Institute for the COVID-19 Genomics UK (COG-UK) Consortium	Jacquelyn Wynn, Mairead Hyland, The Lighthouse Lab in Alderley Park and Alex Alderton, Roberto Amato, Sonia Goncalves, Ewan Harrison, David K. Jackson, Ian Johnston, Dominic Kwiatkowski, Cordelia Langford, John Sillitoe on behalf of the Wellcome Sanger Institute COVID-19 Surveillance Team
EPI_ISL_730785	Lighthouse Lab in Glasgow	Wellcome Sanger Institute for the COVID-19 Genomics UK (COG-UK) Consortium	Harper VanSteenhouse, Yumi Kasai, David Gray, Carol Clugston, Anna Dominiczak and Alex Alderton, Roberto Amato, Sonia Goncalves, Ewan Harrison, David K. Jackson, Ian Johnston, Dominic Kwiatkowski, Cordelia Langford, John Sillitoe on behalf of the Wellcome Sanger Institute COVID-19 Surveillance Team
EPI_ISL_730786, EPI_ISL_730788	Lighthouse Lab in Alderley Park	Wellcome Sanger Institute for the COVID-19 Genomics UK (COG-UK) Consortium	Jacquelyn Wynn, Mairead Hyland, The Lighthouse Lab in Alderley Park and Alex Alderton, Roberto Amato, Sonia Goncalves, Ewan Harrison, David K. Jackson, Ian Johnston, Dominic Kwiatkowski, Cordelia Langford, John Sillitoe on behalf of the Wellcome Sanger Institute COVID-19 Surveillance Team
EPI_ISL_730789, EPI_ISL_730793	Lighthouse Lab in Glasgow	Wellcome Sanger Institute for the COVID-19 Genomics UK (COG-UK) Consortium	Harper VanSteenhouse, Yumi Kasai, David Gray, Carol Clugston, Anna Dominiczak and Alex Alderton, Roberto Amato, Sonia Goncalves, Ewan Harrison, David K. Jackson, Ian Johnston, Dominic Kwiatkowski, Cordelia Langford, John Sillitoe on behalf of the Wellcome Sanger Institute COVID-19 Surveillance Team
EPI_ISL_730794	Lighthouse Lab in Alderley Park	Wellcome Sanger Institute for the COVID-19 Genomics UK (COG-UK) Consortium	Jacquelyn Wynn, Mairead Hyland, The Lighthouse Lab in Alderley Park and Alex Alderton, Roberto Amato, Sonia Goncalves, Ewan Harrison, David K. Jackson, Ian Johnston, Dominic Kwiatkowski, Cordelia Langford, John Sillitoe on behalf of the Wellcome Sanger Institute COVID-19 Surveillance Team
EPI_ISL_730795, EPI_ISL_730796, EPI_ISL_730801, EPI_ISL_730802, EPI_ISL_730803, EPI_ISL_730804, EPI_ISL_730805, EPI_ISL_730806, EPI_ISL_730809, EPI_ISL_730813	Lighthouse Lab in Glasgow	Wellcome Sanger Institute for the COVID-19 Genomics UK (COG-UK) Consortium	Harper VanSteenhouse, Yumi Kasai, David Gray, Carol Clugston, Anna Dominiczak and Alex Alderton, Roberto Amato, Sonia Goncalves, Ewan Harrison, David K. Jackson, Ian Johnston, Dominic Kwiatkowski, Cordelia Langford, John Sillitoe on behalf of the Wellcome Sanger Institute COVID-19 Surveillance Team
EPI_ISL_730814	Lighthouse Lab in Alderley Park	Wellcome Sanger Institute for the COVID-19 Genomics UK (COG-UK) Consortium	Jacquelyn Wynn, Mairead Hyland, The Lighthouse Lab in Alderley Park and Alex Alderton, Roberto Amato, Sonia Goncalves, Ewan Harrison, David K. Jackson, Ian Johnston, Dominic Kwiatkowski, Cordelia Langford, John Sillitoe on behalf of the Wellcome Sanger Institute COVID-19 Surveillance Team
EPI_ISL_730815	Lighthouse Lab in Glasgow	Wellcome Sanger Institute for the COVID-19 Genomics UK (COG-UK) Consortium	Harper VanSteenhouse, Yumi Kasai, David Gray, Carol Clugston, Anna Dominiczak and Alex Alderton, Roberto Amato, Sonia Goncalves, Ewan Harrison, David K. Jackson, Ian Johnston, Dominic Kwiatkowski, Cordelia Langford, John Sillitoe on behalf of the Wellcome Sanger Institute COVID-19 Surveillance

			Team
EPI_ISL_730818, EPI_ISL_730819, EPI_ISL_730823	Lighthouse Lab in Alderley Park	Wellcome Sanger Institute for the COVID-19 Genomics UK (COG-UK) Consortium	Jacquelyn Wynn, Mairead Hyland, The Lighthouse Lab in Alderley Park and Alex Alderton, Roberto Amato, Sonia Goncalves, Ewan Harrison, David K. Jackson, Ian Johnston, Dominic Kwiatkowski, Cordelia Langford, John Sillitoe on behalf of the Wellcome Sanger Institute COVID-19 Surveillance Team
EPI_ISL_730824	Lighthouse Lab in Glasgow	Wellcome Sanger Institute for the COVID-19 Genomics UK (COG-UK) Consortium	Harper VanSteenhouse, Yumi Kasai, David Gray, Carol Clugston, Anna Dominiczak and Alex Alderton, Roberto Amato, Sonia Goncalves, Ewan Harrison, David K. Jackson, Ian Johnston, Dominic Kwiatkowski, Cordelia Langford, John Sillitoe on behalf of the Wellcome Sanger Institute COVID-19 Surveillance Team
EPI_ISL_730825, EPI_ISL_730826	Lighthouse Lab in Alderley Park	Wellcome Sanger Institute for the COVID-19 Genomics UK (COG-UK) Consortium	Jacquelyn Wynn, Mairead Hyland, The Lighthouse Lab in Alderley Park and Alex Alderton, Roberto Amato, Sonia Goncalves, Ewan Harrison, David K. Jackson, Ian Johnston, Dominic Kwiatkowski, Cordelia Langford, John Sillitoe on behalf of the Wellcome Sanger Institute COVID-19 Surveillance Team
EPI_ISL_730828	Lighthouse Lab in Glasgow	Wellcome Sanger Institute for the COVID-19 Genomics UK (COG-UK) Consortium	Harper VanSteenhouse, Yumi Kasai, David Gray, Carol Clugston, Anna Dominiczak and Alex Alderton, Roberto Amato, Sonia Goncalves, Ewan Harrison, David K. Jackson, Ian Johnston, Dominic Kwiatkowski, Cordelia Langford, John Sillitoe on behalf of the Wellcome Sanger Institute COVID-19 Surveillance Team
EPI_ISL_730829, EPI_ISL_730830	Lighthouse Lab in Alderley Park	Wellcome Sanger Institute for the COVID-19 Genomics UK (COG-UK) Consortium	Jacquelyn Wynn, Mairead Hyland, The Lighthouse Lab in Alderley Park and Alex Alderton, Roberto Amato, Sonia Goncalves, Ewan Harrison, David K. Jackson, Ian Johnston, Dominic Kwiatkowski, Cordelia Langford, John Sillitoe on behalf of the Wellcome Sanger Institute COVID-19 Surveillance Team
EPI_ISL_730831, EPI_ISL_730832, EPI_ISL_730833	Lighthouse Lab in Glasgow	Wellcome Sanger Institute for the COVID-19 Genomics UK (COG-UK) Consortium	Harper VanSteenhouse, Yumi Kasai, David Gray, Carol Clugston, Anna Dominiczak and Alex Alderton, Roberto Amato, Sonia Goncalves, Ewan Harrison, David K. Jackson, Ian Johnston, Dominic Kwiatkowski, Cordelia Langford, John Sillitoe on behalf of the Wellcome Sanger Institute COVID-19 Surveillance Team
EPI_ISL_730834, EPI_ISL_730839	Lighthouse Lab in Alderley Park	Wellcome Sanger Institute for the COVID-19 Genomics UK (COG-UK) Consortium	Jacquelyn Wynn, Mairead Hyland, The Lighthouse Lab in Alderley Park and Alex Alderton, Roberto Amato, Sonia Goncalves, Ewan Harrison, David K. Jackson, Ian Johnston, Dominic Kwiatkowski, Cordelia Langford, John Sillitoe on behalf of the Wellcome Sanger Institute COVID-19 Surveillance Team
EPI_ISL_730840	Lighthouse Lab in Glasgow	Wellcome Sanger Institute for the COVID-19 Genomics UK (COG-UK) Consortium	Harper VanSteenhouse, Yumi Kasai, David Gray, Carol Clugston, Anna Dominiczak and Alex Alderton, Roberto Amato, Sonia Goncalves, Ewan Harrison, David K. Jackson, Ian Johnston, Dominic Kwiatkowski, Cordelia Langford, John Sillitoe on behalf of the Wellcome Sanger Institute COVID-19 Surveillance Team
EPI_ISL_730844, EPI_ISL_730845	Lighthouse Lab in Alderley Park	Wellcome Sanger Institute for the COVID-19 Genomics UK (COG-UK) Consortium	Jacquelyn Wynn, Mairead Hyland, The Lighthouse Lab in Alderley Park and Alex Alderton, Roberto Amato, Sonia Goncalves, Ewan Harrison, David K. Jackson, Ian Johnston, Dominic Kwiatkowski, Cordelia Langford, John Sillitoe on behalf of the Wellcome Sanger Institute COVID-19 Surveillance Team
EPI_ISL_730846, EPI_ISL_730847	Lighthouse Lab in Glasgow	Wellcome Sanger Institute for the COVID-19 Genomics UK (COG-UK) Consortium	Harper VanSteenhouse, Yumi Kasai, David Gray, Carol Clugston, Anna Dominiczak and Alex Alderton, Roberto Amato, Sonia Goncalves, Ewan Harrison, David K. Jackson, Ian Johnston, Dominic Kwiatkowski, Cordelia Langford, John Sillitoe on behalf of the Wellcome Sanger Institute COVID-19 Surveillance Team
EPI_ISL_730848	Lighthouse Lab in Alderley Park	Wellcome Sanger Institute for the COVID-19 Genomics UK (COG-UK) Consortium	Jacquelyn Wynn, Mairead Hyland, The Lighthouse Lab in Alderley Park and Alex Alderton, Roberto Amato, Sonia Goncalves, Ewan Harrison, David K. Jackson, Ian Johnston, Dominic Kwiatkowski, Cordelia Langford, John Sillitoe on behalf of the Wellcome Sanger Institute COVID-19 Surveillance Team
EPI_ISL_730849, EPI_ISL_730850	Lighthouse Lab in Glasgow	Wellcome Sanger Institute for the COVID-19 Genomics UK (COG-UK) Consortium	Harper VanSteenhouse, Yumi Kasai, David Gray, Carol Clugston, Anna Dominiczak and Alex Alderton, Roberto Amato, Sonia Goncalves, Ewan Harrison, David K. Jackson, Ian Johnston, Dominic Kwiatkowski, Cordelia Langford, John Sillitoe on behalf of the Wellcome Sanger Institute COVID-19 Surveillance Team
EPI_ISL_730852, EPI_ISL_730854, EPI_ISL_730855	Lighthouse Lab in Alderley Park	Wellcome Sanger Institute for the COVID-19 Genomics UK (COG-UK) Consortium	Jacquelyn Wynn, Mairead Hyland, The Lighthouse Lab in Alderley Park and Alex Alderton, Roberto Amato, Sonia Goncalves, Ewan Harrison, David K. Jackson, Ian Johnston, Dominic Kwiatkowski, Cordelia Langford, John Sillitoe on behalf of the Wellcome Sanger Institute COVID-19 Surveillance Team
EPI_ISL_730858	Lighthouse Lab in Glasgow	Wellcome Sanger Institute for the COVID-19 Genomics UK (COG-UK) Consortium	Harper VanSteenhouse, Yumi Kasai, David Gray, Carol Clugston, Anna Dominiczak and Alex Alderton, Roberto Amato, Sonia Goncalves, Ewan Harrison, David K. Jackson, Ian Johnston, Dominic Kwiatkowski, Cordelia Langford, John Sillitoe on behalf of the Wellcome Sanger Institute COVID-19 Surveillance Team
EPI_ISL_730859	Lighthouse Lab in Alderley Park	Wellcome Sanger Institute for the COVID-19 Genomics UK (COG-UK) Consortium	Jacquelyn Wynn, Mairead Hyland, The Lighthouse Lab in Alderley Park and Alex Alderton, Roberto Amato, Sonia Goncalves, Ewan Harrison, David K. Jackson, Ian Johnston, Dominic Kwiatkowski, Cordelia Langford, John Sillitoe on behalf of the Wellcome Sanger Institute COVID-19 Surveillance Team
EPI_ISL_730860	Lighthouse Lab in Glasgow	Wellcome Sanger Institute for the COVID-19 Genomics UK (COG-UK) Consortium	Harper VanSteenhouse, Yumi Kasai, David Gray, Carol Clugston, Anna Dominiczak and Alex Alderton, Roberto Amato, Sonia Goncalves, Ewan Harrison, David K. Jackson, Ian Johnston, Dominic Kwiatkowski, Cordelia Langford, John Sillitoe on behalf of the Wellcome Sanger Institute COVID-19 Surveillance Team
EPI_ISL_730861	Lighthouse Lab in Alderley Park	Wellcome Sanger Institute for the COVID-19 Genomics UK (COG-UK) Consortium	Jacquelyn Wynn, Mairead Hyland, The Lighthouse Lab in Alderley Park and Alex Alderton, Roberto Amato, Sonia Goncalves, Ewan Harrison, David K. Jackson, Ian Johnston, Dominic Kwiatkowski, Cordelia Langford, John Sillitoe on behalf of the Wellcome Sanger Institute COVID-19 Surveillance Team
EPI_ISL_730862, EPI_ISL_730864	Lighthouse Lab in Glasgow	Wellcome Sanger Institute for the COVID-19 Genomics UK (COG-UK) Consortium	Harper VanSteenhouse, Yumi Kasai, David Gray, Carol Clugston, Anna Dominiczak and Alex Alderton, Roberto Amato, Sonia Goncalves, Ewan Harrison, David K. Jackson, Ian Johnston, Dominic Kwiatkowski, Cordelia Langford, John Sillitoe on behalf of the Wellcome Sanger Institute COVID-19 Surveillance Team
EPI_ISL_730865	Lighthouse Lab in Alderley Park	Wellcome Sanger Institute for the COVID-19 Genomics UK (COG-UK) Consortium	Jacquelyn Wynn, Mairead Hyland, The Lighthouse Lab in Alderley Park and Alex Alderton, Roberto Amato, Sonia Goncalves, Ewan Harrison, David K. Jackson, Ian Johnston, Dominic Kwiatkowski, Cordelia Langford, John Sillitoe on behalf of the Wellcome Sanger Institute COVID-19 Surveillance Team
EPI_ISL_730867, EPI_ISL_730869	Lighthouse Lab in Glasgow	Wellcome Sanger Institute for the COVID-19 Genomics UK (COG-UK) Consortium	Harper VanSteenhouse, Yumi Kasai, David Gray, Carol Clugston, Anna Dominiczak and Alex Alderton, Roberto Amato, Sonia Goncalves, Ewan Harrison, David K. Jackson, Ian Johnston, Dominic Kwiatkowski, Cordelia Langford, John Sillitoe on behalf of the Wellcome Sanger Institute COVID-19 Surveillance Team
EPI_ISL_730870, EPI_ISL_730873	Lighthouse Lab in Alderley Park	Wellcome Sanger Institute for the COVID-19 Genomics UK (COG-UK) Consortium	Jacquelyn Wynn, Mairead Hyland, The Lighthouse Lab in Alderley Park and Alex Alderton, Roberto Amato, Sonia Goncalves, Ewan Harrison, David K. Jackson, Ian Johnston, Dominic Kwiatkowski, Cordelia Langford, John Sillitoe on behalf of the Wellcome Sanger Institute COVID-19 Surveillance Team
EPI_ISL_730875, EPI_ISL_730876, EPI_ISL_730877	Lighthouse Lab in Glasgow	Wellcome Sanger Institute for the COVID-19 Genomics UK (COG-UK) Consortium	Harper VanSteenhouse, Yumi Kasai, David Gray, Carol Clugston, Anna Dominiczak and Alex Alderton, Roberto Amato, Sonia Goncalves, Ewan Harrison, David K. Jackson, Ian Johnston, Dominic Kwiatkowski, Cordelia Langford, John Sillitoe on behalf of the Wellcome Sanger Institute COVID-19 Surveillance Team
EPI_ISL_730878, EPI_ISL_730881, EPI_ISL_730886	Lighthouse Lab in Alderley Park	Wellcome Sanger Institute for the COVID-19 Genomics UK (COG-UK) Consortium	Jacquelyn Wynn, Mairead Hyland, The Lighthouse Lab in Alderley Park and Alex Alderton, Roberto Amato, Sonia Goncalves, Ewan Harrison, David K. Jackson, Ian Johnston, Dominic Kwiatkowski, Cordelia Langford, John Sillitoe on behalf of the Wellcome Sanger Institute COVID-19 Surveillance Team
EPI_ISL_730887, EPI_ISL_730889	Lighthouse Lab in Glasgow	Wellcome Sanger Institute for the COVID-19 Genomics UK (COG-UK) Consortium	Harper VanSteenhouse, Yumi Kasai, David Gray, Carol Clugston, Anna Dominiczak and Alex Alderton, Roberto Amato, Sonia Goncalves, Ewan Harrison, David K. Jackson, Ian Johnston, Dominic Kwiatkowski, Cordelia Langford, John Sillitoe on behalf of the Wellcome Sanger Institute COVID-19 Surveillance Team
EPI_ISL_730890, EPI_ISL_730893	Lighthouse Lab in Alderley Park	Wellcome Sanger Institute for the COVID-19 Genomics UK (COG-UK) Consortium	Jacquelyn Wynn, Mairead Hyland, The Lighthouse Lab in Alderley Park and Alex Alderton, Roberto Amato, Sonia Goncalves, Ewan Harrison, David K. Jackson, Ian Johnston, Dominic Kwiatkowski, Cordelia Langford, John Sillitoe on behalf of the Wellcome Sanger Institute COVID-19 Surveillance Team
EPI_ISL_730894, EPI_ISL_730898	Lighthouse Lab in Glasgow	Wellcome Sanger Institute for the COVID-19 Genomics UK (COG-UK) Consortium	Harper VanSteenhouse, Yumi Kasai, David Gray, Carol Clugston, Anna Dominiczak and Alex Alderton, Roberto Amato, Sonia Goncalves, Ewan Harrison, David K. Jackson, Ian Johnston, Dominic Kwiatkowski, Cordelia Langford, John Sillitoe on behalf of the Wellcome Sanger Institute COVID-19 Surveillance Team
EPI_ISL_730900, EPI_ISL_730902, EPI_ISL_730904	Lighthouse Lab in Alderley Park	Wellcome Sanger Institute for the COVID-19 Genomics UK (COG-UK) Consortium	Jacquelyn Wynn, Mairead Hyland, The Lighthouse Lab in Alderley Park and Alex Alderton, Roberto Amato, Sonia Goncalves, Ewan Harrison, David K. Jackson, Ian Johnston, Dominic Kwiatkowski, Cordelia Langford, John Sillitoe on behalf of the Wellcome Sanger Institute COVID-19 Surveillance Team
EPI_ISL_730908, EPI_ISL_730910, EPI_ISL_730911, EPI_ISL_730913	Lighthouse Lab in Glasgow	Wellcome Sanger Institute for the COVID-19 Genomics UK (COG-UK) Consortium	Harper VanSteenhouse, Yumi Kasai, David Gray, Carol Clugston, Anna Dominiczak and Alex Alderton, Roberto Amato, Sonia Goncalves, Ewan Harrison, David K. Jackson, Ian Johnston, Dominic Kwiatkowski, Cordelia Langford, John Sillitoe on behalf of the Wellcome Sanger Institute COVID-19 Surveillance Team

EPI_ISL_730914, EPI_ISL_730917, EPI_ISL_730918, EPI_ISL_730919, EPI_ISL_730920, EPI_ISL_730922, EPI_ISL_730923, EPI_ISL_730925, EPI_ISL_730926, EPI_ISL_730927, EPI_ISL_730928, EPI_ISL_730930, EPI_ISL_730931, EPI_ISL_730932, EPI_ISL_730933, EPI_ISL_730934, EPI_ISL_730936, EPI_ISL_730937, EPI_ISL_730938, EPI_ISL_730940, EPI_ISL_730941, EPI_ISL_730942, EPI_ISL_730943, EPI_ISL_730944, EPI_ISL_730945, EPI_ISL_730946, EPI_ISL_730947, EPI_ISL_730949, EPI_ISL_730950, EPI_ISL_730951, EPI_ISL_730952, EPI_ISL_730953, EPI_ISL_730954, EPI_ISL_730955, EPI_ISL_730956, EPI_ISL_730957,

EPI_ISL_730958, EPI_ISL_730960, EPI_ISL_730961, EPI_ISL_730962, EPI_ISL_730963, EPI_ISL_730964, EPI_ISL_730965, EPI_ISL_730968, EPI_ISL_730972, EPI_ISL_730974, EPI_ISL_730975, EPI_ISL_730976, EPI_ISL_730977, EPI_ISL_730978, EPI_ISL_730979, EPI_ISL_730981, EPI_ISL_730982, EPI_ISL_730983, EPI_ISL_730984, EPI_ISL_730985, EPI_ISL_730986, EPI_ISL_730988, EPI_ISL_730990, EPI_ISL_730991, EPI_ISL_730992, EPI_ISL_730993, EPI_ISL_730995, EPI_ISL_730996, EPI_ISL_730997, EPI_ISL_730998, EPI_ISL_730999, EPI_ISL_731001, EPI_ISL_731002, EPI_ISL_731003, EPI_ISL_731004, EPI_ISL_731005, EPI_ISL_731007, EPI_ISL_731008, EPI_ISL_731009, EPI_ISL_731010, EPI_ISL_731011, EPI_ISL_731012, EPI_ISL_731013, EPI_ISL_731014, EPI_ISL_731015, EPI_ISL_731016, EPI_ISL_731018, EPI_ISL_731021, EPI_ISL_731022, EPI_ISL_731023, EPI_ISL_731025, EPI_ISL_731028, EPI_ISL_731029, EPI_ISL_731030, EPI_ISL_731031, EPI_ISL_731032, EPI_ISL_731034, EPI_ISL_731036, EPI_ISL_731037, EPI_ISL_731038, EPI_ISL_731039, EPI_ISL_731041, EPI_ISL_731042, EPI_ISL_731043, EPI_ISL_731044, EPI_ISL_731046, EPI_ISL_731047, EPI_ISL_731048, EPI_ISL_731049, EPI_ISL_731051, EPI_ISL_731053, EPI_ISL_731056, EPI_ISL_731058, EPI_ISL_731060, EPI_ISL_731061, EPI_ISL_731062, EPI_ISL_731063, EPI_ISL_731064, EPI_ISL_731067, EPI_ISL_731068, EPI_ISL_731069, EPI_ISL_731070, EPI_ISL_731071, EPI_ISL_731072, EPI_ISL_731073, EPI_ISL_731075, EPI_ISL_731077, EPI_ISL_731078, EPI_ISL_731081, EPI_ISL_731084, EPI_ISL_731085, EPI_ISL_731089, EPI_ISL_731090, EPI_ISL_731091, EPI_ISL_731092, EPI_ISL_731093, EPI_ISL_731095, EPI_ISL_731097, EPI_ISL_731098, EPI_ISL_731099, EPI_ISL_731100, EPI_ISL_731101, EPI_ISL_731102, EPI_ISL_731103, EPI_ISL_731105, EPI_ISL_731108, EPI_ISL_731109, EPI_ISL_731111, EPI_ISL_731112, EPI_ISL_731113, EPI_ISL_731114, EPI_ISL_731115, EPI_ISL_731117, EPI_ISL_731118, EPI_ISL_731121, EPI_ISL_731122, EPI_ISL_731124, EPI_ISL_731125, EPI_ISL_731127, EPI_ISL_731128, EPI_ISL_731132, EPI_ISL_731134, EPI_ISL_731136, EPI_ISL_731138, EPI_ISL_731139, EPI_ISL_731140, EPI_ISL_731142, EPI_ISL_731144, EPI_ISL_731145, EPI_ISL_731146, EPI_ISL_731148, EPI_ISL_731149, EPI_ISL_731150, EPI_ISL_731151, EPI_ISL_731152, EPI_ISL_731155, EPI_ISL_731156, EPI_ISL_731159, EPI_ISL_731162, EPI_ISL_731163, EPI_ISL_731164, EPI_ISL_731165, EPI_ISL_731166, EPI_ISL_731167, EPI_ISL_731168, EPI_ISL_731170, EPI_ISL_731172, EPI_ISL_731173, EPI_ISL_731174, EPI_ISL_731175, EPI_ISL_731176, EPI_ISL_731177, EPI_ISL_731178, EPI_ISL_731179, EPI_ISL_731180, EPI_ISL_731182, EPI_ISL_731184, EPI_ISL_731185, EPI_ISL_731186, EPI_ISL_731187, EPI_ISL_731189, EPI_ISL_731190, EPI_ISL_731191, EPI_ISL_731192, EPI_ISL_731193, EPI_ISL_731194, EPI_ISL_731195, EPI_ISL_731197, EPI_ISL_731198, EPI_ISL_731201, EPI_ISL_731202, EPI_ISL_731203, EPI_ISL_731205, EPI_ISL_731207, EPI_ISL_731208, EPI_ISL_731209, EPI_ISL_731210, EPI_ISL_731211, EPI_ISL_731212, EPI_ISL_731213, EPI_ISL_731216, EPI_ISL_731217, EPI_ISL_731218, EPI_ISL_731219, EPI_ISL_731220, EPI_ISL_731221, EPI_ISL_731222, EPI_ISL_731223, EPI_ISL_731224, EPI_ISL_731225, EPI_ISL_731226, EPI_ISL_731227, EPI_ISL_731228, EPI_ISL_731229, EPI_ISL_731230, EPI_ISL_731231, EPI_ISL_731232, EPI_ISL_731234, EPI_ISL_731236, EPI_ISL_731237, EPI_ISL_731238, EPI_ISL_731239, EPI_ISL_731241, EPI_ISL_731242, EPI_ISL_731243, EPI_ISL_731244, EPI_ISL_731245, EPI_ISL_731246, EPI_ISL_731249, EPI_ISL_731250			
see above	Lighthouse Lab in Alderley Park	Wellcome Sanger Institute for the COVID-19 Genomics UK (COG-UK) Consortium	Jacquelyn Wynn, Mairead Hyland, The Lighthouse Lab in Alderley Park and Alex Alderton, Roberto Amato, Sonia Goncalves, Ewan Harrison, David K. Jackson, Ian Johnston, Dominic Kwiatkowski, Cordelia Langford, John Sillitoe on behalf of the Wellcome Sanger Institute COVID-19 Surveillance Team
EPI_ISL_731255, EPI_ISL_731256, EPI_ISL_731257, EPI_ISL_731258, EPI_ISL_731259, EPI_ISL_731267, EPI_ISL_731271, EPI_ISL_731273, EPI_ISL_731280, EPI_ISL_731281, EPI_ISL_731282, EPI_ISL_731286, EPI_ISL_731287, EPI_ISL_731291, EPI_ISL_731295, EPI_ISL_731297, EPI_ISL_731305, EPI_ISL_731308, EPI_ISL_731309, EPI_ISL_731310, EPI_ISL_731314, EPI_ISL_731319, EPI_ISL_731320, EPI_ISL_731321, EPI_ISL_731322, EPI_ISL_731326, EPI_ISL_731327, EPI_ISL_731330, EPI_ISL_731332, EPI_ISL_731337, EPI_ISL_731338, EPI_ISL_731340, EPI_ISL_731341, EPI_ISL_731345, EPI_ISL_731346, EPI_ISL_731354, EPI_ISL_731355, EPI_ISL_731357, EPI_ISL_731361, EPI_ISL_731367, EPI_ISL_731368, EPI_ISL_731372, EPI_ISL_731373, EPI_ISL_731376, EPI_ISL_731379, EPI_ISL_731380, EPI_ISL_731382, EPI_ISL_731383, EPI_ISL_731386, EPI_ISL_731390, EPI_ISL_731392, EPI_ISL_731397, EPI_ISL_731401, EPI_ISL_731402, EPI_ISL_731403, EPI_ISL_731404, EPI_ISL_731406, EPI_ISL_731407, EPI_ISL_731408, EPI_ISL_731411, EPI_ISL_731414, EPI_ISL_731417, EPI_ISL_731420, EPI_ISL_731425, EPI_ISL_731426, EPI_ISL_731429, EPI_ISL_731431, EPI_ISL_731432, EPI_ISL_731435, EPI_ISL_731437, EPI_ISL_731444, EPI_ISL_731447, EPI_ISL_731449, EPI_ISL_731450, EPI_ISL_731452, EPI_ISL_731453, EPI_ISL_731454, EPI_ISL_731458, EPI_ISL_731469, EPI_ISL_731471, EPI_ISL_731472, EPI_ISL_731476, EPI_ISL_731477, EPI_ISL_731482, EPI_ISL_731484, EPI_ISL_731485, EPI_ISL_731486, EPI_ISL_731488, EPI_ISL_731491, EPI_ISL_731493, EPI_ISL_731494, EPI_ISL_731496, EPI_ISL_731497, EPI_ISL_731499, EPI_ISL_731501, EPI_ISL_731505, EPI_ISL_731507, EPI_ISL_731508, EPI_ISL_731513, EPI_ISL_731517, EPI_ISL_731519, EPI_ISL_731523, EPI_ISL_731536, EPI_ISL_731537, EPI_ISL_731544, EPI_ISL_731548, EPI_ISL_731550, EPI_ISL_731551			
see above	Lighthouse Lab in Glasgow	Wellcome Sanger Institute for the COVID-19 Genomics UK (COG-UK) Consortium	Harper VanSteenhouse, Yumi Kasai, David Gray, Carol Clugston, Anna Dominiczak and Alex Alderton, Roberto Amato, Sonia Goncalves, Ewan Harrison, David K. Jackson, Ian Johnston, Dominic Kwiatkowski, Cordelia Langford, John Sillitoe on behalf of the Wellcome Sanger Institute COVID-19 Surveillance Team
EPI_ISL_731552, EPI_ISL_731553, EPI_ISL_731554, EPI_ISL_731556, EPI_ISL_731557, EPI_ISL_731558, EPI_ISL_731559, EPI_ISL_731560, EPI_ISL_731561, EPI_ISL_731562, EPI_ISL_731563, EPI_ISL_731564, EPI_ISL_731566, EPI_ISL_731567, EPI_ISL_731568, EPI_ISL_731569, EPI_ISL_731572, EPI_ISL_731573, EPI_ISL_731574, EPI_ISL_731575, EPI_ISL_731577, EPI_ISL_731578, EPI_ISL_731579, EPI_ISL_731581, EPI_ISL_731582, EPI_ISL_731583, EPI_ISL_731586, EPI_ISL_731587, EPI_ISL_731589, EPI_ISL_731590, EPI_ISL_731591, EPI_ISL_731593, EPI_ISL_731594, EPI_ISL_731595, EPI_ISL_731596, EPI_ISL_731597, EPI_ISL_731598, EPI_ISL_731600, EPI_ISL_731601, EPI_ISL_731602, EPI_ISL_731603, EPI_ISL_731604, EPI_ISL_731605, EPI_ISL_731606, EPI_ISL_731607, EPI_ISL_731608, EPI_ISL_731609, EPI_ISL_731610, EPI_ISL_731612, EPI_ISL_731613, EPI_ISL_731614, EPI_ISL_731615, EPI_ISL_731616, EPI_ISL_731617, EPI_ISL_731618, EPI_ISL_731619, EPI_ISL_731620, EPI_ISL_731621, EPI_ISL_731622, EPI_ISL_731623, EPI_ISL_731625, EPI_ISL_731627, EPI_ISL_731628, EPI_ISL_731630, EPI_ISL_731631, EPI_ISL_731633, EPI_ISL_731634, EPI_ISL_731636, EPI_ISL_731637, EPI_ISL_731638, EPI_ISL_731642, EPI_ISL_731643, EPI_ISL_731645, EPI_ISL_731646, EPI_ISL_731650, EPI_ISL_731651, EPI_ISL_731652, EPI_ISL_731653, EPI_ISL_731654, EPI_ISL_731657, EPI_ISL_731658, EPI_ISL_731659, EPI_ISL_731660, EPI_ISL_731661, EPI_ISL_731662, EPI_ISL_731663, EPI_ISL_731666, EPI_ISL_731668, EPI_ISL_731669, EPI_ISL_731670, EPI_ISL_731672, EPI_ISL_731673, EPI_ISL_731674, EPI_ISL_731675, EPI_ISL_731676, EPI_ISL_731677, EPI_ISL_731678, EPI_ISL_731679, EPI_ISL_731681, EPI_ISL_731682, EPI_ISL_731683, EPI_ISL_731685, EPI_ISL_731687, EPI_ISL_731689, EPI_ISL_731694, EPI_ISL_731695, EPI_ISL_731696, EPI_ISL_731698, EPI_ISL_731699, EPI_ISL_731700, EPI_ISL_731702, EPI_ISL_731703, EPI_ISL_731704, EPI_ISL_731706, EPI_ISL_731708, EPI_ISL_731710, EPI_ISL_731712, EPI_ISL_731713, EPI_ISL_731715, EPI_ISL_731716, EPI_ISL_731717, EPI_ISL_731718, EPI_ISL_731720, EPI_ISL_731723, EPI_ISL_731725, EPI_ISL_731726, EPI_ISL_731727, EPI_ISL_731728, EPI_ISL_731729, EPI_ISL_731730, EPI_ISL_731732, EPI_ISL_731733, EPI_ISL_731734, EPI_ISL_731735, EPI_ISL_731738, EPI_ISL_731739, EPI_ISL_731740, EPI_ISL_731741, EPI_ISL_731742, EPI_ISL_731743, EPI_ISL_731744, EPI_ISL_731745, EPI_ISL_731746, EPI_ISL_731747, EPI_ISL_731748, EPI_ISL_731749, EPI_ISL_731750, EPI_ISL_731751, EPI_ISL_731752, EPI_ISL_731753, EPI_ISL_731754, EPI_ISL_731755, EPI_ISL_731756, EPI_ISL_731757, EPI_ISL_731758, EPI_ISL_731759, EPI_ISL_731760, EPI_ISL_731761, EPI_ISL_731762, EPI_ISL_731764, EPI_ISL_731765, EPI_ISL_731766, EPI_ISL_731768, EPI_ISL_731769, EPI_ISL_731771, EPI_ISL_731772, EPI_ISL_731773, EPI_ISL_731774, EPI_ISL_731776, EPI_ISL_731777, EPI_ISL_731778, EPI_ISL_731779, EPI_ISL_731780, EPI_ISL_731781, EPI_ISL_731782, EPI_ISL_731783, EPI_ISL_731784, EPI_ISL_731786, EPI_ISL_731789, EPI_ISL_731790, EPI_ISL_731791, EPI_ISL_731793, EPI_ISL_731794, EPI_ISL_731797, EPI_ISL_731798, EPI_ISL_731801, EPI_ISL_731802, EPI_ISL_731803, EPI_ISL_731804, EPI_ISL_731805, EPI_ISL_731807, EPI_ISL_731809, EPI_ISL_731811, EPI_ISL_731812, EPI_ISL_731813, EPI_ISL_731814, EPI_ISL_731815, EPI_ISL_731816, EPI_ISL_731817, EPI_ISL_731818, EPI_ISL_731820, EPI_ISL_731821, EPI_ISL_731822, EPI_ISL_731823, EPI_ISL_731825, EPI_ISL_731826, EPI_ISL_731828, EPI_ISL_731829, EPI_ISL_731830, EPI_ISL_731831, EPI_ISL_731832, EPI_ISL_731833, EPI_ISL_731834, EPI_ISL_731835, EPI_ISL_731837, EPI_ISL_731839, EPI_ISL_731843, EPI_ISL_731844, EPI_ISL_731845, EPI_ISL_731847, EPI_ISL_731848, EPI_ISL_731849, EPI_ISL_731851, EPI_ISL_731852, EPI_ISL_731853, EPI_ISL_731855, EPI_ISL_731856, EPI_ISL_731857, EPI_ISL_731859, EPI_ISL_731860, EPI_ISL_731861, EPI_ISL_731862, EPI_ISL_731864, EPI_ISL_731865, EPI_ISL_731866, EPI_ISL_731867, EPI_ISL_731869, EPI_ISL_731870, EPI_ISL_731871, EPI_ISL_731872, EPI_ISL_731873, EPI_ISL_731876, EPI_ISL_731878, EPI_ISL_731879, EPI_ISL_731880, EPI_ISL_731882, EPI_ISL_731883, EPI_ISL_731886, EPI_ISL_731890			
see above	Lighthouse Lab in Alderley Park	Wellcome Sanger Institute for the COVID-19 Genomics UK (COG-UK) Consortium	Jacquelyn Wynn, Mairead Hyland, The Lighthouse Lab in Alderley Park and Alex Alderton, Roberto Amato, Sonia Goncalves, Ewan Harrison, David K. Jackson, Ian Johnston, Dominic Kwiatkowski, Cordelia Langford, John Sillitoe on behalf of the Wellcome Sanger Institute COVID-19 Surveillance Team
EPI_ISL_732963	Canterbury Health Laboratories	Institute of Environmental Science and Research (ESR)	Xiaoyun Ren, Matt Storey, Nikki Freed, Muhammad Faisal, Jing Wang, Hermes Perez, Anja Werno, Antje van der Linden, Arlo Upton, Chris Mansell, David Hammer, Dragana Drinkovic, Gary McAuliffe, Hana Sofia Andersson, James Ussher, Jill Sherwood, Josh Freeman, Julia Howard, Juliet Elvy, Mary DeAlmeida, Matt Blakiston, Matthew Rogers, Max Bloomfield, Michael Addidle, Michelle Balm, Sally Roberts, Sarah Jefferies, Sharmini Muttaiyah, Susan Morpeth, Susan Taylor, Timothy Blackmore, Vani Sathyendran, Veronica Playle, Virginia Hope, Erasmus Smit, Lauren Jelly, Olin Silander, Joep de Ligt
EPI_ISL_732964, EPI_ISL_732966	LabPLUS	Institute of Environmental Science and Research (ESR)	Xiaoyun Ren, Matt Storey, Nikki Freed, Muhammad Faisal, Jing Wang, Hermes Perez, Anja Werno, Antje van der Linden, Arlo Upton, Chris Mansell, David Hammer, Dragana Drinkovic, Gary McAuliffe, Hana Sofia Andersson, James Ussher, Jill Sherwood, Josh Freeman, Julia Howard, Juliet Elvy, Mary DeAlmeida, Matt Blakiston, Matthew Rogers, Max Bloomfield, Michael Addidle, Michelle Balm, Sally Roberts, Sarah Jefferies, Sharmini Muttaiyah, Susan Morpeth, Susan Taylor, Timothy Blackmore, Vani Sathyendran, Veronica Playle, Virginia Hope, Erasmus Smit, Lauren Jelly, Olin Silander, Joep de Ligt
EPI_ISL_733568	Centre for Health Protection	Hong Kong Department of Health	Alan K.L. Tsang, Peter C.W. Yip, Edman T.K. Lam, Rickjason C.W. Chan, Dominic N.C. Tsang
EPI_ISL_733572, EPI_ISL_733577	Temporary Specimen Collection Centre	Hong Kong Department of Health	Alan K.L. Tsang, Peter C.W. Yip, Edman T.K. Lam, Rickjason C.W. Chan, Dominic N.C. Tsang
EPI_ISL_733916, EPI_ISL_733917, EPI_ISL_733918, EPI_ISL_733919, EPI_ISL_733920, EPI_ISL_733921, EPI_ISL_733922, EPI_ISL_733923, EPI_ISL_733924, EPI_ISL_733925, EPI_ISL_733926, EPI_ISL_733927, EPI_ISL_733929, EPI_ISL_733930, EPI_ISL_733931, EPI_ISL_733932, EPI_ISL_733933, EPI_ISL_733934, EPI_ISL_733935, EPI_ISL_733936, EPI_ISL_733937, EPI_ISL_733938, EPI_ISL_733939, EPI_ISL_733940, EPI_ISL_733941, EPI_ISL_733942, EPI_ISL_733943, EPI_ISL_733944, EPI_ISL_733945, EPI_ISL_733946, EPI_ISL_733947, EPI_ISL_733948, EPI_ISL_733949, EPI_ISL_733951, EPI_ISL_733952, EPI_ISL_733953, EPI_ISL_733954, EPI_ISL_733955, EPI_ISL_733956, EPI_ISL_733957, EPI_ISL_733958, EPI_ISL_733959, EPI_ISL_733960, EPI_ISL_733961, EPI_ISL_733962, EPI_ISL_733963, EPI_ISL_733964, EPI_ISL_733965, EPI_ISL_733966, EPI_ISL_733967, EPI_ISL_733968, EPI_ISL_733969, EPI_ISL_733970, EPI_ISL_733971, EPI_ISL_733972, EPI_ISL_733973, EPI_ISL_733974, EPI_ISL_733975, EPI_ISL_733976, EPI_ISL_733977, EPI_ISL_733978, EPI_ISL_733979, EPI_ISL_733980, EPI_ISL_733981, EPI_ISL_733982, EPI_ISL_733983, EPI_ISL_733984, EPI_ISL_733985, EPI_ISL_733986, EPI_ISL_733987, EPI_ISL_733988, EPI_ISL_733989, EPI_ISL_733990, EPI_ISL_733991, EPI_ISL_733992, EPI_ISL_733993, EPI_ISL_733994, EPI_ISL_733995, EPI_ISL_733996, EPI_ISL_733997, EPI_ISL_733998, EPI_ISL_733999, EPI_ISL_734000, EPI_ISL_734001, EPI_ISL_734002, EPI_ISL_734003, EPI_ISL_734004, EPI_ISL_734005, EPI_ISL_734006, EPI_ISL_734007, EPI_ISL_734008, EPI_ISL_734009, EPI_ISL_734010, EPI_ISL_734011, EPI_ISL_734012, EPI_ISL_734013, EPI_ISL_734014, EPI_ISL_734015, EPI_ISL_734016, EPI_ISL_734017, EPI_ISL_734018, EPI_ISL_734019, EPI_ISL_734020, EPI_ISL_734021, EPI_ISL_734022, EPI_ISL_734023, EPI_ISL_734024, EPI_ISL_734025, EPI_ISL_734026, EPI_ISL_734027, EPI_ISL_734028, EPI_ISL_734029, EPI_ISL_734030, EPI_ISL_734031, EPI_ISL_734032, EPI_ISL_734033, EPI_ISL_734034, EPI_ISL_734035, EPI_ISL_734036, EPI_ISL_734037, EPI_ISL_734038, EPI_ISL_734039, EPI_ISL_734040, EPI_ISL_734042, EPI_ISL_734043, EPI_ISL_734045, EPI_ISL_734046, EPI_ISL_734047, EPI_ISL_734048, EPI_ISL_734049, EPI_ISL_734050, EPI_ISL_734051, EPI_ISL_734052, EPI_ISL_734053, EPI_ISL_734054, EPI_ISL_734055, EPI_ISL_734056, EPI_ISL_734057, EPI_ISL_734058, EPI_ISL_734059, EPI_ISL_734060, EPI_ISL_734061, EPI_ISL_734062, EPI_ISL_734063, EPI_ISL_734064, EPI_ISL_734065, EPI_ISL_734066, EPI_ISL_734067, EPI_ISL_734068, EPI_ISL_734069, EPI_ISL_734070, EPI_ISL_734071, EPI_ISL_734072, EPI_ISL_734073, EPI_ISL_734074, EPI_ISL_734075, EPI_ISL_734076, EPI_ISL_734077, EPI_ISL_734078, EPI_ISL_734079, EPI_ISL_734080, EPI_ISL_734081, EPI_ISL_734082, EPI_ISL_734083, EPI_ISL_734084, EPI_ISL_734085, EPI_ISL_734086, EPI_ISL_734087, EPI_ISL_734088, EPI_ISL_734089, EPI_ISL_734090, EPI_ISL_734091, EPI_ISL_734092, EPI_ISL_734094, EPI_ISL_734095, EPI_ISL_734096, EPI_ISL_734097, EPI_ISL_734098, EPI_ISL_734099, EPI_ISL_734100, EPI_ISL_734101, EPI_ISL_734102, EPI_ISL_734103, EPI_ISL_734104, EPI_ISL_734105, EPI_ISL_734106, EPI_ISL_734107, EPI_ISL_734108, EPI_ISL_734109, EPI_ISL_734110, EPI_ISL_734111, EPI_ISL_734112, EPI_ISL_734113, EPI_ISL_734114, EPI_ISL_734115, EPI_ISL_734116, EPI_ISL_734118, EPI_ISL_734119, EPI_ISL_734120, EPI_ISL_734121, EPI_ISL_734122, EPI_ISL_734123, EPI_ISL_734124, EPI_ISL_734125, EPI_ISL_734126, EPI_ISL_734127, EPI_ISL_734128, EPI_ISL_734129, EPI_ISL_734130, EPI_ISL_734132, EPI_ISL_734133, EPI_ISL_734134, EPI_ISL_734135, EPI_ISL_734136, EPI_ISL_734137, EPI_ISL_734138, EPI_ISL_734139, EPI_ISL_734140, EPI_ISL_734141, EPI_ISL_734142, EPI_ISL_734143, EPI_ISL_734144, EPI_ISL_734145, EPI_ISL_734146, EPI_ISL_734147, EPI_ISL_734148, EPI_ISL_734149, EPI_ISL_734150, EPI_ISL_734151, EPI_ISL_734152, EPI_ISL_734153, EPI_ISL_734154, EPI_ISL_734155, EPI_ISL_734156, EPI_ISL_734157, EPI_ISL_734158, EPI_ISL_734159, EPI_ISL_734160, EPI_ISL_734161, EPI_ISL_734162, EPI_ISL_734163, EPI_ISL_734164, EPI_ISL_734165			
see above	Lighthouse Lab in Glasgow	Wellcome Sanger Institute for the COVID-19 Genomics UK (COG-UK) Consortium	Harper VanSteenhouse, Yumi Kasai, David Gray, Carol Clugston, Anna Dominiczak and Alex Alderton, Roberto Amato, Sonia Goncalves, Ewan Harrison, David K. Jackson, Ian Johnston, Dominic Kwiatkowski, Cordelia Langford, John Sillitoe on behalf of the Wellcome Sanger Institute COVID-19 Surveillance Team
EPI_ISL_735512, EPI_ISL_735513, EPI_ISL_735514, EPI_ISL_735515, EPI_ISL_735516, EPI_ISL_735517, EPI_ISL_735518, EPI_ISL_735519, EPI_ISL_735520, EPI_ISL_735521, EPI_ISL_735522, EPI_ISL_735523, EPI_ISL_735524, EPI_ISL_735525, EPI_ISL_735526, EPI_ISL_735527, EPI_ISL_735528, EPI_ISL_735529, EPI_ISL_735530, EPI_ISL_735531, EPI_ISL_735532, EPI_ISL_735533, EPI_ISL_735534, EPI_ISL_735535, EPI_ISL_735536, EPI_ISL_735537, EPI_ISL_735538, EPI_ISL_735539, EPI_ISL_735540, EPI_ISL_735541, EPI_ISL_735542, EPI_ISL_735543, EPI_ISL_735544, EPI_ISL_735545, EPI_ISL_735546, EPI_ISL_735547, EPI_ISL_735548, EPI_ISL_735549, EPI_ISL_735550, EPI_ISL_735551, EPI_ISL_735552, EPI_ISL_735553, EPI_ISL_735554, EPI_ISL_735555, EPI_ISL_735556, EPI_ISL_735557, EPI_ISL_735558, EPI_ISL_735559, EPI_ISL_735560, EPI_ISL_735561, EPI_ISL_735562, EPI_ISL_735563, EPI_ISL_735564, EPI_ISL_735565, EPI_ISL_735566, EPI_ISL_735567, EPI_ISL_735568, EPI_ISL_735569, EPI_ISL_735570, EPI_ISL_735571, EPI_ISL_735572, EPI_ISL_735573, EPI_ISL_735574, EPI_ISL_735575, EPI_ISL_735576, EPI_ISL_735577, EPI_ISL_735578, EPI_ISL_735579, EPI_ISL_735580, EPI_ISL_735581, EPI_ISL_735582, EPI_ISL_735583, EPI_ISL_735584, EPI_ISL_735585, EPI_ISL_735586, EPI_ISL_735587, EPI_ISL_735588, EPI_ISL_735589, EPI_ISL_735590, EPI_ISL_735591, EPI_ISL_735592, EPI_ISL_735593, EPI_ISL_735594, EPI_ISL_735595, EPI_ISL_735596, EPI_ISL_735597, EPI_ISL_735598, EPI_ISL_735599, EPI_ISL_735600, EPI_ISL_735601, EPI_ISL_735602, EPI_ISL_735603, EPI_ISL_735604, EPI_ISL_735605, EPI_ISL_735606, EPI_ISL_735607, EPI_ISL_735608, EPI_ISL_735609, EPI_ISL_735610, EPI_ISL_735611, EPI_ISL_735612, EPI_ISL_735613, EPI_ISL_735614, EPI_ISL_735615, EPI_ISL_735616, EPI_ISL_735617, EPI_ISL_735618, EPI_ISL_735619, EPI_ISL_735620, EPI_ISL_735621, EPI_ISL_735622, EPI_ISL_735623, EPI_ISL_735624, EPI_ISL_735625, EPI_ISL_735626, EPI_ISL_735627, EPI_ISL_735628, EPI_ISL_735629, EPI_ISL_735630, EPI_ISL_735631, EPI_ISL_735632, EPI_ISL_735633, EPI_ISL_735634, EPI_ISL_735635, EPI_ISL_735636, EPI_ISL_735637, EPI_ISL_735638, EPI_ISL_735639, EPI_ISL_735640, EPI_ISL_735641, EPI_ISL_735642, EPI_ISL_735643, EPI_ISL_735644, EPI_ISL_735645, EPI_ISL_735646, EPI_ISL_735647, EPI_ISL_735648, EPI_ISL_735649, EPI_ISL_735650, EPI_ISL_735651, EPI_ISL_735652, EPI_ISL_735653, EPI_ISL_735654, EPI_ISL_735655, EPI_ISL_735656, EPI_ISL_735657, EPI_ISL_735658, EPI_ISL_735659, EPI_ISL_735660, EPI_ISL_735661, EPI_ISL_735662, EPI_ISL_735663, EPI_ISL_735664, EPI_ISL_735665, EPI_ISL_735666, EPI_ISL_735667, EPI_ISL_735668, EPI_ISL_735669, EPI_ISL_735670, EPI_ISL_735671, EPI_ISL_735672, EPI_ISL_735673, EPI_ISL_735674, EPI_ISL_735675, EPI_ISL_735676, EPI_ISL_735677, EPI_ISL_735678, EPI_ISL_735679, EPI_ISL_735680, EPI_ISL_735681, EPI_ISL_735682, EPI_ISL_735683, EPI_ISL_735684, EPI_ISL_735685, EPI_ISL_735686, EPI_ISL_735687, EPI_ISL_735688, EPI_ISL_735689, EPI_ISL_735690, EPI_ISL_735691, EPI_ISL_735692, EPI_ISL_735693, EPI_ISL_735694, EPI_ISL_735695, EPI_ISL_735696, EPI_ISL_735697, EPI_ISL_735698, EPI_ISL_735699, EPI_ISL_735700, EPI_ISL_735701, EPI_ISL_735702, EPI_ISL_735703, EPI_ISL_735704, EPI_ISL_735705, EPI_ISL_735706, EPI_ISL_735707, EPI_ISL_735708, EPI_ISL_735709, EPI_ISL_735710, EPI_ISL_735711, EPI_ISL_735712, EPI_ISL_735713, EPI_ISL_735714, EPI_ISL_735715, EPI_ISL_735716, EPI_ISL_735717, EPI_ISL_735718, EPI_ISL_735719, EPI_ISL_735720, EPI_ISL_735721, EPI_ISL_735722, EPI_ISL_735723, EPI_ISL_735724, EPI_ISL_735725, EPI_ISL_735726, EPI_ISL_735727, EPI_ISL_735728, EPI_ISL_735729, EPI_ISL_735730, EPI_ISL_735731, EPI_ISL_735732, EPI_ISL_735733, EPI_ISL_735734, EPI_ISL_735735, EPI_ISL_735736, EPI_ISL_735737, EPI_ISL_735738, EPI_ISL_735739, EPI_ISL_735740, EPI_ISL_735741, EPI_ISL_735742, EPI_ISL_735743, EPI_ISL_735744, EPI_ISL_735745, EPI_ISL_735746, EPI_ISL_735747, EPI_ISL_735748, EPI_ISL_735749, EPI_ISL_735750, EPI_ISL_735751, EPI_ISL_735752, EPI_ISL_735753, EPI_ISL_735754, EPI_ISL_735755, EPI_ISL_735756, EPI_ISL_735757, EPI_ISL_735758, EPI_ISL_735759, EPI_ISL_735760, EPI_ISL_735761, EPI_ISL_735762, EPI_ISL_735763, EPI_ISL_735764, EPI_ISL_735765, EPI_ISL_735766, EPI_ISL_735767, EPI_ISL_735768, EPI_ISL_735769, EPI_ISL_735770, EPI_ISL_735771, EPI_ISL_735772, EPI_ISL_735773, EPI_ISL_735774, EPI_ISL_735775, EPI_ISL_735776, EPI_ISL_735777, EPI_ISL_735778, EPI_ISL_735779, EPI_ISL_735780, EPI_ISL_735781,			

EPI_ISL_735782, EPI_ISL_735783, EPI_ISL_735784, EPI_ISL_735785, EPI_ISL_735786, EPI_ISL_735787, EPI_ISL_735788, EPI_ISL_735789, EPI_ISL_735790, EPI_ISL_735791, EPI_ISL_735792, EPI_ISL_735793, EPI_ISL_735794, EPI_ISL_735795, EPI_ISL_735796, EPI_ISL_735797, EPI_ISL_735798, EPI_ISL_735799, EPI_ISL_735800, EPI_ISL_735801, EPI_ISL_735802, EPI_ISL_735803, EPI_ISL_735804, EPI_ISL_735805, EPI_ISL_735806, EPI_ISL_735807, EPI_ISL_735808, EPI_ISL_735809, EPI_ISL_735810, EPI_ISL_735811, EPI_ISL_735812, EPI_ISL_735813, EPI_ISL_735814, EPI_ISL_735815, EPI_ISL_735816, EPI_ISL_735817, EPI_ISL_735818, EPI_ISL_735819, EPI_ISL_735820, EPI_ISL_735821, EPI_ISL_735822, EPI_ISL_735823, EPI_ISL_735824, EPI_ISL_735825, EPI_ISL_735826, EPI_ISL_735827, EPI_ISL_735828, EPI_ISL_735829, EPI_ISL_735830, EPI_ISL_735831, EPI_ISL_735832, EPI_ISL_735833, EPI_ISL_735834, EPI_ISL_735835, EPI_ISL_735836, EPI_ISL_735837, EPI_ISL_735838, EPI_ISL_735839, EPI_ISL_735840, EPI_ISL_735841, EPI_ISL_735842, EPI_ISL_735843, EPI_ISL_735844, EPI_ISL_735845, EPI_ISL_735846, EPI_ISL_735847, EPI_ISL_735848, EPI_ISL_735849, EPI_ISL_735850, EPI_ISL_735851, EPI_ISL_735852, EPI_ISL_735853, EPI_ISL_735854, EPI_ISL_735855, EPI_ISL_735856, EPI_ISL_735857, EPI_ISL_735858, EPI_ISL_735859			
see above	Lighthouse Lab in Milton Keynes	Wellcome Sanger Institute for the COVID-19 Genomics UK (COG-UK) Consortium	The Lighthouse Lab in Milton Keynes and Alex Alderton, Roberto Amato, Sonia Goncalves, Ewan Harrison, David K. Jackson, Ian Johnston, Dominic Kwiatkowski, Cordelia Langford, John Sillitoe on behalf of the Wellcome Sanger Institute COVID-19 Surveillance Team
EPI_ISL_735860, EPI_ISL_735862, EPI_ISL_735863, EPI_ISL_735865, EPI_ISL_735866, EPI_ISL_735869, EPI_ISL_735870, EPI_ISL_735871, EPI_ISL_735872, EPI_ISL_735873, EPI_ISL_735874, EPI_ISL_735875, EPI_ISL_735876, EPI_ISL_735879, EPI_ISL_735883, EPI_ISL_735884, EPI_ISL_735885, EPI_ISL_735890, EPI_ISL_735892, EPI_ISL_735893, EPI_ISL_735895, EPI_ISL_735896, EPI_ISL_735898, EPI_ISL_735903, EPI_ISL_735906, EPI_ISL_735910, EPI_ISL_735911, EPI_ISL_735913, EPI_ISL_735914, EPI_ISL_735915, EPI_ISL_735916, EPI_ISL_735918, EPI_ISL_735919, EPI_ISL_735921, EPI_ISL_735922, EPI_ISL_735925, EPI_ISL_735928, EPI_ISL_735929, EPI_ISL_735933, EPI_ISL_735934, EPI_ISL_735935, EPI_ISL_735940, EPI_ISL_735944, EPI_ISL_735946, EPI_ISL_735947, EPI_ISL_735948, EPI_ISL_735950, EPI_ISL_735953, EPI_ISL_735954, EPI_ISL_735955, EPI_ISL_735957, EPI_ISL_735959, EPI_ISL_735960, EPI_ISL_735962, EPI_ISL_735969, EPI_ISL_735970, EPI_ISL_735971, EPI_ISL_735977, EPI_ISL_735979, EPI_ISL_735984, EPI_ISL_735985, EPI_ISL_735988, EPI_ISL_735989, EPI_ISL_735990, EPI_ISL_735991, EPI_ISL_735994, EPI_ISL_735999, EPI_ISL_736002, EPI_ISL_736004, EPI_ISL_736005, EPI_ISL_736006, EPI_ISL_736009, EPI_ISL_736014, EPI_ISL_736020, EPI_ISL_736022, EPI_ISL_736026, EPI_ISL_736031, EPI_ISL_736032, EPI_ISL_736035, EPI_ISL_736036, EPI_ISL_736040, EPI_ISL_736042, EPI_ISL_736043, EPI_ISL_736044, EPI_ISL_736045, EPI_ISL_736049, EPI_ISL_736053, EPI_ISL_736054, EPI_ISL_736056, EPI_ISL_736058, EPI_ISL_736060, EPI_ISL_736061, EPI_ISL_736062, EPI_ISL_736067, EPI_ISL_736068, EPI_ISL_736069, EPI_ISL_736070, EPI_ISL_736071, EPI_ISL_736073, EPI_ISL_736074, EPI_ISL_736075, EPI_ISL_736076, EPI_ISL_736077, EPI_ISL_736081, EPI_ISL_736082, EPI_ISL_736084, EPI_ISL_736085, EPI_ISL_736086, EPI_ISL_736088, EPI_ISL_736089, EPI_ISL_736094, EPI_ISL_736098, EPI_ISL_736103, EPI_ISL_736106, EPI_ISL_736109, EPI_ISL_736111, EPI_ISL_736112, EPI_ISL_736113, EPI_ISL_736114, EPI_ISL_736116, EPI_ISL_736117, EPI_ISL_736119, EPI_ISL_736120, EPI_ISL_736121, EPI_ISL_736122, EPI_ISL_736123, EPI_ISL_736126, EPI_ISL_736128, EPI_ISL_736129, EPI_ISL_736133, EPI_ISL_736134, EPI_ISL_736136, EPI_ISL_736137, EPI_ISL_736138, EPI_ISL_736139, EPI_ISL_736140, EPI_ISL_736144, EPI_ISL_736145, EPI_ISL_736147, EPI_ISL_736148, EPI_ISL_736150, EPI_ISL_736152, EPI_ISL_736154, EPI_ISL_736155, EPI_ISL_736156, EPI_ISL_736157, EPI_ISL_736160, EPI_ISL_736161, EPI_ISL_736163, EPI_ISL_736164, EPI_ISL_736165, EPI_ISL_736166, EPI_ISL_736170, EPI_ISL_736171, EPI_ISL_736173, EPI_ISL_736174, EPI_ISL_736176, EPI_ISL_736177, EPI_ISL_736179, EPI_ISL_736180, EPI_ISL_736181, EPI_ISL_736185, EPI_ISL_736186, EPI_ISL_736188, EPI_ISL_736192, EPI_ISL_736194, EPI_ISL_736195, EPI_ISL_736196, EPI_ISL_736197, EPI_ISL_736198, EPI_ISL_736200			
see above	Lighthouse Lab in Alderley Park	Wellcome Sanger Institute for the COVID-19 Genomics UK (COG-UK) Consortium	Jacquelyn Wynn, Mairead Hyland, The Lighthouse Lab in Alderley Park and Alex Alderton, Roberto Amato, Sonia Goncalves, Ewan Harrison, David K. Jackson, Ian Johnston, Dominic Kwiatkowski, Cordelia Langford, John Sillitoe on behalf of the Wellcome Sanger Institute COVID-19 Surveillance Team
EPI_ISL_736201, EPI_ISL_736203	Lighthouse Lab in Milton Keynes	Wellcome Sanger Institute for the COVID-19 Genomics UK (COG-UK) Consortium	The Lighthouse Lab in Milton Keynes and Alex Alderton, Roberto Amato, Sonia Goncalves, Ewan Harrison, David K. Jackson, Ian Johnston, Dominic Kwiatkowski, Cordelia Langford, John Sillitoe on behalf of the Wellcome Sanger Institute COVID-19 Surveillance Team
EPI_ISL_736204, EPI_ISL_736205, EPI_ISL_736206	Lighthouse Lab in Glasgow	Wellcome Sanger Institute for the COVID-19 Genomics UK (COG-UK) Consortium	Harper VanSteenhouse, Yumi Kasai, David Gray, Carol Clugston, Anna Dominiczak and Alex Alderton, Roberto Amato, Sonia Goncalves, Ewan Harrison, David K. Jackson, Ian Johnston, Dominic Kwiatkowski, Cordelia Langford, John Sillitoe on behalf of the Wellcome Sanger Institute COVID-19 Surveillance Team
EPI_ISL_736207, EPI_ISL_736208	Lighthouse Lab in Milton Keynes	Wellcome Sanger Institute for the COVID-19 Genomics UK (COG-UK) Consortium	The Lighthouse Lab in Milton Keynes and Alex Alderton, Roberto Amato, Sonia Goncalves, Ewan Harrison, David K. Jackson, Ian Johnston, Dominic Kwiatkowski, Cordelia Langford, John Sillitoe on behalf of the Wellcome Sanger Institute COVID-19 Surveillance Team
EPI_ISL_736209	Lighthouse Lab in Glasgow	Wellcome Sanger Institute for the COVID-19 Genomics UK (COG-UK) Consortium	Harper VanSteenhouse, Yumi Kasai, David Gray, Carol Clugston, Anna Dominiczak and Alex Alderton, Roberto Amato, Sonia Goncalves, Ewan Harrison, David K. Jackson, Ian Johnston, Dominic Kwiatkowski, Cordelia Langford, John Sillitoe on behalf of the Wellcome Sanger Institute COVID-19 Surveillance Team
EPI_ISL_736210, EPI_ISL_736211	Lighthouse Lab in Milton Keynes	Wellcome Sanger Institute for the COVID-19 Genomics UK (COG-UK) Consortium	The Lighthouse Lab in Milton Keynes and Alex Alderton, Roberto Amato, Sonia Goncalves, Ewan Harrison, David K. Jackson, Ian Johnston, Dominic Kwiatkowski, Cordelia Langford, John Sillitoe on behalf of the Wellcome Sanger Institute COVID-19 Surveillance Team
EPI_ISL_736212	Lighthouse Lab in Glasgow	Wellcome Sanger Institute for the COVID-19 Genomics UK (COG-UK) Consortium	Harper VanSteenhouse, Yumi Kasai, David Gray, Carol Clugston, Anna Dominiczak and Alex Alderton, Roberto Amato, Sonia Goncalves, Ewan Harrison, David K. Jackson, Ian Johnston, Dominic Kwiatkowski, Cordelia Langford, John Sillitoe on behalf of the Wellcome Sanger Institute COVID-19 Surveillance Team
EPI_ISL_736213, EPI_ISL_736214, EPI_ISL_736215, EPI_ISL_736216, EPI_ISL_736217, EPI_ISL_736218, EPI_ISL_736219	Lighthouse Lab in Milton Keynes	Wellcome Sanger Institute for the COVID-19 Genomics UK (COG-UK) Consortium	The Lighthouse Lab in Milton Keynes and Alex Alderton, Roberto Amato, Sonia Goncalves, Ewan Harrison, David K. Jackson, Ian Johnston, Dominic Kwiatkowski, Cordelia Langford, John Sillitoe on behalf of the Wellcome Sanger Institute COVID-19 Surveillance Team
EPI_ISL_736220, EPI_ISL_736221, EPI_ISL_736222, EPI_ISL_736223	Lighthouse Lab in Glasgow	Wellcome Sanger Institute for the COVID-19 Genomics UK (COG-UK) Consortium	Harper VanSteenhouse, Yumi Kasai, David Gray, Carol Clugston, Anna Dominiczak and Alex Alderton, Roberto Amato, Sonia Goncalves, Ewan Harrison, David K. Jackson, Ian Johnston, Dominic Kwiatkowski, Cordelia Langford, John Sillitoe on behalf of the Wellcome Sanger Institute COVID-19 Surveillance Team
EPI_ISL_736224, EPI_ISL_736225, EPI_ISL_736226, EPI_ISL_736227, EPI_ISL_736228, EPI_ISL_736229, EPI_ISL_736230	Lighthouse Lab in Milton Keynes	Wellcome Sanger Institute for the COVID-19 Genomics UK (COG-UK) Consortium	The Lighthouse Lab in Milton Keynes and Alex Alderton, Roberto Amato, Sonia Goncalves, Ewan Harrison, David K. Jackson, Ian Johnston, Dominic Kwiatkowski, Cordelia Langford, John Sillitoe on behalf of the Wellcome Sanger Institute COVID-19 Surveillance Team
EPI_ISL_736231	Lighthouse Lab in Glasgow	Wellcome Sanger Institute for the COVID-19 Genomics UK (COG-UK) Consortium	Harper VanSteenhouse, Yumi Kasai, David Gray, Carol Clugston, Anna Dominiczak and Alex Alderton, Roberto Amato, Sonia Goncalves, Ewan Harrison, David K. Jackson, Ian Johnston, Dominic Kwiatkowski, Cordelia Langford, John Sillitoe on behalf of the Wellcome Sanger Institute COVID-19 Surveillance Team
EPI_ISL_736232, EPI_ISL_736233	Lighthouse Lab in Milton Keynes	Wellcome Sanger Institute for the COVID-19 Genomics UK (COG-UK) Consortium	The Lighthouse Lab in Milton Keynes and Alex Alderton, Roberto Amato, Sonia Goncalves, Ewan Harrison, David K. Jackson, Ian Johnston, Dominic Kwiatkowski, Cordelia Langford, John Sillitoe on behalf of the Wellcome Sanger Institute COVID-19 Surveillance Team
EPI_ISL_736234, EPI_ISL_736235, EPI_ISL_736236, EPI_ISL_736237, EPI_ISL_736238, EPI_ISL_736239, EPI_ISL_736241, EPI_ISL_736242, EPI_ISL_736243	Lighthouse Lab in Glasgow	Wellcome Sanger Institute for the COVID-19 Genomics UK (COG-UK) Consortium	Harper VanSteenhouse, Yumi Kasai, David Gray, Carol Clugston, Anna Dominiczak and Alex Alderton, Roberto Amato, Sonia Goncalves, Ewan Harrison, David K. Jackson, Ian Johnston, Dominic Kwiatkowski, Cordelia Langford, John Sillitoe on behalf of the Wellcome Sanger Institute COVID-19 Surveillance Team
EPI_ISL_736244	Lighthouse Lab in Milton Keynes	Wellcome Sanger Institute for the COVID-19 Genomics UK (COG-UK) Consortium	The Lighthouse Lab in Milton Keynes and Alex Alderton, Roberto Amato, Sonia Goncalves, Ewan Harrison, David K. Jackson, Ian Johnston, Dominic Kwiatkowski, Cordelia Langford, John Sillitoe on behalf of the Wellcome Sanger Institute COVID-19 Surveillance Team
EPI_ISL_736245	Lighthouse Lab in Glasgow	Wellcome Sanger Institute for the COVID-19 Genomics UK (COG-UK) Consortium	Harper VanSteenhouse, Yumi Kasai, David Gray, Carol Clugston, Anna Dominiczak and Alex Alderton, Roberto Amato, Sonia Goncalves, Ewan Harrison, David K. Jackson, Ian Johnston, Dominic Kwiatkowski, Cordelia Langford, John Sillitoe on behalf of the Wellcome Sanger Institute COVID-19 Surveillance Team
EPI_ISL_736246, EPI_ISL_736247	Lighthouse Lab in Milton Keynes	Wellcome Sanger Institute for the COVID-19 Genomics UK (COG-UK) Consortium	The Lighthouse Lab in Milton Keynes and Alex Alderton, Roberto Amato, Sonia Goncalves, Ewan Harrison, David K. Jackson, Ian Johnston, Dominic Kwiatkowski, Cordelia Langford, John Sillitoe on behalf of the Wellcome Sanger Institute COVID-19 Surveillance Team
EPI_ISL_736248	Lighthouse Lab in Glasgow	Wellcome Sanger Institute for the COVID-19 Genomics UK (COG-UK) Consortium	Harper VanSteenhouse, Yumi Kasai, David Gray, Carol Clugston, Anna Dominiczak and Alex Alderton, Roberto Amato, Sonia Goncalves, Ewan Harrison, David K. Jackson, Ian Johnston, Dominic Kwiatkowski, Cordelia Langford, John Sillitoe on behalf of the Wellcome Sanger Institute COVID-19 Surveillance Team
EPI_ISL_736249	Lighthouse Lab in Milton Keynes	Wellcome Sanger Institute for the COVID-19 Genomics UK (COG-UK) Consortium	The Lighthouse Lab in Milton Keynes and Alex Alderton, Roberto Amato, Sonia Goncalves, Ewan Harrison, David K. Jackson, Ian Johnston, Dominic Kwiatkowski, Cordelia Langford, John Sillitoe on behalf of the Wellcome Sanger Institute COVID-19 Surveillance Team
EPI_ISL_736250	Lighthouse Lab in Glasgow	Wellcome Sanger Institute for the COVID-19 Genomics UK (COG-UK) Consortium	Harper VanSteenhouse, Yumi Kasai, David Gray, Carol Clugston, Anna Dominiczak and Alex Alderton, Roberto Amato, Sonia Goncalves, Ewan Harrison, David K. Jackson, Ian Johnston, Dominic Kwiatkowski, Cordelia Langford, John Sillitoe on behalf of the Wellcome Sanger Institute COVID-19 Surveillance Team
EPI_ISL_736251, EPI_ISL_736253	Lighthouse Lab in Milton Keynes	Wellcome Sanger Institute for the COVID-19 Genomics UK (COG-UK) Consortium	The Lighthouse Lab in Milton Keynes and Alex Alderton, Roberto Amato, Sonia Goncalves, Ewan Harrison, David K. Jackson, Ian Johnston, Dominic Kwiatkowski, Cordelia Langford, John Sillitoe on behalf of the Wellcome Sanger Institute COVID-19 Surveillance Team
EPI_ISL_736254, EPI_ISL_736255	Lighthouse Lab in Glasgow	Wellcome Sanger Institute for the COVID-19 Genomics UK (COG-UK) Consortium	Harper VanSteenhouse, Yumi Kasai, David Gray, Carol Clugston, Anna Dominiczak and Alex Alderton, Roberto Amato, Sonia Goncalves, Ewan Harrison, David K. Jackson, Ian Johnston, Dominic Kwiatkowski, Cordelia Langford, John Sillitoe on behalf of the Wellcome Sanger Institute COVID-19 Surveillance Team

EPI_ISL_736256	Lighthouse Lab in Milton Keynes	Wellcome Sanger Institute for the COVID-19 Genomics UK (COG-UK) Consortium	The Lighthouse Lab in Milton Keynes and Alex Alderton, Roberto Amato, Sonia Goncalves, Ewan Harrison, David K. Jackson, Ian Johnston, Dominic Kwiatkowski, Cordelia Langford, John Sillitoe on behalf of the Wellcome Sanger Institute COVID-19 Surveillance Team
EPI_ISL_736257, EPI_ISL_736258	Lighthouse Lab in Glasgow	Wellcome Sanger Institute for the COVID-19 Genomics UK (COG-UK) Consortium	Harper VanSteenhouse, Yumi Kasai, David Gray, Carol Clugston, Anna Dominiczak and Alex Alderton, Roberto Amato, Sonia Goncalves, Ewan Harrison, David K. Jackson, Ian Johnston, Dominic Kwiatkowski, Cordelia Langford, John Sillitoe on behalf of the Wellcome Sanger Institute COVID-19 Surveillance Team
EPI_ISL_736259, EPI_ISL_736260	Lighthouse Lab in Milton Keynes	Wellcome Sanger Institute for the COVID-19 Genomics UK (COG-UK) Consortium	The Lighthouse Lab in Milton Keynes and Alex Alderton, Roberto Amato, Sonia Goncalves, Ewan Harrison, David K. Jackson, Ian Johnston, Dominic Kwiatkowski, Cordelia Langford, John Sillitoe on behalf of the Wellcome Sanger Institute COVID-19 Surveillance Team
EPI_ISL_736261	Lighthouse Lab in Glasgow	Wellcome Sanger Institute for the COVID-19 Genomics UK (COG-UK) Consortium	Harper VanSteenhouse, Yumi Kasai, David Gray, Carol Clugston, Anna Dominiczak and Alex Alderton, Roberto Amato, Sonia Goncalves, Ewan Harrison, David K. Jackson, Ian Johnston, Dominic Kwiatkowski, Cordelia Langford, John Sillitoe on behalf of the Wellcome Sanger Institute COVID-19 Surveillance Team
EPI_ISL_736262, EPI_ISL_736263, EPI_ISL_736265	Lighthouse Lab in Milton Keynes	Wellcome Sanger Institute for the COVID-19 Genomics UK (COG-UK) Consortium	The Lighthouse Lab in Milton Keynes and Alex Alderton, Roberto Amato, Sonia Goncalves, Ewan Harrison, David K. Jackson, Ian Johnston, Dominic Kwiatkowski, Cordelia Langford, John Sillitoe on behalf of the Wellcome Sanger Institute COVID-19 Surveillance Team
EPI_ISL_736266	Lighthouse Lab in Glasgow	Wellcome Sanger Institute for the COVID-19 Genomics UK (COG-UK) Consortium	Harper VanSteenhouse, Yumi Kasai, David Gray, Carol Clugston, Anna Dominiczak and Alex Alderton, Roberto Amato, Sonia Goncalves, Ewan Harrison, David K. Jackson, Ian Johnston, Dominic Kwiatkowski, Cordelia Langford, John Sillitoe on behalf of the Wellcome Sanger Institute COVID-19 Surveillance Team
EPI_ISL_736267, EPI_ISL_736268	Lighthouse Lab in Milton Keynes	Wellcome Sanger Institute for the COVID-19 Genomics UK (COG-UK) Consortium	The Lighthouse Lab in Milton Keynes and Alex Alderton, Roberto Amato, Sonia Goncalves, Ewan Harrison, David K. Jackson, Ian Johnston, Dominic Kwiatkowski, Cordelia Langford, John Sillitoe on behalf of the Wellcome Sanger Institute COVID-19 Surveillance Team
EPI_ISL_736269	Lighthouse Lab in Glasgow	Wellcome Sanger Institute for the COVID-19 Genomics UK (COG-UK) Consortium	Harper VanSteenhouse, Yumi Kasai, David Gray, Carol Clugston, Anna Dominiczak and Alex Alderton, Roberto Amato, Sonia Goncalves, Ewan Harrison, David K. Jackson, Ian Johnston, Dominic Kwiatkowski, Cordelia Langford, John Sillitoe on behalf of the Wellcome Sanger Institute COVID-19 Surveillance Team
EPI_ISL_736270	Lighthouse Lab in Milton Keynes	Wellcome Sanger Institute for the COVID-19 Genomics UK (COG-UK) Consortium	The Lighthouse Lab in Milton Keynes and Alex Alderton, Roberto Amato, Sonia Goncalves, Ewan Harrison, David K. Jackson, Ian Johnston, Dominic Kwiatkowski, Cordelia Langford, John Sillitoe on behalf of the Wellcome Sanger Institute COVID-19 Surveillance Team
EPI_ISL_736271	Lighthouse Lab in Glasgow	Wellcome Sanger Institute for the COVID-19 Genomics UK (COG-UK) Consortium	Harper VanSteenhouse, Yumi Kasai, David Gray, Carol Clugston, Anna Dominiczak and Alex Alderton, Roberto Amato, Sonia Goncalves, Ewan Harrison, David K. Jackson, Ian Johnston, Dominic Kwiatkowski, Cordelia Langford, John Sillitoe on behalf of the Wellcome Sanger Institute COVID-19 Surveillance Team
EPI_ISL_736272, EPI_ISL_736273	Lighthouse Lab in Milton Keynes	Wellcome Sanger Institute for the COVID-19 Genomics UK (COG-UK) Consortium	The Lighthouse Lab in Milton Keynes and Alex Alderton, Roberto Amato, Sonia Goncalves, Ewan Harrison, David K. Jackson, Ian Johnston, Dominic Kwiatkowski, Cordelia Langford, John Sillitoe on behalf of the Wellcome Sanger Institute COVID-19 Surveillance Team
EPI_ISL_736275	Lighthouse Lab in Glasgow	Wellcome Sanger Institute for the COVID-19 Genomics UK (COG-UK) Consortium	Harper VanSteenhouse, Yumi Kasai, David Gray, Carol Clugston, Anna Dominiczak and Alex Alderton, Roberto Amato, Sonia Goncalves, Ewan Harrison, David K. Jackson, Ian Johnston, Dominic Kwiatkowski, Cordelia Langford, John Sillitoe on behalf of the Wellcome Sanger Institute COVID-19 Surveillance Team
EPI_ISL_736276	Lighthouse Lab in Milton Keynes	Wellcome Sanger Institute for the COVID-19 Genomics UK (COG-UK) Consortium	The Lighthouse Lab in Milton Keynes and Alex Alderton, Roberto Amato, Sonia Goncalves, Ewan Harrison, David K. Jackson, Ian Johnston, Dominic Kwiatkowski, Cordelia Langford, John Sillitoe on behalf of the Wellcome Sanger Institute COVID-19 Surveillance Team
EPI_ISL_736277, EPI_ISL_736278	Lighthouse Lab in Glasgow	Wellcome Sanger Institute for the COVID-19 Genomics UK (COG-UK) Consortium	Harper VanSteenhouse, Yumi Kasai, David Gray, Carol Clugston, Anna Dominiczak and Alex Alderton, Roberto Amato, Sonia Goncalves, Ewan Harrison, David K. Jackson, Ian Johnston, Dominic Kwiatkowski, Cordelia Langford, John Sillitoe on behalf of the Wellcome Sanger Institute COVID-19 Surveillance Team
EPI_ISL_736279, EPI_ISL_736280, EPI_ISL_736281, EPI_ISL_736282, EPI_ISL_736283	Lighthouse Lab in Milton Keynes	Wellcome Sanger Institute for the COVID-19 Genomics UK (COG-UK) Consortium	The Lighthouse Lab in Milton Keynes and Alex Alderton, Roberto Amato, Sonia Goncalves, Ewan Harrison, David K. Jackson, Ian Johnston, Dominic Kwiatkowski, Cordelia Langford, John Sillitoe on behalf of the Wellcome Sanger Institute COVID-19 Surveillance Team
EPI_ISL_736284	Lighthouse Lab in Glasgow	Wellcome Sanger Institute for the COVID-19 Genomics UK (COG-UK) Consortium	Harper VanSteenhouse, Yumi Kasai, David Gray, Carol Clugston, Anna Dominiczak and Alex Alderton, Roberto Amato, Sonia Goncalves, Ewan Harrison, David K. Jackson, Ian Johnston, Dominic Kwiatkowski, Cordelia Langford, John Sillitoe on behalf of the Wellcome Sanger Institute COVID-19 Surveillance Team
EPI_ISL_736285, EPI_ISL_736286	Lighthouse Lab in Milton Keynes	Wellcome Sanger Institute for the COVID-19 Genomics UK (COG-UK) Consortium	The Lighthouse Lab in Milton Keynes and Alex Alderton, Roberto Amato, Sonia Goncalves, Ewan Harrison, David K. Jackson, Ian Johnston, Dominic Kwiatkowski, Cordelia Langford, John Sillitoe on behalf of the Wellcome Sanger Institute COVID-19 Surveillance Team
EPI_ISL_736287	Lighthouse Lab in Glasgow	Wellcome Sanger Institute for the COVID-19 Genomics UK (COG-UK) Consortium	Harper VanSteenhouse, Yumi Kasai, David Gray, Carol Clugston, Anna Dominiczak and Alex Alderton, Roberto Amato, Sonia Goncalves, Ewan Harrison, David K. Jackson, Ian Johnston, Dominic Kwiatkowski, Cordelia Langford, John Sillitoe on behalf of the Wellcome Sanger Institute COVID-19 Surveillance Team
EPI_ISL_736288	Lighthouse Lab in Milton Keynes	Wellcome Sanger Institute for the COVID-19 Genomics UK (COG-UK) Consortium	The Lighthouse Lab in Milton Keynes and Alex Alderton, Roberto Amato, Sonia Goncalves, Ewan Harrison, David K. Jackson, Ian Johnston, Dominic Kwiatkowski, Cordelia Langford, John Sillitoe on behalf of the Wellcome Sanger Institute COVID-19 Surveillance Team
EPI_ISL_736290	Lighthouse Lab in Glasgow	Wellcome Sanger Institute for the COVID-19 Genomics UK (COG-UK) Consortium	Harper VanSteenhouse, Yumi Kasai, David Gray, Carol Clugston, Anna Dominiczak and Alex Alderton, Roberto Amato, Sonia Goncalves, Ewan Harrison, David K. Jackson, Ian Johnston, Dominic Kwiatkowski, Cordelia Langford, John Sillitoe on behalf of the Wellcome Sanger Institute COVID-19 Surveillance Team
EPI_ISL_736291, EPI_ISL_736294, EPI_ISL_736295, EPI_ISL_736296, EPI_ISL_736297, EPI_ISL_736298, EPI_ISL_736299, EPI_ISL_736300	Lighthouse Lab in Milton Keynes	Wellcome Sanger Institute for the COVID-19 Genomics UK (COG-UK) Consortium	The Lighthouse Lab in Milton Keynes and Alex Alderton, Roberto Amato, Sonia Goncalves, Ewan Harrison, David K. Jackson, Ian Johnston, Dominic Kwiatkowski, Cordelia Langford, John Sillitoe on behalf of the Wellcome Sanger Institute COVID-19 Surveillance Team
EPI_ISL_736301	Lighthouse Lab in Glasgow	Wellcome Sanger Institute for the COVID-19 Genomics UK (COG-UK) Consortium	Harper VanSteenhouse, Yumi Kasai, David Gray, Carol Clugston, Anna Dominiczak and Alex Alderton, Roberto Amato, Sonia Goncalves, Ewan Harrison, David K. Jackson, Ian Johnston, Dominic Kwiatkowski, Cordelia Langford, John Sillitoe on behalf of the Wellcome Sanger Institute COVID-19 Surveillance Team
EPI_ISL_736302	Lighthouse Lab in Milton Keynes	Wellcome Sanger Institute for the COVID-19 Genomics UK (COG-UK) Consortium	The Lighthouse Lab in Milton Keynes and Alex Alderton, Roberto Amato, Sonia Goncalves, Ewan Harrison, David K. Jackson, Ian Johnston, Dominic Kwiatkowski, Cordelia Langford, John Sillitoe on behalf of the Wellcome Sanger Institute COVID-19 Surveillance Team
EPI_ISL_736304	Lighthouse Lab in Glasgow	Wellcome Sanger Institute for the COVID-19 Genomics UK (COG-UK) Consortium	Harper VanSteenhouse, Yumi Kasai, David Gray, Carol Clugston, Anna Dominiczak and Alex Alderton, Roberto Amato, Sonia Goncalves, Ewan Harrison, David K. Jackson, Ian Johnston, Dominic Kwiatkowski, Cordelia Langford, John Sillitoe on behalf of the Wellcome Sanger Institute COVID-19 Surveillance Team
EPI_ISL_736305	Lighthouse Lab in Milton Keynes	Wellcome Sanger Institute for the COVID-19 Genomics UK (COG-UK) Consortium	The Lighthouse Lab in Milton Keynes and Alex Alderton, Roberto Amato, Sonia Goncalves, Ewan Harrison, David K. Jackson, Ian Johnston, Dominic Kwiatkowski, Cordelia Langford, John Sillitoe on behalf of the Wellcome Sanger Institute COVID-19 Surveillance Team
EPI_ISL_736306	Lighthouse Lab in Glasgow	Wellcome Sanger Institute for the COVID-19 Genomics UK (COG-UK) Consortium	Harper VanSteenhouse, Yumi Kasai, David Gray, Carol Clugston, Anna Dominiczak and Alex Alderton, Roberto Amato, Sonia Goncalves, Ewan Harrison, David K. Jackson, Ian Johnston, Dominic Kwiatkowski, Cordelia Langford, John Sillitoe on behalf of the Wellcome Sanger Institute COVID-19 Surveillance Team
EPI_ISL_736307, EPI_ISL_736308	Lighthouse Lab in Milton Keynes	Wellcome Sanger Institute for the COVID-19 Genomics UK (COG-UK) Consortium	The Lighthouse Lab in Milton Keynes and Alex Alderton, Roberto Amato, Sonia Goncalves, Ewan Harrison, David K. Jackson, Ian Johnston, Dominic Kwiatkowski, Cordelia Langford, John Sillitoe on behalf of the Wellcome Sanger Institute COVID-19 Surveillance Team
EPI_ISL_736309	Lighthouse Lab in Glasgow	Wellcome Sanger Institute for the COVID-19 Genomics UK (COG-UK) Consortium	Harper VanSteenhouse, Yumi Kasai, David Gray, Carol Clugston, Anna Dominiczak and Alex Alderton, Roberto Amato, Sonia Goncalves, Ewan Harrison, David K. Jackson, Ian Johnston, Dominic Kwiatkowski, Cordelia Langford, John Sillitoe on behalf of the Wellcome Sanger Institute COVID-19 Surveillance Team

EPI_ISL_736310	Lighthouse Lab in Milton Keynes	Wellcome Sanger Institute for the COVID-19 Genomics UK (COG-UK) Consortium	The Lighthouse Lab in Milton Keynes and Alex Alderton, Roberto Amato, Sonia Goncalves, Ewan Harrison, David K. Jackson, Ian Johnston, Dominic Kwiatkowski, Cordelia Langford, John Sillitoe on behalf of the Wellcome Sanger Institute COVID-19 Surveillance Team
EPI_ISL_736311, EPI_ISL_736312, EPI_ISL_736313	Lighthouse Lab in Glasgow	Wellcome Sanger Institute for the COVID-19 Genomics UK (COG-UK) Consortium	Harper VanSteenhouse, Yumi Kasai, David Gray, Carol Clugston, Anna Dominiczak and Alex Alderton, Roberto Amato, Sonia Goncalves, Ewan Harrison, David K. Jackson, Ian Johnston, Dominic Kwiatkowski, Cordelia Langford, John Sillitoe on behalf of the Wellcome Sanger Institute COVID-19 Surveillance Team
EPI_ISL_736314	Lighthouse Lab in Milton Keynes	Wellcome Sanger Institute for the COVID-19 Genomics UK (COG-UK) Consortium	The Lighthouse Lab in Milton Keynes and Alex Alderton, Roberto Amato, Sonia Goncalves, Ewan Harrison, David K. Jackson, Ian Johnston, Dominic Kwiatkowski, Cordelia Langford, John Sillitoe on behalf of the Wellcome Sanger Institute COVID-19 Surveillance Team
EPI_ISL_736315, EPI_ISL_736316	Lighthouse Lab in Glasgow	Wellcome Sanger Institute for the COVID-19 Genomics UK (COG-UK) Consortium	Harper VanSteenhouse, Yumi Kasai, David Gray, Carol Clugston, Anna Dominiczak and Alex Alderton, Roberto Amato, Sonia Goncalves, Ewan Harrison, David K. Jackson, Ian Johnston, Dominic Kwiatkowski, Cordelia Langford, John Sillitoe on behalf of the Wellcome Sanger Institute COVID-19 Surveillance Team
EPI_ISL_736317	Lighthouse Lab in Milton Keynes	Wellcome Sanger Institute for the COVID-19 Genomics UK (COG-UK) Consortium	The Lighthouse Lab in Milton Keynes and Alex Alderton, Roberto Amato, Sonia Goncalves, Ewan Harrison, David K. Jackson, Ian Johnston, Dominic Kwiatkowski, Cordelia Langford, John Sillitoe on behalf of the Wellcome Sanger Institute COVID-19 Surveillance Team
EPI_ISL_736318	Lighthouse Lab in Glasgow	Wellcome Sanger Institute for the COVID-19 Genomics UK (COG-UK) Consortium	Harper VanSteenhouse, Yumi Kasai, David Gray, Carol Clugston, Anna Dominiczak and Alex Alderton, Roberto Amato, Sonia Goncalves, Ewan Harrison, David K. Jackson, Ian Johnston, Dominic Kwiatkowski, Cordelia Langford, John Sillitoe on behalf of the Wellcome Sanger Institute COVID-19 Surveillance Team
EPI_ISL_736319	Lighthouse Lab in Milton Keynes	Wellcome Sanger Institute for the COVID-19 Genomics UK (COG-UK) Consortium	The Lighthouse Lab in Milton Keynes and Alex Alderton, Roberto Amato, Sonia Goncalves, Ewan Harrison, David K. Jackson, Ian Johnston, Dominic Kwiatkowski, Cordelia Langford, John Sillitoe on behalf of the Wellcome Sanger Institute COVID-19 Surveillance Team
EPI_ISL_736320, EPI_ISL_736321	Lighthouse Lab in Glasgow	Wellcome Sanger Institute for the COVID-19 Genomics UK (COG-UK) Consortium	Harper VanSteenhouse, Yumi Kasai, David Gray, Carol Clugston, Anna Dominiczak and Alex Alderton, Roberto Amato, Sonia Goncalves, Ewan Harrison, David K. Jackson, Ian Johnston, Dominic Kwiatkowski, Cordelia Langford, John Sillitoe on behalf of the Wellcome Sanger Institute COVID-19 Surveillance Team
EPI_ISL_736322	Lighthouse Lab in Milton Keynes	Wellcome Sanger Institute for the COVID-19 Genomics UK (COG-UK) Consortium	The Lighthouse Lab in Milton Keynes and Alex Alderton, Roberto Amato, Sonia Goncalves, Ewan Harrison, David K. Jackson, Ian Johnston, Dominic Kwiatkowski, Cordelia Langford, John Sillitoe on behalf of the Wellcome Sanger Institute COVID-19 Surveillance Team
EPI_ISL_736323	Lighthouse Lab in Glasgow	Wellcome Sanger Institute for the COVID-19 Genomics UK (COG-UK) Consortium	Harper VanSteenhouse, Yumi Kasai, David Gray, Carol Clugston, Anna Dominiczak and Alex Alderton, Roberto Amato, Sonia Goncalves, Ewan Harrison, David K. Jackson, Ian Johnston, Dominic Kwiatkowski, Cordelia Langford, John Sillitoe on behalf of the Wellcome Sanger Institute COVID-19 Surveillance Team
EPI_ISL_736324	Lighthouse Lab in Milton Keynes	Wellcome Sanger Institute for the COVID-19 Genomics UK (COG-UK) Consortium	The Lighthouse Lab in Milton Keynes and Alex Alderton, Roberto Amato, Sonia Goncalves, Ewan Harrison, David K. Jackson, Ian Johnston, Dominic Kwiatkowski, Cordelia Langford, John Sillitoe on behalf of the Wellcome Sanger Institute COVID-19 Surveillance Team
EPI_ISL_736325	Lighthouse Lab in Glasgow	Wellcome Sanger Institute for the COVID-19 Genomics UK (COG-UK) Consortium	Harper VanSteenhouse, Yumi Kasai, David Gray, Carol Clugston, Anna Dominiczak and Alex Alderton, Roberto Amato, Sonia Goncalves, Ewan Harrison, David K. Jackson, Ian Johnston, Dominic Kwiatkowski, Cordelia Langford, John Sillitoe on behalf of the Wellcome Sanger Institute COVID-19 Surveillance Team
EPI_ISL_736326	Lighthouse Lab in Milton Keynes	Wellcome Sanger Institute for the COVID-19 Genomics UK (COG-UK) Consortium	The Lighthouse Lab in Milton Keynes and Alex Alderton, Roberto Amato, Sonia Goncalves, Ewan Harrison, David K. Jackson, Ian Johnston, Dominic Kwiatkowski, Cordelia Langford, John Sillitoe on behalf of the Wellcome Sanger Institute COVID-19 Surveillance Team
EPI_ISL_736327	Lighthouse Lab in Glasgow	Wellcome Sanger Institute for the COVID-19 Genomics UK (COG-UK) Consortium	Harper VanSteenhouse, Yumi Kasai, David Gray, Carol Clugston, Anna Dominiczak and Alex Alderton, Roberto Amato, Sonia Goncalves, Ewan Harrison, David K. Jackson, Ian Johnston, Dominic Kwiatkowski, Cordelia Langford, John Sillitoe on behalf of the Wellcome Sanger Institute COVID-19 Surveillance Team
EPI_ISL_736328, EPI_ISL_736329	Lighthouse Lab in Milton Keynes	Wellcome Sanger Institute for the COVID-19 Genomics UK (COG-UK) Consortium	The Lighthouse Lab in Milton Keynes and Alex Alderton, Roberto Amato, Sonia Goncalves, Ewan Harrison, David K. Jackson, Ian Johnston, Dominic Kwiatkowski, Cordelia Langford, John Sillitoe on behalf of the Wellcome Sanger Institute COVID-19 Surveillance Team
EPI_ISL_736330, EPI_ISL_736331, EPI_ISL_736332	Lighthouse Lab in Glasgow	Wellcome Sanger Institute for the COVID-19 Genomics UK (COG-UK) Consortium	Harper VanSteenhouse, Yumi Kasai, David Gray, Carol Clugston, Anna Dominiczak and Alex Alderton, Roberto Amato, Sonia Goncalves, Ewan Harrison, David K. Jackson, Ian Johnston, Dominic Kwiatkowski, Cordelia Langford, John Sillitoe on behalf of the Wellcome Sanger Institute COVID-19 Surveillance Team
EPI_ISL_736333	Lighthouse Lab in Milton Keynes	Wellcome Sanger Institute for the COVID-19 Genomics UK (COG-UK) Consortium	The Lighthouse Lab in Milton Keynes and Alex Alderton, Roberto Amato, Sonia Goncalves, Ewan Harrison, David K. Jackson, Ian Johnston, Dominic Kwiatkowski, Cordelia Langford, John Sillitoe on behalf of the Wellcome Sanger Institute COVID-19 Surveillance Team
EPI_ISL_736334	Lighthouse Lab in Glasgow	Wellcome Sanger Institute for the COVID-19 Genomics UK (COG-UK) Consortium	Harper VanSteenhouse, Yumi Kasai, David Gray, Carol Clugston, Anna Dominiczak and Alex Alderton, Roberto Amato, Sonia Goncalves, Ewan Harrison, David K. Jackson, Ian Johnston, Dominic Kwiatkowski, Cordelia Langford, John Sillitoe on behalf of the Wellcome Sanger Institute COVID-19 Surveillance Team
EPI_ISL_736335, EPI_ISL_736336, EPI_ISL_736337, EPI_ISL_736338, EPI_ISL_736339, EPI_ISL_736340, EPI_ISL_736341, EPI_ISL_736342	Lighthouse Lab in Milton Keynes	Wellcome Sanger Institute for the COVID-19 Genomics UK (COG-UK) Consortium	The Lighthouse Lab in Milton Keynes and Alex Alderton, Roberto Amato, Sonia Goncalves, Ewan Harrison, David K. Jackson, Ian Johnston, Dominic Kwiatkowski, Cordelia Langford, John Sillitoe on behalf of the Wellcome Sanger Institute COVID-19 Surveillance Team
EPI_ISL_736343	Lighthouse Lab in Glasgow	Wellcome Sanger Institute for the COVID-19 Genomics UK (COG-UK) Consortium	Harper VanSteenhouse, Yumi Kasai, David Gray, Carol Clugston, Anna Dominiczak and Alex Alderton, Roberto Amato, Sonia Goncalves, Ewan Harrison, David K. Jackson, Ian Johnston, Dominic Kwiatkowski, Cordelia Langford, John Sillitoe on behalf of the Wellcome Sanger Institute COVID-19 Surveillance Team
EPI_ISL_736344, EPI_ISL_736345	Lighthouse Lab in Milton Keynes	Wellcome Sanger Institute for the COVID-19 Genomics UK (COG-UK) Consortium	The Lighthouse Lab in Milton Keynes and Alex Alderton, Roberto Amato, Sonia Goncalves, Ewan Harrison, David K. Jackson, Ian Johnston, Dominic Kwiatkowski, Cordelia Langford, John Sillitoe on behalf of the Wellcome Sanger Institute COVID-19 Surveillance Team
EPI_ISL_736346, EPI_ISL_736347	Lighthouse Lab in Glasgow	Wellcome Sanger Institute for the COVID-19 Genomics UK (COG-UK) Consortium	Harper VanSteenhouse, Yumi Kasai, David Gray, Carol Clugston, Anna Dominiczak and Alex Alderton, Roberto Amato, Sonia Goncalves, Ewan Harrison, David K. Jackson, Ian Johnston, Dominic Kwiatkowski, Cordelia Langford, John Sillitoe on behalf of the Wellcome Sanger Institute COVID-19 Surveillance Team
EPI_ISL_736348	Lighthouse Lab in Milton Keynes	Wellcome Sanger Institute for the COVID-19 Genomics UK (COG-UK) Consortium	The Lighthouse Lab in Milton Keynes and Alex Alderton, Roberto Amato, Sonia Goncalves, Ewan Harrison, David K. Jackson, Ian Johnston, Dominic Kwiatkowski, Cordelia Langford, John Sillitoe on behalf of the Wellcome Sanger Institute COVID-19 Surveillance Team
EPI_ISL_736350	Lighthouse Lab in Glasgow	Wellcome Sanger Institute for the COVID-19 Genomics UK (COG-UK) Consortium	Harper VanSteenhouse, Yumi Kasai, David Gray, Carol Clugston, Anna Dominiczak and Alex Alderton, Roberto Amato, Sonia Goncalves, Ewan Harrison, David K. Jackson, Ian Johnston, Dominic Kwiatkowski, Cordelia Langford, John Sillitoe on behalf of the Wellcome Sanger Institute COVID-19 Surveillance Team
EPI_ISL_736351	Lighthouse Lab in Milton Keynes	Wellcome Sanger Institute for the COVID-19 Genomics UK (COG-UK) Consortium	The Lighthouse Lab in Milton Keynes and Alex Alderton, Roberto Amato, Sonia Goncalves, Ewan Harrison, David K. Jackson, Ian Johnston, Dominic Kwiatkowski, Cordelia Langford, John Sillitoe on behalf of the Wellcome Sanger Institute COVID-19 Surveillance Team
EPI_ISL_736352	Lighthouse Lab in Glasgow	Wellcome Sanger Institute for the COVID-19 Genomics UK (COG-UK) Consortium	Harper VanSteenhouse, Yumi Kasai, David Gray, Carol Clugston, Anna Dominiczak and Alex Alderton, Roberto Amato, Sonia Goncalves, Ewan Harrison, David K. Jackson, Ian Johnston, Dominic Kwiatkowski, Cordelia Langford, John Sillitoe on behalf of the Wellcome Sanger Institute COVID-19 Surveillance Team
EPI_ISL_736353, EPI_ISL_736354	Lighthouse Lab in Milton Keynes	Wellcome Sanger Institute for the COVID-19 Genomics UK (COG-UK) Consortium	The Lighthouse Lab in Milton Keynes and Alex Alderton, Roberto Amato, Sonia Goncalves, Ewan Harrison, David K. Jackson, Ian Johnston, Dominic Kwiatkowski, Cordelia Langford, John Sillitoe on behalf of the Wellcome Sanger Institute COVID-19 Surveillance Team
EPI_ISL_736355, EPI_ISL_736356	Lighthouse Lab in Glasgow	Wellcome Sanger Institute for the COVID-19 Genomics UK (COG-UK) Consortium	Harper VanSteenhouse, Yumi Kasai, David Gray, Carol Clugston, Anna Dominiczak and Alex Alderton, Roberto Amato, Sonia Goncalves, Ewan Harrison, David K. Jackson, Ian Johnston, Dominic Kwiatkowski, Cordelia Langford, John Sillitoe on behalf of the Wellcome Sanger Institute COVID-19 Surveillance Team
EPI_ISL_736357	Lighthouse Lab in Milton Keynes	Wellcome Sanger Institute for the COVID-19 Genomics UK	The Lighthouse Lab in Milton Keynes and Alex Alderton, Roberto Amato, Sonia Goncalves, Ewan Harrison, David K. Jackson, Ian Johnston, Dominic

		(COG-UK) Consortium	Kwiatkowski, Cordelia Langford, John Sillitoe on behalf of the Wellcome Sanger Institute COVID-19 Surveillance Team
EPI_ISL_736358	Lighthouse Lab in Glasgow	Wellcome Sanger Institute for the COVID-19 Genomics UK (COG-UK) Consortium	Harper VanSteenhouse, Yumi Kasai, David Gray, Carol Clugston, Anna Dominiczak and Alex Alderton, Roberto Amato, Sonia Goncalves, Ewan Harrison, David K. Jackson, Ian Johnston, Dominic Kwiatkowski, Cordelia Langford, John Sillitoe on behalf of the Wellcome Sanger Institute COVID-19 Surveillance Team
EPI_ISL_736359, EPI_ISL_736360, EPI_ISL_736361	Lighthouse Lab in Milton Keynes	Wellcome Sanger Institute for the COVID-19 Genomics UK (COG-UK) Consortium	The Lighthouse Lab in Milton Keynes and Alex Alderton, Roberto Amato, Sonia Goncalves, Ewan Harrison, David K. Jackson, Ian Johnston, Dominic Kwiatkowski, Cordelia Langford, John Sillitoe on behalf of the Wellcome Sanger Institute COVID-19 Surveillance Team
EPI_ISL_736362	Lighthouse Lab in Glasgow	Wellcome Sanger Institute for the COVID-19 Genomics UK (COG-UK) Consortium	Harper VanSteenhouse, Yumi Kasai, David Gray, Carol Clugston, Anna Dominiczak and Alex Alderton, Roberto Amato, Sonia Goncalves, Ewan Harrison, David K. Jackson, Ian Johnston, Dominic Kwiatkowski, Cordelia Langford, John Sillitoe on behalf of the Wellcome Sanger Institute COVID-19 Surveillance Team
EPI_ISL_736363, EPI_ISL_736364, EPI_ISL_736365, EPI_ISL_736366	Lighthouse Lab in Milton Keynes	Wellcome Sanger Institute for the COVID-19 Genomics UK (COG-UK) Consortium	The Lighthouse Lab in Milton Keynes and Alex Alderton, Roberto Amato, Sonia Goncalves, Ewan Harrison, David K. Jackson, Ian Johnston, Dominic Kwiatkowski, Cordelia Langford, John Sillitoe on behalf of the Wellcome Sanger Institute COVID-19 Surveillance Team
EPI_ISL_736367	Lighthouse Lab in Glasgow	Wellcome Sanger Institute for the COVID-19 Genomics UK (COG-UK) Consortium	Harper VanSteenhouse, Yumi Kasai, David Gray, Carol Clugston, Anna Dominiczak and Alex Alderton, Roberto Amato, Sonia Goncalves, Ewan Harrison, David K. Jackson, Ian Johnston, Dominic Kwiatkowski, Cordelia Langford, John Sillitoe on behalf of the Wellcome Sanger Institute COVID-19 Surveillance Team
EPI_ISL_736368	Lighthouse Lab in Milton Keynes	Wellcome Sanger Institute for the COVID-19 Genomics UK (COG-UK) Consortium	The Lighthouse Lab in Milton Keynes and Alex Alderton, Roberto Amato, Sonia Goncalves, Ewan Harrison, David K. Jackson, Ian Johnston, Dominic Kwiatkowski, Cordelia Langford, John Sillitoe on behalf of the Wellcome Sanger Institute COVID-19 Surveillance Team
EPI_ISL_736370, EPI_ISL_736371	Lighthouse Lab in Glasgow	Wellcome Sanger Institute for the COVID-19 Genomics UK (COG-UK) Consortium	Harper VanSteenhouse, Yumi Kasai, David Gray, Carol Clugston, Anna Dominiczak and Alex Alderton, Roberto Amato, Sonia Goncalves, Ewan Harrison, David K. Jackson, Ian Johnston, Dominic Kwiatkowski, Cordelia Langford, John Sillitoe on behalf of the Wellcome Sanger Institute COVID-19 Surveillance Team
EPI_ISL_736372	Lighthouse Lab in Milton Keynes	Wellcome Sanger Institute for the COVID-19 Genomics UK (COG-UK) Consortium	The Lighthouse Lab in Milton Keynes and Alex Alderton, Roberto Amato, Sonia Goncalves, Ewan Harrison, David K. Jackson, Ian Johnston, Dominic Kwiatkowski, Cordelia Langford, John Sillitoe on behalf of the Wellcome Sanger Institute COVID-19 Surveillance Team
EPI_ISL_736373	Lighthouse Lab in Glasgow	Wellcome Sanger Institute for the COVID-19 Genomics UK (COG-UK) Consortium	Harper VanSteenhouse, Yumi Kasai, David Gray, Carol Clugston, Anna Dominiczak and Alex Alderton, Roberto Amato, Sonia Goncalves, Ewan Harrison, David K. Jackson, Ian Johnston, Dominic Kwiatkowski, Cordelia Langford, John Sillitoe on behalf of the Wellcome Sanger Institute COVID-19 Surveillance Team
EPI_ISL_736374, EPI_ISL_736375	Lighthouse Lab in Milton Keynes	Wellcome Sanger Institute for the COVID-19 Genomics UK (COG-UK) Consortium	The Lighthouse Lab in Milton Keynes and Alex Alderton, Roberto Amato, Sonia Goncalves, Ewan Harrison, David K. Jackson, Ian Johnston, Dominic Kwiatkowski, Cordelia Langford, John Sillitoe on behalf of the Wellcome Sanger Institute COVID-19 Surveillance Team
EPI_ISL_736376	Lighthouse Lab in Glasgow	Wellcome Sanger Institute for the COVID-19 Genomics UK (COG-UK) Consortium	Harper VanSteenhouse, Yumi Kasai, David Gray, Carol Clugston, Anna Dominiczak and Alex Alderton, Roberto Amato, Sonia Goncalves, Ewan Harrison, David K. Jackson, Ian Johnston, Dominic Kwiatkowski, Cordelia Langford, John Sillitoe on behalf of the Wellcome Sanger Institute COVID-19 Surveillance Team
EPI_ISL_736377	Lighthouse Lab in Milton Keynes	Wellcome Sanger Institute for the COVID-19 Genomics UK (COG-UK) Consortium	The Lighthouse Lab in Milton Keynes and Alex Alderton, Roberto Amato, Sonia Goncalves, Ewan Harrison, David K. Jackson, Ian Johnston, Dominic Kwiatkowski, Cordelia Langford, John Sillitoe on behalf of the Wellcome Sanger Institute COVID-19 Surveillance Team
EPI_ISL_736378	Lighthouse Lab in Glasgow	Wellcome Sanger Institute for the COVID-19 Genomics UK (COG-UK) Consortium	Harper VanSteenhouse, Yumi Kasai, David Gray, Carol Clugston, Anna Dominiczak and Alex Alderton, Roberto Amato, Sonia Goncalves, Ewan Harrison, David K. Jackson, Ian Johnston, Dominic Kwiatkowski, Cordelia Langford, John Sillitoe on behalf of the Wellcome Sanger Institute COVID-19 Surveillance Team
EPI_ISL_736379, EPI_ISL_736381	Lighthouse Lab in Milton Keynes	Wellcome Sanger Institute for the COVID-19 Genomics UK (COG-UK) Consortium	The Lighthouse Lab in Milton Keynes and Alex Alderton, Roberto Amato, Sonia Goncalves, Ewan Harrison, David K. Jackson, Ian Johnston, Dominic Kwiatkowski, Cordelia Langford, John Sillitoe on behalf of the Wellcome Sanger Institute COVID-19 Surveillance Team
EPI_ISL_736382, EPI_ISL_736383	Lighthouse Lab in Glasgow	Wellcome Sanger Institute for the COVID-19 Genomics UK (COG-UK) Consortium	Harper VanSteenhouse, Yumi Kasai, David Gray, Carol Clugston, Anna Dominiczak and Alex Alderton, Roberto Amato, Sonia Goncalves, Ewan Harrison, David K. Jackson, Ian Johnston, Dominic Kwiatkowski, Cordelia Langford, John Sillitoe on behalf of the Wellcome Sanger Institute COVID-19 Surveillance Team
EPI_ISL_736384, EPI_ISL_736385	Lighthouse Lab in Milton Keynes	Wellcome Sanger Institute for the COVID-19 Genomics UK (COG-UK) Consortium	The Lighthouse Lab in Milton Keynes and Alex Alderton, Roberto Amato, Sonia Goncalves, Ewan Harrison, David K. Jackson, Ian Johnston, Dominic Kwiatkowski, Cordelia Langford, John Sillitoe on behalf of the Wellcome Sanger Institute COVID-19 Surveillance Team
EPI_ISL_736386	Lighthouse Lab in Glasgow	Wellcome Sanger Institute for the COVID-19 Genomics UK (COG-UK) Consortium	Harper VanSteenhouse, Yumi Kasai, David Gray, Carol Clugston, Anna Dominiczak and Alex Alderton, Roberto Amato, Sonia Goncalves, Ewan Harrison, David K. Jackson, Ian Johnston, Dominic Kwiatkowski, Cordelia Langford, John Sillitoe on behalf of the Wellcome Sanger Institute COVID-19 Surveillance Team
EPI_ISL_736387	Lighthouse Lab in Milton Keynes	Wellcome Sanger Institute for the COVID-19 Genomics UK (COG-UK) Consortium	The Lighthouse Lab in Milton Keynes and Alex Alderton, Roberto Amato, Sonia Goncalves, Ewan Harrison, David K. Jackson, Ian Johnston, Dominic Kwiatkowski, Cordelia Langford, John Sillitoe on behalf of the Wellcome Sanger Institute COVID-19 Surveillance Team
EPI_ISL_736388, EPI_ISL_736389, EPI_ISL_736390, EPI_ISL_736391	Lighthouse Lab in Glasgow	Wellcome Sanger Institute for the COVID-19 Genomics UK (COG-UK) Consortium	Harper VanSteenhouse, Yumi Kasai, David Gray, Carol Clugston, Anna Dominiczak and Alex Alderton, Roberto Amato, Sonia Goncalves, Ewan Harrison, David K. Jackson, Ian Johnston, Dominic Kwiatkowski, Cordelia Langford, John Sillitoe on behalf of the Wellcome Sanger Institute COVID-19 Surveillance Team
EPI_ISL_736393	Lighthouse Lab in Milton Keynes	Wellcome Sanger Institute for the COVID-19 Genomics UK (COG-UK) Consortium	The Lighthouse Lab in Milton Keynes and Alex Alderton, Roberto Amato, Sonia Goncalves, Ewan Harrison, David K. Jackson, Ian Johnston, Dominic Kwiatkowski, Cordelia Langford, John Sillitoe on behalf of the Wellcome Sanger Institute COVID-19 Surveillance Team
EPI_ISL_736394	Lighthouse Lab in Glasgow	Wellcome Sanger Institute for the COVID-19 Genomics UK (COG-UK) Consortium	Harper VanSteenhouse, Yumi Kasai, David Gray, Carol Clugston, Anna Dominiczak and Alex Alderton, Roberto Amato, Sonia Goncalves, Ewan Harrison, David K. Jackson, Ian Johnston, Dominic Kwiatkowski, Cordelia Langford, John Sillitoe on behalf of the Wellcome Sanger Institute COVID-19 Surveillance Team
EPI_ISL_736395, EPI_ISL_736396, EPI_ISL_736397	Lighthouse Lab in Milton Keynes	Wellcome Sanger Institute for the COVID-19 Genomics UK (COG-UK) Consortium	The Lighthouse Lab in Milton Keynes and Alex Alderton, Roberto Amato, Sonia Goncalves, Ewan Harrison, David K. Jackson, Ian Johnston, Dominic Kwiatkowski, Cordelia Langford, John Sillitoe on behalf of the Wellcome Sanger Institute COVID-19 Surveillance Team
EPI_ISL_736398, EPI_ISL_736399, EPI_ISL_736400	Lighthouse Lab in Glasgow	Wellcome Sanger Institute for the COVID-19 Genomics UK (COG-UK) Consortium	Harper VanSteenhouse, Yumi Kasai, David Gray, Carol Clugston, Anna Dominiczak and Alex Alderton, Roberto Amato, Sonia Goncalves, Ewan Harrison, David K. Jackson, Ian Johnston, Dominic Kwiatkowski, Cordelia Langford, John Sillitoe on behalf of the Wellcome Sanger Institute COVID-19 Surveillance Team
EPI_ISL_736401, EPI_ISL_736402, EPI_ISL_736403	Lighthouse Lab in Milton Keynes	Wellcome Sanger Institute for the COVID-19 Genomics UK (COG-UK) Consortium	The Lighthouse Lab in Milton Keynes and Alex Alderton, Roberto Amato, Sonia Goncalves, Ewan Harrison, David K. Jackson, Ian Johnston, Dominic Kwiatkowski, Cordelia Langford, John Sillitoe on behalf of the Wellcome Sanger Institute COVID-19 Surveillance Team
EPI_ISL_736404	Lighthouse Lab in Glasgow	Wellcome Sanger Institute for the COVID-19 Genomics UK (COG-UK) Consortium	Harper VanSteenhouse, Yumi Kasai, David Gray, Carol Clugston, Anna Dominiczak and Alex Alderton, Roberto Amato, Sonia Goncalves, Ewan Harrison, David K. Jackson, Ian Johnston, Dominic Kwiatkowski, Cordelia Langford, John Sillitoe on behalf of the Wellcome Sanger Institute COVID-19 Surveillance Team
EPI_ISL_736405, EPI_ISL_736406, EPI_ISL_736407, EPI_ISL_736409, EPI_ISL_736410	Lighthouse Lab in Milton Keynes	Wellcome Sanger Institute for the COVID-19 Genomics UK (COG-UK) Consortium	The Lighthouse Lab in Milton Keynes and Alex Alderton, Roberto Amato, Sonia Goncalves, Ewan Harrison, David K. Jackson, Ian Johnston, Dominic Kwiatkowski, Cordelia Langford, John Sillitoe on behalf of the Wellcome Sanger Institute COVID-19 Surveillance Team
EPI_ISL_736411, EPI_ISL_736412	Lighthouse Lab in Glasgow	Wellcome Sanger Institute for the COVID-19 Genomics UK (COG-UK) Consortium	Harper VanSteenhouse, Yumi Kasai, David Gray, Carol Clugston, Anna Dominiczak and Alex Alderton, Roberto Amato, Sonia Goncalves, Ewan Harrison, David K. Jackson, Ian Johnston, Dominic Kwiatkowski, Cordelia Langford, John Sillitoe on behalf of the Wellcome Sanger Institute COVID-19 Surveillance Team
EPI_ISL_736413	Lighthouse Lab in Milton Keynes	Wellcome Sanger Institute for the COVID-19 Genomics UK (COG-UK) Consortium	The Lighthouse Lab in Milton Keynes and Alex Alderton, Roberto Amato, Sonia Goncalves, Ewan Harrison, David K. Jackson, Ian Johnston, Dominic Kwiatkowski, Cordelia Langford, John Sillitoe on behalf of the Wellcome Sanger Institute COVID-19 Surveillance Team
EPI_ISL_736414	Lighthouse Lab in Glasgow	Wellcome Sanger Institute for the COVID-19 Genomics UK	Harper VanSteenhouse, Yumi Kasai, David Gray, Carol Clugston, Anna Dominiczak and Alex Alderton, Roberto Amato, Sonia Goncalves, Ewan Harrison,

		(COG-UK) Consortium	David K. Jackson, Ian Johnston, Dominic Kwiatkowski, Cordelia Langford, John Sillitoe on behalf of the Wellcome Sanger Institute COVID-19 Surveillance Team
EPI_ISL_736415	Lighthouse Lab in Milton Keynes	Wellcome Sanger Institute for the COVID-19 Genomics UK (COG-UK) Consortium	The Lighthouse Lab in Milton Keynes and Alex Alderton, Roberto Amato, Sonia Goncalves, Ewan Harrison, David K. Jackson, Ian Johnston, Dominic Kwiatkowski, Cordelia Langford, John Sillitoe on behalf of the Wellcome Sanger Institute COVID-19 Surveillance Team
EPI_ISL_736416, EPI_ISL_736417	Lighthouse Lab in Glasgow	Wellcome Sanger Institute for the COVID-19 Genomics UK (COG-UK) Consortium	Harper VanSteenhouse, Yumi Kasai, David Gray, Carol Clugston, Anna Dominiczak and Alex Alderton, Roberto Amato, Sonia Goncalves, Ewan Harrison, David K. Jackson, Ian Johnston, Dominic Kwiatkowski, Cordelia Langford, John Sillitoe on behalf of the Wellcome Sanger Institute COVID-19 Surveillance Team
EPI_ISL_736418	Lighthouse Lab in Milton Keynes	Wellcome Sanger Institute for the COVID-19 Genomics UK (COG-UK) Consortium	The Lighthouse Lab in Milton Keynes and Alex Alderton, Roberto Amato, Sonia Goncalves, Ewan Harrison, David K. Jackson, Ian Johnston, Dominic Kwiatkowski, Cordelia Langford, John Sillitoe on behalf of the Wellcome Sanger Institute COVID-19 Surveillance Team
EPI_ISL_736419	Lighthouse Lab in Glasgow	Wellcome Sanger Institute for the COVID-19 Genomics UK (COG-UK) Consortium	Harper VanSteenhouse, Yumi Kasai, David Gray, Carol Clugston, Anna Dominiczak and Alex Alderton, Roberto Amato, Sonia Goncalves, Ewan Harrison, David K. Jackson, Ian Johnston, Dominic Kwiatkowski, Cordelia Langford, John Sillitoe on behalf of the Wellcome Sanger Institute COVID-19 Surveillance Team
EPI_ISL_736420, EPI_ISL_736421, EPI_ISL_736422	Lighthouse Lab in Milton Keynes	Wellcome Sanger Institute for the COVID-19 Genomics UK (COG-UK) Consortium	The Lighthouse Lab in Milton Keynes and Alex Alderton, Roberto Amato, Sonia Goncalves, Ewan Harrison, David K. Jackson, Ian Johnston, Dominic Kwiatkowski, Cordelia Langford, John Sillitoe on behalf of the Wellcome Sanger Institute COVID-19 Surveillance Team
EPI_ISL_736423	Lighthouse Lab in Glasgow	Wellcome Sanger Institute for the COVID-19 Genomics UK (COG-UK) Consortium	Harper VanSteenhouse, Yumi Kasai, David Gray, Carol Clugston, Anna Dominiczak and Alex Alderton, Roberto Amato, Sonia Goncalves, Ewan Harrison, David K. Jackson, Ian Johnston, Dominic Kwiatkowski, Cordelia Langford, John Sillitoe on behalf of the Wellcome Sanger Institute COVID-19 Surveillance Team
EPI_ISL_736424	Lighthouse Lab in Milton Keynes	Wellcome Sanger Institute for the COVID-19 Genomics UK (COG-UK) Consortium	The Lighthouse Lab in Milton Keynes and Alex Alderton, Roberto Amato, Sonia Goncalves, Ewan Harrison, David K. Jackson, Ian Johnston, Dominic Kwiatkowski, Cordelia Langford, John Sillitoe on behalf of the Wellcome Sanger Institute COVID-19 Surveillance Team
EPI_ISL_736425, EPI_ISL_736426	Lighthouse Lab in Glasgow	Wellcome Sanger Institute for the COVID-19 Genomics UK (COG-UK) Consortium	Harper VanSteenhouse, Yumi Kasai, David Gray, Carol Clugston, Anna Dominiczak and Alex Alderton, Roberto Amato, Sonia Goncalves, Ewan Harrison, David K. Jackson, Ian Johnston, Dominic Kwiatkowski, Cordelia Langford, John Sillitoe on behalf of the Wellcome Sanger Institute COVID-19 Surveillance Team
EPI_ISL_736427	Lighthouse Lab in Milton Keynes	Wellcome Sanger Institute for the COVID-19 Genomics UK (COG-UK) Consortium	The Lighthouse Lab in Milton Keynes and Alex Alderton, Roberto Amato, Sonia Goncalves, Ewan Harrison, David K. Jackson, Ian Johnston, Dominic Kwiatkowski, Cordelia Langford, John Sillitoe on behalf of the Wellcome Sanger Institute COVID-19 Surveillance Team
EPI_ISL_736428, EPI_ISL_736429, EPI_ISL_736430, EPI_ISL_736431	Lighthouse Lab in Glasgow	Wellcome Sanger Institute for the COVID-19 Genomics UK (COG-UK) Consortium	Harper VanSteenhouse, Yumi Kasai, David Gray, Carol Clugston, Anna Dominiczak and Alex Alderton, Roberto Amato, Sonia Goncalves, Ewan Harrison, David K. Jackson, Ian Johnston, Dominic Kwiatkowski, Cordelia Langford, John Sillitoe on behalf of the Wellcome Sanger Institute COVID-19 Surveillance Team
EPI_ISL_736432, EPI_ISL_736433, EPI_ISL_736434, EPI_ISL_736435, EPI_ISL_736436	Lighthouse Lab in Milton Keynes	Wellcome Sanger Institute for the COVID-19 Genomics UK (COG-UK) Consortium	The Lighthouse Lab in Milton Keynes and Alex Alderton, Roberto Amato, Sonia Goncalves, Ewan Harrison, David K. Jackson, Ian Johnston, Dominic Kwiatkowski, Cordelia Langford, John Sillitoe on behalf of the Wellcome Sanger Institute COVID-19 Surveillance Team
EPI_ISL_736437, EPI_ISL_736438	Lighthouse Lab in Glasgow	Wellcome Sanger Institute for the COVID-19 Genomics UK (COG-UK) Consortium	Harper VanSteenhouse, Yumi Kasai, David Gray, Carol Clugston, Anna Dominiczak and Alex Alderton, Roberto Amato, Sonia Goncalves, Ewan Harrison, David K. Jackson, Ian Johnston, Dominic Kwiatkowski, Cordelia Langford, John Sillitoe on behalf of the Wellcome Sanger Institute COVID-19 Surveillance Team
EPI_ISL_736439, EPI_ISL_736440, EPI_ISL_736441	Lighthouse Lab in Milton Keynes	Wellcome Sanger Institute for the COVID-19 Genomics UK (COG-UK) Consortium	The Lighthouse Lab in Milton Keynes and Alex Alderton, Roberto Amato, Sonia Goncalves, Ewan Harrison, David K. Jackson, Ian Johnston, Dominic Kwiatkowski, Cordelia Langford, John Sillitoe on behalf of the Wellcome Sanger Institute COVID-19 Surveillance Team
EPI_ISL_736442	Lighthouse Lab in Glasgow	Wellcome Sanger Institute for the COVID-19 Genomics UK (COG-UK) Consortium	Harper VanSteenhouse, Yumi Kasai, David Gray, Carol Clugston, Anna Dominiczak and Alex Alderton, Roberto Amato, Sonia Goncalves, Ewan Harrison, David K. Jackson, Ian Johnston, Dominic Kwiatkowski, Cordelia Langford, John Sillitoe on behalf of the Wellcome Sanger Institute COVID-19 Surveillance Team
EPI_ISL_736443	Lighthouse Lab in Milton Keynes	Wellcome Sanger Institute for the COVID-19 Genomics UK (COG-UK) Consortium	The Lighthouse Lab in Milton Keynes and Alex Alderton, Roberto Amato, Sonia Goncalves, Ewan Harrison, David K. Jackson, Ian Johnston, Dominic Kwiatkowski, Cordelia Langford, John Sillitoe on behalf of the Wellcome Sanger Institute COVID-19 Surveillance Team
EPI_ISL_736444, EPI_ISL_736445	Lighthouse Lab in Glasgow	Wellcome Sanger Institute for the COVID-19 Genomics UK (COG-UK) Consortium	Harper VanSteenhouse, Yumi Kasai, David Gray, Carol Clugston, Anna Dominiczak and Alex Alderton, Roberto Amato, Sonia Goncalves, Ewan Harrison, David K. Jackson, Ian Johnston, Dominic Kwiatkowski, Cordelia Langford, John Sillitoe on behalf of the Wellcome Sanger Institute COVID-19 Surveillance Team
EPI_ISL_736446, EPI_ISL_736447, EPI_ISL_736448, EPI_ISL_736450	Lighthouse Lab in Milton Keynes	Wellcome Sanger Institute for the COVID-19 Genomics UK (COG-UK) Consortium	The Lighthouse Lab in Milton Keynes and Alex Alderton, Roberto Amato, Sonia Goncalves, Ewan Harrison, David K. Jackson, Ian Johnston, Dominic Kwiatkowski, Cordelia Langford, John Sillitoe on behalf of the Wellcome Sanger Institute COVID-19 Surveillance Team
EPI_ISL_736451	Lighthouse Lab in Glasgow	Wellcome Sanger Institute for the COVID-19 Genomics UK (COG-UK) Consortium	Harper VanSteenhouse, Yumi Kasai, David Gray, Carol Clugston, Anna Dominiczak and Alex Alderton, Roberto Amato, Sonia Goncalves, Ewan Harrison, David K. Jackson, Ian Johnston, Dominic Kwiatkowski, Cordelia Langford, John Sillitoe on behalf of the Wellcome Sanger Institute COVID-19 Surveillance Team
EPI_ISL_736452, EPI_ISL_736453, EPI_ISL_736454	Lighthouse Lab in Milton Keynes	Wellcome Sanger Institute for the COVID-19 Genomics UK (COG-UK) Consortium	The Lighthouse Lab in Milton Keynes and Alex Alderton, Roberto Amato, Sonia Goncalves, Ewan Harrison, David K. Jackson, Ian Johnston, Dominic Kwiatkowski, Cordelia Langford, John Sillitoe on behalf of the Wellcome Sanger Institute COVID-19 Surveillance Team
EPI_ISL_736455, EPI_ISL_736456	Lighthouse Lab in Glasgow	Wellcome Sanger Institute for the COVID-19 Genomics UK (COG-UK) Consortium	Harper VanSteenhouse, Yumi Kasai, David Gray, Carol Clugston, Anna Dominiczak and Alex Alderton, Roberto Amato, Sonia Goncalves, Ewan Harrison, David K. Jackson, Ian Johnston, Dominic Kwiatkowski, Cordelia Langford, John Sillitoe on behalf of the Wellcome Sanger Institute COVID-19 Surveillance Team
EPI_ISL_736457	Lighthouse Lab in Milton Keynes	Wellcome Sanger Institute for the COVID-19 Genomics UK (COG-UK) Consortium	The Lighthouse Lab in Milton Keynes and Alex Alderton, Roberto Amato, Sonia Goncalves, Ewan Harrison, David K. Jackson, Ian Johnston, Dominic Kwiatkowski, Cordelia Langford, John Sillitoe on behalf of the Wellcome Sanger Institute COVID-19 Surveillance Team
EPI_ISL_736459, EPI_ISL_736460, EPI_ISL_736461	Lighthouse Lab in Glasgow	Wellcome Sanger Institute for the COVID-19 Genomics UK (COG-UK) Consortium	Harper VanSteenhouse, Yumi Kasai, David Gray, Carol Clugston, Anna Dominiczak and Alex Alderton, Roberto Amato, Sonia Goncalves, Ewan Harrison, David K. Jackson, Ian Johnston, Dominic Kwiatkowski, Cordelia Langford, John Sillitoe on behalf of the Wellcome Sanger Institute COVID-19 Surveillance Team
EPI_ISL_736462, EPI_ISL_736463	Lighthouse Lab in Milton Keynes	Wellcome Sanger Institute for the COVID-19 Genomics UK (COG-UK) Consortium	The Lighthouse Lab in Milton Keynes and Alex Alderton, Roberto Amato, Sonia Goncalves, Ewan Harrison, David K. Jackson, Ian Johnston, Dominic Kwiatkowski, Cordelia Langford, John Sillitoe on behalf of the Wellcome Sanger Institute COVID-19 Surveillance Team
EPI_ISL_736464	Lighthouse Lab in Glasgow	Wellcome Sanger Institute for the COVID-19 Genomics UK (COG-UK) Consortium	Harper VanSteenhouse, Yumi Kasai, David Gray, Carol Clugston, Anna Dominiczak and Alex Alderton, Roberto Amato, Sonia Goncalves, Ewan Harrison, David K. Jackson, Ian Johnston, Dominic Kwiatkowski, Cordelia Langford, John Sillitoe on behalf of the Wellcome Sanger Institute COVID-19 Surveillance Team
EPI_ISL_736465	Lighthouse Lab in Milton Keynes	Wellcome Sanger Institute for the COVID-19 Genomics UK (COG-UK) Consortium	The Lighthouse Lab in Milton Keynes and Alex Alderton, Roberto Amato, Sonia Goncalves, Ewan Harrison, David K. Jackson, Ian Johnston, Dominic Kwiatkowski, Cordelia Langford, John Sillitoe on behalf of the Wellcome Sanger Institute COVID-19 Surveillance Team
EPI_ISL_736466	Lighthouse Lab in Glasgow	Wellcome Sanger Institute for the COVID-19 Genomics UK (COG-UK) Consortium	Harper VanSteenhouse, Yumi Kasai, David Gray, Carol Clugston, Anna Dominiczak and Alex Alderton, Roberto Amato, Sonia Goncalves, Ewan Harrison, David K. Jackson, Ian Johnston, Dominic Kwiatkowski, Cordelia Langford, John Sillitoe on behalf of the Wellcome Sanger Institute COVID-19 Surveillance Team
EPI_ISL_736467	Lighthouse Lab in Milton Keynes	Wellcome Sanger Institute for the COVID-19 Genomics UK (COG-UK) Consortium	The Lighthouse Lab in Milton Keynes and Alex Alderton, Roberto Amato, Sonia Goncalves, Ewan Harrison, David K. Jackson, Ian Johnston, Dominic Kwiatkowski, Cordelia Langford, John Sillitoe on behalf of the Wellcome Sanger Institute COVID-19 Surveillance Team
EPI_ISL_736468, EPI_ISL_736470, EPI_ISL_736471	Lighthouse Lab in Glasgow	Wellcome Sanger Institute for the COVID-19 Genomics UK (COG-UK) Consortium	Harper VanSteenhouse, Yumi Kasai, David Gray, Carol Clugston, Anna Dominiczak and Alex Alderton, Roberto Amato, Sonia Goncalves, Ewan Harrison, David K. Jackson, Ian Johnston, Dominic Kwiatkowski, Cordelia Langford, John Sillitoe on behalf of the Wellcome Sanger Institute COVID-19 Surveillance Team

EPI_ISL_736472, EPI_ISL_736473	Lighthouse Lab in Milton Keynes	Wellcome Sanger Institute for the COVID-19 Genomics UK (COG-UK) Consortium	The Lighthouse Lab in Milton Keynes and Alex Alderton, Roberto Amato, Sonia Goncalves, Ewan Harrison, David K. Jackson, Ian Johnston, Dominic Kwiatkowski, Cordelia Langford, John Sillitoe on behalf of the Wellcome Sanger Institute COVID-19 Surveillance Team
EPI_ISL_736474	Lighthouse Lab in Glasgow	Wellcome Sanger Institute for the COVID-19 Genomics UK (COG-UK) Consortium	Harper VanSteenhouse, Yumi Kasai, David Gray, Carol Clugston, Anna Dominiczak and Alex Alderton, Roberto Amato, Sonia Goncalves, Ewan Harrison, David K. Jackson, Ian Johnston, Dominic Kwiatkowski, Cordelia Langford, John Sillitoe on behalf of the Wellcome Sanger Institute COVID-19 Surveillance Team
EPI_ISL_736475	Lighthouse Lab in Milton Keynes	Wellcome Sanger Institute for the COVID-19 Genomics UK (COG-UK) Consortium	The Lighthouse Lab in Milton Keynes and Alex Alderton, Roberto Amato, Sonia Goncalves, Ewan Harrison, David K. Jackson, Ian Johnston, Dominic Kwiatkowski, Cordelia Langford, John Sillitoe on behalf of the Wellcome Sanger Institute COVID-19 Surveillance Team
EPI_ISL_736476, EPI_ISL_736478, EPI_ISL_736479	Lighthouse Lab in Glasgow	Wellcome Sanger Institute for the COVID-19 Genomics UK (COG-UK) Consortium	Harper VanSteenhouse, Yumi Kasai, David Gray, Carol Clugston, Anna Dominiczak and Alex Alderton, Roberto Amato, Sonia Goncalves, Ewan Harrison, David K. Jackson, Ian Johnston, Dominic Kwiatkowski, Cordelia Langford, John Sillitoe on behalf of the Wellcome Sanger Institute COVID-19 Surveillance Team
EPI_ISL_736480	Lighthouse Lab in Milton Keynes	Wellcome Sanger Institute for the COVID-19 Genomics UK (COG-UK) Consortium	The Lighthouse Lab in Milton Keynes and Alex Alderton, Roberto Amato, Sonia Goncalves, Ewan Harrison, David K. Jackson, Ian Johnston, Dominic Kwiatkowski, Cordelia Langford, John Sillitoe on behalf of the Wellcome Sanger Institute COVID-19 Surveillance Team
EPI_ISL_736481, EPI_ISL_736482, EPI_ISL_736484, EPI_ISL_736485, EPI_ISL_736486	Lighthouse Lab in Alderley Park	Wellcome Sanger Institute for the COVID-19 Genomics UK (COG-UK) Consortium	Jacquelyn Wynn, Mairead Hyland, The Lighthouse Lab in Alderley Park and Alex Alderton, Roberto Amato, Sonia Goncalves, Ewan Harrison, David K. Jackson, Ian Johnston, Dominic Kwiatkowski, Cordelia Langford, John Sillitoe on behalf of the Wellcome Sanger Institute COVID-19 Surveillance Team
EPI_ISL_736487	Lighthouse Lab in Milton Keynes	Wellcome Sanger Institute for the COVID-19 Genomics UK (COG-UK) Consortium	The Lighthouse Lab in Milton Keynes and Alex Alderton, Roberto Amato, Sonia Goncalves, Ewan Harrison, David K. Jackson, Ian Johnston, Dominic Kwiatkowski, Cordelia Langford, John Sillitoe on behalf of the Wellcome Sanger Institute COVID-19 Surveillance Team
EPI_ISL_736493, EPI_ISL_736494, EPI_ISL_736495, EPI_ISL_736496	Lighthouse Lab in Alderley Park	Wellcome Sanger Institute for the COVID-19 Genomics UK (COG-UK) Consortium	Jacquelyn Wynn, Mairead Hyland, The Lighthouse Lab in Alderley Park and Alex Alderton, Roberto Amato, Sonia Goncalves, Ewan Harrison, David K. Jackson, Ian Johnston, Dominic Kwiatkowski, Cordelia Langford, John Sillitoe on behalf of the Wellcome Sanger Institute COVID-19 Surveillance Team
EPI_ISL_736497	Lighthouse Lab in Milton Keynes	Wellcome Sanger Institute for the COVID-19 Genomics UK (COG-UK) Consortium	The Lighthouse Lab in Milton Keynes and Alex Alderton, Roberto Amato, Sonia Goncalves, Ewan Harrison, David K. Jackson, Ian Johnston, Dominic Kwiatkowski, Cordelia Langford, John Sillitoe on behalf of the Wellcome Sanger Institute COVID-19 Surveillance Team
EPI_ISL_736498, EPI_ISL_736499, EPI_ISL_736500	Lighthouse Lab in Alderley Park	Wellcome Sanger Institute for the COVID-19 Genomics UK (COG-UK) Consortium	Jacquelyn Wynn, Mairead Hyland, The Lighthouse Lab in Alderley Park and Alex Alderton, Roberto Amato, Sonia Goncalves, Ewan Harrison, David K. Jackson, Ian Johnston, Dominic Kwiatkowski, Cordelia Langford, John Sillitoe on behalf of the Wellcome Sanger Institute COVID-19 Surveillance Team
EPI_ISL_736501, EPI_ISL_736502	Lighthouse Lab in Milton Keynes	Wellcome Sanger Institute for the COVID-19 Genomics UK (COG-UK) Consortium	The Lighthouse Lab in Milton Keynes and Alex Alderton, Roberto Amato, Sonia Goncalves, Ewan Harrison, David K. Jackson, Ian Johnston, Dominic Kwiatkowski, Cordelia Langford, John Sillitoe on behalf of the Wellcome Sanger Institute COVID-19 Surveillance Team
EPI_ISL_736503	Lighthouse Lab in Glasgow	Wellcome Sanger Institute for the COVID-19 Genomics UK (COG-UK) Consortium	Harper VanSteenhouse, Yumi Kasai, David Gray, Carol Clugston, Anna Dominiczak and Alex Alderton, Roberto Amato, Sonia Goncalves, Ewan Harrison, David K. Jackson, Ian Johnston, Dominic Kwiatkowski, Cordelia Langford, John Sillitoe on behalf of the Wellcome Sanger Institute COVID-19 Surveillance Team
EPI_ISL_736504	Lighthouse Lab in Milton Keynes	Wellcome Sanger Institute for the COVID-19 Genomics UK (COG-UK) Consortium	The Lighthouse Lab in Milton Keynes and Alex Alderton, Roberto Amato, Sonia Goncalves, Ewan Harrison, David K. Jackson, Ian Johnston, Dominic Kwiatkowski, Cordelia Langford, John Sillitoe on behalf of the Wellcome Sanger Institute COVID-19 Surveillance Team
EPI_ISL_736505, EPI_ISL_736506, EPI_ISL_736507	Lighthouse Lab in Alderley Park	Wellcome Sanger Institute for the COVID-19 Genomics UK (COG-UK) Consortium	Jacquelyn Wynn, Mairead Hyland, The Lighthouse Lab in Alderley Park and Alex Alderton, Roberto Amato, Sonia Goncalves, Ewan Harrison, David K. Jackson, Ian Johnston, Dominic Kwiatkowski, Cordelia Langford, John Sillitoe on behalf of the Wellcome Sanger Institute COVID-19 Surveillance Team
EPI_ISL_736508	Lighthouse Lab in Milton Keynes	Wellcome Sanger Institute for the COVID-19 Genomics UK (COG-UK) Consortium	The Lighthouse Lab in Milton Keynes and Alex Alderton, Roberto Amato, Sonia Goncalves, Ewan Harrison, David K. Jackson, Ian Johnston, Dominic Kwiatkowski, Cordelia Langford, John Sillitoe on behalf of the Wellcome Sanger Institute COVID-19 Surveillance Team
EPI_ISL_736509, EPI_ISL_736510, EPI_ISL_736511, EPI_ISL_736513	Lighthouse Lab in Alderley Park	Wellcome Sanger Institute for the COVID-19 Genomics UK (COG-UK) Consortium	Jacquelyn Wynn, Mairead Hyland, The Lighthouse Lab in Alderley Park and Alex Alderton, Roberto Amato, Sonia Goncalves, Ewan Harrison, David K. Jackson, Ian Johnston, Dominic Kwiatkowski, Cordelia Langford, John Sillitoe on behalf of the Wellcome Sanger Institute COVID-19 Surveillance Team
EPI_ISL_736514, EPI_ISL_736515	Lighthouse Lab in Milton Keynes	Wellcome Sanger Institute for the COVID-19 Genomics UK (COG-UK) Consortium	The Lighthouse Lab in Milton Keynes and Alex Alderton, Roberto Amato, Sonia Goncalves, Ewan Harrison, David K. Jackson, Ian Johnston, Dominic Kwiatkowski, Cordelia Langford, John Sillitoe on behalf of the Wellcome Sanger Institute COVID-19 Surveillance Team
EPI_ISL_736516, EPI_ISL_736517, EPI_ISL_736518, EPI_ISL_736520, EPI_ISL_736521, EPI_ISL_736522	Lighthouse Lab in Alderley Park	Wellcome Sanger Institute for the COVID-19 Genomics UK (COG-UK) Consortium	Jacquelyn Wynn, Mairead Hyland, The Lighthouse Lab in Alderley Park and Alex Alderton, Roberto Amato, Sonia Goncalves, Ewan Harrison, David K. Jackson, Ian Johnston, Dominic Kwiatkowski, Cordelia Langford, John Sillitoe on behalf of the Wellcome Sanger Institute COVID-19 Surveillance Team
EPI_ISL_736523	Lighthouse Lab in Milton Keynes	Wellcome Sanger Institute for the COVID-19 Genomics UK (COG-UK) Consortium	The Lighthouse Lab in Milton Keynes and Alex Alderton, Roberto Amato, Sonia Goncalves, Ewan Harrison, David K. Jackson, Ian Johnston, Dominic Kwiatkowski, Cordelia Langford, John Sillitoe on behalf of the Wellcome Sanger Institute COVID-19 Surveillance Team
EPI_ISL_736524	Lighthouse Lab in Alderley Park	Wellcome Sanger Institute for the COVID-19 Genomics UK (COG-UK) Consortium	Jacquelyn Wynn, Mairead Hyland, The Lighthouse Lab in Alderley Park and Alex Alderton, Roberto Amato, Sonia Goncalves, Ewan Harrison, David K. Jackson, Ian Johnston, Dominic Kwiatkowski, Cordelia Langford, John Sillitoe on behalf of the Wellcome Sanger Institute COVID-19 Surveillance Team
EPI_ISL_736525	Lighthouse Lab in Milton Keynes	Wellcome Sanger Institute for the COVID-19 Genomics UK (COG-UK) Consortium	The Lighthouse Lab in Milton Keynes and Alex Alderton, Roberto Amato, Sonia Goncalves, Ewan Harrison, David K. Jackson, Ian Johnston, Dominic Kwiatkowski, Cordelia Langford, John Sillitoe on behalf of the Wellcome Sanger Institute COVID-19 Surveillance Team
EPI_ISL_736526	Lighthouse Lab in Alderley Park	Wellcome Sanger Institute for the COVID-19 Genomics UK (COG-UK) Consortium	Jacquelyn Wynn, Mairead Hyland, The Lighthouse Lab in Alderley Park and Alex Alderton, Roberto Amato, Sonia Goncalves, Ewan Harrison, David K. Jackson, Ian Johnston, Dominic Kwiatkowski, Cordelia Langford, John Sillitoe on behalf of the Wellcome Sanger Institute COVID-19 Surveillance Team
EPI_ISL_736527, EPI_ISL_736528	Lighthouse Lab in Milton Keynes	Wellcome Sanger Institute for the COVID-19 Genomics UK (COG-UK) Consortium	The Lighthouse Lab in Milton Keynes and Alex Alderton, Roberto Amato, Sonia Goncalves, Ewan Harrison, David K. Jackson, Ian Johnston, Dominic Kwiatkowski, Cordelia Langford, John Sillitoe on behalf of the Wellcome Sanger Institute COVID-19 Surveillance Team
EPI_ISL_736530, EPI_ISL_736531, EPI_ISL_736534, EPI_ISL_736535	Lighthouse Lab in Alderley Park	Wellcome Sanger Institute for the COVID-19 Genomics UK (COG-UK) Consortium	Jacquelyn Wynn, Mairead Hyland, The Lighthouse Lab in Alderley Park and Alex Alderton, Roberto Amato, Sonia Goncalves, Ewan Harrison, David K. Jackson, Ian Johnston, Dominic Kwiatkowski, Cordelia Langford, John Sillitoe on behalf of the Wellcome Sanger Institute COVID-19 Surveillance Team
EPI_ISL_736536	Lighthouse Lab in Milton Keynes	Wellcome Sanger Institute for the COVID-19 Genomics UK (COG-UK) Consortium	The Lighthouse Lab in Milton Keynes and Alex Alderton, Roberto Amato, Sonia Goncalves, Ewan Harrison, David K. Jackson, Ian Johnston, Dominic Kwiatkowski, Cordelia Langford, John Sillitoe on behalf of the Wellcome Sanger Institute COVID-19 Surveillance Team
EPI_ISL_736537	Lighthouse Lab in Alderley Park	Wellcome Sanger Institute for the COVID-19 Genomics UK (COG-UK) Consortium	Jacquelyn Wynn, Mairead Hyland, The Lighthouse Lab in Alderley Park and Alex Alderton, Roberto Amato, Sonia Goncalves, Ewan Harrison, David K. Jackson, Ian Johnston, Dominic Kwiatkowski, Cordelia Langford, John Sillitoe on behalf of the Wellcome Sanger Institute COVID-19 Surveillance Team
EPI_ISL_736538	Lighthouse Lab in Milton Keynes	Wellcome Sanger Institute for the COVID-19 Genomics UK (COG-UK) Consortium	The Lighthouse Lab in Milton Keynes and Alex Alderton, Roberto Amato, Sonia Goncalves, Ewan Harrison, David K. Jackson, Ian Johnston, Dominic Kwiatkowski, Cordelia Langford, John Sillitoe on behalf of the Wellcome Sanger Institute COVID-19 Surveillance Team
EPI_ISL_736539, EPI_ISL_736540, EPI_ISL_736541	Lighthouse Lab in Alderley Park	Wellcome Sanger Institute for the COVID-19 Genomics UK (COG-UK) Consortium	Jacquelyn Wynn, Mairead Hyland, The Lighthouse Lab in Alderley Park and Alex Alderton, Roberto Amato, Sonia Goncalves, Ewan Harrison, David K. Jackson, Ian Johnston, Dominic Kwiatkowski, Cordelia Langford, John Sillitoe on behalf of the Wellcome Sanger Institute COVID-19 Surveillance Team
EPI_ISL_736542	Lighthouse Lab in Milton Keynes	Wellcome Sanger Institute for the COVID-19 Genomics UK (COG-UK) Consortium	The Lighthouse Lab in Milton Keynes and Alex Alderton, Roberto Amato, Sonia Goncalves, Ewan Harrison, David K. Jackson, Ian Johnston, Dominic Kwiatkowski, Cordelia Langford, John Sillitoe on behalf of the Wellcome Sanger Institute COVID-19 Surveillance Team
EPI_ISL_736543, EPI_ISL_736544, EPI_ISL_736545, EPI_ISL_736546, EPI_ISL_736548	Lighthouse Lab in Alderley Park	Wellcome Sanger Institute for the COVID-19 Genomics UK (COG-UK) Consortium	Jacquelyn Wynn, Mairead Hyland, The Lighthouse Lab in Alderley Park and Alex Alderton, Roberto Amato, Sonia Goncalves, Ewan Harrison, David K. Jackson, Ian Johnston, Dominic Kwiatkowski, Cordelia Langford, John Sillitoe on behalf of the Wellcome Sanger Institute COVID-19 Surveillance Team
EPI_ISL_736549, EPI_ISL_736550, EPI_ISL_736551, EPI_ISL_736552	Lighthouse Lab in Milton Keynes	Wellcome Sanger Institute for the COVID-19 Genomics UK (COG-UK) Consortium	The Lighthouse Lab in Milton Keynes and Alex Alderton, Roberto Amato, Sonia Goncalves, Ewan Harrison, David K. Jackson, Ian Johnston, Dominic Kwiatkowski, Cordelia Langford, John Sillitoe on behalf of the Wellcome Sanger Institute COVID-19 Surveillance Team
EPI_ISL_736553, EPI_ISL_736554, EPI_ISL_736555, EPI_ISL_736557	Lighthouse Lab in Alderley Park	Wellcome Sanger Institute for the COVID-19 Genomics UK (COG-UK) Consortium	Jacquelyn Wynn, Mairead Hyland, The Lighthouse Lab in Alderley Park and Alex Alderton, Roberto Amato, Sonia Goncalves, Ewan Harrison, David K. Jackson, Ian Johnston, Dominic Kwiatkowski, Cordelia Langford, John Sillitoe on behalf of the Wellcome Sanger Institute COVID-19 Surveillance Team
EPI_ISL_736558	Lighthouse Lab in Milton Keynes	Wellcome Sanger Institute for the COVID-19 Genomics UK (COG-UK) Consortium	The Lighthouse Lab in Milton Keynes and Alex Alderton, Roberto Amato, Sonia Goncalves, Ewan Harrison, David K. Jackson, Ian Johnston, Dominic Kwiatkowski, Cordelia Langford, John Sillitoe on behalf of the Wellcome Sanger Institute COVID-19 Surveillance Team

EPI_ISL_736559, EPI_ISL_736560, EPI_ISL_736561, EPI_ISL_736562	Lighthouse Lab in Alderley Park	Wellcome Sanger Institute for the COVID-19 Genomics UK (COG-UK) Consortium	Jacquelyn Wynn, Mairead Hyland, The Lighthouse Lab in Alderley Park and Alex Alderton, Roberto Amato, Sonia Goncalves, Ewan Harrison, David K. Jackson, Ian Johnston, Dominic Kwiatkowski, Cordelia Langford, John Sillitoe on behalf of the Wellcome Sanger Institute COVID-19 Surveillance Team
EPI_ISL_736563	Lighthouse Lab in Milton Keynes	Wellcome Sanger Institute for the COVID-19 Genomics UK (COG-UK) Consortium	The Lighthouse Lab in Milton Keynes and Alex Alderton, Roberto Amato, Sonia Goncalves, Ewan Harrison, David K. Jackson, Ian Johnston, Dominic Kwiatkowski, Cordelia Langford, John Sillitoe on behalf of the Wellcome Sanger Institute COVID-19 Surveillance Team
EPI_ISL_736564	Lighthouse Lab in Alderley Park	Wellcome Sanger Institute for the COVID-19 Genomics UK (COG-UK) Consortium	Jacquelyn Wynn, Mairead Hyland, The Lighthouse Lab in Alderley Park and Alex Alderton, Roberto Amato, Sonia Goncalves, Ewan Harrison, David K. Jackson, Ian Johnston, Dominic Kwiatkowski, Cordelia Langford, John Sillitoe on behalf of the Wellcome Sanger Institute COVID-19 Surveillance Team
EPI_ISL_736565	Lighthouse Lab in Milton Keynes	Wellcome Sanger Institute for the COVID-19 Genomics UK (COG-UK) Consortium	The Lighthouse Lab in Milton Keynes and Alex Alderton, Roberto Amato, Sonia Goncalves, Ewan Harrison, David K. Jackson, Ian Johnston, Dominic Kwiatkowski, Cordelia Langford, John Sillitoe on behalf of the Wellcome Sanger Institute COVID-19 Surveillance Team
EPI_ISL_736566, EPI_ISL_736568, EPI_ISL_736569, EPI_ISL_736570	Lighthouse Lab in Alderley Park	Wellcome Sanger Institute for the COVID-19 Genomics UK (COG-UK) Consortium	Jacquelyn Wynn, Mairead Hyland, The Lighthouse Lab in Alderley Park and Alex Alderton, Roberto Amato, Sonia Goncalves, Ewan Harrison, David K. Jackson, Ian Johnston, Dominic Kwiatkowski, Cordelia Langford, John Sillitoe on behalf of the Wellcome Sanger Institute COVID-19 Surveillance Team
EPI_ISL_736571	Lighthouse Lab in Milton Keynes	Wellcome Sanger Institute for the COVID-19 Genomics UK (COG-UK) Consortium	The Lighthouse Lab in Milton Keynes and Alex Alderton, Roberto Amato, Sonia Goncalves, Ewan Harrison, David K. Jackson, Ian Johnston, Dominic Kwiatkowski, Cordelia Langford, John Sillitoe on behalf of the Wellcome Sanger Institute COVID-19 Surveillance Team
EPI_ISL_736572, EPI_ISL_736574, EPI_ISL_736575, EPI_ISL_736576, EPI_ISL_736577	Lighthouse Lab in Alderley Park	Wellcome Sanger Institute for the COVID-19 Genomics UK (COG-UK) Consortium	Jacquelyn Wynn, Mairead Hyland, The Lighthouse Lab in Alderley Park and Alex Alderton, Roberto Amato, Sonia Goncalves, Ewan Harrison, David K. Jackson, Ian Johnston, Dominic Kwiatkowski, Cordelia Langford, John Sillitoe on behalf of the Wellcome Sanger Institute COVID-19 Surveillance Team
EPI_ISL_736578	Lighthouse Lab in Milton Keynes	Wellcome Sanger Institute for the COVID-19 Genomics UK (COG-UK) Consortium	The Lighthouse Lab in Milton Keynes and Alex Alderton, Roberto Amato, Sonia Goncalves, Ewan Harrison, David K. Jackson, Ian Johnston, Dominic Kwiatkowski, Cordelia Langford, John Sillitoe on behalf of the Wellcome Sanger Institute COVID-19 Surveillance Team
EPI_ISL_736579, EPI_ISL_736581, EPI_ISL_736583, EPI_ISL_736584, EPI_ISL_736585	Lighthouse Lab in Alderley Park	Wellcome Sanger Institute for the COVID-19 Genomics UK (COG-UK) Consortium	Jacquelyn Wynn, Mairead Hyland, The Lighthouse Lab in Alderley Park and Alex Alderton, Roberto Amato, Sonia Goncalves, Ewan Harrison, David K. Jackson, Ian Johnston, Dominic Kwiatkowski, Cordelia Langford, John Sillitoe on behalf of the Wellcome Sanger Institute COVID-19 Surveillance Team
EPI_ISL_736586	Lighthouse Lab in Milton Keynes	Wellcome Sanger Institute for the COVID-19 Genomics UK (COG-UK) Consortium	The Lighthouse Lab in Milton Keynes and Alex Alderton, Roberto Amato, Sonia Goncalves, Ewan Harrison, David K. Jackson, Ian Johnston, Dominic Kwiatkowski, Cordelia Langford, John Sillitoe on behalf of the Wellcome Sanger Institute COVID-19 Surveillance Team
EPI_ISL_736587	Lighthouse Lab in Alderley Park	Wellcome Sanger Institute for the COVID-19 Genomics UK (COG-UK) Consortium	Jacquelyn Wynn, Mairead Hyland, The Lighthouse Lab in Alderley Park and Alex Alderton, Roberto Amato, Sonia Goncalves, Ewan Harrison, David K. Jackson, Ian Johnston, Dominic Kwiatkowski, Cordelia Langford, John Sillitoe on behalf of the Wellcome Sanger Institute COVID-19 Surveillance Team
EPI_ISL_736588	Lighthouse Lab in Milton Keynes	Wellcome Sanger Institute for the COVID-19 Genomics UK (COG-UK) Consortium	The Lighthouse Lab in Milton Keynes and Alex Alderton, Roberto Amato, Sonia Goncalves, Ewan Harrison, David K. Jackson, Ian Johnston, Dominic Kwiatkowski, Cordelia Langford, John Sillitoe on behalf of the Wellcome Sanger Institute COVID-19 Surveillance Team
EPI_ISL_736589	Lighthouse Lab in Alderley Park	Wellcome Sanger Institute for the COVID-19 Genomics UK (COG-UK) Consortium	Jacquelyn Wynn, Mairead Hyland, The Lighthouse Lab in Alderley Park and Alex Alderton, Roberto Amato, Sonia Goncalves, Ewan Harrison, David K. Jackson, Ian Johnston, Dominic Kwiatkowski, Cordelia Langford, John Sillitoe on behalf of the Wellcome Sanger Institute COVID-19 Surveillance Team
EPI_ISL_736590	Lighthouse Lab in Milton Keynes	Wellcome Sanger Institute for the COVID-19 Genomics UK (COG-UK) Consortium	The Lighthouse Lab in Milton Keynes and Alex Alderton, Roberto Amato, Sonia Goncalves, Ewan Harrison, David K. Jackson, Ian Johnston, Dominic Kwiatkowski, Cordelia Langford, John Sillitoe on behalf of the Wellcome Sanger Institute COVID-19 Surveillance Team
EPI_ISL_736591, EPI_ISL_736592	Lighthouse Lab in Alderley Park	Wellcome Sanger Institute for the COVID-19 Genomics UK (COG-UK) Consortium	Jacquelyn Wynn, Mairead Hyland, The Lighthouse Lab in Alderley Park and Alex Alderton, Roberto Amato, Sonia Goncalves, Ewan Harrison, David K. Jackson, Ian Johnston, Dominic Kwiatkowski, Cordelia Langford, John Sillitoe on behalf of the Wellcome Sanger Institute COVID-19 Surveillance Team
EPI_ISL_736593, EPI_ISL_736594	Lighthouse Lab in Milton Keynes	Wellcome Sanger Institute for the COVID-19 Genomics UK (COG-UK) Consortium	The Lighthouse Lab in Milton Keynes and Alex Alderton, Roberto Amato, Sonia Goncalves, Ewan Harrison, David K. Jackson, Ian Johnston, Dominic Kwiatkowski, Cordelia Langford, John Sillitoe on behalf of the Wellcome Sanger Institute COVID-19 Surveillance Team
EPI_ISL_736595, EPI_ISL_736596	Lighthouse Lab in Alderley Park	Wellcome Sanger Institute for the COVID-19 Genomics UK (COG-UK) Consortium	Jacquelyn Wynn, Mairead Hyland, The Lighthouse Lab in Alderley Park and Alex Alderton, Roberto Amato, Sonia Goncalves, Ewan Harrison, David K. Jackson, Ian Johnston, Dominic Kwiatkowski, Cordelia Langford, John Sillitoe on behalf of the Wellcome Sanger Institute COVID-19 Surveillance Team
EPI_ISL_736597, EPI_ISL_736598	Lighthouse Lab in Milton Keynes	Wellcome Sanger Institute for the COVID-19 Genomics UK (COG-UK) Consortium	The Lighthouse Lab in Milton Keynes and Alex Alderton, Roberto Amato, Sonia Goncalves, Ewan Harrison, David K. Jackson, Ian Johnston, Dominic Kwiatkowski, Cordelia Langford, John Sillitoe on behalf of the Wellcome Sanger Institute COVID-19 Surveillance Team
EPI_ISL_736599, EPI_ISL_736600	Lighthouse Lab in Alderley Park	Wellcome Sanger Institute for the COVID-19 Genomics UK (COG-UK) Consortium	Jacquelyn Wynn, Mairead Hyland, The Lighthouse Lab in Alderley Park and Alex Alderton, Roberto Amato, Sonia Goncalves, Ewan Harrison, David K. Jackson, Ian Johnston, Dominic Kwiatkowski, Cordelia Langford, John Sillitoe on behalf of the Wellcome Sanger Institute COVID-19 Surveillance Team
EPI_ISL_736601, EPI_ISL_736602	Lighthouse Lab in Milton Keynes	Wellcome Sanger Institute for the COVID-19 Genomics UK (COG-UK) Consortium	The Lighthouse Lab in Milton Keynes and Alex Alderton, Roberto Amato, Sonia Goncalves, Ewan Harrison, David K. Jackson, Ian Johnston, Dominic Kwiatkowski, Cordelia Langford, John Sillitoe on behalf of the Wellcome Sanger Institute COVID-19 Surveillance Team
EPI_ISL_736603, EPI_ISL_736604, EPI_ISL_736606, EPI_ISL_736607	Lighthouse Lab in Alderley Park	Wellcome Sanger Institute for the COVID-19 Genomics UK (COG-UK) Consortium	Jacquelyn Wynn, Mairead Hyland, The Lighthouse Lab in Alderley Park and Alex Alderton, Roberto Amato, Sonia Goncalves, Ewan Harrison, David K. Jackson, Ian Johnston, Dominic Kwiatkowski, Cordelia Langford, John Sillitoe on behalf of the Wellcome Sanger Institute COVID-19 Surveillance Team
EPI_ISL_736608, EPI_ISL_736609, EPI_ISL_736610	Lighthouse Lab in Milton Keynes	Wellcome Sanger Institute for the COVID-19 Genomics UK (COG-UK) Consortium	The Lighthouse Lab in Milton Keynes and Alex Alderton, Roberto Amato, Sonia Goncalves, Ewan Harrison, David K. Jackson, Ian Johnston, Dominic Kwiatkowski, Cordelia Langford, John Sillitoe on behalf of the Wellcome Sanger Institute COVID-19 Surveillance Team
EPI_ISL_736611, EPI_ISL_736612, EPI_ISL_736613, EPI_ISL_736614, EPI_ISL_736615, EPI_ISL_736616	Lighthouse Lab in Alderley Park	Wellcome Sanger Institute for the COVID-19 Genomics UK (COG-UK) Consortium	Jacquelyn Wynn, Mairead Hyland, The Lighthouse Lab in Alderley Park and Alex Alderton, Roberto Amato, Sonia Goncalves, Ewan Harrison, David K. Jackson, Ian Johnston, Dominic Kwiatkowski, Cordelia Langford, John Sillitoe on behalf of the Wellcome Sanger Institute COVID-19 Surveillance Team
EPI_ISL_736617, EPI_ISL_736618	Lighthouse Lab in Milton Keynes	Wellcome Sanger Institute for the COVID-19 Genomics UK (COG-UK) Consortium	The Lighthouse Lab in Milton Keynes and Alex Alderton, Roberto Amato, Sonia Goncalves, Ewan Harrison, David K. Jackson, Ian Johnston, Dominic Kwiatkowski, Cordelia Langford, John Sillitoe on behalf of the Wellcome Sanger Institute COVID-19 Surveillance Team
EPI_ISL_736619, EPI_ISL_736621	Lighthouse Lab in Alderley Park	Wellcome Sanger Institute for the COVID-19 Genomics UK (COG-UK) Consortium	Jacquelyn Wynn, Mairead Hyland, The Lighthouse Lab in Alderley Park and Alex Alderton, Roberto Amato, Sonia Goncalves, Ewan Harrison, David K. Jackson, Ian Johnston, Dominic Kwiatkowski, Cordelia Langford, John Sillitoe on behalf of the Wellcome Sanger Institute COVID-19 Surveillance Team
EPI_ISL_736622	Lighthouse Lab in Milton Keynes	Wellcome Sanger Institute for the COVID-19 Genomics UK (COG-UK) Consortium	The Lighthouse Lab in Milton Keynes and Alex Alderton, Roberto Amato, Sonia Goncalves, Ewan Harrison, David K. Jackson, Ian Johnston, Dominic Kwiatkowski, Cordelia Langford, John Sillitoe on behalf of the Wellcome Sanger Institute COVID-19 Surveillance Team
EPI_ISL_736623, EPI_ISL_736625, EPI_ISL_736626, EPI_ISL_736629	Lighthouse Lab in Alderley Park	Wellcome Sanger Institute for the COVID-19 Genomics UK (COG-UK) Consortium	Jacquelyn Wynn, Mairead Hyland, The Lighthouse Lab in Alderley Park and Alex Alderton, Roberto Amato, Sonia Goncalves, Ewan Harrison, David K. Jackson, Ian Johnston, Dominic Kwiatkowski, Cordelia Langford, John Sillitoe on behalf of the Wellcome Sanger Institute COVID-19 Surveillance Team
EPI_ISL_736630	Lighthouse Lab in Milton Keynes	Wellcome Sanger Institute for the COVID-19 Genomics UK (COG-UK) Consortium	The Lighthouse Lab in Milton Keynes and Alex Alderton, Roberto Amato, Sonia Goncalves, Ewan Harrison, David K. Jackson, Ian Johnston, Dominic Kwiatkowski, Cordelia Langford, John Sillitoe on behalf of the Wellcome Sanger Institute COVID-19 Surveillance Team
EPI_ISL_736632, EPI_ISL_736634, EPI_ISL_736635, EPI_ISL_736636	Lighthouse Lab in Alderley Park	Wellcome Sanger Institute for the COVID-19 Genomics UK (COG-UK) Consortium	Jacquelyn Wynn, Mairead Hyland, The Lighthouse Lab in Alderley Park and Alex Alderton, Roberto Amato, Sonia Goncalves, Ewan Harrison, David K. Jackson, Ian Johnston, Dominic Kwiatkowski, Cordelia Langford, John Sillitoe on behalf of the Wellcome Sanger Institute COVID-19 Surveillance Team
EPI_ISL_736637	Lighthouse Lab in Milton Keynes	Wellcome Sanger Institute for the COVID-19 Genomics UK (COG-UK) Consortium	The Lighthouse Lab in Milton Keynes and Alex Alderton, Roberto Amato, Sonia Goncalves, Ewan Harrison, David K. Jackson, Ian Johnston, Dominic Kwiatkowski, Cordelia Langford, John Sillitoe on behalf of the Wellcome Sanger Institute COVID-19 Surveillance Team
EPI_ISL_736638, EPI_ISL_736639, EPI_ISL_736640, EPI_ISL_736641, EPI_ISL_736642	Lighthouse Lab in Alderley Park	Wellcome Sanger Institute for the COVID-19 Genomics UK (COG-UK) Consortium	Jacquelyn Wynn, Mairead Hyland, The Lighthouse Lab in Alderley Park and Alex Alderton, Roberto Amato, Sonia Goncalves, Ewan Harrison, David K. Jackson, Ian Johnston, Dominic Kwiatkowski, Cordelia Langford, John Sillitoe on behalf of the Wellcome Sanger Institute COVID-19 Surveillance Team
EPI_ISL_736643	Lighthouse Lab in Milton Keynes	Wellcome Sanger Institute for the COVID-19 Genomics UK (COG-UK) Consortium	The Lighthouse Lab in Milton Keynes and Alex Alderton, Roberto Amato, Sonia Goncalves, Ewan Harrison, David K. Jackson, Ian Johnston, Dominic Kwiatkowski, Cordelia Langford, John Sillitoe on behalf of the Wellcome Sanger Institute COVID-19 Surveillance Team
EPI_ISL_736644, EPI_ISL_736645, EPI_ISL_736647	Lighthouse Lab in Alderley Park	Wellcome Sanger Institute for the COVID-19 Genomics UK (COG-UK) Consortium	Jacquelyn Wynn, Mairead Hyland, The Lighthouse Lab in Alderley Park and Alex Alderton, Roberto Amato, Sonia Goncalves, Ewan Harrison, David K. Jackson, Ian Johnston, Dominic Kwiatkowski, Cordelia Langford, John Sillitoe on behalf of the Wellcome Sanger Institute COVID-19 Surveillance Team
EPI_ISL_736648	Lighthouse Lab in Milton Keynes	Wellcome Sanger Institute for the COVID-19 Genomics UK	The Lighthouse Lab in Milton Keynes and Alex Alderton, Roberto Amato, Sonia Goncalves, Ewan Harrison, David K. Jackson, Ian Johnston, Dominic

		(COG-UK) Consortium	Kwiatkowski, Cordelia Langford, John Sillitoe on behalf of the Wellcome Sanger Institute COVID-19 Surveillance Team
EPI_ISL_736649, EPI_ISL_736651, EPI_ISL_736652, EPI_ISL_736653, EPI_ISL_736654, EPI_ISL_736655, EPI_ISL_736656, EPI_ISL_736658, EPI_ISL_736660, EPI_ISL_736661, EPI_ISL_736662, EPI_ISL_736663, EPI_ISL_736664, EPI_ISL_736665, EPI_ISL_736666, EPI_ISL_736667, EPI_ISL_736669, EPI_ISL_736670, EPI_ISL_736671, EPI_ISL_736673, EPI_ISL_736674			
see above	Lighthouse Lab in Alderley Park	Wellcome Sanger Institute for the COVID-19 Genomics UK (COG-UK) Consortium	Jacquelyn Wynn, Mairead Hyland, The Lighthouse Lab in Alderley Park and Alex Alderton, Roberto Amato, Sonia Goncalves, Ewan Harrison, David K. Jackson, Ian Johnston, Dominic Kwiatkowski, Cordelia Langford, John Sillitoe on behalf of the Wellcome Sanger Institute COVID-19 Surveillance Team
EPI_ISL_736675	Lighthouse Lab in Milton Keynes	Wellcome Sanger Institute for the COVID-19 Genomics UK (COG-UK) Consortium	The Lighthouse Lab in Milton Keynes and Alex Alderton, Roberto Amato, Sonia Goncalves, Ewan Harrison, David K. Jackson, Ian Johnston, Dominic Kwiatkowski, Cordelia Langford, John Sillitoe on behalf of the Wellcome Sanger Institute COVID-19 Surveillance Team
EPI_ISL_736676	Lighthouse Lab in Alderley Park	Wellcome Sanger Institute for the COVID-19 Genomics UK (COG-UK) Consortium	Jacquelyn Wynn, Mairead Hyland, The Lighthouse Lab in Alderley Park and Alex Alderton, Roberto Amato, Sonia Goncalves, Ewan Harrison, David K. Jackson, Ian Johnston, Dominic Kwiatkowski, Cordelia Langford, John Sillitoe on behalf of the Wellcome Sanger Institute COVID-19 Surveillance Team
EPI_ISL_736677	Lighthouse Lab in Milton Keynes	Wellcome Sanger Institute for the COVID-19 Genomics UK (COG-UK) Consortium	The Lighthouse Lab in Milton Keynes and Alex Alderton, Roberto Amato, Sonia Goncalves, Ewan Harrison, David K. Jackson, Ian Johnston, Dominic Kwiatkowski, Cordelia Langford, John Sillitoe on behalf of the Wellcome Sanger Institute COVID-19 Surveillance Team
EPI_ISL_736679, EPI_ISL_736680	Lighthouse Lab in Alderley Park	Wellcome Sanger Institute for the COVID-19 Genomics UK (COG-UK) Consortium	Jacquelyn Wynn, Mairead Hyland, The Lighthouse Lab in Alderley Park and Alex Alderton, Roberto Amato, Sonia Goncalves, Ewan Harrison, David K. Jackson, Ian Johnston, Dominic Kwiatkowski, Cordelia Langford, John Sillitoe on behalf of the Wellcome Sanger Institute COVID-19 Surveillance Team
EPI_ISL_736681, EPI_ISL_736682, EPI_ISL_736683	Lighthouse Lab in Milton Keynes	Wellcome Sanger Institute for the COVID-19 Genomics UK (COG-UK) Consortium	The Lighthouse Lab in Milton Keynes and Alex Alderton, Roberto Amato, Sonia Goncalves, Ewan Harrison, David K. Jackson, Ian Johnston, Dominic Kwiatkowski, Cordelia Langford, John Sillitoe on behalf of the Wellcome Sanger Institute COVID-19 Surveillance Team
EPI_ISL_736684	Lighthouse Lab in Alderley Park	Wellcome Sanger Institute for the COVID-19 Genomics UK (COG-UK) Consortium	Jacquelyn Wynn, Mairead Hyland, The Lighthouse Lab in Alderley Park and Alex Alderton, Roberto Amato, Sonia Goncalves, Ewan Harrison, David K. Jackson, Ian Johnston, Dominic Kwiatkowski, Cordelia Langford, John Sillitoe on behalf of the Wellcome Sanger Institute COVID-19 Surveillance Team
EPI_ISL_736685	Lighthouse Lab in Glasgow	Wellcome Sanger Institute for the COVID-19 Genomics UK (COG-UK) Consortium	Harper VanSteenhouse, Yumi Kasai, David Gray, Carol Clugston, Anna Dominiczak and Alex Alderton, Roberto Amato, Sonia Goncalves, Ewan Harrison, David K. Jackson, Ian Johnston, Dominic Kwiatkowski, Cordelia Langford, John Sillitoe on behalf of the Wellcome Sanger Institute COVID-19 Surveillance Team
EPI_ISL_736687, EPI_ISL_736688, EPI_ISL_736690, EPI_ISL_736692	Lighthouse Lab in Alderley Park	Wellcome Sanger Institute for the COVID-19 Genomics UK (COG-UK) Consortium	Jacquelyn Wynn, Mairead Hyland, The Lighthouse Lab in Alderley Park and Alex Alderton, Roberto Amato, Sonia Goncalves, Ewan Harrison, David K. Jackson, Ian Johnston, Dominic Kwiatkowski, Cordelia Langford, John Sillitoe on behalf of the Wellcome Sanger Institute COVID-19 Surveillance Team
EPI_ISL_736693, EPI_ISL_736696, EPI_ISL_736697	Lighthouse Lab in Milton Keynes	Wellcome Sanger Institute for the COVID-19 Genomics UK (COG-UK) Consortium	The Lighthouse Lab in Milton Keynes and Alex Alderton, Roberto Amato, Sonia Goncalves, Ewan Harrison, David K. Jackson, Ian Johnston, Dominic Kwiatkowski, Cordelia Langford, John Sillitoe on behalf of the Wellcome Sanger Institute COVID-19 Surveillance Team
EPI_ISL_736698	Lighthouse Lab in Alderley Park	Wellcome Sanger Institute for the COVID-19 Genomics UK (COG-UK) Consortium	Jacquelyn Wynn, Mairead Hyland, The Lighthouse Lab in Alderley Park and Alex Alderton, Roberto Amato, Sonia Goncalves, Ewan Harrison, David K. Jackson, Ian Johnston, Dominic Kwiatkowski, Cordelia Langford, John Sillitoe on behalf of the Wellcome Sanger Institute COVID-19 Surveillance Team
EPI_ISL_736699	Lighthouse Lab in Milton Keynes	Wellcome Sanger Institute for the COVID-19 Genomics UK (COG-UK) Consortium	The Lighthouse Lab in Milton Keynes and Alex Alderton, Roberto Amato, Sonia Goncalves, Ewan Harrison, David K. Jackson, Ian Johnston, Dominic Kwiatkowski, Cordelia Langford, John Sillitoe on behalf of the Wellcome Sanger Institute COVID-19 Surveillance Team
EPI_ISL_736701, EPI_ISL_736702	Lighthouse Lab in Alderley Park	Wellcome Sanger Institute for the COVID-19 Genomics UK (COG-UK) Consortium	Jacquelyn Wynn, Mairead Hyland, The Lighthouse Lab in Alderley Park and Alex Alderton, Roberto Amato, Sonia Goncalves, Ewan Harrison, David K. Jackson, Ian Johnston, Dominic Kwiatkowski, Cordelia Langford, John Sillitoe on behalf of the Wellcome Sanger Institute COVID-19 Surveillance Team
EPI_ISL_736703	Lighthouse Lab in Milton Keynes	Wellcome Sanger Institute for the COVID-19 Genomics UK (COG-UK) Consortium	The Lighthouse Lab in Milton Keynes and Alex Alderton, Roberto Amato, Sonia Goncalves, Ewan Harrison, David K. Jackson, Ian Johnston, Dominic Kwiatkowski, Cordelia Langford, John Sillitoe on behalf of the Wellcome Sanger Institute COVID-19 Surveillance Team
EPI_ISL_736704, EPI_ISL_736705, EPI_ISL_736706, EPI_ISL_736707, EPI_ISL_736708, EPI_ISL_736709, EPI_ISL_736710, EPI_ISL_736711	Lighthouse Lab in Alderley Park	Wellcome Sanger Institute for the COVID-19 Genomics UK (COG-UK) Consortium	Jacquelyn Wynn, Mairead Hyland, The Lighthouse Lab in Alderley Park and Alex Alderton, Roberto Amato, Sonia Goncalves, Ewan Harrison, David K. Jackson, Ian Johnston, Dominic Kwiatkowski, Cordelia Langford, John Sillitoe on behalf of the Wellcome Sanger Institute COVID-19 Surveillance Team
EPI_ISL_736712, EPI_ISL_736713	Lighthouse Lab in Milton Keynes	Wellcome Sanger Institute for the COVID-19 Genomics UK (COG-UK) Consortium	The Lighthouse Lab in Milton Keynes and Alex Alderton, Roberto Amato, Sonia Goncalves, Ewan Harrison, David K. Jackson, Ian Johnston, Dominic Kwiatkowski, Cordelia Langford, John Sillitoe on behalf of the Wellcome Sanger Institute COVID-19 Surveillance Team
EPI_ISL_736714, EPI_ISL_736715	Lighthouse Lab in Alderley Park	Wellcome Sanger Institute for the COVID-19 Genomics UK (COG-UK) Consortium	Jacquelyn Wynn, Mairead Hyland, The Lighthouse Lab in Alderley Park and Alex Alderton, Roberto Amato, Sonia Goncalves, Ewan Harrison, David K. Jackson, Ian Johnston, Dominic Kwiatkowski, Cordelia Langford, John Sillitoe on behalf of the Wellcome Sanger Institute COVID-19 Surveillance Team
EPI_ISL_736716	Lighthouse Lab in Milton Keynes	Wellcome Sanger Institute for the COVID-19 Genomics UK (COG-UK) Consortium	The Lighthouse Lab in Milton Keynes and Alex Alderton, Roberto Amato, Sonia Goncalves, Ewan Harrison, David K. Jackson, Ian Johnston, Dominic Kwiatkowski, Cordelia Langford, John Sillitoe on behalf of the Wellcome Sanger Institute COVID-19 Surveillance Team
EPI_ISL_736717, EPI_ISL_736718	Lighthouse Lab in Alderley Park	Wellcome Sanger Institute for the COVID-19 Genomics UK (COG-UK) Consortium	Jacquelyn Wynn, Mairead Hyland, The Lighthouse Lab in Alderley Park and Alex Alderton, Roberto Amato, Sonia Goncalves, Ewan Harrison, David K. Jackson, Ian Johnston, Dominic Kwiatkowski, Cordelia Langford, John Sillitoe on behalf of the Wellcome Sanger Institute COVID-19 Surveillance Team
EPI_ISL_736719	Lighthouse Lab in Milton Keynes	Wellcome Sanger Institute for the COVID-19 Genomics UK (COG-UK) Consortium	The Lighthouse Lab in Milton Keynes and Alex Alderton, Roberto Amato, Sonia Goncalves, Ewan Harrison, David K. Jackson, Ian Johnston, Dominic Kwiatkowski, Cordelia Langford, John Sillitoe on behalf of the Wellcome Sanger Institute COVID-19 Surveillance Team
EPI_ISL_736720	Lighthouse Lab in Alderley Park	Wellcome Sanger Institute for the COVID-19 Genomics UK (COG-UK) Consortium	Jacquelyn Wynn, Mairead Hyland, The Lighthouse Lab in Alderley Park and Alex Alderton, Roberto Amato, Sonia Goncalves, Ewan Harrison, David K. Jackson, Ian Johnston, Dominic Kwiatkowski, Cordelia Langford, John Sillitoe on behalf of the Wellcome Sanger Institute COVID-19 Surveillance Team
EPI_ISL_736721	Lighthouse Lab in Glasgow	Wellcome Sanger Institute for the COVID-19 Genomics UK (COG-UK) Consortium	Harper VanSteenhouse, Yumi Kasai, David Gray, Carol Clugston, Anna Dominiczak and Alex Alderton, Roberto Amato, Sonia Goncalves, Ewan Harrison, David K. Jackson, Ian Johnston, Dominic Kwiatkowski, Cordelia Langford, John Sillitoe on behalf of the Wellcome Sanger Institute COVID-19 Surveillance Team
EPI_ISL_736722	Lighthouse Lab in Alderley Park	Wellcome Sanger Institute for the COVID-19 Genomics UK (COG-UK) Consortium	Jacquelyn Wynn, Mairead Hyland, The Lighthouse Lab in Alderley Park and Alex Alderton, Roberto Amato, Sonia Goncalves, Ewan Harrison, David K. Jackson, Ian Johnston, Dominic Kwiatkowski, Cordelia Langford, John Sillitoe on behalf of the Wellcome Sanger Institute COVID-19 Surveillance Team
EPI_ISL_736723, EPI_ISL_736724, EPI_ISL_736725	Lighthouse Lab in Milton Keynes	Wellcome Sanger Institute for the COVID-19 Genomics UK (COG-UK) Consortium	The Lighthouse Lab in Milton Keynes and Alex Alderton, Roberto Amato, Sonia Goncalves, Ewan Harrison, David K. Jackson, Ian Johnston, Dominic Kwiatkowski, Cordelia Langford, John Sillitoe on behalf of the Wellcome Sanger Institute COVID-19 Surveillance Team
EPI_ISL_736726	Lighthouse Lab in Alderley Park	Wellcome Sanger Institute for the COVID-19 Genomics UK (COG-UK) Consortium	Jacquelyn Wynn, Mairead Hyland, The Lighthouse Lab in Alderley Park and Alex Alderton, Roberto Amato, Sonia Goncalves, Ewan Harrison, David K. Jackson, Ian Johnston, Dominic Kwiatkowski, Cordelia Langford, John Sillitoe on behalf of the Wellcome Sanger Institute COVID-19 Surveillance Team
EPI_ISL_736727, EPI_ISL_736728	Lighthouse Lab in Milton Keynes	Wellcome Sanger Institute for the COVID-19 Genomics UK (COG-UK) Consortium	The Lighthouse Lab in Milton Keynes and Alex Alderton, Roberto Amato, Sonia Goncalves, Ewan Harrison, David K. Jackson, Ian Johnston, Dominic Kwiatkowski, Cordelia Langford, John Sillitoe on behalf of the Wellcome Sanger Institute COVID-19 Surveillance Team
EPI_ISL_736729, EPI_ISL_736730	Lighthouse Lab in Alderley Park	Wellcome Sanger Institute for the COVID-19 Genomics UK (COG-UK) Consortium	Jacquelyn Wynn, Mairead Hyland, The Lighthouse Lab in Alderley Park and Alex Alderton, Roberto Amato, Sonia Goncalves, Ewan Harrison, David K. Jackson, Ian Johnston, Dominic Kwiatkowski, Cordelia Langford, John Sillitoe on behalf of the Wellcome Sanger Institute COVID-19 Surveillance Team
EPI_ISL_736731	Lighthouse Lab in Milton Keynes	Wellcome Sanger Institute for the COVID-19 Genomics UK (COG-UK) Consortium	The Lighthouse Lab in Milton Keynes and Alex Alderton, Roberto Amato, Sonia Goncalves, Ewan Harrison, David K. Jackson, Ian Johnston, Dominic Kwiatkowski, Cordelia Langford, John Sillitoe on behalf of the Wellcome Sanger Institute COVID-19 Surveillance Team
EPI_ISL_736732	Lighthouse Lab in Alderley Park	Wellcome Sanger Institute for the COVID-19 Genomics UK (COG-UK) Consortium	Jacquelyn Wynn, Mairead Hyland, The Lighthouse Lab in Alderley Park and Alex Alderton, Roberto Amato, Sonia Goncalves, Ewan Harrison, David K. Jackson, Ian Johnston, Dominic Kwiatkowski, Cordelia Langford, John Sillitoe on behalf of the Wellcome Sanger Institute COVID-19 Surveillance Team
EPI_ISL_736733	Lighthouse Lab in Milton Keynes	Wellcome Sanger Institute for the COVID-19 Genomics UK (COG-UK) Consortium	The Lighthouse Lab in Milton Keynes and Alex Alderton, Roberto Amato, Sonia Goncalves, Ewan Harrison, David K. Jackson, Ian Johnston, Dominic Kwiatkowski, Cordelia Langford, John Sillitoe on behalf of the Wellcome Sanger Institute COVID-19 Surveillance Team
EPI_ISL_736735	Lighthouse Lab in Alderley Park	Wellcome Sanger Institute for the COVID-19 Genomics UK (COG-UK) Consortium	Jacquelyn Wynn, Mairead Hyland, The Lighthouse Lab in Alderley Park and Alex Alderton, Roberto Amato, Sonia Goncalves, Ewan Harrison, David K. Jackson, Ian Johnston, Dominic Kwiatkowski, Cordelia Langford, John Sillitoe on behalf of the Wellcome Sanger Institute COVID-19 Surveillance Team
EPI_ISL_736738	Lighthouse Lab in Milton Keynes	Wellcome Sanger Institute for the COVID-19 Genomics UK (COG-UK) Consortium	The Lighthouse Lab in Milton Keynes and Alex Alderton, Roberto Amato, Sonia Goncalves, Ewan Harrison, David K. Jackson, Ian Johnston, Dominic Kwiatkowski, Cordelia Langford, John Sillitoe on behalf of the Wellcome Sanger Institute COVID-19 Surveillance Team

EPI_ISL_736739	Lighthouse Lab in Alderley Park	Wellcome Sanger Institute for the COVID-19 Genomics UK (COG-UK) Consortium	Jacquelyn Wynn, Mairead Hyland, The Lighthouse Lab in Alderley Park and Alex Alderton, Roberto Amato, Sonia Goncalves, Ewan Harrison, David K. Jackson, Ian Johnston, Dominic Kwiatkowski, Cordelia Langford, John Sillitoe on behalf of the Wellcome Sanger Institute COVID-19 Surveillance Team
EPI_ISL_736740	Lighthouse Lab in Milton Keynes	Wellcome Sanger Institute for the COVID-19 Genomics UK (COG-UK) Consortium	The Lighthouse Lab in Milton Keynes and Alex Alderton, Roberto Amato, Sonia Goncalves, Ewan Harrison, David K. Jackson, Ian Johnston, Dominic Kwiatkowski, Cordelia Langford, John Sillitoe on behalf of the Wellcome Sanger Institute COVID-19 Surveillance Team
EPI_ISL_736741, EPI_ISL_736742	Lighthouse Lab in Alderley Park	Wellcome Sanger Institute for the COVID-19 Genomics UK (COG-UK) Consortium	Jacquelyn Wynn, Mairead Hyland, The Lighthouse Lab in Alderley Park and Alex Alderton, Roberto Amato, Sonia Goncalves, Ewan Harrison, David K. Jackson, Ian Johnston, Dominic Kwiatkowski, Cordelia Langford, John Sillitoe on behalf of the Wellcome Sanger Institute COVID-19 Surveillance Team
EPI_ISL_736744, EPI_ISL_736746	Lighthouse Lab in Milton Keynes	Wellcome Sanger Institute for the COVID-19 Genomics UK (COG-UK) Consortium	The Lighthouse Lab in Milton Keynes and Alex Alderton, Roberto Amato, Sonia Goncalves, Ewan Harrison, David K. Jackson, Ian Johnston, Dominic Kwiatkowski, Cordelia Langford, John Sillitoe on behalf of the Wellcome Sanger Institute COVID-19 Surveillance Team
EPI_ISL_736747, EPI_ISL_736748, EPI_ISL_736750, EPI_ISL_736751, EPI_ISL_736753, EPI_ISL_736755, EPI_ISL_736756	Lighthouse Lab in Alderley Park	Wellcome Sanger Institute for the COVID-19 Genomics UK (COG-UK) Consortium	Jacquelyn Wynn, Mairead Hyland, The Lighthouse Lab in Alderley Park and Alex Alderton, Roberto Amato, Sonia Goncalves, Ewan Harrison, David K. Jackson, Ian Johnston, Dominic Kwiatkowski, Cordelia Langford, John Sillitoe on behalf of the Wellcome Sanger Institute COVID-19 Surveillance Team
EPI_ISL_736757	Lighthouse Lab in Milton Keynes	Wellcome Sanger Institute for the COVID-19 Genomics UK (COG-UK) Consortium	The Lighthouse Lab in Milton Keynes and Alex Alderton, Roberto Amato, Sonia Goncalves, Ewan Harrison, David K. Jackson, Ian Johnston, Dominic Kwiatkowski, Cordelia Langford, John Sillitoe on behalf of the Wellcome Sanger Institute COVID-19 Surveillance Team
EPI_ISL_736759, EPI_ISL_736760, EPI_ISL_736761, EPI_ISL_736762, EPI_ISL_736763, EPI_ISL_736764, EPI_ISL_736765, EPI_ISL_736766, EPI_ISL_736767, EPI_ISL_736768, EPI_ISL_736769, EPI_ISL_736770, EPI_ISL_736771			
see above	Lighthouse Lab in Alderley Park	Wellcome Sanger Institute for the COVID-19 Genomics UK (COG-UK) Consortium	Jacquelyn Wynn, Mairead Hyland, The Lighthouse Lab in Alderley Park and Alex Alderton, Roberto Amato, Sonia Goncalves, Ewan Harrison, David K. Jackson, Ian Johnston, Dominic Kwiatkowski, Cordelia Langford, John Sillitoe on behalf of the Wellcome Sanger Institute COVID-19 Surveillance Team
EPI_ISL_736774	Lighthouse Lab in Glasgow	Wellcome Sanger Institute for the COVID-19 Genomics UK (COG-UK) Consortium	Harper VanSteenhouse, Yumi Kasai, David Gray, Carol Clugston, Anna Dominiczak and Alex Alderton, Roberto Amato, Sonia Goncalves, Ewan Harrison, David K. Jackson, Ian Johnston, Dominic Kwiatkowski, Cordelia Langford, John Sillitoe on behalf of the Wellcome Sanger Institute COVID-19 Surveillance Team
EPI_ISL_736972	NHLS-IALCH	KRISP, KZN Research Innovation and Sequencing Platform	Giandhari J, Pillay S, Lessells R, ChimukangaraB, Mdlalose K, York D, Khan S, Tegally H, Wilkinson E, de Oliveira T
EPI_ISL_737400, EPI_ISL_737404, EPI_ISL_737408, EPI_ISL_737410, EPI_ISL_737412, EPI_ISL_737420, EPI_ISL_737426, EPI_ISL_737427, EPI_ISL_737438, EPI_ISL_737444, EPI_ISL_737452, EPI_ISL_737453, EPI_ISL_737465, EPI_ISL_737474, EPI_ISL_737476, EPI_ISL_737487, EPI_ISL_737489, EPI_ISL_737490, EPI_ISL_737493, EPI_ISL_737494, EPI_ISL_737499, EPI_ISL_737516, EPI_ISL_737528, EPI_ISL_737529, EPI_ISL_737540, EPI_ISL_737551, EPI_ISL_737552, EPI_ISL_737555, EPI_ISL_737561, EPI_ISL_737562, EPI_ISL_737572, EPI_ISL_737577, EPI_ISL_737588, EPI_ISL_737592, EPI_ISL_737597, EPI_ISL_737602, EPI_ISL_737621, EPI_ISL_737622, EPI_ISL_737640, EPI_ISL_737641, EPI_ISL_737645, EPI_ISL_737646, EPI_ISL_737656, EPI_ISL_737657, EPI_ISL_737666, EPI_ISL_737667, EPI_ISL_737668, EPI_ISL_737669, EPI_ISL_737710, EPI_ISL_737711, EPI_ISL_737712, EPI_ISL_737713, EPI_ISL_737714, EPI_ISL_737715, EPI_ISL_737716, EPI_ISL_737717, EPI_ISL_737718, EPI_ISL_737719, EPI_ISL_737720, EPI_ISL_737721, EPI_ISL_737722, EPI_ISL_737723, EPI_ISL_737724, EPI_ISL_737739, EPI_ISL_737759, EPI_ISL_737760, EPI_ISL_737765, EPI_ISL_737766, EPI_ISL_737780, EPI_ISL_737781, EPI_ISL_737783, EPI_ISL_737800, EPI_ISL_737801, EPI_ISL_737802, EPI_ISL_737803, EPI_ISL_737804, EPI_ISL_737805, EPI_ISL_737806, EPI_ISL_737807, EPI_ISL_737808, EPI_ISL_737809, EPI_ISL_737810, EPI_ISL_737863, EPI_ISL_737864, EPI_ISL_737865, EPI_ISL_737866, EPI_ISL_737868, EPI_ISL_737902, EPI_ISL_737917			
see above	Viollier AG	Department of Biosystems Science and Engineering, ETH Zürich	Chaoran Chen, Sarah Nadeau, Catharine Aquino, Ivan Topolsky, Philipp Jablonski, Lara Fuhrmann, David Dreifuss, Katharina Jahn, Andreia Cabral de Gouvea, Maria Domenica Moccia, Simon Grüter, Timothy Sykes, Lennart Opitz, Griffin White, Laura Neff, Doris Popovic, Andrea Patrignani, Jay Tracy, Ralph Schlapbach, Christiane Beckmann, Maurice Redondo, Olivier Kobel, Christoph Noppen, Sophie Seidel, Noemie Santamaria de Souza, Niko Beerenwinkel, Tanja Stadler
EPI_ISL_738314	Unilabs Laboratory Medicine	Norwegian Institute of Public Health, Department of Virology	Kathrine Stene-Johansen, Kamilla Heddeland Instefjord, Hilde Elshaug, Atiya R Ali,Marie Paulsen Madsen, Rasmus Riis Kopperud, Hilde Vollan, Karoline Bragstad, Olav Hungnes
EPI_ISL_738362, EPI_ISL_738364, EPI_ISL_738366, EPI_ISL_738367, EPI_ISL_738368, EPI_ISL_738370, EPI_ISL_738372, EPI_ISL_738373, EPI_ISL_738375, EPI_ISL_738377, EPI_ISL_738378, EPI_ISL_738379, EPI_ISL_738383, EPI_ISL_738384, EPI_ISL_738385, EPI_ISL_738390, EPI_ISL_738391, EPI_ISL_738393, EPI_ISL_738395, EPI_ISL_738396, EPI_ISL_738397, EPI_ISL_738399, EPI_ISL_738401, EPI_ISL_738402, EPI_ISL_738403, EPI_ISL_738404, EPI_ISL_738405, EPI_ISL_738407, EPI_ISL_738408, EPI_ISL_738410, EPI_ISL_738411, EPI_ISL_738416, EPI_ISL_738418, EPI_ISL_738421, EPI_ISL_738422, EPI_ISL_738423, EPI_ISL_738426, EPI_ISL_738428, EPI_ISL_738429, EPI_ISL_738433, EPI_ISL_738434, EPI_ISL_738438, EPI_ISL_738443, EPI_ISL_738444, EPI_ISL_738445, EPI_ISL_738446, EPI_ISL_738449, EPI_ISL_738451, EPI_ISL_738456, EPI_ISL_738457, EPI_ISL_738462, EPI_ISL_738463, EPI_ISL_738465, EPI_ISL_738468, EPI_ISL_738473, EPI_ISL_738478, EPI_ISL_738481, EPI_ISL_738484, EPI_ISL_738487, EPI_ISL_738490, EPI_ISL_738495, EPI_ISL_738496			
see above	UZ Leuven, National Reference Laboratory for Coronaviruses, Laboratory Medicine, Leuven, Belgium	KU Leuven, Rega Institute, Clinical and Epidemiological Virology	Tony Wawina-Bokalanga, Joan Marti-Carerras, Bert Vanmechelen, Piet Maes
EPI_ISL_739936, EPI_ISL_740160, EPI_ISL_740319	Laboratoire national de santé, Microbiology, Virology	Laboratoire national de santé, Microbiology, Microbial Genomics Platform	Anke Wienecke-Baldacchino, Catherine Ragimbeau,Jessica Tapp, Fatu Djabi, Lise Pignon, Raoul Salmon, Tamir Abdelrahman
EPI_ISL_740869, EPI_ISL_740870	South Eastern Area Laboratory Services (SEALS)	NSW Health Pathology - Institute of Clinical Pathology and Medical Research; Westmead Hospital; University of Sydney	CIDM-PH et al.
EPI_ISL_740995, EPI_ISL_740996, EPI_ISL_740997, EPI_ISL_740998, EPI_ISL_741000, EPI_ISL_741001, EPI_ISL_741002, EPI_ISL_741004, EPI_ISL_741009, EPI_ISL_741011, EPI_ISL_741012			
see above	Department of Pathology, University of Cambridge	COVID-19 Genomics UK (COG-UK) Consortium	Aminu S. Jahun, Yasmin Chaudhry, Grant Hall, Iliana Georgana, Myra Hosmillo, Martin D. Curran, Malte Pinckert, Surendra Parmar, Ian Goodfellow
EPI_ISL_741186, EPI_ISL_741188, EPI_ISL_741189, EPI_ISL_741190, EPI_ISL_741191, EPI_ISL_741192, EPI_ISL_741193, EPI_ISL_741194, EPI_ISL_741195, EPI_ISL_741196, EPI_ISL_741197, EPI_ISL_741198, EPI_ISL_741199, EPI_ISL_741200, EPI_ISL_741201, EPI_ISL_741202, EPI_ISL_741203, EPI_ISL_741204, EPI_ISL_741205, EPI_ISL_741206, EPI_ISL_741208, EPI_ISL_741209, EPI_ISL_741210, EPI_ISL_741211, EPI_ISL_741212, EPI_ISL_741213, EPI_ISL_741214, EPI_ISL_741215, EPI_ISL_741216, EPI_ISL_741217, EPI_ISL_741218, EPI_ISL_741219, EPI_ISL_741220, EPI_ISL_741221, EPI_ISL_741222, EPI_ISL_741223, EPI_ISL_741224, EPI_ISL_741225, EPI_ISL_741226, EPI_ISL_741227, EPI_ISL_741228, EPI_ISL_741229, EPI_ISL_741230, EPI_ISL_741231, EPI_ISL_741232, EPI_ISL_741233, EPI_ISL_741234, EPI_ISL_741235, EPI_ISL_741236, EPI_ISL_741237, EPI_ISL_741238, EPI_ISL_741239, EPI_ISL_741240, EPI_ISL_741241, EPI_ISL_741242, EPI_ISL_741243, EPI_ISL_741244, EPI_ISL_741245, EPI_ISL_741246, EPI_ISL_741247, EPI_ISL_741248, EPI_ISL_741249, EPI_ISL_741250, EPI_ISL_741251, EPI_ISL_741252, EPI_ISL_741253, EPI_ISL_741254, EPI_ISL_741259, EPI_ISL_741260, EPI_ISL_741261, EPI_ISL_741262, EPI_ISL_741263, EPI_ISL_741270, EPI_ISL_741275, EPI_ISL_741277, EPI_ISL_741287, EPI_ISL_741294, EPI_ISL_741296, EPI_ISL_741297, EPI_ISL_741298, EPI_ISL_741299, EPI_ISL_741301, EPI_ISL_741302, EPI_ISL_741315, EPI_ISL_741316, EPI_ISL_741321			
see above	University College London, Great Ormond Street Hospital for Children NHS Foundation Trust, Imperial College Healthcare NHS Trust	COVID-19 Genomics UK (COG-UK) Consortium	Sergi Castellano, Rachel Williams, Mark Kristiansen, Paola Resende Silva, Sunando Roy, Tony Brooks, Helena Tutill, Paola Niola, Patricia Dyal, Charlotte Williams, Leysa Forrest, Yasmin Panchbhaya, Jacqueline Findlay, Samuel Weeks, Julianne Brown, Kathryn Harris, Paul Randell, James Price, Alison Holmes, Judith Breuer
EPI_ISL_741485, EPI_ISL_741532	Quadram Institute Bioscience	COVID-19 Genomics UK (COG-UK) Consortium	Dave J. Baker, Gemma L. Kay, Alp Aydin, Thanh Le-Viet, Steven Rudder, Ana P. Tedim, Anastasia Kolyva, Maria Diaz, Leonardo de Oliveira Martins, Nabil-Fareed Alikhan, Lizzie Meadows, Rachael Stanley, Ngozi Elumogo, Muhammed Yasir, Nicholas M. Thomson, Alexander J Trotter, Rachel Gilroy, Samuel Bloomfield, Claire Stuart, Andrew Bell, Reenesh Prakash, Samir Dervisevic, Alison E. Mather, John Wain, Mark Webber, Andrew J. Page, Justin O'Grady
EPI_ISL_741614, EPI_ISL_741615, EPI_ISL_741616, EPI_ISL_741617, EPI_ISL_741618, EPI_ISL_741619	Queens Medical Centre, Clinical Microbiology Department / DeepSeq Nottingham	COVID-19 Genomics UK (COG-UK) Consortium	Gemma Clark, Wendy Smith, Manjinder Khakh, Vicki M Fleming, Michelle M Lister, Hannah Howson-Wells, Jonathan Ball, Patrick McClure, Joseph Chappell, Theocharis Tsoleridis, Nadine Holmes, Matthew Carlisle, Christopher Moore, Fei Sang, Johnny Debebe, Victoria Wright, Matthew Loose
EPI_ISL_741658, EPI_ISL_741731, EPI_ISL_741732, EPI_ISL_741733, EPI_ISL_741748, EPI_ISL_741749	Centre for Enzyme Innovation, University of Portsmouth / Translational Research Laboratory, Portsmouth Hospitals NHS Trust	COVID-19 Genomics UK (COG-UK) Consortium	Angela Beckett,Yann Bourgeois,Garry Scarlett,Sharon Glaysher,Scott Elliott,Kelly Bicknell,Robert Impey,Allyson Lloyd,Sarah Wyllie,Ethan Butcher,Anoop Chauhan,Samuel Robson
EPI_ISL_741945, EPI_ISL_742009, EPI_ISL_742022, EPI_ISL_742023, EPI_ISL_742028, EPI_ISL_742044, EPI_ISL_742047, EPI_ISL_742055, EPI_ISL_742059, EPI_ISL_742065, EPI_ISL_742066, EPI_ISL_742068, EPI_ISL_742071, EPI_ISL_742075, EPI_ISL_742099, EPI_ISL_742103, EPI_ISL_742106			
see above	Virology Department, Sheffield Teaching Hospitals NHS Foundation Trust/Department of Infection, Immunity and Cardiovascular Disease, The Medical School, University of Sheffield	COVID-19 Genomics UK (COG-UK) Consortium	Thushan de Silva, Matthew Parker, Nikki Smith, Adri Angyal, Rebecca Brown, Luke Green, Rachel Tucker, Paul Parsons, Danielle Groves, Katie Johnson, Laura Carrilero, Alex Keeley, Dave Partridge, Matthew Wyles, Benjamin Lindsey, Mehmet Yavuz, Mohammad Raza, Cariad Evans
EPI_ISL_742177, EPI_ISL_742178, EPI_ISL_742179, EPI_ISL_742181, EPI_ISL_742182, EPI_ISL_742183, EPI_ISL_742184, EPI_ISL_742185, EPI_ISL_742186, EPI_ISL_742187, EPI_ISL_742188, EPI_ISL_742189, EPI_ISL_742190, EPI_ISL_742191, EPI_ISL_742192, EPI_ISL_742193			
see above	Virology Department, Royal Infirmary of Edinburgh, NHS Lothian / School of Biological Sciences, University of Edinburgh / Institute of Genetics and Molecular Medicine, University of Edinburgh	COVID-19 Genomics UK (COG-UK) Consortium	McHugh M, Dewar R, Rooke S, Gallagher M, Balcaza C, O'Toole Á, Scher E, Hill V, McCrone JT, Colquhoun R, Yu X, Jackson B, Rambaut A, Williams TC, Templeton K
EPI_ISL_742546, EPI_ISL_742555, EPI_ISL_742556, EPI_ISL_742557, EPI_ISL_742558, EPI_ISL_742560, EPI_ISL_742562, EPI_ISL_742566, EPI_ISL_742567, EPI_ISL_742568, EPI_ISL_742569, EPI_ISL_742570, EPI_ISL_742571, EPI_ISL_742572, EPI_ISL_742573, EPI_ISL_742574, EPI_ISL_742575, EPI_ISL_742576,			

EPI_ISL_742577, EPI_ISL_742578, EPI_ISL_742579, EPI_ISL_742580, EPI_ISL_742581, EPI_ISL_742582, EPI_ISL_742583, EPI_ISL_742584, EPI_ISL_742647, EPI_ISL_742648, EPI_ISL_742650, EPI_ISL_742652, EPI_ISL_742680, EPI_ISL_742681, EPI_ISL_742682, EPI_ISL_742692, EPI_ISL_742693, EPI_ISL_742698, EPI_ISL_742699, EPI_ISL_742703, EPI_ISL_742706, EPI_ISL_742708, EPI_ISL_742711, EPI_ISL_742712, EPI_ISL_742715, EPI_ISL_742721, EPI_ISL_742722, EPI_ISL_742723, EPI_ISL_742724, EPI_ISL_742725, EPI_ISL_742726, EPI_ISL_742727, EPI_ISL_742728, EPI_ISL_742729, EPI_ISL_742730, EPI_ISL_742731, EPI_ISL_742732, EPI_ISL_742733, EPI_ISL_742734, EPI_ISL_742735, EPI_ISL_742736, EPI_ISL_742737, EPI_ISL_742738, EPI_ISL_742739, EPI_ISL_742740, EPI_ISL_742741, EPI_ISL_742742, EPI_ISL_742743, EPI_ISL_742744, EPI_ISL_742745, EPI_ISL_742746, EPI_ISL_742747, EPI_ISL_742748, EPI_ISL_742749, EPI_ISL_742750, EPI_ISL_742751, EPI_ISL_742752, EPI_ISL_742753, EPI_ISL_742754, EPI_ISL_742755, EPI_ISL_742756, EPI_ISL_742757, EPI_ISL_742758, EPI_ISL_742759, EPI_ISL_742760, EPI_ISL_742761, EPI_ISL_742762, EPI_ISL_742763, EPI_ISL_742764, EPI_ISL_742765, EPI_ISL_742766, EPI_ISL_742767, EPI_ISL_742768, EPI_ISL_742769, EPI_ISL_742770, EPI_ISL_742771, EPI_ISL_742772, EPI_ISL_742773, EPI_ISL_742774, EPI_ISL_742775, EPI_ISL_742776, EPI_ISL_742777, EPI_ISL_742778, EPI_ISL_742779, EPI_ISL_742780, EPI_ISL_742781, EPI_ISL_742782, EPI_ISL_742783, EPI_ISL_742784, EPI_ISL_742785, EPI_ISL_742786, EPI_ISL_742787, EPI_ISL_742788, EPI_ISL_742789, EPI_ISL_742790, EPI_ISL_742791, EPI_ISL_742792, EPI_ISL_742793, EPI_ISL_742794, EPI_ISL_742795, EPI_ISL_742796, EPI_ISL_742797, EPI_ISL_742798, EPI_ISL_742799, EPI_ISL_742800, EPI_ISL_742801, EPI_ISL_742802, EPI_ISL_742803, EPI_ISL_742804, EPI_ISL_742805, EPI_ISL_742806, EPI_ISL_742807, EPI_ISL_742808, EPI_ISL_742809, EPI_ISL_742810, EPI_ISL_742811, EPI_ISL_742812, EPI_ISL_742813, EPI_ISL_742814, EPI_ISL_742815, EPI_ISL_742816, EPI_ISL_742817, EPI_ISL_742818, EPI_ISL_742819, EPI_ISL_742820, EPI_ISL_742821, EPI_ISL_742822, EPI_ISL_742823, EPI_ISL_742824, EPI_ISL_742825, EPI_ISL_742826, EPI_ISL_742827, EPI_ISL_742828, EPI_ISL_742829, EPI_ISL_742830, EPI_ISL_742831, EPI_ISL_742832, EPI_ISL_742833, EPI_ISL_742834, EPI_ISL_742835, EPI_ISL_742836, EPI_ISL_742837, EPI_ISL_743127, EPI_ISL_743146, EPI_ISL_743147, EPI_ISL_743148, EPI_ISL_743149, EPI_ISL_743150, EPI_ISL_743151, EPI_ISL_743152, EPI_ISL_743153, EPI_ISL_743154, EPI_ISL_743155, EPI_ISL_743156, EPI_ISL_743157, EPI_ISL_743158, EPI_ISL_743321, EPI_ISL_743322, EPI_ISL_743326, EPI_ISL_743341, EPI_ISL_743352, EPI_ISL_743365, EPI_ISL_743366, EPI_ISL_743367, EPI_ISL_743368, EPI_ISL_743369, EPI_ISL_743370, EPI_ISL_743371, EPI_ISL_743373, EPI_ISL_743374, EPI_ISL_743375, EPI_ISL_743376, EPI_ISL_743377, EPI_ISL_743378, EPI_ISL_743379, EPI_ISL_743425, EPI_ISL_743426, EPI_ISL_743427, EPI_ISL_743428, EPI_ISL_743429, EPI_ISL_743430, EPI_ISL_743431, EPI_ISL_743433, EPI_ISL_743434, EPI_ISL_743445, EPI_ISL_743446, EPI_ISL_743447, EPI_ISL_743448, EPI_ISL_743449, EPI_ISL_743509, EPI_ISL_743511, EPI_ISL_743512, EPI_ISL_743513, EPI_ISL_743514, EPI_ISL_743515, EPI_ISL_743516, EPI_ISL_743517, EPI_ISL_743518, EPI_ISL_743519, EPI_ISL_743520, EPI_ISL_743521, EPI_ISL_743522, EPI_ISL_743523, EPI_ISL_743524, EPI_ISL_743525, EPI_ISL_743526, EPI_ISL_743527, EPI_ISL_743528, EPI_ISL_743529, EPI_ISL_743530, EPI_ISL_743531, EPI_ISL_743532, EPI_ISL_743533, EPI_ISL_743534, EPI_ISL_743535, EPI_ISL_743536, EPI_ISL_743537, EPI_ISL_743538, EPI_ISL_743539, EPI_ISL_743540, EPI_ISL_743541, EPI_ISL_743542, EPI_ISL_743543, EPI_ISL_743544, EPI_ISL_743545, EPI_ISL_743546, EPI_ISL_743547, EPI_ISL_743548, EPI_ISL_743549, EPI_ISL_743550, EPI_ISL_743551, EPI_ISL_743552, EPI_ISL_743553, EPI_ISL_743554, EPI_ISL_743555, EPI_ISL_743556, EPI_ISL_743557, EPI_ISL_743558, EPI_ISL_743559, EPI_ISL_743560, EPI_ISL_743561, EPI_ISL_743562, EPI_ISL_743563, EPI_ISL_743564, EPI_ISL_743565, EPI_ISL_743566, EPI_ISL_743567, EPI_ISL_743569, EPI_ISL_743570, EPI_ISL_743571, EPI_ISL_743572, EPI_ISL_743573, EPI_ISL_743574, EPI_ISL_743575, EPI_ISL_743576, EPI_ISL_743577, EPI_ISL_743578, EPI_ISL_743579, EPI_ISL_743581, EPI_ISL_743582, EPI_ISL_743583, EPI_ISL_743584, EPI_ISL_743585, EPI_ISL_743586, EPI_ISL_743587, EPI_ISL_743588, EPI_ISL_743589, EPI_ISL_743590, EPI_ISL_743591, EPI_ISL_743596, EPI_ISL_743597, EPI_ISL_743598, EPI_ISL_743599, EPI_ISL_743600, EPI_ISL_743601, EPI_ISL_743602, EPI_ISL_743641, EPI_ISL_743662, EPI_ISL_743663, EPI_ISL_743664, EPI_ISL_743675, EPI_ISL_743678, EPI_ISL_743680, EPI_ISL_743681, EPI_ISL_743682, EPI_ISL_743683, EPI_ISL_743684, EPI_ISL_743685, EPI_ISL_743686, EPI_ISL_743687, EPI_ISL_743688, EPI_ISL_743689, EPI_ISL_743690, EPI_ISL_743691, EPI_ISL_743692, EPI_ISL_743693, EPI_ISL_743694, EPI_ISL_743695, EPI_ISL_743696, EPI_ISL_743697, EPI_ISL_743698, EPI_ISL_743699, EPI_ISL_743700, EPI_ISL_743701, EPI_ISL_743702, EPI_ISL_743703, EPI_ISL_743704, EPI_ISL_743705, EPI_ISL_743706, EPI_ISL_743707, EPI_ISL_743708, EPI_ISL_743709, EPI_ISL_743710, EPI_ISL_743711, EPI_ISL_743712, EPI_ISL_743713, EPI_ISL_743714, EPI_ISL_743715, EPI_ISL_743716, EPI_ISL_743717, EPI_ISL_743718, EPI_ISL_743719, EPI_ISL_743720, EPI_ISL_743721, EPI_ISL_743722, EPI_ISL_743723, EPI_ISL_743724, EPI_ISL_743725, EPI_ISL_743726, EPI_ISL_743727, EPI_ISL_743728, EPI_ISL_743729, EPI_ISL_743730, EPI_ISL_743731, EPI_ISL_743732, EPI_ISL_743733, EPI_ISL_743734, EPI_ISL_743735, EPI_ISL_743736, EPI_ISL_743737, EPI_ISL_743738, EPI_ISL_743739, EPI_ISL_743740, EPI_ISL_743741, EPI_ISL_743742, EPI_ISL_743743, EPI_ISL_743744, EPI_ISL_743745, EPI_ISL_743746, EPI_ISL_743747, EPI_ISL_743748, EPI_ISL_743749, EPI_ISL_743750, EPI_ISL_743751, EPI_ISL_743752, EPI_ISL_743753, EPI_ISL_743754, EPI_ISL_743755, EPI_ISL_743756, EPI_ISL_743757, EPI_ISL_743758, EPI_ISL_743759, EPI_ISL_743760, EPI_ISL_743761, EPI_ISL_743762, EPI_ISL_743763, EPI_ISL_743764, EPI_ISL_743765, EPI_ISL_743766, EPI_ISL_743767, EPI_ISL_743768, EPI_ISL_743769, EPI_ISL_743770, EPI_ISL_743771, EPI_ISL_743772, EPI_ISL_743773, EPI_ISL_743774, EPI_ISL_743775, EPI_ISL_743776, EPI_ISL_743777, EPI_ISL_743778, EPI_ISL_743779, EPI_ISL_743780, EPI_ISL_743781, EPI_ISL_743783, EPI_ISL_743784, EPI_ISL_743786, EPI_ISL_743787, EPI_ISL_743788, EPI_ISL_743789, EPI_ISL_743790, EPI_ISL_743791, EPI_ISL_743792, EPI_ISL_743793, EPI_ISL_743794, EPI_ISL_743795, EPI_ISL_743796, EPI_ISL_743797, EPI_ISL_743798, EPI_ISL_743799, EPI_ISL_743800, EPI_ISL_743801, EPI_ISL_743802, EPI_ISL_743803, EPI_ISL_743804, EPI_ISL_743807, EPI_ISL_743808, EPI_ISL_743809, EPI_ISL_743810, EPI_ISL_743811, EPI_ISL_743812, EPI_ISL_743813, EPI_ISL_743814, EPI_ISL_743815, EPI_ISL_743816, EPI_ISL_743817, EPI_ISL_743818, EPI_ISL_743819, EPI_ISL_743823, EPI_ISL_743824, EPI_ISL_743829, EPI_ISL_743830, EPI_ISL_743834, EPI_ISL_743837, EPI_ISL_743874, EPI_ISL_743875, EPI_ISL_743876, EPI_ISL_743879, EPI_ISL_743880, EPI_ISL_743881, EPI_ISL_743882, EPI_ISL_743883, EPI_ISL_743884, EPI_ISL_743885, EPI_ISL_743890, EPI_ISL_743891, EPI_ISL_743892, EPI_ISL_743893, EPI_ISL_743894, EPI_ISL_743895, EPI_ISL_743896, EPI_ISL_743897, EPI_ISL_743902, EPI_ISL_743937, EPI_ISL_743942, EPI_ISL_743944, EPI_ISL_743952, EPI_ISL_743958, EPI_ISL_743959, EPI_ISL_743960, EPI_ISL_743961, EPI_ISL_743962, EPI_ISL_743963, EPI_ISL_743964, EPI_ISL_743965, EPI_ISL_743966, EPI_ISL_743967, EPI_ISL_743968, EPI_ISL_743969, EPI_ISL_743970, EPI_ISL_743971, EPI_ISL_743972, EPI_ISL_743973, EPI_ISL_743974, EPI_ISL_743975, EPI_ISL_743976, EPI_ISL_743977, EPI_ISL_743978, EPI_ISL_743979, EPI_ISL_743980, EPI_ISL_743981, EPI_ISL_743982, EPI_ISL_743983, EPI_ISL_743984, EPI_ISL_743985, EPI_ISL_743986, EPI_ISL_743988, EPI_ISL_743989, EPI_ISL_743990, EPI_ISL_743991, EPI_ISL_743992, EPI_ISL_743993, EPI_ISL_743994, EPI_ISL_743995, EPI_ISL_743996, EPI_ISL_743997, EPI_ISL_743998, EPI_ISL_743999			
see above	Wales Specialist Virology Centre Sequencing lab: Pathogen Genomics Unit	COVID-19 Genomics UK (COG-UK) Consortium	Catherine Moore, Johnathan Evans, Laura Gifford, Malorie Perry, Simon Cottrell, Angela Marchbank, Alec Birchley, Alexander Adams, Amy Gaskin, Bree Gatica-Wilcox, Jason Coombes, Joel Southgate, Lauren Gilbert, Lee Graham, Nicole Pacchiarini, Sara Kumziene-Summerhayes, Sarah Taylor, Sophie Jones, Sara Rey, Matthew Bull, Joanne Watkins, Sally Corden, Tom Connor
EPI_ISL_744519, EPI_ISL_744793, EPI_ISL_744946	Laboratoire national de santé, Microbiology, Virology	Laboratoire national de santé, Microbiology, Microbial Genomics Platform	Anke Wienecke-Baldacchino, Catherine Ragimbeau,Jessica Tapp, Fatu Djabi, Lise Pignon, Raoul Salmon, Tamir Abdelrahman
EPI_ISL_745056, EPI_ISL_745058, EPI_ISL_745059, EPI_ISL_745060, EPI_ISL_745061, EPI_ISL_745062, EPI_ISL_745063, EPI_ISL_745064, EPI_ISL_745065, EPI_ISL_745066, EPI_ISL_745067, EPI_ISL_745068, EPI_ISL_745069			
see above	Israel Central Virology laboratory	Israel Central Virology laboratory	Neta Zuckerman, Efrat Dahan Bucris, Oran Erster, Michal Mandelboim, Orna Mor, Ella Mendelson
EPI_ISL_745211	Lab voor klinische biologie	Onderzoeksgroep Virologie	Laurens Lambrechts, Nick Vereecke, Marthe Pauwels, Bruno Verhasselt, Linos Vandekerckhove, Hans Nauwynck, Sebastiaan Theuns
EPI_ISL_745213, EPI_ISL_745214, EPI_ISL_745215, EPI_ISL_745216, EPI_ISL_745217, EPI_ISL_745220, EPI_ISL_745221	Lab voor klinische biologie	Onderzoeksgroep Virologie	Nick Vereecke, Laurens Lambrechts, Marthe Pauwels, Bruno Verhasselt, Linos Vandekerckhove, Hans Nauwynck, Sebastiaan Theuns
EPI_ISL_745310, EPI_ISL_745331, EPI_ISL_745378, EPI_ISL_745379, EPI_ISL_745380, EPI_ISL_745381, EPI_ISL_745382, EPI_ISL_745383, EPI_ISL_745384, EPI_ISL_745385, EPI_ISL_745386, EPI_ISL_745387, EPI_ISL_745388			
see above	CNR Virus des Infections Respiratoires - France SUD	CNR Virus des Infections Respiratoires - France SUD	Antonin Bal, Gregory Destras, Claudia Gonzalez, Gwendolyne Burfin, Quentin Semanas, Martine Valette, Bruno Lina, Laurence Josset
EPI_ISL_746827	National Institute for Infectious Diseases, INMI, "L. Spallanzani" IRCCS	National Institute for Infectious Diseases, INMI, "L. Spallanzani" IRCCS	B Bartolini, C.E.M Gruber, M Rueca, F Messina, E Giombini, MR Capobianchi, A Di Caro
EPI_ISL_747498, EPI_ISL_747499, EPI_ISL_747500, EPI_ISL_747501, EPI_ISL_747502, EPI_ISL_747503, EPI_ISL_747504, EPI_ISL_747505, EPI_ISL_747506, EPI_ISL_747507, EPI_ISL_747508, EPI_ISL_747509, EPI_ISL_747510, EPI_ISL_747511, EPI_ISL_747512, EPI_ISL_747513, EPI_ISL_747514, EPI_ISL_747515, EPI_ISL_747516, EPI_ISL_747517, EPI_ISL_747518, EPI_ISL_747519			
see above	Respiratory Virus Unit, National Infection Service, Public Health England	COVID-19 Genomics UK (COG-UK) Consortium	PHE Covid Sequencing Team
EPI_ISL_751802, EPI_ISL_751803, EPI_ISL_751804, EPI_ISL_751805, EPI_ISL_751806, EPI_ISL_751807, EPI_ISL_751808, EPI_ISL_751809, EPI_ISL_751810, EPI_ISL_751811, EPI_ISL_751812, EPI_ISL_751813, EPI_ISL_751814, EPI_ISL_751815, EPI_ISL_751816, EPI_ISL_751817, EPI_ISL_751818, EPI_ISL_751819, EPI_ISL_751820, EPI_ISL_751821, EPI_ISL_751822, EPI_ISL_751823, EPI_ISL_751824, EPI_ISL_751825, EPI_ISL_751826, EPI_ISL_751827, EPI_ISL_751828, EPI_ISL_751829, EPI_ISL_751830, EPI_ISL_751831, EPI_ISL_751832, EPI_ISL_751833, EPI_ISL_751834, EPI_ISL_751835, EPI_ISL_751836, EPI_ISL_751837, EPI_ISL_751838, EPI_ISL_751839, EPI_ISL_751840, EPI_ISL_751841, EPI_ISL_751842, EPI_ISL_751843, EPI_ISL_751844, EPI_ISL_751845, EPI_ISL_751846			
see above	New Mexico Department of Health Scientific Laboratory	New Mexico Department of Health Scientific Laboratory	D'eldra Malone, Ellie Johnson, Anastacia Griego-Fisher
EPI_ISL_752606	Yale Pathology Lab	Grubaugh Lab - Yale School of Public Health	Tara Alpert, Joseph Fauver, Anderson Brito, Chen Liu, Pei Hui, Jianhui Wang, Kien Pham, Nathan Grubaugh
EPI_ISL_753663, EPI_ISL_753669	Clinical virology Laboratory, Children's Hospital Los Angeles	Center for Personalized Medicine, Children's Hospital Los Angeles	Gai et al
EPI_ISL_754142, EPI_ISL_754146, EPI_ISL_754151, EPI_ISL_754167, EPI_ISL_754168, EPI_ISL_754170, EPI_ISL_754171, EPI_ISL_754172	Laboratoire de Microbiologie	National Reference Center for Viruses of Respiratory Infections, Institut Pasteur, Paris	Marion Barbet, Sylvie Behillil, Méline Bizard, Angela Brisebarre, Camille Capel, Etienne Simon-Lorière, Vincent Enouf, Maud Vanpeene, Sylvie van der Werf, Marie-Sarah Fangous
EPI_ISL_754215, EPI_ISL_754216	Wyoming Public Health Laboratory	Wyoming Public Health Laboratory	Noah Hull, Taylor Fearing, Channing Weber, Ashley Norberg, Bailey Bowcutt, and Wanda Manley
EPI_ISL_754543, EPI_ISL_754544, EPI_ISL_754550, EPI_ISL_754551, EPI_ISL_754552, EPI_ISL_754553, EPI_ISL_754554, EPI_ISL_754555, EPI_ISL_754556, EPI_ISL_754557, EPI_ISL_754558, EPI_ISL_754559, EPI_ISL_754560, EPI_ISL_754561, EPI_ISL_754562, EPI_ISL_754563, EPI_ISL_754564, EPI_ISL_754565, EPI_ISL_754566, EPI_ISL_754568, EPI_ISL_754569, EPI_ISL_754570, EPI_ISL_754571, EPI_ISL_754573, EPI_ISL_754574			
see above	Wadsworth Center, New York State Department.of Health	Wadsworth Center, New York State Department.of Health	Kirsten St. George, Daryl M. Lamson, Alexis Russel, Matthew Shudt, Melissa A Leisner, Jonathan Plitnick, Navjot Singh, John Kelly, Sara Griesemer, Erasmus Schneider, Erica Lasek-Nesselquist
EPI_ISL_754629, EPI_ISL_754630, EPI_ISL_754631, EPI_ISL_754632, EPI_ISL_754633, EPI_ISL_754634, EPI_ISL_754635	University of Wisconsin-Madison AIDS Vaccine Research Laboratories	University of Wisconsin-Madison AIDS Vaccine Research Laboratories	Gage Moreno, Katarina Braun, et al. AIDS Vaccine Research Laboratories
EPI_ISL_755146, EPI_ISL_755163, EPI_ISL_755164, EPI_ISL_755165, EPI_ISL_755166, EPI_ISL_755167, EPI_ISL_755168, EPI_ISL_755169, EPI_ISL_755170, EPI_ISL_755174	UCSD EXCITE lab	Andersen lab at Scripps Research	SEARCH Alliance San Diego
EPI_ISL_755494, EPI_ISL_755495, EPI_ISL_755497, EPI_ISL_755498, EPI_ISL_755499, EPI_ISL_755561,	Maine Health and Environmental Testing Laboratory	Tewhey Lab, The Jackson Laboratory	Matluk,N., Dewey,H., Iosue,F., Barter,M., Lynch,R., Munger,H. and Tewhey,R.

EPI_ISL_755562, EPI_ISL_755563, EPI_ISL_755564, EPI_ISL_755565			
EPI_ISL_755573	Center of Advanced Studies and Technology, CAST	Center of Advanced Studies and Technology, CAST	Ferrante,R., Mandatori,D., De Fabritiis,S.
EPI_ISL_755651, EPI_ISL_755653, EPI_ISL_755654	Instituto Adolfo Lutz - Central	Instituto Adolfo Lutz, Interdiciplinary Procedures Center, Strategic Laboratory	Claudio Tavares Sacchi, Claudia Regina Gonçalves, Erica Valessa Ramos Gomes, Karoline Rodrigues Campos
EPI_ISL_755655	Instituto Adolfo Lutz - Regional de Campinas	Instituto Adolfo Lutz, Interdiciplinary Procedures Center, Strategic Laboratory	Claudio Tavares Sacchi, Claudia Regina Gonçalves, Erica Valessa Ramos Gomes, Karoline Rodrigues Campos
EPI_ISL_756191, EPI_ISL_756192, EPI_ISL_756193, EPI_ISL_756194, EPI_ISL_756195, EPI_ISL_756196, EPI_ISL_756197, EPI_ISL_756198, EPI_ISL_756199, EPI_ISL_756200, EPI_ISL_756201, EPI_ISL_756208, EPI_ISL_756209, EPI_ISL_756210, EPI_ISL_756211, EPI_ISL_756212, EPI_ISL_756213, EPI_ISL_756214, EPI_ISL_756215, EPI_ISL_756217, EPI_ISL_756296, EPI_ISL_756297, EPI_ISL_756299, EPI_ISL_756302			
see above	UW Virology Lab	UW Virology Lab	Pavitra Roychoudhury, Hong Xie, Lasata Shrestha, Meei-Li Huang, Keith R Jerome, Alexander Greninger
EPI_ISL_756308, EPI_ISL_756309	The Caribbean Public Health Agency	Carrington Lab, Department of PreClinical Sciences, Faculty of Medical Sciences, The University of the West Indies	Nikita S. D. Sahadeo, Arianne Brown-Jordan, Sarah Hill, Vernie Ramkissoon, Roshan Parasram, Naresh Nandram, Avery Hinds, Jerome Foster, Stanley Giddings, Karla Georges, Marsha Ivey, Rahul Naidu, Risha Singh, SueMin Nathaniel, Rajini Haraksingh, Jaya Jayaraman, Chinna Chinnadurai, Adesh Ramsubhag, Nuno Faria, Oliver Pybus, Christopher Oura, Gabriel Escobar, Christine V. F. Carrington
EPI_ISL_756470	Lighthouse Lab in Alderley Park	Wellcome Sanger Institute for the COVID-19 Genomics UK (COG-UK) Consortium	Jacquelyn Wynn, Mairead Hyland, The Lighthouse Lab in Alderley Park and Alex Alderton, Roberto Amato, Sonia Goncalves, Ewan Harrison, David K. Jackson, Ian Johnston, Dominic Kwiatkowski, Cordelia Langford, John Sillitoe on behalf of the Wellcome Sanger Institute COVID-19 Surveillance Team
EPI_ISL_756846, EPI_ISL_756887, EPI_ISL_756903, EPI_ISL_756946, EPI_ISL_757020	Lighthouse Lab in Milton Keynes	Wellcome Sanger Institute for the COVID-19 Genomics UK (COG-UK) Consortium	The Lighthouse Lab in Milton Keynes and Alex Alderton, Roberto Amato, Sonia Goncalves, Ewan Harrison, David K. Jackson, Ian Johnston, Dominic Kwiatkowski, Cordelia Langford, John Sillitoe on behalf of the Wellcome Sanger Institute COVID-19 Surveillance Team
EPI_ISL_757042, EPI_ISL_757043, EPI_ISL_757044, EPI_ISL_757045, EPI_ISL_757046, EPI_ISL_757047, EPI_ISL_757048, EPI_ISL_757049, EPI_ISL_757050, EPI_ISL_757051, EPI_ISL_757052, EPI_ISL_757053, EPI_ISL_757054, EPI_ISL_757055, EPI_ISL_757056, EPI_ISL_757057, EPI_ISL_757058, EPI_ISL_757059, EPI_ISL_757060, EPI_ISL_757061, EPI_ISL_757062, EPI_ISL_757063, EPI_ISL_757064, EPI_ISL_757065, EPI_ISL_757066, EPI_ISL_757067, EPI_ISL_757068, EPI_ISL_757069, EPI_ISL_757070, EPI_ISL_757071, EPI_ISL_757072, EPI_ISL_757073, EPI_ISL_757074, EPI_ISL_757075, EPI_ISL_757076, EPI_ISL_757077, EPI_ISL_757078, EPI_ISL_757079, EPI_ISL_757080, EPI_ISL_757081, EPI_ISL_757082			
see above	Lighthouse Lab in Glasgow	Wellcome Sanger Institute for the COVID-19 Genomics UK (COG-UK) Consortium	Harper VanSteenhouse, Yumi Kasai, David Gray, Carol Clugston, Anna Dominiczak and Alex Alderton, Roberto Amato, Sonia Goncalves, Ewan Harrison, David K. Jackson, Ian Johnston, Dominic Kwiatkowski, Cordelia Langford, John Sillitoe on behalf of the Wellcome Sanger Institute COVID-19 Surveillance Team
EPI_ISL_757119	Lighthouse Lab in Cambridge	Wellcome Sanger Institute for the COVID-19 Genomics UK (COG-UK) Consortium	Rob Howes, The Lighthouse Lab in Cambridge and Alex Alderton, Roberto Amato, Sonia Goncalves, Ewan Harrison, David K. Jackson, Ian Johnston, Dominic Kwiatkowski, Cordelia Langford, John Sillitoe on behalf of the Wellcome Sanger Institute COVID-19 Surveillance Team
EPI_ISL_757120, EPI_ISL_757121, EPI_ISL_757122, EPI_ISL_757123, EPI_ISL_757124, EPI_ISL_757125, EPI_ISL_757126, EPI_ISL_757127, EPI_ISL_757128, EPI_ISL_757129, EPI_ISL_757130, EPI_ISL_757131, EPI_ISL_757132, EPI_ISL_757133, EPI_ISL_757134, EPI_ISL_757135, EPI_ISL_757136, EPI_ISL_757137, EPI_ISL_757138, EPI_ISL_757139, EPI_ISL_757140, EPI_ISL_757141, EPI_ISL_757142, EPI_ISL_757143, EPI_ISL_757144, EPI_ISL_757145, EPI_ISL_757146, EPI_ISL_757147, EPI_ISL_757148, EPI_ISL_757149, EPI_ISL_757150, EPI_ISL_757151, EPI_ISL_757152, EPI_ISL_757153, EPI_ISL_757154, EPI_ISL_757155, EPI_ISL_757156, EPI_ISL_757157, EPI_ISL_757158, EPI_ISL_757159, EPI_ISL_757160, EPI_ISL_757161, EPI_ISL_757162, EPI_ISL_757163, EPI_ISL_757164, EPI_ISL_757165, EPI_ISL_757166, EPI_ISL_757167, EPI_ISL_757168, EPI_ISL_757169, EPI_ISL_757170, EPI_ISL_757171, EPI_ISL_757172, EPI_ISL_757173, EPI_ISL_757174, EPI_ISL_757175, EPI_ISL_757176, EPI_ISL_757177, EPI_ISL_757178, EPI_ISL_757179, EPI_ISL_757180, EPI_ISL_757181, EPI_ISL_757182, EPI_ISL_757183, EPI_ISL_757184, EPI_ISL_757185, EPI_ISL_757186, EPI_ISL_757225, EPI_ISL_757228, EPI_ISL_757230			
see above	Lighthouse Lab in Glasgow	Wellcome Sanger Institute for the COVID-19 Genomics UK (COG-UK) Consortium	Harper VanSteenhouse, Yumi Kasai, David Gray, Carol Clugston, Anna Dominiczak and Alex Alderton, Roberto Amato, Sonia Goncalves, Ewan Harrison, David K. Jackson, Ian Johnston, Dominic Kwiatkowski, Cordelia Langford, John Sillitoe on behalf of the Wellcome Sanger Institute COVID-19 Surveillance Team
EPI_ISL_760842	Lighthouse Lab in Milton Keynes	Wellcome Sanger Institute for the COVID-19 Genomics UK (COG-UK) Consortium	The Lighthouse Lab in Milton Keynes and Alex Alderton, Roberto Amato, Sonia Goncalves, Ewan Harrison, David K. Jackson, Ian Johnston, Dominic Kwiatkowski, Cordelia Langford, John Sillitoe on behalf of the Wellcome Sanger Institute COVID-19 Surveillance Team
EPI_ISL_760847, EPI_ISL_760977, EPI_ISL_760980, EPI_ISL_761018	Lighthouse Lab in Glasgow	Wellcome Sanger Institute for the COVID-19 Genomics UK (COG-UK) Consortium	Harper VanSteenhouse, Yumi Kasai, David Gray, Carol Clugston, Anna Dominiczak and Alex Alderton, Roberto Amato, Sonia Goncalves, Ewan Harrison, David K. Jackson, Ian Johnston, Dominic Kwiatkowski, Cordelia Langford, John Sillitoe on behalf of the Wellcome Sanger Institute COVID-19 Surveillance Team
EPI_ISL_761059	Lighthouse Lab in Milton Keynes	Wellcome Sanger Institute for the COVID-19 Genomics UK (COG-UK) Consortium	The Lighthouse Lab in Milton Keynes and Alex Alderton, Roberto Amato, Sonia Goncalves, Ewan Harrison, David K. Jackson, Ian Johnston, Dominic Kwiatkowski, Cordelia Langford, John Sillitoe on behalf of the Wellcome Sanger Institute COVID-19 Surveillance Team
EPI_ISL_761096, EPI_ISL_761727	Lighthouse Lab in Alderley Park	Wellcome Sanger Institute for the COVID-19 Genomics UK (COG-UK) Consortium	Jacquelyn Wynn, Mairead Hyland, The Lighthouse Lab in Alderley Park and Alex Alderton, Roberto Amato, Sonia Goncalves, Ewan Harrison, David K. Jackson, Ian Johnston, Dominic Kwiatkowski, Cordelia Langford, John Sillitoe on behalf of the Wellcome Sanger Institute COVID-19 Surveillance Team
EPI_ISL_762237	Lighthouse Lab in Milton Keynes	Wellcome Sanger Institute for the COVID-19 Genomics UK (COG-UK) Consortium	The Lighthouse Lab in Milton Keynes and Alex Alderton, Roberto Amato, Sonia Goncalves, Ewan Harrison, David K. Jackson, Ian Johnston, Dominic Kwiatkowski, Cordelia Langford, John Sillitoe on behalf of the Wellcome Sanger Institute COVID-19 Surveillance Team
EPI_ISL_763003, EPI_ISL_763022, EPI_ISL_763040, EPI_ISL_763041, EPI_ISL_763042, EPI_ISL_763059	Unit 17: Influenza & Other Respiratory Viruses, German National Influenza Center	Project group Epidemiology of Highly Pathogenic Microorganisms, Robert Koch-Institute	Ariane Düx, Andreas Sachse, Grit Schubert, Sébastien Calvignac-Spencer, Fabian Leendertz, Thorsten Wolff, Ralf Dürrwald, Djin-Ye Oh, Marianne Wedde
EPI_ISL_763079, EPI_ISL_763080	Microbiologia e Virologia	Istituto Zooprofilattico Sperimentale delle Venezie	Adelaide Milani, Alessia Schivo, Annalisa Salviato, Erika Giorgia Quaranta, Ambra Pastori, Bianca Zecchin, Alice Fusaro, Isabella Monne, Calogero Terregino, Antonia Ricci
EPI_ISL_763127, EPI_ISL_763175, EPI_ISL_763177, EPI_ISL_763178, EPI_ISL_763179, EPI_ISL_763180, EPI_ISL_763189, EPI_ISL_763194, EPI_ISL_763195, EPI_ISL_763196, EPI_ISL_763197, EPI_ISL_763198, EPI_ISL_763202, EPI_ISL_763241, EPI_ISL_763285, EPI_ISL_763348, EPI_ISL_763356			
see above	Dutch COVID-19 response team	Erasmus Medical Center	Bas Oude Munnink, Reina Sikkema, David Nieuwenhuijse, Irina Chestakova, Anne van der Linden, Marjan Boter, Emmanuelle Munger, Corine GeurtsvanKessel, Annemiek van der Eijk, Richard Molenkamp, Marion Koopmans, on behalf of the Dutch national COVID-19 response team.
EPI_ISL_763390, EPI_ISL_763453, EPI_ISL_763457	University of Exeter	COVID-19 Genomics UK (COG-UK) Consortium	Ben Temperton,Aaron Jeffries,Michelle Michelsen,Joanna Warwick-Dugdale,Audrey Farbos,Robyn Manley,Stephen Michell,Jane Masoli
EPI_ISL_763484, EPI_ISL_763487, EPI_ISL_763519, EPI_ISL_763522	Wales Specialist Virology Centre Sequencing lab: Pathogen Genomics Unit	COVID-19 Genomics UK (COG-UK) Consortium	Catherine Moore, Johnathan Evans, Laura Gifford, Malorie Perry, Simon Cottrell, Angela Marchbank, Alec Birchley, Alexander Adams, Amy Gaskin, Bree Gatica-Wilcox, Jason Coombes, Joel Southgate, Lauren Gilbert, Lee Graham, Nicole Pacchiarini, Sara Kumziene-Summerhayes, Sarah Taylor, Sophie Jones, Sara Rey, Matthew Bull, Joanne Watkins, Sally Corden, Tom Connor
EPI_ISL_763542	Department of Pathology, University of Cambridge	COVID-19 Genomics UK (COG-UK) Consortium	Aminu S. Jahun, Yasmin Chaudhry, Grant Hall, Iliana Georgana, Myra Hosmillo, Martin D. Curran, Malte Pinckert, Surendra Parmar, Ian Goodfellow
EPI_ISL_763561, EPI_ISL_763562	University of Exeter	COVID-19 Genomics UK (COG-UK) Consortium	Ben Temperton,Aaron Jeffries,Michelle Michelsen,Joanna Warwick-Dugdale,Audrey Farbos,Robyn Manley,Stephen Michell,Jane Masoli
EPI_ISL_763566, EPI_ISL_763570, EPI_ISL_763571	Wales Specialist Virology Centre Sequencing lab: Pathogen Genomics Unit	COVID-19 Genomics UK (COG-UK) Consortium	Catherine Moore, Johnathan Evans, Laura Gifford, Malorie Perry, Simon Cottrell, Angela Marchbank, Alec Birchley, Alexander Adams, Amy Gaskin, Bree Gatica-Wilcox, Jason Coombes, Joel Southgate, Lauren Gilbert, Lee Graham, Nicole Pacchiarini, Sara Kumziene-Summerhayes, Sarah Taylor, Sophie Jones, Sara Rey, Matthew Bull, Joanne Watkins, Sally Corden, Tom Connor
EPI_ISL_763611	Centre for Enzyme Innovation, University of Portsmouth / Translational Research Laboratory, Portsmouth Hospitals NHS Trust	COVID-19 Genomics UK (COG-UK) Consortium	Angela Beckett,Yann Bourgeois,Garry Scarlett,Sharon Glaysher,Scott Elliott,Kelly Bicknell,Robert Impey,Allyson Lloyd,Sarah Wyllie,Ethan Butcher,Anoop Chauhan,Samuel Robson
EPI_ISL_763634	Virology Department, Sheffield Teaching Hospitals NHS Foundation Trust/Department of Infection, Immunity and Cardiovascular Disease, The Medical School, University of Sheffield	COVID-19 Genomics UK (COG-UK) Consortium	Thushan de Silva, Matthew Parker, Nikki Smith, Adri Angyal, Rebecca Brown, Luke Green, Rachel Tucker, Paul Parsons, Danielle Groves, Katie Johnson, Laura Carrilero, Alex Keeley, Dave Partridge, Matthew Wyles, Benjamin Lindsey, Mehmet Yavuz, Mohammad Raza, Cariad Evans
EPI_ISL_763637	Department of Pathology, University of Cambridge	COVID-19 Genomics UK (COG-UK) Consortium	Aminu S. Jahun, Yasmin Chaudhry, Grant Hall, Iliana Georgana, Myra Hosmillo, Martin D. Curran, Malte Pinckert, Surendra Parmar, Ian Goodfellow
EPI_ISL_763652	Virology Department, Sheffield Teaching Hospitals NHS Foundation Trust/Department of Infection, Immunity and	COVID-19 Genomics UK (COG-UK) Consortium	Thushan de Silva, Matthew Parker, Nikki Smith, Adri Angyal, Rebecca Brown, Luke Green, Rachel Tucker, Paul Parsons, Danielle Groves, Katie Johnson, Laura Carrilero, Alex Keeley, Dave Partridge, Matthew Wyles, Benjamin Lindsey, Mehmet Yavuz, Mohammad Raza, Cariad Evans

	Cardiovascular Disease, The Medical School, University of Sheffield		
EPI_ISL_763656, EPI_ISL_763664, EPI_ISL_763674, EPI_ISL_763686	Wales Specialist Virology Centre Sequencing lab: Pathogen Genomics Unit	COVID-19 Genomics UK (COG-UK) Consortium	Catherine Moore, Johnathan Evans, Laura Gifford, Malorie Perry, Simon Cottrell, Angela Marchbank, Alec Birchley, Alexander Adams, Amy Gaskin, Bree Gatica-Wilcox, Jason Coombes, Joel Southgate, Lauren Gilbert, Lee Graham, Nicole Pacchiarini, Sara Kumziene-Summerhayes, Sarah Taylor, Sophie Jones, Sara Rey, Matthew Bull, Joanne Watkins, Sally Corden, Tom Connor
EPI_ISL_763696, EPI_ISL_763701, EPI_ISL_763703, EPI_ISL_763704, EPI_ISL_763705, EPI_ISL_763706, EPI_ISL_763707, EPI_ISL_763708	University of Exeter	COVID-19 Genomics UK (COG-UK) Consortium	Ben Temperton,Aaron Jeffries,Michelle Michelsen,Joanna Warwick-Dugdale,Audrey Farbos,Robyn Manley,Stephen Michell,Jane Masoli
EPI_ISL_763732	Virology Department, Royal Infirmary of Edinburgh, NHS Lothian / School of Biological Sciences, University of Edinburgh / Institute of Genetics and Molecular Medicine, University of Edinburgh	COVID-19 Genomics UK (COG-UK) Consortium	McHugh M, Dewar R, Rooke S, Gallagher M, Balcaza C, O'Toole Á, Scher E, Hill V, McCrone JT, Colquhoun R, Yu X, Jackson B, Rambaut A, Williams TC, Templeton K
EPI_ISL_763738, EPI_ISL_763747, EPI_ISL_763768, EPI_ISL_763771, EPI_ISL_763776, EPI_ISL_763786, EPI_ISL_763787	Wales Specialist Virology Centre Sequencing lab: Pathogen Genomics Unit	COVID-19 Genomics UK (COG-UK) Consortium	Catherine Moore, Johnathan Evans, Laura Gifford, Malorie Perry, Simon Cottrell, Angela Marchbank, Alec Birchley, Alexander Adams, Amy Gaskin, Bree Gatica-Wilcox, Jason Coombes, Joel Southgate, Lauren Gilbert, Lee Graham, Nicole Pacchiarini, Sara Kumziene-Summerhayes, Sarah Taylor, Sophie Jones, Sara Rey, Matthew Bull, Joanne Watkins, Sally Corden, Tom Connor
EPI_ISL_763796	Department of Pathology, University of Cambridge	COVID-19 Genomics UK (COG-UK) Consortium	Aminu S. Jahun, Yasmin Chaudhry, Grant Hall, Iliana Georgana, Myra Hosmillo, Martin D. Curran, Malte Pinckert, Surendra Parmar, Ian Goodfellow
EPI_ISL_763820	Wales Specialist Virology Centre Sequencing lab: Pathogen Genomics Unit	COVID-19 Genomics UK (COG-UK) Consortium	Catherine Moore, Johnathan Evans, Laura Gifford, Malorie Perry, Simon Cottrell, Angela Marchbank, Alec Birchley, Alexander Adams, Amy Gaskin, Bree Gatica-Wilcox, Jason Coombes, Joel Southgate, Lauren Gilbert, Lee Graham, Nicole Pacchiarini, Sara Kumziene-Summerhayes, Sarah Taylor, Sophie Jones, Sara Rey, Matthew Bull, Joanne Watkins, Sally Corden, Tom Connor
EPI_ISL_763841, EPI_ISL_763859, EPI_ISL_763861	Oxford Viromics, NDM, University of Oxford; Oxford University Hospitals; Basingstoke and North Hampshire Hospital	COVID-19 Genomics UK (COG-UK) Consortium	Tanya Golubchik, David Bonsall, George Macintyre, Amy Trebes, Mariateresa de Cesare, Catrin Moore, Alex Mobbs, Anita Justice, Robert Shaw, Monique Andersson, Timothy Peto, Emma Wise, Nathan Moore, Jessica Lynch, Nick Cortes, Matilde Mori, Stephen Kidd, David Buck, John Todd, Christophe Fraser
EPI_ISL_763907	University of Exeter	COVID-19 Genomics UK (COG-UK) Consortium	Ben Temperton,Aaron Jeffries,Michelle Michelsen,Joanna Warwick-Dugdale,Audrey Farbos,Robyn Manley,Stephen Michell,Jane Masoli
EPI_ISL_763932, EPI_ISL_763933, EPI_ISL_763942, EPI_ISL_763946, EPI_ISL_763947, EPI_ISL_763948	Oxford Viromics, NDM, University of Oxford; Oxford University Hospitals; Basingstoke and North Hampshire Hospital	COVID-19 Genomics UK (COG-UK) Consortium	Tanya Golubchik, David Bonsall, George Macintyre, Amy Trebes, Mariateresa de Cesare, Catrin Moore, Alex Mobbs, Anita Justice, Robert Shaw, Monique Andersson, Timothy Peto, Emma Wise, Nathan Moore, Jessica Lynch, Nick Cortes, Matilde Mori, Stephen Kidd, David Buck, John Todd, Christophe Fraser
EPI_ISL_763976, EPI_ISL_763977, EPI_ISL_763978, EPI_ISL_763979, EPI_ISL_763980, EPI_ISL_763981, EPI_ISL_763982, EPI_ISL_763989, EPI_ISL_764035	Wales Specialist Virology Centre Sequencing lab: Pathogen Genomics Unit	COVID-19 Genomics UK (COG-UK) Consortium	Catherine Moore, Johnathan Evans, Laura Gifford, Malorie Perry, Simon Cottrell, Angela Marchbank, Alec Birchley, Alexander Adams, Amy Gaskin, Bree Gatica-Wilcox, Jason Coombes, Joel Southgate, Lauren Gilbert, Lee Graham, Nicole Pacchiarini, Sara Kumziene-Summerhayes, Sarah Taylor, Sophie Jones, Sara Rey, Matthew Bull, Joanne Watkins, Sally Corden, Tom Connor
EPI_ISL_764069	Virology Department, Royal Infirmary of Edinburgh, NHS Lothian / School of Biological Sciences, University of Edinburgh / Institute of Genetics and Molecular Medicine, University of Edinburgh	COVID-19 Genomics UK (COG-UK) Consortium	McHugh M, Dewar R, Rooke S, Gallagher M, Balcaza C, O'Toole Á, Scher E, Hill V, McCrone JT, Colquhoun R, Yu X, Jackson B, Rambaut A, Williams TC, Templeton K
EPI_ISL_764077, EPI_ISL_764078, EPI_ISL_764079, EPI_ISL_764086, EPI_ISL_764088, EPI_ISL_764117	Oxford Viromics, NDM, University of Oxford; Oxford University Hospitals; Basingstoke and North Hampshire Hospital	COVID-19 Genomics UK (COG-UK) Consortium	Tanya Golubchik, David Bonsall, George Macintyre, Amy Trebes, Mariateresa de Cesare, Catrin Moore, Alex Mobbs, Anita Justice, Robert Shaw, Monique Andersson, Timothy Peto, Emma Wise, Nathan Moore, Jessica Lynch, Nick Cortes, Matilde Mori, Stephen Kidd, David Buck, John Todd, Christophe Fraser
EPI_ISL_764132, EPI_ISL_764134, EPI_ISL_764135, EPI_ISL_764137	University of Exeter	COVID-19 Genomics UK (COG-UK) Consortium	Ben Temperton,Aaron Jeffries,Michelle Michelsen,Joanna Warwick-Dugdale,Audrey Farbos,Robyn Manley,Stephen Michell,Jane Masoli
EPI_ISL_764147	Virology Department, Royal Infirmary of Edinburgh, NHS Lothian / School of Biological Sciences, University of Edinburgh / Institute of Genetics and Molecular Medicine, University of Edinburgh	COVID-19 Genomics UK (COG-UK) Consortium	McHugh M, Dewar R, Rooke S, Gallagher M, Balcaza C, O'Toole Á, Scher E, Hill V, McCrone JT, Colquhoun R, Yu X, Jackson B, Rambaut A, Williams TC, Templeton K
EPI_ISL_764150, EPI_ISL_764156	Wales Specialist Virology Centre Sequencing lab: Pathogen Genomics Unit	COVID-19 Genomics UK (COG-UK) Consortium	Catherine Moore, Johnathan Evans, Laura Gifford, Malorie Perry, Simon Cottrell, Angela Marchbank, Alec Birchley, Alexander Adams, Amy Gaskin, Bree Gatica-Wilcox, Jason Coombes, Joel Southgate, Lauren Gilbert, Lee Graham, Nicole Pacchiarini, Sara Kumziene-Summerhayes, Sarah Taylor, Sophie Jones, Sara Rey, Matthew Bull, Joanne Watkins, Sally Corden, Tom Connor
EPI_ISL_764165, EPI_ISL_764170, EPI_ISL_764178, EPI_ISL_764182	University of Exeter	COVID-19 Genomics UK (COG-UK) Consortium	Ben Temperton,Aaron Jeffries,Michelle Michelsen,Joanna Warwick-Dugdale,Audrey Farbos,Robyn Manley,Stephen Michell,Jane Masoli
EPI_ISL_764186	Wales Specialist Virology Centre Sequencing lab: Pathogen Genomics Unit	COVID-19 Genomics UK (COG-UK) Consortium	Catherine Moore, Johnathan Evans, Laura Gifford, Malorie Perry, Simon Cottrell, Angela Marchbank, Alec Birchley, Alexander Adams, Amy Gaskin, Bree Gatica-Wilcox, Jason Coombes, Joel Southgate, Lauren Gilbert, Lee Graham, Nicole Pacchiarini, Sara Kumziene-Summerhayes, Sarah Taylor, Sophie Jones, Sara Rey, Matthew Bull, Joanne Watkins, Sally Corden, Tom Connor
EPI_ISL_764198	Virology Department, Royal Infirmary of Edinburgh, NHS Lothian / School of Biological Sciences, University of Edinburgh / Institute of Genetics and Molecular Medicine, University of Edinburgh	COVID-19 Genomics UK (COG-UK) Consortium	McHugh M, Dewar R, Rooke S, Gallagher M, Balcaza C, O'Toole Á, Scher E, Hill V, McCrone JT, Colquhoun R, Yu X, Jackson B, Rambaut A, Williams TC, Templeton K
EPI_ISL_764203	Wales Specialist Virology Centre Sequencing lab: Pathogen Genomics Unit	COVID-19 Genomics UK (COG-UK) Consortium	Catherine Moore, Johnathan Evans, Laura Gifford, Malorie Perry, Simon Cottrell, Angela Marchbank, Alec Birchley, Alexander Adams, Amy Gaskin, Bree Gatica-Wilcox, Jason Coombes, Joel Southgate, Lauren Gilbert, Lee Graham, Nicole Pacchiarini, Sara Kumziene-Summerhayes, Sarah Taylor, Sophie Jones, Sara Rey, Matthew Bull, Joanne Watkins, Sally Corden, Tom Connor
EPI_ISL_764224	University of Exeter	COVID-19 Genomics UK (COG-UK) Consortium	Ben Temperton,Aaron Jeffries,Michelle Michelsen,Joanna Warwick-Dugdale,Audrey Farbos,Robyn Manley,Stephen Michell,Jane Masoli
EPI_ISL_764229, EPI_ISL_764232, EPI_ISL_764234, EPI_ISL_764266	Wales Specialist Virology Centre Sequencing lab: Pathogen Genomics Unit	COVID-19 Genomics UK (COG-UK) Consortium	Catherine Moore, Johnathan Evans, Laura Gifford, Malorie Perry, Simon Cottrell, Angela Marchbank, Alec Birchley, Alexander Adams, Amy Gaskin, Bree Gatica-Wilcox, Jason Coombes, Joel Southgate, Lauren Gilbert, Lee Graham, Nicole Pacchiarini, Sara Kumziene-Summerhayes, Sarah Taylor, Sophie Jones, Sara Rey, Matthew Bull, Joanne Watkins, Sally Corden, Tom Connor
EPI_ISL_764271	Oxford Viromics, NDM, University of Oxford; Oxford University Hospitals; Basingstoke and North Hampshire Hospital	COVID-19 Genomics UK (COG-UK) Consortium	Tanya Golubchik, David Bonsall, George Macintyre, Amy Trebes, Mariateresa de Cesare, Catrin Moore, Alex Mobbs, Anita Justice, Robert Shaw, Monique Andersson, Timothy Peto, Emma Wise, Nathan Moore, Jessica Lynch, Nick Cortes, Matilde Mori, Stephen Kidd, David Buck, John Todd, Christophe Fraser
EPI_ISL_764276, EPI_ISL_764329, EPI_ISL_764330, EPI_ISL_764332, EPI_ISL_764333, EPI_ISL_764334	Department of Pathology, University of Cambridge	COVID-19 Genomics UK (COG-UK) Consortium	Aminu S. Jahun, Yasmin Chaudhry, Grant Hall, Iliana Georgana, Myra Hosmillo, Martin D. Curran, Malte Pinckert, Surendra Parmar, Ian Goodfellow
EPI_ISL_764359	Virology Department, Royal Infirmary of Edinburgh, NHS Lothian / School of Biological Sciences, University of Edinburgh / Institute of Genetics and Molecular Medicine, University of Edinburgh	COVID-19 Genomics UK (COG-UK) Consortium	McHugh M, Dewar R, Rooke S, Gallagher M, Balcaza C, O'Toole Á, Scher E, Hill V, McCrone JT, Colquhoun R, Yu X, Jackson B, Rambaut A, Williams TC, Templeton K
EPI_ISL_764373	University of Exeter	COVID-19 Genomics UK (COG-UK) Consortium	Ben Temperton,Aaron Jeffries,Michelle Michelsen,Joanna Warwick-Dugdale,Audrey Farbos,Robyn Manley,Stephen Michell,Jane Masoli

EPI_ISL_764534, EPI_ISL_764540, EPI_ISL_764541, EPI_ISL_764542, EPI_ISL_764547	Oxford Viromics, NDM, University of Oxford; Oxford University Hospitals; Basingstoke and North Hampshire Hospital	COVID-19 Genomics UK (COG-UK) Consortium	Tanya Golubchik, David Bonsall, George Macintyre, Amy Trebes, Mariateresa de Cesare, Catrin Moore, Alex Mobbs, Anita Justice, Robert Shaw, Monique Andersson, Timothy Peto, Emma Wise, Nathan Moore, Jessica Lynch, Nick Cortes, Matilde Mori, Stephen Kidd, David Buck, John Todd, Christophe Fraser
EPI_ISL_764573, EPI_ISL_764576, EPI_ISL_764581, EPI_ISL_764585, EPI_ISL_764599, EPI_ISL_764600, EPI_ISL_764601, EPI_ISL_764602, EPI_ISL_764603, EPI_ISL_764604, EPI_ISL_764605, EPI_ISL_764606, EPI_ISL_764607, EPI_ISL_764608, EPI_ISL_764609, EPI_ISL_764610, EPI_ISL_764611, EPI_ISL_764612, EPI_ISL_764613, EPI_ISL_764614, EPI_ISL_764615, EPI_ISL_764616, EPI_ISL_764617, EPI_ISL_764618, EPI_ISL_764619, EPI_ISL_764620, EPI_ISL_764621, EPI_ISL_764622, EPI_ISL_764623, EPI_ISL_764625, EPI_ISL_764626, EPI_ISL_764627, EPI_ISL_764628, EPI_ISL_764629, EPI_ISL_764630, EPI_ISL_764631, EPI_ISL_764632, EPI_ISL_764633, EPI_ISL_764634, EPI_ISL_764635, EPI_ISL_764636, EPI_ISL_764637, EPI_ISL_764638, EPI_ISL_764639, EPI_ISL_764640, EPI_ISL_764641, EPI_ISL_764642, EPI_ISL_764643, EPI_ISL_764644, EPI_ISL_764645, EPI_ISL_764646, EPI_ISL_764647, EPI_ISL_764648, EPI_ISL_764649, EPI_ISL_764650, EPI_ISL_764651, EPI_ISL_764652, EPI_ISL_764739, EPI_ISL_764740, EPI_ISL_764805, EPI_ISL_764806, EPI_ISL_764807, EPI_ISL_764808, EPI_ISL_764809, EPI_ISL_764810, EPI_ISL_764811, EPI_ISL_764812, EPI_ISL_764813, EPI_ISL_764814, EPI_ISL_764815, EPI_ISL_764819, EPI_ISL_764820, EPI_ISL_764822, EPI_ISL_764823, EPI_ISL_764825, EPI_ISL_764952, EPI_ISL_764953, EPI_ISL_764954, EPI_ISL_764955, EPI_ISL_764956, EPI_ISL_764957, EPI_ISL_764958, EPI_ISL_764959, EPI_ISL_764960, EPI_ISL_764961, EPI_ISL_764962, EPI_ISL_764963, EPI_ISL_764964, EPI_ISL_764969			
see above	Wales Specialist Virology Centre Sequencing lab: Pathogen Genomics Unit	COVID-19 Genomics UK (COG-UK) Consortium	Catherine Moore, Johnathan Evans, Laura Gifford, Malorie Perry, Simon Cottrell, Angela Marchbank, Alec Birchley, Alexander Adams, Amy Gaskin, Bree Gatica-Wilcox, Jason Coombes, Joel Southgate, Lauren Gilbert, Lee Graham, Nicole Pacchiarini, Sara Kumziene-Summerhayes, Sarah Taylor, Sophie Jones, Sara Rey, Matthew Bull, Joanne Watkins, Sally Corden, Tom Connor
EPI_ISL_765517, EPI_ISL_765520	SARATOGA HOSPITAL LABORATORY	Wadsworth Center, New York State Department.of Health	Kirsten St. George, Daryl M. Lamson, Alexis Russel, Matthew Shudt, Melissa A Leisner, Jonathan Plitnick, Navjot Singh, John Kelly, Sara Griesemer, Erasmus Schneider, Erica Lasek-Nesselquist
EPI_ISL_765895	Unit 17: Influenza & Other Respiratory Viruses, German National Influenza Center	Project group Epidemiology of Highly Pathogenic Microorganisms, Robert Koch-Institute	Ariane Düx, Andreas Sachse, Grit Schubert, Sébastien Calvignac-Spencer, Fabian Leendertz, Thorsten Wolff, Ralf Dürrwald, Djin-Ye Oh, Marianne Wedde
EPI_ISL_766070, EPI_ISL_766079, EPI_ISL_766080, EPI_ISL_766106, EPI_ISL_766107, EPI_ISL_766109, EPI_ISL_766117, EPI_ISL_766118, EPI_ISL_766119, EPI_ISL_766120, EPI_ISL_766127, EPI_ISL_766128, EPI_ISL_766131, EPI_ISL_766133, EPI_ISL_766134, EPI_ISL_766144, EPI_ISL_766158, EPI_ISL_766225, EPI_ISL_766226, EPI_ISL_766230, EPI_ISL_766238, EPI_ISL_766248, EPI_ISL_766249, EPI_ISL_766250, EPI_ISL_766260, EPI_ISL_766261, EPI_ISL_766288, EPI_ISL_766290, EPI_ISL_766293, EPI_ISL_766294, EPI_ISL_766295, EPI_ISL_766296, EPI_ISL_766297, EPI_ISL_766298, EPI_ISL_766299, EPI_ISL_766300, EPI_ISL_766302, EPI_ISL_766304, EPI_ISL_766305, EPI_ISL_766306, EPI_ISL_766307, EPI_ISL_766308, EPI_ISL_766309, EPI_ISL_766311, EPI_ISL_766312, EPI_ISL_766313, EPI_ISL_766315, EPI_ISL_766395, EPI_ISL_766396, EPI_ISL_766399, EPI_ISL_766405, EPI_ISL_766409, EPI_ISL_766425, EPI_ISL_766463, EPI_ISL_766508, EPI_ISL_766562, EPI_ISL_766563			
see above	Respiratory Virus Unit, National Infection Service, Public Health England	COVID-19 Genomics UK (COG-UK) Consortium	PHE Covid Sequencing Team
EPI_ISL_766608	Klinisk mikrobiologi	The Public Health Agency of Sweden	Department of Microbiology, The Public Health Agency of Sweden
EPI_ISL_766632	Klinisk mikrobiologi, Viruslab	The Public Health Agency of Sweden	Department of Microbiology, The Public Health Agency of Sweden
EPI_ISL_766675	Texas Department of State Health Services	Texas Department of State Health Services	Rashmi Tuladhar, Bonnie Oh, Jenny Zhang, Maliha Rahman, Anita Pokharel, Myong Koag, Chung Wang, Rachel Lee, Grace Kubin, Mayela Pedrueza, James Daniel Bonser
EPI_ISL_767121, EPI_ISL_767123, EPI_ISL_767125, EPI_ISL_767128, EPI_ISL_767130, EPI_ISL_767132, EPI_ISL_767133, EPI_ISL_767135, EPI_ISL_767136, EPI_ISL_767137	Lighthouse Lab in Alderley Park	Wellcome Sanger Institute for the COVID-19 Genomics UK (COG-UK) Consortium	Jacquelyn Wynn, Mairead Hyland, The Lighthouse Lab in Alderley Park and Alex Alderton, Roberto Amato, Sonia Goncalves, Ewan Harrison, David K. Jackson, Ian Johnston, Dominic Kwiatkowski, Cordelia Langford, John Sillitoe on behalf of the Wellcome Sanger Institute COVID-19 Surveillance Team
EPI_ISL_767138	Lighthouse Lab in Cambridge	Wellcome Sanger Institute for the COVID-19 Genomics UK (COG-UK) Consortium	Rob Howes, The Lighthouse Lab in Cambridge and Alex Alderton, Roberto Amato, Sonia Goncalves, Ewan Harrison, David K. Jackson, Ian Johnston, Dominic Kwiatkowski, Cordelia Langford, John Sillitoe on behalf of the Wellcome Sanger Institute COVID-19 Surveillance Team
EPI_ISL_767139, EPI_ISL_767140, EPI_ISL_767141, EPI_ISL_767142, EPI_ISL_767143, EPI_ISL_767144, EPI_ISL_767145, EPI_ISL_767146, EPI_ISL_767147, EPI_ISL_767148, EPI_ISL_767149, EPI_ISL_767150, EPI_ISL_767178, EPI_ISL_767216, EPI_ISL_767217, EPI_ISL_767218, EPI_ISL_767219, EPI_ISL_767220, EPI_ISL_767222, EPI_ISL_767223, EPI_ISL_767224, EPI_ISL_767225, EPI_ISL_767226, EPI_ISL_767227, EPI_ISL_767228, EPI_ISL_767229, EPI_ISL_767231, EPI_ISL_767232, EPI_ISL_767233, EPI_ISL_767234, EPI_ISL_767235, EPI_ISL_767236, EPI_ISL_767237, EPI_ISL_767238, EPI_ISL_767277, EPI_ISL_767279, EPI_ISL_767280, EPI_ISL_767281, EPI_ISL_767282, EPI_ISL_767283, EPI_ISL_767285, EPI_ISL_767286, EPI_ISL_767287, EPI_ISL_767288, EPI_ISL_767289, EPI_ISL_767290			
see above	Lighthouse Lab in Alderley Park	Wellcome Sanger Institute for the COVID-19 Genomics UK (COG-UK) Consortium	Jacquelyn Wynn, Mairead Hyland, The Lighthouse Lab in Alderley Park and Alex Alderton, Roberto Amato, Sonia Goncalves, Ewan Harrison, David K. Jackson, Ian Johnston, Dominic Kwiatkowski, Cordelia Langford, John Sillitoe on behalf of the Wellcome Sanger Institute COVID-19 Surveillance Team
EPI_ISL_767352, EPI_ISL_767355	Michigan Department of Health and Human Services, Bureau of Laboratories	Michigan Department of Health and Human Services, Bureau of Laboratories	Blankenship HM, Riner D, Soehnlen MK
EPI_ISL_767452	Wadsworth Center, New York State Department.of Health	Wadsworth Center, New York State Department.of Health	Kirsten St. George, Daryl M. Lamson, Alexis Russel, Matthew Shudt, Melissa A Leisner, Jonathan Plitnick, Navjot Singh, John Kelly, Sara Griesemer, Erasmus Schneider, Erica Lasek-Nesselquist
EPI_ISL_768419, EPI_ISL_768420, EPI_ISL_768421, EPI_ISL_768422, EPI_ISL_768423, EPI_ISL_768424, EPI_ISL_768425, EPI_ISL_768426, EPI_ISL_768427, EPI_ISL_768428, EPI_ISL_768429, EPI_ISL_768430, EPI_ISL_768431, EPI_ISL_768432, EPI_ISL_768433, EPI_ISL_768434, EPI_ISL_768435, EPI_ISL_768436, EPI_ISL_768437, EPI_ISL_768438, EPI_ISL_768439, EPI_ISL_768440, EPI_ISL_768441, EPI_ISL_768442, EPI_ISL_768443, EPI_ISL_768444, EPI_ISL_768445, EPI_ISL_768446, EPI_ISL_768447, EPI_ISL_768448, EPI_ISL_768449, EPI_ISL_768450, EPI_ISL_768451, EPI_ISL_768452, EPI_ISL_768453, EPI_ISL_768454, EPI_ISL_768455, EPI_ISL_768456, EPI_ISL_768457, EPI_ISL_768458, EPI_ISL_768459, EPI_ISL_768460, EPI_ISL_768461, EPI_ISL_768462, EPI_ISL_768463, EPI_ISL_768464, EPI_ISL_768465, EPI_ISL_768466, EPI_ISL_768467, EPI_ISL_768468, EPI_ISL_768469, EPI_ISL_768470, EPI_ISL_768471, EPI_ISL_768472, EPI_ISL_768473, EPI_ISL_768474			
see above	LSUHS Emerging Viral Threat Laboratory	Microbial Genome Sequencing Center	Jeremy P. Kamil, Jennifer L. Carroll, Camille F. Abshire, Maarten Van Diest, Andrew D. Yurochko, Martin J. Sapp, Rona S. Scott, Christopher G. Kevil, Daniel J. Snyder, Vaughn S. Cooper, John A. Vanchiere
EPI_ISL_768804, EPI_ISL_768805, EPI_ISL_768806, EPI_ISL_768807, EPI_ISL_768808, EPI_ISL_768809, EPI_ISL_768810, EPI_ISL_768811, EPI_ISL_768812, EPI_ISL_768813, EPI_ISL_768814			
see above	Respiratory Virus Unit, National Infection Service, Public Health England	COVID-19 Genomics UK (COG-UK) Consortium	PHE Covid Sequencing Team
EPI_ISL_769226, EPI_ISL_769227, EPI_ISL_769228, EPI_ISL_769230, EPI_ISL_769231, EPI_ISL_769232, EPI_ISL_769234, EPI_ISL_769235, EPI_ISL_769237, EPI_ISL_769238, EPI_ISL_769239, EPI_ISL_769240, EPI_ISL_769241, EPI_ISL_769242, EPI_ISL_769247, EPI_ISL_769248, EPI_ISL_769251, EPI_ISL_769253, EPI_ISL_769256, EPI_ISL_769258, EPI_ISL_769262, EPI_ISL_769263, EPI_ISL_769264, EPI_ISL_769265, EPI_ISL_769268, EPI_ISL_769269, EPI_ISL_769270, EPI_ISL_769271, EPI_ISL_769272, EPI_ISL_769274, EPI_ISL_769277, EPI_ISL_769278, EPI_ISL_769279, EPI_ISL_769283, EPI_ISL_769285, EPI_ISL_769287, EPI_ISL_769292, EPI_ISL_769293, EPI_ISL_769295, EPI_ISL_769296, EPI_ISL_769298, EPI_ISL_769299, EPI_ISL_769300, EPI_ISL_769301, EPI_ISL_769302, EPI_ISL_769303, EPI_ISL_769304, EPI_ISL_769305, EPI_ISL_769307, EPI_ISL_769309, EPI_ISL_769311, EPI_ISL_769313, EPI_ISL_769315, EPI_ISL_769317, EPI_ISL_769318, EPI_ISL_769324, EPI_ISL_769326, EPI_ISL_769328, EPI_ISL_769331, EPI_ISL_769332, EPI_ISL_769333, EPI_ISL_769336, EPI_ISL_769337, EPI_ISL_769338, EPI_ISL_769339, EPI_ISL_769340, EPI_ISL_769342, EPI_ISL_769344, EPI_ISL_769346, EPI_ISL_769347, EPI_ISL_769350, EPI_ISL_769351, EPI_ISL_769354, EPI_ISL_769356, EPI_ISL_769357, EPI_ISL_769358, EPI_ISL_769359, EPI_ISL_769362, EPI_ISL_769363, EPI_ISL_769364, EPI_ISL_769366, EPI_ISL_769368, EPI_ISL_769369, EPI_ISL_769371, EPI_ISL_769372, EPI_ISL_769373, EPI_ISL_769375, EPI_ISL_769376, EPI_ISL_769377, EPI_ISL_769378, EPI_ISL_769379, EPI_ISL_769382, EPI_ISL_769386, EPI_ISL_769393, EPI_ISL_769395, EPI_ISL_769397, EPI_ISL_769398, EPI_ISL_769400, EPI_ISL_769403, EPI_ISL_769407, EPI_ISL_769408, EPI_ISL_769409, EPI_ISL_769410, EPI_ISL_769411, EPI_ISL_769414, EPI_ISL_769415, EPI_ISL_769423, EPI_ISL_769424, EPI_ISL_769425, EPI_ISL_769426, EPI_ISL_769429, EPI_ISL_769432, EPI_ISL_769433, EPI_ISL_769435, EPI_ISL_769436, EPI_ISL_769437, EPI_ISL_769438, EPI_ISL_769439, EPI_ISL_769443, EPI_ISL_769444, EPI_ISL_769447, EPI_ISL_769448, EPI_ISL_769451, EPI_ISL_769453, EPI_ISL_769455, EPI_ISL_769456, EPI_ISL_769461, EPI_ISL_769463, EPI_ISL_769464, EPI_ISL_769465, EPI_ISL_769467, EPI_ISL_769472, EPI_ISL_769477, EPI_ISL_769478, EPI_ISL_769479, EPI_ISL_769480, EPI_ISL_769483, EPI_ISL_769484, EPI_ISL_769486, EPI_ISL_769487, EPI_ISL_769488, EPI_ISL_769490, EPI_ISL_769491, EPI_ISL_769492, EPI_ISL_769493, EPI_ISL_769494, EPI_ISL_769495, EPI_ISL_769496, EPI_ISL_769498, EPI_ISL_769505, EPI_ISL_769507, EPI_ISL_769508, EPI_ISL_769509, EPI_ISL_769510, EPI_ISL_769514, EPI_ISL_769515, EPI_ISL_769516, EPI_ISL_769520, EPI_ISL_769521, EPI_ISL_769523, EPI_ISL_769526, EPI_ISL_769527, EPI_ISL_769529, EPI_ISL_769531, EPI_ISL_769532, EPI_ISL_769534, EPI_ISL_769536, EPI_ISL_769537, EPI_ISL_769538, EPI_ISL_769541, EPI_ISL_769544, EPI_ISL_769546, EPI_ISL_769547, EPI_ISL_769548, EPI_ISL_769549, EPI_ISL_769550			
see above	Lighthouse Lab in Milton Keynes	Wellcome Sanger Institute for the COVID-19 Genomics UK (COG-UK) Consortium	The Lighthouse Lab in Milton Keynes and Alex Alderton, Roberto Amato, Sonia Goncalves, Ewan Harrison, David K. Jackson, Ian Johnston, Dominic Kwiatkowski, Cordelia Langford, John Sillitoe on behalf of the Wellcome Sanger Institute COVID-19 Surveillance Team
EPI_ISL_769551	Lighthouse Lab in Glasgow	Wellcome Sanger Institute for the COVID-19 Genomics UK (COG-UK) Consortium	Harper VanSteenhouse, Yumi Kasai, David Gray, Carol Clugston, Anna Dominiczak and Alex Alderton, Roberto Amato, Sonia Goncalves, Ewan Harrison, David K. Jackson, Ian Johnston, Dominic Kwiatkowski, Cordelia Langford, John Sillitoe on behalf of the Wellcome Sanger Institute COVID-19 Surveillance Team
EPI_ISL_769552, EPI_ISL_769553, EPI_ISL_769554, EPI_ISL_769555	Lighthouse Lab in Milton Keynes	Wellcome Sanger Institute for the COVID-19 Genomics UK (COG-UK) Consortium	The Lighthouse Lab in Milton Keynes and Alex Alderton, Roberto Amato, Sonia Goncalves, Ewan Harrison, David K. Jackson, Ian Johnston, Dominic Kwiatkowski, Cordelia Langford, John Sillitoe on behalf of the Wellcome Sanger Institute COVID-19 Surveillance Team
EPI_ISL_769556	Lighthouse Lab in Glasgow	Wellcome Sanger Institute for the COVID-19 Genomics UK (COG-UK) Consortium	Harper VanSteenhouse, Yumi Kasai, David Gray, Carol Clugston, Anna Dominiczak and Alex Alderton, Roberto Amato, Sonia Goncalves, Ewan Harrison, David K. Jackson, Ian Johnston, Dominic Kwiatkowski, Cordelia Langford, John Sillitoe on behalf of the Wellcome Sanger Institute COVID-19 Surveillance Team
EPI_ISL_769557, EPI_ISL_769558, EPI_ISL_769559, EPI_ISL_769560, EPI_ISL_769561, EPI_ISL_769562, EPI_ISL_769563, EPI_ISL_769564, EPI_ISL_769565, EPI_ISL_769566	Lighthouse Lab in Milton Keynes	Wellcome Sanger Institute for the COVID-19 Genomics UK (COG-UK) Consortium	The Lighthouse Lab in Milton Keynes and Alex Alderton, Roberto Amato, Sonia Goncalves, Ewan Harrison, David K. Jackson, Ian Johnston, Dominic Kwiatkowski, Cordelia Langford, John Sillitoe on behalf of the Wellcome Sanger Institute COVID-19 Surveillance Team

EPI_ISL_769567, EPI_ISL_769568	Lighthouse Lab in Glasgow	Wellcome Sanger Institute for the COVID-19 Genomics UK (COG-UK) Consortium	Harper VanSteenhouse, Yumi Kasai, David Gray, Carol Clugston, Anna Dominiczak and Alex Alderton, Roberto Amato, Sonia Goncalves, Ewan Harrison, David K. Jackson, Ian Johnston, Dominic Kwiatkowski, Cordelia Langford, John Sillitoe on behalf of the Wellcome Sanger Institute COVID-19 Surveillance Team
EPI_ISL_769569, EPI_ISL_769570, EPI_ISL_769571, EPI_ISL_769572, EPI_ISL_769573, EPI_ISL_769574, EPI_ISL_769575, EPI_ISL_769576, EPI_ISL_769578, EPI_ISL_769579, EPI_ISL_769580, EPI_ISL_769581, EPI_ISL_769582			
see above	Lighthouse Lab in Milton Keynes	Wellcome Sanger Institute for the COVID-19 Genomics UK (COG-UK) Consortium	The Lighthouse Lab in Milton Keynes and Alex Alderton, Roberto Amato, Sonia Goncalves, Ewan Harrison, David K. Jackson, Ian Johnston, Dominic Kwiatkowski, Cordelia Langford, John Sillitoe on behalf of the Wellcome Sanger Institute COVID-19 Surveillance Team
EPI_ISL_769583	Lighthouse Lab in Glasgow	Wellcome Sanger Institute for the COVID-19 Genomics UK (COG-UK) Consortium	Harper VanSteenhouse, Yumi Kasai, David Gray, Carol Clugston, Anna Dominiczak and Alex Alderton, Roberto Amato, Sonia Goncalves, Ewan Harrison, David K. Jackson, Ian Johnston, Dominic Kwiatkowski, Cordelia Langford, John Sillitoe on behalf of the Wellcome Sanger Institute COVID-19 Surveillance Team
EPI_ISL_769585, EPI_ISL_769586, EPI_ISL_769587, EPI_ISL_769588	Lighthouse Lab in Milton Keynes	Wellcome Sanger Institute for the COVID-19 Genomics UK (COG-UK) Consortium	The Lighthouse Lab in Milton Keynes and Alex Alderton, Roberto Amato, Sonia Goncalves, Ewan Harrison, David K. Jackson, Ian Johnston, Dominic Kwiatkowski, Cordelia Langford, John Sillitoe on behalf of the Wellcome Sanger Institute COVID-19 Surveillance Team
EPI_ISL_769589	Lighthouse Lab in Glasgow	Wellcome Sanger Institute for the COVID-19 Genomics UK (COG-UK) Consortium	Harper VanSteenhouse, Yumi Kasai, David Gray, Carol Clugston, Anna Dominiczak and Alex Alderton, Roberto Amato, Sonia Goncalves, Ewan Harrison, David K. Jackson, Ian Johnston, Dominic Kwiatkowski, Cordelia Langford, John Sillitoe on behalf of the Wellcome Sanger Institute COVID-19 Surveillance Team
EPI_ISL_769591, EPI_ISL_769592, EPI_ISL_769593, EPI_ISL_769595, EPI_ISL_769596, EPI_ISL_769598, EPI_ISL_769599, EPI_ISL_769600, EPI_ISL_769601, EPI_ISL_769602, EPI_ISL_769603, EPI_ISL_769604, EPI_ISL_769605			
see above	Lighthouse Lab in Milton Keynes	Wellcome Sanger Institute for the COVID-19 Genomics UK (COG-UK) Consortium	The Lighthouse Lab in Milton Keynes and Alex Alderton, Roberto Amato, Sonia Goncalves, Ewan Harrison, David K. Jackson, Ian Johnston, Dominic Kwiatkowski, Cordelia Langford, John Sillitoe on behalf of the Wellcome Sanger Institute COVID-19 Surveillance Team
EPI_ISL_769606, EPI_ISL_769607	Lighthouse Lab in Glasgow	Wellcome Sanger Institute for the COVID-19 Genomics UK (COG-UK) Consortium	Harper VanSteenhouse, Yumi Kasai, David Gray, Carol Clugston, Anna Dominiczak and Alex Alderton, Roberto Amato, Sonia Goncalves, Ewan Harrison, David K. Jackson, Ian Johnston, Dominic Kwiatkowski, Cordelia Langford, John Sillitoe on behalf of the Wellcome Sanger Institute COVID-19 Surveillance Team
EPI_ISL_769608	Lighthouse Lab in Milton Keynes	Wellcome Sanger Institute for the COVID-19 Genomics UK (COG-UK) Consortium	The Lighthouse Lab in Milton Keynes and Alex Alderton, Roberto Amato, Sonia Goncalves, Ewan Harrison, David K. Jackson, Ian Johnston, Dominic Kwiatkowski, Cordelia Langford, John Sillitoe on behalf of the Wellcome Sanger Institute COVID-19 Surveillance Team
EPI_ISL_769609	Lighthouse Lab in Glasgow	Wellcome Sanger Institute for the COVID-19 Genomics UK (COG-UK) Consortium	Harper VanSteenhouse, Yumi Kasai, David Gray, Carol Clugston, Anna Dominiczak and Alex Alderton, Roberto Amato, Sonia Goncalves, Ewan Harrison, David K. Jackson, Ian Johnston, Dominic Kwiatkowski, Cordelia Langford, John Sillitoe on behalf of the Wellcome Sanger Institute COVID-19 Surveillance Team
EPI_ISL_769610	Lighthouse Lab in Milton Keynes	Wellcome Sanger Institute for the COVID-19 Genomics UK (COG-UK) Consortium	The Lighthouse Lab in Milton Keynes and Alex Alderton, Roberto Amato, Sonia Goncalves, Ewan Harrison, David K. Jackson, Ian Johnston, Dominic Kwiatkowski, Cordelia Langford, John Sillitoe on behalf of the Wellcome Sanger Institute COVID-19 Surveillance Team
EPI_ISL_769611, EPI_ISL_769612, EPI_ISL_769613, EPI_ISL_769614	Lighthouse Lab in Glasgow	Wellcome Sanger Institute for the COVID-19 Genomics UK (COG-UK) Consortium	Harper VanSteenhouse, Yumi Kasai, David Gray, Carol Clugston, Anna Dominiczak and Alex Alderton, Roberto Amato, Sonia Goncalves, Ewan Harrison, David K. Jackson, Ian Johnston, Dominic Kwiatkowski, Cordelia Langford, John Sillitoe on behalf of the Wellcome Sanger Institute COVID-19 Surveillance Team
EPI_ISL_769615, EPI_ISL_769616	Lighthouse Lab in Milton Keynes	Wellcome Sanger Institute for the COVID-19 Genomics UK (COG-UK) Consortium	The Lighthouse Lab in Milton Keynes and Alex Alderton, Roberto Amato, Sonia Goncalves, Ewan Harrison, David K. Jackson, Ian Johnston, Dominic Kwiatkowski, Cordelia Langford, John Sillitoe on behalf of the Wellcome Sanger Institute COVID-19 Surveillance Team
EPI_ISL_769617	Lighthouse Lab in Glasgow	Wellcome Sanger Institute for the COVID-19 Genomics UK (COG-UK) Consortium	Harper VanSteenhouse, Yumi Kasai, David Gray, Carol Clugston, Anna Dominiczak and Alex Alderton, Roberto Amato, Sonia Goncalves, Ewan Harrison, David K. Jackson, Ian Johnston, Dominic Kwiatkowski, Cordelia Langford, John Sillitoe on behalf of the Wellcome Sanger Institute COVID-19 Surveillance Team
EPI_ISL_769618	Lighthouse Lab in Milton Keynes	Wellcome Sanger Institute for the COVID-19 Genomics UK (COG-UK) Consortium	The Lighthouse Lab in Milton Keynes and Alex Alderton, Roberto Amato, Sonia Goncalves, Ewan Harrison, David K. Jackson, Ian Johnston, Dominic Kwiatkowski, Cordelia Langford, John Sillitoe on behalf of the Wellcome Sanger Institute COVID-19 Surveillance Team
EPI_ISL_769619	Lighthouse Lab in Glasgow	Wellcome Sanger Institute for the COVID-19 Genomics UK (COG-UK) Consortium	Harper VanSteenhouse, Yumi Kasai, David Gray, Carol Clugston, Anna Dominiczak and Alex Alderton, Roberto Amato, Sonia Goncalves, Ewan Harrison, David K. Jackson, Ian Johnston, Dominic Kwiatkowski, Cordelia Langford, John Sillitoe on behalf of the Wellcome Sanger Institute COVID-19 Surveillance Team
EPI_ISL_769620, EPI_ISL_769621, EPI_ISL_769622, EPI_ISL_769623, EPI_ISL_769624	Lighthouse Lab in Milton Keynes	Wellcome Sanger Institute for the COVID-19 Genomics UK (COG-UK) Consortium	The Lighthouse Lab in Milton Keynes and Alex Alderton, Roberto Amato, Sonia Goncalves, Ewan Harrison, David K. Jackson, Ian Johnston, Dominic Kwiatkowski, Cordelia Langford, John Sillitoe on behalf of the Wellcome Sanger Institute COVID-19 Surveillance Team
EPI_ISL_769625	Lighthouse Lab in Glasgow	Wellcome Sanger Institute for the COVID-19 Genomics UK (COG-UK) Consortium	Harper VanSteenhouse, Yumi Kasai, David Gray, Carol Clugston, Anna Dominiczak and Alex Alderton, Roberto Amato, Sonia Goncalves, Ewan Harrison, David K. Jackson, Ian Johnston, Dominic Kwiatkowski, Cordelia Langford, John Sillitoe on behalf of the Wellcome Sanger Institute COVID-19 Surveillance Team
EPI_ISL_769626, EPI_ISL_769627, EPI_ISL_769628, EPI_ISL_769629, EPI_ISL_769630, EPI_ISL_769631, EPI_ISL_769632, EPI_ISL_769633, EPI_ISL_769634, EPI_ISL_769635, EPI_ISL_769636, EPI_ISL_769637, EPI_ISL_769638, EPI_ISL_769639, EPI_ISL_769640, EPI_ISL_769642, EPI_ISL_769643			
see above	Lighthouse Lab in Milton Keynes	Wellcome Sanger Institute for the COVID-19 Genomics UK (COG-UK) Consortium	The Lighthouse Lab in Milton Keynes and Alex Alderton, Roberto Amato, Sonia Goncalves, Ewan Harrison, David K. Jackson, Ian Johnston, Dominic Kwiatkowski, Cordelia Langford, John Sillitoe on behalf of the Wellcome Sanger Institute COVID-19 Surveillance Team
EPI_ISL_769644	Lighthouse Lab in Glasgow	Wellcome Sanger Institute for the COVID-19 Genomics UK (COG-UK) Consortium	Harper VanSteenhouse, Yumi Kasai, David Gray, Carol Clugston, Anna Dominiczak and Alex Alderton, Roberto Amato, Sonia Goncalves, Ewan Harrison, David K. Jackson, Ian Johnston, Dominic Kwiatkowski, Cordelia Langford, John Sillitoe on behalf of the Wellcome Sanger Institute COVID-19 Surveillance Team
EPI_ISL_769645, EPI_ISL_769647	Lighthouse Lab in Milton Keynes	Wellcome Sanger Institute for the COVID-19 Genomics UK (COG-UK) Consortium	The Lighthouse Lab in Milton Keynes and Alex Alderton, Roberto Amato, Sonia Goncalves, Ewan Harrison, David K. Jackson, Ian Johnston, Dominic Kwiatkowski, Cordelia Langford, John Sillitoe on behalf of the Wellcome Sanger Institute COVID-19 Surveillance Team
EPI_ISL_769648	Lighthouse Lab in Glasgow	Wellcome Sanger Institute for the COVID-19 Genomics UK (COG-UK) Consortium	Harper VanSteenhouse, Yumi Kasai, David Gray, Carol Clugston, Anna Dominiczak and Alex Alderton, Roberto Amato, Sonia Goncalves, Ewan Harrison, David K. Jackson, Ian Johnston, Dominic Kwiatkowski, Cordelia Langford, John Sillitoe on behalf of the Wellcome Sanger Institute COVID-19 Surveillance Team
EPI_ISL_769649, EPI_ISL_769650, EPI_ISL_769651	Lighthouse Lab in Milton Keynes	Wellcome Sanger Institute for the COVID-19 Genomics UK (COG-UK) Consortium	The Lighthouse Lab in Milton Keynes and Alex Alderton, Roberto Amato, Sonia Goncalves, Ewan Harrison, David K. Jackson, Ian Johnston, Dominic Kwiatkowski, Cordelia Langford, John Sillitoe on behalf of the Wellcome Sanger Institute COVID-19 Surveillance Team
EPI_ISL_769652	Lighthouse Lab in Glasgow	Wellcome Sanger Institute for the COVID-19 Genomics UK (COG-UK) Consortium	Harper VanSteenhouse, Yumi Kasai, David Gray, Carol Clugston, Anna Dominiczak and Alex Alderton, Roberto Amato, Sonia Goncalves, Ewan Harrison, David K. Jackson, Ian Johnston, Dominic Kwiatkowski, Cordelia Langford, John Sillitoe on behalf of the Wellcome Sanger Institute COVID-19 Surveillance Team
EPI_ISL_769653, EPI_ISL_769654, EPI_ISL_769656, EPI_ISL_769657, EPI_ISL_769658, EPI_ISL_769659, EPI_ISL_769660	Lighthouse Lab in Milton Keynes	Wellcome Sanger Institute for the COVID-19 Genomics UK (COG-UK) Consortium	The Lighthouse Lab in Milton Keynes and Alex Alderton, Roberto Amato, Sonia Goncalves, Ewan Harrison, David K. Jackson, Ian Johnston, Dominic Kwiatkowski, Cordelia Langford, John Sillitoe on behalf of the Wellcome Sanger Institute COVID-19 Surveillance Team
EPI_ISL_769662	Lighthouse Lab in Glasgow	Wellcome Sanger Institute for the COVID-19 Genomics UK (COG-UK) Consortium	Harper VanSteenhouse, Yumi Kasai, David Gray, Carol Clugston, Anna Dominiczak and Alex Alderton, Roberto Amato, Sonia Goncalves, Ewan Harrison, David K. Jackson, Ian Johnston, Dominic Kwiatkowski, Cordelia Langford, John Sillitoe on behalf of the Wellcome Sanger Institute COVID-19 Surveillance Team
EPI_ISL_769663, EPI_ISL_769664, EPI_ISL_769665	Lighthouse Lab in Milton Keynes	Wellcome Sanger Institute for the COVID-19 Genomics UK (COG-UK) Consortium	The Lighthouse Lab in Milton Keynes and Alex Alderton, Roberto Amato, Sonia Goncalves, Ewan Harrison, David K. Jackson, Ian Johnston, Dominic Kwiatkowski, Cordelia Langford, John Sillitoe on behalf of the Wellcome Sanger Institute COVID-19 Surveillance Team
EPI_ISL_769666, EPI_ISL_769667	Lighthouse Lab in Glasgow	Wellcome Sanger Institute for the COVID-19 Genomics UK (COG-UK) Consortium	Harper VanSteenhouse, Yumi Kasai, David Gray, Carol Clugston, Anna Dominiczak and Alex Alderton, Roberto Amato, Sonia Goncalves, Ewan Harrison, David K. Jackson, Ian Johnston, Dominic Kwiatkowski, Cordelia Langford, John Sillitoe on behalf of the Wellcome Sanger Institute COVID-19 Surveillance

			Team
EPI_ISL_769668, EPI_ISL_769669, EPI_ISL_769670, EPI_ISL_769671, EPI_ISL_769672, EPI_ISL_769673, EPI_ISL_769674, EPI_ISL_769675, EPI_ISL_769676	Lighthouse Lab in Milton Keynes	Wellcome Sanger Institute for the COVID-19 Genomics UK (COG-UK) Consortium	The Lighthouse Lab in Milton Keynes and Alex Alderton, Roberto Amato, Sonia Goncalves, Ewan Harrison, David K. Jackson, Ian Johnston, Dominic Kwiatkowski, Cordelia Langford, John Sillitoe on behalf of the Wellcome Sanger Institute COVID-19 Surveillance Team
EPI_ISL_769677	Lighthouse Lab in Glasgow	Wellcome Sanger Institute for the COVID-19 Genomics UK (COG-UK) Consortium	Harper VanSteenhouse, Yumi Kasai, David Gray, Carol Clugston, Anna Dominiczak and Alex Alderton, Roberto Amato, Sonia Goncalves, Ewan Harrison, David K. Jackson, Ian Johnston, Dominic Kwiatkowski, Cordelia Langford, John Sillitoe on behalf of the Wellcome Sanger Institute COVID-19 Surveillance Team
EPI_ISL_769678, EPI_ISL_769679, EPI_ISL_769680, EPI_ISL_769681, EPI_ISL_769682, EPI_ISL_769683, EPI_ISL_769684, EPI_ISL_769685, EPI_ISL_769686	Lighthouse Lab in Milton Keynes	Wellcome Sanger Institute for the COVID-19 Genomics UK (COG-UK) Consortium	The Lighthouse Lab in Milton Keynes and Alex Alderton, Roberto Amato, Sonia Goncalves, Ewan Harrison, David K. Jackson, Ian Johnston, Dominic Kwiatkowski, Cordelia Langford, John Sillitoe on behalf of the Wellcome Sanger Institute COVID-19 Surveillance Team
EPI_ISL_769687	Lighthouse Lab in Glasgow	Wellcome Sanger Institute for the COVID-19 Genomics UK (COG-UK) Consortium	Harper VanSteenhouse, Yumi Kasai, David Gray, Carol Clugston, Anna Dominiczak and Alex Alderton, Roberto Amato, Sonia Goncalves, Ewan Harrison, David K. Jackson, Ian Johnston, Dominic Kwiatkowski, Cordelia Langford, John Sillitoe on behalf of the Wellcome Sanger Institute COVID-19 Surveillance Team
EPI_ISL_769688	Lighthouse Lab in Milton Keynes	Wellcome Sanger Institute for the COVID-19 Genomics UK (COG-UK) Consortium	The Lighthouse Lab in Milton Keynes and Alex Alderton, Roberto Amato, Sonia Goncalves, Ewan Harrison, David K. Jackson, Ian Johnston, Dominic Kwiatkowski, Cordelia Langford, John Sillitoe on behalf of the Wellcome Sanger Institute COVID-19 Surveillance Team
EPI_ISL_769689	Lighthouse Lab in Glasgow	Wellcome Sanger Institute for the COVID-19 Genomics UK (COG-UK) Consortium	Harper VanSteenhouse, Yumi Kasai, David Gray, Carol Clugston, Anna Dominiczak and Alex Alderton, Roberto Amato, Sonia Goncalves, Ewan Harrison, David K. Jackson, Ian Johnston, Dominic Kwiatkowski, Cordelia Langford, John Sillitoe on behalf of the Wellcome Sanger Institute COVID-19 Surveillance Team
EPI_ISL_769690	Lighthouse Lab in Milton Keynes	Wellcome Sanger Institute for the COVID-19 Genomics UK (COG-UK) Consortium	The Lighthouse Lab in Milton Keynes and Alex Alderton, Roberto Amato, Sonia Goncalves, Ewan Harrison, David K. Jackson, Ian Johnston, Dominic Kwiatkowski, Cordelia Langford, John Sillitoe on behalf of the Wellcome Sanger Institute COVID-19 Surveillance Team
EPI_ISL_769691	Lighthouse Lab in Glasgow	Wellcome Sanger Institute for the COVID-19 Genomics UK (COG-UK) Consortium	Harper VanSteenhouse, Yumi Kasai, David Gray, Carol Clugston, Anna Dominiczak and Alex Alderton, Roberto Amato, Sonia Goncalves, Ewan Harrison, David K. Jackson, Ian Johnston, Dominic Kwiatkowski, Cordelia Langford, John Sillitoe on behalf of the Wellcome Sanger Institute COVID-19 Surveillance Team
EPI_ISL_769692, EPI_ISL_769693, EPI_ISL_769694, EPI_ISL_769695, EPI_ISL_769696, EPI_ISL_769697, EPI_ISL_769698, EPI_ISL_769699, EPI_ISL_769700, EPI_ISL_769701, EPI_ISL_769702			
see above	Lighthouse Lab in Milton Keynes	Wellcome Sanger Institute for the COVID-19 Genomics UK (COG-UK) Consortium	The Lighthouse Lab in Milton Keynes and Alex Alderton, Roberto Amato, Sonia Goncalves, Ewan Harrison, David K. Jackson, Ian Johnston, Dominic Kwiatkowski, Cordelia Langford, John Sillitoe on behalf of the Wellcome Sanger Institute COVID-19 Surveillance Team
EPI_ISL_769703	Lighthouse Lab in Glasgow	Wellcome Sanger Institute for the COVID-19 Genomics UK (COG-UK) Consortium	Harper VanSteenhouse, Yumi Kasai, David Gray, Carol Clugston, Anna Dominiczak and Alex Alderton, Roberto Amato, Sonia Goncalves, Ewan Harrison, David K. Jackson, Ian Johnston, Dominic Kwiatkowski, Cordelia Langford, John Sillitoe on behalf of the Wellcome Sanger Institute COVID-19 Surveillance Team
EPI_ISL_769704	Lighthouse Lab in Milton Keynes	Wellcome Sanger Institute for the COVID-19 Genomics UK (COG-UK) Consortium	The Lighthouse Lab in Milton Keynes and Alex Alderton, Roberto Amato, Sonia Goncalves, Ewan Harrison, David K. Jackson, Ian Johnston, Dominic Kwiatkowski, Cordelia Langford, John Sillitoe on behalf of the Wellcome Sanger Institute COVID-19 Surveillance Team
EPI_ISL_769705	Lighthouse Lab in Glasgow	Wellcome Sanger Institute for the COVID-19 Genomics UK (COG-UK) Consortium	Harper VanSteenhouse, Yumi Kasai, David Gray, Carol Clugston, Anna Dominiczak and Alex Alderton, Roberto Amato, Sonia Goncalves, Ewan Harrison, David K. Jackson, Ian Johnston, Dominic Kwiatkowski, Cordelia Langford, John Sillitoe on behalf of the Wellcome Sanger Institute COVID-19 Surveillance Team
EPI_ISL_769706, EPI_ISL_769707, EPI_ISL_769708, EPI_ISL_769709, EPI_ISL_769710	Lighthouse Lab in Milton Keynes	Wellcome Sanger Institute for the COVID-19 Genomics UK (COG-UK) Consortium	The Lighthouse Lab in Milton Keynes and Alex Alderton, Roberto Amato, Sonia Goncalves, Ewan Harrison, David K. Jackson, Ian Johnston, Dominic Kwiatkowski, Cordelia Langford, John Sillitoe on behalf of the Wellcome Sanger Institute COVID-19 Surveillance Team
EPI_ISL_769711	Lighthouse Lab in Glasgow	Wellcome Sanger Institute for the COVID-19 Genomics UK (COG-UK) Consortium	Harper VanSteenhouse, Yumi Kasai, David Gray, Carol Clugston, Anna Dominiczak and Alex Alderton, Roberto Amato, Sonia Goncalves, Ewan Harrison, David K. Jackson, Ian Johnston, Dominic Kwiatkowski, Cordelia Langford, John Sillitoe on behalf of the Wellcome Sanger Institute COVID-19 Surveillance Team
EPI_ISL_769712	Lighthouse Lab in Milton Keynes	Wellcome Sanger Institute for the COVID-19 Genomics UK (COG-UK) Consortium	The Lighthouse Lab in Milton Keynes and Alex Alderton, Roberto Amato, Sonia Goncalves, Ewan Harrison, David K. Jackson, Ian Johnston, Dominic Kwiatkowski, Cordelia Langford, John Sillitoe on behalf of the Wellcome Sanger Institute COVID-19 Surveillance Team
EPI_ISL_769713, EPI_ISL_769714, EPI_ISL_769716	Lighthouse Lab in Glasgow	Wellcome Sanger Institute for the COVID-19 Genomics UK (COG-UK) Consortium	Harper VanSteenhouse, Yumi Kasai, David Gray, Carol Clugston, Anna Dominiczak and Alex Alderton, Roberto Amato, Sonia Goncalves, Ewan Harrison, David K. Jackson, Ian Johnston, Dominic Kwiatkowski, Cordelia Langford, John Sillitoe on behalf of the Wellcome Sanger Institute COVID-19 Surveillance Team
EPI_ISL_769717, EPI_ISL_769718, EPI_ISL_769719	Lighthouse Lab in Milton Keynes	Wellcome Sanger Institute for the COVID-19 Genomics UK (COG-UK) Consortium	The Lighthouse Lab in Milton Keynes and Alex Alderton, Roberto Amato, Sonia Goncalves, Ewan Harrison, David K. Jackson, Ian Johnston, Dominic Kwiatkowski, Cordelia Langford, John Sillitoe on behalf of the Wellcome Sanger Institute COVID-19 Surveillance Team
EPI_ISL_769720	Lighthouse Lab in Glasgow	Wellcome Sanger Institute for the COVID-19 Genomics UK (COG-UK) Consortium	Harper VanSteenhouse, Yumi Kasai, David Gray, Carol Clugston, Anna Dominiczak and Alex Alderton, Roberto Amato, Sonia Goncalves, Ewan Harrison, David K. Jackson, Ian Johnston, Dominic Kwiatkowski, Cordelia Langford, John Sillitoe on behalf of the Wellcome Sanger Institute COVID-19 Surveillance Team
EPI_ISL_769721, EPI_ISL_769722, EPI_ISL_769723	Lighthouse Lab in Milton Keynes	Wellcome Sanger Institute for the COVID-19 Genomics UK (COG-UK) Consortium	The Lighthouse Lab in Milton Keynes and Alex Alderton, Roberto Amato, Sonia Goncalves, Ewan Harrison, David K. Jackson, Ian Johnston, Dominic Kwiatkowski, Cordelia Langford, John Sillitoe on behalf of the Wellcome Sanger Institute COVID-19 Surveillance Team
EPI_ISL_769724	Lighthouse Lab in Glasgow	Wellcome Sanger Institute for the COVID-19 Genomics UK (COG-UK) Consortium	Harper VanSteenhouse, Yumi Kasai, David Gray, Carol Clugston, Anna Dominiczak and Alex Alderton, Roberto Amato, Sonia Goncalves, Ewan Harrison, David K. Jackson, Ian Johnston, Dominic Kwiatkowski, Cordelia Langford, John Sillitoe on behalf of the Wellcome Sanger Institute COVID-19 Surveillance Team
EPI_ISL_769725, EPI_ISL_769726, EPI_ISL_769727, EPI_ISL_769728, EPI_ISL_769729, EPI_ISL_769730, EPI_ISL_769731, EPI_ISL_769732, EPI_ISL_769733, EPI_ISL_769734	Lighthouse Lab in Milton Keynes	Wellcome Sanger Institute for the COVID-19 Genomics UK (COG-UK) Consortium	The Lighthouse Lab in Milton Keynes and Alex Alderton, Roberto Amato, Sonia Goncalves, Ewan Harrison, David K. Jackson, Ian Johnston, Dominic Kwiatkowski, Cordelia Langford, John Sillitoe on behalf of the Wellcome Sanger Institute COVID-19 Surveillance Team
EPI_ISL_769735	Lighthouse Lab in Glasgow	Wellcome Sanger Institute for the COVID-19 Genomics UK (COG-UK) Consortium	Harper VanSteenhouse, Yumi Kasai, David Gray, Carol Clugston, Anna Dominiczak and Alex Alderton, Roberto Amato, Sonia Goncalves, Ewan Harrison, David K. Jackson, Ian Johnston, Dominic Kwiatkowski, Cordelia Langford, John Sillitoe on behalf of the Wellcome Sanger Institute COVID-19 Surveillance Team
EPI_ISL_769736, EPI_ISL_769737, EPI_ISL_769738, EPI_ISL_769739, EPI_ISL_769740	Lighthouse Lab in Milton Keynes	Wellcome Sanger Institute for the COVID-19 Genomics UK (COG-UK) Consortium	The Lighthouse Lab in Milton Keynes and Alex Alderton, Roberto Amato, Sonia Goncalves, Ewan Harrison, David K. Jackson, Ian Johnston, Dominic Kwiatkowski, Cordelia Langford, John Sillitoe on behalf of the Wellcome Sanger Institute COVID-19 Surveillance Team
EPI_ISL_769741	Lighthouse Lab in Glasgow	Wellcome Sanger Institute for the COVID-19 Genomics UK (COG-UK) Consortium	Harper VanSteenhouse, Yumi Kasai, David Gray, Carol Clugston, Anna Dominiczak and Alex Alderton, Roberto Amato, Sonia Goncalves, Ewan Harrison, David K. Jackson, Ian Johnston, Dominic Kwiatkowski, Cordelia Langford, John Sillitoe on behalf of the Wellcome Sanger Institute COVID-19 Surveillance Team
EPI_ISL_769742	Lighthouse Lab in Milton Keynes	Wellcome Sanger Institute for the COVID-19 Genomics UK	The Lighthouse Lab in Milton Keynes and Alex Alderton, Roberto Amato, Sonia Goncalves, Ewan Harrison, David K. Jackson, Ian Johnston, Dominic

		(COG-UK) Consortium	Kwiatkowski, Cordelia Langford, John Sillitoe on behalf of the Wellcome Sanger Institute COVID-19 Surveillance Team
EPI_ISL_769743	Lighthouse Lab in Glasgow	Wellcome Sanger Institute for the COVID-19 Genomics UK (COG-UK) Consortium	Harper VanSteenhouse, Yumi Kasai, David Gray, Carol Clugston, Anna Dominiczak and Alex Alderton, Roberto Amato, Sonia Goncalves, Ewan Harrison, David K. Jackson, Ian Johnston, Dominic Kwiatkowski, Cordelia Langford, John Sillitoe on behalf of the Wellcome Sanger Institute COVID-19 Surveillance Team
EPI_ISL_769745, EPI_ISL_769746, EPI_ISL_769748, EPI_ISL_769749, EPI_ISL_769750	Lighthouse Lab in Milton Keynes	Wellcome Sanger Institute for the COVID-19 Genomics UK (COG-UK) Consortium	The Lighthouse Lab in Milton Keynes and Alex Alderton, Roberto Amato, Sonia Goncalves, Ewan Harrison, David K. Jackson, Ian Johnston, Dominic Kwiatkowski, Cordelia Langford, John Sillitoe on behalf of the Wellcome Sanger Institute COVID-19 Surveillance Team
EPI_ISL_769751, EPI_ISL_769752, EPI_ISL_769753	Lighthouse Lab in Glasgow	Wellcome Sanger Institute for the COVID-19 Genomics UK (COG-UK) Consortium	Harper VanSteenhouse, Yumi Kasai, David Gray, Carol Clugston, Anna Dominiczak and Alex Alderton, Roberto Amato, Sonia Goncalves, Ewan Harrison, David K. Jackson, Ian Johnston, Dominic Kwiatkowski, Cordelia Langford, John Sillitoe on behalf of the Wellcome Sanger Institute COVID-19 Surveillance Team
EPI_ISL_769754, EPI_ISL_769755, EPI_ISL_769756, EPI_ISL_769757	Lighthouse Lab in Milton Keynes	Wellcome Sanger Institute for the COVID-19 Genomics UK (COG-UK) Consortium	The Lighthouse Lab in Milton Keynes and Alex Alderton, Roberto Amato, Sonia Goncalves, Ewan Harrison, David K. Jackson, Ian Johnston, Dominic Kwiatkowski, Cordelia Langford, John Sillitoe on behalf of the Wellcome Sanger Institute COVID-19 Surveillance Team
EPI_ISL_769758, EPI_ISL_769759	Lighthouse Lab in Glasgow	Wellcome Sanger Institute for the COVID-19 Genomics UK (COG-UK) Consortium	Harper VanSteenhouse, Yumi Kasai, David Gray, Carol Clugston, Anna Dominiczak and Alex Alderton, Roberto Amato, Sonia Goncalves, Ewan Harrison, David K. Jackson, Ian Johnston, Dominic Kwiatkowski, Cordelia Langford, John Sillitoe on behalf of the Wellcome Sanger Institute COVID-19 Surveillance Team
EPI_ISL_769760	Lighthouse Lab in Milton Keynes	Wellcome Sanger Institute for the COVID-19 Genomics UK (COG-UK) Consortium	The Lighthouse Lab in Milton Keynes and Alex Alderton, Roberto Amato, Sonia Goncalves, Ewan Harrison, David K. Jackson, Ian Johnston, Dominic Kwiatkowski, Cordelia Langford, John Sillitoe on behalf of the Wellcome Sanger Institute COVID-19 Surveillance Team
EPI_ISL_769761	Lighthouse Lab in Glasgow	Wellcome Sanger Institute for the COVID-19 Genomics UK (COG-UK) Consortium	Harper VanSteenhouse, Yumi Kasai, David Gray, Carol Clugston, Anna Dominiczak and Alex Alderton, Roberto Amato, Sonia Goncalves, Ewan Harrison, David K. Jackson, Ian Johnston, Dominic Kwiatkowski, Cordelia Langford, John Sillitoe on behalf of the Wellcome Sanger Institute COVID-19 Surveillance Team
EPI_ISL_769762, EPI_ISL_769763, EPI_ISL_769765, EPI_ISL_769766, EPI_ISL_769767	Lighthouse Lab in Milton Keynes	Wellcome Sanger Institute for the COVID-19 Genomics UK (COG-UK) Consortium	The Lighthouse Lab in Milton Keynes and Alex Alderton, Roberto Amato, Sonia Goncalves, Ewan Harrison, David K. Jackson, Ian Johnston, Dominic Kwiatkowski, Cordelia Langford, John Sillitoe on behalf of the Wellcome Sanger Institute COVID-19 Surveillance Team
EPI_ISL_769768	Lighthouse Lab in Glasgow	Wellcome Sanger Institute for the COVID-19 Genomics UK (COG-UK) Consortium	Harper VanSteenhouse, Yumi Kasai, David Gray, Carol Clugston, Anna Dominiczak and Alex Alderton, Roberto Amato, Sonia Goncalves, Ewan Harrison, David K. Jackson, Ian Johnston, Dominic Kwiatkowski, Cordelia Langford, John Sillitoe on behalf of the Wellcome Sanger Institute COVID-19 Surveillance Team
EPI_ISL_769769, EPI_ISL_769770, EPI_ISL_769771, EPI_ISL_769772	Lighthouse Lab in Milton Keynes	Wellcome Sanger Institute for the COVID-19 Genomics UK (COG-UK) Consortium	The Lighthouse Lab in Milton Keynes and Alex Alderton, Roberto Amato, Sonia Goncalves, Ewan Harrison, David K. Jackson, Ian Johnston, Dominic Kwiatkowski, Cordelia Langford, John Sillitoe on behalf of the Wellcome Sanger Institute COVID-19 Surveillance Team
EPI_ISL_769773	Lighthouse Lab in Glasgow	Wellcome Sanger Institute for the COVID-19 Genomics UK (COG-UK) Consortium	Harper VanSteenhouse, Yumi Kasai, David Gray, Carol Clugston, Anna Dominiczak and Alex Alderton, Roberto Amato, Sonia Goncalves, Ewan Harrison, David K. Jackson, Ian Johnston, Dominic Kwiatkowski, Cordelia Langford, John Sillitoe on behalf of the Wellcome Sanger Institute COVID-19 Surveillance Team
EPI_ISL_769774, EPI_ISL_769775, EPI_ISL_769777, EPI_ISL_769778, EPI_ISL_769779, EPI_ISL_769780, EPI_ISL_769781, EPI_ISL_769782, EPI_ISL_769783, EPI_ISL_769784, EPI_ISL_769785, EPI_ISL_769786			
see above	Lighthouse Lab in Milton Keynes	Wellcome Sanger Institute for the COVID-19 Genomics UK (COG-UK) Consortium	The Lighthouse Lab in Milton Keynes and Alex Alderton, Roberto Amato, Sonia Goncalves, Ewan Harrison, David K. Jackson, Ian Johnston, Dominic Kwiatkowski, Cordelia Langford, John Sillitoe on behalf of the Wellcome Sanger Institute COVID-19 Surveillance Team
EPI_ISL_769787, EPI_ISL_769788	Lighthouse Lab in Glasgow	Wellcome Sanger Institute for the COVID-19 Genomics UK (COG-UK) Consortium	Harper VanSteenhouse, Yumi Kasai, David Gray, Carol Clugston, Anna Dominiczak and Alex Alderton, Roberto Amato, Sonia Goncalves, Ewan Harrison, David K. Jackson, Ian Johnston, Dominic Kwiatkowski, Cordelia Langford, John Sillitoe on behalf of the Wellcome Sanger Institute COVID-19 Surveillance Team
EPI_ISL_769789, EPI_ISL_769790, EPI_ISL_769791, EPI_ISL_769792, EPI_ISL_769793	Lighthouse Lab in Milton Keynes	Wellcome Sanger Institute for the COVID-19 Genomics UK (COG-UK) Consortium	The Lighthouse Lab in Milton Keynes and Alex Alderton, Roberto Amato, Sonia Goncalves, Ewan Harrison, David K. Jackson, Ian Johnston, Dominic Kwiatkowski, Cordelia Langford, John Sillitoe on behalf of the Wellcome Sanger Institute COVID-19 Surveillance Team
EPI_ISL_769794	Lighthouse Lab in Glasgow	Wellcome Sanger Institute for the COVID-19 Genomics UK (COG-UK) Consortium	Harper VanSteenhouse, Yumi Kasai, David Gray, Carol Clugston, Anna Dominiczak and Alex Alderton, Roberto Amato, Sonia Goncalves, Ewan Harrison, David K. Jackson, Ian Johnston, Dominic Kwiatkowski, Cordelia Langford, John Sillitoe on behalf of the Wellcome Sanger Institute COVID-19 Surveillance Team
EPI_ISL_769795, EPI_ISL_769796, EPI_ISL_769797, EPI_ISL_769798, EPI_ISL_769799, EPI_ISL_769800, EPI_ISL_769801, EPI_ISL_769803, EPI_ISL_769804, EPI_ISL_769805	Lighthouse Lab in Milton Keynes	Wellcome Sanger Institute for the COVID-19 Genomics UK (COG-UK) Consortium	The Lighthouse Lab in Milton Keynes and Alex Alderton, Roberto Amato, Sonia Goncalves, Ewan Harrison, David K. Jackson, Ian Johnston, Dominic Kwiatkowski, Cordelia Langford, John Sillitoe on behalf of the Wellcome Sanger Institute COVID-19 Surveillance Team
EPI_ISL_769806	Lighthouse Lab in Glasgow	Wellcome Sanger Institute for the COVID-19 Genomics UK (COG-UK) Consortium	Harper VanSteenhouse, Yumi Kasai, David Gray, Carol Clugston, Anna Dominiczak and Alex Alderton, Roberto Amato, Sonia Goncalves, Ewan Harrison, David K. Jackson, Ian Johnston, Dominic Kwiatkowski, Cordelia Langford, John Sillitoe on behalf of the Wellcome Sanger Institute COVID-19 Surveillance Team
EPI_ISL_769807, EPI_ISL_769808, EPI_ISL_769809, EPI_ISL_769810, EPI_ISL_769811, EPI_ISL_769812	Lighthouse Lab in Milton Keynes	Wellcome Sanger Institute for the COVID-19 Genomics UK (COG-UK) Consortium	The Lighthouse Lab in Milton Keynes and Alex Alderton, Roberto Amato, Sonia Goncalves, Ewan Harrison, David K. Jackson, Ian Johnston, Dominic Kwiatkowski, Cordelia Langford, John Sillitoe on behalf of the Wellcome Sanger Institute COVID-19 Surveillance Team
EPI_ISL_769813	Lighthouse Lab in Glasgow	Wellcome Sanger Institute for the COVID-19 Genomics UK (COG-UK) Consortium	Harper VanSteenhouse, Yumi Kasai, David Gray, Carol Clugston, Anna Dominiczak and Alex Alderton, Roberto Amato, Sonia Goncalves, Ewan Harrison, David K. Jackson, Ian Johnston, Dominic Kwiatkowski, Cordelia Langford, John Sillitoe on behalf of the Wellcome Sanger Institute COVID-19 Surveillance Team
EPI_ISL_769814	Lighthouse Lab in Milton Keynes	Wellcome Sanger Institute for the COVID-19 Genomics UK (COG-UK) Consortium	The Lighthouse Lab in Milton Keynes and Alex Alderton, Roberto Amato, Sonia Goncalves, Ewan Harrison, David K. Jackson, Ian Johnston, Dominic Kwiatkowski, Cordelia Langford, John Sillitoe on behalf of the Wellcome Sanger Institute COVID-19 Surveillance Team
EPI_ISL_769815	Lighthouse Lab in Glasgow	Wellcome Sanger Institute for the COVID-19 Genomics UK (COG-UK) Consortium	Harper VanSteenhouse, Yumi Kasai, David Gray, Carol Clugston, Anna Dominiczak and Alex Alderton, Roberto Amato, Sonia Goncalves, Ewan Harrison, David K. Jackson, Ian Johnston, Dominic Kwiatkowski, Cordelia Langford, John Sillitoe on behalf of the Wellcome Sanger Institute COVID-19 Surveillance Team
EPI_ISL_769816, EPI_ISL_769817	Lighthouse Lab in Milton Keynes	Wellcome Sanger Institute for the COVID-19 Genomics UK (COG-UK) Consortium	The Lighthouse Lab in Milton Keynes and Alex Alderton, Roberto Amato, Sonia Goncalves, Ewan Harrison, David K. Jackson, Ian Johnston, Dominic Kwiatkowski, Cordelia Langford, John Sillitoe on behalf of the Wellcome Sanger Institute COVID-19 Surveillance Team
EPI_ISL_769818	Lighthouse Lab in Glasgow	Wellcome Sanger Institute for the COVID-19 Genomics UK (COG-UK) Consortium	Harper VanSteenhouse, Yumi Kasai, David Gray, Carol Clugston, Anna Dominiczak and Alex Alderton, Roberto Amato, Sonia Goncalves, Ewan Harrison, David K. Jackson, Ian Johnston, Dominic Kwiatkowski, Cordelia Langford, John Sillitoe on behalf of the Wellcome Sanger Institute COVID-19 Surveillance Team
EPI_ISL_769819, EPI_ISL_769820, EPI_ISL_769821, EPI_ISL_769822, EPI_ISL_769823, EPI_ISL_769824, EPI_ISL_769825	Lighthouse Lab in Milton Keynes	Wellcome Sanger Institute for the COVID-19 Genomics UK (COG-UK) Consortium	The Lighthouse Lab in Milton Keynes and Alex Alderton, Roberto Amato, Sonia Goncalves, Ewan Harrison, David K. Jackson, Ian Johnston, Dominic Kwiatkowski, Cordelia Langford, John Sillitoe on behalf of the Wellcome Sanger Institute COVID-19 Surveillance Team
EPI_ISL_769826	Lighthouse Lab in Glasgow	Wellcome Sanger Institute for the COVID-19 Genomics UK (COG-UK) Consortium	Harper VanSteenhouse, Yumi Kasai, David Gray, Carol Clugston, Anna Dominiczak and Alex Alderton, Roberto Amato, Sonia Goncalves, Ewan Harrison, David K. Jackson, Ian Johnston, Dominic Kwiatkowski, Cordelia Langford, John Sillitoe on behalf of the Wellcome Sanger Institute COVID-19 Surveillance Team

EPI_ISL_769827, EPI_ISL_769828, EPI_ISL_769829, EPI_ISL_769830, EPI_ISL_769831, EPI_ISL_769832, EPI_ISL_769833	Lighthouse Lab in Milton Keynes	Wellcome Sanger Institute for the COVID-19 Genomics UK (COG-UK) Consortium	The Lighthouse Lab in Milton Keynes and Alex Alderton, Roberto Amato, Sonia Goncalves, Ewan Harrison, David K. Jackson, Ian Johnston, Dominic Kwiatkowski, Cordelia Langford, John Sillitoe on behalf of the Wellcome Sanger Institute COVID-19 Surveillance Team
EPI_ISL_769834	Lighthouse Lab in Glasgow	Wellcome Sanger Institute for the COVID-19 Genomics UK (COG-UK) Consortium	Harper VanSteenhouse, Yumi Kasai, David Gray, Carol Clugston, Anna Dominiczak and Alex Alderton, Roberto Amato, Sonia Goncalves, Ewan Harrison, David K. Jackson, Ian Johnston, Dominic Kwiatkowski, Cordelia Langford, John Sillitoe on behalf of the Wellcome Sanger Institute COVID-19 Surveillance Team
EPI_ISL_769835	Lighthouse Lab in Milton Keynes	Wellcome Sanger Institute for the COVID-19 Genomics UK (COG-UK) Consortium	The Lighthouse Lab in Milton Keynes and Alex Alderton, Roberto Amato, Sonia Goncalves, Ewan Harrison, David K. Jackson, Ian Johnston, Dominic Kwiatkowski, Cordelia Langford, John Sillitoe on behalf of the Wellcome Sanger Institute COVID-19 Surveillance Team
EPI_ISL_769836, EPI_ISL_769837	Lighthouse Lab in Glasgow	Wellcome Sanger Institute for the COVID-19 Genomics UK (COG-UK) Consortium	Harper VanSteenhouse, Yumi Kasai, David Gray, Carol Clugston, Anna Dominiczak and Alex Alderton, Roberto Amato, Sonia Goncalves, Ewan Harrison, David K. Jackson, Ian Johnston, Dominic Kwiatkowski, Cordelia Langford, John Sillitoe on behalf of the Wellcome Sanger Institute COVID-19 Surveillance Team
EPI_ISL_769838, EPI_ISL_769839, EPI_ISL_769840, EPI_ISL_769841	Lighthouse Lab in Milton Keynes	Wellcome Sanger Institute for the COVID-19 Genomics UK (COG-UK) Consortium	The Lighthouse Lab in Milton Keynes and Alex Alderton, Roberto Amato, Sonia Goncalves, Ewan Harrison, David K. Jackson, Ian Johnston, Dominic Kwiatkowski, Cordelia Langford, John Sillitoe on behalf of the Wellcome Sanger Institute COVID-19 Surveillance Team
EPI_ISL_769842	Lighthouse Lab in Glasgow	Wellcome Sanger Institute for the COVID-19 Genomics UK (COG-UK) Consortium	Harper VanSteenhouse, Yumi Kasai, David Gray, Carol Clugston, Anna Dominiczak and Alex Alderton, Roberto Amato, Sonia Goncalves, Ewan Harrison, David K. Jackson, Ian Johnston, Dominic Kwiatkowski, Cordelia Langford, John Sillitoe on behalf of the Wellcome Sanger Institute COVID-19 Surveillance Team
EPI_ISL_769843, EPI_ISL_769844, EPI_ISL_769846, EPI_ISL_769847, EPI_ISL_769848, EPI_ISL_769849	Lighthouse Lab in Milton Keynes	Wellcome Sanger Institute for the COVID-19 Genomics UK (COG-UK) Consortium	The Lighthouse Lab in Milton Keynes and Alex Alderton, Roberto Amato, Sonia Goncalves, Ewan Harrison, David K. Jackson, Ian Johnston, Dominic Kwiatkowski, Cordelia Langford, John Sillitoe on behalf of the Wellcome Sanger Institute COVID-19 Surveillance Team
EPI_ISL_769850	Lighthouse Lab in Glasgow	Wellcome Sanger Institute for the COVID-19 Genomics UK (COG-UK) Consortium	Harper VanSteenhouse, Yumi Kasai, David Gray, Carol Clugston, Anna Dominiczak and Alex Alderton, Roberto Amato, Sonia Goncalves, Ewan Harrison, David K. Jackson, Ian Johnston, Dominic Kwiatkowski, Cordelia Langford, John Sillitoe on behalf of the Wellcome Sanger Institute COVID-19 Surveillance Team
EPI_ISL_769851, EPI_ISL_769853, EPI_ISL_769854, EPI_ISL_769855, EPI_ISL_769856, EPI_ISL_769858, EPI_ISL_769859, EPI_ISL_769860	Lighthouse Lab in Milton Keynes	Wellcome Sanger Institute for the COVID-19 Genomics UK (COG-UK) Consortium	The Lighthouse Lab in Milton Keynes and Alex Alderton, Roberto Amato, Sonia Goncalves, Ewan Harrison, David K. Jackson, Ian Johnston, Dominic Kwiatkowski, Cordelia Langford, John Sillitoe on behalf of the Wellcome Sanger Institute COVID-19 Surveillance Team
EPI_ISL_769862	Lighthouse Lab in Glasgow	Wellcome Sanger Institute for the COVID-19 Genomics UK (COG-UK) Consortium	Harper VanSteenhouse, Yumi Kasai, David Gray, Carol Clugston, Anna Dominiczak and Alex Alderton, Roberto Amato, Sonia Goncalves, Ewan Harrison, David K. Jackson, Ian Johnston, Dominic Kwiatkowski, Cordelia Langford, John Sillitoe on behalf of the Wellcome Sanger Institute COVID-19 Surveillance Team
EPI_ISL_769869	Respiratory Virus Unit, National Infection Service, Public Health England	COVID-19 Genomics UK (COG-UK) Consortium	PHE Covid Sequencing Team
EPI_ISL_770688, EPI_ISL_770689, EPI_ISL_770690, EPI_ISL_770691, EPI_ISL_770692, EPI_ISL_770693, EPI_ISL_770694, EPI_ISL_770695, EPI_ISL_770696, EPI_ISL_770697, EPI_ISL_770698, EPI_ISL_770699, EPI_ISL_770700, EPI_ISL_770701, EPI_ISL_770702, EPI_ISL_770703, EPI_ISL_770704, EPI_ISL_770705, EPI_ISL_770706, EPI_ISL_770707, EPI_ISL_770708, EPI_ISL_770709, EPI_ISL_770710, EPI_ISL_770711			
see above	ZOTZ KLIMAS MVZ Düsseldorf-Centrum GbR ÜBAG für Labormedizin, Genetik, Zytologie, Pathologie	Center of Medical Microbiology, Virology, and Hospital Hygiene, University of Duesseldorf	Maximilian Damagnez, Alexander Dilthey, Ashley-Jane Duplessis, Patrick Finzer, Katrin Hoffmann, Torsten Houwaart, Lisanna Hülse, Malte Kohns Vasconcelos, Marek Korencak, Nadine Lübke, Jessica Nicolai, Klaus Pfeffer, Daniel Strelow, Jörg Timm, Andreas Walker, Tobias Wienemann, Rainer Zotz
EPI_ISL_770795, EPI_ISL_770796, EPI_ISL_770798	Minnesota Department of Health, Public Health Laboratory	Minnesota Department of Health, Public Health Laboratory	Alexandra Lorentz, Jacob Garfin, Matt Plumb, and Xiong Wang
EPI_ISL_770815	Vault Health	Minnesota Department of Health, Public Health Laboratory	Alexandra Lorentz, Jacob Garfin, Matt Plumb, and Xiong Wang
EPI_ISL_775270	Akershus University Hospital, Department for Microbiology and Infectious Disease Control	Norwegian Institute of Public Health, Department of Virology	Kathrine Stene-Johansen, Kamilla Heddeland Instefjord, Hilde Elshaug, Atiya R Ali,Marie Paulsen Madsen, Rasmus Riis Kopperud, Hilde Vollan, Karoline Bragstad, Olav Hungnes
EPI_ISL_775410, EPI_ISL_775411, EPI_ISL_775412, EPI_ISL_775413	Oslo University Hospital, Department of Medical Microbiology	Norwegian Institute of Public Health, Department of Virology	Kathrine Stene-Johansen, Kamilla Heddeland Instefjord, Hilde Elshaug, Atiya R Ali,Marie Paulsen Madsen, Rasmus Riis Kopperud, Hilde Vollan, Karoline Bragstad, Olav Hungnes
EPI_ISL_775442	Medical Microbiology Unit, Department for Laboratory Medicine, Drammen Hospital, Vestre Viken Health Trust,	Norwegian Institute of Public Health, Department of Virology	Kathrine Stene-Johansen, Kamilla Heddeland Instefjord, Hilde Elshaug, Atiya R Ali,Marie Paulsen Madsen, Rasmus Riis Kopperud, Hilde Vollan, Karoline Bragstad, Olav Hungnes
EPI_ISL_775518, EPI_ISL_775519, EPI_ISL_775521, EPI_ISL_775522	Hospital of Southern Norway - Kristiansand, Department of Medical Microbiology	Norwegian Institute of Public Health, Department of Virology	Kathrine Stene-Johansen, Kamilla Heddeland Instefjord, Hilde Elshaug, Atiya R Ali,Marie Paulsen Madsen, Rasmus Riis Kopperud, Hilde Vollan, Karoline Bragstad, Olav Hungnes
EPI_ISL_775542, EPI_ISL_775543	Klinisk mikrobiologi, Viruslab	The Public Health Agency of Sweden	Department of Microbiology, The Public Health Agency of Sweden
EPI_ISL_776669, EPI_ISL_776670, EPI_ISL_776708, EPI_ISL_776709, EPI_ISL_776713, EPI_ISL_776715, EPI_ISL_776716, EPI_ISL_776717, EPI_ISL_776718, EPI_ISL_776719, EPI_ISL_776720, EPI_ISL_776721, EPI_ISL_776722, EPI_ISL_776723, EPI_ISL_776724, EPI_ISL_776725, EPI_ISL_776726, EPI_ISL_776727, EPI_ISL_776728, EPI_ISL_776729, EPI_ISL_776742, EPI_ISL_776743, EPI_ISL_776744			
see above	UW Virology Lab	UW Virology Lab	Pavitra Roychoudhury, Hong Xie, Lasata Shrestha, Meei-Li Huang, Keith R Jerome, Alexander Greninger
EPI_ISL_777033, EPI_ISL_777042, EPI_ISL_777053, EPI_ISL_777063	Lighthouse Lab in Alderley Park	Wellcome Sanger Institute for the COVID-19 Genomics UK (COG-UK) Consortium	Jacquelyn Wynn, Mairead Hyland, The Lighthouse Lab in Alderley Park and Alex Alderton, Roberto Amato, Sonia Goncalves, Ewan Harrison, David K. Jackson, Ian Johnston, Dominic Kwiatkowski, Cordelia Langford, John Sillitoe on behalf of the Wellcome Sanger Institute COVID-19 Surveillance Team
EPI_ISL_777064	Lighthouse Lab in Milton Keynes	Wellcome Sanger Institute for the COVID-19 Genomics UK (COG-UK) Consortium	The Lighthouse Lab in Milton Keynes and Alex Alderton, Roberto Amato, Sonia Goncalves, Ewan Harrison, David K. Jackson, Ian Johnston, Dominic Kwiatkowski, Cordelia Langford, John Sillitoe on behalf of the Wellcome Sanger Institute COVID-19 Surveillance Team
EPI_ISL_777067	Lighthouse Lab in Alderley Park	Wellcome Sanger Institute for the COVID-19 Genomics UK (COG-UK) Consortium	Jacquelyn Wynn, Mairead Hyland, The Lighthouse Lab in Alderley Park and Alex Alderton, Roberto Amato, Sonia Goncalves, Ewan Harrison, David K. Jackson, Ian Johnston, Dominic Kwiatkowski, Cordelia Langford, John Sillitoe on behalf of the Wellcome Sanger Institute COVID-19 Surveillance Team
EPI_ISL_777080	Lighthouse Lab in Milton Keynes	Wellcome Sanger Institute for the COVID-19 Genomics UK (COG-UK) Consortium	The Lighthouse Lab in Milton Keynes and Alex Alderton, Roberto Amato, Sonia Goncalves, Ewan Harrison, David K. Jackson, Ian Johnston, Dominic Kwiatkowski, Cordelia Langford, John Sillitoe on behalf of the Wellcome Sanger Institute COVID-19 Surveillance Team
EPI_ISL_777083	Lighthouse Lab in Alderley Park	Wellcome Sanger Institute for the COVID-19 Genomics UK (COG-UK) Consortium	Jacquelyn Wynn, Mairead Hyland, The Lighthouse Lab in Alderley Park and Alex Alderton, Roberto Amato, Sonia Goncalves, Ewan Harrison, David K. Jackson, Ian Johnston, Dominic Kwiatkowski, Cordelia Langford, John Sillitoe on behalf of the Wellcome Sanger Institute COVID-19 Surveillance Team
EPI_ISL_777084, EPI_ISL_777104	Lighthouse Lab in Milton Keynes	Wellcome Sanger Institute for the COVID-19 Genomics UK (COG-UK) Consortium	The Lighthouse Lab in Milton Keynes and Alex Alderton, Roberto Amato, Sonia Goncalves, Ewan Harrison, David K. Jackson, Ian Johnston, Dominic Kwiatkowski, Cordelia Langford, John Sillitoe on behalf of the Wellcome Sanger Institute COVID-19 Surveillance Team
EPI_ISL_777128	Lighthouse Lab in Alderley Park	Wellcome Sanger Institute for the COVID-19 Genomics UK (COG-UK) Consortium	Jacquelyn Wynn, Mairead Hyland, The Lighthouse Lab in Alderley Park and Alex Alderton, Roberto Amato, Sonia Goncalves, Ewan Harrison, David K. Jackson, Ian Johnston, Dominic Kwiatkowski, Cordelia Langford, John Sillitoe on behalf of the Wellcome Sanger Institute COVID-19 Surveillance Team
EPI_ISL_777133	Lighthouse Lab in Cambridge	Wellcome Sanger Institute for the COVID-19 Genomics UK (COG-UK) Consortium	Rob Howes, The Lighthouse Lab in Cambridge and Alex Alderton, Roberto Amato, Sonia Goncalves, Ewan Harrison, David K. Jackson, Ian Johnston, Dominic Kwiatkowski, Cordelia Langford, John Sillitoe on behalf of the Wellcome Sanger Institute COVID-19 Surveillance Team
EPI_ISL_777141	Lighthouse Lab in Milton Keynes	Wellcome Sanger Institute for the COVID-19 Genomics UK (COG-UK) Consortium	The Lighthouse Lab in Milton Keynes and Alex Alderton, Roberto Amato, Sonia Goncalves, Ewan Harrison, David K. Jackson, Ian Johnston, Dominic Kwiatkowski, Cordelia Langford, John Sillitoe on behalf of the Wellcome Sanger Institute COVID-19 Surveillance Team
EPI_ISL_777153, EPI_ISL_777155	Lighthouse Lab in Alderley Park	Wellcome Sanger Institute for the COVID-19 Genomics UK (COG-UK) Consortium	Jacquelyn Wynn, Mairead Hyland, The Lighthouse Lab in Alderley Park and Alex Alderton, Roberto Amato, Sonia Goncalves, Ewan Harrison, David K. Jackson, Ian Johnston, Dominic Kwiatkowski, Cordelia Langford, John Sillitoe on behalf of the Wellcome Sanger Institute COVID-19 Surveillance Team
EPI_ISL_777158	Lighthouse Lab in Milton Keynes	Wellcome Sanger Institute for the COVID-19 Genomics UK	The Lighthouse Lab in Milton Keynes and Alex Alderton, Roberto Amato, Sonia Goncalves, Ewan Harrison, David K. Jackson, Ian Johnston, Dominic

		(COG-UK) Consortium	Kwiatkowski, Cordelia Langford, John Sillitoe on behalf of the Wellcome Sanger Institute COVID-19 Surveillance Team
EPI_ISL_777173, EPI_ISL_777181	Lighthouse Lab in Cambridge	Wellcome Sanger Institute for the COVID-19 Genomics UK (COG-UK) Consortium	Rob Howes, The Lighthouse Lab in Cambridge and Alex Alderton, Roberto Amato, Sonia Goncalves, Ewan Harrison, David K. Jackson, Ian Johnston, Dominic Kwiatkowski, Cordelia Langford, John Sillitoe on behalf of the Wellcome Sanger Institute COVID-19 Surveillance Team
EPI_ISL_777183	Lighthouse Lab in Alderley Park	Wellcome Sanger Institute for the COVID-19 Genomics UK (COG-UK) Consortium	Jacquelyn Wynn, Mairead Hyland, The Lighthouse Lab in Alderley Park and Alex Alderton, Roberto Amato, Sonia Goncalves, Ewan Harrison, David K. Jackson, Ian Johnston, Dominic Kwiatkowski, Cordelia Langford, John Sillitoe on behalf of the Wellcome Sanger Institute COVID-19 Surveillance Team
EPI_ISL_777184, EPI_ISL_777200, EPI_ISL_777207	Lighthouse Lab in Milton Keynes	Wellcome Sanger Institute for the COVID-19 Genomics UK (COG-UK) Consortium	The Lighthouse Lab in Milton Keynes and Alex Alderton, Roberto Amato, Sonia Goncalves, Ewan Harrison, David K. Jackson, Ian Johnston, Dominic Kwiatkowski, Cordelia Langford, John Sillitoe on behalf of the Wellcome Sanger Institute COVID-19 Surveillance Team
EPI_ISL_777211	Lighthouse Lab in Alderley Park	Wellcome Sanger Institute for the COVID-19 Genomics UK (COG-UK) Consortium	Jacquelyn Wynn, Mairead Hyland, The Lighthouse Lab in Alderley Park and Alex Alderton, Roberto Amato, Sonia Goncalves, Ewan Harrison, David K. Jackson, Ian Johnston, Dominic Kwiatkowski, Cordelia Langford, John Sillitoe on behalf of the Wellcome Sanger Institute COVID-19 Surveillance Team
EPI_ISL_777218	Lighthouse Lab in Cambridge	Wellcome Sanger Institute for the COVID-19 Genomics UK (COG-UK) Consortium	Rob Howes, The Lighthouse Lab in Cambridge and Alex Alderton, Roberto Amato, Sonia Goncalves, Ewan Harrison, David K. Jackson, Ian Johnston, Dominic Kwiatkowski, Cordelia Langford, John Sillitoe on behalf of the Wellcome Sanger Institute COVID-19 Surveillance Team
EPI_ISL_777224, EPI_ISL_777229	Lighthouse Lab in Milton Keynes	Wellcome Sanger Institute for the COVID-19 Genomics UK (COG-UK) Consortium	The Lighthouse Lab in Milton Keynes and Alex Alderton, Roberto Amato, Sonia Goncalves, Ewan Harrison, David K. Jackson, Ian Johnston, Dominic Kwiatkowski, Cordelia Langford, John Sillitoe on behalf of the Wellcome Sanger Institute COVID-19 Surveillance Team
EPI_ISL_777244	Lighthouse Lab in Alderley Park	Wellcome Sanger Institute for the COVID-19 Genomics UK (COG-UK) Consortium	Jacquelyn Wynn, Mairead Hyland, The Lighthouse Lab in Alderley Park and Alex Alderton, Roberto Amato, Sonia Goncalves, Ewan Harrison, David K. Jackson, Ian Johnston, Dominic Kwiatkowski, Cordelia Langford, John Sillitoe on behalf of the Wellcome Sanger Institute COVID-19 Surveillance Team
EPI_ISL_777245, EPI_ISL_777269	Lighthouse Lab in Milton Keynes	Wellcome Sanger Institute for the COVID-19 Genomics UK (COG-UK) Consortium	The Lighthouse Lab in Milton Keynes and Alex Alderton, Roberto Amato, Sonia Goncalves, Ewan Harrison, David K. Jackson, Ian Johnston, Dominic Kwiatkowski, Cordelia Langford, John Sillitoe on behalf of the Wellcome Sanger Institute COVID-19 Surveillance Team
EPI_ISL_777270	Lighthouse Lab in Alderley Park	Wellcome Sanger Institute for the COVID-19 Genomics UK (COG-UK) Consortium	Jacquelyn Wynn, Mairead Hyland, The Lighthouse Lab in Alderley Park and Alex Alderton, Roberto Amato, Sonia Goncalves, Ewan Harrison, David K. Jackson, Ian Johnston, Dominic Kwiatkowski, Cordelia Langford, John Sillitoe on behalf of the Wellcome Sanger Institute COVID-19 Surveillance Team
EPI_ISL_777275	Lighthouse Lab in Cambridge	Wellcome Sanger Institute for the COVID-19 Genomics UK (COG-UK) Consortium	Rob Howes, The Lighthouse Lab in Cambridge and Alex Alderton, Roberto Amato, Sonia Goncalves, Ewan Harrison, David K. Jackson, Ian Johnston, Dominic Kwiatkowski, Cordelia Langford, John Sillitoe on behalf of the Wellcome Sanger Institute COVID-19 Surveillance Team
EPI_ISL_777286	Lighthouse Lab in Alderley Park	Wellcome Sanger Institute for the COVID-19 Genomics UK (COG-UK) Consortium	Jacquelyn Wynn, Mairead Hyland, The Lighthouse Lab in Alderley Park and Alex Alderton, Roberto Amato, Sonia Goncalves, Ewan Harrison, David K. Jackson, Ian Johnston, Dominic Kwiatkowski, Cordelia Langford, John Sillitoe on behalf of the Wellcome Sanger Institute COVID-19 Surveillance Team
EPI_ISL_777289	Lighthouse Lab in Cambridge	Wellcome Sanger Institute for the COVID-19 Genomics UK (COG-UK) Consortium	Rob Howes, The Lighthouse Lab in Cambridge and Alex Alderton, Roberto Amato, Sonia Goncalves, Ewan Harrison, David K. Jackson, Ian Johnston, Dominic Kwiatkowski, Cordelia Langford, John Sillitoe on behalf of the Wellcome Sanger Institute COVID-19 Surveillance Team
EPI_ISL_777292	Lighthouse Lab in Alderley Park	Wellcome Sanger Institute for the COVID-19 Genomics UK (COG-UK) Consortium	Jacquelyn Wynn, Mairead Hyland, The Lighthouse Lab in Alderley Park and Alex Alderton, Roberto Amato, Sonia Goncalves, Ewan Harrison, David K. Jackson, Ian Johnston, Dominic Kwiatkowski, Cordelia Langford, John Sillitoe on behalf of the Wellcome Sanger Institute COVID-19 Surveillance Team
EPI_ISL_777293	Lighthouse Lab in Cambridge	Wellcome Sanger Institute for the COVID-19 Genomics UK (COG-UK) Consortium	Rob Howes, The Lighthouse Lab in Cambridge and Alex Alderton, Roberto Amato, Sonia Goncalves, Ewan Harrison, David K. Jackson, Ian Johnston, Dominic Kwiatkowski, Cordelia Langford, John Sillitoe on behalf of the Wellcome Sanger Institute COVID-19 Surveillance Team
EPI_ISL_777303, EPI_ISL_777304, EPI_ISL_777310	Lighthouse Lab in Milton Keynes	Wellcome Sanger Institute for the COVID-19 Genomics UK (COG-UK) Consortium	The Lighthouse Lab in Milton Keynes and Alex Alderton, Roberto Amato, Sonia Goncalves, Ewan Harrison, David K. Jackson, Ian Johnston, Dominic Kwiatkowski, Cordelia Langford, John Sillitoe on behalf of the Wellcome Sanger Institute COVID-19 Surveillance Team
EPI_ISL_777314	Lighthouse Lab in Alderley Park	Wellcome Sanger Institute for the COVID-19 Genomics UK (COG-UK) Consortium	Jacquelyn Wynn, Mairead Hyland, The Lighthouse Lab in Alderley Park and Alex Alderton, Roberto Amato, Sonia Goncalves, Ewan Harrison, David K. Jackson, Ian Johnston, Dominic Kwiatkowski, Cordelia Langford, John Sillitoe on behalf of the Wellcome Sanger Institute COVID-19 Surveillance Team
EPI_ISL_777320	Lighthouse Lab in Cambridge	Wellcome Sanger Institute for the COVID-19 Genomics UK (COG-UK) Consortium	Rob Howes, The Lighthouse Lab in Cambridge and Alex Alderton, Roberto Amato, Sonia Goncalves, Ewan Harrison, David K. Jackson, Ian Johnston, Dominic Kwiatkowski, Cordelia Langford, John Sillitoe on behalf of the Wellcome Sanger Institute COVID-19 Surveillance Team
EPI_ISL_778298, EPI_ISL_778300, EPI_ISL_778304, EPI_ISL_778306, EPI_ISL_778307, EPI_ISL_778308, EPI_ISL_778310, EPI_ISL_778312, EPI_ISL_778313, EPI_ISL_778314, EPI_ISL_778316, EPI_ISL_778317, EPI_ISL_778318, EPI_ISL_778319, EPI_ISL_778320, EPI_ISL_778321, EPI_ISL_778323, EPI_ISL_778326, EPI_ISL_778329, EPI_ISL_778338, EPI_ISL_778339, EPI_ISL_778340, EPI_ISL_778341, EPI_ISL_778343, EPI_ISL_778344			
see above	Lighthouse Lab in Milton Keynes	Wellcome Sanger Institute for the COVID-19 Genomics UK (COG-UK) Consortium	The Lighthouse Lab in Milton Keynes and Alex Alderton, Roberto Amato, Sonia Goncalves, Ewan Harrison, David K. Jackson, Ian Johnston, Dominic Kwiatkowski, Cordelia Langford, John Sillitoe on behalf of the Wellcome Sanger Institute COVID-19 Surveillance Team
EPI_ISL_778347, EPI_ISL_778348	Lighthouse Lab in Alderley Park	Wellcome Sanger Institute for the COVID-19 Genomics UK (COG-UK) Consortium	Jacquelyn Wynn, Mairead Hyland, The Lighthouse Lab in Alderley Park and Alex Alderton, Roberto Amato, Sonia Goncalves, Ewan Harrison, David K. Jackson, Ian Johnston, Dominic Kwiatkowski, Cordelia Langford, John Sillitoe on behalf of the Wellcome Sanger Institute COVID-19 Surveillance Team
EPI_ISL_778349, EPI_ISL_778350, EPI_ISL_778351, EPI_ISL_778354, EPI_ISL_778363, EPI_ISL_778366, EPI_ISL_778367, EPI_ISL_778369, EPI_ISL_778370, EPI_ISL_778371, EPI_ISL_778379, EPI_ISL_778381, EPI_ISL_778383, EPI_ISL_778384, EPI_ISL_778386, EPI_ISL_778387, EPI_ISL_778388, EPI_ISL_778389, EPI_ISL_778391, EPI_ISL_778393, EPI_ISL_778394, EPI_ISL_778395, EPI_ISL_778398, EPI_ISL_778402, EPI_ISL_778405, EPI_ISL_778406, EPI_ISL_778409, EPI_ISL_778413, EPI_ISL_778414, EPI_ISL_778418, EPI_ISL_778425, EPI_ISL_778427, EPI_ISL_778429, EPI_ISL_778431, EPI_ISL_778434, EPI_ISL_778435, EPI_ISL_778436, EPI_ISL_778437, EPI_ISL_778438, EPI_ISL_778439, EPI_ISL_778440, EPI_ISL_778441, EPI_ISL_778442, EPI_ISL_778443, EPI_ISL_778446			
see above	Lighthouse Lab in Milton Keynes	Wellcome Sanger Institute for the COVID-19 Genomics UK (COG-UK) Consortium	The Lighthouse Lab in Milton Keynes and Alex Alderton, Roberto Amato, Sonia Goncalves, Ewan Harrison, David K. Jackson, Ian Johnston, Dominic Kwiatkowski, Cordelia Langford, John Sillitoe on behalf of the Wellcome Sanger Institute COVID-19 Surveillance Team
EPI_ISL_778448	Lighthouse Lab in Alderley Park	Wellcome Sanger Institute for the COVID-19 Genomics UK (COG-UK) Consortium	Jacquelyn Wynn, Mairead Hyland, The Lighthouse Lab in Alderley Park and Alex Alderton, Roberto Amato, Sonia Goncalves, Ewan Harrison, David K. Jackson, Ian Johnston, Dominic Kwiatkowski, Cordelia Langford, John Sillitoe on behalf of the Wellcome Sanger Institute COVID-19 Surveillance Team
EPI_ISL_778449, EPI_ISL_778452, EPI_ISL_778453, EPI_ISL_778456, EPI_ISL_778458, EPI_ISL_778460, EPI_ISL_778464, EPI_ISL_778466, EPI_ISL_778467, EPI_ISL_778468, EPI_ISL_778469, EPI_ISL_778470, EPI_ISL_778474, EPI_ISL_778475, EPI_ISL_778476, EPI_ISL_778477, EPI_ISL_778479, EPI_ISL_778480, EPI_ISL_778481, EPI_ISL_778483, EPI_ISL_778485, EPI_ISL_778486, EPI_ISL_778489, EPI_ISL_778492, EPI_ISL_778493, EPI_ISL_778495, EPI_ISL_778498, EPI_ISL_778501, EPI_ISL_778502, EPI_ISL_778503, EPI_ISL_778505, EPI_ISL_778507, EPI_ISL_778508, EPI_ISL_778509, EPI_ISL_778511, EPI_ISL_778513, EPI_ISL_778514, EPI_ISL_778515, EPI_ISL_778518			
see above	Lighthouse Lab in Milton Keynes	Wellcome Sanger Institute for the COVID-19 Genomics UK (COG-UK) Consortium	The Lighthouse Lab in Milton Keynes and Alex Alderton, Roberto Amato, Sonia Goncalves, Ewan Harrison, David K. Jackson, Ian Johnston, Dominic Kwiatkowski, Cordelia Langford, John Sillitoe on behalf of the Wellcome Sanger Institute COVID-19 Surveillance Team
EPI_ISL_778519	Lighthouse Lab in Alderley Park	Wellcome Sanger Institute for the COVID-19 Genomics UK (COG-UK) Consortium	Jacquelyn Wynn, Mairead Hyland, The Lighthouse Lab in Alderley Park and Alex Alderton, Roberto Amato, Sonia Goncalves, Ewan Harrison, David K. Jackson, Ian Johnston, Dominic Kwiatkowski, Cordelia Langford, John Sillitoe on behalf of the Wellcome Sanger Institute COVID-19 Surveillance Team
EPI_ISL_778520, EPI_ISL_778521, EPI_ISL_778523, EPI_ISL_778526, EPI_ISL_778532, EPI_ISL_778533, EPI_ISL_778535, EPI_ISL_778536, EPI_ISL_778537, EPI_ISL_778538, EPI_ISL_778542, EPI_ISL_778544, EPI_ISL_778545, EPI_ISL_778546, EPI_ISL_778549, EPI_ISL_778550, EPI_ISL_778552, EPI_ISL_778554, EPI_ISL_778555, EPI_ISL_778557, EPI_ISL_778559, EPI_ISL_778561, EPI_ISL_778563			
see above	Lighthouse Lab in Milton Keynes	Wellcome Sanger Institute for the COVID-19 Genomics UK (COG-UK) Consortium	The Lighthouse Lab in Milton Keynes and Alex Alderton, Roberto Amato, Sonia Goncalves, Ewan Harrison, David K. Jackson, Ian Johnston, Dominic Kwiatkowski, Cordelia Langford, John Sillitoe on behalf of the Wellcome Sanger Institute COVID-19 Surveillance Team
EPI_ISL_778564	Lighthouse Lab in Alderley Park	Wellcome Sanger Institute for the COVID-19 Genomics UK (COG-UK) Consortium	Jacquelyn Wynn, Mairead Hyland, The Lighthouse Lab in Alderley Park and Alex Alderton, Roberto Amato, Sonia Goncalves, Ewan Harrison, David K. Jackson, Ian Johnston, Dominic Kwiatkowski, Cordelia Langford, John Sillitoe on behalf of the Wellcome Sanger Institute COVID-19 Surveillance Team
EPI_ISL_778565, EPI_ISL_778566, EPI_ISL_778567, EPI_ISL_778572, EPI_ISL_778573, EPI_ISL_778574, EPI_ISL_778575, EPI_ISL_778576, EPI_ISL_778580, EPI_ISL_778581, EPI_ISL_778582, EPI_ISL_778583, EPI_ISL_778585, EPI_ISL_778587, EPI_ISL_778588, EPI_ISL_778589, EPI_ISL_778590, EPI_ISL_778591, EPI_ISL_778592, EPI_ISL_778595, EPI_ISL_778596, EPI_ISL_778597, EPI_ISL_778600, EPI_ISL_778602, EPI_ISL_778603, EPI_ISL_778604, EPI_ISL_778606, EPI_ISL_778608, EPI_ISL_778609, EPI_ISL_778610, EPI_ISL_778611, EPI_ISL_778613			
see above	Lighthouse Lab in Milton Keynes	Wellcome Sanger Institute for the COVID-19 Genomics UK (COG-UK) Consortium	The Lighthouse Lab in Milton Keynes and Alex Alderton, Roberto Amato, Sonia Goncalves, Ewan Harrison, David K. Jackson, Ian Johnston, Dominic Kwiatkowski, Cordelia Langford, John Sillitoe on behalf of the Wellcome Sanger Institute COVID-19 Surveillance Team
EPI_ISL_778615	Lighthouse Lab in Alderley Park	Wellcome Sanger Institute for the COVID-19 Genomics UK (COG-UK) Consortium	Jacquelyn Wynn, Mairead Hyland, The Lighthouse Lab in Alderley Park and Alex Alderton, Roberto Amato, Sonia Goncalves, Ewan Harrison, David K. Jackson, Ian Johnston, Dominic Kwiatkowski, Cordelia Langford, John Sillitoe on behalf of the Wellcome Sanger Institute COVID-19 Surveillance Team
EPI_ISL_778622, EPI_ISL_778624, EPI_ISL_778625, EPI_ISL_778626, EPI_ISL_778632, EPI_ISL_778633, EPI_ISL_778634, EPI_ISL_778636, EPI_ISL_778637, EPI_ISL_778639, EPI_ISL_778640, EPI_ISL_778641, EPI_ISL_778642, EPI_ISL_778643			
see above	Lighthouse Lab in Milton Keynes	Wellcome Sanger Institute for the COVID-19 Genomics UK (COG-UK) Consortium	The Lighthouse Lab in Milton Keynes and Alex Alderton, Roberto Amato, Sonia Goncalves, Ewan Harrison, David K. Jackson, Ian Johnston, Dominic Kwiatkowski, Cordelia Langford, John Sillitoe on behalf of the Wellcome Sanger Institute COVID-19 Surveillance Team

EPI_ISL_778828, EPI_ISL_778829, EPI_ISL_778831	AIID	Irish Coronavirus Sequencing Consortium-Teagasc Grange	Matthew McCabe, Aljandro Abner Garcia Leon, Fiona Crispie, Calum Walsh, Michael Carr, John Kenny, Paul Cotter, Patrick Mallon, Gabriel Gonzalez
EPI_ISL_778945, EPI_ISL_778946, EPI_ISL_778947, EPI_ISL_778948, EPI_ISL_778949, EPI_ISL_778950, EPI_ISL_778951, EPI_ISL_778952, EPI_ISL_778953, EPI_ISL_778954, EPI_ISL_778955, EPI_ISL_778956, EPI_ISL_778957, EPI_ISL_778958, EPI_ISL_778959, EPI_ISL_778960, EPI_ISL_778961, EPI_ISL_778962, EPI_ISL_778963, EPI_ISL_778964, EPI_ISL_778965, EPI_ISL_778966, EPI_ISL_778967, EPI_ISL_778968, EPI_ISL_778969, EPI_ISL_778970, EPI_ISL_778971, EPI_ISL_778972, EPI_ISL_778973, EPI_ISL_778974, EPI_ISL_778975, EPI_ISL_778976, EPI_ISL_778977, EPI_ISL_778978, EPI_ISL_778979, EPI_ISL_778980, EPI_ISL_778981, EPI_ISL_778982, EPI_ISL_778983, EPI_ISL_778984, EPI_ISL_778985, EPI_ISL_778986, EPI_ISL_778987, EPI_ISL_778988, EPI_ISL_778989, EPI_ISL_778990, EPI_ISL_778991, EPI_ISL_778992, EPI_ISL_778993, EPI_ISL_778994, EPI_ISL_778995, EPI_ISL_778996, EPI_ISL_778997, EPI_ISL_778998, EPI_ISL_778999, EPI_ISL_779000, EPI_ISL_779001, EPI_ISL_779002, EPI_ISL_779003, EPI_ISL_779004, EPI_ISL_779005, EPI_ISL_779006, EPI_ISL_779007, EPI_ISL_779008, EPI_ISL_779009, EPI_ISL_779010, EPI_ISL_779011, EPI_ISL_779012, EPI_ISL_779013, EPI_ISL_779014, EPI_ISL_779015, EPI_ISL_779016, EPI_ISL_779017, EPI_ISL_779018, EPI_ISL_779019, EPI_ISL_779020, EPI_ISL_779021, EPI_ISL_779022, EPI_ISL_779023, EPI_ISL_779024, EPI_ISL_779025, EPI_ISL_779026, EPI_ISL_779027, EPI_ISL_779028, EPI_ISL_779029, EPI_ISL_779030, EPI_ISL_779031, EPI_ISL_779032, EPI_ISL_779033, EPI_ISL_779034, EPI_ISL_779035, EPI_ISL_779036, EPI_ISL_779037, EPI_ISL_779038, EPI_ISL_779039, EPI_ISL_779040, EPI_ISL_779041, EPI_ISL_779042, EPI_ISL_779043, EPI_ISL_779044, EPI_ISL_779045, EPI_ISL_779046, EPI_ISL_779047, EPI_ISL_779048, EPI_ISL_779049, EPI_ISL_779050, EPI_ISL_779051, EPI_ISL_779052, EPI_ISL_779053, EPI_ISL_779054, EPI_ISL_779055, EPI_ISL_779056, EPI_ISL_779057, EPI_ISL_779058, EPI_ISL_779059, EPI_ISL_779060, EPI_ISL_779061, EPI_ISL_779062, EPI_ISL_779063, EPI_ISL_779064, EPI_ISL_779065, EPI_ISL_779066, EPI_ISL_779067, EPI_ISL_779068, EPI_ISL_779069, EPI_ISL_779070, EPI_ISL_779071, EPI_ISL_779072, EPI_ISL_779073, EPI_ISL_779074, EPI_ISL_779075, EPI_ISL_779076, EPI_ISL_779077, EPI_ISL_779078, EPI_ISL_779079, EPI_ISL_779080, EPI_ISL_779081			
see above	LSUHS Emerging Viral Threat Laboratory	Microbial Genome Sequencing Center	Jeremy P. Kamil, Jennifer L. Carroll, Camille F. Abshire, Maarten Van Diest, Andrew D. Yurochko, Martin J. Sapp, Rona S. Scott, Christopher G. Kevil, Daniel J. Snyder, Vaughn S. Cooper, John A. Vanchiere
EPI_ISL_779194	Laboratorio Estatal de Salud Pública de Nuevo León	Laboratorio de Infectología Molecular, Departamento de Bioquímica y Medicina Molecular,Facultad de Medicina - Universidad Autónoma de Nuevo León	Kame A. Galán-Huerta, María F. Herrera-Saldivar, Natalia Martínez-Acuña, Sonia A. Lozano-Sepúlveda, Daniel Arellanos-Soto, Ana M. Rivas-Estilla, Samuel Buentello-Wong, Else del Carmen García-García, Gloria A. Jasso-de-la-Peña, Roberto Montes-de-Oca, Consuelo Treviño-Garza, Manuel E. de-la-O-Cavazos
EPI_ISL_779605, EPI_ISL_779606	Victorian Infectious Diseases Reference Laboratory (VIDRL)	VIDRL and MDU-PHL	Caly L., Seemann T., Sait, M.L., Druce J., Sherry, N.L.
EPI_ISL_779608	Microbiological Diagnostic Unit - Public Health Laboratory (MDU-PHL)	MDU-PHL	Seemann T., Sait, M.L., Sherry, N.L.
EPI_ISL_779609	Victorian Infectious Diseases Reference Laboratory (VIDRL)	VIDRL and MDU-PHL	Caly L., Seemann T., Sait, M.L., Druce J., Sherry, N.L.
EPI_ISL_779851, EPI_ISL_779853, EPI_ISL_779854, EPI_ISL_779856, EPI_ISL_779857, EPI_ISL_779861, EPI_ISL_779864, EPI_ISL_779868, EPI_ISL_779872, EPI_ISL_779878, EPI_ISL_779882, EPI_ISL_779890, EPI_ISL_779895, EPI_ISL_779901, EPI_ISL_779905, EPI_ISL_779911, EPI_ISL_779919			
see above	Servicio de Microbiología, Hospital Universitario Son Espases	SeqCOVID-SPAIN consortium/IBV(CSIC)	Carla López-Causapé, Jordi Reina, Antonio Oliver and SeqCOVID-SPAIN consortium
EPI_ISL_782482, EPI_ISL_782483, EPI_ISL_782484, EPI_ISL_782485, EPI_ISL_782487, EPI_ISL_782488, EPI_ISL_782489, EPI_ISL_782490, EPI_ISL_782491, EPI_ISL_782492, EPI_ISL_782493, EPI_ISL_782495, EPI_ISL_782496, EPI_ISL_782497, EPI_ISL_782498, EPI_ISL_782499, EPI_ISL_782501, EPI_ISL_782502, EPI_ISL_782503, EPI_ISL_782505, EPI_ISL_782506, EPI_ISL_782507, EPI_ISL_782508, EPI_ISL_782509, EPI_ISL_782510, EPI_ISL_782511, EPI_ISL_782513, EPI_ISL_782514, EPI_ISL_782515, EPI_ISL_782516, EPI_ISL_782517, EPI_ISL_782518, EPI_ISL_782519, EPI_ISL_782520, EPI_ISL_782521, EPI_ISL_782522, EPI_ISL_782523, EPI_ISL_782524, EPI_ISL_782525, EPI_ISL_782526, EPI_ISL_782527, EPI_ISL_782528, EPI_ISL_782529, EPI_ISL_782530, EPI_ISL_782531, EPI_ISL_782532, EPI_ISL_782533, EPI_ISL_782534, EPI_ISL_782535, EPI_ISL_782537, EPI_ISL_782539, EPI_ISL_782540, EPI_ISL_782541, EPI_ISL_782542, EPI_ISL_782543, EPI_ISL_782544, EPI_ISL_782545, EPI_ISL_782546, EPI_ISL_782547, EPI_ISL_782548, EPI_ISL_782549, EPI_ISL_782550, EPI_ISL_782551, EPI_ISL_782552, EPI_ISL_782553, EPI_ISL_782554, EPI_ISL_782555, EPI_ISL_782556, EPI_ISL_782557, EPI_ISL_782558, EPI_ISL_782559, EPI_ISL_782560, EPI_ISL_782561, EPI_ISL_782562, EPI_ISL_782563, EPI_ISL_782564, EPI_ISL_782565, EPI_ISL_782566, EPI_ISL_782567, EPI_ISL_782569, EPI_ISL_782570, EPI_ISL_782571, EPI_ISL_782572, EPI_ISL_782573, EPI_ISL_782574, EPI_ISL_782575, EPI_ISL_782576, EPI_ISL_782577, EPI_ISL_782578, EPI_ISL_782579, EPI_ISL_782581, EPI_ISL_782582, EPI_ISL_782583, EPI_ISL_782585, EPI_ISL_782586, EPI_ISL_782587, EPI_ISL_782588, EPI_ISL_782589, EPI_ISL_782590, EPI_ISL_782591, EPI_ISL_782592, EPI_ISL_782593, EPI_ISL_782594, EPI_ISL_782595, EPI_ISL_782597, EPI_ISL_782598, EPI_ISL_782599, EPI_ISL_782600, EPI_ISL_782601, EPI_ISL_782602, EPI_ISL_782603, EPI_ISL_782604, EPI_ISL_782606, EPI_ISL_782607, EPI_ISL_782608, EPI_ISL_782609, EPI_ISL_782610, EPI_ISL_782611, EPI_ISL_782612, EPI_ISL_782613, EPI_ISL_782614, EPI_ISL_782616, EPI_ISL_782617, EPI_ISL_782619, EPI_ISL_782620, EPI_ISL_782621, EPI_ISL_782622, EPI_ISL_782623, EPI_ISL_782625, EPI_ISL_782626, EPI_ISL_782627, EPI_ISL_782628, EPI_ISL_782629, EPI_ISL_782630, EPI_ISL_782632, EPI_ISL_782634, EPI_ISL_782635, EPI_ISL_782636, EPI_ISL_782637, EPI_ISL_782638, EPI_ISL_782639, EPI_ISL_782640, EPI_ISL_782641, EPI_ISL_782642, EPI_ISL_782643, EPI_ISL_782644, EPI_ISL_782645, EPI_ISL_782646, EPI_ISL_782647, EPI_ISL_782648, EPI_ISL_782649, EPI_ISL_782650, EPI_ISL_782651, EPI_ISL_782652, EPI_ISL_782653, EPI_ISL_782654, EPI_ISL_782655, EPI_ISL_782656, EPI_ISL_782657, EPI_ISL_782658, EPI_ISL_782659, EPI_ISL_782660, EPI_ISL_782661, EPI_ISL_782662, EPI_ISL_782663, EPI_ISL_782664, EPI_ISL_782665, EPI_ISL_782666, EPI_ISL_782667, EPI_ISL_782668, EPI_ISL_782669, EPI_ISL_782670, EPI_ISL_782671, EPI_ISL_782672, EPI_ISL_782673, EPI_ISL_782675, EPI_ISL_782676, EPI_ISL_782677, EPI_ISL_782678, EPI_ISL_782680, EPI_ISL_782681, EPI_ISL_782682, EPI_ISL_782683, EPI_ISL_782684, EPI_ISL_782685, EPI_ISL_782686, EPI_ISL_782687, EPI_ISL_782688, EPI_ISL_782690, EPI_ISL_782691, EPI_ISL_782692, EPI_ISL_782693, EPI_ISL_782694, EPI_ISL_782695, EPI_ISL_782696, EPI_ISL_782697, EPI_ISL_782698, EPI_ISL_782699, EPI_ISL_782700, EPI_ISL_782701, EPI_ISL_782702, EPI_ISL_782703, EPI_ISL_782704, EPI_ISL_782705, EPI_ISL_782706, EPI_ISL_782707, EPI_ISL_782708, EPI_ISL_782709, EPI_ISL_782711, EPI_ISL_782712, EPI_ISL_782713, EPI_ISL_782714, EPI_ISL_782715, EPI_ISL_782716, EPI_ISL_782717, EPI_ISL_782718, EPI_ISL_782719, EPI_ISL_782720, EPI_ISL_782721, EPI_ISL_782722, EPI_ISL_782723, EPI_ISL_782724, EPI_ISL_782725, EPI_ISL_782726, EPI_ISL_782727, EPI_ISL_782728, EPI_ISL_782729, EPI_ISL_782730, EPI_ISL_782731, EPI_ISL_782732, EPI_ISL_782733			
see above	Lighthouse Lab in Glasgow	Wellcome Sanger Institute for the COVID-19 Genomics UK (COG-UK) Consortium	Harper VanSteenhouse, Yumi Kasai, David Gray, Carol Clugston, Anna Dominiczak and Alex Alderton, Roberto Amato, Sonia Goncalves, Ewan Harrison, David K. Jackson, Ian Johnston, Dominic Kwiatkowski, Cordelia Langford, John Sillitoe on behalf of the Wellcome Sanger Institute COVID-19 Surveillance Team
EPI_ISL_789045	Klinisk mikrobiologi	The Public Health Agency of Sweden	Department of Microbiology, The Public Health Agency of Sweden
EPI_ISL_790593, EPI_ISL_790596, EPI_ISL_790597, EPI_ISL_790627, EPI_ISL_790648, EPI_ISL_790649, EPI_ISL_790654, EPI_ISL_790655, EPI_ISL_790657, EPI_ISL_790658, EPI_ISL_790659, EPI_ISL_790660, EPI_ISL_790661, EPI_ISL_790662, EPI_ISL_790663, EPI_ISL_790666, EPI_ISL_790668, EPI_ISL_790669, EPI_ISL_790670, EPI_ISL_790671, EPI_ISL_790679, EPI_ISL_790690, EPI_ISL_790691, EPI_ISL_790692, EPI_ISL_790741, EPI_ISL_790792, EPI_ISL_790793, EPI_ISL_790800, EPI_ISL_790810, EPI_ISL_790811, EPI_ISL_790812, EPI_ISL_790818, EPI_ISL_790819, EPI_ISL_790820, EPI_ISL_790821, EPI_ISL_790822, EPI_ISL_790866, EPI_ISL_790867, EPI_ISL_790868, EPI_ISL_790892, EPI_ISL_790900, EPI_ISL_790918, EPI_ISL_790932, EPI_ISL_790952, EPI_ISL_790956, EPI_ISL_790957, EPI_ISL_790958, EPI_ISL_790989, EPI_ISL_790990, EPI_ISL_790991, EPI_ISL_790992, EPI_ISL_790993, EPI_ISL_790994, EPI_ISL_791036, EPI_ISL_791037, EPI_ISL_791042, EPI_ISL_791043, EPI_ISL_791068, EPI_ISL_791069, EPI_ISL_791070, EPI_ISL_791079, EPI_ISL_791082			
see above	Dutch COVID-19 response team	National Institute for Public Health and the Environment (RIVM)	Adam Meijer, Harry Vennema, Jeroen Cremer, Sharon van den Brink, Bas van der Veer, AnneMarie van den Brandt, Florian Zwagemaker, Dennis Schmitz, Chantal Reusken, on behalf of the national COVID-19 response team
EPI_ISL_791208, EPI_ISL_791220	Respiratory Virus Unit, National Infection Service, Public Health England	COVID-19 Genomics UK (COG-UK) Consortium	PHE Covid Sequencing Team
EPI_ISL_791293, EPI_ISL_791294, EPI_ISL_791307, EPI_ISL_791308, EPI_ISL_791324, EPI_ISL_791325, EPI_ISL_791326	National Virus Reference Laboratory	Irish Coronavirus Sequencing Consortium - Teagasc Moorepark	Alejandro Abner Garcia Leon, Paul Cotter, Fiona Crispie, John Kenny, Paddy Mallon, Calum Walsh
EPI_ISL_791996, EPI_ISL_791997, EPI_ISL_791998	CHU - Hôpital Cavale Blanche	National Reference Center for Viruses of Respiratory Infections, Institut Pasteur, Paris	Marion Barbet, Sylvie Behillil, Méline Bizard, Angela Brisebarre, Camille Capel, Etienne Simon-Lorière, Vincent Enouf, Maud Vanpeene, Sylvie van der Werf, Léa Pilorgé
EPI_ISL_792016	Plateforme COVID IDF	National Reference Center for Viruses of Respiratory Infections, Institut Pasteur, Paris	Marion Barbet, Sylvie Behillil, Méline Bizard, Angela Brisebarre, Camille Capel, Etienne Simon-Lorière, Vincent Enouf, Maud Vanpeene, Sylvie van der Werf,Jacques Fourgeaud
EPI_ISL_794725, EPI_ISL_794728	PathWest Laboratory Medicine WA	PathWest Laboratory Medicine WA Microbial Surveillance Unit	PathWest Laboratory Medicine WA Microbial Surveillance Unit
EPI_ISL_794760, EPI_ISL_794761, EPI_ISL_794762, EPI_ISL_794763, EPI_ISL_794764, EPI_ISL_794765, EPI_ISL_794766, EPI_ISL_794767, EPI_ISL_794768, EPI_ISL_794769, EPI_ISL_794770, EPI_ISL_794771, EPI_ISL_794772, EPI_ISL_794773, EPI_ISL_794791, EPI_ISL_794792			
see above	Istituto Zooprofilattico Sperimentale della Puglia e della Basilicata	Istituto Zooprofilattico Sperimentale della Puglia e della Basilicata	Parisi A., Bianco A., Capozzi L., Del Sambro L., Manzulli V, Rondinone V., Pace L., Cipolletta D., Galante D.
EPI_ISL_796658	Dept. of Medical Microbiology, Stavanger University Hospital, Helse Stavanger HF	Norwegian Institute of Public Health, Department of Virology	Kathrine Stene-Johansen, Kamilla Heddeland Instefjord, Hilde Elshaug, Atiya R Ali,Marie Paulsen Madsen, Rasmus Riis Kopperud, Hilde Vollan, Karoline Bragstad, Olav Hungnes
EPI_ISL_796684, EPI_ISL_796685	Norwegian Institute of Public Health, Department of Virology	Norwegian Institute of Public Health, Department of Virology	Kathrine Stene-Johansen, Kamilla Heddeland Instefjord, Hilde Elshaug, Atiya R Ali,Marie Paulsen Madsen, Rasmus Riis Kopperud, Hilde Vollan, Karoline Bragstad, Olav Hungnes
EPI_ISL_796689, EPI_ISL_796690	Nordland Hospital - Bodo, Laboratory Department, Molecular Biology Unit	Norwegian Institute of Public Health, Department of Virology	Kathrine Stene-Johansen, Kamilla Heddeland Instefjord, Hilde Elshaug, Atiya R Ali,Marie Paulsen Madsen, Rasmus Riis Kopperud, Hilde Vollan, Karoline Bragstad, Olav Hungnes
EPI_ISL_796706	Department of Medical Microbiology, St. Olavs hospital	Norwegian Institute of Public Health, Department of Virology	Kathrine Stene-Johansen, Kamilla Heddeland Instefjord, Hilde Elshaug, Atiya R Ali,Marie Paulsen Madsen, Rasmus Riis Kopperud, Hilde Vollan, Karoline Bragstad, Olav Hungnes
EPI_ISL_796707, EPI_ISL_796708, EPI_ISL_796709	University Hospital of Northern Norway, Department for Microbiology and Infectious Disease Control	Norwegian Institute of Public Health, Department of Virology	Kathrine Stene-Johansen, Kamilla Heddeland Instefjord, Hilde Elshaug, Atiya R Ali,Marie Paulsen Madsen, Rasmus Riis Kopperud, Hilde Vollan, Karoline Bragstad, Olav Hungnes
EPI_ISL_796710	Norwegian Institute of Public Health, Department of Virology	Norwegian Institute of Public Health, Department of Virology	Kathrine Stene-Johansen, Kamilla Heddeland Instefjord, Hilde Elshaug, Atiya R Ali,Marie Paulsen Madsen, Rasmus Riis Kopperud, Hilde Vollan, Karoline Bragstad, Olav Hungnes
EPI_ISL_796725, EPI_ISL_796727, EPI_ISL_796728	Department of Medical Microbiology, St. Olavs hospital	Norwegian Institute of Public Health, Department of Virology	Kathrine Stene-Johansen, Kamilla Heddeland Instefjord, Hilde Elshaug, Atiya R Ali,Marie Paulsen Madsen, Rasmus Riis Kopperud, Hilde Vollan, Karoline Bragstad, Olav Hungnes
EPI_ISL_796766, EPI_ISL_796770	Instituto Nacional de Saude (INSA)	Instituto Nacional de Saude (INSA)	Borges et al

EPI_ISL_801179	Lighthouse Lab in Glasgow	Wellcome Sanger Institute for the COVID-19 Genomics UK (COG-UK) Consortium	Harper VanSteenhouse, Yumi Kasai, David Gray, Carol Clugston, Anna Dominiczak and Alex Alderton, Roberto Amato, Sonia Goncalves, Ewan Harrison, David K. Jackson, Ian Johnston, Dominic Kwiatkowski, Cordelia Langford, John Sillitoe on behalf of the Wellcome Sanger Institute COVID-19 Surveillance Team
EPI_ISL_801408, EPI_ISL_801424, EPI_ISL_801425	Dutch COVID-19 response team	Erasmus Medical Center	Bas Oude Munnink, Reina Sikkema, David Nieuwenhuijse, Irina Chestakova, Anne van der Linden, Marjan Boter, Emmanuelle Munger, Corine GeurtsvanKessel, Annemiek van der Eijk, Richard Molenkamp, Marion Koopmans, on behalf of the Dutch national COVID-19 response team.
EPI_ISL_802864, EPI_ISL_802867, EPI_ISL_802868, EPI_ISL_802870, EPI_ISL_802871, EPI_ISL_802873, EPI_ISL_802874, EPI_ISL_802875, EPI_ISL_802877, EPI_ISL_802878, EPI_ISL_802881, EPI_ISL_802882, EPI_ISL_802883, EPI_ISL_802884, EPI_ISL_802885, EPI_ISL_802888, EPI_ISL_802889, EPI_ISL_802890, EPI_ISL_802891, EPI_ISL_802892, EPI_ISL_802895, EPI_ISL_802896, EPI_ISL_802897, EPI_ISL_802899, EPI_ISL_802900, EPI_ISL_802901, EPI_ISL_802903, EPI_ISL_802904, EPI_ISL_802905, EPI_ISL_802907, EPI_ISL_802910, EPI_ISL_802912, EPI_ISL_802913, EPI_ISL_802915, EPI_ISL_802916, EPI_ISL_802918, EPI_ISL_802919, EPI_ISL_802920, EPI_ISL_802922, EPI_ISL_802926, EPI_ISL_802928, EPI_ISL_802930, EPI_ISL_802933, EPI_ISL_802935, EPI_ISL_802937, EPI_ISL_802939, EPI_ISL_802940, EPI_ISL_802942, EPI_ISL_802943, EPI_ISL_802947, EPI_ISL_802950, EPI_ISL_802951, EPI_ISL_802952, EPI_ISL_802955, EPI_ISL_802957, EPI_ISL_802958, EPI_ISL_802962, EPI_ISL_802963, EPI_ISL_802966, EPI_ISL_802969, EPI_ISL_802971, EPI_ISL_802981, EPI_ISL_802982, EPI_ISL_802983, EPI_ISL_802984, EPI_ISL_802985, EPI_ISL_802986, EPI_ISL_802988, EPI_ISL_802989, EPI_ISL_802990, EPI_ISL_802992, EPI_ISL_802993			
see above	Utah Public Health Laboratory	Utah Public Health Laboratory	Erin Young, Kelly Oakeson, Tara Gallagher
EPI_ISL_803890	Robert Koch Institute	Robert Koch Institute	Annika Brinkmann, Janine Michel, Livia Schrick, Steven Uddin, Dominique Seifert, Merle Corty, Lars Schaade, Andreas Nitsche
EPI_ISL_804373, EPI_ISL_804375, EPI_ISL_804377	CHU Purpan - Laboratoire de Virologie - Institut Fédératif de Biologie	CHU Purpan - Laboratoire de Virologie - Institut Fédératif de Biologie	Latour J., Ranger N., Dubois M., Carcenac R., Harter A., Boyer P., Tremeaux P., Izopet J.
EPI_ISL_804625, EPI_ISL_804654, EPI_ISL_804677, EPI_ISL_804706	Michigan Department of Health and Human Services, Bureau of Laboratories	Michigan Department of Health and Human Services, Bureau of Laboratories	Blankenship HM, Riner D, Soehnlen MK
EPI_ISL_804892	DC Public Health Lab/ Dept. of Forensic Sciences	DC Public Health Lab/ Dept. of Forensic Sciences	Scott Nguyen, Elizabeth Zelaya, Connie Maza, Monica Mann, Brittany Hamilton, David Payne, Jocelyn Hauser
EPI_ISL_806845, EPI_ISL_806848, EPI_ISL_806849, EPI_ISL_806850	Alaska State Virology Laboratory	Alaska State Virology Laboratory	Stephanie DeRonde, Lisa Smith, Ph.D., Devin M. Drown, Ph.D., Jack Chen, Ph.D.
EPI_ISL_812362, EPI_ISL_812363, EPI_ISL_812364, EPI_ISL_812365, EPI_ISL_812366, EPI_ISL_812393, EPI_ISL_812394, EPI_ISL_812395, EPI_ISL_812403, EPI_ISL_812405, EPI_ISL_812406			
see above	Utah Public Health Laboratory	Utah Public Health Laboratory	Erin L. Young, Kelly F. Oakeson, Tara Gallagher
EPI_ISL_812421, EPI_ISL_812422	University of Wisconsin-Madison AIDS Vaccine Research Laboratories	University of Wisconsin-Madison AIDS Vaccine Research Laboratories	Gage Moreno, Katarina Braun, et al. AIDS Vaccine Research Laboratories
EPI_ISL_812443, EPI_ISL_812444, EPI_ISL_812445, EPI_ISL_812446, EPI_ISL_812447, EPI_ISL_812448, EPI_ISL_812449, EPI_ISL_812450, EPI_ISL_812451, EPI_ISL_812452, EPI_ISL_812453, EPI_ISL_812454, EPI_ISL_812455, EPI_ISL_812456, EPI_ISL_812457, EPI_ISL_812458, EPI_ISL_812459, EPI_ISL_812460, EPI_ISL_812461, EPI_ISL_812462, EPI_ISL_812463, EPI_ISL_812464, EPI_ISL_812465, EPI_ISL_812466, EPI_ISL_812467, EPI_ISL_812468, EPI_ISL_812469, EPI_ISL_812470, EPI_ISL_812471, EPI_ISL_812472, EPI_ISL_812473, EPI_ISL_812474, EPI_ISL_812475, EPI_ISL_812476, EPI_ISL_812477, EPI_ISL_812478, EPI_ISL_812479, EPI_ISL_812480, EPI_ISL_812483, EPI_ISL_812484, EPI_ISL_812485, EPI_ISL_812486, EPI_ISL_812487, EPI_ISL_812489, EPI_ISL_812490, EPI_ISL_812491, EPI_ISL_812492, EPI_ISL_812493, EPI_ISL_812494, EPI_ISL_812495, EPI_ISL_812496, EPI_ISL_812497, EPI_ISL_812498, EPI_ISL_812499, EPI_ISL_812500, EPI_ISL_812501, EPI_ISL_812502, EPI_ISL_812503, EPI_ISL_812504, EPI_ISL_812505, EPI_ISL_812506, EPI_ISL_812507, EPI_ISL_812508, EPI_ISL_812509, EPI_ISL_812510, EPI_ISL_812511, EPI_ISL_812512, EPI_ISL_812513, EPI_ISL_812514, EPI_ISL_812515, EPI_ISL_812516, EPI_ISL_812517, EPI_ISL_812712, EPI_ISL_812713, EPI_ISL_812714, EPI_ISL_812715, EPI_ISL_812716, EPI_ISL_812717, EPI_ISL_812718, EPI_ISL_812719, EPI_ISL_812720, EPI_ISL_812721			
see above	Laboratorio de Referencia Nacional de Virus Respiratorios, Instituto Nacional de Salud Peru	Laboratorio de Genómica Microbiana, Universidad Peruana Cayetano Heredia	Pablo Tsukayama, Alejandra Dávila-Barclay, Guillermo Salvatierra, Luis González, Pedro E. Romero, Brenda Ayzanoa, Janet Huancachoque, Pool Marcos, Camila Castillo-Vilcahuamán, Oscar Escalante, Priscila Lope, Nancy Rojas
EPI_ISL_812932	Institute for Urban Disease Control and Prevention	COVID-19 Network Investigations (CONI) Alliance	Kamolthip Atsawawaranunt, Elizabeth Batty, Wasun Chantratita, Thanat Chookajorn, Stefan Fernandez, Angkana Huang, Anthony R. Jones, Khajohn Joonsalak, Chonticha Klungtong, Theerarat Kochakarn, Prayuth Kaewmalang, Amornmas Kongklieng, Namfon Kotanan, Krittikorn Kumpornsin, Duangkamon Loesbanluechai, Wudtichai Manasatienkij, Anek Mungaomklang, Bhakbhoom Panthan, Pukkaporn Parnwijitkul, Ekawat Pasomsub, Kingkan Rakmanee, Insee Sensorn, Janjira Thaipadungpanit, Arporn Wangwiwatsin, Treewat Watthanachockchai
EPI_ISL_813037, EPI_ISL_813038, EPI_ISL_813039, EPI_ISL_813040, EPI_ISL_813041, EPI_ISL_813042, EPI_ISL_813043, EPI_ISL_813044, EPI_ISL_813064, EPI_ISL_813065, EPI_ISL_813066, EPI_ISL_813067, EPI_ISL_813068, EPI_ISL_813069, EPI_ISL_813070, EPI_ISL_813072, EPI_ISL_813073, EPI_ISL_813074, EPI_ISL_813075, EPI_ISL_813076, EPI_ISL_813077, EPI_ISL_813078, EPI_ISL_813079, EPI_ISL_813080, EPI_ISL_813081, EPI_ISL_813083, EPI_ISL_813085, EPI_ISL_813086			
see above	University of Birmingham	COVID-19 Genomics UK (COG-UK) Consortium	Institute of Microbiology, University of Birmingham: Claire McMurray, Joanne Stockton, Sam Nicholls, Radoslaw Poplawski, Will Rowe, Josh Quick, Nicholas Loman. University of Birmingham Testing Laboratory: Celina M Whalley, Andrew Bosworth, Charlotte Poxon, Kasun Wanigasooriya, Oliver Pickles, Mike Kidd, Alex Richter, Andrew D Beggs PHE Heartlands Lab: Husam Osman, Andrew Bosworth. Queen Elizabeth Hospital: Anna Casey
EPI_ISL_813189	Department of Pathology, University of Cambridge	COVID-19 Genomics UK (COG-UK) Consortium	Aminu S. Jahun, Yasmin Chaudhry, Grant Hall, Iliana Georgana, Myra Hosmillo, Martin D. Curran, Malte Pinckert, Surendra Parmar, Ian Goodfellow
EPI_ISL_813655	Kettering General Hospital	COVID-19 Genomics UK (COG-UK) Consortium	Patrick McClure, Joseph Chappell, Theocharis Tsoleridis, Jonathan Ball, Nadine Holmes, Matthew Carlisle, Christopher Moore, Fei Sang, Johnny Debebe, Victoria Wright, Matthew Loose
EPI_ISL_813732, EPI_ISL_813733, EPI_ISL_813734, EPI_ISL_813735, EPI_ISL_813736, EPI_ISL_813737, EPI_ISL_813738, EPI_ISL_813739, EPI_ISL_813740, EPI_ISL_813741, EPI_ISL_813742, EPI_ISL_813743, EPI_ISL_813744, EPI_ISL_813745, EPI_ISL_813746, EPI_ISL_813747, EPI_ISL_813748, EPI_ISL_813749, EPI_ISL_813750, EPI_ISL_813764, EPI_ISL_813765, EPI_ISL_813766, EPI_ISL_813767, EPI_ISL_813768, EPI_ISL_813769, EPI_ISL_813770			
see above	Liverpool Clinical Laboratories	COVID-19 Genomics UK (COG-UK) Consortium	Sam Haldenby, Anita Lucaci, Steve Paterson, Julian Hiscox, Alistair Darby, M Almsaud, A Alrezaihi, Muhannad Alruwaili, Stuart D Armstrong, Jones Benjamin, Eleanor G Bentley, Anu Chawla, Jordan J Clark, Angela Cowell, Richard Eccles, Isabel García-Dorival, Matthew Gemmell, Alessandro Gerada, PKF Gilmore, Richard Gregory, Ximeng Han, Catherine Hartley, Margaret Hughes, Miren Iturriza-Gomara, James Johnson, L Luu, Jenifer Manson, Charlotte Nelson, Elaine O'Toole, Cassie Olateju, Rebekah Penrice-Randal , Lucille Rainbow, N.P Randle, Trevor Ian Robinson, Parul Sharma, Ghada T Shawli, James P Stewart, Neil Swainston, Ecaterina Vamos, Joanne Watts, Mark Whitehead
EPI_ISL_813978	University Hospital of Northern Norway, Department for Microbiology and Infectious Disease Control	Norwegian Institute of Public Health, Department of Virology	Kathrine Stene-Johansen, Kamilla Heddeland Instefjord, Hilde Elshaug, Atiya R Ali,Marie Paulsen Madsen, Rasmus Riis Kopperud, Hilde Vollan, Karoline Bragstad, Olav Hungnes
EPI_ISL_814285, EPI_ISL_814286	Liverpool Clinical Laboratories	COVID-19 Genomics UK (COG-UK) Consortium	Sam Haldenby, Anita Lucaci, Steve Paterson, Julian Hiscox, Alistair Darby, M Almsaud, A Alrezaihi, Muhannad Alruwaili, Stuart D Armstrong, Jones Benjamin, Eleanor G Bentley, Anu Chawla, Jordan J Clark, Angela Cowell, Richard Eccles, Isabel García-Dorival, Matthew Gemmell, Alessandro Gerada, PKF Gilmore, Richard Gregory, Ximeng Han, Catherine Hartley, Margaret Hughes, Miren Iturriza-Gomara, James Johnson, L Luu, Jenifer Manson, Charlotte Nelson, Elaine O'Toole, Cassie Olateju, Rebekah Penrice-Randal , Lucille Rainbow, N.P Randle, Trevor Ian Robinson, Parul Sharma, Ghada T Shawli, James P Stewart, Neil Swainston, Ecaterina Vamos, Joanne Watts, Mark Whitehead
EPI_ISL_814320, EPI_ISL_814326, EPI_ISL_814404, EPI_ISL_814405, EPI_ISL_814408, EPI_ISL_814468, EPI_ISL_814492, EPI_ISL_814531, EPI_ISL_814536	Wales Specialist Virology Centre Sequencing lab: Pathogen Genomics Unit	COVID-19 Genomics UK (COG-UK) Consortium	Catherine Moore, Johnathan Evans, Laura Gifford, Malorie Perry, Simon Cottrell, Angela Marchbank, Alec Birchley, Alexander Adams, Amy Gaskin, Bree Gatica-Wilcox, Jason Coombes, Joel Southgate, Lauren Gilbert, Lee Graham, Nicole Pacchiarini, Sara Kumziene-Summerhayes, Sarah Taylor, Sophie Jones, Sara Rey, Matthew Bull, Joanne Watkins, Sally Corden, Tom Connor
EPI_ISL_814552	Liverpool Clinical Laboratories	COVID-19 Genomics UK (COG-UK) Consortium	Sam Haldenby, Anita Lucaci, Steve Paterson, Julian Hiscox, Alistair Darby, M Almsaud, A Alrezaihi, Muhannad Alruwaili, Stuart D Armstrong, Jones Benjamin, Eleanor G Bentley, Anu Chawla, Jordan J Clark, Angela Cowell, Richard Eccles, Isabel García-Dorival, Matthew Gemmell, Alessandro Gerada, PKF Gilmore, Richard Gregory, Ximeng Han, Catherine Hartley, Margaret Hughes, Miren Iturriza-Gomara, James Johnson, L Luu, Jenifer Manson, Charlotte Nelson, Elaine O'Toole, Cassie Olateju, Rebekah Penrice-Randal , Lucille Rainbow, N.P Randle, Trevor Ian Robinson, Parul Sharma, Ghada T Shawli, James P Stewart, Neil Swainston, Ecaterina Vamos, Joanne Watts, Mark Whitehead
EPI_ISL_814702, EPI_ISL_814703, EPI_ISL_814704, EPI_ISL_814717, EPI_ISL_814718, EPI_ISL_814719, EPI_ISL_814720, EPI_ISL_814721, EPI_ISL_814722, EPI_ISL_814723, EPI_ISL_814725, EPI_ISL_814726, EPI_ISL_814753, EPI_ISL_814860, EPI_ISL_814861, EPI_ISL_814862, EPI_ISL_814863, EPI_ISL_814864, EPI_ISL_814865, EPI_ISL_814866, EPI_ISL_814867, EPI_ISL_814868, EPI_ISL_814869, EPI_ISL_814870, EPI_ISL_815036, EPI_ISL_815093, EPI_ISL_815094, EPI_ISL_815095, EPI_ISL_815096, EPI_ISL_815097, EPI_ISL_815098, EPI_ISL_815099, EPI_ISL_815100, EPI_ISL_815101, EPI_ISL_815102, EPI_ISL_815103, EPI_ISL_815104, EPI_ISL_815105			
see above	Wales Specialist Virology Centre Sequencing lab: Pathogen Genomics Unit	COVID-19 Genomics UK (COG-UK) Consortium	Catherine Moore, Johnathan Evans, Laura Gifford, Malorie Perry, Simon Cottrell, Angela Marchbank, Alec Birchley, Alexander Adams, Amy Gaskin, Bree Gatica-Wilcox, Jason Coombes, Joel Southgate, Lauren Gilbert, Lee Graham, Nicole Pacchiarini, Sara Kumziene-Summerhayes, Sarah Taylor, Sophie Jones, Sara Rey, Matthew Bull, Joanne Watkins, Sally Corden, Tom Connor
EPI_ISL_816249, EPI_ISL_816250, EPI_ISL_816254, EPI_ISL_816257, EPI_ISL_816266, EPI_ISL_816278, EPI_ISL_816298, EPI_ISL_816306, EPI_ISL_816346, EPI_ISL_816349, EPI_ISL_816354, EPI_ISL_816360, EPI_ISL_816419, EPI_ISL_816420, EPI_ISL_816422, EPI_ISL_816440, EPI_ISL_816454, EPI_ISL_816459, EPI_ISL_816464, EPI_ISL_816465, EPI_ISL_816466, EPI_ISL_816483, EPI_ISL_816490, EPI_ISL_816515, EPI_ISL_816553, EPI_ISL_816569, EPI_ISL_816579, EPI_ISL_816587, EPI_ISL_816593, EPI_ISL_816594, EPI_ISL_816598, EPI_ISL_816604, EPI_ISL_816610, EPI_ISL_816612, EPI_ISL_816615, EPI_ISL_816628			

see above	Virology Department, Sheffield Teaching Hospitals NHS Foundation Trust/Department of Infection, Immunity and Cardiovascular Disease, The Medical School, University of Sheffield	COVID-19 Genomics UK (COG-UK) Consortium	Thushan de Silva, Matthew Parker, Nikki Smith, Adri Angyal, Rebecca Brown, Luke Green, Rachel Tucker, Paul Parsons, Danielle Groves, Katie Johnson, Laura Carrilero, Alex Keeley, Dave Partridge, Matthew Wyles, Benjamin Lindsey, Mehmet Yavuz, Mohammad Raza, Cariad Evans
EPI_ISL_816894, EPI_ISL_816896, EPI_ISL_816899, EPI_ISL_816905, EPI_ISL_816907, EPI_ISL_816908, EPI_ISL_816912, EPI_ISL_816914, EPI_ISL_816917, EPI_ISL_816919, EPI_ISL_816921, EPI_ISL_816922, EPI_ISL_816926, EPI_ISL_816927, EPI_ISL_816928, EPI_ISL_816929, EPI_ISL_816930, EPI_ISL_816931, EPI_ISL_816932, EPI_ISL_816933, EPI_ISL_816934, EPI_ISL_816935, EPI_ISL_816936, EPI_ISL_816937, EPI_ISL_816938, EPI_ISL_816939, EPI_ISL_816940, EPI_ISL_816941, EPI_ISL_816942, EPI_ISL_816943, EPI_ISL_816944, EPI_ISL_816945, EPI_ISL_816946, EPI_ISL_816947, EPI_ISL_816948, EPI_ISL_816949, EPI_ISL_816950, EPI_ISL_816951, EPI_ISL_816952, EPI_ISL_816953, EPI_ISL_816954, EPI_ISL_816955, EPI_ISL_816956, EPI_ISL_816957, EPI_ISL_816961, EPI_ISL_816963, EPI_ISL_816966, EPI_ISL_816972, EPI_ISL_816974, EPI_ISL_816975, EPI_ISL_816979, EPI_ISL_816981, EPI_ISL_816984, EPI_ISL_816986, EPI_ISL_816988, EPI_ISL_816989, EPI_ISL_816993, EPI_ISL_816994, EPI_ISL_816995, EPI_ISL_816996, EPI_ISL_816997, EPI_ISL_816998, EPI_ISL_816999, EPI_ISL_817000, EPI_ISL_817001, EPI_ISL_817002, EPI_ISL_817003, EPI_ISL_817004, EPI_ISL_817005, EPI_ISL_817006, EPI_ISL_817007, EPI_ISL_817008, EPI_ISL_817009, EPI_ISL_817010, EPI_ISL_817011, EPI_ISL_817012, EPI_ISL_817013, EPI_ISL_817014, EPI_ISL_817015, EPI_ISL_817016, EPI_ISL_817017, EPI_ISL_817018, EPI_ISL_817019, EPI_ISL_817020, EPI_ISL_817021, EPI_ISL_817022, EPI_ISL_817023, EPI_ISL_817024, EPI_ISL_817049, EPI_ISL_817060			
see above	Bioinformatics and Biostatistics Lab, Advanced Sequencing Facility	COVID-19 Genomics UK (COG-UK) Consortium	Aengus Stewart,Jerome Nicod,Chelsea Sawyer,Laura Cubitt,Harshil Patel,Margaret Crawford
EPI_ISL_819575, EPI_ISL_819576	Northumbria University / South Tees Hospitals NHS Foundation Trust / North Cumbria Integrated Care NHS Foundation Trust / North Tees and Hartlepool NHS Foundation Trust / Newcastle Hospitals NHS Foundation Trust	COVID-19 Genomics UK (COG-UK) Consortium	Darren L Smith,Andrew Nelson,Matthew Bashton,Greg R Young,Joshua Loh,John Allan,Mohammad A Tariq,Giles S Holt,Gary Black,Wen C Yew,Lynn Dover,Paul Baker,Steve Liggett,Sarah Essex,Jane Greenaway,Debra Padgett,Clive Graham,Garren Scott,Edward Barton,Emma Swindells,Brendan Payne,Jennifer Collins,Yusri Taha,Gary Eltringham
EPI_ISL_819577	Queens Medical Centre, Clinical Microbiology Department / DeepSeq Nottingham	COVID-19 Genomics UK (COG-UK) Consortium	Gemma Clark, Wendy Smith, Manjinder Khakh, Vicki M Fleming, Michelle M Lister, Hannah Howson-Wells, Jonathan Ball, Patrick McClure, Joseph Chappell, Theocharis Tsoleridis, Nadine Holmes, Matthew Carlisle, Christopher Moore, Fei Sang, Johnny Debebe, Victoria Wright, Matthew Loose
EPI_ISL_820174, EPI_ISL_820346, EPI_ISL_820351, EPI_ISL_820413	Lighthouse Lab in Alderley Park	Wellcome Sanger Institute for the COVID-19 Genomics UK (COG-UK) Consortium	Jacquelyn Wynn, Mairead Hyland, The Lighthouse Lab in Alderley Park and Alex Alderton, Roberto Amato, Sonia Goncalves, Ewan Harrison, David K. Jackson, Ian Johnston, Dominic Kwiatkowski, Cordelia Langford, John Sillitoe on behalf of the Wellcome Sanger Institute COVID-19 Surveillance Team
EPI_ISL_822842, EPI_ISL_822848, EPI_ISL_822849, EPI_ISL_822850, EPI_ISL_822863, EPI_ISL_822869, EPI_ISL_822872, EPI_ISL_822916, EPI_ISL_822927, EPI_ISL_822936, EPI_ISL_822937, EPI_ISL_822977, EPI_ISL_823004, EPI_ISL_823014, EPI_ISL_823057, EPI_ISL_823058, EPI_ISL_823129, EPI_ISL_823162, EPI_ISL_823175, EPI_ISL_823205, EPI_ISL_823208, EPI_ISL_823209, EPI_ISL_823210, EPI_ISL_823211, EPI_ISL_823212, EPI_ISL_823213, EPI_ISL_823214, EPI_ISL_823215, EPI_ISL_823216, EPI_ISL_823217, EPI_ISL_823569, EPI_ISL_823570, EPI_ISL_823571, EPI_ISL_823572, EPI_ISL_823573, EPI_ISL_823574, EPI_ISL_823575, EPI_ISL_823576, EPI_ISL_823577, EPI_ISL_823578, EPI_ISL_823579, EPI_ISL_823580, EPI_ISL_823581, EPI_ISL_823583, EPI_ISL_823584, EPI_ISL_823585, EPI_ISL_823587, EPI_ISL_823588, EPI_ISL_823589, EPI_ISL_823590, EPI_ISL_823591, EPI_ISL_823592, EPI_ISL_823593, EPI_ISL_823594, EPI_ISL_823595, EPI_ISL_823596, EPI_ISL_823597, EPI_ISL_823598, EPI_ISL_823599, EPI_ISL_823600, EPI_ISL_823601, EPI_ISL_823602, EPI_ISL_823603, EPI_ISL_823604, EPI_ISL_823605, EPI_ISL_823606, EPI_ISL_823607, EPI_ISL_823625			
see above	Wales Specialist Virology Centre Sequencing lab: Pathogen Genomics Unit	COVID-19 Genomics UK (COG-UK) Consortium	Catherine Moore, Johnathan Evans, Laura Gifford, Malorie Perry, Simon Cottrell, Angela Marchbank, Alec Birchley, Alexander Adams, Amy Gaskin, Bree Gatica-Wilcox, Jason Coombes, Joel Southgate, Lauren Gilbert, Lee Graham, Nicole Pacchiarini, Sara Kumziene-Summerhayes, Sarah Taylor, Sophie Jones, Sara Rey, Matthew Bull, Joanne Watkins, Sally Corden, Tom Connor
EPI_ISL_823813	DOHMH Jamaica	New York City Public Health Laboratory	Jade Wang, et al.
EPI_ISL_823814	DOHMH Riverside	New York City Public Health Laboratory	Jade Wang, et al.
EPI_ISL_823815, EPI_ISL_823816	DOHMH Chelsea	New York City Public Health Laboratory	Jade Wang, et al.
EPI_ISL_823817, EPI_ISL_823818, EPI_ISL_823819	DOHMH Corona	New York City Public Health Laboratory	Jade Wang, et al.
EPI_ISL_823820	DOHMH PHL	New York City Public Health Laboratory	Jade Wang, et al.
EPI_ISL_823821, EPI_ISL_823822	DOHMH Jamaica	New York City Public Health Laboratory	Jade Wang, et al.
EPI_ISL_823823, EPI_ISL_823824, EPI_ISL_823825	DOHMH Riverside	New York City Public Health Laboratory	Jade Wang, et al.
EPI_ISL_823826, EPI_ISL_823827, EPI_ISL_823828	DOHMH Corona	New York City Public Health Laboratory	Jade Wang, et al.
EPI_ISL_823829, EPI_ISL_823830, EPI_ISL_823831	DOHMH Morrisania	New York City Public Health Laboratory	Jade Wang, et al.
EPI_ISL_823832, EPI_ISL_823833, EPI_ISL_823834	DOHMH Central Harlem	New York City Public Health Laboratory	Jade Wang, et al.
EPI_ISL_823835, EPI_ISL_823836, EPI_ISL_823837	DOHMH Corona	New York City Public Health Laboratory	Jade Wang, et al.
EPI_ISL_823993, EPI_ISL_824034, EPI_ISL_824055	Dutch COVID-19 response team	National Institute for Public Health and the Environment (RIVM)	Adam Meijer, Harry Vennema, Jeroen Cremer, Sharon van den Brink, Bas van der Veer, AnneMarie van den Brandt, Florian Zwagemaker, Dennis Schmitz, Chantal Reusken, on behalf of the national COVID-19 response team
EPI_ISL_824297	DOHMH Jamaica	New York City Public Health Laboratory	Jade Wang, et al.
EPI_ISL_824299	DOHMH Morrisania	New York City Public Health Laboratory	Jade Wang, et al.
EPI_ISL_824300	DOHMH Chelsea	New York City Public Health Laboratory	Jade Wang, et al.
EPI_ISL_824301	DOHMH Crown Heights	New York City Public Health Laboratory	Jade Wang, et al.
EPI_ISL_824302	DOHMH Jamaica	New York City Public Health Laboratory	Jade Wang, et al.
EPI_ISL_824303	DOHMH Morrisania	New York City Public Health Laboratory	Jade Wang, et al.
EPI_ISL_824304, EPI_ISL_824305	DOHMH Jamaica	New York City Public Health Laboratory	Jade Wang, et al.
EPI_ISL_824306, EPI_ISL_824307	DOHMH PHL	New York City Public Health Laboratory	Jade Wang, et al.
EPI_ISL_824308, EPI_ISL_824309	DOHMH Jamaica	New York City Public Health Laboratory	Jade Wang, et al.
EPI_ISL_824310	DOHMH Crown Heights	New York City Public Health Laboratory	Jade Wang, et al.
EPI_ISL_824347, EPI_ISL_824350, EPI_ISL_824353, EPI_ISL_824354, EPI_ISL_824365, EPI_ISL_824367, EPI_ISL_824368, EPI_ISL_824369, EPI_ISL_824380, EPI_ISL_824383	Michigan Department of Health and Human Services, Bureau of Laboratories	Michigan Department of Health and Human Services, Bureau of Laboratories	Blankenship HM, Riner D, Soehnlen MK
EPI_ISL_824401, EPI_ISL_824403	California Department of Public Health	California Department of Public Health	CDPH IDLB COVIDNet
EPI_ISL_824833	Department of Clinical Microbiology	GIGA Medical Genomics	Keith Durkin, Maria Artesi, Sébastien Bontems, Raphaël Boreux, Bouchra Boujemla, Cécile Meex, Pierrette Melin, Marie-Pierre Hayette, Vincent Bours
EPI_ISL_824982	Maryland Public Health Laboratory	Maryland Public Health Laboratory	Maryland Department of Health Laboratories Administration
EPI_ISL_825017, EPI_ISL_825018, EPI_ISL_825019, EPI_ISL_825021, EPI_ISL_825023	Utah Public Health Laboratory, Utah Public Health Laboratory Infectious Disease submission group	Utah Public Health Laboratory, Utah Public Health Laboratory Infectious Disease submission group	Young,E.L., Oakeson,K.F., Gallagher,T.
EPI_ISL_825131, EPI_ISL_825136	NHLS-IALCH	KRISP, KZN Research Innovation and Sequencing Platform	Giandhari J, Pillay S, Lessells R, Mdlalose K, York D, Khan S, Tegally H, Wilkinson E, de Oliveira T
EPI_ISL_825375, EPI_ISL_825376, EPI_ISL_825377	Hospital Universitari Vall d'Hebron - Vall d'Hebron Institut de Recerca	Hospital Universitari Vall d'Hebron	Cristina Andrés, Maria Piñana, Josep F Abril, Damir Garcia-Cehic, Ariadna Rando, Juliana Esperalba, Maria Gema Codina, Carla Castillo, Maria Carmen Martin, Tomàs Pumarola, Josep Quer, Andrés Antón

EPI_ISL_825437	Nigeria Centre For Disease Control	National reference Laboratory, NCDC, Gaduwa, Abuja	Dr Ndodo Nnaemeka, Olusola Anuoluwapo Akanbi, Chimaobi Chukwu, Dr Omoare Adesuyi, Esebanmen Grace, Kingsley Njoku, Anthony Ahumibe, Naidoo Dhamari, Nwando Mba, Dr Chikwe Ihekweazu
EPI_ISL_826275	Sterling Hospital, Memnagar, Ahmedabad	Gujarat Biotechnology Research Centre	Atul K Patel, Nitin Savaliya, Dinesh Kumar, Zuber Saiyed, Labdhi Pandya, Afzal Ansari, Nikha Trivedi, Apurvasinh Puvar, Ramesh Pandit, Janvi Raval, Zarna Patel, Naman Shashtri, Chaitanya Joshi, Madhvi Joshi
EPI_ISL_826276	Sterling Hospital, Memnagar, Ahmedabad	Gujarat Biotechnology Research Centre	Dinesh Kumar, Zuber Saiyed, Labdhi Pandya, Afzal Ansari, Nikha Trivedi, Apurvasinh Puvar, Ramesh Pandit, Janvi Raval, Zarna Patel, Nitin Savaliya, Atul K Patel, Naman Shashtri, Chaitanya Joshi, Madhvi Joshi
EPI_ISL_826465	Lighthouse Lab in Alderley Park	Wellcome Sanger Institute for the COVID-19 Genomics UK (COG-UK) Consortium	Jacquelyn Wynn, Mairead Hyland, The Lighthouse Lab in Alderley Park and Alex Alderton, Roberto Amato, Sonia Goncalves, Ewan Harrison, David K. Jackson, Ian Johnston, Dominic Kwiatkowski, Cordelia Langford, John Sillitoe on behalf of the Wellcome Sanger Institute COVID-19 Surveillance Team
EPI_ISL_827183, EPI_ISL_827184	deCODE genetics	deCODE genetics	Daniel F Gudbjartsson; Agnar Helgason; Hakon Jonsson; Olafur T Magnusson; Pall Melsted; Gudmundur L Norddahl; Jona Saemundsdottir; Asgeir Sigurdsson; Patrick Sulem; Arna B Agustsdottir; Hannes Eggertsson; Berglind Eiriksdottir; Run Fridriksdottir; Elisabet E Gardarsdottir; Gudmundur Georgsson; Olafia S Gretarsdottir; Kjartan R Gudmundsson; Thora R Gunnarsdottir; Arnaldur Gylfason; Hilma Holm; Brynjar O Jensson; Aslaug Jonasdottir; Kamilla S Josefsdottir; Thordur Kristjansson; Droplaug N Magnusdottir; Solvi Rognvaldsson; Louise le Roux; Gudrun Sigmundsdottir; Gardar Sveinbjornsson; Kristin E Sveinsdottir; Maney Sveinsdottir; Emil A Thorarensen; Bjarni Thorbjornsson; Gisli Masson; Ingileif Jonsdottir; Alma Moller; Thorolfur Gudnason; Karl G Kristinsson; Unnur Thorsteinsdottir; Kari Stefansson
EPI_ISL_827782	The National University Hospital of Iceland	deCODE genetics	Daniel F Gudbjartsson; Agnar Helgason; Hakon Jonsson; Olafur T Magnusson; Pall Melsted; Gudmundur L Norddahl; Jona Saemundsdottir; Asgeir Sigurdsson; Patrick Sulem; Arna B Agustsdottir; Hannes Eggertsson; Berglind Eiriksdottir; Run Fridriksdottir; Elisabet E Gardarsdottir; Gudmundur Georgsson; Olafia S Gretarsdottir; Kjartan R Gudmundsson; Thora R Gunnarsdottir; Arnaldur Gylfason; Hilma Holm; Brynjar O Jensson; Aslaug Jonasdottir; Kamilla S Josefsdottir; Thordur Kristjansson; Droplaug N Magnusdottir; Solvi Rognvaldsson; Louise le Roux; Gudrun Sigmundsdottir; Gardar Sveinbjornsson; Kristin E Sveinsdottir; Maney Sveinsdottir; Emil A Thorarensen; Bjarni Thorbjornsson; Gisli Masson; Ingileif Jonsdottir; Alma Moller; Thorolfur Gudnason; Karl G Kristinsson; Unnur Thorsteinsdottir; Kari Stefansson
EPI_ISL_828213, EPI_ISL_828248, EPI_ISL_828252, EPI_ISL_829405, EPI_ISL_829406, EPI_ISL_829407, EPI_ISL_829408, EPI_ISL_829409, EPI_ISL_829411, EPI_ISL_829412, EPI_ISL_829661, EPI_ISL_829663, EPI_ISL_829667, EPI_ISL_829668, EPI_ISL_829669, EPI_ISL_829670, EPI_ISL_829892, EPI_ISL_829910, EPI_ISL_829917, EPI_ISL_829929, EPI_ISL_830066			
see above	deCODE genetics	deCODE genetics	Daniel F Gudbjartsson; Agnar Helgason; Hakon Jonsson; Olafur T Magnusson; Pall Melsted; Gudmundur L Norddahl; Jona Saemundsdottir; Asgeir Sigurdsson; Patrick Sulem; Arna B Agustsdottir; Hannes Eggertsson; Berglind Eiriksdottir; Run Fridriksdottir; Elisabet E Gardarsdottir; Gudmundur Georgsson; Olafia S Gretarsdottir; Kjartan R Gudmundsson; Thora R Gunnarsdottir; Arnaldur Gylfason; Hilma Holm; Brynjar O Jensson; Aslaug Jonasdottir; Kamilla S Josefsdottir; Thordur Kristjansson; Droplaug N Magnusdottir; Solvi Rognvaldsson; Louise le Roux; Gudrun Sigmundsdottir; Gardar Sveinbjornsson; Kristin E Sveinsdottir; Maney Sveinsdottir; Emil A Thorarensen; Bjarni Thorbjornsson; Gisli Masson; Ingileif Jonsdottir; Alma Moller; Thorolfur Gudnason; Karl G Kristinsson; Unnur Thorsteinsdottir; Kari Stefansson
EPI_ISL_830069	The National University Hospital of Iceland	deCODE genetics	Daniel F Gudbjartsson; Agnar Helgason; Hakon Jonsson; Olafur T Magnusson; Pall Melsted; Gudmundur L Norddahl; Jona Saemundsdottir; Asgeir Sigurdsson; Patrick Sulem; Arna B Agustsdottir; Hannes Eggertsson; Berglind Eiriksdottir; Run Fridriksdottir; Elisabet E Gardarsdottir; Gudmundur Georgsson; Olafia S Gretarsdottir; Kjartan R Gudmundsson; Thora R Gunnarsdottir; Arnaldur Gylfason; Hilma Holm; Brynjar O Jensson; Aslaug Jonasdottir; Kamilla S Josefsdottir; Thordur Kristjansson; Droplaug N Magnusdottir; Solvi Rognvaldsson; Louise le Roux; Gudrun Sigmundsdottir; Gardar Sveinbjornsson; Kristin E Sveinsdottir; Maney Sveinsdottir; Emil A Thorarensen; Bjarni Thorbjornsson; Gisli Masson; Ingileif Jonsdottir; Alma Moller; Thorolfur Gudnason; Karl G Kristinsson; Unnur Thorsteinsdottir; Kari Stefansson
EPI_ISL_830071	deCODE genetics	deCODE genetics	Daniel F Gudbjartsson; Agnar Helgason; Hakon Jonsson; Olafur T Magnusson; Pall Melsted; Gudmundur L Norddahl; Jona Saemundsdottir; Asgeir Sigurdsson; Patrick Sulem; Arna B Agustsdottir; Hannes Eggertsson; Berglind Eiriksdottir; Run Fridriksdottir; Elisabet E Gardarsdottir; Gudmundur Georgsson; Olafia S Gretarsdottir; Kjartan R Gudmundsson; Thora R Gunnarsdottir; Arnaldur Gylfason; Hilma Holm; Brynjar O Jensson; Aslaug Jonasdottir; Kamilla S Josefsdottir; Thordur Kristjansson; Droplaug N Magnusdottir; Solvi Rognvaldsson; Louise le Roux; Gudrun Sigmundsdottir; Gardar Sveinbjornsson; Kristin E Sveinsdottir; Maney Sveinsdottir; Emil A Thorarensen; Bjarni Thorbjornsson; Gisli Masson; Ingileif Jonsdottir; Alma Moller; Thorolfur Gudnason; Karl G Kristinsson; Unnur Thorsteinsdottir; Kari Stefansson
EPI_ISL_830072	The National University Hospital of Iceland	deCODE genetics	Daniel F Gudbjartsson; Agnar Helgason; Hakon Jonsson; Olafur T Magnusson; Pall Melsted; Gudmundur L Norddahl; Jona Saemundsdottir; Asgeir Sigurdsson; Patrick Sulem; Arna B Agustsdottir; Hannes Eggertsson; Berglind Eiriksdottir; Run Fridriksdottir; Elisabet E Gardarsdottir; Gudmundur Georgsson; Olafia S Gretarsdottir; Kjartan R Gudmundsson; Thora R Gunnarsdottir; Arnaldur Gylfason; Hilma Holm; Brynjar O Jensson; Aslaug Jonasdottir; Kamilla S Josefsdottir; Thordur Kristjansson; Droplaug N Magnusdottir; Solvi Rognvaldsson; Louise le Roux; Gudrun Sigmundsdottir; Gardar Sveinbjornsson; Kristin E Sveinsdottir; Maney Sveinsdottir; Emil A Thorarensen; Bjarni Thorbjornsson; Gisli Masson; Ingileif Jonsdottir; Alma Moller; Thorolfur Gudnason; Karl G Kristinsson; Unnur Thorsteinsdottir; Kari Stefansson
EPI_ISL_830411, EPI_ISL_830412, EPI_ISL_830416, EPI_ISL_830418, EPI_ISL_830419, EPI_ISL_830420, EPI_ISL_830421, EPI_ISL_830549	deCODE genetics	deCODE genetics	Daniel F Gudbjartsson; Agnar Helgason; Hakon Jonsson; Olafur T Magnusson; Pall Melsted; Gudmundur L Norddahl; Jona Saemundsdottir; Asgeir Sigurdsson; Patrick Sulem; Arna B Agustsdottir; Hannes Eggertsson; Berglind Eiriksdottir; Run Fridriksdottir; Elisabet E Gardarsdottir; Gudmundur Georgsson; Olafia S Gretarsdottir; Kjartan R Gudmundsson; Thora R Gunnarsdottir; Arnaldur Gylfason; Hilma Holm; Brynjar O Jensson; Aslaug Jonasdottir; Kamilla S Josefsdottir; Thordur Kristjansson; Droplaug N Magnusdottir; Solvi Rognvaldsson; Louise le Roux; Gudrun Sigmundsdottir; Gardar Sveinbjornsson; Kristin E Sveinsdottir; Maney Sveinsdottir; Emil A Thorarensen; Bjarni Thorbjornsson; Gisli Masson; Ingileif Jonsdottir; Alma Moller; Thorolfur Gudnason; Karl G Kristinsson; Unnur Thorsteinsdottir; Kari Stefansson
EPI_ISL_831256, EPI_ISL_831280, EPI_ISL_831282, EPI_ISL_831286	Hospital Universitario La Paz (Madrid)	SeqCOVID-SPAIN consortium/IBV(CSIC)	Fernando Lázaro-Perona, María Rodríguez-Tejedor, Elias Dahdouh, Jesús Mingorance and SeqCOVID-SPAIN consortium
EPI_ISL_831648	Santa Clara County Public Health Laboratory	Santa Clara County Public Health Laboratory	Santa Clara County Public Health Department
EPI_ISL_831693, EPI_ISL_831700, EPI_ISL_831754, EPI_ISL_831851, EPI_ISL_831852, EPI_ISL_831853, EPI_ISL_831854, EPI_ISL_831866, EPI_ISL_831867, EPI_ISL_831868, EPI_ISL_831869			
see above	United States Air Force School of Aerospace Medicine	United States Air Force School of Aerospace Medicine	Anthony Fries, Jennifer Meyer, William Gruner, Amanda Javorina, Sarah Purves, Clarise Starr, Elizabeth Macias
EPI_ISL_831894, EPI_ISL_831895	Santa Clara County Public Health Laboratory	Santa Clara County Public Health Laboratory	Santa Clara County Public Health Department
EPI_ISL_831945	Klinisk mikrobiologi, virus F68	The Public Health Agency of Sweden	Department of Microbiology, The Public Health Agency of Sweden
EPI_ISL_831994	TATAA Biocenter	The Public Health Agency of Sweden	Department of Microbiology, The Public Health Agency of Sweden
EPI_ISL_832043	Wyoming Public Health Laboratory	Wyoming Public Health Laboratory	Noah Hull, Taylor Fearing, Lynette Gumbleton, Channing Weber, Ashley Norberg, Bailey Bowcutt, and Wanda Manley
EPI_ISL_832099	Santa Clara County Public Health Laboratory	Santa Clara County Public Health Laboratory	Santa Clara County Public Health Department
EPI_ISL_832170	Hospital	National Reference Center for Viruses of Respiratory Infections, Institut Pasteur, Paris	Marion Barbet, Sylvie Behillil, Méline Bizard, Angela Brisebarre, Camille Capel, Etienne Simon-Lorière, Vincent Enouf, Maud Vanpeene, Sylvie van der Werf, Clémence Guillaume
EPI_ISL_832383, EPI_ISL_832391, EPI_ISL_832398, EPI_ISL_832399, EPI_ISL_832400, EPI_ISL_832401	Santa Clara County Public Health Laboratory	Santa Clara County Public Health Laboratory	Santa Clara County Public Health Department
EPI_ISL_833132	Laboratorio de Ecologia de Doencas Transmissiveis na Amazonia, Instituto Leonidas e Maria Deane - Fiocruz Amazonia	Laboratorio de Ecologia de Doencas Transmissiveis na Amazonia, Instituto Leonidas e Maria Deane - Fiocruz Amazonia	Valdinete Nascimento, Victor Souza, André Corado, Fernanda Nascimento, George Silva, Ágatha Costa, Debora Duarte, Karina Pessoa, Matilde Mejía, Luciana Gonçalves, Maria Júlia Brandão, Michele Jesus, Felipe Naveca
EPI_ISL_833159	Instituto Adolfo Lutz - Central	Instituto Adolfo Lutz, Interdiciplinary Procedures Center, Strategic Laboratory	Claudio Tavares Sacchi, Claudia Regina Gonçalves, Erica Valessa Ramos Gomes, Karoline Rodrigues Campos
EPI_ISL_833160	Instituto Adolfo Lutz - Regional de Santo Andre	Instituto Adolfo Lutz, Interdiciplinary Procedures Center, Strategic Laboratory	Claudio Tavares Sacchi, Claudia Regina Gonçalves, Erica Valessa Ramos Gomes, Karoline Rodrigues Campos

EPI_ISL_837076, EPI_ISL_837186	Respiratory Virus Unit, National Infection Service, Public Health England	COVID-19 Genomics UK (COG-UK) Consortium	PHE Covid Sequencing Team
EPI_ISL_837395, EPI_ISL_837426	National Virus Reference Laboratory	National Virus Reference Laboratory	Michael Carr, Gabriel Gonzalez, Jonathan Dean, Cillian F De Gascun
EPI_ISL_838220, EPI_ISL_838221, EPI_ISL_838284, EPI_ISL_838285, EPI_ISL_838286, EPI_ISL_838287	Virology Department, Royal Infirmary of Edinburgh, NHS Lothian / School of Biological Sciences, University of Edinburgh / Institute of Genetics and Molecular Medicine, University of Edinburgh	COVID-19 Genomics UK (COG-UK) Consortium	McHugh M, Dewar R, Rooke S, Gallagher M, Balcaza C, O'Toole Á, Scher E, Hill V, McCrone JT, Colquhoun R, Yu X, Jackson B, Rambaut A, Williams TC, Templeton K
EPI_ISL_838352, EPI_ISL_838464, EPI_ISL_838465, EPI_ISL_838471, EPI_ISL_838472, EPI_ISL_838481, EPI_ISL_838486, EPI_ISL_838488, EPI_ISL_838490, EPI_ISL_838492, EPI_ISL_838496, EPI_ISL_838665, EPI_ISL_838672			
see above	Liverpool Clinical Laboratories	COVID-19 Genomics UK (COG-UK) Consortium	Sam Haldenby, Anita Lucaci, Steve Paterson, Julian Hiscox, Alistair Darby, M Almsaud, A Alrezaihi, Muhannad Alruwaili, Stuart D Armstrong, Jones Benjamin, Eleanor G Bentley, Anu Chawla, Jordan J Clark, Angela Cowell, Richard Eccles, Isabel García-Dorival, Matthew Gemmell, Alessandro Gerada, PKF Gilmore, Richard Gregory, Ximeng Han, Catherine Hartley, Margaret Hughes, Miren Iturriza-Gomara, James Johnson, L Luu, Jenifer Manson, Charlotte Nelson, Elaine O'Toole, Cassie Olateju, Rebekah Penrice-Randal , Lucille Rainbow, N.P Randle, Trevor Ian Robinson, Parul Sharma, Ghada T Shawli, James P Stewart, Neil Swainston, Ecaterina Vamos, Joanne Watts, Mark Whitehead
EPI_ISL_838673, EPI_ISL_838674, EPI_ISL_838675, EPI_ISL_838676, EPI_ISL_838677, EPI_ISL_838678, EPI_ISL_838679, EPI_ISL_838680, EPI_ISL_838681, EPI_ISL_838682, EPI_ISL_838683, EPI_ISL_838684, EPI_ISL_838685, EPI_ISL_838686, EPI_ISL_838719			
see above	University College London, Great Ormond Street Hospital for Children NHS Foundation Trust, Imperial College Healthcare NHS Trust	COVID-19 Genomics UK (COG-UK) Consortium	Sergi Castellano, Rachel Williams, Mark Kristiansen, Paola Resende Silva, Sunando Roy, Tony Brooks, Helena Tutill, Paola Niola, Patricia Dyal, Charlotte Williams, Leysa Forrest, Yasmin Panchbhaya, Jacqueline Findlay, Samuel Weeks, Julianne Brown, Kathryn Harris, Paul Randell, James Price, Alison Holmes, Judith Breuer
EPI_ISL_839534, EPI_ISL_839535, EPI_ISL_839536, EPI_ISL_839537, EPI_ISL_839538, EPI_ISL_839539, EPI_ISL_839540, EPI_ISL_839541, EPI_ISL_839542, EPI_ISL_839749, EPI_ISL_839750, EPI_ISL_839751, EPI_ISL_839752, EPI_ISL_839753, EPI_ISL_839754, EPI_ISL_839755, EPI_ISL_839756, EPI_ISL_839757, EPI_ISL_839758, EPI_ISL_839759, EPI_ISL_839760, EPI_ISL_839761, EPI_ISL_839762, EPI_ISL_839763, EPI_ISL_839764, EPI_ISL_839765, EPI_ISL_839766, EPI_ISL_839767, EPI_ISL_839768, EPI_ISL_839769, EPI_ISL_839770, EPI_ISL_839771, EPI_ISL_839772, EPI_ISL_839773, EPI_ISL_839774, EPI_ISL_839775, EPI_ISL_839776, EPI_ISL_839777, EPI_ISL_839778, EPI_ISL_839779, EPI_ISL_839780, EPI_ISL_839781, EPI_ISL_839782, EPI_ISL_839783, EPI_ISL_839784, EPI_ISL_839785, EPI_ISL_839786, EPI_ISL_839787, EPI_ISL_839788, EPI_ISL_839789, EPI_ISL_839790, EPI_ISL_839791, EPI_ISL_839792, EPI_ISL_839793, EPI_ISL_839794, EPI_ISL_839795, EPI_ISL_839796, EPI_ISL_839797, EPI_ISL_839798, EPI_ISL_839799, EPI_ISL_839800, EPI_ISL_839801, EPI_ISL_839802, EPI_ISL_839803, EPI_ISL_839804, EPI_ISL_839805, EPI_ISL_839806, EPI_ISL_839807, EPI_ISL_839808, EPI_ISL_839809, EPI_ISL_839810, EPI_ISL_839811, EPI_ISL_839812, EPI_ISL_839813, EPI_ISL_839814, EPI_ISL_839815			
see above	Northumbria University / South Tees Hospitals NHS Foundation Trust / North Cumbria Integrated Care NHS Foundation Trust / North Tees and Hartlepool NHS Foundation Trust / Newcastle Hospitals NHS Foundation Trust	COVID-19 Genomics UK (COG-UK) Consortium	Darren L Smith,Andrew Nelson,Matthew Bashton,Greg R Young,Joshua Loh,John Allan,Mohammad A Tariq,Giles S Holt,Gary Black,Wen C Yew,Lynn Dover,Paul Baker,Steve Liggett,Sarah Essex,Jane Greenaway,Debra Padgett,Clive Graham,Garren Scott,Edward Barton,Emma Swindells,Brendan Payne,Jennifer Collins,Yusri Taha,Gary Eltringham
EPI_ISL_840041, EPI_ISL_840042, EPI_ISL_840043, EPI_ISL_840044, EPI_ISL_840045, EPI_ISL_840046, EPI_ISL_840047	Lincolnshire Hospitals and DeepSeq Nottingham	COVID-19 Genomics UK (COG-UK) Consortium	Nichola Duckworth, Tim Sloan, Sarah Walsh, Jonathan Ball, Patrick McClure, Joeseph Chappell, Nadine Holmes, Matthew Carlisle, Christopher Moore, Fei Sang, Johnny Debebe, Victoria Wright, Matthew Loose
EPI_ISL_840250, EPI_ISL_840252, EPI_ISL_840253, EPI_ISL_840254	Oxford Viromics, NDM, University of Oxford; Oxford University Hospitals; Basingstoke and North Hampshire Hospital	COVID-19 Genomics UK (COG-UK) Consortium	Tanya Golubchik, David Bonsall, George Macintyre, Amy Trebes, Mariateresa de Cesare, Catrin Moore, Alex Mobbs, Anita Justice, Robert Shaw, Monique Andersson, Timothy Peto, Emma Wise, Nathan Moore, Jessica Lynch, Nick Cortes, Matilde Mori, Stephen Kidd, David Buck, John Todd, Christophe Fraser
EPI_ISL_842358, EPI_ISL_842360, EPI_ISL_842363, EPI_ISL_842369, EPI_ISL_842371, EPI_ISL_842372, EPI_ISL_842376, EPI_ISL_842378, EPI_ISL_842381, EPI_ISL_842383, EPI_ISL_842385, EPI_ISL_842386, EPI_ISL_842390, EPI_ISL_842391, EPI_ISL_842392, EPI_ISL_842393, EPI_ISL_842394, EPI_ISL_842395, EPI_ISL_842396, EPI_ISL_842397, EPI_ISL_842398, EPI_ISL_842399, EPI_ISL_842400, EPI_ISL_842401, EPI_ISL_842402, EPI_ISL_842403, EPI_ISL_842404, EPI_ISL_842405, EPI_ISL_842406, EPI_ISL_842407, EPI_ISL_842408, EPI_ISL_842409, EPI_ISL_842410, EPI_ISL_842411, EPI_ISL_842412, EPI_ISL_842413, EPI_ISL_842414, EPI_ISL_842415, EPI_ISL_842416, EPI_ISL_842417, EPI_ISL_842418, EPI_ISL_842419, EPI_ISL_842420, EPI_ISL_842421			
see above	Bioinformatics and Biostatistics Lab, Advanced Sequencing Facility	COVID-19 Genomics UK (COG-UK) Consortium	Aengus Stewart,Jerome Nicod,Chelsea Sawyer,Laura Cubitt,Harshil Patel,Margaret Crawford
EPI_ISL_842680, EPI_ISL_842681, EPI_ISL_842717, EPI_ISL_842718, EPI_ISL_842720, EPI_ISL_842733, EPI_ISL_842735, EPI_ISL_842736, EPI_ISL_842737, EPI_ISL_842738, EPI_ISL_842739, EPI_ISL_842740, EPI_ISL_842741, EPI_ISL_842742, EPI_ISL_842743, EPI_ISL_842744, EPI_ISL_842746, EPI_ISL_842747, EPI_ISL_842749, EPI_ISL_842750, EPI_ISL_842751, EPI_ISL_842752, EPI_ISL_842753, EPI_ISL_842754, EPI_ISL_842755, EPI_ISL_842756, EPI_ISL_842757, EPI_ISL_842758, EPI_ISL_842759, EPI_ISL_842760, EPI_ISL_842761, EPI_ISL_842762, EPI_ISL_842763, EPI_ISL_842764, EPI_ISL_842765, EPI_ISL_842769, EPI_ISL_842770, EPI_ISL_842771, EPI_ISL_842772, EPI_ISL_842773, EPI_ISL_842774, EPI_ISL_842776, EPI_ISL_842778, EPI_ISL_842779, EPI_ISL_842780, EPI_ISL_842781, EPI_ISL_842782, EPI_ISL_842784, EPI_ISL_842786, EPI_ISL_842787, EPI_ISL_842788, EPI_ISL_842789, EPI_ISL_842791			
see above	University College London Hospital	COVID-19 Genomics UK (COG-UK) Consortium	Judith Heaney, Matthew Byott, Catherine Houlihan, Dan Frampton, Stuart Kirk, Moira Spyer and Eleni Nastouli
EPI_ISL_843071, EPI_ISL_843072, EPI_ISL_843073, EPI_ISL_843074, EPI_ISL_843075, EPI_ISL_843076	Barts Health NHS Trust	COVID-19 Genomics UK (COG-UK) Consortium	CUTINO-MOGUEL, Maria-Teresa; HARRINGTON, David; OWOYEMI, Dola; SHYLINI, Raghavendran; BROAD, Claire; KELE, Beatrix
EPI_ISL_845757, EPI_ISL_845758, EPI_ISL_845759	Emory Molecular Diagnostics Laboratory, Emory Healthcare	Piantadosi Lab, Emory Department of Pathology	Ahmed Babiker, Anne Piantadosi
EPI_ISL_846591	Respiratory Virus Unit, National Infection Service, Public Health England	COVID-19 Genomics UK (COG-UK) Consortium	PHE Covid Sequencing Team
EPI_ISL_847518, EPI_ISL_847526, EPI_ISL_847645, EPI_ISL_847646, EPI_ISL_847647, EPI_ISL_847648, EPI_ISL_847783	California Department of Public Health	Chiu Laboratory, University of California, San Francisco	Charles Chiu, Xianding (Wayne) Deng, Candace Wang, Brian Bushnell, Scot Federman, Jill Hacker, Debra Wadford
EPI_ISL_847834	Connecticut Department of Health	Grubaugh Lab - Yale School of Public Health	Tara Alpert, Joseph Fauver, Anderson Brito, Mallery Breban, Anne Wyllie, Chantal Vogels, Mary Petrone, Chaney Kalinich, Isabel Ott, Nathan Grubaugh
EPI_ISL_848426	Illinois Department of Public Health	Gagnon Lab, Southern Illinois University	Keith Gagnon
EPI_ISL_849730	unknown	PHV-FSS	Son Nguyen et al.
EPI_ISL_850668, EPI_ISL_850670	The National Institute of Public Health	State Veterinary Institute Prague	Nagy,A;Jirincova,H;Trnka,D;Vecerova,J;Trinklova,M
EPI_ISL_852566, EPI_ISL_852577, EPI_ISL_852578	Max von Pettenkofer Institute, Virology, National Reference Center for Retroviruses, LMU München	Laboratory for Functional Genome Analysis, Dept. Genomics, Gene Center of the LMU Munich	Max Muenchhoff, Stefan Krebs, Alexander Graf, Oliver Keppler, Helmut Blum
EPI_ISL_853343, EPI_ISL_853344, EPI_ISL_853377, EPI_ISL_853378, EPI_ISL_853379, EPI_ISL_853380, EPI_ISL_853382, EPI_ISL_853386, EPI_ISL_853388	UPMC Clinical Microbiology Laboratory	Microbial Genome Sequencing Center; Microbial Genomic Epidemiology Laboratory	Mustapha M. Mustapha, Jane W. Marsh, Dan Snyder, Marissa P. Griffith, Stephanie L. Mitchell, Vatsala R. Srinivasa, Kady D. Waggle, Chinelo Ezeonwuku, Vaughn S. Cooper, Lee H. Harrison
EPI_ISL_853740, EPI_ISL_853935	Department of Microbiology, University Innsbruck	Bergthaler laboratory, CeMM Research Center for Molecular Medicine of the Austrian Academy of Sciences	Lukas Endler, Alexandra Popa, Benedikt Agerer, Jakob-Wendelin Genger, Alexander Lercher, Anna Schedl, Thomas Penz, Michael Schuster, Jan Laine, Martin Senekowitsch, Christoph Bock, Andreas Bergthaler
EPI_ISL_854436, EPI_ISL_854437	SARATOGA HOSPITAL LABORATORY	Wadsworth Center, New York State Department of Health	Kirsten St. George, Daryl M. Lamson, Alexis Russel, Matthew Shudt, Melissa A Leisner, Jonathan Plitnick, Navjot Singh, John Kelly, Erasmus Schneider, Erica Lasek-Nesselquist
EPI_ISL_855392	California Department of Public Health	Chiu Laboratory, University of California, San Francisco	Charles Chiu, Xianding (Wayne) Deng, Candace Wang, Brian Bushnell, Scot Federman, Jill Hacker, Debra Wadford
EPI_ISL_855420, EPI_ISL_855421, EPI_ISL_855422, EPI_ISL_855423, EPI_ISL_855424, EPI_ISL_855425, EPI_ISL_855428, EPI_ISL_855429	Servicio de Microbiología, Laboratori Clínic Metropolitana Nord. Hospital Universitari Germans Trias i Pujol. Institut d'Investigació en Ciències de la Salut Germans Trias i Pujol (IGTP)	SeqCOVID-SPAIN consortium/IBV(CSIC)	Elisa Martró, Antoni E. Bordoy, Anna Not, Adrián Antuori, Anabel Fernández, Nona Romaní, Verónica Saludes, Cristina Casañ and SeqCOVID-SPAIN consortium
EPI_ISL_855542, EPI_ISL_855547	KEMRI-Wellcome Trust Research	KEMRI-Wellcome Trust Research	Githinji et al

	Programme/KEMRI-CGMR-C Kilifi	Programme/KEMRI-CGMR-C Kilifi	
EPI_ISL_856783, EPI_ISL_856784, EPI_ISL_856786	Servicio Virosis Respiratorias-Departamento Virología-INEI	Instituto Nacional Enfermedades Infecciosas C.G.Malbran	Baumeister E., Avaro M., Benedetti E., Russo M., Dattero ME, Pontoriero A., Cisterna D., Molina V., Perandones C., Tuduri E., Lorenzo F., Poklepovich T., Campos J.
EPI_ISL_857536	Swiss National Reference Centre for Influenza	Swiss National Reference Centre for Influenza	Tim Roloff, Ana Rita Gonçalves, Madlen Stange, Helena MB Seth-Smith, Alfredo Mari, Karoline Leuzinger, Julia Bielicki, Manuel Battegay, Hans Hirsch, Laurent Kaiser, Adrian Egli
EPI_ISL_858409, EPI_ISL_858416, EPI_ISL_858994, EPI_ISL_859001, EPI_ISL_859005, EPI_ISL_859008, EPI_ISL_859018, EPI_ISL_859020, EPI_ISL_859024	Lighthouse Lab in Alderley Park	Wellcome Sanger Institute for the COVID-19 Genomics UK (COG-UK) Consortium	Jacquelyn Wynn, Mairead Hyland, The Lighthouse Lab in Alderley Park and Alex Alderton, Roberto Amato, Sonia Goncalves, Ewan Harrison, David K. Jackson, Ian Johnston, Dominic Kwiatkowski, Cordelia Langford, John Sillitoe on behalf of the Wellcome Sanger Institute COVID-19 Surveillance Team
EPI_ISL_859462, EPI_ISL_859463, EPI_ISL_859465, EPI_ISL_859466, EPI_ISL_859467, EPI_ISL_859468, EPI_ISL_859471, EPI_ISL_859472, EPI_ISL_859477, EPI_ISL_859478, EPI_ISL_859479, EPI_ISL_859480, EPI_ISL_859482, EPI_ISL_859483, EPI_ISL_859484, EPI_ISL_859485, EPI_ISL_859486, EPI_ISL_859487, EPI_ISL_859488, EPI_ISL_859489, EPI_ISL_859490, EPI_ISL_859491, EPI_ISL_859492, EPI_ISL_859493, EPI_ISL_859494, EPI_ISL_859495, EPI_ISL_859496, EPI_ISL_859497, EPI_ISL_859498, EPI_ISL_859499, EPI_ISL_859500, EPI_ISL_859501, EPI_ISL_859502, EPI_ISL_859503, EPI_ISL_859504, EPI_ISL_859505, EPI_ISL_859506, EPI_ISL_859507, EPI_ISL_859508, EPI_ISL_859509, EPI_ISL_859510, EPI_ISL_859511, EPI_ISL_859512, EPI_ISL_859513, EPI_ISL_859514, EPI_ISL_859515, EPI_ISL_859516, EPI_ISL_859517, EPI_ISL_859518, EPI_ISL_859519, EPI_ISL_859520, EPI_ISL_859521, EPI_ISL_859522, EPI_ISL_859523, EPI_ISL_859524, EPI_ISL_859525, EPI_ISL_859526, EPI_ISL_859527, EPI_ISL_859528, EPI_ISL_859529, EPI_ISL_859530, EPI_ISL_859531, EPI_ISL_859532			
see above	Lighthouse Lab in Glasgow	Wellcome Sanger Institute for the COVID-19 Genomics UK (COG-UK) Consortium	Harper VanSteenhouse, Yumi Kasai, David Gray, Carol Clugston, Anna Dominiczak and Alex Alderton, Roberto Amato, Sonia Goncalves, Ewan Harrison, David K. Jackson, Ian Johnston, Dominic Kwiatkowski, Cordelia Langford, John Sillitoe on behalf of the Wellcome Sanger Institute COVID-19 Surveillance Team
EPI_ISL_860174, EPI_ISL_860175, EPI_ISL_860176, EPI_ISL_860177, EPI_ISL_860178, EPI_ISL_860179	Keio University School of Medicine	Keio University School of Medicine	Kenjiro Kosaki, Yuka Iwasaki, Hirotsugu Ishizu, Haruhiko Siomi, Kodai Abe
EPI_ISL_860216, EPI_ISL_860217, EPI_ISL_860237	Medical Microbiology Unit, Department for Laboratory Medicine, Drammen Hospital, Vestre Viken Health Trust,	Norwegian Institute of Public Health, Department of Virology	Kathrine Stene-Johansen, Kamilla Heddeland Instefjord, Hilde Elshaug, Atiya R Ali,Marie Paulsen Madsen, Rasmus Riis Kopperud, Hilde Vollan, Karoline Bragstad, Olav Hungnes
EPI_ISL_860270	Unilabs Laboratory Medicine	Norwegian Institute of Public Health, Department of Virology	Kathrine Stene-Johansen, Kamilla Heddeland Instefjord, Hilde Elshaug, Atiya R Ali,Marie Paulsen Madsen, Rasmus Riis Kopperud, Hilde Vollan, Karoline Bragstad, Olav Hungnes
EPI_ISL_860690	Respiratory Virus Unit, National Infection Service, Public Health England	COVID-19 Genomics UK (COG-UK) Consortium	PHE Covid Sequencing Team
EPI_ISL_860933, EPI_ISL_860934, EPI_ISL_860936	Johns Hopkins Hospital Department of Pathology	Johns Hopkins Hospital Department of Pathology	C. Paul Morris, Chun Huai Luo, Adannaya Amadi, Nicholas Gallagher, Heba H. Mostafa
EPI_ISL_861463, EPI_ISL_861468	UHAS COVID-19 Lab	UHAS COVID-19 Lab	Kwabena O. Duedu, Jones Gyamfi, Reuben Ayivor-Djanie, John O. Gyapong and the UHAS COVID-19 Lab Team
EPI_ISL_861586	Instituto Nacional de Saude (INSA)	Instituto Nacional de Saude (INSA)	Borges et al
EPI_ISL_861781, EPI_ISL_861782, EPI_ISL_861784, EPI_ISL_861785, EPI_ISL_861790, EPI_ISL_861791, EPI_ISL_861796, EPI_ISL_861799, EPI_ISL_861806, EPI_ISL_861810, EPI_ISL_861827, EPI_ISL_861828, EPI_ISL_861829			
see above	Hospital General Universitario Gregorio Marañón	SeqCOVID-SPAIN consortium/IBV(CSIC)	Darío García de Viedma, Laura Pérez-Lago, Pedro J Sola-Campoy, Sergio Buenestado-Serrano, Marta Herranz, Victor Manuel de la Cueva, Julia Suárez, Pilar Catalán, Patricia Muñoz and SeqCOVID-SPAIN consortium
EPI_ISL_861873	LATE - Laboratório de Técnicas Especiais - Hospital Israelita Albert Einstein	LATE - Laboratório de Técnicas Especiais - Hospital Israelita Albert Einstein	Deyvid Amgarten, Fernanda de Mello Malta, Raquel Riyuzo, Ana Paula Moreira Salles, Pedro Henrique Sebe Rodrigues, João Renato Rebello Pinho
EPI_ISL_862251	Respiratory Virus Unit, National Infection Service, Public Health England	COVID-19 Genomics UK (COG-UK) Consortium	PHE Covid Sequencing Team
EPI_ISL_864578	Institute of Medical Microbiology and Hospital Hygiene	Institute of Medical Microbiology and Hospital Hygiene	Prof. Dr. Achim Kaasch, Aljoscha Tersteegen
EPI_ISL_864786, EPI_ISL_864788, EPI_ISL_864793, EPI_ISL_864802, EPI_ISL_864804, EPI_ISL_864806, EPI_ISL_864808, EPI_ISL_864874, EPI_ISL_864882	Department of Pathology, University of Cambridge	COVID-19 Genomics UK (COG-UK) Consortium	Aminu S. Jahun, Yasmin Chaudhry, Grant Hall, Iliana Georgana, Myra Hosmillo, Martin D. Curran, Malte Pinckert, Surendra Parmar, Ian Goodfellow
EPI_ISL_865208, EPI_ISL_865210, EPI_ISL_865255, EPI_ISL_865256, EPI_ISL_865257, EPI_ISL_865258, EPI_ISL_865259, EPI_ISL_865260, EPI_ISL_865262, EPI_ISL_865263, EPI_ISL_865266, EPI_ISL_865267, EPI_ISL_865269, EPI_ISL_865432			
see above	Liverpool Clinical Laboratories	COVID-19 Genomics UK (COG-UK) Consortium	Sam Haldenby, Anita Lucaci, Steve Paterson, Julian Hiscox, Alistair Darby, M Almsaud, A Alrezaihi, Muhannad Alruwaili, Stuart D Armstrong, Jones Benjamin, Eleanor G Bentley, Anu Chawla, Jordan J Clark, Angela Cowell, Richard Eccles, Isabel García-Dorival, Matthew Gemmell, Alessandro Gerada, PKF Gilmore, Richard Gregory, Ximeng Han, Catherine Hartley, Margaret Hughes, Miren Iturriza-Gomara, James Johnson, L Luu, Jenifer Manson, Charlotte Nelson, Elaine O'Toole, Cassie Olateju, Rebekah Penrice-Randal , Lucille Rainbow, N.P Randle, Trevor Ian Robinson, Parul Sharma, Ghada T Shawli, James P Stewart, Neil Swainston, Ecaterina Vamos, Joanne Watts, Mark Whitehead
EPI_ISL_865718, EPI_ISL_865719	University College London, Great Ormond Street Hospital for Children NHS Foundation Trust, Imperial College Healthcare NHS Trust	COVID-19 Genomics UK (COG-UK) Consortium	Sergi Castellano, Rachel Williams, Mark Kristiansen, Paola Resende Silva, Sunando Roy, Tony Brooks, Helena Tutill, Paola Niola, Patricia Dyal, Charlotte Williams, Leysa Forrest, Yasmin Panchbhaya, Jacqueline Findlay, Samuel Weeks, Julianne Brown, Kathryn Harris, Paul Randell, James Price, Alison Holmes, Judith Breuer
EPI_ISL_866065, EPI_ISL_866066, EPI_ISL_866067, EPI_ISL_866107, EPI_ISL_866138	University College London Hospital	COVID-19 Genomics UK (COG-UK) Consortium	Judith Heaney, Matthew Byott, Catherine Houlihan, Dan Frampton, Stuart Kirk, Moira Spyer and Eleni Nastouli
EPI_ISL_866400, EPI_ISL_866402, EPI_ISL_866403, EPI_ISL_866404, EPI_ISL_866405, EPI_ISL_866406, EPI_ISL_866407, EPI_ISL_866408, EPI_ISL_866409, EPI_ISL_866410, EPI_ISL_866411, EPI_ISL_866412, EPI_ISL_866413, EPI_ISL_866423, EPI_ISL_866424, EPI_ISL_866469, EPI_ISL_866470, EPI_ISL_866471, EPI_ISL_866472, EPI_ISL_866473, EPI_ISL_866474, EPI_ISL_866475, EPI_ISL_866478, EPI_ISL_866479			
see above	Northumbria University / South Tees Hospitals NHS Foundation Trust / North Cumbria Integrated Care NHS Foundation Trust / North Tees and Hartlepool NHS Foundation Trust / Newcastle Hospitals NHS Foundation Trust	COVID-19 Genomics UK (COG-UK) Consortium	Darren L Smith,Andrew Nelson,Matthew Bashton,Greg R Young,Joshua Loh,John Allan,Mohammad A Tariq,Giles S Holt,Gary Black,Wen C Yew,Lynn Dover,Paul Baker,Steve Liggett,Sarah Essex,Jane Greenaway,Debra Padgett,Clive Graham,Garren Scott,Edward Barton,Emma Swindells,Brendan Payne,Jennifer Collins,Yusri Taha,Gary Eltringham
EPI_ISL_866581, EPI_ISL_866587, EPI_ISL_866806, EPI_ISL_866815, EPI_ISL_866817	Quadram Institute Bioscience	COVID-19 Genomics UK (COG-UK) Consortium	Dave J. Baker, Gemma L. Kay, Alp Aydin, Thanh Le-Viet, Steven Rudder, Ana P. Tedim, Anastasia Kolyva, Maria Diaz, Leonardo de Oliveira Martins, Nabil-Fareed Alikhan, Lizzie Meadows, Rachael Stanley, Ngozi Elumogo, Muhammed Yasir, Nicholas M. Thomson, Alexander J Trotter, Rachel Gilroy, Samuel Bloomfield, Claire Stuart, Andrew Bell, Reenesh Prakash, Samir Dervisevic, Alison E. Mather, John Wain, Mark Webber, Andrew J. Page, Justin O'Grady
EPI_ISL_866896, EPI_ISL_866897, EPI_ISL_866898, EPI_ISL_866899, EPI_ISL_866900	Queens Medical Centre, Clinical Microbiology Department / DeepSeq Nottingham	COVID-19 Genomics UK (COG-UK) Consortium	Gemma Clark, Wendy Smith, Manjinder Khakh, Vicki M Fleming, Michelle M Lister, Hannah Howson-Wells, Jonathan Ball, Patrick McClure, Joseph Chappell, Theocharis Tsoleridis, Nadine Holmes, Matthew Carlisle, Christopher Moore, Fei Sang, Johnny Debebe, Victoria Wright, Matthew Loose
EPI_ISL_867286, EPI_ISL_867287, EPI_ISL_867288, EPI_ISL_867289, EPI_ISL_867290, EPI_ISL_867291, EPI_ISL_867292, EPI_ISL_867293, EPI_ISL_867294, EPI_ISL_867295, EPI_ISL_867296, EPI_ISL_867297, EPI_ISL_867499			
see above	Originating lab: Wales Specialist Virology Centre Sequencing lab: Pathogen Genomics Unit	Public Health Wales Microbiology Cardiff Wales Specialist Virology Centre	Catherine Moore, Johnathan Evans, Laura Gifford, Malorie Perry, Simon Cottrell, Angela Marchbank, Alec Birchley, Alexander Adams, Amy Gaskin, Bree Gatica-Wilcox, Jason Coombes, Joel Southgate, Lauren Gilbert, Lee Graham, Nicole Pacchiarini, Sara Kumziene-Summerhayes, Sarah Taylor, Sophie Jones, Sara Rey, Matthew Bull, Joanne Watkins, Sally Corden, Tom Connor
EPI_ISL_871921, EPI_ISL_871929	Servicio de Microbiología, Laboratori Clínic Metropolitana	SeqCOVID-SPAIN consortium/IBV(CSIC)	Elisa Martró, Antoni E. Bordoy, Anna Not, Adrián Antuori, Anabel Fernández, Nona Romaní, Verónica Saludes, Cristina Casañ and SeqCOVID-SPAIN

	Nord. Hospital Universitari Germans Trias i Pujol. Institut d'Investigació en Ciències de la Salut Germans Trias i Pujol (IGTP)		consortium
EPI_ISL_872596, EPI_ISL_872597	Department of Laboratory Medicine, National Taiwan University Hospital	Microbial Genomics Core Lab, National Taiwan University Centers of Genomic and Precision Medicine	Shiou-Hwei Yeh, You-Yu Lin, Ya-Yun Lai, Chiao-Ling Li, Shan-Chwen Chang, Pei-Jer Chen, Sui-Yuan Chang
EPI_ISL_872617, EPI_ISL_872618, EPI_ISL_872619	Nigeria Centre for Disease Control (NCDC)	African Centre of Excellence for Genomics of Infectious Diseases (ACEGID), Redeemer's University	Oluniyi P.E. et al
EPI_ISL_872920, EPI_ISL_872921, EPI_ISL_872922, EPI_ISL_872923, EPI_ISL_872924, EPI_ISL_872925, EPI_ISL_872926, EPI_ISL_872970	WHO National Influenza Centre Russian Federation	WHO National Influenza Centre Russian Federation	Andrey Komissarov, Artem Fadeev, Anna Ivanova, Kseniya Komissarova, Dmitry Bazhenov, Mikhail Bakaev, Daria Danilenko, Ksenia Safina, Elena Nabieva, Georgii Bazykin, Dmitry Lioznov
EPI_ISL_873214, EPI_ISL_873216, EPI_ISL_873217, EPI_ISL_873218	M Health Fairview	Minnesota Department of Health, Public Health Laboratory	Alexandra Lorentz, Jacob Garfin, Matt Plumb, and Xiong Wang
EPI_ISL_875555, EPI_ISL_875556	ULSS 2 Treviso	Istituto Zooprofilattico Sperimentale delle Venezie	Adelaide Milani, Alessia Schivo, Annalisa Salviato, Erika Giorgia Quaranta, Ambra Pastori, Bianca Zecchin, Alice Fusaro, Isabella Monne, Calogero Terregino, Antonia Ricci
EPI_ISL_876048, EPI_ISL_876089, EPI_ISL_876105, EPI_ISL_876112, EPI_ISL_876157, EPI_ISL_876194, EPI_ISL_876300, EPI_ISL_876301, EPI_ISL_876302, EPI_ISL_876303, EPI_ISL_876304			
see above	Massachusetts State Public Health Laboratory	Massachusetts State Public Health Laboratory	Andrew Lang, Timelia Fink, Glen Gallagher, Sandra Smole
EPI_ISL_876795, EPI_ISL_876796, EPI_ISL_876797, EPI_ISL_876798, EPI_ISL_876799, EPI_ISL_876800, EPI_ISL_876801, EPI_ISL_876802, EPI_ISL_876803, EPI_ISL_876804, EPI_ISL_876805, EPI_ISL_876806, EPI_ISL_876807, EPI_ISL_876808, EPI_ISL_876809			
see above	Istituto Zooprofilattico Sperimentale della Puglia e della Basilicata	Istituto Zooprofilattico Sperimentale della Puglia e della Basilicata	Parisi A., Bianco A., Capozzi L., Del Sambro L., Manzulli V, Rondinone V., Pace L., Cipolletta D., Galante D.
EPI_ISL_877419	Siti Khodijah Hospital	Institute of Tropical Disease, Universitas Airlangga	Krisnoadi Rahardjo, Aldise M Nastri, Jezzy R Dewantari, Rima R Prasetya, Muhammad Hamdan, Gatot Soegiarto, Laksmi Wulandari, Resti Yudhawati, Yasuko Mori, Soetjipto, Kazufumi Shimizu, Maria I Lusida
EPI_ISL_877420	Darus Syifa' Islamic Hospital	Institute of Tropical Disease, Universitas Airlangga	Aldise M Nastri, Jezzy R Dewantari, Rima R Prasetya, Krisnoadi Rahardjo, HM Faiz, Gatot Soegiarto, Laksmi Wulandari, Resti Yudhawati, Soetjipto, Yasuko Mori, Maria I Lusida, Kazufumi Shimizu
EPI_ISL_877421	Institute of Tropical Disease	Institute of Tropical Disease, Universitas Airlangga	Jezzy R Dewantari, Rima R Prasetya, Krisnoadi Rahardjo, Aldise M Nastri, Gatot Soegiarto, Laksmi Wulandari, Resti Yudhawati, Soetjipto, Yasuko Mori, Maria I Lusida, Kazufumi Shimizu
EPI_ISL_878584, EPI_ISL_878587	San Diego County Public Health Laboratory	Andersen lab at Scripps Research	SEARCH Alliance San Diego with Tracy Basler, Jovan Shephard, Brett Austin
EPI_ISL_878697, EPI_ISL_878757, EPI_ISL_878763, EPI_ISL_878765, EPI_ISL_878776, EPI_ISL_878781, EPI_ISL_880172, EPI_ISL_880181, EPI_ISL_880189, EPI_ISL_880197	Rady's Childrens Hospital	Andersen lab at Scripps Research	SEARCH Alliance San Diego with Nanda Radamchar, David Dimmock, Linda Luo, Christina Clarke, Kathryn Bouic, Teresa Mueller, Denise Malicki
EPI_ISL_882649	Swiss National Reference Centre for Influenza Virology laboratory, CNRI	Swiss National Reference Centre for Influenza Virology laboratory, CNRI	Tim Roloff, Ana Rita Gonçalves, Madlen Stange, Helena MB Seth-Smith, Alfredo Mari, Karoline Leuzinger, Julia Bielicki, Manuel Battegay, Hans Hirsch, Laurent Kaiser, Adrian Egli
EPI_ISL_883009, EPI_ISL_883010	Maryland Public Health Laboratory	Maryland Public Health Laboratory	Maryland Department of Health Laboratories Administration
EPI_ISL_884214	Alaska State Virology Laboratory (Alaska DHHS)	Alaska State Virology Laboratory (Alaska DHHS)	Stephanie DeRonde, Lisa Smith, PhD. Jack Chen, PhD.
EPI_ISL_884871, EPI_ISL_884872, EPI_ISL_884873, EPI_ISL_884900, EPI_ISL_884901, EPI_ISL_884903, EPI_ISL_884904, EPI_ISL_884905, EPI_ISL_884906, EPI_ISL_884907, EPI_ISL_884908, EPI_ISL_884909, EPI_ISL_884910, EPI_ISL_884911, EPI_ISL_884914, EPI_ISL_884915, EPI_ISL_884916, EPI_ISL_884917, EPI_ISL_884937, EPI_ISL_884947, EPI_ISL_884958, EPI_ISL_884959, EPI_ISL_884961, EPI_ISL_884963			
see above	Santa Clara County Public Health Laboratory	Chan-Zuckerberg Biohub	CZB Cliahub Consortium
EPI_ISL_885133, EPI_ISL_885134	California Institute of Technology	Chan-Zuckerberg Biohub	CZB Cliahub Consortium
EPI_ISL_888675	Center of Advanced Studies and Technology, Molecular Genetics Laboratory	Center of Advanced Studies and Technology, Molecular Genetics Laboratory	De Fabritiis Simone, Mandatori Domitilla, Ferrante Rossella
EPI_ISL_888683, EPI_ISL_888684, EPI_ISL_888691, EPI_ISL_888752, EPI_ISL_888753, EPI_ISL_888759, EPI_ISL_888773, EPI_ISL_888778, EPI_ISL_888779, EPI_ISL_888788	KU Leuven, Rega Institute, Clinical and Epidemiological Virology	KU Leuven, Rega Institute, Clinical and Epidemiological Virology	Tony Wawina-Bokalanga, Bert Vanmechelen, Joan Marti-Carerras, Piet Maes
EPI_ISL_888993	RSU Medirossa	Eijkman Institute for Molecular Biology, Ministry of Research and Technology/National Agency for Research and Innovation	Iskandar Adnan, Lydia V. Panggalo, Sukma Oktavianthi, Willy Agustine, Edison Johar, Hidayat Trimarsanto, Frilasita A Yudhaputri, Safarina G Malik, Khin Saw Myint, Amin Soebandrio
EPI_ISL_890253, EPI_ISL_890258, EPI_ISL_890285, EPI_ISL_890289	KU Leuven, Rega Institute, Clinical and Epidemiological Virology	KU Leuven, Rega Institute, Clinical and Epidemiological Virology	Tony Wawina-Bokalanga, Bert Vanmechelen, Joan Marti-Carerras, Piet Maes
EPI_ISL_892215, EPI_ISL_892216	Lighthouse Lab in Alderley Park	Wellcome Sanger Institute for the COVID-19 Genomics UK (COG-UK) Consortium	Jacquelyn Wynn, Mairead Hyland, The Lighthouse Lab in Alderley Park and Alex Alderton, Roberto Amato, Sonia Goncalves, Ewan Harrison, David K. Jackson, Ian Johnston, Dominic Kwiatkowski, Cordelia Langford, John Sillitoe on behalf of the Wellcome Sanger Institute COVID-19 Surveillance Team
EPI_ISL_892270, EPI_ISL_892271, EPI_ISL_892275, EPI_ISL_892277	Servicio de Microbiología Clínica (Complejo Hospitalario de Navarra, Pamplona), Instituto de Investigación Sanitaria de Navarra (IdiSNA)	SeqCOVID-SPAIN consortium/IBV(CSIC)	Carmen Ezpeleta Baquedano, Ana Navascués, Ana Miqueleiz and SeqCOVID-SPAIN consortium
EPI_ISL_892279, EPI_ISL_892314, EPI_ISL_892315, EPI_ISL_892316, EPI_ISL_892317, EPI_ISL_892318, EPI_ISL_892319, EPI_ISL_892320, EPI_ISL_892321, EPI_ISL_892322, EPI_ISL_892323, EPI_ISL_892324			
see above	Servicio de Microbiología. Hospital Universitario Donostia. OSI Donostialdea. Área de Enfermedades Infecciosas, Grupo de Infección Respiratoria y Resistencia Antimicrobiana. Instituto de Investigación Sanitaria Biodonostia	SeqCOVID-SPAIN consortium/IBV(CSIC)	Gustavo Cilla Eguiluz, Milagrosa Montes Ros, Luis Piñeiro Vázquez, Ane Sorrarain, Jose Maria Marimón and SeqCOVID-SPAIN consortium
EPI_ISL_892345, EPI_ISL_892346, EPI_ISL_892347, EPI_ISL_892348, EPI_ISL_892349, EPI_ISL_892350, EPI_ISL_892351, EPI_ISL_892352, EPI_ISL_892353, EPI_ISL_892354, EPI_ISL_892355, EPI_ISL_892356			
see above	Servicio de Microbiología Clínica (Complejo Hospitalario de Navarra, Pamplona), Instituto de Investigación Sanitaria de Navarra (IdiSNA)	SeqCOVID-SPAIN consortium/IBV(CSIC)	Carmen Ezpeleta Baquedano, Ana Navascués, Ana Miqueleiz and SeqCOVID-SPAIN consortium
EPI_ISL_893736, EPI_ISL_893737, EPI_ISL_893738	Massachusetts Department of Public Health	Pathogen Discovery, Respiratory Viruses Branch, Division of Viral Diseases, Centers for Disease Control and Prevention	Yan Li, Anna Montmayeur, Ying Tao, Jing Zhang, Krista Queen, Anna Uehara, Brian Lynch, Rachel Marine, Peter Cook, Clinton R. Paden, Haibin Wang, Suxiang Tong
EPI_ISL_893739	Florida Department of Public Health	Pathogen Discovery, Respiratory Viruses Branch, Division of Viral Diseases, Centers for Disease Control and Prevention	Yan Li, Anna Montmayeur, Ying Tao, Jing Zhang, Krista Queen, Anna Uehara, Brian Lynch, Rachel Marine, Peter Cook, Clinton R. Paden, Haibin Wang, Suxiang Tong
EPI_ISL_896105, EPI_ISL_896117	Viollier AG	University Hospital Basel, Clinical Bacteriology	Tim Roloff, Madlen Stange, Helena MB Seth-Smith, Alfredo Mari, Karoline Leuzinger, Julia Bielicki, Christiane Beckmann, Manuel Battegay, Hans Hirsch, Adrian Egli

EPI_ISL_900569	Hôpital Paris Saint-Joseph	CNR Virus des Infections Respiratoires - France SUD	Antonin Bal, Gregory Destras, Gwendolyne Burfin, Hadrien Règue, Quentin Semanas, Martine Valette, Bruno Lina, Sylvie Larrat, Laurence Josset
EPI_ISL_900572	Hôpital Paris Saint-Joseph	CNR Virus des Infections Respiratoires - France SUD	Antonin Bal, Gregory Destras, Gwendolyne Burfin, Hadrien Règue, Quentin Semanas, Martine Valette, Bruno Lina, Laurence Josset
EPI_ISL_900580, EPI_ISL_900606, EPI_ISL_900607, EPI_ISL_900608, EPI_ISL_900609, EPI_ISL_900663	IZSM	TIGEM	Patrizia Annunziata, Andrea Ballabio, Valentina Bouche, Davide Cacchiarelli (CorrespAuthor), Pellegrino Cerino, Chiara Colantuono, Maria Concetta Cuomo, Denise Di Concilio, Lucio Di Filippo, Antonio Grimaldi, Antonio Limone, Anna Manfredi, Francesco Panariello, Biancamaria Pierri, Marcello Salvi
EPI_ISL_903071	Washington State Department of Health	Seattle Flu Study	Deborah A. Nickerson, Chris D. Frazar, Jover Lee, Benjamin Pelle, Erica Ryke, Matthew Richardson, Amanda Adler, Elisabeth Brandstetter, Peter D. Han, Kairsten Fay, Misja Ilcisin, Kirsten Lacombe, Thomas R. Sibley, Melissa Truong, Caitlin R. Wolf, Romesh Gautom, Geoff Melly, Brian Hiatt, Philip Dykema, Scott Lindquist, Michael Boeckh, Janet A. Englund, Michael Famulare, Barry R. Lutz, Mark J. Rieder, Lea M. Starita, Matthew Thompson, Helen Y. Chu, Jay Shendure, Trevor Bedford
EPI_ISL_903230, EPI_ISL_903242, EPI_ISL_903243, EPI_ISL_903247, EPI_ISL_903249, EPI_ISL_903250	M Health Fairview	Minnesota Department of Health, Public Health Laboratory	Alexandra Lorentz, Jacob Garfin, Matt Plumb, and Xiong Wang
EPI_ISL_903618	MN PHL Division, Minnesota Department of Health	Genomics and Discovery, Respiratory Viruses Branch, Division of Viral Diseases, Centers for Disease Control and Prevention	Krista Queen, Yan Li, Ying Tao, Jing Zhang, Anna Uehara, Anna Montmayeur, Clinton R. Paden, Peter W. Cook, Rachel Marine, Mili Sheth, Jasmine Padilla, Sarah Nobles, Mark Burroughs, Lori Rowe, Haibin Wang, Ben L. Rambo-Martin, Dhwani Batra, Justin Lee, Suxiang Tong
EPI_ISL_903621	IA State Hygienic Laboratory	Genomics and Discovery, Respiratory Viruses Branch, Division of Viral Diseases, Centers for Disease Control and Prevention	Krista Queen, Yan Li, Ying Tao, Jing Zhang, Anna Uehara, Anna Montmayeur, Clinton R. Paden, Peter W. Cook, Rachel Marine, Mili Sheth, Jasmine Padilla, Sarah Nobles, Mark Burroughs, Lori Rowe, Haibin Wang, Ben L. Rambo-Martin, Dhwani Batra, Justin Lee, Suxiang Tong
EPI_ISL_903656	RI State Health Laboratories	Genomics and Discovery, Respiratory Viruses Branch, Division of Viral Diseases, Centers for Disease Control and Prevention	Krista Queen, Yan Li, Ying Tao, Jing Zhang, Anna Uehara, Anna Montmayeur, Clinton R. Paden, Peter W. Cook, Rachel Marine, Mili Sheth, Jasmine Padilla, Sarah Nobles, Mark Burroughs, Lori Rowe, Haibin Wang, Ben L. Rambo-Martin, Dhwani Batra, Justin Lee, Suxiang Tong
EPI_ISL_903693, EPI_ISL_903739	LA Office of Public Health Laboratories	Genomics and Discovery, Respiratory Viruses Branch, Division of Viral Diseases, Centers for Disease Control and Prevention	Krista Queen, Yan Li, Ying Tao, Jing Zhang, Anna Uehara, Anna Montmayeur, Clinton R. Paden, Peter W. Cook, Rachel Marine, Mili Sheth, Jasmine Padilla, Sarah Nobles, Mark Burroughs, Lori Rowe, Haibin Wang, Ben L. Rambo-Martin, Dhwani Batra, Justin Lee, Suxiang Tong
EPI_ISL_903747	NE Public Health Laboratory	Genomics and Discovery, Respiratory Viruses Branch, Division of Viral Diseases, Centers for Disease Control and Prevention	Krista Queen, Yan Li, Ying Tao, Jing Zhang, Anna Uehara, Anna Montmayeur, Clinton R. Paden, Peter W. Cook, Rachel Marine, Mili Sheth, Jasmine Padilla, Sarah Nobles, Mark Burroughs, Lori Rowe, Haibin Wang, Ben L. Rambo-Martin, Dhwani Batra, Justin Lee, Suxiang Tong
EPI_ISL_903760	MN PHL Division, Minnesota Department of Health	Genomics and Discovery, Respiratory Viruses Branch, Division of Viral Diseases, Centers for Disease Control and Prevention	Krista Queen, Yan Li, Ying Tao, Jing Zhang, Anna Uehara, Anna Montmayeur, Clinton R. Paden, Peter W. Cook, Rachel Marine, Mili Sheth, Jasmine Padilla, Sarah Nobles, Mark Burroughs, Lori Rowe, Haibin Wang, Ben L. Rambo-Martin, Dhwani Batra, Justin Lee, Suxiang Tong
EPI_ISL_903776	LA Office of Public Health Laboratories	Genomics and Discovery, Respiratory Viruses Branch, Division of Viral Diseases, Centers for Disease Control and Prevention	Krista Queen, Yan Li, Ying Tao, Jing Zhang, Anna Uehara, Anna Montmayeur, Clinton R. Paden, Peter W. Cook, Rachel Marine, Mili Sheth, Jasmine Padilla, Sarah Nobles, Mark Burroughs, Lori Rowe, Haibin Wang, Ben L. Rambo-Martin, Dhwani Batra, Justin Lee, Suxiang Tong
EPI_ISL_903888	MS Public Health Laboratory	Genomics and Discovery, Respiratory Viruses Branch, Division of Viral Diseases, Centers for Disease Control and Prevention	Krista Queen, Yan Li, Ying Tao, Jing Zhang, Anna Uehara, Anna Montmayeur, Clinton R. Paden, Peter W. Cook, Rachel Marine, Mili Sheth, Jasmine Padilla, Sarah Nobles, Mark Burroughs, Lori Rowe, Haibin Wang, Ben L. Rambo-Martin, Dhwani Batra, Justin Lee, Suxiang Tong
EPI_ISL_903889	TX DSHS, Lab Services Section MC 1947	Genomics and Discovery, Respiratory Viruses Branch, Division of Viral Diseases, Centers for Disease Control and Prevention	Krista Queen, Yan Li, Ying Tao, Jing Zhang, Anna Uehara, Anna Montmayeur, Clinton R. Paden, Peter W. Cook, Rachel Marine, Mili Sheth, Jasmine Padilla, Sarah Nobles, Mark Burroughs, Lori Rowe, Haibin Wang, Ben L. Rambo-Martin, Dhwani Batra, Justin Lee, Suxiang Tong
EPI_ISL_903914, EPI_ISL_903925	RI State Health Laboratories	Genomics and Discovery, Respiratory Viruses Branch, Division of Viral Diseases, Centers for Disease Control and Prevention	Krista Queen, Yan Li, Ying Tao, Jing Zhang, Anna Uehara, Anna Montmayeur, Clinton R. Paden, Peter W. Cook, Rachel Marine, Mili Sheth, Jasmine Padilla, Sarah Nobles, Mark Burroughs, Lori Rowe, Haibin Wang, Ben L. Rambo-Martin, Dhwani Batra, Justin Lee, Suxiang Tong
EPI_ISL_903937	MS Public Health Laboratory	Genomics and Discovery, Respiratory Viruses Branch, Division of Viral Diseases, Centers for Disease Control and Prevention	Krista Queen, Yan Li, Ying Tao, Jing Zhang, Anna Uehara, Anna Montmayeur, Clinton R. Paden, Peter W. Cook, Rachel Marine, Mili Sheth, Jasmine Padilla, Sarah Nobles, Mark Burroughs, Lori Rowe, Haibin Wang, Ben L. Rambo-Martin, Dhwani Batra, Justin Lee, Suxiang Tong
EPI_ISL_903948, EPI_ISL_903961, EPI_ISL_903966	WA State Department of Health	Genomics and Discovery, Respiratory Viruses Branch, Division of Viral Diseases, Centers for Disease Control and Prevention	Krista Queen, Yan Li, Ying Tao, Jing Zhang, Anna Uehara, Anna Montmayeur, Clinton R. Paden, Peter W. Cook, Rachel Marine, Mili Sheth, Jasmine Padilla, Sarah Nobles, Mark Burroughs, Lori Rowe, Haibin Wang, Ben L. Rambo-Martin, Dhwani Batra, Justin Lee, Suxiang Tong
EPI_ISL_904011	Veterinary Specialized Institute Kraljevo	Veterinary Specialized Institute "Kraljevo", Serbia	Vidanovic,D., Tesovic,B., Knezevic,A., Jovanovic,T., Jankovic,M., Sekler,M., Banovic Djeri,B., Petrovic,T., Volkening,J., Afonso,C.
EPI_ISL_904236, EPI_ISL_904240, EPI_ISL_904263, EPI_ISL_904483, EPI_ISL_904484, EPI_ISL_904485, EPI_ISL_904486, EPI_ISL_904487, EPI_ISL_904488, EPI_ISL_904489, EPI_ISL_904490			
see above	Dutch COVID-19 response team	Erasmus Medical Center	Bas Oude Munnink, Reina Sikkema, David Nieuwenhuijse, Irina Chestakova, Anne van der Linden, Marjan Boter, Emmanuelle Munger, Corine GeurtsvanKessel, Annemiek van der Eijk, Richard Molenkamp, Marion Koopmans, on behalf of the Dutch national COVID-19 response team.
EPI_ISL_904768, EPI_ISL_904795, EPI_ISL_905084, EPI_ISL_905086, EPI_ISL_905089, EPI_ISL_905092, EPI_ISL_905093, EPI_ISL_905094, EPI_ISL_905095, EPI_ISL_905131, EPI_ISL_905134, EPI_ISL_905490, EPI_ISL_905607, EPI_ISL_905651, EPI_ISL_905659, EPI_ISL_905689, EPI_ISL_905690, EPI_ISL_905691, EPI_ISL_905692, EPI_ISL_905693			
see above	Dutch COVID-19 response team	National Institute for Public Health and the Environment (RIVM)	Adam Meijer, Harry Vennema, Dirk Eggink, Jeroen Cremer, Sharon van den Brink, Bas van der Veer, AnneMarie van den Brandt, Florian Zwagemaker, Dennis Schmitz, Chantal Reusken, on behalf of the national COVID-19 response team
EPI_ISL_905792, EPI_ISL_905793, EPI_ISL_905794, EPI_ISL_905795, EPI_ISL_905796, EPI_ISL_905797, EPI_ISL_905798, EPI_ISL_905807, EPI_ISL_905808, EPI_ISL_905809, EPI_ISL_905810			
see above	OHSU Lab Services Molecular Microbiology Lab	Oregon SARS-CoV-2 Genome Sequencing Center	Brendan L. O'Connell, Sally Grindstaff, Kayla Carter, Ruth V. Nichols, Alec J. Hirsch, Donna Hansel, Guang Fan, Xuan, Qin, Daniel N. Streblow, William B. Messer, Andrew C. Adey, Benjamin N. Bimber, Brian J. O'Roak
EPI_ISL_906092, EPI_ISL_906097	Child Health Research Foundation	Child Health Research Foundation	Senjuti Saha, Sharmistha Goswami, Afroza Akter Tanni, Syed Muktadir Al Sium, Arif Mohammad Tanmoy, Roly Malaker, Md Hafizur Rahman, Samir K Saha
EPI_ISL_906099	Child Health Research Foundation	Child Health Research Foundation	Senjuti Saha, Afroza Akter Tanni, Sharmistha Goswami, Syed Muktadir Al Sium, Arif Mohammad Tanmoy, Roly Malaker, Md Hafizur Rahman, Samir K Saha
EPI_ISL_906113	Child Health Research Foundation	Child Health Research Foundation	Senjuti Saha, Syed Muktadir Al Sium, Sharmistha Goswami, Afroza Akter Tanni, Arif Mohammad Tanmoy, Roly Malaker, Md Hafizur Rahman, Samir K Saha
EPI_ISL_906114	Child Health Research Foundation	Child Health Research Foundation	Senjuti Saha, Arif Mohammad Tanmoy, Sharmistha Goswami, Afroza Akter Tanni, Syed Muktadir Al Sium, Roly Malaker, Md Hafizur Rahman, Samir K Saha
EPI_ISL_906115	Child Health Research Foundation	Child Health Research Foundation	Senjuti Saha, Syed Muktadir Al Sium, Sharmistha Goswami, Afroza Akter Tanni, Arif Mohammad Tanmoy, Roly Malaker, Md Hafizur Rahman, Samir K Saha
EPI_ISL_906264	University of Wisconsin-Madison AIDS Vaccine Research Laboratories	University of Wisconsin-Madison AIDS Vaccine Research Laboratories	Gage Moreno, Katarina Braun, et al. AIDS Vaccine Research Laboratories
EPI_ISL_906275, EPI_ISL_906276	Nigeria Centre for Disease Control (NCDC)	African Centre of Excellence for Genomics of Infectious Diseases (ACEGID), Redeemer's University	Oluniyi P.E. et al

EPI_ISL_906562, EPI_ISL_906563, EPI_ISL_906564	Maine Health and Environmental Testing Laboratory (Maine HETL)	Tewhey Lab, The Jackson Laboratory	Matluk,N., Dewey,H., Iosue,F., Barter,M., Lynch,R., Munger,H. and Tewhey,R.
EPI_ISL_909969, EPI_ISL_909978, EPI_ISL_909979, EPI_ISL_909980, EPI_ISL_909981, EPI_ISL_909982	A. Krumbholz, Labor Dr. Krause und Kollegen MVZ GmbH, Kiel	Charité Universitätsmedizin Berlin, Institut für Virologie	Victor M Corman, Tobias Bleicker, Julia Tesch, Barbara Mühlemann, Jörn Beheim-Schwarzbach, Talitha Veith, Julia Schneider, Cornelia Schlee, Tomasz Zemojtel, Terry Jones, Christian Drosten
EPI_ISL_910331	Department of Infectious Diseases, Istituto Superiore di Sanità, Rome, Italy; AOUP Paolo Giaccone, Palermo, Italy	Istituto Superiore di Sanità (ISS)	Paola Stefanelli, Angela Di Martino, Alessandra Lo Presti, Stefano Fiore, Fabio Tramuto, Francesco Vitale, Carmelo Maida, Daniela Di Naro, Giulia Randazzo, Manuela Marra, Maria Carollo, Marco Crescenzi
EPI_ISL_910757, EPI_ISL_910758, EPI_ISL_910759, EPI_ISL_910760, EPI_ISL_910761, EPI_ISL_910762, EPI_ISL_910763, EPI_ISL_910764, EPI_ISL_910765, EPI_ISL_910766, EPI_ISL_910769, EPI_ISL_910770, EPI_ISL_910771, EPI_ISL_910772, EPI_ISL_910773, EPI_ISL_910777, EPI_ISL_910778, EPI_ISL_910779, EPI_ISL_910780, EPI_ISL_910781, EPI_ISL_910782, EPI_ISL_910783, EPI_ISL_910784, EPI_ISL_910785, EPI_ISL_910786, EPI_ISL_910787, EPI_ISL_910788, EPI_ISL_910789, EPI_ISL_910790, EPI_ISL_910791, EPI_ISL_910792, EPI_ISL_910793, EPI_ISL_910794, EPI_ISL_910795, EPI_ISL_910796, EPI_ISL_910797, EPI_ISL_910798, EPI_ISL_910801, EPI_ISL_910802, EPI_ISL_910803, EPI_ISL_910804, EPI_ISL_910805, EPI_ISL_910806, EPI_ISL_910807, EPI_ISL_910808, EPI_ISL_910809, EPI_ISL_910810, EPI_ISL_910811, EPI_ISL_910812, EPI_ISL_910813, EPI_ISL_910814, EPI_ISL_910815, EPI_ISL_910816, EPI_ISL_910817, EPI_ISL_910818, EPI_ISL_910819, EPI_ISL_910820, EPI_ISL_910821, EPI_ISL_910822, EPI_ISL_910823, EPI_ISL_910824, EPI_ISL_910825, EPI_ISL_910826, EPI_ISL_910827, EPI_ISL_910828, EPI_ISL_910829, EPI_ISL_910830, EPI_ISL_910831, EPI_ISL_910832, EPI_ISL_910833, EPI_ISL_910834, EPI_ISL_910835, EPI_ISL_910836, EPI_ISL_910837, EPI_ISL_910838, EPI_ISL_910839, EPI_ISL_910840, EPI_ISL_910841, EPI_ISL_910842, EPI_ISL_910843, EPI_ISL_910844, EPI_ISL_910845, EPI_ISL_910846, EPI_ISL_910850, EPI_ISL_910851, EPI_ISL_910852, EPI_ISL_910853, EPI_ISL_910854, EPI_ISL_910855, EPI_ISL_910856, EPI_ISL_910857, EPI_ISL_910858, EPI_ISL_910859, EPI_ISL_910860, EPI_ISL_910861, EPI_ISL_910862, EPI_ISL_910863, EPI_ISL_910864, EPI_ISL_910865, EPI_ISL_910866, EPI_ISL_910867, EPI_ISL_910868, EPI_ISL_910869, EPI_ISL_910870, EPI_ISL_910871, EPI_ISL_910872, EPI_ISL_910873, EPI_ISL_910874, EPI_ISL_910875, EPI_ISL_910876, EPI_ISL_910877, EPI_ISL_910878, EPI_ISL_910879, EPI_ISL_910880, EPI_ISL_910957, EPI_ISL_910958, EPI_ISL_910959, EPI_ISL_910960, EPI_ISL_910961, EPI_ISL_910962, EPI_ISL_910963, EPI_ISL_910964, EPI_ISL_910965, EPI_ISL_910966, EPI_ISL_910967, EPI_ISL_910968, EPI_ISL_910969, EPI_ISL_910970, EPI_ISL_910971, EPI_ISL_910972, EPI_ISL_910973, EPI_ISL_910974, EPI_ISL_910975, EPI_ISL_910976, EPI_ISL_910977, EPI_ISL_910978, EPI_ISL_910979, EPI_ISL_910980, EPI_ISL_910981, EPI_ISL_910982, EPI_ISL_910983, EPI_ISL_910984, EPI_ISL_910985, EPI_ISL_910986, EPI_ISL_910987, EPI_ISL_910988, EPI_ISL_910989, EPI_ISL_910990, EPI_ISL_910991, EPI_ISL_910992, EPI_ISL_910993, EPI_ISL_910994, EPI_ISL_911003			
see above	Laboratoire national de sante, Microbiology, Virology	Laboratoire national de sante, Microbiology, Microbial Genomics Platform	Anke Wienecke-Baldacchino, Catherine Ragimbeau,Jessica Tapp, Fatu Djabi, Lise Pignon, Raoul Salmon, Tamir Abdelrahman
EPI_ISL_911471, EPI_ISL_911472, EPI_ISL_911473, EPI_ISL_911474, EPI_ISL_911475, EPI_ISL_911476, EPI_ISL_911477, EPI_ISL_911478, EPI_ISL_911479, EPI_ISL_911480, EPI_ISL_911481, EPI_ISL_911482, EPI_ISL_911483, EPI_ISL_911484, EPI_ISL_911485, EPI_ISL_911486, EPI_ISL_911487, EPI_ISL_911488, EPI_ISL_911489, EPI_ISL_911490, EPI_ISL_911491, EPI_ISL_911492, EPI_ISL_911493			
see above	Houston Health Dept.	Houston Health Dept.	Ryker Penn, Pamela Brown, Adolpho Lara
EPI_ISL_911515, EPI_ISL_911516, EPI_ISL_911517	Ohio Department of Health Laboratory	Ohio Department of Health Laboratory	Holmes, Jennifer; Eric Brandt, Keoni Omura, Glen McGillivary, Caitlin McDonnell, Jade Mowery, Stephanie Mccracken, Tyler Payne, Kirtana Ramadugu, Erica Leasure, Brent Lee, Kelsey Florek, Heather Blankenship, Quanta Brown, and Tammy Bannerman
EPI_ISL_911694, EPI_ISL_911698	Alaska State Virology Laboratory	Alaska State Virology Laboratory	Stephanie DeRonde, Lisa Smith, Ph.D., Jack Chen, Ph.D.
EPI_ISL_912357, EPI_ISL_912358, EPI_ISL_912359, EPI_ISL_912360, EPI_ISL_912391	Fondation Congolaise pour la recherche medicale (FCRM), Francine Ntoumi	NGS Competence Center Tuebingen, Institut für Medizinische Mikrobiologie und Hygiene, Universitaetsklinikum Tübingen	Angel Angelov
EPI_ISL_912397	Institute for Medical Research, Infectious Disease Research Centre, National Institutes of Health, Ministry of Health Malaysia	Institute for Medical Research, Infectious Disease Research Centre, National Institutes of Health, Ministry of Health Malaysia	Suppiah J, Kamel K, Azizan MA, Thayan R
EPI_ISL_912498, EPI_ISL_912512	NHLS Universitas Academic	UFS Virology	PA Bester, MM Nyaga, P Nthiga, MT Mogotsi, D Goedhals, T de Oliveira
EPI_ISL_913348	Klinisk mikrobiologi	The Public Health Agency of Sweden	Anna-Malin Linde, Maria Lind Karlberg, Carlo Berg, Oskar Karlsson Lindsjo, Sofia Stamouli, Reza Advani, Mattias Haukland, Petra Holmstrom, Noura Walai, Petra Edquist, Mia Brytting, Anna Risberg, Karin Tegmark-Wisell
EPI_ISL_913671, EPI_ISL_913673, EPI_ISL_913674, EPI_ISL_913675	Vault Health	Minnesota Department of Health, Public Health Laboratory	Alexandra Lorentz, Jacob Garfin, Matt Plumb, and Xiong Wang
EPI_ISL_913944, EPI_ISL_913946, EPI_ISL_913947, EPI_ISL_913948, EPI_ISL_913949, EPI_ISL_913951, EPI_ISL_913963, EPI_ISL_913964, EPI_ISL_913976, EPI_ISL_913977, EPI_ISL_913978, EPI_ISL_913981			
see above	Instituto de Diagnostico y Referencia Epidemiologicos INDRE_RNLSP	Instituto de Diagnostico y Referencia Epidemiologicos (INDRE)	Claudia Wong-Arambula, Abril Rodriguez-Maldonado, Fabiola Garces-Ayala, Adnan Araiza-Rodriguez, David Fragoso-Fonseca, Sergio Rangel-Guerrero, Mayra Jimenez-Morales, Nancy Munoz-Hernandez, Natividad Cruz-Ortiz, Tatiana Nunez-Garcia, Gisela Barrera-Badillo, Lucia Hernandez-Rivas, Irma Lopez-Martinez, Ernesto Ramirez-Gonzalez.
EPI_ISL_914715	Maryland Public Health Laboratory (MD PHL)	Maryland Public Health Laboratory (MD PHL)	Maryland Department of Health Laboratories Administration
EPI_ISL_914799, EPI_ISL_914800	AREA DE SALUD SAN FRANCISCO-SAN ANTONIO (COOPESANA)	Inciensa, Instituto Costarricense de Investigación y Enseñanza en Nutrición y Salud	Francisco Duarte, Hebleen Porras, Claudio Soto-Garita, Estela Cordero, Adriana Godínez, Melany Calderón & Mariel López
EPI_ISL_914885	Vilnius university hospital Santaros Klinikos, Center of Laboratory Medicine	Vilnius University Hospital Santaros Klinikos	Ingrida Olendraite, Daniel Naumovas, Rimvydas Norvilas, Dovil Ežerskyt, Justinas Šlikas
EPI_ISL_915359, EPI_ISL_915379, EPI_ISL_915381	Keio University School of Medicine	Keio University School of Medicine	Kenjiro Kosaki, Yuka Iwasaki, Hirotsugu Ishizu, Haruhiko Siomi, Kodai Abe
EPI_ISL_918169	Department of Infectious Diseases and Immunology, National Hospital Organization Nagoya Medical Center	Clinical Research Center, National Hospital Organization Nagoya Medical Center	Yoshihiro Nakata, Hirotaka Ode, Mai Kubota, Masakazu Matsuda, Kazuhiro Matsuoka, Miho Nakasuji, Mikiko Mori, Mayumi Imahashi, Yoshiyuki Yokomaku, Yasumasa Iwatani
EPI_ISL_918579, EPI_ISL_918580, EPI_ISL_918581, EPI_ISL_918582, EPI_ISL_918599, EPI_ISL_918603	University of Birmingham	COVID-19 Genomics UK (COG-UK) Consortium	Institute of Microbiology, University of Birmingham: Claire McMurray, Joanne Stockton, Samuel Nicholls, Radoslaw Poplawski, Will Rowe, Josh Quick, Nicholas Loman. University of Birmingham Testing Laboratory: Celina M Whalley, Andrew Bosworth, Charlotte Poxon, Kasun Wanigasooriya, Oliver Pickles, Mike Kidd, Alex Richter, Andrew D Beggs PHE Heartlands Lab: Husam Osman, Andrew Bosworth. Queen Elizabeth Hospital: Anna Casey
EPI_ISL_919233, EPI_ISL_919235, EPI_ISL_919237, EPI_ISL_919252, EPI_ISL_919261, EPI_ISL_919266, EPI_ISL_919267	West of Scotland Specialist Virology Centre, NHSGGC / MRC-University of Glasgow Centre for Virus Research	COVID-19 Genomics UK (COG-UK) Consortium	Ana da Silva Filipe, Natasha Johnson, Kathy Smollett, Daniel Mair, Stephen Carmichael, Alice Broos, Lily Tong, Jenna Nichols, Kyriaki Nomikou; Sarah McDonald; Richard Orton, Joseph Hughes, Sreenu Vattipally, David L Robertson; Alasdair MacLean, Rory Gunson; Sharif Shaaban, Matthew Holden; Rachel Blacow, Guy Mollett, Kathy Li, James Shepherd, Antonia Ho, Emma Thomson
EPI_ISL_919311, EPI_ISL_919312, EPI_ISL_919367, EPI_ISL_919380, EPI_ISL_919381, EPI_ISL_919388, EPI_ISL_919389, EPI_ISL_919390, EPI_ISL_919391, EPI_ISL_919392, EPI_ISL_919393, EPI_ISL_919396, EPI_ISL_919398			
see above	Virology Department, Royal Infirmary of Edinburgh, NHS Lothian / School of Biological Sciences, University of Edinburgh / Institute of Genetics and Molecular Medicine, University of Edinburgh	COVID-19 Genomics UK (COG-UK) Consortium	McHugh M, Dewar R, Rooke S, Gallagher M, Balcaza C, O'Toole Á, Scher E, Hill V, McCrone JT, Colquhoun R, Yu X, Jackson B, Rambaut A, Williams TC, Templeton K
EPI_ISL_920267, EPI_ISL_920277, EPI_ISL_920322, EPI_ISL_920365, EPI_ISL_920372, EPI_ISL_920459, EPI_ISL_920460, EPI_ISL_920516, EPI_ISL_920576, EPI_ISL_920595	University College London Hospital	COVID-19 Genomics UK (COG-UK) Consortium	Judith Heaney, Matthew Byott, Catherine Houlihan, Dan Frampton, Stuart Kirk, Moira Spyer and Eleni Nastouli
EPI_ISL_922894	Wales Specialist Virology Centre Sequencing lab: Pathogen Genomics Unit	Public Health Wales Microbiology Cardiff Wales Specialist Virology Centre	Catherine Moore, Johnathan Evans, Laura Gifford, Malorie Perry, Simon Cottrell, Angela Marchbank, Alec Birchley, Alexander Adams, Amy Gaskin, Bree Gatica-Wilcox, Jason Coombes, Joel Southgate, Lauren Gilbert, Lee Graham, Nicole Pacchiarini, Sara Kumziene-Summerhayes, Sarah Taylor, Sophie Jones, Sara Rey, Matthew Bull, Joanne Watkins, Sally Corden, Tom Connor
EPI_ISL_925853, EPI_ISL_925857, EPI_ISL_925860, EPI_ISL_925861, EPI_ISL_925862, EPI_ISL_925863, EPI_ISL_925864, EPI_ISL_930567	Nucleic Acid Testing, National Reference Laboratory	GIGA Medical Genomics	Yvan Butera, Keith Durkin, Maria Artesi, Bouchra Boujemla, Robert Rutayisire, Patrick Tuyisenge, Esperence Umumararungu, Sébastien Bontems, Marie-Pierre Hayette, Nathalie Renotte, Swaibu Gatare, Jacob Souopgui, Sabin Nsanzimana, Vincent Bours, Léon Mutesa
EPI_ISL_931393, EPI_ISL_931450	University Hospital Basel, Clinical Virology	University Hospital Basel, Clinical Bacteriology	Tim Roloff, Madlen Stange, Helena MB Seth-Smith, Alfredo Mari, Karoline Leuzinger, Julia Bielicki, Manuel Battegay, Hans Hirsch, Adrian Egli

EPI_ISL_933482, EPI_ISL_933483, EPI_ISL_933484, EPI_ISL_933485, EPI_ISL_933486, EPI_ISL_933487, EPI_ISL_933488, EPI_ISL_933489, EPI_ISL_933490, EPI_ISL_933491	Lighthouse Lab in Milton Keynes	Wellcome Sanger Institute for the COVID-19 Genomics UK (COG-UK) Consortium	The Lighthouse Lab in Milton Keynes and Alex Alderton, Roberto Amato, Sonia Goncalves, Ewan Harrison, David K. Jackson, Ian Johnston, Dominic Kwiatkowski, Cordelia Langford, John Sillitoe on behalf of the Wellcome Sanger Institute COVID-19 Surveillance Team
EPI_ISL_933492	Lighthouse Lab in Glasgow	Wellcome Sanger Institute for the COVID-19 Genomics UK (COG-UK) Consortium	Harper VanSteenhouse, Yumi Kasai, David Gray, Carol Clugston, Anna Dominiczak and Alex Alderton, Roberto Amato, Sonia Goncalves, Ewan Harrison, David K. Jackson, Ian Johnston, Dominic Kwiatkowski, Cordelia Langford, John Sillitoe on behalf of the Wellcome Sanger Institute COVID-19 Surveillance Team
EPI_ISL_933669	Instituto de Diagnostico y Referencia Epidemiologicos INDRE_RNLSP	Instituto de Diagnostico y Referencia Epidemiologicos (INDRE)	Claudia Wong-Arambula, Abril Rodriguez-Maldonado, Fabiola Garces-Ayala, Adnan Araiza-Rodriguez, David Fragoso-Fonseca, Sergio Rangel-Guerrero, Mayra Jimenez-Morales, Nancy Munoz-Hernandez, Natividad Cruz-Ortiz, Tatiana Nunez-Garcia, Gisela Barrera-Badillo, Lucia Hernandez-Rivas, Irma Lopez-Martinez, Ernesto Ramirez-Gonzalez.
EPI_ISL_934061, EPI_ISL_934062, EPI_ISL_934063, EPI_ISL_934064, EPI_ISL_934065, EPI_ISL_934066, EPI_ISL_934067, EPI_ISL_934068, EPI_ISL_934069, EPI_ISL_934070, EPI_ISL_934071, EPI_ISL_934072, EPI_ISL_934073, EPI_ISL_934074, EPI_ISL_934075, EPI_ISL_934076, EPI_ISL_934077, EPI_ISL_934078, EPI_ISL_934079, EPI_ISL_934080, EPI_ISL_934081, EPI_ISL_934082, EPI_ISL_934083, EPI_ISL_934084, EPI_ISL_934085, EPI_ISL_934086, EPI_ISL_934087, EPI_ISL_934088, EPI_ISL_934089, EPI_ISL_934090, EPI_ISL_934091, EPI_ISL_934093, EPI_ISL_934094, EPI_ISL_934095, EPI_ISL_934098, EPI_ISL_934110, EPI_ISL_934111, EPI_ISL_934118, EPI_ISL_934125, EPI_ISL_934126, EPI_ISL_934137, EPI_ISL_934167			
see above	Vilnius university hospital Santaros Klinikos, Center of Laboratory Medicine	Vilnius university hospital Santaros Klinikos, Center of Laboratory Medicine	Ingrida Olendraite, Daniel Naumovas, Rimvydas Norvilas, Dovile Ezerskyte, Justinas Slikas, Gytis Dudas
EPI_ISL_934325, EPI_ISL_934326, EPI_ISL_934339	Klinisk mikrobiologi	The Public Health Agency of Sweden	Anna-Malin Linde, Maria Lind Karlberg, Carlo Berg, Oskar Karlsson Lindsjo, Sofia Stamouli, Reza Advani, Mattias Haukland, Petra Holmstrom, Noura Walai, Petra Edquist, Mia Brytting, Anna Risberg, Karin Tegmark-Wisell
EPI_ISL_935240	KU Leuven, Rega Institute, Clinical and Epidemiological Virology	KU Leuven, Rega Institute, Clinical and Epidemiological Virology	Tony Wawina-Bokalanga, Bert Vanmechelen, Joan Marti-Carerras, Piet Maes
EPI_ISL_936182, EPI_ISL_936183, EPI_ISL_936184, EPI_ISL_936185, EPI_ISL_936186, EPI_ISL_936187, EPI_ISL_936188, EPI_ISL_936189, EPI_ISL_936190, EPI_ISL_936191, EPI_ISL_936192, EPI_ISL_936193, EPI_ISL_936194, EPI_ISL_936195, EPI_ISL_936196, EPI_ISL_936197, EPI_ISL_936198, EPI_ISL_936199, EPI_ISL_936200, EPI_ISL_936201, EPI_ISL_936202, EPI_ISL_936203, EPI_ISL_936204, EPI_ISL_936205, EPI_ISL_936206, EPI_ISL_936207, EPI_ISL_936208, EPI_ISL_936209, EPI_ISL_936210, EPI_ISL_936211			
see above	Houston Health Dept.	Houston Health Dept.	Ryker Penn, Pamela Brown, Adolpho Lara
EPI_ISL_936642, EPI_ISL_936643	Northwestern Memorial Hospital	Ozer Lab	Ramon Lorenzo-Redondo, Lacy M. Simons, Chad J. Achenbach, Lawrence J. Jennings, Michael G. Ison, Judd F. Hultquist, Egon A. Ozer
EPI_ISL_937291, EPI_ISL_937292, EPI_ISL_937293, EPI_ISL_937294, EPI_ISL_937295, EPI_ISL_937296, EPI_ISL_937297, EPI_ISL_937298, EPI_ISL_937299, EPI_ISL_937300, EPI_ISL_937301, EPI_ISL_937302, EPI_ISL_937304, EPI_ISL_937305, EPI_ISL_937306, EPI_ISL_937307, EPI_ISL_937308, EPI_ISL_937309, EPI_ISL_937310, EPI_ISL_937319, EPI_ISL_937320, EPI_ISL_937321, EPI_ISL_937322, EPI_ISL_937323, EPI_ISL_937324, EPI_ISL_937325, EPI_ISL_937327, EPI_ISL_937328, EPI_ISL_937329, EPI_ISL_937339, EPI_ISL_937340, EPI_ISL_937343, EPI_ISL_937344			
see above	Utah Public Health Laboratory	Utah Public Health Laboratory	Erin L. Young, Kelly F. Oakeson, Tara Gallagher
EPI_ISL_940075, EPI_ISL_940076, EPI_ISL_940077, EPI_ISL_940087, EPI_ISL_940093, EPI_ISL_940097, EPI_ISL_940108, EPI_ISL_940109, EPI_ISL_940112, EPI_ISL_940113, EPI_ISL_940116, EPI_ISL_940118, EPI_ISL_940120, EPI_ISL_940127, EPI_ISL_940130, EPI_ISL_940135, EPI_ISL_940136			
see above	Charlotte Maxeke Johannesburg Academic Hospital, National Health Laboratory Services, Gauteng, South Africa	National Institute for Communicable Diseases of the National Health Laboratory Service	Amoako DG, Mohale T, Ntuli N, Mahlangu B, Allam M, Ismail A, Bhiman JN
EPI_ISL_940879	Vaccines and Infectious Diseases Analytics Research Unit (VIDA)	KRISP, KZN Research Innovation and Sequencing Platform	Baillie Vicky, du Plessis Jeanine, Giandhari Jennifer, Pillay Sureshnee, Naidoo Yeshnee, Tegally Houriiyah, de Oliveira Tulio, Madhi Shabir
EPI_ISL_941209, EPI_ISL_941210	Servicio de Microbiología. Hospital Clínico Universitario de Valencia	SeqCOVID-SPAIN consortium/IBV(CSIC)	David Navarro Ortega, Eliseo Albert Vicent, Ignacio Torres and SeqCOVID-SPAIN consortium
EPI_ISL_941251, EPI_ISL_941252, EPI_ISL_941253, EPI_ISL_941254	Virginia DCLS	Virginia DCLS	Virginia DCLS
EPI_ISL_942185, EPI_ISL_942204, EPI_ISL_942205, EPI_ISL_942209, EPI_ISL_942211, EPI_ISL_942213, EPI_ISL_942215, EPI_ISL_942216, EPI_ISL_942217, EPI_ISL_942218, EPI_ISL_942219, EPI_ISL_942220, EPI_ISL_942221, EPI_ISL_942222, EPI_ISL_942223, EPI_ISL_942224, EPI_ISL_942225, EPI_ISL_942226, EPI_ISL_942227, EPI_ISL_942228, EPI_ISL_942229, EPI_ISL_942231, EPI_ISL_942236, EPI_ISL_942237, EPI_ISL_942238, EPI_ISL_942239, EPI_ISL_942240, EPI_ISL_942241, EPI_ISL_942242, EPI_ISL_942243, EPI_ISL_942244, EPI_ISL_942245, EPI_ISL_942246, EPI_ISL_942247			
see above	Wisconsin State Laboratory of Hygiene Communicable Disease Division	Wisconsin State Laboratory of Hygiene Communicable Disease Division	Kelsey R. Florek, Abigail C. Shockey
EPI_ISL_942658, EPI_ISL_942659, EPI_ISL_942662, EPI_ISL_942663, EPI_ISL_942664, EPI_ISL_942665, EPI_ISL_942666, EPI_ISL_942667, EPI_ISL_942668, EPI_ISL_942669, EPI_ISL_942670, EPI_ISL_942671, EPI_ISL_942672, EPI_ISL_942673, EPI_ISL_942674, EPI_ISL_942675, EPI_ISL_942676, EPI_ISL_942677, EPI_ISL_942678, EPI_ISL_942679, EPI_ISL_942680, EPI_ISL_942681, EPI_ISL_942682, EPI_ISL_942683, EPI_ISL_942684, EPI_ISL_942685, EPI_ISL_942686, EPI_ISL_942687, EPI_ISL_942688, EPI_ISL_942689, EPI_ISL_942942			
see above	Gundersen Molecular Diagnostics Laboratory	Kabara Cancer Research Institute	Craig S. Richmond, Paraic A. Kenny
EPI_ISL_942966	General Hospital - Kumanovo	Research Center for Genetic Engineering and Biotechnology "Georgi D. Efremov" , Macedonian Academy of Sciences and Arts	Aleksandar J.Dimovski, Dijana Plasheska-Karanfilska, Predrag Noveski, Gjorgji Bozinovski, Milena Jakimovska
EPI_ISL_942967	General Hospital - Kumanovo	Research Center for Genetic Engineering and Biotechnology "Georgi D. Efremov" , Macedonian Academy of Sciences and Arts	Aleksandar J. Dimovski, Dijana Plasheska-Karanfilska, Predrag Noveski, Gjorgji Bozinovski, Milena Jakimovska
EPI_ISL_943667, EPI_ISL_943756, EPI_ISL_943757, EPI_ISL_943758, EPI_ISL_943759, EPI_ISL_943760, EPI_ISL_943761, EPI_ISL_943762, EPI_ISL_943774, EPI_ISL_943775, EPI_ISL_943776, EPI_ISL_943777, EPI_ISL_943778, EPI_ISL_943779, EPI_ISL_943780, EPI_ISL_943781, EPI_ISL_943782, EPI_ISL_943783, EPI_ISL_943784, EPI_ISL_943785, EPI_ISL_943786, EPI_ISL_943787, EPI_ISL_943788			
see above	Utah Public Health Laboratory	Utah Public Health Laboratory	Erin L. Young, Kelly F. Oakeson, Tara Gallagher
EPI_ISL_945255	Lighthouse Lab in Glasgow	Wellcome Sanger Institute for the COVID-19 Genomics UK (COG-UK) Consortium	Harper VanSteenhouse, Yumi Kasai, David Gray, Carol Clugston, Anna Dominiczak and Alex Alderton, Roberto Amato, Sonia Goncalves, Ewan Harrison, David K. Jackson, Ian Johnston, Dominic Kwiatkowski, Cordelia Langford, John Sillitoe on behalf of the Wellcome Sanger Institute COVID-19 Surveillance Team
EPI_ISL_947298	RSU Medirossa	Eijkman Institute for Molecular Biology, Ministry of Research and Technology/National Agency for Research and Innovation	Iskandar Adnan, Lydia V. Panggalo, Sukma Oktavianthi, Willy Agustine, Edison Johar, Hidayat Trimarsanto, Frilasita A Yudhaputri, Safarina G Malik, Khin Saw Myint, Amin Soebandrio
EPI_ISL_947301	RS Mitra Keluarga Cibubur	Eijkman Institute for Molecular Biology, Ministry of Research and Technology/National Agency for Research and Innovation	Iskandar Adnan, Lydia V. Panggalo, Sukma Oktavianthi, Willy Agustine, Edison Johar, Hidayat Trimarsanto, Frilasita A Yudhaputri, Safarina G Malik, Khin Saw Myint, Amin Soebandrio
EPI_ISL_947304	RSU Harapan Bunda	Eijkman Institute for Molecular Biology, Ministry of Research and Technology/National Agency for Research and Innovation	Iskandar Adnan, Lydia V. Panggalo, Sukma Oktavianthi, Willy Agustine, Edison Johar, Hidayat Trimarsanto, Frilasita A Yudhaputri, Safarina G Malik, Khin Saw Myint, Amin Soebandrio
EPI_ISL_949396, EPI_ISL_949397, EPI_ISL_949398	University of Birmingham	COVID-19 Genomics UK (COG-UK) Consortium	Institute of Microbiology, University of Birmingham: Claire McMurray, Joanne Stockton, Samuel Nicholls, Radoslaw Poplawski, Will Rowe, Josh Quick, Nicholas Loman. University of Birmingham Testing Laboratory: Celina M Whalley, Andrew Bosworth, Charlotte Poxon, Kasun Wanigasooriya, Oliver Pickles, Mike Kidd, Alex Richter, Andrew D Beggs PHE Heartlands Lab: Husam Osman, Andrew Bosworth. Queen Elizabeth Hospital: Anna Casey
EPI_ISL_949618, EPI_ISL_949622, EPI_ISL_949628	NHLS Universitas Academic	UFS Virology	PA Bester, MM Nyaga, P Nthiga, MT Mogotsi, Emmanuel Ogunbayo, D Goedhals, T de Oliveira
EPI_ISL_949786	University College London, Great Ormond Street Hospital for Children NHS Foundation Trust, Imperial College Healthcare NHS Trust	COVID-19 Genomics UK (COG-UK) Consortium	Sergi Castellano, Rachel Williams, Mark Kristiansen, Paola Resende Silva, Sunando Roy, Tony Brooks, Helena Tutill, Paola Niola, Patricia Dyal, Charlotte Williams, Leysa Forrest, Yasmin Panchbhaya, Jacqueline Findlay, Samuel Weeks, Julianne Brown, Kathryn Harris, Paul Randell, James Price, Alison Holmes, Judith Breuer
EPI_ISL_950699	Lincolnshire Hospitals and DeepSeq Nottingham	COVID-19 Genomics UK (COG-UK) Consortium	Nichola Duckworth, Tim Sloan, Sarah Walsh, Jonathan Ball, Patrick McClure, Joeseph Chappell, Nadine Holmes, Matthew Carlisle, Christopher Moore, Fei Sang, Johnny Debebe, Victoria Wright, Matthew Loose

EPI_ISL_955362, EPI_ISL_955363, EPI_ISL_955364, EPI_ISL_955365, EPI_ISL_955366, EPI_ISL_955367, EPI_ISL_955368, EPI_ISL_955369, EPI_ISL_955370, EPI_ISL_955373, EPI_ISL_955374, EPI_ISL_955378, EPI_ISL_955379, EPI_ISL_955380, EPI_ISL_955381, EPI_ISL_955382, EPI_ISL_955383, EPI_ISL_955384, EPI_ISL_955385			
see above	Santa Clara County Public Health Laboratory	Chan-Zuckerberg Biohub	CZB Cliahub Consortium
EPI_ISL_955665, EPI_ISL_955667, EPI_ISL_955668, EPI_ISL_955669, EPI_ISL_955671, EPI_ISL_955672, EPI_ISL_955673	Orange County Public Health Lab	Chan-Zuckerberg Biohub	CZB Cliahub Consortium
EPI_ISL_960414	The National Institute of Public Health	State Veterinary Institute Prague	Nagy,A;Vecerova,J;Cernikova,L;Stara,M;Jirincova,H;Trnka,D
EPI_ISL_960452, EPI_ISL_960456, EPI_ISL_960468, EPI_ISL_960475, EPI_ISL_960476, EPI_ISL_960482, EPI_ISL_960496, EPI_ISL_960497, EPI_ISL_960516, EPI_ISL_960517, EPI_ISL_960518, EPI_ISL_960519, EPI_ISL_960520, EPI_ISL_960521, EPI_ISL_960522, EPI_ISL_960523, EPI_ISL_960524, EPI_ISL_960525, EPI_ISL_960574, EPI_ISL_960575, EPI_ISL_960591, EPI_ISL_960607, EPI_ISL_960608, EPI_ISL_960609, EPI_ISL_960610, EPI_ISL_960611, EPI_ISL_960612, EPI_ISL_960613			
see above	Istituto Zooprofilattico Sperimentale del Mezzogiorno	TIGEM	Patrizia Annunziata, Andrea Ballabio, Valentina Bouche, Davide Cacchiarelli, Pellegrino Cerino, Chiara Colantuono, Maria Concetta Cuomo, Denise Di Concilio, Lucio Di Filippo, Antonio Grimaldi, Antonio Limone, Anna Manfredi, Francesco Panariello, Biancamaria Pierri, Marcello Salvi
EPI_ISL_961380	Toronto Invasive Bacterial Diseases Network	McMaster University	Allison McGeer, Patryk Aftanas, Hooman Derakhshani, Angel Li, Kuganya Nirmalarajah, Emily Panousis, Ahmed Draia, Jalees Nasir, Michael Surette, Samira Mubareka, Andrew G. McArthur
EPI_ISL_961901, EPI_ISL_961924, EPI_ISL_961939, EPI_ISL_961983, EPI_ISL_962080, EPI_ISL_962081, EPI_ISL_962083, EPI_ISL_962085, EPI_ISL_962086, EPI_ISL_962087, EPI_ISL_962088, EPI_ISL_962089, EPI_ISL_962090, EPI_ISL_962092, EPI_ISL_962093, EPI_ISL_962094, EPI_ISL_962095, EPI_ISL_962096, EPI_ISL_962097, EPI_ISL_962098, EPI_ISL_962099, EPI_ISL_962100			
see above	Illinois Department of Public Health	Gagnon Lab, Southern Illinois University	Keith Gagnon
EPI_ISL_962766, EPI_ISL_962767, EPI_ISL_962769, EPI_ISL_962772, EPI_ISL_962773, EPI_ISL_962774, EPI_ISL_962778, EPI_ISL_962779, EPI_ISL_962780, EPI_ISL_962785, EPI_ISL_962786, EPI_ISL_962788, EPI_ISL_962791, EPI_ISL_962792, EPI_ISL_962793, EPI_ISL_962796, EPI_ISL_962797, EPI_ISL_962801, EPI_ISL_962802, EPI_ISL_962803, EPI_ISL_962808, EPI_ISL_962809			
see above	San Diego County Public Health Laboratory	Andersen lab at Scripps Research	SEARCH Alliance San Diego with Tracy Basler, Jovan Shephard, Brett Austin
EPI_ISL_962973	Hospital Universitario de Gran Canaria Dr. Negrín	SeqCOVID-SPAIN consortium/IBV(CSIC)	M. Carmen Pérez González, Francisco J. Chamizo López, Ana Bordes Benítez and SeqCOVID-SPAIN consortium
EPI_ISL_965187, EPI_ISL_965190, EPI_ISL_965221	Virginia Division of Consolidated Laboratory Services	Virginia Division of Consolidated Laboratory Services	Virginia DCLS
EPI_ISL_965538, EPI_ISL_965575, EPI_ISL_965576, EPI_ISL_965599, EPI_ISL_965631, EPI_ISL_965634, EPI_ISL_965635, EPI_ISL_965651, EPI_ISL_965660, EPI_ISL_965664, EPI_ISL_965673, EPI_ISL_965697, EPI_ISL_965698, EPI_ISL_965707, EPI_ISL_965713, EPI_ISL_965740, EPI_ISL_965750, EPI_ISL_965754, EPI_ISL_965789, EPI_ISL_965796, EPI_ISL_965797, EPI_ISL_965803			
see above	Dutch COVID-19 response team	Medical Microbiology, Maastricht University Medical Centre	Jozef Dingemans*, Brian van der Veer*, Erik Beuken, Carmen Reumkens, Lieke van Alphen, Christian Hoebe, Paul Savelkoul
EPI_ISL_966756	Maine HETL	Tewhey Lab, The Jackson Laboratory	Matluk,N., Dewey,H., Iosue,F., Barter,M., Lynch,R., Munger,H. and Tewhey,R.
EPI_ISL_967525	State Laboratories Division, Hawaii State Department of Health	State Laboratories Division, Hawaii State Department of Health	Pamela O'Brien, Drew Kuwazaki, Ayana Garnet, Razvan Sultana, Edward Desmond
EPI_ISL_968822, EPI_ISL_968825, EPI_ISL_968826, EPI_ISL_968827, EPI_ISL_968828, EPI_ISL_968829, EPI_ISL_968844, EPI_ISL_968846, EPI_ISL_968847, EPI_ISL_968849	KEMRI-Wellcome Trust Research Programme/KEMRI-CGMR-C Kilifi	KEMRI-Wellcome Trust Research Programme/KEMRI-CGMR-C Kilifi	Githinji et al
EPI_ISL_976301, EPI_ISL_976302, EPI_ISL_976303, EPI_ISL_976304, EPI_ISL_976306, EPI_ISL_976307, EPI_ISL_976308, EPI_ISL_976309, EPI_ISL_976310, EPI_ISL_976311, EPI_ISL_976312, EPI_ISL_976313, EPI_ISL_976314, EPI_ISL_976315, EPI_ISL_976316, EPI_ISL_976317, EPI_ISL_976318, EPI_ISL_976319, EPI_ISL_976320, EPI_ISL_976321, EPI_ISL_976322, EPI_ISL_976323, EPI_ISL_976324, EPI_ISL_976325, EPI_ISL_976326, EPI_ISL_976327, EPI_ISL_976328, EPI_ISL_976329, EPI_ISL_976330, EPI_ISL_976331, EPI_ISL_976332, EPI_ISL_976333, EPI_ISL_976334, EPI_ISL_976335, EPI_ISL_976336, EPI_ISL_976337, EPI_ISL_976338, EPI_ISL_976339, EPI_ISL_976340, EPI_ISL_976341, EPI_ISL_976342, EPI_ISL_976343, EPI_ISL_976344, EPI_ISL_976345, EPI_ISL_976346, EPI_ISL_976347, EPI_ISL_976348, EPI_ISL_976349, EPI_ISL_976350, EPI_ISL_976351, EPI_ISL_976352, EPI_ISL_976353, EPI_ISL_976354, EPI_ISL_976355, EPI_ISL_976356, EPI_ISL_976357, EPI_ISL_976358, EPI_ISL_976359, EPI_ISL_976360, EPI_ISL_976361, EPI_ISL_976362, EPI_ISL_976363, EPI_ISL_976364, EPI_ISL_976365, EPI_ISL_976366, EPI_ISL_976367, EPI_ISL_976368, EPI_ISL_976369, EPI_ISL_976370, EPI_ISL_976371, EPI_ISL_976372, EPI_ISL_976373, EPI_ISL_976374, EPI_ISL_976375, EPI_ISL_976376, EPI_ISL_976377, EPI_ISL_976378, EPI_ISL_976379, EPI_ISL_976380, EPI_ISL_976381, EPI_ISL_976382, EPI_ISL_976383, EPI_ISL_976384, EPI_ISL_976385, EPI_ISL_976386, EPI_ISL_976387, EPI_ISL_976388, EPI_ISL_976389, EPI_ISL_976390, EPI_ISL_976391, EPI_ISL_976392, EPI_ISL_976393, EPI_ISL_976394, EPI_ISL_976395, EPI_ISL_976396, EPI_ISL_976397, EPI_ISL_976398, EPI_ISL_976399, EPI_ISL_976400, EPI_ISL_976401, EPI_ISL_976402, EPI_ISL_976403, EPI_ISL_976404, EPI_ISL_976405, EPI_ISL_976406, EPI_ISL_976407, EPI_ISL_976408, EPI_ISL_976409, EPI_ISL_976410, EPI_ISL_976411, EPI_ISL_976412, EPI_ISL_976413, EPI_ISL_976414, EPI_ISL_976415, EPI_ISL_976416, EPI_ISL_976417, EPI_ISL_976418, EPI_ISL_976419, EPI_ISL_976420, EPI_ISL_976421, EPI_ISL_976422, EPI_ISL_976423			
see above	BCCDC Public Health Laboratory	BCCDC Public Health Laboratory	Prystajecky Natalie, Linda Hoang, Dan Fornika, John Tyson, Shannon Russell, Kim Macdonald, Kimia Kamelian, Ana Pacagnella, Corrinne Ng, Loretta Janz, Robert Azana Terry Snutch, Mel Krajden
EPI_ISL_977028	Rhode Island Department of Health	Infectious Disease Program, Broad Institute of Harvard and MIT	Lemieux,J.E., Siddle,K.J., Huard,R., King,E., Azevedo,K., Miller,A., Adams,G., Gladden-Young,A., Lagerborg,K., Rudy,M., DeRuff,K., Carter,A., Normandin,E., Bauer,M., Reilly,S., Tomkins-Tinch,C., Loreth,C., Chaluvadi,S., Birren,B.W., Gallagher,G., Smole,S., Park,D.J., MacInnis,B.L., and Sabeti,P.C.
EPI_ISL_977162, EPI_ISL_977167, EPI_ISL_977168, EPI_ISL_977169	Microbiologia e Virologia	Istituto Zooprofilattico Sperimentale delle Venezie	Adelaide Milani, Alessia Schivo, Annalisa Salviato, Erika Giorgia Quaranta, Ambra Pastori, Bianca Zecchin, Alice Fusaro, Isabella Monne, Calogero Terregino, Antonia Ricci
EPI_ISL_977222, EPI_ISL_977223	ULSS 1 Dolomiti	Istituto Zooprofilattico Sperimentale delle Venezie	Adelaide Milani, Alessia Schivo, Annalisa Salviato, Erika Giorgia Quaranta, Ambra Pastori, Bianca Zecchin, Alice Fusaro, Isabella Monne, Calogero Terregino, Antonia Ricci
EPI_ISL_977247, EPI_ISL_977248	ULSS 03 Venezia	Istituto Zooprofilattico Sperimentale delle Venezie	Adelaide Milani, Alessia Schivo, Annalisa Salviato, Erika Giorgia Quaranta, Ambra Pastori, Bianca Zecchin, Alice Fusaro, Isabella Monne, Calogero Terregino, Antonia Ricci
EPI_ISL_978193	Virginia Division of Consolidated Laboratory Services	Virginia Division of Consolidated Laboratory Services	Virginia DCLS
EPI_ISL_979017, EPI_ISL_979018	Santa Clara County Public Health Laboratory	Chan-Zuckerberg Biohub	CZB Cliahub Consortium
EPI_ISL_979162, EPI_ISL_979163, EPI_ISL_979164, EPI_ISL_979165, EPI_ISL_979166, EPI_ISL_979167, EPI_ISL_979168, EPI_ISL_979169, EPI_ISL_979170, EPI_ISL_979171, EPI_ISL_979172			
see above	Humboldt County Public Health Laboratory	Chan-Zuckerberg Biohub	CZB Cliahub Consortium
EPI_ISL_979305, EPI_ISL_979311	Cadham Provincial laboratory	National Microbiology Laboratory (NML)	Anna Majer, Shari Tyson, Grace Seo, Philip Mabon, Elsie Grudeski, Rhiannon Huzarewich, Russell Mandes, Anneliese Landgraff, Jennifer Tanner, Natalie Knox, Morag Graham, Gary Van Domselaar, Paul Van Caeseele, Jared Bullard, David Alexander, Kerry Dust, Nathalie Bastien, Yan Li, Timothy Booth, Darian Hole, Madison Chapel, Kirsten Biggar, CanCOGeN's metadata curation team, Public Health Agency of Canada CanCOGeN team
EPI_ISL_979329	Laboratorio Estatal de Salud Pública de Nuevo León	Laboratorio de Infectología Molecular, Departamento de Bioquímica y Medicina Molecular,Facultad de Medicina - Universidad Autónoma de Nuevo León	Kame A. Galán-Huerta, María F. Herrera-Saldivar, Natalia Martínez-Acuña, Sonia A. Lozano-Sepúlveda, Daniel Arellanos-Soto, Ana M. Rivas-Estilla, Samuel Buentello-Wong, Else del Carmen García-García, Gloria A. Jasso-de-la-Peña, Roberto Montes-de-Oca, Consuelo Treviño-Garza, Manuel E. de-la-O-Cavazos
EPI_ISL_979797	Humboldt County Public Health Laboratory	Chan-Zuckerberg Biohub	CZB Cliahub Consortium
EPI_ISL_981883, EPI_ISL_981884, EPI_ISL_981885, EPI_ISL_981886, EPI_ISL_981887, EPI_ISL_981896, EPI_ISL_981897, EPI_ISL_981898, EPI_ISL_981899, EPI_ISL_981900, EPI_ISL_981901, EPI_ISL_981902			
see above	Microbiology Service, Hospital Universitario Clinico San Cecilio, Granada	Microbiology Service, Hospital Universitario Clinico San Cecilio, Granada	Adolfo de Salazar, Natalia Chueca, Laura Viñuela, Ana Fuentes, Federico García
EPI_ISL_982844	Kentucky State Public Health Lab	Kentucky State Public Health Lab	Stephanie Lunn, Karim George, Joshua Tobias, William Grooms, Vaneet Arora, Matthew Johnson, Rachel Zinner, Rhonda Lucas
EPI_ISL_982845, EPI_ISL_982846	UK Healthcare Clinical Microbiology	Kentucky State Public Health Lab	Stephanie Lunn, Karim George, Joshua Tobias, William Grooms, Vaneet Arora, Matthew Johnson, Rachel Zinner, Rhonda Lucas
