# Supplementary material for: Detection and characterization of the SARS-CoV-2 lineage B.1.526 in New York
Source: Nat Commun. 2021 Aug 9;12:4886. doi: 10.1038/s41467-021-25168-4 (PMC8352861; doi:10.1038/s41467-021-25168-4)
Supplement: Supplementary file 8 — Supplementary Data 4 [file 41467_2021_25168_MOESM8_ESM.zip › GISAID_acknowledements_tables/gisaid_hcov-19_acknowledgement_table_2021_02_13_00-10.pdf]

We gratefully acknowledge the following Authors from the Originating laboratories responsible for obtaining the specimens, as well as the Submitting laboratories where the genome data were generated and shared via GISAID, on which this research is based.

All Submitters of data may be contacted directly via www.gisaid.org

Authors are sorted alphabetically.

Accession ID	Originating Laboratory	Submitting Laboratory	Authors
EPI_ISL_660794, EPI_ISL_661175, EPI_ISL_661176	Respiratory Virus Unit, Microbiology Services Colindale, Public Health England	COVID-19 Genomics UK (COG-UK) Consortium	PHE Covid Sequencing Team
EPI_ISL_661260	Canterbury Health Laboratories	Institute of Environmental Science and Research (ESR)	Xiaoyun Ren, Matt Storey, Nikki Freed, Muhammad Faisal, Jing Wang, Hermes Perez, Anja Werno, Antje van der Linden, Arlo Upton, Chris Mansell, David Hammer, Dragana Drinkovic, Gary McAuliffe, Hana Sofia Andersson, James Ussher, Jill Sherwood, Josh Freeman, Julia Howard, Juliet Elvy, Mary DeAlmeida, Matt Blakiston, Matthew Rogers, Max Bloomfield, Michael Addidle, Michelle Balm, Sally Roberts, Sarah Jefferies, Sharmini Muttaiyah, Susan Morpeth, Susan Taylor, Timothy Blackmore, Vani Sathyendran, Veronica Playle, Virginia Hope, Erasmus Smit, Lauren Jelly, Olin Silander, Joep de Ligt
EPI_ISL_661261, EPI_ISL_661264	LabPLUS	Institute of Environmental Science and Research (ESR)	Xiaoyun Ren, Matt Storey, Nikki Freed, Muhammad Faisal, Jing Wang, Hermes Perez, Anja Werno, Antje van der Linden, Arlo Upton, Chris Mansell, David Hammer, Dragana Drinkovic, Gary McAuliffe, Hana Sofia Andersson, James Ussher, Jill Sherwood, Josh Freeman, Julia Howard, Juliet Elvy, Mary DeAlmeida, Matt Blakiston, Matthew Rogers, Max Bloomfield, Michael Addidle, Michelle Balm, Sally Roberts, Sarah Jefferies, Sharmini Muttaiyah, Susan Morpeth, Susan Taylor, Timothy Blackmore, Vani Sathyendran, Veronica Playle, Virginia Hope, Erasmus Smit, Lauren Jelly, Olin Silander, Joep de Ligt
EPI_ISL_664149, EPI_ISL_664150, EPI_ISL_664326, EPI_ISL_664381, EPI_ISL_664546, EPI_ISL_664591, EPI_ISL_664610, EPI_ISL_665013, EPI_ISL_665014, EPI_ISL_665015, EPI_ISL_665016, EPI_ISL_665017, EPI_ISL_665018, EPI_ISL_665019			
see above	Department of Pathology, University of Cambridge	COVID-19 Genomics UK (COG-UK) Consortium	Aminu S. Jahun, Yasmin Chaudhry, Grant Hall, Iliana Georgana, Myra Hosmillo, Martin D. Curran, Malte Pinckert, Surendra Parmar, Ian Goodfellow
EPI_ISL_665290	Queens Medical Centre, Clinical Microbiology Department / DeepSeq Nottingham	COVID-19 Genomics UK (COG-UK) Consortium	Gemma Clark, Wendy Smith, Manjinder Khakh, Vicki M Fleming, Michelle M Lister, Hannah Howson-Wells, Jonathan Ball, Patrick McClure, Joseph Chappell, Theocharis Tsoleridis, Nadine Holmes, Matthew Carlisle, Christopher Moore, Fei Sang, Johnny Debebe, Victoria Wright, Matthew Loose
EPI_ISL_665295	Quadram Institute Bioscience	COVID-19 Genomics UK (COG-UK) Consortium	Dave J. Baker, Gemma L. Kay, Alp Aydin, Thanh Le-Viet, Steven Rudder, Ana P. Tedim, Anastasia Kolyva, Maria Diaz, Leonardo de Oliveira Martins, Nabil-Fareed Alikhan, Lizzie Meadows, Rachael Stanley, Ngozi Elumogo, Muhammed Yasir, Nicholas M. Thomson, Alexander J Trotter, Rachel Gilroy, Samuel Bloomfield, Claire Stuart, Andrew Bell, Reenesh Prakash, Samir Dervisevic, Alison E. Mather, John Wain, Mark Webber, Andrew J. Page, Justin O'Grady
EPI_ISL_665344, EPI_ISL_665366	Queens Medical Centre, Clinical Microbiology Department / DeepSeq Nottingham	COVID-19 Genomics UK (COG-UK) Consortium	Gemma Clark, Wendy Smith, Manjinder Khakh, Vicki M Fleming, Michelle M Lister, Hannah Howson-Wells, Jonathan Ball, Patrick McClure, Joseph Chappell, Theocharis Tsoleridis, Nadine Holmes, Matthew Carlisle, Christopher Moore, Fei Sang, Johnny Debebe, Victoria Wright, Matthew Loose
EPI_ISL_665377, EPI_ISL_665380, EPI_ISL_665381	Quadram Institute Bioscience	COVID-19 Genomics UK (COG-UK) Consortium	Dave J. Baker, Gemma L. Kay, Alp Aydin, Thanh Le-Viet, Steven Rudder, Ana P. Tedim, Anastasia Kolyva, Maria Diaz, Leonardo de Oliveira Martins, Nabil-Fareed Alikhan, Lizzie Meadows, Rachael Stanley, Ngozi Elumogo, Muhammed Yasir, Nicholas M. Thomson, Alexander J Trotter, Rachel Gilroy, Samuel Bloomfield, Claire Stuart, Andrew Bell, Reenesh Prakash, Samir Dervisevic, Alison E. Mather, John Wain, Mark Webber, Andrew J. Page, Justin O'Grady
EPI_ISL_665415	Queens Medical Centre, Clinical Microbiology Department / DeepSeq Nottingham	COVID-19 Genomics UK (COG-UK) Consortium	Gemma Clark, Wendy Smith, Manjinder Khakh, Vicki M Fleming, Michelle M Lister, Hannah Howson-Wells, Jonathan Ball, Patrick McClure, Joseph Chappell, Theocharis Tsoleridis, Nadine Holmes, Matthew Carlisle, Christopher Moore, Fei Sang, Johnny Debebe, Victoria Wright, Matthew Loose
EPI_ISL_665423, EPI_ISL_665440, EPI_ISL_665450, EPI_ISL_665508	Quadram Institute Bioscience	COVID-19 Genomics UK (COG-UK) Consortium	Dave J. Baker, Gemma L. Kay, Alp Aydin, Thanh Le-Viet, Steven Rudder, Ana P. Tedim, Anastasia Kolyva, Maria Diaz, Leonardo de Oliveira Martins, Nabil-Fareed Alikhan, Lizzie Meadows, Rachael Stanley, Ngozi Elumogo, Muhammed Yasir, Nicholas M. Thomson, Alexander J Trotter, Rachel Gilroy, Samuel Bloomfield, Claire Stuart, Andrew Bell, Reenesh Prakash, Samir Dervisevic, Alison E. Mather, John Wain, Mark Webber, Andrew J. Page, Justin O'Grady
EPI_ISL_665512, EPI_ISL_665513	Queens Medical Centre, Clinical Microbiology Department / DeepSeq Nottingham	COVID-19 Genomics UK (COG-UK) Consortium	Gemma Clark, Wendy Smith, Manjinder Khakh, Vicki M Fleming, Michelle M Lister, Hannah Howson-Wells, Jonathan Ball, Patrick McClure, Joseph Chappell, Theocharis Tsoleridis, Nadine Holmes, Matthew Carlisle, Christopher Moore, Fei Sang, Johnny Debebe, Victoria Wright, Matthew Loose
EPI_ISL_665518	Quadram Institute Bioscience	COVID-19 Genomics UK (COG-UK) Consortium	Dave J. Baker, Gemma L. Kay, Alp Aydin, Thanh Le-Viet, Steven Rudder, Ana P. Tedim, Anastasia Kolyva, Maria Diaz, Leonardo de Oliveira Martins, Nabil-Fareed Alikhan, Lizzie Meadows, Rachael Stanley, Ngozi Elumogo, Muhammed Yasir, Nicholas M. Thomson, Alexander J Trotter, Rachel Gilroy, Samuel Bloomfield, Claire Stuart, Andrew Bell, Reenesh Prakash, Samir Dervisevic, Alison E. Mather, John Wain, Mark Webber, Andrew J. Page, Justin O'Grady
EPI_ISL_665528, EPI_ISL_665545	Queens Medical Centre, Clinical Microbiology Department / DeepSeq Nottingham	COVID-19 Genomics UK (COG-UK) Consortium	Gemma Clark, Wendy Smith, Manjinder Khakh, Vicki M Fleming, Michelle M Lister, Hannah Howson-Wells, Jonathan Ball, Patrick McClure, Joseph Chappell, Theocharis Tsoleridis, Nadine Holmes, Matthew Carlisle, Christopher Moore, Fei Sang, Johnny Debebe, Victoria Wright, Matthew Loose
EPI_ISL_665574, EPI_ISL_665594, EPI_ISL_665596, EPI_ISL_665603	Quadram Institute Bioscience	COVID-19 Genomics UK (COG-UK) Consortium	Dave J. Baker, Gemma L. Kay, Alp Aydin, Thanh Le-Viet, Steven Rudder, Ana P. Tedim, Anastasia Kolyva, Maria Diaz, Leonardo de Oliveira Martins, Nabil-Fareed Alikhan, Lizzie Meadows, Rachael Stanley, Ngozi Elumogo, Muhammed Yasir, Nicholas M. Thomson, Alexander J Trotter, Rachel Gilroy, Samuel Bloomfield, Claire Stuart, Andrew Bell, Reenesh Prakash, Samir Dervisevic, Alison E. Mather, John Wain, Mark Webber, Andrew J. Page, Justin O'Grady
EPI_ISL_665610, EPI_ISL_665623	Queens Medical Centre, Clinical Microbiology Department / DeepSeq Nottingham	COVID-19 Genomics UK (COG-UK) Consortium	Gemma Clark, Wendy Smith, Manjinder Khakh, Vicki M Fleming, Michelle M Lister, Hannah Howson-Wells, Jonathan Ball, Patrick McClure, Joseph Chappell, Theocharis Tsoleridis, Nadine Holmes, Matthew Carlisle, Christopher Moore, Fei Sang, Johnny Debebe, Victoria Wright, Matthew Loose
EPI_ISL_665639, EPI_ISL_665665, EPI_ISL_665689, EPI_ISL_665712, EPI_ISL_665732, EPI_ISL_665737, EPI_ISL_665749, EPI_ISL_665897	Quadram Institute Bioscience	COVID-19 Genomics UK (COG-UK) Consortium	Dave J. Baker, Gemma L. Kay, Alp Aydin, Thanh Le-Viet, Steven Rudder, Ana P. Tedim, Anastasia Kolyva, Maria Diaz, Leonardo de Oliveira Martins, Nabil-Fareed Alikhan, Lizzie Meadows, Rachael Stanley, Ngozi Elumogo, Muhammed Yasir, Nicholas M. Thomson, Alexander J Trotter, Rachel Gilroy, Samuel Bloomfield, Claire Stuart, Andrew Bell, Reenesh Prakash, Samir Dervisevic, Alison E. Mather, John Wain, Mark Webber, Andrew J. Page, Justin O'Grady
EPI_ISL_665909, EPI_ISL_665910, EPI_ISL_665911, EPI_ISL_665912, EPI_ISL_665913, EPI_ISL_665914, EPI_ISL_665915, EPI_ISL_665916, EPI_ISL_665917, EPI_ISL_665918, EPI_ISL_665919, EPI_ISL_665920, EPI_ISL_665921, EPI_ISL_665922, EPI_ISL_665923, EPI_ISL_665924, EPI_ISL_665925, EPI_ISL_665926, EPI_ISL_665927, EPI_ISL_665928, EPI_ISL_665929, EPI_ISL_665930, EPI_ISL_665931, EPI_ISL_665932, EPI_ISL_665933, EPI_ISL_665934, EPI_ISL_665935, EPI_ISL_665936, EPI_ISL_665937, EPI_ISL_665938, EPI_ISL_665939			
see above	Queens Medical Centre, Clinical Microbiology Department / DeepSeq Nottingham	COVID-19 Genomics UK (COG-UK) Consortium	Gemma Clark, Wendy Smith, Manjinder Khakh, Vicki M Fleming, Michelle M Lister, Hannah Howson-Wells, Jonathan Ball, Patrick McClure, Joseph Chappell, Theocharis Tsoleridis, Nadine Holmes, Matthew Carlisle, Christopher Moore, Fei Sang, Johnny Debebe, Victoria Wright, Matthew Loose
EPI_ISL_674863	Lighthouse Lab in Milton Keynes	Wellcome Sanger Institute for the COVID-19 Genomics UK (COG-UK) Consortium	The Lighthouse Lab in Milton Keynes and Alex Alderton, Roberto Amato, Sonia Goncalves, Ewan Harrison, David K. Jackson, Ian Johnston, Dominic Kwiatkowski, Cordelia Langford, John Sillitoe on behalf of the Wellcome Sanger Institute COVID-19 Surveillance Team
EPI_ISL_676491, EPI_ISL_676502, EPI_ISL_676503, EPI_ISL_676504	Klinisk mikrobiologi	The Public Health Agency of Sweden	Department of Microbiology, The Public Health Agency of Sweden
EPI_ISL_678261, EPI_ISL_678262, EPI_ISL_678263, EPI_ISL_678264, EPI_ISL_678265, EPI_ISL_678266, EPI_ISL_678267, EPI_ISL_678268	Communicable Disease Laboratory, Public Health Directorate	Communicable Disease Laboratory, Public Health Directorate	Alwasti,H., Altaif,Z., AlHujairi,Z., AlAbbas,Z.
EPI_ISL_678598	NHLS-IALCH	KRISP, KZN Research Innovation and Sequencing Platform	Giandhari J, Pillay S, Lessells R, ChimukangaraB, Mdlalose K, York D, Khan S, Tegally H, Wilkinson E, de Oliveira T
EPI_ISL_678747, EPI_ISL_678748, EPI_ISL_678754, EPI_ISL_678756, EPI_ISL_678757, EPI_ISL_678758, EPI_ISL_678759, EPI_ISL_678762, EPI_ISL_678763, EPI_ISL_678764, EPI_ISL_678765, EPI_ISL_678766, EPI_ISL_678767, EPI_ISL_678768, EPI_ISL_678769, EPI_ISL_678770, EPI_ISL_678771, EPI_ISL_678772, EPI_ISL_678774, EPI_ISL_678775, EPI_ISL_678776			

see above	Respiratory Virus Unit, Microbiology Services Colindale, Public Health England	COVID-19 Genomics UK (COG-UK) Consortium	PHE Covid Sequencing Team
EPI_ISL_679160, EPI_ISL_679161, EPI_ISL_679193, EPI_ISL_679195	Department of Pathology, University of Cambridge	COVID-19 Genomics UK (COG-UK) Consortium	Aminu S. Jahun, Yasmin Chaudhry, Grant Hall, Iliana Georgana, Myra Hosmillo, Martin D. Curran, Malte Pinckert, Surendra Parmar, Ian Goodfellow
EPI_ISL_679249, EPI_ISL_679250, EPI_ISL_679251, EPI_ISL_679252, EPI_ISL_679253, EPI_ISL_679254, EPI_ISL_679255, EPI_ISL_679256, EPI_ISL_679257, EPI_ISL_679258, EPI_ISL_679259, EPI_ISL_679260, EPI_ISL_679261, EPI_ISL_679262, EPI_ISL_679263, EPI_ISL_679264, EPI_ISL_679265, EPI_ISL_679266, EPI_ISL_679267, EPI_ISL_679268, EPI_ISL_679269, EPI_ISL_679270, EPI_ISL_679271, EPI_ISL_679272, EPI_ISL_679273, EPI_ISL_679274, EPI_ISL_679275, EPI_ISL_679276, EPI_ISL_679277, EPI_ISL_679278, EPI_ISL_679279, EPI_ISL_679280, EPI_ISL_679281, EPI_ISL_679282, EPI_ISL_679283, EPI_ISL_679284, EPI_ISL_679285, EPI_ISL_679286, EPI_ISL_679287, EPI_ISL_679288, EPI_ISL_679289, EPI_ISL_679290, EPI_ISL_679291, EPI_ISL_679295, EPI_ISL_679297, EPI_ISL_679298, EPI_ISL_679299, EPI_ISL_679300, EPI_ISL_679301, EPI_ISL_679302, EPI_ISL_679303, EPI_ISL_679304, EPI_ISL_679305, EPI_ISL_679306, EPI_ISL_679307, EPI_ISL_679312, EPI_ISL_679313, EPI_ISL_679314			
see above	Queens Medical Centre, Clinical Microbiology Department / DeepSeq Nottingham	COVID-19 Genomics UK (COG-UK) Consortium	Gemma Clark, Wendy Smith, Manjinder Khakh, Vicki M Fleming, Michelle M Lister, Hannah Howson-Wells, Jonathan Ball, Patrick McClure, Joseph Chappell, Theocharis Tsoleridis, Nadine Holmes, Matthew Carlisle, Christopher Moore, Fei Sang, Johnny Debebe, Victoria Wright, Matthew Loose
EPI_ISL_679393, EPI_ISL_679394, EPI_ISL_679395, EPI_ISL_679396, EPI_ISL_679397, EPI_ISL_679398, EPI_ISL_679399, EPI_ISL_679400, EPI_ISL_679401, EPI_ISL_679402, EPI_ISL_679403, EPI_ISL_679404, EPI_ISL_679405			
see above	Liverpool Clinical Laboratories	COVID-19 Genomics UK (COG-UK) Consortium	Sam Haldenby, Anita Lucaci, Steve Paterson, Julian Hiscox, Alistair Darby, M Almsaud, A Alrezaihi, Muhannad Alruwaili, Stuart D Armstrong, Jones Benjamin, Eleanor G Bentley, Anu Chawla, Jordan J Clark, Angela Cowell, Richard Eccles, Isabel García-Dorival, Matthew Gemmell, Alessandro Gerada, PKF Gilmore, Richard Gregory, Ximeng Han, Catherine Hartley, Margaret Hughes, Miren Iturriza-Gomara, James Johnson, L Luu, Jenifer Manson, Charlotte Nelson, Elaine O'Toole, Cassie Olateju, Rebekah Penrice-Randal , Lucille Rainbow, N.P Randle, Trevor Ian Robinson, Parul Sharma, Ghada T Shawli, James P Stewart, Neil Swainston, Ecaterina Vamos, Joanne Watts, Mark Whitehead
EPI_ISL_679406, EPI_ISL_679407, EPI_ISL_679422, EPI_ISL_679423, EPI_ISL_679424, EPI_ISL_679425, EPI_ISL_679426, EPI_ISL_679427, EPI_ISL_679428, EPI_ISL_679429, EPI_ISL_679432, EPI_ISL_679433, EPI_ISL_679440, EPI_ISL_679441, EPI_ISL_679442, EPI_ISL_679443, EPI_ISL_679444, EPI_ISL_679445, EPI_ISL_679446			
see above	University College London Hospital	COVID-19 Genomics UK (COG-UK) Consortium	Judith Heaney, Matthew Byott, Catherine Houlihan, Dan Frampton, Stuart Kirk, Moira Spyer and Eleni Nastouli
EPI_ISL_679593, EPI_ISL_679594, EPI_ISL_679595, EPI_ISL_679597, EPI_ISL_679598, EPI_ISL_679599, EPI_ISL_679600, EPI_ISL_679602, EPI_ISL_679603	University College London, Great Ormond Street Hospital for Children NHS Foundation Trust, Imperial College Healthcare NHS Trust	COVID-19 Genomics UK (COG-UK) Consortium	Sergi Castellano, Rachel Williams, Mark Kristiansen, Paola Resende Silva, Sunando Roy, Tony Brooks, Helena Tutill, Paola Niola, Patricia Dyal, Charlotte Williams, Leysa Forrest, Yasmin Panchbhaya, Jacqueline Findlay, Samuel Weeks, Julianne Brown, Kathryn Harris, Paul Randell, James Price, Alison Holmes, Judith Breuer
EPI_ISL_679934	Queens Medical Centre, Clinical Microbiology Department / DeepSeq Nottingham	COVID-19 Genomics UK (COG-UK) Consortium	Gemma Clark, Wendy Smith, Manjinder Khakh, Vicki M Fleming, Michelle M Lister, Hannah Howson-Wells, Jonathan Ball, Patrick McClure, Joseph Chappell, Theocharis Tsoleridis, Nadine Holmes, Matthew Carlisle, Christopher Moore, Fei Sang, Johnny Debebe, Victoria Wright, Matthew Loose
EPI_ISL_679946, EPI_ISL_679947, EPI_ISL_679948, EPI_ISL_679949, EPI_ISL_679950, EPI_ISL_679951, EPI_ISL_679952, EPI_ISL_679953, EPI_ISL_679954, EPI_ISL_679955	Lincolnshire Hospitals and DeepSeq Nottingham	COVID-19 Genomics UK (COG-UK) Consortium	Nichola Duckworth, Tim Sloan, Sarah Walsh, Jonathan Ball, Patrick McClure, Joeseph Chappell, Nadine Holmes, Matthew Carlisle, Christopher Moore, Fei Sang, Johnny Debebe, Victoria Wright, Matthew Loose
EPI_ISL_679971	Centre for Enzyme Innovation, University of Portsmouth / Translational Research Laboratory, Portsmouth Hospitals NHS Trust	COVID-19 Genomics UK (COG-UK) Consortium	Angela Beckett,Yann Bourgeois,Garry Scarlett,Sharon Glaysher,Scott Elliott,Kelly Bicknell,Robert Impey,Allyson Lloyd,Sarah Wyllie,Ethan Butcher,Anoop Chauhan,Samuel Robson
EPI_ISL_680110, EPI_ISL_680111, EPI_ISL_680112, EPI_ISL_680121, EPI_ISL_680135, EPI_ISL_680136, EPI_ISL_680141, EPI_ISL_680144, EPI_ISL_680147, EPI_ISL_680148, EPI_ISL_680150, EPI_ISL_680154, EPI_ISL_680155, EPI_ISL_680158, EPI_ISL_680168, EPI_ISL_680170, EPI_ISL_680171, EPI_ISL_680178, EPI_ISL_680183, EPI_ISL_680194			
see above	Virology Department, Sheffield Teaching Hospitals NHS Foundation Trust/Department of Infection, Immunity and Cardiovascular Disease, The Medical School, University of Sheffield	COVID-19 Genomics UK (COG-UK) Consortium	Thushan de Silva, Matthew Parker, Nikki Smith, Adri Angyal, Rebecca Brown, Luke Green, Rachel Tucker, Paul Parsons, Danielle Groves, Katie Johnson, Laura Carrilero, Alex Keeley, Dave Partridge, Matthew Wyles, Benjamin Lindsey, Mehmet Yavuz, Mohammad Raza, Cariad Evans
EPI_ISL_680464, EPI_ISL_680465, EPI_ISL_680466, EPI_ISL_680467, EPI_ISL_680468, EPI_ISL_680486, EPI_ISL_680487, EPI_ISL_680488, EPI_ISL_680489, EPI_ISL_680490, EPI_ISL_680491, EPI_ISL_680492, EPI_ISL_680493, EPI_ISL_680494			
see above	Virology Department, Royal Infirmary of Edinburgh, NHS Lothian / School of Biological Sciences, University of Edinburgh / Institute of Genetics and Molecular Medicine, University of Edinburgh	COVID-19 Genomics UK (COG-UK) Consortium	McHugh M, Dewar R, Rooke S, Gallagher M, Balcaza C, O'Toole Á, Scher E, Hill V, McCrone JT, Colquhoun R, Yu X, Jackson B, Rambaut A, Williams TC, Templeton K
EPI_ISL_681466	Lighthouse Lab in Milton Keynes	Wellcome Sanger Institute for the COVID-19 Genomics UK (COG-UK) Consortium	The Lighthouse Lab in Milton Keynes and Alex Alderton, Roberto Amato, Sonia Goncalves, Ewan Harrison, David K. Jackson, Ian Johnston, Dominic Kwiatkowski, Cordelia Langford, John Sillitoe on behalf of the Wellcome Sanger Institute COVID-19 Surveillance Team
EPI_ISL_682280, EPI_ISL_682281, EPI_ISL_682282	Middlemore Hospital	Institute of Environmental Science and Research (ESR)	Xiaoyun Ren, Matt Storey, Nikki Freed, Muhammad Faisal, Jing Wang, Hermes Perez, Anja Werno, Antje van der Linden, Arlo Upton, Chris Mansell, David Hammer, Dragana Drinkovic, Gary McAuliffe, Hana Sofia Andersson, James Ussher, Jill Sherwood, Josh Freeman, Julia Howard, Juliet Elvy, Mary DeAlmeida, Matt Blakiston, Matthew Rogers, Max Bloomfield, Michael Addidle, Michelle Balm, Sally Roberts, Sarah Jefferies, Sharmini Muttaiyah, Susan Morpeth, Susan Taylor, Timothy Blackmore, Vani Sathyendran, Veronica Playle, Virginia Hope, Erasmus Smit, Lauren Jelly, Olin Silander, Joep de Ligt
EPI_ISL_682322	Communicable Disease Laboratory, Public Health Directorate	Communicable Disease Laboratory, Public Health Directorate	Alwasti,H., Altaif,Z., AlHujairi,Z., AlAbbas,Z.
EPI_ISL_682353, EPI_ISL_682354, EPI_ISL_682355, EPI_ISL_682356, EPI_ISL_682357, EPI_ISL_682358, EPI_ISL_682359, EPI_ISL_682360, EPI_ISL_682361, EPI_ISL_682362	Department of Virus and Microbiological Special Diagnostics, Statens Serum Institut, Copenhagen, Denmark	Albertsen Lab, Department of Chemistry and Bioscience, Aalborg University, Denmark	Danish Covid-19 Genome Consortium
EPI_ISL_683482, EPI_ISL_683566, EPI_ISL_683567, EPI_ISL_683568, EPI_ISL_683569, EPI_ISL_683570, EPI_ISL_683571, EPI_ISL_683572, EPI_ISL_683576, EPI_ISL_683577, EPI_ISL_683585			
see above	Respiratory Virus Unit, Microbiology Services Colindale, Public Health England	COVID-19 Genomics UK (COG-UK) Consortium	PHE Covid Sequencing Team
EPI_ISL_683754	DOHMH Morrisania	New York City Public Health Laboratory	Jade Wang, et al.
EPI_ISL_683761	DOHMH Corona	New York City Public Health Laboratory	Jade Wang, et al.
EPI_ISL_683762	DOHMH Morrisania	New York City Public Health Laboratory	Jade Wang, et al.
EPI_ISL_683765	DOHMH Chelsea	New York City Public Health Laboratory	Jade Wang, et al.
EPI_ISL_683793, EPI_ISL_683794	DOHMH Corona	New York City Public Health Laboratory	Jade Wang, et al.
EPI_ISL_683795	DOHMH Central Harlem	New York City Public Health Laboratory	Jade Wang, et al.
EPI_ISL_683796	DOHMH Corona	New York City Public Health Laboratory	Jade Wang, et al.
EPI_ISL_683797	DOHMH PHL	New York City Public Health Laboratory	Jade Wang, et al.
EPI_ISL_683798, EPI_ISL_683799, EPI_ISL_683800	DOHMH Corona	New York City Public Health Laboratory	Jade Wang, et al.
EPI_ISL_683801	DOHMH Morrisania	New York City Public Health Laboratory	Jade Wang, et al.
EPI_ISL_683802	DOHMH Corona	New York City Public Health Laboratory	Jade Wang, et al.

EPI_ISL_683803, EPI_ISL_683804, EPI_ISL_683805, EPI_ISL_683806	DOHMH Morrisania	New York City Public Health Laboratory	Jade Wang, et al.
EPI_ISL_683807, EPI_ISL_683808	DOHMH Chelsea	New York City Public Health Laboratory	Jade Wang, et al.
EPI_ISL_686611	Respiratory Virus Unit, Microbiology Services Colindale, Public Health England	COVID-19 Genomics UK (COG-UK) Consortium	PHE Covid Sequencing Team
EPI_ISL_692769, EPI_ISL_692772, EPI_ISL_692773	Massachusetts State Public Health Laboratory	Massachusetts State Public Health Laboratory	Andrew Lang, Timelia Fink, Glen Gallagher, Sandra Smole
EPI_ISL_693304, EPI_ISL_693305, EPI_ISL_693306	Department of Laboratory Medicine, National Taiwan University Hospital	Microbial Genomics Core Lab, National Taiwan University Centers of Genomic and Precision Medicine	Shiou-Hwei Yeh, You-Yu Lin, Ya-Yun Lai, Chiao-Ling Li, Shan-Chwen Chang, Pei-Jer Chen, Sui-Yuan Chang
EPI_ISL_693313, EPI_ISL_693315, EPI_ISL_693316, EPI_ISL_693320, EPI_ISL_693321, EPI_ISL_693324, EPI_ISL_693326, EPI_ISL_693327	National Public Health Laboratory, National Centre for Infectious Diseases	National Public Health Laboratory, National Centre for Infectious Diseases	Tze Minn Mak, Sophie Octavia, Zhenyang Zhou, Lin Cui, Raymond Tzer Pin Lin
EPI_ISL_696453	Pacaltsdorp Clinic wc PAC & NHLS/UCT	KRISP, KZN Research Innovation and Sequencing Platform	Arash Iranzadeh, Deelan Doolabh, Lynn Tyers, Bruna Galvao, Innocent Mudau, Marvin Hsiao, Kruger Marais, Jennifer Giandhari, Sureshnee Pillay, Houriiyah Tegally, Emanuel James San, Tulio de Oliveira, Diana Hardie, Stephen Korsman, Carolyn Williamson
EPI_ISL_696454	Plettenberg Bay Clinic wc PLC & NHLS/UCT	KRISP, KZN Research Innovation and Sequencing Platform	Arash Iranzadeh, Deelan Doolabh, Lynn Tyers, Bruna Galvao, Innocent Mudau, Marvin Hsiao, Kruger Marais, Jennifer Giandhari, Sureshnee Pillay, Houriiyah Tegally, Emanuel James San, Tulio de Oliveira, Diana Hardie, Stephen Korsman, Carolyn Williamson
EPI_ISL_696456	Pacaltsdorp Clinic wc PAC & NHLS/UCT	KRISP, KZN Research Innovation and Sequencing Platform	Arash Iranzadeh, Deelan Doolabh, Lynn Tyers, Bruna Galvao, Innocent Mudau, Marvin Hsiao, Kruger Marais, Jennifer Giandhari, Sureshnee Pillay, Houriiyah Tegally, Emanuel James San, Tulio de Oliveira, Diana Hardie, Stephen Korsman, Carolyn Williamson
EPI_ISL_696458	George Hospital wc GRH & NHLS/UCT	KRISP, KZN Research Innovation and Sequencing Platform	Arash Iranzadeh, Deelan Doolabh, Lynn Tyers, Bruna Galvao, Innocent Mudau, Marvin Hsiao, Kruger Marais, Jennifer Giandhari, Sureshnee Pillay, Houriiyah Tegally, Emanuel James San, Tulio de Oliveira, Diana Hardie, Stephen Korsman, Carolyn Williamson
EPI_ISL_696459	Pacaltsdorp Clinic wc PAC & NHLS/UCT	KRISP, KZN Research Innovation and Sequencing Platform	Arash Iranzadeh, Deelan Doolabh, Lynn Tyers, Bruna Galvao, Innocent Mudau, Marvin Hsiao, Kruger Marais, Jennifer Giandhari, Sureshnee Pillay, Houriiyah Tegally, Emanuel James San, Tulio de Oliveira, Diana Hardie, Stephen Korsman, Carolyn Williamson
EPI_ISL_696460	Kranshoek Clinic wc KSH & NHLS/UCT	KRISP, KZN Research Innovation and Sequencing Platform	Arash Iranzadeh, Deelan Doolabh, Lynn Tyers, Bruna Galvao, Innocent Mudau, Marvin Hsiao, Kruger Marais, Jennifer Giandhari, Sureshnee Pillay, Houriiyah Tegally, Emanuel James San, Tulio de Oliveira, Diana Hardie, Stephen Korsman, Carolyn Williamson
EPI_ISL_696479	Hornlee Clinic wc HLC & NHLS/UCT	KRISP, KZN Research Innovation and Sequencing Platform	Arash Iranzadeh, Deelan Doolabh, Lynn Tyers, Bruna Galvao, Innocent Mudau, Marvin Hsiao, Kruger Marais, Jennifer Giandhari, Sureshnee Pillay, Houriiyah Tegally, Emanuel James San, Tulio de Oliveira, Diana Hardie, Stephen Korsman, Carolyn Williamson
EPI_ISL_696482	Knysna Hospital wc KNY & NHLS/UCT	KRISP, KZN Research Innovation and Sequencing Platform	Arash Iranzadeh, Deelan Doolabh, Lynn Tyers, Bruna Galvao, Innocent Mudau, Marvin Hsiao, Kruger Marais, Jennifer Giandhari, Sureshnee Pillay, Houriiyah Tegally, Emanuel James San, Tulio de Oliveira, Diana Hardie, Stephen Korsman, Carolyn Williamson
EPI_ISL_696483	Hornlee Clinic wc HLC & NHLS/UCT	KRISP, KZN Research Innovation and Sequencing Platform	Arash Iranzadeh, Deelan Doolabh, Lynn Tyers, Bruna Galvao, Innocent Mudau, Marvin Hsiao, Kruger Marais, Jennifer Giandhari, Sureshnee Pillay, Houriiyah Tegally, Emanuel James San, Tulio de Oliveira, Diana Hardie, Stephen Korsman, Carolyn Williamson
EPI_ISL_696484	Khayelethu Clinic wc KLC & NHLS/UCT	KRISP, KZN Research Innovation and Sequencing Platform	Arash Iranzadeh, Deelan Doolabh, Lynn Tyers, Bruna Galvao, Innocent Mudau, Marvin Hsiao, Kruger Marais, Jennifer Giandhari, Sureshnee Pillay, Houriiyah Tegally, Emanuel James San, Tulio de Oliveira, Diana Hardie, Stephen Korsman, Carolyn Williamson
EPI_ISL_696486	Hornlee Clinic wc HLC & NHLS/UCT	KRISP, KZN Research Innovation and Sequencing Platform	Arash Iranzadeh, Deelan Doolabh, Lynn Tyers, Bruna Galvao, Innocent Mudau, Marvin Hsiao, Kruger Marais, Jennifer Giandhari, Sureshnee Pillay, Houriiyah Tegally, Emanuel James San, Tulio de Oliveira, Diana Hardie, Stephen Korsman, Carolyn Williamson
EPI_ISL_696487, EPI_ISL_696488	Sedgefield Clinic wc SGE & NHLS/UCT	KRISP, KZN Research Innovation and Sequencing Platform	Arash Iranzadeh, Deelan Doolabh, Lynn Tyers, Bruna Galvao, Innocent Mudau, Marvin Hsiao, Kruger Marais, Jennifer Giandhari, Sureshnee Pillay, Houriiyah Tegally, Emanuel James San, Tulio de Oliveira, Diana Hardie, Stephen Korsman, Carolyn Williamson
EPI_ISL_696489	New Horizon Clinic wc NZC & NHLS/UCT	KRISP, KZN Research Innovation and Sequencing Platform	Arash Iranzadeh, Deelan Doolabh, Lynn Tyers, Bruna Galvao, Innocent Mudau, Marvin Hsiao, Kruger Marais, Jennifer Giandhari, Sureshnee Pillay, Houriiyah Tegally, Emanuel James San, Tulio de Oliveira, Diana Hardie, Stephen Korsman, Carolyn Williamson
EPI_ISL_696490	Touwsranten Clinic wc TST & NHLS/UCT	KRISP, KZN Research Innovation and Sequencing Platform	Arash Iranzadeh, Deelan Doolabh, Lynn Tyers, Bruna Galvao, Innocent Mudau, Marvin Hsiao, Kruger Marais, Jennifer Giandhari, Sureshnee Pillay, Houriiyah Tegally, Emanuel James San, Tulio de Oliveira, Diana Hardie, Stephen Korsman, Carolyn Williamson
EPI_ISL_699655	1-Laboratory of Microbiology, National Reference Lab, Charles Nicolle Hospital; 2-University of Tunis ElManar, Faculty of Medicine of Tunis, LR99ES09, Tunis, Tunisia	1-Clinical and Experimental Pharmacology Lab, LR16SP02, National Center of Pharmacovigilance, University of Tunis El Manar, Tunis, Tunisia. 2-Neurodegenerative diseases and psychiatric troubles, LR18SP03, Razi Hospital, University of Tunis El Manar, Tunis, Tunisia. 3- Ministry of Health, National Observatory of New and Emerging Diseases, 1006, Tunis, Tunisia	Ilhem Boutiba-Ben Boubaker, Sameh Trabelsi, Nissaf Ben Alaya, Maher Kharrat, Alia Ben Kahla, Jalila Ben Khelil, Salma Abid, Sana Ferjani, Asma Ferjani, Mouna Ben Sassi, Mouna Safer, Guedi Berrabeh,Salwa Mrabet, Hanen ElJebari, Gaies Emna, Riadh Daghfous, Riadh Gouider.

EPI_ISL_701673, EPI_ISL_701674, EPI_ISL_701676, EPI_ISL_701677, EPI_ISL_701678, EPI_ISL_701679, EPI_ISL_701680, EPI_ISL_701682, EPI_ISL_701684, EPI_ISL_701685, EPI_ISL_701686, EPI_ISL_701687, EPI_ISL_701688, EPI_ISL_701689, EPI_ISL_701690, EPI_ISL_701691, EPI_ISL_701692, EPI_ISL_701693, EPI_ISL_701694, EPI_ISL_701695, EPI_ISL_701696, EPI_ISL_701699, EPI_ISL_701700, EPI_ISL_701702, EPI_ISL_701703, EPI_ISL_701704, EPI_ISL_701705, EPI_ISL_701706, EPI_ISL_701707, EPI_ISL_701708, EPI_ISL_701709, EPI_ISL_701710, EPI_ISL_701711, EPI_ISL_701712, EPI_ISL_701715, EPI_ISL_701716, EPI_ISL_701717, EPI_ISL_701718, EPI_ISL_701719, EPI_ISL_701720, EPI_ISL_701721, EPI_ISL_701723, EPI_ISL_701725, EPI_ISL_701727, EPI_ISL_701728, EPI_ISL_701730, EPI_ISL_701731, EPI_ISL_701732, EPI_ISL_701733, EPI_ISL_701734, EPI_ISL_701735, EPI_ISL_701736, EPI_ISL_701738, EPI_ISL_701740, EPI_ISL_701741, EPI_ISL_701742, EPI_ISL_701744, EPI_ISL_701745, EPI_ISL_701747, EPI_ISL_701748, EPI_ISL_701750, EPI_ISL_701751, EPI_ISL_701752, EPI_ISL_701753, EPI_ISL_701755, EPI_ISL_701756, EPI_ISL_701757, EPI_ISL_701758, EPI_ISL_701760, EPI_ISL_701761, EPI_ISL_701763, EPI_ISL_701764, EPI_ISL_701765, EPI_ISL_701766, EPI_ISL_701767, EPI_ISL_701768, EPI_ISL_701769, EPI_ISL_701770, EPI_ISL_701771, EPI_ISL_701772, EPI_ISL_701773, EPI_ISL_701776, EPI_ISL_701777, EPI_ISL_701778, EPI_ISL_701779, EPI_ISL_701780, EPI_ISL_701782, EPI_ISL_701783, EPI_ISL_701784, EPI_ISL_701785, EPI_ISL_701786, EPI_ISL_701787, EPI_ISL_701789, EPI_ISL_701790, EPI_ISL_701791, EPI_ISL_701793, EPI_ISL_701794, EPI_ISL_701796, EPI_ISL_701797, EPI_ISL_701798, EPI_ISL_701799, EPI_ISL_701801, EPI_ISL_701802, EPI_ISL_701804, EPI_ISL_701805, EPI_ISL_701806, EPI_ISL_701807, EPI_ISL_701808, EPI_ISL_701809, EPI_ISL_701810, EPI_ISL_701811, EPI_ISL_701812, EPI_ISL_701813, EPI_ISL_701814, EPI_ISL_701815, EPI_ISL_701816, EPI_ISL_701817, EPI_ISL_701818, EPI_ISL_701820, EPI_ISL_701821, EPI_ISL_701822, EPI_ISL_701823, EPI_ISL_701824, EPI_ISL_701825, EPI_ISL_701826, EPI_ISL_701827, EPI_ISL_701828, EPI_ISL_701830, EPI_ISL_701831, EPI_ISL_701832, EPI_ISL_701833, EPI_ISL_701834, EPI_ISL_701835, EPI_ISL_701836, EPI_ISL_701840, EPI_ISL_701842, EPI_ISL_701844, EPI_ISL_701845, EPI_ISL_701846, EPI_ISL_701849, EPI_ISL_701851, EPI_ISL_701852, EPI_ISL_701854, EPI_ISL_701855, EPI_ISL_701856, EPI_ISL_701857, EPI_ISL_701858, EPI_ISL_701859, EPI_ISL_701860, EPI_ISL_701861, EPI_ISL_701862, EPI_ISL_701863, EPI_ISL_701864, EPI_ISL_701865, EPI_ISL_701866, EPI_ISL_701867, EPI_ISL_701868, EPI_ISL_701869, EPI_ISL_701872, EPI_ISL_701873, EPI_ISL_701874, EPI_ISL_701877, EPI_ISL_701879, EPI_ISL_701880, EPI_ISL_701882, EPI_ISL_701883, EPI_ISL_701884, EPI_ISL_701885, EPI_ISL_701886, EPI_ISL_701887, EPI_ISL_701888, EPI_ISL_701889, EPI_ISL_701890, EPI_ISL_701891, EPI_ISL_701892, EPI_ISL_701893, EPI_ISL_701894, EPI_ISL_701895, EPI_ISL_701896, EPI_ISL_701897, EPI_ISL_701898, EPI_ISL_701899, EPI_ISL_701900, EPI_ISL_701901, EPI_ISL_701902, EPI_ISL_701903, EPI_ISL_701904, EPI_ISL_701905, EPI_ISL_701906, EPI_ISL_701907, EPI_ISL_701908, EPI_ISL_701909, EPI_ISL_701910, EPI_ISL_701911, EPI_ISL_701912, EPI_ISL_701915, EPI_ISL_701917, EPI_ISL_701918, EPI_ISL_701919, EPI_ISL_701921, EPI_ISL_701922, EPI_ISL_701924, EPI_ISL_701925, EPI_ISL_701926, EPI_ISL_701928, EPI_ISL_701929, EPI_ISL_701931, EPI_ISL_701933, EPI_ISL_701934, EPI_ISL_701935, EPI_ISL_701936, EPI_ISL_701937, EPI_ISL_701938, EPI_ISL_701939, EPI_ISL_701940, EPI_ISL_701941, EPI_ISL_701942, EPI_ISL_701944, EPI_ISL_701945, EPI_ISL_701946, EPI_ISL_701948, EPI_ISL_701949, EPI_ISL_701950, EPI_ISL_701951, EPI_ISL_701952, EPI_ISL_701953, EPI_ISL_701954, EPI_ISL_701955, EPI_ISL_701956, EPI_ISL_701957, EPI_ISL_701958, EPI_ISL_701959, EPI_ISL_701960, EPI_ISL_701962, EPI_ISL_701964, EPI_ISL_701965, EPI_ISL_701966, EPI_ISL_701967, EPI_ISL_701968, EPI_ISL_701969, EPI_ISL_701970, EPI_ISL_701971, EPI_ISL_701972, EPI_ISL_701973, EPI_ISL_701974, EPI_ISL_701977, EPI_ISL_701978, EPI_ISL_701979, EPI_ISL_701981, EPI_ISL_701982, EPI_ISL_701983, EPI_ISL_701984, EPI_ISL_701985, EPI_ISL_701986, EPI_ISL_701988, EPI_ISL_701989, EPI_ISL_701990, EPI_ISL_701991, EPI_ISL_701992, EPI_ISL_701995, EPI_ISL_701996, EPI_ISL_701997, EPI_ISL_701998, EPI_ISL_702000, EPI_ISL_702003, EPI_ISL_702004, EPI_ISL_702005, EPI_ISL_702006, EPI_ISL_702007, EPI_ISL_702008, EPI_ISL_702009, EPI_ISL_702010, EPI_ISL_702011, EPI_ISL_702012, EPI_ISL_702013, EPI_ISL_702017, EPI_ISL_702018, EPI_ISL_702019, EPI_ISL_702020, EPI_ISL_702021, EPI_ISL_702022, EPI_ISL_702370, EPI_ISL_702371, EPI_ISL_702372, EPI_ISL_702373, EPI_ISL_702374, EPI_ISL_702375, EPI_ISL_702377, EPI_ISL_702378, EPI_ISL_702379, EPI_ISL_702380, EPI_ISL_702382, EPI_ISL_702383, EPI_ISL_702386, EPI_ISL_702387, EPI_ISL_702388, EPI_ISL_702389, EPI_ISL_702390, EPI_ISL_702391, EPI_ISL_702392, EPI_ISL_702393, EPI_ISL_702394, EPI_ISL_702395, EPI_ISL_702396, EPI_ISL_702397, EPI_ISL_702398, EPI_ISL_702399, EPI_ISL_702400, EPI_ISL_702401, EPI_ISL_702402, EPI_ISL_702403, EPI_ISL_702404, EPI_ISL_702405, EPI_ISL_702406, EPI_ISL_702407, EPI_ISL_702408, EPI_ISL_702409, EPI_ISL_702410, EPI_ISL_702412, EPI_ISL_702414, EPI_ISL_702415, EPI_ISL_702416, EPI_ISL_702417, EPI_ISL_702418, EPI_ISL_702419, EPI_ISL_702420, EPI_ISL_702421, EPI_ISL_702422, EPI_ISL_702425, EPI_ISL_702426, EPI_ISL_702428, EPI_ISL_702429, EPI_ISL_702430, EPI_ISL_702431, EPI_ISL_702432, EPI_ISL_702433, EPI_ISL_702434, EPI_ISL_702435, EPI_ISL_702436, EPI_ISL_702437, EPI_ISL_702439, EPI_ISL_702440, EPI_ISL_702441, EPI_ISL_702442, EPI_ISL_702444, EPI_ISL_702445, EPI_ISL_702446, EPI_ISL_702448, EPI_ISL_702449, EPI_ISL_702450, EPI_ISL_702451, EPI_ISL_702452, EPI_ISL_702454, EPI_ISL_702455, EPI_ISL_702456, EPI_ISL_702457, EPI_ISL_702458, EPI_ISL_702459, EPI_ISL_702460, EPI_ISL_702461, EPI_ISL_702462, EPI_ISL_702463, EPI_ISL_702465, EPI_ISL_702466, EPI_ISL_702467, EPI_ISL_702468, EPI_ISL_702470

see above	Lighthouse Lab in Alderley Park	Wellcome Sanger Institute for the COVID-19 Genomics UK (COG-UK) Consortium	Jacquelyn Wynn, Mairead Hyland, The Lighthouse Lab in Alderley Park and Alex Alderton, Roberto Amato, Sonia Goncalves, Ewan Harrison, David K. Jackson, Ian Johnston, Dominic Kwiatkowski, Cordelia Langford, John Sillitoe on behalf of the Wellcome Sanger Institute COVID-19 Surveillance Team
EPI_ISL_702471	Liverpool Clinical Laboratories	COVID-19 Genomics UK (COG-UK) Consortium	Sam Haldenby, Anita Lucaci, Steve Paterson, Julian Hiscox, Alistair Darby, M Almsaud, A Alrezaihi, Muhannad Alruwaili, Stuart D Armstrong, Jones Benjamin, Eleanor G Bentley, Anu Chawla, Jordan J Clark, Angela Cowell, Richard Eccles, Isabel García-Dorival, Matthew Gemmell, Alessandro Gerada,

			PKF Gilmore, Richard Gregory, Ximeng Han, Catherine Hartley, Margaret Hughes, Miren Iturriza-Gomara, James Johnson, L Luu, Jenifer Manson, Charlotte Nelson, Elaine O'Toole, Cassie Olateju, Rebekah Penrice-Randal , Lucille Rainbow, N.P Randle, Trevor Ian Robinson, Parul Sharma, Ghada T Shawli, James P Stewart, Neil Swainston, Ecaterina Vamos, Joanne Watts, Mark Whitehead
EPI_ISL_702472, EPI_ISL_702473, EPI_ISL_702475	Lighthouse Lab in Alderley Park	Wellcome Sanger Institute for the COVID-19 Genomics UK (COG-UK) Consortium	Jacquelyn Wynn, Mairead Hyland, The Lighthouse Lab in Alderley Park and Alex Alderton, Roberto Amato, Sonia Goncalves, Ewan Harrison, David K. Jackson, Ian Johnston, Dominic Kwiatkowski, Cordelia Langford, John Sillitoe on behalf of the Wellcome Sanger Institute COVID-19 Surveillance Team
EPI_ISL_702476	Virology Department, Sheffield Teaching Hospitals NHS Foundation Trust/Department of Infection, Immunity and Cardiovascular Disease, The Medical School, University of Sheffield	COVID-19 Genomics UK (COG-UK) Consortium	Thushan de Silva, Matthew Parker, Nikki Smith, Adri Angyal, Rebecca Brown, Luke Green, Rachel Tucker, Paul Parsons, Danielle Groves, Katie Johnson, Laura Carrilero, Alex Keeley, Dave Partridge, Matthew Wyles, Benjamin Lindsey, Mehmet Yavuz, Mohammad Raza, Cariad Evans
EPI_ISL_702477, EPI_ISL_702479, EPI_ISL_702480, EPI_ISL_702482, EPI_ISL_702483, EPI_ISL_702485, EPI_ISL_702488	Lighthouse Lab in Alderley Park	Wellcome Sanger Institute for the COVID-19 Genomics UK (COG-UK) Consortium	Jacquelyn Wynn, Mairead Hyland, The Lighthouse Lab in Alderley Park and Alex Alderton, Roberto Amato, Sonia Goncalves, Ewan Harrison, David K. Jackson, Ian Johnston, Dominic Kwiatkowski, Cordelia Langford, John Sillitoe on behalf of the Wellcome Sanger Institute COVID-19 Surveillance Team
EPI_ISL_702489	Department of Pathology, University of Cambridge	COVID-19 Genomics UK (COG-UK) Consortium	Aminu S. Jahun, Yasmin Chaudhry, Grant Hall, Iliana Georgana, Myra Hosmillo, Martin D. Curran, Malte Pinckert, Surendra Parmar, Ian Goodfellow
EPI_ISL_702490, EPI_ISL_702491	Lighthouse Lab in Alderley Park	Wellcome Sanger Institute for the COVID-19 Genomics UK (COG-UK) Consortium	Jacquelyn Wynn, Mairead Hyland, The Lighthouse Lab in Alderley Park and Alex Alderton, Roberto Amato, Sonia Goncalves, Ewan Harrison, David K. Jackson, Ian Johnston, Dominic Kwiatkowski, Cordelia Langford, John Sillitoe on behalf of the Wellcome Sanger Institute COVID-19 Surveillance Team
EPI_ISL_702492	Liverpool Clinical Laboratories	COVID-19 Genomics UK (COG-UK) Consortium	Sam Haldenby, Anita Lucaci, Steve Paterson, Julian Hiscox, Alistair Darby, M Almsaud, A Alrezaihi, Muhannad Alruwaili, Stuart D Armstrong, Jones Benjamin, Eleanor G Bentley, Anu Chawla, Jordan J Clark, Angela Cowell, Richard Eccles, Isabel García-Dorival, Matthew Gemmell, Alessandro Gerada, PKF Gilmore, Richard Gregory, Ximeng Han, Catherine Hartley, Margaret Hughes, Miren Iturriza-Gomara, James Johnson, L Luu, Jenifer Manson, Charlotte Nelson, Elaine O'Toole, Cassie Olateju, Rebekah Penrice-Randal , Lucille Rainbow, N.P Randle, Trevor Ian Robinson, Parul Sharma, Ghada T Shawli, James P Stewart, Neil Swainston, Ecaterina Vamos, Joanne Watts, Mark Whitehead
EPI_ISL_702493, EPI_ISL_702494, EPI_ISL_702496, EPI_ISL_702497, EPI_ISL_702499	Lighthouse Lab in Alderley Park	Wellcome Sanger Institute for the COVID-19 Genomics UK (COG-UK) Consortium	Jacquelyn Wynn, Mairead Hyland, The Lighthouse Lab in Alderley Park and Alex Alderton, Roberto Amato, Sonia Goncalves, Ewan Harrison, David K. Jackson, Ian Johnston, Dominic Kwiatkowski, Cordelia Langford, John Sillitoe on behalf of the Wellcome Sanger Institute COVID-19 Surveillance Team
EPI_ISL_702500	Department of Pathology, University of Cambridge	COVID-19 Genomics UK (COG-UK) Consortium	Aminu S. Jahun, Yasmin Chaudhry, Grant Hall, Iliana Georgana, Myra Hosmillo, Martin D. Curran, Malte Pinckert, Surendra Parmar, Ian Goodfellow
EPI_ISL_702501, EPI_ISL_702502, EPI_ISL_702503, EPI_ISL_702506, EPI_ISL_702508, EPI_ISL_702509	Lighthouse Lab in Alderley Park	Wellcome Sanger Institute for the COVID-19 Genomics UK (COG-UK) Consortium	Jacquelyn Wynn, Mairead Hyland, The Lighthouse Lab in Alderley Park and Alex Alderton, Roberto Amato, Sonia Goncalves, Ewan Harrison, David K. Jackson, Ian Johnston, Dominic Kwiatkowski, Cordelia Langford, John Sillitoe on behalf of the Wellcome Sanger Institute COVID-19 Surveillance Team
EPI_ISL_702510	Liverpool Clinical Laboratories	COVID-19 Genomics UK (COG-UK) Consortium	Sam Haldenby, Anita Lucaci, Steve Paterson, Julian Hiscox, Alistair Darby, M Almsaud, A Alrezaihi, Muhannad Alruwaili, Stuart D Armstrong, Jones Benjamin, Eleanor G Bentley, Anu Chawla, Jordan J Clark, Angela Cowell, Richard Eccles, Isabel García-Dorival, Matthew Gemmell, Alessandro Gerada, PKF Gilmore, Richard Gregory, Ximeng Han, Catherine Hartley, Margaret Hughes, Miren Iturriza-Gomara, James Johnson, L Luu, Jenifer Manson, Charlotte Nelson, Elaine O'Toole, Cassie Olateju, Rebekah Penrice-Randal , Lucille Rainbow, N.P Randle, Trevor Ian Robinson, Parul Sharma, Ghada T Shawli, James P Stewart, Neil Swainston, Ecaterina Vamos, Joanne Watts, Mark Whitehead
EPI_ISL_702511, EPI_ISL_702513, EPI_ISL_702514, EPI_ISL_702516, EPI_ISL_702517	Lighthouse Lab in Alderley Park	Wellcome Sanger Institute for the COVID-19 Genomics UK (COG-UK) Consortium	Jacquelyn Wynn, Mairead Hyland, The Lighthouse Lab in Alderley Park and Alex Alderton, Roberto Amato, Sonia Goncalves, Ewan Harrison, David K. Jackson, Ian Johnston, Dominic Kwiatkowski, Cordelia Langford, John Sillitoe on behalf of the Wellcome Sanger Institute COVID-19 Surveillance Team
EPI_ISL_702518	Virology Department, Sheffield Teaching Hospitals NHS Foundation Trust/Department of Infection, Immunity and Cardiovascular Disease, The Medical School, University of Sheffield	COVID-19 Genomics UK (COG-UK) Consortium	Thushan de Silva, Matthew Parker, Nikki Smith, Adri Angyal, Rebecca Brown, Luke Green, Rachel Tucker, Paul Parsons, Danielle Groves, Katie Johnson, Laura Carrilero, Alex Keeley, Dave Partridge, Matthew Wyles, Benjamin Lindsey, Mehmet Yavuz, Mohammad Raza, Cariad Evans
EPI_ISL_702522, EPI_ISL_702524, EPI_ISL_702526, EPI_ISL_702527, EPI_ISL_702529, EPI_ISL_702530, EPI_ISL_702532, EPI_ISL_702534, EPI_ISL_702535, EPI_ISL_702538, EPI_ISL_702540, EPI_ISL_702542, EPI_ISL_702545, EPI_ISL_702546, EPI_ISL_702548, EPI_ISL_702551, EPI_ISL_702552, EPI_ISL_702554, EPI_ISL_702555, EPI_ISL_702557, EPI_ISL_702559			
see above	Lighthouse Lab in Alderley Park	Wellcome Sanger Institute for the COVID-19 Genomics UK (COG-UK) Consortium	Jacquelyn Wynn, Mairead Hyland, The Lighthouse Lab in Alderley Park and Alex Alderton, Roberto Amato, Sonia Goncalves, Ewan Harrison, David K. Jackson, Ian Johnston, Dominic Kwiatkowski, Cordelia Langford, John Sillitoe on behalf of the Wellcome Sanger Institute COVID-19 Surveillance Team
EPI_ISL_702561	Department of Pathology, University of Cambridge	COVID-19 Genomics UK (COG-UK) Consortium	Aminu S. Jahun, Yasmin Chaudhry, Grant Hall, Iliana Georgana, Myra Hosmillo, Martin D. Curran, Malte Pinckert, Surendra Parmar, Ian Goodfellow
EPI_ISL_702562	University of Exeter	COVID-19 Genomics UK (COG-UK) Consortium	Ben Temperton,Aaron Jeffries,Michelle Michelsen,Joanna Warwick-Dugdale,Audrey Farbos,Robyn Manley,Stephen Michell,Jane Masoli
EPI_ISL_702563, EPI_ISL_702565, EPI_ISL_702567, EPI_ISL_702568, EPI_ISL_702570, EPI_ISL_702572, EPI_ISL_702573	Lighthouse Lab in Alderley Park	Wellcome Sanger Institute for the COVID-19 Genomics UK (COG-UK) Consortium	Jacquelyn Wynn, Mairead Hyland, The Lighthouse Lab in Alderley Park and Alex Alderton, Roberto Amato, Sonia Goncalves, Ewan Harrison, David K. Jackson, Ian Johnston, Dominic Kwiatkowski, Cordelia Langford, John Sillitoe on behalf of the Wellcome Sanger Institute COVID-19 Surveillance Team
EPI_ISL_702574	Liverpool Clinical Laboratories	COVID-19 Genomics UK (COG-UK) Consortium	Sam Haldenby, Anita Lucaci, Steve Paterson, Julian Hiscox, Alistair Darby, M Almsaud, A Alrezaihi, Muhannad Alruwaili, Stuart D Armstrong, Jones Benjamin, Eleanor G Bentley, Anu Chawla, Jordan J Clark, Angela Cowell, Richard Eccles, Isabel García-Dorival, Matthew Gemmell, Alessandro Gerada, PKF Gilmore, Richard Gregory, Ximeng Han, Catherine Hartley, Margaret Hughes, Miren Iturriza-Gomara, James Johnson, L Luu, Jenifer Manson, Charlotte Nelson, Elaine O'Toole, Cassie Olateju, Rebekah Penrice-Randal , Lucille Rainbow, N.P Randle, Trevor Ian Robinson, Parul Sharma, Ghada T Shawli, James P Stewart, Neil Swainston, Ecaterina Vamos, Joanne Watts, Mark Whitehead
EPI_ISL_702575, EPI_ISL_702576	Lighthouse Lab in Alderley Park	Wellcome Sanger Institute for the COVID-19 Genomics UK (COG-UK) Consortium	Jacquelyn Wynn, Mairead Hyland, The Lighthouse Lab in Alderley Park and Alex Alderton, Roberto Amato, Sonia Goncalves, Ewan Harrison, David K. Jackson, Ian Johnston, Dominic Kwiatkowski, Cordelia Langford, John Sillitoe on behalf of the Wellcome Sanger Institute COVID-19 Surveillance Team
EPI_ISL_702577	Liverpool Clinical Laboratories	COVID-19 Genomics UK (COG-UK) Consortium	Sam Haldenby, Anita Lucaci, Steve Paterson, Julian Hiscox, Alistair Darby, M Almsaud, A Alrezaihi, Muhannad Alruwaili, Stuart D Armstrong, Jones Benjamin, Eleanor G Bentley, Anu Chawla, Jordan J Clark, Angela Cowell, Richard Eccles, Isabel García-Dorival, Matthew Gemmell, Alessandro Gerada, PKF Gilmore, Richard Gregory, Ximeng Han, Catherine Hartley, Margaret Hughes, Miren Iturriza-Gomara, James Johnson, L Luu, Jenifer Manson, Charlotte Nelson, Elaine O'Toole, Cassie Olateju, Rebekah Penrice-Randal , Lucille Rainbow, N.P Randle, Trevor Ian Robinson, Parul Sharma, Ghada T Shawli, James P Stewart, Neil Swainston, Ecaterina Vamos, Joanne Watts, Mark Whitehead
EPI_ISL_702578, EPI_ISL_702580, EPI_ISL_702581, EPI_ISL_702583, EPI_ISL_702584, EPI_ISL_702586, EPI_ISL_702588, EPI_ISL_702589, EPI_ISL_702592, EPI_ISL_702593, EPI_ISL_702595, EPI_ISL_702596, EPI_ISL_702598, EPI_ISL_702599, EPI_ISL_702601, EPI_ISL_702603, EPI_ISL_702604, EPI_ISL_702606, EPI_ISL_702608, EPI_ISL_702609, EPI_ISL_702611, EPI_ISL_702613, EPI_ISL_702614, EPI_ISL_702616, EPI_ISL_702617, EPI_ISL_702621, EPI_ISL_702624, EPI_ISL_702625, EPI_ISL_702627, EPI_ISL_702629, EPI_ISL_702630, EPI_ISL_702632			
see above	Lighthouse Lab in Alderley Park	Wellcome Sanger Institute for the COVID-19 Genomics UK (COG-UK) Consortium	Jacquelyn Wynn, Mairead Hyland, The Lighthouse Lab in Alderley Park and Alex Alderton, Roberto Amato, Sonia Goncalves, Ewan Harrison, David K. Jackson, Ian Johnston, Dominic Kwiatkowski, Cordelia Langford, John Sillitoe on behalf of the Wellcome Sanger Institute COVID-19 Surveillance Team
EPI_ISL_702639	Liverpool Clinical Laboratories	COVID-19 Genomics UK (COG-UK) Consortium	Sam Haldenby, Anita Lucaci, Steve Paterson, Julian Hiscox, Alistair Darby, M Almsaud, A Alrezaihi, Muhannad Alruwaili, Stuart D Armstrong, Jones Benjamin, Eleanor G Bentley, Anu Chawla, Jordan J Clark, Angela Cowell, Richard Eccles, Isabel García-Dorival, Matthew Gemmell, Alessandro Gerada, PKF Gilmore, Richard Gregory, Ximeng Han, Catherine Hartley, Margaret Hughes, Miren Iturriza-Gomara, James Johnson, L Luu, Jenifer Manson, Charlotte Nelson, Elaine O'Toole, Cassie Olateju, Rebekah Penrice-Randal , Lucille Rainbow, N.P Randle, Trevor Ian Robinson, Parul Sharma, Ghada T Shawli, James P Stewart, Neil Swainston, Ecaterina Vamos, Joanne Watts, Mark Whitehead
EPI_ISL_702641, EPI_ISL_702643, EPI_ISL_702645, EPI_ISL_702646, EPI_ISL_702648	Lighthouse Lab in Alderley Park	Wellcome Sanger Institute for the COVID-19 Genomics UK (COG-UK) Consortium	Jacquelyn Wynn, Mairead Hyland, The Lighthouse Lab in Alderley Park and Alex Alderton, Roberto Amato, Sonia Goncalves, Ewan Harrison, David K. Jackson, Ian Johnston, Dominic Kwiatkowski, Cordelia Langford, John Sillitoe on behalf of the Wellcome Sanger Institute COVID-19 Surveillance Team
EPI_ISL_702649	Liverpool Clinical Laboratories	COVID-19 Genomics UK (COG-UK) Consortium	Sam Haldenby, Anita Lucaci, Steve Paterson, Julian Hiscox, Alistair Darby, M Almsaud, A Alrezaihi, Muhannad Alruwaili, Stuart D Armstrong, Jones

			Benjamin, Eleanor G Bentley, Anu Chawla, Jordan J Clark, Angela Cowell, Richard Eccles, Isabel García-Dorival, Matthew Gemmell, Alessandro Gerada, PKF Gilmore, Richard Gregory, Ximeng Han, Catherine Hartley, Margaret Hughes, Miren Iturriza-Gomara, James Johnson, L Luu, Jenifer Manson, Charlotte Nelson, Elaine O'Toole, Cassie Olateju, Rebekah Penrice-Randal , Lucille Rainbow, N.P Randle, Trevor Ian Robinson, Parul Sharma, Ghada T Shawli, James P Stewart, Neil Swainston, Ecaterina Vamos, Joanne Watts, Mark Whitehead
EPI_ISL_702650, EPI_ISL_702651, EPI_ISL_702653, EPI_ISL_702654, EPI_ISL_702657, EPI_ISL_702660, EPI_ISL_702662, EPI_ISL_702665, EPI_ISL_702666	Lighthouse Lab in Alderley Park	Wellcome Sanger Institute for the COVID-19 Genomics UK (COG-UK) Consortium	Jacquelyn Wynn, Mairead Hyland, The Lighthouse Lab in Alderley Park and Alex Alderton, Roberto Amato, Sonia Goncalves, Ewan Harrison, David K. Jackson, Ian Johnston, Dominic Kwiatkowski, Cordelia Langford, John Sillitoe on behalf of the Wellcome Sanger Institute COVID-19 Surveillance Team
EPI_ISL_702669	University College London Hospital	COVID-19 Genomics UK (COG-UK) Consortium	Judith Heaney, Matthew Byott, Catherine Houlihan, Dan Frampton, Stuart Kirk, Moira Spyer and Eleni Nastouli
EPI_ISL_702670, EPI_ISL_702671, EPI_ISL_702673, EPI_ISL_702675, EPI_ISL_702676, EPI_ISL_702680, EPI_ISL_702685, EPI_ISL_702686, EPI_ISL_702688, EPI_ISL_702689, EPI_ISL_702691, EPI_ISL_702693, EPI_ISL_702695, EPI_ISL_702696, EPI_ISL_702699, EPI_ISL_702702			
see above	Lighthouse Lab in Alderley Park	Wellcome Sanger Institute for the COVID-19 Genomics UK (COG-UK) Consortium	Jacquelyn Wynn, Mairead Hyland, The Lighthouse Lab in Alderley Park and Alex Alderton, Roberto Amato, Sonia Goncalves, Ewan Harrison, David K. Jackson, Ian Johnston, Dominic Kwiatkowski, Cordelia Langford, John Sillitoe on behalf of the Wellcome Sanger Institute COVID-19 Surveillance Team
EPI_ISL_702703	Department of Pathology, University of Cambridge	COVID-19 Genomics UK (COG-UK) Consortium	Aminu S. Jahun, Yasmin Chaudhry, Grant Hall, Iliana Georgana, Myra Hosmillo, Martin D. Curran, Malte Pinckert, Surendra Parmar, Ian Goodfellow
EPI_ISL_702706, EPI_ISL_702709, EPI_ISL_702714, EPI_ISL_702715, EPI_ISL_702717	Lighthouse Lab in Alderley Park	Wellcome Sanger Institute for the COVID-19 Genomics UK (COG-UK) Consortium	Jacquelyn Wynn, Mairead Hyland, The Lighthouse Lab in Alderley Park and Alex Alderton, Roberto Amato, Sonia Goncalves, Ewan Harrison, David K. Jackson, Ian Johnston, Dominic Kwiatkowski, Cordelia Langford, John Sillitoe on behalf of the Wellcome Sanger Institute COVID-19 Surveillance Team
EPI_ISL_702718	Liverpool Clinical Laboratories	COVID-19 Genomics UK (COG-UK) Consortium	Sam Haldenby, Anita Lucaci, Steve Paterson, Julian Hiscox, Alistair Darby, M Almsaud, A Alrezaihi, Muhannad Alruwaili, Stuart D Armstrong, Jones Benjamin, Eleanor G Bentley, Anu Chawla, Jordan J Clark, Angela Cowell, Richard Eccles, Isabel García-Dorival, Matthew Gemmell, Alessandro Gerada, PKF Gilmore, Richard Gregory, Ximeng Han, Catherine Hartley, Margaret Hughes, Miren Iturriza-Gomara, James Johnson, L Luu, Jenifer Manson, Charlotte Nelson, Elaine O'Toole, Cassie Olateju, Rebekah Penrice-Randal , Lucille Rainbow, N.P Randle, Trevor Ian Robinson, Parul Sharma, Ghada T Shawli, James P Stewart, Neil Swainston, Ecaterina Vamos, Joanne Watts, Mark Whitehead
EPI_ISL_702722, EPI_ISL_702724, EPI_ISL_702727, EPI_ISL_702728, EPI_ISL_702730	Lighthouse Lab in Alderley Park	Wellcome Sanger Institute for the COVID-19 Genomics UK (COG-UK) Consortium	Jacquelyn Wynn, Mairead Hyland, The Lighthouse Lab in Alderley Park and Alex Alderton, Roberto Amato, Sonia Goncalves, Ewan Harrison, David K. Jackson, Ian Johnston, Dominic Kwiatkowski, Cordelia Langford, John Sillitoe on behalf of the Wellcome Sanger Institute COVID-19 Surveillance Team
EPI_ISL_702734	Department of Pathology, University of Cambridge	COVID-19 Genomics UK (COG-UK) Consortium	Aminu S. Jahun, Yasmin Chaudhry, Grant Hall, Iliana Georgana, Myra Hosmillo, Martin D. Curran, Malte Pinckert, Surendra Parmar, Ian Goodfellow
EPI_ISL_702735, EPI_ISL_702737, EPI_ISL_702738, EPI_ISL_702740, EPI_ISL_702743, EPI_ISL_702746	Lighthouse Lab in Alderley Park	Wellcome Sanger Institute for the COVID-19 Genomics UK (COG-UK) Consortium	Jacquelyn Wynn, Mairead Hyland, The Lighthouse Lab in Alderley Park and Alex Alderton, Roberto Amato, Sonia Goncalves, Ewan Harrison, David K. Jackson, Ian Johnston, Dominic Kwiatkowski, Cordelia Langford, John Sillitoe on behalf of the Wellcome Sanger Institute COVID-19 Surveillance Team
EPI_ISL_702747	Northumbria University / South Tees Hospitals NHS Foundation Trust / North Cumbria Integrated Care NHS Foundation Trust / North Tees and Hartlepool NHS Foundation Trust / Newcastle Hospitals NHS Foundation Trust	COVID-19 Genomics UK (COG-UK) Consortium	Darren L Smith,Andrew Nelson,Matthew Bashton,Greg R Young,Joshua Loh,John Allan,Mohammad A Tariq,Giles S Holt,Gary Black,Wen C Yew,Lynn Dover,Paul Baker,Steve Liggett,Sarah Essex,Jane Greenaway,Debra Padgett,Clive Graham,Garren Scott,Edward Barton,Emma Swindells,Brendan Payne,Jennifer Collins,Yusri Taha,Gary Eltringham
EPI_ISL_702748, EPI_ISL_702750, EPI_ISL_702757, EPI_ISL_702759, EPI_ISL_702760	Lighthouse Lab in Alderley Park	Wellcome Sanger Institute for the COVID-19 Genomics UK (COG-UK) Consortium	Jacquelyn Wynn, Mairead Hyland, The Lighthouse Lab in Alderley Park and Alex Alderton, Roberto Amato, Sonia Goncalves, Ewan Harrison, David K. Jackson, Ian Johnston, Dominic Kwiatkowski, Cordelia Langford, John Sillitoe on behalf of the Wellcome Sanger Institute COVID-19 Surveillance Team
EPI_ISL_702761	Virology Department, Sheffield Teaching Hospitals NHS Foundation Trust/Department of Infection, Immunity and Cardiovascular Disease, The Medical School, University of Sheffield	COVID-19 Genomics UK (COG-UK) Consortium	Thushan de Silva, Matthew Parker, Nikki Smith, Adri Angyal, Rebecca Brown, Luke Green, Rachel Tucker, Paul Parsons, Danielle Groves, Katie Johnson, Laura Carrilero, Alex Keeley, Dave Partridge, Matthew Wyles, Benjamin Lindsey, Mehmet Yavuz, Mohammad Raza, Cariad Evans
EPI_ISL_702762, EPI_ISL_702764, EPI_ISL_702765, EPI_ISL_702767, EPI_ISL_702768, EPI_ISL_702770	Lighthouse Lab in Alderley Park	Wellcome Sanger Institute for the COVID-19 Genomics UK (COG-UK) Consortium	Jacquelyn Wynn, Mairead Hyland, The Lighthouse Lab in Alderley Park and Alex Alderton, Roberto Amato, Sonia Goncalves, Ewan Harrison, David K. Jackson, Ian Johnston, Dominic Kwiatkowski, Cordelia Langford, John Sillitoe on behalf of the Wellcome Sanger Institute COVID-19 Surveillance Team
EPI_ISL_702771	West of Scotland Specialist Virology Centre, NHSGGC / MRC-University of Glasgow Centre for Virus Research	COVID-19 Genomics UK (COG-UK) Consortium	Ana da Silva Filipe, Natasha Johnson, Kathy Smollett, Daniel Mair, Stephen Carmichael, Alice Broos, Lily Tong, Jenna Nichols, Kyriaki Nomikou; Sarah McDonald; Richard Orton, Joseph Hughes, Sreenu Vattipally, David L Robertson; Alasdair MacLean, Rory Gunson; Sharif Shaaban, Matthew Holden; Rachel Blacow, Guy Mollett, Kathy Li, James Shepherd, Antonia Ho, Emma Thomson
EPI_ISL_702772, EPI_ISL_702775, EPI_ISL_702777, EPI_ISL_702778, EPI_ISL_702780, EPI_ISL_702781, EPI_ISL_702783, EPI_ISL_702786, EPI_ISL_702788, EPI_ISL_702789	Lighthouse Lab in Alderley Park	Wellcome Sanger Institute for the COVID-19 Genomics UK (COG-UK) Consortium	Jacquelyn Wynn, Mairead Hyland, The Lighthouse Lab in Alderley Park and Alex Alderton, Roberto Amato, Sonia Goncalves, Ewan Harrison, David K. Jackson, Ian Johnston, Dominic Kwiatkowski, Cordelia Langford, John Sillitoe on behalf of the Wellcome Sanger Institute COVID-19 Surveillance Team
EPI_ISL_702790	University of Exeter	COVID-19 Genomics UK (COG-UK) Consortium	Ben Temperton,Aaron Jeffries,Michelle Michelsen,Joanna Warwick-Dugdale,Audrey Farbos,Robyn Manley,Stephen Michell,Jane Masoli
EPI_ISL_702793	Lighthouse Lab in Alderley Park	Wellcome Sanger Institute for the COVID-19 Genomics UK (COG-UK) Consortium	Jacquelyn Wynn, Mairead Hyland, The Lighthouse Lab in Alderley Park and Alex Alderton, Roberto Amato, Sonia Goncalves, Ewan Harrison, David K. Jackson, Ian Johnston, Dominic Kwiatkowski, Cordelia Langford, John Sillitoe on behalf of the Wellcome Sanger Institute COVID-19 Surveillance Team
EPI_ISL_702798	Virology Department, Sheffield Teaching Hospitals NHS Foundation Trust/Department of Infection, Immunity and Cardiovascular Disease, The Medical School, University of Sheffield	COVID-19 Genomics UK (COG-UK) Consortium	Thushan de Silva, Matthew Parker, Nikki Smith, Adri Angyal, Rebecca Brown, Luke Green, Rachel Tucker, Paul Parsons, Danielle Groves, Katie Johnson, Laura Carrilero, Alex Keeley, Dave Partridge, Matthew Wyles, Benjamin Lindsey, Mehmet Yavuz, Mohammad Raza, Cariad Evans
EPI_ISL_702799	Lighthouse Lab in Alderley Park	Wellcome Sanger Institute for the COVID-19 Genomics UK (COG-UK) Consortium	Jacquelyn Wynn, Mairead Hyland, The Lighthouse Lab in Alderley Park and Alex Alderton, Roberto Amato, Sonia Goncalves, Ewan Harrison, David K. Jackson, Ian Johnston, Dominic Kwiatkowski, Cordelia Langford, John Sillitoe on behalf of the Wellcome Sanger Institute COVID-19 Surveillance Team
EPI_ISL_702801	Liverpool Clinical Laboratories	COVID-19 Genomics UK (COG-UK) Consortium	Sam Haldenby, Anita Lucaci, Steve Paterson, Julian Hiscox, Alistair Darby, M Almsaud, A Alrezaihi, Muhannad Alruwaili, Stuart D Armstrong, Jones Benjamin, Eleanor G Bentley, Anu Chawla, Jordan J Clark, Angela Cowell, Richard Eccles, Isabel García-Dorival, Matthew Gemmell, Alessandro Gerada, PKF Gilmore, Richard Gregory, Ximeng Han, Catherine Hartley, Margaret Hughes, Miren Iturriza-Gomara, James Johnson, L Luu, Jenifer Manson, Charlotte Nelson, Elaine O'Toole, Cassie Olateju, Rebekah Penrice-Randal , Lucille Rainbow, N.P Randle, Trevor Ian Robinson, Parul Sharma, Ghada T Shawli, James P Stewart, Neil Swainston, Ecaterina Vamos, Joanne Watts, Mark Whitehead
EPI_ISL_702802	Lighthouse Lab in Alderley Park	Wellcome Sanger Institute for the COVID-19 Genomics UK (COG-UK) Consortium	Jacquelyn Wynn, Mairead Hyland, The Lighthouse Lab in Alderley Park and Alex Alderton, Roberto Amato, Sonia Goncalves, Ewan Harrison, David K. Jackson, Ian Johnston, Dominic Kwiatkowski, Cordelia Langford, John Sillitoe on behalf of the Wellcome Sanger Institute COVID-19 Surveillance Team
EPI_ISL_702803	Northumbria University / South Tees Hospitals NHS Foundation Trust / North Cumbria Integrated Care NHS Foundation Trust / North Tees and Hartlepool NHS Foundation Trust / Newcastle Hospitals NHS Foundation Trust	COVID-19 Genomics UK (COG-UK) Consortium	Darren L Smith,Andrew Nelson,Matthew Bashton,Greg R Young,Joshua Loh,John Allan,Mohammad A Tariq,Giles S Holt,Gary Black,Wen C Yew,Lynn Dover,Paul Baker,Steve Liggett,Sarah Essex,Jane Greenaway,Debra Padgett,Clive Graham,Garren Scott,Edward Barton,Emma Swindells,Brendan Payne,Jennifer Collins,Yusri Taha,Gary Eltringham
EPI_ISL_702805, EPI_ISL_702807, EPI_ISL_702808, EPI_ISL_702810,	Lighthouse Lab in Alderley Park	Wellcome Sanger Institute for the COVID-19 Genomics UK (COG-UK) Consortium	Jacquelyn Wynn, Mairead Hyland, The Lighthouse Lab in Alderley Park and Alex Alderton, Roberto Amato, Sonia Goncalves, Ewan Harrison, David K. Jackson, Ian Johnston, Dominic Kwiatkowski, Cordelia Langford, John Sillitoe on behalf of the Wellcome Sanger Institute COVID-19 Surveillance Team

EPI_ISL_702811, EPI_ISL_702813, EPI_ISL_702814, EPI_ISL_702818, EPI_ISL_702819, EPI_ISL_702822			
EPI_ISL_702826	Liverpool Clinical Laboratories	COVID-19 Genomics UK (COG-UK) Consortium	Sam Haldenby, Anita Lucaci, Steve Paterson, Julian Hiscox, Alistair Darby, M Almsaud, A Alrezaihi, Muhannad Alruwaili, Stuart D Armstrong, Jones Benjamin, Eleanor G Bentley, Anu Chawla, Jordan J Clark, Angela Cowell, Richard Eccles, Isabel García-Dorival, Matthew Gemmell, Alessandro Gerada, PKF Gilmore, Richard Gregory, Ximeng Han, Catherine Hartley, Margaret Hughes, Miren Iturriza-Gomara, James Johnson, L Luu, Jenifer Manson, Charlotte Nelson, Elaine O'Toole, Cassie Olateju, Rebekah Penrice-Randal , Lucille Rainbow, N.P Randle, Trevor Ian Robinson, Parul Sharma, Ghada T Shawli, James P Stewart, Neil Swainston, Ecaterina Vamos, Joanne Watts, Mark Whitehead
EPI_ISL_702829, EPI_ISL_702830, EPI_ISL_702833	Virology Department, Sheffield Teaching Hospitals NHS Foundation Trust/Department of Infection, Immunity and Cardiovascular Disease, The Medical School, University of Sheffield	COVID-19 Genomics UK (COG-UK) Consortium	Thushan de Silva, Matthew Parker, Nikki Smith, Adri Angyal, Rebecca Brown, Luke Green, Rachel Tucker, Paul Parsons, Danielle Groves, Katie Johnson, Laura Carrilero, Alex Keeley, Dave Partridge, Matthew Wyles, Benjamin Lindsey, Mehmet Yavuz, Mohammad Raza, Cariad Evans
EPI_ISL_702844	University of Exeter	COVID-19 Genomics UK (COG-UK) Consortium	Ben Temperton,Aaron Jeffries,Michelle Michelsen,Joanna Warwick-Dugdale,Audrey Farbos,Robyn Manley,Stephen Michell,Jane Masoli
EPI_ISL_702845	Liverpool Clinical Laboratories	COVID-19 Genomics UK (COG-UK) Consortium	Sam Haldenby, Anita Lucaci, Steve Paterson, Julian Hiscox, Alistair Darby, M Almsaud, A Alrezaihi, Muhannad Alruwaili, Stuart D Armstrong, Jones Benjamin, Eleanor G Bentley, Anu Chawla, Jordan J Clark, Angela Cowell, Richard Eccles, Isabel García-Dorival, Matthew Gemmell, Alessandro Gerada, PKF Gilmore, Richard Gregory, Ximeng Han, Catherine Hartley, Margaret Hughes, Miren Iturriza-Gomara, James Johnson, L Luu, Jenifer Manson, Charlotte Nelson, Elaine O'Toole, Cassie Olateju, Rebekah Penrice-Randal , Lucille Rainbow, N.P Randle, Trevor Ian Robinson, Parul Sharma, Ghada T Shawli, James P Stewart, Neil Swainston, Ecaterina Vamos, Joanne Watts, Mark Whitehead
EPI_ISL_702848, EPI_ISL_702876	Department of Pathology, University of Cambridge	COVID-19 Genomics UK (COG-UK) Consortium	Aminu S. Jahun, Yasmin Chaudhry, Grant Hall, Iliana Georgana, Myra Hosmillo, Martin D. Curran, Malte Pinckert, Surendra Parmar, Ian Goodfellow
EPI_ISL_702880	Virology Department, Sheffield Teaching Hospitals NHS Foundation Trust/Department of Infection, Immunity and Cardiovascular Disease, The Medical School, University of Sheffield	COVID-19 Genomics UK (COG-UK) Consortium	Thushan de Silva, Matthew Parker, Nikki Smith, Adri Angyal, Rebecca Brown, Luke Green, Rachel Tucker, Paul Parsons, Danielle Groves, Katie Johnson, Laura Carrilero, Alex Keeley, Dave Partridge, Matthew Wyles, Benjamin Lindsey, Mehmet Yavuz, Mohammad Raza, Cariad Evans
EPI_ISL_702882	Quadram Institute Bioscience	COVID-19 Genomics UK (COG-UK) Consortium	Dave J. Baker, Gemma L. Kay, Alp Aydin, Thanh Le-Viet, Steven Rudder, Ana P. Tedim, Anastasia Kolyva, Maria Diaz, Leonardo de Oliveira Martins, Nabil-Fareed Alikhan, Lizzie Meadows, Rachael Stanley, Ngozi Elumogo, Muhammed Yasir, Nicholas M. Thomson, Alexander J Trotter, Rachel Gilroy, Samuel Bloomfield, Claire Stuart, Andrew Bell, Reenesh Prakash, Samir Dervisevic, Alison E. Mather, John Wain, Mark Webber, Andrew J. Page, Justin O'Grady
EPI_ISL_702883, EPI_ISL_702886, EPI_ISL_702891	Department of Pathology, University of Cambridge	COVID-19 Genomics UK (COG-UK) Consortium	Aminu S. Jahun, Yasmin Chaudhry, Grant Hall, Iliana Georgana, Myra Hosmillo, Martin D. Curran, Malte Pinckert, Surendra Parmar, Ian Goodfellow
EPI_ISL_702893	West of Scotland Specialist Virology Centre, NHSGGC / MRC-University of Glasgow Centre for Virus Research	COVID-19 Genomics UK (COG-UK) Consortium	Ana da Silva Filipe, Natasha Johnson, Kathy Smollett, Daniel Mair, Stephen Carmichael, Alice Broos, Lily Tong, Jenna Nichols, Kyriaki Nomikou; Sarah McDonald; Richard Orton, Joseph Hughes, Sreenu Vattipally, David L Robertson; Alasdair MacLean, Rory Gunson; Sharif Shaaban, Matthew Holden; Rachel Blacow, Guy Mollett, Kathy Li, James Shepherd, Antonia Ho, Emma Thomson
EPI_ISL_702898, EPI_ISL_702899, EPI_ISL_702901, EPI_ISL_702903, EPI_ISL_702904	Lighthouse Lab in Alderley Park	Wellcome Sanger Institute for the COVID-19 Genomics UK (COG-UK) Consortium	Jacquelyn Wynn, Mairead Hyland, The Lighthouse Lab in Alderley Park and Alex Alderton, Roberto Amato, Sonia Goncalves, Ewan Harrison, David K. Jackson, Ian Johnston, Dominic Kwiatkowski, Cordelia Langford, John Sillitoe on behalf of the Wellcome Sanger Institute COVID-19 Surveillance Team
EPI_ISL_702907	Liverpool Clinical Laboratories	COVID-19 Genomics UK (COG-UK) Consortium	Sam Haldenby, Anita Lucaci, Steve Paterson, Julian Hiscox, Alistair Darby, M Almsaud, A Alrezaihi, Muhannad Alruwaili, Stuart D Armstrong, Jones Benjamin, Eleanor G Bentley, Anu Chawla, Jordan J Clark, Angela Cowell, Richard Eccles, Isabel García-Dorival, Matthew Gemmell, Alessandro Gerada, PKF Gilmore, Richard Gregory, Ximeng Han, Catherine Hartley, Margaret Hughes, Miren Iturriza-Gomara, James Johnson, L Luu, Jenifer Manson, Charlotte Nelson, Elaine O'Toole, Cassie Olateju, Rebekah Penrice-Randal , Lucille Rainbow, N.P Randle, Trevor Ian Robinson, Parul Sharma, Ghada T Shawli, James P Stewart, Neil Swainston, Ecaterina Vamos, Joanne Watts, Mark Whitehead
EPI_ISL_702909, EPI_ISL_702912, EPI_ISL_702914, EPI_ISL_702919, EPI_ISL_702921, EPI_ISL_702924, EPI_ISL_702926, EPI_ISL_702931, EPI_ISL_702933, EPI_ISL_702934, EPI_ISL_702936, EPI_ISL_702938, EPI_ISL_702943, EPI_ISL_702945, EPI_ISL_702946, EPI_ISL_702948, EPI_ISL_702951, EPI_ISL_702955, EPI_ISL_702957, EPI_ISL_702958, EPI_ISL_702960, EPI_ISL_702962			
see above	Lighthouse Lab in Alderley Park	Wellcome Sanger Institute for the COVID-19 Genomics UK (COG-UK) Consortium	Jacquelyn Wynn, Mairead Hyland, The Lighthouse Lab in Alderley Park and Alex Alderton, Roberto Amato, Sonia Goncalves, Ewan Harrison, David K. Jackson, Ian Johnston, Dominic Kwiatkowski, Cordelia Langford, John Sillitoe on behalf of the Wellcome Sanger Institute COVID-19 Surveillance Team
EPI_ISL_702964	Virology Department, Sheffield Teaching Hospitals NHS Foundation Trust/Department of Infection, Immunity and Cardiovascular Disease, The Medical School, University of Sheffield	COVID-19 Genomics UK (COG-UK) Consortium	Thushan de Silva, Matthew Parker, Nikki Smith, Adri Angyal, Rebecca Brown, Luke Green, Rachel Tucker, Paul Parsons, Danielle Groves, Katie Johnson, Laura Carrilero, Alex Keeley, Dave Partridge, Matthew Wyles, Benjamin Lindsey, Mehmet Yavuz, Mohammad Raza, Cariad Evans
EPI_ISL_702973, EPI_ISL_702975, EPI_ISL_702978, EPI_ISL_702984, EPI_ISL_702989	Lighthouse Lab in Alderley Park	Wellcome Sanger Institute for the COVID-19 Genomics UK (COG-UK) Consortium	Jacquelyn Wynn, Mairead Hyland, The Lighthouse Lab in Alderley Park and Alex Alderton, Roberto Amato, Sonia Goncalves, Ewan Harrison, David K. Jackson, Ian Johnston, Dominic Kwiatkowski, Cordelia Langford, John Sillitoe on behalf of the Wellcome Sanger Institute COVID-19 Surveillance Team
EPI_ISL_702994	University of Exeter	COVID-19 Genomics UK (COG-UK) Consortium	Ben Temperton,Aaron Jeffries,Michelle Michelsen,Joanna Warwick-Dugdale,Audrey Farbos,Robyn Manley,Stephen Michell,Jane Masoli
EPI_ISL_702995, EPI_ISL_702998, EPI_ISL_703000, EPI_ISL_703001, EPI_ISL_703003, EPI_ISL_703004, EPI_ISL_703006, EPI_ISL_703008, EPI_ISL_703009, EPI_ISL_703011, EPI_ISL_703014, EPI_ISL_703016, EPI_ISL_703017			
see above	Lighthouse Lab in Alderley Park	Wellcome Sanger Institute for the COVID-19 Genomics UK (COG-UK) Consortium	Jacquelyn Wynn, Mairead Hyland, The Lighthouse Lab in Alderley Park and Alex Alderton, Roberto Amato, Sonia Goncalves, Ewan Harrison, David K. Jackson, Ian Johnston, Dominic Kwiatkowski, Cordelia Langford, John Sillitoe on behalf of the Wellcome Sanger Institute COVID-19 Surveillance Team
EPI_ISL_703018	Virology Department, Sheffield Teaching Hospitals NHS Foundation Trust/Department of Infection, Immunity and Cardiovascular Disease, The Medical School, University of Sheffield	COVID-19 Genomics UK (COG-UK) Consortium	Thushan de Silva, Matthew Parker, Nikki Smith, Adri Angyal, Rebecca Brown, Luke Green, Rachel Tucker, Paul Parsons, Danielle Groves, Katie Johnson, Laura Carrilero, Alex Keeley, Dave Partridge, Matthew Wyles, Benjamin Lindsey, Mehmet Yavuz, Mohammad Raza, Cariad Evans
EPI_ISL_703019	Lighthouse Lab in Alderley Park	Wellcome Sanger Institute for the COVID-19 Genomics UK (COG-UK) Consortium	Jacquelyn Wynn, Mairead Hyland, The Lighthouse Lab in Alderley Park and Alex Alderton, Roberto Amato, Sonia Goncalves, Ewan Harrison, David K. Jackson, Ian Johnston, Dominic Kwiatkowski, Cordelia Langford, John Sillitoe on behalf of the Wellcome Sanger Institute COVID-19 Surveillance Team
EPI_ISL_703020	Virology Department, Sheffield Teaching Hospitals NHS Foundation Trust/Department of Infection, Immunity and Cardiovascular Disease, The Medical School, University of Sheffield	COVID-19 Genomics UK (COG-UK) Consortium	Thushan de Silva, Matthew Parker, Nikki Smith, Adri Angyal, Rebecca Brown, Luke Green, Rachel Tucker, Paul Parsons, Danielle Groves, Katie Johnson, Laura Carrilero, Alex Keeley, Dave Partridge, Matthew Wyles, Benjamin Lindsey, Mehmet Yavuz, Mohammad Raza, Cariad Evans
EPI_ISL_703024, EPI_ISL_703025	Lighthouse Lab in Alderley Park	Wellcome Sanger Institute for the COVID-19 Genomics UK (COG-UK) Consortium	Jacquelyn Wynn, Mairead Hyland, The Lighthouse Lab in Alderley Park and Alex Alderton, Roberto Amato, Sonia Goncalves, Ewan Harrison, David K. Jackson, Ian Johnston, Dominic Kwiatkowski, Cordelia Langford, John Sillitoe on behalf of the Wellcome Sanger Institute COVID-19 Surveillance Team
EPI_ISL_703026	Virology Department, Sheffield Teaching Hospitals NHS Foundation Trust/Department of Infection, Immunity and Cardiovascular Disease, The Medical School, University of Sheffield	COVID-19 Genomics UK (COG-UK) Consortium	Thushan de Silva, Matthew Parker, Nikki Smith, Adri Angyal, Rebecca Brown, Luke Green, Rachel Tucker, Paul Parsons, Danielle Groves, Katie Johnson, Laura Carrilero, Alex Keeley, Dave Partridge, Matthew Wyles, Benjamin Lindsey, Mehmet Yavuz, Mohammad Raza, Cariad Evans
EPI_ISL_703028	Lighthouse Lab in Alderley Park	Wellcome Sanger Institute for the COVID-19 Genomics UK (COG-UK) Consortium	Jacquelyn Wynn, Mairead Hyland, The Lighthouse Lab in Alderley Park and Alex Alderton, Roberto Amato, Sonia Goncalves, Ewan Harrison, David K. Jackson, Ian Johnston, Dominic Kwiatkowski, Cordelia Langford, John Sillitoe on behalf of the Wellcome Sanger Institute COVID-19 Surveillance Team
EPI_ISL_703029	Virology Department, Sheffield Teaching Hospitals NHS Foundation Trust/Department of Infection, Immunity and	COVID-19 Genomics UK (COG-UK) Consortium	Thushan de Silva, Matthew Parker, Nikki Smith, Adri Angyal, Rebecca Brown, Luke Green, Rachel Tucker, Paul Parsons, Danielle Groves, Katie Johnson, Laura Carrilero, Alex Keeley, Dave Partridge, Matthew Wyles, Benjamin Lindsey, Mehmet Yavuz, Mohammad Raza, Cariad Evans

	Cardiovascular Disease, The Medical School, University of Sheffield		
EPI_ISL_703031	Lighthouse Lab in Alderley Park	Wellcome Sanger Institute for the COVID-19 Genomics UK (COG-UK) Consortium	Jacquelyn Wynn, Mairead Hyland, The Lighthouse Lab in Alderley Park and Alex Alderton, Roberto Amato, Sonia Goncalves, Ewan Harrison, David K. Jackson, Ian Johnston, Dominic Kwiatkowski, Cordelia Langford, John Sillitoe on behalf of the Wellcome Sanger Institute COVID-19 Surveillance Team
EPI_ISL_703032	Department of Pathology, University of Cambridge	COVID-19 Genomics UK (COG-UK) Consortium	Aminu S. Jahun, Yasmin Chaudhry, Grant Hall, Iliana Georgana, Myra Hosmillo, Martin D. Curran, Malte Pinckert, Surendra Parmar, Ian Goodfellow
EPI_ISL_703037, EPI_ISL_703041, EPI_ISL_703042, EPI_ISL_703045, EPI_ISL_703047, EPI_ISL_703050, EPI_ISL_703052, EPI_ISL_703060, EPI_ISL_703062, EPI_ISL_703063, EPI_ISL_703065, EPI_ISL_703067, EPI_ISL_703068, EPI_ISL_703070, EPI_ISL_703072, EPI_ISL_703073			
see above	Lighthouse Lab in Alderley Park	Wellcome Sanger Institute for the COVID-19 Genomics UK (COG-UK) Consortium	Jacquelyn Wynn, Mairead Hyland, The Lighthouse Lab in Alderley Park and Alex Alderton, Roberto Amato, Sonia Goncalves, Ewan Harrison, David K. Jackson, Ian Johnston, Dominic Kwiatkowski, Cordelia Langford, John Sillitoe on behalf of the Wellcome Sanger Institute COVID-19 Surveillance Team
EPI_ISL_703076	Department of Pathology, University of Cambridge	COVID-19 Genomics UK (COG-UK) Consortium	Aminu S. Jahun, Yasmin Chaudhry, Grant Hall, Iliana Georgana, Myra Hosmillo, Martin D. Curran, Malte Pinckert, Surendra Parmar, Ian Goodfellow
EPI_ISL_703078, EPI_ISL_703079, EPI_ISL_703083, EPI_ISL_703084, EPI_ISL_703086, EPI_ISL_703091, EPI_ISL_703092, EPI_ISL_703095, EPI_ISL_703097, EPI_ISL_703098, EPI_ISL_703100, EPI_ISL_703103, EPI_ISL_703104, EPI_ISL_703106, EPI_ISL_703108			
see above	Lighthouse Lab in Alderley Park	Wellcome Sanger Institute for the COVID-19 Genomics UK (COG-UK) Consortium	Jacquelyn Wynn, Mairead Hyland, The Lighthouse Lab in Alderley Park and Alex Alderton, Roberto Amato, Sonia Goncalves, Ewan Harrison, David K. Jackson, Ian Johnston, Dominic Kwiatkowski, Cordelia Langford, John Sillitoe on behalf of the Wellcome Sanger Institute COVID-19 Surveillance Team
EPI_ISL_703113	Quadram Institute Bioscience	COVID-19 Genomics UK (COG-UK) Consortium	Dave J. Baker, Gemma L. Kay, Alp Aydin, Thanh Le-Viet, Steven Rudder, Ana P. Tedim, Anastasia Kolyva, Maria Diaz, Leonardo de Oliveira Martins, Nabil-Fareed Alikhan, Lizzie Meadows, Rachael Stanley, Ngozi Elumogo, Muhammed Yasir, Nicholas M. Thomson, Alexander J Trotter, Rachel Gilroy, Samuel Bloomfield, Claire Stuart, Andrew Bell, Reenesh Prakash, Samir Dervisevic, Alison E. Mather, John Wain, Mark Webber, Andrew J. Page, Justin O'Grady
EPI_ISL_703118	Department of Pathology, University of Cambridge	COVID-19 Genomics UK (COG-UK) Consortium	Aminu S. Jahun, Yasmin Chaudhry, Grant Hall, Iliana Georgana, Myra Hosmillo, Martin D. Curran, Malte Pinckert, Surendra Parmar, Ian Goodfellow
EPI_ISL_703120, EPI_ISL_703122, EPI_ISL_703123, EPI_ISL_703126	Lighthouse Lab in Alderley Park	Wellcome Sanger Institute for the COVID-19 Genomics UK (COG-UK) Consortium	Jacquelyn Wynn, Mairead Hyland, The Lighthouse Lab in Alderley Park and Alex Alderton, Roberto Amato, Sonia Goncalves, Ewan Harrison, David K. Jackson, Ian Johnston, Dominic Kwiatkowski, Cordelia Langford, John Sillitoe on behalf of the Wellcome Sanger Institute COVID-19 Surveillance Team
EPI_ISL_703127	Department of Pathology, University of Cambridge	COVID-19 Genomics UK (COG-UK) Consortium	Aminu S. Jahun, Yasmin Chaudhry, Grant Hall, Iliana Georgana, Myra Hosmillo, Martin D. Curran, Malte Pinckert, Surendra Parmar, Ian Goodfellow
EPI_ISL_703128, EPI_ISL_703130, EPI_ISL_703131, EPI_ISL_703135, EPI_ISL_703138	Lighthouse Lab in Alderley Park	Wellcome Sanger Institute for the COVID-19 Genomics UK (COG-UK) Consortium	Jacquelyn Wynn, Mairead Hyland, The Lighthouse Lab in Alderley Park and Alex Alderton, Roberto Amato, Sonia Goncalves, Ewan Harrison, David K. Jackson, Ian Johnston, Dominic Kwiatkowski, Cordelia Langford, John Sillitoe on behalf of the Wellcome Sanger Institute COVID-19 Surveillance Team
EPI_ISL_703141	West of Scotland Specialist Virology Centre, NHSGGC / MRC-University of Glasgow Centre for Virus Research	COVID-19 Genomics UK (COG-UK) Consortium	Ana da Silva Filipe, Natasha Johnson, Kathy Smollett, Daniel Mair, Stephen Carmichael, Alice Broos, Lily Tong, Jenna Nichols, Kyriaki Nomikou; Sarah McDonald; Richard Orton, Joseph Hughes, Sreenu Vattipally, David L Robertson; Alasdair MacLean, Rory Gunson; Sharif Shaaban, Matthew Holden; Rachel Blacow, Guy Mollett, Kathy Li, James Shepherd, Antonia Ho, Emma Thomson
EPI_ISL_703142, EPI_ISL_703143, EPI_ISL_703146	Lighthouse Lab in Alderley Park	Wellcome Sanger Institute for the COVID-19 Genomics UK (COG-UK) Consortium	Jacquelyn Wynn, Mairead Hyland, The Lighthouse Lab in Alderley Park and Alex Alderton, Roberto Amato, Sonia Goncalves, Ewan Harrison, David K. Jackson, Ian Johnston, Dominic Kwiatkowski, Cordelia Langford, John Sillitoe on behalf of the Wellcome Sanger Institute COVID-19 Surveillance Team
EPI_ISL_703147	Liverpool Clinical Laboratories	COVID-19 Genomics UK (COG-UK) Consortium	Sam Haldenby, Anita Lucaci, Steve Paterson, Julian Hiscox, Alistair Darby, M Almsaud, A Alrezaihi, Muhannad Alruwaili, Stuart D Armstrong, Jones Benjamin, Eleanor G Bentley, Anu Chawla, Jordan J Clark, Angela Cowell, Richard Eccles, Isabel García-Dorival, Matthew Gemmell, Alessandro Gerada, PKF Gilmore, Richard Gregory, Ximeng Han, Catherine Hartley, Margaret Hughes, Miren Iturriza-Gomara, James Johnson, L Luu, Jenifer Manson, Charlotte Nelson, Elaine O'Toole, Cassie Olateju, Rebekah Penrice-Randal , Lucille Rainbow, N.P Randle, Trevor Ian Robinson, Parul Sharma, Ghada T Shawli, James P Stewart, Neil Swainston, Ecaterina Vamos, Joanne Watts, Mark Whitehead
EPI_ISL_703149, EPI_ISL_703153, EPI_ISL_703154, EPI_ISL_703156, EPI_ISL_703159, EPI_ISL_703160, EPI_ISL_703161	Lighthouse Lab in Alderley Park	Wellcome Sanger Institute for the COVID-19 Genomics UK (COG-UK) Consortium	Jacquelyn Wynn, Mairead Hyland, The Lighthouse Lab in Alderley Park and Alex Alderton, Roberto Amato, Sonia Goncalves, Ewan Harrison, David K. Jackson, Ian Johnston, Dominic Kwiatkowski, Cordelia Langford, John Sillitoe on behalf of the Wellcome Sanger Institute COVID-19 Surveillance Team
EPI_ISL_703162	Liverpool Clinical Laboratories	COVID-19 Genomics UK (COG-UK) Consortium	Sam Haldenby, Anita Lucaci, Steve Paterson, Julian Hiscox, Alistair Darby, M Almsaud, A Alrezaihi, Muhannad Alruwaili, Stuart D Armstrong, Jones Benjamin, Eleanor G Bentley, Anu Chawla, Jordan J Clark, Angela Cowell, Richard Eccles, Isabel García-Dorival, Matthew Gemmell, Alessandro Gerada, PKF Gilmore, Richard Gregory, Ximeng Han, Catherine Hartley, Margaret Hughes, Miren Iturriza-Gomara, James Johnson, L Luu, Jenifer Manson, Charlotte Nelson, Elaine O'Toole, Cassie Olateju, Rebekah Penrice-Randal , Lucille Rainbow, N.P Randle, Trevor Ian Robinson, Parul Sharma, Ghada T Shawli, James P Stewart, Neil Swainston, Ecaterina Vamos, Joanne Watts, Mark Whitehead
EPI_ISL_703163, EPI_ISL_703169, EPI_ISL_703173, EPI_ISL_703183	Lighthouse Lab in Alderley Park	Wellcome Sanger Institute for the COVID-19 Genomics UK (COG-UK) Consortium	Jacquelyn Wynn, Mairead Hyland, The Lighthouse Lab in Alderley Park and Alex Alderton, Roberto Amato, Sonia Goncalves, Ewan Harrison, David K. Jackson, Ian Johnston, Dominic Kwiatkowski, Cordelia Langford, John Sillitoe on behalf of the Wellcome Sanger Institute COVID-19 Surveillance Team
EPI_ISL_703184	Liverpool Clinical Laboratories	COVID-19 Genomics UK (COG-UK) Consortium	Sam Haldenby, Anita Lucaci, Steve Paterson, Julian Hiscox, Alistair Darby, M Almsaud, A Alrezaihi, Muhannad Alruwaili, Stuart D Armstrong, Jones Benjamin, Eleanor G Bentley, Anu Chawla, Jordan J Clark, Angela Cowell, Richard Eccles, Isabel García-Dorival, Matthew Gemmell, Alessandro Gerada, PKF Gilmore, Richard Gregory, Ximeng Han, Catherine Hartley, Margaret Hughes, Miren Iturriza-Gomara, James Johnson, L Luu, Jenifer Manson, Charlotte Nelson, Elaine O'Toole, Cassie Olateju, Rebekah Penrice-Randal , Lucille Rainbow, N.P Randle, Trevor Ian Robinson, Parul Sharma, Ghada T Shawli, James P Stewart, Neil Swainston, Ecaterina Vamos, Joanne Watts, Mark Whitehead
EPI_ISL_703186	Lighthouse Lab in Alderley Park	Wellcome Sanger Institute for the COVID-19 Genomics UK (COG-UK) Consortium	Jacquelyn Wynn, Mairead Hyland, The Lighthouse Lab in Alderley Park and Alex Alderton, Roberto Amato, Sonia Goncalves, Ewan Harrison, David K. Jackson, Ian Johnston, Dominic Kwiatkowski, Cordelia Langford, John Sillitoe on behalf of the Wellcome Sanger Institute COVID-19 Surveillance Team
EPI_ISL_703187	Quadram Institute Bioscience	COVID-19 Genomics UK (COG-UK) Consortium	Dave J. Baker, Gemma L. Kay, Alp Aydin, Thanh Le-Viet, Steven Rudder, Ana P. Tedim, Anastasia Kolyva, Maria Diaz, Leonardo de Oliveira Martins, Nabil-Fareed Alikhan, Lizzie Meadows, Rachael Stanley, Ngozi Elumogo, Muhammed Yasir, Nicholas M. Thomson, Alexander J Trotter, Rachel Gilroy, Samuel Bloomfield, Claire Stuart, Andrew Bell, Reenesh Prakash, Samir Dervisevic, Alison E. Mather, John Wain, Mark Webber, Andrew J. Page, Justin O'Grady
EPI_ISL_703188, EPI_ISL_703193, EPI_ISL_703197, EPI_ISL_703201, EPI_ISL_703204, EPI_ISL_703207	Lighthouse Lab in Alderley Park	Wellcome Sanger Institute for the COVID-19 Genomics UK (COG-UK) Consortium	Jacquelyn Wynn, Mairead Hyland, The Lighthouse Lab in Alderley Park and Alex Alderton, Roberto Amato, Sonia Goncalves, Ewan Harrison, David K. Jackson, Ian Johnston, Dominic Kwiatkowski, Cordelia Langford, John Sillitoe on behalf of the Wellcome Sanger Institute COVID-19 Surveillance Team
EPI_ISL_703208	Liverpool Clinical Laboratories	COVID-19 Genomics UK (COG-UK) Consortium	Sam Haldenby, Anita Lucaci, Steve Paterson, Julian Hiscox, Alistair Darby, M Almsaud, A Alrezaihi, Muhannad Alruwaili, Stuart D Armstrong, Jones Benjamin, Eleanor G Bentley, Anu Chawla, Jordan J Clark, Angela Cowell, Richard Eccles, Isabel García-Dorival, Matthew Gemmell, Alessandro Gerada, PKF Gilmore, Richard Gregory, Ximeng Han, Catherine Hartley, Margaret Hughes, Miren Iturriza-Gomara, James Johnson, L Luu, Jenifer Manson, Charlotte Nelson, Elaine O'Toole, Cassie Olateju, Rebekah Penrice-Randal , Lucille Rainbow, N.P Randle, Trevor Ian Robinson, Parul Sharma, Ghada T Shawli, James P Stewart, Neil Swainston, Ecaterina Vamos, Joanne Watts, Mark Whitehead
EPI_ISL_703209, EPI_ISL_703210, EPI_ISL_703212	Lighthouse Lab in Alderley Park	Wellcome Sanger Institute for the COVID-19 Genomics UK (COG-UK) Consortium	Jacquelyn Wynn, Mairead Hyland, The Lighthouse Lab in Alderley Park and Alex Alderton, Roberto Amato, Sonia Goncalves, Ewan Harrison, David K. Jackson, Ian Johnston, Dominic Kwiatkowski, Cordelia Langford, John Sillitoe on behalf of the Wellcome Sanger Institute COVID-19 Surveillance Team
EPI_ISL_703213	Department of Pathology, University of Cambridge	COVID-19 Genomics UK (COG-UK) Consortium	Aminu S. Jahun, Yasmin Chaudhry, Grant Hall, Iliana Georgana, Myra Hosmillo, Martin D. Curran, Malte Pinckert, Surendra Parmar, Ian Goodfellow
EPI_ISL_703215, EPI_ISL_703217, EPI_ISL_703220, EPI_ISL_703221	Lighthouse Lab in Alderley Park	Wellcome Sanger Institute for the COVID-19 Genomics UK (COG-UK) Consortium	Jacquelyn Wynn, Mairead Hyland, The Lighthouse Lab in Alderley Park and Alex Alderton, Roberto Amato, Sonia Goncalves, Ewan Harrison, David K. Jackson, Ian Johnston, Dominic Kwiatkowski, Cordelia Langford, John Sillitoe on behalf of the Wellcome Sanger Institute COVID-19 Surveillance Team
EPI_ISL_703222	University College London Hospital	COVID-19 Genomics UK (COG-UK) Consortium	Judith Heaney, Matthew Byott, Catherine Houlihan, Dan Frampton, Stuart Kirk, Moira Spyer and Eleni Nastouli
EPI_ISL_703224, EPI_ISL_703226, EPI_ISL_703228, EPI_ISL_703231, EPI_ISL_703232, EPI_ISL_703237	Lighthouse Lab in Alderley Park	Wellcome Sanger Institute for the COVID-19 Genomics UK (COG-UK) Consortium	Jacquelyn Wynn, Mairead Hyland, The Lighthouse Lab in Alderley Park and Alex Alderton, Roberto Amato, Sonia Goncalves, Ewan Harrison, David K. Jackson, Ian Johnston, Dominic Kwiatkowski, Cordelia Langford, John Sillitoe on behalf of the Wellcome Sanger Institute COVID-19 Surveillance Team
EPI_ISL_703238	Department of Pathology, University of Cambridge	COVID-19 Genomics UK (COG-UK) Consortium	Aminu S. Jahun, Yasmin Chaudhry, Grant Hall, Iliana Georgana, Myra Hosmillo, Martin D. Curran, Malte Pinckert, Surendra Parmar, Ian Goodfellow

EPI_ISL_703240	West of Scotland Specialist Virology Centre, NHSGGC / MRC-University of Glasgow Centre for Virus Research	COVID-19 Genomics UK (COG-UK) Consortium	Ana da Silva Filipe, Natasha Johnson, Kathy Smollett, Daniel Mair, Stephen Carmichael, Alice Broos, Lily Tong, Jenna Nichols, Kyriaki Nomikou; Sarah McDonald; Richard Orton, Joseph Hughes, Sreenu Vattipally, David L Robertson; Alasdair MacLean, Rory Gunson; Sharif Shaaban, Matthew Holden; Rachel Blacow, Guy Mollett, Kathy Li, James Shepherd, Antonia Ho, Emma Thomson
EPI_ISL_703241, EPI_ISL_703242	Lighthouse Lab in Alderley Park	Wellcome Sanger Institute for the COVID-19 Genomics UK (COG-UK) Consortium	Jacquelyn Wynn, Mairead Hyland, The Lighthouse Lab in Alderley Park and Alex Alderton, Roberto Amato, Sonia Goncalves, Ewan Harrison, David K. Jackson, Ian Johnston, Dominic Kwiatkowski, Cordelia Langford, John Sillitoe on behalf of the Wellcome Sanger Institute COVID-19 Surveillance Team
EPI_ISL_703243	West of Scotland Specialist Virology Centre, NHSGGC / MRC-University of Glasgow Centre for Virus Research	COVID-19 Genomics UK (COG-UK) Consortium	Ana da Silva Filipe, Natasha Johnson, Kathy Smollett, Daniel Mair, Stephen Carmichael, Alice Broos, Lily Tong, Jenna Nichols, Kyriaki Nomikou; Sarah McDonald; Richard Orton, Joseph Hughes, Sreenu Vattipally, David L Robertson; Alasdair MacLean, Rory Gunson; Sharif Shaaban, Matthew Holden; Rachel Blacow, Guy Mollett, Kathy Li, James Shepherd, Antonia Ho, Emma Thomson
EPI_ISL_703245, EPI_ISL_703247, EPI_ISL_703248, EPI_ISL_703250, EPI_ISL_703252, EPI_ISL_703253, EPI_ISL_703255, EPI_ISL_703257, EPI_ISL_703258, EPI_ISL_703260	Lighthouse Lab in Alderley Park	Wellcome Sanger Institute for the COVID-19 Genomics UK (COG-UK) Consortium	Jacquelyn Wynn, Mairead Hyland, The Lighthouse Lab in Alderley Park and Alex Alderton, Roberto Amato, Sonia Goncalves, Ewan Harrison, David K. Jackson, Ian Johnston, Dominic Kwiatkowski, Cordelia Langford, John Sillitoe on behalf of the Wellcome Sanger Institute COVID-19 Surveillance Team
EPI_ISL_703261	Liverpool Clinical Laboratories	COVID-19 Genomics UK (COG-UK) Consortium	Sam Haldenby, Anita Lucaci, Steve Paterson, Julian Hiscox, Alistair Darby, M Almsaud, A Alrezaihi, Muhannad Alruwaili, Stuart D Armstrong, Jones Benjamin, Eleanor G Bentley, Anu Chawla, Jordan J Clark, Angela Cowell, Richard Eccles, Isabel García-Dorival, Matthew Gemmell, Alessandro Gerada, PKF Gilmore, Richard Gregory, Ximeng Han, Catherine Hartley, Margaret Hughes, Miren Iturriza-Gomara, James Johnson, L Luu, Jenifer Manson, Charlotte Nelson, Elaine O'Toole, Cassie Olateju, Rebekah Penrice-Randal , Lucille Rainbow, N.P Randle, Trevor Ian Robinson, Parul Sharma, Ghada T Shawli, James P Stewart, Neil Swainston, Ecaterina Vamos, Joanne Watts, Mark Whitehead
EPI_ISL_703265, EPI_ISL_703266, EPI_ISL_703268, EPI_ISL_703271, EPI_ISL_703274	Lighthouse Lab in Alderley Park	Wellcome Sanger Institute for the COVID-19 Genomics UK (COG-UK) Consortium	Jacquelyn Wynn, Mairead Hyland, The Lighthouse Lab in Alderley Park and Alex Alderton, Roberto Amato, Sonia Goncalves, Ewan Harrison, David K. Jackson, Ian Johnston, Dominic Kwiatkowski, Cordelia Langford, John Sillitoe on behalf of the Wellcome Sanger Institute COVID-19 Surveillance Team
EPI_ISL_703275	Liverpool Clinical Laboratories	COVID-19 Genomics UK (COG-UK) Consortium	Sam Haldenby, Anita Lucaci, Steve Paterson, Julian Hiscox, Alistair Darby, M Almsaud, A Alrezaihi, Muhannad Alruwaili, Stuart D Armstrong, Jones Benjamin, Eleanor G Bentley, Anu Chawla, Jordan J Clark, Angela Cowell, Richard Eccles, Isabel García-Dorival, Matthew Gemmell, Alessandro Gerada, PKF Gilmore, Richard Gregory, Ximeng Han, Catherine Hartley, Margaret Hughes, Miren Iturriza-Gomara, James Johnson, L Luu, Jenifer Manson, Charlotte Nelson, Elaine O'Toole, Cassie Olateju, Rebekah Penrice-Randal , Lucille Rainbow, N.P Randle, Trevor Ian Robinson, Parul Sharma, Ghada T Shawli, James P Stewart, Neil Swainston, Ecaterina Vamos, Joanne Watts, Mark Whitehead
EPI_ISL_703276, EPI_ISL_703277, EPI_ISL_703282	Lighthouse Lab in Alderley Park	Wellcome Sanger Institute for the COVID-19 Genomics UK (COG-UK) Consortium	Jacquelyn Wynn, Mairead Hyland, The Lighthouse Lab in Alderley Park and Alex Alderton, Roberto Amato, Sonia Goncalves, Ewan Harrison, David K. Jackson, Ian Johnston, Dominic Kwiatkowski, Cordelia Langford, John Sillitoe on behalf of the Wellcome Sanger Institute COVID-19 Surveillance Team
EPI_ISL_703286	Lighthouse Lab in Glasgow	Wellcome Sanger Institute for the COVID-19 Genomics UK (COG-UK) Consortium	Harper VanSteenhouse, Yumi Kasai, David Gray, Carol Clugston, Anna Dominiczak and Alex Alderton, Roberto Amato, Sonia Goncalves, Ewan Harrison, David K. Jackson, Ian Johnston, Dominic Kwiatkowski, Cordelia Langford, John Sillitoe on behalf of the Wellcome Sanger Institute COVID-19 Surveillance Team
EPI_ISL_703289	Liverpool Clinical Laboratories	COVID-19 Genomics UK (COG-UK) Consortium	Sam Haldenby, Anita Lucaci, Steve Paterson, Julian Hiscox, Alistair Darby, M Almsaud, A Alrezaihi, Muhannad Alruwaili, Stuart D Armstrong, Jones Benjamin, Eleanor G Bentley, Anu Chawla, Jordan J Clark, Angela Cowell, Richard Eccles, Isabel García-Dorival, Matthew Gemmell, Alessandro Gerada, PKF Gilmore, Richard Gregory, Ximeng Han, Catherine Hartley, Margaret Hughes, Miren Iturriza-Gomara, James Johnson, L Luu, Jenifer Manson, Charlotte Nelson, Elaine O'Toole, Cassie Olateju, Rebekah Penrice-Randal , Lucille Rainbow, N.P Randle, Trevor Ian Robinson, Parul Sharma, Ghada T Shawli, James P Stewart, Neil Swainston, Ecaterina Vamos, Joanne Watts, Mark Whitehead
EPI_ISL_703293, EPI_ISL_703294, EPI_ISL_703298, EPI_ISL_703301, EPI_ISL_703306, EPI_ISL_703307, EPI_ISL_703311, EPI_ISL_703312, EPI_ISL_703314, EPI_ISL_703317, EPI_ISL_703318, EPI_ISL_703322, EPI_ISL_703326, EPI_ISL_703328, EPI_ISL_703329, EPI_ISL_703333, EPI_ISL_703336, EPI_ISL_703339, EPI_ISL_703341, EPI_ISL_703342, EPI_ISL_703344			
see above	Lighthouse Lab in Alderley Park	Wellcome Sanger Institute for the COVID-19 Genomics UK (COG-UK) Consortium	Jacquelyn Wynn, Mairead Hyland, The Lighthouse Lab in Alderley Park and Alex Alderton, Roberto Amato, Sonia Goncalves, Ewan Harrison, David K. Jackson, Ian Johnston, Dominic Kwiatkowski, Cordelia Langford, John Sillitoe on behalf of the Wellcome Sanger Institute COVID-19 Surveillance Team
EPI_ISL_703345	Lighthouse Lab in Glasgow	Wellcome Sanger Institute for the COVID-19 Genomics UK (COG-UK) Consortium	Harper VanSteenhouse, Yumi Kasai, David Gray, Carol Clugston, Anna Dominiczak and Alex Alderton, Roberto Amato, Sonia Goncalves, Ewan Harrison, David K. Jackson, Ian Johnston, Dominic Kwiatkowski, Cordelia Langford, John Sillitoe on behalf of the Wellcome Sanger Institute COVID-19 Surveillance Team
EPI_ISL_703347	Lighthouse Lab in Alderley Park	Wellcome Sanger Institute for the COVID-19 Genomics UK (COG-UK) Consortium	Jacquelyn Wynn, Mairead Hyland, The Lighthouse Lab in Alderley Park and Alex Alderton, Roberto Amato, Sonia Goncalves, Ewan Harrison, David K. Jackson, Ian Johnston, Dominic Kwiatkowski, Cordelia Langford, John Sillitoe on behalf of the Wellcome Sanger Institute COVID-19 Surveillance Team
EPI_ISL_703353	Liverpool Clinical Laboratories	COVID-19 Genomics UK (COG-UK) Consortium	Sam Haldenby, Anita Lucaci, Steve Paterson, Julian Hiscox, Alistair Darby, M Almsaud, A Alrezaihi, Muhannad Alruwaili, Stuart D Armstrong, Jones Benjamin, Eleanor G Bentley, Anu Chawla, Jordan J Clark, Angela Cowell, Richard Eccles, Isabel García-Dorival, Matthew Gemmell, Alessandro Gerada, PKF Gilmore, Richard Gregory, Ximeng Han, Catherine Hartley, Margaret Hughes, Miren Iturriza-Gomara, James Johnson, L Luu, Jenifer Manson, Charlotte Nelson, Elaine O'Toole, Cassie Olateju, Rebekah Penrice-Randal , Lucille Rainbow, N.P Randle, Trevor Ian Robinson, Parul Sharma, Ghada T Shawli, James P Stewart, Neil Swainston, Ecaterina Vamos, Joanne Watts, Mark Whitehead
EPI_ISL_703357, EPI_ISL_703363, EPI_ISL_703365	Lighthouse Lab in Alderley Park	Wellcome Sanger Institute for the COVID-19 Genomics UK (COG-UK) Consortium	Jacquelyn Wynn, Mairead Hyland, The Lighthouse Lab in Alderley Park and Alex Alderton, Roberto Amato, Sonia Goncalves, Ewan Harrison, David K. Jackson, Ian Johnston, Dominic Kwiatkowski, Cordelia Langford, John Sillitoe on behalf of the Wellcome Sanger Institute COVID-19 Surveillance Team
EPI_ISL_703366	Lighthouse Lab in Glasgow	Wellcome Sanger Institute for the COVID-19 Genomics UK (COG-UK) Consortium	Harper VanSteenhouse, Yumi Kasai, David Gray, Carol Clugston, Anna Dominiczak and Alex Alderton, Roberto Amato, Sonia Goncalves, Ewan Harrison, David K. Jackson, Ian Johnston, Dominic Kwiatkowski, Cordelia Langford, John Sillitoe on behalf of the Wellcome Sanger Institute COVID-19 Surveillance Team
EPI_ISL_703368, EPI_ISL_703373, EPI_ISL_703374, EPI_ISL_703376	Lighthouse Lab in Alderley Park	Wellcome Sanger Institute for the COVID-19 Genomics UK (COG-UK) Consortium	Jacquelyn Wynn, Mairead Hyland, The Lighthouse Lab in Alderley Park and Alex Alderton, Roberto Amato, Sonia Goncalves, Ewan Harrison, David K. Jackson, Ian Johnston, Dominic Kwiatkowski, Cordelia Langford, John Sillitoe on behalf of the Wellcome Sanger Institute COVID-19 Surveillance Team
EPI_ISL_703380	Liverpool Clinical Laboratories	COVID-19 Genomics UK (COG-UK) Consortium	Sam Haldenby, Anita Lucaci, Steve Paterson, Julian Hiscox, Alistair Darby, M Almsaud, A Alrezaihi, Muhannad Alruwaili, Stuart D Armstrong, Jones Benjamin, Eleanor G Bentley, Anu Chawla, Jordan J Clark, Angela Cowell, Richard Eccles, Isabel García-Dorival, Matthew Gemmell, Alessandro Gerada, PKF Gilmore, Richard Gregory, Ximeng Han, Catherine Hartley, Margaret Hughes, Miren Iturriza-Gomara, James Johnson, L Luu, Jenifer Manson, Charlotte Nelson, Elaine O'Toole, Cassie Olateju, Rebekah Penrice-Randal , Lucille Rainbow, N.P Randle, Trevor Ian Robinson, Parul Sharma, Ghada T Shawli, James P Stewart, Neil Swainston, Ecaterina Vamos, Joanne Watts, Mark Whitehead
EPI_ISL_703386, EPI_ISL_703388, EPI_ISL_703394, EPI_ISL_703396, EPI_ISL_703397	Lighthouse Lab in Alderley Park	Wellcome Sanger Institute for the COVID-19 Genomics UK (COG-UK) Consortium	Jacquelyn Wynn, Mairead Hyland, The Lighthouse Lab in Alderley Park and Alex Alderton, Roberto Amato, Sonia Goncalves, Ewan Harrison, David K. Jackson, Ian Johnston, Dominic Kwiatkowski, Cordelia Langford, John Sillitoe on behalf of the Wellcome Sanger Institute COVID-19 Surveillance Team
EPI_ISL_703399, EPI_ISL_703401	Lighthouse Lab in Glasgow	Wellcome Sanger Institute for the COVID-19 Genomics UK (COG-UK) Consortium	Harper VanSteenhouse, Yumi Kasai, David Gray, Carol Clugston, Anna Dominiczak and Alex Alderton, Roberto Amato, Sonia Goncalves, Ewan Harrison, David K. Jackson, Ian Johnston, Dominic Kwiatkowski, Cordelia Langford, John Sillitoe on behalf of the Wellcome Sanger Institute COVID-19 Surveillance Team
EPI_ISL_703402, EPI_ISL_703404, EPI_ISL_703405, EPI_ISL_703413, EPI_ISL_703416, EPI_ISL_703418, EPI_ISL_703420	Lighthouse Lab in Alderley Park	Wellcome Sanger Institute for the COVID-19 Genomics UK (COG-UK) Consortium	Jacquelyn Wynn, Mairead Hyland, The Lighthouse Lab in Alderley Park and Alex Alderton, Roberto Amato, Sonia Goncalves, Ewan Harrison, David K. Jackson, Ian Johnston, Dominic Kwiatkowski, Cordelia Langford, John Sillitoe on behalf of the Wellcome Sanger Institute COVID-19 Surveillance Team
EPI_ISL_703424	Quadram Institute Bioscience	COVID-19 Genomics UK (COG-UK) Consortium	Dave J. Baker, Gemma L. Kay, Alp Aydin, Thanh Le-Viet, Steven Rudder, Ana P. Tedim, Anastasia Kolyva, Maria Diaz, Leonardo de Oliveira Martins, Nabil-Fareed Alikhan, Lizzie Meadows, Rachael Stanley, Ngozi Elumogo, Muhammed Yasir, Nicholas M. Thomson, Alexander J Trotter, Rachel Gilroy, Samuel Bloomfield, Claire Stuart, Andrew Bell, Reenesh Prakash, Samir Dervisevic, Alison E. Mather, John Wain, Mark Webber, Andrew J. Page, Justin O'Grady

EPI_ISL_703425, EPI_ISL_703433, EPI_ISL_703434, EPI_ISL_703436, EPI_ISL_703437, EPI_ISL_703441	Lighthouse Lab in Alderley Park	Wellcome Sanger Institute for the COVID-19 Genomics UK (COG-UK) Consortium	Jacquelyn Wynn, Mairead Hyland, The Lighthouse Lab in Alderley Park and Alex Alderton, Roberto Amato, Sonia Goncalves, Ewan Harrison, David K. Jackson, Ian Johnston, Dominic Kwiatkowski, Cordelia Langford, John Sillitoe on behalf of the Wellcome Sanger Institute COVID-19 Surveillance Team
EPI_ISL_703445	Lighthouse Lab in Glasgow	Wellcome Sanger Institute for the COVID-19 Genomics UK (COG-UK) Consortium	Harper VanSteenhouse, Yumi Kasai, David Gray, Carol Clugston, Anna Dominiczak and Alex Alderton, Roberto Amato, Sonia Goncalves, Ewan Harrison, David K. Jackson, Ian Johnston, Dominic Kwiatkowski, Cordelia Langford, John Sillitoe on behalf of the Wellcome Sanger Institute COVID-19 Surveillance Team
EPI_ISL_703448, EPI_ISL_703450	Lighthouse Lab in Alderley Park	Wellcome Sanger Institute for the COVID-19 Genomics UK (COG-UK) Consortium	Jacquelyn Wynn, Mairead Hyland, The Lighthouse Lab in Alderley Park and Alex Alderton, Roberto Amato, Sonia Goncalves, Ewan Harrison, David K. Jackson, Ian Johnston, Dominic Kwiatkowski, Cordelia Langford, John Sillitoe on behalf of the Wellcome Sanger Institute COVID-19 Surveillance Team
EPI_ISL_703451	Department of Pathology, University of Cambridge	COVID-19 Genomics UK (COG-UK) Consortium	Aminu S. Jahun, Yasmin Chaudhry, Grant Hall, Iliana Georgana, Myra Hosmillo, Martin D. Curran, Malte Pinckert, Surendra Parmar, Ian Goodfellow
EPI_ISL_703452, EPI_ISL_703455	Lighthouse Lab in Alderley Park	Wellcome Sanger Institute for the COVID-19 Genomics UK (COG-UK) Consortium	Jacquelyn Wynn, Mairead Hyland, The Lighthouse Lab in Alderley Park and Alex Alderton, Roberto Amato, Sonia Goncalves, Ewan Harrison, David K. Jackson, Ian Johnston, Dominic Kwiatkowski, Cordelia Langford, John Sillitoe on behalf of the Wellcome Sanger Institute COVID-19 Surveillance Team
EPI_ISL_703457	Department of Pathology, University of Cambridge	COVID-19 Genomics UK (COG-UK) Consortium	Aminu S. Jahun, Yasmin Chaudhry, Grant Hall, Iliana Georgana, Myra Hosmillo, Martin D. Curran, Malte Pinckert, Surendra Parmar, Ian Goodfellow
EPI_ISL_703460, EPI_ISL_703461, EPI_ISL_703464	Lighthouse Lab in Alderley Park	Wellcome Sanger Institute for the COVID-19 Genomics UK (COG-UK) Consortium	Jacquelyn Wynn, Mairead Hyland, The Lighthouse Lab in Alderley Park and Alex Alderton, Roberto Amato, Sonia Goncalves, Ewan Harrison, David K. Jackson, Ian Johnston, Dominic Kwiatkowski, Cordelia Langford, John Sillitoe on behalf of the Wellcome Sanger Institute COVID-19 Surveillance Team
EPI_ISL_703466	Lighthouse Lab in Glasgow	Wellcome Sanger Institute for the COVID-19 Genomics UK (COG-UK) Consortium	Harper VanSteenhouse, Yumi Kasai, David Gray, Carol Clugston, Anna Dominiczak and Alex Alderton, Roberto Amato, Sonia Goncalves, Ewan Harrison, David K. Jackson, Ian Johnston, Dominic Kwiatkowski, Cordelia Langford, John Sillitoe on behalf of the Wellcome Sanger Institute COVID-19 Surveillance Team
EPI_ISL_703467, EPI_ISL_703470, EPI_ISL_703475	Lighthouse Lab in Alderley Park	Wellcome Sanger Institute for the COVID-19 Genomics UK (COG-UK) Consortium	Jacquelyn Wynn, Mairead Hyland, The Lighthouse Lab in Alderley Park and Alex Alderton, Roberto Amato, Sonia Goncalves, Ewan Harrison, David K. Jackson, Ian Johnston, Dominic Kwiatkowski, Cordelia Langford, John Sillitoe on behalf of the Wellcome Sanger Institute COVID-19 Surveillance Team
EPI_ISL_703476	Department of Pathology, University of Cambridge	COVID-19 Genomics UK (COG-UK) Consortium	Aminu S. Jahun, Yasmin Chaudhry, Grant Hall, Iliana Georgana, Myra Hosmillo, Martin D. Curran, Malte Pinckert, Surendra Parmar, Ian Goodfellow
EPI_ISL_703477, EPI_ISL_703478	Lighthouse Lab in Alderley Park	Wellcome Sanger Institute for the COVID-19 Genomics UK (COG-UK) Consortium	Jacquelyn Wynn, Mairead Hyland, The Lighthouse Lab in Alderley Park and Alex Alderton, Roberto Amato, Sonia Goncalves, Ewan Harrison, David K. Jackson, Ian Johnston, Dominic Kwiatkowski, Cordelia Langford, John Sillitoe on behalf of the Wellcome Sanger Institute COVID-19 Surveillance Team
EPI_ISL_703479	Department of Pathology, University of Cambridge	COVID-19 Genomics UK (COG-UK) Consortium	Aminu S. Jahun, Yasmin Chaudhry, Grant Hall, Iliana Georgana, Myra Hosmillo, Martin D. Curran, Malte Pinckert, Surendra Parmar, Ian Goodfellow
EPI_ISL_703480, EPI_ISL_703483, EPI_ISL_703485	Lighthouse Lab in Alderley Park	Wellcome Sanger Institute for the COVID-19 Genomics UK (COG-UK) Consortium	Jacquelyn Wynn, Mairead Hyland, The Lighthouse Lab in Alderley Park and Alex Alderton, Roberto Amato, Sonia Goncalves, Ewan Harrison, David K. Jackson, Ian Johnston, Dominic Kwiatkowski, Cordelia Langford, John Sillitoe on behalf of the Wellcome Sanger Institute COVID-19 Surveillance Team
EPI_ISL_703487	Northumbria University / South Tees Hospitals NHS Foundation Trust / North Cumbria Integrated Care NHS Foundation Trust / North Tees and Hartlepool NHS Foundation Trust / Newcastle Hospitals NHS Foundation Trust	COVID-19 Genomics UK (COG-UK) Consortium	Darren L Smith,Andrew Nelson,Matthew Bashton,Greg R Young,Joshua Loh,John Allan,Mohammad A Tariq,Giles S Holt,Gary Black,Wen C Yew,Lynn Dover,Paul Baker,Steve Liggett,Sarah Essex,Jane Greenaway,Debra Padgett,Clive Graham,Garren Scott,Edward Barton,Emma Swindells,Brendan Payne,Jennifer Collins,Yusri Taha,Gary Eltringham
EPI_ISL_703488, EPI_ISL_703490, EPI_ISL_703493, EPI_ISL_703494, EPI_ISL_703498	Lighthouse Lab in Alderley Park	Wellcome Sanger Institute for the COVID-19 Genomics UK (COG-UK) Consortium	Jacquelyn Wynn, Mairead Hyland, The Lighthouse Lab in Alderley Park and Alex Alderton, Roberto Amato, Sonia Goncalves, Ewan Harrison, David K. Jackson, Ian Johnston, Dominic Kwiatkowski, Cordelia Langford, John Sillitoe on behalf of the Wellcome Sanger Institute COVID-19 Surveillance Team
EPI_ISL_703501	Lighthouse Lab in Glasgow	Wellcome Sanger Institute for the COVID-19 Genomics UK (COG-UK) Consortium	Harper VanSteenhouse, Yumi Kasai, David Gray, Carol Clugston, Anna Dominiczak and Alex Alderton, Roberto Amato, Sonia Goncalves, Ewan Harrison, David K. Jackson, Ian Johnston, Dominic Kwiatkowski, Cordelia Langford, John Sillitoe on behalf of the Wellcome Sanger Institute COVID-19 Surveillance Team
EPI_ISL_703503, EPI_ISL_703504	Lighthouse Lab in Alderley Park	Wellcome Sanger Institute for the COVID-19 Genomics UK (COG-UK) Consortium	Jacquelyn Wynn, Mairead Hyland, The Lighthouse Lab in Alderley Park and Alex Alderton, Roberto Amato, Sonia Goncalves, Ewan Harrison, David K. Jackson, Ian Johnston, Dominic Kwiatkowski, Cordelia Langford, John Sillitoe on behalf of the Wellcome Sanger Institute COVID-19 Surveillance Team
EPI_ISL_703505	Virology Department, Sheffield Teaching Hospitals NHS Foundation Trust/Department of Infection, Immunity and Cardiovascular Disease, The Medical School, University of Sheffield	COVID-19 Genomics UK (COG-UK) Consortium	Thushan de Silva, Matthew Parker, Nikki Smith, Adri Angyal, Rebecca Brown, Luke Green, Rachel Tucker, Paul Parsons, Danielle Groves, Katie Johnson, Laura Carrilero, Alex Keeley, Dave Partridge, Matthew Wyles, Benjamin Lindsey, Mehmet Yavuz, Mohammad Raza, Cariad Evans
EPI_ISL_703507	Lighthouse Lab in Alderley Park	Wellcome Sanger Institute for the COVID-19 Genomics UK (COG-UK) Consortium	Jacquelyn Wynn, Mairead Hyland, The Lighthouse Lab in Alderley Park and Alex Alderton, Roberto Amato, Sonia Goncalves, Ewan Harrison, David K. Jackson, Ian Johnston, Dominic Kwiatkowski, Cordelia Langford, John Sillitoe on behalf of the Wellcome Sanger Institute COVID-19 Surveillance Team
EPI_ISL_703508	Department of Pathology, University of Cambridge	COVID-19 Genomics UK (COG-UK) Consortium	Aminu S. Jahun, Yasmin Chaudhry, Grant Hall, Iliana Georgana, Myra Hosmillo, Martin D. Curran, Malte Pinckert, Surendra Parmar, Ian Goodfellow
EPI_ISL_703509, EPI_ISL_703518, EPI_ISL_703520, EPI_ISL_703523	Lighthouse Lab in Alderley Park	Wellcome Sanger Institute for the COVID-19 Genomics UK (COG-UK) Consortium	Jacquelyn Wynn, Mairead Hyland, The Lighthouse Lab in Alderley Park and Alex Alderton, Roberto Amato, Sonia Goncalves, Ewan Harrison, David K. Jackson, Ian Johnston, Dominic Kwiatkowski, Cordelia Langford, John Sillitoe on behalf of the Wellcome Sanger Institute COVID-19 Surveillance Team
EPI_ISL_703524	Department of Pathology, University of Cambridge	COVID-19 Genomics UK (COG-UK) Consortium	Aminu S. Jahun, Yasmin Chaudhry, Grant Hall, Iliana Georgana, Myra Hosmillo, Martin D. Curran, Malte Pinckert, Surendra Parmar, Ian Goodfellow
EPI_ISL_703526	Lighthouse Lab in Alderley Park	Wellcome Sanger Institute for the COVID-19 Genomics UK (COG-UK) Consortium	Jacquelyn Wynn, Mairead Hyland, The Lighthouse Lab in Alderley Park and Alex Alderton, Roberto Amato, Sonia Goncalves, Ewan Harrison, David K. Jackson, Ian Johnston, Dominic Kwiatkowski, Cordelia Langford, John Sillitoe on behalf of the Wellcome Sanger Institute COVID-19 Surveillance Team
EPI_ISL_703527	Liverpool Clinical Laboratories	COVID-19 Genomics UK (COG-UK) Consortium	Sam Haldenby, Anita Lucaci, Steve Paterson, Julian Hiscox, Alistair Darby, M Almsaud, A Alrezaihi, Muhannad Alruwaili, Stuart D Armstrong, Jones Benjamin, Eleanor G Bentley, Anu Chawla, Jordan J Clark, Angela Cowell, Richard Eccles, Isabel García-Dorival, Matthew Gemmell, Alessandro Gerada, PKF Gilmore, Richard Gregory, Ximeng Han, Catherine Hartley, Margaret Hughes, Miren Iturriza-Gomara, James Johnson, L Luu, Jenifer Manson, Charlotte Nelson, Elaine O'Toole, Cassie Olateju, Rebekah Penrice-Randal , Lucille Rainbow, N.P Randle, Trevor Ian Robinson, Parul Sharma, Ghada T Shawli, James P Stewart, Neil Swainston, Ecaterina Vamos, Joanne Watts, Mark Whitehead
EPI_ISL_703528, EPI_ISL_703531, EPI_ISL_703532, EPI_ISL_703536, EPI_ISL_703539, EPI_ISL_703543, EPI_ISL_703544, EPI_ISL_703547, EPI_ISL_703549	Lighthouse Lab in Alderley Park	Wellcome Sanger Institute for the COVID-19 Genomics UK (COG-UK) Consortium	Jacquelyn Wynn, Mairead Hyland, The Lighthouse Lab in Alderley Park and Alex Alderton, Roberto Amato, Sonia Goncalves, Ewan Harrison, David K. Jackson, Ian Johnston, Dominic Kwiatkowski, Cordelia Langford, John Sillitoe on behalf of the Wellcome Sanger Institute COVID-19 Surveillance Team
EPI_ISL_703550	Lighthouse Lab in Glasgow	Wellcome Sanger Institute for the COVID-19 Genomics UK (COG-UK) Consortium	Harper VanSteenhouse, Yumi Kasai, David Gray, Carol Clugston, Anna Dominiczak and Alex Alderton, Roberto Amato, Sonia Goncalves, Ewan Harrison, David K. Jackson, Ian Johnston, Dominic Kwiatkowski, Cordelia Langford, John Sillitoe on behalf of the Wellcome Sanger Institute COVID-19 Surveillance Team
EPI_ISL_703552, EPI_ISL_703555, EPI_ISL_703558	Lighthouse Lab in Alderley Park	Wellcome Sanger Institute for the COVID-19 Genomics UK (COG-UK) Consortium	Jacquelyn Wynn, Mairead Hyland, The Lighthouse Lab in Alderley Park and Alex Alderton, Roberto Amato, Sonia Goncalves, Ewan Harrison, David K. Jackson, Ian Johnston, Dominic Kwiatkowski, Cordelia Langford, John Sillitoe on behalf of the Wellcome Sanger Institute COVID-19 Surveillance Team
EPI_ISL_703560	Lighthouse Lab in Glasgow	Wellcome Sanger Institute for the COVID-19 Genomics UK (COG-UK) Consortium	Harper VanSteenhouse, Yumi Kasai, David Gray, Carol Clugston, Anna Dominiczak and Alex Alderton, Roberto Amato, Sonia Goncalves, Ewan Harrison, David K. Jackson, Ian Johnston, Dominic Kwiatkowski, Cordelia Langford, John Sillitoe on behalf of the Wellcome Sanger Institute COVID-19 Surveillance Team
EPI_ISL_703563, EPI_ISL_703565, EPI_ISL_703566, EPI_ISL_703568	Lighthouse Lab in Alderley Park	Wellcome Sanger Institute for the COVID-19 Genomics UK (COG-UK) Consortium	Jacquelyn Wynn, Mairead Hyland, The Lighthouse Lab in Alderley Park and Alex Alderton, Roberto Amato, Sonia Goncalves, Ewan Harrison, David K. Jackson, Ian Johnston, Dominic Kwiatkowski, Cordelia Langford, John Sillitoe on behalf of the Wellcome Sanger Institute COVID-19 Surveillance Team
EPI_ISL_703569	Virology Department, Sheffield Teaching Hospitals NHS Foundation Trust/Department of Infection, Immunity and Cardiovascular Disease, The Medical School, University of Sheffield	COVID-19 Genomics UK (COG-UK) Consortium	Thushan de Silva, Matthew Parker, Nikki Smith, Adri Angyal, Rebecca Brown, Luke Green, Rachel Tucker, Paul Parsons, Danielle Groves, Katie Johnson, Laura Carrilero, Alex Keeley, Dave Partridge, Matthew Wyles, Benjamin Lindsey, Mehmet Yavuz, Mohammad Raza, Cariad Evans

EPI_ISL_703570, EPI_ISL_703571, EPI_ISL_703573, EPI_ISL_703574	Lighthouse Lab in Alderley Park	Wellcome Sanger Institute for the COVID-19 Genomics UK (COG-UK) Consortium	Jacquelyn Wynn, Mairead Hyland, The Lighthouse Lab in Alderley Park and Alex Alderton, Roberto Amato, Sonia Goncalves, Ewan Harrison, David K. Jackson, Ian Johnston, Dominic Kwiatkowski, Cordelia Langford, John Sillitoe on behalf of the Wellcome Sanger Institute COVID-19 Surveillance Team
EPI_ISL_703575	Virology Department, Sheffield Teaching Hospitals NHS Foundation Trust/Department of Infection, Immunity and Cardiovascular Disease, The Medical School, University of Sheffield	COVID-19 Genomics UK (COG-UK) Consortium	Thushan de Silva, Matthew Parker, Nikki Smith, Adri Angyal, Rebecca Brown, Luke Green, Rachel Tucker, Paul Parsons, Danielle Groves, Katie Johnson, Laura Carrilero, Alex Keeley, Dave Partridge, Matthew Wyles, Benjamin Lindsey, Mehmet Yavuz, Mohammad Raza, Cariad Evans
EPI_ISL_703576, EPI_ISL_703580, EPI_ISL_703581	Lighthouse Lab in Alderley Park	Wellcome Sanger Institute for the COVID-19 Genomics UK (COG-UK) Consortium	Jacquelyn Wynn, Mairead Hyland, The Lighthouse Lab in Alderley Park and Alex Alderton, Roberto Amato, Sonia Goncalves, Ewan Harrison, David K. Jackson, Ian Johnston, Dominic Kwiatkowski, Cordelia Langford, John Sillitoe on behalf of the Wellcome Sanger Institute COVID-19 Surveillance Team
EPI_ISL_703582	Virology Department, Sheffield Teaching Hospitals NHS Foundation Trust/Department of Infection, Immunity and Cardiovascular Disease, The Medical School, University of Sheffield	COVID-19 Genomics UK (COG-UK) Consortium	Thushan de Silva, Matthew Parker, Nikki Smith, Adri Angyal, Rebecca Brown, Luke Green, Rachel Tucker, Paul Parsons, Danielle Groves, Katie Johnson, Laura Carrilero, Alex Keeley, Dave Partridge, Matthew Wyles, Benjamin Lindsey, Mehmet Yavuz, Mohammad Raza, Cariad Evans
EPI_ISL_703584	Lighthouse Lab in Glasgow	Wellcome Sanger Institute for the COVID-19 Genomics UK (COG-UK) Consortium	Harper VanSteenhouse, Yumi Kasai, David Gray, Carol Clugston, Anna Dominiczak and Alex Alderton, Roberto Amato, Sonia Goncalves, Ewan Harrison, David K. Jackson, Ian Johnston, Dominic Kwiatkowski, Cordelia Langford, John Sillitoe on behalf of the Wellcome Sanger Institute COVID-19 Surveillance Team
EPI_ISL_703585	Virology Department, Sheffield Teaching Hospitals NHS Foundation Trust/Department of Infection, Immunity and Cardiovascular Disease, The Medical School, University of Sheffield	COVID-19 Genomics UK (COG-UK) Consortium	Thushan de Silva, Matthew Parker, Nikki Smith, Adri Angyal, Rebecca Brown, Luke Green, Rachel Tucker, Paul Parsons, Danielle Groves, Katie Johnson, Laura Carrilero, Alex Keeley, Dave Partridge, Matthew Wyles, Benjamin Lindsey, Mehmet Yavuz, Mohammad Raza, Cariad Evans
EPI_ISL_703586	Lighthouse Lab in Alderley Park	Wellcome Sanger Institute for the COVID-19 Genomics UK (COG-UK) Consortium	Jacquelyn Wynn, Mairead Hyland, The Lighthouse Lab in Alderley Park and Alex Alderton, Roberto Amato, Sonia Goncalves, Ewan Harrison, David K. Jackson, Ian Johnston, Dominic Kwiatkowski, Cordelia Langford, John Sillitoe on behalf of the Wellcome Sanger Institute COVID-19 Surveillance Team
EPI_ISL_703587	Virology Department, Sheffield Teaching Hospitals NHS Foundation Trust/Department of Infection, Immunity and Cardiovascular Disease, The Medical School, University of Sheffield	COVID-19 Genomics UK (COG-UK) Consortium	Thushan de Silva, Matthew Parker, Nikki Smith, Adri Angyal, Rebecca Brown, Luke Green, Rachel Tucker, Paul Parsons, Danielle Groves, Katie Johnson, Laura Carrilero, Alex Keeley, Dave Partridge, Matthew Wyles, Benjamin Lindsey, Mehmet Yavuz, Mohammad Raza, Cariad Evans
EPI_ISL_703588, EPI_ISL_703589	Lighthouse Lab in Alderley Park	Wellcome Sanger Institute for the COVID-19 Genomics UK (COG-UK) Consortium	Jacquelyn Wynn, Mairead Hyland, The Lighthouse Lab in Alderley Park and Alex Alderton, Roberto Amato, Sonia Goncalves, Ewan Harrison, David K. Jackson, Ian Johnston, Dominic Kwiatkowski, Cordelia Langford, John Sillitoe on behalf of the Wellcome Sanger Institute COVID-19 Surveillance Team
EPI_ISL_703590	Quadram Institute Bioscience	COVID-19 Genomics UK (COG-UK) Consortium	Dave J. Baker, Gemma L. Kay, Alp Aydin, Thanh Le-Viet, Steven Rudder, Ana P. Tedim, Anastasia Kolyva, Maria Diaz, Leonardo de Oliveira Martins, Nabil-Fareed Alikhan, Lizzie Meadows, Rachael Stanley, Ngozi Elumogo, Muhammed Yasir, Nicholas M. Thomson, Alexander J Trotter, Rachel Gilroy, Samuel Bloomfield, Claire Stuart, Andrew Bell, Reenesh Prakash, Samir Dervisevic, Alison E. Mather, John Wain, Mark Webber, Andrew J. Page, Justin O'Grady
EPI_ISL_703591, EPI_ISL_703592	Lighthouse Lab in Alderley Park	Wellcome Sanger Institute for the COVID-19 Genomics UK (COG-UK) Consortium	Jacquelyn Wynn, Mairead Hyland, The Lighthouse Lab in Alderley Park and Alex Alderton, Roberto Amato, Sonia Goncalves, Ewan Harrison, David K. Jackson, Ian Johnston, Dominic Kwiatkowski, Cordelia Langford, John Sillitoe on behalf of the Wellcome Sanger Institute COVID-19 Surveillance Team
EPI_ISL_703593	Virology Department, Sheffield Teaching Hospitals NHS Foundation Trust/Department of Infection, Immunity and Cardiovascular Disease, The Medical School, University of Sheffield	COVID-19 Genomics UK (COG-UK) Consortium	Thushan de Silva, Matthew Parker, Nikki Smith, Adri Angyal, Rebecca Brown, Luke Green, Rachel Tucker, Paul Parsons, Danielle Groves, Katie Johnson, Laura Carrilero, Alex Keeley, Dave Partridge, Matthew Wyles, Benjamin Lindsey, Mehmet Yavuz, Mohammad Raza, Cariad Evans
EPI_ISL_703595, EPI_ISL_703597, EPI_ISL_703602, EPI_ISL_703603, EPI_ISL_703605, EPI_ISL_703613, EPI_ISL_703615, EPI_ISL_703618	Lighthouse Lab in Alderley Park	Wellcome Sanger Institute for the COVID-19 Genomics UK (COG-UK) Consortium	Jacquelyn Wynn, Mairead Hyland, The Lighthouse Lab in Alderley Park and Alex Alderton, Roberto Amato, Sonia Goncalves, Ewan Harrison, David K. Jackson, Ian Johnston, Dominic Kwiatkowski, Cordelia Langford, John Sillitoe on behalf of the Wellcome Sanger Institute COVID-19 Surveillance Team
EPI_ISL_703619	Department of Pathology, University of Cambridge	COVID-19 Genomics UK (COG-UK) Consortium	Aminu S. Jahun, Yasmin Chaudhry, Grant Hall, Iliana Georgana, Myra Hosmillo, Martin D. Curran, Malte Pinckert, Surendra Parmar, Ian Goodfellow
EPI_ISL_703620, EPI_ISL_703623, EPI_ISL_703625, EPI_ISL_703628	Lighthouse Lab in Alderley Park	Wellcome Sanger Institute for the COVID-19 Genomics UK (COG-UK) Consortium	Jacquelyn Wynn, Mairead Hyland, The Lighthouse Lab in Alderley Park and Alex Alderton, Roberto Amato, Sonia Goncalves, Ewan Harrison, David K. Jackson, Ian Johnston, Dominic Kwiatkowski, Cordelia Langford, John Sillitoe on behalf of the Wellcome Sanger Institute COVID-19 Surveillance Team
EPI_ISL_703629	Department of Pathology, University of Cambridge	COVID-19 Genomics UK (COG-UK) Consortium	Aminu S. Jahun, Yasmin Chaudhry, Grant Hall, Iliana Georgana, Myra Hosmillo, Martin D. Curran, Malte Pinckert, Surendra Parmar, Ian Goodfellow
EPI_ISL_703630	Lighthouse Lab in Alderley Park	Wellcome Sanger Institute for the COVID-19 Genomics UK (COG-UK) Consortium	Jacquelyn Wynn, Mairead Hyland, The Lighthouse Lab in Alderley Park and Alex Alderton, Roberto Amato, Sonia Goncalves, Ewan Harrison, David K. Jackson, Ian Johnston, Dominic Kwiatkowski, Cordelia Langford, John Sillitoe on behalf of the Wellcome Sanger Institute COVID-19 Surveillance Team
EPI_ISL_703632, EPI_ISL_703635	Department of Pathology, University of Cambridge	COVID-19 Genomics UK (COG-UK) Consortium	Aminu S. Jahun, Yasmin Chaudhry, Grant Hall, Iliana Georgana, Myra Hosmillo, Martin D. Curran, Malte Pinckert, Surendra Parmar, Ian Goodfellow
EPI_ISL_703636	Virology Department, Sheffield Teaching Hospitals NHS Foundation Trust/Department of Infection, Immunity and Cardiovascular Disease, The Medical School, University of Sheffield	COVID-19 Genomics UK (COG-UK) Consortium	Thushan de Silva, Matthew Parker, Nikki Smith, Adri Angyal, Rebecca Brown, Luke Green, Rachel Tucker, Paul Parsons, Danielle Groves, Katie Johnson, Laura Carrilero, Alex Keeley, Dave Partridge, Matthew Wyles, Benjamin Lindsey, Mehmet Yavuz, Mohammad Raza, Cariad Evans
EPI_ISL_703637, EPI_ISL_703638	Lighthouse Lab in Alderley Park	Wellcome Sanger Institute for the COVID-19 Genomics UK (COG-UK) Consortium	Jacquelyn Wynn, Mairead Hyland, The Lighthouse Lab in Alderley Park and Alex Alderton, Roberto Amato, Sonia Goncalves, Ewan Harrison, David K. Jackson, Ian Johnston, Dominic Kwiatkowski, Cordelia Langford, John Sillitoe on behalf of the Wellcome Sanger Institute COVID-19 Surveillance Team
EPI_ISL_703639	Virology Department, Sheffield Teaching Hospitals NHS Foundation Trust/Department of Infection, Immunity and Cardiovascular Disease, The Medical School, University of Sheffield	COVID-19 Genomics UK (COG-UK) Consortium	Thushan de Silva, Matthew Parker, Nikki Smith, Adri Angyal, Rebecca Brown, Luke Green, Rachel Tucker, Paul Parsons, Danielle Groves, Katie Johnson, Laura Carrilero, Alex Keeley, Dave Partridge, Matthew Wyles, Benjamin Lindsey, Mehmet Yavuz, Mohammad Raza, Cariad Evans
EPI_ISL_703646, EPI_ISL_703648	Lighthouse Lab in Alderley Park	Wellcome Sanger Institute for the COVID-19 Genomics UK (COG-UK) Consortium	Jacquelyn Wynn, Mairead Hyland, The Lighthouse Lab in Alderley Park and Alex Alderton, Roberto Amato, Sonia Goncalves, Ewan Harrison, David K. Jackson, Ian Johnston, Dominic Kwiatkowski, Cordelia Langford, John Sillitoe on behalf of the Wellcome Sanger Institute COVID-19 Surveillance Team
EPI_ISL_703650, EPI_ISL_703659	Lighthouse Lab in Glasgow	Wellcome Sanger Institute for the COVID-19 Genomics UK (COG-UK) Consortium	Harper VanSteenhouse, Yumi Kasai, David Gray, Carol Clugston, Anna Dominiczak and Alex Alderton, Roberto Amato, Sonia Goncalves, Ewan Harrison, David K. Jackson, Ian Johnston, Dominic Kwiatkowski, Cordelia Langford, John Sillitoe on behalf of the Wellcome Sanger Institute COVID-19 Surveillance Team
EPI_ISL_703662, EPI_ISL_703664, EPI_ISL_703665, EPI_ISL_703667, EPI_ISL_703670, EPI_ISL_703675, EPI_ISL_703678	Lighthouse Lab in Alderley Park	Wellcome Sanger Institute for the COVID-19 Genomics UK (COG-UK) Consortium	Jacquelyn Wynn, Mairead Hyland, The Lighthouse Lab in Alderley Park and Alex Alderton, Roberto Amato, Sonia Goncalves, Ewan Harrison, David K. Jackson, Ian Johnston, Dominic Kwiatkowski, Cordelia Langford, John Sillitoe on behalf of the Wellcome Sanger Institute COVID-19 Surveillance Team
EPI_ISL_703679	Department of Pathology, University of Cambridge	COVID-19 Genomics UK (COG-UK) Consortium	Aminu S. Jahun, Yasmin Chaudhry, Grant Hall, Iliana Georgana, Myra Hosmillo, Martin D. Curran, Malte Pinckert, Surendra Parmar, Ian Goodfellow
EPI_ISL_703680, EPI_ISL_703681, EPI_ISL_703683, EPI_ISL_703686, EPI_ISL_703688, EPI_ISL_703689, EPI_ISL_703691, EPI_ISL_703692	Lighthouse Lab in Alderley Park	Wellcome Sanger Institute for the COVID-19 Genomics UK (COG-UK) Consortium	Jacquelyn Wynn, Mairead Hyland, The Lighthouse Lab in Alderley Park and Alex Alderton, Roberto Amato, Sonia Goncalves, Ewan Harrison, David K. Jackson, Ian Johnston, Dominic Kwiatkowski, Cordelia Langford, John Sillitoe on behalf of the Wellcome Sanger Institute COVID-19 Surveillance Team
EPI_ISL_703693	Department of Pathology, University of Cambridge	COVID-19 Genomics UK (COG-UK) Consortium	Aminu S. Jahun, Yasmin Chaudhry, Grant Hall, Iliana Georgana, Myra Hosmillo, Martin D. Curran, Malte Pinckert, Surendra Parmar, Ian Goodfellow
EPI_ISL_703696	Lighthouse Lab in Alderley Park	Wellcome Sanger Institute for the COVID-19 Genomics UK	Jacquelyn Wynn, Mairead Hyland, The Lighthouse Lab in Alderley Park and Alex Alderton, Roberto Amato, Sonia Goncalves, Ewan Harrison, David K.

		(COG-UK) Consortium	Jackson, Ian Johnston, Dominic Kwiatkowski, Cordelia Langford, John Sillitoe on behalf of the Wellcome Sanger Institute COVID-19 Surveillance Team
EPI_ISL_703697	Lighthouse Lab in Glasgow	Wellcome Sanger Institute for the COVID-19 Genomics UK (COG-UK) Consortium	Harper VanSteenhouse, Yumi Kasai, David Gray, Carol Clugston, Anna Dominiczak and Alex Alderton, Roberto Amato, Sonia Goncalves, Ewan Harrison, David K. Jackson, Ian Johnston, Dominic Kwiatkowski, Cordelia Langford, John Sillitoe on behalf of the Wellcome Sanger Institute COVID-19 Surveillance Team
EPI_ISL_703699, EPI_ISL_703702, EPI_ISL_703705, EPI_ISL_703707	Lighthouse Lab in Alderley Park	Wellcome Sanger Institute for the COVID-19 Genomics UK (COG-UK) Consortium	Jacquelyn Wynn, Mairead Hyland, The Lighthouse Lab in Alderley Park and Alex Alderton, Roberto Amato, Sonia Goncalves, Ewan Harrison, David K. Jackson, Ian Johnston, Dominic Kwiatkowski, Cordelia Langford, John Sillitoe on behalf of the Wellcome Sanger Institute COVID-19 Surveillance Team
EPI_ISL_703708, EPI_ISL_703709, EPI_ISL_703710, EPI_ISL_703713, EPI_ISL_703716	Department of Pathology, University of Cambridge	COVID-19 Genomics UK (COG-UK) Consortium	Aminu S. Jahun, Yasmin Chaudhry, Grant Hall, Iliana Georgana, Myra Hosmillo, Martin D. Curran, Malte Pinckert, Surendra Parmar, Ian Goodfellow
EPI_ISL_703723	Virology Department, Sheffield Teaching Hospitals NHS Foundation Trust/Department of Infection, Immunity and Cardiovascular Disease, The Medical School, University of Sheffield	COVID-19 Genomics UK (COG-UK) Consortium	Thushan de Silva, Matthew Parker, Nikki Smith, Adri Angyal, Rebecca Brown, Luke Green, Rachel Tucker, Paul Parsons, Danielle Groves, Katie Johnson, Laura Carrilero, Alex Keeley, Dave Partridge, Matthew Wyles, Benjamin Lindsey, Mehmet Yavuz, Mohammad Raza, Cariad Evans
EPI_ISL_703724	Department of Pathology, University of Cambridge	COVID-19 Genomics UK (COG-UK) Consortium	Aminu S. Jahun, Yasmin Chaudhry, Grant Hall, Iliana Georgana, Myra Hosmillo, Martin D. Curran, Malte Pinckert, Surendra Parmar, Ian Goodfellow
EPI_ISL_703738	Liverpool Clinical Laboratories	COVID-19 Genomics UK (COG-UK) Consortium	Sam Haldenby, Anita Lucaci, Steve Paterson, Julian Hiscox, Alistair Darby, M Almsaud, A Alrezaihi, Muhannad Alruwaili, Stuart D Armstrong, Jones Benjamin, Eleanor G Bentley, Anu Chawla, Jordan J Clark, Angela Cowell, Richard Eccles, Isabel García-Dorival, Matthew Gemmell, Alessandro Gerada, PKF Gilmore, Richard Gregory, Ximeng Han, Catherine Hartley, Margaret Hughes, Miren Iturriza-Gomara, James Johnson, L Luu, Jenifer Manson, Charlotte Nelson, Elaine O'Toole, Cassie Olateju, Rebekah Penrice-Randal , Lucille Rainbow, N.P Randle, Trevor Ian Robinson, Parul Sharma, Ghada T Shawli, James P Stewart, Neil Swainston, Ecaterina Vamos, Joanne Watts, Mark Whitehead
EPI_ISL_703741, EPI_ISL_703742	Department of Pathology, University of Cambridge	COVID-19 Genomics UK (COG-UK) Consortium	Aminu S. Jahun, Yasmin Chaudhry, Grant Hall, Iliana Georgana, Myra Hosmillo, Martin D. Curran, Malte Pinckert, Surendra Parmar, Ian Goodfellow
EPI_ISL_703743	West of Scotland Specialist Virology Centre, NHSGGC / MRC-University of Glasgow Centre for Virus Research	COVID-19 Genomics UK (COG-UK) Consortium	Ana da Silva Filipe, Natasha Johnson, Kathy Smollett, Daniel Mair, Stephen Carmichael, Alice Broos, Lily Tong, Jenna Nichols, Kyriaki Nomikou; Sarah McDonald; Richard Orton, Joseph Hughes, Sreenu Vattipally, David L Robertson; Alasdair MacLean, Rory Gunson; Sharif Shaaban, Matthew Holden; Rachel Blacow, Guy Mollett, Kathy Li, James Shepherd, Antonia Ho, Emma Thomson
EPI_ISL_703747	Department of Pathology, University of Cambridge	COVID-19 Genomics UK (COG-UK) Consortium	Aminu S. Jahun, Yasmin Chaudhry, Grant Hall, Iliana Georgana, Myra Hosmillo, Martin D. Curran, Malte Pinckert, Surendra Parmar, Ian Goodfellow
EPI_ISL_703750, EPI_ISL_703751, EPI_ISL_703752	West of Scotland Specialist Virology Centre, NHSGGC / MRC-University of Glasgow Centre for Virus Research	COVID-19 Genomics UK (COG-UK) Consortium	Ana da Silva Filipe, Natasha Johnson, Kathy Smollett, Daniel Mair, Stephen Carmichael, Alice Broos, Lily Tong, Jenna Nichols, Kyriaki Nomikou; Sarah McDonald; Richard Orton, Joseph Hughes, Sreenu Vattipally, David L Robertson; Alasdair MacLean, Rory Gunson; Sharif Shaaban, Matthew Holden; Rachel Blacow, Guy Mollett, Kathy Li, James Shepherd, Antonia Ho, Emma Thomson
EPI_ISL_703759, EPI_ISL_703762	Liverpool Clinical Laboratories	COVID-19 Genomics UK (COG-UK) Consortium	Sam Haldenby, Anita Lucaci, Steve Paterson, Julian Hiscox, Alistair Darby, M Almsaud, A Alrezaihi, Muhannad Alruwaili, Stuart D Armstrong, Jones Benjamin, Eleanor G Bentley, Anu Chawla, Jordan J Clark, Angela Cowell, Richard Eccles, Isabel García-Dorival, Matthew Gemmell, Alessandro Gerada, PKF Gilmore, Richard Gregory, Ximeng Han, Catherine Hartley, Margaret Hughes, Miren Iturriza-Gomara, James Johnson, L Luu, Jenifer Manson, Charlotte Nelson, Elaine O'Toole, Cassie Olateju, Rebekah Penrice-Randal , Lucille Rainbow, N.P Randle, Trevor Ian Robinson, Parul Sharma, Ghada T Shawli, James P Stewart, Neil Swainston, Ecaterina Vamos, Joanne Watts, Mark Whitehead
EPI_ISL_703769	Quadram Institute Bioscience	COVID-19 Genomics UK (COG-UK) Consortium	Dave J. Baker, Gemma L. Kay, Alp Aydin, Thanh Le-Viet, Steven Rudder, Ana P. Tedim, Anastasia Kolyva, Maria Diaz, Leonardo de Oliveira Martins, Nabil-Fareed Alikhan, Lizzie Meadows, Rachael Stanley, Ngozi Elumogo, Muhammed Yasir, Nicholas M. Thomson, Alexander J Trotter, Rachel Gilroy, Samuel Bloomfield, Claire Stuart, Andrew Bell, Reenesh Prakash, Samir Dervisevic, Alison E. Mather, John Wain, Mark Webber, Andrew J. Page, Justin O'Grady
EPI_ISL_703773	West of Scotland Specialist Virology Centre, NHSGGC / MRC-University of Glasgow Centre for Virus Research	COVID-19 Genomics UK (COG-UK) Consortium	Ana da Silva Filipe, Natasha Johnson, Kathy Smollett, Daniel Mair, Stephen Carmichael, Alice Broos, Lily Tong, Jenna Nichols, Kyriaki Nomikou; Sarah McDonald; Richard Orton, Joseph Hughes, Sreenu Vattipally, David L Robertson; Alasdair MacLean, Rory Gunson; Sharif Shaaban, Matthew Holden; Rachel Blacow, Guy Mollett, Kathy Li, James Shepherd, Antonia Ho, Emma Thomson
EPI_ISL_703777, EPI_ISL_703778, EPI_ISL_703780, EPI_ISL_703781, EPI_ISL_703783, EPI_ISL_703785, EPI_ISL_703788, EPI_ISL_703791, EPI_ISL_703793, EPI_ISL_703795, EPI_ISL_703796, EPI_ISL_703798, EPI_ISL_703803, EPI_ISL_703804, EPI_ISL_703809, EPI_ISL_703812			
see above	Lighthouse Lab in Alderley Park	Wellcome Sanger Institute for the COVID-19 Genomics UK (COG-UK) Consortium	Jacquelyn Wynn, Mairead Hyland, The Lighthouse Lab in Alderley Park and Alex Alderton, Roberto Amato, Sonia Goncalves, Ewan Harrison, David K. Jackson, Ian Johnston, Dominic Kwiatkowski, Cordelia Langford, John Sillitoe on behalf of the Wellcome Sanger Institute COVID-19 Surveillance Team
EPI_ISL_703813	Virology Department, Royal Infirmary of Edinburgh, NHS Lothian / School of Biological Sciences, University of Edinburgh / Institute of Genetics and Molecular Medicine, University of Edinburgh	COVID-19 Genomics UK (COG-UK) Consortium	McHugh M, Dewar R, Rooke S, Gallagher M, Balcaza C, O'Toole Á, Scher E, Hill V, McCrone JT, Colquhoun R, Yu X, Jackson B, Rambaut A, Williams TC, Templeton K
EPI_ISL_703814, EPI_ISL_703817	Lighthouse Lab in Alderley Park	Wellcome Sanger Institute for the COVID-19 Genomics UK (COG-UK) Consortium	Jacquelyn Wynn, Mairead Hyland, The Lighthouse Lab in Alderley Park and Alex Alderton, Roberto Amato, Sonia Goncalves, Ewan Harrison, David K. Jackson, Ian Johnston, Dominic Kwiatkowski, Cordelia Langford, John Sillitoe on behalf of the Wellcome Sanger Institute COVID-19 Surveillance Team
EPI_ISL_703821, EPI_ISL_703823	Liverpool Clinical Laboratories	COVID-19 Genomics UK (COG-UK) Consortium	Sam Haldenby, Anita Lucaci, Steve Paterson, Julian Hiscox, Alistair Darby, M Almsaud, A Alrezaihi, Muhannad Alruwaili, Stuart D Armstrong, Jones Benjamin, Eleanor G Bentley, Anu Chawla, Jordan J Clark, Angela Cowell, Richard Eccles, Isabel García-Dorival, Matthew Gemmell, Alessandro Gerada, PKF Gilmore, Richard Gregory, Ximeng Han, Catherine Hartley, Margaret Hughes, Miren Iturriza-Gomara, James Johnson, L Luu, Jenifer Manson, Charlotte Nelson, Elaine O'Toole, Cassie Olateju, Rebekah Penrice-Randal , Lucille Rainbow, N.P Randle, Trevor Ian Robinson, Parul Sharma, Ghada T Shawli, James P Stewart, Neil Swainston, Ecaterina Vamos, Joanne Watts, Mark Whitehead
EPI_ISL_703842	West of Scotland Specialist Virology Centre, NHSGGC / MRC-University of Glasgow Centre for Virus Research	COVID-19 Genomics UK (COG-UK) Consortium	Ana da Silva Filipe, Natasha Johnson, Kathy Smollett, Daniel Mair, Stephen Carmichael, Alice Broos, Lily Tong, Jenna Nichols, Kyriaki Nomikou; Sarah McDonald; Richard Orton, Joseph Hughes, Sreenu Vattipally, David L Robertson; Alasdair MacLean, Rory Gunson; Sharif Shaaban, Matthew Holden; Rachel Blacow, Guy Mollett, Kathy Li, James Shepherd, Antonia Ho, Emma Thomson
EPI_ISL_703876, EPI_ISL_703913, EPI_ISL_704006	Liverpool Clinical Laboratories	COVID-19 Genomics UK (COG-UK) Consortium	Sam Haldenby, Anita Lucaci, Steve Paterson, Julian Hiscox, Alistair Darby, M Almsaud, A Alrezaihi, Muhannad Alruwaili, Stuart D Armstrong, Jones Benjamin, Eleanor G Bentley, Anu Chawla, Jordan J Clark, Angela Cowell, Richard Eccles, Isabel García-Dorival, Matthew Gemmell, Alessandro Gerada, PKF Gilmore, Richard Gregory, Ximeng Han, Catherine Hartley, Margaret Hughes, Miren Iturriza-Gomara, James Johnson, L Luu, Jenifer Manson, Charlotte Nelson, Elaine O'Toole, Cassie Olateju, Rebekah Penrice-Randal , Lucille Rainbow, N.P Randle, Trevor Ian Robinson, Parul Sharma, Ghada T Shawli, James P Stewart, Neil Swainston, Ecaterina Vamos, Joanne Watts, Mark Whitehead
EPI_ISL_704060	Department of Pathology, University of Cambridge	COVID-19 Genomics UK (COG-UK) Consortium	Aminu S. Jahun, Yasmin Chaudhry, Grant Hall, Iliana Georgana, Myra Hosmillo, Martin D. Curran, Malte Pinckert, Surendra Parmar, Ian Goodfellow
EPI_ISL_704100	Quadram Institute Bioscience	COVID-19 Genomics UK (COG-UK) Consortium	Dave J. Baker, Gemma L. Kay, Alp Aydin, Thanh Le-Viet, Steven Rudder, Ana P. Tedim, Anastasia Kolyva, Maria Diaz, Leonardo de Oliveira Martins, Nabil-Fareed Alikhan, Lizzie Meadows, Rachael Stanley, Ngozi Elumogo, Muhammed Yasir, Nicholas M. Thomson, Alexander J Trotter, Rachel Gilroy, Samuel Bloomfield, Claire Stuart, Andrew Bell, Reenesh Prakash, Samir Dervisevic, Alison E. Mather, John Wain, Mark Webber, Andrew J. Page, Justin O'Grady
EPI_ISL_704102	University College London Hospital	COVID-19 Genomics UK (COG-UK) Consortium	Judith Heaney, Matthew Byott, Catherine Houlihan, Dan Frampton, Stuart Kirk, Moira Spyer and Eleni Nastouli
EPI_ISL_704115	Department of Pathology, University of Cambridge	COVID-19 Genomics UK (COG-UK) Consortium	Aminu S. Jahun, Yasmin Chaudhry, Grant Hall, Iliana Georgana, Myra Hosmillo, Martin D. Curran, Malte Pinckert, Surendra Parmar, Ian Goodfellow
EPI_ISL_704131, EPI_ISL_704137, EPI_ISL_704153, EPI_ISL_704156	Liverpool Clinical Laboratories	COVID-19 Genomics UK (COG-UK) Consortium	Sam Haldenby, Anita Lucaci, Steve Paterson, Julian Hiscox, Alistair Darby, M Almsaud, A Alrezaihi, Muhannad Alruwaili, Stuart D Armstrong, Jones Benjamin, Eleanor G Bentley, Anu Chawla, Jordan J Clark, Angela Cowell, Richard Eccles, Isabel García-Dorival, Matthew Gemmell, Alessandro Gerada, PKF Gilmore, Richard Gregory, Ximeng Han, Catherine Hartley, Margaret Hughes, Miren Iturriza-Gomara, James Johnson, L Luu, Jenifer Manson, Charlotte Nelson, Elaine O'Toole, Cassie Olateju, Rebekah Penrice-Randal , Lucille Rainbow, N.P Randle, Trevor Ian Robinson, Parul Sharma, Ghada T Shawli, James P Stewart, Neil Swainston, Ecaterina Vamos, Joanne Watts, Mark Whitehead
EPI_ISL_704190	Virology Department, Sheffield Teaching Hospitals NHS	COVID-19 Genomics UK (COG-UK) Consortium	Thushan de Silva, Matthew Parker, Nikki Smith, Adri Angyal, Rebecca Brown, Luke Green, Rachel Tucker, Paul Parsons, Danielle Groves, Katie Johnson,

	Foundation Trust/Department of Infection, Immunity and Cardiovascular Disease, The Medical School, University of Sheffield		Laura Carrilero, Alex Keeley, Dave Partridge, Matthew Wyles, Benjamin Lindsey, Mehmet Yavuz, Mohammad Raza, Cariad Evans
EPI_ISL_704198	Northumbria University / South Tees Hospitals NHS Foundation Trust / North Cumbria Integrated Care NHS Foundation Trust / North Tees and Hartlepool NHS Foundation Trust / Newcastle Hospitals NHS Foundation Trust	COVID-19 Genomics UK (COG-UK) Consortium	Darren L Smith,Andrew Nelson,Matthew Bashton,Greg R Young,Joshua Loh,John Allan,Mohammad A Tariq,Giles S Holt,Gary Black,Wen C Yew,Lynn Dover,Paul Baker,Steve Liggett,Sarah Essex,Jane Greenaway,Debra Padgett,Clive Graham,Garren Scott,Edward Barton,Emma Swindells,Brendan Payne,Jennifer Collins,Yusri Taha,Gary Eltringham
EPI_ISL_704203	Department of Pathology, University of Cambridge	COVID-19 Genomics UK (COG-UK) Consortium	Aminu S. Jahun, Yasmin Chaudhry, Grant Hall, Iliana Georgana, Myra Hosmillo, Martin D. Curran, Malte Pinckert, Surendra Parmar, Ian Goodfellow
EPI_ISL_704228	West of Scotland Specialist Virology Centre, NHSGGC / MRC-University of Glasgow Centre for Virus Research	COVID-19 Genomics UK (COG-UK) Consortium	Ana da Silva Filipe, Natasha Johnson, Kathy Smollett, Daniel Mair, Stephen Carmichael, Alice Broos, Lily Tong, Jenna Nichols, Kyriaki Nomikou; Sarah McDonald; Richard Orton, Joseph Hughes, Sreenu Vattipally, David L Robertson; Alasdair MacLean, Rory Gunson; Sharif Shaaban, Matthew Holden; Rachel Blacow, Guy Mollett, Kathy Li, James Shepherd, Antonia Ho, Emma Thomson
EPI_ISL_704247, EPI_ISL_704250, EPI_ISL_704252, EPI_ISL_704255, EPI_ISL_704257, EPI_ISL_704269, EPI_ISL_704289, EPI_ISL_704291, EPI_ISL_704294, EPI_ISL_704297, EPI_ISL_704299, EPI_ISL_704302, EPI_ISL_704305, EPI_ISL_704307, EPI_ISL_704309, EPI_ISL_704328, EPI_ISL_704331, EPI_ISL_704333, EPI_ISL_704336, EPI_ISL_704339, EPI_ISL_704341, EPI_ISL_704344, EPI_ISL_704347, EPI_ISL_704350, EPI_ISL_704353, EPI_ISL_704356, EPI_ISL_704359, EPI_ISL_704361, EPI_ISL_704364, EPI_ISL_704366, EPI_ISL_704369, EPI_ISL_704374, EPI_ISL_704377, EPI_ISL_704379, EPI_ISL_704384			
see above	Department of Pathology, University of Cambridge	COVID-19 Genomics UK (COG-UK) Consortium	Aminu S. Jahun, Yasmin Chaudhry, Grant Hall, Iliana Georgana, Myra Hosmillo, Martin D. Curran, Malte Pinckert, Surendra Parmar, Ian Goodfellow
EPI_ISL_704422, EPI_ISL_704425	Northumbria University / South Tees Hospitals NHS Foundation Trust / North Cumbria Integrated Care NHS Foundation Trust / North Tees and Hartlepool NHS Foundation Trust / Newcastle Hospitals NHS Foundation Trust	COVID-19 Genomics UK (COG-UK) Consortium	Darren L Smith,Andrew Nelson,Matthew Bashton,Greg R Young,Joshua Loh,John Allan,Mohammad A Tariq,Giles S Holt,Gary Black,Wen C Yew,Lynn Dover,Paul Baker,Steve Liggett,Sarah Essex,Jane Greenaway,Debra Padgett,Clive Graham,Garren Scott,Edward Barton,Emma Swindells,Brendan Payne,Jennifer Collins,Yusri Taha,Gary Eltringham
EPI_ISL_704478, EPI_ISL_704481, EPI_ISL_704484, EPI_ISL_704486, EPI_ISL_704489, EPI_ISL_704492, EPI_ISL_704494, EPI_ISL_704497, EPI_ISL_704499, EPI_ISL_704510, EPI_ISL_704513, EPI_ISL_704516, EPI_ISL_704518, EPI_ISL_704521, EPI_ISL_704524, EPI_ISL_704526, EPI_ISL_704529, EPI_ISL_704532, EPI_ISL_704535, EPI_ISL_704537, EPI_ISL_704540, EPI_ISL_704543, EPI_ISL_704545, EPI_ISL_704548, EPI_ISL_704551, EPI_ISL_704554, EPI_ISL_704557			
see above	Department of Pathology, University of Cambridge	COVID-19 Genomics UK (COG-UK) Consortium	Aminu S. Jahun, Yasmin Chaudhry, Grant Hall, Iliana Georgana, Myra Hosmillo, Martin D. Curran, Malte Pinckert, Surendra Parmar, Ian Goodfellow
EPI_ISL_704562	Liverpool Clinical Laboratories	COVID-19 Genomics UK (COG-UK) Consortium	Sam Haldenby, Anita Lucaci, Steve Paterson, Julian Hiscox, Alistair Darby, M Almsaud, A Alrezaihi, Muhannad Alruwaili, Stuart D Armstrong, Jones Benjamin, Eleanor G Bentley, Anu Chawla, Jordan J Clark, Angela Cowell, Richard Eccles, Isabel García-Dorival, Matthew Gemmell, Alessandro Gerada, PKF Gilmore, Richard Gregory, Ximeng Han, Catherine Hartley, Margaret Hughes, Miren Iturriza-Gomara, James Johnson, L Luu, Jenifer Manson, Charlotte Nelson, Elaine O'Toole, Cassie Olateju, Rebekah Penrice-Randal , Lucille Rainbow, N.P Randle, Trevor Ian Robinson, Parul Sharma, Ghada T Shawli, James P Stewart, Neil Swainston, Ecaterina Vamos, Joanne Watts, Mark Whitehead
EPI_ISL_704575, EPI_ISL_704576	Northumbria University / South Tees Hospitals NHS Foundation Trust / North Cumbria Integrated Care NHS Foundation Trust / North Tees and Hartlepool NHS Foundation Trust / Newcastle Hospitals NHS Foundation Trust	COVID-19 Genomics UK (COG-UK) Consortium	Darren L Smith,Andrew Nelson,Matthew Bashton,Greg R Young,Joshua Loh,John Allan,Mohammad A Tariq,Giles S Holt,Gary Black,Wen C Yew,Lynn Dover,Paul Baker,Steve Liggett,Sarah Essex,Jane Greenaway,Debra Padgett,Clive Graham,Garren Scott,Edward Barton,Emma Swindells,Brendan Payne,Jennifer Collins,Yusri Taha,Gary Eltringham
EPI_ISL_704580, EPI_ISL_704598, EPI_ISL_704599	Quadram Institute Bioscience	COVID-19 Genomics UK (COG-UK) Consortium	Dave J. Baker, Gemma L. Kay, Alp Aydin, Thanh Le-Viet, Steven Rudder, Ana P. Tedim, Anastasia Kolyva, Maria Diaz, Leonardo de Oliveira Martins, Nabil-Fareed Alikhan, Lizzie Meadows, Rachael Stanley, Ngozi Elumogo, Muhammed Yasir, Nicholas M. Thomson, Alexander J Trotter, Rachel Gilroy, Samuel Bloomfield, Claire Stuart, Andrew Bell, Reenesh Prakash, Samir Dervisevic, Alison E. Mather, John Wain, Mark Webber, Andrew J. Page, Justin O'Grady
EPI_ISL_704600, EPI_ISL_704601, EPI_ISL_704602, EPI_ISL_704603, EPI_ISL_704604	Virology Department, Sheffield Teaching Hospitals NHS Foundation Trust/Department of Infection, Immunity and Cardiovascular Disease, The Medical School, University of Sheffield	COVID-19 Genomics UK (COG-UK) Consortium	Thushan de Silva, Matthew Parker, Nikki Smith, Adri Angyal, Rebecca Brown, Luke Green, Rachel Tucker, Paul Parsons, Danielle Groves, Katie Johnson, Laura Carrilero, Alex Keeley, Dave Partridge, Matthew Wyles, Benjamin Lindsey, Mehmet Yavuz, Mohammad Raza, Cariad Evans
EPI_ISL_704606, EPI_ISL_704617, EPI_ISL_704618	Department of Pathology, University of Cambridge	COVID-19 Genomics UK (COG-UK) Consortium	Aminu S. Jahun, Yasmin Chaudhry, Grant Hall, Iliana Georgana, Myra Hosmillo, Martin D. Curran, Malte Pinckert, Surendra Parmar, Ian Goodfellow
EPI_ISL_704619, EPI_ISL_704620, EPI_ISL_704621, EPI_ISL_704622, EPI_ISL_704623, EPI_ISL_704624, EPI_ISL_704625, EPI_ISL_704626, EPI_ISL_704627, EPI_ISL_704629, EPI_ISL_704630, EPI_ISL_704631, EPI_ISL_704632, EPI_ISL_704633, EPI_ISL_704634, EPI_ISL_704635			
see above	Quadram Institute Bioscience	COVID-19 Genomics UK (COG-UK) Consortium	Dave J. Baker, Gemma L. Kay, Alp Aydin, Thanh Le-Viet, Steven Rudder, Ana P. Tedim, Anastasia Kolyva, Maria Diaz, Leonardo de Oliveira Martins, Nabil-Fareed Alikhan, Lizzie Meadows, Rachael Stanley, Ngozi Elumogo, Muhammed Yasir, Nicholas M. Thomson, Alexander J Trotter, Rachel Gilroy, Samuel Bloomfield, Claire Stuart, Andrew Bell, Reenesh Prakash, Samir Dervisevic, Alison E. Mather, John Wain, Mark Webber, Andrew J. Page, Justin O'Grady
EPI_ISL_704636, EPI_ISL_704637, EPI_ISL_704638, EPI_ISL_704639	Virology Department, Sheffield Teaching Hospitals NHS Foundation Trust/Department of Infection, Immunity and Cardiovascular Disease, The Medical School, University of Sheffield	COVID-19 Genomics UK (COG-UK) Consortium	Thushan de Silva, Matthew Parker, Nikki Smith, Adri Angyal, Rebecca Brown, Luke Green, Rachel Tucker, Paul Parsons, Danielle Groves, Katie Johnson, Laura Carrilero, Alex Keeley, Dave Partridge, Matthew Wyles, Benjamin Lindsey, Mehmet Yavuz, Mohammad Raza, Cariad Evans
EPI_ISL_704640, EPI_ISL_704641, EPI_ISL_704643, EPI_ISL_704646, EPI_ISL_704648, EPI_ISL_704651, EPI_ISL_704654, EPI_ISL_704656, EPI_ISL_704659, EPI_ISL_704662, EPI_ISL_704664			
see above	Department of Pathology, University of Cambridge	COVID-19 Genomics UK (COG-UK) Consortium	Aminu S. Jahun, Yasmin Chaudhry, Grant Hall, Iliana Georgana, Myra Hosmillo, Martin D. Curran, Malte Pinckert, Surendra Parmar, Ian Goodfellow
EPI_ISL_704688, EPI_ISL_704689, EPI_ISL_704691, EPI_ISL_704693, EPI_ISL_704694, EPI_ISL_704696, EPI_ISL_704697, EPI_ISL_704699, EPI_ISL_704700, EPI_ISL_704702, EPI_ISL_704704, EPI_ISL_704705, EPI_ISL_704707, EPI_ISL_704708, EPI_ISL_704710, EPI_ISL_704712, EPI_ISL_704713, EPI_ISL_704715, EPI_ISL_704717, EPI_ISL_704718, EPI_ISL_704720, EPI_ISL_704721, EPI_ISL_704723, EPI_ISL_704724, EPI_ISL_704726, EPI_ISL_704727, EPI_ISL_704729, EPI_ISL_704730, EPI_ISL_704732, EPI_ISL_704733, EPI_ISL_704735, EPI_ISL_704737, EPI_ISL_704738, EPI_ISL_704740, EPI_ISL_704742, EPI_ISL_704743, EPI_ISL_704745, EPI_ISL_704747, EPI_ISL_704748, EPI_ISL_704751, EPI_ISL_704753, EPI_ISL_704755, EPI_ISL_704756, EPI_ISL_704758, EPI_ISL_704760, EPI_ISL_704761			
see above	Lighthouse Lab in Cambridge	Wellcome Sanger Institute for the COVID-19 Genomics UK (COG-UK) Consortium	Rob Howes, The Lighthouse Lab in Cambridge and Alex Alderton, Roberto Amato, Sonia Goncalves, Ewan Harrison, David K. Jackson, Ian Johnston, Dominic Kwiatkowski, Cordelia Langford, John Sillitoe on behalf of the Wellcome Sanger Institute COVID-19 Surveillance Team
EPI_ISL_704762	Quadram Institute Bioscience	COVID-19 Genomics UK (COG-UK) Consortium	Dave J. Baker, Gemma L. Kay, Alp Aydin, Thanh Le-Viet, Steven Rudder, Ana P. Tedim, Anastasia Kolyva, Maria Diaz, Leonardo de Oliveira Martins, Nabil-Fareed Alikhan, Lizzie Meadows, Rachael Stanley, Ngozi Elumogo, Muhammed Yasir, Nicholas M. Thomson, Alexander J Trotter, Rachel Gilroy, Samuel Bloomfield, Claire Stuart, Andrew Bell, Reenesh Prakash, Samir Dervisevic, Alison E. Mather, John Wain, Mark Webber, Andrew J. Page, Justin O'Grady
EPI_ISL_704763, EPI_ISL_704764	Lighthouse Lab in Cambridge	Wellcome Sanger Institute for the COVID-19 Genomics UK (COG-UK) Consortium	Rob Howes, The Lighthouse Lab in Cambridge and Alex Alderton, Roberto Amato, Sonia Goncalves, Ewan Harrison, David K. Jackson, Ian Johnston, Dominic Kwiatkowski, Cordelia Langford, John Sillitoe on behalf of the Wellcome Sanger Institute COVID-19 Surveillance Team
EPI_ISL_704765	Quadram Institute Bioscience	COVID-19 Genomics UK (COG-UK) Consortium	Dave J. Baker, Gemma L. Kay, Alp Aydin, Thanh Le-Viet, Steven Rudder, Ana P. Tedim, Anastasia Kolyva, Maria Diaz, Leonardo de Oliveira Martins, Nabil-Fareed Alikhan, Lizzie Meadows, Rachael Stanley, Ngozi Elumogo, Muhammed Yasir, Nicholas M. Thomson, Alexander J Trotter, Rachel Gilroy, Samuel Bloomfield, Claire Stuart, Andrew Bell, Reenesh Prakash, Samir Dervisevic, Alison E. Mather, John Wain, Mark Webber, Andrew J. Page, Justin O'Grady
EPI_ISL_704766, EPI_ISL_704767	Lighthouse Lab in Cambridge	Wellcome Sanger Institute for the COVID-19 Genomics UK (COG-UK) Consortium	Rob Howes, The Lighthouse Lab in Cambridge and Alex Alderton, Roberto Amato, Sonia Goncalves, Ewan Harrison, David K. Jackson, Ian Johnston, Dominic Kwiatkowski, Cordelia Langford, John Sillitoe on behalf of the Wellcome Sanger Institute COVID-19 Surveillance Team
EPI_ISL_704768	Quadram Institute Bioscience	COVID-19 Genomics UK (COG-UK) Consortium	Dave J. Baker, Gemma L. Kay, Alp Aydin, Thanh Le-Viet, Steven Rudder, Ana P. Tedim, Anastasia Kolyva, Maria Diaz, Leonardo de Oliveira Martins, Nabil-Fareed Alikhan, Lizzie Meadows, Rachael Stanley, Ngozi Elumogo, Muhammed Yasir, Nicholas M. Thomson, Alexander J Trotter, Rachel Gilroy, Samuel Bloomfield, Claire Stuart, Andrew Bell, Reenesh Prakash, Samir Dervisevic, Alison E. Mather, John Wain, Mark Webber, Andrew J. Page, Justin O'Grady
EPI_ISL_704769	Lighthouse Lab in Cambridge	Wellcome Sanger Institute for the COVID-19 Genomics UK (COG-UK) Consortium	Rob Howes, The Lighthouse Lab in Cambridge and Alex Alderton, Roberto Amato, Sonia Goncalves, Ewan Harrison, David K. Jackson, Ian Johnston, Dominic Kwiatkowski, Cordelia Langford, John Sillitoe on behalf of the Wellcome Sanger Institute COVID-19 Surveillance Team

EPI_ISL_704770	Quadram Institute Bioscience	COVID-19 Genomics UK (COG-UK) Consortium	Dave J. Baker, Gemma L. Kay, Alp Aydin, Thanh Le-Viet, Steven Rudder, Ana P. Tedim, Anastasia Kolyva, Maria Diaz, Leonardo de Oliveira Martins, Nabil-Fareed Alikhan, Lizzie Meadows, Rachael Stanley, Ngozi Elumogo, Muhammed Yasir, Nicholas M. Thomson, Alexander J Trotter, Rachel Gilroy, Samuel Bloomfield, Claire Stuart, Andrew Bell, Reenesh Prakash, Samir Dervisevic, Alison E. Mather, John Wain, Mark Webber, Andrew J. Page, Justin O'Grady
EPI_ISL_704771, EPI_ISL_704773, EPI_ISL_704774, EPI_ISL_704776, EPI_ISL_704778, EPI_ISL_704779, EPI_ISL_704781, EPI_ISL_704782, EPI_ISL_704784, EPI_ISL_704785, EPI_ISL_704787, EPI_ISL_704789, EPI_ISL_704790, EPI_ISL_704792, EPI_ISL_704793, EPI_ISL_704795, EPI_ISL_704796, EPI_ISL_704798, EPI_ISL_704800, EPI_ISL_704801, EPI_ISL_704803, EPI_ISL_704805, EPI_ISL_704806, EPI_ISL_704808, EPI_ISL_704810, EPI_ISL_704811, EPI_ISL_704813, EPI_ISL_704814, EPI_ISL_704816, EPI_ISL_704818, EPI_ISL_704819			
see above	Lighthouse Lab in Cambridge	Wellcome Sanger Institute for the COVID-19 Genomics UK (COG-UK) Consortium	Rob Howes, The Lighthouse Lab in Cambridge and Alex Alderton, Roberto Amato, Sonia Goncalves, Ewan Harrison, David K. Jackson, Ian Johnston, Dominic Kwiatkowski, Cordelia Langford, John Sillitoe on behalf of the Wellcome Sanger Institute COVID-19 Surveillance Team
EPI_ISL_704820	Quadram Institute Bioscience	COVID-19 Genomics UK (COG-UK) Consortium	Dave J. Baker, Gemma L. Kay, Alp Aydin, Thanh Le-Viet, Steven Rudder, Ana P. Tedim, Anastasia Kolyva, Maria Diaz, Leonardo de Oliveira Martins, Nabil-Fareed Alikhan, Lizzie Meadows, Rachael Stanley, Ngozi Elumogo, Muhammed Yasir, Nicholas M. Thomson, Alexander J Trotter, Rachel Gilroy, Samuel Bloomfield, Claire Stuart, Andrew Bell, Reenesh Prakash, Samir Dervisevic, Alison E. Mather, John Wain, Mark Webber, Andrew J. Page, Justin O'Grady
EPI_ISL_704821, EPI_ISL_704822	Lighthouse Lab in Cambridge	Wellcome Sanger Institute for the COVID-19 Genomics UK (COG-UK) Consortium	Rob Howes, The Lighthouse Lab in Cambridge and Alex Alderton, Roberto Amato, Sonia Goncalves, Ewan Harrison, David K. Jackson, Ian Johnston, Dominic Kwiatkowski, Cordelia Langford, John Sillitoe on behalf of the Wellcome Sanger Institute COVID-19 Surveillance Team
EPI_ISL_704823	Quadram Institute Bioscience	COVID-19 Genomics UK (COG-UK) Consortium	Dave J. Baker, Gemma L. Kay, Alp Aydin, Thanh Le-Viet, Steven Rudder, Ana P. Tedim, Anastasia Kolyva, Maria Diaz, Leonardo de Oliveira Martins, Nabil-Fareed Alikhan, Lizzie Meadows, Rachael Stanley, Ngozi Elumogo, Muhammed Yasir, Nicholas M. Thomson, Alexander J Trotter, Rachel Gilroy, Samuel Bloomfield, Claire Stuart, Andrew Bell, Reenesh Prakash, Samir Dervisevic, Alison E. Mather, John Wain, Mark Webber, Andrew J. Page, Justin O'Grady
EPI_ISL_704824, EPI_ISL_704825	Lighthouse Lab in Cambridge	Wellcome Sanger Institute for the COVID-19 Genomics UK (COG-UK) Consortium	Rob Howes, The Lighthouse Lab in Cambridge and Alex Alderton, Roberto Amato, Sonia Goncalves, Ewan Harrison, David K. Jackson, Ian Johnston, Dominic Kwiatkowski, Cordelia Langford, John Sillitoe on behalf of the Wellcome Sanger Institute COVID-19 Surveillance Team
EPI_ISL_704826	Quadram Institute Bioscience	COVID-19 Genomics UK (COG-UK) Consortium	Dave J. Baker, Gemma L. Kay, Alp Aydin, Thanh Le-Viet, Steven Rudder, Ana P. Tedim, Anastasia Kolyva, Maria Diaz, Leonardo de Oliveira Martins, Nabil-Fareed Alikhan, Lizzie Meadows, Rachael Stanley, Ngozi Elumogo, Muhammed Yasir, Nicholas M. Thomson, Alexander J Trotter, Rachel Gilroy, Samuel Bloomfield, Claire Stuart, Andrew Bell, Reenesh Prakash, Samir Dervisevic, Alison E. Mather, John Wain, Mark Webber, Andrew J. Page, Justin O'Grady
EPI_ISL_704827	Lighthouse Lab in Cambridge	Wellcome Sanger Institute for the COVID-19 Genomics UK (COG-UK) Consortium	Rob Howes, The Lighthouse Lab in Cambridge and Alex Alderton, Roberto Amato, Sonia Goncalves, Ewan Harrison, David K. Jackson, Ian Johnston, Dominic Kwiatkowski, Cordelia Langford, John Sillitoe on behalf of the Wellcome Sanger Institute COVID-19 Surveillance Team
EPI_ISL_704828	Virology Department, Sheffield Teaching Hospitals NHS Foundation Trust/Department of Infection, Immunity and Cardiovascular Disease, The Medical School, University of Sheffield	COVID-19 Genomics UK (COG-UK) Consortium	Thushan de Silva, Matthew Parker, Nikki Smith, Adri Angyal, Rebecca Brown, Luke Green, Rachel Tucker, Paul Parsons, Danielle Groves, Katie Johnson, Laura Carrilero, Alex Keeley, Dave Partridge, Matthew Wyles, Benjamin Lindsey, Mehmet Yavuz, Mohammad Raza, Cariad Evans
EPI_ISL_704829, EPI_ISL_704830	Lighthouse Lab in Cambridge	Wellcome Sanger Institute for the COVID-19 Genomics UK (COG-UK) Consortium	Rob Howes, The Lighthouse Lab in Cambridge and Alex Alderton, Roberto Amato, Sonia Goncalves, Ewan Harrison, David K. Jackson, Ian Johnston, Dominic Kwiatkowski, Cordelia Langford, John Sillitoe on behalf of the Wellcome Sanger Institute COVID-19 Surveillance Team
EPI_ISL_704831	Quadram Institute Bioscience	COVID-19 Genomics UK (COG-UK) Consortium	Dave J. Baker, Gemma L. Kay, Alp Aydin, Thanh Le-Viet, Steven Rudder, Ana P. Tedim, Anastasia Kolyva, Maria Diaz, Leonardo de Oliveira Martins, Nabil-Fareed Alikhan, Lizzie Meadows, Rachael Stanley, Ngozi Elumogo, Muhammed Yasir, Nicholas M. Thomson, Alexander J Trotter, Rachel Gilroy, Samuel Bloomfield, Claire Stuart, Andrew Bell, Reenesh Prakash, Samir Dervisevic, Alison E. Mather, John Wain, Mark Webber, Andrew J. Page, Justin O'Grady
EPI_ISL_704832, EPI_ISL_704833	Lighthouse Lab in Cambridge	Wellcome Sanger Institute for the COVID-19 Genomics UK (COG-UK) Consortium	Rob Howes, The Lighthouse Lab in Cambridge and Alex Alderton, Roberto Amato, Sonia Goncalves, Ewan Harrison, David K. Jackson, Ian Johnston, Dominic Kwiatkowski, Cordelia Langford, John Sillitoe on behalf of the Wellcome Sanger Institute COVID-19 Surveillance Team
EPI_ISL_704834	Department of Pathology, University of Cambridge	COVID-19 Genomics UK (COG-UK) Consortium	Aminu S. Jahun, Yasmin Chaudhry, Grant Hall, Iliana Georgana, Myra Hosmillo, Martin D. Curran, Malte Pinckert, Surendra Parmar, Ian Goodfellow
EPI_ISL_704835, EPI_ISL_704837, EPI_ISL_704838, EPI_ISL_704840, EPI_ISL_704841	Lighthouse Lab in Cambridge	Wellcome Sanger Institute for the COVID-19 Genomics UK (COG-UK) Consortium	Rob Howes, The Lighthouse Lab in Cambridge and Alex Alderton, Roberto Amato, Sonia Goncalves, Ewan Harrison, David K. Jackson, Ian Johnston, Dominic Kwiatkowski, Cordelia Langford, John Sillitoe on behalf of the Wellcome Sanger Institute COVID-19 Surveillance Team
EPI_ISL_704842	Quadram Institute Bioscience	COVID-19 Genomics UK (COG-UK) Consortium	Dave J. Baker, Gemma L. Kay, Alp Aydin, Thanh Le-Viet, Steven Rudder, Ana P. Tedim, Anastasia Kolyva, Maria Diaz, Leonardo de Oliveira Martins, Nabil-Fareed Alikhan, Lizzie Meadows, Rachael Stanley, Ngozi Elumogo, Muhammed Yasir, Nicholas M. Thomson, Alexander J Trotter, Rachel Gilroy, Samuel Bloomfield, Claire Stuart, Andrew Bell, Reenesh Prakash, Samir Dervisevic, Alison E. Mather, John Wain, Mark Webber, Andrew J. Page, Justin O'Grady
EPI_ISL_704843, EPI_ISL_704844, EPI_ISL_704846, EPI_ISL_704848, EPI_ISL_704849, EPI_ISL_704851, EPI_ISL_704852, EPI_ISL_704854, EPI_ISL_704856, EPI_ISL_704857, EPI_ISL_704859, EPI_ISL_704860, EPI_ISL_704862, EPI_ISL_704863, EPI_ISL_704865, EPI_ISL_704868, EPI_ISL_704870, EPI_ISL_704871, EPI_ISL_704873, EPI_ISL_704875, EPI_ISL_704876, EPI_ISL_704878, EPI_ISL_704880, EPI_ISL_704881, EPI_ISL_704883, EPI_ISL_704884, EPI_ISL_704886, EPI_ISL_704888, EPI_ISL_704889, EPI_ISL_704891, EPI_ISL_704893, EPI_ISL_704894, EPI_ISL_704896, EPI_ISL_704898, EPI_ISL_704899, EPI_ISL_704901, EPI_ISL_704902			
see above	Lighthouse Lab in Cambridge	Wellcome Sanger Institute for the COVID-19 Genomics UK (COG-UK) Consortium	Rob Howes, The Lighthouse Lab in Cambridge and Alex Alderton, Roberto Amato, Sonia Goncalves, Ewan Harrison, David K. Jackson, Ian Johnston, Dominic Kwiatkowski, Cordelia Langford, John Sillitoe on behalf of the Wellcome Sanger Institute COVID-19 Surveillance Team
EPI_ISL_704903	Liverpool Clinical Laboratories	COVID-19 Genomics UK (COG-UK) Consortium	Sam Haldenby, Anita Lucaci, Steve Paterson, Julian Hiscox, Alistair Darby, M Almsaud, A Alrezaihi, Muhannad Alruwaili, Stuart D Armstrong, Jones Benjamin, Eleanor G Bentley, Anu Chawla, Jordan J Clark, Angela Cowell, Richard Eccles, Isabel García-Dorival, Matthew Gemmell, Alessandro Gerada, PKF Gilmore, Richard Gregory, Ximeng Han, Catherine Hartley, Margaret Hughes, Miren Iturriza-Gomara, James Johnson, L Luu, Jenifer Manson, Charlotte Nelson, Elaine O'Toole, Cassie Olateju, Rebekah Penrice-Randal , Lucille Rainbow, N.P Randle, Trevor Ian Robinson, Parul Sharma, Ghada T Shawli, James P Stewart, Neil Swainston, Ecaterina Vamos, Joanne Watts, Mark Whitehead
EPI_ISL_704904, EPI_ISL_704906, EPI_ISL_704907, EPI_ISL_704909, EPI_ISL_704910, EPI_ISL_704912, EPI_ISL_704913	Lighthouse Lab in Cambridge	Wellcome Sanger Institute for the COVID-19 Genomics UK (COG-UK) Consortium	Rob Howes, The Lighthouse Lab in Cambridge and Alex Alderton, Roberto Amato, Sonia Goncalves, Ewan Harrison, David K. Jackson, Ian Johnston, Dominic Kwiatkowski, Cordelia Langford, John Sillitoe on behalf of the Wellcome Sanger Institute COVID-19 Surveillance Team
EPI_ISL_704914	Quadram Institute Bioscience	COVID-19 Genomics UK (COG-UK) Consortium	Dave J. Baker, Gemma L. Kay, Alp Aydin, Thanh Le-Viet, Steven Rudder, Ana P. Tedim, Anastasia Kolyva, Maria Diaz, Leonardo de Oliveira Martins, Nabil-Fareed Alikhan, Lizzie Meadows, Rachael Stanley, Ngozi Elumogo, Muhammed Yasir, Nicholas M. Thomson, Alexander J Trotter, Rachel Gilroy, Samuel Bloomfield, Claire Stuart, Andrew Bell, Reenesh Prakash, Samir Dervisevic, Alison E. Mather, John Wain, Mark Webber, Andrew J. Page, Justin O'Grady
EPI_ISL_704915, EPI_ISL_704917, EPI_ISL_704919, EPI_ISL_704920, EPI_ISL_704922, EPI_ISL_704924, EPI_ISL_704925, EPI_ISL_704927, EPI_ISL_704928, EPI_ISL_704930, EPI_ISL_704933, EPI_ISL_704935, EPI_ISL_704936, EPI_ISL_704938, EPI_ISL_704940, EPI_ISL_704941, EPI_ISL_704943, EPI_ISL_704944, EPI_ISL_704946, EPI_ISL_704948, EPI_ISL_704949, EPI_ISL_704951, EPI_ISL_704953, EPI_ISL_704954, EPI_ISL_704956, EPI_ISL_704958, EPI_ISL_704959, EPI_ISL_704961, EPI_ISL_704962, EPI_ISL_704964, EPI_ISL_704966, EPI_ISL_704967, EPI_ISL_704969, EPI_ISL_704971, EPI_ISL_704972, EPI_ISL_704974, EPI_ISL_704975			
see above	Lighthouse Lab in Cambridge	Wellcome Sanger Institute for the COVID-19 Genomics UK (COG-UK) Consortium	Rob Howes, The Lighthouse Lab in Cambridge and Alex Alderton, Roberto Amato, Sonia Goncalves, Ewan Harrison, David K. Jackson, Ian Johnston, Dominic Kwiatkowski, Cordelia Langford, John Sillitoe on behalf of the Wellcome Sanger Institute COVID-19 Surveillance Team
EPI_ISL_704976	Northumbria University / South Tees Hospitals NHS Foundation Trust / North Cumbria Integrated Care NHS Foundation Trust / North Tees and Hartlepool NHS Foundation Trust / Newcastle Hospitals NHS Foundation Trust	COVID-19 Genomics UK (COG-UK) Consortium	Darren L Smith,Andrew Nelson,Matthew Bashton,Greg R Young,Joshua Loh,John Allan,Mohammad A Tariq,Giles S Holt,Gary Black,Wen C Yew,Lynn Dover,Paul Baker,Steve Liggett,Sarah Essex,Jane Greenaway,Debra Padgett,Clive Graham,Garren Scott,Edward Barton,Emma Swindells,Brendan Payne,Jennifer Collins,Yusri Taha,Gary Eltringham
EPI_ISL_704977, EPI_ISL_704979, EPI_ISL_704980, EPI_ISL_704982	Lighthouse Lab in Cambridge	Wellcome Sanger Institute for the COVID-19 Genomics UK (COG-UK) Consortium	Rob Howes, The Lighthouse Lab in Cambridge and Alex Alderton, Roberto Amato, Sonia Goncalves, Ewan Harrison, David K. Jackson, Ian Johnston, Dominic Kwiatkowski, Cordelia Langford, John Sillitoe on behalf of the Wellcome Sanger Institute COVID-19 Surveillance Team

EPI_ISL_704983	Liverpool Clinical Laboratories	COVID-19 Genomics UK (COG-UK) Consortium	Sam Haldenby, Anita Lucaci, Steve Paterson, Julian Hiscox, Alistair Darby, M Almsaud, A Alrezaihi, Muhannad Alruwaili, Stuart D Armstrong, Jones Benjamin, Eleanor G Bentley, Anu Chawla, Jordan J Clark, Angela Cowell, Richard Eccles, Isabel García-Dorival, Matthew Gemmell, Alessandro Gerada, PKF Gilmore, Richard Gregory, Ximeng Han, Catherine Hartley, Margaret Hughes, Miren Iturriza-Gomara, James Johnson, L Luu, Jenifer Manson, Charlotte Nelson, Elaine O'Toole, Cassie Olateju, Rebekah Penrice-Randal , Lucille Rainbow, N.P Randle, Trevor Ian Robinson, Parul Sharma, Ghada T Shawli, James P Stewart, Neil Swainston, Ecaterina Vamos, Joanne Watts, Mark Whitehead
EPI_ISL_704984, EPI_ISL_704985, EPI_ISL_704987, EPI_ISL_704988	Lighthouse Lab in Cambridge	Wellcome Sanger Institute for the COVID-19 Genomics UK (COG-UK) Consortium	Rob Howes, The Lighthouse Lab in Cambridge and Alex Alderton, Roberto Amato, Sonia Goncalves, Ewan Harrison, David K. Jackson, Ian Johnston, Dominic Kwiatkowski, Cordelia Langford, John Sillitoe on behalf of the Wellcome Sanger Institute COVID-19 Surveillance Team
EPI_ISL_704989	Liverpool Clinical Laboratories	COVID-19 Genomics UK (COG-UK) Consortium	Sam Haldenby, Anita Lucaci, Steve Paterson, Julian Hiscox, Alistair Darby, M Almsaud, A Alrezaihi, Muhannad Alruwaili, Stuart D Armstrong, Jones Benjamin, Eleanor G Bentley, Anu Chawla, Jordan J Clark, Angela Cowell, Richard Eccles, Isabel García-Dorival, Matthew Gemmell, Alessandro Gerada, PKF Gilmore, Richard Gregory, Ximeng Han, Catherine Hartley, Margaret Hughes, Miren Iturriza-Gomara, James Johnson, L Luu, Jenifer Manson, Charlotte Nelson, Elaine O'Toole, Cassie Olateju, Rebekah Penrice-Randal , Lucille Rainbow, N.P Randle, Trevor Ian Robinson, Parul Sharma, Ghada T Shawli, James P Stewart, Neil Swainston, Ecaterina Vamos, Joanne Watts, Mark Whitehead
EPI_ISL_704990, EPI_ISL_704992, EPI_ISL_704993, EPI_ISL_704995, EPI_ISL_704996	Lighthouse Lab in Cambridge	Wellcome Sanger Institute for the COVID-19 Genomics UK (COG-UK) Consortium	Rob Howes, The Lighthouse Lab in Cambridge and Alex Alderton, Roberto Amato, Sonia Goncalves, Ewan Harrison, David K. Jackson, Ian Johnston, Dominic Kwiatkowski, Cordelia Langford, John Sillitoe on behalf of the Wellcome Sanger Institute COVID-19 Surveillance Team
EPI_ISL_704997	University College London, Great Ormond Street Hospital for Children NHS Foundation Trust, Imperial College Healthcare NHS Trust	COVID-19 Genomics UK (COG-UK) Consortium	Sergi Castellano, Rachel Williams, Mark Kristiansen, Paola Resende Silva, Sunando Roy, Tony Brooks, Helena Tutill, Paola Niola, Patricia Dyal, Charlotte Williams, Leysa Forrest, Yasmin Panchbhaya, Jacqueline Findlay, Samuel Weeks, Julianne Brown, Kathryn Harris, Paul Randell, James Price, Alison Holmes, Judith Breuer
EPI_ISL_704998	Lighthouse Lab in Cambridge	Wellcome Sanger Institute for the COVID-19 Genomics UK (COG-UK) Consortium	Rob Howes, The Lighthouse Lab in Cambridge and Alex Alderton, Roberto Amato, Sonia Goncalves, Ewan Harrison, David K. Jackson, Ian Johnston, Dominic Kwiatkowski, Cordelia Langford, John Sillitoe on behalf of the Wellcome Sanger Institute COVID-19 Surveillance Team
EPI_ISL_704999	University College London, Great Ormond Street Hospital for Children NHS Foundation Trust, Imperial College Healthcare NHS Trust	COVID-19 Genomics UK (COG-UK) Consortium	Sergi Castellano, Rachel Williams, Mark Kristiansen, Paola Resende Silva, Sunando Roy, Tony Brooks, Helena Tutill, Paola Niola, Patricia Dyal, Charlotte Williams, Leysa Forrest, Yasmin Panchbhaya, Jacqueline Findlay, Samuel Weeks, Julianne Brown, Kathryn Harris, Paul Randell, James Price, Alison Holmes, Judith Breuer
EPI_ISL_705000, EPI_ISL_705001, EPI_ISL_705003, EPI_ISL_705004	Lighthouse Lab in Cambridge	Wellcome Sanger Institute for the COVID-19 Genomics UK (COG-UK) Consortium	Rob Howes, The Lighthouse Lab in Cambridge and Alex Alderton, Roberto Amato, Sonia Goncalves, Ewan Harrison, David K. Jackson, Ian Johnston, Dominic Kwiatkowski, Cordelia Langford, John Sillitoe on behalf of the Wellcome Sanger Institute COVID-19 Surveillance Team
EPI_ISL_705005	Liverpool Clinical Laboratories	COVID-19 Genomics UK (COG-UK) Consortium	Sam Haldenby, Anita Lucaci, Steve Paterson, Julian Hiscox, Alistair Darby, M Almsaud, A Alrezaihi, Muhannad Alruwaili, Stuart D Armstrong, Jones Benjamin, Eleanor G Bentley, Anu Chawla, Jordan J Clark, Angela Cowell, Richard Eccles, Isabel García-Dorival, Matthew Gemmell, Alessandro Gerada, PKF Gilmore, Richard Gregory, Ximeng Han, Catherine Hartley, Margaret Hughes, Miren Iturriza-Gomara, James Johnson, L Luu, Jenifer Manson, Charlotte Nelson, Elaine O'Toole, Cassie Olateju, Rebekah Penrice-Randal , Lucille Rainbow, N.P Randle, Trevor Ian Robinson, Parul Sharma, Ghada T Shawli, James P Stewart, Neil Swainston, Ecaterina Vamos, Joanne Watts, Mark Whitehead
EPI_ISL_705006, EPI_ISL_705008, EPI_ISL_705009, EPI_ISL_705011, EPI_ISL_705012, EPI_ISL_705014, EPI_ISL_705016, EPI_ISL_705017, EPI_ISL_705019, EPI_ISL_705020, EPI_ISL_705022, EPI_ISL_705023, EPI_ISL_705025, EPI_ISL_705027, EPI_ISL_705028, EPI_ISL_705029, EPI_ISL_705031, EPI_ISL_705033, EPI_ISL_705034, EPI_ISL_705036, EPI_ISL_705037, EPI_ISL_705039, EPI_ISL_705041, EPI_ISL_705042, EPI_ISL_705044, EPI_ISL_705045, EPI_ISL_705047, EPI_ISL_705049, EPI_ISL_705050, EPI_ISL_705052, EPI_ISL_705053, EPI_ISL_705055, EPI_ISL_705057, EPI_ISL_705058			
see above	Lighthouse Lab in Cambridge	Wellcome Sanger Institute for the COVID-19 Genomics UK (COG-UK) Consortium	Rob Howes, The Lighthouse Lab in Cambridge and Alex Alderton, Roberto Amato, Sonia Goncalves, Ewan Harrison, David K. Jackson, Ian Johnston, Dominic Kwiatkowski, Cordelia Langford, John Sillitoe on behalf of the Wellcome Sanger Institute COVID-19 Surveillance Team
EPI_ISL_705059	Liverpool Clinical Laboratories	COVID-19 Genomics UK (COG-UK) Consortium	Sam Haldenby, Anita Lucaci, Steve Paterson, Julian Hiscox, Alistair Darby, M Almsaud, A Alrezaihi, Muhannad Alruwaili, Stuart D Armstrong, Jones Benjamin, Eleanor G Bentley, Anu Chawla, Jordan J Clark, Angela Cowell, Richard Eccles, Isabel García-Dorival, Matthew Gemmell, Alessandro Gerada, PKF Gilmore, Richard Gregory, Ximeng Han, Catherine Hartley, Margaret Hughes, Miren Iturriza-Gomara, James Johnson, L Luu, Jenifer Manson, Charlotte Nelson, Elaine O'Toole, Cassie Olateju, Rebekah Penrice-Randal , Lucille Rainbow, N.P Randle, Trevor Ian Robinson, Parul Sharma, Ghada T Shawli, James P Stewart, Neil Swainston, Ecaterina Vamos, Joanne Watts, Mark Whitehead
EPI_ISL_705060, EPI_ISL_705063, EPI_ISL_705065, EPI_ISL_705066	Lighthouse Lab in Cambridge	Wellcome Sanger Institute for the COVID-19 Genomics UK (COG-UK) Consortium	Rob Howes, The Lighthouse Lab in Cambridge and Alex Alderton, Roberto Amato, Sonia Goncalves, Ewan Harrison, David K. Jackson, Ian Johnston, Dominic Kwiatkowski, Cordelia Langford, John Sillitoe on behalf of the Wellcome Sanger Institute COVID-19 Surveillance Team
EPI_ISL_705067	Northumbria University / South Tees Hospitals NHS Foundation Trust / North Cumbria Integrated Care NHS Foundation Trust / North Tees and Hartlepool NHS Foundation Trust / Newcastle Hospitals NHS Foundation Trust	COVID-19 Genomics UK (COG-UK) Consortium	Darren L Smith,Andrew Nelson,Matthew Bashton,Greg R Young,Joshua Loh,John Allan,Mohammad A Tariq,Giles S Holt,Gary Black,Wen C Yew,Lynn Dover,Paul Baker,Steve Liggett,Sarah Essex,Jane Greenaway,Debra Padgett,Clive Graham,Garren Scott,Edward Barton,Emma Swindells,Brendan Payne,Jennifer Collins,Yusri Taha,Gary Eltringham
EPI_ISL_705068, EPI_ISL_705069, EPI_ISL_705071	Lighthouse Lab in Cambridge	Wellcome Sanger Institute for the COVID-19 Genomics UK (COG-UK) Consortium	Rob Howes, The Lighthouse Lab in Cambridge and Alex Alderton, Roberto Amato, Sonia Goncalves, Ewan Harrison, David K. Jackson, Ian Johnston, Dominic Kwiatkowski, Cordelia Langford, John Sillitoe on behalf of the Wellcome Sanger Institute COVID-19 Surveillance Team
EPI_ISL_705072	University College London, Great Ormond Street Hospital for Children NHS Foundation Trust, Imperial College Healthcare NHS Trust	COVID-19 Genomics UK (COG-UK) Consortium	Sergi Castellano, Rachel Williams, Mark Kristiansen, Paola Resende Silva, Sunando Roy, Tony Brooks, Helena Tutill, Paola Niola, Patricia Dyal, Charlotte Williams, Leysa Forrest, Yasmin Panchbhaya, Jacqueline Findlay, Samuel Weeks, Julianne Brown, Kathryn Harris, Paul Randell, James Price, Alison Holmes, Judith Breuer
EPI_ISL_705073, EPI_ISL_705074, EPI_ISL_705076, EPI_ISL_705077, EPI_ISL_705079, EPI_ISL_705080	Lighthouse Lab in Cambridge	Wellcome Sanger Institute for the COVID-19 Genomics UK (COG-UK) Consortium	Rob Howes, The Lighthouse Lab in Cambridge and Alex Alderton, Roberto Amato, Sonia Goncalves, Ewan Harrison, David K. Jackson, Ian Johnston, Dominic Kwiatkowski, Cordelia Langford, John Sillitoe on behalf of the Wellcome Sanger Institute COVID-19 Surveillance Team
EPI_ISL_705081	Department of Pathology, University of Cambridge	COVID-19 Genomics UK (COG-UK) Consortium	Aminu S. Jahun, Yasmin Chaudhry, Grant Hall, Iliana Georgana, Myra Hosmillo, Martin D. Curran, Malte Pinckert, Surendra Parmar, Ian Goodfellow
EPI_ISL_705082, EPI_ISL_705084, EPI_ISL_705085, EPI_ISL_705087, EPI_ISL_705089, EPI_ISL_705090, EPI_ISL_705092, EPI_ISL_705093, EPI_ISL_705095, EPI_ISL_705097, EPI_ISL_705098, EPI_ISL_705100, EPI_ISL_705101, EPI_ISL_705103, EPI_ISL_705106, EPI_ISL_705107, EPI_ISL_705109			
see above	Lighthouse Lab in Cambridge	Wellcome Sanger Institute for the COVID-19 Genomics UK (COG-UK) Consortium	Rob Howes, The Lighthouse Lab in Cambridge and Alex Alderton, Roberto Amato, Sonia Goncalves, Ewan Harrison, David K. Jackson, Ian Johnston, Dominic Kwiatkowski, Cordelia Langford, John Sillitoe on behalf of the Wellcome Sanger Institute COVID-19 Surveillance Team
EPI_ISL_705110	Department of Pathology, University of Cambridge	COVID-19 Genomics UK (COG-UK) Consortium	Aminu S. Jahun, Yasmin Chaudhry, Grant Hall, Iliana Georgana, Myra Hosmillo, Martin D. Curran, Malte Pinckert, Surendra Parmar, Ian Goodfellow
EPI_ISL_705111, EPI_ISL_705112, EPI_ISL_705114, EPI_ISL_705116, EPI_ISL_705117, EPI_ISL_705119, EPI_ISL_705120, EPI_ISL_705122, EPI_ISL_705123, EPI_ISL_705125, EPI_ISL_705127, EPI_ISL_705128, EPI_ISL_705130, EPI_ISL_705131, EPI_ISL_705133			
see above	Lighthouse Lab in Cambridge	Wellcome Sanger Institute for the COVID-19 Genomics UK (COG-UK) Consortium	Rob Howes, The Lighthouse Lab in Cambridge and Alex Alderton, Roberto Amato, Sonia Goncalves, Ewan Harrison, David K. Jackson, Ian Johnston, Dominic Kwiatkowski, Cordelia Langford, John Sillitoe on behalf of the Wellcome Sanger Institute COVID-19 Surveillance Team
EPI_ISL_705134	University of Exeter	COVID-19 Genomics UK (COG-UK) Consortium	Ben Temperton,Aaron Jeffries,Michelle Michelsen,Joanna Warwick-Dugdale,Audrey Farbos,Robyn Manley,Stephen Michell,Jane Masoli
EPI_ISL_705135, EPI_ISL_705136, EPI_ISL_705138, EPI_ISL_705139, EPI_ISL_705141, EPI_ISL_705142, EPI_ISL_705144, EPI_ISL_705146, EPI_ISL_705147, EPI_ISL_705149	Lighthouse Lab in Cambridge	Wellcome Sanger Institute for the COVID-19 Genomics UK (COG-UK) Consortium	Rob Howes, The Lighthouse Lab in Cambridge and Alex Alderton, Roberto Amato, Sonia Goncalves, Ewan Harrison, David K. Jackson, Ian Johnston, Dominic Kwiatkowski, Cordelia Langford, John Sillitoe on behalf of the Wellcome Sanger Institute COVID-19 Surveillance Team
EPI_ISL_705150	Liverpool Clinical Laboratories	COVID-19 Genomics UK (COG-UK) Consortium	Sam Haldenby, Anita Lucaci, Steve Paterson, Julian Hiscox, Alistair Darby, M Almsaud, A Alrezaihi, Muhannad Alruwaili, Stuart D Armstrong, Jones Benjamin, Eleanor G Bentley, Anu Chawla, Jordan J Clark, Angela Cowell, Richard Eccles, Isabel García-Dorival, Matthew Gemmell, Alessandro Gerada, PKF Gilmore, Richard Gregory, Ximeng Han, Catherine Hartley, Margaret Hughes, Miren Iturriza-Gomara, James Johnson, L Luu, Jenifer Manson, Charlotte Nelson, Elaine O'Toole, Cassie Olateju, Rebekah Penrice-Randal , Lucille Rainbow, N.P Randle, Trevor Ian Robinson, Parul Sharma, Ghada T Shawli, James P Stewart, Neil Swainston, Ecaterina Vamos, Joanne Watts, Mark Whitehead
EPI_ISL_705151, EPI_ISL_705152, EPI_ISL_705154, EPI_ISL_705155,	Lighthouse Lab in Cambridge	Wellcome Sanger Institute for the COVID-19 Genomics UK (COG-UK) Consortium	Rob Howes, The Lighthouse Lab in Cambridge and Alex Alderton, Roberto Amato, Sonia Goncalves, Ewan Harrison, David K. Jackson, Ian Johnston, Dominic Kwiatkowski, Cordelia Langford, John Sillitoe on behalf of the Wellcome Sanger Institute COVID-19 Surveillance Team

EPI_ISL_705157, EPI_ISL_705159, EPI_ISL_705160, EPI_ISL_705162			
EPI_ISL_705163	Virology Department, Sheffield Teaching Hospitals NHS Foundation Trust/Department of Infection, Immunity and Cardiovascular Disease, The Medical School, University of Sheffield	COVID-19 Genomics UK (COG-UK) Consortium	Thushan de Silva, Matthew Parker, Nikki Smith, Adri Angyal, Rebecca Brown, Luke Green, Rachel Tucker, Paul Parsons, Danielle Groves, Katie Johnson, Laura Carrilero, Alex Keeley, Dave Partridge, Matthew Wyles, Benjamin Lindsey, Mehmet Yavuz, Mohammad Raza, Cariad Evans
EPI_ISL_705164, EPI_ISL_705165, EPI_ISL_705167, EPI_ISL_705169, EPI_ISL_705170, EPI_ISL_705172, EPI_ISL_705173, EPI_ISL_705175, EPI_ISL_705177, EPI_ISL_705178, EPI_ISL_705180, EPI_ISL_705181, EPI_ISL_705183, EPI_ISL_705185, EPI_ISL_705186, EPI_ISL_705188, EPI_ISL_705189			
see above	Lighthouse Lab in Cambridge	Wellcome Sanger Institute for the COVID-19 Genomics UK (COG-UK) Consortium	Rob Howes, The Lighthouse Lab in Cambridge and Alex Alderton, Roberto Amato, Sonia Goncalves, Ewan Harrison, David K. Jackson, Ian Johnston, Dominic Kwiatkowski, Cordelia Langford, John Sillitoe on behalf of the Wellcome Sanger Institute COVID-19 Surveillance Team
EPI_ISL_705195	Virology Department, Sheffield Teaching Hospitals NHS Foundation Trust/Department of Infection, Immunity and Cardiovascular Disease, The Medical School, University of Sheffield	COVID-19 Genomics UK (COG-UK) Consortium	Thushan de Silva, Matthew Parker, Nikki Smith, Adri Angyal, Rebecca Brown, Luke Green, Rachel Tucker, Paul Parsons, Danielle Groves, Katie Johnson, Laura Carrilero, Alex Keeley, Dave Partridge, Matthew Wyles, Benjamin Lindsey, Mehmet Yavuz, Mohammad Raza, Cariad Evans
EPI_ISL_705201	Northumbria University / South Tees Hospitals NHS Foundation Trust / North Cumbria Integrated Care NHS Foundation Trust / North Tees and Hartlepool NHS Foundation Trust / Newcastle Hospitals NHS Foundation Trust	COVID-19 Genomics UK (COG-UK) Consortium	Darren L Smith,Andrew Nelson,Matthew Bashton,Greg R Young,Joshua Loh,John Allan,Mohammad A Tariq,Giles S Holt,Gary Black,Wen C Yew,Lynn Dover,Paul Baker,Steve Liggett,Sarah Essex,Jane Greenaway,Debra Padgett,Clive Graham,Garren Scott,Edward Barton,Emma Swindells,Brendan Payne,Jennifer Collins,Yusri Taha,Gary Eltringham
EPI_ISL_705211	Quadram Institute Bioscience	COVID-19 Genomics UK (COG-UK) Consortium	Dave J. Baker, Gemma L. Kay, Alp Aydin, Thanh Le-Viet, Steven Rudder, Ana P. Tedim, Anastasia Kolyva, Maria Diaz, Leonardo de Oliveira Martins, Nabil-Fareed Alikhan, Lizzie Meadows, Rachael Stanley, Ngozi Elumogo, Muhammed Yasir, Nicholas M. Thomson, Alexander J Trotter, Rachel Gilroy, Samuel Bloomfield, Claire Stuart, Andrew Bell, Reenesh Prakash, Samir Dervisevic, Alison E. Mather, John Wain, Mark Webber, Andrew J. Page, Justin O'Grady
EPI_ISL_705215, EPI_ISL_705216	University College London, Great Ormond Street Hospital for Children NHS Foundation Trust, Imperial College Healthcare NHS Trust	COVID-19 Genomics UK (COG-UK) Consortium	Sergi Castellano, Rachel Williams, Mark Kristiansen, Paola Resende Silva, Sunando Roy, Tony Brooks, Helena Tutill, Paola Niola, Patricia Dyal, Charlotte Williams, Leysa Forrest, Yasmin Panchbhaya, Jacqueline Findlay, Samuel Weeks, Julianne Brown, Kathryn Harris, Paul Randell, James Price, Alison Holmes, Judith Breuer
EPI_ISL_705217	Department of Pathology, University of Cambridge	COVID-19 Genomics UK (COG-UK) Consortium	Aminu S. Jahun, Yasmin Chaudhry, Grant Hall, Iliana Georgana, Myra Hosmillo, Martin D. Curran, Malte Pinckert, Surendra Parmar, Ian Goodfellow
EPI_ISL_705228	Quadram Institute Bioscience	COVID-19 Genomics UK (COG-UK) Consortium	Dave J. Baker, Gemma L. Kay, Alp Aydin, Thanh Le-Viet, Steven Rudder, Ana P. Tedim, Anastasia Kolyva, Maria Diaz, Leonardo de Oliveira Martins, Nabil-Fareed Alikhan, Lizzie Meadows, Rachael Stanley, Ngozi Elumogo, Muhammed Yasir, Nicholas M. Thomson, Alexander J Trotter, Rachel Gilroy, Samuel Bloomfield, Claire Stuart, Andrew Bell, Reenesh Prakash, Samir Dervisevic, Alison E. Mather, John Wain, Mark Webber, Andrew J. Page, Justin O'Grady
EPI_ISL_705237	University of Exeter	COVID-19 Genomics UK (COG-UK) Consortium	Ben Temperton,Aaron Jeffries,Michelle Michelsen,Joanna Warwick-Dugdale,Audrey Farbos,Robyn Manley,Stephen Michell,Jane Masoli
EPI_ISL_705241, EPI_ISL_705248	Liverpool Clinical Laboratories	COVID-19 Genomics UK (COG-UK) Consortium	Sam Haldenby, Anita Lucaci, Steve Paterson, Julian Hiscox, Alistair Darby, M Almsaud, A Alrezaihi, Muhannad Alruwaili, Stuart D Armstrong, Jones Benjamin, Eleanor G Bentley, Anu Chawla, Jordan J Clark, Angela Cowell, Richard Eccles, Isabel García-Dorival, Matthew Gemmell, Alessandro Gerada, PKF Gilmore, Richard Gregory, Ximeng Han, Catherine Hartley, Margaret Hughes, Miren Iturriza-Gomara, James Johnson, L Luu, Jenifer Manson, Charlotte Nelson, Elaine O'Toole, Cassie Olateju, Rebekah Penrice-Randal , Lucille Rainbow, N.P Randle, Trevor Ian Robinson, Parul Sharma, Ghada T Shawli, James P Stewart, Neil Swainston, Ecaterina Vamos, Joanne Watts, Mark Whitehead
EPI_ISL_705254	University of Exeter	COVID-19 Genomics UK (COG-UK) Consortium	Ben Temperton,Aaron Jeffries,Michelle Michelsen,Joanna Warwick-Dugdale,Audrey Farbos,Robyn Manley,Stephen Michell,Jane Masoli
EPI_ISL_705262	Quadram Institute Bioscience	COVID-19 Genomics UK (COG-UK) Consortium	Dave J. Baker, Gemma L. Kay, Alp Aydin, Thanh Le-Viet, Steven Rudder, Ana P. Tedim, Anastasia Kolyva, Maria Diaz, Leonardo de Oliveira Martins, Nabil-Fareed Alikhan, Lizzie Meadows, Rachael Stanley, Ngozi Elumogo, Muhammed Yasir, Nicholas M. Thomson, Alexander J Trotter, Rachel Gilroy, Samuel Bloomfield, Claire Stuart, Andrew Bell, Reenesh Prakash, Samir Dervisevic, Alison E. Mather, John Wain, Mark Webber, Andrew J. Page, Justin O'Grady
EPI_ISL_705269	Liverpool Clinical Laboratories	COVID-19 Genomics UK (COG-UK) Consortium	Sam Haldenby, Anita Lucaci, Steve Paterson, Julian Hiscox, Alistair Darby, M Almsaud, A Alrezaihi, Muhannad Alruwaili, Stuart D Armstrong, Jones Benjamin, Eleanor G Bentley, Anu Chawla, Jordan J Clark, Angela Cowell, Richard Eccles, Isabel García-Dorival, Matthew Gemmell, Alessandro Gerada, PKF Gilmore, Richard Gregory, Ximeng Han, Catherine Hartley, Margaret Hughes, Miren Iturriza-Gomara, James Johnson, L Luu, Jenifer Manson, Charlotte Nelson, Elaine O'Toole, Cassie Olateju, Rebekah Penrice-Randal , Lucille Rainbow, N.P Randle, Trevor Ian Robinson, Parul Sharma, Ghada T Shawli, James P Stewart, Neil Swainston, Ecaterina Vamos, Joanne Watts, Mark Whitehead
EPI_ISL_705270	Virology Department, Sheffield Teaching Hospitals NHS Foundation Trust/Department of Infection, Immunity and Cardiovascular Disease, The Medical School, University of Sheffield	COVID-19 Genomics UK (COG-UK) Consortium	Thushan de Silva, Matthew Parker, Nikki Smith, Adri Angyal, Rebecca Brown, Luke Green, Rachel Tucker, Paul Parsons, Danielle Groves, Katie Johnson, Laura Carrilero, Alex Keeley, Dave Partridge, Matthew Wyles, Benjamin Lindsey, Mehmet Yavuz, Mohammad Raza, Cariad Evans
EPI_ISL_705273	Northumbria University / South Tees Hospitals NHS Foundation Trust / North Cumbria Integrated Care NHS Foundation Trust / North Tees and Hartlepool NHS Foundation Trust / Newcastle Hospitals NHS Foundation Trust	COVID-19 Genomics UK (COG-UK) Consortium	Darren L Smith,Andrew Nelson,Matthew Bashton,Greg R Young,Joshua Loh,John Allan,Mohammad A Tariq,Giles S Holt,Gary Black,Wen C Yew,Lynn Dover,Paul Baker,Steve Liggett,Sarah Essex,Jane Greenaway,Debra Padgett,Clive Graham,Garren Scott,Edward Barton,Emma Swindells,Brendan Payne,Jennifer Collins,Yusri Taha,Gary Eltringham
EPI_ISL_705274	Liverpool Clinical Laboratories	COVID-19 Genomics UK (COG-UK) Consortium	Sam Haldenby, Anita Lucaci, Steve Paterson, Julian Hiscox, Alistair Darby, M Almsaud, A Alrezaihi, Muhannad Alruwaili, Stuart D Armstrong, Jones Benjamin, Eleanor G Bentley, Anu Chawla, Jordan J Clark, Angela Cowell, Richard Eccles, Isabel García-Dorival, Matthew Gemmell, Alessandro Gerada, PKF Gilmore, Richard Gregory, Ximeng Han, Catherine Hartley, Margaret Hughes, Miren Iturriza-Gomara, James Johnson, L Luu, Jenifer Manson, Charlotte Nelson, Elaine O'Toole, Cassie Olateju, Rebekah Penrice-Randal , Lucille Rainbow, N.P Randle, Trevor Ian Robinson, Parul Sharma, Ghada T Shawli, James P Stewart, Neil Swainston, Ecaterina Vamos, Joanne Watts, Mark Whitehead
EPI_ISL_705278	University College London, Great Ormond Street Hospital for Children NHS Foundation Trust, Imperial College Healthcare NHS Trust	COVID-19 Genomics UK (COG-UK) Consortium	Sergi Castellano, Rachel Williams, Mark Kristiansen, Paola Resende Silva, Sunando Roy, Tony Brooks, Helena Tutill, Paola Niola, Patricia Dyal, Charlotte Williams, Leysa Forrest, Yasmin Panchbhaya, Jacqueline Findlay, Samuel Weeks, Julianne Brown, Kathryn Harris, Paul Randell, James Price, Alison Holmes, Judith Breuer
EPI_ISL_705286	Liverpool Clinical Laboratories	COVID-19 Genomics UK (COG-UK) Consortium	Sam Haldenby, Anita Lucaci, Steve Paterson, Julian Hiscox, Alistair Darby, M Almsaud, A Alrezaihi, Muhannad Alruwaili, Stuart D Armstrong, Jones Benjamin, Eleanor G Bentley, Anu Chawla, Jordan J Clark, Angela Cowell, Richard Eccles, Isabel García-Dorival, Matthew Gemmell, Alessandro Gerada, PKF Gilmore, Richard Gregory, Ximeng Han, Catherine Hartley, Margaret Hughes, Miren Iturriza-Gomara, James Johnson, L Luu, Jenifer Manson, Charlotte Nelson, Elaine O'Toole, Cassie Olateju, Rebekah Penrice-Randal , Lucille Rainbow, N.P Randle, Trevor Ian Robinson, Parul Sharma, Ghada T Shawli, James P Stewart, Neil Swainston, Ecaterina Vamos, Joanne Watts, Mark Whitehead
EPI_ISL_705289	Department of Pathology, University of Cambridge	COVID-19 Genomics UK (COG-UK) Consortium	Aminu S. Jahun, Yasmin Chaudhry, Grant Hall, Iliana Georgana, Myra Hosmillo, Martin D. Curran, Malte Pinckert, Surendra Parmar, Ian Goodfellow
EPI_ISL_705299	Liverpool Clinical Laboratories	COVID-19 Genomics UK (COG-UK) Consortium	Sam Haldenby, Anita Lucaci, Steve Paterson, Julian Hiscox, Alistair Darby, M Almsaud, A Alrezaihi, Muhannad Alruwaili, Stuart D Armstrong, Jones Benjamin, Eleanor G Bentley, Anu Chawla, Jordan J Clark, Angela Cowell, Richard Eccles, Isabel García-Dorival, Matthew Gemmell, Alessandro Gerada, PKF Gilmore, Richard Gregory, Ximeng Han, Catherine Hartley, Margaret Hughes, Miren Iturriza-Gomara, James Johnson, L Luu, Jenifer Manson, Charlotte Nelson, Elaine O'Toole, Cassie Olateju, Rebekah Penrice-Randal , Lucille Rainbow, N.P Randle, Trevor Ian Robinson, Parul Sharma, Ghada T Shawli, James P Stewart, Neil Swainston, Ecaterina Vamos, Joanne Watts, Mark Whitehead
EPI_ISL_705300	West of Scotland Specialist Virology Centre, NHSGGC / MRC-University of Glasgow Centre for Virus Research	COVID-19 Genomics UK (COG-UK) Consortium	Ana da Silva Filipe, Natasha Johnson, Kathy Smollett, Daniel Mair, Stephen Carmichael, Alice Broos, Lily Tong, Jenna Nichols, Kyriaki Nomikou; Sarah McDonald; Richard Orton, Joseph Hughes, Sreenu Vattipally, David L Robertson; Alasdair MacLean, Rory Gunson; Sharif Shaaban, Matthew Holden; Rachel Blacow, Guy Mollett, Kathy Li, James Shepherd, Antonia Ho, Emma Thomson
EPI_ISL_705313	Liverpool Clinical Laboratories	COVID-19 Genomics UK (COG-UK) Consortium	Sam Haldenby, Anita Lucaci, Steve Paterson, Julian Hiscox, Alistair Darby, M Almsaud, A Alrezaihi, Muhannad Alruwaili, Stuart D Armstrong, Jones

			Benjamin, Eleanor G Bentley, Anu Chawla, Jordan J Clark, Angela Cowell, Richard Eccles, Isabel García-Dorival, Matthew Gemmell, Alessandro Gerada, PKF Gilmore, Richard Gregory, Ximeng Han, Catherine Hartley, Margaret Hughes, Miren Iturriza-Gomara, James Johnson, L Luu, Jenifer Manson, Charlotte Nelson, Elaine O'Toole, Cassie Olateju, Rebekah Penrice-Randal , Lucille Rainbow, N.P Randle, Trevor Ian Robinson, Parul Sharma, Ghada T Shawli, James P Stewart, Neil Swainston, Ecaterina Vamos, Joanne Watts, Mark Whitehead
EPI_ISL_705317, EPI_ISL_705319	Virology Department, Sheffield Teaching Hospitals NHS Foundation Trust/Department of Infection, Immunity and Cardiovascular Disease, The Medical School, University of Sheffield	COVID-19 Genomics UK (COG-UK) Consortium	Thushan de Silva, Matthew Parker, Nikki Smith, Adri Angyal, Rebecca Brown, Luke Green, Rachel Tucker, Paul Parsons, Danielle Groves, Katie Johnson, Laura Carrilero, Alex Keeley, Dave Partridge, Matthew Wyles, Benjamin Lindsey, Mehmet Yavuz, Mohammad Raza, Cariad Evans
EPI_ISL_705334	Liverpool Clinical Laboratories	COVID-19 Genomics UK (COG-UK) Consortium	Sam Haldenby, Anita Lucaci, Steve Paterson, Julian Hiscox, Alistair Darby, M Almsaud, A Alrezaihi, Muhannad Alruwaili, Stuart D Armstrong, Jones Benjamin, Eleanor G Bentley, Anu Chawla, Jordan J Clark, Angela Cowell, Richard Eccles, Isabel García-Dorival, Matthew Gemmell, Alessandro Gerada, PKF Gilmore, Richard Gregory, Ximeng Han, Catherine Hartley, Margaret Hughes, Miren Iturriza-Gomara, James Johnson, L Luu, Jenifer Manson, Charlotte Nelson, Elaine O'Toole, Cassie Olateju, Rebekah Penrice-Randal , Lucille Rainbow, N.P Randle, Trevor Ian Robinson, Parul Sharma, Ghada T Shawli, James P Stewart, Neil Swainston, Ecaterina Vamos, Joanne Watts, Mark Whitehead
EPI_ISL_705335	Virology Department, Sheffield Teaching Hospitals NHS Foundation Trust/Department of Infection, Immunity and Cardiovascular Disease, The Medical School, University of Sheffield	COVID-19 Genomics UK (COG-UK) Consortium	Thushan de Silva, Matthew Parker, Nikki Smith, Adri Angyal, Rebecca Brown, Luke Green, Rachel Tucker, Paul Parsons, Danielle Groves, Katie Johnson, Laura Carrilero, Alex Keeley, Dave Partridge, Matthew Wyles, Benjamin Lindsey, Mehmet Yavuz, Mohammad Raza, Cariad Evans
EPI_ISL_705339	Department of Pathology, University of Cambridge	COVID-19 Genomics UK (COG-UK) Consortium	Aminu S. Jahun, Yasmin Chaudhry, Grant Hall, Iliana Georgana, Myra Hosmillo, Martin D. Curran, Malte Pinckert, Surendra Parmar, Ian Goodfellow
EPI_ISL_705356	Northumbria University / South Tees Hospitals NHS Foundation Trust / North Cumbria Integrated Care NHS Foundation Trust / North Tees and Hartlepool NHS Foundation Trust / Newcastle Hospitals NHS Foundation Trust	COVID-19 Genomics UK (COG-UK) Consortium	Darren L Smith,Andrew Nelson,Matthew Bashton,Greg R Young,Joshua Loh,John Allan,Mohammad A Tariq,Giles S Holt,Gary Black,Wen C Yew,Lynn Dover,Paul Baker,Steve Liggett,Sarah Essex,Jane Greenaway,Debra Padgett,Clive Graham,Garren Scott,Edward Barton,Emma Swindells,Brendan Payne,Jennifer Collins,Yusri Taha,Gary Eltringham
EPI_ISL_705371	Virology Department, Sheffield Teaching Hospitals NHS Foundation Trust/Department of Infection, Immunity and Cardiovascular Disease, The Medical School, University of Sheffield	COVID-19 Genomics UK (COG-UK) Consortium	Thushan de Silva, Matthew Parker, Nikki Smith, Adri Angyal, Rebecca Brown, Luke Green, Rachel Tucker, Paul Parsons, Danielle Groves, Katie Johnson, Laura Carrilero, Alex Keeley, Dave Partridge, Matthew Wyles, Benjamin Lindsey, Mehmet Yavuz, Mohammad Raza, Cariad Evans
EPI_ISL_705373	University of Exeter	COVID-19 Genomics UK (COG-UK) Consortium	Ben Temperton,Aaron Jeffries,Michelle Michelsen,Joanna Warwick-Dugdale,Audrey Farbos,Robyn Manley,Stephen Michell,Jane Masoli
EPI_ISL_705375	Quadram Institute Bioscience	COVID-19 Genomics UK (COG-UK) Consortium	Dave J. Baker, Gemma L. Kay, Alp Aydin, Thanh Le-Viet, Steven Rudder, Ana P. Tedim, Anastasia Kolyva, Maria Diaz, Leonardo de Oliveira Martins, Nabil-Fareed Alikhan, Lizzie Meadows, Rachael Stanley, Ngozi Elumogo, Muhammed Yasir, Nicholas M. Thomson, Alexander J Trotter, Rachel Gilroy, Samuel Bloomfield, Claire Stuart, Andrew Bell, Reenesh Prakash, Samir Dervisevic, Alison E. Mather, John Wain, Mark Webber, Andrew J. Page, Justin O'Grady
EPI_ISL_705376	Department of Pathology, University of Cambridge	COVID-19 Genomics UK (COG-UK) Consortium	Aminu S. Jahun, Yasmin Chaudhry, Grant Hall, Iliana Georgana, Myra Hosmillo, Martin D. Curran, Malte Pinckert, Surendra Parmar, Ian Goodfellow
EPI_ISL_705383	University of Exeter	COVID-19 Genomics UK (COG-UK) Consortium	Ben Temperton,Aaron Jeffries,Michelle Michelsen,Joanna Warwick-Dugdale,Audrey Farbos,Robyn Manley,Stephen Michell,Jane Masoli
EPI_ISL_705398	Liverpool Clinical Laboratories	COVID-19 Genomics UK (COG-UK) Consortium	Sam Haldenby, Anita Lucaci, Steve Paterson, Julian Hiscox, Alistair Darby, M Almsaud, A Alrezaihi, Muhannad Alruwaili, Stuart D Armstrong, Jones Benjamin, Eleanor G Bentley, Anu Chawla, Jordan J Clark, Angela Cowell, Richard Eccles, Isabel García-Dorival, Matthew Gemmell, Alessandro Gerada, PKF Gilmore, Richard Gregory, Ximeng Han, Catherine Hartley, Margaret Hughes, Miren Iturriza-Gomara, James Johnson, L Luu, Jenifer Manson, Charlotte Nelson, Elaine O'Toole, Cassie Olateju, Rebekah Penrice-Randal , Lucille Rainbow, N.P Randle, Trevor Ian Robinson, Parul Sharma, Ghada T Shawli, James P Stewart, Neil Swainston, Ecaterina Vamos, Joanne Watts, Mark Whitehead
EPI_ISL_705405	Virology Department, Sheffield Teaching Hospitals NHS Foundation Trust/Department of Infection, Immunity and Cardiovascular Disease, The Medical School, University of Sheffield	COVID-19 Genomics UK (COG-UK) Consortium	Thushan de Silva, Matthew Parker, Nikki Smith, Adri Angyal, Rebecca Brown, Luke Green, Rachel Tucker, Paul Parsons, Danielle Groves, Katie Johnson, Laura Carrilero, Alex Keeley, Dave Partridge, Matthew Wyles, Benjamin Lindsey, Mehmet Yavuz, Mohammad Raza, Cariad Evans
EPI_ISL_705410	Department of Pathology, University of Cambridge	COVID-19 Genomics UK (COG-UK) Consortium	Aminu S. Jahun, Yasmin Chaudhry, Grant Hall, Iliana Georgana, Myra Hosmillo, Martin D. Curran, Malte Pinckert, Surendra Parmar, Ian Goodfellow
EPI_ISL_705471	Liverpool Clinical Laboratories	COVID-19 Genomics UK (COG-UK) Consortium	Sam Haldenby, Anita Lucaci, Steve Paterson, Julian Hiscox, Alistair Darby, M Almsaud, A Alrezaihi, Muhannad Alruwaili, Stuart D Armstrong, Jones Benjamin, Eleanor G Bentley, Anu Chawla, Jordan J Clark, Angela Cowell, Richard Eccles, Isabel García-Dorival, Matthew Gemmell, Alessandro Gerada, PKF Gilmore, Richard Gregory, Ximeng Han, Catherine Hartley, Margaret Hughes, Miren Iturriza-Gomara, James Johnson, L Luu, Jenifer Manson, Charlotte Nelson, Elaine O'Toole, Cassie Olateju, Rebekah Penrice-Randal , Lucille Rainbow, N.P Randle, Trevor Ian Robinson, Parul Sharma, Ghada T Shawli, James P Stewart, Neil Swainston, Ecaterina Vamos, Joanne Watts, Mark Whitehead
EPI_ISL_705472	University College London Hospital	COVID-19 Genomics UK (COG-UK) Consortium	Judith Heaney, Matthew Byott, Catherine Houlihan, Dan Frampton, Stuart Kirk, Moira Spyer and Eleni Nastouli
EPI_ISL_705486	Northumbria University / South Tees Hospitals NHS Foundation Trust / North Cumbria Integrated Care NHS Foundation Trust / North Tees and Hartlepool NHS Foundation Trust / Newcastle Hospitals NHS Foundation Trust	COVID-19 Genomics UK (COG-UK) Consortium	Darren L Smith,Andrew Nelson,Matthew Bashton,Greg R Young,Joshua Loh,John Allan,Mohammad A Tariq,Giles S Holt,Gary Black,Wen C Yew,Lynn Dover,Paul Baker,Steve Liggett,Sarah Essex,Jane Greenaway,Debra Padgett,Clive Graham,Garren Scott,Edward Barton,Emma Swindells,Brendan Payne,Jennifer Collins,Yusri Taha,Gary Eltringham
EPI_ISL_705487	Queens Medical Centre, Clinical Microbiology Department / DeepSeq Nottingham	COVID-19 Genomics UK (COG-UK) Consortium	Gemma Clark, Wendy Smith, Manjinder Khakh, Vicki M Fleming, Michelle M Lister, Hannah Howson-Wells, Jonathan Ball, Patrick McClure, Joseph Chappell, Theocharis Tsoleridis, Nadine Holmes, Matthew Carlisle, Christopher Moore, Fei Sang, Johnny Debebe, Victoria Wright, Matthew Loose
EPI_ISL_705491	Quadram Institute Bioscience	COVID-19 Genomics UK (COG-UK) Consortium	Dave J. Baker, Gemma L. Kay, Alp Aydin, Thanh Le-Viet, Steven Rudder, Ana P. Tedim, Anastasia Kolyva, Maria Diaz, Leonardo de Oliveira Martins, Nabil-Fareed Alikhan, Lizzie Meadows, Rachael Stanley, Ngozi Elumogo, Muhammed Yasir, Nicholas M. Thomson, Alexander J Trotter, Rachel Gilroy, Samuel Bloomfield, Claire Stuart, Andrew Bell, Reenesh Prakash, Samir Dervisevic, Alison E. Mather, John Wain, Mark Webber, Andrew J. Page, Justin O'Grady
EPI_ISL_705494, EPI_ISL_705495	Northumbria University / South Tees Hospitals NHS Foundation Trust / North Cumbria Integrated Care NHS Foundation Trust / North Tees and Hartlepool NHS Foundation Trust / Newcastle Hospitals NHS Foundation Trust	COVID-19 Genomics UK (COG-UK) Consortium	Darren L Smith,Andrew Nelson,Matthew Bashton,Greg R Young,Joshua Loh,John Allan,Mohammad A Tariq,Giles S Holt,Gary Black,Wen C Yew,Lynn Dover,Paul Baker,Steve Liggett,Sarah Essex,Jane Greenaway,Debra Padgett,Clive Graham,Garren Scott,Edward Barton,Emma Swindells,Brendan Payne,Jennifer Collins,Yusri Taha,Gary Eltringham
EPI_ISL_705551, EPI_ISL_705557, EPI_ISL_705559, EPI_ISL_705561, EPI_ISL_705562, EPI_ISL_705563, EPI_ISL_705564, EPI_ISL_705565, EPI_ISL_705566	Department of Pathology, University of Cambridge	COVID-19 Genomics UK (COG-UK) Consortium	Aminu S. Jahun, Yasmin Chaudhry, Grant Hall, Iliana Georgana, Myra Hosmillo, Martin D. Curran, Malte Pinckert, Surendra Parmar, Ian Goodfellow
EPI_ISL_705567, EPI_ISL_705568, EPI_ISL_705569, EPI_ISL_705570, EPI_ISL_705571, EPI_ISL_705573, EPI_ISL_705574, EPI_ISL_705575, EPI_ISL_705576, EPI_ISL_705577, EPI_ISL_705578, EPI_ISL_705579, EPI_ISL_705580, EPI_ISL_705581, EPI_ISL_705582, EPI_ISL_705583, EPI_ISL_705584, EPI_ISL_705585, EPI_ISL_705586, EPI_ISL_705587, EPI_ISL_705588, EPI_ISL_705589, EPI_ISL_705590, EPI_ISL_705591, EPI_ISL_705592, EPI_ISL_705595, EPI_ISL_705596, EPI_ISL_705597, EPI_ISL_705598, EPI_ISL_705599, EPI_ISL_705600, EPI_ISL_705601, EPI_ISL_705602, EPI_ISL_705603, EPI_ISL_705604, EPI_ISL_705605, EPI_ISL_705606, EPI_ISL_705607, EPI_ISL_705608, EPI_ISL_705609, EPI_ISL_705610, EPI_ISL_705611, EPI_ISL_705612			
see above	Quadram Institute Bioscience	COVID-19 Genomics UK (COG-UK) Consortium	Dave J. Baker, Gemma L. Kay, Alp Aydin, Thanh Le-Viet, Steven Rudder, Ana P. Tedim, Anastasia Kolyva, Maria Diaz, Leonardo de Oliveira Martins, Nabil-Fareed Alikhan, Lizzie Meadows, Rachael Stanley, Ngozi Elumogo, Muhammed Yasir, Nicholas M. Thomson, Alexander J Trotter, Rachel Gilroy, Samuel Bloomfield, Claire Stuart, Andrew Bell, Reenesh Prakash, Samir Dervisevic, Alison E. Mather, John Wain, Mark Webber, Andrew J. Page, Justin O'Grady
EPI_ISL_705613, EPI_ISL_705614	Queens Medical Centre, Clinical Microbiology Department /	COVID-19 Genomics UK (COG-UK) Consortium	Gemma Clark, Wendy Smith, Manjinder Khakh, Vicki M Fleming, Michelle M Lister, Hannah Howson-Wells, Jonathan Ball, Patrick McClure, Joseph

	DeepSeq Nottingham		Chappell, Theocharis Tsoleridis, Nadine Holmes, Matthew Carlisle, Christopher Moore, Fei Sang, Johnny Debebe, Victoria Wright, Matthew Loose
EPI_ISL_705615, EPI_ISL_705616, EPI_ISL_705617, EPI_ISL_705618, EPI_ISL_705619, EPI_ISL_705620, EPI_ISL_705621, EPI_ISL_705622, EPI_ISL_705623, EPI_ISL_705624, EPI_ISL_705625			
see above	Virology Department, Sheffield Teaching Hospitals NHS Foundation Trust/Department of Infection, Immunity and Cardiovascular Disease, The Medical School, University of Sheffield	COVID-19 Genomics UK (COG-UK) Consortium	Thushan de Silva, Matthew Parker, Nikki Smith, Adri Angyal, Rebecca Brown, Luke Green, Rachel Tucker, Paul Parsons, Danielle Groves, Katie Johnson, Laura Carrilero, Alex Keeley, Dave Partridge, Matthew Wyles, Benjamin Lindsey, Mehmet Yavuz, Mohammad Raza, Cariad Evans
EPI_ISL_705629, EPI_ISL_705630, EPI_ISL_705631, EPI_ISL_705632, EPI_ISL_705633, EPI_ISL_705634, EPI_ISL_705635	Department of Pathology, University of Cambridge	COVID-19 Genomics UK (COG-UK) Consortium	Aminu S. Jahun, Yasmin Chaudhry, Grant Hall, Iliana Georgana, Myra Hosmillo, Martin D. Curran, Malte Pinckert, Surendra Parmar, Ian Goodfellow
EPI_ISL_705667, EPI_ISL_705668, EPI_ISL_705669, EPI_ISL_705670, EPI_ISL_705671, EPI_ISL_705672, EPI_ISL_705673, EPI_ISL_705674, EPI_ISL_705675, EPI_ISL_705676, EPI_ISL_705677, EPI_ISL_705678, EPI_ISL_705679, EPI_ISL_705680, EPI_ISL_705681, EPI_ISL_705682, EPI_ISL_705683, EPI_ISL_705684, EPI_ISL_705685, EPI_ISL_705686, EPI_ISL_705687, EPI_ISL_705688, EPI_ISL_705689, EPI_ISL_705690, EPI_ISL_705691, EPI_ISL_705692, EPI_ISL_705693, EPI_ISL_705694, EPI_ISL_705695, EPI_ISL_705696, EPI_ISL_705697, EPI_ISL_705701, EPI_ISL_705709, EPI_ISL_705729			
see above	West of Scotland Specialist Virology Centre, NHSGGC / MRC-University of Glasgow Centre for Virus Research	COVID-19 Genomics UK (COG-UK) Consortium	Ana da Silva Filipe, Natasha Johnson, Kathy Smollett, Daniel Mair, Stephen Carmichael, Alice Broos, Lily Tong, Jenna Nichols, Kyriaki Nomikou; Sarah McDonald; Richard Orton, Joseph Hughes, Sreenu Vattipally, David L Robertson; Alasdair MacLean, Rory Gunson; Sharif Shaaban, Matthew Holden; Rachel Blacow, Guy Mollett, Kathy Li, James Shepherd, Antonia Ho, Emma Thomson
EPI_ISL_705737, EPI_ISL_705738	Virology Department, Royal Infirmary of Edinburgh, NHS Lothian / School of Biological Sciences, University of Edinburgh / Institute of Genetics and Molecular Medicine, University of Edinburgh	COVID-19 Genomics UK (COG-UK) Consortium	McHugh M, Dewar R, Rooke S, Gallagher M, Balcaza C, O'Toole Á, Scher E, Hill V, McCrone JT, Colquhoun R, Yu X, Jackson B, Rambaut A, Williams TC, Templeton K
EPI_ISL_705809, EPI_ISL_705811, EPI_ISL_705813, EPI_ISL_705814, EPI_ISL_705815, EPI_ISL_705816, EPI_ISL_705817, EPI_ISL_705819, EPI_ISL_705820, EPI_ISL_705821, EPI_ISL_705822, EPI_ISL_705823, EPI_ISL_705826, EPI_ISL_705829, EPI_ISL_705831, EPI_ISL_705836, EPI_ISL_705838, EPI_ISL_705859, EPI_ISL_705860, EPI_ISL_705861, EPI_ISL_705862, EPI_ISL_705863, EPI_ISL_705864, EPI_ISL_705925			
see above	Liverpool Clinical Laboratories	COVID-19 Genomics UK (COG-UK) Consortium	Sam Haldenby, Anita Lucaci, Steve Paterson, Julian Hiscox, Alistair Darby, M Almsaud, A Alrezaihi, Muhannad Alruwaili, Stuart D Armstrong, Jones Benjamin, Eleanor G Bentley, Anu Chawla, Jordan J Clark, Angela Cowell, Richard Eccles, Isabel García-Dorival, Matthew Gemmell, Alessandro Gerada, PKF Gilmore, Richard Gregory, Ximeng Han, Catherine Hartley, Margaret Hughes, Miren Iturriza-Gomara, James Johnson, L Luu, Jenifer Manson, Charlotte Nelson, Elaine O'Toole, Cassie Olateju, Rebekah Penrice-Randal , Lucille Rainbow, N.P Randle, Trevor Ian Robinson, Parul Sharma, Ghada T Shawli, James P Stewart, Neil Swainston, Ecaterina Vamos, Joanne Watts, Mark Whitehead
EPI_ISL_705942, EPI_ISL_705943, EPI_ISL_705944	University College London Hospital	COVID-19 Genomics UK (COG-UK) Consortium	Judith Heaney, Matthew Byott, Catherine Houlihan, Dan Frampton, Stuart Kirk, Moira Spyer and Eleni Nastouli
EPI_ISL_706320, EPI_ISL_706321, EPI_ISL_706323, EPI_ISL_706324, EPI_ISL_706325, EPI_ISL_706326, EPI_ISL_706327, EPI_ISL_706328, EPI_ISL_706329, EPI_ISL_706330	Northumbria University / South Tees Hospitals NHS Foundation Trust / North Cumbria Integrated Care NHS Foundation Trust / North Tees and Hartlepool NHS Foundation Trust / Newcastle Hospitals NHS Foundation Trust	COVID-19 Genomics UK (COG-UK) Consortium	Darren L Smith,Andrew Nelson,Matthew Bashton,Greg R Young,Joshua Loh,John Allan,Mohammad A Tariq,Giles S Holt,Gary Black,Wen C Yew,Lynn Dover,Paul Baker,Steve Liggett,Sarah Essex,Jane Greenaway,Debra Padgett,Clive Graham,Garren Scott,Edward Barton,Emma Swindells,Brendan Payne,Jennifer Collins,Yusri Taha,Gary Eltringham
EPI_ISL_706361, EPI_ISL_706362, EPI_ISL_706408	Quadram Institute Bioscience	COVID-19 Genomics UK (COG-UK) Consortium	Dave J. Baker, Gemma L. Kay, Alp Aydin, Thanh Le-Viet, Steven Rudder, Ana P. Tedim, Anastasia Kolyva, Maria Diaz, Leonardo de Oliveira Martins, Nabil-Fareed Alikhan, Lizzie Meadows, Rachael Stanley, Ngozi Elumogo, Muhammed Yasir, Nicholas M. Thomson, Alexander J Trotter, Rachel Gilroy, Samuel Bloomfield, Claire Stuart, Andrew Bell, Reenesh Prakash, Samir Dervisevic, Alison E. Mather, John Wain, Mark Webber, Andrew J. Page, Justin O'Grady
EPI_ISL_706420, EPI_ISL_706421	Queens Medical Centre, Clinical Microbiology Department / DeepSeq Nottingham	COVID-19 Genomics UK (COG-UK) Consortium	Gemma Clark, Wendy Smith, Manjinder Khakh, Vicki M Fleming, Michelle M Lister, Hannah Howson-Wells, Jonathan Ball, Patrick McClure, Joseph Chappell, Theocharis Tsoleridis, Nadine Holmes, Matthew Carlisle, Christopher Moore, Fei Sang, Johnny Debebe, Victoria Wright, Matthew Loose
EPI_ISL_706463	Wales Specialist Virology Centre Sequencing lab: Pathogen Genomics Unit	COVID-19 Genomics UK (COG-UK) Consortium	Catherine Moore, Johnathan Evans, Laura Gifford, Malorie Perry, Simon Cottrell, Angela Marchbank, Alec Birchley, Alexander Adams, Amy Gaskin, Bree Gatica-Wilcox, Jason Coombes, Joel Southgate, Lauren Gilbert, Lee Graham, Nicole Pacchiarini, Sara Kumziene-Summerhayes, Sarah Taylor, Sophie Jones, Sara Rey, Matthew Bull, Joanne Watkins, Sally Corden, Tom Connor
EPI_ISL_706983	Virology Department, Sheffield Teaching Hospitals NHS Foundation Trust/Department of Infection, Immunity and Cardiovascular Disease, The Medical School, University of Sheffield	COVID-19 Genomics UK (COG-UK) Consortium	Thushan de Silva, Matthew Parker, Nikki Smith, Adri Angyal, Rebecca Brown, Luke Green, Rachel Tucker, Paul Parsons, Danielle Groves, Katie Johnson, Laura Carrilero, Alex Keeley, Dave Partridge, Matthew Wyles, Benjamin Lindsey, Mehmet Yavuz, Mohammad Raza, Cariad Evans
EPI_ISL_707697, EPI_ISL_707698, EPI_ISL_707699, EPI_ISL_707700	1-Laboratory of Microbiology, National Reference Lab, Charles Nicolle Hospital; 2-University of Tunis ElManar, Faculty of Medicine of Tunis, LR99ES09, Tunis, Tunisia	1-Clinical and Experimental Pharmacology Lab, LR16SP02, National Center of Pharmacovigilance, University of Tunis El Manar, Tunis, Tunisia. 2-Neurodegenerative diseases and psychiatric troubles, LR18SP03, Razi Hospital, University of Tunis El Manar, Tunis, Tunisia. 3- Ministry of Health, National Observatory of New and Emerging Diseases, 1006, Tunis, Tunisia	Ilhem Boutiba-Ben Boubaker, Sameh Trabelsi, Nissaf Ben Alaya, Maher Kharrat, Alia Ben Kahla, Jalila Ben Khelil, Salma Abid, Sana Ferjani, Mouna Ben Sassi, Mouna Safer, Zaineb Hamzaoui, Habiba Ben Romdhane, Souissi Amira, Rouaa Ben Othman, Hanen El Jebari, Asma Ferjani, Gaies Emna, Riadh Daghfous, Riadh Guider.
EPI_ISL_708485, EPI_ISL_708486, EPI_ISL_708488	Minnesota Department of Health, Public Health Laboratory	Minnesota Department of Health, Public Health Laboratory	Alexandra Lorentz, Jacob Garfin, Matt Plumb, and Xiong Wang
EPI_ISL_708530	Secretaria Municipal de Saude de Fernandópolis	Instituto Adolfo Lutz, Interdiciplinary Procedures Center, Strategic Laboratory	Claudio Tavares Sacchi, Claudia Regina Gonçalves, Erica Valessa Ramos Gomes, Carlos Henrique Camargo, Karoline Rodrigues Campos, Fernanda Modesto Tolentino Binhardi, Maricelia Navarro Pinheiro Flores, Marcia Maria Costa Nunes Soares, Janaina Other Martins Montanha
EPI_ISL_710424, EPI_ISL_710427, EPI_ISL_710428	Los Angeles County PHL	Los Angeles County PHL	P. Hemarajata et al.
EPI_ISL_710577	Vardcentralen Brinken	The Public Health Agency of Sweden	Department of Microbiology, The Public Health Agency of Sweden
EPI_ISL_710612	Capio Bro VC	The Public Health Agency of Sweden	Department of Microbiology, The Public Health Agency of Sweden
EPI_ISL_710699, EPI_ISL_710700, EPI_ISL_710701, EPI_ISL_710702, EPI_ISL_710703, EPI_ISL_710704, EPI_ISL_710705, EPI_ISL_710706, EPI_ISL_710707, EPI_ISL_710708, EPI_ISL_710709, EPI_ISL_710710, EPI_ISL_710711, EPI_ISL_710712, EPI_ISL_710713, EPI_ISL_710714, EPI_ISL_710715, EPI_ISL_710716, EPI_ISL_710717, EPI_ISL_710718, EPI_ISL_710719, EPI_ISL_710720, EPI_ISL_710721, EPI_ISL_710722, EPI_ISL_710723, EPI_ISL_710724, EPI_ISL_710725, EPI_ISL_710726, EPI_ISL_710727, EPI_ISL_710728, EPI_ISL_710729, EPI_ISL_710730, EPI_ISL_710731, EPI_ISL_710732, EPI_ISL_710733, EPI_ISL_710734, EPI_ISL_710735, EPI_ISL_710736, EPI_ISL_710737, EPI_ISL_710738, EPI_ISL_710739, EPI_ISL_710740, EPI_ISL_710741, EPI_ISL_710742, EPI_ISL_710743, EPI_ISL_710744, EPI_ISL_710745, EPI_ISL_710746, EPI_ISL_710747, EPI_ISL_710749, EPI_ISL_710750, EPI_ISL_710751, EPI_ISL_710752			
see above	Lighthouse Lab in Alderley Park	Wellcome Sanger Institute for the COVID-19 Genomics UK (COG-UK) Consortium	Jacquelyn Wynn, Mairead Hyland, The Lighthouse Lab in Alderley Park and Alex Alderton, Roberto Amato, Sonia Goncalves, Ewan Harrison, David K. Jackson, Ian Johnston, Dominic Kwiatkowski, Cordelia Langford, John Sillitoe on behalf of the Wellcome Sanger Institute COVID-19 Surveillance Team
EPI_ISL_710821, EPI_ISL_710822, EPI_ISL_710823, EPI_ISL_710824, EPI_ISL_710825, EPI_ISL_710826, EPI_ISL_710827, EPI_ISL_710828, EPI_ISL_710829	Lighthouse Lab in Cambridge	Wellcome Sanger Institute for the COVID-19 Genomics UK (COG-UK) Consortium	Rob Howes, The Lighthouse Lab in Cambridge and Alex Alderton, Roberto Amato, Sonia Goncalves, Ewan Harrison, David K. Jackson, Ian Johnston, Dominic Kwiatkowski, Cordelia Langford, John Sillitoe on behalf of the Wellcome Sanger Institute COVID-19 Surveillance Team
EPI_ISL_711067, EPI_ISL_711074, EPI_ISL_711076, EPI_ISL_711077, EPI_ISL_711078, EPI_ISL_711149, EPI_ISL_711150, EPI_ISL_711151, EPI_ISL_711152, EPI_ISL_711153, EPI_ISL_711154, EPI_ISL_711155, EPI_ISL_711156, EPI_ISL_711157, EPI_ISL_711158, EPI_ISL_711159, EPI_ISL_711160, EPI_ISL_711161, EPI_ISL_711162, EPI_ISL_711163, EPI_ISL_711164, EPI_ISL_711165, EPI_ISL_711166, EPI_ISL_711167, EPI_ISL_711168, EPI_ISL_711169, EPI_ISL_711170, EPI_ISL_711171, EPI_ISL_711172, EPI_ISL_711173, EPI_ISL_711174, EPI_ISL_711175, EPI_ISL_711177, EPI_ISL_711178, EPI_ISL_711179, EPI_ISL_711180, EPI_ISL_711181, EPI_ISL_711185, EPI_ISL_711190, EPI_ISL_711191, EPI_ISL_711192, EPI_ISL_711194, EPI_ISL_711195, EPI_ISL_711198, EPI_ISL_711199, EPI_ISL_711201, EPI_ISL_711207, EPI_ISL_711208, EPI_ISL_711209, EPI_ISL_711210, EPI_ISL_711211, EPI_ISL_711212, EPI_ISL_711213, EPI_ISL_711216, EPI_ISL_711217, EPI_ISL_711218, EPI_ISL_711220, EPI_ISL_711228, EPI_ISL_711232, EPI_ISL_711256, EPI_ISL_711257, EPI_ISL_711258, EPI_ISL_711259, EPI_ISL_711260, EPI_ISL_711261, EPI_ISL_711262, EPI_ISL_711263, EPI_ISL_711264, EPI_ISL_711265, EPI_ISL_711268, EPI_ISL_711271, EPI_ISL_711272,			

EPI_ISL_711273, EPI_ISL_711274, EPI_ISL_711276, EPI_ISL_711277, EPI_ISL_711281, EPI_ISL_711295, EPI_ISL_711296, EPI_ISL_711297, EPI_ISL_711298, EPI_ISL_711361, EPI_ISL_711362, EPI_ISL_711363, EPI_ISL_711364, EPI_ISL_711365, EPI_ISL_711366, EPI_ISL_711367, EPI_ISL_711368, EPI_ISL_711369, EPI_ISL_711370, EPI_ISL_711371, EPI_ISL_711372, EPI_ISL_711373, EPI_ISL_711374, EPI_ISL_711375, EPI_ISL_711376, EPI_ISL_711377, EPI_ISL_711378, EPI_ISL_711379, EPI_ISL_711380, EPI_ISL_711381, EPI_ISL_711382, EPI_ISL_711383, EPI_ISL_711390, EPI_ISL_711391, EPI_ISL_711392, EPI_ISL_711395, EPI_ISL_711396, EPI_ISL_711397, EPI_ISL_711407, EPI_ISL_711408, EPI_ISL_711411, EPI_ISL_711429, EPI_ISL_711430, EPI_ISL_711438, EPI_ISL_711439, EPI_ISL_711440, EPI_ISL_711441, EPI_ISL_711442, EPI_ISL_711443, EPI_ISL_711444, EPI_ISL_711850, EPI_ISL_711851, EPI_ISL_711852, EPI_ISL_711853, EPI_ISL_711854, EPI_ISL_711855, EPI_ISL_711856, EPI_ISL_711857, EPI_ISL_711858, EPI_ISL_711859, EPI_ISL_711860, EPI_ISL_711861, EPI_ISL_711862, EPI_ISL_711863, EPI_ISL_711864, EPI_ISL_711865, EPI_ISL_711866, EPI_ISL_711867, EPI_ISL_711868, EPI_ISL_711869, EPI_ISL_711870, EPI_ISL_711871, EPI_ISL_711872, EPI_ISL_711873, EPI_ISL_711874, EPI_ISL_711875, EPI_ISL_711876, EPI_ISL_711877, EPI_ISL_711878, EPI_ISL_711879, EPI_ISL_711880, EPI_ISL_711881, EPI_ISL_711882, EPI_ISL_711883, EPI_ISL_711884, EPI_ISL_711885, EPI_ISL_711886, EPI_ISL_711887, EPI_ISL_711888, EPI_ISL_711889, EPI_ISL_711890, EPI_ISL_711891, EPI_ISL_711892, EPI_ISL_711893, EPI_ISL_711894, EPI_ISL_711895, EPI_ISL_711896, EPI_ISL_711897, EPI_ISL_711898, EPI_ISL_711899, EPI_ISL_711900, EPI_ISL_711901, EPI_ISL_711902, EPI_ISL_711903, EPI_ISL_711904, EPI_ISL_711905, EPI_ISL_711906, EPI_ISL_711907, EPI_ISL_711908, EPI_ISL_711909, EPI_ISL_711910, EPI_ISL_711911, EPI_ISL_711912, EPI_ISL_711913, EPI_ISL_711914, EPI_ISL_711915, EPI_ISL_711916, EPI_ISL_711917, EPI_ISL_711918, EPI_ISL_711919, EPI_ISL_711920, EPI_ISL_711921, EPI_ISL_711922, EPI_ISL_711923, EPI_ISL_711924, EPI_ISL_711925, EPI_ISL_711926, EPI_ISL_711927, EPI_ISL_711928, EPI_ISL_711929, EPI_ISL_711930, EPI_ISL_711931, EPI_ISL_711932, EPI_ISL_711933, EPI_ISL_711934, EPI_ISL_711935, EPI_ISL_711936, EPI_ISL_711937, EPI_ISL_711938, EPI_ISL_711939, EPI_ISL_711940, EPI_ISL_711941, EPI_ISL_711942, EPI_ISL_711943, EPI_ISL_711944, EPI_ISL_711945, EPI_ISL_711946, EPI_ISL_711947, EPI_ISL_711948, EPI_ISL_711949, EPI_ISL_711950, EPI_ISL_711951, EPI_ISL_711952, EPI_ISL_711953, EPI_ISL_711954, EPI_ISL_711955, EPI_ISL_711956, EPI_ISL_711957, EPI_ISL_711958, EPI_ISL_711959, EPI_ISL_711960, EPI_ISL_711961, EPI_ISL_711962, EPI_ISL_711963, EPI_ISL_711964, EPI_ISL_711965, EPI_ISL_711966, EPI_ISL_711967, EPI_ISL_711968, EPI_ISL_711969, EPI_ISL_711970, EPI_ISL_711971, EPI_ISL_711972, EPI_ISL_711973, EPI_ISL_711974, EPI_ISL_711975, EPI_ISL_711976, EPI_ISL_711977, EPI_ISL_711978, EPI_ISL_711979, EPI_ISL_711980, EPI_ISL_711981, EPI_ISL_711982, EPI_ISL_711983, EPI_ISL_711984, EPI_ISL_711985, EPI_ISL_711986, EPI_ISL_711987, EPI_ISL_711988, EPI_ISL_711989, EPI_ISL_711990, EPI_ISL_711991, EPI_ISL_711992, EPI_ISL_711993, EPI_ISL_711994, EPI_ISL_711995, EPI_ISL_711996, EPI_ISL_711997, EPI_ISL_711998, EPI_ISL_711999, EPI_ISL_712000, EPI_ISL_712001, EPI_ISL_712002, EPI_ISL_712003, EPI_ISL_712004, EPI_ISL_712005, EPI_ISL_712006, EPI_ISL_712007, EPI_ISL_712008, EPI_ISL_712009, EPI_ISL_712010, EPI_ISL_712011, EPI_ISL_712012, EPI_ISL_712013, EPI_ISL_712014, EPI_ISL_712015, EPI_ISL_712016, EPI_ISL_712017, EPI_ISL_712018, EPI_ISL_712019, EPI_ISL_712020, EPI_ISL_712021, EPI_ISL_712022, EPI_ISL_712023, EPI_ISL_712024, EPI_ISL_712025, EPI_ISL_712026, EPI_ISL_712027, EPI_ISL_712028, EPI_ISL_712029, EPI_ISL_712030, EPI_ISL_712031, EPI_ISL_712032, EPI_ISL_712033, EPI_ISL_712034, EPI_ISL_712035, EPI_ISL_712036, EPI_ISL_712037, EPI_ISL_712038, EPI_ISL_712039, EPI_ISL_712040, EPI_ISL_712041, EPI_ISL_712042, EPI_ISL_712043, EPI_ISL_712044, EPI_ISL_712045, EPI_ISL_712046, EPI_ISL_712047, EPI_ISL_712048, EPI_ISL_712049, EPI_ISL_712050, EPI_ISL_712051, EPI_ISL_712052, EPI_ISL_712053, EPI_ISL_712054, EPI_ISL_712055, EPI_ISL_712056, EPI_ISL_712057, EPI_ISL_712058, EPI_ISL_712059, EPI_ISL_713098, EPI_ISL_713099, EPI_ISL_713100, EPI_ISL_713101, EPI_ISL_713102, EPI_ISL_713103, EPI_ISL_713104, EPI_ISL_713105, EPI_ISL_713106, EPI_ISL_713107, EPI_ISL_713108, EPI_ISL_713109, EPI_ISL_713110, EPI_ISL_713111, EPI_ISL_713112, EPI_ISL_713113, EPI_ISL_713114, EPI_ISL_713115, EPI_ISL_713116, EPI_ISL_713117, EPI_ISL_713118, EPI_ISL_713119, EPI_ISL_713120, EPI_ISL_713121, EPI_ISL_713122, EPI_ISL_713123, EPI_ISL_713124, EPI_ISL_713125, EPI_ISL_713126, EPI_ISL_713127, EPI_ISL_713128, EPI_ISL_713129, EPI_ISL_713130, EPI_ISL_713131, EPI_ISL_713132, EPI_ISL_713133, EPI_ISL_713134, EPI_ISL_713135, EPI_ISL_713136, EPI_ISL_713137, EPI_ISL_713138, EPI_ISL_713139, EPI_ISL_713140, EPI_ISL_713141, EPI_ISL_713142, EPI_ISL_713143, EPI_ISL_713144, EPI_ISL_713145, EPI_ISL_713146, EPI_ISL_713147, EPI_ISL_713148, EPI_ISL_713149, EPI_ISL_713150, EPI_ISL_713151, EPI_ISL_713152, EPI_ISL_713153, EPI_ISL_713154, EPI_ISL_713155, EPI_ISL_713156, EPI_ISL_713157, EPI_ISL_713158, EPI_ISL_713159, EPI_ISL_713160, EPI_ISL_713161, EPI_ISL_713162, EPI_ISL_713163, EPI_ISL_713164, EPI_ISL_713165, EPI_ISL_713166, EPI_ISL_713167, EPI_ISL_713168, EPI_ISL_713169, EPI_ISL_713170, EPI_ISL_713171, EPI_ISL_713172, EPI_ISL_713173, EPI_ISL_713174, EPI_ISL_713175, EPI_ISL_713176, EPI_ISL_713177, EPI_ISL_713178, EPI_ISL_713179, EPI_ISL_713180, EPI_ISL_713181, EPI_ISL_713182, EPI_ISL_713183, EPI_ISL_713184, EPI_ISL_713185, EPI_ISL_713186, EPI_ISL_713187, EPI_ISL_713188, EPI_ISL_713189, EPI_ISL_713190, EPI_ISL_713191, EPI_ISL_713192, EPI_ISL_713193, EPI_ISL_713194, EPI_ISL_713195, EPI_ISL_713196, EPI_ISL_713197, EPI_ISL_713198, EPI_ISL_713199, EPI_ISL_713200, EPI_ISL_713201, EPI_ISL_713202, EPI_ISL_713203, EPI_ISL_713204, EPI_ISL_713205, EPI_ISL_713206, EPI_ISL_713207, EPI_ISL_713208, EPI_ISL_713209, EPI_ISL_713210, EPI_ISL_713211, EPI_ISL_713212, EPI_ISL_713213, EPI_ISL_713214, EPI_ISL_713215, EPI_ISL_713216, EPI_ISL_713217, EPI_ISL_713218, EPI_ISL_713219, EPI_ISL_713220, EPI_ISL_713221, EPI_ISL_713222, EPI_ISL_713223, EPI_ISL_713224, EPI_ISL_713225, EPI_ISL_713226, EPI_ISL_713227, EPI_ISL_713228, EPI_ISL_713229, EPI_ISL_713230, EPI_ISL_713231, EPI_ISL_713232, EPI_ISL_713233, EPI_ISL_713234, EPI_ISL_713235, EPI_ISL_713236, EPI_ISL_713237, EPI_ISL_713238, EPI_ISL_713239, EPI_ISL_713240, EPI_ISL_713241, EPI_ISL_713242, EPI_ISL_713243, EPI_ISL_713244, EPI_ISL_713245, EPI_ISL_713246, EPI_ISL_713247, EPI_ISL_713248, EPI_ISL_713249, EPI_ISL_713250, EPI_ISL_713251, EPI_ISL_713252, EPI_ISL_713253, EPI_ISL_713254, EPI_ISL_713255, EPI_ISL_713256, EPI_ISL_713257, EPI_ISL_713258, EPI_ISL_713259, EPI_ISL_713260, EPI_ISL_713261, EPI_ISL_713262, EPI_ISL_713263, EPI_ISL_713264, EPI_ISL_713265, EPI_ISL_713266, EPI_ISL_713267, EPI_ISL_713268, EPI_ISL_713269, EPI_ISL_713270, EPI_ISL_713271, EPI_ISL_713272, EPI_ISL_713273, EPI_ISL_713274, EPI_ISL_713275, EPI_ISL_713276, EPI_ISL_713277, EPI_ISL_713278, EPI_ISL_713279, EPI_ISL_713280, EPI_ISL_713281, EPI_ISL_713282, EPI_ISL_713283, EPI_ISL_713284, EPI_ISL_713285, EPI_ISL_713286, EPI_ISL_713287, EPI_ISL_713288, EPI_ISL_713289, EPI_ISL_713290, EPI_ISL_713291, EPI_ISL_713292, EPI_ISL_713293, EPI_ISL_713294, EPI_ISL_713295, EPI_ISL_713296, EPI_ISL_713297, EPI_ISL_713298, EPI_ISL_713299, EPI_ISL_713300, EPI_ISL_713301, EPI_ISL_713302, EPI_ISL_713303, EPI_ISL_713304, EPI_ISL_713305, EPI_ISL_713306, EPI_ISL_713307, EPI_ISL_713308, EPI_ISL_713309, EPI_ISL_713310, EPI_ISL_713311, EPI_ISL_713312, EPI_ISL_713313, EPI_ISL_713314, EPI_ISL_713315, EPI_ISL_713316, EPI_ISL_713317, EPI_ISL_713318, EPI_ISL_713319, EPI_ISL_713320, EPI_ISL_713321, EPI_ISL_713322, EPI_ISL_713323, EPI_ISL_713324, EPI_ISL_713325, EPI_ISL_713326, EPI_ISL_713327, EPI_ISL_713328, EPI_ISL_713329, EPI_ISL_713330, EPI_ISL_713331, EPI_ISL_713332, EPI_ISL_713333, EPI_ISL_713334, EPI_ISL_713335, EPI_ISL_713336, EPI_ISL_713337, EPI_ISL_713338, EPI_ISL_713339, EPI_ISL_713340, EPI_ISL_713341, EPI_ISL_713342, EPI_ISL_713343, EPI_ISL_713344, EPI_ISL_713345, EPI_ISL_713346, EPI_ISL_713347, EPI_ISL_713348, EPI_ISL_713349, EPI_ISL_713350, EPI_ISL_713351, EPI_ISL_713352, EPI_ISL_713353, EPI_ISL_713354, EPI_ISL_713355, EPI_ISL_713356, EPI_ISL_713357, EPI_ISL_713358, EPI_ISL_713359, EPI_ISL_713360, EPI_ISL_713361, EPI_ISL_713362, EPI_ISL_713363, EPI_ISL_713364, EPI_ISL_713365, EPI_ISL_713366, EPI_ISL_713367, EPI_ISL_713368, EPI_ISL_713369, EPI_ISL_713370, EPI_ISL_713371, EPI_ISL_713372, EPI_ISL_713373, EPI_ISL_713374, EPI_ISL_713375, EPI_ISL_713376, EPI_ISL_713377, EPI_ISL_713378, EPI_ISL_713379, EPI_ISL_713380, EPI_ISL_713381, EPI_ISL_713382, EPI_ISL_713383, EPI_ISL_713384, EPI_ISL_713385, EPI_ISL_713386, EPI_ISL_713387, EPI_ISL_713388, EPI_ISL_713389, EPI_ISL_713390, EPI_ISL_713391, EPI_ISL_713392, EPI_ISL_713393, EPI_ISL_713394, EPI_ISL_713395, EPI_ISL_713396, EPI_ISL_713397, EPI_ISL_713398, EPI_ISL_713399, EPI_ISL_713400, EPI_ISL_713401, EPI_ISL_713402, EPI_ISL_713403, EPI_ISL_713404, EPI_ISL_713405, EPI_ISL_713406, EPI_ISL_713407, EPI_ISL_713408, EPI_ISL_713409, EPI_ISL_713410, EPI_ISL_713411, EPI_ISL_713412, EPI_ISL_713413, EPI_ISL_713414, EPI_ISL_713415, EPI_ISL_713416, EPI_ISL_713417, EPI_ISL_713418, EPI_ISL_713419, EPI_ISL_713420, EPI_ISL_713421, EPI_ISL_713422, EPI_ISL_713423, EPI_ISL_713424, EPI_ISL_713425, EPI_ISL_713426, EPI_ISL_713427, EPI_ISL_713428, EPI_ISL_713429, EPI_ISL_713430, EPI_ISL_713431, EPI_ISL_713432, EPI_ISL_713433, EPI_ISL_713434, EPI_ISL_713435, EPI_ISL_713436, EPI_ISL_713437, EPI_ISL_713438, EPI_ISL_713439, EPI_ISL_713440, EPI_ISL_713441, EPI_ISL_713442, EPI_ISL_713443, EPI_ISL_713444, EPI_ISL_713445, EPI_ISL_713446, EPI_ISL_713447, EPI_ISL_713448, EPI_ISL_713449, EPI_ISL_713450, EPI_ISL_713451, EPI_ISL_713452, EPI_ISL_713453, EPI_ISL_713454, EPI_ISL_713455, EPI_ISL_713456, EPI_ISL_713457, EPI_ISL_713458, EPI_ISL_713459, EPI_ISL_713460, EPI_ISL_713461, EPI_ISL_713462, EPI_ISL_713463, EPI_ISL_713464, EPI_ISL_713465, EPI_ISL_713466, EPI_ISL_713467, EPI_ISL_713468, EPI_ISL_713469, EPI_ISL_713470, EPI_ISL_713471, EPI_ISL_713472, EPI_ISL_713473, EPI_ISL_713474, EPI_ISL_713475, EPI_ISL_713476, EPI_ISL_713477, EPI_ISL_713478, EPI_ISL_713479, EPI_ISL_713480, EPI_ISL_713481, EPI_ISL_713482, EPI_ISL_713483, EPI_ISL_713484, EPI_ISL_713485, EPI_ISL_713486, EPI_ISL_713487, EPI_ISL_713488, EPI_ISL_713489, EPI_ISL_713490, EPI_ISL_713491, EPI_ISL_713492, EPI_ISL_713493, EPI_ISL_713494, EPI_ISL_713495, EPI_ISL_713496, EPI_ISL_713497, EPI_ISL_713498, EPI_ISL_713499, EPI_ISL_713500, EPI_ISL_713501, EPI_ISL_713502, EPI_ISL_713503, EPI_ISL_713504, EPI_ISL_713505, EPI_ISL_713506, EPI_ISL_713507, EPI_ISL_713508, EPI_ISL_713509, EPI_ISL_713510, EPI_ISL_713511, EPI_ISL_713512, EPI_ISL_713513, EPI_ISL_713514, EPI_ISL_713515, EPI_ISL_713516, EPI_ISL_713517, EPI_ISL_713518, EPI_ISL_713519, EPI_ISL_713520, EPI_ISL_713521, EPI_ISL_713522, EPI_ISL_713523, EPI_ISL_713524, EPI_ISL_713525, EPI_ISL_713526, EPI_ISL_713527, EPI_ISL_713528, EPI_ISL_713529, EPI_ISL_713530, EPI_ISL_713531, EPI_ISL_713532, EPI_ISL_713533, EPI_ISL_713534, EPI_ISL_713535, EPI_ISL_713536, EPI_ISL_713537, EPI_ISL_713538, EPI_ISL_713539, EPI_ISL_713540, EPI_ISL_713541, EPI_ISL_713542, EPI_ISL_713543, EPI_ISL_713544, EPI_ISL_713545, EPI_ISL_713546, EPI_ISL_713547, EPI_ISL_713548, EPI_ISL_713549, EPI_ISL_713550, EPI_ISL_713551, EPI_ISL_713552, EPI_ISL_713553, EPI_ISL_713554, EPI_ISL_713555, EPI_ISL_713556, EPI_ISL_713557, EPI_ISL_713558, EPI_ISL_713559, EPI_ISL_713560, EPI_ISL_713561, EPI_ISL_713562, EPI_ISL_713563, EPI_ISL_713564, EPI_ISL_713565, EPI_ISL_713566, EPI_ISL_713567, EPI_ISL_713568, EPI_ISL_713569, EPI_ISL_713570, EPI_ISL_713571, EPI_ISL_713572, EPI_ISL_713573, EPI_ISL_713574, EPI_ISL_713575, EPI_ISL_713576, EPI_ISL_713577, EPI_ISL_713578, EPI_ISL_713579, EPI_ISL_713580, EPI_ISL_713581, EPI_ISL_713582, EPI_ISL_713583, EPI_ISL_713584, EPI_ISL_713585, EPI_ISL_713586, EPI_ISL_713587, EPI_ISL_713588, EPI_ISL_713589, EPI_ISL_713590, EPI_ISL_713591, EPI_ISL_713592, EPI_ISL_713593, EPI_ISL_713594, EPI_ISL_713595, EPI_ISL_713596, EPI_ISL_713597, EPI_ISL_713598, EPI_ISL_713599, EPI_ISL_713600, EPI_ISL_713601, EPI_ISL_713602, EPI_ISL_713603, EPI_ISL_713604, EPI_ISL_713605, EPI_ISL_713606, EPI_ISL_713607, EPI_ISL_713608, EPI_ISL_713609, EPI_ISL_713610, EPI_ISL_713611, EPI_ISL_713612, EPI_ISL_713613, EPI_ISL_713614, EPI_ISL_713615, EPI_ISL_713616, EPI_ISL_713617, EPI_ISL_713618, EPI_ISL_713619, EPI_ISL_713620, EPI_ISL_713621, EPI_ISL_713622, EPI_ISL_713623, EPI_ISL_713624, EPI_ISL_713625, EPI_ISL_713626, EPI_ISL_713627, EPI_ISL_713628, EPI_ISL_713629, EPI_ISL_713630, EPI_ISL_713631, EPI_ISL_713632, EPI_ISL_713633, EPI_ISL_713634, EPI_ISL_713635, EPI_ISL_713636, EPI_ISL_713637, EPI_ISL_713638, EPI_ISL_713639, EPI_ISL_713640, EPI_ISL_713641, EPI_ISL_713642, EPI_ISL_713643, EPI_ISL_713644, EPI_ISL_713645, EPI_ISL_713646, EPI_ISL_713647, EPI_ISL_713648, EPI_ISL_713649, EPI_ISL_713650, EPI_ISL_713651, EPI_ISL_713652, EPI_ISL_713653, EPI_ISL_713654, EPI_ISL_713655, EPI_ISL_713656, EPI_ISL_713657, EPI_ISL_713658, EPI_ISL_713659, EPI_ISL_713660, EPI_ISL_713661, EPI_ISL_713662, EPI_ISL_713663, EPI_ISL_713664, EPI_ISL_713665, EPI_ISL_713666, EPI_ISL_713667, EPI_ISL_713668, EPI_ISL_713669, EPI_ISL_713670, EPI_ISL_713671, EPI_ISL_713672, EPI_ISL_713673, EPI_ISL_713674, EPI_ISL_713675, EPI_ISL_713676, EPI_ISL_713677, EPI_ISL_713678, EPI_ISL_713679, EPI_ISL_713680, EPI_ISL_713681, EPI_ISL_713682, EPI_ISL_713683, EPI_ISL_713684, EPI_ISL_713685, EPI_ISL_713686, EPI_ISL_713687, EPI_ISL_713688, EPI_ISL_713689, EPI_ISL_713690, EPI_ISL_713691, EPI_ISL_713692, EPI_ISL_713693, EPI_ISL_713694, EPI_ISL_713695, EPI_ISL_713696, EPI_ISL_713697, EPI_ISL_713698, EPI_ISL_713699, EPI_ISL_713700, EPI_ISL_713701, EPI_ISL_713702, EPI_ISL_713703, EPI_ISL_713704, EPI_ISL_713705, EPI_ISL_713706, EPI_ISL_713707, EPI_ISL_713708, EPI_ISL_713709, EPI_ISL_713710, EPI_ISL_713711, EPI_ISL_713712, EPI_ISL_713713, EPI_ISL_713714, EPI_ISL_713715, EPI_ISL_713716, EPI_ISL_713717, EPI_ISL_713718, EPI_ISL_713719, EPI_ISL_713720, EPI_ISL_713721, EPI_ISL_713722, EPI_ISL_713723, EPI_ISL_713724, EPI_ISL_713725, EPI_ISL_713726, EPI_ISL_713727, EPI_ISL_713728, EPI_ISL_713729, EPI_ISL_713730, EPI_ISL_713731, EPI_ISL_713732, EPI_ISL_713733, EPI_ISL_713734, EPI_ISL_713735, EPI_ISL_713736, EPI_ISL_713737, EPI_ISL_713738, EPI_ISL_713739, EPI_ISL_713740, EPI_ISL_713741, EPI_ISL_713742, EPI_ISL_713743, EPI_ISL_713744, EPI_ISL_713745, EPI_ISL_713746, EPI_ISL_713747, EPI_ISL_713748, EPI_ISL_713749, EPI_ISL_713750, EPI_ISL_713751, EPI_ISL_713752, EPI_ISL_713753, EPI_ISL_713754, EPI_ISL_713755, EPI_ISL_713756, EPI_ISL_713757, EPI_ISL_713758, EPI_ISL_713759, EPI_ISL_713760, EPI_ISL_713761, EPI_ISL_713762, EPI_ISL_713763, EPI_ISL_713764, EPI_ISL_713765, EPI_ISL_713766, EPI_ISL_713767, EPI_ISL_713768, EPI_ISL_713769, EPI_ISL_713770, EPI_ISL_713771, EPI_ISL_713772, EPI_ISL_713773, EPI_ISL_713774, EPI_ISL_713775, EPI_ISL_713776, EPI_ISL_713777, EPI_ISL_713778, EPI_ISL_713779, EPI_ISL_713780, EPI_ISL_713781, EPI_ISL_713782, EPI_ISL_713783, EPI_ISL_713784, EPI_ISL_713785, EPI_ISL_713786, EPI_ISL_713787, EPI_ISL_713788, EPI_ISL_713789, EPI_ISL_713790, EPI_ISL_713791, EPI_ISL_713792, EPI_ISL_713793, EPI_ISL_713794, EPI_ISL_713795, EPI_ISL_713796, EPI_ISL_713797, EPI_ISL_713798, EPI_ISL_713799, EPI_ISL_713800, EPI_ISL_713801, EPI_ISL_713802, EPI_ISL_713803, EPI_ISL_713804, EPI_ISL_713805, EPI_ISL_713806, EPI_ISL_713807, EPI_ISL_713808, EPI_ISL_713809, EPI_ISL_713810, EPI_ISL_713811, EPI_ISL_713812, EPI_ISL_713813, EPI_ISL_713814, EPI_ISL_713815, EPI_ISL_713816, EPI_ISL_713817, EPI_ISL_713818, EPI_ISL_713819, EPI_ISL_713820, EPI_ISL_713821, EPI_ISL_713822, EPI_ISL_713823, EPI_ISL_713824, EPI_ISL_713825, EPI_ISL_713826, EPI_ISL_713827, EPI_ISL_713828, EPI_ISL_713829, EPI_ISL_713830, EPI_ISL_713831, EPI_ISL_713832, EPI_ISL_713833, EPI_ISL_713834, EPI_ISL_713835, EPI_ISL_713836, EPI_ISL_713837, EPI_ISL_713838, EPI_ISL_713839, EPI_ISL_713840, EPI_ISL_713841, EPI_ISL_713842, EPI_ISL_713843, EPI_ISL_713844, EPI_ISL_713845, EPI_ISL_713846, EPI_ISL_713847, EPI_ISL_713848, EPI_ISL_713849, EPI_ISL_713850, EPI_ISL_713851, EPI_ISL_713852, EPI_ISL_713853, EPI_ISL_713854, EPI_ISL_713855, EPI_ISL_713856, EPI_ISL_713857, EPI_ISL_713858, EPI_ISL_713859, EPI_ISL_713860, EPI_ISL_713861, EPI_ISL_713862, EPI_ISL_713863, EPI_ISL_713864, EPI_ISL_713865, EPI_ISL_713866, EPI_ISL_713867, EPI_ISL_713868, EPI_ISL_713869, EPI_ISL_713870, EPI_ISL_713871, EPI_ISL_713872, EPI_ISL_713873, EPI_ISL_713874, EPI_ISL_713875, EPI_ISL_713876, EPI_ISL_713877, EPI_ISL_713878, EPI_ISL_713879, EPI_ISL_713880, EPI_ISL_713881, EPI_ISL_713882, EPI_ISL_713883, EPI_ISL_713884, EPI_ISL_713885, EPI_ISL_713886, EPI_ISL_713887, EPI_ISL_713888, EPI_ISL_713889, EPI_ISL_713890, EPI_ISL_713891, EPI_ISL_713892, EPI_ISL_713893, EPI_ISL_713894, EPI_ISL_713895, EPI_ISL_713896, EPI_ISL_713897, EPI_ISL_713898, EPI_ISL_713899, EPI_ISL_713900, EPI_ISL_713901, EPI_ISL_713902, EPI_ISL_713903, EPI_ISL_713904, EPI_ISL_713905, EPI_ISL_713906, EPI_ISL_713907, EPI_ISL_713908, EPI_ISL_713909, EPI_ISL_713910, EPI_ISL_713911, EPI_ISL_713912, EPI_ISL_713913, EPI_ISL_713914, EPI_ISL_713915, EPI_ISL_713916, EPI_ISL_713917, EPI_ISL_713918, EPI_ISL_713919, EPI_ISL_713920, EPI_ISL_713921, EPI_ISL_713922, EPI_ISL_713923, EPI_ISL_713924, EPI_ISL_713925, EPI_ISL_713926, EPI_ISL_713927, EPI_ISL_713928, EPI_ISL_713929, EPI_ISL_713930, EPI_ISL_713931, EPI_ISL_713932, EPI_ISL_713933, EPI_ISL_713934, EPI_ISL_713935, EPI_ISL_713936, EPI_ISL_713937, EPI_ISL_713938, EPI_ISL_713939, EPI_ISL_713940, EPI_ISL_713941, EPI_ISL_713942, EPI_ISL_713943, EPI_ISL_713944, EPI_ISL_713945, EPI_ISL_713946, EPI_ISL_713947, EPI_ISL_713948, EPI_ISL_713949, EPI_ISL_713950, EPI_ISL_713951, EPI_ISL_713952, EPI_ISL_713953, EPI_ISL_713954, EPI_ISL_713955, EPI_ISL_713956, EPI_ISL_713957, EPI_ISL_713958, EPI_ISL_713959, EPI_ISL_713960, EPI_ISL_713961, EPI_ISL_713962, EPI_ISL_713963, EPI_ISL_713964, EPI_ISL_713965, EPI_ISL_713966, EPI_ISL_713967, EPI_ISL_713968, EPI_ISL_713969, EPI_ISL_713970, EPI_ISL_713971, EPI_ISL_713972, EPI_ISL_713973, EPI_ISL_713974, EPI_ISL_713975, EPI_ISL_713976, EPI_ISL_713977, EPI_ISL_713978, EPI_ISL_713979, EPI_ISL_713980, EPI_ISL_713981, EPI_ISL_713982, EPI_ISL_713983, EPI_ISL_713984, EPI_ISL_713985, EPI_ISL_713986, EPI_ISL_713987, EPI_ISL_713988, EPI_ISL_713989, EPI_ISL_713990, EPI_ISL_713991, EPI_ISL_713992, EPI_ISL_713993, EPI_ISL_713994, EPI_ISL_713995, EPI_ISL_713996, EPI_ISL_713997, EPI_ISL_713998, EPI_ISL_713999, EPI_ISL_714000, EPI_ISL_714001, EPI_ISL_714002, EPI_ISL_714003, EPI_ISL_714004, EPI_ISL_714005, EPI_ISL_714006, EPI_ISL_714007, EPI_ISL_714008, EPI_ISL_714009, EPI_ISL_714010, EPI_ISL_714011, EPI_ISL_714012, EPI_ISL_714013, EPI_ISL_714014, EPI_ISL_714015, EPI_ISL_714016, EPI_ISL_714017, EPI_ISL_714018, EPI_ISL_714019, EPI_ISL_714020, EPI_ISL_714021, EPI_ISL_714022, EPI_ISL_714023, EPI_ISL_714024, EPI_ISL_714025, EPI_ISL_714026, EPI_ISL_714027, EPI_ISL_714028, EPI_ISL_714029, EPI_ISL_714030, EPI_ISL_714031, EPI_ISL_714032, EPI_ISL_714033, EPI_ISL_714034, EPI_ISL_714035, EPI_ISL_714036, EPI_ISL_714037, EPI_ISL_714038, EPI_ISL_714039, EPI_ISL_714040, EPI_ISL_714041, EPI_ISL_714042, EPI_ISL_714043, EPI_ISL_714044, EPI_ISL_714045, EPI_ISL_714046, EPI_ISL_714047, EPI_ISL_714048, EPI_ISL_714049, EPI_ISL_714050, EPI_ISL_714051, EPI_ISL_714052, EPI_ISL_714053, EPI_ISL_714054, EPI_ISL_714055, EPI_ISL_714056, EPI_ISL_714057, EPI_ISL_714058, EPI_ISL_714059, EPI_ISL_714060, EPI_ISL_714061, EPI_ISL_714062, EPI_ISL_714063, EPI_ISL_714064, EPI_ISL_714065, EPI_ISL_714066, EPI_ISL_714067, EPI_ISL_714068, EPI_ISL_714069, EPI_ISL_714070, EPI_ISL_714071, EPI_ISL_714072, EPI_ISL_714073, EPI_ISL_714074, EPI_ISL_714075, EPI_ISL_714076, EPI_ISL_714077, EPI_ISL_714078, EPI_ISL_714079, EPI_ISL_714080, EPI_ISL_714081, EPI_ISL_714082, EPI_ISL_714083, EPI_ISL_714084, EPI_ISL_714085, EPI_ISL_714086, EPI_ISL_714087, EPI_ISL_714088, EPI_ISL_714089, EPI_ISL_714090, EPI_ISL_714091, EPI_ISL_714092, EPI_ISL_714093, EPI_ISL_714094, EPI_ISL_714095, EPI_ISL_714096, EPI_ISL_714097, EPI_ISL_714099, EPI_ISL_714115, EPI_ISL_714116, EPI_ISL_714117, EPI_ISL_714118, EPI_ISL_714119, EPI_ISL_714120, EPI_ISL_714121, EPI_ISL_714122, EPI_ISL_714123, EPI_ISL_714182, EPI_ISL_714196, EPI_ISL_714197, EPI_ISL_714198, EPI_ISL_714222, EPI_ISL_714235, EPI_ISL_714236, EPI_ISL_714240, EPI_ISL_714241, EPI_ISL_714254, EPI_ISL_714259, EPI_ISL_714260, EPI_ISL_714264, EPI_ISL_714272, EPI_ISL_714276, EPI_ISL_714279, EPI_ISL_714289, EPI_ISL_714300, EPI_ISL_714301, EPI_ISL_714302, EPI_ISL_714303, EPI_ISL_714304, EPI_ISL_714305, EPI_ISL_714306, EPI_ISL_714307, EPI_ISL_714308, EPI_ISL_714309, EPI_ISL_714310, EPI_ISL_714311, EPI_ISL_714312, EPI_ISL_714313, EPI_ISL_714314, EPI_ISL_714315, EPI_ISL_714316, EPI_ISL_714317, EPI_ISL_714318, EPI_ISL_714319, EPI_ISL_714366, EPI_ISL_714369, EPI_ISL_714385, EPI_ISL_714386, EPI_ISL_714387, EPI_ISL_714388, EPI_ISL_714389, EPI_ISL_714390, EPI_ISL_714391, EPI_ISL_714416, EPI_ISL_714450, EPI_ISL_714462, EPI_ISL_714463, EPI_ISL_714464, EPI_ISL_714478, EPI_ISL_714479, EPI_ISL_714480, EPI_ISL_714481, EPI_ISL_714482, EPI_ISL_714483, EPI_ISL_714484, EPI_ISL_714485, EPI_ISL_714486, EPI_ISL_714487, EPI_ISL_714488, EPI_ISL_714489, EPI_ISL_714490, EPI_ISL_714491, EPI_ISL_714492, EPI_ISL_714493, EPI_ISL_714494, EPI_ISL_714495, EPI_ISL_714496, EPI_ISL_714497, EPI_ISL_714498, EPI_ISL_714499, EPI_ISL_714500, EPI_ISL_714501, EPI_ISL_714502, EPI_ISL_714503, EPI_ISL_714504, EPI_ISL_714505, EPI_ISL_714506, EPI_ISL_714507, EPI_ISL_714508, EPI_ISL_714509, EPI_ISL_714510, EPI_ISL_714511, EPI_ISL_714512, EPI_ISL_714513, EPI_ISL_714514, EPI_ISL_714515, EPI_ISL_714516, EPI_ISL_714517, EPI_ISL_714518, EPI_ISL_714519, EPI_ISL_714520, EPI_ISL_714521, EPI_ISL_714522, EPI_ISL_714523, EPI_ISL_714524, EPI_ISL_714525, EPI_ISL_714526, EPI_ISL_714527, EPI_ISL_714528, EPI_ISL_714529, EPI_ISL_714530, EPI_ISL_714531, EPI_ISL_714532, EPI_ISL_714533, EPI_ISL_714534, EPI_ISL_714535, EPI_ISL_714536, EPI_ISL_714537, EPI_ISL_714538, EPI_ISL_714539, EPI_ISL_714540, EPI_ISL_714541, EPI_ISL_714542, EPI_ISL_714543, EPI_ISL_714544, EPI_ISL_714545, EPI_ISL_714546, EPI_ISL_714547, EPI_ISL_714548, EPI_ISL_714549, EPI_ISL_714550, EPI_ISL_714551, EPI_ISL_714552, EPI_ISL_714553, EPI_ISL_714554, EPI_ISL_714555, EPI_ISL_714556, EPI_ISL_714557, EPI_ISL_714558, EPI_ISL_714559, EPI_ISL_714560, EPI_ISL_714561, EPI_ISL_714562, EPI_ISL_714563, EPI_ISL_714564, EPI_ISL_714565			
see above	Department of Virus and Microbiological Special Diagnostics, Statens Serum Institut, Copenhagen, Denmark	Albertsen Lab, Department of Chemistry and Bioscience, Aalborg University, Denmark	Danish Covid-19 Genome Consortium
EPI_ISL_717979	Laboratory of Microbiology and Infectious Diseases, Faculty of Veterinary Medicine, Aristotle University of Thessaloniki, University Campus, 541 24, Thessaloniki, Greece.	Laboratory of Biology, Department of Medicine, Democritus University of Thrace, Alexandroupolis, Greece	Dovrolis N., Chaintoutis S., Dimitriou M., Bampali M., Dovas C., Karakasiliotis I.
EPI_ISL_717980, EPI_ISL_718009, EPI_ISL_718011, EPI_ISL_718013	Lab voor klinische biologie	Onderzoeksgroep Virologie	Laurens Lambrechts, Nick Vereecke, Marthe Pauwels, Bruno Verhasselt, Linos Vandekerckhove, Hans Nauwynck, Sebastiaan Theuns

EPI_ISL_718050, EPI_ISL_718059	ZOTZ KLIMAS MVZ Düsseldorf-Centrum GbR ÜBAG für Labormedizin, Genetik, Zytologie, Pathologie	Center of Medical Microbiology, Virology, and Hospital Hygiene, University of Duesseldorf	Maximilian Damagnez, Alexander Dilthey, Ashley-Jane Duplessis, Patrick Finzer, Katrin Hoffmann, Torsten Houwaart, Lisanna Hülse, Malte Kohns Vasconcelos, Marek Korencak, Nadine Lübke, Jessica Nicolai, Klaus Pfeffer, Daniel Strelow, Jörg Timm, Andreas Walker, Tobias Wienemann, Rainer Zotz
EPI_ISL_718202	Ministry of Health Hospitals	Institute of Health and Community Medicine	David Perera, Ooi Mong How, Chua Hock Hin, Tonnii Sia Loong Loong, Wong Jyn Shan, Wong Kiing Aik, Chan Chia Jui
EPI_ISL_718250	Institute of Virology, Biomedical Research Center of the Slovak Academy of Sciences, Bratislava	Faculty of Natural Sciences, Comenius University, Bratislava	Kristína Boršová, Viktória Hodorová, Broa Brejová, Viktória abanová, Sabina Fumaová Havlíková, Juraj Kopáek, Martina Liková, ubomíra Lukáiková, Martina Neboháová, Monika Sláviková, Tomáš Vina, Boris Klempa, Jozef Nosek
EPI_ISL_721185, EPI_ISL_721186, EPI_ISL_721187, EPI_ISL_721188, EPI_ISL_721189, EPI_ISL_721190, EPI_ISL_721191, EPI_ISL_721192, EPI_ISL_721193, EPI_ISL_721194, EPI_ISL_721195, EPI_ISL_721196, EPI_ISL_721197, EPI_ISL_721198, EPI_ISL_721199, EPI_ISL_721200, EPI_ISL_721201, EPI_ISL_721202, EPI_ISL_721203, EPI_ISL_721204, EPI_ISL_721205, EPI_ISL_721206, EPI_ISL_721207, EPI_ISL_721208, EPI_ISL_721209, EPI_ISL_721210, EPI_ISL_721211, EPI_ISL_721212, EPI_ISL_721213, EPI_ISL_721214, EPI_ISL_721215, EPI_ISL_721216, EPI_ISL_721217, EPI_ISL_721218, EPI_ISL_721219, EPI_ISL_721220, EPI_ISL_721221, EPI_ISL_721222, EPI_ISL_721223, EPI_ISL_721224, EPI_ISL_721225, EPI_ISL_721226, EPI_ISL_721227, EPI_ISL_721228, EPI_ISL_721229, EPI_ISL_721230, EPI_ISL_721231, EPI_ISL_721232, EPI_ISL_721233, EPI_ISL_721234, EPI_ISL_721235, EPI_ISL_721236, EPI_ISL_721237, EPI_ISL_721238, EPI_ISL_721239, EPI_ISL_721240, EPI_ISL_721241, EPI_ISL_721242, EPI_ISL_721243, EPI_ISL_721244, EPI_ISL_721245, EPI_ISL_721246, EPI_ISL_721247, EPI_ISL_721248, EPI_ISL_721249, EPI_ISL_721250, EPI_ISL_721251, EPI_ISL_721252, EPI_ISL_721253, EPI_ISL_721254, EPI_ISL_721255, EPI_ISL_721256, EPI_ISL_721257, EPI_ISL_721258, EPI_ISL_721259, EPI_ISL_721260, EPI_ISL_721261, EPI_ISL_721262, EPI_ISL_721263, EPI_ISL_721264, EPI_ISL_721265, EPI_ISL_721266, EPI_ISL_721267, EPI_ISL_721268, EPI_ISL_721269, EPI_ISL_721270, EPI_ISL_721271, EPI_ISL_721272, EPI_ISL_721273, EPI_ISL_721274, EPI_ISL_721275, EPI_ISL_721276, EPI_ISL_721277, EPI_ISL_721278, EPI_ISL_721279, EPI_ISL_721280, EPI_ISL_721281, EPI_ISL_721282, EPI_ISL_721283, EPI_ISL_721284, EPI_ISL_721285, EPI_ISL_721286, EPI_ISL_721287, EPI_ISL_721288, EPI_ISL_721289, EPI_ISL_721290, EPI_ISL_721291, EPI_ISL_721292, EPI_ISL_721293, EPI_ISL_721294, EPI_ISL_721295, EPI_ISL_721296, EPI_ISL_721297, EPI_ISL_721298, EPI_ISL_721299, EPI_ISL_721300, EPI_ISL_721301, EPI_ISL_721302, EPI_ISL_721303, EPI_ISL_721304, EPI_ISL_721305, EPI_ISL_721307, EPI_ISL_721308, EPI_ISL_721309, EPI_ISL_721310, EPI_ISL_721311, EPI_ISL_721312, EPI_ISL_721313, EPI_ISL_721314, EPI_ISL_721315, EPI_ISL_721316, EPI_ISL_721317, EPI_ISL_721318, EPI_ISL_721319, EPI_ISL_721320, EPI_ISL_721321, EPI_ISL_721322, EPI_ISL_721323, EPI_ISL_721324, EPI_ISL_721325, EPI_ISL_721326, EPI_ISL_721327, EPI_ISL_721328, EPI_ISL_721329, EPI_ISL_721330, EPI_ISL_721331, EPI_ISL_721332, EPI_ISL_721333, EPI_ISL_721334, EPI_ISL_721335, EPI_ISL_721336, EPI_ISL_721337, EPI_ISL_721338, EPI_ISL_721339, EPI_ISL_721340, EPI_ISL_721341, EPI_ISL_721342, EPI_ISL_721343, EPI_ISL_721344, EPI_ISL_721345, EPI_ISL_721346, EPI_ISL_721347, EPI_ISL_721348, EPI_ISL_721349, EPI_ISL_721350, EPI_ISL_721351, EPI_ISL_721352, EPI_ISL_721353, EPI_ISL_721354, EPI_ISL_721355, EPI_ISL_721356, EPI_ISL_721357, EPI_ISL_721358, EPI_ISL_721359, EPI_ISL_721360, EPI_ISL_721361, EPI_ISL_721362, EPI_ISL_721363, EPI_ISL_721364, EPI_ISL_721365, EPI_ISL_721366, EPI_ISL_721367, EPI_ISL_721368, EPI_ISL_721369, EPI_ISL_721371, EPI_ISL_721372, EPI_ISL_721373, EPI_ISL_721374, EPI_ISL_721375, EPI_ISL_721376, EPI_ISL_721377, EPI_ISL_721378, EPI_ISL_721379, EPI_ISL_721380, EPI_ISL_721381, EPI_ISL_721382, EPI_ISL_721383, EPI_ISL_721384, EPI_ISL_721385, EPI_ISL_721386, EPI_ISL_721387, EPI_ISL_721388, EPI_ISL_721389, EPI_ISL_721390, EPI_ISL_721391, EPI_ISL_721392, EPI_ISL_721393, EPI_ISL_721394, EPI_ISL_721395, EPI_ISL_721396, EPI_ISL_721397, EPI_ISL_721398, EPI_ISL_721399, EPI_ISL_721400, EPI_ISL_721401, EPI_ISL_721402, EPI_ISL_721403, EPI_ISL_721404, EPI_ISL_721405, EPI_ISL_721406, EPI_ISL_721407, EPI_ISL_721408, EPI_ISL_721409, EPI_ISL_721410, EPI_ISL_721411, EPI_ISL_721412, EPI_ISL_721413, EPI_ISL_721414, EPI_ISL_721415, EPI_ISL_721416, EPI_ISL_721417, EPI_ISL_721418, EPI_ISL_721419, EPI_ISL_721420, EPI_ISL_721421, EPI_ISL_721422, EPI_ISL_721423, EPI_ISL_721424, EPI_ISL_721425, EPI_ISL_721426, EPI_ISL_721427, EPI_ISL_721428, EPI_ISL_721429, EPI_ISL_721430, EPI_ISL_721431, EPI_ISL_721432, EPI_ISL_721433, EPI_ISL_721434, EPI_ISL_721435, EPI_ISL_721436, EPI_ISL_721437, EPI_ISL_721438, EPI_ISL_721439, EPI_ISL_721440, EPI_ISL_721441, EPI_ISL_721442, EPI_ISL_721443, EPI_ISL_721444, EPI_ISL_721445, EPI_ISL_721446, EPI_ISL_721447, EPI_ISL_721448, EPI_ISL_721449, EPI_ISL_721450, EPI_ISL_721451, EPI_ISL_721452, EPI_ISL_721453, EPI_ISL_721454, EPI_ISL_721455, EPI_ISL_721457, EPI_ISL_721458, EPI_ISL_721459, EPI_ISL_721461, EPI_ISL_721462, EPI_ISL_721463, EPI_ISL_721464, EPI_ISL_721465, EPI_ISL_721466, EPI_ISL_721467, EPI_ISL_721468, EPI_ISL_721469, EPI_ISL_721470, EPI_ISL_721471, EPI_ISL_721472, EPI_ISL_721473, EPI_ISL_721474, EPI_ISL_721475, EPI_ISL_721476, EPI_ISL_721477, EPI_ISL_721478, EPI_ISL_721479, EPI_ISL_721480, EPI_ISL_721481, EPI_ISL_721482, EPI_ISL_721483, EPI_ISL_721484, EPI_ISL_721485, EPI_ISL_721486, EPI_ISL_721487, EPI_ISL_721488, EPI_ISL_721489, EPI_ISL_721490, EPI_ISL_721491, EPI_ISL_721492, EPI_ISL_721493, EPI_ISL_721494, EPI_ISL_721495, EPI_ISL_721496, EPI_ISL_721497, EPI_ISL_721498, EPI_ISL_721499, EPI_ISL_721500, EPI_ISL_721501, EPI_ISL_721502, EPI_ISL_721503, EPI_ISL_721504, EPI_ISL_721505, EPI_ISL_721506, EPI_ISL_721507, EPI_ISL_721508, EPI_ISL_721509, EPI_ISL_721510, EPI_ISL_721511, EPI_ISL_721512, EPI_ISL_721513, EPI_ISL_721514, EPI_ISL_721515, EPI_ISL_721516			
see above	Lighthouse Lab in Alderley Park	Wellcome Sanger Institute for the COVID-19 Genomics UK (COG-UK) Consortium	Jacquelyn Wynn, Mairead Hyland, The Lighthouse Lab in Alderley Park and Alex Alderton, Roberto Amato, Sonia Goncalves, Ewan Harrison, David K. Jackson, Ian Johnston, Dominic Kwiatkowski, Cordelia Langford, John Sillitoe on behalf of the Wellcome Sanger Institute COVID-19 Surveillance Team
EPI_ISL_721626	National Institute for Infectious Diseases, INMI, "L. Spallanzani" IRCCS	National Institute for Infectious Diseases, INMI, "L. Spallanzani" IRCCS	M Rueca, B Bartolini, C.E.M Gruber, F Messina, E Giombini, A Di Caro, MR Capobianchi
EPI_ISL_721627	National Institute for Infectious Diseases, INMI, "L. Spallanzani" IRCCS	National Institute for Infectious Diseases, INMI, "L. Spallanzani" IRCCS	F Messina, E Giombini, M Rueca, B Bartolini, C.E.M Gruber, MR Capobianchi, A Di Caro
EPI_ISL_721671, EPI_ISL_721677, EPI_ISL_721679, EPI_ISL_721682, EPI_ISL_721684, EPI_ISL_721691, EPI_ISL_721693, EPI_ISL_721696, EPI_ISL_721701, EPI_ISL_721711, EPI_ISL_721712, EPI_ISL_721713, EPI_ISL_721714, EPI_ISL_721715, EPI_ISL_721757, EPI_ISL_721758, EPI_ISL_721759, EPI_ISL_721760, EPI_ISL_721767, EPI_ISL_721768, EPI_ISL_721769, EPI_ISL_721770, EPI_ISL_721778, EPI_ISL_721779, EPI_ISL_721780, EPI_ISL_721781, EPI_ISL_721782, EPI_ISL_721783, EPI_ISL_721784, EPI_ISL_721785, EPI_ISL_721786, EPI_ISL_721788, EPI_ISL_721789, EPI_ISL_721790, EPI_ISL_721791, EPI_ISL_721792, EPI_ISL_721793, EPI_ISL_721794, EPI_ISL_721795, EPI_ISL_721796, EPI_ISL_721797, EPI_ISL_721798, EPI_ISL_721799, EPI_ISL_721800, EPI_ISL_721801, EPI_ISL_721802, EPI_ISL_721803, EPI_ISL_721804, EPI_ISL_721805, EPI_ISL_721813, EPI_ISL_721814, EPI_ISL_721826, EPI_ISL_721844, EPI_ISL_721845, EPI_ISL_721846, EPI_ISL_721847, EPI_ISL_721858, EPI_ISL_721859, EPI_ISL_721860, EPI_ISL_721861, EPI_ISL_721862, EPI_ISL_721863, EPI_ISL_721864, EPI_ISL_721869, EPI_ISL_721870, EPI_ISL_721871, EPI_ISL_721872, EPI_ISL_721873, EPI_ISL_721874, EPI_ISL_721885, EPI_ISL_721886, EPI_ISL_721887, EPI_ISL_721915, EPI_ISL_721916, EPI_ISL_721940, EPI_ISL_721942, EPI_ISL_721943, EPI_ISL_721944, EPI_ISL_721945, EPI_ISL_721946, EPI_ISL_721947, EPI_ISL_721948, EPI_ISL_721949, EPI_ISL_721950, EPI_ISL_721951, EPI_ISL_721952, EPI_ISL_721953, EPI_ISL_721954, EPI_ISL_721955, EPI_ISL_721956, EPI_ISL_721957, EPI_ISL_721958			
see above	Viollier AG	Department of Biosystems Science and Engineering, ETH Zürich	Christian Beisel
EPI_ISL_722201	Vitalis Mostar	Alea Genetic Center	Salihefendic L., Pecar D., Konjhodzic R.
EPI_ISL_722209	Dom Zdravlja Sarajevo	Alea Genetic Center	Salihefendic L., Pecar D., Konjhodzic R.
EPI_ISL_722290, EPI_ISL_722328, EPI_ISL_722334, EPI_ISL_722351, EPI_ISL_722363, EPI_ISL_722388, EPI_ISL_722406, EPI_ISL_722682, EPI_ISL_722683, EPI_ISL_722684, EPI_ISL_722685, EPI_ISL_722686			
see above	Dutch COVID-19 response team	Erasmus Medical Center	Bas Oude Munnink, Reina Sikkema, David Nieuwenhuijse, Irina Chestakova, Anne van der Linden, Marjan Boter, Emmanuelle Munger, Corine GeurtsvanKessel, Annemiek van der Eijk, Richard Molenkamp, Marion Koopmans, on behalf of the Dutch national COVID-19 response team.
EPI_ISL_722910	Istituto Zooprofilattico Sperimentale della Puglia e della Basilicata	Istituto Zooprofilattico Sperimentale della Puglia e della Basilicata	Parisi A., Bianco A., Capozzi L., Del Sambro L., Manzulli V, Rondinone V., Pace L., Cipolletta D., Galante D.
EPI_ISL_723124, EPI_ISL_723125, EPI_ISL_723126	Minnesota Department of Health, Public Health Laboratory	Minnesota Department of Health, Public Health Laboratory	Alexandra Lorentz, Jacob Garfin, Matt Plumb, and Xiong Wang
EPI_ISL_723150	Vault Health	Minnesota Department of Health, Public Health Laboratory	Alexandra Lorentz, Jacob Garfin, Matt Plumb, and Xiong Wang
EPI_ISL_723156, EPI_ISL_723200, EPI_ISL_723202, EPI_ISL_723203, EPI_ISL_723204, EPI_ISL_723205, EPI_ISL_723206, EPI_ISL_723207, EPI_ISL_723208, EPI_ISL_723209, EPI_ISL_723210, EPI_ISL_723211, EPI_ISL_723213, EPI_ISL_723214, EPI_ISL_723215, EPI_ISL_723216, EPI_ISL_723217, EPI_ISL_723220, EPI_ISL_723226, EPI_ISL_723227, EPI_ISL_723229, EPI_ISL_723230, EPI_ISL_723231, EPI_ISL_723266, EPI_ISL_723267, EPI_ISL_723268, EPI_ISL_723289, EPI_ISL_723316, EPI_ISL_723317, EPI_ISL_723323, EPI_ISL_723356, EPI_ISL_723358, EPI_ISL_723359, EPI_ISL_723383, EPI_ISL_723394, EPI_ISL_723395, EPI_ISL_723396, EPI_ISL_723397, EPI_ISL_723412, EPI_ISL_723413, EPI_ISL_723423, EPI_ISL_723429, EPI_ISL_723441, EPI_ISL_723442, EPI_ISL_723443, EPI_ISL_723445, EPI_ISL_723449, EPI_ISL_723450, EPI_ISL_723451			
see above	Dutch COVID-19 response team	National Institute for Public Health and the Environment (RIVM)	Adam Meijer, Harry Vennema, Jeroen Cremer, Sharon van den Brink, Bas van der Veer, AnneMarie van den Brandt, Florian Zwagemaker, Dennis Schmitz, Chantal Reusken, on behalf of the national COVID-19 response team
EPI_ISL_723480, EPI_ISL_723481, EPI_ISL_723482, EPI_ISL_723483, EPI_ISL_723484, EPI_ISL_723485, EPI_ISL_723486, EPI_ISL_723487, EPI_ISL_723488, EPI_ISL_723489, EPI_ISL_723490, EPI_ISL_723491			
see above	Virginia DCLS	Virginia DCLS	Virginia DCLS
EPI_ISL_723852, EPI_ISL_723854, EPI_ISL_723855, EPI_ISL_723856	Department of Pathology, University of Cambridge	COVID-19 Genomics UK (COG-UK) Consortium	Aminu S. Jahun, Yasmin Chaudhry, Grant Hall, Iliana Georgana, Myra Hosmillo, Martin D. Curran, Malte Pinckert, Surendra Parmar, Ian Goodfellow
EPI_ISL_724064, EPI_ISL_724065, EPI_ISL_724066, EPI_ISL_724067, EPI_ISL_724068, EPI_ISL_724069, EPI_ISL_724070, EPI_ISL_724071, EPI_ISL_724072, EPI_ISL_724073, EPI_ISL_724074, EPI_ISL_724075, EPI_ISL_724076, EPI_ISL_724077, EPI_ISL_724078, EPI_ISL_724079, EPI_ISL_724080, EPI_ISL_724081, EPI_ISL_724082, EPI_ISL_724083, EPI_ISL_724084, EPI_ISL_724085, EPI_ISL_724086, EPI_ISL_724087, EPI_ISL_724088, EPI_ISL_724089, EPI_ISL_724090, EPI_ISL_724091, EPI_ISL_724092, EPI_ISL_724093, EPI_ISL_724094, EPI_ISL_724095, EPI_ISL_724096, EPI_ISL_724097, EPI_ISL_724098, EPI_ISL_724099, EPI_ISL_724100, EPI_ISL_724101, EPI_ISL_724102, EPI_ISL_724103, EPI_ISL_724104, EPI_ISL_724105, EPI_ISL_724106, EPI_ISL_724107, EPI_ISL_724108, EPI_ISL_724109, EPI_ISL_724110, EPI_ISL_724111, EPI_ISL_724112, EPI_ISL_724113, EPI_ISL_724114, EPI_ISL_724115, EPI_ISL_724116, EPI_ISL_724117, EPI_ISL_724118, EPI_ISL_724119, EPI_ISL_724120, EPI_ISL_724121, EPI_ISL_724122, EPI_ISL_724123			
see above	Northumbria University / South Tees Hospitals NHS Foundation Trust / North Cumbria Integrated Care NHS Foundation Trust / North Tees and Hartlepool NHS Foundation Trust / Newcastle Hospitals NHS Foundation Trust	COVID-19 Genomics UK (COG-UK) Consortium	Darren L Smith,Andrew Nelson,Matthew Bashton,Greg R Young,Joshua Loh,John Allan,Mohammad A Tariq,Giles S Holt,Gary Black,Wen C Yew,Lynn Dover,Paul Baker,Steve Liggett,Sarah Essex,Jane Greenaway,Debra Padgett,Clive Graham,Garren Scott,Edward Barton,Emma Swindells,Brendan Payne,Jennifer Collins,Yusri Taha,Gary Eltringham
EPI_ISL_724124, EPI_ISL_724130, EPI_ISL_724131, EPI_ISL_724132, EPI_ISL_724133, EPI_ISL_724134, EPI_ISL_724135, EPI_ISL_724136, EPI_ISL_724137, EPI_ISL_724138, EPI_ISL_724139, EPI_ISL_724140, EPI_ISL_724141, EPI_ISL_724142, EPI_ISL_724143, EPI_ISL_724144, EPI_ISL_724145, EPI_ISL_724146, EPI_ISL_724147, EPI_ISL_724151, EPI_ISL_724152, EPI_ISL_724153, EPI_ISL_724154, EPI_ISL_724155, EPI_ISL_724156, EPI_ISL_724157, EPI_ISL_724158, EPI_ISL_724159, EPI_ISL_724160, EPI_ISL_724161, EPI_ISL_724162, EPI_ISL_724163, EPI_ISL_724164, EPI_ISL_724165, EPI_ISL_724166, EPI_ISL_724167, EPI_ISL_724168, EPI_ISL_724169, EPI_ISL_724170, EPI_ISL_724171, EPI_ISL_724172, EPI_ISL_724173, EPI_ISL_724174, EPI_ISL_724175, EPI_ISL_724176, EPI_ISL_724177, EPI_ISL_724178, EPI_ISL_724179, EPI_ISL_724180, EPI_ISL_724181, EPI_ISL_724182, EPI_ISL_724183, EPI_ISL_724184, EPI_ISL_724185, EPI_ISL_724186, EPI_ISL_724187, EPI_ISL_724189, EPI_ISL_724190, EPI_ISL_724191, EPI_ISL_724192, EPI_ISL_724193, EPI_ISL_724194, EPI_ISL_724195, EPI_ISL_724196, EPI_ISL_724197, EPI_ISL_724198, EPI_ISL_724199			
see above	Quadram Institute Bioscience	COVID-19 Genomics UK (COG-UK) Consortium	Dave J. Baker, Gemma L. Kay, Alp Aydin, Thanh Le-Viet, Steven Rudder, Ana P. Tedim, Anastasia Kolyva, Maria Diaz, Leonardo de Oliveira Martins, Nabil-Fareed Alikhan, Lizzie Meadows, Rachael Stanley, Ngozi Elumogo, Muhammed Yasir, Nicholas M. Thomson, Alexander J Trotter, Rachel Gilroy, Samuel Bloomfield, Claire Stuart, Andrew Bell, Reenesh Prakash, Samir Dervisevic, Alison E. Mather, John Wain, Mark Webber, Andrew J. Page, Justin O'Grady
EPI_ISL_724222	Virology Department, Royal Infirmary of Edinburgh, NHS Lothian / School of Biological Sciences, University of	COVID-19 Genomics UK (COG-UK) Consortium	McHugh M, Dewar R, Rooke S, Gallagher M, Balcaza C, O'Toole Á, Scher E, Hill V, McCrone JT, Colquhoun R, Yu X, Jackson B, Rambaut A, Williams TC, Templeton K

	Edinburgh / Institute of Genetics and Molecular Medicine, University of Edinburgh		
EPI_ISL_724397, EPI_ISL_724398, EPI_ISL_724400, EPI_ISL_724402, EPI_ISL_724403, EPI_ISL_724404, EPI_ISL_724405, EPI_ISL_724407, EPI_ISL_724418, EPI_ISL_724424, EPI_ISL_724427, EPI_ISL_724430, EPI_ISL_724433, EPI_ISL_724435, EPI_ISL_724438, EPI_ISL_724467, EPI_ISL_724468, EPI_ISL_724469, EPI_ISL_724470, EPI_ISL_724471, EPI_ISL_724472, EPI_ISL_724473, EPI_ISL_724474, EPI_ISL_724475, EPI_ISL_724476, EPI_ISL_724478, EPI_ISL_724480, EPI_ISL_724481, EPI_ISL_724482, EPI_ISL_724484, EPI_ISL_724486, EPI_ISL_724487, EPI_ISL_724488, EPI_ISL_724489, EPI_ISL_724490, EPI_ISL_724491, EPI_ISL_724492, EPI_ISL_724494, EPI_ISL_724495			
see above	Liverpool Clinical Laboratories	COVID-19 Genomics UK (COG-UK) Consortium	Sam Haldenby, Anita Lucaci, Steve Paterson, Julian Hiscox, Alistair Darby, M Almsaud, A Alrezaihi, Muhannad Alruwaili, Stuart D Armstrong, Jones Benjamin, Eleanor G Bentley, Anu Chawla, Jordan J Clark, Angela Cowell, Richard Eccles, Isabel García-Dorival, Matthew Gemmell, Alessandro Gerada, PKF Gilmore, Richard Gregory, Ximeng Han, Catherine Hartley, Margaret Hughes, Miren Iturriza-Gomara, James Johnson, L Luu, Jenifer Manson, Charlotte Nelson, Elaine O'Toole, Cassie Olateju, Rebekah Penrice-Randal , Lucille Rainbow, N.P Randle, Trevor Ian Robinson, Parul Sharma, Ghada T Shawli, James P Stewart, Neil Swainston, Ecaterina Vamos, Joanne Watts, Mark Whitehead
EPI_ISL_724542, EPI_ISL_724543, EPI_ISL_724544, EPI_ISL_724545, EPI_ISL_724546, EPI_ISL_724547, EPI_ISL_724548, EPI_ISL_724550, EPI_ISL_724551, EPI_ISL_724552, EPI_ISL_724553, EPI_ISL_724554, EPI_ISL_724555, EPI_ISL_724556, EPI_ISL_724557, EPI_ISL_724558, EPI_ISL_724559, EPI_ISL_724560, EPI_ISL_724561, EPI_ISL_724562, EPI_ISL_724563, EPI_ISL_724564, EPI_ISL_724565, EPI_ISL_724566, EPI_ISL_724567, EPI_ISL_724568, EPI_ISL_724569, EPI_ISL_724570, EPI_ISL_724571, EPI_ISL_724572, EPI_ISL_724573, EPI_ISL_724574, EPI_ISL_724575, EPI_ISL_724580, EPI_ISL_724581, EPI_ISL_724582			
see above	University College London, Great Ormond Street Hospital for Children NHS Foundation Trust, Imperial College Healthcare NHS Trust	COVID-19 Genomics UK (COG-UK) Consortium	Sergi Castellano, Rachel Williams, Mark Kristiansen, Paola Resende Silva, Sunando Roy, Tony Brooks, Helena Tutill, Paola Niola, Patricia Dyal, Charlotte Williams, Leysa Forrest, Yasmin Panchbhaya, Jacqueline Findlay, Samuel Weeks, Julianne Brown, Kathryn Harris, Paul Randell, James Price, Alison Holmes, Judith Breuer
EPI_ISL_724927, EPI_ISL_724930, EPI_ISL_724932, EPI_ISL_724933, EPI_ISL_724934, EPI_ISL_724935, EPI_ISL_724936, EPI_ISL_724937, EPI_ISL_724938, EPI_ISL_724939, EPI_ISL_724940, EPI_ISL_724941, EPI_ISL_724942, EPI_ISL_724943, EPI_ISL_724944, EPI_ISL_724945, EPI_ISL_724946, EPI_ISL_724947, EPI_ISL_724948, EPI_ISL_724949, EPI_ISL_724950, EPI_ISL_724951, EPI_ISL_724952, EPI_ISL_724970, EPI_ISL_725012, EPI_ISL_725013, EPI_ISL_725014, EPI_ISL_725015, EPI_ISL_725016, EPI_ISL_725017			
see above	Northumbria University / South Tees Hospitals NHS Foundation Trust / North Cumbria Integrated Care NHS Foundation Trust / North Tees and Hartlepool NHS Foundation Trust / Newcastle Hospitals NHS Foundation Trust	COVID-19 Genomics UK (COG-UK) Consortium	Darren L Smith,Andrew Nelson,Matthew Bashton,Greg R Young,Joshua Loh,John Allan,Mohammad A Tariq,Giles S Holt,Gary Black,Wen C Yew,Lynn Dover,Paul Baker,Steve Liggett,Sarah Essex,Jane Greenaway,Debra Padgett,Clive Graham,Garren Scott,Edward Barton,Emma Swindells,Brendan Payne,Jennifer Collins,Yusri Taha,Gary Eltringham
EPI_ISL_725192, EPI_ISL_725201, EPI_ISL_725205, EPI_ISL_725206, EPI_ISL_725210, EPI_ISL_725220, EPI_ISL_725223, EPI_ISL_725224, EPI_ISL_725225, EPI_ISL_725226, EPI_ISL_725229, EPI_ISL_725232, EPI_ISL_725234, EPI_ISL_725235, EPI_ISL_725236, EPI_ISL_725243, EPI_ISL_725244, EPI_ISL_725253, EPI_ISL_725259, EPI_ISL_725284, EPI_ISL_725288			
see above	Quadram Institute Bioscience	COVID-19 Genomics UK (COG-UK) Consortium	Dave J. Baker, Gemma L. Kay, Alp Aydin, Thanh Le-Viet, Steven Rudder, Ana P. Tedim, Anastasia Kolyva, Maria Diaz, Leonardo de Oliveira Martins, Nabil-Fareed Alikhan, Lizzie Meadows, Rachael Stanley, Ngozi Elumogo, Muhammed Yasir, Nicholas M. Thomson, Alexander J Trotter, Rachel Gilroy, Samuel Bloomfield, Claire Stuart, Andrew Bell, Reenesh Prakash, Samir Dervisevic, Alison E. Mather, John Wain, Mark Webber, Andrew J. Page, Justin O'Grady
EPI_ISL_725519	Queens Medical Centre, Clinical Microbiology Department / DeepSeq Nottingham	COVID-19 Genomics UK (COG-UK) Consortium	Gemma Clark, Wendy Smith, Manjinder Khakh, Vicki M Fleming, Michelle M Lister, Hannah Howson-Wells, Jonathan Ball, Patrick McClure, Joseph Chappell, Theocharis Tsoleridis, Nadine Holmes, Matthew Carlisle, Christopher Moore, Fei Sang, Johnny Debebe, Victoria Wright, Matthew Loose
EPI_ISL_725677, EPI_ISL_725678, EPI_ISL_725680, EPI_ISL_725681, EPI_ISL_725682, EPI_ISL_725683, EPI_ISL_725684, EPI_ISL_725685, EPI_ISL_725716, EPI_ISL_725719, EPI_ISL_725725, EPI_ISL_725727, EPI_ISL_725728, EPI_ISL_725729, EPI_ISL_725730, EPI_ISL_725731, EPI_ISL_725732, EPI_ISL_725733, EPI_ISL_725734, EPI_ISL_725735, EPI_ISL_725736, EPI_ISL_725737, EPI_ISL_725738, EPI_ISL_725983, EPI_ISL_726000, EPI_ISL_726001, EPI_ISL_726007, EPI_ISL_726008, EPI_ISL_726009, EPI_ISL_726022, EPI_ISL_726023, EPI_ISL_726024, EPI_ISL_726026, EPI_ISL_726027, EPI_ISL_726028, EPI_ISL_726029, EPI_ISL_726030, EPI_ISL_726031, EPI_ISL_726107, EPI_ISL_726111, EPI_ISL_726120, EPI_ISL_726123, EPI_ISL_726129, EPI_ISL_726131, EPI_ISL_726132, EPI_ISL_726133, EPI_ISL_726134, EPI_ISL_726135, EPI_ISL_726136, EPI_ISL_726137, EPI_ISL_726138, EPI_ISL_726139, EPI_ISL_726140, EPI_ISL_726141, EPI_ISL_726142, EPI_ISL_726143, EPI_ISL_726144, EPI_ISL_726145, EPI_ISL_726146, EPI_ISL_726147, EPI_ISL_726148, EPI_ISL_726149, EPI_ISL_726150, EPI_ISL_726151, EPI_ISL_726152, EPI_ISL_726153, EPI_ISL_726154, EPI_ISL_726155, EPI_ISL_726156, EPI_ISL_726157, EPI_ISL_726158, EPI_ISL_726159, EPI_ISL_726160, EPI_ISL_726161, EPI_ISL_726162, EPI_ISL_726163, EPI_ISL_726164, EPI_ISL_726165, EPI_ISL_726166, EPI_ISL_726167, EPI_ISL_726168, EPI_ISL_726169, EPI_ISL_726170, EPI_ISL_726171, EPI_ISL_726172, EPI_ISL_726173, EPI_ISL_726174, EPI_ISL_726175, EPI_ISL_726176, EPI_ISL_726177, EPI_ISL_726178, EPI_ISL_726179, EPI_ISL_726180, EPI_ISL_726181, EPI_ISL_726182, EPI_ISL_726183, EPI_ISL_726184, EPI_ISL_726185, EPI_ISL_726186, EPI_ISL_726187, EPI_ISL_726188, EPI_ISL_726189, EPI_ISL_726190, EPI_ISL_726191, EPI_ISL_726192, EPI_ISL_726193, EPI_ISL_726194, EPI_ISL_726195, EPI_ISL_726196, EPI_ISL_726197, EPI_ISL_726198, EPI_ISL_726199, EPI_ISL_726200, EPI_ISL_726201, EPI_ISL_726202, EPI_ISL_726203, EPI_ISL_726204, EPI_ISL_726205, EPI_ISL_726206, EPI_ISL_726207, EPI_ISL_726209, EPI_ISL_726210, EPI_ISL_726211, EPI_ISL_726214, EPI_ISL_726215, EPI_ISL_726216, EPI_ISL_726218, EPI_ISL_726219, EPI_ISL_726222, EPI_ISL_726223, EPI_ISL_726224, EPI_ISL_726225, EPI_ISL_726226, EPI_ISL_726227, EPI_ISL_726228, EPI_ISL_726229, EPI_ISL_726230, EPI_ISL_726231, EPI_ISL_726232, EPI_ISL_726233, EPI_ISL_726234, EPI_ISL_726235, EPI_ISL_726242, EPI_ISL_726243, EPI_ISL_726244, EPI_ISL_726245, EPI_ISL_726246, EPI_ISL_726247, EPI_ISL_726248, EPI_ISL_726249, EPI_ISL_726250, EPI_ISL_726252, EPI_ISL_726257, EPI_ISL_726258, EPI_ISL_726259, EPI_ISL_726260, EPI_ISL_726261, EPI_ISL_726262, EPI_ISL_726263, EPI_ISL_726264, EPI_ISL_726265, EPI_ISL_726268, EPI_ISL_726269, EPI_ISL_726270, EPI_ISL_726271, EPI_ISL_726276, EPI_ISL_726277, EPI_ISL_726278, EPI_ISL_726279, EPI_ISL_726305, EPI_ISL_726307, EPI_ISL_726311, EPI_ISL_726312, EPI_ISL_726313, EPI_ISL_726314, EPI_ISL_726315, EPI_ISL_726316, EPI_ISL_726317, EPI_ISL_726318, EPI_ISL_726319, EPI_ISL_726320, EPI_ISL_726321, EPI_ISL_726322, EPI_ISL_726323, EPI_ISL_726324, EPI_ISL_726325, EPI_ISL_726326, EPI_ISL_726327, EPI_ISL_726328, EPI_ISL_726329, EPI_ISL_726330, EPI_ISL_726331, EPI_ISL_726332, EPI_ISL_726333, EPI_ISL_726334, EPI_ISL_726335, EPI_ISL_726336, EPI_ISL_726337, EPI_ISL_726338, EPI_ISL_726339, EPI_ISL_726340, EPI_ISL_726341, EPI_ISL_726342, EPI_ISL_726343, EPI_ISL_726344, EPI_ISL_726345, EPI_ISL_726346, EPI_ISL_726347, EPI_ISL_726348, EPI_ISL_726349, EPI_ISL_726350, EPI_ISL_726351, EPI_ISL_726352, EPI_ISL_726353, EPI_ISL_726354, EPI_ISL_726355, EPI_ISL_726356, EPI_ISL_726358, EPI_ISL_726359, EPI_ISL_726360, EPI_ISL_726361, EPI_ISL_726362, EPI_ISL_726363, EPI_ISL_726364, EPI_ISL_726365, EPI_ISL_726366, EPI_ISL_726367, EPI_ISL_726368, EPI_ISL_726369, EPI_ISL_726370, EPI_ISL_726371, EPI_ISL_726372, EPI_ISL_726373, EPI_ISL_726374, EPI_ISL_726375, EPI_ISL_726376, EPI_ISL_726377, EPI_ISL_726378, EPI_ISL_726379, EPI_ISL_726380, EPI_ISL_726381, EPI_ISL_726382, EPI_ISL_726383, EPI_ISL_726384, EPI_ISL_726385, EPI_ISL_726386, EPI_ISL_726387, EPI_ISL_726388, EPI_ISL_726389, EPI_ISL_726390, EPI_ISL_726391, EPI_ISL_726392, EPI_ISL_726393, EPI_ISL_726394, EPI_ISL_726395, EPI_ISL_726396, EPI_ISL_726397, EPI_ISL_726398, EPI_ISL_726399, EPI_ISL_726401, EPI_ISL_726499, EPI_ISL_726500, EPI_ISL_726501, EPI_ISL_726502, EPI_ISL_726503, EPI_ISL_726504, EPI_ISL_726505, EPI_ISL_726506, EPI_ISL_726507, EPI_ISL_726508, EPI_ISL_726520, EPI_ISL_726521, EPI_ISL_726523, EPI_ISL_726524, EPI_ISL_726525, EPI_ISL_726526, EPI_ISL_726538, EPI_ISL_726539, EPI_ISL_726540, EPI_ISL_726541, EPI_ISL_726542, EPI_ISL_726546, EPI_ISL_726547, EPI_ISL_726548, EPI_ISL_726555, EPI_ISL_726556, EPI_ISL_726557, EPI_ISL_726558, EPI_ISL_726559, EPI_ISL_726567, EPI_ISL_726568, EPI_ISL_726572, EPI_ISL_726573, EPI_ISL_726574, EPI_ISL_726575, EPI_ISL_726576, EPI_ISL_726577, EPI_ISL_726578, EPI_ISL_726579, EPI_ISL_726580, EPI_ISL_726581, EPI_ISL_726582, EPI_ISL_726583, EPI_ISL_726584, EPI_ISL_726585, EPI_ISL_726587, EPI_ISL_726589, EPI_ISL_726591, EPI_ISL_726594, EPI_ISL_726599, EPI_ISL_726601, EPI_ISL_726603, EPI_ISL_726608, EPI_ISL_726612, EPI_ISL_726614, EPI_ISL_726617, EPI_ISL_726620, EPI_ISL_726621, EPI_ISL_726622, EPI_ISL_726623, EPI_ISL_726624, EPI_ISL_726625, EPI_ISL_726626, EPI_ISL_726627, EPI_ISL_726628, EPI_ISL_726629, EPI_ISL_726630, EPI_ISL_726631, EPI_ISL_726632, EPI_ISL_726633, EPI_ISL_726634, EPI_ISL_726635, EPI_ISL_726636, EPI_ISL_726637, EPI_ISL_726638, EPI_ISL_726639, EPI_ISL_726640, EPI_ISL_726641, EPI_ISL_726642, EPI_ISL_726643, EPI_ISL_726644, EPI_ISL_726645, EPI_ISL_726646, EPI_ISL_726647, EPI_ISL_726648, EPI_ISL_726649, EPI_ISL_726650, EPI_ISL_726651, EPI_ISL_726652, EPI_ISL_726653, EPI_ISL_726654, EPI_ISL_726655, EPI_ISL_726656, EPI_ISL_726657, EPI_ISL_726658, EPI_ISL_726659, EPI_ISL_726660, EPI_ISL_726661, EPI_ISL_726662, EPI_ISL_726663, EPI_ISL_726664, EPI_ISL_726665, EPI_ISL_726666, EPI_ISL_726667, EPI_ISL_726668, EPI_ISL_726670, EPI_ISL_726671, EPI_ISL_726672, EPI_ISL_726673, EPI_ISL_726674, EPI_ISL_726675, EPI_ISL_726676, EPI_ISL_726677, EPI_ISL_726678, EPI_ISL_726679, EPI_ISL_726680, EPI_ISL_726681, EPI_ISL_726682, EPI_ISL_726683, EPI_ISL_726684, EPI_ISL_726685, EPI_ISL_726686, EPI_ISL_726687, EPI_ISL_726688, EPI_ISL_726689, EPI_ISL_726691, EPI_ISL_726692, EPI_ISL_726693, EPI_ISL_726694, EPI_ISL_726695, EPI_ISL_726697, EPI_ISL_726698, EPI_ISL_726699, EPI_ISL_726700, EPI_ISL_726701, EPI_ISL_726702, EPI_ISL_726706, EPI_ISL_726707, EPI_ISL_726708, EPI_ISL_726709, EPI_ISL_726710, EPI_ISL_726711, EPI_ISL_726713, EPI_ISL_726714, EPI_ISL_726865, EPI_ISL_726866, EPI_ISL_726867, EPI_ISL_726868, EPI_ISL_726869, EPI_ISL_726870, EPI_ISL_726871, EPI_ISL_726872, EPI_ISL_726873, EPI_ISL_726874, EPI_ISL_726875, EPI_ISL_726876, EPI_ISL_726877, EPI_ISL_726878, EPI_ISL_726879, EPI_ISL_726880, EPI_ISL_726881, EPI_ISL_726882, EPI_ISL_726883, EPI_ISL_726884, EPI_ISL_726885, EPI_ISL_726886, EPI_ISL_726887, EPI_ISL_726888, EPI_ISL_726889, EPI_ISL_726890, EPI_ISL_726891, EPI_ISL_726892, EPI_ISL_726893, EPI_ISL_726894, EPI_ISL_726895, EPI_ISL_726896, EPI_ISL_726897, EPI_ISL_726898, EPI_ISL_726899, EPI_ISL_726900, EPI_ISL_726901, EPI_ISL_726902, EPI_ISL_726903, EPI_ISL_726904, EPI_ISL_726905, EPI_ISL_726906, EPI_ISL_726907, EPI_ISL_726908, EPI_ISL_726909, EPI_ISL_726910, EPI_ISL_726911, EPI_ISL_726912, EPI_ISL_726913, EPI_ISL_726914, EPI_ISL_726915, EPI_ISL_726916, EPI_ISL_726917, EPI_ISL_726918, EPI_ISL_726919, EPI_ISL_726920, EPI_ISL_726921, EPI_ISL_726922, EPI_ISL_726923, EPI_ISL_726924, EPI_ISL_726925, EPI_ISL_726926, EPI_ISL_726927, EPI_ISL_726928, EPI_ISL_726929, EPI_ISL_726930, EPI_ISL_726931, EPI_ISL_726932, EPI_ISL_726933, EPI_ISL_726934, EPI_ISL_726935, EPI_ISL_726936, EPI_ISL_726937, EPI_ISL_726938, EPI_ISL_726939, EPI_ISL_726940, EPI_ISL_726941, EPI_ISL_726942, EPI_ISL_727103, EPI_ISL_727104, EPI_ISL_727105, EPI_ISL_727106, EPI_ISL_727107, EPI_ISL_727109, EPI_ISL_727113, EPI_ISL_727114, EPI_ISL_727181, EPI_ISL_727182, EPI_ISL_727183, EPI_ISL_727185			
see above	Wales Specialist Virology Centre Sequencing lab: Pathogen Genomics Unit	COVID-19 Genomics UK (COG-UK) Consortium	Catherine Moore, Johnathan Evans, Laura Gifford, Malorie Perry, Simon Cottrell, Angela Marchbank, Alec Birchley, Alexander Adams, Amy Gaskin, Bree Gatica-Wilcox, Jason Coombes, Joel Southgate, Lauren Gilbert, Lee Graham, Nicole Pacchiarini, Sara Kumziene-Summerhayes, Sarah Taylor, Sophie Jones, Sara Rey, Matthew Bull, Joanne Watkins, Sally Corden, Tom Connor
EPI_ISL_727908, EPI_ISL_727917, EPI_ISL_727978, EPI_ISL_727981, EPI_ISL_727996	Virology Department, Sheffield Teaching Hospitals NHS Foundation Trust/Department of Infection, Immunity and Cardiovascular Disease, The Medical School, University of Sheffield	COVID-19 Genomics UK (COG-UK) Consortium	Thushan de Silva, Matthew Parker, Nikki Smith, Adri Angyal, Rebecca Brown, Luke Green, Rachel Tucker, Paul Parsons, Danielle Groves, Katie Johnson, Laura Carrilero, Alex Keeley, Dave Partridge, Matthew Wyles, Benjamin Lindsey, Mehmet Yavuz, Mohammad Raza, Cariad Evans
EPI_ISL_728058, EPI_ISL_728061, EPI_ISL_728062, EPI_ISL_728063, EPI_ISL_728090	University of Wisconsin-Madison AIDS Vaccine Research Laboratories	University of Wisconsin-Madison AIDS Vaccine Research Laboratories	Gage Moreno, Katarina Braun, et al. AIDS Vaccine Research Laboratories
EPI_ISL_728534, EPI_ISL_728535, EPI_ISL_728536, EPI_ISL_728537, EPI_ISL_728538, EPI_ISL_728539, EPI_ISL_728540, EPI_ISL_728541, EPI_ISL_728542, EPI_ISL_728543, EPI_ISL_728544, EPI_ISL_728545			
see above	CNR Virus des Infections Respiratoires - France SUD	CNR Virus des Infections Respiratoires - France SUD	Antonin Bal, Gregory Destras, Claudia Gonzalez, Gwendolyne Burfin, Quentin Semanas, Martine Valette, Bruno Lina, Laurence Josset
EPI_ISL_728674, EPI_ISL_728675, EPI_ISL_728685, EPI_ISL_728709, EPI_ISL_728710, EPI_ISL_728711, EPI_ISL_728712, EPI_ISL_728727, EPI_ISL_728734, EPI_ISL_728735, EPI_ISL_728748, EPI_ISL_728749, EPI_ISL_728750, EPI_ISL_728751, EPI_ISL_728752, EPI_ISL_728757			
see above	Dutch COVID-19 response team	National Institute for Public Health and the Environment (RIVM)	Adam Meijer, Harry Vennema, Jeroen Cremer, Sharon van den Brink, Bas van der Veer, AnneMarie van den Brandt, Florian Zwagemaker, Dennis Schmitz, Chantal Reusken, on behalf of the national COVID-19 response team
EPI_ISL_729385, EPI_ISL_729684	A. Krumbholz, Labor Dr. Krause und Kollegen MVZ GmbH, Kiel	Charité Universitätsmedizin Berlin, Institut für Virologie	Victor M Corman, Barbara Mühlemann, Jörn Beheim-Schwarzbach, Talitha Veith, Julia Schneider, Terry Jones, Christian Drosten
EPI_ISL_731900, EPI_ISL_731929, EPI_ISL_731930	Instituto Nacional de Saude (INSA) and Instituto Gulbenkian de Ciencia (IGC)	Instituto Nacional de Saude (INSA) and Instituto Gulbenkian de Ciencia (IGC)	Borges et al
EPI_ISL_732111, EPI_ISL_732112,	Instituto Nacional de Saude (INSA)	Instituto Nacional de Saude (INSA)	Borges et al

EPI_ISL_732113			
EPI_ISL_732186, EPI_ISL_732187, EPI_ISL_732188, EPI_ISL_732241, EPI_ISL_732242, EPI_ISL_732243, EPI_ISL_732244, EPI_ISL_732245	Instituto Nacional de Saude (INSA) and Instituto Gulbenkian de Ciencia (IGC)	Instituto Nacional de Saude (INSA) and Instituto Gulbenkian de Ciencia (IGC)	Borges et al
EPI_ISL_732376, EPI_ISL_732377, EPI_ISL_732387, EPI_ISL_732392, EPI_ISL_732400, EPI_ISL_732428, EPI_ISL_732435, EPI_ISL_732443, EPI_ISL_732447, EPI_ISL_732510, EPI_ISL_732511, EPI_ISL_732512, EPI_ISL_732513			
see above	National Virus Reference Laboratory	National Virus Reference Laboratory	Michael Carr, Gabriel Gonzalez, Jonathan Dean, Daniel Hare, Cillian F De Gascun
EPI_ISL_732689, EPI_ISL_732690	CNR Virus des Infections Respiratoires - France SUD	CNR Virus des Infections Respiratoires - France SUD	Antonin Bal, Gregory Destras, Claudia Gonzalez, Gwendolyne Burfin, Quentin Semanas, Martine Valette, Bruno Lina, Laurence Josset
EPI_ISL_733462, EPI_ISL_733463, EPI_ISL_733465, EPI_ISL_733468, EPI_ISL_733470, EPI_ISL_733471, EPI_ISL_733472, EPI_ISL_733474	WHO National Influenza Centre Russian Federation	WHO National Influenza Centre Russian Federation	Andrey Komissarov, Artem Fadeev, Anna Ivanova, Kseniya Komissarova, Dmitry Bazhenov, Daria Danilenko, Ksenia Safina, Elena Nabieva, Georgii Bazykin, Dmitry Lioznov
EPI_ISL_733496	University Hospital Zürich	Institute of Medical Virology, University of Zurich	Stefan Schmutz, Verena Kufner, Maryam Zaheri, Gabriela Ziltener, Thomas Scheier, Jürg Böni, Michael Huber, Alexandra Trkola
EPI_ISL_734177, EPI_ISL_734178, EPI_ISL_734179, EPI_ISL_734193, EPI_ISL_734194, EPI_ISL_734195, EPI_ISL_734196, EPI_ISL_734197, EPI_ISL_734198, EPI_ISL_734226, EPI_ISL_734227, EPI_ISL_734228, EPI_ISL_734229, EPI_ISL_734231			
see above	Virginia Division of Consolidated Laboratory Services (DCLS)	Virginia Division of Consolidated Laboratory Services (DCLS)	Virginia DCLS
EPI_ISL_735493	Cox's Bazar Medicila College	Central Biological Research Laboratory and Department of Biochemistry and Molecular Biology Central Biological Research Laboratory and Department of Biochemistry and Molecular Biology	Robiul Hasan Bhuiyan, Md. Imranul Hoq, Md. Khondakar Raziur Rahman, Imam Hossen, Sajib Rudra, Md. Arif Hossain, Shanta Paul, Md. Omer Faruq, H. M. Abdullah Al Masud, Mohammad Omar Faruque
EPI_ISL_735496	Chattogram Veterinary and Animal Sciences University	Central Biological Research Laboratory and Department of Biochemistry and Molecular Biology	Md. Imranul Hoq, Robiul Hasan Bhuiyan, Md. Khondakar Raziur Rahman, Imam Hossen, Sajib Rudra, Md. Arif Hossain, Shanta Paul, Md. Omer Faruq, Mohammad Omar Faruque, H. M. Abdullah Al Masud
EPI_ISL_735497	Chattogram Veterinary and Animal Sciences University	Central Biological Research Laboratory and Department of Biochemistry and Molecular Biology	Mohammad Omar Faruque, H. M. Abdullah Al Masud, Imam Hossen, Md. Khondakar Raziur Rahman, Sajib Rudra, Md. Arif Hossain, Shanta Paul, Md. Omer Faruq, Robiul Hasan Bhuiyan, Md. Imranul Hoq
EPI_ISL_736957, EPI_ISL_736958, EPI_ISL_736959	NHLS-IALCH	KRISP, KZN Research Innovation and Sequencing Platform	Giandhari J, Pillay S, Lessells R, ChimukangaraB, Mdlalose K, York D, Khan S, Tegally H, Wilkinson E, de Oliveira T
EPI_ISL_737103, EPI_ISL_737115, EPI_ISL_737125, EPI_ISL_737131, EPI_ISL_737134, EPI_ISL_737135, EPI_ISL_737141, EPI_ISL_737142, EPI_ISL_737143, EPI_ISL_737144, EPI_ISL_737146, EPI_ISL_737147, EPI_ISL_737148, EPI_ISL_737151, EPI_ISL_737156, EPI_ISL_737157, EPI_ISL_737167, EPI_ISL_737175, EPI_ISL_737183, EPI_ISL_737194, EPI_ISL_737196, EPI_ISL_737286, EPI_ISL_737288, EPI_ISL_737292, EPI_ISL_737294, EPI_ISL_737303, EPI_ISL_737304			
see above	Michigan Department of Health and Human Services, Bureau of Laboratories	Michigan Department of Health and Human Services, Bureau of Laboratories	Blankenship HM, Riner D, Soehnlen MK
EPI_ISL_738505	Humboldt County Public Health Laboratory	Chan-Zuckerberg Biohub	CZB Cliahub Consortium
EPI_ISL_738508, EPI_ISL_738525, EPI_ISL_738533, EPI_ISL_738534, EPI_ISL_738543, EPI_ISL_738549, EPI_ISL_738551, EPI_ISL_738552, EPI_ISL_738558, EPI_ISL_738582, EPI_ISL_738583, EPI_ISL_738589, EPI_ISL_738608, EPI_ISL_738611, EPI_ISL_738631, EPI_ISL_738643			
see above	Santa Clara County Public Health Laboratory	Chan-Zuckerberg Biohub	CZB Cliahub Consortium
EPI_ISL_738647, EPI_ISL_738649	Humboldt County Public Health Laboratory	Chan-Zuckerberg Biohub	CZB Cliahub Consortium
EPI_ISL_738650	Santa Clara County Public Health Laboratory	Chan-Zuckerberg Biohub	CZB Cliahub Consortium
EPI_ISL_738651	Humboldt County Public Health Laboratory	Chan-Zuckerberg Biohub	CZB Cliahub Consortium
EPI_ISL_738652, EPI_ISL_738653, EPI_ISL_738657, EPI_ISL_738661, EPI_ISL_738663, EPI_ISL_738689, EPI_ISL_738697	Santa Clara County Public Health Laboratory	Chan-Zuckerberg Biohub	CZB Cliahub Consortium
EPI_ISL_738723	Renegade	Chan-Zuckerberg Biohub	CZB Cliahub Consortium
EPI_ISL_738746, EPI_ISL_738763, EPI_ISL_738772, EPI_ISL_738789	Santa Clara County Public Health Laboratory	Chan-Zuckerberg Biohub	CZB Cliahub Consortium
EPI_ISL_738790	Orange County Public Health Lab	Chan-Zuckerberg Biohub	CZB Cliahub Consortium
EPI_ISL_738791, EPI_ISL_738794, EPI_ISL_738803	Santa Clara County Public Health Laboratory	Chan-Zuckerberg Biohub	CZB Cliahub Consortium
EPI_ISL_738809	Humboldt County Public Health Laboratory	Chan-Zuckerberg Biohub	CZB Cliahub Consortium
EPI_ISL_738812	Santa Clara County Public Health Laboratory	Chan-Zuckerberg Biohub	CZB Cliahub Consortium
EPI_ISL_738854	Humboldt County Public Health Laboratory	Chan-Zuckerberg Biohub	CZB Cliahub Consortium
EPI_ISL_738883, EPI_ISL_738889, EPI_ISL_738906, EPI_ISL_738909	Santa Clara County Public Health Laboratory	Chan-Zuckerberg Biohub	CZB Cliahub Consortium
EPI_ISL_738929	Humboldt County Public Health Laboratory	Chan-Zuckerberg Biohub	CZB Cliahub Consortium
EPI_ISL_738931, EPI_ISL_738933, EPI_ISL_738941, EPI_ISL_738942, EPI_ISL_738948	Santa Clara County Public Health Laboratory	Chan-Zuckerberg Biohub	CZB Cliahub Consortium
EPI_ISL_738958	Humboldt County Public Health Laboratory	Chan-Zuckerberg Biohub	CZB Cliahub Consortium
EPI_ISL_738971, EPI_ISL_738984, EPI_ISL_738987	Santa Clara County Public Health Laboratory	Chan-Zuckerberg Biohub	CZB Cliahub Consortium
EPI_ISL_738991	Orange County Public Health Lab	Chan-Zuckerberg Biohub	CZB Cliahub Consortium
EPI_ISL_738999	Humboldt County Public Health Laboratory	Chan-Zuckerberg Biohub	CZB Cliahub Consortium
EPI_ISL_739008, EPI_ISL_739012, EPI_ISL_739021, EPI_ISL_739041, EPI_ISL_739065, EPI_ISL_739070	Santa Clara County Public Health Laboratory	Chan-Zuckerberg Biohub	CZB Cliahub Consortium
EPI_ISL_739072	Orange County Public Health Lab	Chan-Zuckerberg Biohub	CZB Cliahub Consortium
EPI_ISL_739085	Santa Clara County Public Health Laboratory	Chan-Zuckerberg Biohub	CZB Cliahub Consortium
EPI_ISL_739106	Humboldt County Public Health Laboratory	Chan-Zuckerberg Biohub	CZB Cliahub Consortium
EPI_ISL_739117, EPI_ISL_739121	Santa Clara County Public Health Laboratory	Chan-Zuckerberg Biohub	CZB Cliahub Consortium
EPI_ISL_739126	Humboldt County Public Health Laboratory	Chan-Zuckerberg Biohub	CZB Cliahub Consortium

EPI_ISL_739128, EPI_ISL_739129	Santa Clara County Public Health Laboratory	Chan-Zuckerberg Biohub	CZB Cliahub Consortium
EPI_ISL_739131	Humboldt County Public Health Laboratory	Chan-Zuckerberg Biohub	CZB Cliahub Consortium
EPI_ISL_739138	Santa Clara County Public Health Laboratory	Chan-Zuckerberg Biohub	CZB Cliahub Consortium
EPI_ISL_739155, EPI_ISL_739159	Humboldt County Public Health Laboratory	Chan-Zuckerberg Biohub	CZB Cliahub Consortium
EPI_ISL_739180	Santa Clara County Public Health Laboratory	Chan-Zuckerberg Biohub	CZB Cliahub Consortium
EPI_ISL_739183	Humboldt County Public Health Laboratory	Chan-Zuckerberg Biohub	CZB Cliahub Consortium
EPI_ISL_739187	Santa Clara County Public Health Laboratory	Chan-Zuckerberg Biohub	CZB Cliahub Consortium
EPI_ISL_739198	Humboldt County Public Health Laboratory	Chan-Zuckerberg Biohub	CZB Cliahub Consortium
EPI_ISL_739219, EPI_ISL_739234	Santa Clara County Public Health Laboratory	Chan-Zuckerberg Biohub	CZB Cliahub Consortium
EPI_ISL_739235	Humboldt County Public Health Laboratory	Chan-Zuckerberg Biohub	CZB Cliahub Consortium
EPI_ISL_739237	Santa Clara County Public Health Laboratory	Chan-Zuckerberg Biohub	CZB Cliahub Consortium
EPI_ISL_739250	Humboldt County Public Health Laboratory	Chan-Zuckerberg Biohub	CZB Cliahub Consortium
EPI_ISL_739252, EPI_ISL_739254	Santa Clara County Public Health Laboratory	Chan-Zuckerberg Biohub	CZB Cliahub Consortium
EPI_ISL_739269	Humboldt County Public Health Laboratory	Chan-Zuckerberg Biohub	CZB Cliahub Consortium
EPI_ISL_739273, EPI_ISL_739278	Santa Clara County Public Health Laboratory	Chan-Zuckerberg Biohub	CZB Cliahub Consortium
EPI_ISL_739299	Humboldt County Public Health Laboratory	Chan-Zuckerberg Biohub	CZB Cliahub Consortium
EPI_ISL_739310, EPI_ISL_739360, EPI_ISL_739377, EPI_ISL_739382	Santa Clara County Public Health Laboratory	Chan-Zuckerberg Biohub	CZB Cliahub Consortium
EPI_ISL_739394	Renegade	Chan-Zuckerberg Biohub	CZB Cliahub Consortium
EPI_ISL_739413, EPI_ISL_739416	Santa Clara County Public Health Laboratory	Chan-Zuckerberg Biohub	CZB Cliahub Consortium
EPI_ISL_739418	Humboldt County Public Health Laboratory	Chan-Zuckerberg Biohub	CZB Cliahub Consortium
EPI_ISL_739431	Santa Clara County Public Health Laboratory	Chan-Zuckerberg Biohub	CZB Cliahub Consortium
EPI_ISL_739441	Orange County Public Health Lab	Chan-Zuckerberg Biohub	CZB Cliahub Consortium
EPI_ISL_739443	Santa Clara County Public Health Laboratory	Chan-Zuckerberg Biohub	CZB Cliahub Consortium
EPI_ISL_739445	Orange County Public Health Lab	Chan-Zuckerberg Biohub	CZB Cliahub Consortium
EPI_ISL_739452	Humboldt County Public Health Laboratory	Chan-Zuckerberg Biohub	CZB Cliahub Consortium
EPI_ISL_739458, EPI_ISL_739491, EPI_ISL_739498	Santa Clara County Public Health Laboratory	Chan-Zuckerberg Biohub	CZB Cliahub Consortium
EPI_ISL_739499	Humboldt County Public Health Laboratory	Chan-Zuckerberg Biohub	CZB Cliahub Consortium
EPI_ISL_739501, EPI_ISL_739508, EPI_ISL_739509, EPI_ISL_739520, EPI_ISL_739537, EPI_ISL_739549	Santa Clara County Public Health Laboratory	Chan-Zuckerberg Biohub	CZB Cliahub Consortium
EPI_ISL_739559, EPI_ISL_739581	Orange County Public Health Lab	Chan-Zuckerberg Biohub	CZB Cliahub Consortium
EPI_ISL_739585	Humboldt County Public Health Laboratory	Chan-Zuckerberg Biohub	CZB Cliahub Consortium
EPI_ISL_739594, EPI_ISL_739597, EPI_ISL_739602	Santa Clara County Public Health Laboratory	Chan-Zuckerberg Biohub	CZB Cliahub Consortium
EPI_ISL_739607	Orange County Public Health Lab	Chan-Zuckerberg Biohub	CZB Cliahub Consortium
EPI_ISL_739608	Humboldt County Public Health Laboratory	Chan-Zuckerberg Biohub	CZB Cliahub Consortium
EPI_ISL_739626, EPI_ISL_739629, EPI_ISL_739647	Santa Clara County Public Health Laboratory	Chan-Zuckerberg Biohub	CZB Cliahub Consortium
EPI_ISL_739672	Instituto Nacional de Salud, Bogotá, Colombia	Instituto Nacional de Salud, Bogotá, Colombia	Katherine Laiton-Donato, Diego A. Álvarez-Díaz, Carlos Franco-Muñoz, Mauricio Pacheco-Montealegre, Jonathan Reales, Diego Andrés Prada, Sheryl Corchuelo, Magdalena Weisner, Martha Lucia Ospina Martinez, Marcela Mercado-Reyes
EPI_ISL_739694, EPI_ISL_739697, EPI_ISL_739721, EPI_ISL_739724, EPI_ISL_739730, EPI_ISL_739740, EPI_ISL_739764, EPI_ISL_739771, EPI_ISL_739846, EPI_ISL_739857, EPI_ISL_739858, EPI_ISL_739900, EPI_ISL_739901, EPI_ISL_739921, EPI_ISL_739966, EPI_ISL_739972, EPI_ISL_739976, EPI_ISL_739984, EPI_ISL_739991, EPI_ISL_740012, EPI_ISL_740072, EPI_ISL_740094, EPI_ISL_740119, EPI_ISL_740158, EPI_ISL_740167, EPI_ISL_740212, EPI_ISL_740213, EPI_ISL_740236, EPI_ISL_740284, EPI_ISL_740299, EPI_ISL_740309, EPI_ISL_740313, EPI_ISL_740323, EPI_ISL_740335, EPI_ISL_740339, EPI_ISL_740341, EPI_ISL_740351, EPI_ISL_740365, EPI_ISL_740368, EPI_ISL_740376, EPI_ISL_740378, EPI_ISL_740380, EPI_ISL_740389, EPI_ISL_740392, EPI_ISL_740393, EPI_ISL_740395, EPI_ISL_740416, EPI_ISL_740420, EPI_ISL_740421, EPI_ISL_740428, EPI_ISL_740437, EPI_ISL_740439, EPI_ISL_740463, EPI_ISL_740466, EPI_ISL_740472, EPI_ISL_740478, EPI_ISL_740494, EPI_ISL_740505, EPI_ISL_740523, EPI_ISL_740529, EPI_ISL_740541			
see above	Laboratoire national de santé, Microbiology, Virology	Laboratoire national de santé, Microbiology, Microbial Genomics Platform	Anke Wienecke-Baldacchino, Catherine Ragimbeau,Jessica Tapp, Fatu Djabi, Lise Pignon, Raoul Salmon, Tamir Abdelrahman
EPI_ISL_741759	Centre for Enzyme Innovation, University of Portsmouth / Translational Research Laboratory, Portsmouth Hospitals NHS Trust	COVID-19 Genomics UK (COG-UK) Consortium	Angela Beckett,Yann Bourgeois,Garry Scarlett,Sharon Glaysher,Scott Elliott,Kelly Bicknell,Robert Impey,Allyson Lloyd,Sarah Wyllie,Ethan Butcher,Anoop Chauhan,Samuel Robson
EPI_ISL_742174	Regional Virus Laboratory, Belfast Health and Social Care Trust	COVID-19 Genomics UK (COG-UK) Consortium	Conall McCaughey, James McKenna, Tanya Curran, Susan Feeney, Alison Watt, Ciara Cox, Mairead Connor, Zoltan Molnar, David Simpson, Derek Fairley
EPI_ISL_743611, EPI_ISL_743612, EPI_ISL_743613	Wales Specialist Virology Centre Sequencing lab: Pathogen Genomics Unit	COVID-19 Genomics UK (COG-UK) Consortium	Catherine Moore, Johnathan Evans, Laura Gifford, Malorie Perry, Simon Cottrell, Angela Marchbank, Alec Birchley, Alexander Adams, Amy Gaskin, Bree Gatica-Wilcox, Jason Coombes, Joel Southgate, Lauren Gilbert, Lee Graham, Nicole Pacchiarini, Sara Kumziene-Summerhayes, Sarah Taylor, Sophie Jones, Sara Rey, Matthew Bull, Joanne Watkins, Sally Corden, Tom Connor
EPI_ISL_744151, EPI_ISL_744176, EPI_ISL_744183, EPI_ISL_744210, EPI_ISL_744230, EPI_ISL_744273, EPI_ISL_744328, EPI_ISL_744330, EPI_ISL_744333, EPI_ISL_744335, EPI_ISL_744338, EPI_ISL_744342, EPI_ISL_744360, EPI_ISL_744377, EPI_ISL_744468, EPI_ISL_744479, EPI_ISL_744485, EPI_ISL_744504, EPI_ISL_744508, EPI_ISL_744518, EPI_ISL_744523, EPI_ISL_744542, EPI_ISL_744554, EPI_ISL_744558, EPI_ISL_744564, EPI_ISL_744576, EPI_ISL_744580, EPI_ISL_744606, EPI_ISL_744608, EPI_ISL_744689, EPI_ISL_744717, EPI_ISL_744728, EPI_ISL_744730, EPI_ISL_744743, EPI_ISL_744747, EPI_ISL_744786, EPI_ISL_744787, EPI_ISL_744817, EPI_ISL_744838, EPI_ISL_744839, EPI_ISL_744840, EPI_ISL_744850, EPI_ISL_744856, EPI_ISL_744871, EPI_ISL_744884, EPI_ISL_744905, EPI_ISL_744915, EPI_ISL_744927, EPI_ISL_744930, EPI_ISL_744931, EPI_ISL_744944, EPI_ISL_744959, EPI_ISL_744965, EPI_ISL_744966, EPI_ISL_744980, EPI_ISL_744991, EPI_ISL_745008, EPI_ISL_745009			
see above	Laboratoire national de santé, Microbiology, Virology	Laboratoire national de santé, Microbiology, Microbial Genomics Platform	Anke Wienecke-Baldacchino, Catherine Ragimbeau,Jessica Tapp, Fatu Djabi, Lise Pignon, Raoul Salmon, Tamir Abdelrahman
EPI_ISL_745335	CHU Clermont-Ferrand	CNR Virus des Infections Respiratoires - France SUD	Antonin Bal, Gregory Destras, Gwendolyne Burfin, Hadrien Règue, Quentin Semanas, Martine Valette, Bruno Lina, Christine Archimbaud, Amélie Brebion, Hélène Chabrolles, Martine Chambon, Audrey Mirand, Christel Regagnon, Maxime Bisseux, Patricia Combes, Cécile Henquell, Laurence Josset
EPI_ISL_746839, EPI_ISL_746853, EPI_ISL_746899, EPI_ISL_746914,	Utah Public Health Laboratory	Utah Public Health Laboratory	Erin Young, Kelly Oakeson, Tara Gallagher

EPI_ISL_746963, EPI_ISL_746974, EPI_ISL_746979			
EPI_ISL_747168, EPI_ISL_747170, EPI_ISL_747171, EPI_ISL_747181, EPI_ISL_747182	Respiratory Viruses Branch, Centers for Disease Control and Prevention	Respiratory Viruses Branch, Centers for Disease Control and Prevention	Queen,K., Li,Y., Tao,Y., Uehara,A., Montmayeur,A., Paden,C.R., Cook,P.W., Marine,R., Sheth,M., Wang,H., Lee,J., Tong,S.
EPI_ISL_747391, EPI_ISL_747398, EPI_ISL_747399, EPI_ISL_747408, EPI_ISL_747425, EPI_ISL_747426, EPI_ISL_747427, EPI_ISL_747428, EPI_ISL_747429, EPI_ISL_747438, EPI_ISL_747458			
see above	Division of Emerging Infectious Diseases, Bureau of Infectious Diseases Diagnosis Control, Korea Disease Control and Prevention Agency	Division of Emerging Infectious Diseases, Bureau of Infectious Diseases Diagnosis Control, Korea Disease Control and Prevention Agency	Ae Kyung Park, Il-Hwan Kim, Heui Man Kim, Jeong-Min Kim, Namjoo Lee, Chaeyoung Lee, Sang Hee Woo, Eun-Jin Kim
EPI_ISL_747471, EPI_ISL_747472, EPI_ISL_747473, EPI_ISL_747474, EPI_ISL_747475	ULSS9 Distretto di Bussolengo	Istituto Zooprofilattico Sperimentale delle Venezie	Adelaide Milani, Alessia Schivo, Annalisa Salviato, Erika Giorgia Quaranta, Ambra Pastori, Bianca Zecchin, Alice Fusaro, Isabella Monne, Calogero Terregino, Antonia Ricci
EPI_ISL_747531, EPI_ISL_747585, EPI_ISL_747586, EPI_ISL_747587, EPI_ISL_747598, EPI_ISL_747609, EPI_ISL_747629, EPI_ISL_747630, EPI_ISL_747631, EPI_ISL_747680, EPI_ISL_748099, EPI_ISL_748100, EPI_ISL_748101, EPI_ISL_748102, EPI_ISL_748103, EPI_ISL_748104, EPI_ISL_748105, EPI_ISL_748106, EPI_ISL_748107, EPI_ISL_748108, EPI_ISL_748109, EPI_ISL_748110, EPI_ISL_748111, EPI_ISL_748112			
see above	Department of Virus and Microbiological Special Diagnostics, Statens Serum Institut, Copenhagen, Denmark	Albertsen Lab, Department of Chemistry and Bioscience, Aalborg University, Denmark	Danish Covid-19 Genome Consortium
EPI_ISL_751458, EPI_ISL_751459, EPI_ISL_751460, EPI_ISL_751464, EPI_ISL_751473	CHU Purpan - Laboratoire de Virologie - Institut Fédératif de Biologie	CHU Purpan - Laboratoire de Virologie - Institut Fédératif de Biologie	Latour J., Ranger N., Dubois M., Carcenac R., Harter A., Boyer P., Tremeaux P., Izopet J.
EPI_ISL_751562	NYSDOH Wadsworth Center, Virology Lab	Genomics and Discovery, Respiratory Viruses Branch, Division of Viral Diseases, Centers for Disease Control and Prevention	Krista Queen, Yan Li, Ying Tao, Jing Zhang, Anna Uehara, Anna Montmayeur, Clinton R. Paden, Peter W. Cook,Rachel Marine, Mili Sheth, Haibin Wang, Justin Lee, Suxiang Tong
EPI_ISL_751577	MD DOH Laboratories Administration	Genomics and Discovery, Respiratory Viruses Branch, Division of Viral Diseases, Centers for Disease Control and Prevention	Krista Queen, Yan Li, Ying Tao, Jing Zhang, Anna Uehara, Anna Montmayeur, Clinton R. Paden, Peter W. Cook,Rachel Marine, Mili Sheth, Haibin Wang, Justin Lee, Suxiang Tong
EPI_ISL_751581	NYSDOH Wadsworth Center, Virology Lab	Genomics and Discovery, Respiratory Viruses Branch, Division of Viral Diseases, Centers for Disease Control and Prevention	Krista Queen, Yan Li, Ying Tao, Jing Zhang, Anna Uehara, Anna Montmayeur, Clinton R. Paden, Peter W. Cook,Rachel Marine, Mili Sheth, Haibin Wang, Justin Lee, Suxiang Tong
EPI_ISL_751606, EPI_ISL_751607	MD DOH Laboratories Administration	Genomics and Discovery, Respiratory Viruses Branch, Division of Viral Diseases, Centers for Disease Control and Prevention	Krista Queen, Yan Li, Ying Tao, Jing Zhang, Anna Uehara, Anna Montmayeur, Clinton R. Paden, Peter W. Cook,Rachel Marine, Mili Sheth, Haibin Wang, Justin Lee, Suxiang Tong
EPI_ISL_751638	NE Public Health Laboratory	Genomics and Discovery, Respiratory Viruses Branch, Division of Viral Diseases, Centers for Disease Control and Prevention	Krista Queen, Yan Li, Ying Tao, Jing Zhang, Anna Uehara, Anna Montmayeur, Clinton R. Paden, Peter W. Cook,Rachel Marine, Mili Sheth, Haibin Wang, Justin Lee, Suxiang Tong
EPI_ISL_751649, EPI_ISL_751650	CT-Dr. Katherine A. Kelley State Public Health Lab	Genomics and Discovery, Respiratory Viruses Branch, Division of Viral Diseases, Centers for Disease Control and Prevention	Krista Queen, Yan Li, Ying Tao, Jing Zhang, Anna Uehara, Anna Montmayeur, Clinton R. Paden, Peter W. Cook,Rachel Marine, Mili Sheth, Haibin Wang, Justin Lee, Suxiang Tong
EPI_ISL_751673	HI Dept. of Health, State Laboratories Division	Genomics and Discovery, Respiratory Viruses Branch, Division of Viral Diseases, Centers for Disease Control and Prevention	Krista Queen, Yan Li, Ying Tao, Jing Zhang, Anna Uehara, Anna Montmayeur, Clinton R. Paden, Peter W. Cook,Rachel Marine, Mili Sheth, Haibin Wang, Justin Lee, Suxiang Tong
EPI_ISL_751702	MD DOH Laboratories Administration	Genomics and Discovery, Respiratory Viruses Branch, Division of Viral Diseases, Centers for Disease Control and Prevention	Krista Queen, Yan Li, Ying Tao, Jing Zhang, Anna Uehara, Anna Montmayeur, Clinton R. Paden, Peter W. Cook,Rachel Marine, Mili Sheth, Haibin Wang, Justin Lee, Suxiang Tong
EPI_ISL_751709	NJ Public Health and Environmental Laboratories	Genomics and Discovery, Respiratory Viruses Branch, Division of Viral Diseases, Centers for Disease Control and Prevention	Krista Queen, Yan Li, Ying Tao, Jing Zhang, Anna Uehara, Anna Montmayeur, Clinton R. Paden, Peter W. Cook,Rachel Marine, Mili Sheth, Haibin Wang, Justin Lee, Suxiang Tong
EPI_ISL_751725	MD DOH Laboratories Administration	Genomics and Discovery, Respiratory Viruses Branch, Division of Viral Diseases, Centers for Disease Control and Prevention	Krista Queen, Yan Li, Ying Tao, Jing Zhang, Anna Uehara, Anna Montmayeur, Clinton R. Paden, Peter W. Cook,Rachel Marine, Mili Sheth, Haibin Wang, Justin Lee, Suxiang Tong
EPI_ISL_751767	HI Dept. of Health, State Laboratories Division	Genomics and Discovery, Respiratory Viruses Branch, Division of Viral Diseases, Centers for Disease Control and Prevention	Krista Queen, Yan Li, Ying Tao, Jing Zhang, Anna Uehara, Anna Montmayeur, Clinton R. Paden, Peter W. Cook,Rachel Marine, Mili Sheth, Haibin Wang, Justin Lee, Suxiang Tong
EPI_ISL_751787	TX DSHS, Lab Services Section MC 1947	Genomics and Discovery, Respiratory Viruses Branch, Division of Viral Diseases, Centers for Disease Control and Prevention	Krista Queen, Yan Li, Ying Tao, Jing Zhang, Anna Uehara, Anna Montmayeur, Clinton R. Paden, Peter W. Cook,Rachel Marine, Mili Sheth, Haibin Wang, Justin Lee, Suxiang Tong
EPI_ISL_751788	OH Department of Health Laboratory	Genomics and Discovery, Respiratory Viruses Branch, Division of Viral Diseases, Centers for Disease Control and Prevention	Krista Queen, Yan Li, Ying Tao, Jing Zhang, Anna Uehara, Anna Montmayeur, Clinton R. Paden, Peter W. Cook,Rachel Marine, Mili Sheth, Haibin Wang, Justin Lee, Suxiang Tong
EPI_ISL_752531, EPI_ISL_752532, EPI_ISL_752533, EPI_ISL_752534, EPI_ISL_752535, EPI_ISL_752536, EPI_ISL_752537, EPI_ISL_752538, EPI_ISL_752539, EPI_ISL_752540, EPI_ISL_752541, EPI_ISL_752542, EPI_ISL_752543, EPI_ISL_752544, EPI_ISL_752545, EPI_ISL_752546, EPI_ISL_752547, EPI_ISL_752548, EPI_ISL_752549, EPI_ISL_752550, EPI_ISL_752551, EPI_ISL_752552, EPI_ISL_752553, EPI_ISL_752554, EPI_ISL_752555, EPI_ISL_752556, EPI_ISL_752557, EPI_ISL_752558, EPI_ISL_752559, EPI_ISL_752560, EPI_ISL_752561, EPI_ISL_752562, EPI_ISL_752563, EPI_ISL_752564, EPI_ISL_752565, EPI_ISL_752566, EPI_ISL_752567, EPI_ISL_752568, EPI_ISL_752569, EPI_ISL_752570, EPI_ISL_752571, EPI_ISL_752572, EPI_ISL_752573, EPI_ISL_752574, EPI_ISL_752575, EPI_ISL_752576, EPI_ISL_752577, EPI_ISL_752578, EPI_ISL_752579, EPI_ISL_752580, EPI_ISL_752581, EPI_ISL_752582, EPI_ISL_752593			
see above	National Virus Reference Laboratory	National Virus Reference Laboratory	Michael Carr, Gabriel Gonzalez, Jonathan Dean, Daniel Hare, Cillian F De Gascun
EPI_ISL_753116, EPI_ISL_753117, EPI_ISL_753118, EPI_ISL_753119, EPI_ISL_753120, EPI_ISL_753129, EPI_ISL_753130, EPI_ISL_753131, EPI_ISL_753132, EPI_ISL_753133, EPI_ISL_753134, EPI_ISL_753135, EPI_ISL_753136, EPI_ISL_753137, EPI_ISL_753138, EPI_ISL_753139, EPI_ISL_753140, EPI_ISL_753141, EPI_ISL_753142, EPI_ISL_753143, EPI_ISL_753144, EPI_ISL_753145, EPI_ISL_753146, EPI_ISL_753154, EPI_ISL_753155, EPI_ISL_753156, EPI_ISL_753157, EPI_ISL_753158, EPI_ISL_753159, EPI_ISL_753160, EPI_ISL_753161, EPI_ISL_753162, EPI_ISL_753163, EPI_ISL_753164, EPI_ISL_753165, EPI_ISL_753166, EPI_ISL_753167, EPI_ISL_753168, EPI_ISL_753169, EPI_ISL_753170, EPI_ISL_753171, EPI_ISL_753172, EPI_ISL_753173			
see above	State Laboratories Division, Hawaii State Department of Health	State Laboratories Division, Hawaii State Department of Health	Pamela O'Brien, Sabrina Diemert, Drew Kuwazaki, Razvan Sultana, Edward Desmond
EPI_ISL_753393, EPI_ISL_753395, EPI_ISL_753396, EPI_ISL_753397, EPI_ISL_753398, EPI_ISL_753399, EPI_ISL_753400, EPI_ISL_753401, EPI_ISL_753402, EPI_ISL_753409, EPI_ISL_753410, EPI_ISL_753411, EPI_ISL_753412			
see above	Clinical virology Laboratory, Children's Hospital Los Angeles	Center for Personalized Medicine, Children's Hospital Los Angeles	Gai et al
EPI_ISL_753741, EPI_ISL_753828, EPI_ISL_753829, EPI_ISL_753830	Charité Universitätsmedizin Berlin, Institut für Virologie/Labor Berlin	Charité Universitätsmedizin Berlin, Institut für Virologie	Victor M Corman, Jörn Beheim-Schwarzbach, Barbara Mühlemann, Julia Schneider, Talitha Veith, Terry Jones, Christian Drosten
EPI_ISL_754143, EPI_ISL_754144,	Laboratoire de Microbiologie	National Reference Center for Viruses of Respiratory	Marion Barbet, Sylvie Behillil, Méline Bizard, Angela Brisebarre, Camille Capel, Etienne Simon-Lorière, Vincent Enouf, Maud Vanpeene, Sylvie van der

EPI_ISL_754152, EPI_ISL_754153, EPI_ISL_754154		Infections, Institut Pasteur, Paris	Werf, Marie-Sarah Fangous
EPI_ISL_754233	The Republican Research and Practical Center for Epidemiology and Microbiology (RRPCEM)	WHO National Influenza Centre Russian Federation	Elena Gasich, Kirill Bulda, Anatoly Krasko, Andrey Komissarov, Artem Fadeev, Anna Ivanova, Kseniya Komissarova, Dmitry Bazhenov, Daria Danilenko, Ksenia Safina, Elena Nabieva, Georgii Bazykin, Dmitry Lioznov
EPI_ISL_754405, EPI_ISL_754406, EPI_ISL_754477, EPI_ISL_754478, EPI_ISL_754479, EPI_ISL_754480, EPI_ISL_754481, EPI_ISL_754488, EPI_ISL_754587, EPI_ISL_754588, EPI_ISL_754589, EPI_ISL_754590, EPI_ISL_754591, EPI_ISL_754592, EPI_ISL_754593, EPI_ISL_754594, EPI_ISL_754595, EPI_ISL_754596, EPI_ISL_754597, EPI_ISL_754598, EPI_ISL_754599, EPI_ISL_754600, EPI_ISL_754601, EPI_ISL_754602, EPI_ISL_754603, EPI_ISL_754604, EPI_ISL_754605, EPI_ISL_754606, EPI_ISL_754607, EPI_ISL_754608, EPI_ISL_754609, EPI_ISL_754610, EPI_ISL_754611, EPI_ISL_754612, EPI_ISL_754613, EPI_ISL_754614, EPI_ISL_754615, EPI_ISL_754616, EPI_ISL_754617, EPI_ISL_754618			
see above	Wadsworth Center, New York State Department.of Health	Wadsworth Center, New York State Department.of Health	Kirsten St. George, Daryl M. Lamson, Alexis Russel, Matthew Shudt, Melissa A Leisner, Jonathan Plitnick, Navjot Singh, John Kelly, Sara Griesemer, Erasmus Schneider, Erica Lasek-Nesselquist
EPI_ISL_755135, EPI_ISL_755272, EPI_ISL_755281, EPI_ISL_755284, EPI_ISL_755299	San Diego County Public Health Laboratory	Andersen lab at Scripps Research	SEARCH Alliance San Diego with Tracy Basler, Jovan Shephard, Brett Austin
EPI_ISL_755474, EPI_ISL_755475, EPI_ISL_755476	Maine Health and Environmental Testing Laboratory	Tewhey Lab, The Jackson Laboratory	Matluk,N., Dewey,H., Iosue,F., Barter,M., Lynch,R., Munger,H. and Tewhey,R.
EPI_ISL_756279	State Laboratories Division, Hawaii State Department of Health	State Laboratories Division, Hawaii State Department of Health	Pamela O'Brien, Sabrina Diemert, Drew Kuwazaki, Razvan Sultana, Edward Desmond
EPI_ISL_759978, EPI_ISL_759979, EPI_ISL_759980	Medical Microbiology Unit, Department for Laboratory Medicine, Drammen Hospital, Vestre Viken Health Trust,	Norwegian Institute of Public Health, Department of Virology	Kathrine Stene-Johansen, Kamilla Heddeland Instefjord, Hilde Elshaug, Marie Paulsen Madsen, Rasmus Riis Kopperud, Hilde Vollan, Karoline Bragstad, Olav Hungnes
EPI_ISL_759981	Norwegian Institute of Public Health, Department of Virology	Norwegian Institute of Public Health, Department of Virology	Kathrine Stene-Johansen, Kamilla Heddeland Instefjord, Hilde Elshaug, Marie Paulsen Madsen, Rasmus Riis Kopperud, Hilde Vollan, Karoline Bragstad, Olav Hungnes
EPI_ISL_760490	Lighthouse Lab in Glasgow	Wellcome Sanger Institute for the COVID-19 Genomics UK (COG-UK) Consortium	Harper VanSteenhouse, Yumi Kasai, David Gray, Carol Clugston, Anna Dominiczak and Alex Alderton, Roberto Amato, Sonia Goncalves, Ewan Harrison, David K. Jackson, Ian Johnston, Dominic Kwiatkowski, Cordelia Langford, John Sillitoe on behalf of the Wellcome Sanger Institute COVID-19 Surveillance Team
EPI_ISL_763026, EPI_ISL_763030, EPI_ISL_763051	Unit 17: Influenza & Other Respiratory Viruses, German National Influenza Center	Project group Epidemiology of Highly Pathogenic Microorganisms, Robert Koch-Institute	Ariane Düx, Andreas Sachse, Grit Schubert, Sébastien Calvignac-Spencer, Fabian Leendertz, Thorsten Wolff, Ralf Dürrwald, Djin-Ye Oh, Marianne Wedde
EPI_ISL_763100, EPI_ISL_763101, EPI_ISL_763102, EPI_ISL_763103, EPI_ISL_763112, EPI_ISL_763113, EPI_ISL_763114, EPI_ISL_763115, EPI_ISL_763116, EPI_ISL_763117, EPI_ISL_763118, EPI_ISL_763164, EPI_ISL_763165, EPI_ISL_763166, EPI_ISL_763167, EPI_ISL_763171, EPI_ISL_763236, EPI_ISL_763275, EPI_ISL_763276, EPI_ISL_763277, EPI_ISL_763313			
see above	Dutch COVID-19 response team	Erasmus Medical Center	Bas Oude Munnink, Reina Sikkema, David Nieuwenhuijse, Irina Chestakova, Anne van der Linden, Marjan Boter, Emmanuelle Munger, Corine GeurtsvanKessel, Annemiek van der Eijk, Richard Molenkamp, Marion Koopmans, on behalf of the Dutch national COVID-19 response team.
EPI_ISL_763332, EPI_ISL_763343	Florida Bureau of Public Health Laboratories	Florida Bureau of Public Health Laboratories	Sarah Schmedes, Jason Blanton
EPI_ISL_763359	Dutch COVID-19 response team	Erasmus Medical Center	Bas Oude Munnink, Reina Sikkema, David Nieuwenhuijse, Irina Chestakova, Anne van der Linden, Marjan Boter, Emmanuelle Munger, Corine GeurtsvanKessel, Annemiek van der Eijk, Richard Molenkamp, Marion Koopmans, on behalf of the Dutch national COVID-19 response team.
EPI_ISL_764360	Virology Department, Royal Infirmary of Edinburgh, NHS Lothian / School of Biological Sciences, University of Edinburgh / Institute of Genetics and Molecular Medicine, University of Edinburgh	COVID-19 Genomics UK (COG-UK) Consortium	McHugh M, Dewar R, Rooke S, Gallagher M, Balcaza C, O'Toole Á, Scher E, Hill V, McCrone JT, Colquhoun R, Yu X, Jackson B, Rambaut A, Williams TC, Templeton K
EPI_ISL_765217	Instituto Nacional de Saude (INSA) and Instituto Gulbenkian de Ciencia (IGC)	Instituto Nacional de Saude (INSA) and Instituto Gulbenkian de Ciencia (IGC)	Borges et al
EPI_ISL_765779, EPI_ISL_765780, EPI_ISL_765854, EPI_ISL_765855, EPI_ISL_765856, EPI_ISL_765857, EPI_ISL_765858, EPI_ISL_765859, EPI_ISL_765860, EPI_ISL_765861, EPI_ISL_765862			
see above	Massachusetts General Hospital	Infectious Disease Program, Broad Institute of Harvard and MIT	Lemieux,J.E., Siddle,K.J., Shaw,B., Adams,G., Pierce,V., Turbett,S., Anahtar,M., Branda,J., Slater,D., Harris,J., Lin,A.E., Gladden-Young,A., Lagerborg,K., Rudy,M., DeRuff,K., Carter,A., Normandin,E., Bauer,M., Reilly,S., Tomkins-Tinch,C., Loreth,C., Chaluvadi,S., Neumann,A., Cusick,C., Chapman,S.B., Gnirke,A., Flowers,K., Cerrato,F., Birren,B.W., Gallagher,G., Smole,S., Park,D.J., MacInnis,B.L., Ryan,E., LaRocque,R., Rosenberg,E. and Sabeti,P.C.
EPI_ISL_766276, EPI_ISL_766277	Respiratory Virus Unit, National Infection Service, Public Health England	COVID-19 Genomics UK (COG-UK) Consortium	PHE Covid Sequencing Team
EPI_ISL_766617, EPI_ISL_766618, EPI_ISL_766620	Klinisk mikrobiologi	The Public Health Agency of Sweden	Department of Microbiology, The Public Health Agency of Sweden
EPI_ISL_766642	Vardcentralen Brinken	The Public Health Agency of Sweden	Department of Microbiology, The Public Health Agency of Sweden
EPI_ISL_766659, EPI_ISL_766660, EPI_ISL_766680	Texas Department of State Health Services	Texas Department of State Health Services	Rashmi Tuladhar, Bonnie Oh, Jenny Zhang, Maliha Rahman, Anita Pokharel, Myong Koag, Chung Wang, Rachel Lee, Grace Kubin, Mayela Pedrueza, James Daniel Bonser
EPI_ISL_766976, EPI_ISL_766993, EPI_ISL_766999	Delaware Public Health Laboratory	Delaware Public Health Laboratory	Gregory Hovan
EPI_ISL_767319, EPI_ISL_767320, EPI_ISL_767321, EPI_ISL_767322, EPI_ISL_767323, EPI_ISL_767324, EPI_ISL_767325, EPI_ISL_767326, EPI_ISL_767327, EPI_ISL_767328, EPI_ISL_767329, EPI_ISL_767330, EPI_ISL_767331, EPI_ISL_767332, EPI_ISL_767333			
see above	Lighthouse Lab in Alderley Park	Wellcome Sanger Institute for the COVID-19 Genomics UK (COG-UK) Consortium	Jacquelyn Wynn, Mairead Hyland, The Lighthouse Lab in Alderley Park and Alex Alderton, Roberto Amato, Sonia Goncalves, Ewan Harrison, David K. Jackson, Ian Johnston, Dominic Kwiatkowski, Cordelia Langford, John Sillitoe on behalf of the Wellcome Sanger Institute COVID-19 Surveillance Team
EPI_ISL_767582, EPI_ISL_767590, EPI_ISL_767626, EPI_ISL_767627	WHITE PLAINS HOSPITAL CENTER LABORATORY	Wadsworth Center, New York State Department.of Health	Kirsten St. George, Daryl M. Lamson, Alexis Russel, Matthew Shudt, Melissa A Leisner, Jonathan Plitnick, Navjot Singh, John Kelly, Sara Griesemer, Erasmus Schneider, Erica Lasek-Nesselquist
EPI_ISL_767851, EPI_ISL_767852	South Eastern Area Laboratory Services (SEALS)	NSW Health Pathology - Institute of Clinical Pathology and Medical Research; Westmead Hospital; University of Sydney	CIDM-PH et al.
EPI_ISL_767858, EPI_ISL_767859, EPI_ISL_767860	Sydney South West Pathology Service (SSWPS) - Royal Prince Alfred Hospital - NSW Health Pathology	NSW Health Pathology - Institute of Clinical Pathology and Medical Research; Westmead Hospital; University of Sydney	CIDM-PH et al.
EPI_ISL_768353, EPI_ISL_768354, EPI_ISL_768355, EPI_ISL_768356	LSUHS Emerging Viral Threat Laboratory	Microbial Genome Sequencing Center	Jeremy P. Kamil, Jennifer L. Carroll, Camille F. Abshire, Maarten Van Diest, Andrew D. Yurochko, Martin J. Sapp, Rona S. Scott, Christopher G. Kevil, Daniel J. Snyder, Vaughn S. Cooper, John A. Vanchiere
EPI_ISL_770022	Area De Salud Perez Zeledon	Inciensa, Instituto Costarricense de Investigación y Enseñanza en Nutrición y Salud	Francisco Duarte, Hebleen Porras, Claudio Soto-Garita, Estela Cordero, Adriana Godínez, Melany Calderón & Mariel López
EPI_ISL_770067, EPI_ISL_770068, EPI_ISL_770069, EPI_ISL_770086, EPI_ISL_770087, EPI_ISL_770088, EPI_ISL_770089, EPI_ISL_770090, EPI_ISL_770091, EPI_ISL_770092, EPI_ISL_770093, EPI_ISL_770108, EPI_ISL_770128			
see above	Wyoming Public Health Laboratory	Wyoming Public Health Laboratory	Noah Hull, Taylor Fearing, Lynette Gumbleton, Channing Weber, Ashley Norberg, Bailey Bowcutt, and Wanda Manley
EPI_ISL_770555, EPI_ISL_770556, EPI_ISL_770573, EPI_ISL_770574, EPI_ISL_770575, EPI_ISL_770576	Laboratório de Microbiologia Molecular - Universidade FEEVALE	Bioinformatics Laboratory / LNCC	Felipe Benites, Fernando Rosado Spilki, Alana Witt Hansen, Juliane Deise Fleck, Juliana Schons, Meriane Demoliner, Ana Karolina Eisen Antunes, Fagner Henrique Heldt, Larissa Mallmann, Bruna Hermann, Ana Luiza Ziulkoski, Vyctoria Goes, Karoline Schallenberger, Matheus Nunes Weber, Paula Rodrigues de Almeida, Alessandra Pavan Lamarca da Silva, Ronaldo da Silva F Jr , Luiz G P de Almeida, Alexandra L Gerber , Ana Paula de C Guimarães,Ana Tereza R de Vasconcelos
EPI_ISL_770658, EPI_ISL_770673	WHO National Influenza Centre Russian Federation	WHO National Influenza Centre Russian Federation	Andrey Komissarov, Artem Fadeev, Anna Ivanova, Kseniya Komissarova, Dmitry Bazhenov, Daria Danilenko, Ksenia Safina, Elena Nabieva, Georgii

			Bazykin, Dmitry Lioznov
EPI_ISL_771157, EPI_ISL_771160, EPI_ISL_771162, EPI_ISL_771165, EPI_ISL_771167, EPI_ISL_771168, EPI_ISL_771169, EPI_ISL_771170, EPI_ISL_771173, EPI_ISL_771176, EPI_ISL_771179, EPI_ISL_771180, EPI_ISL_771182, EPI_ISL_771183, EPI_ISL_771191, EPI_ISL_771192, EPI_ISL_771193, EPI_ISL_771198, EPI_ISL_771199, EPI_ISL_771201, EPI_ISL_771208, EPI_ISL_771212, EPI_ISL_771222, EPI_ISL_771291, EPI_ISL_771300, EPI_ISL_771301, EPI_ISL_771303, EPI_ISL_771304, EPI_ISL_771305, EPI_ISL_771306, EPI_ISL_771308, EPI_ISL_771309, EPI_ISL_771310, EPI_ISL_771311, EPI_ISL_771312, EPI_ISL_771313, EPI_ISL_771314, EPI_ISL_771315, EPI_ISL_771316, EPI_ISL_771317, EPI_ISL_771318, EPI_ISL_771319, EPI_ISL_771320, EPI_ISL_771321, EPI_ISL_771322, EPI_ISL_771323, EPI_ISL_771324, EPI_ISL_771325, EPI_ISL_771326, EPI_ISL_771327, EPI_ISL_771328, EPI_ISL_771330, EPI_ISL_771333, EPI_ISL_771334, EPI_ISL_771351, EPI_ISL_771352			
see above	Colorado Department of Public Health and Environment	Colorado Department of Puplic Health and Environment	Laura Bankers, Molly C. Hetherington-Rauth, Diana Ir, Shannon Ely, Shannon R. Matzinger, Sarah Elizabeth Totten, Emily A. Travanty
EPI_ISL_775286	Nordland Hospital - Bodo, Laboratory Department, Molecular Biology Unit	Norwegian Institute of Public Health, Department of Virology	Kathrine Stene-Johansen, Kamilla Heddeland Instefjord, Hilde Elshaug, Atiya R Ali,Marie Paulsen Madsen, Rasmus Riis Kopperud, Hilde Vollan, Karoline Bragstad, Olav Hungnes
EPI_ISL_775306	Department of Medical Microbiology, St. Olavs hospital	Norwegian Institute of Public Health, Department of Virology	Kathrine Stene-Johansen, Kamilla Heddeland Instefjord, Hilde Elshaug, Atiya R Ali,Marie Paulsen Madsen, Rasmus Riis Kopperud, Hilde Vollan, Karoline Bragstad, Olav Hungnes
EPI_ISL_775348	Medical Microbiology Unit, Department for Laboratory Medicine, Drammen Hospital, Vestre Viken Health Trust,	Norwegian Institute of Public Health, Department of Virology	Kathrine Stene-Johansen, Kamilla Heddeland Instefjord, Hilde Elshaug, Atiya R Ali,Marie Paulsen Madsen, Rasmus Riis Kopperud, Hilde Vollan, Karoline Bragstad, Olav Hungnes
EPI_ISL_775358, EPI_ISL_775359, EPI_ISL_775360, EPI_ISL_775361	Unilabs Laboratory Medicine	Norwegian Institute of Public Health, Department of Virology	Kathrine Stene-Johansen, Kamilla Heddeland Instefjord, Hilde Elshaug, Atiya R Ali,Marie Paulsen Madsen, Rasmus Riis Kopperud, Hilde Vollan, Karoline Bragstad, Olav Hungnes
EPI_ISL_775374, EPI_ISL_775376, EPI_ISL_775398	Nordland Hospital - Bodo, Laboratory Department, Molecular Biology Unit	Norwegian Institute of Public Health, Department of Virology	Kathrine Stene-Johansen, Kamilla Heddeland Instefjord, Hilde Elshaug, Atiya R Ali,Marie Paulsen Madsen, Rasmus Riis Kopperud, Hilde Vollan, Karoline Bragstad, Olav Hungnes
EPI_ISL_775488	Furst Medical Laboratory	Norwegian Institute of Public Health, Department of Virology	Kathrine Stene-Johansen, Kamilla Heddeland Instefjord, Hilde Elshaug, Atiya R Ali,Marie Paulsen Madsen, Rasmus Riis Kopperud, Hilde Vollan, Karoline Bragstad, Olav Hungnes
EPI_ISL_775493, EPI_ISL_775494	Department of Medical Microbiology, St. Olavs hospital	Norwegian Institute of Public Health, Department of Virology	Kathrine Stene-Johansen, Kamilla Heddeland Instefjord, Hilde Elshaug, Atiya R Ali,Marie Paulsen Madsen, Rasmus Riis Kopperud, Hilde Vollan, Karoline Bragstad, Olav Hungnes
EPI_ISL_778843	Servicio Virosis Respiratorias-Departamento Virología-INEI	Instituto Nacional Enfermedades Infecciosas C.G.Malbran	Baumeister E., Avaro M., Benedetti E., Russo M., Dattero ME, Pontoriero A., Cisterna D., Molina V., Perandones C., Tuduri E., Lorenzo F., Poklepovich T., Campos J.
EPI_ISL_779155, EPI_ISL_779156	Laboratório de Microbiologia Molecular - Universidade FEEVALE	Bioinformatics Laboratory / LNCC	Felipe Benites, Fernando Rosado Spilki, Alana Witt Hansen, Juliane Deise Fleck, Juliana Schons, Meriane Demoliner, Ana Karolina Eisen Antunes, Fagner Henrique Heldt, Larissa Mallmann, Bruna Hermann, Ana Luiza Ziulkoski, Vyctoria Goes, Karoline Schallenberger, Matheus Nunes Weber, Paula Rodrigues de Almeida, Alessandra Pavan Lamarca da Silva, Ronaldo da Silva F Jr , Luiz G P de Almeida, Alexandra L Gerber , Ana Paula de C Guimarães,Ana Tereza R de Vasconcelos
EPI_ISL_779198	Laboratorio Estatal de Salud Pública de Nuevo León	Laboratorio de Infectología Molecular, Departamento de Bioquímica y Medicina Molecular,Facultad de Medicina - Universidad Autónoma de Nuevo León	Kame A. Galán-Huerta, María F. Herrera-Saldivar, Natalia Martínez-Acuña, Sonia A. Lozano-Sepúlveda, Daniel Arellanos-Soto, Ana M. Rivas-Estilla, Samuel Buentello-Wong, Else del Carmen García-García, Gloria A. Jasso-de-la-Peña, Roberto Montes-de-Oca, Consuelo Treviño-Garza, Manuel E. de-la-O-Cavazos
EPI_ISL_779403	Institute of Virology, Biomedical Research Center of the Slovak Academy of Sciences, Bratislava	Faculty of Natural Sciences, Comenius University, Bratislava	Viktória Hodorová, Kristína Boršová, Broa Brejová, Viktória abanová, Sabina Fumaová Havlíková, Juraj Kopáek, Martina Liková, ubomíra Lukáiková, Martina Neboháová, Monika Sláviková, Tomáš Vina, Jozef Nosek, Boris Klempa
EPI_ISL_779956, EPI_ISL_779957, EPI_ISL_779958, EPI_ISL_779960, EPI_ISL_779962, EPI_ISL_779964, EPI_ISL_779965, EPI_ISL_779966, EPI_ISL_779967, EPI_ISL_779968, EPI_ISL_779974, EPI_ISL_779977, EPI_ISL_779978, EPI_ISL_779979, EPI_ISL_779980, EPI_ISL_779981, EPI_ISL_779982, EPI_ISL_779983, EPI_ISL_779984, EPI_ISL_779985, EPI_ISL_779986, EPI_ISL_779987, EPI_ISL_779988, EPI_ISL_779989, EPI_ISL_779990, EPI_ISL_779991, EPI_ISL_779992, EPI_ISL_779993, EPI_ISL_779994, EPI_ISL_779995, EPI_ISL_779996, EPI_ISL_779997, EPI_ISL_779998, EPI_ISL_779999, EPI_ISL_780000, EPI_ISL_780001, EPI_ISL_780002, EPI_ISL_780003, EPI_ISL_780004, EPI_ISL_780005, EPI_ISL_780006, EPI_ISL_780007, EPI_ISL_780008, EPI_ISL_780009, EPI_ISL_780010, EPI_ISL_780011, EPI_ISL_780033, EPI_ISL_780049, EPI_ISL_780061, EPI_ISL_780069			
see above	Hospital General Universitario Gregorio Marañón	SeqCOVID-SPAIN consortium/IBV(CSIC)	Darío García de Viedma, Laura Pérez-Lago, Marta Herranz, Jon Sicilia, Julia Suárez, Pilar Catalán, Patricia Muñoz and SeqCOVID-SPAIN consortium
EPI_ISL_783538, EPI_ISL_783544, EPI_ISL_783550, EPI_ISL_783552, EPI_ISL_783560, EPI_ISL_783566, EPI_ISL_783571, EPI_ISL_783594, EPI_ISL_783595, EPI_ISL_783597, EPI_ISL_783603, EPI_ISL_783604, EPI_ISL_783605, EPI_ISL_783607, EPI_ISL_783613, EPI_ISL_783621, EPI_ISL_783622, EPI_ISL_783623, EPI_ISL_783624, EPI_ISL_783626, EPI_ISL_783629, EPI_ISL_783631, EPI_ISL_783632, EPI_ISL_783633, EPI_ISL_783634, EPI_ISL_783636, EPI_ISL_783637, EPI_ISL_783638, EPI_ISL_783639, EPI_ISL_783640, EPI_ISL_783642, EPI_ISL_783643, EPI_ISL_783645, EPI_ISL_783646, EPI_ISL_783648, EPI_ISL_783650, EPI_ISL_783654, EPI_ISL_783655, EPI_ISL_783656, EPI_ISL_783657, EPI_ISL_783658, EPI_ISL_783659, EPI_ISL_783660, EPI_ISL_783661, EPI_ISL_783662, EPI_ISL_783663, EPI_ISL_783664, EPI_ISL_783665, EPI_ISL_783666, EPI_ISL_783668, EPI_ISL_783670, EPI_ISL_783672, EPI_ISL_783673, EPI_ISL_783675, EPI_ISL_783676, EPI_ISL_783678, EPI_ISL_783681, EPI_ISL_783688, EPI_ISL_783689, EPI_ISL_783697, EPI_ISL_783704, EPI_ISL_783711, EPI_ISL_783712, EPI_ISL_783713, EPI_ISL_783720, EPI_ISL_783723, EPI_ISL_783724, EPI_ISL_783725, EPI_ISL_783734, EPI_ISL_783735, EPI_ISL_783954, EPI_ISL_783956, EPI_ISL_783959, EPI_ISL_783960, EPI_ISL_783962, EPI_ISL_783965, EPI_ISL_783966, EPI_ISL_783968, EPI_ISL_783974, EPI_ISL_783987, EPI_ISL_783988, EPI_ISL_783995, EPI_ISL_784007, EPI_ISL_784015, EPI_ISL_784018, EPI_ISL_784020, EPI_ISL_784023, EPI_ISL_784028, EPI_ISL_784029, EPI_ISL_784033, EPI_ISL_784034, EPI_ISL_784038, EPI_ISL_784041, EPI_ISL_784050, EPI_ISL_784051, EPI_ISL_784053, EPI_ISL_784055, EPI_ISL_784058, EPI_ISL_784061, EPI_ISL_784063, EPI_ISL_784065, EPI_ISL_784067, EPI_ISL_784068, EPI_ISL_784075, EPI_ISL_784079, EPI_ISL_784082, EPI_ISL_784083, EPI_ISL_784084, EPI_ISL_784091, EPI_ISL_784093, EPI_ISL_784094, EPI_ISL_784096, EPI_ISL_784097, EPI_ISL_784101, EPI_ISL_784103, EPI_ISL_784105, EPI_ISL_784107, EPI_ISL_784108, EPI_ISL_784116, EPI_ISL_784121, EPI_ISL_784122, EPI_ISL_784124, EPI_ISL_784126, EPI_ISL_784129, EPI_ISL_784130, EPI_ISL_784131, EPI_ISL_784132, EPI_ISL_784137, EPI_ISL_784138, EPI_ISL_784141, EPI_ISL_784142, EPI_ISL_784143, EPI_ISL_784144, EPI_ISL_784145, EPI_ISL_784148, EPI_ISL_784157, EPI_ISL_784160, EPI_ISL_784161, EPI_ISL_784166, EPI_ISL_784167, EPI_ISL_784168, EPI_ISL_784169, EPI_ISL_784172, EPI_ISL_784173, EPI_ISL_784176, EPI_ISL_784179, EPI_ISL_784181, EPI_ISL_784183, EPI_ISL_784707, EPI_ISL_784773, EPI_ISL_784775, EPI_ISL_784777, EPI_ISL_784779, EPI_ISL_784781, EPI_ISL_784782, EPI_ISL_784786, EPI_ISL_784790, EPI_ISL_784794, EPI_ISL_784802, EPI_ISL_784813, EPI_ISL_784814, EPI_ISL_784815, EPI_ISL_784817, EPI_ISL_784818, EPI_ISL_784824, EPI_ISL_784826, EPI_ISL_784828, EPI_ISL_784830, EPI_ISL_784832, EPI_ISL_784839, EPI_ISL_784841, EPI_ISL_784842, EPI_ISL_784849, EPI_ISL_784852, EPI_ISL_784854, EPI_ISL_784857, EPI_ISL_784860, EPI_ISL_784861, EPI_ISL_784862, EPI_ISL_784865, EPI_ISL_784866, EPI_ISL_784871, EPI_ISL_784876, EPI_ISL_784877, EPI_ISL_784880, EPI_ISL_784882, EPI_ISL_784884, EPI_ISL_784886, EPI_ISL_784888, EPI_ISL_784889, EPI_ISL_784890, EPI_ISL_784891, EPI_ISL_784893, EPI_ISL_784894, EPI_ISL_784898, EPI_ISL_784900, EPI_ISL_784906, EPI_ISL_784907, EPI_ISL_784909, EPI_ISL_784910, EPI_ISL_784914, EPI_ISL_784917, EPI_ISL_784918, EPI_ISL_784920, EPI_ISL_784922, EPI_ISL_784924, EPI_ISL_784929, EPI_ISL_784930, EPI_ISL_784931, EPI_ISL_784932, EPI_ISL_784934, EPI_ISL_784935, EPI_ISL_784939, EPI_ISL_784945, EPI_ISL_784951, EPI_ISL_784952, EPI_ISL_784954, EPI_ISL_784955, EPI_ISL_784957, EPI_ISL_784959, EPI_ISL_784961, EPI_ISL_784963, EPI_ISL_784965, EPI_ISL_784966, EPI_ISL_784968, EPI_ISL_784969, EPI_ISL_784970, EPI_ISL_784971, EPI_ISL_784972, EPI_ISL_784973, EPI_ISL_784974, EPI_ISL_784976, EPI_ISL_784978, EPI_ISL_784979, EPI_ISL_784980, EPI_ISL_784981, EPI_ISL_784982, EPI_ISL_784983, EPI_ISL_784984, EPI_ISL_784985, EPI_ISL_784986, EPI_ISL_784988, EPI_ISL_784989, EPI_ISL_784990, EPI_ISL_784991, EPI_ISL_784993, EPI_ISL_784994, EPI_ISL_784995, EPI_ISL_784996, EPI_ISL_784997, EPI_ISL_784999, EPI_ISL_785001, EPI_ISL_785002, EPI_ISL_785003, EPI_ISL_785004, EPI_ISL_785005, EPI_ISL_785006, EPI_ISL_785008, EPI_ISL_785009, EPI_ISL_785010, EPI_ISL_785011, EPI_ISL_785014, EPI_ISL_785015, EPI_ISL_785017, EPI_ISL_785018, EPI_ISL_785019, EPI_ISL_785020, EPI_ISL_785021, EPI_ISL_785022, EPI_ISL_785024, EPI_ISL_785029, EPI_ISL_785030, EPI_ISL_785031, EPI_ISL_785032, EPI_ISL_785033, EPI_ISL_785035, EPI_ISL_785036, EPI_ISL_785037, EPI_ISL_785039, EPI_ISL_785040, EPI_ISL_785041, EPI_ISL_785042, EPI_ISL_785043, EPI_ISL_785044, EPI_ISL_785045, EPI_ISL_785046, EPI_ISL_785047, EPI_ISL_785053, EPI_ISL_785054, EPI_ISL_785055, EPI_ISL_785056, EPI_ISL_785057, EPI_ISL_785058, EPI_ISL_785059, EPI_ISL_785060, EPI_ISL_785061, EPI_ISL_785062, EPI_ISL_785063, EPI_ISL_785064, EPI_ISL_785065, EPI_ISL_785066, EPI_ISL_785067, EPI_ISL_785068, EPI_ISL_785069, EPI_ISL_785070, EPI_ISL_785071, EPI_ISL_785072, EPI_ISL_785073, EPI_ISL_785075, EPI_ISL_785078, EPI_ISL_785079, EPI_ISL_785080, EPI_ISL_785081, EPI_ISL_785082, EPI_ISL_785083, EPI_ISL_785084, EPI_ISL_785086, EPI_ISL_785087, EPI_ISL_785088, EPI_ISL_785089, EPI_ISL_785090, EPI_ISL_785091, EPI_ISL_785092, EPI_ISL_785093, EPI_ISL_785094, EPI_ISL_785095, EPI_ISL_785096, EPI_ISL_785097, EPI_ISL_785098, EPI_ISL_785099, EPI_ISL_785100, EPI_ISL_785103, EPI_ISL_785104, EPI_ISL_785105, EPI_ISL_785107, EPI_ISL_785108, EPI_ISL_785109, EPI_ISL_785110, EPI_ISL_785112, EPI_ISL_785114, EPI_ISL_785115, EPI_ISL_785116, EPI_ISL_785117, EPI_ISL_785118, EPI_ISL_785119, EPI_ISL_785120, EPI_ISL_785123, EPI_ISL_785124, EPI_ISL_785125, EPI_ISL_785126, EPI_ISL_785127, EPI_ISL_785128, EPI_ISL_785129, EPI_ISL_785132, EPI_ISL_785133, EPI_ISL_785134, EPI_ISL_785135, EPI_ISL_785136, EPI_ISL_785137, EPI_ISL_785138, EPI_ISL_785144, EPI_ISL_785155, EPI_ISL_785162, EPI_ISL_785172, EPI_ISL_785184, EPI_ISL_785185, EPI_ISL_785192, EPI_ISL_785194, EPI_ISL_785200, EPI_ISL_785201, EPI_ISL_785280, EPI_ISL_785288			
see above	Houston Methodist Hospital	Houston Methodist Hospital	S. Wesley Long, Randall J. Olsen, Paul A. Christensen, David W. Bernard, James J. Davis, Maulik Shukla, Marcus Nguyen, Matthew Ojeda Saavedra, Prasanti Yerramilli, Layne Pruitt, Sishir Subedi, Heather Hendrickson, and James M. Musser
EPI_ISL_788985	Institute of Virology, Biomedical Research Center of the Slovak Academy of Sciences, Bratislava	Faculty of Natural Sciences, Comenius University, Bratislava	Broa Brejová, Viktória abanová, Kristína Boršová, Viktória Hodorová, Sabina Fumaová Havlíková, Juraj Kopáek, Martina Liková, ubomíra Lukáiková, Martina Neboháová, Monika Sláviková, Alena Košálová, Peter Sabaka, Tomáš Vina, Jozef Nosek, Boris Klempa
EPI_ISL_790695, EPI_ISL_790697	Dutch COVID-19 response team	National Institute for Public Health and the Environment (RIVM)	Adam Meijer, Harry Vennema, Jeroen Cremer, Sharon van den Brink, Bas van der Veer, AnneMarie van den Brandt, Florian Zwagemaker, Dennis Schmitz, Chantal Reusken, on behalf of the national COVID-19 response team
EPI_ISL_791299	National Virus Reference Laboratory	Irish Coronavirus Sequencing Consortium - Teagasc Moorepark	Alejandro Abner Garcia Leon, Paul Cotter, Fiona Crispie, John Kenny, Paddy Mallon, Calum Walsh
EPI_ISL_791343, EPI_ISL_791350, EPI_ISL_791392, EPI_ISL_791393, EPI_ISL_791395, EPI_ISL_791404, EPI_ISL_791405, EPI_ISL_791408, EPI_ISL_791412, EPI_ISL_791414	Johns Hopkins Hospital Department of Pathology	Johns Hopkins Hospital Department of Pathology	C. Paul Morris, Chun Huai Luo, Heba H. Mostafa
EPI_ISL_791990	Institute of Virology, Biomedical Research Center of the Slovak Academy of Sciences, Bratislava	Faculty of Natural Sciences, Comenius University, Bratislava	Viktória abanová, Kristína Boršová, Broa Brejová, Viktória Hodorová, Sabina Fumaová Havlíková, Juraj Kopáek, Martina Liková, ubomíra Lukáiková, Martina Neboháová, Monika Sláviková, Alena Košálová, Peter Sabaka, Tomáš Vina, Jozef Nosek, Boris Klempa
EPI_ISL_791992	CHU - Hôpital Cavale Blanche	National Reference Center for Viruses of Respiratory Infections, Institut Pasteur, Paris	Marion Barbet, Sylvie Behillil, Méline Bizard, Angela Brisebarre, Camille Capel, Etienne Simon-Lorière, Vincent Enouf, Maud Vanpeene, Sylvie van der Werf, Léa Pilorgé
EPI_ISL_792639, EPI_ISL_792641	LACEN-AL	Laboratory of Respiratory Viruses and Measles, Oswaldo Cruz Institute, FIOCRUZ	Paola Resende, Luciana Appolinario, Fernando Motta, Anna Carolina Paixao, Ana Carolina Mendonca, Anderson Brandao Leite, Marilda Siqueira

EPI_ISL_792650, EPI_ISL_792651, EPI_ISL_792654	LACEN-PR	Laboratory of Respiratory Viruses and Measles, Oswaldo Cruz Institute, FIOCRUZ	Paola Resende, Luciana Appolinario, Fernando Motta, Anna Carolina Paixao, Ana Carolina Mendonca, Maria do Carmo Debur, Irina Nastassja Riediger, Marilda Siqueira
EPI_ISL_792688, EPI_ISL_792695	The National Institute of Public Health	State Veterinary Institute Prague	Nagy,A;Jirincova,H;Trnka,D;Vecerova,J
EPI_ISL_793011, EPI_ISL_794589, EPI_ISL_794590, EPI_ISL_794591, EPI_ISL_795010	Department of Virus and Microbiological Special Diagnostics, Statens Serum Institut, Copenhagen, Denmark	Albertsen Lab, Department of Chemistry and Bioscience, Aalborg University, Denmark	Danish Covid-19 Genome Consortium
EPI_ISL_800885, EPI_ISL_800886, EPI_ISL_800887, EPI_ISL_800888, EPI_ISL_800889, EPI_ISL_800890, EPI_ISL_800891, EPI_ISL_800892, EPI_ISL_800893, EPI_ISL_800894, EPI_ISL_800895, EPI_ISL_800896, EPI_ISL_800898, EPI_ISL_800899, EPI_ISL_800900, EPI_ISL_800901, EPI_ISL_800902, EPI_ISL_800903, EPI_ISL_800904, EPI_ISL_800905, EPI_ISL_800906, EPI_ISL_800907, EPI_ISL_800908, EPI_ISL_800909, EPI_ISL_800910, EPI_ISL_800911, EPI_ISL_800912, EPI_ISL_800913, EPI_ISL_800914, EPI_ISL_800915, EPI_ISL_800916, EPI_ISL_800917, EPI_ISL_800918, EPI_ISL_800919, EPI_ISL_800921, EPI_ISL_800922, EPI_ISL_800923, EPI_ISL_800925, EPI_ISL_800926, EPI_ISL_800927, EPI_ISL_800928, EPI_ISL_800929, EPI_ISL_800930, EPI_ISL_800931, EPI_ISL_800932, EPI_ISL_800933, EPI_ISL_800934, EPI_ISL_800935, EPI_ISL_800936, EPI_ISL_800937, EPI_ISL_800938, EPI_ISL_800939, EPI_ISL_800940, EPI_ISL_800941, EPI_ISL_800942, EPI_ISL_800943, EPI_ISL_800944, EPI_ISL_800945, EPI_ISL_800946, EPI_ISL_800947, EPI_ISL_800948, EPI_ISL_800949, EPI_ISL_800950, EPI_ISL_800951, EPI_ISL_800952, EPI_ISL_800953, EPI_ISL_800954, EPI_ISL_800955, EPI_ISL_800956, EPI_ISL_800957, EPI_ISL_800958, EPI_ISL_800959, EPI_ISL_800960, EPI_ISL_800961, EPI_ISL_800962, EPI_ISL_800963, EPI_ISL_800964, EPI_ISL_800965, EPI_ISL_800966, EPI_ISL_800968, EPI_ISL_800969, EPI_ISL_800970, EPI_ISL_800972, EPI_ISL_800973, EPI_ISL_800974, EPI_ISL_800975, EPI_ISL_800976, EPI_ISL_800977, EPI_ISL_800978, EPI_ISL_800979, EPI_ISL_800980, EPI_ISL_800981, EPI_ISL_800982, EPI_ISL_800983, EPI_ISL_800984, EPI_ISL_800985, EPI_ISL_800986, EPI_ISL_800987, EPI_ISL_800988, EPI_ISL_800989, EPI_ISL_800990, EPI_ISL_800991, EPI_ISL_800992, EPI_ISL_800993, EPI_ISL_800994, EPI_ISL_800996, EPI_ISL_800997, EPI_ISL_800998, EPI_ISL_800999, EPI_ISL_801000, EPI_ISL_801001, EPI_ISL_801003, EPI_ISL_801004, EPI_ISL_801005, EPI_ISL_801006, EPI_ISL_801007, EPI_ISL_801008, EPI_ISL_801009, EPI_ISL_801010, EPI_ISL_801011, EPI_ISL_801012, EPI_ISL_801013, EPI_ISL_801014, EPI_ISL_801015, EPI_ISL_801016, EPI_ISL_801017, EPI_ISL_801018, EPI_ISL_801019, EPI_ISL_801020, EPI_ISL_801021, EPI_ISL_801022, EPI_ISL_801023, EPI_ISL_801024, EPI_ISL_801025, EPI_ISL_801026, EPI_ISL_801027, EPI_ISL_801028, EPI_ISL_801029, EPI_ISL_801030, EPI_ISL_801031, EPI_ISL_801032, EPI_ISL_801033, EPI_ISL_801034, EPI_ISL_801035, EPI_ISL_801036, EPI_ISL_801037, EPI_ISL_801038, EPI_ISL_801039, EPI_ISL_801040, EPI_ISL_801041, EPI_ISL_801042, EPI_ISL_801043, EPI_ISL_801044, EPI_ISL_801045, EPI_ISL_801046, EPI_ISL_801047, EPI_ISL_801048, EPI_ISL_801049, EPI_ISL_801050, EPI_ISL_801051, EPI_ISL_801052, EPI_ISL_801053, EPI_ISL_801054, EPI_ISL_801056, EPI_ISL_801057, EPI_ISL_801058, EPI_ISL_801059, EPI_ISL_801060, EPI_ISL_801061, EPI_ISL_801062, EPI_ISL_801064, EPI_ISL_801065, EPI_ISL_801066, EPI_ISL_801067, EPI_ISL_801068, EPI_ISL_801069, EPI_ISL_801070, EPI_ISL_801071, EPI_ISL_801072, EPI_ISL_801073, EPI_ISL_801074, EPI_ISL_801075, EPI_ISL_801076, EPI_ISL_801077, EPI_ISL_801078, EPI_ISL_801079, EPI_ISL_801080, EPI_ISL_801081, EPI_ISL_801082, EPI_ISL_801083, EPI_ISL_801084, EPI_ISL_801085, EPI_ISL_801086, EPI_ISL_801087, EPI_ISL_801088, EPI_ISL_801089, EPI_ISL_801090, EPI_ISL_801091, EPI_ISL_801092, EPI_ISL_801093, EPI_ISL_801094, EPI_ISL_801095, EPI_ISL_801096, EPI_ISL_801097, EPI_ISL_801098, EPI_ISL_801099, EPI_ISL_801100, EPI_ISL_801101, EPI_ISL_801102, EPI_ISL_801103, EPI_ISL_801104, EPI_ISL_801105, EPI_ISL_801106, EPI_ISL_801107, EPI_ISL_801108, EPI_ISL_801109, EPI_ISL_801111, EPI_ISL_801112, EPI_ISL_801113, EPI_ISL_801114, EPI_ISL_801115, EPI_ISL_801116, EPI_ISL_801117, EPI_ISL_801118, EPI_ISL_801119, EPI_ISL_801120, EPI_ISL_801121, EPI_ISL_801122, EPI_ISL_801123, EPI_ISL_801124, EPI_ISL_801126, EPI_ISL_801127, EPI_ISL_801128, EPI_ISL_801129, EPI_ISL_801130, EPI_ISL_801131, EPI_ISL_801132, EPI_ISL_801133, EPI_ISL_801134, EPI_ISL_801135, EPI_ISL_801136, EPI_ISL_801137, EPI_ISL_801138, EPI_ISL_801139, EPI_ISL_801140, EPI_ISL_801141, EPI_ISL_801142, EPI_ISL_801143, EPI_ISL_801144, EPI_ISL_801145, EPI_ISL_801146, EPI_ISL_801147, EPI_ISL_801149, EPI_ISL_801150, EPI_ISL_801151, EPI_ISL_801152, EPI_ISL_801153, EPI_ISL_801154, EPI_ISL_801155, EPI_ISL_801156, EPI_ISL_801157, EPI_ISL_801158, EPI_ISL_801159, EPI_ISL_801160, EPI_ISL_801161, EPI_ISL_801162, EPI_ISL_801163, EPI_ISL_801164, EPI_ISL_801165, EPI_ISL_801166, EPI_ISL_801167, EPI_ISL_801168, EPI_ISL_801169, EPI_ISL_801170, EPI_ISL_801171, EPI_ISL_801172, EPI_ISL_801173, EPI_ISL_801174, EPI_ISL_801175, EPI_ISL_801176, EPI_ISL_801177, EPI_ISL_801178			
see above	Lighthouse Lab in Cambridge	Wellcome Sanger Institute for the COVID-19 Genomics UK (COG-UK) Consortium	Rob Howes, The Lighthouse Lab in Cambridge and Alex Alderton, Roberto Amato, Sonia Goncalves, Ewan Harrison, David K. Jackson, Ian Johnston, Dominic Kwiatkowski, Cordelia Langford, John Sillitoe on behalf of the Wellcome Sanger Institute COVID-19 Surveillance Team
EPI_ISL_802567	Essentia Health-St. Mary's Medical Center	Minnesota Department of Health, Public Health Laboratory	Alexandra Lorentz, Jacob Garfin, Matt Plumb, and Xiong Wang
EPI_ISL_802853, EPI_ISL_802854	Vilnius University Hospital Santaros Klinikos, Vilnius University	Institute of Biotechnology, Life Sciences Center, Vilnius University	Emilija Vasiliunaite, Milda Norkiene, Albertas Timinskas, Alma Gedvilaite, Aurelija Zvirbliene, Daniel Naumovas, Laimonas Griskevicius
EPI_ISL_804600, EPI_ISL_804605, EPI_ISL_804616, EPI_ISL_804628, EPI_ISL_804630, EPI_ISL_804633, EPI_ISL_804638, EPI_ISL_804643, EPI_ISL_804652, EPI_ISL_804659, EPI_ISL_804665, EPI_ISL_804670, EPI_ISL_804676, EPI_ISL_804681, EPI_ISL_804683, EPI_ISL_804686, EPI_ISL_804691, EPI_ISL_804695, EPI_ISL_804696, EPI_ISL_804697, EPI_ISL_804700, EPI_ISL_804711, EPI_ISL_804761, EPI_ISL_804762, EPI_ISL_804763, EPI_ISL_804764, EPI_ISL_804765, EPI_ISL_804766, EPI_ISL_804768, EPI_ISL_804769, EPI_ISL_804773, EPI_ISL_804774, EPI_ISL_804775, EPI_ISL_804776, EPI_ISL_804777, EPI_ISL_804778, EPI_ISL_804780, EPI_ISL_804782, EPI_ISL_804783, EPI_ISL_804787, EPI_ISL_804788, EPI_ISL_804789, EPI_ISL_804791, EPI_ISL_804794, EPI_ISL_804795, EPI_ISL_804796, EPI_ISL_804797, EPI_ISL_804801, EPI_ISL_804802, EPI_ISL_804803, EPI_ISL_804804, EPI_ISL_804805, EPI_ISL_804807, EPI_ISL_804809, EPI_ISL_804810, EPI_ISL_804811			
see above	Michigan Department of Health and Human Services, Bureau of Laboratories	Michigan Department of Health and Human Services, Bureau of Laboratories	Blankenship HM, Riner D, Soehnlen MK
EPI_ISL_806816, EPI_ISL_806817, EPI_ISL_806818	Alaska State Virology Laboratory	Alaska State Virology Laboratory	Stephanie DeRonde, Lisa Smith, Ph.D., Devin M. Drown, Ph.D., Jack Chen, Ph.D.
EPI_ISL_807094, EPI_ISL_807108, EPI_ISL_807113, EPI_ISL_807114, EPI_ISL_807116, EPI_ISL_807117, EPI_ISL_807118, EPI_ISL_807119, EPI_ISL_807120, EPI_ISL_807121, EPI_ISL_807122, EPI_ISL_807123, EPI_ISL_807124, EPI_ISL_807125, EPI_ISL_807126, EPI_ISL_807127, EPI_ISL_807128, EPI_ISL_807129, EPI_ISL_807130, EPI_ISL_807131, EPI_ISL_807132, EPI_ISL_807133, EPI_ISL_807134, EPI_ISL_807135, EPI_ISL_807136, EPI_ISL_807137, EPI_ISL_807138, EPI_ISL_807139, EPI_ISL_807140, EPI_ISL_807141, EPI_ISL_807142, EPI_ISL_807143, EPI_ISL_807144, EPI_ISL_807145, EPI_ISL_807146, EPI_ISL_807147			
see above	Washington State Department of Health	Seattle Flu Study	Deborah A. Nickerson, Chris D. Frazar, Jover Lee, Benjamin Pelle, Matthew Richardson, Amanda Adler, Elisabeth Brandstetter, Peter D. Han, Kairsten Fay, Misja Ilcisin, Kirsten Lacombe, Thomas R. Sibley, Melissa Truong, Caitlin R. Wolf, Romesh Gautom, Geoff Melly, Brian Hiatt, Philip Dykema, Scott Lindquist, Michael Boeckh, Janet A. Englund, Michael Famulare, Barry R. Lutz, Mark J. Rieder, Lea M. Starita, Matthew Thompson, Helen Y. Chu, Jay Shendure, Trevor Bedford
EPI_ISL_814321, EPI_ISL_814343, EPI_ISL_814695, EPI_ISL_814764, EPI_ISL_814765, EPI_ISL_814766, EPI_ISL_814767, EPI_ISL_814768, EPI_ISL_814769, EPI_ISL_814770, EPI_ISL_814771, EPI_ISL_814772, EPI_ISL_814773, EPI_ISL_814774, EPI_ISL_814775, EPI_ISL_814776, EPI_ISL_814777			
see above	Wales Specialist Virology Centre Sequencing lab: Pathogen Genomics Unit	COVID-19 Genomics UK (COG-UK) Consortium	Catherine Moore, Johnathan Evans, Laura Gifford, Malorie Perry, Simon Cottrell, Angela Marchbank, Alec Birchley, Alexander Adams, Amy Gaskin, Bree Gatica-Wilcox, Jason Coombes, Joel Southgate, Lauren Gilbert, Lee Graham, Nicole Pacchiarini, Sara Kumziene-Summerhayes, Sarah Taylor, Sophie Jones, Sara Rey, Matthew Bull, Joanne Watkins, Sally Corden, Tom Connor
EPI_ISL_815525, EPI_ISL_815530, EPI_ISL_815599, EPI_ISL_815640, EPI_ISL_815654, EPI_ISL_815660, EPI_ISL_816073, EPI_ISL_816074, EPI_ISL_816075, EPI_ISL_816076, EPI_ISL_816077, EPI_ISL_816078, EPI_ISL_816079, EPI_ISL_816080, EPI_ISL_816081, EPI_ISL_816082, EPI_ISL_816083, EPI_ISL_816084, EPI_ISL_816085, EPI_ISL_816086, EPI_ISL_816087, EPI_ISL_816088, EPI_ISL_816089, EPI_ISL_816090, EPI_ISL_816091, EPI_ISL_816092, EPI_ISL_816093, EPI_ISL_816094			
see above	Department of Virus and Microbiological Special Diagnostics, Statens Serum Institut, Copenhagen, Denmark	Albertsen Lab, Department of Chemistry and Bioscience, Aalborg University, Denmark	Danish Covid-19 Genome Consortium
EPI_ISL_819462, EPI_ISL_819463	University College London, Great Ormond Street Hospital for Children NHS Foundation Trust, Imperial College Healthcare NHS Trust	COVID-19 Genomics UK (COG-UK) Consortium	Sergi Castellano, Rachel Williams, Mark Kristiansen, Paola Resende Silva, Sunando Roy, Tony Brooks, Helena Tutill, Paola Niola, Patricia Dyal, Charlotte Williams, Leysa Forrest, Yasmin Panchbhaya, Jacqueline Findlay, Samuel Weeks, Julianne Brown, Kathryn Harris, Paul Randell, James Price, Alison Holmes, Judith Breuer
EPI_ISL_823973	Department of Homeless Services	New York City Public Health Laboratory	Jade Wang, et al.
EPI_ISL_824000	Dutch COVID-19 response team	National Institute for Public Health and the Environment (RIVM)	Adam Meijer, Harry Vennema, Jeroen Cremer, Sharon van den Brink, Bas van der Veer, AnneMarie van den Brandt, Florian Zwagemaker, Dennis Schmitz, Chantal Reusken, on behalf of the national COVID-19 response team
EPI_ISL_824334	Michigan Department of Health and Human Services, Bureau of Laboratories	Michigan Department of Health and Human Services, Bureau of Laboratories	Blankenship HM, Riner D, Soehnlen MK
EPI_ISL_824395, EPI_ISL_824399	California Department of Public Health	California Department of Public Health	CDPH IDLB COVIDNet
EPI_ISL_824517, EPI_ISL_824518	New Mexico Department of Health Scientific Laboratory	New Mexico Department of Health Scientific Laboratory	Ellie Johnson, Anastacia Griego-Fisher, D'eldra Malone
EPI_ISL_824659, EPI_ISL_824670, EPI_ISL_824682, EPI_ISL_824694, EPI_ISL_824705, EPI_ISL_824706, EPI_ISL_824717, EPI_ISL_824718, EPI_ISL_824729, EPI_ISL_824740, EPI_ISL_824741			
see above	Cedars-Sinai Medical Center, Department of Pathology & Laboratory Medicine, Molecular Pathology Laboratory	Cedars-Sinai Medical Center, Molecular Pathology Laboratory of Department of Pathology & Laboratory Medicine and Genomic Core	Wenjuan Zhang, Brian Davis, Stephanie Chen, Jorge Mario Sincuir Martinez, Jasmine T Plummer, Eric Vail
EPI_ISL_825034	B.J. Medical College and Civil hospital, Ahmedabad	Gujarat Biotechnology Research Centre	Kamlesh J Upadhyay, Zarna Patel, Nitin Savaliya, Dinesh Kumar, Zuber Saiyed, Labdhi Pandya, Afzal Ansari, Nikha Trivedi, Apurvasinh Puvar, Ramesh Pandit, Janvi Raval, Pranay Shah, Nilima Shah, Sanjay Kapadia, Dipa Kinariwala, Chaitanya Joshi, Madhvi Joshi
EPI_ISL_825090, EPI_ISL_825091, EPI_ISL_825093, EPI_ISL_825094, EPI_ISL_825095, EPI_ISL_825100, EPI_ISL_825102, EPI_ISL_825103, EPI_ISL_825105, EPI_ISL_825121, EPI_ISL_825122, EPI_ISL_825123			
see above	NHLS-IALCH	KRISP, KZN Research Innovation and Sequencing Platform	Giandhari J, Pillay S, Lessells R, Mdlalose K, York D, Khan S, Tegally H, Wilkinson E, de Oliveira T
EPI_ISL_825334, EPI_ISL_825361, EPI_ISL_825362, EPI_ISL_825363	Hospital Universitari Vall d'Hebron - Vall d'Hebron Institut de Rercerca	Hospital Universitari Vall d'Hebron	Cristina Andrés, Maria Piñana, Josep F Abril, Damir Garcia-Cehic, Ariadna Rando, Juliana Esperalba, Maria Gema Codina, Carla Castillo, Maria Carmen Martin, Tomàs Pumarola, Josep Quer, Andrés Antón
EPI_ISL_825552, EPI_ISL_826281	Respiratory Virus Unit, National Infection Service, Public Health England	COVID-19 Genomics UK (COG-UK) Consortium	PHE Covid Sequencing Team
EPI_ISL_826879, EPI_ISL_826882, EPI_ISL_827068, EPI_ISL_827662,	deCODE genetics	deCODE genetics	Daniel F Gudbjartsson; Agnar Helgason; Hakon Jonsson; Olafur T Magnusson; Pall Melsted; Gudmundur L Norddahl; Jona Saemundsdottir; Asgeir Sigurdsson; Patrick Sulem; Arna B Agustsdottir; Hannes Eggertsson; Berglind Eiriksdottir; Run Fridriksdottir; Elisabet E Gardarsdottir; Gudmundur

EPI_ISL_827826, EPI_ISL_828173, EPI_ISL_828179, EPI_ISL_828233, EPI_ISL_828249, EPI_ISL_828321			Georgsson; Olafia S Gretarsdottir; Kjartan R Gudmundsson; Thora R Gunnarsdottir; Arnaldur Gylfason; Hilma Holm; Brynjar O Jensson; Aslaug Jonasdottir; Kamilla S Josefsdottir; Thordur Kristjansson; Droplaug N Magnusdottir; Solvi Rognvaldsson; Louise le Roux; Gudrun Sigmundsdottir; Gardar Sveinbjornsson; Kristin E Sveinsdottir; Maney Sveinsdottir; Emil A Thorarensen; Bjarni Thorbjornsson; Gisli Masson; Ingileif Jonsdottir; Alma Moller; Thorolfur Gudnason; Karl G Kristinsson; Unnur Thorsteinsdottir; Kari Stefansson
EPI_ISL_828483	The National University Hospital of Iceland	deCODE genetics	Daniel F Gudbjartsson; Agnar Helgason; Hakon Jonsson; Olafur T Magnusson; Pall Melsted; Gudmundur L Norddahl; Jona Saemundsdottir; Asgeir Sigurdsson; Patrick Sulem; Arna B Agustsdottir; Hannes Eggertsson; Berglind Eiriksdottir; Run Fridriksdottir; Elisabet E Gardarsdottir; Gudmundur Georgsson; Olafia S Gretarsdottir; Kjartan R Gudmundsson; Thora R Gunnarsdottir; Arnaldur Gylfason; Hilma Holm; Brynjar O Jensson; Aslaug Jonasdottir; Kamilla S Josefsdottir; Thordur Kristjansson; Droplaug N Magnusdottir; Solvi Rognvaldsson; Louise le Roux; Gudrun Sigmundsdottir; Gardar Sveinbjornsson; Kristin E Sveinsdottir; Maney Sveinsdottir; Emil A Thorarensen; Bjarni Thorbjornsson; Gisli Masson; Ingileif Jonsdottir; Alma Moller; Thorolfur Gudnason; Karl G Kristinsson; Unnur Thorsteinsdottir; Kari Stefansson
EPI_ISL_828920, EPI_ISL_829051, EPI_ISL_829052, EPI_ISL_829137, EPI_ISL_829138, EPI_ISL_829145, EPI_ISL_829148, EPI_ISL_829150, EPI_ISL_829151, EPI_ISL_829176, EPI_ISL_829177, EPI_ISL_829178, EPI_ISL_829182, EPI_ISL_829184, EPI_ISL_829185, EPI_ISL_829684, EPI_ISL_829904, EPI_ISL_829905, EPI_ISL_829906, EPI_ISL_829908, EPI_ISL_829911, EPI_ISL_829912, EPI_ISL_829913, EPI_ISL_830174, EPI_ISL_830175			
see above	deCODE genetics	deCODE genetics	Daniel F Gudbjartsson; Agnar Helgason; Hakon Jonsson; Olafur T Magnusson; Pall Melsted; Gudmundur L Norddahl; Jona Saemundsdottir; Asgeir Sigurdsson; Patrick Sulem; Arna B Agustsdottir; Hannes Eggertsson; Berglind Eiriksdottir; Run Fridriksdottir; Elisabet E Gardarsdottir; Gudmundur Georgsson; Olafia S Gretarsdottir; Kjartan R Gudmundsson; Thora R Gunnarsdottir; Arnaldur Gylfason; Hilma Holm; Brynjar O Jensson; Aslaug Jonasdottir; Kamilla S Josefsdottir; Thordur Kristjansson; Droplaug N Magnusdottir; Solvi Rognvaldsson; Louise le Roux; Gudrun Sigmundsdottir; Gardar Sveinbjornsson; Kristin E Sveinsdottir; Maney Sveinsdottir; Emil A Thorarensen; Bjarni Thorbjornsson; Gisli Masson; Ingileif Jonsdottir; Alma Moller; Thorolfur Gudnason; Karl G Kristinsson; Unnur Thorsteinsdottir; Kari Stefansson
EPI_ISL_831701, EPI_ISL_831708, EPI_ISL_831709, EPI_ISL_831713, EPI_ISL_831743, EPI_ISL_831825, EPI_ISL_831826, EPI_ISL_831827, EPI_ISL_831828, EPI_ISL_831830, EPI_ISL_831831			
see above	United States Air Force School of Aerospace Medicine	United States Air Force School of Aerospace Medicine	Anthony Fries, Jennifer Meyer, William Gruner, Amanda Javorina, Sarah Purves, Clarise Starr, Elizabeth Macias
EPI_ISL_832032, EPI_ISL_832033, EPI_ISL_832034	Wyoming Public Health Laboratory	Wyoming Public Health Laboratory	Noah Hull, Taylor Fearing, Lynette Gumbleton, Channing Weber, Ashley Norberg, Bailey Bowcutt, and Wanda Manley
EPI_ISL_832146, EPI_ISL_832147, EPI_ISL_832148	Hospital	National Reference Center for Viruses of Respiratory Infections, Institut Pasteur, Paris	Marion Barbet, Sylvie Behillil, Méline Bizard, Angela Brisebarre, Camille Capel, Etienne Simon-Lorière, Vincent Enouf, Maud Vanpeene, Sylvie van der Werf, Léa Pilorge
EPI_ISL_832781, EPI_ISL_832782, EPI_ISL_832794, EPI_ISL_832795, EPI_ISL_832796, EPI_ISL_832797, EPI_ISL_832798, EPI_ISL_832799, EPI_ISL_832800, EPI_ISL_832811, EPI_ISL_832812			
see above	OHSU Lab Services Molecular Microbiology Lab	Oregon SARS-CoV-2 Genome Sequencing Center	Brendan L. O'Connell, Ruth V. Nichols, Sally Grindstaff, Alec J. Hirsch, Donna Hansel, Guang Fan, Daniel N. Streblow, William B. Messer, Andrew C. Adey, Benjamin N. Bimber, Brian J. O'Roak
EPI_ISL_833455	SC (UCO) Igiene e Sanità Pubblica (funzione integrata con SC Microbiologia e Virologia) e Laboratory of Molecular Virology of the International Centre for Genetic Engineering and Biotechnology (ICGEB)	ARGO Laboratorio Genomica ed Epigenomica	Licastro D, Dal Monego S, Degasperi M, Marcello A, D'Agaro P
EPI_ISL_833458, EPI_ISL_833475, EPI_ISL_833476, EPI_ISL_833479	CHU Purpan - Laboratoire de Virologie - Institut Fédératif de Biologie	CHU Purpan - Laboratoire de Virologie - Institut Fédératif de Biologie	Latour J., Ranger N., Dubois M., Carcenac R., Harter A., Boyer P., Tremeaux P., Izopet J.
EPI_ISL_838281	Virology Department, Royal Infirmary of Edinburgh, NHS Lothian / School of Biological Sciences, University of Edinburgh / Institute of Genetics and Molecular Medicine, University of Edinburgh	COVID-19 Genomics UK (COG-UK) Consortium	McHugh M, Dewar R, Rooke S, Gallagher M, Balcaza C, O'Toole Á, Scher E, Hill V, McCrone JT, Colquhoun R, Yu X, Jackson B, Rambaut A, Williams TC, Templeton K
EPI_ISL_838344, EPI_ISL_838345, EPI_ISL_838346	Queens Medical Centre, Clinical Microbiology Department / DeepSeq Nottingham	COVID-19 Genomics UK (COG-UK) Consortium	Gemma Clark, Wendy Smith, Manjinder Khakh, Vicki M Fleming, Michelle M Lister, Hannah Howson-Wells, Jonathan Ball, Patrick McClure, Joseph Chappell, Theocharis Tsoleridis, Nadine Holmes, Matthew Carlisle, Christopher Moore, Fei Sang, Johnny Debebe, Victoria Wright, Matthew Loose
EPI_ISL_840188, EPI_ISL_840189, EPI_ISL_840204, EPI_ISL_840205, EPI_ISL_840206, EPI_ISL_840216, EPI_ISL_840217	Oxford Viromics, NDM, University of Oxford; Oxford University Hospitals; Basingstoke and North Hampshire Hospital	COVID-19 Genomics UK (COG-UK) Consortium	Tanya Golubchik, David Bonsall, George Macintyre, Amy Trebes, Mariateresa de Cesare, Catrin Moore, Alex Mobbs, Anita Justice, Robert Shaw, Monique Andersson, Timothy Peto, Emma Wise, Nathan Moore, Jessica Lynch, Nick Cortes, Matilde Mori, Stephen Kidd, David Buck, John Todd, Christophe Fraser
EPI_ISL_842916, EPI_ISL_842917, EPI_ISL_842918, EPI_ISL_842925, EPI_ISL_842926, EPI_ISL_842927, EPI_ISL_842928, EPI_ISL_842929, EPI_ISL_842930, EPI_ISL_842931, EPI_ISL_842933, EPI_ISL_842934, EPI_ISL_842935, EPI_ISL_842936, EPI_ISL_842943, EPI_ISL_842944, EPI_ISL_842946, EPI_ISL_842947, EPI_ISL_842948, EPI_ISL_842949, EPI_ISL_842953, EPI_ISL_842956, EPI_ISL_842963, EPI_ISL_842964, EPI_ISL_842965, EPI_ISL_842967, EPI_ISL_842968, EPI_ISL_842984, EPI_ISL_842985, EPI_ISL_842986, EPI_ISL_842987, EPI_ISL_842988, EPI_ISL_842989, EPI_ISL_842990, EPI_ISL_842991, EPI_ISL_842992, EPI_ISL_842993, EPI_ISL_842995, EPI_ISL_842996, EPI_ISL_842997, EPI_ISL_842998, EPI_ISL_842999, EPI_ISL_843001, EPI_ISL_843002, EPI_ISL_843003, EPI_ISL_843004			
see above	Barts Health NHS Trust	COVID-19 Genomics UK (COG-UK) Consortium	CUTINO-MOGUEL, Maria-Teresa; HARRINGTON, David; OWOYEMI, Dola; SHYLINI, Raghavendran; BROAD, Claire; KELE, Beatrix
EPI_ISL_844204, EPI_ISL_844223, EPI_ISL_844229, EPI_ISL_844239, EPI_ISL_844292, EPI_ISL_844293, EPI_ISL_844294, EPI_ISL_844296, EPI_ISL_844528, EPI_ISL_844529, EPI_ISL_844530, EPI_ISL_844531, EPI_ISL_844532, EPI_ISL_844533, EPI_ISL_844534, EPI_ISL_844535, EPI_ISL_844536, EPI_ISL_844537, EPI_ISL_844538			
see above	Department of Virus and Microbiological Special Diagnostics, Statens Serum Institut, Copenhagen, Denmark	Albertsen Lab, Department of Chemistry and Bioscience, Aalborg University, Denmark	Danish Covid-19 Genome Consortium
EPI_ISL_847516, EPI_ISL_847521, EPI_ISL_847523, EPI_ISL_847552, EPI_ISL_847554, EPI_ISL_847561, EPI_ISL_847573, EPI_ISL_847588, EPI_ISL_847674, EPI_ISL_847675, EPI_ISL_847676, EPI_ISL_847677, EPI_ISL_847678, EPI_ISL_847679, EPI_ISL_847680, EPI_ISL_847681, EPI_ISL_847682, EPI_ISL_847683, EPI_ISL_847684, EPI_ISL_847685, EPI_ISL_847686, EPI_ISL_847687, EPI_ISL_847688, EPI_ISL_847689, EPI_ISL_847690, EPI_ISL_847691, EPI_ISL_847692, EPI_ISL_847693, EPI_ISL_847694, EPI_ISL_847706, EPI_ISL_847709, EPI_ISL_847748, EPI_ISL_847804, EPI_ISL_847805, EPI_ISL_847806, EPI_ISL_847807, EPI_ISL_847808, EPI_ISL_847809, EPI_ISL_847810, EPI_ISL_847811, EPI_ISL_847812, EPI_ISL_847813, EPI_ISL_847814, EPI_ISL_847815			
see above	California Department of Public Health	Chiu Laboratory, University of California, San Francisco	Charles Chiu, Xianding (Wayne) Deng, Candace Wang, Brian Bushnell, Scot Federman, Jill Hacker, Debra Wadford
EPI_ISL_848231	Illinois Department of Public Health	Gagnon Lab, Southern Illinois University	Keith Gagnon
EPI_ISL_848734, EPI_ISL_848735, EPI_ISL_848736, EPI_ISL_848737, EPI_ISL_848738, EPI_ISL_848739, EPI_ISL_848740, EPI_ISL_848741, EPI_ISL_848742, EPI_ISL_848743, EPI_ISL_848744, EPI_ISL_848745, EPI_ISL_848746, EPI_ISL_848747, EPI_ISL_848748, EPI_ISL_848749, EPI_ISL_848750, EPI_ISL_848751, EPI_ISL_848752, EPI_ISL_848753, EPI_ISL_848754, EPI_ISL_848755, EPI_ISL_848756, EPI_ISL_848757			
see above	Florida Bureau of Public Health Laboratories	Florida Bureau of Public Health Laboratories	Sarah Schmedes, Jason Blanton
EPI_ISL_849421, EPI_ISL_849448, EPI_ISL_849518, EPI_ISL_849519, EPI_ISL_849520, EPI_ISL_849575, EPI_ISL_849618	Washington State Department of Health	Seattle Flu Study	Deborah A. Nickerson, Chris D. Frazar, Jover Lee, Benjamin Pelle, Matthew Richardson, Amanda Adler, Elisabeth Brandstetter, Peter D. Han, Kairsten Fay, Misja Ilcisin, Kirsten Lacombe, Thomas R. Sibley, Melissa Truong, Caitlin R. Wolf, Romesh Gautom, Geoff Melly, Brian Hiatt, Philip Dykema, Scott Lindquist, Michael Boeckh, Janet A. Englund, Michael Famulare, Barry R. Lutz, Mark J. Rieder, Lea M. Starita, Matthew Thompson, Helen Y. Chu, Jay Shendure, Trevor Bedford
EPI_ISL_850019, EPI_ISL_850020, EPI_ISL_850021, EPI_ISL_850022, EPI_ISL_850023, EPI_ISL_850024, EPI_ISL_850025, EPI_ISL_850026, EPI_ISL_850027, EPI_ISL_850028, EPI_ISL_850029, EPI_ISL_850030, EPI_ISL_850031, EPI_ISL_850032, EPI_ISL_850033, EPI_ISL_850034, EPI_ISL_850035, EPI_ISL_850036, EPI_ISL_850037, EPI_ISL_850038			
see above	Renegade	Chan-Zuckerberg Biohub	CZB Cliahub Consortium
EPI_ISL_850663	The National Institute of Public Health	State Veterinary Institute Prague	Nagy,A;Jirincova,H;Trnka,D;Vecerova,J;Trinklova,M
EPI_ISL_853336	UPMC Clinical Microbiology Laboratory	Microbial Genome Sequencing Center; Microbial Genomic Epidemiology Laboratory	Mustapha M. Mustapha, Jane W. Marsh, Dan Snyder, Marissa P. Griffith, Stephanie L. Mitchell, Vatsala R. Srinivasa, Kady D. Waggle, Chinelo Ezeonwuku, Vaughn S. Cooper, Lee H. Harrison
EPI_ISL_853733, EPI_ISL_853779, EPI_ISL_853780	Austrian Agency for Health and Food Safety (AGES)	Bergthaler laboratory, CeMM Research Center for Molecular Medicine of the Austrian Academy of Sciences	Lukas Endler, Alexandra Popa, Benedikt Agerer, Jakob-Wendelin Genger, Alexander Lercher, Anna Schedl, Thomas Penz, Michael Schuster, Jan Laine, Martin Senekowitsch, Christoph Bock, Andreas Bergthaler
EPI_ISL_853948	Department of Microbiology, University Innsbruck	Bergthaler laboratory, CeMM Research Center for Molecular Medicine of the Austrian Academy of Sciences	Lukas Endler, Alexandra Popa, Benedikt Agerer, Jakob-Wendelin Genger, Alexander Lercher, Anna Schedl, Thomas Penz, Michael Schuster, Jan Laine, Martin Senekowitsch, Christoph Bock, Andreas Bergthaler

EPI_ISL_853976, EPI_ISL_854113, EPI_ISL_854114, EPI_ISL_854115, EPI_ISL_854116, EPI_ISL_854117, EPI_ISL_854118, EPI_ISL_854119, EPI_ISL_854120, EPI_ISL_854121, EPI_ISL_854243, EPI_ISL_854285, EPI_ISL_854297			
see above	Austrian Agency for Health and Food Safety (AGES)	Bergthaler laboratory, CeMM Research Center for Molecular Medicine of the Austrian Academy of Sciences	Lukas Endler, Alexandra Popa, Benedikt Agerer, Jakob-Wendelin Genger, Alexander Lercher, Anna Schedl, Thomas Penz, Michael Schuster, Jan Laine, Martin Senekowitsch, Christoph Bock, Andreas Bergthaler
EPI_ISL_856761, EPI_ISL_856762, EPI_ISL_856763	Servicio Virosis Respiratorias-Departamento Virología-INEI	Instituto Nacional Enfermedades Infecciosas C.G.Malbran	Baumeister E., Avaro M., Benedetti E., Russo M., Dattero ME, Pontoriero A., Cisterna D., Molina V., Perandones C., Tuduri E., Lorenzo F., Poklepovich T., Campos J.
EPI_ISL_857521, EPI_ISL_857522, EPI_ISL_857523	Swiss National Reference Centre for Influenza	Swiss National Reference Centre for Influenza	Tim Roloff, Ana Rita Gonçalves, Madlen Stange, Helena MB Seth-Smith, Alfredo Mari, Karoline Leuzinger, Julia Bielicki, Manuel Battegay, Hans Hirsch, Laurent Kaiser, Adrian Egli
EPI_ISL_859554, EPI_ISL_859556, EPI_ISL_859557, EPI_ISL_859558, EPI_ISL_859559	Lighthouse Lab in Cambridge	Wellcome Sanger Institute for the COVID-19 Genomics UK (COG-UK) Consortium	Rob Howes, The Lighthouse Lab in Cambridge and Alex Alderton, Roberto Amato, Sonia Goncalves, Ewan Harrison, David K. Jackson, Ian Johnston, Dominic Kwiatkowski, Cordelia Langford, John Sillitoe on behalf of the Wellcome Sanger Institute COVID-19 Surveillance Team
EPI_ISL_859854, EPI_ISL_859855, EPI_ISL_859856, EPI_ISL_859857, EPI_ISL_859858, EPI_ISL_859859, EPI_ISL_859860, EPI_ISL_859861, EPI_ISL_859862, EPI_ISL_859863, EPI_ISL_859864, EPI_ISL_859876, EPI_ISL_859877, EPI_ISL_859878, EPI_ISL_859879, EPI_ISL_859880, EPI_ISL_859881, EPI_ISL_859904, EPI_ISL_859905, EPI_ISL_859906, EPI_ISL_859907, EPI_ISL_859908, EPI_ISL_859909, EPI_ISL_859910, EPI_ISL_859911, EPI_ISL_859912, EPI_ISL_859913, EPI_ISL_859920, EPI_ISL_859921, EPI_ISL_859922, EPI_ISL_859923, EPI_ISL_859934, EPI_ISL_859935			
see above	BTC, Khalifa University	BTC, Khalifa University	Al Safar et al
EPI_ISL_860161, EPI_ISL_860164	Keio University School of Medicine	Keio University School of Medicine	Kenjiro Kosaki, Yuka Iwasaki, Hirotsugu Ishizu, Haruhiko Siomi, Kodai Abe
EPI_ISL_861460	UHAS COVID-19 Lab	UHAS COVID-19 Lab	Kwabena O. Duedu, Jones Gyamfi, Reuben Ayivor-Djanie, John O. Gyapong and the UHAS COVID-19 Lab Team
EPI_ISL_861868, EPI_ISL_861881, EPI_ISL_861883, EPI_ISL_861894, EPI_ISL_861903, EPI_ISL_861904, EPI_ISL_861908, EPI_ISL_861909	LATE - Laboratório de Técnicas Especiais - Hospital Israelita Albert Einstein	LATE - Laboratório de Técnicas Especiais - Hospital Israelita Albert Einstein	Deyvid Amgarten, Fernanda de Mello Malta, Raquel Riyuzo, Ana Paula Moreira Salles, Pedro Henrique Sebe Rodrigues, João Renato Rebello Pinho
EPI_ISL_861916, EPI_ISL_861917, EPI_ISL_861918, EPI_ISL_861919, EPI_ISL_861920, EPI_ISL_861921, EPI_ISL_861922, EPI_ISL_861923, EPI_ISL_861924	OHSU Lab Services Molecular Microbiology Lab	Oregon SARS-CoV-2 Genome Sequencing Center	Brendan L. O'Connell, Sally Grindstaff, Kayla Carter, Ruth V. Nichols, Alec J. Hirsch, Donna Hansel, Guang Fan, Xuan Qin, Daniel N. Streblow, William B. Messer, Andrew C. Adey, Benjamin N. Bimber, Brian J. O'Roak
EPI_ISL_864570, EPI_ISL_864571	Institute of Medical Microbiology and Hospital Hygiene	Institute of Medical Microbiology and Hospital Hygiene	Prof. Dr. Achim Kaasch, Aljoscha Tersteegen
EPI_ISL_868760, EPI_ISL_868763, EPI_ISL_868766, EPI_ISL_868772, EPI_ISL_868773, EPI_ISL_868776, EPI_ISL_868780, EPI_ISL_868781, EPI_ISL_868782, EPI_ISL_868783, EPI_ISL_868784, EPI_ISL_868788			
see above	Bioinformatics and Biostatistics Lab, Advanced Sequencing Facility	COVID-19 Genomics UK (COG-UK) Consortium	Aengus Stewart,Jerome Nicod,Chelsea Sawyer,Laura Cubitt,Harshil Patel,Margaret Crawford
EPI_ISL_869250, EPI_ISL_869262, EPI_ISL_869263, EPI_ISL_869264	Krasnoyarsk Regional Clinical Hospital	WHO National Influenza Centre Russian Federation	Andrey Komissarov, Artem Fadeev, Anna Ivanova, Kseniya Komissarova, Dmitry Bazhenov, Mikhail Bakaev, Daria Danilenko, Ksenia Safina, Elena Nabieva, Georgii Bazykin, Dmitry Lioznov
EPI_ISL_871783, EPI_ISL_871784, EPI_ISL_871785	Department of Virus and Microbiological Special Diagnostics, Statens Serum Institut, Copenhagen, Denmark	Aalborg University	Danish Covid-19 Genome Consortium
EPI_ISL_872570	Krasnoyarsk Regional Clinical Hospital	WHO National Influenza Centre Russian Federation	Andrey Komissarov, Artem Fadeev, Anna Ivanova, Kseniya Komissarova, Dmitry Bazhenov, Mikhail Bakaev, Daria Danilenko, Ksenia Safina, Elena Nabieva, Georgii Bazykin, Dmitry Lioznov
EPI_ISL_872697, EPI_ISL_872698	Rhode Island Department of Health	Infectious Disease Program, Broad Institute of Harvard and MIT	Lemieux,J.E., Siddle,K.J., Huard,R., King,E., Azevedo,K., Miller,A., Adams,G., Gladden-Young,A., Lagerborg,K., Rudy,M., DeRuff,K., Carter,A., Normandin,E., Bauer,M., Reilly,S., Tomkins-Tinch,C., Loreth,C., Chaluvadi,S., Birren,B.W., Gallagher,G., Smole,S., Park,D.J., MacInnis,B.L., and Sabeti,P.C.
EPI_ISL_873126, EPI_ISL_873127, EPI_ISL_873128, EPI_ISL_873131, EPI_ISL_873136, EPI_ISL_873137, EPI_ISL_873141, EPI_ISL_873142, EPI_ISL_873145	University of Michigan Clinical Microbiology Laboratory	Lauring Lab, University of Michigan, Department of Microbiology and Immunology	Valesano
EPI_ISL_875553	ULSS 2 Treviso	Istituto Zooprofilattico Sperimentale delle Venezie	Adelaide Milani, Alessia Schivo, Annalisa Salviato, Erika Giorgia Quaranta, Ambra Pastori, Bianca Zecchin, Alice Fusaro, Isabella Monne, Calogero Terregino, Antonia Ricci
EPI_ISL_876074, EPI_ISL_876104, EPI_ISL_876113, EPI_ISL_876119, EPI_ISL_876135, EPI_ISL_876138, EPI_ISL_876187, EPI_ISL_876322, EPI_ISL_876323, EPI_ISL_876324	Massachusetts State Public Health Laboratory	Massachusetts State Public Health Laboratory	Andrew Lang, Timelia Fink, Glen Gallagher, Sandra Smole
EPI_ISL_876523, EPI_ISL_876527, EPI_ISL_876529, EPI_ISL_876530, EPI_ISL_876555, EPI_ISL_876556, EPI_ISL_876557, EPI_ISL_876558, EPI_ISL_876559, EPI_ISL_876560, EPI_ISL_876561, EPI_ISL_876562, EPI_ISL_876563, EPI_ISL_876564, EPI_ISL_876565, EPI_ISL_876566			
see above	Florida Bureau of Public Health Laboratories	Florida Bureau of Public Health Laboratories	Sarah Schmedes, Jason Blanton
EPI_ISL_878276, EPI_ISL_878279, EPI_ISL_878300, EPI_ISL_878303, EPI_ISL_878308, EPI_ISL_878319, EPI_ISL_878322, EPI_ISL_878330, EPI_ISL_878336, EPI_ISL_878345, EPI_ISL_878346, EPI_ISL_878351, EPI_ISL_878362, EPI_ISL_878365, EPI_ISL_878382, EPI_ISL_878400, EPI_ISL_878401, EPI_ISL_878402, EPI_ISL_878404, EPI_ISL_878406, EPI_ISL_878408, EPI_ISL_878416, EPI_ISL_878418, EPI_ISL_878423, EPI_ISL_878426, EPI_ISL_878434, EPI_ISL_878437			
see above	San Diego County Public Health Laboratory	Andersen lab at Scripps Research	SEARCH Alliance San Diego with Tracy Basler, Jovan Shephard, Brett Austin
EPI_ISL_878544	Robert Garry lab	Andersen lab at Scripps Research	Allison Smither, Gilberto Sabino-Santos, Patricia Snarski, Lilia Melnik, Antoinette Bell, Kaylynn Genemaras, Arnaud Drouin, Dahlene Fusco, Robert Garry with SEARCH Alliance San Diego
EPI_ISL_878648, EPI_ISL_878661, EPI_ISL_878705	Rady's Childrens Hospital	Andersen lab at Scripps Research	SEARCH Alliance San Diego with Nanda Radamchar, David Dimmock, Linda Luo, Christina Clarke, Kathryn Bouic, Teresa Mueller, Denise Malicki
EPI_ISL_882658	Secretaria Municipal de Saude	Instituto Adolfo Lutz, Interdiciplinary Procedures Center, Strategic Laboratory	Claudio Tavares Sacchi, Claudia Regina Gonçalves, Erica Valessa Ramos Gomes, Karoline Rodrigues Campos
EPI_ISL_887164	Massachusetts General Hospital	Infectious Disease Program, Broad Institute of Harvard and MIT	Lemieux,J.E., Siddle,K.J., Shaw,B., Adams,G., Pierce,V., Turbett,S., Anahtar,M., Branda,J., Slater,D., Harris,J., Lin,A.E., Gladden-Young,A., Lagerborg,K., Rudy,M., DeRuff,K., Carter,A., Normandin,E., Bauer,M., Reilly,S., Tomkins-Tinch,C., Loreth,C., Chaluvadi,S., Neumann,A., Cusick,C., Chapman,S.B., Gnirke,A., Flowers,K., Cerrato,F., Birren,B.W., Gallagher,G., Smole,S., Park,D.J., MacInnis,B.L., Ryan,E., LaRocque,R., Rosenberg,E. and Sabeti,P.C.
EPI_ISL_887425, EPI_ISL_887461, EPI_ISL_887462, EPI_ISL_887463, EPI_ISL_887465	Instituto Nacional de Saude (INS), Mozambique	KRISP, KZN Research Innovation and Sequencing Platform	Nalia Ismael, Nadia Sitoe, Paulo Arnaldo, Nedio Mabunda, Giandhari J, Pillay S, Tegally H, Wilkinson E, de Oliveira T
EPI_ISL_887589	SC Dept of Health and Env. Control-Bureau of Laboratories	Centers for Disease Control and Prevention Division of Viral Diseases, Pathogen Discovery	Yan Li, Anna Montmayeur, Ying Tao, Jing Zhang, Krista Queen, Anna Uehara, Rachel Marine, Peter Cook, Clinton R. Paden, Haibin Wang, Suxiang Tong
EPI_ISL_888990	RS Pondok Indah Bintaro Jaya	Eijkman Institute for Molecular Biology, Ministry of Research and Technology/National Agency for Research and Innovation	Frilasita A Yudhaputri, Hidayat Trimarsanto, Iskandar Adnan, Lydia V. Panggalo, Sukma Oktavianthi, Willy Agustine, Edison Johar, Safarina G Malik, Khin Saw Myint, Amin Soebandrio
EPI_ISL_890297	KU Leuven, Rega Institute, Clinical and Epidemiological	KU Leuven, Rega Institute, Clinical and Epidemiological	Tony Wawina-Bokalanga, Bert Vanmechelen, Joan Marti-Carerras, Piet Maes

	Virology	Virology	
EPI_ISL_891221	University of Medicine and Pharmacy of Craiova	"Stefan cel Mare" University Metagenomics Lab	Lobiuc Andrei, Gheorghita Roxana
EPI_ISL_891229	Suceava County Emergency Hospital	"Stefan cel Mare" University Metagenomics Lab	Lobiuc Andrei, Gheorghita Roxana
EPI_ISL_893721, EPI_ISL_893722, EPI_ISL_893723	Oregon State Public Health Laboratory	Pathogen Discovery, Respiratory Viruses Branch, Division of Viral Diseases, Centers for Disease Control and Prevention	Ying Tao, Yan Li, Jing Zhang, Krista Queen, Anna Uehara, Peter Cook, Clinton R. Paden, Haibin Wang, Suxiang Tong
EPI_ISL_896206	MEPHI, Aix Marseille University	MEPHI, Aix Marseille University	Anthony LEVASSEUR
EPI_ISL_896376	University of Medicine and Pharmacy of Craiova	"Stefan cel Mare" University Metagenomics Lab	Lobiuc Andrei, Gheorghita Roxana
EPI_ISL_896378, EPI_ISL_896379	Suceava County Emergency Hospital	"Stefan cel Mare" University Metagenomics Lab	Lobiuc Andrei, Gheorghita Roxana
EPI_ISL_903002, EPI_ISL_903004, EPI_ISL_903005, EPI_ISL_903006	Seattle Flu Study	Seattle Flu Study	Deborah A. Nickerson, Chris D. Frazar, Jover Lee, Benjamin Pelle, Erica Ryke, Matthew Richardson, Amanda Adler, Elisabeth Brandstetter, Peter D. Han, Kairsten Fay, Misja Ilcisin, Kirsten Lacombe, Thomas R. Sibley, Melissa Truong, Caitlin R. Wolf, Michael Boeckh, Janet A. Englund, Michael Famulare, Barry R. Lutz, Mark J. Rieder, Lea M. Starita, Matthew Thompson, Jay Shendure, Trevor Bedford, Helen Y. Chu
EPI_ISL_903613, EPI_ISL_903627	LA Office of Public Health Laboratories	Genomics and Discovery, Respiratory Viruses Branch, Division of Viral Diseases, Centers for Disease Control and Prevention	Krista Queen, Yan Li, Ying Tao, Jing Zhang, Anna Uehara, Anna Montmayeur, Clinton R. Paden, Peter W. Cook, Rachel Marine, Mili Sheth, Jasmine Padilla, Sarah Nobles, Mark Burroughs, Lori Rowe, Haibin Wang, Ben L. Rambo-Martin, Dhwani Batra, Justin Lee, Suxiang Tong
EPI_ISL_903632	NC State Laboratory of Public Health	Genomics and Discovery, Respiratory Viruses Branch, Division of Viral Diseases, Centers for Disease Control and Prevention	Krista Queen, Yan Li, Ying Tao, Jing Zhang, Anna Uehara, Anna Montmayeur, Clinton R. Paden, Peter W. Cook, Rachel Marine, Mili Sheth, Jasmine Padilla, Sarah Nobles, Mark Burroughs, Lori Rowe, Haibin Wang, Ben L. Rambo-Martin, Dhwani Batra, Justin Lee, Suxiang Tong
EPI_ISL_903720, EPI_ISL_903765, EPI_ISL_903906	LA Office of Public Health Laboratories	Genomics and Discovery, Respiratory Viruses Branch, Division of Viral Diseases, Centers for Disease Control and Prevention	Krista Queen, Yan Li, Ying Tao, Jing Zhang, Anna Uehara, Anna Montmayeur, Clinton R. Paden, Peter W. Cook, Rachel Marine, Mili Sheth, Jasmine Padilla, Sarah Nobles, Mark Burroughs, Lori Rowe, Haibin Wang, Ben L. Rambo-Martin, Dhwani Batra, Justin Lee, Suxiang Tong
EPI_ISL_903954, EPI_ISL_903960, EPI_ISL_903972, EPI_ISL_903975, EPI_ISL_903978	WA State Department of Health	Genomics and Discovery, Respiratory Viruses Branch, Division of Viral Diseases, Centers for Disease Control and Prevention	Krista Queen, Yan Li, Ying Tao, Jing Zhang, Anna Uehara, Anna Montmayeur, Clinton R. Paden, Peter W. Cook, Rachel Marine, Mili Sheth, Jasmine Padilla, Sarah Nobles, Mark Burroughs, Lori Rowe, Haibin Wang, Ben L. Rambo-Martin, Dhwani Batra, Justin Lee, Suxiang Tong
EPI_ISL_904736	Dutch COVID-19 response team	National Institute for Public Health and the Environment (RIVM)	Adam Meijer, Harry Vennema, Dirk Eggink, Jeroen Cremer, Sharon van den Brink, Bas van der Veer, AnneMarie van den Brandt, Florian Zwagemaker, Dennis Schmitz, Chantal Reusken, on behalf of the national COVID-19 response team
EPI_ISL_904941, EPI_ISL_904949, EPI_ISL_904950, EPI_ISL_904954	Vilnius University Hospital Santaros Klinikos, Vilnius University	Institute of Biotechnology, Life Sciences Center, Vilnius University	Emilija Vasiliunaite, Milda Norkiene, Albertas Timinskas, Alma Gedvilaite, Aurelija Zvirbliene, Daniel Naumovas, Laimonas Griskevicius
EPI_ISL_906096	Child Health Research Foundation	Child Health Research Foundation	Senjuti Saha, Afroza Akter Tanni, Sharmistha Goswami, Syed Muktadir Al Sium, Arif Mohammad Tanmoy, Roly Malaker, Md Hafizur Rahman, Samir K Saha
EPI_ISL_909958, EPI_ISL_909962	Apollo Hospitals	CSIR-Centre for Cellular and Molecular Biology	Onkar Kulkarni, Suneetha Narreddy, Lamuk Zaveri, Irawathy Goud, Sofia Banu, Payel Mukherjee, Karthik Bharadwaj Tallapaka, Divya Tej Sowpati
EPI_ISL_911679	Alaska State Virology Laboratory	Alaska State Virology Laboratory	Stephanie DeRonde, Lisa Smith, Ph.D., Jack Chen, Ph.D.
EPI_ISL_912465, EPI_ISL_912505, EPI_ISL_912511, EPI_ISL_912527	NHLS Universitas Academic	UFS Virology	PA Bester, MM Nyaga, P Nthiga, MT Mogotsi, D Goedhals, T de Oliveira
EPI_ISL_913934, EPI_ISL_913935, EPI_ISL_913970	Instituto de Diagnostico y Referencia Epidemiologicos INDRE_RNLSP	Instituto de Diagnostico y Referencia Epidemiologicos (INDRE)	Claudia Wong-Arambula, Abril Rodriguez-Maldonado, Fabiola Garces-Ayala, Adnan Araiza-Rodriguez, David Fragoso-Fonseca, Sergio Rangel-Guerrero, Mayra Jimenez-Morales, Nancy Munoz-Hernandez, Natividad Cruz-Ortiz, Tatiana Nunez-Garcia, Gisela Barrera-Badillo, Lucia Hernandez-Rivas, Irma Lopez-Martinez, Ernesto Ramirez-Gonzalez.
EPI_ISL_914882	Vilnius university hospital Santaros Klinikos, Center of Laboratory Medicine	Vilnius University Hospital Santaros Klinikos	Ingrida Olendraite, Daniel Naumovas, Rimvydas Norvilas, Dovil Ežerskyt, Justinas Šlikas
EPI_ISL_915367, EPI_ISL_915370, EPI_ISL_915413, EPI_ISL_915414	Keio University School of Medicine	Keio University School of Medicine	Kenjiro Kosaki, Yuka Iwasaki, Hirotsugu Ishizu, Haruhiko Siomi, Kodai Abe

EPI_ISL_915440, EPI_ISL_915441, EPI_ISL_915442, EPI_ISL_915443, EPI_ISL_915444, EPI_ISL_915445, EPI_ISL_915446, EPI_ISL_915447, EPI_ISL_915448, EPI_ISL_915449, EPI_ISL_915450, EPI_ISL_915451, EPI_ISL_915452, EPI_ISL_915453, EPI_ISL_915454, EPI_ISL_915455, EPI_ISL_915456, EPI_ISL_915457, EPI_ISL_915458, EPI_ISL_915459, EPI_ISL_915460, EPI_ISL_915461, EPI_ISL_915462, EPI_ISL_915463, EPI_ISL_915464, EPI_ISL_915465, EPI_ISL_915466, EPI_ISL_915467, EPI_ISL_915468, EPI_ISL_915469, EPI_ISL_915470, EPI_ISL_915471, EPI_ISL_915472, EPI_ISL_915473, EPI_ISL_915474, EPI_ISL_915475, EPI_ISL_915476, EPI_ISL_915477, EPI_ISL_915478, EPI_ISL_915479, EPI_ISL_915480, EPI_ISL_915481, EPI_ISL_915482, EPI_ISL_915483, EPI_ISL_915484, EPI_ISL_915485, EPI_ISL_915486, EPI_ISL_915487, EPI_ISL_915488, EPI_ISL_915489, EPI_ISL_915490, EPI_ISL_915491, EPI_ISL_915492, EPI_ISL_915493, EPI_ISL_915494, EPI_ISL_915495, EPI_ISL_915496, EPI_ISL_915497, EPI_ISL_915498, EPI_ISL_915499, EPI_ISL_915500, EPI_ISL_915501, EPI_ISL_915502, EPI_ISL_915503, EPI_ISL_915504, EPI_ISL_915505, EPI_ISL_915506, EPI_ISL_915507, EPI_ISL_915508, EPI_ISL_915509, EPI_ISL_915510, EPI_ISL_915511, EPI_ISL_915512, EPI_ISL_915513, EPI_ISL_915514, EPI_ISL_915515, EPI_ISL_915516, EPI_ISL_915517, EPI_ISL_915518, EPI_ISL_915519, EPI_ISL_915520, EPI_ISL_915521, EPI_ISL_915522, EPI_ISL_915523, EPI_ISL_915524, EPI_ISL_915525, EPI_ISL_915526, EPI_ISL_915527, EPI_ISL_915528, EPI_ISL_915529, EPI_ISL_915530, EPI_ISL_915531, EPI_ISL_915532, EPI_ISL_915533, EPI_ISL_915534, EPI_ISL_915535, EPI_ISL_915536, EPI_ISL_915537, EPI_ISL_915538, EPI_ISL_915539, EPI_ISL_915540, EPI_ISL_915541, EPI_ISL_915542, EPI_ISL_915543, EPI_ISL_915544, EPI_ISL_915545, EPI_ISL_915546, EPI_ISL_915547, EPI_ISL_915548, EPI_ISL_915549, EPI_ISL_915550, EPI_ISL_915551, EPI_ISL_915552, EPI_ISL_915553, EPI_ISL_915554, EPI_ISL_915555, EPI_ISL_915556, EPI_ISL_915557, EPI_ISL_915558, EPI_ISL_915559, EPI_ISL_915560, EPI_ISL_915561, EPI_ISL_915562, EPI_ISL_915563, EPI_ISL_915564, EPI_ISL_915565, EPI_ISL_915566, EPI_ISL_915567, EPI_ISL_915568, EPI_ISL_915569, EPI_ISL_915570, EPI_ISL_915571, EPI_ISL_915572, EPI_ISL_915573, EPI_ISL_915574, EPI_ISL_915575, EPI_ISL_915576, EPI_ISL_915577, EPI_ISL_915578, EPI_ISL_915579, EPI_ISL_915580, EPI_ISL_915581, EPI_ISL_915582, EPI_ISL_915583, EPI_ISL_915584, EPI_ISL_915585, EPI_ISL_915586, EPI_ISL_915587, EPI_ISL_915588, EPI_ISL_915589, EPI_ISL_915590, EPI_ISL_915591, EPI_ISL_915592, EPI_ISL_915593, EPI_ISL_915594, EPI_ISL_915595, EPI_ISL_915596, EPI_ISL_915597, EPI_ISL_915598, EPI_ISL_915599, EPI_ISL_915600, EPI_ISL_915601, EPI_ISL_915602, EPI_ISL_915603, EPI_ISL_915604, EPI_ISL_915605, EPI_ISL_915606, EPI_ISL_915607, EPI_ISL_915608, EPI_ISL_915609, EPI_ISL_915610, EPI_ISL_915611, EPI_ISL_915612, EPI_ISL_915613, EPI_ISL_915614, EPI_ISL_915615, EPI_ISL_915616, EPI_ISL_915617, EPI_ISL_915618, EPI_ISL_915619, EPI_ISL_915620, EPI_ISL_915621, EPI_ISL_915622, EPI_ISL_915623, EPI_ISL_915624, EPI_ISL_915625, EPI_ISL_915626, EPI_ISL_915627, EPI_ISL_915628, EPI_ISL_915629, EPI_ISL_915630, EPI_ISL_915631, EPI_ISL_915632, EPI_ISL_915633, EPI_ISL_915634, EPI_ISL_915635, EPI_ISL_915636, EPI_ISL_915637, EPI_ISL_915638, EPI_ISL_915639, EPI_ISL_915640, EPI_ISL_915641, EPI_ISL_915642, EPI_ISL_915643, EPI_ISL_915644, EPI_ISL_915645, EPI_ISL_915646, EPI_ISL_915647, EPI_ISL_915648, EPI_ISL_915649, EPI_ISL_915650, EPI_ISL_915651, EPI_ISL_915652, EPI_ISL_915653, EPI_ISL_915654, EPI_ISL_915655, EPI_ISL_915657, EPI_ISL_915658, EPI_ISL_915659, EPI_ISL_915660, EPI_ISL_915661, EPI_ISL_915662, EPI_ISL_915663, EPI_ISL_915664, EPI_ISL_915665, EPI_ISL_915666, EPI_ISL_915667, EPI_ISL_915668, EPI_ISL_915669, EPI_ISL_915670, EPI_ISL_915671, EPI_ISL_915672, EPI_ISL_915673, EPI_ISL_915674, EPI_ISL_915675, EPI_ISL_915676, EPI_ISL_915677, EPI_ISL_915678, EPI_ISL_915679, EPI_ISL_915680, EPI_ISL_915681, EPI_ISL_915682, EPI_ISL_915683, EPI_ISL_915684, EPI_ISL_915685, EPI_ISL_915686, EPI_ISL_915687, EPI_ISL_915688, EPI_ISL_915689, EPI_ISL_915690, EPI_ISL_915691, EPI_ISL_915692, EPI_ISL_915693, EPI_ISL_915694, EPI_ISL_915695, EPI_ISL_915696, EPI_ISL_915697, EPI_ISL_915698, EPI_ISL_915699, EPI_ISL_915700, EPI_ISL_915701, EPI_ISL_915702, EPI_ISL_915703, EPI_ISL_915704, EPI_ISL_915705, EPI_ISL_915706, EPI_ISL_915707, EPI_ISL_915708, EPI_ISL_915709, EPI_ISL_915710, EPI_ISL_915711, EPI_ISL_915712, EPI_ISL_915713, EPI_ISL_915714, EPI_ISL_915715, EPI_ISL_915716, EPI_ISL_915717, EPI_ISL_915718, EPI_ISL_915719, EPI_ISL_915720, EPI_ISL_915721, EPI_ISL_915722, EPI_ISL_915723, EPI_ISL_915724, EPI_ISL_915725, EPI_ISL_915726, EPI_ISL_915727, EPI_ISL_915728, EPI_ISL_915729, EPI_ISL_915730, EPI_ISL_915731, EPI_ISL_915732, EPI_ISL_915733, EPI_ISL_915734, EPI_ISL_915735, EPI_ISL_915736, EPI_ISL_915737, EPI_ISL_915738, EPI_ISL_915739, EPI_ISL_915740, EPI_ISL_915741, EPI_ISL_915742, EPI_ISL_915743, EPI_ISL_915744, EPI_ISL_915745, EPI_ISL_915746, EPI_ISL_915747, EPI_ISL_915748, EPI_ISL_915749, EPI_ISL_915750, EPI_ISL_915751, EPI_ISL_915752, EPI_ISL_915753, EPI_ISL_915754, EPI_ISL_915755, EPI_ISL_915756, EPI_ISL_915757, EPI_ISL_915758, EPI_ISL_915759, EPI_ISL_915760, EPI_ISL_915761, EPI_ISL_915762, EPI_ISL_915763, EPI_ISL_915764, EPI_ISL_915765, EPI_ISL_915766, EPI_ISL_915767, EPI_ISL_915768, EPI_ISL_915769, EPI_ISL_915770, EPI_ISL_915771, EPI_ISL_915772, EPI_ISL_915773, EPI_ISL_915774, EPI_ISL_915775, EPI_ISL_915776, EPI_ISL_915777, EPI_ISL_915778, EPI_ISL_915779, EPI_ISL_915780, EPI_ISL_915781, EPI_ISL_915782, EPI_ISL_915783, EPI_ISL_915784, EPI_ISL_915785, EPI_ISL_915786, EPI_ISL_915787, EPI_ISL_915788, EPI_ISL_915789, EPI_ISL_915790, EPI_ISL_915791, EPI_ISL_915792, EPI_ISL_915793, EPI_ISL_915794, EPI_ISL_915795, EPI_ISL_915796, EPI_ISL_915797, EPI_ISL_915798, EPI_ISL_915799, EPI_ISL_915800, EPI_ISL_915801, EPI_ISL_915802, EPI_ISL_915803, EPI_ISL_915804, EPI_ISL_915805, EPI_ISL_915806, EPI_ISL_915807, EPI_ISL_915808, EPI_ISL_915809, EPI_ISL_915810, EPI_ISL_915811, EPI_ISL_915812, EPI_ISL_915813, EPI_ISL_915814, EPI_ISL_915815, EPI_ISL_915816, EPI_ISL_915817, EPI_ISL_915818, EPI_ISL_915819, EPI_ISL_915820, EPI_ISL_915821, EPI_ISL_915822, EPI_ISL_915823, EPI_ISL_915824, EPI_ISL_915825, EPI_ISL_915826, EPI_ISL_915827, EPI_ISL_915828, EPI_ISL_915829, EPI_ISL_915830, EPI_ISL_915831, EPI_ISL_915832, EPI_ISL_915833, EPI_ISL_915834, EPI_ISL_915835, EPI_ISL_915836, EPI_ISL_915837, EPI_ISL_915838, EPI_ISL_915839, EPI_ISL_915840, EPI_ISL_915841, EPI_ISL_915842, EPI_ISL_915843, EPI_ISL_915844, EPI_ISL_915845, EPI_ISL_915846, EPI_ISL_915847, EPI_ISL_915848, EPI_ISL_915849, EPI_ISL_915850, EPI_ISL_915851, EPI_ISL_915852, EPI_ISL_915853, EPI_ISL_915854, EPI_ISL_915855, EPI_ISL_915856, EPI_ISL_915857, EPI_ISL_915858, EPI_ISL_915859, EPI_ISL_915860, EPI_ISL_915861, EPI_ISL_915862, EPI_ISL_915863, EPI_ISL_915864, EPI_ISL_915865, EPI_ISL_915866, EPI_ISL_915867, EPI_ISL_915868, EPI_ISL_915869, EPI_ISL_915870, EPI_ISL_915871, EPI_ISL_915872, EPI_ISL_915873, EPI_ISL_915874, EPI_ISL_915875, EPI_ISL_915876, EPI_ISL_915877, EPI_ISL_915878, EPI_ISL_915879, EPI_ISL_915880, EPI_ISL_915881, EPI_ISL_915882, EPI_ISL_915883, EPI_ISL_915884, EPI_ISL_915885, EPI_ISL_915886, EPI_ISL_915887, EPI_ISL_915888, EPI_ISL_915889, EPI_ISL_915890, EPI_ISL_915891, EPI_ISL_915892, EPI_ISL_915893, EPI_ISL_915894, EPI_ISL_915895, EPI_ISL_915896, EPI_ISL_915897, EPI_ISL_915898, EPI_ISL_915899, EPI_ISL_915900, EPI_ISL_915901, EPI_ISL_915902, EPI_ISL_915903, EPI_ISL_915904, EPI_ISL_915905, EPI_ISL_915906, EPI_ISL_915907, EPI_ISL_915908, EPI_ISL_915909, EPI_ISL_915910, EPI_ISL_915912, EPI_ISL_915913, EPI_ISL_915914, EPI_ISL_915915, EPI_ISL_915916, EPI_ISL_915917, EPI_ISL_915918, EPI_ISL_915919, EPI_ISL_915920, EPI_ISL_915921, EPI_ISL_915922, EPI_ISL_915923, EPI_ISL_915924, EPI_ISL_915925, EPI_ISL_915926, EPI_ISL_915927, EPI_ISL_915928, EPI_ISL_915929, EPI_ISL_915930, EPI_ISL_915931, EPI_ISL_915932, EPI_ISL_915933, EPI_ISL_915934, EPI_ISL_915935, EPI_ISL_915936, EPI_ISL_915937, EPI_ISL_915938, EPI_ISL_915939, EPI_ISL_915940, EPI_ISL_915941, EPI_ISL_915942, EPI_ISL_915943, EPI_ISL_915944, EPI_ISL_915945, EPI_ISL_915946, EPI_ISL_915947, EPI_ISL_915948, EPI_ISL_915949, EPI_ISL_915950, EPI_ISL_915951, EPI_ISL_915952, EPI_ISL_915953, EPI_ISL_915954, EPI_ISL_915955, EPI_ISL_915956, EPI_ISL_915957, EPI_ISL_915958, EPI_ISL_915959, EPI_ISL_915960, EPI_ISL_915961, EPI_ISL_915962, EPI_ISL_915963, EPI_ISL_915964, EPI_ISL_915965, EPI_ISL_915966, EPI_ISL_915967, EPI_ISL_915968, EPI_ISL_915969, EPI_ISL_915970, EPI_ISL_915971, EPI_ISL_915972, EPI_ISL_915973, EPI_ISL_915974, EPI_ISL_915975, EPI_ISL_915976, EPI_ISL_915977, EPI_ISL_915978, EPI_ISL_915979, EPI_ISL_915980, EPI_ISL_915981, EPI_ISL_915982, EPI_ISL_915983, EPI_ISL_915984, EPI_ISL_915985, EPI_ISL_915986, EPI_ISL_915987, EPI_ISL_915988, EPI_ISL_915989, EPI_ISL_915990, EPI_ISL_915991, EPI_ISL_915992, EPI_ISL_915993, EPI_ISL_915994, EPI_ISL_915995, EPI_ISL_915996, EPI_ISL_915997, EPI_ISL_915998, EPI_ISL_915999, EPI_ISL_916000, EPI_ISL_916001, EPI_ISL_916002, EPI_ISL_916003, EPI_ISL_916004, EPI_ISL_916005, EPI_ISL_916006, EPI_ISL_916007, EPI_ISL_916008, EPI_ISL_916009, EPI_ISL_916010, EPI_ISL_916011, EPI_ISL_916012, EPI_ISL_916013, EPI_ISL_916014, EPI_ISL_916015, EPI_ISL_916016, EPI_ISL_916017, EPI_ISL_916018, EPI_ISL_916019, EPI_ISL_916020, EPI_ISL_916021, EPI_ISL_916022, EPI_ISL_916023, EPI_ISL_916024, EPI_ISL_916025, EPI_ISL_916026, EPI_ISL_916027, EPI_ISL_916028, EPI_ISL_916029, EPI_ISL_916030, EPI_ISL_916031, EPI_ISL_916032, EPI_ISL_916033, EPI_ISL_916034, EPI_ISL_916035, EPI_ISL_916036, EPI_ISL_916037, EPI_ISL_916038, EPI_ISL_916039, EPI_ISL_916040, EPI_ISL_916041, EPI_ISL_916042, EPI_ISL_916043, EPI_ISL_916044, EPI_ISL_916045, EPI_ISL_916046, EPI_ISL_916047, EPI_ISL_916048, EPI_ISL_916049, EPI_ISL_916050, EPI_ISL_916051, EPI_ISL_916052, EPI_ISL_916053, EPI_ISL_916054, EPI_ISL_916055, EPI_ISL_916056, EPI_ISL_916057, EPI_ISL_916058, EPI_ISL_916059, EPI_ISL_916060, EPI_ISL_916061, EPI_ISL_916062, EPI_ISL_916063, EPI_ISL_916064, EPI_ISL_916065, EPI_ISL_916066, EPI_ISL_916067, EPI_ISL_916069, EPI_ISL_916070, EPI_ISL_916072, EPI_ISL_916073, EPI_ISL_916074, EPI_ISL_916077, EPI_ISL_916078, EPI_ISL_916081, EPI_ISL_916083, EPI_ISL_916084, EPI_ISL_916085, EPI_ISL_916089, EPI_ISL_916090, EPI_ISL_916093, EPI_ISL_916095, EPI_ISL_916096, EPI_ISL_916097, EPI_ISL_916101, EPI_ISL_916102, EPI_ISL_916103, EPI_ISL_916104, EPI_ISL_916112, EPI_ISL_916114, EPI_ISL_916115, EPI_ISL_916116, EPI_ISL_916117, EPI_ISL_916118, EPI_ISL_916119, EPI_ISL_916120, EPI_ISL_916122, EPI_ISL_916123, EPI_ISL_916125, EPI_ISL_916126, EPI_ISL_916128, EPI_ISL_916130, EPI_ISL_916133, EPI_ISL_916135, EPI_ISL_916136, EPI_ISL_916138, EPI_ISL_916139,

EPI_ISL_916140, EPI_ISL_916141, EPI_ISL_916142, EPI_ISL_916143, EPI_ISL_916146, EPI_ISL_916148, EPI_ISL_916149, EPI_ISL_916150, EPI_ISL_916153, EPI_ISL_916155, EPI_ISL_916156, EPI_ISL_916157, EPI_ISL_916158, EPI_ISL_916160, EPI_ISL_916163, EPI_ISL_916168, EPI_ISL_916170, EPI_ISL_916172, EPI_ISL_916173, EPI_ISL_916175, EPI_ISL_916176, EPI_ISL_916177, EPI_ISL_916178, EPI_ISL_916179			
see above	Lighthouse Lab in Alderley Park	Wellcome Sanger Institute for the COVID-19 Genomics UK (COG-UK) Consortium	Jacquelyn Wynn, Mairead Hyland, The Lighthouse Lab in Alderley Park and Alex Alderton, Roberto Amato, Sonia Goncalves, Ewan Harrison, David K. Jackson, Ian Johnston, Dominic Kwiatkowski, Cordelia Langford, John Sillitoe on behalf of the Wellcome Sanger Institute COVID-19 Surveillance Team
EPI_ISL_918516	LACEN - Laboratório Central de Saúde Pública do Para	Evandro Chagas Institute	Santos, M.C.; Silva, A.M.; Junior, W.D.C.; Barbagelata, L.S.; Ferreira, J.A.; Sousa, E.M.A.; da Silva, P.S.; Pinheiro, K.C.; L.C.; Sousa Junior, E.C.
EPI_ISL_918569, EPI_ISL_918570, EPI_ISL_918571, EPI_ISL_918572, EPI_ISL_918573, EPI_ISL_918589, EPI_ISL_918591, EPI_ISL_918592, EPI_ISL_918593, EPI_ISL_918594, EPI_ISL_918595			
see above	University of Birmingham	COVID-19 Genomics UK (COG-UK) Consortium	Institute of Microbiology, University of Birmingham: Claire McMurray, Joanne Stockton, Samuel Nicholls, Radoslaw Poplawski, Will Rowe, Josh Quick, Nicholas Loman. University of Birmingham Testing Laboratory: Celina M Whalley, Andrew Bosworth, Charlotte Poxon, Kasun Wanigasooriya, Oliver Pickles, Mike Kidd, Alex Richter, Andrew D Beggs PHE Heartlands Lab: Husam Osman, Andrew Bosworth. Queen Elizabeth Hospital: Anna Casey
EPI_ISL_919272	West of Scotland Specialist Virology Centre, NHSGGC / MRC-University of Glasgow Centre for Virus Research	COVID-19 Genomics UK (COG-UK) Consortium	Ana da Silva Filipe, Natasha Johnson, Kathy Smollett, Daniel Mair, Stephen Carmichael, Alice Broos, Lily Tong, Jenna Nichols, Kyriaki Nomikou; Sarah McDonald; Richard Orton, Joseph Hughes, Sreenu Vattipally, David L Robertson; Alasdair MacLean, Rory Gunson; Sharif Shaaban, Matthew Holden; Rachel Blacow, Guy Mollett, Kathy Li, James Shepherd, Antonia Ho, Emma Thomson
EPI_ISL_920482	University College London Hospital	COVID-19 Genomics UK (COG-UK) Consortium	Judith Heaney, Matthew Byott, Catherine Houlihan, Dan Frampton, Stuart Kirk, Moira Spyer and Eleni Nastouli
EPI_ISL_924783, EPI_ISL_924784, EPI_ISL_924785, EPI_ISL_924786, EPI_ISL_924789, EPI_ISL_924790, EPI_ISL_924791, EPI_ISL_924792, EPI_ISL_924793	Bioinformatics and Biostatistics Lab, Advanced Sequencing Facility	COVID-19 Genomics UK (COG-UK) Consortium	Aengus Stewart,Jerome Nicod,Chelsea Sawyer,Laura Cubitt,Harshil Patel,Margaret Crawford
EPI_ISL_925496	Department of Clinical Microbiology	GIGA Medical Genomics	Keith Durkin, Maria Artesi, Sébastien Bontems, Raphaël Boreux, Bouchra Boujemla, Cécile Meex, Pierrette Melin, Marie-Pierre Hayette, Vincent Bours
EPI_ISL_926137, EPI_ISL_926166, EPI_ISL_926339, EPI_ISL_926776, EPI_ISL_926882, EPI_ISL_927025, EPI_ISL_927325, EPI_ISL_927352, EPI_ISL_927461, EPI_ISL_927491, EPI_ISL_927502, EPI_ISL_927662, EPI_ISL_927843, EPI_ISL_927872, EPI_ISL_927895, EPI_ISL_927932, EPI_ISL_928073, EPI_ISL_928106, EPI_ISL_928112, EPI_ISL_928148, EPI_ISL_928208, EPI_ISL_928240, EPI_ISL_928261, EPI_ISL_928316, EPI_ISL_928322, EPI_ISL_928335, EPI_ISL_928696, EPI_ISL_928718, EPI_ISL_928939, EPI_ISL_929027, EPI_ISL_929116, EPI_ISL_929357, EPI_ISL_929467, EPI_ISL_929695, EPI_ISL_929937, EPI_ISL_929995, EPI_ISL_930105, EPI_ISL_930111, EPI_ISL_930265, EPI_ISL_930431, EPI_ISL_930434			
see above	Department of Virus and Microbiological Special Diagnostics, Statens Serum Institut, Copenhagen, Denmark	Aalborg University	Danish Covid-19 Genome Consortium
EPI_ISL_931363, EPI_ISL_931364, EPI_ISL_931389	University Hospital Basel, Clinical Virology	University Hospital Basel, Clinical Bacteriology	Tim Roloff, Madlen Stange, Helena MB Seth-Smith, Alfredo Mari, Karoline Leuzinger, Julia Bielicki, Manuel Battegay, Hans Hirsch, Adrian Egli
EPI_ISL_933516, EPI_ISL_933518	Instituto Nacional de Medicina Genomica	Instituto Nacional de Medicina Genomica	Hidalgo-Miranda A, Mendoza-Vargas A, Reyes-Grajeda JP, Cisneros-Villanueva M, Cedro-Tanda A,Peñaloza-Figueroa F, Herrera-Montalvo LA
EPI_ISL_933547, EPI_ISL_933548, EPI_ISL_933549, EPI_ISL_933550, EPI_ISL_933551, EPI_ISL_933552, EPI_ISL_933553, EPI_ISL_933558	Arizona State Public Health Laboratory	Arizona State Public Health Laboratory	Trung Huynh, Jessica Escobar, Katherine Fullerton, Nobuko Fukushima, Stacy White, Linda Getsinger, Victor Waddell
EPI_ISL_933668	Instituto de Diagnostico y Referencia Epidemiologicos INDRE_RNLSP	Instituto de Diagnostico y Referencia Epidemiologicos (INDRE)	Claudia Wong-Arambula, Abril Rodriguez-Maldonado, Fabiola Garces-Ayala, Adnan Araiza-Rodriguez, David Fragoso-Fonseca, Sergio Rangel-Guerrero, Mayra Jimenez-Morales, Nancy Munoz-Hernandez, Natividad Cruz-Ortiz, Tatiana Nunez-Garcia, Gisela Barrera-Badillo, Lucia Hernandez-Rivas, Irma Lopez-Martinez, Ernesto Ramirez-Gonzalez.
EPI_ISL_935464, EPI_ISL_935465	Florida Bureau of Public Health Laboratories	Florida Bureau of Public Health Laboratories	Sarah Schmedes, Jason Blanton
EPI_ISL_936489	Hospital Raja Perempuan Zainab II	Institute for Medical Research, Infectious Disease Research Centre, National Institutes of Health, Ministry of Health Malaysia	Suppiah J, Kamel K, Azizan MA, Thayan R
EPI_ISL_936619, EPI_ISL_936620, EPI_ISL_936621, EPI_ISL_936623, EPI_ISL_936624, EPI_ISL_937015	Northwestern Memorial Hospital	Ozer Lab	Ramon Lorenzo-Redondo, Lacy M. Simons, Chad J. Achenbach, Lawrence J. Jennings, Michael G. Ison, Judd F. Hultquist, Egon A. Ozer
EPI_ISL_940187, EPI_ISL_940501	Hôpital Bichat Claude Bernard, Laboratoire de Virologie	IAME UMR1137 Inserm, Université de Paris, Hôpital Bichat	Antoine Bridier-Nahmias, Amélie Recoing, Quentin Le Hingrat, Lena Daniel, Siham Hamri, Gilles Collin, Alexandre Storto, Mélanie Bertine, Charlotte Charpentier, Nadhira Houhou-Fidouh, Diane Descamps, Benoit Visseaux
EPI_ISL_941276, EPI_ISL_941278	Nigeria Centre for Disease Control (NCDC)	African Centre of Excellence for Genomics of Infectious Diseases (ACEGID), Redeemer's University	Oluniyi P.E. et al
EPI_ISL_941909, EPI_ISL_941912	Virginia DCLS	Virginia DCLS	Virginia DCLS
EPI_ISL_941930, EPI_ISL_941934, EPI_ISL_941939	Florida Bureau of Public Health Laboratories	Florida Bureau of Public Health Laboratories	Sarah Schmedes, Jason Blanton
EPI_ISL_942403	Virginia DCLS	Virginia DCLS	Virginia DCLS
EPI_ISL_942480, EPI_ISL_942481	Gundersen Molecular Diagnostics Laboratory	Kabara Cancer Research Institute	Craig S. Richmond, Paraic A. Kenny
EPI_ISL_942482, EPI_ISL_942483, EPI_ISL_942484, EPI_ISL_942485, EPI_ISL_942486, EPI_ISL_942487, EPI_ISL_942488, EPI_ISL_942489, EPI_ISL_942490, EPI_ISL_942491, EPI_ISL_942492, EPI_ISL_942493, EPI_ISL_942494			
see above	Gundersen Clinical Microbiology Laboratory	Kabara Cancer Research Institute	Craig S. Richmond, Paraic A. Kenny
EPI_ISL_942495, EPI_ISL_942496, EPI_ISL_942497, EPI_ISL_942498, EPI_ISL_942499, EPI_ISL_942500, EPI_ISL_942501, EPI_ISL_942503	Gundersen Molecular Diagnostics Laboratory	Kabara Cancer Research Institute	Craig S. Richmond, Paraic A. Kenny
EPI_ISL_942941	Gundersen Clinical Microbiology Laboratory	Kabara Cancer Research Institute	Craig S. Richmond, Paraic A. Kenny
EPI_ISL_943604, EPI_ISL_943607, EPI_ISL_943609, EPI_ISL_943611, EPI_ISL_943613	Lacen_RS	State Center for Health Surveillance. Rio Grande do Sul State Secretary of Health	Aline Campos, Amanda da Silva, Anelise Schaurich, Claudia Dornelles, Cynthia Molina, Fernanda Godinho, Lara Crescente, Leticia Garay, Regina Barcellos, Richard Salvato, Tatiana Gregianini, Vagner Fonseca
EPI_ISL_951788	Originating lab: Wales Specialist Virology Centre Sequencing lab: Pathogen Genomics Unit	Public Health Wales Microbiology Cardiff Wales Specialist Virology Centre	Catherine Moore, Johnathan Evans, Laura Gifford, Malorie Perry, Simon Cottrell, Angela Marchbank, Alec Birchley, Alexander Adams, Amy Gaskin, Bree Gatica-Wilcox, Jason Coombes, Joel Southgate, Lauren Gilbert, Lee Graham, Nicole Pacchiarini, Sara Kumziene-Summerhayes, Sarah Taylor, Sophie Jones, Sara Rey, Matthew Bull, Joanne Watkins, Sally Corden, Tom Connor
EPI_ISL_952404	Centre for Enzyme Innovation, University of Portsmouth / Translational Research Laboratory, Portsmouth Hospitals NHS Trust	COVID-19 Genomics UK (COG-UK) Consortium	Angela Beckett,Salman Goudarzi,Christopher Fearn,Kate Cook,Katie Loveson,Sharon Glaysher,Scott Elliott,Samuel Robson
EPI_ISL_955167, EPI_ISL_955168	Institute for Biocides and Medical Ecology	Institute of microbiology and Immunology, Faculty of Medicine, University of Belgrade	Knezevic,A., Jankovic,M., Vidanovic,D., Milicevic,O., Tesovic,B., Sekler,M., Jovanovic,T.
EPI_ISL_955192, EPI_ISL_955193, EPI_ISL_955196	Instituto Nacional de Medicina Genomica	Instituto Nacional de Medicina Genomica	Hidalgo-Miranda A, Mendoza-Vargas A, Reyes-Grajeda JP, Cisneros-Villanueva M, Cedro-Tanda A,Peñaloza-Figueroa F, Herrera-Montalvo LA
EPI_ISL_955337, EPI_ISL_955338,	UC Davis- Department of Pathology and Laboratory Medicine	Chan-Zuckerberg Biohub	CZB Cliahub Consortium

EPI_ISL_955339, EPI_ISL_955340			
EPI_ISL_955945, EPI_ISL_955956, EPI_ISL_955957, EPI_ISL_955958, EPI_ISL_955959, EPI_ISL_955979, EPI_ISL_955980, EPI_ISL_959483, EPI_ISL_959484, EPI_ISL_959507, EPI_ISL_959508, EPI_ISL_959509, EPI_ISL_959510, EPI_ISL_959511, EPI_ISL_959512, EPI_ISL_959513			
see above	Division of Emerging Infectious Diseases, Bureau of Infectious Diseases Diagnosis Control, Korea Disease Control and Prevention Agency	Division of Emerging Infectious Diseases, Bureau of Infectious Diseases Diagnosis Control, Korea Disease Control and Prevention Agency	Ae Kyung Park, Il-Hwan Kim, Heui Man Kim, Jeong-Min Kim, Namjoo Lee, Chae Young Lee, Sang Hee Woo, Eun-Jin Kim
EPI_ISL_960340, EPI_ISL_960358, EPI_ISL_960359, EPI_ISL_960376	University of Wisconsin-Madison AIDS Vaccine Research Laboratories	University of Wisconsin-Madison AIDS Vaccine Research Laboratories	Gage Moreno, Katarina Braun, et al. AIDS Vaccine Research Laboratories
EPI_ISL_960556, EPI_ISL_960581, EPI_ISL_960647	Istituto Zooprofilattico Sperimentale del Mezzogiorno	TIGEM	Patrizia Annunziata, Andrea Ballabio, Valentina Bouche, Davide Cacchiarelli, Pellegrino Cerino, Chiara Colantuono, Maria Concetta Cuomo, Denise Di Concilio, Lucio Di Filippo, Antonio Grimaldi, Antonio Limone, Anna Manfredi, Francesco Panariello, Biancamaria Pierri, Marcello Salvi
EPI_ISL_962224, EPI_ISL_962225, EPI_ISL_962226, EPI_ISL_962227, EPI_ISL_962228, EPI_ISL_962229, EPI_ISL_962230, EPI_ISL_962231, EPI_ISL_962232, EPI_ISL_962233, EPI_ISL_962234, EPI_ISL_962235, EPI_ISL_962236, EPI_ISL_962237, EPI_ISL_962238, EPI_ISL_962239, EPI_ISL_962240, EPI_ISL_962241, EPI_ISL_962242, EPI_ISL_962243, EPI_ISL_962244, EPI_ISL_962260, EPI_ISL_962261			
see above	Seattle Flu Study	Seattle Flu Study	Deborah A. Nickerson, Chris D. Frazar, Jover Lee, Benjamin Pelle, Erica Ryke, Matthew Richardson, Amanda Adler, Elisabeth Brandstetter, Peter D. Han, Kairsten Fay, Misja Ilcisin, Kirsten Lacombe, Thomas R. Sibley, Melissa Truong, Caitlin R. Wolf, Karen Cowgill, Stephanie Schrag, Jeff Duchin, Michael Boeckh, Janet A. Englund, Michael Famulare, Barry R. Lutz, Mark J. Rieder, Lea M. Starita, Matthew Thompson, Helen Y. Chu, Trevor Bedford, Jay Shendure
EPI_ISL_965345, EPI_ISL_965389, EPI_ISL_965390	University of Liège COVID-19 testing center	GIGA Medical Genomics	Keith Durkin, Maria Artesi, Bouchra Boujemla, Emmanuel André, Marc Van Ranst, Fabrice Bureau, Laurent Gillet, Wouter Coppieters, Vincent Bours
EPI_ISL_965535, EPI_ISL_965554, EPI_ISL_965611, EPI_ISL_965702, EPI_ISL_965703, EPI_ISL_965790	Dutch COVID-19 response team	Medical Microbiology, Maastricht University Medical Centre	Jozef Dingemans*, Brian van der Veer*, Erik Beuken, Carmen Reumkens, Lieke van Alphen, Christian Hoebe, Paul Savelkoul
EPI_ISL_968205, EPI_ISL_968206, EPI_ISL_968207, EPI_ISL_968208, EPI_ISL_968209, EPI_ISL_968210, EPI_ISL_968211	Arizona State Public Health Laboratory	Arizona State Public Health Laboratory	Trung Huynh, Jessica Escobar, Katherine Fullerton, Nobuko Fukushima, Stacy White, Linda Getsinger, Victor Waddell
EPI_ISL_968842, EPI_ISL_968930	KEMRI-Wellcome Trust Research Programme/KEMRI-CGMR-C Kilifi	KEMRI-Wellcome Trust Research Programme/KEMRI-CGMR-C Kilifi	Githinji et al
EPI_ISL_975661, EPI_ISL_975662, EPI_ISL_975663, EPI_ISL_975664, EPI_ISL_975665, EPI_ISL_975666, EPI_ISL_975667, EPI_ISL_975668, EPI_ISL_975669, EPI_ISL_975670, EPI_ISL_975671, EPI_ISL_975672, EPI_ISL_975673, EPI_ISL_975674, EPI_ISL_975675, EPI_ISL_975676, EPI_ISL_975677, EPI_ISL_975678, EPI_ISL_975679, EPI_ISL_975680, EPI_ISL_975681, EPI_ISL_975682, EPI_ISL_975683, EPI_ISL_975684, EPI_ISL_975685, EPI_ISL_975686, EPI_ISL_975687, EPI_ISL_975688, EPI_ISL_975689, EPI_ISL_975690, EPI_ISL_975691, EPI_ISL_975692, EPI_ISL_975693, EPI_ISL_975694, EPI_ISL_975695, EPI_ISL_975696, EPI_ISL_975697, EPI_ISL_975698, EPI_ISL_975699, EPI_ISL_975700, EPI_ISL_975701, EPI_ISL_975702, EPI_ISL_975703, EPI_ISL_975704, EPI_ISL_975705, EPI_ISL_975706, EPI_ISL_975707, EPI_ISL_975708, EPI_ISL_975709, EPI_ISL_975710, EPI_ISL_975711, EPI_ISL_975712, EPI_ISL_975713, EPI_ISL_975714, EPI_ISL_975715, EPI_ISL_975716, EPI_ISL_975717, EPI_ISL_975718, EPI_ISL_975719, EPI_ISL_975720, EPI_ISL_975721, EPI_ISL_975722, EPI_ISL_975723, EPI_ISL_975724, EPI_ISL_975725, EPI_ISL_975726, EPI_ISL_975727, EPI_ISL_975728, EPI_ISL_975729, EPI_ISL_975730, EPI_ISL_975731, EPI_ISL_975732, EPI_ISL_975733, EPI_ISL_975734, EPI_ISL_975735, EPI_ISL_975736, EPI_ISL_975737, EPI_ISL_975738, EPI_ISL_975739, EPI_ISL_975740, EPI_ISL_975741, EPI_ISL_975742, EPI_ISL_975743, EPI_ISL_975744, EPI_ISL_975745, EPI_ISL_975746, EPI_ISL_975747, EPI_ISL_975748, EPI_ISL_975749, EPI_ISL_975750, EPI_ISL_975751, EPI_ISL_975752, EPI_ISL_975753, EPI_ISL_975754, EPI_ISL_975755, EPI_ISL_975756, EPI_ISL_975757, EPI_ISL_975758, EPI_ISL_975759, EPI_ISL_975760, EPI_ISL_975761, EPI_ISL_975762, EPI_ISL_975763, EPI_ISL_975764, EPI_ISL_975765, EPI_ISL_975766, EPI_ISL_975767, EPI_ISL_975768, EPI_ISL_975769, EPI_ISL_975770, EPI_ISL_975771, EPI_ISL_975772, EPI_ISL_975773, EPI_ISL_975774, EPI_ISL_975776, EPI_ISL_975777, EPI_ISL_975778, EPI_ISL_975779, EPI_ISL_975780, EPI_ISL_975781, EPI_ISL_975782, EPI_ISL_975783, EPI_ISL_975784, EPI_ISL_975785, EPI_ISL_975786, EPI_ISL_975787, EPI_ISL_975788, EPI_ISL_975789, EPI_ISL_975790, EPI_ISL_975791, EPI_ISL_975792, EPI_ISL_975793, EPI_ISL_975794			
see above	BCCDC Public Health Laboratory	BCCDC Public Health Laboratory	Prystajecky Natalie, Linda Hoang, Dan Fornika, John Tyson, Shannon Russell, Kim Macdonald, Kimia Kamelian, Ana Pacagnella, Corrinne Ng, Loretta Janz, Robert Azana Terry Snutch, Mel Krajden
EPI_ISL_977154	ULSS 2 Treviso	Istituto Zooprofilattico Sperimentale delle Venezie	Adelaide Milani, Alessia Schivo, Annalisa Salviato, Erika Giorgia Quaranta, Ambra Pastori, Bianca Zecchin, Alice Fusaro, Isabella Monne, Calogero Terregino, Antonia Ricci
EPI_ISL_977177, EPI_ISL_977178, EPI_ISL_977179	Microbiologia e Virologia	Istituto Zooprofilattico Sperimentale delle Venezie	Adelaide Milani, Alessia Schivo, Annalisa Salviato, Erika Giorgia Quaranta, Ambra Pastori, Bianca Zecchin, Alice Fusaro, Isabella Monne, Calogero Terregino, Antonia Ricci
EPI_ISL_977188, EPI_ISL_977189, EPI_ISL_977192, EPI_ISL_977193	ULSS 2 Treviso	Istituto Zooprofilattico Sperimentale delle Venezie	Adelaide Milani, Alessia Schivo, Annalisa Salviato, Erika Giorgia Quaranta, Ambra Pastori, Bianca Zecchin, Alice Fusaro, Isabella Monne, Calogero Terregino, Antonia Ricci
EPI_ISL_977225, EPI_ISL_977226	ULSS 1 Dolomiti	Istituto Zooprofilattico Sperimentale delle Venezie	Adelaide Milani, Alessia Schivo, Annalisa Salviato, Erika Giorgia Quaranta, Ambra Pastori, Bianca Zecchin, Alice Fusaro, Isabella Monne, Calogero Terregino, Antonia Ricci
