# Supplementary material for: Detection and characterization of the SARS-CoV-2 lineage B.1.526 in New York
Source: Nat Commun. 2021 Aug 9;12:4886. doi: 10.1038/s41467-021-25168-4 (PMC8352861; doi:10.1038/s41467-021-25168-4)
Supplement: Supplementary file 8 — Supplementary Data 4 [file 41467_2021_25168_MOESM8_ESM.zip › GISAID_acknowledements_tables/gisaid_hcov-19_acknowledgement_table_2021_02_13_00-12.pdf]

We gratefully acknowledge the following Authors from the Originating laboratories responsible for obtaining the specimens, as well as the Submitting laboratories where the genome data were generated and shared via GISAID, on which this research is based.

All Submitters of data may be contacted directly via www.gisaid.org

Authors are sorted alphabetically.

Accession ID	Originating Laboratory	Submitting Laboratory	Authors
EPI_ISL_672574	Histopath	NSW Health Pathology - Institute of Clinical Pathology and Medical Research; Westmead Hospital; University of Sydney	CIDM-PH et al.
EPI_ISL_678386	Area of Virology, Serology and Virology Division (SAViD), New South Wales Health Pathology Randwick	Virology Research Laboratory; Area of Virology, Serology and Virology Division (SAViD), New South Wales Health Pathology Randwick	Foster, C.; Au, J.; Ruiz Silva, M.; Deveson, I.; Bull, R.; Van Hal, S.; Rawlinson, W.
EPI_ISL_678830	Respiratory Virus Unit, Microbiology Services Colindale, Public Health England	COVID-19 Genomics UK (COG-UK) Consortium	PHE Covid Sequencing Team
EPI_ISL_679945	Queens Medical Centre, Clinical Microbiology Department / DeepSeq Nottingham	COVID-19 Genomics UK (COG-UK) Consortium	Gemma Clark, Wendy Smith, Manjinder Khakh, Vicki M Fleming, Michelle M Lister, Hannah Howson-Wells, Jonathan Ball, Patrick McClure, Joseph Chappell, Theocharis Tsoleridis, Nadine Holmes, Matthew Carlisle, Christopher Moore, Fei Sang, Johnny Debebe, Victoria Wright, Matthew Loose
EPI_ISL_680459, EPI_ISL_680460	West of Scotland Specialist Virology Centre, NHSGGC / MRC-University of Glasgow Centre for Virus Research	COVID-19 Genomics UK (COG-UK) Consortium	Ana da Silva Filipe, Natasha Johnson, Kathy Smollett, Daniel Mair, Stephen Carmichael, Alice Broos, Lily Tong, Jenna Nichols, Kyriaki Nomikou; Sarah McDonald; Richard Orton, Joseph Hughes, Sreenu Vattipally, David L Robertson; Alasdair MacLean, Rory Gunson; Sharif Shaaban, Matthew Holden; Rachel Blacow, Guy Mollett, Kathy Li, James Shepherd, Antonia Ho, Emma Thomson
EPI_ISL_682296	Canterbury Health Laboratories	Institute of Environmental Science and Research (ESR)	Xiaoyun Ren, Matt Storey, Nikki Freed, Muhammad Faisal, Jing Wang, Hermes Perez, Anja Werno, Antje van der Linden, Arlo Upton, Chris Mansell, David Hammer, Dragana Drinkovic, Gary McAuliffe, Hana Sofia Andersson, James Ussher, Jill Sherwood, Josh Freeman, Julia Howard, Juliet Elvy, Mary DeAlmeida, Matt Blakiston, Matthew Rogers, Max Bloomfield, Michael Addidle, Michelle Balm, Sally Roberts, Sarah Jefferies, Sharmini Muttaiyah, Susan Morpeth, Susan Taylor, Timothy Blackmore, Vani Sathyendran, Veronica Playle, Virginia Hope, Erasmus Smit, Lauren Jelly, Olin Silander, Joep de Ligt
EPI_ISL_684006, EPI_ISL_684007, EPI_ISL_684008, EPI_ISL_684009, EPI_ISL_684010, EPI_ISL_684011, EPI_ISL_684012, EPI_ISL_684013, EPI_ISL_684015, EPI_ISL_684016, EPI_ISL_684017, EPI_ISL_684018, EPI_ISL_684019, EPI_ISL_684020, EPI_ISL_684021, EPI_ISL_684022, EPI_ISL_684023, EPI_ISL_684024, EPI_ISL_684025, EPI_ISL_684026, EPI_ISL_684027			
see above	Utah Public Health Laboratory	Utah Public Health Laboratory	Erin Young, Kelly Oakeson
EPI_ISL_692770, EPI_ISL_692771	Massachusetts State Public Health Laboratory	Massachusetts State Public Health Laboratory	Andrew Lang, Timelia Fink, Glen Gallagher, Sandra Smole
EPI_ISL_693322, EPI_ISL_693330	National Public Health Laboratory, National Centre for Infectious Diseases	National Public Health Laboratory, National Centre for Infectious Diseases	Tze Minn Mak, Sophie Octavia, Zhenyang Zhou, Lin Cui, Raymond Tzer Pin Lin
EPI_ISL_697798, EPI_ISL_697799	Institute of Microbiology, Universidad San Francisco de Quito	Institute of Microbiology, Universidad San Francisco de Quito	Belén Prado-Vivar, Sully Márquez, Juan José Guadalupe, Monica Becerra-Wong, Diana Zambrano, Fredy Loor, Juan Zuñiga, Edison Chavez, Bernardo Gutiérrez, Verónica Barragán, Patricio Rojas-Silva, Gabriel Trueba, Michelle Grunauer, Paúl Cárdenas
EPI_ISL_697800	Institute of Microbiology, Universidad San Francisco de Quito	Institute of Microbiology, Universidad San Francisco de Quito	Belén Prado-Vivar, Sully Márquez, Juan José Guadalupe, Monica Becerra-Wong, Maureen Mosquera, Bernardo Gutiérrez, Verónica Barragán, Patricio Rojas-Silva, Gabriel Trueba, Michelle Grunauer, Paúl Cárdenas
EPI_ISL_702478	Quadram Institute Bioscience	COVID-19 Genomics UK (COG-UK) Consortium	Dave J. Baker, Gemma L. Kay, Alp Aydin, Thanh Le-Viet, Steven Rudder, Ana P. Tedim, Anastasia Kolyva, Maria Diaz, Leonardo de Oliveira Martins, Nabil-Fareed Alikhan, Lizzie Meadows, Rachael Stanley, Ngozi Elumogo, Muhammed Yasir, Nicholas M. Thomson, Alexander J Trotter, Rachel Gilroy, Samuel Bloomfield, Claire Stuart, Andrew Bell, Reenesh Prakash, Samir Dervisevic, Alison E. Mather, John Wain, Mark Webber, Andrew J. Page, Justin O'Grady
EPI_ISL_702515, EPI_ISL_702558	Liverpool Clinical Laboratories	COVID-19 Genomics UK (COG-UK) Consortium	Sam Haldenby, Anita Lucaci, Steve Paterson, Julian Hiscox, Alistair Darby, M Almsaud, A Alrezaihi, Muhannad Alruwaili, Stuart D Armstrong, Jones Benjamin, Eleanor G Bentley, Anu Chawla, Jordan J Clark, Angela Cowell, Richard Eccles, Isabel García-Dorival, Matthew Gemmell, Alessandro Gerada, PKF Gilmore, Richard Gregory, Ximeng Han, Catherine Hartley, Margaret Hughes, Miren Iturriza-Gomara, James Johnson, L Luu, Jenifer Manson, Charlotte Nelson, Elaine O'Toole, Cassie Olateju, Rebekah Penrice-Randal , Lucille Rainbow, N.P Randle, Trevor Ian Robinson, Parul Sharma, Ghada T Shawli, James P Stewart, Neil Swainston, Ecaterina Vamos, Joanne Watts, Mark Whitehead
EPI_ISL_702607	Virology Department, Sheffield Teaching Hospitals NHS Foundation Trust/Department of Infection, Immunity and Cardiovascular Disease, The Medical School, University of Sheffield	COVID-19 Genomics UK (COG-UK) Consortium	Thushan de Silva, Matthew Parker, Nikki Smith, Adri Angyal, Rebecca Brown, Luke Green, Rachel Tucker, Paul Parsons, Danielle Groves, Katie Johnson, Laura Carrilero, Alex Keeley, Dave Partridge, Matthew Wyles, Benjamin Lindsey, Mehmet Yavuz, Mohammad Raza, Cariad Evans
EPI_ISL_702652	Quadram Institute Bioscience	COVID-19 Genomics UK (COG-UK) Consortium	Dave J. Baker, Gemma L. Kay, Alp Aydin, Thanh Le-Viet, Steven Rudder, Ana P. Tedim, Anastasia Kolyva, Maria Diaz, Leonardo de Oliveira Martins, Nabil-Fareed Alikhan, Lizzie Meadows, Rachael Stanley, Ngozi Elumogo, Muhammed Yasir, Nicholas M. Thomson, Alexander J Trotter, Rachel Gilroy, Samuel Bloomfield, Claire Stuart, Andrew Bell, Reenesh Prakash, Samir Dervisevic, Alison E. Mather, John Wain, Mark Webber, Andrew J. Page, Justin O'Grady
EPI_ISL_702661	University College London, Great Ormond Street Hospital for Children NHS Foundation Trust, Imperial College Healthcare NHS Trust	COVID-19 Genomics UK (COG-UK) Consortium	Sergi Castellano, Rachel Williams, Mark Kristiansen, Paola Resende Silva, Sunando Roy, Tony Brooks, Helena Tutill, Paola Niola, Patricia Dyal, Charlotte Williams, Leysa Forrest, Yasmin Panchbhaya, Jacqueline Findlay, Samuel Weeks, Julianne Brown, Kathryn Harris, Paul Randell, James Price, Alison Holmes, Judith Breuer
EPI_ISL_702694, EPI_ISL_702749, EPI_ISL_702825, EPI_ISL_702840	Quadram Institute Bioscience	COVID-19 Genomics UK (COG-UK) Consortium	Dave J. Baker, Gemma L. Kay, Alp Aydin, Thanh Le-Viet, Steven Rudder, Ana P. Tedim, Anastasia Kolyva, Maria Diaz, Leonardo de Oliveira Martins, Nabil-Fareed Alikhan, Lizzie Meadows, Rachael Stanley, Ngozi Elumogo, Muhammed Yasir, Nicholas M. Thomson, Alexander J Trotter, Rachel Gilroy, Samuel Bloomfield, Claire Stuart, Andrew Bell, Reenesh Prakash, Samir Dervisevic, Alison E. Mather, John Wain, Mark Webber, Andrew J. Page, Justin O'Grady
EPI_ISL_702852	Virology Department, Sheffield Teaching Hospitals NHS Foundation Trust/Department of Infection, Immunity and Cardiovascular Disease, The Medical School, University of Sheffield	COVID-19 Genomics UK (COG-UK) Consortium	Thushan de Silva, Matthew Parker, Nikki Smith, Adri Angyal, Rebecca Brown, Luke Green, Rachel Tucker, Paul Parsons, Danielle Groves, Katie Johnson, Laura Carrilero, Alex Keeley, Dave Partridge, Matthew Wyles, Benjamin Lindsey, Mehmet Yavuz, Mohammad Raza, Cariad Evans
EPI_ISL_702873	Quadram Institute Bioscience	COVID-19 Genomics UK (COG-UK) Consortium	Dave J. Baker, Gemma L. Kay, Alp Aydin, Thanh Le-Viet, Steven Rudder, Ana P. Tedim, Anastasia Kolyva, Maria Diaz, Leonardo de Oliveira Martins, Nabil-Fareed Alikhan, Lizzie Meadows, Rachael Stanley, Ngozi Elumogo, Muhammed Yasir, Nicholas M. Thomson, Alexander J Trotter, Rachel Gilroy, Samuel Bloomfield, Claire Stuart, Andrew Bell, Reenesh Prakash, Samir Dervisevic, Alison E. Mather, John Wain, Mark Webber, Andrew J. Page, Justin O'Grady
EPI_ISL_703069, EPI_ISL_703107	University College London, Great Ormond Street Hospital for Children NHS Foundation Trust, Imperial College Healthcare NHS Trust	COVID-19 Genomics UK (COG-UK) Consortium	Sergi Castellano, Rachel Williams, Mark Kristiansen, Paola Resende Silva, Sunando Roy, Tony Brooks, Helena Tutill, Paola Niola, Patricia Dyal, Charlotte Williams, Leysa Forrest, Yasmin Panchbhaya, Jacqueline Findlay, Samuel Weeks, Julianne Brown, Kathryn Harris, Paul Randell, James Price, Alison Holmes, Judith Breuer
EPI_ISL_703133	Virology Department, Sheffield Teaching Hospitals NHS Foundation Trust/Department of Infection, Immunity and Cardiovascular Disease, The Medical School, University of Sheffield	COVID-19 Genomics UK (COG-UK) Consortium	Thushan de Silva, Matthew Parker, Nikki Smith, Adri Angyal, Rebecca Brown, Luke Green, Rachel Tucker, Paul Parsons, Danielle Groves, Katie Johnson, Laura Carrilero, Alex Keeley, Dave Partridge, Matthew Wyles, Benjamin Lindsey, Mehmet Yavuz, Mohammad Raza, Cariad Evans

EPI_ISL_703136	West of Scotland Specialist Virology Centre, NHSGGC / MRC-University of Glasgow Centre for Virus Research	COVID-19 Genomics UK (COG-UK) Consortium	Ana da Silva Filipe, Natasha Johnson, Kathy Smollett, Daniel Mair, Stephen Carmichael, Alice Broos, Lily Tong, Jenna Nichols, Kyriaki Nomikou; Sarah McDonald; Richard Orton, Joseph Hughes, Sreenu Vattipally, David L Robertson; Alasdair MacLean, Rory Gunson; Sharif Shaaban, Matthew Holden; Rachel Blacow, Guy Mollett, Kathy Li, James Shepherd, Antonia Ho, Emma Thomson
EPI_ISL_703295	Quadram Institute Bioscience	COVID-19 Genomics UK (COG-UK) Consortium	Dave J. Baker, Gemma L. Kay, Alp Aydin, Thanh Le-Viet, Steven Rudder, Ana P. Tedim, Anastasia Kolyva, Maria Diaz, Leonardo de Oliveira Martins, Nabil-Fareed Alikhan, Lizzie Meadows, Rachael Stanley, Ngozi Elumogo, Muhammed Yasir, Nicholas M. Thomson, Alexander J Trotter, Rachel Gilroy, Samuel Bloomfield, Claire Stuart, Andrew Bell, Reenesh Prakash, Samir Dervisevic, Alison E. Mather, John Wain, Mark Webber, Andrew J. Page, Justin O'Grady
EPI_ISL_703310	Liverpool Clinical Laboratories	COVID-19 Genomics UK (COG-UK) Consortium	Sam Haldenby, Anita Lucaci, Steve Paterson, Julian Hiscox, Alistair Darby, M Almsaud, A Alrezaihi, Muhannad Alruwaili, Stuart D Armstrong, Jones Benjamin, Eleanor G Bentley, Anu Chawla, Jordan J Clark, Angela Cowell, Richard Eccles, Isabel García-Dorival, Matthew Gemmell, Alessandro Gerada, PKF Gilmore, Richard Gregory, Ximeng Han, Catherine Hartley, Margaret Hughes, Miren Iturriza-Gomara, James Johnson, L Luu, Jenifer Manson, Charlotte Nelson, Elaine O'Toole, Cassie Olateju, Rebekah Penrice-Randal , Lucille Rainbow, N.P Randle, Trevor Ian Robinson, Parul Sharma, Ghada T Shawli, James P Stewart, Neil Swainston, Ecaterina Vamos, Joanne Watts, Mark Whitehead
EPI_ISL_703369	University College London Hospital	COVID-19 Genomics UK (COG-UK) Consortium	Judith Heaney, Matthew Byott, Catherine Houlihan, Dan Frampton, Stuart Kirk, Moira Spyer and Eleni Nastouli
EPI_ISL_703409	Liverpool Clinical Laboratories	COVID-19 Genomics UK (COG-UK) Consortium	Sam Haldenby, Anita Lucaci, Steve Paterson, Julian Hiscox, Alistair Darby, M Almsaud, A Alrezaihi, Muhannad Alruwaili, Stuart D Armstrong, Jones Benjamin, Eleanor G Bentley, Anu Chawla, Jordan J Clark, Angela Cowell, Richard Eccles, Isabel García-Dorival, Matthew Gemmell, Alessandro Gerada, PKF Gilmore, Richard Gregory, Ximeng Han, Catherine Hartley, Margaret Hughes, Miren Iturriza-Gomara, James Johnson, L Luu, Jenifer Manson, Charlotte Nelson, Elaine O'Toole, Cassie Olateju, Rebekah Penrice-Randal , Lucille Rainbow, N.P Randle, Trevor Ian Robinson, Parul Sharma, Ghada T Shawli, James P Stewart, Neil Swainston, Ecaterina Vamos, Joanne Watts, Mark Whitehead
EPI_ISL_703414	Virology Department, Royal Infirmary of Edinburgh, NHS Lothian / School of Biological Sciences, University of Edinburgh / Institute of Genetics and Molecular Medicine, University of Edinburgh	COVID-19 Genomics UK (COG-UK) Consortium	McHugh M, Dewar R, Rooke S, Gallagher M, Balcaza C, O'Toole Á, Scher E, Hill V, McCrone JT, Colquhoun R, Yu X, Jackson B, Rambaut A, Williams TC, Templeton K
EPI_ISL_703611	Liverpool Clinical Laboratories	COVID-19 Genomics UK (COG-UK) Consortium	Sam Haldenby, Anita Lucaci, Steve Paterson, Julian Hiscox, Alistair Darby, M Almsaud, A Alrezaihi, Muhannad Alruwaili, Stuart D Armstrong, Jones Benjamin, Eleanor G Bentley, Anu Chawla, Jordan J Clark, Angela Cowell, Richard Eccles, Isabel García-Dorival, Matthew Gemmell, Alessandro Gerada, PKF Gilmore, Richard Gregory, Ximeng Han, Catherine Hartley, Margaret Hughes, Miren Iturriza-Gomara, James Johnson, L Luu, Jenifer Manson, Charlotte Nelson, Elaine O'Toole, Cassie Olateju, Rebekah Penrice-Randal , Lucille Rainbow, N.P Randle, Trevor Ian Robinson, Parul Sharma, Ghada T Shawli, James P Stewart, Neil Swainston, Ecaterina Vamos, Joanne Watts, Mark Whitehead
EPI_ISL_703649	Virology Department, Sheffield Teaching Hospitals NHS Foundation Trust/Department of Infection, Immunity and Cardiovascular Disease, The Medical School, University of Sheffield	COVID-19 Genomics UK (COG-UK) Consortium	Thushan de Silva, Matthew Parker, Nikki Smith, Adri Angyal, Rebecca Brown, Luke Green, Rachel Tucker, Paul Parsons, Danielle Groves, Katie Johnson, Laura Carrilero, Alex Keeley, Dave Partridge, Matthew Wyles, Benjamin Lindsey, Mehmet Yavuz, Mohammad Raza, Cariad Evans
EPI_ISL_703758	West of Scotland Specialist Virology Centre, NHSGGC / MRC-University of Glasgow Centre for Virus Research	COVID-19 Genomics UK (COG-UK) Consortium	Ana da Silva Filipe, Natasha Johnson, Kathy Smollett, Daniel Mair, Stephen Carmichael, Alice Broos, Lily Tong, Jenna Nichols, Kyriaki Nomikou; Sarah McDonald; Richard Orton, Joseph Hughes, Sreenu Vattipally, David L Robertson; Alasdair MacLean, Rory Gunson; Sharif Shaaban, Matthew Holden; Rachel Blacow, Guy Mollett, Kathy Li, James Shepherd, Antonia Ho, Emma Thomson
EPI_ISL_703765	Virology Department, Sheffield Teaching Hospitals NHS Foundation Trust/Department of Infection, Immunity and Cardiovascular Disease, The Medical School, University of Sheffield	COVID-19 Genomics UK (COG-UK) Consortium	Thushan de Silva, Matthew Parker, Nikki Smith, Adri Angyal, Rebecca Brown, Luke Green, Rachel Tucker, Paul Parsons, Danielle Groves, Katie Johnson, Laura Carrilero, Alex Keeley, Dave Partridge, Matthew Wyles, Benjamin Lindsey, Mehmet Yavuz, Mohammad Raza, Cariad Evans
EPI_ISL_703886	University College London Hospital	COVID-19 Genomics UK (COG-UK) Consortium	Judith Heaney, Matthew Byott, Catherine Houlihan, Dan Frampton, Stuart Kirk, Moira Spyer and Eleni Nastouli
EPI_ISL_703927	West of Scotland Specialist Virology Centre, NHSGGC / MRC-University of Glasgow Centre for Virus Research	COVID-19 Genomics UK (COG-UK) Consortium	Ana da Silva Filipe, Natasha Johnson, Kathy Smollett, Daniel Mair, Stephen Carmichael, Alice Broos, Lily Tong, Jenna Nichols, Kyriaki Nomikou; Sarah McDonald; Richard Orton, Joseph Hughes, Sreenu Vattipally, David L Robertson; Alasdair MacLean, Rory Gunson; Sharif Shaaban, Matthew Holden; Rachel Blacow, Guy Mollett, Kathy Li, James Shepherd, Antonia Ho, Emma Thomson
EPI_ISL_703982, EPI_ISL_704070	Quadram Institute Bioscience	COVID-19 Genomics UK (COG-UK) Consortium	Dave J. Baker, Gemma L. Kay, Alp Aydin, Thanh Le-Viet, Steven Rudder, Ana P. Tedim, Anastasia Kolyva, Maria Diaz, Leonardo de Oliveira Martins, Nabil-Fareed Alikhan, Lizzie Meadows, Rachael Stanley, Ngozi Elumogo, Muhammed Yasir, Nicholas M. Thomson, Alexander J Trotter, Rachel Gilroy, Samuel Bloomfield, Claire Stuart, Andrew Bell, Reenesh Prakash, Samir Dervisevic, Alison E. Mather, John Wain, Mark Webber, Andrew J. Page, Justin O'Grady
EPI_ISL_704105	University College London Hospital	COVID-19 Genomics UK (COG-UK) Consortium	Judith Heaney, Matthew Byott, Catherine Houlihan, Dan Frampton, Stuart Kirk, Moira Spyer and Eleni Nastouli
EPI_ISL_704142, EPI_ISL_704145, EPI_ISL_704148, EPI_ISL_704176, EPI_ISL_704217, EPI_ISL_704233, EPI_ISL_704238	Quadram Institute Bioscience	COVID-19 Genomics UK (COG-UK) Consortium	Dave J. Baker, Gemma L. Kay, Alp Aydin, Thanh Le-Viet, Steven Rudder, Ana P. Tedim, Anastasia Kolyva, Maria Diaz, Leonardo de Oliveira Martins, Nabil-Fareed Alikhan, Lizzie Meadows, Rachael Stanley, Ngozi Elumogo, Muhammed Yasir, Nicholas M. Thomson, Alexander J Trotter, Rachel Gilroy, Samuel Bloomfield, Claire Stuart, Andrew Bell, Reenesh Prakash, Samir Dervisevic, Alison E. Mather, John Wain, Mark Webber, Andrew J. Page, Justin O'Grady
EPI_ISL_704596, EPI_ISL_704597	West of Scotland Specialist Virology Centre, NHSGGC / MRC-University of Glasgow Centre for Virus Research	COVID-19 Genomics UK (COG-UK) Consortium	Ana da Silva Filipe, Natasha Johnson, Kathy Smollett, Daniel Mair, Stephen Carmichael, Alice Broos, Lily Tong, Jenna Nichols, Kyriaki Nomikou; Sarah McDonald; Richard Orton, Joseph Hughes, Sreenu Vattipally, David L Robertson; Alasdair MacLean, Rory Gunson; Sharif Shaaban, Matthew Holden; Rachel Blacow, Guy Mollett, Kathy Li, James Shepherd, Antonia Ho, Emma Thomson
EPI_ISL_704608	University College London Hospital	COVID-19 Genomics UK (COG-UK) Consortium	Judith Heaney, Matthew Byott, Catherine Houlihan, Dan Frampton, Stuart Kirk, Moira Spyer and Eleni Nastouli
EPI_ISL_704609	Quadram Institute Bioscience	COVID-19 Genomics UK (COG-UK) Consortium	Dave J. Baker, Gemma L. Kay, Alp Aydin, Thanh Le-Viet, Steven Rudder, Ana P. Tedim, Anastasia Kolyva, Maria Diaz, Leonardo de Oliveira Martins, Nabil-Fareed Alikhan, Lizzie Meadows, Rachael Stanley, Ngozi Elumogo, Muhammed Yasir, Nicholas M. Thomson, Alexander J Trotter, Rachel Gilroy, Samuel Bloomfield, Claire Stuart, Andrew Bell, Reenesh Prakash, Samir Dervisevic, Alison E. Mather, John Wain, Mark Webber, Andrew J. Page, Justin O'Grady
EPI_ISL_704669, EPI_ISL_704674	University College London Hospital	COVID-19 Genomics UK (COG-UK) Consortium	Judith Heaney, Matthew Byott, Catherine Houlihan, Dan Frampton, Stuart Kirk, Moira Spyer and Eleni Nastouli
EPI_ISL_704709, EPI_ISL_704711, EPI_ISL_704714, EPI_ISL_704716, EPI_ISL_704728, EPI_ISL_704731, EPI_ISL_704734, EPI_ISL_704736, EPI_ISL_704739, EPI_ISL_704746, EPI_ISL_704788, EPI_ISL_704791, EPI_ISL_704794			
see above	Quadram Institute Bioscience	COVID-19 Genomics UK (COG-UK) Consortium	Dave J. Baker, Gemma L. Kay, Alp Aydin, Thanh Le-Viet, Steven Rudder, Ana P. Tedim, Anastasia Kolyva, Maria Diaz, Leonardo de Oliveira Martins, Nabil-Fareed Alikhan, Lizzie Meadows, Rachael Stanley, Ngozi Elumogo, Muhammed Yasir, Nicholas M. Thomson, Alexander J Trotter, Rachel Gilroy, Samuel Bloomfield, Claire Stuart, Andrew Bell, Reenesh Prakash, Samir Dervisevic, Alison E. Mather, John Wain, Mark Webber, Andrew J. Page, Justin O'Grady
EPI_ISL_704804, EPI_ISL_704807	West of Scotland Specialist Virology Centre, NHSGGC / MRC-University of Glasgow Centre for Virus Research	COVID-19 Genomics UK (COG-UK) Consortium	Ana da Silva Filipe, Natasha Johnson, Kathy Smollett, Daniel Mair, Stephen Carmichael, Alice Broos, Lily Tong, Jenna Nichols, Kyriaki Nomikou; Sarah McDonald; Richard Orton, Joseph Hughes, Sreenu Vattipally, David L Robertson; Alasdair MacLean, Rory Gunson; Sharif Shaaban, Matthew Holden; Rachel Blacow, Guy Mollett, Kathy Li, James Shepherd, Antonia Ho, Emma Thomson
EPI_ISL_704850, EPI_ISL_704853, EPI_ISL_704887, EPI_ISL_704890, EPI_ISL_704923, EPI_ISL_704926, EPI_ISL_704929	Quadram Institute Bioscience	COVID-19 Genomics UK (COG-UK) Consortium	Dave J. Baker, Gemma L. Kay, Alp Aydin, Thanh Le-Viet, Steven Rudder, Ana P. Tedim, Anastasia Kolyva, Maria Diaz, Leonardo de Oliveira Martins, Nabil-Fareed Alikhan, Lizzie Meadows, Rachael Stanley, Ngozi Elumogo, Muhammed Yasir, Nicholas M. Thomson, Alexander J Trotter, Rachel Gilroy, Samuel Bloomfield, Claire Stuart, Andrew Bell, Reenesh Prakash, Samir Dervisevic, Alison E. Mather, John Wain, Mark Webber, Andrew J. Page, Justin O'Grady
EPI_ISL_704963	University College London, Great Ormond Street Hospital for Children NHS Foundation Trust, Imperial College Healthcare NHS Trust	COVID-19 Genomics UK (COG-UK) Consortium	Sergi Castellano, Rachel Williams, Mark Kristiansen, Paola Resende Silva, Sunando Roy, Tony Brooks, Helena Tutill, Paola Niola, Patricia Dyal, Charlotte Williams, Leysa Forrest, Yasmin Panchbhaya, Jacqueline Findlay, Samuel Weeks, Julianne Brown, Kathryn Harris, Paul Randell, James Price, Alison Holmes, Judith Breuer

EPI_ISL_705002	West of Scotland Specialist Virology Centre, NHSGGC / MRC-University of Glasgow Centre for Virus Research	COVID-19 Genomics UK (COG-UK) Consortium	Ana da Silva Filipe, Natasha Johnson, Kathy Smollett, Daniel Mair, Stephen Carmichael, Alice Broos, Lily Tong, Jenna Nichols, Kyriaki Nomikou; Sarah McDonald; Richard Orton, Joseph Hughes, Sreenu Vattipally, David L Robertson; Alasdair MacLean, Rory Gunson; Sharif Shaaban, Matthew Holden; Rachel Blacow, Guy Mollett, Kathy Li, James Shepherd, Antonia Ho, Emma Thomson
EPI_ISL_705091	Quadram Institute Bioscience	COVID-19 Genomics UK (COG-UK) Consortium	Dave J. Baker, Gemma L. Kay, Alp Aydin, Thanh Le-Viet, Steven Rudder, Ana P. Tedim, Anastasia Kolyva, Maria Diaz, Leonardo de Oliveira Martins, Nabil-Fareed Alikhan, Lizzie Meadows, Rachael Stanley, Ngozi Elumogo, Muhammed Yasir, Nicholas M. Thomson, Alexander J Trotter, Rachel Gilroy, Samuel Bloomfield, Claire Stuart, Andrew Bell, Reenesh Prakash, Samir Dervisevic, Alison E. Mather, John Wain, Mark Webber, Andrew J. Page, Justin O'Grady
EPI_ISL_705129, EPI_ISL_705220	Virology Department, Sheffield Teaching Hospitals NHS Foundation Trust/Department of Infection, Immunity and Cardiovascular Disease, The Medical School, University of Sheffield	COVID-19 Genomics UK (COG-UK) Consortium	Thushan de Silva, Matthew Parker, Nikki Smith, Adri Angyal, Rebecca Brown, Luke Green, Rachel Tucker, Paul Parsons, Danielle Groves, Katie Johnson, Laura Carrilero, Alex Keeley, Dave Partridge, Matthew Wyles, Benjamin Lindsey, Mehmet Yavuz, Mohammad Raza, Cariad Evans
EPI_ISL_705232	University College London, Great Ormond Street Hospital for Children NHS Foundation Trust, Imperial College Healthcare NHS Trust	COVID-19 Genomics UK (COG-UK) Consortium	Sergi Castellano, Rachel Williams, Mark Kristiansen, Paola Resende Silva, Sunando Roy, Tony Brooks, Helena Tutill, Paola Niola, Patricia Dyal, Charlotte Williams, Leysa Forrest, Yasmin Panchbhaya, Jacqueline Findlay, Samuel Weeks, Julianne Brown, Kathryn Harris, Paul Randell, James Price, Alison Holmes, Judith Breuer
EPI_ISL_705245	Liverpool Clinical Laboratories	COVID-19 Genomics UK (COG-UK) Consortium	Sam Haldenby, Anita Lucaci, Steve Paterson, Julian Hiscox, Alistair Darby, M Almsaud, A Alrezaihi, Muhannad Alruwaili, Stuart D Armstrong, Jones Benjamin, Eleanor G Bentley, Anu Chawla, Jordan J Clark, Angela Cowell, Richard Eccles, Isabel García-Dorival, Matthew Gemmell, Alessandro Gerada, PKF Gilmore, Richard Gregory, Ximeng Han, Catherine Hartley, Margaret Hughes, Miren Iturriza-Gomara, James Johnson, L Luu, Jenifer Manson, Charlotte Nelson, Elaine O'Toole, Cassie Olateju, Rebekah Penrice-Randal , Lucille Rainbow, N.P Randle, Trevor Ian Robinson, Parul Sharma, Ghada T Shawli, James P Stewart, Neil Swainston, Ecaterina Vamos, Joanne Watts, Mark Whitehead
EPI_ISL_705255, EPI_ISL_705256, EPI_ISL_705261	Quadram Institute Bioscience	COVID-19 Genomics UK (COG-UK) Consortium	Dave J. Baker, Gemma L. Kay, Alp Aydin, Thanh Le-Viet, Steven Rudder, Ana P. Tedim, Anastasia Kolyva, Maria Diaz, Leonardo de Oliveira Martins, Nabil-Fareed Alikhan, Lizzie Meadows, Rachael Stanley, Ngozi Elumogo, Muhammed Yasir, Nicholas M. Thomson, Alexander J Trotter, Rachel Gilroy, Samuel Bloomfield, Claire Stuart, Andrew Bell, Reenesh Prakash, Samir Dervisevic, Alison E. Mather, John Wain, Mark Webber, Andrew J. Page, Justin O'Grady
EPI_ISL_705285, EPI_ISL_705297	University College London, Great Ormond Street Hospital for Children NHS Foundation Trust, Imperial College Healthcare NHS Trust	COVID-19 Genomics UK (COG-UK) Consortium	Sergi Castellano, Rachel Williams, Mark Kristiansen, Paola Resende Silva, Sunando Roy, Tony Brooks, Helena Tutill, Paola Niola, Patricia Dyal, Charlotte Williams, Leysa Forrest, Yasmin Panchbhaya, Jacqueline Findlay, Samuel Weeks, Julianne Brown, Kathryn Harris, Paul Randell, James Price, Alison Holmes, Judith Breuer
EPI_ISL_705345, EPI_ISL_705394	Liverpool Clinical Laboratories	COVID-19 Genomics UK (COG-UK) Consortium	Sam Haldenby, Anita Lucaci, Steve Paterson, Julian Hiscox, Alistair Darby, M Almsaud, A Alrezaihi, Muhannad Alruwaili, Stuart D Armstrong, Jones Benjamin, Eleanor G Bentley, Anu Chawla, Jordan J Clark, Angela Cowell, Richard Eccles, Isabel García-Dorival, Matthew Gemmell, Alessandro Gerada, PKF Gilmore, Richard Gregory, Ximeng Han, Catherine Hartley, Margaret Hughes, Miren Iturriza-Gomara, James Johnson, L Luu, Jenifer Manson, Charlotte Nelson, Elaine O'Toole, Cassie Olateju, Rebekah Penrice-Randal , Lucille Rainbow, N.P Randle, Trevor Ian Robinson, Parul Sharma, Ghada T Shawli, James P Stewart, Neil Swainston, Ecaterina Vamos, Joanne Watts, Mark Whitehead
EPI_ISL_705431	Quadram Institute Bioscience	COVID-19 Genomics UK (COG-UK) Consortium	Dave J. Baker, Gemma L. Kay, Alp Aydin, Thanh Le-Viet, Steven Rudder, Ana P. Tedim, Anastasia Kolyva, Maria Diaz, Leonardo de Oliveira Martins, Nabil-Fareed Alikhan, Lizzie Meadows, Rachael Stanley, Ngozi Elumogo, Muhammed Yasir, Nicholas M. Thomson, Alexander J Trotter, Rachel Gilroy, Samuel Bloomfield, Claire Stuart, Andrew Bell, Reenesh Prakash, Samir Dervisevic, Alison E. Mather, John Wain, Mark Webber, Andrew J. Page, Justin O'Grady
EPI_ISL_705492	Virology Department, Royal Infirmary of Edinburgh, NHS Lothian / School of Biological Sciences, University of Edinburgh / Institute of Genetics and Molecular Medicine, University of Edinburgh	COVID-19 Genomics UK (COG-UK) Consortium	McHugh M, Dewar R, Rooke S, Gallagher M, Balcaza C, O'Toole Á, Scher E, Hill V, McCrone JT, Colquhoun R, Yu X, Jackson B, Rambaut A, Williams TC, Templeton K
EPI_ISL_705715, EPI_ISL_705717, EPI_ISL_705721, EPI_ISL_705723, EPI_ISL_705724, EPI_ISL_705725, EPI_ISL_705728	West of Scotland Specialist Virology Centre, NHSGGC / MRC-University of Glasgow Centre for Virus Research	COVID-19 Genomics UK (COG-UK) Consortium	Ana da Silva Filipe, Natasha Johnson, Kathy Smollett, Daniel Mair, Stephen Carmichael, Alice Broos, Lily Tong, Jenna Nichols, Kyriaki Nomikou; Sarah McDonald; Richard Orton, Joseph Hughes, Sreenu Vattipally, David L Robertson; Alasdair MacLean, Rory Gunson; Sharif Shaaban, Matthew Holden; Rachel Blacow, Guy Mollett, Kathy Li, James Shepherd, Antonia Ho, Emma Thomson
EPI_ISL_705777, EPI_ISL_705780, EPI_ISL_705781, EPI_ISL_705782, EPI_ISL_705783, EPI_ISL_705784, EPI_ISL_705785, EPI_ISL_705786, EPI_ISL_705787, EPI_ISL_705788, EPI_ISL_705789, EPI_ISL_705790, EPI_ISL_705791, EPI_ISL_705792			
see above	Virology Department, Royal Infirmary of Edinburgh, NHS Lothian / School of Biological Sciences, University of Edinburgh / Institute of Genetics and Molecular Medicine, University of Edinburgh	COVID-19 Genomics UK (COG-UK) Consortium	McHugh M, Dewar R, Rooke S, Gallagher M, Balcaza C, O'Toole Á, Scher E, Hill V, McCrone JT, Colquhoun R, Yu X, Jackson B, Rambaut A, Williams TC, Templeton K
EPI_ISL_705928	Liverpool Clinical Laboratories	COVID-19 Genomics UK (COG-UK) Consortium	Sam Haldenby, Anita Lucaci, Steve Paterson, Julian Hiscox, Alistair Darby, M Almsaud, A Alrezaihi, Muhannad Alruwaili, Stuart D Armstrong, Jones Benjamin, Eleanor G Bentley, Anu Chawla, Jordan J Clark, Angela Cowell, Richard Eccles, Isabel García-Dorival, Matthew Gemmell, Alessandro Gerada, PKF Gilmore, Richard Gregory, Ximeng Han, Catherine Hartley, Margaret Hughes, Miren Iturriza-Gomara, James Johnson, L Luu, Jenifer Manson, Charlotte Nelson, Elaine O'Toole, Cassie Olateju, Rebekah Penrice-Randal , Lucille Rainbow, N.P Randle, Trevor Ian Robinson, Parul Sharma, Ghada T Shawli, James P Stewart, Neil Swainston, Ecaterina Vamos, Joanne Watts, Mark Whitehead
EPI_ISL_705957, EPI_ISL_705959	University College London Hospital	COVID-19 Genomics UK (COG-UK) Consortium	Judith Heaney, Matthew Byott, Catherine Houlihan, Dan Frampton, Stuart Kirk, Moira Spyer and Eleni Nastouli
EPI_ISL_706357, EPI_ISL_706358, EPI_ISL_706360, EPI_ISL_706367, EPI_ISL_706368, EPI_ISL_706369, EPI_ISL_706370, EPI_ISL_706371, EPI_ISL_706372, EPI_ISL_706373, EPI_ISL_706374, EPI_ISL_706375, EPI_ISL_706376, EPI_ISL_706377, EPI_ISL_706378, EPI_ISL_706379, EPI_ISL_706381, EPI_ISL_706382, EPI_ISL_706397, EPI_ISL_706398, EPI_ISL_706399, EPI_ISL_706401, EPI_ISL_706403, EPI_ISL_706404, EPI_ISL_706405, EPI_ISL_706406, EPI_ISL_706407, EPI_ISL_706409, EPI_ISL_706410, EPI_ISL_706411, EPI_ISL_706413, EPI_ISL_706415, EPI_ISL_706416, EPI_ISL_706418, EPI_ISL_706419			
see above	Quadram Institute Bioscience	COVID-19 Genomics UK (COG-UK) Consortium	Dave J. Baker, Gemma L. Kay, Alp Aydin, Thanh Le-Viet, Steven Rudder, Ana P. Tedim, Anastasia Kolyva, Maria Diaz, Leonardo de Oliveira Martins, Nabil-Fareed Alikhan, Lizzie Meadows, Rachael Stanley, Ngozi Elumogo, Muhammed Yasir, Nicholas M. Thomson, Alexander J Trotter, Rachel Gilroy, Samuel Bloomfield, Claire Stuart, Andrew Bell, Reenesh Prakash, Samir Dervisevic, Alison E. Mather, John Wain, Mark Webber, Andrew J. Page, Justin O'Grady
EPI_ISL_706429, EPI_ISL_706430	Queens Medical Centre, Clinical Microbiology Department / DeepSeq Nottingham	COVID-19 Genomics UK (COG-UK) Consortium	Gemma Clark, Wendy Smith, Manjinder Khakh, Vicki M Fleming, Michelle M Lister, Hannah Howson-Wells, Jonathan Ball, Patrick McClure, Joseph Chappell, Theocharis Tsoleridis, Nadine Holmes, Matthew Carlisle, Christopher Moore, Fei Sang, Johnny Debebe, Victoria Wright, Matthew Loose
EPI_ISL_706975, EPI_ISL_706978, EPI_ISL_706990, EPI_ISL_707004	Virology Department, Sheffield Teaching Hospitals NHS Foundation Trust/Department of Infection, Immunity and Cardiovascular Disease, The Medical School, University of Sheffield	COVID-19 Genomics UK (COG-UK) Consortium	Thushan de Silva, Matthew Parker, Nikki Smith, Adri Angyal, Rebecca Brown, Luke Green, Rachel Tucker, Paul Parsons, Danielle Groves, Katie Johnson, Laura Carrilero, Alex Keeley, Dave Partridge, Matthew Wyles, Benjamin Lindsey, Mehmet Yavuz, Mohammad Raza, Cariad Evans
EPI_ISL_707708	Institute for Urban Disease Control and Prevention	COVID-19 Network Investigations (CONI) Alliance	Kamolthip Atsawawaranunt, Elizabeth Batty, Wasun Chantratita, Thanat Chookajorn, Stefan Fernandez, Angkana Huang, Anthony R. Jones, Khajohn Joonsalak, Prayuth Kaewmalang, Amornmas Kongklieng, Chonticha Klungtong, Theerarat Kochakarn, Namfon Kotanan, Krittikorn Kumpornsin, Wudtichai Manasatienkij, Anek Mungaomklang, Bhakbhoom Panthan, Ekawat Pasomsub, Pukkaporn Parnwijitkul, Kingkan Rakmanee, Insee Sensorn, Janjira Thaipadungpanit, Arporn Wangwiwatsin,Treewat Watthanachockchai
EPI_ISL_707795, EPI_ISL_707796, EPI_ISL_707797, EPI_ISL_707798	LabPLUS	Institute of Environmental Science and Research (ESR)	Xiaoyun Ren, Matt Storey, Nikki Freed, Muhammad Faisal, Jing Wang, Hermes Perez, Anja Werno, Antje van der Linden, Arlo Upton, Chris Mansell, David Hammer, Dragana Drinkovic, Gary McAuliffe, Hana Sofia Andersson, James Ussher, Jill Sherwood, Josh Freeman, Julia Howard, Juliet Elvy, Mary DeAlmeida, Matt Blakiston, Matthew Rogers, Max Bloomfield, Michael Addidle, Michelle Balm, Sally Roberts, Sarah Jefferies, Sharmini Muttaiyah, Susan Morpeth, Susan Taylor, Timothy Blackmore, Vani Sathyendran, Veronica Playle, Virginia Hope, Erasmus Smit, Lauren Jelly, Olin Silander, Joep de Ligt
EPI_ISL_708493, EPI_ISL_708494, EPI_ISL_708495, EPI_ISL_708496, EPI_ISL_708497, EPI_ISL_708498,	Minnesota Department of Health, Public Health Laboratory	Minnesota Department of Health, Public Health Laboratory	Alexandra Lorentz, Jacob Garfin, Matt Plumb, and Xiong Wang

EPI_ISL_708499, EPI_ISL_708503			
EPI_ISL_708793, EPI_ISL_708795	PathWest Laboratory Medicine WA	PathWest Laboratory Medicine WA Microbial Surveillance Unit	PathWest Laboratory Medicine WA Microbial Surveillance Unit
EPI_ISL_708844, EPI_ISL_708847, EPI_ISL_708848, EPI_ISL_708849, EPI_ISL_708850	Lighthouse Lab in Milton Keynes	Wellcome Sanger Institute for the COVID-19 Genomics UK (COG-UK) Consortium	The Lighthouse Lab in Milton Keynes and Alex Alderton, Roberto Amato, Sonia Goncalves, Ewan Harrison, David K. Jackson, Ian Johnston, Dominic Kwiatkowski, Cordelia Langford, John Sillitoe on behalf of the Wellcome Sanger Institute COVID-19 Surveillance Team
EPI_ISL_708851	Lighthouse Lab in Alderley Park	Wellcome Sanger Institute for the COVID-19 Genomics UK (COG-UK) Consortium	Jacquelyn Wynn, Mairead Hyland, The Lighthouse Lab in Alderley Park and Alex Alderton, Roberto Amato, Sonia Goncalves, Ewan Harrison, David K. Jackson, Ian Johnston, Dominic Kwiatkowski, Cordelia Langford, John Sillitoe on behalf of the Wellcome Sanger Institute COVID-19 Surveillance Team
EPI_ISL_708856, EPI_ISL_708857, EPI_ISL_708858, EPI_ISL_708862, EPI_ISL_708869, EPI_ISL_708871	Lighthouse Lab in Milton Keynes	Wellcome Sanger Institute for the COVID-19 Genomics UK (COG-UK) Consortium	The Lighthouse Lab in Milton Keynes and Alex Alderton, Roberto Amato, Sonia Goncalves, Ewan Harrison, David K. Jackson, Ian Johnston, Dominic Kwiatkowski, Cordelia Langford, John Sillitoe on behalf of the Wellcome Sanger Institute COVID-19 Surveillance Team
EPI_ISL_708873, EPI_ISL_708875	Lighthouse Lab in Alderley Park	Wellcome Sanger Institute for the COVID-19 Genomics UK (COG-UK) Consortium	Jacquelyn Wynn, Mairead Hyland, The Lighthouse Lab in Alderley Park and Alex Alderton, Roberto Amato, Sonia Goncalves, Ewan Harrison, David K. Jackson, Ian Johnston, Dominic Kwiatkowski, Cordelia Langford, John Sillitoe on behalf of the Wellcome Sanger Institute COVID-19 Surveillance Team
EPI_ISL_708879	Lighthouse Lab in Milton Keynes	Wellcome Sanger Institute for the COVID-19 Genomics UK (COG-UK) Consortium	The Lighthouse Lab in Milton Keynes and Alex Alderton, Roberto Amato, Sonia Goncalves, Ewan Harrison, David K. Jackson, Ian Johnston, Dominic Kwiatkowski, Cordelia Langford, John Sillitoe on behalf of the Wellcome Sanger Institute COVID-19 Surveillance Team
EPI_ISL_708880	Lighthouse Lab in Alderley Park	Wellcome Sanger Institute for the COVID-19 Genomics UK (COG-UK) Consortium	Jacquelyn Wynn, Mairead Hyland, The Lighthouse Lab in Alderley Park and Alex Alderton, Roberto Amato, Sonia Goncalves, Ewan Harrison, David K. Jackson, Ian Johnston, Dominic Kwiatkowski, Cordelia Langford, John Sillitoe on behalf of the Wellcome Sanger Institute COVID-19 Surveillance Team
EPI_ISL_708881, EPI_ISL_708883	Lighthouse Lab in Milton Keynes	Wellcome Sanger Institute for the COVID-19 Genomics UK (COG-UK) Consortium	The Lighthouse Lab in Milton Keynes and Alex Alderton, Roberto Amato, Sonia Goncalves, Ewan Harrison, David K. Jackson, Ian Johnston, Dominic Kwiatkowski, Cordelia Langford, John Sillitoe on behalf of the Wellcome Sanger Institute COVID-19 Surveillance Team
EPI_ISL_708885, EPI_ISL_708886	Lighthouse Lab in Alderley Park	Wellcome Sanger Institute for the COVID-19 Genomics UK (COG-UK) Consortium	Jacquelyn Wynn, Mairead Hyland, The Lighthouse Lab in Alderley Park and Alex Alderton, Roberto Amato, Sonia Goncalves, Ewan Harrison, David K. Jackson, Ian Johnston, Dominic Kwiatkowski, Cordelia Langford, John Sillitoe on behalf of the Wellcome Sanger Institute COVID-19 Surveillance Team
EPI_ISL_708889	Lighthouse Lab in Milton Keynes	Wellcome Sanger Institute for the COVID-19 Genomics UK (COG-UK) Consortium	The Lighthouse Lab in Milton Keynes and Alex Alderton, Roberto Amato, Sonia Goncalves, Ewan Harrison, David K. Jackson, Ian Johnston, Dominic Kwiatkowski, Cordelia Langford, John Sillitoe on behalf of the Wellcome Sanger Institute COVID-19 Surveillance Team
EPI_ISL_708893	Lighthouse Lab in Glasgow	Wellcome Sanger Institute for the COVID-19 Genomics UK (COG-UK) Consortium	Harper VanSteenhouse, Yumi Kasai, David Gray, Carol Clugston, Anna Dominiczak and Alex Alderton, Roberto Amato, Sonia Goncalves, Ewan Harrison, David K. Jackson, Ian Johnston, Dominic Kwiatkowski, Cordelia Langford, John Sillitoe on behalf of the Wellcome Sanger Institute COVID-19 Surveillance Team
EPI_ISL_708894	Lighthouse Lab in Alderley Park	Wellcome Sanger Institute for the COVID-19 Genomics UK (COG-UK) Consortium	Jacquelyn Wynn, Mairead Hyland, The Lighthouse Lab in Alderley Park and Alex Alderton, Roberto Amato, Sonia Goncalves, Ewan Harrison, David K. Jackson, Ian Johnston, Dominic Kwiatkowski, Cordelia Langford, John Sillitoe on behalf of the Wellcome Sanger Institute COVID-19 Surveillance Team
EPI_ISL_708896, EPI_ISL_708897, EPI_ISL_708898	Lighthouse Lab in Milton Keynes	Wellcome Sanger Institute for the COVID-19 Genomics UK (COG-UK) Consortium	The Lighthouse Lab in Milton Keynes and Alex Alderton, Roberto Amato, Sonia Goncalves, Ewan Harrison, David K. Jackson, Ian Johnston, Dominic Kwiatkowski, Cordelia Langford, John Sillitoe on behalf of the Wellcome Sanger Institute COVID-19 Surveillance Team
EPI_ISL_708899	Lighthouse Lab in Alderley Park	Wellcome Sanger Institute for the COVID-19 Genomics UK (COG-UK) Consortium	Jacquelyn Wynn, Mairead Hyland, The Lighthouse Lab in Alderley Park and Alex Alderton, Roberto Amato, Sonia Goncalves, Ewan Harrison, David K. Jackson, Ian Johnston, Dominic Kwiatkowski, Cordelia Langford, John Sillitoe on behalf of the Wellcome Sanger Institute COVID-19 Surveillance Team
EPI_ISL_708900, EPI_ISL_708901, EPI_ISL_708908, EPI_ISL_708912, EPI_ISL_708913	Lighthouse Lab in Milton Keynes	Wellcome Sanger Institute for the COVID-19 Genomics UK (COG-UK) Consortium	The Lighthouse Lab in Milton Keynes and Alex Alderton, Roberto Amato, Sonia Goncalves, Ewan Harrison, David K. Jackson, Ian Johnston, Dominic Kwiatkowski, Cordelia Langford, John Sillitoe on behalf of the Wellcome Sanger Institute COVID-19 Surveillance Team
EPI_ISL_708915	Lighthouse Lab in Alderley Park	Wellcome Sanger Institute for the COVID-19 Genomics UK (COG-UK) Consortium	Jacquelyn Wynn, Mairead Hyland, The Lighthouse Lab in Alderley Park and Alex Alderton, Roberto Amato, Sonia Goncalves, Ewan Harrison, David K. Jackson, Ian Johnston, Dominic Kwiatkowski, Cordelia Langford, John Sillitoe on behalf of the Wellcome Sanger Institute COVID-19 Surveillance Team
EPI_ISL_708916	Lighthouse Lab in Milton Keynes	Wellcome Sanger Institute for the COVID-19 Genomics UK (COG-UK) Consortium	The Lighthouse Lab in Milton Keynes and Alex Alderton, Roberto Amato, Sonia Goncalves, Ewan Harrison, David K. Jackson, Ian Johnston, Dominic Kwiatkowski, Cordelia Langford, John Sillitoe on behalf of the Wellcome Sanger Institute COVID-19 Surveillance Team
EPI_ISL_708918	Lighthouse Lab in Alderley Park	Wellcome Sanger Institute for the COVID-19 Genomics UK (COG-UK) Consortium	Jacquelyn Wynn, Mairead Hyland, The Lighthouse Lab in Alderley Park and Alex Alderton, Roberto Amato, Sonia Goncalves, Ewan Harrison, David K. Jackson, Ian Johnston, Dominic Kwiatkowski, Cordelia Langford, John Sillitoe on behalf of the Wellcome Sanger Institute COVID-19 Surveillance Team
EPI_ISL_708920	Lighthouse Lab in Milton Keynes	Wellcome Sanger Institute for the COVID-19 Genomics UK (COG-UK) Consortium	The Lighthouse Lab in Milton Keynes and Alex Alderton, Roberto Amato, Sonia Goncalves, Ewan Harrison, David K. Jackson, Ian Johnston, Dominic Kwiatkowski, Cordelia Langford, John Sillitoe on behalf of the Wellcome Sanger Institute COVID-19 Surveillance Team
EPI_ISL_708921, EPI_ISL_708925	Lighthouse Lab in Glasgow	Wellcome Sanger Institute for the COVID-19 Genomics UK (COG-UK) Consortium	Harper VanSteenhouse, Yumi Kasai, David Gray, Carol Clugston, Anna Dominiczak and Alex Alderton, Roberto Amato, Sonia Goncalves, Ewan Harrison, David K. Jackson, Ian Johnston, Dominic Kwiatkowski, Cordelia Langford, John Sillitoe on behalf of the Wellcome Sanger Institute COVID-19 Surveillance Team
EPI_ISL_708926, EPI_ISL_708927	Lighthouse Lab in Alderley Park	Wellcome Sanger Institute for the COVID-19 Genomics UK (COG-UK) Consortium	Jacquelyn Wynn, Mairead Hyland, The Lighthouse Lab in Alderley Park and Alex Alderton, Roberto Amato, Sonia Goncalves, Ewan Harrison, David K. Jackson, Ian Johnston, Dominic Kwiatkowski, Cordelia Langford, John Sillitoe on behalf of the Wellcome Sanger Institute COVID-19 Surveillance Team
EPI_ISL_708928, EPI_ISL_708930	Lighthouse Lab in Milton Keynes	Wellcome Sanger Institute for the COVID-19 Genomics UK (COG-UK) Consortium	The Lighthouse Lab in Milton Keynes and Alex Alderton, Roberto Amato, Sonia Goncalves, Ewan Harrison, David K. Jackson, Ian Johnston, Dominic Kwiatkowski, Cordelia Langford, John Sillitoe on behalf of the Wellcome Sanger Institute COVID-19 Surveillance Team
EPI_ISL_708934, EPI_ISL_708936, EPI_ISL_708938, EPI_ISL_708939	Lighthouse Lab in Alderley Park	Wellcome Sanger Institute for the COVID-19 Genomics UK (COG-UK) Consortium	Jacquelyn Wynn, Mairead Hyland, The Lighthouse Lab in Alderley Park and Alex Alderton, Roberto Amato, Sonia Goncalves, Ewan Harrison, David K. Jackson, Ian Johnston, Dominic Kwiatkowski, Cordelia Langford, John Sillitoe on behalf of the Wellcome Sanger Institute COVID-19 Surveillance Team
EPI_ISL_708941, EPI_ISL_708945, EPI_ISL_708948, EPI_ISL_708951	Lighthouse Lab in Milton Keynes	Wellcome Sanger Institute for the COVID-19 Genomics UK (COG-UK) Consortium	The Lighthouse Lab in Milton Keynes and Alex Alderton, Roberto Amato, Sonia Goncalves, Ewan Harrison, David K. Jackson, Ian Johnston, Dominic Kwiatkowski, Cordelia Langford, John Sillitoe on behalf of the Wellcome Sanger Institute COVID-19 Surveillance Team
EPI_ISL_708952	Lighthouse Lab in Alderley Park	Wellcome Sanger Institute for the COVID-19 Genomics UK (COG-UK) Consortium	Jacquelyn Wynn, Mairead Hyland, The Lighthouse Lab in Alderley Park and Alex Alderton, Roberto Amato, Sonia Goncalves, Ewan Harrison, David K. Jackson, Ian Johnston, Dominic Kwiatkowski, Cordelia Langford, John Sillitoe on behalf of the Wellcome Sanger Institute COVID-19 Surveillance Team
EPI_ISL_708958, EPI_ISL_708963, EPI_ISL_708964	Lighthouse Lab in Milton Keynes	Wellcome Sanger Institute for the COVID-19 Genomics UK (COG-UK) Consortium	The Lighthouse Lab in Milton Keynes and Alex Alderton, Roberto Amato, Sonia Goncalves, Ewan Harrison, David K. Jackson, Ian Johnston, Dominic Kwiatkowski, Cordelia Langford, John Sillitoe on behalf of the Wellcome Sanger Institute COVID-19 Surveillance Team
EPI_ISL_708967	Lighthouse Lab in Alderley Park	Wellcome Sanger Institute for the COVID-19 Genomics UK (COG-UK) Consortium	Jacquelyn Wynn, Mairead Hyland, The Lighthouse Lab in Alderley Park and Alex Alderton, Roberto Amato, Sonia Goncalves, Ewan Harrison, David K. Jackson, Ian Johnston, Dominic Kwiatkowski, Cordelia Langford, John Sillitoe on behalf of the Wellcome Sanger Institute COVID-19 Surveillance Team
EPI_ISL_708969	Lighthouse Lab in Milton Keynes	Wellcome Sanger Institute for the COVID-19 Genomics UK (COG-UK) Consortium	The Lighthouse Lab in Milton Keynes and Alex Alderton, Roberto Amato, Sonia Goncalves, Ewan Harrison, David K. Jackson, Ian Johnston, Dominic Kwiatkowski, Cordelia Langford, John Sillitoe on behalf of the Wellcome Sanger Institute COVID-19 Surveillance Team
EPI_ISL_708970, EPI_ISL_708971	Lighthouse Lab in Alderley Park	Wellcome Sanger Institute for the COVID-19 Genomics UK (COG-UK) Consortium	Jacquelyn Wynn, Mairead Hyland, The Lighthouse Lab in Alderley Park and Alex Alderton, Roberto Amato, Sonia Goncalves, Ewan Harrison, David K. Jackson, Ian Johnston, Dominic Kwiatkowski, Cordelia Langford, John Sillitoe on behalf of the Wellcome Sanger Institute COVID-19 Surveillance Team
EPI_ISL_708975, EPI_ISL_708979	Lighthouse Lab in Milton Keynes	Wellcome Sanger Institute for the COVID-19 Genomics UK (COG-UK) Consortium	The Lighthouse Lab in Milton Keynes and Alex Alderton, Roberto Amato, Sonia Goncalves, Ewan Harrison, David K. Jackson, Ian Johnston, Dominic Kwiatkowski, Cordelia Langford, John Sillitoe on behalf of the Wellcome Sanger Institute COVID-19 Surveillance Team
EPI_ISL_708981	Lighthouse Lab in Alderley Park	Wellcome Sanger Institute for the COVID-19 Genomics UK (COG-UK) Consortium	Jacquelyn Wynn, Mairead Hyland, The Lighthouse Lab in Alderley Park and Alex Alderton, Roberto Amato, Sonia Goncalves, Ewan Harrison, David K. Jackson, Ian Johnston, Dominic Kwiatkowski, Cordelia Langford, John Sillitoe on behalf of the Wellcome Sanger Institute COVID-19 Surveillance Team
EPI_ISL_708983, EPI_ISL_708984	Lighthouse Lab in Milton Keynes	Wellcome Sanger Institute for the COVID-19 Genomics UK (COG-UK) Consortium	The Lighthouse Lab in Milton Keynes and Alex Alderton, Roberto Amato, Sonia Goncalves, Ewan Harrison, David K. Jackson, Ian Johnston, Dominic Kwiatkowski, Cordelia Langford, John Sillitoe on behalf of the Wellcome Sanger Institute COVID-19 Surveillance Team
EPI_ISL_708985	Lighthouse Lab in Glasgow	Wellcome Sanger Institute for the COVID-19 Genomics UK (COG-UK) Consortium	Harper VanSteenhouse, Yumi Kasai, David Gray, Carol Clugston, Anna Dominiczak and Alex Alderton, Roberto Amato, Sonia Goncalves, Ewan Harrison, David K. Jackson, Ian Johnston, Dominic Kwiatkowski, Cordelia Langford, John Sillitoe on behalf of the Wellcome Sanger Institute COVID-19 Surveillance

			Team
EPI_ISL_708986	Lighthouse Lab in Milton Keynes	Wellcome Sanger Institute for the COVID-19 Genomics UK (COG-UK) Consortium	The Lighthouse Lab in Milton Keynes and Alex Alderton, Roberto Amato, Sonia Goncalves, Ewan Harrison, David K. Jackson, Ian Johnston, Dominic Kwiatkowski, Cordelia Langford, John Sillitoe on behalf of the Wellcome Sanger Institute COVID-19 Surveillance Team
EPI_ISL_709480	Lighthouse Lab in Alderley Park	Wellcome Sanger Institute for the COVID-19 Genomics UK (COG-UK) Consortium	Jacquelyn Wynn, Mairead Hyland, The Lighthouse Lab in Alderley Park and Alex Alderton, Roberto Amato, Sonia Goncalves, Ewan Harrison, David K. Jackson, Ian Johnston, Dominic Kwiatkowski, Cordelia Langford, John Sillitoe on behalf of the Wellcome Sanger Institute COVID-19 Surveillance Team
EPI_ISL_709800	Lighthouse Lab in Glasgow	Wellcome Sanger Institute for the COVID-19 Genomics UK (COG-UK) Consortium	Harper VanSteenhouse, Yumi Kasai, David Gray, Carol Clugston, Anna Dominiczak and Alex Alderton, Roberto Amato, Sonia Goncalves, Ewan Harrison, David K. Jackson, Ian Johnston, Dominic Kwiatkowski, Cordelia Langford, John Sillitoe on behalf of the Wellcome Sanger Institute COVID-19 Surveillance Team
EPI_ISL_709804	Lighthouse Lab in Cambridge	Wellcome Sanger Institute for the COVID-19 Genomics UK (COG-UK) Consortium	Rob Howes, The Lighthouse Lab in Cambridge and Alex Alderton, Roberto Amato, Sonia Goncalves, Ewan Harrison, David K. Jackson, Ian Johnston, Dominic Kwiatkowski, Cordelia Langford, John Sillitoe on behalf of the Wellcome Sanger Institute COVID-19 Surveillance Team
EPI_ISL_709806, EPI_ISL_709809, EPI_ISL_709811, EPI_ISL_709812, EPI_ISL_709816, EPI_ISL_709818, EPI_ISL_709820	Lighthouse Lab in Glasgow	Wellcome Sanger Institute for the COVID-19 Genomics UK (COG-UK) Consortium	Harper VanSteenhouse, Yumi Kasai, David Gray, Carol Clugston, Anna Dominiczak and Alex Alderton, Roberto Amato, Sonia Goncalves, Ewan Harrison, David K. Jackson, Ian Johnston, Dominic Kwiatkowski, Cordelia Langford, John Sillitoe on behalf of the Wellcome Sanger Institute COVID-19 Surveillance Team
EPI_ISL_709821	Lighthouse Lab in Cambridge	Wellcome Sanger Institute for the COVID-19 Genomics UK (COG-UK) Consortium	Rob Howes, The Lighthouse Lab in Cambridge and Alex Alderton, Roberto Amato, Sonia Goncalves, Ewan Harrison, David K. Jackson, Ian Johnston, Dominic Kwiatkowski, Cordelia Langford, John Sillitoe on behalf of the Wellcome Sanger Institute COVID-19 Surveillance Team
EPI_ISL_709826	Lighthouse Lab in Glasgow	Wellcome Sanger Institute for the COVID-19 Genomics UK (COG-UK) Consortium	Harper VanSteenhouse, Yumi Kasai, David Gray, Carol Clugston, Anna Dominiczak and Alex Alderton, Roberto Amato, Sonia Goncalves, Ewan Harrison, David K. Jackson, Ian Johnston, Dominic Kwiatkowski, Cordelia Langford, John Sillitoe on behalf of the Wellcome Sanger Institute COVID-19 Surveillance Team
EPI_ISL_709827	Lighthouse Lab in Cambridge	Wellcome Sanger Institute for the COVID-19 Genomics UK (COG-UK) Consortium	Rob Howes, The Lighthouse Lab in Cambridge and Alex Alderton, Roberto Amato, Sonia Goncalves, Ewan Harrison, David K. Jackson, Ian Johnston, Dominic Kwiatkowski, Cordelia Langford, John Sillitoe on behalf of the Wellcome Sanger Institute COVID-19 Surveillance Team
EPI_ISL_709831, EPI_ISL_709832, EPI_ISL_709834, EPI_ISL_709835	Lighthouse Lab in Glasgow	Wellcome Sanger Institute for the COVID-19 Genomics UK (COG-UK) Consortium	Harper VanSteenhouse, Yumi Kasai, David Gray, Carol Clugston, Anna Dominiczak and Alex Alderton, Roberto Amato, Sonia Goncalves, Ewan Harrison, David K. Jackson, Ian Johnston, Dominic Kwiatkowski, Cordelia Langford, John Sillitoe on behalf of the Wellcome Sanger Institute COVID-19 Surveillance Team
EPI_ISL_709836	Lighthouse Lab in Cambridge	Wellcome Sanger Institute for the COVID-19 Genomics UK (COG-UK) Consortium	Rob Howes, The Lighthouse Lab in Cambridge and Alex Alderton, Roberto Amato, Sonia Goncalves, Ewan Harrison, David K. Jackson, Ian Johnston, Dominic Kwiatkowski, Cordelia Langford, John Sillitoe on behalf of the Wellcome Sanger Institute COVID-19 Surveillance Team
EPI_ISL_709840, EPI_ISL_709845, EPI_ISL_709848, EPI_ISL_709849, EPI_ISL_709851	Lighthouse Lab in Glasgow	Wellcome Sanger Institute for the COVID-19 Genomics UK (COG-UK) Consortium	Harper VanSteenhouse, Yumi Kasai, David Gray, Carol Clugston, Anna Dominiczak and Alex Alderton, Roberto Amato, Sonia Goncalves, Ewan Harrison, David K. Jackson, Ian Johnston, Dominic Kwiatkowski, Cordelia Langford, John Sillitoe on behalf of the Wellcome Sanger Institute COVID-19 Surveillance Team
EPI_ISL_709854	Lighthouse Lab in Cambridge	Wellcome Sanger Institute for the COVID-19 Genomics UK (COG-UK) Consortium	Rob Howes, The Lighthouse Lab in Cambridge and Alex Alderton, Roberto Amato, Sonia Goncalves, Ewan Harrison, David K. Jackson, Ian Johnston, Dominic Kwiatkowski, Cordelia Langford, John Sillitoe on behalf of the Wellcome Sanger Institute COVID-19 Surveillance Team
EPI_ISL_709855, EPI_ISL_709857, EPI_ISL_709858, EPI_ISL_709859, EPI_ISL_709861, EPI_ISL_709862, EPI_ISL_709875	Lighthouse Lab in Glasgow	Wellcome Sanger Institute for the COVID-19 Genomics UK (COG-UK) Consortium	Harper VanSteenhouse, Yumi Kasai, David Gray, Carol Clugston, Anna Dominiczak and Alex Alderton, Roberto Amato, Sonia Goncalves, Ewan Harrison, David K. Jackson, Ian Johnston, Dominic Kwiatkowski, Cordelia Langford, John Sillitoe on behalf of the Wellcome Sanger Institute COVID-19 Surveillance Team
EPI_ISL_709948	Microbiology, Department of Pathology, St. Bernard's Hospital, Gibraltar Health Authority	Respiratory Virus Unit, National Infection Service, Public Health England	PHE Covid Sequencing Team, Dr Nicholas Cortes (Gibraltar), Charlotte Gillborn-Jones (Gibraltar)
EPI_ISL_709949	Respiratory Virus Unit, National Infection Service, Public Health England	COVID-19 Genomics UK (COG-UK) Consortium	PHE Covid Sequencing Team
EPI_ISL_709950, EPI_ISL_709951, EPI_ISL_709952, EPI_ISL_709953, EPI_ISL_709954, EPI_ISL_709955, EPI_ISL_709956, EPI_ISL_709957	Microbiology, Department of Pathology, St. Bernard's Hospital, Gibraltar Health Authority	Respiratory Virus Unit, National Infection Service, Public Health England	PHE Covid Sequencing Team, Dr Nicholas Cortes (Gibraltar), Charlotte Gillborn-Jones (Gibraltar)
EPI_ISL_709958	Respiratory Virus Unit, National Infection Service, Public Health England	COVID-19 Genomics UK (COG-UK) Consortium	PHE Covid Sequencing Team
EPI_ISL_709959, EPI_ISL_709960, EPI_ISL_709961, EPI_ISL_709962, EPI_ISL_709963, EPI_ISL_709964, EPI_ISL_709965, EPI_ISL_709966, EPI_ISL_709967, EPI_ISL_709968, EPI_ISL_709969, EPI_ISL_709970, EPI_ISL_709971, EPI_ISL_709972, EPI_ISL_709973, EPI_ISL_709974, EPI_ISL_709975, EPI_ISL_709976, EPI_ISL_709977, EPI_ISL_709978, EPI_ISL_709979, EPI_ISL_709980, EPI_ISL_709981, EPI_ISL_709982, EPI_ISL_709983, EPI_ISL_709984, EPI_ISL_709985, EPI_ISL_709986, EPI_ISL_709987, EPI_ISL_709988, EPI_ISL_709989, EPI_ISL_709990, EPI_ISL_709991, EPI_ISL_709992, EPI_ISL_709993, EPI_ISL_709994, EPI_ISL_709995, EPI_ISL_709996, EPI_ISL_709997, EPI_ISL_709998, EPI_ISL_709999, EPI_ISL_710000, EPI_ISL_710001, EPI_ISL_710002, EPI_ISL_710003, EPI_ISL_710004, EPI_ISL_710005, EPI_ISL_710006, EPI_ISL_710007, EPI_ISL_710008, EPI_ISL_710009, EPI_ISL_710010, EPI_ISL_710011, EPI_ISL_710012, EPI_ISL_710013, EPI_ISL_710014, EPI_ISL_710015, EPI_ISL_710016, EPI_ISL_710017, EPI_ISL_710018, EPI_ISL_710019, EPI_ISL_710020, EPI_ISL_710021, EPI_ISL_710022, EPI_ISL_710023, EPI_ISL_710024, EPI_ISL_710025, EPI_ISL_710026, EPI_ISL_710027, EPI_ISL_710028, EPI_ISL_710029, EPI_ISL_710030, EPI_ISL_710031, EPI_ISL_710032, EPI_ISL_710033			
see above	Microbiology, Department of Pathology, St. Bernard's Hospital, Gibraltar Health Authority	Respiratory Virus Unit, National Infection Service, Public Health England	PHE Covid Sequencing Team, Dr Nicholas Cortes (Gibraltar), Charlotte Gillborn-Jones (Gibraltar)
EPI_ISL_710125, EPI_ISL_710127, EPI_ISL_710128	South Eastern Area Laboratory Services (SEALS)	CIDM-PH et al.	CIDM-PH et al.
EPI_ISL_710485, EPI_ISL_710486	Utah Public Health Laboratory, Utah Public Health Laboratory Infectious Disease submission group	Utah Public Health Laboratory, Utah Public Health Laboratory Infectious Disease submission group	Young,E.L., Oakeson,K.F.
EPI_ISL_710535, EPI_ISL_710536	Microbiology, Department of Pathology, St. Bernard's Hospital, Gibraltar Health Authority	Respiratory Virus Unit, National Infection Service, Public Health England	PHE Covid Sequencing Team, Dr Nicholas Cortes (Gibraltar), Charlotte Gillborn-Jones (Gibraltar)
EPI_ISL_710616, EPI_ISL_710617, EPI_ISL_710625, EPI_ISL_710628, EPI_ISL_710636, EPI_ISL_710645, EPI_ISL_710648	Lighthouse Lab in Alderley Park	Wellcome Sanger Institute for the COVID-19 Genomics UK (COG-UK) Consortium	Jacquelyn Wynn, Mairead Hyland, The Lighthouse Lab in Alderley Park and Alex Alderton, Roberto Amato, Sonia Goncalves, Ewan Harrison, David K. Jackson, Ian Johnston, Dominic Kwiatkowski, Cordelia Langford, John Sillitoe on behalf of the Wellcome Sanger Institute COVID-19 Surveillance Team
EPI_ISL_711060, EPI_ISL_711061, EPI_ISL_711062, EPI_ISL_711063, EPI_ISL_711064, EPI_ISL_711065, EPI_ISL_711066, EPI_ISL_711068, EPI_ISL_711069, EPI_ISL_711070, EPI_ISL_711071, EPI_ISL_711072, EPI_ISL_711073, EPI_ISL_711075, EPI_ISL_711079, EPI_ISL_711080, EPI_ISL_711081, EPI_ISL_711082, EPI_ISL_711083, EPI_ISL_711084, EPI_ISL_711085, EPI_ISL_711086, EPI_ISL_711087, EPI_ISL_711088, EPI_ISL_711089, EPI_ISL_711090, EPI_ISL_711091, EPI_ISL_711092, EPI_ISL_711093, EPI_ISL_711094, EPI_ISL_711095, EPI_ISL_711096, EPI_ISL_711097, EPI_ISL_711098, EPI_ISL_711099, EPI_ISL_711100, EPI_ISL_711101, EPI_ISL_711102, EPI_ISL_711103, EPI_ISL_711104, EPI_ISL_711105, EPI_ISL_711106, EPI_ISL_711107, EPI_ISL_711108, EPI_ISL_711109, EPI_ISL_711110, EPI_ISL_711111, EPI_ISL_711112, EPI_ISL_711113, EPI_ISL_711114, EPI_ISL_711115, EPI_ISL_711116, EPI_ISL_711117, EPI_ISL_711118, EPI_ISL_711119, EPI_ISL_711120, EPI_ISL_711121, EPI_ISL_711122, EPI_ISL_711123, EPI_ISL_711124, EPI_ISL_711125, EPI_ISL_711126, EPI_ISL_711127, EPI_ISL_711128, EPI_ISL_711129, EPI_ISL_711130, EPI_ISL_711131, EPI_ISL_711132, EPI_ISL_711133, EPI_ISL_711134, EPI_ISL_711135, EPI_ISL_711136, EPI_ISL_711137, EPI_ISL_711138, EPI_ISL_711139, EPI_ISL_711140, EPI_ISL_711141, EPI_ISL_711142, EPI_ISL_711143, EPI_ISL_711144, EPI_ISL_711145, EPI_ISL_711146, EPI_ISL_711147, EPI_ISL_711148, EPI_ISL_711176, EPI_ISL_711182, EPI_ISL_711183, EPI_ISL_711184, EPI_ISL_711186, EPI_ISL_711187, EPI_ISL_711188, EPI_ISL_711189, EPI_ISL_711193, EPI_ISL_711196, EPI_ISL_711197, EPI_ISL_711200, EPI_ISL_711202, EPI_ISL_711203, EPI_ISL_711204, EPI_ISL_711205, EPI_ISL_711206, EPI_ISL_711214, EPI_ISL_711215, EPI_ISL_711219, EPI_ISL_711221, EPI_ISL_711222, EPI_ISL_711223, EPI_ISL_711224, EPI_ISL_711225, EPI_ISL_711226, EPI_ISL_711227, EPI_ISL_711229, EPI_ISL_711230, EPI_ISL_711231, EPI_ISL_711233, EPI_ISL_711234, EPI_ISL_711235, EPI_ISL_711236, EPI_ISL_711237, EPI_ISL_711238, EPI_ISL_711239, EPI_ISL_711240, EPI_ISL_711241, EPI_ISL_711242, EPI_ISL_711243, EPI_ISL_711244, EPI_ISL_711245, EPI_ISL_711246, EPI_ISL_711247, EPI_ISL_711248, EPI_ISL_711249, EPI_ISL_711250, EPI_ISL_711251, EPI_ISL_711252, EPI_ISL_711253, EPI_ISL_711254, EPI_ISL_711255, EPI_ISL_711266, EPI_ISL_711267, EPI_ISL_711269, EPI_ISL_711270, EPI_ISL_711275, EPI_ISL_711278, EPI_ISL_711279, EPI_ISL_711280, EPI_ISL_711282, EPI_ISL_711283, EPI_ISL_711284, EPI_ISL_711285, EPI_ISL_711286, EPI_ISL_711287, EPI_ISL_711288, EPI_ISL_711289, EPI_ISL_711290, EPI_ISL_711291, EPI_ISL_711292, EPI_ISL_711293, EPI_ISL_711294, EPI_ISL_711299, EPI_ISL_711300, EPI_ISL_711301, EPI_ISL_711302, EPI_ISL_711303, EPI_ISL_711304, EPI_ISL_711305, EPI_ISL_711306, EPI_ISL_711307, EPI_ISL_711308, EPI_ISL_711309, EPI_ISL_711310, EPI_ISL_711311, EPI_ISL_711312, EPI_ISL_711313, EPI_ISL_711314, EPI_ISL_711315, EPI_ISL_711316, EPI_ISL_711317, EPI_ISL_711318, EPI_ISL_711319, EPI_ISL_711320, EPI_ISL_711321, EPI_ISL_711322, EPI_ISL_711323, EPI_ISL_711324, EPI_ISL_711325, EPI_ISL_711326, EPI_ISL_711327, EPI_ISL_711328, EPI_ISL_711329, EPI_ISL_711330, EPI_ISL_711331, EPI_ISL_711332, EPI_ISL_711333, EPI_ISL_711334, EPI_ISL_711335, EPI_ISL_711336, EPI_ISL_711337, EPI_ISL_711338, EPI_ISL_711339, EPI_ISL_711340, EPI_ISL_711341, EPI_ISL_711342, EPI_ISL_711343, EPI_ISL_711344, EPI_ISL_711345, EPI_ISL_711346, EPI_ISL_711347, EPI_ISL_711348, EPI_ISL_711349, EPI_ISL_711350, EPI_ISL_711351, EPI_ISL_711352, EPI_ISL_711353, EPI_ISL_711354, EPI_ISL_711355, EPI_ISL_711356, EPI_ISL_711357, EPI_ISL_711358, EPI_ISL_711359, EPI_ISL_711360, EPI_ISL_711384, EPI_ISL_711385, EPI_ISL_711386, EPI_ISL_711387, EPI_ISL_711388, EPI_ISL_711389, EPI_ISL_711393, EPI_ISL_711394, EPI_ISL_711398, EPI_ISL_711399, EPI_ISL_711400, EPI_ISL_711401, EPI_ISL_711402, EPI_ISL_711403,			

EPI_ISL_711404, EPI_ISL_711405, EPI_ISL_711406, EPI_ISL_711409, EPI_ISL_711410, EPI_ISL_711412, EPI_ISL_711413, EPI_ISL_711414, EPI_ISL_711415, EPI_ISL_711416, EPI_ISL_711417, EPI_ISL_711418, EPI_ISL_711419, EPI_ISL_711420, EPI_ISL_711421, EPI_ISL_711422, EPI_ISL_711423, EPI_ISL_711424, EPI_ISL_711425, EPI_ISL_711426, EPI_ISL_711427, EPI_ISL_711428, EPI_ISL_711431, EPI_ISL_711432, EPI_ISL_711433, EPI_ISL_711434, EPI_ISL_711435, EPI_ISL_711436, EPI_ISL_711437, EPI_ISL_711445, EPI_ISL_711446, EPI_ISL_711447, EPI_ISL_711448, EPI_ISL_711449, EPI_ISL_711450, EPI_ISL_711451, EPI_ISL_711452, EPI_ISL_711453, EPI_ISL_711454, EPI_ISL_711455, EPI_ISL_711456, EPI_ISL_711457, EPI_ISL_711458, EPI_ISL_711459, EPI_ISL_711460, EPI_ISL_711461, EPI_ISL_711462, EPI_ISL_711463, EPI_ISL_711464, EPI_ISL_711465, EPI_ISL_711466, EPI_ISL_711467, EPI_ISL_711468, EPI_ISL_711469, EPI_ISL_711470, EPI_ISL_711471, EPI_ISL_711472, EPI_ISL_711473, EPI_ISL_711474, EPI_ISL_711475, EPI_ISL_711476, EPI_ISL_711477, EPI_ISL_711478, EPI_ISL_711479, EPI_ISL_711480, EPI_ISL_711481, EPI_ISL_711482, EPI_ISL_711483, EPI_ISL_711484, EPI_ISL_711485, EPI_ISL_711486, EPI_ISL_711487, EPI_ISL_711488, EPI_ISL_711489, EPI_ISL_711490, EPI_ISL_711491, EPI_ISL_711492, EPI_ISL_711493, EPI_ISL_711494, EPI_ISL_711495, EPI_ISL_711496, EPI_ISL_711497, EPI_ISL_711498, EPI_ISL_711499, EPI_ISL_711500, EPI_ISL_711501, EPI_ISL_711502, EPI_ISL_711503, EPI_ISL_711504, EPI_ISL_711505, EPI_ISL_711506, EPI_ISL_711507, EPI_ISL_711508, EPI_ISL_711509, EPI_ISL_711510, EPI_ISL_711511, EPI_ISL_711512, EPI_ISL_711513, EPI_ISL_711514, EPI_ISL_711515, EPI_ISL_711516, EPI_ISL_711517, EPI_ISL_711518, EPI_ISL_711519, EPI_ISL_711520, EPI_ISL_711521, EPI_ISL_711522, EPI_ISL_711523, EPI_ISL_711524, EPI_ISL_711525, EPI_ISL_711526, EPI_ISL_711527, EPI_ISL_711528, EPI_ISL_711529, EPI_ISL_711530, EPI_ISL_711531, EPI_ISL_711532, EPI_ISL_711533, EPI_ISL_711534, EPI_ISL_711535, EPI_ISL_711536, EPI_ISL_711537, EPI_ISL_711538, EPI_ISL_711539, EPI_ISL_711540, EPI_ISL_711541, EPI_ISL_711542, EPI_ISL_711543, EPI_ISL_711544, EPI_ISL_711545, EPI_ISL_711546, EPI_ISL_711547, EPI_ISL_711548, EPI_ISL_711549, EPI_ISL_711550, EPI_ISL_711551, EPI_ISL_711552, EPI_ISL_711553, EPI_ISL_711554, EPI_ISL_711555, EPI_ISL_711556, EPI_ISL_711557, EPI_ISL_711558, EPI_ISL_711559, EPI_ISL_711560, EPI_ISL_711561, EPI_ISL_711562, EPI_ISL_711563, EPI_ISL_711564, EPI_ISL_711565, EPI_ISL_711566, EPI_ISL_711567, EPI_ISL_711568, EPI_ISL_711569, EPI_ISL_711570, EPI_ISL_711571, EPI_ISL_711572, EPI_ISL_711573, EPI_ISL_711574, EPI_ISL_711575, EPI_ISL_711576, EPI_ISL_711577, EPI_ISL_711578, EPI_ISL_711579, EPI_ISL_711580, EPI_ISL_711581, EPI_ISL_711582, EPI_ISL_711583, EPI_ISL_711584, EPI_ISL_711585, EPI_ISL_711586, EPI_ISL_711587, EPI_ISL_711588, EPI_ISL_711589, EPI_ISL_711590, EPI_ISL_711591, EPI_ISL_711592, EPI_ISL_711593, EPI_ISL_711594, EPI_ISL_711595, EPI_ISL_711596, EPI_ISL_711597, EPI_ISL_711598, EPI_ISL_711599, EPI_ISL_711600, EPI_ISL_711601, EPI_ISL_711602, EPI_ISL_711603, EPI_ISL_711604, EPI_ISL_711605, EPI_ISL_711606, EPI_ISL_711607, EPI_ISL_711608, EPI_ISL_711609, EPI_ISL_711610, EPI_ISL_711611, EPI_ISL_711612, EPI_ISL_711613, EPI_ISL_711614, EPI_ISL_711615, EPI_ISL_711616, EPI_ISL_711617, EPI_ISL_711618, EPI_ISL_711619, EPI_ISL_711620, EPI_ISL_711621, EPI_ISL_711622, EPI_ISL_711623, EPI_ISL_711624, EPI_ISL_711625, EPI_ISL_711626, EPI_ISL_711627, EPI_ISL_711628, EPI_ISL_711629, EPI_ISL_711630, EPI_ISL_711631, EPI_ISL_711632, EPI_ISL_711633, EPI_ISL_711634, EPI_ISL_711635, EPI_ISL_711636, EPI_ISL_711637, EPI_ISL_711638, EPI_ISL_711639, EPI_ISL_711640, EPI_ISL_711641, EPI_ISL_711642, EPI_ISL_711643, EPI_ISL_711644, EPI_ISL_711645, EPI_ISL_711646, EPI_ISL_711647, EPI_ISL_711648, EPI_ISL_711649, EPI_ISL_711650, EPI_ISL_711651, EPI_ISL_711652, EPI_ISL_711653, EPI_ISL_711654, EPI_ISL_711655, EPI_ISL_711656, EPI_ISL_711657, EPI_ISL_711658, EPI_ISL_711659, EPI_ISL_711660, EPI_ISL_711661, EPI_ISL_711662, EPI_ISL_711663, EPI_ISL_711664, EPI_ISL_711665, EPI_ISL_711666, EPI_ISL_711667, EPI_ISL_711668, EPI_ISL_711669, EPI_ISL_711670, EPI_ISL_711671, EPI_ISL_711672, EPI_ISL_711673, EPI_ISL_711674, EPI_ISL_711675, EPI_ISL_711676, EPI_ISL_711677, EPI_ISL_711678, EPI_ISL_711679, EPI_ISL_711680, EPI_ISL_711681, EPI_ISL_711682, EPI_ISL_711683, EPI_ISL_711684, EPI_ISL_711685, EPI_ISL_711686, EPI_ISL_711687, EPI_ISL_711688, EPI_ISL_711689, EPI_ISL_711690, EPI_ISL_711691, EPI_ISL_711692, EPI_ISL_711693, EPI_ISL_711694, EPI_ISL_711695, EPI_ISL_711696, EPI_ISL_711697, EPI_ISL_711698, EPI_ISL_711699, EPI_ISL_711700, EPI_ISL_711701, EPI_ISL_711702, EPI_ISL_711703, EPI_ISL_711704, EPI_ISL_711705, EPI_ISL_711706, EPI_ISL_711707, EPI_ISL_711708, EPI_ISL_711709, EPI_ISL_711710, EPI_ISL_711711, EPI_ISL_711712, EPI_ISL_711713, EPI_ISL_711714, EPI_ISL_711715, EPI_ISL_711716, EPI_ISL_711717, EPI_ISL_711718, EPI_ISL_711719, EPI_ISL_711720, EPI_ISL_711721, EPI_ISL_711722, EPI_ISL_711723, EPI_ISL_711724, EPI_ISL_711725, EPI_ISL_711726, EPI_ISL_711727, EPI_ISL_711728, EPI_ISL_711729, EPI_ISL_711730, EPI_ISL_711731, EPI_ISL_711732, EPI_ISL_711733, EPI_ISL_711734, EPI_ISL_711735, EPI_ISL_711736, EPI_ISL_711737, EPI_ISL_711738, EPI_ISL_711739, EPI_ISL_711740, EPI_ISL_711741, EPI_ISL_711742, EPI_ISL_711743, EPI_ISL_711744, EPI_ISL_711745, EPI_ISL_711746, EPI_ISL_711747, EPI_ISL_711748, EPI_ISL_711749, EPI_ISL_711750, EPI_ISL_711751, EPI_ISL_711752, EPI_ISL_711753, EPI_ISL_711754, EPI_ISL_711755, EPI_ISL_711756, EPI_ISL_711757, EPI_ISL_711758, EPI_ISL_711759, EPI_ISL_711760, EPI_ISL_711761, EPI_ISL_711762, EPI_ISL_711763, EPI_ISL_711764, EPI_ISL_711765, EPI_ISL_711766, EPI_ISL_711767, EPI_ISL_711768, EPI_ISL_711769, EPI_ISL_711770, EPI_ISL_711771, EPI_ISL_711772, EPI_ISL_711773, EPI_ISL_711774, EPI_ISL_711775, EPI_ISL_711776, EPI_ISL_711777, EPI_ISL_711778, EPI_ISL_711779, EPI_ISL_711780, EPI_ISL_711781, EPI_ISL_711782, EPI_ISL_711783, EPI_ISL_711784, EPI_ISL_711785, EPI_ISL_711786, EPI_ISL_711787, EPI_ISL_711788, EPI_ISL_711789, EPI_ISL_711790, EPI_ISL_711791, EPI_ISL_711792, EPI_ISL_711793, EPI_ISL_711794, EPI_ISL_711795, EPI_ISL_711796, EPI_ISL_711797, EPI_ISL_711798, EPI_ISL_711799, EPI_ISL_711800, EPI_ISL_711801, EPI_ISL_711802, EPI_ISL_711803, EPI_ISL_711804, EPI_ISL_711805, EPI_ISL_711806, EPI_ISL_711807, EPI_ISL_711808, EPI_ISL_711809, EPI_ISL_711810, EPI_ISL_711811, EPI_ISL_711812, EPI_ISL_711813, EPI_ISL_711814, EPI_ISL_711815, EPI_ISL_711816, EPI_ISL_711817, EPI_ISL_711818, EPI_ISL_711819, EPI_ISL_711820, EPI_ISL_711821, EPI_ISL_711822, EPI_ISL_711823, EPI_ISL_711824, EPI_ISL_711825, EPI_ISL_711826, EPI_ISL_711827, EPI_ISL_711828, EPI_ISL_711829, EPI_ISL_711830, EPI_ISL_711831, EPI_ISL_711832, EPI_ISL_711833, EPI_ISL_711834, EPI_ISL_711835, EPI_ISL_711836, EPI_ISL_711837, EPI_ISL_711838, EPI_ISL_711839, EPI_ISL_711840, EPI_ISL_711841, EPI_ISL_711842, EPI_ISL_711843, EPI_ISL_711844, EPI_ISL_711845, EPI_ISL_711846, EPI_ISL_711847, EPI_ISL_711848, EPI_ISL_711849, EPI_ISL_712097, EPI_ISL_712098, EPI_ISL_712101, EPI_ISL_712102, EPI_ISL_712103, EPI_ISL_712104, EPI_ISL_712105, EPI_ISL_712106, EPI_ISL_712107, EPI_ISL_712108, EPI_ISL_712109, EPI_ISL_712110, EPI_ISL_712111, EPI_ISL_712112, EPI_ISL_712113, EPI_ISL_712114, EPI_ISL_712115, EPI_ISL_712116, EPI_ISL_712117, EPI_ISL_712118, EPI_ISL_712129, EPI_ISL_712130, EPI_ISL_712131, EPI_ISL_712132, EPI_ISL_712133, EPI_ISL_712134, EPI_ISL_712135, EPI_ISL_712136, EPI_ISL_712137, EPI_ISL_712138, EPI_ISL_712139, EPI_ISL_712140, EPI_ISL_712141, EPI_ISL_712142, EPI_ISL_712143, EPI_ISL_712144, EPI_ISL_712145, EPI_ISL_712146, EPI_ISL_712147, EPI_ISL_712148, EPI_ISL_712149, EPI_ISL_712150, EPI_ISL_712151, EPI_ISL_712152, EPI_ISL_712153, EPI_ISL_712154, EPI_ISL_712155, EPI_ISL_712156, EPI_ISL_712157, EPI_ISL_712158, EPI_ISL_712159, EPI_ISL_712160, EPI_ISL_712161, EPI_ISL_712162, EPI_ISL_712163, EPI_ISL_712164, EPI_ISL_712165, EPI_ISL_712166, EPI_ISL_712167, EPI_ISL_712168, EPI_ISL_712169, EPI_ISL_712170, EPI_ISL_712171, EPI_ISL_712172, EPI_ISL_712173, EPI_ISL_712174, EPI_ISL_712175, EPI_ISL_712176, EPI_ISL_712177, EPI_ISL_712178, EPI_ISL_712179, EPI_ISL_712180, EPI_ISL_712181, EPI_ISL_712182, EPI_ISL_712183, EPI_ISL_712184, EPI_ISL_712185, EPI_ISL_712186, EPI_ISL_712187, EPI_ISL_712188, EPI_ISL_712189, EPI_ISL_712190, EPI_ISL_712191, EPI_ISL_712192, EPI_ISL_712193, EPI_ISL_712194, EPI_ISL_712195, EPI_ISL_712196, EPI_ISL_712197, EPI_ISL_712198, EPI_ISL_712199, EPI_ISL_712200, EPI_ISL_712201, EPI_ISL_712202, EPI_ISL_712203, EPI_ISL_712204, EPI_ISL_712205, EPI_ISL_712206, EPI_ISL_712207, EPI_ISL_712208, EPI_ISL_712209, EPI_ISL_712210, EPI_ISL_712211, EPI_ISL_712212, EPI_ISL_712213, EPI_ISL_712214, EPI_ISL_712215, EPI_ISL_712216, EPI_ISL_712217, EPI_ISL_712218, EPI_ISL_712219, EPI_ISL_712220, EPI_ISL_712221, EPI_ISL_712222, EPI_ISL_712223, EPI_ISL_712224, EPI_ISL_712225, EPI_ISL_712226, EPI_ISL_712227, EPI_ISL_712228, EPI_ISL_712229, EPI_ISL_712230, EPI_ISL_712231, EPI_ISL_712232, EPI_ISL_712233, EPI_ISL_712234, EPI_ISL_712235, EPI_ISL_712236, EPI_ISL_712237, EPI_ISL_712238, EPI_ISL_712239, EPI_ISL_712240, EPI_ISL_712241, EPI_ISL_712242, EPI_ISL_712243, EPI_ISL_712244, EPI_ISL_712245, EPI_ISL_712246, EPI_ISL_712247, EPI_ISL_712248, EPI_ISL_712249, EPI_ISL_712254, EPI_ISL_712255, EPI_ISL_712256, EPI_ISL_712257, EPI_ISL_712258, EPI_ISL_712259, EPI_ISL_712260, EPI_ISL_712261, EPI_ISL_712262, EPI_ISL_712263, EPI_ISL_712264, EPI_ISL_712265, EPI_ISL_712266, EPI_ISL_712267, EPI_ISL_712268, EPI_ISL_712269, EPI_ISL_712270, EPI_ISL_712271, EPI_ISL_712272, EPI_ISL_712273, EPI_ISL_712274, EPI_ISL_712275, EPI_ISL_712276, EPI_ISL_712277, EPI_ISL_712278, EPI_ISL_712279, EPI_ISL_712280, EPI_ISL_712281, EPI_ISL_712282, EPI_ISL_712283, EPI_ISL_712284, EPI_ISL_712285, EPI_ISL_712286, EPI_ISL_712287, EPI_ISL_712288, EPI_ISL_712289, EPI_ISL_712290, EPI_ISL_712291, EPI_ISL_712292, EPI_ISL_712293, EPI_ISL_712294, EPI_ISL_712296, EPI_ISL_712297, EPI_ISL_712298, EPI_ISL_712299, EPI_ISL_712300, EPI_ISL_712301, EPI_ISL_712302, EPI_ISL_712303, EPI_ISL_712304, EPI_ISL_712305, EPI_ISL_712306, EPI_ISL_712307, EPI_ISL_712308, EPI_ISL_712309, EPI_ISL_712310, EPI_ISL_712311, EPI_ISL_712312, EPI_ISL_712313, EPI_ISL_712314, EPI_ISL_712315, EPI_ISL_712316, EPI_ISL_712317, EPI_ISL_712318, EPI_ISL_712319, EPI_ISL_712320, EPI_ISL_712322, EPI_ISL_712323, EPI_ISL_712324, EPI_ISL_712325, EPI_ISL_712326, EPI_ISL_712327, EPI_ISL_712328, EPI_ISL_712329, EPI_ISL_712330, EPI_ISL_712331, EPI_ISL_712332, EPI_ISL_712333, EPI_ISL_712334, EPI_ISL_712335, EPI_ISL_712336, EPI_ISL_712337, EPI_ISL_712338, EPI_ISL_712339, EPI_ISL_712347, EPI_ISL_712348, EPI_ISL_712349, EPI_ISL_712350, EPI_ISL_712351, EPI_ISL_712352, EPI_ISL_712353, EPI_ISL_712354, EPI_ISL_712355, EPI_ISL_712356, EPI_ISL_712357, EPI_ISL_712358, EPI_ISL_712359, EPI_ISL_712360, EPI_ISL_712361, EPI_ISL_712362, EPI_ISL_712363, EPI_ISL_712364, EPI_ISL_712365, EPI_ISL_712366, EPI_ISL_712367, EPI_ISL_712368, EPI_ISL_712369, EPI_ISL_712370, EPI_ISL_712371, EPI_ISL_712372, EPI_ISL_712373, EPI_ISL_712374, EPI_ISL_712375, EPI_ISL_712376, EPI_ISL_712377, EPI_ISL_712378, EPI_ISL_712379, EPI_ISL_712380, EPI_ISL_712381, EPI_ISL_712382, EPI_ISL_712383, EPI_ISL_712384, EPI_ISL_712385, EPI_ISL_712386, EPI_ISL_712387, EPI_ISL_712388, EPI_ISL_712389, EPI_ISL_712390, EPI_ISL_712391, EPI_ISL_712392, EPI_ISL_712393, EPI_ISL_712394, EPI_ISL_712395, EPI_ISL_712396, EPI_ISL_712397, EPI_ISL_712398, EPI_ISL_712399, EPI_ISL_712400, EPI_ISL_712401, EPI_ISL_712402, EPI_ISL_712403, EPI_ISL_712404, EPI_ISL_712405, EPI_ISL_712406, EPI_ISL_712407, EPI_ISL_712408, EPI_ISL_712409, EPI_ISL_712410, EPI_ISL_712411, EPI_ISL_712412, EPI_ISL_712413, EPI_ISL_712414, EPI_ISL_712415, EPI_ISL_712416, EPI_ISL_712418, EPI_ISL_712419, EPI_ISL_712420, EPI_ISL_712421, EPI_ISL_712422, EPI_ISL_712423, EPI_ISL_712424, EPI_ISL_712425, EPI_ISL_712426, EPI_ISL_712427, EPI_ISL_712428, EPI_ISL_712429, EPI_ISL_712430, EPI_ISL_712432, EPI_ISL_712433, EPI_ISL_712434, EPI_ISL_712435, EPI_ISL_712436, EPI_ISL_712437, EPI_ISL_712438, EPI_ISL_712440, EPI_ISL_712441, EPI_ISL_712442, EPI_ISL_712443, EPI_ISL_712444, EPI_ISL_712445, EPI_ISL_712449, EPI_ISL_712450, EPI_ISL_712451, EPI_ISL_712452, EPI_ISL_712453, EPI_ISL_712454, EPI_ISL_712531, EPI_ISL_712532, EPI_ISL_712533, EPI_ISL_712534, EPI_ISL_712535, EPI_ISL_712536, EPI_ISL_712537, EPI_ISL_712538, EPI_ISL_712539, EPI_ISL_712540, EPI_ISL_712541, EPI_ISL_712542, EPI_ISL_712543, EPI_ISL_712544, EPI_ISL_712545, EPI_ISL_712546, EPI_ISL_712547, EPI_ISL_712548, EPI_ISL_712549, EPI_ISL_712550, EPI_ISL_712551, EPI_ISL_712552, EPI_ISL_712553, EPI_ISL_712554, EPI_ISL_712555, EPI_ISL_712556, EPI_ISL_712557, EPI_ISL_712558, EPI_ISL_712559, EPI_ISL_712560, EPI_ISL_712561, EPI_ISL_712562, EPI_ISL_712563, EPI_ISL_712564, EPI_ISL_712565, EPI_ISL_712566, EPI_ISL_712567, EPI_ISL_712569, EPI_ISL_712570, EPI_ISL_712571, EPI_ISL_712572, EPI_ISL_712573, EPI_ISL_712574, EPI_ISL_712575, EPI_ISL_712576, EPI_ISL_712577, EPI_ISL_712578, EPI_ISL_712579, EPI_ISL_712580, EPI_ISL_712581, EPI_ISL_712582, EPI_ISL_712583, EPI_ISL_712584, EPI_ISL_712585, EPI_ISL_712586, EPI_ISL_712587, EPI_ISL_712588, EPI_ISL_712589, EPI_ISL_712590, EPI_ISL_712591, EPI_ISL_712592, EPI_ISL_712593, EPI_ISL_712594, EPI_ISL_712595, EPI_ISL_712596, EPI_ISL_712597, EPI_ISL_712598, EPI_ISL_712599, EPI_ISL_712600, EPI_ISL_712601, EPI_ISL_712602, EPI_ISL_712603, EPI_ISL_712604, EPI_ISL_712605, EPI_ISL_712606, EPI_ISL_712607, EPI_ISL_712608, EPI_ISL_712609, EPI_ISL_712610, EPI_ISL_712611, EPI_ISL_712612, EPI_ISL_712613, EPI_ISL_712614, EPI_ISL_712615, EPI_ISL_712616, EPI_ISL_712617, EPI_ISL_712618, EPI_ISL_712619, EPI_ISL_712620, EPI_ISL_712621, EPI_ISL_712622, EPI_ISL_712623, EPI_ISL_712624, EPI_ISL_712625, EPI_ISL_712626, EPI_ISL_712627, EPI_ISL_712628, EPI_ISL_712629, EPI_ISL_712630, EPI_ISL_712631, EPI_ISL_712632, EPI_ISL_712633, EPI_ISL_712634, EPI_ISL_712635, EPI_ISL_712636, EPI_ISL_712637, EPI_ISL_712638, EPI_ISL_712639, EPI_ISL_712640, EPI_ISL_712641, EPI_ISL_712642, EPI_ISL_712643, EPI_ISL_712644, EPI_ISL_712645, EPI_ISL_712646, EPI_ISL_712647, EPI_ISL_712648, EPI_ISL_712649, EPI_ISL_712650, EPI_ISL_712651, EPI_ISL_712652, EPI_ISL_712653, EPI_ISL_712654, EPI_ISL_712655, EPI_ISL_712656, EPI_ISL_712657, EPI_ISL_712658, EPI_ISL_712659, EPI_ISL_712660, EPI_ISL_712661, EPI_ISL_712662, EPI_ISL_712663, EPI_ISL_712664, EPI_ISL_712665, EPI_ISL_712666, EPI_ISL_712667, EPI_ISL_712668, EPI_ISL_712669, EPI_ISL_712670, EPI_ISL_712671, EPI_ISL_712672, EPI_ISL_712673, EPI_ISL_712674, EPI_ISL_712675, EPI_ISL_712676, EPI_ISL_712677, EPI_ISL_712678, EPI_ISL_712679, EPI_ISL_712680, EPI_ISL_712681, EPI_ISL_712682, EPI_ISL_712683, EPI_ISL_712684, EPI_ISL_712685, EPI_ISL_712686, EPI_ISL_712687, EPI_ISL_712688, EPI_ISL_712689, EPI_ISL_712690, EPI_ISL_712691, EPI_ISL_712692, EPI_ISL_712693, EPI_ISL_712694, EPI_ISL_712695, EPI_ISL_712696, EPI_ISL_712697, EPI_ISL_712698, EPI_ISL_712699, EPI_ISL_712700, EPI_ISL_712701, EPI_ISL_712702, EPI_ISL_712703, EPI_ISL_712704, EPI_ISL_712705, EPI_ISL_712706, EPI_ISL_712707, EPI_ISL_712708, EPI_ISL_712709, EPI_ISL_712710, EPI_ISL_712711, EPI_ISL_712712, EPI_ISL_712713, EPI_ISL_712714, EPI_ISL_712715, EPI_ISL_712716, EPI_ISL_712717, EPI_ISL_712718, EPI_ISL_712719, EPI_ISL_712720, EPI_ISL_712721, EPI_ISL_712722, EPI_ISL_712723, EPI_ISL_712724, EPI_ISL_712725, EPI_ISL_712726, EPI_ISL_712727, EPI_ISL_712728, EPI_ISL_712729, EPI_ISL_712730, EPI_ISL_712731, EPI_ISL_712732, EPI_ISL_712733, EPI_ISL_712734, EPI_ISL_712735, EPI_ISL_712736, EPI_ISL_712737, EPI_ISL_712738, EPI_ISL_712739, EPI_ISL_712740, EPI_ISL_712741, EPI_ISL_712742, EPI_ISL_712743, EPI_ISL_712744, EPI_ISL_712745, EPI_ISL_712746, EPI_ISL_712747, EPI_ISL_712748, EPI_ISL_712749, EPI_ISL_712750, EPI_ISL_712751, EPI_ISL_712752, EPI_ISL_712753, EPI_ISL_712754, EPI_ISL_712755, EPI_ISL_712756, EPI_ISL_712757, EPI_ISL_712758, EPI_ISL_712759, EPI_ISL_712760, EPI_ISL_712761, EPI_ISL_712762, EPI_ISL_712763, EPI_ISL_712764, EPI_ISL_712765, EPI_ISL_712766, EPI_ISL_712767, EPI_ISL_712768, EPI_ISL_712769, EPI_ISL_712770, EPI_ISL_712771, EPI_ISL_712772, EPI_ISL_712773, EPI_ISL_712774, EPI_ISL_712775, EPI_ISL_712776, EPI_ISL_712777, EPI_ISL_712778, EPI_ISL_712779, EPI_ISL_712780, EPI_ISL_712781, EPI_ISL_712782, EPI_ISL_712783, EPI_ISL_712784, EPI_ISL_712785, EPI_ISL_712786, EPI_ISL_712787, EPI_ISL_712788, EPI_ISL_712789, EPI_ISL_712790, EPI_ISL_712791, EPI_ISL_712792, EPI_ISL_712793, EPI_ISL_712794, EPI_ISL_712795, EPI_ISL_712796, EPI_ISL_712797, EPI_ISL_712798, EPI_ISL_712799, EPI_ISL_712800, EPI_ISL_712801, EPI_ISL_712802, EPI_ISL_712803, EPI_ISL_712804, EPI_ISL_712805, EPI_ISL_712806, EPI_ISL_712807, EPI_ISL_712808, EPI_ISL_712809, EPI_ISL_712810, EPI_ISL_712811, EPI_ISL_712812, EPI_ISL_712813, EPI_ISL_712814, EPI_ISL_712815, EPI_ISL_712816, EPI_ISL_712817, EPI_ISL_712818, EPI_ISL_712819, EPI_ISL_712820, EPI_ISL_712821, EPI_ISL_712822, EPI_ISL_712823, EPI_ISL_712824, EPI_ISL_712825, EPI_ISL_712826, EPI_ISL_712827, EPI_ISL_712828, EPI_ISL_712829, EPI_ISL_712830, EPI_ISL_712831, EPI_ISL_712832, EPI_ISL_712833, EPI_ISL_712834, EPI_ISL_712835, EPI_ISL_712836, EPI_ISL_712837, EPI_ISL_712838, EPI_ISL_712839, EPI_ISL_712840, EPI_ISL_712841, EPI_ISL_712842, EPI_ISL_712843, EPI_ISL_712844, EPI_ISL_712845, EPI_ISL_712846, EPI_ISL_712847, EPI_ISL_712848, EPI_ISL_712849, EPI_ISL_712850, EPI_ISL_712851, EPI_ISL_712852, EPI_ISL_712853, EPI_ISL_712854, EPI_ISL_712855, EPI_ISL_712856, EPI_ISL_712857, EPI_ISL_712858, EPI_ISL_712859, EPI_ISL_712860, EPI_ISL_712861, EPI_ISL_712862, EPI_ISL_712863, EPI_ISL_712864, EPI_ISL_712865, EPI_ISL_712866, EPI_ISL_712867, EPI_ISL_712868, EPI_ISL_712869, EPI_ISL_712870, EPI_ISL_712871, EPI_ISL_712872, EPI_ISL_712873, EPI_ISL_712874, EPI_ISL_712875, EPI_ISL_712876, EPI_ISL_712877, EPI_ISL_712878, EPI_ISL_712879, EPI_ISL_712880, EPI_ISL_712881, EPI_ISL_712882, EPI_ISL_712883, EPI_ISL_712884, EPI_ISL_712885, EPI_ISL_712886, EPI_ISL_712887, EPI_ISL_712888, EPI_ISL_712889, EPI_ISL_712890, EPI_ISL_712891, EPI_ISL_712892, EPI_ISL_712893, EPI_ISL_712894, EPI_ISL_712895, EPI_ISL_712896, EPI_ISL_712897, EPI_ISL_712898, EPI_ISL_712899, EPI_ISL_712900, EPI_ISL_712901, EPI_ISL_712902, EPI_ISL_712903, EPI_ISL_712904, EPI_ISL_712905, EPI_ISL_712906, EPI_ISL_712907, EPI_ISL_712908, EPI_ISL_712909, EPI_ISL_712910, EPI_ISL_712911, EPI_ISL_712912, EPI_ISL_712913, EPI_ISL_712914, EPI_ISL_712915, EPI_ISL_712916, EPI_ISL_712917, EPI_ISL_712918, EPI_ISL_712919, EPI_ISL_712920, EPI_ISL_712921, EPI_ISL_712922, EPI_ISL_712923, EPI_ISL_712924, EPI_ISL_712925, EPI_ISL_712926, EPI_ISL_712927, EPI_ISL_712928, EPI_ISL_712929, EPI_ISL_712930, EPI_ISL_712931, EPI_ISL_712932, EPI_ISL_712933, EPI_ISL_712934, EPI_ISL_712935, EPI_ISL_712936, EPI_ISL_712937, EPI_ISL_712938, EPI_ISL_712939, EPI_ISL_712940, EPI_ISL_712941, EPI_ISL_712942, EPI_ISL_712943, EPI_ISL_712944, EPI_ISL_712945, EPI_ISL_712946, EPI_ISL_712947, EPI_ISL_712948, EPI_ISL_712949, EPI_ISL_712950, EPI_ISL_712951, EPI_ISL_712952, EPI_ISL_712953, EPI_ISL_712954, EPI_ISL_712955, EPI_ISL_712956, EPI_ISL_712957, EPI_ISL_712958, EPI_ISL_712959, EPI_ISL_712960, EPI_ISL_712961, EPI_ISL_712962, EPI_ISL_712963, EPI_ISL_712964, EPI_ISL_712965, EPI_ISL_712966, EPI_ISL_712967, EPI_ISL_712968, EPI_ISL_712969, EPI_ISL_712970, EPI_ISL_712971, EPI_ISL_712972, EPI_ISL_712973, EPI_ISL_712974, EPI_ISL_712975, EPI_ISL_712976, EPI_ISL_712977, EPI_ISL_712978, EPI_ISL_712979, EPI_ISL_712980, EPI_ISL_712981, EPI_ISL_712982, EPI_ISL_712983, EPI_ISL_712984, EPI_ISL_712985, EPI_ISL_712986, EPI_ISL_712987, EPI_ISL_712988, EPI_ISL_712989, EPI_ISL_712990, EPI_ISL_712991, EPI_ISL_712992, EPI_ISL_712993, EPI_ISL_712994, EPI_ISL_712995, EPI_ISL_712996, EPI_ISL_712997, EPI_ISL_712998, EPI_ISL_712999, EPI_ISL_713000, EPI_ISL_713001, EPI_ISL_713002, EPI_ISL_713003, EPI_ISL_713004, EPI_ISL_713005, EPI_ISL_713006, EPI_ISL_713007, EPI_ISL_713008, EPI_ISL_713009, EPI_ISL_713010, EPI_ISL_713011, EPI_ISL_713012, EPI_ISL_713013, EPI_ISL_713014, EPI_ISL_713015, EPI_ISL_713016, EPI_ISL_713017, EPI_ISL_713018, EPI_ISL_713019, EPI_ISL_713020, EPI_ISL_713021, EPI_ISL_713022, EPI_ISL_713023, EPI_ISL_713024, EPI_ISL_713025, EPI_ISL_713026, EPI_ISL_713027, EPI_ISL_713028, EPI_ISL_713029, EPI_ISL_713030, EPI_ISL_713031, EPI_ISL_713032, EPI_ISL_713033, EPI_ISL_713034, EPI_ISL_713035, EPI_ISL_713036, EPI_ISL_713037, EPI_ISL_713038, EPI_ISL_713039, EPI_ISL_713040, EPI_ISL_713041, EPI_ISL_713042, EPI_ISL_713043, EPI_ISL_713044, EPI_ISL_713045, EPI_ISL_713046, EPI_ISL_713047, EPI_ISL_713048, EPI_ISL_713049, EPI_ISL_713050, EPI_ISL_713051, EPI_ISL_713052, EPI_ISL_713053, EPI_ISL_713054, EPI_ISL_713055, EPI_ISL_713056, EPI_ISL_713057, EPI_ISL_713058, EPI_ISL_713059, EPI_ISL_713060, EPI_ISL_713061, EPI_ISL_713062, EPI_ISL_713063, EPI_ISL_713064, EPI_ISL_713065, EPI_ISL_713066, EPI_ISL_713067, EPI_ISL_713068, EPI_ISL_713069, EPI_ISL_713070, EPI_ISL_713071, EPI_ISL_713072, EPI_ISL_713073, EPI_ISL_713074, EPI_ISL_713075, EPI_ISL_713076, EPI_ISL_713077, EPI_ISL_713078, EPI_ISL_713079, EPI_ISL_713080, EPI_ISL_713081, EPI_ISL_713082, EPI_ISL_713083, EPI_ISL_713084, EPI_ISL_713085, EPI_ISL_713086, EPI_ISL_713087, EPI_ISL_713088, EPI_ISL_713089, EPI_ISL_713090, EPI_ISL_713091, EPI_ISL_713092, EPI_ISL_713093, EPI_ISL_713094, EPI_ISL_713095, EPI_ISL_713096, EPI_ISL_713097

see above	Department of Virus and Microbiological Special Diagnostics, Statens Serum Institut, Copenhagen, Denmark	Albertsen Lab, Department of Chemistry and Bioscience, Aalborg University, Denmark	Danish Covid-19 Genome Consortium
EPI_ISL_717982, EPI_ISL_717986	Lab voor klinische biologie	Onderzoeksgroep Virologie	Nick Vereecke, Laurens Lambrechts, Marthe Pauwels, Bruno Verhasselt, Linos Vandekerckhove, Hans Nauwynck, Sebastiaan Theuns
EPI_ISL_718024	Lab voor klinische biologie	Onderzoeksgroep Virologie	Laurens Lambrechts, Nick Vereecke, Marthe Pauwels, Bruno Verhasselt, Linos Vandekerckhove, Hans Nauwynck, Sebastiaan Theuns
EPI_ISL_718026	Lab voor klinische biologie	Onderzoeksgroep Virologie	Nick Vereecke, Laurens Lambrechts, Marthe Pauwels, Bruno Verhasselt, Linos Vandekerckhove, Hans Nauwynck, Sebastiaan Theuns
EPI_ISL_718252	Institute of Virology, Biomedical Research Center of the Slovak Academy of Sciences, Bratislava	Faculty of Natural Sciences, Comenius University, Bratislava	Kristína Boršová, Viktória Hodorová, Broa Brejová, Viktória abanová, Sabina Fumaová Havlíková, Juraj Kopáek, Martina Liková, ubomíra Lukáiková, Martina Neboháová, Monika Sláviková, Tomáš Vina, Boris Klempa, Jozef Nosek
EPI_ISL_718253	Institute of Virology, Biomedical Research Center of the Slovak Academy of Sciences, Bratislava	Faculty of Natural Sciences, Comenius University, Bratislava	Viktória Hodorová, Kristína Boršová, Broa Brejová, Viktória abanová, Sabina Fumaová Havlíková, Juraj Kopáek, Martina Liková, ubomíra Lukáiková, Martina Neboháová, Monika Sláviková, Tomáš Vina, Jozef Nosek, Boris Klempa
EPI_ISL_718254	Institute of Virology, Biomedical Research Center of the	Faculty of Natural Sciences, Comenius University, Bratislava	Kristína Boršová, Viktória Hodorová, Broa Brejová, Viktória abanová, Sabina Fumaová Havlíková, Juraj Kopáek, Martina Liková, ubomíra Lukáiková,

	Slovak Academy of Sciences, Bratislava		Martina Neboháová, Monika Sláviková, Tomáš Vina, Boris Klempa, Jozef Nosek
EPI_ISL_718255, EPI_ISL_718256	Institute of Virology, Biomedical Research Center of the Slovak Academy of Sciences, Bratislava	Faculty of Natural Sciences, Comenius University, Bratislava	Broa Brejová, Viktória Hodorová, Kristína Boršová, Viktória abanová, Sabina Fumaová Havlíková, Juraj Kopáek, Martina Liková, ubomíra Lukáiková, Martina Neboháová, Monika Sláviková, Tomáš Vina, Jozef Nosek, Boris Klempa
EPI_ISL_720669, EPI_ISL_720670, EPI_ISL_720675, EPI_ISL_720676, EPI_ISL_720677, EPI_ISL_720679, EPI_ISL_720680, EPI_ISL_720681, EPI_ISL_720682, EPI_ISL_720687, EPI_ISL_720692, EPI_ISL_720696, EPI_ISL_720698, EPI_ISL_720699, EPI_ISL_720701, EPI_ISL_720703, EPI_ISL_720705, EPI_ISL_720706, EPI_ISL_720708, EPI_ISL_720710, EPI_ISL_720712, EPI_ISL_720714, EPI_ISL_720716, EPI_ISL_720717, EPI_ISL_720718, EPI_ISL_720720, EPI_ISL_720721, EPI_ISL_720723, EPI_ISL_720724, EPI_ISL_720725, EPI_ISL_720727, EPI_ISL_720729, EPI_ISL_720730, EPI_ISL_720731, EPI_ISL_720735, EPI_ISL_720736, EPI_ISL_720739, EPI_ISL_720742, EPI_ISL_720743, EPI_ISL_720744, EPI_ISL_720746, EPI_ISL_720747, EPI_ISL_720748, EPI_ISL_720751, EPI_ISL_720753, EPI_ISL_720754, EPI_ISL_720758, EPI_ISL_720759, EPI_ISL_720760, EPI_ISL_720763, EPI_ISL_720768, EPI_ISL_720772, EPI_ISL_720774, EPI_ISL_720775, EPI_ISL_720778, EPI_ISL_720781, EPI_ISL_720782, EPI_ISL_720785, EPI_ISL_720789, EPI_ISL_720791, EPI_ISL_720794, EPI_ISL_720797, EPI_ISL_720798, EPI_ISL_720799, EPI_ISL_720800, EPI_ISL_720801, EPI_ISL_720804, EPI_ISL_720812, EPI_ISL_720813, EPI_ISL_720815, EPI_ISL_720817, EPI_ISL_720818, EPI_ISL_720820, EPI_ISL_720822, EPI_ISL_720824, EPI_ISL_720826, EPI_ISL_720829, EPI_ISL_720830, EPI_ISL_720832, EPI_ISL_720835, EPI_ISL_720838, EPI_ISL_720840, EPI_ISL_720843, EPI_ISL_720844, EPI_ISL_720847, EPI_ISL_720852, EPI_ISL_720854, EPI_ISL_720855, EPI_ISL_720859, EPI_ISL_720861, EPI_ISL_720863			
see above	Lighthouse Lab in Milton Keynes	Wellcome Sanger Institute for the COVID-19 Genomics UK (COG-UK) Consortium	The Lighthouse Lab in Milton Keynes and Alex Alderton, Roberto Amato, Sonia Goncalves, Ewan Harrison, David K. Jackson, Ian Johnston, Dominic Kwiatkowski, Cordelia Langford, John Sillitoe on behalf of the Wellcome Sanger Institute COVID-19 Surveillance Team
EPI_ISL_720864	Lighthouse Lab in Alderley Park	Wellcome Sanger Institute for the COVID-19 Genomics UK (COG-UK) Consortium	Jacquelyn Wynn, Mairead Hyland, The Lighthouse Lab in Alderley Park and Alex Alderton, Roberto Amato, Sonia Goncalves, Ewan Harrison, David K. Jackson, Ian Johnston, Dominic Kwiatkowski, Cordelia Langford, John Sillitoe on behalf of the Wellcome Sanger Institute COVID-19 Surveillance Team
EPI_ISL_720865, EPI_ISL_720866, EPI_ISL_720868, EPI_ISL_720873, EPI_ISL_720876, EPI_ISL_720878, EPI_ISL_720879, EPI_ISL_720881, EPI_ISL_720882, EPI_ISL_720883, EPI_ISL_720884, EPI_ISL_720887, EPI_ISL_720890, EPI_ISL_720892, EPI_ISL_720895, EPI_ISL_720899, EPI_ISL_720901, EPI_ISL_720905, EPI_ISL_720906, EPI_ISL_720908, EPI_ISL_721526, EPI_ISL_721527			
see above	Lighthouse Lab in Milton Keynes	Wellcome Sanger Institute for the COVID-19 Genomics UK (COG-UK) Consortium	The Lighthouse Lab in Milton Keynes and Alex Alderton, Roberto Amato, Sonia Goncalves, Ewan Harrison, David K. Jackson, Ian Johnston, Dominic Kwiatkowski, Cordelia Langford, John Sillitoe on behalf of the Wellcome Sanger Institute COVID-19 Surveillance Team
EPI_ISL_721568, EPI_ISL_721573, EPI_ISL_721575, EPI_ISL_721598, EPI_ISL_721599, EPI_ISL_721600, EPI_ISL_721611, EPI_ISL_721612, EPI_ISL_721613, EPI_ISL_721614, EPI_ISL_721615, EPI_ISL_721616, EPI_ISL_721617, EPI_ISL_721622, EPI_ISL_721623			
see above	Pathogen Genomics Center, National Institute of Infectious Diseases	Pathogen Genomics Center, National Institute of Infectious Diseases	Tsuyoshi Sekizuka, Kentaro Itokawa, Rina Tanaka, Masanori Hashino, Makoto Kuroda
EPI_ISL_721670, EPI_ISL_721675, EPI_ISL_721695, EPI_ISL_721702, EPI_ISL_721741, EPI_ISL_721772, EPI_ISL_721818, EPI_ISL_721819, EPI_ISL_721820, EPI_ISL_721821, EPI_ISL_721822, EPI_ISL_721840, EPI_ISL_721841, EPI_ISL_721842, EPI_ISL_721851, EPI_ISL_721852, EPI_ISL_721853, EPI_ISL_721889, EPI_ISL_721890, EPI_ISL_721891, EPI_ISL_721892, EPI_ISL_721893, EPI_ISL_721894, EPI_ISL_721895, EPI_ISL_721896, EPI_ISL_721901, EPI_ISL_721902, EPI_ISL_721906, EPI_ISL_721907, EPI_ISL_721908, EPI_ISL_721928, EPI_ISL_721966, EPI_ISL_721967, EPI_ISL_721968, EPI_ISL_721969, EPI_ISL_721970			
see above	Viollier AG	Department of Biosystems Science and Engineering, ETH Zürich	Christian Beisel
EPI_ISL_722338, EPI_ISL_722372, EPI_ISL_722384, EPI_ISL_722454, EPI_ISL_722711, EPI_ISL_722712, EPI_ISL_722713, EPI_ISL_722758, EPI_ISL_722759, EPI_ISL_722760, EPI_ISL_722761, EPI_ISL_722762, EPI_ISL_722784, EPI_ISL_722785, EPI_ISL_722786			
see above	Dutch COVID-19 response team	Erasmus Medical Center	Bas Oude Munnink, Reina Sikkema, David Nieuwenhuijse, Irina Chestakova, Anne van der Linden, Marjan Boter, Emmanuelle Munger, Corine GeurtsvanKessel, Annemiek van der Eijk, Richard Molenkamp, Marion Koopmans, on behalf of the Dutch national COVID-19 response team.
EPI_ISL_722926, EPI_ISL_722927, EPI_ISL_722928, EPI_ISL_722929, EPI_ISL_722930, EPI_ISL_722931, EPI_ISL_722932, EPI_ISL_722933, EPI_ISL_722935, EPI_ISL_722944, EPI_ISL_722945, EPI_ISL_722946, EPI_ISL_722947, EPI_ISL_722948, EPI_ISL_722949, EPI_ISL_722950, EPI_ISL_722951, EPI_ISL_722952, EPI_ISL_722953, EPI_ISL_722954, EPI_ISL_722955, EPI_ISL_722956, EPI_ISL_722957, EPI_ISL_722958, EPI_ISL_722959, EPI_ISL_722960, EPI_ISL_722961, EPI_ISL_722962, EPI_ISL_722963, EPI_ISL_722964, EPI_ISL_722965, EPI_ISL_722966			
see above	Department of Clinical Microbiology	GIGA Medical Genomics	Keith Durkin, Maria Artesi, Sébastien Bontems, Raphaël Boreux, Bouchra Boujemla, Cécile Meex, Pierrette Melin, Marie-Pierre Hayette, Vincent Bours
EPI_ISL_722967, EPI_ISL_722968, EPI_ISL_722969, EPI_ISL_722970, EPI_ISL_722976, EPI_ISL_722977, EPI_ISL_722978, EPI_ISL_723008, EPI_ISL_723009, EPI_ISL_723010, EPI_ISL_723011, EPI_ISL_723012, EPI_ISL_723013, EPI_ISL_723014, EPI_ISL_723022, EPI_ISL_723023, EPI_ISL_723024			
see above	Respiratory Virus Unit, National Infection Service, Public Health England	COVID-19 Genomics UK (COG-UK) Consortium	PHE Covid Sequencing Team
EPI_ISL_723137	Minnesota Department of Health, Public Health Laboratory	Minnesota Department of Health, Public Health Laboratory	Alexandra Lorentz, Jacob Garfin, Matt Plumb, and Xiong Wang
EPI_ISL_723155, EPI_ISL_723158, EPI_ISL_723159, EPI_ISL_723161, EPI_ISL_723162, EPI_ISL_723163, EPI_ISL_723165, EPI_ISL_723169, EPI_ISL_723175, EPI_ISL_723176, EPI_ISL_723177, EPI_ISL_723201, EPI_ISL_723252, EPI_ISL_723277, EPI_ISL_723278, EPI_ISL_723279, EPI_ISL_723312, EPI_ISL_723319, EPI_ISL_723328, EPI_ISL_723332, EPI_ISL_723334, EPI_ISL_723335, EPI_ISL_723336, EPI_ISL_723337, EPI_ISL_723338, EPI_ISL_723339, EPI_ISL_723346, EPI_ISL_723354, EPI_ISL_723355, EPI_ISL_723366, EPI_ISL_723367, EPI_ISL_723368, EPI_ISL_723369, EPI_ISL_723381, EPI_ISL_723382, EPI_ISL_723384, EPI_ISL_723386, EPI_ISL_723387, EPI_ISL_723388, EPI_ISL_723389, EPI_ISL_723390, EPI_ISL_723391, EPI_ISL_723392, EPI_ISL_723393, EPI_ISL_723446, EPI_ISL_723447, EPI_ISL_723454, EPI_ISL_723466, EPI_ISL_723467			
see above	Dutch COVID-19 response team	National Institute for Public Health and the Environment (RIVM)	Adam Meijer, Harry Vennema, Jeroen Cremer, Sharon van den Brink, Bas van der Veer, AnneMarie van den Brandt, Florian Zwagemaker, Dennis Schmitz, Chantal Reusken, on behalf of the national COVID-19 response team
EPI_ISL_723886, EPI_ISL_723887, EPI_ISL_723888, EPI_ISL_723916, EPI_ISL_723920	Department of Pathology, University of Cambridge	COVID-19 Genomics UK (COG-UK) Consortium	Aminu S. Jahun, Yasmin Chaudhry, Grant Hall, Iliana Georgana, Myra Hosmillo, Martin D. Curran, Malte Pinckert, Surendra Parmar, Ian Goodfellow
EPI_ISL_724210, EPI_ISL_724211	West of Scotland Specialist Virology Centre, NHSGGC / MRC-University of Glasgow Centre for Virus Research	COVID-19 Genomics UK (COG-UK) Consortium	Ana da Silva Filipe, Natasha Johnson, Kathy Smollett, Daniel Mair, Stephen Carmichael, Alice Broos, Lily Tong, Jenna Nichols, Kyriaki Nomikou; Sarah McDonald; Richard Orton, Joseph Hughes, Sreenu Vattipally, David L Robertson; Alasdair MacLean, Rory Gunson; Sharif Shaaban, Matthew Holden; Rachel Blacow, Guy Mollett, Kathy Li, James Shepherd, Antonia Ho, Emma Thomson
EPI_ISL_724268, EPI_ISL_724276, EPI_ISL_724277, EPI_ISL_724278, EPI_ISL_724279, EPI_ISL_724280, EPI_ISL_724281, EPI_ISL_724282, EPI_ISL_724283, EPI_ISL_724284, EPI_ISL_724285, EPI_ISL_724286, EPI_ISL_724287, EPI_ISL_724288, EPI_ISL_724290, EPI_ISL_724292, EPI_ISL_724293			
see above	University of Exeter	COVID-19 Genomics UK (COG-UK) Consortium	Ben Temperton,Aaron Jeffries,Michelle Michelsen,Joanna Warwick-Dugdale,Audrey Farbos,Robyn Manley,Stephen Michell,Jane Masoli
EPI_ISL_724349, EPI_ISL_724350, EPI_ISL_724352, EPI_ISL_724353	Liverpool Clinical Laboratories	COVID-19 Genomics UK (COG-UK) Consortium	Sam Haldenby, Anita Lucaci, Steve Paterson, Julian Hiscox, Alistair Darby, M Almsaud, A Alrezaihi, Muhannad Alruwaili, Stuart D Armstrong, Jones Benjamin, Eleanor G Bentley, Anu Chawla, Jordan J Clark, Angela Cowell, Richard Eccles, Isabel García-Dorival, Matthew Gemmell, Alessandro Gerada, PKF Gilmore, Richard Gregory, Ximeng Han, Catherine Hartley, Margaret Hughes, Miren Iturriza-Gomara, James Johnson, L Luu, Jenifer Manson, Charlotte Nelson, Elaine O'Toole, Cassie Olateju, Rebekah Penrice-Randal , Lucille Rainbow, N.P Randle, Trevor Ian Robinson, Parul Sharma, Ghada T Shawli, James P Stewart, Neil Swainston, Ecaterina Vamos, Joanne Watts, Mark Whitehead
EPI_ISL_724597	University College London, Great Ormond Street Hospital for Children NHS Foundation Trust, Imperial College Healthcare NHS Trust	COVID-19 Genomics UK (COG-UK) Consortium	Sergi Castellano, Rachel Williams, Mark Kristiansen, Paola Resende Silva, Sunando Roy, Tony Brooks, Helena Tutill, Paola Niola, Patricia Dyal, Charlotte Williams, Leysa Forrest, Yasmin Panchbhaya, Jacqueline Findlay, Samuel Weeks, Julianne Brown, Kathryn Harris, Paul Randell, James Price, Alison Holmes, Judith Breuer
EPI_ISL_724837, EPI_ISL_724841, EPI_ISL_724842, EPI_ISL_724848, EPI_ISL_724849, EPI_ISL_724850, EPI_ISL_724882, EPI_ISL_724883, EPI_ISL_724884, EPI_ISL_724887, EPI_ISL_724888, EPI_ISL_724889, EPI_ISL_724890, EPI_ISL_724891, EPI_ISL_724892, EPI_ISL_724893, EPI_ISL_724894			
see above	Northumbria University / South Tees Hospitals NHS Foundation Trust / North Cumbria Integrated Care NHS Foundation Trust / North Tees and Hartlepool NHS Foundation Trust / Newcastle Hospitals NHS Foundation Trust	COVID-19 Genomics UK (COG-UK) Consortium	Darren L Smith,Andrew Nelson,Matthew Bashton,Greg R Young,Joshua Loh,John Allan,Mohammad A Tariq,Giles S Holt,Gary Black,Wen C Yew,Lynn Dover,Paul Baker,Steve Liggett,Sarah Essex,Jane Greenaway,Debra Padgett,Clive Graham,Garren Scott,Edward Barton,Emma Swindells,Brendan Payne,Jennifer Collins,Yusri Taha,Gary Eltringham
EPI_ISL_725380, EPI_ISL_725381, EPI_ISL_725382, EPI_ISL_725383, EPI_ISL_725384, EPI_ISL_725385, EPI_ISL_725386, EPI_ISL_725387, EPI_ISL_725388, EPI_ISL_725389, EPI_ISL_725390, EPI_ISL_725391, EPI_ISL_725392, EPI_ISL_725393, EPI_ISL_725394, EPI_ISL_725395, EPI_ISL_725396, EPI_ISL_725397, EPI_ISL_725398, EPI_ISL_725399, EPI_ISL_725400, EPI_ISL_725401, EPI_ISL_725402, EPI_ISL_725403, EPI_ISL_725404			
see above	Quadram Institute Bioscience	COVID-19 Genomics UK (COG-UK) Consortium	Dave J. Baker, Gemma L. Kay, Alp Aydin, Thanh Le-Viet, Steven Rudder, Ana P. Tedim, Anastasia Kolyva, Maria Diaz, Leonardo de Oliveira Martins, Nabil-Fareed Alikhan, Lizzie Meadows, Rachael Stanley, Ngozi Elumogo, Muhammed Yasir, Nicholas M. Thomson, Alexander J Trotter, Rachel Gilroy, Samuel Bloomfield, Claire Stuart, Andrew Bell, Reenesh Prakash, Samir Dervisevic, Alison E. Mather, John Wain, Mark Webber, Andrew J. Page, Justin O'Grady
EPI_ISL_725538, EPI_ISL_725539	Queens Medical Centre, Clinical Microbiology Department / DeepSeq Nottingham	COVID-19 Genomics UK (COG-UK) Consortium	Gemma Clark, Wendy Smith, Manjinder Khakh, Vicki M Fleming, Michelle M Lister, Hannah Howson-Wells, Jonathan Ball, Patrick McClure, Joseph Chappell, Theocharis Tsoleridis, Nadine Holmes, Matthew Carlisle, Christopher Moore, Fei Sang, Johnny Debebe, Victoria Wright, Matthew Loose
EPI_ISL_725671, EPI_ISL_725701, EPI_ISL_725709, EPI_ISL_725710, EPI_ISL_725893, EPI_ISL_725895, EPI_ISL_725919, EPI_ISL_725955, EPI_ISL_725956, EPI_ISL_725957, EPI_ISL_725958, EPI_ISL_725959, EPI_ISL_725960, EPI_ISL_725961, EPI_ISL_725962, EPI_ISL_725963, EPI_ISL_725964, EPI_ISL_725965, EPI_ISL_725966, EPI_ISL_725967, EPI_ISL_725968, EPI_ISL_725969, EPI_ISL_725970, EPI_ISL_725971, EPI_ISL_725972, EPI_ISL_725973, EPI_ISL_725974, EPI_ISL_725975, EPI_ISL_725976, EPI_ISL_725977, EPI_ISL_725978, EPI_ISL_725979, EPI_ISL_725980, EPI_ISL_725981, EPI_ISL_726838, EPI_ISL_726840, EPI_ISL_726841, EPI_ISL_726842, EPI_ISL_726843, EPI_ISL_726844, EPI_ISL_726845, EPI_ISL_726846, EPI_ISL_726847, EPI_ISL_726848, EPI_ISL_726849, EPI_ISL_726850, EPI_ISL_726851, EPI_ISL_726852, EPI_ISL_726853, EPI_ISL_726854, EPI_ISL_726855, EPI_ISL_726856, EPI_ISL_726857, EPI_ISL_726858, EPI_ISL_726859, EPI_ISL_726860, EPI_ISL_726861, EPI_ISL_726862, EPI_ISL_726864, EPI_ISL_726961, EPI_ISL_726963, EPI_ISL_726964, EPI_ISL_726965, EPI_ISL_726966, EPI_ISL_726967, EPI_ISL_726968, EPI_ISL_726969, EPI_ISL_726970, EPI_ISL_726971, EPI_ISL_726972, EPI_ISL_726973, EPI_ISL_726974,			

EPI_ISL_726975, EPI_ISL_726976, EPI_ISL_726977, EPI_ISL_726978, EPI_ISL_726979, EPI_ISL_726980, EPI_ISL_726981, EPI_ISL_726982, EPI_ISL_726983, EPI_ISL_726984, EPI_ISL_726985, EPI_ISL_726986, EPI_ISL_726987, EPI_ISL_726988, EPI_ISL_726989, EPI_ISL_726990, EPI_ISL_726991, EPI_ISL_726992, EPI_ISL_726993, EPI_ISL_726994, EPI_ISL_726995, EPI_ISL_726996, EPI_ISL_726997, EPI_ISL_726998, EPI_ISL_726999, EPI_ISL_727000, EPI_ISL_727001, EPI_ISL_727002, EPI_ISL_727003, EPI_ISL_727004, EPI_ISL_727005, EPI_ISL_727006, EPI_ISL_727007, EPI_ISL_727008, EPI_ISL_727009, EPI_ISL_727010, EPI_ISL_727011, EPI_ISL_727012, EPI_ISL_727013, EPI_ISL_727014, EPI_ISL_727015, EPI_ISL_727016, EPI_ISL_727017, EPI_ISL_727018, EPI_ISL_727019, EPI_ISL_727020, EPI_ISL_727021, EPI_ISL_727022, EPI_ISL_727023, EPI_ISL_727024, EPI_ISL_727025, EPI_ISL_727026, EPI_ISL_727027, EPI_ISL_727028, EPI_ISL_727029, EPI_ISL_727030, EPI_ISL_727031, EPI_ISL_727032, EPI_ISL_727033, EPI_ISL_727034, EPI_ISL_727035, EPI_ISL_727036, EPI_ISL_727037, EPI_ISL_727038, EPI_ISL_727039, EPI_ISL_727040, EPI_ISL_727041, EPI_ISL_727042, EPI_ISL_727043, EPI_ISL_727044, EPI_ISL_727045, EPI_ISL_727046, EPI_ISL_727047, EPI_ISL_727048, EPI_ISL_727049, EPI_ISL_727050, EPI_ISL_727051, EPI_ISL_727052, EPI_ISL_727053, EPI_ISL_727054, EPI_ISL_727055, EPI_ISL_727056, EPI_ISL_727057, EPI_ISL_727058, EPI_ISL_727059, EPI_ISL_727060, EPI_ISL_727061, EPI_ISL_727062, EPI_ISL_727063, EPI_ISL_727064, EPI_ISL_727065, EPI_ISL_727066, EPI_ISL_727067, EPI_ISL_727068, EPI_ISL_727069, EPI_ISL_727070, EPI_ISL_727071, EPI_ISL_727072, EPI_ISL_727073, EPI_ISL_727074, EPI_ISL_727075, EPI_ISL_727076, EPI_ISL_727077, EPI_ISL_727078, EPI_ISL_727079, EPI_ISL_727080, EPI_ISL_727081, EPI_ISL_727082, EPI_ISL_727083, EPI_ISL_727084, EPI_ISL_727085, EPI_ISL_727086, EPI_ISL_727087, EPI_ISL_727088, EPI_ISL_727089, EPI_ISL_727090, EPI_ISL_727091, EPI_ISL_727092, EPI_ISL_727093, EPI_ISL_727094, EPI_ISL_727095, EPI_ISL_727096, EPI_ISL_727097, EPI_ISL_727101, EPI_ISL_727118, EPI_ISL_727119, EPI_ISL_727120, EPI_ISL_727121, EPI_ISL_727147, EPI_ISL_727148, EPI_ISL_727149, EPI_ISL_727178, EPI_ISL_727179, EPI_ISL_727187, EPI_ISL_727223, EPI_ISL_727224, EPI_ISL_727225, EPI_ISL_727226, EPI_ISL_727227, EPI_ISL_727228, EPI_ISL_727229, EPI_ISL_727230, EPI_ISL_727231, EPI_ISL_727232, EPI_ISL_727233, EPI_ISL_727234, EPI_ISL_727235, EPI_ISL_727236, EPI_ISL_727238, EPI_ISL_727239, EPI_ISL_727240, EPI_ISL_727241, EPI_ISL_727242, EPI_ISL_727243, EPI_ISL_727245, EPI_ISL_727246, EPI_ISL_727247, EPI_ISL_727248, EPI_ISL_727249, EPI_ISL_727250, EPI_ISL_727251, EPI_ISL_727252, EPI_ISL_727253, EPI_ISL_727254, EPI_ISL_727257, EPI_ISL_727258, EPI_ISL_727259, EPI_ISL_727260, EPI_ISL_727261, EPI_ISL_727262, EPI_ISL_727263, EPI_ISL_727264, EPI_ISL_727265, EPI_ISL_727266, EPI_ISL_727267, EPI_ISL_727268, EPI_ISL_727269, EPI_ISL_727270, EPI_ISL_727271, EPI_ISL_727272, EPI_ISL_727273, EPI_ISL_727274, EPI_ISL_727275, EPI_ISL_727277, EPI_ISL_727278, EPI_ISL_727280, EPI_ISL_727417, EPI_ISL_727419, EPI_ISL_727421, EPI_ISL_727422, EPI_ISL_727423, EPI_ISL_727485, EPI_ISL_727486, EPI_ISL_727487, EPI_ISL_727488, EPI_ISL_727489, EPI_ISL_727490, EPI_ISL_727491, EPI_ISL_727492, EPI_ISL_727493, EPI_ISL_727494, EPI_ISL_727495, EPI_ISL_727496, EPI_ISL_727497, EPI_ISL_727498, EPI_ISL_727499, EPI_ISL_727500, EPI_ISL_727501, EPI_ISL_727502, EPI_ISL_727503, EPI_ISL_727504, EPI_ISL_727505, EPI_ISL_727506, EPI_ISL_727507, EPI_ISL_727508, EPI_ISL_727509, EPI_ISL_727510, EPI_ISL_727511, EPI_ISL_727512, EPI_ISL_727513, EPI_ISL_727514, EPI_ISL_727515, EPI_ISL_727516, EPI_ISL_727517, EPI_ISL_727518, EPI_ISL_727519, EPI_ISL_727520, EPI_ISL_727521, EPI_ISL_727522, EPI_ISL_727523, EPI_ISL_727524, EPI_ISL_727525, EPI_ISL_727526, EPI_ISL_727527, EPI_ISL_727528, EPI_ISL_727529, EPI_ISL_727530, EPI_ISL_727531, EPI_ISL_727532, EPI_ISL_727533, EPI_ISL_727534, EPI_ISL_727535, EPI_ISL_727536, EPI_ISL_727537, EPI_ISL_727538, EPI_ISL_727539, EPI_ISL_727540, EPI_ISL_727541, EPI_ISL_727542, EPI_ISL_727543, EPI_ISL_727544, EPI_ISL_727545, EPI_ISL_727546, EPI_ISL_727547, EPI_ISL_727548, EPI_ISL_727549, EPI_ISL_727550, EPI_ISL_727551, EPI_ISL_727553, EPI_ISL_727554, EPI_ISL_727555, EPI_ISL_727556, EPI_ISL_727557, EPI_ISL_727558, EPI_ISL_727559, EPI_ISL_727560, EPI_ISL_727561, EPI_ISL_727562, EPI_ISL_727563, EPI_ISL_727564, EPI_ISL_727565, EPI_ISL_727566, EPI_ISL_727567, EPI_ISL_727568, EPI_ISL_727569, EPI_ISL_727570, EPI_ISL_727571, EPI_ISL_727572, EPI_ISL_727573, EPI_ISL_727574, EPI_ISL_727575, EPI_ISL_727576, EPI_ISL_727577, EPI_ISL_727578, EPI_ISL_727579, EPI_ISL_727580, EPI_ISL_727581, EPI_ISL_727582, EPI_ISL_727583, EPI_ISL_727584, EPI_ISL_727585, EPI_ISL_727586, EPI_ISL_727587, EPI_ISL_727588, EPI_ISL_727589, EPI_ISL_727590, EPI_ISL_727591, EPI_ISL_727592, EPI_ISL_727593, EPI_ISL_727594, EPI_ISL_727595, EPI_ISL_727596, EPI_ISL_727597, EPI_ISL_727598, EPI_ISL_727599, EPI_ISL_727600, EPI_ISL_727601, EPI_ISL_727602, EPI_ISL_727603, EPI_ISL_727604, EPI_ISL_727605, EPI_ISL_727606, EPI_ISL_727607, EPI_ISL_727608, EPI_ISL_727609, EPI_ISL_727610, EPI_ISL_727611, EPI_ISL_727612, EPI_ISL_727613, EPI_ISL_727615, EPI_ISL_727616, EPI_ISL_727617, EPI_ISL_727618, EPI_ISL_727619, EPI_ISL_727620, EPI_ISL_727621			
see above	Wales Specialist Virology Centre Sequencing lab: Pathogen Genomics Unit	COVID-19 Genomics UK (COG-UK) Consortium	Catherine Moore, Johnathan Evans, Laura Gifford, Malorie Perry, Simon Cottrell, Angela Marchbank, Alec Birchley, Alexander Adams, Amy Gaskin, Bree Gatica-Wilcox, Jason Coombes, Joel Southgate, Lauren Gilbert, Lee Graham, Nicole Pacchiarini, Sara Kumziene-Summerhayes, Sarah Taylor, Sophie Jones, Sara Rey, Matthew Bull, Joanne Watkins, Sally Corden, Tom Connor
EPI_ISL_727863, EPI_ISL_727869, EPI_ISL_727875, EPI_ISL_727888, EPI_ISL_727892, EPI_ISL_727903, EPI_ISL_727918, EPI_ISL_727929, EPI_ISL_727971, EPI_ISL_727983, EPI_ISL_727985, EPI_ISL_727986			
see above	Virology Department, Sheffield Teaching Hospitals NHS Foundation Trust/Department of Infection, Immunity and Cardiovascular Disease, The Medical School, University of Sheffield	COVID-19 Genomics UK (COG-UK) Consortium	Thushan de Silva, Matthew Parker, Nikki Smith, Adri Angyal, Rebecca Brown, Luke Green, Rachel Tucker, Paul Parsons, Danielle Groves, Katie Johnson, Laura Carrilero, Alex Keeley, Dave Partridge, Matthew Wyles, Benjamin Lindsey, Mehmet Yavuz, Mohammad Raza, Cariad Evans
EPI_ISL_728188	National Public Health Laboratory, National Centre for Infectious Diseases	National Public Health Laboratory, National Centre for Infectious Diseases	Tze Minn Mak, Sophie Octavia, Zhenyang Zhou, Lin Cui, Raymond Tzer Pin Lin
EPI_ISL_728204	Institute of Microbiology, Universidad San Francisco de Quito	Institute of Microbiology, Universidad San Francisco de Quito	Sully Márquez, Belén Prado-Vivar, Juan José Guadalupe, Monica Becerra-Wong, Bernardo Gutiérrez, Tania Guayasamin, Patricio Reyes, Verónica Barragán, Patricio Rojas-Silva, Gabriel Trueba, Michelle Grunauer, Paúl Cárdenas
EPI_ISL_728279	National Institute for Infectious Diseases, INMI, "L. Spallanzani" IRCCS	National Institute for Infectious Diseases, INMI, "L. Spallanzani" IRCCS	E Giombini, C.E.M Gruber, M Rueca, B Bartolini, F Messina, A Di Caro, MR Capobianchi
EPI_ISL_728280	National Institute for Infectious Diseases, INMI, "L. Spallanzani" IRCCS	National Institute for Infectious Diseases, INMI, "L. Spallanzani" IRCCS	F Messina, M Rueca, B Bartolini, C.E.M Gruber, E Giombini, MR Capobianchi, A Di Caro
EPI_ISL_728281	National Institute for Infectious Diseases, INMI, "L. Spallanzani" IRCCS	National Institute for Infectious Diseases, INMI, "L. Spallanzani" IRCCS	B Bartolini, M Rueca, C.E.M Gruber, F Messina, E Giombini, A Di Caro, MR Capobianchi
EPI_ISL_728282	National Institute for Infectious Diseases, INMI, "L. Spallanzani" IRCCS	National Institute for Infectious Diseases, INMI, "L. Spallanzani" IRCCS	M. Rueca, C.E.M Gruber, B Bartolini, F Messina, E Giombini, A Di Caro, MR Capobianchi
EPI_ISL_728283	National Institute for Infectious Diseases, INMI, "L. Spallanzani" IRCCS	National Institute for Infectious Diseases, INMI, "L. Spallanzani" IRCCS	B Bartolini, C.E.M Gruber, M Rueca, F Messina, E Giombini, MR Capobianchi, A Di Caro
EPI_ISL_728284	National Institute for Infectious Diseases, INMI, "L. Spallanzani" IRCCS	National Institute for Infectious Diseases, INMI, "L. Spallanzani" IRCCS	M Rueca, B Bartolini, C.E.M Gruber, F Messina, E Giombini, A Di Caro, MR Capobianchi
EPI_ISL_728285	National Institute for Infectious Diseases, INMI, "L. Spallanzani" IRCCS	National Institute for Infectious Diseases, INMI, "L. Spallanzani" IRCCS	F Messina, E Giombini, M Rueca, B Bartolini, C.E.M Gruber, MR Capobianchi, A Di Caro
EPI_ISL_728287	National Institute for Infectious Diseases, INMI, "L. Spallanzani" IRCCS	National Institute for Infectious Diseases, INMI, "L. Spallanzani" IRCCS	C.E.M Gruber, F Messina, M Rueca, B Bartolini, E Giombini, MR Capobianchi, A Di Caro
EPI_ISL_728324, EPI_ISL_728331, EPI_ISL_728333	Jena University Hospital, Institute for Infectious Diseases and Infection Control	Institute of infectious medicine & hospital hygiene, CaSe-Group	Spott, Riccardo; Marquet, Mike; Pletz, Matthias W.; Brandt, Christian
EPI_ISL_728334, EPI_ISL_728336, EPI_ISL_728339	Laboratoire Biolife	Laboratoire de Biotechnologie	Mouna Ouadghiri, Tarik Aanniz, Mohammed Walid Chemao Elfihri, Mohamed Chenaoui, Hanae Dakka, Afaf Alaoui, Otmane Touzani, Bouchra Belfquih, Lahcen belyamani, Saaid Amzazi and Azeddine Ibrahimi
EPI_ISL_728340	Laboratoire Biolife	Laboratoire de Biotechnologie	Mouna Ouadghiri, Tarik Aanniz, Mohammed Walid Chemao Elfihri, Mohamed Chenaoui, Hanae Dakka, Afaf Alaoui, Otmane Touzani, Bouchra Belfquih, Lahcen belyamani, Saaid Amzazi and Azeddine Ibrahim
EPI_ISL_728342, EPI_ISL_728344, EPI_ISL_728347, EPI_ISL_728349, EPI_ISL_728352, EPI_ISL_728353, EPI_ISL_728355, EPI_ISL_728359, EPI_ISL_728360, EPI_ISL_728363, EPI_ISL_728366, EPI_ISL_728367			
see above	Laboratoire Biolife	Laboratoire de Biotechnologie	Mouna Ouadghiri, Tarik Aanniz, Mohammed Walid Chemao Elfihri, Mohamed Chenaoui, Hanae Dakka, Afaf Alaoui, Otmane Touzani, Bouchra Belfquih, Lahcen belyamani, Saaid Amzazi and Azeddine Ibrahimi
EPI_ISL_728635, EPI_ISL_728636, EPI_ISL_728637, EPI_ISL_728640, EPI_ISL_728641, EPI_ISL_728643, EPI_ISL_728645, EPI_ISL_728646, EPI_ISL_728647, EPI_ISL_728648, EPI_ISL_728696, EPI_ISL_728745			
see above	Dutch COVID-19 response team	National Institute for Public Health and the Environment (RIVM)	Adam Meijer, Harry Vennema, Jeroen Cremer, Sharon van den Brink, Bas van der Veer, AnneMarie van den Brandt, Florian Zwagemaker, Dennis Schmitz, Chantal Reusken, on behalf of the national COVID-19 response team
EPI_ISL_728765, EPI_ISL_728768, EPI_ISL_728770, EPI_ISL_728783, EPI_ISL_728813, EPI_ISL_728885, EPI_ISL_728892, EPI_ISL_728896, EPI_ISL_728900, EPI_ISL_728907, EPI_ISL_728914, EPI_ISL_728916, EPI_ISL_728919, EPI_ISL_728922, EPI_ISL_728924, EPI_ISL_728928, EPI_ISL_728932, EPI_ISL_728939, EPI_ISL_728942, EPI_ISL_728945, EPI_ISL_728947, EPI_ISL_728948, EPI_ISL_728955, EPI_ISL_728956, EPI_ISL_728957, EPI_ISL_728963, EPI_ISL_728978, EPI_ISL_728995, EPI_ISL_729002, EPI_ISL_729011, EPI_ISL_729019, EPI_ISL_729032, EPI_ISL_729035, EPI_ISL_729036, EPI_ISL_729040, EPI_ISL_729041, EPI_ISL_729068			
see above	Viollier AG	Department of Biosystems Science and Engineering, ETH Zürich	Chaoran Chen, Sarah Nadeau, Catharine Aquino, Ivan Topolsky, Pedro Ferreira, Philipp Jablonski, Susana Posada-Céspedes, Andreia Cabral de Gouvea, Maria Domenica Moccia, Simon Grüter, Timothy Sykes, Lennart Opitz, Ralph Schlapbach, Christiane Beckmann, Maurice Redondo, Olivier Kobel, Christoph Noppen, Sophie Seidel, Noemie Santamaria de Souza, Niko Beerenwinkel, Tanja Stadler
EPI_ISL_729347, EPI_ISL_729392, EPI_ISL_729416, EPI_ISL_729449, EPI_ISL_729450, EPI_ISL_729451, EPI_ISL_729452, EPI_ISL_729453, EPI_ISL_729454, EPI_ISL_729455, EPI_ISL_729456, EPI_ISL_729459, EPI_ISL_729460, EPI_ISL_729463, EPI_ISL_729697, EPI_ISL_729698, EPI_ISL_729699, EPI_ISL_729700, EPI_ISL_729701, EPI_ISL_729702, EPI_ISL_729703, EPI_ISL_729705, EPI_ISL_729706, EPI_ISL_729707, EPI_ISL_729708, EPI_ISL_729709, EPI_ISL_729710, EPI_ISL_729711, EPI_ISL_729712, EPI_ISL_729713, EPI_ISL_729714, EPI_ISL_729715, EPI_ISL_729716, EPI_ISL_729717, EPI_ISL_729719, EPI_ISL_729721, EPI_ISL_729722, EPI_ISL_729728, EPI_ISL_729729, EPI_ISL_729730, EPI_ISL_729731, EPI_ISL_729732, EPI_ISL_729733			
see above	A. Krumbholz, Labor Dr. Krause und Kollegen MVZ GmbH, Kiel	Charité Universitätsmedizin Berlin, Institut für Virologie	Victor M Corman, Barbara Mühlemann, Jörn Beheim-Schwarzbach, Talitha Veith, Julia Schneider, Terry Jones, Christian Drosten
EPI_ISL_730303, EPI_ISL_730312, EPI_ISL_730319, EPI_ISL_730325, EPI_ISL_730332, EPI_ISL_730340, EPI_ISL_730349	San Diego County Public Health Laboratory	Andersen lab at Scripps Research	SEARCH Alliance San Diego with Tracy Basler, Jovan Shephard, Brett Austin

EPI_ISL_731891, EPI_ISL_731892, EPI_ISL_731893	Lighthouse Lab in Milton Keynes	Wellcome Sanger Institute for the COVID-19 Genomics UK (COG-UK) Consortium	The Lighthouse Lab in Milton Keynes and Alex Alderton, Roberto Amato, Sonia Goncalves, Ewan Harrison, David K. Jackson, Ian Johnston, Dominic Kwiatkowski, Cordelia Langford, John Sillitoe on behalf of the Wellcome Sanger Institute COVID-19 Surveillance Team
EPI_ISL_732114, EPI_ISL_732115, EPI_ISL_732116, EPI_ISL_732117	Instituto Nacional de Saude (INSA)	Instituto Nacional de Saude (INSA)	Borges et al
EPI_ISL_732426, EPI_ISL_732433	National Virus Reference Laboratory	National Virus Reference Laboratory	Michael Carr, Gabriel Gonzalez, Jonathan Dean, Daniel Hare, Cillian F De Gascun
EPI_ISL_732567, EPI_ISL_732570, EPI_ISL_732572, EPI_ISL_732576, EPI_ISL_732577, EPI_ISL_732578, EPI_ISL_732579, EPI_ISL_732580, EPI_ISL_732581, EPI_ISL_732582, EPI_ISL_732583, EPI_ISL_732584, EPI_ISL_732597, EPI_ISL_732598, EPI_ISL_732600, EPI_ISL_732612, EPI_ISL_732613, EPI_ISL_732614, EPI_ISL_732615			
see above	Department of Virology and Immunology, University of Helsinki and Helsinki University Hospital, Huslab Finland	Department of Virology, Faculty of Medicine, University of Helsinki, Helsinki, Finland	Teemu Smura, Ravi Kant, Phuoc Truong, Hussein Alburkat, Hannimari Kallio-Kokko, Jenni Virtanen, Maija Suvanto, Sari Hannula, Harri Kangas, Pekka Ellonen, Olli Vapalahti
EPI_ISL_733499	1-Laboratory of Microbiology, National Reference Lab, Charles Nicolle Hospital; 2-University of Tunis ElManar, Faculty of Medicine of Tunis, LR99ES09, Tunis, Tunisia	1-Clinical and Experimental Pharmacology Lab, LR16SP02, National Center of Pharmacovigilance, University of Tunis El Manar, Tunis, Tunisia. 2-Neurodegenerative diseases and psychiatric troubles, LR18SP03, Razi Hospital, University of Tunis El Manar, Tunis, Tunisia. 3- Ministry of Health, National Observatory of New and Emerging Diseases, 1006, Tunis, Tunisia	Ilhem Boutiba-Ben Boubaker, Sameh Trabelsi, Nissaf Ben Alaya, Maher Kharrat, Alia Ben Kahla, Jalila Ben Khelil, Salma Abid, Sana Ferjani, Mouna Ben Sassi, Mouna Safer, Guedi Ali Barreh, Habiba Ben Romdhane, Souissi Amira, Sarra Chamman, Hanen El Jebari, Asma Ferjani, Gaies Emna, Riadh Daghfous, Riadh Guider.
EPI_ISL_733576	Temporary Specimen Collection Centre	Hong Kong Department of Health	Alan K.L. Tsang, Peter C.W. Yip, Edman T.K. Lam, Rickjason C.W. Chan, Dominic N.C. Tsang
EPI_ISL_733606, EPI_ISL_733753, EPI_ISL_733883, EPI_ISL_733899, EPI_ISL_733906, EPI_ISL_733907	Lighthouse Lab in Cambridge	Wellcome Sanger Institute for the COVID-19 Genomics UK (COG-UK) Consortium	Rob Howes, The Lighthouse Lab in Cambridge and Alex Alderton, Roberto Amato, Sonia Goncalves, Ewan Harrison, David K. Jackson, Ian Johnston, Dominic Kwiatkowski, Cordelia Langford, John Sillitoe on behalf of the Wellcome Sanger Institute COVID-19 Surveillance Team
EPI_ISL_735236, EPI_ISL_735237, EPI_ISL_735238, EPI_ISL_735239, EPI_ISL_735240	UZ Leuven, National Reference Laboratory for Coronaviruses, Laboratory Medicine, Leuven, Belgium	KU Leuven, Rega Institute, Clinical and Epidemiological Virology	Tony Wawina-Bokalanga, Joan Marti-Carerras, Bert Vanmechelen, Piet Maes
EPI_ISL_735439, EPI_ISL_735440, EPI_ISL_735441, EPI_ISL_735442, EPI_ISL_735443	Pathogen Genomics Center, National Institute of Infectious Diseases	Pathogen Genomics Center, National Institute of Infectious Diseases	Tsuyoshi Sekizuka, Kentaro Itokawa, Rina Tanaka, Masanori Hashino, Makoto Kuroda
EPI_ISL_735500	University of Chittagong	Central Biological Research Laboratory and Department of Biochemistry and Molecular Biology	H. M. Abdullah Al Masud, Mohammad Omar Faruque, Sajib Rudra, Md. Khondakar Raziur Rahman, Imam Hossen, Md. Arif Hossain, Shanta Paul, Md. Omer Faruq, Md. Imranul Hoq, Robiul Hasan Bhuiyan
EPI_ISL_736891, EPI_ISL_736892, EPI_ISL_736893, EPI_ISL_736894, EPI_ISL_736895, EPI_ISL_736896, EPI_ISL_736897, EPI_ISL_736898, EPI_ISL_736899, EPI_ISL_736900, EPI_ISL_736901, EPI_ISL_736902, EPI_ISL_736903, EPI_ISL_736904, EPI_ISL_736905, EPI_ISL_736906, EPI_ISL_736907, EPI_ISL_736908, EPI_ISL_736909, EPI_ISL_736910, EPI_ISL_736911, EPI_ISL_736912, EPI_ISL_736913, EPI_ISL_736914, EPI_ISL_736915, EPI_ISL_736916, EPI_ISL_736917, EPI_ISL_736918, EPI_ISL_736919, EPI_ISL_736920, EPI_ISL_736921, EPI_ISL_736922, EPI_ISL_736923, EPI_ISL_736924			
see above	Pathogen Genomics Center, National Institute of Infectious Diseases	Pathogen Genomics Center, National Institute of Infectious Diseases	Tsuyoshi Sekizuka, Kentaro Itokawa, Rina Tanaka, Masanori Hashino, Makoto Kuroda
EPI_ISL_736969	NHLS-IALCH	KRISP, KZN Research Innovation and Sequencing Platform	Giandhari J, Pillay S, Lessells R, ChimukangaraB, Mdlalose K, York D, Khan S, Tegally H, Wilkinson E, de Oliveira T
EPI_ISL_737100, EPI_ISL_737101, EPI_ISL_737102, EPI_ISL_737107, EPI_ISL_737111, EPI_ISL_737112, EPI_ISL_737113, EPI_ISL_737114, EPI_ISL_737117, EPI_ISL_737118, EPI_ISL_737120, EPI_ISL_737121, EPI_ISL_737123, EPI_ISL_737127, EPI_ISL_737128, EPI_ISL_737129, EPI_ISL_737132, EPI_ISL_737145, EPI_ISL_737150, EPI_ISL_737152, EPI_ISL_737162, EPI_ISL_737165, EPI_ISL_737168, EPI_ISL_737181, EPI_ISL_737198			
see above	Michigan Department of Health and Human Services, Bureau of Laboratories	Michigan Department of Health and Human Services, Bureau of Laboratories	Blankenship HM, Riner D, Soehnlen MK
EPI_ISL_737208	Pathogen Genomics Center, National Institute of Infectious Diseases	Pathogen Genomics Center, National Institute of Infectious Diseases	Tsuyoshi Sekizuka, Kentaro Itokawa, Rina Tanaka, Masanori Hashino, Makoto Kuroda
EPI_ISL_737243, EPI_ISL_737244	Los Angeles County PHL	Los Angeles County PHL	P. Hemarajata et al.
EPI_ISL_737605, EPI_ISL_737637, EPI_ISL_737638, EPI_ISL_737661, EPI_ISL_737747, EPI_ISL_737748, EPI_ISL_737749, EPI_ISL_737750, EPI_ISL_737758, EPI_ISL_737762, EPI_ISL_737816, EPI_ISL_737871, EPI_ISL_737872, EPI_ISL_737873, EPI_ISL_737874, EPI_ISL_737907, EPI_ISL_737925, EPI_ISL_737926, EPI_ISL_737927, EPI_ISL_737928, EPI_ISL_737929			
see above	Viollier AG	Department of Biosystems Science and Engineering, ETH Zürich	Chaoran Chen, Sarah Nadeau, Catharine Aquino, Ivan Topolsky, Philipp Jablonski, Lara Fuhrmann, David Dreifuss, Katharina Jahn, Andreia Cabral de Gouvea, Maria Domenica Moccia, Simon Grüter, Timothy Sykes, Lennart Opitz, Griffin White, Laura Neff, Doris Popovic, Andrea Patrignani, Jay Tracy, Ralph Schlapbach, Christiane Beckmann, Maurice Redondo, Olivier Kobel, Christoph Noppen, Sophie Seidel, Noemie Santamaria de Souza, Niko Beerenwinkel, Tanja Stadler
EPI_ISL_738507	Orange County Public Health Lab	Chan-Zuckerberg Biohub	CZB Cliahub Consortium
EPI_ISL_738521, EPI_ISL_738542, EPI_ISL_738550, EPI_ISL_738555, EPI_ISL_738557	Santa Clara County Public Health Laboratory	Chan-Zuckerberg Biohub	CZB Cliahub Consortium
EPI_ISL_738567	Renegade	Chan-Zuckerberg Biohub	CZB Cliahub Consortium
EPI_ISL_738569, EPI_ISL_738570	Orange County Public Health Lab	Chan-Zuckerberg Biohub	CZB Cliahub Consortium
EPI_ISL_738574	Renegade	Chan-Zuckerberg Biohub	CZB Cliahub Consortium
EPI_ISL_738579	Santa Clara County Public Health Laboratory	Chan-Zuckerberg Biohub	CZB Cliahub Consortium
EPI_ISL_738588	Orange County Public Health Lab	Chan-Zuckerberg Biohub	CZB Cliahub Consortium
EPI_ISL_738591	Renegade	Chan-Zuckerberg Biohub	CZB Cliahub Consortium
EPI_ISL_738620, EPI_ISL_738624, EPI_ISL_738630, EPI_ISL_738634, EPI_ISL_738635, EPI_ISL_738636, EPI_ISL_738637, EPI_ISL_738638, EPI_ISL_738639, EPI_ISL_738640, EPI_ISL_738641, EPI_ISL_738646			
see above	Orange County Public Health Lab	Chan-Zuckerberg Biohub	CZB Cliahub Consortium
EPI_ISL_738662	Renegade	Chan-Zuckerberg Biohub	CZB Cliahub Consortium
EPI_ISL_738696	Santa Clara County Public Health Laboratory	Chan-Zuckerberg Biohub	CZB Cliahub Consortium
EPI_ISL_738712, EPI_ISL_738725	Renegade	Chan-Zuckerberg Biohub	CZB Cliahub Consortium
EPI_ISL_738747, EPI_ISL_738750	Santa Clara County Public Health Laboratory	Chan-Zuckerberg Biohub	CZB Cliahub Consortium
EPI_ISL_738758	Orange County Public Health Lab	Chan-Zuckerberg Biohub	CZB Cliahub Consortium
EPI_ISL_738770	Renegade	Chan-Zuckerberg Biohub	CZB Cliahub Consortium
EPI_ISL_738784	Orange County Public Health Lab	Chan-Zuckerberg Biohub	CZB Cliahub Consortium
EPI_ISL_738805	Santa Clara County Public Health Laboratory	Chan-Zuckerberg Biohub	CZB Cliahub Consortium
EPI_ISL_738816	Orange County Public Health Lab	Chan-Zuckerberg Biohub	CZB Cliahub Consortium
EPI_ISL_738834	Santa Clara County Public Health Laboratory	Chan-Zuckerberg Biohub	CZB Cliahub Consortium

EPI_ISL_738851	Renegade	Chan-Zuckerberg Biohub	CZB Cliahub Consortium
EPI_ISL_738855	Santa Clara County Public Health Laboratory	Chan-Zuckerberg Biohub	CZB Cliahub Consortium
EPI_ISL_738874, EPI_ISL_738878, EPI_ISL_738949	Orange County Public Health Lab	Chan-Zuckerberg Biohub	CZB Cliahub Consortium
EPI_ISL_738955	Santa Clara County Public Health Laboratory	Chan-Zuckerberg Biohub	CZB Cliahub Consortium
EPI_ISL_738977	Renegade	Chan-Zuckerberg Biohub	CZB Cliahub Consortium
EPI_ISL_738978, EPI_ISL_738981, EPI_ISL_739002	Orange County Public Health Lab	Chan-Zuckerberg Biohub	CZB Cliahub Consortium
EPI_ISL_739014	Renegade	Chan-Zuckerberg Biohub	CZB Cliahub Consortium
EPI_ISL_739027, EPI_ISL_739037, EPI_ISL_739040, EPI_ISL_739050, EPI_ISL_739053, EPI_ISL_739058, EPI_ISL_739068, EPI_ISL_739087, EPI_ISL_739104	Santa Clara County Public Health Laboratory	Chan-Zuckerberg Biohub	CZB Cliahub Consortium
EPI_ISL_739116	Orange County Public Health Lab	Chan-Zuckerberg Biohub	CZB Cliahub Consortium
EPI_ISL_739141, EPI_ISL_739166, EPI_ISL_739186	Santa Clara County Public Health Laboratory	Chan-Zuckerberg Biohub	CZB Cliahub Consortium
EPI_ISL_739259	Orange County Public Health Lab	Chan-Zuckerberg Biohub	CZB Cliahub Consortium
EPI_ISL_739263, EPI_ISL_739298, EPI_ISL_739319	Santa Clara County Public Health Laboratory	Chan-Zuckerberg Biohub	CZB Cliahub Consortium
EPI_ISL_739341	Orange County Public Health Lab	Chan-Zuckerberg Biohub	CZB Cliahub Consortium
EPI_ISL_739344	Santa Clara County Public Health Laboratory	Chan-Zuckerberg Biohub	CZB Cliahub Consortium
EPI_ISL_739348	Renegade	Chan-Zuckerberg Biohub	CZB Cliahub Consortium
EPI_ISL_739354	Santa Clara County Public Health Laboratory	Chan-Zuckerberg Biohub	CZB Cliahub Consortium
EPI_ISL_739355	Renegade	Chan-Zuckerberg Biohub	CZB Cliahub Consortium
EPI_ISL_739370, EPI_ISL_739371, EPI_ISL_739398, EPI_ISL_739419	Santa Clara County Public Health Laboratory	Chan-Zuckerberg Biohub	CZB Cliahub Consortium
EPI_ISL_739462	Orange County Public Health Lab	Chan-Zuckerberg Biohub	CZB Cliahub Consortium
EPI_ISL_739467, EPI_ISL_739478	Renegade	Chan-Zuckerberg Biohub	CZB Cliahub Consortium
EPI_ISL_739485, EPI_ISL_739487	Santa Clara County Public Health Laboratory	Chan-Zuckerberg Biohub	CZB Cliahub Consortium
EPI_ISL_739496	Orange County Public Health Lab	Chan-Zuckerberg Biohub	CZB Cliahub Consortium
EPI_ISL_739505	Santa Clara County Public Health Laboratory	Chan-Zuckerberg Biohub	CZB Cliahub Consortium
EPI_ISL_739524	Orange County Public Health Lab	Chan-Zuckerberg Biohub	CZB Cliahub Consortium
EPI_ISL_739548	Santa Clara County Public Health Laboratory	Chan-Zuckerberg Biohub	CZB Cliahub Consortium
EPI_ISL_739609	Orange County Public Health Lab	Chan-Zuckerberg Biohub	CZB Cliahub Consortium
EPI_ISL_739617, EPI_ISL_739650, EPI_ISL_739653	Santa Clara County Public Health Laboratory	Chan-Zuckerberg Biohub	CZB Cliahub Consortium
EPI_ISL_739662	BCCDC Public Health Laboratory	BCCDC Public Health Laboratory	Prystajecky Natalie, Linda Hoang, Dan Fornika, Shannon Russell, Kim Macdonald, Kimia Kamelian, John Tyson, Inna Sekirov, Mel Krajden
EPI_ISL_740863, EPI_ISL_740865	South Eastern Area Laboratory Services (SEALS)	NSW Health Pathology - Institute of Clinical Pathology and Medical Research; Westmead Hospital; University of Sydney	CIDM-PH et al.
EPI_ISL_740955	Department of Pathology, University of Cambridge	COVID-19 Genomics UK (COG-UK) Consortium	Aminu S. Jahun, Yasmin Chaudhry, Grant Hall, Iliana Georgana, Myra Hosmillo, Martin D. Curran, Malte Pinckert, Surendra Parmar, Ian Goodfellow
EPI_ISL_741256, EPI_ISL_741257, EPI_ISL_741258	University College London, Great Ormond Street Hospital for Children NHS Foundation Trust, Imperial College Healthcare NHS Trust	COVID-19 Genomics UK (COG-UK) Consortium	Sergi Castellano, Rachel Williams, Mark Kristiansen, Paola Resende Silva, Sunando Roy, Tony Brooks, Helena Tutill, Paola Niola, Patricia Dyal, Charlotte Williams, Leysa Forrest, Yasmin Panchbhaya, Jacqueline Findlay, Samuel Weeks, Julianne Brown, Kathryn Harris, Paul Randell, James Price, Alison Holmes, Judith Breuer
EPI_ISL_741425, EPI_ISL_741429, EPI_ISL_741430, EPI_ISL_741431, EPI_ISL_741432, EPI_ISL_741433, EPI_ISL_741434, EPI_ISL_741435	Northumbria University / South Tees Hospitals NHS Foundation Trust / North Cumbria Integrated Care NHS Foundation Trust / North Tees and Hartlepool NHS Foundation Trust / Newcastle Hospitals NHS Foundation Trust	COVID-19 Genomics UK (COG-UK) Consortium	Darren L Smith,Andrew Nelson,Matthew Bashton,Greg R Young,Joshua Loh,John Allan,Mohammad A Tariq,Giles S Holt,Gary Black,Wen C Yew,Lynn Dover,Paul Baker,Steve Liggett,Sarah Essex,Jane Greenaway,Debra Padgett,Clive Graham,Garren Scott,Edward Barton,Emma Swindells,Brendan Payne,Jennifer Collins,Yusri Taha,Gary Eltringham
EPI_ISL_741611, EPI_ISL_741612	Queens Medical Centre, Clinical Microbiology Department / DeepSeq Nottingham	COVID-19 Genomics UK (COG-UK) Consortium	Gemma Clark, Wendy Smith, Manjinder Khakh, Vicki M Fleming, Michelle M Lister, Hannah Howson-Wells, Jonathan Ball, Patrick McClure, Joseph Chappell, Theocharis Tsoleridis, Nadine Holmes, Matthew Carlisle, Christopher Moore, Fei Sang, Johnny Debebe, Victoria Wright, Matthew Loose
EPI_ISL_741737, EPI_ISL_741754	Centre for Enzyme Innovation, University of Portsmouth / Translational Research Laboratory, Portsmouth Hospitals NHS Trust	COVID-19 Genomics UK (COG-UK) Consortium	Angela Beckett,Yann Bourgeois,Garry Scarlett,Sharon Glaysher,Scott Elliott,Kelly Bicknell,Robert Impey,Allyson Lloyd,Sarah Wyllie,Ethan Butcher,Anoop Chauhan,Samuel Robson
EPI_ISL_741944, EPI_ISL_741957, EPI_ISL_741963, EPI_ISL_741980, EPI_ISL_741997, EPI_ISL_741998, EPI_ISL_742026, EPI_ISL_742032, EPI_ISL_742037, EPI_ISL_742073, EPI_ISL_742087, EPI_ISL_742102			
see above	Virology Department, Sheffield Teaching Hospitals NHS Foundation Trust/Department of Infection, Immunity and Cardiovascular Disease, The Medical School, University of Sheffield	COVID-19 Genomics UK (COG-UK) Consortium	Thushan de Silva, Matthew Parker, Nikki Smith, Adri Angyal, Rebecca Brown, Luke Green, Rachel Tucker, Paul Parsons, Danielle Groves, Katie Johnson, Laura Carrilero, Alex Keeley, Dave Partridge, Matthew Wyles, Benjamin Lindsey, Mehmet Yavuz, Mohammad Raza, Cariad Evans
EPI_ISL_742175	Virology Department, Royal Infirmary of Edinburgh, NHS Lothian / School of Biological Sciences, University of Edinburgh / Institute of Genetics and Molecular Medicine, University of Edinburgh	COVID-19 Genomics UK (COG-UK) Consortium	McHugh M, Dewar R, Rooke S, Gallagher M, Balcaza C, O'Toole Á, Scher E, Hill V, McCrone JT, Colquhoun R, Yu X, Jackson B, Rambaut A, Williams TC, Templeton K
EPI_ISL_742267, EPI_ISL_742268, EPI_ISL_742351, EPI_ISL_742353, EPI_ISL_742354, EPI_ISL_742355, EPI_ISL_742841, EPI_ISL_742842, EPI_ISL_742843, EPI_ISL_742844, EPI_ISL_742845, EPI_ISL_742846, EPI_ISL_742890, EPI_ISL_742891, EPI_ISL_742892, EPI_ISL_742893, EPI_ISL_742894, EPI_ISL_742895, EPI_ISL_742896, EPI_ISL_742897, EPI_ISL_742898, EPI_ISL_742899, EPI_ISL_742900, EPI_ISL_742901, EPI_ISL_742902, EPI_ISL_742903, EPI_ISL_742904, EPI_ISL_742905, EPI_ISL_742906, EPI_ISL_743118, EPI_ISL_743124, EPI_ISL_743125, EPI_ISL_743132, EPI_ISL_743133, EPI_ISL_743134, EPI_ISL_743135, EPI_ISL_743166, EPI_ISL_743167, EPI_ISL_743168, EPI_ISL_743169, EPI_ISL_743170, EPI_ISL_743171, EPI_ISL_743172, EPI_ISL_743173, EPI_ISL_743342, EPI_ISL_743343, EPI_ISL_743353, EPI_ISL_743354, EPI_ISL_743358, EPI_ISL_743359, EPI_ISL_743360, EPI_ISL_743361, EPI_ISL_743382, EPI_ISL_743383, EPI_ISL_743384, EPI_ISL_743385, EPI_ISL_743386, EPI_ISL_743387, EPI_ISL_743388, EPI_ISL_743389, EPI_ISL_743390, EPI_ISL_743391, EPI_ISL_743392, EPI_ISL_743393, EPI_ISL_743394, EPI_ISL_743395, EPI_ISL_743396, EPI_ISL_743398, EPI_ISL_743399, EPI_ISL_743400, EPI_ISL_743401, EPI_ISL_743402, EPI_ISL_743403, EPI_ISL_743404, EPI_ISL_743405			
see above	Wales Specialist Virology Centre Sequencing lab: Pathogen Genomics Unit	COVID-19 Genomics UK (COG-UK) Consortium	Catherine Moore, Johnathan Evans, Laura Gifford, Malorie Perry, Simon Cottrell, Angela Marchbank, Alec Birchley, Alexander Adams, Amy Gaskin, Bree Gatica-Wilcox, Jason Coombes, Joel Southgate, Lauren Gilbert, Lee Graham, Nicole Pacchiarini, Sara Kumziene-Summerhayes, Sarah Taylor, Sophie

			Jones, Sara Rey, Matthew Bull, Joanne Watkins, Sally Corden, Tom Connor
EPI_ISL_745034, EPI_ISL_745035, EPI_ISL_745036, EPI_ISL_745037, EPI_ISL_745038	Israel Central Virology laboratory	Israel Central Virology laboratory	Neta Zuckerman, Efrat Dahan Bucris, Oran Erster, Michal Mandelboim, Orna Mor, Ella Mendelson
EPI_ISL_745113	Dr Ivan Toms Clinic wc IVT	National Health Laboratory Service (NHLS), Tygerberg	Susan Engelbrecht, Kayla Delaney, Bronwyn Kleinhans, Houriiyah Tegally, Eduan Wilkindon, Gert van Zyl, Wolfgang Preiser, Tulio de Oliveira
EPI_ISL_745121	Calvinia Hospital	National Health Laboratory Service (NHLS), Tygerberg	Susan Engelbrecht, Kayla Delaney, Bronwyn Kleinhans, Houriiyah Tegally, Eduan Wilkindon, Gert van Zyl, Wolfgang Preiser, Tulio de Oliveira
EPI_ISL_745124	Laetitia Bam CHC	National Health Laboratory Service (NHLS), Tygerberg	Susan Engelbrecht, Kayla Delaney, Bronwyn Kleinhans, Houriiyah Tegally, Eduan Wilkindon, Gert van Zyl, Wolfgang Preiser, Tulio de Oliveira
EPI_ISL_745126	Okiep CHC	National Health Laboratory Service (NHLS), Tygerberg	Susan Engelbrecht, Kayla Delaney, Bronwyn Kleinhans, Houriiyah Tegally, Eduan Wilkindon, Gert van Zyl, Wolfgang Preiser, Tulio de Oliveira
EPI_ISL_745129	Port Nolloth Hospital	National Health Laboratory Service (NHLS), Tygerberg	Susan Engelbrecht, Kayla Delaney, Bronwyn Kleinhans, Houriiyah Tegally, Eduan Wilkindon, Gert van Zyl, Wolfgang Preiser, Tulio de Oliveira
EPI_ISL_745137	Edameni Clinic	National Health Laboratory Service (NHLS), Tygerberg	Susan Engelbrecht, Kayla Delaney, Bronwyn Kleinhans, Houriiyah Tegally, Eduan Wilkindon, Gert van Zyl, Wolfgang Preiser, Tulio de Oliveira
EPI_ISL_745138	Dr Van Niekerk Hospital	National Health Laboratory Service (NHLS), Tygerberg	Susan Engelbrecht, Kayla Delaney, Bronwyn Kleinhans, Houriiyah Tegally, Eduan Wilkindon, Gert van Zyl, Wolfgang Preiser, Tulio de Oliveira
EPI_ISL_745142, EPI_ISL_745143	Nomangesi Jayiya Clinic	National Health Laboratory Service (NHLS), Tygerberg	Susan Engelbrecht, Kayla Delaney, Bronwyn Kleinhans, Houriiyah Tegally, Eduan Wilkindon, Gert van Zyl, Wolfgang Preiser, Tulio de Oliveira
EPI_ISL_745151	Edameni Clinic	National Health Laboratory Service (NHLS), Tygerberg	Susan Engelbrecht, Kayla Delaney, Bronwyn Kleinhans, Houriiyah Tegally, Eduan Wilkindon, Gert van Zyl, Wolfgang Preiser, Tulio de Oliveira
EPI_ISL_745152	Laetitia Bam CHC	National Health Laboratory Service (NHLS), Tygerberg	Susan Engelbrecht, Kayla Delaney, Bronwyn Kleinhans, Houriiyah Tegally, Eduan Wilkindon, Gert van Zyl, Wolfgang Preiser, Tulio de Oliveira
EPI_ISL_745154	Dr Ivan Toms Clinic wc IVT	National Health Laboratory Service (NHLS), Tygerberg	Susan Engelbrecht, Kayla Delaney, Bronwyn Kleinhans, Houriiyah Tegally, Eduan Wilkindon, Gert van Zyl, Wolfgang Preiser, Tulio de Oliveira
EPI_ISL_745155	Langebaan Clinic wc LBC	National Health Laboratory Service (NHLS), Tygerberg	Susan Engelbrecht, Kayla Delaney, Bronwyn Kleinhans, Houriiyah Tegally, Eduan Wilkindon, Gert van Zyl, Wolfgang Preiser, Tulio de Oliveira
EPI_ISL_745156	SAS Saldanha	National Health Laboratory Service (NHLS), Tygerberg	Susan Engelbrecht, Kayla Delaney, Bronwyn Kleinhans, Houriiyah Tegally, Eduan Wilkindon, Gert van Zyl, Wolfgang Preiser, Tulio de Oliveira
EPI_ISL_745202	Lab voor klinische biologie	Onderzoeksgroep Virologie	Laurens Lambrechts, Nick Vereecke, Marthe Pauwels, Bruno Verhasselt, Linos Vandekerckhove, Hans Nauwynck, Sebastiaan Theuns
EPI_ISL_745317, EPI_ISL_745326, EPI_ISL_745327, EPI_ISL_745328, EPI_ISL_745329	CNR Virus des Infections Respiratoires - France SUD	CNR Virus des Infections Respiratoires - France SUD	Antonin Bal, Gregory Destras, Claudia Gonzalez, Gwendolyne Burfin, Quentin Semanas, Martine Valette, Bruno Lina, Laurence Josset
EPI_ISL_745336	CHU Clermont-Ferrand	CNR Virus des Infections Respiratoires - France SUD	Antonin Bal, Gregory Destras, Gwendolyne Burfin, Hadrien Règue, Quentin Semanas, Martine Valette, Bruno Lina, Christine Archimbaud, Amélie Brebion, Hélène Chabrolles, Martine Chambon, Audrey Mirand, Christel Regagnon, Maxime Bisseux, Patricia Combes, Cécile Henquell, Laurence Josset
EPI_ISL_745346, EPI_ISL_745347, EPI_ISL_745348, EPI_ISL_745349, EPI_ISL_745350, EPI_ISL_745351, EPI_ISL_745352, EPI_ISL_745353, EPI_ISL_745354, EPI_ISL_745355, EPI_ISL_745356			
see above	CNR Virus des Infections Respiratoires - France SUD	CNR Virus des Infections Respiratoires - France SUD	Antonin Bal, Gregory Destras, Claudia Gonzalez, Gwendolyne Burfin, Quentin Semanas, Martine Valette, Bruno Lina, Laurence Josset
EPI_ISL_745400	DOHMH Central Harlem	New York City Public Health Laboratory	Jade Wang, et al.
EPI_ISL_745401	DOHMH Corona	New York City Public Health Laboratory	Jade Wang, et al.
EPI_ISL_745402	DOHMH Jamaica	New York City Public Health Laboratory	Jade Wang, et al.
EPI_ISL_745403	DOHMH Corona	New York City Public Health Laboratory	Jade Wang, et al.
EPI_ISL_745405	DOHMH Jamaica	New York City Public Health Laboratory	Jade Wang, et al.
EPI_ISL_745406	DOHMH Morrisania	New York City Public Health Laboratory	Jade Wang, et al.
EPI_ISL_745408	DOHMH Riverside	New York City Public Health Laboratory	Jade Wang, et al.
EPI_ISL_745409	DOHMH Corona	New York City Public Health Laboratory	Jade Wang, et al.
EPI_ISL_745410	DOHMH Chelsea	New York City Public Health Laboratory	Jade Wang, et al.
EPI_ISL_745411	DOHMH Corona	New York City Public Health Laboratory	Jade Wang, et al.
EPI_ISL_745412	DOHMH Morrisania	New York City Public Health Laboratory	Jade Wang, et al.
EPI_ISL_745420	DOHMH Crown Heights	New York City Public Health Laboratory	Jade Wang, et al.
EPI_ISL_745421, EPI_ISL_745422	DOHMH Chelsea	New York City Public Health Laboratory	Jade Wang, et al.
EPI_ISL_745423, EPI_ISL_745424	DOHMH Corona	New York City Public Health Laboratory	Jade Wang, et al.
EPI_ISL_745425	DOHMH Central Harlem	New York City Public Health Laboratory	Jade Wang, et al.
EPI_ISL_745426, EPI_ISL_745427	DOHMH Corona	New York City Public Health Laboratory	Jade Wang, et al.
EPI_ISL_745428, EPI_ISL_745429	DOHMH Riverside	New York City Public Health Laboratory	Jade Wang, et al.
EPI_ISL_745430, EPI_ISL_745431, EPI_ISL_745432, EPI_ISL_745433	DOHMH Morrisania	New York City Public Health Laboratory	Jade Wang, et al.
EPI_ISL_745434, EPI_ISL_745435	DOHMH Corona	New York City Public Health Laboratory	Jade Wang, et al.
EPI_ISL_745436	DOHMH Chelsea	New York City Public Health Laboratory	Jade Wang, et al.
EPI_ISL_745437, EPI_ISL_745438	DOHMH PHL	New York City Public Health Laboratory	Jade Wang, et al.
EPI_ISL_745439	DOHMH Riverside	New York City Public Health Laboratory	Jade Wang, et al.
EPI_ISL_745440	DOHMH Jamaica	New York City Public Health Laboratory	Jade Wang, et al.
EPI_ISL_745445	DOHMH Crown Heights	New York City Public Health Laboratory	Jade Wang, et al.
EPI_ISL_745446, EPI_ISL_745447	DOHMH Corona	New York City Public Health Laboratory	Jade Wang, et al.
EPI_ISL_745448	DOHMH Crown Heights	New York City Public Health Laboratory	Jade Wang, et al.
EPI_ISL_745449, EPI_ISL_745450	DOHMH Morrisania	New York City Public Health Laboratory	Jade Wang, et al.
EPI_ISL_745451	DOHMH Central Harlem	New York City Public Health Laboratory	Jade Wang, et al.
EPI_ISL_745452	DOHMH Crown Heights	New York City Public Health Laboratory	Jade Wang, et al.
EPI_ISL_745453	DOHMH Chelsea	New York City Public Health Laboratory	Jade Wang, et al.
EPI_ISL_745454	DOHMH Crown Heights	New York City Public Health Laboratory	Jade Wang, et al.
EPI_ISL_745455	DOHMH Corona	New York City Public Health Laboratory	Jade Wang, et al.
EPI_ISL_745456	DOHMH PHL	New York City Public Health Laboratory	Jade Wang, et al.
EPI_ISL_745457	DOHMH Chelsea	New York City Public Health Laboratory	Jade Wang, et al.
EPI_ISL_745458, EPI_ISL_745459,	DOHMH Corona	New York City Public Health Laboratory	Jade Wang, et al.

EPI_ISL_745460, EPI_ISL_745461, EPI_ISL_745462			
EPI_ISL_745463	DOHMH Fort Greene	New York City Public Health Laboratory	Jade Wang, et al.
EPI_ISL_745464	DOHMH Corona	New York City Public Health Laboratory	Jade Wang, et al.
EPI_ISL_745465	DOHMH PHL	New York City Public Health Laboratory	Jade Wang, et al.
EPI_ISL_745466, EPI_ISL_745467	DOHMH Corona	New York City Public Health Laboratory	Jade Wang, et al.
EPI_ISL_745468	DOHMH Riverside	New York City Public Health Laboratory	Jade Wang, et al.
EPI_ISL_745469	DOHMH Central Harlem	New York City Public Health Laboratory	Jade Wang, et al.
EPI_ISL_746390, EPI_ISL_746398, EPI_ISL_746402, EPI_ISL_746403, EPI_ISL_746406, EPI_ISL_746413, EPI_ISL_746416, EPI_ISL_746422, EPI_ISL_746426, EPI_ISL_746428, EPI_ISL_746430, EPI_ISL_746431, EPI_ISL_746432, EPI_ISL_746434, EPI_ISL_746440, EPI_ISL_746446, EPI_ISL_746454, EPI_ISL_746455, EPI_ISL_746460, EPI_ISL_746461, EPI_ISL_746463, EPI_ISL_746466, EPI_ISL_746469, EPI_ISL_746837, EPI_ISL_746838, EPI_ISL_746841, EPI_ISL_746842, EPI_ISL_746843, EPI_ISL_746846, EPI_ISL_746847, EPI_ISL_746848, EPI_ISL_746849, EPI_ISL_746852, EPI_ISL_746854, EPI_ISL_746858, EPI_ISL_746860, EPI_ISL_746861, EPI_ISL_746864, EPI_ISL_746865, EPI_ISL_746868, EPI_ISL_746870, EPI_ISL_746873, EPI_ISL_746875, EPI_ISL_746876, EPI_ISL_746877, EPI_ISL_746884, EPI_ISL_746889, EPI_ISL_746891, EPI_ISL_746892, EPI_ISL_746893, EPI_ISL_746896, EPI_ISL_746897, EPI_ISL_746900, EPI_ISL_746901, EPI_ISL_746902, EPI_ISL_746903, EPI_ISL_746906, EPI_ISL_746912, EPI_ISL_746915, EPI_ISL_746916, EPI_ISL_746917, EPI_ISL_746918, EPI_ISL_746924, EPI_ISL_746926, EPI_ISL_746927, EPI_ISL_746928, EPI_ISL_746929, EPI_ISL_746931, EPI_ISL_746932, EPI_ISL_746934, EPI_ISL_746941, EPI_ISL_746942, EPI_ISL_746945, EPI_ISL_746949, EPI_ISL_746956, EPI_ISL_746957, EPI_ISL_746959, EPI_ISL_746960, EPI_ISL_746961, EPI_ISL_746962, EPI_ISL_746967, EPI_ISL_746968, EPI_ISL_746971, EPI_ISL_746973, EPI_ISL_746975, EPI_ISL_746977, EPI_ISL_746978, EPI_ISL_746980, EPI_ISL_746981, EPI_ISL_746985, EPI_ISL_746987, EPI_ISL_746988, EPI_ISL_746989, EPI_ISL_746990, EPI_ISL_746991, EPI_ISL_746993, EPI_ISL_746994, EPI_ISL_746995, EPI_ISL_747015, EPI_ISL_747016, EPI_ISL_747017, EPI_ISL_747018, EPI_ISL_747019, EPI_ISL_747024, EPI_ISL_747025, EPI_ISL_747026, EPI_ISL_747027, EPI_ISL_747028			
see above	Utah Public Health Laboratory	Utah Public Health Laboratory	Erin Young, Kelly Oakeson, Tara Gallagher
EPI_ISL_747464, EPI_ISL_747483	Ospedale San Bonifacio	Istituto Zooprofilattico Sperimentale delle Venezie	Adelaide Milani, Alessia Schivo, Annalisa Salviato, Erika Giorgia Quaranta, Ambra Pastori, Bianca Zecchin, Alice Fusaro, Isabella Monne, Calogero Terregino, Antonia Ricci
EPI_ISL_747490, EPI_ISL_747491, EPI_ISL_747493, EPI_ISL_747494, EPI_ISL_747495, EPI_ISL_747496, EPI_ISL_747497	Respiratory Virus Unit, National Infection Service, Public Health England	COVID-19 Genomics UK (COG-UK) Consortium	PHE Covid Sequencing Team
EPI_ISL_747530, EPI_ISL_747532, EPI_ISL_747534, EPI_ISL_747537, EPI_ISL_747538, EPI_ISL_747539, EPI_ISL_747577, EPI_ISL_747578, EPI_ISL_747579, EPI_ISL_747580, EPI_ISL_747581, EPI_ISL_747582, EPI_ISL_747583, EPI_ISL_747584, EPI_ISL_747596, EPI_ISL_747603, EPI_ISL_747606, EPI_ISL_747624, EPI_ISL_747625, EPI_ISL_747626, EPI_ISL_747627, EPI_ISL_747628, EPI_ISL_747633, EPI_ISL_747634, EPI_ISL_747635, EPI_ISL_747641, EPI_ISL_747646, EPI_ISL_747650, EPI_ISL_747654, EPI_ISL_747657, EPI_ISL_747675, EPI_ISL_747676, EPI_ISL_747677, EPI_ISL_747678, EPI_ISL_747679, EPI_ISL_747709, EPI_ISL_747710, EPI_ISL_747711, EPI_ISL_747712, EPI_ISL_747713, EPI_ISL_747714, EPI_ISL_747715, EPI_ISL_747716, EPI_ISL_747717, EPI_ISL_747718, EPI_ISL_747719, EPI_ISL_747724, EPI_ISL_747725, EPI_ISL_747726, EPI_ISL_747727, EPI_ISL_747730, EPI_ISL_747735, EPI_ISL_747736, EPI_ISL_747737, EPI_ISL_747738, EPI_ISL_747755, EPI_ISL_748021, EPI_ISL_748022, EPI_ISL_748023, EPI_ISL_748024, EPI_ISL_748025, EPI_ISL_748026, EPI_ISL_748027, EPI_ISL_748028, EPI_ISL_748029, EPI_ISL_748030, EPI_ISL_748031, EPI_ISL_748032, EPI_ISL_748033, EPI_ISL_748034, EPI_ISL_748035, EPI_ISL_748036, EPI_ISL_748037, EPI_ISL_748038, EPI_ISL_748039, EPI_ISL_748040, EPI_ISL_748041, EPI_ISL_748042, EPI_ISL_748043, EPI_ISL_748044, EPI_ISL_748045, EPI_ISL_748046, EPI_ISL_748047, EPI_ISL_748048, EPI_ISL_748049, EPI_ISL_748050, EPI_ISL_748051, EPI_ISL_748052, EPI_ISL_748053, EPI_ISL_748054, EPI_ISL_748055, EPI_ISL_748056, EPI_ISL_748057, EPI_ISL_748058, EPI_ISL_748059, EPI_ISL_748060, EPI_ISL_748061, EPI_ISL_748062, EPI_ISL_748063, EPI_ISL_748064, EPI_ISL_748065, EPI_ISL_748066, EPI_ISL_748067, EPI_ISL_748068, EPI_ISL_748069, EPI_ISL_748070, EPI_ISL_748071, EPI_ISL_748072, EPI_ISL_748073, EPI_ISL_748074, EPI_ISL_748075, EPI_ISL_748076, EPI_ISL_748077, EPI_ISL_748078, EPI_ISL_748079, EPI_ISL_748080, EPI_ISL_748081, EPI_ISL_748082, EPI_ISL_748083, EPI_ISL_748084, EPI_ISL_748085, EPI_ISL_748086, EPI_ISL_748087, EPI_ISL_748088, EPI_ISL_748089, EPI_ISL_748090, EPI_ISL_748091, EPI_ISL_748092, EPI_ISL_748093, EPI_ISL_748094, EPI_ISL_748095, EPI_ISL_748096, EPI_ISL_748097, EPI_ISL_748098			
see above	Department of Virus and Microbiological Special Diagnostics, Statens Serum Institut, Copenhagen, Denmark	Albertsen Lab, Department of Chemistry and Bioscience, Aalborg University, Denmark	Danish Covid-19 Genome Consortium
EPI_ISL_751270	Devki Devi Foundation, a unit of Max Healthcare	CSIR-IGIB/Max	Rajesh Pandey#, Samreen Siddiqui, Janani Srinivasa Vasudevan, Akshay Kanakan, Ranjeet Maurya, Uzma Shamim, Bansidhar Tarai, Akansha Tyagi, Mitali Mukerji, Poonam Das, Sujeet Jha, Mohammed Faruq, Anurag Agrawal
EPI_ISL_751542	SC Dept of Health and Env. Control-Bureau of Laboratories	Genomics and Discovery, Respiratory Viruses Branch, Division of Viral Diseases, Centers for Disease Control and Prevention	Krista Queen, Yan Li, Ying Tao, Jing Zhang, Anna Uehara, Anna Montmayeur, Clinton R. Paden, Peter W. Cook,Rachel Marine, Mili Sheth, Haibin Wang, Justin Lee, Suxiang Tong
EPI_ISL_751549	MS Public Health Laboratory	Genomics and Discovery, Respiratory Viruses Branch, Division of Viral Diseases, Centers for Disease Control and Prevention	Krista Queen, Yan Li, Ying Tao, Jing Zhang, Anna Uehara, Anna Montmayeur, Clinton R. Paden, Peter W. Cook,Rachel Marine, Mili Sheth, Haibin Wang, Justin Lee, Suxiang Tong
EPI_ISL_751550	SC Dept of Health and Env. Control-Bureau of Laboratories	Genomics and Discovery, Respiratory Viruses Branch, Division of Viral Diseases, Centers for Disease Control and Prevention	Krista Queen, Yan Li, Ying Tao, Jing Zhang, Anna Uehara, Anna Montmayeur, Clinton R. Paden, Peter W. Cook,Rachel Marine, Mili Sheth, Haibin Wang, Justin Lee, Suxiang Tong
EPI_ISL_751559	MD DOH Laboratories Administration	Genomics and Discovery, Respiratory Viruses Branch, Division of Viral Diseases, Centers for Disease Control and Prevention	Krista Queen, Yan Li, Ying Tao, Jing Zhang, Anna Uehara, Anna Montmayeur, Clinton R. Paden, Peter W. Cook,Rachel Marine, Mili Sheth, Haibin Wang, Justin Lee, Suxiang Tong
EPI_ISL_751566, EPI_ISL_751589	WVDHHR - Office of Laboratory Services	Genomics and Discovery, Respiratory Viruses Branch, Division of Viral Diseases, Centers for Disease Control and Prevention	Krista Queen, Yan Li, Ying Tao, Jing Zhang, Anna Uehara, Anna Montmayeur, Clinton R. Paden, Peter W. Cook,Rachel Marine, Mili Sheth, Haibin Wang, Justin Lee, Suxiang Tong
EPI_ISL_751611	SC Dept of Health and Env. Control-Bureau of Laboratories	Genomics and Discovery, Respiratory Viruses Branch, Division of Viral Diseases, Centers for Disease Control and Prevention	Krista Queen, Yan Li, Ying Tao, Jing Zhang, Anna Uehara, Anna Montmayeur, Clinton R. Paden, Peter W. Cook,Rachel Marine, Mili Sheth, Haibin Wang, Justin Lee, Suxiang Tong
EPI_ISL_751615	OR State PHL-Virology/Immunology Section	Genomics and Discovery, Respiratory Viruses Branch, Division of Viral Diseases, Centers for Disease Control and Prevention	Krista Queen, Yan Li, Ying Tao, Jing Zhang, Anna Uehara, Anna Montmayeur, Clinton R. Paden, Peter W. Cook,Rachel Marine, Mili Sheth, Haibin Wang, Justin Lee, Suxiang Tong
EPI_ISL_751617	TX DSHS, Lab Services Section MC 1947	Genomics and Discovery, Respiratory Viruses Branch, Division of Viral Diseases, Centers for Disease Control and Prevention	Krista Queen, Yan Li, Ying Tao, Jing Zhang, Anna Uehara, Anna Montmayeur, Clinton R. Paden, Peter W. Cook,Rachel Marine, Mili Sheth, Haibin Wang, Justin Lee, Suxiang Tong
EPI_ISL_751620	LA Office of Public Health Laboratories	Genomics and Discovery, Respiratory Viruses Branch, Division of Viral Diseases, Centers for Disease Control and Prevention	Krista Queen, Yan Li, Ying Tao, Jing Zhang, Anna Uehara, Anna Montmayeur, Clinton R. Paden, Peter W. Cook,Rachel Marine, Mili Sheth, Haibin Wang, Justin Lee, Suxiang Tong
EPI_ISL_751633	MT Public Health Laboratory	Genomics and Discovery, Respiratory Viruses Branch, Division of Viral Diseases, Centers for Disease Control and Prevention	Krista Queen, Yan Li, Ying Tao, Jing Zhang, Anna Uehara, Anna Montmayeur, Clinton R. Paden, Peter W. Cook,Rachel Marine, Mili Sheth, Haibin Wang, Justin Lee, Suxiang Tong
EPI_ISL_751634	MO State Public Health Laboratory	Genomics and Discovery, Respiratory Viruses Branch, Division of Viral Diseases, Centers for Disease Control and Prevention	Krista Queen, Yan Li, Ying Tao, Jing Zhang, Anna Uehara, Anna Montmayeur, Clinton R. Paden, Peter W. Cook,Rachel Marine, Mili Sheth, Haibin Wang, Justin Lee, Suxiang Tong
EPI_ISL_751640	MT Public Health Laboratory	Genomics and Discovery, Respiratory Viruses Branch, Division of Viral Diseases, Centers for Disease Control and Prevention	Krista Queen, Yan Li, Ying Tao, Jing Zhang, Anna Uehara, Anna Montmayeur, Clinton R. Paden, Peter W. Cook,Rachel Marine, Mili Sheth, Haibin Wang, Justin Lee, Suxiang Tong
EPI_ISL_751645	TX DSHS, Lab Services Section MC 1947	Genomics and Discovery, Respiratory Viruses Branch, Division of Viral Diseases, Centers for Disease Control and Prevention	Krista Queen, Yan Li, Ying Tao, Jing Zhang, Anna Uehara, Anna Montmayeur, Clinton R. Paden, Peter W. Cook,Rachel Marine, Mili Sheth, Haibin Wang, Justin Lee, Suxiang Tong
EPI_ISL_751655	MN PHL Division, Minnesota Department of Health	Genomics and Discovery, Respiratory Viruses Branch, Division of Viral Diseases, Centers for Disease Control and	Krista Queen, Yan Li, Ying Tao, Jing Zhang, Anna Uehara, Anna Montmayeur, Clinton R. Paden, Peter W. Cook,Rachel Marine, Mili Sheth, Haibin Wang, Justin Lee, Suxiang Tong

		Prevention	
EPI_ISL_751661	MS Public Health Laboratory	Genomics and Discovery, Respiratory Viruses Branch, Division of Viral Diseases, Centers for Disease Control and Prevention	Krista Queen, Yan Li, Ying Tao, Jing Zhang, Anna Uehara, Anna Montmayeur, Clinton R. Paden, Peter W. Cook,Rachel Marine, Mili Sheth, Haibin Wang, Justin Lee, Suxiang Tong
EPI_ISL_751664, EPI_ISL_751665	SC Dept of Health and Env. Control-Bureau of Laboratories	Genomics and Discovery, Respiratory Viruses Branch, Division of Viral Diseases, Centers for Disease Control and Prevention	Krista Queen, Yan Li, Ying Tao, Jing Zhang, Anna Uehara, Anna Montmayeur, Clinton R. Paden, Peter W. Cook,Rachel Marine, Mili Sheth, Haibin Wang, Justin Lee, Suxiang Tong
EPI_ISL_751693	OR State PHL-Virology/Immunology Section	Genomics and Discovery, Respiratory Viruses Branch, Division of Viral Diseases, Centers for Disease Control and Prevention	Krista Queen, Yan Li, Ying Tao, Jing Zhang, Anna Uehara, Anna Montmayeur, Clinton R. Paden, Peter W. Cook,Rachel Marine, Mili Sheth, Haibin Wang, Justin Lee, Suxiang Tong
EPI_ISL_751696	LA Office of Public Health Laboratories	Genomics and Discovery, Respiratory Viruses Branch, Division of Viral Diseases, Centers for Disease Control and Prevention	Krista Queen, Yan Li, Ying Tao, Jing Zhang, Anna Uehara, Anna Montmayeur, Clinton R. Paden, Peter W. Cook,Rachel Marine, Mili Sheth, Haibin Wang, Justin Lee, Suxiang Tong
EPI_ISL_751703	MD DOH Laboratories Administration	Genomics and Discovery, Respiratory Viruses Branch, Division of Viral Diseases, Centers for Disease Control and Prevention	Krista Queen, Yan Li, Ying Tao, Jing Zhang, Anna Uehara, Anna Montmayeur, Clinton R. Paden, Peter W. Cook,Rachel Marine, Mili Sheth, Haibin Wang, Justin Lee, Suxiang Tong
EPI_ISL_751713	MT Public Health Laboratory	Genomics and Discovery, Respiratory Viruses Branch, Division of Viral Diseases, Centers for Disease Control and Prevention	Krista Queen, Yan Li, Ying Tao, Jing Zhang, Anna Uehara, Anna Montmayeur, Clinton R. Paden, Peter W. Cook,Rachel Marine, Mili Sheth, Haibin Wang, Justin Lee, Suxiang Tong
EPI_ISL_751718	SC Dept of Health and Env. Control-Bureau of Laboratories	Genomics and Discovery, Respiratory Viruses Branch, Division of Viral Diseases, Centers for Disease Control and Prevention	Krista Queen, Yan Li, Ying Tao, Jing Zhang, Anna Uehara, Anna Montmayeur, Clinton R. Paden, Peter W. Cook,Rachel Marine, Mili Sheth, Haibin Wang, Justin Lee, Suxiang Tong
EPI_ISL_751728	CO Dept. of Public Health and Environment, Lab Services Division	Genomics and Discovery, Respiratory Viruses Branch, Division of Viral Diseases, Centers for Disease Control and Prevention	Krista Queen, Yan Li, Ying Tao, Jing Zhang, Anna Uehara, Anna Montmayeur, Clinton R. Paden, Peter W. Cook,Rachel Marine, Mili Sheth, Haibin Wang, Justin Lee, Suxiang Tong
EPI_ISL_751740	MO State Public Health Laboratory	Genomics and Discovery, Respiratory Viruses Branch, Division of Viral Diseases, Centers for Disease Control and Prevention	Krista Queen, Yan Li, Ying Tao, Jing Zhang, Anna Uehara, Anna Montmayeur, Clinton R. Paden, Peter W. Cook,Rachel Marine, Mili Sheth, Haibin Wang, Justin Lee, Suxiang Tong
EPI_ISL_751744	SC Dept of Health and Env. Control-Bureau of Laboratories	Genomics and Discovery, Respiratory Viruses Branch, Division of Viral Diseases, Centers for Disease Control and Prevention	Krista Queen, Yan Li, Ying Tao, Jing Zhang, Anna Uehara, Anna Montmayeur, Clinton R. Paden, Peter W. Cook,Rachel Marine, Mili Sheth, Haibin Wang, Justin Lee, Suxiang Tong
EPI_ISL_751746	WVDHHR - Office of Laboratory Services	Genomics and Discovery, Respiratory Viruses Branch, Division of Viral Diseases, Centers for Disease Control and Prevention	Krista Queen, Yan Li, Ying Tao, Jing Zhang, Anna Uehara, Anna Montmayeur, Clinton R. Paden, Peter W. Cook,Rachel Marine, Mili Sheth, Haibin Wang, Justin Lee, Suxiang Tong
EPI_ISL_751749	TX DSHS, Lab Services Section MC 1947	Genomics and Discovery, Respiratory Viruses Branch, Division of Viral Diseases, Centers for Disease Control and Prevention	Krista Queen, Yan Li, Ying Tao, Jing Zhang, Anna Uehara, Anna Montmayeur, Clinton R. Paden, Peter W. Cook,Rachel Marine, Mili Sheth, Haibin Wang, Justin Lee, Suxiang Tong
EPI_ISL_751752	LA Office of Public Health Laboratories	Genomics and Discovery, Respiratory Viruses Branch, Division of Viral Diseases, Centers for Disease Control and Prevention	Krista Queen, Yan Li, Ying Tao, Jing Zhang, Anna Uehara, Anna Montmayeur, Clinton R. Paden, Peter W. Cook,Rachel Marine, Mili Sheth, Haibin Wang, Justin Lee, Suxiang Tong
EPI_ISL_751763	CO Dept. of Public Health and Environment, Lab Services Division	Genomics and Discovery, Respiratory Viruses Branch, Division of Viral Diseases, Centers for Disease Control and Prevention	Krista Queen, Yan Li, Ying Tao, Jing Zhang, Anna Uehara, Anna Montmayeur, Clinton R. Paden, Peter W. Cook,Rachel Marine, Mili Sheth, Haibin Wang, Justin Lee, Suxiang Tong
EPI_ISL_751771	MD DOH Laboratories Administration	Genomics and Discovery, Respiratory Viruses Branch, Division of Viral Diseases, Centers for Disease Control and Prevention	Krista Queen, Yan Li, Ying Tao, Jing Zhang, Anna Uehara, Anna Montmayeur, Clinton R. Paden, Peter W. Cook,Rachel Marine, Mili Sheth, Haibin Wang, Justin Lee, Suxiang Tong
EPI_ISL_751772	HI Dept. of Health, State Laboratories Division	Genomics and Discovery, Respiratory Viruses Branch, Division of Viral Diseases, Centers for Disease Control and Prevention	Krista Queen, Yan Li, Ying Tao, Jing Zhang, Anna Uehara, Anna Montmayeur, Clinton R. Paden, Peter W. Cook,Rachel Marine, Mili Sheth, Haibin Wang, Justin Lee, Suxiang Tong
EPI_ISL_751775	MT Public Health Laboratory	Genomics and Discovery, Respiratory Viruses Branch, Division of Viral Diseases, Centers for Disease Control and Prevention	Krista Queen, Yan Li, Ying Tao, Jing Zhang, Anna Uehara, Anna Montmayeur, Clinton R. Paden, Peter W. Cook,Rachel Marine, Mili Sheth, Haibin Wang, Justin Lee, Suxiang Tong
EPI_ISL_751780	ID Bureau of Laboratories	Genomics and Discovery, Respiratory Viruses Branch, Division of Viral Diseases, Centers for Disease Control and Prevention	Krista Queen, Yan Li, Ying Tao, Jing Zhang, Anna Uehara, Anna Montmayeur, Clinton R. Paden, Peter W. Cook,Rachel Marine, Mili Sheth, Haibin Wang, Justin Lee, Suxiang Tong
EPI_ISL_751785, EPI_ISL_751786	WVDHHR - Office of Laboratory Services	Genomics and Discovery, Respiratory Viruses Branch, Division of Viral Diseases, Centers for Disease Control and Prevention	Krista Queen, Yan Li, Ying Tao, Jing Zhang, Anna Uehara, Anna Montmayeur, Clinton R. Paden, Peter W. Cook,Rachel Marine, Mili Sheth, Haibin Wang, Justin Lee, Suxiang Tong
EPI_ISL_751790	RI State Health Laboratories	Genomics and Discovery, Respiratory Viruses Branch, Division of Viral Diseases, Centers for Disease Control and Prevention	Krista Queen, Yan Li, Ying Tao, Jing Zhang, Anna Uehara, Anna Montmayeur, Clinton R. Paden, Peter W. Cook,Rachel Marine, Mili Sheth, Haibin Wang, Justin Lee, Suxiang Tong
EPI_ISL_751794	KY State Public Health Lab	Genomics and Discovery, Respiratory Viruses Branch, Division of Viral Diseases, Centers for Disease Control and Prevention	Krista Queen, Yan Li, Ying Tao, Jing Zhang, Anna Uehara, Anna Montmayeur, Clinton R. Paden, Peter W. Cook,Rachel Marine, Mili Sheth, Haibin Wang, Justin Lee, Suxiang Tong
EPI_ISL_751796, EPI_ISL_751797, EPI_ISL_751798	Public Health Ontario Laboratory	Public Health Ontario Laboratory	Vanessa G Allen, Philip Banh, Richard de Borja, Yao Chen, Alireza Eshaghi, Nahuel Fittipaldi, Christine Frantz, Jonathan B Gubbay, Jennifer L Guthrie, Lawrence Heisler, Esha Joshi, Michael Laszloffy, Aimin Li, Michael CY Li, Dean Maxwell, Sandeep Nagra, Samir N Patel, Heather Rilkoff, Jared Simpson, Karthikeyan Sivaraman, Yogi Sundaravadanam, Sarah Teatero, Andre Villegas, Sandra Zittermann
EPI_ISL_753176, EPI_ISL_753177, EPI_ISL_753178, EPI_ISL_753179, EPI_ISL_753180, EPI_ISL_753181, EPI_ISL_753182, EPI_ISL_753183, EPI_ISL_753184, EPI_ISL_753185, EPI_ISL_753186, EPI_ISL_753187, EPI_ISL_753188, EPI_ISL_753189, EPI_ISL_753190, EPI_ISL_753191, EPI_ISL_753200, EPI_ISL_753201, EPI_ISL_753202, EPI_ISL_753203, EPI_ISL_753204, EPI_ISL_753205, EPI_ISL_753206, EPI_ISL_753207, EPI_ISL_753208, EPI_ISL_753209, EPI_ISL_753210, EPI_ISL_753211, EPI_ISL_753212, EPI_ISL_753213, EPI_ISL_753216, EPI_ISL_753217, EPI_ISL_753218, EPI_ISL_753219, EPI_ISL_753220, EPI_ISL_753240			
see above	State Laboratories Division, Hawaii State Department of Health	State Laboratories Division, Hawaii State Department of Health	Pamela O'Brien, Sabrina Diemert, Drew Kuwazaki, Razvan Sultana, Edward Desmond
EPI_ISL_753413, EPI_ISL_753414, EPI_ISL_753415, EPI_ISL_753416, EPI_ISL_753417, EPI_ISL_753418, EPI_ISL_753419, EPI_ISL_753420, EPI_ISL_753421, EPI_ISL_753422, EPI_ISL_753423, EPI_ISL_753424, EPI_ISL_753425, EPI_ISL_753426, EPI_ISL_753427, EPI_ISL_753428, EPI_ISL_753429, EPI_ISL_753430, EPI_ISL_753431, EPI_ISL_753432, EPI_ISL_753433, EPI_ISL_753434, EPI_ISL_753435, EPI_ISL_753436, EPI_ISL_753437, EPI_ISL_753438, EPI_ISL_753439, EPI_ISL_753440, EPI_ISL_753441, EPI_ISL_753442, EPI_ISL_753443, EPI_ISL_753444, EPI_ISL_753445, EPI_ISL_753446, EPI_ISL_753447, EPI_ISL_753448, EPI_ISL_753449, EPI_ISL_753450, EPI_ISL_753451, EPI_ISL_753452, EPI_ISL_753453, EPI_ISL_753454, EPI_ISL_753455, EPI_ISL_753456, EPI_ISL_753457, EPI_ISL_753458, EPI_ISL_753459, EPI_ISL_753460, EPI_ISL_753461, EPI_ISL_753462, EPI_ISL_753463, EPI_ISL_753464, EPI_ISL_753465, EPI_ISL_753466, EPI_ISL_753467, EPI_ISL_753468, EPI_ISL_753469, EPI_ISL_753470, EPI_ISL_753471, EPI_ISL_753472, EPI_ISL_753473, EPI_ISL_753474, EPI_ISL_753475, EPI_ISL_753476, EPI_ISL_753477, EPI_ISL_753478, EPI_ISL_753479, EPI_ISL_753480, EPI_ISL_753481, EPI_ISL_753482, EPI_ISL_753483, EPI_ISL_753484,			

EPI_ISL_753485, EPI_ISL_753486, EPI_ISL_753487, EPI_ISL_753488, EPI_ISL_753489, EPI_ISL_753490, EPI_ISL_753491, EPI_ISL_753492, EPI_ISL_753493, EPI_ISL_753494, EPI_ISL_753495, EPI_ISL_753496, EPI_ISL_753497, EPI_ISL_753498, EPI_ISL_753499, EPI_ISL_753500, EPI_ISL_753501, EPI_ISL_753502, EPI_ISL_753503, EPI_ISL_753504, EPI_ISL_753505, EPI_ISL_753506, EPI_ISL_753507, EPI_ISL_753508, EPI_ISL_753509, EPI_ISL_753510, EPI_ISL_753511, EPI_ISL_753512, EPI_ISL_753513, EPI_ISL_753514, EPI_ISL_753515, EPI_ISL_753516, EPI_ISL_753517, EPI_ISL_753518, EPI_ISL_753519, EPI_ISL_753520, EPI_ISL_753521, EPI_ISL_753522, EPI_ISL_753523, EPI_ISL_753524, EPI_ISL_753525, EPI_ISL_753526, EPI_ISL_753534, EPI_ISL_753535, EPI_ISL_753536, EPI_ISL_753537, EPI_ISL_753538, EPI_ISL_753539, EPI_ISL_753540, EPI_ISL_753541, EPI_ISL_753542, EPI_ISL_753543, EPI_ISL_753544, EPI_ISL_753545, EPI_ISL_753546, EPI_ISL_753547, EPI_ISL_753548, EPI_ISL_753549, EPI_ISL_753550, EPI_ISL_753551, EPI_ISL_753552, EPI_ISL_753553, EPI_ISL_753554, EPI_ISL_753555, EPI_ISL_753556, EPI_ISL_753557, EPI_ISL_753558, EPI_ISL_753559, EPI_ISL_753560, EPI_ISL_753561, EPI_ISL_753562, EPI_ISL_753563, EPI_ISL_753564, EPI_ISL_753565, EPI_ISL_753566, EPI_ISL_753567, EPI_ISL_753568, EPI_ISL_753569, EPI_ISL_753570, EPI_ISL_753571, EPI_ISL_753572, EPI_ISL_753573, EPI_ISL_753574, EPI_ISL_753575, EPI_ISL_753576, EPI_ISL_753577, EPI_ISL_753578, EPI_ISL_753579, EPI_ISL_753580, EPI_ISL_753581, EPI_ISL_753582, EPI_ISL_753583, EPI_ISL_753584, EPI_ISL_753585, EPI_ISL_753586, EPI_ISL_753587, EPI_ISL_753588, EPI_ISL_753589, EPI_ISL_753590, EPI_ISL_753591, EPI_ISL_753592, EPI_ISL_753593, EPI_ISL_753594, EPI_ISL_753624, EPI_ISL_753625, EPI_ISL_753627, EPI_ISL_753628, EPI_ISL_753630, EPI_ISL_753633, EPI_ISL_753634, EPI_ISL_753646, EPI_ISL_753653			
see above	Clinical virology Laboratory, Children's Hospital Los Angeles	Center for Personalized Medicine, Children's Hospital Los Angeles	Gai et al
EPI_ISL_753697	Specialized Lab for COVID-19 Detection, Department of Genetic Engineering and Biotechnology	Specialized Lab for COVID-19 Detection, Department of Genetic Engineering and Biotechnology	Shamsul H. Prodhan, Md. Asraful Jahan, Hammadul Hoque, Nurnabi Azad Jewel, Md. Nazmul Hasan, Hafiz Al Ashad, Rahatul Islam, Salman Ahmed
EPI_ISL_753700	Specialized Lab for COVID-19 Detection, Department of Genetic Engineering and Biotechnology	Specialized Lab for COVID-19 Detection, Department of Genetic Engineering and Biotechnology	Hammadul Hoque, Ajit Ghosh, Nurnabi Azad Jewel, Md. Nazmul Hasan, Shamsul H. Prodhan, Amit Kumar Mondal, Fahmid H. Bhuiyan, Md. Rakib Wazed Nayon
EPI_ISL_753701	Specialized Lab for COVID-19 Detection, Department of Genetic Engineering and Biotechnology	Specialized Lab for COVID-19 Detection, Department of Genetic Engineering and Biotechnology	Nurnabi Azad Jewel, Ziaul Faruque Joy , Md. Nazmul Hasan, Shamsul H. Prodhan, Hammadul Hoque , Md. Mobarok Karim, Shahrear Arefin, Md. Tahsin Khan
EPI_ISL_754059	Specialized Lab for COVID-19 Detection, Department of Genetic Engineering and Biotechnology	Specialized Lab for COVID-19 Detection, Department of Genetic Engineering and Biotechnology	Md. Nazmul Hasan, Md. Akkas Ali, Shamsul H. Prodhan, Hammadul Hoque , Nurnabi Azad Jewel, Md Mohsin Bapary, Diptha Chakraborthy
EPI_ISL_754109	Specialized Lab for COVID-19 Detection, Department of Genetic Engineering and Biotechnology	Specialized Lab for COVID-19 Detection, Department of Genetic Engineering and Biotechnology	Hammadul Hoque , Nurnabi Azad Jewel, Md. Nazmul Hasan, Shamsul H. Prodhan, Md. Mosarof Hossen, Md. Mashiur Rahaman
EPI_ISL_754148, EPI_ISL_754156, EPI_ISL_754157, EPI_ISL_754158, EPI_ISL_754160, EPI_ISL_754164	Laboratoire de Microbiologie	National Reference Center for Viruses of Respiratory Infections, Institut Pasteur, Paris	Marion Barbet, Sylvie Behillil, Méline Bizard, Angela Brisebarre, Camille Capel, Etienne Simon-Lorière, Vincent Enouf, Maud Vanpeene, Sylvie van der Werf, Marie-Sarah Fangous
EPI_ISL_754176	Specialized Lab for COVID-19 Detection, Department of Genetic Engineering and Biotechnology	Specialized Lab for COVID-19 Detection, Department of Genetic Engineering and Biotechnology	Shamsul H. Prodhan, Hammadul Hoque, Nurnabi Azad Jewel, Md. Nazmul Hasan, Md. Mainul Hossain Bakul, Md. Rabiul Awal
EPI_ISL_754242, EPI_ISL_754244, EPI_ISL_754248, EPI_ISL_754249, EPI_ISL_754250, EPI_ISL_754258, EPI_ISL_754260, EPI_ISL_754290, EPI_ISL_754298, EPI_ISL_754299, EPI_ISL_754300, EPI_ISL_754301, EPI_ISL_754304, EPI_ISL_754339, EPI_ISL_754340, EPI_ISL_754341, EPI_ISL_754342, EPI_ISL_754344, EPI_ISL_754345, EPI_ISL_754346, EPI_ISL_754347, EPI_ISL_754351, EPI_ISL_754354, EPI_ISL_754355			
see above	Respiratory Virus Unit, National Infection Service, Public Health England	COVID-19 Genomics UK (COG-UK) Consortium	PHE Covid Sequencing Team
EPI_ISL_754388, EPI_ISL_754389	Wyoming Public Health Laboratory	Wyoming Public Health Laboratory	Noah Hull, Taylor Fearing, Channing Weber, Ashley Norberg, Bailey Bowcutt, and Wanda Manley
EPI_ISL_754395, EPI_ISL_754396, EPI_ISL_754397, EPI_ISL_754398, EPI_ISL_754403, EPI_ISL_754407, EPI_ISL_754409, EPI_ISL_754410, EPI_ISL_754411, EPI_ISL_754412, EPI_ISL_754413, EPI_ISL_754414, EPI_ISL_754415, EPI_ISL_754416, EPI_ISL_754417, EPI_ISL_754418, EPI_ISL_754419, EPI_ISL_754420, EPI_ISL_754421, EPI_ISL_754422, EPI_ISL_754423, EPI_ISL_754424, EPI_ISL_754425, EPI_ISL_754426, EPI_ISL_754427, EPI_ISL_754428, EPI_ISL_754429, EPI_ISL_754430, EPI_ISL_754431, EPI_ISL_754432, EPI_ISL_754433, EPI_ISL_754434, EPI_ISL_754435, EPI_ISL_754436, EPI_ISL_754437, EPI_ISL_754438, EPI_ISL_754505, EPI_ISL_754506, EPI_ISL_754507, EPI_ISL_754508, EPI_ISL_754509			
see above	Wadsworth Center, New York State Department.of Health	Wadsworth Center, New York State Department.of Health	Kirsten St. George, Daryl M. Lamson, Alexis Russel, Matthew Shudt, Melissa A Leisner, Jonathan Plitnick, Navjot Singh, John Kelly, Sara Griesemer, Erasmus Schneider, Erica Lasek-Nesselquist
EPI_ISL_754657, EPI_ISL_754658	University of Wisconsin-Madison AIDS Vaccine Research Laboratories	University of Wisconsin-Madison AIDS Vaccine Research Laboratories	Gage Moreno, Katarina Braun, et al. AIDS Vaccine Research Laboratories
EPI_ISL_754772, EPI_ISL_754775	Emory Molecular Diagnostics Laboratory, Emory Healthcare	Piantadosi Lab, Emory Department of Pathology	Ahmed Babiker, Anne Piantadosi
EPI_ISL_755132, EPI_ISL_755138	San Diego County Public Health Laboratory	Andersen lab at Scripps Research	SEARCH Alliance San Diego with Tracy Basler, Jovan Shephard, Brett Austin
EPI_ISL_755198, EPI_ISL_755199, EPI_ISL_755200, EPI_ISL_755201, EPI_ISL_755202, EPI_ISL_755203, EPI_ISL_755204, EPI_ISL_755205, EPI_ISL_755209, EPI_ISL_755216, EPI_ISL_755217, EPI_ISL_755218, EPI_ISL_755219, EPI_ISL_755220, EPI_ISL_755226, EPI_ISL_755227, EPI_ISL_755228, EPI_ISL_755230, EPI_ISL_755231			
see above	UCSD EXCITE lab	Andersen lab at Scripps Research	SEARCH Alliance San Diego
EPI_ISL_755279, EPI_ISL_755283, EPI_ISL_755285, EPI_ISL_755286, EPI_ISL_755289, EPI_ISL_755294	San Diego County Public Health Laboratory	Andersen lab at Scripps Research	SEARCH Alliance San Diego with Tracy Basler, Jovan Shephard, Brett Austin
EPI_ISL_755434, EPI_ISL_755435, EPI_ISL_755436, EPI_ISL_755437, EPI_ISL_755438, EPI_ISL_755439, EPI_ISL_755440, EPI_ISL_755441, EPI_ISL_755442, EPI_ISL_755443, EPI_ISL_755444, EPI_ISL_755445, EPI_ISL_755446, EPI_ISL_755447, EPI_ISL_755448, EPI_ISL_755449, EPI_ISL_755450, EPI_ISL_755451, EPI_ISL_755452, EPI_ISL_755453, EPI_ISL_755454, EPI_ISL_755455, EPI_ISL_755482, EPI_ISL_755483, EPI_ISL_755484, EPI_ISL_755485, EPI_ISL_755486, EPI_ISL_755487, EPI_ISL_755488			
see above	Maine Health and Environmental Testing Laboratory	Tewhey Lab, The Jackson Laboratory	Matluk,N., Dewey,H., Iosue,F., Barter,M., Lynch,R., Munger,H. and Tewhey,R.
EPI_ISL_755579	Utah Public Health Laboratory, Utah Public Health Laboratory Infectious Disease submission group	Utah Public Health Laboratory, Utah Public Health Laboratory Infectious Disease submission group	Gallagher,T., Young,E.L., Oakeson,K.F.
EPI_ISL_755641	Instituto Adolfo Lutz - Regional de Santo Andre	Instituto Adolfo Lutz, Interdiciplinary Procedures Center, Strategic Laboratory	Claudio Tavares Sacchi, Claudia Regina Gonçalves, Erica Valessa Ramos Gomes, Karoline Rodrigues Campos
EPI_ISL_755803	Toronto Invasive Bacterial Diseases Network	McMaster University	Allison McGeer, Patryk Aftanas, Hooman Derakhshani, Angel Li, Kuganya Nirmalarajah, Emily Panousis, Ahmed Draia, Jalees Nasir, Michael Surette, Samira Mubareka, Andrew G. McArthur
EPI_ISL_756126, EPI_ISL_756127, EPI_ISL_756128, EPI_ISL_756129, EPI_ISL_756130, EPI_ISL_756131, EPI_ISL_756132, EPI_ISL_756133, EPI_ISL_756134, EPI_ISL_756164, EPI_ISL_756184, EPI_ISL_756185, EPI_ISL_756186			
see above	Wyoming Public Health Laboratory	Wyoming Public Health Laboratory	Noah Hull, Taylor Fearing, Channing Weber, Ashley Norberg, Bailey Bowcutt, and Wanda Manley
EPI_ISL_756281	State Laboratories Division, Hawaii State Department of Health	State Laboratories Division, Hawaii State Department of Health	Pamela O'Brien, Sabrina Diemert, Drew Kuwazaki, Razvan Sultana, Edward Desmond
EPI_ISL_756765, EPI_ISL_756860, EPI_ISL_756865, EPI_ISL_756876, EPI_ISL_756925, EPI_ISL_757014	Lighthouse Lab in Milton Keynes	Wellcome Sanger Institute for the COVID-19 Genomics UK (COG-UK) Consortium	The Lighthouse Lab in Milton Keynes and Alex Alderton, Roberto Amato, Sonia Goncalves, Ewan Harrison, David K. Jackson, Ian Johnston, Dominic Kwiatkowski, Cordelia Langford, John Sillitoe on behalf of the Wellcome Sanger Institute COVID-19 Surveillance Team
EPI_ISL_757699	Department of Virus and Microbiological Special Diagnostics, Statens Serum Institut, Copenhagen, Denmark	Albertsen Lab, Department of Chemistry and Bioscience, Aalborg University, Denmark	Danish Covid-19 Genome Consortium
EPI_ISL_759758	University of Wisconsin-Madison AIDS Vaccine Research Laboratories	University of Wisconsin-Madison AIDS Vaccine Research Laboratories	Gage Moreno, Katarina Braun, et al. AIDS Vaccine Research Laboratories
EPI_ISL_759985	Furst Medical Laboratory	Norwegian Institute of Public Health, Department of Virology	Kathrine Stene-Johansen, Kamilla Heddeland Instefjord, Hilde Elshaug, Marie Paulsen Madsen, Rasmus Riis Kopperud, Hilde Vollan, Karoline Bragstad, Olav Hungnes
EPI_ISL_759986, EPI_ISL_759987	University Hospital of Northern Norway, Department for Microbiology and Infectious Disease Control	Norwegian Institute of Public Health, Department of Virology	Kathrine Stene-Johansen, Kamilla Heddeland Instefjord, Hilde Elshaug, Marie Paulsen Madsen, Rasmus Riis Kopperud, Hilde Vollan, Karoline Bragstad, Olav Hungnes
EPI_ISL_759988	Dept. of Medical Microbiology, Stavanger University Hospital, Helse Stavanger HF	Norwegian Institute of Public Health, Department of Virology	Kathrine Stene-Johansen, Kamilla Heddeland Instefjord, Hilde Elshaug, Marie Paulsen Madsen, Rasmus Riis Kopperud, Hilde Vollan, Karoline Bragstad, Olav Hungnes
EPI_ISL_760247	Pasteur Institute in Ho Chi Minh city	Department of Microbiology and Immunology - Pasteur	Lan Trong Phan, Manh Huy Dao, Hang Thi Thu Pham, Nhung Pham Hong Vu, Hieu Minh Le, Thang Minh Cao, Loan Thi Kim Huynh, Long Thanh Nguyen,

		Institute in Ho Chi Minh city	Anh Hoang Nguyen, Hieu Trung Nguyen, Thao Thi Ngoc Nguyen, Quang Duy Pham, Quang Chan Luong, Thuong Vu Nguyen
EPI_ISL_760813, EPI_ISL_760833, EPI_ISL_760858, EPI_ISL_760859	Lighthouse Lab in Cambridge	Wellcome Sanger Institute for the COVID-19 Genomics UK (COG-UK) Consortium	Rob Howes, The Lighthouse Lab in Cambridge and Alex Alderton, Roberto Amato, Sonia Goncalves, Ewan Harrison, David K. Jackson, Ian Johnston, Dominic Kwiatkowski, Cordelia Langford, John Sillitoe on behalf of the Wellcome Sanger Institute COVID-19 Surveillance Team
EPI_ISL_760876	Lighthouse Lab in Glasgow	Wellcome Sanger Institute for the COVID-19 Genomics UK (COG-UK) Consortium	Harper VanSteenhouse, Yumi Kasai, David Gray, Carol Clugston, Anna Dominiczak and Alex Alderton, Roberto Amato, Sonia Goncalves, Ewan Harrison, David K. Jackson, Ian Johnston, Dominic Kwiatkowski, Cordelia Langford, John Sillitoe on behalf of the Wellcome Sanger Institute COVID-19 Surveillance Team
EPI_ISL_760881	Lighthouse Lab in Milton Keynes	Wellcome Sanger Institute for the COVID-19 Genomics UK (COG-UK) Consortium	The Lighthouse Lab in Milton Keynes and Alex Alderton, Roberto Amato, Sonia Goncalves, Ewan Harrison, David K. Jackson, Ian Johnston, Dominic Kwiatkowski, Cordelia Langford, John Sillitoe on behalf of the Wellcome Sanger Institute COVID-19 Surveillance Team
EPI_ISL_760888	Lighthouse Lab in Alderley Park	Wellcome Sanger Institute for the COVID-19 Genomics UK (COG-UK) Consortium	Jacquelyn Wynn, Mairead Hyland, The Lighthouse Lab in Alderley Park and Alex Alderton, Roberto Amato, Sonia Goncalves, Ewan Harrison, David K. Jackson, Ian Johnston, Dominic Kwiatkowski, Cordelia Langford, John Sillitoe on behalf of the Wellcome Sanger Institute COVID-19 Surveillance Team
EPI_ISL_760943, EPI_ISL_760975	Lighthouse Lab in Milton Keynes	Wellcome Sanger Institute for the COVID-19 Genomics UK (COG-UK) Consortium	The Lighthouse Lab in Milton Keynes and Alex Alderton, Roberto Amato, Sonia Goncalves, Ewan Harrison, David K. Jackson, Ian Johnston, Dominic Kwiatkowski, Cordelia Langford, John Sillitoe on behalf of the Wellcome Sanger Institute COVID-19 Surveillance Team
EPI_ISL_760976	Lighthouse Lab in Cambridge	Wellcome Sanger Institute for the COVID-19 Genomics UK (COG-UK) Consortium	Rob Howes, The Lighthouse Lab in Cambridge and Alex Alderton, Roberto Amato, Sonia Goncalves, Ewan Harrison, David K. Jackson, Ian Johnston, Dominic Kwiatkowski, Cordelia Langford, John Sillitoe on behalf of the Wellcome Sanger Institute COVID-19 Surveillance Team
EPI_ISL_761023, EPI_ISL_761039	Lighthouse Lab in Milton Keynes	Wellcome Sanger Institute for the COVID-19 Genomics UK (COG-UK) Consortium	The Lighthouse Lab in Milton Keynes and Alex Alderton, Roberto Amato, Sonia Goncalves, Ewan Harrison, David K. Jackson, Ian Johnston, Dominic Kwiatkowski, Cordelia Langford, John Sillitoe on behalf of the Wellcome Sanger Institute COVID-19 Surveillance Team
EPI_ISL_761042	Lighthouse Lab in Alderley Park	Wellcome Sanger Institute for the COVID-19 Genomics UK (COG-UK) Consortium	Jacquelyn Wynn, Mairead Hyland, The Lighthouse Lab in Alderley Park and Alex Alderton, Roberto Amato, Sonia Goncalves, Ewan Harrison, David K. Jackson, Ian Johnston, Dominic Kwiatkowski, Cordelia Langford, John Sillitoe on behalf of the Wellcome Sanger Institute COVID-19 Surveillance Team
EPI_ISL_761043, EPI_ISL_761067	Lighthouse Lab in Glasgow	Wellcome Sanger Institute for the COVID-19 Genomics UK (COG-UK) Consortium	Harper VanSteenhouse, Yumi Kasai, David Gray, Carol Clugston, Anna Dominiczak and Alex Alderton, Roberto Amato, Sonia Goncalves, Ewan Harrison, David K. Jackson, Ian Johnston, Dominic Kwiatkowski, Cordelia Langford, John Sillitoe on behalf of the Wellcome Sanger Institute COVID-19 Surveillance Team
EPI_ISL_763001, EPI_ISL_763007, EPI_ISL_763020, EPI_ISL_763025, EPI_ISL_763027, EPI_ISL_763028, EPI_ISL_763033, EPI_ISL_763034, EPI_ISL_763035, EPI_ISL_763047, EPI_ISL_763048, EPI_ISL_763049, EPI_ISL_763050			
see above	Unit 17: Influenza & Other Respiratory Viruses, German National Influenza Center	Project group Epidemiology of Highly Pathogenic Microorganisms, Robert Koch-Institute	Ariane Düx, Andreas Sachse, Grit Schubert, Sébastien Calvignac-Spencer, Fabian Leendertz, Thorsten Wolff, Ralf Dürrwald, Djin-Ye Oh, Marianne Wedde
EPI_ISL_763344, EPI_ISL_763345, EPI_ISL_763346	Florida Bureau of Public Health Laboratories	Florida Bureau of Public Health Laboratories	Sarah Schmedes, Jason Blanton
EPI_ISL_763375, EPI_ISL_763438, EPI_ISL_763569, EPI_ISL_763593, EPI_ISL_763641, EPI_ISL_763792, EPI_ISL_763793, EPI_ISL_763794, EPI_ISL_763815, EPI_ISL_763872, EPI_ISL_764159, EPI_ISL_764171, EPI_ISL_764174, EPI_ISL_764176, EPI_ISL_764177, EPI_ISL_764191, EPI_ISL_764209, EPI_ISL_764281, EPI_ISL_764284, EPI_ISL_764285, EPI_ISL_764286, EPI_ISL_764287, EPI_ISL_764288, EPI_ISL_764289, EPI_ISL_764290, EPI_ISL_764291, EPI_ISL_764292, EPI_ISL_764293, EPI_ISL_764294, EPI_ISL_764295, EPI_ISL_764296, EPI_ISL_764297, EPI_ISL_764298, EPI_ISL_764299, EPI_ISL_764300, EPI_ISL_764301, EPI_ISL_764305, EPI_ISL_764307, EPI_ISL_764309, EPI_ISL_764312, EPI_ISL_764313, EPI_ISL_764314, EPI_ISL_764322, EPI_ISL_764323, EPI_ISL_764324, EPI_ISL_764325, EPI_ISL_764326			
see above	Department of Pathology, University of Cambridge	COVID-19 Genomics UK (COG-UK) Consortium	Aminu S. Jahun, Yasmin Chaudhry, Grant Hall, Iliana Georgana, Myra Hosmillo, Martin D. Curran, Malte Pinckert, Surendra Parmar, Ian Goodfellow
EPI_ISL_764766	Wales Specialist Virology Centre Sequencing lab: Pathogen Genomics Unit	COVID-19 Genomics UK (COG-UK) Consortium	Catherine Moore, Johnathan Evans, Laura Gifford, Malorie Perry, Simon Cottrell, Angela Marchbank, Alec Birchley, Alexander Adams, Amy Gaskin, Bree Gatica-Wilcox, Jason Coombes, Joel Southgate, Lauren Gilbert, Lee Graham, Nicole Pacchiarini, Sara Kumziene-Summerhayes, Sarah Taylor, Sophie Jones, Sara Rey, Matthew Bull, Joanne Watkins, Sally Corden, Tom Connor
EPI_ISL_765882, EPI_ISL_765883, EPI_ISL_765884, EPI_ISL_765885, EPI_ISL_765886, EPI_ISL_765887, EPI_ISL_765888, EPI_ISL_765889, EPI_ISL_765890, EPI_ISL_765891, EPI_ISL_765892			
see above	Massachusetts General Hospital	Infectious Disease Program, Broad Institute of Harvard and MIT	Lemieux,J.E., Siddle,K.J., Shaw,B., Adams,G., Pierce,V., Turbett,S., Anahtar,M., Branda,J., Slater,D., Harris,J., Lin,A.E., Gladden-Young,A., Lagerborg,K., Rudy,M., DeRuff,K., Carter,A., Normandin,E., Bauer,M., Reilly,S., Tomkins-Tinch,C., Loreth,C., Chaluvadi,S., Neumann,A., Cusick,C., Chapman,S.B., Gnirke,A., Flowers,K., Cerrato,F., Birren,B.W., Gallagher,G., Smole,S., Park,D.J., MacInnis,B.L., Ryan,E., LaRocque,R., Rosenberg,E. and Sabeti,P.C.
EPI_ISL_766028	Hannover Medical School, Institute of Virology	Hannover Medical School, Institute of Virology	Lars Steinbrück
EPI_ISL_766055	Department of Virology	Department of Virology	Massab Umair, Muhammad Salman, Sana Tamim, Nazish Badar, Zaira Rehman, Adnan Khurshid, Samee Ullah, Abdul Ahad, Hamza Ahmed, Aamer Ikram
EPI_ISL_766064, EPI_ISL_766239, EPI_ISL_766279, EPI_ISL_766280	Respiratory Virus Unit, National Infection Service, Public Health England	COVID-19 Genomics UK (COG-UK) Consortium	PHE Covid Sequencing Team
EPI_ISL_766576	ULSS 8 Berica	Istituto Zooprofilattico Sperimentale delle Venezie	Adelaide Milani, Alessia Schivo, Annalisa Salviato, Erika Giorgia Quaranta, Ambra Pastori, Bianca Zecchin, Alice Fusaro, Isabella Monne, Calogero Terregino, Antonia Ricci
EPI_ISL_766591	Halsomedicinskt Center	The Public Health Agency of Sweden	Department of Microbiology, The Public Health Agency of Sweden
EPI_ISL_766661, EPI_ISL_766666, EPI_ISL_766674, EPI_ISL_766734, EPI_ISL_766751, EPI_ISL_766759	Texas Department of State Health Services	Texas Department of State Health Services	Rashmi Tuladhar, Bonnie Oh, Jenny Zhang, Maliha Rahman, Anita Pokharel, Myong Koag, Chung Wang, Rachel Lee, Grace Kubin, Mayela Pedrueza, James Daniel Bonser
EPI_ISL_766972, EPI_ISL_766973, EPI_ISL_766974, EPI_ISL_766978, EPI_ISL_766979, EPI_ISL_766981, EPI_ISL_766982, EPI_ISL_766983, EPI_ISL_766985, EPI_ISL_766986, EPI_ISL_766987, EPI_ISL_766990, EPI_ISL_766991, EPI_ISL_766992, EPI_ISL_766994, EPI_ISL_766997, EPI_ISL_767002, EPI_ISL_767007, EPI_ISL_767009, EPI_ISL_767010, EPI_ISL_767011, EPI_ISL_767012			
see above	Delaware Public Health Laboratory	Delaware Public Health Laboratory	Gregory Hovan
EPI_ISL_767291, EPI_ISL_767299, EPI_ISL_767318	Lighthouse Lab in Alderley Park	Wellcome Sanger Institute for the COVID-19 Genomics UK (COG-UK) Consortium	Jacquelyn Wynn, Mairead Hyland, The Lighthouse Lab in Alderley Park and Alex Alderton, Roberto Amato, Sonia Goncalves, Ewan Harrison, David K. Jackson, Ian Johnston, Dominic Kwiatkowski, Cordelia Langford, John Sillitoe on behalf of the Wellcome Sanger Institute COVID-19 Surveillance Team
EPI_ISL_767583, EPI_ISL_767588, EPI_ISL_767591, EPI_ISL_767592, EPI_ISL_767593, EPI_ISL_767596, EPI_ISL_767597, EPI_ISL_767629, EPI_ISL_767630, EPI_ISL_767631, EPI_ISL_767632, EPI_ISL_767633, EPI_ISL_767634, EPI_ISL_767635, EPI_ISL_767636, EPI_ISL_767637, EPI_ISL_767638, EPI_ISL_767639, EPI_ISL_767640, EPI_ISL_767641, EPI_ISL_767642, EPI_ISL_767643, EPI_ISL_767644, EPI_ISL_767659, EPI_ISL_767660, EPI_ISL_767661, EPI_ISL_767662, EPI_ISL_767663, EPI_ISL_767664			
see above	MEMORIAL SLOAN KETTERING CANCER CENTER	Wadsworth Center, New York State Department.of Health	Kirsten St. George, Daryl M. Lamson, Alexis Russel, Matthew Shudt, Melissa A Leisner, Jonathan Plitnick, Navjot Singh, John Kelly, Sara Griesemer, Erasmus Schneider, Erica Lasek-Nesselquist
EPI_ISL_767854, EPI_ISL_767855, EPI_ISL_767856	Sydney South West Pathology Service (SSWPS) - Royal Prince Alfred Hospital - NSW Health Pathology	NSW Health Pathology - Institute of Clinical Pathology and Medical Research; Westmead Hospital; University of Sydney	CIDM-PH et al.
EPI_ISL_768357, EPI_ISL_768358, EPI_ISL_768359, EPI_ISL_768360, EPI_ISL_768361, EPI_ISL_768362, EPI_ISL_768363, EPI_ISL_768364, EPI_ISL_768365, EPI_ISL_768366, EPI_ISL_768367, EPI_ISL_768368, EPI_ISL_768369, EPI_ISL_768370, EPI_ISL_768371, EPI_ISL_768372			
see above	LSUHS Emerging Viral Threat Laboratory	Microbial Genome Sequencing Center	Jeremy P. Kamil, Jennifer L. Carroll, Camille F. Abshire, Maarten Van Diest, Andrew D. Yurochko, Martin J. Sapp, Rona S. Scott, Christopher G. Kevil, Daniel J. Snyder, Vaughn S. Cooper, John A. Vanchiere
EPI_ISL_768521	Regional medical sciences center 6 Chonburi	National Institute of Health, Department of Medical Sciences, Ministry of Public Health, Thailand	Pilailuk Okada; Siripaporn Phuygun; Sittiporn Parnmen; Ratana Tacharoenmuang; Pakorn Piromtong; Natchaya Khiadsang; Thanutsapa Thanadachakul; Warawan Wongboot; sirikanda wimol; Sunthareeya Waicharoen;
EPI_ISL_768526, EPI_ISL_768527	Department of Virology I, National Institute of Infectious Diseases	Pathogen Genomics Center, National Institute of Infectious Diseases	Tsuyoshi Sekizuka, Shuetsu Fukushi, Souichi Yamada, Kentaro Itokawa, Rina Tanaka, Masanori Hashino, Makoto Kuroda
EPI_ISL_768528, EPI_ISL_768614	Regional Medical Sciences Center 1/1 Chiang Rai	National Institute of Health, Department of Medical Sciences, Ministry of Public Health, Thailand	Pilailuk Okada; Siripaporn Phuygun; Sittiporn Parnmen; Ratana Tacharoenmuang; Pakorn Piromtong; Natchaya Khiadsang; Thanutsapa Thanadachakul; Warawan Wongboot; sirikanda wimol; Sunthareeya Waicharoen;
EPI_ISL_768640, EPI_ISL_768641, EPI_ISL_768642, EPI_ISL_768655, EPI_ISL_768656, EPI_ISL_768657, EPI_ISL_768658, EPI_ISL_768661, EPI_ISL_768662, EPI_ISL_768663, EPI_ISL_768664, EPI_ISL_768665, EPI_ISL_768666, EPI_ISL_768668, EPI_ISL_768669, EPI_ISL_768670, EPI_ISL_768671, EPI_ISL_768672, EPI_ISL_768673, EPI_ISL_768674, EPI_ISL_768675, EPI_ISL_768676, EPI_ISL_768677, EPI_ISL_768678, EPI_ISL_768679, EPI_ISL_768680, EPI_ISL_768681, EPI_ISL_768682, EPI_ISL_768683, EPI_ISL_768684, EPI_ISL_768685, EPI_ISL_768686, EPI_ISL_768687, EPI_ISL_768688, EPI_ISL_768689, EPI_ISL_768690, EPI_ISL_768691, EPI_ISL_768692, EPI_ISL_768693, EPI_ISL_768694, EPI_ISL_768695, EPI_ISL_768696, EPI_ISL_768697, EPI_ISL_768698, EPI_ISL_768699, EPI_ISL_768700, EPI_ISL_768701, EPI_ISL_768703, EPI_ISL_768704, EPI_ISL_768705, EPI_ISL_768706, EPI_ISL_768707, EPI_ISL_768708, EPI_ISL_768709,			

EPI_ISL_768710, EPI_ISL_768711, EPI_ISL_768712, EPI_ISL_768713, EPI_ISL_768714, EPI_ISL_768715, EPI_ISL_768716, EPI_ISL_768717, EPI_ISL_768718, EPI_ISL_768719, EPI_ISL_768720, EPI_ISL_768721, EPI_ISL_768722, EPI_ISL_768723, EPI_ISL_768724			
see above	Pathogen Genomics Center, National Institute of Infectious Diseases	Pathogen Genomics Center, National Institute of Infectious Diseases	Tsuyoshi Sekizuka, Kentaro Itokawa, Rina Tanaka, Masanori Hashino, Makoto Kuroda
EPI_ISL_768725	Jena University Hospital, Institute for Infectious Diseases and Infection Control	Institute of infectious medicine & hospital hygiene, CaSe-Group	Spott, Riccardo; Marquet, Mike; Pletz, Matthias W.; Brandt, Christian
EPI_ISL_768831, EPI_ISL_768832, EPI_ISL_768834, EPI_ISL_768835, EPI_ISL_768836, EPI_ISL_768837	Laboratoire Biolife	Laboratoire de Biotechnologie	Mouna Ouadghiri, Tarik Aanniz, Mohammed Walid Chemao Elfihri, Mohamed Chenaoui, Hanae Dakka, Afaf Alaoui, Otmane Touzani, Amina Benouda, Bouchra Belfquih, Lahcen belyamani, Saaid Amzazi and Azeddine Ibrahimi
EPI_ISL_770070, EPI_ISL_770113, EPI_ISL_770114, EPI_ISL_770115, EPI_ISL_770116, EPI_ISL_770117, EPI_ISL_770118, EPI_ISL_770119, EPI_ISL_770120	Wyoming Public Health Laboratory	Wyoming Public Health Laboratory	Noah Hull, Taylor Fearing, Lynette Gumbleton, Channing Weber, Ashley Norberg, Bailey Bowcutt, and Wanda Manley
EPI_ISL_770542, EPI_ISL_770543, EPI_ISL_770544, EPI_ISL_770545, EPI_ISL_770546, EPI_ISL_770547, EPI_ISL_770548, EPI_ISL_770549	Private lab	Lithuanian University of Health Sciences, Molecular cardiology lab.	Lukas Zemaitis, Ingrida Olendrait, Arnoldas Pautienius, Kamile Tamusauskaite, Dovydas Gecys, Laura Pareckaite, Vaiva Lesauskaite, Astra Vitkauskiene
EPI_ISL_770630	Laboratório de Microbiologia Molecular - Universidade FEEVALE	Bioinformatics Laboratory / LNCC	Felipe Benites, Fernando Rosado Spilki, Alana Witt Hansen, Juliane Deise Fleck, Juliana Schons, Meriane Demoliner, Ana Karolina Eisen Antunes, Fagner Henrique Heldt, Larissa Mallmann, Bruna Hermann, Ana Luiza Ziulkoski, Vyctoria Goes, Karoline Schallenberger, Matheus Nunes Weber, Paula Rodrigues de Almeida, Alessandra Pavan Lamarca da Silva, Ronaldo da Silva F Jr , Luiz G P de Almeida, Alexandra L Gerber , Ana Paula de C Guimarães,Ana Tereza R de Vasconcelos
EPI_ISL_770634, EPI_ISL_770636, EPI_ISL_770637, EPI_ISL_770639, EPI_ISL_770640, EPI_ISL_770641, EPI_ISL_770643, EPI_ISL_770644, EPI_ISL_770645, EPI_ISL_770646, EPI_ISL_770653, EPI_ISL_770660, EPI_ISL_770666, EPI_ISL_770669, EPI_ISL_770675, EPI_ISL_770676, EPI_ISL_770677, EPI_ISL_770678, EPI_ISL_770679, EPI_ISL_770680, EPI_ISL_770681, EPI_ISL_770682, EPI_ISL_770683, EPI_ISL_770684, EPI_ISL_770685, EPI_ISL_770686, EPI_ISL_770687			
see above	WHO National Influenza Centre Russian Federation	WHO National Influenza Centre Russian Federation	Andrey Komissarov, Artem Fadeev, Anna Ivanova, Kseniya Komissarova, Dmitry Bazhenov, Daria Danilenko, Ksenia Safina, Elena Nabieva, Georgii Bazykin, Dmitry Lioznov
EPI_ISL_770715, EPI_ISL_770717, EPI_ISL_770718, EPI_ISL_770719	ZOTZ KLIMAS MVZ Düsseldorf-Centrum GbR ÜBAG für Labormedizin, Genetik, Zytologie, Pathologie	Center of Medical Microbiology, Virology, and Hospital Hygiene, University of Duesseldorf	Maximilian Damagnez, Alexander Dilthey, Ashley-Jane Duplessis, Patrick Finzer, Katrin Hoffmann, Torsten Houwaart, Lisanna Hülse, Malte Kohns Vasconcelos, Marek Korencak, Nadine Lübke, Jessica Nicolai, Klaus Pfeffer, Daniel Strelow, Jörg Timm, Andreas Walker, Tobias Wienemann, Rainer Zotz
EPI_ISL_770783, EPI_ISL_770784, EPI_ISL_770785, EPI_ISL_770786, EPI_ISL_770787, EPI_ISL_770788, EPI_ISL_770789	Minnesota Department of Health, Public Health Laboratory	Minnesota Department of Health, Public Health Laboratory	Alexandra Lorentz, Jacob Garfin, Matt Plumb, and Xiong Wang
EPI_ISL_770804, EPI_ISL_770805, EPI_ISL_770806, EPI_ISL_770807, EPI_ISL_770814	Vault Health	Minnesota Department of Health, Public Health Laboratory	Alexandra Lorentz, Jacob Garfin, Matt Plumb, and Xiong Wang
EPI_ISL_770848, EPI_ISL_770854, EPI_ISL_770862, EPI_ISL_770866, EPI_ISL_770867, EPI_ISL_770869, EPI_ISL_770874, EPI_ISL_770875, EPI_ISL_770878, EPI_ISL_770879, EPI_ISL_770892, EPI_ISL_770893, EPI_ISL_770894, EPI_ISL_770896, EPI_ISL_770898, EPI_ISL_770899, EPI_ISL_770900, EPI_ISL_770906, EPI_ISL_770907, EPI_ISL_770908, EPI_ISL_770909, EPI_ISL_770910, EPI_ISL_770911, EPI_ISL_770912, EPI_ISL_770913, EPI_ISL_770914, EPI_ISL_770930, EPI_ISL_770931, EPI_ISL_770932, EPI_ISL_770933, EPI_ISL_770934, EPI_ISL_770935, EPI_ISL_770936, EPI_ISL_770937, EPI_ISL_770938, EPI_ISL_770939, EPI_ISL_770940, EPI_ISL_770941, EPI_ISL_770942, EPI_ISL_770943, EPI_ISL_770944, EPI_ISL_770945, EPI_ISL_770946, EPI_ISL_770947, EPI_ISL_770948, EPI_ISL_770949, EPI_ISL_770950, EPI_ISL_770951, EPI_ISL_770952, EPI_ISL_770953, EPI_ISL_770954, EPI_ISL_770955, EPI_ISL_770956, EPI_ISL_770957, EPI_ISL_770958, EPI_ISL_770959, EPI_ISL_770960, EPI_ISL_770961, EPI_ISL_770962, EPI_ISL_770963, EPI_ISL_770964, EPI_ISL_770965, EPI_ISL_770966, EPI_ISL_770967, EPI_ISL_770968, EPI_ISL_770969, EPI_ISL_770970, EPI_ISL_770971, EPI_ISL_770972, EPI_ISL_770973, EPI_ISL_770974, EPI_ISL_770975, EPI_ISL_770976, EPI_ISL_770977, EPI_ISL_770978, EPI_ISL_770979, EPI_ISL_770985, EPI_ISL_770986, EPI_ISL_770987, EPI_ISL_770988, EPI_ISL_770989, EPI_ISL_770991, EPI_ISL_770992, EPI_ISL_770993, EPI_ISL_770994, EPI_ISL_770995, EPI_ISL_770996, EPI_ISL_770997, EPI_ISL_770998, EPI_ISL_770999, EPI_ISL_771000, EPI_ISL_771001, EPI_ISL_771002, EPI_ISL_771003, EPI_ISL_771004, EPI_ISL_771005, EPI_ISL_771006, EPI_ISL_771007, EPI_ISL_771008, EPI_ISL_771009, EPI_ISL_771010, EPI_ISL_771011, EPI_ISL_771012, EPI_ISL_771013, EPI_ISL_771014, EPI_ISL_771015, EPI_ISL_771016, EPI_ISL_771017, EPI_ISL_771018, EPI_ISL_771019, EPI_ISL_771020, EPI_ISL_771022, EPI_ISL_771024, EPI_ISL_771025, EPI_ISL_771030, EPI_ISL_771031, EPI_ISL_771032, EPI_ISL_771033, EPI_ISL_771034, EPI_ISL_771035, EPI_ISL_771036, EPI_ISL_771037, EPI_ISL_771038			
see above	Laboratoire national de santé, Microbiology, Virology	Laboratoire national de santé, Microbiology, Microbial Genomics Platform	Anke Wienecke-Baldacchino, Catherine Ragimbeau,Jessica Tapp, Fatu Djabi, Lise Pignon, Raoul Salmon, Tamir Abdelrahman
EPI_ISL_775223, EPI_ISL_775224, EPI_ISL_775226, EPI_ISL_775247, EPI_ISL_775249, EPI_ISL_775254	Laboratoire Biolife	Laboratoire de Biotechnologie	Mouna Ouadghiri, Tarik Aanniz, Mohammed Walid Chemao Elfihri, Mohamed Chenaoui, Hanae Dakka, Afaf Alaoui, Otmane Touzani, Amina Benouda, Bouchra Belfquih, Lahcen belyamani, Saaid Amzazi and Azeddine Ibrahimi
EPI_ISL_775285, EPI_ISL_775371	Hospital of Southern Norway - Kristiansand, Department of Medical Microbiology	Norwegian Institute of Public Health, Department of Virology	Kathrine Stene-Johansen, Kamilla Heddeland Instefjord, Hilde Elshaug, Atiya R Ali,Marie Paulsen Madsen, Rasmus Riis Kopperud, Hilde Vollan, Karoline Bragstad, Olav Hungnes
EPI_ISL_775385	Unilabs Laboratory Medicine	Norwegian Institute of Public Health, Department of Virology	Kathrine Stene-Johansen, Kamilla Heddeland Instefjord, Hilde Elshaug, Atiya R Ali,Marie Paulsen Madsen, Rasmus Riis Kopperud, Hilde Vollan, Karoline Bragstad, Olav Hungnes
EPI_ISL_775400, EPI_ISL_775402	Nordland Hospital - Bodo, Laboratory Department, Molecular Biology Unit	Norwegian Institute of Public Health, Department of Virology	Kathrine Stene-Johansen, Kamilla Heddeland Instefjord, Hilde Elshaug, Atiya R Ali,Marie Paulsen Madsen, Rasmus Riis Kopperud, Hilde Vollan, Karoline Bragstad, Olav Hungnes
EPI_ISL_775417, EPI_ISL_775418, EPI_ISL_775419, EPI_ISL_775420, EPI_ISL_775421, EPI_ISL_775422, EPI_ISL_775423	Oslo University Hospital, Department of Medical Microbiology	Norwegian Institute of Public Health, Department of Virology	Kathrine Stene-Johansen, Kamilla Heddeland Instefjord, Hilde Elshaug, Atiya R Ali,Marie Paulsen Madsen, Rasmus Riis Kopperud, Hilde Vollan, Karoline Bragstad, Olav Hungnes
EPI_ISL_775492	Department of Medical Microbiology, St. Olavs hospital	Norwegian Institute of Public Health, Department of Virology	Kathrine Stene-Johansen, Kamilla Heddeland Instefjord, Hilde Elshaug, Atiya R Ali,Marie Paulsen Madsen, Rasmus Riis Kopperud, Hilde Vollan, Karoline Bragstad, Olav Hungnes
EPI_ISL_776768	Instituto Adolfo Lutz - Regional de Aracatuba	Instituto Adolfo Lutz, Interdiciplinary Procedures Center, Strategic Laboratory	Claudio Tavares Sacchi, Claudia Regina Gonçalves, Erica Valessa Ramos Gomes, Karoline Rodrigues Campos
EPI_ISL_776769	Instituto Adolfo Lutz - Regional de Santo Andre	Instituto Adolfo Lutz, Interdiciplinary Procedures Center, Strategic Laboratory	Claudio Tavares Sacchi, Claudia Regina Gonçalves, Erica Valessa Ramos Gomes, Karoline Rodrigues Campos
EPI_ISL_777056	Lighthouse Lab in Milton Keynes	Wellcome Sanger Institute for the COVID-19 Genomics UK (COG-UK) Consortium	The Lighthouse Lab in Milton Keynes and Alex Alderton, Roberto Amato, Sonia Goncalves, Ewan Harrison, David K. Jackson, Ian Johnston, Dominic Kwiatkowski, Cordelia Langford, John Sillitoe on behalf of the Wellcome Sanger Institute COVID-19 Surveillance Team
EPI_ISL_778825, EPI_ISL_778826, EPI_ISL_778827, EPI_ISL_778836	AIID	Irish Coronavirus Sequencing Consortium-Teagasc Grange	Matthew McCabe, Aljandro Abner Garcia Leon, Fiona Crispie, Calum Walsh, Michael Carr, John Kenny, Paul Cotter, Patrick Mallon, Gabriel Gonzalez
EPI_ISL_778871, EPI_ISL_778872, EPI_ISL_778873, EPI_ISL_778874, EPI_ISL_778875, EPI_ISL_778876, EPI_ISL_778877, EPI_ISL_778891, EPI_ISL_778893, EPI_ISL_778894, EPI_ISL_778895, EPI_ISL_778896, EPI_ISL_778897, EPI_ISL_778898, EPI_ISL_778899, EPI_ISL_778900, EPI_ISL_778901, EPI_ISL_778902, EPI_ISL_778903, EPI_ISL_778904, EPI_ISL_778905, EPI_ISL_778906, EPI_ISL_778907, EPI_ISL_778908, EPI_ISL_778909, EPI_ISL_778910, EPI_ISL_778911, EPI_ISL_778912, EPI_ISL_778913, EPI_ISL_778914, EPI_ISL_778915, EPI_ISL_778916, EPI_ISL_778917			
see above	Maryland Public Health Laboratory	Maryland Public Health Laboratory	Maryland Department of Health Laboratories Administration
EPI_ISL_778918	LSUHS Emerging Viral Threat Laboratory	Microbial Genome Sequencing Center	Jeremy P. Kamil, Jennifer L. Carroll, Camille F. Abshire, Maarten Van Diest, Andrew D. Yurochko, Martin J. Sapp, Rona S. Scott, Christopher G. Kevil, Daniel J. Snyder, Vaughn S. Cooper, John A. Vanchiere
EPI_ISL_779135	Wyoming Public Health Laboratory	Wyoming Public Health Laboratory	Noah Hull, Taylor Fearing, Lynette Gumbleton, Channing Weber, Ashley Norberg, Bailey Bowcutt, and Wanda Manley
EPI_ISL_779205, EPI_ISL_779206, EPI_ISL_779207, EPI_ISL_779208, EPI_ISL_779209, EPI_ISL_779210, EPI_ISL_779211, EPI_ISL_779212, EPI_ISL_779213, EPI_ISL_779214, EPI_ISL_779215, EPI_ISL_779216, EPI_ISL_779217, EPI_ISL_779232, EPI_ISL_779233, EPI_ISL_779234, EPI_ISL_779235, EPI_ISL_779236,			

EPI_ISL_779237, EPI_ISL_779238			
see above	Pathogen Genomics Center, National Institute of Infectious Diseases	Pathogen Genomics Center, National Institute of Infectious Diseases	Tsuyoshi Sekizuka, Kentaro Itokawa, Rina Tanaka, Masanori Hashino, Makoto Kuroda
EPI_ISL_779291, EPI_ISL_779292, EPI_ISL_779293, EPI_ISL_779294, EPI_ISL_779295, EPI_ISL_779297, EPI_ISL_779298, EPI_ISL_779299, EPI_ISL_779300, EPI_ISL_779301, EPI_ISL_779303, EPI_ISL_779304, EPI_ISL_779305, EPI_ISL_779306, EPI_ISL_779307, EPI_ISL_779308, EPI_ISL_779309, EPI_ISL_779312, EPI_ISL_779313, EPI_ISL_779314, EPI_ISL_779317			
see above	Utah Public Health Laboratory, Utah Public Health Laboratory Infectious Disease submission group	Utah Public Health Laboratory, Utah Public Health Laboratory Infectious Disease submission group	Gallagher,T., Young,E.L., Oakeson,K.F.
EPI_ISL_779697, EPI_ISL_779698	Pathogen Genomics Center, National Institute of Infectious Diseases	Pathogen Genomics Center, National Institute of Infectious Diseases	Tsuyoshi Sekizuka, Kentaro Itokawa, Rina Tanaka, Masanori Hashino, Makoto Kuroda
EPI_ISL_779871, EPI_ISL_779875, EPI_ISL_779881, EPI_ISL_779886, EPI_ISL_779893, EPI_ISL_779899, EPI_ISL_779916	Servicio de Microbiología, Hospital Universitario Son Espases	SeqCOVID-SPAIN consortium/IBV(CSIC)	Carla López-Causapé, Jordi Reina, Antonio Oliver and SeqCOVID-SPAIN consortium
EPI_ISL_783543, EPI_ISL_783561, EPI_ISL_783798, EPI_ISL_783808, EPI_ISL_783809, EPI_ISL_783810, EPI_ISL_783811, EPI_ISL_783812, EPI_ISL_783813, EPI_ISL_783815, EPI_ISL_783816, EPI_ISL_783817, EPI_ISL_783818, EPI_ISL_783819, EPI_ISL_783821, EPI_ISL_783822, EPI_ISL_783823, EPI_ISL_783824, EPI_ISL_783825, EPI_ISL_783826, EPI_ISL_783827, EPI_ISL_783828, EPI_ISL_783829, EPI_ISL_783830, EPI_ISL_783831, EPI_ISL_783835, EPI_ISL_783836, EPI_ISL_783837, EPI_ISL_783838, EPI_ISL_783839, EPI_ISL_783841, EPI_ISL_783842, EPI_ISL_783843, EPI_ISL_783844, EPI_ISL_783846, EPI_ISL_783847, EPI_ISL_783848, EPI_ISL_783849, EPI_ISL_783851, EPI_ISL_783852, EPI_ISL_783854, EPI_ISL_783855, EPI_ISL_783856, EPI_ISL_783858, EPI_ISL_783859, EPI_ISL_783860, EPI_ISL_783861, EPI_ISL_783864, EPI_ISL_783865, EPI_ISL_783866, EPI_ISL_783867, EPI_ISL_783868, EPI_ISL_783875, EPI_ISL_783876, EPI_ISL_783877, EPI_ISL_783878, EPI_ISL_783884, EPI_ISL_783885, EPI_ISL_783886, EPI_ISL_783887, EPI_ISL_783888, EPI_ISL_783895, EPI_ISL_783896, EPI_ISL_783897, EPI_ISL_783904, EPI_ISL_783905, EPI_ISL_783906, EPI_ISL_783907, EPI_ISL_783916, EPI_ISL_783917, EPI_ISL_783918, EPI_ISL_783925, EPI_ISL_783926, EPI_ISL_783927, EPI_ISL_783935, EPI_ISL_783936, EPI_ISL_783937, EPI_ISL_783938, EPI_ISL_783939, EPI_ISL_784031, EPI_ISL_784032, EPI_ISL_784047, EPI_ISL_784074, EPI_ISL_784139, EPI_ISL_784147			
see above	Houston Methodist Hospital	Houston Methodist Hospital	S. Wesley Long, Randall J. Olsen, Paul A. Christensen, David W. Bernard, James J. Davis, Maulik Shukla, Marcus Nguyen, Matthew Ojeda Saavedra, Prasanti Yerramilli, Layne Pruitt, Sishir Subedi, Heather Hendrickson, and James M. Musser
EPI_ISL_788984, EPI_ISL_788986	Institute of Virology, Biomedical Research Center of the Slovak Academy of Sciences, Bratislava	Faculty of Natural Sciences, Comenius University, Bratislava	Broa Brejová, Viktória abanová, Kristína Boršová, Viktória Hodorová, Sabina Fumaová Havlíková, Juraj Kopáek, Martina Liková, ubomíra Lukáiková, Martina Neboháová, Monika Sláviková, Alena Košálová, Peter Sabaka, Tomáš Vina, Jozef Nosek, Boris Klempa
EPI_ISL_788987	Institute of Virology, Biomedical Research Center of the Slovak Academy of Sciences, Bratislava	Faculty of Natural Sciences, Comenius University, Bratislava	Viktória abanová, Kristína Boršová, Broa Brejová, Viktória Hodorová, Sabina Fumaová Havlíková, Juraj Kopáek, Martina Liková, ubomíra Lukáiková, Martina Neboháová, Monika Sláviková, Alena Košálová, Peter Sabaka, Tomáš Vina, Jozef Nosek, Boris Klempa
EPI_ISL_788988	Institute of Virology, Biomedical Research Center of the Slovak Academy of Sciences, Bratislava	Faculty of Natural Sciences, Comenius University, Bratislava	Kristína Boršová, Viktória abanová, Broa Brejová, Viktória Hodorová, Sabina Fumaová Havlíková, Juraj Kopáek, Martina Liková, ubomíra Lukáiková, Martina Neboháová, Monika Sláviková, Tomáš Vina, Boris Klempa, Jozef Nosek
EPI_ISL_791067	Dutch COVID-19 response team	National Institute for Public Health and the Environment (RIVM)	Adam Meijer, Harry Vennema, Jeroen Cremer, Sharon van den Brink, Bas van der Veer, AnneMarie van den Brandt, Florian Zwagemaker, Dennis Schmitz, Chantal Reusken, on behalf of the national COVID-19 response team
EPI_ISL_791281, EPI_ISL_791288, EPI_ISL_791289, EPI_ISL_791303, EPI_ISL_791304, EPI_ISL_791305, EPI_ISL_791319, EPI_ISL_791320	National Virus Reference Laboratory	Irish Coronavirus Sequencing Consortium - Teagasc Moorepark	Alejandro Abner Garcia Leon, Paul Cotter, Fiona Crispie, John Kenny, Paddy Mallon, Calum Walsh
EPI_ISL_791351, EPI_ISL_791391, EPI_ISL_791401	Johns Hopkins Hospital Department of Pathology	Johns Hopkins Hospital Department of Pathology	C. Paul Morris, Chun Huai Luo, Heba H. Mostafa
EPI_ISL_792549	Centre for Dengue Research and AICBU, Department of Immunology and Molecular Medicine	Centre for Dengue Research and AICBU, Department of Immunology and Molecular Medicine	Chandima Jeewandara, Deshni Jayathilaka, Dinuka Ariyaratne, Diyanath Ranasinghe, Laksiri Gomes, Gathsaurie Neelika Malavige
EPI_ISL_792697, EPI_ISL_792702	The National Institute of Public Health	State Veterinary Institute Prague	Nagy,A;Jirincova,H;Trnka,D;Vecerova,J
EPI_ISL_792838, EPI_ISL_793010	Department of Virus and Microbiological Special Diagnostics, Statens Serum Institut, Copenhagen, Denmark	Albertsen Lab, Department of Chemistry and Bioscience, Aalborg University, Denmark	Danish Covid-19 Genome Consortium
EPI_ISL_794321, EPI_ISL_794322, EPI_ISL_794323	UZ Leuven, National Reference Laboratory for Coronaviruses, Laboratory Medicine, Leuven, Belgium	KU Leuven, Rega Institute, Clinical and Epidemiological Virology	Tony Wawina-Bokalanga, Joan Marti-Carerras, Bert Vanmechelen, Piet Maes
EPI_ISL_794337, EPI_ISL_794338, EPI_ISL_794342, EPI_ISL_794352, EPI_ISL_794370, EPI_ISL_794372, EPI_ISL_794373, EPI_ISL_794374, EPI_ISL_794378, EPI_ISL_794412, EPI_ISL_794413, EPI_ISL_794414, EPI_ISL_794415, EPI_ISL_794416, EPI_ISL_794417, EPI_ISL_794418, EPI_ISL_794419, EPI_ISL_794424, EPI_ISL_794431, EPI_ISL_794435, EPI_ISL_794437, EPI_ISL_794440, EPI_ISL_794441, EPI_ISL_794555, EPI_ISL_794556, EPI_ISL_794557, EPI_ISL_794558, EPI_ISL_794559, EPI_ISL_794560, EPI_ISL_794561, EPI_ISL_794562, EPI_ISL_794563, EPI_ISL_794564, EPI_ISL_794565, EPI_ISL_794566, EPI_ISL_794567, EPI_ISL_794568, EPI_ISL_794569, EPI_ISL_794570, EPI_ISL_794571, EPI_ISL_794572, EPI_ISL_794573, EPI_ISL_794574, EPI_ISL_794575, EPI_ISL_794576, EPI_ISL_794577, EPI_ISL_794578, EPI_ISL_794579, EPI_ISL_794580, EPI_ISL_794581, EPI_ISL_794582, EPI_ISL_794583, EPI_ISL_794584, EPI_ISL_794585, EPI_ISL_794586, EPI_ISL_794587, EPI_ISL_794588			
see above	Department of Virus and Microbiological Special Diagnostics, Statens Serum Institut, Copenhagen, Denmark	Albertsen Lab, Department of Chemistry and Bioscience, Aalborg University, Denmark	Danish Covid-19 Genome Consortium
EPI_ISL_794634	Biology, MCL	Biology, MCL	Seadawy,M.G., Shamel,M.D., EL-hosieny,M.F., Gad,A.F., EL-harty,B.S. and EL-Safty,A.S.
EPI_ISL_794721	PathWest Laboratory Medicine WA	PathWest Laboratory Medicine WA Microbial Surveillance Unit	PathWest Laboratory Medicine WA Microbial Surveillance Unit
EPI_ISL_794829, EPI_ISL_794858, EPI_ISL_794859, EPI_ISL_794870, EPI_ISL_794902, EPI_ISL_795008, EPI_ISL_795009	Department of Virus and Microbiological Special Diagnostics, Statens Serum Institut, Copenhagen, Denmark	Albertsen Lab, Department of Chemistry and Bioscience, Aalborg University, Denmark	Danish Covid-19 Genome Consortium
EPI_ISL_796673	Unilabs Laboratory Medicine	Norwegian Institute of Public Health, Department of Virology	Kathrine Stene-Johansen, Kamilla Heddeland Instefjord, Hilde Elshaug, Atiya R Ali,Marie Paulsen Madsen, Rasmus Riis Kopperud, Hilde Vollan, Karoline Bragstad, Olav Hungnes
EPI_ISL_796779	Instituto Nacional de Saude (INSA)	Instituto Nacional de Saude (INSA)	Borges et al
EPI_ISL_801414, EPI_ISL_801427, EPI_ISL_801428, EPI_ISL_801451, EPI_ISL_801478, EPI_ISL_801480	Dutch COVID-19 response team	Erasmus Medical Center	Bas Oude Munnink, Reina Sikkema, David Nieuwenhuijse, Irina Chestakova, Anne van der Linden, Marjan Boter, Emmanuelle Munger, Corine GeurtsvanKessel, Annemiek van der Eijk, Richard Molenkamp, Marion Koopmans, on behalf of the Dutch national COVID-19 response team.
EPI_ISL_802438	Wadsworth Center, New York State Department.of Health	Wadsworth Center, New York State Department.of Health	Kirsten St. George, Daryl M. Lamson, Alexis Russel, Matthew Shudt, Melissa A Leisner, Jonathan Plitnick, Navjot Singh, John Kelly, Sara Griesemer, Erasmus Schneider, Erica Lasek-Nesselquist
EPI_ISL_802571	Essentia Health-St. Mary's Medical Center	Minnesota Department of Health, Public Health Laboratory	Alexandra Lorentz, Jacob Garfin, Matt Plumb, and Xiong Wang
EPI_ISL_803699	Wisconsin State Laboratory of Hygiene Communicable Disease Division	Wisconsin State Laboratory of Hygiene Communicable Disease Division	Kelsey R. Florek, Abigail C. Shockey
EPI_ISL_803920, EPI_ISL_803940, EPI_ISL_803941, EPI_ISL_803942, EPI_ISL_803943, EPI_ISL_803944, EPI_ISL_803945, EPI_ISL_803946, EPI_ISL_803947, EPI_ISL_803948, EPI_ISL_803949			
see above	Pathogen Genomics Center, National Institute of Infectious Diseases	Pathogen Genomics Center, National Institute of Infectious Diseases	Tsuyoshi Sekizuka, Kentaro Itokawa, Rina Tanaka, Masanori Hashino, Makoto Kuroda
EPI_ISL_804007, EPI_ISL_804008	Department of Virology I, National Institute of Infectious Diseases	Pathogen Genomics Center, National Institute of Infectious Diseases	Tsuyoshi Sekizuka, Shuetsu Fukushi, Souichi Yamada, Kentaro Itokawa, Rina Tanaka, Masanori Hashino, Makoto Kuroda
EPI_ISL_804226, EPI_ISL_804265	Respiratory Virus Unit, National Infection Service, Public Health England	COVID-19 Genomics UK (COG-UK) Consortium	PHE Covid Sequencing Team

EPI_ISL_804374	CHU Purpan - Laboratoire de Virologie - Institut Fédératif de Biologie	CHU Purpan - Laboratoire de Virologie - Institut Fédératif de Biologie	Latour J., Ranger N., Dubois M., Carcenac R., Harter A., Boyer P., Tremeaux P., Izopet J.
EPI_ISL_806830, EPI_ISL_806831, EPI_ISL_806834, EPI_ISL_806835, EPI_ISL_806836, EPI_ISL_806840, EPI_ISL_806847	Alaska State Virology Laboratory	Alaska State Virology Laboratory	Stephanie DeRonde, Lisa Smith, Ph.D., Devin M. Drown, Ph.D., Jack Chen, Ph.D.
EPI_ISL_806998, EPI_ISL_807006, EPI_ISL_807018, EPI_ISL_807022	Washington State Department of Health	Seattle Flu Study	Deborah A. Nickerson, Chris D. Frazar, Jover Lee, Benjamin Pelle, Matthew Richardson, Amanda Adler, Elisabeth Brandstetter, Peter D. Han, Kairsten Fay, Misja Ilcisin, Kirsten Lacombe, Thomas R. Sibley, Melissa Truong, Caitlin R. Wolf, Romesh Gautom, Geoff Melly, Brian Hiatt, Philip Dykema, Scott Lindquist, Michael Boeckh, Janet A. Englund, Michael Famulare, Barry R. Lutz, Mark J. Rieder, Lea M. Starita, Matthew Thompson, Helen Y. Chu, Jay Shendure, Trevor Bedford
EPI_ISL_812350, EPI_ISL_812351	Santa Clara County Public Health Laboratory	Santa Clara County Public Health Laboratory	Santa Clara County Public Health Department
EPI_ISL_812404	Utah Public Health Laboratory	Utah Public Health Laboratory	Erin L. Young, Kelly F. Oakeson, Tara Gallagher
EPI_ISL_812488	Laboratorio de Referencia Nacional de Virus Respiratorios, Instituto Nacional de Salud Peru	Laboratorio de Genómica Microbiana, Universidad Peruana Cayetano Heredia	Pablo Tsukayama, Alejandra Dávila-Barclay, Guillermo Salvatierra, Luis González, Pedro E. Romero, Brenda Ayzanoa, Janet Huancachoque, Pool Marcos, Camila Castillo-Vilcahuamán, Oscar Escalante, Priscila Lope, Nancy Rojas
EPI_ISL_813706, EPI_ISL_813710, EPI_ISL_813712, EPI_ISL_813715, EPI_ISL_813716, EPI_ISL_813717, EPI_ISL_813718, EPI_ISL_813719, EPI_ISL_813720, EPI_ISL_813721, EPI_ISL_813722, EPI_ISL_813752, EPI_ISL_813754, EPI_ISL_813763			
see above	Liverpool Clinical Laboratories	COVID-19 Genomics UK (COG-UK) Consortium	Sam Haldenby, Anita Lucaci, Steve Paterson, Julian Hiscox, Alistair Darby, M Almsaud, A Alrezaihi, Muhannad Alruwaili, Stuart D Armstrong, Jones Benjamin, Eleanor G Bentley, Anu Chawla, Jordan J Clark, Angela Cowell, Richard Eccles, Isabel García-Dorival, Matthew Gemmell, Alessandro Gerada, PKF Gilmore, Richard Gregory, Ximeng Han, Catherine Hartley, Margaret Hughes, Miren Iturriza-Gomara, James Johnson, L Luu, Jenifer Manson, Charlotte Nelson, Elaine O'Toole, Cassie Olateju, Rebekah Penrice-Randal , Lucille Rainbow, N.P Randle, Trevor Ian Robinson, Parul Sharma, Ghada T Shawli, James P Stewart, Neil Swainston, Ecaterina Vamos, Joanne Watts, Mark Whitehead
EPI_ISL_814297, EPI_ISL_814324, EPI_ISL_814346, EPI_ISL_814390, EPI_ISL_814399, EPI_ISL_814472, EPI_ISL_814514, EPI_ISL_814516	Wales Specialist Virology Centre Sequencing lab: Pathogen Genomics Unit	COVID-19 Genomics UK (COG-UK) Consortium	Catherine Moore, Johnathan Evans, Laura Gifford, Malorie Perry, Simon Cottrell, Angela Marchbank, Alec Birchley, Alexander Adams, Amy Gaskin, Bree Gatica-Wilcox, Jason Coombes, Joel Southgate, Lauren Gilbert, Lee Graham, Nicole Pacchiarini, Sara Kumziene-Summerhayes, Sarah Taylor, Sophie Jones, Sara Rey, Matthew Bull, Joanne Watkins, Sally Corden, Tom Connor
EPI_ISL_814533	Virology Department, Royal Infirmary of Edinburgh, NHS Lothian / School of Biological Sciences, University of Edinburgh / Institute of Genetics and Molecular Medicine, University of Edinburgh	COVID-19 Genomics UK (COG-UK) Consortium	McHugh M, Dewar R, Rooke S, Gallagher M, Balcaza C, O'Toole Á, Scher E, Hill V, McCrone JT, Colquhoun R, Yu X, Jackson B, Rambaut A, Williams TC, Templeton K
EPI_ISL_814562, EPI_ISL_814563, EPI_ISL_814564, EPI_ISL_814565, EPI_ISL_814576, EPI_ISL_814577, EPI_ISL_814603	Wales Specialist Virology Centre Sequencing lab: Pathogen Genomics Unit	COVID-19 Genomics UK (COG-UK) Consortium	Catherine Moore, Johnathan Evans, Laura Gifford, Malorie Perry, Simon Cottrell, Angela Marchbank, Alec Birchley, Alexander Adams, Amy Gaskin, Bree Gatica-Wilcox, Jason Coombes, Joel Southgate, Lauren Gilbert, Lee Graham, Nicole Pacchiarini, Sara Kumziene-Summerhayes, Sarah Taylor, Sophie Jones, Sara Rey, Matthew Bull, Joanne Watkins, Sally Corden, Tom Connor
EPI_ISL_814665, EPI_ISL_814666, EPI_ISL_814667, EPI_ISL_814668, EPI_ISL_814669, EPI_ISL_814670	Virology Department, Royal Infirmary of Edinburgh, NHS Lothian / School of Biological Sciences, University of Edinburgh / Institute of Genetics and Molecular Medicine, University of Edinburgh	COVID-19 Genomics UK (COG-UK) Consortium	McHugh M, Dewar R, Rooke S, Gallagher M, Balcaza C, O'Toole Á, Scher E, Hill V, McCrone JT, Colquhoun R, Yu X, Jackson B, Rambaut A, Williams TC, Templeton K
EPI_ISL_814754, EPI_ISL_814755, EPI_ISL_814794, EPI_ISL_814795, EPI_ISL_814800, EPI_ISL_814801, EPI_ISL_814802, EPI_ISL_814803, EPI_ISL_814804, EPI_ISL_814805, EPI_ISL_814806, EPI_ISL_814807, EPI_ISL_814808, EPI_ISL_814809, EPI_ISL_814810, EPI_ISL_814811, EPI_ISL_814812, EPI_ISL_814813, EPI_ISL_814814, EPI_ISL_814815, EPI_ISL_814816, EPI_ISL_814817, EPI_ISL_814818, EPI_ISL_814819, EPI_ISL_814820, EPI_ISL_814821, EPI_ISL_814822, EPI_ISL_814823, EPI_ISL_814824, EPI_ISL_814825, EPI_ISL_814826, EPI_ISL_814827, EPI_ISL_814828, EPI_ISL_814829, EPI_ISL_814830, EPI_ISL_814831, EPI_ISL_814832, EPI_ISL_814833, EPI_ISL_814834, EPI_ISL_814835, EPI_ISL_814837, EPI_ISL_814838, EPI_ISL_814839, EPI_ISL_814840, EPI_ISL_814841, EPI_ISL_814842, EPI_ISL_814843, EPI_ISL_814844, EPI_ISL_814845, EPI_ISL_814846, EPI_ISL_814847, EPI_ISL_814848, EPI_ISL_814849, EPI_ISL_814850, EPI_ISL_814851, EPI_ISL_814852, EPI_ISL_814853, EPI_ISL_814854, EPI_ISL_814855, EPI_ISL_814856, EPI_ISL_814857, EPI_ISL_814858, EPI_ISL_814859			
see above	Wales Specialist Virology Centre Sequencing lab: Pathogen Genomics Unit	COVID-19 Genomics UK (COG-UK) Consortium	Catherine Moore, Johnathan Evans, Laura Gifford, Malorie Perry, Simon Cottrell, Angela Marchbank, Alec Birchley, Alexander Adams, Amy Gaskin, Bree Gatica-Wilcox, Jason Coombes, Joel Southgate, Lauren Gilbert, Lee Graham, Nicole Pacchiarini, Sara Kumziene-Summerhayes, Sarah Taylor, Sophie Jones, Sara Rey, Matthew Bull, Joanne Watkins, Sally Corden, Tom Connor
EPI_ISL_815597, EPI_ISL_815598, EPI_ISL_816067, EPI_ISL_816068, EPI_ISL_816069, EPI_ISL_816070, EPI_ISL_816071, EPI_ISL_816072	Department of Virus and Microbiological Special Diagnostics, Statens Serum Institut, Copenhagen, Denmark	Albertsen Lab, Department of Chemistry and Bioscience, Aalborg University, Denmark	Danish Covid-19 Genome Consortium
EPI_ISL_816241, EPI_ISL_816295, EPI_ISL_816370, EPI_ISL_816428, EPI_ISL_816443, EPI_ISL_816512, EPI_ISL_816522, EPI_ISL_816551	Virology Department, Sheffield Teaching Hospitals NHS Foundation Trust/Department of Infection, Immunity and Cardiovascular Disease, The Medical School, University of Sheffield	COVID-19 Genomics UK (COG-UK) Consortium	Thushan de Silva, Matthew Parker, Nikki Smith, Adri Angyal, Rebecca Brown, Luke Green, Rachel Tucker, Paul Parsons, Danielle Groves, Katie Johnson, Laura Carrilero, Alex Keeley, Dave Partridge, Matthew Wyles, Benjamin Lindsey, Mehmet Yavuz, Mohammad Raza, Cariad Evans
EPI_ISL_819505	University College London, Great Ormond Street Hospital for Children NHS Foundation Trust, Imperial College Healthcare NHS Trust	COVID-19 Genomics UK (COG-UK) Consortium	Sergi Castellano, Rachel Williams, Mark Kristiansen, Paola Resende Silva, Sunando Roy, Tony Brooks, Helena Tutill, Paola Niola, Patricia Dyal, Charlotte Williams, Leysa Forrest, Yasmin Panchbhaya, Jacqueline Findlay, Samuel Weeks, Julianne Brown, Kathryn Harris, Paul Randell, James Price, Alison Holmes, Judith Breuer
EPI_ISL_819506, EPI_ISL_819507	Lincolnshire Hospitals and DeepSeq Nottingham	COVID-19 Genomics UK (COG-UK) Consortium	Nichola Duckworth, Tim Sloan, Sarah Walsh, Jonathan Ball, Patrick McClure, Joeseph Chappell, Nadine Holmes, Matthew Carlisle, Christopher Moore, Fei Sang, Johnny Debebe, Victoria Wright, Matthew Loose
EPI_ISL_819508, EPI_ISL_819509, EPI_ISL_819510, EPI_ISL_819511, EPI_ISL_819512, EPI_ISL_819513, EPI_ISL_819514, EPI_ISL_819515, EPI_ISL_819516, EPI_ISL_819517, EPI_ISL_819518, EPI_ISL_819519			
see above	University College London, Great Ormond Street Hospital for Children NHS Foundation Trust, Imperial College Healthcare NHS Trust	COVID-19 Genomics UK (COG-UK) Consortium	Sergi Castellano, Rachel Williams, Mark Kristiansen, Paola Resende Silva, Sunando Roy, Tony Brooks, Helena Tutill, Paola Niola, Patricia Dyal, Charlotte Williams, Leysa Forrest, Yasmin Panchbhaya, Jacqueline Findlay, Samuel Weeks, Julianne Brown, Kathryn Harris, Paul Randell, James Price, Alison Holmes, Judith Breuer
EPI_ISL_819520, EPI_ISL_819521, EPI_ISL_819522, EPI_ISL_819523	Lincolnshire Hospitals and DeepSeq Nottingham	COVID-19 Genomics UK (COG-UK) Consortium	Nichola Duckworth, Tim Sloan, Sarah Walsh, Jonathan Ball, Patrick McClure, Joeseph Chappell, Nadine Holmes, Matthew Carlisle, Christopher Moore, Fei Sang, Johnny Debebe, Victoria Wright, Matthew Loose
EPI_ISL_819524, EPI_ISL_819525, EPI_ISL_819526, EPI_ISL_819527, EPI_ISL_819528	University College London, Great Ormond Street Hospital for Children NHS Foundation Trust, Imperial College Healthcare NHS Trust	COVID-19 Genomics UK (COG-UK) Consortium	Sergi Castellano, Rachel Williams, Mark Kristiansen, Paola Resende Silva, Sunando Roy, Tony Brooks, Helena Tutill, Paola Niola, Patricia Dyal, Charlotte Williams, Leysa Forrest, Yasmin Panchbhaya, Jacqueline Findlay, Samuel Weeks, Julianne Brown, Kathryn Harris, Paul Randell, James Price, Alison Holmes, Judith Breuer
EPI_ISL_819529	Lincolnshire Hospitals and DeepSeq Nottingham	COVID-19 Genomics UK (COG-UK) Consortium	Nichola Duckworth, Tim Sloan, Sarah Walsh, Jonathan Ball, Patrick McClure, Joeseph Chappell, Nadine Holmes, Matthew Carlisle, Christopher Moore, Fei Sang, Johnny Debebe, Victoria Wright, Matthew Loose
EPI_ISL_819530, EPI_ISL_819531	University College London, Great Ormond Street Hospital for Children NHS Foundation Trust, Imperial College Healthcare NHS Trust	COVID-19 Genomics UK (COG-UK) Consortium	Sergi Castellano, Rachel Williams, Mark Kristiansen, Paola Resende Silva, Sunando Roy, Tony Brooks, Helena Tutill, Paola Niola, Patricia Dyal, Charlotte Williams, Leysa Forrest, Yasmin Panchbhaya, Jacqueline Findlay, Samuel Weeks, Julianne Brown, Kathryn Harris, Paul Randell, James Price, Alison Holmes, Judith Breuer
EPI_ISL_819532	Lincolnshire Hospitals and DeepSeq Nottingham	COVID-19 Genomics UK (COG-UK) Consortium	Nichola Duckworth, Tim Sloan, Sarah Walsh, Jonathan Ball, Patrick McClure, Joeseph Chappell, Nadine Holmes, Matthew Carlisle, Christopher Moore, Fei Sang, Johnny Debebe, Victoria Wright, Matthew Loose
EPI_ISL_819533, EPI_ISL_819534, EPI_ISL_819535, EPI_ISL_819536, EPI_ISL_819537, EPI_ISL_819538, EPI_ISL_819539, EPI_ISL_819540, EPI_ISL_819541, EPI_ISL_819542, EPI_ISL_819543, EPI_ISL_819544, EPI_ISL_819545, EPI_ISL_819546, EPI_ISL_819547, EPI_ISL_819548, EPI_ISL_819549			
see above	University College London, Great Ormond Street Hospital for	COVID-19 Genomics UK (COG-UK) Consortium	Sergi Castellano, Rachel Williams, Mark Kristiansen, Paola Resende Silva, Sunando Roy, Tony Brooks, Helena Tutill, Paola Niola, Patricia Dyal, Charlotte

	Children NHS Foundation Trust, Imperial College Healthcare NHS Trust		Williams, Leysa Forrest, Yasmin Panchbhaya, Jacqueline Findlay, Samuel Weeks, Julianne Brown, Kathryn Harris, Paul Randell, James Price, Alison Holmes, Judith Breuer
EPI_ISL_822093	Lighthouse Lab in Alderley Park	Wellcome Sanger Institute for the COVID-19 Genomics UK (COG-UK) Consortium	Jacquelyn Wynn, Mairead Hyland, The Lighthouse Lab in Alderley Park and Alex Alderton, Roberto Amato, Sonia Goncalves, Ewan Harrison, David K. Jackson, Ian Johnston, Dominic Kwiatkowski, Cordelia Langford, John Sillitoe on behalf of the Wellcome Sanger Institute COVID-19 Surveillance Team
EPI_ISL_823967	DOHMH Corona	New York City Public Health Laboratory	Jade Wang, et al.
EPI_ISL_823968, EPI_ISL_823969	DOHMH Jamaica	New York City Public Health Laboratory	Jade Wang, et al.
EPI_ISL_824004, EPI_ISL_824005, EPI_ISL_824007, EPI_ISL_824009, EPI_ISL_824011, EPI_ISL_824012, EPI_ISL_824014, EPI_ISL_824018, EPI_ISL_824126, EPI_ISL_824127, EPI_ISL_824131			
see above	Dutch COVID-19 response team	National Institute for Public Health and the Environment (RIVM)	Adam Meijer, Harry Vennema, Jeroen Cremer, Sharon van den Brink, Bas van der Veer, AnneMarie van den Brandt, Florian Zwagemaker, Dennis Schmitz, Chantal Reusken, on behalf of the national COVID-19 response team
EPI_ISL_824335, EPI_ISL_824344, EPI_ISL_824351, EPI_ISL_824352, EPI_ISL_824355, EPI_ISL_824356, EPI_ISL_824357, EPI_ISL_824366, EPI_ISL_824371, EPI_ISL_824374, EPI_ISL_824375, EPI_ISL_824376, EPI_ISL_824379, EPI_ISL_824382			
see above	Michigan Department of Health and Human Services, Bureau of Laboratories	Michigan Department of Health and Human Services, Bureau of Laboratories	Blankenship HM, Riner D, Soehnlen MK
EPI_ISL_824396, EPI_ISL_824397	California Department of Public Health	California Department of Public Health	CDPH IDLB COVIDNet
EPI_ISL_825084, EPI_ISL_825086, EPI_ISL_825087, EPI_ISL_825088, EPI_ISL_825089	Indian Council of Medical Research-National Institute of Virology,Maximum Containment Laboratory	Indian Council of Medical Research-National Institute of Virology,Maximum Containment Laboratory	Pragya D Yadav, Dimpal A Nyayanit, Rima R Sahay, Prasad Sarkale, Jayshri Pethani, Savita Patil, Shrikant Baradkar, Varsha Potdar, Deepak Y Patil
EPI_ISL_825333, EPI_ISL_825335, EPI_ISL_825366, EPI_ISL_825367, EPI_ISL_825368, EPI_ISL_825369, EPI_ISL_825370, EPI_ISL_825371	Hospital Universitari Vall d'Hebron - Vall d'Hebron Institut de Rercerca	Hospital Universitari Vall d'Hebron	Cristina Andrés, Maria Piñana, Josep F Abril, Damir Garcia-Cehic, Ariadna Rando, Juliana Esperalba, Maria Gema Codina, Carla Castillo, Maria Carmen Martin, Tomàs Pumarola, Josep Quer, Andrés Antón
EPI_ISL_826554	Texas Department of State Health Services	Texas Department of State Health Services	Rashmi Tuladhar, Bonnie Oh, Jenny Zhang, Maliha Rahman, Anita Pokharel, Myong Koag, Chung Wang, Rachel Lee, Grace Kubin, Mayela Pedrueza, James Daniel Bonser
EPI_ISL_826881, EPI_ISL_826892, EPI_ISL_827078, EPI_ISL_827152, EPI_ISL_827170, EPI_ISL_827434	deCODE genetics	deCODE genetics	Daniel F Gudbjartsson; Agnar Helgason; Hakon Jonsson; Olafur T Magnusson; Pall Melsted; Gudmundur L Norddahl; Jona Saemundsdottir; Asgeir Sigurdsson; Patrick Sulem; Arna B Agustsdottir; Hannes Eggertsson; Berglind Eiriksdottir; Run Fridriksdottir; Elisabet E Gardarsdottir; Gudmundur Georgsson; Olafia S Gretarsdottir; Kjartan R Gudmundsson; Thora R Gunnarsdottir; Arnaldur Gylfason; Hilma Holm; Brynjar O Jensson; Aslaug Jonasdottir; Kamilla S Josefsdottir; Thordur Kristjansson; Droplaug N Magnusdottir; Solvi Rognvaldsson; Louise le Roux; Gudrun Sigmundsdottir; Gardar Sveinbjornsson; Kristin E Sveinsdottir; Maney Sveinsdottir; Emil A Thorarensen; Bjarni Thorbjornsson; Gisli Masson; Ingileif Jonsdottir; Alma Moller; Thorolfur Gudnason; Karl G Kristinsson; Unnur Thorsteinsdottir; Kari Stefansson
EPI_ISL_827692	The National University Hospital of Iceland	deCODE genetics	Daniel F Gudbjartsson; Agnar Helgason; Hakon Jonsson; Olafur T Magnusson; Pall Melsted; Gudmundur L Norddahl; Jona Saemundsdottir; Asgeir Sigurdsson; Patrick Sulem; Arna B Agustsdottir; Hannes Eggertsson; Berglind Eiriksdottir; Run Fridriksdottir; Elisabet E Gardarsdottir; Gudmundur Georgsson; Olafia S Gretarsdottir; Kjartan R Gudmundsson; Thora R Gunnarsdottir; Arnaldur Gylfason; Hilma Holm; Brynjar O Jensson; Aslaug Jonasdottir; Kamilla S Josefsdottir; Thordur Kristjansson; Droplaug N Magnusdottir; Solvi Rognvaldsson; Louise le Roux; Gudrun Sigmundsdottir; Gardar Sveinbjornsson; Kristin E Sveinsdottir; Maney Sveinsdottir; Emil A Thorarensen; Bjarni Thorbjornsson; Gisli Masson; Ingileif Jonsdottir; Alma Moller; Thorolfur Gudnason; Karl G Kristinsson; Unnur Thorsteinsdottir; Kari Stefansson
EPI_ISL_827858, EPI_ISL_827912, EPI_ISL_827920, EPI_ISL_827926, EPI_ISL_828050, EPI_ISL_828208, EPI_ISL_828520, EPI_ISL_828521, EPI_ISL_828524, EPI_ISL_828535, EPI_ISL_828536, EPI_ISL_828632, EPI_ISL_828860, EPI_ISL_829056, EPI_ISL_829209, EPI_ISL_829210, EPI_ISL_829211, EPI_ISL_829212, EPI_ISL_829213, EPI_ISL_829214, EPI_ISL_829215, EPI_ISL_829216, EPI_ISL_829329, EPI_ISL_829330, EPI_ISL_829331, EPI_ISL_829332, EPI_ISL_829333, EPI_ISL_829334			
see above	deCODE genetics	deCODE genetics	Daniel F Gudbjartsson; Agnar Helgason; Hakon Jonsson; Olafur T Magnusson; Pall Melsted; Gudmundur L Norddahl; Jona Saemundsdottir; Asgeir Sigurdsson; Patrick Sulem; Arna B Agustsdottir; Hannes Eggertsson; Berglind Eiriksdottir; Run Fridriksdottir; Elisabet E Gardarsdottir; Gudmundur Georgsson; Olafia S Gretarsdottir; Kjartan R Gudmundsson; Thora R Gunnarsdottir; Arnaldur Gylfason; Hilma Holm; Brynjar O Jensson; Aslaug Jonasdottir; Kamilla S Josefsdottir; Thordur Kristjansson; Droplaug N Magnusdottir; Solvi Rognvaldsson; Louise le Roux; Gudrun Sigmundsdottir; Gardar Sveinbjornsson; Kristin E Sveinsdottir; Maney Sveinsdottir; Emil A Thorarensen; Bjarni Thorbjornsson; Gisli Masson; Ingileif Jonsdottir; Alma Moller; Thorolfur Gudnason; Karl G Kristinsson; Unnur Thorsteinsdottir; Kari Stefansson
EPI_ISL_829607	The National University Hospital of Iceland	deCODE genetics	Daniel F Gudbjartsson; Agnar Helgason; Hakon Jonsson; Olafur T Magnusson; Pall Melsted; Gudmundur L Norddahl; Jona Saemundsdottir; Asgeir Sigurdsson; Patrick Sulem; Arna B Agustsdottir; Hannes Eggertsson; Berglind Eiriksdottir; Run Fridriksdottir; Elisabet E Gardarsdottir; Gudmundur Georgsson; Olafia S Gretarsdottir; Kjartan R Gudmundsson; Thora R Gunnarsdottir; Arnaldur Gylfason; Hilma Holm; Brynjar O Jensson; Aslaug Jonasdottir; Kamilla S Josefsdottir; Thordur Kristjansson; Droplaug N Magnusdottir; Solvi Rognvaldsson; Louise le Roux; Gudrun Sigmundsdottir; Gardar Sveinbjornsson; Kristin E Sveinsdottir; Maney Sveinsdottir; Emil A Thorarensen; Bjarni Thorbjornsson; Gisli Masson; Ingileif Jonsdottir; Alma Moller; Thorolfur Gudnason; Karl G Kristinsson; Unnur Thorsteinsdottir; Kari Stefansson
EPI_ISL_829742, EPI_ISL_829966, EPI_ISL_829968, EPI_ISL_829969, EPI_ISL_829974, EPI_ISL_830251	deCODE genetics	deCODE genetics	Daniel F Gudbjartsson; Agnar Helgason; Hakon Jonsson; Olafur T Magnusson; Pall Melsted; Gudmundur L Norddahl; Jona Saemundsdottir; Asgeir Sigurdsson; Patrick Sulem; Arna B Agustsdottir; Hannes Eggertsson; Berglind Eiriksdottir; Run Fridriksdottir; Elisabet E Gardarsdottir; Gudmundur Georgsson; Olafia S Gretarsdottir; Kjartan R Gudmundsson; Thora R Gunnarsdottir; Arnaldur Gylfason; Hilma Holm; Brynjar O Jensson; Aslaug Jonasdottir; Kamilla S Josefsdottir; Thordur Kristjansson; Droplaug N Magnusdottir; Solvi Rognvaldsson; Louise le Roux; Gudrun Sigmundsdottir; Gardar Sveinbjornsson; Kristin E Sveinsdottir; Maney Sveinsdottir; Emil A Thorarensen; Bjarni Thorbjornsson; Gisli Masson; Ingileif Jonsdottir; Alma Moller; Thorolfur Gudnason; Karl G Kristinsson; Unnur Thorsteinsdottir; Kari Stefansson
EPI_ISL_830254	The National University Hospital of Iceland	deCODE genetics	Daniel F Gudbjartsson; Agnar Helgason; Hakon Jonsson; Olafur T Magnusson; Pall Melsted; Gudmundur L Norddahl; Jona Saemundsdottir; Asgeir Sigurdsson; Patrick Sulem; Arna B Agustsdottir; Hannes Eggertsson; Berglind Eiriksdottir; Run Fridriksdottir; Elisabet E Gardarsdottir; Gudmundur Georgsson; Olafia S Gretarsdottir; Kjartan R Gudmundsson; Thora R Gunnarsdottir; Arnaldur Gylfason; Hilma Holm; Brynjar O Jensson; Aslaug Jonasdottir; Kamilla S Josefsdottir; Thordur Kristjansson; Droplaug N Magnusdottir; Solvi Rognvaldsson; Louise le Roux; Gudrun Sigmundsdottir; Gardar Sveinbjornsson; Kristin E Sveinsdottir; Maney Sveinsdottir; Emil A Thorarensen; Bjarni Thorbjornsson; Gisli Masson; Ingileif Jonsdottir; Alma Moller; Thorolfur Gudnason; Karl G Kristinsson; Unnur Thorsteinsdottir; Kari Stefansson
EPI_ISL_830256, EPI_ISL_830260, EPI_ISL_830262	deCODE genetics	deCODE genetics	Daniel F Gudbjartsson; Agnar Helgason; Hakon Jonsson; Olafur T Magnusson; Pall Melsted; Gudmundur L Norddahl; Jona Saemundsdottir; Asgeir Sigurdsson; Patrick Sulem; Arna B Agustsdottir; Hannes Eggertsson; Berglind Eiriksdottir; Run Fridriksdottir; Elisabet E Gardarsdottir; Gudmundur Georgsson; Olafia S Gretarsdottir; Kjartan R Gudmundsson; Thora R Gunnarsdottir; Arnaldur Gylfason; Hilma Holm; Brynjar O Jensson; Aslaug Jonasdottir; Kamilla S Josefsdottir; Thordur Kristjansson; Droplaug N Magnusdottir; Solvi Rognvaldsson; Louise le Roux; Gudrun Sigmundsdottir; Gardar Sveinbjornsson; Kristin E Sveinsdottir; Maney Sveinsdottir; Emil A Thorarensen; Bjarni Thorbjornsson; Gisli Masson; Ingileif Jonsdottir; Alma Moller; Thorolfur Gudnason; Karl G Kristinsson; Unnur Thorsteinsdottir; Kari Stefansson
EPI_ISL_830268	The National University Hospital of Iceland	deCODE genetics	Daniel F Gudbjartsson; Agnar Helgason; Hakon Jonsson; Olafur T Magnusson; Pall Melsted; Gudmundur L Norddahl; Jona Saemundsdottir; Asgeir Sigurdsson; Patrick Sulem; Arna B Agustsdottir; Hannes Eggertsson; Berglind Eiriksdottir; Run Fridriksdottir; Elisabet E Gardarsdottir; Gudmundur Georgsson; Olafia S Gretarsdottir; Kjartan R Gudmundsson; Thora R Gunnarsdottir; Arnaldur Gylfason; Hilma Holm; Brynjar O Jensson; Aslaug Jonasdottir; Kamilla S Josefsdottir; Thordur Kristjansson; Droplaug N Magnusdottir; Solvi Rognvaldsson; Louise le Roux; Gudrun Sigmundsdottir; Gardar Sveinbjornsson; Kristin E Sveinsdottir; Maney Sveinsdottir; Emil A Thorarensen; Bjarni Thorbjornsson; Gisli Masson; Ingileif Jonsdottir; Alma Moller; Thorolfur Gudnason; Karl G Kristinsson; Unnur Thorsteinsdottir; Kari Stefansson
EPI_ISL_831420, EPI_ISL_831421, EPI_ISL_831422	Labor Dr. Wisplinghoff - Köln	Robert Koch Institute, Influenza and respiratory viruses FG17 & Bioinformatics MF1, Berlin, Germany	Dr. R. Grosser, Stephan Fuchs, Stefan Kroeger, Marianne Wedde, Oliver Drechsel, Aleksandar Radonic, Rene Kmiecinski, Ralf Duerrwald, Thorsten Wolff
EPI_ISL_831423	Labor Dr. Krause & Kollegen MVZ GmbH	Robert Koch Institute, Influenza and respiratory viruses FG17 & Bioinformatics MF1, Berlin, Germany	Dr. Lorentz, Stephan Fuchs, Stefan Kroeger, Marianne Wedde, Oliver Drechsel, Aleksandar Radonic, Rene Kmiecinski, Ralf Duerrwald, Thorsten Wolff

EPI_ISL_831424, EPI_ISL_831425	Labor Dr. Wisplinghoff - Köln	Robert Koch Institute, Influenza and respiratory viruses FG17 & Bioinformatics MF1, Berlin, Germany	Dr. R. Grosser, Stephan Fuchs, Stefan Kroeger, Marianne Wedde, Oliver Drechsel, Aleksandar Radonic, Rene Kmiecinski, Ralf Duerrwald, Thorsten Wolff
EPI_ISL_831426	Labor Dr. Krause & Kollegen MVZ GmbH	Robert Koch Institute, Influenza and respiratory viruses FG17 & Bioinformatics MF1, Berlin, Germany	Dr. Lorentz, Stephan Fuchs, Stefan Kroeger, Marianne Wedde, Oliver Drechsel, Aleksandar Radonic, Rene Kmiecinski, Ralf Duerrwald, Thorsten Wolff
EPI_ISL_831662, EPI_ISL_831684, EPI_ISL_831686	Santa Clara County Public Health Laboratory	Santa Clara County Public Health Laboratory	Santa Clara County Public Health Department
EPI_ISL_831697, EPI_ISL_831706, EPI_ISL_831715, EPI_ISL_831730, EPI_ISL_831731, EPI_ISL_831736, EPI_ISL_831741, EPI_ISL_831834, EPI_ISL_831835, EPI_ISL_831836, EPI_ISL_831837, EPI_ISL_831838, EPI_ISL_831839, EPI_ISL_831840, EPI_ISL_831843, EPI_ISL_831844, EPI_ISL_831845, EPI_ISL_831855, EPI_ISL_831856, EPI_ISL_831857, EPI_ISL_831858, EPI_ISL_831859, EPI_ISL_831860, EPI_ISL_831864			
see above	United States Air Force School of Aerospace Medicine	United States Air Force School of Aerospace Medicine	Anthony Fries, Jennifer Meyer, William Gruner, Amanda Javorina, Sarah Purves, Clarise Starr, Elizabeth Macias
EPI_ISL_832156, EPI_ISL_832160, EPI_ISL_832161, EPI_ISL_832162	Hospital	National Reference Center for Viruses of Respiratory Infections, Institut Pasteur, Paris	Marion Barbet, Sylvie Behillil, Méline Bizard, Angela Brisebarre, Camille Capel, Etienne Simon-Lorière, Vincent Enouf, Maud Vanpeene, Sylvie van der Werf, Léa Pilorge
EPI_ISL_832814	OHSU Lab Services Molecular Microbiology Lab	Oregon SARS-CoV-2 Genome Sequencing Center	Brendan L. O'Connell, Ruth V. Nichols, Sally Grindstaff, Alec J. Hirsch, Donna Hansel, Guang Fan, Daniel N. Streblow, William B. Messer, Andrew C. Adey, Benjamin N. Bimber, Brian J. O'Roak
EPI_ISL_833131	Laboratorio de Ecologia de Doencas Transmissiveis na Amazonia, Instituto Leonidas e Maria Deane - Fiocruz Amazonia	Laboratorio de Ecologia de Doencas Transmissiveis na Amazonia, Instituto Leonidas e Maria Deane - Fiocruz Amazonia	Valdinete Nascimento, Victor Souza, André Corado, Fernanda Nascimento, George Silva, Ágatha Costa, Debora Duarte, Karina Pessoa, Matilde Mejía, Luciana Gonçalves, Maria Júlia Brandão, Michele Jesus, Felipe Naveca
EPI_ISL_833158	Instituto Adolfo Lutz - Regional de Santo Andre	Instituto Adolfo Lutz, Interdiciplinary Procedures Center, Strategic Laboratory	Claudio Tavares Sacchi, Claudia Regina Gonçalves, Erica Valessa Ramos Gomes, Karoline Rodrigues Campos
EPI_ISL_833343	Buenavista Rural Health Unit	Research Institute for Tropical Medicine	Hannah Leah Morito, Othoniel Jan Onza, John Leonard Chan, Ma Angelica Tujan, Francisco Gerardo Polotan, Inez Andrea Medado, Kirstyn Brunker, Edelwisa Mercado, Daria Manalo, Catalino Demetria
EPI_ISL_833356, EPI_ISL_833357, EPI_ISL_833358, EPI_ISL_833359, EPI_ISL_833360, EPI_ISL_833361, EPI_ISL_833362, EPI_ISL_833363	Microbiology Division, South Carolina Department of Health and Environmental Control (SC DHEC)	Microbiology Division, South Carolina Department of Health and Environmental Control (SC DHEC)	Flores,H., Freeman,J.
EPI_ISL_835474	Lighthouse Lab in Alderley Park	Wellcome Sanger Institute for the COVID-19 Genomics UK (COG-UK) Consortium	Jacquelyn Wynn, Mairead Hyland, The Lighthouse Lab in Alderley Park and Alex Alderton, Roberto Amato, Sonia Goncalves, Ewan Harrison, David K. Jackson, Ian Johnston, Dominic Kwiatkowski, Cordelia Langford, John Sillitoe on behalf of the Wellcome Sanger Institute COVID-19 Surveillance Team
EPI_ISL_837107, EPI_ISL_837108, EPI_ISL_837109, EPI_ISL_837110, EPI_ISL_837111, EPI_ISL_837198	Respiratory Virus Unit, National Infection Service, Public Health England	COVID-19 Genomics UK (COG-UK) Consortium	PHE Covid Sequencing Team
EPI_ISL_837450, EPI_ISL_837451, EPI_ISL_837452, EPI_ISL_837453, EPI_ISL_837454, EPI_ISL_837455, EPI_ISL_837456, EPI_ISL_837481, EPI_ISL_837482	Istituto Zooprofilattico Sperimentale del Mezzogiorno	TIGEM	Patrizia Annunziata, Andrea Ballabio, Valentina Bouche, Davide Cacchiarelli (CorrespAuthor), Pellegrino Cerino, Chiara Colantuono, Lucio Di Filippo, Antonio Grimaldi, Antonio Limone, Gabriella Loconte, Anna Manfredi, Francesco Panariello, Biancamaria Pierri, Marcello Salvi, Lucia Vassallo
EPI_ISL_838065, EPI_ISL_838073, EPI_ISL_838074, EPI_ISL_838075, EPI_ISL_838076, EPI_ISL_838077, EPI_ISL_838078, EPI_ISL_838079	West of Scotland Specialist Virology Centre, NHSGGC / MRC-University of Glasgow Centre for Virus Research	COVID-19 Genomics UK (COG-UK) Consortium	Ana da Silva Filipe, Natasha Johnson, Kathy Smollett, Daniel Mair, Stephen Carmichael, Alice Broos, Lily Tong, Jenna Nichols, Kyriaki Nomikou; Sarah McDonald; Richard Orton, Joseph Hughes, Sreenu Vattipally, David L Robertson; Alasdair MacLean, Rory Gunson; Sharif Shaaban, Matthew Holden; Rachel Blacow, Guy Mollett, Kathy Li, James Shepherd, Antonia Ho, Emma Thomson
EPI_ISL_838215	Virology Department, Royal Infirmary of Edinburgh, NHS Lothian / School of Biological Sciences, University of Edinburgh / Institute of Genetics and Molecular Medicine, University of Edinburgh	COVID-19 Genomics UK (COG-UK) Consortium	McHugh M, Dewar R, Rooke S, Gallagher M, Balcaza C, O'Toole Á, Scher E, Hill V, McCrone JT, Colquhoun R, Yu X, Jackson B, Rambaut A, Williams TC, Templeton K
EPI_ISL_838353, EPI_ISL_838354, EPI_ISL_838355, EPI_ISL_838356, EPI_ISL_838357, EPI_ISL_838358, EPI_ISL_838359, EPI_ISL_838361, EPI_ISL_838362, EPI_ISL_838363, EPI_ISL_838364, EPI_ISL_838366, EPI_ISL_838367, EPI_ISL_838368, EPI_ISL_838369, EPI_ISL_838372, EPI_ISL_838376, EPI_ISL_838377, EPI_ISL_838378, EPI_ISL_838379, EPI_ISL_838380, EPI_ISL_838382, EPI_ISL_838383, EPI_ISL_838384, EPI_ISL_838385, EPI_ISL_838386, EPI_ISL_838388, EPI_ISL_838393, EPI_ISL_838394, EPI_ISL_838395, EPI_ISL_838398, EPI_ISL_838399, EPI_ISL_838401, EPI_ISL_838405, EPI_ISL_838422, EPI_ISL_838424, EPI_ISL_838427, EPI_ISL_838440, EPI_ISL_838446, EPI_ISL_838453, EPI_ISL_838454			
see above	Liverpool Clinical Laboratories	COVID-19 Genomics UK (COG-UK) Consortium	Sam Haldenby, Anita Lucaci, Steve Paterson, Julian Hiscox, Alistair Darby, M Almsaud, A Alrezaihi, Muhannad Alruwaili, Stuart D Armstrong, Jones Benjamin, Eleanor G Bentley, Anu Chawla, Jordan J Clark, Angela Cowell, Richard Eccles, Isabel García-Dorival, Matthew Gemmell, Alessandro Gerada, PKF Gilmore, Richard Gregory, Ximeng Han, Catherine Hartley, Margaret Hughes, Miren Iturriza-Gomara, James Johnson, L Luu, Jenifer Manson, Charlotte Nelson, Elaine O'Toole, Cassie Olateju, Rebekah Penrice-Randal , Lucille Rainbow, N.P Randle, Trevor Ian Robinson, Parul Sharma, Ghada T Shawli, James P Stewart, Neil Swainston, Ecaterina Vamos, Joanne Watts, Mark Whitehead
EPI_ISL_839338, EPI_ISL_839341	University College London, Great Ormond Street Hospital for Children NHS Foundation Trust, Imperial College Healthcare NHS Trust	COVID-19 Genomics UK (COG-UK) Consortium	Sergi Castellano, Rachel Williams, Mark Kristiansen, Paola Resende Silva, Sunando Roy, Tony Brooks, Helena Tutill, Paola Niola, Patricia Dyal, Charlotte Williams, Leysa Forrest, Yasmin Panchbhaya, Jacqueline Findlay, Samuel Weeks, Julianne Brown, Kathryn Harris, Paul Randell, James Price, Alison Holmes, Judith Breuer
EPI_ISL_839987	Queens Medical Centre, Clinical Microbiology Department / DeepSeq Nottingham	COVID-19 Genomics UK (COG-UK) Consortium	Gemma Clark, Wendy Smith, Manjinder Khakh, Vicki M Fleming, Michelle M Lister, Hannah Howson-Wells, Jonathan Ball, Patrick McClure, Joseph Chappell, Theocharis Tsoleridis, Nadine Holmes, Matthew Carlisle, Christopher Moore, Fei Sang, Johnny Debebe, Victoria Wright, Matthew Loose
EPI_ISL_840019, EPI_ISL_840020, EPI_ISL_840021, EPI_ISL_840025	Lincolnshire Hospitals and DeepSeq Nottingham	COVID-19 Genomics UK (COG-UK) Consortium	Nichola Duckworth, Tim Sloan, Sarah Walsh, Jonathan Ball, Patrick McClure, Joeseph Chappell, Nadine Holmes, Matthew Carlisle, Christopher Moore, Fei Sang, Johnny Debebe, Victoria Wright, Matthew Loose
EPI_ISL_840222, EPI_ISL_840223, EPI_ISL_840224	Oxford Viromics, NDM, University of Oxford; Oxford University Hospitals; Basingstoke and North Hampshire Hospital	COVID-19 Genomics UK (COG-UK) Consortium	Tanya Golubchik, David Bonsall, George Macintyre, Amy Trebes, Mariateresa de Cesare, Catrin Moore, Alex Mobbs, Anita Justice, Robert Shaw, Monique Andersson, Timothy Peto, Emma Wise, Nathan Moore, Jessica Lynch, Nick Cortes, Matilde Mori, Stephen Kidd, David Buck, John Todd, Christophe Fraser
EPI_ISL_841503, EPI_ISL_841529, EPI_ISL_841562, EPI_ISL_841573, EPI_ISL_841574, EPI_ISL_841575, EPI_ISL_841576, EPI_ISL_841577, EPI_ISL_841578	Originating lab: Wales Specialist Virology Centre Sequencing lab: Pathogen Genomics Unit	Public Health Wales Microbiology Cardiff Wales Specialist Virology Centre	Catherine Moore, Johnathan Evans, Laura Gifford, Malorie Perry, Simon Cottrell, Angela Marchbank, Alec Birchley, Alexander Adams, Amy Gaskin, Bree Gatica-Wilcox, Jason Coombes, Joel Southgate, Lauren Gilbert, Lee Graham, Nicole Pacchiarini, Sara Kumziene-Summerhayes, Sarah Taylor, Sophie Jones, Sara Rey, Matthew Bull, Joanne Watkins, Sally Corden, Tom Connor
EPI_ISL_842662, EPI_ISL_842696	University College London Hospital	COVID-19 Genomics UK (COG-UK) Consortium	Judith Heaney, Matthew Byott, Catherine Houlihan, Dan Frampton, Stuart Kirk, Moira Spyer and Eleni Nastouli
EPI_ISL_843008, EPI_ISL_843009, EPI_ISL_843012, EPI_ISL_843025, EPI_ISL_843026, EPI_ISL_843027, EPI_ISL_843028, EPI_ISL_843029, EPI_ISL_843030, EPI_ISL_843031, EPI_ISL_843038, EPI_ISL_843040, EPI_ISL_843042, EPI_ISL_843043, EPI_ISL_843045, EPI_ISL_843047, EPI_ISL_843067, EPI_ISL_843068, EPI_ISL_843070			
see above	Barts Health NHS Trust	COVID-19 Genomics UK (COG-UK) Consortium	CUTINO-MOGUEL, Maria-Teresa; HARRINGTON, David; OWOYEMI, Dola; SHYLINI, Raghavendran; BROAD, Claire; KELE, Beatrix
EPI_ISL_844220, EPI_ISL_844221, EPI_ISL_844222, EPI_ISL_844228, EPI_ISL_844232, EPI_ISL_844233, EPI_ISL_844236, EPI_ISL_844238, EPI_ISL_844251, EPI_ISL_844252, EPI_ISL_844263, EPI_ISL_844264, EPI_ISL_844266, EPI_ISL_844269, EPI_ISL_844287, EPI_ISL_844288, EPI_ISL_844289, EPI_ISL_844290, EPI_ISL_844291, EPI_ISL_844297, EPI_ISL_844302, EPI_ISL_844303, EPI_ISL_844304, EPI_ISL_844305, EPI_ISL_844462, EPI_ISL_844463, EPI_ISL_844464, EPI_ISL_844465, EPI_ISL_844466, EPI_ISL_844467, EPI_ISL_844468, EPI_ISL_844469, EPI_ISL_844470, EPI_ISL_844471, EPI_ISL_844472, EPI_ISL_844473, EPI_ISL_844474, EPI_ISL_844475, EPI_ISL_844476, EPI_ISL_844477, EPI_ISL_844478, EPI_ISL_844479, EPI_ISL_844480, EPI_ISL_844481, EPI_ISL_844482, EPI_ISL_844483, EPI_ISL_844484, EPI_ISL_844485, EPI_ISL_844486, EPI_ISL_844487, EPI_ISL_844488, EPI_ISL_844489, EPI_ISL_844490, EPI_ISL_844491, EPI_ISL_844492, EPI_ISL_844493, EPI_ISL_844494, EPI_ISL_844495, EPI_ISL_844496, EPI_ISL_844497, EPI_ISL_844498, EPI_ISL_844499, EPI_ISL_844500, EPI_ISL_844501, EPI_ISL_844502, EPI_ISL_844503, EPI_ISL_844504, EPI_ISL_844505, EPI_ISL_844506, EPI_ISL_844507, EPI_ISL_844508, EPI_ISL_844509, EPI_ISL_844510, EPI_ISL_844511, EPI_ISL_844512, EPI_ISL_844513, EPI_ISL_844514, EPI_ISL_844515, EPI_ISL_844516, EPI_ISL_844517, EPI_ISL_844518, EPI_ISL_844519, EPI_ISL_844520, EPI_ISL_844521, EPI_ISL_844522, EPI_ISL_844523, EPI_ISL_844524, EPI_ISL_844525, EPI_ISL_844526, EPI_ISL_844527			
see above	Department of Virus and Microbiological Special Diagnostics,	Albertsen Lab, Department of Chemistry and Bioscience,	Danish Covid-19 Genome Consortium

	Statens Serum Institut, Copenhagen, Denmark	Aalborg University, Denmark	
EPI_ISL_845563	National Public Health Laboratory, Cameroon	African Centre of Excellence for Genomics of Infectious Diseases (ACEGID), Redeemer's University	Oluniyi P.E. et al
EPI_ISL_845680, EPI_ISL_845681, EPI_ISL_845682, EPI_ISL_845683, EPI_ISL_845684, EPI_ISL_845685, EPI_ISL_845686, EPI_ISL_845687, EPI_ISL_845688, EPI_ISL_845689, EPI_ISL_845690, EPI_ISL_845691, EPI_ISL_845692, EPI_ISL_845693, EPI_ISL_845694, EPI_ISL_845695, EPI_ISL_845696, EPI_ISL_845697, EPI_ISL_845698, EPI_ISL_845699, EPI_ISL_845700, EPI_ISL_845701, EPI_ISL_845702, EPI_ISL_845703, EPI_ISL_845704, EPI_ISL_845705, EPI_ISL_845706, EPI_ISL_845707, EPI_ISL_845708, EPI_ISL_845709, EPI_ISL_845710, EPI_ISL_845711, EPI_ISL_845712, EPI_ISL_845713, EPI_ISL_845714			
see above	Ontario's COVID-19 Genomics Rapid Response Coalition	McMaster University	Allison McGeer, Patryk Aftanas, Hooman Derakhshani, Angel Li, Kuganya Nirmalarajah, Emily Panousis, Ahmed Draia, Jalees Nasir, Michael Surette, Samira Mubareka, Andrew G. McArthur
EPI_ISL_845794	South Eastern Area Laboratory Services (SEALS)	NSW Health Pathology - Institute of Clinical Pathology and Medical Research; Westmead Hospital; University of Sydney	CIDM-PH et al.
EPI_ISL_846588	Respiratory Virus Unit, National Infection Service, Public Health England	COVID-19 Genomics UK (COG-UK) Consortium	PHE Covid Sequencing Team
EPI_ISL_847179	Department of Virus and Microbiological Special Diagnostics, Statens Serum Institut, Copenhagen, Denmark	Albertsen Lab, Department of Chemistry and Bioscience, Aalborg University, Denmark	Danish Covid-19 Genome Consortium
EPI_ISL_847558, EPI_ISL_847560, EPI_ISL_847566, EPI_ISL_847571, EPI_ISL_847583, EPI_ISL_847664, EPI_ISL_847665, EPI_ISL_847666, EPI_ISL_847667, EPI_ISL_847668, EPI_ISL_847669, EPI_ISL_847670, EPI_ISL_847671, EPI_ISL_847715, EPI_ISL_847749, EPI_ISL_847797, EPI_ISL_847798, EPI_ISL_847799, EPI_ISL_847800, EPI_ISL_847801, EPI_ISL_847802			
see above	California Department of Public Health	Chiu Laboratory, University of California, San Francisco	Charles Chiu, Xianding (Wayne) Deng, Candace Wang, Brian Bushnell, Scot Federman, Jill Hacker, Debra Wadford
EPI_ISL_847980, EPI_ISL_847981, EPI_ISL_847985, EPI_ISL_847986, EPI_ISL_848005, EPI_ISL_848007, EPI_ISL_848008, EPI_ISL_848015, EPI_ISL_848017, EPI_ISL_848018, EPI_ISL_848019, EPI_ISL_848024, EPI_ISL_848025, EPI_ISL_848026, EPI_ISL_848027, EPI_ISL_848028, EPI_ISL_848029, EPI_ISL_848030, EPI_ISL_848031, EPI_ISL_848032, EPI_ISL_848033, EPI_ISL_848034, EPI_ISL_848035, EPI_ISL_848036, EPI_ISL_848045, EPI_ISL_848047, EPI_ISL_848054, EPI_ISL_848059, EPI_ISL_848060, EPI_ISL_848061			
see above	Michigan Department of Health and Human Services, Bureau of Laboratories	Michigan Department of Health and Human Services, Bureau of Laboratories	Blankenship HM, Riner D, Soehnlen MK
EPI_ISL_848223, EPI_ISL_848227, EPI_ISL_848228, EPI_ISL_848236, EPI_ISL_848414, EPI_ISL_848415, EPI_ISL_848416, EPI_ISL_848417, EPI_ISL_848418, EPI_ISL_848419, EPI_ISL_848420, EPI_ISL_848421, EPI_ISL_848422, EPI_ISL_848423, EPI_ISL_848424, EPI_ISL_848439, EPI_ISL_848447, EPI_ISL_848462, EPI_ISL_848530, EPI_ISL_848531, EPI_ISL_848532, EPI_ISL_848533, EPI_ISL_848534			
see above	Illinois Department of Public Health	Gagnon Lab, Southern Illinois University	Keith Gagnon
EPI_ISL_848674, EPI_ISL_848675, EPI_ISL_848676, EPI_ISL_848677, EPI_ISL_848678, EPI_ISL_848679, EPI_ISL_848680, EPI_ISL_848681, EPI_ISL_848682, EPI_ISL_848683, EPI_ISL_848684, EPI_ISL_848685, EPI_ISL_848686, EPI_ISL_848687, EPI_ISL_848688, EPI_ISL_848689, EPI_ISL_848690, EPI_ISL_848691, EPI_ISL_848692, EPI_ISL_848693, EPI_ISL_848694, EPI_ISL_848695, EPI_ISL_848696, EPI_ISL_848697, EPI_ISL_848698, EPI_ISL_848699, EPI_ISL_848700, EPI_ISL_848701, EPI_ISL_848702, EPI_ISL_848703, EPI_ISL_848704, EPI_ISL_848705, EPI_ISL_848706, EPI_ISL_848707, EPI_ISL_848708, EPI_ISL_848709, EPI_ISL_848710, EPI_ISL_848711, EPI_ISL_848712, EPI_ISL_848713, EPI_ISL_848714, EPI_ISL_848715, EPI_ISL_848716			
see above	Florida Bureau of Public Health Laboratories	Florida Bureau of Public Health Laboratories	Sarah Schmedes, Jason Blanton
EPI_ISL_849387, EPI_ISL_849390, EPI_ISL_849392, EPI_ISL_849396, EPI_ISL_849397, EPI_ISL_849399, EPI_ISL_849400, EPI_ISL_849401, EPI_ISL_849402, EPI_ISL_849405, EPI_ISL_849414, EPI_ISL_849419, EPI_ISL_849423, EPI_ISL_849432, EPI_ISL_849439, EPI_ISL_849461, EPI_ISL_849463, EPI_ISL_849526, EPI_ISL_849527, EPI_ISL_849528, EPI_ISL_849529, EPI_ISL_849530, EPI_ISL_849531, EPI_ISL_849563, EPI_ISL_849573, EPI_ISL_849574, EPI_ISL_849587, EPI_ISL_849624, EPI_ISL_849625, EPI_ISL_849626			
see above	Washington State Department of Health	Seattle Flu Study	Deborah A. Nickerson, Chris D. Frazar, Jover Lee, Benjamin Pelle, Matthew Richardson, Amanda Adler, Elisabeth Brandstetter, Peter D. Han, Kairsten Fay, Misja Ilcisin, Kirsten Lacombe, Thomas R. Sibley, Melissa Truong, Caitlin R. Wolf, Romesh Gautom, Geoff Melly, Brian Hiatt, Philip Dykema, Scott Lindquist, Michael Boeckh, Janet A. Englund, Michael Famulare, Barry R. Lutz, Mark J. Rieder, Lea M. Starita, Matthew Thompson, Helen Y. Chu, Jay Shendure, Trevor Bedford
EPI_ISL_849953, EPI_ISL_849954, EPI_ISL_850012, EPI_ISL_850013, EPI_ISL_850014, EPI_ISL_850015, EPI_ISL_850016, EPI_ISL_850017, EPI_ISL_850018	Santa Clara County Public Health Laboratory	Chan-Zuckerberg Biohub	CZB Cliahub Consortium
EPI_ISL_850114, EPI_ISL_850115, EPI_ISL_850116, EPI_ISL_850117, EPI_ISL_850118, EPI_ISL_850119, EPI_ISL_850120, EPI_ISL_850121, EPI_ISL_850122, EPI_ISL_850123, EPI_ISL_850124, EPI_ISL_850125, EPI_ISL_850126, EPI_ISL_850127, EPI_ISL_850128, EPI_ISL_850129, EPI_ISL_850130, EPI_ISL_850131, EPI_ISL_850132, EPI_ISL_850133, EPI_ISL_850134, EPI_ISL_850135, EPI_ISL_850136, EPI_ISL_850137, EPI_ISL_850138, EPI_ISL_850139, EPI_ISL_850140, EPI_ISL_850141, EPI_ISL_850142, EPI_ISL_850143, EPI_ISL_850144, EPI_ISL_850145, EPI_ISL_850146, EPI_ISL_850147, EPI_ISL_850148, EPI_ISL_850149, EPI_ISL_850150, EPI_ISL_850151, EPI_ISL_850152, EPI_ISL_850153, EPI_ISL_850154, EPI_ISL_850155, EPI_ISL_850156, EPI_ISL_850157, EPI_ISL_850158, EPI_ISL_850159, EPI_ISL_850160, EPI_ISL_850161, EPI_ISL_850162, EPI_ISL_850163, EPI_ISL_850164, EPI_ISL_850165, EPI_ISL_850166, EPI_ISL_850167, EPI_ISL_850168, EPI_ISL_850169, EPI_ISL_850170, EPI_ISL_850171, EPI_ISL_850172, EPI_ISL_850173, EPI_ISL_850174, EPI_ISL_850175, EPI_ISL_850179			
see above	Renegade	Chan-Zuckerberg Biohub	CZB Cliahub Consortium
EPI_ISL_852602, EPI_ISL_852603	Max von Pettenkofer Institute, Virology, National Reference Center for Retroviruses, LMU München	Laboratory for Functional Genome Analysis, Dept. Genomics, Gene Center of the LMU Munich	Max Muenchhoff, Stefan Krebs, Alexander Graf, Oliver Keppler, Helmut Blum
EPI_ISL_852953	Hospital General Universitario Gregorio Marañón	SeqCOVID-SPAIN consortium/IBV(CSIC)	Darío García de Viedma, Laura Pérez-Lago, Pedro J Sola-Campoy, Sergio Buenestado-Serrano, Marta Herranz, Victor Manuel de la Cueva, Julia Suárez, Pilar Catalán, Patricia Muñoz and SeqCOVID-SPAIN consortium
EPI_ISL_853330, EPI_ISL_853333, EPI_ISL_853334, EPI_ISL_853337, EPI_ISL_853339, EPI_ISL_853340	UPMC Clinical Microbiology Laboratory	Microbial Genome Sequencing Center; Microbial Genomic Epidemiology Laboratory	Mustapha M. Mustapha, Jane W. Marsh, Dan Snyder, Marissa P. Griffith, Stephanie L. Mitchell, Vatsala R. Srinivasa, Kady D. Waggle, Chinelo Ezeonwuku, Vaughn S. Cooper, Lee H. Harrison
EPI_ISL_853417, EPI_ISL_853418, EPI_ISL_853419, EPI_ISL_853420, EPI_ISL_853421, EPI_ISL_853422, EPI_ISL_853423, EPI_ISL_853424, EPI_ISL_853425, EPI_ISL_853428, EPI_ISL_853429, EPI_ISL_853430, EPI_ISL_853431, EPI_ISL_853432, EPI_ISL_853433, EPI_ISL_853434, EPI_ISL_853435, EPI_ISL_853436, EPI_ISL_853437, EPI_ISL_853438, EPI_ISL_853439, EPI_ISL_853440, EPI_ISL_853441, EPI_ISL_853442, EPI_ISL_853443, EPI_ISL_853444, EPI_ISL_853445, EPI_ISL_853446, EPI_ISL_853447, EPI_ISL_853448, EPI_ISL_853449, EPI_ISL_853450, EPI_ISL_853451, EPI_ISL_853452, EPI_ISL_853453, EPI_ISL_853454, EPI_ISL_853455, EPI_ISL_853456, EPI_ISL_853457, EPI_ISL_853458, EPI_ISL_853459, EPI_ISL_853460, EPI_ISL_853461, EPI_ISL_853462, EPI_ISL_853463, EPI_ISL_853464, EPI_ISL_853465, EPI_ISL_853466, EPI_ISL_853467, EPI_ISL_853468, EPI_ISL_853469, EPI_ISL_853470, EPI_ISL_853471, EPI_ISL_853472, EPI_ISL_853473, EPI_ISL_853474, EPI_ISL_853475, EPI_ISL_853476, EPI_ISL_853477, EPI_ISL_853478, EPI_ISL_853479, EPI_ISL_853480, EPI_ISL_853481, EPI_ISL_853482, EPI_ISL_853483, EPI_ISL_853484, EPI_ISL_853485, EPI_ISL_853486, EPI_ISL_853487, EPI_ISL_853488, EPI_ISL_853489, EPI_ISL_853490, EPI_ISL_853491, EPI_ISL_853492, EPI_ISL_853493, EPI_ISL_853494, EPI_ISL_853495, EPI_ISL_853496, EPI_ISL_853497, EPI_ISL_853498, EPI_ISL_853499, EPI_ISL_853500, EPI_ISL_853501, EPI_ISL_853502, EPI_ISL_853503, EPI_ISL_853504, EPI_ISL_853505, EPI_ISL_853506, EPI_ISL_853507, EPI_ISL_853508, EPI_ISL_853509, EPI_ISL_853510, EPI_ISL_853511, EPI_ISL_853512, EPI_ISL_853513, EPI_ISL_853514, EPI_ISL_853515, EPI_ISL_853516, EPI_ISL_853517, EPI_ISL_853518, EPI_ISL_853519, EPI_ISL_853520, EPI_ISL_853521, EPI_ISL_853522, EPI_ISL_853523, EPI_ISL_853524, EPI_ISL_853525, EPI_ISL_853526, EPI_ISL_853527, EPI_ISL_853528, EPI_ISL_853529, EPI_ISL_853530, EPI_ISL_853531, EPI_ISL_853532, EPI_ISL_853533, EPI_ISL_853534, EPI_ISL_853535, EPI_ISL_853536, EPI_ISL_853537, EPI_ISL_853538, EPI_ISL_853539, EPI_ISL_853540, EPI_ISL_853541, EPI_ISL_853542, EPI_ISL_853543, EPI_ISL_853544, EPI_ISL_853545, EPI_ISL_853560, EPI_ISL_853561, EPI_ISL_853562, EPI_ISL_853563, EPI_ISL_853564, EPI_ISL_853565			
see above	Santa Clara County Public Health Laboratory	Chan-Zuckerberg Biohub	CZB Cliahub Consortium
EPI_ISL_853814, EPI_ISL_853930, EPI_ISL_853934, EPI_ISL_853940	Department of Microbiology, University Innsbruck	Bergthaler laboratory, CeMM Research Center for Molecular Medicine of the Austrian Academy of Sciences	Lukas Endler, Alexandra Popa, Benedikt Agerer, Jakob-Wendelin Genger, Alexander Lercher, Anna Schedl, Thomas Penz, Michael Schuster, Jan Laine, Martin Senekowitsch, Christoph Bock, Andreas Bergthaler
EPI_ISL_854440	SARATOGA HOSPITAL LABORATORY	Wadsworth Center, New York State Department of Health	Kirsten St. George, Daryl M. Lamson, Alexis Russel, Matthew Shudt, Melissa A Leisner, Jonathan Plitnick, Navjot Singh, John Kelly, Erasmus Schneider, Erica Lasek-Nesselquist
EPI_ISL_854625, EPI_ISL_854626, EPI_ISL_854627, EPI_ISL_854628, EPI_ISL_854629, EPI_ISL_854630, EPI_ISL_854631, EPI_ISL_854632, EPI_ISL_854633, EPI_ISL_854634, EPI_ISL_854635, EPI_ISL_854636, EPI_ISL_854637, EPI_ISL_854638, EPI_ISL_854639, EPI_ISL_854640, EPI_ISL_854641, EPI_ISL_854642, EPI_ISL_854643, EPI_ISL_854644, EPI_ISL_854645, EPI_ISL_854646, EPI_ISL_854647, EPI_ISL_854648, EPI_ISL_854649, EPI_ISL_854650, EPI_ISL_854651, EPI_ISL_854652, EPI_ISL_854653, EPI_ISL_854654, EPI_ISL_854655, EPI_ISL_854656, EPI_ISL_854657, EPI_ISL_854658, EPI_ISL_854659, EPI_ISL_854660, EPI_ISL_854661, EPI_ISL_854662, EPI_ISL_854663, EPI_ISL_854664, EPI_ISL_854665, EPI_ISL_854666, EPI_ISL_854667, EPI_ISL_854668, EPI_ISL_854669, EPI_ISL_854670, EPI_ISL_854671, EPI_ISL_854672, EPI_ISL_854673, EPI_ISL_854674, EPI_ISL_854675, EPI_ISL_854676, EPI_ISL_854677, EPI_ISL_854678, EPI_ISL_854679, EPI_ISL_854680, EPI_ISL_854681, EPI_ISL_854682, EPI_ISL_854683, EPI_ISL_854684, EPI_ISL_854685, EPI_ISL_854686, EPI_ISL_854687, EPI_ISL_854688, EPI_ISL_854689, EPI_ISL_854690, EPI_ISL_854691, EPI_ISL_854692, EPI_ISL_854693, EPI_ISL_854694, EPI_ISL_854695, EPI_ISL_854696, EPI_ISL_854697, EPI_ISL_854698, EPI_ISL_854699, EPI_ISL_854700, EPI_ISL_854701, EPI_ISL_854702, EPI_ISL_854703, EPI_ISL_854704, EPI_ISL_854705, EPI_ISL_854706, EPI_ISL_854707, EPI_ISL_854708, EPI_ISL_854709, EPI_ISL_854710, EPI_ISL_854711, EPI_ISL_854712, EPI_ISL_854713, EPI_ISL_854716, EPI_ISL_854717, EPI_ISL_854718, EPI_ISL_854719, EPI_ISL_854720, EPI_ISL_854721, EPI_ISL_854722, EPI_ISL_854723, EPI_ISL_854724, EPI_ISL_854725, EPI_ISL_854726, EPI_ISL_854727, EPI_ISL_854728, EPI_ISL_854729, EPI_ISL_854730, EPI_ISL_854731, EPI_ISL_854732, EPI_ISL_854733, EPI_ISL_854734, EPI_ISL_854735, EPI_ISL_854737, EPI_ISL_854738, EPI_ISL_854739, EPI_ISL_854740, EPI_ISL_854741, EPI_ISL_854742, EPI_ISL_854743, EPI_ISL_854744			
see above	Public Health Ontario Laboratory	Public Health Ontario Laboratory	Vanessa G Allen, Philip Banh, Yao Chen, Richard de Borja, Alireza Eshaghi, Nahuel Fittipaldi, Christine Frantz, Jonathan B Gubbay, Jennifer L Guthrie, Lawrence Heisler, Esha Joshi, Michael Laszloffy, Aimin Li, Michael CY Li, Dean Maxwell, Sandeep Nagra, Samir N Patel, Jared Simpson, Karthikeyan Sivaraman, Ashleigh Sullivan, Yogi Sundaravadanam, Sarah Teatero, Matthew Watson, Andre Villegas, Sandra Zittermann

EPI_ISL_856772, EPI_ISL_856773	Servicio Virosis Respiratorias-Departamento Virología-INEI	Instituto Nacional Enfermedades Infecciosas C.G.Malbran	Baumeister E., Avaro M., Benedetti E., Russo M., Dattero ME, Pontoriero A., Cisterna D., Molina V., Perandones C., Tuduri E., Lorenzo F., Poklepovich T., Campos J.
EPI_ISL_857500, EPI_ISL_857501, EPI_ISL_857502, EPI_ISL_857503, EPI_ISL_857504	Swiss National Reference Centre for Influenza	Swiss National Reference Centre for Influenza	Ana Rita Goncalves,Samuel Cordey, Laurent Kaiser, Lorenzo Cerutti, Henri Pegeot, Melyssa Elies, Keith Harshman, Ioannis Xenarios, Emmanouil Dermitzakis
EPI_ISL_857527	Swiss National Reference Centre for Influenza	Swiss National Reference Centre for Influenza	Tim Roloff, Ana Rita Gonçalves, Madlen Stange, Helena MB Seth-Smith, Alfredo Mari, Karoline Leuzinger, Julia Bielicki, Manuel Battegay, Hans Hirsch, Laurent Kaiser, Adrian Egli
EPI_ISL_859931, EPI_ISL_859932, EPI_ISL_859933, EPI_ISL_860090, EPI_ISL_860091	BTC, Khalifa University	BTC, Khalifa University	Al Safar et al
EPI_ISL_860804	WHO/Minsk	Charité Universitätsmedizin Berlin, Institut für Virologie	Victor M Corman, Barbara Mühlemann, Jörn Beheim-Schwarzbach, Talitha Veith, Julia Tesch, Tobias Bleicker, Julia Schneider, Shmialiova Natallia, Sivets Natallia, Terry Jones, Christian Drosten
EPI_ISL_861780	Hospital General Universitario Gregorio Marañón	SeqCOVID-SPAIN consortium/IBV(CSIC)	Darío García de Viedma, Laura Pérez-Lago, Pedro J Sola-Campoy, Sergio Buenestado-Serrano, Marta Herranz, Victor Manuel de la Cueva, Julia Suárez, Pilar Catalán, Patricia Muñoz and SeqCOVID-SPAIN consortium
EPI_ISL_862588	Hospital Clínic	Instituto de Salud Carlos III	Iglesias-Caballero, M.Camarero, S. Molinero Calamita, M. González-Esguevillas, M. Pozo, F. Casas, I. Jiménez, P. Jiménez, M. Zaballos, A. Monzón, S. Varona, S. Juliá, M. Cuesta, I. Marcos, M.A.
EPI_ISL_864577	Institute of Medical Microbiology and Hospital Hygiene	Institute of Medical Microbiology and Hospital Hygiene	Prof. Dr. Achim Kaasch, Aljoscha Tersteegen
EPI_ISL_865708, EPI_ISL_865709, EPI_ISL_865710, EPI_ISL_865711, EPI_ISL_865712	University College London, Great Ormond Street Hospital for Children NHS Foundation Trust, Imperial College Healthcare NHS Trust	COVID-19 Genomics UK (COG-UK) Consortium	Sergi Castellano, Rachel Williams, Mark Kristiansen, Paola Resende Silva, Sunando Roy, Tony Brooks, Helena Tutill, Paola Niola, Patricia Dyal, Charlotte Williams, Leysa Forrest, Yasmin Panchbhaya, Jacqueline Findlay, Samuel Weeks, Julianne Brown, Kathryn Harris, Paul Randell, James Price, Alison Holmes, Judith Breuer
EPI_ISL_866453	Northumbria University / South Tees Hospitals NHS Foundation Trust / North Cumbria Integrated Care NHS Foundation Trust / North Tees and Hartlepool NHS Foundation Trust / Newcastle Hospitals NHS Foundation Trust	COVID-19 Genomics UK (COG-UK) Consortium	Darren L Smith,Andrew Nelson,Matthew Bashton,Greg R Young,Joshua Loh,John Allan,Mohammad A Tariq,Giles S Holt,Gary Black,Wen C Yew,Lynn Dover,Paul Baker,Steve Liggett,Sarah Essex,Jane Greenaway,Debra Padgett,Clive Graham,Garren Scott,Edward Barton,Emma Swindells,Brendan Payne,Jennifer Collins,Yusri Taha,Gary Eltringham
EPI_ISL_866804, EPI_ISL_866813, EPI_ISL_866827	Quadram Institute Bioscience	COVID-19 Genomics UK (COG-UK) Consortium	Dave J. Baker, Gemma L. Kay, Alp Aydin, Thanh Le-Viet, Steven Rudder, Ana P. Tedim, Anastasia Kolyva, Maria Diaz, Leonardo de Oliveira Martins, Nabil-Fareed Alikhan, Lizzie Meadows, Rachael Stanley, Ngozi Elumogo, Muhammed Yasir, Nicholas M. Thomson, Alexander J Trotter, Rachel Gilroy, Samuel Bloomfield, Claire Stuart, Andrew Bell, Reenesh Prakash, Samir Dervisevic, Alison E. Mather, John Wain, Mark Webber, Andrew J. Page, Justin O'Grady
EPI_ISL_867272, EPI_ISL_867273, EPI_ISL_867274, EPI_ISL_867275, EPI_ISL_867276, EPI_ISL_867277, EPI_ISL_867427	Originating lab: Wales Specialist Virology Centre Sequencing lab: Pathogen Genomics Unit	Public Health Wales Microbiology Cardiff Wales Specialist Virology Centre	Catherine Moore, Johnathan Evans, Laura Gifford, Malorie Perry, Simon Cottrell, Angela Marchbank, Alec Birchley, Alexander Adams, Amy Gaskin, Bree Gatica-Wilcox, Jason Coombes, Joel Southgate, Lauren Gilbert, Lee Graham, Nicole Pacchiarini, Sara Kumziene-Summerhayes, Sarah Taylor, Sophie Jones, Sara Rey, Matthew Bull, Joanne Watkins, Sally Corden, Tom Connor
EPI_ISL_871781, EPI_ISL_871782	Department of Virus and Microbiological Special Diagnostics, Statens Serum Institut, Copenhagen, Denmark	Aalborg University	Danish Covid-19 Genome Consortium
EPI_ISL_872571	Kamchatka Regional Children's Infectious Diseases Hospital	WHO National Influenza Centre Russian Federation	Andrey Komissarov, Artem Fadeev, Anna Ivanova, Kseniya Komissarova, Dmitry Bazhenov, Mikhail Bakaev, Daria Danilenko, Ksenia Safina, Elena Nabieva, Georgii Bazykin, Dmitry Lioznov
EPI_ISL_872611, EPI_ISL_872612	Nigeria Centre for Disease Control (NCDC)	African Centre of Excellence for Genomics of Infectious Diseases (ACEGID), Redeemer's University	Oluniyi P.E. et al
EPI_ISL_872644, EPI_ISL_872648, EPI_ISL_872654, EPI_ISL_872667, EPI_ISL_872673, EPI_ISL_872677	University of Massachusetts Medical School	Infectious Disease Program, Broad Institute of Harvard and MIT	Lemieux,J.E., Siddle,K.J., Ward,D., Ellison,R., Adams,G., Gladden-Young,A., Lagerborg,K., Rudy,M., DeRuff,K., Carter,A., Normandin,E., Bauer,M., Reilly,S., Tomkins-Tinch,C., Loreth,C., Chaluvadi,S., Birren,B.W., Gallagher,G., Smole,S., Park,D.J., MacInnis,B.L., and Sabeti,P.C.
EPI_ISL_872704, EPI_ISL_872705, EPI_ISL_872706, EPI_ISL_872707, EPI_ISL_872708, EPI_ISL_872709, EPI_ISL_872710, EPI_ISL_872711, EPI_ISL_872712, EPI_ISL_872713, EPI_ISL_872714, EPI_ISL_872715, EPI_ISL_872716, EPI_ISL_872718, EPI_ISL_872719, EPI_ISL_872721, EPI_ISL_872723			
see above	Rhode Island Department of Health	Infectious Disease Program, Broad Institute of Harvard and MIT	Lemieux,J.E., Siddle,K.J., Huard,R., King,E., Azevedo,K., Miller,A., Adams,G., Gladden-Young,A., Lagerborg,K., Rudy,M., DeRuff,K., Carter,A., Normandin,E., Bauer,M., Reilly,S., Tomkins-Tinch,C., Loreth,C., Chaluvadi,S., Birren,B.W., Gallagher,G., Smole,S., Park,D.J., MacInnis,B.L., and Sabeti,P.C.
EPI_ISL_872918	WHO National Influenza Centre Russian Federation	WHO National Influenza Centre Russian Federation	Andrey Komissarov, Artem Fadeev, Anna Ivanova, Kseniya Komissarova, Dmitry Bazhenov, Mikhail Bakaev, Daria Danilenko, Ksenia Safina, Elena Nabieva, Georgii Bazykin, Dmitry Lioznov
EPI_ISL_873124, EPI_ISL_873129, EPI_ISL_873130, EPI_ISL_873133, EPI_ISL_873134, EPI_ISL_873139, EPI_ISL_873147	University of Michigan Clinical Microbiology Laboratory	Lauring Lab, University of Michigan, Department of Microbiology and Immunology	Valesano
EPI_ISL_873211, EPI_ISL_873212	M Health Fairview	Minnesota Department of Health, Public Health Laboratory	Alexandra Lorentz, Jacob Garfin, Matt Plumb, and Xiong Wang
EPI_ISL_875690, EPI_ISL_875691, EPI_ISL_875692, EPI_ISL_875693, EPI_ISL_875694, EPI_ISL_875695, EPI_ISL_875696, EPI_ISL_875697, EPI_ISL_875698, EPI_ISL_875699, EPI_ISL_875700, EPI_ISL_875701, EPI_ISL_875702, EPI_ISL_875703, EPI_ISL_875704, EPI_ISL_875705, EPI_ISL_875706, EPI_ISL_875707, EPI_ISL_875708, EPI_ISL_875709, EPI_ISL_875710, EPI_ISL_875711, EPI_ISL_875712, EPI_ISL_875713, EPI_ISL_875714, EPI_ISL_875715, EPI_ISL_875716, EPI_ISL_875717, EPI_ISL_875718, EPI_ISL_875719, EPI_ISL_875720, EPI_ISL_875721, EPI_ISL_875722, EPI_ISL_875723, EPI_ISL_875724, EPI_ISL_875725, EPI_ISL_875726, EPI_ISL_875727, EPI_ISL_875728, EPI_ISL_875729, EPI_ISL_875730, EPI_ISL_875731, EPI_ISL_875732, EPI_ISL_875733, EPI_ISL_875734, EPI_ISL_875735, EPI_ISL_875736, EPI_ISL_875737, EPI_ISL_875738, EPI_ISL_875739, EPI_ISL_875740, EPI_ISL_875741, EPI_ISL_875742, EPI_ISL_875743, EPI_ISL_875744, EPI_ISL_875745, EPI_ISL_875746, EPI_ISL_875747, EPI_ISL_875748, EPI_ISL_875749, EPI_ISL_875750, EPI_ISL_875751, EPI_ISL_875752, EPI_ISL_875753, EPI_ISL_875754, EPI_ISL_875755, EPI_ISL_875756, EPI_ISL_875757, EPI_ISL_875758, EPI_ISL_875759, EPI_ISL_875760, EPI_ISL_875761, EPI_ISL_875762, EPI_ISL_875763, EPI_ISL_875764, EPI_ISL_875765, EPI_ISL_875766, EPI_ISL_875767, EPI_ISL_875768, EPI_ISL_875769, EPI_ISL_875770, EPI_ISL_875771, EPI_ISL_875772, EPI_ISL_875773, EPI_ISL_875774, EPI_ISL_875775, EPI_ISL_875776, EPI_ISL_875777, EPI_ISL_875778, EPI_ISL_875779, EPI_ISL_875780, EPI_ISL_875781, EPI_ISL_875782, EPI_ISL_875783, EPI_ISL_875784, EPI_ISL_875785, EPI_ISL_875786, EPI_ISL_875787, EPI_ISL_875788, EPI_ISL_875789, EPI_ISL_875790, EPI_ISL_875791, EPI_ISL_875792, EPI_ISL_875793, EPI_ISL_875794, EPI_ISL_875795, EPI_ISL_875796, EPI_ISL_875797, EPI_ISL_875798, EPI_ISL_875799, EPI_ISL_875800, EPI_ISL_875801, EPI_ISL_875802, EPI_ISL_875803, EPI_ISL_875804, EPI_ISL_875805, EPI_ISL_875806, EPI_ISL_875807, EPI_ISL_875808, EPI_ISL_875809, EPI_ISL_875810, EPI_ISL_875811, EPI_ISL_875812, EPI_ISL_875813, EPI_ISL_875816, EPI_ISL_875817, EPI_ISL_875818, EPI_ISL_875819, EPI_ISL_875820, EPI_ISL_875821, EPI_ISL_875822, EPI_ISL_875823, EPI_ISL_875824, EPI_ISL_875825, EPI_ISL_875826, EPI_ISL_875827, EPI_ISL_875828, EPI_ISL_875829, EPI_ISL_875830, EPI_ISL_875831, EPI_ISL_875832, EPI_ISL_875833, EPI_ISL_875834, EPI_ISL_875835, EPI_ISL_875836, EPI_ISL_875837, EPI_ISL_875838, EPI_ISL_875839, EPI_ISL_875840, EPI_ISL_875841, EPI_ISL_875842, EPI_ISL_875843, EPI_ISL_875844, EPI_ISL_875845, EPI_ISL_875846, EPI_ISL_875847, EPI_ISL_875848, EPI_ISL_875849, EPI_ISL_875850, EPI_ISL_875851, EPI_ISL_875852, EPI_ISL_875853, EPI_ISL_875854, EPI_ISL_875855, EPI_ISL_875856, EPI_ISL_875857, EPI_ISL_875858, EPI_ISL_875859, EPI_ISL_875860, EPI_ISL_875861, EPI_ISL_875862, EPI_ISL_875863, EPI_ISL_875864, EPI_ISL_875865, EPI_ISL_875866, EPI_ISL_875867, EPI_ISL_875868, EPI_ISL_875869, EPI_ISL_875870			
see above	Public Health Ontario Laboratory	Public Health Ontario Laboratory	Vanessa G Allen, Philip Banh, Yao Chen, Richard de Borja, Alireza Eshaghi, Nahuel Fittipaldi, Christine Frantz, Jonathan B Gubbay, Jennifer L Guthrie, Lawrence Heisler, Esha Joshi, Michael Laszloffy, Aimin Li, Michael CY Li, Dean Maxwell, Sandeep Nagra, Samir N Patel, Jared Simpson, Karthikeyan Sivaraman, Ashleigh Sullivan, Yogi Sundaravadanam, Sarah Teatero, Matthew Watson, Andre Villegas, Sandra Zittermann
EPI_ISL_876177, EPI_ISL_876192, EPI_ISL_876317	Massachusetts State Public Health Laboratory	Massachusetts State Public Health Laboratory	Andrew Lang, Timelia Fink, Glen Gallagher, Sandra Smole
EPI_ISL_876549, EPI_ISL_876550, EPI_ISL_876551	Florida Bureau of Public Health Laboratories	Florida Bureau of Public Health Laboratories	Sarah Schmedes, Jason Blanton
EPI_ISL_878157, EPI_ISL_878167, EPI_ISL_878170, EPI_ISL_878180, EPI_ISL_878183, EPI_ISL_878191, EPI_ISL_878202, EPI_ISL_878205, EPI_ISL_878212, EPI_ISL_878215, EPI_ISL_878226, EPI_ISL_878229, EPI_ISL_878232, EPI_ISL_878239, EPI_ISL_878248, EPI_ISL_878250, EPI_ISL_878253, EPI_ISL_878258, EPI_ISL_878260, EPI_ISL_878263, EPI_ISL_878274, EPI_ISL_878355, EPI_ISL_878360, EPI_ISL_878361, EPI_ISL_878367, EPI_ISL_878371, EPI_ISL_878374, EPI_ISL_878381, EPI_ISL_878386, EPI_ISL_878392, EPI_ISL_878393, EPI_ISL_878398			
see above	San Diego County Public Health Laboratory	Andersen lab at Scripps Research	SEARCH Alliance San Diego with Tracy Basler, Jovan Shephard, Brett Austin
EPI_ISL_878557	Robert Garry lab	Andersen lab at Scripps Research	Allison Smither, Gilberto Sabino-Santos, Patricia Snarski, Lilia Melnik, Antoinette Bell, Kaylynn Genemaras, Arnaud Drouin, Dahlene Fusco, Robert Garry with SEARCH Alliance San Diego

EPI_ISL_878674, EPI_ISL_878742	Rady's Childrens Hospital	Andersen lab at Scripps Research	SEARCH Alliance San Diego with Nanda Radamchar, David Dimmock, Linda Luo, Christina Clarke, Kathryn Bouic, Teresa Mueller, Denise Malicki
EPI_ISL_879926, EPI_ISL_879932, EPI_ISL_879934, EPI_ISL_879942, EPI_ISL_879947, EPI_ISL_879950, EPI_ISL_879953, EPI_ISL_879959, EPI_ISL_879969, EPI_ISL_879975, EPI_ISL_879977, EPI_ISL_879990, EPI_ISL_879995, EPI_ISL_880003, EPI_ISL_880017, EPI_ISL_880022			
see above	San Diego County Public Health Laboratory	Andersen lab at Scripps Research	SEARCH Alliance San Diego with Tracy Basler, Jovan Shephard, Brett Austin
EPI_ISL_882333	Lighthouse Lab in Alderley Park	Wellcome Sanger Institute for the COVID-19 Genomics UK (COG-UK) Consortium	Jacquelyn Wynn, Mairead Hyland, The Lighthouse Lab in Alderley Park and Alex Alderton, Roberto Amato, Sonia Goncalves, Ewan Harrison, David K. Jackson, Ian Johnston, Dominic Kwiatkowski, Cordelia Langford, John Sillitoe on behalf of the Wellcome Sanger Institute COVID-19 Surveillance Team
EPI_ISL_882648	Swiss National Reference Centre for Influenza Virology laboratory, CNRI	Swiss National Reference Centre for Influenza Virology laboratory, CNRI	Tim Roloff, Ana Rita Gonçalves, Madlen Stange, Helena MB Seth-Smith, Alfredo Mari, Karoline Leuzinger, Julia Bielicki, Manuel Battegay, Hans Hirsch, Laurent Kaiser, Adrian Egli
EPI_ISL_882764	1.AO Universitaria 'S. Giovanni di Dio e Ruggi D'Aragona, Scuola Medica Salernitana' Hospital / 2.UOC di Virologia e Microbiologia, Università della Campania 'L. Vanvitelli' / 3.AO Universitaria 'Federico II' Napoli Hospital / 4.AORN 'San Giuseppe Moscati' Avellino Hospital / 5.AO 'San Pio - presidio G. Rummo' Benevento Hospital / 6.AO 'Sant'Anna e San Sebastiano' Caserta Hospital / 7.PO 'Maria Santissima Addolorata' Eboli Hospital / 8.Biogem Istituto di Ricerche Genetiche	1. Genome Research Center for Health (CRGS) / 2. Laboratory of Molecular Medicine and Genomics(LMMGe) / 3. Center for Research in Pure and Applied Mathematics (CRMPA)	Giorgio Giurato, Francesca Rizzo, Alessandro Weisz, Gianluigi Franci, Giovanni Nassa, Pasquale Pagliano, Roberta Tarallo, Elena Alexandrova, Ylenia D'Agostino, Carlo Ferravante, Jessica Lamberti, Viola Melone, Domenico Memoli, Valeria Mirici Cappa, Domenico Palumbo, Giovanni Pecoraro, Assunta Sellitto, Oriana Strianese, Ilaria Terenzi, Giuseppe Fenza, Aniello Gentile, Antonello Saccomanno, Sonia Amabile, Teresa Rocco, Annamaria Salvati, Emilia Vaccaro, Massimiliano Galdiero, Michele Cennamo, Giuseppe Portella, Maria Grazia Foti, Mariarosaria Ingino, Maria Landi, Maurizio Fumi, Vincenzo Rocco, Rita Greco, Vittoria Letizia, Arnolfo Petruzziello, Maddalena Schioppa, Gregorio Goffredi, Francesca Marciano, Michele Caraglia, Alessia Cossu, Marianna Scrima
EPI_ISL_883196, EPI_ISL_883198, EPI_ISL_883199, EPI_ISL_883200, EPI_ISL_883201, EPI_ISL_883202, EPI_ISL_883203, EPI_ISL_883204, EPI_ISL_883205, EPI_ISL_883206, EPI_ISL_883207, EPI_ISL_883208, EPI_ISL_883209, EPI_ISL_883210, EPI_ISL_883211, EPI_ISL_883212, EPI_ISL_883213, EPI_ISL_883214, EPI_ISL_883215, EPI_ISL_883216, EPI_ISL_883217, EPI_ISL_883218, EPI_ISL_883219, EPI_ISL_883220, EPI_ISL_883221, EPI_ISL_883222, EPI_ISL_883223, EPI_ISL_883224, EPI_ISL_883225, EPI_ISL_883227, EPI_ISL_883228, EPI_ISL_883229, EPI_ISL_883230, EPI_ISL_883231, EPI_ISL_883232, EPI_ISL_883233, EPI_ISL_883234, EPI_ISL_883235, EPI_ISL_883236, EPI_ISL_883237, EPI_ISL_883238, EPI_ISL_883239, EPI_ISL_883240, EPI_ISL_883241, EPI_ISL_883242, EPI_ISL_883243, EPI_ISL_883244, EPI_ISL_883245, EPI_ISL_883246, EPI_ISL_883247, EPI_ISL_883248, EPI_ISL_883249, EPI_ISL_883250, EPI_ISL_883251, EPI_ISL_883252, EPI_ISL_883253, EPI_ISL_883254, EPI_ISL_883255, EPI_ISL_883256, EPI_ISL_883257, EPI_ISL_883258, EPI_ISL_883259, EPI_ISL_883260, EPI_ISL_883261, EPI_ISL_883262, EPI_ISL_883263, EPI_ISL_883264, EPI_ISL_883265, EPI_ISL_883266, EPI_ISL_883267, EPI_ISL_883269, EPI_ISL_883270, EPI_ISL_883271, EPI_ISL_883272, EPI_ISL_883273, EPI_ISL_883274, EPI_ISL_883275, EPI_ISL_883276, EPI_ISL_883277, EPI_ISL_883278, EPI_ISL_883279, EPI_ISL_883280, EPI_ISL_883281, EPI_ISL_883282, EPI_ISL_883283, EPI_ISL_883284, EPI_ISL_883501, EPI_ISL_883502, EPI_ISL_883503, EPI_ISL_883504, EPI_ISL_883505, EPI_ISL_883506, EPI_ISL_883507, EPI_ISL_883508, EPI_ISL_883509, EPI_ISL_883510, EPI_ISL_883511, EPI_ISL_883512, EPI_ISL_883513, EPI_ISL_883514, EPI_ISL_883515, EPI_ISL_883516, EPI_ISL_883517, EPI_ISL_883519, EPI_ISL_883520, EPI_ISL_883521, EPI_ISL_883522, EPI_ISL_883523, EPI_ISL_883524, EPI_ISL_883525, EPI_ISL_883526, EPI_ISL_883527, EPI_ISL_883528, EPI_ISL_883529, EPI_ISL_883530, EPI_ISL_883531, EPI_ISL_883532, EPI_ISL_883533, EPI_ISL_883534, EPI_ISL_883535, EPI_ISL_883536, EPI_ISL_883537, EPI_ISL_883538, EPI_ISL_883539, EPI_ISL_883540, EPI_ISL_883541, EPI_ISL_883542, EPI_ISL_883543, EPI_ISL_883544, EPI_ISL_883545, EPI_ISL_883546, EPI_ISL_883547, EPI_ISL_883548, EPI_ISL_883549, EPI_ISL_883550, EPI_ISL_883551, EPI_ISL_883552, EPI_ISL_883553, EPI_ISL_883554, EPI_ISL_883555, EPI_ISL_883556, EPI_ISL_883557, EPI_ISL_883558, EPI_ISL_883559, EPI_ISL_883560, EPI_ISL_883561, EPI_ISL_883562, EPI_ISL_883563, EPI_ISL_883564, EPI_ISL_883565, EPI_ISL_883566, EPI_ISL_883567, EPI_ISL_883568, EPI_ISL_883569, EPI_ISL_883570, EPI_ISL_883571, EPI_ISL_883572, EPI_ISL_883573, EPI_ISL_883574, EPI_ISL_883575, EPI_ISL_883576, EPI_ISL_883577, EPI_ISL_883578, EPI_ISL_883579, EPI_ISL_883580, EPI_ISL_883581, EPI_ISL_883582, EPI_ISL_883583, EPI_ISL_883584, EPI_ISL_883585, EPI_ISL_883586, EPI_ISL_883587, EPI_ISL_883588, EPI_ISL_883589, EPI_ISL_883590, EPI_ISL_883591, EPI_ISL_883592, EPI_ISL_883593, EPI_ISL_883594, EPI_ISL_883595, EPI_ISL_883596, EPI_ISL_883597, EPI_ISL_883598, EPI_ISL_883599, EPI_ISL_883600, EPI_ISL_883601, EPI_ISL_883602, EPI_ISL_883603, EPI_ISL_883604, EPI_ISL_883605, EPI_ISL_883606, EPI_ISL_883607, EPI_ISL_883608, EPI_ISL_883609, EPI_ISL_883610, EPI_ISL_883611, EPI_ISL_883612, EPI_ISL_883613, EPI_ISL_883614, EPI_ISL_883615, EPI_ISL_883616, EPI_ISL_883617, EPI_ISL_883618, EPI_ISL_883619, EPI_ISL_883620, EPI_ISL_883621, EPI_ISL_883622, EPI_ISL_883623, EPI_ISL_883624, EPI_ISL_883625, EPI_ISL_883626, EPI_ISL_883627, EPI_ISL_883628, EPI_ISL_883629, EPI_ISL_883630, EPI_ISL_883631, EPI_ISL_883632, EPI_ISL_883633, EPI_ISL_883634, EPI_ISL_883635, EPI_ISL_883636, EPI_ISL_883637, EPI_ISL_883638, EPI_ISL_883639, EPI_ISL_883640, EPI_ISL_883641, EPI_ISL_883642, EPI_ISL_883643, EPI_ISL_883644, EPI_ISL_883645, EPI_ISL_883646, EPI_ISL_883647, EPI_ISL_883648, EPI_ISL_883649, EPI_ISL_883650, EPI_ISL_883656, EPI_ISL_883657, EPI_ISL_883658, EPI_ISL_883659, EPI_ISL_883660, EPI_ISL_883661, EPI_ISL_883662, EPI_ISL_883663, EPI_ISL_883664, EPI_ISL_883665, EPI_ISL_883666, EPI_ISL_883667, EPI_ISL_883677, EPI_ISL_883678, EPI_ISL_883679, EPI_ISL_883680, EPI_ISL_883681, EPI_ISL_883686, EPI_ISL_883687, EPI_ISL_883688, EPI_ISL_883689, EPI_ISL_883690, EPI_ISL_883691, EPI_ISL_883692, EPI_ISL_883693, EPI_ISL_883695, EPI_ISL_883723, EPI_ISL_883724, EPI_ISL_883725, EPI_ISL_883741, EPI_ISL_883742, EPI_ISL_883743, EPI_ISL_883755, EPI_ISL_883757			
see above	Eurofins Diatherix	Hudsonalpha Genome Sequencing Center	Jane Grimwood, Melissa Williams, Lori H. Handley, Joshua Stough, Leslie Malone, Stefan Brzezinski, Ada Stewart, Teresa Jones, Jenell Webber, John Lovell, Jennifer Cart, and Jeremy Schmutz
EPI_ISL_884088	Wazed Mia Science Research Center, Jahangirnagar University in collaboration with GRMDRC, Dhaka and Invent Technologies Ltd, Dhaka, Bangladesh	COVID Research Cell (CRC), Wazed Miah Science Research Center	Md. Abdul Matin, Bishajit Sarkar, Md. Asad Ullah, Yusha Araf, Nihad Adnan, Abdullah Al Nahid, Rashedul Islam, Mohammad Shahedur Rahman
EPI_ISL_884216, EPI_ISL_884219, EPI_ISL_884220	Alaska State Virology Laboratory (Alaska DHHS)	Alaska State Virology Laboratory (Alaska DHHS)	Stephanie DeRonde, Lisa Smith, PhD. Jack Chen, PhD.
EPI_ISL_884295	Microbiology Division, South Carolina Department of Health and Environmental Control Public Health Laboratory (SC DHEC PHL)	Microbiology Division, South Carolina Department of Health and Environmental Control Public Health Laboratory (SC DHEC PHL)	Haley Flores, Jessica Freeman
EPI_ISL_884884, EPI_ISL_884885, EPI_ISL_884887, EPI_ISL_884888, EPI_ISL_884898	Santa Clara County Public Health Laboratory	Chan-Zuckerberg Biohub	CZB Cliahub Consortium
EPI_ISL_885135, EPI_ISL_885136	California Institute of Technology	Chan-Zuckerberg Biohub	CZB Cliahub Consortium
EPI_ISL_888685, EPI_ISL_888755, EPI_ISL_888756	KU Leuven, Rega Institute, Clinical and Epidemiological Virology	KU Leuven, Rega Institute, Clinical and Epidemiological Virology	Tony Wawina-Bokalanga, Bert Vanmechelen, Joan Marti-Carerras, Piet Maes
EPI_ISL_888842, EPI_ISL_888876	Michigan Department of Health and Human Services, Bureau of Laboratories	Michigan Department of Health and Human Services, Bureau of Laboratories	Blankenship HM, Riner D, Soehnlen MK
EPI_ISL_888992	RS Mitra Keluarga Gading Serpong	Eijkman Institute for Molecular Biology, Ministry of Research and Technology/National Agency for Research and Innovation	Hidayat Trimarsanto, Iskandar Adnan, Lydia V. Panggalo, Sukma Oktavianthi, Willy Agustine, Edison Johar, Frilasita A Yudhaputri, Safarina G Malik, Khin Saw Myint, Amin Soebandrio
EPI_ISL_889021	RSIA Kenari Graha Medika	Eijkman Institute for Molecular Biology, Ministry of Research and Technology/National Agency for Research and Innovation	Hidayat Trimarsanto, Iskandar Adnan, Lydia V. Panggalo, Sukma Oktavianthi, Willy Agustine, Edison Johar, Frilasita A Yudhaputri, Safarina G Malik, Khin Saw Myint, Amin Soebandrio
EPI_ISL_889022	RS Anna	Eijkman Institute for Molecular Biology, Ministry of Research and Technology/National Agency for Research and Innovation	Hidayat Trimarsanto, Iskandar Adnan, Lydia V. Panggalo, Sukma Oktavianthi, Willy Agustine, Edison Johar, Frilasita A Yudhaputri, Safarina G Malik, Khin Saw Myint, Amin Soebandrio
EPI_ISL_889542, EPI_ISL_890174, EPI_ISL_890175, EPI_ISL_890176, EPI_ISL_890178, EPI_ISL_890179, EPI_ISL_890180	Ohio Department of Health Laboratory	Ohio Department of Health Laboratory	Holmes, Jennifer; Eric Brandt, Keoni Omura, Glen McGillivary, Caitlin McDonnell, Jade Mowery, Stephanie Mccracken, Tyler Payne, Kirtana Ramadugu, Erica Leasure, Brent Lee, Kelsey Florek, Heather Blankenship, Quanta Brown, and Tammy Bannerman
EPI_ISL_890185	Academic Hospital of Gadjah Mada University (RSA UGM)	Genetics Working Group (Pokja Genetik) Faculty of Medicine, Public Health and Nursing Universitas Gadjah Mada (FK-KMK UGM); Disease Investigation Center Wates Ministry of Agriculture Indonesia; Department of Microbiology FK-KMK UGM; Laboratorium Diagnostik Yayasan Tahija World Mosquito Program (WMP) Yogyakarta Center for Tropical Medicine FK-KMK UGM; Integrated Research center FK-KMK UGM; Department of Computer Science and Electronics FMIPA UGM	Gunadi, Hendra Wibawa, Marcellus, Mohamad S. Hakim, Edwin W. Daniwijaya, Ludhang P. Rizki, Endah Supriyati, Eggi Arguni, Titik Nuryastuti, Tri Wibawa, Dwi AA Nugrahaningsih, Afiahayati, Siswanto, Kristy Iskandar, Nungki Anggorowati, Aditya Rifqi Fauzi, Fadil Fahri
EPI_ISL_890301, EPI_ISL_890302	KU Leuven, Rega Institute, Clinical and Epidemiological Virology	KU Leuven, Rega Institute, Clinical and Epidemiological Virology	Tony Wawina-Bokalanga, Bert Vanmechelen, Joan Marti-Carerras, Piet Maes
EPI_ISL_890907, EPI_ISL_890908, EPI_ISL_890909, EPI_ISL_890913, EPI_ISL_890914, EPI_ISL_890915, EPI_ISL_890916, EPI_ISL_890917, EPI_ISL_890918	Seattle Flu Study	Seattle Flu Study	Deborah A. Nickerson, Chris D. Frazar, Jover Lee, Benjamin Pelle, Erica Ryke, Matthew Richardson, Amanda Adler, Elisabeth Brandstetter, Peter D. Han, Kairsten Fay, Misja Ilcisin, Kirsten Lacombe, Thomas R. Sibley, Melissa Truong, Caitlin R. Wolf, Karen Cowgill, Stephanie Schrag, Jeff Duchin, Michael Boeckh, Janet A. Englund, Michael Famulare, Barry R. Lutz, Mark J. Rieder, Lea M. Starita, Matthew Thompson, Helen Y. Chu, Trevor Bedford, Jay Shendure
EPI_ISL_890919, EPI_ISL_890920	Seattle Flu Study	Seattle Flu Study	Deborah A. Nickerson, Chris D. Frazar, Jover Lee, Benjamin Pelle, Erica Ryke, Matthew Richardson, Amanda Adler, Elisabeth Brandstetter, Peter D. Han,

			Kairsten Fay, Misja Ilcisin, Kirsten Lacombe, Thomas R. Sibley, Melissa Truong, Caitlin R. Wolf, Michael Boeckh, Janet A. Englund, Michael Famulare, Barry R. Lutz, Mark J. Rieder, Lea M. Starita, Matthew Thompson, Jay Shendure, Trevor Bedford, Helen Y. Chu
EPI_ISL_890921, EPI_ISL_890922, EPI_ISL_890923, EPI_ISL_890924, EPI_ISL_890926	Seattle Flu Study	Seattle Flu Study	Deborah A. Nickerson, Chris D. Frazar, Jover Lee, Benjamin Pelle, Erica Ryke, Matthew Richardson, Amanda Adler, Elisabeth Brandstetter, Peter D. Han, Kairsten Fay, Misja Ilcisin, Kirsten Lacombe, Thomas R. Sibley, Melissa Truong, Caitlin R. Wolf, Karen Cowgill, Stephanie Schrag, Jeff Duchin, Michael Boeckh, Janet A. Englund, Michael Famulare, Barry R. Lutz, Mark J. Rieder, Lea M. Starita, Matthew Thompson, Helen Y. Chu, Trevor Bedford, Jay Shendure
EPI_ISL_890956	Seattle Flu Study	Seattle Flu Study	Deborah A. Nickerson, Chris D. Frazar, Jover Lee, Benjamin Pelle, Erica Ryke, Matthew Richardson, Amanda Adler, Elisabeth Brandstetter, Peter D. Han, Kairsten Fay, Misja Ilcisin, Kirsten Lacombe, Thomas R. Sibley, Melissa Truong, Caitlin R. Wolf, Michael Boeckh, Janet A. Englund, Michael Famulare, Barry R. Lutz, Mark J. Rieder, Lea M. Starita, Matthew Thompson, Jay Shendure, Trevor Bedford, Helen Y. Chu
EPI_ISL_892280, EPI_ISL_892281, EPI_ISL_892282, EPI_ISL_892283, EPI_ISL_892284, EPI_ISL_892285, EPI_ISL_892286, EPI_ISL_892287	Servicio de Microbiología. Hospital Universitario Donostia. OSI Donostialdea. Área de Enfermedades Infecciosas, Grupo de Infección Respiratoria y Resistencia Antimicrobiana. Instituto de Investigación Sanitaria Biodonostia	SeqCOVID-SPAIN consortium/IBV(CSIC)	Gustavo Cilla Eguiluz, Milagrosa Montes Ros, Luis Piñeiro Vázquez, Ane Sorrarain, Jose Maria Marimón and SeqCOVID-SPAIN consortium
EPI_ISL_892325, EPI_ISL_892326, EPI_ISL_892327, EPI_ISL_892328, EPI_ISL_892329, EPI_ISL_892330, EPI_ISL_892331, EPI_ISL_892332	Servicio de Microbiología Clínica (Complejo Hospitalario de Navarra, Pamplona), Instituto de Investigación Sanitaria de Navarra (IdiSNA)	SeqCOVID-SPAIN consortium/IBV(CSIC)	Carmen Ezpeleta Baquedano, Ana Navascués, Ana Miqueleiz and SeqCOVID-SPAIN consortium
EPI_ISL_892369, EPI_ISL_892370, EPI_ISL_892371, EPI_ISL_892372, EPI_ISL_892378, EPI_ISL_892379, EPI_ISL_892380, EPI_ISL_892381, EPI_ISL_892382, EPI_ISL_892383, EPI_ISL_892384, EPI_ISL_892385, EPI_ISL_892386, EPI_ISL_892387, EPI_ISL_892388, EPI_ISL_892389, EPI_ISL_892390, EPI_ISL_892391, EPI_ISL_892392, EPI_ISL_892393, EPI_ISL_892394, EPI_ISL_892395, EPI_ISL_892396, EPI_ISL_892397, EPI_ISL_892398, EPI_ISL_892399, EPI_ISL_892400, EPI_ISL_892401, EPI_ISL_892402, EPI_ISL_892403, EPI_ISL_892404, EPI_ISL_892405, EPI_ISL_892406, EPI_ISL_892415, EPI_ISL_892416, EPI_ISL_892417, EPI_ISL_892418, EPI_ISL_892419, EPI_ISL_892420, EPI_ISL_892421, EPI_ISL_892422, EPI_ISL_892423, EPI_ISL_892424, EPI_ISL_892425, EPI_ISL_892426, EPI_ISL_892427, EPI_ISL_892428, EPI_ISL_892429, EPI_ISL_892430, EPI_ISL_892431, EPI_ISL_892432, EPI_ISL_892433, EPI_ISL_892434, EPI_ISL_892435, EPI_ISL_892436, EPI_ISL_892437, EPI_ISL_892438, EPI_ISL_892439, EPI_ISL_892440, EPI_ISL_892441, EPI_ISL_892442, EPI_ISL_892443, EPI_ISL_892444, EPI_ISL_892445, EPI_ISL_892446, EPI_ISL_892447, EPI_ISL_892448, EPI_ISL_892449, EPI_ISL_892450, EPI_ISL_892451, EPI_ISL_892452, EPI_ISL_892453, EPI_ISL_892454, EPI_ISL_892455, EPI_ISL_892456, EPI_ISL_892457, EPI_ISL_892458, EPI_ISL_892459, EPI_ISL_892460, EPI_ISL_892461, EPI_ISL_892462, EPI_ISL_892463, EPI_ISL_892464, EPI_ISL_892465, EPI_ISL_892466, EPI_ISL_892467, EPI_ISL_892468, EPI_ISL_892469, EPI_ISL_892470, EPI_ISL_892471, EPI_ISL_892472, EPI_ISL_892473, EPI_ISL_892474, EPI_ISL_892475, EPI_ISL_892476, EPI_ISL_892477, EPI_ISL_892478, EPI_ISL_892479, EPI_ISL_892480, EPI_ISL_892481, EPI_ISL_892482, EPI_ISL_892483, EPI_ISL_892484, EPI_ISL_892485, EPI_ISL_892486, EPI_ISL_892487, EPI_ISL_892488, EPI_ISL_892489, EPI_ISL_892490, EPI_ISL_892491, EPI_ISL_892492, EPI_ISL_892493, EPI_ISL_892494, EPI_ISL_892495, EPI_ISL_892496, EPI_ISL_892497, EPI_ISL_892498, EPI_ISL_892499, EPI_ISL_892500, EPI_ISL_892501, EPI_ISL_892502, EPI_ISL_892503, EPI_ISL_892504, EPI_ISL_892505, EPI_ISL_892506, EPI_ISL_892507, EPI_ISL_892508, EPI_ISL_892509, EPI_ISL_892510, EPI_ISL_892511, EPI_ISL_892512, EPI_ISL_892513, EPI_ISL_892514, EPI_ISL_892534, EPI_ISL_892535, EPI_ISL_892536, EPI_ISL_892537, EPI_ISL_892538, EPI_ISL_892539, EPI_ISL_892540, EPI_ISL_892541, EPI_ISL_892542, EPI_ISL_892543, EPI_ISL_892544, EPI_ISL_892545, EPI_ISL_892546, EPI_ISL_892547, EPI_ISL_892548, EPI_ISL_892549, EPI_ISL_892550, EPI_ISL_892551, EPI_ISL_892552, EPI_ISL_892553, EPI_ISL_892554, EPI_ISL_892555, EPI_ISL_892556, EPI_ISL_892557, EPI_ISL_892558, EPI_ISL_892559, EPI_ISL_892560			
see above	Pathogen Genomics Center, National Institute of Infectious Diseases	Pathogen Genomics Center, National Institute of Infectious Diseases	Tsuyoshi Sekizuka, Kentaro Itokawa, Rina Tanaka, Masanori Hashino, Makoto Kuroda
EPI_ISL_892561, EPI_ISL_892562, EPI_ISL_892563, EPI_ISL_892564, EPI_ISL_892565	Osaka Institute of Public Health, Morinomiya Center	Pathogen Genomics Center, National Institute of Infectious Diseases	Tsuyoshi Sekizuka, Kentaro Itokawa, Rina Tanaka, Masanori Hashino, Makoto Kuroda
EPI_ISL_892585, EPI_ISL_892586, EPI_ISL_892587, EPI_ISL_892588, EPI_ISL_892611, EPI_ISL_892612, EPI_ISL_892613, EPI_ISL_892614, EPI_ISL_892615, EPI_ISL_892616, EPI_ISL_892617, EPI_ISL_892618, EPI_ISL_892619, EPI_ISL_892620, EPI_ISL_892621, EPI_ISL_892622, EPI_ISL_892623, EPI_ISL_892624, EPI_ISL_892625, EPI_ISL_892626, EPI_ISL_892627, EPI_ISL_892628, EPI_ISL_892629, EPI_ISL_892630, EPI_ISL_892631, EPI_ISL_892632, EPI_ISL_892633, EPI_ISL_892634, EPI_ISL_892635, EPI_ISL_892636, EPI_ISL_892637, EPI_ISL_892638, EPI_ISL_892639, EPI_ISL_892640, EPI_ISL_892641, EPI_ISL_892642, EPI_ISL_892643, EPI_ISL_892644, EPI_ISL_892645, EPI_ISL_892646, EPI_ISL_892647, EPI_ISL_892648, EPI_ISL_892649, EPI_ISL_892650, EPI_ISL_892651, EPI_ISL_892652, EPI_ISL_892653, EPI_ISL_892654, EPI_ISL_892655, EPI_ISL_892656, EPI_ISL_892657, EPI_ISL_892658, EPI_ISL_892659, EPI_ISL_892660, EPI_ISL_892661, EPI_ISL_892662, EPI_ISL_892663, EPI_ISL_892664, EPI_ISL_892665, EPI_ISL_892666, EPI_ISL_892667, EPI_ISL_892668, EPI_ISL_892669, EPI_ISL_892670, EPI_ISL_892671, EPI_ISL_892672, EPI_ISL_892673, EPI_ISL_892674, EPI_ISL_892675, EPI_ISL_892676, EPI_ISL_892677, EPI_ISL_892678, EPI_ISL_892679, EPI_ISL_892680, EPI_ISL_892681, EPI_ISL_892682, EPI_ISL_892683, EPI_ISL_892684, EPI_ISL_892685, EPI_ISL_892686, EPI_ISL_892687, EPI_ISL_892688, EPI_ISL_892689, EPI_ISL_892690, EPI_ISL_892691, EPI_ISL_892692, EPI_ISL_892693, EPI_ISL_892694, EPI_ISL_892695, EPI_ISL_892696, EPI_ISL_892697, EPI_ISL_892698, EPI_ISL_892699, EPI_ISL_892701, EPI_ISL_892702, EPI_ISL_892703, EPI_ISL_892704, EPI_ISL_893242, EPI_ISL_893243, EPI_ISL_893244, EPI_ISL_893245, EPI_ISL_893246, EPI_ISL_893247, EPI_ISL_893248, EPI_ISL_893249, EPI_ISL_893250, EPI_ISL_893251, EPI_ISL_893255, EPI_ISL_893256, EPI_ISL_893257, EPI_ISL_893258, EPI_ISL_893259, EPI_ISL_893260, EPI_ISL_893261, EPI_ISL_893262, EPI_ISL_893263, EPI_ISL_893264, EPI_ISL_893265, EPI_ISL_893266, EPI_ISL_893281, EPI_ISL_893282, EPI_ISL_893283, EPI_ISL_893284, EPI_ISL_893285, EPI_ISL_893286, EPI_ISL_893287, EPI_ISL_893288, EPI_ISL_893289, EPI_ISL_893290, EPI_ISL_893291, EPI_ISL_893292, EPI_ISL_893293, EPI_ISL_893294, EPI_ISL_893295, EPI_ISL_893296, EPI_ISL_893297, EPI_ISL_893298, EPI_ISL_893299, EPI_ISL_893300, EPI_ISL_893301, EPI_ISL_893302, EPI_ISL_893303, EPI_ISL_893304, EPI_ISL_893305, EPI_ISL_893306, EPI_ISL_893307, EPI_ISL_893308, EPI_ISL_893309, EPI_ISL_893310, EPI_ISL_893311, EPI_ISL_893312, EPI_ISL_893313, EPI_ISL_893314, EPI_ISL_893315, EPI_ISL_893316, EPI_ISL_893317, EPI_ISL_893318, EPI_ISL_893319, EPI_ISL_893320, EPI_ISL_893321, EPI_ISL_893322, EPI_ISL_893323, EPI_ISL_893324, EPI_ISL_893325, EPI_ISL_893326, EPI_ISL_893327, EPI_ISL_893328, EPI_ISL_893329, EPI_ISL_893330, EPI_ISL_893331, EPI_ISL_893332, EPI_ISL_893333, EPI_ISL_893334, EPI_ISL_893335, EPI_ISL_893336, EPI_ISL_893337, EPI_ISL_893338, EPI_ISL_893339, EPI_ISL_893340, EPI_ISL_893341, EPI_ISL_893342, EPI_ISL_893343, EPI_ISL_893344, EPI_ISL_893345, EPI_ISL_893346, EPI_ISL_893347, EPI_ISL_893348, EPI_ISL_893349, EPI_ISL_893350, EPI_ISL_893351, EPI_ISL_893352, EPI_ISL_893353, EPI_ISL_893354, EPI_ISL_893355, EPI_ISL_893356, EPI_ISL_893357, EPI_ISL_893358, EPI_ISL_893359, EPI_ISL_893360, EPI_ISL_893361, EPI_ISL_893362, EPI_ISL_893363, EPI_ISL_893364, EPI_ISL_893365, EPI_ISL_893366			
see above	Pathogen Genomics Center, National Institute of Infectious Diseases	Pathogen Genomics Center, National Institute of Infectious Diseases	Tsuyoshi Sekizuka, Kentaro Itokawa, Rina Tanaka, Masanori Hashino, Makoto Kuroda
EPI_ISL_893371, EPI_ISL_893608, EPI_ISL_893609, EPI_ISL_893610, EPI_ISL_893611, EPI_ISL_893612, EPI_ISL_893613, EPI_ISL_893614, EPI_ISL_893615, EPI_ISL_893616, EPI_ISL_893617, EPI_ISL_893618, EPI_ISL_893619, EPI_ISL_893620, EPI_ISL_893621, EPI_ISL_893622, EPI_ISL_893623, EPI_ISL_893624, EPI_ISL_893625, EPI_ISL_893626, EPI_ISL_893627, EPI_ISL_893628, EPI_ISL_893629, EPI_ISL_893630, EPI_ISL_893631, EPI_ISL_893632, EPI_ISL_893633, EPI_ISL_893634, EPI_ISL_893635, EPI_ISL_893636, EPI_ISL_893637, EPI_ISL_893638, EPI_ISL_893639, EPI_ISL_893640, EPI_ISL_893641, EPI_ISL_893642, EPI_ISL_893643, EPI_ISL_893644, EPI_ISL_893645, EPI_ISL_893646, EPI_ISL_893647, EPI_ISL_893648, EPI_ISL_893649, EPI_ISL_893650, EPI_ISL_893651, EPI_ISL_893652, EPI_ISL_893653, EPI_ISL_893654, EPI_ISL_893655, EPI_ISL_893656, EPI_ISL_893657, EPI_ISL_893658, EPI_ISL_893659, EPI_ISL_893660, EPI_ISL_893661, EPI_ISL_893662, EPI_ISL_893663, EPI_ISL_893664, EPI_ISL_893665, EPI_ISL_893666, EPI_ISL_893667, EPI_ISL_893668, EPI_ISL_893669, EPI_ISL_893670, EPI_ISL_893671, EPI_ISL_893672, EPI_ISL_893677, EPI_ISL_893678, EPI_ISL_893679, EPI_ISL_893680, EPI_ISL_893681, EPI_ISL_893682, EPI_ISL_893683, EPI_ISL_893684, EPI_ISL_893685, EPI_ISL_893686, EPI_ISL_893687, EPI_ISL_893688, EPI_ISL_893689, EPI_ISL_893690, EPI_ISL_893691, EPI_ISL_893692, EPI_ISL_893693, EPI_ISL_893694, EPI_ISL_893695, EPI_ISL_893696, EPI_ISL_893697, EPI_ISL_893698, EPI_ISL_893699, EPI_ISL_893700, EPI_ISL_893701, EPI_ISL_893702, EPI_ISL_893703, EPI_ISL_893704, EPI_ISL_893705, EPI_ISL_893706, EPI_ISL_893707, EPI_ISL_893708, EPI_ISL_893709, EPI_ISL_893710, EPI_ISL_893711, EPI_ISL_893712, EPI_ISL_893713, EPI_ISL_893714, EPI_ISL_893715, EPI_ISL_893716, EPI_ISL_893717, EPI_ISL_893718, EPI_ISL_893719			
see above	Department of Infectious Diseases, Kobe Institute of Health	Department of Infectious Diseases, Kobe Institute of Health	Ryohei Nomoto, Tomotada Iwamoto, Tsuyoshi Sekizuka, Kentaro Itokawa, Rina Tanaka, Masanori Hashino, Makoto Kuroda
EPI_ISL_894278, EPI_ISL_894279	Fukuoka Institute of Health and Environmental Sciences	Pathogen Genomics Center, National Institute of Infectious Diseases	Tsuyoshi Sekizuka, Kentaro Itokawa, Rina Tanaka, Masanori Hashino, Makoto Kuroda
EPI_ISL_894281, EPI_ISL_894282, EPI_ISL_894283, EPI_ISL_894284, EPI_ISL_894285, EPI_ISL_894286, EPI_ISL_894289, EPI_ISL_894292, EPI_ISL_894293, EPI_ISL_894294, EPI_ISL_894295, EPI_ISL_894298, EPI_ISL_894299, EPI_ISL_894305, EPI_ISL_894306, EPI_ISL_894307, EPI_ISL_894308, EPI_ISL_894309, EPI_ISL_894310, EPI_ISL_894311, EPI_ISL_894312, EPI_ISL_894313, EPI_ISL_894314, EPI_ISL_894315, EPI_ISL_894316, EPI_ISL_894323, EPI_ISL_894324, EPI_ISL_894325, EPI_ISL_894326, EPI_ISL_894327, EPI_ISL_894328, EPI_ISL_894329, EPI_ISL_894333, EPI_ISL_894334, EPI_ISL_894335, EPI_ISL_894336, EPI_ISL_894337, EPI_ISL_894338, EPI_ISL_894339, EPI_ISL_894340, EPI_ISL_894341, EPI_ISL_894342, EPI_ISL_894343, EPI_ISL_894344, EPI_ISL_894345, EPI_ISL_894346, EPI_ISL_894347, EPI_ISL_894348, EPI_ISL_894349, EPI_ISL_894350, EPI_ISL_894351, EPI_ISL_894352, EPI_ISL_894353			
see above	Pathogen Genomics Center, National Institute of Infectious Diseases	Pathogen Genomics Center, National Institute of Infectious Diseases	Tsuyoshi Sekizuka, Kentaro Itokawa, Rina Tanaka, Masanori Hashino, Makoto Kuroda
EPI_ISL_894354, EPI_ISL_894355, EPI_ISL_894356, EPI_ISL_894357, EPI_ISL_894358, EPI_ISL_894359, EPI_ISL_894360, EPI_ISL_894361, EPI_ISL_894362, EPI_ISL_894363	Department of Infectious Diseases, Kobe Institute of Health	Pathogen Genomics Center, National Institute of Infectious Diseases	Tsuyoshi Sekizuka, Kentaro Itokawa, Rina Tanaka, Masanori Hashino, Makoto Kuroda
EPI_ISL_894624, EPI_ISL_894625, EPI_ISL_894626, EPI_ISL_894627, EPI_ISL_894628, EPI_ISL_894629, EPI_ISL_894630, EPI_ISL_894631, EPI_ISL_894632, EPI_ISL_894633, EPI_ISL_894634, EPI_ISL_894635, EPI_ISL_894636, EPI_ISL_894637, EPI_ISL_894638, EPI_ISL_894639, EPI_ISL_894640, EPI_ISL_894641, EPI_ISL_894642, EPI_ISL_894643, EPI_ISL_894644, EPI_ISL_894645, EPI_ISL_894646, EPI_ISL_894647, EPI_ISL_894648, EPI_ISL_894649, EPI_ISL_894650, EPI_ISL_894651, EPI_ISL_894652, EPI_ISL_894653, EPI_ISL_894654, EPI_ISL_894655, EPI_ISL_894656, EPI_ISL_894657, EPI_ISL_894658, EPI_ISL_894659, EPI_ISL_894660, EPI_ISL_894661, EPI_ISL_894662, EPI_ISL_894663, EPI_ISL_894664, EPI_ISL_894665, EPI_ISL_894666, EPI_ISL_894667, EPI_ISL_894668, EPI_ISL_894669, EPI_ISL_894670, EPI_ISL_894671			
see above	Pathogen Genomics Center, National Institute of Infectious Diseases	Pathogen Genomics Center, National Institute of Infectious Diseases	Tsuyoshi Sekizuka, Kentaro Itokawa, Rina Tanaka, Masanori Hashino, Makoto Kuroda
EPI_ISL_894695, EPI_ISL_894696, EPI_ISL_894697, EPI_ISL_894698, EPI_ISL_894699, EPI_ISL_894700, EPI_ISL_894701, EPI_ISL_894702, EPI_ISL_894703, EPI_ISL_894704, EPI_ISL_894705, EPI_ISL_894706, EPI_ISL_894707, EPI_ISL_894708, EPI_ISL_894709, EPI_ISL_894710, EPI_ISL_894711, EPI_ISL_894712, EPI_ISL_894713, EPI_ISL_894714, EPI_ISL_894715, EPI_ISL_894716, EPI_ISL_894717, EPI_ISL_894718, EPI_ISL_894719, EPI_ISL_894720, EPI_ISL_894721, EPI_ISL_894722, EPI_ISL_894723, EPI_ISL_894724, EPI_ISL_894725, EPI_ISL_894726, EPI_ISL_894727, EPI_ISL_894728, EPI_ISL_894729, EPI_ISL_894730, EPI_ISL_894731, EPI_ISL_894732, EPI_ISL_894733, EPI_ISL_894734, EPI_ISL_894735, EPI_ISL_894736, EPI_ISL_894737, EPI_ISL_894738, EPI_ISL_894739, EPI_ISL_894740, EPI_ISL_894741, EPI_ISL_894742, EPI_ISL_894743, EPI_ISL_894744, EPI_ISL_894745, EPI_ISL_894746, EPI_ISL_894747, EPI_ISL_894748, EPI_ISL_894749, EPI_ISL_894750, EPI_ISL_894751, EPI_ISL_894752, EPI_ISL_894753, EPI_ISL_894754, EPI_ISL_894755, EPI_ISL_894756, EPI_ISL_894757, EPI_ISL_894758, EPI_ISL_894759, EPI_ISL_894760, EPI_ISL_894761, EPI_ISL_894762, EPI_ISL_894763, EPI_ISL_894764, EPI_ISL_894765, EPI_ISL_894766, EPI_ISL_894767, EPI_ISL_894768, EPI_ISL_894769, EPI_ISL_894770, EPI_ISL_894771, EPI_ISL_894772, EPI_ISL_894773, EPI_ISL_894774, EPI_ISL_894775, EPI_ISL_894776, EPI_ISL_894777, EPI_ISL_894778, EPI_ISL_894779, EPI_ISL_894780, EPI_ISL_894781, EPI_ISL_894782, EPI_ISL_894783, EPI_ISL_894816			
see above	Fukuoka Institute of Health and Environmental Sciences	Pathogen Genomics Center, National Institute of Infectious Diseases	Tsuyoshi Sekizuka, Kentaro Itokawa, Rina Tanaka, Masanori Hashino, Makoto Kuroda

EPI_ISL_894820, EPI_ISL_894821, EPI_ISL_894822, EPI_ISL_894823, EPI_ISL_894824, EPI_ISL_894825, EPI_ISL_894826, EPI_ISL_894827, EPI_ISL_894828, EPI_ISL_894829, EPI_ISL_894830, EPI_ISL_894831, EPI_ISL_894832, EPI_ISL_894833, EPI_ISL_894834, EPI_ISL_894835, EPI_ISL_894836, EPI_ISL_894837, EPI_ISL_894838, EPI_ISL_894839, EPI_ISL_894840, EPI_ISL_894841, EPI_ISL_894842, EPI_ISL_894843, EPI_ISL_894844, EPI_ISL_894845, EPI_ISL_894846, EPI_ISL_894847, EPI_ISL_894848, EPI_ISL_894849, EPI_ISL_894850, EPI_ISL_894851, EPI_ISL_894852, EPI_ISL_894853, EPI_ISL_894854, EPI_ISL_894855, EPI_ISL_894856, EPI_ISL_894857, EPI_ISL_894858, EPI_ISL_894859, EPI_ISL_894860, EPI_ISL_894861, EPI_ISL_894862, EPI_ISL_894863, EPI_ISL_894864, EPI_ISL_894865, EPI_ISL_894866, EPI_ISL_894867, EPI_ISL_894868, EPI_ISL_894869, EPI_ISL_894870, EPI_ISL_894871, EPI_ISL_894872, EPI_ISL_894873, EPI_ISL_894874, EPI_ISL_894875, EPI_ISL_894876, EPI_ISL_894877, EPI_ISL_894878, EPI_ISL_894879, EPI_ISL_894880, EPI_ISL_894881, EPI_ISL_894882, EPI_ISL_894883, EPI_ISL_894884, EPI_ISL_894885, EPI_ISL_894886, EPI_ISL_894887, EPI_ISL_894888, EPI_ISL_894913, EPI_ISL_894914, EPI_ISL_894915, EPI_ISL_894916, EPI_ISL_894917, EPI_ISL_894918, EPI_ISL_894919, EPI_ISL_894920, EPI_ISL_894921, EPI_ISL_894922, EPI_ISL_894923, EPI_ISL_894924, EPI_ISL_894925, EPI_ISL_894926, EPI_ISL_894927, EPI_ISL_894928, EPI_ISL_894929, EPI_ISL_894930, EPI_ISL_894931, EPI_ISL_894933, EPI_ISL_894934, EPI_ISL_894935, EPI_ISL_894936, EPI_ISL_894937, EPI_ISL_894938, EPI_ISL_894939, EPI_ISL_894940, EPI_ISL_894941, EPI_ISL_894942, EPI_ISL_894959, EPI_ISL_894960, EPI_ISL_894961, EPI_ISL_894962, EPI_ISL_894963			
see above	Pathogen Genomics Center, National Institute of Infectious Diseases	Pathogen Genomics Center, National Institute of Infectious Diseases	Tsuyoshi Sekizuka, Kentaro Itokawa, Rina Tanaka, Masanori Hashino, Makoto Kuroda
EPI_ISL_895013	Niigata Prefectural Institute of Public Health and Environmental Sciences	Pathogen Genomics Center, National Institute of Infectious Diseases	Tsuyoshi Sekizuka, Kentaro Itokawa, Rina Tanaka, Masanori Hashino, Makoto Kuroda
EPI_ISL_895023, EPI_ISL_895024, EPI_ISL_895025, EPI_ISL_895026, EPI_ISL_895027, EPI_ISL_895028, EPI_ISL_895029, EPI_ISL_895030, EPI_ISL_895031, EPI_ISL_895032, EPI_ISL_895033, EPI_ISL_895034, EPI_ISL_895035, EPI_ISL_895036, EPI_ISL_895037, EPI_ISL_895038, EPI_ISL_895039, EPI_ISL_895040, EPI_ISL_895041, EPI_ISL_895042, EPI_ISL_895043, EPI_ISL_895044, EPI_ISL_895045, EPI_ISL_895046, EPI_ISL_895047, EPI_ISL_895048, EPI_ISL_895049, EPI_ISL_895050, EPI_ISL_895051, EPI_ISL_895052, EPI_ISL_895053, EPI_ISL_895054, EPI_ISL_895055, EPI_ISL_895056, EPI_ISL_895057, EPI_ISL_895058, EPI_ISL_895059, EPI_ISL_895060, EPI_ISL_895061, EPI_ISL_895062, EPI_ISL_895063, EPI_ISL_895064, EPI_ISL_895065, EPI_ISL_895066, EPI_ISL_895067, EPI_ISL_895068, EPI_ISL_895069, EPI_ISL_895089, EPI_ISL_895090, EPI_ISL_895091, EPI_ISL_895092, EPI_ISL_895094			
see above	Department of Infectious Diseases, Kobe Institute of Health	Pathogen Genomics Center, National Institute of Infectious Diseases	Tsuyoshi Sekizuka, Kentaro Itokawa, Rina Tanaka, Masanori Hashino, Makoto Kuroda
EPI_ISL_895151, EPI_ISL_895152, EPI_ISL_895153, EPI_ISL_895154, EPI_ISL_895155, EPI_ISL_895156, EPI_ISL_895157, EPI_ISL_895158, EPI_ISL_895159, EPI_ISL_895266, EPI_ISL_895267, EPI_ISL_895268, EPI_ISL_895269, EPI_ISL_895270, EPI_ISL_895271, EPI_ISL_895272, EPI_ISL_895273, EPI_ISL_895274, EPI_ISL_895275, EPI_ISL_895276, EPI_ISL_895277, EPI_ISL_895278, EPI_ISL_895279, EPI_ISL_895280, EPI_ISL_895281, EPI_ISL_895282, EPI_ISL_895283, EPI_ISL_895284, EPI_ISL_895285, EPI_ISL_895286, EPI_ISL_895287, EPI_ISL_895288, EPI_ISL_895290, EPI_ISL_895291, EPI_ISL_895292, EPI_ISL_895293, EPI_ISL_895294, EPI_ISL_895295, EPI_ISL_895296, EPI_ISL_895297, EPI_ISL_895298, EPI_ISL_895299, EPI_ISL_895300, EPI_ISL_895301, EPI_ISL_895302, EPI_ISL_895303, EPI_ISL_895304, EPI_ISL_895305, EPI_ISL_895306, EPI_ISL_895307, EPI_ISL_895308, EPI_ISL_895309, EPI_ISL_895310, EPI_ISL_895311, EPI_ISL_895312, EPI_ISL_895313, EPI_ISL_895314, EPI_ISL_895315, EPI_ISL_895316, EPI_ISL_895317, EPI_ISL_895318, EPI_ISL_895319, EPI_ISL_895320, EPI_ISL_895321, EPI_ISL_895322, EPI_ISL_895323, EPI_ISL_895324, EPI_ISL_895325, EPI_ISL_895326, EPI_ISL_895327, EPI_ISL_895328, EPI_ISL_895329, EPI_ISL_895330, EPI_ISL_895331, EPI_ISL_895332, EPI_ISL_895333, EPI_ISL_895334, EPI_ISL_895335, EPI_ISL_895336, EPI_ISL_895337, EPI_ISL_895338, EPI_ISL_895339, EPI_ISL_895340, EPI_ISL_895341, EPI_ISL_895342, EPI_ISL_895343, EPI_ISL_895344, EPI_ISL_895345, EPI_ISL_895346, EPI_ISL_895347, EPI_ISL_895348, EPI_ISL_895349, EPI_ISL_895350, EPI_ISL_895351, EPI_ISL_895352, EPI_ISL_895353, EPI_ISL_895354, EPI_ISL_895355, EPI_ISL_895356, EPI_ISL_895357, EPI_ISL_895358, EPI_ISL_895359, EPI_ISL_895360, EPI_ISL_895361, EPI_ISL_895362, EPI_ISL_895363, EPI_ISL_895364, EPI_ISL_895365, EPI_ISL_895366, EPI_ISL_895367, EPI_ISL_895368, EPI_ISL_895369, EPI_ISL_895370, EPI_ISL_895371, EPI_ISL_895372, EPI_ISL_895373, EPI_ISL_895374, EPI_ISL_895375, EPI_ISL_895376, EPI_ISL_895377, EPI_ISL_895378, EPI_ISL_895379, EPI_ISL_895380, EPI_ISL_895381, EPI_ISL_895382, EPI_ISL_895383, EPI_ISL_895384, EPI_ISL_895385, EPI_ISL_895386, EPI_ISL_895387, EPI_ISL_895388, EPI_ISL_895389, EPI_ISL_895390, EPI_ISL_895391, EPI_ISL_895392, EPI_ISL_895393, EPI_ISL_895394, EPI_ISL_895395, EPI_ISL_895467, EPI_ISL_895468, EPI_ISL_895469, EPI_ISL_895470, EPI_ISL_895471, EPI_ISL_895472, EPI_ISL_895473, EPI_ISL_895474, EPI_ISL_895475, EPI_ISL_895476, EPI_ISL_895477, EPI_ISL_895478, EPI_ISL_895479, EPI_ISL_895480, EPI_ISL_895481, EPI_ISL_895482, EPI_ISL_895483, EPI_ISL_895484, EPI_ISL_895485, EPI_ISL_895486, EPI_ISL_895488, EPI_ISL_895489, EPI_ISL_895508, EPI_ISL_895509, EPI_ISL_895510, EPI_ISL_895511, EPI_ISL_895512, EPI_ISL_895513, EPI_ISL_895514, EPI_ISL_895515, EPI_ISL_895516, EPI_ISL_895517, EPI_ISL_895533, EPI_ISL_895534, EPI_ISL_895535, EPI_ISL_895567, EPI_ISL_895568, EPI_ISL_895569, EPI_ISL_895570, EPI_ISL_895571, EPI_ISL_895572, EPI_ISL_895573, EPI_ISL_895574, EPI_ISL_895575, EPI_ISL_895576, EPI_ISL_895577, EPI_ISL_895578, EPI_ISL_895579, EPI_ISL_895580, EPI_ISL_895581, EPI_ISL_895582, EPI_ISL_895583, EPI_ISL_895584, EPI_ISL_895585, EPI_ISL_895586, EPI_ISL_895587, EPI_ISL_895588, EPI_ISL_895589, EPI_ISL_895590, EPI_ISL_895591, EPI_ISL_895592, EPI_ISL_895593, EPI_ISL_895594, EPI_ISL_895595, EPI_ISL_895596, EPI_ISL_895597, EPI_ISL_895598, EPI_ISL_895599, EPI_ISL_895600, EPI_ISL_895601, EPI_ISL_895602, EPI_ISL_895603, EPI_ISL_895604, EPI_ISL_895605, EPI_ISL_895606, EPI_ISL_895607, EPI_ISL_895658, EPI_ISL_895659, EPI_ISL_895660, EPI_ISL_895661, EPI_ISL_895662, EPI_ISL_895663, EPI_ISL_895664, EPI_ISL_895665, EPI_ISL_895666, EPI_ISL_895667, EPI_ISL_895668, EPI_ISL_895669, EPI_ISL_895670, EPI_ISL_895671, EPI_ISL_895672, EPI_ISL_895673, EPI_ISL_895674, EPI_ISL_895675, EPI_ISL_895676, EPI_ISL_895677, EPI_ISL_895678, EPI_ISL_895679, EPI_ISL_895680, EPI_ISL_895681, EPI_ISL_895682, EPI_ISL_895683, EPI_ISL_895684, EPI_ISL_895685, EPI_ISL_895686, EPI_ISL_895687, EPI_ISL_895688, EPI_ISL_895689, EPI_ISL_895690, EPI_ISL_895691, EPI_ISL_895692, EPI_ISL_895693, EPI_ISL_895694, EPI_ISL_895695, EPI_ISL_895696, EPI_ISL_895697, EPI_ISL_895698, EPI_ISL_895699, EPI_ISL_895700, EPI_ISL_895701, EPI_ISL_895702, EPI_ISL_895703, EPI_ISL_895704, EPI_ISL_895705, EPI_ISL_895706, EPI_ISL_895707, EPI_ISL_895708, EPI_ISL_895709, EPI_ISL_895710, EPI_ISL_895711, EPI_ISL_895712, EPI_ISL_895713, EPI_ISL_895714, EPI_ISL_895715, EPI_ISL_895716, EPI_ISL_895717, EPI_ISL_895718, EPI_ISL_895719, EPI_ISL_895720, EPI_ISL_895721, EPI_ISL_895722, EPI_ISL_895723, EPI_ISL_895724, EPI_ISL_895725, EPI_ISL_895726, EPI_ISL_895849, EPI_ISL_895850, EPI_ISL_895851, EPI_ISL_895852, EPI_ISL_895853, EPI_ISL_895854, EPI_ISL_895855, EPI_ISL_895856, EPI_ISL_895857, EPI_ISL_895858, EPI_ISL_895859, EPI_ISL_895860, EPI_ISL_895861, EPI_ISL_895862, EPI_ISL_895863, EPI_ISL_895864, EPI_ISL_895865, EPI_ISL_895866, EPI_ISL_895867, EPI_ISL_895868, EPI_ISL_895869, EPI_ISL_895870, EPI_ISL_895871, EPI_ISL_895872, EPI_ISL_895873, EPI_ISL_895874, EPI_ISL_895875, EPI_ISL_895876, EPI_ISL_895877, EPI_ISL_895878, EPI_ISL_895879, EPI_ISL_895880, EPI_ISL_895881, EPI_ISL_895882, EPI_ISL_895883, EPI_ISL_895884, EPI_ISL_895885, EPI_ISL_895886, EPI_ISL_895887, EPI_ISL_895888, EPI_ISL_895889, EPI_ISL_895890, EPI_ISL_895891, EPI_ISL_895892, EPI_ISL_895893, EPI_ISL_895894, EPI_ISL_895895, EPI_ISL_895896, EPI_ISL_895897, EPI_ISL_895898, EPI_ISL_895899, EPI_ISL_895900, EPI_ISL_895901, EPI_ISL_895902, EPI_ISL_895903, EPI_ISL_895904, EPI_ISL_895905, EPI_ISL_895906, EPI_ISL_895907, EPI_ISL_895908, EPI_ISL_895909, EPI_ISL_895910, EPI_ISL_895911, EPI_ISL_895912, EPI_ISL_895913, EPI_ISL_895914, EPI_ISL_895915, EPI_ISL_895916, EPI_ISL_895917, EPI_ISL_895918, EPI_ISL_895919, EPI_ISL_895920, EPI_ISL_895921, EPI_ISL_895922, EPI_ISL_895923, EPI_ISL_895924, EPI_ISL_895925, EPI_ISL_895926, EPI_ISL_895927, EPI_ISL_895928, EPI_ISL_895929, EPI_ISL_895930, EPI_ISL_895931, EPI_ISL_895932, EPI_ISL_895933, EPI_ISL_895934, EPI_ISL_895935, EPI_ISL_895936, EPI_ISL_895937, EPI_ISL_895938, EPI_ISL_895939, EPI_ISL_895940, EPI_ISL_895941, EPI_ISL_895942, EPI_ISL_895943, EPI_ISL_895944, EPI_ISL_895945, EPI_ISL_895946, EPI_ISL_895947, EPI_ISL_895948, EPI_ISL_895949, EPI_ISL_895950, EPI_ISL_895951, EPI_ISL_895952, EPI_ISL_895953, EPI_ISL_895954, EPI_ISL_895955, EPI_ISL_895956, EPI_ISL_895957, EPI_ISL_895958, EPI_ISL_895959, EPI_ISL_895960, EPI_ISL_895961, EPI_ISL_895962, EPI_ISL_895963, EPI_ISL_895964, EPI_ISL_895965, EPI_ISL_895966, EPI_ISL_895967, EPI_ISL_895968, EPI_ISL_895969, EPI_ISL_895970, EPI_ISL_895971, EPI_ISL_895972, EPI_ISL_895973, EPI_ISL_895974, EPI_ISL_895975, EPI_ISL_895976, EPI_ISL_895977, EPI_ISL_895978			
see above	Pathogen Genomics Center, National Institute of Infectious Diseases	Pathogen Genomics Center, National Institute of Infectious Diseases	Tsuyoshi Sekizuka, Kentaro Itokawa, Rina Tanaka, Masanori Hashino, Makoto Kuroda
EPI_ISL_895989, EPI_ISL_895990, EPI_ISL_895991, EPI_ISL_895992, EPI_ISL_895993, EPI_ISL_895994, EPI_ISL_895995, EPI_ISL_895996, EPI_ISL_895997, EPI_ISL_895998, EPI_ISL_895999, EPI_ISL_896000, EPI_ISL_896001, EPI_ISL_896002, EPI_ISL_896003, EPI_ISL_896004, EPI_ISL_896005, EPI_ISL_896006, EPI_ISL_896007, EPI_ISL_896008, EPI_ISL_896009, EPI_ISL_896010, EPI_ISL_896011, EPI_ISL_896012			
see above	Tokyo Metropolitan Institute of Public Health	Pathogen Genomics Center, National Institute of Infectious Diseases	Tsuyoshi Sekizuka, Kentaro Itokawa, Rina Tanaka, Masanori Hashino, Makoto Kuroda
EPI_ISL_896064, EPI_ISL_896065, EPI_ISL_896066, EPI_ISL_896067, EPI_ISL_896068	Pathogen Genomics Center, National Institute of Infectious Diseases	Pathogen Genomics Center, National Institute of Infectious Diseases	Tsuyoshi Sekizuka, Kentaro Itokawa, Rina Tanaka, Masanori Hashino, Makoto Kuroda
EPI_ISL_896093	Viollier AG	University Hospital Basel, Clinical Bacteriology	Tim Roloff, Madlen Stange, Helena MB Seth-Smith, Alfredo Mari, Karoline Leuzinger, Julia Bielicki, Christiane Beckmann, Manuel Battegay, Hans Hirsch, Adrian Egli
EPI_ISL_896184, EPI_ISL_896207, EPI_ISL_896208	MEPHI, Aix Marseille University	MEPHI, Aix Marseille University	Anthony LEVASSEUR
EPI_ISL_897097, EPI_ISL_897135, EPI_ISL_897136, EPI_ISL_897137, EPI_ISL_897138, EPI_ISL_897248, EPI_ISL_897249, EPI_ISL_897250, EPI_ISL_897252, EPI_ISL_897253, EPI_ISL_897254, EPI_ISL_897255, EPI_ISL_897256, EPI_ISL_897257, EPI_ISL_897258, EPI_ISL_897259, EPI_ISL_897260, EPI_ISL_897261, EPI_ISL_897262, EPI_ISL_897263, EPI_ISL_897264, EPI_ISL_897265, EPI_ISL_897266, EPI_ISL_897267, EPI_ISL_897268, EPI_ISL_897269, EPI_ISL_897270, EPI_ISL_897271, EPI_ISL_897272, EPI_ISL_897313, EPI_ISL_897314, EPI_ISL_897315, EPI_ISL_897434, EPI_ISL_897435, EPI_ISL_897436, EPI_ISL_897437, EPI_ISL_897438, EPI_ISL_897439, EPI_ISL_897440, EPI_ISL_897441, EPI_ISL_897442, EPI_ISL_897443, EPI_ISL_897444			
see above	Pathogen Genomics Center, National Institute of Infectious Diseases	Pathogen Genomics Center, National Institute of Infectious Diseases	Tsuyoshi Sekizuka, Kentaro Itokawa, Rina Tanaka, Masanori Hashino, Makoto Kuroda
EPI_ISL_900173, EPI_ISL_900229, EPI_ISL_900273, EPI_ISL_900477	MEPHI, Aix Marseille University	MEPHI, Aix Marseille University	Anthony LEVASSEUR
EPI_ISL_902933, EPI_ISL_902934, EPI_ISL_902949	Maryland Public Health Laboratory	Maryland Public Health Laboratory	Maryland Department of Health Laboratories Administration
EPI_ISL_903013, EPI_ISL_903014, EPI_ISL_903015, EPI_ISL_903016, EPI_ISL_903017, EPI_ISL_903018, EPI_ISL_903019	Seattle Flu Study	Seattle Flu Study	Deborah A. Nickerson, Chris D. Frazar, Jover Lee, Benjamin Pelle, Erica Ryke, Matthew Richardson, Amanda Adler, Elisabeth Brandstetter, Peter D. Han, Kairsten Fay, Misja Ilcisin, Kirsten Lacombe, Thomas R. Sibley, Melissa Truong, Caitlin R. Wolf, Michael Boeckh, Janet A. Englund, Michael Famulare, Barry R. Lutz, Mark J. Rieder, Lea M. Starita, Matthew Thompson, Jay Shendure, Trevor Bedford, Helen Y. Chu
EPI_ISL_903568, EPI_ISL_903569	NC State Laboratory of Public Health	Genomics and Discovery, Respiratory Viruses Branch, Division of Viral Diseases, Centers for Disease Control and Prevention	Krista Queen, Yan Li, Ying Tao, Jing Zhang, Anna Uehara, Anna Montmayeur, Clinton R. Paden, Peter W. Cook, Rachel Marine, Mili Sheth, Jasmine Padilla, Sarah Nobles, Mark Burroughs, Lori Rowe, Haibin Wang, Ben L. Rambo-Martin, Dhwani Batra, Justin Lee, Suxiang Tong
EPI_ISL_903598, EPI_ISL_903617	UT-Unified State Labs: Public Health Utah DOH	Genomics and Discovery, Respiratory Viruses Branch, Division of Viral Diseases, Centers for Disease Control and Prevention	Krista Queen, Yan Li, Ying Tao, Jing Zhang, Anna Uehara, Anna Montmayeur, Clinton R. Paden, Peter W. Cook, Rachel Marine, Mili Sheth, Jasmine Padilla, Sarah Nobles, Mark Burroughs, Lori Rowe, Haibin Wang, Ben L. Rambo-Martin, Dhwani Batra, Justin Lee, Suxiang Tong
EPI_ISL_903684	WI State Laboratory of Hygiene	Genomics and Discovery, Respiratory Viruses Branch, Division of Viral Diseases, Centers for Disease Control and Prevention	Krista Queen, Yan Li, Ying Tao, Jing Zhang, Anna Uehara, Anna Montmayeur, Clinton R. Paden, Peter W. Cook, Rachel Marine, Mili Sheth, Jasmine Padilla, Sarah Nobles, Mark Burroughs, Lori Rowe, Haibin Wang, Ben L. Rambo-Martin, Dhwani Batra, Justin Lee, Suxiang Tong
EPI_ISL_903722	OK Public Health Laboratory, Oklahoma State DOH	Genomics and Discovery, Respiratory Viruses Branch, Division of Viral Diseases, Centers for Disease Control and Prevention	Krista Queen, Yan Li, Ying Tao, Jing Zhang, Anna Uehara, Anna Montmayeur, Clinton R. Paden, Peter W. Cook, Rachel Marine, Mili Sheth, Jasmine Padilla, Sarah Nobles, Mark Burroughs, Lori Rowe, Haibin Wang, Ben L. Rambo-Martin, Dhwani Batra, Justin Lee, Suxiang Tong
EPI_ISL_903741	VA-Division of Consolidated Laboratory Services	Genomics and Discovery, Respiratory Viruses Branch,	Krista Queen, Yan Li, Ying Tao, Jing Zhang, Anna Uehara, Anna Montmayeur, Clinton R. Paden, Peter W. Cook, Rachel Marine, Mili Sheth, Jasmine

		Division of Viral Diseases, Centers for Disease Control and Prevention	Padilla, Sarah Nobles, Mark Burroughs, Lori Rowe, Haibin Wang, Ben L. Rambo-Martin, Dhwani Batra, Justin Lee, Suxiang Tong
EPI_ISL_903756, EPI_ISL_903758	OK Public Health Laboratory, Oklahoma State DOH	Genomics and Discovery, Respiratory Viruses Branch, Division of Viral Diseases, Centers for Disease Control and Prevention	Krista Queen, Yan Li, Ying Tao, Jing Zhang, Anna Uehara, Anna Montmayeur, Clinton R. Paden, Peter W. Cook, Rachel Marine, Mili Sheth, Jasmine Padilla, Sarah Nobles, Mark Burroughs, Lori Rowe, Haibin Wang, Ben L. Rambo-Martin, Dhwani Batra, Justin Lee, Suxiang Tong
EPI_ISL_903766	UT-Unified State Labs: Public Health Utah DOH	Genomics and Discovery, Respiratory Viruses Branch, Division of Viral Diseases, Centers for Disease Control and Prevention	Krista Queen, Yan Li, Ying Tao, Jing Zhang, Anna Uehara, Anna Montmayeur, Clinton R. Paden, Peter W. Cook, Rachel Marine, Mili Sheth, Jasmine Padilla, Sarah Nobles, Mark Burroughs, Lori Rowe, Haibin Wang, Ben L. Rambo-Martin, Dhwani Batra, Justin Lee, Suxiang Tong
EPI_ISL_903770	OK Public Health Laboratory, Oklahoma State DOH	Genomics and Discovery, Respiratory Viruses Branch, Division of Viral Diseases, Centers for Disease Control and Prevention	Krista Queen, Yan Li, Ying Tao, Jing Zhang, Anna Uehara, Anna Montmayeur, Clinton R. Paden, Peter W. Cook, Rachel Marine, Mili Sheth, Jasmine Padilla, Sarah Nobles, Mark Burroughs, Lori Rowe, Haibin Wang, Ben L. Rambo-Martin, Dhwani Batra, Justin Lee, Suxiang Tong
EPI_ISL_903771	VA-Division of Consolidated Laboratory Services	Genomics and Discovery, Respiratory Viruses Branch, Division of Viral Diseases, Centers for Disease Control and Prevention	Krista Queen, Yan Li, Ying Tao, Jing Zhang, Anna Uehara, Anna Montmayeur, Clinton R. Paden, Peter W. Cook, Rachel Marine, Mili Sheth, Jasmine Padilla, Sarah Nobles, Mark Burroughs, Lori Rowe, Haibin Wang, Ben L. Rambo-Martin, Dhwani Batra, Justin Lee, Suxiang Tong
EPI_ISL_903783	OK Public Health Laboratory, Oklahoma State DOH	Genomics and Discovery, Respiratory Viruses Branch, Division of Viral Diseases, Centers for Disease Control and Prevention	Krista Queen, Yan Li, Ying Tao, Jing Zhang, Anna Uehara, Anna Montmayeur, Clinton R. Paden, Peter W. Cook, Rachel Marine, Mili Sheth, Jasmine Padilla, Sarah Nobles, Mark Burroughs, Lori Rowe, Haibin Wang, Ben L. Rambo-Martin, Dhwani Batra, Justin Lee, Suxiang Tong
EPI_ISL_903792	VA-Division of Consolidated Laboratory Services	Genomics and Discovery, Respiratory Viruses Branch, Division of Viral Diseases, Centers for Disease Control and Prevention	Krista Queen, Yan Li, Ying Tao, Jing Zhang, Anna Uehara, Anna Montmayeur, Clinton R. Paden, Peter W. Cook, Rachel Marine, Mili Sheth, Jasmine Padilla, Sarah Nobles, Mark Burroughs, Lori Rowe, Haibin Wang, Ben L. Rambo-Martin, Dhwani Batra, Justin Lee, Suxiang Tong
EPI_ISL_903801	WI State Laboratory of Hygiene	Genomics and Discovery, Respiratory Viruses Branch, Division of Viral Diseases, Centers for Disease Control and Prevention	Krista Queen, Yan Li, Ying Tao, Jing Zhang, Anna Uehara, Anna Montmayeur, Clinton R. Paden, Peter W. Cook, Rachel Marine, Mili Sheth, Jasmine Padilla, Sarah Nobles, Mark Burroughs, Lori Rowe, Haibin Wang, Ben L. Rambo-Martin, Dhwani Batra, Justin Lee, Suxiang Tong
EPI_ISL_903830	VA-Division of Consolidated Laboratory Services	Genomics and Discovery, Respiratory Viruses Branch, Division of Viral Diseases, Centers for Disease Control and Prevention	Krista Queen, Yan Li, Ying Tao, Jing Zhang, Anna Uehara, Anna Montmayeur, Clinton R. Paden, Peter W. Cook, Rachel Marine, Mili Sheth, Jasmine Padilla, Sarah Nobles, Mark Burroughs, Lori Rowe, Haibin Wang, Ben L. Rambo-Martin, Dhwani Batra, Justin Lee, Suxiang Tong
EPI_ISL_903841	UT-Unified State Labs: Public Health Utah DOH	Genomics and Discovery, Respiratory Viruses Branch, Division of Viral Diseases, Centers for Disease Control and Prevention	Krista Queen, Yan Li, Ying Tao, Jing Zhang, Anna Uehara, Anna Montmayeur, Clinton R. Paden, Peter W. Cook, Rachel Marine, Mili Sheth, Jasmine Padilla, Sarah Nobles, Mark Burroughs, Lori Rowe, Haibin Wang, Ben L. Rambo-Martin, Dhwani Batra, Justin Lee, Suxiang Tong
EPI_ISL_903846	WI State Laboratory of Hygiene	Genomics and Discovery, Respiratory Viruses Branch, Division of Viral Diseases, Centers for Disease Control and Prevention	Krista Queen, Yan Li, Ying Tao, Jing Zhang, Anna Uehara, Anna Montmayeur, Clinton R. Paden, Peter W. Cook, Rachel Marine, Mili Sheth, Jasmine Padilla, Sarah Nobles, Mark Burroughs, Lori Rowe, Haibin Wang, Ben L. Rambo-Martin, Dhwani Batra, Justin Lee, Suxiang Tong
EPI_ISL_903891	UT-Unified State Labs: Public Health Utah DOH	Genomics and Discovery, Respiratory Viruses Branch, Division of Viral Diseases, Centers for Disease Control and Prevention	Krista Queen, Yan Li, Ying Tao, Jing Zhang, Anna Uehara, Anna Montmayeur, Clinton R. Paden, Peter W. Cook, Rachel Marine, Mili Sheth, Jasmine Padilla, Sarah Nobles, Mark Burroughs, Lori Rowe, Haibin Wang, Ben L. Rambo-Martin, Dhwani Batra, Justin Lee, Suxiang Tong
EPI_ISL_903903	WI State Laboratory of Hygiene	Genomics and Discovery, Respiratory Viruses Branch, Division of Viral Diseases, Centers for Disease Control and Prevention	Krista Queen, Yan Li, Ying Tao, Jing Zhang, Anna Uehara, Anna Montmayeur, Clinton R. Paden, Peter W. Cook, Rachel Marine, Mili Sheth, Jasmine Padilla, Sarah Nobles, Mark Burroughs, Lori Rowe, Haibin Wang, Ben L. Rambo-Martin, Dhwani Batra, Justin Lee, Suxiang Tong
EPI_ISL_903910, EPI_ISL_903938	UT-Unified State Labs: Public Health Utah DOH	Genomics and Discovery, Respiratory Viruses Branch, Division of Viral Diseases, Centers for Disease Control and Prevention	Krista Queen, Yan Li, Ying Tao, Jing Zhang, Anna Uehara, Anna Montmayeur, Clinton R. Paden, Peter W. Cook, Rachel Marine, Mili Sheth, Jasmine Padilla, Sarah Nobles, Mark Burroughs, Lori Rowe, Haibin Wang, Ben L. Rambo-Martin, Dhwani Batra, Justin Lee, Suxiang Tong
EPI_ISL_903940	WI State Laboratory of Hygiene	Genomics and Discovery, Respiratory Viruses Branch, Division of Viral Diseases, Centers for Disease Control and Prevention	Krista Queen, Yan Li, Ying Tao, Jing Zhang, Anna Uehara, Anna Montmayeur, Clinton R. Paden, Peter W. Cook, Rachel Marine, Mili Sheth, Jasmine Padilla, Sarah Nobles, Mark Burroughs, Lori Rowe, Haibin Wang, Ben L. Rambo-Martin, Dhwani Batra, Justin Lee, Suxiang Tong
EPI_ISL_903951, EPI_ISL_903953	MN PHL Division, Minnesota Department of Health	Genomics and Discovery, Respiratory Viruses Branch, Division of Viral Diseases, Centers for Disease Control and Prevention	Krista Queen, Yan Li, Ying Tao, Jing Zhang, Anna Uehara, Anna Montmayeur, Clinton R. Paden, Peter W. Cook, Rachel Marine, Mili Sheth, Jasmine Padilla, Sarah Nobles, Mark Burroughs, Lori Rowe, Haibin Wang, Ben L. Rambo-Martin, Dhwani Batra, Justin Lee, Suxiang Tong
EPI_ISL_904140, EPI_ISL_904176, EPI_ISL_904475, EPI_ISL_904476, EPI_ISL_904477, EPI_ISL_904478	Dutch COVID-19 response team	Erasmus Medical Center	Bas Oude Munnink, Reina Sikkema, David Nieuwenhuijse, Irina Chestakova, Anne van der Linden, Marjan Boter, Emmanuelle Munger, Corine GeurtsvanKessel, Annemiek van der Eijk, Richard Molenkamp, Marion Koopmans, on behalf of the Dutch national COVID-19 response team.
EPI_ISL_904861, EPI_ISL_904864, EPI_ISL_905080, EPI_ISL_905083	Dutch COVID-19 response team	National Institute for Public Health and the Environment (RIVM)	Adam Meijer, Harry Vennema, Dirk Eggink, Jeroen Cremer, Sharon van den Brink, Bas van der Veer, AnneMarie van den Brandt, Florian Zwagemaker, Dennis Schmitz, Chantal Reusken, on behalf of the national COVID-19 response team
EPI_ISL_906082	Child Health Research Foundation	Child Health Research Foundation	Senjuti Saha, Arif Mohammad Tanmoy, Sharmistha Goswami, Afroza Akter Tanni, Syed Muktadir Al Sium, Roly Malaker, Md Hafizur Rahman, Samir K Saha
EPI_ISL_906085, EPI_ISL_906089	Child Health Research Foundation	Child Health Research Foundation	Senjuti Saha, Sharmistha Goswami, Afroza Akter Tanni, Syed Muktadir Al Sium, Arif Mohammad Tanmoy, Roly Malaker, Md Hafizur Rahman, Samir K Saha
EPI_ISL_906090	Child Health Research Foundation	Child Health Research Foundation	Senjuti Saha, Afroza Akter Tanni, Sharmistha Goswami, Syed Muktadir Al Sium, Arif Mohammad Tanmoy, Roly Malaker, Md Hafizur Rahman, Samir K Saha
EPI_ISL_906094, EPI_ISL_906102	Child Health Research Foundation	Child Health Research Foundation	Senjuti Saha, Syed Muktadir Al Sium, Sharmistha Goswami, Afroza Akter Tanni, Arif Mohammad Tanmoy, Roly Malaker, Md Hafizur Rahman, Samir K Saha
EPI_ISL_906104	Child Health Research Foundation	Child Health Research Foundation	Senjuti Saha, Arif Mohammad Tanmoy, Sharmistha Goswami, Afroza Akter Tanni, Syed Muktadir Al Sium, Roly Malaker, Md Hafizur Rahman, Samir K Saha
EPI_ISL_906107	Child Health Research Foundation	Child Health Research Foundation	Senjuti Saha, Afroza Akter Tanni, Sharmistha Goswami, Syed Muktadir Al Sium, Arif Mohammad Tanmoy, Roly Malaker, Md Hafizur Rahman, Samir K Saha
EPI_ISL_906259, EPI_ISL_906260, EPI_ISL_906261	University of Wisconsin-Madison AIDS Vaccine Research Laboratories	University of Wisconsin-Madison AIDS Vaccine Research Laboratories	Gage Moreno, Katarina Braun, et al. AIDS Vaccine Research Laboratories
EPI_ISL_906285	Nigeria Centre for Disease Control (NCDC)	African Centre of Excellence for Genomics of Infectious Diseases (ACEGID), Redeemer's University	Oluniyi P.E. et al
EPI_ISL_906523	Department of Infectious Diseases, Kobe Institute of Health	Pathogen Genomics Center, National Institute of Infectious Diseases	Tsuyoshi Sekizuka, Kentaro Itokawa, Rina Tanaka, Masanori Hashino, Makoto Kuroda
EPI_ISL_906526	Pathogen Genomics Center, National Institute of Infectious Diseases	Pathogen Genomics Center, National Institute of Infectious Diseases	Tsuyoshi Sekizuka, Kentaro Itokawa, Rina Tanaka, Masanori Hashino, Makoto Kuroda
EPI_ISL_906533, EPI_ISL_906542	Laboratorio de Virologia-Instituto Nacional de Salud	Instituto Nacional de Salud- Dirección de Investigación en	Katherine Laiton-Donato, Diego A. Álvarez-Díaz, Carlos Franco-Muñoz, Mauricio Pacheco-Montealegre, Héctor Alejandro Ruiz-Moreno, Maria T.

		Salud Pública, Universidad de los Andes- Applied genomics research group, Vicerrectoria de Investigación y Creación, Universidad de los Andes- Systems and Computing Engineering Department	Herrera-Sepúlveda, Diego Andrés Prada, Jhonnatan Reales-González, Sheryll Corchuelo, Julian Naizaque, Gerardo Santamaría Jorge Duitama, Laura Natalia Gonzalez, Jorge Ivan Diaz, Silvia Restrepo-Restrepo, Magdalena Wiesner, Martha Lucia Ospina Martinez, Marcela Mercado-Reyes
EPI_ISL_910360, EPI_ISL_910386, EPI_ISL_910387, EPI_ISL_910388, EPI_ISL_910389, EPI_ISL_910457, EPI_ISL_910458, EPI_ISL_910459, EPI_ISL_910460, EPI_ISL_910461, EPI_ISL_910462, EPI_ISL_910647, EPI_ISL_910700, EPI_ISL_910701, EPI_ISL_910702, EPI_ISL_910703, EPI_ISL_910705, EPI_ISL_910714, EPI_ISL_910715, EPI_ISL_910716, EPI_ISL_910717, EPI_ISL_910718, EPI_ISL_910719, EPI_ISL_910720, EPI_ISL_910723, EPI_ISL_910774, EPI_ISL_910775, EPI_ISL_910776, EPI_ISL_911235			
see above	Laboratoire national de sante, Microbiology, Virology	Laboratoire national de sante, Microbiology, Microbial Genomics Platform	Anke Wienecke-Baldacchino, Catherine Ragimbeau,Jessica Tapp, Fatu Djabi, Lise Pignon, Raoul Salmon, Tamir Abdelrahman
EPI_ISL_911532, EPI_ISL_911533, EPI_ISL_911534, EPI_ISL_911535, EPI_ISL_911536, EPI_ISL_911537, EPI_ISL_911538, EPI_ISL_911539, EPI_ISL_911540, EPI_ISL_911541, EPI_ISL_911542, EPI_ISL_911543, EPI_ISL_911544, EPI_ISL_911546, EPI_ISL_911547			
see above	CSIR-IGIB	CSIR-IGIB	Rajesh Pandey, Akshay Kanakan, Janani SV, Ranjeet Maurya, Aparna S Murali, Shweta Sahni, Azka Khan, Partha Chattopadhyay, Priti Devi, Priyanka Mehta, Anil Kumar, Nisha Rawat
EPI_ISL_911677, EPI_ISL_911680, EPI_ISL_911682, EPI_ISL_911700, EPI_ISL_911703	Alaska State Virology Laboratory	Alaska State Virology Laboratory	Stephanie DeRonde, Lisa Smith, Ph.D., Jack Chen, Ph.D.
EPI_ISL_912467, EPI_ISL_912489, EPI_ISL_912515, EPI_ISL_912523, EPI_ISL_912530	NHLS Universitas Academic	UFS Virology	PA Bester, MM Nyaga, P Nthiga, MT Mogotsi, D Goedhals, T de Oliveira
EPI_ISL_913306, EPI_ISL_913462	Klinisk mikrobiologi	The Public Health Agency of Sweden	Anna-Malin Linde, Maria Lind Karlberg, Carlo Berg, Oskar Karlsson Lindsjo, Sofia Stamouli, Reza Advani, Mattias Haukland, Petra Holmstrom, Noura Walai, Petra Edquist, Mia Brytting, Anna Risberg, Karin Tegmark-Wisell
EPI_ISL_914794	AREA DE SALUD BUENOS AIRES	Inciensa, Instituto Costarricense de Investigación y Enseñanza en Nutrición y Salud	Francisco Duarte, Hebleen Porras, Claudio Soto-Garita, Estela Cordero, Adriana Godínez, Melany Calderón & Mariel López
EPI_ISL_914795	HOSPITAL DR. FERNANDO ESCALANTE PRADILLA	Inciensa, Instituto Costarricense de Investigación y Enseñanza en Nutrición y Salud	Francisco Duarte, Hebleen Porras, Claudio Soto-Garita, Estela Cordero, Adriana Godínez, Melany Calderón & Mariel López
EPI_ISL_915374, EPI_ISL_915377, EPI_ISL_915415, EPI_ISL_915416	Keio University School of Medicine	Keio University School of Medicine	Kenjiro Kosaki, Yuka Iwasaki, Hirotsugu Ishizu, Haruhiko Siomi, Kodai Abe
EPI_ISL_918167	Department of Infectious Diseases and Immunology, National Hospital Organization Nagoya Medical Center	Clinical Research Center, National Hospital Organization Nagoya Medical Center	Yoshihiro Nakata, Hirotaka Ode, Mai Kubota, Masakazu Matsuda, Kazuhiro Matsuoka, Miho Nakasuji, Mikiko Mori, Mayumi Imahashi, Yoshiyuki Yokomaku, Yasumasa Iwatani
EPI_ISL_918359, EPI_ISL_918365	Virology Unit, Institut Pasteur du Cambodge	Virology Unit, Institut Pasteur du Cambodge	Sokhoun Yann, Ly Sovann, Kraing Sidonn, Yi Sengdoeurn, Chin Savuth, Chau Darapheak, Etienne Simon-Loriere, Veasna Duong, Erik A Karlsson
EPI_ISL_918578, EPI_ISL_918636, EPI_ISL_918637, EPI_ISL_918638, EPI_ISL_918639, EPI_ISL_918640, EPI_ISL_918641, EPI_ISL_918642, EPI_ISL_918643, EPI_ISL_918644, EPI_ISL_918645, EPI_ISL_918646, EPI_ISL_918648, EPI_ISL_918649, EPI_ISL_918650, EPI_ISL_918651, EPI_ISL_918652, EPI_ISL_918653, EPI_ISL_918654, EPI_ISL_918655, EPI_ISL_918658, EPI_ISL_918659, EPI_ISL_918660, EPI_ISL_918661, EPI_ISL_918662, EPI_ISL_918663, EPI_ISL_918664, EPI_ISL_918685			
see above	University of Birmingham	COVID-19 Genomics UK (COG-UK) Consortium	Institute of Microbiology, University of Birmingham: Claire McMurray, Joanne Stockton, Samuel Nicholls, Radoslaw Poplawski, Will Rowe, Josh Quick, Nicholas Loman. University of Birmingham Testing Laboratory: Celina M Whalley, Andrew Bosworth, Charlotte Poxon, Kasun Wanigasooriya, Oliver Pickles, Mike Kidd, Alex Richter, Andrew D Beggs PHE Heartlands Lab: Husam Osman, Andrew Bosworth. Queen Elizabeth Hospital: Anna Casey
EPI_ISL_919271	West of Scotland Specialist Virology Centre, NHSGGC / MRC-University of Glasgow Centre for Virus Research	COVID-19 Genomics UK (COG-UK) Consortium	Ana da Silva Filipe, Natasha Johnson, Kathy Smollett, Daniel Mair, Stephen Carmichael, Alice Broos, Lily Tong, Jenna Nichols, Kyriaki Nomikou; Sarah McDonald; Richard Orton, Joseph Hughes, Sreenu Vattipally, David L Robertson; Alasdair MacLean, Rory Gunson; Sharif Shaaban, Matthew Holden; Rachel Blacow, Guy Mollett, Kathy Li, James Shepherd, Antonia Ho, Emma Thomson
EPI_ISL_919348	Virology Department, Royal Infirmary of Edinburgh, NHS Lothian / School of Biological Sciences, University of Edinburgh / Institute of Genetics and Molecular Medicine, University of Edinburgh	COVID-19 Genomics UK (COG-UK) Consortium	McHugh M, Dewar R, Rooke S, Gallagher M, Balcaza C, O'Toole Á, Scher E, Hill V, McCrone JT, Colquhoun R, Yu X, Jackson B, Rambaut A, Williams TC, Templeton K
EPI_ISL_919445	Liverpool Clinical Laboratories	COVID-19 Genomics UK (COG-UK) Consortium	Sam Haldenby, Anita Lucaci, Steve Paterson, Julian Hiscox, Alistair Darby, M Almsaud, A Alrezaihi, Muhannad Alruwaili, Stuart D Armstrong, Jones Benjamin, Eleanor G Bentley, Anu Chawla, Jordan J Clark, Angela Cowell, Richard Eccles, Isabel García-Dorival, Matthew Gemmell, Alessandro Gerada, PKF Gilmore, Richard Gregory, Ximeng Han, Catherine Hartley, Margaret Hughes, Miren Iturriza-Gomara, James Johnson, L Luu, Jenifer Manson, Charlotte Nelson, Elaine O'Toole, Cassie Olateju, Rebekah Penrice-Randal , Lucille Rainbow, N.P Randle, Trevor Ian Robinson, Parul Sharma, Ghada T Shawli, James P Stewart, Neil Swainston, Ecaterina Vamos, Joanne Watts, Mark Whitehead
EPI_ISL_920223	University College London Hospital	COVID-19 Genomics UK (COG-UK) Consortium	Judith Heaney, Matthew Byott, Catherine Houlihan, Dan Frampton, Stuart Kirk, Moira Spyer and Eleni Nastouli
EPI_ISL_923221, EPI_ISL_923222	Centre for Enzyme Innovation, University of Portsmouth / Translational Research Laboratory, Portsmouth Hospitals NHS Trust	COVID-19 Genomics UK (COG-UK) Consortium	Angela Beckett,Salman Goudarzi,Christopher Fearn,Kate Cook,Katie Loveson,Sharon Glaysher,Scott Elliott,Samuel Robson
EPI_ISL_924141	Virology Department, Sheffield Teaching Hospitals NHS Foundation Trust/Department of Infection, Immunity and Cardiovascular Disease, The Medical School, University of Sheffield	COVID-19 Genomics UK (COG-UK) Consortium	Thushan de Silva, Matthew Parker, Nikki Smith, Adri Angyal, Rebecca Brown, Luke Green, Rachel Tucker, Paul Parsons, Danielle Groves, Katie Johnson, Laura Carrilero, Alex Keeley, Dave Partridge, Matthew Wyles, Benjamin Lindsey, Mehmet Yavuz, Mohammad Raza, Cariad Evans
EPI_ISL_925151, EPI_ISL_925152, EPI_ISL_925153, EPI_ISL_925154, EPI_ISL_925155, EPI_ISL_925156, EPI_ISL_925157, EPI_ISL_925158, EPI_ISL_925159, EPI_ISL_925164, EPI_ISL_925165, EPI_ISL_925166			
see above	Virginia DCLS	Virginia DCLS	Virginia DCLS
EPI_ISL_925539, EPI_ISL_925597, EPI_ISL_925598, EPI_ISL_925599, EPI_ISL_925600, EPI_ISL_925601, EPI_ISL_925602, EPI_ISL_925603, EPI_ISL_925604, EPI_ISL_925605, EPI_ISL_925606, EPI_ISL_925607, EPI_ISL_925608, EPI_ISL_925609, EPI_ISL_925610, EPI_ISL_925611, EPI_ISL_925612, EPI_ISL_925613, EPI_ISL_925614, EPI_ISL_925615, EPI_ISL_925616, EPI_ISL_925617, EPI_ISL_925618, EPI_ISL_925619, EPI_ISL_925620, EPI_ISL_925621, EPI_ISL_925622, EPI_ISL_925623, EPI_ISL_925624, EPI_ISL_925625, EPI_ISL_925626, EPI_ISL_925627, EPI_ISL_925628, EPI_ISL_925629, EPI_ISL_925630, EPI_ISL_925631, EPI_ISL_925632, EPI_ISL_925633, EPI_ISL_925634, EPI_ISL_925635, EPI_ISL_925636, EPI_ISL_925637, EPI_ISL_925638, EPI_ISL_925639, EPI_ISL_925640, EPI_ISL_925641, EPI_ISL_925642, EPI_ISL_925643, EPI_ISL_925644, EPI_ISL_925645, EPI_ISL_925646, EPI_ISL_925647, EPI_ISL_925648, EPI_ISL_925649, EPI_ISL_925650, EPI_ISL_925651, EPI_ISL_925652, EPI_ISL_925653, EPI_ISL_925654, EPI_ISL_925655, EPI_ISL_925656, EPI_ISL_925657, EPI_ISL_925658, EPI_ISL_925659, EPI_ISL_925660, EPI_ISL_925661, EPI_ISL_925662, EPI_ISL_925663, EPI_ISL_925664, EPI_ISL_925665, EPI_ISL_925666, EPI_ISL_925667, EPI_ISL_925668, EPI_ISL_925669, EPI_ISL_925670, EPI_ISL_925671, EPI_ISL_925672, EPI_ISL_925673, EPI_ISL_925674, EPI_ISL_925675, EPI_ISL_925676, EPI_ISL_925677, EPI_ISL_925701, EPI_ISL_925702, EPI_ISL_925703, EPI_ISL_925704, EPI_ISL_925705, EPI_ISL_925706, EPI_ISL_925707, EPI_ISL_925708, EPI_ISL_925709, EPI_ISL_925710, EPI_ISL_925711, EPI_ISL_925712, EPI_ISL_925713, EPI_ISL_925714, EPI_ISL_925715, EPI_ISL_925716, EPI_ISL_925717, EPI_ISL_925718, EPI_ISL_925719, EPI_ISL_925720, EPI_ISL_925721, EPI_ISL_925722, EPI_ISL_925723, EPI_ISL_925724, EPI_ISL_925725, EPI_ISL_925730, EPI_ISL_925763			
see above	Public Health Ontario Laboratory	Public Health Ontario Laboratory	Vanessa G Allen, Philip Banh, Yao Chen, Richard de Borja, Alireza Eshaghi, Nahuel Fittipaldi, Christine Frantz, Jonathan B Gubbay, Jennifer L Guthrie, Lawrence Heisler, Esha Joshi, Michael Laszloffy, Aimin Li, Michael CY Li, Dean Maxwell, Sandeep Nagra, Samir N Patel, Jared Simpson, Karthikeyan Sivaraman, Ashleigh Sullivan, Yogi Sundaravadanam, Sarah Teatero, Matthew Watson, Andre Villegas, Sandra Zittermann
EPI_ISL_925979, EPI_ISL_926193, EPI_ISL_926252, EPI_ISL_926292, EPI_ISL_926323, EPI_ISL_926334, EPI_ISL_926515, EPI_ISL_926536, EPI_ISL_926697, EPI_ISL_926878, EPI_ISL_926995, EPI_ISL_927223, EPI_ISL_927239, EPI_ISL_927808, EPI_ISL_927824, EPI_ISL_928081, EPI_ISL_928230, EPI_ISL_928687, EPI_ISL_928888, EPI_ISL_928988, EPI_ISL_929006, EPI_ISL_929069, EPI_ISL_929355, EPI_ISL_929513, EPI_ISL_929658, EPI_ISL_929662, EPI_ISL_929738, EPI_ISL_929781, EPI_ISL_929790, EPI_ISL_929830, EPI_ISL_930065, EPI_ISL_930158, EPI_ISL_930178, EPI_ISL_930180, EPI_ISL_930212, EPI_ISL_930324, EPI_ISL_930347, EPI_ISL_930458, EPI_ISL_930497, EPI_ISL_930518, EPI_ISL_930523			
see above	Department of Virus and Microbiological Special Diagnostics, Statens Serum Institut, Copenhagen, Denmark	Aalborg University	Danish Covid-19 Genome Consortium
EPI_ISL_931367, EPI_ISL_931368, EPI_ISL_931369	University Hospital Basel, Clinical Virology	University Hospital Basel, Clinical Bacteriology	Tim Roloff, Madlen Stange, Helena MB Seth-Smith, Alfredo Mari, Karoline Leuzinger, Julia Bielicki, Manuel Battegay, Hans Hirsch, Adrian Egli
EPI_ISL_933496, EPI_ISL_933497, EPI_ISL_933498, EPI_ISL_933499, EPI_ISL_933500, EPI_ISL_933501, EPI_ISL_933502, EPI_ISL_933503, EPI_ISL_933504, EPI_ISL_933505, EPI_ISL_933506, EPI_ISL_933507, EPI_ISL_933508, EPI_ISL_933509			

see above	Lighthouse Lab in Milton Keynes	Wellcome Sanger Institute for the COVID-19 Genomics UK (COG-UK) Consortium	The Lighthouse Lab in Milton Keynes and Alex Alderton, Roberto Amato, Sonia Goncalves, Ewan Harrison, David K. Jackson, Ian Johnston, Dominic Kwiatkowski, Cordelia Langford, John Sillitoe on behalf of the Wellcome Sanger Institute COVID-19 Surveillance Team
EPI_ISL_933519, EPI_ISL_933520, EPI_ISL_933521, EPI_ISL_933522, EPI_ISL_933523, EPI_ISL_933524, EPI_ISL_933525, EPI_ISL_933526, EPI_ISL_933527, EPI_ISL_933528, EPI_ISL_933529, EPI_ISL_933530, EPI_ISL_933531			
see above	Instituto Nacional de Medicina Genomica	Instituto Nacional de Medicina Genomica	Hidalgo-Miranda A, Mendoza-Vargas A, Reyes-Grajeda JP, Cisneros-Villanueva M, Cedro-Tanda A,Peñaloza-Figueroa F, Herrera-Montalvo LA
EPI_ISL_933554, EPI_ISL_933555, EPI_ISL_933556, EPI_ISL_933557, EPI_ISL_933580, EPI_ISL_933581, EPI_ISL_933582, EPI_ISL_933583, EPI_ISL_933584, EPI_ISL_933585, EPI_ISL_933586, EPI_ISL_933587, EPI_ISL_933588, EPI_ISL_933589, EPI_ISL_933590, EPI_ISL_933591, EPI_ISL_933592, EPI_ISL_933593, EPI_ISL_933595			
see above	Arizona State Public Health Laboratory	Arizona State Public Health Laboratory	Trung Huynh, Jessica Escobar, Katherine Fullerton, Nobuko Fukushima, Stacy White, Linda Getsinger, Victor Waddell
EPI_ISL_933805, EPI_ISL_933806, EPI_ISL_933807, EPI_ISL_933808, EPI_ISL_933809, EPI_ISL_933810, EPI_ISL_933811, EPI_ISL_933812, EPI_ISL_933813, EPI_ISL_933814, EPI_ISL_933815, EPI_ISL_933816, EPI_ISL_933817, EPI_ISL_933818, EPI_ISL_933819, EPI_ISL_933820, EPI_ISL_933821, EPI_ISL_933822, EPI_ISL_933823, EPI_ISL_933824, EPI_ISL_933825, EPI_ISL_933826, EPI_ISL_933827, EPI_ISL_933828, EPI_ISL_933829, EPI_ISL_933830, EPI_ISL_933831, EPI_ISL_933832, EPI_ISL_933833, EPI_ISL_933834, EPI_ISL_933835, EPI_ISL_933836, EPI_ISL_933837, EPI_ISL_933838, EPI_ISL_933839, EPI_ISL_933840, EPI_ISL_933841, EPI_ISL_933842, EPI_ISL_933843, EPI_ISL_933844, EPI_ISL_933845, EPI_ISL_933846, EPI_ISL_933847, EPI_ISL_933848, EPI_ISL_933849, EPI_ISL_933850, EPI_ISL_933851, EPI_ISL_933852, EPI_ISL_933853, EPI_ISL_933854, EPI_ISL_933855, EPI_ISL_933856, EPI_ISL_933857, EPI_ISL_933858, EPI_ISL_933859, EPI_ISL_933860, EPI_ISL_933861, EPI_ISL_933862, EPI_ISL_933863, EPI_ISL_933864, EPI_ISL_933865, EPI_ISL_933866, EPI_ISL_933867, EPI_ISL_933868, EPI_ISL_933869, EPI_ISL_933870, EPI_ISL_933871, EPI_ISL_933872, EPI_ISL_933873, EPI_ISL_933874, EPI_ISL_933875, EPI_ISL_933876, EPI_ISL_933877, EPI_ISL_933941, EPI_ISL_933942, EPI_ISL_933943, EPI_ISL_933947			
see above	Vilnius university hospital Santaros Klinikos, Center of Laboratory Medicine	Vilnius university hospital Santaros Klinikos, Center of Laboratory Medicine	Ingrida Olendraite, Daniel Naumovas, Rimvydas Norvilas, Dovile Ezerskyte, Justinas Slikas, Gytis Dudas
EPI_ISL_935215	KU Leuven, Rega Institute, Clinical and Epidemiological Virology	KU Leuven, Rega Institute, Clinical and Epidemiological Virology	Tony Wawina-Bokalanga, Bert Vanmechelen, Joan Marti-Carerras, Piet Maes
EPI_ISL_935466, EPI_ISL_935467, EPI_ISL_935468, EPI_ISL_935469, EPI_ISL_935470, EPI_ISL_935471, EPI_ISL_935472, EPI_ISL_935473, EPI_ISL_935474, EPI_ISL_935475, EPI_ISL_935476, EPI_ISL_935477, EPI_ISL_935478, EPI_ISL_935479, EPI_ISL_935480, EPI_ISL_935481, EPI_ISL_935482, EPI_ISL_935483, EPI_ISL_935484, EPI_ISL_935485, EPI_ISL_935486, EPI_ISL_935487, EPI_ISL_935488, EPI_ISL_935489, EPI_ISL_935490, EPI_ISL_935491, EPI_ISL_935492, EPI_ISL_935493, EPI_ISL_935494			
see above	Florida Bureau of Public Health Laboratories	Florida Bureau of Public Health Laboratories	Sarah Schmedes, Jason Blanton
EPI_ISL_936632	Northwestern Memorial Hospital	Ozer Lab	Ramon Lorenzo-Redondo, Lacy M. Simons, Chad J. Achenbach, Lawrence J. Jennings, Michael G. Ison, Judd F. Hultquist, Egon A. Ozer
EPI_ISL_940153, EPI_ISL_940154	NHLS Universitas Academic	UFS Virology	PA Bester, MM Nyaga, P Nthiga, MT Mogotsi, D Goedhals, T de Oliveira
EPI_ISL_940259, EPI_ISL_940262, EPI_ISL_940263, EPI_ISL_940275	Hôpital Bichat Claude Bernard, Laboratoire de Virologie	IAME UMR1137 Inserm, Université de Paris, Hôpital Bichat	Antoine Bridier-Nahmias, Amélie Recoing, Quentin Le Hingrat, Lena Daniel, Siham Hamri, Gilles Collin, Alexandre Storto, Mélanie Bertine, Charlotte Charpentier, Nadhira Houhou-Fidouh, Diane Descamps, Benoit Visseaux
EPI_ISL_940694, EPI_ISL_940699, EPI_ISL_940702, EPI_ISL_940707	National Laboratory for Health, Environment and Food	National Laboratory for Health, Environment and Food	Aleksander Mahnic, Sandra Janezic, Maja Rupnik
EPI_ISL_940830, EPI_ISL_940831, EPI_ISL_941918, EPI_ISL_941919, EPI_ISL_941920	Virginia DCLS	Virginia DCLS	Virginia DCLS
EPI_ISL_942097, EPI_ISL_942098, EPI_ISL_942099, EPI_ISL_942100, EPI_ISL_942101, EPI_ISL_942102, EPI_ISL_942103, EPI_ISL_942104, EPI_ISL_942105, EPI_ISL_942106, EPI_ISL_942107, EPI_ISL_942108, EPI_ISL_942120, EPI_ISL_942127, EPI_ISL_942143, EPI_ISL_942144, EPI_ISL_942145, EPI_ISL_942146			
see above	Wisconsin State Laboratory of Hygiene Communicable Disease Division	Wisconsin State Laboratory of Hygiene Communicable Disease Division	Kelsey R. Florek, Abigail C. Shockey
EPI_ISL_942544, EPI_ISL_942545, EPI_ISL_942546, EPI_ISL_942547, EPI_ISL_942548, EPI_ISL_942549, EPI_ISL_942550, EPI_ISL_942551, EPI_ISL_942552, EPI_ISL_942553, EPI_ISL_942554, EPI_ISL_942555, EPI_ISL_942556, EPI_ISL_942557, EPI_ISL_942558, EPI_ISL_942559, EPI_ISL_942560, EPI_ISL_942561, EPI_ISL_942562, EPI_ISL_942563, EPI_ISL_942564, EPI_ISL_942565, EPI_ISL_942566, EPI_ISL_942567, EPI_ISL_942568, EPI_ISL_942569, EPI_ISL_942570, EPI_ISL_942571, EPI_ISL_942572, EPI_ISL_942573, EPI_ISL_942574, EPI_ISL_942575, EPI_ISL_942576, EPI_ISL_942577, EPI_ISL_942578, EPI_ISL_942579, EPI_ISL_942580, EPI_ISL_942581, EPI_ISL_942582			
see above	Gundersen Molecular Diagnostics Laboratory	Kabara Cancer Research Institute	Craig S. Richmond, Paraic A. Kenny
EPI_ISL_944596	Connecticut Department of Public Health	Grubaugh Lab - Yale School of Public Health	Joseph Fauver, Tara Alpert, Anderson Brito, Mallery Breban, Anne Wyllie, Chantal Vogels, Mary Petrone, Annie Watkins, Chaney Kalinich, Isabel Ott, Nathan Grubaugh
EPI_ISL_947275	RS Umum Mulia Pajajaran	Eijkman Institute for Molecular Biology, Ministry of Research and Technology/National Agency for Research and Innovation	Frilasita A Yudhaputri, Hidayat Trimarsanto, Iskandar Adnan, Lydia V. Panggalo, Sukma Oktavianthi, Willy Agustine, Edison Johar, Safarina G Malik, Khin Saw Myint, Amin Soebandrio
EPI_ISL_947278	RS Umum Ciputra Hospital Citragarden City	Eijkman Institute for Molecular Biology, Ministry of Research and Technology/National Agency for Research and Innovation	Hidayat Trimarsanto, Iskandar Adnan, Lydia V. Panggalo, Sukma Oktavianthi, Willy Agustine, Edison Johar, Frilasita A Yudhaputri, Safarina G Malik, Khin Saw Myint, Amin Soebandrio
EPI_ISL_947281	RSU Medika Dramaga	Eijkman Institute for Molecular Biology, Ministry of Research and Technology/National Agency for Research and Innovation	Hidayat Trimarsanto, Iskandar Adnan, Lydia V. Panggalo, Sukma Oktavianthi, Willy Agustine, Edison Johar, Frilasita A Yudhaputri, Safarina G Malik, Khin Saw Myint, Amin Soebandrio
EPI_ISL_947287	RSIA PKU Muhammadiyah	Eijkman Institute for Molecular Biology, Ministry of Research and Technology/National Agency for Research and Innovation	Hidayat Trimarsanto, Iskandar Adnan, Lydia V. Panggalo, Sukma Oktavianthi, Willy Agustine, Edison Johar, Frilasita A Yudhaputri, Safarina G Malik, Khin Saw Myint, Amin Soebandrio
EPI_ISL_947290	RS Hermina Bitung	Eijkman Institute for Molecular Biology, Ministry of Research and Technology/National Agency for Research and Innovation	Hidayat Trimarsanto, Iskandar Adnan, Lydia V. Panggalo, Sukma Oktavianthi, Willy Agustine, Edison Johar, Frilasita A Yudhaputri, Safarina G Malik, Khin Saw Myint, Amin Soebandrio
EPI_ISL_949391	University of Birmingham	COVID-19 Genomics UK (COG-UK) Consortium	Institute of Microbiology, University of Birmingham: Claire McMurray, Joanne Stockton, Samuel Nicholls, Radoslaw Poplawski, Will Rowe, Josh Quick, Nicholas Loman. University of Birmingham Testing Laboratory: Celina M Whalley, Andrew Bosworth, Charlotte Poxon, Kasun Wanigasooriya, Oliver Pickles, Mike Kidd, Alex Richter, Andrew D Beggs PHE Heartlands Lab: Husam Osman, Andrew Bosworth. Queen Elizabeth Hospital: Anna Casey
EPI_ISL_949631, EPI_ISL_949633, EPI_ISL_949635, EPI_ISL_949636	West of Scotland Specialist Virology Centre, NHSGGC / MRC-University of Glasgow Centre for Virus Research	COVID-19 Genomics UK (COG-UK) Consortium	Ana da Silva Filipe, Natasha Johnson, Kathy Smollett, Daniel Mair, Stephen Carmichael, Alice Broos, Lily Tong, Jenna Nichols, Kyriaki Nomikou; Sarah McDonald; Richard Orton, Joseph Hughes, Sreenu Vattipally, David L Robertson; Alasdair MacLean, Rory Gunson; Sharif Shaaban, Matthew Holden; Rachel Blacow, Guy Mollett, Kathy Li, James Shepherd, Antonia Ho, Emma Thomson
EPI_ISL_955511, EPI_ISL_955523, EPI_ISL_955524, EPI_ISL_955525, EPI_ISL_955526, EPI_ISL_955527, EPI_ISL_955528, EPI_ISL_955529, EPI_ISL_955530, EPI_ISL_955531, EPI_ISL_955532, EPI_ISL_955534, EPI_ISL_955535, EPI_ISL_955536, EPI_ISL_955537, EPI_ISL_955538, EPI_ISL_955539, EPI_ISL_955540, EPI_ISL_955541, EPI_ISL_955542, EPI_ISL_955543, EPI_ISL_955544, EPI_ISL_955545, EPI_ISL_955546, EPI_ISL_955547, EPI_ISL_955548, EPI_ISL_955549, EPI_ISL_955550, EPI_ISL_955551, EPI_ISL_955552, EPI_ISL_955553, EPI_ISL_955554, EPI_ISL_955555, EPI_ISL_955556, EPI_ISL_955557, EPI_ISL_955558, EPI_ISL_955559, EPI_ISL_955560			
see above	Humboldt County Public Health Laboratory	Chan-Zuckerberg Biohub	CZB Cliahub Consortium
EPI_ISL_955568, EPI_ISL_955569, EPI_ISL_955570, EPI_ISL_955571	Santa Clara County Public Health Laboratory	Chan-Zuckerberg Biohub	CZB Cliahub Consortium
EPI_ISL_955632	Orange County Public Health Lab	Chan-Zuckerberg Biohub	CZB Cliahub Consortium
EPI_ISL_956323	Laboratory Medicine	Department of Laboratory Medicine, Lin-Kou Chang Gung Memorial Hospital, Taoyuan, Taiwan	Kuo-Chien Tsao, Yu-Nong Gong, Shu-Li Yang, Yi-Chun Liu, Chung-Guei Huang, Mei-Jen Hsiao, Po-Wei Huang, Cheng-Ta Yang, Cheng-Hsun Chiu, Peng-Nien Huang, Kuo-Ming Lee, Guang-Wu Chen, Shin-Ru Shih
EPI_ISL_959494, EPI_ISL_959495, EPI_ISL_959496, EPI_ISL_959529, EPI_ISL_959530, EPI_ISL_959531, EPI_ISL_959532, EPI_ISL_959533, EPI_ISL_959534, EPI_ISL_959535	Division of Emerging Infectious Diseases, Bureau of Infectious Diseases Diagnosis Control, Korea Disease Control and Prevention Agency	Division of Emerging Infectious Diseases, Bureau of Infectious Diseases Diagnosis Control, Korea Disease Control and Prevention Agency	Ae Kyung Park, Il-Hwan Kim, Heui Man Kim, Jeong-Min Kim, Namjoo Lee, Chae Young Lee, Sang Hee Woo, Eun-Jin Kim
EPI_ISL_960097, EPI_ISL_960098	Mitchells Plain Hospital wc MPH	National Health Laboratory Service/UCT	Arash Iranzadeh, Deelan Doolabh, Lynn Tyers, Bruna Galvao, Innocent Mudau, Marvin Hsiao, Kruger Marais, Diana Hardie, Stephen Korsman, Carolyn Williamson
EPI_ISL_960099	Victoria Hospital wc VHW	National Health Laboratory Service/UCT	Arash Iranzadeh, Deelan Doolabh, Lynn Tyers, Bruna Galvao, Innocent Mudau, Marvin Hsiao, Kruger Marais, Diana Hardie, Stephen Korsman, Carolyn Williamson

EPI_ISL_960100	Mitchells Plain Hospital wc MPH	National Health Laboratory Service/UCT	Arash Iranzadeh, Deelan Doolabh, Lynn Tyers, Bruna Galvao, Innocent Mudau, Marvin Hsiao, Kruger Marais, Diana Hardie, Stephen Korsman, Carolyn Williamson
EPI_ISL_960101	Victoria Hospital wc VHW	National Health Laboratory Service/UCT	Arash Iranzadeh, Deelan Doolabh, Lynn Tyers, Bruna Galvao, Innocent Mudau, Marvin Hsiao, Kruger Marais, Diana Hardie, Stephen Korsman, Carolyn Williamson
EPI_ISL_960102, EPI_ISL_960103	Groote Schuur Hospital wc GSH	National Health Laboratory Service/UCT	Arash Iranzadeh, Deelan Doolabh, Lynn Tyers, Bruna Galvao, Innocent Mudau, Marvin Hsiao, Kruger Marais, Diana Hardie, Stephen Korsman, Carolyn Williamson
EPI_ISL_960104	Mowbray Maternity Hospital wc MMH	National Health Laboratory Service/UCT	Arash Iranzadeh, Deelan Doolabh, Lynn Tyers, Bruna Galvao, Innocent Mudau, Marvin Hsiao, Kruger Marais, Diana Hardie, Stephen Korsman, Carolyn Williamson
EPI_ISL_960105	False Bay Hospital wc FBH	National Health Laboratory Service/UCT	Arash Iranzadeh, Deelan Doolabh, Lynn Tyers, Bruna Galvao, Innocent Mudau, Marvin Hsiao, Kruger Marais, Diana Hardie, Stephen Korsman, Carolyn Williamson
EPI_ISL_960106	Red Cross Children's Hospital wc RXH	National Health Laboratory Service/UCT	Arash Iranzadeh, Deelan Doolabh, Lynn Tyers, Bruna Galvao, Innocent Mudau, Marvin Hsiao, Kruger Marais, Diana Hardie, Stephen Korsman, Carolyn Williamson
EPI_ISL_960107	Groote Schuur Hospital wc GSH	National Health Laboratory Service/UCT	Arash Iranzadeh, Deelan Doolabh, Lynn Tyers, Bruna Galvao, Innocent Mudau, Marvin Hsiao, Kruger Marais, Diana Hardie, Stephen Korsman, Carolyn Williamson
EPI_ISL_960108	Victoria Hospital wc VHW	National Health Laboratory Service/UCT	Arash Iranzadeh, Deelan Doolabh, Lynn Tyers, Bruna Galvao, Innocent Mudau, Marvin Hsiao, Kruger Marais, Diana Hardie, Stephen Korsman, Carolyn Williamson
EPI_ISL_960109	Groote Schuur Hospital wc GSH	National Health Laboratory Service/UCT	Arash Iranzadeh, Deelan Doolabh, Lynn Tyers, Bruna Galvao, Innocent Mudau, Marvin Hsiao, Kruger Marais, Diana Hardie, Stephen Korsman, Carolyn Williamson
EPI_ISL_960110	False Bay Hospital wc FBH	National Health Laboratory Service/UCT	Arash Iranzadeh, Deelan Doolabh, Lynn Tyers, Bruna Galvao, Innocent Mudau, Marvin Hsiao, Kruger Marais, Diana Hardie, Stephen Korsman, Carolyn Williamson
EPI_ISL_960111	Pelican Park CDC wc PAX	National Health Laboratory Service/UCT	Arash Iranzadeh, Deelan Doolabh, Lynn Tyers, Bruna Galvao, Innocent Mudau, Marvin Hsiao, Kruger Marais, Diana Hardie, Stephen Korsman, Carolyn Williamson
EPI_ISL_961364	Toronto Invasive Bacterial Diseases Network	McMaster University	Allison McGeer, Patryk Aftanas, Hooman Derakhshani, Angel Li, Kuganya Nirmalarajah, Emily Panousis, Ahmed Draia, Jalees Nasir, Michael Surette, Samira Mubareka, Andrew G. McArthur
EPI_ISL_962263	Seattle Flu Study	Seattle Flu Study	Deborah A. Nickerson, Chris D. Frazar, Jover Lee, Benjamin Pelle, Erica Ryke, Matthew Richardson, Amanda Adler, Elisabeth Brandstetter, Peter D. Han, Kairsten Fay, Misja Ilcisin, Kirsten Lacombe, Thomas R. Sibley, Melissa Truong, Caitlin R. Wolf, Karen Cowgill, Stephanie Schrag, Jeff Duchin, Michael Boeckh, Janet A. Englund, Michael Famulare, Barry R. Lutz, Mark J. Rieder, Lea M. Starita, Matthew Thompson, Helen Y. Chu, Trevor Bedford, Jay Shendure
EPI_ISL_962522	Tokyo Metropolitan Institute of Public Health	Tokyo Metropolitan Institute of Public Health	Masaki Hayashi, Takako Yamazaki, Mami Nagashima,Takushi Fujiwara, Takashi Chiba, Kenji Sadamasu
EPI_ISL_962609, EPI_ISL_962614, EPI_ISL_962616, EPI_ISL_962617	San Diego County Public Health Laboratory	Andersen lab at Scripps Research	SEARCH Alliance San Diego with Tracy Basler, Jovan Shephard, Brett Austin
EPI_ISL_965553, EPI_ISL_965577, EPI_ISL_965580, EPI_ISL_965590, EPI_ISL_965639, EPI_ISL_965688, EPI_ISL_965689, EPI_ISL_965705, EPI_ISL_965714, EPI_ISL_965769, EPI_ISL_965799			
see above	Dutch COVID-19 response team	Medical Microbiology, Maastricht University Medical Centre	Jozef Dingemans*, Brian van der Veer*, Erik Beuken, Carmen Reumkens, Lieke van Alphen, Christian Hoebe, Paul Savelkoul
EPI_ISL_971260, EPI_ISL_972244, EPI_ISL_973148	Department of Virus and Microbiological Special Diagnostics, Statens Serum Institut, Copenhagen, Denmark	Aalborg University	Danish Covid-19 Genome Consortium
EPI_ISL_975973, EPI_ISL_975974, EPI_ISL_975975, EPI_ISL_975976, EPI_ISL_975977, EPI_ISL_975978, EPI_ISL_975979, EPI_ISL_975980, EPI_ISL_975981, EPI_ISL_975982, EPI_ISL_975983, EPI_ISL_975984, EPI_ISL_975985, EPI_ISL_975986, EPI_ISL_975987, EPI_ISL_975988, EPI_ISL_975989, EPI_ISL_975990, EPI_ISL_975991, EPI_ISL_975992, EPI_ISL_975993, EPI_ISL_975994, EPI_ISL_975995, EPI_ISL_975996, EPI_ISL_975997, EPI_ISL_975998, EPI_ISL_975999, EPI_ISL_976000, EPI_ISL_976001, EPI_ISL_976002, EPI_ISL_976003, EPI_ISL_976004, EPI_ISL_976005, EPI_ISL_976006, EPI_ISL_976007, EPI_ISL_976008, EPI_ISL_976009, EPI_ISL_976010, EPI_ISL_976011, EPI_ISL_976012, EPI_ISL_976013, EPI_ISL_976014, EPI_ISL_976015, EPI_ISL_976016, EPI_ISL_976017, EPI_ISL_976018, EPI_ISL_976019, EPI_ISL_976020, EPI_ISL_976021, EPI_ISL_976022, EPI_ISL_976023, EPI_ISL_976024, EPI_ISL_976025, EPI_ISL_976026, EPI_ISL_976027, EPI_ISL_976028, EPI_ISL_976029, EPI_ISL_976030, EPI_ISL_976031, EPI_ISL_976032, EPI_ISL_976033, EPI_ISL_976034, EPI_ISL_976035, EPI_ISL_976036, EPI_ISL_976037, EPI_ISL_976038, EPI_ISL_976039, EPI_ISL_976040, EPI_ISL_976041, EPI_ISL_976042, EPI_ISL_976043, EPI_ISL_976044, EPI_ISL_976045, EPI_ISL_976046, EPI_ISL_976047, EPI_ISL_976048, EPI_ISL_976049, EPI_ISL_976050, EPI_ISL_976051, EPI_ISL_976052, EPI_ISL_976053, EPI_ISL_976054, EPI_ISL_976055, EPI_ISL_976056, EPI_ISL_976057, EPI_ISL_976058, EPI_ISL_976059, EPI_ISL_976060, EPI_ISL_976061, EPI_ISL_976062, EPI_ISL_976063			
see above	BCCDC Public Health Laboratory	BCCDC Public Health Laboratory	Prystajecky Natalie, Linda Hoang, Dan Fornika, John Tyson, Shannon Russell, Kim Macdonald, Kimia Kamelian, Ana Pacagnella, Corrinne Ng, Loretta Janz, Robert Azana Terry Snutch, Mel Krajden
EPI_ISL_977204	ULSS 7 Pedemontana - Distretto 1	Istituto Zooprofilattico Sperimentale delle Venezie	Adelaide Milani, Alessia Schivo, Annalisa Salviato, Erika Giorgia Quaranta, Ambra Pastori, Bianca Zecchin, Alice Fusaro, Isabella Monne, Calogero Terregino, Antonia Ricci
EPI_ISL_977352, EPI_ISL_977353, EPI_ISL_977354	University of Zambia, School of Veterinary Medicine	UNZAVET and PATH	Mulenga Mwenda-Chimfwembe, Ngonda Saasa, Daniel Bridges
EPI_ISL_977999, EPI_ISL_978000, EPI_ISL_978002, EPI_ISL_978004	Chiu Laboratory, University of California, San Francisco	Chiu Laboratory, University of California, San Francisco	Charles Chiu, Xianding (Wayne) Deng, Candace Wang, Venice Servellita, Jill Hacker, Debra Wadford
EPI_ISL_979247	Institute of Microbiology and Immunology, Faculty of Medicine, University of Ljubljana	Institute of Microbiology and Immunology, Faculty of Medicine, University of Ljubljana	Samo Zakotnik, Tomaž Mark Zorec, Matic Brvar, Doroteja Vlaj, Patricija Pozvek,Špela Pleh, Miša Korva, Mario Poljak, Tatjana Avši - Županc
EPI_ISL_979716, EPI_ISL_979717	Humboldt County Public Health Laboratory	Chan-Zuckerberg Biohub	CZB Cliahub Consortium
EPI_ISL_981850, EPI_ISL_981851, EPI_ISL_981852, EPI_ISL_981853, EPI_ISL_981854, EPI_ISL_981855, EPI_ISL_981856, EPI_ISL_981857, EPI_ISL_981858, EPI_ISL_981859, EPI_ISL_981860, EPI_ISL_981861, EPI_ISL_981862, EPI_ISL_981863			
see above	Microbiology Service, Hospital Universitario Clinico San Cecilio, Granada	Microbiology Service, Hospital Universitario Clinico San Cecilio, Granada	Adolfo de Salazar, Natalia Chueca, Laura Viñuela, Ana Fuentes, Federico García
EPI_ISL_982116, EPI_ISL_982117, EPI_ISL_982119, EPI_ISL_982122, EPI_ISL_982124, EPI_ISL_982126, EPI_ISL_982129, EPI_ISL_982150, EPI_ISL_982151, EPI_ISL_982152, EPI_ISL_982157, EPI_ISL_982160, EPI_ISL_982163, EPI_ISL_982167, EPI_ISL_982168, EPI_ISL_982172, EPI_ISL_982173, EPI_ISL_982184, EPI_ISL_982185, EPI_ISL_982186, EPI_ISL_982187, EPI_ISL_982188, EPI_ISL_982189, EPI_ISL_982190, EPI_ISL_982191, EPI_ISL_982192, EPI_ISL_982193, EPI_ISL_982194, EPI_ISL_982195, EPI_ISL_982196, EPI_ISL_982197, EPI_ISL_982198, EPI_ISL_982199			
see above	Hôpital Henri Mondor	Department of Virology, Henri Mondor University Hospital, Assistance Publique Hôpitaux de Paris, Université Paris-Est Créteil, INSERM U955	Christophe Rodriguez, Slim Fourati, Vanessa Demontant, Guillaume Gricourt, Melissa N'Debi, Alexandre Soulier, Elisabeth Trawinski, Jean-Michel Pawlotsky
EPI_ISL_982776, EPI_ISL_982777	Kentucky State Public Health Lab	Kentucky State Public Health Lab	Stephanie Lunn, Karim George, Joshua Tobias, William Grooms, Vaneet Arora, Matthew Johnson, Rachel Zinner, Rhonda Lucas
EPI_ISL_983861, EPI_ISL_983862	Colorado Department of Public Health and Environment	Colorado Department of Puplic Health and Environment	Laura Bankers, Molly C. Hetherington-Rauth, Diana Ir, Shannon Ely, Shannon R. Matzinger, Sarah Elizabeth Totten, Emily A. Travanty
