# Supplementary material for: Detection and characterization of the SARS-CoV-2 lineage B.1.526 in New York
Source: Nat Commun. 2021 Aug 9;12:4886. doi: 10.1038/s41467-021-25168-4 (PMC8352861; doi:10.1038/s41467-021-25168-4)
Supplement: Supplementary file 8 — Supplementary Data 4 [file 41467_2021_25168_MOESM8_ESM.zip › GISAID_acknowledements_tables/gisaid_hcov-19_acknowledgement_table_2021_02_12_16-7.pdf]

We gratefully acknowledge the following Authors from the Originating laboratories responsible for obtaining the specimens, as well as the Submitting laboratories where the genome data were generated and shared via GISAID, on which this research is based.

All Submitters of data may be contacted directly via www.gisaid.org

Authors are sorted alphabetically.

Accession ID	Originating Laboratory	Submitting Laboratory	Authors
EPI_ISL_420144	Department for Virology, Molecular Biology and Genome Research, R. G. Lugar Center for Public Health Research, National Center for Disease Control and Public Health (NCDC) of Georgia.	Department for Virology, Molecular Biology and Genome Research, R. G. Lugar Center for Public Health Research, National Center for Disease Control and Public Health (NCDC) of Georgia.	Gvantsa Chanturia, Ann Machablishvili, Nato Kotaria, Marine Murtskhvaladze, Lela Sabadze, Mari Gavashelidze, Ana Papkiauri, Meri Pantsulaia, Gvantsa Brachveli, Tata Imnadze, Tamar Jashiashvili, Tea Tevdoradze, Ketevan Sidamonidze, Ekaterine Khmaladze, Ekaterine Zhghenti, Roena Sukhiashvili, Mariam Zakalashvili, Lela Urushadze, Magda Dgebuadze, Giorgi Tomashvili, Davit Tsaguria, Ekaterine Zangaladze, Nino Berishvili, Adam Kotorashvili, Maia Alkhazashvili, Irma Burjanadze, Anna Kasradze, Khatuna Zakhashvili, Paata Imnadze, Amiran Gamkrelidze.
EPI_ISL_420769	Respiratory Virus Unit, Microbiology Services Colindale, Public Health England	Respiratory Virus Unit, Microbiology Services Colindale, Public Health England	Monica Galiano, Shahjahan Miah, Angie Lackenby, Omolola Akinbami, Tiina Talts, Leena Bhaw, Richard Myers, Steven Platt, Kirstin Edwards, Jonathan Hubb, Joanna Ellis, Maria Zambon
EPI_ISL_421184, EPI_ISL_421190, EPI_ISL_421193, EPI_ISL_421197, EPI_ISL_421199, EPI_ISL_421200, EPI_ISL_421201, EPI_ISL_421204, EPI_ISL_421205, EPI_ISL_421206, EPI_ISL_421207, EPI_ISL_421209, EPI_ISL_421210, EPI_ISL_421211, EPI_ISL_421212, EPI_ISL_421213			
see above	Department of Clinical Microbiology	GIGA Medical Genomics	Keith Durkin, Maria Artesi, Sébastien Bontems, Raphaël Boreux, Cécile Meex, Pierrette Melin, Marie-Pierre Hayette, Vincent Bours.
EPI_ISL_421573, EPI_ISL_421574, EPI_ISL_421575, EPI_ISL_421576	Molecular Diagnostic Services	KRISP, KZN Research Innovation and Sequencing Platform	Giandhari J, Pillay S, Ngcapu S, Samsunder N, Lessells R, Chimukangara B, Deforche K, Tegally H, Wilkinson E, de Oliveira T
EPI_ISL_421660	The Ohio State University	The Ohio State University-James Molecular Lab at Polaris	Huolin Tu, Matthew Avenarius, Preeti Panchioli, Sean Caruthers, Joan-Miquel Balada-Llasat, Jason Garee, Matt Hunt, Xiaokang Pan, Dan Jones
EPI_ISL_421675	The Ohio State University Wexner Medical Center	The Ohio State University James Molecular lab	Huolin Tu, Sean Caruthers, Matthew Avenarius, Joan-Miquel Balada-Llasat, Matthew Hunt, Preeti Panchioli, Xiaokang Pen, Jason Garee, Pam Snyder, Dan Jones
EPI_ISL_421931, EPI_ISL_421932, EPI_ISL_421933, EPI_ISL_421934, EPI_ISL_421935, EPI_ISL_421936, EPI_ISL_421939, EPI_ISL_421941, EPI_ISL_421942, EPI_ISL_421943, EPI_ISL_421944, EPI_ISL_421945, EPI_ISL_421946, EPI_ISL_421947, EPI_ISL_421948, EPI_ISL_421949, EPI_ISL_421951, EPI_ISL_421952, EPI_ISL_421953, EPI_ISL_421956, EPI_ISL_421958, EPI_ISL_421959, EPI_ISL_421960, EPI_ISL_421961, EPI_ISL_421962, EPI_ISL_421963, EPI_ISL_421964, EPI_ISL_421965, EPI_ISL_421966, EPI_ISL_421967, EPI_ISL_421968, EPI_ISL_421969, EPI_ISL_421970, EPI_ISL_421971, EPI_ISL_421972, EPI_ISL_421973, EPI_ISL_421975, EPI_ISL_421977, EPI_ISL_421978, EPI_ISL_421979, EPI_ISL_421980, EPI_ISL_421981, EPI_ISL_421982, EPI_ISL_421983, EPI_ISL_421984, EPI_ISL_421985, EPI_ISL_421987, EPI_ISL_421988, EPI_ISL_421990, EPI_ISL_421991, EPI_ISL_421992, EPI_ISL_421993, EPI_ISL_421994, EPI_ISL_421995, EPI_ISL_421996, EPI_ISL_421997, EPI_ISL_421999, EPI_ISL_422003, EPI_ISL_422004, EPI_ISL_422005, EPI_ISL_422007			
see above	Respiratory Virus Unit, Microbiology Services Colindale, Public Health England	Respiratory Virus Unit, Microbiology Services Colindale, Public Health England	Monica Galiano, Shahjahan Miah, Angie Lackenby, Omolola Akinbami, Tiina Talts, Leena Bhaw, Richard Myers, Steven Platt, Kirstin Edwards, Jonathan Hubb, Joanna Ellis, Maria Zambon
EPI_ISL_422105	Wales Specialist Virology Centre	Public Health Wales Microbiology Cardiff	Catherine Moore, Johnathan Evans, Malorie Perry, Simon Cottrell, Alec Birchley, Alexander Adams, Amy Gaskin, Bree Gatica-Wilcox, Jason Coombes, Lauren Gilbert, Lee Graham, Nicole Pacchiarini, Sara Kumziene-Summerhayes, Sarah Taylor, Sophie Jones, Sara Rey, Matthew Bull, Joanne Watkins, Sally Corden, Tom Connor
EPI_ISL_422401	NMIMR, Department of Virology	WACCBIP, University of Ghana	Joyce M. Ngoi, Bright Adu, Collins M. Morang'a, Selassie Kumordjie, Miriam Eshun, Linda Boatemaa, Vanessa Magnussen, Erasmus Kotey, Fred Tei-Maya, Dominic S. Y. Amuzu, Peter Quashie, Augustina Arjarquah, Ivy Asante, Evelyn Bonney, George B. Kyei, Kofi Bonney, Abraham Kwabena Anang, Gordon A. Awandare, William Ampofo
EPI_ISL_422431, EPI_ISL_422432, EPI_ISL_422433, EPI_ISL_422434, EPI_ISL_422435	National Public Health Laboratory, National Centre for Infectious Diseases	National Public Health Laboratory, National Centre for Infectious Diseases	Mak TM, Octavia S, Cui L, Lin RTP
EPI_ISL_422453	Gundersen Molecular Diagnostics Laboratory	Kabara Cancer Research Institute	Craig S. Richmond & Paraic A. Kenny
EPI_ISL_422567, EPI_ISL_422587, EPI_ISL_422588, EPI_ISL_422589, EPI_ISL_422590, EPI_ISL_422591, EPI_ISL_422592, EPI_ISL_422593, EPI_ISL_422594, EPI_ISL_422595, EPI_ISL_422596, EPI_ISL_422597, EPI_ISL_422598, EPI_ISL_422599, EPI_ISL_422600, EPI_ISL_422601, EPI_ISL_422602, EPI_ISL_422603, EPI_ISL_422604, EPI_ISL_422605, EPI_ISL_422606, EPI_ISL_422607, EPI_ISL_422608, EPI_ISL_422609, EPI_ISL_422610, EPI_ISL_422611, EPI_ISL_422612, EPI_ISL_422613, EPI_ISL_422614, EPI_ISL_422615, EPI_ISL_422628, EPI_ISL_422629, EPI_ISL_422630, EPI_ISL_422631, EPI_ISL_422633, EPI_ISL_422634, EPI_ISL_422635			
see above	Dutch COVID-19 response team	Erasmus Medical Center	Bas Oude Munnink, David Nieuwenhuijse, Reina Sikkema, Claudia Schapendonk, Irina Chestakova, Anne van der Linden, Theo Bestebroer, Stefan van Nieuwkoop, Mark Pronk, Pascal Lexmond, Corien Swaan, Manon Haverkate, Madelief Mollers, Mart Stein, Sandra Kengne Kamga Mobou, Jeroen van Kampen, Jolanda Voermans, Aura Timen, Corine GeurtsvanKessel, Annemiek van der Eijk, Richard Molenkamp, Marion Koopmans, on behalf of the Dutch national COVID-19 response team.
EPI_ISL_423427, EPI_ISL_423429, EPI_ISL_423430, EPI_ISL_423448, EPI_ISL_423454, EPI_ISL_423469, EPI_ISL_423472, EPI_ISL_423473, EPI_ISL_423474, EPI_ISL_423479, EPI_ISL_423480, EPI_ISL_423481, EPI_ISL_423482, EPI_ISL_423483, EPI_ISL_423484, EPI_ISL_423485, EPI_ISL_423486, EPI_ISL_423487, EPI_ISL_423488, EPI_ISL_423489, EPI_ISL_423490, EPI_ISL_423491, EPI_ISL_423492, EPI_ISL_423493, EPI_ISL_423494, EPI_ISL_423497, EPI_ISL_423498, EPI_ISL_423499, EPI_ISL_423500, EPI_ISL_423501, EPI_ISL_423502, EPI_ISL_423503, EPI_ISL_423504, EPI_ISL_423508, EPI_ISL_423509, EPI_ISL_423510, EPI_ISL_423511, EPI_ISL_423512, EPI_ISL_423534, EPI_ISL_423535, EPI_ISL_423536, EPI_ISL_423537, EPI_ISL_423538, EPI_ISL_423539, EPI_ISL_423540, EPI_ISL_423541, EPI_ISL_423545, EPI_ISL_423546, EPI_ISL_423547, EPI_ISL_423548, EPI_ISL_423549, EPI_ISL_423550, EPI_ISL_423551, EPI_ISL_423552, EPI_ISL_423555, EPI_ISL_423557, EPI_ISL_423558, EPI_ISL_423559, EPI_ISL_423560, EPI_ISL_423561, EPI_ISL_423562, EPI_ISL_423563, EPI_ISL_423564, EPI_ISL_423565, EPI_ISL_423566, EPI_ISL_423567, EPI_ISL_423568, EPI_ISL_423569, EPI_ISL_423570, EPI_ISL_423572, EPI_ISL_423573, EPI_ISL_423574, EPI_ISL_423575, EPI_ISL_423576, EPI_ISL_423577, EPI_ISL_423578, EPI_ISL_423579, EPI_ISL_423582, EPI_ISL_423583, EPI_ISL_423584			
see above	Respiratory Virus Unit, Microbiology Services Colindale, Public Health England	Respiratory Virus Unit, Microbiology Services Colindale, Public Health England	Monica Galiano, Shahjahan Miah, Angie Lackenby, Omolola Akinbami, Tiina Talts, Leena Bhaw, Richard Myers, Steven Platt, Kirstin Edwards, Jonathan Hubb, Joanna Ellis, Maria Zambon
EPI_ISL_424929, EPI_ISL_424930, EPI_ISL_424931, EPI_ISL_424932, EPI_ISL_424933, EPI_ISL_424934, EPI_ISL_424935, EPI_ISL_424936, EPI_ISL_424937, EPI_ISL_424938, EPI_ISL_424939, EPI_ISL_424940, EPI_ISL_424941, EPI_ISL_424942, EPI_ISL_424943, EPI_ISL_424944, EPI_ISL_424945, EPI_ISL_424946, EPI_ISL_424947, EPI_ISL_424948, EPI_ISL_424949, EPI_ISL_424950, EPI_ISL_424951, EPI_ISL_424952, EPI_ISL_424953, EPI_ISL_424954, EPI_ISL_424955, EPI_ISL_424956, EPI_ISL_424957, EPI_ISL_424958, EPI_ISL_424959, EPI_ISL_424960, EPI_ISL_424961, EPI_ISL_424962, EPI_ISL_424963, EPI_ISL_424964, EPI_ISL_424965, EPI_ISL_424966, EPI_ISL_424967, EPI_ISL_424968			
see above	NYU Langone Health	Departments of Pathology and Medicine, New York University School of Medicine	Maria Aguero-Rosenfeld, Brendan Belovarac, Margaret Black, Ludovic Boytard, John Cadley, Paolo Cotzia, John Chen, Dacia Dimartino, Xiaojun Feng, Tatyana Gindin, Adriana Heguy, Megan Hogan, Emily Huang, George Jour, Andrew Lytle, Christian Marier, Matthew T. Maurano, Mark J. Mulligan, Peter Meyn, Iman Osman, Jared Pinnell, Sitharam Ramaswami, Amy Rapkiewicz, Marie Samanovic-Golden, Antonio Serrano, Guomiao Shen, Matija Snuderl, Theodore Vougiouklakis, Nick Vulpescu, Gael Westby, Paul Zappile, Yutong Zhang
EPI_ISL_425146, EPI_ISL_425148, EPI_ISL_425153, EPI_ISL_425155, EPI_ISL_425158, EPI_ISL_425164, EPI_ISL_425167, EPI_ISL_425168, EPI_ISL_425170, EPI_ISL_425172	University of Wisconsin-Madison AIDS Vaccine Research Laboratories	University of Wisconsin-Madison AIDS Vaccine Research Laboratories	Gage Moreno, Katarina Braun, et al. AIDS Vaccine Research Laboratories
EPI_ISL_425239, EPI_ISL_425240, EPI_ISL_425241, EPI_ISL_425242, EPI_ISL_425243, EPI_ISL_425244, EPI_ISL_425245, EPI_ISL_425246, EPI_ISL_425249, EPI_ISL_425251, EPI_ISL_425252, EPI_ISL_425255, EPI_ISL_425256, EPI_ISL_425262, EPI_ISL_425263, EPI_ISL_425264, EPI_ISL_425265, EPI_ISL_425266, EPI_ISL_425267, EPI_ISL_425268, EPI_ISL_425269, EPI_ISL_425270, EPI_ISL_425271, EPI_ISL_425272, EPI_ISL_425275, EPI_ISL_425276, EPI_ISL_425278, EPI_ISL_425424, EPI_ISL_425426, EPI_ISL_425427, EPI_ISL_425428, EPI_ISL_425432, EPI_ISL_425434, EPI_ISL_425435, EPI_ISL_425436, EPI_ISL_425437, EPI_ISL_425438, EPI_ISL_425441, EPI_ISL_425442, EPI_ISL_425443, EPI_ISL_425444, EPI_ISL_425445, EPI_ISL_425446, EPI_ISL_425448, EPI_ISL_425449, EPI_ISL_425450, EPI_ISL_425451, EPI_ISL_425452, EPI_ISL_425453, EPI_ISL_425454, EPI_ISL_425455, EPI_ISL_425456, EPI_ISL_425457, EPI_ISL_425458, EPI_ISL_425459, EPI_ISL_425460			
see above	Department of Pathology, University of Cambridge	COVID-19 Genomics UK (COG-UK) Consortium	Luke W Meredith, M. Estee Torok , Myra Hosmillo, William L. Hamilton, Martin D. Curran, Theresa Feltwell, Anna Yakovleva, Charlotte J. Houldcroft, Aminu S. Jahun, Sarah L. Caddy, Ian Goodfellow
EPI_ISL_426007, EPI_ISL_426008, EPI_ISL_426009, EPI_ISL_426010, EPI_ISL_426011, EPI_ISL_426012, EPI_ISL_426013, EPI_ISL_426014, EPI_ISL_426015, EPI_ISL_426016, EPI_ISL_426017, EPI_ISL_426018, EPI_ISL_426019, EPI_ISL_426020, EPI_ISL_426021, EPI_ISL_426022, EPI_ISL_426023, EPI_ISL_426024			
see above	Virology Department, Royal Infirmary of Edinburgh, NHS Lothian / School of Biological Sciences, University of	COVID-19 Genomics UK (COG-UK) Consortium	McHugh M, Dewar R, Rooke S, Gallagher M, Balcaza C, O'Toole A, Hill V, McCrone JT, Colquhoun R, Yu X, Jackson B, Scher E, Rambaut A, Williams TC, Templeton K

	Edinburgh / Institute of Genetics and Molecular Medicine, University of Edinburgh		
EPI_ISL_426134, EPI_ISL_426136, EPI_ISL_426137	UW Virology Lab	UW Virology Lab	Pavitra Roychoudhury, Hong Xie, Keith Jerome, Alexander Greninger
EPI_ISL_426464, EPI_ISL_426465, EPI_ISL_426466, EPI_ISL_426467, EPI_ISL_426468, EPI_ISL_426470	Virginia DCLS	Virginia DCLS	Virginia DCLS
EPI_ISL_426556, EPI_ISL_426557	TGen North	TGen North	Jolene Bowers, Megan Folkerts, Darrin Lemmer, Dave Engelthaler
EPI_ISL_426561, EPI_ISL_426562, EPI_ISL_426563, EPI_ISL_426564, EPI_ISL_426565, EPI_ISL_426566, EPI_ISL_426567	AZ SPHL, Arizona Department of Health Services	TGen North	Jolene Bowers, Megan Folkerts, Darrin Lemmer, Dave Engelthaler
EPI_ISL_426617, EPI_ISL_426618, EPI_ISL_426619, EPI_ISL_426620, EPI_ISL_426621, EPI_ISL_426622, EPI_ISL_426623, EPI_ISL_426624, EPI_ISL_426625, EPI_ISL_426626	NYU Langone Health	Departments of Pathology and Medicine, New York University School of Medicine	Maria Aguero-Rosenfeld, Brendan Belovarac, Margaret Black, Ludovic Boytard, John Cadley, Paolo Cotzia, John Chen, Dacia Dimartino, Xiaojun Feng, Tatyana Gindin, Emily Guzman, Adriana Heguy, Megan Hogan, Emily Huang, George Jour, Andrew Lytle, Christian Marier, Matthew T. Maurano, Mark J. Mulligan, Peter Meyn, Iman Osman, Jared Pinnell, Vanessa Raabe, Sitharam Ramaswami, Amy Rapkiewicz, Marie Samanovic-Golden, Antonio Serrano, Guomiao Shen, Matija Snuderl, Theodore Vougiouklakis, Nick Vulpescu, Gael Westby, Paul Zappile, Yutong Zhang
EPI_ISL_426887, EPI_ISL_426888	Motol University Hospital	Institute of Applied Biotechnologies a.s.	Petr Brož, Jan Geryk, Petr Klempt, Martin Kašný, Adam Novotný, Kateina Kvapilová, Pavel Devínek, Petr Kvapil, Milan Macek
EPI_ISL_427025, EPI_ISL_427027, EPI_ISL_427034, EPI_ISL_427053	Victorian Infectious Diseases Reference Laboratory (VIDRL)	Microbiological Diagnostic Unit Public Health Laboratory and Victorian Infectious Diseases Reference Laboratory, Doherty Institute	Caly L., Seemann T., Sait, M., Schultz M., Druce J., Sherry, N.
EPI_ISL_427063, EPI_ISL_427064, EPI_ISL_427065, EPI_ISL_427066, EPI_ISL_427067, EPI_ISL_427068, EPI_ISL_427069, EPI_ISL_427070, EPI_ISL_427071, EPI_ISL_427072, EPI_ISL_427073, EPI_ISL_427074, EPI_ISL_427075, EPI_ISL_427076, EPI_ISL_427077, EPI_ISL_427078			
see above	Microbiological Diagnostic Unit Public Health Laboratory	Microbiological Diagnostic Unit Public Health Laboratory	Seemann T., Schultz M., Sait, M., Sherry, N.
EPI_ISL_427084, EPI_ISL_427160	Victorian Infectious Diseases Reference Laboratory (VIDRL)	Microbiological Diagnostic Unit Public Health Laboratory and Victorian Infectious Diseases Reference Laboratory, Doherty Institute	Caly L., Seemann T., Sait, M., Schultz M., Druce J., Sherry, N.
EPI_ISL_427163, EPI_ISL_427164, EPI_ISL_427173, EPI_ISL_427222, EPI_ISL_427223, EPI_ISL_427238, EPI_ISL_427239, EPI_ISL_427240, EPI_ISL_427241, EPI_ISL_427242, EPI_ISL_427243, EPI_ISL_427244, EPI_ISL_427245, EPI_ISL_427246, EPI_ISL_427247, EPI_ISL_427248, EPI_ISL_427249, EPI_ISL_427250, EPI_ISL_427251, EPI_ISL_427252, EPI_ISL_427253, EPI_ISL_427254, EPI_ISL_427255, EPI_ISL_427256, EPI_ISL_427257, EPI_ISL_427258, EPI_ISL_427259, EPI_ISL_427260, EPI_ISL_427261, EPI_ISL_427263, EPI_ISL_427265, EPI_ISL_427269, EPI_ISL_427270			
see above	UW Virology Lab	UW Virology Lab	Pavitra Roychoudhury, Hong Xie, Keith Jerome, Alexander Greninger
EPI_ISL_427288	The Ohio State University	The Ohio State University-James Molecular Lab at Polaris	Huolin Tu, Preeti Panchioli, Jason Garee, Matthew Hunt, Joan-Miquel Balada-Llasat, Erica Vincent, Weiqiang Zhao, Dan Jones
EPI_ISL_427289	The Ohio State University	The Ohio State University-James Molecular Lab at Polaris	Huolin Tu, Joan-Miquel Balada-Llasat, Jason Garee, Matthew Hunt, Preeti Pancholi, Erica Vincent, Xiaokang Zhao, Dan Jones
EPI_ISL_427290	The Ohio State University	The Ohio State University-James Molecular Lab at Polaris	Huolin Tu, Jason Garee, Matthew Hunt, Joan-Miquel Balada-Llasat, Preeti Pancholi, Erica Vincent, Rongqin Ren, Dan Jones
EPI_ISL_427291	The Ohio State University	The Ohio State University-James Molecular Lab at Polaris	Huolin Tu, Matthew Hunt, Preeti Pancholi, Jason Garee, Joan-Miquel Balada-Llasat, Erica Vincent, Weiqiang Zhao, Dan Jones
EPI_ISL_427340, EPI_ISL_427342, EPI_ISL_427343, EPI_ISL_427347	Department of Clinical Microbiology	GIGA Medical Genomics	Keith Durkin, Maria Artesi, Sébastien Bontems, Raphaël Boreux, Cécile Meex, Pierrette Melin, Marie-Pierre Hayette, Vincent Bours.
EPI_ISL_427469, EPI_ISL_427470, EPI_ISL_427471, EPI_ISL_427472, EPI_ISL_427473, EPI_ISL_427474, EPI_ISL_427475, EPI_ISL_427476, EPI_ISL_427477, EPI_ISL_427478, EPI_ISL_427479, EPI_ISL_427480, EPI_ISL_427481, EPI_ISL_427482, EPI_ISL_427483, EPI_ISL_427484, EPI_ISL_427485, EPI_ISL_427486, EPI_ISL_427487, EPI_ISL_427488, EPI_ISL_427489, EPI_ISL_427490, EPI_ISL_427491, EPI_ISL_427492, EPI_ISL_427493, EPI_ISL_427494, EPI_ISL_427495, EPI_ISL_427496, EPI_ISL_427497, EPI_ISL_427498, EPI_ISL_427499, EPI_ISL_427500, EPI_ISL_427501, EPI_ISL_427524, EPI_ISL_427525, EPI_ISL_427627			
see above	NYU Langone Health	Departments of Pathology and Medicine, New York University School of Medicine	Maria Aguero-Rosenfeld, Brendan Belovarac, Margaret Black, Ludovic Boytard, John Cadley, Paolo Cotzia, John Chen, Dacia Dimartino, Xiaojun Feng, Tatyana Gindin, Emily Guzman, Adriana Heguy, Megan Hogan, Emily Huang, George Jour, Andrew Lytle, Christian Marier, Matthew T. Maurano, Mark J. Mulligan, Peter Meyn, Iman Osman, Jared Pinnell, Vanessa Raabe, Sitharam Ramaswami, Amy Rapkiewicz, Marie Samanovic-Golden, Antonio Serrano, Guomiao Shen, Matija Snuderl, Theodore Vougiouklakis, Nick Vulpescu, Gael Westby, Paul Zappile, Yutong Zhang
EPI_ISL_428380, EPI_ISL_428387, EPI_ISL_428391	Yale COVID-19 Biorepository	Grubaugh Lab - Yale School of Public Health	Joseph Fauver, Tara Alpert, Anderson Brito, Anne Wyllie, Chantal Vogels, Mary Petrone, Chaney Kalinich, Isabel Ott, Arnau Casanovas, Catherine Muenker, Adam Moore, Alice Lu, Maria Tokuyama, Patrick Wong, Peiwen Lu, Saad Omer, Richard Martinello, Allison Nelson, Shelli Farhadian, Akiko Iwasaki, Charlese Dela Cruz, Albert Ko, Nathan Grubaugh
EPI_ISL_428673	Centre for Dengue Research	Centre for Dengue Research	Chandima Jeewandara, Dinuka Ariyaratne, Laksiri Gomes, Deshni Jayathilaka, Diyanath Ranasinghe, Ananda Wijewickrama, Eranga Narangoda, Damayanthi Idampitiya, Neelika Malavige
EPI_ISL_428728	University of Wisconsin-Madison AIDS Vaccine Research Laboratories	University of Wisconsin-Madison AIDS Vaccine Research Laboratories	Gage Moreno, Katarina Braun, et al. AIDS Vaccine Research Laboratories
EPI_ISL_428878	State Research Center of Virology and Biotechnology VECTOR, Department of Collection of Microorganisms	State Research Center of Virology and Biotechnology VECTOR, Department of Collection of Microorganisms	Sergey A. Bodnev, Oleg V. Pyankov, Tatyana V. Tregubchak, Alexander N. Shvalov, Elena V. Gavrilova, Rinat A. Maksyutov
EPI_ISL_428907, EPI_ISL_428910, EPI_ISL_428911	State Research Center of Virology and Biotechnology VECTOR, Department of Collection of Microorganisms	State Research Center of Virology and Biotechnology VECTOR, Department of Collection of Microorganisms	Oleg V. Pyankov, Sergey A. Bodnev, Tatyana V. Tregubchak, Alexander N. Shvalov, Elena V. Gavrilova, Rinat A. Maksyutov
EPI_ISL_428939, EPI_ISL_428943, EPI_ISL_428944, EPI_ISL_428950, EPI_ISL_428952, EPI_ISL_428955, EPI_ISL_428956, EPI_ISL_428959	Laboratoire National de Sante, Microbiology, Virology	Laboratoire National de Sante, Microbiology, Epidemiology and Microbial Genomics	Anke Wienecke-Baldacchino, Ardashel Latsuzbaia, Jessica Tapp, Catherine Ragimbeau, Guillaume Fournier, Tamir Abdelrahman, Trung Nguyen Nguyen, Joel Mossong
EPI_ISL_429020, EPI_ISL_429023, EPI_ISL_429025, EPI_ISL_429026, EPI_ISL_429055, EPI_ISL_429060, EPI_ISL_429067, EPI_ISL_429072	UCSF Clinical Microbiology Laboratory	Chan-Zuckerberg Biohub	CZB Cliahub Consortium
EPI_ISL_429598, EPI_ISL_429600, EPI_ISL_429605, EPI_ISL_429606, EPI_ISL_429607, EPI_ISL_429608, EPI_ISL_429609, EPI_ISL_429611, EPI_ISL_429648	UW Virology Lab	UW Virology Lab	Pavitra Roychoudhury, Hong Xie, Keith Jerome, Alexander Greninger
EPI_ISL_429717, EPI_ISL_429721, EPI_ISL_429722, EPI_ISL_429731, EPI_ISL_429786, EPI_ISL_429789, EPI_ISL_429794	Laboratoire National de Sante, Microbiology, Virology	Laboratoire National de Sante, Microbiology, Epidemiology and Microbial Genomics	Anke Wienecke-Baldacchino, Ardashel Latsuzbaia, Jessica Tapp, Catherine Ragimbeau, Guillaume Fournier, Tamir Abdelrahman, Trung Nguyen Nguyen, Joel Mossong
EPI_ISL_429989	Virginia DCLS	Virginia DCLS	Virginia DCLS
EPI_ISL_430040, EPI_ISL_430045, EPI_ISL_430050	Utah Public Health Laboratory	Utah Public Health Laboratory	Erin Young, Kelly Oakeson

EPI_ISL_430121, EPI_ISL_430125, EPI_ISL_430130, EPI_ISL_430147, EPI_ISL_430149, EPI_ISL_430153	Seattle Flu Study	Seattle Flu Study	Chu et al
EPI_ISL_430351, EPI_ISL_430352, EPI_ISL_430353	NYU Langone Health	Departments of Pathology and Medicine, New York University School of Medicine	Maria Aguero-Rosenfeld, Brendan Belovarac, Margaret Black, Ludovic Boytard, John Cadley, Paolo Cotzia, John Chen, Dacia Dimartino, Xiaojun Feng, Tatyana Gindin, Emily Guzman, Adriana Heguy, Megan Hogan, Emily Huang, George Jour, Lawrence H. Lin, Raven Luther, Andrew Lytle, Christian Marier, Matthew T. Maurano, Mark J. Mulligan, Peter Meyn, Raquel Ordonez Ciriza, Iman Osman, Jared Pinnell, Vanessa Raabe, Sitharam Ramaswami, Amy Rapkiewicz, Andre M. Ribeiro-dos-Santos, Marie Samanovic-Golden, Antonio Serrano, Guomiao Shen, Matija Snuderl, Theodore Vougiouklakis, Nick Vulpescu, Gael Westby, Paul Zappile, Yutong Zhang
EPI_ISL_430473, EPI_ISL_430474, EPI_ISL_430475, EPI_ISL_430476, EPI_ISL_430477, EPI_ISL_430478, EPI_ISL_430479, EPI_ISL_430480, EPI_ISL_430481, EPI_ISL_430482, EPI_ISL_430483, EPI_ISL_430484, EPI_ISL_430485, EPI_ISL_430486, EPI_ISL_430487, EPI_ISL_430488, EPI_ISL_430489, EPI_ISL_430490, EPI_ISL_430491, EPI_ISL_430492, EPI_ISL_430493, EPI_ISL_430498, EPI_ISL_430499, EPI_ISL_430500, EPI_ISL_430501, EPI_ISL_430502, EPI_ISL_430503, EPI_ISL_430504, EPI_ISL_430505, EPI_ISL_430506, EPI_ISL_430507, EPI_ISL_430508, EPI_ISL_430509, EPI_ISL_430513, EPI_ISL_430514, EPI_ISL_430515, EPI_ISL_430516, EPI_ISL_430517, EPI_ISL_430518, EPI_ISL_430519, EPI_ISL_430522, EPI_ISL_430523, EPI_ISL_430527, EPI_ISL_430544, EPI_ISL_430547, EPI_ISL_430548, EPI_ISL_430551, EPI_ISL_430553, EPI_ISL_430555, EPI_ISL_430556, EPI_ISL_430559, EPI_ISL_430560			
see above	Victorian Infectious Diseases Reference Laboratory (VIDRL)	Microbiological Diagnostic Unit Public Health Laboratory and Victorian Infectious Diseases Reference Laboratory, The Peter Doherty Institute for Infection and Immunity	Caly L., Seemann T., Sait, M., Schultz M., Druce J., Sherry, N.
EPI_ISL_430801	Laboratorio de Virología del Hospital de Niños Dr. Ricardo Gutierrez	Área de Secuenciación del Laboratorio de Virología del Hospital de Niños Dr. Ricardo Gutierrez on behalf of 'Proyecto Argentino Interinstitucional de genomica de SARS-CoV-2' (PAIS Consortium)	Nabaes Jodar, MS; Goya, S; Natale, MI; Lusso, S; Gravis, E; Mistchenko, AS; Valinotto, LE; Viegas, M.
EPI_ISL_430802	Departamento de Biología y genética molecular, IACA Laboratorios.	Área de Secuenciación del Laboratorio de Virología del Hospital de Niños Dr. Ricardo Gutierrez on behalf of 'Proyecto Argentino Interinstitucional de genomica de SARS-CoV-2' (PAIS Consortium)	Nabaes Jodar, MS; Goya, S; Natale, MI; Lusso, S; Tittarelli, E; Suárez, A; Masciovecchio MV; Streitenberger ER; Mistchenko, AS; Valinotto, LE; Viegas, M.
EPI_ISL_430803, EPI_ISL_430804	Laboratorio de Virología del Hospital de Niños Dr. Ricardo Gutierrez	Área de Secuenciación del Laboratorio de Virología del Hospital de Niños Dr. Ricardo Gutierrez on behalf of 'Proyecto Argentino Interinstitucional de genomica de SARS-CoV-2' (PAIS Consortium)	Nabaes Jodar, MS; Goya, S; Natale, MI; Lusso, S; Gravis, E; Mistchenko, AS; Valinotto, LE; Viegas, M.
EPI_ISL_430805, EPI_ISL_430806	Departamento de Biología y genética molecular, IACA Laboratorios.	Área de Secuenciación del Laboratorio de Virología del Hospital de Niños Dr. Ricardo Gutierrez on behalf of 'Proyecto Argentino Interinstitucional de genomica de SARS-CoV-2' (PAIS Consortium)	Nabaes Jodar, MS; Goya, S; Natale, MI; Lusso, S; Tittarelli, E; Suárez, A; Masciovecchio MV; Streitenberger ER; Mistchenko, AS; Valinotto, LE; Viegas, M.
EPI_ISL_430846	General Intensive Care Unit, Raymond Poincaré Hospital (AP-HP), Lab Inflammation & Infection, U1173 University Paris Saclay-UVSQ/INSERM, Garches, France.	Institut Pasteur, Laboratory for Urgent Response to biological Threats	Annane Djillali, Vanhomwegen Jessica, Caro Valérie, Manuguerra Jean-Claude
EPI_ISL_430868, EPI_ISL_430869, EPI_ISL_430873, EPI_ISL_430874, EPI_ISL_430876, EPI_ISL_430899, EPI_ISL_430933, EPI_ISL_430934, EPI_ISL_430935, EPI_ISL_430936, EPI_ISL_430937, EPI_ISL_430938, EPI_ISL_430939, EPI_ISL_430941, EPI_ISL_430942, EPI_ISL_430951, EPI_ISL_430953, EPI_ISL_430954, EPI_ISL_430955, EPI_ISL_430956, EPI_ISL_430957, EPI_ISL_430958, EPI_ISL_430960, EPI_ISL_430961, EPI_ISL_430962, EPI_ISL_430963, EPI_ISL_430965, EPI_ISL_430968, EPI_ISL_430970, EPI_ISL_430972, EPI_ISL_430973			
see above	UW Virology Lab	UW Virology Lab	Pavitra Roychoudhury, Hong Xie, Keith Jerome, Alexander Greninger
EPI_ISL_431902, EPI_ISL_431903, EPI_ISL_431905, EPI_ISL_431908, EPI_ISL_431910, EPI_ISL_431912, EPI_ISL_431916, EPI_ISL_431917, EPI_ISL_431922, EPI_ISL_431923, EPI_ISL_431931, EPI_ISL_431936, EPI_ISL_431937, EPI_ISL_431939, EPI_ISL_431940, EPI_ISL_431941, EPI_ISL_431944, EPI_ISL_431954, EPI_ISL_431956, EPI_ISL_431957, EPI_ISL_431958, EPI_ISL_431959, EPI_ISL_431962, EPI_ISL_431972, EPI_ISL_431975, EPI_ISL_431976, EPI_ISL_431985, EPI_ISL_431989, EPI_ISL_431990, EPI_ISL_431991, EPI_ISL_431992, EPI_ISL_431994, EPI_ISL_432001, EPI_ISL_432003, EPI_ISL_432006, EPI_ISL_432007, EPI_ISL_432008, EPI_ISL_432011, EPI_ISL_432013, EPI_ISL_432014, EPI_ISL_432016, EPI_ISL_432022, EPI_ISL_432024, EPI_ISL_432028, EPI_ISL_432032, EPI_ISL_432033, EPI_ISL_432035, EPI_ISL_432036, EPI_ISL_432039, EPI_ISL_432040, EPI_ISL_432041, EPI_ISL_432045, EPI_ISL_432048, EPI_ISL_432049, EPI_ISL_432052, EPI_ISL_432053, EPI_ISL_432054, EPI_ISL_432057, EPI_ISL_432065, EPI_ISL_432066, EPI_ISL_432070, EPI_ISL_432072, EPI_ISL_432075, EPI_ISL_432078, EPI_ISL_432080, EPI_ISL_432081, EPI_ISL_432082, EPI_ISL_432086, EPI_ISL_432092, EPI_ISL_432093, EPI_ISL_432096, EPI_ISL_432099, EPI_ISL_432106, EPI_ISL_432108, EPI_ISL_432119, EPI_ISL_432131, EPI_ISL_432134, EPI_ISL_432136, EPI_ISL_432142, EPI_ISL_432143, EPI_ISL_432158, EPI_ISL_432173, EPI_ISL_432176, EPI_ISL_432185, EPI_ISL_432192, EPI_ISL_432199, EPI_ISL_432200, EPI_ISL_432206, EPI_ISL_432208, EPI_ISL_432210, EPI_ISL_432212, EPI_ISL_432214, EPI_ISL_432217, EPI_ISL_432218, EPI_ISL_432221, EPI_ISL_432223, EPI_ISL_432224, EPI_ISL_432232, EPI_ISL_432233, EPI_ISL_432236, EPI_ISL_432238, EPI_ISL_432239, EPI_ISL_432240, EPI_ISL_432242, EPI_ISL_432245, EPI_ISL_432254, EPI_ISL_432256, EPI_ISL_432258, EPI_ISL_432260, EPI_ISL_432270, EPI_ISL_432277, EPI_ISL_432278, EPI_ISL_432282, EPI_ISL_432285, EPI_ISL_432287, EPI_ISL_432288, EPI_ISL_432289, EPI_ISL_432298, EPI_ISL_432299, EPI_ISL_432302, EPI_ISL_432303, EPI_ISL_432306, EPI_ISL_432307, EPI_ISL_432310, EPI_ISL_432312, EPI_ISL_432314, EPI_ISL_432316, EPI_ISL_432322, EPI_ISL_432325, EPI_ISL_432327, EPI_ISL_432328, EPI_ISL_432329, EPI_ISL_432335, EPI_ISL_432340, EPI_ISL_432346, EPI_ISL_432349, EPI_ISL_432350, EPI_ISL_432351, EPI_ISL_432355, EPI_ISL_432356, EPI_ISL_432362, EPI_ISL_432365, EPI_ISL_432366, EPI_ISL_432370, EPI_ISL_432371, EPI_ISL_432374, EPI_ISL_432380, EPI_ISL_432381, EPI_ISL_432385, EPI_ISL_432389, EPI_ISL_432394, EPI_ISL_432407, EPI_ISL_432408, EPI_ISL_432412, EPI_ISL_432413, EPI_ISL_432416, EPI_ISL_432417, EPI_ISL_432425, EPI_ISL_432426, EPI_ISL_432427, EPI_ISL_432429, EPI_ISL_432430, EPI_ISL_432431, EPI_ISL_432434, EPI_ISL_432435, EPI_ISL_432437, EPI_ISL_432438, EPI_ISL_432441, EPI_ISL_432445, EPI_ISL_432446, EPI_ISL_432448, EPI_ISL_432450			
see above	Wales Specialist Virology Centre	Public Health Wales Microbiology Cardiff	Catherine Moore, Johnathan Evans, Malorie Perry, Simon Cottrell, Alec Birchley, Alexander Adams, Amy Gaskin, Bree Gatica-Wilcox, Jason Coombes, Lauren Gilbert, Lee Graham, Nicole Pacchiarini, Sara Kumziene-Summerhayes, Sarah Taylor, Sophie Jones, Sara Rey, Matthew Bull, Joanne Watkins, Sally Corden, Tom Connor
EPI_ISL_432454, EPI_ISL_432466, EPI_ISL_432467, EPI_ISL_432484, EPI_ISL_432494, EPI_ISL_432496, EPI_ISL_432504, EPI_ISL_432505, EPI_ISL_432507, EPI_ISL_432512, EPI_ISL_432518, EPI_ISL_432522, EPI_ISL_432529, EPI_ISL_432531, EPI_ISL_432533, EPI_ISL_432542, EPI_ISL_432547, EPI_ISL_432555, EPI_ISL_432571, EPI_ISL_432573, EPI_ISL_432579, EPI_ISL_432584, EPI_ISL_432595, EPI_ISL_432606, EPI_ISL_432614, EPI_ISL_432617, EPI_ISL_432626, EPI_ISL_432633, EPI_ISL_432654, EPI_ISL_432657, EPI_ISL_432666, EPI_ISL_432714, EPI_ISL_432727, EPI_ISL_432732, EPI_ISL_432733, EPI_ISL_432736, EPI_ISL_432738, EPI_ISL_432744, EPI_ISL_432745, EPI_ISL_432751, EPI_ISL_432755, EPI_ISL_432763, EPI_ISL_432764, EPI_ISL_432765, EPI_ISL_432772, EPI_ISL_432775, EPI_ISL_432787, EPI_ISL_432790, EPI_ISL_432792, EPI_ISL_432802, EPI_ISL_432809, EPI_ISL_432812, EPI_ISL_432816, EPI_ISL_432818, EPI_ISL_432824, EPI_ISL_432825, EPI_ISL_432842, EPI_ISL_432849, EPI_ISL_432851, EPI_ISL_432855, EPI_ISL_432857, EPI_ISL_432859, EPI_ISL_432861			
see above	Virology Department, Sheffield Teaching Hospitals NHS Foundation Trust / Virology Department, Sheffield Teaching Hospitals NHS Foundation Trust	COVID-19 Genomics UK (COG-UK) Consortium	Thushan de Silva, Matthew Parker,Adri Angyal, Rebecca Brown, Luke Green, Rachel Tucker, Paul Parsons, Danielle Groves, Alex Keeley, Dave Partridge, Matthew Wyles, Benjamin Lindsey, Mehmet Yavuz, Mohammad Raza, Cariad Evans
EPI_ISL_432949, EPI_ISL_432950, EPI_ISL_432962, EPI_ISL_432963, EPI_ISL_432965, EPI_ISL_432966, EPI_ISL_432967, EPI_ISL_432968, EPI_ISL_432969, EPI_ISL_432970, EPI_ISL_432971, EPI_ISL_432972, EPI_ISL_432973, EPI_ISL_432974, EPI_ISL_432975, EPI_ISL_432976, EPI_ISL_432977, EPI_ISL_432978, EPI_ISL_432979, EPI_ISL_432980, EPI_ISL_432981, EPI_ISL_432982, EPI_ISL_432983, EPI_ISL_432984, EPI_ISL_432985, EPI_ISL_432986, EPI_ISL_432987, EPI_ISL_432988, EPI_ISL_432989, EPI_ISL_432990, EPI_ISL_432991, EPI_ISL_432992, EPI_ISL_432994			
see above	Queens Medical Centre, Clinical Microbiology Department / DeepSeq Nottingham	COVID-19 Genomics UK (COG-UK) Consortium	Gemma Clark, Wendy Smith, Manjinder Khakh, Hannah Howson-Wells, Jonathan Ball, Patrick McClure, Joseph Chappell, Theocharis Tsoleridis, Nadine Holmes, Matthew Carlisle, Christopher Moore, Fei Sang, Johnny Debebe, Victoria Wright, Matthew Loose
EPI_ISL_433200, EPI_ISL_433201, EPI_ISL_433202, EPI_ISL_433203, EPI_ISL_433207, EPI_ISL_433208, EPI_ISL_433209, EPI_ISL_433210, EPI_ISL_433211, EPI_ISL_433212, EPI_ISL_433214, EPI_ISL_433216, EPI_ISL_433217, EPI_ISL_433218, EPI_ISL_433220, EPI_ISL_433224, EPI_ISL_433225, EPI_ISL_433226, EPI_ISL_433227			
see above	Virology Department, Royal Infirmary of Edinburgh, NHS Lothian / School of Biological Sciences, University of Edinburgh / Institute of Genetics and Molecular Medicine, University of Edinburgh	COVID-19 Genomics UK (COG-UK) Consortium	McHugh M, Dewar R, Rooke S, Gallagher M, Balcaza C, O'Toole A, Hill V, McCrone JT, Colquhoun R, Yu X, Jackson B, Rambaut A, Williams TC, Templeton K
EPI_ISL_433524, EPI_ISL_433525, EPI_ISL_433526, EPI_ISL_433527, EPI_ISL_433528, EPI_ISL_433529, EPI_ISL_433530, EPI_ISL_433533, EPI_ISL_433539, EPI_ISL_433540, EPI_ISL_433541, EPI_ISL_433542, EPI_ISL_433543, EPI_ISL_433544, EPI_ISL_433545, EPI_ISL_433546, EPI_ISL_433547, EPI_ISL_433548, EPI_ISL_433549, EPI_ISL_433550, EPI_ISL_433551, EPI_ISL_433552, EPI_ISL_433553, EPI_ISL_433554, EPI_ISL_433555, EPI_ISL_433556, EPI_ISL_433557, EPI_ISL_433558, EPI_ISL_433559, EPI_ISL_433560, EPI_ISL_433561, EPI_ISL_433562, EPI_ISL_433563, EPI_ISL_433564, EPI_ISL_433565, EPI_ISL_433566, EPI_ISL_433567, EPI_ISL_433568, EPI_ISL_433569, EPI_ISL_433570, EPI_ISL_433571, EPI_ISL_433572, EPI_ISL_433573, EPI_ISL_433574, EPI_ISL_433575, EPI_ISL_433577, EPI_ISL_433578, EPI_ISL_433579, EPI_ISL_433582, EPI_ISL_433584, EPI_ISL_433586, EPI_ISL_433588, EPI_ISL_433589, EPI_ISL_433593, EPI_ISL_433599			
see above	West of Scotland Specialist Virology Centre, NHSGGC / MRC-University of Glasgow Centre for Virus Research	COVID-19 Genomics UK (COG-UK) Consortium	Ana da Silva Filipe, Natasha Johnson, Kathy Smollett, Daniel Mair, Stephen Carmichael, Lily Tong, Jenna Nichols, Elihu Aranday-Cortes, Kirstyn Brunker, Yasmin Parr, Kyriaki Nomikou; Sarah McDonald, Marc Niebel, Patawee Asamaphan; Richard Orton, Joseph Hughes, Sreenu Vattipally, David L Robertson; Alasdair MacLean, Rory Gunson; Kathy Li, Natasha Jesudason, Rajiv Shah, James Shepherd, Antonia Ho, Emma Thomson
EPI_ISL_433684, EPI_ISL_433688, EPI_ISL_433689, EPI_ISL_433691, EPI_ISL_433692, EPI_ISL_433693, EPI_ISL_433694, EPI_ISL_433696, EPI_ISL_433697, EPI_ISL_433698, EPI_ISL_433699			
see above	Department of Pathology, University of Cambridge	COVID-19 Genomics UK (COG-UK) Consortium	Luke W Meredith, M. Estee Torok , Myra Hosmillo, William L. Hamilton, Martin D. Curran, Theresa Feltwell, Grant Hall, Anna Yakovleva, Fahad A Khokhar, Charlotte J. Houldcroft, Laura G Caller, Aminu S. Jahun, Sarah L. Caddy, Ian Goodfellow

EPI_ISL_434065, EPI_ISL_434070, EPI_ISL_434072, EPI_ISL_434076, EPI_ISL_434078, EPI_ISL_434080, EPI_ISL_434082, EPI_ISL_434084, EPI_ISL_434086, EPI_ISL_434089, EPI_ISL_434097, EPI_ISL_434107, EPI_ISL_434108, EPI_ISL_434196, EPI_ISL_434197, EPI_ISL_434198, EPI_ISL_434200, EPI_ISL_434201, EPI_ISL_434204, EPI_ISL_434205, EPI_ISL_434206, EPI_ISL_434207, EPI_ISL_434208, EPI_ISL_434209, EPI_ISL_434210, EPI_ISL_434211, EPI_ISL_434212, EPI_ISL_434214, EPI_ISL_434215, EPI_ISL_434225, EPI_ISL_434226, EPI_ISL_434234, EPI_ISL_434235, EPI_ISL_434238, EPI_ISL_434245, EPI_ISL_434246, EPI_ISL_434247, EPI_ISL_434248, EPI_ISL_434250, EPI_ISL_434253, EPI_ISL_434254, EPI_ISL_434255, EPI_ISL_434256, EPI_ISL_434257, EPI_ISL_434258, EPI_ISL_434259, EPI_ISL_434264, EPI_ISL_434266, EPI_ISL_434272, EPI_ISL_434273, EPI_ISL_434275, EPI_ISL_434276, EPI_ISL_434280, EPI_ISL_434282, EPI_ISL_434284, EPI_ISL_434285, EPI_ISL_434286, EPI_ISL_434287, EPI_ISL_434288, EPI_ISL_434298, EPI_ISL_434305, EPI_ISL_434308, EPI_ISL_434313, EPI_ISL_434314, EPI_ISL_434317, EPI_ISL_434323, EPI_ISL_434328, EPI_ISL_434334, EPI_ISL_434335, EPI_ISL_434342, EPI_ISL_434346			
see above	Washington State Department of Health	Seattle Flu Study	Chu et al
EPI_ISL_434368, EPI_ISL_434371	Hospital AZ Rivierenland	Institute of Tropical Medicine	Philippe Selhorst, Colin Anthony
EPI_ISL_434520, EPI_ISL_434521, EPI_ISL_434522, EPI_ISL_434529, EPI_ISL_434530, EPI_ISL_434531, EPI_ISL_434532	Robert Garry lab	Andersen lab at Scripps Research	Allison Smither, Gilberto Sabino-Santos, Patricia Snarski, Lilia Melnik, Antoinette Bell, Kaylynn Genemaras, Arnaud Drouin, Dahlene Fusco, Robert Garry with SEARCH Alliance San Diego
EPI_ISL_434552, EPI_ISL_434553	Puerto Rico Department of Health	Centers for Disease Control and Prevention, Dengue Branch	Gilberto A. Santiago, Glenda Gonzalez, Betzabel Flores, Keyla Charriez, Fabiola Cruz, Chaney Kalinich, Joseph Fauver, Jessica I. Falcon, Nathan Grubaugh, Jorge L. Munoz-Jordan
EPI_ISL_434657	Kristianstadkliniken	The Public Health Agency of Sweden	Mia Settergren Hammer, Oskar Karlsson Lindsjo, Maria Lind Karlberg, Anna-Malin Linde, Olov Svartstrom, Anna Risberg, Theresa Enkirch, Mia Brytting, Karin Tegmark-Wisell
EPI_ISL_434941, EPI_ISL_434943, EPI_ISL_434964, EPI_ISL_434966, EPI_ISL_434970, EPI_ISL_434971, EPI_ISL_434972, EPI_ISL_434975, EPI_ISL_434976, EPI_ISL_434979, EPI_ISL_434980, EPI_ISL_434981, EPI_ISL_434982, EPI_ISL_434983, EPI_ISL_434984, EPI_ISL_434985, EPI_ISL_434986, EPI_ISL_434987, EPI_ISL_434988, EPI_ISL_434990, EPI_ISL_434991, EPI_ISL_434992, EPI_ISL_434994, EPI_ISL_434995, EPI_ISL_434997, EPI_ISL_434998, EPI_ISL_434999, EPI_ISL_435000, EPI_ISL_435001, EPI_ISL_435002, EPI_ISL_435003, EPI_ISL_435004, EPI_ISL_435005, EPI_ISL_435006, EPI_ISL_435007, EPI_ISL_435008, EPI_ISL_435009			
see above	Houston Methodist Hospital	Houston Methodist Hospital	S. Wesley Long, Randall J. Olsen, Paul A. Christensen, David W. Bernard, James J. Davis, Maulik Shukla, Marcus Nguyen, Matthew Ojeda Saavedra, Concepcion C. Cantu, Prasanti Yerramilli, Layne Pruitt, Sishir Subedi, Heather Hendrickson, Ghazaleh Eskandari, Muthiah Kumaraswami, Jason S. McLellan, Hakon Jonsson, Kari Stefansson, and James M. Musser
EPI_ISL_435343, EPI_ISL_435344, EPI_ISL_435345, EPI_ISL_435346, EPI_ISL_435347	Laboratoire de microbiologie, Hopital de Verdun	Smith Laboratory, Centre de Recherche CHU Sainte-Justine	Martin Smith, Marieke Rozendaal, Ivan Pavlov
EPI_ISL_435374	Utah Public Health Laboratory	Utah Public Health Laboratory	Erin Young, Kelly Oakeson
EPI_ISL_435445, EPI_ISL_435447, EPI_ISL_435465, EPI_ISL_435467	Robert Garry lab	Andersen lab at Scripps Research	Allison Smither, Gilberto Sabino-Santos, Patricia Snarski, Lilia Melnik, Antoinette Bell, Kaylynn Genemaras, Arnaud Drouin, Dahlene Fusco, Robert Garry with SEARCH Alliance San Diego
EPI_ISL_435489	NYU Langone Health	Departments of Pathology and Medicine, New York University School of Medicine	Maria Aguero-Rosenfeld, Brendan Belovarac, Margaret Black, Ludovic Boytard, John Cadley, Paolo Cotzia, John Chen, Dacia Dimartino, Xiaojun Feng, Tatyana Gindin, Emily Guzman, Adriana Heguy, Megan Hogan, Emily Huang, George Jour, Lawrence H. Lin, Raven Luther, Andrew Lytle, Christian Marier, Matthew T. Maurano, Mark J. Mulligan, Peter Meyn, Raquel Ordonez Ciriza, Iman Osman, Jared Pinnell, Vanessa Raabe, Sitharam Ramaswami, Amy Rapkiewicz, Andre M. Ribeiro-dos-Santos, Marie Samanovic-Golden, Antonio Serrano, Guomiao Shen, Matija Snuderl, Theodore Vougiouklakis, Nick Vulpescu, Gael Westby, Paul Zappile, Yutong Zhang
EPI_ISL_435679, EPI_ISL_435680	National Public Health Laboratory, National Centre for Infectious Diseases	National Public Health Laboratory, National Centre for Infectious Diseases	Mak Tze Minn, Octavia Sophie, Chavatte Jean-Marc, Cui Lin, Lin Raymond Tzer Pin
EPI_ISL_436046	US VI Department of Health	Pathogen Discovery, Respiratory Viruses Branch, Division of Viral Diseases, Centers for Disease Control and Prevention	Ying Tao, Jing Zhang, Krista Queen, Yan Li, Anna Uehara, Clinton R. Paden, Haibin Wang, Zachary Weiner, Bettina Bankamp, Suxiang Tong
EPI_ISL_436107	TSGH-CP molecular lab	TSGH-CP molecular lab	Cherng-Lih Perng, Ming-Jr JIAN, Chih-Kai Chang, Jung-Chung Lin, Kuo-Ming Yeh, Chien-Wen Chen, Sheng-Kang Chiu, Hsing-Yi Chung, Shih-Hung Tsai, Kuo-Sheng Hung, Tien-Yao Chang, Feng-Yee Chang, Hung-Sheng Shang
EPI_ISL_436414, EPI_ISL_436415, EPI_ISL_436416, EPI_ISL_436417, EPI_ISL_436418, EPI_ISL_436419, EPI_ISL_436420, EPI_ISL_436421, EPI_ISL_436422	National Centre for Disease control (NCDC)	NCDC/CSIR-IGIB	Pramod Kumar#, Rajesh Pandey#, Pooja Sharma, Mahesh S Dhar, Vivekanand A, Bharathram Uppili, Himanshu Vashisht, Saruchi Wadhwa, Nishu Tyagi, Uma Sharma, Priyanka Singh, Hemlata Lall, Meena Datta, Poonam Gupta, Nidhi Saini, Aarti Tewari, Bibhash Nandi, Dhirendra Kumar, Satyabrata Bag, Varun Jaiswal, Hema Gogia, Preeti Madan, Simrita Singh, Prateek Singh, Debasis Dash, Mitali Mukerji, Manju Bala, Sandhya Kabra, Sujeet Singh, Mohammed Faruq, Anurag Agrawal*, Partha Rakshit*
EPI_ISL_436567, EPI_ISL_436568, EPI_ISL_436569, EPI_ISL_436570, EPI_ISL_436633, EPI_ISL_436634	University of Wisconsin-Madison AIDS Vaccine Research Laboratories	University of Wisconsin-Madison AIDS Vaccine Research Laboratories	Gage Moreno, Katarina Braun, et al. AIDS Vaccine Research Laboratories
EPI_ISL_436641, EPI_ISL_436642, EPI_ISL_436643, EPI_ISL_436645, EPI_ISL_436673, EPI_ISL_436674, EPI_ISL_436675, EPI_ISL_436676	County of Santa Clara Public Health Department	Chan-Zuckerberg Biohub	CZB Cliahub Consortium
EPI_ISL_436684, EPI_ISL_436685	KRISP, KZN Research Innovation and Sequencing Platform	KRISP, KZN Research Innovation and Sequencing Platform	Giandhari J, Pillay S, Lessells R, Chimukangara B, Deforche K, Tegally H, Wilkinson E, de Oliveira T
EPI_ISL_437043, EPI_ISL_437044	County of Santa Clara Public Health	Chan-Zuckerberg Biohub	CZB Cliahub Consortium
EPI_ISL_437192	Mitra Keluarga Kelapa Gading Hospital	Eijkman Institute for Molecular Biology, Ministry of Research and Technology/National Agency for Research and Innovation	Edison Johar, Frilasita A Yudhaputri, Hidayat Trimarsanto, David H Muljono, Safarina G Malik, Khin Saw Myint, Amin Soebandrio
EPI_ISL_437199, EPI_ISL_437200	Diagnostic- and Research Institute of Pathology, Medical University of Graz	Diagnostic- and Research Institute of Pathology, Medical University of Graz	Karl Kashofer, Peter Regitnig, Martin Zacharias, Gregor Gorkiewicz
EPI_ISL_437225, EPI_ISL_437232, EPI_ISL_437277, EPI_ISL_437278, EPI_ISL_437279, EPI_ISL_437280, EPI_ISL_437281, EPI_ISL_437282	Max von Pettenkofer Institute, Virology, National Reference Center for Retroviruses, LMU München	Laboratory for Functional Genome Analysis, Dept. Genomics, Gene Center of the LMU Munich	Max Muenchhoff, Stefan Krebs, Alexander Graf, Oliver Keppler, Helmut Blum
EPI_ISL_437363	Minnesota Department of Health, Public Health Laboratory	Minnesota Department of Health, Public Health Laboratory	Matt Plumb, Jacob Garfin, and Xiong Wang
EPI_ISL_437399	Virginia DCLS	Virginia DCLS	Virginia DCLS
EPI_ISL_437475	Pathogen Genomics Lab King Abdullah University of Science and Technology(KAUST)	Pathogen Genomics Lab King Abdullah University of Science and Technology(KAUST)	Sharif Hala,Raeece Naeem,Sara Mfarrej,Arnab Pain
EPI_ISL_437547, EPI_ISL_437548	Robert Garry lab	Andersen lab at Scripps Research	Allison Smither, Gilberto Sabino-Santos, Patricia Snarski, Lilia Melnik, Antoinette Bell, Kaylynn Genemaras, Arnaud Drouin, Dahlene Fusco, Robert Garry with SEARCH Alliance San Diego
EPI_ISL_437627, EPI_ISL_437628, EPI_ISL_437629, EPI_ISL_437630, EPI_ISL_437633, EPI_ISL_437634, EPI_ISL_437636, EPI_ISL_437682, EPI_ISL_437683	Department of Virus and Microbiological Special Diagnostics, Statens Serum Institut, Copenhagen, Denmark, Artillerivej 5, 2300 Copenahgen S	Albertsen lab, Department of Chemistry and Bioscience, Aalborg University, Denmark	Rasmus Kirkegaard
EPI_ISL_437684, EPI_ISL_437685, EPI_ISL_437686, EPI_ISL_437687, EPI_ISL_437688	UCD National Virus Reference Laboratory	UCD National Virus Reference Laboratory	Michael J. Carr, Gabriel Gonzalez, Brendan Crowley, Cillian F De Gascun

EPI_ISL_437734, EPI_ISL_437735	Pathogen Genomics Lab King Abdullah University of Science and Technology(KAUST)	Pathogen Genomics Lab King Abdullah University of Science and Technology(KAUST)	Sharif Hala,Fadwa Alofi,Afrah Alsomali, Asim Khogeer, Sara Mfarrej, Khaled Algithami,Raeece Naeem, Amit Kumar Subudhi,Fathia Ben-Rached, Rahul Salunke, Anwar Hashem, Naif Almontashiri, Arnab Pain
EPI_ISL_437800	Virginia DCLS	Virginia DCLS	Virginia DCLS
EPI_ISL_437813, EPI_ISL_437814, EPI_ISL_437815, EPI_ISL_437816, EPI_ISL_437817, EPI_ISL_437818, EPI_ISL_437819, EPI_ISL_437820, EPI_ISL_437821, EPI_ISL_437822, EPI_ISL_437823, EPI_ISL_437824, EPI_ISL_437827, EPI_ISL_437829			
see above	UW Virology Lab	UW Virology Lab	Pavitra Roychoudhury, Hong Xie, Keith Jerome, Alexander Greninger
EPI_ISL_437873	Alaska State Virology Laboratory	Alaska State Virology Laboratory	Jack Chen, Ph.D.
EPI_ISL_437910, EPI_ISL_437911	Laboratory of Microbiology, Medical School, National and Kapodistrian University of Athens	Laboratory of Biology, Department of Medicine, Democritus University of Thrace	Kassela K., Dovrolis,N., Bampali,M., Gatzidou,E., Froukala,E., Stavropoulou,A., Veletza,S., Tsakris,A., Spanakis,N. and Karakasiliotis,I.
EPI_ISL_438121	Center for Virology, Medical University of Vienna	Bergthaler laboratory, CeMM Research Center for Molecular Medicine of the Austrian Academy of Sciences	Alexandra Popa, Benedikt Agerer, Henrique Colaco, Lukas Endler, Jakob-Wendelin Genger, Alexander Lercher, Mark Smyth, Thomas Penz, Michael Schuster, Jan Laine, Martin Senekowitsch, Judith Aberle, Stephan Aberle, Elisabeth Puchhammer-Stoeckl, Manfred Nairz, Guenter Weiss, Wegene Borena, Dorothee von Laer, Christoph Bock, Andreas Bergthaler
EPI_ISL_438140	Seattle Flu Study	Seattle Flu Study	Chu et al
EPI_ISL_438242	Johns Hopkins Hospital Department of Pathology	Johns Hopkins Hospital Department of Pathology	Peter M. Thielen, Thomas Mehoke, Shirlee Wohl, Srividya Ramakrishnan, Melanie Kirsche, Amanda Ernlund, Oluwaseun Falade-Nwulia, Timothy Gilpatrick, Paul Morris, Norah Sadowski, Nidia Trovao, Victoria Gniazdowski, Michael Schatz, Stuart C. Ray, Winston Timp, Heba Mostafa
EPI_ISL_438248, EPI_ISL_438249, EPI_ISL_438250, EPI_ISL_438252, EPI_ISL_438254, EPI_ISL_438264, EPI_ISL_438265, EPI_ISL_438279, EPI_ISL_438282, EPI_ISL_438291, EPI_ISL_438316, EPI_ISL_438340, EPI_ISL_438381, EPI_ISL_438382, EPI_ISL_438391, EPI_ISL_438398, EPI_ISL_438419, EPI_ISL_438431, EPI_ISL_438450, EPI_ISL_438459, EPI_ISL_438470, EPI_ISL_438473, EPI_ISL_438476, EPI_ISL_438483, EPI_ISL_438489, EPI_ISL_438507, EPI_ISL_438509, EPI_ISL_438510, EPI_ISL_438511, EPI_ISL_438514, EPI_ISL_438515, EPI_ISL_438517, EPI_ISL_438519, EPI_ISL_438520, EPI_ISL_438523, EPI_ISL_438524, EPI_ISL_438525, EPI_ISL_438527, EPI_ISL_438528, EPI_ISL_438530, EPI_ISL_438531, EPI_ISL_438532, EPI_ISL_438533, EPI_ISL_438534, EPI_ISL_438535, EPI_ISL_438536, EPI_ISL_438537, EPI_ISL_438539, EPI_ISL_438540, EPI_ISL_438542, EPI_ISL_438544, EPI_ISL_438545			
see above	Department of Pathology, University of Cambridge	Wellcome Sanger Institute for the COVID-19 Genomics UK (COG-UK) consortium	Luke W Meredith, M. Estée Török , Myra Hosmillo, William L. Hamilton, Martin D. Curran, Theresa Feltwell, Grant Hall, Anna Yakovleva, Fahad A Khokhar, Charlotte J. Houldcroft, Laura G Caller, Aminu S. Jahun, Sarah L. Caddy, Ian Goodfellow, Alex Alderton, Roberto Amato, Sonia Goncalves, Ewan Harrison, David K. Jackson, Ian Johnston, Dominic Kwiatkowski, Cordelia Langford, John Sillitoe on behalf of the Wellcome Sanger Institute COVID-19 Surveillance Team (http://www.sanger.ac.uk/covid-team)
EPI_ISL_438774, EPI_ISL_438777, EPI_ISL_438778, EPI_ISL_438779, EPI_ISL_438780, EPI_ISL_438781, EPI_ISL_438783, EPI_ISL_438784, EPI_ISL_438785, EPI_ISL_438787, EPI_ISL_438788, EPI_ISL_438789, EPI_ISL_438790, EPI_ISL_438791, EPI_ISL_438792, EPI_ISL_438793, EPI_ISL_438794, EPI_ISL_438795, EPI_ISL_438796, EPI_ISL_438797, EPI_ISL_438798, EPI_ISL_438799, EPI_ISL_438800, EPI_ISL_438801, EPI_ISL_438802, EPI_ISL_438803, EPI_ISL_438804, EPI_ISL_438807, EPI_ISL_438808, EPI_ISL_438809, EPI_ISL_438810, EPI_ISL_438812, EPI_ISL_438813, EPI_ISL_438814, EPI_ISL_438815, EPI_ISL_438818, EPI_ISL_438819, EPI_ISL_438821, EPI_ISL_438822, EPI_ISL_438823			
see above	West of Scotland Specialist Virology Centre, NHSGGC / MRC-University of Glasgow Centre for Virus Research	COVID-19 Genomics UK (COG-UK) Consortium	Ana da Silva Filipe, Natasha Johnson, Kathy Smollett, Daniel Mair, Stephen Carmichael, Lily Tong, Jenna Nichols, Elihu Aranday-Cortes, Kirstyn Brunker, Yasmin Parr, Kyriaki Nomikou; Sarah McDonald, Marc Niebel, Patawee Asamaphan; Richard Orton, Joseph Hughes, Sreenu Vattipally, David L Robertson; Alasdair MacLean, Rory Gunson; Kathy Li, Natasha Jesudason, Rajiv Shah, James Shepherd, Antonia Ho, Emma Thomson
EPI_ISL_439368, EPI_ISL_439369, EPI_ISL_439370, EPI_ISL_439371, EPI_ISL_439372, EPI_ISL_439373, EPI_ISL_439374, EPI_ISL_439375, EPI_ISL_439376, EPI_ISL_439377, EPI_ISL_439378, EPI_ISL_439380, EPI_ISL_439381, EPI_ISL_439382, EPI_ISL_439386, EPI_ISL_439387, EPI_ISL_439388, EPI_ISL_439389, EPI_ISL_439390, EPI_ISL_439391, EPI_ISL_439392, EPI_ISL_439393, EPI_ISL_439395, EPI_ISL_439397, EPI_ISL_439399, EPI_ISL_439400, EPI_ISL_439401, EPI_ISL_439404, EPI_ISL_439405, EPI_ISL_439406, EPI_ISL_439409, EPI_ISL_439412, EPI_ISL_439413, EPI_ISL_439414, EPI_ISL_439415, EPI_ISL_439416, EPI_ISL_439417, EPI_ISL_439418, EPI_ISL_439420, EPI_ISL_439423, EPI_ISL_439424, EPI_ISL_439425, EPI_ISL_439426, EPI_ISL_439428, EPI_ISL_439429, EPI_ISL_439430, EPI_ISL_439431, EPI_ISL_439433, EPI_ISL_439434, EPI_ISL_439435, EPI_ISL_439436, EPI_ISL_439437, EPI_ISL_439438, EPI_ISL_439439, EPI_ISL_439440, EPI_ISL_439441, EPI_ISL_439443, EPI_ISL_439444, EPI_ISL_439445, EPI_ISL_439446, EPI_ISL_439447, EPI_ISL_439449, EPI_ISL_439451, EPI_ISL_439452, EPI_ISL_439453, EPI_ISL_439454, EPI_ISL_439455, EPI_ISL_439456, EPI_ISL_439459, EPI_ISL_439460, EPI_ISL_439462, EPI_ISL_439463, EPI_ISL_439464, EPI_ISL_439466, EPI_ISL_439468, EPI_ISL_439470, EPI_ISL_439472, EPI_ISL_439473, EPI_ISL_439474, EPI_ISL_439477, EPI_ISL_439478, EPI_ISL_439479, EPI_ISL_439480, EPI_ISL_439481, EPI_ISL_439483, EPI_ISL_439484, EPI_ISL_439485, EPI_ISL_439487, EPI_ISL_439488, EPI_ISL_439489, EPI_ISL_439490, EPI_ISL_439492, EPI_ISL_439494, EPI_ISL_439495, EPI_ISL_439496, EPI_ISL_439497, EPI_ISL_439498, EPI_ISL_439501, EPI_ISL_439502, EPI_ISL_439503, EPI_ISL_439504, EPI_ISL_439509, EPI_ISL_439510, EPI_ISL_439511, EPI_ISL_439512, EPI_ISL_439513, EPI_ISL_439514, EPI_ISL_439516, EPI_ISL_439517, EPI_ISL_439519, EPI_ISL_439521, EPI_ISL_439524, EPI_ISL_439525, EPI_ISL_439526, EPI_ISL_439528, EPI_ISL_439529, EPI_ISL_439531, EPI_ISL_439536, EPI_ISL_439563, EPI_ISL_439571, EPI_ISL_439578, EPI_ISL_439599, EPI_ISL_439601, EPI_ISL_439607, EPI_ISL_439612, EPI_ISL_439617, EPI_ISL_439619, EPI_ISL_439620, EPI_ISL_439637, EPI_ISL_439647, EPI_ISL_439650, EPI_ISL_439659, EPI_ISL_439866, EPI_ISL_439870, EPI_ISL_439880, EPI_ISL_439901, EPI_ISL_439903, EPI_ISL_439927, EPI_ISL_439929, EPI_ISL_439946, EPI_ISL_439950			
see above	Department of Pathology, University of Cambridge	Wellcome Sanger Institute for the COVID-19 Genomics UK (COG-UK) consortium	Luke W Meredith, M. Estée Török , Myra Hosmillo, William L. Hamilton, Martin D. Curran, Theresa Feltwell, Grant Hall, Anna Yakovleva, Fahad A Khokhar, Charlotte J. Houldcroft, Laura G Caller, Aminu S. Jahun, Sarah L. Caddy, Ian Goodfellow, Alex Alderton, Roberto Amato, Sonia Goncalves, Ewan Harrison, David K. Jackson, Ian Johnston, Dominic Kwiatkowski, Cordelia Langford, John Sillitoe on behalf of the Wellcome Sanger Institute COVID-19 Surveillance Team (http://www.sanger.ac.uk/covid-team)
EPI_ISL_439998, EPI_ISL_440029, EPI_ISL_440114, EPI_ISL_440158, EPI_ISL_440294	PHE South West Regional Laboratory, National Infection Service	Wellcome Sanger Institute for the COVID-19 Genomics UK (COG-UK) consortium	Stephanie Hutchings, Hannah Pymont, Dr Peter Muir, Barry Vipond, Rich Hopes, Alex Alderton, Roberto Amato, Sonia Goncalves, Ewan Harrison, David K. Jackson, Ian Johnston, Dominic Kwiatkowski, Cordelia Langford, John Sillitoe on behalf of the Wellcome Sanger Institute COVID-19 Surveillance Team (http://www.sanger.ac.uk/covid-team)
EPI_ISL_440564, EPI_ISL_440568, EPI_ISL_440575, EPI_ISL_440601, EPI_ISL_440602, EPI_ISL_440613, EPI_ISL_440617, EPI_ISL_440620	Department of Pathology, University of Cambridge	Wellcome Sanger Institute for the COVID-19 Genomics UK (COG-UK) consortium	Luke W Meredith, M. Estée Török , Myra Hosmillo, William L. Hamilton, Martin D. Curran, Theresa Feltwell, Grant Hall, Anna Yakovleva, Fahad A Khokhar, Charlotte J. Houldcroft, Laura G Caller, Aminu S. Jahun, Sarah L. Caddy, Ian Goodfellow, Alex Alderton, Roberto Amato, Sonia Goncalves, Ewan Harrison, David K. Jackson, Ian Johnston, Dominic Kwiatkowski, Cordelia Langford, John Sillitoe on behalf of the Wellcome Sanger Institute COVID-19 Surveillance Team (http://www.sanger.ac.uk/covid-team)
EPI_ISL_440662, EPI_ISL_440669, EPI_ISL_440678, EPI_ISL_440694, EPI_ISL_440696, EPI_ISL_440700, EPI_ISL_440703, EPI_ISL_440720, EPI_ISL_440736, EPI_ISL_440765, EPI_ISL_440779, EPI_ISL_440786, EPI_ISL_440809			
see above	PHE South West Regional Laboratory, National Infection Service	Wellcome Sanger Institute for the COVID-19 Genomics UK (COG-UK) consortium	Stephanie Hutchings, Hannah Pymont, Dr Peter Muir, Barry Vipond, Rich Hopes, Alex Alderton, Roberto Amato, Sonia Goncalves, Ewan Harrison, David K. Jackson, Ian Johnston, Dominic Kwiatkowski, Cordelia Langford, John Sillitoe on behalf of the Wellcome Sanger Institute COVID-19 Surveillance Team (http://www.sanger.ac.uk/covid-team)
EPI_ISL_440810, EPI_ISL_440811, EPI_ISL_440813, EPI_ISL_440815, EPI_ISL_440817, EPI_ISL_440818, EPI_ISL_440819, EPI_ISL_440820, EPI_ISL_440821, EPI_ISL_440822, EPI_ISL_440823, EPI_ISL_440824, EPI_ISL_440825, EPI_ISL_440826, EPI_ISL_440827, EPI_ISL_440828, EPI_ISL_440829, EPI_ISL_440831, EPI_ISL_440832, EPI_ISL_440836, EPI_ISL_440837, EPI_ISL_440838, EPI_ISL_440839, EPI_ISL_440840, EPI_ISL_440841, EPI_ISL_440842, EPI_ISL_440844, EPI_ISL_440845, EPI_ISL_440846, EPI_ISL_440848, EPI_ISL_440849, EPI_ISL_440850, EPI_ISL_440851			
see above	Department of Pathology, University of Cambridge	Wellcome Sanger Institute for the COVID-19 Genomics UK (COG-UK) consortium	Luke W Meredith, M. Estée Török , Myra Hosmillo, William L. Hamilton, Martin D. Curran, Theresa Feltwell, Grant Hall, Anna Yakovleva, Fahad A Khokhar, Charlotte J. Houldcroft, Laura G Caller, Aminu S. Jahun, Sarah L. Caddy, Ian Goodfellow, Alex Alderton, Roberto Amato, Sonia Goncalves, Ewan Harrison, David K. Jackson, Ian Johnston, Dominic Kwiatkowski, Cordelia Langford, John Sillitoe on behalf of the Wellcome Sanger Institute COVID-19 Surveillance Team (http://www.sanger.ac.uk/covid-team)
EPI_ISL_440971, EPI_ISL_440972, EPI_ISL_440973, EPI_ISL_440974, EPI_ISL_440975, EPI_ISL_440976, EPI_ISL_440977, EPI_ISL_440978, EPI_ISL_440979, EPI_ISL_440980, EPI_ISL_440981, EPI_ISL_440982, EPI_ISL_440983, EPI_ISL_440984, EPI_ISL_440985, EPI_ISL_440986, EPI_ISL_440987, EPI_ISL_440988, EPI_ISL_440989, EPI_ISL_440990			
see above	University College London, Great Ormond Street Hospital for Children NHS Foundation Trust, Imperial College Healthcare NHS Trust	COVID-19 Genomics UK (COG-UK) Consortium	Sergi Castellano, Rachel Williams, Mark Kristiansen, Paola Resende Silva, Sunando Roy, Tony Brooks, Helena Tutill, Paola Niola, Patricia Dyal, Charlotte Williams, Leysa Forrest, Yasmin Panchbhaya, Jacqueline Findlay, Sam Weeks, Julianne Brown, Kathryn Harris, Paul Randell, James Price, Alison Holmes, Judith Breuer
EPI_ISL_441053, EPI_ISL_441065, EPI_ISL_441068, EPI_ISL_441071, EPI_ISL_441076, EPI_ISL_441077, EPI_ISL_441082, EPI_ISL_441090, EPI_ISL_441093, EPI_ISL_441103, EPI_ISL_441107, EPI_ISL_441108, EPI_ISL_441110, EPI_ISL_441112, EPI_ISL_441118, EPI_ISL_441120, EPI_ISL_441121, EPI_ISL_441123, EPI_ISL_441125, EPI_ISL_441127, EPI_ISL_441137, EPI_ISL_441144, EPI_ISL_441145, EPI_ISL_441146, EPI_ISL_441149, EPI_ISL_441152, EPI_ISL_441153, EPI_ISL_441160, EPI_ISL_441162, EPI_ISL_441163, EPI_ISL_441166, EPI_ISL_441167, EPI_ISL_441173, EPI_ISL_441179, EPI_ISL_441182, EPI_ISL_441185, EPI_ISL_441187, EPI_ISL_441204, EPI_ISL_441211, EPI_ISL_441227, EPI_ISL_441229, EPI_ISL_441235, EPI_ISL_441241, EPI_ISL_441243, EPI_ISL_441246, EPI_ISL_441248, EPI_ISL_441250, EPI_ISL_441254, EPI_ISL_441256, EPI_ISL_441257, EPI_ISL_441264, EPI_ISL_441274, EPI_ISL_441283, EPI_ISL_441290, EPI_ISL_441291, EPI_ISL_441293, EPI_ISL_441296, EPI_ISL_441302, EPI_ISL_441303, EPI_ISL_441304, EPI_ISL_441307, EPI_ISL_441312, EPI_ISL_441321, EPI_ISL_441325, EPI_ISL_441328, EPI_ISL_441331, EPI_ISL_441339, EPI_ISL_441341			
see above	Department of Pathology, University of Cambridge	Wellcome Sanger Institute for the COVID-19 Genomics UK (COG-UK) consortium	Luke W Meredith, M. Estée Török , Myra Hosmillo, William L. Hamilton, Martin D. Curran, Theresa Feltwell, Grant Hall, Anna Yakovleva, Fahad A Khokhar, Charlotte J. Houldcroft, Laura G Caller, Aminu S. Jahun, Sarah L. Caddy, Ian Goodfellow, Alex Alderton, Roberto Amato, Sonia Goncalves, Ewan Harrison, David K. Jackson, Ian Johnston, Dominic Kwiatkowski, Cordelia Langford, John Sillitoe on behalf of the Wellcome Sanger Institute COVID-19 Surveillance Team (http://www.sanger.ac.uk/covid-team)
EPI_ISL_441659, EPI_ISL_441670, EPI_ISL_441704, EPI_ISL_441717, EPI_ISL_441733, EPI_ISL_441766, EPI_ISL_441775	Regional Virus Laboratory, Belfast Health and Social Care Trust	Wellcome Sanger Institute for the COVID-19 Genomics UK (COG-UK) consortium	Conall McCaughey, James McKenna, Tanya Curran, Susan Feeney, Alison Watt, Ciara Cox, Mairead Connor, Zoltan Molnar, David Simpson, Derek Fairley, Alex Alderton, Roberto Amato, Sonia Goncalves, Ewan Harrison, David K. Jackson, Ian Johnston, Dominic Kwiatkowski, Cordelia Langford, John Sillitoe on behalf of the Wellcome Sanger Institute COVID-19 Surveillance Team (http://www.sanger.ac.uk/covid-team)
EPI_ISL_441783, EPI_ISL_441788, EPI_ISL_441789, EPI_ISL_441790, EPI_ISL_441795, EPI_ISL_441796, EPI_ISL_441797, EPI_ISL_441803, EPI_ISL_441805, EPI_ISL_441809, EPI_ISL_441811, EPI_ISL_441816, EPI_ISL_441818, EPI_ISL_441819, EPI_ISL_441831, EPI_ISL_441835, EPI_ISL_441836, EPI_ISL_441837,			

EPI_ISL_441840, EPI_ISL_441841, EPI_ISL_441842, EPI_ISL_441844			
see above	Department of Pathology, University of Cambridge	Wellcome Sanger Institute for the COVID-19 Genomics UK (COG-UK) consortium	Luke W Meredith, M. Estée Török , Myra Hosmillo, William L. Hamilton, Martin D. Curran, Theresa Feltwell, Grant Hall, Anna Yakovleva, Fahad A Khokhar, Charlotte J. Houldcroft, Laura G Caller, Aminu S. Jahun, Sarah L. Caddy, Ian Goodfellow, Alex Alderton, Roberto Amato, Sonia Goncalves, Ewan Harrison, David K. Jackson, Ian Johnston, Dominic Kwiatkowski, Cordelia Langford, John Sillitoe on behalf of the Wellcome Sanger Institute COVID-19 Surveillance Team (http://www.sanger.ac.uk/covid-team)
EPI_ISL_441914, EPI_ISL_441934, EPI_ISL_441963, EPI_ISL_441973	Virology Department, Sheffield Teaching Hospitals NHS Foundation Trust/Department of Infection, Immunity and Cardiovascular Disease, The Medical School, University of Sheffield	COVID-19 Genomics UK (COG-UK) Consortium	Thushan de Silva, Matthew Parker, Nikki Smith, Adri Angyal, Rebecca Brown, Luke Green, Rachel Tucker, Paul Parsons, Danielle Groves, Katie Johnson, Laura Carrilero, Alex Keeley, Dave Partridge, Matthew Wyles, Benjamin Lindsey, Mehmet Yavuz, Mohammad Raza, Cariad Evans
EPI_ISL_442322	Department of Pathology, University of Cambridge	Wellcome Sanger Institute for the COVID-19 Genomics UK (COG-UK) consortium	Luke W Meredith, M. Estée Török , Myra Hosmillo, William L. Hamilton, Martin D. Curran, Theresa Feltwell, Grant Hall, Anna Yakovleva, Fahad A Khokhar, Charlotte J. Houldcroft, Laura G Caller, Aminu S. Jahun, Sarah L. Caddy, Ian Goodfellow, Alex Alderton, Roberto Amato, Sonia Goncalves, Ewan Harrison, David K. Jackson, Ian Johnston, Dominic Kwiatkowski, Cordelia Langford, John Sillitoe on behalf of the Wellcome Sanger Institute COVID-19 Surveillance Team (http://www.sanger.ac.uk/covid-team)
EPI_ISL_442348, EPI_ISL_442363, EPI_ISL_442374, EPI_ISL_442435, EPI_ISL_442461, EPI_ISL_442484	Virology Department, Sheffield Teaching Hospitals NHS Foundation Trust/Department of Infection, Immunity and Cardiovascular Disease, The Medical School, University of Sheffield	COVID-19 Genomics UK (COG-UK) Consortium	Thushan de Silva, Matthew Parker, Nikki Smith, Adri Angyal, Rebecca Brown, Luke Green, Rachel Tucker, Paul Parsons, Danielle Groves, Katie Johnson, Laura Carrilero, Alex Keeley, Dave Partridge, Matthew Wyles, Benjamin Lindsey, Mehmet Yavuz, Mohammad Raza, Cariad Evans
EPI_ISL_443184, EPI_ISL_443186	UW Virology Lab	UW Virology Lab	Pavitra Roychoudhury, Hong Xie, Keith Jerome, Alexander Greninger
EPI_ISL_443255	M Health Fairview	University of Minnesota Genomics Center	Daryl M. Gohl, John Garbe, Patrick Grady, Jerry Daniel, Ray Watson, Benjamin Auch, Andrew Nelson, Sophia Yohe, and Kenneth B. Beckman
EPI_ISL_443279, EPI_ISL_443280	CHU - Hôpital Cavale Blanche - Labo. de Virologie	National Reference Center for Viruses of Respiratory Infections, Institut Pasteur, Paris	Mélanie Albert, Marion Barbet, Sylvie Behillil, Méline Bizard, Angela Brisebarre, Flora Donati, Etienne Simon-Lorière, Vincent Enouf, Maud Vanpeene, Sylvie van der Werf, Léa Pilorge
EPI_ISL_443290	CHRU Pontchaillou - Laboratoire de Virologie	National Reference Center for Viruses of Respiratory Infections, Institut Pasteur, Paris	Mélanie Albert, Marion Barbet, Sylvie Behillil, Méline Bizard, Angela Brisebarre, Flora Donati, Etienne Simon-Lorière, Vincent Enouf, Maud Vanpeene, Sylvie van der Werf, Gisèle Lagathu
EPI_ISL_443304	Résidence Esterel	National Reference Center for Viruses of Respiratory Infections, Institut Pasteur, Paris	Mélanie Albert, Marion Barbet, Sylvie Behillil, Méline Bizard, Angela Brisebarre, Flora Donati, Etienne Simon-Lorière, Vincent Enouf, Maud Vanpeene, Sylvie van der Werf
EPI_ISL_443762, EPI_ISL_443791, EPI_ISL_443827, EPI_ISL_443829, EPI_ISL_443841, EPI_ISL_443855, EPI_ISL_443894	PHE South West Regional Laboratory, National Infection Service	Wellcome Sanger Institute for the COVID-19 Genomics UK (COG-UK) consortium	Stephanie Hutchings, Hannah Pymont, Dr Peter Muir, Barry Vipond, Rich Hopes; and Alex Alderton, Roberto Amato, Sonia Goncalves, Ewan Harrison, David K. Jackson, Ian Johnston, Dominic Kwiatkowski, Cordelia Langford, John Sillitoe on behalf of the Wellcome Sanger Institute COVID-19 Surveillance Team (http://www.sanger.ac.uk/covid-team)
EPI_ISL_444662, EPI_ISL_444663, EPI_ISL_444669, EPI_ISL_444673, EPI_ISL_444676, EPI_ISL_444677, EPI_ISL_444689, EPI_ISL_444706, EPI_ISL_444708, EPI_ISL_444709, EPI_ISL_444710, EPI_ISL_444728, EPI_ISL_444729, EPI_ISL_444731, EPI_ISL_444733			
see above	NYU Langone Health	Departments of Pathology and Medicine, New York University School of Medicine	Maria Aguero-Rosenfeld, Brendan Belovarac, Margaret Black, Ludovic Boytard, John Cadley, Paolo Cotzia, John Chen, Dacia Dimartino, Xiaojun Feng, Tatyana Gindin, Emily Guzman, Adriana Heguy, Megan Hogan, Emily Huang, George Jour, Alireza Khodadadi-Jamayran, Lawrence H. Lin, Raven Luther, Andrew Lytle, Christian Marier, Matthew T. Maurano, Mark J. Mulligan, Peter Meyn, Raquel Ordonez Ciriza, Iman Osman, Jared Pinnell, Vanessa Raabe, Sitharam Ramaswami, Amy Rapkiewicz, Andre M. Ribeiro-dos-Santos, Marie Samanovic-Golden, Antonio Serrano, Guomiao Shen, Matija Snuderl, Theodore Vougiouklakis, Nick Vulpescu, Gael Westby, Paul Zappile, Yutong Zhang
EPI_ISL_444847, EPI_ISL_444848, EPI_ISL_444954, EPI_ISL_444956, EPI_ISL_444958, EPI_ISL_444959, EPI_ISL_444960, EPI_ISL_444964, EPI_ISL_444968	Department of Virus and Microbiological Special Diagnostics, Statens Serum Institut, Copenhagen, Denmark, Artillerivej 5, 2300 Copenahgen S	Albertsen lab, Department of Chemistry and Bioscience, Aalborg University, Denmark	Rasmus Kirkegaard
EPI_ISL_445116	UC San Diego Center for Advanced Laboratory Medicine	Andersen lab at Scripps Research	SEARCH Alliance San Diego with David Pride, Ji H Shin
EPI_ISL_445119, EPI_ISL_445121, EPI_ISL_445122, EPI_ISL_445123, EPI_ISL_445125, EPI_ISL_445126, EPI_ISL_445127, EPI_ISL_445128, EPI_ISL_445130, EPI_ISL_445131, EPI_ISL_445133, EPI_ISL_445134, EPI_ISL_445135, EPI_ISL_445136, EPI_ISL_445137, EPI_ISL_445138, EPI_ISL_445139, EPI_ISL_445140, EPI_ISL_445141, EPI_ISL_445142, EPI_ISL_445143, EPI_ISL_445144, EPI_ISL_445145			
see above	Robert Garry lab	Andersen lab at Scripps Research	Allison Smither, Gilberto Sabino-Santos, Patricia Snarski, Lilia Melnik, Antoinette Bell, Kaylynn Genemaras, Arnaud Drouin, Dahlene Fusco, Robert Garry with SEARCH Alliance San Diego
EPI_ISL_445173, EPI_ISL_445174, EPI_ISL_445177, EPI_ISL_445179	UCSF Clinical Microbiology Laboratory	Chan-Zuckerberg Biohub	CZB Cliahub Consortium
EPI_ISL_445226	Saroledens Familjelakare	The Public Health Agency of Sweden	Katarina Jarbur, Oskar Karlsson Lindsjo, Maria Lind Karlberg, Anna-Malin Linde, Olov Svartstrom, Anna Risberg, Theresa Enkirch, Mia Brytting, Karin Tegmark-Wisell
EPI_ISL_445227	Uppsala Narakut Aleris	The Public Health Agency of Sweden	Annika Nilsson, Oskar Karlsson Lindsjo, Maria Lind Karlberg, Anna-Malin Linde, Olov Svartstrom, Anna Risberg, Theresa Enkirch, Mia Brytting, Karin Tegmark-Wisell
EPI_ISL_445228	Ulltuna Vardcentral	The Public Health Agency of Sweden	Heidi Lindback, Oskar Karlsson Lindsjo, Maria Lind Karlberg, Anna-Malin Linde, Olov Svartstrom, Anna Risberg, Theresa Enkirch, Mia Brytting, Karin Tegmark-Wisell
EPI_ISL_445229	Narhalsan Backa vardcentral	The Public Health Agency of Sweden	Mats Olsson, Oskar Karlsson Lindsjo, Maria Lind Karlberg, Anna-Malin Linde, Olov Svartstrom, Anna Risberg, Theresa Enkirch, Mia Brytting, Karin Tegmark-Wisell
EPI_ISL_445349, EPI_ISL_445350	HOSPITAL SAN JUAN DE DIOS	Instituto de Salud Publica de Chile	Andrés E Castillo, Bárbara Parra,Paz Tapia, Jaime Lagos, Loredana Arata, Alejandra Acevedo, Winston Andrade, Gabriel Leal, Carolina Tambley, Patricia Bustos, Rodrigo Fasce, Jorge Fernandez
EPI_ISL_445352	HOSPITAL DEL PROFESOR	Instituto de Salud Publica de Chile	Andrés E Castillo, Bárbara Parra,Paz Tapia, Jaime Lagos, Loredana Arata, Alejandra Acevedo, Winston Andrade, Gabriel Leal, Carolina Tambley, Patricia Bustos, Rodrigo Fasce, Jorge Fernandez
EPI_ISL_445381, EPI_ISL_445384, EPI_ISL_445385, EPI_ISL_445386, EPI_ISL_445387, EPI_ISL_445388, EPI_ISL_445397, EPI_ISL_445418, EPI_ISL_445419, EPI_ISL_445420, EPI_ISL_445421, EPI_ISL_445464, EPI_ISL_445465, EPI_ISL_445466, EPI_ISL_445518, EPI_ISL_445525, EPI_ISL_445568, EPI_ISL_445748, EPI_ISL_445782, EPI_ISL_445875, EPI_ISL_445876, EPI_ISL_445877, EPI_ISL_445878, EPI_ISL_445879, EPI_ISL_445880, EPI_ISL_445881, EPI_ISL_445882, EPI_ISL_445883, EPI_ISL_445884, EPI_ISL_445885, EPI_ISL_445886, EPI_ISL_445887, EPI_ISL_445888, EPI_ISL_445889, EPI_ISL_445890, EPI_ISL_445891, EPI_ISL_445892, EPI_ISL_445893, EPI_ISL_445894, EPI_ISL_445895, EPI_ISL_445896, EPI_ISL_445897, EPI_ISL_445898, EPI_ISL_445899, EPI_ISL_445900, EPI_ISL_445901, EPI_ISL_445902, EPI_ISL_445903, EPI_ISL_445904, EPI_ISL_445905, EPI_ISL_445906, EPI_ISL_445907, EPI_ISL_445908, EPI_ISL_445909, EPI_ISL_445910, EPI_ISL_445911, EPI_ISL_445912, EPI_ISL_445913, EPI_ISL_445914, EPI_ISL_445915, EPI_ISL_445916, EPI_ISL_445917, EPI_ISL_445918, EPI_ISL_445919, EPI_ISL_445920, EPI_ISL_445921, EPI_ISL_445922, EPI_ISL_445923, EPI_ISL_445924, EPI_ISL_445925, EPI_ISL_445926, EPI_ISL_445927, EPI_ISL_445928, EPI_ISL_445929, EPI_ISL_445930, EPI_ISL_445931, EPI_ISL_445932, EPI_ISL_445933, EPI_ISL_445934, EPI_ISL_445935, EPI_ISL_445936, EPI_ISL_445937, EPI_ISL_445938, EPI_ISL_445939, EPI_ISL_445940, EPI_ISL_445941, EPI_ISL_445942, EPI_ISL_445943, EPI_ISL_445944, EPI_ISL_445945, EPI_ISL_445946, EPI_ISL_445947, EPI_ISL_445948, EPI_ISL_445949, EPI_ISL_445950, EPI_ISL_445951, EPI_ISL_445952, EPI_ISL_445953, EPI_ISL_445954, EPI_ISL_445955, EPI_ISL_446271			
see above	Wales Specialist Virology Centre	Public Health Wales Microbiology Cardiff	Catherine Moore, Johnathan Evans, Laura Gifford, Malorie Perry, Simon Cottrell, Alec Birchley, Alexander Adams, Amy Gaskin, Bree Gatica-Wilcox, Jason Coombes, Lauren Gilbert, Lee Graham, Nicole Pacchiarini, Sara Kumziene-Summerhayes, Sarah Taylor, Sophie Jones, Sara Rey, Matthew Bull, Joanne Watkins, Sally Corden, Tom Connor
EPI_ISL_447015, EPI_ISL_447016, EPI_ISL_447017, EPI_ISL_447018, EPI_ISL_447019, EPI_ISL_447020, EPI_ISL_447021	Ramathibodi Hospital	COVID-19 Network Investigations (CONI) Alliance	Elizabeth Batty, Wasun Chantratita, Thanat Chookajorn, Stefan Fernandez, Angkana Huang, Anthony R. Jones, Khajohn Joonsalak, Chonticha Klungtong, Theerarat Kochakarn, Namfon Kotanan, Krittikorn Kumpornsin, Wudtichai Manasatienkij, Bhakbhoom Panthan, Ekawat Pasomsub, Kingkan Rakmanee, Insee Sensorn, Janjira Thaipadungpanit, Arporn Wangwiwatsin,Treewat Watthanachockchai
EPI_ISL_447090	Michigan Department of Health and Human Services, Bureau of Laboratories	Michigan Department of Health and Human Services, Bureau of Laboratories	Blankenship HM, Riner D, Soehnlen MK

EPI_ISL_447265, EPI_ISL_447266	Microbiology laboratory, Assuta Ashdod University-Affiliated Hospital	Stern Lab	Stern Lab
EPI_ISL_447317	Clinical Virology Laboratory, Soroka Medical Center and the Faculty of Health Sciences, Ben-Gurion University of the Negev	Stern Lab	Stern Lab
EPI_ISL_447359, EPI_ISL_447360, EPI_ISL_447361, EPI_ISL_447362, EPI_ISL_447363, EPI_ISL_447364, EPI_ISL_447365, EPI_ISL_447366, EPI_ISL_447367, EPI_ISL_447368, EPI_ISL_447369, EPI_ISL_447370, EPI_ISL_447371, EPI_ISL_447372, EPI_ISL_447373, EPI_ISL_447374, EPI_ISL_447375, EPI_ISL_447376, EPI_ISL_447377, EPI_ISL_447378, EPI_ISL_447379, EPI_ISL_447380, EPI_ISL_447381, EPI_ISL_447382			
see above	Clinical Virology Unit, Hadassah Hebrew University Medical Center	Stern Lab	Stern Lab
EPI_ISL_447383, EPI_ISL_447384, EPI_ISL_447403, EPI_ISL_447404, EPI_ISL_447405, EPI_ISL_447406	Clinical Microbiology Laboratory, The Baruch Padeh Medical Center, Poriya	Stern Lab	Stern Lab
EPI_ISL_447516	Servicio de Microbiologia. Hospital Clinico Universitario de Valencia	Sequencing and Bioinformatics Service and Molecular Epidemiology Research Group. FISABIO-Public Health	Lidia Ruiz Roldan, Neris Garcia-Gonzalez, Inma Galán Vendrell, Sandra Carbo, Loreto Ferrús Abad, Paula Ruiz-Hueso, Mariana Reyes-Prieto, Vicente Soriano Chirona, Ivan Ansari, Llúcia Martínez-Priego, Giuseppe 'Auria, David Navarro, Eliseo Albert, Maria Alma Bracho, Fernando Gonzalez-Candelas
EPI_ISL_447517	Servicio de Microbiologia. Hospital Clinico Universitario de Valencia	Sequencing and Bioinformatics Service and Molecular Epidemiology Research Group. FISABIO-Public Health	Neris Garcia-Gonzalez, Inma Galán Vendrell, Sandra Carbo, Loreto Ferrús Abad, Paula Ruiz-Hueso, Mariana Reyes-Prieto, Vicente Soriano Chirona, Ivan Ansari, Llúcia Martínez-Priego, Giuseppe 'Auria, David Navarro, Eliseo Albert, Maria Alma Bracho, Lidia Ruiz Roldan, Fernando Gonzalez-Candelas
EPI_ISL_447518	Servicio de Microbiologia. Hospital Clinico Universitario de Valencia	Sequencing and Bioinformatics Service and Molecular Epidemiology Research Group. FISABIO-Public Health	Inma Galán Vendrell, Sandra Carbo, Loreto Ferrús Abad, Paula Ruiz-Hueso, Mariana Reyes-Prieto, Vicente Soriano Chirona, Ivan Ansari, Llúcia Martínez-Priego, Giuseppe 'Auria, David Navarro, Eliseo Albert, Maria Alma Bracho, Lidia Ruiz Roldan, Neris Garcia-Gonzalez, Fernando Gonzalez-Candelas
EPI_ISL_447519	Servicio de Microbiologia. Hospital Clinico Universitario de Valencia	Sequencing and Bioinformatics Service and Molecular Epidemiology Research Group. FISABIO-Public Health	Sandra Carbo, Loreto Ferrús Abad, Paula Ruiz-Hueso, Mariana Reyes-Prieto, Vicente Soriano Chirona, Ivan Ansari, Llúcia Martínez-Priego, Giuseppe 'Auria, David Navarro, Eliseo Albert, Maria Alma Bracho, Lidia Ruiz Roldan, Neris Garcia-Gonzalez, Inma Galán Vendrell, Fernando Gonzalez-Candelas
EPI_ISL_447520	Servicio de Microbiologia. Hospital Clinico Universitario de Valencia	Sequencing and Bioinformatics Service and Molecular Epidemiology Research Group. FISABIO-Public Health	Loreto Ferrús Abad, Paula Ruiz-Hueso, Mariana Reyes-Prieto, Vicente Soriano Chirona, Ivan Ansari, Llúcia Martínez-Priego, Giuseppe 'Auria, David Navarro, Eliseo Albert, Maria Alma Bracho, Lidia Ruiz Roldan, Neris Garcia-Gonzalez, Inma Galán Vendrell, Sandra Carbo, Fernando Gonzalez-Candelas
EPI_ISL_447521	Servicio de Microbiologia. Hospital Clinico Universitario de Valencia	Sequencing and Bioinformatics Service and Molecular Epidemiology Research Group. FISABIO-Public Health	Paula Ruiz-Hueso, Mariana Reyes-Prieto, Vicente Soriano Chirona, Ivan Ansari, Llúcia Martínez-Priego, Giuseppe 'Auria, David Navarro, Eliseo Albert, Maria Alma Bracho, Lidia Ruiz Roldan, Neris Garcia-Gonzalez, Inma Galán Vendrell, Sandra Carbo, Loreto Ferrús Abad, Fernando Gonzalez-Candelas
EPI_ISL_447522	Servicio de Microbiologia. Hospital Clinico Universitario de Valencia	Sequencing and Bioinformatics Service and Molecular Epidemiology Research Group. FISABIO-Public Health	Vicente Soriano Chirona, Ivan Ansari, Llúcia Martínez-Priego, Giuseppe 'Auria, David Navarro, Eliseo Albert, Maria Alma Bracho, Lidia Ruiz Roldan, Neris Garcia-Gonzalez, Inma Galán Vendrell, Sandra Carbo, Loreto Ferrús Abad, Paula Ruiz-Hueso, Mariana Reyes-Prieto, Fernando Gonzalez-Candelas
EPI_ISL_447523	Servicio de Microbiologia. Hospital Clinico Universitario de Valencia	Sequencing and Bioinformatics Service and Molecular Epidemiology Research Group. FISABIO-Public Health	Giuseppe 'Auria, David Navarro, Eliseo Albert, Maria Alma Bracho, Lidia Ruiz Roldan, Neris Garcia-Gonzalez, Inma Galán Vendrell, Sandra Carbo, Loreto Ferrús Abad, Paula Ruiz-Hueso, Mariana Reyes-Prieto, Vicente Soriano Chirona, Ivan Ansari, Llúcia Martínez-Priego, Fernando Gonzalez-Candelas
EPI_ISL_447524	Servicio de Microbiologia. Hospital Clinico Universitario de Valencia	Sequencing and Bioinformatics Service and Molecular Epidemiology Research Group. FISABIO-Public Health	Llúcia Martínez-Priego, Giuseppe 'Auria, David Navarro, Eliseo Albert, Maria Alma Bracho, Lidia Ruiz Roldan, Neris Garcia-Gonzalez, Inma Galán Vendrell, Sandra Carbo, Loreto Ferrús Abad, Paula Ruiz-Hueso, Mariana Reyes-Prieto, Vicente Soriano Chirona, Ivan Ansari, Fernando Gonzalez-Candelas
EPI_ISL_447525	Servicio de Microbiologia. Hospital Clinico Universitario de Valencia	Sequencing and Bioinformatics Service and Molecular Epidemiology Research Group. FISABIO-Public Health	David Navarro, Eliseo Albert, Maria Alma Bracho, Griselda De Marco, Lidia Ruiz Roldan, Neris Garcia-Gonzalez, Inma Galán Vendrell, Sandra Carbo, Loreto Ferrús Abad, Paula Ruiz-Hueso, Mariana Reyes-Prieto, Vicente Soriano Chirona, Ivan Ansari, Llúcia Martínez-Priego, Giuseppe 'Auria, Fernando Gonzalez-Candelas
EPI_ISL_447526	Servicio de Microbiologia. Hospital Clinico Universitario de Valencia	Sequencing and Bioinformatics Service and Molecular Epidemiology Research Group. FISABIO-Public Health	Eliseo Albert, Maria Alma Bracho, Griselda De Marco, Lidia Ruiz Roldan, Neris Garcia-Gonzalez, Inma Galán Vendrell, Sandra Carbo, Loreto Ferrús Abad, Paula Ruiz-Hueso, Mariana Reyes-Prieto, Vicente Soriano Chirona, Ivan Ansari, Llúcia Martínez-Priego, Giuseppe 'Auria, David Navarro, Fernando Gonzalez-Candelas
EPI_ISL_447527	Servicio de Microbiologia. Hospital Clinico Universitario de Valencia	Sequencing and Bioinformatics Service and Molecular Epidemiology Research Group. FISABIO-Public Health	Maria Alma Bracho, Griselda De Marco, Lidia Ruiz Roldan, Neris Garcia-Gonzalez, Inma Galán Vendrell, Sandra Carbo, Loreto Ferrús Abad, Paula Ruiz-Hueso, Mariana Reyes-Prieto, Vicente Soriano Chirona, Ivan Ansari, Llúcia Martínez-Priego, Giuseppe 'Auria, David Navarro, Eliseo Albert, Fernando Gonzalez-Candelas
EPI_ISL_447528	Servicio de Microbiologia. Hospital Clinico Universitario de Valencia	Sequencing and Bioinformatics Service and Molecular Epidemiology Research Group. FISABIO-Public Health	Griselda De Marco, Lidia Ruiz Roldan, Neris Garcia-Gonzalez, Inma Galán Vendrell, Sandra Carbo, Loreto Ferrús Abad, Paula Ruiz-Hueso, Mariana Reyes-Prieto, Vicente Soriano Chirona, Ivan Ansari, Llúcia Martínez-Priego, Giuseppe 'Auria, David Navarro, Eliseo Albert, Maria Alma Bracho, Fernando Gonzalez-Candelas
EPI_ISL_447529	Servicio de Microbiologia. Hospital Clinico Universitario de Valencia	Sequencing and Bioinformatics Service and Molecular Epidemiology Research Group. FISABIO-Public Health	Lidia Ruiz Roldan, Neris Garcia-Gonzalez, Inma Galán Vendrell, Sandra Carbo, Loreto Ferrús Abad, Paula Ruiz-Hueso, Mariana Reyes-Prieto, Vicente Soriano Chirona, Ivan Ansari, Llúcia Martínez-Priego, Giuseppe 'Auria, David Navarro, Eliseo Albert, Maria Alma Bracho, Fernando Gonzalez-Candelas
EPI_ISL_447530	Servicio de Microbiologia. Hospital Clinico Universitario de Valencia	Sequencing and Bioinformatics Service and Molecular Epidemiology Research Group. FISABIO-Public Health	Neris Garcia-Gonzalez, Inma Galán Vendrell, Sandra Carbo, Loreto Ferrús Abad, Paula Ruiz-Hueso, Mariana Reyes-Prieto, Vicente Soriano Chirona, Ivan Ansari, Llúcia Martínez-Priego, Giuseppe 'Auria, David Navarro, Eliseo Albert, Maria Alma Bracho, Lidia Ruiz Roldan, Fernando Gonzalez-Candelas
EPI_ISL_447531	Servicio de Microbiologia. Hospital Clinico Universitario de Valencia	Sequencing and Bioinformatics Service and Molecular Epidemiology Research Group. FISABIO-Public Health	Inma Galán Vendrell, Sandra Carbo, Loreto Ferrús Abad, Paula Ruiz-Hueso, Mariana Reyes-Prieto, Vicente Soriano Chirona, Ivan Ansari, Llúcia Martínez-Priego, Giuseppe 'Auria, David Navarro, Eliseo Albert, Maria Alma Bracho, Lidia Ruiz Roldan, Neris Garcia-Gonzalez, Fernando Gonzalez-Candelas
EPI_ISL_447559	CSIR-Centre for Cellular and Molecular Biology	CSIR-Centre for Cellular and Molecular Biology	Sofia Banu, Payel Mukherjee, Priya Singh, Dhiviya Vedagiri, Divya Gupta, Vishal Sah, Santosh Kumar Kuncha, Krishnan Harinivas Harshan, Archana Bharadwaj Siva, Karthik Bharadwaj Tallapaka, Shagufta Khan, Lamuk Zaveri, Namami Gaur, Sakshi Shambhavi, Tulasi Nagabandi, Purushotham Vodnala, Rakesh K Mishra, Divya Tej Sowpati
EPI_ISL_447560	CSIR-Centre for Cellular and Molecular Biology	CSIR-Centre for Cellular and Molecular Biology	Payel Mukherjee, Sofia Banu, Priya Singh, Dhiviya Vedagiri, Divya Gupta, Vishal Sah, Santosh Kumar Kuncha, Krishnan Harinivas Harshan, Archana Bharadwaj Siva, Karthik Bharadwaj Tallapaka, Shagufta Khan, Lamuk Zaveri, Namami Gaur, Sakshi Shambhavi, Tulasi Nagabandi, Purushotham Vodnala, Rakesh K Mishra, Divya Tej Sowpati
EPI_ISL_447561, EPI_ISL_447562	CSIR-Centre for Cellular and Molecular Biology	CSIR-Centre for Cellular and Molecular Biology	Shagufta Khan, Lamuk Zaveri, Namami Gaur, Sakshi Shambhavi, Tulasi Nagabandi, Purushotham Vodnala, Payel Mukherjee, Sofia Banu, Priya Singh, Dhiviya Vedagiri, Divya Gupta, Vishal Sah, Santosh Kumar Kuncha, Krishnan Harinivas Harshan, Archana Bharadwaj Siva, Karthik Bharadwaj Tallapaka, Rakesh K Mishra, Divya Tej Sowpati
EPI_ISL_447563	CSIR-Centre for Cellular and Molecular Biology	CSIR-Centre for Cellular and Molecular Biology	Sakshi Shambhavi, Lamuk Zaveri, Shagufta Khan, Namami Gaur, Tulasi Nagabandi, Purushotham Vodnala, Payel Mukherjee, Sofia Banu, Priya Singh, Dhiviya Vedagiri, Divya Gupta, Vishal Sah, Santosh Kumar Kuncha, Krishnan Harinivas Harshan, Archana Bharadwaj Siva, Karthik Bharadwaj Tallapaka, Rakesh K Mishra, Divya Tej Sowpati
EPI_ISL_447564, EPI_ISL_447565, EPI_ISL_447566	CSIR-Centre for Cellular and Molecular Biology	CSIR-Centre for Cellular and Molecular Biology	Sofia Banu, Payel Mukherjee, Priya Singh, Dhiviya Vedagiri, Divya Gupta, Vishal Sah, Santosh Kumar Kuncha, Krishnan Harinivas Harshan, Archana Bharadwaj Siva, Karthik Bharadwaj Tallapaka, Shagufta Khan, Lamuk Zaveri, Namami Gaur, Sakshi Shambhavi, Tulasi Nagabandi, Purushotham Vodnala, Rakesh K Mishra, Divya Tej Sowpati
EPI_ISL_447567	CSIR-Centre for Cellular and Molecular Biology	CSIR-Centre for Cellular and Molecular Biology	Payel Mukherjee, Sofia Banu, Priya Singh, Dhiviya Vedagiri, Divya Gupta, Vishal Sah, Santosh Kumar Kuncha, Krishnan Harinivas Harshan, Archana Bharadwaj Siva, Karthik Bharadwaj Tallapaka, Shagufta Khan, Lamuk Zaveri, Namami Gaur, Sakshi Shambhavi, Tulasi Nagabandi, Purushotham Vodnala, Rakesh K Mishra, Divya Tej Sowpati
EPI_ISL_447568, EPI_ISL_447569	CSIR-Centre for Cellular and Molecular Biology	CSIR-Centre for Cellular and Molecular Biology	Shagufta Khan, Lamuk Zaveri, Namami Gaur, Sakshi Shambhavi, Tulasi Nagabandi, Purushotham Vodnala, Payel Mukherjee, Sofia Banu, Priya Singh, Dhiviya Vedagiri, Divya Gupta, Vishal Sah, Santosh Kumar Kuncha, Krishnan Harinivas Harshan, Archana Bharadwaj Siva, Karthik Bharadwaj Tallapaka, Rakesh K Mishra, Divya Tej Sowpati
EPI_ISL_447570, EPI_ISL_447571, EPI_ISL_447572	CSIR-Centre for Cellular and Molecular Biology	CSIR-Centre for Cellular and Molecular Biology	Lamuk Zaveri, Shagufta Khan, Namami Gaur, Sakshi Shambhavi, Tulasi Nagabandi, Purushotham Vodnala, Payel Mukherjee, Sofia Banu, Priya Singh, Dhiviya Vedagiri, Divya Gupta, Vishal Sah, Santosh Kumar Kuncha, Krishnan Harinivas Harshan, Archana Bharadwaj Siva, Karthik Bharadwaj Tallapaka,

			Rakesh K Mishra, Divya Tej Sowpati
EPI_ISL_447573	CSIR-Centre for Cellular and Molecular Biology	CSIR-Centre for Cellular and Molecular Biology	Sakshi Shambhavi, Lamuk Zaveri, Shagufta Khan, Namami Gaur, Tulasi Nagabandi, Purushotham Vodnala, Payel Mukherjee, Sofia Banu, Priya Singh, Dhiviya Vedagiri, Divya Gupta, Vishal Sah, Santosh Kumar Kuncha, Krishnan Harinivas Harshan, Archana Bharadwaj Siva, Karthik Bharadwaj Tallapaka, Rakesh K Mishra, Divya Tej Sowpati
EPI_ISL_447574	CSIR-Centre for Cellular and Molecular Biology	CSIR-Centre for Cellular and Molecular Biology	Namami Gaur, Sakshi Shambhavi, Lamuk Zaveri, Shagufta Khan, Tulasi Nagabandi, Purushotham Vodnala, Payel Mukherjee, Sofia Banu, Priya Singh, Dhiviya Vedagiri, Divya Gupta, Vishal Sah, Santosh Kumar Kuncha, Krishnan Harinivas Harshan, Archana Bharadwaj Siva, Karthik Bharadwaj Tallapaka, Rakesh K Mishra, Divya Tej Sowpati
EPI_ISL_447593	TSGH-CP molecular lab	TSGH-CP molecular lab	Cherng-Lih Perng, Ming-Jr JIAN, Chih-Kai Chang, Jung-Chung Lin, Kuo-Ming Yeh, Chien-Wen Chen, Sheng-Kang Chiu, Hsing-Yi Chung, Shih-Hung Tsai, Kuo-Sheng Hung, Tien-Yao Chang, Feng-Yee Chang, Hung-Sheng Shang
EPI_ISL_447754	Grupo de Investigaciones Microbiológicas-UR (GIMUR), Departamento de Biología, Facultad de Ciencias Naturales, Universidad del Rosario, Bogotá, Colombia	Grupo de Investigaciones Microbiológicas-UR (GIMUR), Departamento de Biología, Facultad de Ciencias Naturales, Universidad del Rosario, Bogotá, Colombia Instituto Nacional de Salud, Bogotá, Colombia Icahn School of Medicine at Mount Sinai, New York, USA	Juan David Ramírez, Carolina Florez, Marina Muñoz, Carolina Hernandez, Adriana Castillo, Sergio Castañeda, Nathalia Ballesteros, David Martínez, Laura Vega, Jesús E. Jaimes, Sergio Gomez, Angelica Rico, Lisseth Pardo, Esther C. Barros, Martha L. Ospina, Aníbal A. Teherán, Ana S. Gonzalez-Reiche, Matthew M. Hernandez, Emilia Mia Sordillo, Viviana Simon, Harm van Bakel, Alberto Paniz-Mondolfi
EPI_ISL_447755, EPI_ISL_447756, EPI_ISL_447757, EPI_ISL_447758, EPI_ISL_447759, EPI_ISL_447760, EPI_ISL_447761, EPI_ISL_447762, EPI_ISL_447763, EPI_ISL_447764, EPI_ISL_447765, EPI_ISL_447766, EPI_ISL_447767, EPI_ISL_447768, EPI_ISL_447769, EPI_ISL_447771, EPI_ISL_447772, EPI_ISL_447774, EPI_ISL_447775, EPI_ISL_447776, EPI_ISL_447777, EPI_ISL_447778, EPI_ISL_447779, EPI_ISL_447780, EPI_ISL_447781			
see above	Instituto Nacional de Salud, Bogotá, Colombia	Grupo de Investigaciones Microbiológicas-UR (GIMUR), Departamento de Biología, Facultad de Ciencias Naturales, Universidad del Rosario, Bogotá, Colombia Instituto Nacional de Salud, Bogotá, Colombia Icahn School of Medicine at Mount Sinai, New York, USA	Juan David Ramírez, Carolina Florez, Marina Muñoz, Carolina Hernandez, Adriana Castillo, Sergio Castañeda, Nathalia Ballesteros, David Martínez, Laura Vega, Jesús E. Jaimes, Sergio Gomez, Angelica Rico, Lisseth Pardo, Esther C. Barros, Martha L. Ospina, Aníbal A. Teherán, Ana S. Gonzalez-Reiche, Matthew M. Hernandez, Emilia Mia Sordillo, Viviana Simon, Harm van Bakel, Alberto Paniz-Mondolfi
EPI_ISL_447835	unknown	Department of Medicine	Kassela,K., Dovrolis,N., Bampali,M., Gatzidou,E., Froukala,E., Stavropoulou,A., Veletza,S., Tsakris,A., Spanakis,N. and Karakasiliotis,I.
EPI_ISL_447847	CSIR-Centre for Cellular and Molecular Biology	CSIR-Centre for Cellular and Molecular Biology	Payel Mukherjee, Sofia Banu, Priya Singh, Dhiviya Vedagiri, Divya Gupta, Vishal Sah, Santosh Kumar Kuncha, Krishnan Harinivas Harshan, Archana Bharadwaj Siva, Karthik Bharadwaj Tallapaka, Shagufta Khan, Lamuk Zaveri, Namami Gaur, Sakshi Shambhavi, Tulasi Nagabandi, Purushotham Vodnala, Rakesh K Mishra, Divya Tej Sowpati
EPI_ISL_447848	CSIR-Centre for Cellular and Molecular Biology	CSIR-Centre for Cellular and Molecular Biology	Sofia Banu, Payel Mukherjee, Priya Singh, Dhiviya Vedagiri, Divya Gupta, Vishal Sah, Santosh Kumar Kuncha, Krishnan Harinivas Harshan, Archana Bharadwaj Siva, Karthik Bharadwaj Tallapaka, Shagufta Khan, Lamuk Zaveri, Namami Gaur, Sakshi Shambhavi, Tulasi Nagabandi, Purushotham Vodnala, Rakesh K Mishra, Divya Tej Sowpati
EPI_ISL_447849, EPI_ISL_447850	CSIR-Centre for Cellular and Molecular Biology	CSIR-Centre for Cellular and Molecular Biology	Shagufta Khan, Lamuk Zaveri, Namami Gaur, Sakshi Shambhavi, Tulasi Nagabandi, Purushotham Vodnala, Payel Mukherjee, Sofia Banu, Priya Singh, Dhiviya Vedagiri, Divya Gupta, Vishal Sah, Santosh Kumar Kuncha, Krishnan Harinivas Harshan, Archana Bharadwaj Siva, Karthik Bharadwaj Tallapaka, Rakesh K Mishra, Divya Tej Sowpati
EPI_ISL_447851, EPI_ISL_447852	CSIR-Centre for Cellular and Molecular Biology	CSIR-Centre for Cellular and Molecular Biology	Lamuk Zaveri, Shagufta Khan, Namami Gaur, Sakshi Shambhavi, Tulasi Nagabandi, Purushotham Vodnala, Payel Mukherjee, Sofia Banu, Priya Singh, Dhiviya Vedagiri, Divya Gupta, Vishal Sah, Santosh Kumar Kuncha, Krishnan Harinivas Harshan, Archana Bharadwaj Siva, Karthik Bharadwaj Tallapaka, Rakesh K Mishra, Divya Tej Sowpati
EPI_ISL_447853	CSIR-Centre for Cellular and Molecular Biology	CSIR-Centre for Cellular and Molecular Biology	Namami Gaur, Sakshi Shambhavi, Lamuk Zaveri, Shagufta Khan, Tulasi Nagabandi, Purushotham Vodnala, Payel Mukherjee, Sofia Banu, Priya Singh, Dhiviya Vedagiri, Divya Gupta, Vishal Sah, Santosh Kumar Kuncha, Krishnan Harinivas Harshan, Archana Bharadwaj Siva, Karthik Bharadwaj Tallapaka, Rakesh K Mishra, Divya Tej Sowpati
EPI_ISL_447862	CSIR-Centre for Cellular and Molecular Biology	CSIR-Centre for Cellular and Molecular Biology	Payel Mukherjee, Sofia Banu, Priya Singh, Dhiviya Vedagiri, Divya Gupta, Vishal Sah, Santosh Kumar Kuncha, Krishnan Harinivas Harshan, Archana Bharadwaj Siva, Karthik Bharadwaj Tallapaka, Shagufta Khan, Lamuk Zaveri, Namami Gaur, Sakshi Shambhavi, Tulasi Nagabandi, Purushotham Vodnala, Rakesh K Mishra, Divya Tej Sowpati
EPI_ISL_447889, EPI_ISL_447891	University of California, Davis	Chan-Zuckerberg Biohub	CZB Cliahub Consortium
EPI_ISL_448116	West of Scotland Specialist Virology Centre, NHSGGC / MRC-University of Glasgow Centre for Virus Research	COVID-19 Genomics UK (COG-UK) Consortium	Ana da Silva Filipe, Natasha Johnson, Kathy Smollett, Daniel Mair, Stephen Carmichael, Lily Tong, Jenna Nichols, Elihu Aranday-Cortes, Kirstyn Brunker, Yasmin Parr, Kyriaki Nomikou, Sarah McDonald, Marc Niebel, Patawee Asamaphan, Richard Orton, Joseph Hughes, Sreenu Vattipally, David L Robertson, Alasdair MacLean, Rory Gunson, Kathy Li, Natasha Jesudason, Rajiv Shah, James Shepherd, Antonia Ho, Emma Thomson
EPI_ISL_448894	Virology Laboratory, Castle Hill Hospital, Hull University Teaching Hospitals NHS Trust/Department of Infection, Immunity and Cardiovascular Disease, The Medical School, University of Sheffield	COVID-19 Genomics UK (COG-UK) Consortium	Thushan de Silva, Matthew Parker, Nikki Smith, Adri Angyal, Rebecca Brown, Luke Green, Rachel Tucker, Paul Parsons, Danielle Groves, Katie Johnson, Laura Carrilero, Alex Keeley, Dave Partridge, Matthew Wyles, Benjamin Lindsey, Mehmet Yavuz, Mohammad Raza, Cariad Evans
EPI_ISL_449381, EPI_ISL_449382, EPI_ISL_449383, EPI_ISL_449384, EPI_ISL_449385, EPI_ISL_449386, EPI_ISL_449387, EPI_ISL_449388, EPI_ISL_449389, EPI_ISL_449448, EPI_ISL_449449, EPI_ISL_449450, EPI_ISL_449451, EPI_ISL_449452, EPI_ISL_449504, EPI_ISL_449505, EPI_ISL_449506, EPI_ISL_449507, EPI_ISL_449508, EPI_ISL_449509, EPI_ISL_449510, EPI_ISL_449511, EPI_ISL_449512, EPI_ISL_449513, EPI_ISL_449514, EPI_ISL_449515, EPI_ISL_449517, EPI_ISL_449518, EPI_ISL_449519, EPI_ISL_449520, EPI_ISL_449521, EPI_ISL_449522, EPI_ISL_449523, EPI_ISL_449530, EPI_ISL_449531, EPI_ISL_449532, EPI_ISL_449534, EPI_ISL_449536, EPI_ISL_449540, EPI_ISL_449542, EPI_ISL_449543, EPI_ISL_449567, EPI_ISL_449568, EPI_ISL_449569, EPI_ISL_449570, EPI_ISL_449571, EPI_ISL_449572, EPI_ISL_449573, EPI_ISL_449574, EPI_ISL_449576, EPI_ISL_449577, EPI_ISL_449578, EPI_ISL_449579			
see above	Liverpool Clinical Laboratories	COVID-19 Genomics UK (COG-UK) Consortium	Sam Haldenby, Anita Lucaci, Steve Paterson, Julian Hiscox, Alistair Darby, M Almsaud, A Alrezaihi, Muhannad Alruwaili, Stuart D Armstrong, Jones Benjamin , Eleanor G Bentley, Anu Chawla, Jordan J Clark, Angela Cowell, Richard Eccles, Isabel García-Dorival, Matthew Gemmell, Alessandro Gerada, PKF Gilmore, Richard Gregory, Ximeng Han, Catherine Hartley, Margaret Hughes, Miren Iturriza-Gomara, James Johnson, L Luu, Jenifer Manson , Charlotte Nelson, Elaine O'Toole, Cassie Olateju, Rebekah Penrice-Randal , Lucille Rainbow, N.P Randle, Trevor Ian Robinson, Parul Sharma, Ghada T Shawli, James P Stewart , Neil Swainston, Ecaterina Vamos, Joanne Watts, Mark Whitehead
EPI_ISL_449790	Dept. of Medical Microbiology, Stavanger University Hospital, Helse Stavanger HF	Norwegian Institute of Public Health, Department of Virology	Kathrine Stene-Johansen, Kamilla Heddeland Instefjord, Hilde Elshaug, Rasmus Riis Kopperud, Karoline Bragstad, Olav Hungnes
EPI_ISL_449800	HOSPITAL SAN JUAN DE DIOS	Instituto de Salud Publica de Chile	Andrés E Castillo, Bárbara Parra,Paz Tapia, Jaime Lagos, Loredana Arata, Alejandra Acevedo, Winston Andrade, Gabriel Leal, Carolina Tambley, Patricia Bustos, Rodrigo Fasce, Jorge Fernandez
EPI_ISL_449811, EPI_ISL_449812, EPI_ISL_449813	Utah Public Health Laboratory	Utah Public Health Laboratory	Erin Young, Kelly Oakeson
EPI_ISL_450217, EPI_ISL_450218, EPI_ISL_450219, EPI_ISL_450220, EPI_ISL_450221, EPI_ISL_450222, EPI_ISL_450223, EPI_ISL_450224, EPI_ISL_450225, EPI_ISL_450226, EPI_ISL_450227, EPI_ISL_450228, EPI_ISL_450229, EPI_ISL_450230			
see above	unknown	Hamadan University of Medical Sciences	Teimoori,A., Azizi Jallilian,F., Ansari,N., Jamehdor,S., Nazari,A., Saadat,N., Mazaheri,Z., Zanjani,M.
EPI_ISL_450231	Robert Garry lab	Andersen lab at Scripps Research	Allison Smither, Gilberto Sabino-Santos, Patricia Snarski, Lilia Melnik, Antoinette Bell, Kaylynn Genemaras, Arnaud Drouin, Dahlene Fusco, Robert Garry with SEARCH Alliance San Diego
EPI_ISL_450299, EPI_ISL_450301	National Institute for Communicable Diseases of the National Health Laboratory Service	National Institute for Communicable Diseases of the National Health Laboratory Service	Allam M, Ismail A, Khumalo Z, Kwenda S, van Heusden P, Mtshali P, Mnyameni F, Mohale T, Subramoney K, Bhiman JN
EPI_ISL_450326	CSIR-Centre for Cellular and Molecular Biology	CSIR-Centre for Cellular and Molecular Biology	Payel Mukherjee, Sofia Banu, Priya Singh, Dhiviya Vedagiri, Divya Gupta, Vishal Sah, Santosh Kumar Kuncha, Krishnan Harinivas Harshan, Archana Bharadwaj Siva, Karthik Bharadwaj Tallapaka, Shagufta Khan, Lamuk Zaveri, Namami Gaur, Sakshi Shambhavi, Tulasi Nagabandi, Purushotham Vodnala,G. Aditya Kumar, Koushick Sivakumar, Pooja Ramesh Gupta, Rajan Kumar Jha, Shraddha Vijay Lahoti, Deepak Kumar, Devi Prasad Vijayashankara, Disha Nanda, Divya Das, Jotin Gogoi, Manish Bhattacharjee, Rakesh K Mishra, Divya Tej Sowpati
EPI_ISL_450327	CSIR-Centre for Cellular and Molecular Biology	CSIR-Centre for Cellular and Molecular Biology	Sofia Banu, Payel Mukherjee, Priya Singh, Dhiviya Vedagiri, Divya Gupta, Vishal Sah, Santosh Kumar Kuncha, Krishnan Harinivas Harshan, Archana Bharadwaj Siva, Karthik Bharadwaj Tallapaka, Shagufta Khan, Lamuk Zaveri, Namami Gaur, Sakshi Shambhavi, Tulasi Nagabandi, Purushotham Vodnala,

			Disha Nanda, Divya Das, Jotin Gogoi, Manish Bhattacharjee, Ravi Prasad Mukku, Renu Sudhakar, Somesh Gorde, Gangumala Srinivas Reddy, Sujoy Deb, Swati Bayyana, Zeba Rizvi, Rakesh K Mishra, Divya Tej Sowpati
EPI_ISL_450328	CSIR-Centre for Cellular and Molecular Biology	CSIR-Centre for Cellular and Molecular Biology	Shagufta Khan, Lamuk Zaveri, Namami Gaur, Sakshi Shambhavi, Tulasi Nagabandi, Purushotham Vodnala, Payel Mukherjee, Sofia Banu, Priya Singh, Dhiviya Vedagiri, Divya Gupta, Vishal Sah, Santosh Kumar Kuncha, Krishnan Harinivas Harshan, Archana Bharadwaj Siva, Karthik Bharadwaj Tallapaka, Zeba Rizvi, Zuberwasim Sayyad, Kakade Aishwarya Arun, Amrutha H C, Ananga Ghosh, Kezia J Ann, Radhika Khandelwal, Roshan Maku Venkata, Shemin Mansuri, Sonu Uday, Sudipta Mondal, Rakesh K Mishra, Divya Tej Sowpati
EPI_ISL_450329	CSIR-Centre for Cellular and Molecular Biology	CSIR-Centre for Cellular and Molecular Biology	Namami Gaur, Sakshi Shambhavi, Lamuk Zaveri, Shagufta Khan, Tulasi Nagabandi, Purushotham Vodnala, Payel Mukherjee, Sofia Banu, Priya Singh, Dhiviya Vedagiri, Divya Gupta, Vishal Sah, Santosh Kumar Kuncha, Krishnan Harinivas Harshan, Archana Bharadwaj Siva, Karthik Bharadwaj Tallapaka, Sonu Uday, Sudipta Mondal, Annapoorna P Karthyayani, Debabrata Jana, Debrya Saha, Gokulan C G, Gunjan Purohit, Hanuman Tulashiram Kale, Pankaj Kumar, Prachand Issarapu, Preethi Jampala Rakesh K Mishra, Divya Tej Sowpati
EPI_ISL_450331	CSIR-Centre for Cellular and Molecular Biology	CSIR-Centre for Cellular and Molecular Biology	Tulasi Nagabandi, Namami Gaur, Sakshi Shambhavi, Lamuk Zaveri, Shagufta Khan, Purushotham Vodnala, Payel Mukherjee, Sofia Banu, Priya Singh, Dhiviya Vedagiri, Divya Gupta, Vishal Sah, Santosh Kumar Kuncha, Krishnan Harinivas Harshan, Archana Bharadwaj Siva, Karthik Bharadwaj Tallapaka,G. Aditya Kumar, Koushick Sivakumar, Pooja Ramesh Gupta, Rajan Kumar Jha, Shraddha Vijay Lahoti, Deepak Kumar, Devi Prasad Vijayashankara, Disha Nanda, Divya Das, Jotin Gogoi, Manish Bhattacharjee, Rakesh K Mishra, Divya Tej Sowpati
EPI_ISL_450332	CSIR-Centre for Cellular and Molecular Biology	CSIR-Centre for Cellular and Molecular Biology	Payel Mukherjee, Sofia Banu, Priya Singh, Dhiviya Vedagiri, Divya Gupta, Vishal Sah, Santosh Kumar Kuncha, Krishnan Harinivas Harshan, Archana Bharadwaj Siva, Karthik Bharadwaj Tallapaka, Shagufta Khan, Lamuk Zaveri, Namami Gaur, Sakshi Shambhavi, Tulasi Nagabandi, Purushotham Vodnala,G. Aditya Kumar, Koushick Sivakumar, Pooja Ramesh Gupta, Rajan Kumar Jha, Shraddha Vijay Lahoti, Deepak Kumar, Devi Prasad Vijayashankara, Disha Nanda, Divya Das, Jotin Gogoi, Manish Bhattacharjee, Rakesh K Mishra, Divya Tej Sowpati
EPI_ISL_450401	NYU Langone Health	Departments of Pathology and Medicine, New York University School of Medicine	Maria Aguero-Rosenfeld, Brendan Belovarac, Margaret Black, Ludovic Boytard, John Cadley, Paolo Cotzia, John Chen, Dacia Dimartino, Xiaojun Feng, Tatyana Gindin, Emily Guzman, Adriana Heguy, Megan Hogan, Emily Huang, George Jour, Alireza Khodadadi-Jamayran, Lawrence H. Lin, Raven Luther, Andrew Lytle, Christian Marier, Matthew T. Maurano, Mark J. Mulligan, Peter Meyn, Raquel Ordonez Ciriza, Iman Osman, Jared Pinnell, Vanessa Raabe, Sitharam Ramaswami, Amy Rapkiewicz, Andre M. Ribeiro-dos-Santos, Marie Samanovic-Golden, Antonio Serrano, Guomiao Shen, Matija Snuderl, Theodore Vougiouklakis, Nick Vulpescu, Gael Westby, Paul Zappile, Yutong Zhang
EPI_ISL_450406	Molecular Diagnostics, Antech Diagnostics	Molecular Diagnostics, Antech Diagnostics	Leutenegger,C.M., Lozoya,C.E., Tereski,J.L., Wyler,D. and Moroff,S.
EPI_ISL_450443	Institute for Forensic Medicine, Faculty of Medicine, University of Belgrade	Institute for Forensic Medicine, Faculty of Medicine, University of Belgrade	Vidanovic,D., Skadric,I.R., Dordevic,N., Tolic,A., Tesovic,B., Sekler,M., Dmitric,M., Debeljak,Z., Zarkovic,A., Kolarevic,M., Petrovic,T. and Baskic,D.
EPI_ISL_450486, EPI_ISL_450487	unknown	Data Science	Carroll,T.D., Tran,N.K., Cohen,S.H., Miller,C.J.
EPI_ISL_450529, EPI_ISL_450530	Hematology Laboratory, Section of Molecular Diagnostics, University Clinical Centre, Medical University of Gdansk	Department of Virology, Faculty of Medicine, University of Helsinki, Helsinki, Finland	Maciej Grzybek, Marlena Robakowska, Aneta Szulc, Olli Vapalahti, Teemu Smura
EPI_ISL_450708, EPI_ISL_450721	University of Wisconsin-Madison AIDS Vaccine Research Laboratories	University of Wisconsin-Madison AIDS Vaccine Research Laboratories	Gage Moreno, Katarina Braun, et al. AIDS Vaccine Research Laboratories
EPI_ISL_450731, EPI_ISL_450732	Hospital AZ Rivierenland	Institute of Tropical Medicine	Philippe Selhorst, Colin Anthony
EPI_ISL_450756, EPI_ISL_450757, EPI_ISL_450758, EPI_ISL_450769	Minnesota Department of Health, Public Health Laboratory	Minnesota Department of Health, Public Health Laboratory	Matt Plumb, Jacob Garfin, and Xiong Wang
EPI_ISL_450800	AR Dept. of Health-Public Health Lab	Pathogen Discovery, Respiratory Viruses Branch, Division of Viral Diseases, Centers for Disease Control and Prevention	Yan Li, Anna Montmayeur, Ying Tao, Krista Queen, Jing Zhang, Anna Uehara, Clinton R. Paden, Rachel Marine, Haibin Wang, Zachary Weiner, Bettina Bankamp, Suxiang Tong
EPI_ISL_450802	PA Department of Health, Bureau of Laboratories	Pathogen Discovery, Respiratory Viruses Branch, Division of Viral Diseases, Centers for Disease Control and Prevention	Yan Li, Anna Montmayeur, Ying Tao, Krista Queen, Jing Zhang, Anna Uehara, Clinton R. Paden, Rachel Marine, Haibin Wang, Zachary Weiner, Bettina Bankamp, Suxiang Tong
EPI_ISL_450808	Saroledens Familjelakare	The Public Health Agency of Sweden	Katarina Jarbur, Anna-Malin Linde, Maria Lind Karlberg, Oskar Karlsson Lindsjo, Olov Svartstrom, Anna Risberg, Theresa Enkirch, Mia Brytting, Karin Tegmark-Wisell
EPI_ISL_450809	Kungsholmsdoktorn	The Public Health Agency of Sweden	Linus Hammar, Anna-Malin Linde, Maria Lind Karlberg, Oskar Karlsson Lindsjo, Olov Svartstrom, Anna Risberg, Theresa Enkirch, Mia Brytting, Karin Tegmark-Wisell
EPI_ISL_450810	Saroledens Familjelakare	The Public Health Agency of Sweden	Katarina Jarbur, Anna-Malin Linde, Maria Lind Karlberg, Oskar Karlsson Lindsjo, Olov Svartstrom, Anna Risberg, Theresa Enkirch, Mia Brytting, Karin Tegmark-Wisell
EPI_ISL_450901, EPI_ISL_450941, EPI_ISL_450981, EPI_ISL_451021, EPI_ISL_451061	Center of Excellence in Clinical Virology	Center of Excellence in Clinical Virology	Puenpa,J., Chansaenroj,J., Nilyanimit,P., Auphimai,C., Yorsaeng,R., Suwannakarn,K., Poovorawan,Y.
EPI_ISL_451109, EPI_ISL_451110, EPI_ISL_451111, EPI_ISL_451112, EPI_ISL_451113, EPI_ISL_451114, EPI_ISL_451115, EPI_ISL_451116, EPI_ISL_451117, EPI_ISL_451118, EPI_ISL_451119, EPI_ISL_451120, EPI_ISL_451121, EPI_ISL_451122, EPI_ISL_451123, EPI_ISL_451124, EPI_ISL_451125, EPI_ISL_451126, EPI_ISL_451127, EPI_ISL_451143			
see above	SA Pathology	SA Pathology	Lex Leong, Chuan Kok Lim, Mark Turra, Ivan Bastian, Geoff Higgins
EPI_ISL_451165, EPI_ISL_451166, EPI_ISL_451167	Lab voor klinische biologie	Onderzoeksgroep Virologie	Laurens Lambrechts, Nick Vereecke, Marthe Pauwels, Jozefien De Clercq, Bruno Verhasselt, Linos Vandekerckhove, Hans Nauwynck, Sebastiaan Theuns
EPI_ISL_451496, EPI_ISL_451538, EPI_ISL_451546, EPI_ISL_451547	Pathology West - NSW Health Pathology	NSW Health Pathology - Institute of Clinical Pathology and Medical Research; Westmead Hospital; University of Sydney	CIDM-PH et al.
EPI_ISL_451549	Pathology Sydney South West - NSW Health Pathology	NSW Health Pathology - Institute of Clinical Pathology and Medical Research; Westmead Hospital; University of Sydney	CIDM-PH et al.
EPI_ISL_451553	Medlab Pathology	NSW Health Pathology - Institute of Clinical Pathology and Medical Research; Westmead Hospital; University of Sydney	CIDM-PH et al.
EPI_ISL_451557	Pathology West - NSW Health Pathology	NSW Health Pathology - Institute of Clinical Pathology and Medical Research; Westmead Hospital; University of Sydney	CIDM-PH et al.
EPI_ISL_451564	Pathology Sydney South West - NSW Health Pathology	NSW Health Pathology - Institute of Clinical Pathology and Medical Research; Westmead Hospital; University of Sydney	CIDM-PH et al.
EPI_ISL_451570, EPI_ISL_451577, EPI_ISL_451580	Pathology West - NSW Health Pathology	NSW Health Pathology - Institute of Clinical Pathology and Medical Research; Westmead Hospital; University of Sydney	CIDM-PH et al.
EPI_ISL_451581	Pathology Sydney South West - NSW Health Pathology	NSW Health Pathology - Institute of Clinical Pathology and Medical Research; Westmead Hospital; University of Sydney	CIDM-PH et al.
EPI_ISL_451582	Pathology West - NSW Health Pathology	NSW Health Pathology - Institute of Clinical Pathology and Medical Research; Westmead Hospital; University of Sydney	CIDM-PH et al.
EPI_ISL_451591	ACT pathology	NSW Health Pathology - Institute of Clinical Pathology and Medical Research; Westmead Hospital; University of Sydney	CIDM-PH et al.
EPI_ISL_451599	Australian Clinical Labs	NSW Health Pathology - Institute of Clinical Pathology and Medical Research; Westmead Hospital; University of Sydney	CIDM-PH et al.

EPI_ISL_451603	Pathology Sydney South West - NSW Health Pathology	NSW Health Pathology - Institute of Clinical Pathology and Medical Research; Westmead Hospital; University of Sydney	CIDM-PH et al.
EPI_ISL_451606	Pathology West - NSW Health Pathology	NSW Health Pathology - Institute of Clinical Pathology and Medical Research; Westmead Hospital; University of Sydney	CIDM-PH et al.
EPI_ISL_451805, EPI_ISL_451806, EPI_ISL_451807, EPI_ISL_451808, EPI_ISL_451809, EPI_ISL_451810, EPI_ISL_451811, EPI_ISL_451812, EPI_ISL_451813, EPI_ISL_451814, EPI_ISL_451815, EPI_ISL_451816, EPI_ISL_451817, EPI_ISL_451818, EPI_ISL_451819, EPI_ISL_451820, EPI_ISL_451821, EPI_ISL_451822, EPI_ISL_451823, EPI_ISL_451824, EPI_ISL_451825, EPI_ISL_451826, EPI_ISL_451827, EPI_ISL_451828, EPI_ISL_451829, EPI_ISL_451830, EPI_ISL_451831, EPI_ISL_451832, EPI_ISL_451833, EPI_ISL_451834, EPI_ISL_451835, EPI_ISL_451836, EPI_ISL_451837, EPI_ISL_451838, EPI_ISL_451839, EPI_ISL_451840, EPI_ISL_451841, EPI_ISL_451842, EPI_ISL_451843, EPI_ISL_451844, EPI_ISL_451846, EPI_ISL_451847, EPI_ISL_451848, EPI_ISL_451849, EPI_ISL_451850, EPI_ISL_451851, EPI_ISL_451852, EPI_ISL_451853, EPI_ISL_451854, EPI_ISL_451855			
see above	Viollier AG	Department of Biosystems Science and Engineering, ETH Zürich	Christian Beisel, Sarah Nadeau, Ivan Topolsky, Pedro Ferreira, Philipp Jablonski, Susana Posada-Céspedes, Tobias Schär, Ina Nissen, Natascha Santacroce, Elodie Burcklen, Christiane Beckmann, Maurice Redondo, Olivier Kobel, Christoph Noppen, Sophie Seidel, Noemie Santamaria de Souza, Niko Beerenwinkel, Tanja Stadler
EPI_ISL_451944, EPI_ISL_451945	Max von Pettenkofer Institute, Virology, National Reference Center for Retroviruses, LMU München	Laboratory for Functional Genome Analysis, Dept. Genomics, Gene Center of the LMU Munich	Max Muenchhoff, Stefan Krebs, Alexander Graf, Oliver Keppler, Helmut Blum
EPI_ISL_451957	Molecular Pathology Division, Department of Pathology, Hong Kong Sanatorium & Hospital	Molecular Pathology Division, Department of Pathology, Hong Kong Sanatorium & Hospital	Chun Hang AU, Wai Sing CHAN, Ho Yin LAM, Dona N. HO, Simon Y.M. LAM, Jonpaul S.T. ZEE, Tsun Leung CHAN, Edmond S.K. MA
EPI_ISL_451959	Alaska State Virology Laboratory	Alaska State Virology Laboratory	DeRonde,S., Deuling,H. and Chen,J.
EPI_ISL_451960	VI-US Virgin Islands Department of Health	Centers for Disease Control and Prevention	Queen,K., Li,Y., Montmayeur,A., Tao,Y., Zhang,J., Uehara,A., Paden,C.R., Marine,R., Wang,H., Padilla,J., Lee,J., Weiner,Z., Bankamp,B. and Tong,S.
EPI_ISL_451971, EPI_ISL_451973, EPI_ISL_451975, EPI_ISL_451978, EPI_ISL_451979, EPI_ISL_451987	1. ViroGenetics - BSL3 Laboratory of Virology, Maopolska Centre of Biotechnology, Jagiellonian University; 2. II Department of Internal Medicine, Faculty of Medicine, Jagiellonian University Medical College; 3. DIAGNOSTYKA Ltd.	1. ViroGenetics - BSL3 Laboratory of Virology, Maopolska Centre of Biotechnology, Jagiellonian University; 2. II Department of Internal Medicine, Faculty of Medicine, Jagiellonian University Medical College.	Marek Sanak, Marcin Surmiak, Maria Gsecka-Czapla, Wojciech Branicki, Pawe P abaj, Marta Rogalska-Kupiec, Jakub Swadba, Krzysztof Pyr
EPI_ISL_452148	CUB Hopital Erasme Laboratoire d'Anatomie Pathologique	CUB Hopital Erasme Laboratoire d'Anatomie Pathologique	Isabelle Salmon, Nicky D'Haene
EPI_ISL_452187, EPI_ISL_452188, EPI_ISL_452189	ULSS9 Distretto di Bussolengo	Istituto Zooprofilattico Sperimentale delle Venezie	Adelaide Milani, Alessia Schivo, Annalisa Salviato, Erika Giorgia Quaranta, Ambra Pastori, Bianca Zecchin, Alice Fusaro, Isabella Monne, Calogero Terregino, Antonia Ricci
EPI_ISL_452234	Din Klinik	The Public Health Agency of Sweden	Helene Warnborg, Anna-Malin Linde, Maria Lind Karlberg, Oskar Karlsson Lindsjo, Olov Svartstrom, Anna Risberg, Theresa Enkirch, Mia Brytting, Karin Tegmark-Wisell
EPI_ISL_452308	Michigan Department of Health and Human Services, Bureau of Laboratories	Michigan Department of Health and Human Services, Bureau of Laboratories	Blankenship HM, Riner D, Soehnlen MK
EPI_ISL_452371	Servicio de Microbiología. HRU de Málaga. Servicio Andaluz de Salud	SeqCOVID-SPAIN consortium/IBV(CSIC)	Inmaculada de Toro Peinado, Maria Concepción Mediavilla Gradolph, Begoña Palop Borrás and SeqCOVID-SPAIN consortium
EPI_ISL_452609, EPI_ISL_452610, EPI_ISL_452611, EPI_ISL_452612, EPI_ISL_452613, EPI_ISL_452614, EPI_ISL_452615, EPI_ISL_452616	Servicio de Microbiología y Parasitología clínica. UCEIMP. Hospital Universitario Virgen del Rocío/IBIS/CSIC/US.	SeqCOVID-SPAIN consortium/IBV(CSIC)	Guillermo Martí-n Gutiérrez, Ángel Rodrí-guez Villodres, Lidia Gálvez Benitez, Verónica González Galán, Javier Aznar Martí-n and SeqCOVID-SPAIN consortium
EPI_ISL_452809	Virginia DCLS	Virginia DCLS	Virginia DCLS
EPI_ISL_453006, EPI_ISL_453007	West of Scotland Specialist Virology Centre, NHSGGC / MRC-University of Glasgow Centre for Virus Research	COVID-19 Genomics UK (COG-UK) Consortium	Ana da Silva Filipe, Natasha Johnson, Kathy Smollett, Daniel Mair, Stephen Carmichael, Lily Tong, Jenna Nichols, Elihu Aranday-Cortes, Kirstyn Brunker, Yasmin Parr, Kyriaki Nomikou; Sarah McDonald, Marc Niebel, Patawee Asamaphan; Richard Orton, Joseph Hughes, Sreenu Vattipally, David L Robertson; Alasdair MacLean, Rory Gunson; Kathy Li, Natasha Jesudason, Rajiv Shah, James Shepherd, Antonia Ho, Emma Thomson
EPI_ISL_453157, EPI_ISL_453164, EPI_ISL_453165, EPI_ISL_453166, EPI_ISL_453186, EPI_ISL_453187, EPI_ISL_453188, EPI_ISL_453190	Virology Department, Royal Infirmary of Edinburgh, NHS Lothian / School of Biological Sciences, University of Edinburgh / Institute of Genetics and Molecular Medicine, University of Edinburgh	COVID-19 Genomics UK (COG-UK) Consortium	McHugh M, Dewar R, Rooke S, Gallagher M, Balcaza C, O'Toole Á, Scher E, Hill V, McCrone JT, Colquhoun R, Yu X, Jackson B, Rambaut A, Williams TC, Templeton K
EPI_ISL_453504, EPI_ISL_453505, EPI_ISL_453506	Northumbria University / South Tees Hospitals NHS Foundation Trust / North Cumbria Integrated Care NHS Foundation Trust / North Tees and Hartlepool NHS Foundation Trust / Newcastle Hospitals NHS Foundation Trust	COVID-19 Genomics UK (COG-UK) Consortium	Darren L Smith,Andrew Nelson,Matthew Bashton,Greg R Young,Joshua Loh,John Allan,Mohammad A Tariq,Giles S Holt,Gary Black,Wen C Yew,Lynn Dover ,Paul Baker,Steve Liggett,Sarah Essex,Jane Greenaway ,Debra Padgett,Clive Graham,Garren Scott,Edward Barton ,Emma Swindells ,Brendan Payne,Jennifer Collins,Yusri Taha,Gary Eltringham
EPI_ISL_453938, EPI_ISL_453939, EPI_ISL_453940, EPI_ISL_453941, EPI_ISL_453942, EPI_ISL_453943, EPI_ISL_453944, EPI_ISL_453945, EPI_ISL_453946, EPI_ISL_453947, EPI_ISL_453948, EPI_ISL_453949, EPI_ISL_453950, EPI_ISL_453951, EPI_ISL_453952, EPI_ISL_453953, EPI_ISL_453954, EPI_ISL_453955, EPI_ISL_453956, EPI_ISL_453957, EPI_ISL_453958, EPI_ISL_453959, EPI_ISL_454153, EPI_ISL_454154, EPI_ISL_454155, EPI_ISL_454156, EPI_ISL_454157, EPI_ISL_454174, EPI_ISL_454208, EPI_ISL_454211, EPI_ISL_454212, EPI_ISL_454350			
see above	unknown	Instituto Nacional de Saude (INSA)	Borges et al
EPI_ISL_454356, EPI_ISL_454368, EPI_ISL_454370, EPI_ISL_454372, EPI_ISL_454373, EPI_ISL_454374, EPI_ISL_454376, EPI_ISL_454378, EPI_ISL_454380, EPI_ISL_454381, EPI_ISL_454382, EPI_ISL_454383, EPI_ISL_454384			
see above	UPMC Clinical Microbiology Laboratory	Microbial Genome Sequencing Center, Microbial Genomic Epidemiological Laboratory	Mustapha M. Mustapha, Jane W. Marsh, Dan Snyder, Marissa P. Griffith, Stephanie L. Mitchell, Vatsala R. Srinivasa, Kady D. Waggle, Chinelo Ezeonwuku, Vaughn S. Cooper, Lee H. Harrison
EPI_ISL_454540, EPI_ISL_454543, EPI_ISL_454565	NIV Influenza	NIV Influenza	Potdar V
EPI_ISL_454605	Institute for Public Health	Laboratory for advanced genomics	Filip Roki, Lovro Trgovec-Greif, Neven Sui, Tomislav Rukavina, Igor Jurak, Oliver Vugrek
EPI_ISL_454638, EPI_ISL_454639, EPI_ISL_454640	Humboldt County Public Health Laboratory	Chan-Zuckerberg Biohub	CZB Cliahub Consortium
EPI_ISL_454775, EPI_ISL_454777, EPI_ISL_454793	Dutch COVID-19 response team	National Institute for Public Health and the Environment (RIVM)	Adam Meijer, Harry Vennema, Jeroen Cremer, Sharon van den Brink, Pieter Overduin, Florian Zwagemaker, Dennis Schmitz, Chantal Reusken, on behalf of the national COVID-19 response team
EPI_ISL_454809, EPI_ISL_454810, EPI_ISL_454811, EPI_ISL_454812, EPI_ISL_454813, EPI_ISL_454829	Dirk Dittmer	Dirk Dittmer	Bailey,A.G., Caro-Vegas,C.P., Dittmer,D., Eason,A.B., Juarez,A., Landis,J.T., McNamara,R.P., Miller,M.B., Moorad,R., Pluta,L.J., Seltzer,T.A., Thompson,C., Vahrson,W., Villamor,F.
EPI_ISL_455036	South Eastern Area Laboratory Services	NSW Health Pathology - Institute of Clinical Pathology and Medical Research; Westmead Hospital; University of Sydney	CIDM-PH et al.
EPI_ISL_455052	Douglas Hanly Moir Pathology	NSW Health Pathology - Institute of Clinical Pathology and Medical Research; Westmead Hospital; University of Sydney	CIDM-PH et al.
EPI_ISL_455054	South Eastern Area Laboratory Services	NSW Health Pathology - Institute of Clinical Pathology and Medical Research; Westmead Hospital; University of Sydney	CIDM-PH et al.
EPI_ISL_455065	Douglas Hanly Moir Pathology	NSW Health Pathology - Institute of Clinical Pathology and Medical Research; Westmead Hospital; University of Sydney	CIDM-PH et al.
EPI_ISL_455081, EPI_ISL_455082	South Eastern Area Laboratory Services	NSW Health Pathology - Institute of Clinical Pathology and Medical Research; Westmead Hospital; University of Sydney	CIDM-PH et al.

EPI_ISL_455100	Orsa VC	The Public Health Agency of Sweden	Anna-Karin Lundqvist, Anna-Malin Linde, Maria Lind Karlberg, Oskar Karlsson Lindsjo, Olov Svartstrom, Anna Risberg, Theresa Enkirch, Mia Brytting, Karin Tegmark-Wisell
EPI_ISL_455112, EPI_ISL_455113, EPI_ISL_455114, EPI_ISL_455115, EPI_ISL_455116, EPI_ISL_455117, EPI_ISL_455118, EPI_ISL_455119, EPI_ISL_455196, EPI_ISL_455204, EPI_ISL_455222, EPI_ISL_455249, EPI_ISL_455258, EPI_ISL_455264, EPI_ISL_455280, EPI_ISL_455285, EPI_ISL_455287			
see above	Dutch COVID-19 response team	Erasmus Medical Center	Bas Oude Munnink, David Nieuwenhuijse, Reina Sikkema, Claudia Schapendonk, Irina Chestakova, Anne van der Linden, Theo Bestebroer, Stefan van Nieuwkoop, Mark Pronk, Pascal Lexmond, Corien Swaan, Manon Haverkate, Madelief Mollers, Mart Stein, Sandra Kengne Kamga Mobou, Jeroen van Kampen, Jolanda Voermans, Aura Timen, Corine GeurtsvanKessel, Annemiek van der Eijk, Richard Molenkamp, Marion Koopmans, on behalf of the Dutch national COVID-19 response team.
EPI_ISL_455441, EPI_ISL_455443, EPI_ISL_455448	1. ViroGenetics - BSL3 Laboratory of Virology, Maopolska Centre of Biotechnology, Jagiellonian University; 2. II Department of Internal Medicine, Faculty of Medicine, Jagiellonian University Medical College; 3. Narodowy Instytut Zdrowia Publicznego - Pastwowy Zakad Higieny (NIZP-PZH)	1. ViroGenetics - BSL3 Laboratory of Virology, Maopolska Centre of Biotechnology, Jagiellonian University; 2. II Department of Internal Medicine, Faculty of Medicine, Jagiellonian University Medical College; 3. Narodowy Instytut Zdrowia Publicznego - Pastwowy Zakad Higieny (NIZP-PZH).	Katarzyna Pancer, Marek Sanak, Aleksandra A. Zasada, Magdalena Rzeczkowska, Tomasz Wokowicz, Katarzyna Zacharczuk, Agnieszka Koakowska-Kulesza, Katarzyna Owczarek, Aleksandra Milewska, Natalia Wolaniuk, Ewelina Hallman-Szeliska, Pawe P abaj, Wojciech Branicki, Krzysztof Pyr
EPI_ISL_455598, EPI_ISL_455599, EPI_ISL_455600, EPI_ISL_455601	SA Pathology	VPRL	Beard, MR., Van Der Hoek, K., Lim, C.K., Leong, L.E.X., Coldbeck-Shackley, R., Shue, B., Kirby, E., Merrett, J., Llamas, B.
EPI_ISL_455636, EPI_ISL_455639	KRISP, KZN Research Innovation and Sequencing Platform	KRISP, KZN Research Innovation and Sequencing Platform	Giandhari J, Pillay S, Lessells R, Chimukangara B, Deforche K, Tegally H, Wilkinson E, de Oliveira T
EPI_ISL_455641	ICMR-National Institute of Cholera and Enteric Diseases	National Institute of Biomedical Genomics	Arindam Maitra, Mamta Chawla Sarkar, Sreedhar Chinnaswamy, Hasina Banu, Ananya Chatterjee, Shanta Dutta, Saumitra Das
EPI_ISL_455704, EPI_ISL_455705	National Hospital of Tropical Diseases	Oxford University Clinical Research Unit, Hanoi, Vietnam	Nguyen Thi Tam, Van Dinh Trang, Nguyen Thu Trang, Nguyen Thi Ngoc Diep, Le Nguyen Minh Hoa, Pham Ngoc Thach, H. Rogier van Doorn, on behalf of the OUCRU COVID-19 research group
EPI_ISL_455721	Servicio de Microbiologia. Hospital Clinico Universitario de Valencia	Sequencing and Bioinformatics Service and Molecular Epidemiology Research Group. FISABIO-Public Health, and SeqCOVID-Spain Consortium	Sandra Carbo, Loreto Ferrús Abad, Paula Ruiz-Hueso, Mariana Reyes-Prieto, Vicente Soriano Chirona, Ivan Ansari, Llúcia Martínez-Priego, Giuseppe 'Auria, David Navarro, Eliseo Albert, Maria Alma Bracho, Lidia Ruiz Roldan, Neris Garcia-Gonzalez, Inma Galán Vendrell, Fernando Gonzalez-Candelas
EPI_ISL_455723	Servicio de Microbiologia. Hospital Clinico Universitario de Valencia	Sequencing and Bioinformatics Service and Molecular Epidemiology Research Group. FISABIO-Public Health, and SeqCOVID-Spain Consortium	Vicente Soriano Chirona, Ivan Ansari, Llúcia Martínez-Priego, Giuseppe 'Auria, David Navarro, Eliseo Albert, Maria Alma Bracho, Lidia Ruiz Roldan, Neris Garcia-Gonzalez, Inma Galán Vendrell, Sandra Carbo, Loreto Ferrús Abad, Paula Ruiz-Hueso, Mariana Reyes-Prieto, Fernando Gonzalez-Candelas
EPI_ISL_455724	Servicio de Microbiologia. Hospital Clinico Universitario de Valencia	Sequencing and Bioinformatics Service and Molecular Epidemiology Research Group. FISABIO-Public Health, and SeqCOVID-Spain Consortium	Loreto Ferrús Abad, Paula Ruiz-Hueso, Mariana Reyes-Prieto, Vicente Soriano Chirona, Ivan Ansari, Llúcia Martínez-Priego, Giuseppe 'Auria, David Navarro, Eliseo Albert, Maria Alma Bracho, Lidia Ruiz Roldan, Neris Garcia-Gonzalez, Inma Galán Vendrell, Sandra Carbo, Fernando Gonzalez-Candelas
EPI_ISL_455725	Servicio de Microbiologia. Hospital Clinico Universitario de Valencia	Sequencing and Bioinformatics Service and Molecular Epidemiology Research Group. FISABIO-Public Health, and SeqCOVID-Spain Consortium	Eliseo Albert, Maria Alma Bracho, Griselda De Marco, Lidia Ruiz Roldan, Neris Garcia-Gonzalez, Inma Galán Vendrell, Sandra Carbo, Loreto Ferrús Abad, Paula Ruiz-Hueso, Mariana Reyes-Prieto, Vicente Soriano Chirona, Ivan Ansari, Llúcia Martínez-Priego, Giuseppe 'Auria, David Navarro, Fernando Gonzalez-Candelas
EPI_ISL_455726	Servicio de Microbiologia. Hospital Clinico Universitario de Valencia	Sequencing and Bioinformatics Service and Molecular Epidemiology Research Group. FISABIO-Public Health, and SeqCOVID-Spain Consortium	Inma Galán Vendrell, Sandra Carbo, Loreto Ferrús Abad, Paula Ruiz-Hueso, Mariana Reyes-Prieto, Vicente Soriano Chirona, Ivan Ansari, Llúcia Martínez-Priego, Giuseppe 'Auria, David Navarro, Eliseo Albert, Maria Alma Bracho, Lidia Ruiz Roldan, Neris Garcia-Gonzalez, Fernando Gonzalez-Candelas
EPI_ISL_455727	Servicio de Microbiologia. Hospital Clinico Universitario de Valencia	Sequencing and Bioinformatics Service and Molecular Epidemiology Research Group. FISABIO-Public Health, and SeqCOVID-Spain Consortium	David Navarro, Eliseo Albert, Maria Alma Bracho, Griselda De Marco, Lidia Ruiz Roldan, Neris Garcia-Gonzalez, Inma Galán Vendrell, Sandra Carbo, Loreto Ferrús Abad, Paula Ruiz-Hueso, Mariana Reyes-Prieto, Vicente Soriano Chirona, Ivan Ansari, Llúcia Martínez-Priego, Giuseppe 'Auria, Fernando Gonzalez-Candelas
EPI_ISL_455728	Servicio de Microbiologia. Hospital Clinico Universitario de Valencia	Sequencing and Bioinformatics Service and Molecular Epidemiology Research Group. FISABIO-Public Health, and SeqCOVID-Spain Consortium	Maria Alma Bracho, Griselda De Marco, Lidia Ruiz Roldan, Neris Garcia-Gonzalez, Inma Galán Vendrell, Sandra Carbo, Loreto Ferrús Abad, Paula Ruiz-Hueso, Mariana Reyes-Prieto, Vicente Soriano Chirona, Ivan Ansari, Llúcia Martínez-Priego, Giuseppe 'Auria, David Navarro, Eliseo Albert, Fernando Gonzalez-Candelas
EPI_ISL_455729	Servicio de Microbiologia. Hospital Clinico Universitario de Valencia	Sequencing and Bioinformatics Service and Molecular Epidemiology Research Group. FISABIO-Public Health, and SeqCOVID-Spain Consortium	Sandra Carbo, Loreto Ferrús Abad, Paula Ruiz-Hueso, Mariana Reyes-Prieto, Vicente Soriano Chirona, Ivan Ansari, Llúcia Martínez-Priego, Giuseppe 'Auria, David Navarro, Eliseo Albert, Maria Alma Bracho, Lidia Ruiz Roldan, Neris Garcia-Gonzalez, Inma Galán Vendrell, Fernando Gonzalez-Candelas
EPI_ISL_455730, EPI_ISL_455731	Servicio de Microbiologia. Hospital Clinico Universitario de Valencia	Sequencing and Bioinformatics Service and Molecular Epidemiology Research Group. FISABIO-Public Health, and SeqCOVID-Spain Consortium	Mariana Reyes-Prieto, Vicente Soriano Chirona, Ivan Ansari, Llúcia Martínez-Priego, Giuseppe 'Auria, David Navarro, Eliseo Albert, Maria Alma Bracho, Lidia Ruiz Roldan, Neris Garcia-Gonzalez, Inma Galán Vendrell, Sandra Carbo, Loreto Ferrús Abad, Paula Ruiz-Hueso, Fernando Gonzalez-Candelas
EPI_ISL_455732	Servicio de Microbiologia. Hospital Clinico Universitario de Valencia	Sequencing and Bioinformatics Service and Molecular Epidemiology Research Group. FISABIO-Public Health, and SeqCOVID-Spain Consortium	Lidia Ruiz Roldan, Neris Garcia-Gonzalez, Inma Galán Vendrell, Sandra Carbo, Loreto Ferrús Abad, Paula Ruiz-Hueso, Mariana Reyes-Prieto, Vicente Soriano Chirona, Ivan Ansari, Llúcia Martínez-Priego, Giuseppe 'Auria, David Navarro, Eliseo Albert, Maria Alma Bracho, Fernando Gonzalez-Candelas
EPI_ISL_455733	Servicio de Microbiologia. Hospital Clinico Universitario de Valencia	Sequencing and Bioinformatics Service and Molecular Epidemiology Research Group. FISABIO-Public Health, and SeqCOVID-Spain Consortium	Loreto Ferrús Abad, Paula Ruiz-Hueso, Mariana Reyes-Prieto, Vicente Soriano Chirona, Ivan Ansari, Llúcia Martínez-Priego, Giuseppe 'Auria, David Navarro, Eliseo Albert, Maria Alma Bracho, Lidia Ruiz Roldan, Neris Garcia-Gonzalez, Inma Galán Vendrell, Sandra Carbo, Fernando Gonzalez-Candelas
EPI_ISL_455734, EPI_ISL_455737	Servicio de Microbiologia. Hospital Clinico Universitario de Valencia	Sequencing and Bioinformatics Service and Molecular Epidemiology Research Group. FISABIO-Public Health, and SeqCOVID-Spain Consortium	Griselda De Marco, Lidia Ruiz Roldan, Neris Garcia-Gonzalez, Inma Galán Vendrell, Sandra Carbo, Loreto Ferrús Abad, Paula Ruiz-Hueso, Mariana Reyes-Prieto, Vicente Soriano Chirona, Ivan Ansari, Llúcia Martínez-Priego, Giuseppe 'Auria, David Navarro, Eliseo Albert, Maria Alma Bracho, Fernando Gonzalez-Candelas
EPI_ISL_455738	Servicio de Microbiologia. Hospital Clinico Universitario de Valencia	Sequencing and Bioinformatics Service and Molecular Epidemiology Research Group. FISABIO-Public Health, and SeqCOVID-Spain Consortium	Maria Alma Bracho, Griselda De Marco, Lidia Ruiz Roldan, Neris Garcia-Gonzalez, Inma Galán Vendrell, Sandra Carbo, Loreto Ferrús Abad, Paula Ruiz-Hueso, Mariana Reyes-Prieto, Vicente Soriano Chirona, Ivan Ansari, Llúcia Martínez-Priego, Giuseppe 'Auria, David Navarro, Eliseo Albert, Fernando Gonzalez-Candelas
EPI_ISL_455739	Servicio de Microbiologia. Hospital Clinico Universitario de Valencia	Sequencing and Bioinformatics Service and Molecular Epidemiology Research Group. FISABIO-Public Health, and SeqCOVID-Spain Consortium	Lidia Ruiz Roldan, Neris Garcia-Gonzalez, Inma Galán Vendrell, Sandra Carbo, Loreto Ferrús Abad, Paula Ruiz-Hueso, Mariana Reyes-Prieto, Vicente Soriano Chirona, Ivan Ansari, Llúcia Martínez-Priego, Giuseppe 'Auria, David Navarro, Eliseo Albert, Maria Alma Bracho, Fernando Gonzalez-Candelas
EPI_ISL_455744	Servicio de Microbiologia. Hospital Clinico Universitario de Valencia	Sequencing and Bioinformatics Service and Molecular Epidemiology Research Group. FISABIO-Public Health, and SeqCOVID-Spain Consortium	Llúcia Martínez-Priego, Giuseppe 'Auria, David Navarro, Eliseo Albert, Maria Alma Bracho, Lidia Ruiz Roldan, Neris Garcia-Gonzalez, Inma Galán Vendrell, Sandra Carbo, Loreto Ferrús Abad, Paula Ruiz-Hueso, Mariana Reyes-Prieto, Vicente Soriano Chirona, Ivan Ansari, Fernando Gonzalez-Candelas
EPI_ISL_455994, EPI_ISL_455995, EPI_ISL_455996, EPI_ISL_455997, EPI_ISL_455998, EPI_ISL_455999	LSUHS Emerging Viral Threat Laboratory	Microbial Genome Sequencing Center	John A. Vanchiere, Jeremy P. Kamil, Rona S. Scott, Camille F. Abshire, Abida Siddiqa, Byeong-Jae Lee, Chan-ki Min, Md Maksudul Alam, Monica Gestal-Cartele, Edna Ondari, Adam Greer, Malgorzata Bienkowska-Haba, Katarzyna Zwolinska, Michelle M. Arnold, Jason M. Bodily, Andrew D. Yurochko, Paul M. Weinberger, Christopher G. Kevil, Martin J. Sapp, Daniel J. Snyder, Vaughn S. Cooper
EPI_ISL_456000, EPI_ISL_456001, EPI_ISL_456002	LSUHS Emerging Viral Threat Laboratory	Microbial Genome Sequencing Center	Rona S. Scott, Jeremy P. Kamil, John A. Vanchiere, Camille F. Abshire, Abida Siddiqa, Byeong-Jae Lee, Chan-ki Min, Md Maksudul Alam, Monica Gestal-Cartele, Edna Ondari, Adam Greer, Malgorzata Bienkowska-Haba, Katarzyna Zwolinska, Michelle M. Arnold, Jason M. Bodily, Andrew D. Yurochko, Paul M. Weinberger, Christopher G. Kevil, Martin J. Sapp, Daniel J. Snyder, Vaughn S. Cooper
EPI_ISL_456072, EPI_ISL_456073, EPI_ISL_456074	Laboratory of Respiratory Viruses and Measles, Oswaldo Cruz Institute, FIOCRUZ	Laboratory of Respiratory Viruses and Measles, Oswaldo Cruz Institute, FIOCRUZ	Paola Resende, Luciana Appolinario, Fernando Motta, Aline Mattos, Milene Miranda, Cristiana Garcia, Braulia Caetano, Maria Ogrzewalska, Jonathan Lopes, Marilda Siqueira
EPI_ISL_456147, EPI_ISL_456152	Instituto Nacional de Salud - Unidad de Secuenciación y Análisis Genómico	Instituto Nacional de Salud, Universidad Cooperativa de Colombia, Instituto Alexander von Humboldt, Imperial College-London, London School of Hygiene & Tropical	Katherine Laiton-Donato, Diego A. Álvarez-Díaz, Carlos Franco-Muñoz, Jose A. Usme-Ciro, Gloria Puerto, Nicolas D. Franco-Sierra, Mailyn A.Gonzalez, Zulma M. Cucunubá, Christian Julian VillabonaArenas, Liz Villabona-Arenas, Sussy Echeverria, Astrid C. Flórez, Sergio Gomez-Rangel, Luz Dary Rodriguez, Juliana Barbosa, Erika Ospitia, Diana Marcela Walteros-Acero, Martha Lucia Ospina Martinez, Marcela Mercado-Reyes.

		Medicine	
EPI_ISL_456287, EPI_ISL_456292, EPI_ISL_456295, EPI_ISL_456296, EPI_ISL_456297, EPI_ISL_456298, EPI_ISL_456299	Southern Community Labs Dunedin	Institute of Environmental Science and Research (ESR)	Matt Storey, Xiaoyun Ren, Anja Werno, Antje van der Linden, Arlo Upton, Chris Mansell, David Hammer, Dragana Drinkovic, Erasmus Smit, Gary McAuliffe, Hana Sofia Andersson, James Ussher, Jill Sherwood, Josh Freeman, Julia Howard, Juliet Elvy, Mary DeAlmeida, Matt Blakiston, Matthew Rogers, Max Bloomfield, Michael Addidle, Michelle Balm, Sally Roberts, Sarah Jefferies, Sharmini Muttaiyah, Susan Morpeth, Susan Taylor, Timothy Blackmore, Vani Sathyendran, Veronica Playle, Virginia Hope, Erasmus Smit, Lauren Jelly, Joep de Ligt
EPI_ISL_456300	LabPLUS	Institute of Environmental Science and Research (ESR)	Matt Storey, Xiaoyun Ren, Anja Werno, Antje van der Linden, Arlo Upton, Chris Mansell, David Hammer, Dragana Drinkovic, Erasmus Smit, Gary McAuliffe, Hana Sofia Andersson, James Ussher, Jill Sherwood, Josh Freeman, Julia Howard, Juliet Elvy, Mary DeAlmeida, Matt Blakiston, Matthew Rogers, Max Bloomfield, Michael Addidle, Michelle Balm, Sally Roberts, Sarah Jefferies, Sharmini Muttaiyah, Susan Morpeth, Susan Taylor, Timothy Blackmore, Vani Sathyendran, Veronica Playle, Virginia Hope, Erasmus Smit, Lauren Jelly, Joep de Ligt
EPI_ISL_456304, EPI_ISL_456307, EPI_ISL_456308, EPI_ISL_456316	Southern Community Labs Dunedin	Institute of Environmental Science and Research (ESR)	Matt Storey, Xiaoyun Ren, Anja Werno, Antje van der Linden, Arlo Upton, Chris Mansell, David Hammer, Dragana Drinkovic, Erasmus Smit, Gary McAuliffe, Hana Sofia Andersson, James Ussher, Jill Sherwood, Josh Freeman, Julia Howard, Juliet Elvy, Mary DeAlmeida, Matt Blakiston, Matthew Rogers, Max Bloomfield, Michael Addidle, Michelle Balm, Sally Roberts, Sarah Jefferies, Sharmini Muttaiyah, Susan Morpeth, Susan Taylor, Timothy Blackmore, Vani Sathyendran, Veronica Playle, Virginia Hope, Erasmus Smit, Lauren Jelly, Joep de Ligt
EPI_ISL_456322	Canterbury Health Laboratories	Institute of Environmental Science and Research (ESR)	Matt Storey, Xiaoyun Ren, Anja Werno, Antje van der Linden, Arlo Upton, Chris Mansell, David Hammer, Dragana Drinkovic, Erasmus Smit, Gary McAuliffe, Hana Sofia Andersson, James Ussher, Jill Sherwood, Josh Freeman, Julia Howard, Juliet Elvy, Mary DeAlmeida, Matt Blakiston, Matthew Rogers, Max Bloomfield, Michael Addidle, Michelle Balm, Sally Roberts, Sarah Jefferies, Sharmini Muttaiyah, Susan Morpeth, Susan Taylor, Timothy Blackmore, Vani Sathyendran, Veronica Playle, Virginia Hope, Erasmus Smit, Lauren Jelly, Joep de Ligt
EPI_ISL_456324, EPI_ISL_456325, EPI_ISL_456326, EPI_ISL_456327, EPI_ISL_456328, EPI_ISL_456329, EPI_ISL_456330, EPI_ISL_456331, EPI_ISL_456332, EPI_ISL_456333, EPI_ISL_456334, EPI_ISL_456335, EPI_ISL_456336, EPI_ISL_456337, EPI_ISL_456338, EPI_ISL_456339, EPI_ISL_456340, EPI_ISL_456341, EPI_ISL_456342, EPI_ISL_456343			
see above	Wellington SCL	Institute of Environmental Science and Research (ESR)	Matt Storey, Xiaoyun Ren, Anja Werno, Antje van der Linden, Arlo Upton, Chris Mansell, David Hammer, Dragana Drinkovic, Erasmus Smit, Gary McAuliffe, Hana Sofia Andersson, James Ussher, Jill Sherwood, Josh Freeman, Julia Howard, Juliet Elvy, Mary DeAlmeida, Matt Blakiston, Matthew Rogers, Max Bloomfield, Michael Addidle, Michelle Balm, Sally Roberts, Sarah Jefferies, Sharmini Muttaiyah, Susan Morpeth, Susan Taylor, Timothy Blackmore, Vani Sathyendran, Veronica Playle, Virginia Hope, Erasmus Smit, Lauren Jelly, Joep de Ligt
EPI_ISL_456404	unknown	Research Center Of Tropical and Infectious Of Medical Sciences	Mollaei,H.R., Aghaei-Afshar,A., Kalantar-Neyestanaki,D., Tabatabaeifar,F., Morones Ramirez,J.R.
EPI_ISL_456406	unknown	Research Center Of Tropical and Infectious Of Medical Sciences	Mollaei,H.R., Aghaei-Afshar,A., Kalantar-Neyestanaki,D., Morones Ramirez,J.R.
EPI_ISL_456899, EPI_ISL_456900, EPI_ISL_456904, EPI_ISL_456950, EPI_ISL_456953, EPI_ISL_456954, EPI_ISL_456955, EPI_ISL_457012, EPI_ISL_457013, EPI_ISL_457014, EPI_ISL_457015			
see above	Virology Department, Royal Infirmary of Edinburgh, NHS Lothian / School of Biological Sciences, University of Edinburgh / Institute of Genetics and Molecular Medicine, University of Edinburgh	COVID-19 Genomics UK (COG-UK) Consortium	McHugh M, Dewar R, Rooke S, Gallagher M, Balcaza C, O'Toole Á, Scher E, Hill V, McCrone JT, Colquhoun R, Yu X, Jackson B, Rambaut A, Williams TC, Templeton K
EPI_ISL_457052, EPI_ISL_457089, EPI_ISL_457101, EPI_ISL_457126, EPI_ISL_457134, EPI_ISL_457139, EPI_ISL_457150, EPI_ISL_457181	University of Exeter	COVID-19 Genomics UK (COG-UK) Consortium	Ben Temperton,Aaron Jeffries,Michelle Michelsen,Joanna Warwick-Dugdale,Audrey Farbos,Robyn Manley,Stephen Michell,Jane Masoli
EPI_ISL_457608, EPI_ISL_457627, EPI_ISL_457630, EPI_ISL_457638, EPI_ISL_457642, EPI_ISL_457659, EPI_ISL_457668, EPI_ISL_457673, EPI_ISL_457680, EPI_ISL_457682	Virology Department, Sheffield Teaching Hospitals NHS Foundation Trust/Department of Infection, Immunity and Cardiovascular Disease, The Medical School, University of Sheffield	COVID-19 Genomics UK (COG-UK) Consortium	Thushan de Silva, Matthew Parker, Nikki Smith, Adri Angyal, Rebecca Brown, Luke Green, Rachel Tucker, Paul Parsons, Danielle Groves, Katie Johnson, Laura Carrilero, Alex Keeley, Dave Partridge, Matthew Wyles, Benjamin Lindsey, Mehmet Yavuz, Mohammad Raza, Cariad Evans
EPI_ISL_457758, EPI_ISL_457759	Johns Hopkins Hospital Department of Pathology	Johns Hopkins Hospital Department of Pathology	Peter M. Thielen, Thomas Mehoke, Shirlee Wohl, Srividya Ramakrishnan, Melanie Kirsche, Amanda Ernlund, Craig Howser, Kristina Zudock, Oluwaseun Falade-Nwulia, Norah Sadowski, Paul Morris, Mark Hopkins, Yunfan Fan, Nidia Trovao, Victoria Gniazdowski, Michael C. Schatz, Stuart C. Ray, Winston Timp, Heba H. Mostafa
EPI_ISL_457855	KEMRI-CGMR-C	KEMRI-Wellcome Trust Research Programme/KEMRI-CGMR-C Kilifi	Githinji G. et al 2020
EPI_ISL_457964	Laboratorio de Biología Molecular Asociación Española Primera en Salud	Departments of Pathology and Medicine, New York University School of Medicine	Maria Victoria Elizondo, Maria Noel Zubillaga, Gonzalo Manrique, Paul Zappile, Gael Westby, Matthew T Maurano, Christian Marier, Adriana Heguy
EPI_ISL_458001, EPI_ISL_458002, EPI_ISL_458003, EPI_ISL_458004, EPI_ISL_458005, EPI_ISL_458006	Dirk Dittmer	Dirk Dittmer	Bailey,A.G., Caro-Vegas,C.P., Dittmer,D., Eason,A.B., Juarez,A., Landis,J.T., McNamara,R.P., Miller,M.B., Moorad,R., Pluta,L.J., Seltzer,T.A., Thompson,C., Vahrson,W., Villamor,F.
EPI_ISL_458012, EPI_ISL_458013, EPI_ISL_458015	Department of Food Safety, Nutrition and Veterinary public health, Istituto Superiore di Sanita'	Department of Food Safety, Nutrition and Veterinary public health, Istituto Superiore di Sanita'	La Rosa,G., Iaconelli,M., Mancini,P., Bonanno Ferraro,G., Veneri,C., Bonadonna,L., Lucentini,L., Suffredini,E.
EPI_ISL_458022	NYU Langone Health	Departments of Pathology and Medicine, New York University School of Medicine	Maria Aguero-Rosenfeld, Brendan Belovarac, Margaret Black, Ludovic Boytard, John Cadley, Paolo Cotzia, John Chen, Dacia Dimartino, Xiaojun Feng, Tatyana Gindin, Emily Guzman, Adriana Heguy, Megan Hogan, Emily Huang, George Jour, Alireza Khodadadi-Jamayran, Lawrence H. Lin, Raven Luther, Andrew Lytle, Christian Marier, Matthew T. Maurano, Mark J. Mulligan, Peter Meyn, Raquel Ordonez Ciriza, Iman Osman, Jared Pinnell, Vanessa Raabe, Sitharam Ramaswami, Amy Rapkiewicz, Andre M. Ribeiro-dos-Santos, Marie Samanovic-Golden, Antonio Serrano, Guomiao Shen, Matija Snuderl, Theodore Vougiouklakis, Nick Vulpescu, Gael Westby, Paul Zappile, Yutong Zhang
EPI_ISL_458025	Hospital for Tropical Diseases	COVID-19 Network Investigations (CONI) Alliance	Elizabeth Batty, Nantarat Chantawat, Wasun Chantratita, Thanat Chookajorn, Stefan Fernandez, Angkana Huang, Weena Janwitthayanan, Akanitt Jittmittraphap, Anthony R. Jones, Khajohn Joonsalak, Chonticha Klungtong, Theerarat Kochakarn, Namfon Kotanan, Krittikorn Kumpornsin, Pornsawan Leaungwutiwong, Wudtichai Manasatienkij, Bhakbhoom Panthan, Ekawat Pasomsub, Kingkan Rakmanee, Insee Sensorn, Janjira Thaipadungpanit, Arporn Wangwiwatsin,Treewat Watthanachockchai
EPI_ISL_458071	CSIR-Centre for Cellular and Molecular Biology	CSIR-Centre for Cellular and Molecular Biology	Sakshi Shambhavi, Lamuk Zaveri, Shagufta Khan, Namami Gaur, Tulasi Nagabandi, Purushotham Vodnala, Payel Mukherjee, Sofia Banu, Priya Singh, Dhiviya Vedagiri, Divya Gupta, Vishal Sah, Santosh Kumar Kuncha, Krishnan Harinivas Harshan, Archana Bharadwaj Siva, Karthik Bharadwaj Tallapaka,Nikhil Hajirnis, Pratheusa Maccha, M Soujanya Reddy,G. Aditya Kumar, Koushick Sivakumar, Rakesh K Mishra, Divya Tej Sowpati
EPI_ISL_458233, EPI_ISL_458234	KU Leuven, Rega Institute, Clinical and Epidemiological Virology	KU Leuven, Rega Institute, Clinical and Epidemiological Virology	Tony Wawina-Bokalanga, Bert Vanmechelen, Joan Marti-Carerras, Piet Maes
EPI_ISL_458262	Scripps Medical Laboratory	Andersen lab at Scripps Research	SEARCH Alliance San Diego with Michael Quigley, Ellen Stefanski, Ian Mchardy
EPI_ISL_458291, EPI_ISL_458294, EPI_ISL_458295, EPI_ISL_458296, EPI_ISL_458297	Dirk Dittmer	Dirk Dittmer	Bailey,A.G., Caro-Vegas,C.P., Dittmer,D., Eason,A.B., Juarez,A., Landis,J.T., McNamara,R.P., Miller,M.B., Moorad,R., Pluta,L.J., Seltzer,T.A., Thompson,C., Vahrson,W., Villamor,F.
EPI_ISL_458302, EPI_ISL_458340, EPI_ISL_458357, EPI_ISL_458365, EPI_ISL_458372, EPI_ISL_458378, EPI_ISL_458417, EPI_ISL_458425, EPI_ISL_458426, EPI_ISL_458430, EPI_ISL_458433, EPI_ISL_458436, EPI_ISL_458440, EPI_ISL_458448, EPI_ISL_458455, EPI_ISL_458499, EPI_ISL_458503			
see above	PHE South West Regional Laboratory, National Infection Service	Wellcome Sanger Institute for the COVID-19 Genomics UK (COG-UK) consortium	Stephanie Hutchings, Hannah Pymont, Dr Peter Muir, Barry Vipond, Rich Hopes; and Alex Alderton, Roberto Amato, Sonia Goncalves, Ewan Harrison, David K. Jackson, Ian Johnston, Dominic Kwiatkowski, Cordelia Langford, John Sillitoe on behalf of the Wellcome Sanger Institute COVID-19 Surveillance Team (http://www.sanger.ac.uk/covid-team)

EPI_ISL_458581, EPI_ISL_458582, EPI_ISL_458585, EPI_ISL_458587, EPI_ISL_458589, EPI_ISL_458590, EPI_ISL_458598, EPI_ISL_458599, EPI_ISL_458605, EPI_ISL_458608, EPI_ISL_458616, EPI_ISL_458620, EPI_ISL_458624, EPI_ISL_458627, EPI_ISL_458630, EPI_ISL_458635, EPI_ISL_458637, EPI_ISL_458643, EPI_ISL_458647, EPI_ISL_458648, EPI_ISL_458649, EPI_ISL_458655, EPI_ISL_458656, EPI_ISL_458658, EPI_ISL_458664, EPI_ISL_458665, EPI_ISL_458671, EPI_ISL_458673, EPI_ISL_458675, EPI_ISL_458676, EPI_ISL_458679, EPI_ISL_458681, EPI_ISL_458683, EPI_ISL_458689, EPI_ISL_458692, EPI_ISL_458695, EPI_ISL_458702, EPI_ISL_458706			
see above	NU-OMICS DNA Sequencing research facility, Northumbria University	Wellcome Sanger Institute for the COVID-19 Genomics UK (COG-UK) consortium	Chris Duncan, Sheia Waugh, Shirelle Burton-Fanning, Gary Eltringham, Jennifer Collins, Brendan Payne, Yusri Taha, Emma Swindells, Jane Greenaway, Edward Barton, Garren Scott, Debra Padgett, Clive Graham, Sarah Essex, Steve Ligget, Paul Baker, Lynn Dover, Wen Yew, Gary Black, John Allan, Joshua Loh, Greg Young, Matthew Bashton, Andrew Nelson, Darren Smith and Alex Alderton, Roberto Amato, Sonia Goncalves, Ewan Harrison, David K. Jackson, Ian Johnston, Dominic Kwiatkowski, Cordelia Langford, John Sillitoe on behalf of the Wellcome Sanger Institute COVID-19 Surveillance Team (http://www.sanger.ac.uk/covid-team)
EPI_ISL_458712	NU-OMICS DNA Sequencing research facility, Northumbria University	Wellcome Sanger Institute for the COVID-19 Genomics UK (COG-UK) Consortium	Chris Duncan, Sheia Waugh, Shirelle Burton-Fanning, Gary Eltringham, Jennifer Collins, Brendan Payne, Yusri Taha, Emma Swindells, Jane Greenaway, Edward Barton, Garren Scott, Debra Padgett, Clive Graham, Sarah Essex, Steve Ligget, Paul Baker, Lynn Dover, Wen Yew, Gary Black, John Allan, Joshua Loh, Greg Young, Matthew Bashton, Andrew Nelson, Darren Smith and Alex Alderton, Roberto Amato, Sonia Goncalves, Ewan Harrison, David K. Jackson, Ian Johnston, Dominic Kwiatkowski, Cordelia Langford, John Sillitoe on behalf of the Wellcome Sanger Institute COVID-19 Surveillance Team
EPI_ISL_458714, EPI_ISL_458716	NU-OMICS DNA Sequencing research facility, Northumbria University	Wellcome Sanger Institute for the COVID-19 Genomics UK (COG-UK) consortium	Chris Duncan, Sheia Waugh, Shirelle Burton-Fanning, Gary Eltringham, Jennifer Collins, Brendan Payne, Yusri Taha, Emma Swindells, Jane Greenaway, Edward Barton, Garren Scott, Debra Padgett, Clive Graham, Sarah Essex, Steve Ligget, Paul Baker, Lynn Dover, Wen Yew, Gary Black, John Allan, Joshua Loh, Greg Young, Matthew Bashton, Andrew Nelson, Darren Smith and Alex Alderton, Roberto Amato, Sonia Goncalves, Ewan Harrison, David K. Jackson, Ian Johnston, Dominic Kwiatkowski, Cordelia Langford, John Sillitoe on behalf of the Wellcome Sanger Institute COVID-19 Surveillance Team (http://www.sanger.ac.uk/covid-team)
EPI_ISL_458770, EPI_ISL_458778, EPI_ISL_458801, EPI_ISL_458802, EPI_ISL_458821, EPI_ISL_458823, EPI_ISL_458825, EPI_ISL_458828, EPI_ISL_458830, EPI_ISL_458837, EPI_ISL_458841, EPI_ISL_458851, EPI_ISL_458855, EPI_ISL_458862, EPI_ISL_458875, EPI_ISL_458895, EPI_ISL_458899, EPI_ISL_458902, EPI_ISL_458904			
see above	PHE South West Regional Laboratory, National Infection Service	Wellcome Sanger Institute for the COVID-19 Genomics UK (COG-UK) consortium	Stephanie Hutchings, Hannah Pymont, Dr Peter Muir, Barry Vipond, Rich Hopes; and Alex Alderton, Roberto Amato, Sonia Goncalves, Ewan Harrison, David K. Jackson, Ian Johnston, Dominic Kwiatkowski, Cordelia Langford, John Sillitoe on behalf of the Wellcome Sanger Institute COVID-19 Surveillance Team (http://www.sanger.ac.uk/covid-team)
EPI_ISL_459957	Institute for Medical Research, Infectious Disease Research Centre, National Institutes of Health, Minis	Institute for Medical Research, Infectious Disease Research Centre, National Institutes of Health, Minis	Suppiah J, Mohd-Zawawi Z, Kamel KA, Ellan K, Kalyanasundram J, Mohd-Zain R, Thayan R
EPI_ISL_459962, EPI_ISL_459963	Centogene AG	Centogene AG	Prof. Dr. Peter Bauer, Dr. Krishna Kumar Kandaswamy
EPI_ISL_460010, EPI_ISL_460011, EPI_ISL_460012, EPI_ISL_460013, EPI_ISL_460014, EPI_ISL_460015, EPI_ISL_460016, EPI_ISL_460017, EPI_ISL_460018, EPI_ISL_460020, EPI_ISL_460021, EPI_ISL_460022, EPI_ISL_460023, EPI_ISL_460024, EPI_ISL_460025, EPI_ISL_460026, EPI_ISL_460027, EPI_ISL_460028, EPI_ISL_460030			
see above	Michigan Department of Health and Human Services, Bureau of Laboratories	Michigan Department of Health and Human Services, Bureau of Laboratories	Blankenship HM, Riner D, Soehnlen MK
EPI_ISL_460098, EPI_ISL_460100, EPI_ISL_460120, EPI_ISL_460131, EPI_ISL_460133, EPI_ISL_460141, EPI_ISL_460144, EPI_ISL_460148, EPI_ISL_460151, EPI_ISL_460155, EPI_ISL_460157, EPI_ISL_460160, EPI_ISL_460164, EPI_ISL_460169, EPI_ISL_460171, EPI_ISL_460172, EPI_ISL_460181, EPI_ISL_460193, EPI_ISL_460195, EPI_ISL_460196, EPI_ISL_460198, EPI_ISL_460210, EPI_ISL_460234, EPI_ISL_460243, EPI_ISL_460249, EPI_ISL_460255, EPI_ISL_460260, EPI_ISL_460265, EPI_ISL_460274, EPI_ISL_460282, EPI_ISL_460287, EPI_ISL_460294, EPI_ISL_460308, EPI_ISL_460342, EPI_ISL_460352, EPI_ISL_460379, EPI_ISL_460408, EPI_ISL_460416, EPI_ISL_460430, EPI_ISL_460432, EPI_ISL_460461, EPI_ISL_460463, EPI_ISL_460464			
see above	Massachusetts General Hospital	Infectious Disease Program, Broad Institute of Harvard and MIT	Lemieux,J.E., Siddle,K.J., Shaw,B., Adams,G., Pierce,V., Turbett,S., Anahtar,M., Branda,J., Slater,D., Harris,J., Lin,A.E., Gladden-Young,A., Lagerborg,K., Rudy,M., DeRuff,K., Carter,A., Normandin,E., Bauer,M., Reilly,S., Tomkins-Tinch,C., Loreth,C., Chaluvadi,S., Neumann,A., Cusick,C., Chapman,S.B., Gnirke,A., Flowers,K., Cerrato,F., Birren,B.W., Gallagher,G., Smole,S., Park,D.J., MacInnis,B.L., Ryan,E., LaRocque,R., Rosenberg,E., Sabeti,P.C.
EPI_ISL_460623, EPI_ISL_460625, EPI_ISL_460626, EPI_ISL_460627	UW Virology Lab	UW Virology Lab	Pavitra Roychoudhury, Amin Addetia, Hong Xie, Lasata Shrestha, Truong Nguyen, Meei-Li Huang, Keith Jerome, Alexander Greninger
EPI_ISL_460638, EPI_ISL_460675, EPI_ISL_460685, EPI_ISL_460688, EPI_ISL_460719, EPI_ISL_460791, EPI_ISL_460796, EPI_ISL_460797, EPI_ISL_460798, EPI_ISL_461076, EPI_ISL_461077, EPI_ISL_461078, EPI_ISL_461079, EPI_ISL_461080, EPI_ISL_461081, EPI_ISL_461082, EPI_ISL_461128, EPI_ISL_461164, EPI_ISL_461250, EPI_ISL_461261, EPI_ISL_461337			
see above	Dutch COVID-19 response team	Erasmus Medical Center	Bas Oude Munnink, David Nieuwenhuijse, Reina Sikkema, Claudia Schapendonk, Irina Chestakova, Anne van der Linden, Theo Bestebroer, Stefan van Nieuwkoop, Mark Pronk, Pascal Lexmond, Corien Swaan, Manon Haverkate, Madelief Mollers, Mart Stein, Sandra Kengne Kamga Mobou, Jeroen van Kampen, Jolanda Voermans, Aura Timen, Corine GeurtsvanKessel, Annemiek van der Eijk, Richard Molenkamp, Marion Koopmans, on behalf of the Dutch national COVID-19 response team.
EPI_ISL_461423, EPI_ISL_461443, EPI_ISL_461444, EPI_ISL_461445	UW Virology Lab	UW Virology Lab	Pavitra Roychoudhury, Amin Addetia, Hong Xie, Lasata Shrestha, Truong Nguyen, Meei-Li Huang, Keith Jerome, Alexander Greninger
EPI_ISL_461732, EPI_ISL_461733, EPI_ISL_461734, EPI_ISL_461761	Virology Department, Royal Infirmary of Edinburgh, NHS Lothian / School of Biological Sciences, University of Edinburgh / Institute of Genetics and Molecular Medicine, University of Edinburgh	COVID-19 Genomics UK (COG-UK) Consortium	McHugh M, Dewar R, Rooke S, Gallagher M, Balcaza C, O'Toole Á, Scher E, Hill V, McCrone JT, Colquhoun R, Yu X, Jackson B, Rambaut A, Williams TC, Templeton K
EPI_ISL_461773, EPI_ISL_461774, EPI_ISL_461775, EPI_ISL_461776, EPI_ISL_461777, EPI_ISL_461778, EPI_ISL_461779, EPI_ISL_461780, EPI_ISL_461781, EPI_ISL_461783	Regional Virus Laboratory, Belfast Health and Social Care Trust	COVID-19 Genomics UK (COG-UK) Consortium	Conall McCaughey, James McKenna, Tanya Curran, Susan Feeney, Alison Watt, Ciara Cox, Mairead Connor, Zoltan Molnar, David Simpson, Derek Fairley
EPI_ISL_462150	Molecular diagnostic laboratory of Federal Budget Institution of Science "Central Research Institute of Epidemiology" of The Federal Service on Customers' Rights Protection and Human Well-being Surveillance	Group of Genomics and Postgenomic Technologies of Central Research Institute of Epidemilology	Speranskaya AS, Kaptelova VV, Samoilov AE, Korneenko EV, Sizova TV, Tivanova EV, Shipulina OY, Akimkin VG
EPI_ISL_462213, EPI_ISL_462214, EPI_ISL_462215, EPI_ISL_462216, EPI_ISL_462218, EPI_ISL_462220, EPI_ISL_462222, EPI_ISL_462225, EPI_ISL_462226, EPI_ISL_462227, EPI_ISL_462229, EPI_ISL_462230, EPI_ISL_462231, EPI_ISL_462232, EPI_ISL_462233			
see above	KU Leuven, Rega Institute, Clinical and Epidemiological Virology	KU Leuven, Rega Institute, Clinical and Epidemiological Virology	Tony Wawina-Bokalanga, Bert Vanmechelen, Joan Marti-Carerras, Piet Maes
EPI_ISL_462335, EPI_ISL_462350, EPI_ISL_462368, EPI_ISL_462418	National Public Health Laboratory, National Centre for Infectious Diseases	National Public Health Laboratory, National Centre for Infectious Diseases	Mak TM, Octavia S, Chavatte JM, Cui L, Lin RTP
EPI_ISL_462434	unknown	Laboratory Diagnostic	Vidanovic,D., Tesovic,B., Banovic Djeri,B., Knezevic,A., Jankovic,M., Sekler,M., Dmitric,M., Petrovic,T., Volkening,J., Afonso,C.L.
EPI_ISL_462439, EPI_ISL_462440, EPI_ISL_462441, EPI_ISL_462442, EPI_ISL_462443, EPI_ISL_462444, EPI_ISL_462445	unknown	Ryota Kumagai Tokyo Metropolitan Institute of Public Health	Asakura,H., Kumagai,R., Yoshida,I., Nagashima,M., Chiba,T., Sadamasu,K.
EPI_ISL_462472	Clinical Center, University of Sarajevo	Charite Universitatsmedizin Berlin, Institute of Virology	Victor M Corman, Jorn Beheim-Schwarzbach, Barbara Muehlemann, Talitha Veith, Julia Schneider, Terry Jones, Amela Dedeic-Ljubovic, Irma Salimovic-Besic, Suzana Arapcic, Almedina Hadzihasanovic-Moro, Selma Mutevelic, Christian Drosten
EPI_ISL_463004, EPI_ISL_463006	unknown	Clinical virology	Fares,W., Triki,H.
EPI_ISL_463151, EPI_ISL_463152, EPI_ISL_463153, EPI_ISL_463154, EPI_ISL_463155, EPI_ISL_463156, EPI_ISL_463157, EPI_ISL_463158, EPI_ISL_463159, EPI_ISL_463160, EPI_ISL_463161, EPI_ISL_463162, EPI_ISL_463163, EPI_ISL_463164, EPI_ISL_463165, EPI_ISL_463166, EPI_ISL_463167, EPI_ISL_463168, EPI_ISL_463169, EPI_ISL_463170, EPI_ISL_463171, EPI_ISL_463172, EPI_ISL_463173, EPI_ISL_463174			
see above	Yale Clinical Virology Laboratory	Grubaugh Lab - Yale School of Public Health	Joseph Fauver, Tara Alpert, Anderson Brito, Anne Wyllie, Chantal Vogels, Mary Petrone, Cole Jensen, Chaney Kalinich, Isabel Ott, Arnau Casanovas,

			Catherine Muenker, Adam Moore, Alice Lu, Maria Tokuyama, Patrick Wong, Peiwen Lu, Saad Omer, Richard Martinello, Allison Nelson, Shelli Farhadian, Akiko Iwasaki, Charlese Dela Cruz, Albert Ko, Nathan Grubaugh
EPI_ISL_463745	Department of Molecular Virology, Cyprus Institute of Neurology and Genetics	Department of Molecular Virology, Cyprus Institute of Neurology and Genetics	Jan Richter, George Krashias, Christina Tryfonos, Stavros Bashiardes, Dana Koptides, Christina Christodoulou
EPI_ISL_463928, EPI_ISL_463933, EPI_ISL_463940, EPI_ISL_463952	Laboratoire de microbiologie, Hopital de Verdun	Smith Laboratory, Centre de Recherche CHU Sainte-Justine	Martin Smith, Marieke Rozendaal, Ivan Pavlov
EPI_ISL_463986	Toronto Invasive Bacterial Diseases Network	McMaster University	Allison McGeer, Patryk Aftanas, Angel Li, Kuganya Nirmalarajah, Samira Mubareka, Andrew G. McArthur
EPI_ISL_464000, EPI_ISL_464005, EPI_ISL_464016, EPI_ISL_464032, EPI_ISL_464035, EPI_ISL_464044, EPI_ISL_464045, EPI_ISL_464048, EPI_ISL_464055, EPI_ISL_464060	Unity Health Toronto	Ontario Institute for Cancer Research	Ramzi Fattouh,Larissa M. Matukas,Mark Downing,Annette Gower,Karel Boissinot,Samira Mubareka,TIBDN,Ilinca Lungu,Bernard Lam,Jeremy Johns,Paul Krzyzanowski,Richard de Borja,Philip Zuzarte,Jared Simpson
EPI_ISL_465894, EPI_ISL_465907, EPI_ISL_465919, EPI_ISL_465922, EPI_ISL_465971, EPI_ISL_465973, EPI_ISL_465975, EPI_ISL_465982, EPI_ISL_465983, EPI_ISL_465998, EPI_ISL_465999, EPI_ISL_466000, EPI_ISL_466001, EPI_ISL_466002, EPI_ISL_466003, EPI_ISL_466004, EPI_ISL_466007, EPI_ISL_466008, EPI_ISL_466009, EPI_ISL_466010, EPI_ISL_466011, EPI_ISL_466012, EPI_ISL_466013, EPI_ISL_466014, EPI_ISL_466015, EPI_ISL_466016, EPI_ISL_466017, EPI_ISL_466018, EPI_ISL_466019, EPI_ISL_466020, EPI_ISL_466021, EPI_ISL_466022, EPI_ISL_466023, EPI_ISL_466024, EPI_ISL_466025, EPI_ISL_466026, EPI_ISL_466027, EPI_ISL_466028, EPI_ISL_466029, EPI_ISL_466030, EPI_ISL_466031, EPI_ISL_466032, EPI_ISL_466033, EPI_ISL_466034, EPI_ISL_466035, EPI_ISL_466038, EPI_ISL_466039, EPI_ISL_466040, EPI_ISL_466041, EPI_ISL_466043, EPI_ISL_466044, EPI_ISL_466045, EPI_ISL_466047, EPI_ISL_466051, EPI_ISL_466052, EPI_ISL_466053, EPI_ISL_466054, EPI_ISL_466055, EPI_ISL_466057, EPI_ISL_466062, EPI_ISL_466064, EPI_ISL_466065, EPI_ISL_466072, EPI_ISL_466106, EPI_ISL_466108, EPI_ISL_466109, EPI_ISL_466110, EPI_ISL_466111, EPI_ISL_466112, EPI_ISL_466113, EPI_ISL_466114, EPI_ISL_466115, EPI_ISL_466116, EPI_ISL_466117, EPI_ISL_466118, EPI_ISL_466119, EPI_ISL_466121, EPI_ISL_466122, EPI_ISL_466123, EPI_ISL_466127, EPI_ISL_466128, EPI_ISL_466129, EPI_ISL_466130, EPI_ISL_466131, EPI_ISL_466132, EPI_ISL_466133, EPI_ISL_466134, EPI_ISL_466135, EPI_ISL_466136, EPI_ISL_466137, EPI_ISL_466139, EPI_ISL_466140, EPI_ISL_466143, EPI_ISL_466146, EPI_ISL_466147, EPI_ISL_466148, EPI_ISL_466149, EPI_ISL_466229, EPI_ISL_466230, EPI_ISL_466239			
see above	Respiratory Virus Unit, Microbiology Services Colindale, Public Health England	Respiratory Virus Unit, Microbiology Services Colindale, Public Health England	PHE Covid Sequencing Team
EPI_ISL_466696, EPI_ISL_466697, EPI_ISL_466698, EPI_ISL_466699, EPI_ISL_466700, EPI_ISL_466701, EPI_ISL_466702, EPI_ISL_466703, EPI_ISL_466704, EPI_ISL_466705, EPI_ISL_466706, EPI_ISL_466707, EPI_ISL_466708, EPI_ISL_466709, EPI_ISL_466710, EPI_ISL_466711, EPI_ISL_466712, EPI_ISL_466713, EPI_ISL_466714, EPI_ISL_466715, EPI_ISL_466716, EPI_ISL_466717, EPI_ISL_466718, EPI_ISL_466719, EPI_ISL_466720, EPI_ISL_466721, EPI_ISL_466722, EPI_ISL_466723, EPI_ISL_466724, EPI_ISL_466725, EPI_ISL_466726, EPI_ISL_466727, EPI_ISL_466728, EPI_ISL_466729, EPI_ISL_466730, EPI_ISL_466731, EPI_ISL_466732, EPI_ISL_466733, EPI_ISL_466734, EPI_ISL_466735, EPI_ISL_466736, EPI_ISL_466737, EPI_ISL_466738, EPI_ISL_466739, EPI_ISL_466740, EPI_ISL_466741, EPI_ISL_466742, EPI_ISL_466743, EPI_ISL_466744, EPI_ISL_466745, EPI_ISL_466746, EPI_ISL_466747, EPI_ISL_466748, EPI_ISL_466749, EPI_ISL_466750, EPI_ISL_466751, EPI_ISL_466752, EPI_ISL_466753, EPI_ISL_466754, EPI_ISL_466755, EPI_ISL_466756, EPI_ISL_466757, EPI_ISL_466758, EPI_ISL_466759, EPI_ISL_466760, EPI_ISL_466761, EPI_ISL_466762, EPI_ISL_466763, EPI_ISL_466764, EPI_ISL_466765, EPI_ISL_466766, EPI_ISL_466767, EPI_ISL_466768, EPI_ISL_466769, EPI_ISL_466770, EPI_ISL_466771, EPI_ISL_466772, EPI_ISL_466773, EPI_ISL_466774, EPI_ISL_466775, EPI_ISL_466776, EPI_ISL_466777, EPI_ISL_466778, EPI_ISL_466779, EPI_ISL_466780, EPI_ISL_466781, EPI_ISL_466782, EPI_ISL_466783, EPI_ISL_466784, EPI_ISL_466785, EPI_ISL_466786, EPI_ISL_466787, EPI_ISL_466788, EPI_ISL_466789, EPI_ISL_466790, EPI_ISL_466791, EPI_ISL_466792, EPI_ISL_466793, EPI_ISL_466794, EPI_ISL_466795, EPI_ISL_466796, EPI_ISL_466797, EPI_ISL_466798, EPI_ISL_466799, EPI_ISL_466800, EPI_ISL_466801, EPI_ISL_466802, EPI_ISL_466803, EPI_ISL_466804, EPI_ISL_466805, EPI_ISL_466806, EPI_ISL_466807, EPI_ISL_466808, EPI_ISL_466809, EPI_ISL_466810, EPI_ISL_466811, EPI_ISL_466812, EPI_ISL_466813, EPI_ISL_466814, EPI_ISL_466815, EPI_ISL_466816, EPI_ISL_466817, EPI_ISL_466818, EPI_ISL_466819, EPI_ISL_466820, EPI_ISL_466821, EPI_ISL_466822, EPI_ISL_466823, EPI_ISL_466824, EPI_ISL_466825, EPI_ISL_466826, EPI_ISL_466827, EPI_ISL_466828, EPI_ISL_466829, EPI_ISL_466830, EPI_ISL_466831, EPI_ISL_466832, EPI_ISL_466833, EPI_ISL_466834, EPI_ISL_466835, EPI_ISL_466836, EPI_ISL_466837, EPI_ISL_466838			
see above	BCCDC Public Health Laboratory	BCCDC Public Health Laboratory	Richard Harrigan, Hope Lapointe, Jinny Choi, Kimia Kamelian, John Tyson,Terry Snutch, Linda Hoang, Inna Sekirov, Paul Levett, Mel Krajden, Natalie Prystajecky
EPI_ISL_466873	Centre for Clinical Infection and Diagnostics Research and Genomics Innovation Unit	Respiratory Virus Unit, Microbiology Services Colindale, Public Health England	PHE Covid Sequencing Team, Chloe Fisher, Luke Snell, Gaia Nebbia, Ali Awan
EPI_ISL_467059, EPI_ISL_467063	Hospital Universitario Virgen de las Nieves de Granada-SAS	SeqCOVID-SPAIN consortium/IBV(CSIC)	Mercedes Pérez Ruiz, Sara Sanbonmatsu Gámez, Irene Pedrosa Corral, José M. Navarro-Marí and SeqCOVID-SPAIN consortium
EPI_ISL_467187, EPI_ISL_467188, EPI_ISL_467189, EPI_ISL_467193, EPI_ISL_467196, EPI_ISL_467197, EPI_ISL_467201, EPI_ISL_467204, EPI_ISL_467205, EPI_ISL_467206, EPI_ISL_467208, EPI_ISL_467209, EPI_ISL_467213, EPI_ISL_467214, EPI_ISL_467215, EPI_ISL_467218, EPI_ISL_467221, EPI_ISL_467222, EPI_ISL_467223, EPI_ISL_467225, EPI_ISL_467226, EPI_ISL_467228, EPI_ISL_467232, EPI_ISL_467239, EPI_ISL_467244, EPI_ISL_467245, EPI_ISL_467247, EPI_ISL_467250, EPI_ISL_467253, EPI_ISL_467254, EPI_ISL_467255			
see above	Hospital General Universitario Gregorio Marañón	SeqCOVID-SPAIN consortium/IBV(CSIC)	Laura Pérez-Lago, Marta Herranz, Jon Sicilia, Julia Suárez, Pilar Catalán, Patricia Muñoz, Darío García de Viedma and SeqCOVID-SPAIN consortium
EPI_ISL_467303, EPI_ISL_467305, EPI_ISL_467306, EPI_ISL_467307, EPI_ISL_467308, EPI_ISL_467309, EPI_ISL_467310, EPI_ISL_467311, EPI_ISL_467312, EPI_ISL_467313, EPI_ISL_467314, EPI_ISL_467315, EPI_ISL_467316, EPI_ISL_467317, EPI_ISL_467318, EPI_ISL_467319, EPI_ISL_467320, EPI_ISL_467321, EPI_ISL_467322, EPI_ISL_467323, EPI_ISL_467324, EPI_ISL_467325, EPI_ISL_467326, EPI_ISL_467327, EPI_ISL_467328, EPI_ISL_467329, EPI_ISL_467330, EPI_ISL_467331, EPI_ISL_467332, EPI_ISL_467333, EPI_ISL_467334, EPI_ISL_467335, EPI_ISL_467336, EPI_ISL_467337, EPI_ISL_467338, EPI_ISL_467339, EPI_ISL_467340, EPI_ISL_467341, EPI_ISL_467342, EPI_ISL_467343, EPI_ISL_467423, EPI_ISL_467424, EPI_ISL_467425, EPI_ISL_467426, EPI_ISL_467427, EPI_ISL_467428, EPI_ISL_467429			
see above	BCCDC Public Health Laboratory	BCCDC Public Health Laboratory	Richard Harrigan, Hope Lapointe, Jinny Choi, Kimia Kamelian, John Tyson,Terry Snutch, Linda Hoang, Inna Sekirov, Paul Levett, Mel Krajden, Natalie Prystajecky
EPI_ISL_467436, EPI_ISL_467437, EPI_ISL_467440	NHLS-IALCH	KRISP, KZN Research Innovation and Sequencing Platform	Giandhari J, Pillay S, Lessells R, Chimukangara B, Mdlalose K, York D, Khan S, Tegally H, Wilkinson E, de Oliveira T
EPI_ISL_467448	Molecular Diagnostics Services (MDS)	KRISP, KZN Research Innovation and Sequencing Platform	Giandhari J, Pillay S, Lessells R, Chimukangara B, Mdlalose K, York D, Khan S, Tegally H, Wilkinson E, de Oliveira T
EPI_ISL_467454, EPI_ISL_467461, EPI_ISL_467462, EPI_ISL_467463, EPI_ISL_467464, EPI_ISL_467465, EPI_ISL_467470, EPI_ISL_467471, EPI_ISL_467472, EPI_ISL_467473, EPI_ISL_467474			
see above	AMPATH-DBN	KRISP, KZN Research Innovation and Sequencing Platform	Giandhari J, Pillay S, Lessells R, Chimukangara B, Mdlalose K, York D, Khan S, Tegally H, Wilkinson E, de Oliveira T
EPI_ISL_467539, EPI_ISL_467540, EPI_ISL_467541, EPI_ISL_467542, EPI_ISL_467543, EPI_ISL_467544, EPI_ISL_467545, EPI_ISL_467546, EPI_ISL_467547, EPI_ISL_467629, EPI_ISL_467630, EPI_ISL_467631, EPI_ISL_467632, EPI_ISL_467633, EPI_ISL_467634, EPI_ISL_467635, EPI_ISL_467636, EPI_ISL_467637, EPI_ISL_467638, EPI_ISL_467639, EPI_ISL_467640			
see above	New Mexico Department of Health Scientific Laboratory Division	Center for Global Health, University of New Mexico Health Sciences Center	Daryl Domman, Kurt Schwalm, Twila Kunde, Joseph Hicks, Michael Edwards, Darrell Dinwiddie
EPI_ISL_467774	Molecular diagnostic laboratory of Federal Budget Institution of Science "Central Research Institute of Epidemiology" of The Federal Service on Customers' Rights Protection and Human Well-being Surveillance	Group of Genomics and Postgenomic Technologies of Central Research Institute of Epidemilology	Speranskaya AS, Kaptelova VV, Samoilov AE, Korneenko EV, Sizova TV, Tivanova EV, Shipulina OY, Akimkin VG
EPI_ISL_467993, EPI_ISL_467999, EPI_ISL_468005, EPI_ISL_468006, EPI_ISL_468010, EPI_ISL_468035, EPI_ISL_468040, EPI_ISL_468041, EPI_ISL_468043	SA Pathology	SA Pathology	Lex Leong, Chuan Kok Lim, Mark Turra, Ivan Bastian, Geoff Higgins
EPI_ISL_468359	Alameda County Public Health Lab	Chan-Zuckerberg Biohub	CZB Cliahub Consortium
EPI_ISL_468453, EPI_ISL_468454, EPI_ISL_468455, EPI_ISL_468456, EPI_ISL_468457, EPI_ISL_468458	Humboldt County Public Health Laboratory	Chan-Zuckerberg Biohub	CZB Cliahub Consortium
EPI_ISL_468498	Ventura County Public Health Lab	Chan-Zuckerberg Biohub	CZB Cliahub Consortium
EPI_ISL_468528, EPI_ISL_468529, EPI_ISL_468531	San Joaquin County Public Health Lab	Chan-Zuckerberg Biohub	CZB Cliahub Consortium
EPI_ISL_468620, EPI_ISL_468621, EPI_ISL_468622	Contra Costa Public Health Lab	Chan-Zuckerberg Biohub	CZB Cliahub Consortium
EPI_ISL_468657, EPI_ISL_468658, EPI_ISL_468659, EPI_ISL_468660, EPI_ISL_468661, EPI_ISL_468662, EPI_ISL_468663, EPI_ISL_468664, EPI_ISL_468665, EPI_ISL_468666, EPI_ISL_468667, EPI_ISL_468668, EPI_ISL_468669, EPI_ISL_468670, EPI_ISL_468671, EPI_ISL_468672, EPI_ISL_468673, EPI_ISL_468674, EPI_ISL_468675, EPI_ISL_468676, EPI_ISL_468677, EPI_ISL_468678, EPI_ISL_468679, EPI_ISL_468680, EPI_ISL_468681, EPI_ISL_468682, EPI_ISL_468683, EPI_ISL_468684, EPI_ISL_468685, EPI_ISL_468686, EPI_ISL_468687, EPI_ISL_468688, EPI_ISL_468689, EPI_ISL_468690, EPI_ISL_468691, EPI_ISL_468692, EPI_ISL_468693, EPI_ISL_468694, EPI_ISL_468695, EPI_ISL_468696, EPI_ISL_468697, EPI_ISL_468698, EPI_ISL_468699, EPI_ISL_468700			
see above	BCCDC Public Health Laboratory	BCCDC Public Health Laboratory	Richard Harrigan, Hope Lapointe, Jinny Choi, Kimia Kamelian, John Tyson,Terry Snutch, Linda Hoang, Inna Sekirov, Paul Levett, Mel Krajden, Natalie Prystajecky

EPI_ISL_469054	LNR National Reference Laboratory, Mohammed VI University of Health Sciences	Medical Biotechnology Laboratory, Rabat Medical and Pharmacy School, Mohammed The Vth University in Rabat	Meriem LAAMARTI, Souad KARTTI, Rokaia LAAMARTI , M.W. CHEMAO-ELFIHRI, Loubna ALLAM, Mouna OUADGHIRI, Imane SMYEJ, Jalila RAHOUI, Houda BENRAHMA, Jalil El Atar, Idrissa Diawara, Rachid EL JAOUDI, Laila SBABOU, Chakib NEJJARI, Saaid AMZAZI, Rachid MENTAG, Lahcen BELYAMANI and Azeddine IBRAHIMI
EPI_ISL_469098, EPI_ISL_469103, EPI_ISL_469104, EPI_ISL_469121	National Public Health Laboratory, National Centre for Infectious Diseases	National Public Health Laboratory, National Centre for Infectious Diseases	Mak TM, Octavia S, Chavatte JM, Cui L, Lin RTP
EPI_ISL_469171, EPI_ISL_469172, EPI_ISL_469173, EPI_ISL_469174, EPI_ISL_469175, EPI_ISL_469176, EPI_ISL_469177, EPI_ISL_469178, EPI_ISL_469179, EPI_ISL_469180, EPI_ISL_469181, EPI_ISL_469182, EPI_ISL_469183, EPI_ISL_469184, EPI_ISL_469185, EPI_ISL_469186, EPI_ISL_469187, EPI_ISL_469188			
see above	Yale Clinical Virology Laboratory	Grubaugh Lab - Yale School of Public Health	Joseph Fauver, Tara Alpert, Anderson Brito, Anne Wyllie, Chantal Vogels, Mary Petrone, Cole Jensen, Chaney Kalinich, Isabel Ott, Arnau Casanovas, Catherine Muenker, Adam Moore, Alice Lu, Maria Tokuyama, Patrick Wong, Peiwen Lu, Saad Omer, Richard Martinello, Allison Nelson, Shelli Farhadian, Akiko Iwasaki, Charlese Dela Cruz, Albert Ko, Nathan Grubaugh
EPI_ISL_469209, EPI_ISL_469210, EPI_ISL_469211, EPI_ISL_469212, EPI_ISL_469213, EPI_ISL_469214, EPI_ISL_469215, EPI_ISL_469216, EPI_ISL_469217, EPI_ISL_469218, EPI_ISL_469219, EPI_ISL_469220, EPI_ISL_469221, EPI_ISL_469222, EPI_ISL_469223			
see above	BCCDC Public Health Laboratory	BCCDC Public Health Laboratory	Richard Harrigan, Hope Lapointe, Jinny Choi, Kimia Kamelian, John Tyson,Terry Snutch, Linda Hoang, Inna Sekirov, Paul Levett, Mel Krajden, Natalie Prystajecky
EPI_ISL_469279	Mohammed Bin Rashid University of Medicine and Health Sciences	Al Jalila Genomics Center	Ahmad Abou Tayoun, Tom Loney, Hamda Khansaheb, Sathishkumar Ramaswamy, Divinlal Harilal, Zulfa Omar Deesi, Rupa Murthy Varghese, Hanan Al Suwaidi, Abdulmajeed Alkhaja, Mohammed Uddin, Rifat Hamoudi, Rabih Halwani, Abiola Catherine Senok, Qutayba Hamid, Norbert Nowotny, Alawi Alsheikh-Ali
EPI_ISL_469289	Keio University Hospital	Keio University Hospital	Kenjiro Kosaki
EPI_ISL_469541, EPI_ISL_469548, EPI_ISL_469551, EPI_ISL_469553, EPI_ISL_469556, EPI_ISL_469561, EPI_ISL_469565, EPI_ISL_469568, EPI_ISL_469573, EPI_ISL_469577, EPI_ISL_469594, EPI_ISL_469597, EPI_ISL_469601, EPI_ISL_469603, EPI_ISL_469609, EPI_ISL_469617, EPI_ISL_469620, EPI_ISL_469634, EPI_ISL_469642, EPI_ISL_469645, EPI_ISL_469648, EPI_ISL_469650, EPI_ISL_469662, EPI_ISL_469670, EPI_ISL_469681, EPI_ISL_469698, EPI_ISL_469708, EPI_ISL_469751, EPI_ISL_469771, EPI_ISL_469778, EPI_ISL_469784, EPI_ISL_469793, EPI_ISL_469800			
see above	PHE South West Regional Laboratory, National Infection Service	Wellcome Sanger Institute for the COVID-19 Genomics UK (COG-UK) consortium	Stephanie Hutchings, Hannah Pymont, Dr Peter Muir, Barry Vipond, Rich Hopes; and Alex Alderton, Roberto Amato, Sonia Goncalves, Ewan Harrison, David K. Jackson, Ian Johnston, Dominic Kwiatkowski, Cordelia Langford, John Sillitoe on behalf of the Wellcome Sanger Institute COVID-19 Surveillance Team (http://www.sanger.ac.uk/covid-team)
EPI_ISL_469915	NU-OMICS DNA Sequencing research facility, Northumbria University	Wellcome Sanger Institute for the COVID-19 Genomics UK (COG-UK) consortium	Chris Duncan, Sheia Waugh, Shirelle Burton-Fanning, Gary Eltringham, Jennifer Collins, Brendan Payne, Yusri Taha, Emma Swindells, Jane Greenaway, Edward Barton, Garren Scott, Debra Padgett, Clive Graham, Sarah Essex, Steve Ligget, Paul Baker, Lynn Dover, Wen Yew, Gary Black, John Allan, Joshua Loh, Greg Young, Matthew Bashton, Andrew Nelson, Darren Smith and Alex Alderton, Roberto Amato, Sonia Goncalves, Ewan Harrison, David K. Jackson, Ian Johnston, Dominic Kwiatkowski, Cordelia Langford, John Sillitoe on behalf of the Wellcome Sanger Institute COVID-19 Surveillance Team (http://www.sanger.ac.uk/covid-team)
EPI_ISL_469931, EPI_ISL_469939, EPI_ISL_469944, EPI_ISL_469950, EPI_ISL_469955, EPI_ISL_469962, EPI_ISL_469978, EPI_ISL_469986, EPI_ISL_469997, EPI_ISL_469998, EPI_ISL_469999, EPI_ISL_470010			
see above	NHSGGC West of Scotland Specialist Virology Centre / MRC-University of Glasgow Centre for Virus Research	Wellcome Sanger Institute for the COVID-19 Genomics UK (COG-UK) consortium	Ana da Silva Filipe, Natasha Johnson, Kathy Smollett, Daniel Mair, Stephen Carmichael, Lily Tong, Jenna Nichols, Elihu Aranday-Cortes, Kirstyn Brunker, Yasmin Parr, Kyriaki Nomikou; Sarah McDonald, Marc Niebel, Patawee Asamaphan; Richard Orton, Joseph Hughes, Sreenu Vattipally, David L Robertson; Alasdair MacLean, Rory Gunson; Kathy Li, Natasha Jesudason, Rajiv Shah, James Shepherd, Antonia Ho, Alice Broos, Emma Thomson and Alex Alderton, Roberto Amato, Sonia Goncalves, Ewan Harrison, David K. Jackson, Ian Johnston, Dominic Kwiatkowski, Cordelia Langford, John Sillitoe on behalf of the Wellcome Sanger Institute COVID-19 Surveillance Team (http://www.sanger.ac.uk/covid-team)
EPI_ISL_470534	Regional Virus Laboratory, Belfast Health and Social Care Trust	Wellcome Sanger Institute for the COVID-19 Genomics UK (COG-UK) consortium	Conall McCaughey, James McKenna, Tanya Curran, Susan Feeney, Alison Watt, Ciara Cox, Mairead Connor, Zoltan Molnar, David Simpson, Derek Fairley; and Alex Alderton, Roberto Amato, Sonia Goncalves, Ewan Harrison, David K. Jackson, Ian Johnston, Dominic Kwiatkowski, Cordelia Langford, John Sillitoe on behalf of the Wellcome Sanger Institute COVID-19 Surveillance Team (http://www.sanger.ac.uk/covid-team)
EPI_ISL_470568, EPI_ISL_470569, EPI_ISL_470600, EPI_ISL_470601, EPI_ISL_470602, EPI_ISL_470603	Hermes Pardini	Bioinformatics Laboratory / LNCC	Alexandra Gerber, Ana Paula Guimarães, Luiz Gonzaga Paula de Almeida, Ronaldo da Silva Francisco Junior, Mariane Talon, Filipe Romero, Átila Duque Rossi, Terezinha Marta Pereira, working group UFRJ, Jaqueline Goes de Jesus, Ingra Morales Claro, Ester Cerdeira Sabino, Nuno Rodrigues Faria, CADDE-group, Laboratorio Hermes Pardini, Laboratorio Simile, working group UFMG, Amilcar Tanuri, Carolina Voloch, Renato Santana Aguiar e Ana Tereza Vasconcelos
EPI_ISL_470621, EPI_ISL_470622	Laboratorio de Virologia Molecular / UFRJ	Bioinformatics Laboratory / LNCC	Alexandra Gerber, Ana Paula Guimarães, Luiz Gonzaga Paula de Almeida, Ronaldo da Silva Francisco Junior, Mariane Talon, Filipe Romero, Átila Duque Rossi, Terezinha Marta Pereira, working group UFRJ, Jaqueline Goes de Jesus, Ingra Morales Claro, Ester Cerdeira Sabino, Nuno Rodrigues Faria, CADDE-group, Laboratorio Hermes Pardini, Laboratorio Simile, working group UFMG, Amilcar Tanuri, Carolina Voloch, Renato Santana Aguiar e Ana Tereza Vasconcelos
EPI_ISL_470830, EPI_ISL_470842, EPI_ISL_470843, EPI_ISL_470866	PathWest Laboratory Medicine WA	PathWest Laboratory Medicine WA	Chisha Sikazwe, Jurissa Lang, Avram Levy, David Smith and David Speers
EPI_ISL_471182, EPI_ISL_471223, EPI_ISL_471266	Wisconsin State Laboratory of Hygiene Communicable Disease Division	Wisconsin State Laboratory of Hygiene Communicable Disease Division	Kelsey R. Florek, Abigail C. Shockey
EPI_ISL_471456	Centre de Virologie des Maladies Tropicales	Functional Genomic Platform/Service Analyses Biologique/UATRS/ Centre National Pour la Recherche Scientifique Et Technique (CNRST)	Hicham EL ANNAZ, Elmostafa EL FAHIME, Marouane MELLOUL, Youssef AKHOUAD, Mly Abdelaziz ELALAOUI, Ahmed REGGAD, Sanaa ALAOUI-Amine, Rachid ABI, Rida TAGAJDID, Zohour KASMY, Safae ELKOCHRI, Nadia TOUIL, Farida HILALI, Abdelkader LAATIRIS, Abdelillah LARAQUI, Tahar BAJJOU , Yassine SEKHSOKH , Idriss-Amine LAHLOU, Mostafa ELOUENNASS, Khalid ENNIBI
EPI_ISL_471552	Hospital Sancta Maggiore	Instituto Adolfo Lutz, Interdiciplinary Procedures Center, Strategic Laboratory	Claudio Tavares Sacchi, Claudia Regina Gonçalves, Erica Valessa Ramos Gomes
EPI_ISL_471957, EPI_ISL_471959, EPI_ISL_471960, EPI_ISL_471963	University of Exeter	COVID-19 Genomics UK (COG-UK) Consortium	Ben Temperton,Aaron Jeffries,Michelle Michelsen,Joanna Warwick-Dugdale,Audrey Farbos,Robyn Manley,Stephen Michell,Jane Masoli
EPI_ISL_472136, EPI_ISL_472137, EPI_ISL_472138, EPI_ISL_472139, EPI_ISL_472140, EPI_ISL_472141, EPI_ISL_472142, EPI_ISL_472143, EPI_ISL_472144, EPI_ISL_472146	Regional Virus Laboratory, Belfast Health and Social Care Trust	COVID-19 Genomics UK (COG-UK) Consortium	Conall McCaughey, James McKenna, Tanya Curran, Susan Feeney, Alison Watt, Ciara Cox, Mairead Connor, Zoltan Molnar, David Simpson, Derek Fairley
EPI_ISL_472246, EPI_ISL_472262	Northumbria University / South Tees Hospitals NHS Foundation Trust / North Cumbria Integrated Care NHS Foundation Trust / North Tees and Hartlepool NHS Foundation Trust / Newcastle Hospitals NHS Foundation Trust	COVID-19 Genomics UK (COG-UK) Consortium	Darren L Smith,Andrew Nelson,Matthew Bashton,Greg R Young,Joshua Loh,John Allan,Mohammad A Tariq,Giles S Holt,Gary Black,Wen C Yew,Lynn Dover,Paul Baker,Steve Liggett,Sarah Essex,Jane Greenaway,Debra Padgett,Clive Graham,Garren Scott,Edward Barton,Emma Swindells,Brendan Payne,Jennifer Collins,Yusri Taha,Gary Eltringham
EPI_ISL_472432, EPI_ISL_472434, EPI_ISL_472435, EPI_ISL_472456, EPI_ISL_472458, EPI_ISL_472478, EPI_ISL_472499, EPI_ISL_472521, EPI_ISL_472526, EPI_ISL_472527, EPI_ISL_472530, EPI_ISL_472533, EPI_ISL_472539, EPI_ISL_472552, EPI_ISL_472565, EPI_ISL_472569, EPI_ISL_472572, EPI_ISL_472578, EPI_ISL_472591, EPI_ISL_472592, EPI_ISL_472593, EPI_ISL_472600, EPI_ISL_472619, EPI_ISL_472632, EPI_ISL_472654, EPI_ISL_472690, EPI_ISL_472704, EPI_ISL_472709, EPI_ISL_472714, EPI_ISL_473244, EPI_ISL_474234, EPI_ISL_474238, EPI_ISL_474257, EPI_ISL_474309, EPI_ISL_474310, EPI_ISL_474311, EPI_ISL_474312, EPI_ISL_474314			
see above	Wales Specialist Virology Centre Sequencing lab: Pathogen Genomics Unit	COVID-19 Genomics UK (COG-UK) Consortium	Catherine Moore, Johnathan Evans, Laura Gifford, Malorie Perry, Simon Cottrell, Angela Marchbank, Alec Birchley, Alexander Adams, Amy Gaskin, Bree Gatica-Wilcox, Jason Coombes, Joel Southgate, Lauren Gilbert, Lee Graham, Nicole Pacchiarini, Sara Kumziene-Summerhayes, Sarah Taylor, Sophie Jones, Sara Rey, Matthew Bull, Joanne Watkins, Sally Corden, Tom Connor
EPI_ISL_474798, EPI_ISL_474799, EPI_ISL_474806	Complejo Hospitalario Universitario de Albacete	SeqCOVID-SPAIN consortium/IBV(CSIC)	Encarnacion Simarro Córdoba, Julia Lozano Serra, Lorena Robles Fonseca , Monica Parra Grandes, Caridad Sainz de Baranda Camino and SeqCOVID-SPAIN consortium
EPI_ISL_474832, EPI_ISL_474835	Hospital Universitario Virgen de las Nieves de Granada-SAS	SeqCOVID-SPAIN consortium/IBV(CSIC)	Mercedes Pérez Ruiz, Sara Sanbonmatsu Gámez, Irene Pedrosa Corral, José M. Navarro-Marí and SeqCOVID-SPAIN consortium
EPI_ISL_474837	Complejo Hospitalario Universitario de Albacete	SeqCOVID-SPAIN consortium/IBV(CSIC)	Encarnacion Simarro Córdoba, Julia Lozano Serra, Lorena Robles Fonseca , Monica Parra Grandes, Caridad Sainz de Baranda Camino and SeqCOVID-SPAIN consortium

EPI_ISL_474846, EPI_ISL_474856, EPI_ISL_474884, EPI_ISL_474885, EPI_ISL_474887, EPI_ISL_474888, EPI_ISL_474898, EPI_ISL_474899	Hospital Universitario Virgen de las Nieves de Granada-SAS	SeqCOVID-SPAIN consortium/IBV(CSIC)	Mercedes Pérez Ruiz, Sara Sanbonmatsu Gámez, Irene Pedrosa Corral, José M. Navarro-Marí and SeqCOVID-SPAIN consortium
EPI_ISL_474904	Complejo Hospitalario Universitario de Albacete	SeqCOVID-SPAIN consortium/IBV(CSIC)	Encarnacion Simarro Córdoba, Julia Lozano Serra, Lorena Robles Fonseca , Monica Parra Grandes, Caridad Sainz de Baranda Camino and SeqCOVID-SPAIN consortium
EPI_ISL_474908, EPI_ISL_474931, EPI_ISL_474943, EPI_ISL_474944	Hospital Universitario Virgen de las Nieves de Granada-SAS	SeqCOVID-SPAIN consortium/IBV(CSIC)	Mercedes Pérez Ruiz, Sara Sanbonmatsu Gámez, Irene Pedrosa Corral, José M. Navarro-Marí and SeqCOVID-SPAIN consortium
EPI_ISL_474952, EPI_ISL_474953, EPI_ISL_474955	Complejo Hospitalario Universitario de Albacete	SeqCOVID-SPAIN consortium/IBV(CSIC)	Encarnacion Simarro Córdoba, Julia Lozano Serra, Lorena Robles Fonseca , Monica Parra Grandes, Caridad Sainz de Baranda Camino and SeqCOVID-SPAIN consortium
EPI_ISL_474962, EPI_ISL_474968	Israel Central Virology laboratory	Israel Central Virology laboratory	Neta Zuckerman, Efrat Dahan Bucris, Oran Erster, Ella Mendelson, Michal Mandelboim
EPI_ISL_475116, EPI_ISL_475117	Halmstad klinisk mikrobiologi	The Public Health Agency of Sweden	Oskar Karlsson Lindsjo, Maria Lind Karlberg, Mattias Haukland, Reza Advani, Olov Svartstrom, Anna-Malin Linde, Sandra Broddesson, Petra Edquist, Shamam Muradrasoli, Anna Risberg, Karin Tegmark-Wisell
EPI_ISL_475118	Uppsala klinisk mikrobiologi	The Public Health Agency of Sweden	Oskar Karlsson Lindsjo, Maria Lind Karlberg, Mattias Haukland, Reza Advani, Olov Svartstrom, Anna-Malin Linde, Sandra Broddesson, Petra Edquist, Shamam Muradrasoli, Anna Risberg, Karin Tegmark-Wisell
EPI_ISL_475146, EPI_ISL_475147, EPI_ISL_475148	Klinisk mikrobiologi Vasternorrland	The Public Health Agency of Sweden	Oskar Karlsson Lindsjo, Maria Lind Karlberg, Mattias Haukland, Reza Advani, Olov Svartstrom, Anna-Malin Linde, Sandra Broddesson, Petra Edquist, Shamam Muradrasoli, Anna Risberg, Karin Tegmark-Wisell
EPI_ISL_475279, EPI_ISL_475280, EPI_ISL_475285, EPI_ISL_475289, EPI_ISL_475297, EPI_ISL_475299	Centre for Enzyme Innovation, University of Portsmouth / Translational Research Laboratory, Portsmouth Hospitals NHS Trust	COVID-19 Genomics UK (COG-UK) Consortium	Angela Beckett,Yann Bourgeois,Garry Scarlett,Sharon Glaysher,Scott Elliott,Kelly Bicknell,Robert Impey,Allyson Lloyd,Sarah Wyllie,Ethan Butcher,Anoop Chauhan,Samuel Robson
EPI_ISL_475585, EPI_ISL_475642, EPI_ISL_475646, EPI_ISL_475654, EPI_ISL_475655, EPI_ISL_475656, EPI_ISL_475657, EPI_ISL_475661, EPI_ISL_475666, EPI_ISL_475669, EPI_ISL_475670, EPI_ISL_475671, EPI_ISL_475674, EPI_ISL_475675, EPI_ISL_475676			
see above	Cedars-Sinai Medical Center, Department of Pathology & Laboratory Medicine, Molecular Pathology Laboratory	Cedars-Sinai Medical Center, Molecular Pathology Laboratory of Department of Pathology & Laboratory Medicine and Genomic Core	Wenjuan Zhang, John Paul Govindavari, Brian Davis, Stephanie Chen, Jong Taek Kim, Jianbo Song, Jean Lopategui, Jasmine T Plummer, Eric Vail
EPI_ISL_475792, EPI_ISL_475793, EPI_ISL_475794, EPI_ISL_475795, EPI_ISL_475796, EPI_ISL_475797, EPI_ISL_475798, EPI_ISL_475799	Center for Virology, Medical University of Vienna	Bergthaler laboratory, CeMM Research Center for Molecular Medicine of the Austrian Academy of Sciences	Alexandra Popa, Benedikt Agerer, Henrique Colaco, Lukas Endler, Jakob-Wendelin Genger, Alexander Lercher, Mark Smyth, Thomas Penz, Michael Schuster, Jan Laine, Martin Senekowitsch, Judith Aberle, Stephan Aberle, Peter Hufnagl, Daniela Schmid, Franz Allerberger, Elisabeth Puchhammer-Stoeckl, Manfred Nairz, Guenter Weiss, Gregor Hörmann, Kinga Rigler-Hohenwarter, Rainer Gattringer, Wegene Borena, Dorothee von Laer, Christoph Bock, Andreas Bergthaler
EPI_ISL_475822, EPI_ISL_475823, EPI_ISL_475824, EPI_ISL_475825, EPI_ISL_475826, EPI_ISL_475827	Institut für Virologie am Department für Hygiene, Mikrobiologie und Public Health	Bergthaler laboratory, CeMM Research Center for Molecular Medicine of the Austrian Academy of Sciences	Alexandra Popa, Benedikt Agerer, Henrique Colaco, Lukas Endler, Jakob-Wendelin Genger, Alexander Lercher, Mark Smyth, Thomas Penz, Michael Schuster, Jan Laine, Martin Senekowitsch, Judith Aberle, Stephan Aberle, Peter Hufnagl, Daniela Schmid, Franz Allerberger, Elisabeth Puchhammer-Stoeckl, Manfred Nairz, Guenter Weiss, Gregor Hörmann, Kinga Rigler-Hohenwarter, Rainer Gattringer, Wegene Borena, Dorothee von Laer, Christoph Bock, Andreas Bergthaler
EPI_ISL_475892, EPI_ISL_475893	Zentralinstitut für medizinische und chemische Labordiagnostik, Universitätskliniken Insbruck	Bergthaler laboratory, CeMM Research Center for Molecular Medicine of the Austrian Academy of Sciences	Alexandra Popa, Benedikt Agerer, Henrique Colaco, Lukas Endler, Jakob-Wendelin Genger, Alexander Lercher, Mark Smyth, Thomas Penz, Michael Schuster, Jan Laine, Martin Senekowitsch, Judith Aberle, Stephan Aberle, Peter Hufnagl, Daniela Schmid, Franz Allerberger, Elisabeth Puchhammer-Stoeckl, Manfred Nairz, Guenter Weiss, Gregor Hörmann, Kinga Rigler-Hohenwarter, Rainer Gattringer, Wegene Borena, Dorothee von Laer, Christoph Bock, Andreas Bergthaler
EPI_ISL_475911, EPI_ISL_475914	Klinikum Wels-Grieskirchen	Bergthaler laboratory, CeMM Research Center for Molecular Medicine of the Austrian Academy of Sciences	Alexandra Popa, Benedikt Agerer, Henrique Colaco, Lukas Endler, Jakob-Wendelin Genger, Alexander Lercher, Mark Smyth, Thomas Penz, Michael Schuster, Jan Laine, Martin Senekowitsch, Judith Aberle, Stephan Aberle, Peter Hufnagl, Daniela Schmid, Franz Allerberger, Elisabeth Puchhammer-Stoeckl, Manfred Nairz, Guenter Weiss, Gregor Hörmann, Kinga Rigler-Hohenwarter, Rainer Gattringer, Wegene Borena, Dorothee von Laer, Christoph Bock, Andreas Bergthaler
EPI_ISL_475923	Institut für Virologie am Department für Hygiene, Mikrobiologie und Public Health	Bergthaler laboratory, CeMM Research Center for Molecular Medicine of the Austrian Academy of Sciences	Alexandra Popa, Benedikt Agerer, Henrique Colaco, Lukas Endler, Jakob-Wendelin Genger, Alexander Lercher, Mark Smyth, Thomas Penz, Michael Schuster, Jan Laine, Martin Senekowitsch, Judith Aberle, Stephan Aberle, Peter Hufnagl, Daniela Schmid, Franz Allerberger, Elisabeth Puchhammer-Stoeckl, Manfred Nairz, Guenter Weiss, Gregor Hörmann, Kinga Rigler-Hohenwarter, Rainer Gattringer, Wegene Borena, Dorothee von Laer, Christoph Bock, Andreas Bergthaler
EPI_ISL_476023	Defence Research & Development Establishment (DRDE)	Defence Research & Development Establishment (DRDE)	Shashi Sharma, Paban Kumar Dash, Sushil Kumar Sharma, Ambuj Shrivastava, Jyoti S. Kumar
EPI_ISL_476161, EPI_ISL_476164	Laboratório de Patologia Clínica - UNICAMP	Laboratório de Estudos de Vírus Emergentes - UNICAMP	José Luiz Proença-Modena, Magnun Nueldo Nunes dos Santos, Angelica Schreiber, Julia Forato,Camila Simeoni, Marcilio Jorge Fumagalli, Mariene Ribeiro Amorim, Darlan da Silva Candido, Nuno Rodrigues Faria, Julien Theze, Luiz Gonzaga,Jaqueline Goes Jesus e William Marciel de Souza
EPI_ISL_476302, EPI_ISL_476303, EPI_ISL_476304, EPI_ISL_476305, EPI_ISL_476306, EPI_ISL_476307, EPI_ISL_476308, EPI_ISL_476309, EPI_ISL_476310, EPI_ISL_476311, EPI_ISL_476312, EPI_ISL_476313, EPI_ISL_476314, EPI_ISL_476315, EPI_ISL_476316, EPI_ISL_476317, EPI_ISL_476318, EPI_ISL_476319, EPI_ISL_476320, EPI_ISL_476323, EPI_ISL_476324, EPI_ISL_476325, EPI_ISL_476326, EPI_ISL_476354			
see above	DB Diagnósticos do Brasil	Instituto de Medicina Tropical da Univesidade de São Paulo	Samples: Nelson Gaburo Jr; Sequencing: Ingra Morales Claro, Jaqueline Goes de Jesus, Erika Regina Manuli, Flavia Cristina da Silva Sales, Thais de Moura Coletti, Camila Alves Maia da Silva, Mariana Severo Ramundo, Giula Magalhaes Ferreira, Darlan da Silva Candido, Julien Theze, Nuno Faria, Ester Sabino
EPI_ISL_476372, EPI_ISL_476373	Hospital da Clínicas da Faculdade de Medicina da Universidade de São Paulo	Instituto de Medicina Tropical da Univesidade de São Paulo	Samples: Ingra Morales Claro, Erika Regina Manuli, Cecilia Salete Alencar, Carolina S. Lazar, Silvia F. Costa; Sequencing: Ingra Morales Claro, Jaqueline Goes de Jesus, Erika Regina Manuli, Flavia Cristina da Silva Sales, Thais de Moura Coletti, Camila Alves Maia da Silva, Mariana Severo Ramundo, Giula Magalhaes Ferreira, Darlan da Silva Candido, Julien Theze, Nuno Faria, Ester Sabino
EPI_ISL_476539, EPI_ISL_476540, EPI_ISL_476541, EPI_ISL_476542, EPI_ISL_476543, EPI_ISL_476544, EPI_ISL_476545, EPI_ISL_476546, EPI_ISL_476547, EPI_ISL_476548, EPI_ISL_476549, EPI_ISL_476550, EPI_ISL_476551, EPI_ISL_476552, EPI_ISL_476553, EPI_ISL_476554, EPI_ISL_476555, EPI_ISL_476556, EPI_ISL_476557			
see above	Yale Clinical Virology Laboratory	Grubaugh Lab - Yale School of Public Health	Joseph Fauver, Tara Alpert, Anderson Brito, Anne Wyllie, Chantal Vogels, Mary Petrone, Cole Jensen, Chaney Kalinich, Isabel Ott, Arnau Casanovas, Catherine Muenker, Adam Moore, Alice Lu, Maria Tokuyama, Patrick Wong, Peiwen Lu, Saad Omer, Richard Martinello, Allison Nelson, Shelli Farhadian, Akiko Iwasaki, Charlese Dela Cruz, Albert Ko, Nathan Grubaugh
EPI_ISL_476792	Stanford clinical virology lab	Chan-Zuckerberg Biohub	Benjamin Pinksy, Katharine Walter, Victoria N. Parikh, John Gorzynski, Hannah N. DeJong, Matthew T. Wheeler, Jason Andrews, Manuel Rivas, Carlos Bustamante, Euan Ashley, with CZB Cliahub Consortium
EPI_ISL_476846	Defence Research & Development Establishment (DRDE)	Defence Research & Development Establishment (DRDE)	Shashi Sharma, Paban Kumar Dash, Sushil Kumar Sharma, Ambuj Shrivastava, Jyoti S. Kumar
EPI_ISL_477017, EPI_ISL_477018, EPI_ISL_477019, EPI_ISL_477020, EPI_ISL_477021, EPI_ISL_477022, EPI_ISL_477023, EPI_ISL_477024, EPI_ISL_477025, EPI_ISL_477026, EPI_ISL_477027, EPI_ISL_477028, EPI_ISL_477029, EPI_ISL_477030, EPI_ISL_477031, EPI_ISL_477032, EPI_ISL_477033, EPI_ISL_477034, EPI_ISL_477035, EPI_ISL_477036, EPI_ISL_477037, EPI_ISL_477038, EPI_ISL_477064, EPI_ISL_477076, EPI_ISL_477090, EPI_ISL_477091, EPI_ISL_477092, EPI_ISL_477093, EPI_ISL_477094, EPI_ISL_477095, EPI_ISL_477096, EPI_ISL_477097, EPI_ISL_477098, EPI_ISL_477099, EPI_ISL_477100, EPI_ISL_477101, EPI_ISL_477102, EPI_ISL_477103, EPI_ISL_477104, EPI_ISL_477105, EPI_ISL_477106, EPI_ISL_477107, EPI_ISL_477108, EPI_ISL_477109, EPI_ISL_477110, EPI_ISL_477111, EPI_ISL_477112, EPI_ISL_477113, EPI_ISL_477114, EPI_ISL_477115, EPI_ISL_477116, EPI_ISL_477117, EPI_ISL_477118, EPI_ISL_477119			
see above	BCCDC Public Health Laboratory	BCCDC Public Health Laboratory	Richard Harrigan, Hope Lapointe, Jinny Choi, Kimia Kamelian, John Tyson,Terry Snutch, Linda Hoang, Inna Sekirov, Paul Levett, Mel Krajden, Natalie Prystajecky
EPI_ISL_477706	UW Virology Lab	UW Virology Lab	Pavitra Roychoudhury, Hong Xie, Lasata Shrestha, Amin Addetia, Truong Nguyen, Victoria M Rachleff, Meei-Li Huang, Keith R Jerome, Alexander Greninger
EPI_ISL_477821	West of Scotland Specialist Virology Centre, NHSGGC / MRC-University of Glasgow Centre for Virus Research	COVID-19 Genomics UK (COG-UK) Consortium	Ana da Silva Filipe, Natasha Johnson, Kathy Smollett, Daniel Mair, Stephen Carmichael, Lily Tong, Jenna Nichols, Elihu Aranday-Cortes, Kirstyn Brunker, Yasmin Parr, Alice Broos, Kyriaki Nomikou; Sarah McDonald, Marc Niebel, Patawee Asamaphan; Richard Orton, Joseph Hughes, Sreenu Vattipally, David L Robertson; Alasdair MacLean, Rory Gunson; Kathy Li, Natasha Jesudason, Rajiv Shah, James Shepherd, Antonia Ho, Emma Thomson

EPI_ISL_478417, EPI_ISL_478418, EPI_ISL_478419, EPI_ISL_478420, EPI_ISL_478421, EPI_ISL_478422, EPI_ISL_478423, EPI_ISL_478424, EPI_ISL_478425, EPI_ISL_478426, EPI_ISL_478427, EPI_ISL_478428, EPI_ISL_478429, EPI_ISL_478430, EPI_ISL_478431			
see above	University College London, Great Ormond Street Hospital for Children NHS Foundation Trust, Imperial College Healthcare NHS Trust	COVID-19 Genomics UK (COG-UK) Consortium	Sergi Castellano, Rachel Williams, Mark Kristiansen, Paola Resende Silva, Sunando Roy, Tony Brooks, Helena Tutill, Paola Niola, Patricia Dyal, Charlotte Williams, Leysa Forrest, Yasmin Panchbhaya, Jacqueline Findlay, Samuel Weeks, Julianne Brown, Kathryn Harris, Paul Randell, James Price, Alison Holmes, Judith Breuer
EPI_ISL_478496, EPI_ISL_478497, EPI_ISL_478498, EPI_ISL_478500, EPI_ISL_478501, EPI_ISL_478502, EPI_ISL_478503, EPI_ISL_478504, EPI_ISL_478505, EPI_ISL_478506, EPI_ISL_478507, EPI_ISL_478508, EPI_ISL_478509, EPI_ISL_478510, EPI_ISL_478511, EPI_ISL_478633, EPI_ISL_478635, EPI_ISL_478636, EPI_ISL_478637, EPI_ISL_478638, EPI_ISL_478663, EPI_ISL_478664, EPI_ISL_478665, EPI_ISL_478666, EPI_ISL_478667			
see above	Northumbria University / South Tees Hospitals NHS Foundation Trust / North Cumbria Integrated Care NHS Foundation Trust / North Tees and Hartlepool NHS Foundation Trust / Newcastle Hospitals NHS Foundation Trust	COVID-19 Genomics UK (COG-UK) Consortium	Darren L Smith,Andrew Nelson,Matthew Bashton,Greg R Young,Joshua Loh,John Allan,Mohammad A Tariq,Giles S Holt,Gary Black,Wen C Yew,Lynn Dover,Paul Baker,Steve Liggett,Sarah Essex,Jane Greenaway,Debra Padgett,Clive Graham,Garren Scott,Edward Barton,Emma Swindells,Brendan Payne,Jennifer Collins,Yusri Taha,Gary Eltringham
EPI_ISL_478673	Pathology North - Royal North Shore Hospital - NSW Health Pathology	NSW Health Pathology - Institute of Clinical Pathology and Medical Research; Westmead Hospital; University of Sydney	CIDM-PH et al.
EPI_ISL_478700, EPI_ISL_478701	South Eastern Area Laboratory Services (SEALS)	NSW Health Pathology - Institute of Clinical Pathology and Medical Research; Westmead Hospital; University of Sydney	CIDM-PH et al.
EPI_ISL_479620, EPI_ISL_479621, EPI_ISL_479622, EPI_ISL_479623	Molecular diagnostic laboratory of Federal Budget Institution of Science "Central Research Institute of Epidemiology" of The Federal Service on Customers' Rights Protection and Human Well-being Surveillance	Group of Genomics and Postgenomic Technologies of Central Research Institute of Epidemilology	Speranskaya AS, Kaptelova VV,Valdokhina AV, Bulanenko VP, Samoilov AE, Korneenko EV, Sizova TV, Tivanova EV, Shipulina OY, Akimkin VG
EPI_ISL_479648, EPI_ISL_479649, EPI_ISL_479650	Dr. Georges-L.-Dumont University Hospital Centre	National Microbiology Laboratory	Anna Majer, Shari Tyson, Grace Seo, Kristyn Burak, Philip Mabon, Elsie Grudeski, Rhiannon Huzarewich, Russell Mandes, Jennifer Tanner, Natalie Knox, Morag Graham, Gary Van Domselaar, Richard Garceau, Guillaume Desnoyers, Nathalie Bastien, Yan Li, Timothy Booth
EPI_ISL_479673	Center for Genomics and System Biology, New York University	Center for Genomics and System Biology, New York University	Roder,A., Banakis,S., Johnson,K., Khalfan,M., Borenstein,E.S., Samanovic,M., Cornelius,A., Herati,R., Ulrich,R., Fleming,A., Kottkamp,A., Raabe,V., Mulligan,M.J., Gresham,D., Ghedin,E.
EPI_ISL_479676, EPI_ISL_479677, EPI_ISL_479678, EPI_ISL_479679, EPI_ISL_479680, EPI_ISL_479681, EPI_ISL_479682, EPI_ISL_479683, EPI_ISL_479684, EPI_ISL_479685	unknown	Contact:Hiroyuki Asakura Tokyo Metropolitan Institute of Public Health, Department of Microbiology	Asakura,H., Yoshida,I., Kumagai,R., Nagashima,M., Chiba,T., Sadamasu,K.
EPI_ISL_480009, EPI_ISL_480010	Chiba Prefectural Institute of Public Health	Pathogen Genomics Center, National Institute of Infectious Diseases	Tsuyoshi Sekizuka, Masakatsu Taira, Kentaro Itokawa, Rina Tanaka, Masanori Hashino, Hajime Kamiya, Motoi Suzuki, Makoto Kuroda
EPI_ISL_480026, EPI_ISL_480027, EPI_ISL_480028, EPI_ISL_480029	Ibaraki Prefectural Institute of Public Health	Pathogen Genomics Center, National Institute of Infectious Diseases	Tsuyoshi Sekizuka, Keiko Goto, Kentaro Itokawa, Rina Tanaka, Masanori Hashino, Hajime Kamiya, Motoi Suzuki, Makoto Kuroda
EPI_ISL_480067, EPI_ISL_480068, EPI_ISL_480069	Sakai City Institute of Public Health	Pathogen Genomics Center, National Institute of Infectious Diseases	Tsuyoshi Sekizuka, Tatsuya Miyoshi, Kentaro Itokawa, Rina Tanaka, Masanori Hashino, Hajime Kamiya, Motoi Suzuki, Makoto Kuroda
EPI_ISL_480081	Shizuoka City Institute of Environmental Sciences and Public Health	Pathogen Genomics Center, National Institute of Infectious Diseases	Tsuyoshi Sekizuka, Takaharu Maehata,Sou Okamura,Yuji Kanazawa,Kenji Yagi, Kentaro Itokawa, Rina Tanaka, Masanori Hashino, Hajime Kamiya, Motoi Suzuki, Makoto Kuroda
EPI_ISL_480090	Department of Infectious Diseases, Kobe Institute of Health	Pathogen Genomics Center, National Institute of Infectious Diseases	Tsuyoshi Sekizuka, Ryohei Nomoto, Kentaro Itokawa, Rina Tanaka, Masanori Hashino, Hajime Kamiya, Motoi Suzuki, Makoto Kuroda
EPI_ISL_480125, EPI_ISL_480127, EPI_ISL_480128, EPI_ISL_480129, EPI_ISL_480130, EPI_ISL_480131, EPI_ISL_480132, EPI_ISL_480133, EPI_ISL_480134	Fukui Prefectural Institute of Public Health and Environmental Science	Pathogen Genomics Center, National Institute of Infectious Diseases	Tsuyoshi Sekizuka, Miho Toho, Kentaro Itokawa, Rina Tanaka, Masanori Hashino, Hajime Kamiya, Motoi Suzuki, Makoto Kuroda
EPI_ISL_480177	Gunma Prefectural Institute of Public Health and Environmental Sciences	Pathogen Genomics Center, National Institute of Infectious Diseases	Tsuyoshi Sekizuka, Hiroyuki Tsukagoshi, Kentaro Itokawa, Rina Tanaka, Masanori Hashino, Hajime Kamiya, Motoi Suzuki, Makoto Kuroda
EPI_ISL_480182	Ibaraki Prefectural Institute of Public Health	Pathogen Genomics Center, National Institute of Infectious Diseases	Tsuyoshi Sekizuka, Keiko Goto, Kentaro Itokawa, Rina Tanaka, Masanori Hashino, Hajime Kamiya, Motoi Suzuki, Makoto Kuroda
EPI_ISL_480190, EPI_ISL_480191, EPI_ISL_480192, EPI_ISL_480193, EPI_ISL_480194, EPI_ISL_480195	Ota Health Center Welfare Section	Pathogen Genomics Center, National Institute of Infectious Diseases	Tsuyoshi Sekizuka, Chika Takahashi, Kentaro Itokawa, Rina Tanaka, Masanori Hashino, Hajime Kamiya, Motoi Suzuki, Makoto Kuroda
EPI_ISL_480199, EPI_ISL_480200, EPI_ISL_480201, EPI_ISL_480202	Toyama Institute of Health	Pathogen Genomics Center, National Institute of Infectious Diseases	Tsuyoshi Sekizuka, Masae Itamochi, Kazunori Oishi, Kentaro Itokawa, Rina Tanaka, Masanori Hashino, Hajime Kamiya, Motoi Suzuki, Makoto Kuroda
EPI_ISL_480221	Koshigaya City Public Health Center	Pathogen Genomics Center, National Institute of Infectious Diseases	Tsuyoshi Sekizuka, Yuka Furui, Aya Tamura, Kyohei Sakata, Takumi Daimon, Yoko Togawa, Yoshiko Hamada, Kentaro Itokawa, Rina Tanaka, Masanori Hashino, Hajime Kamiya, Motoi Suzuki, Makoto Kuroda
EPI_ISL_480320	Hospital Clínica Bíblica	Charité Virology-University of Costa Rica	Andres Moreira-Soto, Eugenia Corrales-Aguilar, Ignacio Postigo-Hidalgo, Karla Sofía Gutiérrez, Jan Felix Drexler
EPI_ISL_480326	Hospital Nacional de Niños	Charité Virology-University of Costa Rica	Andres Moreira-Soto, Eugenia Corrales-Aguilar, Ignacio Postigo-Hidalgo, Cristian Pérez Corrales, Andrei Montero Bonilla, Jan Felix Drexler
EPI_ISL_480391, EPI_ISL_480392, EPI_ISL_480393	University of Wisconsin-Madison AIDS Vaccine Research Laboratories	University of Wisconsin-Madison AIDS Vaccine Research Laboratories	Gage Moreno, Katarina Braun, et al. AIDS Vaccine Research Laboratories
EPI_ISL_480570, EPI_ISL_480572	Victorian Infectious Diseases Reference Laboratory (VIDRL)	VIDRL and MDU-PHL	Caly L., Seemann T., Sait, M., Schultz M., Druce J., Sherry, N.
EPI_ISL_480615	Microbiological Diagnostic Unit - Public Health Laboratory (MDU-PHL)	MDU-PHL	Seemann T., Schultz M., Sait, M., Sherry, N.
EPI_ISL_480783, EPI_ISL_480786, EPI_ISL_480787, EPI_ISL_480788, EPI_ISL_480789	Institut Pasteur Dakar	Institut Pasteur de Dakar	Ndongo Dia, Moussa Moise Diagne, Mamadou Diop, Marie Henriette Dior Ndione, Mamadou Malado Jallow, Safietou Sanke, Ousmane Faye, Amadou Alpha Sall.
EPI_ISL_480801, EPI_ISL_480802, EPI_ISL_480804, EPI_ISL_480806, EPI_ISL_480807, EPI_ISL_480808, EPI_ISL_480809, EPI_ISL_480810, EPI_ISL_480811, EPI_ISL_480812, EPI_ISL_480813, EPI_ISL_480814, EPI_ISL_480816			
see above	Florida Bureau of Public Health Laboratories	Florida Bureau of Public Health Laboratories	Sarah Schmedes, Jason Blanton
EPI_ISL_480961	ISGlobal, Institut de Salut Global de Barcelona	SeqCOVID-SPAIN consortium/IBV(CSIC)	Alfredo Mayor, Alberto L Garcia-Basteiro, Carlota Dobaño, Gemma Moncunill, Pau Cisteró and SeqCOVID-SPAIN consortium
EPI_ISL_481254	Department of Emerging Infectious Diseases, Institute of Tropical Medicine, Nagasaki University	Department of Emerging Infectious Diseases, Institute of Tropical Medicine, Nagasaki University	Jiro Yasuda, Rokusuke Yoshikawa, Yuichiro Furusato, Haruka Abe
EPI_ISL_481265, EPI_ISL_481266, EPI_ISL_481267, EPI_ISL_481268, EPI_ISL_481269, EPI_ISL_481270	Maryland Department of Health	Maryland Department of Health	Keller,E.
EPI_ISL_481284	Department of Experimental Modeling and Pathogenesis of	Department of Experimental Modeling and Pathogenesis of	Sobolev,I.A., Shanshin,D.V., Chepurnov,A.A., Kononova,J.V., Bondar,A.A., Alekseev,A.Y. and Shestopalov,A.M.

	Infectious Diseases, Federal Research Center of Fundamental and Translational Medicine	Infectious Diseases, Federal Research Center of Fundamental and Translational Medicine	
EPI_ISL_481744, EPI_ISL_481745, EPI_ISL_481746, EPI_ISL_481749	Dr. Georges-L.-Dumont University Hospital Centre	National Microbiology Laboratory	Anna Majer, Shari Tyson, Grace Seo, Kristyn Burak, Philip Mabon, Elsie Grudeski, Rhiannon Huzarewich, Russell Mandes, Jennifer Tanner, Natalie Knox, Morag Graham, Gary Van Domselaar, Richard Garceau, Guillaume Desnoyers, Nathalie Bastien, Yan Li, Timothy Booth
EPI_ISL_482292, EPI_ISL_482293, EPI_ISL_482298, EPI_ISL_482299, EPI_ISL_482300, EPI_ISL_482301	Providence St. Joseph Health Molecular Genomics Laboratory	Providence St. Joseph Health Molecular Genomics Laboratory	Alexa K Dowdell, Brian D Piening, Fred L Robinson, Carlo B Bifulco, Mary Campbell
EPI_ISL_482733, EPI_ISL_482734	LNR National Reference Laboratory, Mohammed VI University of Health Sciences	Medical Biotechnology Laboratory, Rabat Medical and Pharmacy School, Mohammed The Vth University in Rabat	Meriem LAAMARTI, Souad KARTTI, Rokia LAAMARTI , M.W. CHEMAO-ELFIHRI, Loubna ALLAM, Mouna OUADGHIRI, Imane SMYEJ, Jalila RAHOUI, Houda BENRHAMA, Jalil El ATAR, Idrissa DIAWARA, Rachid EL JAOUDI, Laila SBABOU, Chakib NEJJARI, Saaid AMZAZI, Rachid MENTAG, Lahcen BELYAMANI and Azeddine IBRAHIMI
EPI_ISL_483072, EPI_ISL_483086, EPI_ISL_483091	SA Pathology	SA Pathology	Lex Leong, Chuan Kok Lim, Mark Turra, Ivan Bastian, Geoff Higgins
EPI_ISL_483167, EPI_ISL_483170, EPI_ISL_483171, EPI_ISL_483172, EPI_ISL_483177, EPI_ISL_483179, EPI_ISL_483183, EPI_ISL_483188, EPI_ISL_483189, EPI_ISL_483190, EPI_ISL_483193, EPI_ISL_483196, EPI_ISL_483197, EPI_ISL_483317, EPI_ISL_483367, EPI_ISL_483368, EPI_ISL_483370, EPI_ISL_483376, EPI_ISL_483379, EPI_ISL_483386, EPI_ISL_483388, EPI_ISL_483392, EPI_ISL_483434, EPI_ISL_483439, EPI_ISL_483441, EPI_ISL_483442, EPI_ISL_483443, EPI_ISL_483447			
see above	UC San Diego Center for Advanced Laboratory Medicine	Andersen lab at Scripps Research	SEARCH Alliance San Diego with David Pride, Ji H Shin
EPI_ISL_483621	National Public Health Laboratory, National Centre for Infectious Diseases	National Public Health Laboratory, National Centre for Infectious Diseases	Mak TM, Octavia S, Zhou Z, Chavatte JM, Cui L, Lin RTP
EPI_ISL_484153, EPI_ISL_484156, EPI_ISL_484158, EPI_ISL_484160, EPI_ISL_484162, EPI_ISL_484164, EPI_ISL_484165, EPI_ISL_484166, EPI_ISL_484167, EPI_ISL_484169, EPI_ISL_484170, EPI_ISL_484171, EPI_ISL_484172, EPI_ISL_484173, EPI_ISL_484175, EPI_ISL_484176, EPI_ISL_484177, EPI_ISL_484178, EPI_ISL_484179, EPI_ISL_484180, EPI_ISL_484181, EPI_ISL_484182, EPI_ISL_484202, EPI_ISL_484203, EPI_ISL_484204, EPI_ISL_484205, EPI_ISL_484206, EPI_ISL_484207, EPI_ISL_484208, EPI_ISL_484209, EPI_ISL_484210, EPI_ISL_484211, EPI_ISL_484212, EPI_ISL_484213, EPI_ISL_484214, EPI_ISL_484215, EPI_ISL_484216, EPI_ISL_484217			
see above	Centre for Clinical Infection and Diagnostics Research and Genomics Innovation Unit, Guy's and St. Thomas' NHS Trust	COVID-19 Genomics UK (COG-UK) Consortium	Chloe Fisher, Luke Snell, Penny Cliff, Rahul Batra, Jonathan Edgeworth, Ali Raza Awan
EPI_ISL_484415, EPI_ISL_484416, EPI_ISL_484417	Centre for Enzyme Innovation, University of Portsmouth / Translational Research Laboratory, Portsmouth Hospitals NHS Trust	COVID-19 Genomics UK (COG-UK) Consortium	Angela Beckett,Yann Bourgeois,Garry Scarlett,Sharon Glaysher,Scott Elliott,Kelly Bicknell,Robert Impey,Allyson Lloyd,Sarah Wyllie,Ethan Butcher,Anoop Chauhan,Samuel Robson
EPI_ISL_484710, EPI_ISL_484713, EPI_ISL_484717, EPI_ISL_484718, EPI_ISL_484723, EPI_ISL_484742, EPI_ISL_484745, EPI_ISL_484760, EPI_ISL_484762, EPI_ISL_484787, EPI_ISL_484788, EPI_ISL_484793, EPI_ISL_484794, EPI_ISL_484801, EPI_ISL_484806			
see above	University of Michigan Clinical Microbiology Laboratory	Lauring Lab, University of Michigan, Department of Microbiology and Immunology	Valesano et al.
EPI_ISL_485388, EPI_ISL_485394, EPI_ISL_485395, EPI_ISL_485396	University of Ulsan College of Medicine and Asan Medical Center	University of Ulsan College of Medicine and Asan Medical Center	Kuenyoul Park, Jaewoong Lee, Kihyun Lee, Jiwon Jung, Sung-Han Kim, Jina Lee, Mauricio Chalita, Seok-Hwan Yoon, Jongsik Chun, Kyu-Hwa Hur, Heungsup Sung, Mi-Na Kim, and Hae Kyung Lee
EPI_ISL_485871	Virginia DCLS	Virginia DCLS	Virginia DCLS
EPI_ISL_485987	UW Virology Lab	UW Virology Lab	Pavitra Roychoudhury, Hong Xie, Lasata Shrestha, Amin Addetia, Truong Nguyen, Victoria M Rachleff, Meei-Li Huang, Keith R Jerome, Alexander Greninger
EPI_ISL_486442, EPI_ISL_486443, EPI_ISL_486444, EPI_ISL_486445, EPI_ISL_486446, EPI_ISL_486447, EPI_ISL_486448, EPI_ISL_486449, EPI_ISL_486450, EPI_ISL_486451, EPI_ISL_486452, EPI_ISL_486453, EPI_ISL_486454, EPI_ISL_486455, EPI_ISL_486456, EPI_ISL_486457, EPI_ISL_486458, EPI_ISL_486459, EPI_ISL_486460, EPI_ISL_486461, EPI_ISL_486462, EPI_ISL_486463, EPI_ISL_486464, EPI_ISL_486465, EPI_ISL_486466, EPI_ISL_486467, EPI_ISL_486468, EPI_ISL_486469, EPI_ISL_486470, EPI_ISL_486471, EPI_ISL_486472, EPI_ISL_486473, EPI_ISL_486474, EPI_ISL_486475, EPI_ISL_486476, EPI_ISL_486477, EPI_ISL_486478, EPI_ISL_486479, EPI_ISL_486480, EPI_ISL_486481, EPI_ISL_486482, EPI_ISL_486483, EPI_ISL_486484, EPI_ISL_486485, EPI_ISL_486486, EPI_ISL_486487, EPI_ISL_486488, EPI_ISL_486489, EPI_ISL_486490, EPI_ISL_486491, EPI_ISL_486492, EPI_ISL_486493, EPI_ISL_486494, EPI_ISL_486495, EPI_ISL_486496, EPI_ISL_486497, EPI_ISL_486498, EPI_ISL_486499, EPI_ISL_486500			
see above	Viollier AG	Department of Biosystems Science and Engineering, ETH Zürich	Christian Beisel, Sarah Nadeau, Ivan Topolsky, Pedro Ferreira, Philipp Jablonski, Susana Posada-Céspedes, Tobias Schär, Ina Nissen, Natascha Santacroce, Elodie Burcklen, Christiane Beckmann, Maurice Redondo, Olivier Kobel, Christoph Noppen, Sophie Seidel, Noemie Santamaria de Souza, Niko Beerenwinkel, Tanja Stadler
EPI_ISL_486658	Microbiology, Virology and Biemergency Laboratory-ASST FBF Sacco	Microbiology, Virology and Biemergency Laboratory-ASST FBF Sacco	Romeri F, Comandatore F, Mancon A, Micheli V, Rimoldi SG
EPI_ISL_486820, EPI_ISL_486821, EPI_ISL_486822, EPI_ISL_486823, EPI_ISL_486824, EPI_ISL_486825, EPI_ISL_486826, EPI_ISL_486827, EPI_ISL_486828, EPI_ISL_486829	Molecular diagnostic laboratory of Federal Budget Institution of Science "Central Research Institute of Epidemiology" of The Federal Service on Customers' Rights Protection and Human Well-being Surveillance	Group of Genomics and Postgenomic Technologies of Central Research Institute of Epidemilology	Speranskaya AS, Kaptelova VV, Valdokhina AV, Bulanenko VP, Samoilov AE, Korneenko EV, Tivanova EV, Shipulina OY, Akimkin VG
EPI_ISL_486890, EPI_ISL_486891, EPI_ISL_486892, EPI_ISL_486893, EPI_ISL_486894, EPI_ISL_486895, EPI_ISL_486896	Tokyo Metoropolitan Institute of Public Health	Tokyo Metoropolitan Institute of Public Health	Asakura,H., Yoshida,I., Kumagai,R., Nagashima,M., Chiba,T., Sadamasu,K.
EPI_ISL_486912, EPI_ISL_486913, EPI_ISL_486914	Maryland Department of Health	Maryland Department of Health	Keller,E.
EPI_ISL_487684, EPI_ISL_487703, EPI_ISL_487707, EPI_ISL_487710, EPI_ISL_487721, EPI_ISL_487789, EPI_ISL_487805, EPI_ISL_487809, EPI_ISL_487812, EPI_ISL_487815, EPI_ISL_487817, EPI_ISL_487845, EPI_ISL_487856, EPI_ISL_487934, EPI_ISL_487991			
see above	Virology Department, Royal Infirmary of Edinburgh, NHS Lothian / School of Biological Sciences, University of Edinburgh	Wellcome Sanger Institute for the COVID-19 Genomics UK (COG-UK) consortium	McHugh M, Dewar R, Rooke S, O'Toole Á, Scher E, Hill V, McCrone JT, Colquhoun R, Yu X, Jackson B, Rambaut A, Templeton K and Alex Alderton, Roberto Amato, Sonia Goncalves, Ewan Harrison, David K. Jackson, Ian Johnston, Dominic Kwiatkowski, Cordelia Langford, John Sillitoe on behalf of the Wellcome Sanger Institute COVID-19 Surveillance Team (http://www.sanger.ac.uk/covid-team)
EPI_ISL_488214, EPI_ISL_488410	PHE South West Regional Laboratory, National Infection Service	Wellcome Sanger Institute for the COVID-19 Genomics UK (COG-UK) consortium	Stephanie Hutchings, Hannah Pymont, Dr Peter Muir, Barry Vipond, Rich Hopes; and Alex Alderton, Roberto Amato, Sonia Goncalves, Ewan Harrison, David K. Jackson, Ian Johnston, Dominic Kwiatkowski, Cordelia Langford, John Sillitoe on behalf of the Wellcome Sanger Institute COVID-19 Surveillance Team (http://www.sanger.ac.uk/covid-team)
EPI_ISL_488465, EPI_ISL_488477, EPI_ISL_488488, EPI_ISL_488496, EPI_ISL_488501, EPI_ISL_488516, EPI_ISL_488522, EPI_ISL_488523, EPI_ISL_488524, EPI_ISL_488526, EPI_ISL_488529, EPI_ISL_488531, EPI_ISL_488533, EPI_ISL_488534, EPI_ISL_488537, EPI_ISL_488539, EPI_ISL_488540, EPI_ISL_488545, EPI_ISL_488546, EPI_ISL_488553, EPI_ISL_488558, EPI_ISL_488559, EPI_ISL_488561, EPI_ISL_488566, EPI_ISL_488568, EPI_ISL_488569, EPI_ISL_488577, EPI_ISL_488583, EPI_ISL_488597, EPI_ISL_488604, EPI_ISL_488607, EPI_ISL_488615, EPI_ISL_488623, EPI_ISL_488625, EPI_ISL_488642, EPI_ISL_488643, EPI_ISL_488645, EPI_ISL_488649, EPI_ISL_488651, EPI_ISL_488652, EPI_ISL_488653, EPI_ISL_488660, EPI_ISL_488663, EPI_ISL_488665, EPI_ISL_488668, EPI_ISL_488672, EPI_ISL_488677, EPI_ISL_488684, EPI_ISL_488691, EPI_ISL_488692, EPI_ISL_488693, EPI_ISL_488696, EPI_ISL_488702, EPI_ISL_488703, EPI_ISL_488705, EPI_ISL_488708, EPI_ISL_488709, EPI_ISL_488711, EPI_ISL_488712, EPI_ISL_488721, EPI_ISL_488735, EPI_ISL_488736, EPI_ISL_488738, EPI_ISL_488741			
see above	NU-OMICS DNA Sequencing research facility, Northumbria University	Wellcome Sanger Institute for the COVID-19 Genomics UK (COG-UK) consortium	Chris Duncan, Sheia Waugh, Shirelle Burton-Fanning, Gary Eltringham, Jennifer Collins, Brendan Payne, Yusri Taha, Emma Swindells, Jane Greenaway, Edward Barton, Garren Scott, Debra Padgett, Clive Graham, Sarah Essex, Steve Ligget, Paul Baker, Lynn Dover, Wen Yew, Gary Black, John Allan, Joshua Loh, Greg Young, Matthew Bashton, Andrew Nelson, Darren Smith and Alex Alderton, Roberto Amato, Sonia Goncalves, Ewan Harrison, David K. Jackson, Ian Johnston, Dominic Kwiatkowski, Cordelia Langford, John Sillitoe on behalf of the Wellcome Sanger Institute COVID-19 Surveillance Team (http://www.sanger.ac.uk/covid-team)
EPI_ISL_488884, EPI_ISL_488886, EPI_ISL_488895, EPI_ISL_488897, EPI_ISL_488902, EPI_ISL_488909, EPI_ISL_488915, EPI_ISL_488918, EPI_ISL_488930, EPI_ISL_488931, EPI_ISL_488940, EPI_ISL_488945, EPI_ISL_488957, EPI_ISL_488961, EPI_ISL_488965, EPI_ISL_488970, EPI_ISL_488973, EPI_ISL_488995, EPI_ISL_489010, EPI_ISL_489016, EPI_ISL_489025, EPI_ISL_489027, EPI_ISL_489028, EPI_ISL_489030, EPI_ISL_489041			
see above	Virology Department, Royal Infirmary of Edinburgh, NHS Lothian / School of Biological Sciences, University of	Wellcome Sanger Institute for the COVID-19 Genomics UK (COG-UK) consortium	McHugh M, Dewar R, Rooke S, O'Toole Á, Scher E, Hill V, McCrone JT, Colquhoun R, Yu X, Jackson B, Rambaut A, Templeton K and Alex Alderton, Roberto Amato, Sonia Goncalves, Ewan Harrison, David K. Jackson, Ian Johnston, Dominic Kwiatkowski, Cordelia Langford, John Sillitoe on behalf of the

	Edinburgh		Wellcome Sanger Institute COVID-19 Surveillance Team (http://www.sanger.ac.uk/covid-team)
EPI_ISL_490031	South Eastern Area Laboratory Services (SEALS)	NSW Health Pathology - Institute of Clinical Pathology and Medical Research; Westmead Hospital; University of Sydney	CIDM-PH et al.
EPI_ISL_490058	National Public Health Laboratory, National Centre for Infectious Diseases	National Public Health Laboratory, National Centre for Infectious Diseases	Mak TM, Octavia S, Zhou Z, Chavatte JM, Cui L, Lin RTP
EPI_ISL_490105	South Eastern Area Laboratory Services (SEALS)	NSW Health Pathology - Institute of Clinical Pathology and Medical Research; Westmead Hospital; University of Sydney	CIDM-PH et al.
EPI_ISL_490210, EPI_ISL_490211, EPI_ISL_490212, EPI_ISL_490213, EPI_ISL_490214, EPI_ISL_490215, EPI_ISL_490216, EPI_ISL_490217, EPI_ISL_490218, EPI_ISL_490219, EPI_ISL_490220, EPI_ISL_490221, EPI_ISL_490222, EPI_ISL_490223			
see above	Quest Diagnostics	Q Squared Solutions - QRTP facility	Victor J Weigman
EPI_ISL_491118	The National Institute of Public Health	The National Institute of Public Health and State Veterinary Institute Prague	Nagy,A;Jirincova,H;Novakova,L;Trnka,D;Vecerova,J
EPI_ISL_491459, EPI_ISL_491460, EPI_ISL_491461, EPI_ISL_491462, EPI_ISL_491464	Laboratorio de Referencia Nacional de Virus Respiratorio. Instituto Nacional de Salud Perú	Laboratorio de Referencia Nacional de Biotecnología y Biología Molecular. Instituto Nacional de Salud Perú	Carlos Padilla Rojas, Karolyn Vega Chozo, Priscila Lope Pari, Omar Caceres Rey, Marco Galarza Perez, Maribel Huaringa Nuñez, Johanna Balbuena Torrez, Henri Bailon Calderon, Nancy Rojas Serrano
EPI_ISL_491504, EPI_ISL_491558, EPI_ISL_491574, EPI_ISL_491605, EPI_ISL_491668, EPI_ISL_491689	Virology Department, Royal Infirmary of Edinburgh, NHS Lothian / School of Biological Sciences, University of Edinburgh	Wellcome Sanger Institute for the COVID-19 Genomics UK (COG-UK) consortium	McHugh M, Dewar R, Rooke S, O'Toole Á, Scher E, Hill V, McCrone JT, Colquhoun R, Yu X, Jackson B, Rambaut A, Templeton K and Alex Alderton, Roberto Amato, Sonia Goncalves, Ewan Harrison, David K. Jackson, Ian Johnston, Dominic Kwiatkowski, Cordelia Langford, John Sillitoe on behalf of the Wellcome Sanger Institute COVID-19 Surveillance Team (http://www.sanger.ac.uk/covid-team)
EPI_ISL_491920	Naval Infectious Diseases Diagnostic Laboratory	Naval Medical Research Center Biological Defense Research Directorate	Logan Voegtly, Regina Cer, Lindsay Glang, Victor Sugiharto, Francisco Malgon Bautista, Hua Wei Chen, Dessiree Pena-Gomez, Megan Schilling, Adrian Paskey, Kyle Long, Mark Simons, Kimberly Bishop-Lilly
EPI_ISL_492068	1. ViroGenetics - BSL3 Laboratory of Virology, Maopolska Centre of Biotechnology, Jagiellonian University; 2. II Department of Internal Medicine, Faculty of Medicine, Jagiellonian University Medical College; 3. Narodowy Instytut Zdrowia Publicznego - Pastwowy Zakad Higieny (NIZP-PZH)	1. ViroGenetics - BSL3 Laboratory of Virology, Maopolska Centre of Biotechnology, Jagiellonian University; 2. II Department of Internal Medicine, Faculty of Medicine, Jagiellonian University Medical College; 3. Narodowy Instytut Zdrowia Publicznego - Pastwowy Zakad Higieny (NIZP-PZH).	Katarzyna Pancer, Marek Sanak, Aleksandra A. Zasada, Magdalena Rzeczkowska, Tomasz Wokowicz, Katarzyna Zacharczuk, Agnieszka Koakowska-Kulesza, Katarzyna Owczarek, Aleksandra Milewska, Natalia Wolaniuk, Ewelina Hallman-Szeliska, Pawe P abaj, Wojciech Branicki, Krzysztof Pyr
EPI_ISL_492152, EPI_ISL_492166	SA Pathology	SA Pathology	Lex Leong, Chuan Kok Lim, Mark Turra, Ivan Bastian, Geoff Higgins
EPI_ISL_492214, EPI_ISL_492216, EPI_ISL_492224, EPI_ISL_492241, EPI_ISL_492263, EPI_ISL_492285, EPI_ISL_492291, EPI_ISL_492309, EPI_ISL_492313, EPI_ISL_492333, EPI_ISL_492341, EPI_ISL_492364, EPI_ISL_492371, EPI_ISL_492376, EPI_ISL_492407, EPI_ISL_492414, EPI_ISL_492415, EPI_ISL_492425, EPI_ISL_492444			
see above	PHE South West Regional Laboratory, National Infection Service	Wellcome Sanger Institute for the COVID-19 Genomics UK (COG-UK) consortium	Stephanie Hutchings, Hannah Pymont, Dr Peter Muir, Barry Vipond, Rich Hopes; and Alex Alderton, Roberto Amato, Sonia Goncalves, Ewan Harrison, David K. Jackson, Ian Johnston, Dominic Kwiatkowski, Cordelia Langford, John Sillitoe on behalf of the Wellcome Sanger Institute COVID-19 Surveillance Team (http://www.sanger.ac.uk/covid-team)
EPI_ISL_492852	Royal Free Hospital / Health Services Laboratories	Wellcome Sanger Institute for the COVID-19 Genomics UK (COG-UK) consortium	Tanzina Haque, Tabitha Mahungu, Dianne Irish, Cate Goodlad, Jenny Cross, Judith Heaney and Alex Alderton, Roberto Amato, Sonia Goncalves, Ewan Harrison, David K. Jackson, Ian Johnston, Dominic Kwiatkowski, Cordelia Langford, John Sillitoe on behalf of the Wellcome Sanger Institute COVID-19 Surveillance Team (http://www.sanger.ac.uk/covid-team)
EPI_ISL_493062, EPI_ISL_493063	Wyoming Public Health Laboratory	Wyoming Public Health Laboratory	Noah Hull, Rob Christensen, Jim Mildenberger, Joel Sevinsky, Cari Sloma, and Wanda Manley
EPI_ISL_493646, EPI_ISL_493647, EPI_ISL_493653, EPI_ISL_493656, EPI_ISL_493662	Centre for Enzyme Innovation, University of Portsmouth / Translational Research Laboratory, Portsmouth Hospitals NHS Trust	COVID-19 Genomics UK (COG-UK) Consortium	Angela Beckett,Yann Bourgeois,Garry Scarlett,Sharon Glaysher,Scott Elliott,Kelly Bicknell,Robert Impey,Allyson Lloyd,Sarah Wyllie,Ethan Butcher,Anoop Chauhan,Samuel Robson
EPI_ISL_494185, EPI_ISL_494188, EPI_ISL_494198, EPI_ISL_494209, EPI_ISL_494213, EPI_ISL_494218, EPI_ISL_494231, EPI_ISL_494239, EPI_ISL_494253, EPI_ISL_494276, EPI_ISL_494285, EPI_ISL_494294, EPI_ISL_494296, EPI_ISL_494317, EPI_ISL_494344, EPI_ISL_494345, EPI_ISL_494358, EPI_ISL_494364			
see above	Wales Specialist Virology Centre Sequencing lab: Pathogen Genomics Unit	COVID-19 Genomics UK (COG-UK) Consortium	Catherine Moore, Johnathan Evans, Laura Gifford, Malorie Perry, Simon Cottrell, Angela Marchbank, Alec Birchley, Alexander Adams, Amy Gaskin, Bree Gatica-Wilcox, Jason Coombes, Joel Southgate, Lauren Gilbert, Lee Graham, Nicole Pacchiarini, Sara Kumziene-Summerhayes, Sarah Taylor, Sophie Jones, Sara Rey, Matthew Bull, Joanne Watkins, Sally Corden, Tom Connor
EPI_ISL_494395, EPI_ISL_494400, EPI_ISL_494408, EPI_ISL_494412, EPI_ISL_494420, EPI_ISL_494572, EPI_ISL_494622, EPI_ISL_494623	San Diego County Public Health Laboratory	Andersen lab at Scripps Research	SEARCH Alliance San Diego with Tracy Basler, Jovan Shephard, Brett Austin
EPI_ISL_494667	Scripps Medical Laboratory	Andersen lab at Scripps Research	SEARCH Alliance San Diego with Michael Quigley, Ellen Stefanski, Ian Mchardy
EPI_ISL_494715, EPI_ISL_494717, EPI_ISL_494719, EPI_ISL_494720, EPI_ISL_494725, EPI_ISL_494736, EPI_ISL_494739, EPI_ISL_494742	San Diego County Public Health Laboratory	Andersen lab at Scripps Research	SEARCH Alliance San Diego with Tracy Basler, Jovan Shephard, Brett Austin
EPI_ISL_495374, EPI_ISL_495379	Florida Bureau of Public Health Laboratories	Florida Bureau of Public Health Laboratories	Sarah Schmedes, Jason Blanton
EPI_ISL_495564, EPI_ISL_495570	University of Michigan Clinical Microbiology Laboratory	Lauring Lab, University of Michigan, Department of Microbiology and Immunology	Valesano et al.
EPI_ISL_496786, EPI_ISL_496787, EPI_ISL_496788, EPI_ISL_496789, EPI_ISL_496790, EPI_ISL_496791, EPI_ISL_496792, EPI_ISL_496793, EPI_ISL_496794, EPI_ISL_496795, EPI_ISL_496796, EPI_ISL_496797, EPI_ISL_496798, EPI_ISL_496799, EPI_ISL_496800, EPI_ISL_496801, EPI_ISL_496802, EPI_ISL_496803, EPI_ISL_496804, EPI_ISL_496805, EPI_ISL_496806, EPI_ISL_496807, EPI_ISL_496808, EPI_ISL_496809			
see above	Gorgas Memorial Laboratory of Health Studies	Gorgas Memorial Laboratory of Health Studies	Danilo Franco, Claudia Gonzalez Sandra Lopez-Verges, Alexander A Martinez
EPI_ISL_497818, EPI_ISL_497858	Department of Microbiology, The University of Hong Kong	Department of Microbiology, The University of Hong Kong	Kelvin K.W. To, Kwok-Yung Yuen
EPI_ISL_497872	UW Virology Lab	UW Virology Lab	Pavitra Roychoudhury, Amin Addetia, Hong Xie, Lasata Shrestha, Truong Nguyen, Meei-Li Huang, Keith Jerome, Alexander Greninger
EPI_ISL_498159, EPI_ISL_498160, EPI_ISL_498163, EPI_ISL_498166	Instituto Nacional de Salud, Bogotá, Colombia	Instituto Nacional de Salud, Bogotá, Colombia	Katherine Laiton-Donato, Diego A. Álvarez-Díaz, Carlos Franco-Muñoz, Jonathan Reales, Diego Andrés Prada, Jose A. Usme-Ciro, Nicolas D. Franco-Sierra, Zulma M. Cucunubá, Christian Julian VillabonaArenas, Liz Villabona-Arenas, Sussy Echeverria, Astrid C. Flórez, Carolina Ferro, Diana Marcela Walteros-Acero, Franklin Prieto, Carlos Andrés Durán, Martha Lucia Ospina Martinez, Marcela Mercado-Reyes
EPI_ISL_498191, EPI_ISL_498192	OUCRU	OUCRU	Nguyen Van Vinh Chau, Nguyen Thi Thu Hong, Nguyen Thi Han Ny, Le Nguyen Truc Nhu, Nghiem My Ngoc, Vo Thanh Lam, Nguyen Thanh Dung, Lam Minh Yen, Ngo Ngoc Quang Minh, Le Manh Hung, Nguyen Tri Dung, Dinh Nguyen Huy Man, Lam Anh Nguyet, Tran Chanh Xuan, Tran Tinh Hien, Nguyen Thanh Phong, Tran Nguyen Hoang Tu, Tran Tan Thanh, Nguyen Thanh Truong, Nguyen Tan Binh, Tang Chi Thuong, Guy Thwaites, and Le Van Tan, for OUCRU COVID-19 research group*
EPI_ISL_498508, EPI_ISL_498524, EPI_ISL_498526, EPI_ISL_498530	ACT Pathology	Schwessinger Lab	Ashley Jones, Benjamin Schwessinger, Robert Lanfear, Robyn N Hall, Megan McDonald, Ming-Dao Chia, Kevin Murray, Craig Kennedy, Karina Kennedy
EPI_ISL_499281	Centre for Enzyme Innovation, University of Portsmouth / Translational Research Laboratory, Portsmouth Hospitals NHS Trust	COVID-19 Genomics UK (COG-UK) Consortium	Angela Beckett,Yann Bourgeois,Garry Scarlett,Sharon Glaysher,Scott Elliott,Kelly Bicknell,Robert Impey,Allyson Lloyd,Sarah Wyllie,Ethan Butcher,Anoop Chauhan,Samuel Robson
EPI_ISL_499333	Virology Department, Sheffield Teaching Hospitals NHS Foundation Trust/Department of Infection, Immunity and	COVID-19 Genomics UK (COG-UK) Consortium	Thushan de Silva, Matthew Parker, Nikki Smith, Adri Angyal, Rebecca Brown, Luke Green, Rachel Tucker, Paul Parsons, Danielle Groves, Katie Johnson, Laura Carrilero, Alex Keeley, Dave Partridge, Matthew Wyles, Benjamin Lindsey, Mehmet Yavuz, Mohammad Raza, Cariad Evans

	Cardiovascular Disease, The Medical School, University of Sheffield		
EPI_ISL_499358, EPI_ISL_499369, EPI_ISL_499374, EPI_ISL_499403, EPI_ISL_499413, EPI_ISL_499429, EPI_ISL_499433, EPI_ISL_499442, EPI_ISL_499447, EPI_ISL_499451	Wales Specialist Virology Centre Sequencing lab: Pathogen Genomics Unit	COVID-19 Genomics UK (COG-UK) Consortium	Catherine Moore, Johnathan Evans, Laura Gifford, Malorie Perry, Simon Cottrell, Angela Marchbank, Alec Birchley, Alexander Adams, Amy Gaskin, Bree Gatica-Wilcox, Jason Coombes, Joel Southgate, Lauren Gilbert, Lee Graham, Nicole Pacchiarini, Sara Kumziene-Summerhayes, Sarah Taylor, Sophie Jones, Sara Rey, Matthew Bull, Joanne Watkins, Sally Corden, Tom Connor
EPI_ISL_500371, EPI_ISL_500389, EPI_ISL_500426, EPI_ISL_500448, EPI_ISL_500456, EPI_ISL_500457	Centro de Investigación Biomédica de La Rioja - Hospital San Pedro Logroño	SeqCOVID-SPAIN consortium/IBV(CSIC)	María de Toro, José Manuel Azcona Gutiérrez, María Pilar Bea Escudero, Miriam Blasco Alberdi and SeqCOVID-SPAIN consortium
EPI_ISL_500832	Virginia DCLS	Virginia DCLS	Virginia DCLS
EPI_ISL_501191, EPI_ISL_501206, EPI_ISL_501207, EPI_ISL_501208, EPI_ISL_501209, EPI_ISL_501210	Department of Medical Microbiology, University Malaya Medical Centre	Department of Medical Microbiology, Faculty of Medicine, University of Malaya	Yoong Min CHONG, Jennifer Chong, I-Ching SAM, Yoke Fun CHAN, University Malaya Medical Centre COVID Team
EPI_ISL_501246, EPI_ISL_501251	Hellenic Pasteur Institute, National Influenza Reference laboratory of Southern Greece & Unit of Bioinformatics and Applied Genomics	Hellenic Pasteur Institute, National Influenza Reference laboratory of Southern Greece & Unit of Bioinformatics and Applied Genomics	Vasiliki Pogka, Timokratis Karamitros, Athanasios Kossyvakis, Antonios Kalliaropoulos, Horefti Elina, Evangelidou Maria, Androniki Voulgari-Kokota, Aspasia Kontou, Andreas Mentis
EPI_ISL_506987, EPI_ISL_506990	Division of Viral Diseases, Center for Laboratory Control of Infectious Diseases, Korea Centers for Diseases Control and Prevention	Division of Viral Diseases, Center for Laboratory Control of Infectious Diseases, Korea Centers for Diseases Control and Prevention	Jeong-Min Kim, Yoon-Seok Chung, Namjoo Lee, Sang Hee Woo, Hye-Jun Jo, Heui Man Kim, Jun-Sub Kim, Dong Hyun Song, Daesang Lee, Seong Tae Jeong, Myung Guk Han
EPI_ISL_507025, EPI_ISL_507026	unknown	Infectious Diseases Research, King Abdullah International Medical Research Center (KAIMRC)	Alghoribi,M.F.
EPI_ISL_507079, EPI_ISL_507080	University College London Hospital	COVID-19 Genomics UK (COG-UK) Consortium	Judith Heaney, Matthew Byott, Catherine Houlihan, Dan Frampton, Stuart Kirk, Moira Spyer and Eleni Nastouli
EPI_ISL_507135	Centre for Enzyme Innovation, University of Portsmouth / Translational Research Laboratory, Portsmouth Hospitals NHS Trust	COVID-19 Genomics UK (COG-UK) Consortium	Angela Beckett,Yann Bourgeois,Garry Scarlett,Sharon Glaysher,Scott Elliott,Kelly Bicknell,Robert Impey,Allyson Lloyd,Sarah Wyllie,Ethan Butcher,Anoop Chauhan,Samuel Robson
EPI_ISL_507431, EPI_ISL_507447, EPI_ISL_507448, EPI_ISL_507450, EPI_ISL_507452, EPI_ISL_507455, EPI_ISL_507457, EPI_ISL_507460, EPI_ISL_507461, EPI_ISL_507462, EPI_ISL_507463, EPI_ISL_507470, EPI_ISL_507471, EPI_ISL_507560, EPI_ISL_507564, EPI_ISL_507566, EPI_ISL_507567, EPI_ISL_507570, EPI_ISL_507572, EPI_ISL_507573, EPI_ISL_507575, EPI_ISL_507578, EPI_ISL_507580, EPI_ISL_507584, EPI_ISL_507585, EPI_ISL_507586, EPI_ISL_507595, EPI_ISL_507597, EPI_ISL_507600, EPI_ISL_507601, EPI_ISL_507602, EPI_ISL_507604, EPI_ISL_507609, EPI_ISL_507612, EPI_ISL_507613, EPI_ISL_507614, EPI_ISL_507619, EPI_ISL_507804, EPI_ISL_507812, EPI_ISL_507813, EPI_ISL_507826, EPI_ISL_507835, EPI_ISL_507837, EPI_ISL_507840, EPI_ISL_507841, EPI_ISL_507843, EPI_ISL_507847, EPI_ISL_507849, EPI_ISL_507853, EPI_ISL_507863, EPI_ISL_507880, EPI_ISL_507884, EPI_ISL_507904, EPI_ISL_507913, EPI_ISL_507920, EPI_ISL_507921, EPI_ISL_507922, EPI_ISL_507931			
see above	Michigan Department of Health and Human Services, Bureau of Laboratories	Michigan Department of Health and Human Services, Bureau of Laboratories	Blankenship HM, Riner D, Soehnlen MK
EPI_ISL_508145	SA Pathology	SA Pathology	Lex Leong, Chuan Kok Lim, Mark Turra, Ivan Bastian, Geoff Higgins
EPI_ISL_508368, EPI_ISL_508376	Institute of Post Graduate Medical Education & Research	National Institute of Biomedical Genomics	Arindam Maitra, Aritra Biswas, Jayeeta Haldar, Raja Ray, Monimoy Banerjee, Saumitra Das
EPI_ISL_508745, EPI_ISL_508746, EPI_ISL_508747, EPI_ISL_508748, EPI_ISL_508749	Florida Bureau of Public Health Laboratories	Florida Bureau of Public Health Laboratories	Sarah Schmedes, Jason Blanton
EPI_ISL_508959, EPI_ISL_508960	Centre Hospitalier Saint Joseph Saint Luc	CNR Virus des Infections Respiratoires - France SUD	Antonin Bal, Gregory Destras, Gwendolyne Burfin, Solenne Brun, Carine Moustaud, Raphaelle Lamy, Alexandre Gaymard, Maude Bouscambert-Duchamp, Florence Morfin-Sherpa, Martine Valette, Bruno Lina, Laurence Josset
EPI_ISL_508961, EPI_ISL_508962, EPI_ISL_508963, EPI_ISL_508976	CNR Virus des Infections Respiratoires - France SUD	CNR Virus des Infections Respiratoires - France SUD	Antonin Bal, Gregory Destras, Gwendolyne Burfin, Solenne Brun, Carine Moustaud, Raphaelle Lamy, Alexandre Gaymard, Maude Bouscambert-Duchamp, Florence Morfin-Sherpa, Martine Valette, Bruno Lina, Laurence Josset
EPI_ISL_509011, EPI_ISL_509013, EPI_ISL_509014	Institut des Agents Infectieux (IAI), Hospices Civils de Lyon	CNR Virus des Infections Respiratoires - France SUD	Antonin Bal, Gregory Destras, Gwendolyne Burfin, Solenne Brun, Carine Moustaud, Raphaelle Lamy, Alexandre Gaymard, Maude Bouscambert-Duchamp, Florence Morfin-Sherpa, Martine Valette, Bruno Lina, Laurence Josset
EPI_ISL_509015	Centre Hospitalier de Villefranche	CNR Virus des Infections Respiratoires - France SUD	Antonin Bal, Gregory Destras, Gwendolyne Burfin, Solenne Brun, Carine Moustaud, Raphaelle Lamy, Alexandre Gaymard, Maude Bouscambert-Duchamp, Florence Morfin-Sherpa, Martine Valette, Bruno Lina, Laurence Josset
EPI_ISL_509016	Centre Hospitalier du Haut-Bugey	CNR Virus des Infections Respiratoires - France SUD	Antonin Bal, Gregory Destras, Gwendolyne Burfin, Solenne Brun, Carine Moustaud, Raphaelle Lamy, Alexandre Gaymard, Maude Bouscambert-Duchamp, Florence Morfin-Sherpa, Martine Valette, Bruno Lina, Laurence Josset
EPI_ISL_509587, EPI_ISL_509588, EPI_ISL_509589, EPI_ISL_509590, EPI_ISL_509591, EPI_ISL_509597, EPI_ISL_509598, EPI_ISL_509600	Utah Public Health Laboratory	Utah Public Health Laboratory	Heidi Butz, Erin Young, Kelly Oakeson
EPI_ISL_509769, EPI_ISL_509772, EPI_ISL_509773, EPI_ISL_509774	Florida Bureau of Public Health Laboratories	Florida Bureau of Public Health Laboratories	Sarah Schmedes, Jason Blanton
EPI_ISL_510071	Instituto de Investigaciones Biomédicas de Barcelona (CSIC), Hospital Clinic i Provincial de Barcelona, Instituto de Biomedicina de Valencia (CSIC), Hospital de Sant Pau	SeqCOVID-SPAIN consortium/IBV(CSIC)	Anna M. Planas, Mª Angeles Marcos, Miguel J. Martínez, Andrea Vergara, Alex Soriano, Jordi Pérez Tur, Israel Fernández Cadenas and SeqCOVID-SPAIN consortium
EPI_ISL_510119, EPI_ISL_510134, EPI_ISL_510141, EPI_ISL_510239, EPI_ISL_510240, EPI_ISL_510241, EPI_ISL_510242	Hospital General Universitario Gregorio Marañón	SeqCOVID-SPAIN consortium/IBV(CSIC)	Laura Pérez-Lago, Marta Herranz, Jon Sicilia, Julia Suárez, Pilar Catalán, Patricia Muñoz, Darío García de Viedma and SeqCOVID-SPAIN consortium
EPI_ISL_510464	Instituto de Investigaciones Biomédicas de Barcelona (CSIC), Hospital Clinic i Provincial de Barcelona, Instituto de Biomedicina de Valencia (CSIC), Hospital de Sant Pau	SeqCOVID-SPAIN consortium/IBV(CSIC)	Anna M. Planas, Mª Angeles Marcos, Miguel J. Martínez, Andrea Vergara, Alex Soriano, Jordi Pérez Tur, Israel Fernández Cadenas and SeqCOVID-SPAIN consortium
EPI_ISL_510530	UMR 190 - Faculte de medecine, UMR 'Emergence des Pathologies Virales' (EPV: Aix-Marseille University - IRD 190 - Inserm 1207 - EHE	UMR 190 - Faculte de medecine, UMR 'Emergence des Pathologies Virales' (EPV: Aix-Marseille University - IRD 190 - Inserm 1207 - EHE	Durand, G.A., Piorkowski,G., Pommier De Santi,V., De Laval,F., Leparc Goffart,I., Gilles,M., Geulen,M., Peduzzi,F., Le Flem,F.-X.
EPI_ISL_510537	Microbiology Division, SC DHEC	Microbiology Division, SC DHEC	Flores,H.
EPI_ISL_510909, EPI_ISL_510910, EPI_ISL_510911, EPI_ISL_510912, EPI_ISL_510927, EPI_ISL_510946, EPI_ISL_511028, EPI_ISL_511178	Instituto Nacional de Saude (INSA)	Instituto Nacional de Saude (INSA)	Borges et al
EPI_ISL_511192, EPI_ISL_511193, EPI_ISL_511194, EPI_ISL_511195, EPI_ISL_511196, EPI_ISL_511197, EPI_ISL_511198, EPI_ISL_511199, EPI_ISL_511200, EPI_ISL_511201, EPI_ISL_511202, EPI_ISL_511203, EPI_ISL_511204, EPI_ISL_511205, EPI_ISL_511206, EPI_ISL_511207, EPI_ISL_511208, EPI_ISL_511209			
see above	Instituto Nacional de Saude (INSA) and Instituto Gulbenkian	Instituto Nacional de Saude (INSA) and Instituto Gulbenkian	Borges et al

	de Ciencia (IGC)	de Ciencia (IGC)	
EPI_ISL_511362, EPI_ISL_511398, EPI_ISL_511431, EPI_ISL_511432, EPI_ISL_511435, EPI_ISL_511445, EPI_ISL_511451, EPI_ISL_511456, EPI_ISL_511470, EPI_ISL_511529, EPI_ISL_511656, EPI_ISL_511663, EPI_ISL_511668, EPI_ISL_511669, EPI_ISL_511675, EPI_ISL_511676, EPI_ISL_511687, EPI_ISL_511688, EPI_ISL_511689, EPI_ISL_511705, EPI_ISL_511707, EPI_ISL_511737, EPI_ISL_511738, EPI_ISL_511739, EPI_ISL_511740, EPI_ISL_511741, EPI_ISL_511742, EPI_ISL_511743, EPI_ISL_511744, EPI_ISL_511745, EPI_ISL_511746, EPI_ISL_511747			
see above	Instituto Nacional de Saude (INSA)	Instituto Nacional de Saude (INSA)	Borges et al
EPI_ISL_511865, EPI_ISL_511873	Johns Hopkins Hospital Department of Pathology	Johns Hopkins Hospital Department of Pathology	Peter M. Thielen, Thomas Mehoke, Shirlee Wohl, Srividya Ramakrishnan, Melanie Kirsche, Amanda Ernlund, Craig Howser, Kristina Zudock, Oluwaseun Falade-Nwulia, Norah Sadowski, Paul Morris, Mark Hopkins, Yunfan Fan, Nidia Trovao, Victoria Gniazdowski, Michael C. Schatz, Stuart C. Ray, Winston Timp, Heba H. Mostafa
EPI_ISL_511900	Institute of Post Graduate Medical Education & Research	National Institute of Biomedical Genomics - DBT's PAN-INDIA 1000 SARS--CoV-2 RNA Genome Sequencing Consortium	Arindam Maitra, Aritra Biswas, Jayeeta Haldar, Raja Ray, Monimoy Banerjee, Saumitra Das
EPI_ISL_512393, EPI_ISL_512394, EPI_ISL_512395, EPI_ISL_512396, EPI_ISL_512397, EPI_ISL_512398, EPI_ISL_512399, EPI_ISL_512400, EPI_ISL_512405	Centre for Enzyme Innovation, University of Portsmouth / Translational Research Laboratory, Portsmouth Hospitals NHS Trust	COVID-19 Genomics UK (COG-UK) Consortium	Angela Beckett,Yann Bourgeois,Garry Scarlett,Sharon Glaysher,Scott Elliott,Kelly Bicknell,Robert Impey,Allyson Lloyd,Sarah Wyllie,Ethan Butcher,Anoop Chauhan,Samuel Robson
EPI_ISL_512437	West of Scotland Specialist Virology Centre, NHSGGC / MRC-University of Glasgow Centre for Virus Research	COVID-19 Genomics UK (COG-UK) Consortium	Ana da Silva Filipe, Natasha Johnson, Kathy Smollett, Daniel Mair, Stephen Carmichael, Lily Tong, Jenna Nichols, Elihu Aranday-Cortes, Kirstyn Brunker, Yasmin Parr, Alice Broos, Kyriaki Nomikou; Sarah McDonald, Marc Niebel, Patawee Asamaphan; Richard Orton, Joseph Hughes, Sreenu Vattipally, David L Robertson; Alasdair MacLean, Rory Gunson; Kathy Li, Natasha Jesudason, Rajiv Shah, James Shepherd, Antonia Ho, Emma Thomson
EPI_ISL_512482	Wales Specialist Virology Centre Sequencing lab: Pathogen Genomics Unit	COVID-19 Genomics UK (COG-UK) Consortium	Catherine Moore, Johnathan Evans, Laura Gifford, Malorie Perry, Simon Cottrell, Angela Marchbank, Alec Birchley, Alexander Adams, Amy Gaskin, Bree Gatica-Wilcox, Jason Coombes, Joel Southgate, Lauren Gilbert, Lee Graham, Nicole Pacchiarini, Sara Kumziene-Summerhayes, Sarah Taylor, Sophie Jones, Sara Rey, Matthew Bull, Joanne Watkins, Sally Corden, Tom Connor
EPI_ISL_512745, EPI_ISL_512746	PathWest Laboratory Medicine WA	PathWest Laboratory Medicine WA Microbial Surveillance Unit	PathWest Laboratory Medicine WA Microbial Surveillance Unit
EPI_ISL_512793, EPI_ISL_512799, EPI_ISL_512800, EPI_ISL_512809	Public Health, United States Air Force School of Aerospace Medicine	Public Health, United States Air Force School of Aerospace Medicine	Fries,A.C., Purves,S.M., Meyer,J.R., Javorina,A.K., Connors,B.C., Macias,E.A., Lambert,A.W., Chapleau,R.R., Starr,C.R.
EPI_ISL_512812	Kenema Government Hospital, Ministry of Health and Sanitation	Kenema Government Hospital, Ministry of Health and Sanitation	Goba,A., Momoh,M., Sandi,J., Tomkins-Tinch,C., Siddle,K., Mehta,S., Oluniyi,P., Jalloh,S., Park,D., Andersen,K., Garry,R., Happi,C., Grant,D., Olawoye,I.
EPI_ISL_512928, EPI_ISL_512929	Pathogen Genomics Lab King Abdullah University of Science and Technology(KAUST)	Pathogen Genomics Lab King Abdullah University of Science and Technology(KAUST)	Fadwa Alofi, Sharif Hala, Rahul P Salunke, Sara Mfarrej, Amit Kumar Subudhi, Fathia Ben Rached, Amanda, Luke, Afrah Alsomali, Asim Khogeer, Jumana Taha, Abdulaziz Alahmadi, Kahled Algithami, Raeece Naeem, Anwar Hashem, Naif Almontashiri, Arnab Pain
EPI_ISL_512989, EPI_ISL_512993, EPI_ISL_512994, EPI_ISL_513000, EPI_ISL_513001	Pathogen Genomics Lab King Abdullah University of Science and Technology(KAUST)	Pathogen Genomics Lab King Abdullah University of Science and Technology(KAUST)	Amit Kumar Subudhi, Rahul P Salunke, Sara Mfarrej, Sharif Hala, Fadwa Alofi, Fathia Ben Rached, Afrah Alsomali, Asim Khogeer, Nashwa Al-khotani, Raeece Naeem, Anwar Hashem, Naif Almontashiri, Arnab Pain
EPI_ISL_513121, EPI_ISL_513122, EPI_ISL_513123, EPI_ISL_513124, EPI_ISL_513125, EPI_ISL_513126	Pathogen Genomics Lab King Abdullah University of Science and Technology(KAUST)	Pathogen Genomics Lab King Abdullah University of Science and Technology(KAUST)	Sharif Hala, Fadwa Alofi, Sara Mfarrej, Amit Kumar Subudhi, Rahul P Salunke, Fathia Ben Rached, Amanda Ooi, Luke Esau, Afrah Alsomali, Asim Khogeer, Jumana Taha, Abdulaziz Alahmadi, Kahled Algithami, Raeece Naeem, Anwar Hashem, Naif Almontashiri, Arnab Pain
EPI_ISL_513182, EPI_ISL_513183, EPI_ISL_513184, EPI_ISL_513185, EPI_ISL_513186, EPI_ISL_513187, EPI_ISL_513188, EPI_ISL_513189, EPI_ISL_513232, EPI_ISL_513233	Pathogen Genomics Lab King Abdullah University of Science and Technology(KAUST)	Pathogen Genomics Lab King Abdullah University of Science and Technology(KAUST)	Amit Kumar Subudhi, Rahul P Salunke, Sara Mfarrej, Sharif Hala, Fadwa Alofi, Fathia Ben Rached, Afrah Alsomali, Asim Khogeer, Nashwa Al-khotani, Raeece Naeem, Anwar Hashem, Naif Almontashiri, Arnab Pain
EPI_ISL_513632	University of Washington Virology Lab	University of Washington Virology Lab	Pavitra Roychoudhury, Hong Xie, Lasata Shrestha, Amin Addetia, Truong Nguyen, Victoria M Rachleff, Meei-Li Huang, Keith R Jerome, Alexander Greninger
EPI_ISL_514264	Laboratorio de Referencia Nacional de Virus Respiratorio. Instituto Nacional de Salud. Perú	Laboratorio de Referencia Nacional de Biotecnologia y Biologia Molecular.Instituto Nacional de Salud.Perú	Carlos Padilla Rojas, Karolyn Vega Chozo, Priscila Lope Pari, Omar Caceres Rey, Marco Galarza Perez, Maribel Huaringa Nuñez, Johanna Balbuena Torrez, Henri Bailon Calderon, Nancy Rojas Serrano
EPI_ISL_514653, EPI_ISL_514654, EPI_ISL_514655, EPI_ISL_514656	Mayo Clinic & Mayo Clinic Laboratories	Minnesota Department of Health, Public Health Laboratory	Matt Plumb, Jacob Garfin, and Xiong Wang
EPI_ISL_514657	M Health Fairview St. Joseph's Hospital	Minnesota Department of Health, Public Health Laboratory	Matt Plumb, Jacob Garfin, and Xiong Wang
EPI_ISL_515272, EPI_ISL_515273, EPI_ISL_515274, EPI_ISL_515275, EPI_ISL_515276, EPI_ISL_515278	University of Washington Virology Lab	University of Washington Virology Lab	Pavitra Roychoudhury, Hong Xie, Lasata Shrestha, Amin Addetia, Truong Nguyen, Victoria M Rachleff, Meei-Li Huang, Keith R Jerome, Alexander Greninger
EPI_ISL_515345	Nevada State Public Health Laboratory	Nevada State Public Health Laboratory	Richard Tillett, Joel R. Sevinsky, Paul Hartley, Heather Kerwin, David Jackson, Subhash C. Verma, Cyprian Rossetto, Andrew Gorzalski, Chris Laverdure, Natalie Crawford, Stephanie Van Hooser, and Mark Pandori
EPI_ISL_515522	UPA 24HS de Itatiba	Instituto Adolfo Lutz, Interdiciplinary Procedures Center, Strategic Laboratory	Claudio Tavares Sacchi, Claudia Regina Gonçalves, Erica Valessa Ramos Gomes
EPI_ISL_515524	PS Municipal Dr Lauro Ribas Braga	Instituto Adolfo Lutz, Interdiciplinary Procedures Center, Strategic Laboratory	Claudio Tavares Sacchi, Claudia Regina Gonçalves, Erica Valessa Ramos Gomes
EPI_ISL_515543	Serviço de Vigilância Sanitária e Epidemiológica	Instituto Adolfo Lutz, Interdiciplinary Procedures Center, Strategic Laboratory	Claudio Tavares Sacchi, Claudia Regina Gonçalves, Erica Valessa Ramos Gomes
EPI_ISL_516075	BIMS	Department of Neurovirology, National Institute of Mental Health and Neuroscience (NIMHANS)	Chitra Pattabiraman,Vijayalakshmi Reddy, Harsha PK, Risha Rasheed, Pramada Prasad, Shafeeq S Hameed, Manjunatha Venkataswamy, Anita Desai, Ravi Vasanthapuram
EPI_ISL_516197, EPI_ISL_516226, EPI_ISL_516227, EPI_ISL_516228, EPI_ISL_516229, EPI_ISL_516230, EPI_ISL_516231, EPI_ISL_516232, EPI_ISL_516233, EPI_ISL_516234, EPI_ISL_516236, EPI_ISL_516237, EPI_ISL_516239, EPI_ISL_516240, EPI_ISL_516241, EPI_ISL_516365, EPI_ISL_516366, EPI_ISL_516367, EPI_ISL_516368, EPI_ISL_516369, EPI_ISL_516370, EPI_ISL_516371, EPI_ISL_516372, EPI_ISL_516373, EPI_ISL_516374, EPI_ISL_516375, EPI_ISL_516380			
see above	Michigan Department of Health and Human Services, Bureau of Laboratories	Michigan Department of Health and Human Services, Bureau of Laboratories	Blankenship HM, Riner D, Soehnlen MK
EPI_ISL_516945	King Georges Medical University	CSIR-National Botanical Research Institute	Priti Prasad, Shantanu Prakash, Kishan Sahu, Babita Singh, Suruchi Shukla, Hricha Mishra, Danish Nasar Khan , Om Prakash, MLB Bhatt, SK Barik, Mehar H.Asif,Samir V. Sawant,Amita Jain, Sumit Kr. Bag
EPI_ISL_517713	Laboratorio de Referencia Nacional de Virus Respiratorio. Centro Nacional de Salud Publica. Instituto Nacional de Salud Peru.	Laboratorio de Referencia Nacional de Biotecnología y Biología Molecular. Centro Nacional de Salud Publica. Instituto Nacional de Salud Peru.	Carlos Padilla Rojas, Karolyn Vega Chozo, Priscila Lope Pari, Omar Caceres Rey, Marco Galarza Perez, Maribel Huaringa Nuñez, Johanna Balbuena Torres, Henri Bailon Calderon, Nancy Rojas Serrano.
EPI_ISL_520726, EPI_ISL_520727, EPI_ISL_520728	Mohammed Bin Rashid University of Medicine and Health Sciences	Al Jalila Genomics Center	Ahmad Abou Tayoun, Tom Loney, Hamda Khansaheb, Sathishkumar Ramaswamy, Divinlal Harilal, Zulfa Omar Deesi, Rupa Murthy Varghese, Hanan Al Suwaidi, Abdulmajeed Alkhaja, Mohammed Uddin, Rifat Hamoudi, Rabih Halwani, Abiola Catherine Senok, Qutayba Hamid, Norbert Nowotny, Alawi Alsheikh-Ali
EPI_ISL_521867, EPI_ISL_521868	Victorian Infectious Diseases Reference Laboratory (VIDRL)	VIDRL and MDU-PHL	Caly L., Seemann T., Sait, M., Schultz M., Druce J., Sherry, N.
EPI_ISL_521910	Microbiological Diagnostic Unit - Public Health Laboratory	MDU-PHL	Seemann T., Schultz M., Sait, M., Sherry, N.

	(MDU-PHL)		
EPI_ISL_522549	Félix Guyon Hospital	UMR PIMIT Université de La Réunion	David Wilkinson, Camille Lebarbenchon, Patrick Mavingui
EPI_ISL_522577, EPI_ISL_522657, EPI_ISL_522761	Royal Hobart Hospital Microbiology Department	MDU-PHL	Cooley L., van Haeften R., Seemann T., Sait M., Schultz, M.B., Sherry N.
EPI_ISL_523476	Dutch COVID-19 response team	Erasmus Medical Center	Bas Oude Munnink, David Nieuwenhuijse, Reina Sikkema, Claudia Schapendonk, Irina Chestakova, Anne van der Linden, Theo Bestebroer, Stefan van Nieuwkoop, Mark Pronk, Pascal Lexmond, Corien Swaan, Manon Haverkate, Madelief Mollers, Mart Stein, Sandra Kengne Kamga Mobou, Jeroen van Kampen, Jolanda Voermans, Aura Timen, Corine GeurtsvanKessel, Annemiek van der Eijk, Richard Molenkamp, Marion Koopmans, on behalf of the Dutch national COVID-19 response team.
EPI_ISL_523936, EPI_ISL_523945, EPI_ISL_523946	Center of Medical Microbiology, Virology, and Hospital Hygiene, University of Duesseldorf	Center of Medical Microbiology, Virology, and Hospital Hygiene, University of Duesseldorf	Maximilian Damagnez, Alexander Dilthey, Torsten Houwaart, Malte Kohns Vasconcelos, Marek Korencak, Jessica Nicolai, Klaus Pfeffer, Hendrik Streeck, Daniel Strelow, Jörg Timm, Andreas Walker, Tobias Wienemann
EPI_ISL_523956	Hospital Regional de Assis	Instituto Adolfo Lutz, Interdiciplinary Procedures Center, Strategic Laboratory	Claudio Tavares Sacchi, Claudia Regina Gonçalves, Erica Valessa Ramos Gomes
EPI_ISL_523969	Hospital Sao Paulo de Ensino da Unifesp	Instituto Adolfo Lutz, Interdiciplinary Procedures Center, Strategic Laboratory	Claudio Tavares Sacchi, Claudia Regina Gonçalves, Erica Valessa Ramos Gomes
EPI_ISL_524450, EPI_ISL_524451, EPI_ISL_524452, EPI_ISL_524453, EPI_ISL_524454	Microbiology & Immunology, University of North Carolina	Microbiology & Immunology, University of North Carolina	Bailey,A.G., Caro-Vegas,C.P., Dittmer,D., Eason,A.B., Juarez,A., Landis,J.T., McNamara,R.P., Miller,M.B., Moorad,R., Pluta,L.J., Seltzer,T.A., Thompson,C., Vahrson,W., Villamor,F.
EPI_ISL_524473	Laboratorio de Referencia Nacional de Virus Respiratorio. Centro Nacional de Salud Publica. Instituto Nacional de Salud Peru.	Laboratorio de Referencia Nacional de Biotecnología y Biología Molecular. Centro Nacional de Salud Publica. Instituto Nacional de Salud Peru.	Carlos Padilla Rojas, Karolyn Vega Chozo, Priscila Lope Pari, Omar Caceres Rey, Marco Galarza Perez, Maribel Huaringa Nuñez, Johanna Balbuena Torres, Henri Bailon Calderon, Nancy Rojas Serrano.
EPI_ISL_525472	Institute of Clinical Microbiology and Hygiene, University Hospital Regensburg	Institute of Clinical Microbiology and Hygiene, University Hospital Regensburg	Hiergeist, A.
EPI_ISL_525479	Centre for Dengue Research	Centre for Dengue Research	Chandima Jeewandara, Deshni Jayathilaka, Dinuka Ariyaratne, Laksiri Gomes, Diyanath Ranasinghe, Ananda Wijewickrama, Eranga Narangoda, Damayanthi Idampitiya, Gathsaurie Neelika Malavige
EPI_ISL_525643, EPI_ISL_525644, EPI_ISL_525645, EPI_ISL_525646, EPI_ISL_525647, EPI_ISL_525656	Wadsworth Center, New York State Department of Health	Wadsworth Center, New York State Department of Health	Kirsten St. George, Daryl M. Lamson, Sara Griesemer, Jonathan Plitnick, Navjot Singh, Matthew D. Shudt, Erica Lasek-Nesselquist
EPI_ISL_525772, EPI_ISL_525773, EPI_ISL_525774, EPI_ISL_525775, EPI_ISL_525776, EPI_ISL_525777, EPI_ISL_525778	Texas Department of State Health Services	Texas Department of State Health Services	Jenny Zhang, Rashmi Tuladhar, Bonnie Oh, Maliha Rahman, Anita Pokharel, Myong Koag, Chun Wang, Rachel Lee, Grace Kubin
EPI_ISL_526451, EPI_ISL_526452, EPI_ISL_526453, EPI_ISL_526454, EPI_ISL_526455, EPI_ISL_526456	West of Scotland Specialist Virology Centre, NHSGGC / MRC-University of Glasgow Centre for Virus Research	COVID-19 Genomics UK (COG-UK) Consortium	Ana da Silva Filipe, Natasha Johnson, Kathy Smollett, Daniel Mair, Stephen Carmichael, Lily Tong, Jenna Nichols, Elihu Aranday-Cortes, Kirstyn Brunker, Yasmin Parr, Alice Broos, Kyriaki Nomikou; Sarah McDonald, Marc Niebel, Patawee Asamaphan; Richard Orton, Joseph Hughes, Sreenu Vattipally, David L Robertson; Alasdair MacLean, Rory Gunson; Kathy Li, Natasha Jesudason, Rajiv Shah, James Shepherd, Antonia Ho, Emma Thomson
EPI_ISL_526470	Virology Department, Royal Infirmary of Edinburgh, NHS Lothian / School of Biological Sciences, University of Edinburgh / Institute of Genetics and Molecular Medicine, University of Edinburgh	COVID-19 Genomics UK (COG-UK) Consortium	McHugh M, Dewar R, Rooke S, Gallagher M, Balcaza C, O'Toole Á, Scher E, Hill V, McCrone JT, Colquhoun R, Yu X, Jackson B, Rambaut A, Williams TC, Templeton K
EPI_ISL_526827, EPI_ISL_526828, EPI_ISL_526829, EPI_ISL_526830, EPI_ISL_526831, EPI_ISL_526832, EPI_ISL_526833, EPI_ISL_526834, EPI_ISL_526835	Virginia DCLS	Virginia DCLS	Virginia DCLS
EPI_ISL_527878	Nigeria Centre for Disease Control (NCDC)	African Centre of Excellence for Genomics of Infectious Diseases (ACEGID), Redeemer's University, Ede, Osun State, Nigeria	Oluniyi P.E. et al
EPI_ISL_528636	National Genomics Core-Center for DNA Fingerprinting and Diagnostics	National Genomics Core- Center for DNA Fingerprinting and Diagnostics (NGC-CDFD)- DBT's PAN-INDIA-1000 Genome consortium	Heena Shah, G Shashikanth, Bala Pratyusha, Vinay Donipadi, K.Manohar, Mudhumohan Rao, Shruti Dasgupta, Kandali Sreethi Sreenivasulu Reddy, Chandra Shekhar Singh, Sunke Vijayakumar, R Lakshmi Vaishna, Jenige Aravindh Kumar, Muthulakshmi, V Naga Sailaja, R Harinarayanan, Rashna Bhandari, Murali Dharan Bashyam, Debashish Mitra, Divya Vashisht, Ashwin Dalal
EPI_ISL_528990	Ospedale Civile Maria SS. dello Splendore	Istitituto Zooprofilattico Sperimentale dell'Abruzzo e Molise "G.Caporale"	Lorusso A, Marcacci M, Di Domenico M, Curini V, Ancora M, Cammà C, Rinaldi A, Mangone I, Di Pasquale A, Puglia I, Savini G.
EPI_ISL_529009	Ospedale Civile S. Liberatore-Atri	Istitituto Zooprofilattico Sperimentale dell'Abruzzo e Molise "G.Caporale"	Lorusso A, Marcacci M, Di Domenico M, Curini V, Ancora M, Cammà C, Rinaldi A, Mangone I, Di Pasquale A, Puglia I, Savini G.
EPI_ISL_529162	Department of Immunology, The Scripps Research Institute	Andersen lab at Scripps Research	Allison Smither, Gilberto Sabino-Santos, Patricia Snarski, Lilia Melnik, Antoinette Bell, Kaylynn Genemaras, Arnaud Drouin, Dahlene Fusco, Robert Garry with SEARCH Alliance San Diego
EPI_ISL_529176	South Carolina Department of Health and Environmental Control	South Carolina Department of Health and Environmental Control	Haley V. Flores
EPI_ISL_529972, EPI_ISL_529973	Hospital Universitario 12 de Octubre	Hospital Universitario 12 de Octubre	Raúl Recio, Sara González, Esther Viedma, Elias Dahdouh, Fernando Lázaro, Natalia Stella, Julio García, Juan Carlos Galán, Rafael Cantón, Mª Dolores Folgueira, Rafael Delgado, Jesús Mingorance
EPI_ISL_530005, EPI_ISL_530006	Hospital Universitario 12 de Octubre	Hospital Universitario 12 de Octubre	Sara González, Esther Viedma, Raúl Recio, Elias Dahdouh, Fernando Lázaro, Natalia Stella, Julio García, Juan Carlos Galán, Rafael Cantón, Mª Dolores Folgueira, Rafael Delgado, Jesús Mingorance
EPI_ISL_530220, EPI_ISL_530222, EPI_ISL_530223	Minnesota Department of Health, Public Health Laboratory	Minnesota Department of Health, Public Health Laboratory	Matt Plumb, Jacob Garfin, and Xiong Wang
EPI_ISL_530226, EPI_ISL_530227, EPI_ISL_530228, EPI_ISL_530229, EPI_ISL_530230	Queensland Health Forensic and Scientific Services, Public Health Virology	Public Health Virology Laboratory, Forensic and Scientific Services, Queensland Health	Son Nguyen et al
EPI_ISL_534346, EPI_ISL_534347, EPI_ISL_534348, EPI_ISL_534349	Molecular diagnostic laboratory of Federal Budget Institution of Science "Central Research Institute of Epidemiology" of The Federal Service on Customers' Rights Protection and Human Well-being Surveillance	Group of Genomics and Postgenomic Technologies of Central Research Institute of Epidemilology	Speranskaya AS, Kaptelova VV, Valdokhina AV, Bulanenko VP, Samoilov AE, Korneenko EV, Tivanova EV, Shipulina OY, Akimkin VG

EPI_ISL_534817, EPI_ISL_534821, EPI_ISL_534824, EPI_ISL_534841, EPI_ISL_534852, EPI_ISL_534868, EPI_ISL_534883, EPI_ISL_534884, EPI_ISL_534899, EPI_ISL_534904, EPI_ISL_534918, EPI_ISL_534924, EPI_ISL_534948, EPI_ISL_534954, EPI_ISL_534955, EPI_ISL_534971, EPI_ISL_534980, EPI_ISL_534991, EPI_ISL_534999, EPI_ISL_535001, EPI_ISL_535006, EPI_ISL_535010, EPI_ISL_535016

see above	Oxford Viromics, NDM, University of Oxford; Oxford University	COVID-19 Genomics UK (COG-UK) Consortium	Tanya Golubchik, David Bonsall, George Macintyre, Amy Trebes, Mariateresa de Cesare, Catrin Moore, Alex Mobbs, Anita Justice, Robert Shaw, Monique

	Hospitals; Basingstoke and North Hampshire Hospital		Andersson, Timothy Peto, Emma Wise, Nathan Moore, Jessica Lynch, Nick Cortes, Matilde Mori, Stephen Kidd, David Buck, John Todd, Christophe Fraser
EPI_ISL_536368, EPI_ISL_536369, EPI_ISL_536370	Centre hospitalier Anna-Laberge	Laboratoire de santé publique du Québec	Sandrine Moreira, Ioannis Ragoussis, Guillaume Bourque, Jesse Shapiro, Mark Lathrop and Michel Roger
EPI_ISL_536371, EPI_ISL_536372, EPI_ISL_536373	Hôpital Charles-LeMoyne	Laboratoire de santé publique du Québec	Sandrine Moreira, Ioannis Ragoussis, Guillaume Bourque, Jesse Shapiro, Mark Lathrop and Michel Roger
EPI_ISL_536374	Hôpital Pierre-Boucher	Laboratoire de santé publique du Québec	Sandrine Moreira, Ioannis Ragoussis, Guillaume Bourque, Jesse Shapiro, Mark Lathrop and Michel Roger
EPI_ISL_536376, EPI_ISL_536377, EPI_ISL_536378	Centre hospitalier Anna-Laberge	Laboratoire de santé publique du Québec	Sandrine Moreira, Ioannis Ragoussis, Guillaume Bourque, Jesse Shapiro, Mark Lathrop and Michel Roger
EPI_ISL_536379	Hôpital Honoré-Mercier	Laboratoire de santé publique du Québec	Sandrine Moreira, Ioannis Ragoussis, Guillaume Bourque, Jesse Shapiro, Mark Lathrop and Michel Roger
EPI_ISL_536380	Hôpital Pierre-Boucher	Laboratoire de santé publique du Québec	Sandrine Moreira, Ioannis Ragoussis, Guillaume Bourque, Jesse Shapiro, Mark Lathrop and Michel Roger
EPI_ISL_536381	Centre Hospitalier Régional de Lanaudière	Laboratoire de santé publique du Québec	Sandrine Moreira, Ioannis Ragoussis, Guillaume Bourque, Jesse Shapiro, Mark Lathrop and Michel Roger
EPI_ISL_536382, EPI_ISL_536383	CSSS Haut-Richelieu/Rouville (Hôpital)	Laboratoire de santé publique du Québec	Sandrine Moreira, Ioannis Ragoussis, Guillaume Bourque, Jesse Shapiro, Mark Lathrop and Michel Roger
EPI_ISL_536384	Hôpital de Gatineau	Laboratoire de santé publique du Québec	Sandrine Moreira, Ioannis Ragoussis, Guillaume Bourque, Jesse Shapiro, Mark Lathrop and Michel Roger
EPI_ISL_536385, EPI_ISL_536386, EPI_ISL_536387	Hôpital de Hull	Laboratoire de santé publique du Québec	Sandrine Moreira, Ioannis Ragoussis, Guillaume Bourque, Jesse Shapiro, Mark Lathrop and Michel Roger
EPI_ISL_536388, EPI_ISL_536389	Hôpital Pierre-Boucher	Laboratoire de santé publique du Québec	Sandrine Moreira, Ioannis Ragoussis, Guillaume Bourque, Jesse Shapiro, Mark Lathrop and Michel Roger
EPI_ISL_536390	Hôpital du Suroît	Laboratoire de santé publique du Québec	Sandrine Moreira, Ioannis Ragoussis, Guillaume Bourque, Jesse Shapiro, Mark Lathrop and Michel Roger
EPI_ISL_536391	Hôpital Pierre-Boucher	Laboratoire de santé publique du Québec	Sandrine Moreira, Ioannis Ragoussis, Guillaume Bourque, Jesse Shapiro, Mark Lathrop and Michel Roger
EPI_ISL_536392, EPI_ISL_536393, EPI_ISL_536394	Hôpital de Hull	Laboratoire de santé publique du Québec	Sandrine Moreira, Ioannis Ragoussis, Guillaume Bourque, Jesse Shapiro, Mark Lathrop and Michel Roger
EPI_ISL_536395	Hôpital Pierre-Boucher	Laboratoire de santé publique du Québec	Sandrine Moreira, Ioannis Ragoussis, Guillaume Bourque, Jesse Shapiro, Mark Lathrop and Michel Roger
EPI_ISL_536396	Hôpital de Hull	Laboratoire de santé publique du Québec	Sandrine Moreira, Ioannis Ragoussis, Guillaume Bourque, Jesse Shapiro, Mark Lathrop and Michel Roger
EPI_ISL_536397	Hôpital Pierre-Boucher	Laboratoire de santé publique du Québec	Sandrine Moreira, Ioannis Ragoussis, Guillaume Bourque, Jesse Shapiro, Mark Lathrop and Michel Roger
EPI_ISL_536523, EPI_ISL_536524, EPI_ISL_536525, EPI_ISL_536526, EPI_ISL_536528, EPI_ISL_536529, EPI_ISL_536530, EPI_ISL_536562, EPI_ISL_536563, EPI_ISL_536564	Instituto Nacional de Salud	Laboratorio de Infecciones Respiratorias Agudas	Eduardo Juscamayta Lopez, David Tarazona, Faviola Valdivia Guerrero, Nancy Rojas Serrano, Dennis Carhuaricra, Lenin Maturrano Hernandez, Ronnie Gavilan Chavez
EPI_ISL_537288, EPI_ISL_537289, EPI_ISL_537290, EPI_ISL_537291, EPI_ISL_537292, EPI_ISL_537293, EPI_ISL_537294, EPI_ISL_537295	Universidad de León	SeqCOVID-SPAIN consortium/IBV(CSIC)	Ana Carvajal, Vicente Martín, Héctor Argüello, Juan M. Fregeneda, Tania Fernández-Villa, Antonio J. Molina and SeqCOVID-SPAIN consortium
EPI_ISL_537393, EPI_ISL_537394, EPI_ISL_537401, EPI_ISL_537438	Centro de Investigación Biomédica de La Rioja - Hospital San Pedro Logroño	SeqCOVID-SPAIN consortium/IBV(CSIC)	María de Toro, José Manuel Azcona Gutiérrez, María Pilar Bea Escudero, Miriam Blasco Alberdi and SeqCOVID-SPAIN consortium
EPI_ISL_537496, EPI_ISL_537497, EPI_ISL_537573, EPI_ISL_537574, EPI_ISL_537591, EPI_ISL_537592, EPI_ISL_537593	UCLA Pathology Clinical Microbiology Lab	Kruglyak Lab	Guo et al.
EPI_ISL_537719	Hospital Universitario de Gran Canaria Dr. Negrín	SeqCOVID-SPAIN consortium/IBV(CSIC)	M. Carmen Pérez González, Francisco J. Chamizo López, Ana Bordes Benítez and SeqCOVID-SPAIN consortium
EPI_ISL_537857, EPI_ISL_537858, EPI_ISL_537869, EPI_ISL_537870	Centro de Investigación Biomédica de La Rioja - Hospital San Pedro Logroño	SeqCOVID-SPAIN consortium/IBV(CSIC)	María de Toro, José Manuel Azcona Gutiérrez, María Pilar Bea Escudero, Miriam Blasco Alberdi and SeqCOVID-SPAIN consortium
EPI_ISL_537994, EPI_ISL_537995	Servicio de Microbiología. Hospital General Universitario de Castellón	SeqCOVID-SPAIN consortium/IBV(CSIC)	Rosario Moreno, María Dolores Tirado and SeqCOVID-SPAIN consortium
EPI_ISL_538070, EPI_ISL_538071, EPI_ISL_538072, EPI_ISL_538073, EPI_ISL_538074, EPI_ISL_538075, EPI_ISL_538076, EPI_ISL_538077, EPI_ISL_538078, EPI_ISL_538079, EPI_ISL_538080, EPI_ISL_538081			
see above	Servicio de Microbiología. Hospital Universitario Donostia. OSI Donostialdea. Área de Enfermedades Infecciosas, Grupo de Infección Respiratoria y Resistencia Antimicrobiana. Instituto de Investigación Sanitaria Biodonostia	SeqCOVID-SPAIN consortium/IBV(CSIC)	Gustavo Cilla, Milagrosa Montes, Luis Piñeiro, Jose Maria Marimón and SeqCOVID-SPAIN consortium
EPI_ISL_538158, EPI_ISL_538159, EPI_ISL_538160, EPI_ISL_538163, EPI_ISL_538166, EPI_ISL_538167, EPI_ISL_538168, EPI_ISL_538169, EPI_ISL_538170, EPI_ISL_538171	Servicio de Microbiología y Parasitología clínica. UCEIMP. Hospital Universitario Virgen del Rocío/IBIS/CSIC/US	SeqCOVID-SPAIN consortium/IBV(CSIC)	Guillermo Martín Gutiérrez, Ángel Rodríguez Villodres, Lidia Gálvez Benitez, Verónica González Galán, Javier Aznar Martín and SeqCOVID-SPAIN consortium
EPI_ISL_538336, EPI_ISL_538337, EPI_ISL_538338, EPI_ISL_538339, EPI_ISL_538340, EPI_ISL_538341, EPI_ISL_538342, EPI_ISL_538343	Kingston Health Sciences Centre / Queen's University	Ontario Institute for Cancer Research	Prameet M. Sheth, Calvin Sjaarda, Robert Colautti, Katya Douchant, Ilinca Lungu, Bernard Lam, Paul Krzyzanowski, Michael Laszloffy, Lawrence E Heisler, Richard de Borja, Jared T. Simpson
EPI_ISL_538465, EPI_ISL_538466, EPI_ISL_538467, EPI_ISL_538468	Department of Laboratory Medicine, Tan Tock Seng Hospital	Department of Laboratory Medicine, Tan Tock Seng Hospital	Chen YYC, Zair X, Lim JX, Li C, Tang WY, Maurer-Stroh S, Barkham TMS, Nagarajan N, Sessions OM
EPI_ISL_538613, EPI_ISL_538615, EPI_ISL_538616	Servicio de Microbiología. Hospital General Universitario de Castellón	SeqCOVID-SPAIN consortium/IBV(CSIC)	Rosario Moreno, María Dolores Tirado and SeqCOVID-SPAIN consortium
EPI_ISL_538688	Hospital Universitario Virgen de las Nieves de Granada-SAS	SeqCOVID-SPAIN consortium/IBV(CSIC)	Mercedes Pérez Ruiz, Sara Sanbonmatsu Gámez, Irene Pedrosa Corral, José M. Navarro-Marí and SeqCOVID-SPAIN consortium
EPI_ISL_538791, EPI_ISL_538816, EPI_ISL_538820, EPI_ISL_538832, EPI_ISL_538844, EPI_ISL_538847, EPI_ISL_538884, EPI_ISL_538901, EPI_ISL_538979, EPI_ISL_538983, EPI_ISL_538986, EPI_ISL_538988, EPI_ISL_538989, EPI_ISL_538990, EPI_ISL_539002, EPI_ISL_539004, EPI_ISL_539009, EPI_ISL_539023, EPI_ISL_539034, EPI_ISL_539047, EPI_ISL_539051, EPI_ISL_539068, EPI_ISL_539069, EPI_ISL_539072, EPI_ISL_539088, EPI_ISL_539090, EPI_ISL_539095, EPI_ISL_539099, EPI_ISL_539111, EPI_ISL_539112, EPI_ISL_539118, EPI_ISL_539119, EPI_ISL_539134, EPI_ISL_539137, EPI_ISL_539139			
see above	Leeds Teaching Hospitals NHS Trust and Public Health England, National Infection Service (Leeds laboratory)	Wellcome Sanger Institute for the COVID-19 Genomics UK (COG-UK) consortium	Louissa Macfarlane-Smith, Holli Carden, Katherine L. Harper, Antony Hale and Alex Alderton, Roberto Amato, Sonia Goncalves, Ewan Harrison, David K. Jackson, Ian Johnston, Dominic Kwiatkowski, Cordelia Langford, John Sillitoe on behalf of the Wellcome Sanger Institute COVID-19 Surveillance Team
EPI_ISL_539152	Leeds Teaching Hospitals NHS Trust and Public Health England, National Infection Service (Leeds laboratory)	Wellcome Sanger Institute for the COVID-19 Genomics UK (COG-UK) Consortium	Louissa Macfarlane-Smith, Holli Carden, Katherine L. Harper, Antony Hale and Alex Alderton, Roberto Amato, Sonia Goncalves, Ewan Harrison, David K. Jackson, Ian Johnston, Dominic Kwiatkowski, Cordelia Langford, John Sillitoe on behalf of the Wellcome Sanger Institute COVID-19 Surveillance Team
EPI_ISL_539163, EPI_ISL_539171, EPI_ISL_539176, EPI_ISL_539194,	Leeds Teaching Hospitals NHS Trust and Public Health England, National Infection Service (Leeds laboratory)	Wellcome Sanger Institute for the COVID-19 Genomics UK (COG-UK) consortium	Louissa Macfarlane-Smith, Holli Carden, Katherine L. Harper, Antony Hale and Alex Alderton, Roberto Amato, Sonia Goncalves, Ewan Harrison, David K. Jackson, Ian Johnston, Dominic Kwiatkowski, Cordelia Langford, John Sillitoe on behalf of the Wellcome Sanger Institute COVID-19 Surveillance Team

EPI_ISL_539203, EPI_ISL_539219			
EPI_ISL_539246, EPI_ISL_539247, EPI_ISL_539248	Hospital Universitario de La Ribera (Alzira, València)	SeqCOVID-SPAIN consortium/IBV(CSIC)	Olalla Martínez Macias, Julia González and SeqCOVID-SPAIN consortium
EPI_ISL_539306, EPI_ISL_539308, EPI_ISL_539309, EPI_ISL_539317, EPI_ISL_539318, EPI_ISL_539319, EPI_ISL_539320	KWR Watercycle Research Institute	Erasmus Medical Center	Ray Izquierdo-Lara, Goffe Elsinga, Leo Heijnen, Bas B. Oude Munnink, Claudia M. E. Schapendonk, David Nieuwenhuijse, Matthijs Kon, Lu Lu, Frank M. Aarestrup, Samantha Lycett, Gertjan Medema, Marion P.G. Koopmans, Miranda de Graaf
EPI_ISL_539494, EPI_ISL_539777	The National Institute of Public Health	State Veterinary Institute Prague	Nagy,A;Jirincova,H;Novakova,L;Trnka,D;Vecerova,J
EPI_ISL_541037	Hospital Clínico Universitario de Santiago de Compostela	SeqCOVID-SPAIN consortium/Institute of Biomedicine of Valencia, IBV-CSIC	José Javier Costa Alcalde, Antonio Aguilera Guirao, Mª Luisa Pérez del Molino Bernal, Amparo Coira Nieto, Gema Barbeito Castiñeiras, Rocio Trastoy Pena and SeqCOVID-SPAIN consortium
EPI_ISL_541761, EPI_ISL_541770	Barts Health NHS Trust	Wellcome Sanger Institute for the COVID-19 Genomics UK (COG-UK) consortium	Teresa Cutino-Moguel, Mark Hopkins, Beatrix Kele, David Harrington and Alex Alderton, Roberto Amato, Sonia Goncalves, Ewan Harrison, David K. Jackson, Ian Johnston, Dominic Kwiatkowski, Cordelia Langford, John Sillitoe on behalf of the Wellcome Sanger Institute COVID-19 Surveillance Team
EPI_ISL_541953	Servicio de Microbiología, Hospital Universitario Son Espases	SeqCOVID-SPAIN consortium/IBV(CSIC)	Carla López-Causapé, Jordi Reina, Antonio Oliver and SeqCOVID-SPAIN consortium
EPI_ISL_542389, EPI_ISL_542390, EPI_ISL_542392	San Matteo Hospital Pavia	Dep. Of Oncology and Hemato-Oncology University of Milan	Claudia Alteri, Valeria Cento, Antonio Piralla, Valentino Costabile, Monica Tallarita, Luna Colagrossi, Silvia Renica, Federica Giardina, Federica Novazzi, Stefano Gaiarsa, Elisa Matarazzo, Maria Antonello, Chiara Vismara, Roberto Fumagalli, Oscar Massimiliano Epis, Massimo Puoti, Carlo Federico Perno, Fausto Baldanti
EPI_ISL_542571, EPI_ISL_542573, EPI_ISL_542581, EPI_ISL_542582, EPI_ISL_542585, EPI_ISL_542587, EPI_ISL_542590, EPI_ISL_542599, EPI_ISL_542610, EPI_ISL_542692, EPI_ISL_542801, EPI_ISL_544456, EPI_ISL_544465, EPI_ISL_544471, EPI_ISL_544479, EPI_ISL_545240, EPI_ISL_545867, EPI_ISL_545882			
see above	Houston Methodist Hospital	Houston Methodist Hospital	S. Wesley Long, Randall J. Olsen, Paul A. Christensen, David W. Bernard, James J. Davis, Maulik Shukla, Marcus Nguyen, Matthew Ojeda Saavedra, Concepcion C. Cantu, Prasanti Yerramilli, Layne Pruitt, Sishir Subedi, Hung-Che Kuo, Heather Hendrickson, Ghazaleh Eskandari, Hoang A. T. Nguyen, J. Hunter Long, Muthiah Kumaraswami, Jule Goike, Daniel Boutz, Jimmy Gollihar, Jason S. McLellan, Chia-Wei Chou, Kamyab Javanmardi, Ilya J. Finkelstein, and James M. Musser
EPI_ISL_545955	Laboratorio de Infecciones Respiratorias Agudas. Centro Nacional de Salud Publica,Instituto Nacional de Salud	Laboratorio de Infecciones Respiratorias Agudas. Centro Nacional de Salud Publica,Instituto Nacional de Salud	Juscamayta,E.
EPI_ISL_546738	Houston Methodist Hospital	Houston Methodist Hospital	S. Wesley Long, Randall J. Olsen, Paul A. Christensen, David W. Bernard, James J. Davis, Maulik Shukla, Marcus Nguyen, Matthew Ojeda Saavedra, Concepcion C. Cantu, Prasanti Yerramilli, Layne Pruitt, Sishir Subedi, Hung-Che Kuo, Heather Hendrickson, Ghazaleh Eskandari, Hoang A. T. Nguyen, J. Hunter Long, Muthiah Kumaraswami, Jule Goike, Daniel Boutz, Jimmy Gollihar, Jason S. McLellan, Chia-Wei Chou, Kamyab Javanmardi, Ilya J. Finkelstein, and James M. Musser
EPI_ISL_547494, EPI_ISL_547505, EPI_ISL_547534	Dutch COVID-19 response team	National Institute for Public Health and the Environment (RIVM)	Adam Meijer, Harry Vennema, Jeroen Cremer, Sharon van den Brink, Bas van der Veer, AnneMarie van den Brandt, Florian Zwagemaker, Dennis Schmitz, Chantal Reusken, on behalf of the national COVID-19 response team
EPI_ISL_548364	Ventura County Public Health Lab	Chan-Zuckerberg Biohub	CZB Cliahub Consortium
EPI_ISL_548781, EPI_ISL_548931	Public Health Ontario Laboratory	Public Health Ontario Laboratory	Vanessa G Allen, Philip Banh, Richard de Borja, Yao Chen, Alireza Eshaghi, Nahuel Fittipaldi, Christine Frantz, Jonathan B Gubbay, Jennifer L Guthrie, Lawrence Heisler, Esha Joshi, Michael Laszloffy, Aimin Li, Michael CY Li, Dean Maxwell, Sandeep Nagra, Samir N Patel, Heather Rilkoff, Jared Simpson, Karthikeyan Sivaraman, Yogi Sundaravadanam, Sarah Teatero, Andre Villegas, Sandra Zittermann
EPI_ISL_549016, EPI_ISL_549021	KWR Watercycle Research Institute	Erasmus Medical Center	Ray Izquierdo-Lara, Goffe Elsinga, Leo Heijnen, Bas B. Oude Munnink, Claudia M. E. Schapendonk, David Nieuwenhuijse, Matthijs Kon, Lu Lu, Frank M. Aarestrup, Samantha Lycett, Gertjan Medema, Marion P.G. Koopmans, Miranda de Graaf
EPI_ISL_549336, EPI_ISL_549349, EPI_ISL_549353, EPI_ISL_549368, EPI_ISL_549369, EPI_ISL_549370, EPI_ISL_549373, EPI_ISL_549377, EPI_ISL_549378, EPI_ISL_549379, EPI_ISL_549383, EPI_ISL_549477, EPI_ISL_549482, EPI_ISL_549483, EPI_ISL_549484, EPI_ISL_549485, EPI_ISL_549489, EPI_ISL_549490			
see above	Quadram Institute Bioscience	COVID-19 Genomics UK (COG-UK) Consortium	Dave J. Baker, Gemma L. Kay, Alp Aydin, Thanh Le-Viet, Steven Rudder, Ana P. Tedim, Anastasia Kolyva, Maria Diaz, Leonardo de Oliveira Martins, Nabil-Fareed Alikhan, Lizzie Meadows, Rachael Stanley, Ngozi Elumogo, Muhammed Yasir, Nicholas M. Thomson, Alexander J Trotter, Rachel Gilroy, Samuel Bloomfield, Claire Stuart, Andrew Bell, Reenesh Prakash, Samir Dervisevic, Alison E. Mather, John Wain, Mark Webber, Andrew J. Page, Justin O'Grady
EPI_ISL_560569	hôpital	National Reference Center for Viruses of Respiratory Infections, Institut Pasteur, Paris	Sylvie Behillil, Fabiana Gambaro, Etienne Simon-Lorière, Vincent Enouf, Maud Vanpeene, Sylvie van der Werf
EPI_ISL_560582, EPI_ISL_560583	Hopital	National Reference Center for Viruses of Respiratory Infections, Institut Pasteur, Paris	Sylvie Behillil, Fabiana Gambaro, Etienne Simon-Lorière, Vincent Enouf, Maud Vanpeene, Sylvie van der Werf
EPI_ISL_560596	hopital	National Reference Center for Viruses of Respiratory Infections, Institut Pasteur, Paris	Sylvie Behillil, Fabiana Gambaro, Etienne Simon-Lorière, Vincent Enouf, Maud Vanpeene, Sylvie van der Werf
EPI_ISL_560739	Centre for Clinical Infection and Diagnostics Research and Genomics Innovation Unit, Guy's and St. Thomas' NHS Trust	Centre for Clinical Infection and Diagnostics Research and Genomics Innovation Unit, Guy's and St. Thomas' NHS Trust	Chloe Fisher, Luke Snell, Rahul Batra, Jonathan Edgeworth, Ali Raza Awan
EPI_ISL_560743, EPI_ISL_560744, EPI_ISL_560745	Minnesota Department of Health, Public Health Laboratory	Minnesota Department of Health, Public Health Laboratory	Matt Plumb, Jacob Garfin, and Xiong Wang
EPI_ISL_560841, EPI_ISL_560847, EPI_ISL_560861	Utah Public Health Laboratory	Utah Public Health Laboratory	Erin Young, Kelly Oakeson
EPI_ISL_562588	Victorian Infectious Diseases Reference Laboratory (VIDRL)	VIDRL and MDU-PHL	Caly, L., Seemann, T., Sait, M., Schultz, M. B., Druce J., Sherry, N.
EPI_ISL_565930, EPI_ISL_565931, EPI_ISL_565953, EPI_ISL_565973, EPI_ISL_565974, EPI_ISL_565978, EPI_ISL_565997, EPI_ISL_566002, EPI_ISL_566004, EPI_ISL_566011	Michigan Department of Health and Human Services, Bureau of Laboratories	Michigan Department of Health and Human Services, Bureau of Laboratories	Blankenship HM, Riner D, Soehnlen MK
EPI_ISL_568558	Department of Infectious Diseases and Immunology, National Hospital Organization Nagoya Medical Center	Clinical Research Center, National Hospital Organization Nagoya Medical Center	Yoshihiro Nakata, Hirotaka Ode, Mai Kubota, Masakazu Matsuda, Kazuhiro Matsuoka, Nakasuji Miho, Mikiko Mori, Mayumi Imahashi, Yoshiyuki Yokomaku, Yasumasa Iwatani
EPI_ISL_569418, EPI_ISL_569419, EPI_ISL_569420, EPI_ISL_569423, EPI_ISL_569424, EPI_ISL_569425, EPI_ISL_569426, EPI_ISL_569427, EPI_ISL_569428, EPI_ISL_569429, EPI_ISL_569430, EPI_ISL_569431, EPI_ISL_569432, EPI_ISL_569433, EPI_ISL_569434, EPI_ISL_569435, EPI_ISL_569436, EPI_ISL_569437, EPI_ISL_569438, EPI_ISL_569439, EPI_ISL_569440, EPI_ISL_569441, EPI_ISL_569442, EPI_ISL_569443, EPI_ISL_569444, EPI_ISL_569445, EPI_ISL_569446, EPI_ISL_569447, EPI_ISL_569448, EPI_ISL_569449, EPI_ISL_569451, EPI_ISL_569452, EPI_ISL_569453, EPI_ISL_569454, EPI_ISL_569455, EPI_ISL_569456, EPI_ISL_569457, EPI_ISL_569458, EPI_ISL_569459, EPI_ISL_569460, EPI_ISL_569461, EPI_ISL_569462, EPI_ISL_569463, EPI_ISL_569464			
see above	MEPHI, Aix Marseille University	MEPHI, Aix Marseille University	Anthony LEVASSEUR
EPI_ISL_569864, EPI_ISL_569868, EPI_ISL_569869, EPI_ISL_569870, EPI_ISL_569871, EPI_ISL_569872, EPI_ISL_569873, EPI_ISL_569874, EPI_ISL_569875, EPI_ISL_569876, EPI_ISL_569877, EPI_ISL_569878, EPI_ISL_569879, EPI_ISL_569880, EPI_ISL_569881, EPI_ISL_569882			
see above	Amedeo di savoia	Crosetto lab, Karolinska Institutet, SciLifeLab	Michele Simonetti, Maria Grazia Milia, Luuk Harbers, Ning Zhang, Anna Sapino, Valeria Ghisetti, Nicola Crosetto
EPI_ISL_570045, EPI_ISL_570046, EPI_ISL_570051, EPI_ISL_570082, EPI_ISL_570083, EPI_ISL_570084, EPI_ISL_570085, EPI_ISL_570086, EPI_ISL_570087, EPI_ISL_570088, EPI_ISL_570089, EPI_ISL_570092, EPI_ISL_570093, EPI_ISL_570094, EPI_ISL_570095, EPI_ISL_570099, EPI_ISL_570100, EPI_ISL_570104, EPI_ISL_570105, EPI_ISL_570106, EPI_ISL_570107, EPI_ISL_570109, EPI_ISL_570110			
see above	UW Virology Lab	UW Virology Lab	Pavitra Roychoudhury, Hong Xie, Lasata Shrestha, Amin Addetia, Victoria M Rachleff, Meei-Li Huang, Keith R Jerome, Alexander Greninger
EPI_ISL_574541, EPI_ISL_574542	National Public Health Laboratory, National Centre for Infectious Diseases	National Public Health Laboratory, National Centre for Infectious Diseases	Tze Minn Mak, Sophie Octavia, Zhenyang Zhou, Lin Cui, Raymond Tzer Pin Lin

EPI_ISL_574591, EPI_ISL_574592	Hospital Domingos Leonardo Ceravolo Presidente Prudente	Instituto Adolfo Lutz, Interdiciplinary Procedures Center, Strategic Laboratory	Claudio Tavares Sacchi, Claudia Regina Gonçalves, Erica Valessa Ramos Gomes, Karoline Rodrigues Campos
EPI_ISL_574843, EPI_ISL_574844, EPI_ISL_574845, EPI_ISL_574846	Institute for Infectious Diseases, University of Bern	Institute for Infectious Diseases, University of Bern	Michel C Koch, Christian Baumann, Miguel A Terrazos Miani, Cora Sägesser, Stephen L Leib, Peter Keller, Franziska Suter-Riniker, Alban Ramette
EPI_ISL_575038, EPI_ISL_575039, EPI_ISL_575040, EPI_ISL_575041, EPI_ISL_575044, EPI_ISL_575045, EPI_ISL_575046, EPI_ISL_575047, EPI_ISL_575057, EPI_ISL_575058, EPI_ISL_575059, EPI_ISL_575105, EPI_ISL_575106, EPI_ISL_575107, EPI_ISL_575108			
see above	Utah Public Health Laboratory	Utah Public Health Laboratory	Erin Young, Kelly Oakeson
EPI_ISL_577742	Institute of Virology, Biomedical Research Center of the Slovak Academy of Sciences, Bratislava	Faculty of Natural Sciences, Comenius University, Bratislava	Broa Brejová, Viktória Hodorová, Kristína Boršová, Viktória abanová, Dominika Friová, Sabina Fumaová Havlíková, Juraj Kopáek, Martina Liková, ubomíra Lukáiková, Martina Neboháová, Monika Sláviková, Edita Staroová, Elena Tichá, Tomáš Vina, Jozef Nosek, Boris Klempa
EPI_ISL_578084, EPI_ISL_578094, EPI_ISL_578130, EPI_ISL_578131, EPI_ISL_578132, EPI_ISL_578133, EPI_ISL_578134, EPI_ISL_578135	University of Michigan Clinical Microbiology Laboratory	Lauring Lab, University of Michigan, Department of Microbiology and Immunology	Valesano
EPI_ISL_578335, EPI_ISL_578336, EPI_ISL_578337, EPI_ISL_578383, EPI_ISL_578384, EPI_ISL_578385, EPI_ISL_578386, EPI_ISL_578388, EPI_ISL_578389, EPI_ISL_578390, EPI_ISL_578391, EPI_ISL_578392, EPI_ISL_578393, EPI_ISL_578394, EPI_ISL_578395, EPI_ISL_578396, EPI_ISL_578397			
see above	Wisconsin State Laboratory of Hygiene Communicable Disease Division	Wisconsin State Laboratory of Hygiene Communicable Disease Division	Kelsey R. Florek, Abigail C. Shockey
EPI_ISL_579087, EPI_ISL_579088, EPI_ISL_579089	Canterbury Health Laboratories	Institute of Environmental Science and Research (ESR)	Xiaoyun Ren, Matt Storey, Nikki Freed, Muhammad Faisal, Jing Wang, Hermes Perez, Anja Werno, Antje van der Linden, Arlo Upton, Chris Mansell, David Hammer, Dragana Drinkovic, Gary McAuliffe, Hana Sofia Andersson, James Ussher, Jill Sherwood, Josh Freeman, Julia Howard, Juliet Elvy, Mary DeAlmeida, Matt Blakiston, Matthew Rogers, Max Bloomfield, Michael Addidle, Michelle Balm, Sally Roberts, Sarah Jefferies, Sharmini Muttaiyah, Susan Morpeth, Susan Taylor, Timothy Blackmore, Vani Sathyendran, Veronica Playle, Virginia Hope, Erasmus Smit, Lauren Jelly, Olin Silander, Joep de Ligt
EPI_ISL_579153, EPI_ISL_579155, EPI_ISL_579157, EPI_ISL_579158, EPI_ISL_579163, EPI_ISL_579174	Southern Community Labs Dunedin	Institute of Environmental Science and Research (ESR)	Xiaoyun Ren, Matt Storey, Nikki Freed, Muhammad Faisal, Jing Wang, Hermes Perez, Anja Werno, Antje van der Linden, Arlo Upton, Chris Mansell, David Hammer, Dragana Drinkovic, Gary McAuliffe, Hana Sofia Andersson, James Ussher, Jill Sherwood, Josh Freeman, Julia Howard, Juliet Elvy, Mary DeAlmeida, Matt Blakiston, Matthew Rogers, Max Bloomfield, Michael Addidle, Michelle Balm, Sally Roberts, Sarah Jefferies, Sharmini Muttaiyah, Susan Morpeth, Susan Taylor, Timothy Blackmore, Vani Sathyendran, Veronica Playle, Virginia Hope, Erasmus Smit, Lauren Jelly, Olin Silander, Joep de Ligt
EPI_ISL_579229	Middlemore Hospital	Institute of Environmental Science and Research (ESR)	Xiaoyun Ren, Matt Storey, Nikki Freed, Muhammad Faisal, Jing Wang, Hermes Perez, Anja Werno, Antje van der Linden, Arlo Upton, Chris Mansell, David Hammer, Dragana Drinkovic, Gary McAuliffe, Hana Sofia Andersson, James Ussher, Jill Sherwood, Josh Freeman, Julia Howard, Juliet Elvy, Mary DeAlmeida, Matt Blakiston, Matthew Rogers, Max Bloomfield, Michael Addidle, Michelle Balm, Sally Roberts, Sarah Jefferies, Sharmini Muttaiyah, Susan Morpeth, Susan Taylor, Timothy Blackmore, Vani Sathyendran, Veronica Playle, Virginia Hope, Erasmus Smit, Lauren Jelly, Olin Silander, Joep de Ligt
EPI_ISL_579418	Wellington SCL (WN)	Institute of Environmental Science and Research (ESR)	Xiaoyun Ren, Matt Storey, Nikki Freed, Muhammad Faisal, Jing Wang, Hermes Perez, Anja Werno, Antje van der Linden, Arlo Upton, Chris Mansell, David Hammer, Dragana Drinkovic, Gary McAuliffe, Hana Sofia Andersson, James Ussher, Jill Sherwood, Josh Freeman, Julia Howard, Juliet Elvy, Mary DeAlmeida, Matt Blakiston, Matthew Rogers, Max Bloomfield, Michael Addidle, Michelle Balm, Sally Roberts, Sarah Jefferies, Sharmini Muttaiyah, Susan Morpeth, Susan Taylor, Timothy Blackmore, Vani Sathyendran, Veronica Playle, Virginia Hope, Erasmus Smit, Lauren Jelly, Olin Silander, Joep de Ligt
EPI_ISL_579487, EPI_ISL_579488, EPI_ISL_579489, EPI_ISL_579490, EPI_ISL_579491, EPI_ISL_579492, EPI_ISL_579493, EPI_ISL_579494, EPI_ISL_579495	Canterbury Health Laboratories	Institute of Environmental Science and Research (ESR)	Xiaoyun Ren, Matt Storey, Nikki Freed, Muhammad Faisal, Jing Wang, Hermes Perez, Anja Werno, Antje van der Linden, Arlo Upton, Chris Mansell, David Hammer, Dragana Drinkovic, Gary McAuliffe, Hana Sofia Andersson, James Ussher, Jill Sherwood, Josh Freeman, Julia Howard, Juliet Elvy, Mary DeAlmeida, Matt Blakiston, Matthew Rogers, Max Bloomfield, Michael Addidle, Michelle Balm, Sally Roberts, Sarah Jefferies, Sharmini Muttaiyah, Susan Morpeth, Susan Taylor, Timothy Blackmore, Vani Sathyendran, Veronica Playle, Virginia Hope, Erasmus Smit, Lauren Jelly, Olin Silander, Joep de Ligt
EPI_ISL_579594, EPI_ISL_579595, EPI_ISL_579596, EPI_ISL_579597, EPI_ISL_579598	QEII Health Sciences Centre	National Microbiology Laboratory (NML)	Anna Majer, Shari Tyson, Grace Seo, Philip Mabon, Darian Hole, Elsie Grudeski, Rhiannon Huzarewich, Russell Mandes, Anneliese Landgraff, Jennifer Tanner, Natalie Knox, Morag Graham, Gary Van Domselaar, Todd Hatchette, Jason LeBlanc, Nathalie Bastien, Yan Li, Timothy Booth, CanCOGeN's metadata curation team, Public Health Agency of Canada's CanCOGeN team
EPI_ISL_581462	Fondation Congolaise pour la recherche medicale (FCRM)	NGS Competence Center Tübingen, Institut für Medizinische Mikrobiologie und Hygiene, Universitätsklinikum Tübingen	Angel Angelov
EPI_ISL_581482, EPI_ISL_581483	Medizinische Klinik Innere Medizin I, Universitätsklinikum Tübingen	NGS Competence Center Tübingen, Institut für Medizinische Mikrobiologie und Hygiene, Universitätsklinikum Tübingen	Angel Angelov
EPI_ISL_581486	Fondation Congolaise pour la recherche medicale (FCRM)	NGS Competence Center Tübingen, Institut für Medizinische Mikrobiologie und Hygiene, Universitätsklinikum Tübingen	Angel Angelov
EPI_ISL_581668, EPI_ISL_581669, EPI_ISL_581670, EPI_ISL_581671, EPI_ISL_581672, EPI_ISL_581673, EPI_ISL_581674, EPI_ISL_581813, EPI_ISL_581814, EPI_ISL_581815, EPI_ISL_581816			
see above	University Hospital Basel, Clinical Virology	University Hospital Basel, Clinical Bacteriology	Madlen Stange, Alfredo Mari, Tim Roloff, Helena MB Seth-Smith, Michael Schweitzer, Myrta Brunner, Karoline Leuzinger, Kirstine K. Soegaard, Alexander Gensch, Sarah Tschudin-Sutter, Simon Fuchs, Julia Bielicki, Hans Pargger, Martin Siegemund, Christian Nickel, Roland Bingisser, Michael Osthoff, Stefano Bassetti, Rita Schneider-Sliwa, Manuel Battegay, Hans Hirsch, Adrian Egli
EPI_ISL_582242, EPI_ISL_582244, EPI_ISL_582256, EPI_ISL_582257, EPI_ISL_582258, EPI_ISL_582273, EPI_ISL_582278, EPI_ISL_582308, EPI_ISL_582354	Cadham Provincial Laboratory	National Microbiology Laboratory (NML)	Anna Majer, Shari Tyson, Grace Seo, Philip Mabon, Elsie Grudeski, Rhiannon Huzarewich, Russell Mandes, Anneliese Landgraff, Jennifer Tanner, Natalie Knox, Morag Graham, Gary Van Domselaar, Paul Van Caeseele, Jared Bullard, David Alexander, Kerry Dust, Nathalie Bastien, Yan Li, Timothy Booth, Darian Hole, Madison Chapel, CanCOGeN's metadata curation team, Public Health Agency of Canada CanCOGeN team
EPI_ISL_582521	unknown	Instituto Nacional de Saude (INSA)	Borges et al
EPI_ISL_583472	Memorial Sloan Kettering Cancer Center	van Bakel Laboratory, Genetics and Genomics Sciences, Icahn School of Medicine at Mount Sinai	Teresa Aydillo, Ana S. Gonzalez-Reiche, Sadaf Aslam, Adriana van de Guchte, Zenab Khan, Ajay Obla, Jayeeta Dutta, Harm van Bakel, Judith Aberg, Adolfo García-Sastre, Gunjan Shah, Tobias Hohl, Genovefa Papanicolaou, Miguel-Angel Perales, Kent Sepkowitz, Ngolela Esther Babady, and Mini Kamboj
EPI_ISL_583570	Center for Virology, Medical University of Vienna	Bergthaler laboratory, CeMM Research Center for Molecular Medicine of the Austrian Academy of Sciences	Alexandra Popa, Benedikt Agerer, Henrique Colaco, Lukas Endler, Jakob-Wendelin Genger, Alexander Lercher, Mark Smyth, Thomas Penz, Michael Schuster, Jan Laine, Martin Senekowitsch, Judith Aberle, Stephan Aberle, Peter Hufnagl, Daniela Schmid, Franz Allerberger, Elisabeth Puchhammer-Stoeckl, Manfred Nairz, Guenter Weiss, Gregor Hörmann, Kinga Rigler-Hohenwarter, Rainer Gattringer, Wegene Borena, Dorothee von Laer, Gernot Walder, Peter Obrist, Christian Paar, Sabine Sussitz-Rack, Gunther Vogl, Adi Steinrigl, Christoph Bock, Andreas Bergthaler
EPI_ISL_583589	Institute for Medical and Chemical Laboratory Diagnostics, Kepler Universitätsklinikum	Bergthaler laboratory, CeMM Research Center for Molecular Medicine of the Austrian Academy of Sciences	Alexandra Popa, Benedikt Agerer, Henrique Colaco, Lukas Endler, Jakob-Wendelin Genger, Alexander Lercher, Mark Smyth, Thomas Penz, Michael Schuster, Jan Laine, Martin Senekowitsch, Judith Aberle, Stephan Aberle, Peter Hufnagl, Daniela Schmid, Franz Allerberger, Elisabeth Puchhammer-Stoeckl, Manfred Nairz, Guenter Weiss, Gregor Hörmann, Kinga Rigler-Hohenwarter, Rainer Gattringer, Wegene Borena, Dorothee von Laer, Gernot Walder, Peter Obrist, Christian Paar, Sabine Sussitz-Rack, Gunther Vogl, Adi Steinrigl, Christoph Bock, Andreas Bergthaler
EPI_ISL_583599	Zentralinstitut für medizinische und chemische Labordiagnostik, Universitätskliniken Insbruck	Bergthaler laboratory, CeMM Research Center for Molecular Medicine of the Austrian Academy of Sciences	Alexandra Popa, Benedikt Agerer, Henrique Colaco, Lukas Endler, Jakob-Wendelin Genger, Alexander Lercher, Mark Smyth, Thomas Penz, Michael Schuster, Jan Laine, Martin Senekowitsch, Judith Aberle, Stephan Aberle, Peter Hufnagl, Daniela Schmid, Franz Allerberger, Elisabeth Puchhammer-Stoeckl, Manfred Nairz, Guenter Weiss, Gregor Hörmann, Kinga Rigler-Hohenwarter, Rainer Gattringer, Wegene Borena, Dorothee von Laer, Gernot Walder, Peter Obrist, Christian Paar, Sabine Sussitz-Rack, Gunther Vogl, Adi Steinrigl, Christoph Bock, Andreas Bergthaler
EPI_ISL_583633, EPI_ISL_583640, EPI_ISL_583648, EPI_ISL_583649, EPI_ISL_583658, EPI_ISL_583678, EPI_ISL_583686	Austrian Agency for Health and Food Safety (AGES)	Bergthaler laboratory, CeMM Research Center for Molecular Medicine of the Austrian Academy of Sciences	Alexandra Popa, Benedikt Agerer, Henrique Colaco, Lukas Endler, Jakob-Wendelin Genger, Alexander Lercher, Mark Smyth, Thomas Penz, Michael Schuster, Jan Laine, Martin Senekowitsch, Judith Aberle, Stephan Aberle, Peter Hufnagl, Daniela Schmid, Franz Allerberger, Elisabeth Puchhammer-Stoeckl, Manfred Nairz, Guenter Weiss, Gregor Hörmann, Kinga Rigler-Hohenwarter, Rainer Gattringer, Wegene Borena, Dorothee von Laer, Gernot Walder, Peter Obrist, Christian Paar, Sabine Sussitz-Rack, Gunther Vogl, Adi Steinrigl, Christoph Bock, Andreas Bergthaler

EPI_ISL_583756, EPI_ISL_583757, EPI_ISL_583758, EPI_ISL_583759, EPI_ISL_583760, EPI_ISL_583815, EPI_ISL_583816, EPI_ISL_583817, EPI_ISL_583818, EPI_ISL_583819, EPI_ISL_583820, EPI_ISL_583821, EPI_ISL_583822			
see above	Dr. Gernot Walder GmbH	Bergthaler laboratory, CeMM Research Center for Molecular Medicine of the Austrian Academy of Sciences	Alexandra Popa, Benedikt Agerer, Henrique Colaco, Lukas Endler, Jakob-Wendelin Genger, Alexander Lercher, Mark Smyth, Thomas Penz, Michael Schuster, Jan Laine, Martin Senekowitsch, Judith Aberle, Stephan Aberle, Peter Hufnagl, Daniela Schmid, Franz Allerberger, Elisabeth Puchhammer-Stoeckl, Manfred Nairz, Guenter Weiss, Gregor Hörmann, Kinga Rigler-Hohenwarter, Rainer Gattringer, Wegene Borena, Dorothee von Laer, Gernot Walder, Peter Obrist, Christian Paar, Sabine Sussitz-Rack, Gunther Vogl, Adi Steinrigl, Christoph Bock, Andreas Bergthaler
EPI_ISL_584079	The National Institute of Public Health	State Veterinary Institute Prague	Nagy,A;Jirincova,H;Novakova,L;Trnka,D;Vecerova,J
EPI_ISL_585093, EPI_ISL_585130, EPI_ISL_585132, EPI_ISL_585134, EPI_ISL_585144, EPI_ISL_585145, EPI_ISL_585146, EPI_ISL_585147, EPI_ISL_585148, EPI_ISL_585149, EPI_ISL_585151, EPI_ISL_585152, EPI_ISL_585153, EPI_ISL_585174, EPI_ISL_585175, EPI_ISL_585176, EPI_ISL_585177, EPI_ISL_585178, EPI_ISL_585179, EPI_ISL_585180, EPI_ISL_585181, EPI_ISL_585182, EPI_ISL_585183, EPI_ISL_585184, EPI_ISL_585246, EPI_ISL_585248, EPI_ISL_585250, EPI_ISL_585252, EPI_ISL_585254, EPI_ISL_585256			
see above	Regional Virus Laboratory, Belfast Health and Social Care Trust	COVID-19 Genomics UK (COG-UK) Consortium	Conall McCaughey, James McKenna, Tanya Curran, Susan Feeney, Alison Watt, Ciara Cox, Mairead Connor, Zoltan Molnar, David Simpson, Derek Fairley
EPI_ISL_586408, EPI_ISL_586429, EPI_ISL_586439, EPI_ISL_586450, EPI_ISL_586451, EPI_ISL_586454, EPI_ISL_586457, EPI_ISL_586462, EPI_ISL_586464, EPI_ISL_586465	Toronto Invasive Bacterial Diseases Network	McMaster University	Allison McGeer, Patryk Aftanas, Hooman Derakhshani, Angel Li, Kuganya Nirmalarajah, Emily Panousis, Ahmed Draia, Jalees Nasir, Michael Surette, Samira Mubareka, Andrew G. McArthur
EPI_ISL_591081, EPI_ISL_591082, EPI_ISL_591093	Department of Pathology, University of Cambridge	Wellcome Sanger Institute for the COVID-19 Genomics UK (COG-UK) consortium	Luke W Meredith, M. Estée Török , Myra Hosmillo, William L. Hamilton, Martin D. Curran, Theresa Feltwell, Grant Hall, Anna Yakovleva, Fahad A Khokhar, Charlotte J. Houldcroft, Laura G Caller, Aminu S. Jahun, Sarah L. Caddy, Ian Goodfellow; and Alex Alderton, Roberto Amato, Sonia Goncalves, Ewan Harrison, David K. Jackson, Ian Johnston, Dominic Kwiatkowski, Cordelia Langford, John Sillitoe on behalf of the Wellcome Sanger Institute COVID-19 Surveillance Team
EPI_ISL_591135, EPI_ISL_591142, EPI_ISL_591144, EPI_ISL_591145, EPI_ISL_591146, EPI_ISL_591147, EPI_ISL_591149, EPI_ISL_591151, EPI_ISL_591153, EPI_ISL_591161, EPI_ISL_591162, EPI_ISL_591163, EPI_ISL_591167, EPI_ISL_591171, EPI_ISL_591172, EPI_ISL_591173, EPI_ISL_591174, EPI_ISL_591213, EPI_ISL_591214, EPI_ISL_591215, EPI_ISL_591217, EPI_ISL_591218, EPI_ISL_591219, EPI_ISL_591221, EPI_ISL_591226, EPI_ISL_591227, EPI_ISL_591228, EPI_ISL_591229, EPI_ISL_591230, EPI_ISL_591231, EPI_ISL_591232, EPI_ISL_591233, EPI_ISL_591234, EPI_ISL_591235, EPI_ISL_591236, EPI_ISL_591239, EPI_ISL_591241, EPI_ISL_591242, EPI_ISL_591243, EPI_ISL_591244, EPI_ISL_591248, EPI_ISL_591250, EPI_ISL_591251, EPI_ISL_591252			
see above	Toronto Invasive Bacterial Diseases Network	McMaster University	Allison McGeer, Patryk Aftanas, Hooman Derakhshani, Angel Li, Kuganya Nirmalarajah, Emily Panousis, Ahmed Draia, Jalees Nasir, Michael Surette, Samira Mubareka, Andrew G. McArthur
EPI_ISL_591316, EPI_ISL_591317, EPI_ISL_591318, EPI_ISL_591319, EPI_ISL_591324	Virology, Iran University of Medical Sciences	Virology, Iran University of Medical Sciences	Keyvani,H., Ranjbar,Mm., Keyvani,F., Soleimani,S.
EPI_ISL_591351, EPI_ISL_591361, EPI_ISL_591362, EPI_ISL_591363, EPI_ISL_591364, EPI_ISL_591365, EPI_ISL_591366, EPI_ISL_591367, EPI_ISL_591368, EPI_ISL_591369, EPI_ISL_591377, EPI_ISL_591378			
see above	Pathogen Genomics Center, National Institute of Infectious Diseases	Pathogen Genomics Center, National Institute of Infectious Diseases	Tsuyoshi Sekizuka, Kentaro Itokawa, Rina Tanaka, Masanori Hashino, Makoto Kuroda
EPI_ISL_591479, EPI_ISL_591480, EPI_ISL_591481, EPI_ISL_591482, EPI_ISL_591483	Pathogen Genomics Center, National Institute of Infectious Diseases	Pathogen Genomics Center, National Institute of Infectious Diseases	Tsuyoshi Sekizuka, Kentaro Itokawa, Rina Tanaka, Masanori Hashino, Hajime Kamiya, Tomoe Shimada, Makoto Kuroda
EPI_ISL_591536	Pathogen Genomics Center, National Institute of Infectious Diseases	Pathogen Genomics Center, National Institute of Infectious Diseases	Tsuyoshi Sekizuka, Kentaro Itokawa, Rina Tanaka, Masanori Hashino, Makoto Kuroda
EPI_ISL_593613, EPI_ISL_593633	unknown	Public Health Virology Laboratory, Forensic and Scientific Services (PHV-FSS)	Son Nguyen et al.
EPI_ISL_593957, EPI_ISL_593958, EPI_ISL_593959, EPI_ISL_593960, EPI_ISL_593961, EPI_ISL_593962, EPI_ISL_593963, EPI_ISL_593964, EPI_ISL_593966, EPI_ISL_593967, EPI_ISL_593968, EPI_ISL_593969, EPI_ISL_593970, EPI_ISL_593971, EPI_ISL_593973, EPI_ISL_593974, EPI_ISL_593975			
see above	Delaware Public Health Lab	Delaware Public Health Lab	Gregory Hovan
EPI_ISL_596378, EPI_ISL_596380, EPI_ISL_596381, EPI_ISL_596383	Virology, Iran University of Medical Sciences	Virology, Iran University of Medical Sciences	Keyvani,H., Ranjbar,Mm., Keyvani,F., Soleimani,S.
EPI_ISL_596513, EPI_ISL_596515, EPI_ISL_596516, EPI_ISL_596517, EPI_ISL_596518	Palestinian Ministry of Health	Molecular Genetics Lab	Nouar Qutob, Zaidoun Salah, Damien Richard, Hisham Darwish, Husam Sallam, Issa Shtayeh, Osama Najjar, Mahmoud Ruzayqat, Dana Najjar, Francois Balloux, Lucy van Dorp
EPI_ISL_596656, EPI_ISL_596657, EPI_ISL_596665, EPI_ISL_596675	St.Vincent's University Hospital	St.Vincent's University Hospital	Mary Lucey, Guerrino Macori, Niamh Mullane, Una Sutton-Fitzpatrick, Gabriel Gonzalez, Suzie Coughlan, Aisling Purcell, Lynda Fenelon, Séamus Fanning, Kirsten Schaffer
EPI_ISL_596677, EPI_ISL_596684, EPI_ISL_596707, EPI_ISL_596754, EPI_ISL_596755, EPI_ISL_596761, EPI_ISL_596776, EPI_ISL_596794, EPI_ISL_596796, EPI_ISL_596797, EPI_ISL_596798, EPI_ISL_596812, EPI_ISL_596816, EPI_ISL_596825, EPI_ISL_596849, EPI_ISL_596852, EPI_ISL_596870			
see above	PathWest Laboratory Medicine WA	PathWest Laboratory Medicine WA Microbial Surveillance Unit	PathWest Laboratory Medicine WA Microbial Surveillance Unit
EPI_ISL_602282, EPI_ISL_602283, EPI_ISL_602287	Evangelisches Klinikum Bethel, Institut für Laboratoriumsmedizin, Mikrobiologie und Hygiene	Bielefeld University	David Brandt, Tobias Busche, Markus Haak, Jörn Kalinowski, Levin-Joe Klages, Christiane Scherer, Alexander Sczyrba, Marina Simunovic, Svenja Vinke
EPI_ISL_602467	Institute for Virology, University Hospital Essen	Center of Medical Microbiology, Virology, and Hospital Hygiene, University of Duesseldorf	Olympia E. Anastasiou, Ulf Dittmer, Maximilian Damagnez, Alexander Dilthey, Torsten Houwaart, Lisanna Hülse, Malte Kohns Vasconcelos, Nadine Lübke, Jessica Nicolai, Klaus Pfeffer, Daniel Strelow, Jörg Timm, Andreas Walker, Tobias Wienemann
EPI_ISL_605058, EPI_ISL_605061, EPI_ISL_605062, EPI_ISL_605063, EPI_ISL_605065, EPI_ISL_605066, EPI_ISL_605072, EPI_ISL_605078	National Virus Reference Laboratory	Irish Coronavirus Sequencing Consortium - Helixworks	Sachin Chalapati, Conor Crosbie, Nimesh Pinnamaneni
EPI_ISL_605793	Department of Experimental Modeling and Pathogenesis of Infectious Diseases	WHO National Influenza Centre Russian Federation	Andrey Komissarov, Artem Fadeev, Anna Ivanova, Kseniya Komissarova, Sobolev I.A., Alekseev A.Yu., Chepurnov A.A., Kononova Yu.V., Shestopalov A.M.
EPI_ISL_605794	Department of Experimental Modeling and Pathogenesis of Infectious Diseases	WHO National Influenza Centre Russian Federation	Andrey Komissarov, Artem Fadeev, Anna Ivanova, Kseniya Komissarova, Sobolev I.A., Alekseev A.Yu., Kononova Yu.V., Shestopalov A.M.
EPI_ISL_605795, EPI_ISL_605796	Department of Experimental Modeling and Pathogenesis of Infectious Diseases	WHO National Influenza Centre Russian Federation	Andrey Komissarov, Artem Fadeev, Anna Ivanova, Kseniya Komissarova, Sobolev I.A., Alekseev A.Yu., Chepurnov A.A., Kononova Yu.V., Shestopalov A.M.
EPI_ISL_605803, EPI_ISL_605811, EPI_ISL_605812, EPI_ISL_605816	Clinical Virology Laboratory, Institute of Liver and Biliary Sciences	ILBS - IGIB	Ekta Gupta, Sheetalnath Rooge, Abhishek Padhi, Reshu Agarwal, Jaswinder Singh Maras, Shridhar Sivasubbu, Vinod Scaria, Shvetank Sharma
EPI_ISL_605868	PathWest Laboratory Medicine WA	PathWest Laboratory Medicine WA Microbial Surveillance Unit	PathWest Laboratory Medicine WA Microbial Surveillance Unit
EPI_ISL_610237, EPI_ISL_610238	Molecular diagnostic laboratory of Federal Budget Institution of Science "Central Research Institute of Epidemiology" of The Federal Service on Customers' Rights Protection and Human Well-being Surveillance	Group of Genomics and Postgenomic Technologies of Central Research Institute of Epidemilology	Samoilov AE, Kaptelova VV, Bukharina AY, Speranskaya AS, Tivanova EV, Shipulina OY, Akimkin VG
EPI_ISL_611653, EPI_ISL_611672, EPI_ISL_611845, EPI_ISL_611853, EPI_ISL_612017, EPI_ISL_612191, EPI_ISL_612416, EPI_ISL_612419, EPI_ISL_612422, EPI_ISL_612425, EPI_ISL_612428, EPI_ISL_612429, EPI_ISL_612430, EPI_ISL_612431			
see above	Liverpool Clinical Laboratories	COVID-19 Genomics UK (COG-UK) Consortium	Sam Haldenby, Anita Lucaci, Steve Paterson, Julian Hiscox, Alistair Darby, M Almsaud, A Alrezaihi, Muhannad Alruwaili, Stuart D Armstrong, Jones Benjamin, Eleanor G Bentley, Anu Chawla, Jordan J Clark, Angela Cowell, Richard Eccles, Isabel García-Dorival, Matthew Gemmell, Alessandro Gerada,

			PKF Gilmore, Richard Gregory, Ximeng Han, Catherine Hartley, Margaret Hughes, Miren Iturriza-Gomara, James Johnson, L Luu, Jenifer Manson, Charlotte Nelson, Elaine O'Toole, Cassie Olateju, Rebekah Penrice-Randal , Lucille Rainbow, N.P Randle, Trevor Ian Robinson, Parul Sharma, Ghada T Shawli, James P Stewart, Neil Swainston, Ecaterina Vamos, Joanne Watts, Mark Whitehead
EPI_ISL_613445	Institut Pasteur de la Guadeloupe	Institut Pasteur de la Guadeloupe	Marion Barbet, Sylvie Behillil, Méline Bizard, Angela Brisebarre, Camille Capel, Etienne Simon-Lorière, Vincent Enouf, Maud Vanpeene, Sylvie van der Werf, Stéphanie Guyomard, Sébastien Breurec, Antoine Talarmin
EPI_ISL_613563	Laboratory of Molecular Biology, Blood Center of Ribeirão Preto	Laboratory of Molecular Biology, Blood Center of Ribeirão Preto, Faculty of Medicine of Ribeirão Preto, University of São Paulo	Svetoslav N Slavov, Marta Giovanetti, Vagner Fonseca, Elaine V Santos, Evandra S Rodrigues, Talita Adelino, Joilson Xavier, Glauco de Carvalho Pereira, Aparecida Y Yamamoto, Diego Villa Clé, Rodrigo T Calado; Dimas T Covas, Luiz CJ Alcantara, Simone Kashima
EPI_ISL_613711, EPI_ISL_613951, EPI_ISL_613965, EPI_ISL_614011, EPI_ISL_614156	Laboratory of Molecular Biology, Blood Center of Ribeirão Preto, Faculty of Medicine of Ribeirão Preto, University of São Paulo	Laboratory of Molecular Biology, Blood Center of Ribeirão Preto, Faculty of Medicine of Ribeirão Preto, University of São Paulo	Svetoslav N Slavov, Marta Giovanetti, Vagner Fonseca, Elaine V Santos, Evandra S Rodrigues, Talita Adelino, Joilson Xavier, Glauco de Carvalho Pereira, Aparecida Y Yamamoto, Diego Villa Clé, Rodrigo T Calado; Dimas T Covas, Luiz CJ Alcantara, Simone Kashima
EPI_ISL_622788, EPI_ISL_622800, EPI_ISL_622801	Canterbury Health Laboratories	Institute of Environmental Science and Research (ESR)	Xiaoyun Ren, Matt Storey, Nikki Freed, Muhammad Faisal, Jing Wang, Hermes Perez, Anja Werno, Antje van der Linden, Arlo Upton, Chris Mansell, David Hammer, Dragana Drinkovic, Gary McAuliffe, Hana Sofia Andersson, James Ussher, Jill Sherwood, Josh Freeman, Julia Howard, Juliet Elvy, Mary DeAlmeida, Matt Blakiston, Matthew Rogers, Max Bloomfield, Michael Addidle, Michelle Balm, Sally Roberts, Sarah Jefferies, Sharmini Muttaiyah, Susan Morpeth, Susan Taylor, Timothy Blackmore, Vani Sathyendran, Veronica Playle, Virginia Hope, Erasmus Smit, Lauren Jelly, Olin Silander, Joep de Ligt
EPI_ISL_623126	Laboratorio de Virologia Molecular / UFRJ	Bioinformatics Laboratory / LNCC	Carolina M Voloch, Ronaldo S Francisco Jr, Luiz G P de Almeida, Otavio J. Brustolini, Cynthia C Cardoso, Alexandra L Gerber, Ana Paula de C Guimarães, Diana Mariani, Covid19-UFRJ Workgroup, Luís Cristóvão Pôrto, Renato S Aguiar, Terezinha M P P Castiñeiras, Orlando C. Ferreira, Amilcar Tanuri, Ana Tereza R de Vasconcelos
EPI_ISL_623181, EPI_ISL_623182	Utah Public Health Laboratory	Utah Public Health Laboratory	Erin Young, Kelly Oakeson
EPI_ISL_626228, EPI_ISL_626229	Institute for Virology, University Hospital Essen	Center of Medical Microbiology, Virology, and Hospital Hygiene, University of Duesseldorf	Olympia E. Anastasiou, Ulf Dittmer, Maximilian Damagnez, Alexander Dilthey, Torsten Houwaart, Lisanna Hülse, Malte Kohns Vasconcelos, Nadine Lübke, Jessica Nicolai, Klaus Pfeffer, Daniel Strelow, Jörg Timm, Andreas Walker, Tobias Wienemann
EPI_ISL_626513, EPI_ISL_626514, EPI_ISL_626515, EPI_ISL_626516	Northwestern Memorial Hospital	Ozer Lab	Ramon Lorenzo-Redondo, Hannah H. Nam, Scott C. Roberts, Lacy M. Simons, Chad J. Achenbach, Lawrence J. Jennings, Chao Qi, Alan R. Hauser, Michael G. Ison, Judd F. Hultquist, Egon A. Ozer
EPI_ISL_629096	Laboratoire du Centre Hospitalier Annecy Genevois	CNR Virus des Infections Respiratoires - France SUD	Antonin Bal, Gregory Destras, Gwendolyne Burfin, Hadrien Règue, Quentin Semanas, Martine Valette, Bruno Lina, Hélène Petitprez, Bruno Chanzy, Laurence Josset
EPI_ISL_631390	Wisconsin State Laboratory of Hygiene Communicable Disease Division	Wisconsin State Laboratory of Hygiene Communicable Disease Division	Kelsey R. Florek, Abigail C. Shockey
EPI_ISL_631613	Jamaica Hospital Medical Center	New York City Public Health Laboratory	Jade Wang, et al.
EPI_ISL_631614	Flushing Hospital Medical Center	New York City Public Health Laboratory	Jade Wang, et al.
EPI_ISL_631615	Jamaica Hospital Medical Center	New York City Public Health Laboratory	Jade Wang, et al.
EPI_ISL_631616	Flushing Hospital Medical Center	New York City Public Health Laboratory	Jade Wang, et al.
EPI_ISL_631617, EPI_ISL_631762	OCME Office Of Chief Medical Examiner	New York City Public Health Laboratory	Jade Wang, et al.
EPI_ISL_631904	Jamaica Hospital Medical Center	New York City Public Health Laboratory	Jade Wang, et al.
EPI_ISL_631905	Richmond University Medical Center	New York City Public Health Laboratory	Jade Wang, et al.
EPI_ISL_631906	Flushing Hospital Medical Center	New York City Public Health Laboratory	Jade Wang, et al.
EPI_ISL_631917	St Barnabas Hospital	New York City Public Health Laboratory	Jade Wang, et al.
EPI_ISL_631918	Flushing Hospital Medical Center	New York City Public Health Laboratory	Jade Wang, et al.
EPI_ISL_631919, EPI_ISL_631920	Richmond University Medical Center	New York City Public Health Laboratory	Jade Wang, et al.
EPI_ISL_631953	NYC HH Lincoln Medical And Mental Health Center	New York City Public Health Laboratory	Jade Wang, et al.
EPI_ISL_631954, EPI_ISL_631992, EPI_ISL_632031, EPI_ISL_632032, EPI_ISL_632033	Richmond University Medical Center	New York City Public Health Laboratory	Jade Wang, et al.
EPI_ISL_632220	Flushing Hospital Medical Center	New York City Public Health Laboratory	Jade Wang, et al.
EPI_ISL_632866, EPI_ISL_632867, EPI_ISL_632889	Idaho Bureau of Laboratories	Center for Global Health, University of New Mexico Health Sciences Center	Daryl Domman, Kurt Schwalm, Matthew Burns, Robert Voermans, Christopher Ball, Darrell Dinwiddie
EPI_ISL_632908	Genomic Sciences, Rehman Medical Institute	Genomic Sciences, Rehman Medical Institute	Ali,J., Afridi,U.K., Haider,S.A., Sabiha,B., Jan,H. and Jehanzeb,V.
EPI_ISL_634829	Laboratoire de virologie, CHU de Grenoble - CS 10217 - 38043 Grenoble cedex 21	CNR Virus des Infections Respiratoires - France SUD	Antonin Bal, Gregory Destras, Gwendolyne Burfin, Hadrien Règue, Quentin Semanas, Martine Valette, Bruno Lina, Sylvie Larrat, Laurence Josset
EPI_ISL_634830	Laboratoire de virologie, CHU de Grenoble - CS 10217 - 38043 Grenoble cedex 22	CNR Virus des Infections Respiratoires - France SUD	Antonin Bal, Gregory Destras, Gwendolyne Burfin, Hadrien Règue, Quentin Semanas, Martine Valette, Bruno Lina, Sylvie Larrat, Laurence Josset
EPI_ISL_634836	Laboratoire de virologie, CHU de Grenoble - CS 10217 - 38043 Grenoble cedex 28	CNR Virus des Infections Respiratoires - France SUD	Antonin Bal, Gregory Destras, Gwendolyne Burfin, Hadrien Règue, Quentin Semanas, Martine Valette, Bruno Lina, Sylvie Larrat, Laurence Josset
EPI_ISL_635278, EPI_ISL_635280, EPI_ISL_635281, EPI_ISL_635283, EPI_ISL_635284	Institute of Microbiology and Immunology, Faculty of Medicine, University of Ljubljana	Institute of Microbiology and Immunology, Faculty of Medicine, University of Ljubljana	Tomaž Mark Zorec, Samo Zakotnik, Miša Korva, Tatjana Avši - Županc, Mario Poljak
EPI_ISL_636542, EPI_ISL_636565	Dutch COVID-19 response team	National Institute for Public Health and the Environment (RIVM)	Adam Meijer, Harry Vennema, Jeroen Cremer, Sharon van den Brink, Bas van der Veer, AnneMarie van den Brandt, Florian Zwagemaker, Dennis Schmitz, Chantal Reusken, on behalf of the national COVID-19 response team
EPI_ISL_636946, EPI_ISL_636947	Public Health Ontario Laboratory	Public Health Ontario Laboratory	Vanessa G Allen, Philip Banh, Richard de Borja, Yao Chen, Alireza Eshaghi, Nahuel Fittipaldi, Christine Frantz, Jonathan B Gubbay, Jennifer L Guthrie, Lawrence Heisler, Esha Joshi, Michael Laszloffy, Aimin Li, Michael CY Li, Dean Maxwell, Sandeep Nagra, Samir N Patel, Heather Rilkoff, Jared Simpson, Karthikeyan Sivaraman, Yogi Sundaravadanam, Sarah Teatero, Andre Villegas, Sandra Zittermann
EPI_ISL_636969, EPI_ISL_636970	Etlik Veterinary Control Central Research Institute	Etlik Veterinary Control Central Research Institute	Sabri Hacioglu, Ahu Pakdemirli, Dilek Dulger, Erdem Danyer, Ummu Sena Sari, Cevdet Yarali, Ozcan Yildirim
EPI_ISL_636990	Department of Infectious Diseases and Immunology, National Hospital Organization Nagoya Medical Center	Clinical Research Center, National Hospital Organization Nagoya Medical Center	Yoshihiro Nakata, Hirotaka Ode, Mai Kubota, Masakazu Matsuda, Kazuhiro Matsuoka, Miho Nakasuji, Mikiko Mori, Mayumi Imahashi, Yoshiyuki Yokomaku, Yasumasa Iwatani
EPI_ISL_637333	Wales Specialist Virology Centre Sequencing lab: Pathogen Genomics Unit	COVID-19 Genomics UK (COG-UK) Consortium	Catherine Moore, Johnathan Evans, Laura Gifford, Malorie Perry, Simon Cottrell, Angela Marchbank, Alec Birchley, Alexander Adams, Amy Gaskin, Bree Gatica-Wilcox, Jason Coombes, Joel Southgate, Lauren Gilbert, Lee Graham, Nicole Pacchiarini, Sara Kumziene-Summerhayes, Sarah Taylor, Sophie Jones, Sara Rey, Matthew Bull, Joanne Watkins, Sally Corden, Tom Connor
EPI_ISL_639727, EPI_ISL_639728	Public Health Ontario Laboratory	Public Health Ontario Laboratory	Vanessa G Allen, Philip Banh, Richard de Borja, Yao Chen, Alireza Eshaghi, Nahuel Fittipaldi, Christine Frantz, Jonathan B Gubbay, Jennifer L Guthrie, Lawrence Heisler, Esha Joshi, Michael Laszloffy, Aimin Li, Michael CY Li, Dean Maxwell, Sandeep Nagra, Samir N Patel, Heather Rilkoff, Jared Simpson, Karthikeyan Sivaraman, Yogi Sundaravadanam, Sarah Teatero, Andre Villegas, Sandra Zittermann

EPI_ISL_639755, EPI_ISL_639758, EPI_ISL_639759, EPI_ISL_639760, EPI_ISL_639761, EPI_ISL_639762, EPI_ISL_639816, EPI_ISL_639817	unknown	Public Health Virology Laboratory, Forensic and Scientific Services (PHV-FSS)	Son Nguyen et al.
EPI_ISL_640044	District 6 CDC wc DSI	NHLS/UCT	Arash Iranzadeh, Deelan Doolabh, Lynn Tyers, Bruna Galvao, Innocent Mudau, Marvin Hsiao, Kruger Marais, Diana Hardie, Stephen Korsman, Carolyn Williamson
EPI_ISL_640045	Groote Schuur Hospital wc GSH	NHLS/UCT	Arash Iranzadeh, Deelan Doolabh, Lynn Tyers, Bruna Galvao, Innocent Mudau, Marvin Hsiao, Kruger Marais, Diana Hardie, Stephen Korsman, Carolyn Williamson
EPI_ISL_641309, EPI_ISL_641310	unknown	Public Health Virology Laboratory, Forensic and Scientific Services (PHV-FSS)	Son Nguyen et al.
EPI_ISL_641319	Environmental and Global Health, University of Florida	Environmental and Global Health, University of Florida	Elbadry,M.A., Subramaniam,K., Waltzek,T.B., Loeb,J.C., Stephenson,C.J., Lauzardo,M., Morris,J.G., Lednicky,J.A.
EPI_ISL_644214, EPI_ISL_644225, EPI_ISL_644227, EPI_ISL_644228	CEPHR / Vincent's Hospital	Irish Coronavirus Sequencing Consortium - National Virus Reference Laboratory	Michael Carr, Gabriel Gonzalez, Alejandro Abner Garcia Leon, Patrick Mallon
EPI_ISL_644675	CHU de Limoges	CNR Virus des Infections Respiratoires - France SUD	Antonin Bal, Gregory Destras, Gwendolyne Burfin, Hadrien Règue, Quentin Semanas, Martine Valette, Bruno Lina, Sylvie Rogez, Laurence Josset
EPI_ISL_644753, EPI_ISL_644757, EPI_ISL_644772, EPI_ISL_644773, EPI_ISL_644774, EPI_ISL_644781, EPI_ISL_644783, EPI_ISL_644784, EPI_ISL_644785, EPI_ISL_644786, EPI_ISL_644787, EPI_ISL_644788, EPI_ISL_644789, EPI_ISL_644822			
see above	National Microbiology Reference Laboratory	Quadram Institute Bioscience	Thanh Le Viet, Andrew J. Page, Justin O'Grady, Gemma Kay, David Baker, Gaetan Thilliez, Ana-Victoria Gutierrez, Robert Kingsley, Leonardo de Oliveira Martins, Sekesai Zinyowera, Tatenda Takawira, Muchaneta Mugabe, Gibson Mhlanga, Portia Manangazira, Andrew Tarupiwa, Hlanai Gumbo, Agnes Juru, Charles Nyagupe, Alexander Goredema, Isaac Phiri, Barbra Murwira, Beuty Makamure, Tapfumanei Mashe
EPI_ISL_645182	CHU de Limoges	CNR Virus des Infections Respiratoires - France SUD	Antonin Bal, Gregory Destras, Gwendolyne Burfin, Hadrien Règue, Quentin Semanas, Martine Valette, Bruno Lina, Sylvie Rogez, Laurence Josset
EPI_ISL_648030, EPI_ISL_648031, EPI_ISL_648032, EPI_ISL_648033, EPI_ISL_648041	MS Public Health Laboratory	Pathogen Discovery, Respiratory Viruses Branch, Division of Viral Diseases, Centers for Disease Control and Prevention	Yan Li, Jing Zhang, Ying Tao, Brian Lynch, Krista Queen, Anna Montmayeur, Anna Uehara, Clinton R. Paden, Rachel Marine, Haibin Wang, Suxiang Tong
EPI_ISL_648219, EPI_ISL_648220, EPI_ISL_648221, EPI_ISL_648222, EPI_ISL_648223, EPI_ISL_648224, EPI_ISL_648225, EPI_ISL_648226, EPI_ISL_648227, EPI_ISL_648228, EPI_ISL_648229, EPI_ISL_648230, EPI_ISL_648231, EPI_ISL_648232, EPI_ISL_648233, EPI_ISL_648234, EPI_ISL_648236, EPI_ISL_648237, EPI_ISL_648238, EPI_ISL_648239, EPI_ISL_648240, EPI_ISL_648241, EPI_ISL_648242, EPI_ISL_648243, EPI_ISL_648244, EPI_ISL_648245, EPI_ISL_648246, EPI_ISL_648247, EPI_ISL_648248, EPI_ISL_648249, EPI_ISL_648250, EPI_ISL_648251, EPI_ISL_648252, EPI_ISL_648253, EPI_ISL_648254, EPI_ISL_648255, EPI_ISL_648256, EPI_ISL_648257, EPI_ISL_648258, EPI_ISL_648259, EPI_ISL_648260, EPI_ISL_648261, EPI_ISL_648262, EPI_ISL_648263			
see above	Texas Department of State Health Services	Texas Department of State Health Services	Rashmi Tuladhar, Bonnie Oh, Jenny Zhang, Maliha Rahman, Anita Pokharel, Myong Koag, Chung Wang, Rachel Lee, Grace Kubin, Mayela Pedrueza, James Daniel Bonser
EPI_ISL_648555, EPI_ISL_648556	Utah Public Health Laboratory	Utah Public Health Laboratory	Erin Young, Kelly Oakeson
EPI_ISL_649176, EPI_ISL_649177, EPI_ISL_649178, EPI_ISL_649179	CHU Nantes	CNR Virus des Infections Respiratoires - France SUD	Antonin Bal, Louise Castain, Gregory Destras, Gwendolyne Burfin, Hadrien Règue, Quentin Semanas, Martine Valette, Bruno Lina, Celine Bressollette, Laurence Josset
EPI_ISL_653185, EPI_ISL_653186	Florida Bureau of Public Health Laboratories	Florida Bureau of Public Health Laboratories	Sarah Schmedes, Jason Blanton
EPI_ISL_654625, EPI_ISL_654663, EPI_ISL_654664, EPI_ISL_654665, EPI_ISL_654666, EPI_ISL_654667	Servicio de Microbiología, Hospital Universitario Central de Asturias	SeqCOVID-SPAIN consortium/IBV(CSIC)	Cristián Castelló Abietar, Jose A. Boga, Susana Rojo-Alba, Marta Elena Álvarez-Argüelles, Santiago Melón and SeqCOVID-SPAIN consortium
EPI_ISL_654881	Pasteur Institute in Ho Chi Minh city	Department of Microbiology and Immunology - Pasteur Institute in Ho Chi Minh city	Lng Chn Quang, ào Huy Mnh, Phm Th Thu Hng, V Phm Hng Nhung, Cao Minh Thng, Hunh Th Kim Loan, Nguyn Hoàng Quân, Hunh Phng Tho, Hoàng Nh ào, Nguyn Trung Hiu, Nguyn Hoàng Anh, Nguyn Thu Ngc, Phm Th Nhung, Nguyn Th Ngc Tho, ng Thanh Giang, Nguyn Th Thanh Thng, Hoàng Minh, Nguyn Th Phng Thúy, Nguyn Thanh Long, Phm Duy Quang, Hoàng Quc Cng, Nguyn V Thng, Phan Trng Lân
EPI_ISL_654900, EPI_ISL_654901, EPI_ISL_654902, EPI_ISL_654903, EPI_ISL_654904, EPI_ISL_654905, EPI_ISL_654906, EPI_ISL_654907, EPI_ISL_654908, EPI_ISL_654909, EPI_ISL_654910, EPI_ISL_654913			
see above	Texas Department of State Health Services	Texas Department of State Health Services	Rashmi Tuladhar, Bonnie Oh, Jenny Zhang, Maliha Rahman, Anita Pokharel, Myong Koag, Chung Wang, Rachel Lee, Grace Kubin, Mayela Pedrueza, James Daniel Bonser
EPI_ISL_657450, EPI_ISL_657452, EPI_ISL_657455, EPI_ISL_657458	Servicio de Microbiología. Hospital General Universitario de Castellón	SeqCOVID-SPAIN consortium/IBV(CSIC)	Rosario Moreno Muñoz, María Dolores Tirado Balaguer and SeqCOVID-SPAIN consortium
EPI_ISL_660378, EPI_ISL_660383	Orebro klinisk mikrobiologi	The Public Health Agency of Sweden	Anna-Malin Linde, Maria Lind Karlberg, Mattias Haukland, Reza Advani, Olov Svartstrom, Oskar Karlsson Lindsjo, Sandra Broddesson, Petra Edquist, Mia Brytting, Anna Risberg, Karin Tegmark-Wisell
EPI_ISL_660395, EPI_ISL_660396, EPI_ISL_660397, EPI_ISL_660398, EPI_ISL_660399	Klinsisk mikrobiologi Linkoping	The Public Health Agency of Sweden	Anna-Malin Linde, Maria Lind Karlberg, Mattias Haukland, Reza Advani, Olov Svartstrom, Oskar Karlsson Lindsjo, Sandra Broddesson, Petra Edquist, Mia Brytting, Anna Risberg, Karin Tegmark-Wisell
EPI_ISL_660737	Unité des Virus Émergents	CNR Virus des Infections Respiratoires - France SUD	Antonin Bal, Gregory Destras, Gwendolyne Burfin, Hadrien Règue, Quentin Semanas, Martine Valette, Bruno Lina, Laetitia Ninove, Léa Luciani, Antoine Nougairède, Laurence Josset
EPI_ISL_661200	Scientific Veterinary Institute Novi Sad	Veterinary Specialized Institute "Kraljevo", Serbia	Vidanovic,D., Tesovic,B., Knezevic,A., Jovanovic,T., Jankovic,M., Sekler,M., Banovic Djeri,B., Petrovic,T., Volkening,J., Afonso,C.
EPI_ISL_663264, EPI_ISL_663265, EPI_ISL_663266, EPI_ISL_663267, EPI_ISL_663268, EPI_ISL_663269, EPI_ISL_663270	CHU Nantes	CNR Virus des Infections Respiratoires - France SUD	Antonin Bal, Louise Castain, Gregory Destras, Gwendolyne Burfin, Hadrien Règue, Quentin Semanas, Martine Valette, Bruno Lina, Virginie Ferré, Celine Bressollette, Laurence Josset
EPI_ISL_664188, EPI_ISL_664196, EPI_ISL_664236, EPI_ISL_664651, EPI_ISL_664728, EPI_ISL_665207	University College London Hospital	COVID-19 Genomics UK (COG-UK) Consortium	Judith Heaney, Matthew Byott, Catherine Houlihan, Dan Frampton, Stuart Kirk, Moira Spyer and Eleni Nastouli
EPI_ISL_666662, EPI_ISL_666663, EPI_ISL_666664, EPI_ISL_666665	Laboratoire du Centre Hospitalier Annecy Genevois	CNR Virus des Infections Respiratoires - France SUD	Antonin Bal, Gregory Destras, Gwendolyne Burfin, Hadrien Règue, Quentin Semanas, Martine Valette, Bruno Lina, Hélène Petitprez, Bruno Chanzy, Laurence Josset
EPI_ISL_666694	National Virus Reference Lab (NVRL)	Irish Coronavirus Sequencing Consortium-Teagasc Grange	Matthew McCabe, Calum Walsh, Fiona Crispie, Paul Cotter, Michael Carr, Aljandro Abner Garcia Leon
EPI_ISL_671379, EPI_ISL_671380	National Virus Reference Laboratory	Irish Coronavirus Sequencing Consortium - Teagasc Moorepark	Calum Walsh, Fiona Crispie, John Kenny, Paul Cotter
EPI_ISL_672172, EPI_ISL_672184	The Ashley Laboratory, Stanford University	Chan-Zuckerberg Biohub	CZB Cliahub Consortium
EPI_ISL_672540, EPI_ISL_672541, EPI_ISL_672550, EPI_ISL_672552, EPI_ISL_672553, EPI_ISL_672554, EPI_ISL_672555, EPI_ISL_672556, EPI_ISL_672558, EPI_ISL_672559	Utah Public Health Laboratory	Utah Public Health Laboratory	Erin Young, Kelly Oakeson
EPI_ISL_672672, EPI_ISL_672686	DB Diagnosticos do Brasil	Laboratório de Parasitologia Médica - Instituto de Medicina Tropical - Universidade de São Paulo	Brazil-UK Centre for Arbovirus Discovery Diagnosis Genomics and Epidemiology (CADDE) Genomic Network - Instituto de Medicina Tropical
EPI_ISL_676576	Scientific Veterinary Institute Novi Sad	Veterinary Specialized Institute "Kraljevo", Serbia	Vidanovic,D., Tesovic,B., Knezevic,A., Jovanovic,T., Jankovic,M., Sekler,M., Banovic Djeri,B., Petrovic,T., Volkening,J., Afonso,C.

EPI_ISL_676655, EPI_ISL_676792, EPI_ISL_676794, EPI_ISL_676795, EPI_ISL_676796	Wadsworth Center, New York State Department.of Health	Wadsworth Center, New York State Department.of Health	Kirsten St. George, Daryl M. Lamson, Alexis Russel, Jonathan Plitnick, Navjot Singh, John Kelly, Sara Griesemer, Erasmus Schneider, Erica Lasek-Nesselquist
EPI_ISL_677944	Pathogen Genomics Lab King Abdullah University of Science and Technology(KAUST)	Pathogen Genomics Lab King Abdullah University of Science and Technology(KAUST)	Sara Mfarrej, Olga Douvropoulou, Raushan Nugmanova, Sharif Hala, Raeece Naeem, Afrah Alsomali, Fadwa Alofi, Asim Khogeer, Afrah Alsomali, Jumana Taha, Abdulaziz Alahmadi, Kahled Algithami, Anwar Hashem, Naif Almontashiri, Arnab Pain
EPI_ISL_677946	Pathogen Genomics Lab King Abdullah University of Science and Technology(KAUST)	Pathogen Genomics Lab King Abdullah University of Science and Technology(KAUST)	Fathia Ben Rached, Sharif Hala, Amit Kumar Subudhi, Sara Mfarrej, Raeece Naeem, Rahul P Salunke, Fadwa Alofi, Afrah Alsomali, Asim Khogeer, Jumana Taha, Abdulaziz Alahmadi, Kahled Algithami, Anwar Hashem, Naif Almontashiri, Arnab Pain
EPI_ISL_677947	Pathogen Genomics Lab King Abdullah University of Science and Technology(KAUST)	Pathogen Genomics Lab King Abdullah University of Science and Technology(KAUST)	Sara Mfarrej, Amanda Ooi, Luke Esau, Sharif Hala, Raeece Naeem, Fadwa Alofi, Afrah Alsomali, Asim Khogeer, Jumana Taha, Abdulaziz Alahmadi, Kahled Algithami, Anwar Hashem, Naif Almontashiri, Arnab Pain
EPI_ISL_677955	Pathogen Genomics Lab King Abdullah University of Science and Technology(KAUST)	Pathogen Genomics Lab King Abdullah University of Science and Technology(KAUST)	Sara Mfarrej, Olga Douvropoulou, Raushan Nugmanova, Sharif Hala, Raeece Naeem, Amanda Ooi, Luke Esau, Fadwa Alofi, Afrah Alsomali, Asim Khogeer, Jumana Taha, Abdulaziz Alahmadi, Kahled Algithami, Anwar Hashem, Naif Almontashiri, Arnab Pain
EPI_ISL_677958, EPI_ISL_677960, EPI_ISL_677963	Pathogen Genomics Lab King Abdullah University of Science and Technology(KAUST)	Pathogen Genomics Lab King Abdullah University of Science and Technology(KAUST)	Fathia Ben Rached, Sharif Hala, Amit Kumar Subudhi, Sara Mfarrej, Raeece Naeem, Rahul P Salunke, Fadwa Alofi, Afrah Alsomali, Asim Khogeer, Jumana Taha, Abdulaziz Alahmadi, Kahled Algithami, Anwar Hashem, Naif Almontashiri, Arnab Pain
EPI_ISL_677966	Pathogen Genomics Lab King Abdullah University of Science and Technology(KAUST)	Pathogen Genomics Lab King Abdullah University of Science and Technology(KAUST)	Sara Mfarrej, Amanda Ooi, Luke Esau, Sharif Hala, Raeece Naeem, Fadwa Alofi, Afrah Alsomali, Asim Khogeer, Jumana Taha, Abdulaziz Alahmadi, Kahled Algithami, Anwar Hashem, Naif Almontashiri, Arnab Pain
EPI_ISL_677968, EPI_ISL_677970	Pathogen Genomics Lab King Abdullah University of Science and Technology(KAUST)	Pathogen Genomics Lab King Abdullah University of Science and Technology(KAUST)	Fathia Ben Rached, Sharif Hala, Amit Kumar Subudhi, Sara Mfarrej, Raeece Naeem, Rahul P Salunke, Fadwa Alofi, Afrah Alsomali, Asim Khogeer, Jumana Taha, Abdulaziz Alahmadi, Kahled Algithami, Anwar Hashem, Naif Almontashiri, Arnab Pain
EPI_ISL_677973	Pathogen Genomics Lab King Abdullah University of Science and Technology(KAUST)	Pathogen Genomics Lab King Abdullah University of Science and Technology(KAUST)	Sara Mfarrej, Amanda Ooi, Luke Esau, Sharif Hala, Raeece Naeem, Fadwa Alofi, Afrah Alsomali, Asim Khogeer, Jumana Taha, Abdulaziz Alahmadi, Kahled Algithami, Anwar Hashem, Naif Almontashiri, Arnab Pain
EPI_ISL_678026	Pathogen Genomics Lab King Abdullah University of Science and Technology(KAUST)	Pathogen Genomics Lab King Abdullah University of Science and Technology(KAUST)	Sharif Hala, Sara Mfarrej, Raeece Naeem, Amit Kumar Subudhi, Rahul P Salunke, Fadwa Alofi, Asim Khogeer, Afrah Alsomali, Jumana Taha, Abdulaziz Alahmadi, Kahled Algithami, Anwar Hashem, Naif Almontashiri, Arnab Pain
EPI_ISL_678028	Pathogen Genomics Lab King Abdullah University of Science and Technology(KAUST)	Pathogen Genomics Lab King Abdullah University of Science and Technology(KAUST)	Fathia Ben Rached, Sharif Hala, Amit Kumar Subudhi, Sara Mfarrej, Raeece Naeem, Rahul P Salunke, Fadwa Alofi, Afrah Alsomali, Asim Khogeer, Jumana Taha, Abdulaziz Alahmadi, Kahled Algithami, Anwar Hashem, Naif Almontashiri, Arnab Pain
EPI_ISL_678029	Pathogen Genomics Lab King Abdullah University of Science and Technology(KAUST)	Pathogen Genomics Lab King Abdullah University of Science and Technology(KAUST)	Sharif Hala, Sara Mfarrej, Raushan Nugmanova, Olga Douvropoulou, Raeece Naeem, Fadwa Alofi, Asim Khogeer, Afrah Alsomali, Jumana Taha, Abdulaziz Alahmadi, Kahled Algithami, Anwar Hashem, Naif Almontashiri, Arnab Pain
EPI_ISL_678035, EPI_ISL_678036, EPI_ISL_678038	Pathogen Genomics Lab King Abdullah University of Science and Technology(KAUST)	Pathogen Genomics Lab King Abdullah University of Science and Technology(KAUST)	Sara Mfarrej, Amanda Ooi, Luke Esau, Sharif Hala, Raeece Naeem, Fadwa Alofi, Afrah Alsomali, Asim Khogeer, Jumana Taha, Abdulaziz Alahmadi, Kahled Algithami, Anwar Hashem, Naif Almontashiri, Arnab Pain
EPI_ISL_678234	Pathogen Genomics Lab King Abdullah University of Science and Technology(KAUST)	Pathogen Genomics Lab King Abdullah University of Science and Technology(KAUST)	Sharif Hala, Sara Mfarrej, Raeece Naeem, Amit Kumar Subudhi, Rahul P Salunke, Fadwa Alofi, Asim Khogeer, Afrah Alsomali, Jumana Taha, Abdulaziz Alahmadi, Kahled Algithami, Anwar Hashem, Naif Almontashiri, Arnab Pain
EPI_ISL_678235	Pathogen Genomics Lab King Abdullah University of Science and Technology(KAUST)	Pathogen Genomics Lab King Abdullah University of Science and Technology(KAUST)	Fathia Ben Rached, Sharif Hala, Amit Kumar Subudhi, Sara Mfarrej, Raeece Naeem, Rahul P Salunke, Fadwa Alofi, Afrah Alsomali, Asim Khogeer, Jumana Taha, Abdulaziz Alahmadi, Kahled Algithami, Anwar Hashem, Naif Almontashiri, Arnab Pain
EPI_ISL_678502	CNR Virus des Infections Respiratoires - France SUD	CNR Virus des Infections Respiratoires - France SUD	Antonin Bal, Gregory Destras, Gwendolyne Burfin, Solenne Brun, Martine Valette, Bruno Lina, Laurence Josset
EPI_ISL_680283, EPI_ISL_680284, EPI_ISL_680285	Regional Virus Laboratory, Belfast Health and Social Care Trust	COVID-19 Genomics UK (COG-UK) Consortium	Conall McCaughey, James McKenna, Tanya Curran, Susan Feeney, Alison Watt, Ciara Cox, Mairead Connor, Zoltan Molnar, David Simpson, Derek Fairley
EPI_ISL_681721, EPI_ISL_681728, EPI_ISL_681731, EPI_ISL_681752, EPI_ISL_681754, EPI_ISL_681759, EPI_ISL_681760, EPI_ISL_681764, EPI_ISL_681767	University Hospital Limerick	Irish Coronavirus Sequencing Consortium - Teagasc Moorepark	Paul Cotter, Fiona Crispie, John Kenny, Calum Walsh
EPI_ISL_681920, EPI_ISL_682057	National Virus Reference Laboratory	Irish Coronavirus Sequencing Consortium - Teagasc Moorepark	Paul Cotter, Fiona Crispie, John Kenny, Matthew McCabe, Calum Walsh
EPI_ISL_684166, EPI_ISL_684167, EPI_ISL_684174, EPI_ISL_684175, EPI_ISL_684176, EPI_ISL_684177, EPI_ISL_684178, EPI_ISL_684179, EPI_ISL_684180, EPI_ISL_684181, EPI_ISL_684182, EPI_ISL_684183, EPI_ISL_684184, EPI_ISL_684185, EPI_ISL_684186, EPI_ISL_684187, EPI_ISL_684188, EPI_ISL_684189, EPI_ISL_684190, EPI_ISL_684191, EPI_ISL_684192, EPI_ISL_684193, EPI_ISL_684194, EPI_ISL_684195, EPI_ISL_684196, EPI_ISL_684197, EPI_ISL_684198, EPI_ISL_684199, EPI_ISL_684200, EPI_ISL_684201, EPI_ISL_684202, EPI_ISL_684203, EPI_ISL_684204, EPI_ISL_684205, EPI_ISL_684206, EPI_ISL_684207, EPI_ISL_684208, EPI_ISL_684209, EPI_ISL_684210, EPI_ISL_684211, EPI_ISL_684212, EPI_ISL_684213, EPI_ISL_684214, EPI_ISL_684215, EPI_ISL_684216, EPI_ISL_684217, EPI_ISL_684218			
see above	Pathogen Genomics Center, National Institute of Infectious Diseases	Pathogen Genomics Center, National Institute of Infectious Diseases	Tsuyoshi Sekizuka, Kentaro Itokawa, Rina Tanaka, Masanori Hashino, Makoto Kuroda
EPI_ISL_684220, EPI_ISL_684221, EPI_ISL_684222, EPI_ISL_684223, EPI_ISL_684224, EPI_ISL_684225, EPI_ISL_684226, EPI_ISL_684227, EPI_ISL_684228, EPI_ISL_684229, EPI_ISL_684230, EPI_ISL_684231, EPI_ISL_684232, EPI_ISL_684233, EPI_ISL_684234, EPI_ISL_684235, EPI_ISL_684236, EPI_ISL_684237, EPI_ISL_684238, EPI_ISL_684239, EPI_ISL_684240, EPI_ISL_684241, EPI_ISL_684242, EPI_ISL_684243, EPI_ISL_684244, EPI_ISL_684245, EPI_ISL_684246, EPI_ISL_684247, EPI_ISL_684248, EPI_ISL_684249, EPI_ISL_684250, EPI_ISL_684251, EPI_ISL_684252, EPI_ISL_684253, EPI_ISL_684254, EPI_ISL_684255, EPI_ISL_684256, EPI_ISL_684257, EPI_ISL_684258, EPI_ISL_684259, EPI_ISL_684260, EPI_ISL_684261, EPI_ISL_684262, EPI_ISL_684263, EPI_ISL_684264, EPI_ISL_684265, EPI_ISL_684266, EPI_ISL_684267, EPI_ISL_684268, EPI_ISL_684269, EPI_ISL_684270, EPI_ISL_684271, EPI_ISL_684272, EPI_ISL_684273, EPI_ISL_684274, EPI_ISL_684275, EPI_ISL_684276, EPI_ISL_684277, EPI_ISL_684278, EPI_ISL_684279, EPI_ISL_684280, EPI_ISL_684281, EPI_ISL_684282, EPI_ISL_684283, EPI_ISL_684284, EPI_ISL_684285, EPI_ISL_684286, EPI_ISL_684287, EPI_ISL_684288			
see above	Gunma Prefectural Institute of Public Health and Environmental Sciences	Pathogen Genomics Center, National Institute of Infectious Diseases	Tsuyoshi Sekizuka, Kentaro Itokawa, Rina Tanaka, Masanori Hashino, Makoto Kuroda
EPI_ISL_684289, EPI_ISL_684290, EPI_ISL_684291, EPI_ISL_684292, EPI_ISL_684293, EPI_ISL_684294, EPI_ISL_684295, EPI_ISL_684297, EPI_ISL_684298, EPI_ISL_684299, EPI_ISL_684300, EPI_ISL_684301, EPI_ISL_684302, EPI_ISL_684303, EPI_ISL_684305, EPI_ISL_684306, EPI_ISL_684307, EPI_ISL_684308, EPI_ISL_684309, EPI_ISL_684310, EPI_ISL_684311, EPI_ISL_684312, EPI_ISL_684313, EPI_ISL_684314, EPI_ISL_684315, EPI_ISL_684316, EPI_ISL_684317, EPI_ISL_684318, EPI_ISL_684319, EPI_ISL_684320, EPI_ISL_684321, EPI_ISL_684322, EPI_ISL_684323, EPI_ISL_684324			
see above	Pathogen Genomics Center, National Institute of Infectious Diseases	Pathogen Genomics Center, National Institute of Infectious Diseases	Tsuyoshi Sekizuka, Kentaro Itokawa, Rina Tanaka, Masanori Hashino, Makoto Kuroda
EPI_ISL_684325, EPI_ISL_684326, EPI_ISL_684327, EPI_ISL_684328, EPI_ISL_684329, EPI_ISL_684330, EPI_ISL_684331, EPI_ISL_684332, EPI_ISL_684333, EPI_ISL_684334, EPI_ISL_684335, EPI_ISL_684336, EPI_ISL_684337, EPI_ISL_684338			
see above	Gunma Prefectural Institute of Public Health and Environmental Sciences	Pathogen Genomics Center, National Institute of Infectious Diseases	Tsuyoshi Sekizuka, Kentaro Itokawa, Rina Tanaka, Masanori Hashino, Makoto Kuroda
EPI_ISL_684339, EPI_ISL_684340, EPI_ISL_684341, EPI_ISL_684342, EPI_ISL_684343, EPI_ISL_684344, EPI_ISL_684345, EPI_ISL_684346, EPI_ISL_684347, EPI_ISL_684348, EPI_ISL_684349, EPI_ISL_684350, EPI_ISL_684351, EPI_ISL_684352, EPI_ISL_684353, EPI_ISL_684354, EPI_ISL_684355, EPI_ISL_684356, EPI_ISL_684357, EPI_ISL_684358, EPI_ISL_684359, EPI_ISL_684360, EPI_ISL_684362, EPI_ISL_684363, EPI_ISL_684364, EPI_ISL_684365, EPI_ISL_684366, EPI_ISL_684367, EPI_ISL_684368, EPI_ISL_684369, EPI_ISL_684385, EPI_ISL_684386, EPI_ISL_684387, EPI_ISL_684388, EPI_ISL_684389, EPI_ISL_684390, EPI_ISL_684391, EPI_ISL_684392, EPI_ISL_684393, EPI_ISL_684394, EPI_ISL_684395, EPI_ISL_684396, EPI_ISL_684397, EPI_ISL_684398, EPI_ISL_684399, EPI_ISL_684400, EPI_ISL_684401, EPI_ISL_684402, EPI_ISL_684403, EPI_ISL_684404, EPI_ISL_684405, EPI_ISL_684406, EPI_ISL_684407			
see above	Pathogen Genomics Center, National Institute of Infectious Diseases	Pathogen Genomics Center, National Institute of Infectious Diseases	Tsuyoshi Sekizuka, Kentaro Itokawa, Rina Tanaka, Masanori Hashino, Makoto Kuroda
EPI_ISL_684408, EPI_ISL_684409, EPI_ISL_684410, EPI_ISL_684411, EPI_ISL_684412, EPI_ISL_684413, EPI_ISL_684414, EPI_ISL_684415, EPI_ISL_684416, EPI_ISL_684417, EPI_ISL_684418, EPI_ISL_684419, EPI_ISL_684420, EPI_ISL_684423, EPI_ISL_684424, EPI_ISL_684425, EPI_ISL_684426, EPI_ISL_684427, EPI_ISL_684428, EPI_ISL_684430, EPI_ISL_684431, EPI_ISL_684432, EPI_ISL_684433, EPI_ISL_684440, EPI_ISL_684441, EPI_ISL_684442, EPI_ISL_684443, EPI_ISL_684444, EPI_ISL_684445, EPI_ISL_684446, EPI_ISL_684447, EPI_ISL_684448, EPI_ISL_684449, EPI_ISL_684450, EPI_ISL_684451, EPI_ISL_684452, EPI_ISL_684453, EPI_ISL_684454, EPI_ISL_684455, EPI_ISL_684456, EPI_ISL_684457, EPI_ISL_684458, EPI_ISL_684459, EPI_ISL_684460, EPI_ISL_684461, EPI_ISL_684462, EPI_ISL_684463, EPI_ISL_684464, EPI_ISL_684465, EPI_ISL_684466, EPI_ISL_684467, EPI_ISL_684468, EPI_ISL_684469, EPI_ISL_684470			
see above	Fukuoka Institute of Health and Environmental Sciences	Pathogen Genomics Center, National Institute of Infectious Diseases	Tsuyoshi Sekizuka, Kentaro Itokawa, Rina Tanaka, Masanori Hashino, Makoto Kuroda
EPI_ISL_684484, EPI_ISL_684485, EPI_ISL_684489, EPI_ISL_684490, EPI_ISL_684491, EPI_ISL_684494, EPI_ISL_684495, EPI_ISL_684496, EPI_ISL_684497, EPI_ISL_684498, EPI_ISL_684499, EPI_ISL_684502, EPI_ISL_684503, EPI_ISL_684504, EPI_ISL_684505			
see above	Pathogen Genomics Center, National Institute of Infectious Diseases	Pathogen Genomics Center, National Institute of Infectious Diseases	Tsuyoshi Sekizuka, Kentaro Itokawa, Rina Tanaka, Masanori Hashino, Makoto Kuroda

EPI_ISL_684515, EPI_ISL_684516, EPI_ISL_684518	Tokyo Metropolitan Institute of Public Health	Pathogen Genomics Center, National Institute of Infectious Diseases	Tsuyoshi Sekizuka, Kentaro Itokawa, Rina Tanaka, Masanori Hashino, Makoto Kuroda
EPI_ISL_684523, EPI_ISL_684524, EPI_ISL_684525, EPI_ISL_684526, EPI_ISL_684527, EPI_ISL_684528, EPI_ISL_684529, EPI_ISL_684530, EPI_ISL_684531, EPI_ISL_684532, EPI_ISL_684533, EPI_ISL_684534, EPI_ISL_684535, EPI_ISL_684536, EPI_ISL_684537, EPI_ISL_684538, EPI_ISL_684539, EPI_ISL_684540, EPI_ISL_684541, EPI_ISL_684542, EPI_ISL_684543, EPI_ISL_684544, EPI_ISL_684545, EPI_ISL_684546, EPI_ISL_684547, EPI_ISL_684548, EPI_ISL_684549, EPI_ISL_684550, EPI_ISL_684551, EPI_ISL_684552, EPI_ISL_684553, EPI_ISL_684554, EPI_ISL_684555			
see above	Pathogen Genomics Center, National Institute of Infectious Diseases	Pathogen Genomics Center, National Institute of Infectious Diseases	Tsuyoshi Sekizuka, Kentaro Itokawa, Rina Tanaka, Masanori Hashino, Makoto Kuroda
EPI_ISL_684557	Tokyo Metropolitan Institute of Public Health	Pathogen Genomics Center, National Institute of Infectious Diseases	Tsuyoshi Sekizuka, Kentaro Itokawa, Rina Tanaka, Masanori Hashino, Makoto Kuroda
EPI_ISL_684558, EPI_ISL_684559, EPI_ISL_684560, EPI_ISL_684561, EPI_ISL_684562, EPI_ISL_684563, EPI_ISL_684564, EPI_ISL_684565	Pathogen Genomics Center, National Institute of Infectious Diseases	Pathogen Genomics Center, National Institute of Infectious Diseases	Tsuyoshi Sekizuka, Kentaro Itokawa, Rina Tanaka, Masanori Hashino, Makoto Kuroda
EPI_ISL_684566, EPI_ISL_684567, EPI_ISL_684568, EPI_ISL_684569, EPI_ISL_684570, EPI_ISL_684571, EPI_ISL_684572, EPI_ISL_684573, EPI_ISL_684574, EPI_ISL_684575, EPI_ISL_684576, EPI_ISL_684577			
see above	Gunma Prefectural Institute of Public Health and Environmental Sciences	Pathogen Genomics Center, National Institute of Infectious Diseases	Tsuyoshi Sekizuka, Kentaro Itokawa, Rina Tanaka, Masanori Hashino, Makoto Kuroda
EPI_ISL_684578, EPI_ISL_684579, EPI_ISL_684580, EPI_ISL_684581, EPI_ISL_684582, EPI_ISL_684583, EPI_ISL_684584, EPI_ISL_684585, EPI_ISL_684586, EPI_ISL_684587, EPI_ISL_684588, EPI_ISL_684589, EPI_ISL_684590, EPI_ISL_684591, EPI_ISL_684592, EPI_ISL_684593, EPI_ISL_684594, EPI_ISL_684595, EPI_ISL_684596, EPI_ISL_684597, EPI_ISL_684598, EPI_ISL_684599, EPI_ISL_684600, EPI_ISL_684601, EPI_ISL_684602, EPI_ISL_684603, EPI_ISL_684604, EPI_ISL_684605, EPI_ISL_684606, EPI_ISL_684607, EPI_ISL_684608, EPI_ISL_684609, EPI_ISL_684610, EPI_ISL_684611, EPI_ISL_684612, EPI_ISL_684613, EPI_ISL_684614			
see above	Pathogen Genomics Center, National Institute of Infectious Diseases	Pathogen Genomics Center, National Institute of Infectious Diseases	Tsuyoshi Sekizuka, Kentaro Itokawa, Rina Tanaka, Masanori Hashino, Makoto Kuroda
EPI_ISL_684632, EPI_ISL_684633	Tokyo Metropolitan Institute of Public Health	Pathogen Genomics Center, National Institute of Infectious Diseases	Tsuyoshi Sekizuka, Kentaro Itokawa, Rina Tanaka, Masanori Hashino, Makoto Kuroda
EPI_ISL_684634, EPI_ISL_684635, EPI_ISL_684636, EPI_ISL_684637, EPI_ISL_684638, EPI_ISL_684639, EPI_ISL_684640, EPI_ISL_684641, EPI_ISL_684642, EPI_ISL_684643, EPI_ISL_684644, EPI_ISL_684645, EPI_ISL_684646, EPI_ISL_684647, EPI_ISL_684648, EPI_ISL_684649, EPI_ISL_684650, EPI_ISL_684651, EPI_ISL_684652, EPI_ISL_684653, EPI_ISL_684654, EPI_ISL_684655, EPI_ISL_684656, EPI_ISL_684657, EPI_ISL_684658, EPI_ISL_684659, EPI_ISL_684660, EPI_ISL_684661, EPI_ISL_684662, EPI_ISL_684663, EPI_ISL_684664, EPI_ISL_684665, EPI_ISL_684666, EPI_ISL_684667, EPI_ISL_684668, EPI_ISL_684669, EPI_ISL_684670, EPI_ISL_684671, EPI_ISL_684672, EPI_ISL_684673, EPI_ISL_684674, EPI_ISL_684675, EPI_ISL_684676, EPI_ISL_684677, EPI_ISL_684678, EPI_ISL_684679, EPI_ISL_684680, EPI_ISL_684681, EPI_ISL_684682, EPI_ISL_684683, EPI_ISL_684684, EPI_ISL_684685, EPI_ISL_684686, EPI_ISL_684687, EPI_ISL_684688, EPI_ISL_684689, EPI_ISL_684690, EPI_ISL_684691, EPI_ISL_684692			
see above	Fukuoka Institute of Health and Environmental Sciences	Pathogen Genomics Center, National Institute of Infectious Diseases	Tsuyoshi Sekizuka, Kentaro Itokawa, Rina Tanaka, Masanori Hashino, Makoto Kuroda
EPI_ISL_684695, EPI_ISL_684696, EPI_ISL_684697, EPI_ISL_684698, EPI_ISL_684699, EPI_ISL_684700, EPI_ISL_684701, EPI_ISL_684702, EPI_ISL_684703, EPI_ISL_684704, EPI_ISL_684705, EPI_ISL_684706, EPI_ISL_684707, EPI_ISL_684708, EPI_ISL_684709, EPI_ISL_684710, EPI_ISL_684711, EPI_ISL_684712, EPI_ISL_684713, EPI_ISL_684714, EPI_ISL_684715, EPI_ISL_684719, EPI_ISL_684720, EPI_ISL_684721, EPI_ISL_684722			
see above	Pathogen Genomics Center, National Institute of Infectious Diseases	Pathogen Genomics Center, National Institute of Infectious Diseases	Tsuyoshi Sekizuka, Kentaro Itokawa, Rina Tanaka, Masanori Hashino, Makoto Kuroda
EPI_ISL_684723, EPI_ISL_684724, EPI_ISL_684725, EPI_ISL_684726, EPI_ISL_684727	Fukuoka Institute of Health and Environmental Sciences	Pathogen Genomics Center, National Institute of Infectious Diseases	Tsuyoshi Sekizuka, Kentaro Itokawa, Rina Tanaka, Masanori Hashino, Makoto Kuroda
EPI_ISL_684728, EPI_ISL_684729, EPI_ISL_684730, EPI_ISL_684731, EPI_ISL_684732, EPI_ISL_684733, EPI_ISL_684734, EPI_ISL_684735	Tokyo Metropolitan Institute of Public Health	Pathogen Genomics Center, National Institute of Infectious Diseases	Tsuyoshi Sekizuka, Kentaro Itokawa, Rina Tanaka, Masanori Hashino, Makoto Kuroda
EPI_ISL_684737, EPI_ISL_684738, EPI_ISL_684739, EPI_ISL_684740, EPI_ISL_684741, EPI_ISL_684742, EPI_ISL_684743, EPI_ISL_684744, EPI_ISL_684745, EPI_ISL_684746, EPI_ISL_684747, EPI_ISL_684748, EPI_ISL_684749, EPI_ISL_684750, EPI_ISL_684751, EPI_ISL_684752, EPI_ISL_684753, EPI_ISL_684754, EPI_ISL_684755, EPI_ISL_684756, EPI_ISL_684757, EPI_ISL_684758, EPI_ISL_684759, EPI_ISL_684760, EPI_ISL_684761, EPI_ISL_684764, EPI_ISL_684765, EPI_ISL_684766, EPI_ISL_684767, EPI_ISL_684768, EPI_ISL_684769, EPI_ISL_684770, EPI_ISL_684771, EPI_ISL_684772, EPI_ISL_684773, EPI_ISL_684774, EPI_ISL_684775, EPI_ISL_684776, EPI_ISL_684777, EPI_ISL_684778, EPI_ISL_684779, EPI_ISL_684780, EPI_ISL_684781, EPI_ISL_684782, EPI_ISL_684783, EPI_ISL_684784, EPI_ISL_684785, EPI_ISL_684786, EPI_ISL_684787, EPI_ISL_684788, EPI_ISL_684789, EPI_ISL_684790, EPI_ISL_684791, EPI_ISL_684792, EPI_ISL_684793, EPI_ISL_684794, EPI_ISL_684795, EPI_ISL_684796, EPI_ISL_684797			
see above	Pathogen Genomics Center, National Institute of Infectious Diseases	Pathogen Genomics Center, National Institute of Infectious Diseases	Tsuyoshi Sekizuka, Kentaro Itokawa, Rina Tanaka, Masanori Hashino, Makoto Kuroda
EPI_ISL_684800, EPI_ISL_684801, EPI_ISL_684802, EPI_ISL_684803, EPI_ISL_684804, EPI_ISL_684805, EPI_ISL_684806, EPI_ISL_684807, EPI_ISL_684808, EPI_ISL_684809, EPI_ISL_684810, EPI_ISL_684811, EPI_ISL_684812, EPI_ISL_684813, EPI_ISL_684814, EPI_ISL_684815, EPI_ISL_684816, EPI_ISL_684817, EPI_ISL_684818, EPI_ISL_684819, EPI_ISL_684820, EPI_ISL_684821, EPI_ISL_684822, EPI_ISL_684823, EPI_ISL_684824, EPI_ISL_684825, EPI_ISL_684826, EPI_ISL_684827, EPI_ISL_684828, EPI_ISL_684829, EPI_ISL_684830, EPI_ISL_684831, EPI_ISL_684832, EPI_ISL_684833, EPI_ISL_684834, EPI_ISL_684835, EPI_ISL_684836, EPI_ISL_684837, EPI_ISL_684838, EPI_ISL_684839, EPI_ISL_684840, EPI_ISL_684841, EPI_ISL_684842, EPI_ISL_684843, EPI_ISL_684844, EPI_ISL_684845, EPI_ISL_684846, EPI_ISL_684847, EPI_ISL_684848, EPI_ISL_684849, EPI_ISL_684850, EPI_ISL_684851, EPI_ISL_684852, EPI_ISL_684853, EPI_ISL_684854, EPI_ISL_684855, EPI_ISL_684856			
see above	Yamagata Prefectural Institute of Public Health	Pathogen Genomics Center, National Institute of Infectious Diseases	Tsuyoshi Sekizuka, Kentaro Itokawa, Rina Tanaka, Masanori Hashino, Makoto Kuroda
EPI_ISL_684910, EPI_ISL_684911, EPI_ISL_684912, EPI_ISL_684913, EPI_ISL_684914, EPI_ISL_684915, EPI_ISL_684916, EPI_ISL_684917, EPI_ISL_684918, EPI_ISL_684919, EPI_ISL_684920, EPI_ISL_684921, EPI_ISL_684922, EPI_ISL_684923, EPI_ISL_684924, EPI_ISL_684925, EPI_ISL_684926, EPI_ISL_684927, EPI_ISL_684928, EPI_ISL_684929, EPI_ISL_684930, EPI_ISL_684931, EPI_ISL_684932, EPI_ISL_684933, EPI_ISL_684934, EPI_ISL_684935, EPI_ISL_684936, EPI_ISL_684937, EPI_ISL_684938, EPI_ISL_684939, EPI_ISL_684940, EPI_ISL_684941, EPI_ISL_684942, EPI_ISL_684943, EPI_ISL_684944, EPI_ISL_684945, EPI_ISL_684946, EPI_ISL_684947, EPI_ISL_684948, EPI_ISL_684949, EPI_ISL_684950, EPI_ISL_684951, EPI_ISL_684952, EPI_ISL_684953, EPI_ISL_684954, EPI_ISL_684955, EPI_ISL_684956, EPI_ISL_684957, EPI_ISL_684958, EPI_ISL_684959, EPI_ISL_684960, EPI_ISL_684961, EPI_ISL_684962, EPI_ISL_684963, EPI_ISL_684964, EPI_ISL_684965, EPI_ISL_684966, EPI_ISL_684967, EPI_ISL_684968, EPI_ISL_684969, EPI_ISL_684970, EPI_ISL_684971, EPI_ISL_684972, EPI_ISL_684973, EPI_ISL_684974, EPI_ISL_684975, EPI_ISL_684976, EPI_ISL_684977, EPI_ISL_684978, EPI_ISL_684979, EPI_ISL_684980, EPI_ISL_684981, EPI_ISL_684982, EPI_ISL_684983, EPI_ISL_684984, EPI_ISL_684985, EPI_ISL_684986, EPI_ISL_684987, EPI_ISL_684988, EPI_ISL_684989, EPI_ISL_684990, EPI_ISL_684991, EPI_ISL_684992, EPI_ISL_684993, EPI_ISL_684994, EPI_ISL_684995, EPI_ISL_684996, EPI_ISL_684997, EPI_ISL_684998, EPI_ISL_684999, EPI_ISL_685000, EPI_ISL_685001, EPI_ISL_685002, EPI_ISL_685003, EPI_ISL_685004, EPI_ISL_685005, EPI_ISL_685006, EPI_ISL_685007, EPI_ISL_685008, EPI_ISL_685009, EPI_ISL_685010, EPI_ISL_685011, EPI_ISL_685012, EPI_ISL_685013, EPI_ISL_685014, EPI_ISL_685015, EPI_ISL_685016, EPI_ISL_685017, EPI_ISL_685018, EPI_ISL_685019, EPI_ISL_685020, EPI_ISL_685021, EPI_ISL_685022, EPI_ISL_685023, EPI_ISL_685024, EPI_ISL_685025, EPI_ISL_685026, EPI_ISL_685027, EPI_ISL_685028, EPI_ISL_685029, EPI_ISL_685030, EPI_ISL_685031, EPI_ISL_685032, EPI_ISL_685033, EPI_ISL_685034, EPI_ISL_685035, EPI_ISL_685036, EPI_ISL_685037, EPI_ISL_685038, EPI_ISL_685039, EPI_ISL_685040, EPI_ISL_685041, EPI_ISL_685042, EPI_ISL_685043, EPI_ISL_685044, EPI_ISL_685045, EPI_ISL_685046, EPI_ISL_685047, EPI_ISL_685048, EPI_ISL_685049, EPI_ISL_685050, EPI_ISL_685051, EPI_ISL_685052, EPI_ISL_685053, EPI_ISL_685054, EPI_ISL_685055, EPI_ISL_685056, EPI_ISL_685057, EPI_ISL_685058, EPI_ISL_685059, EPI_ISL_685060, EPI_ISL_685061, EPI_ISL_685062, EPI_ISL_685063, EPI_ISL_685064, EPI_ISL_685065, EPI_ISL_685066, EPI_ISL_685067, EPI_ISL_685068, EPI_ISL_685069, EPI_ISL_685070, EPI_ISL_685071, EPI_ISL_685072, EPI_ISL_685073, EPI_ISL_685074, EPI_ISL_685075, EPI_ISL_685076, EPI_ISL_685077, EPI_ISL_685078, EPI_ISL_685079, EPI_ISL_685080, EPI_ISL_685081, EPI_ISL_685082, EPI_ISL_685083, EPI_ISL_685084, EPI_ISL_685085, EPI_ISL_685086, EPI_ISL_685087, EPI_ISL_685088, EPI_ISL_685089, EPI_ISL_685090, EPI_ISL_685091, EPI_ISL_685092, EPI_ISL_685093, EPI_ISL_685094, EPI_ISL_685095, EPI_ISL_685096, EPI_ISL_685097, EPI_ISL_685098, EPI_ISL_685099, EPI_ISL_685100, EPI_ISL_685101, EPI_ISL_685102, EPI_ISL_685103, EPI_ISL_685104, EPI_ISL_685105, EPI_ISL_685106, EPI_ISL_685107, EPI_ISL_685108, EPI_ISL_685109, EPI_ISL_685110, EPI_ISL_685111, EPI_ISL_685112, EPI_ISL_685113, EPI_ISL_685114, EPI_ISL_685115, EPI_ISL_685116, EPI_ISL_685117, EPI_ISL_685118, EPI_ISL_685119, EPI_ISL_685120, EPI_ISL_685121, EPI_ISL_685122, EPI_ISL_685123, EPI_ISL_685124, EPI_ISL_685125, EPI_ISL_685126, EPI_ISL_685127, EPI_ISL_685128, EPI_ISL_685129, EPI_ISL_685130, EPI_ISL_685131			
see above	Saitama Prefectural Institute of Public Health	Pathogen Genomics Center, National Institute of Infectious Diseases	Tsuyoshi Sekizuka, Kentaro Itokawa, Rina Tanaka, Masanori Hashino, Makoto Kuroda
EPI_ISL_685136, EPI_ISL_685137, EPI_ISL_685138, EPI_ISL_685139, EPI_ISL_685140, EPI_ISL_685141, EPI_ISL_685142, EPI_ISL_685143, EPI_ISL_685144, EPI_ISL_685145, EPI_ISL_685146, EPI_ISL_685147, EPI_ISL_685148, EPI_ISL_685149, EPI_ISL_685150, EPI_ISL_685151, EPI_ISL_685152, EPI_ISL_685153, EPI_ISL_685154, EPI_ISL_685155, EPI_ISL_685156, EPI_ISL_685157, EPI_ISL_685158, EPI_ISL_685159, EPI_ISL_685160, EPI_ISL_685161, EPI_ISL_685162, EPI_ISL_685163, EPI_ISL_685164, EPI_ISL_685165, EPI_ISL_685166, EPI_ISL_685167, EPI_ISL_685168, EPI_ISL_685169, EPI_ISL_685170, EPI_ISL_685171, EPI_ISL_685172, EPI_ISL_685173, EPI_ISL_685174, EPI_ISL_685175, EPI_ISL_685176, EPI_ISL_685177, EPI_ISL_685178, EPI_ISL_685179, EPI_ISL_685180, EPI_ISL_685181, EPI_ISL_685182, EPI_ISL_685183, EPI_ISL_685184, EPI_ISL_685185, EPI_ISL_685186, EPI_ISL_685187, EPI_ISL_685188, EPI_ISL_685189, EPI_ISL_685190, EPI_ISL_685191, EPI_ISL_685192, EPI_ISL_685193, EPI_ISL_685194, EPI_ISL_685195, EPI_ISL_685196, EPI_ISL_685197, EPI_ISL_685198, EPI_ISL_685199, EPI_ISL_685200, EPI_ISL_685201, EPI_ISL_685202, EPI_ISL_685204, EPI_ISL_685205, EPI_ISL_685206, EPI_ISL_685207, EPI_ISL_685208, EPI_ISL_685209, EPI_ISL_685210, EPI_ISL_685211, EPI_ISL_685212, EPI_ISL_685213, EPI_ISL_685214, EPI_ISL_685215, EPI_ISL_685216, EPI_ISL_685217, EPI_ISL_685218, EPI_ISL_685219			
see above	Pathogen Genomics Center, National Institute of Infectious Diseases	Pathogen Genomics Center, National Institute of Infectious Diseases	Tsuyoshi Sekizuka, Kentaro Itokawa, Rina Tanaka, Masanori Hashino, Makoto Kuroda
EPI_ISL_685221, EPI_ISL_685222, EPI_ISL_685223, EPI_ISL_685224, EPI_ISL_685225, EPI_ISL_685226, EPI_ISL_685227, EPI_ISL_685228, EPI_ISL_685229, EPI_ISL_685230, EPI_ISL_685231, EPI_ISL_685232, EPI_ISL_685233, EPI_ISL_685234, EPI_ISL_685235, EPI_ISL_685236, EPI_ISL_685237			

see above	Tokyo Metropolitan Institute of Public Health	Pathogen Genomics Center, National Institute of Infectious Diseases	Tsuyoshi Sekizuka, Kentaro Itokawa, Rina Tanaka, Masanori Hashino, Makoto Kuroda
EPI_ISL_685244, EPI_ISL_685245, EPI_ISL_685246, EPI_ISL_685247, EPI_ISL_685248, EPI_ISL_685249, EPI_ISL_685250, EPI_ISL_685251, EPI_ISL_685252, EPI_ISL_685253, EPI_ISL_685254, EPI_ISL_685255, EPI_ISL_685256, EPI_ISL_685257, EPI_ISL_685258, EPI_ISL_685259, EPI_ISL_685260, EPI_ISL_685261, EPI_ISL_685262, EPI_ISL_685263, EPI_ISL_685264, EPI_ISL_685265, EPI_ISL_685266, EPI_ISL_685267, EPI_ISL_685268, EPI_ISL_685269, EPI_ISL_685270, EPI_ISL_685271, EPI_ISL_685272, EPI_ISL_685273, EPI_ISL_685274, EPI_ISL_685275, EPI_ISL_685276, EPI_ISL_685277, EPI_ISL_685278			
see above	Pathogen Genomics Center, National Institute of Infectious Diseases	Pathogen Genomics Center, National Institute of Infectious Diseases	Tsuyoshi Sekizuka, Kentaro Itokawa, Rina Tanaka, Masanori Hashino, Makoto Kuroda
EPI_ISL_685279, EPI_ISL_685280	Gunma Prefectural Institute of Public Health and Environmental Sciences	Pathogen Genomics Center, National Institute of Infectious Diseases	Tsuyoshi Sekizuka, Kentaro Itokawa, Rina Tanaka, Masanori Hashino, Makoto Kuroda
EPI_ISL_685282	Pathogen Genomics Center, National Institute of Infectious Diseases	Pathogen Genomics Center, National Institute of Infectious Diseases	Tsuyoshi Sekizuka, Kentaro Itokawa, Rina Tanaka, Masanori Hashino, Makoto Kuroda
EPI_ISL_685299, EPI_ISL_685300, EPI_ISL_685301, EPI_ISL_685302, EPI_ISL_685303, EPI_ISL_685304, EPI_ISL_685305, EPI_ISL_685306, EPI_ISL_685307, EPI_ISL_685308, EPI_ISL_685309, EPI_ISL_685310			
see above	Niigata Prefectural Institute of Public Health and Environmental Sciences	Pathogen Genomics Center, National Institute of Infectious Diseases	Tsuyoshi Sekizuka, Kentaro Itokawa, Rina Tanaka, Masanori Hashino, Makoto Kuroda
EPI_ISL_685313, EPI_ISL_685314, EPI_ISL_685315, EPI_ISL_685316, EPI_ISL_685317, EPI_ISL_685318, EPI_ISL_685319, EPI_ISL_685320, EPI_ISL_685321, EPI_ISL_685322, EPI_ISL_685323, EPI_ISL_685324, EPI_ISL_685325, EPI_ISL_685326, EPI_ISL_685327, EPI_ISL_685328, EPI_ISL_685329, EPI_ISL_685330, EPI_ISL_685331, EPI_ISL_685332, EPI_ISL_685333, EPI_ISL_685334, EPI_ISL_685335, EPI_ISL_685336, EPI_ISL_685337, EPI_ISL_685338, EPI_ISL_685339, EPI_ISL_685340, EPI_ISL_685341, EPI_ISL_685342, EPI_ISL_685343, EPI_ISL_685354, EPI_ISL_685355, EPI_ISL_685356, EPI_ISL_685357, EPI_ISL_685358, EPI_ISL_685359, EPI_ISL_685360, EPI_ISL_685361, EPI_ISL_685362, EPI_ISL_685363, EPI_ISL_685364, EPI_ISL_685365, EPI_ISL_685366, EPI_ISL_685367, EPI_ISL_685368, EPI_ISL_685369, EPI_ISL_685370, EPI_ISL_685371, EPI_ISL_685372, EPI_ISL_685373, EPI_ISL_685374, EPI_ISL_685375, EPI_ISL_685376, EPI_ISL_685377, EPI_ISL_685378, EPI_ISL_685379, EPI_ISL_685380, EPI_ISL_685381, EPI_ISL_685382, EPI_ISL_685383, EPI_ISL_685384, EPI_ISL_685385, EPI_ISL_685386, EPI_ISL_685387, EPI_ISL_685388, EPI_ISL_685389			
see above	Pathogen Genomics Center, National Institute of Infectious Diseases	Pathogen Genomics Center, National Institute of Infectious Diseases	Tsuyoshi Sekizuka, Kentaro Itokawa, Rina Tanaka, Masanori Hashino, Makoto Kuroda
EPI_ISL_685407, EPI_ISL_685408, EPI_ISL_685409, EPI_ISL_685410, EPI_ISL_685411, EPI_ISL_685412, EPI_ISL_685413, EPI_ISL_685414, EPI_ISL_685415, EPI_ISL_685416, EPI_ISL_685417, EPI_ISL_685418, EPI_ISL_685419, EPI_ISL_685420, EPI_ISL_685421, EPI_ISL_685422, EPI_ISL_685423, EPI_ISL_685424, EPI_ISL_685425, EPI_ISL_685426, EPI_ISL_685427, EPI_ISL_685428, EPI_ISL_685429, EPI_ISL_685430, EPI_ISL_685431, EPI_ISL_685432, EPI_ISL_685433, EPI_ISL_685434, EPI_ISL_685435, EPI_ISL_685436, EPI_ISL_685437, EPI_ISL_685438, EPI_ISL_685439, EPI_ISL_685440, EPI_ISL_685441, EPI_ISL_685442, EPI_ISL_685443, EPI_ISL_685444, EPI_ISL_685445, EPI_ISL_685446, EPI_ISL_685447, EPI_ISL_685448, EPI_ISL_685449, EPI_ISL_685450, EPI_ISL_685451, EPI_ISL_685452, EPI_ISL_685453			
see above	Tokyo Metropolitan Institute of Public Health	Pathogen Genomics Center, National Institute of Infectious Diseases	Tsuyoshi Sekizuka, Kentaro Itokawa, Rina Tanaka, Masanori Hashino, Makoto Kuroda
EPI_ISL_685498, EPI_ISL_685499, EPI_ISL_685500, EPI_ISL_685501, EPI_ISL_685503, EPI_ISL_685504	Yamagata Prefectural Institute of Public Health	Pathogen Genomics Center, National Institute of Infectious Diseases	Tsuyoshi Sekizuka, Kentaro Itokawa, Rina Tanaka, Masanori Hashino, Makoto Kuroda
EPI_ISL_685506, EPI_ISL_685507, EPI_ISL_685508, EPI_ISL_685509, EPI_ISL_685510, EPI_ISL_685511, EPI_ISL_685512, EPI_ISL_685513, EPI_ISL_685514, EPI_ISL_685515, EPI_ISL_685516, EPI_ISL_685517, EPI_ISL_685518, EPI_ISL_685519, EPI_ISL_685520, EPI_ISL_685528, EPI_ISL_685529, EPI_ISL_685530, EPI_ISL_685531, EPI_ISL_685532, EPI_ISL_685533, EPI_ISL_685534, EPI_ISL_685535, EPI_ISL_685536, EPI_ISL_685537, EPI_ISL_685538, EPI_ISL_685539, EPI_ISL_685540, EPI_ISL_685541, EPI_ISL_685542, EPI_ISL_685543, EPI_ISL_685544, EPI_ISL_685545, EPI_ISL_685546, EPI_ISL_685547, EPI_ISL_685548, EPI_ISL_685549, EPI_ISL_685550, EPI_ISL_685551, EPI_ISL_685552, EPI_ISL_685553, EPI_ISL_685554, EPI_ISL_685555, EPI_ISL_685556, EPI_ISL_685557, EPI_ISL_685558, EPI_ISL_685559, EPI_ISL_685560, EPI_ISL_685561, EPI_ISL_685562, EPI_ISL_685563, EPI_ISL_685564, EPI_ISL_685565, EPI_ISL_685566, EPI_ISL_685567, EPI_ISL_685568, EPI_ISL_685569, EPI_ISL_685570, EPI_ISL_685571, EPI_ISL_685572, EPI_ISL_685573, EPI_ISL_685587, EPI_ISL_685588, EPI_ISL_685589, EPI_ISL_685590, EPI_ISL_685591, EPI_ISL_685592, EPI_ISL_685593, EPI_ISL_685594, EPI_ISL_685595, EPI_ISL_685596, EPI_ISL_685597, EPI_ISL_685598, EPI_ISL_685599, EPI_ISL_685600, EPI_ISL_685601, EPI_ISL_685602, EPI_ISL_685603			
see above	Pathogen Genomics Center, National Institute of Infectious Diseases	Pathogen Genomics Center, National Institute of Infectious Diseases	Tsuyoshi Sekizuka, Kentaro Itokawa, Rina Tanaka, Masanori Hashino, Makoto Kuroda
EPI_ISL_685607, EPI_ISL_685608, EPI_ISL_685609, EPI_ISL_685610, EPI_ISL_685611, EPI_ISL_685612, EPI_ISL_685613, EPI_ISL_685614, EPI_ISL_685615, EPI_ISL_685616	Tokyo Metropolitan Institute of Public Health	Pathogen Genomics Center, National Institute of Infectious Diseases	Tsuyoshi Sekizuka, Kentaro Itokawa, Rina Tanaka, Masanori Hashino, Makoto Kuroda
EPI_ISL_685623, EPI_ISL_685624, EPI_ISL_685625, EPI_ISL_685626, EPI_ISL_685627, EPI_ISL_685628, EPI_ISL_685629, EPI_ISL_685630, EPI_ISL_685631, EPI_ISL_685632, EPI_ISL_685633, EPI_ISL_685634, EPI_ISL_685635, EPI_ISL_685636, EPI_ISL_685637, EPI_ISL_685649, EPI_ISL_685650, EPI_ISL_685651, EPI_ISL_685652, EPI_ISL_685653, EPI_ISL_685654, EPI_ISL_685655, EPI_ISL_685656, EPI_ISL_685657, EPI_ISL_685658, EPI_ISL_685659, EPI_ISL_685660, EPI_ISL_685661, EPI_ISL_685662, EPI_ISL_685663, EPI_ISL_685664, EPI_ISL_685665			
see above	Pathogen Genomics Center, National Institute of Infectious Diseases	Pathogen Genomics Center, National Institute of Infectious Diseases	Tsuyoshi Sekizuka, Kentaro Itokawa, Rina Tanaka, Masanori Hashino, Makoto Kuroda
EPI_ISL_685666, EPI_ISL_685667, EPI_ISL_685668, EPI_ISL_685669, EPI_ISL_685670, EPI_ISL_685671, EPI_ISL_685672, EPI_ISL_685673, EPI_ISL_685674, EPI_ISL_685675, EPI_ISL_685676, EPI_ISL_685677, EPI_ISL_685678, EPI_ISL_685679, EPI_ISL_685680, EPI_ISL_685681, EPI_ISL_685682, EPI_ISL_685683, EPI_ISL_685684, EPI_ISL_685685, EPI_ISL_685686, EPI_ISL_685687, EPI_ISL_685688, EPI_ISL_685689, EPI_ISL_685690, EPI_ISL_685691, EPI_ISL_685692, EPI_ISL_685693, EPI_ISL_685694			
see above	Tokyo Metropolitan Institute of Public Health	Pathogen Genomics Center, National Institute of Infectious Diseases	Tsuyoshi Sekizuka, Kentaro Itokawa, Rina Tanaka, Masanori Hashino, Makoto Kuroda
EPI_ISL_685698, EPI_ISL_685699, EPI_ISL_685701, EPI_ISL_685702, EPI_ISL_685703, EPI_ISL_685704, EPI_ISL_685705, EPI_ISL_685706, EPI_ISL_685707, EPI_ISL_685708, EPI_ISL_685709, EPI_ISL_685710, EPI_ISL_685711, EPI_ISL_685734, EPI_ISL_685735, EPI_ISL_685736, EPI_ISL_685737, EPI_ISL_685738, EPI_ISL_685739, EPI_ISL_685740, EPI_ISL_685741, EPI_ISL_685742, EPI_ISL_685743, EPI_ISL_685746, EPI_ISL_685747, EPI_ISL_685748, EPI_ISL_685749, EPI_ISL_685750, EPI_ISL_685751, EPI_ISL_685752, EPI_ISL_685753, EPI_ISL_685754, EPI_ISL_685755, EPI_ISL_685756, EPI_ISL_685757, EPI_ISL_685758, EPI_ISL_685759, EPI_ISL_685760, EPI_ISL_685761, EPI_ISL_685762, EPI_ISL_685763, EPI_ISL_685764, EPI_ISL_685765, EPI_ISL_685766, EPI_ISL_685767, EPI_ISL_685768, EPI_ISL_685769, EPI_ISL_685770, EPI_ISL_685771, EPI_ISL_685772, EPI_ISL_685773, EPI_ISL_685774, EPI_ISL_685786, EPI_ISL_685787, EPI_ISL_685788, EPI_ISL_685789, EPI_ISL_685790, EPI_ISL_685791, EPI_ISL_685792, EPI_ISL_685793, EPI_ISL_685794, EPI_ISL_685795, EPI_ISL_685796, EPI_ISL_685797, EPI_ISL_685798, EPI_ISL_685799, EPI_ISL_685800, EPI_ISL_685801, EPI_ISL_685802, EPI_ISL_685803, EPI_ISL_685804, EPI_ISL_685805, EPI_ISL_685806, EPI_ISL_685807, EPI_ISL_685808, EPI_ISL_685809, EPI_ISL_685810, EPI_ISL_685811, EPI_ISL_685812, EPI_ISL_685813, EPI_ISL_685814, EPI_ISL_685815, EPI_ISL_685816, EPI_ISL_685817, EPI_ISL_685818, EPI_ISL_685819, EPI_ISL_685820, EPI_ISL_685821, EPI_ISL_685822, EPI_ISL_685823, EPI_ISL_685824, EPI_ISL_685825, EPI_ISL_685826, EPI_ISL_685827, EPI_ISL_685828, EPI_ISL_685829, EPI_ISL_685830, EPI_ISL_685831, EPI_ISL_685832, EPI_ISL_685833, EPI_ISL_685834, EPI_ISL_685835, EPI_ISL_685836, EPI_ISL_685837, EPI_ISL_685838, EPI_ISL_685839, EPI_ISL_685840, EPI_ISL_685841, EPI_ISL_685842, EPI_ISL_685843, EPI_ISL_685844, EPI_ISL_685845, EPI_ISL_685846, EPI_ISL_685847, EPI_ISL_685848, EPI_ISL_685849, EPI_ISL_685850, EPI_ISL_685851, EPI_ISL_685852, EPI_ISL_685853, EPI_ISL_685854, EPI_ISL_685855, EPI_ISL_685856, EPI_ISL_685857, EPI_ISL_685858, EPI_ISL_685859, EPI_ISL_685860, EPI_ISL_685861, EPI_ISL_685862, EPI_ISL_685863, EPI_ISL_685864, EPI_ISL_685865, EPI_ISL_685866, EPI_ISL_685867, EPI_ISL_685868, EPI_ISL_685869, EPI_ISL_685870, EPI_ISL_685871, EPI_ISL_685872, EPI_ISL_685873, EPI_ISL_685874, EPI_ISL_685875, EPI_ISL_685876, EPI_ISL_685877, EPI_ISL_685878, EPI_ISL_685879, EPI_ISL_685880, EPI_ISL_685881, EPI_ISL_685882, EPI_ISL_685883, EPI_ISL_685884, EPI_ISL_685885, EPI_ISL_685886, EPI_ISL_685887, EPI_ISL_685888, EPI_ISL_685889, EPI_ISL_685890, EPI_ISL_685891, EPI_ISL_685892, EPI_ISL_685893, EPI_ISL_685894, EPI_ISL_685895, EPI_ISL_685896, EPI_ISL_685897, EPI_ISL_685898, EPI_ISL_685899, EPI_ISL_685900, EPI_ISL_685901, EPI_ISL_685902, EPI_ISL_685903, EPI_ISL_685906, EPI_ISL_685907, EPI_ISL_685908			
see above	Pathogen Genomics Center, National Institute of Infectious Diseases	Pathogen Genomics Center, National Institute of Infectious Diseases	Tsuyoshi Sekizuka, Kentaro Itokawa, Rina Tanaka, Masanori Hashino, Makoto Kuroda
EPI_ISL_685918, EPI_ISL_685919, EPI_ISL_685920, EPI_ISL_685921	Fukuoka Institute of Health and Environmental Sciences	Pathogen Genomics Center, National Institute of Infectious Diseases	Tsuyoshi Sekizuka, Kentaro Itokawa, Rina Tanaka, Masanori Hashino, Makoto Kuroda
EPI_ISL_685925, EPI_ISL_685926, EPI_ISL_685927, EPI_ISL_685929, EPI_ISL_685931, EPI_ISL_685932, EPI_ISL_685933, EPI_ISL_685934, EPI_ISL_685935, EPI_ISL_685936, EPI_ISL_685947, EPI_ISL_685953, EPI_ISL_685954, EPI_ISL_685955, EPI_ISL_685957, EPI_ISL_685958, EPI_ISL_685959, EPI_ISL_685960, EPI_ISL_685961			
see above	Pathogen Genomics Center, National Institute of Infectious Diseases	Pathogen Genomics Center, National Institute of Infectious Diseases	Tsuyoshi Sekizuka, Kentaro Itokawa, Rina Tanaka, Masanori Hashino, Makoto Kuroda
EPI_ISL_685987	Tokyo Metropolitan Institute of Public Health	Pathogen Genomics Center, National Institute of Infectious Diseases	Tsuyoshi Sekizuka, Kentaro Itokawa, Rina Tanaka, Masanori Hashino, Makoto Kuroda
EPI_ISL_685992, EPI_ISL_685993, EPI_ISL_685994, EPI_ISL_685995, EPI_ISL_685996, EPI_ISL_685997, EPI_ISL_685998, EPI_ISL_685999, EPI_ISL_686000, EPI_ISL_686001, EPI_ISL_686002, EPI_ISL_686003, EPI_ISL_686004, EPI_ISL_686005, EPI_ISL_686006, EPI_ISL_686007, EPI_ISL_686008, EPI_ISL_686009, EPI_ISL_686010, EPI_ISL_686011, EPI_ISL_686012, EPI_ISL_686013, EPI_ISL_686014, EPI_ISL_686015, EPI_ISL_686016, EPI_ISL_686017, EPI_ISL_686018, EPI_ISL_686019, EPI_ISL_686020, EPI_ISL_686025, EPI_ISL_686026, EPI_ISL_686027, EPI_ISL_686028, EPI_ISL_686029, EPI_ISL_686030, EPI_ISL_686031, EPI_ISL_686032, EPI_ISL_686033, EPI_ISL_686034, EPI_ISL_686035, EPI_ISL_686036, EPI_ISL_686037, EPI_ISL_686044, EPI_ISL_686045, EPI_ISL_686046, EPI_ISL_686047, EPI_ISL_686048, EPI_ISL_686049, EPI_ISL_686050, EPI_ISL_686051, EPI_ISL_686052, EPI_ISL_686053, EPI_ISL_686054, EPI_ISL_686055, EPI_ISL_686056, EPI_ISL_686057, EPI_ISL_686058, EPI_ISL_686059, EPI_ISL_686060, EPI_ISL_686061, EPI_ISL_686062, EPI_ISL_686063, EPI_ISL_686064, EPI_ISL_686065, EPI_ISL_686066, EPI_ISL_686067, EPI_ISL_686068, EPI_ISL_686069, EPI_ISL_686070, EPI_ISL_686071, EPI_ISL_686072, EPI_ISL_686073			
see above	Pathogen Genomics Center, National Institute of Infectious Diseases	Pathogen Genomics Center, National Institute of Infectious Diseases	Tsuyoshi Sekizuka, Kentaro Itokawa, Rina Tanaka, Masanori Hashino, Makoto Kuroda
EPI_ISL_686083, EPI_ISL_686084, EPI_ISL_686085, EPI_ISL_686086, EPI_ISL_686087, EPI_ISL_686088, EPI_ISL_686089, EPI_ISL_686090, EPI_ISL_686091, EPI_ISL_686092, EPI_ISL_686093, EPI_ISL_686094, EPI_ISL_686095, EPI_ISL_686096, EPI_ISL_686097, EPI_ISL_686098, EPI_ISL_686099, EPI_ISL_686100,			

EPI_ISL_686101, EPI_ISL_686102, EPI_ISL_686103, EPI_ISL_686104, EPI_ISL_686105, EPI_ISL_686106, EPI_ISL_686107			
see above	Saitama Prefectural Institute of Public Health	Pathogen Genomics Center, National Institute of Infectious Diseases	Tsuyoshi Sekizuka, Kentaro Itokawa, Rina Tanaka, Masanori Hashino, Makoto Kuroda
EPI_ISL_686849, EPI_ISL_686850, EPI_ISL_686851, EPI_ISL_686852, EPI_ISL_686853, EPI_ISL_686854, EPI_ISL_686855, EPI_ISL_686856, EPI_ISL_686857, EPI_ISL_686858, EPI_ISL_686859, EPI_ISL_686860, EPI_ISL_686861, EPI_ISL_686862, EPI_ISL_686863, EPI_ISL_686864, EPI_ISL_686865, EPI_ISL_686866, EPI_ISL_686867, EPI_ISL_686868, EPI_ISL_686869, EPI_ISL_686870, EPI_ISL_686871, EPI_ISL_686872, EPI_ISL_686873, EPI_ISL_687618, EPI_ISL_687921, EPI_ISL_687922, EPI_ISL_687924, EPI_ISL_687925, EPI_ISL_687926, EPI_ISL_687927, EPI_ISL_687928, EPI_ISL_687929, EPI_ISL_687930, EPI_ISL_688084, EPI_ISL_688085, EPI_ISL_688762, EPI_ISL_688763, EPI_ISL_688764, EPI_ISL_688765, EPI_ISL_688766, EPI_ISL_688767, EPI_ISL_688768, EPI_ISL_688769, EPI_ISL_688770, EPI_ISL_688771, EPI_ISL_688772, EPI_ISL_688773, EPI_ISL_688774, EPI_ISL_688775, EPI_ISL_688776, EPI_ISL_688777, EPI_ISL_688778, EPI_ISL_688779, EPI_ISL_688780, EPI_ISL_688781, EPI_ISL_688782, EPI_ISL_688783, EPI_ISL_688784, EPI_ISL_688785, EPI_ISL_688786, EPI_ISL_688787, EPI_ISL_688788, EPI_ISL_688789, EPI_ISL_688790, EPI_ISL_688791, EPI_ISL_688792, EPI_ISL_688793, EPI_ISL_688794, EPI_ISL_688795, EPI_ISL_688796, EPI_ISL_688797, EPI_ISL_688798, EPI_ISL_688799, EPI_ISL_688800, EPI_ISL_688801, EPI_ISL_688802, EPI_ISL_688803, EPI_ISL_688804, EPI_ISL_688805, EPI_ISL_688806, EPI_ISL_688807, EPI_ISL_688808, EPI_ISL_688809, EPI_ISL_688810, EPI_ISL_688811, EPI_ISL_690614, EPI_ISL_690615, EPI_ISL_690617, EPI_ISL_690620, EPI_ISL_690621, EPI_ISL_690622, EPI_ISL_690623, EPI_ISL_690625			
see above	Pathogen Genomics Center, National Institute of Infectious Diseases	Pathogen Genomics Center, National Institute of Infectious Diseases	Tsuyoshi Sekizuka, Kentaro Itokawa, Rina Tanaka, Masanori Hashino, Makoto Kuroda
EPI_ISL_690626, EPI_ISL_690627, EPI_ISL_690628	Sakai City Institute of Public Health	Pathogen Genomics Center, National Institute of Infectious Diseases	Tsuyoshi Sekizuka, Kentaro Itokawa, Rina Tanaka, Masanori Hashino, Makoto Kuroda
EPI_ISL_690629	Pathogen Genomics Center, National Institute of Infectious Diseases	Pathogen Genomics Center, National Institute of Infectious Diseases	Tsuyoshi Sekizuka, Kentaro Itokawa, Rina Tanaka, Masanori Hashino, Makoto Kuroda
EPI_ISL_690630, EPI_ISL_690631	Saitama Prefectural Institute of Public Health	Pathogen Genomics Center, National Institute of Infectious Diseases	Tsuyoshi Sekizuka, Kentaro Itokawa, Rina Tanaka, Masanori Hashino, Makoto Kuroda
EPI_ISL_690636, EPI_ISL_690637	Pathogen Genomics Center, National Institute of Infectious Diseases	Pathogen Genomics Center, National Institute of Infectious Diseases	Tsuyoshi Sekizuka, Kentaro Itokawa, Rina Tanaka, Masanori Hashino, Makoto Kuroda
EPI_ISL_690641, EPI_ISL_690642, EPI_ISL_690643, EPI_ISL_690644, EPI_ISL_690645, EPI_ISL_690646, EPI_ISL_690647, EPI_ISL_690648, EPI_ISL_690649, EPI_ISL_690650, EPI_ISL_690651, EPI_ISL_690652, EPI_ISL_690653, EPI_ISL_690654, EPI_ISL_690655, EPI_ISL_690657			
see above	Saitama Prefectural Institute of Public Health	Pathogen Genomics Center, National Institute of Infectious Diseases	Tsuyoshi Sekizuka, Kentaro Itokawa, Rina Tanaka, Masanori Hashino, Makoto Kuroda
EPI_ISL_690684, EPI_ISL_690685, EPI_ISL_690686, EPI_ISL_690711, EPI_ISL_690712, EPI_ISL_690713, EPI_ISL_690714, EPI_ISL_690715, EPI_ISL_690716, EPI_ISL_690717, EPI_ISL_690718, EPI_ISL_690719, EPI_ISL_690720, EPI_ISL_690721, EPI_ISL_690722, EPI_ISL_690723, EPI_ISL_690724, EPI_ISL_690725, EPI_ISL_690726, EPI_ISL_690727, EPI_ISL_690728, EPI_ISL_690729, EPI_ISL_690730, EPI_ISL_690731, EPI_ISL_690732, EPI_ISL_690733, EPI_ISL_690734, EPI_ISL_690735, EPI_ISL_690736, EPI_ISL_690737, EPI_ISL_690738, EPI_ISL_690739, EPI_ISL_690740, EPI_ISL_690752, EPI_ISL_690753, EPI_ISL_690754, EPI_ISL_690755, EPI_ISL_690756, EPI_ISL_690757, EPI_ISL_690758, EPI_ISL_690759, EPI_ISL_690760, EPI_ISL_690761, EPI_ISL_690762, EPI_ISL_690763, EPI_ISL_690764, EPI_ISL_690765, EPI_ISL_690766, EPI_ISL_690767, EPI_ISL_690768, EPI_ISL_690769, EPI_ISL_690770, EPI_ISL_690771, EPI_ISL_690772, EPI_ISL_690773, EPI_ISL_690774, EPI_ISL_690775, EPI_ISL_690776, EPI_ISL_690777, EPI_ISL_690779, EPI_ISL_690780, EPI_ISL_690781, EPI_ISL_690782, EPI_ISL_690783, EPI_ISL_690784, EPI_ISL_690785, EPI_ISL_690786, EPI_ISL_690787, EPI_ISL_690788, EPI_ISL_690789, EPI_ISL_690790, EPI_ISL_690791			
see above	Pathogen Genomics Center, National Institute of Infectious Diseases	Pathogen Genomics Center, National Institute of Infectious Diseases	Tsuyoshi Sekizuka, Kentaro Itokawa, Rina Tanaka, Masanori Hashino, Makoto Kuroda
EPI_ISL_690792, EPI_ISL_690793	Tokyo Metropolitan Institute of Public Health	Pathogen Genomics Center, National Institute of Infectious Diseases	Tsuyoshi Sekizuka, Kentaro Itokawa, Rina Tanaka, Masanori Hashino, Makoto Kuroda
EPI_ISL_690827, EPI_ISL_690828, EPI_ISL_690829, EPI_ISL_690830, EPI_ISL_690831, EPI_ISL_690832, EPI_ISL_690833, EPI_ISL_690834	Kanagawa Prefectural Institute of Public Health	Pathogen Genomics Center, National Institute of Infectious Diseases	Tsuyoshi Sekizuka, Kentaro Itokawa, Rina Tanaka, Masanori Hashino, Makoto Kuroda
EPI_ISL_690837, EPI_ISL_690838, EPI_ISL_690839, EPI_ISL_690840, EPI_ISL_690841	Sakai City Institute of Public Health	Pathogen Genomics Center, National Institute of Infectious Diseases	Tsuyoshi Sekizuka, Kentaro Itokawa, Rina Tanaka, Masanori Hashino, Makoto Kuroda
EPI_ISL_690874, EPI_ISL_690875, EPI_ISL_690876, EPI_ISL_690877, EPI_ISL_690878, EPI_ISL_690879, EPI_ISL_690880, EPI_ISL_690881, EPI_ISL_690882, EPI_ISL_690883, EPI_ISL_690884, EPI_ISL_690885, EPI_ISL_690886, EPI_ISL_690887, EPI_ISL_690888, EPI_ISL_690889, EPI_ISL_690890, EPI_ISL_690891, EPI_ISL_690892, EPI_ISL_690893, EPI_ISL_690894, EPI_ISL_690895, EPI_ISL_690896, EPI_ISL_690897, EPI_ISL_690898, EPI_ISL_690899, EPI_ISL_690900, EPI_ISL_690901, EPI_ISL_690909, EPI_ISL_690910, EPI_ISL_690911, EPI_ISL_690912, EPI_ISL_690913, EPI_ISL_690914, EPI_ISL_690915, EPI_ISL_690916, EPI_ISL_690917, EPI_ISL_690918, EPI_ISL_690919, EPI_ISL_690920, EPI_ISL_690921, EPI_ISL_690922, EPI_ISL_690923, EPI_ISL_690924, EPI_ISL_690925, EPI_ISL_690926, EPI_ISL_690927, EPI_ISL_690928, EPI_ISL_690929, EPI_ISL_690930, EPI_ISL_690931, EPI_ISL_690932, EPI_ISL_690933, EPI_ISL_690934, EPI_ISL_690935, EPI_ISL_690936, EPI_ISL_690937, EPI_ISL_690938, EPI_ISL_690939, EPI_ISL_690940, EPI_ISL_690941, EPI_ISL_690942, EPI_ISL_690943, EPI_ISL_690944, EPI_ISL_690945, EPI_ISL_690946, EPI_ISL_690947, EPI_ISL_690948, EPI_ISL_690949, EPI_ISL_690950, EPI_ISL_690951, EPI_ISL_690952, EPI_ISL_690953, EPI_ISL_690954, EPI_ISL_690955, EPI_ISL_690956, EPI_ISL_690957, EPI_ISL_690958, EPI_ISL_690959, EPI_ISL_690960, EPI_ISL_690961, EPI_ISL_690962, EPI_ISL_690963, EPI_ISL_690964, EPI_ISL_690965, EPI_ISL_690966, EPI_ISL_690967, EPI_ISL_690968, EPI_ISL_690969, EPI_ISL_690970, EPI_ISL_690971, EPI_ISL_690972, EPI_ISL_690978, EPI_ISL_690979, EPI_ISL_690980			
see above	Pathogen Genomics Center, National Institute of Infectious Diseases	Pathogen Genomics Center, National Institute of Infectious Diseases	Tsuyoshi Sekizuka, Kentaro Itokawa, Rina Tanaka, Masanori Hashino, Makoto Kuroda
EPI_ISL_690982, EPI_ISL_690983, EPI_ISL_690984, EPI_ISL_690985, EPI_ISL_690986	Fukuoka Institute of Health and Environmental Sciences	Pathogen Genomics Center, National Institute of Infectious Diseases	Tsuyoshi Sekizuka, Kentaro Itokawa, Rina Tanaka, Masanori Hashino, Makoto Kuroda
EPI_ISL_690991, EPI_ISL_690992, EPI_ISL_690993, EPI_ISL_690994, EPI_ISL_690995, EPI_ISL_690996, EPI_ISL_690997, EPI_ISL_690998, EPI_ISL_690999, EPI_ISL_691000, EPI_ISL_691001, EPI_ISL_691002, EPI_ISL_691003, EPI_ISL_691004, EPI_ISL_691005, EPI_ISL_691006, EPI_ISL_691007, EPI_ISL_691008, EPI_ISL_691009, EPI_ISL_691010, EPI_ISL_691011, EPI_ISL_691033, EPI_ISL_691034, EPI_ISL_691035, EPI_ISL_691036, EPI_ISL_691037, EPI_ISL_691038, EPI_ISL_691039, EPI_ISL_691040, EPI_ISL_691041, EPI_ISL_691042, EPI_ISL_691043, EPI_ISL_691044, EPI_ISL_691045, EPI_ISL_691046, EPI_ISL_691047, EPI_ISL_691048, EPI_ISL_691049, EPI_ISL_691050, EPI_ISL_691051, EPI_ISL_691052, EPI_ISL_691053, EPI_ISL_691054, EPI_ISL_691055, EPI_ISL_691056, EPI_ISL_691057, EPI_ISL_691058, EPI_ISL_691059, EPI_ISL_691060, EPI_ISL_691061, EPI_ISL_691062, EPI_ISL_691063, EPI_ISL_691064, EPI_ISL_691065, EPI_ISL_691066, EPI_ISL_691067, EPI_ISL_691068, EPI_ISL_691069, EPI_ISL_691070, EPI_ISL_691071, EPI_ISL_691072, EPI_ISL_691073, EPI_ISL_691074, EPI_ISL_691075, EPI_ISL_691076, EPI_ISL_691077, EPI_ISL_691078, EPI_ISL_691079, EPI_ISL_691080, EPI_ISL_691081, EPI_ISL_691082, EPI_ISL_691083, EPI_ISL_691084, EPI_ISL_691085, EPI_ISL_691086, EPI_ISL_691087, EPI_ISL_691088, EPI_ISL_691089, EPI_ISL_691090, EPI_ISL_691091, EPI_ISL_691092, EPI_ISL_691093, EPI_ISL_691094, EPI_ISL_691095, EPI_ISL_691096, EPI_ISL_691097, EPI_ISL_691098, EPI_ISL_691099, EPI_ISL_691100, EPI_ISL_691101, EPI_ISL_691102, EPI_ISL_691103, EPI_ISL_691104, EPI_ISL_691109, EPI_ISL_691110, EPI_ISL_691111, EPI_ISL_691112, EPI_ISL_691113, EPI_ISL_691114, EPI_ISL_691115, EPI_ISL_691116, EPI_ISL_691117, EPI_ISL_691118, EPI_ISL_691119, EPI_ISL_691120, EPI_ISL_691121, EPI_ISL_691122, EPI_ISL_691123, EPI_ISL_691124, EPI_ISL_691125, EPI_ISL_691126, EPI_ISL_691127, EPI_ISL_691128, EPI_ISL_691129, EPI_ISL_691130, EPI_ISL_691131, EPI_ISL_691132, EPI_ISL_691133, EPI_ISL_691134, EPI_ISL_691135, EPI_ISL_691136, EPI_ISL_691137, EPI_ISL_691138, EPI_ISL_691139, EPI_ISL_691140, EPI_ISL_691141, EPI_ISL_691142, EPI_ISL_691143, EPI_ISL_691144, EPI_ISL_691145, EPI_ISL_691146, EPI_ISL_691147, EPI_ISL_691148, EPI_ISL_691149, EPI_ISL_691150, EPI_ISL_691151, EPI_ISL_691152, EPI_ISL_691153, EPI_ISL_691154, EPI_ISL_691155, EPI_ISL_691156, EPI_ISL_691157, EPI_ISL_691158, EPI_ISL_691159, EPI_ISL_691160, EPI_ISL_691161, EPI_ISL_691162, EPI_ISL_691163, EPI_ISL_691164, EPI_ISL_691165, EPI_ISL_691166, EPI_ISL_691167, EPI_ISL_691168, EPI_ISL_691169, EPI_ISL_691170, EPI_ISL_691171, EPI_ISL_691172, EPI_ISL_691173, EPI_ISL_691174, EPI_ISL_691175, EPI_ISL_691176, EPI_ISL_691177, EPI_ISL_691178, EPI_ISL_691179, EPI_ISL_691180, EPI_ISL_691181, EPI_ISL_691182, EPI_ISL_691183, EPI_ISL_691184, EPI_ISL_691185, EPI_ISL_691186, EPI_ISL_691187, EPI_ISL_691188, EPI_ISL_691189, EPI_ISL_691190, EPI_ISL_691191, EPI_ISL_691192, EPI_ISL_691193, EPI_ISL_691194, EPI_ISL_691195, EPI_ISL_691196, EPI_ISL_691197, EPI_ISL_691198, EPI_ISL_691199, EPI_ISL_691200, EPI_ISL_691201, EPI_ISL_691202, EPI_ISL_691203, EPI_ISL_691204, EPI_ISL_691205, EPI_ISL_691206, EPI_ISL_691207, EPI_ISL_691208, EPI_ISL_691209, EPI_ISL_691210, EPI_ISL_691211, EPI_ISL_691212, EPI_ISL_691213, EPI_ISL_691214, EPI_ISL_691215, EPI_ISL_691216, EPI_ISL_691217, EPI_ISL_691218, EPI_ISL_691219, EPI_ISL_691220, EPI_ISL_691221, EPI_ISL_691222, EPI_ISL_691223, EPI_ISL_691224, EPI_ISL_691225, EPI_ISL_691228, EPI_ISL_691229, EPI_ISL_691230, EPI_ISL_691231, EPI_ISL_691243, EPI_ISL_691244, EPI_ISL_691245, EPI_ISL_691246, EPI_ISL_691247, EPI_ISL_691249, EPI_ISL_691250, EPI_ISL_691251, EPI_ISL_691257, EPI_ISL_691258, EPI_ISL_691300, EPI_ISL_691301, EPI_ISL_691302, EPI_ISL_691303, EPI_ISL_691304, EPI_ISL_691305, EPI_ISL_691306, EPI_ISL_691307, EPI_ISL_691308, EPI_ISL_691309, EPI_ISL_691310, EPI_ISL_691311, EPI_ISL_691312, EPI_ISL_691313, EPI_ISL_691314, EPI_ISL_691315, EPI_ISL_691316, EPI_ISL_691317, EPI_ISL_691318, EPI_ISL_691319, EPI_ISL_691320, EPI_ISL_691321, EPI_ISL_691322, EPI_ISL_691323, EPI_ISL_691324, EPI_ISL_691325, EPI_ISL_691326, EPI_ISL_691327, EPI_ISL_691328, EPI_ISL_691329, EPI_ISL_691330, EPI_ISL_691331, EPI_ISL_691332, EPI_ISL_691333, EPI_ISL_691334, EPI_ISL_691335, EPI_ISL_691336, EPI_ISL_691337, EPI_ISL_691338, EPI_ISL_691339, EPI_ISL_691340, EPI_ISL_691341, EPI_ISL_691342, EPI_ISL_691343, EPI_ISL_691344, EPI_ISL_691345, EPI_ISL_691346, EPI_ISL_691347, EPI_ISL_691348, EPI_ISL_691349, EPI_ISL_691350, EPI_ISL_691351, EPI_ISL_691352, EPI_ISL_691353, EPI_ISL_691354, EPI_ISL_691355, EPI_ISL_691356, EPI_ISL_691357, EPI_ISL_691358, EPI_ISL_691359, EPI_ISL_691360, EPI_ISL_691361, EPI_ISL_691362, EPI_ISL_691363, EPI_ISL_691384, EPI_ISL_691385, EPI_ISL_691396, EPI_ISL_691397, EPI_ISL_691398, EPI_ISL_691399, EPI_ISL_691400, EPI_ISL_691401, EPI_ISL_691402, EPI_ISL_691403, EPI_ISL_691404, EPI_ISL_691405, EPI_ISL_691406, EPI_ISL_691407, EPI_ISL_691408, EPI_ISL_691409, EPI_ISL_691410, EPI_ISL_691411, EPI_ISL_691412, EPI_ISL_691413, EPI_ISL_691414, EPI_ISL_691415, EPI_ISL_691416, EPI_ISL_691417, EPI_ISL_691418, EPI_ISL_691419, EPI_ISL_691420, EPI_ISL_691421, EPI_ISL_691422, EPI_ISL_691423, EPI_ISL_691424, EPI_ISL_691425, EPI_ISL_691426, EPI_ISL_691427, EPI_ISL_691428, EPI_ISL_691429, EPI_ISL_691430, EPI_ISL_691431, EPI_ISL_691432, EPI_ISL_691433, EPI_ISL_691434, EPI_ISL_691435, EPI_ISL_691436, EPI_ISL_691437, EPI_ISL_691438, EPI_ISL_691439, EPI_ISL_691440, EPI_ISL_691441, EPI_ISL_691442, EPI_ISL_691443, EPI_ISL_691444, EPI_ISL_691445, EPI_ISL_691446, EPI_ISL_691447, EPI_ISL_691448, EPI_ISL_691449, EPI_ISL_691450, EPI_ISL_691451, EPI_ISL_691452, EPI_ISL_691453, EPI_ISL_691454, EPI_ISL_691455, EPI_ISL_691456, EPI_ISL_691457, EPI_ISL_691458, EPI_ISL_691459, EPI_ISL_691460, EPI_ISL_691461, EPI_ISL_691462, EPI_ISL_691463, EPI_ISL_691464, EPI_ISL_691465, EPI_ISL_691466, EPI_ISL_691467, EPI_ISL_691468, EPI_ISL_691469, EPI_ISL_691470, EPI_ISL_691471, EPI_ISL_691472, EPI_ISL_691473, EPI_ISL_691474, EPI_ISL_691475, EPI_ISL_691476, EPI_ISL_691477, EPI_ISL_691478, EPI_ISL_691479, EPI_ISL_691480, EPI_ISL_691481, EPI_ISL_691482, EPI_ISL_691483, EPI_ISL_691484, EPI_ISL_691485, EPI_ISL_691486, EPI_ISL_691487, EPI_ISL_691488, EPI_ISL_691489, EPI_ISL_691490, EPI_ISL_691491, EPI_ISL_691492, EPI_ISL_691493, EPI_ISL_691494, EPI_ISL_691495, EPI_ISL_691496, EPI_ISL_691497, EPI_ISL_691498, EPI_ISL_691499, EPI_ISL_691507, EPI_ISL_691508			
see above	Pathogen Genomics Center, National Institute of Infectious Diseases	Pathogen Genomics Center, National Institute of Infectious Diseases	Tsuyoshi Sekizuka, Kentaro Itokawa, Rina Tanaka, Masanori Hashino, Makoto Kuroda
EPI_ISL_691509, EPI_ISL_691510, EPI_ISL_691511, EPI_ISL_691512, EPI_ISL_691513, EPI_ISL_691514, EPI_ISL_691515, EPI_ISL_691516, EPI_ISL_691517, EPI_ISL_691518, EPI_ISL_691519, EPI_ISL_691520, EPI_ISL_691521, EPI_ISL_691522, EPI_ISL_691523, EPI_ISL_691524, EPI_ISL_691525, EPI_ISL_691526, EPI_ISL_691527, EPI_ISL_691528, EPI_ISL_691529, EPI_ISL_691530, EPI_ISL_691531, EPI_ISL_691532, EPI_ISL_691533, EPI_ISL_691534, EPI_ISL_691535, EPI_ISL_691536, EPI_ISL_691537, EPI_ISL_691538, EPI_ISL_691539, EPI_ISL_691540, EPI_ISL_691541, EPI_ISL_691542			
see above	Sakai City Institute of Public Health	Pathogen Genomics Center, National Institute of Infectious Diseases	Tsuyoshi Sekizuka, Kentaro Itokawa, Rina Tanaka, Masanori Hashino, Makoto Kuroda

EPI_ISL_691757, EPI_ISL_691758, EPI_ISL_691767, EPI_ISL_691872, EPI_ISL_691873, EPI_ISL_691874, EPI_ISL_691875, EPI_ISL_691876, EPI_ISL_691877, EPI_ISL_691878, EPI_ISL_691879, EPI_ISL_691880, EPI_ISL_691881, EPI_ISL_691882, EPI_ISL_691883, EPI_ISL_691884, EPI_ISL_691885, EPI_ISL_691886, EPI_ISL_691887, EPI_ISL_691888, EPI_ISL_691889, EPI_ISL_691890, EPI_ISL_691891, EPI_ISL_691892, EPI_ISL_691893, EPI_ISL_691894, EPI_ISL_691895, EPI_ISL_691896, EPI_ISL_691897, EPI_ISL_691898, EPI_ISL_691899, EPI_ISL_691900, EPI_ISL_691901, EPI_ISL_691902, EPI_ISL_691903, EPI_ISL_691904, EPI_ISL_691905, EPI_ISL_691906, EPI_ISL_691907, EPI_ISL_691908, EPI_ISL_691909, EPI_ISL_691910, EPI_ISL_691911, EPI_ISL_691912, EPI_ISL_691913, EPI_ISL_691914, EPI_ISL_691915, EPI_ISL_691916, EPI_ISL_691917, EPI_ISL_691918, EPI_ISL_691919, EPI_ISL_691920, EPI_ISL_691921, EPI_ISL_691922, EPI_ISL_691923, EPI_ISL_691924, EPI_ISL_691925, EPI_ISL_691926, EPI_ISL_691927, EPI_ISL_691928, EPI_ISL_691929, EPI_ISL_691930, EPI_ISL_691931, EPI_ISL_691932, EPI_ISL_691933, EPI_ISL_691934, EPI_ISL_691935, EPI_ISL_691936, EPI_ISL_691937, EPI_ISL_691938, EPI_ISL_691939, EPI_ISL_691940, EPI_ISL_691941, EPI_ISL_691942, EPI_ISL_691943, EPI_ISL_691944, EPI_ISL_691945, EPI_ISL_691946, EPI_ISL_691947, EPI_ISL_691948, EPI_ISL_691949, EPI_ISL_691950, EPI_ISL_691951, EPI_ISL_691952, EPI_ISL_691953, EPI_ISL_691954, EPI_ISL_691955, EPI_ISL_691956, EPI_ISL_691957, EPI_ISL_691958, EPI_ISL_691959, EPI_ISL_691960, EPI_ISL_691961, EPI_ISL_691962, EPI_ISL_691963, EPI_ISL_691964, EPI_ISL_691965, EPI_ISL_691966, EPI_ISL_691967, EPI_ISL_691968, EPI_ISL_691969, EPI_ISL_691970, EPI_ISL_691971, EPI_ISL_691972, EPI_ISL_691973, EPI_ISL_691974, EPI_ISL_691975, EPI_ISL_691976, EPI_ISL_691977, EPI_ISL_691978, EPI_ISL_691979, EPI_ISL_691980, EPI_ISL_691981, EPI_ISL_691982, EPI_ISL_691983, EPI_ISL_691984, EPI_ISL_691985, EPI_ISL_691986, EPI_ISL_691987, EPI_ISL_691988, EPI_ISL_691989, EPI_ISL_691990, EPI_ISL_691991, EPI_ISL_691992, EPI_ISL_691993, EPI_ISL_691994, EPI_ISL_691995, EPI_ISL_691996, EPI_ISL_691997, EPI_ISL_691998, EPI_ISL_691999, EPI_ISL_692000, EPI_ISL_692001, EPI_ISL_692002, EPI_ISL_692003, EPI_ISL_692004, EPI_ISL_692005, EPI_ISL_692006, EPI_ISL_692007, EPI_ISL_692008, EPI_ISL_692009, EPI_ISL_692010, EPI_ISL_692011, EPI_ISL_692012, EPI_ISL_692013, EPI_ISL_692014, EPI_ISL_692015, EPI_ISL_692016, EPI_ISL_692017, EPI_ISL_692018, EPI_ISL_692019, EPI_ISL_692020, EPI_ISL_692021, EPI_ISL_692022, EPI_ISL_692023, EPI_ISL_692024, EPI_ISL_692025, EPI_ISL_692026, EPI_ISL_692027, EPI_ISL_692028, EPI_ISL_692029, EPI_ISL_692030, EPI_ISL_692031, EPI_ISL_692032, EPI_ISL_692033, EPI_ISL_692034, EPI_ISL_692035, EPI_ISL_692036, EPI_ISL_692037, EPI_ISL_692038, EPI_ISL_692039, EPI_ISL_692040, EPI_ISL_692041, EPI_ISL_692042, EPI_ISL_692043, EPI_ISL_692197

see above	Pathogen Genomics Center, National Institute of Infectious Diseases	Pathogen Genomics Center, National Institute of Infectious Diseases	Tsuyoshi Sekizuka, Kentaro Itokawa, Rina Tanaka, Masanori Hashino, Makoto Kuroda
EPI_ISL_693201	Hospital Sao Paulo de Ensino da Unifesp	Instituto Adolfo Lutz, Interdiciplinary Procedures Center, Strategic Laboratory	Claudio Tavares Sacchi, Claudia Regina Gonçalves, Erica Valessa Ramos Gomes, Karoline Rodrigues Campos
EPI_ISL_693202	Pronto Socorro Municipal Prof. Joao Catarin Mezomo	Instituto Adolfo Lutz, Interdiciplinary Procedures Center, Strategic Laboratory	Claudio Tavares Sacchi, Claudia Regina Gonçalves, Erica Valessa Ramos Gomes, Karoline Rodrigues Campos
EPI_ISL_693287	unknown	Public Health Virology Laboratory, Forensic and Scientific Services (PHV-FSS)	Son Nguyen et al.
EPI_ISL_694055	AZ SPHL, Arizona Department of Health Services	TGen North	Jolene Bowers, Megan Folkerts, Chris French, Hayley Yaglom, Ashlyn Pfeiffer, Darrin Lemmer, Dave Engelthaler, The Arizona COVID Genomics Union (ACGU)
EPI_ISL_696015, EPI_ISL_696017, EPI_ISL_696019, EPI_ISL_696022, EPI_ISL_696023, EPI_ISL_696350, EPI_ISL_696379	Sonora Quest Laboratories, Laboratory Sciences of Arizona	TGen North	Jolene Bowers, Megan Folkerts, Chris French, Hayley Yaglom, Ashlyn Pfeiffer, Darrin Lemmer, Dave Engelthaler, The Arizona COVID Genomics Union (ACGU)
EPI_ISL_699641	Sydney South West Pathology Service (SSWPS) - Liverpool Hospital - NSW Health Pathology	NSW Health Pathology - Institute of Clinical Pathology and Medical Research; Westmead Hospital; University of Sydney	CIDM-PH et al.
EPI_ISL_700361	Laboratoire de virologie, CHU de Grenoble - CS 10217 - 38043 Grenoble cedex 22	CNR Virus des Infections Respiratoires - France SUD	Antonin Bal, Gregory Destras, Gwendolyne Burfin, Hadrien Règue, Quentin Semanas, Martine Valette, Bruno Lina, Sylvie Larrat, Laurence Josset
EPI_ISL_707792	1-Laboratory of Microbiology, National Reference Lab, Charles Nicolle Hospital; 2-University of Tunis ElManar, Faculty of Medicine of Tunis, LR99ES09, Tunis, Tunisia	1-Clinical and Experimental Pharmacology Lab, LR16SP02, National Center of Pharmacovigilance, University of Tunis El Manar, Tunis, Tunisia. 2-Neurodegenerative diseases and psychiatric troubles, LR18SP03, Razi Hospital, University of Tunis El Manar, Tunis, Tunisia. 3- Ministry of Health, National Observatory of New and Emerging Diseases, 1006, Tunis, Tunisia	Ilhem Boutiba-Ben Boubaker, Sameh Trabelsi, Nissaf Ben Alaya, Maher Kharrat, Alia Ben Kahla, Jalila Ben Khelil, Salma Abid, Sana Ferjani, Mouna Ben Sassi, Mouna Safer, Awatef El MOussi, Habiba Ben Romdhane, Souissi Amira, Ines Mdini, Hanen El Jebari, Asma Ferjani, Gaies Emna, Riadh Daghfous, Riadh Gouider.
EPI_ISL_707960, EPI_ISL_707961, EPI_ISL_707962, EPI_ISL_708016, EPI_ISL_708017	Virology, Universitätsklinikum des Saarlandes	Epigenetics, Saarland University	Kathrin Kattler, Markus Vogelgesang, Stefan Lohse, Sascha Tierling, Sigrun Smola, Jörn Walter
EPI_ISL_708741, EPI_ISL_708744, EPI_ISL_708765, EPI_ISL_708766, EPI_ISL_708772, EPI_ISL_708775, EPI_ISL_708782, EPI_ISL_708784	PathWest Laboratory Medicine WA	PathWest Laboratory Medicine WA Microbial Surveillance Unit	PathWest Laboratory Medicine WA Microbial Surveillance Unit
EPI_ISL_718140, EPI_ISL_718142, EPI_ISL_718150, EPI_ISL_718152, EPI_ISL_718157, EPI_ISL_718162, EPI_ISL_718163, EPI_ISL_718164	Ministry of Health Hospitals	Institute of Health and Community Medicine	David Perera, Ooi Mong How, Chua Hock Hin, Tonnii Sia Loong Loong, Wong Jyn Shan, Wong Kiing Aik, Chan Chia Jui
EPI_ISL_722208	Triemli Hospital Zurich	Institute of Medical Virology, University of Zurich	Stefan Schmutz, Verena Kufner, Maryam Zaheri, Gabriela Ziltener, Gerhard Eich, Daniel Röösli, Jürg Böni, Michael Huber, Alexandra Trkola
EPI_ISL_723105, EPI_ISL_723106, EPI_ISL_723107	Institute of Medical Genetics and Applied Genomics	Institute of Medical Genetics and Applied Genomics	Caspar Gross, Tina Ganzenmüller, Siri Göpel, Michaela Pogoda, Daniela Bezdan, Michael Sonnabend, Angel Angelov, Nicolas Casadei, Stephan Ossowski, Thomas Iftner, Michael Bitzer
EPI_ISL_728221, EPI_ISL_728222, EPI_ISL_728223, EPI_ISL_728224, EPI_ISL_728235, EPI_ISL_728236, EPI_ISL_728237	LNR National Reference Laboratory, Mohammed VI University of Health Sciences	Medical Biotechnology Laboratory, Rabat Medical and Pharmacy School, Mohammed The Vth University in Rabat	Souad KARTTI, Housna ARROUCHI, Loubna ALLAM, Mouneem Essabbar, Mouna OUADGHIRI, Tarek Aanniz, Nabila Soara, Adib Ghassan, Saaid AMZAZI, Lahcen BELYAMANI and Azeddine IBRAHIMI
EPI_ISL_728358	Servicio de Microbiología. Hospital Clínico Universitario de Valencia	SeqCOVID-SPAIN consortium/IBV(CSIC)	David Navarro Ortega, Eliseo Albert Vicent, Ignacio Torres and SeqCOVID-SPAIN consortium
EPI_ISL_729405, EPI_ISL_729534, EPI_ISL_729548, EPI_ISL_729558	Charité Universitätsmedizin Berlin, Institut für Virologie/Labor Berlin	Charité Universitätsmedizin Berlin, Institut für Virologie	Victor M Corman, Barbara Mühlemann, Jörn Beheim-Schwarzbach, Talitha Veith, Julia Schneider, Terry Jones, Christian Drosten
EPI_ISL_730153	Scripps Medical Laboratory	Andersen lab at Scripps Research	SEARCH Alliance San Diego with Michael Quigley, Ellen Stefanski, Ian Mchardy
EPI_ISL_732552	Bundeswehr Institute of Microbiology	Bundeswehr Institute of Microbiology	Markus Antwerpen, Alexandra Rehn, Mathias Walter, Malena Bestehorn-Willmann, Sabine Zange, Enrico Georgi, Roman Wölfel
EPI_ISL_734526, EPI_ISL_734527, EPI_ISL_734528, EPI_ISL_734529, EPI_ISL_734530	UZ Leuven, National Reference Laboratory for Coronaviruses, Laboratory Medicine, Leuven, Belgium	KU Leuven, Rega Institute, Clinical and Epidemiological Virology	Tony Wawina-Bokalanga, Joan Marti-Carerras, Bert Vanmechelen, Piet Maes
EPI_ISL_738286, EPI_ISL_738287, EPI_ISL_738288, EPI_ISL_738300, EPI_ISL_738301, EPI_ISL_738302, EPI_ISL_738303, EPI_ISL_738304, EPI_ISL_738305, EPI_ISL_738306	Connecticut Veterans' Affairs Hospital	Grubaugh Lab - Yale School of Public Health	Joseph Fauver, Tara Alpert, Chantal Vogels, Mary Petrone, Isabel Ott, Ellen Foxman, Shaili Gupta, Danielle Plank, Nathan Grubaugh
EPI_ISL_751212	Pathogen Genomics Lab King Abdullah University of Science and Technology(KAUST)	Pathogen Genomics Lab King Abdullah University of Science and Technology(KAUST)	Sharif Hala, Sara Mfarrej, Raushan Nugmanova, Olga Douvropoulou, Rahul P Salunke, Raeece Naeem, Fadwa Alofi, Asim Khogeer, Afrah Alsomali, Jumana Taha, Abdulaziz Alahmadi, Kahled Algithami, Anwar Hashem, Naif Almontashiri, Arnab Pain
EPI_ISL_751321, EPI_ISL_751328, EPI_ISL_751427, EPI_ISL_751428	IRCCS Sacro Cuore Don Calabria Hospital, Department of Infectious, Tropical Diseases & Microbiology	University of Verona, Department of Biotechnology	Antonio Mori, Michela Deiana, Elena Pomari, Chiara Piubelli; Giulia Lopatriello, Luca Marcolungo, Cristina Beltrami, Chiara Degli Esposti, Emanuela Cosentino, Massimo Delledonne

EPI_ISL_753839, EPI_ISL_753840, EPI_ISL_753846, EPI_ISL_753847, EPI_ISL_753848, EPI_ISL_753850, EPI_ISL_753851, EPI_ISL_753929, EPI_ISL_753936, EPI_ISL_753937, EPI_ISL_753945

see above	Charité Universitätsmedizin Berlin, Institut für Virologie/Labor Berlin	Charité Universitätsmedizin Berlin, Institut für Virologie	Victor M Corman, Jörn Beheim-Schwarzbach, Barbara Mühlemann, Julia Schneider, Talitha Veith, Terry Jones, Christian Drosten

EPI_ISL_755878, EPI_ISL_755879, EPI_ISL_755880	Toronto Invasive Bacterial Diseases Network	McMaster University	Allison McGeer, Patryk Aftanas, Hooman Derakhshani, Angel Li, Kuganya Nirmalarajah, Emily Panousis, Ahmed Draia, Jalees Nasir, Michael Surette, Samira Mubareka, Andrew G. McArthur
EPI_ISL_759967	RSUD Dr. Iskak Tulungagung	Institute of Tropical Disease, Universitas Airlangga	Rima R Prasetya, Krisnoadi Rahardjo, Aldise M Nastri, Jezzy R Dewantari, Supriyanto Dharmoredjo, Gatot Soegiarto, Laksmi Wulandari, Resti Yudhawati, Soetjipto, Yasuko Mori, Maria I Lusida, Kazufumi Shimizu
EPI_ISL_763065	1-Laboratory of Microbiology, National Reference Lab, Charles Nicolle Hospital; 2-University of Tunis ElManar, Faculty of Medicine of Tunis, LR99ES09, Tunis, Tunisia	1-Clinical and Experimental Pharmacology Lab, LR16SP02, National Center of Pharmacovigilance, University of Tunis El Manar, Tunis, Tunisia. 2-Neurodegenerative diseases and psychiatric troubles, LR18SP03, Razi Hospital, University of Tunis El Manar, Tunis, Tunisia. 3- Ministry of Health, National Observatory of New and Emerging Diseases, 1006, Tunis, Tunisia	Ilhem Boutiba-Ben Boubaker, Sameh Trabelsi, Nissaf Ben Alaya, Maher Kharrat, Alia Ben Kahla, Jalila Ben Khelil, Salma Abid, Sana Ferjani, Mouna Ben Sassi, Mouna Safer, Awatef El Moussi, Habiba Ben Romdhane, Souissi Amira, Ines Mdini, Hanen El Jebari, Asma Ferjani, Gaies Emna, Riadh Daghfous, Riadh Gouider.
EPI_ISL_763092	Jena University Hospital, Institute for Infectious Diseases and Infection Control	Institute of infectious medicine & hospital hygiene, CaSe-Group	Spott, Riccardo; Marquet, Mike; Pletz, Matthias W.; Brandt, Christian
EPI_ISL_763351	University Hospital Limerick	Irish Coronavirus Sequencing Consortium - Teagasc Moorepark	Paul Cotter, Fiona Crispie, John Kenny, Calum Walsh
EPI_ISL_770829	Toronto Invasive Bacterial Diseases Network	McMaster University	Allison McGeer, Patryk Aftanas, Hooman Derakhshani, Angel Li, Kuganya Nirmalarajah, Emily Panousis, Ahmed Draia, Jalees Nasir, Michael Surette, Samira Mubareka, Andrew G. McArthur
EPI_ISL_775243, EPI_ISL_775244	INT Fondazione Pascale	INT Fondazione Pascale	INT Fondazione Pascale
EPI_ISL_776753, EPI_ISL_776754	Instituto Adolfo Lutz - Central	Instituto Adolfo Lutz, Interdiciplinary Procedures Center, Strategic Laboratory	Claudio Tavares Sacchi, Claudia Regina Gonçalves, Erica Valessa Ramos Gomes, Karoline Rodrigues Campos
EPI_ISL_778686, EPI_ISL_778687, EPI_ISL_778688, EPI_ISL_778689, EPI_ISL_778690	Istituto Zooprofilattico Sperimentale del Mezzogiorno	TIGEM	Patrizia Annunziata, Andrea Ballabio, Valentina Bouche, Davide Cacchiarelli (CorrespAuthor), Pellegrino Cerino, Chiara Colantuono, Lucio Di Filippo, Antonio Grimaldi, Antonio Limone, Gabriella Loconte, Anna Manfredi, Francesco Panariello, Biancamaria Pierri, Marcello Salvi, Lucia Vassallo
EPI_ISL_791522, EPI_ISL_791523, EPI_ISL_791524, EPI_ISL_791525, EPI_ISL_791526	Massachusetts State Public Health Laboratory	Infectious Disease Program, Broad Institute of Harvard and MIT	Lemieux,J.E., Siddle,K.J., Shaw,B., Adams,G., Pierce,V., Turbett,S., Anahtar,M., Branda,J., Slater,D., Harris,J., Lin,A.E., Gladden-Young,A., Lagerborg,K., Rudy,M., DeRuff,K., Carter,A., Normandin,E., Bauer,M., Reilly,S., Tomkins-Tinch,C., Loreth,C., Chaluvadi,S., Neumann,A., Cusick,C., Chapman,S.B., Gnirke,A., Flowers,K., Cerrato,F., Birren,B.W., Gallagher,G., Smole,S., Park,D.J., MacInnis,B.L., Ryan,E., LaRocque,R., Rosenberg,E. and Sabeti,P.C.
EPI_ISL_792092, EPI_ISL_792094	UW Virology Lab	UW Virology Lab	Pavitra Roychoudhury, Hong Xie, Lasata Shrestha, Meei-Li Huang, Keith R Jerome, Alexander Greninger
EPI_ISL_792103	Instituto Adolfo Lutz - Regional de Santo Andre	Instituto Adolfo Lutz, Interdiciplinary Procedures Center, Strategic Laboratory	Claudio Tavares Sacchi, Claudia Regina Gonçalves, Erica Valessa Ramos Gomes, Karoline Rodrigues Campos
EPI_ISL_792107	Instituto Adolfo Lutz - Central	Instituto Adolfo Lutz, Interdiciplinary Procedures Center, Strategic Laboratory	Claudio Tavares Sacchi, Claudia Regina Gonçalves, Erica Valessa Ramos Gomes, Karoline Rodrigues Campos
EPI_ISL_792364	Laboratorio de Virologia - HIEAyC San Juan de Dios	Área de Secuenciación del Laboratorio de Virología del Hospital de Niños Dr. Ricardo Gutierrez on behalf of 'Proyecto Argentino Interinstitucional de genomica de SARS-CoV-2' (PAIS Consortium)	Nabaes Jodar, MS; Goya, S; Natale, MI; Lusso, S; Ferioli, M; Colmeiro, M; Gatelli, A; Ercole, R; Valinotto, LE; Viegas, M.
EPI_ISL_792424	Laboratorio de Inmunología del Hospital Perrando e Instituto de Medicina Regional de la UNNE	Instituto de Biotecnología, IABIMO (CONICET), Instituto de Virología, IVIT(CONICET), Instituto de Patobiología, IPVET(CONICET), CICVyA, INTA on behalf of 'Proyecto Argentino Interinstitucional de genomica de SARS-CoV-2' (PAIS Consortium)	König, GA; Peralta, AV; Distéfano, AJ; Zavallo, D; Muñoz Hidalgo, MG; Vera, PA; Fass, M; Farber, MD; Cacciabué, MPD; Pedroarias, VC; Lozano Calderón, LC; Bengoa Luoni, S; Asurmendi, S; Foussal, MD; Deluca, G; Ayala, NA; Gómez, MV; Giusiano, G; Lucero, H; Marin, M; Lescano, L; Cayré, A; Paniego, NB; Rivarola, M; Puebla, AF; Viegas, M.
EPI_ISL_792455, EPI_ISL_792468, EPI_ISL_792469, EPI_ISL_792473	Laboratorio del Hospital Regional Ushuaia Gdor. Ernesto Campos	Hospital Regional Ushuaia - Centro Austral De Investigaciones Científicas - Universidad Nacional De Tierra Del Fuego on behalf of 'Proyecto Argentino Interinstitucional de genomica de SARS-CoV-2' (PAIS Consortium)	Ceballos, SG; Nardi, CF; Gramundi, ID; Gallego, F; De Roccis, CA; Castro, G; Cáceres, SB; Yulan, CB; Boutureira, MF.
EPI_ISL_792485, EPI_ISL_792486	Laboratorio Central Mg. Luis Alfredo Pianciola	Hospital Regional Ushuaia - Centro Austral De Investigaciones Científicas - Universidad Nacional De Tierra Del Fuego on behalf of 'Proyecto Argentino Interinstitucional de genomica de SARS-CoV-2' (PAIS Consortium)	Ceballos, SG; Nardi, CF; Gramundi, ID; Gallego, F; Pinto, C; Ziehm, MC; Mazzeo, M; Pianciola, L.
EPI_ISL_792528, EPI_ISL_792533, EPI_ISL_792537	Laboratorio Central, Ministerio de Salud Córdoba	Instituto de Patologia Vegetal (CIAP-INTA) on behalf of 'Proyecto Argentino Interinstitucional de genomica de SARS-CoV-2' (PAIS Consortium)	Fernández, FD; Debat, HJ., Re, V; Pisano, MB; Castro, G.; Barbas, G.
EPI_ISL_793013	Laboratorio de Virología del Hospital de Niños Dr. Ricardo Gutierrez	Área de Secuenciación del Laboratorio de Virología del Hospital de Niños Dr. Ricardo Gutierrez on behalf of 'Proyecto Argentino Interinstitucional de genomica de SARS-CoV-2' (PAIS Consortium)	Nabaes Jodar, MS; Goya, S; Natale, MI; Lusso, S; Gravis, E; Mistchenko, AS; Valinotto, LE; Viegas, M.
EPI_ISL_794745	Istituto Zooprofilattico Sperimentale della Puglia e della Basilicata	Istituto Zooprofilattico Sperimentale della Puglia e della Basilicata	Parisi A., Bianco A., Capozzi L., Del Sambro L., Manzulli V, Rondinone V., Pace L., Cipolletta D., Galante D.
EPI_ISL_796069, EPI_ISL_796100, EPI_ISL_796110, EPI_ISL_796113	Servicio de Microbiología. Hospital General Universitario de Castellón	SeqCOVID-SPAIN consortium/IBV(CSIC)	Rosario Moreno Muñoz, María Dolores Tirado Balaguer and SeqCOVID-SPAIN consortium
EPI_ISL_801582	Laboratory of Molecular Virology, Pontificia Universidad Católica de Chile	MSHS Pathogen Surveillance Program	Leonardo I. Almonacid, Ana S. Gonzalez-Reiche, Matthew M. Hernandez, Jorge Levican, Tamara García-Salum, Zenab Khan, Adriana van De Guchte, Ajay Obla, Jayeeta Dutta, Bremy Alburquerque, Eileen Serrano, Erick Salinas, Hala Alshammary, Juan Soto, Shwetha Hara Sridhar, Ying-Chih Wang, Melissa Smith, Robert Sebra, Adolfo Garcia-Sastre, Edward C. Holmes, Viviana Simon, Harm van Bakel, Rafael A. Medina
EPI_ISL_801670, EPI_ISL_801675, EPI_ISL_801676, EPI_ISL_801677, EPI_ISL_801678, EPI_ISL_801679, EPI_ISL_801680, EPI_ISL_801681, EPI_ISL_801682, EPI_ISL_801683	Laboratory of Molecular Virology, Pontificia Universidad Católica de Chile	MSHS Pathogen Surveillance Program	Leonardo I. Almonacid, Ana S. Gonzalez-Reiche, Matthew M. Hernandez, Jorge Levican, Ana Maria Contreras, Carlos Palma, Tamara García-Salum, Zenab Khan, Adriana van de Guchte, Ajay Obla, Jayeeta Dutta, Bremy Alburquerque, Eileen Serrano, Constanza Maldonado, M. Belen Leyton, Erick Salinas, Hala Alshammary, Juan Soto, Shwetha Hara Sridhar, Ying-Chih Wang, Melissa Smith, Robert Sebra, Marcela Ferres, Adolfo Garcia-Sastre, Edward C. Holmes, Viviana Simon, Harm van Bakel, Rafael A. Medina.
EPI_ISL_801877, EPI_ISL_801906, EPI_ISL_801935, EPI_ISL_801981, EPI_ISL_801982, EPI_ISL_801983, EPI_ISL_801984, EPI_ISL_801985, EPI_ISL_801986	MSHS Clinical Microbiology Laboratories	MSHS Pathogen Surveillance Program	Ana S. Gonzalez-Reiche, Hala Alshammary, Mitchell J. Sullivan, Brianne Ciferri, Ajay Obla, Angela Amoako, Mahmoud Awawda, Elena Hirsch, Ashley S. Salimbangon, Levy Sominsky, Katherine Beach, Kayla Russo, Charles Gleason, Shelcie Fabre, Giulio Kleiner, Zenab Khan, Bremy Alburquerque, Adriana van de Guchte, Komal Srivastava, Matthew M. Hernandez, Jayeeta Dutta, Denise Jurczyszak, Emily Ferreri, Rachel Chernet, Nancy Francoeur, Betsaida Salom Melo, Irina Oussenko, Gintaras Deikus, Juan Soto, Shwetha Hara Sridhar, Ying-Chih Wang, Kathryn Twyman, Andrew Kasarskis, Deena R. Altman, Robert Sebra, Adolfo García-Sastre, Marta Luksza, Gopi Patel, Sarah Schaefer, Melissa Gitman, Michael D. Nowak, Alberto Paniz-Mondolfi, Emilia Mia Sordillo, Viviana Simon, Harm van Bakel
EPI_ISL_804380, EPI_ISL_804396, EPI_ISL_804399, EPI_ISL_804409,	Dutch COVID-19 response team	National Institute for Public Health and the Environment (RIVM)	Adam Meijer, Harry Vennema, Dirk Eggink, Matthijs Welkers, Jeroen Cremer, Sharon van den Brink, Bas van der Veer, AnneMarie van den Brandt, Florian Zwagemaker, Dennis Schmitz, Chantal Reusken, on behalf of the national COVID-19 response team

EPI_ISL_804424, EPI_ISL_804432, EPI_ISL_804456, EPI_ISL_804458, EPI_ISL_804462			
EPI_ISL_806092, EPI_ISL_806094, EPI_ISL_806172, EPI_ISL_806224, EPI_ISL_806290, EPI_ISL_806321, EPI_ISL_806372, EPI_ISL_806404, EPI_ISL_806417	Alberta Precision Labs (APL)	Alberta Precision Labs (APL)	Gordon P, Lam LG, Pabbaraju K, Wong A, Ma R, Li V, Melin A, Tipples G, Berenger B, Zelyas N, Kellner J, Bernier F, Chui L, Croxen M
EPI_ISL_806532, EPI_ISL_806538	Charité Universitätsmedizin Berlin, Institut für Virologie/Labor Berlin	Charité Universitätsmedizin Berlin, Institut für Virologie	Victor M Corman, Jörn Beheim-Schwarzbach, Barbara Mühlemann, Julia Schneider, Talitha Veith, Cornelia Schlee, Tomasz Zemojtel, Terry Jones, Christian Drosten
EPI_ISL_812314, EPI_ISL_812323, EPI_ISL_812333, EPI_ISL_812344, EPI_ISL_812345	United States Air Force School of Aerospace Medicine	United States Air Force School of Aerospace Medicine	Anthony Fries, Jennifer Meyer, Amanda Javorina, Sarah Purves, William Gruner, Clarise Starr, Elizabeth Macias
EPI_ISL_812967	University Clinical Research Center, University of Sciences	University Clinical Research Center, University of Sciences	Diarra,B., Kone,A., Guindo,I., Bane,S., Diakite,M., Dao,S., Iknane,A.A., Doumbia,S.
EPI_ISL_819293, EPI_ISL_819315, EPI_ISL_819316, EPI_ISL_819317	Hospital Universitari Vall d'Hebron - Vall d'Hebron Institut de Rercerca	Hospital Universitari Vall d'Hebron	Cristina Andrés, Maria Piñana, Josep F Abril, Damir Garcia-Cehic, Ariadna Rando, Juliana Esperalba, Maria Gema Codina, Carla Castillo, Maria Carmen Martin, Tomàs Pumarola, Josep Quer, Andrés Antón
EPI_ISL_831334, EPI_ISL_831335, EPI_ISL_831336, EPI_ISL_831337, EPI_ISL_831338, EPI_ISL_831339, EPI_ISL_831340, EPI_ISL_831341, EPI_ISL_831342, EPI_ISL_831343, EPI_ISL_831344, EPI_ISL_831345, EPI_ISL_831346, EPI_ISL_831347, EPI_ISL_831348, EPI_ISL_831349, EPI_ISL_831350, EPI_ISL_831351, EPI_ISL_831352, EPI_ISL_831353, EPI_ISL_831354, EPI_ISL_831355, EPI_ISL_831356, EPI_ISL_831357, EPI_ISL_831358, EPI_ISL_831359, EPI_ISL_831360, EPI_ISL_832082, EPI_ISL_832083, EPI_ISL_832084, EPI_ISL_832085, EPI_ISL_832086, EPI_ISL_832087, EPI_ISL_832088, EPI_ISL_832089, EPI_ISL_832091, EPI_ISL_832092, EPI_ISL_832093, EPI_ISL_832094			
see above	Clinical Molecular Microbiology Laboratory, UNC Hospitals	Jeremy Wang	Jeremy Wang, Alexander Rubinsteyn, Cecilia Thompson, Clark Cunningham, Kaylee Gentry, Jonathan Juliano, Benjamin Vincent, Melissa Miller, Robert Hagan, Corbin Jones
EPI_ISL_833576	Veterinary Specialized Instute "Nis"	Veterinary Specialized Institute "Nis", Serbia	Vidanovic,D., Tesovic,B., Manic,M., Petrovic,M.,Knezevic,A., Jovanovic,T., Jankovic,M., Sekler,M., Banovic Djeri,B., Petrovic,T., Volkening,J., Afonso,C.
EPI_ISL_837576	Centro Nacional de Enfermedades Tropicales (CENETROP)	Laboratory of Respiratory Viruses and Measles, Oswaldo Cruz Institute, FIOCRUZ	Paola Resende, Roxana Loayza, Cinthia Avila, Luciana Appolinario, Fernando Motta, Anna Carolina Paixao, Ana Carolina Mendonca, Marilda Siqueira
EPI_ISL_837621, EPI_ISL_837622, EPI_ISL_837623	Instituto Nacional de Enfermedades Respiratorias (INER)	Instituto Nacional de Enfermedades Respiratorias (INER)	Celia Boukadida, Margarita Matías-Florentino, Alma Rincón-Rubio, Hector Esteban Paz-Juárez, Olivia Briceño, Edgar Sevilla-Reyes, Fidencio Mejía-Nepomuceno, Mario Mújica-Sánchez, Eduardo Becerril-Vargas, José Arturo Martínez-Orozco, Alejandra Hernández-Terán, Jorge Salas-Hernández, Santiago Ávila-Ríos, Joel Armando Vázquez-Pérez
EPI_ISL_842830, EPI_ISL_842831	Barts Health NHS Trust	COVID-19 Genomics UK (COG-UK) Consortium	CUTINO-MOGUEL, Maria-Teresa; HARRINGTON, David; OWOYEMI, Dola; SHYLINI, Raghavendran; BROAD, Claire; KELE, Beatrix
EPI_ISL_848624	Evandro Chagas Institute	Evandro Chagas Institute	Santos, M.C.; Silva, A.M.; Junior, W.D.C.; Barbagelata, L.S.; Ferreira, J.A.; Sousa, E.M.A.; da Silva, P.S.; Pinheiro, K.C.; L.C.; Sousa Junior, E.C.
EPI_ISL_852763	Institute of Virology, Medical Center, University of Freiburg, Freiburg, Germany	Institute of Virology, Clinial Virus Genomics, Medical Center, University of Freiburg, Freiburg, Germany	Jonas Fuchs, Lisa Kern, Sandra Reuter, Hajo Grundmann, Marcus Panning
EPI_ISL_856720	Infectious Diseases Unit, Department of Internal Medicine, Azienda Ospedaliera-Universitaria di Padova	Laboratory of Infectious Diseases, Department of Biomedical and Clinical Sciences L. Sacco, University of Milan	Anna Maria Cattelan, Lolita Sasset, Davide Leoni, Alessia Lai, Annalisa Bergna, Carla Della Ventura, Claudia Balotta, Massimo Galli, Gianguglielmo Zehnder on behalf of SARS-CoV-2 ITALIAN RESEARCH ENTERPRISE-(SCIRE) Collaborative Group
EPI_ISL_861915	LATE - Laboratório de Técnicas Especiais - Hospital Israelita Albert Einstein	LATE - Laboratório de Técnicas Especiais - Hospital Israelita Albert Einstein	Deyvid Amgarten, Fernanda de Mello Malta, Raquel Riyuzo, Ana Paula Moreira Salles, Pedro Henrique Sebe Rodrigues, João Renato Rebello Pinho
EPI_ISL_862849	Ehime Prefectural Central Hospital	Fujita health University, School of Medicine, Department of Microbiology	Masahiro suzuki, Yohei Doi
EPI_ISL_862853	Tosei General Hospital	Fujita health University, School of Medicine, Department of Microbiology	Masahiro suzuki, Yohei Doi
EPI_ISL_862857	Fujita Health University Bantane Hospital	Fujita health University, School of Medicine, Department of Microbiology	Masahiro suzuki, Yohei Doi
EPI_ISL_862860	Nara Medical University Hospital	Fujita health University, School of Medicine, Department of Microbiology	Masahiro suzuki, Yohei Doi
EPI_ISL_872089	Instituto de Diagnostico y Referencia Epidemiologicos (INDRE)	Instituto de Diagnostico y Referencia Epidemiologicos (INDRE)	Abril Rodriguez-Maldonado, Gisela Barrera-Badillo ,Claudia Wong-Arambula , Natividad Cruz-Ortiz, Tatiana Nunez-Garcia, Dayanira Arellano-Suarez, Fabiola Garces-Ayala, Adnan-Araiza Rodríguez, Edgar Mendieta-Condado, Lucia Hernandez-Rivas, Irma Lopez-Martinez, Ernesto Ramirez-Gonzalez.
EPI_ISL_877014, EPI_ISL_877015	1.AO Universitaria 'S. Giovanni di Dio e Ruggi D'Aragona, Scuola Medica Salernitana' Hospital / 2.UOC di Virologia e Microbiologia, Università della Campania 'L. Vanvitelli' / 3.AO Universitaria 'Federico II' Napoli Hospital / 4.AORN 'San Giuseppe Moscati' Avellino Hospital / 5.AO 'San Pio - presidio G. Rummo' Benevento Hospital / 6.AO 'Sant'Anna e San Sebastiano' Caserta Hospital / 7.PO 'Maria Santissima Addolorata' Eboli Hospital / 8.Biogem Istituto di Ricerche Genetiche	1. Genome Research Center for Health (CRGS) / 2. Laboratory of Molecular Medicine and Genomics(LMMGe) / 3. Center for Research in Pure and Applied Mathematics (CRMPA)	Giorgio Giurato (Corresponding Author), Francesca Rizzo (Corresponding Author), Alessandro Weisz (Corresponding Author), Gianluigi Franci, Giovanni Nassa, Pasquale Pagliano, Roberta Tarallo, Elena Alexandrova, Ylenia D'Agostino, Carlo Ferravante, Jessica Lamberti, Viola Melone, Domenico Memoli, Valeria Mirici Cappa, Domenico Palumbo, Giovanni Pecoraro, Assunta Sellitto, Oriana Strianese, Ilaria Terenzi, Giuseppe Fenza, Aniello Gentile, Antonello Saccomanno, Sonia Amabile, Teresa Rocco, Annamaria Salvati, Emilia Vaccaro, Massimiliano Galdiero, Michele Cennamo, Giuseppe Portella, Maria Grazia Foti, Mariarosaria Ingino, Maria Landi, Maurizio Fumi, Vincenzo Rocco, Rita Greco, Vittoria Letizia, Arnolfo Petruzziello, Maddalena Schioppa, Gregorio Goffredi, Francesca Marciano, Michele Caraglia, Alessia Cossu, Marianna Scrima
EPI_ISL_877592, EPI_ISL_877761	Clinical Molecular Microbiology Laboratory, UNC Hospital	Dirk Dittmer	Razia Moorad , Justin T. Landis , Brent A. Eason, Melissa B. Miller, Linda Pluta, Dirk Dittmer, Angelica Juarez, Cecilia Thompson , Cameroon Grant, Evelyn Hoffman, Patricio Cano, Jason Wong, Carolina Caro-Vegas, Blossom Damania.
EPI_ISL_878539, EPI_ISL_878541	Robert Garry lab	Andersen lab at Scripps Research	Allison Smither, Gilberto Sabino-Santos, Patricia Snarski, Lilia Melnik, Antoinette Bell, Kaylynn Genemaras, Arnaud Drouin, Dahlene Fusco, Robert Garry with SEARCH Alliance San Diego
EPI_ISL_883038, EPI_ISL_883039, EPI_ISL_883040, EPI_ISL_883041, EPI_ISL_883042, EPI_ISL_883043, EPI_ISL_883044, EPI_ISL_883045, EPI_ISL_883046, EPI_ISL_883047, EPI_ISL_883048, EPI_ISL_883049, EPI_ISL_883062, EPI_ISL_883063, EPI_ISL_883064, EPI_ISL_883065			
see above	Kansas Health and Environmental Lab	Kansas Health and Environmental Lab	Mike Grose, Carissa Robertson, Ben Olsen, and Phil Adam
EPI_ISL_884556, EPI_ISL_884557	Molecular Microbiology & Immunology, University of Missouri	Molecular Microbiology & Immunology, University of Missouri	Tang,C.Y., Li,T., Hang,J., Lidl,G.M., Wan,X.-F.
EPI_ISL_887230, EPI_ISL_887246, EPI_ISL_887261, EPI_ISL_887275, EPI_ISL_887289, EPI_ISL_887338, EPI_ISL_887347, EPI_ISL_887364, EPI_ISL_887389, EPI_ISL_887393, EPI_ISL_887396, EPI_ISL_887399, EPI_ISL_887411, EPI_ISL_887414			
see above	Protzer Lab	Protzer Lab, Gagneur Lab, Robert Koch Institut	Ulrike Protzer, Dieter Hoffmann, Eva Schulte, Andrea Theumer, Oliver Drechsel, Max von Kleist,Aleksandar Radonic,Stephan Fuchs, Alexander Karollus, Julien Gagneur
EPI_ISL_887506	1.AO Universitaria 'S. Giovanni di Dio e Ruggi D'Aragona, Scuola Medica Salernitana' Hospital / 2.UOC di Virologia e Microbiologia, Università della Campania 'L. Vanvitelli' / 3.AO Universitaria 'Federico II' Napoli Hospital / 4.AORN 'San Giuseppe Moscati' Avellino Hospital / 5.AO 'San Pio - presidio G. Rummo' Benevento Hospital / 6.AO 'Sant'Anna e San Sebastiano' Caserta Hospital / 7.PO 'Maria Santissima Addolorata' Eboli Hospital / 8.Biogem Istituto di Ricerche Genetiche	1. Genome Research Center for Health (CRGS) / 2. Laboratory of Molecular Medicine and Genomics(LMMGe) / 3. Center for Research in Pure and Applied Mathematics (CRMPA)	Giorgio Giurato, Francesca Rizzo, Alessandro Weisz, Gianluigi Franci, Giovanni Nassa, Pasquale Pagliano, Roberta Tarallo, Elena Alexandrova, Ylenia D'Agostino, Carlo Ferravante, Jessica Lamberti, Viola Melone, Domenico Memoli, Valeria Mirici Cappa, Domenico Palumbo, Giovanni Pecoraro, Assunta Sellitto, Oriana Strianese, Ilaria Terenzi, Giuseppe Fenza, Aniello Gentile, Antonello Saccomanno, Sonia Amabile, Teresa Rocco, Annamaria Salvati, Emilia Vaccaro, Massimiliano Galdiero, Michele Cennamo, Giuseppe Portella, Maria Grazia Foti, Mariarosaria Ingino, Maria Landi, Maurizio Fumi, Vincenzo Rocco, Rita Greco, Vittoria Letizia, Arnolfo Petruzziello, Maddalena Schioppa, Gregorio Goffredi, Francesca Marciano, Michele Caraglia, Alessia Cossu, Marianna Scrima

EPI_ISL_889008	RS Sentra Medika Cibinong	Eijkman Institute for Molecular Biology, Ministry of Research and Technology/National Agency for Research and Innovation	Edison Johar, Frilasita A Yudhaputri, Hidayat Trimarsanto, Iskandar Adnan, Lydia V. Panggalo, Sukma Oktavianthi, Willy Agustine, Safarina G Malik, Khin Saw Myint, Amin Soebandrio
EPI_ISL_889381, EPI_ISL_889382, EPI_ISL_889383	Olomouc University Hospital	Institute of Applied Biotechnologies a.s.	Petr Klempt, Ondej Brzo, Martin Kašný, Kateina Kvapilová, Marián Hajdúch, Petr Kvapil
EPI_ISL_889808, EPI_ISL_889815, EPI_ISL_889822, EPI_ISL_889829, EPI_ISL_889833, EPI_ISL_889835, EPI_ISL_889837, EPI_ISL_889839, EPI_ISL_889840, EPI_ISL_889842, EPI_ISL_889843, EPI_ISL_889845, EPI_ISL_889848, EPI_ISL_889849, EPI_ISL_889850, EPI_ISL_889851, EPI_ISL_889852, EPI_ISL_889853, EPI_ISL_889854, EPI_ISL_889855, EPI_ISL_889856, EPI_ISL_889860, EPI_ISL_889862, EPI_ISL_889863, EPI_ISL_889864, EPI_ISL_889865, EPI_ISL_889866, EPI_ISL_889872, EPI_ISL_889889, EPI_ISL_889906, EPI_ISL_889907, EPI_ISL_889913, EPI_ISL_889914, EPI_ISL_889918, EPI_ISL_889919, EPI_ISL_889920, EPI_ISL_889927, EPI_ISL_889928, EPI_ISL_889929, EPI_ISL_889930, EPI_ISL_889931, EPI_ISL_889932, EPI_ISL_889933, EPI_ISL_889934, EPI_ISL_889936, EPI_ISL_889942, EPI_ISL_889943, EPI_ISL_889944, EPI_ISL_889945, EPI_ISL_889946, EPI_ISL_889954, EPI_ISL_889956, EPI_ISL_889959, EPI_ISL_889960, EPI_ISL_889974, EPI_ISL_889975, EPI_ISL_889980, EPI_ISL_889981, EPI_ISL_889982, EPI_ISL_889988, EPI_ISL_889989, EPI_ISL_889992, EPI_ISL_889993, EPI_ISL_889994, EPI_ISL_889995, EPI_ISL_889996, EPI_ISL_889997, EPI_ISL_889998, EPI_ISL_889999, EPI_ISL_890003, EPI_ISL_890005, EPI_ISL_890007, EPI_ISL_890015, EPI_ISL_890016, EPI_ISL_890017, EPI_ISL_890018, EPI_ISL_890019, EPI_ISL_890020, EPI_ISL_890021, EPI_ISL_890022, EPI_ISL_890023, EPI_ISL_890024, EPI_ISL_890025, EPI_ISL_890026, EPI_ISL_890027, EPI_ISL_890030, EPI_ISL_890031, EPI_ISL_890032			
see above	Laboratoire de santé publique du Québec	Laboratoire de santé publique du Québec	Sandrine Moreira, Ioannis Ragoussis, Guillaume Bourque, Jesse Shapiro, Mark Lathrop and Michel Roger on behalf of the CoVSeQ research group
EPI_ISL_890851	Laboratoire de santé publique du Québec	Laboratoire de santé publique du Québec	Sandrine Moreira, Ioannis Ragoussis, Guillaume Bourque, Jesse Shapiro, Mark Lathrop and Michel Roger on behalf of the CoVSeQ research group (http://covseq.ca/researchgroup)
EPI_ISL_892515, EPI_ISL_892516, EPI_ISL_892517, EPI_ISL_892518, EPI_ISL_892519, EPI_ISL_892520, EPI_ISL_892521, EPI_ISL_892522, EPI_ISL_892523, EPI_ISL_892524, EPI_ISL_892525, EPI_ISL_892526, EPI_ISL_892527, EPI_ISL_892528, EPI_ISL_892529, EPI_ISL_892530, EPI_ISL_892531, EPI_ISL_892532, EPI_ISL_892533			
see above	Osaka Institute of Public Health, Morinomiya Center	Pathogen Genomics Center, National Institute of Infectious Diseases	Tsuyoshi Sekizuka, Kentaro Itokawa, Rina Tanaka, Masanori Hashino, Makoto Kuroda
EPI_ISL_895749, EPI_ISL_895774, EPI_ISL_895782, EPI_ISL_895804, EPI_ISL_895812, EPI_ISL_895820, EPI_ISL_895821	Molecular biology division, Institute of Clinical Biochemistry and Diagnostics, Charles University, Faculty of Medicine in Hradec Králové and University Hospital Hradec Králové	Molecular biology division, Institute of Clinical Biochemistry and Diagnostics, Charles University, Faculty of Medicine in Hradec Králové and University Hospital Hradec Králové	Helena Kovaíková, Petr Brož, Ivana Baranová, Kateina Hrochová, Tereza Baková, Jitka Novotná, Kateina Pehlíková, Vladimír Palika. Cooperation project with BioVendor-R&D and bioinformatics company BIOXSYS s r.o.
EPI_ISL_896572, EPI_ISL_896573, EPI_ISL_896574, EPI_ISL_896575	Department of Medical Research	DMR	Myat Htut Nyunt
EPI_ISL_897529, EPI_ISL_897530, EPI_ISL_897531, EPI_ISL_897532, EPI_ISL_897533, EPI_ISL_897534, EPI_ISL_897535, EPI_ISL_897536, EPI_ISL_898569, EPI_ISL_898570, EPI_ISL_898571, EPI_ISL_898572, EPI_ISL_898573, EPI_ISL_898574, EPI_ISL_898575, EPI_ISL_898576, EPI_ISL_898577, EPI_ISL_898578, EPI_ISL_898579, EPI_ISL_898580, EPI_ISL_898581, EPI_ISL_898582, EPI_ISL_898583, EPI_ISL_898584			
see above	Pathogen Genomics Center, National Institute of Infectious Diseases	Pathogen Genomics Center, National Institute of Infectious Diseases	Tsuyoshi Sekizuka, Kentaro Itokawa, Rina Tanaka, Masanori Hashino, Makoto Kuroda
EPI_ISL_900117, EPI_ISL_900142, EPI_ISL_900197, EPI_ISL_900240, EPI_ISL_900264, EPI_ISL_900286, EPI_ISL_900292, EPI_ISL_900327, EPI_ISL_900329	MEPHI, Aix Marseille University	MEPHI, Aix Marseille University	Anthony LEVASSEUR
EPI_ISL_901227, EPI_ISL_901228, EPI_ISL_901229, EPI_ISL_901230, EPI_ISL_901231, EPI_ISL_901232, EPI_ISL_901233, EPI_ISL_901234, EPI_ISL_901235, EPI_ISL_901236, EPI_ISL_901237, EPI_ISL_901238, EPI_ISL_901239, EPI_ISL_901240, EPI_ISL_901241, EPI_ISL_901242, EPI_ISL_901243, EPI_ISL_901244, EPI_ISL_901245, EPI_ISL_901246, EPI_ISL_901247, EPI_ISL_901248, EPI_ISL_901249, EPI_ISL_901250, EPI_ISL_901328, EPI_ISL_901329, EPI_ISL_901330, EPI_ISL_901331, EPI_ISL_901332, EPI_ISL_901333, EPI_ISL_901334, EPI_ISL_901335, EPI_ISL_901336, EPI_ISL_901337, EPI_ISL_901338, EPI_ISL_901339, EPI_ISL_901340, EPI_ISL_901341, EPI_ISL_901342, EPI_ISL_901343, EPI_ISL_901344, EPI_ISL_901345, EPI_ISL_901346, EPI_ISL_901347, EPI_ISL_901348, EPI_ISL_901349, EPI_ISL_901350, EPI_ISL_901351, EPI_ISL_901352, EPI_ISL_901353, EPI_ISL_901354, EPI_ISL_901355, EPI_ISL_901356, EPI_ISL_901364, EPI_ISL_901365, EPI_ISL_901366, EPI_ISL_901367, EPI_ISL_902308, EPI_ISL_902473, EPI_ISL_902475, EPI_ISL_902476			
see above	Pathogen Genomics Center, National Institute of Infectious Diseases	Pathogen Genomics Center, National Institute of Infectious Diseases	Tsuyoshi Sekizuka, Kentaro Itokawa, Rina Tanaka, Masanori Hashino, Makoto Kuroda
EPI_ISL_904019	DB Diagnosticos do Brasil	Laboratório de Parasitologia Médica - Instituto de Medicina Tropical - Universidade de São Paulo	Brazil-UK Centre for Arbovirus Discovery Diagnosis Genomics and Epidemiology (CADDE) Genomic Network - Instituto de Medicina Tropical
EPI_ISL_906476	Pathogen Genomics Center, National Institute of Infectious Diseases	Pathogen Genomics Center, National Institute of Infectious Diseases	Tsuyoshi Sekizuka, Kentaro Itokawa, Rina Tanaka, Masanori Hashino, Makoto Kuroda
EPI_ISL_906769, EPI_ISL_906770, EPI_ISL_906771	Dr. Leonard A. Miller Centre for Health Services	National Microbiology Laboratory (NML)	Anna Majer, Shari Tyson, Grace Seo, Philip Mabon, Elsie Grudeski, Rhiannon Huzarewich, Russell Mandes, Anneliese Landgraff, Jennifer Tanner, Natalie Knox, Morag Graham, Gary Van Domselaar, Robert Needle, Yang Yu, Laura Gilbert, George Zahariadis, Nathalie Bastien, Yan Li, Timothy Booth, Darian Hole, Madison Chapel, Kerri Smith, CanCOGeN's metadata curation team, Public Health Agency of Canada CanCOGeN team
EPI_ISL_914997	QEII Health Sciences Centre	National Microbiology Laboratory (NML)	Anna Majer, Shari Tyson, Grace Seo, Philip Mabon, Elsie Grudeski, Rhiannon Huzarewich, Russell Mandes, Anneliese Landgraff, Jennifer Tanner, Natalie Knox, Morag Graham, Gary Van Domselaar, Todd Hatchette, Jason LeBlanc, Janice Pettipas, Dan Gaston, Nathalie Bastien, Yan Li, Timothy Booth, Darian Hole, Madison Chapel, CanCOGeN's metadata curation team, Public Health Agency of Canada CanCOGeN team
EPI_ISL_925537, EPI_ISL_925543, EPI_ISL_925544, EPI_ISL_925545, EPI_ISL_925546, EPI_ISL_925547, EPI_ISL_925548, EPI_ISL_925549, EPI_ISL_925550, EPI_ISL_925551, EPI_ISL_925552, EPI_ISL_925553, EPI_ISL_925554, EPI_ISL_925555, EPI_ISL_925556, EPI_ISL_925557, EPI_ISL_925558, EPI_ISL_925559, EPI_ISL_925560, EPI_ISL_925561, EPI_ISL_925562, EPI_ISL_925563, EPI_ISL_925564, EPI_ISL_925565, EPI_ISL_925566, EPI_ISL_925567, EPI_ISL_925568, EPI_ISL_925569, EPI_ISL_925570, EPI_ISL_925571, EPI_ISL_925572, EPI_ISL_925573, EPI_ISL_925574, EPI_ISL_925575, EPI_ISL_925576, EPI_ISL_925577, EPI_ISL_925578, EPI_ISL_925579, EPI_ISL_925580, EPI_ISL_925581, EPI_ISL_925582, EPI_ISL_925583, EPI_ISL_925678, EPI_ISL_925679, EPI_ISL_925680, EPI_ISL_925681, EPI_ISL_925682, EPI_ISL_925683, EPI_ISL_925684, EPI_ISL_925685, EPI_ISL_925686, EPI_ISL_925687, EPI_ISL_925688, EPI_ISL_925689, EPI_ISL_925690, EPI_ISL_925691, EPI_ISL_925692, EPI_ISL_925693, EPI_ISL_925694, EPI_ISL_925695, EPI_ISL_925696, EPI_ISL_925697, EPI_ISL_925726, EPI_ISL_925727, EPI_ISL_925731, EPI_ISL_925732, EPI_ISL_925733, EPI_ISL_925734, EPI_ISL_925735, EPI_ISL_925736, EPI_ISL_925737, EPI_ISL_925738, EPI_ISL_925739, EPI_ISL_925740, EPI_ISL_925741, EPI_ISL_925742, EPI_ISL_925743, EPI_ISL_925744, EPI_ISL_925745, EPI_ISL_925746, EPI_ISL_925747, EPI_ISL_925751, EPI_ISL_925752, EPI_ISL_925753, EPI_ISL_925754, EPI_ISL_925755, EPI_ISL_925756, EPI_ISL_925757, EPI_ISL_925758, EPI_ISL_925759, EPI_ISL_925760, EPI_ISL_925761			
see above	Public Health Ontario Laboratory	Public Health Ontario Laboratory	Vanessa G Allen, Philip Banh, Yao Chen, Richard de Borja, Alireza Eshaghi, Nahuel Fittipaldi, Christine Frantz, Jonathan B Gubbay, Jennifer L Guthrie, Lawrence Heisler, Esha Joshi, Michael Laszloffy, Aimin Li, Michael CY Li, Dean Maxwell, Sandeep Nagra, Samir N Patel, Jared Simpson, Karthikeyan Sivaraman, Ashleigh Sullivan, Yogi Sundaravadanam, Sarah Teatero, Matthew Watson, Andre Villegas, Sandra Zittermann
EPI_ISL_933618, EPI_ISL_933619	Toronto Invasive Bacterial Diseases Network	McMaster University	Allison McGeer, Patryk Aftanas, Hooman Derakhshani, Angel Li, Kuganya Nirmalarajah, Emily Panousis, Ahmed Draia, Jalees Nasir, Michael Surette, Samira Mubareka, Andrew G. McArthur
EPI_ISL_934422, EPI_ISL_934423	1.AO Universitaria 'S. Giovanni di Dio e Ruggi D'Aragona, Scuola Medica Salernitana' Hospital / 2.UOC di Virologia e Microbiologia, Università della Campania 'L. Vanvitelli' / 3.AO Universitaria 'Federico II' Napoli Hospital / 4.AORN 'San Giuseppe Moscati' Avellino Hospital / 5.AO 'San Pio - presidio G. Rummo' Benevento Hospital / 6.AO 'Sant'Anna e San Sebastiano' Caserta Hospital / 7.PO 'Maria Santissima Addolorata' Eboli Hospital / 8.Biogem Istituto di Ricerche Genetiche	1. Genome Research Center for Health (CRGS) / 2. Laboratory of Molecular Medicine and Genomics(LMMGe) / 3. Center for Research in Pure and Applied Mathematics (CRMPA)	Giorgio Giurato (Corresponding Author), Francesca Rizzo (Corresponding Author), Alessandro Weisz (Corresponding Author), Gianluigi Franci, Giovanni Nassa, Pasquale Pagliano, Roberta Tarallo, Elena Alexandrova, Ylenia D'Agostino, Carlo Ferravante, Jessica Lamberti, Viola Melone, Domenico Memoli, Valeria Mirici Cappa, Domenico Palumbo, Giovanni Pecoraro, Assunta Sellitto, Oriana Strianese, Ilaria Terenzi, Giuseppe Fenza, Aniello Gentile, Antonello Saccomanno, Sonia Amabile, Teresa Rocco, Annamaria Salvati, Emilia Vaccaro, Massimiliano Galdiero, Michele Cennamo, Giuseppe Portella, Maria Grazia Foti, Mariarosaria Ingino, Maria Landi, Maurizio Fumi, Vincenzo Rocco, Rita Greco, Vittoria Letizia, Arnolfo Petruzziello, Maddalena Schioppa, Gregorio Goffredi, Francesca Marciano, Michele Caraglia, Alessia Cossu, Marianna Scrima, Edmondo Adorisio, Morena D'Avenia, Michela Iacobellis, Rosanna Piluscio, Giorgio Dirani, Vittorio Sambri, Simona Semprini, Silvia Zanoli, Francesco Curcio, Stefania Marzinotto, Andreina Baj, Fausto Sessa.
EPI_ISL_939660, EPI_ISL_939661	Division of Infectious Diseases, University Hospital Zürich	Institute of Medical Virology, University of Zurich	Stefan Schmutz, Maryam Zaheri, Verena Kufner, Annette Audigé, Maria Grünberg, Kevin Steiner, Jon Huder, Cyril Shah, Riccarda Capaul, Guido Bloemberg, Jürg Böni, Michael Huber, Alexandra Trkola
EPI_ISL_940163, EPI_ISL_940164, EPI_ISL_940166, EPI_ISL_940167, EPI_ISL_940168, EPI_ISL_940169, EPI_ISL_940170, EPI_ISL_940183, EPI_ISL_940184, EPI_ISL_940242, EPI_ISL_940247, EPI_ISL_940248, EPI_ISL_940249, EPI_ISL_940250, EPI_ISL_940251, EPI_ISL_940252, EPI_ISL_940253, EPI_ISL_940254, EPI_ISL_940255, EPI_ISL_940256, EPI_ISL_940257, EPI_ISL_940352, EPI_ISL_940353, EPI_ISL_940357, EPI_ISL_940358, EPI_ISL_940359, EPI_ISL_940361, EPI_ISL_940362, EPI_ISL_940363, EPI_ISL_940364, EPI_ISL_940370			
see above	Hôpital Bichat Claude Bernard, Laboratoire de Virologie	IAME UMR1137 Inserm, Université de Paris, Hôpital Bichat	Antoine Bridier-Nahmias, Amélie Recoing, Quentin Le Hingrat, Lena Daniel, Siham Hamri, Gilles Collin, Alexandre Storto, Mélanie Bertine, Charlotte Charpentier, Nadhira Houhou-Fidouh, Diane Descamps, Benoit Visseaux
EPI_ISL_949240	Departamento de Microbiología, CDB, Hospital Clínic, Barcelona	SeqCOVID-SPAIN consortium/IBV(CSIC)	Andrea Vergara, Mikel Martínez, Elisa Rubio, Jéssica Navero, Aida Peiró and SeqCOVID-SPAIN consortium

EPI_ISL_949345	University of Birmingham	COVID-19 Genomics UK (COG-UK) Consortium	Institute of Microbiology, University of Birmingham: Claire McMurray, Joanne Stockton, Samuel Nicholls, Radoslaw Poplawski, Will Rowe, Josh Quick, Nicholas Loman. University of Birmingham Testing Laboratory: Celina M Whalley, Andrew Bosworth, Charlotte Poxon, Kasun Wanigasooriya, Oliver Pickles, Mike Kidd, Alex Richter, Andrew D Beggs PHE Heartlands Lab: Husam Osman, Andrew Bosworth. Queen Elizabeth Hospital: Anna Casey
EPI_ISL_954727, EPI_ISL_954728, EPI_ISL_954729, EPI_ISL_954730, EPI_ISL_954731, EPI_ISL_954732, EPI_ISL_954733, EPI_ISL_954734, EPI_ISL_954735, EPI_ISL_954736, EPI_ISL_954737, EPI_ISL_954738, EPI_ISL_954739, EPI_ISL_954740, EPI_ISL_954741, EPI_ISL_954742, EPI_ISL_954743, EPI_ISL_954744			
see above	Laboratoire de santé publique du Québec	Laboratoire de santé publique du Québec	Sandrine Moreira, Ioannis Ragoussis, Guillaume Bourque, Jesse Shapiro, Mark Lathrop and Michel Roger on behalf of the CoVSeQ research group (http://covseq.ca/researchgroup)
EPI_ISL_955257	HGZ 32 VILLA COAPA	BIOBANCO / COCTI	Borja-Aburto VH, Grajales-Muñiz C, Santacruz Tinoco CE, Rojas-Mendoza T, Ochoa Carrera LA, Sánchez A, Grande R, Isa P, Taboada B, López S, Arias C, Muñoz-Medina JE
EPI_ISL_955982, EPI_ISL_955983, EPI_ISL_955984, EPI_ISL_955985, EPI_ISL_955987, EPI_ISL_955989, EPI_ISL_955991, EPI_ISL_955992, EPI_ISL_955993, EPI_ISL_955994, EPI_ISL_955995, EPI_ISL_955996, EPI_ISL_955997, EPI_ISL_955998, EPI_ISL_955999, EPI_ISL_956000, EPI_ISL_956005, EPI_ISL_956007, EPI_ISL_956008, EPI_ISL_956010, EPI_ISL_956011, EPI_ISL_956012, EPI_ISL_956013, EPI_ISL_956015, EPI_ISL_956016, EPI_ISL_956018, EPI_ISL_956019, EPI_ISL_956022, EPI_ISL_956024, EPI_ISL_956029, EPI_ISL_956033, EPI_ISL_956034, EPI_ISL_956036, EPI_ISL_956039, EPI_ISL_956041, EPI_ISL_956046, EPI_ISL_956047, EPI_ISL_956048, EPI_ISL_956049, EPI_ISL_956051, EPI_ISL_956052, EPI_ISL_956053, EPI_ISL_956056, EPI_ISL_956061, EPI_ISL_956062, EPI_ISL_956070, EPI_ISL_956074, EPI_ISL_956075, EPI_ISL_956076, EPI_ISL_956080, EPI_ISL_956081, EPI_ISL_956082, EPI_ISL_956083, EPI_ISL_956084, EPI_ISL_956086, EPI_ISL_956089, EPI_ISL_956090, EPI_ISL_956092, EPI_ISL_956093, EPI_ISL_956097, EPI_ISL_956098, EPI_ISL_956100, EPI_ISL_956102, EPI_ISL_956104, EPI_ISL_956105, EPI_ISL_956107, EPI_ISL_956109, EPI_ISL_956113, EPI_ISL_956115, EPI_ISL_956120, EPI_ISL_956121, EPI_ISL_956123, EPI_ISL_956124, EPI_ISL_956125, EPI_ISL_956126, EPI_ISL_956130, EPI_ISL_956131, EPI_ISL_956132, EPI_ISL_956133, EPI_ISL_956137, EPI_ISL_956141, EPI_ISL_956144, EPI_ISL_956145, EPI_ISL_956149, EPI_ISL_956152, EPI_ISL_956153, EPI_ISL_956154, EPI_ISL_956155, EPI_ISL_956157, EPI_ISL_956158, EPI_ISL_956159, EPI_ISL_956160, EPI_ISL_956161, EPI_ISL_956163, EPI_ISL_956165, EPI_ISL_956166, EPI_ISL_956167, EPI_ISL_956170, EPI_ISL_956172, EPI_ISL_956174, EPI_ISL_956176, EPI_ISL_956177, EPI_ISL_956178, EPI_ISL_956179, EPI_ISL_956180, EPI_ISL_956181, EPI_ISL_956182, EPI_ISL_956183, EPI_ISL_956184, EPI_ISL_956185, EPI_ISL_956186, EPI_ISL_956187, EPI_ISL_956188, EPI_ISL_956189, EPI_ISL_956190, EPI_ISL_956192, EPI_ISL_956193, EPI_ISL_956194, EPI_ISL_956195, EPI_ISL_956196, EPI_ISL_956199, EPI_ISL_956200, EPI_ISL_956203, EPI_ISL_956205, EPI_ISL_956207, EPI_ISL_956209, EPI_ISL_956210, EPI_ISL_956211, EPI_ISL_956212, EPI_ISL_956213, EPI_ISL_956214, EPI_ISL_956216, EPI_ISL_956217, EPI_ISL_956218, EPI_ISL_956219, EPI_ISL_956220, EPI_ISL_956221, EPI_ISL_956222, EPI_ISL_956224, EPI_ISL_956225, EPI_ISL_956227, EPI_ISL_956228, EPI_ISL_956229, EPI_ISL_956230, EPI_ISL_956231, EPI_ISL_956232, EPI_ISL_956234, EPI_ISL_956236, EPI_ISL_956237, EPI_ISL_956238, EPI_ISL_956239, EPI_ISL_956240, EPI_ISL_956242, EPI_ISL_956243, EPI_ISL_956244, EPI_ISL_956246, EPI_ISL_956247, EPI_ISL_956248, EPI_ISL_956250, EPI_ISL_956253, EPI_ISL_956254, EPI_ISL_956255, EPI_ISL_956256, EPI_ISL_956257, EPI_ISL_956258, EPI_ISL_956259, EPI_ISL_956260, EPI_ISL_956262, EPI_ISL_956268			
see above	Pathology and Laboratory Medicine Institute, Cleveland Clinic, Ohio, USA	Pathology and Laboratory Medicine Institute, Cleveland Clinic, Ohio, USA	Frank P. Esper, Yu-Wei Cheng, Thamali M. Adhikari, Zheng Jin Tu, Daniel H. Farkas, Gary Procop, Jennifer Ko, Timothy A. Chan, Jing Li, Brian P. Rubin
EPI_ISL_960309	NLZOH, Laboratory for Virology	NLZOH, Laboratory for Virology	Katarina Prosenc (Laboratory for Virology), Cesare Camma (IZSAM), Erik Alm (ECDC)
EPI_ISL_961290, EPI_ISL_961292, EPI_ISL_961293, EPI_ISL_961294, EPI_ISL_961295, EPI_ISL_961296, EPI_ISL_961297, EPI_ISL_961298, EPI_ISL_961299, EPI_ISL_961300, EPI_ISL_961301, EPI_ISL_961302, EPI_ISL_961303, EPI_ISL_961304, EPI_ISL_961305, EPI_ISL_961306, EPI_ISL_961307, EPI_ISL_961308, EPI_ISL_961309, EPI_ISL_961310, EPI_ISL_961311, EPI_ISL_961312, EPI_ISL_961313, EPI_ISL_961314, EPI_ISL_961315			
see above	Biotia	Biotia	Dorottya Nagy-Szakal, Mara Couto-Rodriguez, Heather Wells, Joseph Barrows, Marilyne Debieu, Kristin Butcher, Siyuan Chen, Agnes Berki, Courteny Hager, Robert Boorstein, Mariah Taylor, Colleen Jonsson, Christopher Mason, Niamh O'Hara
EPI_ISL_961669	Hôpital Georges L. Dumont	National Microbiology Laboratory (NML)	Anna Majer, Shari Tyson, Grace Seo, Philip Mabon, Elsie Grudeski, Rhiannon Huzarewich, Russell Mandes, Anneliese Landgraff, Jennifer Tanner, Natalie Knox, Morag Graham, Gary Van Domselaar, Richard Garceau, Guillaume Desnoyers, Nathalie Bastien, Yan Li, Timothy Booth, Darian Hole, Madison Chapel, Kirsten Biggar, CanCOGeN's metadata curation team, Public Health Agency of Canada CanCOGeN team
EPI_ISL_964879	Institute of Virology Ulm University Medical Center Prof. Dr. Thomas Stamminger	Münch Lab / Ulm University Medical Center Kirchhoff Lab / Ulm University Medical Center Sparrer Lab / Ulm University Medical Center Blum Lab / LMU Munich	Thomas Stamminger, Helmut Blum, Stefan Krebs, Alexander Graf, Rüdiger Gross, Janis Müller, Carina Conzelmann, Jan Münch, Frank Kirchhoff, Konstantin Sparrer
EPI_ISL_965583	Dutch COVID-19 response team	Medical Microbiology, Maastricht University Medical Centre	Jozef Dingemans*, Brian van der Veer*, Erik Beuken, Carmen Reumkens, Lieke van Alphen, Christian Hoebe, Paul Savelkoul
EPI_ISL_965983, EPI_ISL_965999, EPI_ISL_966001, EPI_ISL_966010, EPI_ISL_966015, EPI_ISL_966018, EPI_ISL_966024, EPI_ISL_966026, EPI_ISL_966031, EPI_ISL_966176, EPI_ISL_966177, EPI_ISL_966178, EPI_ISL_966181, EPI_ISL_966183, EPI_ISL_966185, EPI_ISL_966186, EPI_ISL_966187, EPI_ISL_966188, EPI_ISL_966189, EPI_ISL_966190, EPI_ISL_966191, EPI_ISL_966192, EPI_ISL_966193, EPI_ISL_966194, EPI_ISL_966195, EPI_ISL_966196, EPI_ISL_966197, EPI_ISL_966198, EPI_ISL_966199, EPI_ISL_966200, EPI_ISL_966201, EPI_ISL_966202, EPI_ISL_966203, EPI_ISL_966205, EPI_ISL_966206, EPI_ISL_966207, EPI_ISL_966208, EPI_ISL_966209, EPI_ISL_966210, EPI_ISL_966211, EPI_ISL_966212, EPI_ISL_966213, EPI_ISL_966214, EPI_ISL_966215, EPI_ISL_966216, EPI_ISL_966217, EPI_ISL_966218, EPI_ISL_966219, EPI_ISL_966220, EPI_ISL_966221, EPI_ISL_966222, EPI_ISL_966223, EPI_ISL_966224, EPI_ISL_966225, EPI_ISL_966226, EPI_ISL_966236, EPI_ISL_966237, EPI_ISL_966239, EPI_ISL_966240, EPI_ISL_966242, EPI_ISL_966243, EPI_ISL_966244, EPI_ISL_966247, EPI_ISL_966248, EPI_ISL_966249, EPI_ISL_966250, EPI_ISL_966251, EPI_ISL_966252, EPI_ISL_966254, EPI_ISL_966255, EPI_ISL_966256, EPI_ISL_966257, EPI_ISL_966258, EPI_ISL_966259			
see above	Public Health Ontario Laboratory	Public Health Ontario Laboratory	Vanessa G Allen, Philip Banh, Yao Chen, Richard de Borja, Alireza Eshaghi, Nahuel Fittipaldi, Christine Frantz, Jonathan B Gubbay, Jennifer L Guthrie, Lawrence Heisler, Esha Joshi, Michael Laszloffy, Aimin Li, Michael CY Li, Dean Maxwell, Sandeep Nagra, Samir N Patel, Jared Simpson, Karthikeyan Sivaraman, Ashleigh Sullivan, Yogi Sundaravadanam, Sarah Teatero, Andre Villegas, Matthew Watson, Sandra Zittermann
EPI_ISL_968174	Clinical Molecular Microbiology Laboratory, UNC Hospital	Dirk Dittmer	Justin T. Landis , Razia Moorad , Brent A. Eason, Melissa B. Miller, Linda Pluta, Dirk Dittmer, Angelica Juarez, Cecilia Thompson, Shawn Hawken, Cameroon Grant, Evelyn Hoffman, Patricio Cano, Jason Wong, Carolina Caro-Vegas, Ryan McNamara, Blossom Damania.
EPI_ISL_978494	Central Public Health Laboratory - LACEN -Bahia, Salvador, Brazil	Central Public Health Laboratory - LACEN -Bahia, Salvador, Brazil	Stephane Tosta, Luciana Oliveira, Vanessa Nardy,Patrícia Cajado,Marcela Gómez, Breno Dominguez, Jaqueline Gomes, Vagner Fonseca,Marta Giovanetti,Luiz Alcantara, Felicidade Pereira, Arabela Leal
